# Assessing the causal relationships between circulating metabolic biomarkers and breast cancer by using mendelian randomization

**DOI:** 10.3389/fgene.2024.1448748

**Published:** 2024-12-18

**Authors:** Bowen Wang, Yue Ling, Hui Zhang, Ming Yang

**Affiliations:** ^1^ Department of Breast Surgery, General Surgery Center, First Hospital of Jilin University, Changchun, Jilin, China; ^2^ Department of Ophthalmology, The Second Hospital of Jilin University, Changchun, Jilin, China

**Keywords:** circulating metabolic biomarkers, metabolite, lipoprotein, lipids, mendelian randomization, breast cancer

## Abstract

**Objective:**

Previous studies have established a causal relationship between metabolites and breast cancer (BC), but the underlying mechanisms remain unclear. Thus, we aimed to investigate the genetic relationship between metabolites and BC, including its subtypes, using Mendelian randomization (MR) analysis.

**Methods:**

Utilizing the latest and most comprehensive summary statistics from genome-wide association studies we conducted an Mendelian randomization study. Data on 233 metabolites, used as exposure variables, were obtained from a study involving 136,016 participants. BC data, used as outcome variables, were sourced from a study comprising 122,977 cases and 105,974 controls. We used the inverse-variance weighted method as the primary approach, along with three supplementary methods, to assess the causal relationship. We also used Cochran’s Q test to detect heterogeneity and MR-Egger regression to examine the presence of horizontal pleiotropy.

**Results:**

Upon analyzing 233 metabolites across 11 classes in relation to BC, we found six classes of metabolites (fatty acids glycerides and phospholipids, lipoprotein subclasses, lipids, apolipoproteins, and lipoprotein particle size) associated with overall BC. Five classes of metabolites (fatty acids glycerides and phospholipids, lipoprotein subclasses, lipids, and lipoprotein particle size) were related to estrogen receptor (ER) + BC, and eight classes of metabolites (fatty acids, amino acids, glycerides and phospholipids, lipoprotein subclasses, lipids, apolipoproteins, glycolysis-related metabolites, and lipoprotein particle size) were linked to ER- BC.

**Conclusion:**

Our study demonstrates a genetic causal relationship between most metabolites and BC, confirming the link between these factors. This research provides a significant foundation for the prevention and treatment of BC.

## 1 Introduction

Breast cancer (BC) remains the most common cancer among women globally. In 2024, the United States is expected to see 313,510 new cases—2,790 in men and 310,720 in women—and about 42,780 deaths, with 530 involving men and 42,250 women (Siegel et al., 2024). Key risk factors for BC include gender, with women being at higher risk, advancing age, and genetic predispositions. Additional risk factors encompass obesity, alcohol consumption, tobacco use, and hormone replacement therapy (Britt et al., 2020). Symptoms of BC can vary but often include the presence of lumps, changes in breast shape or size, skin indentations, or redness, although early stages may be asymptomatic. The management of BC typically involves a combination of surgical intervention, radiation therapy, and pharmacotherapy, with the choice of treatment depending on the cancer subtype and stage (Li et al., 2022). BC can be classified into estrogen receptor (ER) positive and ER negative types based on the presence of ER in the cancer cells. Approximately 60%–75% of BC are ER + BC, while the remaining 25%–40% are ER- BC(Haibe-Kains et al., 2012). ER- BC tends to grow faster and have a worse short-term prognosis. They often recur within the first few years after treatment, making their management more challenging. Even with the emergence of many novel methods (Faris et al., 2021; Salam et al., 2023), early detection and treatment are critical for improving survival rates.

In recent years, circulating metabolic biomarkers have gained significant attention for their role in understanding metabolic processes and disease mechanisms (Gieger et al., 2008; Illig et al., 2010). These biomarkers include a diverse array of fatty acids, amino acids, lipoprotein subclasses, apolipoproteins, lipids, glycerides and phospholipids, glycolysis-related metabolites, inflammation and lipoprotein particle size. These biomarkers provide detailed insights into the metabolic state of the human body. They play crucial roles in fundamental metabolic activities and are closely linked with various diseases, such as diabetes, and metabolic syndrome. Metabolites are crucial components of metabolic pathways that can influence cancer development and progression. Changes in lipid metabolism have a significant role in BC by affecting cell membrane composition, signaling pathways, and energy production (Zipinotti dos Santos et al., 2023). Understanding the relationship between circulating metabolic biomarkers and BC is essential for uncovering potential metabolic pathways involved in cancer development. Identifying causal links can provide insights into how metabolic alterations influence BC risk. Additionally, this knowledge may inform personalized prevention strategies and therapeutic interventions targeting metabolic processes. Therefore, investigating these relationships is vital for advancing BC research and improving patient outcomes. Several observational studies have investigated the association between metabolic biomarkers and BC. For instance, an observational study by Jennifer C. Melvin et al., involving 1,824 Swedish women diagnosed with BC, reported a modest positive association between serum glucose and the ApoB/ApoA-1 ratio with BC severity (Melvin et al., 2017). This finding suggests that these factors do not significantly contribute to the association between obesity and BC severity. Additionally, a case-control study by Julia Debik et al., which included 1,199 case-control pairs from the Trøndelag Health Study (HUNT), found that several lipoprotein subfractions, particularly VLDL subfractions, were inversely linked with the BC in premenopausal women (Debik et al., 2022). Meanwhile, another study demonstrated that, compared to healthy women, serum TC, LDL-C, and TG levels were significantly elevated in BC patients, while HDL-C levels were significantly reduced, suggesting that lipids may play an important role in the development of BC (Faris et al., 2023). This indicates that alterations in lipid metabolism may occur well in advance of diagnosis of BC. However, there are few observational studies on other types of metabolites, highlighting the need for new approaches to investigate the relationship between metabolites and BC. However, observational studies have limitations, such as the inability to establish the direction of causality and the potential for reverse causation (Sheehan et al., 2008; Sobczyk et al., 2023).

Mendelian randomization (MR) is an epidemiological method that uses genetic variants as instrumental variables (IVs) to determine causal relationships between risk factors and health outcome (Shin et al., 2014; Murphy et al., 2020). By leveraging the random assortment of genes from parents to offspring, MR minimizes reverse causation and confounding which are common issues in observation study (Davies et al., 2018). The primary advantage of MR is its ability to provide more robust causal inferences, similar to randomized controlled trials, but using observational data. This method has been successfully used to investigate the causal effects of various exposures on diseases, offering valuable insights into potential mechanisms and therapeutic targets. By elucidating whether specific metabolic profiles influence BC risk, this study can inform targeted prevention strategies and potential metabolic interventions for BC.

## 2 Materials and methods

### 2.1 Study design and MR assumptions

**FIGURE 1:**
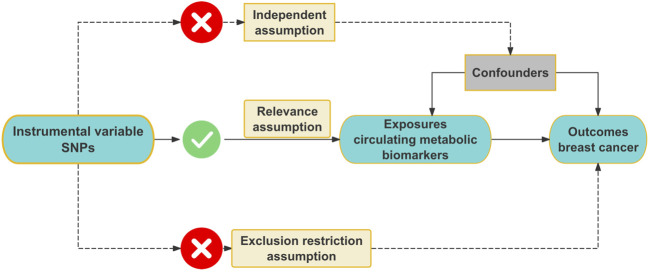
Flowchart illustrating the MR process and its core assumptions.

We conducted this MR study to investigate the association between circulating metabolic biomarkers and BC. MR analysis must adhere to three core assumptions: the relevance assumption, the independence assumption, and the exclusion restriction assumption (Bowden and Holmes, 2019). Firstly, the relevance assumption requires that the chosen IVs are strongly correlated with the circulating metabolic biomarkers. Secondly, the independence assumption stipulates that the IVs must not influence BC through any confounding factors that could affect the development of BC. Lastly, the exclusion restriction assumption asserts that the IVs do not directly cause the development of BC. The illustration of the MR process is provided in Figure 1. In this study, we first adhered to the three core assumptions of MR analysis. To address weak IVs, we applied the *F-statistic* to exclude IVs with an *F-value* less than 10, ensuring the robustness of our analysis. Additionally, we mitigated potential bias due to linkage disequilibrium by retaining independent IVs. After obtaining the filtered IVs, we conducted analyses using four distinct MR methods: IVW, MR-Egger regression, weighted median, and weighted mode method, to ensure the robustness and consistency of the results. Following the preliminary analysis, we performed various sensitivity analyses to assess the reliability of the findings. First, Cochran’s Q test was used to assess heterogeneity and determine the consistency of effects across different IVs. Second, MR-Egger regression and MR-PRESSO were applied to detect horizontal pleiotropy, ruling out the possibility that the IVs directly influenced the outcome. Furthermore, we conducted reverse MR analysis to check for reverse causality, ensuring the validity of the causal direction. Finally, we performed leave-one-out analysis by removing each IV one at a time and reanalyzing the data to verify that the results were not driven by any single IV. To assess potential publication bias, we utilized a funnel plot, ensuring the accuracy and impartiality of the results. Through this comprehensive series of sensitivity analyses and robustness checks, we arrived at reliable causal inference outcomes.

### 2.2 Data sources for exposure and outcome data

In this MR study, circulating metabolic biomarkers were selected as exposures, and BC including ER + BC and ER- BC as outcomes. Data on 233 circulating metabolic biomarkers were obtained from the study conducted by Minna et al. (Karjalainen et al., 2024). A total of 136,016 participants from 33 cohorts participated in this study and the authors finally identified more than 400 independent loci and assigned probable causal genes at two-thirds of these using manual curation of plausible biological candidates. The GWAS IDs of the data we used in this study were GCST90301941–GCST90302173 from the GWAS Catalog (https://www.ebi.ac.uk/gwas/) (Supplementary Table S1). Most metabolites have the unit mmol/l, with specific units for each metabolite detailed in Supplementary Table S17. For the outcomes, BC was categorized into three groups: overall BC, ER + BC, and ER- BC. BC genetic association data were sourced from the Breast Cancer Association Consortium (BCAC). This study, conducted by Kyriaki et al., included 122,977 cases and 105,974 controls of European ancestry, combining genetic data from iCOGS, Oncoarray, and GWAS meta-analysis (Michailidou et al., 2017). Detailed information about the data sources is provided in Table 1.

**TABLE 1:** Detailed information about the data sources of BC.

Year	Trait	Consortium	Author	Sample size	Number of SNPs
2017	Breast cancer (combined Oncoarray; iCOGS; GWAS meta analysis)	BCAC	Michailidou K	228,951	10,680,257
2017	ER + Breast cancer (combined Oncoarray; iCOGS; GWAS meta analysis)	BCAC	Michailidou K	175,475	10,680,257
2017	ER- Breast cancer (combined Oncoarray; iCOGS; GWAS meta analysis)	BCAC	Michailidou K	127,442	10,680,257

### 2.3 Selection of instrumental variable

During this process, we selected single nucleotide polymorphisms (SNPs) as IVs. To satisfy the relevance assumption, we set the *p-value* threshold for IVs at genome-wide significance (*p* < 1 × 10⁻⁸). Additionally, we chose *R* ^*2*^ < 0.001 and a distance of 10,000 kb to avoid linkage disequilibrium with other SNPs. We calculated the *F-statistic* to assess the validity of the association between the IVs and the exposure (Levin et al., 2020). The formula for the *F-statistic* is $F=\frac{R^{2}\times \left(N-2\right)}{1-R^{2}}$ , $R^{2}=2\times MAF\times \left(1-MAF\right)\times {beta}^{2}$ . We excluded SNPs with an *F-statistic* less than 10, as they are considered weak IVs. The final set of SNPs is strongly correlated with the outcome. Details of the SNPs selected for this MR analysis are listed in Supplementary Tables 11–13.

## 3 Statistical analysis

In this MR analysis, four methods were used to determine the causal relationship between circulating metabolic biomarkers and BC, including the inverse-variance weighted (IVW) method, the weighted mode method, the weighted median method, and the MR-Egger method. The IVW method, a fixed-effect meta-analysis model, is the primary approach used to combine ratio estimates for each variant into a single causal estimate (Burgess et al., 2015). Weighted median method, weighted mode, and method MR-egger method are complements to the IVW method. The false discovery rate (FDR) method is used in MR studies to control for multiple comparisons, ensuring that findings are robust despite the testing of many interrelated phenotypes (Benjamini and Hochberg, 1995). By setting an FDR threshold of 5%, the method helps confirm the significance of observed effects, adjusting for the potential of false positives due to the correlation among phenotypes. Furthermore, Cochran’s Q test was employed to assess heterogeneity among the IVs. A *p-value* greater than 0.05 indicated the absence of heterogeneity, justifying the use of the fixed-effects IVW method. Conversely, a *p-value* less than 0.05 suggested the presence of heterogeneity, necessitating the application of the random-effects IVW method. In addition, odds Ratio (OR) and confidence interval (CI) were used to determine the effect of exposure on outcomes as protective or risk factors. MR-Egger regression is employed to detect horizontal pleiotropy. A *p-value* greater than 0.05 indicates the absence of horizontal pleiotropy. We use MR-PRESSO to detect outliers of SNPs and remove these. We use the Steiger test to detect whether there is an inverse causality, and *p* less than 0.05 indicates that there is no inverse causality. Sensitivity analysis included leave-one-out analysis, funnel plots, forest plots, and scatter plots in this MR analysis. Leave-one-out analysis is used to observe the stability of causal relationships.

In this study, R software 4.4.0 (https://www.R-project.org) with MRPRESSO (1.0), MendelianRandomization (version 0.10.0), and TwoSampleMR (version 0.5.8) packages were used to perform the above analysis.

## 4 Results

233 circulating metabolic biomarkers have been classified into 11 categories: lipoprotein subclasses, lipids, inflammation, fluid balance, ketone bodies, glycolysis-related metabolites, amino acids, fatty acids, glycerides and phospholipids, apolipoproteins, and lipoprotein particle size. Eight of these categories were causally linked to all BC types. Six categories—fatty acids glycerides and phospholipids, lipoprotein subclasses, lipids, apolipoproteins, and lipoprotein particle size—showed a causal association with overall BC, whereas five categories—fatty acids glycerides and phospholipids, lipoprotein subclasses, lipids, and lipoprotein particle size—were associated with ER + BC, and another eight categories (fatty acids, amino acids, glycerides and phospholipids, lipoprotein subclasses, lipids, apolipoproteins, glycolysis-related metabolites, and lipoprotein particle size) are related to ER- BC. This finding suggests that variations in the concentrations of different types of circulating metabolites may contribute to the promotion or inhibition of BC development.

### 4.1 Causal effects of 233 circulating metabolic biomarkers and overall BC

**FIGURE 2:**
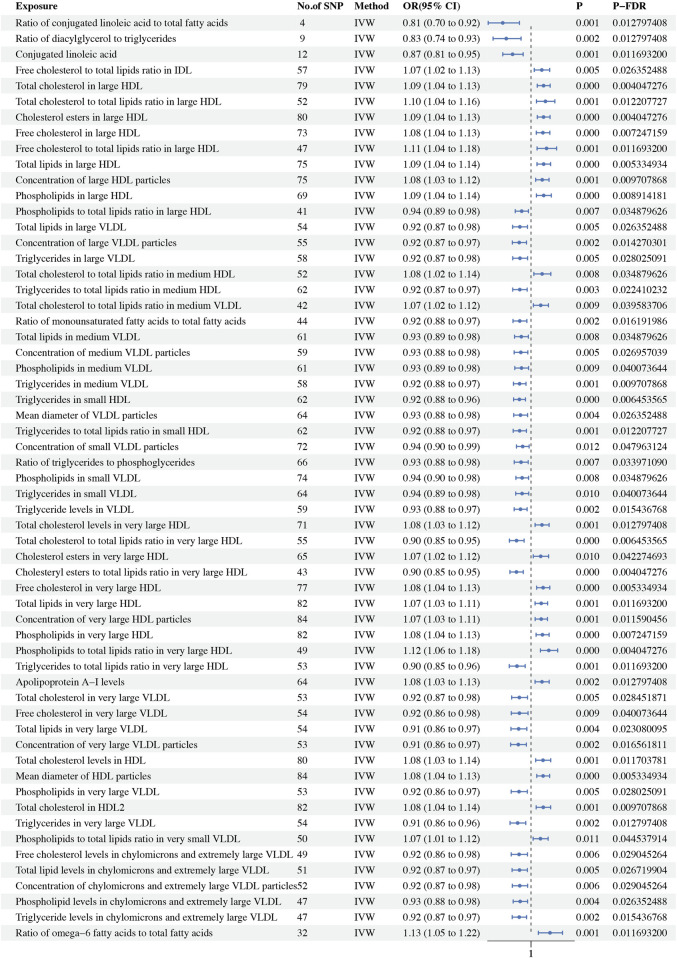
Forest plot showing the OR and 95% CI for overall breast cancer.

In this MR study, a comprehensive analysis of 223 metabolic biomarkers was conducted to investigate their potential causal associations with BC risk. Among these, 59 biomarkers demonstrated significant causal relationships with BC, with 35 acting as protective factors (OR < 1) and 24 as risk factors (OR > 1). The metabolites associated with BC were categorized into six major groups: fatty acids, lipids, glycerides and phospholipids, apolipoproteins, lipoprotein subclasses, and lipoprotein particle size. Notably, within the Lipoprotein subclasses category, 48 biomarkers were identified as having causal associations. Of these, 29 were protective factors, including ratio of phospholipids to total lipids in large HDL (*P-fdr*: 0.035, OR: 0.936, 95%CI: 0.891–0.982), total lipids in large VLDL (*P-fdr*: 0.026, OR: 0.923, 95%CI: 0.874–0.976), concentration of large VLDL particles (*P-fdr*: 0.014, OR: 0.919, 95%CI: 0.871–0.969), triglycerides in large VLDL (*P-fdr*: 0.028, OR: 0.924, 95%CI: 0.874–0.977), the ratio of triglycerides to total lipids in medium HDL (*P-fdr*: 0.022, OR: 0.923, 95%CI: 0.874–0.974), total lipids in medium VLDL (*P-fdr*: 0.035, OR: 0.933, 95%CI: 0.887–0.982), concentration of medium VLDL particles (*P-fdr*: 0.027, OR: 0.930, 95%CI: 0.884–0.978), phospholipids in medium VLDL (*P-fdr*: 0.040, OR: 0.935, 95%CI: 0.888–0.984), triglycerides in medium VLDL (*P-fdr*: 0.010, OR: 0.921, 95%CI: 0.879–0.965), triglycerides in small HDL (*P-fdr*: 0.006, OR: 0.920, 95%CI: 0.880–0.962), triglycerides ratio of small HDL to total lipids (*P-fdr*: 0.012, OR: 0.922, 95%CI: 0.877–0.968), concentration of small VLDL particles (*P-fdr*: 0.048, OR: 0.943, 95%CI: 0.900–0.987), phospholipids in small VLDL (*P-fdr*: 0.035, OR: 0.939, 95%CI: 0.896–0.983), triglycerides in small VLDL (*P-fdr*: 0.040, OR: 0.939, 95%CI: 0.895–0.985), triglyceride levels in VLDL (*P-fdr*: 0.015, OR: 0.927, 95%CI: 0.884–0.973), ratio of total cholesterol to total lipids in very large HDL (*P-fdr*: 0.006, OR: 0.900, 95%CI: 0.851–0.952), ratio of cholesteryl esters to total lipids in very large HDL (*P-fdr*: 0.004, OR: 0.899, 95%CI: 0.855–0.947), ratio of triglycerides to total lipids in very large HDL (*P-fdr*: 0.012, OR: 0.903, 95%CI: 0.850–0.960), total cholesterol in very large VLDL (*P-fdr*: 0.028, OR: 0.920, 95%CI: 0.867–0.976), free cholesterol in very large VLDL (*P-fdr*: 0.040, OR: 0.920, 95%CI: 0.864–0.980), total lipids in very large VLDL (*P-fdr* = 0.023, OR: 0.915, 95%CI: 0.862–0.971), concentration of very large VLDL particles (*P-fdr*: 0.017, OR: 0.912, 95%CI: 0.860–0.968), phospholipids in very large VLDL (*P-fdr*: 0.028, OR: 0.917, 95%CI: 0.863–0.975), triglycerides in very large VLDL (*P-fdr*: 0.013, OR: 0.908, 95%CI: 0.855–0.964), free cholesterol levels in chylomicrons and extremely large VLDL (*P-fdr*: 0.029, OR: 0.918, 95%CI: 0.863–0.976), total lipid levels in chylomicrons and extremely large VLDL (*P-fdr*: 0.027, OR: 0.921, 95%CI: 0.869–0.975), concentration of chylomicrons and extremely large VLDL particles (*P-fdr*: 0.029, OR: 0.922, 95%CI: 0.871–0.977), phospholipid levels in chylomicrons and extremely large VLDL (*P-fdr*: 0.026, OR: 0.925, 95%CI: 0.877–0.976) and triglyceride levels in chylomicrons and extremely large VLDL (*P-fdr*: 0.015, OR: 0.919, 95%CI: 0.870–0.970). Conversely, 19 biomarkers were identified as risk factors, including free cholesterol to total lipids ratio in IDL (*P-fdr*: 0.026, OR: 1.074, 95%CI: 1.023–1.129), total cholesterol in large HDL (*P-fdr*: 0.004, OR: 1.087, 95%CI: 1.043–1.132), ratio of total cholesterol to total lipids in large HDL (*P-fdr*: 0.012, OR: 1.100, 95%CI: 1.038–1.165), cholesterol esters in large HDL (*P-fdr*: 0.004, OR: 1.086, 95%CI: 1.043–1.131), free cholesterol in large HDL (*P-fdr*: 0.007, OR: 1.082, 95%CI: 1.036–1.130), ratio of free cholesterol to total lipids in large HDL (*P-fdr*: 0.012, OR: 1.107, 95%CI: 1.042–1.177), total lipids in large HDL (*P-fdr*: 0.005, OR: 1.087, 95%CI: 1.041–1.135), concentration of large HDL particles (*P-fdr*: 0.010, OR: 1.077, 95%CI: 1.032–1.125), phospholipids in large HDL (*P-fdr*: 0.009, OR: 1.087, 95%CI: 1.038–1.140), ratio of total cholesterol to total lipids in medium HDL (*P-fdr*: 0.035, OR: 1.077, 95%CI: 1.020–1.137), ratio of medium VLDL cholesterol to medium VLDL lipids (*P-fdr*: 0.040, OR: 1.067, 95%CI: 1.016–1.120), total cholesterol levels in very large HDL (*P-fdr*: 0.013, OR: 1.075, 95%CI: 1.028–1.125), cholesterol esters in very large HDL (*P-fdr*: 0.042, OR: 1.067, 95%CI: 1.015–1.122), free cholesterol in very large HDL (*P-fdr*: 0.005, OR: 1.084, 95%CI: 1.039–1.130), total lipids in very large HDL (*P-fdr*: 0.012, OR: 1.070, 95%CI: 1.028–1.115), concentration of very large HDL particles (*P-fdr*: 0.012, OR: 1.070, 95%CI: 1.029–1.113), phospholipids in very large HDL (*P-fdr*: 0.007, OR: 1.079, 95%CI: 1.035–1.125), ratio of phospholipids to total lipids in very large HDL (*P-fdr*: 0.004, OR: 1.123, 95%CI: 1.065–1.184) and ratio of phospholipids to total lipids in very small VLDL (*P-fdr*: 0.045, OR: 1.065, 95%CI: 1.014–1.118). In the lipid category, both total cholesterol levels in HDL (OR: 1.083, 95%CI: 1.032–1.136, *P-fdr*: 0.012) and total cholesterol in HDL2 (*P-fdr*: 0.010, OR: 1.085, 95%CI: 1.036–1.136) were identified as risk factors for BC. Within the glycerides and phospholipids category, the ratio of diacylglycerol to triglycerides (*P-fdr*: 0.013, OR: 0.829, 95%CI: 0.738–0.931) and the ratio of triglycerides to phosphoglycerides (*P-fdr*: 0.034, OR: 0.931, 95%CI: 0.884–0.981) were recognized as protective factors. Among the apolipoproteins, elevated apolipoprotein A-I levels (*P-fdr*: 0.013, OR: 1.076, 95%CI: 1.028-1.126) were linked to a higher risk of BC. In the fatty acids category, three biomarkers, namely, ratio of conjugated linoleic acid to total fatty acids (*P-fdr*: 0.013, OR: 0.805, 95%CI: 0.705–0.920), conjugated linoleic acid (*P-fdr*: 0.012, OR: 0.873, 95%CI: 0.805–0.946) and ratio of monounsaturated fatty acids to total fatty acids (*P-fdr*: 0.016, OR: 0.922, 95%CI: 0.876–0.972) were found to be protective factors. However, the ratio of omega-6 fatty acids to total fatty acids (*P-fdr*: 0.012, OR: 1.131, 95%CI: 1.051–1.218) was associated with increased risk. In the category of lipoprotein particle size, the mean diameter of VLDL particles (*P-fdr*: 0.026, OR: 0.927, 95%CI: 0.881–0.977) was found to be protective, whereas the mean diameter of HDL particles (*P-fdr*: 0.005, OR: 1.083, 95%CI: 1.040–1.129) posed an increased risk. Sensitivity analyses indicated significant heterogeneity in 56 of the metabolic biomarkers. Consequently, the IVW method was employed for analysis. Additionally, the Steiger test did not detect any reverse causality (Supplementary Table S14). No pleiotropy was detected in the study. Detailed findings on heterogeneity and pleiotropy are presented in Supplementary Tables S5, 8, and the causal effects of the circulating metabolic biomarkers on overall breast cancer risk are depicted in Figure 2. The results are shown in Table 2.

**TABLE 2:** Causal effects of 233 circulating metabolic biomarkers and BC.

Exposure	Outcome	Method	nsnp	b	se	Or	pval	p_fdr
Ratio of conjugated linoleic acid to total fatty acids	Breast cancer	IVW	4	−0.216673334	0.068095147	0.805192956	0.00146302	0.012797408
Ratio of diacylglycerol to triglycerides	Breast cancer	IVW	9	−0.187237837	0.05930465	0.829246488	0.00159281	0.012797408
Conjugated linoleic acid	Breast cancer	IVW	12	−0.13603266	0.041009329	0.872814126	0.000909522	0.0116932
Cholesteryl esters to total lipids ratio in very large HDL	Breast cancer	IVW	43	−0.105953996	0.026041371	0.899466026	4.72803E-05	0.004047276
Total cholesterol to total lipids ratio in very large HDL	Breast cancer	IVW	55	−0.105032845	0.028674002	0.900294952	0.000249279	0.006453565
Triglycerides to total lipids ratio in very large HDL	Breast cancer	IVW	53	−0.101755688	0.030973769	0.9032502	0.001019023	0.0116932
Triglycerides in very large VLDL	Breast cancer	IVW	54	−0.096507778	0.030422678	0.908002835	0.001512724	0.012797408
Concentration of very large VLDL particles	Breast cancer	IVW	53	−0.091780695	0.03025496	0.912305201	0.002416745	0.016561811
Total lipids in very large VLDL	Breast cancer	IVW	54	−0.089023938	0.030548337	0.914823674	0.003566023	0.023080095
Phospholipids in very large VLDL	Breast cancer	IVW	53	−0.08630994	0.030950078	0.917309876	0.005292292	0.028025091
Free cholesterol levels in chylomicrons and extremely large VLDL	Breast cancer	IVW	49	−0.085787698	0.031132418	0.917789059	0.005858916	0.029045264
Triglyceride levels in chylomicrons and extremely large VLDL	Breast cancer	IVW	47	−0.084908368	0.027561538	0.918596453	0.002065262	0.015436768
Concentration of large VLDL particles	Breast cancer	IVW	55	−0.084628182	0.027165058	0.918853867	0.001837378	0.014270301
Total cholesterol in very large VLDL	Breast cancer	IVW	53	−0.083898046	0.030217347	0.919525	0.005494997	0.028451871
Triglycerides in small HDL	Breast cancer	IVW	62	−0.083552301	0.022683977	0.919842977	0.000230218	0.006453565
Free cholesterol in very large VLDL	Breast cancer	IVW	54	−0.08338935	0.032059256	0.919992879	0.00929252	0.040073644
Total lipid levels in chylomicrons and extremely large VLDL	Breast cancer	IVW	51	−0.082681078	0.029249155	0.920644715	0.004701786	0.026719904
Triglycerides in medium VLDL	Breast cancer	IVW	58	−0.082074421	0.023925182	0.9212034	0.000602555	0.009707868
Triglycerides to total lipids ratio in small HDL	Breast cancer	IVW	62	−0.081628257	0.025307021	0.921614499	0.001257448	0.012207727
Ratio of monounsaturated fatty acids to total fatty acids	Breast cancer	IVW	44	−0.080758163	0.026483633	0.922416739	0.002293286	0.016191986
Concentration of chylomicrons and extremely large VLDL particles	Breast cancer	IVW	52	−0.080670967	0.029213107	0.922497174	0.005754272	0.029045264
Triglycerides to total lipids ratio in medium HDL	Breast cancer	IVW	62	−0.080625576	0.027497173	0.922539048	0.003366344	0.022410232
Total lipids in large VLDL	Breast cancer	IVW	54	−0.079991434	0.028174881	0.923124253	0.004524032	0.026352488
Triglycerides in large VLDL	Breast cancer	IVW	58	−0.079123566	0.028307832	0.923925751	0.00518817	0.028025091
Phospholipid levels in chylomicrons and extremely large VLDL	Breast cancer	IVW	47	−0.077694727	0.027318669	0.925246836	0.004454883	0.026352488
Mean diameter of VLDL particles	Breast cancer	IVW	64	−0.075352447	0.026317819	0.927416563	0.004194225	0.026352488
Triglyceride levels in VLDL	Breast cancer	IVW	59	−0.07530692	0.024506989	0.927458787	0.002120071	0.015436768
Concentration of medium VLDL particles	Breast cancer	IVW	59	−0.072784804	0.02584484	0.929800897	0.004859209	0.026957039
Ratio of triglycerides to phosphoglycerides	Breast cancer	IVW	66	−0.071261046	0.026423089	0.931218769	0.006998336	0.03397109
Total lipids in medium VLDL	Breast cancer	IVW	61	−0.069346647	0.026003687	0.933003201	0.00765771	0.034879626
Phospholipids in medium VLDL	Breast cancer	IVW	61	−0.067525853	0.026027636	0.934703556	0.009475878	0.040073644
Phospholipids to total lipids ratio in large HDL	Breast cancer	IVW	41	−0.066370723	0.024806425	0.935783883	0.00746077	0.034879626
Triglycerides in small VLDL	Breast cancer	IVW	64	−0.063442011	0.024506452	0.938528542	0.009631434	0.040073644
Phospholipids in small VLDL	Breast cancer	IVW	74	−0.063301744	0.023786154	0.938660196	0.007784294	0.034879626
Concentration of small VLDL particles	Breast cancer	IVW	72	−0.059178186	0.023596727	0.942538807	0.012145169	0.047963124
Phospholipids to total lipids ratio in very small VLDL	Breast cancer	IVW	50	0.063012037	0.024808333	1.065039659	0.011086691	0.044537914
Total cholesterol to total lipids ratio in medium VLDL	Breast cancer	IVW	42	0.064941761	0.024863786	1.067096876	0.009004019	0.039583706
Cholesterol esters in very large HDL	Breast cancer	IVW	65	0.065036635	0.025363485	1.067198121	0.010341878	0.042274693
Concentration of very large HDL particles	Breast cancer	IVW	84	0.067840501	0.020225461	1.0701946	0.000795911	0.011590456
Total lipids in very large HDL	Breast cancer	IVW	82	0.068105414	0.020574254	1.070478146	0.00093221	0.0116932
Free cholesterol to total lipids ratio in IDL	Breast cancer	IVW	57	0.071785318	0.025269964	1.07442466	0.004500943	0.026352488
Total cholesterol levels in very large HDL	Breast cancer	IVW	71	0.072772387	0.022887702	1.075485714	0.001475085	0.012797408
Apolipoprotein A-I levels	Breast cancer	IVW	64	0.07305185	0.023113164	1.075786315	0.001574356	0.012797408
Total cholesterol to total lipids ratio in medium HDL	Breast cancer	IVW	52	0.073853032	0.027713454	1.076648561	0.007701577	0.034879626
Concentration of large HDL particles	Breast cancer	IVW	75	0.074616292	0.021814187	1.077470637	0.00062497	0.009707868
Phospholipids in very large HDL	Breast cancer	IVW	82	0.076124281	0.021257188	1.079096677	0.000342141	0.007247159
Free cholesterol in large HDL	Breast cancer	IVW	73	0.079035353	0.022043842	1.082242582	0.000336599	0.007247159
Total cholesterol levels in HDL	Breast cancer	IVW	80	0.079740699	0.024443009	1.083006207	0.001105078	0.011703781
Mean diameter of HDL particles	Breast cancer	IVW	84	0.080120491	0.020880579	1.083417602	0.000124505	0.005334934
Free cholesterol in very large HDL	Breast cancer	IVW	77	0.080246838	0.021259802	1.083554497	0.000160277	0.005334934
Total cholesterol in HDL2	Breast cancer	IVW	82	0.081150545	0.023491243	1.084534155	0.00055131	0.009707868
Cholesterol esters in large HDL	Breast cancer	IVW	80	0.082961018	0.020632826	1.086499454	5.79943E-05	0.004047276
Total cholesterol in large HDL	Breast cancer	IVW	79	0.083124214	0.020895689	1.086676781	6.94811E-05	0.004047276
Total lipids in large HDL	Breast cancer	IVW	75	0.083739745	0.02202084	1.08734587	0.000143099	0.005334934
Phospholipids in large HDL	Breast cancer	IVW	69	0.083873975	0.023939707	1.087491834	0.000459099	0.008914181
Total cholesterol to total lipids ratio in large HDL	Breast cancer	IVW	52	0.094939487	0.029356716	1.099592313	0.001220714	0.012207727
Free cholesterol to total lipids ratio in large HDL	Breast cancer	IVW	47	0.101917068	0.031038972	1.107291638	0.001025196	0.0116932
Phospholipids to total lipids ratio in very large HDL	Breast cancer	IVW	49	0.115904545	0.027010679	1.122888682	1.77819E-05	0.004047276
Ratio of omega-6 fatty acids to total fatty acids	Breast cancer	IVW	32	0.123157088	0.03759684	1.131062083	0.001053894	0.0116932

### 4.2 Causal effects of 233 circulating metabolic biomarkers and ER + BC

**FIGURE 3:**
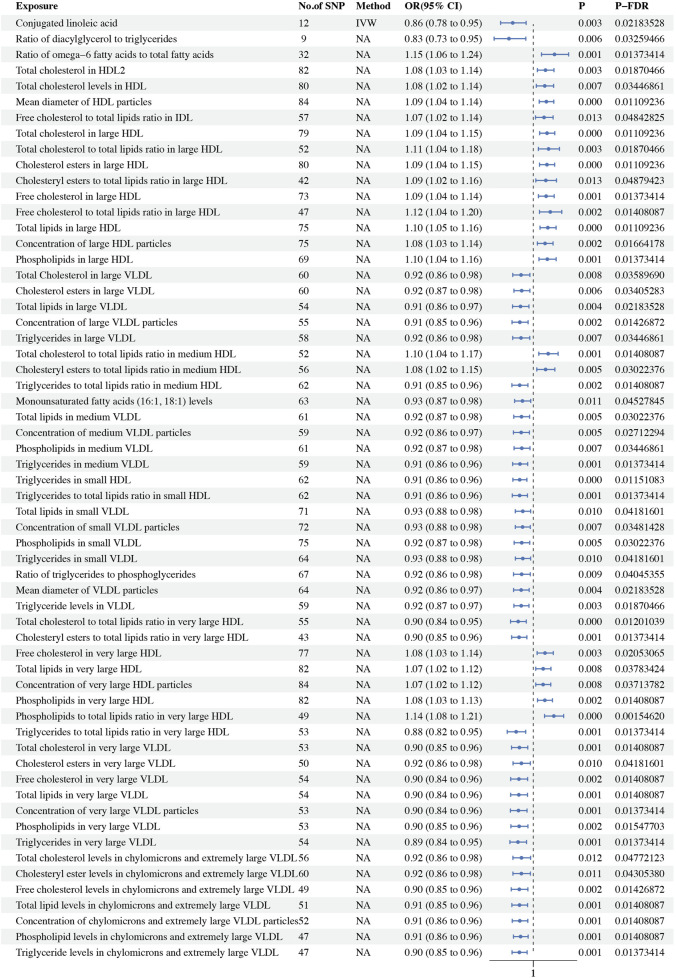
Forest plot showing the OR and 95% CI for ER+ breast cancer.

In our study on ER + BC, we identified a total of 60 circulating metabolic biomarkers with causal relationships to BC. Among these, 39 were found to be protective factors against BC (OR < 1), while 21 were identified as risk factors (OR > 1). Specifically, within the lipoprotein subclasses, 34 biomarkers including total cholesterol in large VLDL (*P-fdr*: 0.036, OR: 0.916, 95%CI: 0.859–0.977), cholesterol esters in large VLDL (*P-fdr*: 0.034, OR: 0.920, 95%CI: 0.867–0.977), total lipids in large VLDL (*P-fdr*: 0.022, OR: 0.912, 95%CI: 0.858–0.970), concentration of large VLDL particles (*P-fdr*: 0.014, OR: 0.908, 95%CI: 0.854–0.964), triglycerides in large VLDL (*P-fdr*: 0.034, OR: 0.916, 95%CI: 0.859–0.976), the ratio of triglycerides to total lipids in medium HDL (*P-fdr*: 0.014, OR: 0.907, 95%CI: 0.853–0.963), total lipids in medium VLDL (*P-fdr*: 0.030, OR: 0.919, 95%CI: 0.866–0.975), concentration of medium VLDL particles (*P-fdr*: 0.027, OR: 0.917, 95%CI: 0.864–0.974), phospholipids in medium VLDL (*P-fdr*: 0.034, OR: 0.921, 95%CI: 0.868–0.978), triglycerides in medium VLDL (*P-fdr*: 0.014, OR: 0.909, 95%CI: 0.858–0.962), triglycerides in small HDL (*P-fdr*: 0.012, OR: 0.907, 95%CI: 0.860–0.956), triglycerides ratio of small HDL to total lipids (*P-fdr*: 0.014, OR: 0.906, 95%CI: 0.856–0.960), total lipids in small VLDL (*P-fdr*: 0.042, OR: 0.928, 95%CI: 0.877–0.982), concentration of small VLDL particles (*P-fdr*: 0.035, OR: 0.927, 95%CI: 0.877–0.980), phospholipids in small VLDL (*P-fdr*: 0.030, OR: 0.921, 95%CI: 0.869–0.976), triglycerides in small VLDL (*P-fdr*: 0.042, OR: 0.928, 95%CI: 0.877–0.982), triglyceride levels in VLDL (*P-fdr*: 0.019, OR: 0.917, 95%CI: 0.867–0.971), ratio of total cholesterol to total lipids in very large HDL (*P-fdr*: 0.012, OR: 0.896, 95%CI: 0.844–0.952), ratio of cholesteryl esters to total lipids in very large HDL (*P-fdr*: 0.014, OR: 0.902, 95%CI: 0.852–0.956), ratio of triglycerides to total lipids in very large HDL (*P-fdr*: 0.014, OR: 0.882, 95%CI: 0.821–0.948), total cholesterol in very large VLDL (*P-fdr*: 0.014, OR: 0.905, 95%CI: 0.851–0.962), cholesterol esters in very large VLDL (*P-fdr*: 0.042, OR: 0.919, 95%CI: 0.861–0.980), free cholesterol in very large VLDL (*P-fdr*: 0.014, OR: 0.901, 95%CI: 0.845–0.962), total lipids in very large VLDL (*P-fdr*: 0.014, OR: 0.901, 95%CI: 0.845–0.960), concentration of very large VLDL particles (*P-fdr*: 0.014, OR: 0.898, 95%CI: 0.843–0.957), phospholipids in very large VLDL (*P-fdr*: 0.015, OR: 0.903, 95%CI: 0.846–0.963), triglycerides in very large VLDL (*P-fdr*: 0.014, OR: 0.895, 95%CI: 0.840–0.954), total cholesterol levels in chylomicrons and extremely large VLDL (*P-fdr*: 0.048, OR: 0.919, 95%CI: 0.860–0.982), cholesteryl ester levels in chylomicrons and extremely large VLDL (*P-fdr*: 0.043, OR: 0.918, 95%CI: 0.860–0.980), free cholesterol levels in chylomicrons and extremely large VLDL (*P-fdr*: 0.014, OR: 0.904, 95%CI: 0.849–0.963), total lipid levels in chylomicrons and extremely large VLDL (*P-fdr*: 0.014, OR: 0.905, 95%CI: 0.853–0.961), concentration of chylomicrons and extremely large VLDL particles (*P-fdr*: 0.014, OR: 0.907, 95%CI: 0.856–0.962), phospholipid levels in chylomicrons and extremely large VLDL (*P-fdr*: 0.014, OR: 0.913, 95%CI: 0.864–0.965) and triglyceride levels in chylomicrons and extremely large VLDL (*P-fdr*: 0.014, OR: 0.905, 95%CI: 0.854–0.958) exhibited a negative association with BC, and 17 biomarkers including free cholesterol to total lipids ratio in IDL (*P-fdr*: 0.048, OR: 1.074, 95%CI: 1.015–1.136), total cholesterol in large HDL (*P-fdr*: 0.011, OR: 1.095, 95%CI: 1.044–1.148), ratio of total cholesterol to total lipids in large HDL (*P-fdr*: 0.019, OR: 1.105, 95%CI: 1.035–1.180), cholesterol esters in large HDL (*P-fdr*: 0.011, OR: 1.094, 95%CI: 1.043–1.147), ratio of cholesteryl esters to total lipids in large HDL (*P-fdr*: 0.049, OR: 1.086, 95%CI: 1.018–1.159), free cholesterol in large HDL (*P-fdr*: 0.014, OR: 1.088, 95%CI: 1.036–1.144), ratio of free cholesterol to total lipids in large HDL (*P-fdr*: 0.014, OR: 1.117, 95%CI: 1.043–1.196), total lipids in large HDL (*P-fdr*: 0.011, OR: 1.100, 95%CI: 1.046–1.157), concentration of large HDL particles (*P-fdr*: 0.017, OR: 1.081, 95%CI: 1.028–1.136), phospholipids in large HDL (*P-fdr*: 0.014, OR: 1.097, 95%CI: 1.039–1.157), ratio of total cholesterol to total lipids in medium HDL (*P-fdr*: 0.014, OR: 1.102, 95%CI: 1.038–1.171), ratio of cholesteryl esters to total lipids in medium HDL (*P-fdr*: 0.030, OR: 1.085, 95%CI: 1.024–1.148), free cholesterol in very large HDL (*P-fdr*: 0.021, OR: 1.081, 95%CI: 1.027–1.138), total lipids in very large HDL (*P-fdr*: 0.038, OR: 1.068, 95%CI: 1.017–1.122), concentration of very large HDL particles (*P-fdr*: 0.037, OR: 1.068, 95%CI: 1.017–1.120), phospholipids in very large HDL (*P-fdr*: 0.014, OR: 1.079, 95%CI: 1.030–1.132) and ratio of phospholipids to total lipids in very large HDL (*P-fdr*: 0.002, OR: 1.141, 95%CI: 1.078–1.209) showed a positive association. Regarding lipids, total cholesterol in HDL2 (*P-fdr*: 0.019, OR: 1.084, 95%CI: 1.028–1.142) and total cholesterol levels in HDL (*P-fdr*: 0.034, OR: 1.079, 95%CI: 1.021–1.140) were determined to be risk factors for ER + BC. Within the category of glycerides and phospholipids, ratio of diacylglycerol to triglycerides (*P-fdr*: 0.033, OR: 0.833, 95%CI: 0.731–0.949), ratio of triglycerides to phosphoglycerides (*P-fdr*: 0.040, OR: 0.920, 95%CI: 0.864–0.979) were identified as protective factors against ER + BC. In the fatty acids category, conjugated linoleic acid (*P-fdr*: 0.022, OR: 0.859, 95%CI: 0.776–0.951), monounsaturated fatty acids (16:1, 18:1) levels (*P-fdr*: 0.045, OR: 0.926, 95%CI: 0.872–0.983) was found to be a protective factor for ER + BC, whereas the ratio of omega-6 fatty acids to total fatty acids (*P-fdr*: 0.014, OR: 1.146, 95%CI: 1.057–1.243) was identified as a risk factor. For lipoprotein particle size, the mean diameter of VLDL particles (*P-fdr*: 0.022, OR: 0.917, 95%CI: 0.865–0.972) was found to be a protective factor for ER + BC, while the mean diameter of HDL particles (*P-fdr*: 0.011, OR: 1.090, 95%CI: 1.041–1.141) was a risk factor. Our analysis identified 58 instances of heterogeneity but did not reveal any evidence of horizontal pleiotropy. Additionally, the Steiger test did not detect any reverse causality (Supplementary Table S15). Detailed findings on heterogeneity and pleiotropy are presented in Supplementary Tables 6, 10, and the causal effects of the circulating metabolic biomarkers on ER + BC risk are depicted in Figure 3. The results are shown in Table 3.

**TABLE 3:** Causal effects of 233 circulating metabolic biomarkers and ER + BC.

Exposure	Outcome	Method	nsnp	b	se	Or	pval	p_fdr
Conjugated linoleic acid	ER + Breast cancer	IVW	12	−0.152018589	0.051941283	0.858972313	0.00342536	0.021835285
Ratio of diacylglycerol to triglycerides	ER + Breast cancer	IVW	9	−0.182864055	0.066569975	0.832881374	0.006015324	0.032594661
Ratio of omega-6 fatty acids to total fatty acids	ER + Breast cancer	IVW	32	0.136318993	0.041435008	1.146047416	0.001002061	0.013734136
Total cholesterol in HDL2	ER + Breast cancer	IVW	82	0.080548283	0.026760599	1.083881179	0.002612837	0.018704655
Total cholesterol levels in HDL	ER + Breast cancer	IVW	80	0.076018527	0.028118715	1.078982564	0.006861653	0.034468614
Mean diameter of HDL particles	ER + Breast cancer	IVW	84	0.086139223	0.023440504	1.089958065	0.000238033	0.01109236
Free cholesterol to total lipids ratio in IDL	ER + Breast cancer	IVW	57	0.07154162	0.028700792	1.074162857	0.012678641	0.048428251
Total cholesterol in large HDL	ER + Breast cancer	IVW	79	0.09042932	0.024206075	1.094644135	0.000187111	0.01109236
Total cholesterol to total lipids ratio in large HDL	ER + Breast cancer	IVW	52	0.100065484	0.033325784	1.105243291	0.002676449	0.018704655
Cholesterol esters in large HDL	ER + Breast cancer	IVW	80	0.089820853	0.024217597	1.093978283	0.000208155	0.01109236
Cholesteryl esters to total lipids ratio in large HDL	ER + Breast cancer	IVW	42	0.082697537	0.033289251	1.08621322	0.012983871	0.048794226
Free cholesterol in large HDL	ER + Breast cancer	IVW	73	0.084666586	0.025358543	1.088354134	0.000841473	0.013734136
Free cholesterol to total lipids ratio in large HDL	ER + Breast cancer	IVW	47	0.110509811	0.034959687	1.116847306	0.001571939	0.014080872
Total lipids in large HDL	ER + Breast cancer	IVW	75	0.095434541	0.02560649	1.100136806	0.000193798	0.01109236
Concentration of large HDL particles	ER + Breast cancer	IVW	75	0.077750095	0.025409396	1.080852515	0.002214143	0.016641785
Phospholipids in large HDL	ER + Breast cancer	IVW	69	0.092153673	0.027431001	1.096533317	0.000780916	0.013734136
Total Cholesterol in large VLDL	ER + Breast cancer	IVW	60	−0.087587452	0.032784684	0.91613875	0.007549134	0.035896902
Cholesterol esters in large VLDL	ER + Breast cancer	IVW	60	−0.082904306	0.030423844	0.920439223	0.006430577	0.034052827
Total lipids in large VLDL	ER + Breast cancer	IVW	54	−0.091647052	0.031443817	0.912427132	0.003561119	0.021835285
Concentration of large VLDL particles	ER + Breast cancer	IVW	55	−0.096893479	0.030976792	0.907652685	0.001760374	0.014268722
Triglycerides in large VLDL	ER + Breast cancer	IVW	58	−0.088174959	0.032668379	0.91560067	0.006952896	0.034468614
Total cholesterol to total lipids ratio in medium HDL	ER + Breast cancer	IVW	52	0.097533205	0.030717487	1.102448047	0.001497494	0.014080872
Cholesteryl esters to total lipids ratio in medium HDL	ER + Breast cancer	IVW	56	0.081277329	0.029163412	1.084671666	0.005320468	0.03022376
Triglycerides to total lipids ratio in medium HDL	ER + Breast cancer	IVW	62	−0.098000868	0.030974328	0.906648117	0.001556497	0.014080872
Monounsaturated fatty acids (16:1, 18:1) levels	ER + Breast cancer	IVW	63	−0.077206818	0.030538425	0.925698382	0.011465359	0.045278451
Total lipids in medium VLDL	ER + Breast cancer	IVW	61	−0.08447001	0.030286801	0.918999216	0.005287041	0.03022376
Concentration of medium VLDL particles	ER + Breast cancer	IVW	59	−0.086592772	0.03051203	0.917050468	0.004539891	0.027122936
Phospholipids in medium VLDL	ER + Breast cancer	IVW	61	−0.08209343	0.030278922	0.921185888	0.006703206	0.034468614
Triglycerides in medium VLDL	ER + Breast cancer	IVW	59	−0.095842198	0.029013337	0.908607385	0.000955254	0.013734136
Triglycerides in small HDL	ER + Breast cancer	IVW	62	−0.097788898	0.027025362	0.90684032	0.000296416	0.011510825
Triglycerides to total lipids ratio in small HDL	ER + Breast cancer	IVW	62	−0.098269967	0.02925778	0.906404172	0.00078292	0.013734136
Total lipids in small VLDL	ER + Breast cancer	IVW	71	−0.074724735	0.029010997	0.927998897	0.010002634	0.04181601
Concentration of small VLDL particles	ER + Breast cancer	IVW	72	−0.07619846	0.028339749	0.926632289	0.007172041	0.03481428
Phospholipids in small VLDL	ER + Breast cancer	IVW	75	−0.082570028	0.029709211	0.920746957	0.00544806	0.03022376
Triglycerides in small VLDL	ER + Breast cancer	IVW	64	−0.07460138	0.028809352	0.928113377	0.009611966	0.04181601
Ratio of triglycerides to phosphoglycerides	ER + Breast cancer	IVW	67	−0.083195769	0.031863783	0.920170989	0.00902826	0.040453547
Mean diameter of VLDL particles	ER + Breast cancer	IVW	64	−0.086740887	0.029738259	0.91691465	0.003536297	0.021835285
Triglyceride levels in VLDL	ER + Breast cancer	IVW	59	−0.086304883	0.028800235	0.917314515	0.002729435	0.018704655
Total cholesterol to total lipids ratio in very large HDL	ER + Breast cancer	IVW	55	−0.109490345	0.030693707	0.896290818	0.000360827	0.012010388
Cholesteryl esters to total lipids ratio in very large HDL	ER + Breast cancer	IVW	43	−0.102691979	0.029526845	0.90240489	0.000505322	0.013734136
Free cholesterol in very large HDL	ER + Breast cancer	IVW	77	0.077537482	0.026201835	1.080622736	0.003084002	0.020530645
Total lipids in very large HDL	ER + Breast cancer	IVW	82	0.065838065	0.024935073	1.068053748	0.008281314	0.037834238
Concentration of very large HDL particles	ER + Breast cancer	IVW	84	0.065352865	0.024630232	1.067535654	0.007969489	0.037137819
Phospholipids in very large HDL	ER + Breast cancer	IVW	82	0.076484908	0.024187666	1.079485898	0.001566115	0.014080872
Phospholipids to total lipids ratio in very large HDL	ER + Breast cancer	IVW	49	0.132249905	0.029355991	1.141393523	6.64E-06	0.0015462
Triglycerides to total lipids ratio in very large HDL	ER + Breast cancer	IVW	53	−0.125175294	0.036711351	0.88234222	0.000650305	0.013734136
Total cholesterol in very large VLDL	ER + Breast cancer	IVW	53	−0.099959509	0.031419658	0.904874057	0.001465489	0.014080872
Cholesterol esters in very large VLDL	ER + Breast cancer	IVW	50	−0.084979515	0.032980524	0.9185311	0.009976075	0.04181601
Free cholesterol in very large VLDL	ER + Breast cancer	IVW	54	−0.103873465	0.0329738	0.901339341	0.001631689	0.014080872
Total lipids in very large VLDL	ER + Breast cancer	IVW	54	−0.104542657	0.032692882	0.900736374	0.001385191	0.014080872
Concentration of very large VLDL particles	ER + Breast cancer	IVW	53	−0.107293349	0.032395575	0.89826213	0.000926397	0.013734136
Phospholipids in very large VLDL	ER + Breast cancer	IVW	53	−0.102021077	0.033002531	0.903010519	0.00199275	0.015477027
Triglycerides in very large VLDL	ER + Breast cancer	IVW	54	−0.111092154	0.032541852	0.89485628	0.000640579	0.013734136
Total cholesterol levels in chylomicrons and extremely large VLDL	ER + Breast cancer	IVW	56	−0.084373319	0.033698874	0.919088079	0.012288728	0.047721227
Cholesteryl ester levels in chylomicrons and extremely large VLDL	ER + Breast cancer	IVW	60	−0.085110527	0.033274384	0.91841077	0.010532475	0.043053803
Free cholesterol levels in chylomicrons and extremely large VLDL	ER + Breast cancer	IVW	49	−0.100844322	0.032266568	0.904073766	0.001775935	0.014268722
Total lipid levels in chylomicrons and extremely large VLDL	ER + Breast cancer	IVW	51	−0.099274121	0.030419863	0.905494459	0.001100593	0.014080872
Concentration of chylomicrons and extremely large VLDL particles	ER + Breast cancer	IVW	52	−0.097100329	0.030000775	0.907464957	0.001209654	0.014080872
Phospholipid levels in chylomicrons and extremely large VLDL	ER + Breast cancer	IVW	47	−0.091026997	0.028145875	0.912993062	0.001220203	0.014080872
Triglyceride levels in chylomicrons and extremely large VLDL	ER + Breast cancer	IVW	47	−0.100272307	0.029158167	0.904591058	0.000584062	0.013734136

### 4.3 Causal effects of 233 circulating metabolic biomarkers and ER- BC

**FIGURE 4:**
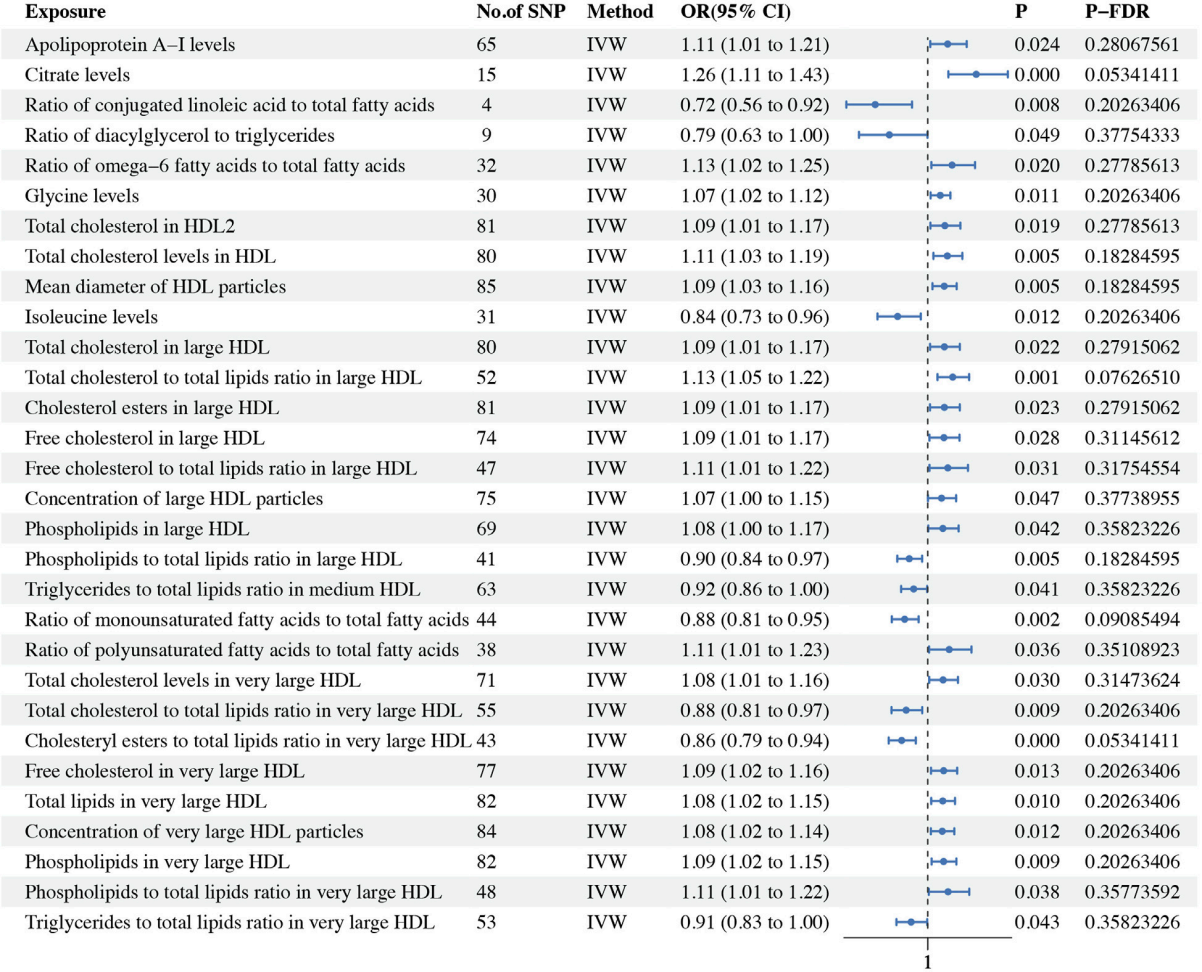
Forest plot showing the OR and 95% CI for ER− breast cancer.

Using the IVW method as the primary analytical approach, we found 30 metabolites with causal relationships to ER- BC, although these did not pass the Bonferroni correction. Among these 30 metabolites, 21 were risk factors for ER- BC, and 9 were protective factors. Within the lipoprotein subclasses, there were 18 metabolites in total, with 5 metabolites including ratio of phospholipids to total lipids in large HDL (*P*: 0.005, OR: 0.901, 95%CI: 0.837–0.970), the ratio of triglycerides to total lipids in medium HDL (*P*: 0.041, OR: 0.924, 95%CI: 0.857–0.997), ratio of total cholesterol to total lipids in very large HDL (*P*: 0.009, OR: 0.884, 95%CI: 0.805–0.970), ratio of cholesteryl esters to total lipids in very large HDL (*P*: 0.0005, OR: 0.860, 95%CI: 0.791–0.936) and ratio of triglycerides to total lipids in very large HDL (*P*: 0.043, OR: 0.911, 95%CI: 0.832–0.997) identified as protective factors, and 13 metabolites including total cholesterol in large HDL (*P*: 0.022, OR: 1.089, 95%CI: 1.012–1.172), ratio of total cholesterol to total lipids in large HDL (*P*: 0.001, OR: 1.134, 95%CI: 1.052–1.222), cholesterol esters in large HDL (*P*: 0.023, OR: 1.089, 95%CI: 1.012–1.172), free cholesterol in large HDL (*P*: 0.028, OR: 1.087, 95%CI: 1.009–1.170), ratio of free cholesterol to total lipids in large HDL (*P*: 0.031, OR: 1.108, 95%CI: 1.009–1.216), concentration of large HDL particles (*P*: 0.047, OR: 1.074, 95%CI: 1.001–1.152), phospholipids in large HDL (*P*: 0.042, OR: 1.081, 95%CI: 1.003–1.165), total cholesterol levels in very large HDL (*P*: 0.030, OR: 1.082, 95%CI: 1.008–1.162), free cholesterol in very large HDL (*P*: 0.013, OR: 1.085, 95%CI: 1.017–1.158), total lipids in very large HDL (*P*: 0.010, OR: 1.080, 95%CI: 1.018–1.146), concentration of very large HDL particles (*p* = 0.012, OR: 1.078, 95%CI: 1.017–1.142), phospholipids in very large HDL (*P*: 0.009, OR: 1.085, 95%CI: 1.021–1.154) and ratio of phospholipids to total lipids in very large HDL (*P*: 0.038, OR: 1.109, 95%CI: 1.006–1.223) identified as risk factors. In the lipids category, total cholesterol in HDL2 (*P*: 0.019, OR: 1.092, 95%CI: 1.014–1.175) and total cholesterol levels in HDL (*P*: 0.005, OR: 1.105, 95%CI: 1.030–1.185) were protective factors for ER- BC. For glycerides and phospholipids, the ratio of diacylglycerol to triglycerides (*P*: 0.049, OR: 0.794, 95%CI: 0.631–0.999) was a protective factor for ER- BC. Within the apolipoproteins, the apolipoprotein A-I levels (*P*: 0.024, OR: 1.107, 95%CI: 1.013–1.209) were identified as a risk factor for ER- BC. In the fatty acids category, the ratio of conjugated linoleic acid to total fatty acids (*P*: 0.008, OR: 0.718, 95%CI: 0.563–0.916), ratio of monounsaturated fatty acids to total fatty acids (*p* = 0.002, OR: 0.876, 95%CI: 0.807–0.951) were protective factors for ER- BC, whereas the ratio of omega-6 fatty acids to total fatty acids (*P*: 0.020, OR: 1.130, 95%CI: 1.019–1.253) and the ratio of polyunsaturated fatty acids to total fatty acids (*P*: 0.036, OR: 1.115, 95%CI: 1.007–1.234) were risk factors. Among glycolysis-related metabolites, the citrate levels (*P*: 0.0003, OR: 1.260, 95%CI: 1.111–1.429) were found to be a risk factor for ER- BC. In the amino acids category, the isoleucine levels (*P*: 0.012, OR: 0.838, 95%CI: 0.730–0.962) were protective factors, while the glycine levels (*P*: 0.011, OR: 1.067, 95%CI: 1.015–1.121) were risk factors. For lipoprotein particle size, the mean diameter of HDL particles (*P*: 0.005, OR: 1.088, 95%CI: 1.025–1.155) was identified as a risk factor for ER- BC. Nineteen instances of heterogeneity were detected (Supplementary Table S7). Two instances of horizontal pleiotropy were identified using the MR-Egger method: the ratio of diacylglycerol to triglycerides (*P*: 0.049, OR: 0.794, 95%CI: 0.631–0.999) and the ratio of phospholipids to total lipids in very large HDL (*P*: 0.038, OR: 1.109, 95%CI: 1.006–1.223) (Supplementary Table S10). Consequently, these two metabolites were excluded from the analysis. No other horizontal pleiotropy was detected. Additionally, the Steiger test did not identify any reverse causality (Supplementary Table S16). The causal effects of the metabolites on ER- BC risk are depicted in Figure 4. The results are shown in Table 4.

**TABLE 4:** Causal effects of 233 circulating metabolic biomarkers and ER- BC.

Exposure	Outcome	Method	nsnp	b	se	Or	pval	p_fdr
Apolipoprotein A-I levels	ER- Breast cancer	IVW	65	0.101481929	0.04499003	1.106809917	0.024092327	0.280675611
Citrate levels	ER- Breast cancer	IVW	15	0.230998983	0.064346766	1.259857958	0.000330794	0.053414107
Ratio of conjugated linoleic acid to total fatty acids	ER- Breast cancer	IVW	4	−0.33110009	0.123918613	0.718133287	0.007541959	0.202634062
Ratio of diacylglycerol to triglycerides	ER- Breast cancer	IVW	9	−0.23084858	0.117063758	0.793859663	0.048610729	0.377543329
Ratio of omega-6 fatty acids to total fatty acids	ER- Breast cancer	IVW	32	0.122489849	0.052768668	1.130307646	0.020272765	0.277856133
Glycine levels	ER- Breast cancer	IVW	30	0.064817735	0.025354082	1.066964537	0.010573079	0.202634062
Total cholesterol in HDL2	ER- Breast cancer	IVW	81	0.087638076	0.037473307	1.091592977	0.019351997	0.277856133
Total cholesterol levels in HDL	ER- Breast cancer	IVW	80	0.099890228	0.03583987	1.105049608	0.005317782	0.182845947
Mean diameter of HDL particles	ER- Breast cancer	IVW	85	0.084587951	0.030414876	1.088268554	0.00541688	0.182845947
Isoleucine levels	ER- Breast cancer	IVW	31	−0.176229065	0.070337826	0.838425907	0.012228999	0.202634062
Total cholesterol in large HDL	ER- Breast cancer	IVW	80	0.085429699	0.037365184	1.089184988	0.022234087	0.279150624
Total cholesterol to total lipids ratio in large HDL	ER- Breast cancer	IVW	52	0.125618185	0.038116399	1.133849165	0.000981954	0.076265099
Cholesterol esters in large HDL	ER- Breast cancer	IVW	81	0.085239749	0.037428814	1.088978117	0.022763356	0.279150624
Free cholesterol in large HDL	ER- Breast cancer	IVW	74	0.083014104	0.037797414	1.086557134	0.028071152	0.311456118
Free cholesterol to total lipids ratio in large HDL	ER- Breast cancer	IVW	47	0.102214584	0.047483001	1.107621124	0.031345697	0.317545541
Concentration of large HDL particles	ER- Breast cancer	IVW	75	0.071274635	0.035878429	1.07387611	0.046971231	0.377389547
Phospholipids in large HDL	ER- Breast cancer	IVW	69	0.077904523	0.038389895	1.081019442	0.042427982	0.358232258
Phospholipids to total lipids ratio in large HDL	ER- Breast cancer	IVW	41	−0.104633162	0.037684039	0.900654857	0.005493226	0.182845947
Triglycerides to total lipids ratio in medium HDL	ER- Breast cancer	IVW	63	−0.078636259	0.038478966	0.924376096	0.040991426	0.358232258
Ratio of monounsaturated fatty acids to total fatty acids	ER- Breast cancer	IVW	44	−0.13225597	0.041809065	0.876116707	0.001559741	0.090854936
Ratio of polyunsaturated fatty acids to total fatty acids	ER- Breast cancer	IVW	38	0.108822844	0.051942044	1.11496481	0.036163698	0.35108923
Total cholesterol levels in very large HDL	ER- Breast cancer	IVW	71	0.078902216	0.036296335	1.082098505	0.029717585	0.314736244
Total cholesterol to total lipids ratio in very large HDL	ER- Breast cancer	IVW	55	−0.123318648	0.047490353	0.883981939	0.00941215	0.202634062
Cholesteryl esters to total lipids ratio in very large HDL	ER- Breast cancer	IVW	43	−0.150748731	0.043023074	0.860063779	0.00045849	0.053414107
Free cholesterol in very large HDL	ER- Breast cancer	IVW	77	0.081791125	0.03294661	1.085229109	0.013045111	0.202634062
Total lipids in very large HDL	ER- Breast cancer	IVW	82	0.077309139	0.03009746	1.080376011	0.010210237	0.202634062
Concentration of very large HDL particles	ER- Breast cancer	IVW	84	0.074796148	0.029737804	1.077664444	0.011896916	0.202634062
Phospholipids in very large HDL	ER- Breast cancer	IVW	82	0.081606723	0.031255038	1.085029009	0.009027942	0.202634062
Phospholipids to total lipids ratio in very large HDL	ER- Breast cancer	IVW	48	0.103264604	0.049868605	1.108784759	0.038383683	0.357735923
Triglycerides to total lipids ratio in very large HDL	ER- Breast cancer	IVW	53	−0.093454801	0.046190878	0.910779183	0.04304937	0.358232258

## 5 Discussion

In our study, we utilized MR analysis to investigate the causal relationships between 233 circulating metabolic biomarkers and BC at a genetic level. We identified 59 circulating metabolic biomarkers that exhibit a causal relationship with overall BC following FDR method. Additionally, 60 circulating metabolic biomarkers were found to have a causal association with ER + BC, also confirmed through Bonferroni correction. Furthermore, 30 circulating metabolic biomarkers demonstrated a causal link with ER- BC via the IVW method, although they did not pass the Bonferroni correction. As far as we are aware, this is the pioneering study that employs MR analysis to examine the causal links between 233 circulating metabolic biomarkers and BC at the genetic level. In discussing the relationship between circulating metabolic biomarkers and BC, numerous studies have indicated that specific metabolic biomarkers are significantly associated with the onset, progression, and prognosis of BC. For example, research has shown that plasma levels of Transforming Growth Factor β1 are significantly elevated in BC individuals and correlate with treatment response and risk of recurrence (Veyssière et al., 2022). Additionally, serum amino acid metabolism, particularly levels of serine and glutamine, has been linked with drug resistance and overall survival in BC patients (Wang et al., 2023).

In our investigation, we established a causal relationship at the genetic level between 233 circulating metabolic biomarkers—including lipoproteins, glycerides and phospholipids, apolipoproteins, and lipoprotein particle size—and all types of BC. This evidence highlights the significant genetic interactions involving metabolic biomarkers in the pathogenesis of BC, providing a robust basis for the development of targeted therapies and preventive measures.

In our study, we observed a significant correlation between lipoprotein levels and the incidence of BC. Lipoproteins play a significant role in cancer through their influence on lipid metabolism and antioxidative activities. Similarly, evidence from previous observational studies aligns with our MR findings. A case-control study involving 1,199 pairs by Julia Debik et al., demonstrated that various lipoprotein subfractions, especially VLDL subfractions, were inversely linked with BC risk in premenopausal women. (Debik et al., 2022). Moreover, lipid metabolism is deeply intertwined with tumorigenesis. Changes in lipid metabolism may affect several cancer-related pathways including inflammation, oxidative stress, and angiogenesis (Mazzuferi et al., 2021; Allegra et al., 2024). The relationship between lipoprotein levels and BC risk has been extensively studied, with various findings suggesting a complex interaction. For instance, an MR study led by Johnson et al. found that elevated levels of both LDL and HDL cholesterol are linked with a higher risk of BC. This study underlines the potential causal relationship of lipoprotein levels on breast cancer, emphasizing the need for further investigation into the underlying mechanisms and potential therapeutic targets for modifying these lipid levels (Johnson et al., 2020). Another study conducted by Debik et al. reported that specific subfractions of VLDL particles, including VLDL-2, VLDL-3, and VLDL-4, are inversely associated with the risk of BC in premenopausal women. This study underscores significant metabolic changes in the endogenous lipid pathway that can appear long before a breast cancer diagnosis (Debik et al., 2021). These findings suggest that while some lipoproteins may increase risk, others could potentially have a protective effect. This complexity underscores the importance of detailed lipid profiling in understanding breast cancer risk and developing targeted interventions.

Glycerides and phospholipids, essential components of cell membranes, are significantly altered in cancerous tissues compared to normal tissues. In exploring the causal relationship between glycerides and phospholipids, and BC, it is essential to consider various biochemical and genetic factors that may contribute to disease progression. Research indicates that glycerides and phospholipids may play significant roles in the development of BC. A study by Lin et al. explored how alterations in glyceride and phospholipid metabolism might influence BC risk. Their findings suggested that disruptions in these lipid pathways could be linked to the development of BC, proposing a potential mechanism involving lipid metabolism in cancer progression (Au Yeung and Gill, 2023). Another significant piece of research by Kim et al. investigated the relationship between blood levels of certain phospholipids and BC risk. They observed that elevated levels of specific phospholipids were linked to an increased risk of BC (Kerber et al., 2008). These insights underline the complexity of lipid-related pathways in BC and highlight the potential for targeting these mechanisms in therapeutic strategies. As such, understanding the interplay of genetic factors and lipid metabolism could open new avenues for personalized medicine strategies in BC treatment and prevention.

Apolipoproteins are involved in the management of lipid transport and metabolism and have shown varying impacts on BC based on their structure and function. The relationship between apolipoproteins and BC is increasingly recognized in scientific literature, highlighting their roles in both promoting and inhibiting tumor growth depending on their specific types and interactions within the body. For instance, the distribution of apolipoproteins in different lipoprotein fractions appears associated with the severity of BC, suggesting their potential utility in understanding disease progression (Bobin-Dubigeon et al., 2022). Moreover, certain apolipoproteins like ApoA1 and ApoE have shown potential as biomarkers in male BC due to their upregulated levels in serum, which correlates with disease presence and progression (He et al., 2022). Studies have highlighted how the dysregulation of apolipoproteins can influence the onset of BC, with different apolipoproteins playing distinct roles in cancer cell dynamics. For example, ApoC1 is found to promote breast tumorigenesis by altering cell adhesion and migration processes, while ApoM has inhibitory effects on cancer cell invasion and proliferation, potentially by its interaction with vitamin D receptors (He et al., 2022). These studies underline the importance of apolipoproteins in BC, indicating that their modulation could offer new avenues for targeted therapies and diagnostics. As research continues to unravel the specific mechanisms by which apolipoproteins influence cancer biology, their potential as therapeutic targets and diagnostic markers will become increasingly important in managing BC.

Lipoprotein particle size refers to the dimensions of lipoproteins in the blood, which vary in size and composition. These particles, such as HDL and LDL, play roles in transporting cholesterol. Research indicates a connection between the sizes and levels of these particles, particularly HDL, and cancer risk (Pedersen et al., 2020). The relationship between lipoprotein particle size and BC risk appears to be nuanced, with different subclasses of lipoproteins demonstrating varied associations with cancer risk. Research using MR analysis and nuclear magnetic resonance (NMR) has indicated causal links between certain lipoprotein traits and BC risk. Notably, LDL receptor expression in BC cells, such as in certain ER-cell lines, suggests a potential role in lipid uptake and storage which may influence tumor characteristics and progression (Okekunle et al., 2022).

Consistent with our experimental findings, several studies have shown that elevated LDL levels are associated with increased malignancy in breast cancer, potentially through mechanisms related to lipid metabolism (Yuan et al., 2023). One plausible mechanism involves the LDL receptor, a common receptor for LDL. After binding to LDL, LDLR facilitates its internalization into the cell via endocytosis, thereby enhancing fatty acid metabolism in cancer cells. Additional studies have demonstrated that LDLR is highly expressed in BC, contributing to elevated blood cholesterol levels in patients and correlating with poor prognosis in those with breast cancer (de Gonzalo-Calvo et al., 2015; Pires et al., 2012). The overexpression of LDLR increases the uptake of circulating LDL by BC cells, promoting increased metabolic activity and further enhancing the malignancy of the cancer (Rodrigues dos Santos et al., 2023). Moreover, another mechanism that may promote breast cancer development is related to epithelial-mesenchymal transition (EMT). LDL uptake can lead to elevated cholesterol levels in cancer cells, increasing the production of 27-hydroxycholesterol, which has been shown to stimulate both cancer cell proliferation and EMT in BC cells (Cruz et al., 2010; Sierralta et al., 2011). These findings suggest that lipid metabolism plays a crucial role in BC progression.

Previous analyses have already examined the relationship between metabolites and BC. For example, the study by Xiaosheng Zhu et al. analyzed 249 plasma metabolites from the UK Biobank, finding that total cholesterol in HDL and LDL lipid levels were associated with both ER+ and ER- BC (Zhu et al., 2024). However, our study did not find a relationship between acetate and BC, which was confirmed in their experiments. Addressing this issue requires a larger database and more detailed subgroup analyses. Additionally, another MR study indicated that elevated HDL-C (OR-1.08) might increase the risk of BC (Zhou et al., 2023). However, this study did not further analyze the relationship between specific components of HDL and BC. These discrepancies highlight the need for further research to better understand the complex relationship between metabolites and BC.

In our MR study, we successfully leveraged genetic proxies to elucidate the causal relationships between 233 circulating metabolic biomarkers and BC, including its subtypes ER + BC and ER- BC. Our approach has several advantages as follows. Firstly, by utilizing genetic variants as instruments in MR analysis, we significantly reduced the impact of confounding factors that typically affect observational studies. This approach enables a more precise understanding of the causal effects of metabolites on BC risk. Secondly, we analyzed 233 circulating metabolic biomarkers, providing a broad and detailed panorama of circulating metabolic biomarkers’ role in BC. This comprehensive approach helps in identifying potential biomarkers and therapeutic targets.

Our experimental results have significant clinical implications. Firstly, these biomarkers can be used for the early diagnosis and risk prediction of BC. Since we have identified several metabolic biomarkers significantly associated with BC, future developments in blood-based testing tools could enable more precise risk assessment and personalized screening strategies. For instance, elevated levels of certain metabolic biomarkers may serve as warning signs for high-risk individuals, aiding clinicians in adopting more proactive monitoring and preventive measures. Secondly, these metabolic biomarkers may serve as potential therapeutic targets. By targeting the metabolic pathways causally linked to breast cancer, new treatment strategies could be developed. Particularly, the biomarkers identified in our study could help formulate tailored treatment plans for different subtypes of BC, such as ER+ and ER-subtypes. In summary, this research not only provides new insights into the metabolic mechanisms of breast cancer but also offers potential avenues for future clinical applications and translational research.

While our MR study offers valuable insights into the causal links between circulating metabolic biomarkers and BC, it is important to acknowledge a few limitations. The genetic instruments used might not always be strong enough, which could compromise the reliability of our causal inferences, especially if these variants explain only a minimal part of the variation in lipid levels. Additionally, despite employing MR-Egger to check for pleiotropy, the issue of horizontal pleiotropy remains, where genetic variants might influence multiple traits through unrelated pathways, potentially skewing the results. Unfortunately, in this experiment, the ancestry of the population for the metabolite data was diverse, yet for the outcome analysis, we only selected individuals of European descent. We searched through numerous databases, and the metabolite GWAS database we used was the most comprehensive one we could find. However, this may introduce bias into the results of the study. Moreover, the generalizability of our findings could be limited, as they may not apply to different populations due to variations in genetic background, lifestyle, or environmental factors that influence lipid metabolism and BC risk. This necessitates cautious interpretation and underscores the importance of validating our results across diverse groups. At the last, there are multiple classification methods for BC, including the commonly recognized subtypes: Luminal A, Luminal B, HER2-overexpressing, and TNBC. Additionally, BC can also be categorized based on ER expression levels into ER-negative BC and ER-low BC. These molecular subtypes differ significantly in terms of characteristics, prognosis, and aggressiveness. However, publicly available databases currently lack SNP data with such detailed classifications. Therefore, this study focuses on exploring the relationship between metabolites and these two BC subtypes based on ER expression levels. Access to more detailed classification data in the future would allow for further subtype-specific research, deepening our understanding of the relationship between metabolites and BC.

## 6 Conclusion

Our MR study offers new insights into the relationship between metabolites and BC. In our investigation, most metabolites were found to have a causal relationship with the occurrence of BC. Specifically, the six classes of metabolites—fatty acids glycerides and phospholipids, lipoprotein subclasses, lipids, apolipoproteins, and lipoprotein particle size—showed causal associations with overall BC. Five classes of metabolites—fatty acids glycerides and phospholipids, lipoprotein subclasses, lipids, and lipoprotein particle size—were associated with ER + BC, while eight classes of metabolites—fatty acids, amino acids, glycerides and phospholipids, lipoprotein subclasses, lipids, apolipoproteins, glycolysis-related metabolites, and lipoprotein particle size—were linked to ER- BC. This study enhances our insight into the association between metabolites and BC, offering a significant basis for future research and potential prevention strategies for BC.

## Data Availability

The datasets presented in this study can be found in online repositories. The names of the repository/repositories and accession number(s) can be found in the article/Supplementary Material.
