# Supplementary material for: Assessing the causal relationships between circulating metabolic biomarkers and breast cancer by using mendelian randomization
Source: Front Genet. 2024 Dec 18;15:1448748. doi: 10.3389/fgene.2024.1448748 (PMC11688392; doi:10.3389/fgene.2024.1448748)
Supplement: Supplementary file 1 [file DataSheet7.pdf]

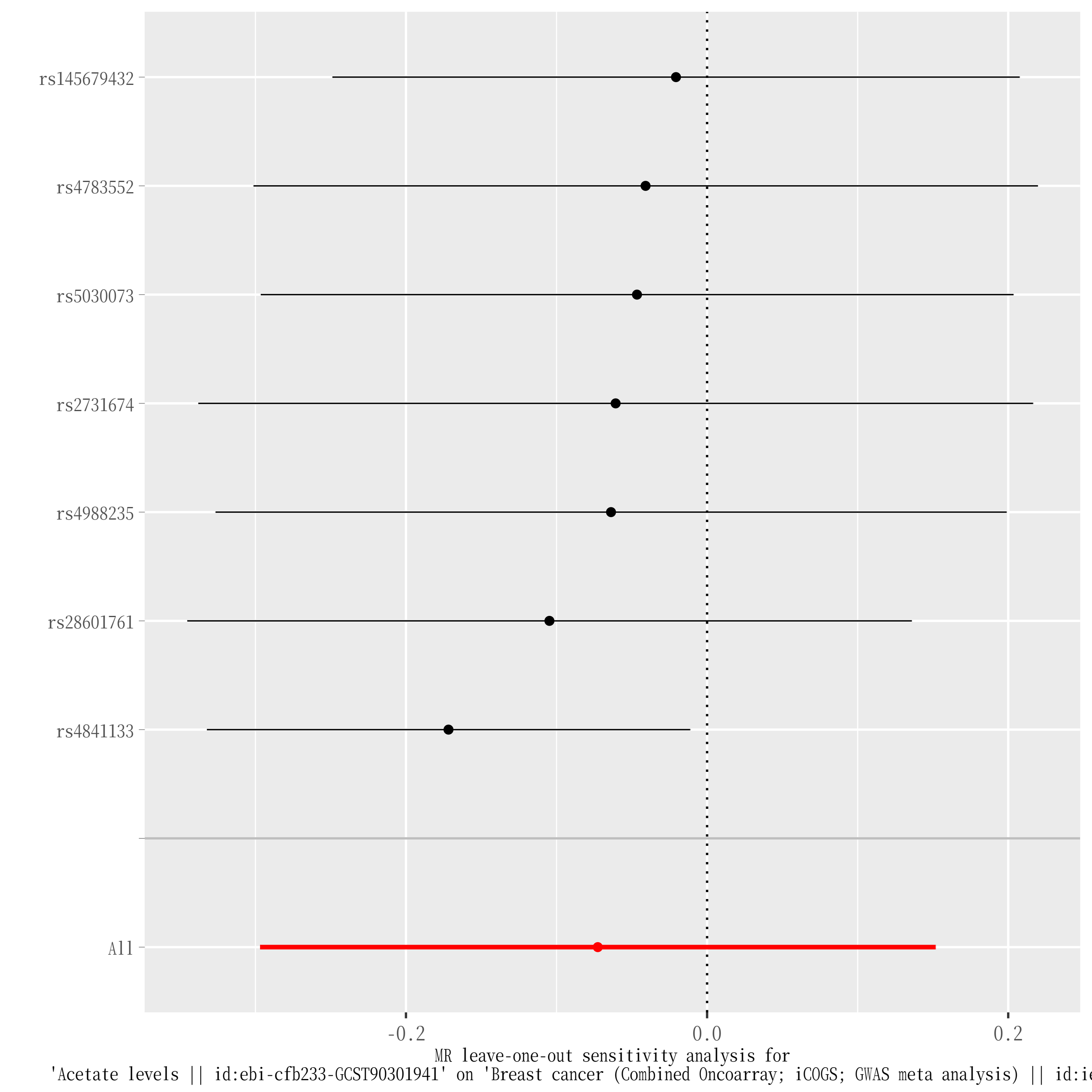
rs145679432
rs4783552
rs5030073
rs2731674
rs4988235
rs28601761
rs4841133
All
-0.2
0.0
0.2
MR leave-one-out sensitivity analysis for
'Acetate levels || id:ebi-cfb233-GCST90301941' on 'Breast cancer (Combined Oncoarray; iCOGS; GWAS meta analysis) || id:ie

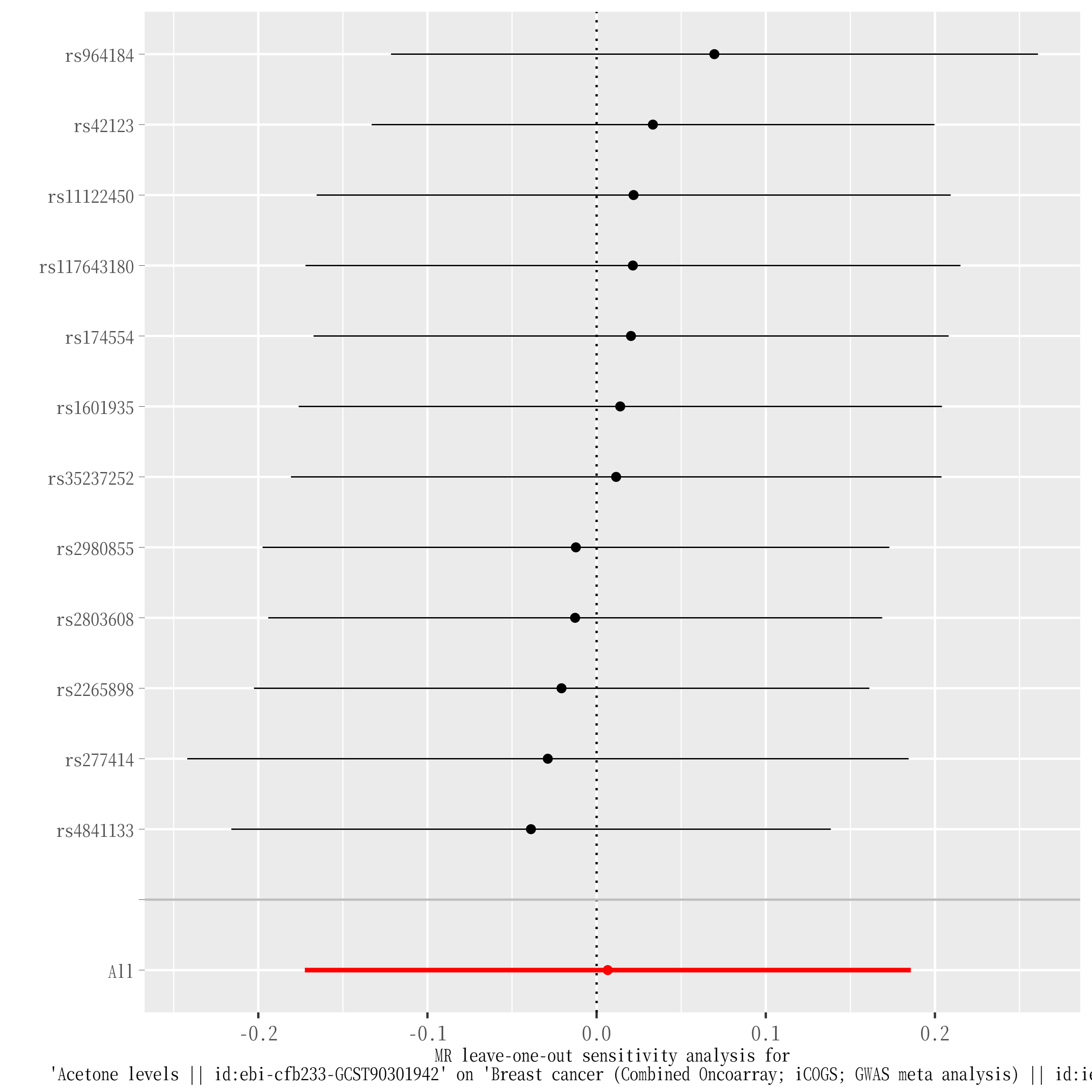
rs964184
rs42123
rs11122450
rs117643180
rs174554
rs1601935
rs35237252
rs2980855
rs2803608
rs2265898
rs277414
rs4841133
All
-0.2
-0.1
0.0
0.1
0.2
MR leave-one-out sensitivity analysis for
'Acetone levels || id:ebi-cfb233-GCST90301942' on 'Breast cancer (Combined Oncoarray; iCOGS; GWAS meta analysis) || id:ie

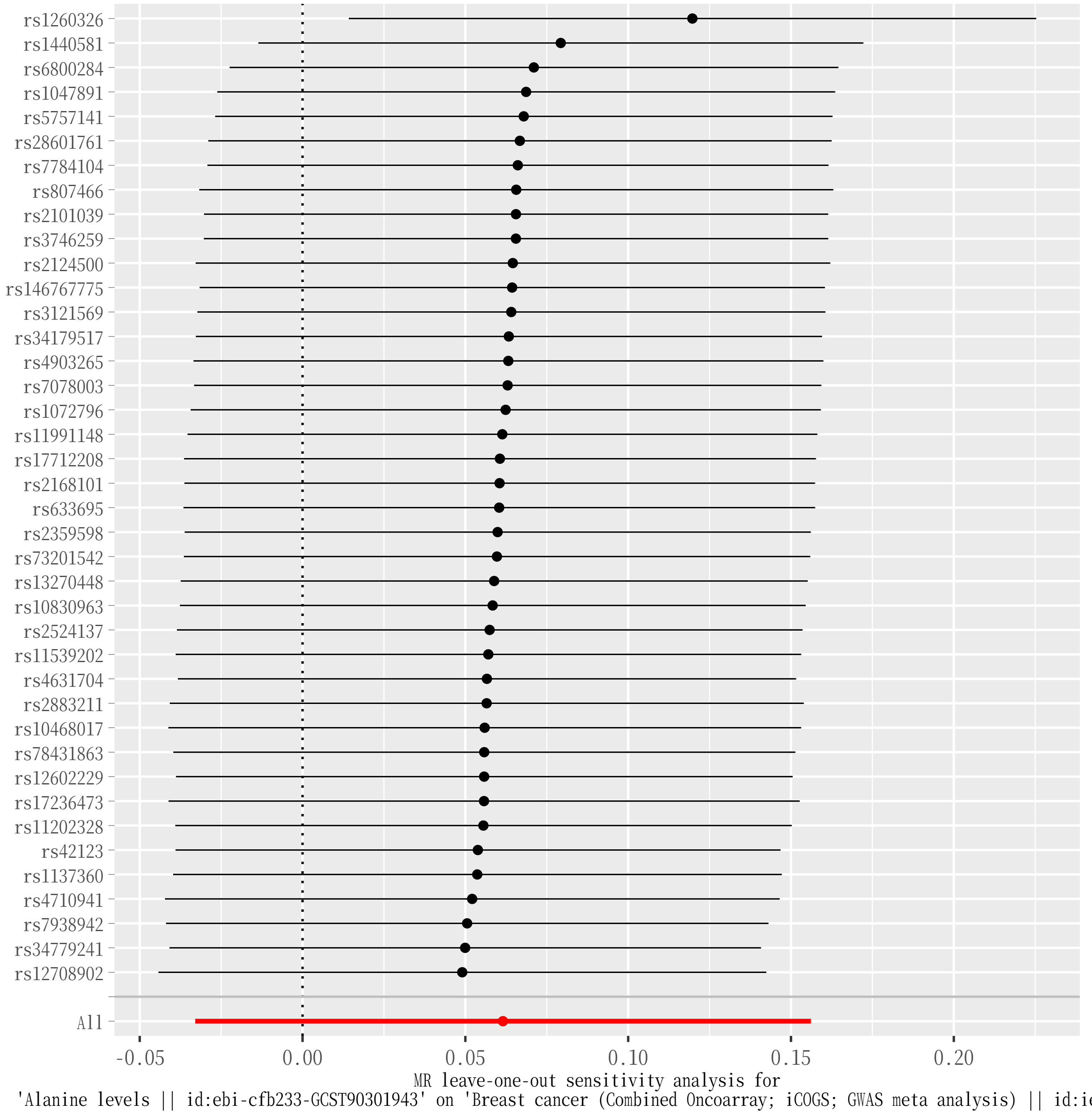

rs1260326
rs1440581
rs6800284
rs1047891
rs5757141
rs28601761
rs7784104
rs807466
rs2101039
rs3746259
rs2124500
rs146767775
rs3121569
rs34179517
rs4903265
rs7078003
rs1072796
rs11991148
rs17712208
rs2168101
rs633695
rs2359598
rs73201542
rs13270448
rs10830963
rs2524137
rs11539202
rs4631704
rs2883211
rs10468017
rs78431863
rs12602229
rs17236473
rs11202328
rs42123
rs1137360
rs4710941
rs7938942
rs34779241
rs12708902
All
-0.05
0.00
0.05
0.10
0.15
0.20
MR leave-one-out sensitivity analysis for
'Alanine levels || id:ebi-cfb233-GCST90301943' on 'Breast cancer (Combined Oncoarray; iCOGS; GWAS meta analysis) || id:i

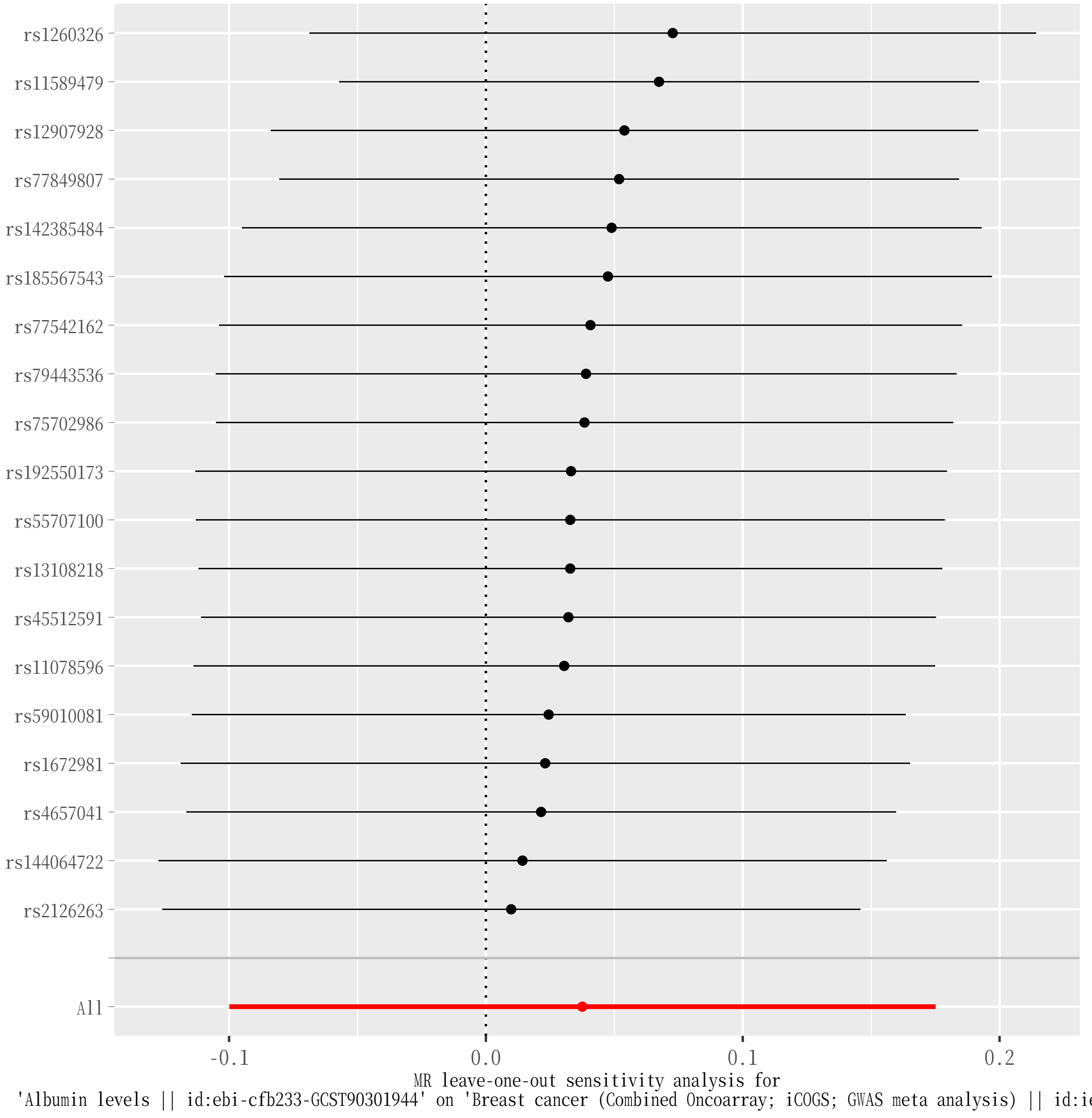
rs1260326
rs11589479
rs12907928
rs77849807
rs142385484
rs185567543
rs77542162
rs79443536
rs75702986
rs192550173
rs55707100
rs13108218
rs45512591
rs11078596
rs59010081
rs1672981
rs4657041
rs144064722
rs2126263
All
-0.1
0.0
0.1
0.2
MR leave-one-out sensitivity analysis for
'Albumin levels || id:ebi-cfb233-GCST90301944' on 'Breast cancer (Combined Oncoarray; iCOGS; GWAS meta analysis) || id:i

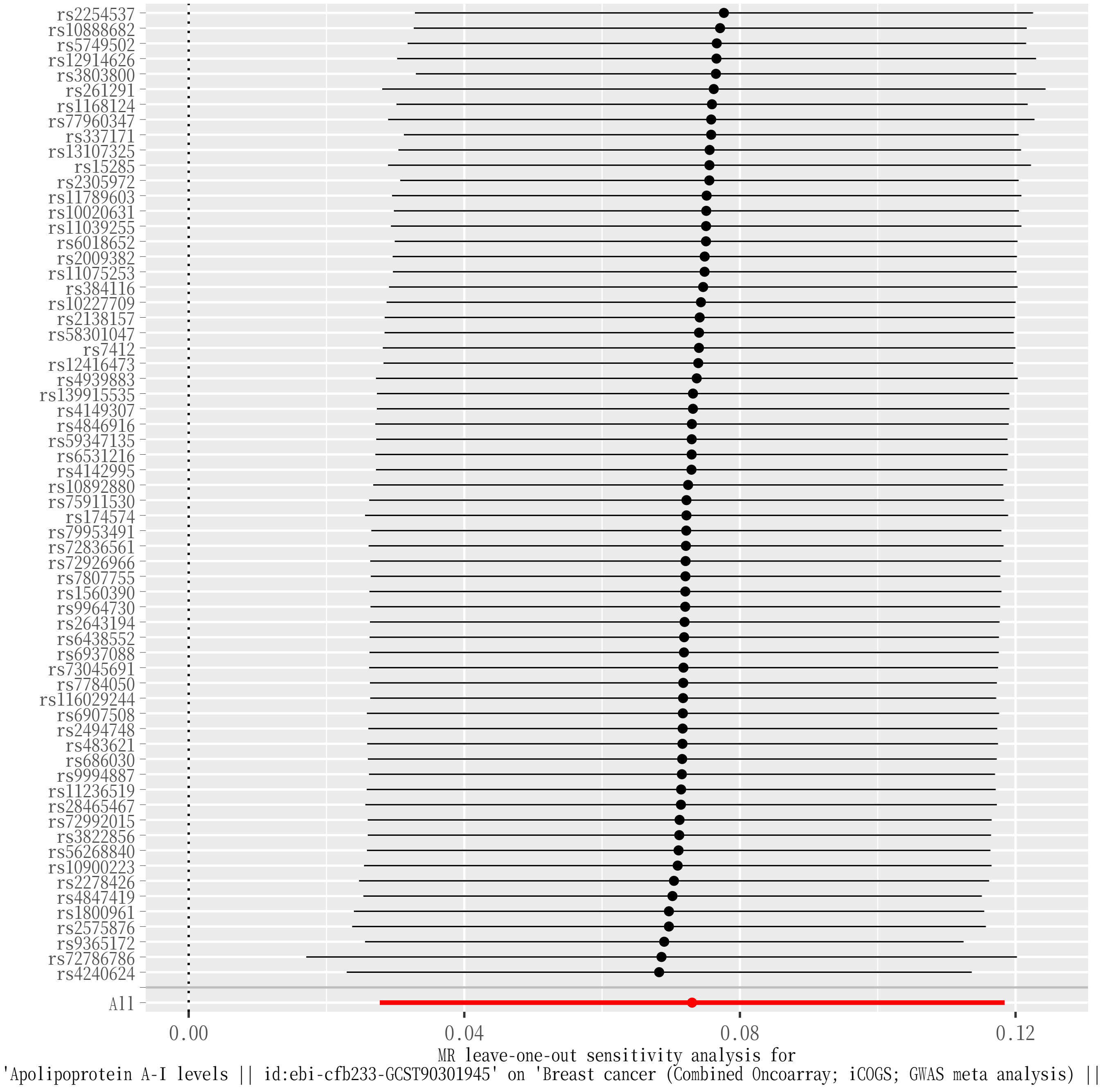
rs2254537
rs10888682
rs5749502
rs12914626
rs3803800
rs261291
rs1168124
rs77960347
rs337171
rs13107325
rs15285
rs2305972
rs11789603
rs10020631
rs11039255
rs6018652
rs2009382
rs11075253
rs384116
rs10227709
rs2138157
rs58301047
rs7412
rs12416473
rs4939883
rs139915535
rs4149307
rs4846916
rs59347135
rs6531216
rs4142995
rs10892880
rs75911530
rs174574
rs79953491
rs72836561
rs72926966
rs7807755
rs1560390
rs9964730
rs2643194
rs6438552
rs6937088
rs73045691
rs7784050
rs116029244
rs6907508
rs2494748
rs483621
rs686030
rs9994887
rs11236519
rs28465467
rs72992015
rs3822856
rs56268840
rs10900223
rs2278426
rs4847419
rs1800961
rs2575876
rs9365172
rs72786786
rs4240624
All
0.00
0.04
0.08
0.12
MR leave-one-out sensitivity analysis for
'Apolipoprotein A-I levels || id:ebi-cfb233-GCST90301945' on 'Breast cancer (Combined Oncoarray; iCOGS; GWAS meta analysis) ||

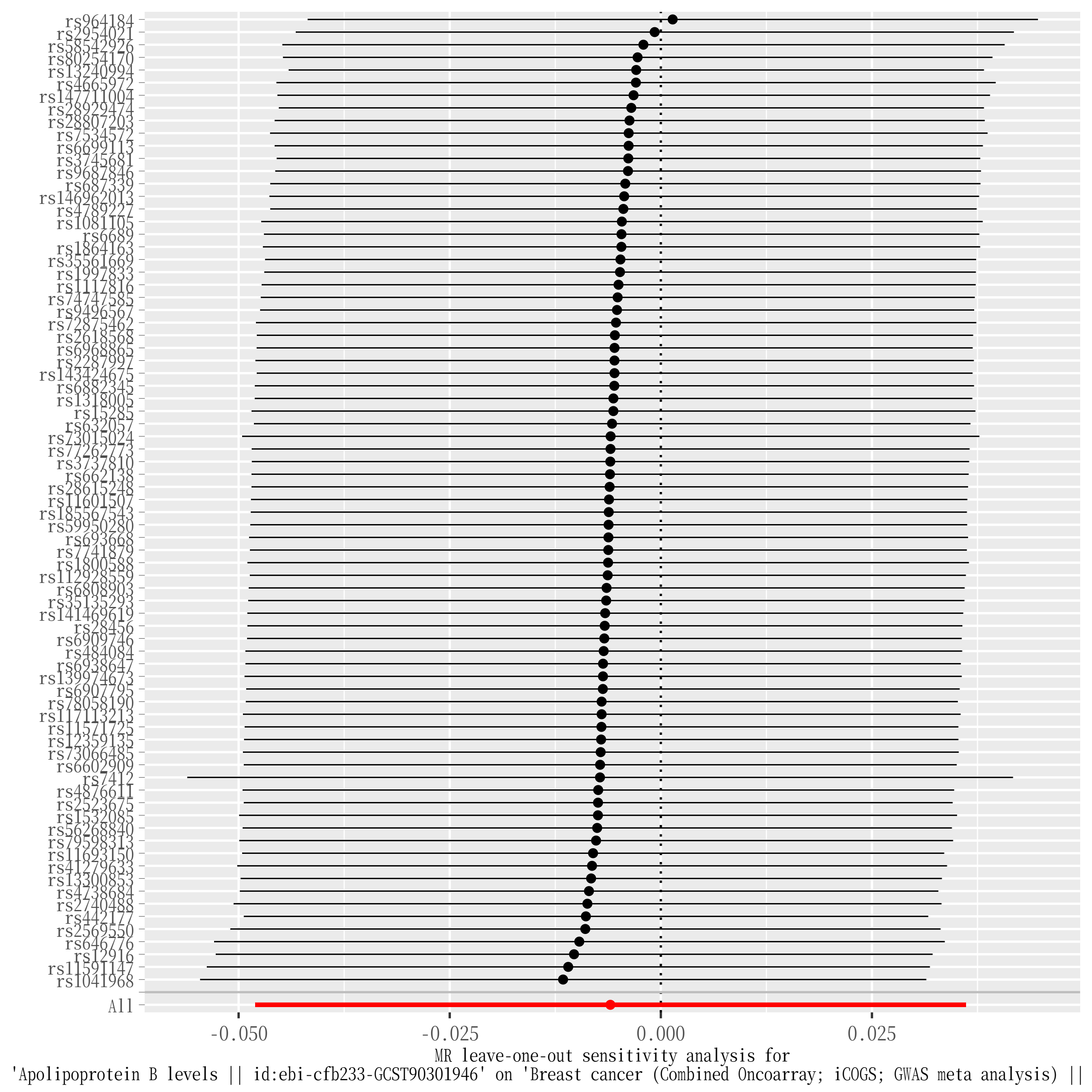
rs964184
rs2954021
rs58542926
rs80254170
rs13240994
rs4665972
rs147711004
rs28929474
rs28807203
rs7534572
rs6699113
rs3745681
rs9687846
rs687339
rs146962013
rs4789227
rs1081105
rs6689
rs1864163
rs35561669
rs1997833
rs1117816
rs74747585
rs9496567
rs72875462
rs2618568
rs6968865
rs2287997
rs143424675
rs6882345
rs1318005
rs15285
rs632057
rs73015024
rs77262773
rs3737810
rs662138
rs28615248
rs11601507
rs185567543
rs59950280
rs693668
rs7741879
rs1800588
rs112928559
rs6808903
rs35135293
rs141469619
rs28456
rs6909746
rs484084
rs6938647
rs139974673
rs6907795
rs78058190
rs117113213
rs11571725
rs12359135
rs73066485
rs6602909
rs7412
rs4876611
rs2523675
rs1532085
rs56268840
rs79598313
rs11623150
rs41279633
rs13300853
rs4738684
rs2740488
rs442177
rs2569550
rs646776
rs12916
rs11591147
rs1041968
All
-0.050
-0.025
0.000
0.025
MR leave-one-out sensitivity analysis for
'Apolipoprotein B levels || id:ebi-cfb233-GCST90301946' on 'Breast cancer (Combined Oncoarray; iCOGS; GWAS meta analysis) ||

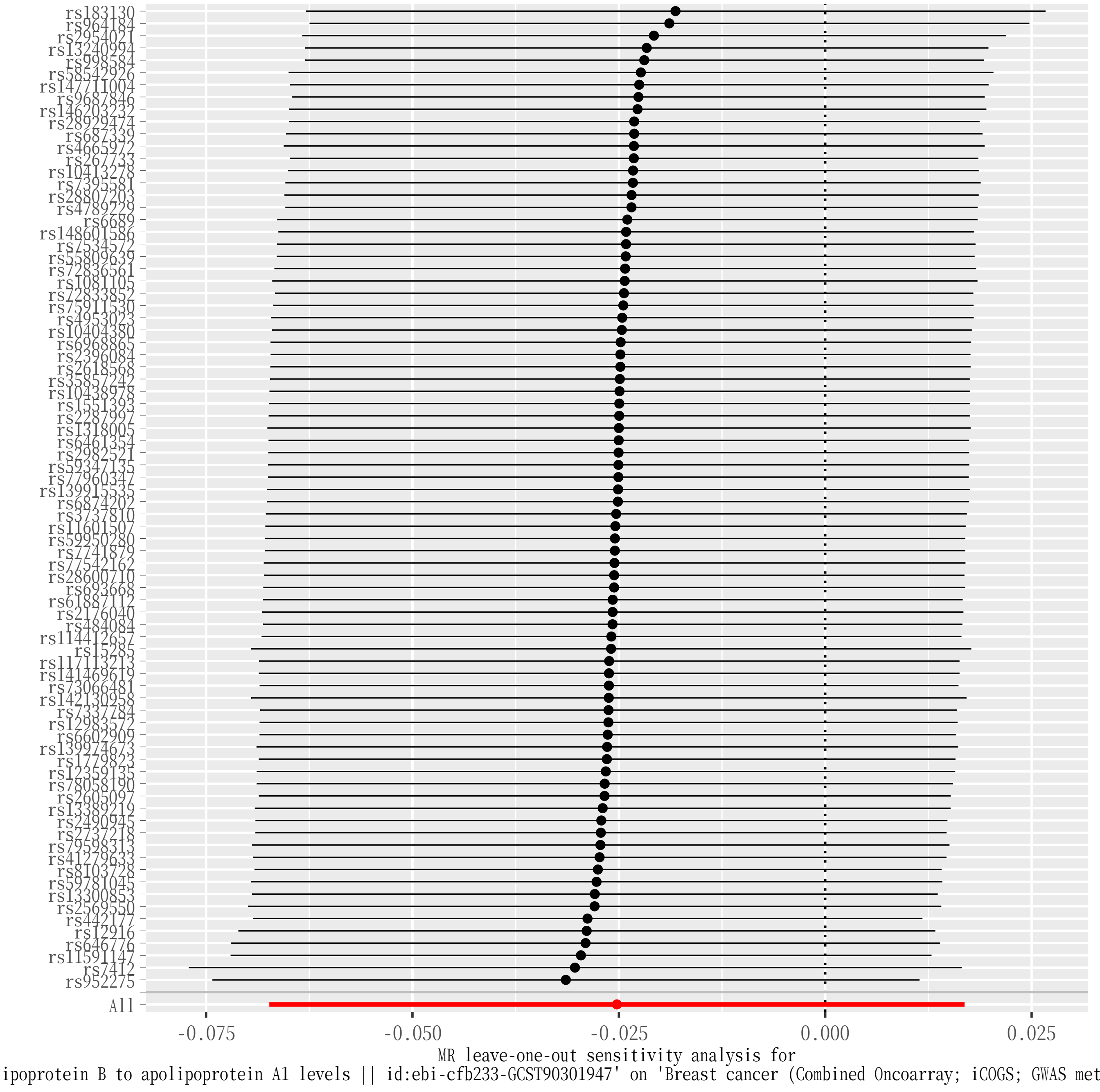

rs183130
rs964184
rs2954021
rs13240294
rs998584
rs58542926
rs147711004
rs9687846
rs146203232
rs28929474
rs687339
rs4665972
rs267733
rs10413278
rs7395581
rs28807203
rs4789229
rs6689
rs148601586
rs7534572
rs55809639
rs72836561
rs1081105
rs72833852
rs75911530
rs4953023
rs10404380
rs6968865
rs2396084
rs2618568
rs35857242
rs10438978
rs1551393
rs2287997
rs1318005
rs6461354
rs2982521
rs59347135
rs77960347
rs139915535
rs6874202
rs3737810
rs11601507
rs59950280
rs7741879
rs77542162
rs28600710
rs693668
rs61887112
rs2176040
rs484084
rs114412657
rs15285
rs117113213
rs141469619
rs73066481
rs142130958
rs7337784
rs12983572
rs6602909
rs139974673
rs1779823
rs12359135
rs78058190
rs2605097
rs13389219
rs2490945
rs2737218
rs79528313
rs41279633
rs8103728
rs59781045
rs13300853
rs2569550
rs442177
rs12916
rs646776
rs11591147
rs7412
rs952275
All
-0.075
-0.050
-0.025
0.000
0.025
MR leave-one-out sensitivity analysis for
ipoprotein B to apolipoprotein A1 levels || id:ebi-cfb233-GCST90301947' on 'Breast cancer (Combined Oncoarray; iCOGS; GWAS met

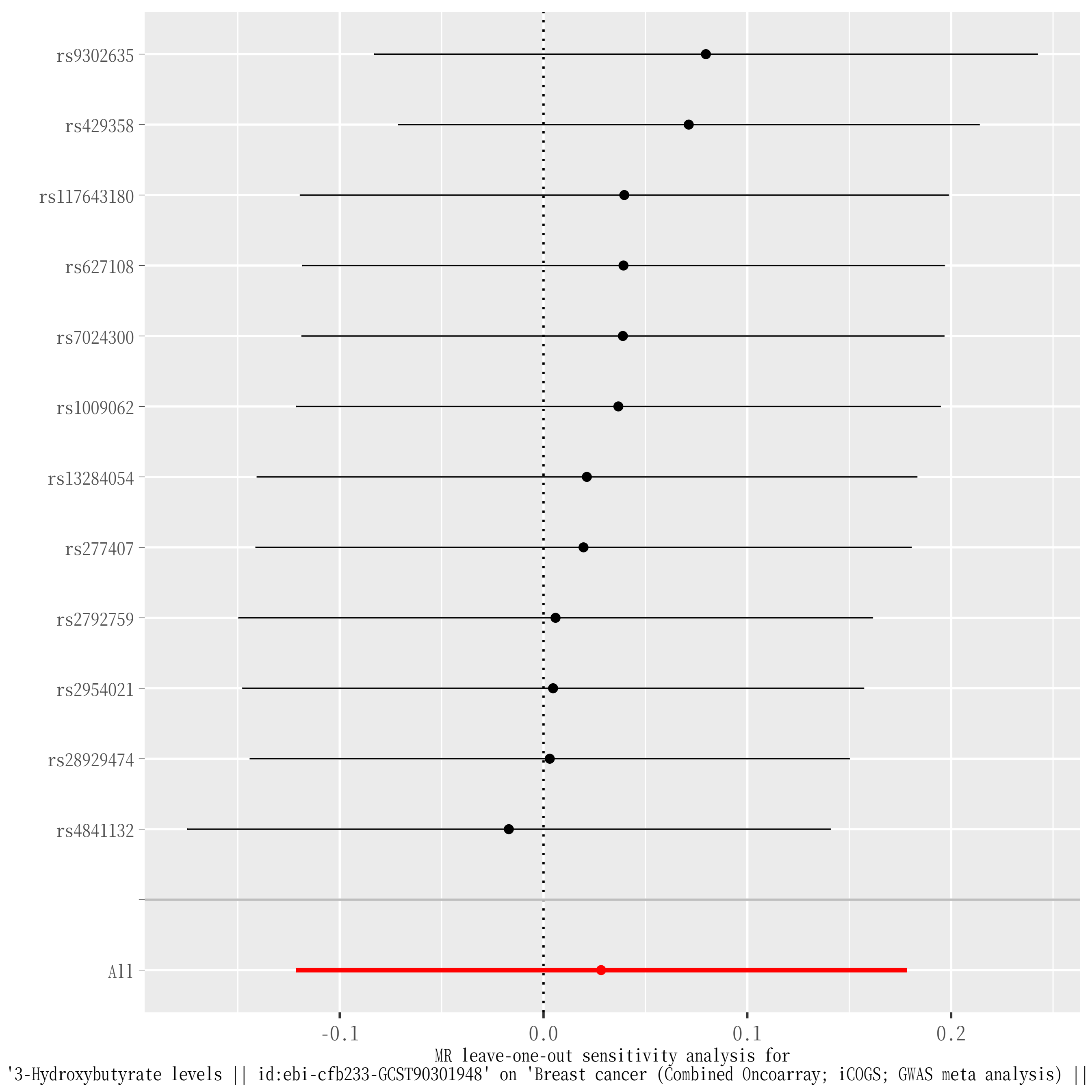
rs9302635
rs429358
rs117643180
rs627108
rs7024300
rs1009062
rs13284054
rs277407
rs2792759
rs2954021
rs28929474
rs4841132
All
-0.1
0.0
0.1
0.2
MR leave-one-out sensitivity analysis for
'3-Hydroxybutyrate levels || id:ebi-cfb233-GCST90301948' on 'Breast cancer (Combined Oncoarray; iCOGS; GWAS meta analysis) ||

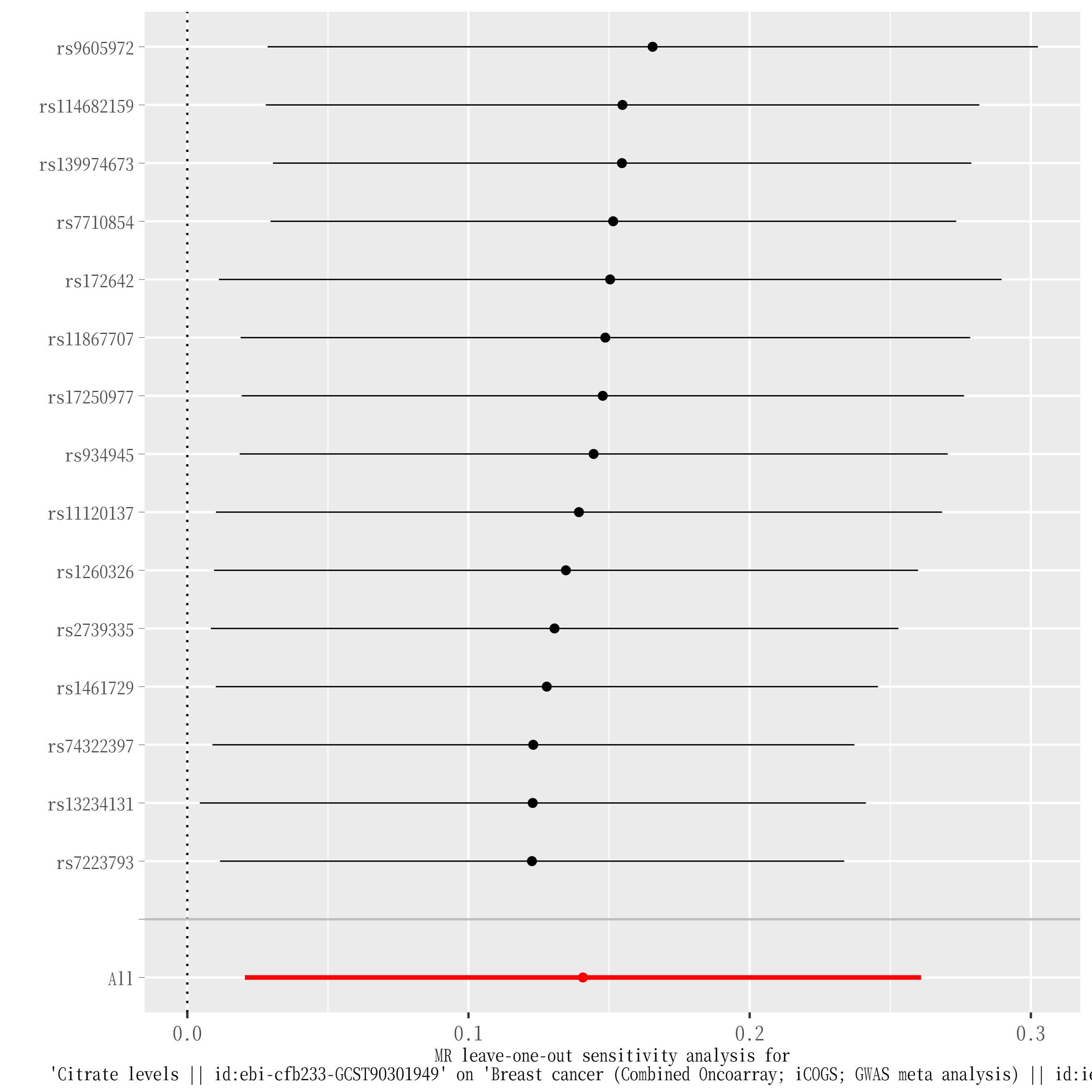

rs9605972
rs114682159
rs139974673
rs7710854
rs172642
rs11867707
rs17250977
rs934945
rs11120137
rs1260326
rs2739335
rs1461729
rs74322397
rs13234131
rs7223793
All
0.0
0.1
0.2
0.3
MR leave-one-out sensitivity analysis for
'Citrate levels || id:ebi-cfb233-GCST90301949' on 'Breast cancer (Combined Oncoarray; iCOGS; GWAS meta analysis) || id:ie

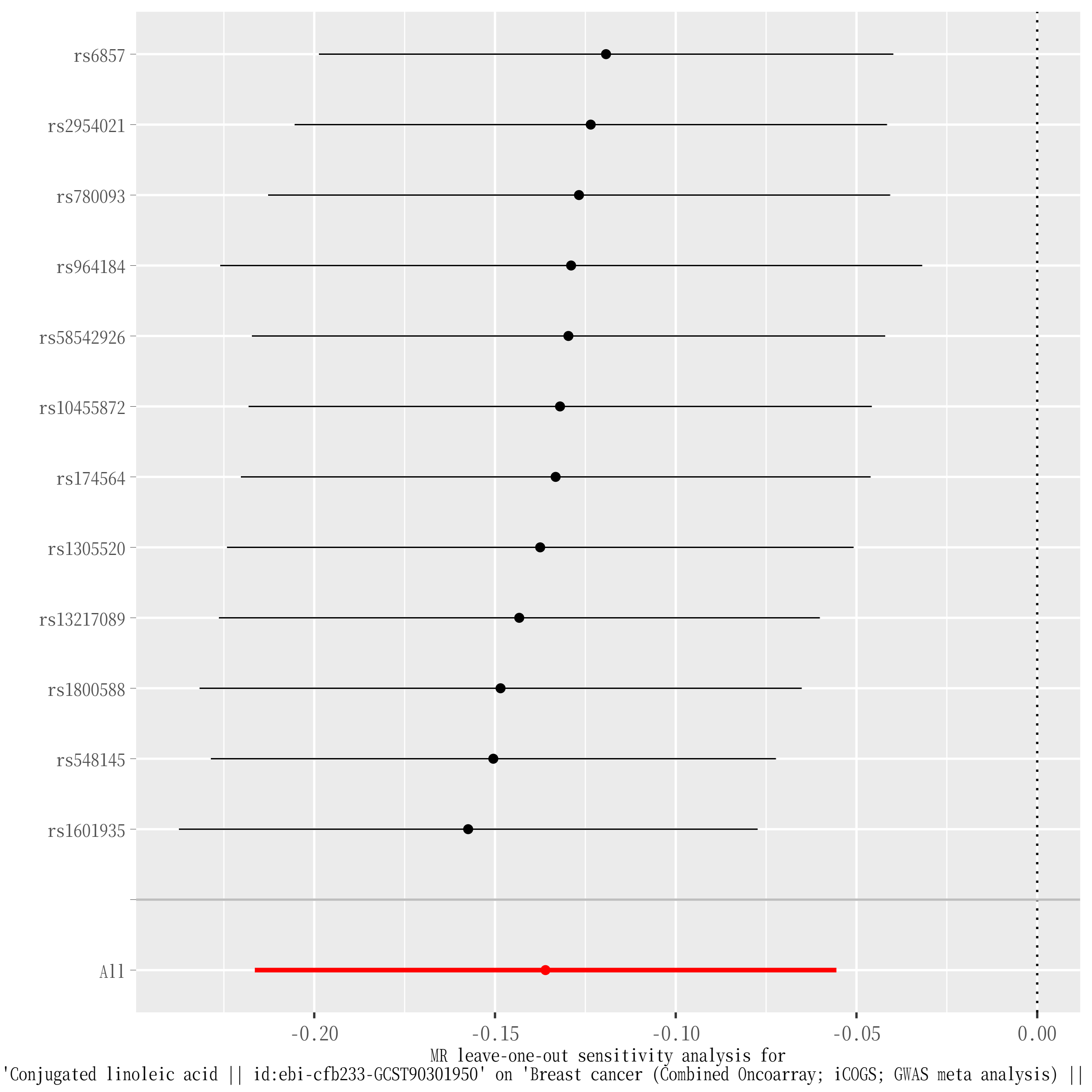
rs6857
rs2954021
rs780093
rs964184
rs58542926
rs10455872
rs174564
rs1305520
rs13217089
rs1800588
rs548145
rs1601935
All
-0.20
-0.15
-0.10
-0.05
0.00
MR leave-one-out sensitivity analysis for
'Conjugated linoleic acid || id:ebi-cfb233-GCST90301950' on 'Breast cancer (Combined Oncoarray; iCOGS; GWAS meta analysis) ||

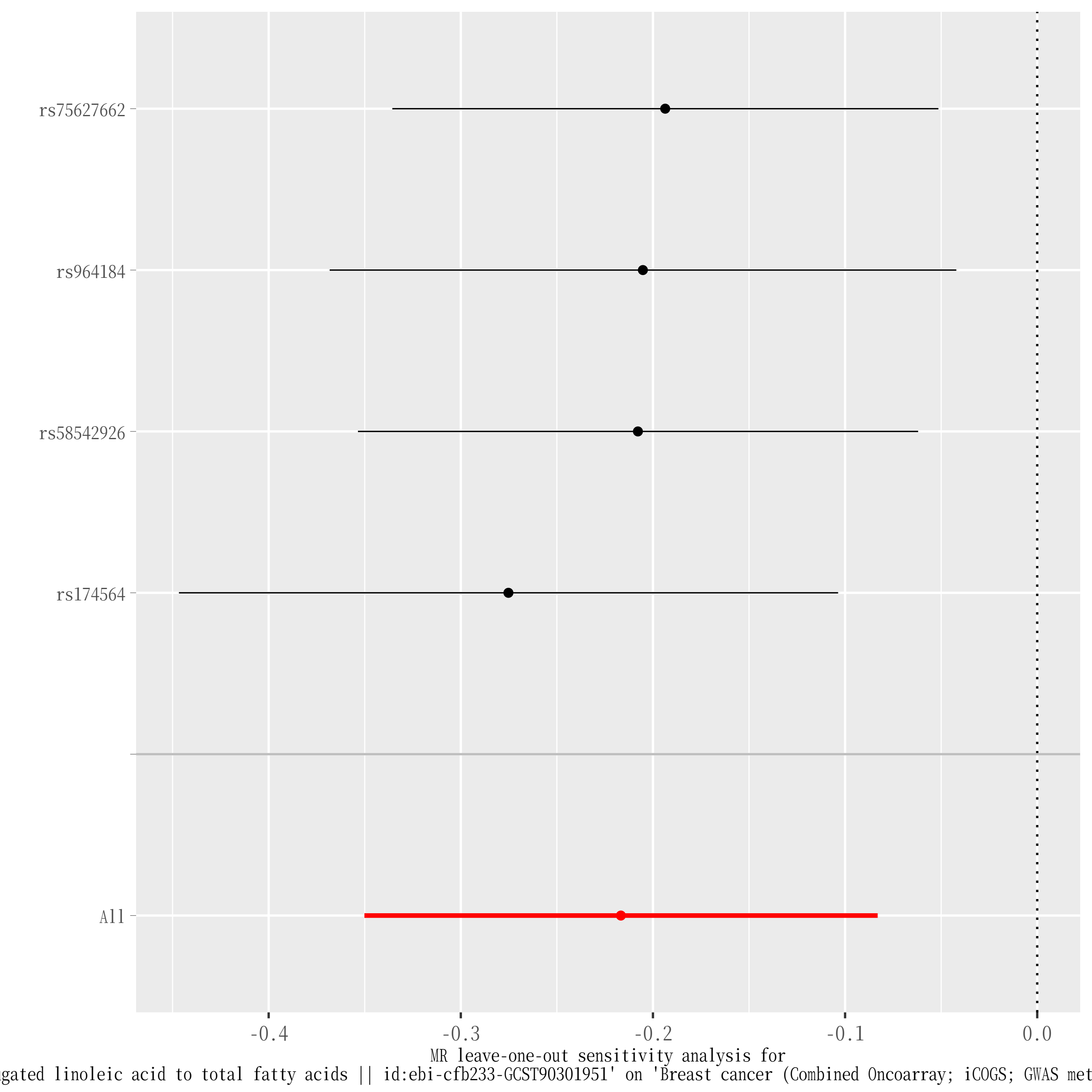
rs75627662
rs964184
rs58542926
rs174564
All
-0.4
-0.3
-0.2
-0.1
0.0
MR leave-one-out sensitivity analysis for
gated linoleic acid to total fatty acids || id:ebi-cfb233-GCST90301951' on 'Breast cancer (Combined Oncoarray; iCOGS; GWAS met

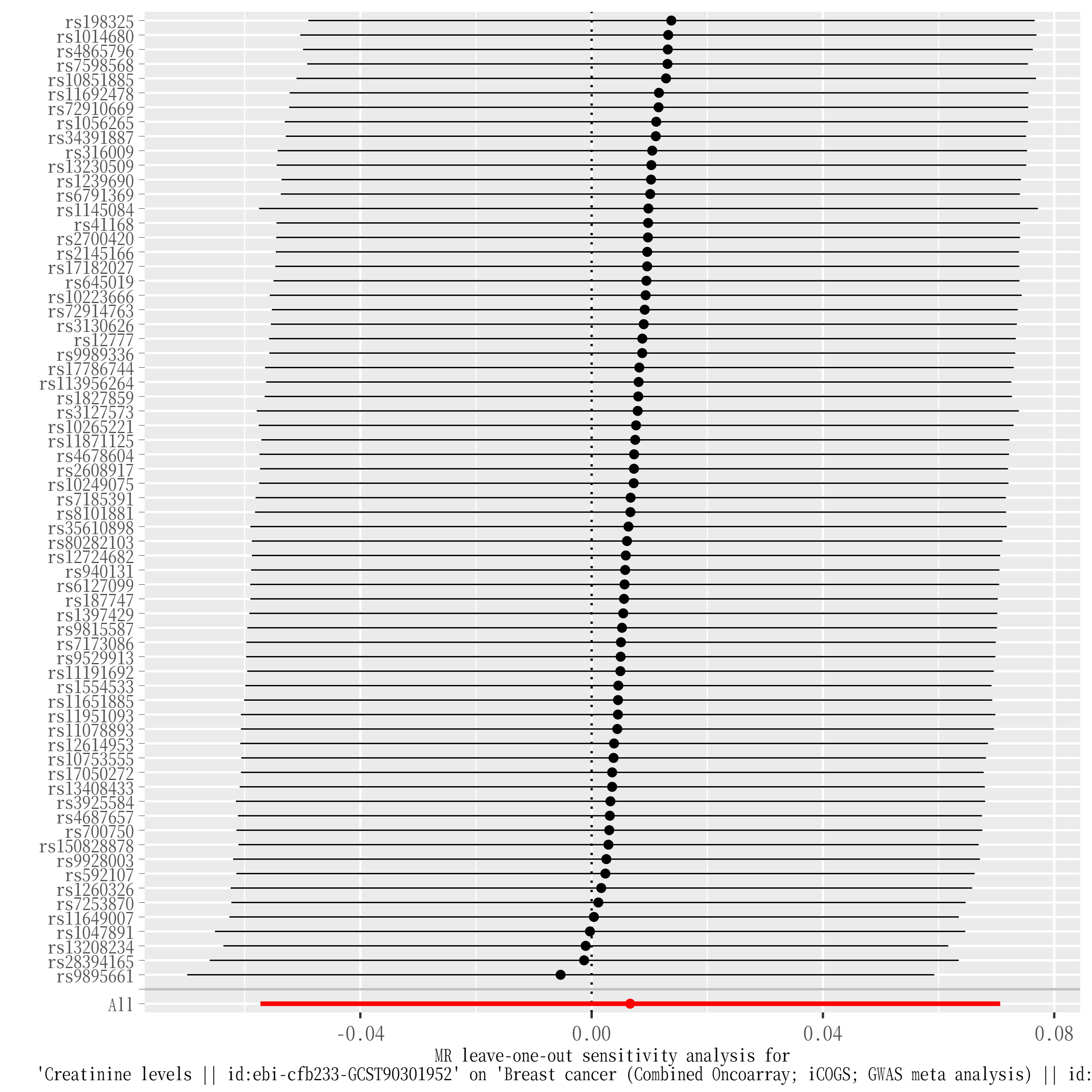

rs198325
rs1014680
rs4865796
rs7598568
rs10851885
rs11692478
rs72910669
rs1056265
rs34391887
rs316009
rs13230509
rs1239690
rs6791369
rs1145084
rs41168
rs2700420
rs2145166
rs17182027
rs645019
rs10223666
rs72914763
rs3130626
rs12777
rs9989336
rs17786744
rs113956264
rs1827859
rs3127573
rs10265221
rs11871125
rs4678604
rs2608917
rs10249075
rs7185391
rs8101881
rs35610898
rs80282103
rs12724682
rs940131
rs6127099
rs187747
rs1397429
rs9815587
rs7173086
rs9529913
rs11191692
rs1554533
rs11651885
rs11951093
rs11078893
rs12614953
rs10753555
rs17050272
rs13408433
rs3925584
rs4687657
rs700750
rs150828878
rs9928003
rs592107
rs1260326
rs7253870
rs11649007
rs1047891
rs13208234
rs28394165
rs9895661
All
-0.04
0.00
0.04
0.08
MR leave-one-out sensitivity analysis for
'Creatinine levels || id:ebi-cfb233-GCST90301952' on 'Breast cancer (Combined Oncoarray; iCOGS; GWAS meta analysis) || id:

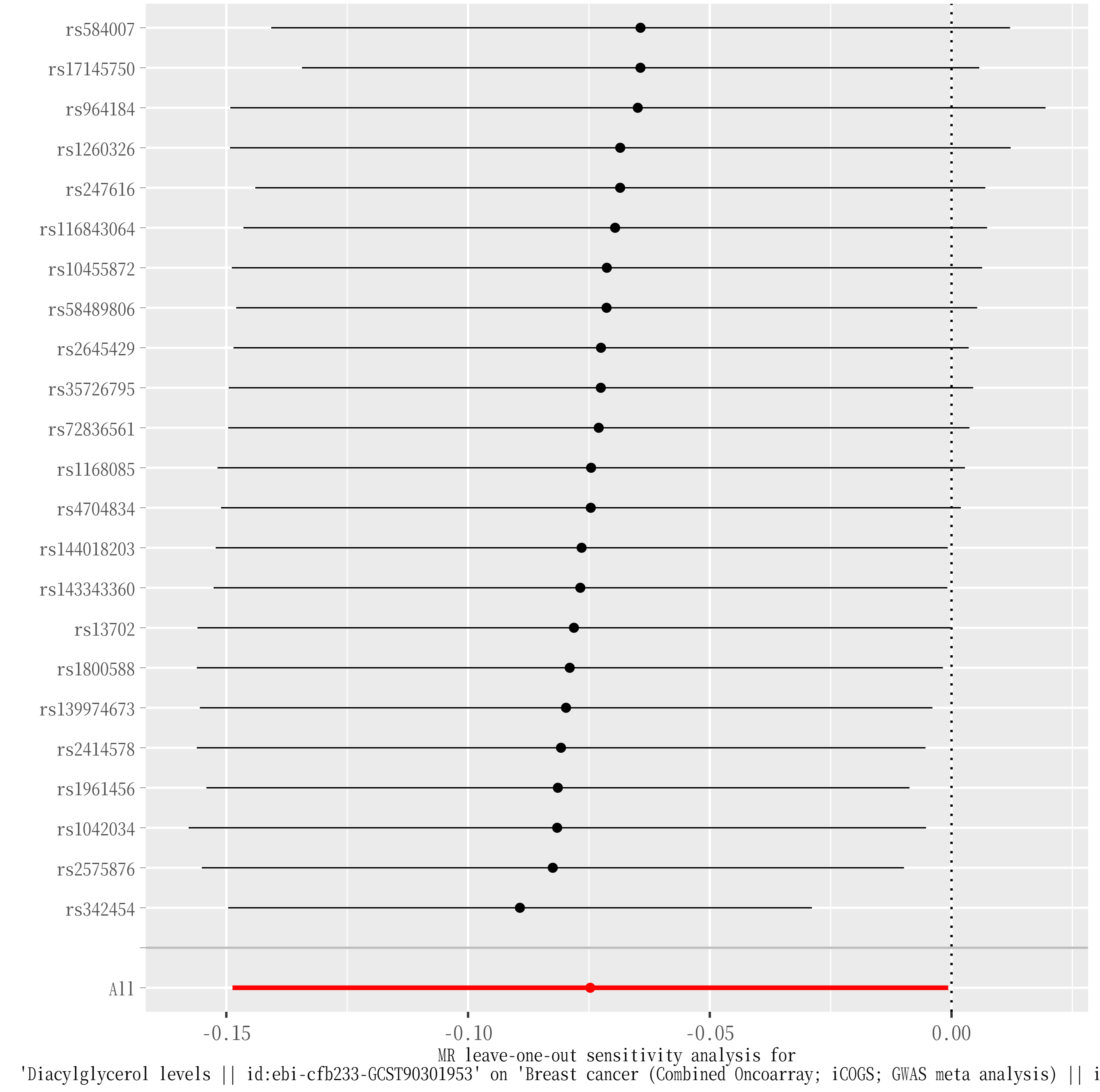

rs584007
rs17145750
rs964184
rs1260326
rs247616
rs116843064
rs10455872
rs58489806
rs2645429
rs35726795
rs72836561
rs1168085
rs4704834
rs144018203
rs143343360
rs13702
rs1800588
rs139974673
rs2414578
rs1961456
rs1042034
rs2575876
rs342454
All
-0.15
-0.10
-0.05
0.00
MR leave-one-out sensitivity analysis for
'Diacylglycerol levels || id:ebi-cfb233-GCST90301953' on 'Breast cancer (Combined Oncoarray; iCOGS; GWAS meta analysis) || i

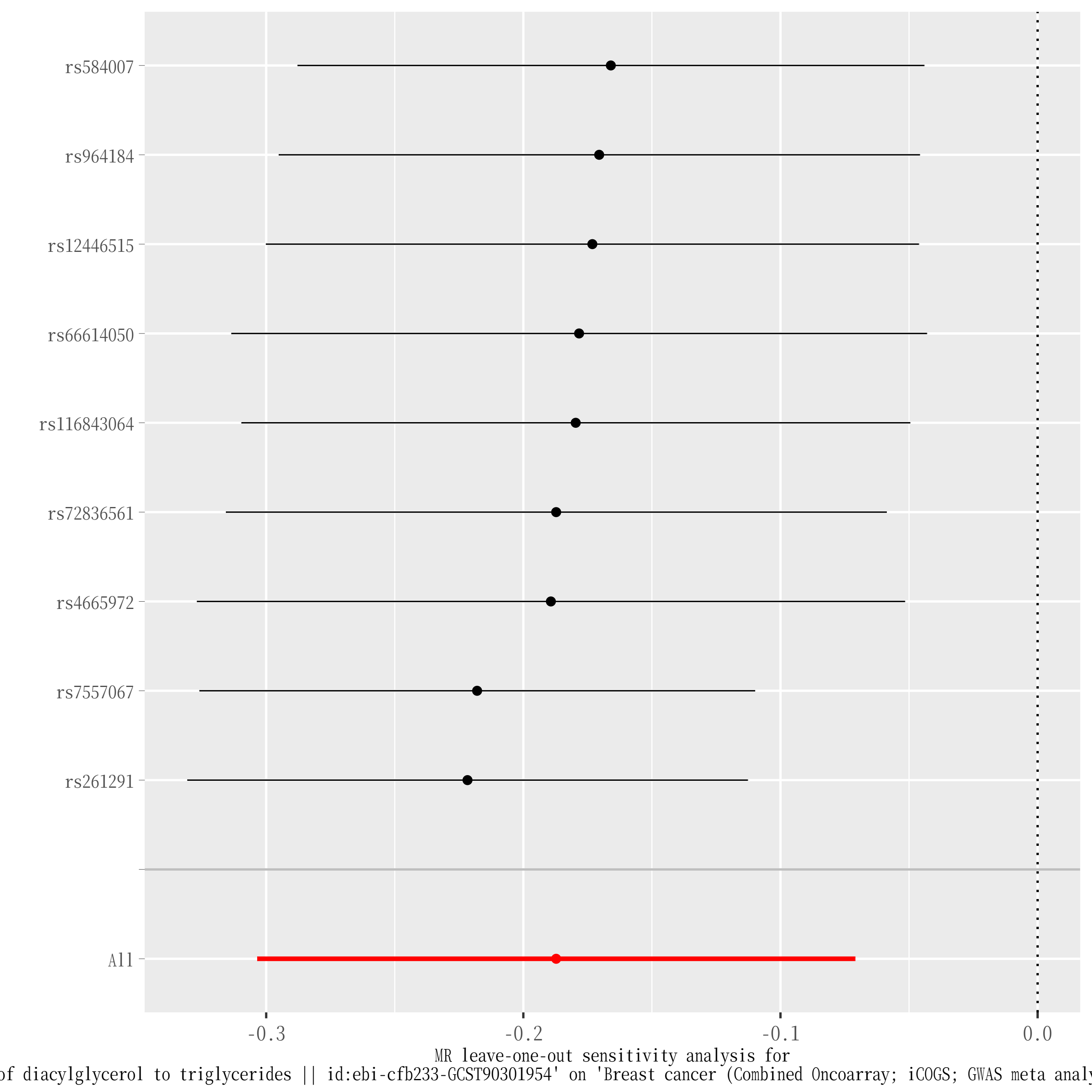

rs584007
rs964184
rs12446515
rs66614050
rs116843064
rs72836561
rs4665972
rs7557067
rs261291
All
-0.3
-0.2
-0.1
0.0
MR leave-one-out sensitivity analysis for
of diacylglycerol to triglycerides || id:ebi-cfb233-GCST90301954' on 'Breast cancer (Combined Oncoarray; iCOGS; GWAS meta analy

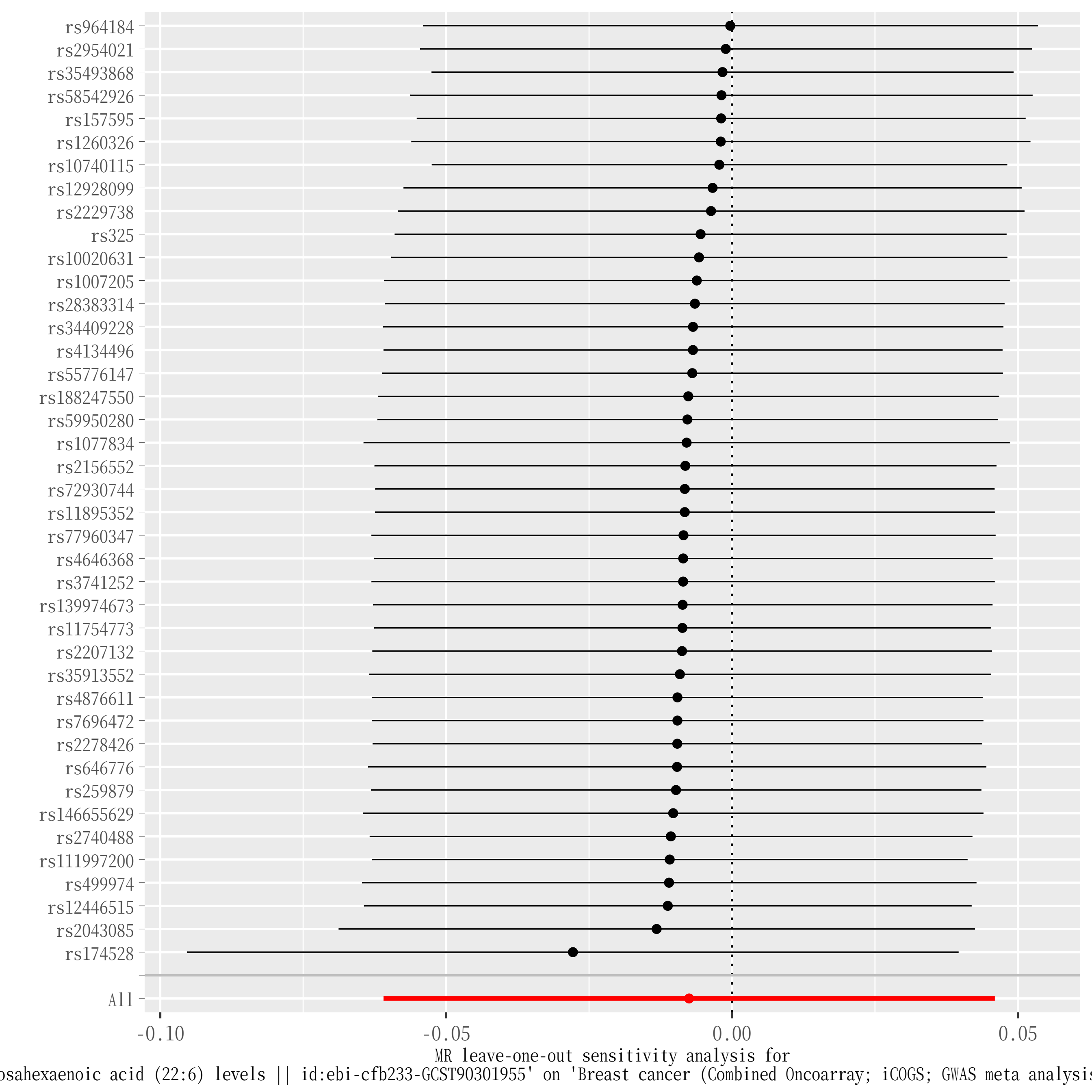
rs964184
rs2954021
rs35493868
rs58542926
rs157595
rs1260326
rs10740115
rs12928099
rs2229738
rs325
rs10020631
rs1007205
rs28383314
rs34409228
rs4134496
rs55776147
rs188247550
rs59950280
rs1077834
rs2156552
rs72930744
rs11895352
rs77960347
rs4646368
rs3741252
rs139974673
rs11754773
rs2207132
rs35913552
rs4876611
rs7696472
rs2278426
rs646776
rs259879
rs146655629
rs2740488
rs111997200
rs499974
rs12446515
rs2043085
rs174528
All
-0.10
-0.05
0.00
0.05
MR leave-one-out sensitivity analysis for
osahexaenoic acid (22:6) levels || id:ebi-cfb233-GCST90301955' on 'Breast cancer (Combined Oncoarray; iCOGS; GWAS meta analysis

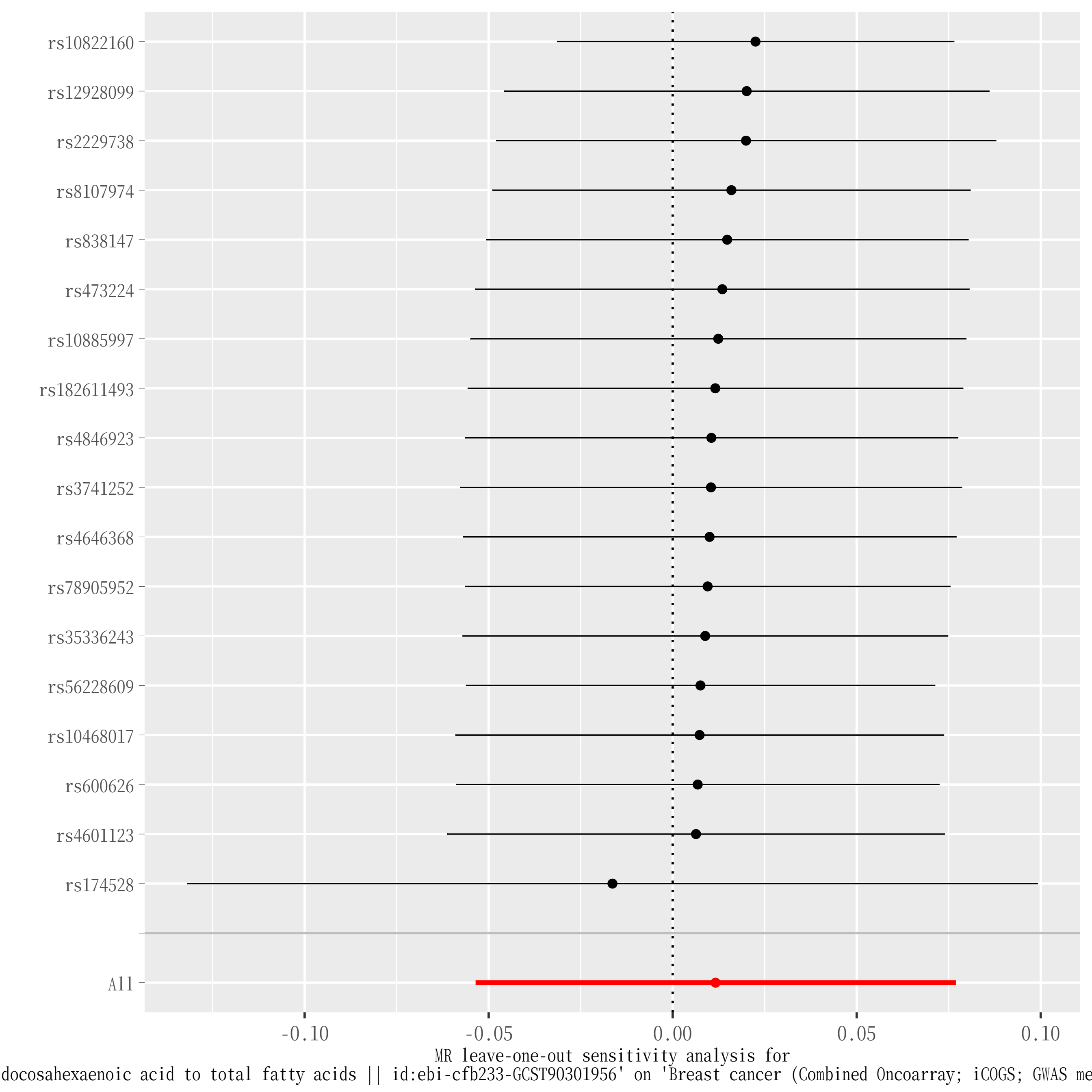

rs10822160
rs12928099
rs2229738
rs8107974
rs838147
rs473224
rs10885997
rs182611493
rs4846923
rs3741252
rs4646368
rs78905952
rs35336243
rs56228609
rs10468017
rs600626
rs4601123
rs174528
All
-0.10
-0.05
0.00
0.05
0.10
MR leave-one-out sensitivity analysis for
docosahexaenoic acid to total fatty acids || id:ebi-cfb233-GCST90301956' on 'Breast cancer (Combined Oncoarray; iCOGS; GWAS me

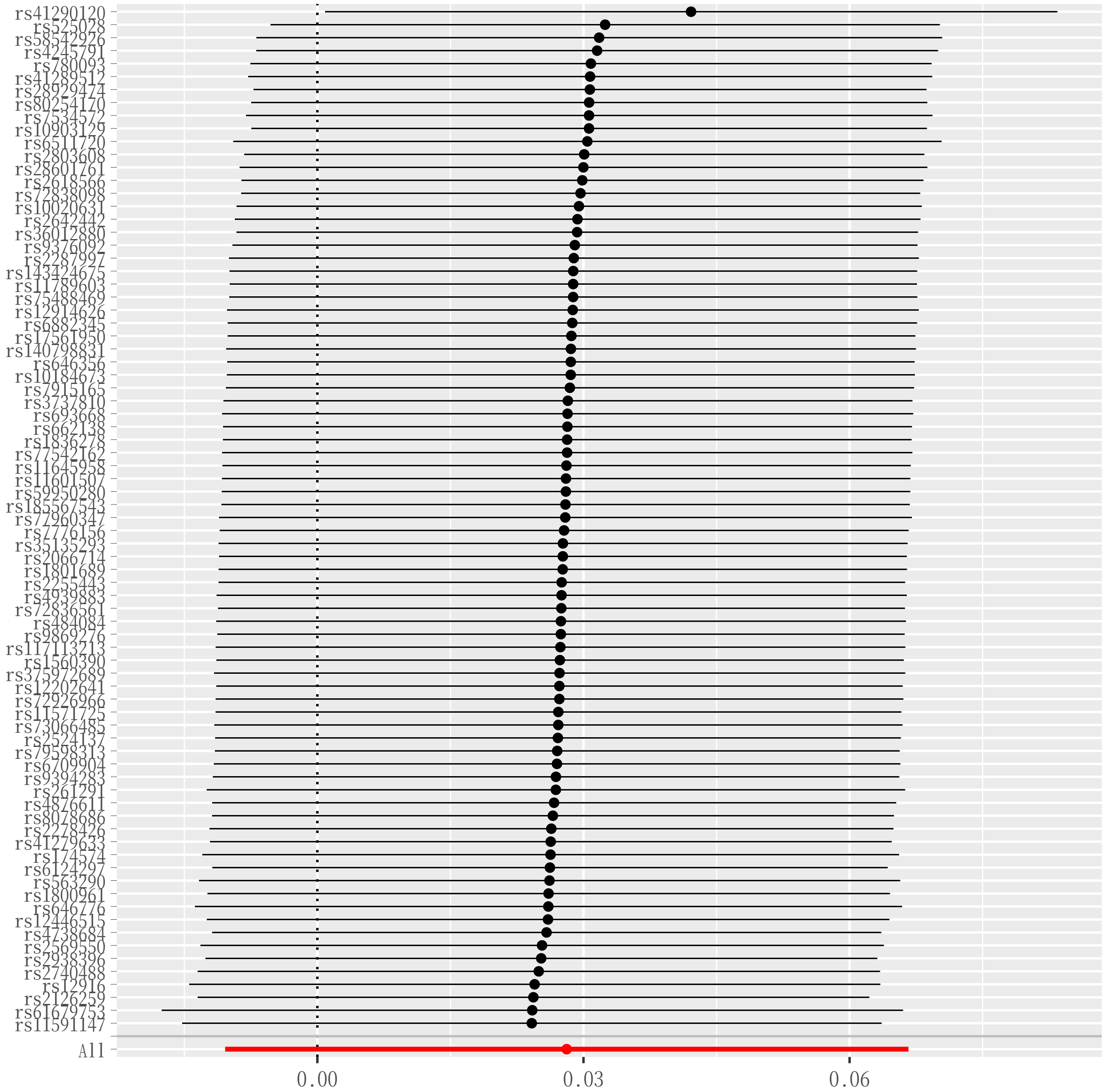

MR leave-one-out sensitivity analysis for
Esterified cholesterol levels || id:ebi-cfb233-GCST90301957' on 'Breast cancer (Combined Oncoarray; iCOGS; GWAS meta analysis)

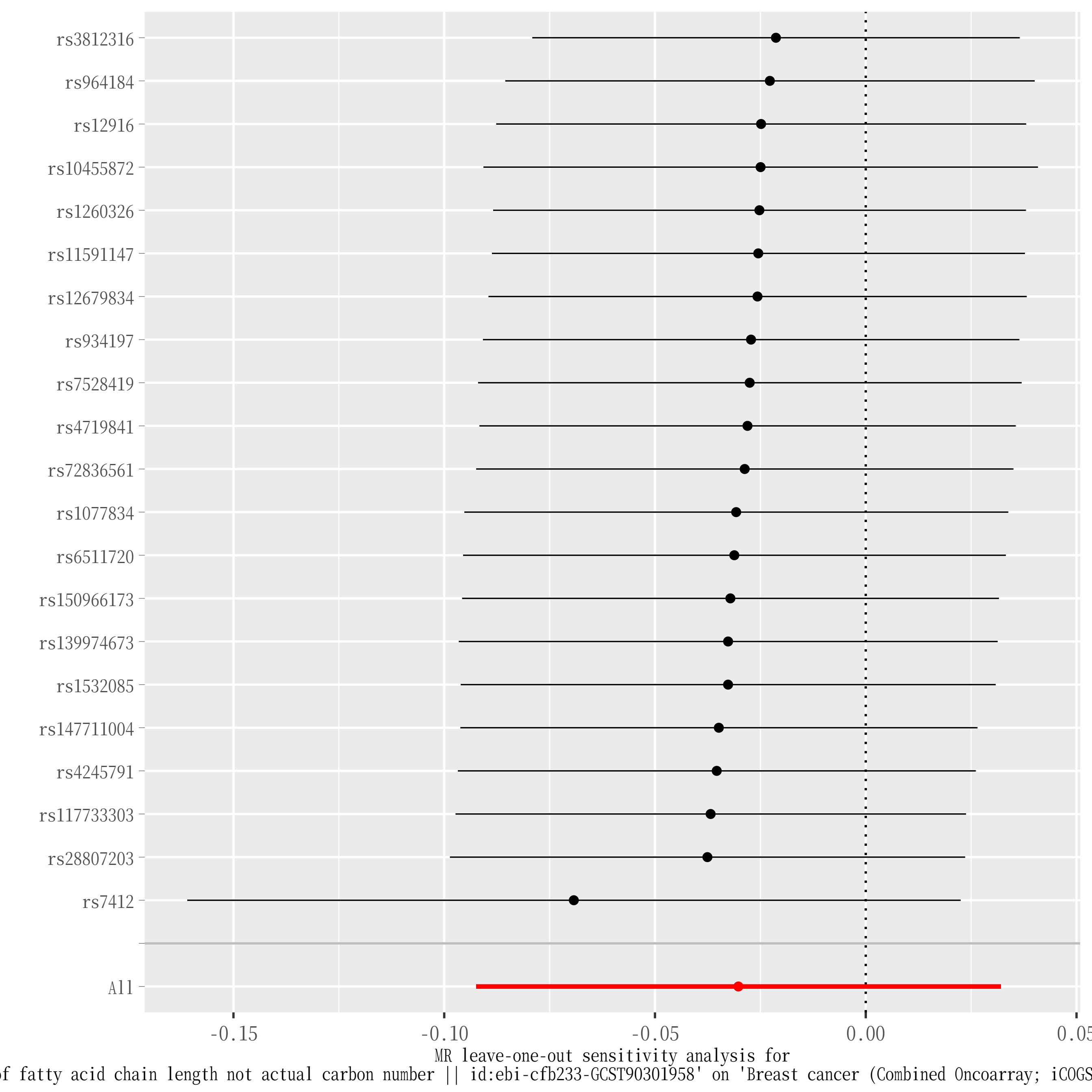
rs3812316
rs964184
rs12916
rs10455872
rs1260326
rs11591147
rs12679834
rs934197
rs7528419
rs4719841
rs72836561
rs1077834
rs6511720
rs150966173
rs139974673
rs1532085
rs147711004
rs4245791
rs117733303
rs28807203
rs7412
All
-0.15
-0.10
-0.05
0.00
0.05
MR leave-one-out sensitivity analysis for
of fatty acid chain length not actual carbon number || id:ebi-cfb233-GCST90301958' on 'Breast cancer (Combined Oncoarray; iCOGS

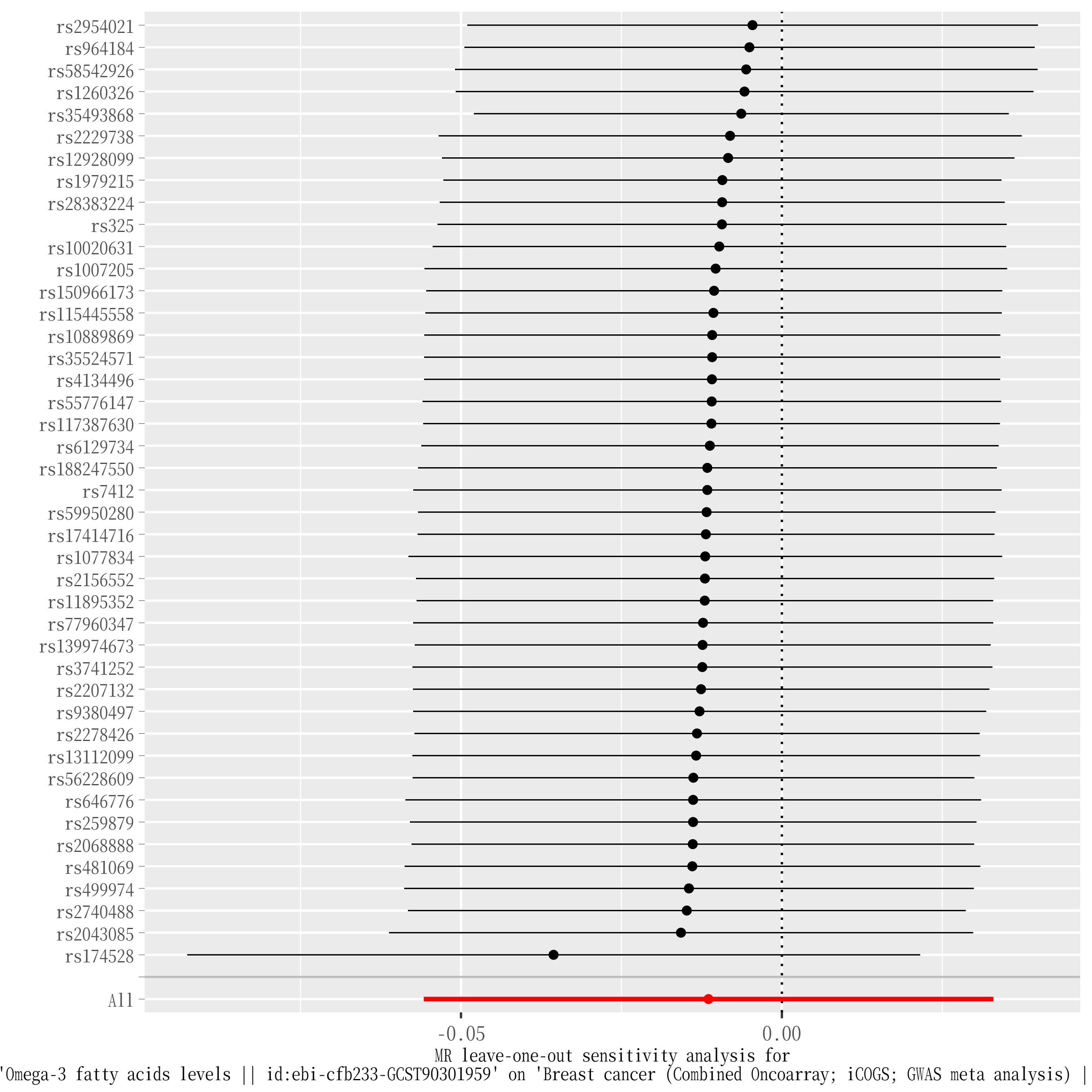

rs2954021
rs964184
rs58542926
rs1260326
rs35493868
rs2229738
rs12928099
rs1979215
rs28383224
rs325
rs10020631
rs1007205
rs150966173
rs115445558
rs10889869
rs35524571
rs4134496
rs55776147
rs117387630
rs6129734
rs188247550
rs7412
rs59950280
rs17414716
rs1077834
rs2156552
rs11895352
rs77960347
rs139974673
rs3741252
rs2207132
rs9380497
rs2278426
rs13112099
rs56228609
rs646776
rs259879
rs2068888
rs481069
rs499974
rs2740488
rs2043085
rs174528
All
-0.05
0.00
MR leave-one-out sensitivity analysis for
'Omega-3 fatty acids levels || id:ebi-cfb233-GCST90301959' on 'Breast cancer (Combined Oncoarray; iCOGS; GWAS meta analysis) |

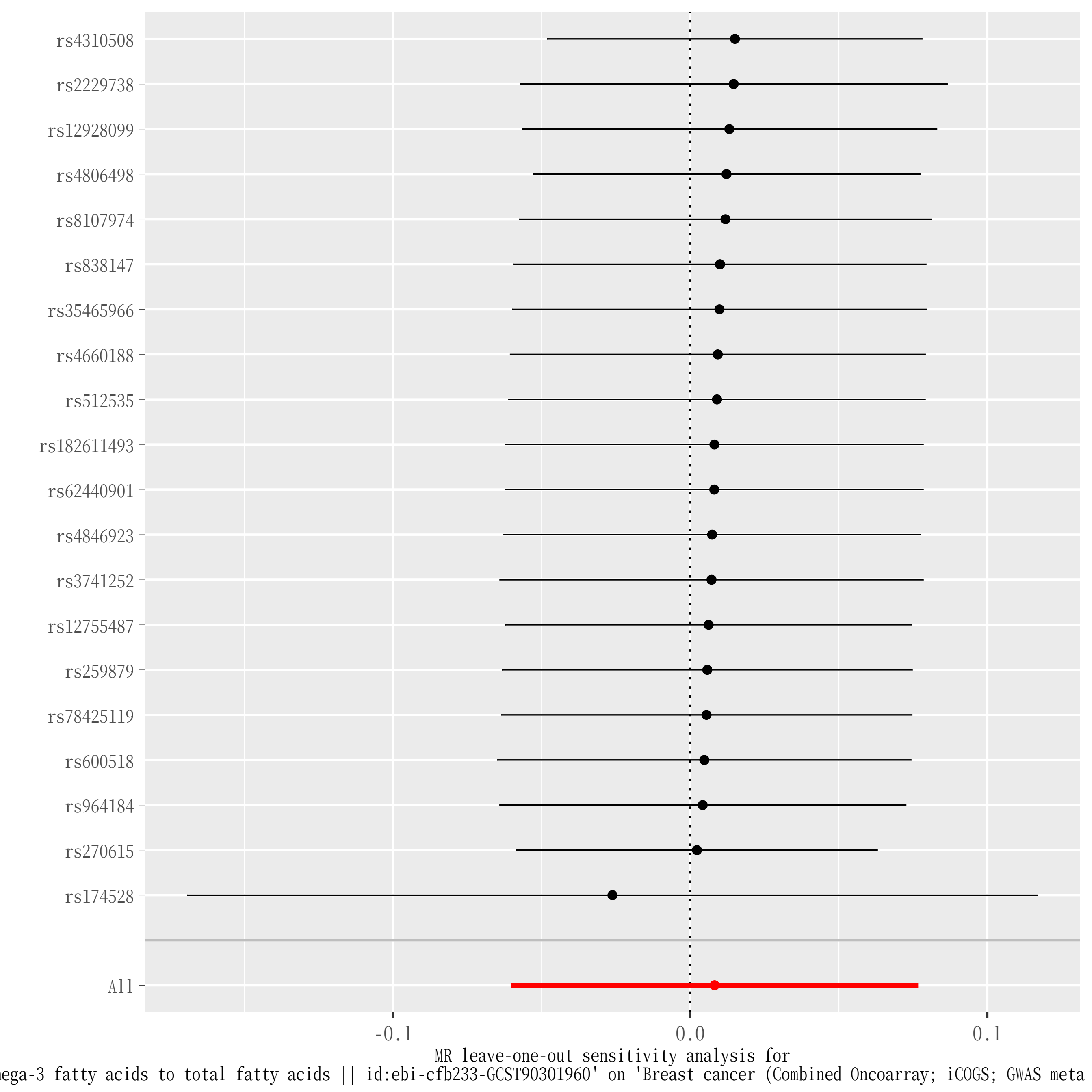
rs4310508
rs2229738
rs12928099
rs4806498
rs8107974
rs838147
rs35465966
rs4660188
rs512535
rs182611493
rs62440901
rs4846923
rs3741252
rs12755487
rs259879
rs78425119
rs600518
rs964184
rs270615
rs174528
All
-0.1
0.0
0.1
MR leave-one-out sensitivity analysis for
ega-3 fatty acids to total fatty acids || id:ebi-cfb233-GCST90301960' on 'Breast cancer (Combined Oncoarray; iCOGS; GWAS meta

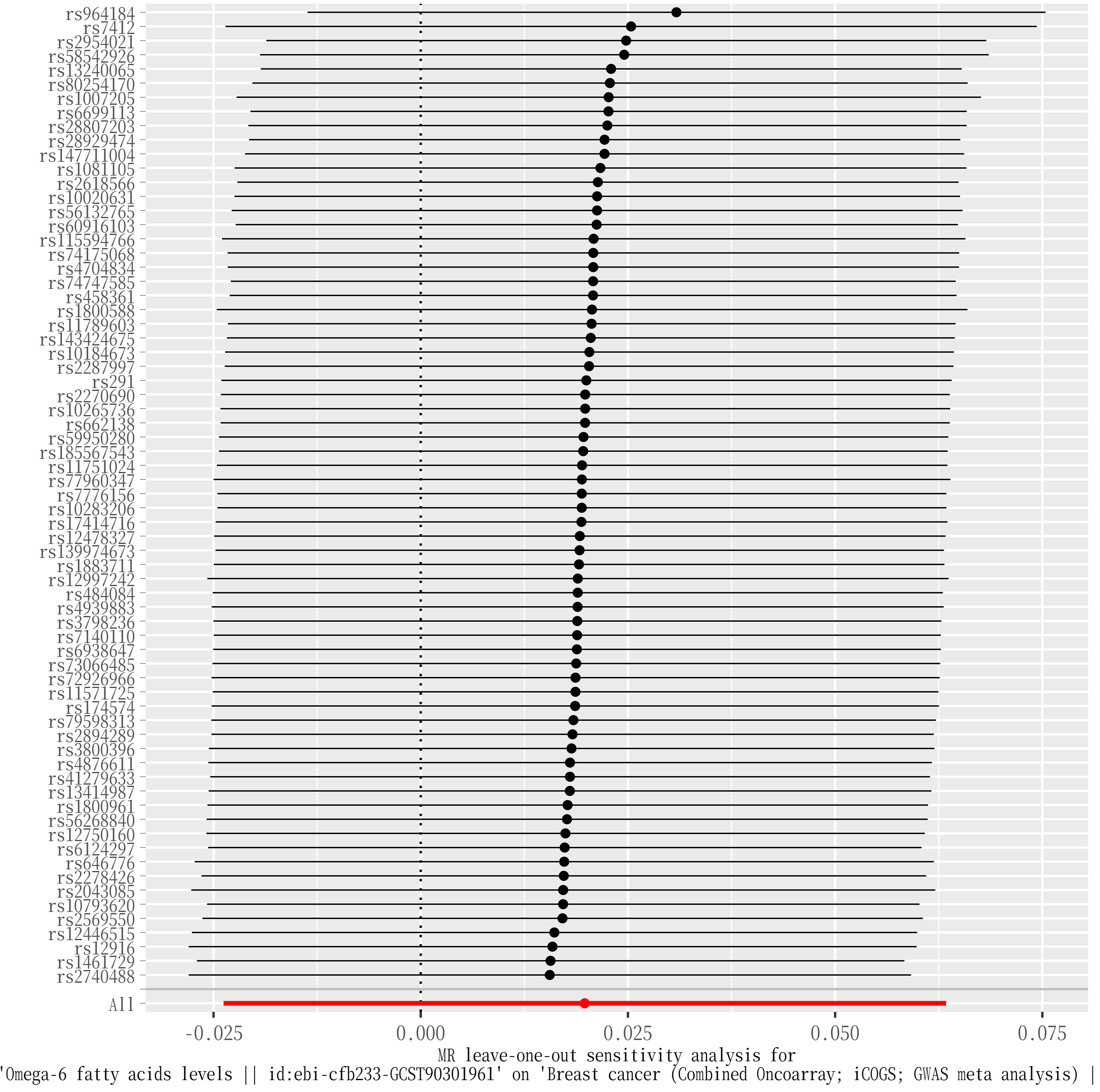
rs964184
rs7412
rs2954021
rs58542926
rs13240065
rs80254170
rs1007205
rs6699113
rs28807203
rs28929474
rs147711004
rs1081105
rs2618566
rs10020631
rs56132765
rs60916103
rs115594766
rs74175068
rs4704834
rs74747585
rs458361
rs1800588
rs11789603
rs143424675
rs10184673
rs2287997
rs291
rs2270690
rs10265736
rs662138
rs59950280
rs185567543
rs11751024
rs77960347
rs7776156
rs10283206
rs17414716
rs12478327
rs139974673
rs1883711
rs12997242
rs484084
rs4939883
rs3798236
rs7140110
rs6938647
rs73066485
rs72926966
rs11571725
rs174574
rs79598313
rs2894289
rs3800396
rs4876611
rs41279633
rs13414987
rs1800961
rs56268840
rs12750160
rs6124297
rs646776
rs2278426
rs2043085
rs10793620
rs2569550
rs12446515
rs12916
rs1461729
rs2740488
All
-0.025
0.000
0.025
0.050
0.075
MR leave-one-out sensitivity analysis for
'Omega-6 fatty acids levels || id:ebi-cfb233-GCST90301961' on 'Breast cancer (Combined Oncoarray; iCOGS; GWAS meta analysis) |

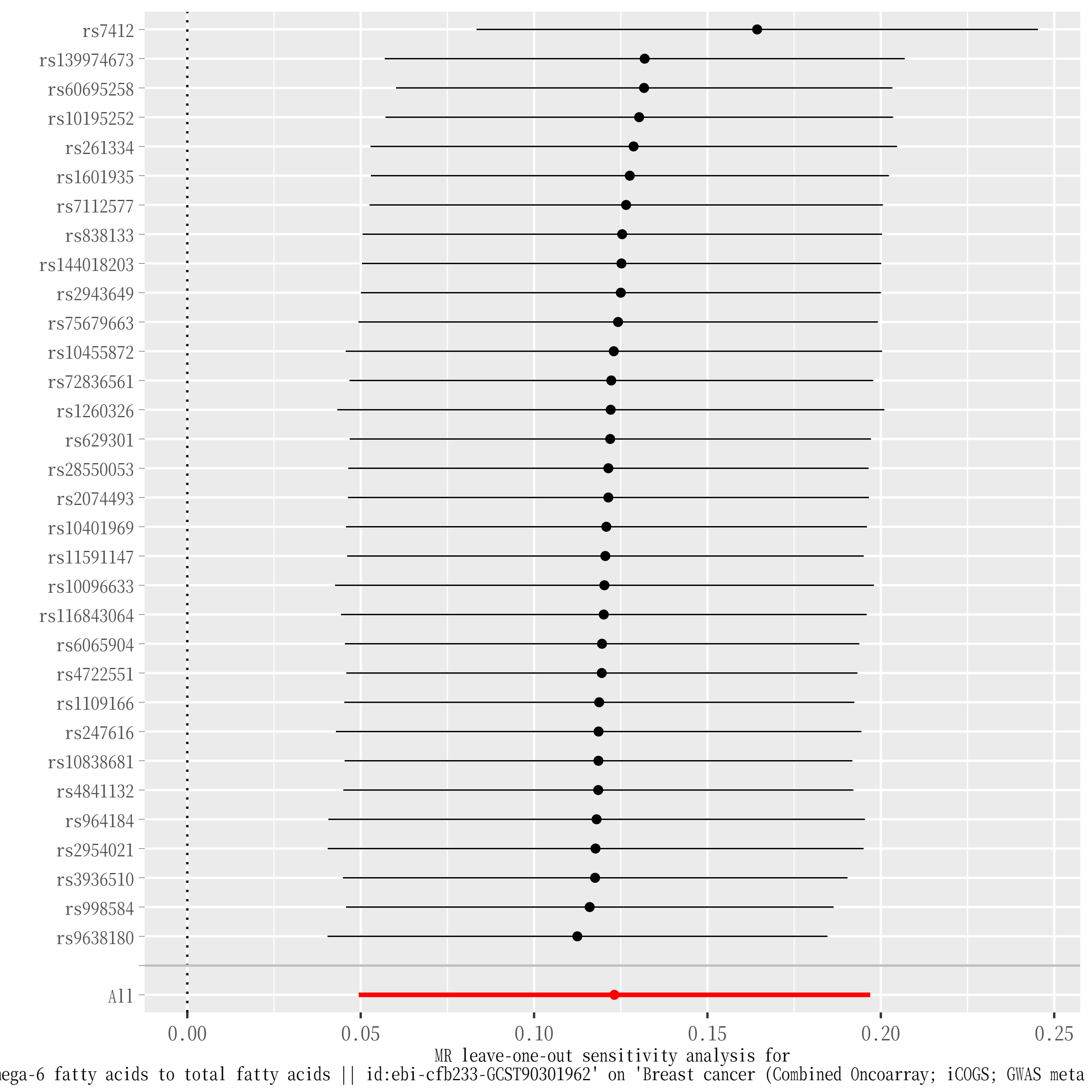

rs7412
rs139974673
rs60695258
rs10195252
rs261334
rs1601935
rs7112577
rs838133
rs144018203
rs2943649
rs75679663
rs10455872
rs72836561
rs1260326
rs629301
rs28550053
rs2074493
rs10401969
rs11591147
rs10096633
rs116843064
rs6065904
rs4722551
rs1109166
rs247616
rs10838681
rs4841132
rs964184
rs2954021
rs3936510
rs998584
rs9638180
All
0.00
0.05
0.10
0.15
0.20
0.25
MR leave-one-out sensitivity analysis for
nega-6 fatty acids to total fatty acids || id:ebi-cfb233-GCST90301962' on 'Breast cancer (Combined Oncoarray; iCOGS; GWAS meta

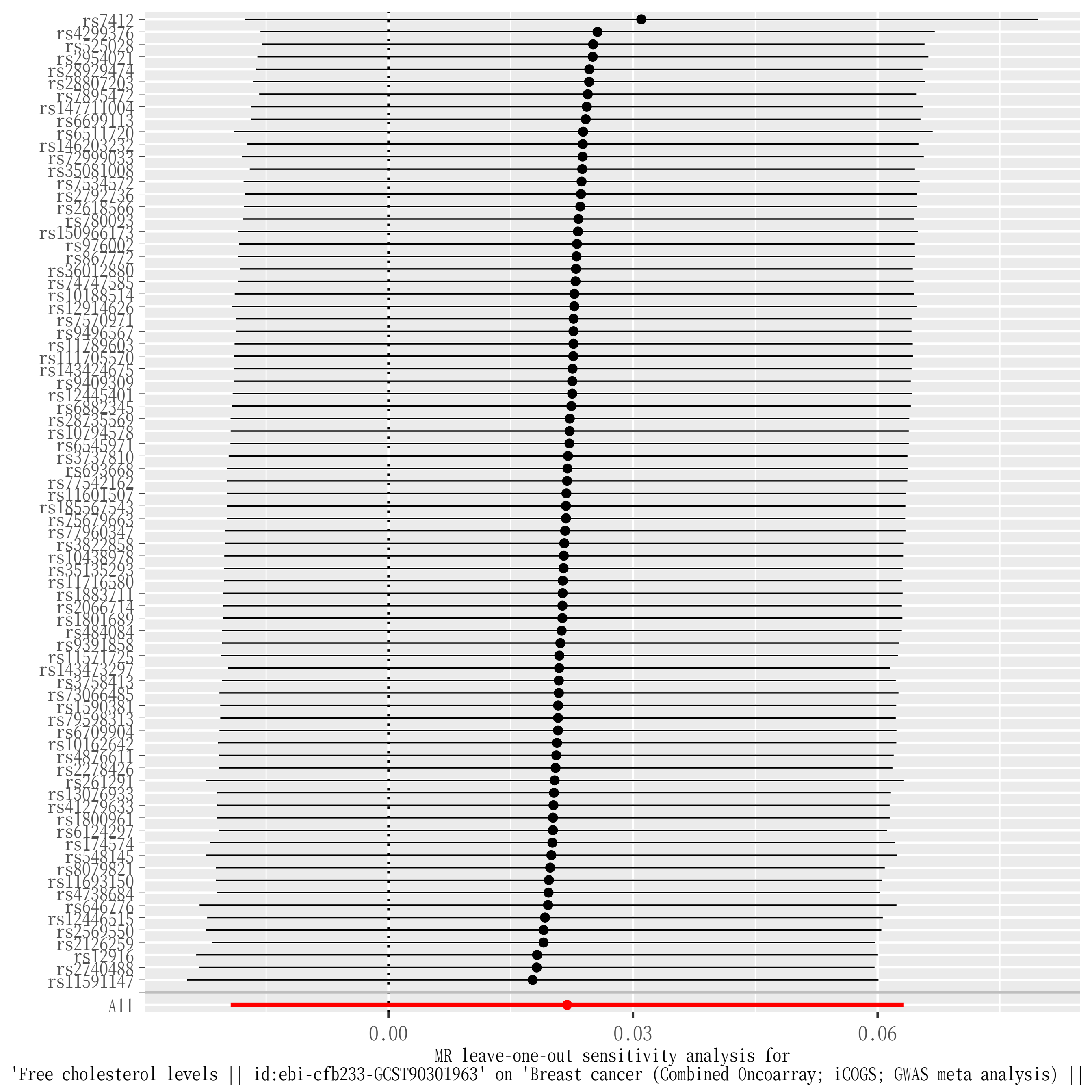
rs7412
rs4299376
rs525028
rs2954021
rs28929474
rs28807203
rs7895472
rs147711004
rs6699113
rs6511720
rs146203232
rs72999033
rs35081008
rs7534572
rs2792736
rs2618566
rs780093
rs150966173
rs976002
rs867772
rs36012880
rs74747585
rs10188514
rs12914626
rs7570971
rs9496567
rs11789603
rs111705570
rs143424675
rs9409309
rs12445401
rs6882345
rs28735569
rs10794578
rs6545971
rs3737810
rs693668
rs77542162
rs11601507
rs185567543
rs75679663
rs77960347
rs3822858
rs10438978
rs35135293
rs11716580
rs1883711
rs2066714
rs1801689
rs484084
rs9391858
rs11571725
rs143473297
rs3758413
rs73066485
rs1590381
rs79598313
rs6709904
rs10162642
rs4876611
rs2278426
rs261291
rs13076933
rs41279633
rs1800961
rs6124297
rs174574
rs548145
rs8079821
rs11693150
rs4738684
rs646776
rs12446515
rs2569550
rs2126259
rs12916
rs2740488
rs11591147
All
0.00
0.03
0.06
MR leave-one-out sensitivity analysis for
'Free cholesterol levels || id:ebi-cfb233-GCST90301963' on 'Breast cancer (Combined Oncoarray; iCOGS; GWAS meta analysis) ||

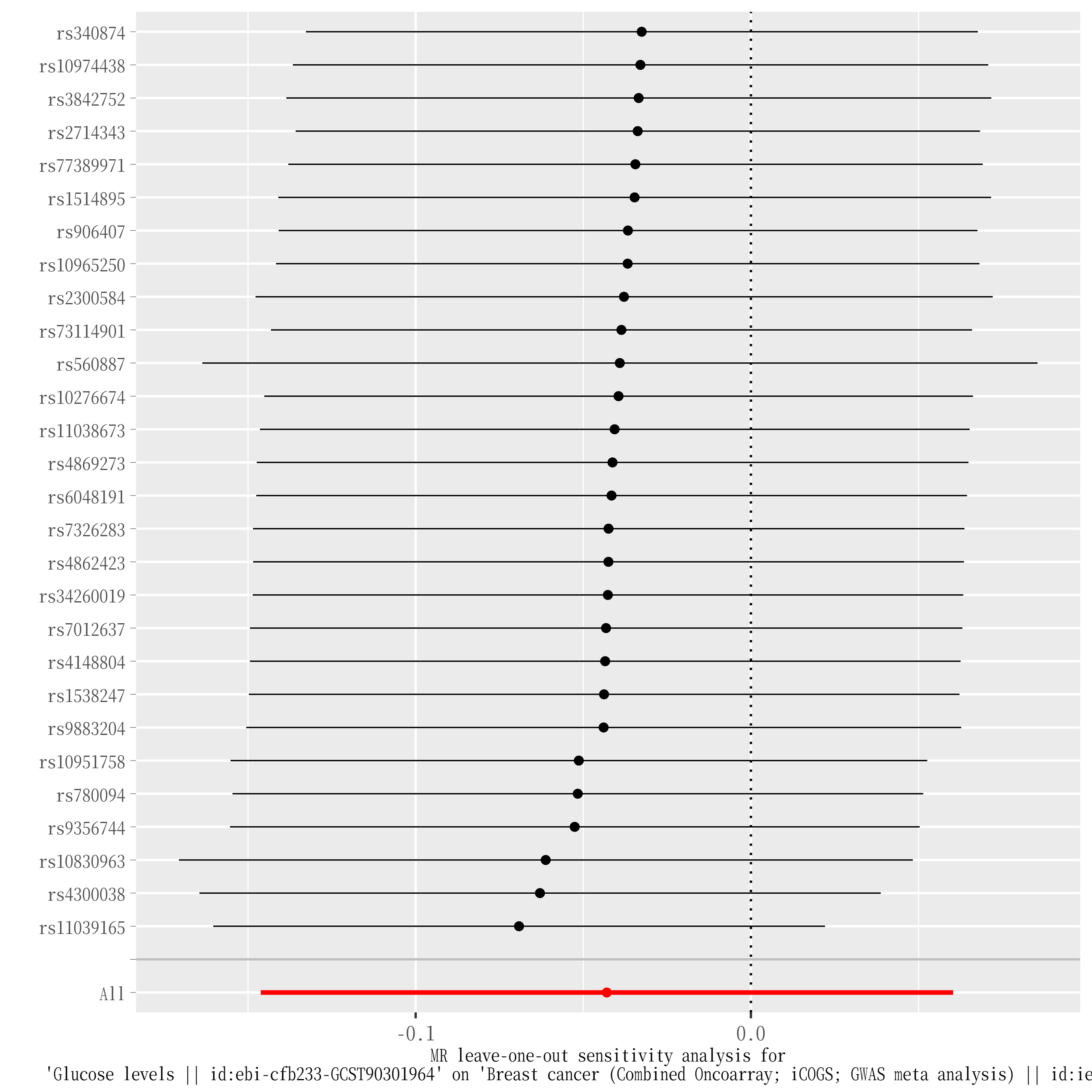
rs340874
rs10974438
rs3842752
rs2714343
rs77389971
rs1514895
rs906407
rs10965250
rs2300584
rs73114901
rs560887
rs10276674
rs11038673
rs4869273
rs6048191
rs7326283
rs4862423
rs34260019
rs7012637
rs4148804
rs1538247
rs9883204
rs10951758
rs780094
rs9356744
rs10830963
rs4300038
rs11039165
All
-0.1
0.0
MR leave-one-out sensitivity analysis for
'Glucose levels || id:ebi-cfb233-GCST90301964' on 'Breast cancer (Combined Oncoarray; iCOGS; GWAS meta analysis) || id:ie

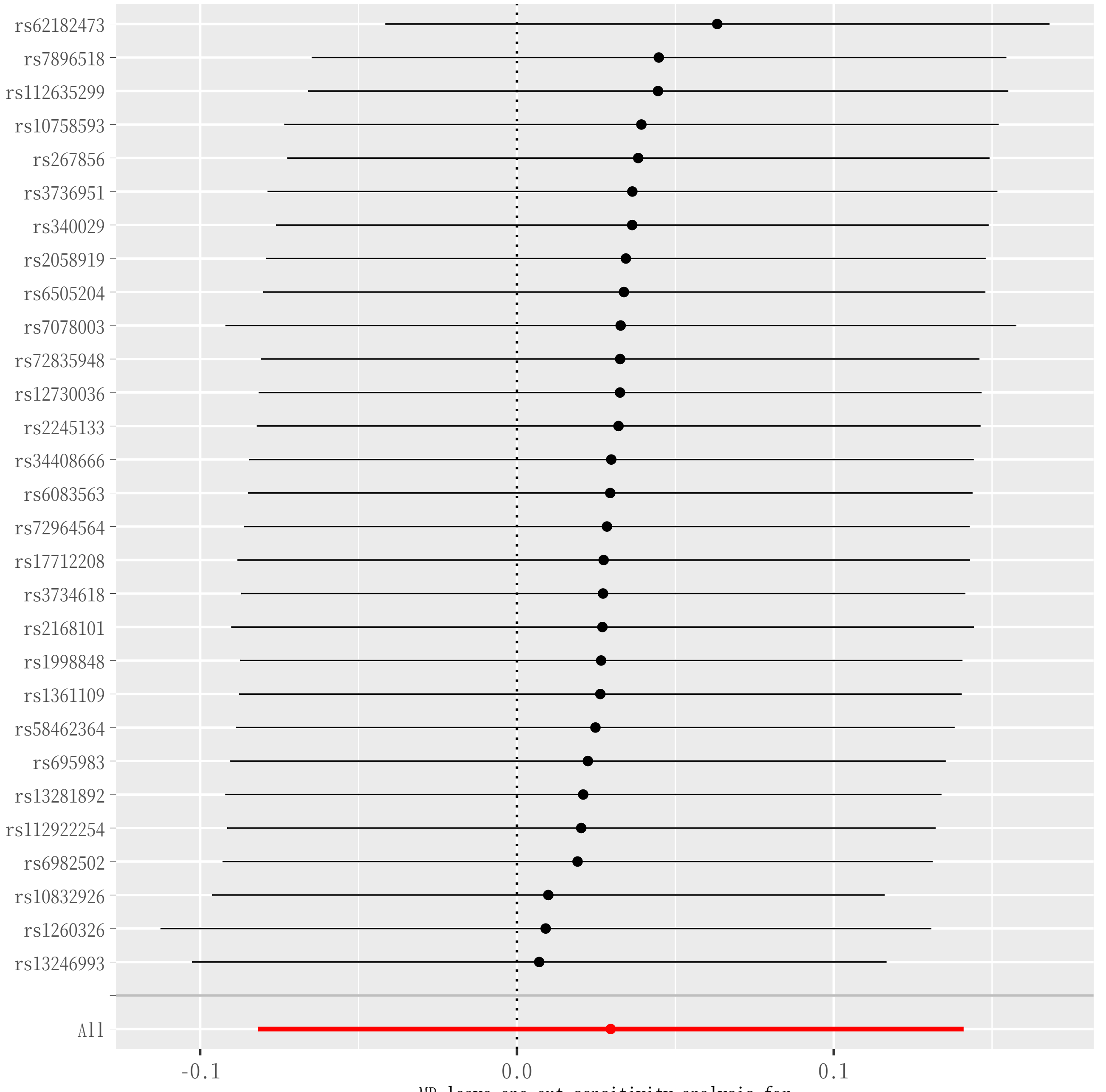

MR leave-one-out sensitivity analysis for
'Glutamine levels || id:ebi-cfb233-GCST90301965' on 'Breast cancer (Combined Oncoarray; iCOGS; GWAS meta analysis) || id:

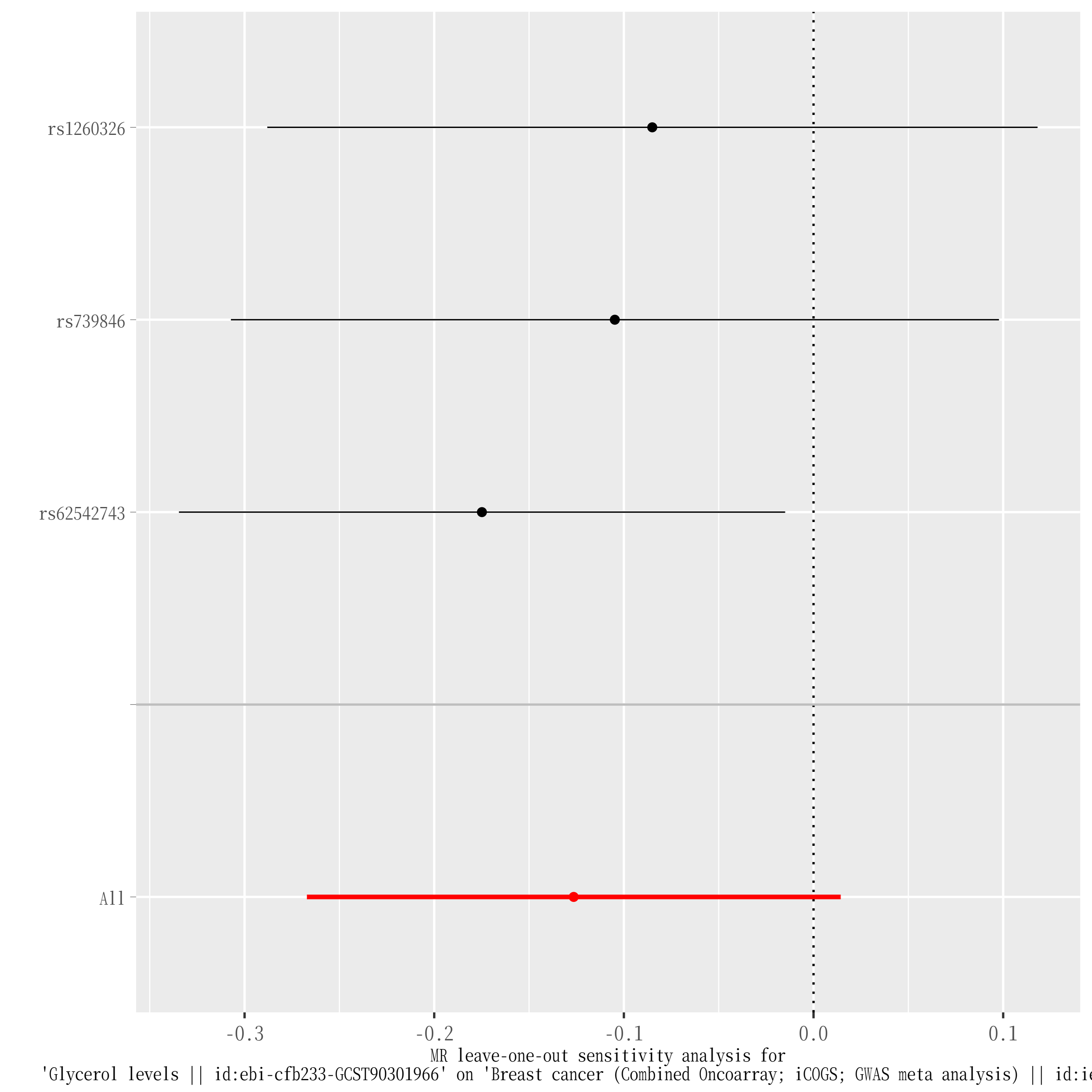
rs1260326
rs739846
rs62542743
All
-0.3
-0.2
-0.1
0.0
0.1
MR leave-one-out sensitivity analysis for
'Glycerol levels || id:ebi-cfb233-GCST90301966' on 'Breast cancer (Combined Oncoarray; iCOGS; GWAS meta analysis) || id:ie

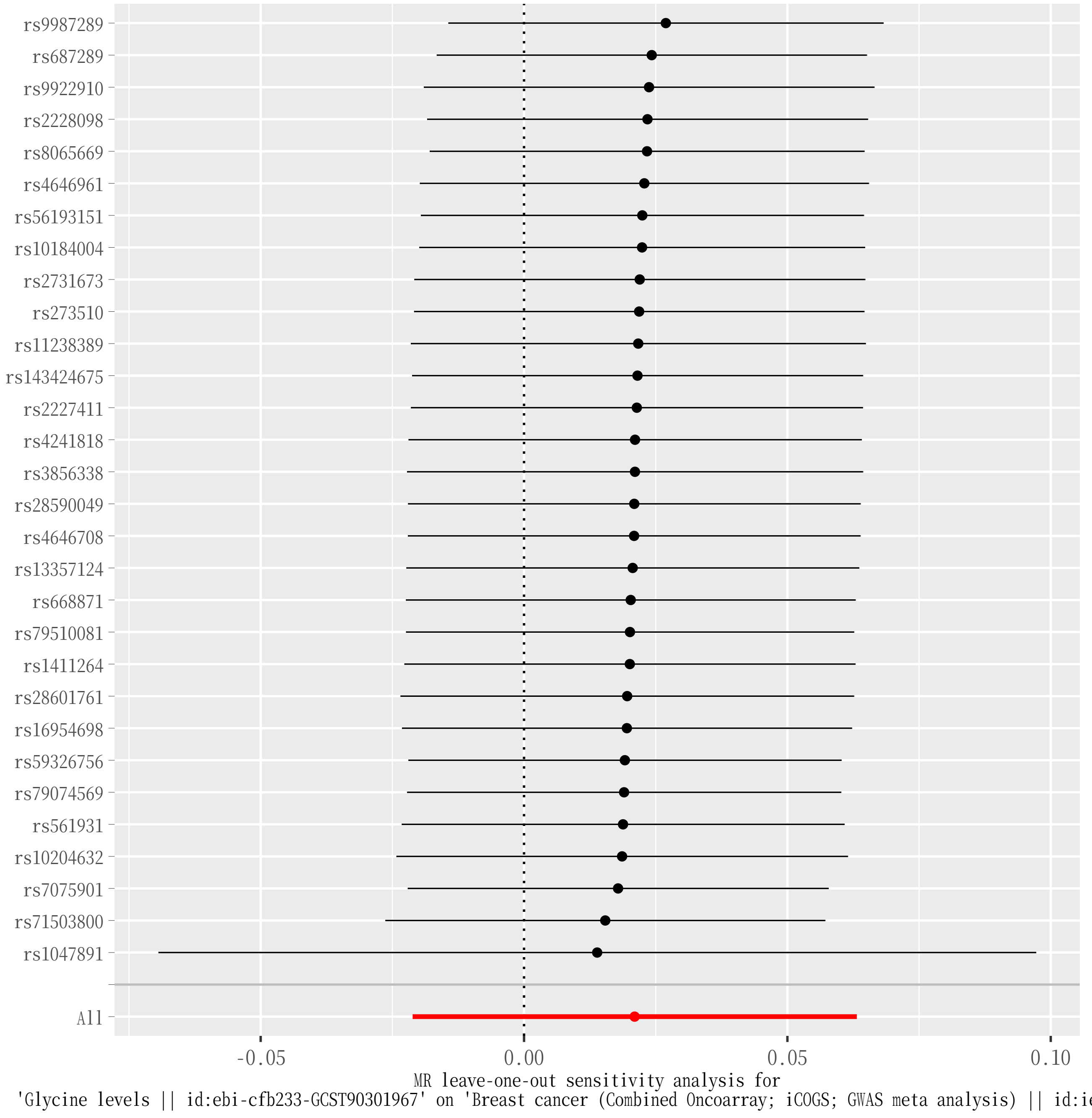
rs9987289
rs687289
rs9922910
rs2228098
rs8065669
rs4646961
rs56193151
rs10184004
rs2731673
rs273510
rs11238389
rs143424675
rs2227411
rs4241818
rs3856338
rs28590049
rs4646708
rs13357124
rs668871
rs79510081
rs1411264
rs28601761
rs16954698
rs59326756
rs79074569
rs561931
rs10204632
rs7075901
rs71503800
rs1047891
All
-0.05
0.00
0.05
0.10
MR leave-one-out sensitivity analysis for
'Glycine levels || id:ebi-cfb233-GCST90301967' on 'Breast cancer (Combined Oncoarray; iCOGS; GWAS meta analysis) || id:i

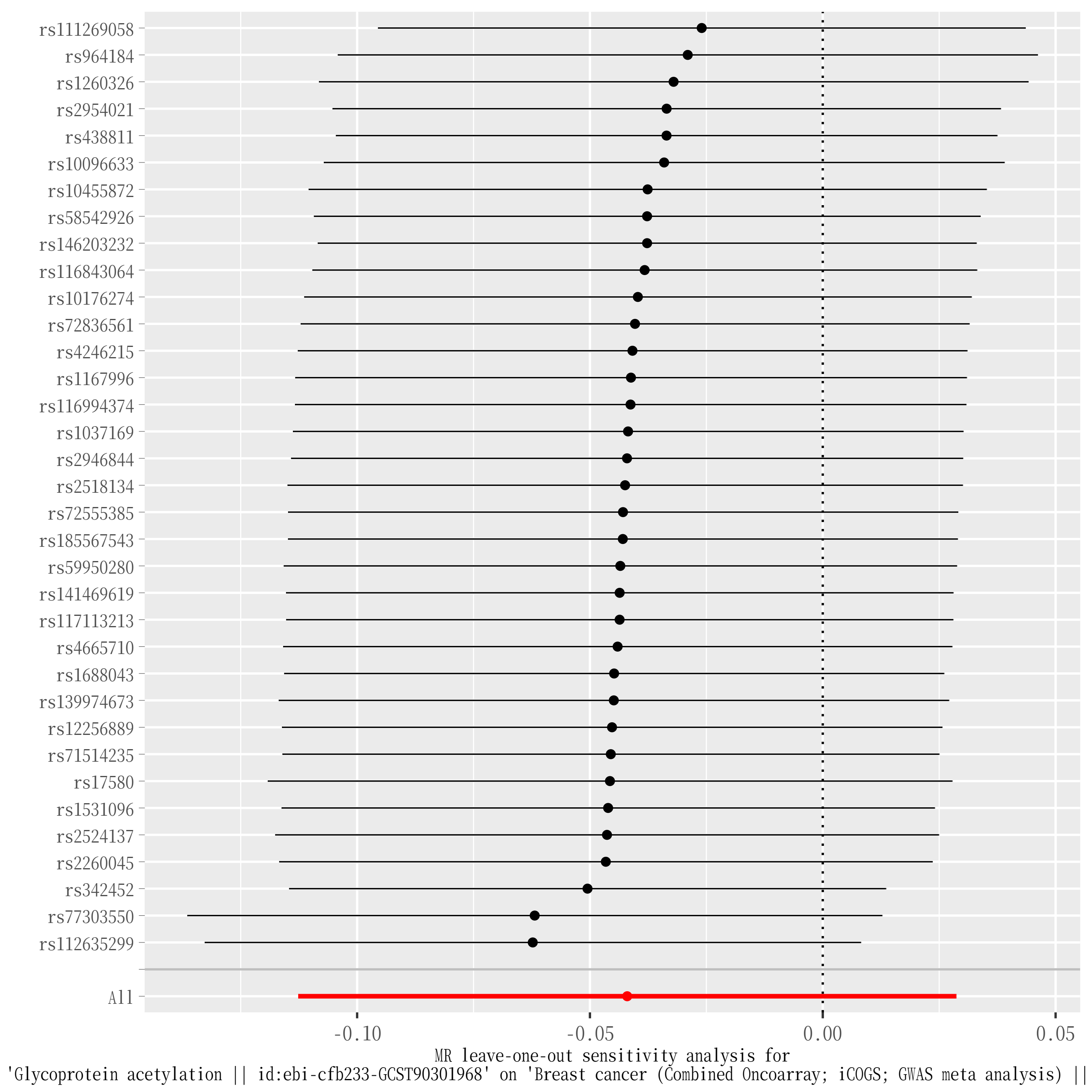
rs111269058
rs964184
rs1260326
rs2954021
rs438811
rs10096633
rs10455872
rs58542926
rs146203232
rs116843064
rs10176274
rs72836561
rs4246215
rs1167996
rs116994374
rs1037169
rs2946844
rs2518134
rs72555385
rs185567543
rs59950280
rs141469619
rs117113213
rs4665710
rs1688043
rs139974673
rs12256889
rs71514235
rs17580
rs1531096
rs2524137
rs2260045
rs342452
rs77303550
rs112635299
All
-0.10
-0.05
0.00
0.05
MR leave-one-out sensitivity analysis for
'Glycoprotein acetylation || id:ebi-cfb233-GCST90301968' on 'Breast cancer (Combined Oncoarray; iCOGS; GWAS meta analysis) ||

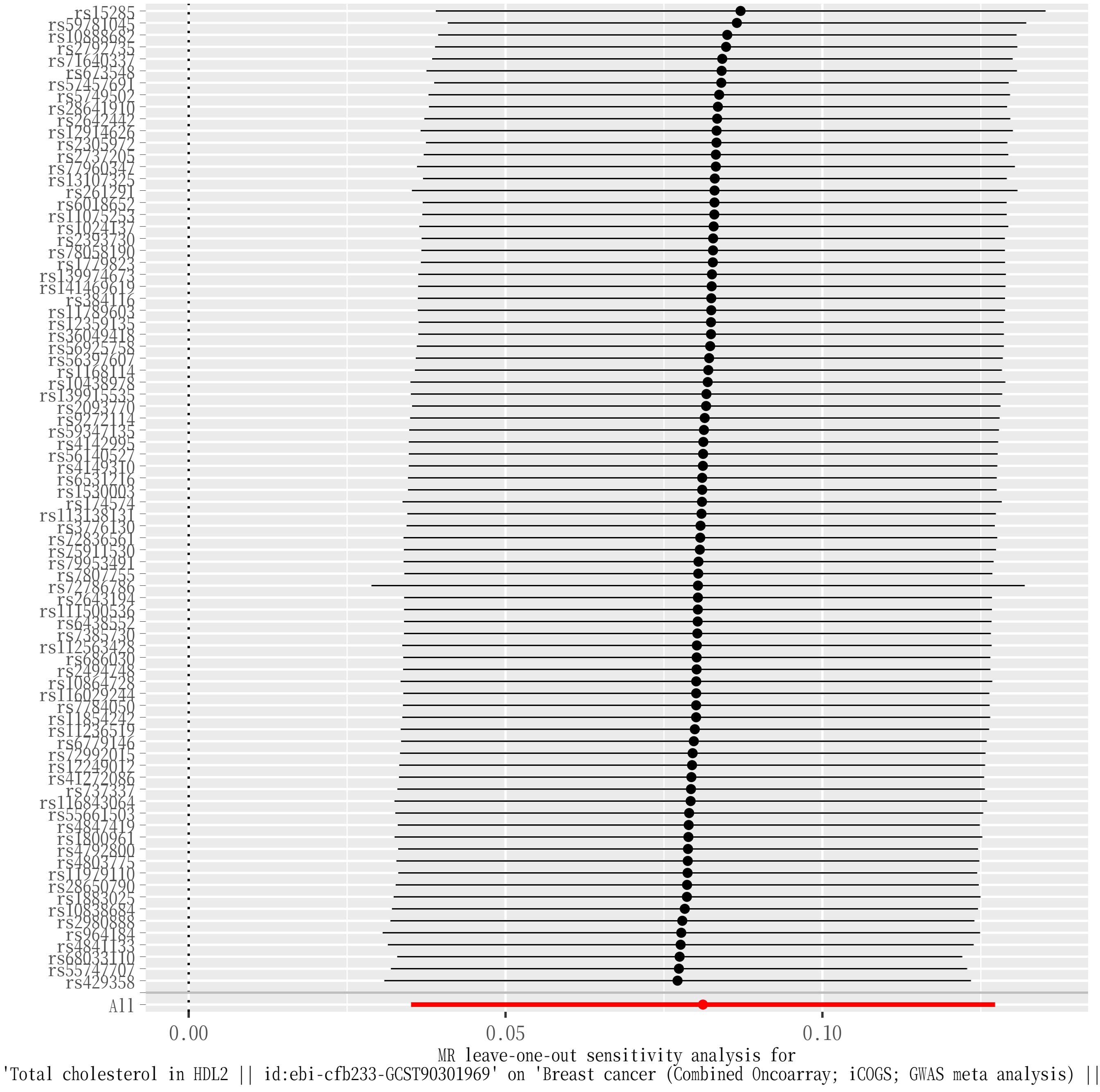
rs15285
rs59781045
rs10888682
rs2792735
rs71640337
rs673548
rs57457691
rs5749502
rs28641910
rs2642442
rs12914626
rs2305972
rs2737205
rs77960347
rs13107325
rs261291
rs6018652
rs11075253
rs1024137
rs2323730
rs78058190
rs1779823
rs139974673
rs141469619
rs384116
rs11789603
rs12359135
rs36042418
rs56925758
rs56397607
rs1168114
rs10438978
rs139915535
rs2093770
rs9272114
rs59347135
rs4142995
rs56140527
rs4149310
rs6531216
rs1530003
rs174574
rs113138131
rs3776130
rs72836561
rs75911530
rs79953491
rs7807755
rs72786786
rs2643194
rs111500536
rs6438552
rs7385730
rs112563428
rs686030
rs2494748
rs10864728
rs116029244
rs7784050
rs11854242
rs11236519
rs6779146
rs72992015
rs12249012
rs41272086
rs737337
rs116843064
rs55661503
rs4847419
rs1800961
rs4792800
rs4803775
rs11979110
rs28650790
rs1883025
rs10838684
rs2980888
rs964184
rs4841133
rs68033110
rs55747707
rs429358
All
0.00
0.05
0.10
MR leave-one-out sensitivity analysis for
'Total cholesterol in HDL2 || id:ebi-cfb233-GCST90301969' on 'Breast cancer (Combined Oncoarray; iCOGS; GWAS meta analysis) ||

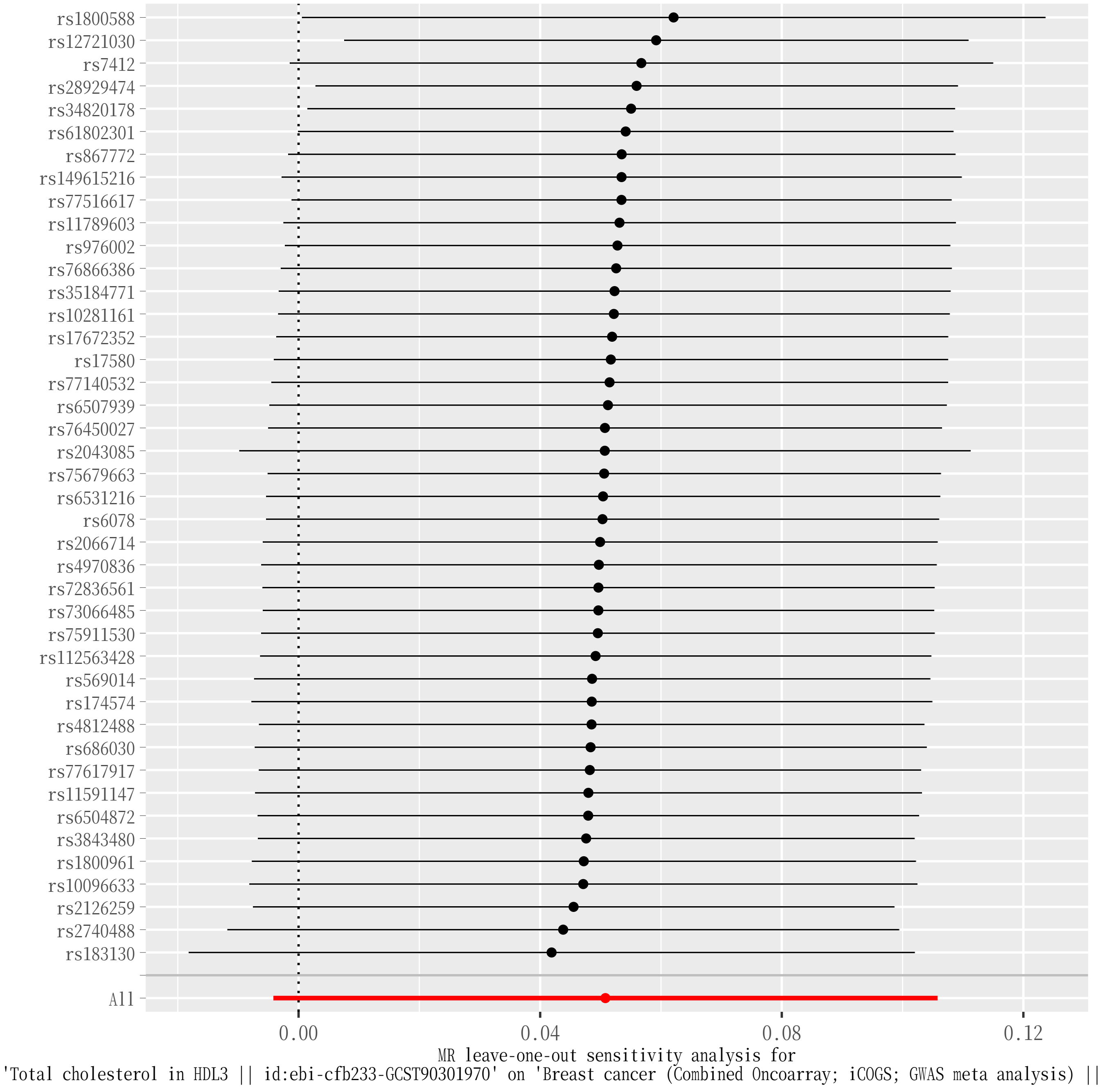

rs1800588
rs12721030
rs7412
rs28929474
rs34820178
rs61802301
rs867772
rs149615216
rs77516617
rs11789603
rs976002
rs76866386
rs35184771
rs10281161
rs17672352
rs17580
rs77140532
rs6507939
rs76450027
rs2043085
rs75679663
rs6531216
rs6078
rs2066714
rs4970836
rs72836561
rs73066485
rs75911530
rs112563428
rs569014
rs174574
rs4812488
rs686030
rs77617917
rs11591147
rs6504872
rs3843480
rs1800961
rs10096633
rs2126259
rs2740488
rs183130
All
0.00
0.04
0.08
0.12
MR leave-one-out sensitivity analysis for
'Total cholesterol in HDL3 || id:ebi-cfb233-GCST90301970' on 'Breast cancer (Combined Oncoarray; iCOGS; GWAS meta analysis) ||

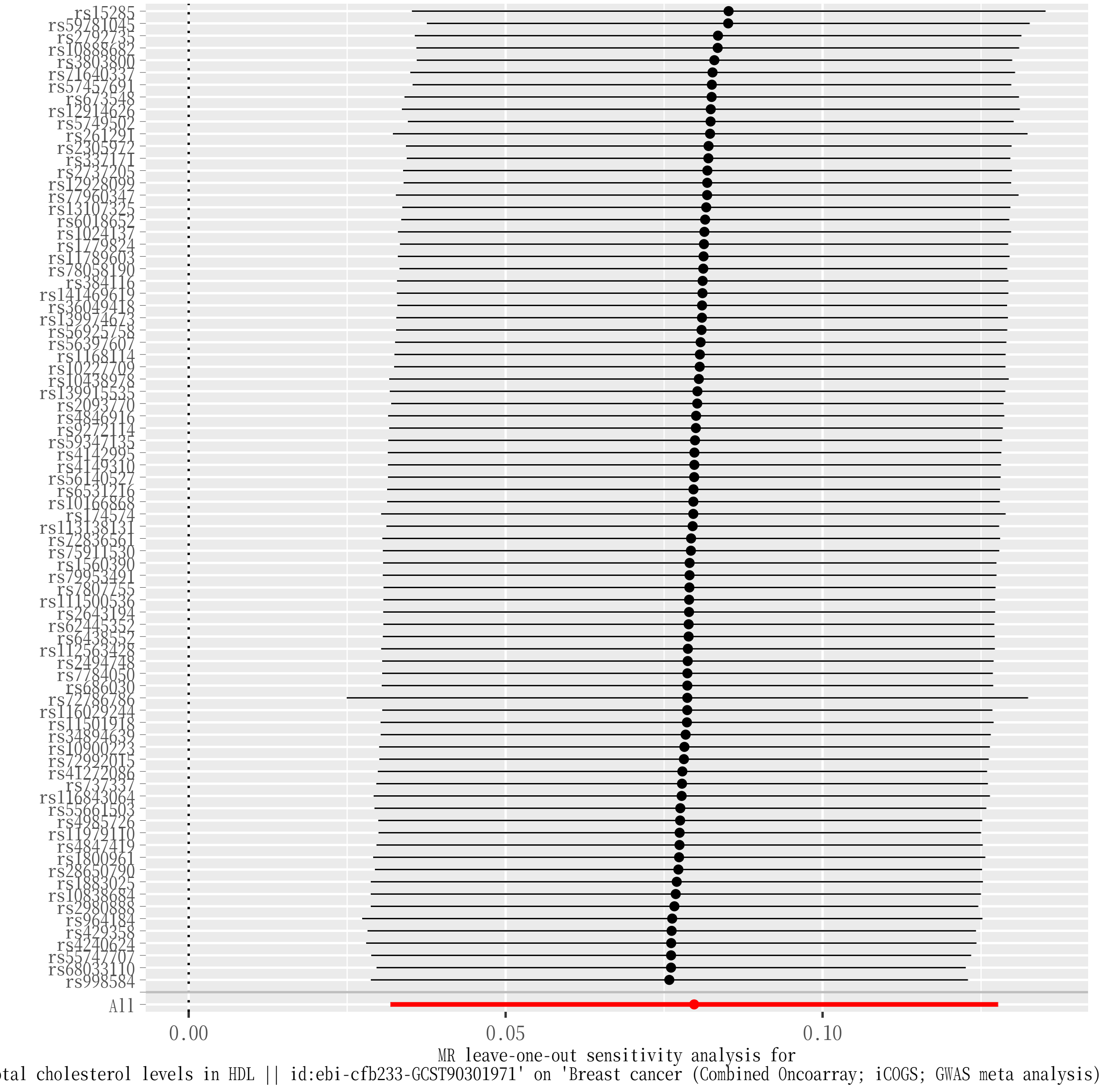
rs15285
rs59781045
rs2792735
rs10888682
rs3803800
rs71640337
rs57457691
rs673548
rs12914626
rs5749502
rs261291
rs2305972
rs332171
rs2737205
rs12928099
rs77960347
rs13107325
rs6018652
rs1024137
rs1779824
rs11789603
rs78058190
rs384116
rs141469619
rs36049418
rs139974673
rs56925758
rs56397607
rs1168114
rs10227709
rs10438978
rs139915535
rs2093770
rs4846916
rs9272114
rs59347135
rs4142995
rs4149310
rs56140527
rs6531216
rs10166868
rs174574
rs113138131
rs72836561
rs75911530
rs1560390
rs79953491
rs7807755
rs111500536
rs2643194
rs62445352
rs6438552
rs112563428
rs2494748
rs7784050
rs686030
rs72786786
rs116029244
rs11501918
rs34894639
rs10900223
rs72992015
rs41272086
rs737337
rs116843064
rs55661503
rs4985726
rs11979110
rs4847419
rs1800961
rs28650790
rs1883025
rs10838684
rs2980888
rs964184
rs429358
rs4240624
rs55747707
rs68033110
rs998584
All
0.00
0.05
0.10
MR leave-one-out sensitivity analysis for
otal cholesterol levels in HDL || id:ebi-cfb233-GCST90301971' on 'Breast cancer (Combined Oncoarray; iCOGS; GWAS meta analysis)

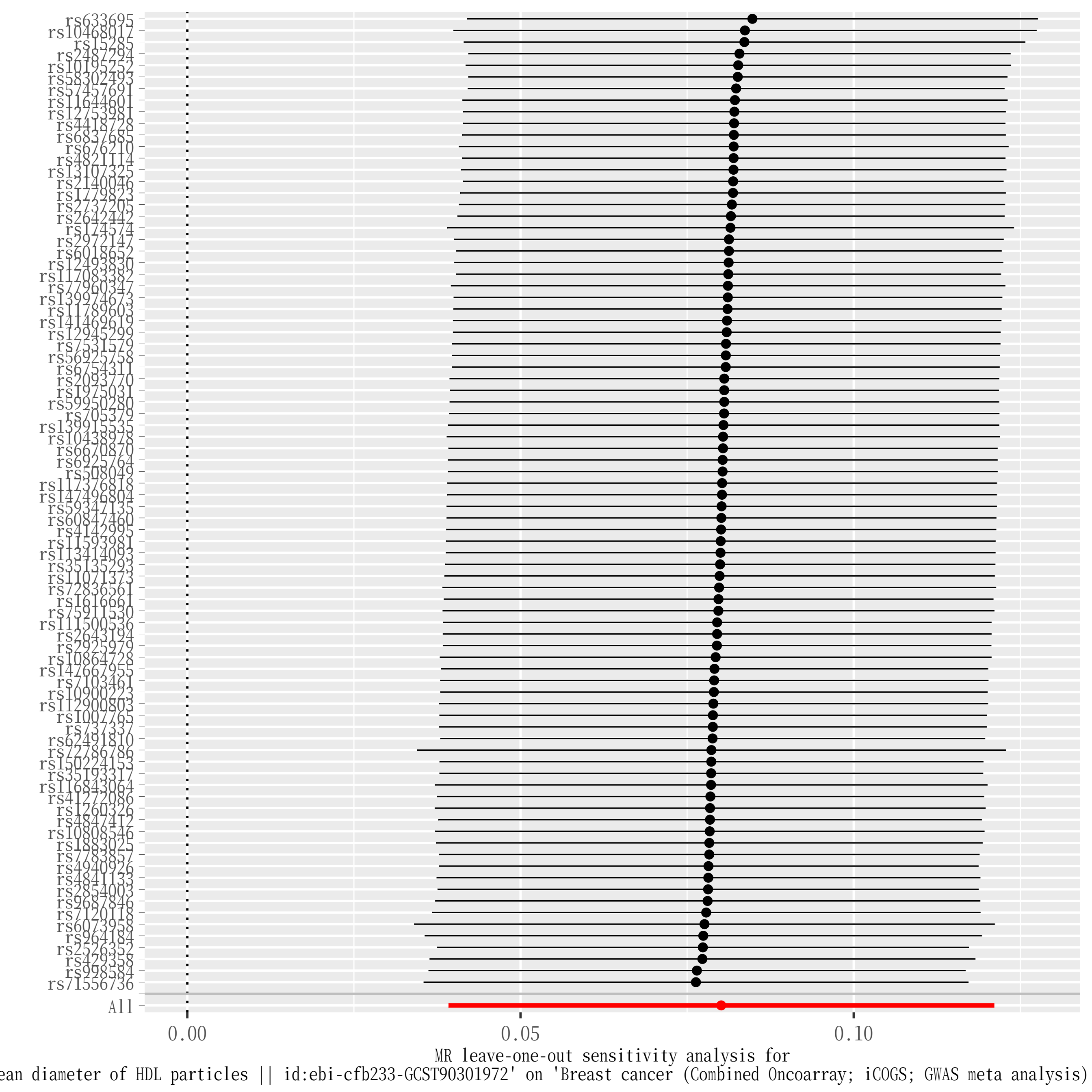

rs633695
rs10468017
rs15285
rs2487794
rs10195252
rs58302493
rs57457691
rs11644601
rs12753981
rs4418728
rs6837685
rs676210
rs4821114
rs13107325
rs2140046
rs1779823
rs2737205
rs2642442
rs174574
rs2972147
rs6018652
rs12493830
rs117083382
rs77960347
rs139974673
rs11789603
rs141469619
rs12945299
rs7531579
rs56925758
rs6754311
rs2093770
rs1975031
rs59950280
rs705379
rs139915535
rs10438978
rs6670870
rs6925764
rs508049
rs117376818
rs147496804
rs59347135
rs60847460
rs4142995
rs11593981
rs113414093
rs35135293
rs11071373
rs72836561
rs1616661
rs75911530
rs111500536
rs2643194
rs2925979
rs10864728
rs147667955
rs7103461
rs10900223
rs112900803
rs1007765
rs737337
rs62491810
rs72786786
rs150224153
rs35193317
rs116843064
rs41272086
rs1260326
rs4847412
rs10808546
rs1883025
rs7783857
rs4940926
rs4841133
rs2854003
rs9687846
rs7120118
rs6073958
rs964184
rs2526352
rs429358
rs998584
rs71556736
All
0.00
0.05
0.10
MR leave-one-out sensitivity analysis for
ean diameter of HDL particles || id:ebi-cfb233-GCST90301972' on 'Breast cancer (Combined Oncoarray; iCOGS; GWAS meta analysis)

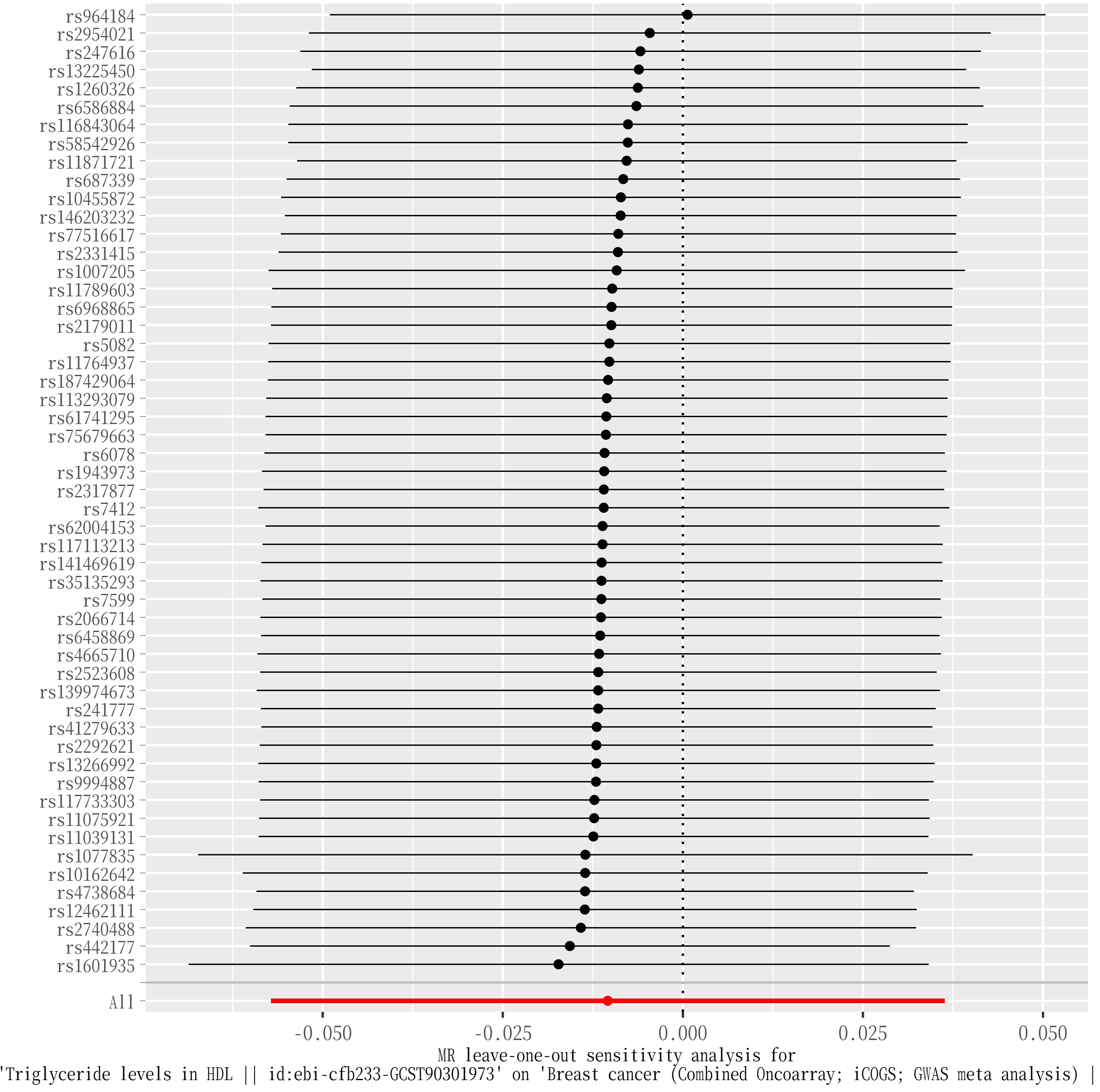
rs964184
rs2954021
rs247616
rs13225450
rs1260326
rs6586884
rs116843064
rs58542926
rs11871721
rs687339
rs10455872
rs146203232
rs77516617
rs2331415
rs1007205
rs11789603
rs6968865
rs2179011
rs5082
rs11764937
rs187429064
rs113293079
rs61741295
rs75679663
rs6078
rs1943973
rs2317877
rs7412
rs62004153
rs117113213
rs141469619
rs35135293
rs7599
rs2066714
rs6458869
rs4665710
rs2523608
rs139974673
rs241777
rs41279633
rs2292621
rs13266992
rs9994887
rs117733303
rs11075921
rs11039131
rs1077835
rs10162642
rs4738684
rs12462111
rs2740488
rs442177
rs1601935
All
-0.050
-0.025
0.000
0.025
0.050
MR leave-one-out sensitivity analysis for
'Triglyceride levels in HDL || id:ebi-cfb233-GCST90301973' on 'Breast cancer (Combined Oncoarray; iCOGS; GWAS meta analysis) |

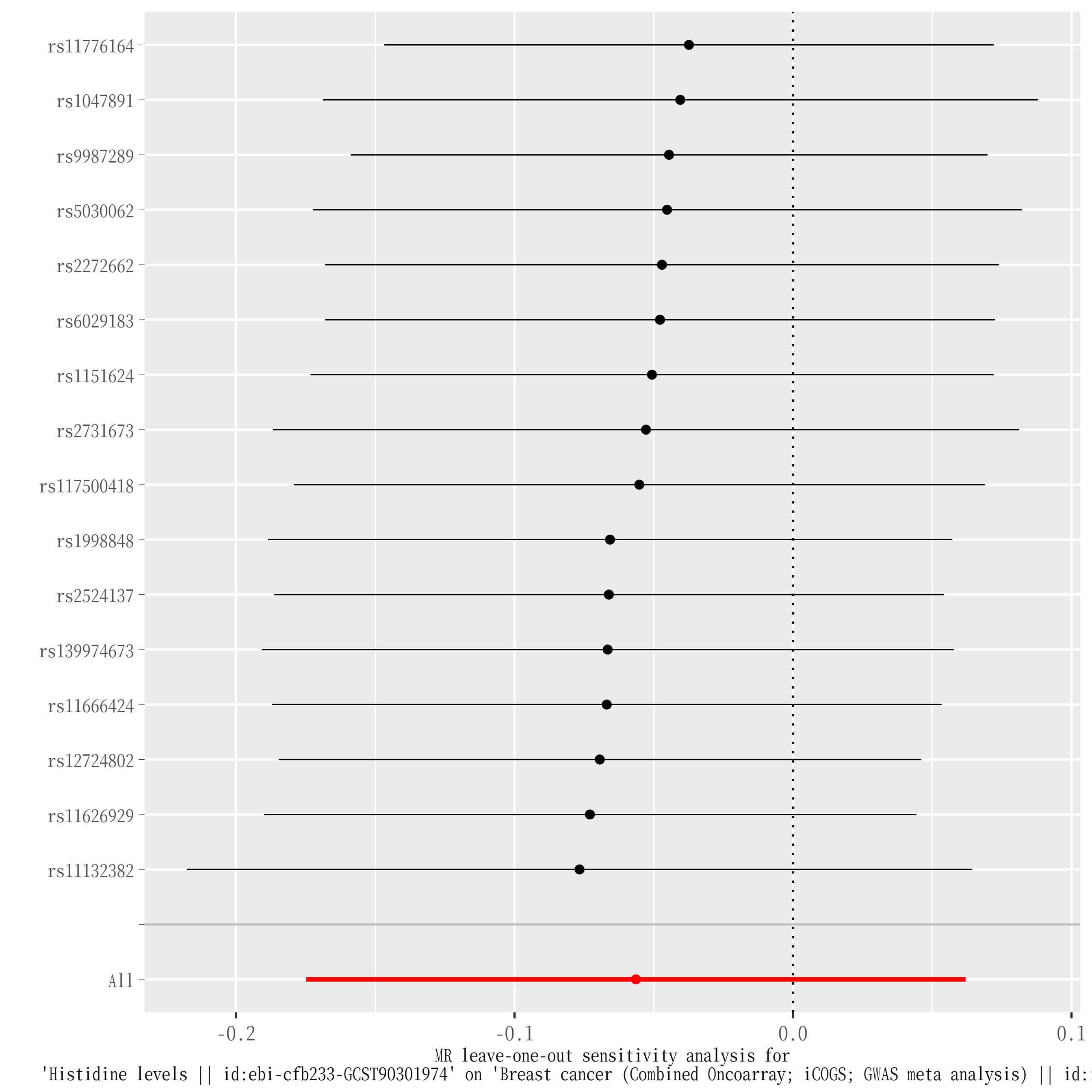
rs11776164
rs1047891
rs9987289
rs5030062
rs2272662
rs6029183
rs1151624
rs2731673
rs117500418
rs1998848
rs2524137
rs139974673
rs11666424
rs12724802
rs11626929
rs11132382
All
-0.2
-0.1
0.0
0.1
MR leave-one-out sensitivity analysis for
'Histidine levels || id:ebi-cfb233-GCST90301974' on 'Breast cancer (Combined Oncoarray; iCOGS; GWAS meta analysis) || id:

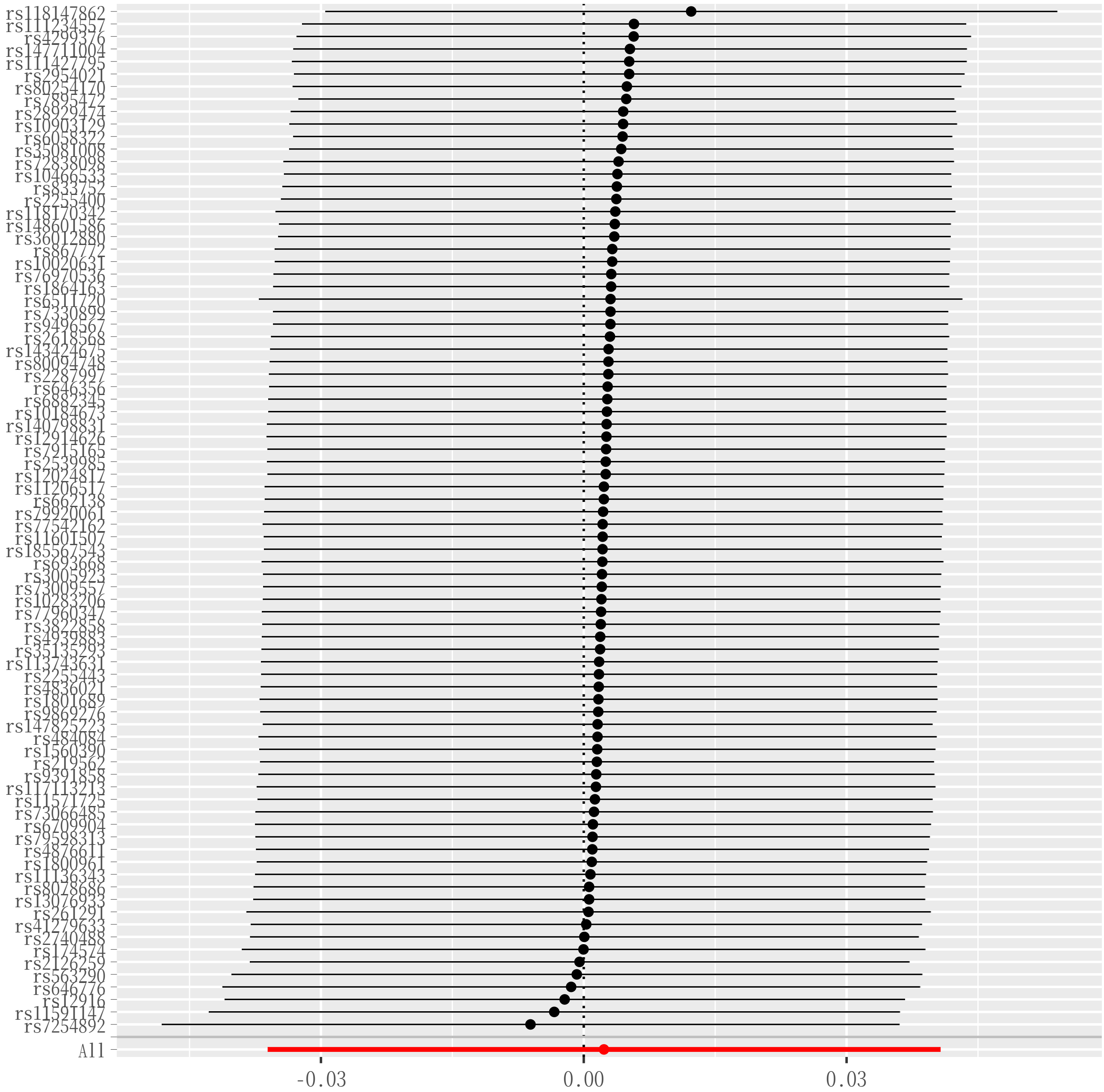

MR leave-one-out sensitivity analysis for
'Total Cholesterol in IDL || id:ebi-cfb233-GCST90301975' on 'Breast cancer (Combined Oncoarray; iCOGS; GWAS meta analysis) ||

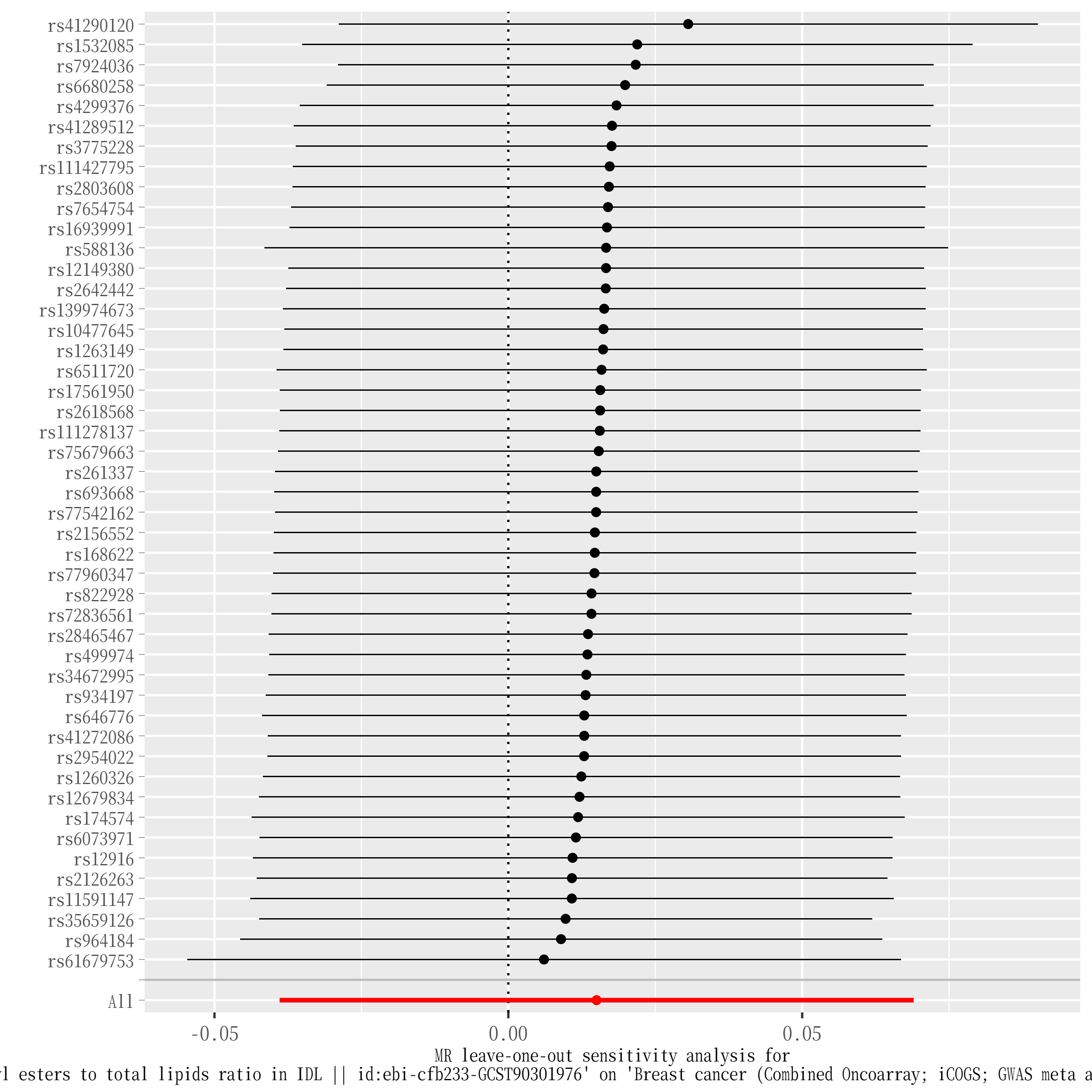
rs41290120
rs1532085
rs7924036
rs6680258
rs4299376
rs41289512
rs3775228
rs111427795
rs2803608
rs7654754
rs16939991
rs588136
rs12149380
rs2642442
rs139974673
rs10477645
rs1263149
rs6511720
rs17561950
rs2618568
rs111278137
rs75679663
rs261337
rs693668
rs77542162
rs2156552
rs168622
rs77960347
rs822928
rs72836561
rs28465467
rs499974
rs34672995
rs934197
rs646776
rs41272086
rs2954022
rs1260326
rs12679834
rs174574
rs6073971
rs12916
rs2126263
rs11591147
rs35659126
rs964184
rs61679753
All
-0.05
0.00
0.05
MR leave-one-out sensitivity analysis for
l esters to total lipids ratio in IDL || id:ebi-cfb233-GCST90301976' on 'Breast cancer (Combined Oncoarray; iCOGS; GWAS meta a

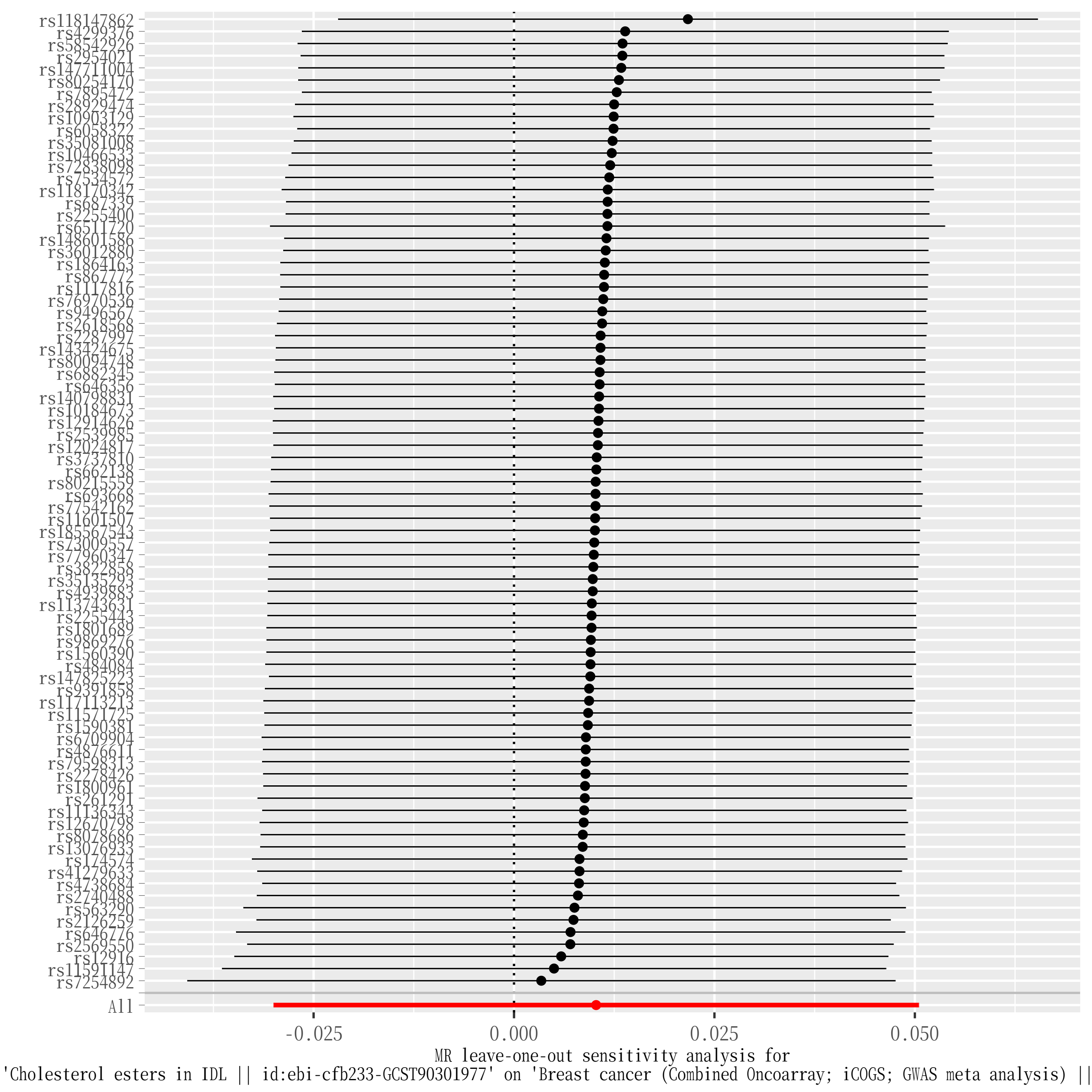
rs118147862
rs4299376
rs58542926
rs2954021
rs147711004
rs80254170
rs7895472
rs28929474
rs10903129
rs6058322
rs35081008
rs10466533
rs72838028
rs7534572
rs118170342
rs687339
rs2255400
rs6511720
rs148601586
rs36012880
rs1864163
rs867772
rs1117816
rs76970536
rs9496567
rs2618568
rs2287997
rs143424675
rs80094748
rs6882345
rs646356
rs140798831
rs10184673
rs12914626
rs2539985
rs12024817
rs3737810
rs662138
rs80215559
rs693668
rs77542162
rs11601507
rs185567543
rs73009557
rs77960347
rs3822858
rs35135293
rs4939883
rs113743631
rs2255443
rs1801689
rs9869276
rs1560390
rs484084
rs147825223
rs9391858
rs117113213
rs11571725
rs1590381
rs6709904
rs4876611
rs79598313
rs2278426
rs1800961
rs261291
rs11136343
rs12670798
rs8078686
rs13076933
rs174574
rs41279633
rs4738684
rs2740488
rs563290
rs2126259
rs646776
rs2569550
rs12916
rs11591147
rs7254892
All
-0.025
0.000
0.025
0.050
MR leave-one-out sensitivity analysis for
'Cholesterol esters in IDL || id:ebi-cfb233-GCST90301977' on 'Breast cancer (Combined Oncoarray; iCOGS; GWAS meta analysis) ||

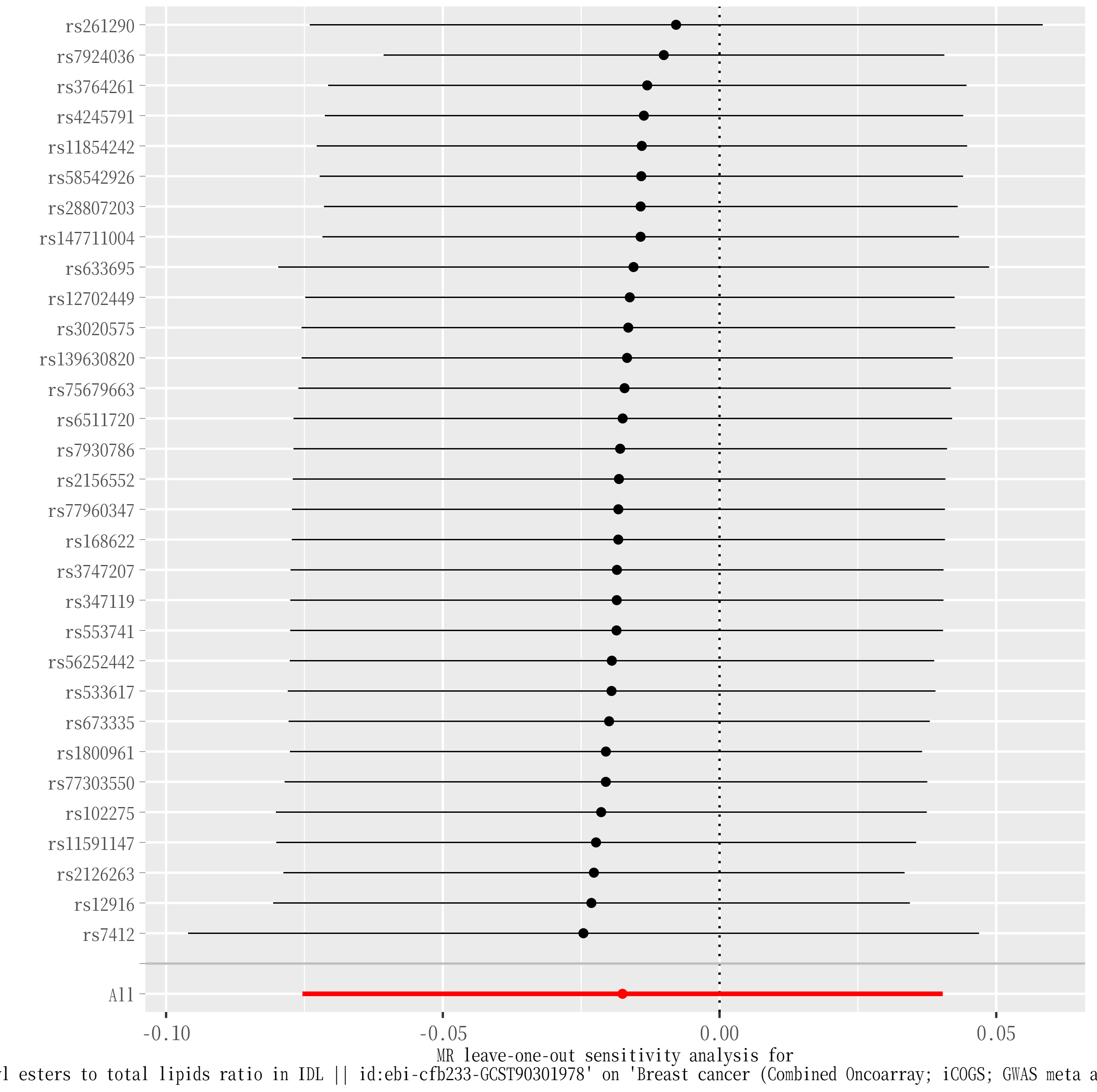

rs261290
rs7924036
rs3764261
rs4245791
rs11854242
rs58542926
rs28807203
rs147711004
rs633695
rs12702449
rs3020575
rs139630820
rs75679663
rs6511720
rs7930786
rs2156552
rs77960347
rs168622
rs3747207
rs347119
rs553741
rs56252442
rs533617
rs673335
rs1800961
rs77303550
rs102275
rs11591147
rs2126263
rs12916
rs7412
All
-0.10
-0.05
0.00
0.05
MR leave-one-out sensitivity analysis for
l esters to total lipids ratio in IDL || id:ebi-cfb233-GCST90301978' on 'Breast cancer (Combined Oncoarray; iCOGS; GWAS meta a

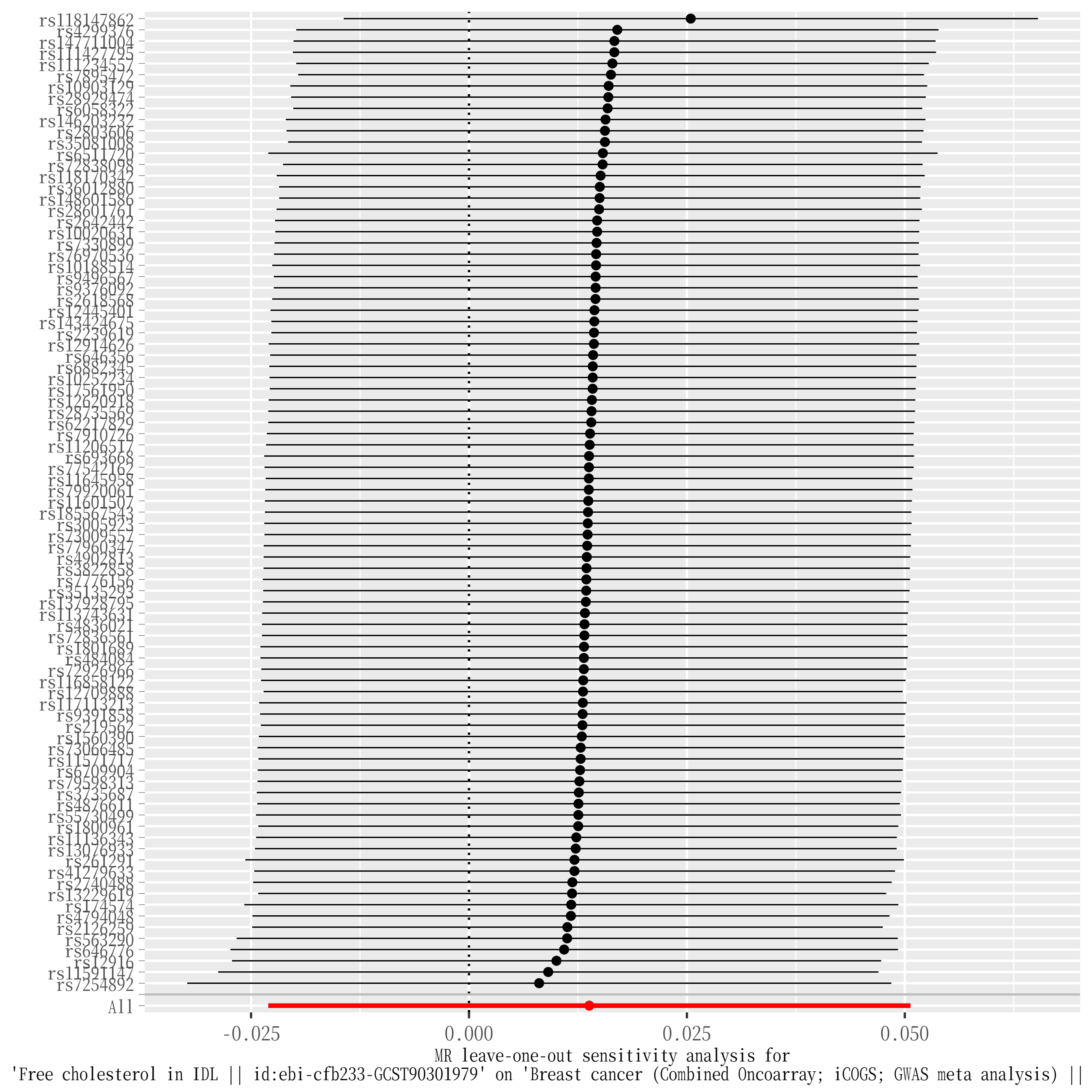

rs118147862
rs4299376
rs147711004
rs111427795
rs111234557
rs7895472
rs10903129
rs28929474
rs6058322
rs146203232
rs2803606
rs35081008
rs6511720
rs72838098
rs118170342
rs36012880
rs148601586
rs28601761
rs2642442
rs10020631
rs7330899
rs76970536
rs10188514
rs9496567
rs9376092
rs2618568
rs12445401
rs143424675
rs2239619
rs12914626
rs646356
rs6882345
rs10252234
rs17561950
rs12620918
rs28735569
rs62217829
rs7910726
rs11206517
rs693668
rs77542162
rs11645958
rs79920061
rs11601507
rs185567543
rs3005923
rs73009557
rs77960347
rs4902813
rs3822858
rs2776156
rs35135293
rs137928795
rs113743631
rs4836021
rs72836561
rs1801689
rs484084
rs72926966
rs116858122
rs12709888
rs117113213
rs9391858
rs219562
rs1560390
rs73066485
rs11571717
rs6709904
rs79598313
rs3735687
rs4876611
rs55730499
rs1800961
rs11136343
rs13076933
rs261291
rs41279633
rs2740488
rs13229619
rs174574
rs4794048
rs2126259
rs563290
rs646776
rs12916
rs11591147
rs7254892
All
-0.025
0.000
0.025
0.050
MR leave-one-out sensitivity analysis for
'Free cholesterol in IDL || id:ebi-cfb233-GCST90301979' on 'Breast cancer (Combined Oncoarray; iCOGS; GWAS meta analysis) ||

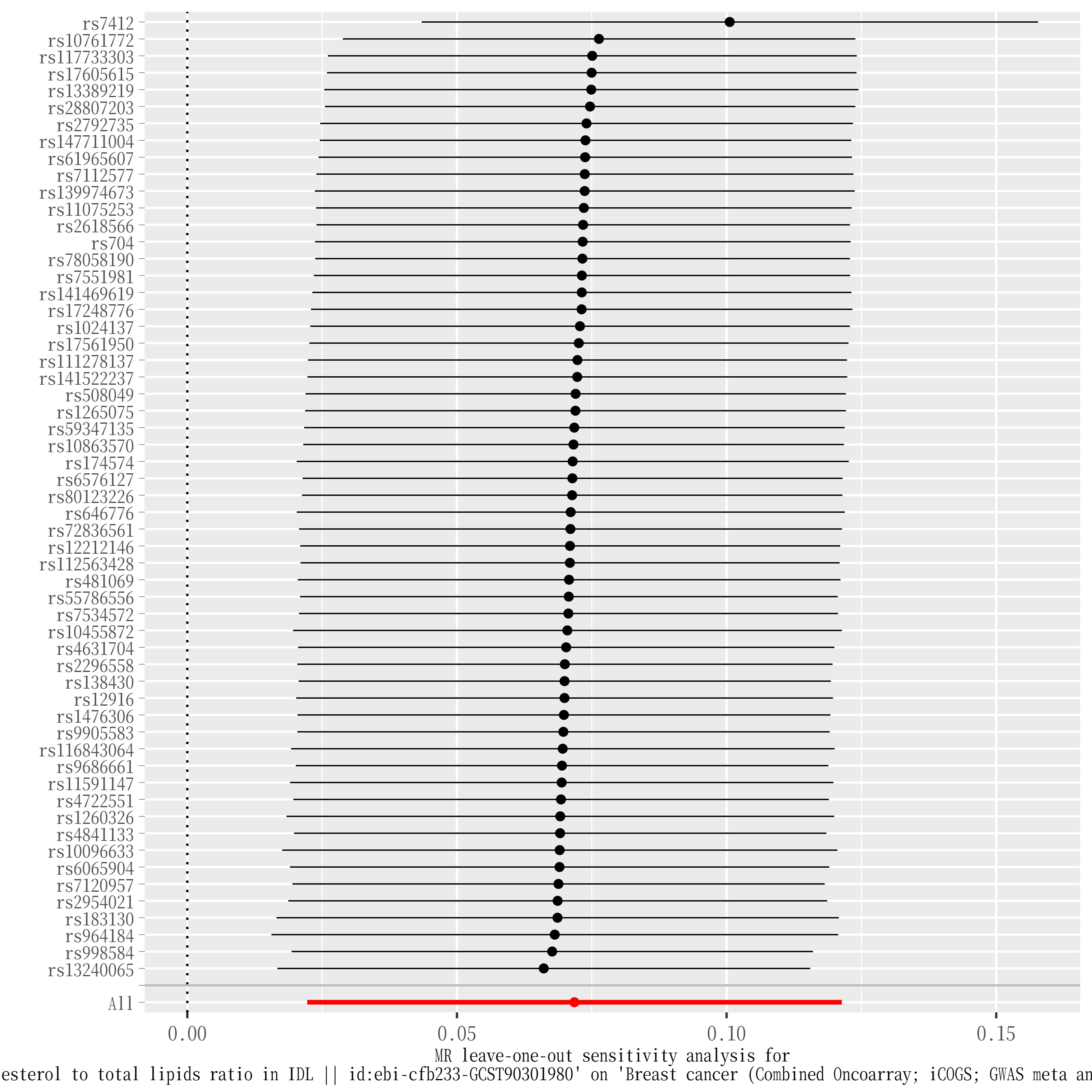

rs7412
rs10761772
rs117733303
rs17605615
rs13389219
rs28807203
rs2792735
rs147711004
rs61965607
rs7112577
rs139974673
rs11075253
rs2618566
rs704
rs78058190
rs7551981
rs141469619
rs17248776
rs1024137
rs17561950
rs111278137
rs141522237
rs508049
rs1265075
rs59347135
rs10863570
rs174574
rs6576127
rs80123226
rs646776
rs72836561
rs12212146
rs112563428
rs481069
rs55786556
rs7534572
rs10455872
rs4631704
rs2296558
rs138430
rs12916
rs1476306
rs9905583
rs116843064
rs9686661
rs11591147
rs4722551
rs1260326
rs4841133
rs10096633
rs6065904
rs7120957
rs2954021
rs183130
rs964184
rs998584
rs13240065
All
0.00
0.05
0.10
0.15
MR leave-one-out sensitivity analysis for
esterol to total lipids ratio in IDL || id:ebi-cfb233-GCST90301980' on 'Breast cancer (Combined Oncoarray; iCOGS; GWAS meta an

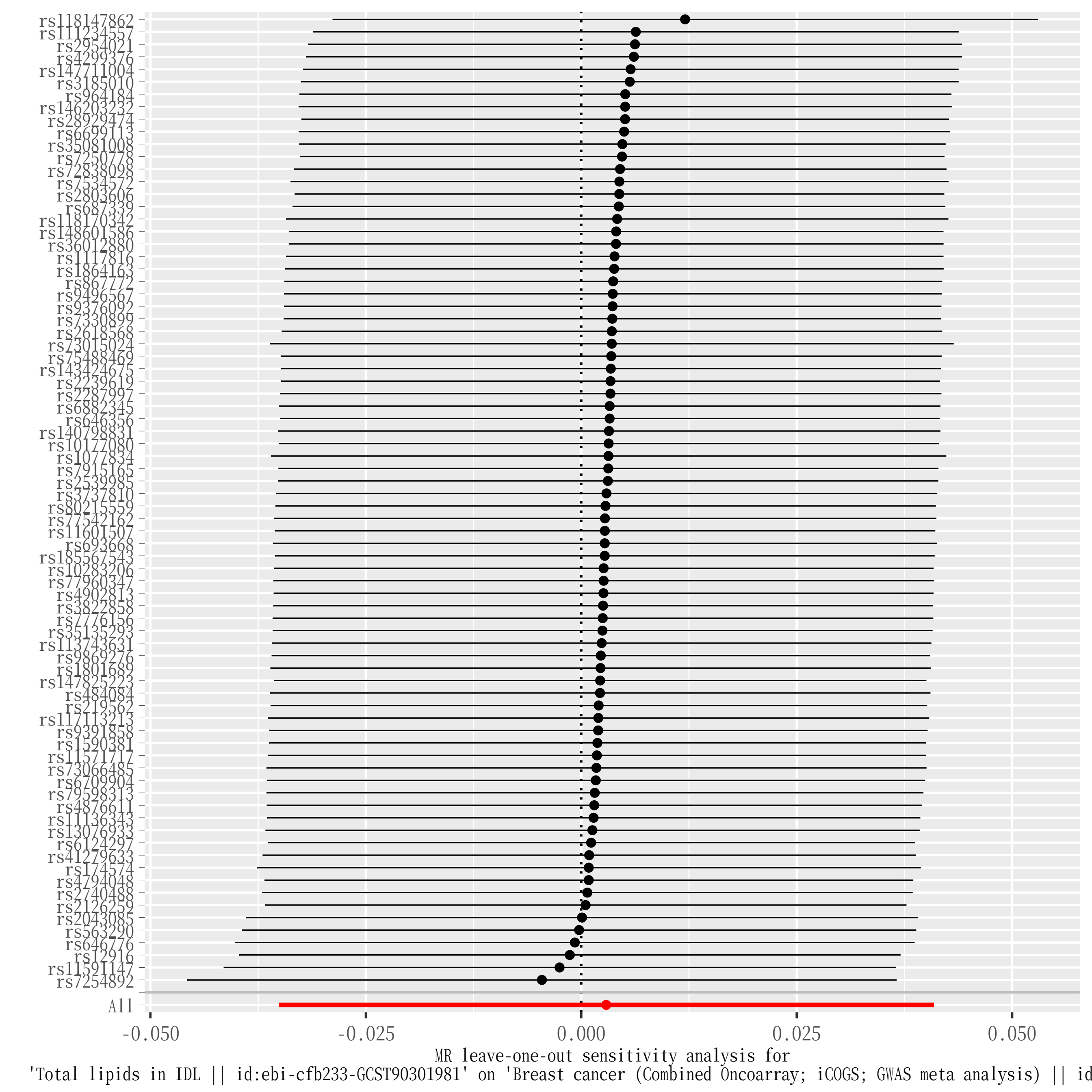
rs118147862
rs111234557
rs2954021
rs4299376
rs147711004
rs3185010
rs964184
rs146203232
rs28929474
rs6699113
rs35081008
rs7250778
rs72838098
rs7534572
rs2803606
rs687339
rs118170342
rs148601586
rs36012880
rs1117816
rs1864163
rs867772
rs9496567
rs9376092
rs7330899
rs2618568
rs73015024
rs75488469
rs143424675
rs2239619
rs2287997
rs6882345
rs646356
rs140798831
rs10177080
rs1077834
rs7915165
rs2539985
rs3737810
rs80215559
rs77542162
rs11601507
rs693668
rs185567543
rs10283206
rs77960347
rs4902813
rs3822858
rs7776156
rs35135293
rs113743631
rs9869276
rs1801689
rs147825223
rs484084
rs219562
rs117113213
rs9391858
rs1520381
rs11571717
rs73066485
rs6709904
rs79598313
rs4876611
rs11136343
rs13076933
rs6124297
rs41279633
rs174574
rs4794048
rs2740488
rs2126259
rs2043085
rs563290
rs646776
rs12916
rs11591147
rs7254892
All
-0.050
-0.025
0.000
0.025
0.050
MR leave-one-out sensitivity analysis for
'Total lipids in IDL || id:ebi-cfb233-GCST90301981' on 'Breast cancer (Combined Oncoarray; iCOGS; GWAS meta analysis) || id

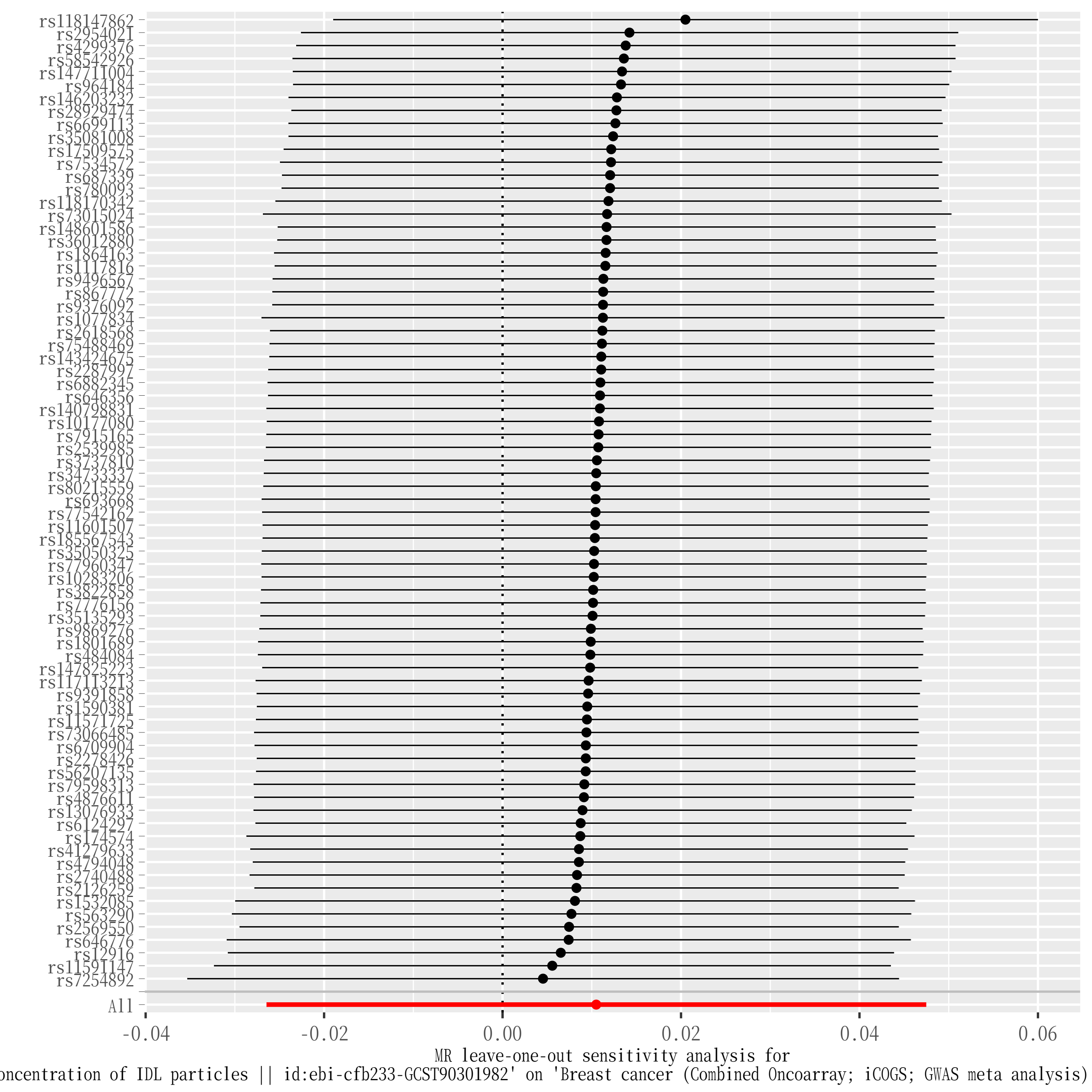
rs118147862
rs2954021
rs4299376
rs58542926
rs147711004
rs964184
rs146203232
rs28929474
rs6699113
rs35081008
rs17509575
rs7534572
rs687339
rs780093
rs118170342
rs73015024
rs148601586
rs36012880
rs1864163
rs1117816
rs9496567
rs867772
rs9376092
rs1077834
rs2618568
rs75488469
rs143424675
rs2287997
rs6882345
rs646356
rs140798831
rs10177080
rs7915165
rs2539985
rs3737810
rs34733337
rs80215559
rs693668
rs77542162
rs11601507
rs185567543
rs35050325
rs77960347
rs10283206
rs3822858
rs7776156
rs35135293
rs9869276
rs1801689
rs484084
rs147825223
rs117113213
rs9391858
rs1590381
rs11571725
rs73066485
rs6709904
rs2278426
rs56207135
rs79598313
rs4876611
rs13076933
rs6124297
rs174574
rs41279633
rs4794048
rs2740488
rs2126259
rs1532085
rs563290
rs2569550
rs646776
rs12916
rs11591147
rs7254892
All
-0.04
-0.02
0.00
0.02
0.04
0.06
MR leave-one-out sensitivity analysis for
oncentration of IDL particles || id:ebi-cfb233-GCST90301982' on 'Breast cancer (Combined Oncoarray; iCOGS; GWAS meta analysis)

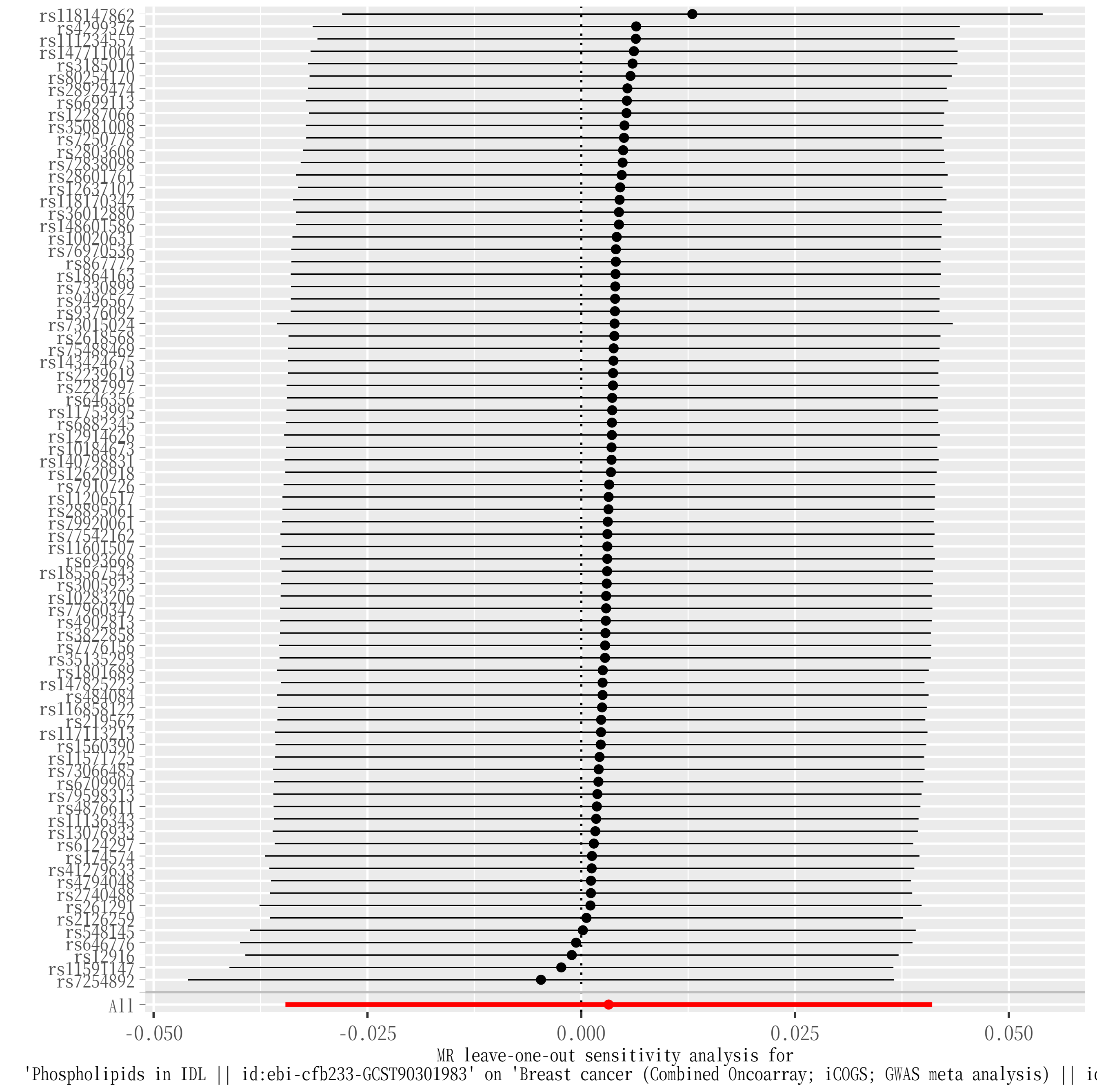
rs118147862
rs4299376
rs11234557
rs147711004
rs3185010
rs80254170
rs28929474
rs6699113
rs12287066
rs35081008
rs7250778
rs2803606
rs72838098
rs28601761
rs12637102
rs118170347
rs36012880
rs148601586
rs10020631
rs76970536
rs867772
rs1864163
rs7330899
rs9496567
rs9376092
rs73015024
rs2618568
rs75488469
rs143424675
rs2239619
rs2287997
rs646356
rs11753995
rs6882345
rs12914626
rs10184673
rs140798831
rs12620918
rs7910726
rs11206517
rs28895061
rs79920061
rs77542162
rs11601507
rs693668
rs185567543
rs3005923
rs10283206
rs77960347
rs4902813
rs3822858
rs7776156
rs35135293
rs1801689
rs147825223
rs484084
rs116858122
rs219562
rs117113213
rs1560390
rs11571725
rs73066485
rs6709904
rs79598313
rs4876611
rs11136343
rs13076933
rs6124297
rs174574
rs41279633
rs4794048
rs2740488
rs261291
rs2126259
rs548145
rs646776
rs12916
rs11591147
rs7254892
All
-0.050
-0.025
0.000
0.025
0.050
MR leave-one-out sensitivity analysis for
'Phospholipids in IDL || id:ebi-cfb233-GCST90301983' on 'Breast cancer (Combined Oncoarray; iCOGS; GWAS meta analysis) || ic

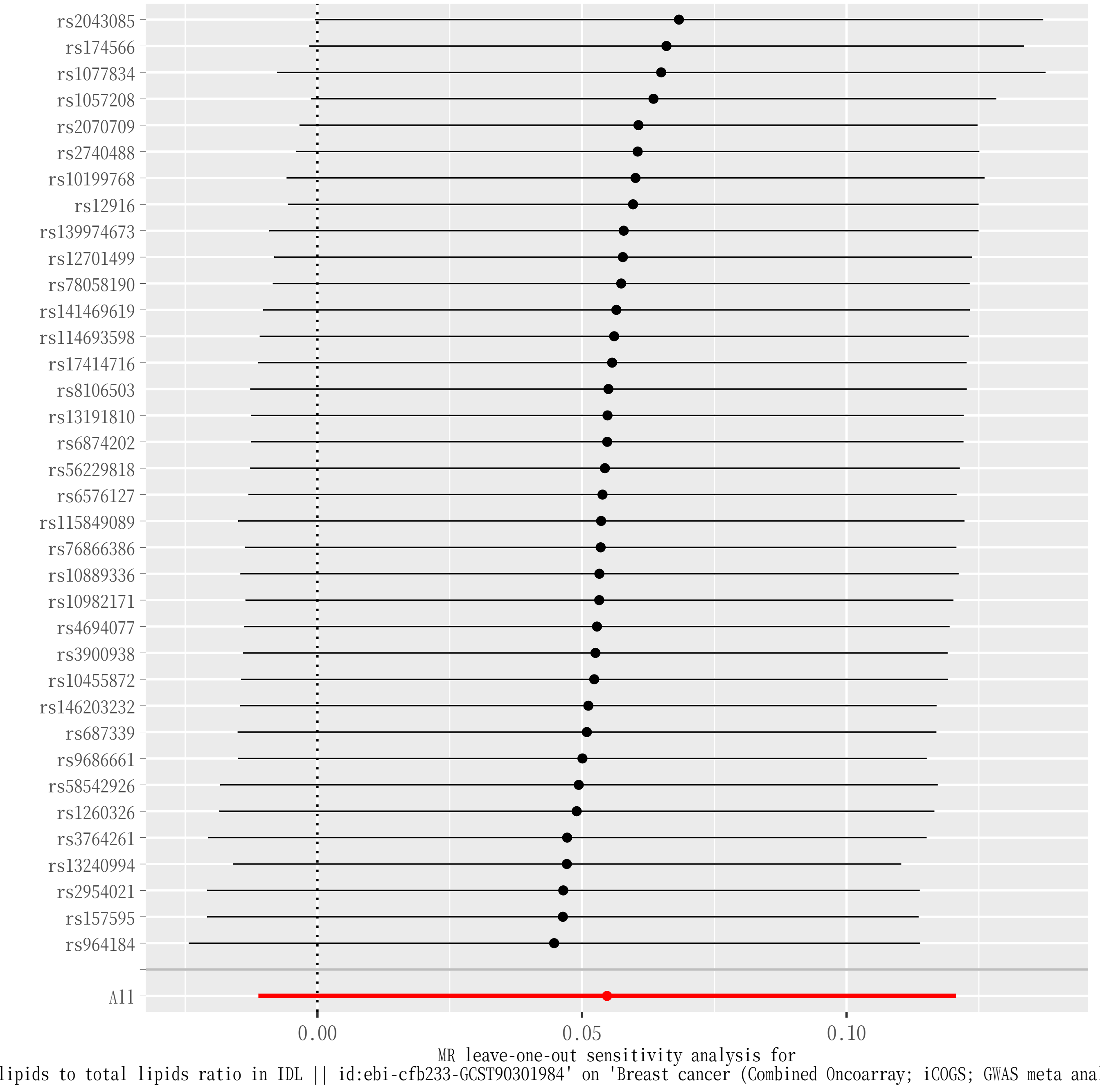
rs2043085
rs174566
rs1077834
rs1057208
rs2070709
rs2740488
rs10199768
rs12916
rs139974673
rs12701499
rs78058190
rs141469619
rs114693598
rs17414716
rs8106503
rs13191810
rs6874202
rs56229818
rs6576127
rs115849089
rs76866386
rs10889336
rs10982171
rs4694077
rs3900938
rs10455872
rs146203232
rs687339
rs9686661
rs58542926
rs1260326
rs3764261
rs13240994
rs2954021
rs157595
rs964184
All
0.00
0.05
0.10
MR leave-one-out sensitivity analysis for
lipids to total lipids ratio in IDL || id:ebi-cfb233-GCST90301984' on 'Breast cancer (Combined Oncoarray; iCOGS; GWAS meta anal

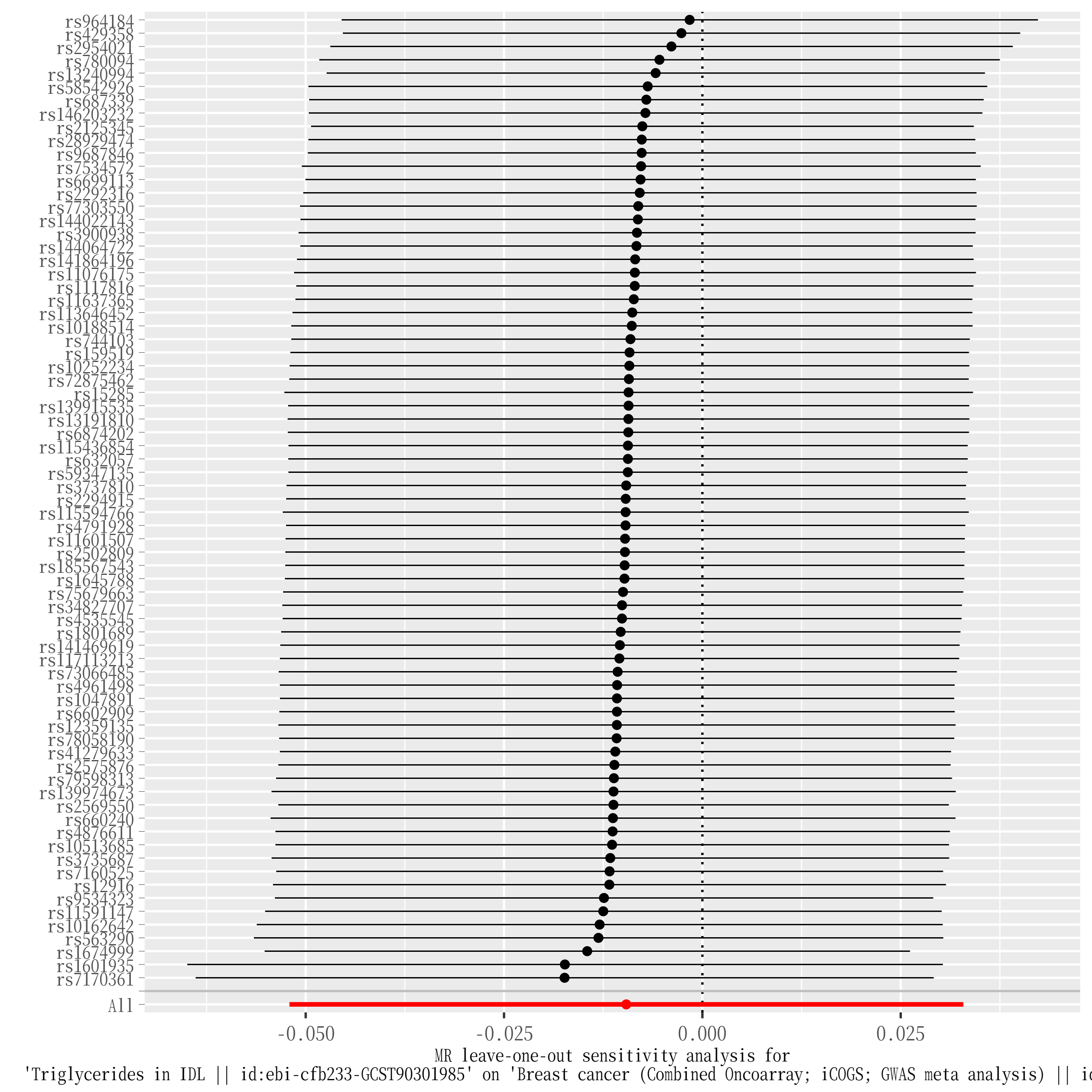

rs964184
rs429358
rs2954021
rs780094
rs13240994
rs58542926
rs687339
rs146203232
rs2125345
rs28929474
rs9687846
rs7534572
rs6699113
rs2292316
rs77303550
rs144022143
rs3900938
rs144064722
rs141864196
rs11076175
rs1117816
rs11637365
rs113646452
rs10188514
rs744103
rs159519
rs10252234
rs72875462
rs15285
rs139915535
rs13191810
rs6874202
rs115436854
rs632057
rs59347135
rs3737810
rs2294915
rs115594766
rs4791928
rs11601507
rs2502809
rs185567543
rs1645788
rs75679663
rs34827707
rs4535545
rs1801689
rs141469619
rs117113213
rs73066485
rs4961498
rs1047891
rs6602909
rs12359135
rs78058190
rs41279633
rs2575876
rs79598313
rs139974673
rs2569550
rs660240
rs4876611
rs10513685
rs3735687
rs7160525
rs12916
rs9534323
rs11591147
rs10162642
rs563290
rs1674999
rs1601935
rs7170361
All
-0.050
-0.025
0.000
0.025
MR leave-one-out sensitivity analysis for
'Triglycerides in IDL || id:ebi-cfb233-GCST90301985' on 'Breast cancer (Combined Oncoarray; iCOGS; GWAS meta analysis) || id

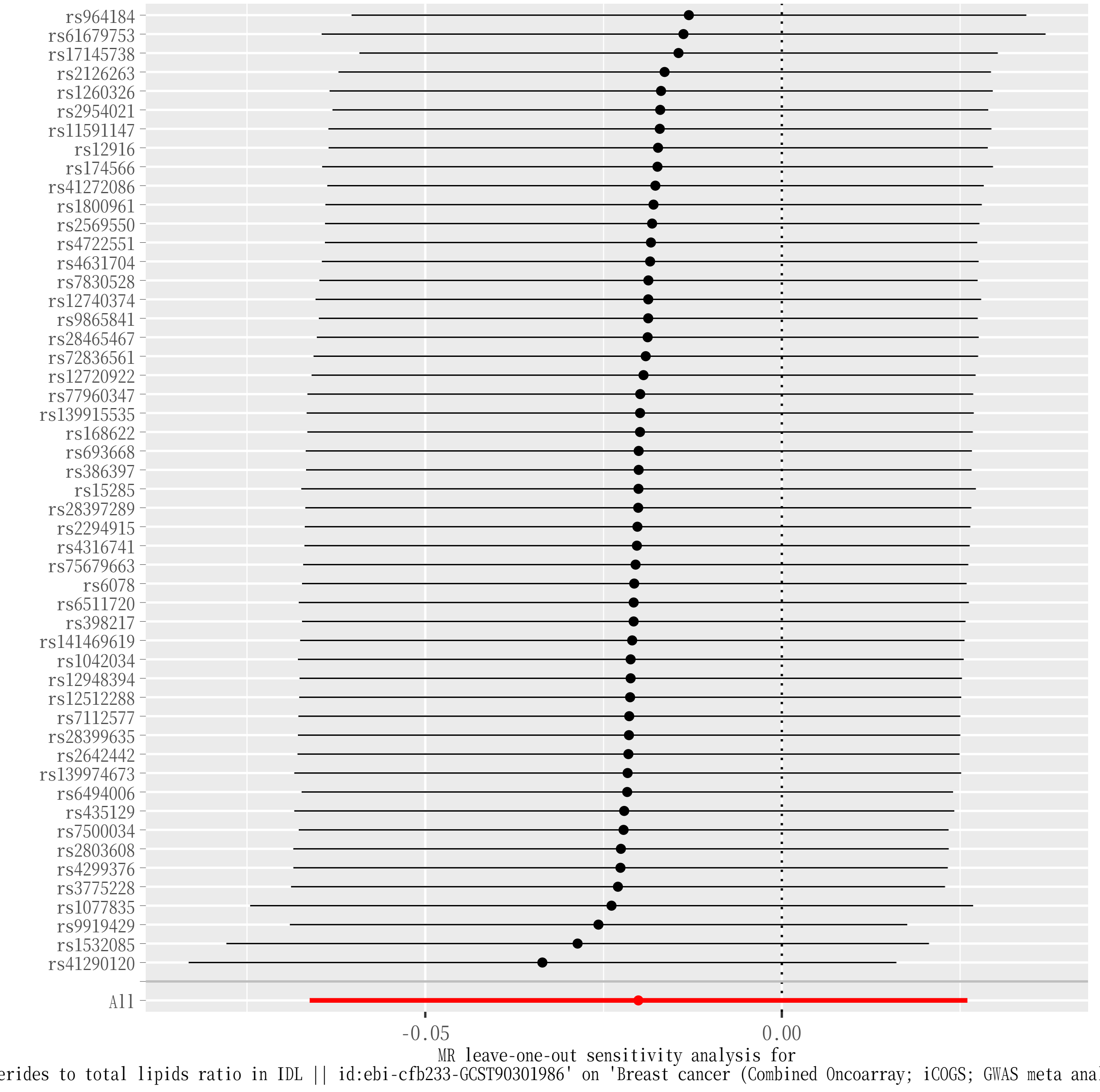
rs964184
rs61679753
rs17145738
rs2126263
rs1260326
rs2954021
rs11591147
rs12916
rs174566
rs41272086
rs1800961
rs2569550
rs4722551
rs4631704
rs7830528
rs12740374
rs9865841
rs28465467
rs72836561
rs12720922
rs77960347
rs139915535
rs168622
rs693668
rs386397
rs15285
rs28397289
rs2294915
rs4316741
rs75679663
rs6078
rs6511720
rs398217
rs141469619
rs1042034
rs12948394
rs12512288
rs7112577
rs28399635
rs2642442
rs139974673
rs6494006
rs435129
rs7500034
rs2803608
rs4299376
rs3775228
rs1077835
rs9919429
rs1532085
rs41290120
All
-0.05
0.00
MR leave-one-out sensitivity analysis for
erides to total lipids ratio in IDL || id:ebi-cfb233-GCST90301986' on 'Breast cancer (Combined Oncoarray; iCOGS; GWAS meta anal

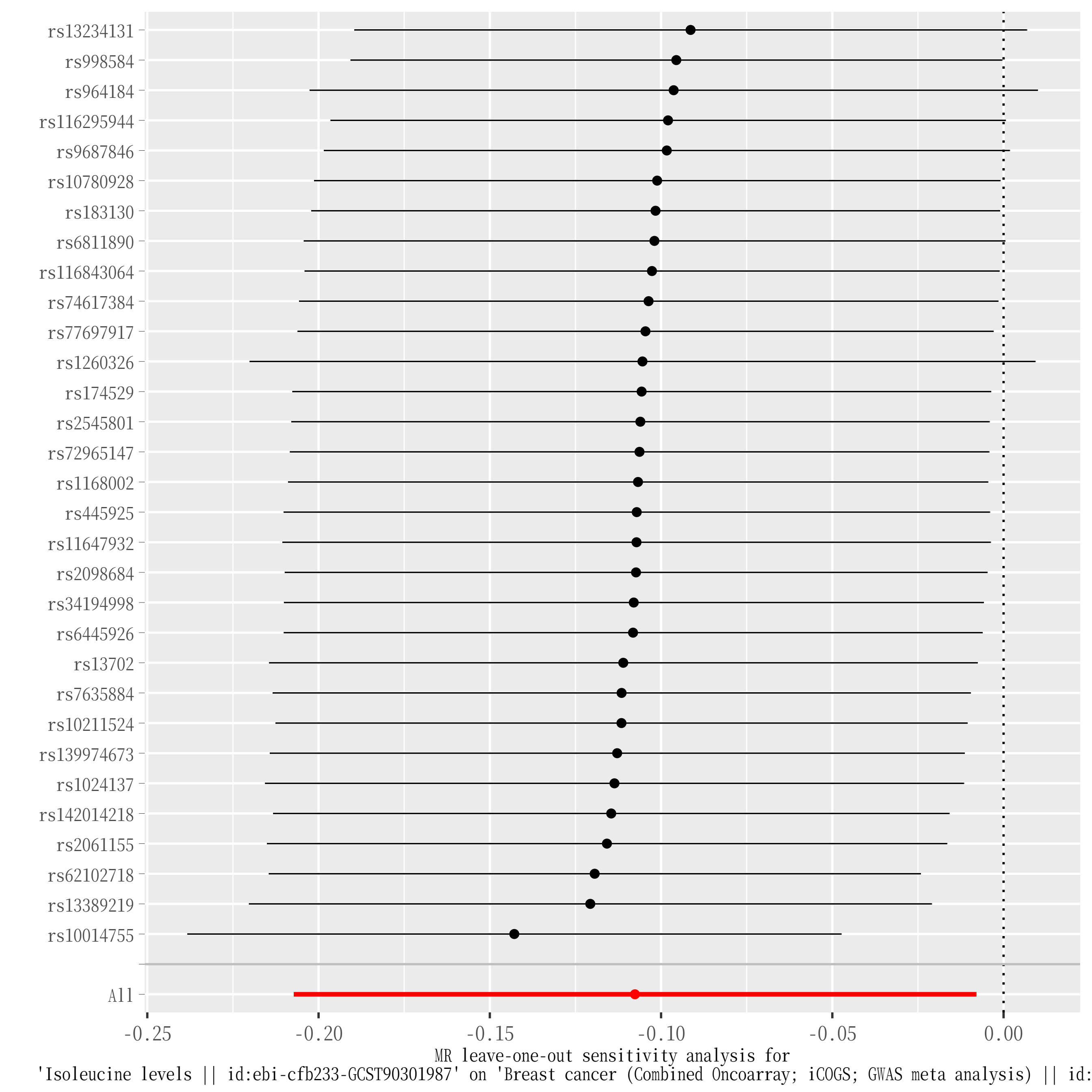
rs13234131
rs998584
rs964184
rs116295944
rs9687846
rs10780928
rs183130
rs6811890
rs116843064
rs74617384
rs77697917
rs1260326
rs174529
rs2545801
rs72965147
rs1168002
rs445925
rs11647932
rs2098684
rs34194998
rs6445926
rs13702
rs7635884
rs10211524
rs139974673
rs1024137
rs142014218
rs2061155
rs62102718
rs13389219
rs10014755
All
-0.25
-0.20
-0.15
-0.10
-0.05
0.00
MR leave-one-out sensitivity analysis for
'Isoleucine levels || id:ebi-cfb233-GCST90301987' on 'Breast cancer (Combined Oncoarray; iCOGS; GWAS meta analysis) || id:

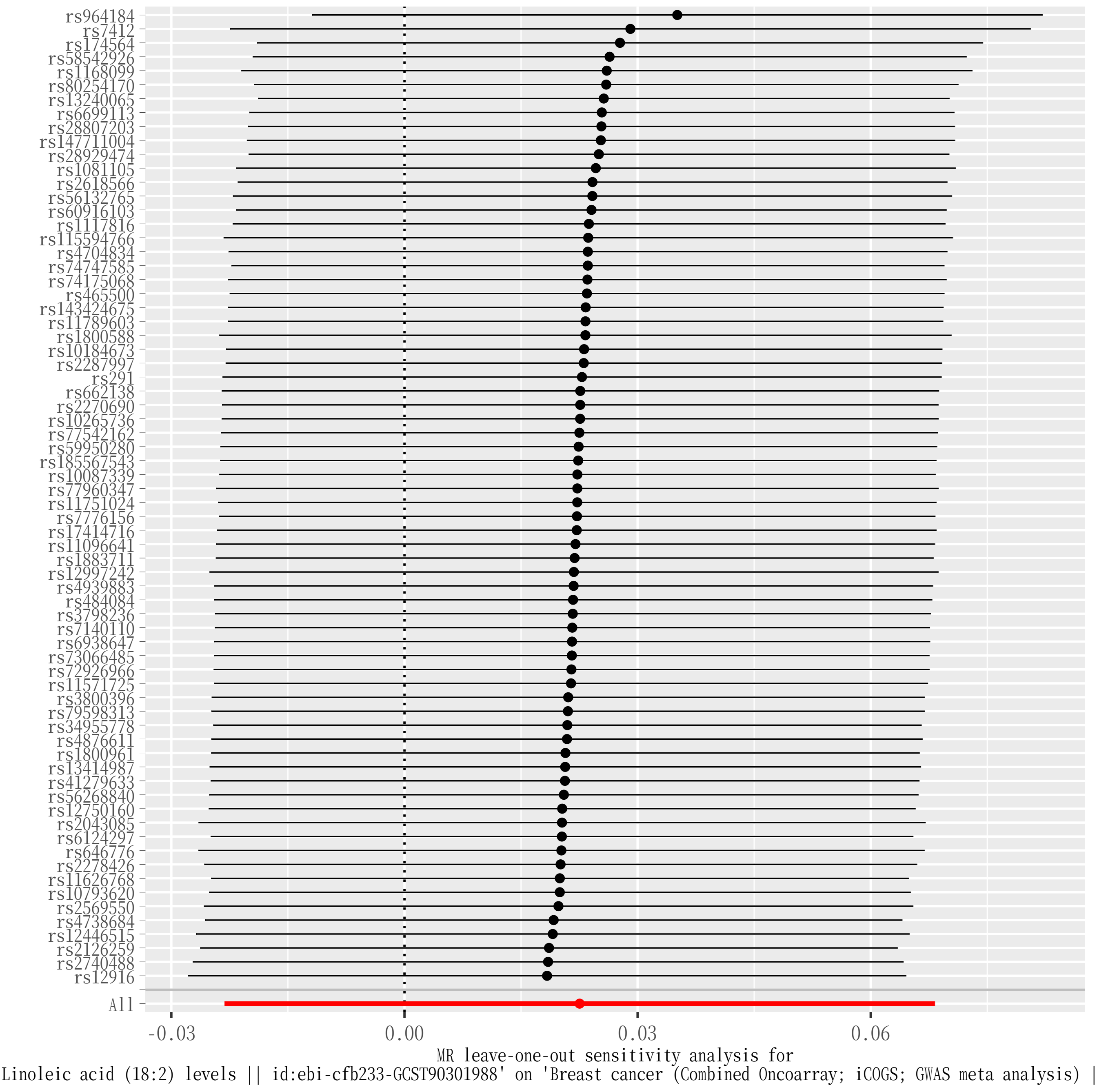

rs964184
rs7412
rs174564
rs58542926
rs1168099
rs80254170
rs13240065
rs6699113
rs28807203
rs147711004
rs28929474
rs1081105
rs2618566
rs56132765
rs60916103
rs1117816
rs115594766
rs4704834
rs74747585
rs74175068
rs465500
rs143424675
rs11789603
rs1800588
rs10184673
rs2287997
rs291
rs662138
rs2270690
rs10265736
rs77542162
rs59950280
rs185567543
rs10087339
rs77960347
rs11751024
rs7776156
rs17414716
rs11096641
rs1883711
rs12997242
rs4939883
rs484084
rs3798236
rs7140110
rs6938647
rs73066485
rs72926966
rs11571725
rs3800396
rs79598313
rs34955778
rs4876611
rs1800961
rs13414987
rs41279633
rs56268840
rs12750160
rs2043085
rs6124297
rs646776
rs2278426
rs11626768
rs10793620
rs2569550
rs4738684
rs12446515
rs2126259
rs2740488
rs12916
All
-0.03
0.00
0.03
0.06
MR leave-one-out sensitivity analysis for
Linoleic acid (18:2) levels || id:ebi-cfb233-GCST90301988' on 'Breast cancer (Combined Oncoarray; iCOGS; GWAS meta analysis) |

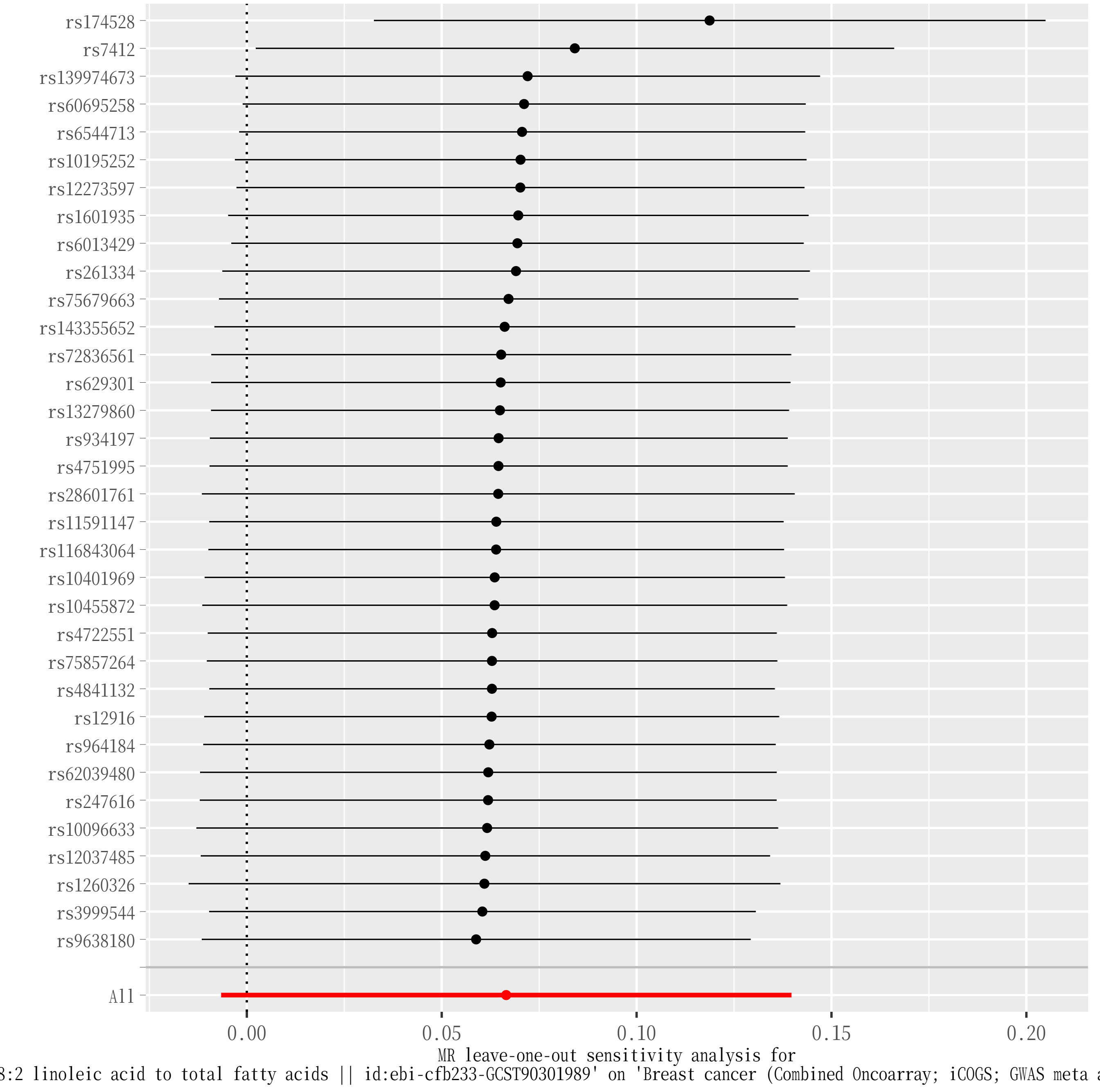
rs174528
rs7412
rs139974673
rs60695258
rs6544713
rs10195252
rs12273597
rs1601935
rs6013429
rs261334
rs75679663
rs143355652
rs72836561
rs629301
rs13279860
rs934197
rs4751995
rs28601761
rs11591147
rs116843064
rs10401969
rs10455872
rs4722551
rs75857264
rs4841132
rs12916
rs964184
rs62039480
rs247616
rs10096633
rs12037485
rs1260326
rs3999544
rs9638180
All
0.00
0.05
0.10
0.15
0.20
MR leave-one-out sensitivity analysis for
8:2 linoleic acid to total fatty acids || id:ebi-cfb233-GCST90301989' on 'Breast cancer (Combined Oncoarray; iCOGS; GWAS meta a

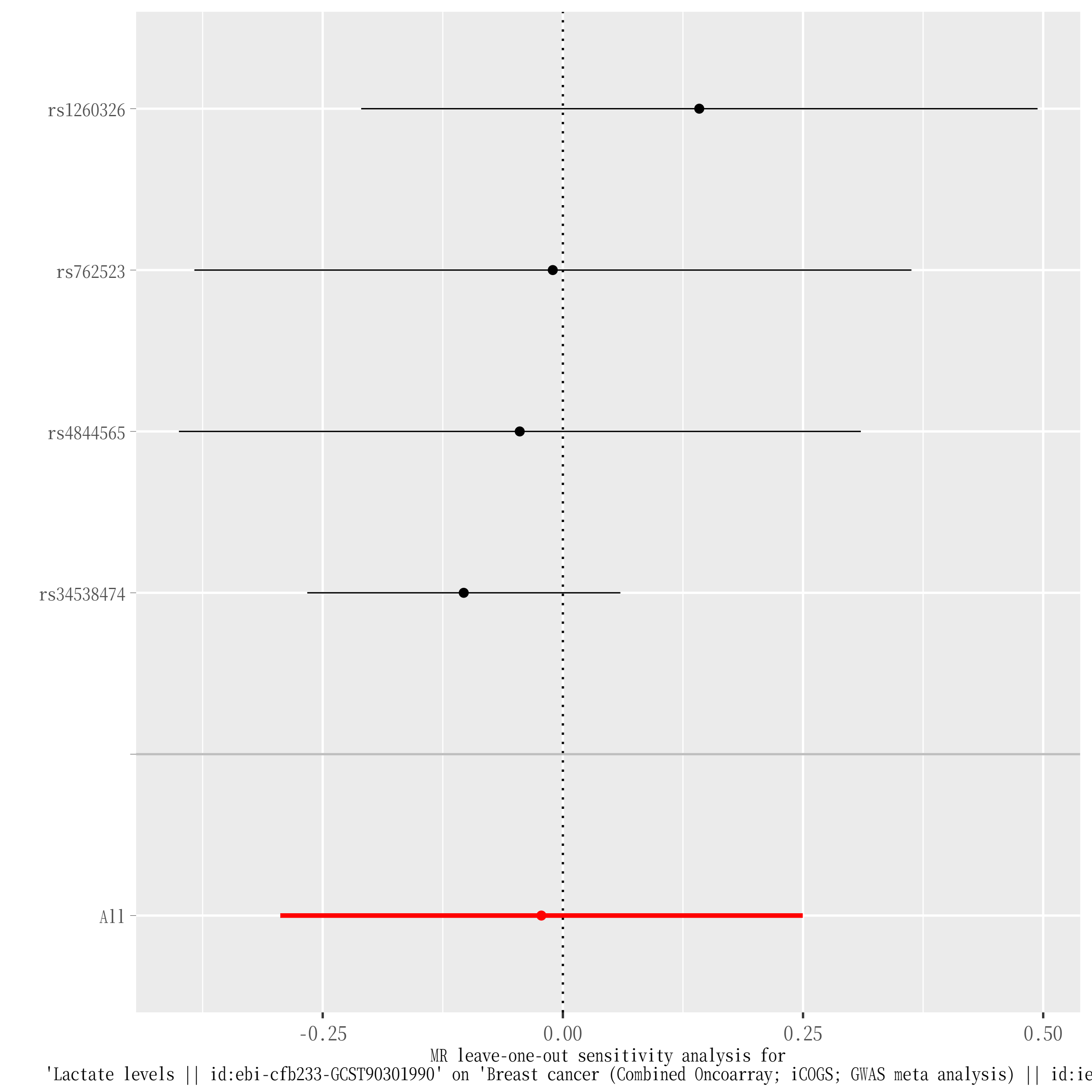

rs1260326
rs762523
rs4844565
rs34538474
All
-0.25
0.00
0.25
0.50
MR leave-one-out sensitivity analysis for
'Lactate levels || id:ebi-cfb233-GCST90301990' on 'Breast cancer (Combined Oncoarray; iCOGS; GWAS meta analysis) || id:ie

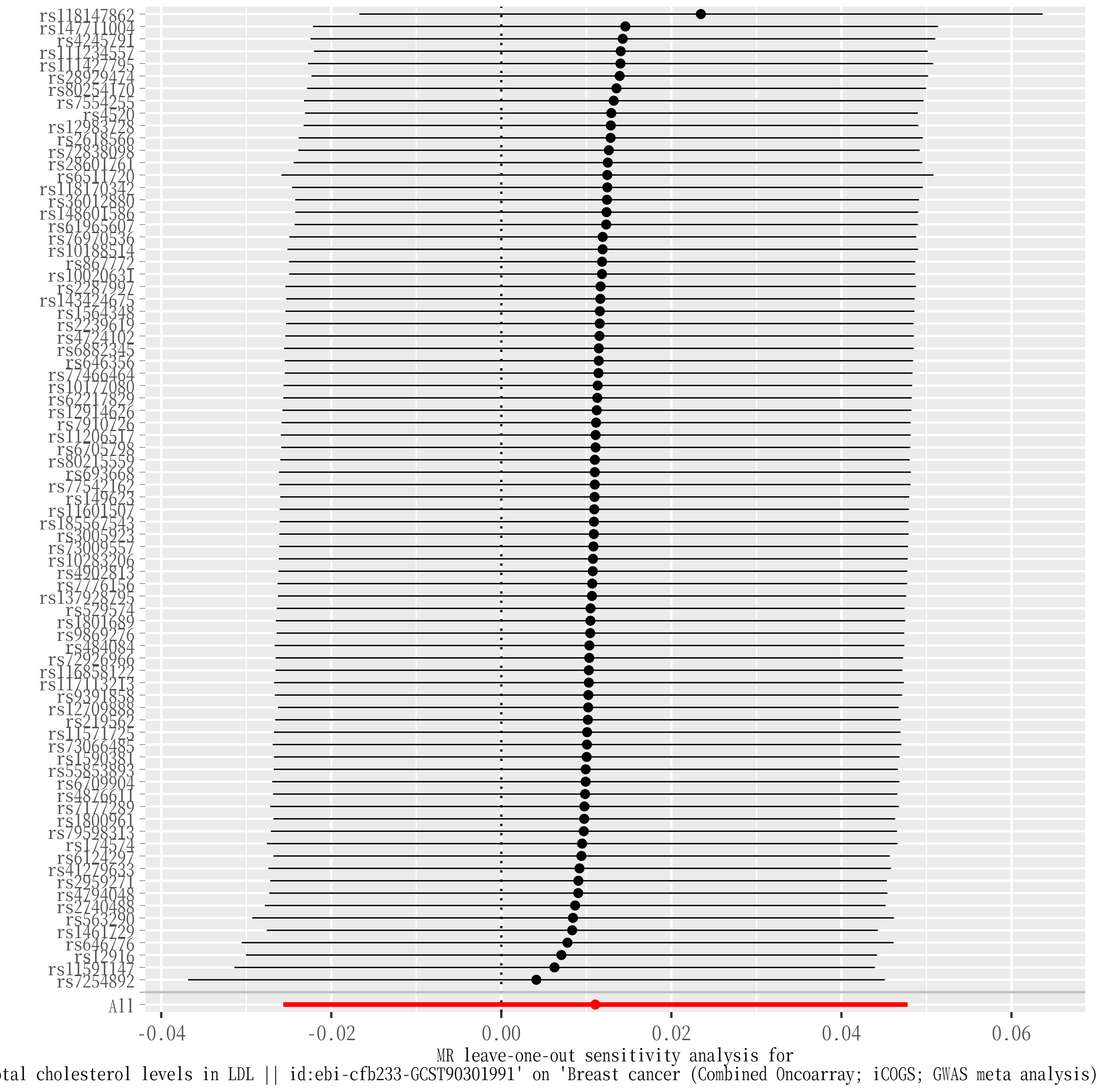
rs118147862
rs147711004
rs4245791
rs111234557
rs111427795
rs28929474
rs80254170
rs7554255
rs4520
rs12983728
rs2618566
rs72838098
rs28601761
rs6511720
rs118170342
rs36012880
rs148601586
rs61965607
rs76970536
rs10188514
rs867772
rs10020631
rs2287997
rs143424675
rs1564348
rs2239619
rs4724102
rs6882345
rs646356
rs77466464
rs10177080
rs62217829
rs12914626
rs7910726
rs11206517
rs6705728
rs80215559
rs693668
rs77542162
rs149623
rs11601507
rs185567543
rs3005923
rs73009557
rs10283206
rs4902813
rs2776156
rs137928795
rs529574
rs1801689
rs9869276
rs484084
rs72926966
rs116858122
rs117113213
rs9391858
rs12709888
rs219562
rs11571725
rs73066485
rs1590381
rs55853893
rs6709904
rs4876611
rs7177289
rs1800961
rs79598313
rs174574
rs6124297
rs41279633
rs2959271
rs4794048
rs2740488
rs563290
rs1461729
rs646776
rs12916
rs11591147
rs7254892
All
-0.04
-0.02
0.00
0.02
0.04
0.06
MR leave-one-out sensitivity analysis for
otal cholesterol levels in LDL || id:ebi-cfb233-GCST90301991' on 'Breast cancer (Combined Oncoarray; iCOGS; GWAS meta analysis)

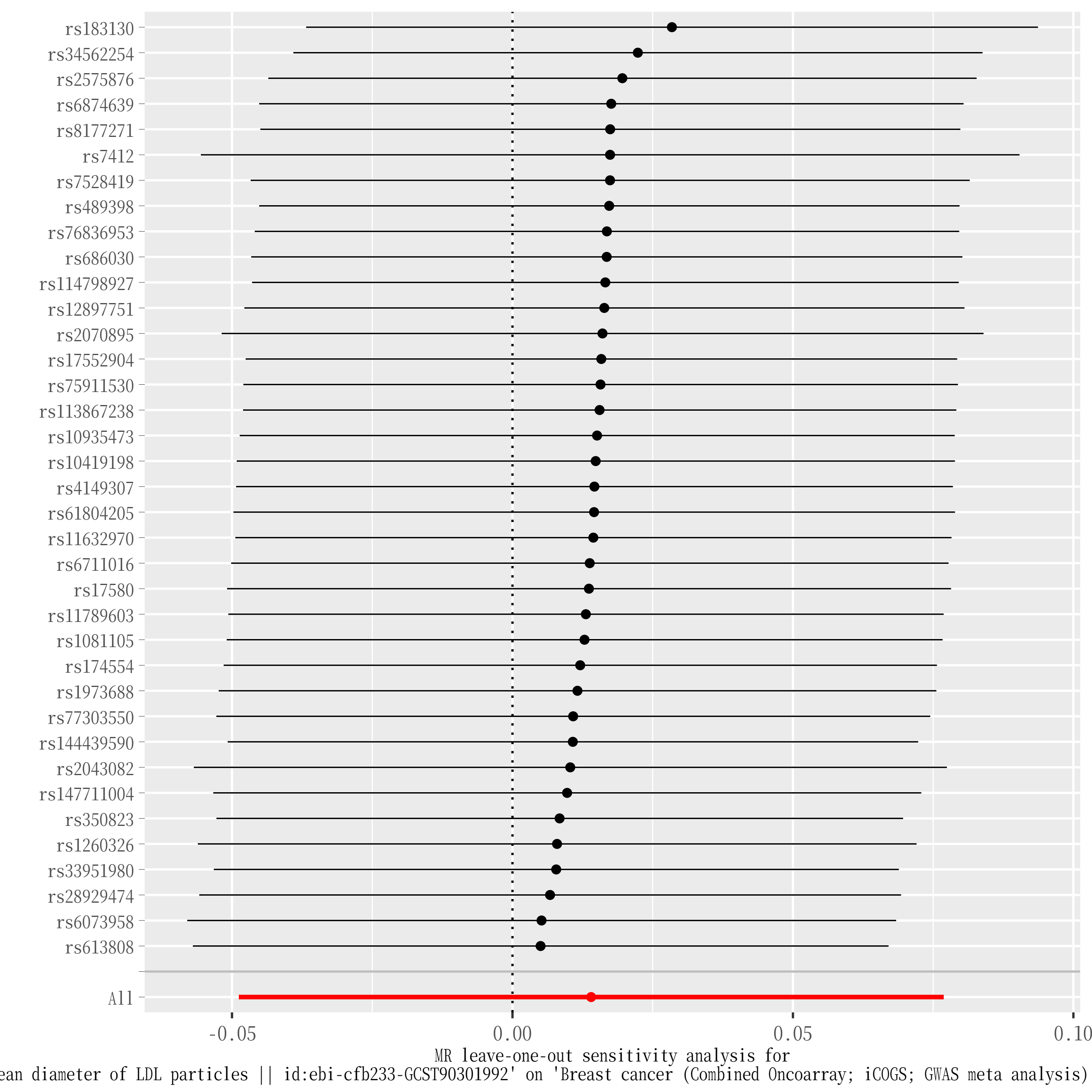

rs183130
rs34562254
rs2575876
rs6874639
rs8177271
rs7412
rs7528419
rs489398
rs76836953
rs686030
rs114798927
rs12897751
rs2070895
rs17552904
rs75911530
rs113867238
rs10935473
rs10419198
rs4149307
rs61804205
rs11632970
rs6711016
rs17580
rs11789603
rs1081105
rs174554
rs1973688
rs77303550
rs144439590
rs2043082
rs147711004
rs350823
rs1260326
rs33951980
rs28929474
rs6073958
rs613808
All
-0.05
0.00
0.05
0.10
MR leave-one-out sensitivity analysis for
ean diameter of LDL particles || id:ebi-cfb233-GCST90301992' on 'Breast cancer (Combined Oncoarray; iCOGS; GWAS meta analysis)

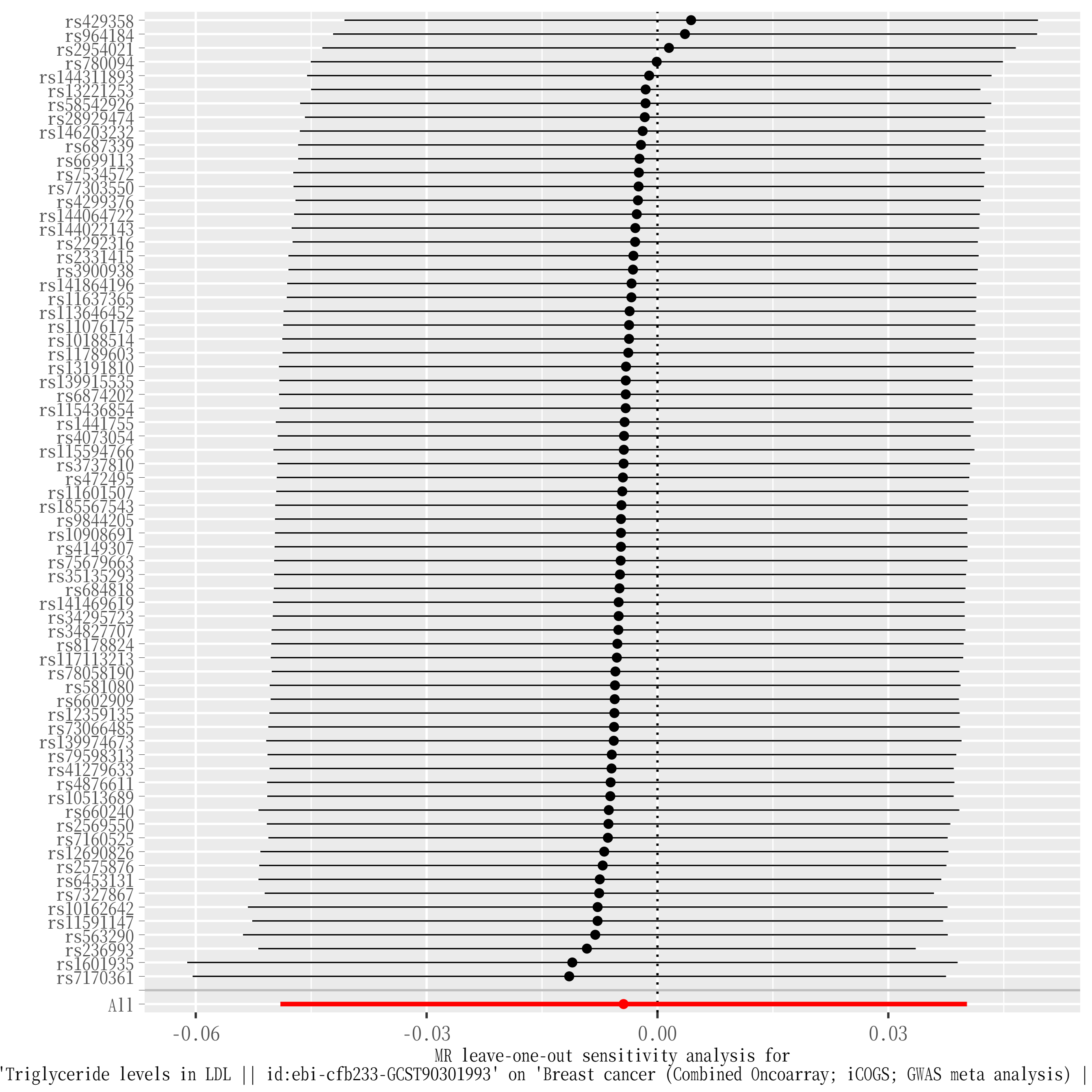

rs429358
rs964184
rs2954021
rs780094
rs144311893
rs13221253
rs58542926
rs28929474
rs146203232
rs687339
rs6699113
rs7534572
rs77303550
rs4299376
rs144064722
rs144022143
rs2292316
rs2331415
rs3900938
rs141864196
rs11637365
rs113646452
rs11076175
rs10188514
rs11789603
rs13191810
rs139915535
rs6874202
rs115436854
rs1441755
rs4073054
rs115594766
rs3737810
rs472495
rs11601507
rs185567543
rs9844205
rs10908691
rs4149307
rs75679663
rs35135293
rs684818
rs141469619
rs34295723
rs34827707
rs8178824
rs117113213
rs78058190
rs581080
rs6602909
rs12359135
rs73066485
rs139974673
rs79598313
rs41279633
rs4876611
rs10513689
rs660240
rs2569550
rs7160525
rs12690826
rs2575876
rs6453131
rs7327867
rs10162642
rs11591147
rs563290
rs236993
rs1601935
rs7170361
All
-0.06
-0.03
0.00
0.03
MR leave-one-out sensitivity analysis for
'Triglyceride levels in LDL || id:ebi-cfb233-GCST90301993' on 'Breast cancer (Combined Oncoarray; iCOGS; GWAS meta analysis) |

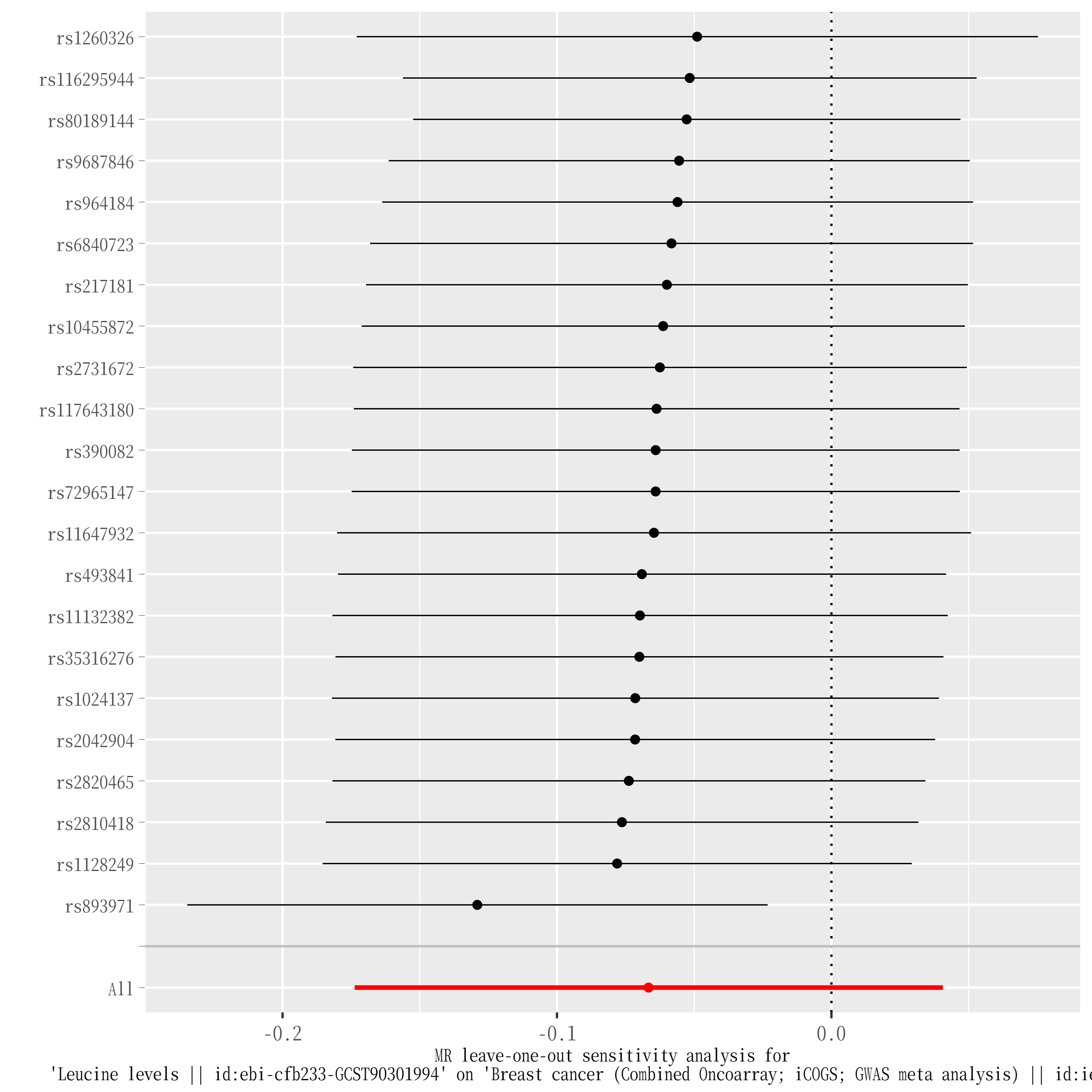
rs1260326
rs116295944
rs80189144
rs9687846
rs964184
rs6840723
rs217181
rs10455872
rs2731672
rs117643180
rs390082
rs72965147
rs11647932
rs493841
rs11132382
rs35316276
rs1024137
rs2042904
rs2820465
rs2810418
rs1128249
rs893971
All
-0.2
-0.1
0.0
MR leave-one-out sensitivity analysis for
'Leucine levels || id:ebi-cfb233-GCST90301994' on 'Breast cancer (Combined Oncoarray; iCOGS; GWAS meta analysis) || id:i

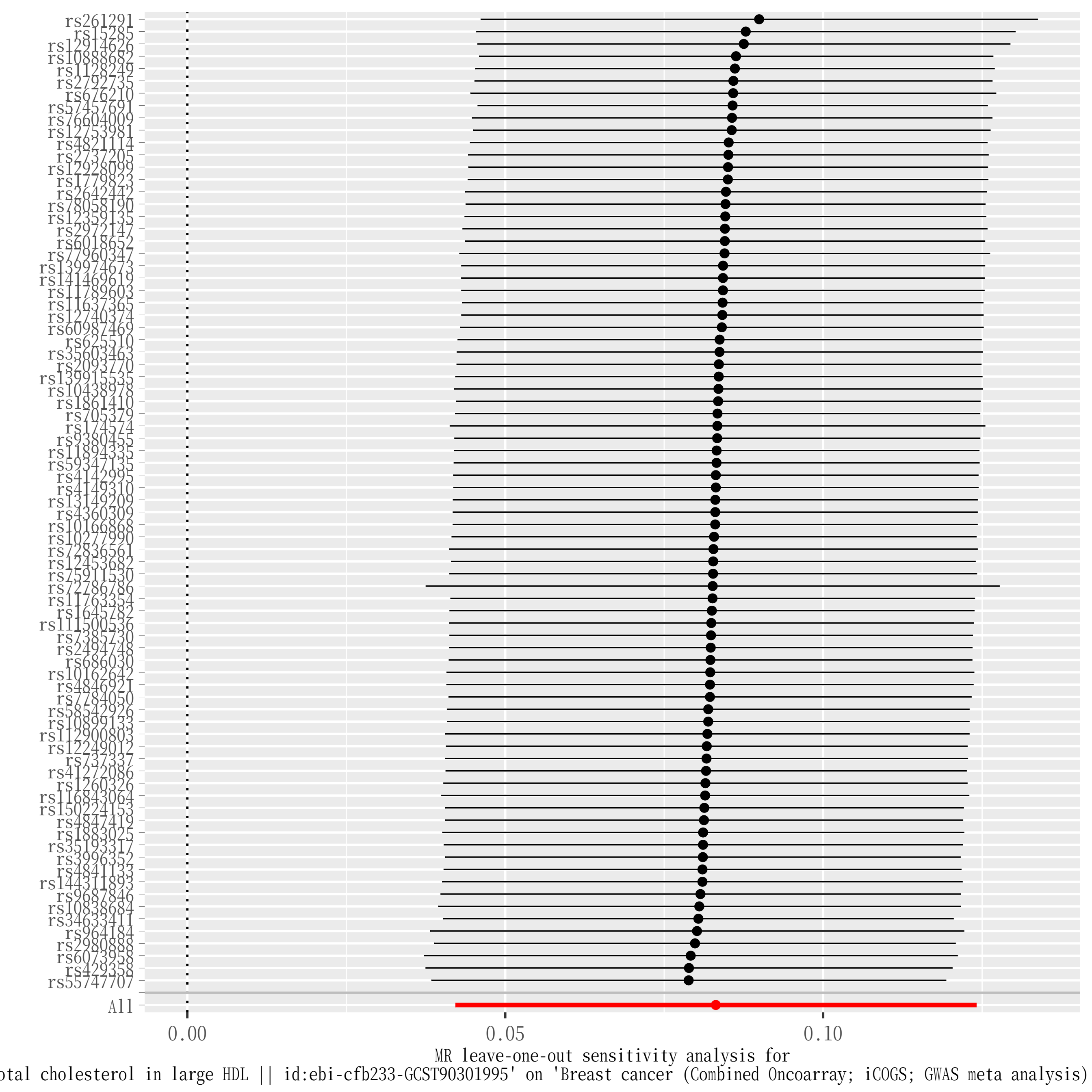

rs261291
rs15285
rs12914626
rs10888682
rs1128249
rs2792735
rs676210
rs57457691
rs76604009
rs12753981
rs4821114
rs2737205
rs12928099
rs1779823
rs2642442
rs78058190
rs12359135
rs2972147
rs6018652
rs77960347
rs139974673
rs141469619
rs11789603
rs11637365
rs12740374
rs60987469
rs625510
rs35603463
rs2093770
rs139915535
rs10438978
rs1861410
rs705379
rs174574
rs9380455
rs11894335
rs59347135
rs4142995
rs4149310
rs13149209
rs4360309
rs10166868
rs10277990
rs72836561
rs12453682
rs75911530
rs72786786
rs11763354
rs1645782
rs111500536
rs7385730
rs2494748
rs686030
rs10162642
rs4846921
rs7784050
rs58542926
rs10899133
rs112900803
rs12249012
rs737337
rs41272086
rs1260326
rs116843064
rs150224153
rs4847419
rs1883025
rs35193317
rs3996352
rs4841133
rs144311893
rs9687846
rs10838684
rs34633411
rs964184
rs2980888
rs6073958
rs429358
rs55747707
All
0.00
0.05
0.10
MR leave-one-out sensitivity analysis for
'Total cholesterol in large HDL || id:ebi-cfb233-GCST90301995' on 'Breast cancer (Combined Oncoarray; iCOGS; GWAS meta analysis)

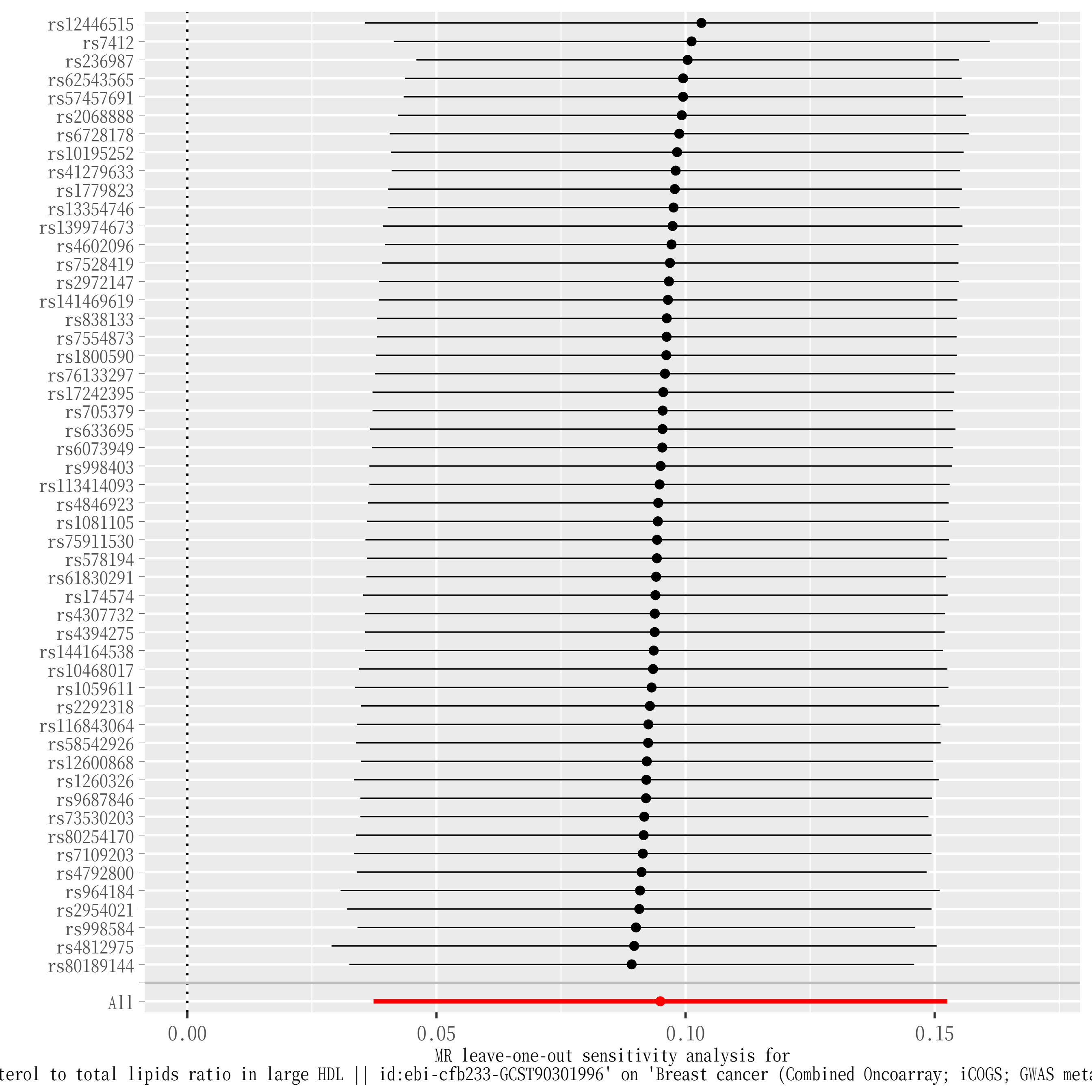

rs12446515
rs7412
rs236987
rs62543565
rs57457691
rs2068888
rs6728178
rs10195252
rs41279633
rs1779823
rs13354746
rs139974673
rs4602096
rs7528419
rs2972147
rs141469619
rs838133
rs7554873
rs1800590
rs76133297
rs17242395
rs705379
rs633695
rs6073949
rs998403
rs113414093
rs4846923
rs1081105
rs75911530
rs578194
rs61830291
rs174574
rs4307732
rs4394275
rs144164538
rs10468017
rs1059611
rs2292318
rs116843064
rs58542926
rs12600868
rs1260326
rs9687846
rs73530203
rs80254170
rs7109203
rs4792800
rs964184
rs2954021
rs998584
rs4812975
rs80189144
All
0.00
0.05
0.10
0.15
MR leave-one-out sensitivity analysis for
terol to total lipids ratio in large HDL || id:ebi-cfb233-GCST90301996' on 'Breast cancer (Combined Oncoarray; iCOGS; GWAS meta

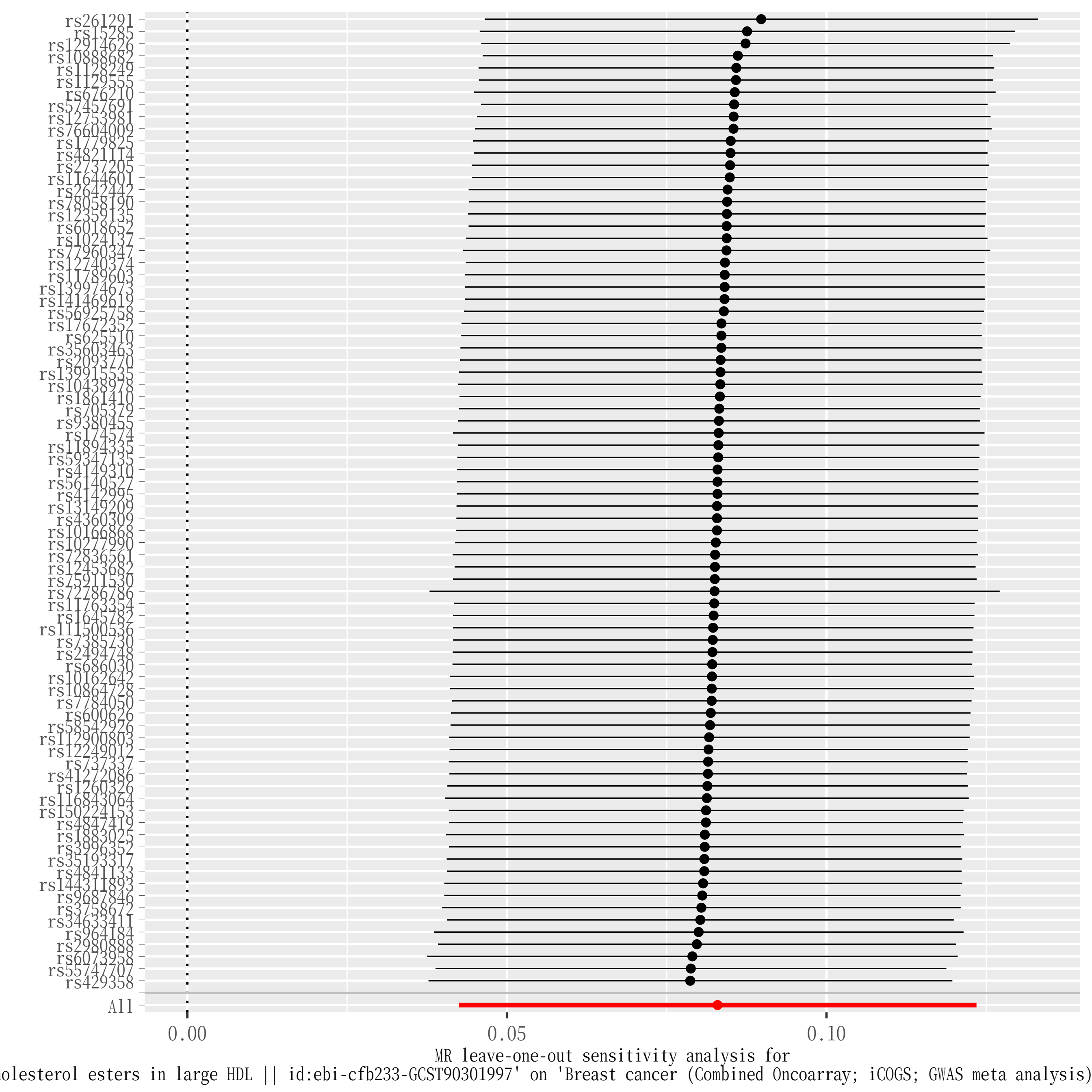
rs261291
rs15285
rs12914626
rs10888682
rs1128249
rs1129555
rs676210
rs57457691
rs12753981
rs76604009
rs1779825
rs4821114
rs2737205
rs11644601
rs2642442
rs78058190
rs12359135
rs6018652
rs1024137
rs77960347
rs12740374
rs11789603
rs139974673
rs141469619
rs56925758
rs17672352
rs625510
rs35603463
rs2093770
rs139915535
rs10438978
rs1861410
rs705379
rs9380455
rs174574
rs11894335
rs59347135
rs4149310
rs56140527
rs4142995
rs13149209
rs4360309
rs10166868
rs10277990
rs72836561
rs12453682
rs75911530
rs72786786
rs11763354
rs1645782
rs111500536
rs7385730
rs2494748
rs686030
rs10162642
rs10864728
rs7784050
rs600626
rs58542926
rs112900803
rs12249012
rs737337
rs41272086
rs1260326
rs116843064
rs150224153
rs4847419
rs1883025
rs3996352
rs35193317
rs4841133
rs144311893
rs9687846
rs3758672
rs34633411
rs964184
rs2980888
rs6073958
rs55747707
rs429358
All
0.00
0.05
0.10
MR leave-one-out sensitivity analysis for
holesterol esters in large HDL || id:ebi-cfb233-GCST90301997' on 'Breast cancer (Combined Oncoarray; iCOGS; GWAS meta analysis)

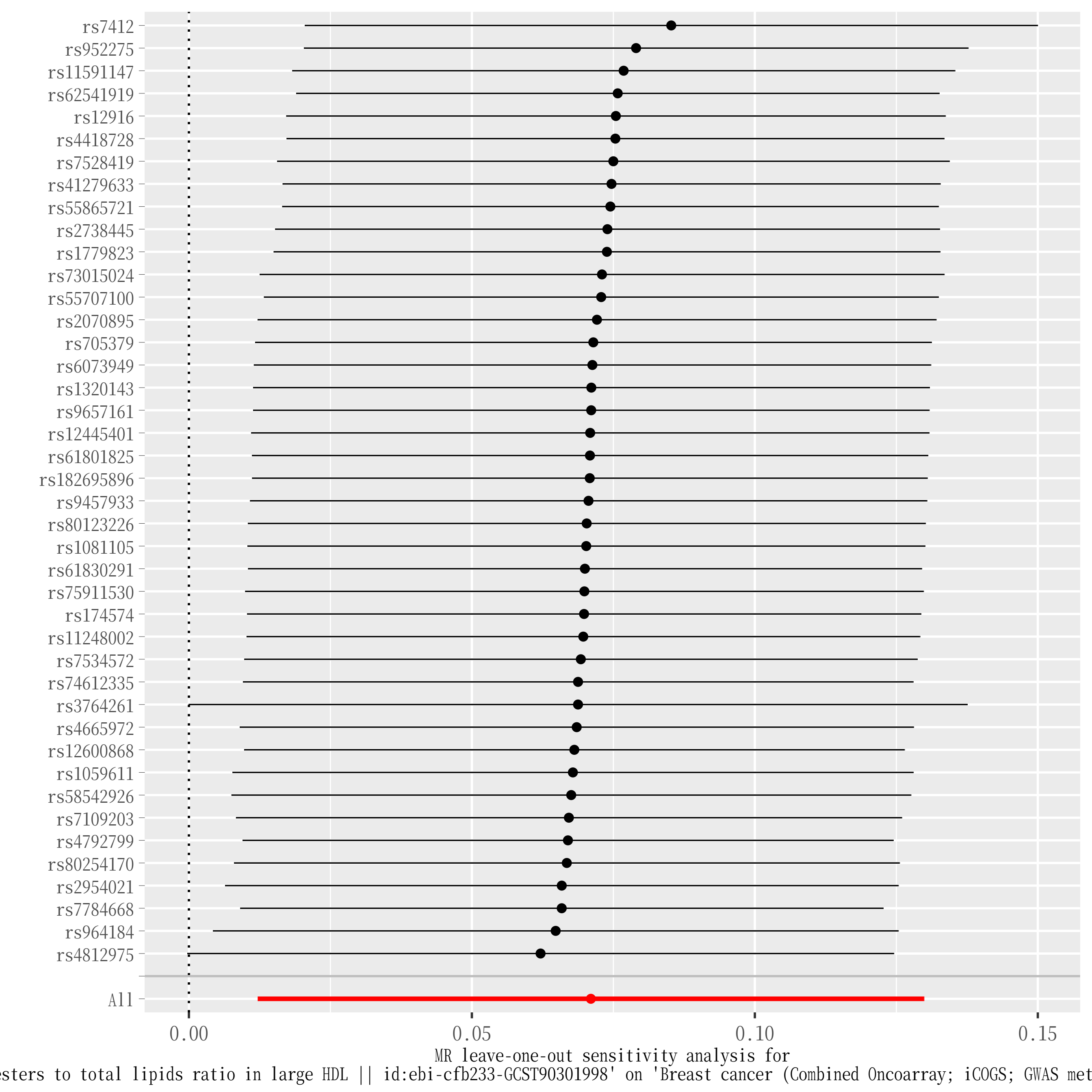

rs7412
rs952275
rs11591147
rs62541919
rs12916
rs4418728
rs7528419
rs41279633
rs55865721
rs2738445
rs1779823
rs73015024
rs55707100
rs2070895
rs705379
rs6073949
rs1320143
rs9657161
rs12445401
rs61801825
rs182695896
rs9457933
rs80123226
rs1081105
rs61830291
rs75911530
rs174574
rs11248002
rs7534572
rs74612335
rs3764261
rs4665972
rs12600868
rs1059611
rs58542926
rs7109203
rs4792799
rs80254170
rs2954021
rs7784668
rs964184
rs4812975
All
0.00
0.05
0.10
0.15
MR leave-one-out sensitivity analysis for
esters to total lipids ratio in large HDL || id:ebi-cfb233-GCST90301998' on 'Breast cancer (Combined Oncoarray; iCOGS; GWAS met

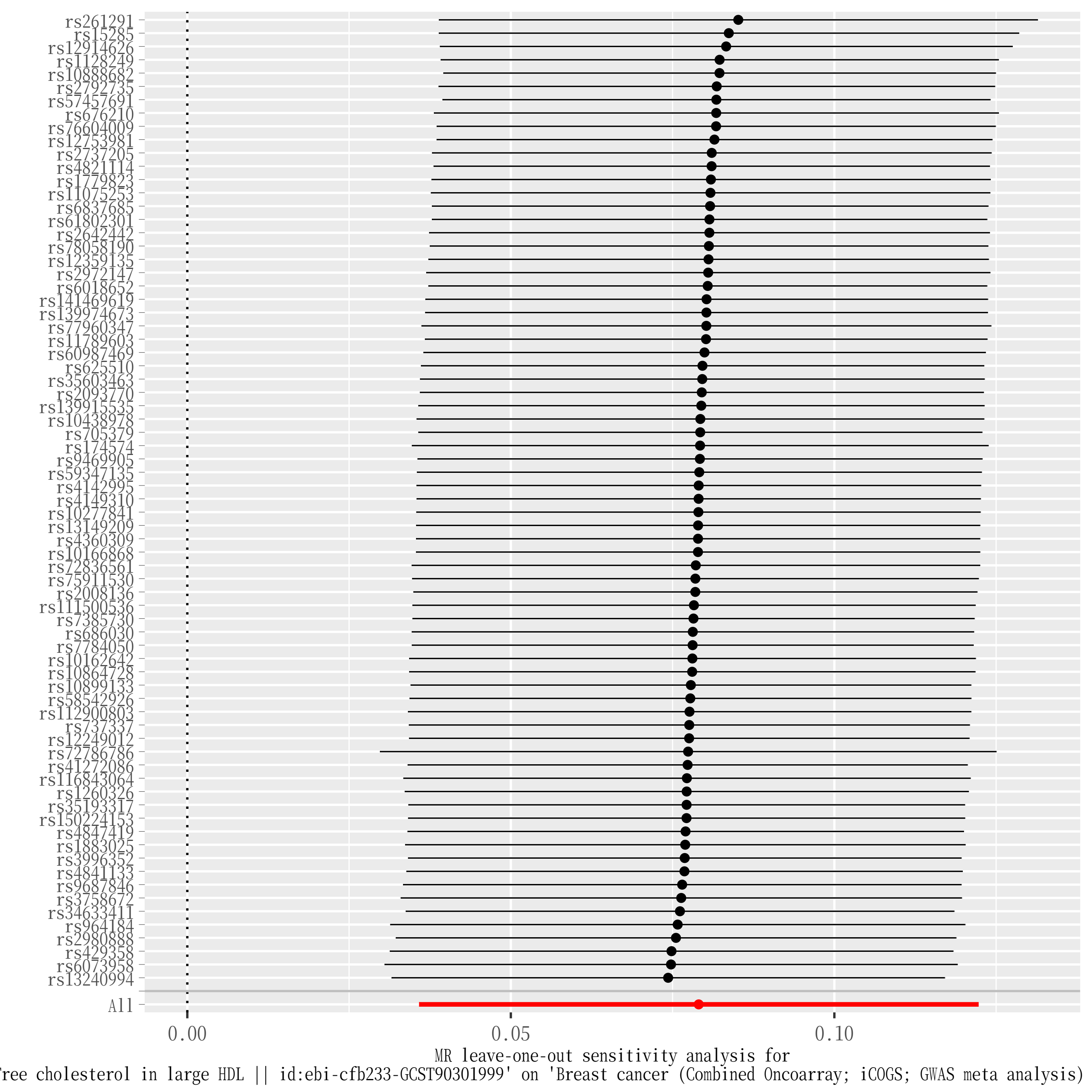
rs261291
rs15285
rs12914626
rs1128249
rs10888682
rs2792735
rs57457691
rs676210
rs76604009
rs12753981
rs2737205
rs4821114
rs1779823
rs11075253
rs6837685
rs61802301
rs2642442
rs78058190
rs12359135
rs2972147
rs6018652
rs141469619
rs139974673
rs77960347
rs11789603
rs60987469
rs625510
rs35603463
rs2093770
rs139915535
rs10438978
rs705379
rs174574
rs9469905
rs59347135
rs4142995
rs4149310
rs10277841
rs13149209
rs4360309
rs10166868
rs72836561
rs75911530
rs2008136
rs111500536
rs7385730
rs686030
rs7784050
rs10162642
rs10864728
rs10899133
rs58542926
rs112900803
rs737337
rs12249012
rs72786786
rs41272086
rs116843064
rs1260326
rs35193317
rs150224153
rs4847419
rs1883025
rs3996352
rs4841133
rs9687846
rs3758672
rs34633411
rs964184
rs2980888
rs429358
rs6073958
rs13240994
All
0.00
0.05
0.10
MR leave-one-out sensitivity analysis for
'Free cholesterol in large HDL || id:ebi-cfb233-GCST90301999' on 'Breast cancer (Combined Oncoarray; iCOGS; GWAS meta analysis)'

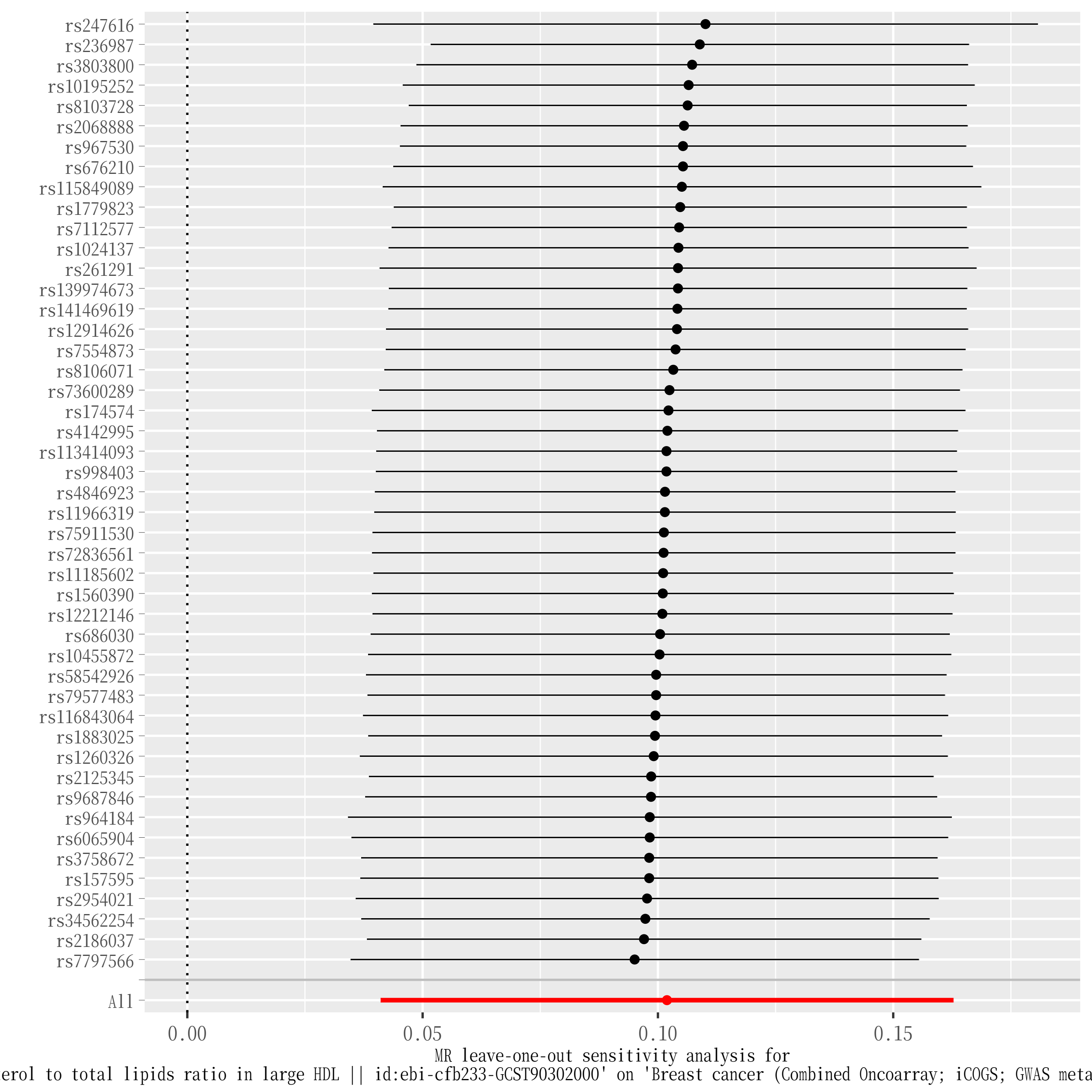
rs247616
rs236987
rs3803800
rs10195252
rs8103728
rs2068888
rs967530
rs676210
rs115849089
rs1779823
rs7112577
rs1024137
rs261291
rs139974673
rs141469619
rs12914626
rs7554873
rs8106071
rs73600289
rs174574
rs4142995
rs113414093
rs998403
rs4846923
rs11966319
rs75911530
rs72836561
rs11185602
rs1560390
rs12212146
rs686030
rs10455872
rs58542926
rs79577483
rs116843064
rs1883025
rs1260326
rs2125345
rs9687846
rs964184
rs6065904
rs3758672
rs157595
rs2954021
rs34562254
rs2186037
rs7797566
All
0.00
0.05
0.10
0.15
MR leave-one-out sensitivity analysis for
erol to total lipids ratio in large HDL || id:ebi-cfb233-GCST90302000' on 'Breast cancer (Combined Oncoarray; iCOGS; GWAS meta

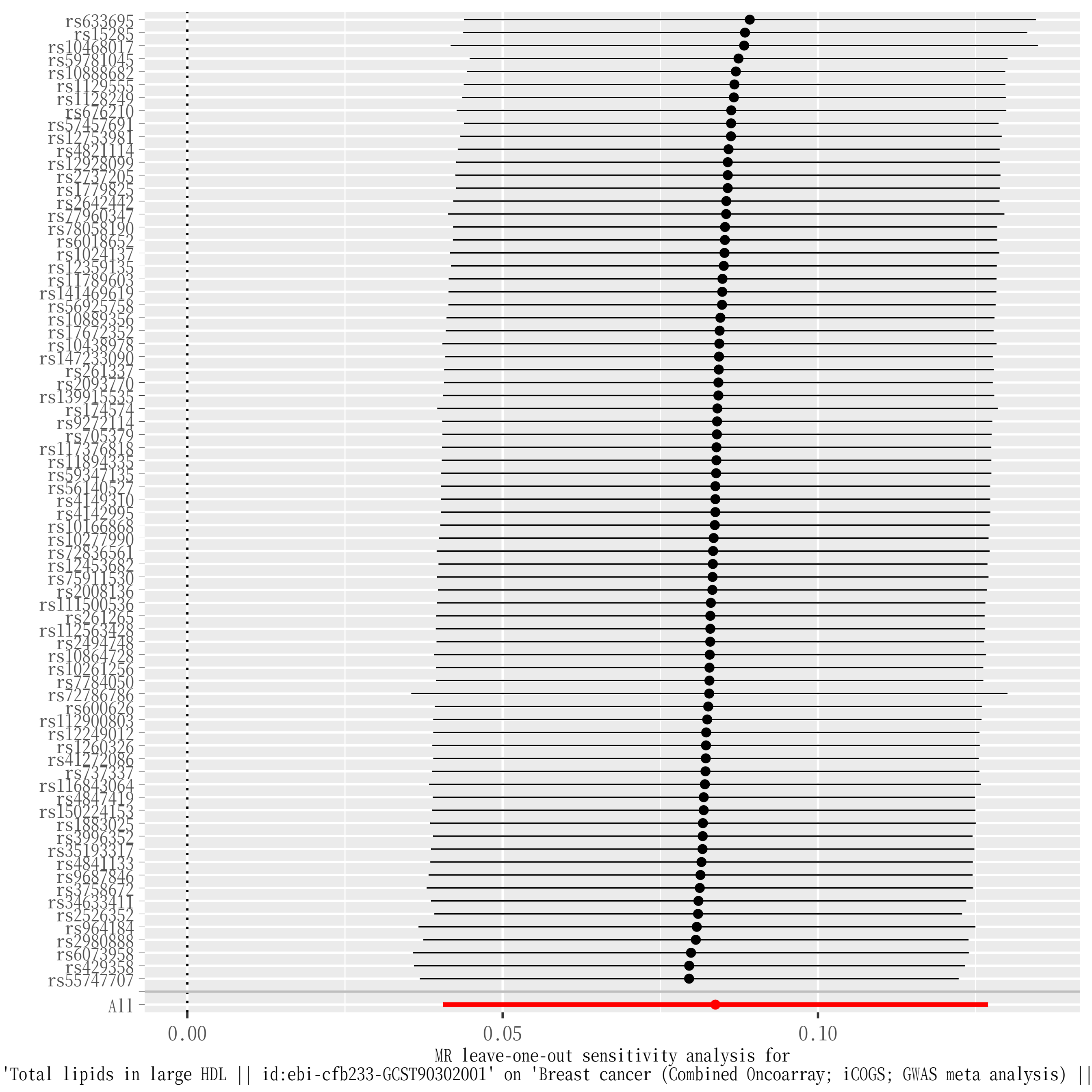
rs633695
rs15285
rs10468017
rs59781045
rs10888682
rs1129555
rs1128249
rs676210
rs57457691
rs12753981
rs4821114
rs12928099
rs2737205
rs1779825
rs2642442
rs77960347
rs78058190
rs6018652
rs1024137
rs12359135
rs11789603
rs141469619
rs56925758
rs10889356
rs17672352
rs10438978
rs147233090
rs261337
rs2093770
rs139915535
rs174574
rs9272114
rs705379
rs117376818
rs11894335
rs59347135
rs56140527
rs4149310
rs4142995
rs10166868
rs10277990
rs72836561
rs12453682
rs75911530
rs2008136
rs111500536
rs261265
rs112563428
rs2494748
rs10864728
rs10261256
rs7784050
rs72786786
rs600626
rs112900803
rs12249012
rs1260326
rs41272086
rs737337
rs116843064
rs4847419
rs150224153
rs1883025
rs3996352
rs35193317
rs4841133
rs9687846
rs3758672
rs34633411
rs2526352
rs964184
rs2980888
rs6073958
rs429358
rs55747707
All
0.00
0.05
0.10
MR leave-one-out sensitivity analysis for
'Total lipids in large HDL || id:ebi-cfb233-GCST90302001' on 'Breast cancer (Combined Oncoarray; iCOGS; GWAS meta analysis) ||

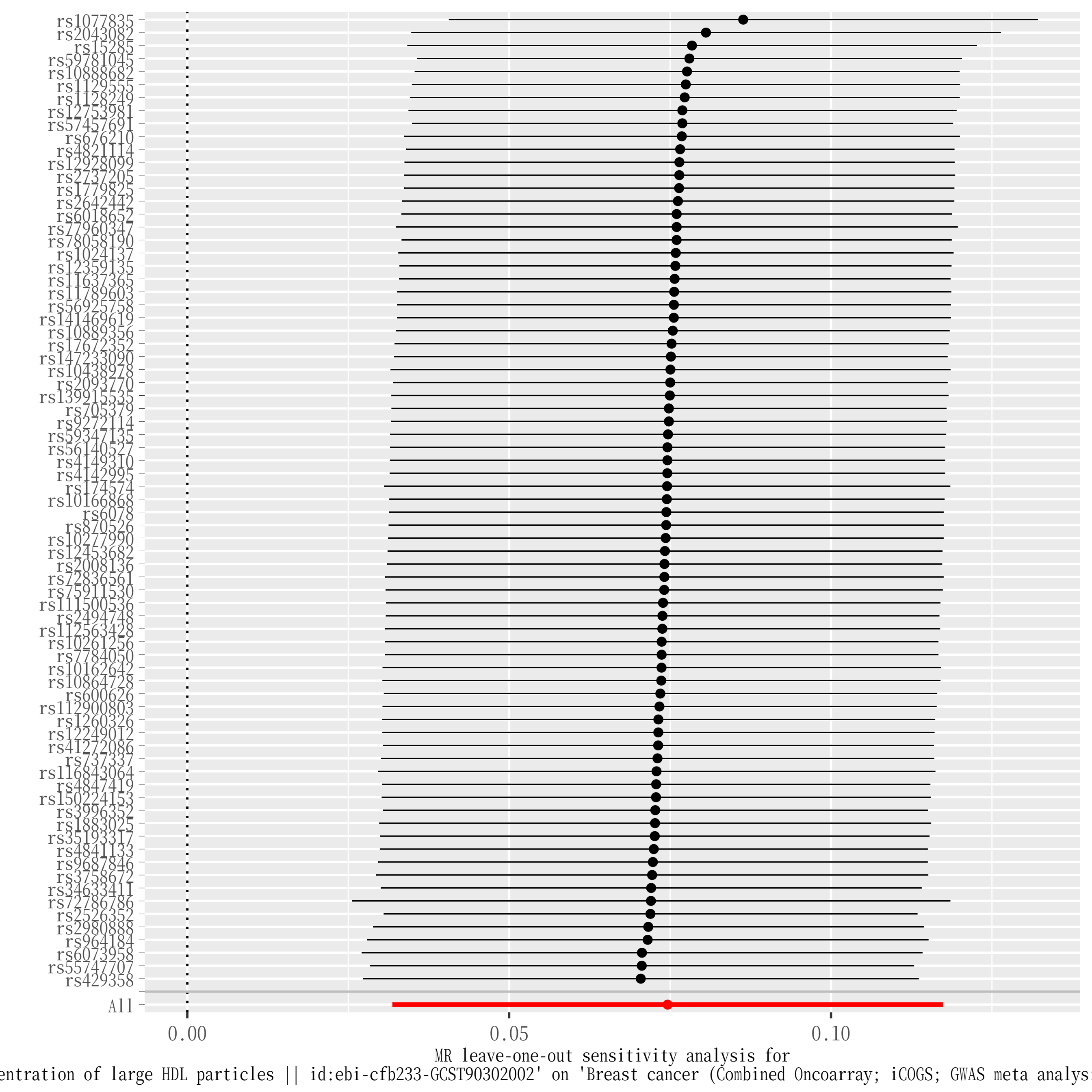
rs1077835
rs2043082
rs15285
rs59781045
rs10888682
rs1129555
rs1128249
rs12753981
rs57457691
rs676210
rs4821114
rs12928099
rs2737205
rs1779825
rs2642442
rs6018652
rs77960347
rs78058190
rs1024137
rs12359135
rs11637365
rs11789603
rs56925758
rs141469619
rs10889356
rs17672352
rs147233090
rs10438978
rs2093770
rs139915535
rs705379
rs9272114
rs59347135
rs56140527
rs4149310
rs4142995
rs174574
rs10166868
rs6078
rs870526
rs10277990
rs12453682
rs2008136
rs72836561
rs75911530
rs111500536
rs2494748
rs112563428
rs10261256
rs7784050
rs10162642
rs10864728
rs600626
rs112900803
rs1260326
rs12249012
rs41272086
rs737337
rs116843064
rs4847419
rs150224153
rs3996352
rs1883025
rs35193317
rs4841133
rs9687846
rs3758672
rs34633411
rs72786786
rs2526352
rs2980888
rs964184
rs6073958
rs55747707
rs429358
All
0.00
0.05
0.10
MR leave-one-out sensitivity analysis for
entration of large HDL particles || id:ebi-cfb233-GCST90302002' on 'Breast cancer (Combined Oncoarray; iCOGS; GWAS meta analys

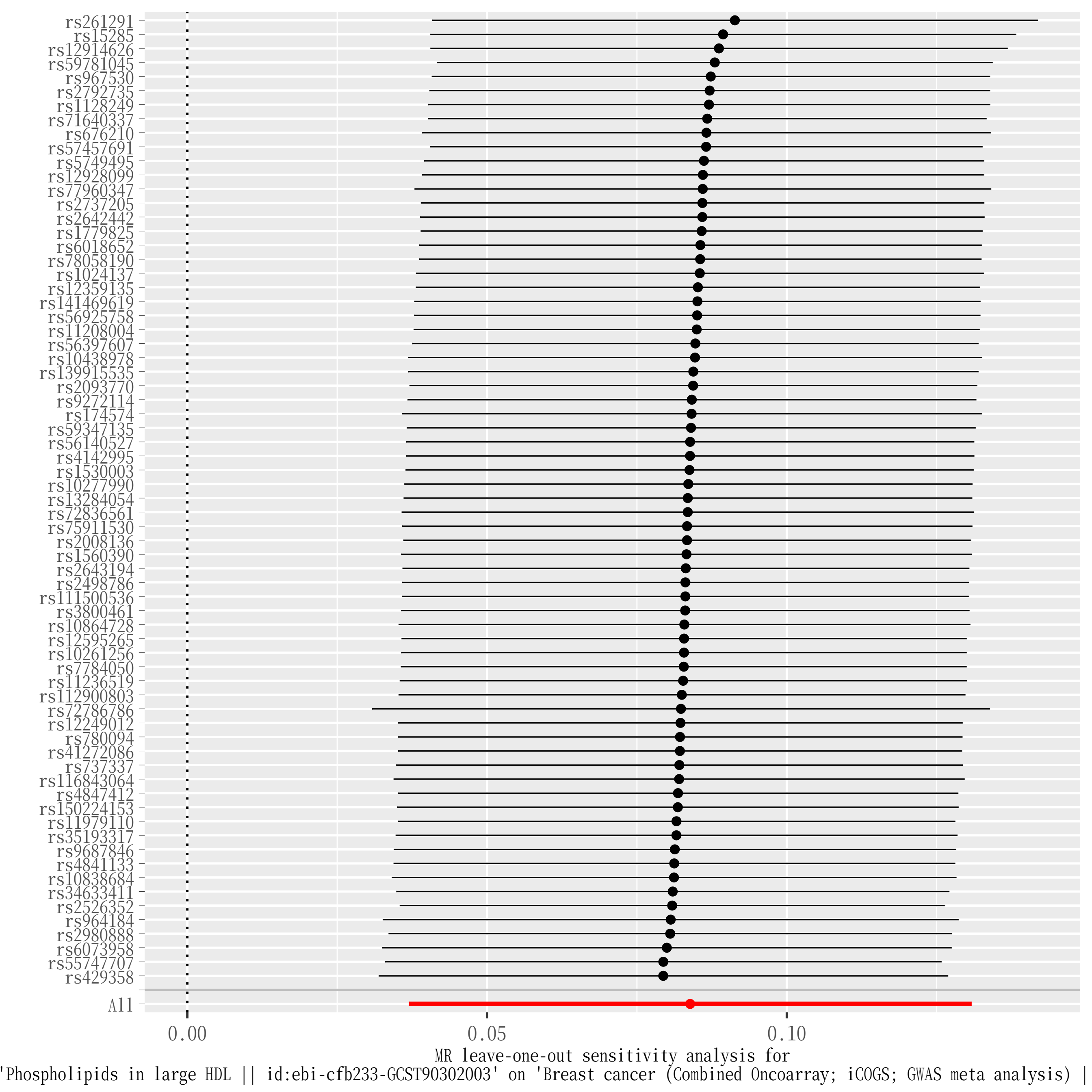

rs261291
rs15285
rs12914626
rs59781045
rs967530
rs2792735
rs1128249
rs71640337
rs676210
rs57457691
rs5749495
rs12928099
rs77960347
rs2737205
rs2642442
rs1779825
rs6018652
rs78058190
rs1024137
rs12359135
rs141469619
rs56925758
rs11208004
rs56397607
rs10438978
rs139915535
rs2093770
rs9272114
rs174574
rs59347135
rs56140527
rs4142995
rs1530003
rs10277990
rs13284054
rs72836561
rs75911530
rs2008136
rs1560390
rs2643194
rs2498786
rs111500536
rs3800461
rs10864728
rs12595265
rs10261256
rs7784050
rs11236519
rs112900803
rs72786786
rs12249012
rs780094
rs41272086
rs737337
rs116843064
rs4847412
rs150224153
rs11979110
rs35193317
rs9687846
rs4841133
rs10838684
rs34633411
rs2526352
rs964184
rs2980888
rs6073958
rs55747707
rs429358
All
0.00
0.05
0.10
MR leave-one-out sensitivity analysis for
'Phospholipids in large HDL || id:ebi-cfb233-GCST90302003' on 'Breast cancer (Combined Oncoarray; iCOGS; GWAS meta analysis) |

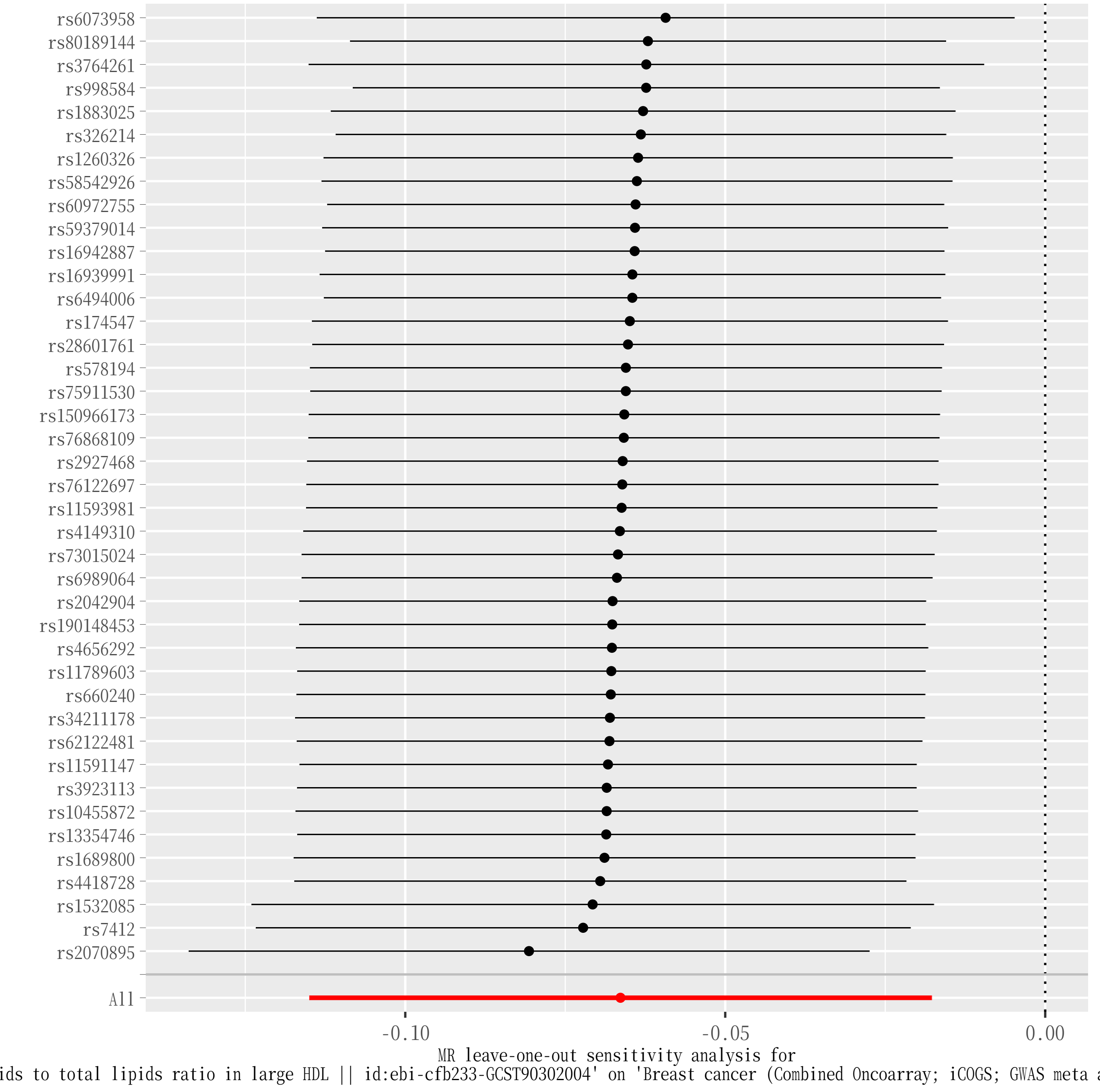

rs6073958
rs80189144
rs3764261
rs998584
rs1883025
rs326214
rs1260326
rs58542926
rs60972755
rs59379014
rs16942887
rs16939991
rs6494006
rs174547
rs28601761
rs578194
rs75911530
rs150966173
rs76868109
rs2927468
rs76122697
rs11593981
rs4149310
rs73015024
rs6989064
rs2042904
rs190148453
rs4656292
rs11789603
rs660240
rs34211178
rs62122481
rs11591147
rs3923113
rs10455872
rs13354746
rs1689800
rs4418728
rs1532085
rs7412
rs2070895
All
-0.10
-0.05
0.00
MR leave-one-out sensitivity analysis for
ids to total lipids ratio in large HDL || id:ebi-cfb233-GCST90302004' on 'Breast cancer (Combined Oncoarray; iCOGS; GWAS meta a

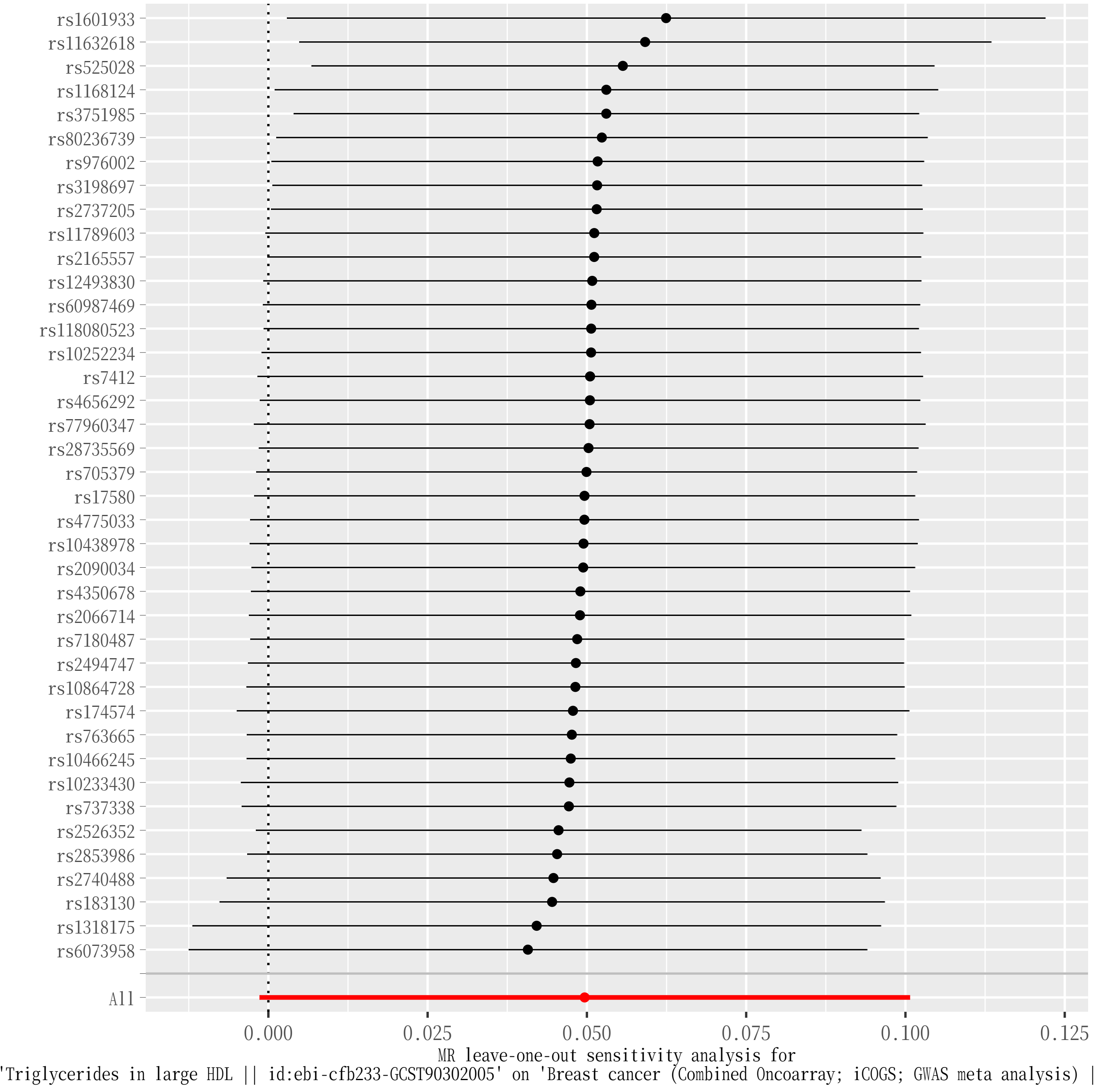
rs1601933
rs11632618
rs525028
rs1168124
rs3751985
rs80236739
rs976002
rs3198697
rs2737205
rs11789603
rs2165557
rs12493830
rs60987469
rs118080523
rs10252234
rs7412
rs4656292
rs77960347
rs28735569
rs705379
rs17580
rs4775033
rs10438978
rs2090034
rs4350678
rs2066714
rs7180487
rs2494747
rs10864728
rs174574
rs763665
rs10466245
rs10233430
rs737338
rs2526352
rs2853986
rs2740488
rs183130
rs1318175
rs6073958
All
0.000
0.025
0.050
0.075
0.100
0.125
MR leave-one-out sensitivity analysis for
'Triglycerides in large HDL || id:ebi-cfb233-GCST90302005' on 'Breast cancer (Combined Oncoarray; iCOGS; GWAS meta analysis) |

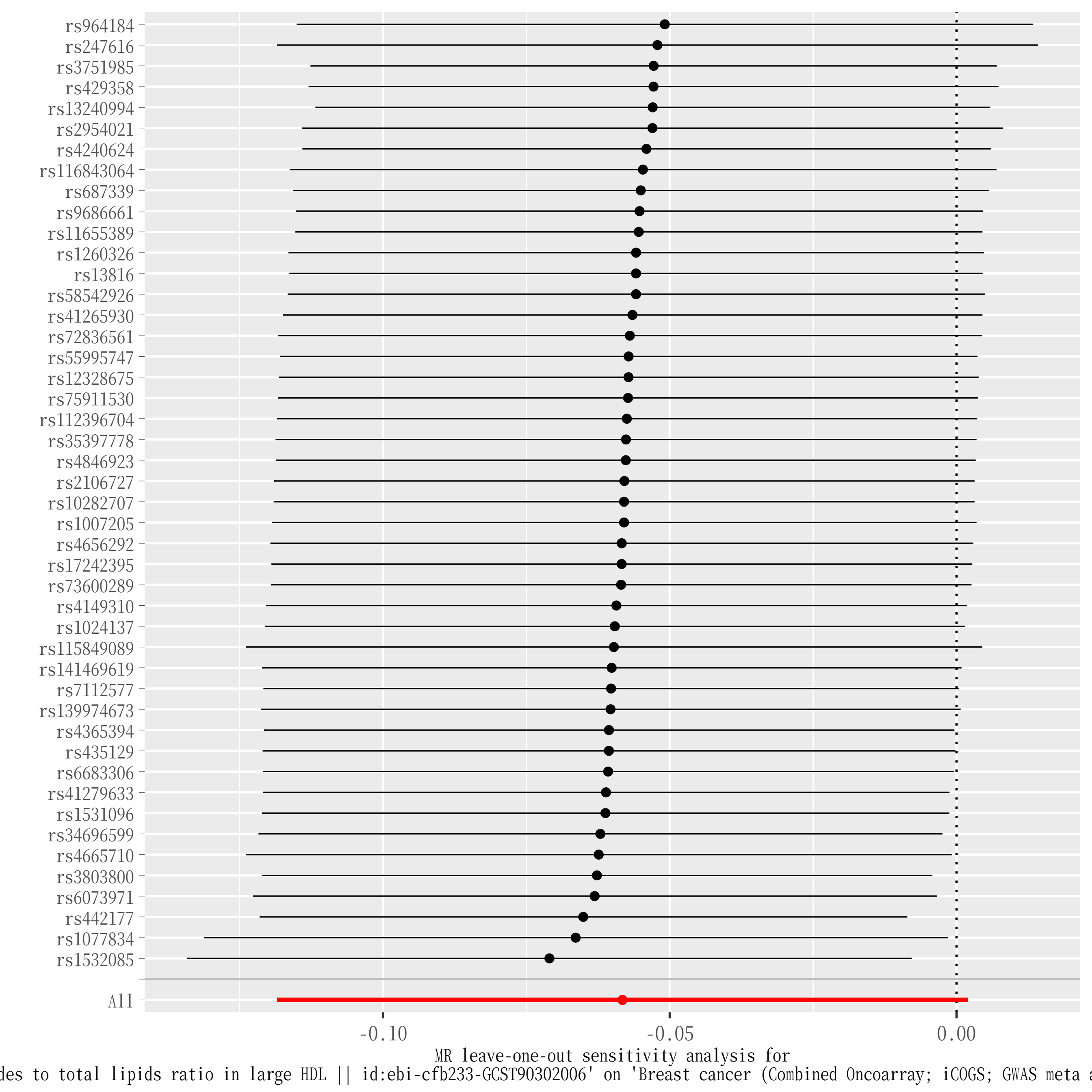
rs964184
rs247616
rs3751985
rs429358
rs13240994
rs2954021
rs4240624
rs116843064
rs687339
rs9686661
rs11655389
rs1260326
rs13816
rs58542926
rs41265930
rs72836561
rs55995747
rs12328675
rs75911530
rs112396704
rs35397778
rs4846923
rs2106727
rs10282707
rs1007205
rs4656292
rs17242395
rs73600289
rs4149310
rs1024137
rs115849089
rs141469619
rs7112577
rs139974673
rs4365394
rs435129
rs6683306
rs41279633
rs1531096
rs34696599
rs4665710
rs3803800
rs6073971
rs442177
rs1077834
rs1532085
All
-0.10
-0.05
0.00
MR leave-one-out sensitivity analysis for
des to total lipids ratio in large HDL || id:ebi-cfb233-GCST90302006' on 'Breast cancer (Combined Oncoarray; iCOGS; GWAS meta a

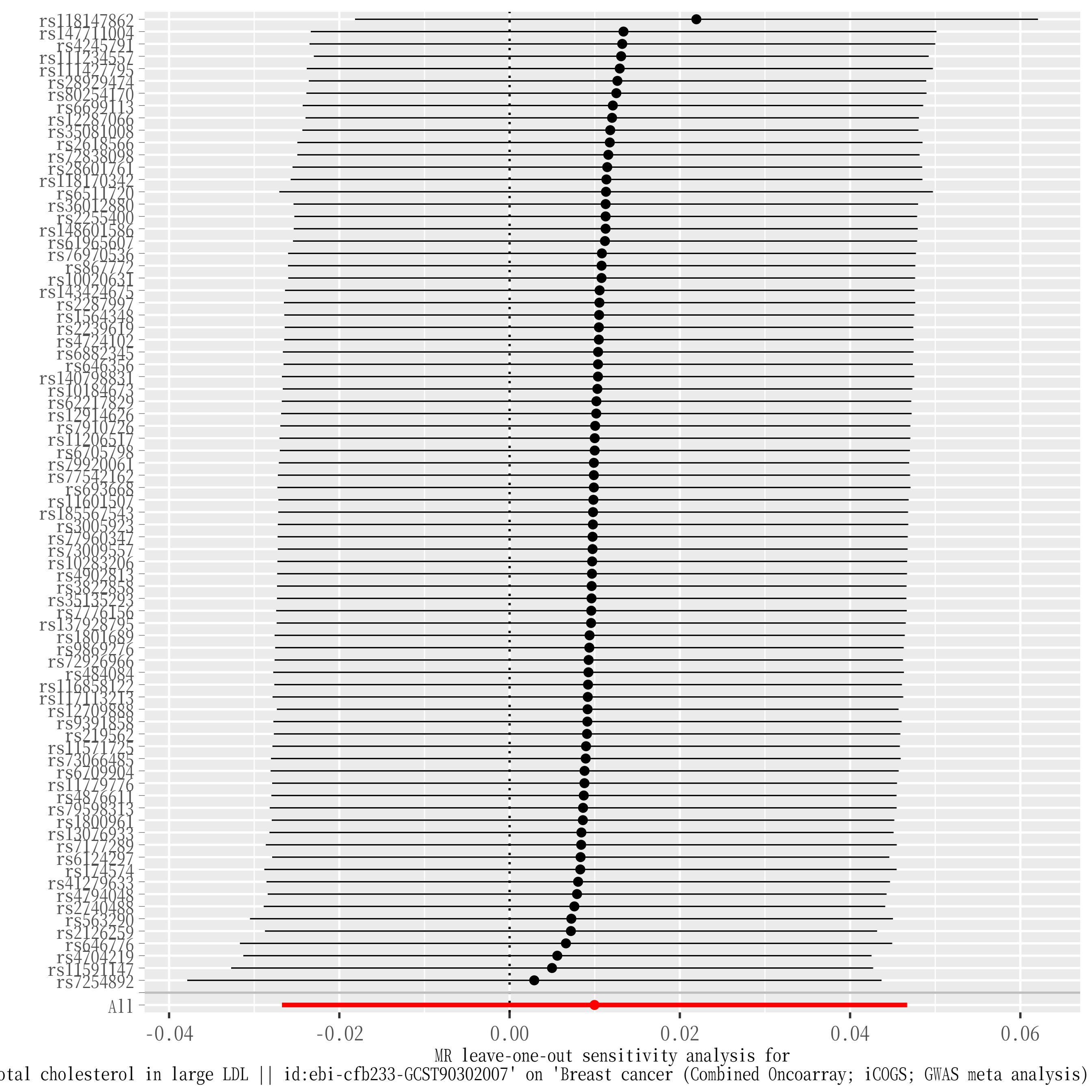

rs118147862
rs147711004
rs4245791
rs111234557
rs111427795
rs28929474
rs80254170
rs6699113
rs12287066
rs35081008
rs2618566
rs72838098
rs28601761
rs118170342
rs6511720
rs36012880
rs2255400
rs148601586
rs61965607
rs76970536
rs867772
rs10020631
rs143424675
rs2287997
rs1564348
rs2239619
rs4724102
rs6882345
rs646356
rs140798831
rs10184673
rs62217829
rs12914626
rs7910726
rs11206517
rs6705798
rs79920061
rs77542162
rs693668
rs11601507
rs185567543
rs3005923
rs77960347
rs73009557
rs10283206
rs4902813
rs3822858
rs35135293
rs7776156
rs137928795
rs1801689
rs9869276
rs72926966
rs484084
rs116858122
rs117113213
rs12709888
rs9391858
rs219562
rs11571725
rs73066485
rs6709904
rs11779776
rs4876611
rs79598313
rs1800961
rs13076933
rs7177289
rs6124297
rs174574
rs41279633
rs4794048
rs2740488
rs563290
rs2126259
rs646776
rs4704219
rs11591147
rs7254892
All
-0.04
-0.02
0.00
0.02
0.04
0.06
MR leave-one-out sensitivity analysis for
otal cholesterol in large LDL || id:ebi-cfb233-GCST90302007' on 'Breast cancer (Combined Oncoarray; iCOGS; GWAS meta analysis)

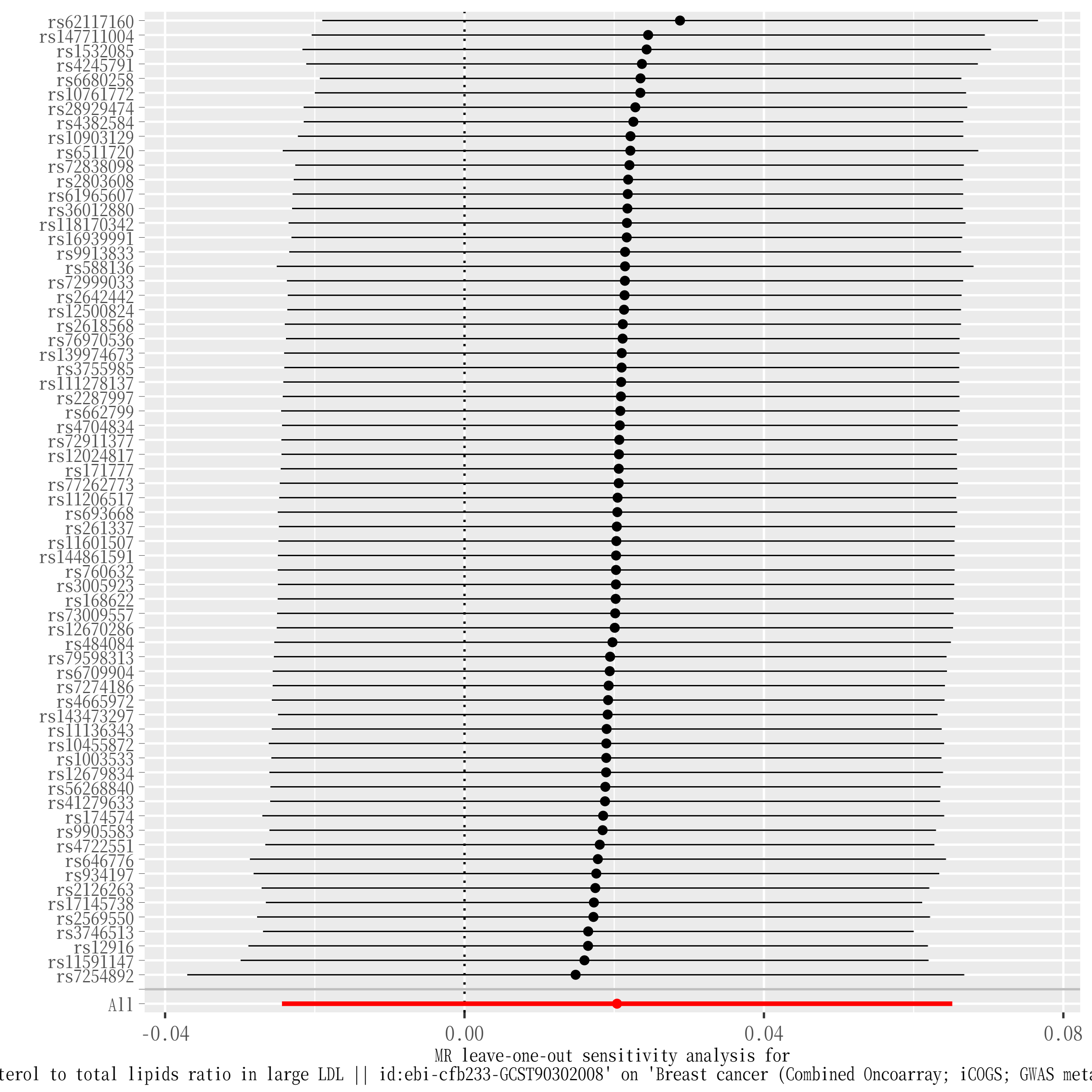
rs62117160
rs147711004
rs1532085
rs4245791
rs6680258
rs10761772
rs28929474
rs4382584
rs10903129
rs6511720
rs72838098
rs2803608
rs61965607
rs36012880
rs118170342
rs16939991
rs9913833
rs588136
rs72999033
rs2642442
rs12500824
rs2618568
rs76970536
rs139974673
rs3755985
rs111278137
rs2287997
rs662799
rs4704834
rs72911377
rs12024817
rs171777
rs77262773
rs11206517
rs693668
rs261337
rs11601507
rs144861591
rs760632
rs3005923
rs168622
rs73009557
rs12670286
rs484084
rs79598313
rs6709904
rs7274186
rs4665972
rs143473297
rs11136343
rs10455872
rs1003533
rs12679834
rs56268840
rs41279633
rs174574
rs9905583
rs4722551
rs646776
rs934197
rs2126263
rs17145738
rs2569550
rs3746513
rs12916
rs11591147
rs7254892
All
-0.04
0.00
0.04
0.08
MR leave-one-out sensitivity analysis for
terol to total lipids ratio in large LDL || id:ebi-cfb233-GCST90302008' on 'Breast cancer (Combined Oncoarray; iCOGS; GWAS meta

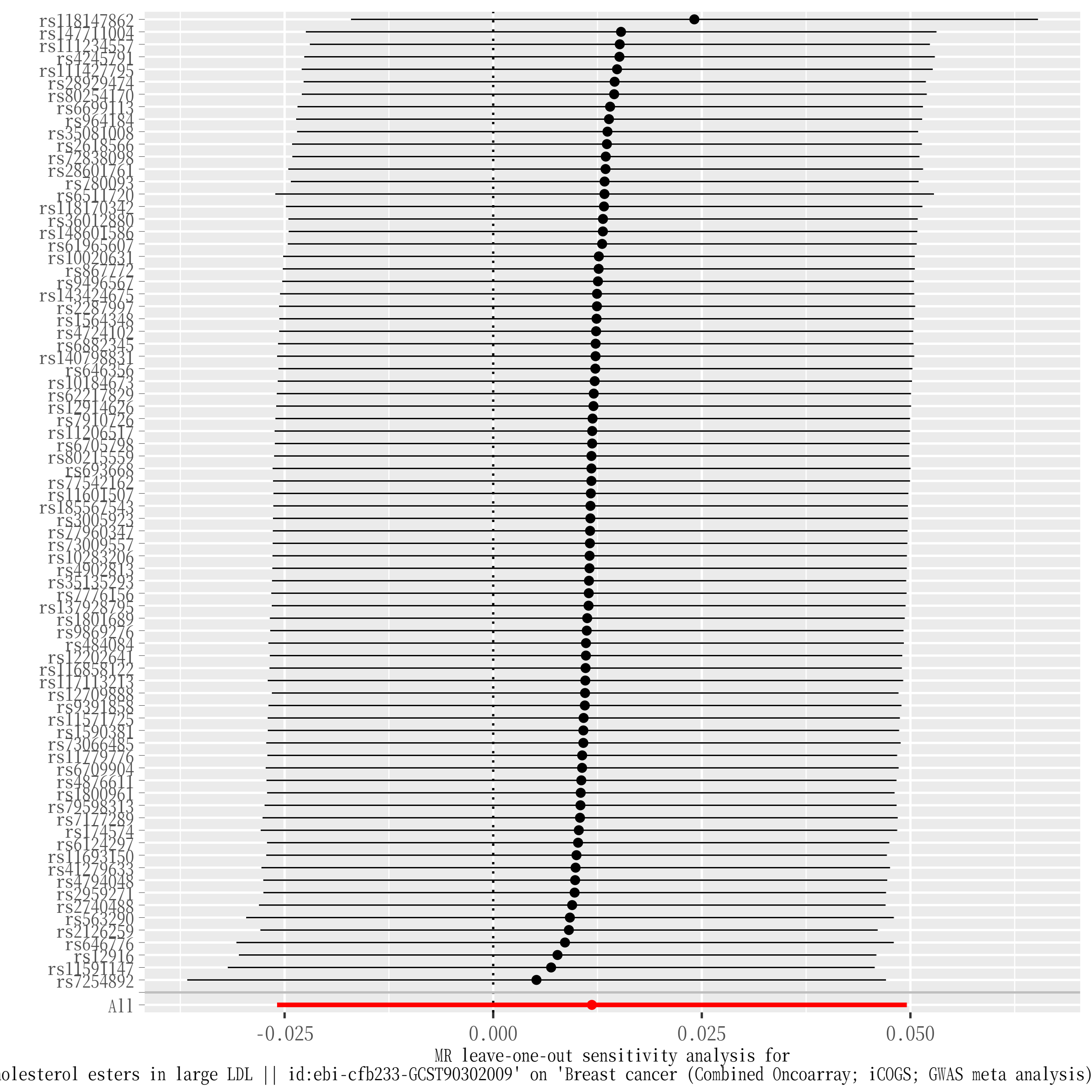

rs118147862
rs147711004
rs111234557
rs4245791
rs111427795
rs28929474
rs80254170
rs6699113
rs964184
rs35081008
rs2618566
rs72838098
rs28601761
rs780093
rs6511720
rs118170342
rs36012880
rs148601586
rs61965607
rs10020631
rs867772
rs9496567
rs143424675
rs2287997
rs1564348
rs4724102
rs6882345
rs140798831
rs646356
rs10184673
rs62217829
rs12914626
rs7910726
rs11206517
rs6705798
rs80215559
rs693668
rs77542162
rs11601507
rs185567543
rs3005923
rs77960347
rs73009557
rs10283206
rs4902813
rs35135293
rs7776156
rs137928795
rs1801689
rs9869276
rs484084
rs12202641
rs116858122
rs117113213
rs12709888
rs9321858
rs11571725
rs1590381
rs73066485
rs11779776
rs6709904
rs4876611
rs1800961
rs79598313
rs7177289
rs174574
rs6124297
rs11693150
rs41279633
rs4794048
rs2959271
rs2740488
rs563290
rs2126259
rs646776
rs12916
rs11591147
rs7254892
All
-0.025
0.000
0.025
0.050
MR leave-one-out sensitivity analysis for
olesterol esters in large LDL || id:ebi-cfb233-GCST90302009' on 'Breast cancer (Combined Oncoarray; iCOGS; GWAS meta analysis)

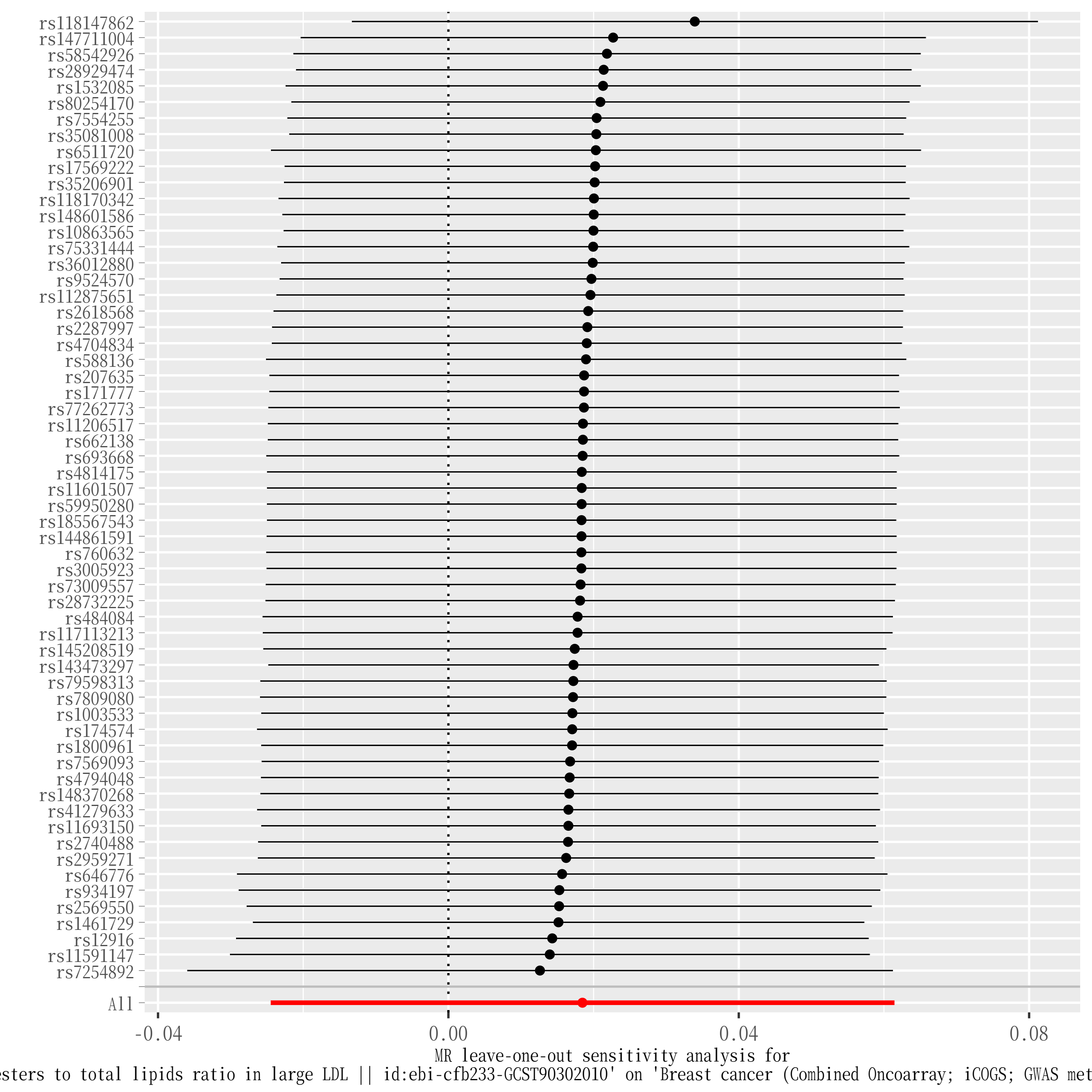

rs118147862
rs147711004
rs58542926
rs28929474
rs1532085
rs80254170
rs7554255
rs35081008
rs6511720
rs17569222
rs35206901
rs118170342
rs148601586
rs10863565
rs75331444
rs36012880
rs9524570
rs112875651
rs2618568
rs2287997
rs4704834
rs588136
rs207635
rs171777
rs77262773
rs11206517
rs662138
rs693668
rs4814175
rs11601507
rs59950280
rs185567543
rs144861591
rs760632
rs3005923
rs73009557
rs28732225
rs484084
rs117113213
rs145208519
rs143473297
rs79598313
rs7809080
rs1003533
rs174574
rs1800961
rs7569093
rs4794048
rs148370268
rs41279633
rs11693150
rs2740488
rs2959271
rs646776
rs934197
rs2569550
rs1461729
rs12916
rs11591147
rs7254892
All
-0.04
0.00
0.04
0.08
MR leave-one-out sensitivity analysis for
esters to total lipids ratio in large LDL || id:ebi-cfb233-GCST90302010' on 'Breast cancer (Combined Oncoarray; iCOGS; GWAS met

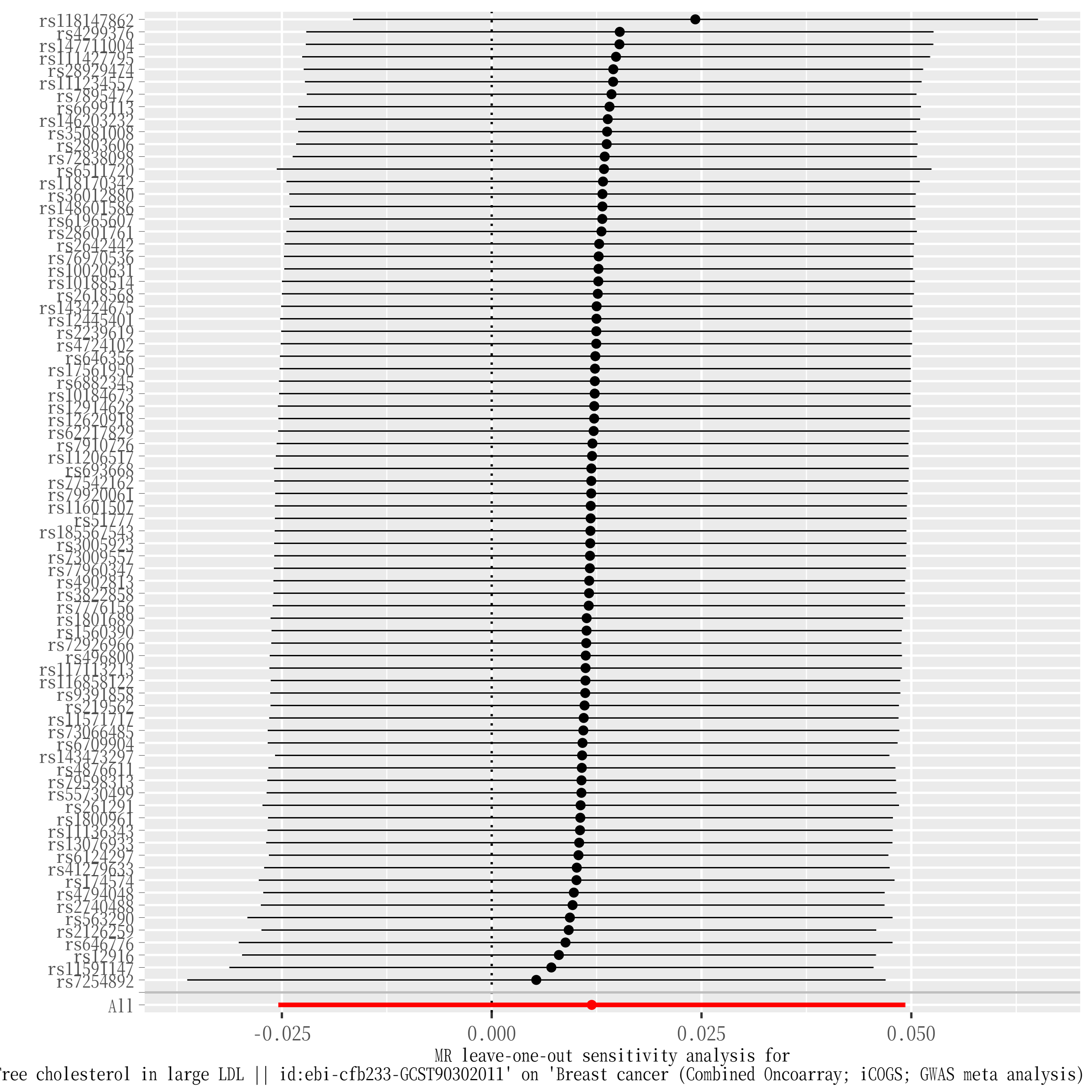
rs118147862
rs4299376
rs147711004
rs111427795
rs28929474
rs111234557
rs7895472
rs6699113
rs146203232
rs35081008
rs2803606
rs72838098
rs6511720
rs118170342
rs36012880
rs148601586
rs61965607
rs28601761
rs2642442
rs76970536
rs10020631
rs10188514
rs2618568
rs143424675
rs12445401
rs2239619
rs4724102
rs646356
rs17561950
rs6882345
rs10184673
rs12914626
rs12620918
rs62217829
rs7910726
rs11206517
rs693668
rs77542162
rs79920061
rs11601507
rs51777
rs185567543
rs3005923
rs73009557
rs77960347
rs4902813
rs3822858
rs7776156
rs1801689
rs1560390
rs72926966
rs496800
rs117113213
rs116858122
rs9391858
rs219562
rs11571717
rs73066485
rs6709904
rs143473297
rs4876611
rs79598313
rs55730499
rs261291
rs1800961
rs11136343
rs13076933
rs6124297
rs41279633
rs174574
rs4794048
rs2740488
rs563290
rs2126259
rs646776
rs12916
rs11591147
rs7254892
All
-0.025
0.000
0.025
0.050
MR leave-one-out sensitivity analysis for
ree cholesterol in large LDL || id:ebi-cfb233-GCST90302011' on 'Breast cancer (Combined Oncoarray; iCOGS; GWAS meta analysis)

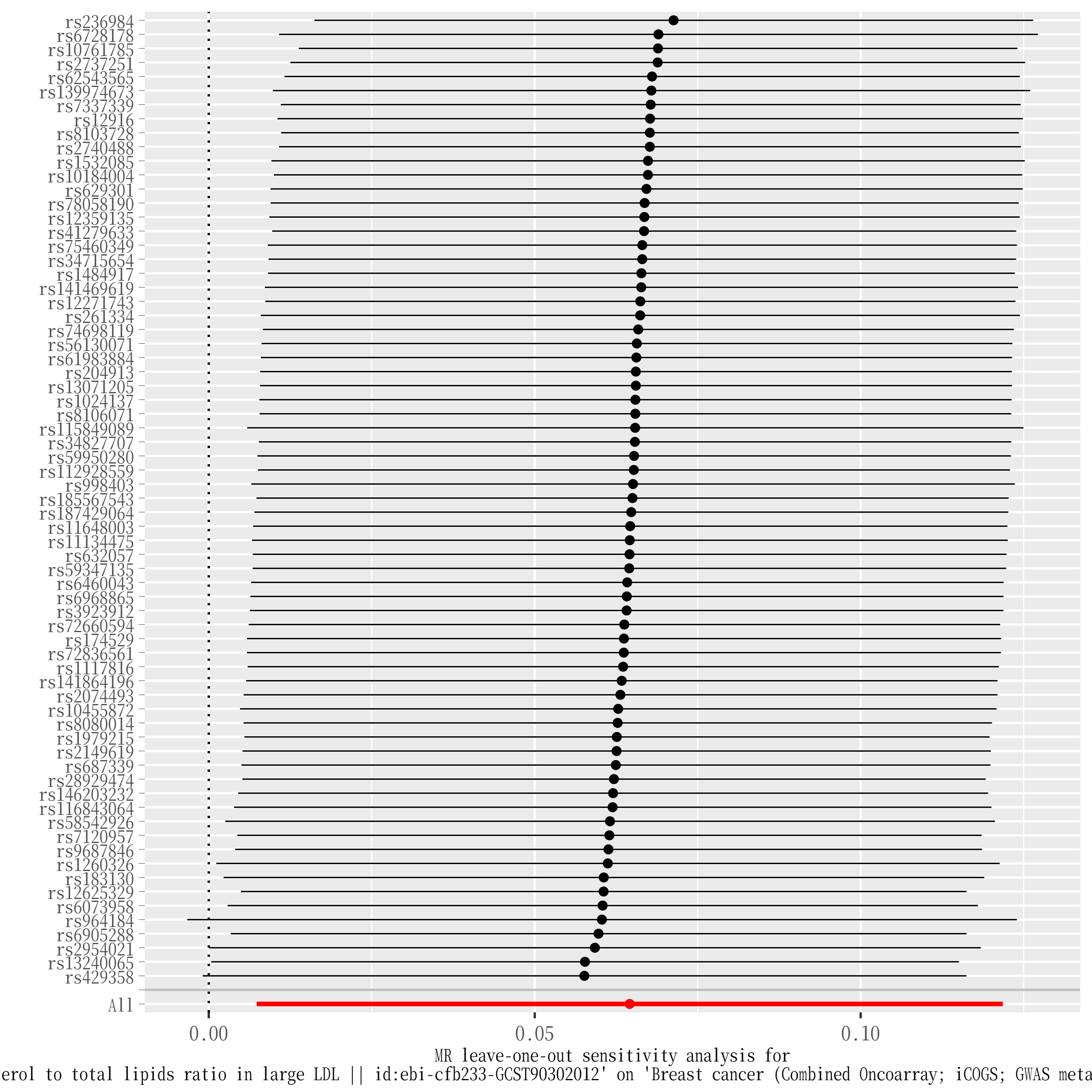

rs236984
rs6728178
rs10761785
rs2737251
rs62543565
rs139974673
rs7337339
rs12916
rs8103728
rs2740488
rs1532085
rs10184004
rs629301
rs78058190
rs12359135
rs41279633
rs75460349
rs34715654
rs1484917
rs141469619
rs12271743
rs261334
rs74698119
rs56130071
rs61983884
rs204913
rs13071205
rs1024137
rs8106071
rs115849089
rs34827707
rs59950280
rs112928559
rs998403
rs185567543
rs187429064
rs11648003
rs11134475
rs632057
rs59347135
rs6460043
rs6968865
rs3923912
rs72660594
rs174529
rs72836561
rs1117816
rs141864196
rs2074493
rs10455872
rs8080014
rs1979215
rs2149619
rs687339
rs28929474
rs146203232
rs116843064
rs58542926
rs7120957
rs9687846
rs1260326
rs183130
rs12625329
rs6073958
rs964184
rs6905288
rs2954021
rs13240065
rs429358
All
0.00
0.05
0.10
MR leave-one-out sensitivity analysis for
erol to total lipids ratio in large LDL || id:ebi-cfb233-GCST90302012' on 'Breast cancer (Combined Oncoarray; iCOGS; GWAS meta

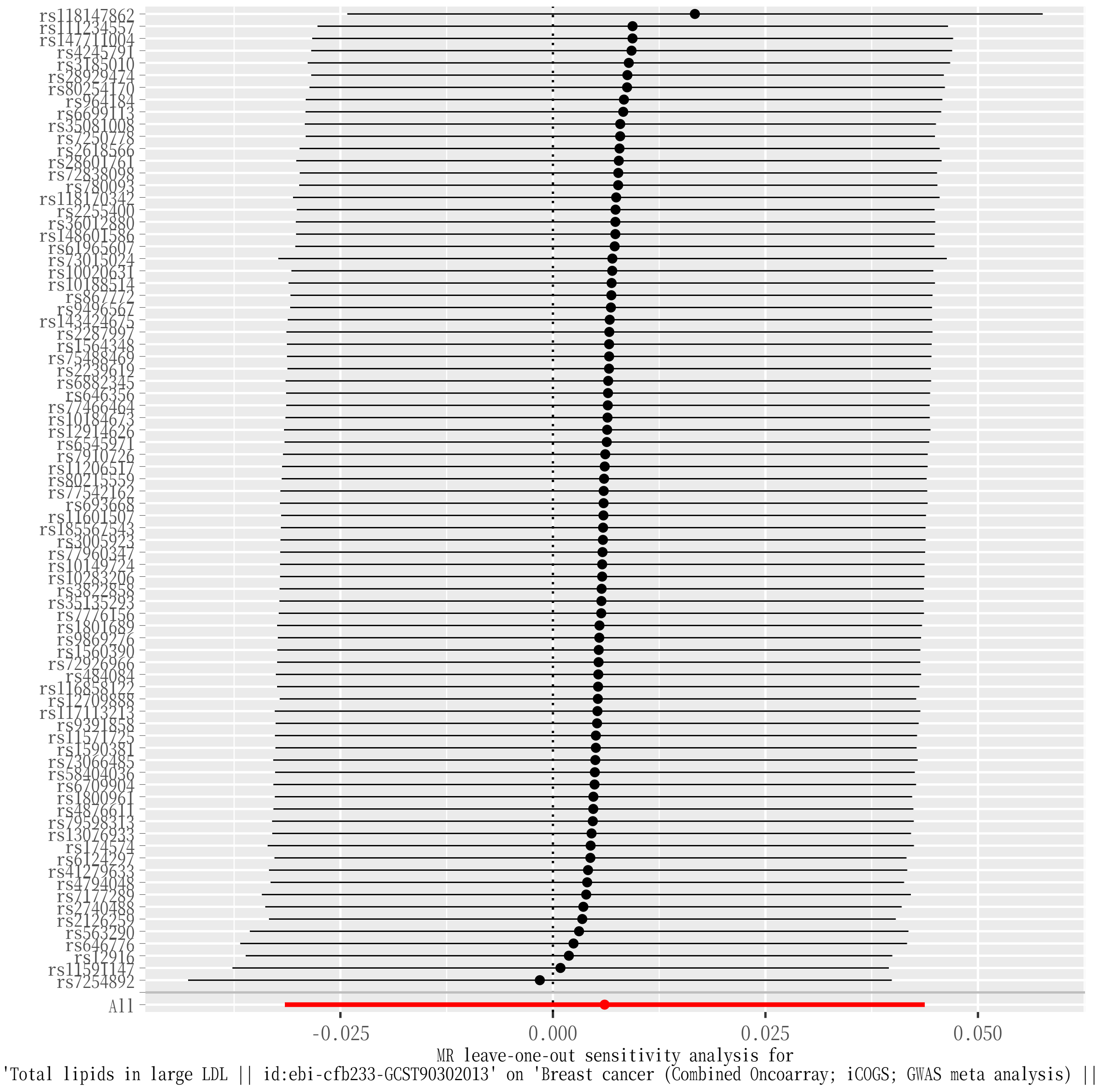

All
-0.025
0.000
0.025
0.050
MR leave-one-out sensitivity analysis for
'Total lipids in large LDL || id:ebi-cfb233-GCST90302013' on 'Breast cancer (Combined Oncoarray; iCOGS; GWAS meta analysis) ||

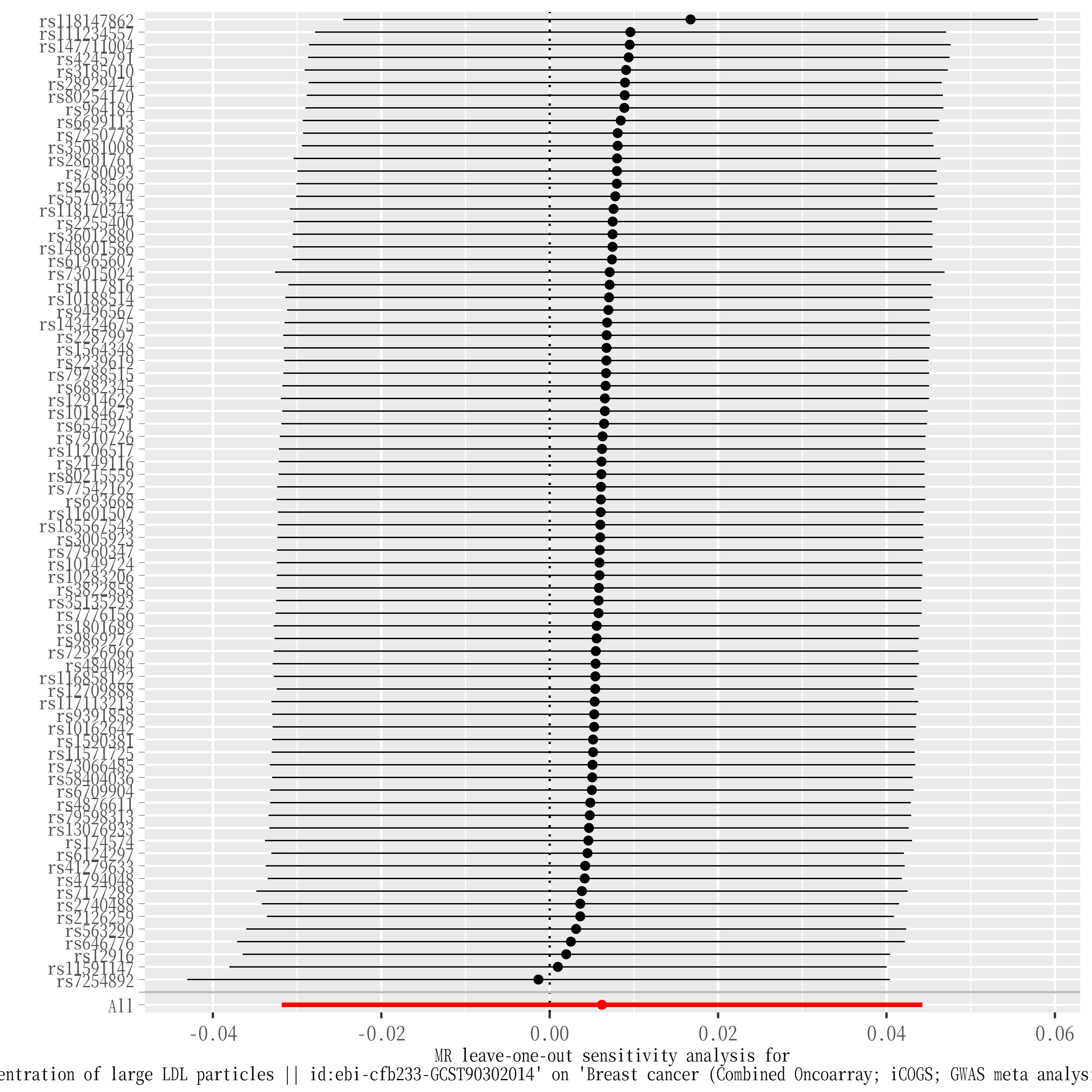

rs118147862
rs111234557
rs147711004
rs4245791
rs3185010
rs28929474
rs80254170
rs964184
rs6699113
rs7250778
rs35081008
rs28601761
rs780093
rs2618566
rs55703214
rs118170342
rs2255400
rs36012880
rs148601586
rs61965607
rs73015024
rs1117816
rs10188514
rs9496567
rs143424675
rs2287997
rs1564348
rs2239619
rs79788515
rs6882345
rs12914626
rs10184673
rs6545971
rs7910726
rs11206517
rs2149116
rs80215559
rs77542162
rs693668
rs11601507
rs185567543
rs3005923
rs77960347
rs10149724
rs10283206
rs3822858
rs35135293
rs7776156
rs1801689
rs9869276
rs72926966
rs484084
rs116858122
rs12709888
rs117113213
rs9391858
rs10162642
rs1590381
rs11571725
rs73066485
rs58404036
rs6709904
rs4876611
rs79528313
rs13076933
rs174574
rs6124297
rs41279633
rs4794048
rs7177289
rs2740488
rs2126259
rs563290
rs646776
rs12916
rs115911147
rs7254892
All
-0.04
-0.02
0.00
0.02
0.04
0.06
MR leave-one-out sensitivity analysis for
entration of large LDL particles || id:ebi-cfb233-GCST90302014' on 'Breast cancer (Combined Oncoarray; iCOGS; GWAS meta analys

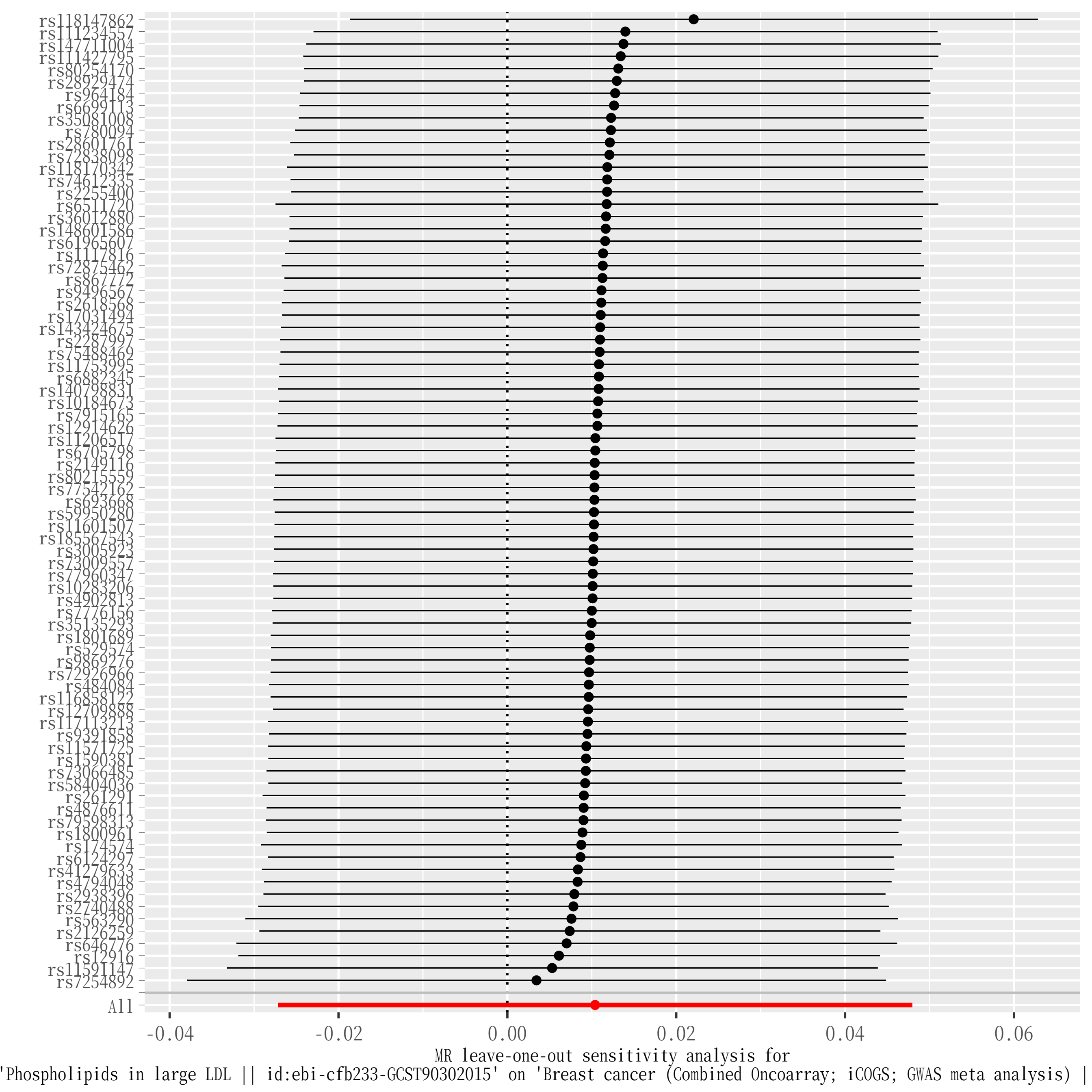
rs118147862
rs111234557
rs147711004
rs111427795
rs80254170
rs28929474
rs964184
rs6699113
rs35081008
rs780094
rs28601761
rs72838098
rs118170342
rs74612335
rs2255400
rs6511720
rs36012880
rs148601586
rs61965607
rs1117816
rs72875462
rs867772
rs9496567
rs2618568
rs17031494
rs143424675
rs2287997
rs75488469
rs11753995
rs6882345
rs140798831
rs10184673
rs7915165
rs12914626
rs11206517
rs6705798
rs2149116
rs80215559
rs77542162
rs693668
rs59950280
rs11601507
rs185567543
rs3005923
rs73009557
rs77960347
rs10283206
rs4902813
rs7776156
rs35135293
rs1801689
rs529574
rs9869276
rs72926966
rs484084
rs116858122
rs12709888
rs117113213
rs9321858
rs11571725
rs1590381
rs73066485
rs58404036
rs261291
rs4876611
rs79598313
rs1800961
rs174574
rs6124297
rs41279633
rs4794048
rs2938396
rs2740488
rs563290
rs2126259
rs646776
rs12916
rs11591147
rs7254892
All
-0.04
-0.02
0.00
0.02
0.04
0.06
MR leave-one-out sensitivity analysis for
'Phospholipids in large LDL || id:ebi-cfb233-GCST90302015' on 'Breast cancer (Combined Oncoarray; iCOGS; GWAS meta analysis) |

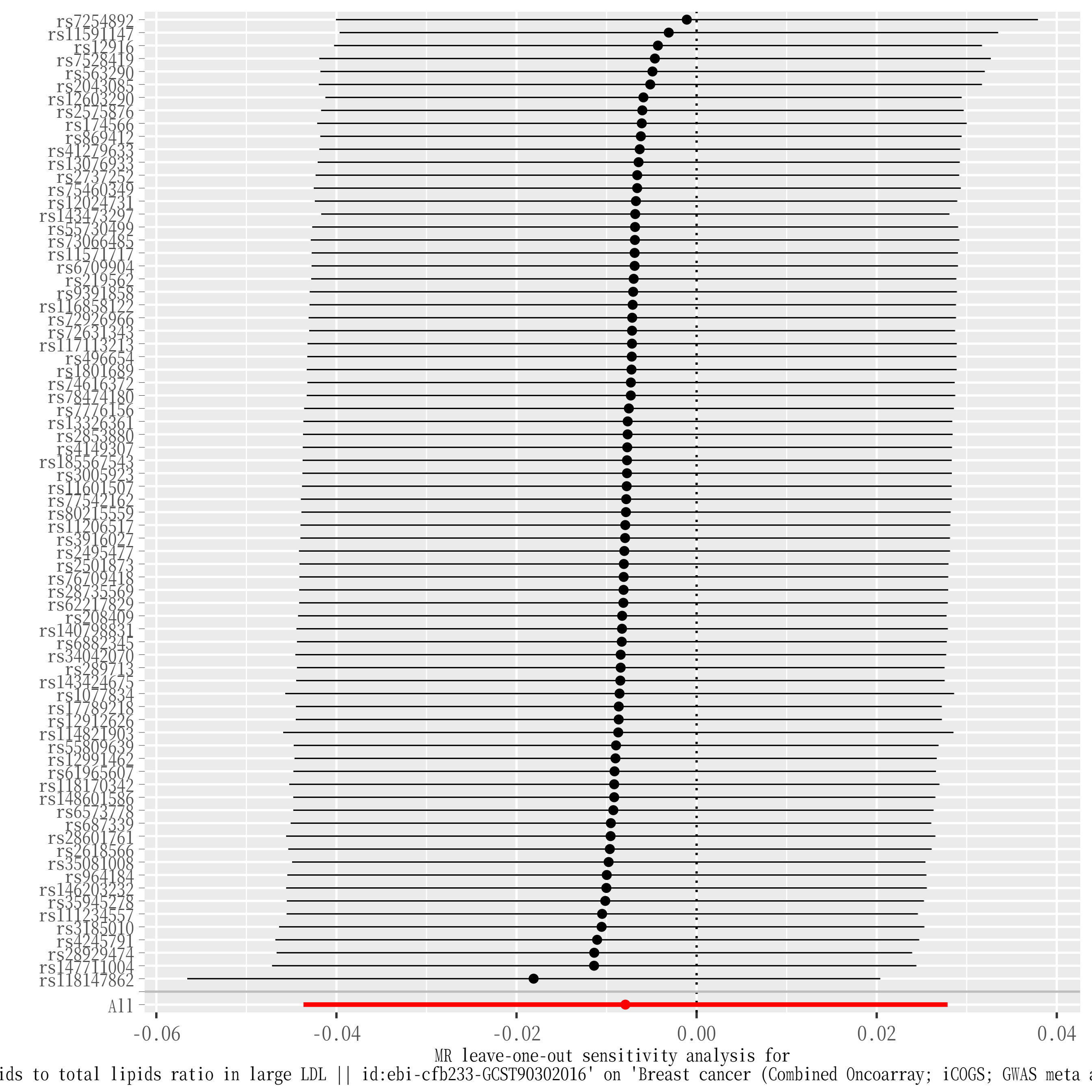
rs7254892
rs11591147
rs12916
rs7528419
rs563290
rs2043085
rs12603290
rs2575876
rs174566
rs869412
rs41279633
rs13076933
rs2737252
rs75460349
rs12024731
rs143473297
rs55730499
rs73066485
rs11571717
rs6709904
rs219562
rs9391858
rs116858122
rs72926966
rs72631343
rs117113213
rs496654
rs1801689
rs74616372
rs78474180
rs7776156
rs13326361
rs2853880
rs4149307
rs185567543
rs3005923
rs11601507
rs77542162
rs80215559
rs11206517
rs3916027
rs2495477
rs2501873
rs76709418
rs28735569
rs62217829
rs208409
rs140798831
rs6882345
rs34042070
rs289713
rs143424675
rs1077834
rs17789218
rs12912626
rs114821903
rs55809639
rs12991462
rs61965607
rs118170342
rs148601586
rs6573778
rs687339
rs28601761
rs2618566
rs35081008
rs964184
rs146203232
rs35945278
rs111234557
rs3185010
rs4245791
rs28929474
rs147711004
rs118147862
All
-0.06
-0.04
-0.02
0.00
0.02
0.04
MR leave-one-out sensitivity analysis for
ids to total lipids ratio in large LDL || id:ebi-cfb233-GCST90302016' on 'Breast cancer (Combined Oncoarray; iCOGS; GWAS meta a

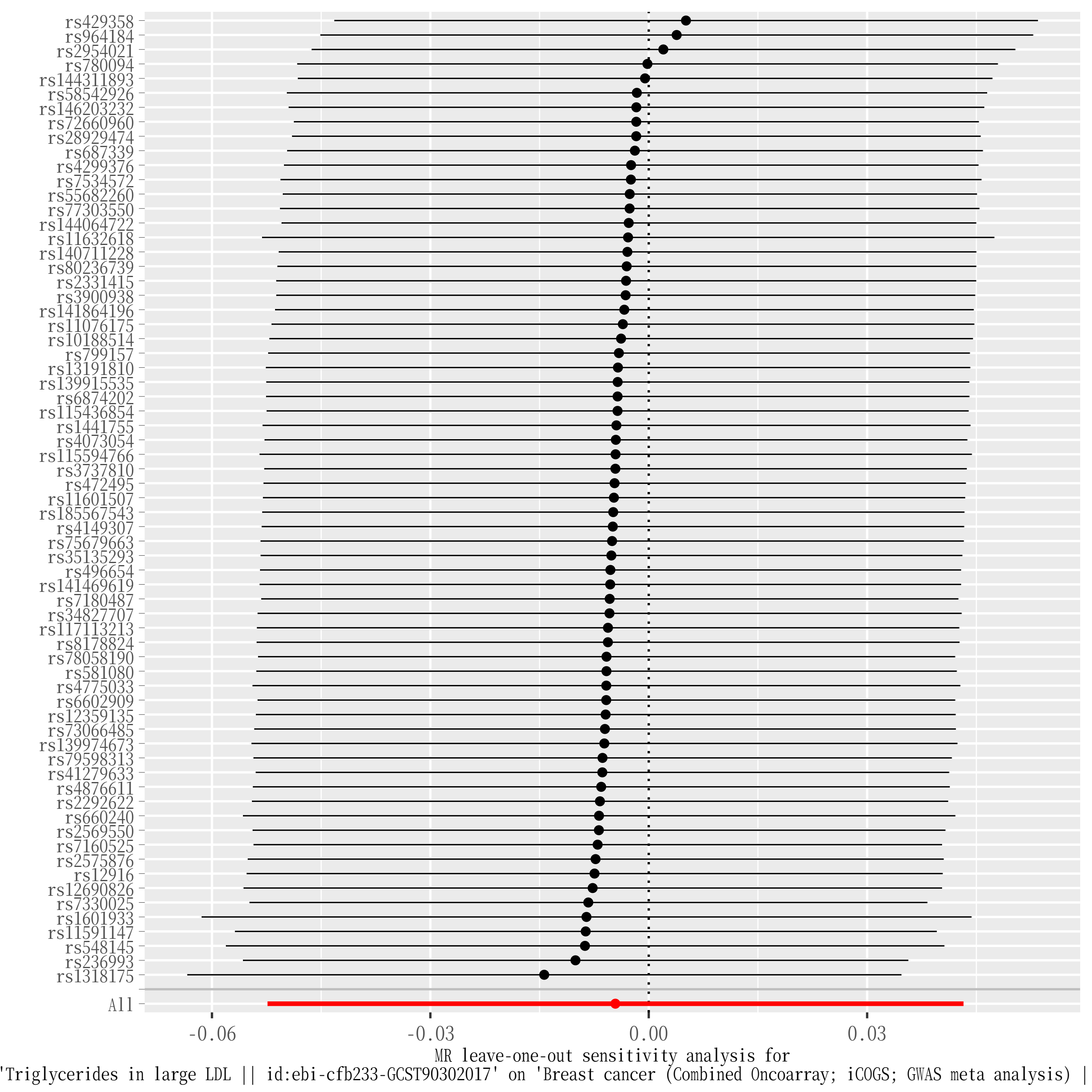

rs429358
rs964184
rs2954021
rs780094
rs144311893
rs58542926
rs146203232
rs72660960
rs28929474
rs687339
rs4299376
rs7534572
rs55682260
rs77303550
rs144064722
rs11632618
rs140711228
rs80236739
rs2331415
rs3900938
rs141864196
rs11076175
rs10188514
rs799157
rs13191810
rs139915535
rs6874202
rs115436854
rs1441755
rs4073054
rs115594766
rs3737810
rs472495
rs11601507
rs185567543
rs4149307
rs75679663
rs35135293
rs496654
rs141469619
rs7180487
rs34827707
rs117113213
rs8178824
rs78058190
rs581080
rs4775033
rs6602909
rs12359135
rs73066485
rs139974673
rs79598313
rs41279633
rs4876611
rs2292622
rs660240
rs2569550
rs7160525
rs2575876
rs12916
rs12690826
rs7330025
rs1601933
rs11591147
rs548145
rs236993
rs1318175
All
-0.06
-0.03
0.00
0.03
MR leave-one-out sensitivity analysis for
'Triglycerides in large LDL || id:ebi-cfb233-GCST90302017' on 'Breast cancer (Combined Oncoarray; iCOGS; GWAS meta analysis) |

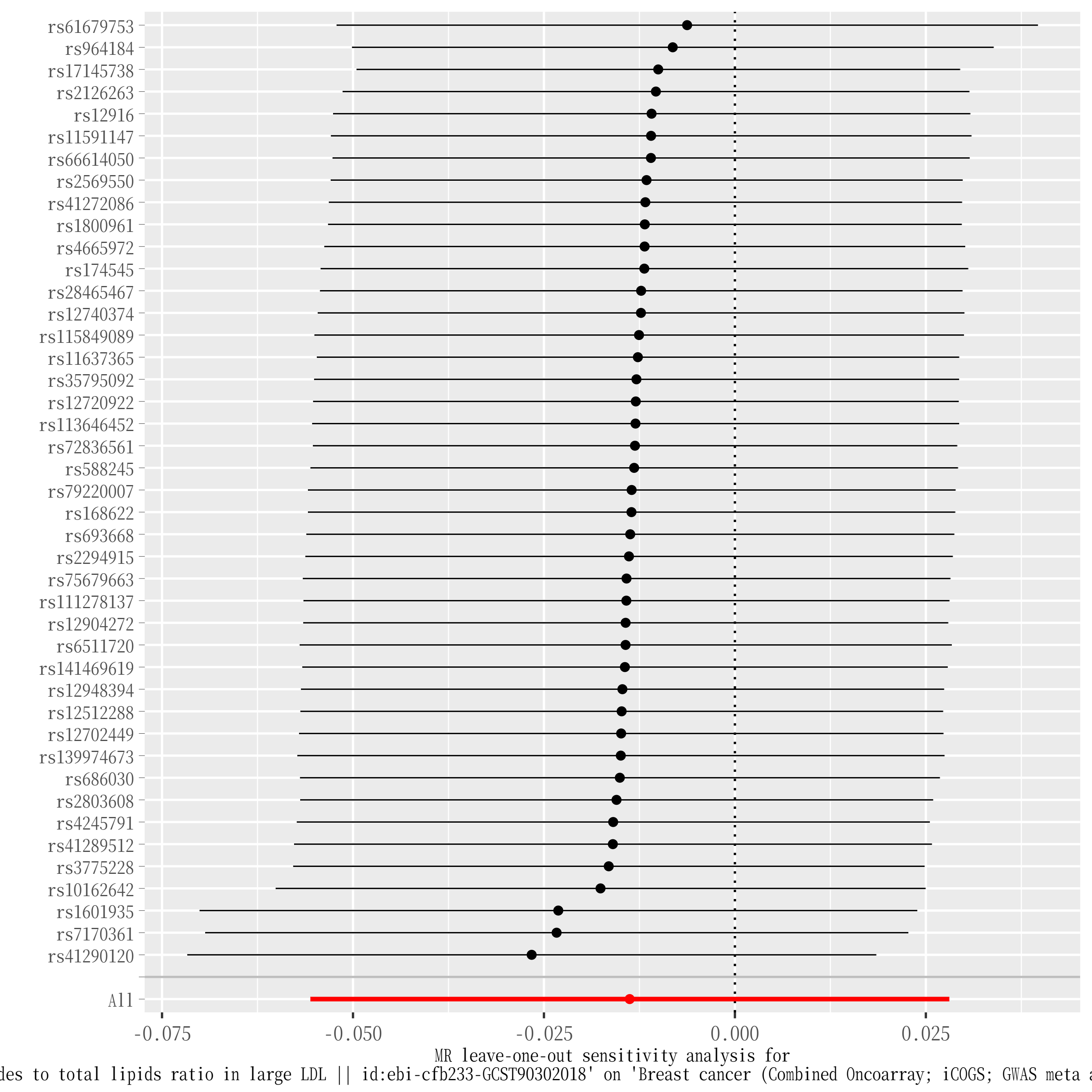
rs61679753
rs964184
rs17145738
rs2126263
rs12916
rs11591147
rs66614050
rs2569550
rs41272086
rs1800961
rs4665972
rs174545
rs28465467
rs12740374
rs115849089
rs11637365
rs35795092
rs12720922
rs113646452
rs72836561
rs588245
rs79220007
rs168622
rs693668
rs2294915
rs75679663
rs111278137
rs12904272
rs6511720
rs141469619
rs12948394
rs12512288
rs12702449
rs139974673
rs686030
rs2803608
rs4245791
rs41289512
rs3775228
rs10162642
rs1601935
rs7170361
rs41290120
All
-0.075
-0.050
-0.025
0.000
0.025
MR leave-one-out sensitivity analysis for
des to total lipids ratio in large LDL || id:ebi-cfb233-GCST90302018' on 'Breast cancer (Combined Oncoarray; iCOGS; GWAS meta a

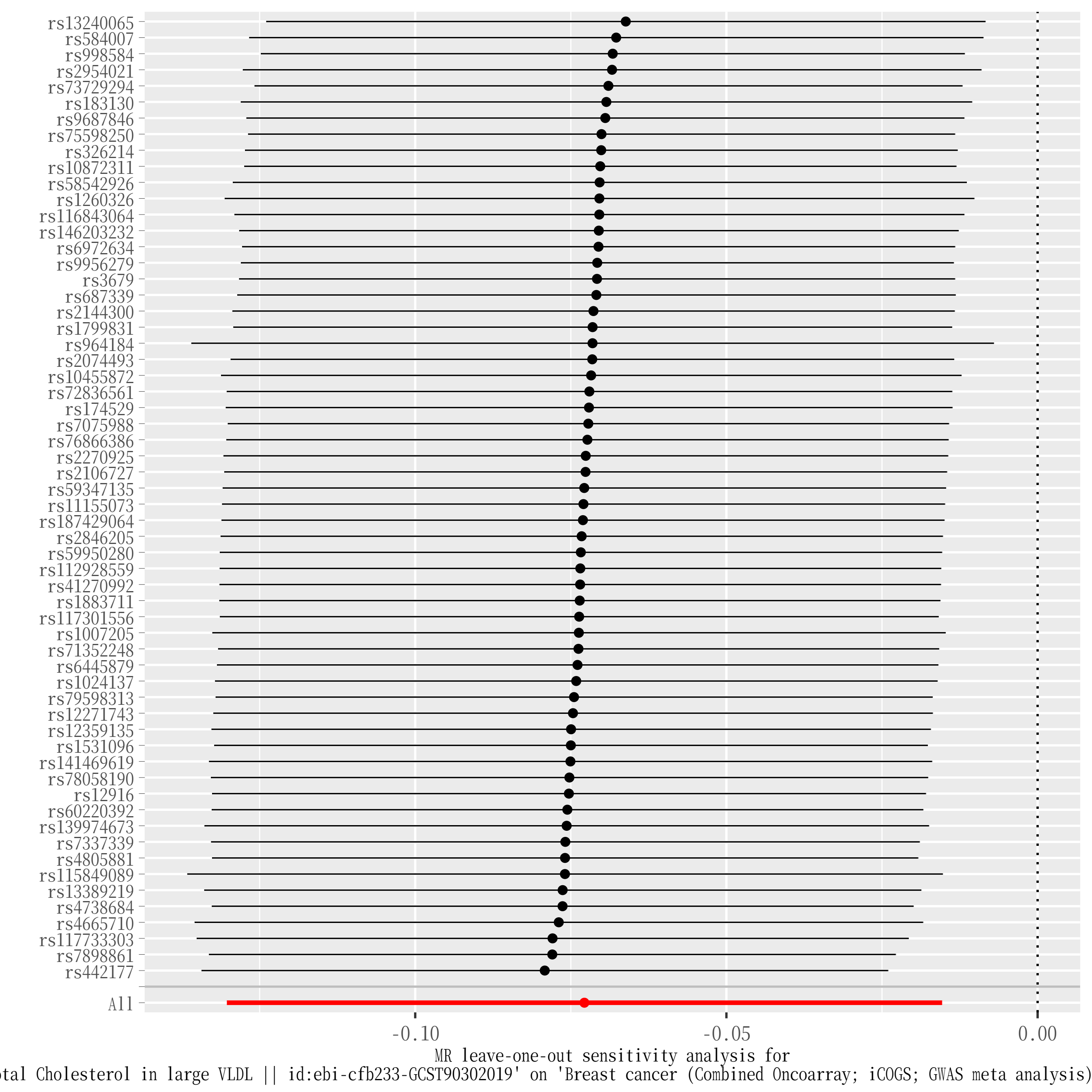

rs13240065
rs584007
rs998584
rs2954021
rs73729294
rs183130
rs9687846
rs75598250
rs326214
rs10872311
rs58542926
rs1260326
rs116843064
rs146203232
rs6972634
rs9956279
rs3679
rs687339
rs2144300
rs1799831
rs964184
rs2074493
rs10455872
rs72836561
rs174529
rs7075988
rs76866386
rs2270925
rs2106727
rs59347135
rs11155073
rs187429064
rs2846205
rs59950280
rs112928559
rs41270992
rs1883711
rs117301556
rs1007205
rs71352248
rs6445879
rs1024137
rs79598313
rs12271743
rs12359135
rs1531096
rs141469619
rs78058190
rs12916
rs60220392
rs139974673
rs7337339
rs4805881
rs115849089
rs13389219
rs4738684
rs4665710
rs117733303
rs7898861
rs442177
All
-0.10
-0.05
0.00
MR leave-one-out sensitivity analysis for
otal Cholesterol in large VLDL || id:ebi-cfb233-GCST90302019' on 'Breast cancer (Combined Oncoarray; iCOGS; GWAS meta analysis)

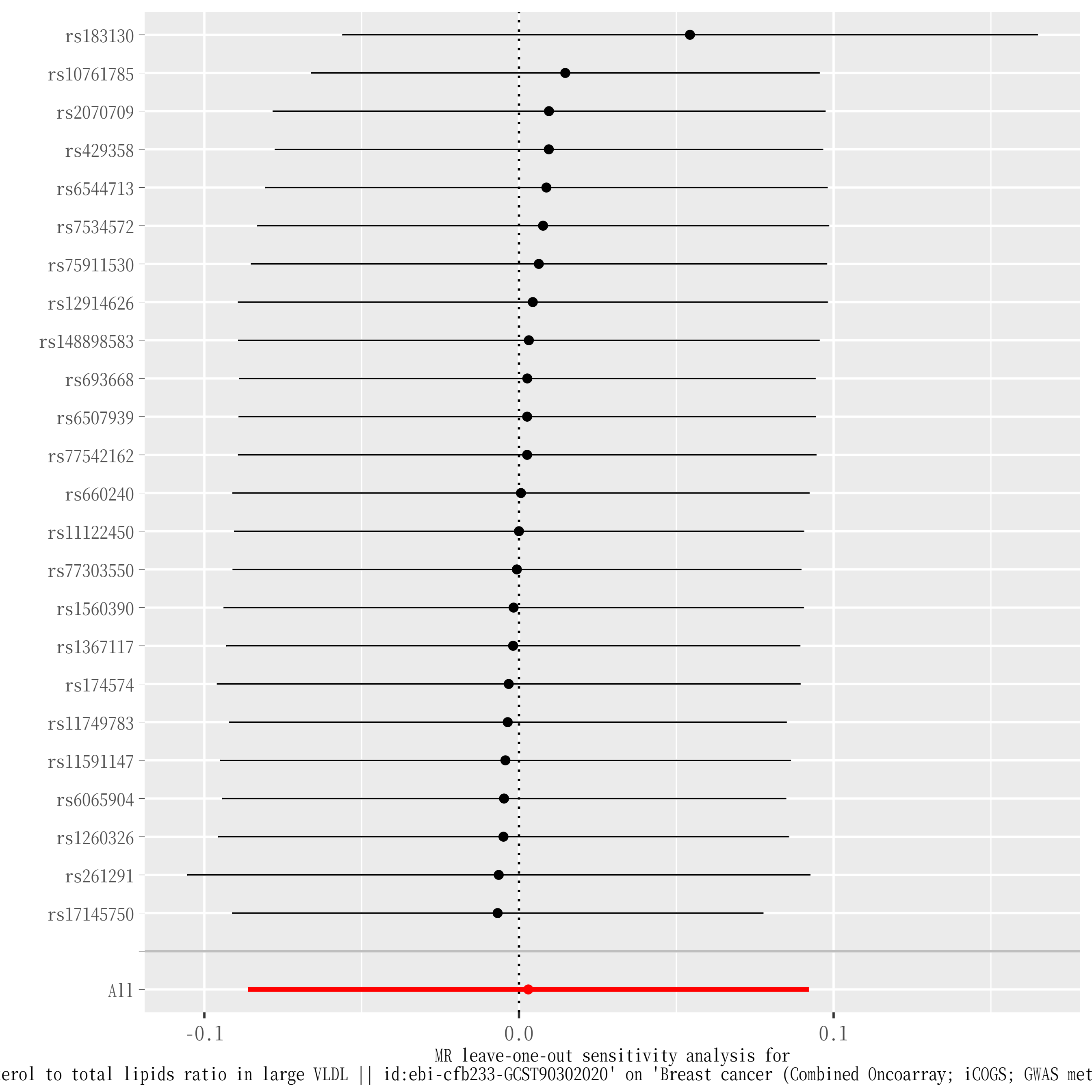

rs183130
rs10761785
rs2070709
rs429358
rs6544713
rs7534572
rs75911530
rs12914626
rs148898583
rs693668
rs6507939
rs77542162
rs660240
rs11122450
rs77303550
rs1560390
rs1367117
rs174574
rs11749783
rs11591147
rs6065904
rs1260326
rs261291
rs17145750
All
-0.1
0.0
0.1
MR leave-one-out sensitivity analysis for
erol to total lipids ratio in large VLDL || id:ebi-cfb233-GCST90302020' on 'Breast cancer (Combined Oncoarray; iCOGS; GWAS met

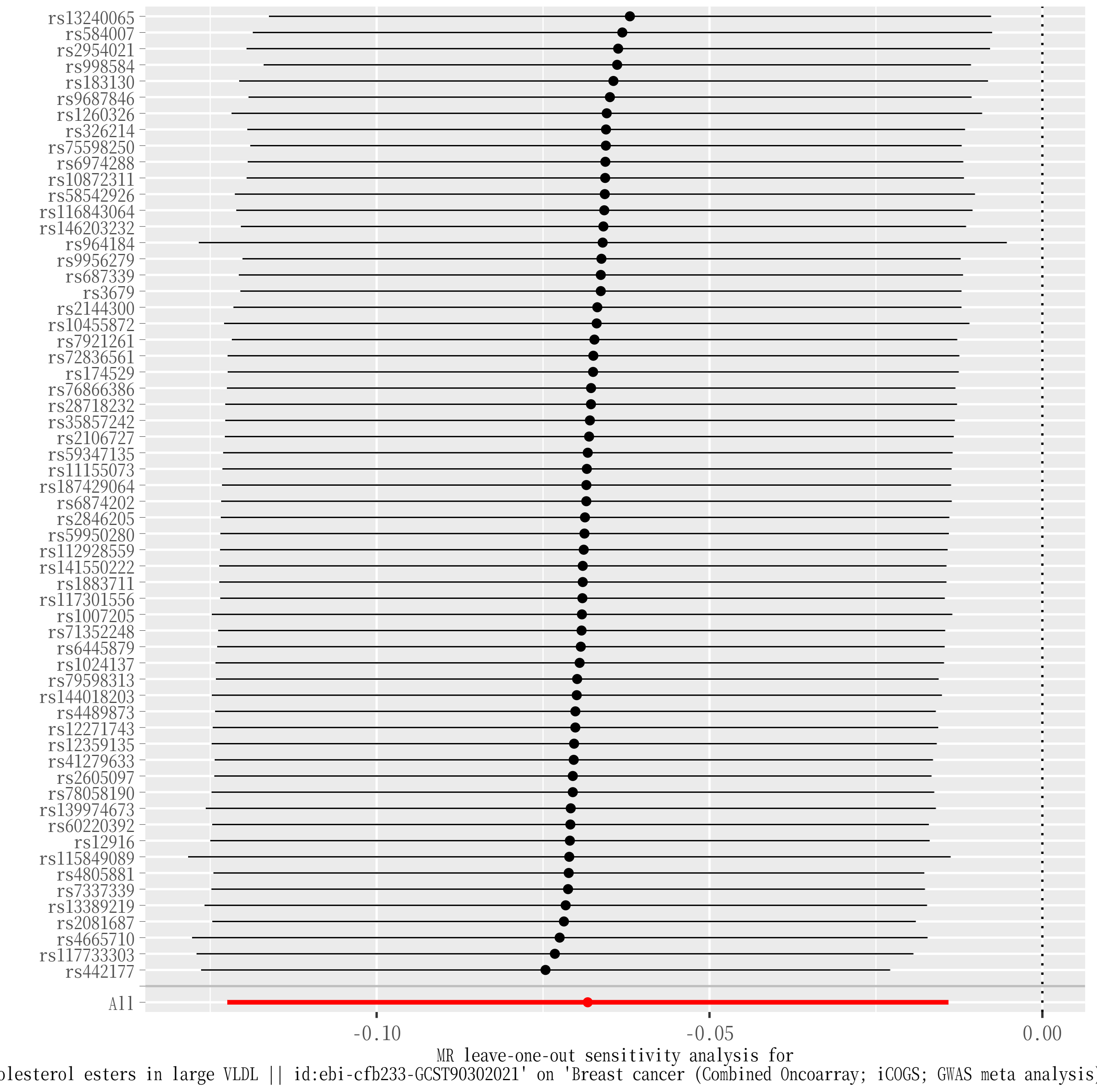
rs13240065
rs584007
rs2954021
rs998584
rs183130
rs9687846
rs1260326
rs326214
rs75598250
rs6974288
rs10872311
rs58542926
rs116843064
rs146203232
rs964184
rs9956279
rs687339
rs3679
rs2144300
rs10455872
rs7921261
rs72836561
rs174529
rs76866386
rs28718232
rs35857242
rs2106727
rs59347135
rs11155073
rs187429064
rs6874202
rs2846205
rs59950280
rs112928559
rs141550222
rs1883711
rs117301556
rs1007205
rs71352248
rs6445879
rs1024137
rs79598313
rs144018203
rs4489873
rs12271743
rs12359135
rs41279633
rs2605097
rs78058190
rs139974673
rs60220392
rs12916
rs115849089
rs4805881
rs7337339
rs13389219
rs2081687
rs4665710
rs117733303
rs442177
All
-0.10
-0.05
0.00
MR leave-one-out sensitivity analysis for
olesterol esters in large VLDL || id:ebi-cfb233-GCST90302021' on 'Breast cancer (Combined Oncoarray; iCOGS; GWAS meta analysis)

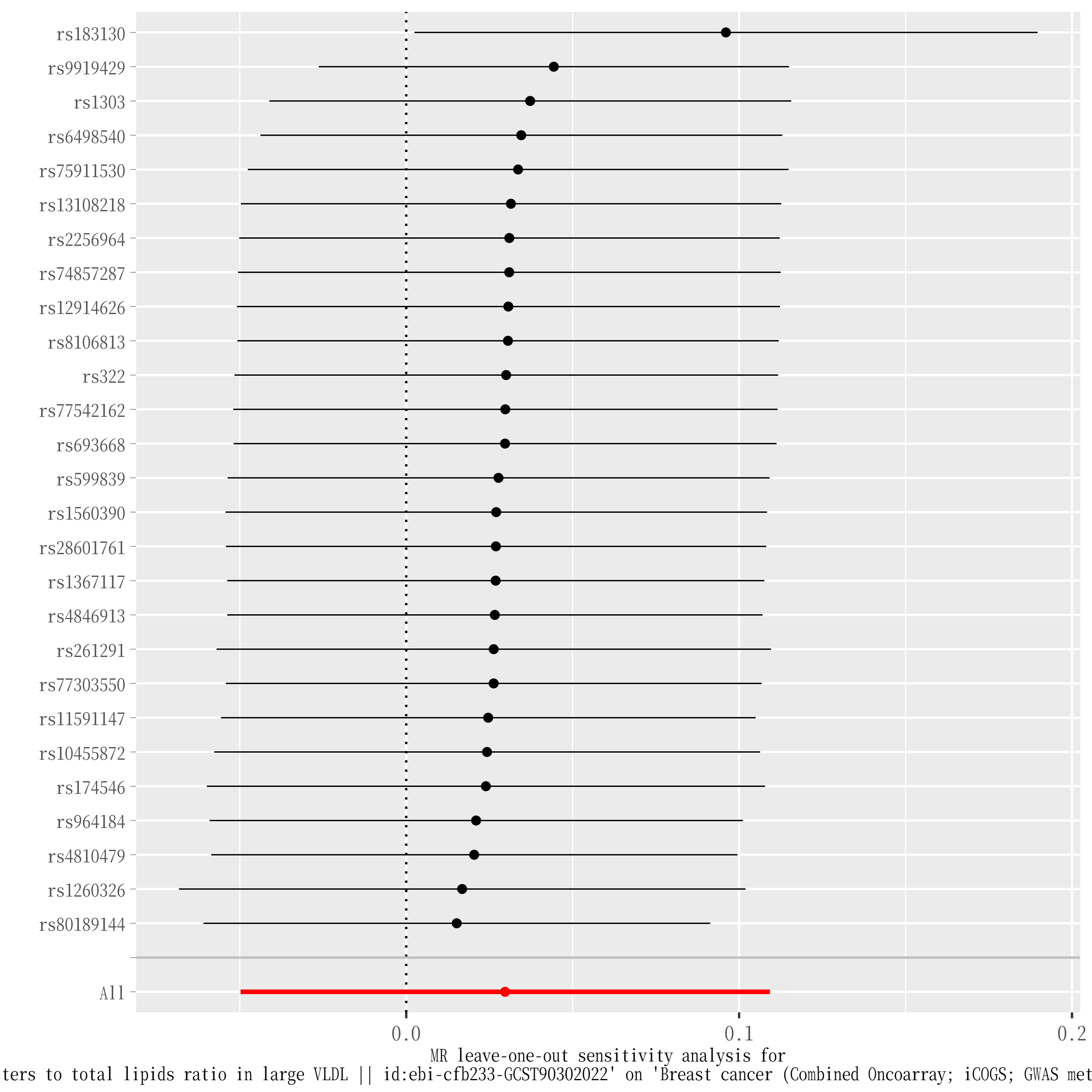

rs183130
rs9919429
rs1303
rs6498540
rs75911530
rs13108218
rs2256964
rs74857287
rs12914626
rs8106813
rs322
rs77542162
rs693668
rs599839
rs1560390
rs28601761
rs1367117
rs4846913
rs261291
rs77303550
rs11591147
rs10455872
rs174546
rs964184
rs4810479
rs1260326
rs80189144
All
0.0
0.1
0.2
MR leave-one-out sensitivity analysis for
ters to total lipids ratio in large VLDL || id:ebi-cfb233-GCST90302022' on 'Breast cancer (Combined Oncoarray; iCOGS; GWAS met

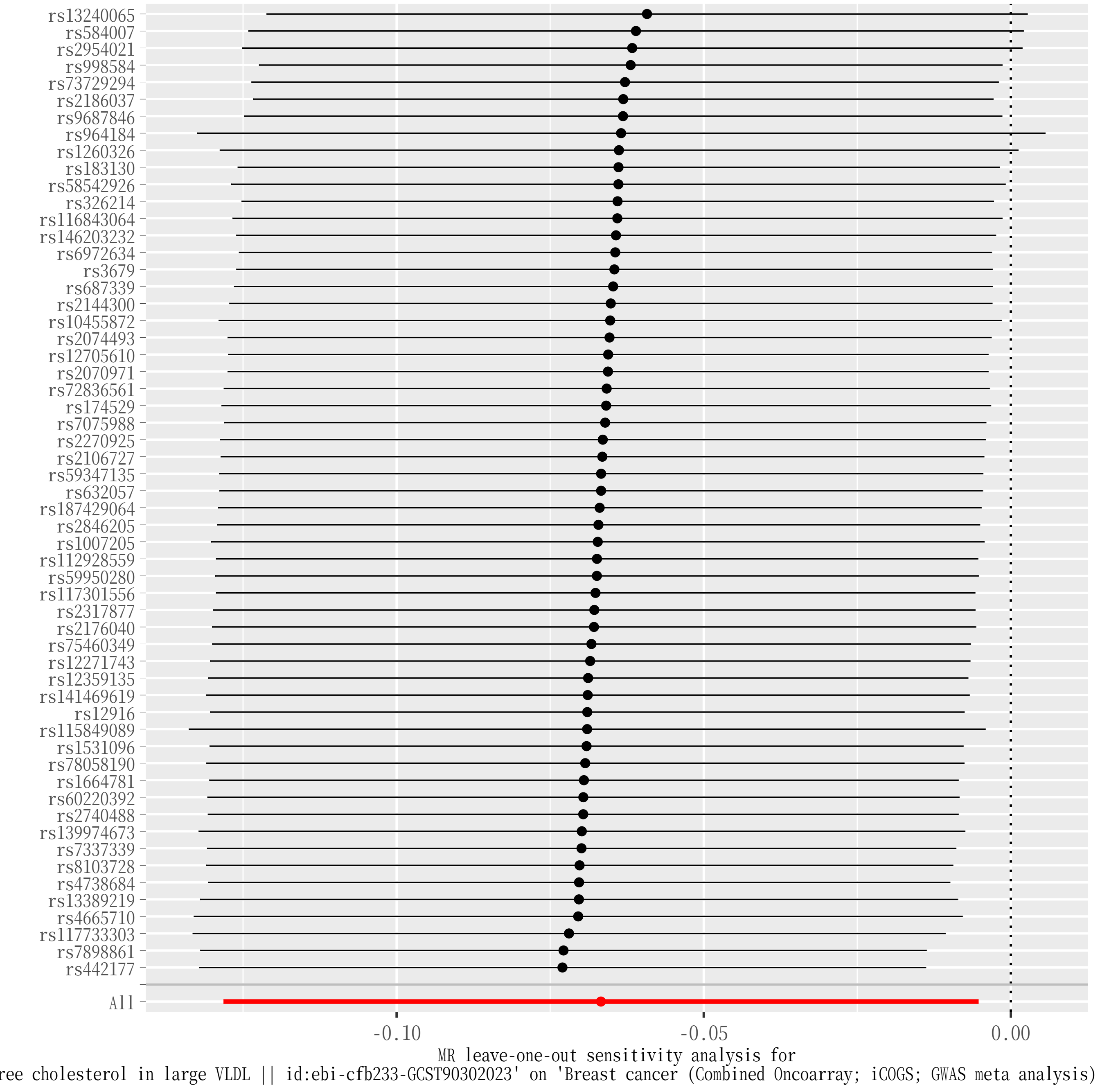
rs13240065
rs584007
rs2954021
rs998584
rs73729294
rs2186037
rs9687846
rs964184
rs1260326
rs183130
rs58542926
rs326214
rs116843064
rs146203232
rs6972634
rs3679
rs687339
rs2144300
rs10455872
rs2074493
rs12705610
rs2070971
rs72836561
rs174529
rs7075988
rs2270925
rs2106727
rs59347135
rs632057
rs187429064
rs2846205
rs1007205
rs112928559
rs59950280
rs117301556
rs2317877
rs2176040
rs75460349
rs12271743
rs12359135
rs141469619
rs12916
rs115849089
rs1531096
rs78058190
rs1664781
rs60220392
rs2740488
rs139974673
rs7337339
rs8103728
rs4738684
rs13389219
rs4665710
rs117733303
rs7898861
rs442177
All
-0.10
-0.05
0.00
MR leave-one-out sensitivity analysis for
ree cholesterol in large VLDL || id:ebi-cfb233-GCST90302023' on 'Breast cancer (Combined Oncoarray; iCOGS; GWAS meta analysis)

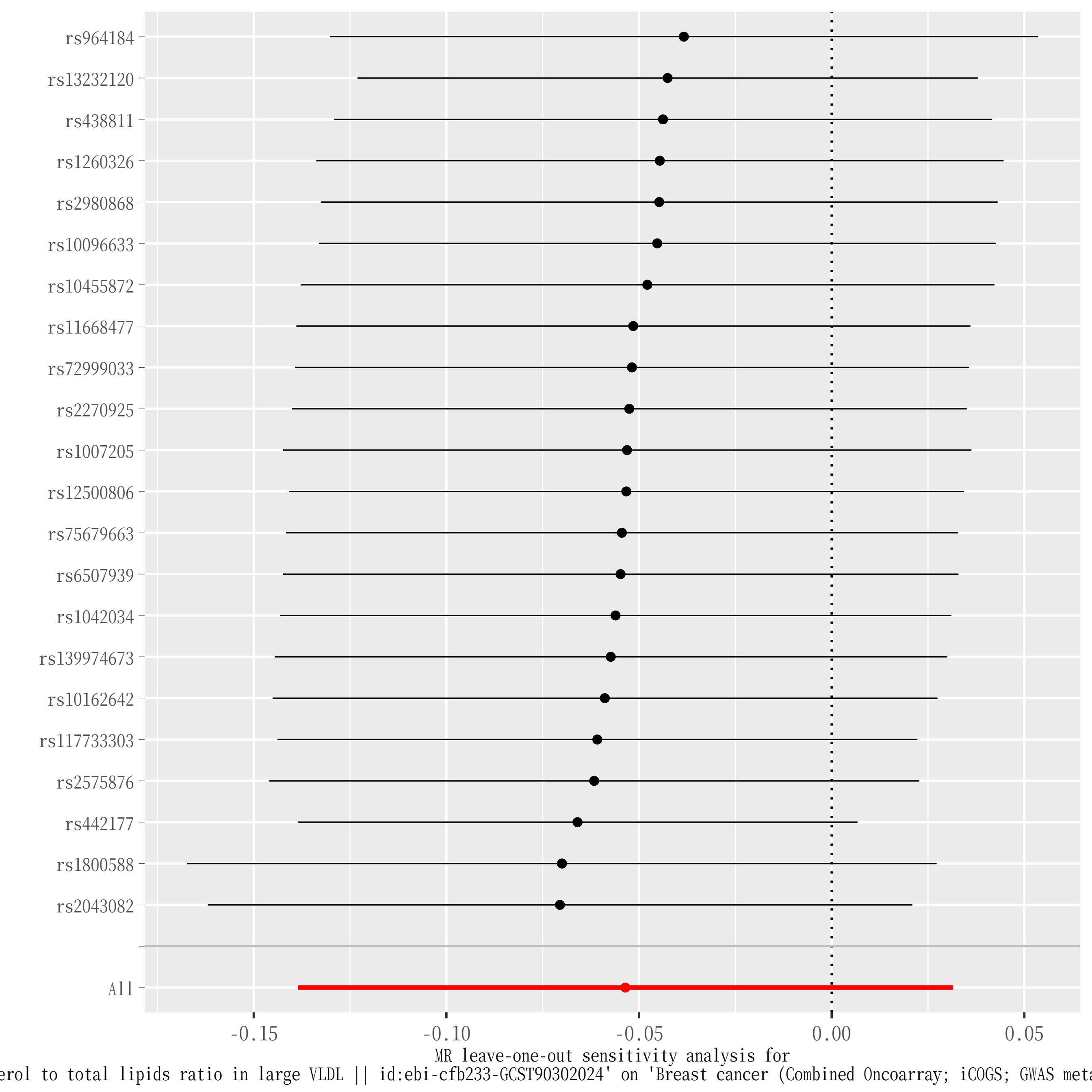
rs964184
rs13232120
rs438811
rs1260326
rs2980868
rs10096633
rs10455872
rs11668477
rs72999033
rs2270925
rs1007205
rs12500806
rs75679663
rs6507939
rs1042034
rs139974673
rs10162642
rs117733303
rs2575876
rs442177
rs1800588
rs2043082
All
-0.15
-0.10
-0.05
0.00
0.05
MR leave-one-out sensitivity analysis for
erol to total lipids ratio in large VLDL || id:ebi-cfb233-GCST90302024' on 'Breast cancer (Combined Oncoarray; iCOGS; GWAS meta

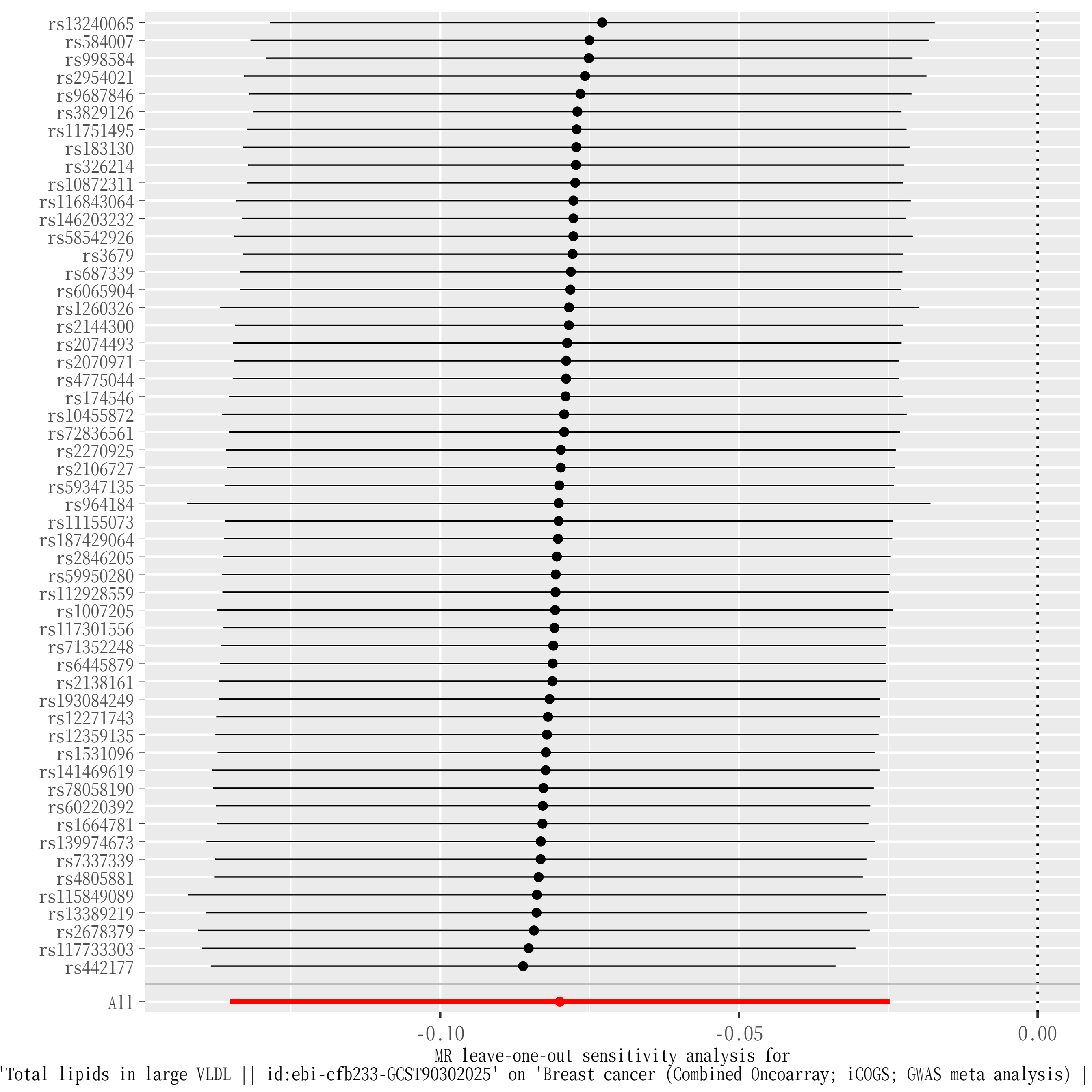
rs13240065
rs584007
rs998584
rs2954021
rs9687846
rs3829126
rs11751495
rs183130
rs326214
rs10872311
rs116843064
rs146203232
rs58542926
rs3679
rs687339
rs6065904
rs1260326
rs2144300
rs2074493
rs2070971
rs4775044
rs174546
rs10455872
rs72836561
rs2270925
rs2106727
rs59347135
rs964184
rs11155073
rs187429064
rs2846205
rs59950280
rs112928559
rs1007205
rs117301556
rs71352248
rs6445879
rs2138161
rs193084249
rs12271743
rs12359135
rs1531096
rs141469619
rs78058190
rs60220392
rs1664781
rs139974673
rs7337339
rs4805881
rs115849089
rs13389219
rs2678379
rs117733303
rs442177
All
-0.10
-0.05
0.00
MR leave-one-out sensitivity analysis for
'Total lipids in large VLDL || id:ebi-cfb233-GCST90302025' on 'Breast cancer (Combined Oncoarray; iCOGS; GWAS meta analysis) |

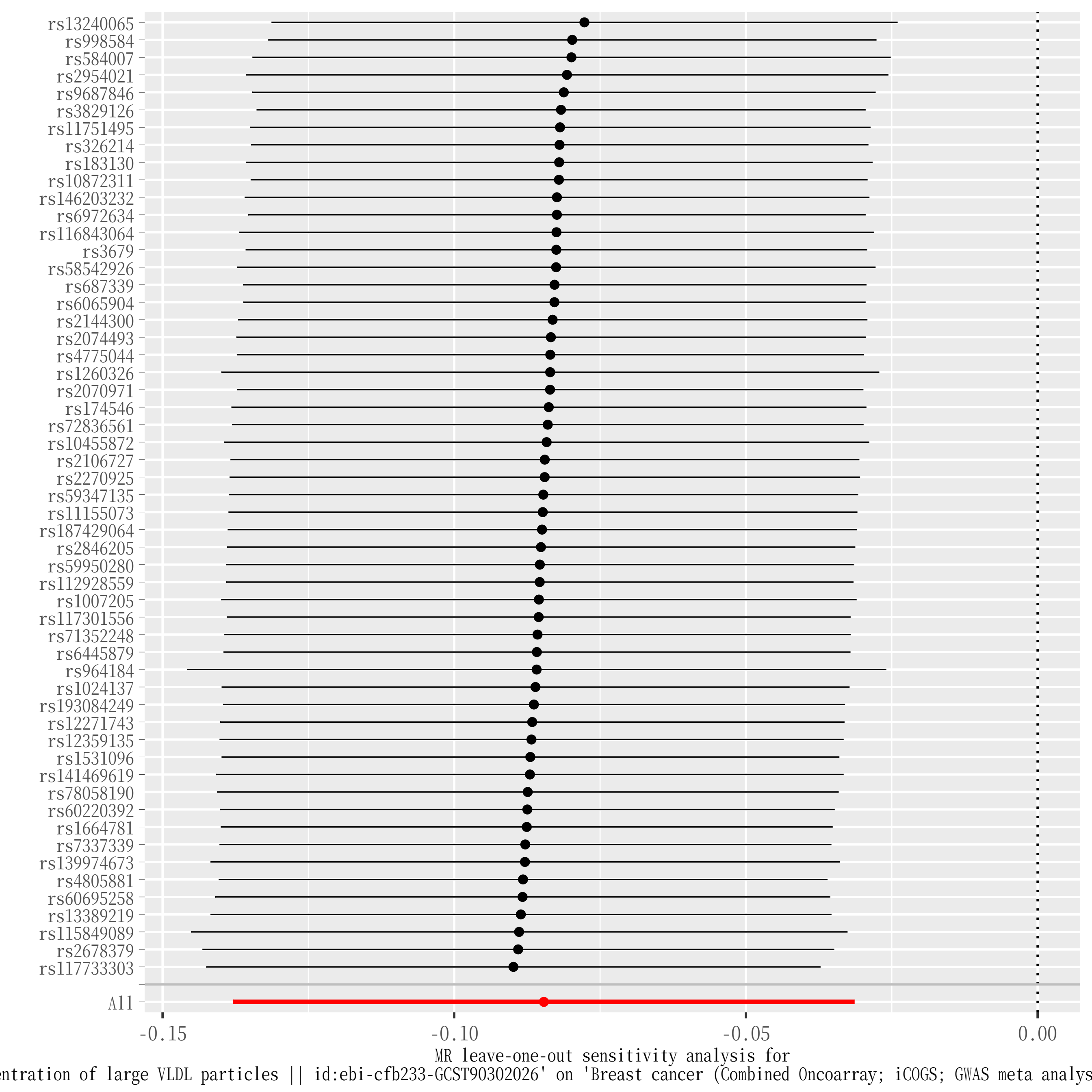

rs13240065
rs998584
rs584007
rs2954021
rs9687846
rs3829126
rs11751495
rs326214
rs183130
rs10872311
rs146203232
rs6972634
rs116843064
rs3679
rs58542926
rs687339
rs6065904
rs2144300
rs2074493
rs4775044
rs1260326
rs2070971
rs174546
rs72836561
rs10455872
rs2106727
rs2270925
rs59347135
rs11155073
rs187429064
rs2846205
rs59950280
rs112928559
rs1007205
rs117301556
rs71352248
rs6445879
rs964184
rs1024137
rs193084249
rs12271743
rs12359135
rs1531096
rs141469619
rs78058190
rs60220392
rs1664781
rs7337339
rs139974673
rs4805881
rs60695258
rs13389219
rs115849089
rs2678379
rs117733303
All
-0.15
-0.10
-0.05
0.00
MR leave-one-out sensitivity analysis for
entration of large VLDL particles || id:ebi-cfb233-GCST90302026' on 'Breast cancer (Combined Oncoarray; iCOGS; GWAS meta analys

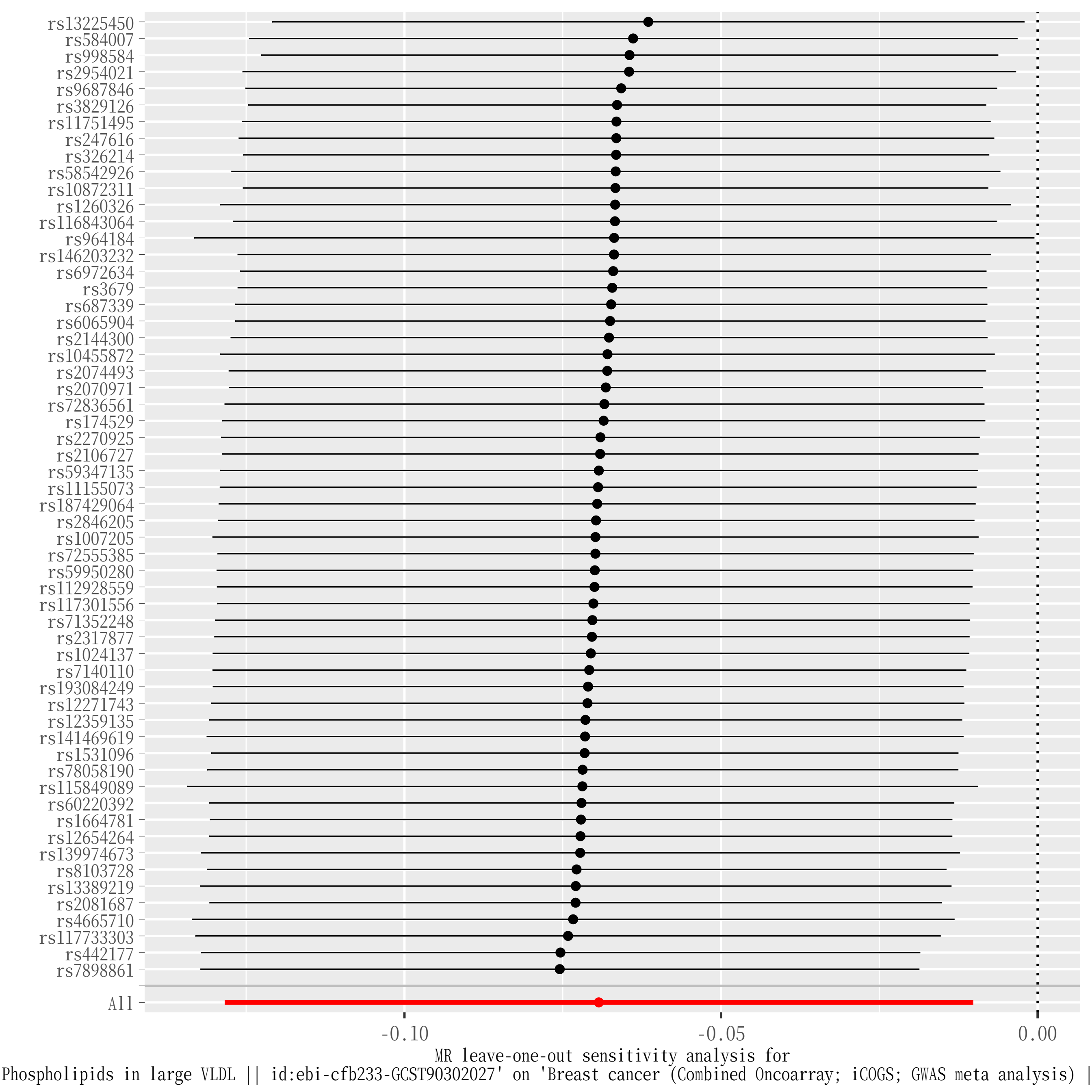
rs13225450
rs584007
rs998584
rs2954021
rs9687846
rs3829126
rs11751495
rs247616
rs326214
rs58542926
rs10872311
rs1260326
rs116843064
rs964184
rs146203232
rs6972634
rs3679
rs687339
rs6065904
rs2144300
rs10455872
rs2074493
rs2070971
rs72836561
rs174529
rs2270925
rs2106727
rs59347135
rs11155073
rs187429064
rs2846205
rs1007205
rs72555385
rs59950280
rs112928559
rs117301556
rs71352248
rs2317877
rs1024137
rs7140110
rs193084249
rs12271743
rs12359135
rs141469619
rs1531096
rs78058190
rs115849089
rs60220392
rs1664781
rs12654264
rs139974673
rs8103728
rs13389219
rs2081687
rs4665710
rs117733303
rs442177
rs7898861
All
-0.10
-0.05
0.00
MR leave-one-out sensitivity analysis for
Phospholipids in large VLDL || id:ebi-cfb233-GCST90302027' on 'Breast cancer (Combined Oncoarray; iCOGS; GWAS meta analysis) |

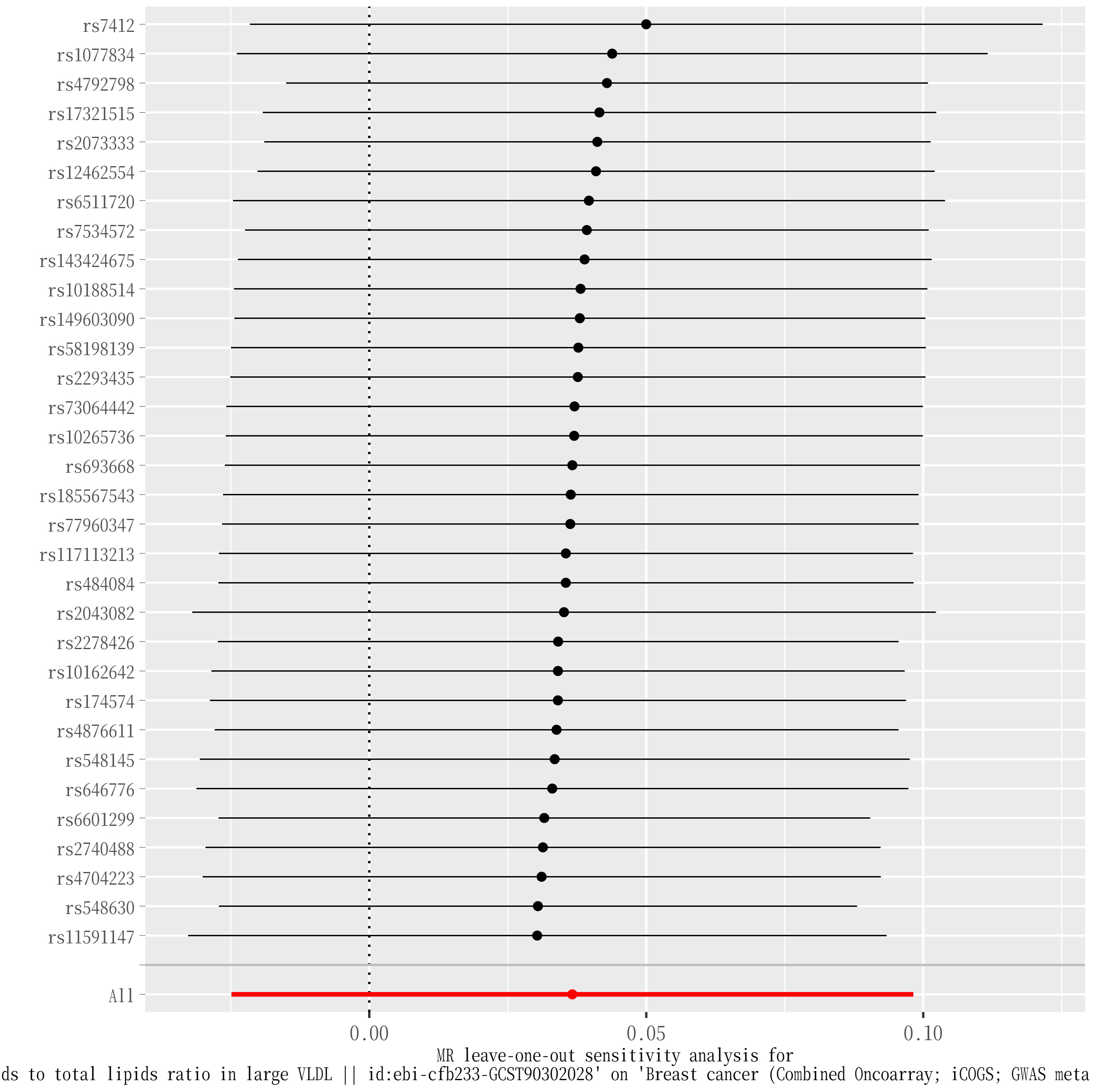

rs7412
rs1077834
rs4792798
rs17321515
rs2073333
rs12462554
rs6511720
rs7534572
rs143424675
rs10188514
rs149603090
rs58198139
rs2293435
rs73064442
rs10265736
rs693668
rs185567543
rs77960347
rs117113213
rs484084
rs2043082
rs2278426
rs10162642
rs174574
rs4876611
rs548145
rs646776
rs6601299
rs2740488
rs4704223
rs548630
rs11591147
All
0.00
0.05
0.10
MR leave-one-out sensitivity analysis for
ds to total lipids ratio in large VLDL || id:ebi-cfb233-GCST90302028' on 'Breast cancer (Combined Oncoarray; iCOGS; GWAS meta

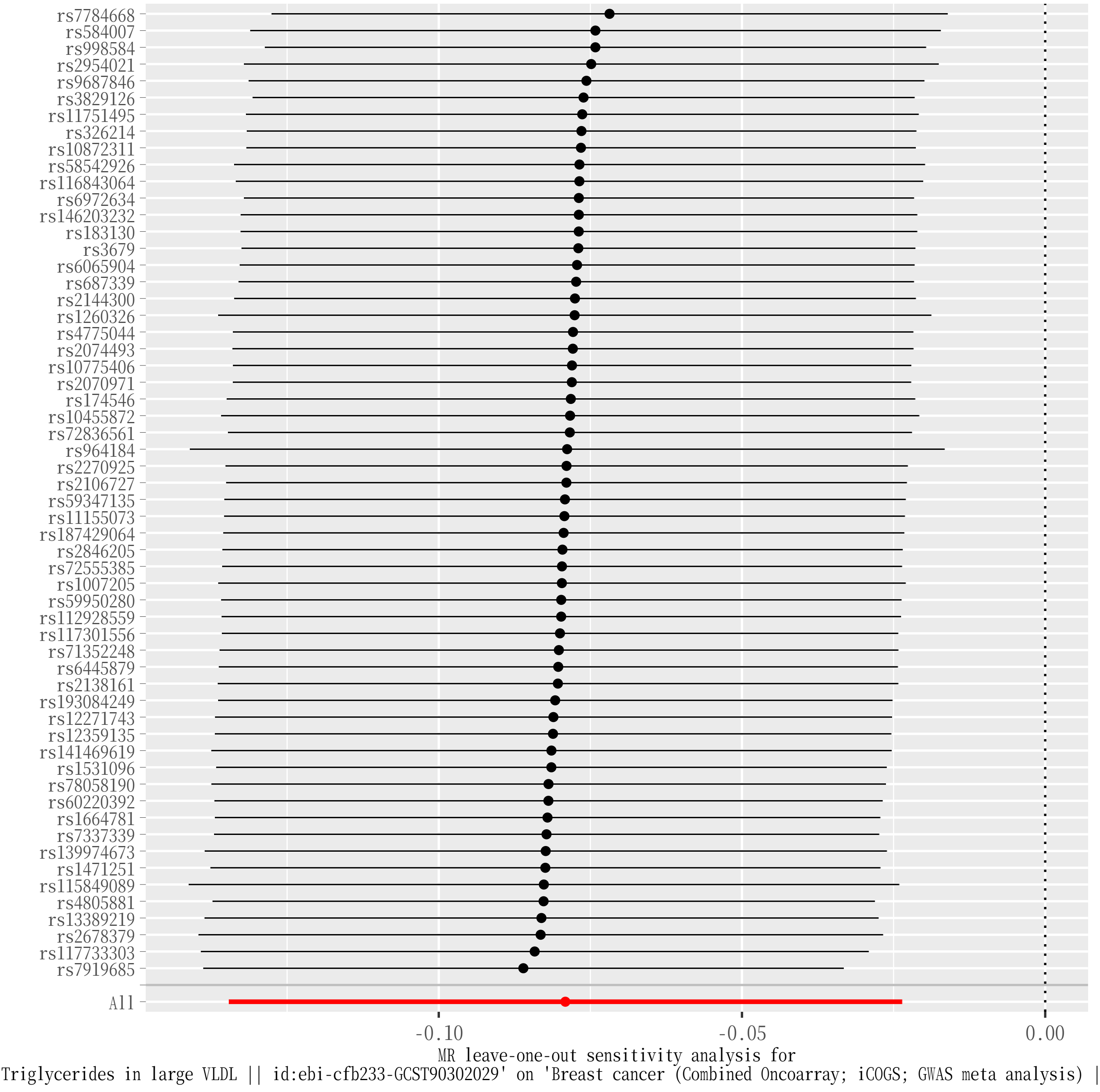
rs7784668
rs584007
rs998584
rs2954021
rs9687846
rs3829126
rs11751495
rs326214
rs10872311
rs58542926
rs116843064
rs6972634
rs146203232
rs183130
rs3679
rs6065904
rs687339
rs2144300
rs1260326
rs4775044
rs2074493
rs10775406
rs2070971
rs174546
rs10455872
rs72836561
rs964184
rs2270925
rs2106727
rs59347135
rs11155073
rs187429064
rs2846205
rs72555385
rs1007205
rs59950280
rs112928559
rs117301556
rs71352248
rs6445879
rs2138161
rs193084249
rs12271743
rs12359135
rs141469619
rs1531096
rs78058190
rs60220392
rs1664781
rs7337339
rs139974673
rs1471251
rs115849089
rs4805881
rs13389219
rs2678379
rs117733303
rs7919685
All
-0.10
-0.05
0.00
MR leave-one-out sensitivity analysis for
Triglycerides in large VLDL || id:ebi-cfb233-GCST90302029' on 'Breast cancer (Combined Oncoarray; iCOGS; GWAS meta analysis) |

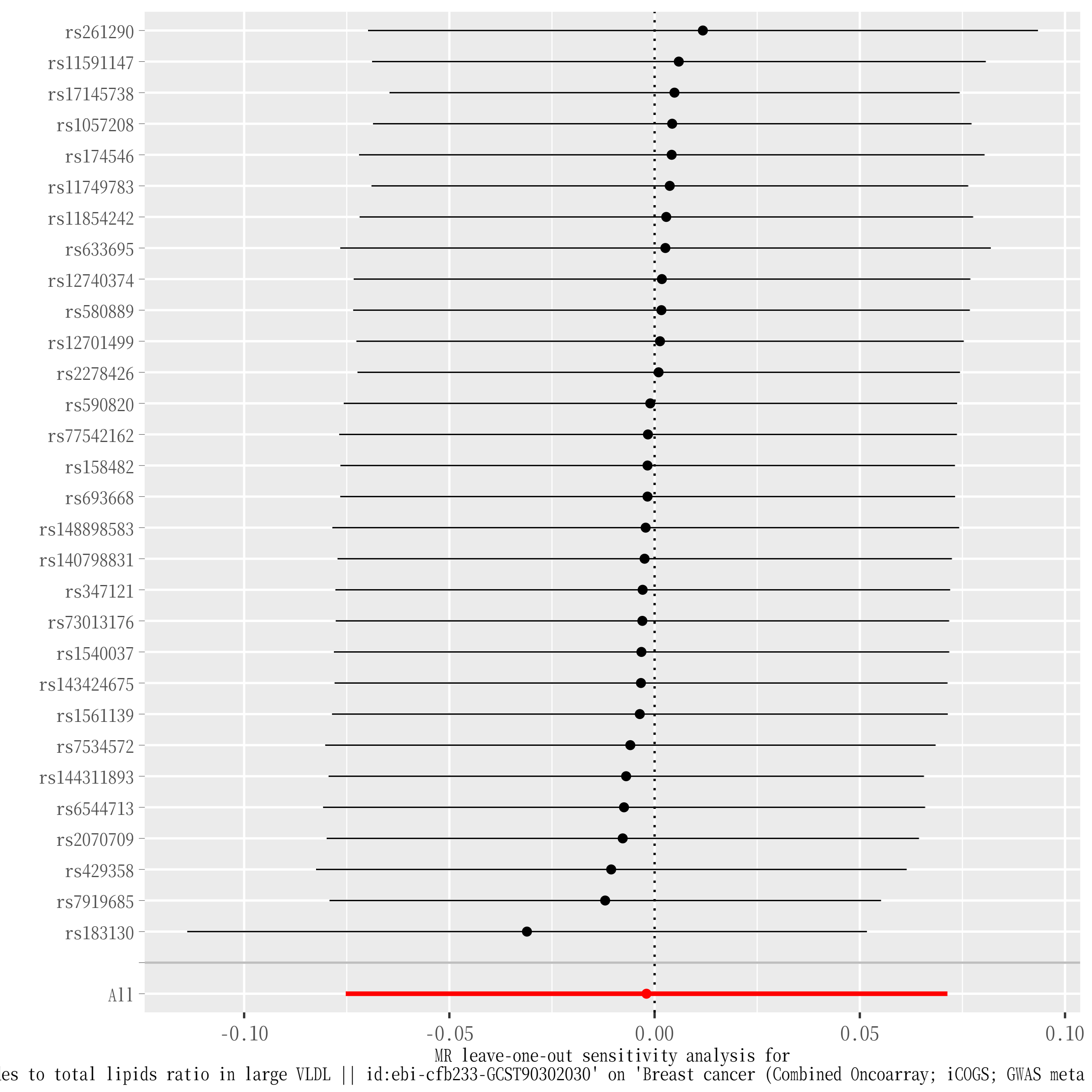
rs261290
rs11591147
rs17145738
rs1057208
rs174546
rs11749783
rs11854242
rs633695
rs12740374
rs580889
rs12701499
rs2278426
rs590820
rs77542162
rs158482
rs693668
rs148898583
rs140798831
rs347121
rs73013176
rs1540037
rs143424675
rs1561139
rs7534572
rs144311893
rs6544713
rs2070709
rs429358
rs7919685
rs183130
All
-0.10
-0.05
0.00
0.05
0.10
MR leave-one-out sensitivity analysis for
les to total lipids ratio in large VLDL || id:ebi-cfb233-GCST90302030' on 'Breast cancer (Combined Oncoarray; iCOGS; GWAS meta

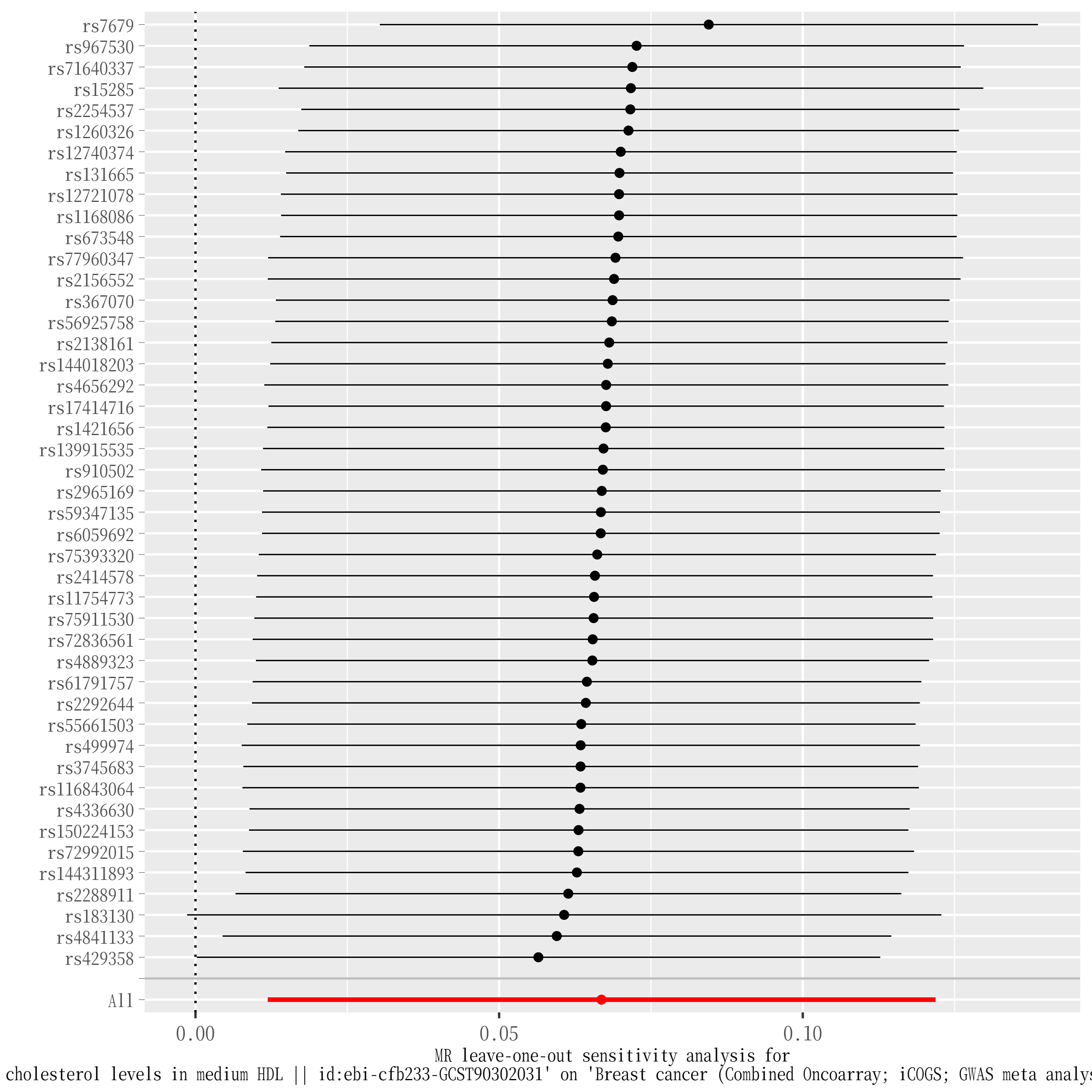
rs7679
rs967530
rs71640337
rs15285
rs2254537
rs1260326
rs12740374
rs131665
rs12721078
rs1168086
rs673548
rs77960347
rs2156552
rs367070
rs56925758
rs2138161
rs144018203
rs4656292
rs17414716
rs1421656
rs139915535
rs910502
rs2965169
rs59347135
rs6059692
rs75393320
rs2414578
rs11754773
rs75911530
rs72836561
rs4889323
rs61791757
rs2292644
rs55661503
rs499974
rs3745683
rs116843064
rs4336630
rs150224153
rs72992015
rs144311893
rs2288911
rs183130
rs4841133
rs429358
All
0.00
0.05
0.10
MR leave-one-out sensitivity analysis for
cholesterol levels in medium HDL || id:ebi-cfb233-GCST90302031' on 'Breast cancer (Combined Oncoarray; iCOGS; GWAS meta analys

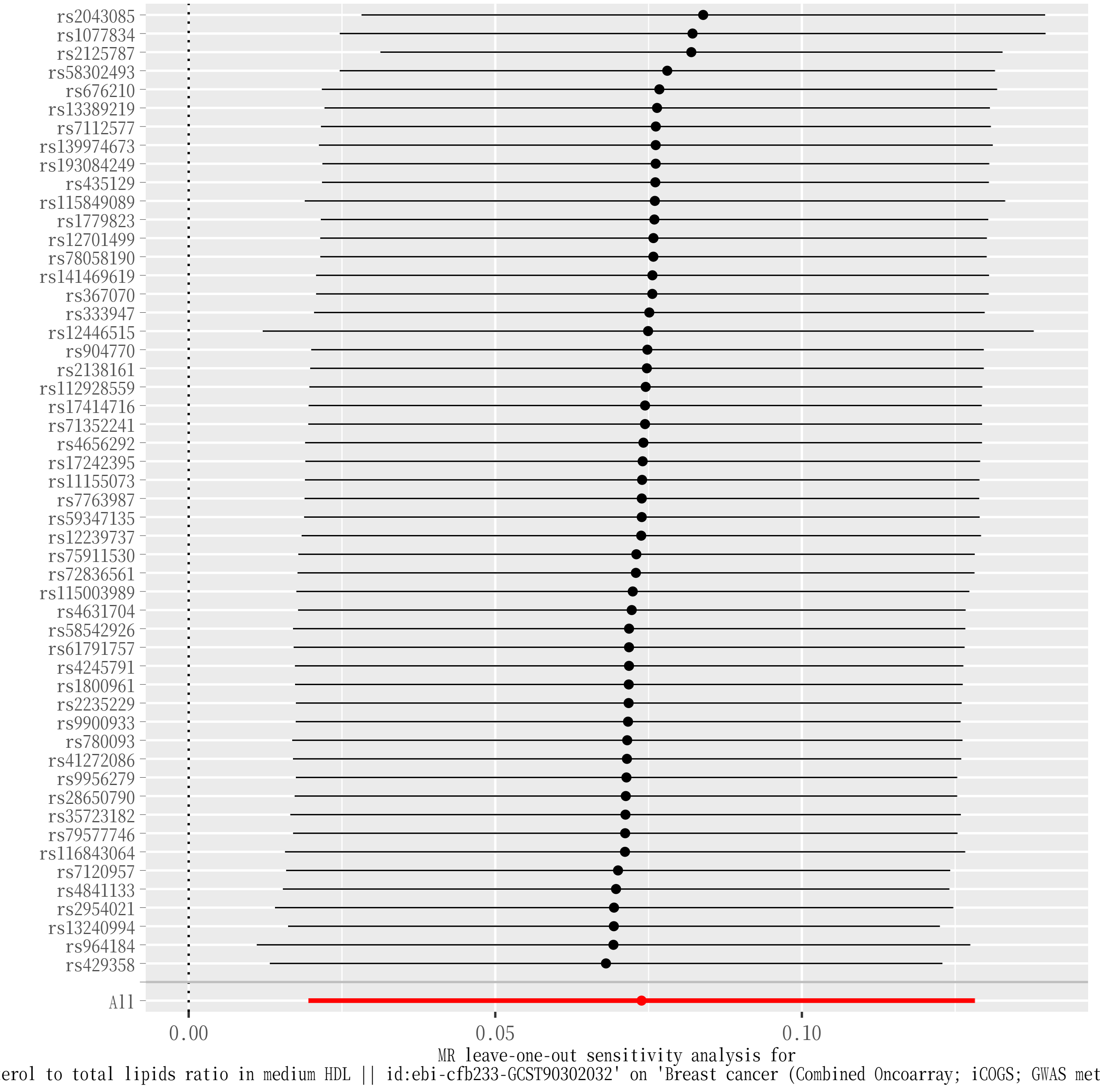

rs2043085
rs1077834
rs2125787
rs58302493
rs676210
rs13389219
rs7112577
rs139974673
rs193084249
rs435129
rs115849089
rs1779823
rs12701499
rs78058190
rs141469619
rs367070
rs333947
rs12446515
rs904770
rs2138161
rs112928559
rs17414716
rs71352241
rs4656292
rs17242395
rs11155073
rs7763987
rs59347135
rs12239737
rs75911530
rs72836561
rs115003989
rs4631704
rs58542926
rs61791757
rs4245791
rs1800961
rs2235229
rs9900933
rs780093
rs41272086
rs9956279
rs28650790
rs35723182
rs79577746
rs116843064
rs7120957
rs4841133
rs2954021
rs13240994
rs964184
rs429358
All
0.00
0.05
0.10
MR leave-one-out sensitivity analysis for
erol to total lipids ratio in medium HDL || id:ebi-cfb233-GCST90302032' on 'Breast cancer (Combined Oncoarray; iCOGS; GWAS met

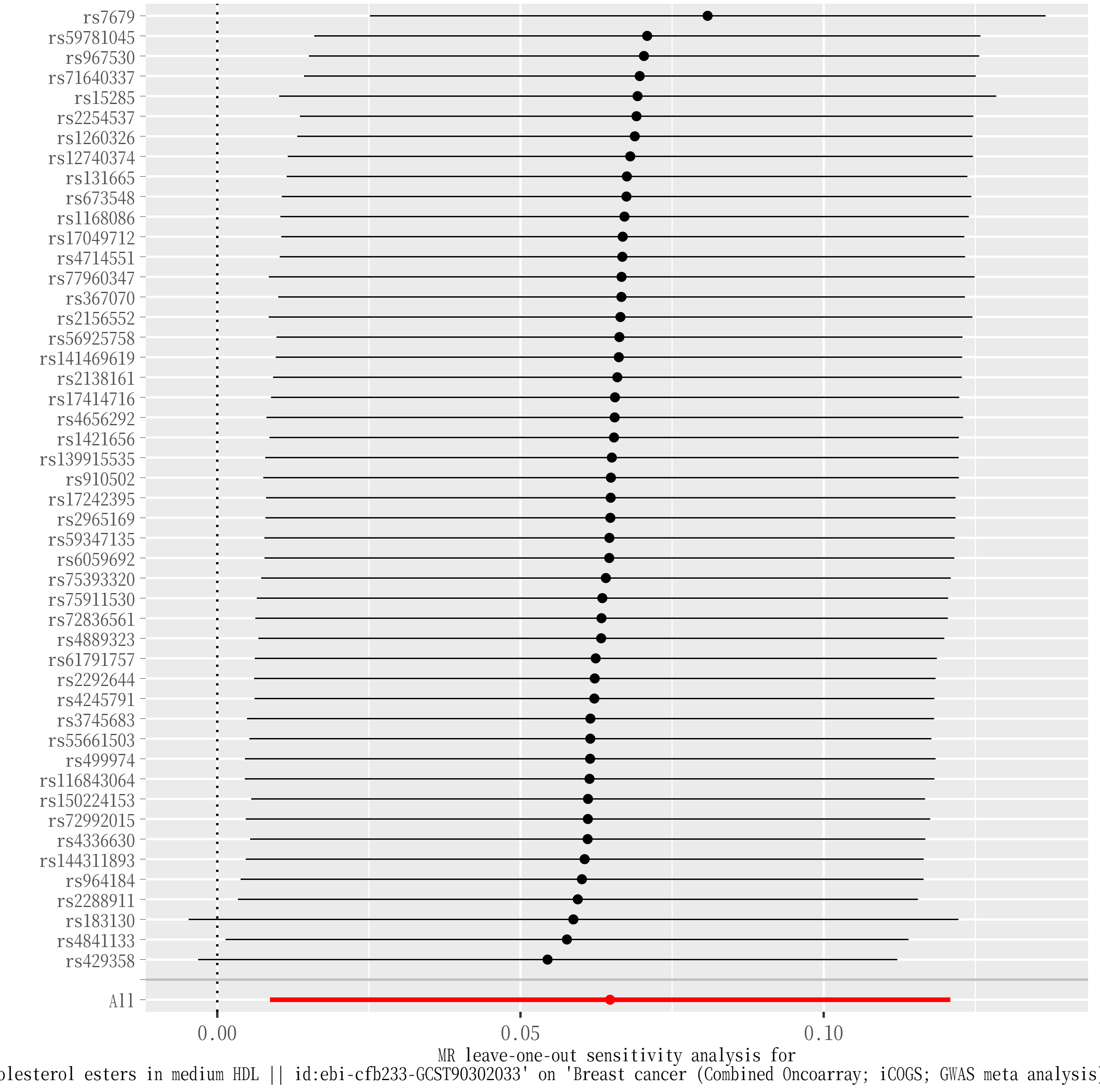
rs7679
rs59781045
rs967530
rs71640337
rs15285
rs2254537
rs1260326
rs12740374
rs131665
rs673548
rs1168086
rs17049712
rs4714551
rs77960347
rs367070
rs2156552
rs56925758
rs141469619
rs2138161
rs17414716
rs4656292
rs1421656
rs139915535
rs910502
rs17242395
rs2965169
rs59347135
rs6059692
rs75393320
rs75911530
rs72836561
rs4889323
rs61791757
rs2292644
rs4245791
rs3745683
rs55661503
rs499974
rs116843064
rs150224153
rs72992015
rs4336630
rs144311893
rs964184
rs2288911
rs183130
rs4841133
rs429358
All
0.00
0.05
0.10
MR leave-one-out sensitivity analysis for
olesterol esters in medium HDL || id:ebi-cfb233-GCST90302033' on 'Breast cancer (Combined Oncoarray; iCOGS; GWAS meta analysis)

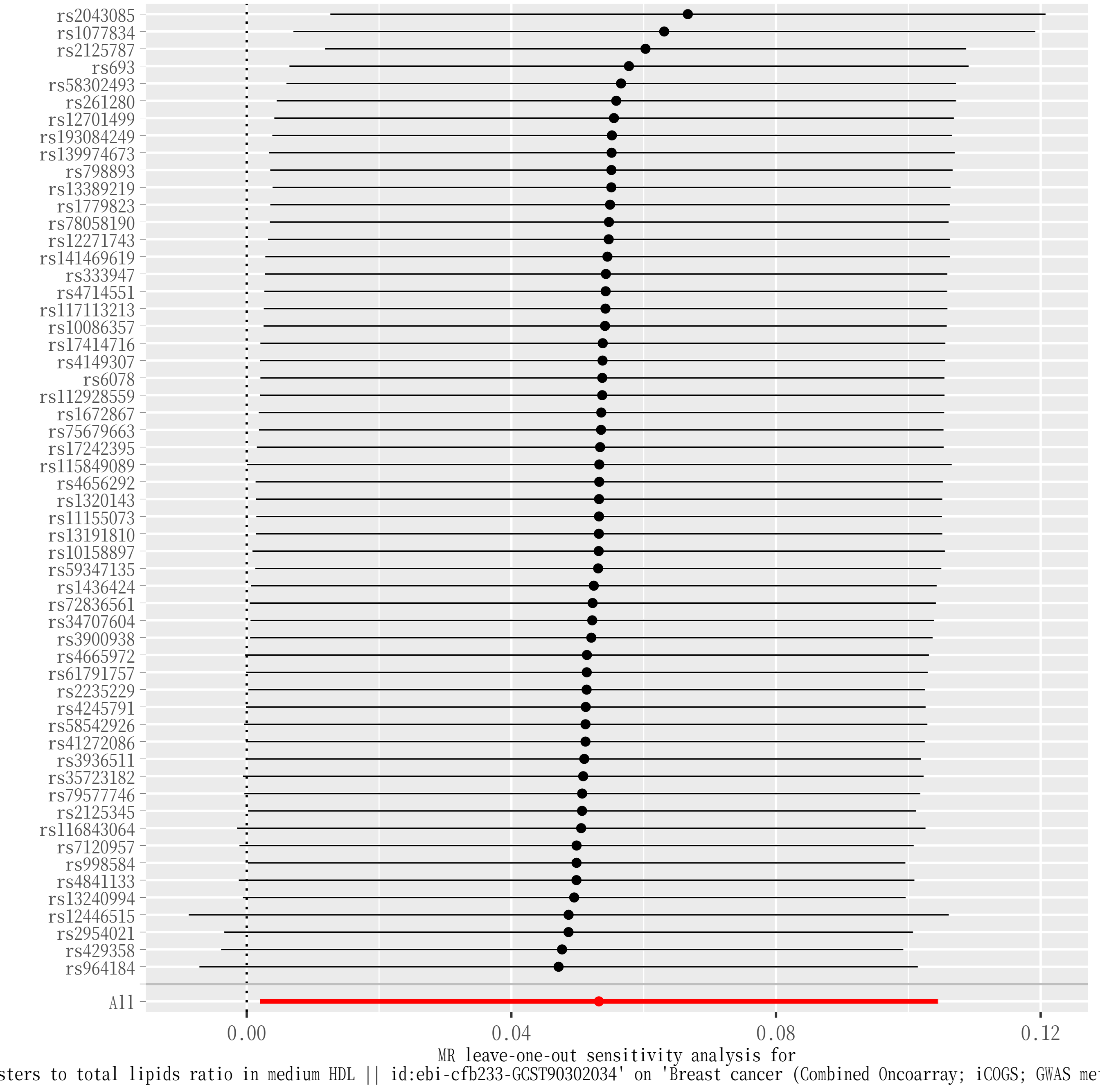
rs2043085
rs1077834
rs2125787
rs693
rs58302493
rs261280
rs12701499
rs193084249
rs139974673
rs798893
rs13389219
rs1779823
rs78058190
rs12271743
rs141469619
rs333947
rs4714551
rs117113213
rs10086357
rs17414716
rs4149307
rs6078
rs112928559
rs1672867
rs75679663
rs17242395
rs115849089
rs4656292
rs1320143
rs11155073
rs13191810
rs10158897
rs59347135
rs1436424
rs72836561
rs34707604
rs3900938
rs4665972
rs61791757
rs2235229
rs4245791
rs58542926
rs41272086
rs3936511
rs35723182
rs79577746
rs2125345
rs116843064
rs7120957
rs998584
rs4841133
rs13240994
rs12446515
rs2954021
rs429358
rs964184
All
0.00
0.04
0.08
0.12
MR leave-one-out sensitivity analysis for
sters to total lipids ratio in medium HDL || id:ebi-cfb233-GCST90302034' on 'Breast cancer (Combined Oncoarray; iCOGS; GWAS me

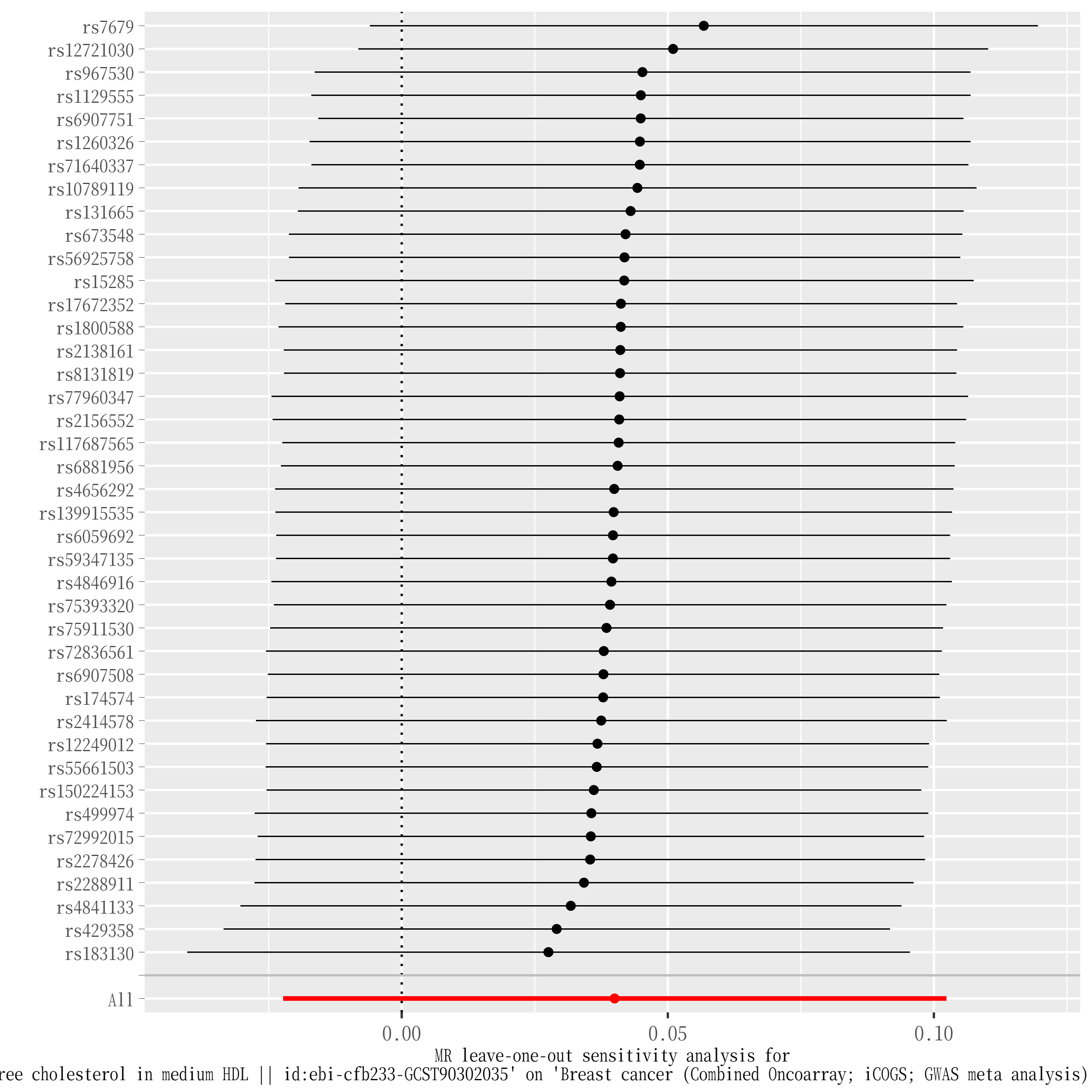
rs7679
rs12721030
rs967530
rs1129555
rs6907751
rs1260326
rs71640337
rs10789119
rs131665
rs673548
rs56925758
rs15285
rs17672352
rs1800588
rs2138161
rs8131819
rs77960347
rs2156552
rs117687565
rs6881956
rs4656292
rs139915535
rs6059692
rs59347135
rs4846916
rs75393320
rs75911530
rs72836561
rs6907508
rs174574
rs2414578
rs12249012
rs55661503
rs150224153
rs499974
rs72992015
rs2278426
rs2288911
rs4841133
rs429358
rs183130
All
0.00
0.05
0.10
MR leave-one-out sensitivity analysis for
ree cholesterol in medium HDL || id:ebi-cfb233-GCST90302035' on 'Breast cancer (Combined Oncoarray; iCOGS; GWAS meta analysis)

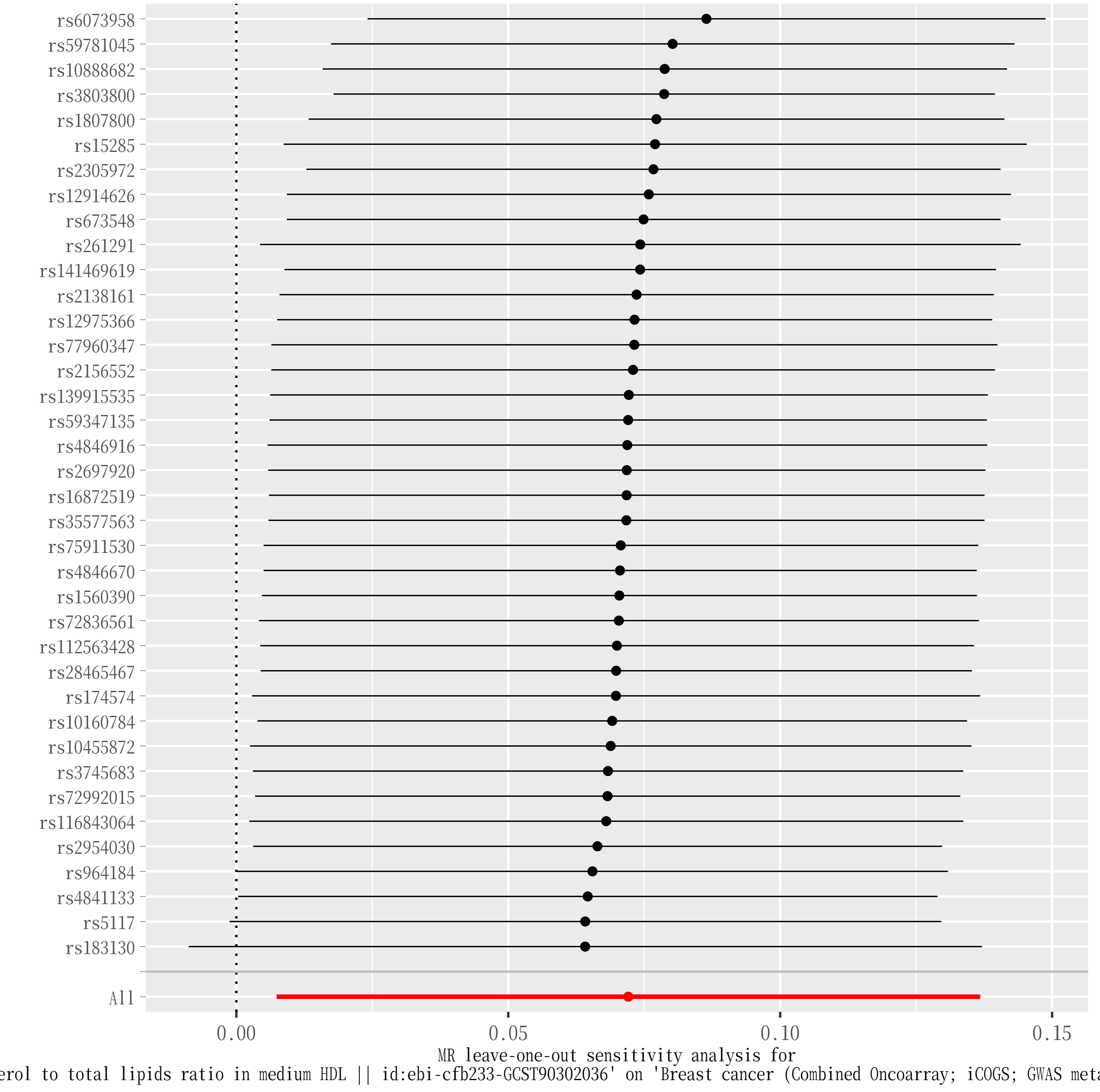
rs6073958
rs59781045
rs10888682
rs3803800
rs1807800
rs15285
rs2305972
rs12914626
rs673548
rs261291
rs141469619
rs2138161
rs12975366
rs77960347
rs2156552
rs139915535
rs59347135
rs4846916
rs2697920
rs16872519
rs35577563
rs75911530
rs4846670
rs1560390
rs72836561
rs112563428
rs28465467
rs174574
rs10160784
rs10455872
rs3745683
rs72992015
rs116843064
rs2954030
rs964184
rs4841133
rs5117
rs183130
All
0.00
0.05
0.10
0.15
MR leave-one-out sensitivity analysis for
erol to total lipids ratio in medium HDL || id:ebi-cfb233-GCST90302036' on 'Breast cancer (Combined Oncoarray; iCOGS; GWAS meta

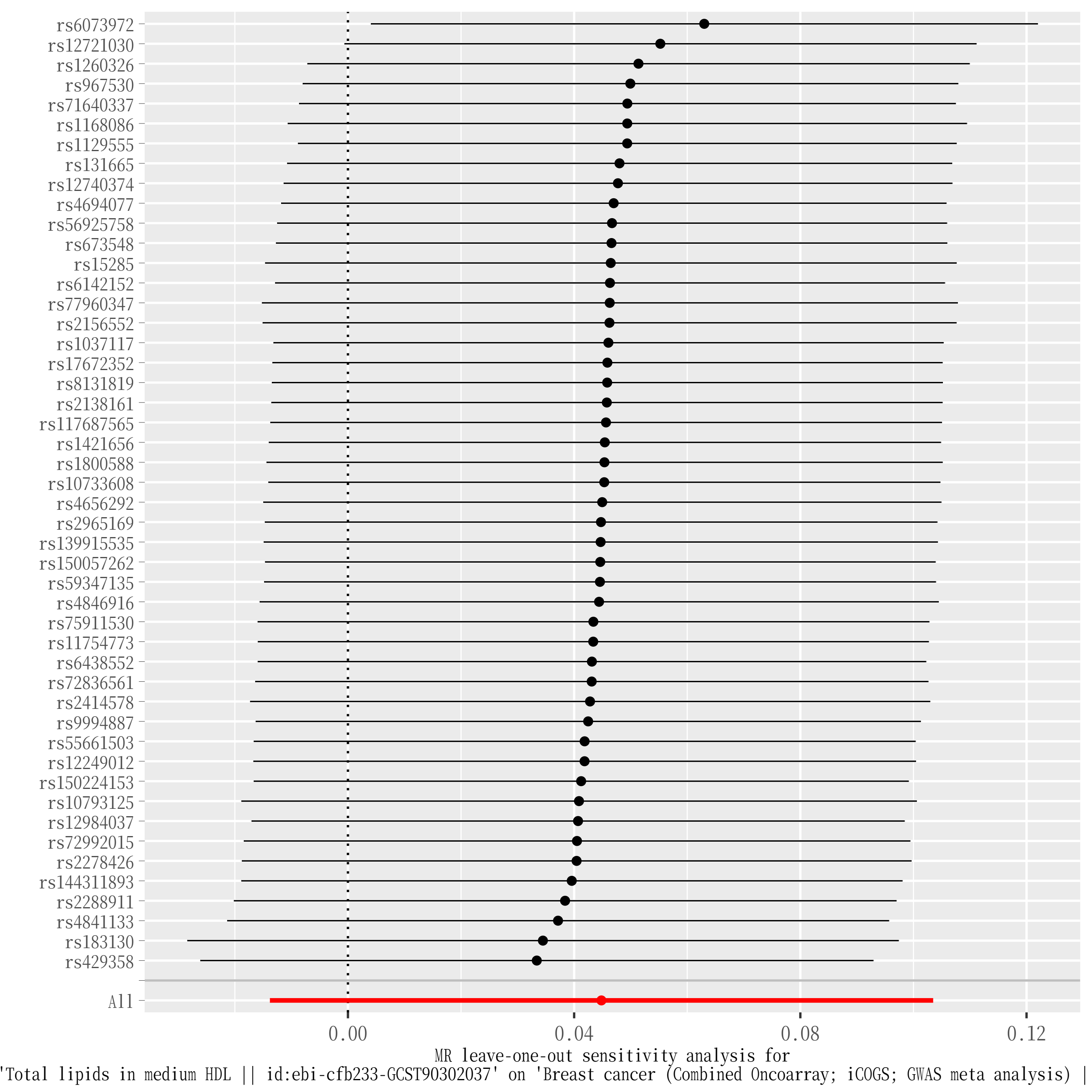

rs6073972
rs12721030
rs1260326
rs967530
rs71640337
rs1168086
rs1129555
rs131665
rs12740374
rs4694077
rs56925758
rs673548
rs15285
rs6142152
rs77960347
rs2156552
rs1037117
rs17672352
rs8131819
rs2138161
rs117687565
rs1421656
rs1800588
rs10733608
rs4656292
rs2965169
rs139915535
rs150057262
rs59347135
rs4846916
rs75911530
rs11754773
rs6438552
rs72836561
rs2414578
rs9994887
rs55661503
rs12249012
rs150224153
rs10793125
rs12984037
rs72992015
rs2278426
rs144311893
rs2288911
rs4841133
rs183130
rs429358
All
0.00
0.04
0.08
0.12
MR leave-one-out sensitivity analysis for
'Total lipids in medium HDL || id:ebi-cfb233-GCST90302037' on 'Breast cancer (Combined Oncoarray; iCOGS; GWAS meta analysis) |

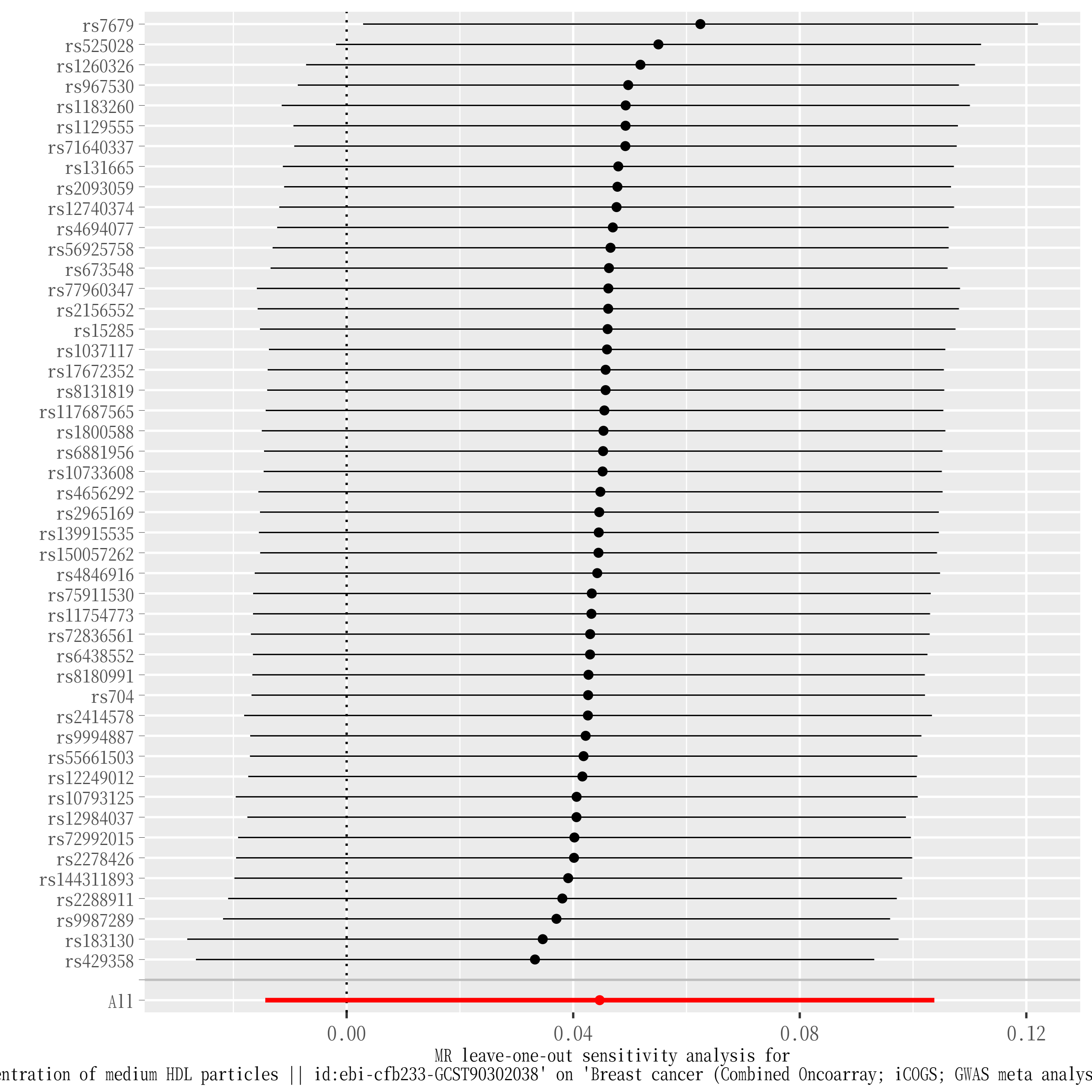
rs7679
rs525028
rs1260326
rs967530
rs1183260
rs1129555
rs71640337
rs131665
rs2093059
rs12740374
rs4694077
rs56925758
rs673548
rs77960347
rs2156552
rs15285
rs1037117
rs17672352
rs8131819
rs117687565
rs1800588
rs6881956
rs10733608
rs4656292
rs2965169
rs139915535
rs150057262
rs4846916
rs75911530
rs11754773
rs72836561
rs6438552
rs8180991
rs704
rs2414578
rs9994887
rs55661503
rs12249012
rs10793125
rs12984037
rs72992015
rs2278426
rs144311893
rs2288911
rs9987289
rs183130
rs429358
All
0.00
0.04
0.08
0.12
MR leave-one-out sensitivity analysis for
entration of medium HDL particles || id:ebi-cfb233-GCST90302038' on 'Breast cancer (Combined Oncoarray; iCOGS; GWAS meta analys

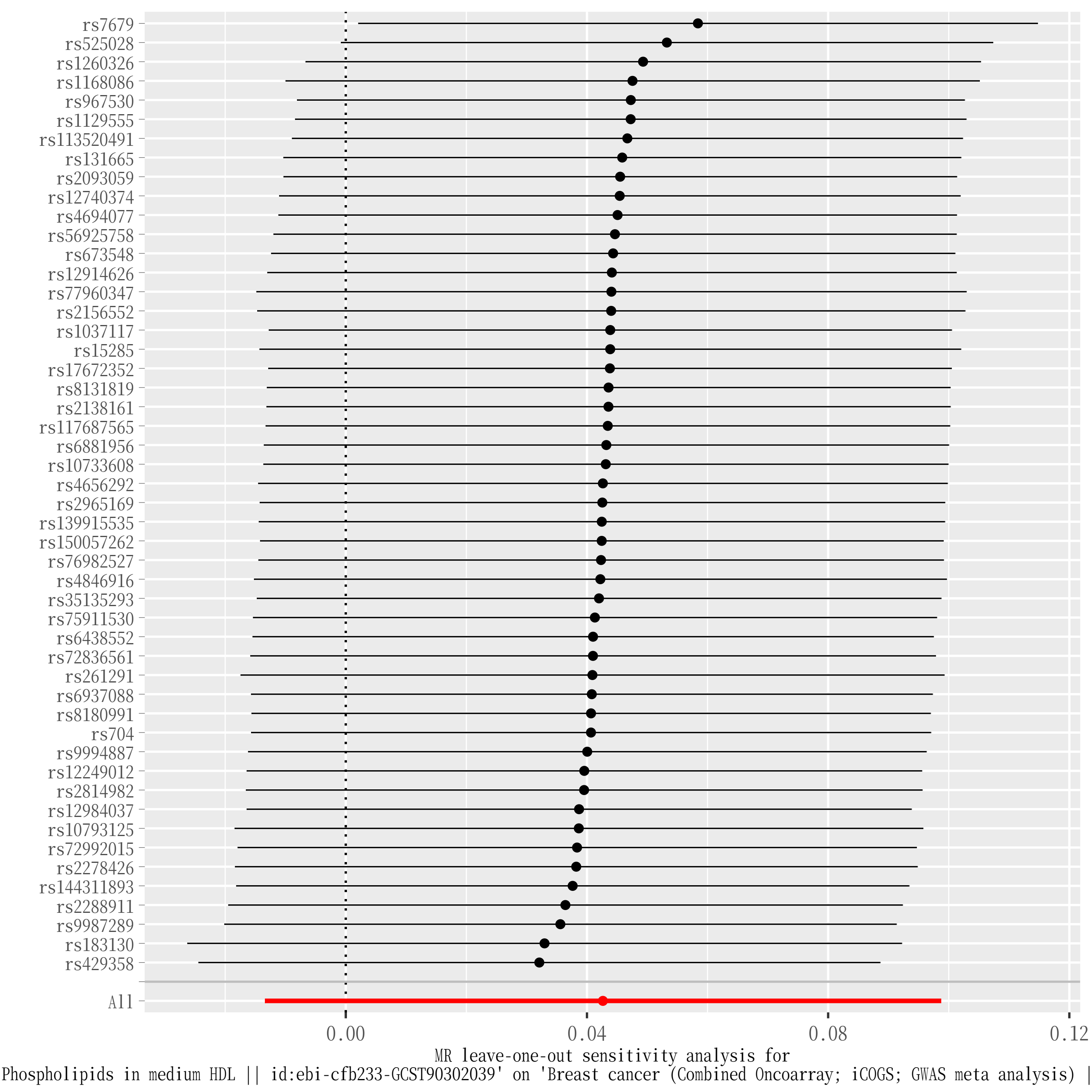

rs7679
rs525028
rs1260326
rs1168086
rs967530
rs1129555
rs113520491
rs131665
rs2093059
rs12740374
rs4694077
rs56925758
rs673548
rs12914626
rs77960347
rs2156552
rs1037117
rs15285
rs17672352
rs8131819
rs2138161
rs117687565
rs6881956
rs10733608
rs4656292
rs2965169
rs139915535
rs150057262
rs76982527
rs4846916
rs35135293
rs75911530
rs6438552
rs72836561
rs261291
rs6937088
rs8180991
rs704
rs9994887
rs12249012
rs2814982
rs12984037
rs10793125
rs72992015
rs2278426
rs144311893
rs2288911
rs9987289
rs183130
rs429358
All
0.00
0.04
0.08
0.12
MR leave-one-out sensitivity analysis for
Phospholipids in medium HDL || id:ebi-cfb233-GCST90302039' on 'Breast cancer (Combined Oncoarray; iCOGS; GWAS meta analysis) |

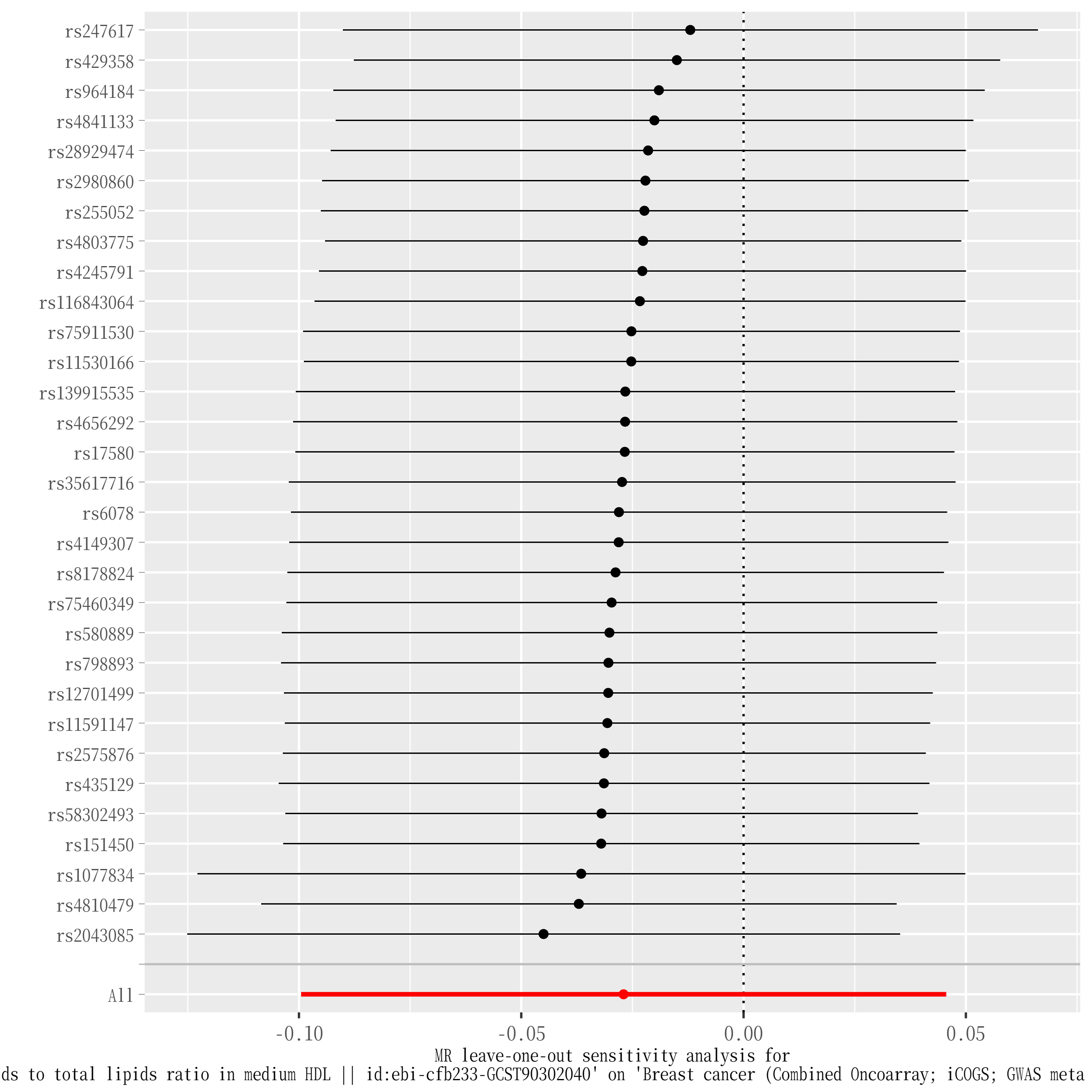
rs247617
rs429358
rs964184
rs4841133
rs28929474
rs2980860
rs255052
rs4803775
rs4245791
rs116843064
rs75911530
rs11530166
rs139915535
rs4656292
rs17580
rs35617716
rs6078
rs4149307
rs8178824
rs75460349
rs580889
rs798893
rs12701499
rs11591147
rs2575876
rs435129
rs58302493
rs151450
rs1077834
rs4810479
rs2043085
All
-0.10
-0.05
0.00
0.05
MR leave-one-out sensitivity analysis for
ds to total lipids ratio in medium HDL || id:ebi-cfb233-GCST90302040' on 'Breast cancer (Combined Oncoarray; iCOGS; GWAS meta

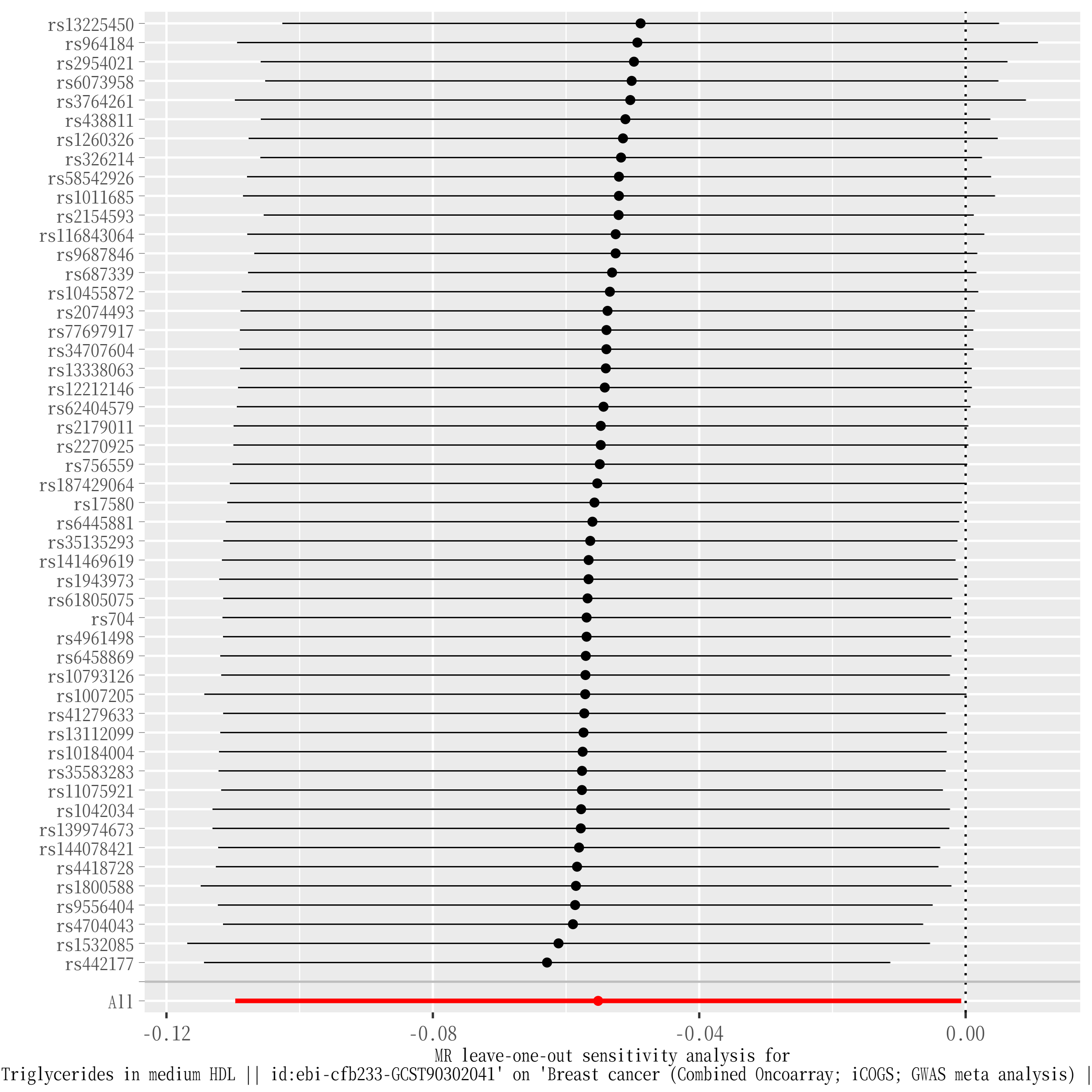
rs13225450
rs964184
rs2954021
rs6073958
rs3764261
rs438811
rs1260326
rs326214
rs58542926
rs1011685
rs2154593
rs116843064
rs9687846
rs687339
rs10455872
rs2074493
rs77697917
rs34707604
rs13338063
rs12212146
rs62404579
rs2179011
rs2270925
rs756559
rs187429064
rs17580
rs6445881
rs35135293
rs141469619
rs1943973
rs61805075
rs704
rs4961498
rs6458869
rs10793126
rs1007205
rs41279633
rs13112099
rs10184004
rs35583283
rs11075921
rs1042034
rs139974673
rs144078421
rs4418728
rs1800588
rs9556404
rs4704043
rs1532085
rs442177
All
-0.12
-0.08
-0.04
0.00
MR leave-one-out sensitivity analysis for
Triglycerides in medium HDL || id:ebi-cfb233-GCST90302041' on 'Breast cancer (Combined Oncoarray; iCOGS; GWAS meta analysis) |

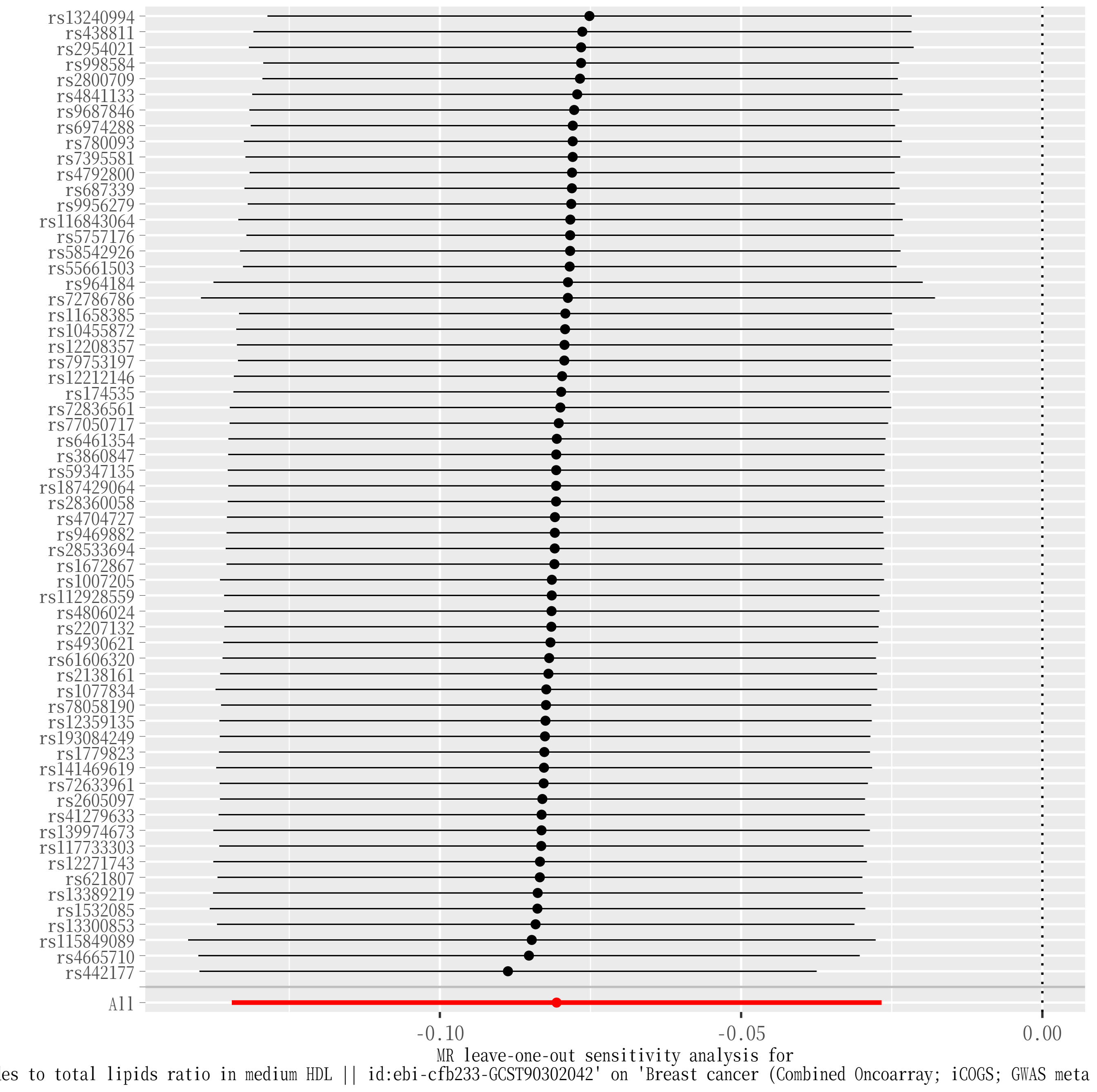
rs13240994
rs438811
rs2954021
rs998584
rs2800709
rs4841133
rs9687846
rs6974288
rs780093
rs7395581
rs4792800
rs687339
rs9956279
rs116843064
rs5757176
rs58542926
rs55661503
rs964184
rs72786786
rs11658385
rs10455872
rs12208357
rs79753197
rs12212146
rs174535
rs72836561
rs77050717
rs6461354
rs3860847
rs59347135
rs187429064
rs28360058
rs4704727
rs9469882
rs28533694
rs1672867
rs1007205
rs112928559
rs4806024
rs2207132
rs4930621
rs61606320
rs2138161
rs1077834
rs78058190
rs12359135
rs193084249
rs1779823
rs141469619
rs72633961
rs2605097
rs41279633
rs139974673
rs117733303
rs12271743
rs621807
rs13389219
rs1532085
rs13300853
rs115849089
rs4665710
rs442177
All
-0.10
-0.05
0.00
MR leave-one-out sensitivity analysis for
les to total lipids ratio in medium HDL || id:ebi-cfb233-GCST90302042' on 'Breast cancer (Combined Oncoarray; iCOGS; GWAS meta

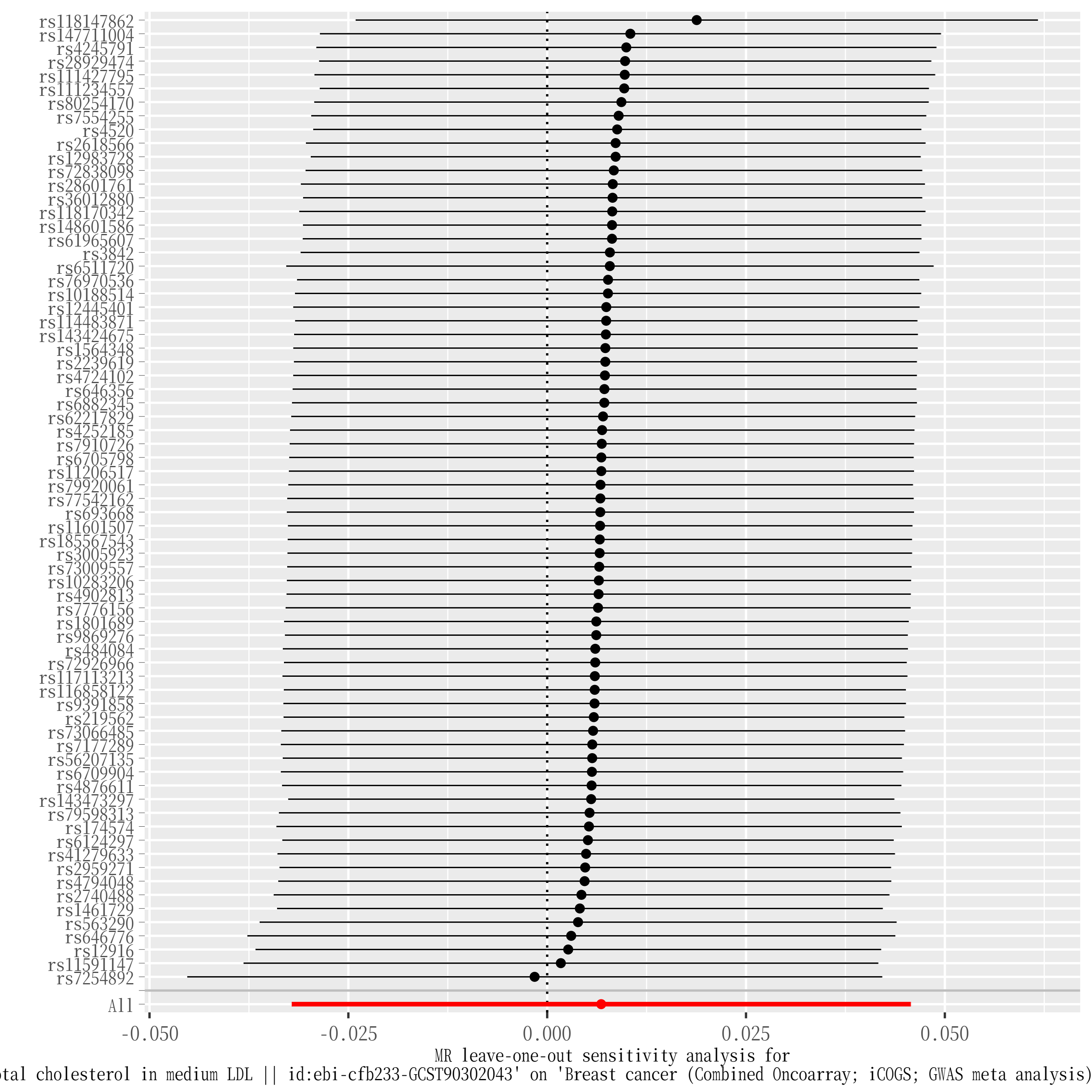
rs118147862
rs147711004
rs4245791
rs28929474
rs111427795
rs111234557
rs80254170
rs7554255
rs4520
rs2618566
rs12983728
rs72838098
rs28601761
rs36012880
rs118170342
rs148601586
rs61965607
rs3842
rs6511720
rs76970536
rs10188514
rs12445401
rs114483871
rs143424675
rs1564348
rs2239619
rs4724102
rs646356
rs6882345
rs62217829
rs4252185
rs7910726
rs6705798
rs11206517
rs79920061
rs77542162
rs693668
rs11601507
rs185567543
rs3005923
rs73009557
rs10283206
rs4902813
rs7776156
rs1801689
rs9869276
rs484084
rs72926966
rs117113213
rs116858122
rs9391858
rs219562
rs73066485
rs7177289
rs56207135
rs6709904
rs4876611
rs143473297
rs79598313
rs174574
rs6124297
rs41279633
rs2959271
rs4794048
rs2740488
rs1461729
rs563290
rs646776
rs12916
rs11591147
rs7254892
All
-0.050
-0.025
0.000
0.025
0.050
MR leave-one-out sensitivity analysis for
otal cholesterol in medium LDL || id:ebi-cfb233-GCST90302043' on 'Breast cancer (Combined Oncoarray; iCOGS; GWAS meta analysis)

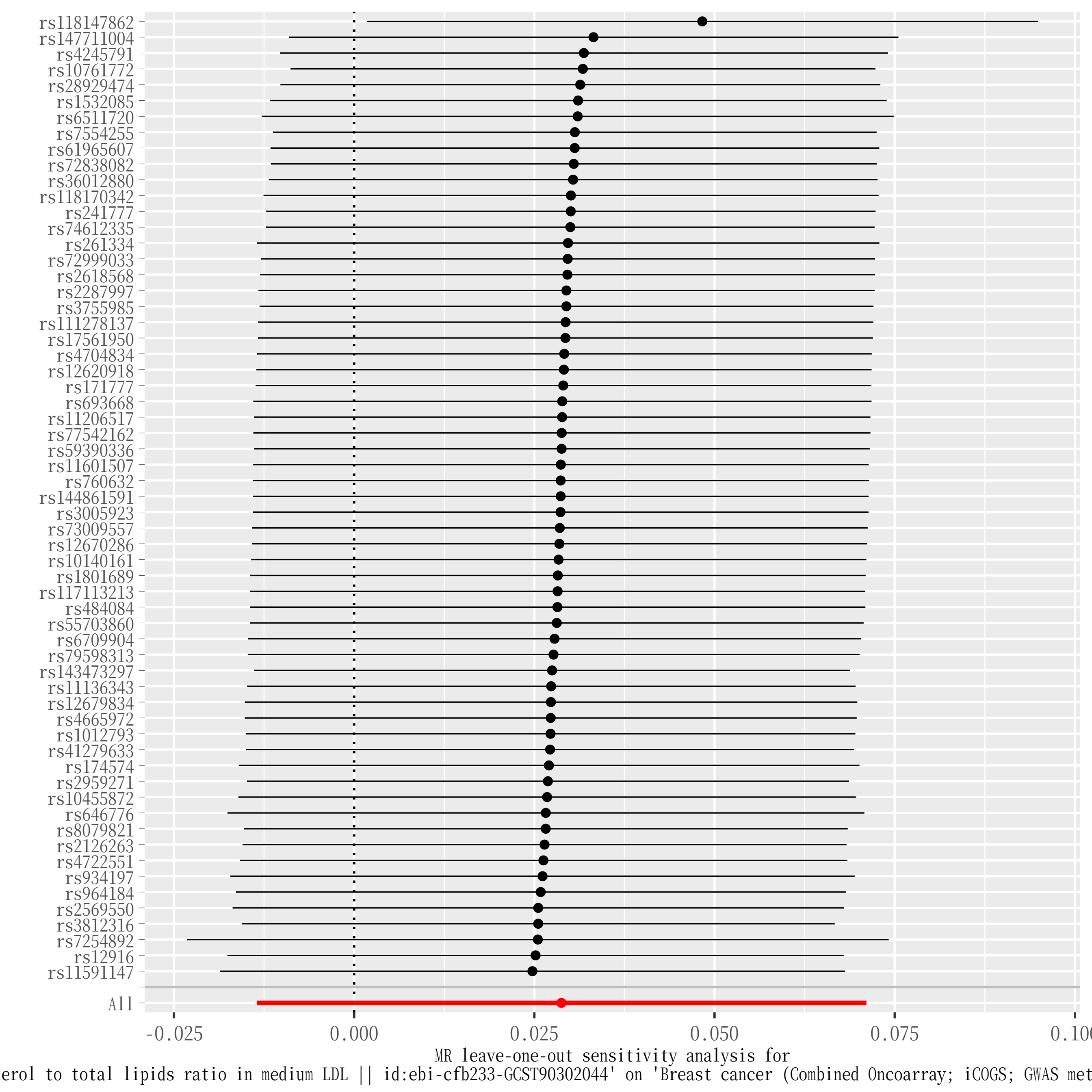
rs118147862
rs147711004
rs4245791
rs10761772
rs28929474
rs1532085
rs6511720
rs7554255
rs61965607
rs72838082
rs36012880
rs118170342
rs241777
rs74612335
rs261334
rs72999033
rs2618568
rs2287997
rs3755985
rs111278137
rs17561950
rs4704834
rs12620918
rs171777
rs693668
rs11206517
rs77542162
rs59390336
rs11601507
rs760632
rs144861591
rs3005923
rs73009557
rs12670286
rs10140161
rs1801689
rs117113213
rs484084
rs55703860
rs6709904
rs79598313
rs143473297
rs11136343
rs12679834
rs4665972
rs1012793
rs41279633
rs174574
rs2959271
rs10455872
rs646776
rs8079821
rs2126263
rs4722551
rs934197
rs964184
rs2569550
rs3812316
rs7254892
rs12916
rs11591147
All
-0.025
0.000
0.025
0.050
0.075
0.100
MR leave-one-out sensitivity analysis for
erol to total lipids ratio in medium LDL || id:ebi-cfb233-GCST90302044' on 'Breast cancer (Combined Oncoarray; iCOGS; GWAS met

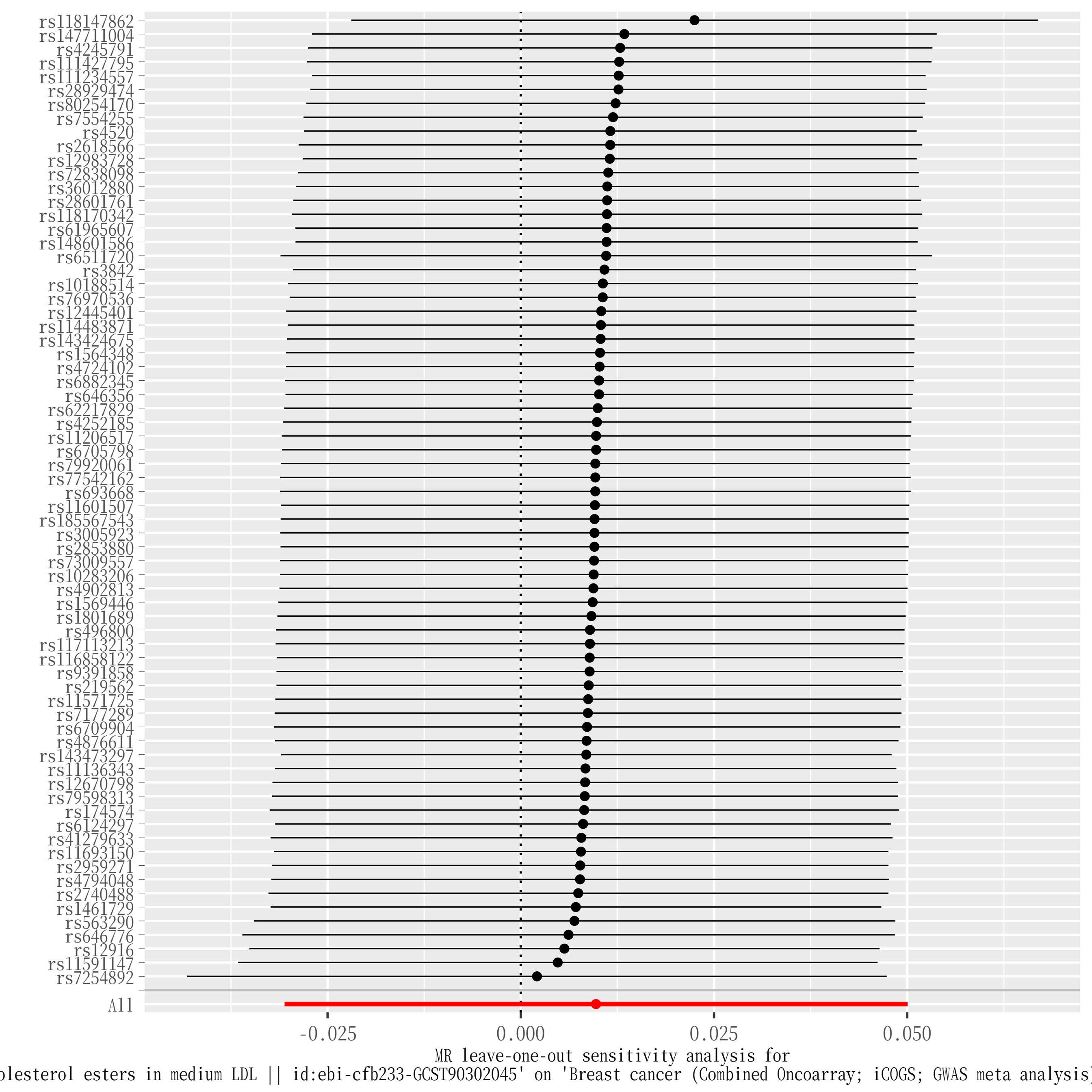

rs118147862
rs147711004
rs4245791
rs111427795
rs111234557
rs28929474
rs80254170
rs7554255
rs4520
rs2618566
rs12983728
rs72838098
rs36012880
rs28601761
rs118170342
rs61965607
rs148601586
rs6511720
rs3842
rs10188514
rs76970536
rs12445401
rs114483871
rs143424675
rs1564348
rs4724102
rs6882345
rs646356
rs62217829
rs4252185
rs11206517
rs6705798
rs79920061
rs77542162
rs693668
rs11601507
rs185567543
rs3005923
rs2853880
rs73009557
rs10283206
rs4902813
rs1569446
rs1801689
rs496800
rs117113213
rs116858122
rs9391858
rs219562
rs11571725
rs7177289
rs6709904
rs4876611
rs143473297
rs11136343
rs12670798
rs79598313
rs174574
rs6124297
rs41279633
rs11693150
rs2959271
rs4794048
rs2740488
rs1461729
rs563290
rs646776
rs12916
rs11591147
rs7254892
All
-0.025
0.000
0.025
0.050
MR leave-one-out sensitivity analysis for
olesterol esters in medium LDL || id:ebi-cfb233-GCST90302045' on 'Breast cancer (Combined Oncoarray; iCOGS; GWAS meta analysis)

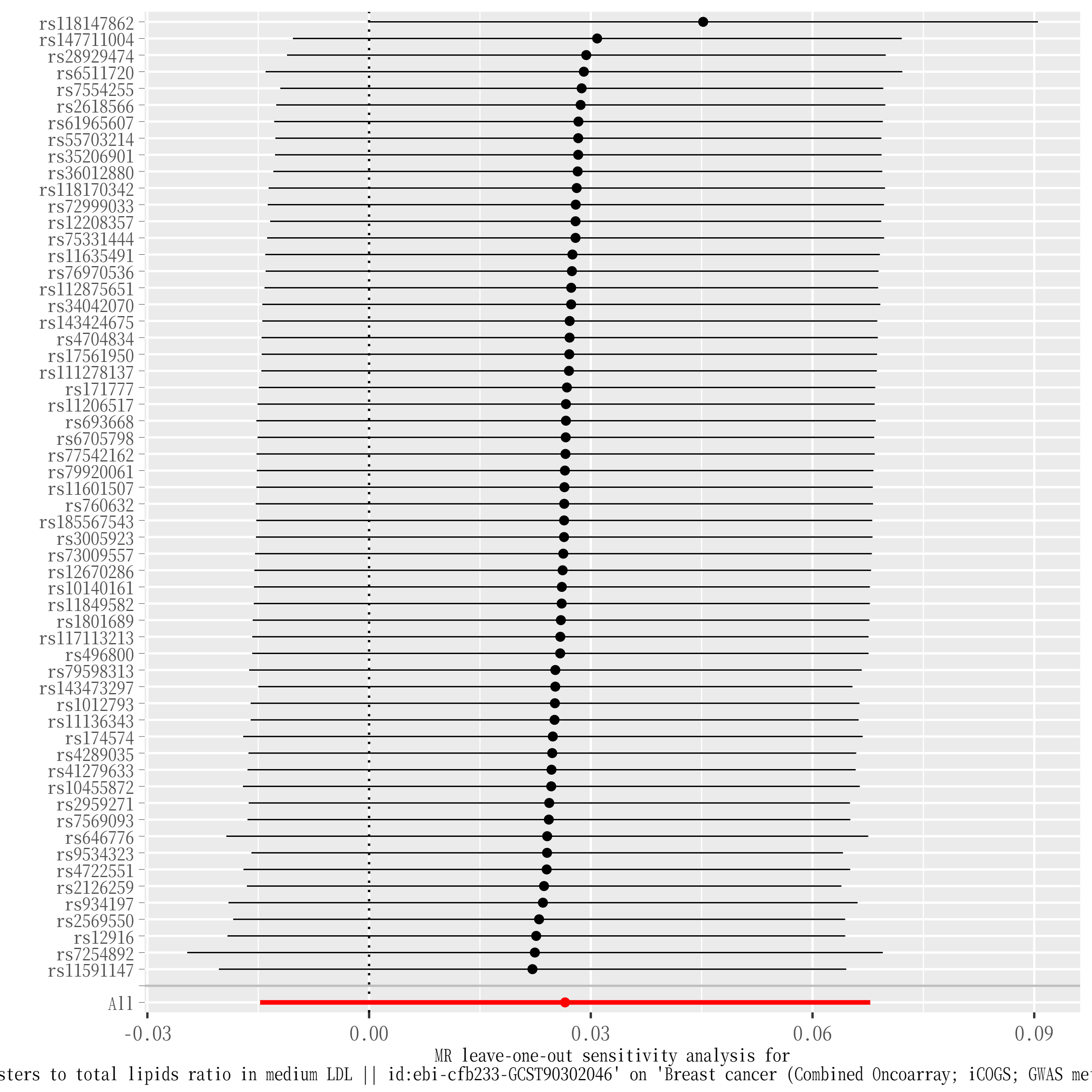

rs118147862
rs147711004
rs28929474
rs6511720
rs7554255
rs2618566
rs61965607
rs55703214
rs35206901
rs36012880
rs118170342
rs72999033
rs12208357
rs75331444
rs11635491
rs76970536
rs112875651
rs34042070
rs143424675
rs4704834
rs17561950
rs111278137
rs171777
rs11206517
rs693668
rs6705798
rs77542162
rs79920061
rs11601507
rs760632
rs185567543
rs3005923
rs73009557
rs12670286
rs10140161
rs11849582
rs1801689
rs117113213
rs496800
rs79598313
rs143473297
rs1012793
rs11136343
rs174574
rs4289035
rs41279633
rs10455872
rs2959271
rs7569093
rs646776
rs9534323
rs4722551
rs2126259
rs934197
rs2569550
rs12916
rs7254892
rs11591147
All
-0.03
0.00
0.03
0.06
0.09
MR leave-one-out sensitivity analysis for
sters to total lipids ratio in medium LDL || id:ebi-cfb233-GCST90302046' on 'Breast cancer (Combined Oncoarray; iCOGS; GWAS me

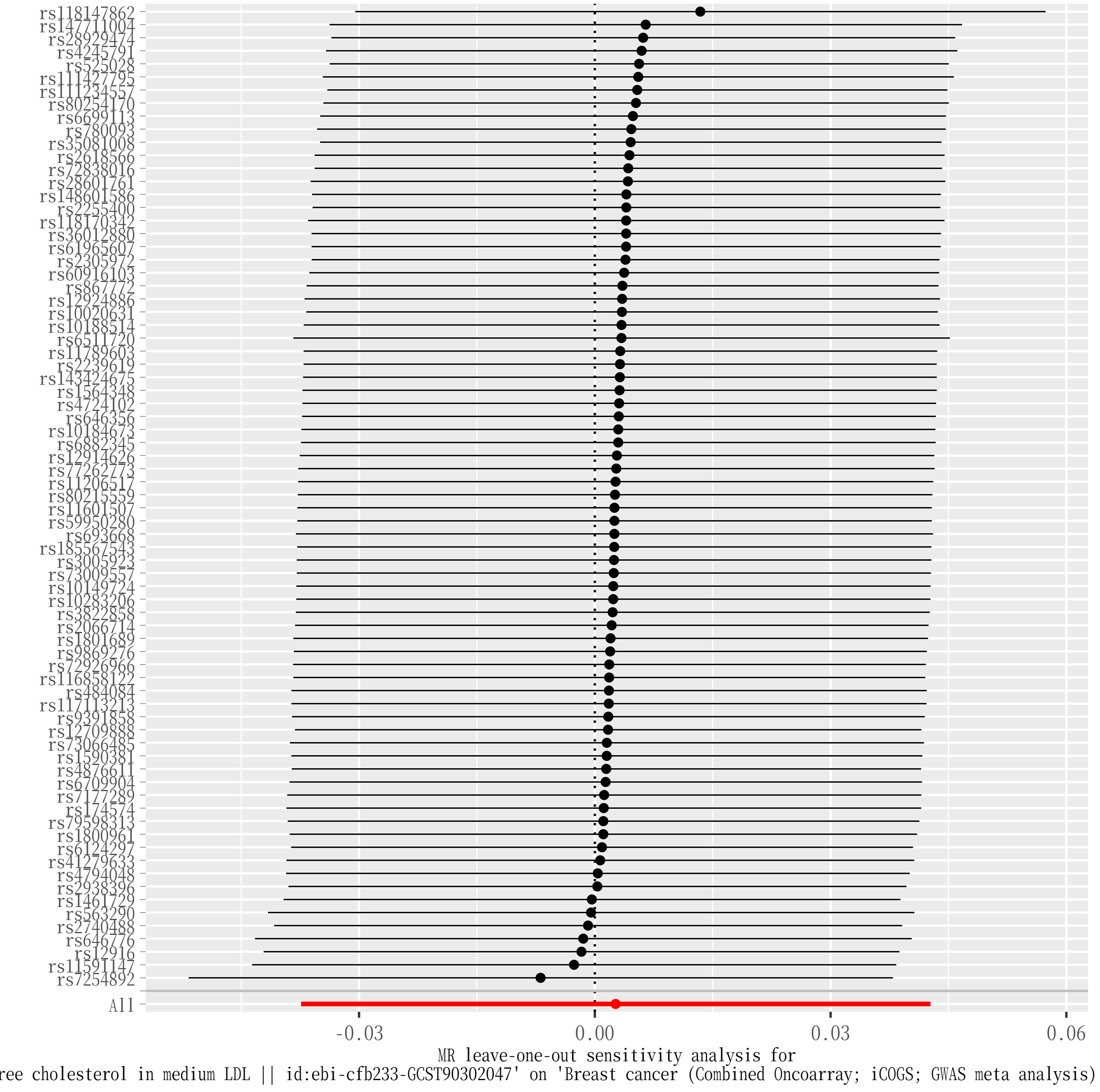

rs118147862
rs147711004
rs28929474
rs4245791
rs525028
rs111427795
rs111234557
rs80254170
rs6699113
rs780093
rs35081008
rs2618566
rs72838016
rs28601761
rs148601586
rs2255400
rs118170342
rs36012880
rs61965607
rs2305972
rs60916103
rs867772
rs12924886
rs10020631
rs10188514
rs6511720
rs11789603
rs2239619
rs143424675
rs1564348
rs4724102
rs646356
rs10184673
rs6882345
rs12914626
rs77262773
rs11206517
rs80215559
rs11601507
rs59950280
rs693668
rs185567543
rs3005923
rs73009557
rs10149724
rs10283206
rs3822858
rs2066714
rs1801689
rs9869276
rs72926966
rs116858122
rs484084
rs117113213
rs9391858
rs12709888
rs73066485
rs1590381
rs4876611
rs6709904
rs7177289
rs174574
rs79598313
rs1800961
rs6124297
rs41279633
rs4794048
rs2938396
rs1461729
rs563290
rs2740488
rs646776
rs12916
rs11591147
rs7254892
All
-0.03
0.00
0.03
0.06
MR leave-one-out sensitivity analysis for
ree cholesterol in medium LDL || id:ebi-cfb233-GCST90302047' on 'Breast cancer (Combined Oncoarray; iCOGS; GWAS meta analysis)

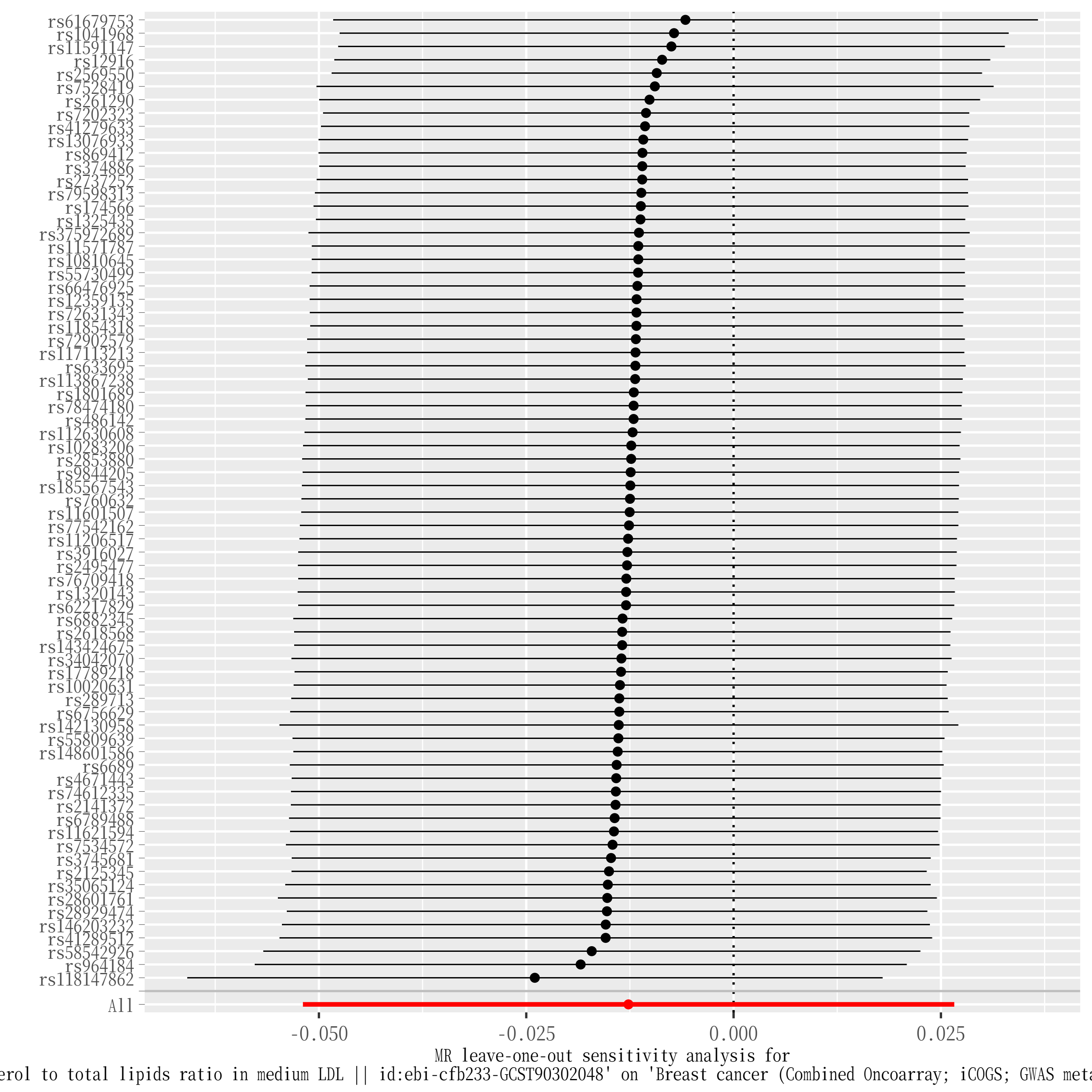
rs61679753
rs1041968
rs11591147
rs12916
rs2569550
rs7528419
rs261290
rs7202323
rs41279633
rs13076933
rs869412
rs374886
rs2737252
rs79598313
rs174566
rs1325435
rs375972689
rs11571787
rs10810645
rs55730499
rs66476925
rs12359135
rs72631343
rs11854318
rs72902579
rs117113213
rs633695
rs113867238
rs1801689
rs78474180
rs486142
rs112630608
rs10283206
rs2853880
rs9844205
rs185567543
rs760632
rs11601507
rs77542162
rs11206517
rs3916027
rs2495477
rs76709418
rs1320143
rs62217829
rs6882345
rs2618568
rs143424675
rs34042070
rs17789218
rs10020631
rs289713
rs6756629
rs142130958
rs55809639
rs148601586
rs6689
rs4671443
rs74612335
rs2141372
rs6789488
rs11621594
rs7534572
rs3745681
rs2125345
rs35065124
rs28601761
rs28929474
rs146203232
rs41289512
rs58542926
rs964184
rs118147862
All
-0.050
-0.025
0.000
0.025
MR leave-one-out sensitivity analysis for
erol to total lipids ratio in medium LDL || id:ebi-cfb233-GCST90302048' on 'Breast cancer (Combined Oncoarray; iCOGS; GWAS meta

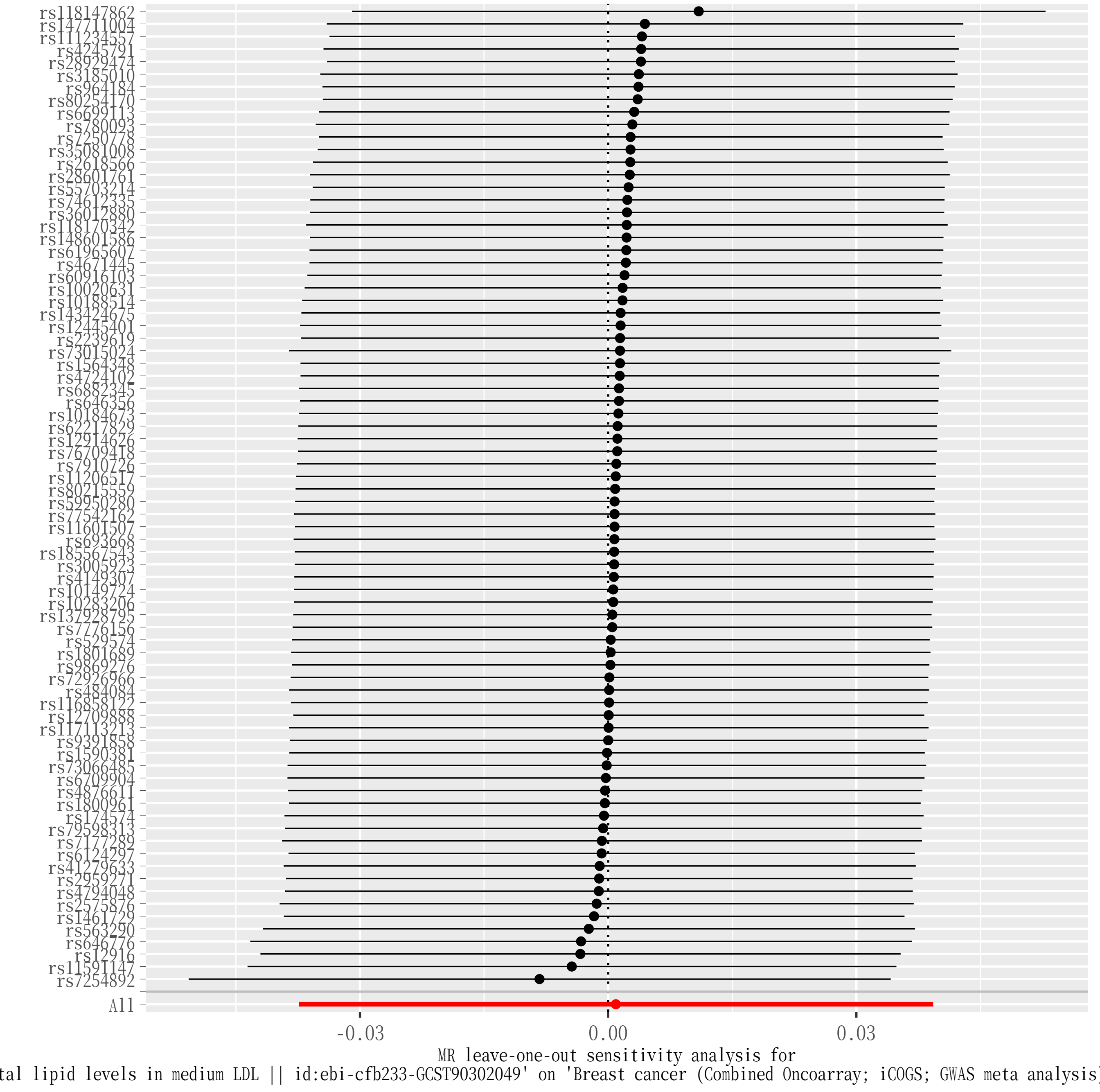

rs118147862
rs147711004
rs111234557
rs4245791
rs28929474
rs3185010
rs964184
rs80254170
rs6699113
rs780093
rs7250778
rs35081008
rs2618566
rs28601761
rs55703214
rs74612335
rs36012880
rs118170342
rs148601586
rs61965607
rs4671445
rs60916103
rs10020631
rs10188514
rs143424675
rs12445401
rs2239619
rs73015024
rs1564348
rs4724102
rs6882345
rs646356
rs10184673
rs62217829
rs12914626
rs76709418
rs7910726
rs11206517
rs80215559
rs59950280
rs77542162
rs11601507
rs693668
rs185567543
rs3005923
rs4149307
rs10149724
rs10283206
rs137928795
rs7776156
rs529574
rs1801689
rs9869276
rs72926966
rs484084
rs116858122
rs12709888
rs117113213
rs9391858
rs1590381
rs73066485
rs6709904
rs4876611
rs1800961
rs174574
rs79598313
rs7177289
rs6124297
rs41279633
rs2959271
rs4794048
rs2575876
rs1461729
rs563290
rs646776
rs12916
rs115911147
rs7254892
All
-0.03
0.00
0.03
MR leave-one-out sensitivity analysis for
tal lipid levels in medium LDL || id:ebi-cfb233-GCST90302049' on 'Breast cancer (Combined Oncoarray; iCOGS; GWAS meta analysis)

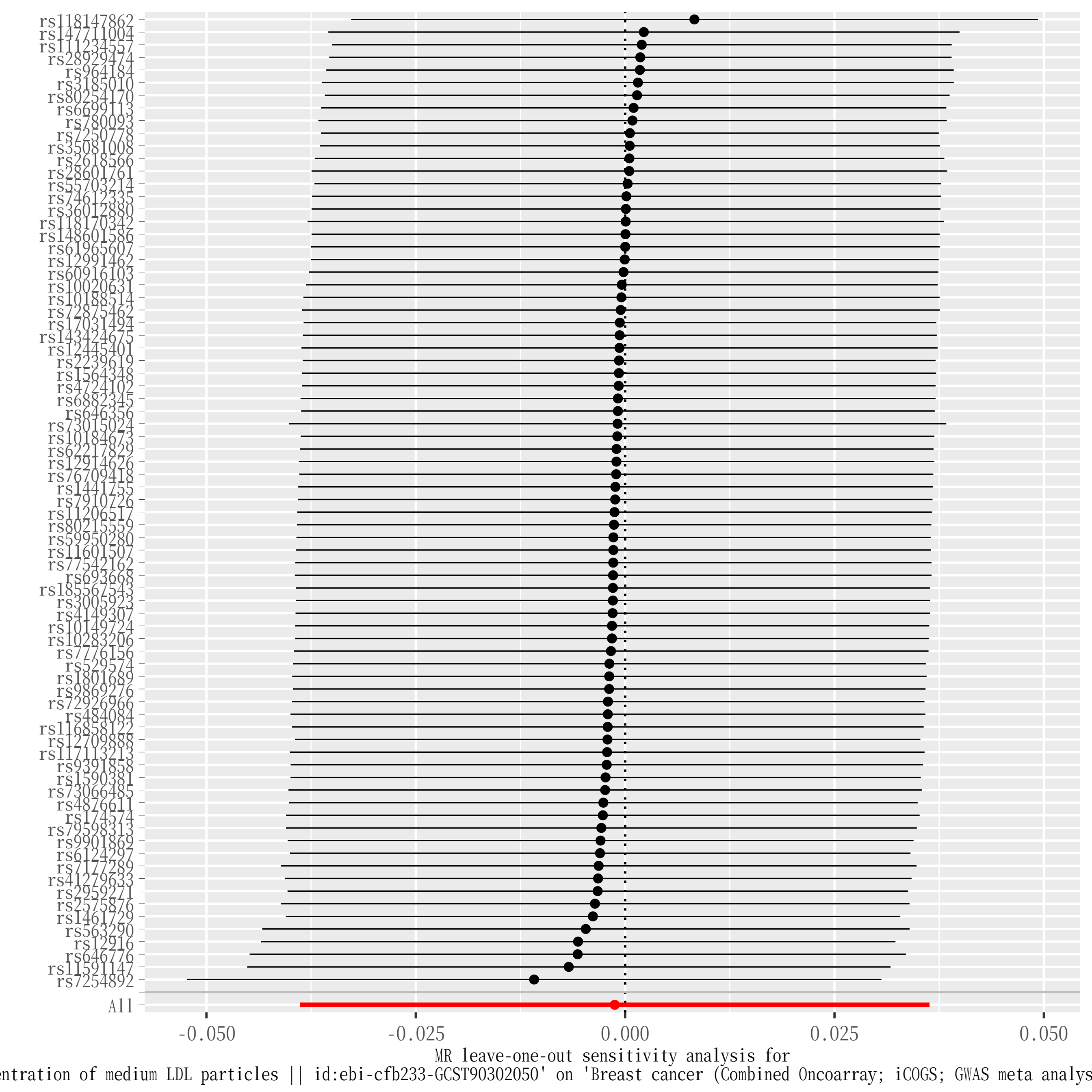
rs118147862
rs147711004
rs111234557
rs28929474
rs964184
rs3185010
rs80254170
rs6699113
rs780093
rs7250778
rs35081008
rs2618566
rs28601761
rs55703214
rs74612335
rs36012880
rs118170342
rs148601586
rs61965607
rs12991462
rs60916103
rs10020631
rs10188514
rs72875462
rs17031494
rs143424675
rs12445401
rs2239619
rs1564348
rs4724102
rs6882345
rs646356
rs73015024
rs10184673
rs62217829
rs12914626
rs76709418
rs1441755
rs7910726
rs11206517
rs80215559
rs59950280
rs11601507
rs77542162
rs693668
rs185567543
rs3005923
rs4149307
rs10149724
rs10283206
rs7776156
rs529574
rs1801689
rs9869276
rs72926966
rs484084
rs116858122
rs12709888
rs117113213
rs9391858
rs1590381
rs73066485
rs4876611
rs174574
rs79598313
rs9901869
rs6124297
rs7177289
rs41279633
rs2959271
rs2575876
rs1461729
rs563290
rs12916
rs646776
rs11591147
rs7254892
All
-0.050
-0.025
0.000
0.025
0.050
MR leave-one-out sensitivity analysis for
entration of medium LDL particles || id:ebi-cfb233-GCST90302050' on 'Breast cancer (Combined Oncoarray; iCOGS; GWAS meta analys

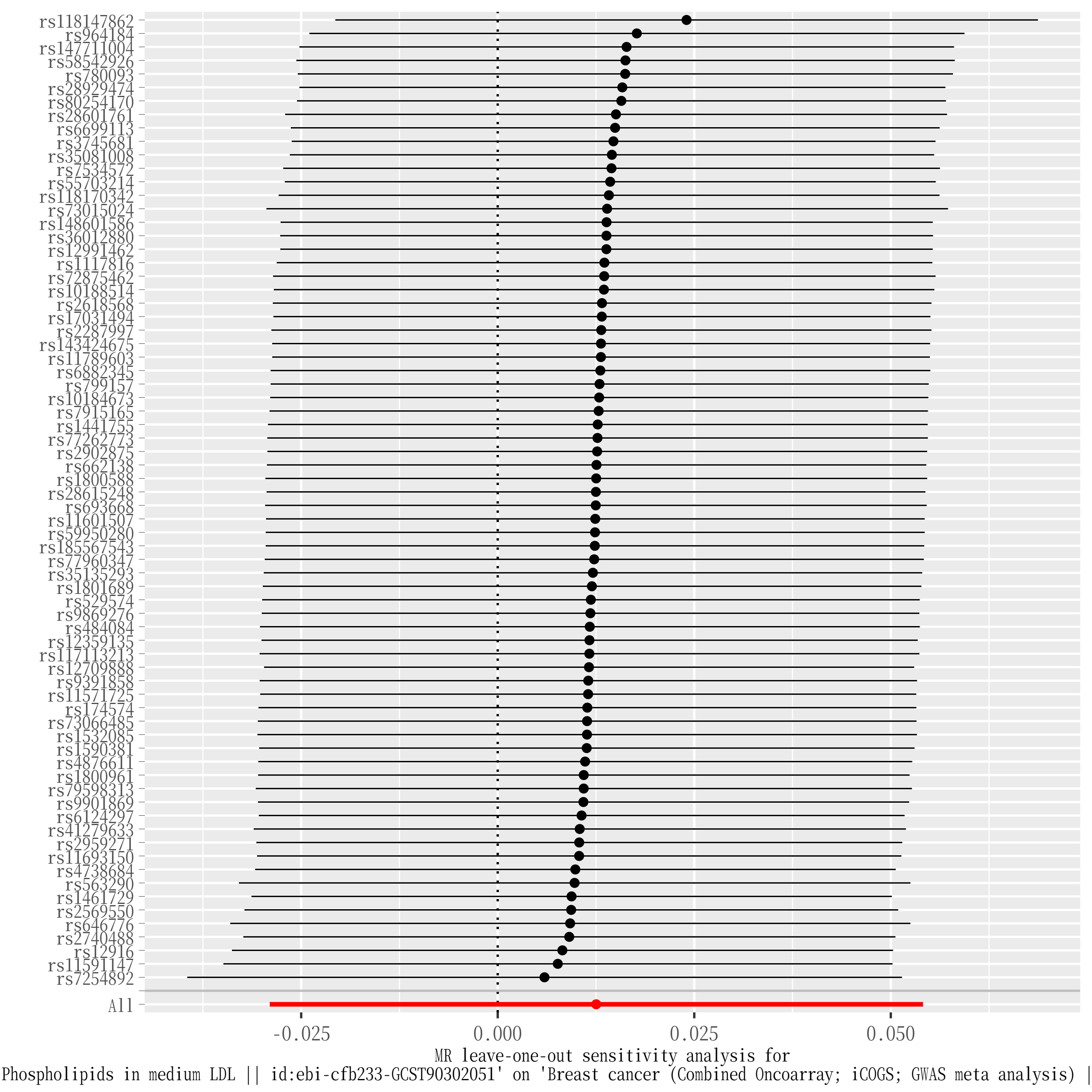

rs118147862
rs964184
rs147711004
rs58542926
rs780093
rs28929474
rs80254170
rs28601761
rs6699113
rs3745681
rs35081008
rs7534572
rs55703214
rs118170342
rs73015024
rs148601586
rs36012880
rs12991462
rs1117816
rs72875462
rs10188514
rs2618568
rs17031494
rs2287997
rs143424675
rs11789603
rs6882345
rs799157
rs10184673
rs7915165
rs1441755
rs77262773
rs2902875
rs662138
rs1800588
rs28615248
rs693668
rs11601507
rs59950280
rs185567543
rs77960347
rs35135293
rs1801689
rs529574
rs9869276
rs484084
rs12359135
rs117113213
rs12709888
rs9391858
rs11571725
rs174574
rs73066485
rs1532085
rs1590381
rs4876611
rs1800961
rs79598313
rs9901869
rs6124297
rs41279633
rs2959271
rs11693150
rs4738684
rs563290
rs1461729
rs2569550
rs646776
rs2740488
rs12916
rs11591147
rs7254892
All
-0.025
0.000
0.025
0.050
MR leave-one-out sensitivity analysis for
Phospholipids in medium LDL || id:ebi-cfb233-GCST90302051' on 'Breast cancer (Combined Oncoarray; iCOGS; GWAS meta analysis) |

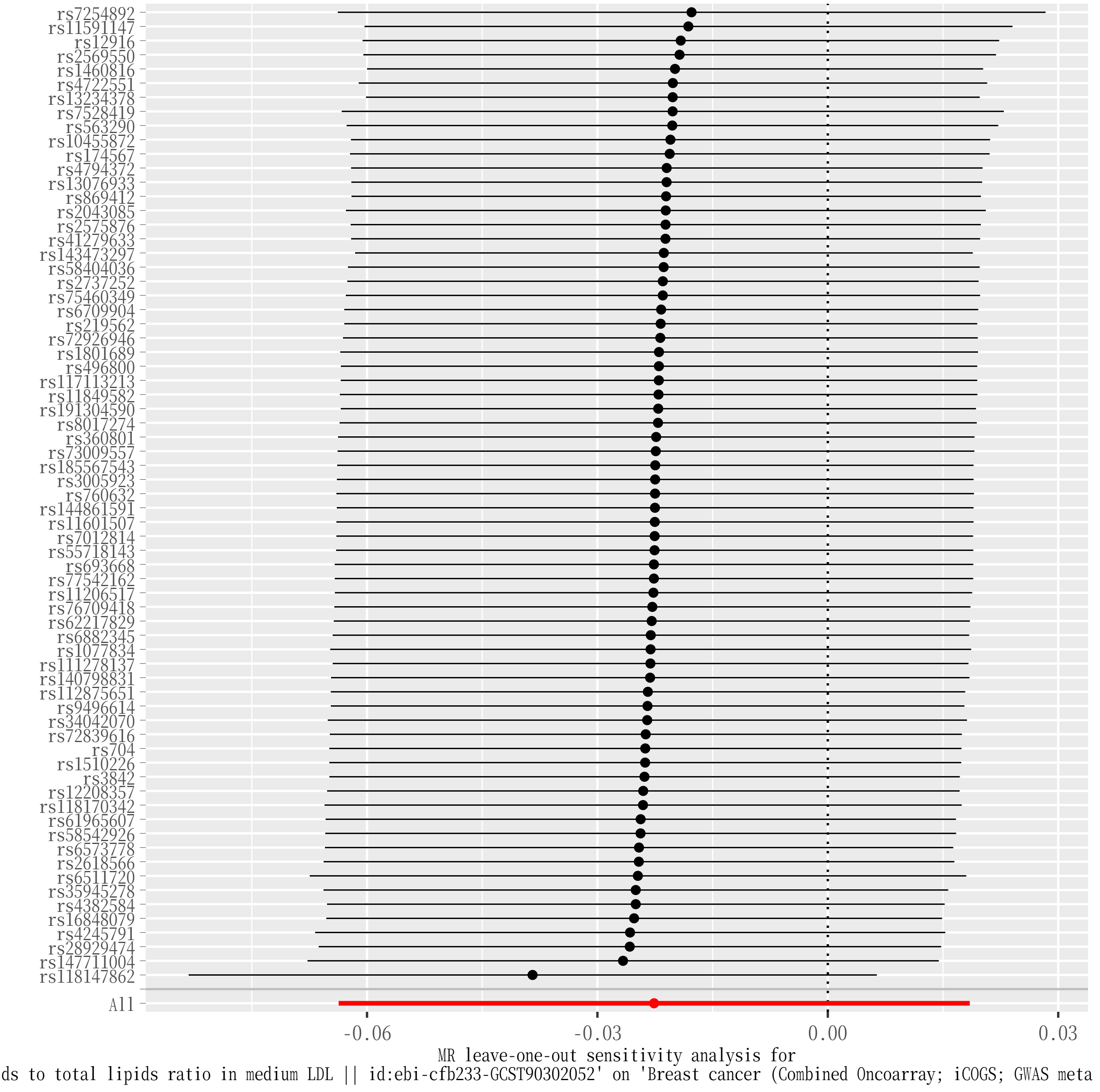
rs7254892
rs11591147
rs12916
rs2569550
rs1460816
rs4722551
rs13234378
rs7528419
rs563290
rs10455872
rs174567
rs4794372
rs13076933
rs869412
rs2043085
rs2575876
rs41279633
rs143473297
rs58404036
rs2737252
rs75460349
rs6709904
rs219562
rs72926946
rs1801689
rs496800
rs117113213
rs11849582
rs191304590
rs8017274
rs360801
rs73009557
rs185567543
rs3005923
rs760632
rs144861591
rs11601507
rs7012814
rs55718143
rs693668
rs77542162
rs11206517
rs76709418
rs62217829
rs6882345
rs1077834
rs111278137
rs140798831
rs112875651
rs9496614
rs34042070
rs72839616
rs704
rs1510226
rs3842
rs12208357
rs118170342
rs61965607
rs58542926
rs6573778
rs2618566
rs6511720
rs35945278
rs4382584
rs16848079
rs4245791
rs28929474
rs147711004
rs118147862
All
-0.06
-0.03
0.00
0.03
MR leave-one-out sensitivity analysis for
ds to total lipids ratio in medium LDL || id:ebi-cfb233-GCST90302052' on 'Breast cancer (Combined Oncoarray; iCOGS; GWAS meta

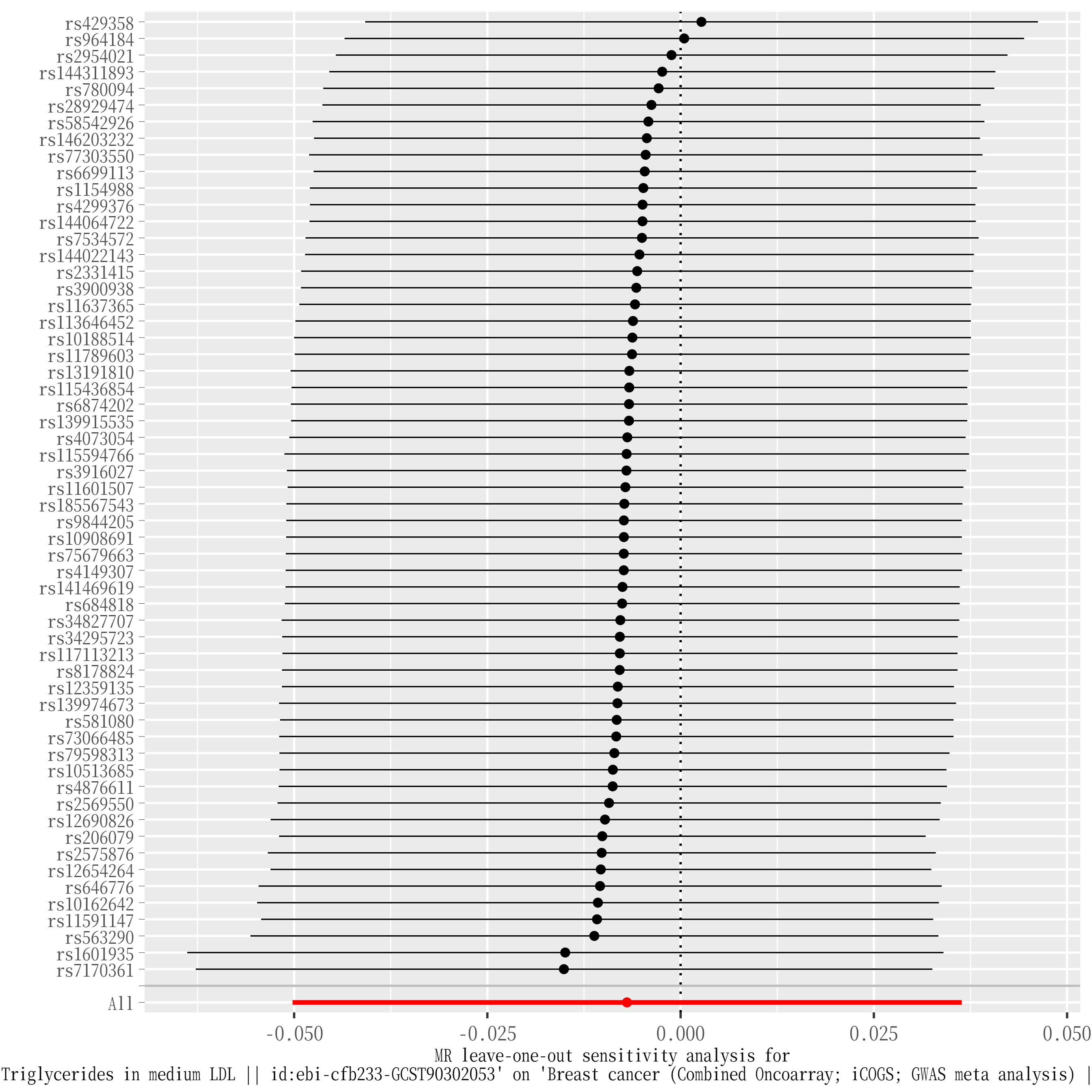

rs429358
rs964184
rs2954021
rs144311893
rs780094
rs28929474
rs58542926
rs146203232
rs77303550
rs6699113
rs1154988
rs4299376
rs144064722
rs7534572
rs144022143
rs2331415
rs3900938
rs11637365
rs113646452
rs10188514
rs11789603
rs13191810
rs115436854
rs6874202
rs139915535
rs4073054
rs115594766
rs3916027
rs11601507
rs185567543
rs9844205
rs10908691
rs75679663
rs4149307
rs141469619
rs684818
rs34827707
rs34295723
rs117113213
rs8178824
rs12359135
rs139974673
rs581080
rs73066485
rs79598313
rs10513685
rs4876611
rs2569550
rs12690826
rs206079
rs2575876
rs12654264
rs646776
rs10162642
rs11591147
rs563290
rs1601935
rs7170361
All
-0.050
-0.025
0.000
0.025
0.050
MR leave-one-out sensitivity analysis for
'Triglycerides in medium LDL || id:ebi-cfb233-GCST90302053' on 'Breast cancer (Combined Oncoarray; iCOGS; GWAS meta analysis) ||

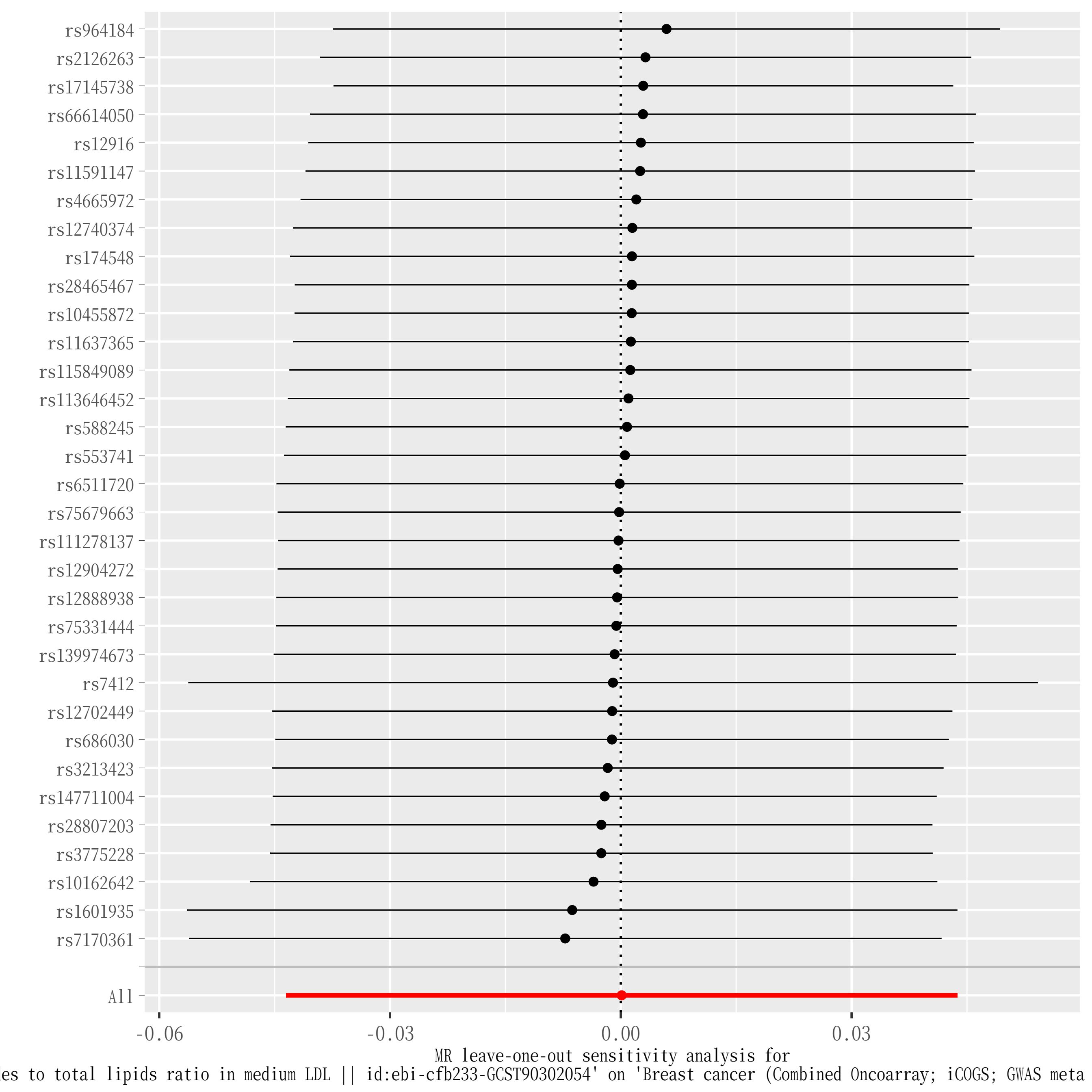
rs964184
rs2126263
rs17145738
rs66614050
rs12916
rs11591147
rs4665972
rs12740374
rs174548
rs28465467
rs10455872
rs11637365
rs115849089
rs113646452
rs588245
rs553741
rs6511720
rs75679663
rs111278137
rs12904272
rs12888938
rs75331444
rs139974673
rs7412
rs12702449
rs686030
rs3213423
rs147711004
rs28807203
rs3775228
rs10162642
rs1601935
rs7170361
All
-0.06
-0.03
0.00
0.03
MR leave-one-out sensitivity analysis for
les to total lipids ratio in medium LDL || id:ebi-cfb233-GCST90302054' on 'Breast cancer (Combined Oncoarray; iCOGS; GWAS meta

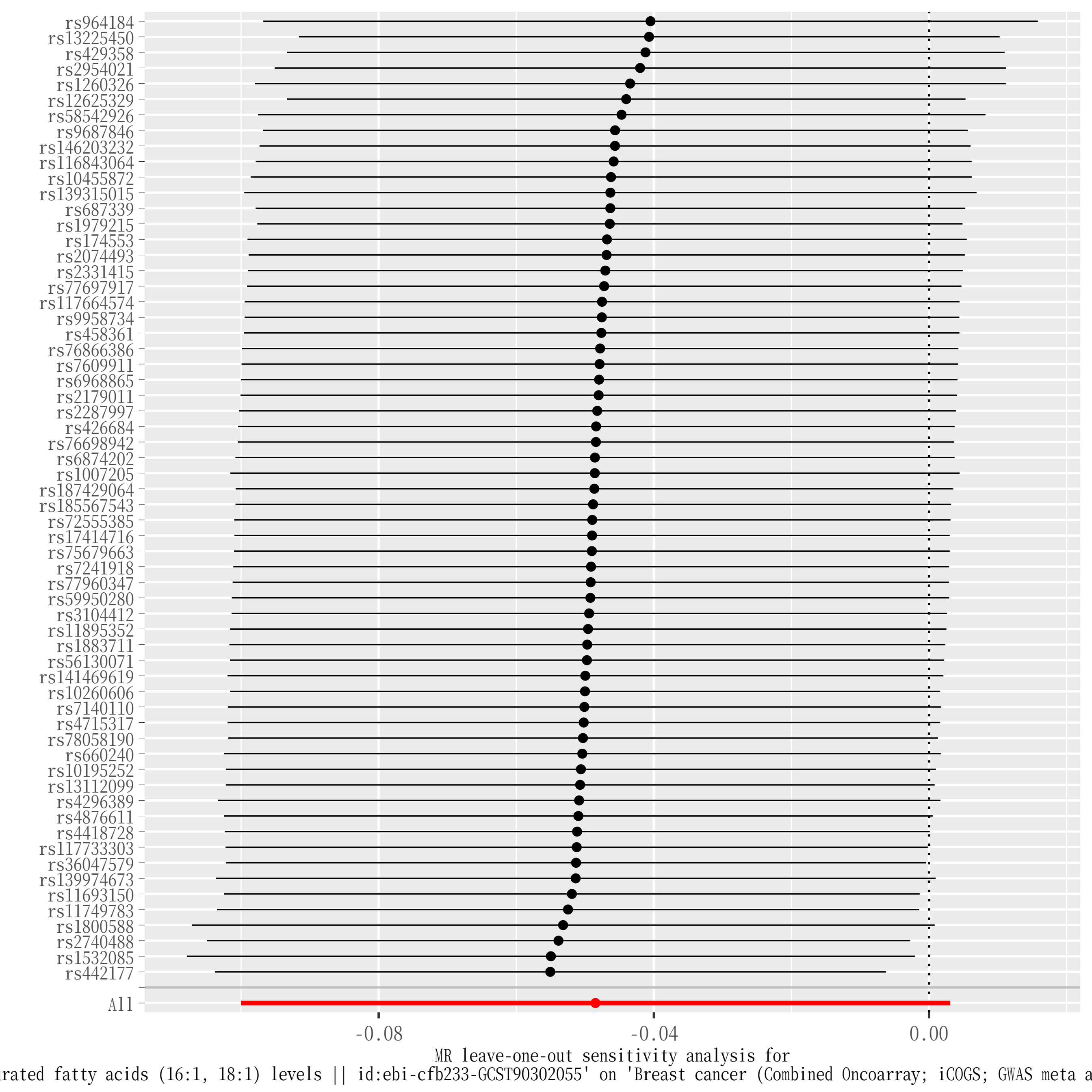
rs964184
rs13225450
rs429358
rs2954021
rs1260326
rs12625329
rs58542926
rs9687846
rs146203232
rs116843064
rs10455872
rs139315015
rs687339
rs1979215
rs174553
rs2074493
rs2331415
rs77697917
rs117664574
rs9958734
rs458361
rs76866386
rs7609911
rs6968865
rs2179011
rs2287997
rs426684
rs76698942
rs6874202
rs1007205
rs187429064
rs185567543
rs72555385
rs17414716
rs75679663
rs7241918
rs77960347
rs59950280
rs3104412
rs11895352
rs1883711
rs56130071
rs141469619
rs10260606
rs7140110
rs4715317
rs78058190
rs660240
rs10195252
rs13112099
rs4296389
rs4876611
rs4418728
rs117733303
rs36047579
rs139974673
rs11693150
rs11749783
rs1800588
rs2740488
rs1532085
rs442177
All
-0.08
-0.04
0.00
MR leave-one-out sensitivity analysis for
rated fatty acids (16:1, 18:1) levels || id:ebi-cfb233-GCST90302055' on 'Breast cancer (Combined Oncoarray; iCOGS; GWAS meta a

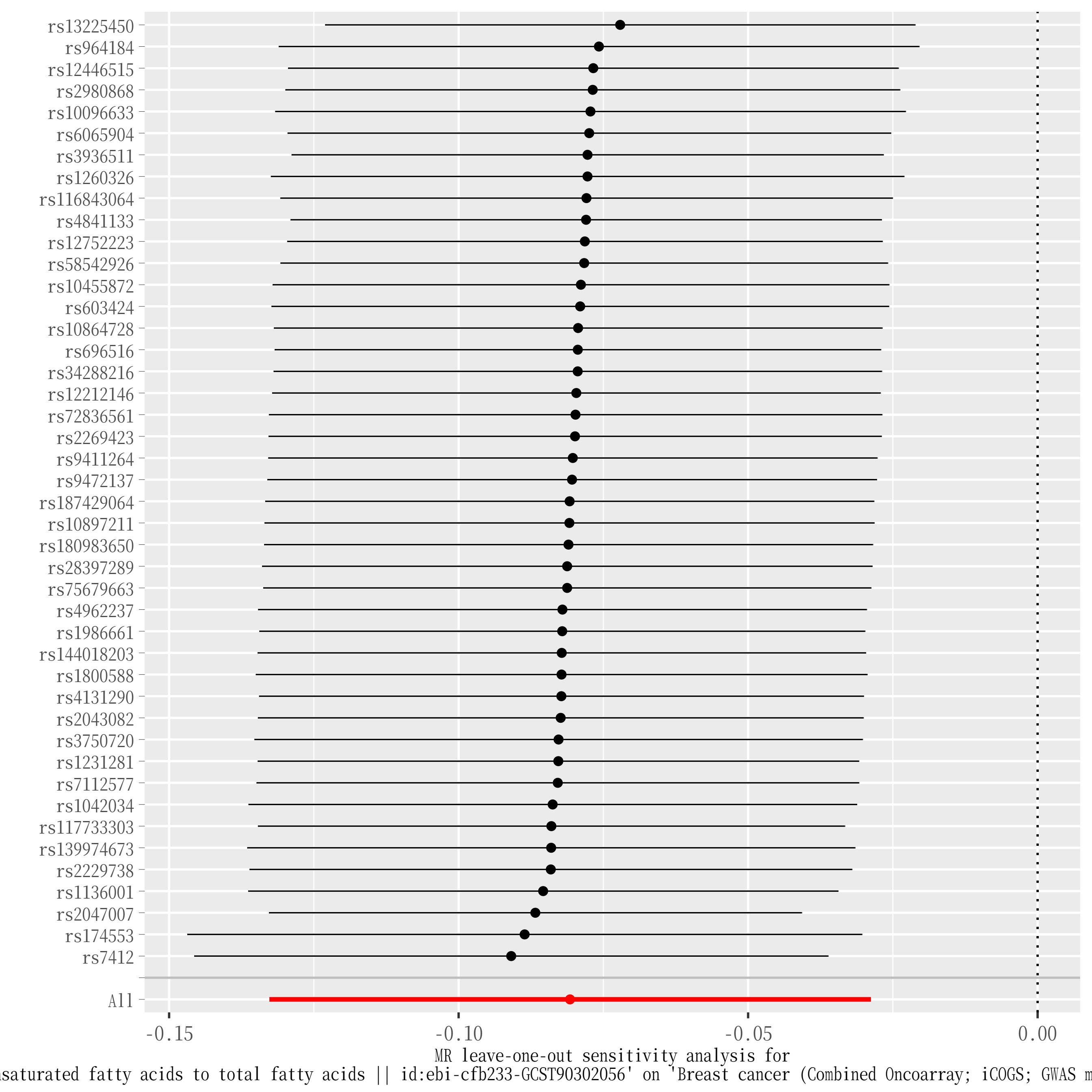

rs13225450
rs964184
rs12446515
rs2980868
rs10096633
rs6065904
rs3936511
rs1260326
rs116843064
rs4841133
rs12752223
rs58542926
rs10455872
rs603424
rs10864728
rs696516
rs34288216
rs12212146
rs72836561
rs2269423
rs9411264
rs9472137
rs187429064
rs10897211
rs180983650
rs28397289
rs75679663
rs4962237
rs1986661
rs144018203
rs1800588
rs4131290
rs2043082
rs3750720
rs1231281
rs7112577
rs1042034
rs117733303
rs139974673
rs2229738
rs1136001
rs2047007
rs174553
rs7412
All
-0.15
-0.10
-0.05
0.00
MR leave-one-out sensitivity analysis for
nsaturated fatty acids to total fatty acids || id:ebi-cfb233-GCST90302056' on 'Breast cancer (Combined Oncoarray; iCOGS; GWAS m

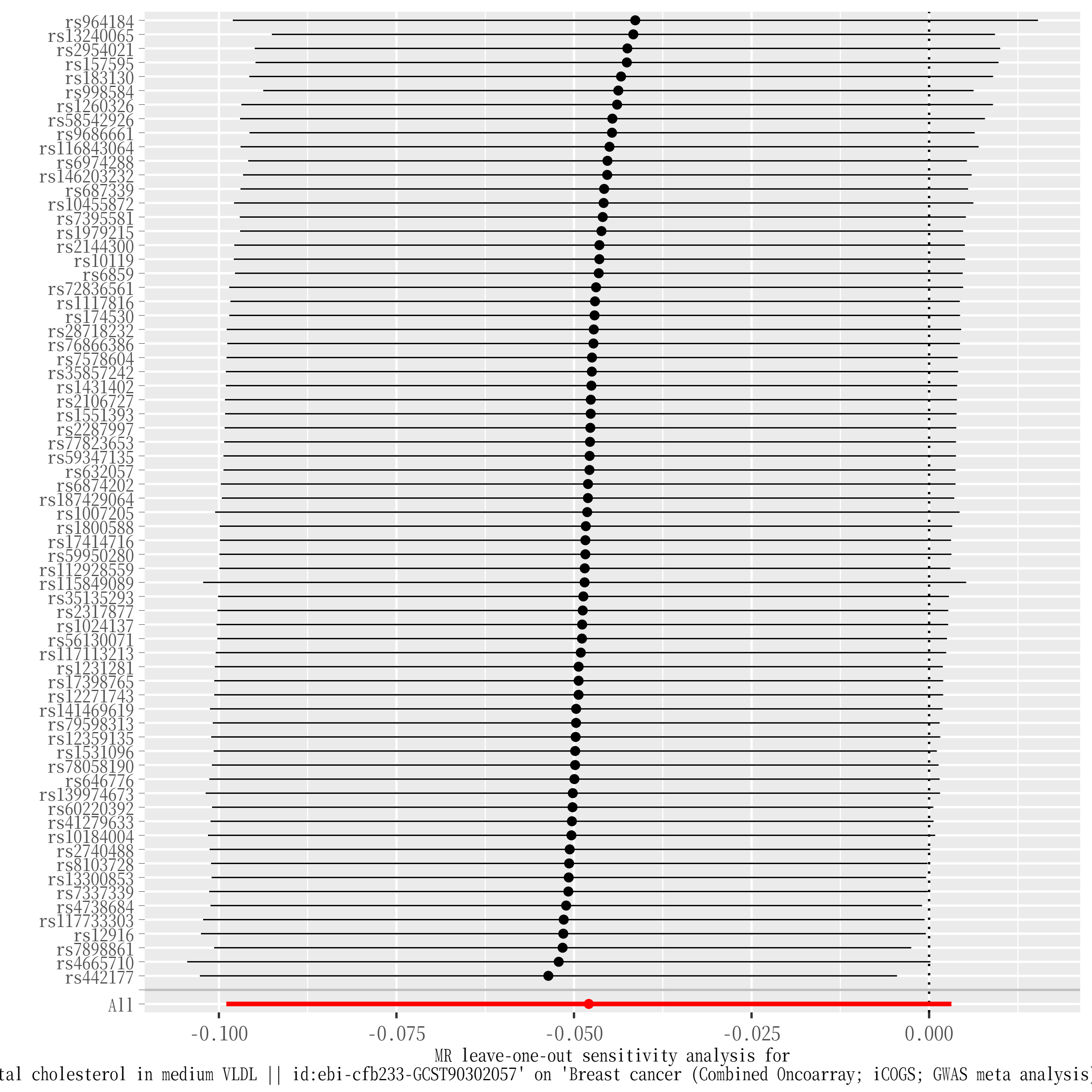
rs964184
rs13240065
rs2954021
rs157595
rs183130
rs998584
rs1260326
rs58542926
rs9686661
rs116843064
rs6974288
rs146203232
rs687339
rs10455872
rs7395581
rs1979215
rs2144300
rs10119
rs6859
rs72836561
rs1117816
rs174530
rs28718232
rs76866386
rs7578604
rs35857242
rs1431402
rs2106727
rs1551393
rs2287997
rs77823653
rs59347135
rs632057
rs6874202
rs187429064
rs1007205
rs1800588
rs17414716
rs59950280
rs112928559
rs115849089
rs35135293
rs2317877
rs1024137
rs56130071
rs117113213
rs1231281
rs17398765
rs12271743
rs141469619
rs79598313
rs12359135
rs1531096
rs78058190
rs646776
rs139974673
rs60220392
rs41279633
rs10184004
rs2740488
rs8103728
rs13300853
rs7337339
rs4738684
rs117733303
rs12916
rs7898861
rs4665710
rs442177
All
-0.100
-0.075
-0.050
-0.025
0.000
MR leave-one-out sensitivity analysis for
tal cholesterol in medium VLDL || id:ebi-cfb233-GCST90302057' on 'Breast cancer (Combined Oncoarray; iCOGS; GWAS meta analysis)

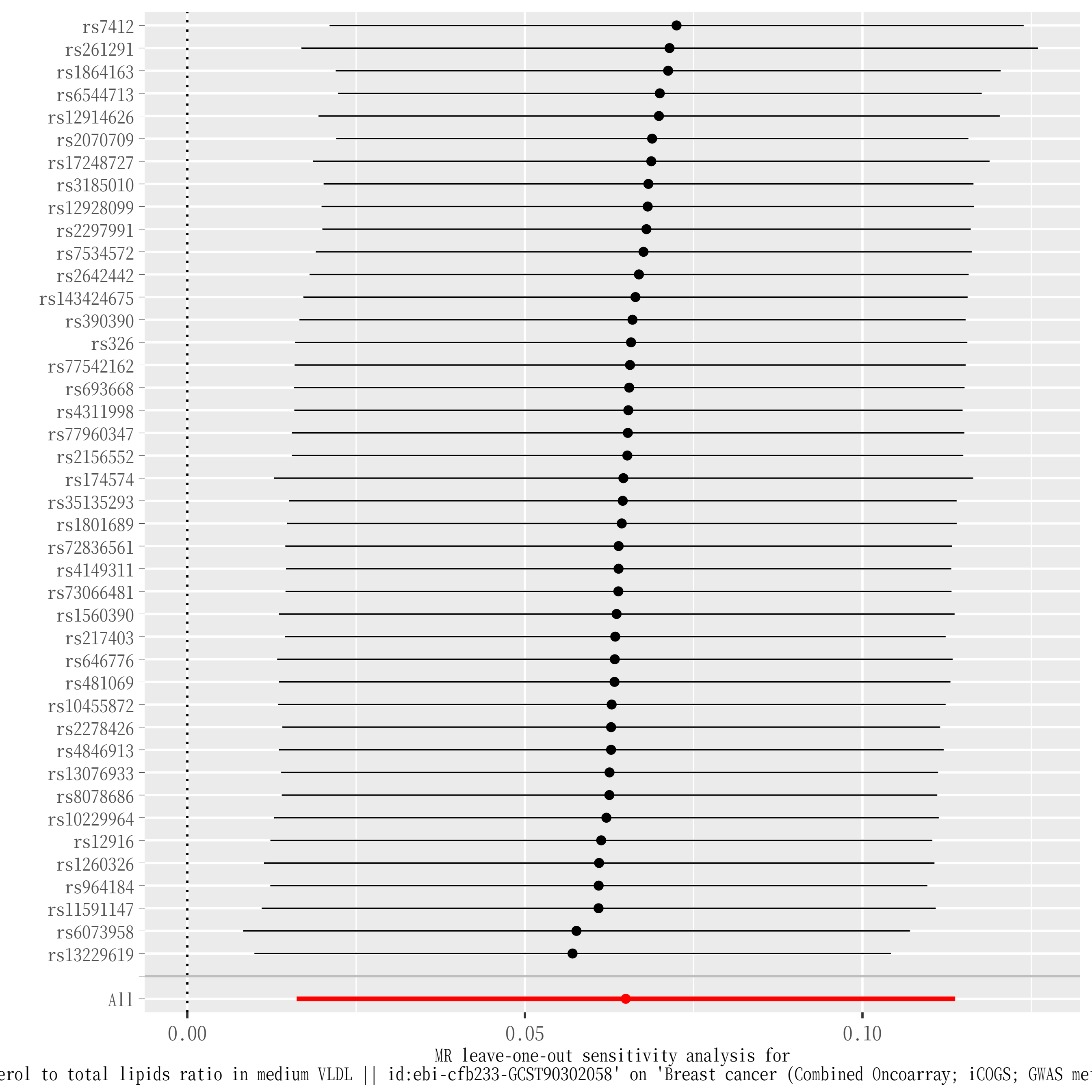
rs7412
rs261291
rs1864163
rs6544713
rs12914626
rs2070709
rs17248727
rs3185010
rs12928099
rs2297991
rs7534572
rs2642442
rs143424675
rs390390
rs326
rs77542162
rs693668
rs4311998
rs77960347
rs2156552
rs174574
rs35135293
rs1801689
rs72836561
rs4149311
rs73066481
rs1560390
rs217403
rs646776
rs481069
rs10455872
rs2278426
rs4846913
rs13076933
rs8078686
rs10229964
rs12916
rs1260326
rs964184
rs11591147
rs6073958
rs13229619
All
0.00
0.05
0.10
MR leave-one-out sensitivity analysis for
erol to total lipids ratio in medium VLDL || id:ebi-cfb233-GCST90302058' on 'Breast cancer (Combined Oncoarray; iCOGS; GWAS me

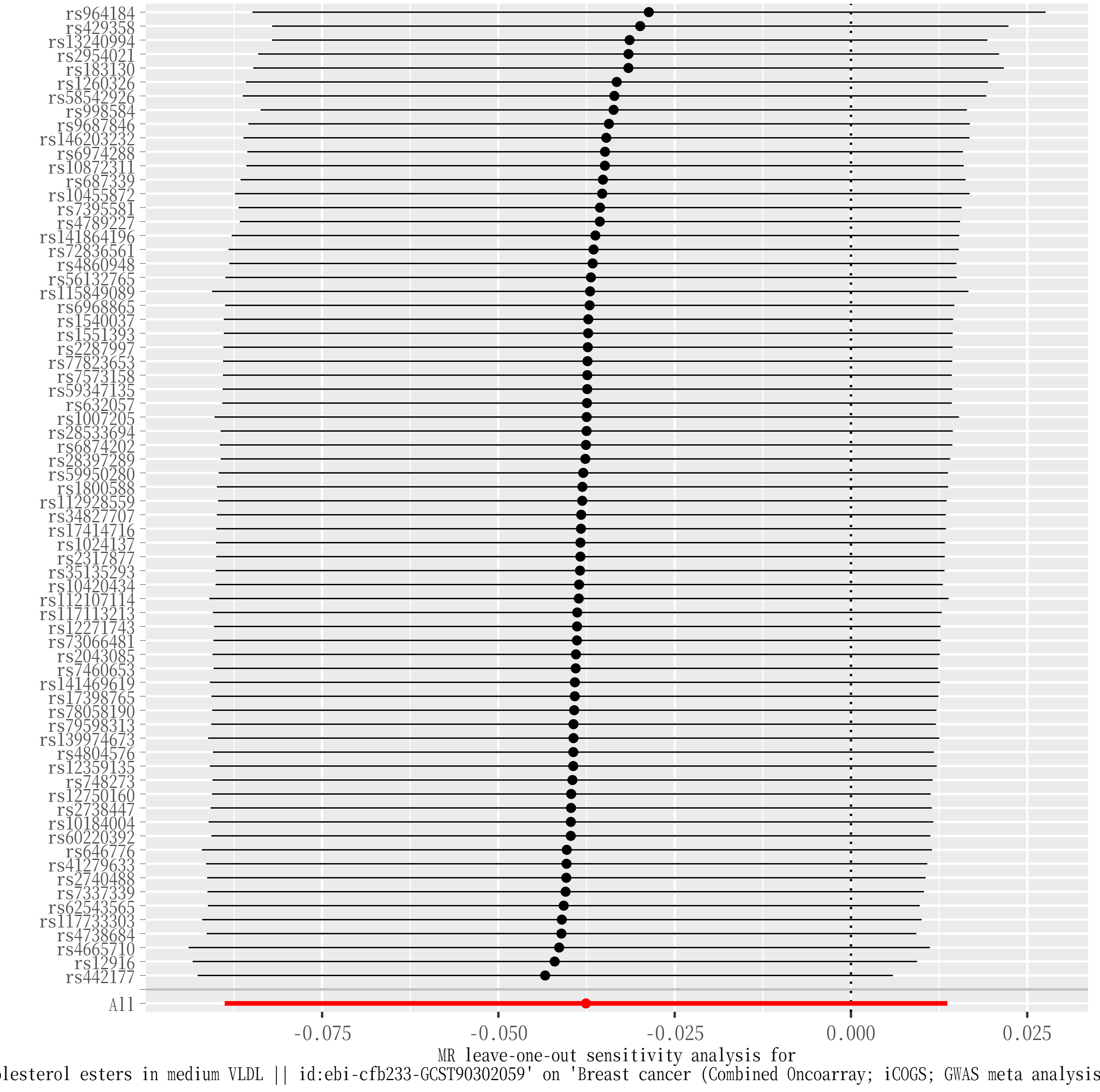

rs964184
rs429358
rs13240994
rs2954021
rs183130
rs1260326
rs58542926
rs998584
rs9687846
rs146203232
rs6974288
rs10872311
rs687339
rs10455872
rs7395581
rs4789227
rs141864196
rs72836561
rs4860948
rs56132765
rs115849089
rs6968865
rs1540037
rs1551393
rs2287997
rs77823653
rs7573158
rs59347135
rs632057
rs1007205
rs28533694
rs6874202
rs28397289
rs59950280
rs1800588
rs112928559
rs34827707
rs17414716
rs1024137
rs2317877
rs35135293
rs10420434
rs112107114
rs117113213
rs12271743
rs73066481
rs2043085
rs7460653
rs141469619
rs17398765
rs78058190
rs79598313
rs139974673
rs4804576
rs12359135
rs748273
rs12750160
rs2738447
rs10184004
rs60220392
rs646776
rs41279633
rs2740488
rs7337339
rs62543565
rs117733303
rs4738684
rs4665710
rs12916
rs442177
All
-0.075
-0.050
-0.025
0.000
0.025
MR leave-one-out sensitivity analysis for
lesterol esters in medium VLDL || id:ebi-cfb233-GCST90302059' on 'Breast cancer (Combined Oncoarray; iCOGS; GWAS meta analysis

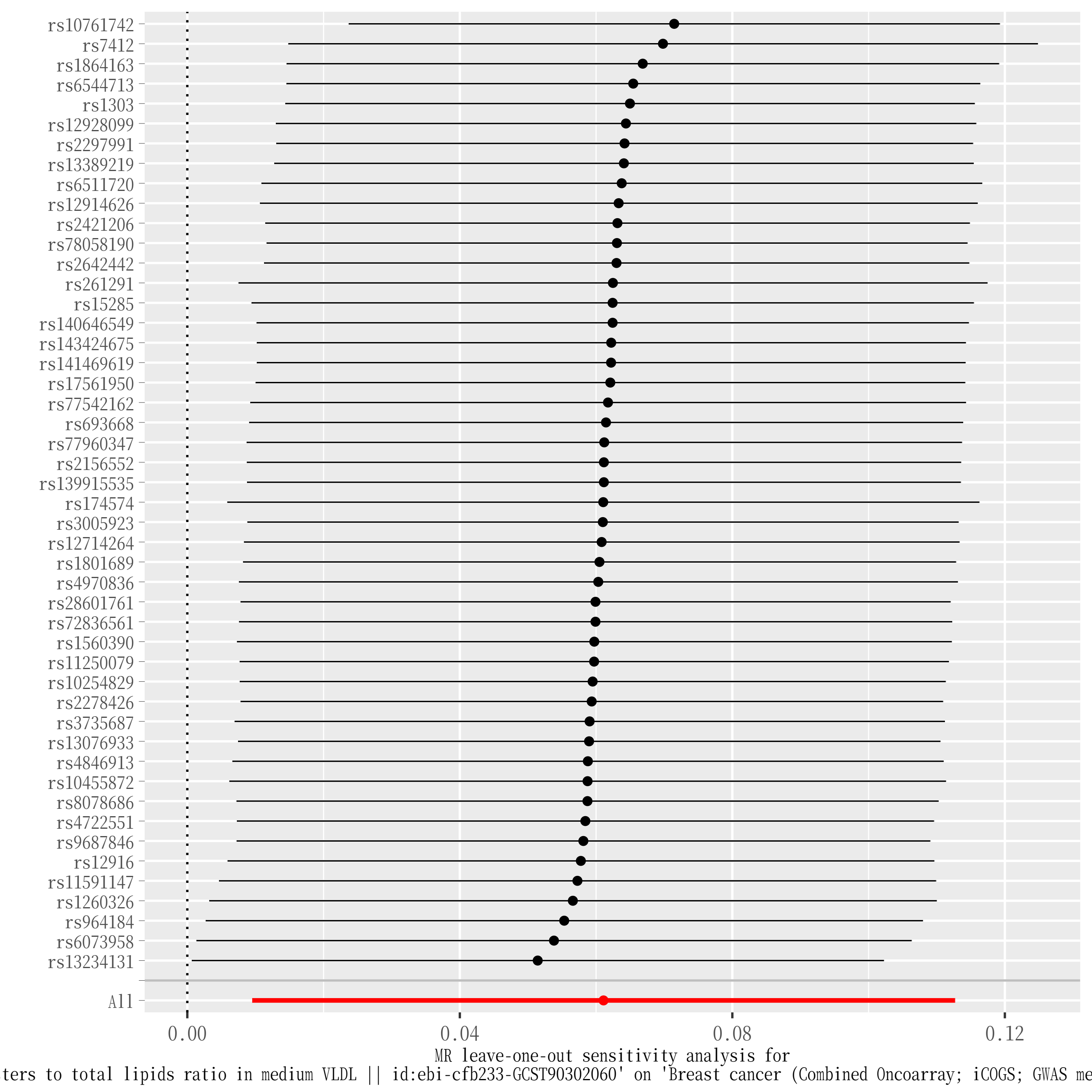

rs10761742
rs7412
rs1864163
rs6544713
rs1303
rs12928099
rs2297991
rs13389219
rs6511720
rs12914626
rs2421206
rs78058190
rs2642442
rs261291
rs15285
rs140646549
rs143424675
rs141469619
rs17561950
rs77542162
rs693668
rs77960347
rs2156552
rs139915535
rs174574
rs3005923
rs12714264
rs1801689
rs4970836
rs28601761
rs72836561
rs1560390
rs11250079
rs10254829
rs2278426
rs3735687
rs13076933
rs4846913
rs10455872
rs8078686
rs4722551
rs9687846
rs12916
rs11591147
rs1260326
rs964184
rs6073958
rs13234131
All
0.00
0.04
0.08
0.12
MR leave-one-out sensitivity analysis for
ters to total lipids ratio in medium VLDL || id:ebi-cfb233-GCST90302060' on 'Breast cancer (Combined Oncoarray; iCOGS; GWAS me

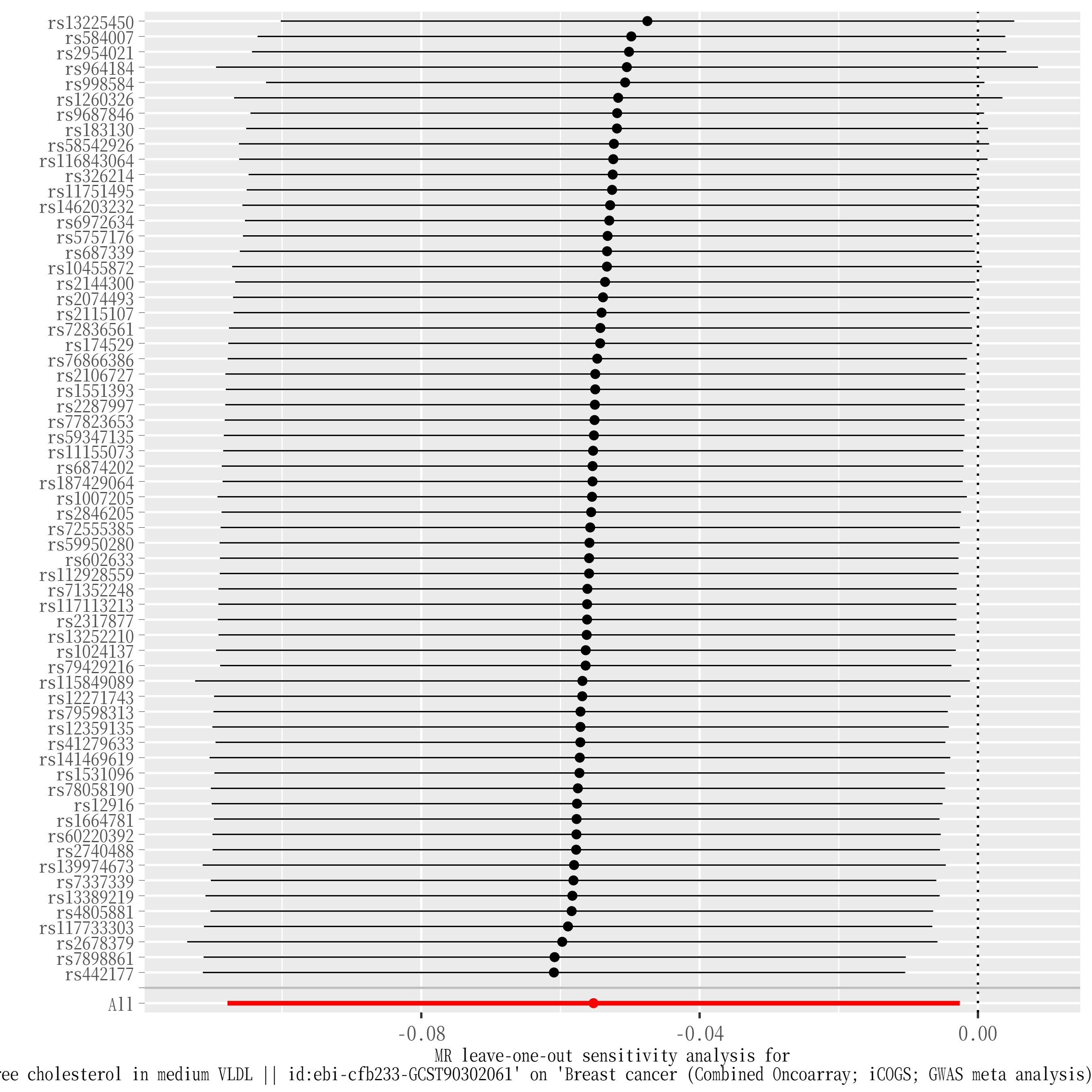
rs13225450
rs584007
rs2954021
rs964184
rs998584
rs1260326
rs9687846
rs183130
rs58542926
rs116843064
rs326214
rs11751495
rs146203232
rs6972634
rs5757176
rs687339
rs10455872
rs2144300
rs2074493
rs2115107
rs72836561
rs174529
rs76866386
rs2106727
rs1551393
rs2287997
rs77823653
rs59347135
rs11155073
rs6874202
rs187429064
rs1007205
rs2846205
rs72555385
rs59950280
rs602633
rs112928559
rs71352248
rs117113213
rs2317877
rs13252210
rs1024137
rs79429216
rs115849089
rs12271743
rs79598313
rs12359135
rs41279633
rs141469619
rs1531096
rs78058190
rs12916
rs1664781
rs60220392
rs2740488
rs139974673
rs7337339
rs13389219
rs4805881
rs117733303
rs2678379
rs7898861
rs442177
All
-0.08
-0.04
0.00
MR leave-one-out sensitivity analysis for
ree cholesterol in medium VLDL || id:ebi-cfb233-GCST90302061' on 'Breast cancer (Combined Oncoarray; iCOGS; GWAS meta analysis)

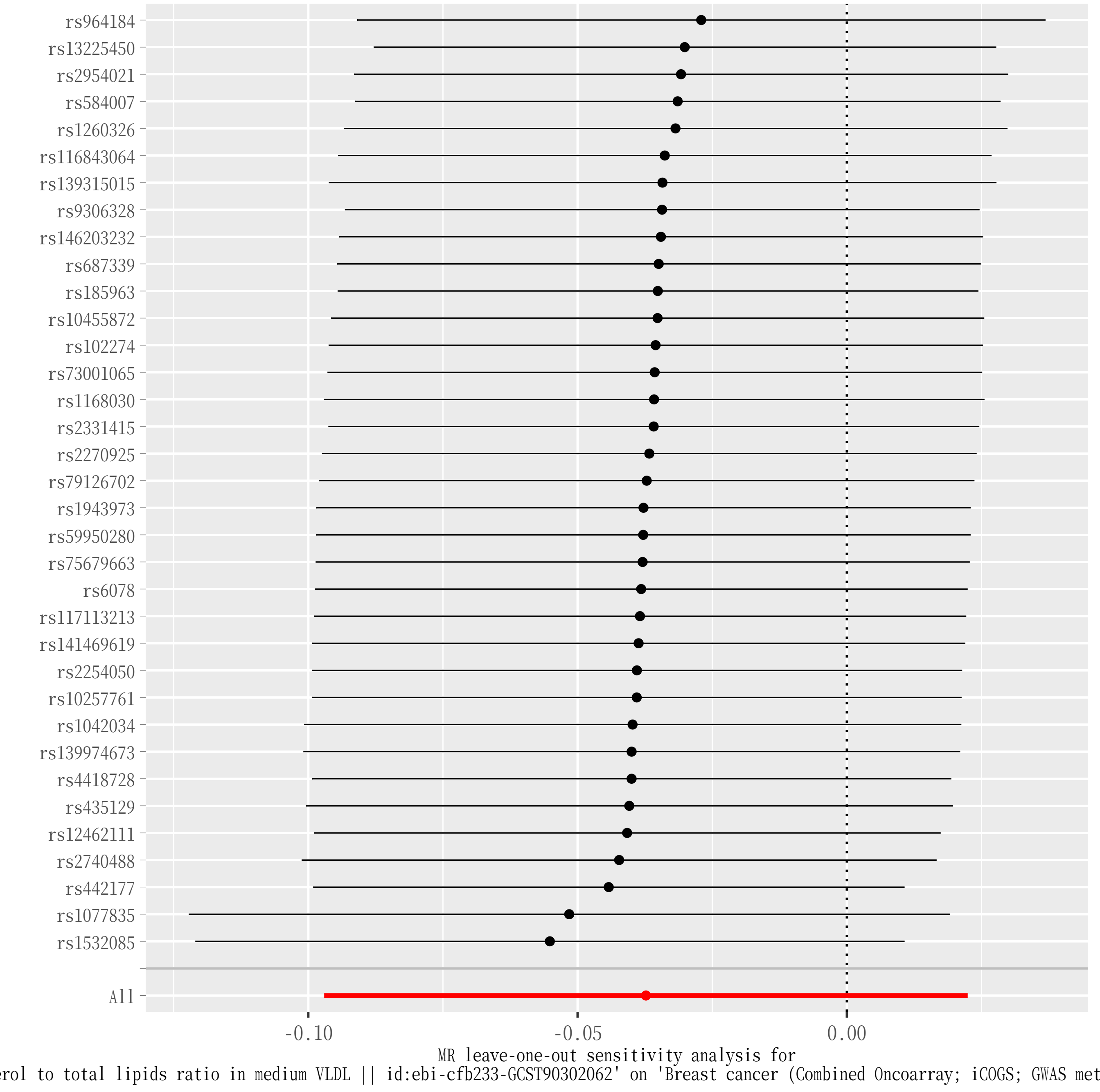
rs964184
rs13225450
rs2954021
rs584007
rs1260326
rs116843064
rs139315015
rs9306328
rs146203232
rs687339
rs185963
rs10455872
rs102274
rs73001065
rs1168030
rs2331415
rs2270925
rs79126702
rs1943973
rs59950280
rs75679663
rs6078
rs117113213
rs141469619
rs2254050
rs10257761
rs1042034
rs139974673
rs4418728
rs435129
rs12462111
rs2740488
rs442177
rs1077835
rs1532085
All
-0.10
-0.05
0.00
MR leave-one-out sensitivity analysis for
erol to total lipids ratio in medium VLDL || id:ebi-cfb233-GCST90302062' on 'Breast cancer (Combined Oncoarray; iCOGS; GWAS met

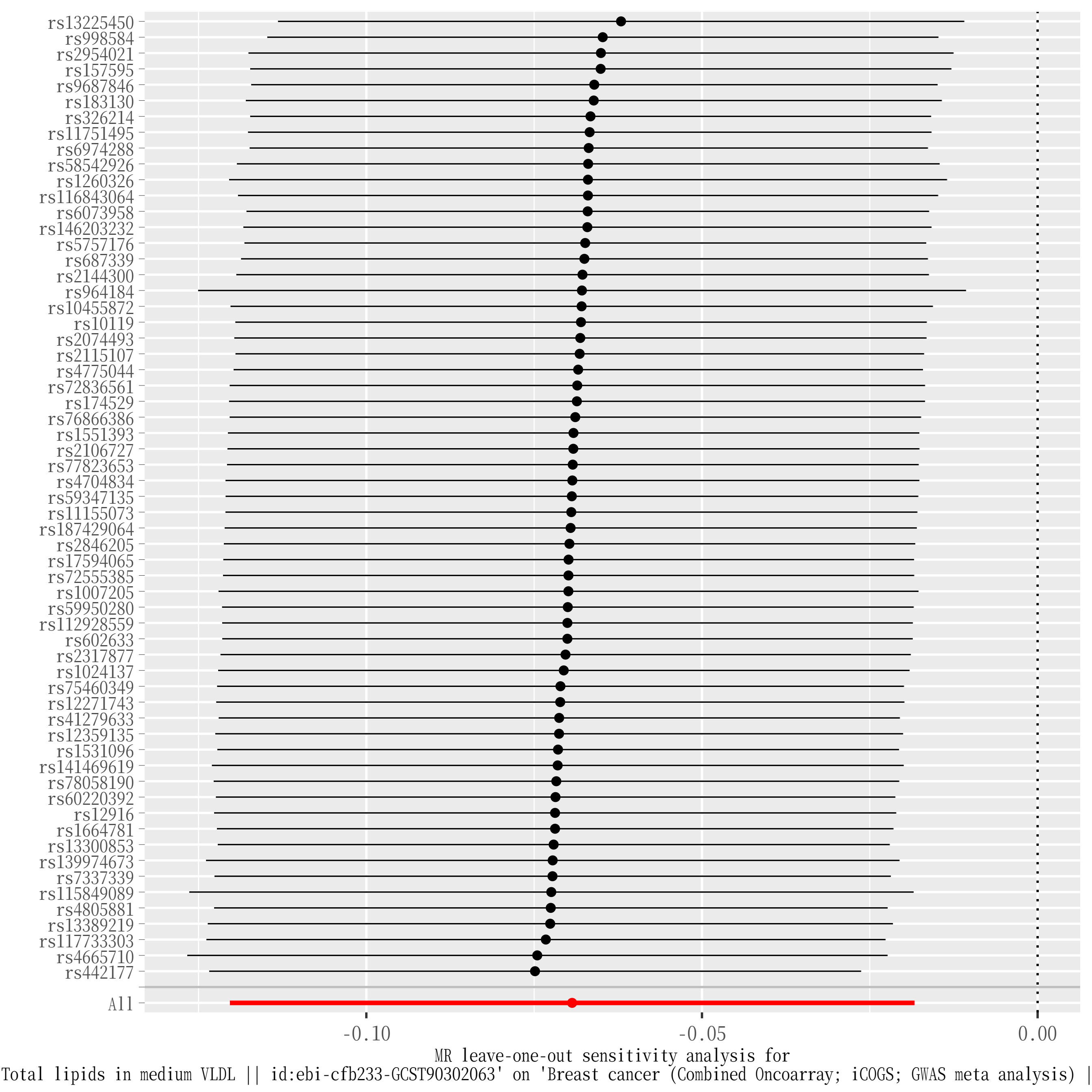

rs13225450
rs998584
rs2954021
rs157595
rs9687846
rs183130
rs326214
rs11751495
rs6974288
rs58542926
rs1260326
rs116843064
rs6073958
rs146203232
rs5757176
rs687339
rs2144300
rs964184
rs10455872
rs10119
rs2074493
rs2115107
rs4775044
rs72836561
rs174529
rs76866386
rs1551393
rs2106727
rs77823653
rs4704834
rs59347135
rs11155073
rs187429064
rs2846205
rs17594065
rs72555385
rs1007205
rs59950280
rs112928559
rs602633
rs2317877
rs1024137
rs75460349
rs12271743
rs41279633
rs12359135
rs1531096
rs141469619
rs78058190
rs60220392
rs12916
rs1664781
rs13300853
rs139974673
rs7337339
rs115849089
rs4805881
rs13389219
rs117733303
rs4665710
rs442177
All
-0.10
-0.05
0.00
MR leave-one-out sensitivity analysis for
Total lipids in medium VLDL || id:ebi-cfb233-GCST90302063' on 'Breast cancer (Combined Oncoarray; iCOGS; GWAS meta analysis) |

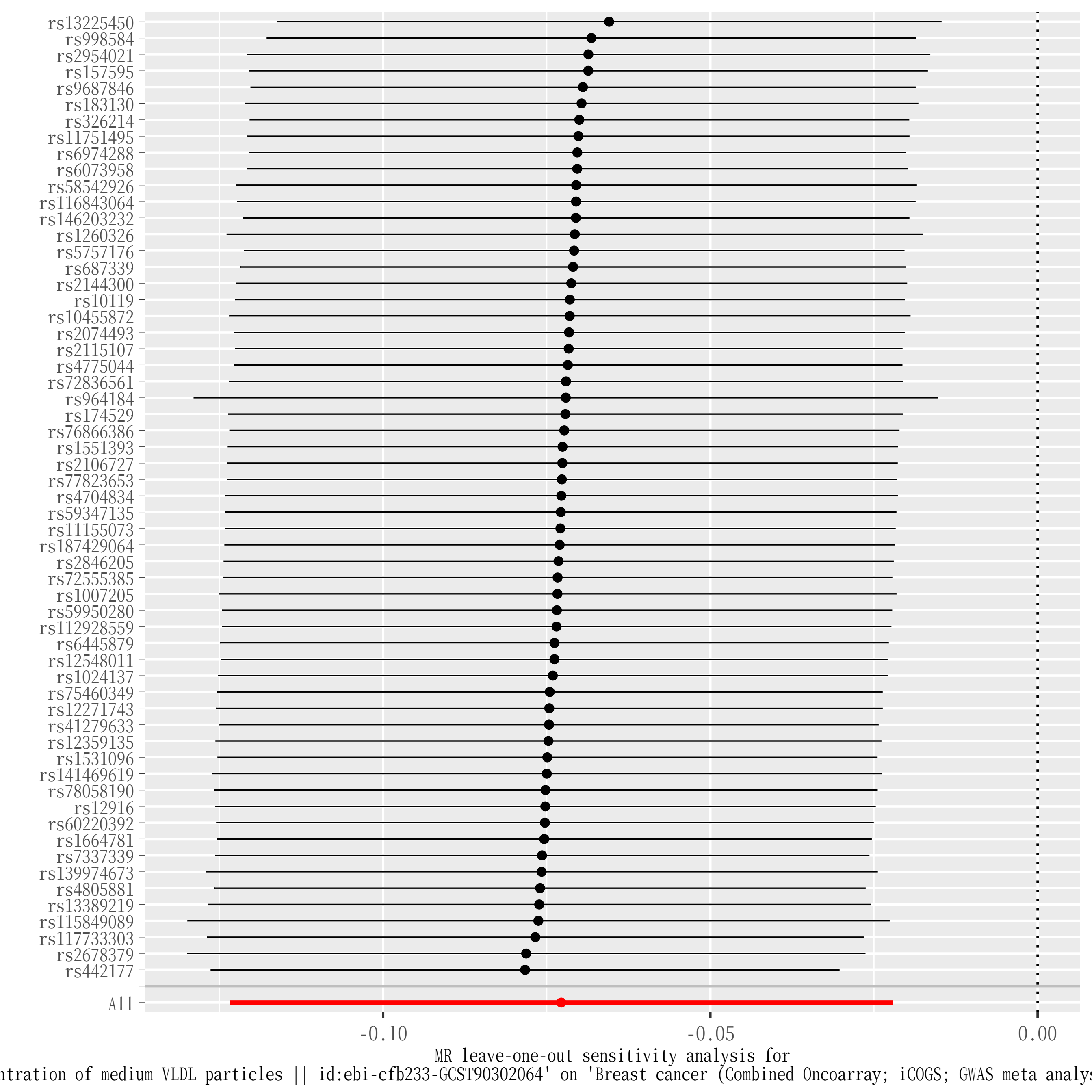

rs13225450
rs998584
rs2954021
rs157595
rs9687846
rs183130
rs326214
rs11751495
rs6974288
rs6073958
rs58542926
rs116843064
rs146203232
rs1260326
rs5757176
rs687339
rs2144300
rs10119
rs10455872
rs2074493
rs2115107
rs4775044
rs72836561
rs964184
rs174529
rs76866386
rs1551393
rs2106727
rs77823653
rs4704834
rs59347135
rs11155073
rs187429064
rs2846205
rs72555385
rs1007205
rs59950280
rs112928559
rs6445879
rs12548011
rs1024137
rs75460349
rs12271743
rs41279633
rs12359135
rs1531096
rs141469619
rs78058190
rs12916
rs60220392
rs1664781
rs7337339
rs139974673
rs4805881
rs13389219
rs115849089
rs117733303
rs2678379
rs442177
All
-0.10
-0.05
0.00
MR leave-one-out sensitivity analysis for
ntration of medium VLDL particles || id:ebi-cfb233-GCST90302064' on 'Breast cancer (Combined Oncoarray; iCOGS; GWAS meta analys

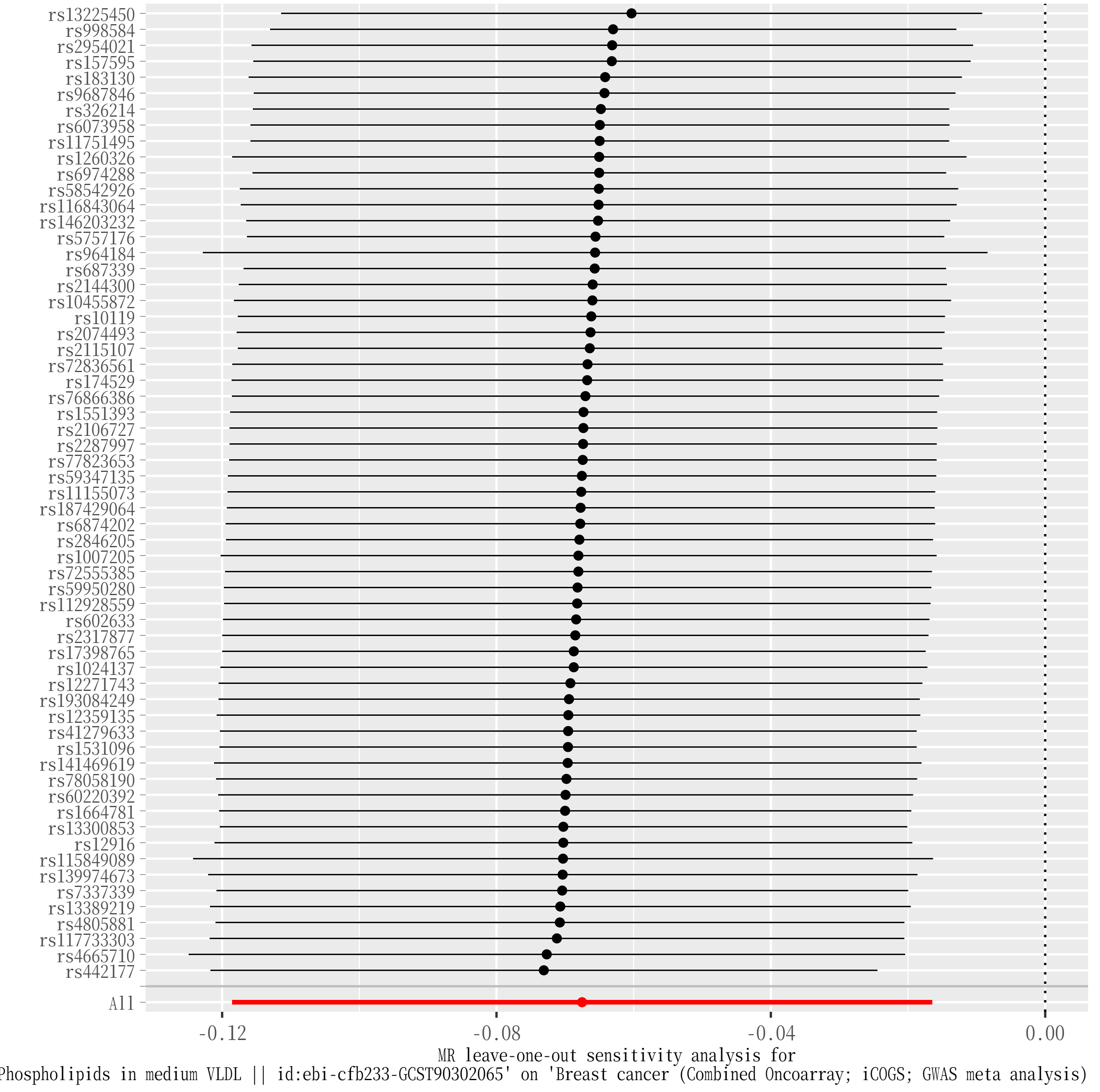

rs13225450
rs998584
rs2954021
rs157595
rs183130
rs9687846
rs326214
rs6073958
rs11751495
rs1260326
rs6974288
rs58542926
rs116843064
rs146203232
rs5757176
rs964184
rs687339
rs2144300
rs10455872
rs10119
rs2074493
rs2115107
rs72836561
rs174529
rs76866386
rs1551393
rs2106727
rs2287997
rs77823653
rs59347135
rs11155073
rs187429064
rs6874202
rs2846205
rs1007205
rs72555385
rs59950280
rs112928559
rs602633
rs2317877
rs17398765
rs1024137
rs12271743
rs193084249
rs12359135
rs41279633
rs1531096
rs141469619
rs78058190
rs60220392
rs1664781
rs13300853
rs12916
rs115849089
rs139974673
rs7337339
rs13389219
rs4805881
rs117733303
rs4665710
rs442177
All
-0.12
-0.08
-0.04
0.00
MR leave-one-out sensitivity analysis for
Phospholipids in medium VLDL || id:ebi-cfb233-GCST90302065' on 'Breast cancer (Combined Oncoarray; iCOGS; GWAS meta analysis)

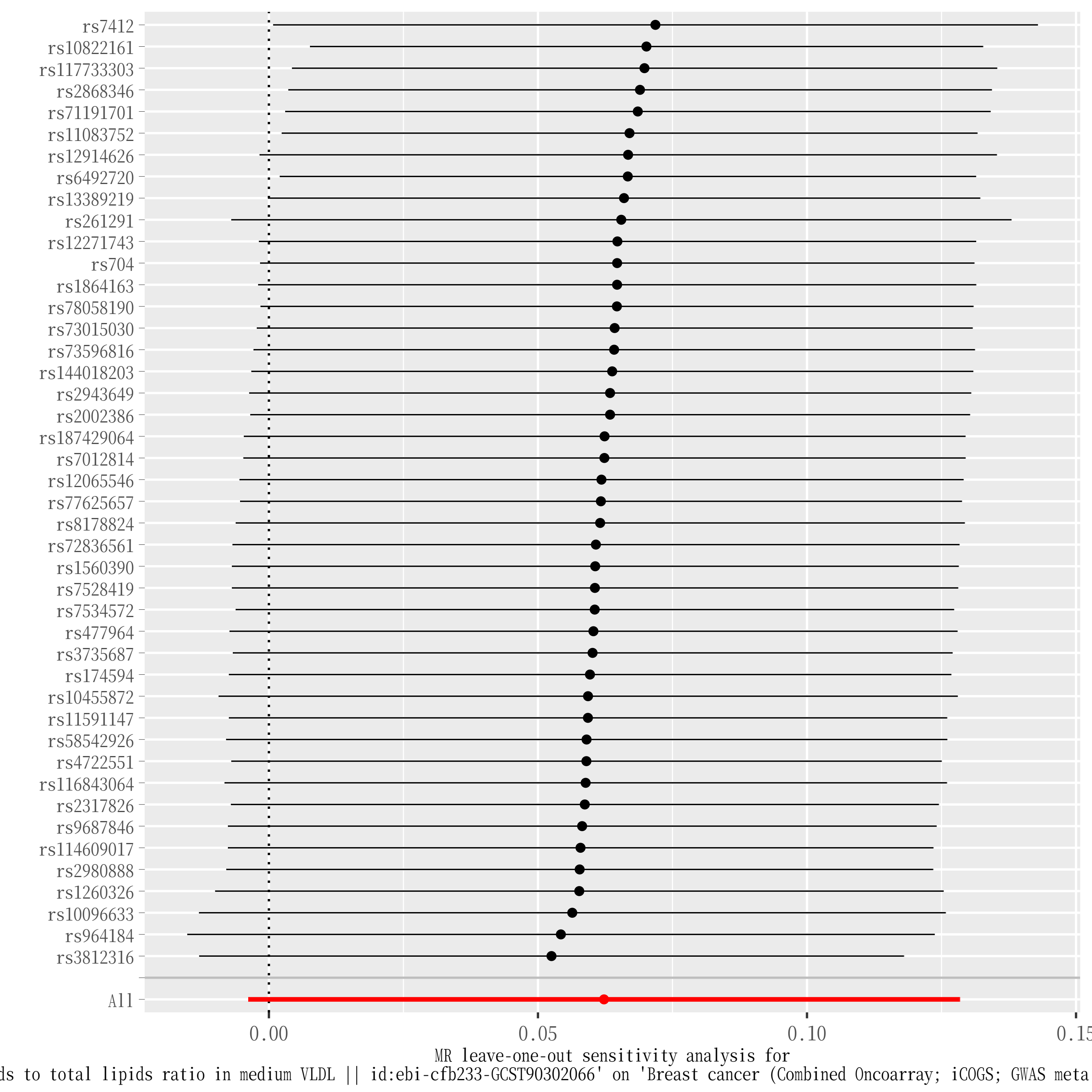
rs7412
rs10822161
rs117733303
rs2868346
rs71191701
rs11083752
rs12914626
rs6492720
rs13389219
rs261291
rs12271743
rs704
rs1864163
rs78058190
rs73015030
rs73596816
rs144018203
rs2943649
rs2002386
rs187429064
rs7012814
rs12065546
rs77625657
rs8178824
rs72836561
rs1560390
rs7528419
rs7534572
rs477964
rs3735687
rs174594
rs10455872
rs11591147
rs58542926
rs4722551
rs116843064
rs2317826
rs9687846
rs114609017
rs2980888
rs1260326
rs10096633
rs964184
rs3812316
All
0.00
0.05
0.10
0.15
MR leave-one-out sensitivity analysis for
ds to total lipids ratio in medium VLDL || id:ebi-cfb233-GCST90302066' on 'Breast cancer (Combined Oncoarray; iCOGS; GWAS meta

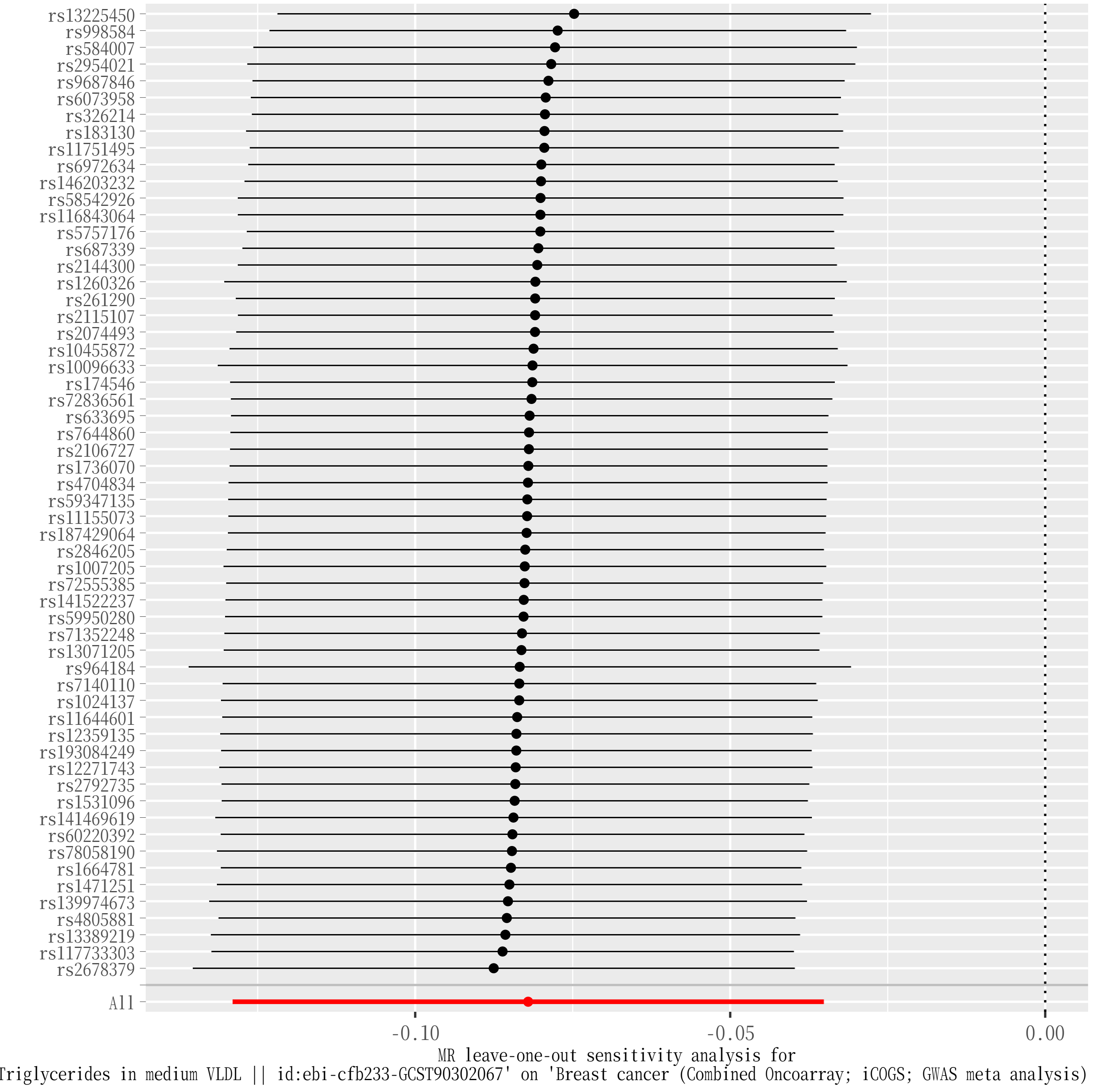
rs13225450
rs998584
rs584007
rs2954021
rs9687846
rs6073958
rs326214
rs183130
rs11751495
rs6972634
rs146203232
rs58542926
rs116843064
rs5757176
rs687339
rs2144300
rs1260326
rs261290
rs2115107
rs2074493
rs10455872
rs10096633
rs174546
rs72836561
rs633695
rs7644860
rs2106727
rs1736070
rs4704834
rs59347135
rs11155073
rs187429064
rs2846205
rs1007205
rs72555385
rs141522237
rs59950280
rs71352248
rs13071205
rs964184
rs7140110
rs1024137
rs11644601
rs12359135
rs193084249
rs12271743
rs2792735
rs1531096
rs141469619
rs60220392
rs78058190
rs1664781
rs1471251
rs139974673
rs4805881
rs13389219
rs117733303
rs2678379
All
-0.10
-0.05
0.00
MR leave-one-out sensitivity analysis for
'Triglycerides in medium VLDL || id:ebi-cfb233-GCST90302067' on 'Breast cancer (Combined Oncoarray; iCOGS; GWAS meta analysis)'

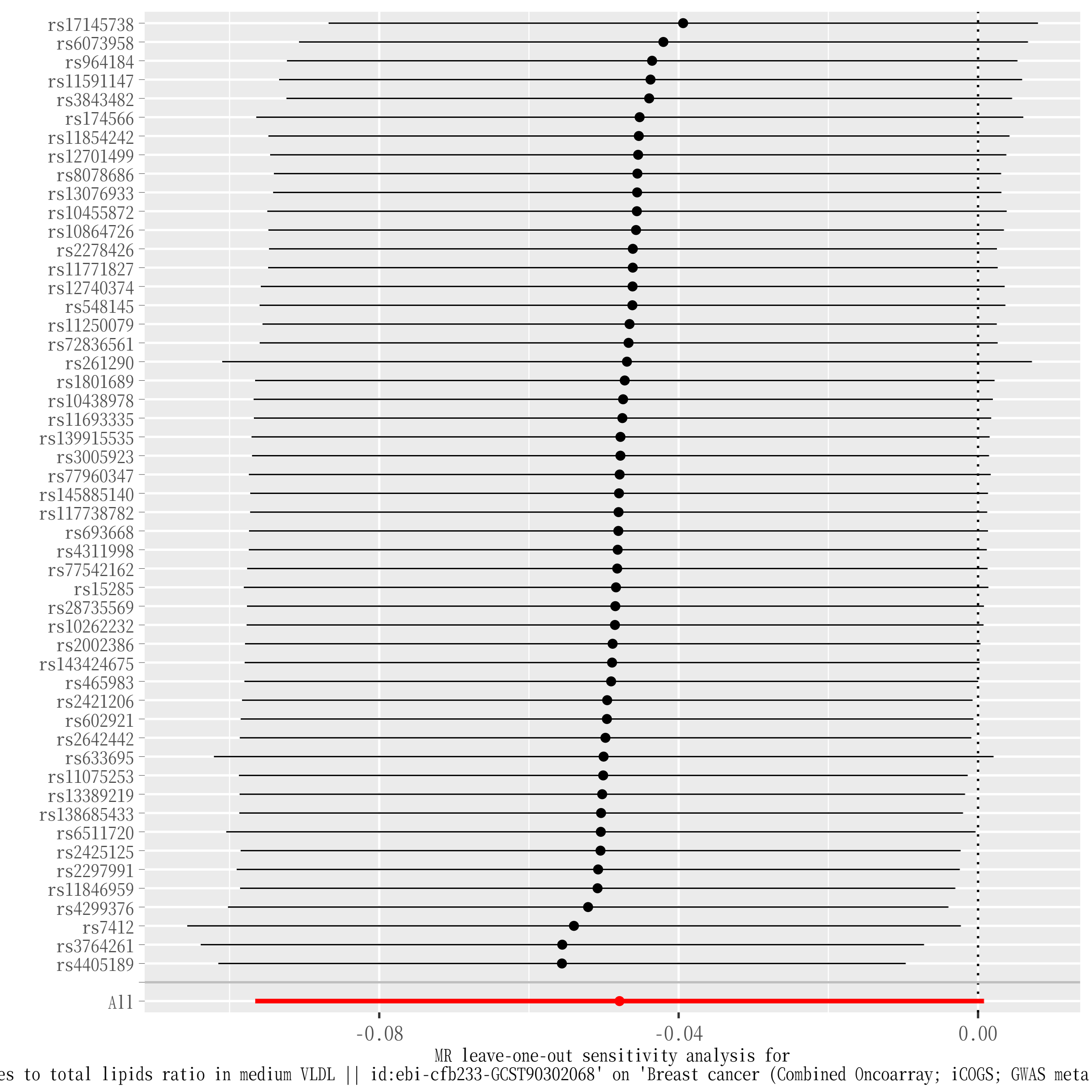
rs17145738
rs6073958
rs964184
rs11591147
rs3843482
rs174566
rs11854242
rs12701499
rs8078686
rs13076933
rs10455872
rs10864726
rs2278426
rs11771827
rs12740374
rs548145
rs11250079
rs72836561
rs261290
rs1801689
rs10438978
rs11693335
rs139915535
rs3005923
rs77960347
rs145885140
rs117738782
rs693668
rs4311998
rs77542162
rs15285
rs28735569
rs10262232
rs2002386
rs143424675
rs465983
rs2421206
rs602921
rs2642442
rs633695
rs11075253
rs13389219
rs138685433
rs6511720
rs2425125
rs2297991
rs11846959
rs4299376
rs7412
rs3764261
rs4405189
All
-0.08
-0.04
0.00
MR leave-one-out sensitivity analysis for
es to total lipids ratio in medium VLDL || id:ebi-cfb233-GCST90302068' on 'Breast cancer (Combined Oncoarray; iCOGS; GWAS meta

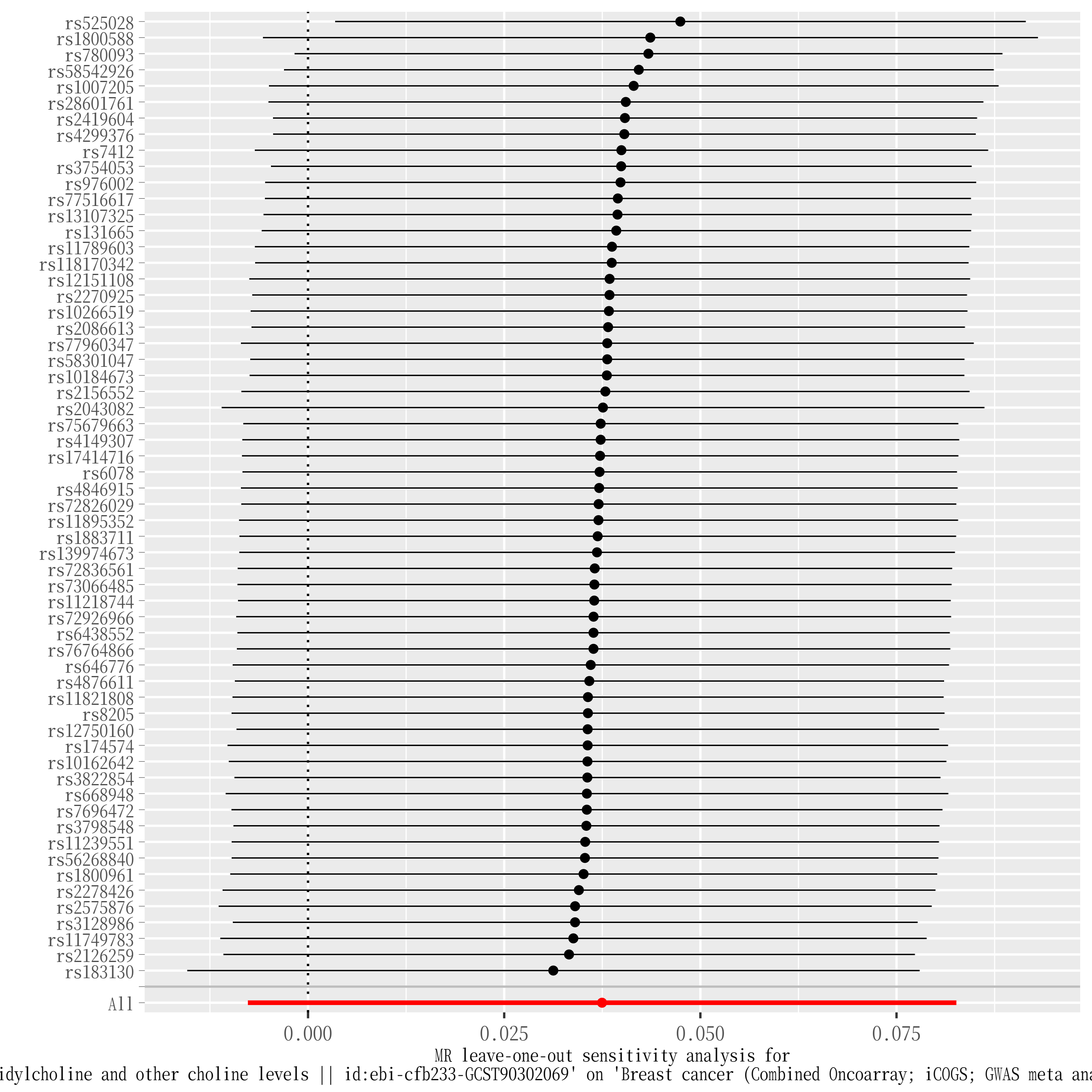
rs525028
rs1800588
rs780093
rs58542926
rs1007205
rs28601761
rs2419604
rs4299376
rs7412
rs3754053
rs976002
rs77516617
rs13107325
rs131665
rs11789603
rs118170342
rs12151108
rs2270925
rs10266519
rs2086613
rs77960347
rs58301047
rs10184673
rs2156552
rs2043082
rs75679663
rs4149307
rs17414716
rs6078
rs4846915
rs72826029
rs11895352
rs1883711
rs139974673
rs72836561
rs73066485
rs11218744
rs72926966
rs6438552
rs76764866
rs646776
rs4876611
rs11821808
rs8205
rs12750160
rs174574
rs10162642
rs3822854
rs668948
rs7696472
rs3798548
rs11239551
rs56268840
rs1800961
rs2278426
rs2575876
rs3128986
rs11749783
rs2126259
rs183130
All
0.000
0.025
0.050
0.075
MR leave-one-out sensitivity analysis for
idylcholine and other choline levels || id:ebi-cfb233-GCST90302069' on 'Breast cancer (Combined Oncoarray; iCOGS; GWAS meta ana

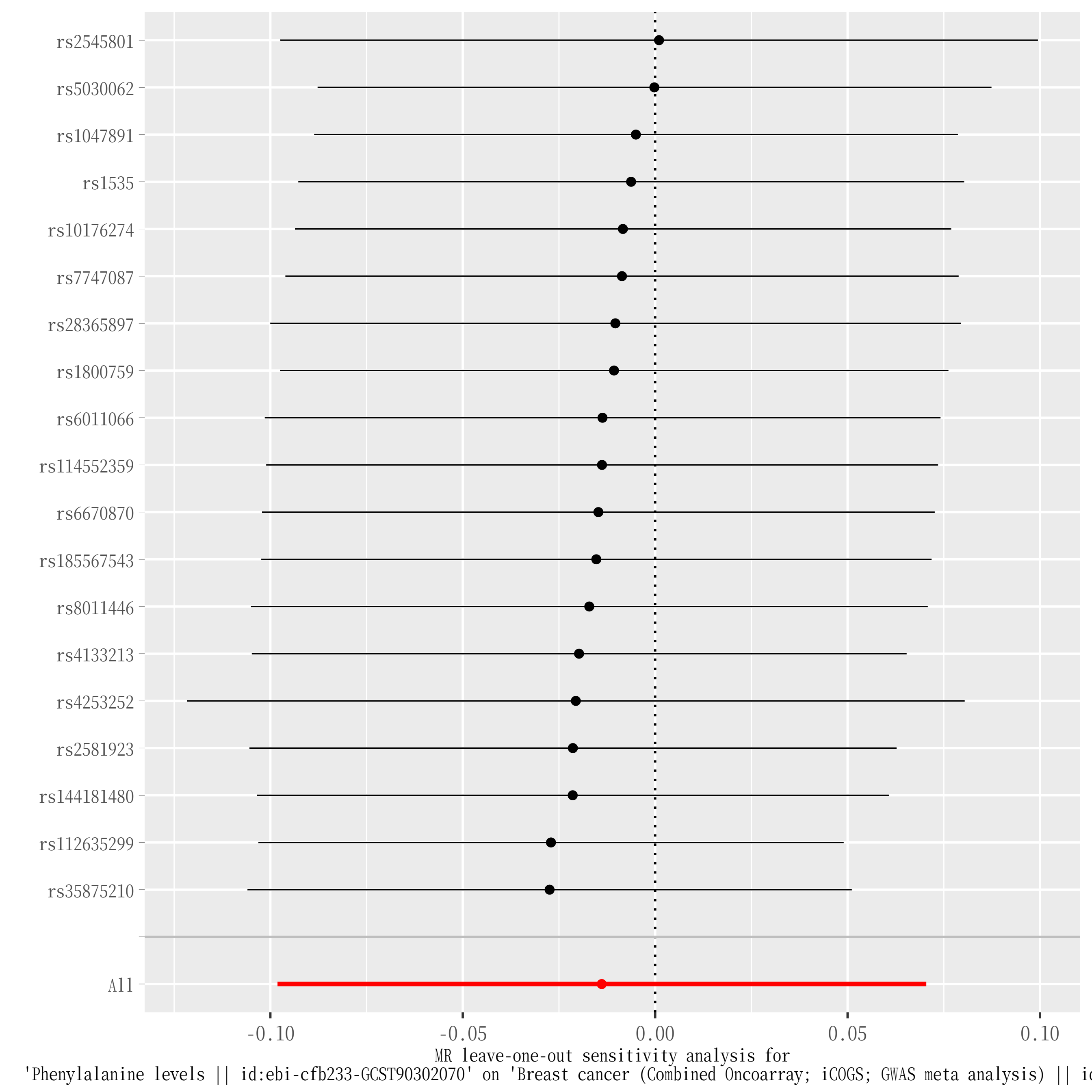

rs2545801
rs5030062
rs1047891
rs1535
rs10176274
rs7747087
rs28365897
rs1800759
rs6011066
rs114552359
rs6670870
rs185567543
rs8011446
rs4133213
rs4253252
rs2581923
rs144181480
rs112635299
rs35875210
All
-0.10
-0.05
0.00
0.05
0.10
MR leave-one-out sensitivity analysis for
'Phenylalanine levels || id:ebi-cfb233-GCST90302070' on 'Breast cancer (Combined Oncoarray; iCOGS; GWAS meta analysis) || id

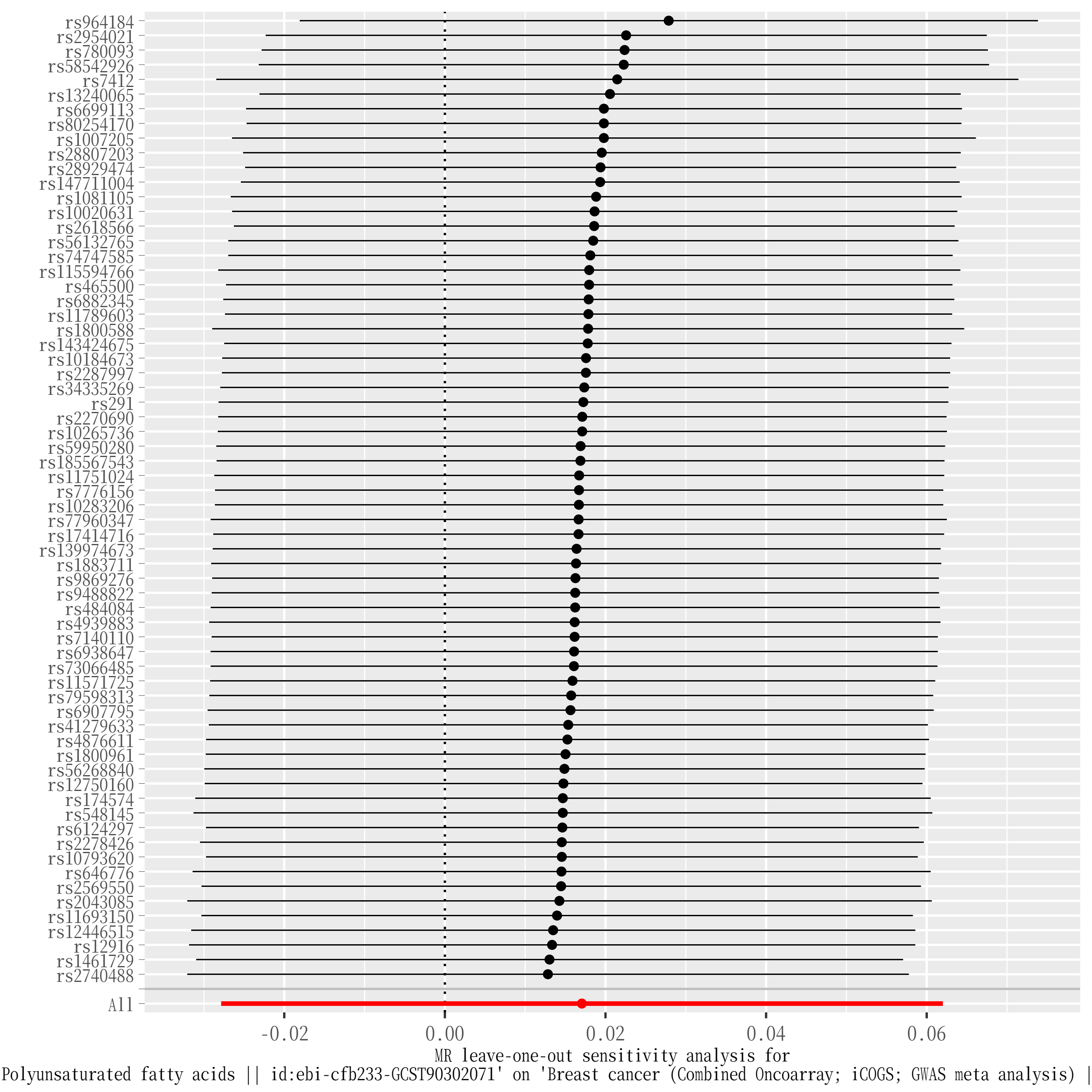

rs964184
rs2954021
rs780093
rs58542926
rs7412
rs13240065
rs6699113
rs80254170
rs1007205
rs28807203
rs28929474
rs147711004
rs1081105
rs10020631
rs2618566
rs56132765
rs74747585
rs115594766
rs465500
rs6882345
rs11789603
rs1800588
rs143424675
rs10184673
rs2287997
rs34335269
rs291
rs2270690
rs10265736
rs59950280
rs185567543
rs11751024
rs7776156
rs10283206
rs77960347
rs17414716
rs139974673
rs1883711
rs9869276
rs9488822
rs484084
rs4939883
rs7140110
rs6938647
rs73066485
rs11571725
rs79598313
rs6907795
rs41279633
rs4876611
rs1800961
rs56268840
rs12750160
rs174574
rs548145
rs6124297
rs2278426
rs10793620
rs646776
rs2569550
rs2043085
rs11693150
rs12446515
rs12916
rs1461729
rs2740488
All
-0.02
0.00
0.02
0.04
0.06
MR leave-one-out sensitivity analysis for
Polyunsaturated fatty acids || id:ebi-cfb233-GCST90302071' on 'Breast cancer (Combined Oncoarray; iCOGS; GWAS meta analysis) |

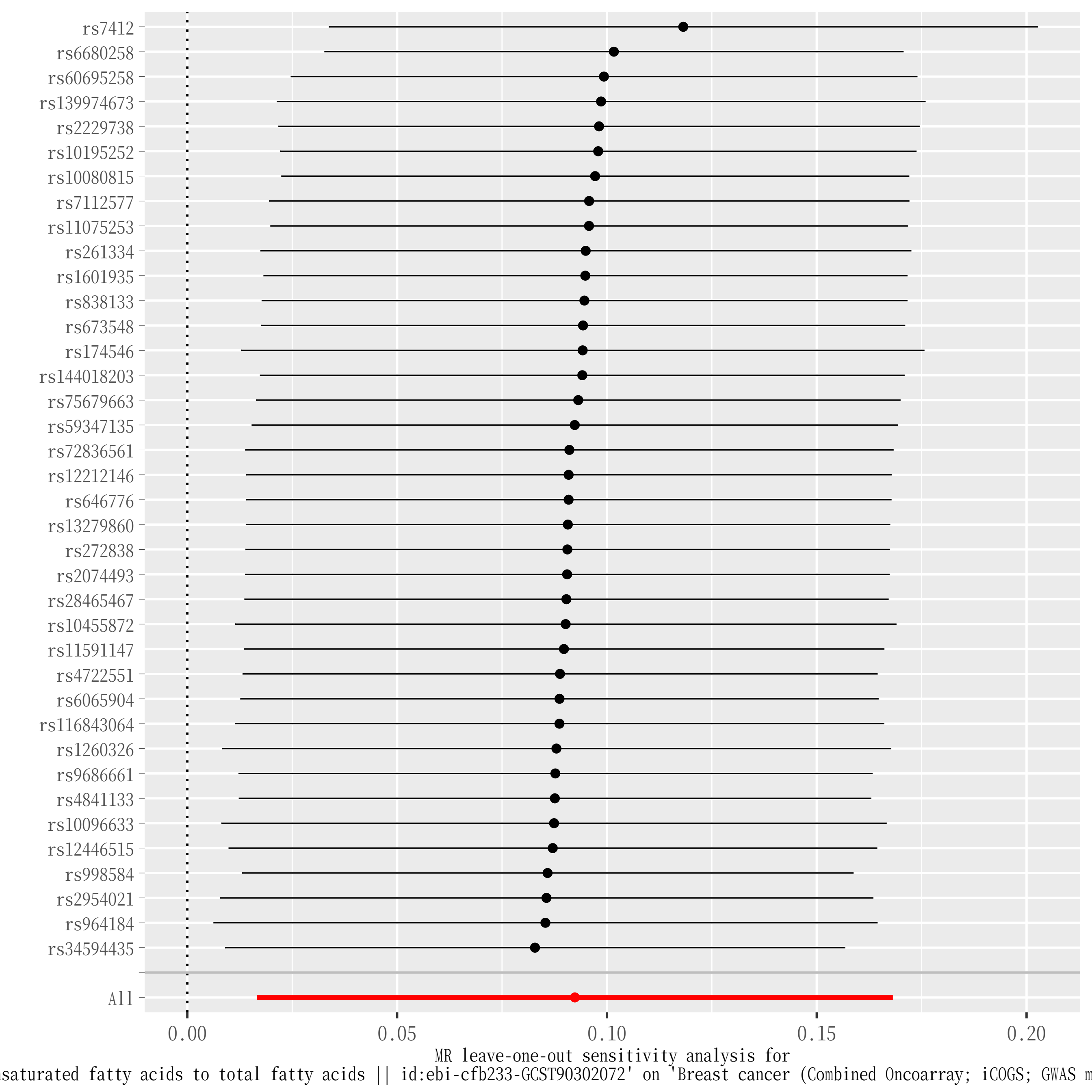

rs7412
rs6680258
rs60695258
rs139974673
rs2229738
rs10195252
rs10080815
rs7112577
rs11075253
rs261334
rs1601935
rs838133
rs673548
rs174546
rs144018203
rs75679663
rs59347135
rs72836561
rs12212146
rs646776
rs13279860
rs272838
rs2074493
rs28465467
rs10455872
rs11591147
rs4722551
rs6065904
rs116843064
rs1260326
rs9686661
rs4841133
rs10096633
rs12446515
rs998584
rs2954021
rs964184
rs34594435
All
0.00
0.05
0.10
0.15
0.20
MR leave-one-out sensitivity analysis for
nsaturated fatty acids to total fatty acids || id:ebi-cfb233-GCST90302072' on 'Breast cancer (Combined Oncoarray; iCOGS; GWAS m

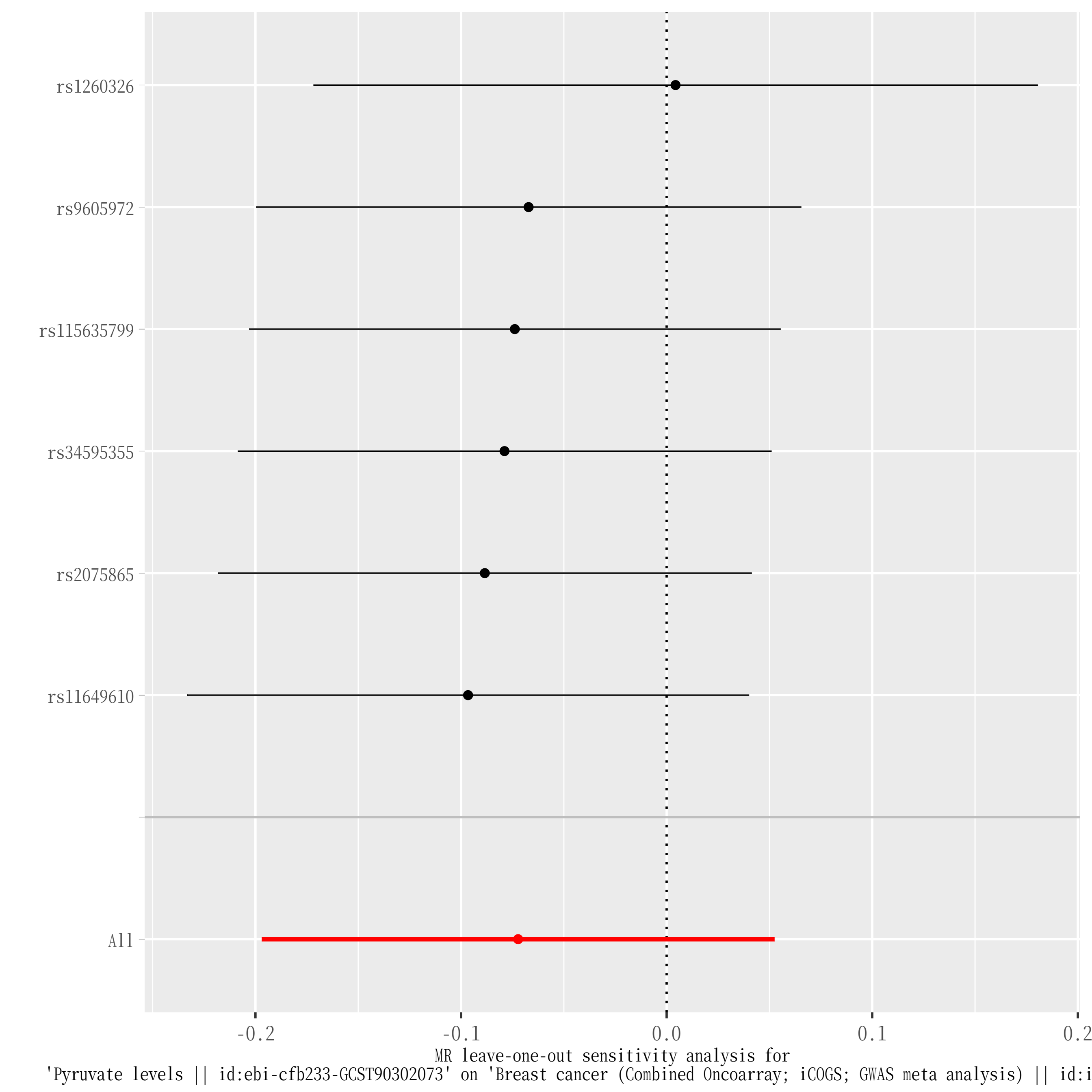
rs1260326
rs9605972
rs115635799
rs34595355
rs2075865
rs11649610
All
-0.2
-0.1
0.0
0.1
0.2
MR leave-one-out sensitivity analysis for
'Pyruvate levels || id:ebi-cfb233-GCST90302073' on 'Breast cancer (Combined Oncoarray; iCOGS; GWAS meta analysis) || id:i

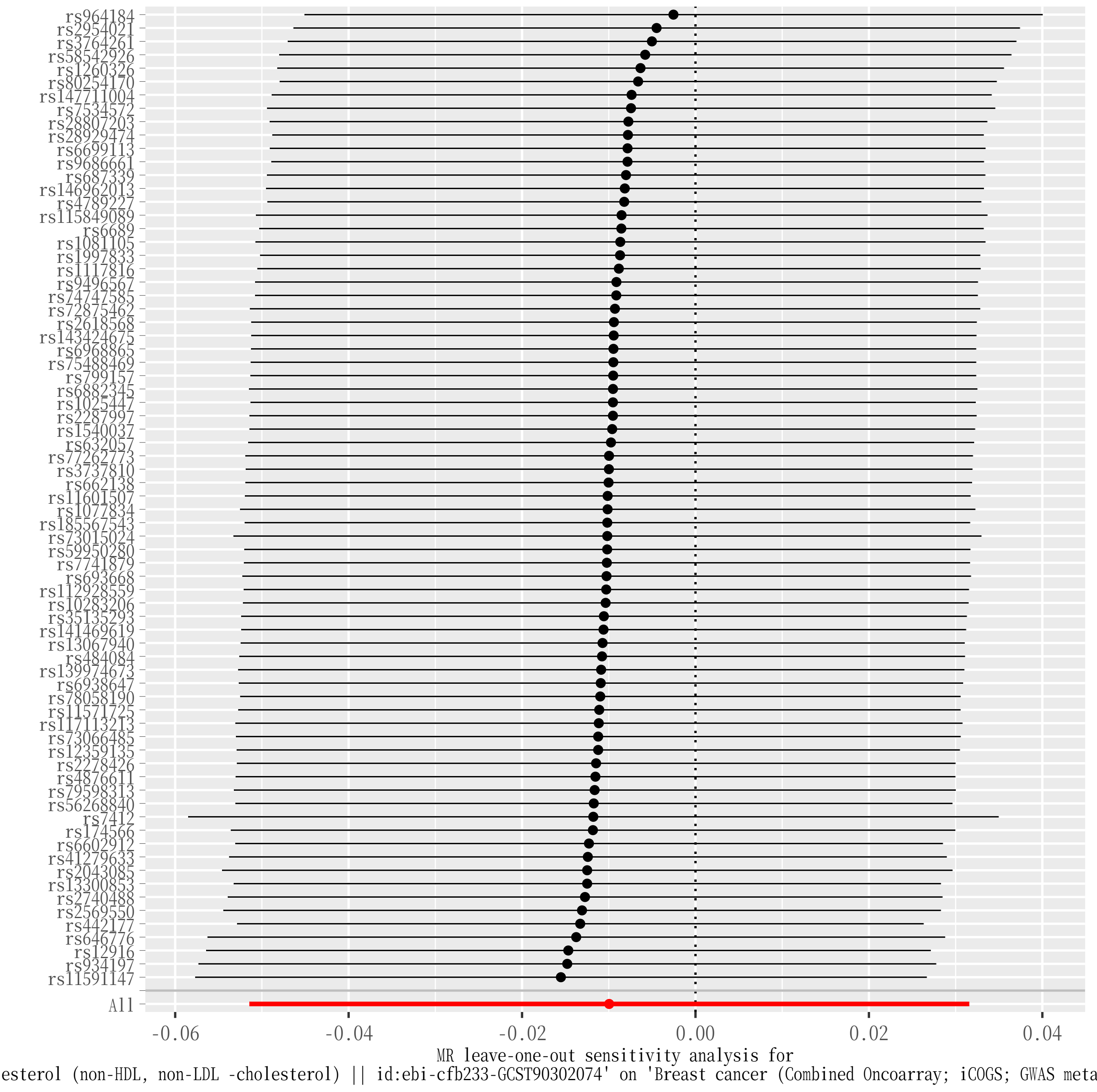
rs964184
rs2954021
rs3764261
rs58542926
rs1260326
rs80254170
rs147711004
rs7534572
rs28807203
rs28929474
rs6699113
rs9686661
rs687339
rs146962013
rs4789227
rs115849089
rs6689
rs1081105
rs1997833
rs1117816
rs9496567
rs74747585
rs72875462
rs2618568
rs143424675
rs6968865
rs75488469
rs799157
rs6882345
rs1025447
rs2287997
rs1540037
rs632057
rs77262773
rs3737810
rs662138
rs11601507
rs1077834
rs185567543
rs73015024
rs59950280
rs7741879
rs693668
rs112928559
rs10283206
rs35135293
rs141469619
rs13067940
rs484084
rs139974673
rs6938647
rs78058190
rs11571725
rs117113213
rs73066485
rs12359135
rs2278426
rs4876611
rs79598313
rs56268840
rs7412
rs174566
rs6602912
rs41279633
rs2043085
rs13300853
rs2740488
rs2569550
rs442177
rs646776
rs12916
rs934197
rs11591147
All
-0.06
-0.04
-0.02
0.00
0.02
0.04
MR leave-one-out sensitivity analysis for
esterol (non-HDL, non-LDL -cholesterol) || id:ebi-cfb233-GCST90302074' on 'Breast cancer (Combined Oncoarray; iCOGS; GWAS meta

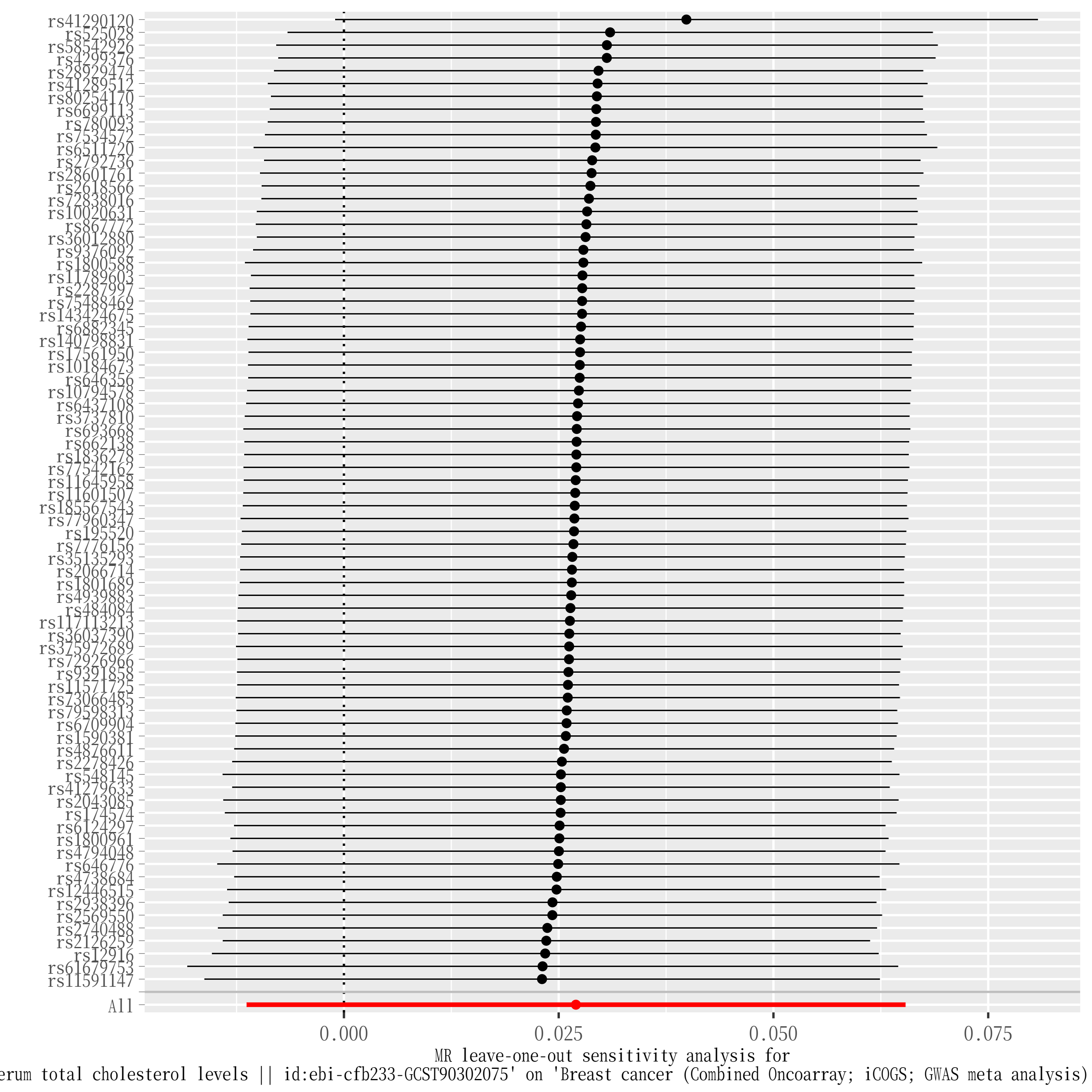
rs41290120
rs525028
rs58542926
rs4299376
rs28929474
rs41289512
rs80254170
rs6699113
rs780093
rs7534572
rs6511720
rs2792736
rs28601761
rs2618566
rs72838016
rs10020631
rs867772
rs36012880
rs9376092
rs1800588
rs11789603
rs2287997
rs75488469
rs143424675
rs6882345
rs140798831
rs17561950
rs10184673
rs646356
rs10794578
rs6437108
rs3737810
rs693668
rs662138
rs1836278
rs77542162
rs11645958
rs11601507
rs185567543
rs77960347
rs195520
rs7776156
rs35135293
rs2066714
rs1801689
rs4939883
rs484084
rs117113213
rs36037390
rs375972689
rs72926966
rs9321858
rs11571725
rs73066485
rs79598313
rs6709904
rs1590381
rs4876611
rs2278426
rs548145
rs41279633
rs2043085
rs174574
rs6124297
rs1800961
rs4794048
rs646776
rs4738684
rs12446515
rs2938396
rs2569550
rs2740488
rs2126259
rs12916
rs61679753
rs11591147
All
0.000
0.025
0.050
0.075
MR leave-one-out sensitivity analysis for
erum total cholesterol levels || id:ebi-cfb233-GCST90302075' on 'Breast cancer (Combined Oncoarray; iCOGS; GWAS meta analysis)

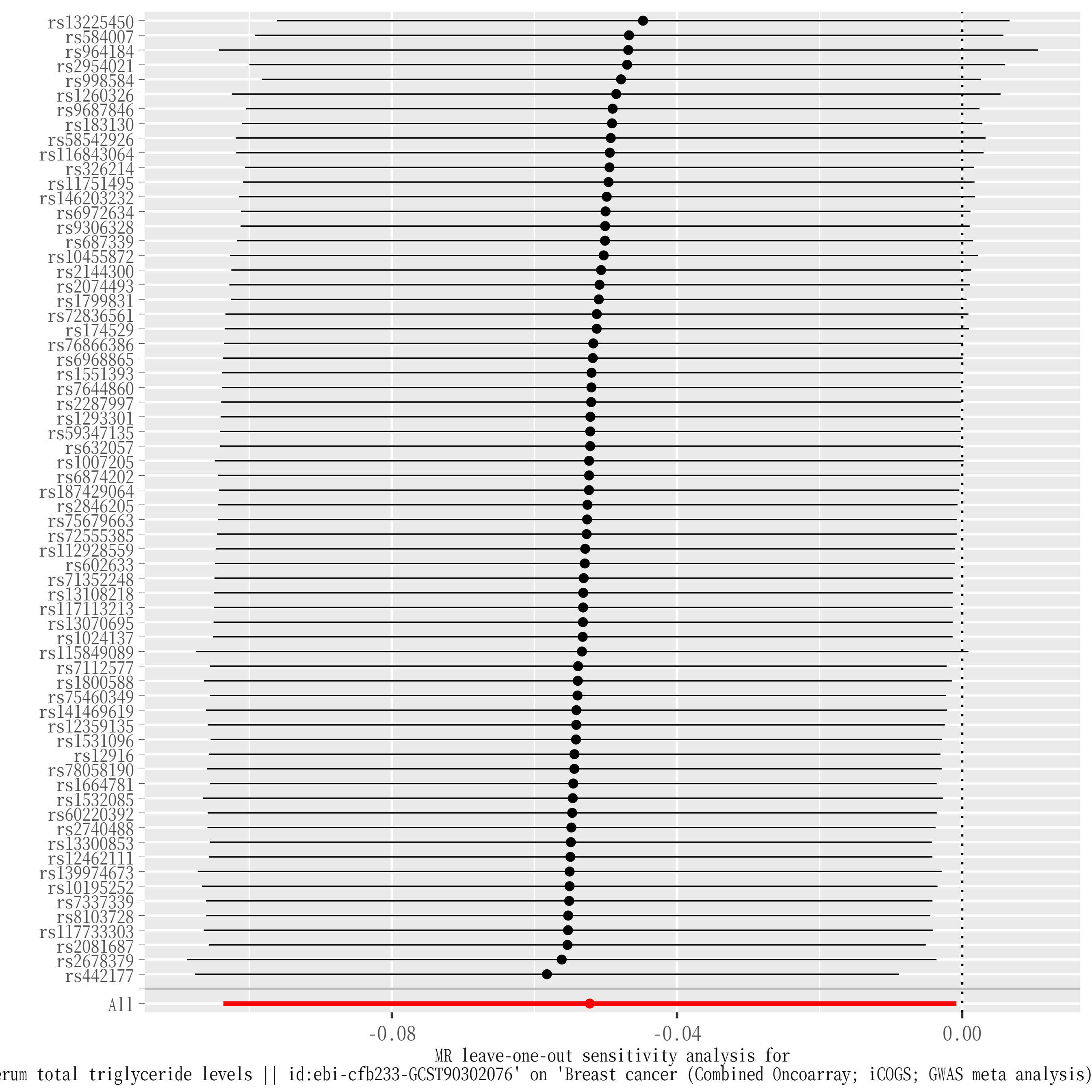
rs13225450
rs584007
rs964184
rs2954021
rs998584
rs1260326
rs9687846
rs183130
rs58542926
rs116843064
rs326214
rs11751495
rs146203232
rs6972634
rs9306328
rs687339
rs10455872
rs2144300
rs2074493
rs1799831
rs72836561
rs174529
rs76866386
rs6968865
rs1551393
rs7644860
rs2287997
rs1293301
rs59347135
rs632057
rs1007205
rs6874202
rs187429064
rs2846205
rs75679663
rs72555385
rs112928559
rs602633
rs71352248
rs13108218
rs117113213
rs13070695
rs1024137
rs115849089
rs7112577
rs1800588
rs75460349
rs141469619
rs12359135
rs1531096
rs12916
rs78058190
rs1664781
rs1532085
rs60220392
rs2740488
rs13300853
rs12462111
rs139974673
rs10195252
rs7337339
rs8103728
rs117733303
rs2081687
rs2678379
rs442177
All
-0.08
-0.04
0.00
MR leave-one-out sensitivity analysis for
erum total triglyceride levels || id:ebi-cfb233-GCST90302076' on 'Breast cancer (Combined Oncoarray; iCOGS; GWAS meta analysis)

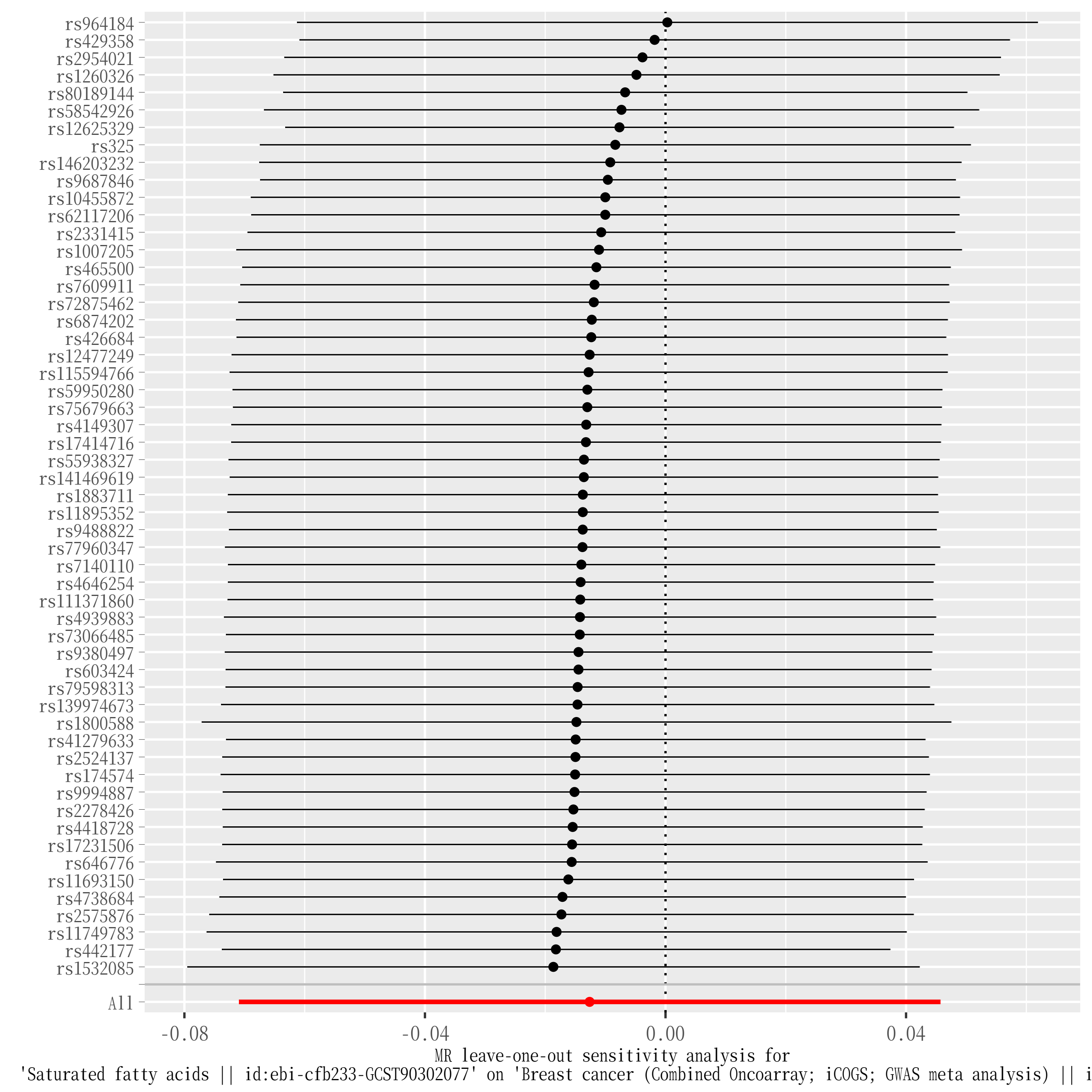
rs964184
rs429358
rs2954021
rs1260326
rs80189144
rs58542926
rs12625329
rs325
rs146203232
rs9687846
rs10455872
rs62117206
rs2331415
rs1007205
rs465500
rs7609911
rs72875462
rs6874202
rs426684
rs12477249
rs115594766
rs59950280
rs75679663
rs4149307
rs17414716
rs55938327
rs141469619
rs1883711
rs11895352
rs9488822
rs77960347
rs7140110
rs4646254
rs111371860
rs4939883
rs73066485
rs9380497
rs603424
rs79598313
rs139974673
rs1800588
rs41279633
rs2524137
rs174574
rs9994887
rs2278426
rs4418728
rs17231506
rs646776
rs11693150
rs4738684
rs2575876
rs11749783
rs442177
rs1532085
All
-0.08
-0.04
0.00
0.04
MR leave-one-out sensitivity analysis for
'Saturated fatty acids || id:ebi-cfb233-GCST90302077' on 'Breast cancer (Combined Oncoarray; iCOGS; GWAS meta analysis) || i

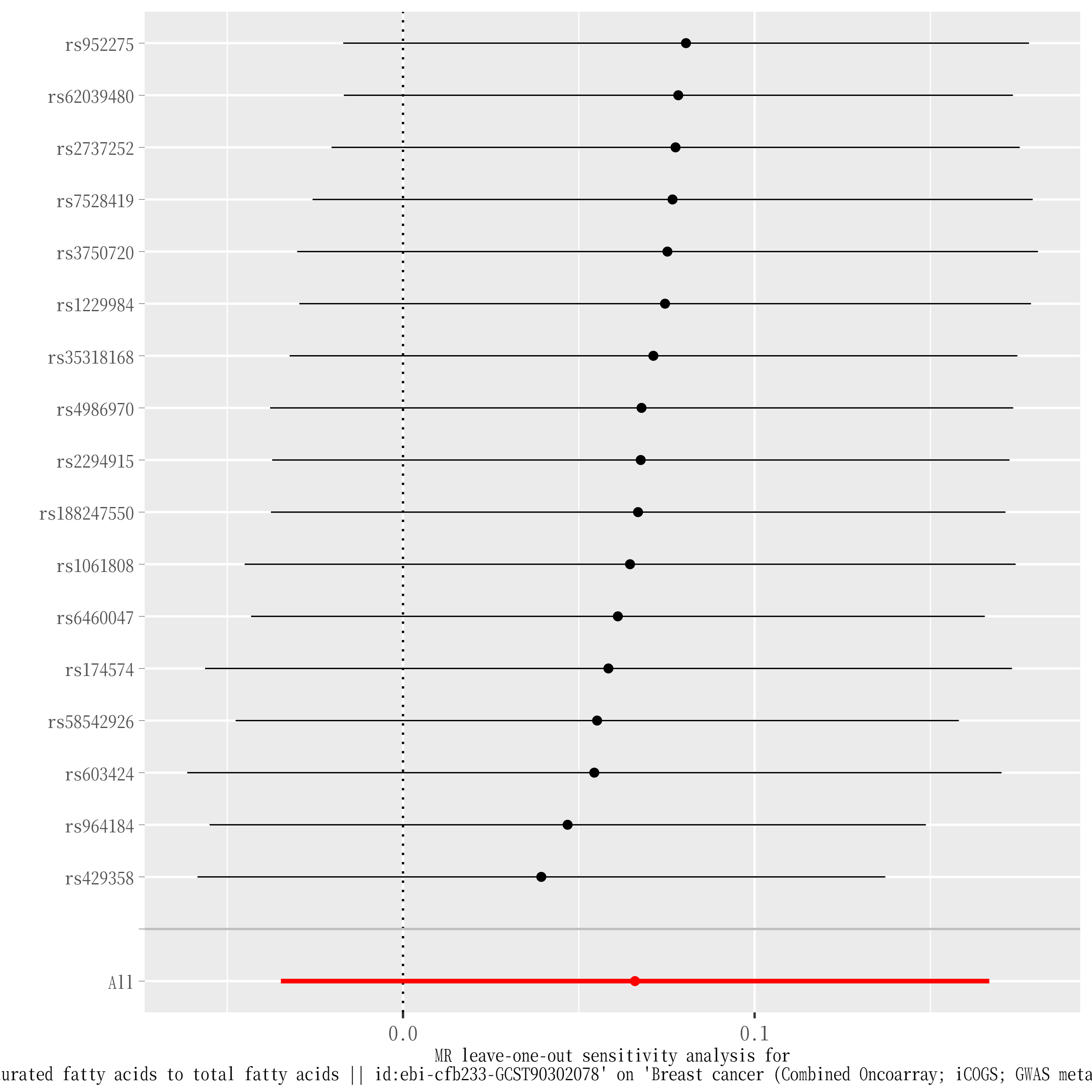
rs952275
rs62039480
rs2737252
rs7528419
rs3750720
rs1229984
rs35318168
rs4986970
rs2294915
rs188247550
rs1061808
rs6460047
rs174574
rs58542926
rs603424
rs964184
rs429358
All
0.0
0.1
MR leave-one-out sensitivity analysis for
urated fatty acids to total fatty acids || id:ebi-cfb233-GCST90302078' on 'Breast cancer (Combined Oncoarray; iCOGS; GWAS meta

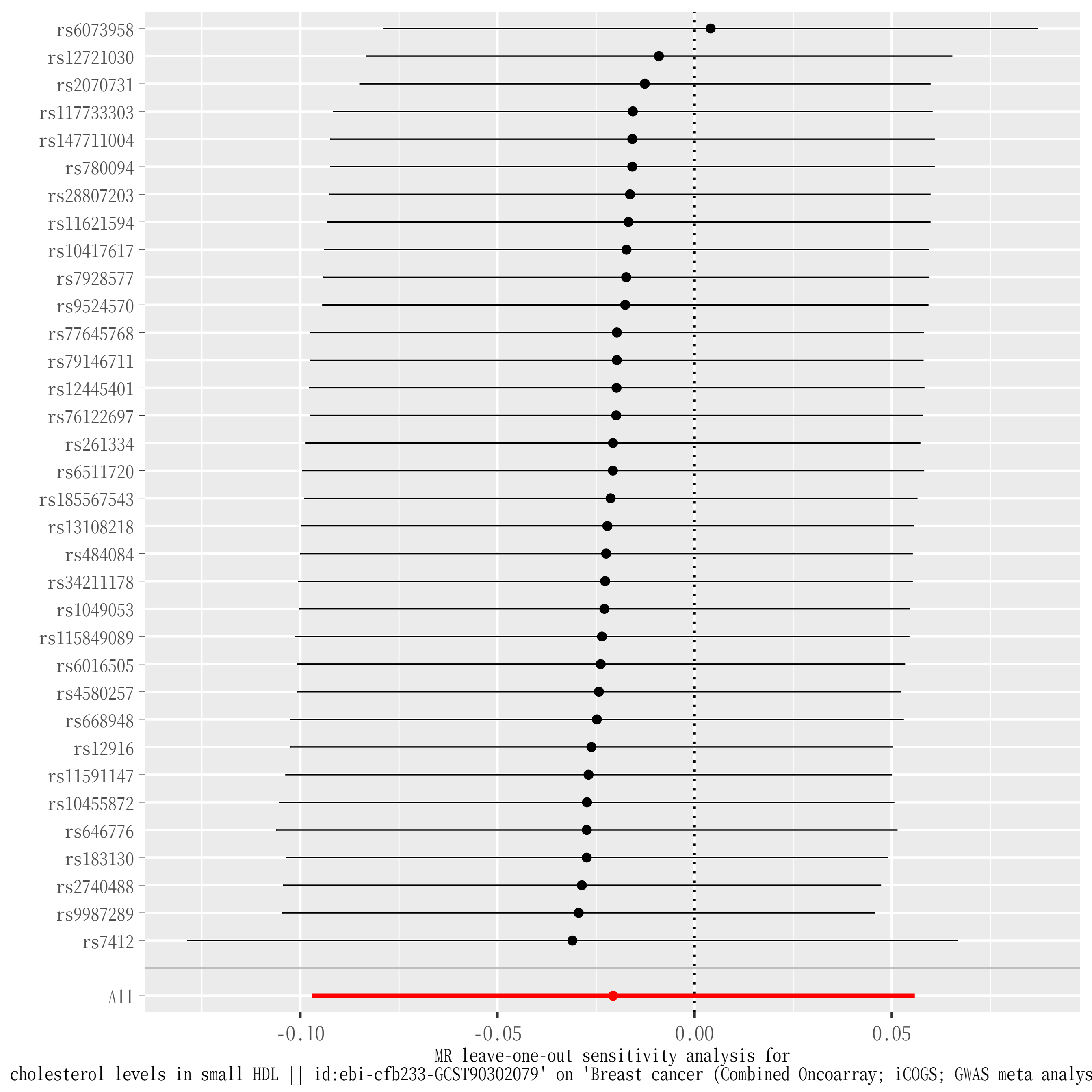
rs6073958
rs12721030
rs2070731
rs117733303
rs147711004
rs780094
rs28807203
rs11621594
rs10417617
rs7928577
rs9524570
rs77645768
rs79146711
rs12445401
rs76122697
rs261334
rs6511720
rs185567543
rs13108218
rs484084
rs34211178
rs1049053
rs115849089
rs6016505
rs4580257
rs668948
rs12916
rs11591147
rs10455872
rs646776
rs183130
rs2740488
rs9987289
rs7412
All
-0.10
-0.05
0.00
0.05
MR leave-one-out sensitivity analysis for
cholesterol levels in small HDL || id:ebi-cfb233-GCST90302079' on 'Breast cancer (Combined Oncoarray; iCOGS; GWAS meta analys

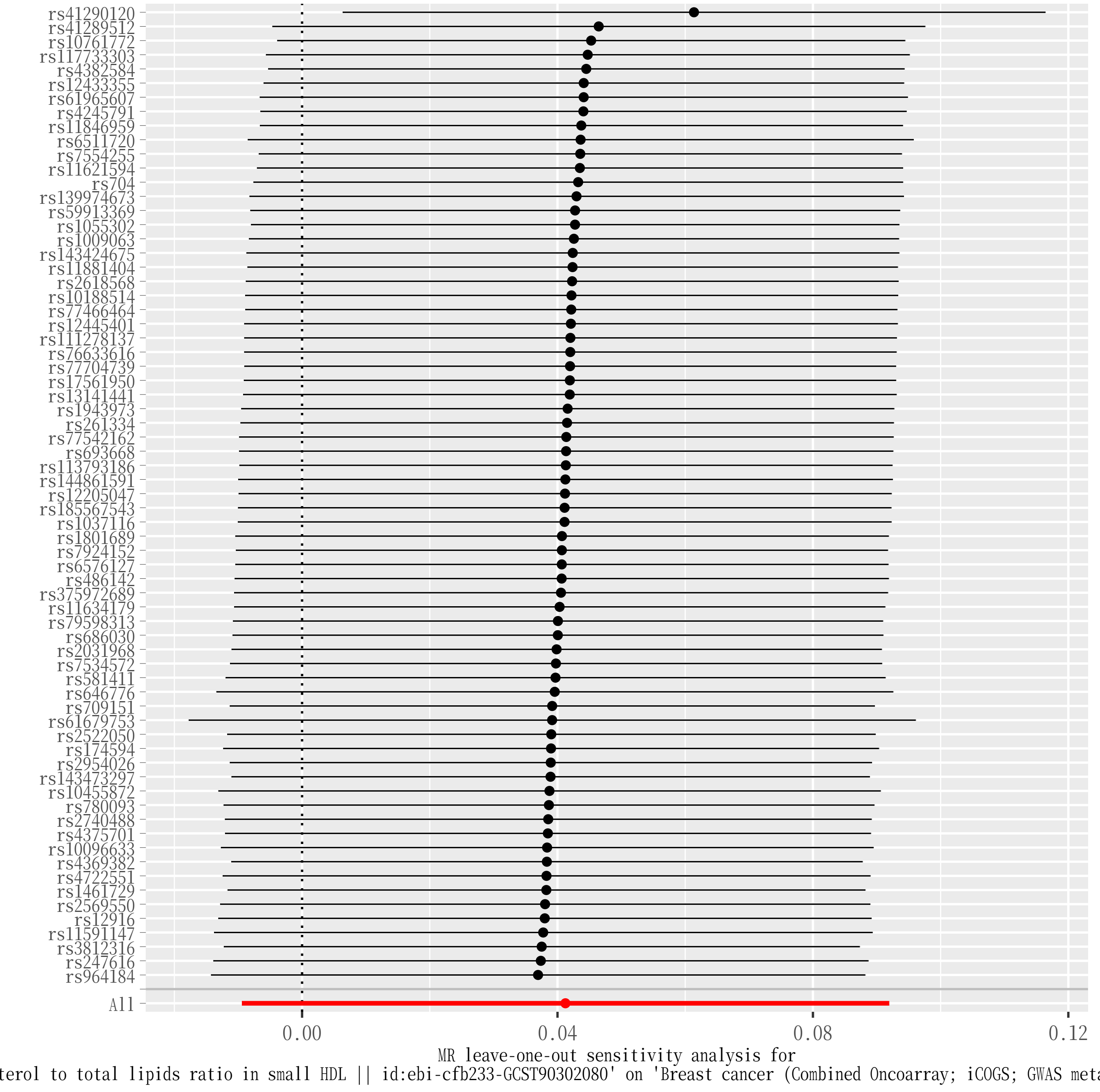

rs41290120
rs41289512
rs10761772
rs117733303
rs4382584
rs12433355
rs61965607
rs4245791
rs11846959
rs6511720
rs7554255
rs11621594
rs704
rs139974673
rs59913369
rs1055302
rs1009063
rs143424675
rs11881404
rs2618568
rs10188514
rs77466464
rs12445401
rs111278137
rs76633616
rs77704739
rs17561950
rs13141441
rs1943973
rs261334
rs77542162
rs693668
rs113793186
rs144861591
rs12205047
rs185567543
rs1037116
rs1801689
rs7924152
rs6576127
rs486142
rs375972689
rs11634179
rs79598313
rs686030
rs2031968
rs7534572
rs581411
rs646776
rs709151
rs61679753
rs2522050
rs174594
rs2954026
rs143473297
rs10455872
rs780093
rs2740488
rs4375701
rs10096633
rs4369382
rs4722551
rs1461729
rs2569550
rs12916
rs11591147
rs3812316
rs247616
rs964184
All
0.00
0.04
0.08
0.12
MR leave-one-out sensitivity analysis for
terol to total lipids ratio in small HDL || id:ebi-cfb233-GCST90302080' on 'Breast cancer (Combined Oncoarray; iCOGS; GWAS meta

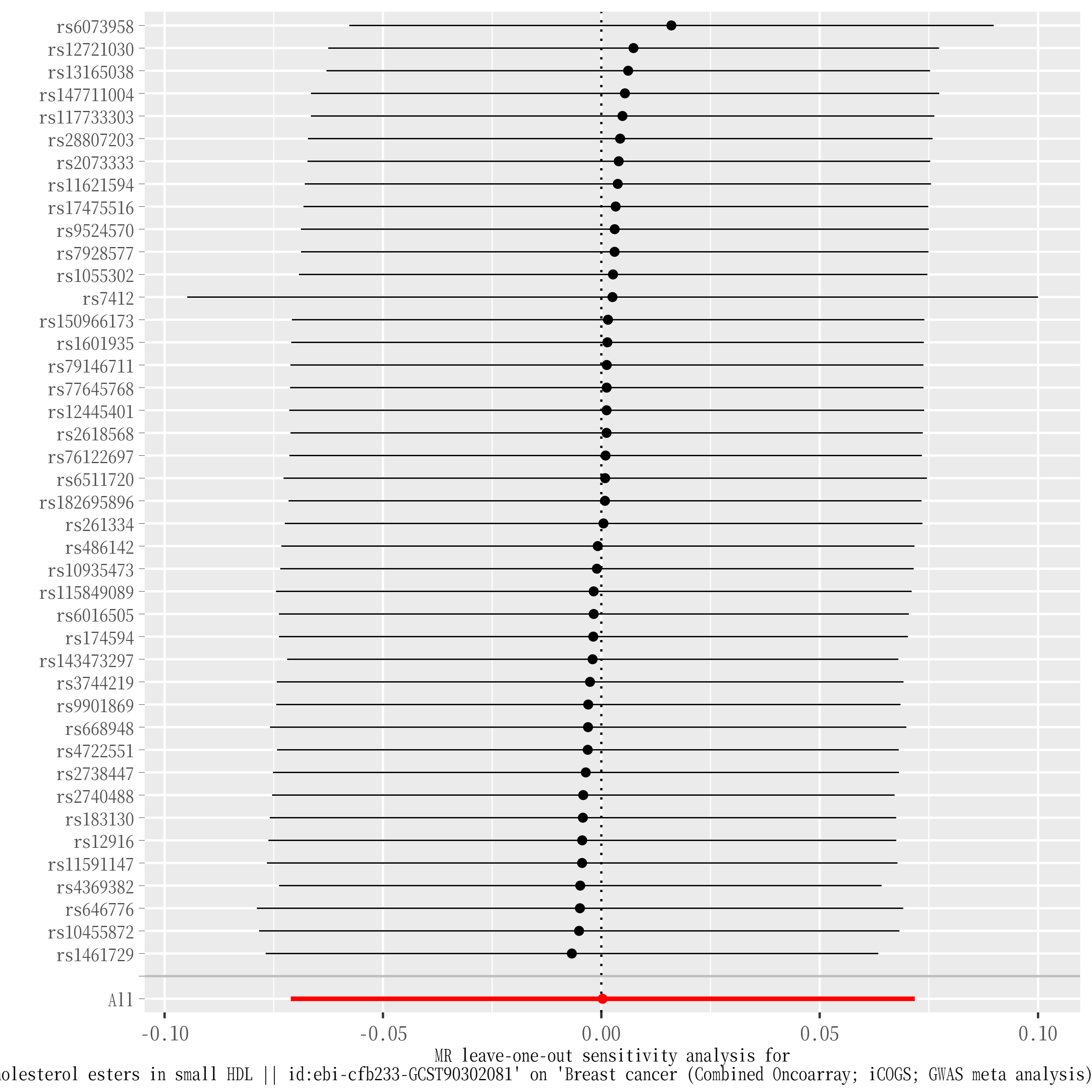

rs6073958
rs12721030
rs13165038
rs147711004
rs117733303
rs28807203
rs2073333
rs11621594
rs17475516
rs9524570
rs7928577
rs1055302
rs7412
rs150966173
rs1601935
rs79146711
rs77645768
rs12445401
rs2618568
rs76122697
rs6511720
rs182695896
rs261334
rs486142
rs10935473
rs115849089
rs6016505
rs174594
rs143473297
rs3744219
rs9901869
rs668948
rs4722551
rs2738447
rs2740488
rs183130
rs12916
rs11591147
rs4369382
rs646776
rs10455872
rs1461729
All
-0.10
-0.05
0.00
0.05
0.10
MR leave-one-out sensitivity analysis for
olesterol esters in small HDL || id:ebi-cfb233-GCST90302081' on 'Breast cancer (Combined Oncoarray; iCOGS; GWAS meta analysis)

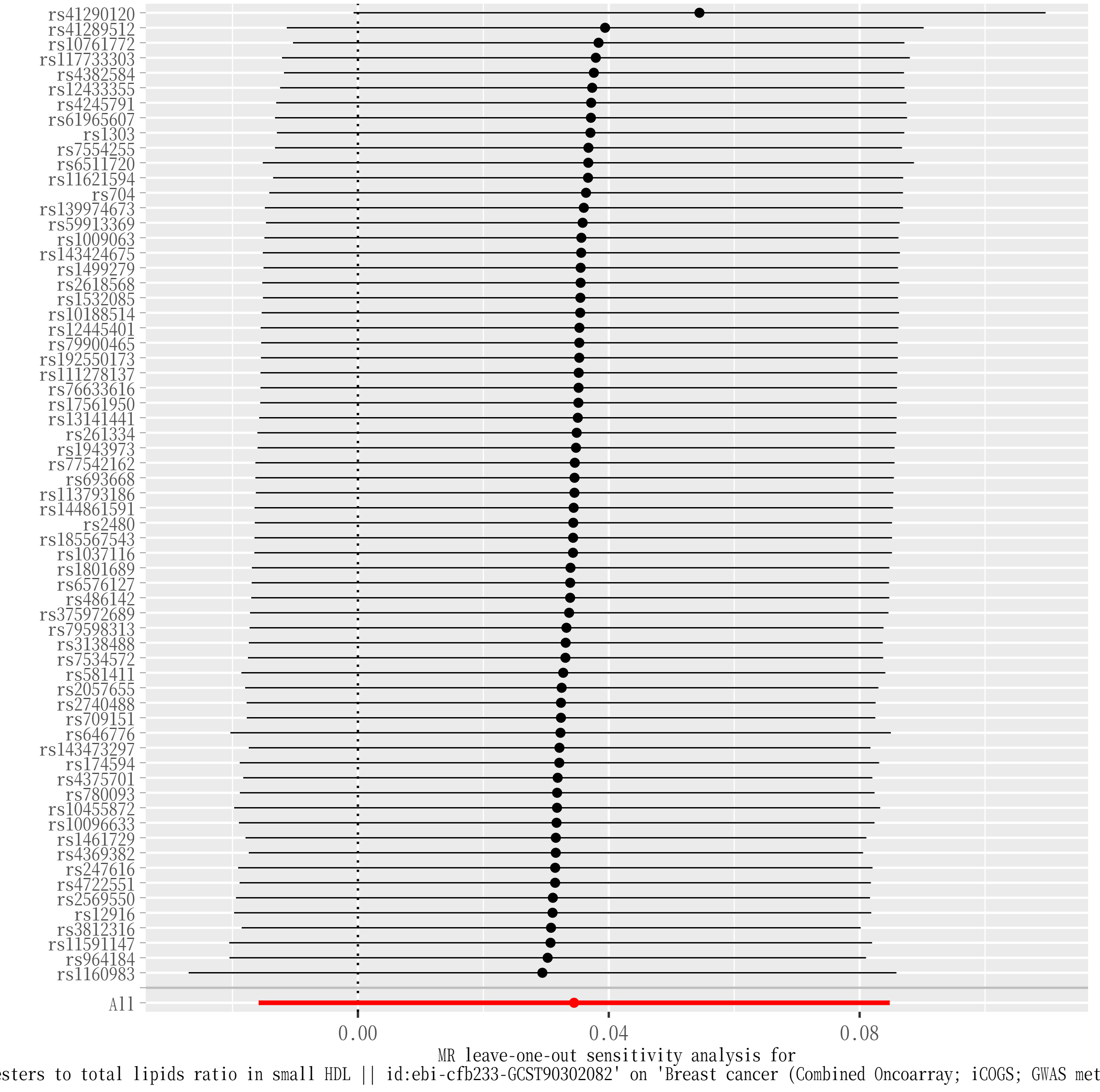

rs41290120
rs41289512
rs10761772
rs117733303
rs4382584
rs12433355
rs4245791
rs61965607
rs1303
rs7554255
rs6511720
rs11621594
rs704
rs139974673
rs59913369
rs1009063
rs143424675
rs1499279
rs2618568
rs1532085
rs10188514
rs12445401
rs79900465
rs192550173
rs111278137
rs76633616
rs17561950
rs13141441
rs261334
rs1943973
rs77542162
rs693668
rs113793186
rs144861591
rs2480
rs185567543
rs1037116
rs1801689
rs6576127
rs486142
rs375972689
rs79598313
rs3138488
rs7534572
rs581411
rs2057655
rs2740488
rs709151
rs646776
rs143473297
rs174594
rs4375701
rs780093
rs10455872
rs10096633
rs1461729
rs4369382
rs247616
rs4722551
rs2569550
rs12916
rs3812316
rs11591147
rs964184
rs1160983
All
0.00
0.04
0.08
MR leave-one-out sensitivity analysis for
esters to total lipids ratio in small HDL || id:ebi-cfb233-GCST90302082' on 'Breast cancer (Combined Oncoarray; iCOGS; GWAS met

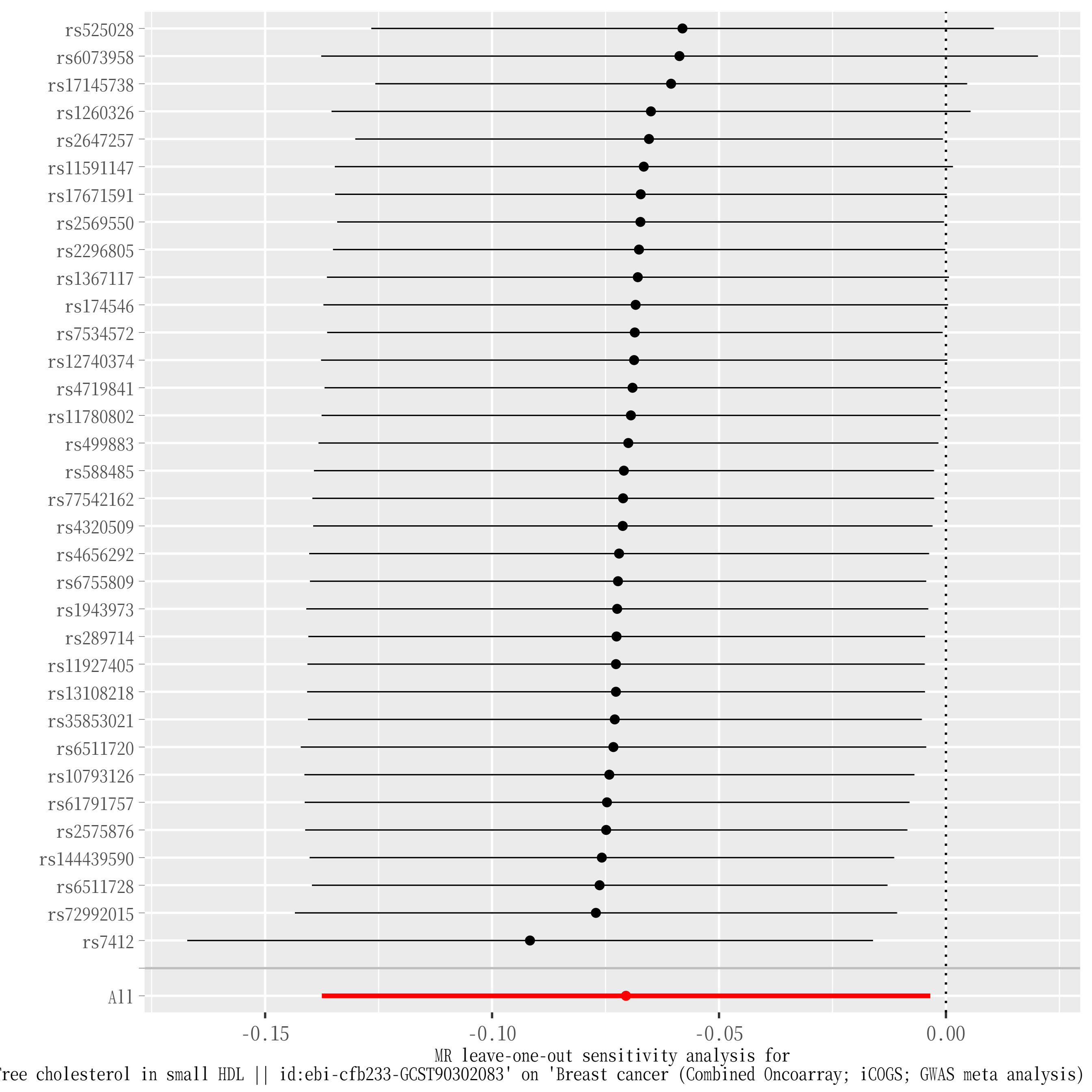
rs525028
rs6073958
rs17145738
rs1260326
rs2647257
rs11591147
rs17671591
rs2569550
rs2296805
rs1367117
rs174546
rs7534572
rs12740374
rs4719841
rs11780802
rs499883
rs588485
rs77542162
rs4320509
rs4656292
rs6755809
rs1943973
rs289714
rs11927405
rs13108218
rs35853021
rs6511720
rs10793126
rs61791757
rs2575876
rs144439590
rs6511728
rs72992015
rs7412
All
-0.15
-0.10
-0.05
0.00
MR leave-one-out sensitivity analysis for
ree cholesterol in small HDL || id:ebi-cfb233-GCST90302083' on 'Breast cancer (Combined Oncoarray; iCOGS; GWAS meta analysis)'

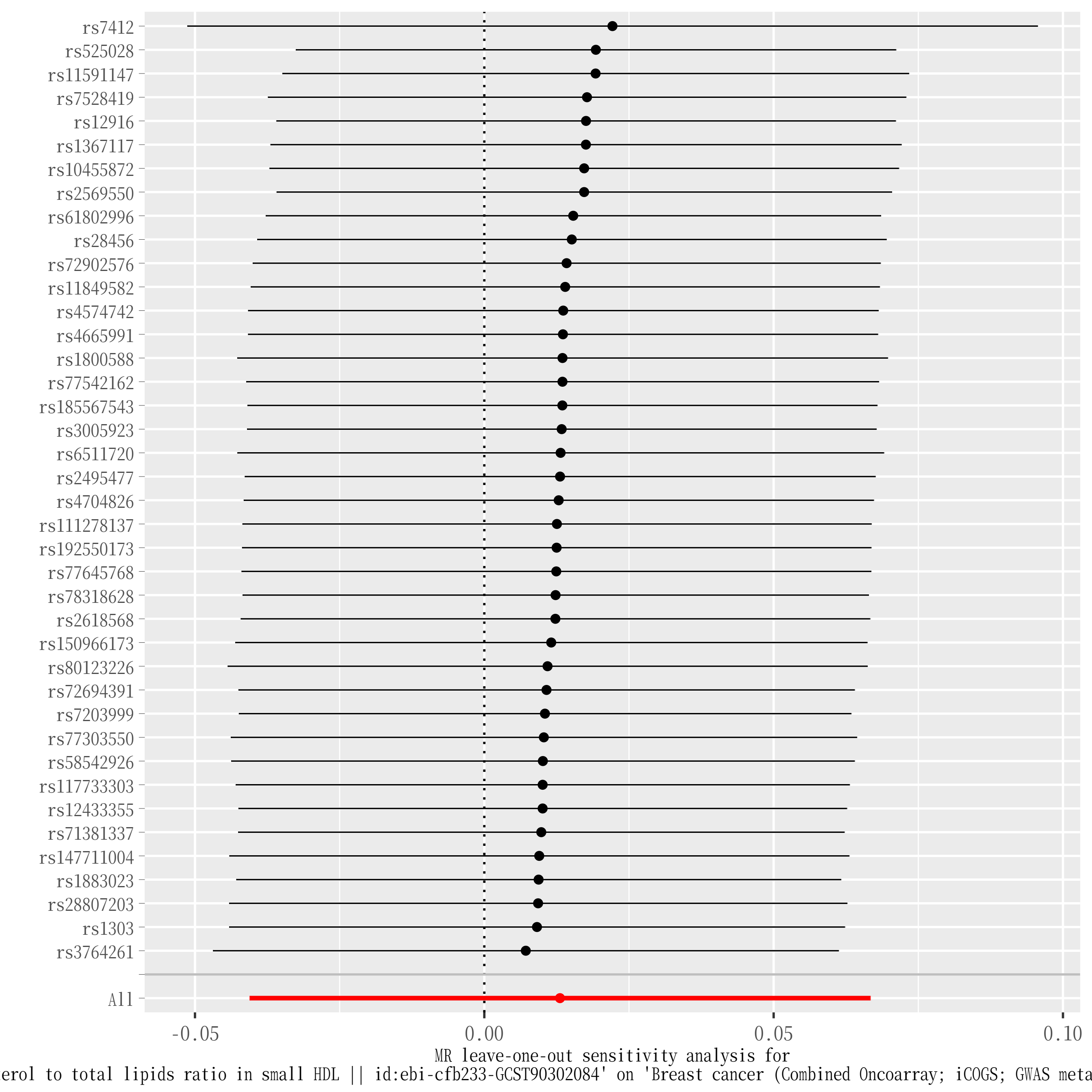
rs7412
rs525028
rs11591147
rs7528419
rs12916
rs1367117
rs10455872
rs2569550
rs61802996
rs28456
rs72902576
rs11849582
rs4574742
rs4665991
rs1800588
rs77542162
rs185567543
rs3005923
rs6511720
rs2495477
rs4704826
rs111278137
rs192550173
rs77645768
rs78318628
rs2618568
rs150966173
rs80123226
rs72694391
rs7203999
rs77303550
rs58542926
rs117733303
rs12433355
rs71381337
rs147711004
rs1883023
rs28807203
rs1303
rs3764261
All
-0.05
0.00
0.05
0.10
MR leave-one-out sensitivity analysis for
erol to total lipids ratio in small HDL || id:ebi-cfb233-GCST90302084' on 'Breast cancer (Combined Oncoarray; iCOGS; GWAS meta

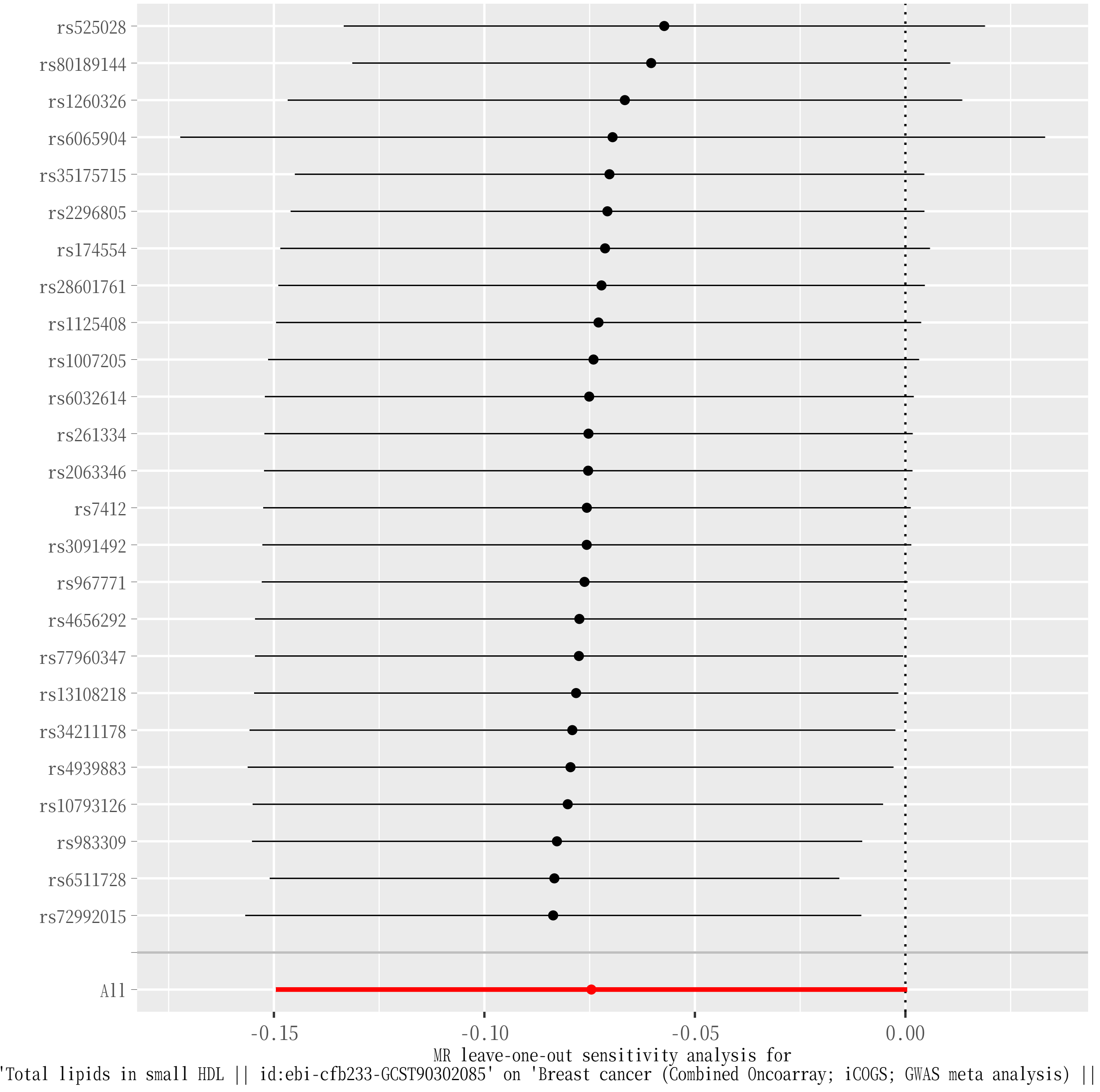
rs525028
rs80189144
rs1260326
rs6065904
rs35175715
rs2296805
rs174554
rs28601761
rs1125408
rs1007205
rs6032614
rs261334
rs2063346
rs7412
rs3091492
rs967771
rs4656292
rs77960347
rs13108218
rs34211178
rs4939883
rs10793126
rs983309
rs6511728
rs72992015
All
-0.15
-0.10
-0.05
0.00
MR leave-one-out sensitivity analysis for
'Total lipids in small HDL || id:ebi-cfb233-GCST90302085' on 'Breast cancer (Combined Oncoarray; iCOGS; GWAS meta analysis) ||

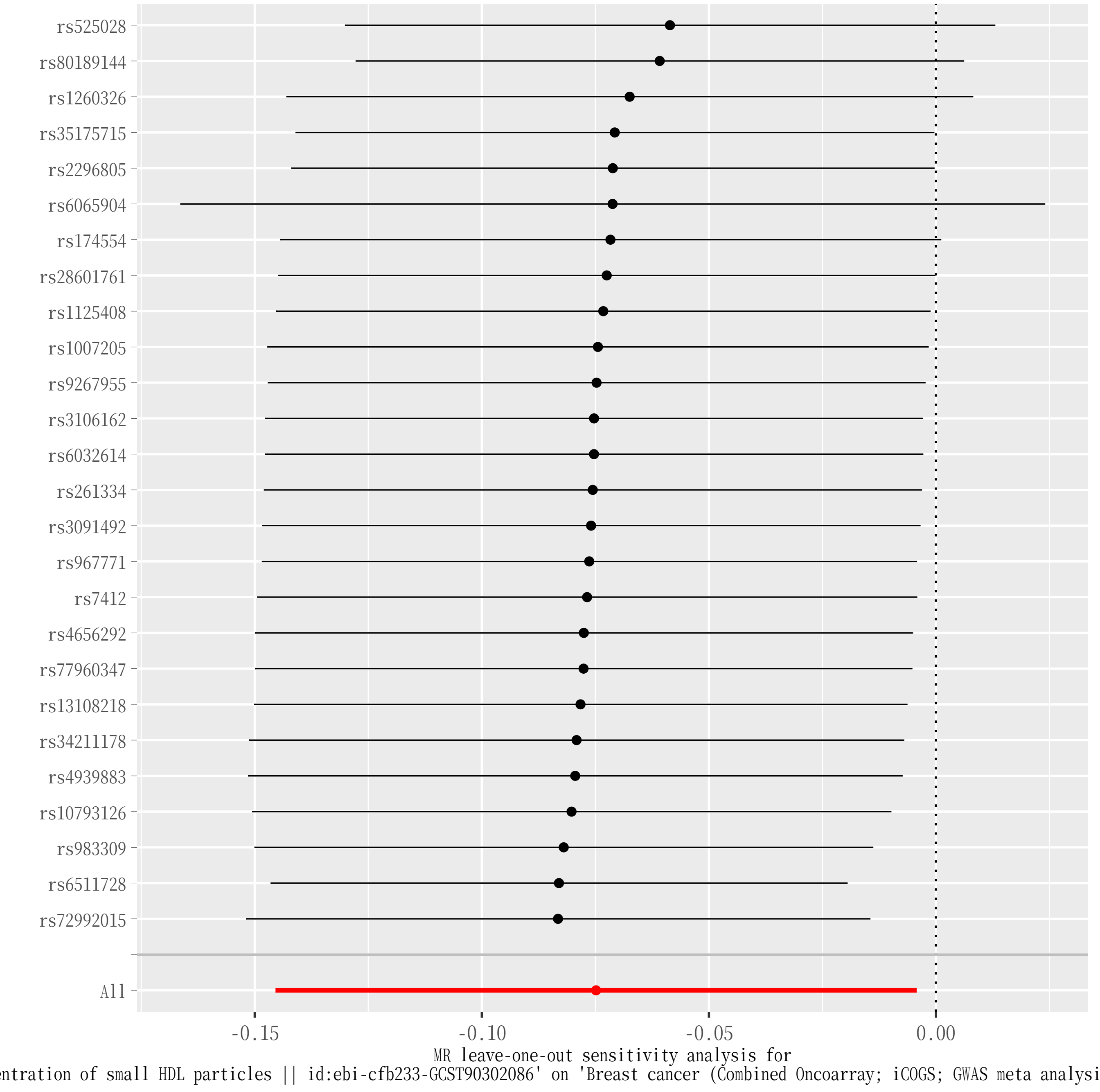

rs525028
rs80189144
rs1260326
rs35175715
rs2296805
rs6065904
rs174554
rs28601761
rs1125408
rs1007205
rs9267955
rs3106162
rs6032614
rs261334
rs3091492
rs967771
rs7412
rs4656292
rs77960347
rs13108218
rs34211178
rs4939883
rs10793126
rs983309
rs6511728
rs72992015
All
-0.15
-0.10
-0.05
0.00
MR leave-one-out sensitivity analysis for
entration of small HDL particles || id:ebi-cfb233-GCST90302086' on 'Breast cancer (Combined Oncoarray; iCOGS; GWAS meta analysi

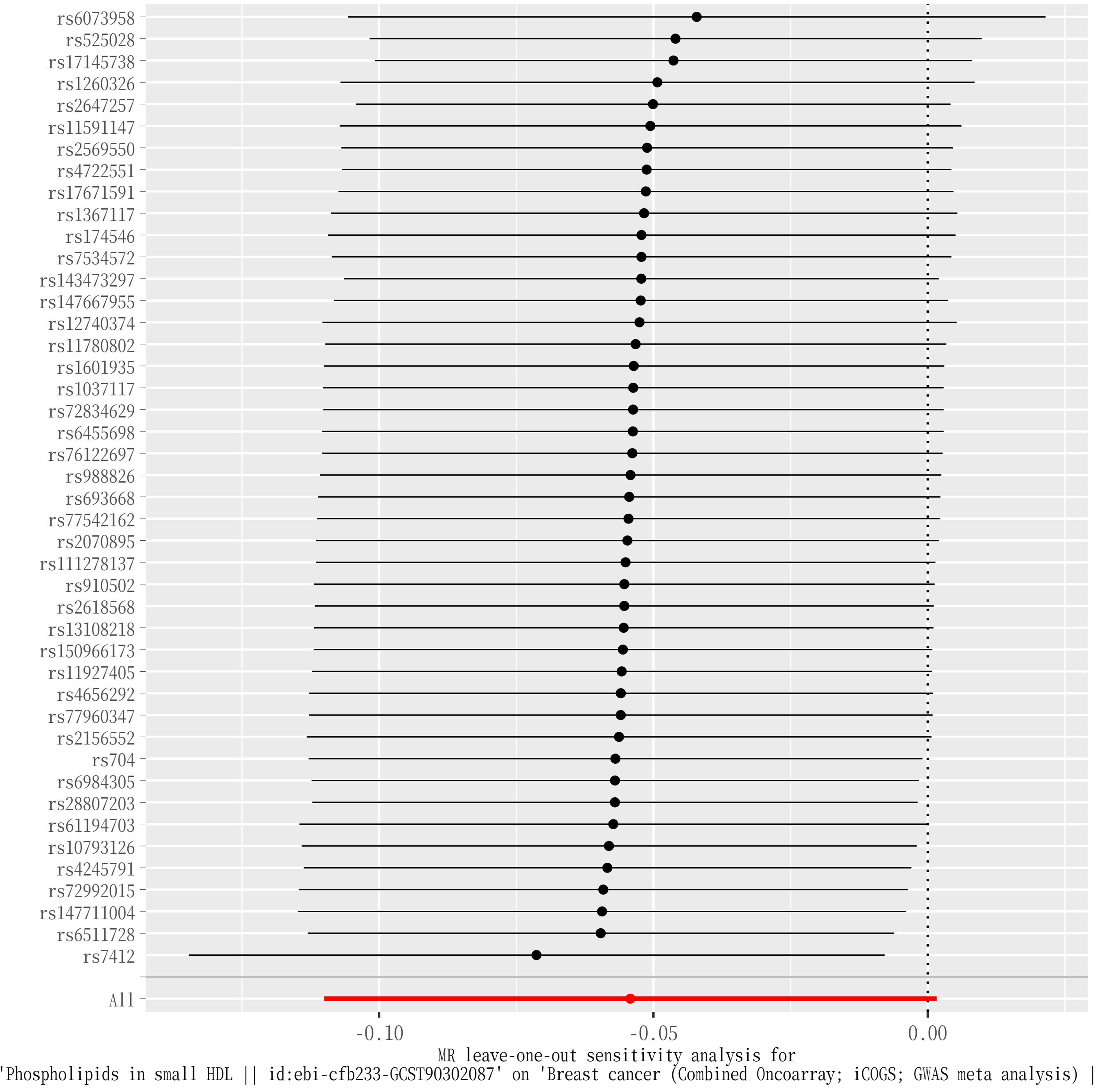
rs6073958
rs525028
rs17145738
rs1260326
rs2647257
rs11591147
rs2569550
rs4722551
rs17671591
rs1367117
rs174546
rs7534572
rs143473297
rs147667955
rs12740374
rs11780802
rs1601935
rs1037117
rs72834629
rs6455698
rs76122697
rs988826
rs693668
rs77542162
rs2070895
rs111278137
rs910502
rs2618568
rs13108218
rs150966173
rs11927405
rs4656292
rs77960347
rs2156552
rs704
rs6984305
rs28807203
rs61194703
rs10793126
rs4245791
rs72992015
rs147711004
rs6511728
rs7412
All
-0.10
-0.05
0.00
MR leave-one-out sensitivity analysis for
'Phospholipids in small HDL || id:ebi-cfb233-GCST90302087' on 'Breast cancer (Combined Oncoarray; iCOGS; GWAS meta analysis) |

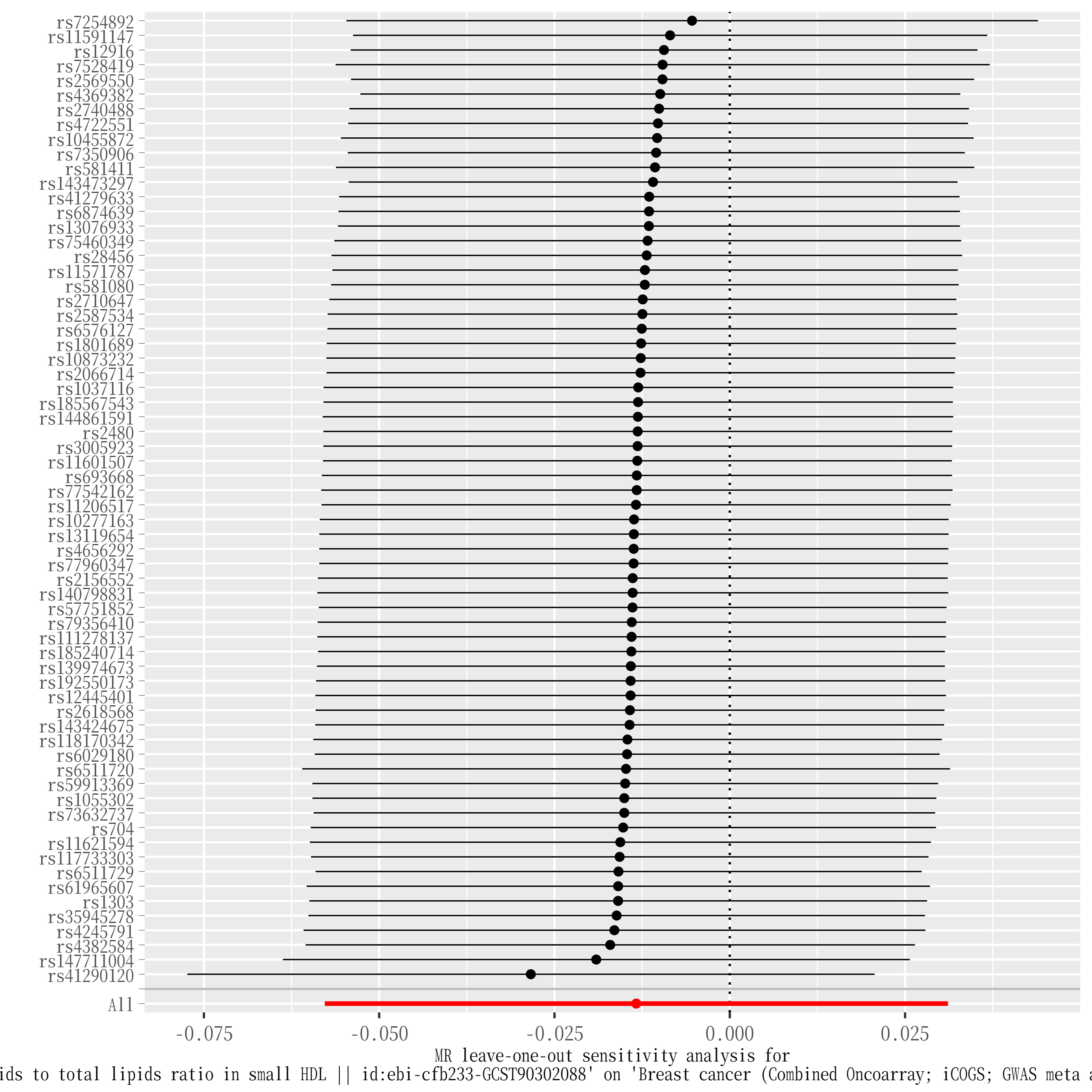
rs7254892
rs11591147
rs12916
rs7528419
rs2569550
rs4369382
rs2740488
rs4722551
rs10455872
rs7350906
rs581411
rs143473297
rs41279633
rs6874639
rs13076933
rs75460349
rs28456
rs11571787
rs581080
rs2710647
rs2587534
rs6576127
rs1801689
rs10873232
rs2066714
rs1037116
rs185567543
rs144861591
rs2480
rs3005923
rs11601507
rs693668
rs77542162
rs11206517
rs10277163
rs13119654
rs4656292
rs77960347
rs2156552
rs140798831
rs57751852
rs79356410
rs111278137
rs185240714
rs139974673
rs192550173
rs12445401
rs2618568
rs143424675
rs118170342
rs6029180
rs6511720
rs59913369
rs1055302
rs73632737
rs704
rs11621594
rs117733303
rs6511729
rs61965607
rs1303
rs35945278
rs4245791
rs4382584
rs147711004
rs41290120
All
-0.075
-0.050
-0.025
0.000
0.025
MR leave-one-out sensitivity analysis for
ids to total lipids ratio in small HDL || id:ebi-cfb233-GCST90302088' on 'Breast cancer (Combined Oncoarray; iCOGS; GWAS meta a

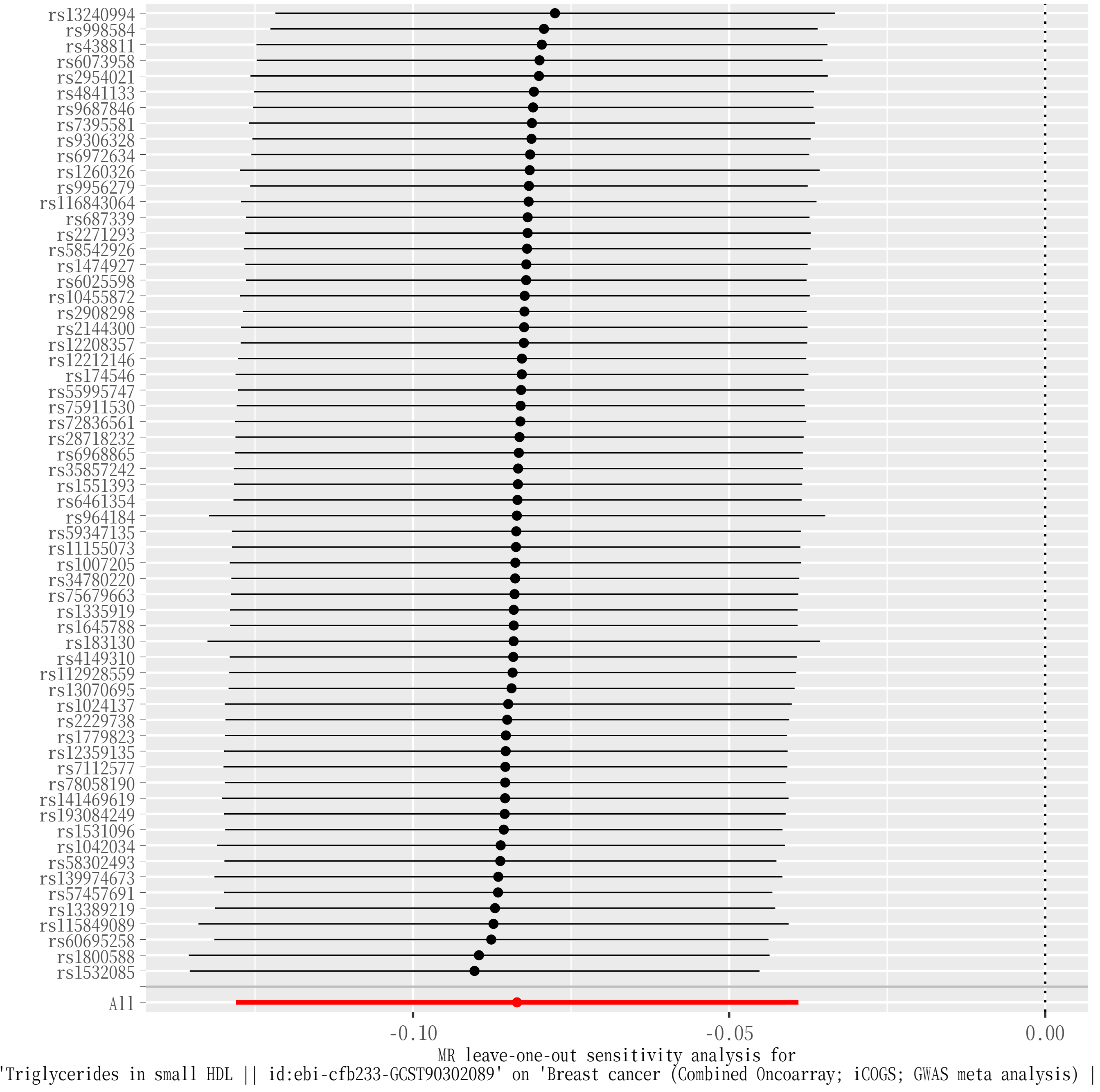
rs13240994
rs998584
rs438811
rs6073958
rs2954021
rs4841133
rs9687846
rs7395581
rs9306328
rs6972634
rs1260326
rs9956279
rs116843064
rs687339
rs2271293
rs58542926
rs1474927
rs6025598
rs10455872
rs2908298
rs2144300
rs12208357
rs12212146
rs174546
rs55995747
rs75911530
rs72836561
rs28718232
rs6968865
rs35857242
rs1551393
rs6461354
rs964184
rs59347135
rs11155073
rs1007205
rs34780220
rs75679663
rs1335919
rs1645788
rs183130
rs4149310
rs112928559
rs13070695
rs1024137
rs2229738
rs1779823
rs12359135
rs7112577
rs78058190
rs141469619
rs193084249
rs1531096
rs1042034
rs58302493
rs139974673
rs57457691
rs13389219
rs115849089
rs60695258
rs1800588
rs1532085
All
-0.10
-0.05
0.00
MR leave-one-out sensitivity analysis for
'Triglycerides in small HDL || id:ebi-cfb233-GCST90302089' on 'Breast cancer (Combined Oncoarray; iCOGS; GWAS meta analysis) |

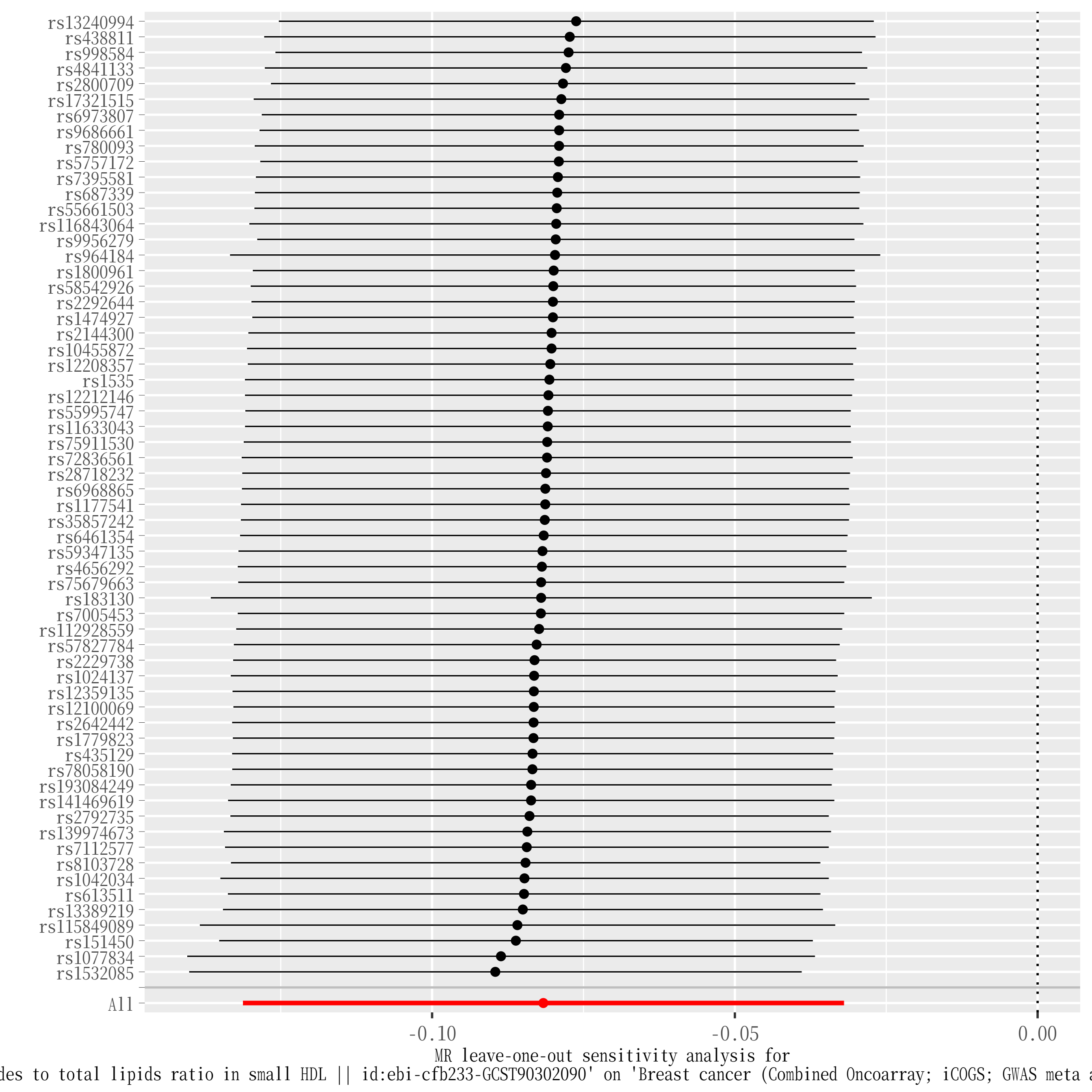
rs13240994
rs438811
rs998584
rs4841133
rs2800709
rs17321515
rs6973807
rs9686661
rs780093
rs5757172
rs7395581
rs687339
rs55661503
rs116843064
rs9956279
rs964184
rs1800961
rs58542926
rs2292644
rs1474927
rs2144300
rs10455872
rs12208357
rs1535
rs12212146
rs55995747
rs11633043
rs75911530
rs72836561
rs28718232
rs6968865
rs1177541
rs35857242
rs6461354
rs59347135
rs4656292
rs75679663
rs183130
rs7005453
rs112928559
rs57827784
rs2229738
rs1024137
rs12359135
rs12100069
rs2642442
rs1779823
rs435129
rs78058190
rs193084249
rs141469619
rs2792735
rs139974673
rs7112577
rs8103728
rs1042034
rs613511
rs13389219
rs115849089
rs151450
rs1077834
rs1532085
All
-0.10
-0.05
0.00
MR leave-one-out sensitivity analysis for
des to total lipids ratio in small HDL || id:ebi-cfb233-GCST90302090' on 'Breast cancer (Combined Oncoarray; iCOGS; GWAS meta a

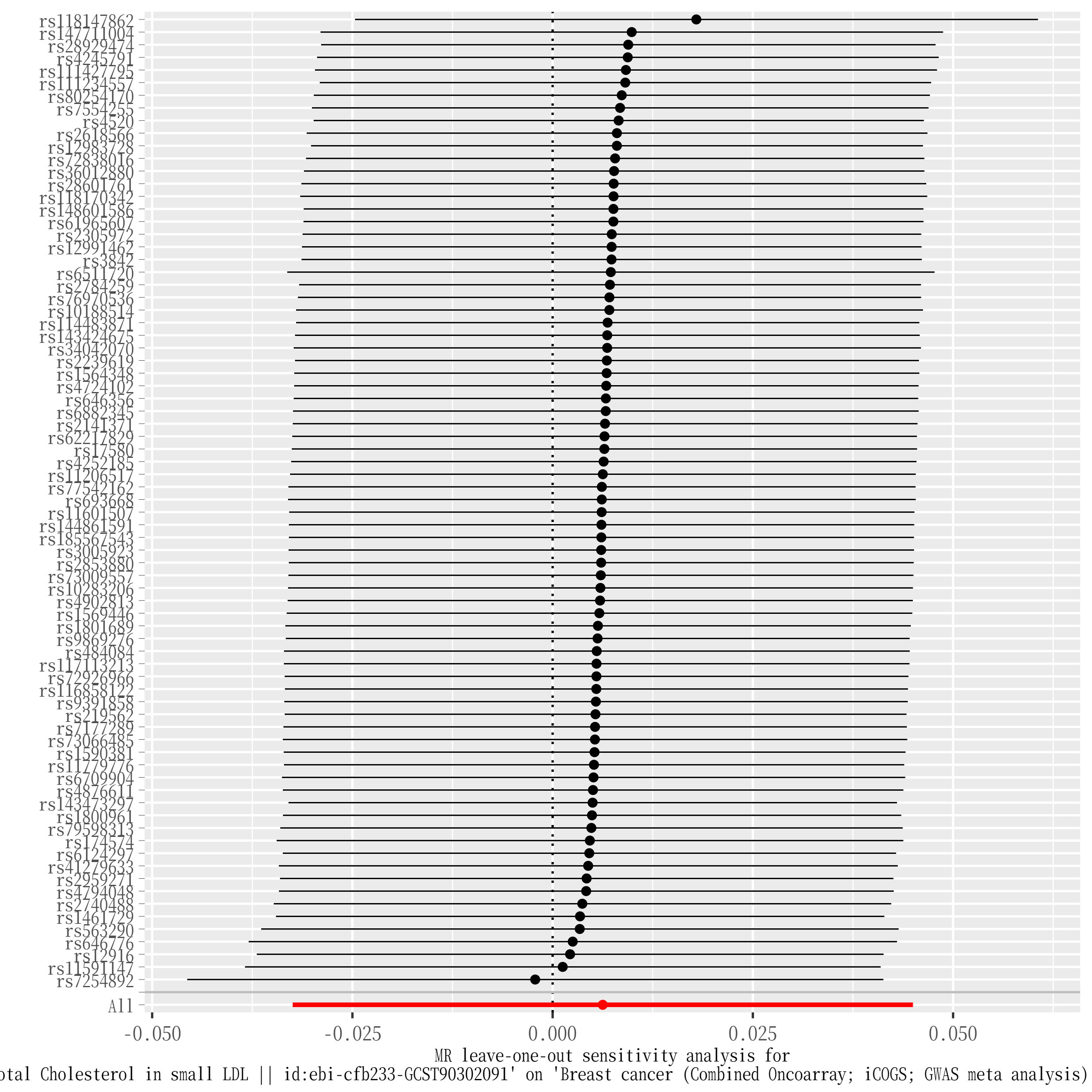
rs118147862
rs147711004
rs28929474
rs4245791
rs111427795
rs111234557
rs80254170
rs7554255
rs4520
rs2618566
rs12983728
rs72838016
rs36012880
rs28601761
rs118170342
rs148601586
rs61965607
rs2305972
rs12991462
rs3842
rs6511720
rs2784259
rs76970536
rs10188514
rs114483871
rs143424675
rs34042070
rs2239619
rs1564348
rs4724102
rs646356
rs6882345
rs2141371
rs62217829
rs17580
rs4252185
rs11206517
rs77542162
rs693668
rs11601507
rs144861591
rs185567543
rs3005923
rs2853880
rs73009557
rs10283206
rs4902813
rs1569446
rs1801689
rs9869276
rs484084
rs117113213
rs72926966
rs116858122
rs9391858
rs219562
rs7177289
rs73066485
rs1520381
rs11779776
rs6709904
rs4876611
rs143473297
rs1800961
rs79598313
rs174574
rs6124297
rs41279633
rs2959271
rs4794048
rs2740488
rs1461729
rs563290
rs646776
rs12916
rs11591147
rs7254892
All
-0.050
-0.025
0.000
0.025
0.050
MR leave-one-out sensitivity analysis for
otal Cholesterol in small LDL || id:ebi-cfb233-GCST90302091' on 'Breast cancer (Combined Oncoarray; iCOGS; GWAS meta analysis)

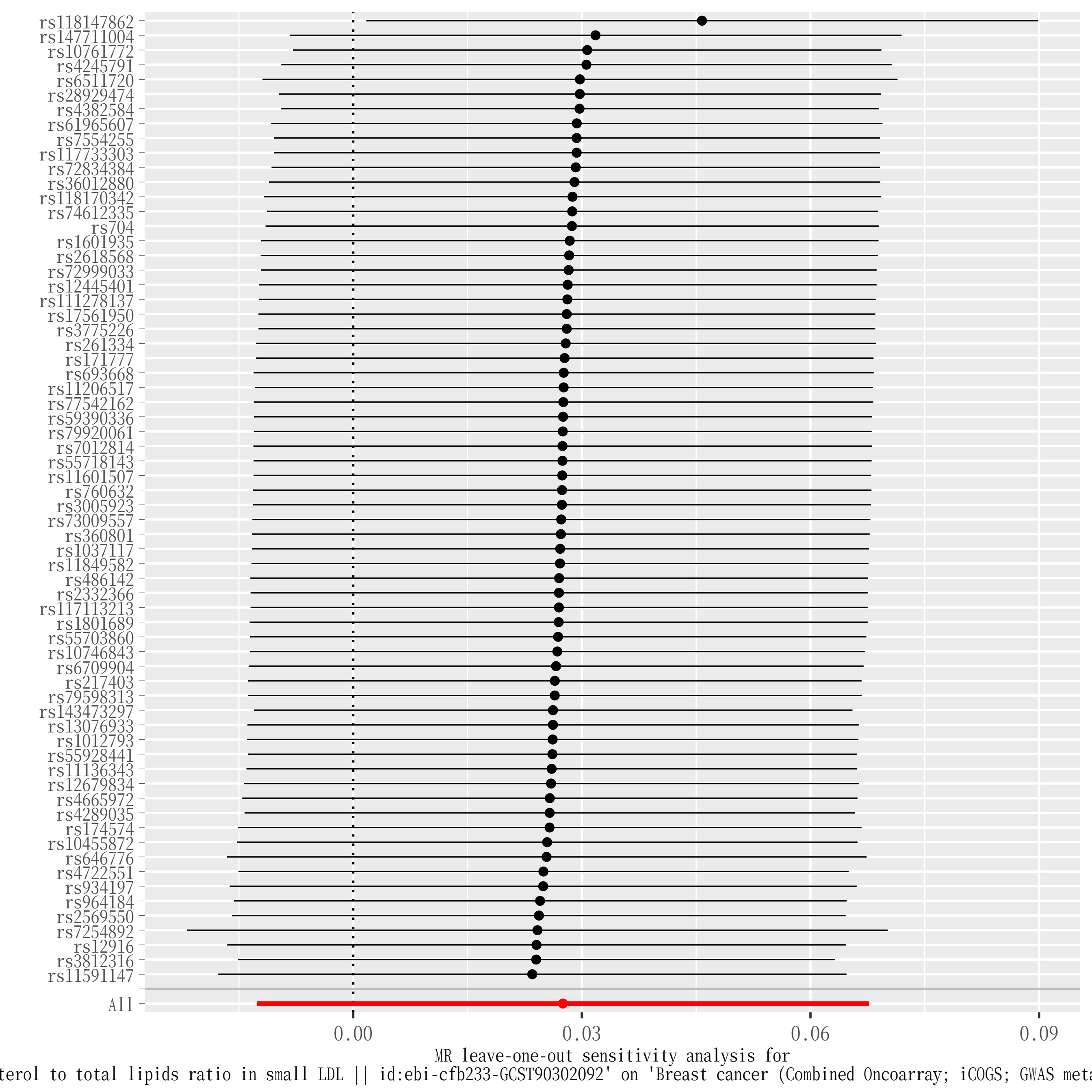

rs118147862
rs147711004
rs10761772
rs4245791
rs6511720
rs28929474
rs4382584
rs61965607
rs7554255
rs117733303
rs72834384
rs36012880
rs118170342
rs74612335
rs704
rs1601935
rs2618568
rs72999033
rs12445401
rs111278137
rs17561950
rs3775226
rs261334
rs171777
rs693668
rs11206517
rs77542162
rs59390336
rs79920061
rs7012814
rs55718143
rs11601507
rs760632
rs3005923
rs73009557
rs360801
rs1037117
rs11849582
rs486142
rs2332366
rs117113213
rs1801689
rs55703860
rs10746843
rs6709904
rs217403
rs79598313
rs143473297
rs13076933
rs1012793
rs55928441
rs11136343
rs12679834
rs4665972
rs4289035
rs174574
rs10455872
rs646776
rs4722551
rs934197
rs964184
rs2569550
rs7254892
rs12916
rs3812316
rs11591147
All
0.00
0.03
0.06
0.09
MR leave-one-out sensitivity analysis for
terol to total lipids ratio in small LDL || id:ebi-cfb233-GCST90302092' on 'Breast cancer (Combined Oncoarray; iCOGS; GWAS meta

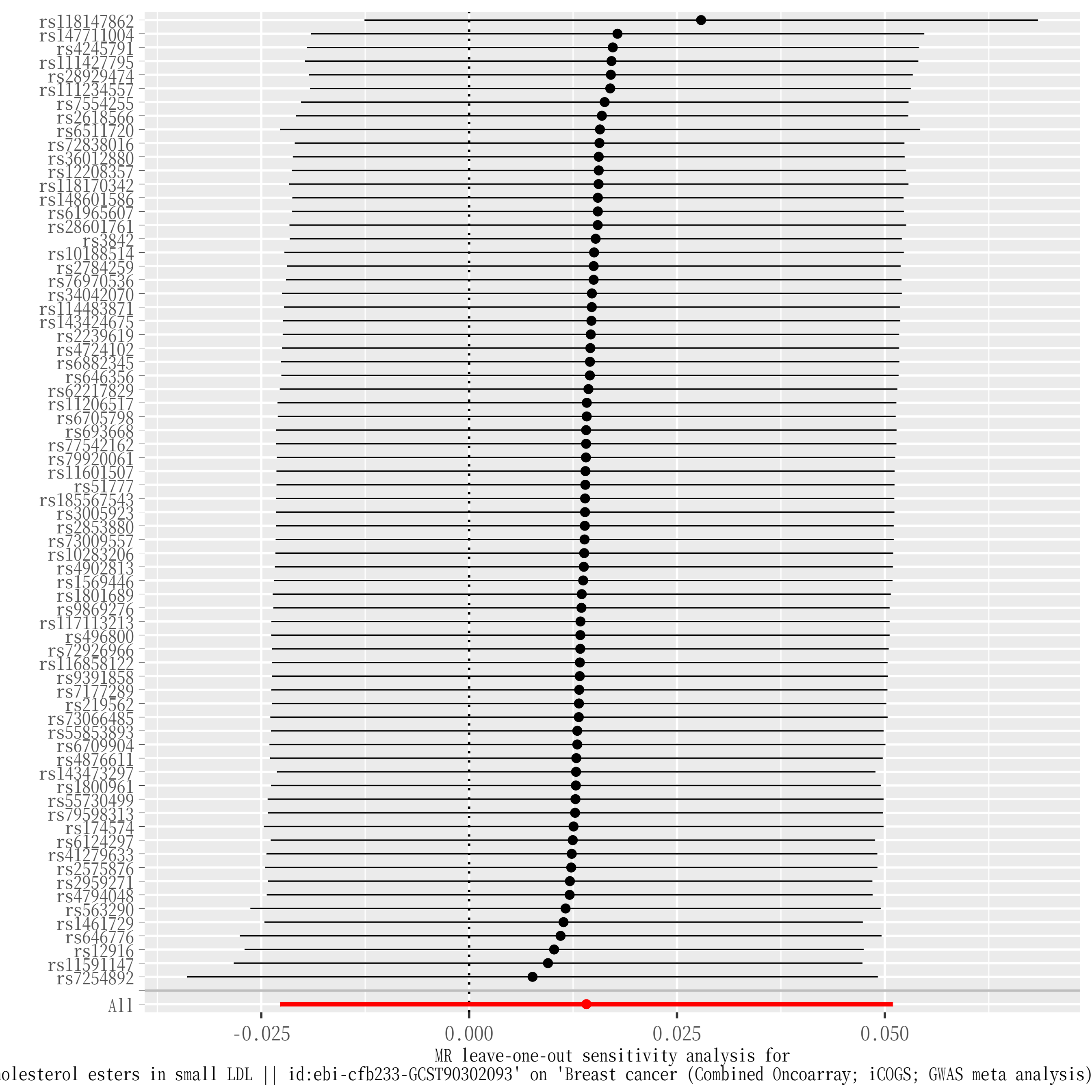

rs118147862
rs147711004
rs4245791
rs111427795
rs28929474
rs111234557
rs7554255
rs2618566
rs6511720
rs72838016
rs36012880
rs12208357
rs118170342
rs148601586
rs61965607
rs28601761
rs3842
rs10188514
rs2784259
rs76970536
rs34042070
rs114483871
rs143424675
rs2239619
rs4724102
rs6882345
rs646356
rs62217829
rs11206517
rs6705798
rs693668
rs77542162
rs79920061
rs11601507
rs51777
rs185567543
rs3005923
rs2853880
rs73009557
rs10283206
rs4902813
rs1569446
rs1801689
rs9869276
rs117113213
rs496800
rs72926966
rs116858122
rs9391858
rs7177289
rs219562
rs73066485
rs55853893
rs6709904
rs4876611
rs143473297
rs1800961
rs55730499
rs79598313
rs174574
rs6124297
rs41279633
rs2575876
rs2959271
rs4794048
rs563290
rs1461729
rs646776
rs12916
rs11591147
rs7254892
All
-0.025
0.000
0.025
0.050
MR leave-one-out sensitivity analysis for
holesterol esters in small LDL || id:ebi-cfb233-GCST90302093' on 'Breast cancer (Combined Oncoarray; iCOGS; GWAS meta analysis)

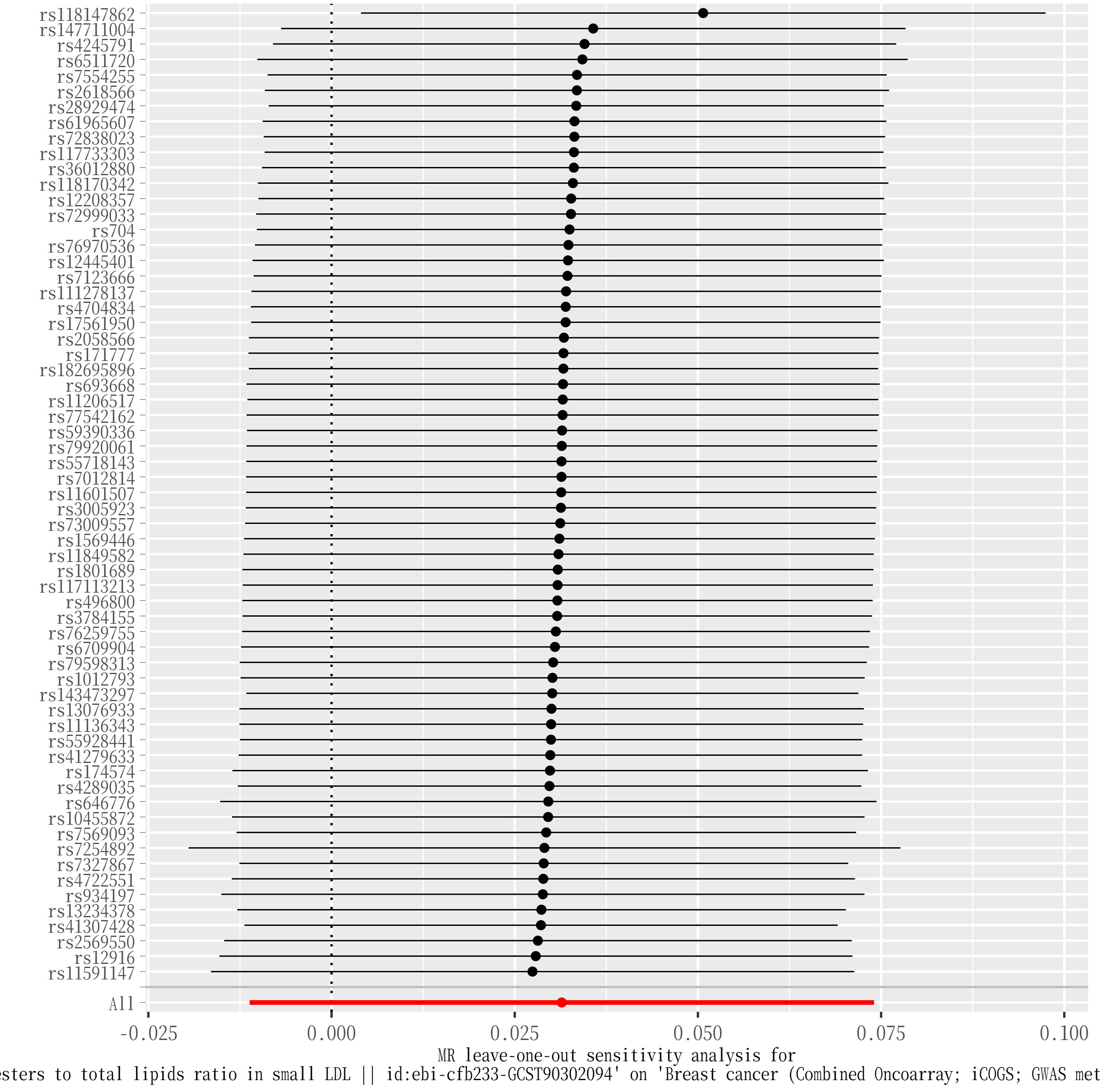
rs118147862
rs147711004
rs4245791
rs6511720
rs7554255
rs2618566
rs28929474
rs61965607
rs72838023
rs117733303
rs36012880
rs118170342
rs12208357
rs72999033
rs704
rs76970536
rs12445401
rs7123666
rs111278137
rs4704834
rs17561950
rs2058566
rs171777
rs182695896
rs693668
rs11206517
rs77542162
rs59390336
rs79920061
rs55718143
rs7012814
rs11601507
rs3005923
rs73009557
rs1569446
rs11849582
rs1801689
rs117113213
rs496800
rs3784155
rs76259755
rs6709904
rs79598313
rs1012793
rs143473297
rs13076933
rs11136343
rs55928441
rs41279633
rs174574
rs4289035
rs646776
rs10455872
rs7569093
rs7254892
rs7327867
rs4722551
rs934197
rs13234378
rs41307428
rs2569550
rs12916
rs11591147
All
-0.025
0.000
0.025
0.050
0.075
0.100
MR leave-one-out sensitivity analysis for
esters to total lipids ratio in small LDL || id:ebi-cfb233-GCST90302094' on 'Breast cancer (Combined Oncoarray; iCOGS; GWAS met

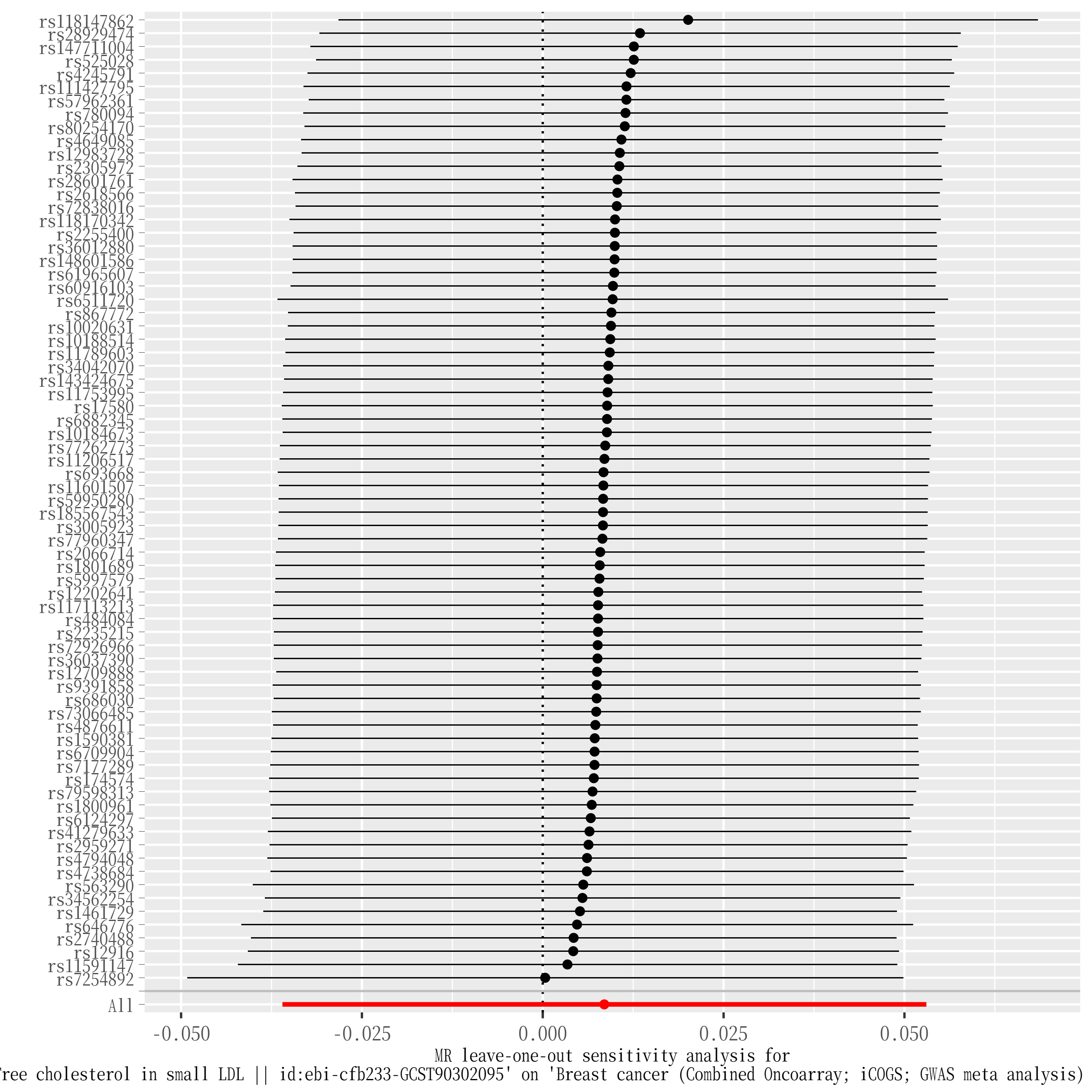

rs118147862
rs28929474
rs147711004
rs525028
rs4245791
rs111427795
rs57962361
rs780094
rs80254170
rs4649085
rs12983728
rs2305972
rs28601761
rs2618566
rs72838016
rs118170342
rs2255400
rs36012880
rs148601586
rs61965607
rs60916103
rs6511720
rs867772
rs10020631
rs10188514
rs11789603
rs34042070
rs143424675
rs11753995
rs17580
rs6882345
rs10184673
rs77262773
rs11206517
rs693668
rs11601507
rs59950280
rs185567543
rs3005923
rs77960347
rs2066714
rs1801689
rs5997579
rs12202641
rs117113213
rs484084
rs2235215
rs72926966
rs36037390
rs12709888
rs9391858
rs686030
rs73066485
rs4876611
rs1590381
rs6709904
rs7177289
rs174574
rs79598313
rs1800961
rs6124297
rs41279633
rs2959271
rs4794048
rs4738684
rs563290
rs34562254
rs1461729
rs646776
rs2740488
rs12916
rs11591147
rs7254892
All
-0.050
-0.025
0.000
0.025
0.050
MR leave-one-out sensitivity analysis for
Free cholesterol in small LDL || id:ebi-cfb233-GCST90302095' on 'Breast cancer (Combined Oncoarray; iCOGS; GWAS meta analysis)'

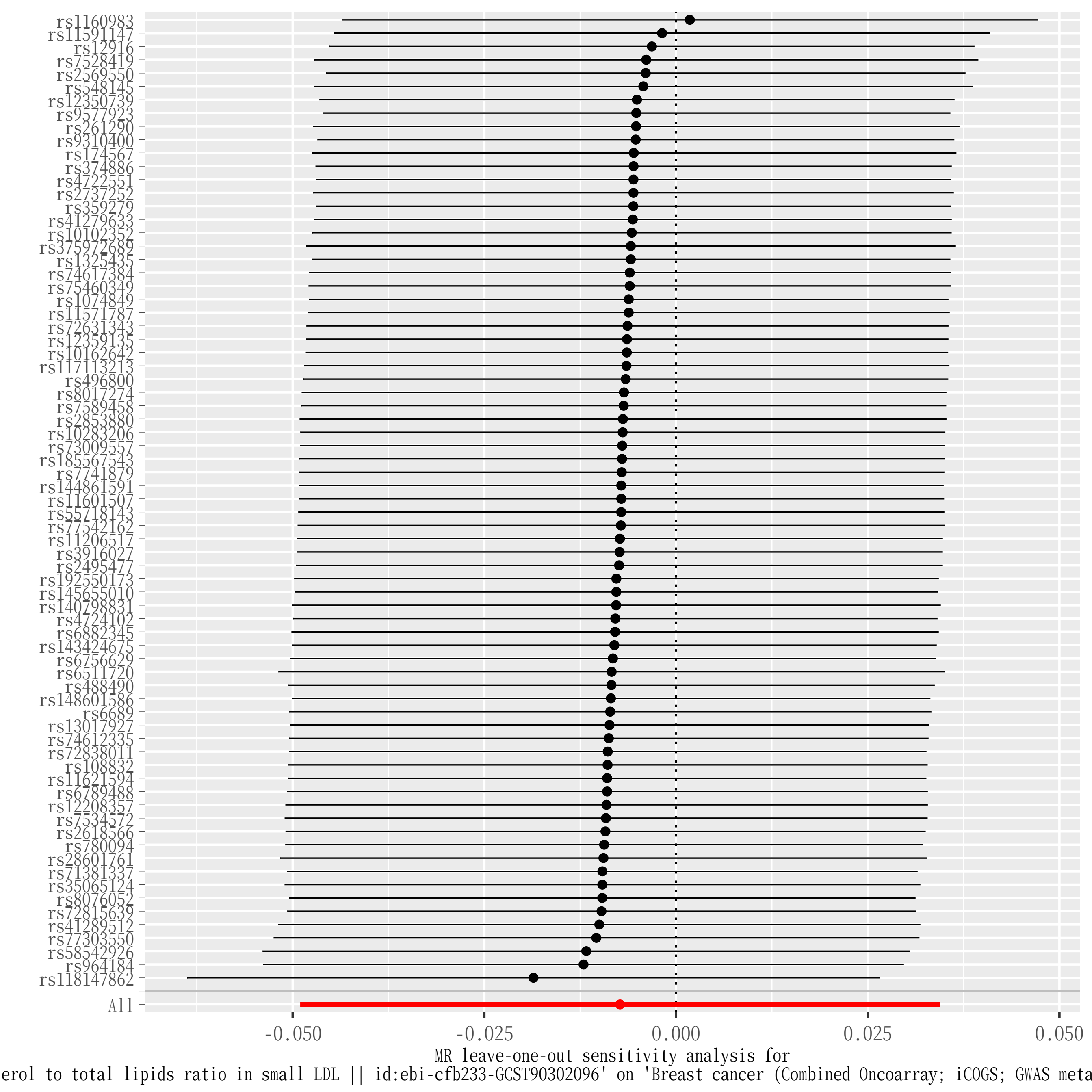
rs1160983
rs11591147
rs12916
rs7528419
rs2569550
rs548145
rs12350739
rs9577923
rs261290
rs9310400
rs174567
rs374886
rs4722551
rs2737252
rs359279
rs41279633
rs10102352
rs375972689
rs1325435
rs74617384
rs75460349
rs1074849
rs11571787
rs72631343
rs12359135
rs10162642
rs117113213
rs496800
rs8017274
rs7589458
rs2853880
rs10283206
rs73009557
rs185567543
rs7741879
rs144861591
rs11601507
rs55718143
rs77542162
rs11206517
rs3916027
rs2495477
rs192550173
rs145655010
rs140798831
rs4724102
rs6882345
rs143424675
rs6756629
rs6511720
rs488490
rs148601586
rs6689
rs13017927
rs74612335
rs72838011
rs108832
rs11621594
rs6789488
rs12208357
rs7534572
rs2618566
rs780094
rs28601761
rs71381337
rs35065124
rs8076052
rs72815639
rs41289512
rs77303550
rs58542926
rs964184
rs118147862
All
-0.050
-0.025
0.000
0.025
0.050
MR leave-one-out sensitivity analysis for
erol to total lipids ratio in small LDL || id:ebi-cfb233-GCST90302096' on 'Breast cancer (Combined Oncoarray; iCOGS; GWAS meta

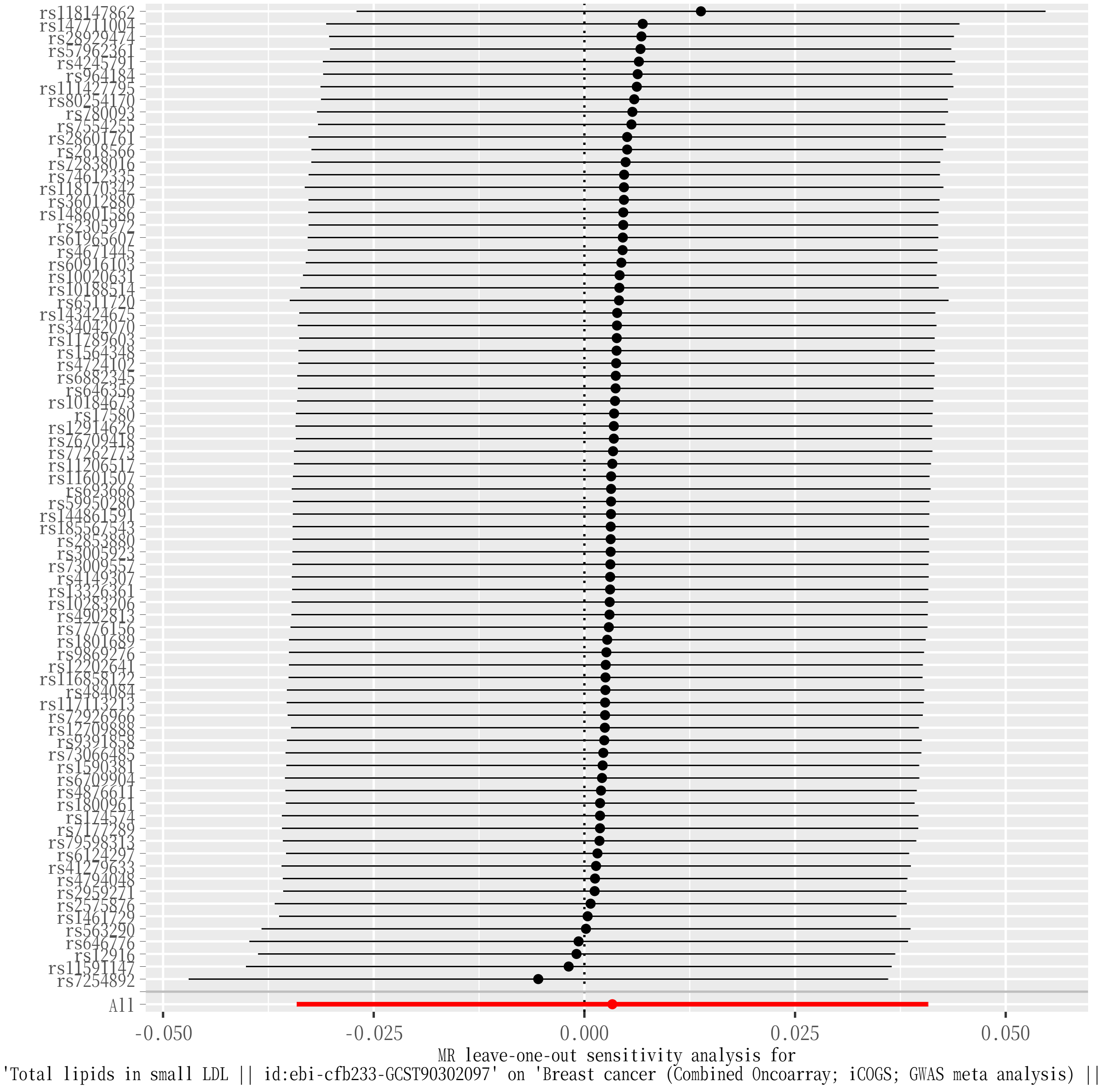
rs118147862
rs147711004
rs28929474
rs57962361
rs4245791
rs964184
rs111427725
rs80254170
rs780093
rs7554255
rs28601761
rs2618566
rs72838016
rs74612335
rs118170342
rs36012880
rs148601586
rs2305972
rs61965607
rs4671445
rs60916103
rs10020631
rs10188514
rs6511720
rs143424675
rs34042070
rs11789603
rs1564348
rs4724102
rs6882345
rs646356
rs10184673
rs17580
rs12914626
rs76709418
rs77262773
rs11206517
rs11601507
rs693668
rs59950280
rs144861591
rs185567543
rs2853880
rs3005923
rs73009557
rs4149307
rs13326361
rs10283206
rs4902813
rs7776156
rs1801689
rs9869276
rs12202641
rs116858122
rs484084
rs117113213
rs72926966
rs12709888
rs9321858
rs73066485
rs1590381
rs6709904
rs4876611
rs1800961
rs174574
rs7177289
rs79598313
rs6124297
rs41279633
rs4794048
rs2959271
rs2575876
rs1461729
rs563290
rs646776
rs12916
rs11591147
rs7254892
All
-0.050
-0.025
0.000
0.025
0.050
MR leave-one-out sensitivity analysis for
'Total lipids in small LDL || id:ebi-cfb233-GCST90302097' on 'Breast cancer (Combined Oncoarray; iCOGS; GWAS meta analysis) ||

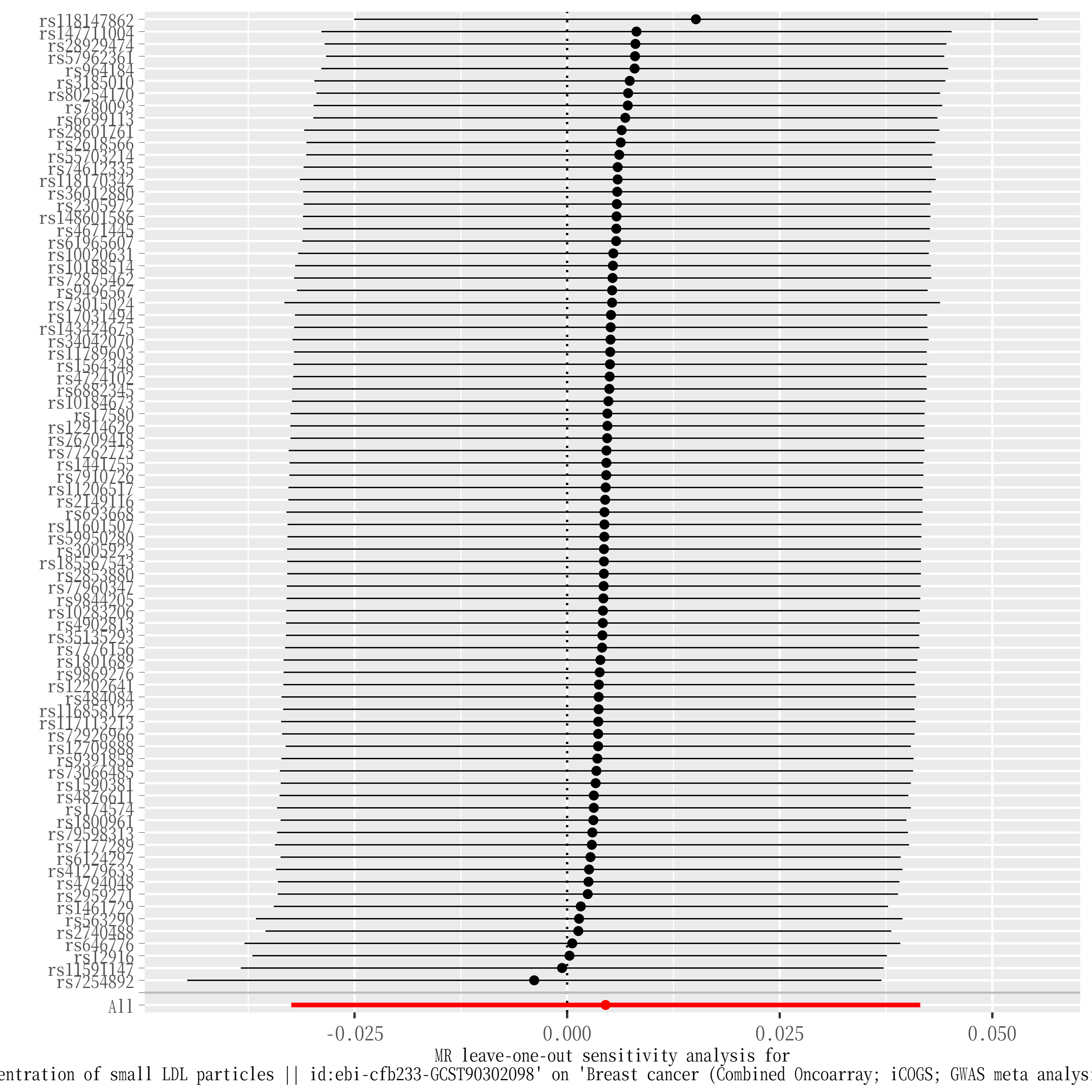
rs118147862
rs147711004
rs28929474
rs57962361
rs964184
rs3185010
rs80254170
rs780093
rs6699113
rs28601761
rs2618566
rs55703214
rs74612335
rs118170342
rs36012880
rs2305972
rs148601586
rs4671445
rs61965607
rs10020631
rs10188514
rs72875462
rs9496567
rs73015024
rs17031494
rs143424675
rs34042070
rs11789603
rs1564348
rs4724102
rs6882345
rs10184673
rs17580
rs12914626
rs76709418
rs77262773
rs1441755
rs7910726
rs11206517
rs2149116
rs693668
rs11601507
rs59950280
rs3005923
rs185567543
rs2853880
rs77960347
rs9844205
rs10283206
rs4902813
rs35135293
rs7776156
rs1801689
rs9869276
rs12202641
rs484084
rs116858122
rs117113213
rs72926966
rs12709888
rs9391858
rs73066485
rs1520381
rs4876611
rs174574
rs1800961
rs79598313
rs7177289
rs6124297
rs41279633
rs4794048
rs2959271
rs1461729
rs563290
rs2740488
rs646776
rs12916
rs11591147
rs7254892
All
-0.025
0.000
0.025
0.050
MR leave-one-out sensitivity analysis for
entration of small LDL particles || id:ebi-cfb233-GCST90302098' on 'Breast cancer (Combined Oncoarray; iCOGS; GWAS meta analys

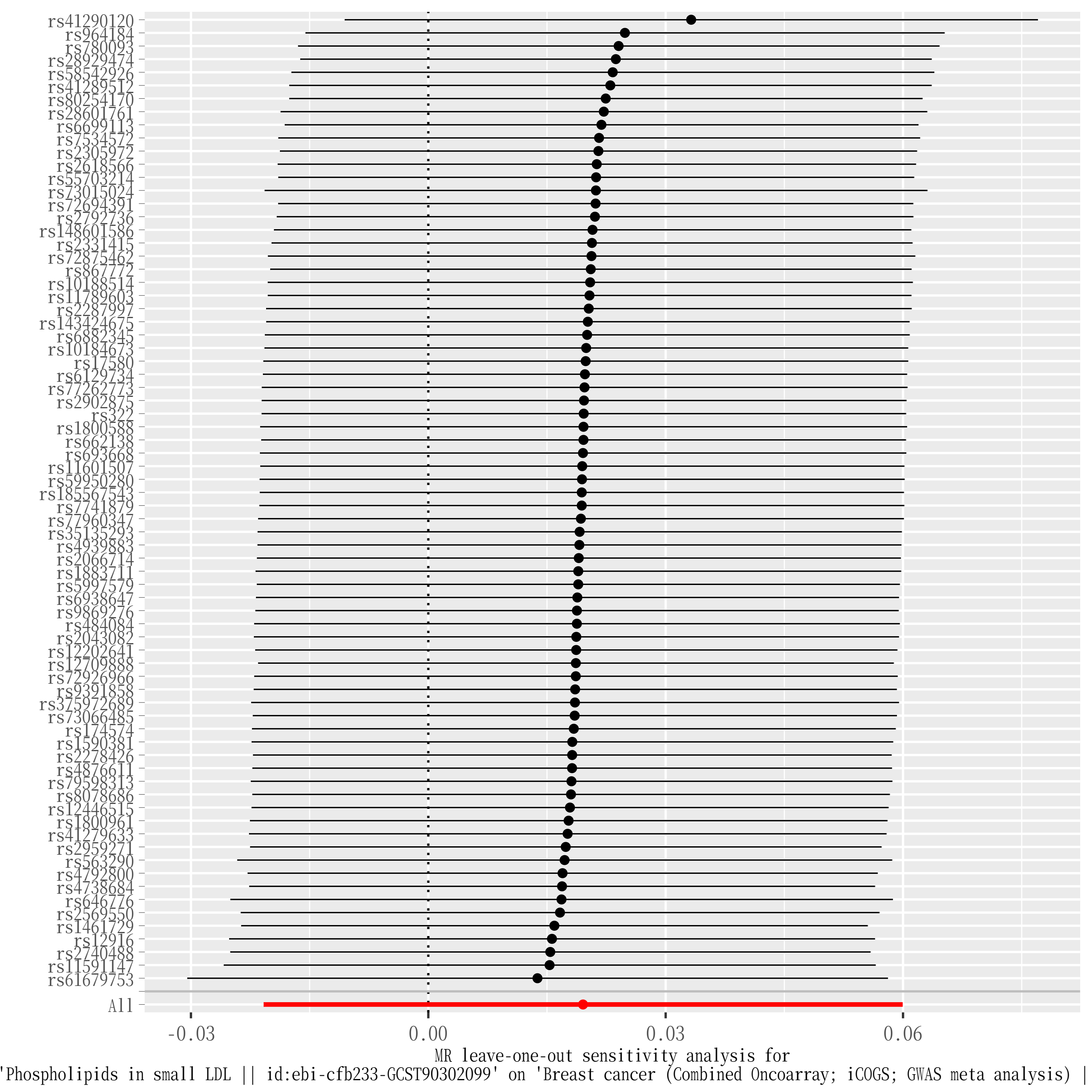
rs41290120
rs964184
rs780093
rs28929474
rs58542926
rs41289512
rs80254170
rs28601761
rs6699113
rs7534572
rs2305972
rs2618566
rs55703214
rs73015024
rs72694391
rs2792736
rs148601586
rs2331415
rs72875462
rs867772
rs10188514
rs11789603
rs2287997
rs143424675
rs6882345
rs10184673
rs17580
rs6129734
rs77262773
rs2902875
rs322
rs1800588
rs662138
rs693668
rs11601507
rs59950280
rs185567543
rs7741879
rs77960347
rs35135293
rs4939883
rs2066714
rs1883711
rs5997579
rs6938647
rs9869276
rs484084
rs2043082
rs12202641
rs12709888
rs72926966
rs9391858
rs375972689
rs73066485
rs174574
rs1590381
rs2278426
rs4876611
rs79598313
rs8078686
rs12446515
rs1800961
rs41279633
rs2959271
rs563290
rs4792800
rs4738684
rs646776
rs2569550
rs1461729
rs12916
rs2740488
rs11591147
rs61679753
All
-0.03
0.00
0.03
0.06
MR leave-one-out sensitivity analysis for
'Phospholipids in small LDL || id:ebi-cfb233-GCST90302099' on 'Breast cancer (Combined Oncoarray; iCOGS; GWAS meta analysis) ||

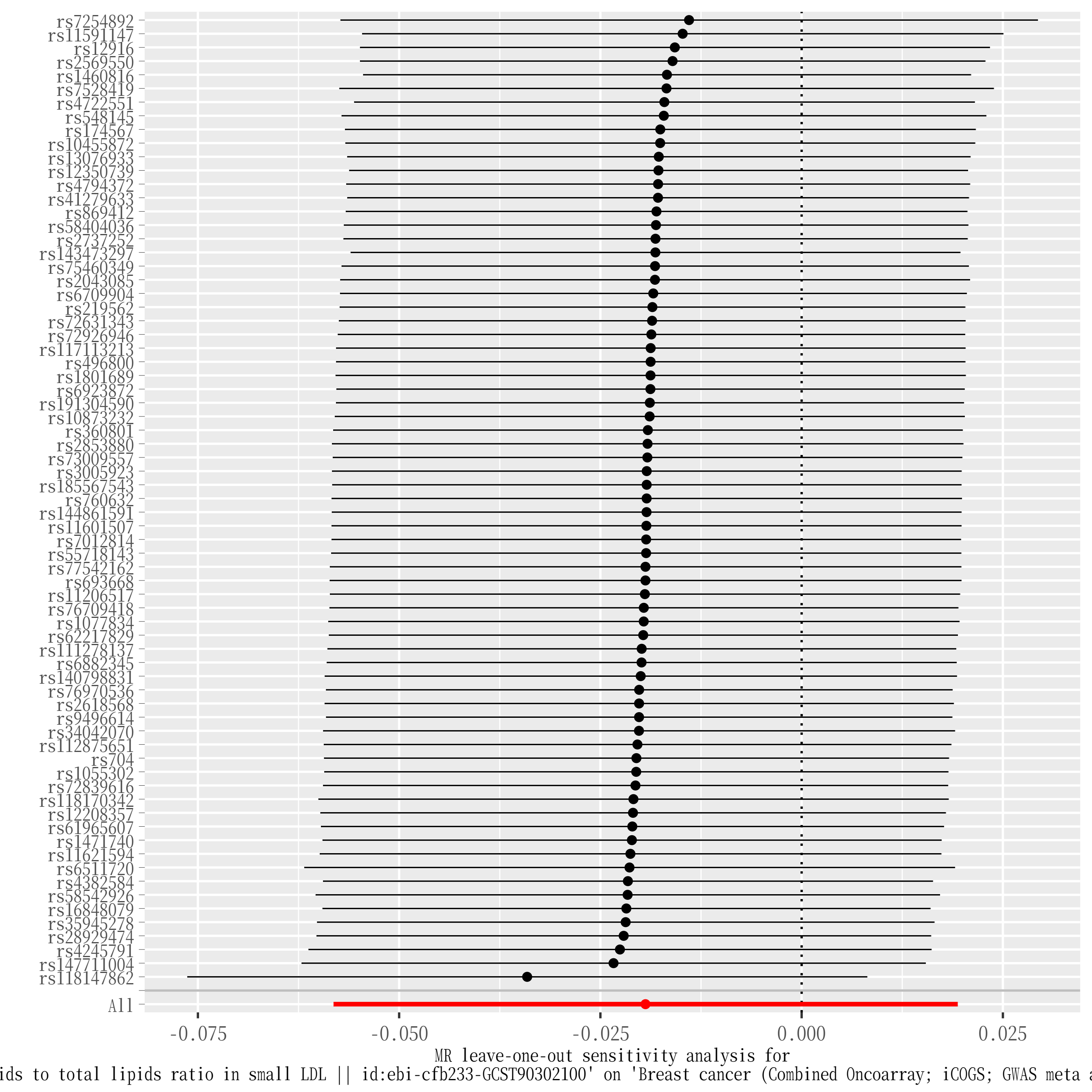

rs7254892
rs11591147
rs12916
rs2569550
rs1460816
rs7528419
rs4722551
rs548145
rs174567
rs10455872
rs13076933
rs12350739
rs4794372
rs41279633
rs869412
rs58404036
rs2737252
rs143473297
rs75460349
rs2043085
rs6709904
rs219562
rs72631343
rs72926946
rs117113213
rs496800
rs1801689
rs6923872
rs191304590
rs10873232
rs360801
rs2853880
rs73009557
rs3005923
rs185567543
rs760632
rs144861591
rs11601507
rs7012814
rs55718143
rs77542162
rs693668
rs11206517
rs76709418
rs1077834
rs62217829
rs111278137
rs6882345
rs140798831
rs76970536
rs2618568
rs9496614
rs34042070
rs112875651
rs704
rs1055302
rs72839616
rs118170342
rs12208357
rs61965607
rs1471740
rs11621594
rs6511720
rs4382584
rs58542926
rs16848079
rs35945278
rs28929474
rs4245791
rs147711004
rs118147862
All
-0.075
-0.050
-0.025
0.000
0.025
MR leave-one-out sensitivity analysis for
ids to total lipids ratio in small LDL || id:ebi-cfb233-GCST90302100' on 'Breast cancer (Combined Oncoarray; iCOGS; GWAS meta a

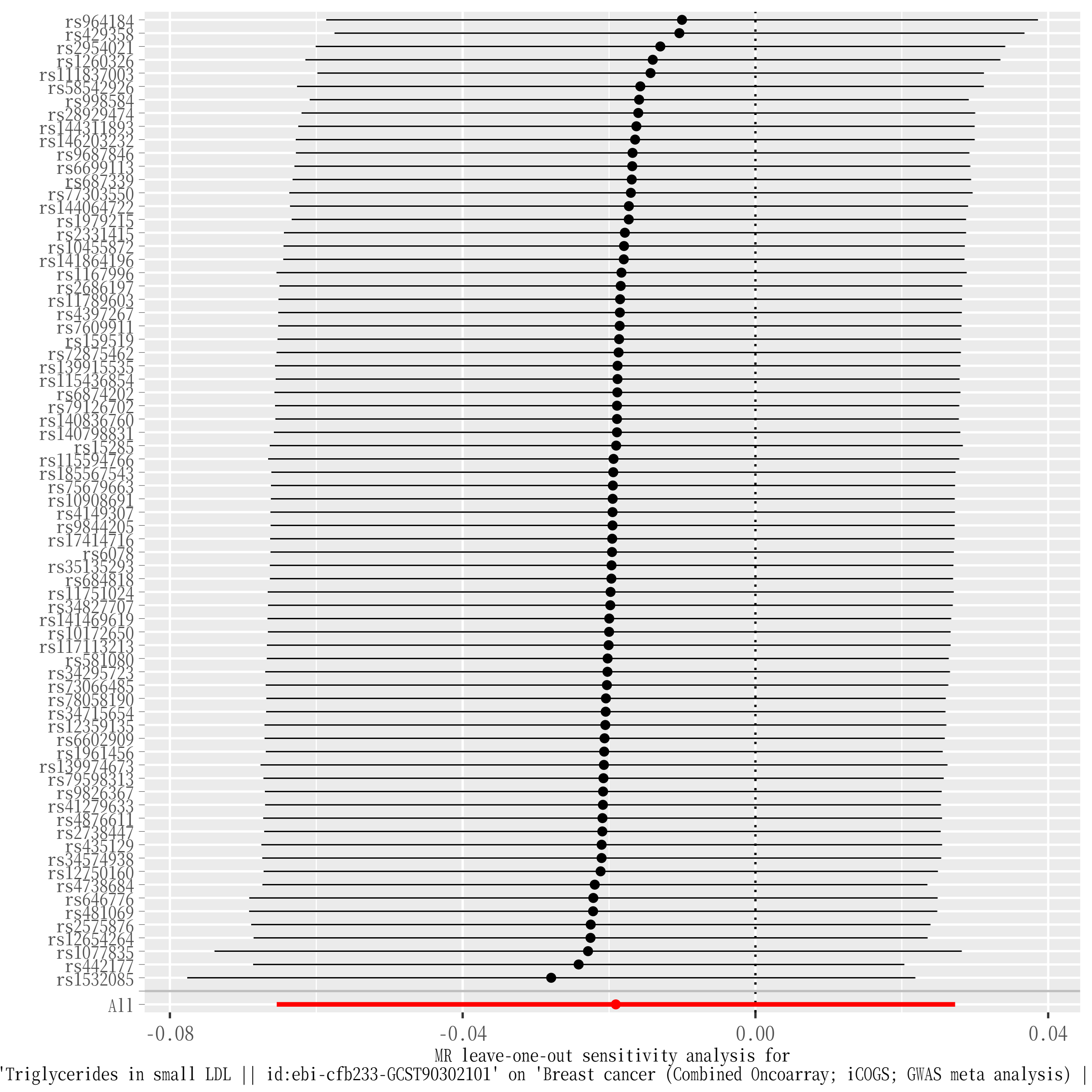

rs964184
rs429358
rs2954021
rs1260326
rs111837003
rs58542926
rs998584
rs28929474
rs144311893
rs146203232
rs9687846
rs6699113
rs687339
rs77303550
rs144064722
rs1979215
rs2331415
rs10455872
rs141864196
rs1167996
rs2686197
rs11789603
rs4397267
rs7609911
rs159519
rs72875462
rs139915535
rs115436854
rs6874202
rs79126702
rs140836760
rs140798831
rs15285
rs115594766
rs185567543
rs75679663
rs10908691
rs4149307
rs9844205
rs17414716
rs6078
rs35135293
rs684818
rs11751024
rs34827707
rs141469619
rs10172650
rs117113213
rs581080
rs34295723
rs73066485
rs78058190
rs34715654
rs12359135
rs6602909
rs1961456
rs139974673
rs79598313
rs9826367
rs41279633
rs4876611
rs2738447
rs435129
rs34574938
rs12750160
rs4738684
rs646776
rs481069
rs2575876
rs12654264
rs1077835
rs442177
rs1532085
All
-0.08
-0.04
0.00
0.04
MR leave-one-out sensitivity analysis for
'Triglycerides in small LDL || id:ebi-cfb233-GCST90302101' on 'Breast cancer (Combined Oncoarray; iCOGS; GWAS meta analysis) |

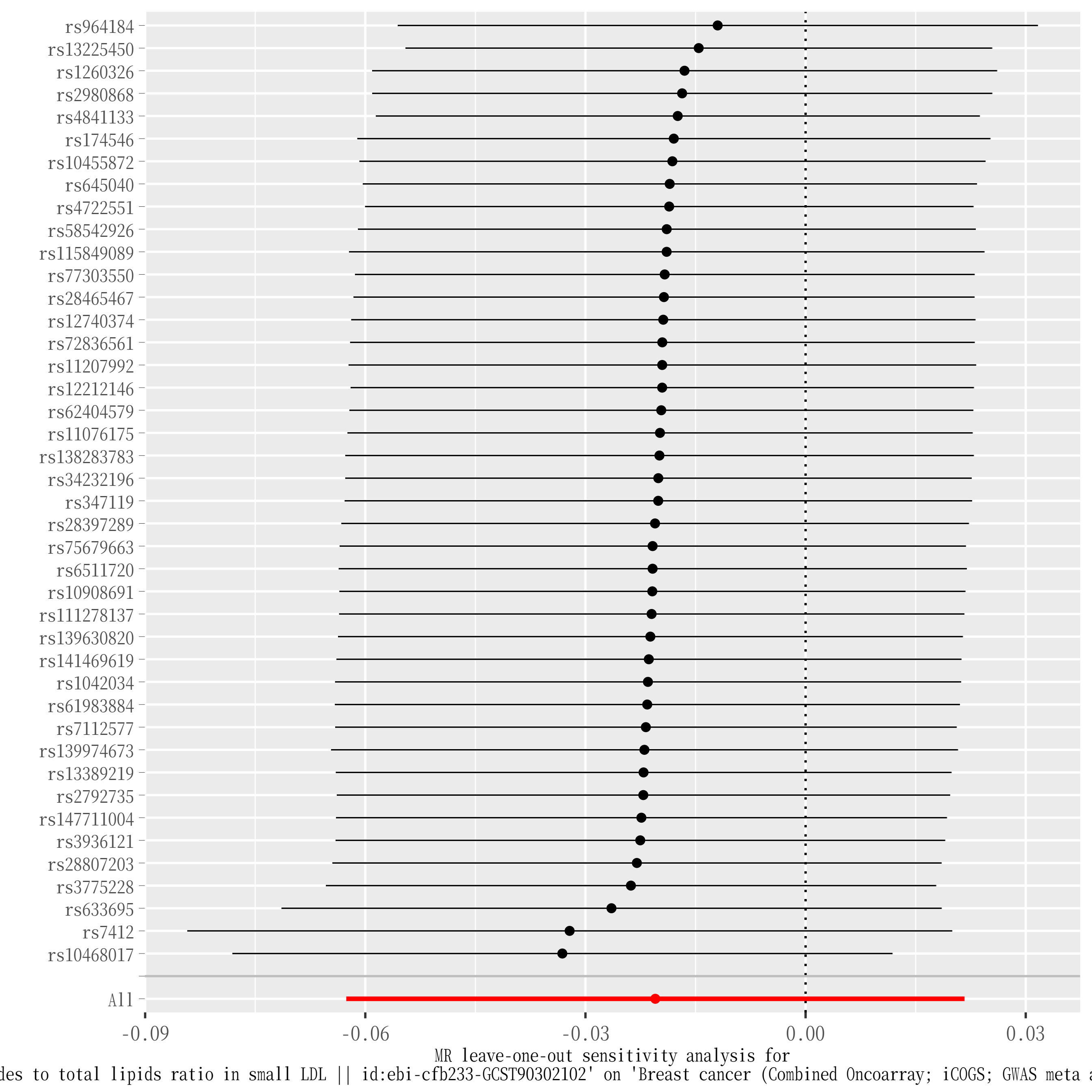
rs964184
rs13225450
rs1260326
rs2980868
rs4841133
rs174546
rs10455872
rs645040
rs4722551
rs58542926
rs115849089
rs77303550
rs28465467
rs12740374
rs72836561
rs11207992
rs12212146
rs62404579
rs11076175
rs138283783
rs34232196
rs347119
rs28397289
rs75679663
rs6511720
rs10908691
rs111278137
rs139630820
rs141469619
rs1042034
rs61983884
rs7112577
rs139974673
rs13389219
rs2792735
rs147711004
rs3936121
rs28807203
rs3775228
rs633695
rs7412
rs10468017
All
-0.09
-0.06
-0.03
0.00
0.03
MR leave-one-out sensitivity analysis for
des to total lipids ratio in small LDL || id:ebi-cfb233-GCST90302102' on 'Breast cancer (Combined Oncoarray; iCOGS; GWAS meta a

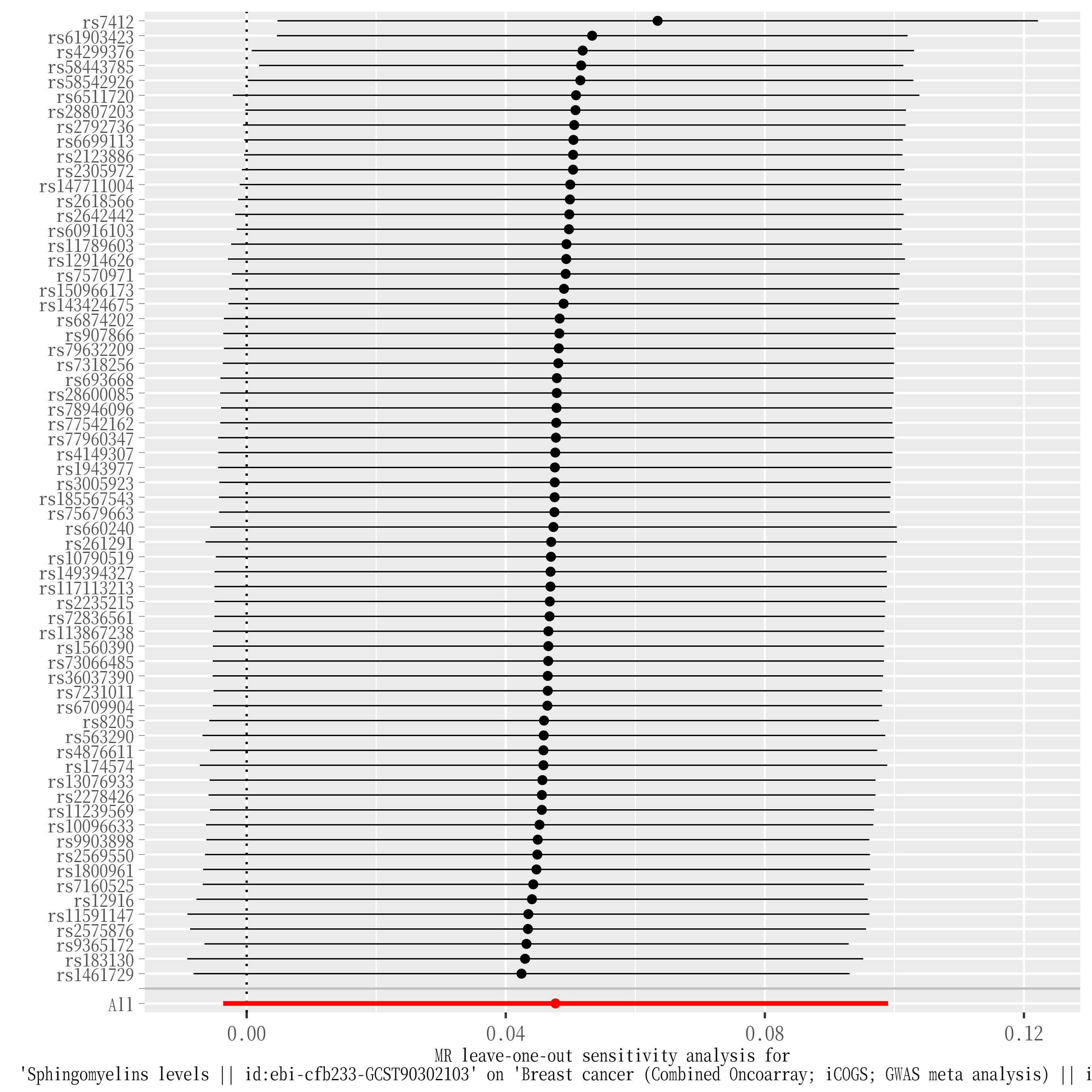

rs7412
rs61903423
rs4299376
rs58443785
rs58542926
rs6511720
rs28807203
rs2792736
rs6699113
rs2123886
rs2305972
rs147711004
rs2618566
rs2642442
rs60916103
rs11789603
rs12914626
rs7570971
rs150966173
rs143424675
rs6874202
rs907866
rs79632209
rs7318256
rs693668
rs28600085
rs78946096
rs77542162
rs77960347
rs4149307
rs1943977
rs3005923
rs185567543
rs75679663
rs660240
rs261291
rs10790519
rs149394327
rs117113213
rs2235215
rs72836561
rs113867238
rs1560390
rs73066485
rs36037390
rs7231011
rs6709904
rs8205
rs563290
rs4876611
rs174574
rs13076933
rs2278426
rs11239569
rs10096633
rs9903898
rs2569550
rs1800961
rs7160525
rs12916
rs11591147
rs2575876
rs9365172
rs183130
rs1461729
All
0.00
0.04
0.08
0.12
MR leave-one-out sensitivity analysis for
'Sphingomyelins levels || id:ebi-cfb233-GCST90302103' on 'Breast cancer (Combined Oncoarray; iCOGS; GWAS meta analysis) || i

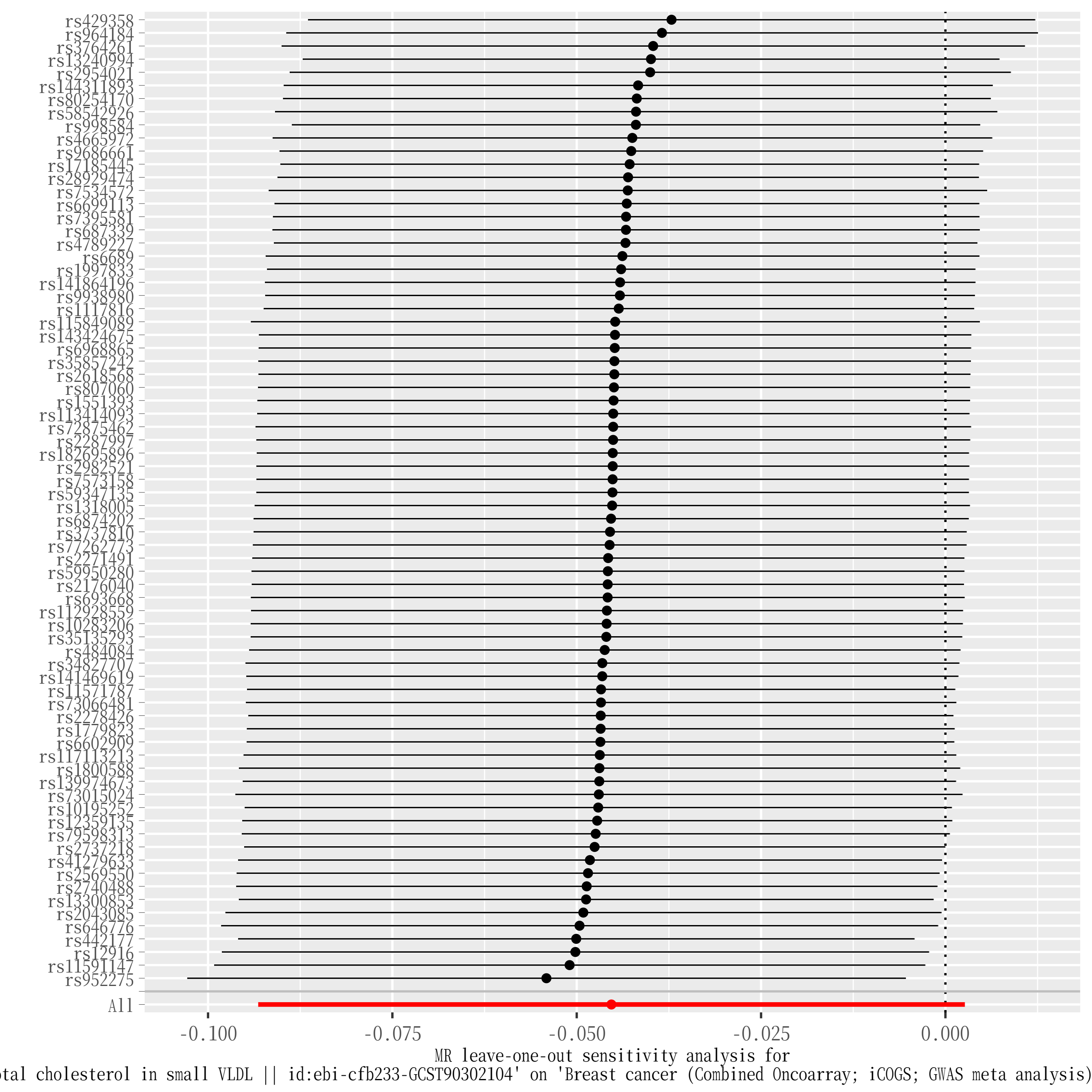

rs429358
rs964184
rs3764261
rs13240994
rs2954021
rs144311893
rs80254170
rs58542926
rs998584
rs4665972
rs9686661
rs17185445
rs28929474
rs7534572
rs6699113
rs7395581
rs687339
rs4789227
rs6689
rs1997833
rs141864196
rs9938980
rs1117816
rs115849089
rs143424675
rs6968865
rs35857242
rs2618568
rs807060
rs1551393
rs113414093
rs72875462
rs2287997
rs182695896
rs2982521
rs7573158
rs59347135
rs1318005
rs6874202
rs3737810
rs77262773
rs2271491
rs59950280
rs2176040
rs693668
rs112928559
rs10283206
rs35135293
rs484084
rs34827707
rs141469619
rs11571787
rs73066481
rs2278426
rs1779823
rs6602909
rs117113213
rs1800588
rs139974673
rs73015024
rs10195252
rs12359135
rs79598313
rs2737218
rs41279633
rs2569550
rs2740488
rs13300853
rs2043085
rs646776
rs442177
rs12916
rs11591147
rs952275
All
-0.100
-0.075
-0.050
-0.025
0.000
MR leave-one-out sensitivity analysis for
otal cholesterol in small VLDL || id:ebi-cfb233-GCST90302104' on 'Breast cancer (Combined Oncoarray; iCOGS; GWAS meta analysis)

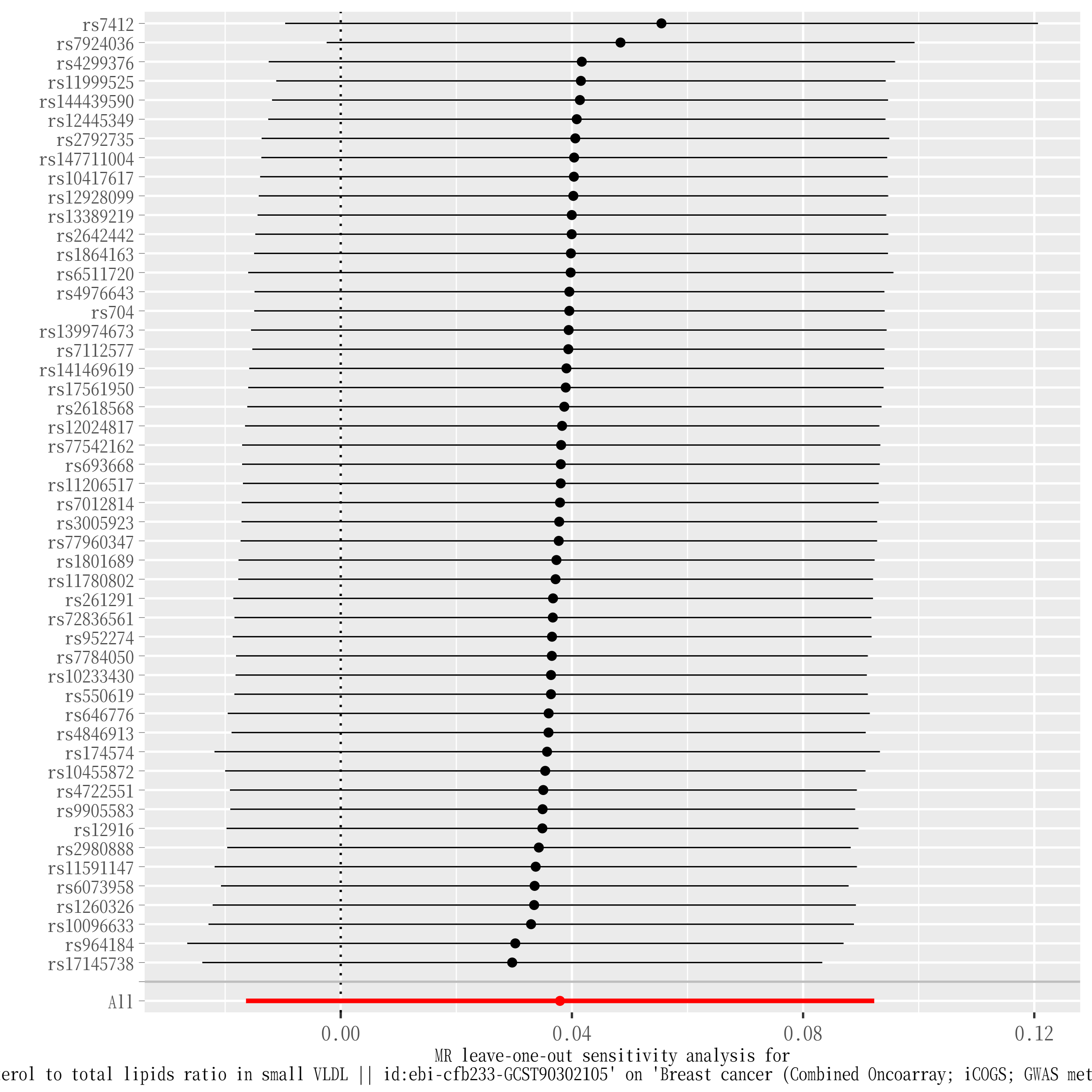
rs7412
rs7924036
rs4299376
rs11999525
rs144439590
rs12445349
rs2792735
rs147711004
rs10417617
rs12928099
rs13389219
rs2642442
rs1864163
rs6511720
rs4976643
rs704
rs139974673
rs7112577
rs141469619
rs17561950
rs2618568
rs12024817
rs77542162
rs693668
rs11206517
rs7012814
rs3005923
rs77960347
rs1801689
rs11780802
rs261291
rs72836561
rs952274
rs7784050
rs10233430
rs550619
rs646776
rs4846913
rs174574
rs10455872
rs4722551
rs9905583
rs12916
rs2980888
rs11591147
rs6073958
rs1260326
rs10096633
rs964184
rs17145738
All
0.00
0.04
0.08
0.12
MR leave-one-out sensitivity analysis for
erol to total lipids ratio in small VLDL || id:ebi-cfb233-GCST90302105' on 'Breast cancer (Combined Oncoarray; iCOGS; GWAS met

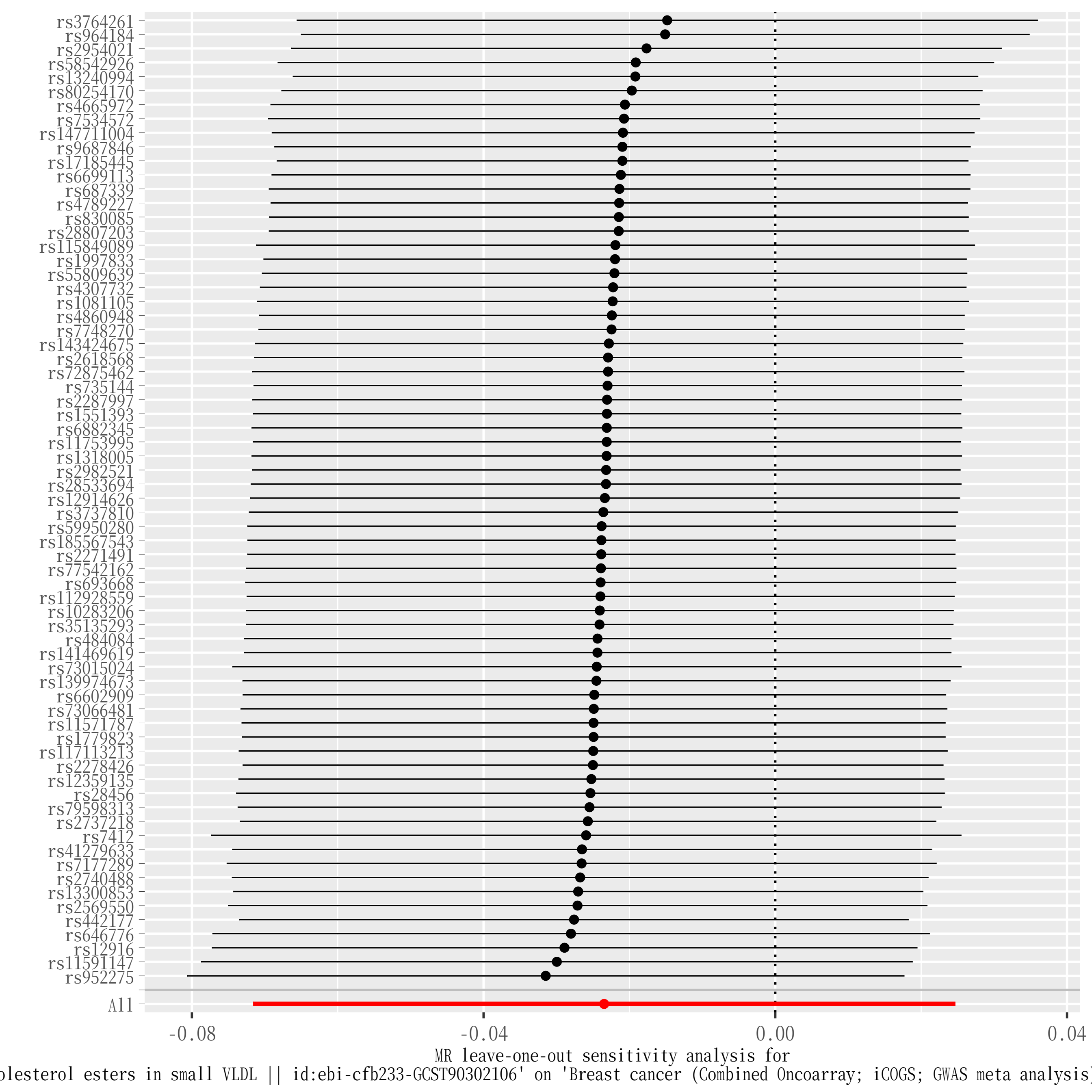

rs3764261
rs964184
rs2954021
rs58542926
rs13240994
rs80254170
rs4665972
rs7534572
rs147711004
rs9687846
rs17185445
rs6699113
rs687339
rs4789227
rs830085
rs28807203
rs115849089
rs1997833
rs55809639
rs4307732
rs1081105
rs4860948
rs7748270
rs143424675
rs2618568
rs72875462
rs735144
rs2287997
rs1551393
rs6882345
rs11753995
rs1318005
rs2982521
rs28533694
rs12914626
rs3737810
rs59950280
rs185567543
rs2271491
rs77542162
rs693668
rs112928559
rs10283206
rs35135293
rs484084
rs141469619
rs73015024
rs139974673
rs6602909
rs73066481
rs11571787
rs1779823
rs117113213
rs2278426
rs12359135
rs28456
rs79598313
rs2737218
rs7412
rs41279633
rs7177289
rs2740488
rs13300853
rs2569550
rs442177
rs646776
rs12916
rs11591147
rs952275
All
-0.08
-0.04
0.00
0.04
MR leave-one-out sensitivity analysis for
olesterol esters in small VLDL || id:ebi-cfb233-GCST90302106' on 'Breast cancer (Combined Oncoarray; iCOGS; GWAS meta analysis)

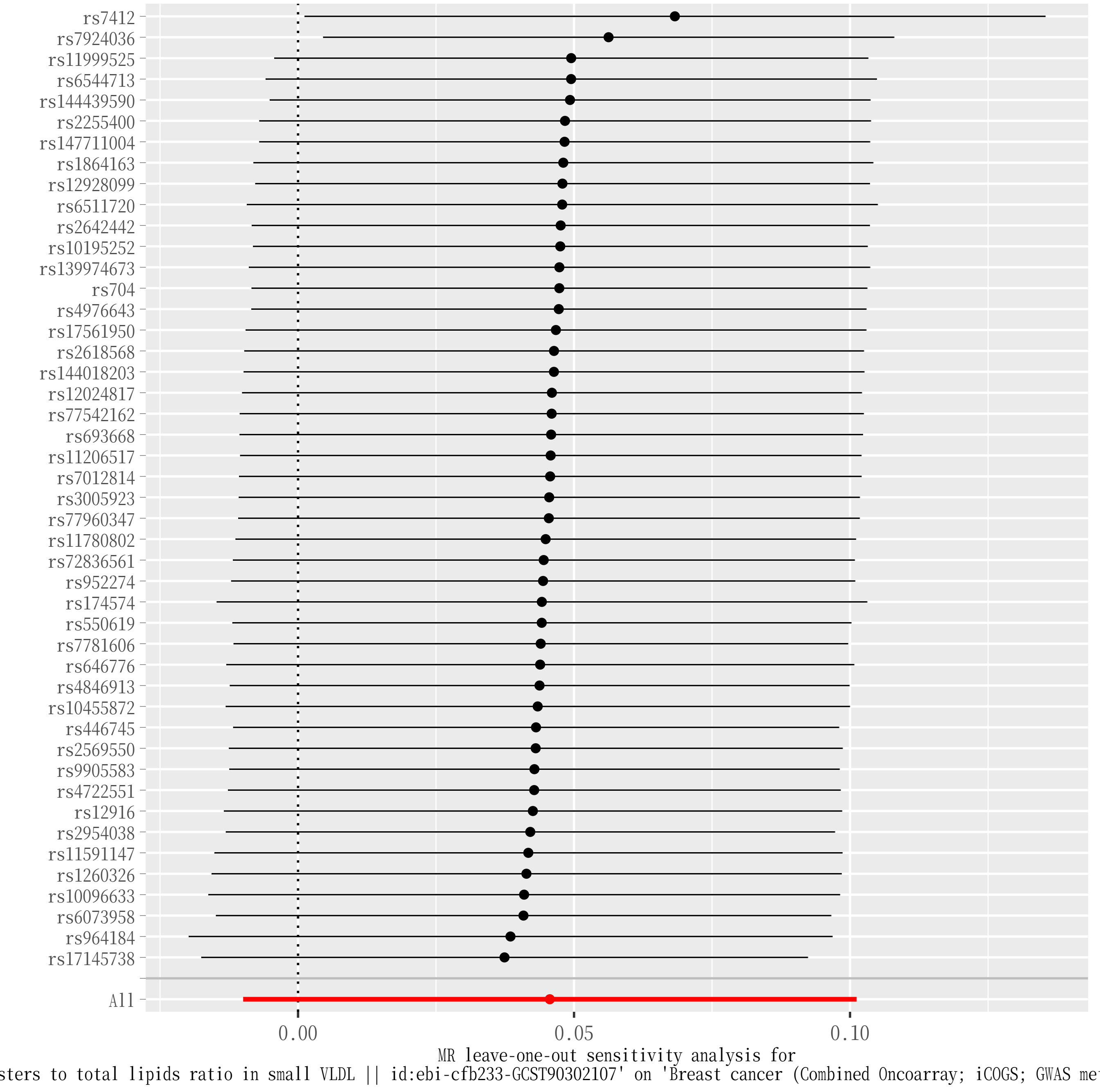
rs7412
rs7924036
rs11999525
rs6544713
rs144439590
rs2255400
rs147711004
rs1864163
rs12928099
rs6511720
rs2642442
rs10195252
rs139974673
rs704
rs4976643
rs17561950
rs2618568
rs144018203
rs12024817
rs77542162
rs693668
rs11206517
rs7012814
rs3005923
rs77960347
rs11780802
rs72836561
rs952274
rs174574
rs550619
rs7781606
rs646776
rs4846913
rs10455872
rs446745
rs2569550
rs9905583
rs4722551
rs12916
rs2954038
rs11591147
rs1260326
rs10096633
rs6073958
rs964184
rs17145738
All
0.00
0.05
0.10
MR leave-one-out sensitivity analysis for
sters to total lipids ratio in small VLDL || id:ebi-cfb233-GCST90302107' on 'Breast cancer (Combined Oncoarray; iCOGS; GWAS me

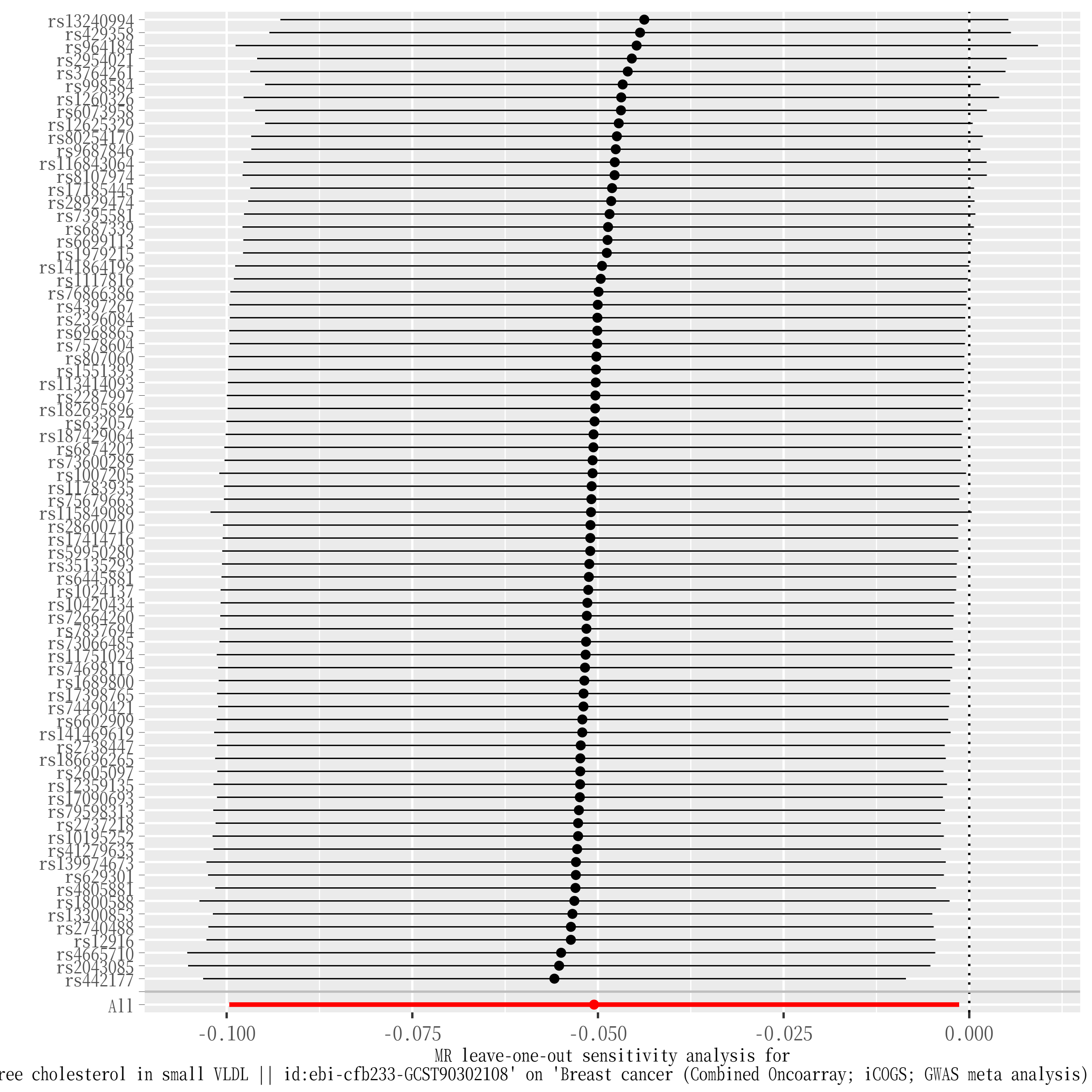
rs13240994
rs429358
rs964184
rs2954021
rs3764261
rs998584
rs1260326
rs6073958
rs12625329
rs80254170
rs9687846
rs116843064
rs8107974
rs17185445
rs28929474
rs7395581
rs687339
rs6699113
rs1979215
rs141864196
rs1117816
rs76866386
rs4397267
rs2396084
rs6968865
rs7578604
rs807060
rs1551393
rs113414093
rs2287997
rs182695896
rs632057
rs187429064
rs6874202
rs73600289
rs1007205
rs11783935
rs75679663
rs115849089
rs28600710
rs17414716
rs59950280
rs35135293
rs6445881
rs1024137
rs10420434
rs72664260
rs7837694
rs73066485
rs11751024
rs74698119
rs1689800
rs17398765
rs74490421
rs6602909
rs141469619
rs2738447
rs186696265
rs2605097
rs12359135
rs17090693
rs79598313
rs2737218
rs10195252
rs41279633
rs139974673
rs629301
rs4805881
rs1800588
rs13300853
rs2740488
rs12916
rs4665710
rs2043085
rs442177
All
-0.100
-0.075
-0.050
-0.025
0.000
MR leave-one-out sensitivity analysis for
ree cholesterol in small VLDL || id:ebi-cfb233-GCST90302108' on 'Breast cancer (Combined Oncoarray; iCOGS; GWAS meta analysis)

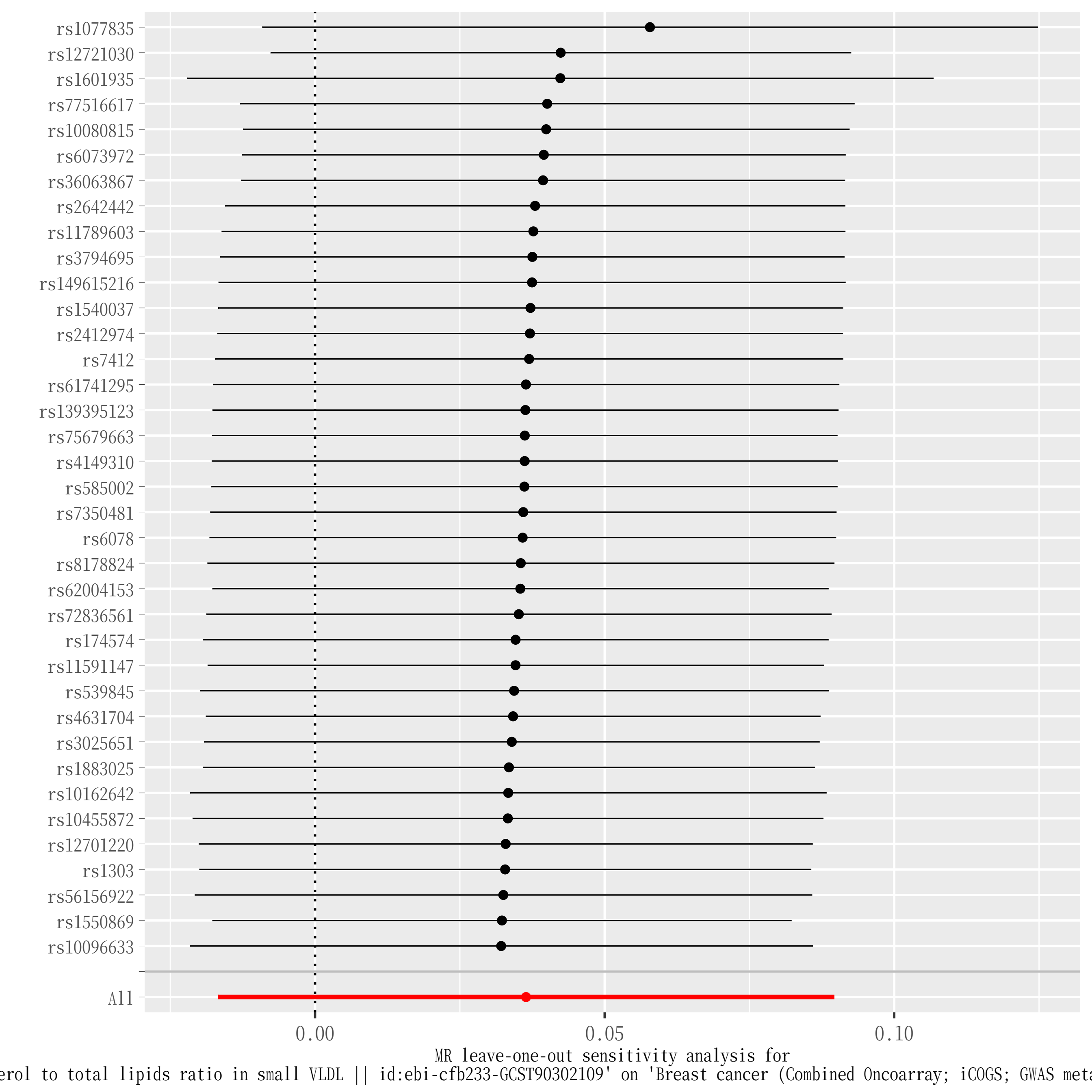
rs1077835
rs12721030
rs1601935
rs77516617
rs10080815
rs6073972
rs36063867
rs2642442
rs11789603
rs3794695
rs149615216
rs1540037
rs2412974
rs7412
rs61741295
rs139395123
rs75679663
rs4149310
rs585002
rs7350481
rs6078
rs8178824
rs62004153
rs72836561
rs174574
rs11591147
rs539845
rs4631704
rs3025651
rs1883025
rs10162642
rs10455872
rs12701220
rs1303
rs56156922
rs1550869
rs10096633
All
0.00
0.05
0.10
MR leave-one-out sensitivity analysis for
erol to total lipids ratio in small VLDL || id:ebi-cfb233-GCST90302109' on 'Breast cancer (Combined Oncoarray; iCOGS; GWAS meta

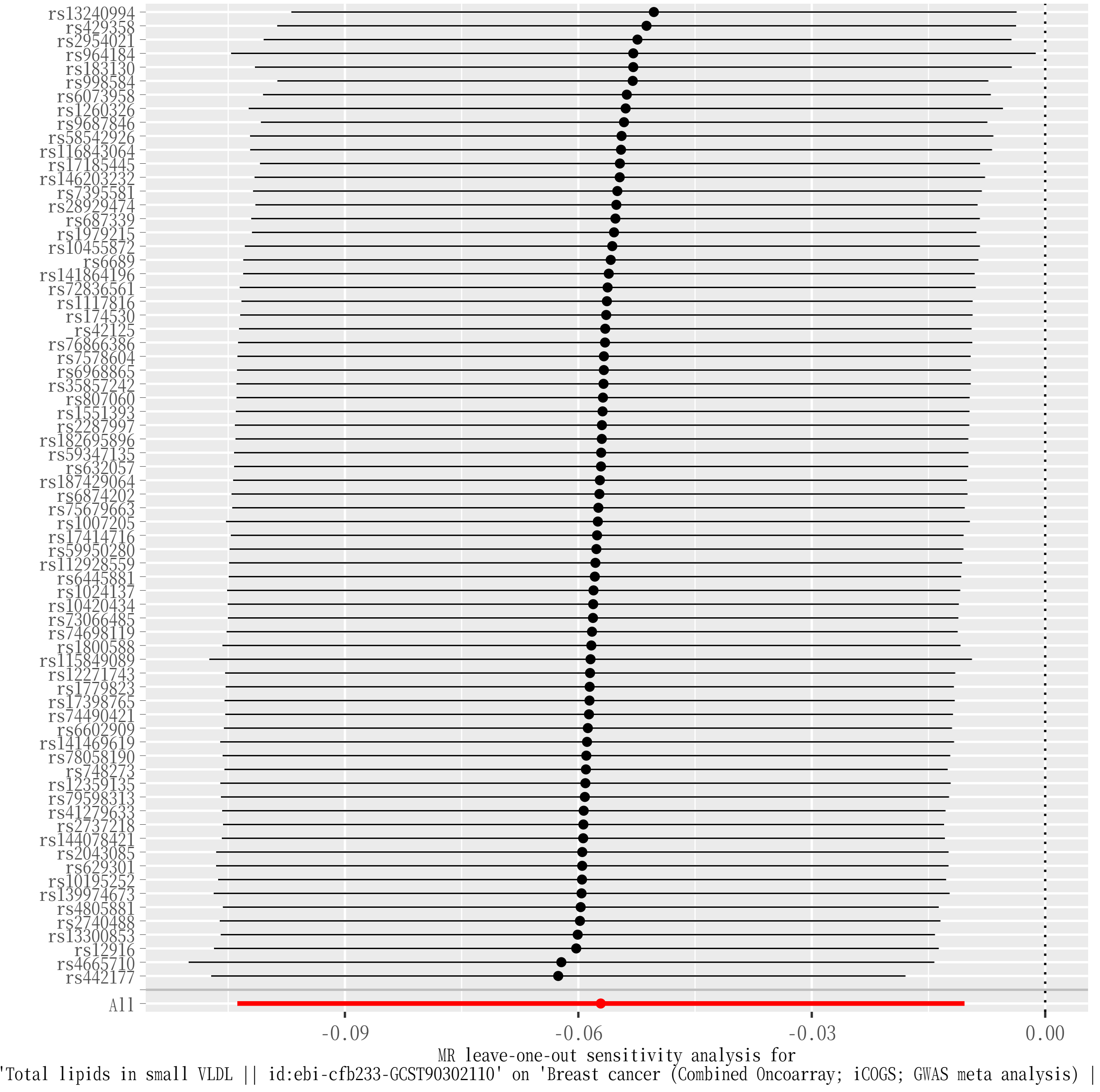
rs13240994
rs429358
rs2954021
rs964184
rs183130
rs998584
rs6073958
rs1260326
rs9687846
rs58542926
rs116843064
rs17185445
rs146203232
rs7395581
rs28929474
rs687339
rs1979215
rs10455872
rs6689
rs141864196
rs72836561
rs1117816
rs174530
rs42125
rs76866386
rs7578604
rs6968865
rs35857242
rs807060
rs1551393
rs2287997
rs182695896
rs59347135
rs632057
rs187429064
rs6874202
rs75679663
rs1007205
rs17414716
rs59950280
rs112928559
rs6445881
rs1024137
rs10420434
rs73066485
rs74698119
rs1800588
rs115849089
rs12271743
rs1779823
rs17398765
rs74490421
rs6602909
rs141469619
rs78058190
rs748273
rs12359135
rs79598313
rs41279633
rs2737218
rs144078421
rs2043085
rs629301
rs10195252
rs139974673
rs4805881
rs2740488
rs13300853
rs12916
rs4665710
rs442177
All
-0.09
-0.06
-0.03
0.00
MR leave-one-out sensitivity analysis for
'Total lipids in small VLDL || id:ebi-cfb233-GCST90302110' on 'Breast cancer (Combined Oncoarray; iCOGS; GWAS meta analysis) |

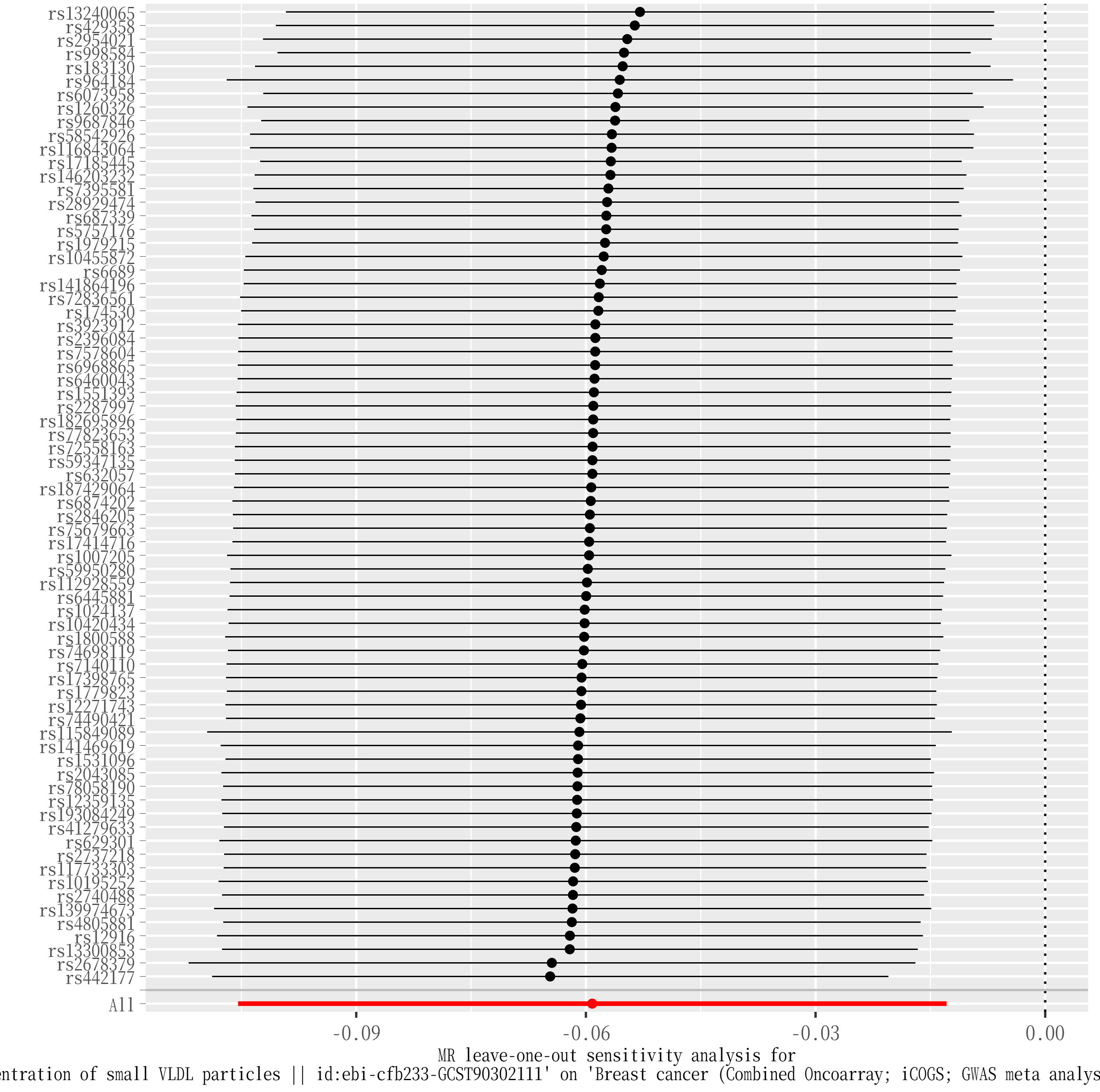

rs13240065
rs429358
rs2954021
rs998584
rs183130
rs964184
rs6073958
rs1260326
rs9687846
rs58542926
rs116843064
rs17185445
rs146203232
rs7395581
rs28929474
rs687339
rs5757176
rs1979215
rs10455872
rs6689
rs141864196
rs72836561
rs174530
rs3923912
rs2396084
rs7578604
rs6968865
rs6460043
rs1551393
rs2287997
rs182695896
rs77823653
rs72558163
rs59347135
rs632057
rs187429064
rs6874202
rs2846205
rs75679663
rs17414716
rs1007205
rs59950280
rs112928559
rs6445881
rs1024137
rs10420434
rs1800588
rs74698119
rs7140110
rs17398765
rs1779823
rs12271743
rs74490421
rs115849089
rs141469619
rs1531096
rs2043085
rs78058190
rs12359135
rs193084249
rs41279633
rs629301
rs2737218
rs117733303
rs10195252
rs2740488
rs139974673
rs4805881
rs12916
rs13300853
rs2678379
rs442177
All
-0.09
-0.06
-0.03
0.00
MR leave-one-out sensitivity analysis for
entration of small VLDL particles || id:ebi-cfb233-GCST90302111' on 'Breast cancer (Combined Oncoarray; iCOGS; GWAS meta analys

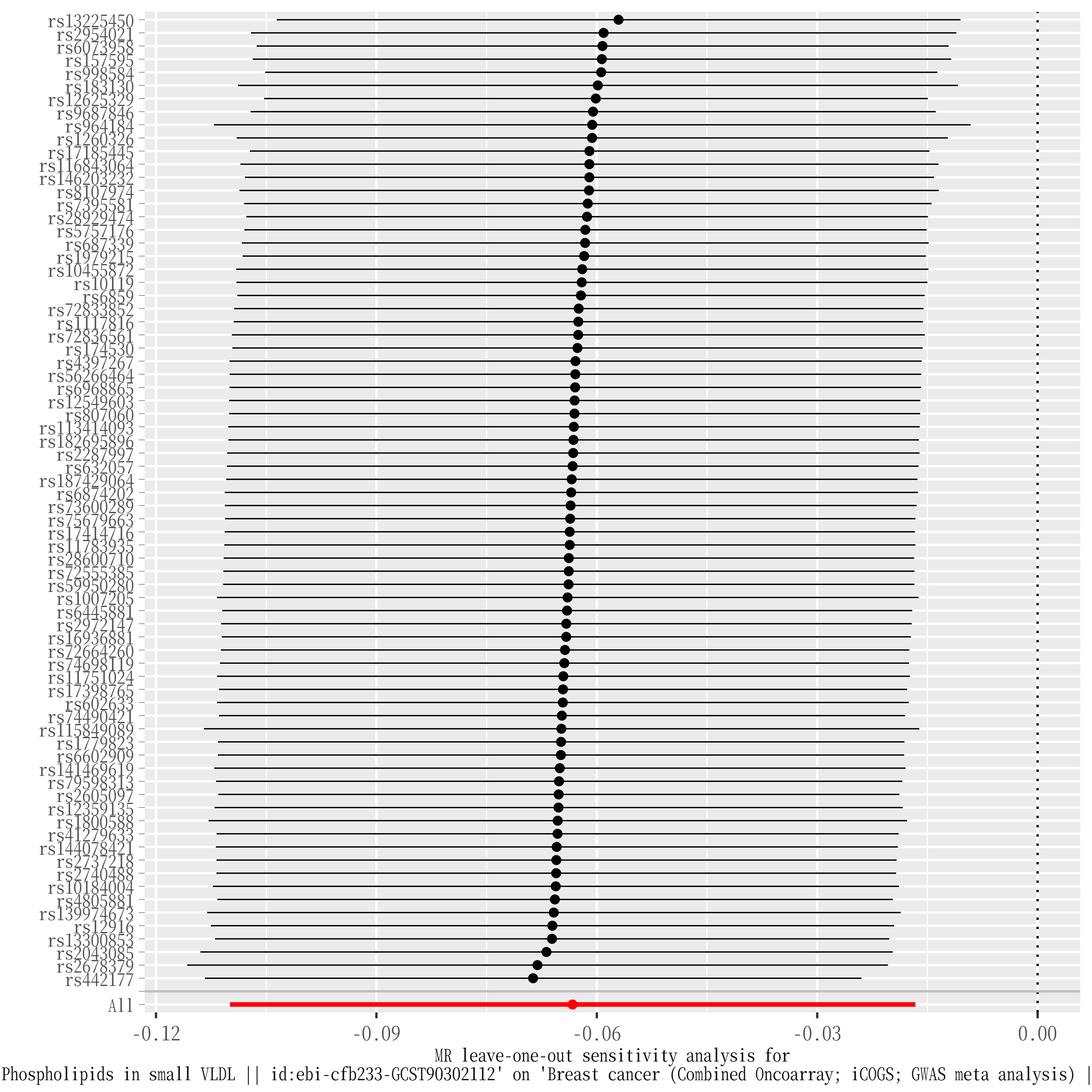
rs13225450
rs2954021
rs6073958
rs157595
rs998584
rs183130
rs12625329
rs9687846
rs964184
rs1260326
rs17185445
rs116843064
rs146203232
rs8107974
rs7395581
rs28929474
rs5757176
rs687339
rs1979215
rs10455872
rs10119
rs6859
rs72833852
rs1117816
rs72836561
rs174530
rs4397267
rs56266464
rs6968865
rs12549603
rs807060
rs113414093
rs182695896
rs2287997
rs632057
rs187429064
rs6874202
rs73600289
rs75679663
rs17414716
rs11783935
rs28600710
rs72555385
rs59950280
rs1007205
rs6445881
rs2972147
rs16936881
rs72664260
rs74698119
rs11751024
rs17398765
rs602633
rs74490421
rs115849089
rs1779823
rs6602909
rs141469619
rs79598313
rs2605097
rs12359135
rs1800588
rs41279633
rs144078421
rs2737218
rs2740488
rs10184004
rs4805881
rs139974673
rs12916
rs13300853
rs2043085
rs2678379
rs442177
All
-0.12
-0.09
-0.06
-0.03
0.00
MR leave-one-out sensitivity analysis for
Phospholipids in small VLDL || id:ebi-cfb233-GCST90302112' on 'Breast cancer (Combined Oncoarray; iCOGS; GWAS meta analysis) |

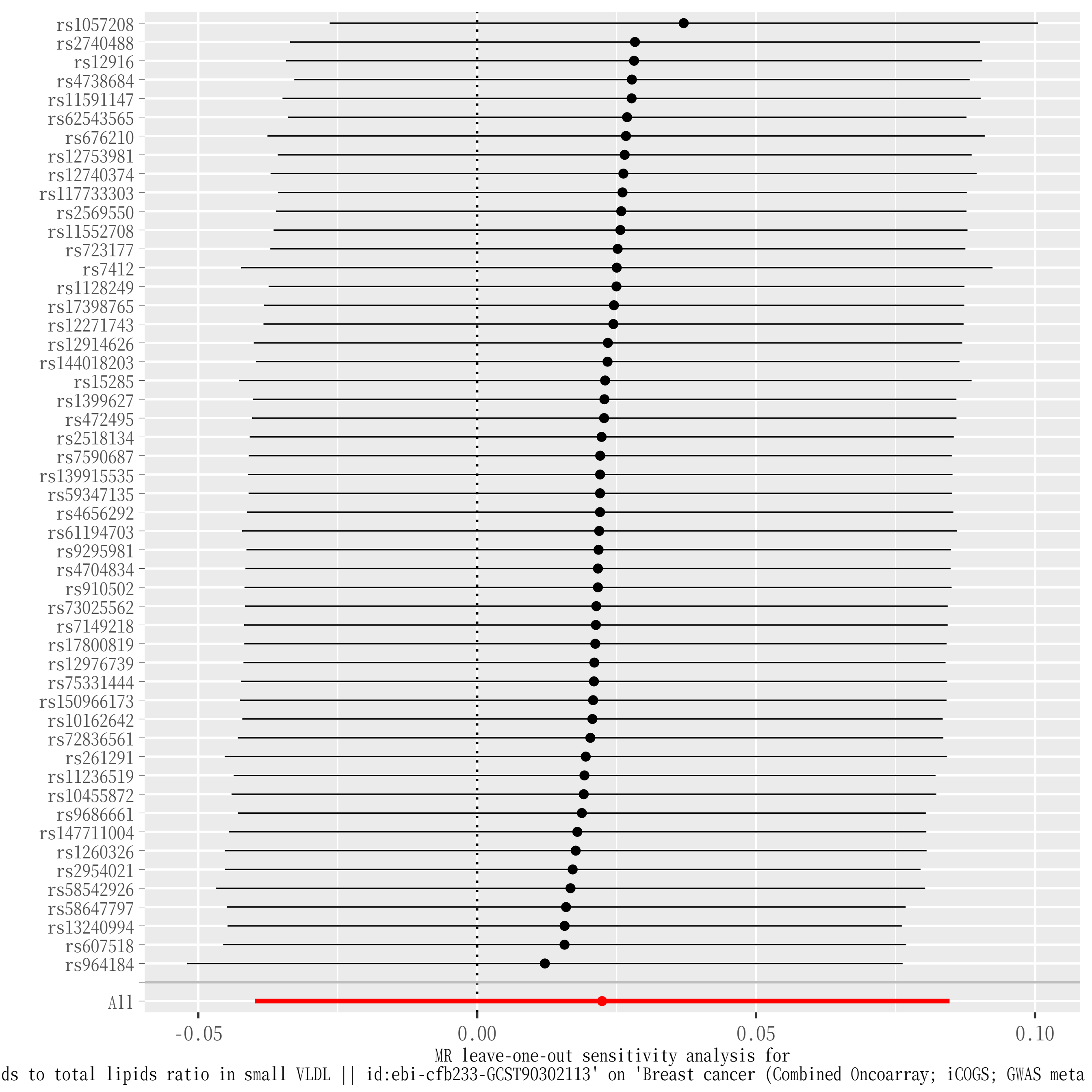
rs1057208
rs2740488
rs12916
rs4738684
rs11591147
rs62543565
rs676210
rs12753981
rs12740374
rs117733303
rs2569550
rs11552708
rs723177
rs7412
rs1128249
rs17398765
rs12271743
rs12914626
rs144018203
rs15285
rs1399627
rs472495
rs2518134
rs7590687
rs139915535
rs59347135
rs4656292
rs61194703
rs9295981
rs4704834
rs910502
rs73025562
rs7149218
rs17800819
rs12976739
rs75331444
rs150966173
rs10162642
rs72836561
rs261291
rs11236519
rs10455872
rs9686661
rs147711004
rs1260326
rs2954021
rs58542926
rs58647797
rs13240994
rs607518
rs964184
All
-0.05
0.00
0.05
0.10
MR leave-one-out sensitivity analysis for
ds to total lipids ratio in small VLDL || id:ebi-cfb233-GCST90302113' on 'Breast cancer (Combined Oncoarray; iCOGS; GWAS meta

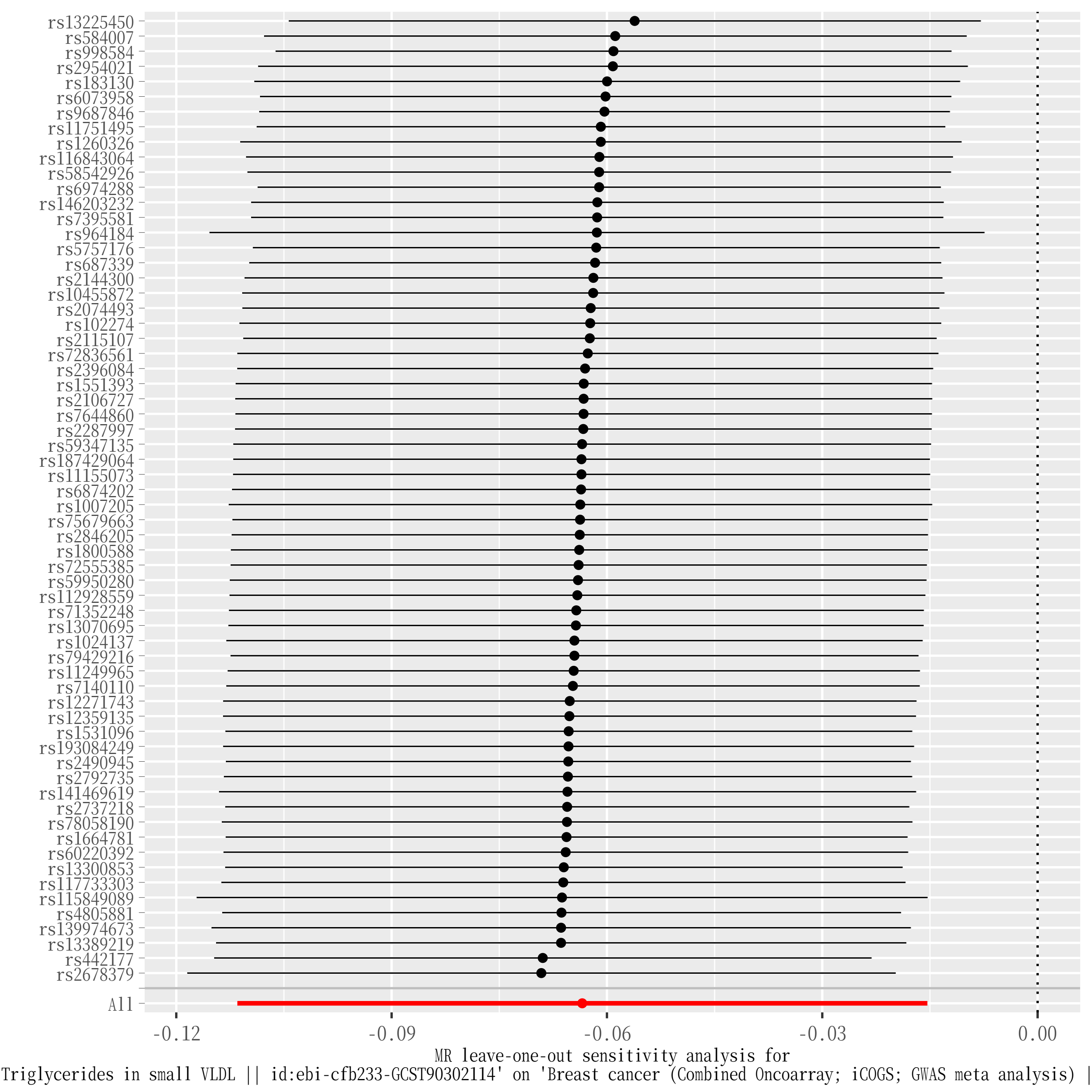
rs13225450
rs584007
rs998584
rs2954021
rs183130
rs6073958
rs9687846
rs11751495
rs1260326
rs116843064
rs58542926
rs6974288
rs146203232
rs7395581
rs964184
rs5757176
rs687339
rs2144300
rs10455872
rs2074493
rs102274
rs2115107
rs72836561
rs2396084
rs1551393
rs2106727
rs7644860
rs2287997
rs59347135
rs187429064
rs11155073
rs6874202
rs1007205
rs75679663
rs2846205
rs1800588
rs72555385
rs59950280
rs112928559
rs71352248
rs13070695
rs1024137
rs79429216
rs11249965
rs7140110
rs12271743
rs12359135
rs1531096
rs193084249
rs2490945
rs2792735
rs141469619
rs2737218
rs78058190
rs1664781
rs60220392
rs13300853
rs117733303
rs115849089
rs4805881
rs139974673
rs13389219
rs442177
rs2678379
All
-0.12
-0.09
-0.06
-0.03
0.00
MR leave-one-out sensitivity analysis for
Triglycerides in small VLDL || id:ebi-cfb233-GCST90302114' on 'Breast cancer (Combined Oncoarray; iCOGS; GWAS meta analysis) |

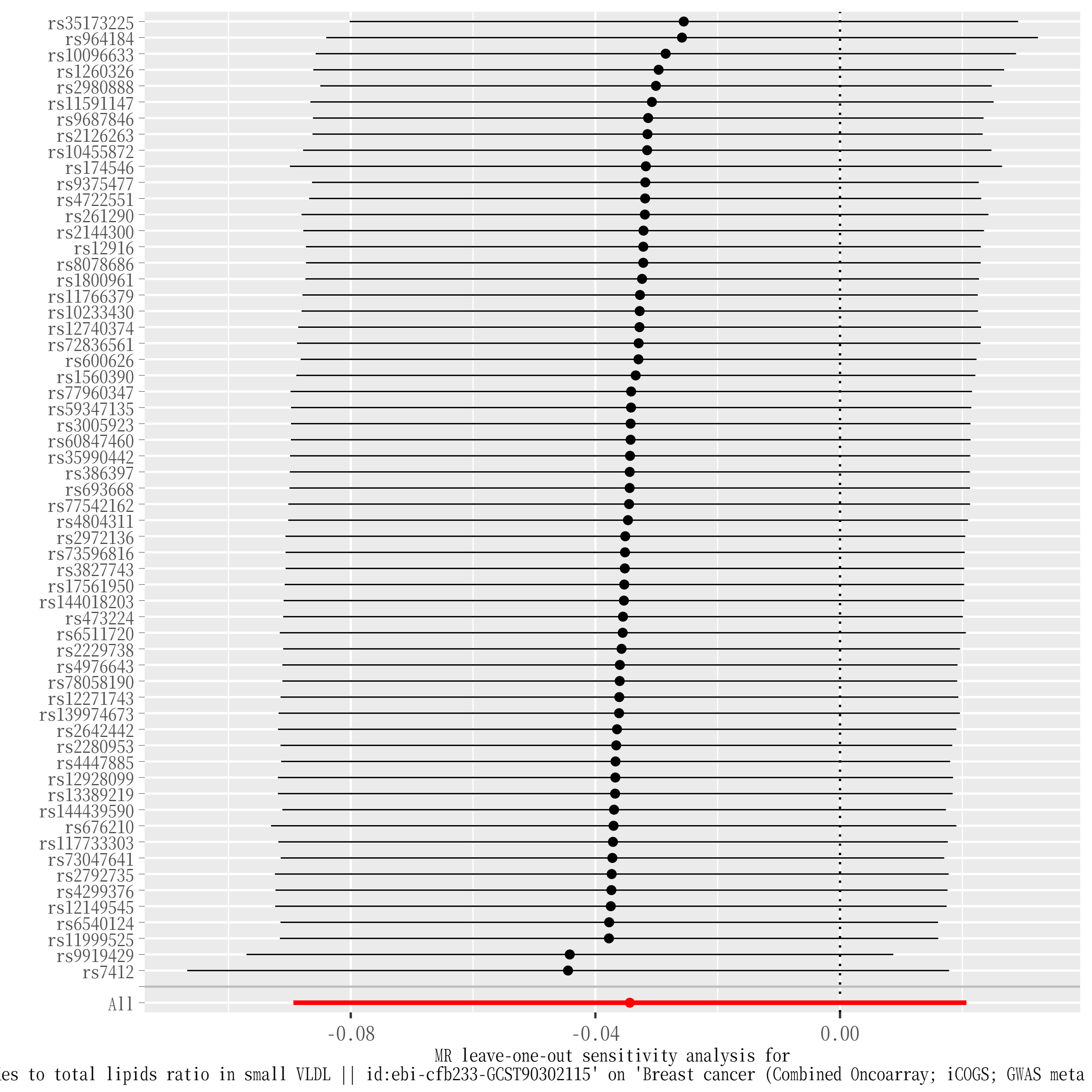
rs35173225
rs964184
rs10096633
rs1260326
rs2980888
rs11591147
rs9687846
rs2126263
rs10455872
rs174546
rs9375477
rs4722551
rs261290
rs2144300
rs12916
rs8078686
rs1800961
rs11766379
rs10233430
rs12740374
rs72836561
rs600626
rs1560390
rs77960347
rs59347135
rs3005923
rs60847460
rs35990442
rs386397
rs693668
rs77542162
rs4804311
rs2972136
rs73596816
rs3827743
rs17561950
rs144018203
rs473224
rs6511720
rs2229738
rs4976643
rs78058190
rs12271743
rs139974673
rs2642442
rs2280953
rs4447885
rs12928099
rs13389219
rs144439590
rs676210
rs117733303
rs73047641
rs2792735
rs4299376
rs12149545
rs6540124
rs11999525
rs9919429
rs7412
All
-0.08
-0.04
0.00
MR leave-one-out sensitivity analysis for
les to total lipids ratio in small VLDL || id:ebi-cfb233-GCST90302115' on 'Breast cancer (Combined Oncoarray; iCOGS; GWAS meta

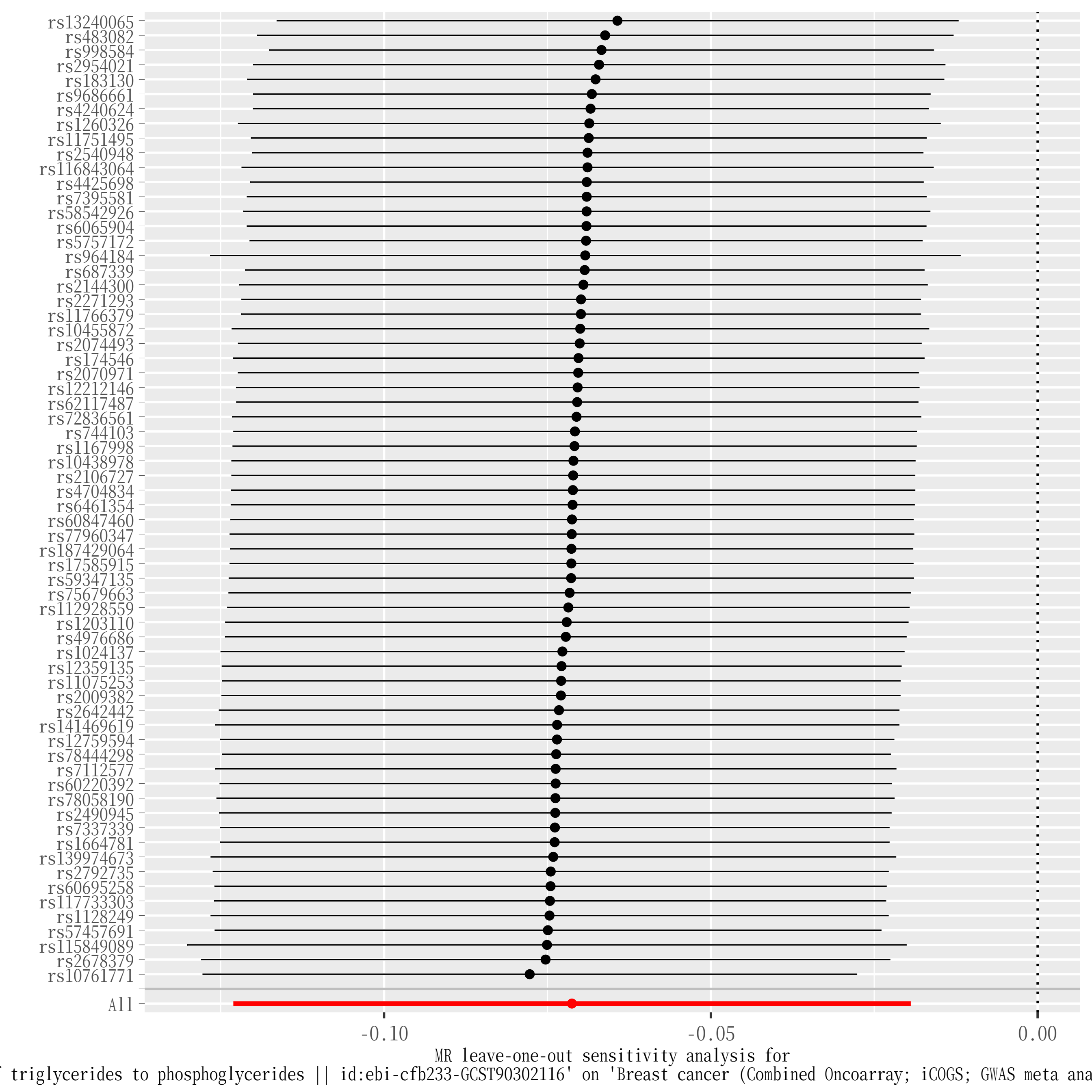
rs13240065
rs483082
rs998584
rs2954021
rs183130
rs9686661
rs4240624
rs1260326
rs11751495
rs2540948
rs116843064
rs4425698
rs7395581
rs58542926
rs6065904
rs5757172
rs964184
rs687339
rs2144300
rs2271293
rs11766379
rs10455872
rs2074493
rs174546
rs2070971
rs12212146
rs62117487
rs72836561
rs744103
rs1167998
rs10438978
rs2106727
rs4704834
rs6461354
rs60847460
rs77960347
rs187429064
rs17585915
rs59347135
rs75679663
rs112928559
rs1203110
rs4976686
rs1024137
rs12359135
rs11075253
rs2009382
rs2642442
rs141469619
rs12759594
rs78444298
rs7112577
rs60220392
rs78058190
rs2490945
rs7337339
rs1664781
rs139974673
rs2792735
rs60695258
rs117733303
rs1128249
rs57457691
rs115849089
rs2678379
rs10761771
All
-0.10
-0.05
0.00
MR leave-one-out sensitivity analysis for
' triglycerides to phosphoglycerides || id:ebi-cfb233-GCST90302116' on 'Breast cancer (Combined Oncoarray; iCOGS; GWAS meta ana

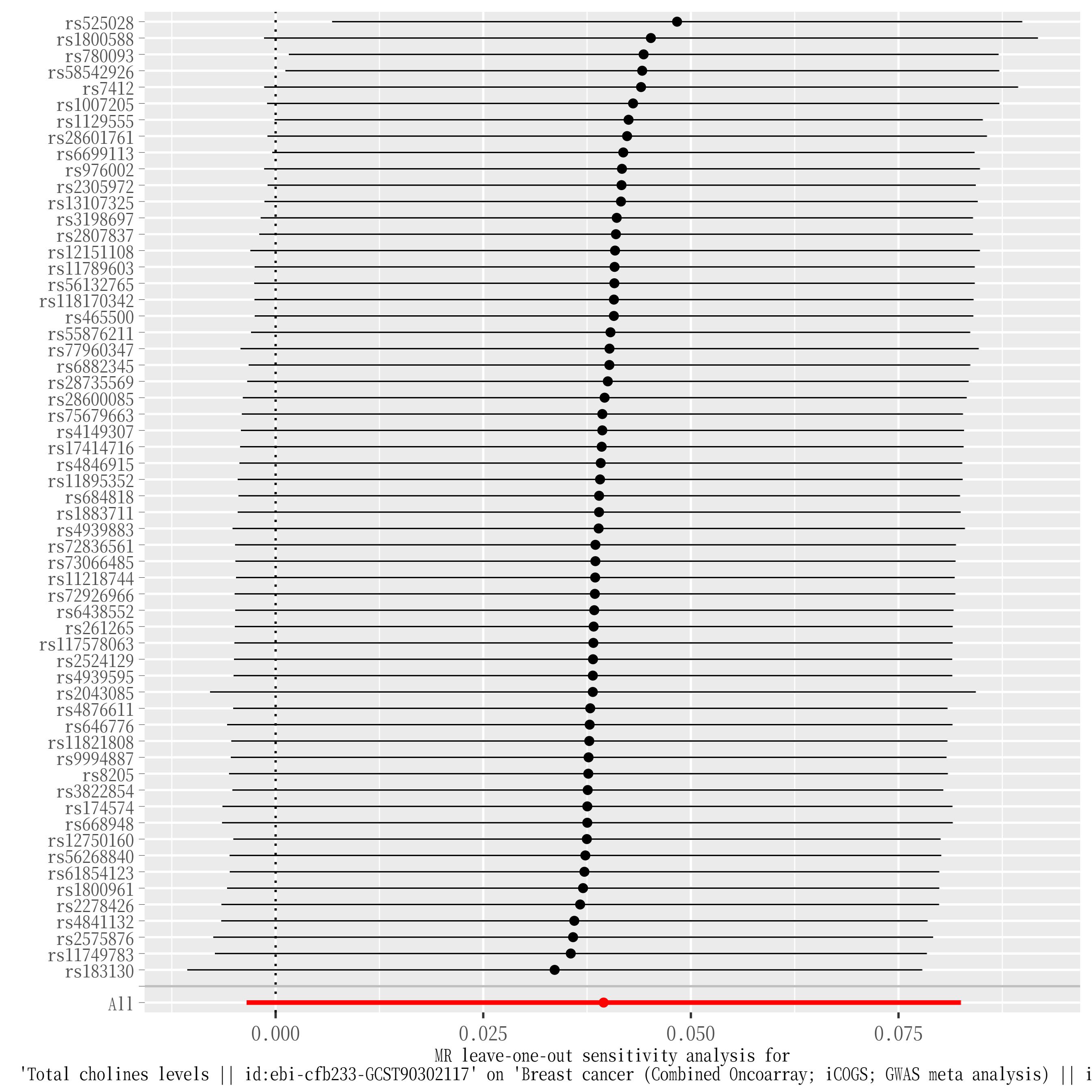

rs525028
rs1800588
rs780093
rs58542926
rs7412
rs1007205
rs1129555
rs28601761
rs6699113
rs976002
rs2305972
rs13107325
rs3198697
rs2807837
rs12151108
rs11789603
rs56132765
rs118170342
rs465500
rs55876211
rs77960347
rs6882345
rs28735569
rs28600085
rs75679663
rs4149307
rs17414716
rs4846915
rs11895352
rs684818
rs1883711
rs4939883
rs72836561
rs73066485
rs11218744
rs72926966
rs6438552
rs261265
rs117578063
rs2524129
rs4939595
rs2043085
rs4876611
rs646776
rs11821808
rs9994887
rs8205
rs3822854
rs174574
rs668948
rs12750160
rs56268840
rs61854123
rs1800961
rs2278426
rs4841132
rs2575876
rs11749783
rs183130
All
0.000
0.025
0.050
0.075
MR leave-one-out sensitivity analysis for
'Total cholines levels || id:ebi-cfb233-GCST90302117' on 'Breast cancer (Combined Oncoarray; iCOGS; GWAS meta analysis) || i

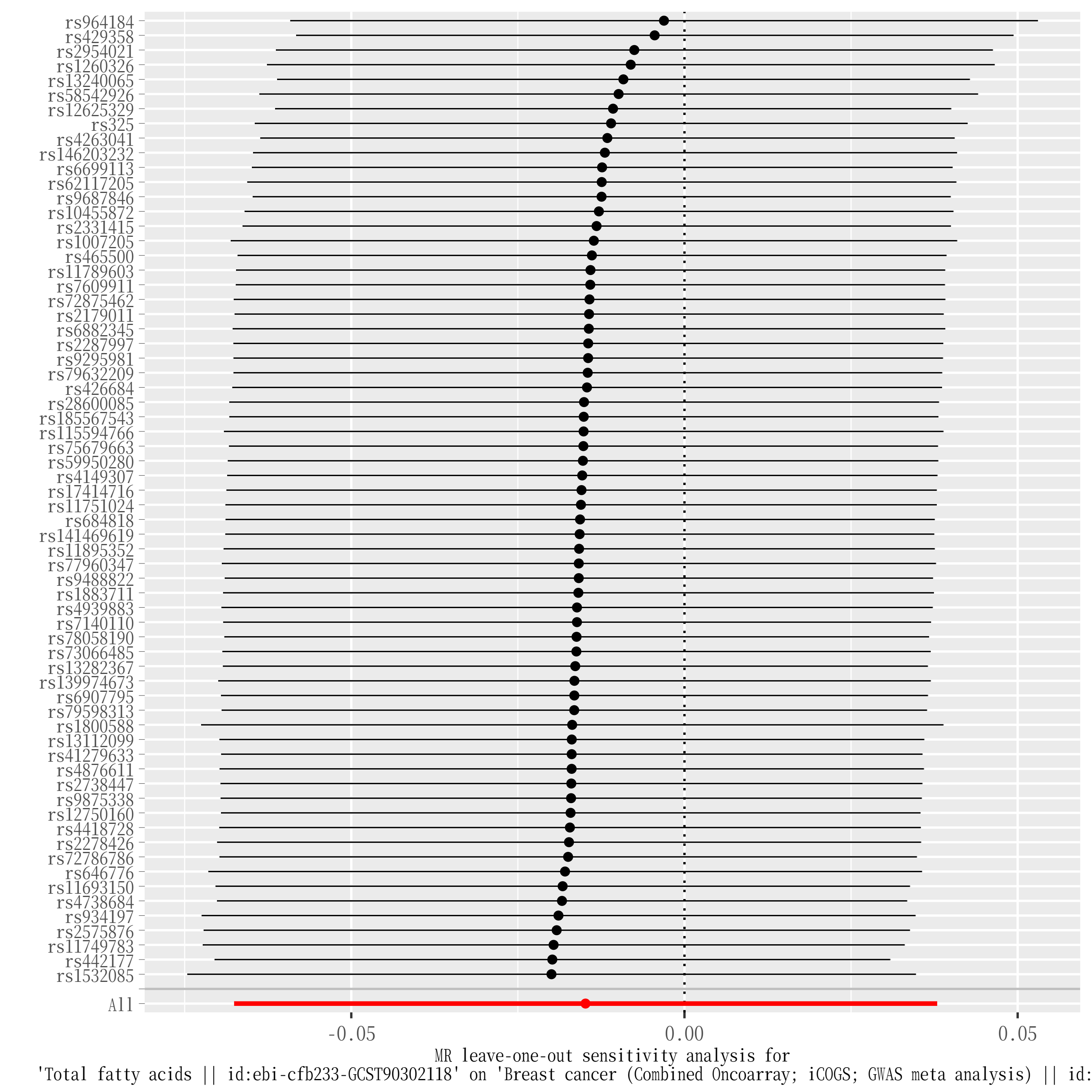
rs964184
rs429358
rs2954021
rs1260326
rs13240065
rs58542926
rs12625329
rs325
rs4263041
rs146203232
rs6699113
rs62117205
rs9687846
rs10455872
rs2331415
rs1007205
rs465500
rs11789603
rs7609911
rs72875462
rs2179011
rs6882345
rs2287997
rs9295981
rs79632209
rs426684
rs28600085
rs185567543
rs115594766
rs75679663
rs59950280
rs4149307
rs17414716
rs11751024
rs684818
rs141469619
rs11895352
rs77960347
rs9488822
rs1883711
rs4939883
rs7140110
rs78058190
rs73066485
rs13282367
rs139974673
rs6907795
rs79598313
rs1800588
rs13112099
rs41279633
rs4876611
rs2738447
rs9875338
rs12750160
rs4418728
rs2278426
rs72786786
rs646776
rs11693150
rs4738684
rs934197
rs2575876
rs11749783
rs442177
rs1532085
All
-0.05
0.00
0.05
MR leave-one-out sensitivity analysis for
'Total fatty acids || id:ebi-cfb233-GCST90302118' on 'Breast cancer (Combined Oncoarray; iCOGS; GWAS meta analysis) || id:

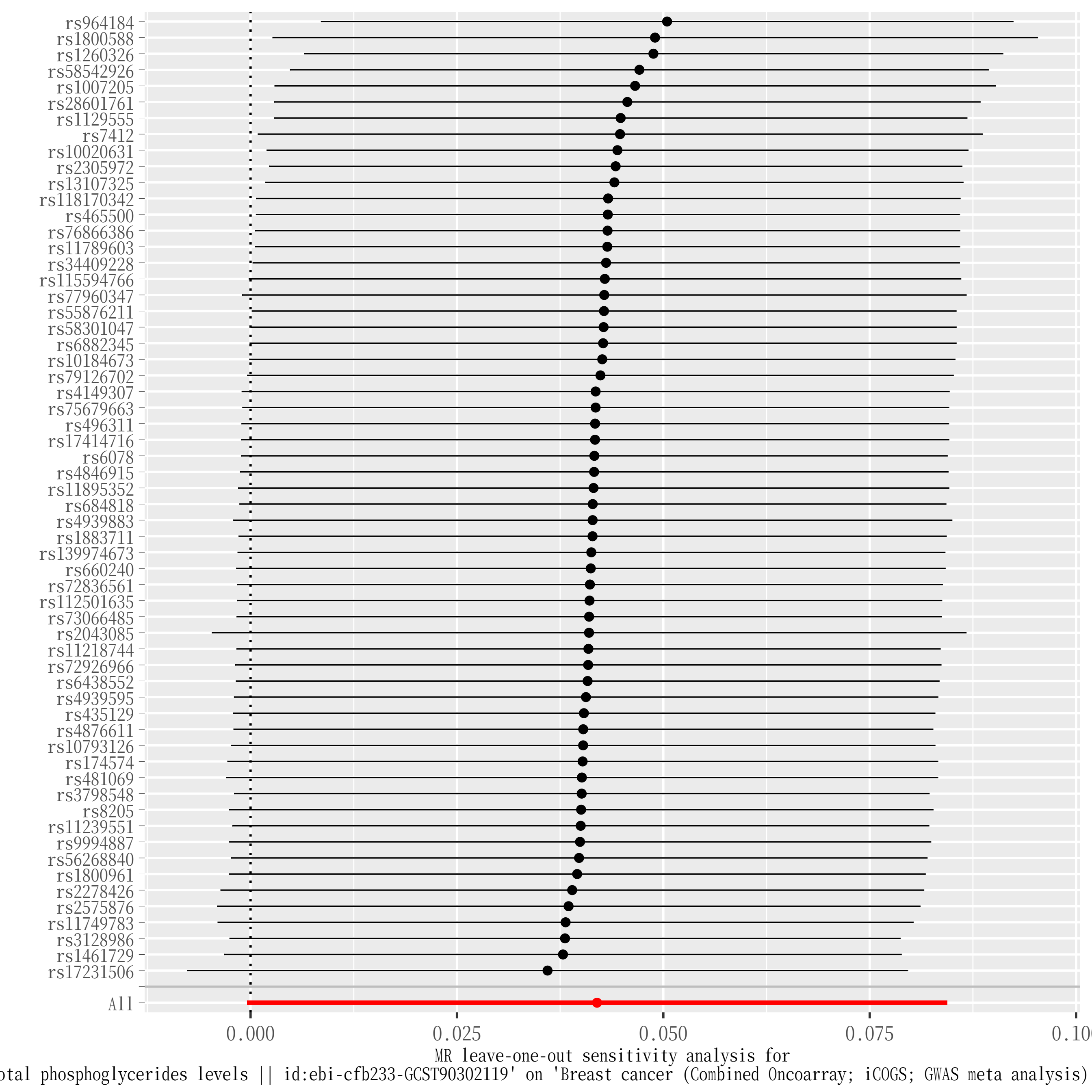
rs964184
rs1800588
rs1260326
rs58542926
rs1007205
rs28601761
rs1129555
rs7412
rs10020631
rs2305972
rs13107325
rs118170342
rs465500
rs76866386
rs11789603
rs34409228
rs115594766
rs77960347
rs55876211
rs58301047
rs6882345
rs10184673
rs79126702
rs4149307
rs75679663
rs496311
rs17414716
rs6078
rs4846915
rs11895352
rs684818
rs4939883
rs1883711
rs139974673
rs660240
rs72836561
rs112501635
rs73066485
rs2043085
rs11218744
rs72926966
rs6438552
rs4939595
rs435129
rs4876611
rs10793126
rs174574
rs481069
rs3798548
rs8205
rs11239551
rs9994887
rs56268840
rs1800961
rs2278426
rs2575876
rs11749783
rs3128986
rs1461729
rs17231506
All
0.000
0.025
0.050
0.075
0.10
MR leave-one-out sensitivity analysis for
otal phosphoglycerides levels || id:ebi-cfb233-GCST90302119' on 'Breast cancer (Combined Oncoarray; iCOGS; GWAS meta analysis)

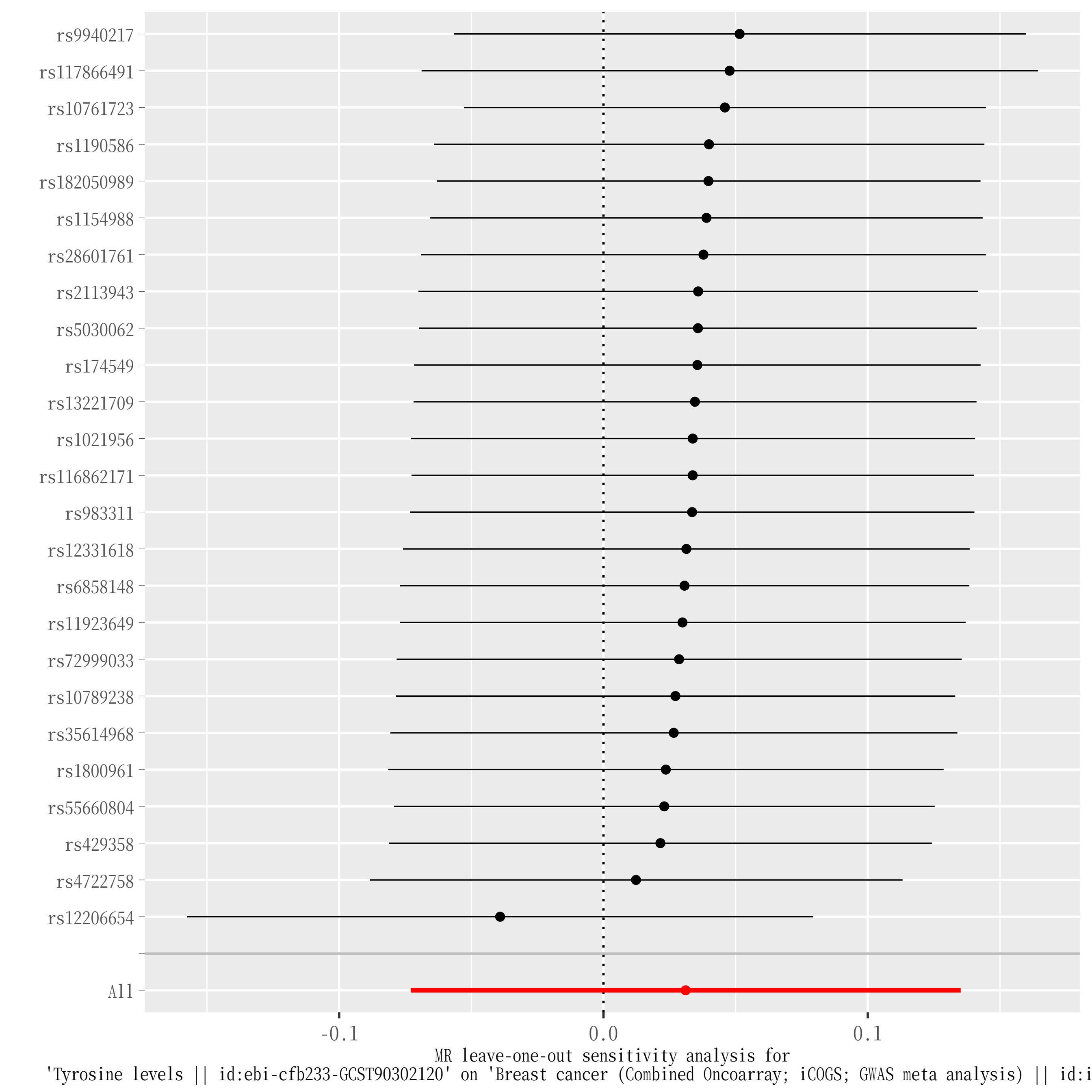

rs9940217
rs117866491
rs10761723
rs1190586
rs182050989
rs1154988
rs28601761
rs2113943
rs5030062
rs174549
rs13221709
rs1021956
rs116862171
rs983311
rs12331618
rs6858148
rs11923649
rs72999033
rs10789238
rs35614968
rs1800961
rs55660804
rs429358
rs4722758
rs12206654
All
-0.1
0.0
0.1
MR leave-one-out sensitivity analysis for
'Tyrosine levels || id:ebi-cfb233-GCST90302120' on 'Breast cancer (Combined Oncoarray; iCOGS; GWAS meta analysis) || id:i

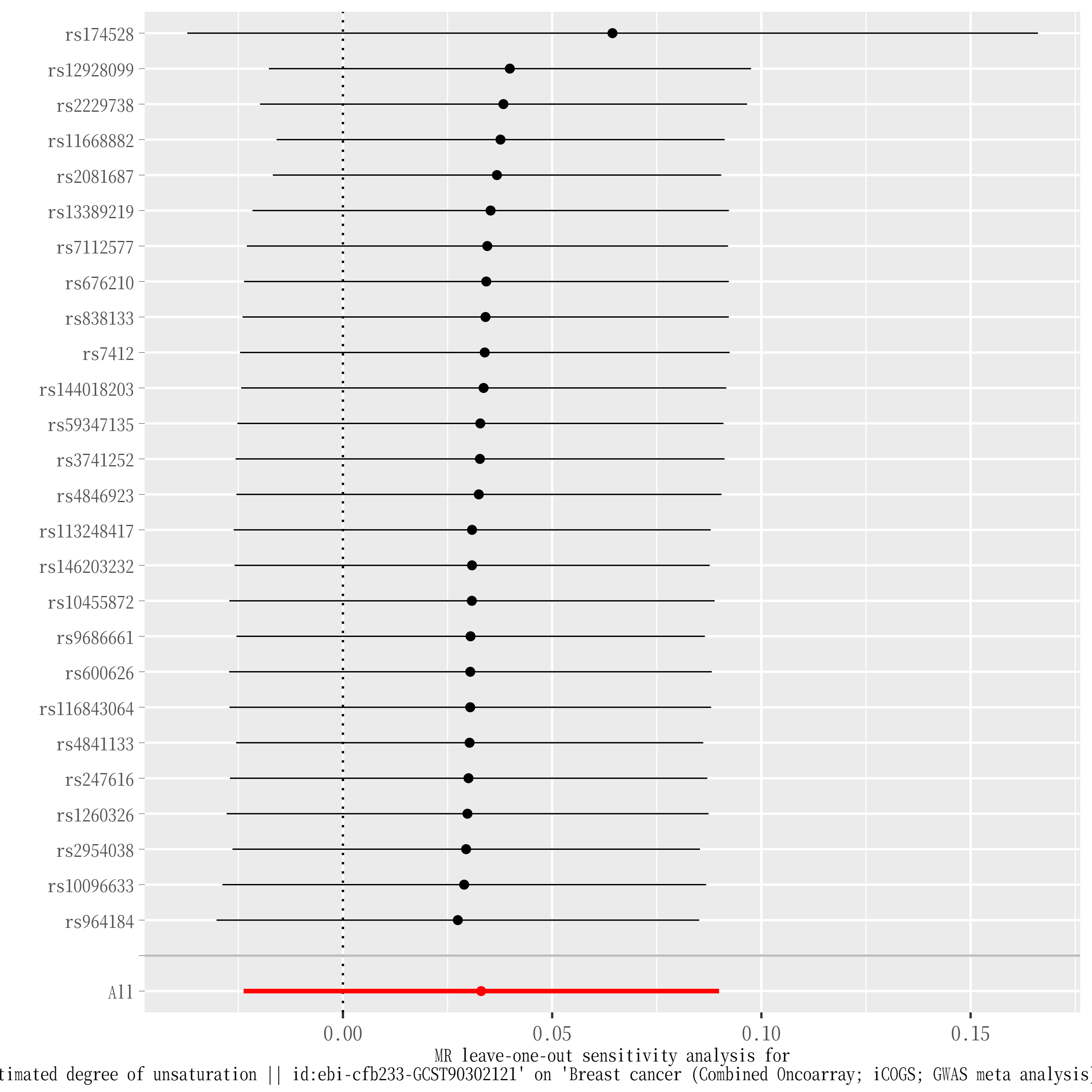
rs174528
rs12928099
rs2229738
rs11668882
rs2081687
rs13389219
rs7112577
rs676210
rs838133
rs7412
rs144018203
rs59347135
rs3741252
rs4846923
rs113248417
rs146203232
rs10455872
rs9686661
rs600626
rs116843064
rs4841133
rs247616
rs1260326
rs2954038
rs10096633
rs964184
All
0.00
0.05
0.10
0.15
MR leave-one-out sensitivity analysis for
timated degree of unsaturation || id:ebi-cfb233-GCST90302121' on 'Breast cancer (Combined Oncoarray; iCOGS; GWAS meta analysis)

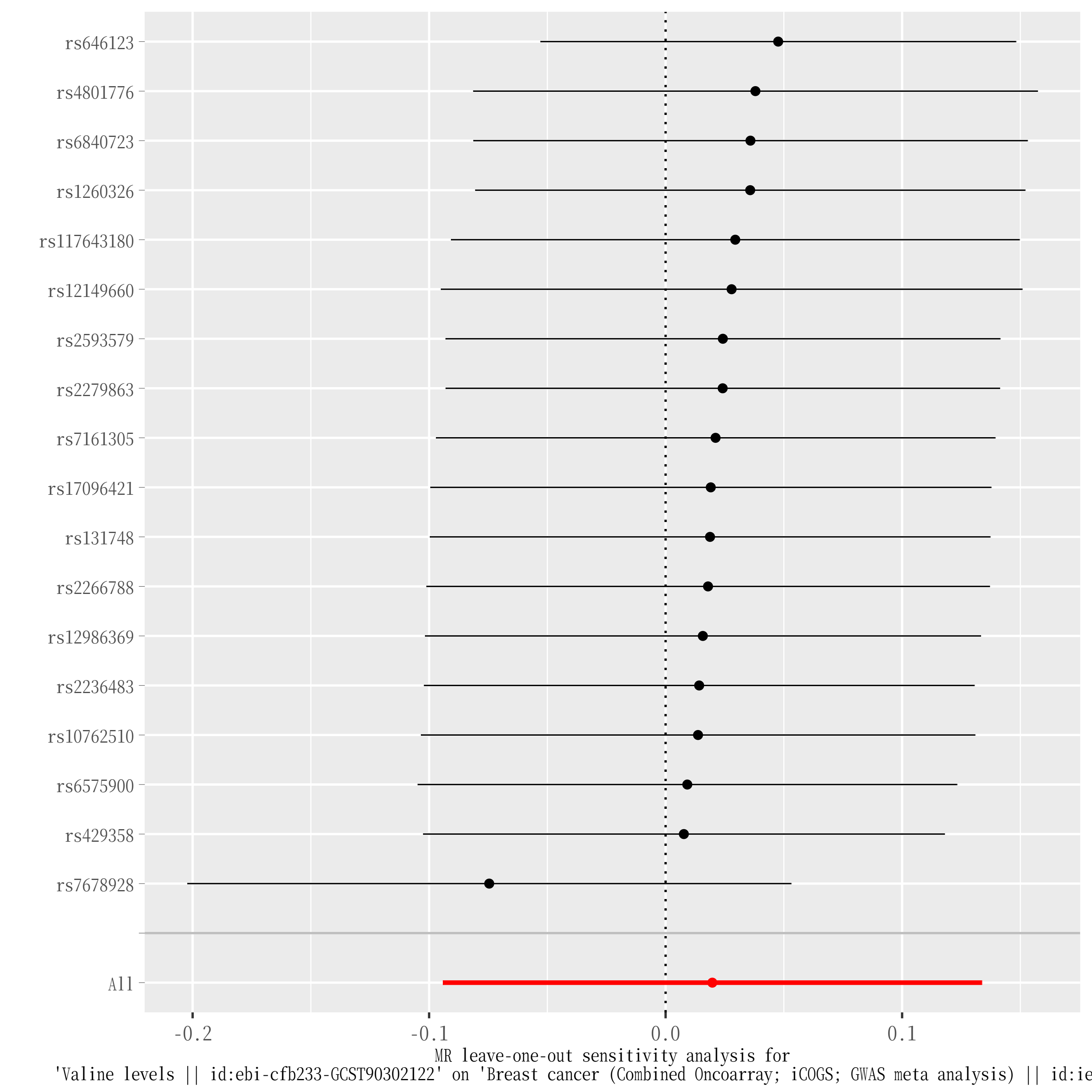
rs646123
rs4801776
rs6840723
rs1260326
rs117643180
rs12149660
rs2593579
rs2279863
rs7161305
rs17096421
rs131748
rs2266788
rs12986369
rs2236483
rs10762510
rs6575900
rs429358
rs7678928
All
-0.2
-0.1
0.0
0.1
MR leave-one-out sensitivity analysis for
'Valine levels || id:ebi-cfb233-GCST90302122' on 'Breast cancer (Combined Oncoarray; iCOGS; GWAS meta analysis) || id:ie

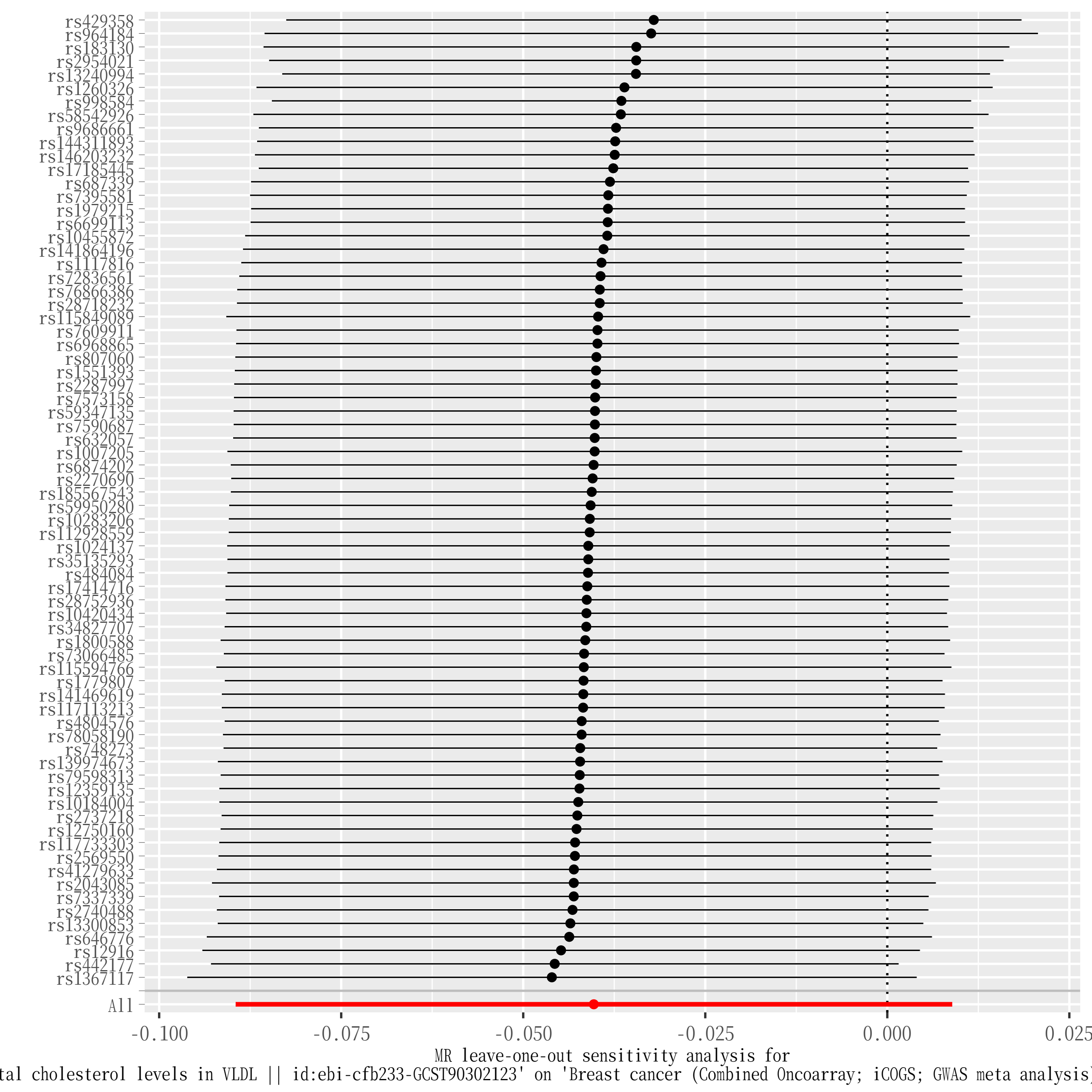
rs429358
rs964184
rs183130
rs2954021
rs13240994
rs1260326
rs998584
rs58542926
rs9686661
rs144311893
rs146203232
rs17185445
rs687339
rs7395581
rs1979215
rs6699113
rs10455872
rs141864196
rs1117816
rs72836561
rs76866386
rs28718232
rs115849089
rs7609911
rs6968865
rs807060
rs1551393
rs2287997
rs7573158
rs59347135
rs7590687
rs632057
rs1007205
rs6874202
rs2270690
rs185567543
rs59950280
rs10283206
rs112928559
rs1024137
rs35135293
rs484084
rs17414716
rs28752936
rs10420434
rs34827707
rs1800588
rs73066485
rs115594766
rs1779807
rs141469619
rs117113213
rs4804576
rs78058190
rs748273
rs139974673
rs79598313
rs12359135
rs10184004
rs2737218
rs12750160
rs117733303
rs2569550
rs41279633
rs2043085
rs7337339
rs2740488
rs13300853
rs646776
rs12916
rs442177
rs1367117
All
-0.100
-0.075
-0.050
-0.025
0.000
0.025
MR leave-one-out sensitivity analysis for
tal cholesterol levels in VLDL || id:ebi-cfb233-GCST90302123' on 'Breast cancer (Combined Oncoarray; iCOGS; GWAS meta analysis)

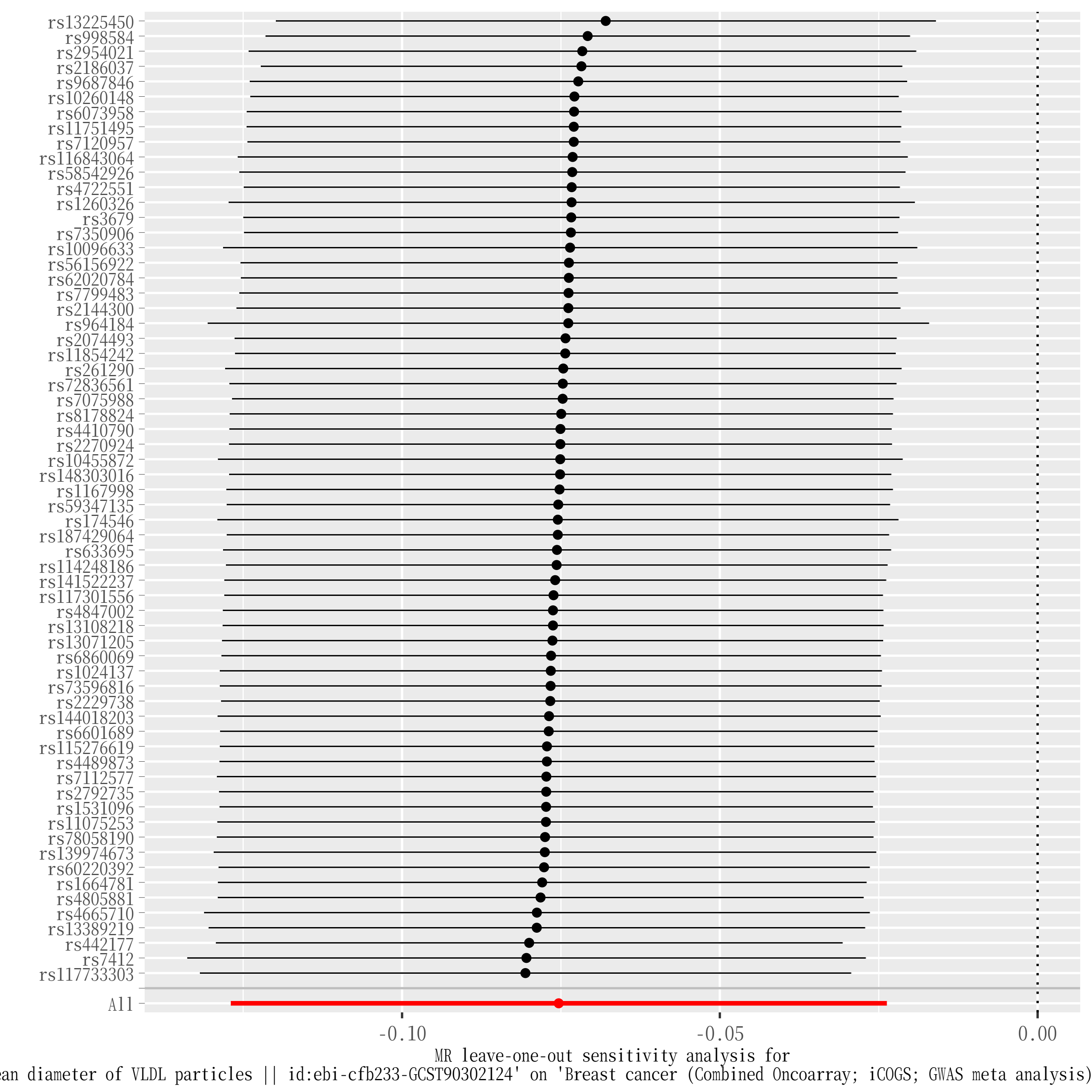
rs13225450
rs998584
rs2954021
rs2186037
rs9687846
rs10260148
rs6073958
rs11751495
rs7120957
rs116843064
rs58542926
rs4722551
rs1260326
rs3679
rs7350906
rs10096633
rs56156922
rs62020784
rs7799483
rs2144300
rs964184
rs2074493
rs11854242
rs261290
rs72836561
rs7075988
rs8178824
rs4410790
rs2270924
rs10455872
rs148303016
rs1167998
rs59347135
rs174546
rs187429064
rs633695
rs114248186
rs141522237
rs117301556
rs4847002
rs13108218
rs13071205
rs6860069
rs1024137
rs73596816
rs2229738
rs144018203
rs6601689
rs115276619
rs4489873
rs7112577
rs2792735
rs1531096
rs11075253
rs78058190
rs139974673
rs60220392
rs1664781
rs4805881
rs4665710
rs13389219
rs442177
rs7412
rs117733303
All
-0.10
-0.05
0.00
MR leave-one-out sensitivity analysis for
ean diameter of VLDL particles || id:ebi-cfb233-GCST90302124' on 'Breast cancer (Combined Oncoarray; iCOGS; GWAS meta analysis)'

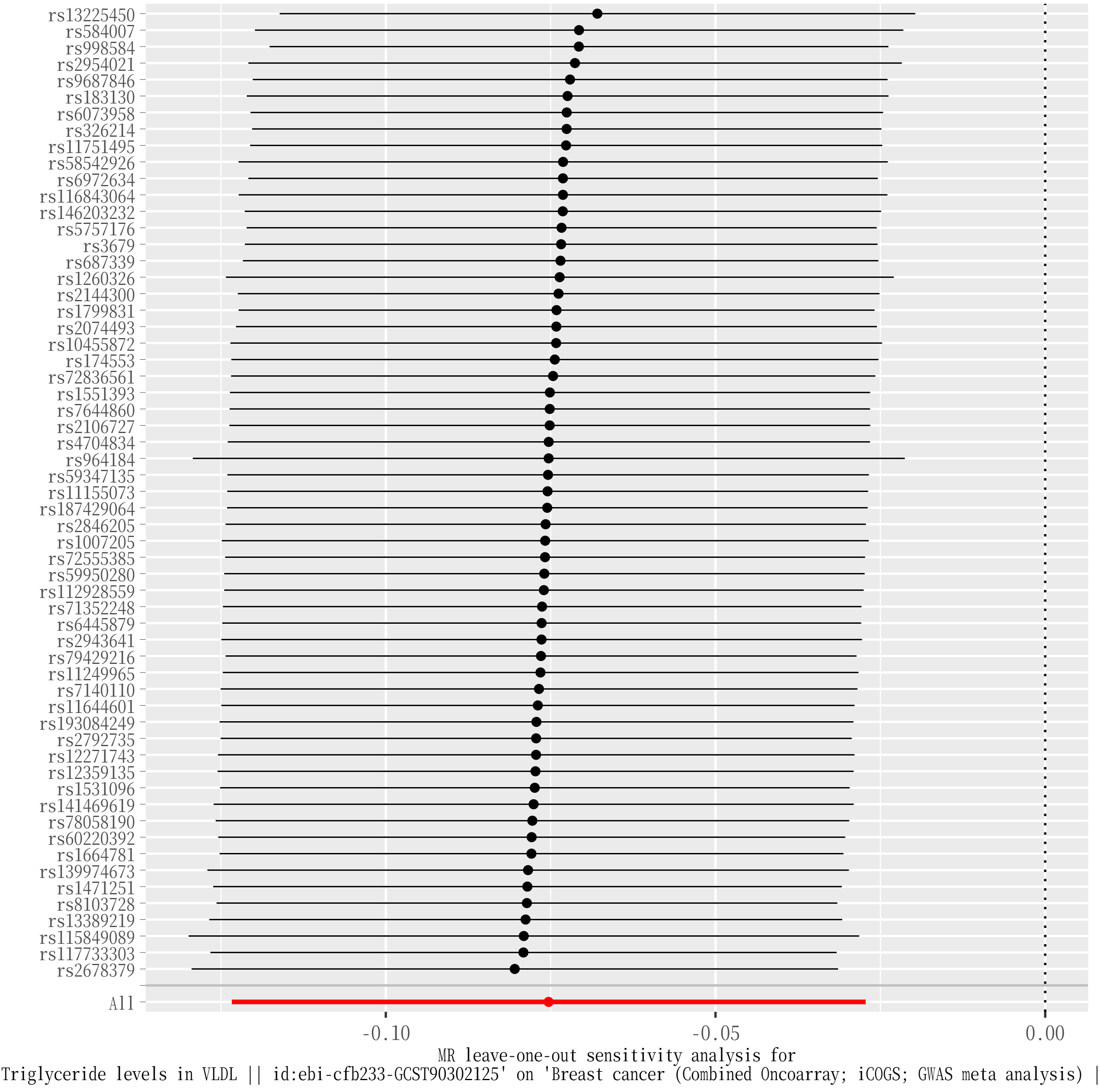

rs13225450
rs584007
rs998584
rs2954021
rs9687846
rs183130
rs6073958
rs326214
rs11751495
rs58542926
rs6972634
rs116843064
rs146203232
rs5757176
rs3679
rs687339
rs1260326
rs2144300
rs1799831
rs2074493
rs10455872
rs174553
rs72836561
rs1551393
rs7644860
rs2106727
rs4704834
rs964184
rs59347135
rs11155073
rs187429064
rs2846205
rs1007205
rs72555385
rs59950280
rs112928559
rs71352248
rs6445879
rs2943641
rs79429216
rs11249965
rs7140110
rs11644601
rs193084249
rs2792735
rs12271743
rs12359135
rs1531096
rs141469619
rs78058190
rs60220392
rs1664781
rs139974673
rs1471251
rs8103728
rs13389219
rs115849089
rs117733303
rs2678379
All
-0.10
-0.05
0.00
MR leave-one-out sensitivity analysis for
Triglyceride levels in VLDL || id:ebi-cfb233-GCST90302125' on 'Breast cancer (Combined Oncoarray; iCOGS; GWAS meta analysis) |

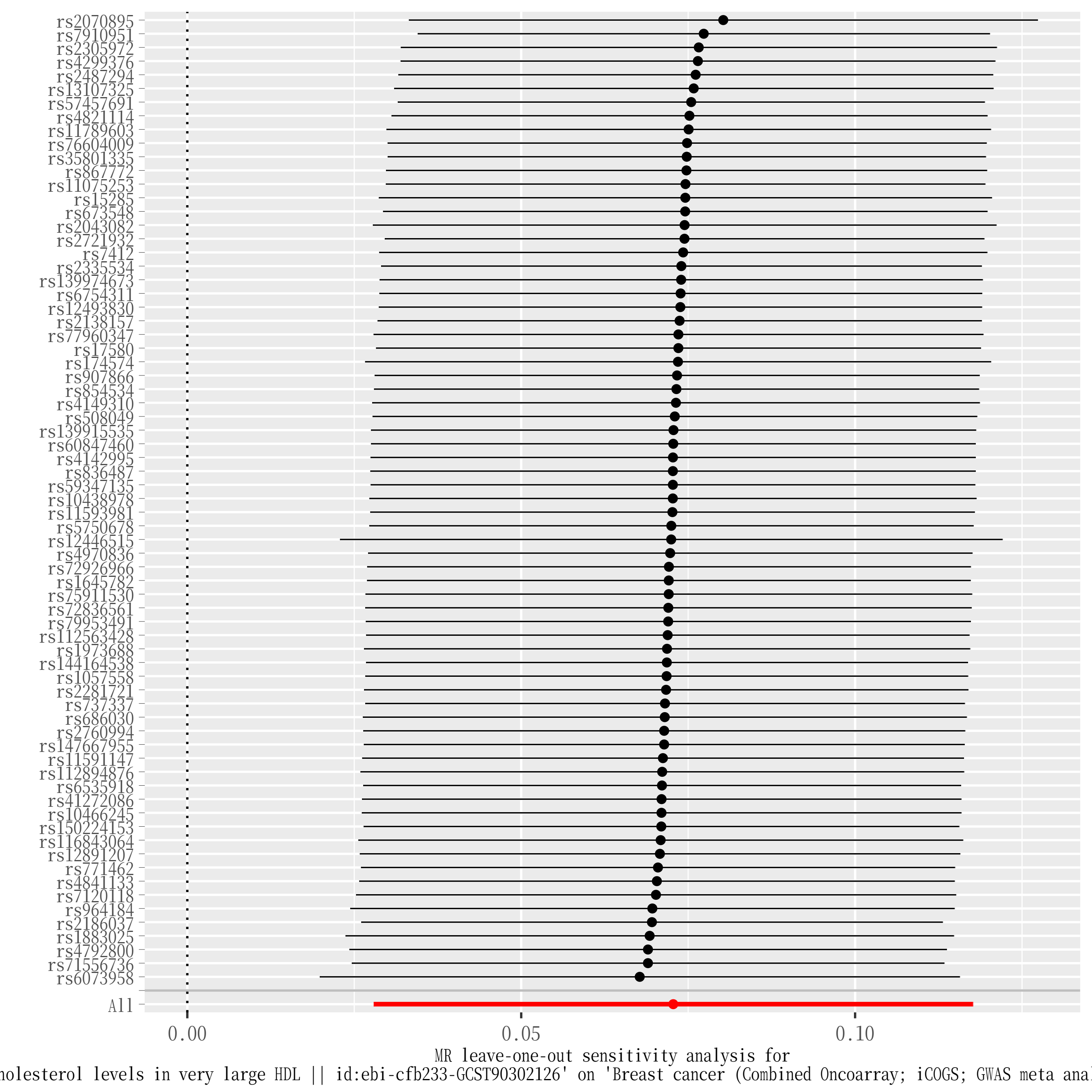

rs2070895
rs7910951
rs2305972
rs4299376
rs2487294
rs13107325
rs57457691
rs4821114
rs11789603
rs76604009
rs35801335
rs867772
rs11075253
rs15285
rs673548
rs2043082
rs2721932
rs7412
rs2335534
rs139974673
rs6754311
rs12493830
rs2138157
rs77960347
rs17580
rs174574
rs907866
rs854534
rs4149310
rs508049
rs139915535
rs60847460
rs4142995
rs836487
rs59347135
rs10438978
rs11593981
rs5750678
rs12446515
rs4970836
rs72926966
rs1645782
rs75911530
rs72836561
rs79953491
rs112563428
rs1973688
rs144164538
rs1057558
rs2281721
rs737337
rs686030
rs2760994
rs147667955
rs11591147
rs112894876
rs6535918
rs41272086
rs10466245
rs150224153
rs116843064
rs12891207
rs771462
rs4841133
rs7120118
rs964184
rs2186037
rs1883025
rs4792800
rs71556736
rs6073958
All
0.00
0.05
0.10
MR leave-one-out sensitivity analysis for
holesterol levels in very large HDL || id:ebi-cfb233-GCST90302126' on 'Breast cancer (Combined Oncoarray; iCOGS; GWAS meta anal

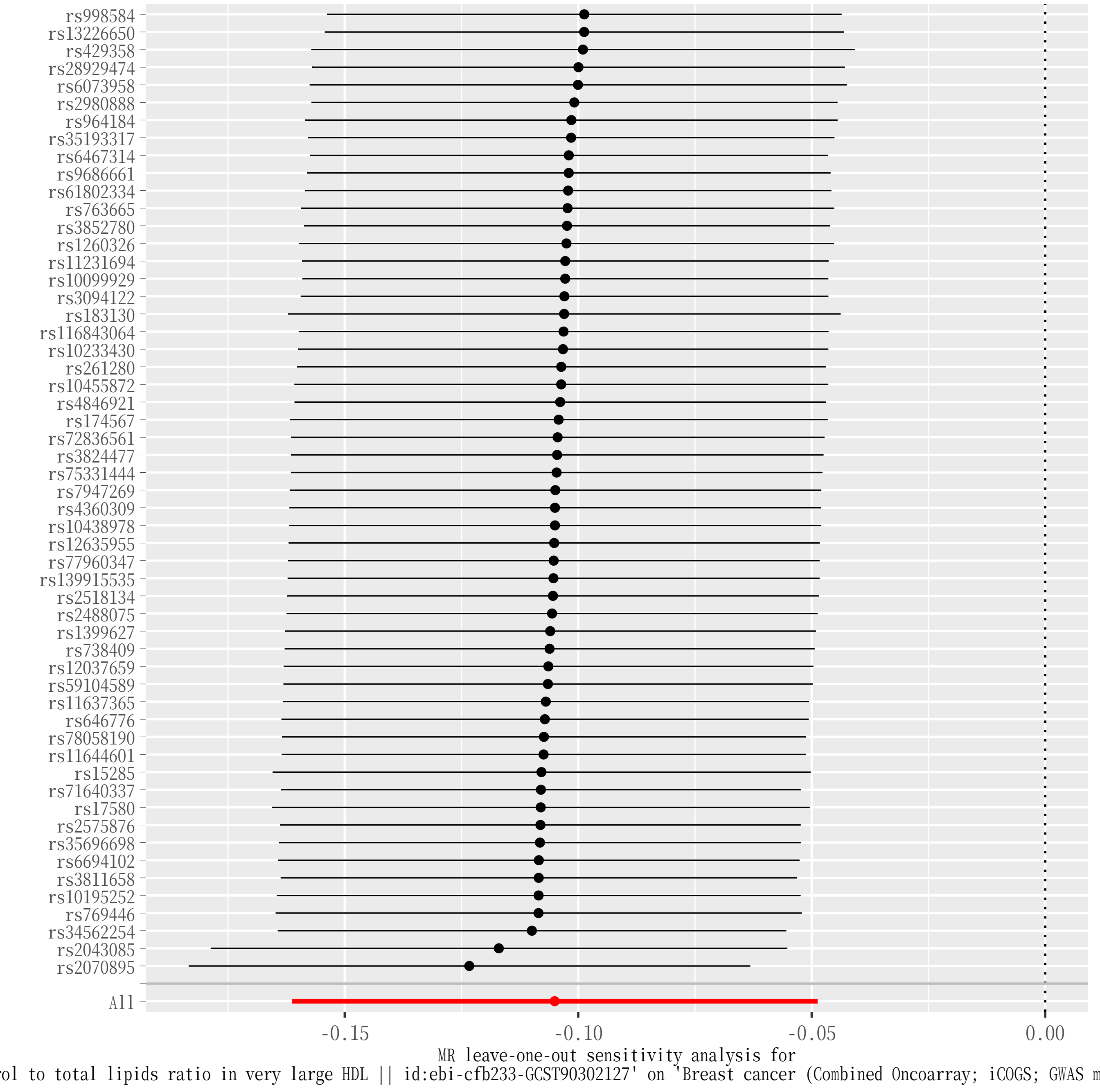

rs998584
rs13226650
rs429358
rs28929474
rs6073958
rs2980888
rs964184
rs35193317
rs6467314
rs9686661
rs61802334
rs763665
rs3852780
rs1260326
rs11231694
rs10099929
rs3094122
rs183130
rs116843064
rs10233430
rs261280
rs10455872
rs4846921
rs174567
rs72836561
rs3824477
rs75331444
rs7947269
rs4360309
rs10438978
rs12635955
rs77960347
rs139915535
rs2518134
rs2488075
rs1399627
rs738409
rs12037659
rs59104589
rs11637365
rs646776
rs78058190
rs11644601
rs15285
rs71640337
rs17580
rs2575876
rs35696698
rs6694102
rs3811658
rs10195252
rs769446
rs34562254
rs2043085
rs2070895
All
-0.15
-0.10
-0.05
0.00
MR leave-one-out sensitivity analysis for
rol to total lipids ratio in very large HDL || id:ebi-cfb233-GCST90302127' on 'Breast cancer (Combined Oncoarray; iCOGS; GWAS m

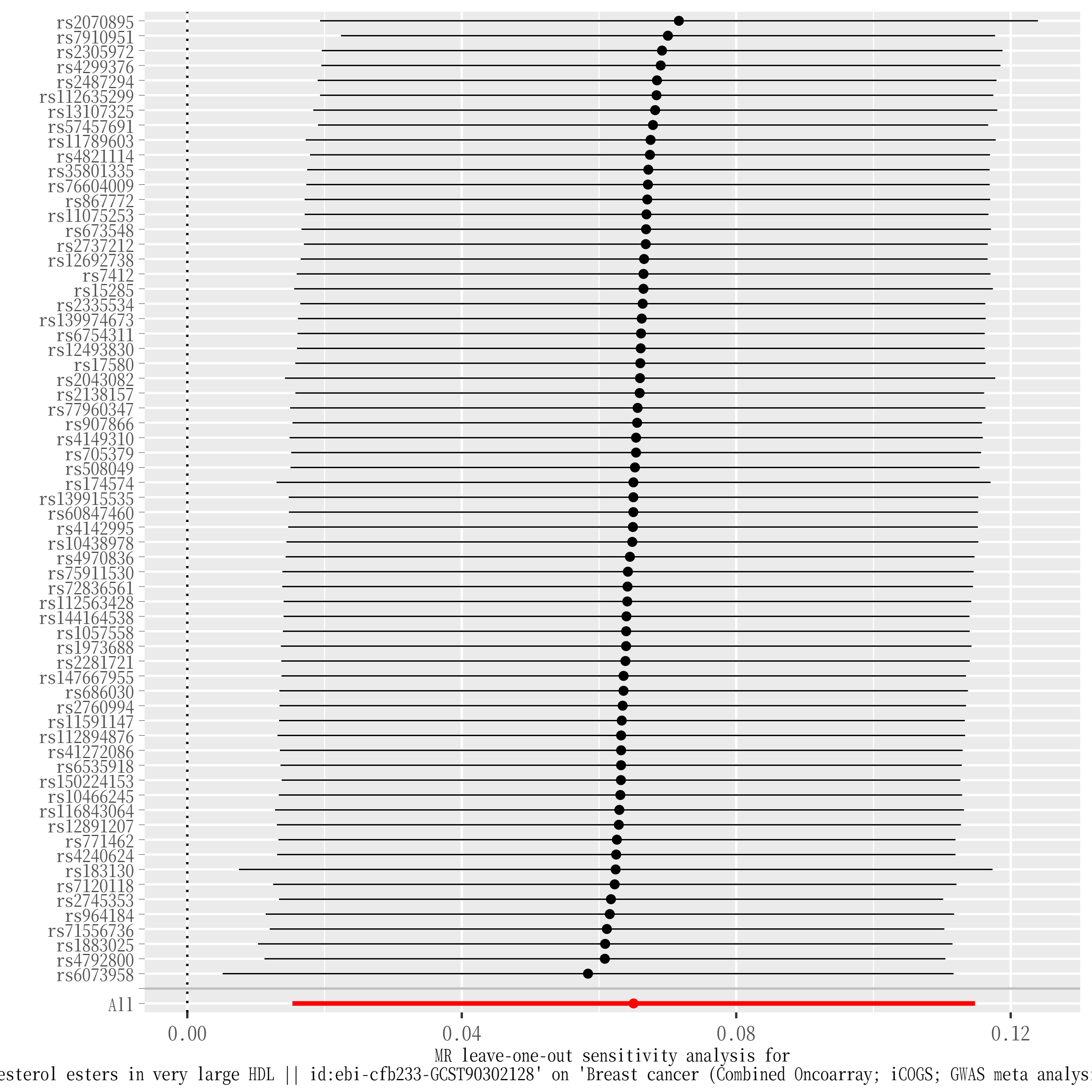

rs2070895
rs7910951
rs2305972
rs4299376
rs2487294
rs112635299
rs13107325
rs57457691
rs11789603
rs4821114
rs35801335
rs76604009
rs867772
rs11075253
rs673548
rs2737212
rs12692738
rs7412
rs15285
rs2335534
rs139974673
rs6754311
rs12493830
rs17580
rs2043082
rs2138157
rs77960347
rs907866
rs4149310
rs705379
rs508049
rs174574
rs139915535
rs60847460
rs4142995
rs10438978
rs4970836
rs75911530
rs72836561
rs112563428
rs144164538
rs1057558
rs1973688
rs2281721
rs147667955
rs686030
rs2760994
rs11591147
rs112894876
rs41272086
rs6535918
rs150224153
rs10466245
rs116843064
rs12891207
rs771462
rs4240624
rs183130
rs7120118
rs2745353
rs964184
rs71556736
rs1883025
rs4792800
rs6073958
All
0.00
0.04
0.08
0.12
MR leave-one-out sensitivity analysis for
esterol esters in very large HDL || id:ebi-cfb233-GCST90302128' on 'Breast cancer (Combined Oncoarray; iCOGS; GWAS meta analys

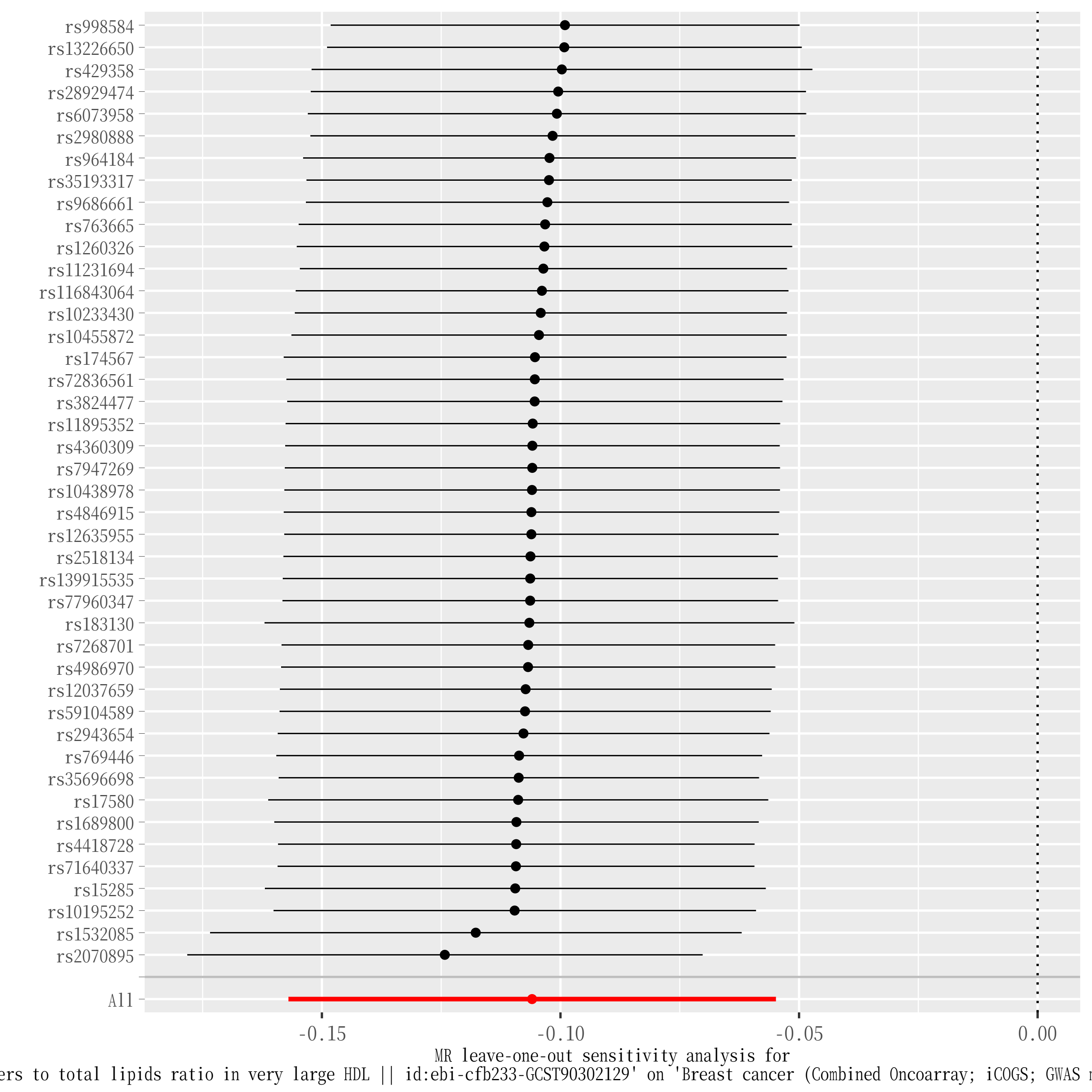

rs998584
rs13226650
rs429358
rs28929474
rs6073958
rs2980888
rs964184
rs35193317
rs9686661
rs763665
rs1260326
rs11231694
rs116843064
rs10233430
rs10455872
rs174567
rs72836561
rs3824477
rs11895352
rs4360309
rs7947269
rs10438978
rs4846915
rs12635955
rs2518134
rs139915535
rs77960347
rs183130
rs7268701
rs4986970
rs12037659
rs59104589
rs2943654
rs769446
rs35696698
rs17580
rs1689800
rs4418728
rs71640337
rs15285
rs10195252
rs1532085
rs2070895
All
-0.15
-0.10
-0.05
0.00
MR leave-one-out sensitivity analysis for
ers to total lipids ratio in very large HDL || id:ebi-cfb233-GCST90302129' on 'Breast cancer (Combined Oncoarray; iCOGS; GWAS r

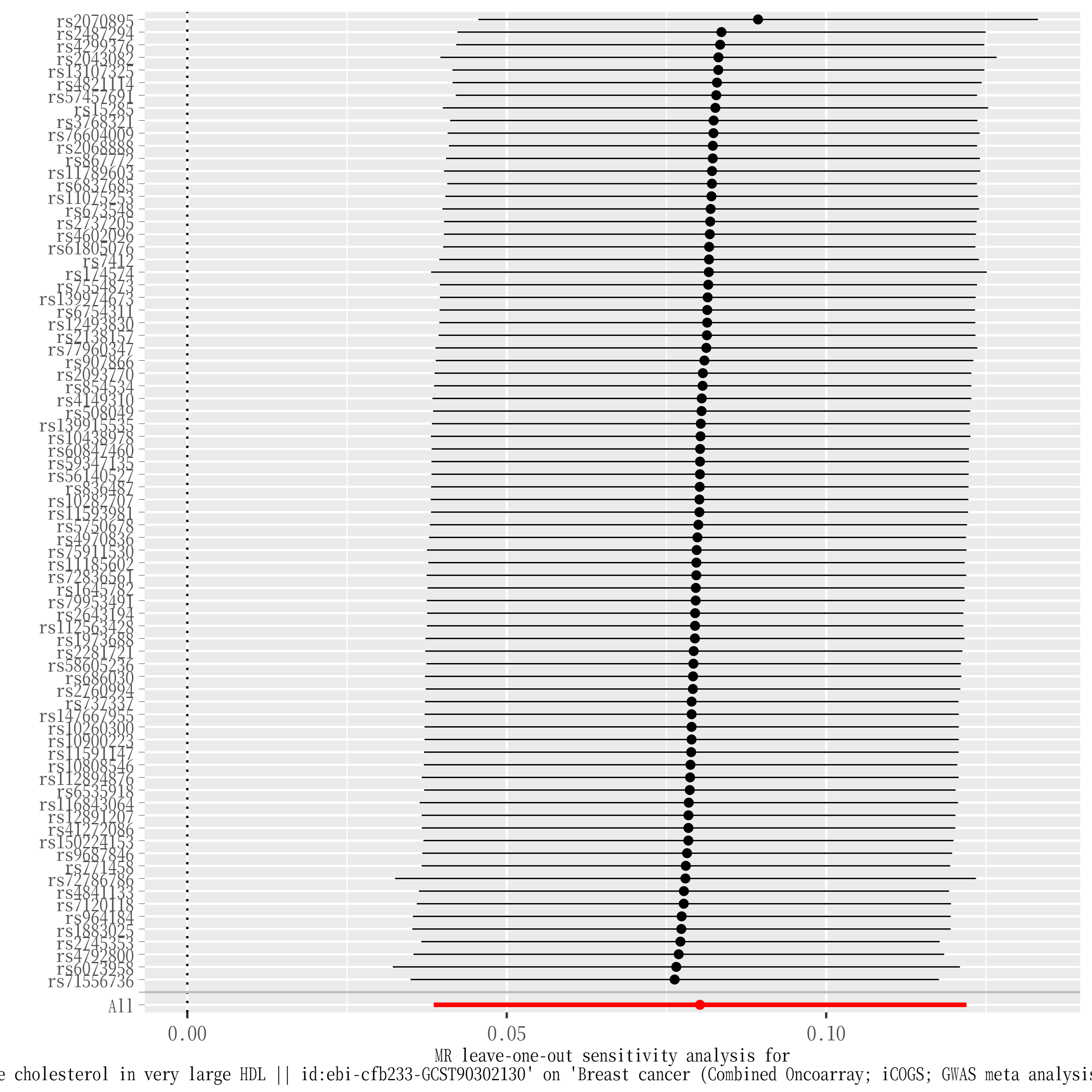

rs2070895
rs2487294
rs4299376
rs2043082
rs13107325
rs4821114
rs57457691
rs15285
rs3768321
rs76604009
rs2068888
rs867772
rs11789603
rs6837685
rs11075253
rs673548
rs2737205
rs4602096
rs61805076
rs7412
rs174574
rs7554873
rs139974673
rs6754311
rs12493830
rs2138157
rs77960347
rs907866
rs2093770
rs854534
rs4149310
rs508049
rs139915535
rs10438978
rs60847460
rs59347135
rs56140527
rs836487
rs10282707
rs11593981
rs5750678
rs4970836
rs75911530
rs11185602
rs72836561
rs1645782
rs79953491
rs2643194
rs112563428
rs1973688
rs2281721
rs58605236
rs686030
rs2760994
rs737337
rs147667955
rs10260300
rs10900223
rs11591147
rs10808546
rs112894876
rs6535918
rs116843064
rs12891207
rs41272086
rs150224153
rs9687846
rs771458
rs72786786
rs4841133
rs7120118
rs964184
rs1883025
rs2745353
rs4792800
rs6073958
rs71556736
All
0.00
0.05
0.10
MR leave-one-out sensitivity analysis for
e cholesterol in very large HDL || id:ebi-cfb233-GCST90302130' on 'Breast cancer (Combined Oncoarray; iCOGS; GWAS meta analysis

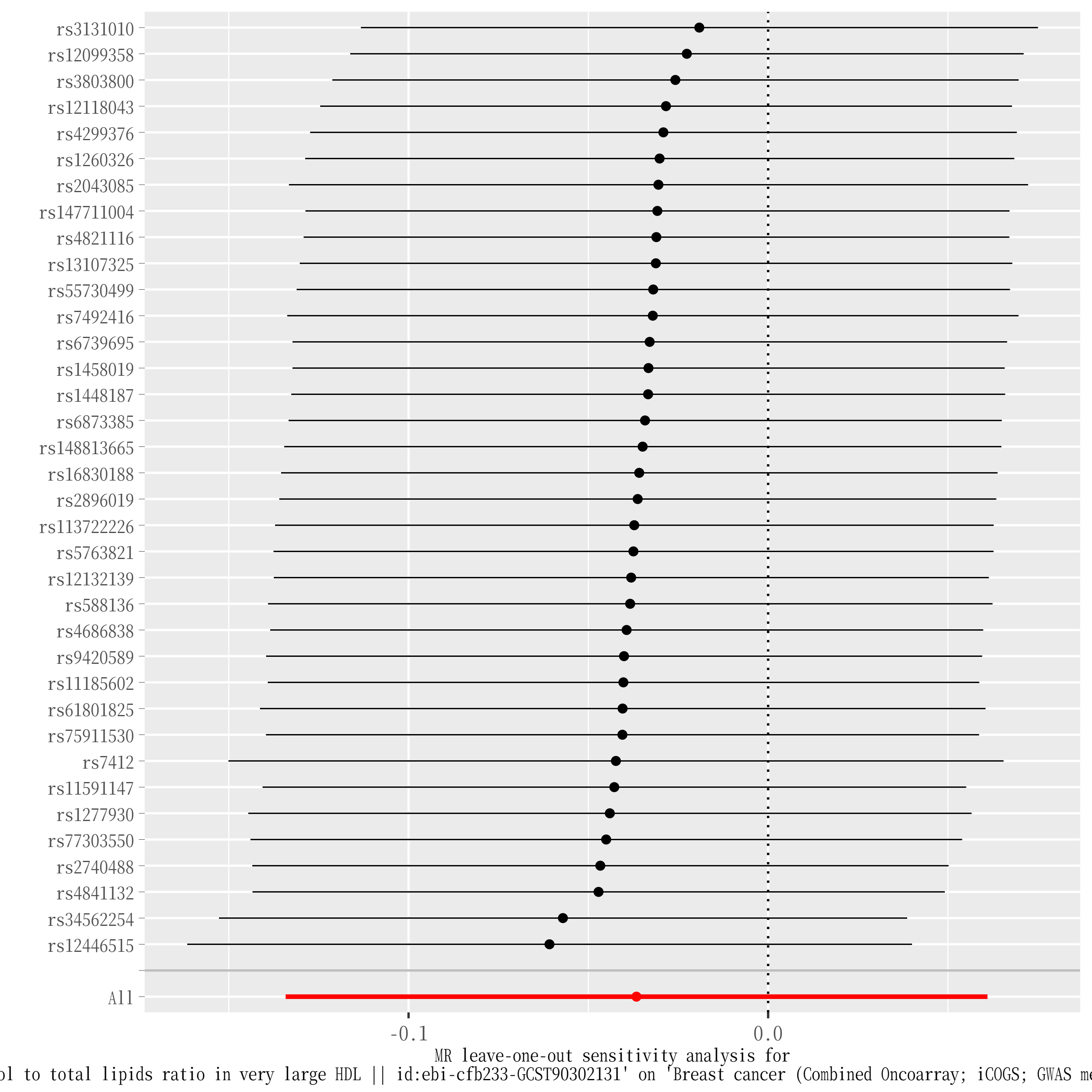

rs3131010
rs12099358
rs3803800
rs12118043
rs4299376
rs1260326
rs2043085
rs147711004
rs4821116
rs13107325
rs55730499
rs7492416
rs6739695
rs1458019
rs1448187
rs6873385
rs148813665
rs16830188
rs2896019
rs113722226
rs5763821
rs12132139
rs588136
rs4686838
rs9420589
rs11185602
rs61801825
rs75911530
rs7412
rs11591147
rs1277930
rs77303550
rs2740488
rs4841132
rs34562254
rs12446515
All
-0.1
0.0
MR leave-one-out sensitivity analysis for
ol to total lipids ratio in very large HDL || id:ebi-cfb233-GCST90302131' on 'Breast cancer (Combined Oncoarray; iCOGS; GWAS me

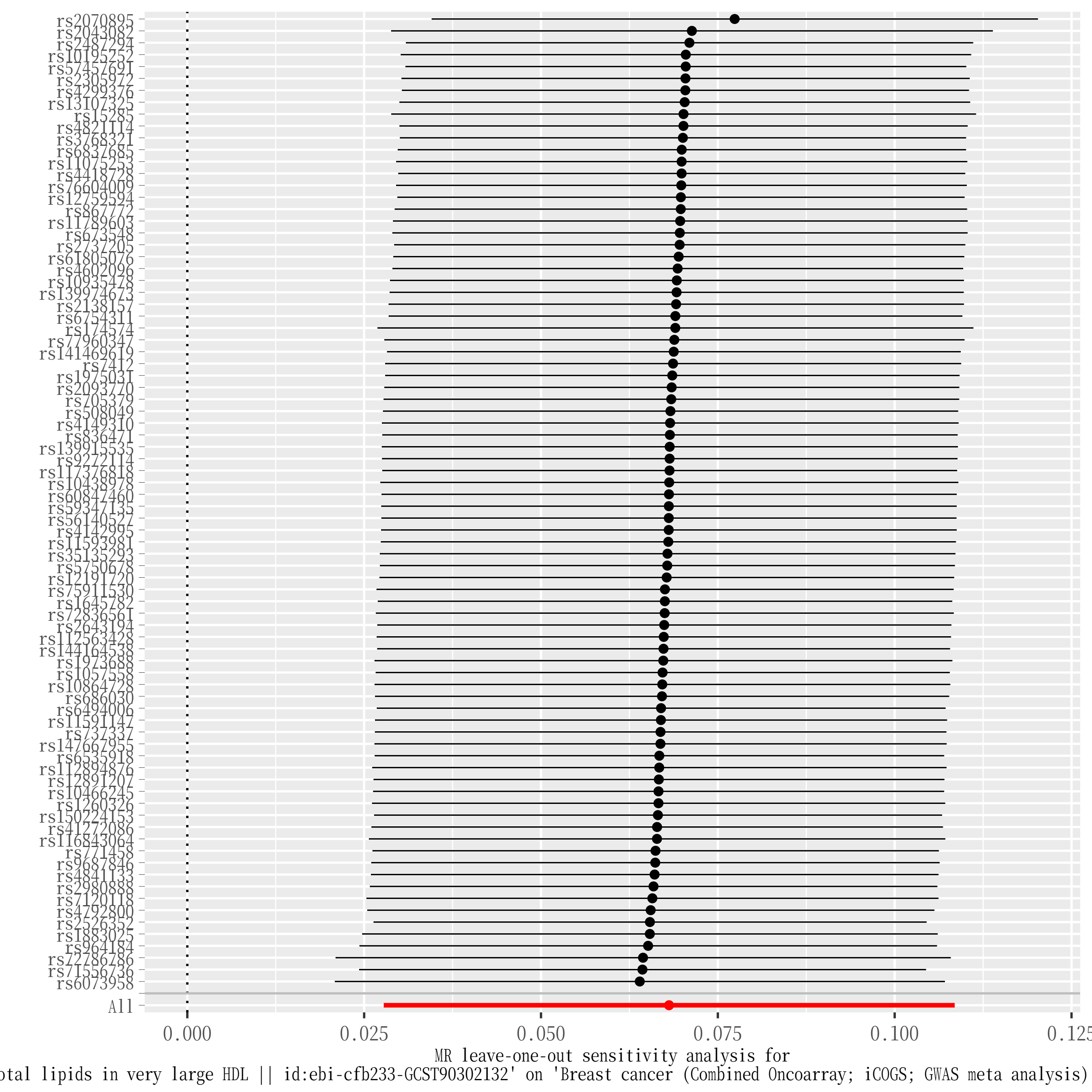
rs2070895
rs2043082
rs2487294
rs10195252
rs57457691
rs2305972
rs4299376
rs13107325
rs15285
rs4821114
rs3768321
rs6837685
rs11075253
rs4418728
rs76604009
rs12759524
rs867772
rs11789603
rs673548
rs2737205
rs61805076
rs4602096
rs10935478
rs139974673
rs2138157
rs6754311
rs174574
rs77960347
rs141469619
rs7412
rs1975031
rs2093770
rs705379
rs508049
rs4149310
rs836471
rs139915535
rs9272114
rs117376818
rs10438978
rs60847460
rs59347135
rs56140527
rs4142995
rs11593981
rs35135293
rs5750678
rs12191720
rs75911530
rs1645782
rs72836561
rs2643194
rs112563428
rs144164538
rs1973688
rs1057558
rs10864728
rs686030
rs6494006
rs11591147
rs737337
rs147667955
rs6535918
rs112894876
rs12891207
rs10466245
rs1260326
rs150224153
rs41272086
rs116843064
rs771458
rs9687846
rs4841133
rs2980888
rs7120118
rs4792800
rs2526352
rs1883025
rs964184
rs72786786
rs71556736
rs6073958
All
0.000
0.025
0.050
0.075
0.100
0.12
MR leave-one-out sensitivity analysis for
otal lipids in very large HDL || id:ebi-cfb233-GCST90302132' on 'Breast cancer (Combined Oncoarray; iCOGS; GWAS meta analysis)

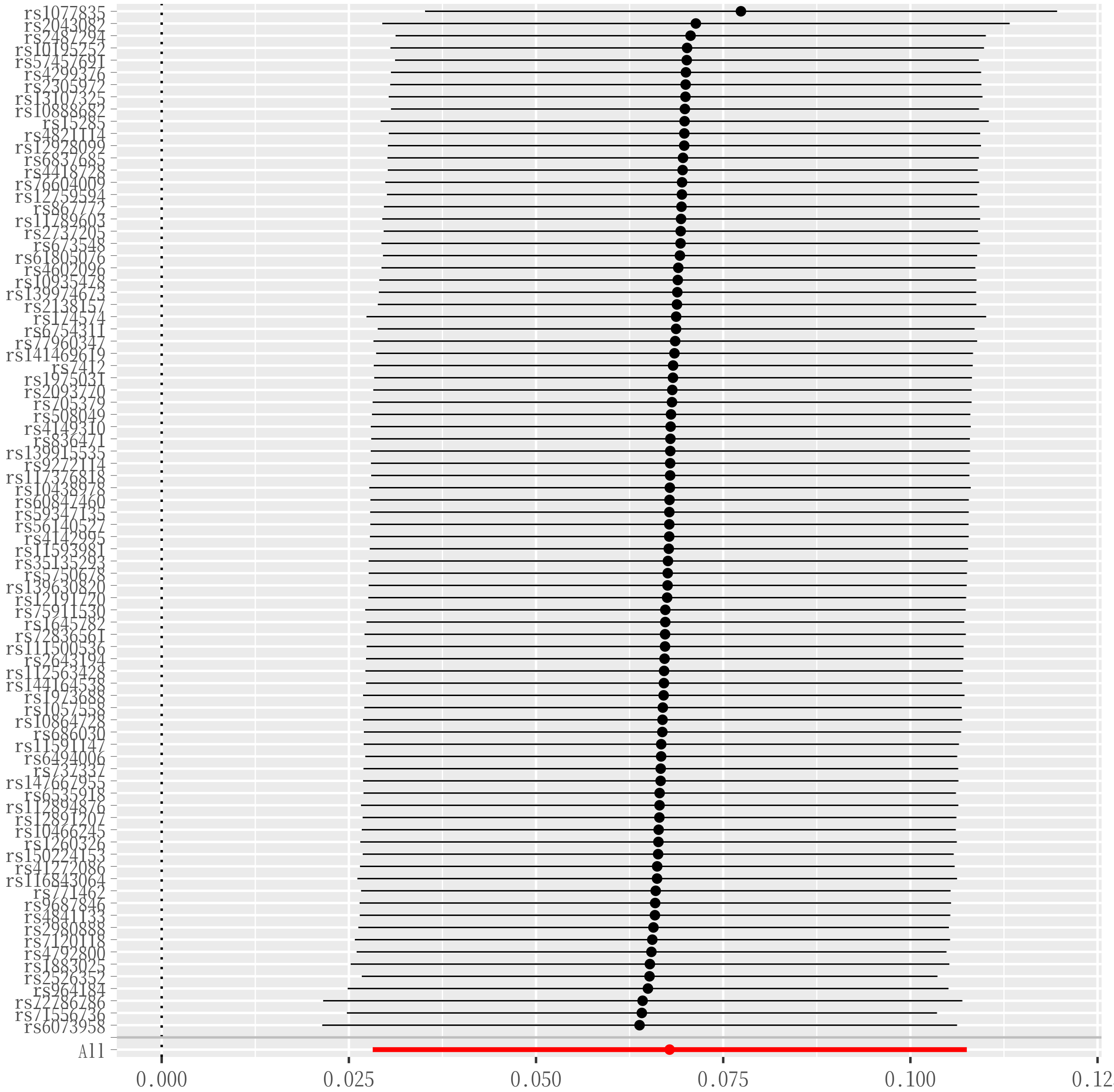

MR leave-one-out sensitivity analysis for
ration of very large HDL particles || id:ebi-cfb233-GCST90302133' on 'Breast cancer (Combined Oncoarray; iCOGS; GWAS meta anal

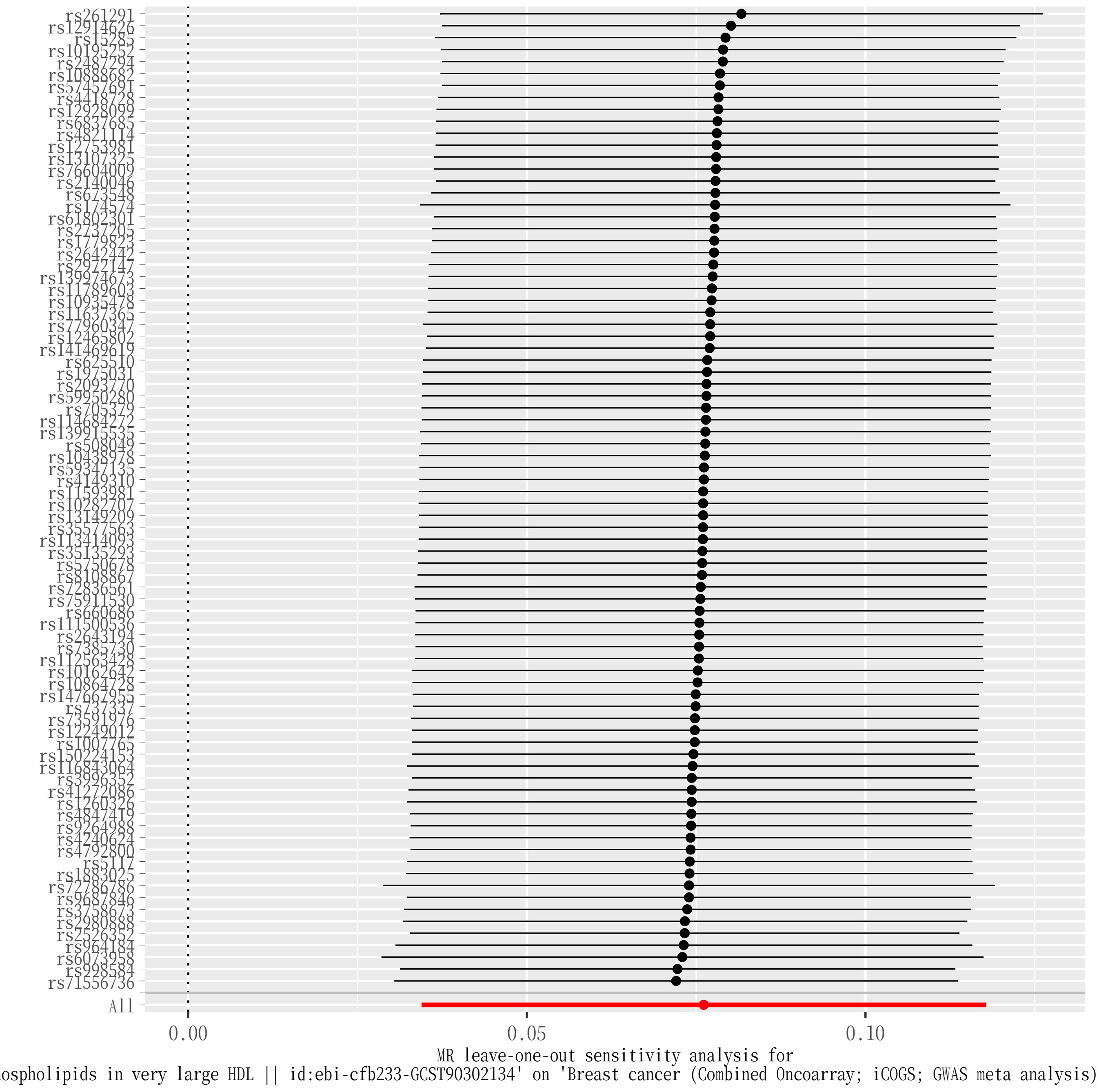

rs261291
rs12914626
rs15285
rs10195252
rs2487294
rs10888682
rs57457691
rs4418728
rs12928099
rs6837685
rs4821114
rs12753981
rs13107325
rs76604009
rs2140046
rs673548
rs174574
rs61802301
rs2737205
rs1779823
rs2642442
rs2972147
rs139974673
rs11789603
rs10935478
rs11637365
rs77960347
rs12465802
rs141469619
rs625510
rs1975031
rs2093770
rs59950280
rs705379
rs114684272
rs139915535
rs508049
rs10438978
rs59347135
rs4149310
rs11593981
rs10282707
rs13149209
rs35577563
rs113414093
rs35135293
rs5750678
rs8108867
rs72836561
rs75911530
rs660686
rs111500536
rs2643194
rs7385730
rs112563428
rs10162642
rs10864728
rs147667955
rs737337
rs73591976
rs12249012
rs1007765
rs150224153
rs116843064
rs3996352
rs41272086
rs1260326
rs4847419
rs9264988
rs4240624
rs4792800
rs5117
rs1883025
rs72786786
rs9687846
rs3758673
rs2980888
rs2526352
rs964184
rs6073958
rs998584
rs71556736
All
0.00
0.05
0.10
MR leave-one-out sensitivity analysis for
ospholipids in very large HDL || id:ebi-cfb233-GCST90302134' on 'Breast cancer (Combined Oncoarray; iCOGS; GWAS meta analysis)

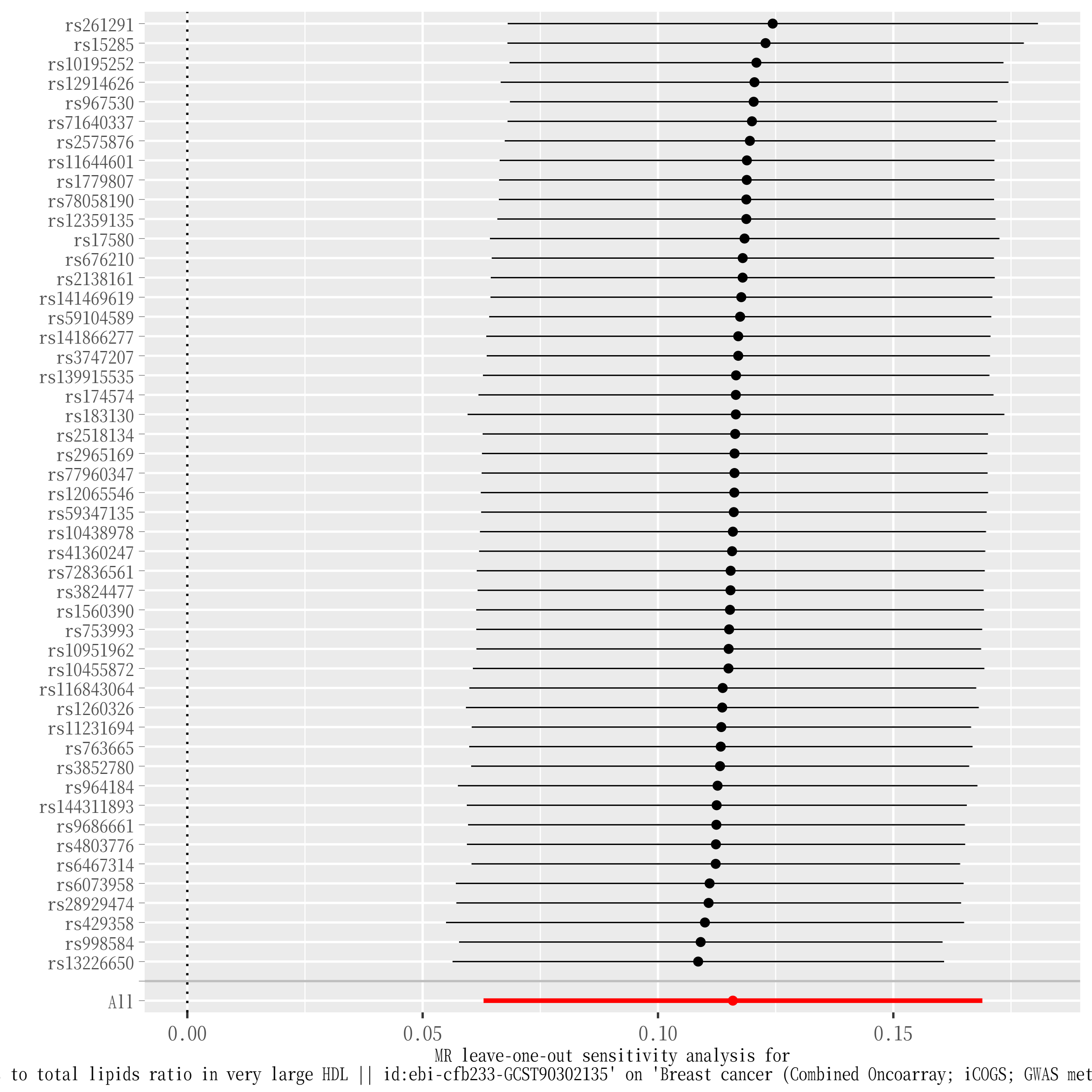
rs261291
rs15285
rs10195252
rs12914626
rs967530
rs71640337
rs2575876
rs11644601
rs1779807
rs78058190
rs12359135
rs17580
rs676210
rs2138161
rs141469619
rs59104589
rs141866277
rs3747207
rs139915535
rs174574
rs183130
rs2518134
rs2965169
rs77960347
rs12065546
rs59347135
rs10438978
rs41360247
rs72836561
rs3824477
rs1560390
rs753993
rs10951962
rs10455872
rs116843064
rs1260326
rs11231694
rs763665
rs3852780
rs964184
rs144311893
rs9686661
rs4803776
rs6467314
rs6073958
rs28929474
rs429358
rs998584
rs13226650
All
0.00
0.05
0.10
0.15
MR leave-one-out sensitivity analysis for
to total lipids ratio in very large HDL || id:ebi-cfb233-GCST90302135' on 'Breast cancer (Combined Oncoarray; iCOGS; GWAS met

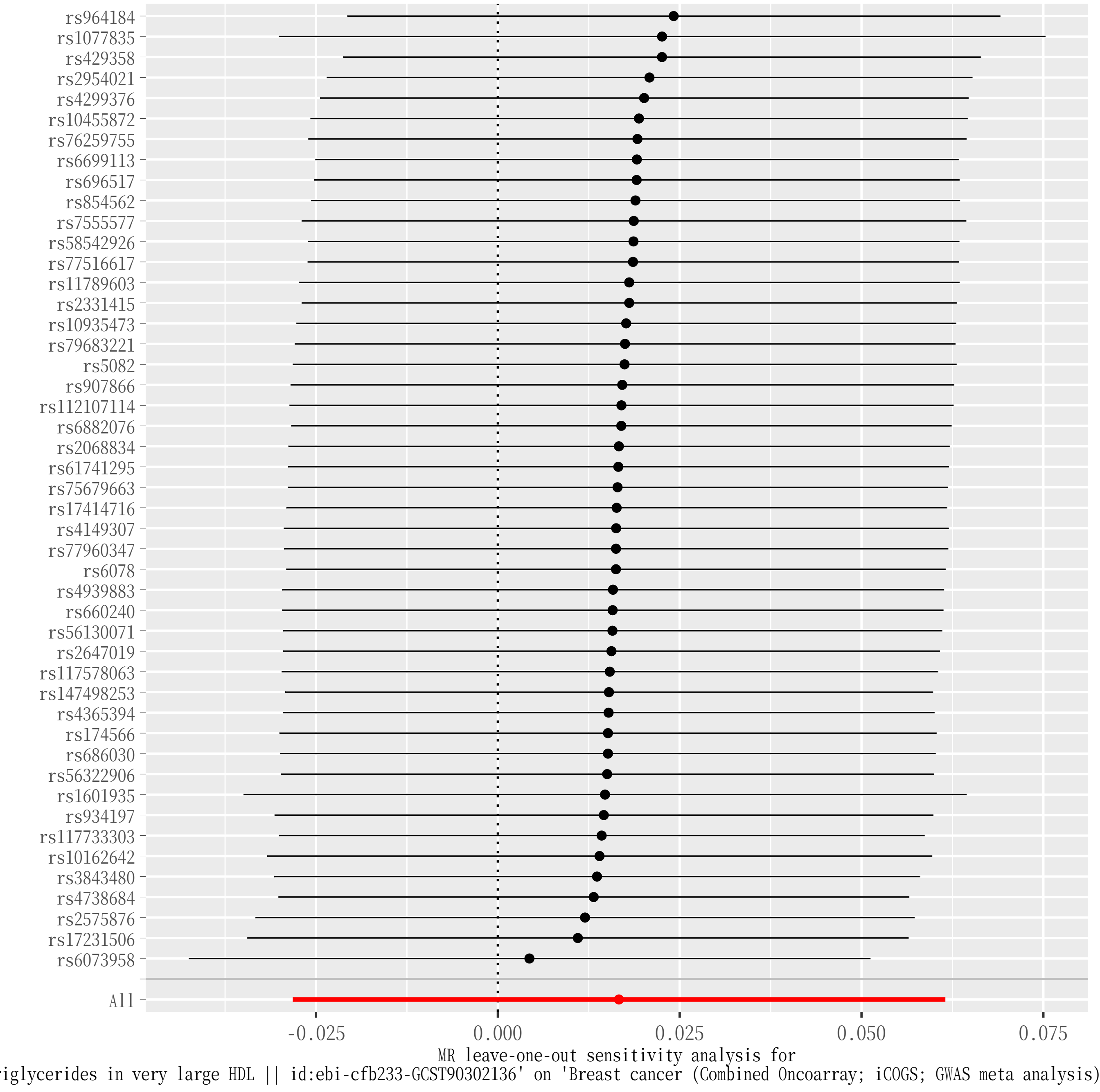
rs964184
rs1077835
rs429358
rs2954021
rs4299376
rs10455872
rs76259755
rs6699113
rs696517
rs854562
rs7555577
rs58542926
rs77516617
rs11789603
rs2331415
rs10935473
rs79683221
rs5082
rs907866
rs112107114
rs6882076
rs2068834
rs61741295
rs75679663
rs17414716
rs4149307
rs77960347
rs6078
rs4939883
rs660240
rs56130071
rs2647019
rs117578063
rs147498253
rs4365394
rs174566
rs686030
rs56322906
rs1601935
rs934197
rs117733303
rs10162642
rs3843480
rs4738684
rs2575876
rs17231506
rs6073958
All
-0.025
0.000
0.025
0.050
0.075
MR leave-one-out sensitivity analysis for
riglycerides in very large HDL || id:ebi-cfb233-GCST90302136' on 'Breast cancer (Combined Oncoarray; iCOGS; GWAS meta analysis)

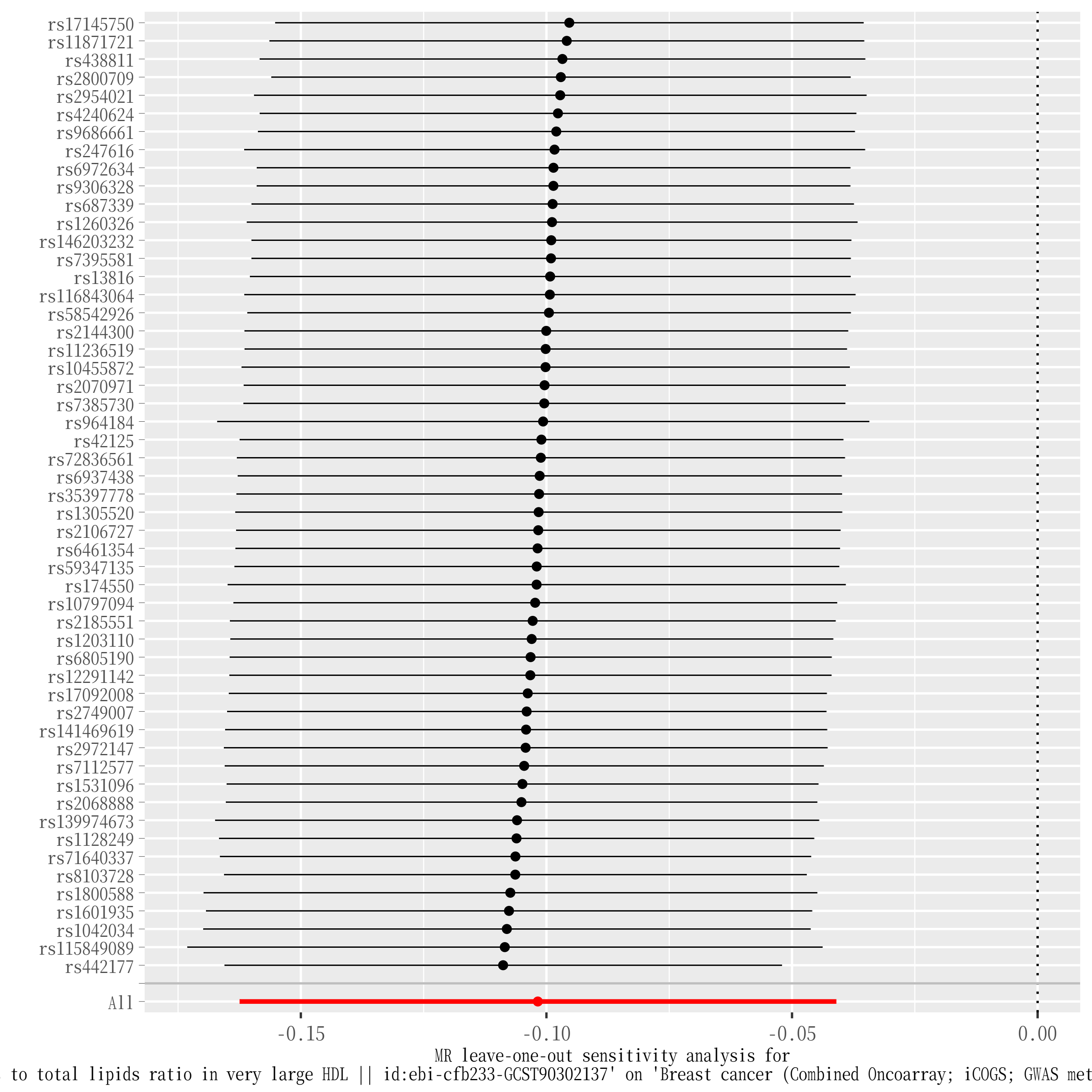
rs17145750
rs11871721
rs438811
rs2800709
rs2954021
rs4240624
rs9686661
rs247616
rs6972634
rs9306328
rs687339
rs1260326
rs146203232
rs7395581
rs13816
rs116843064
rs58542926
rs2144300
rs11236519
rs10455872
rs2070971
rs7385730
rs964184
rs42125
rs72836561
rs6937438
rs35397778
rs1305520
rs2106727
rs6461354
rs59347135
rs174550
rs10797094
rs2185551
rs1203110
rs6805190
rs12291142
rs17092008
rs2749007
rs141469619
rs2972147
rs7112577
rs1531096
rs2068888
rs139974673
rs1128249
rs71640337
rs8103728
rs1800588
rs1601935
rs1042034
rs115849089
rs442177
All
-0.15
-0.10
-0.05
0.00
MR leave-one-out sensitivity analysis for
to total lipids ratio in very large HDL || id:ebi-cfb233-GCST90302137' on 'Breast cancer (Combined Oncoarray; iCOGS; GWAS met

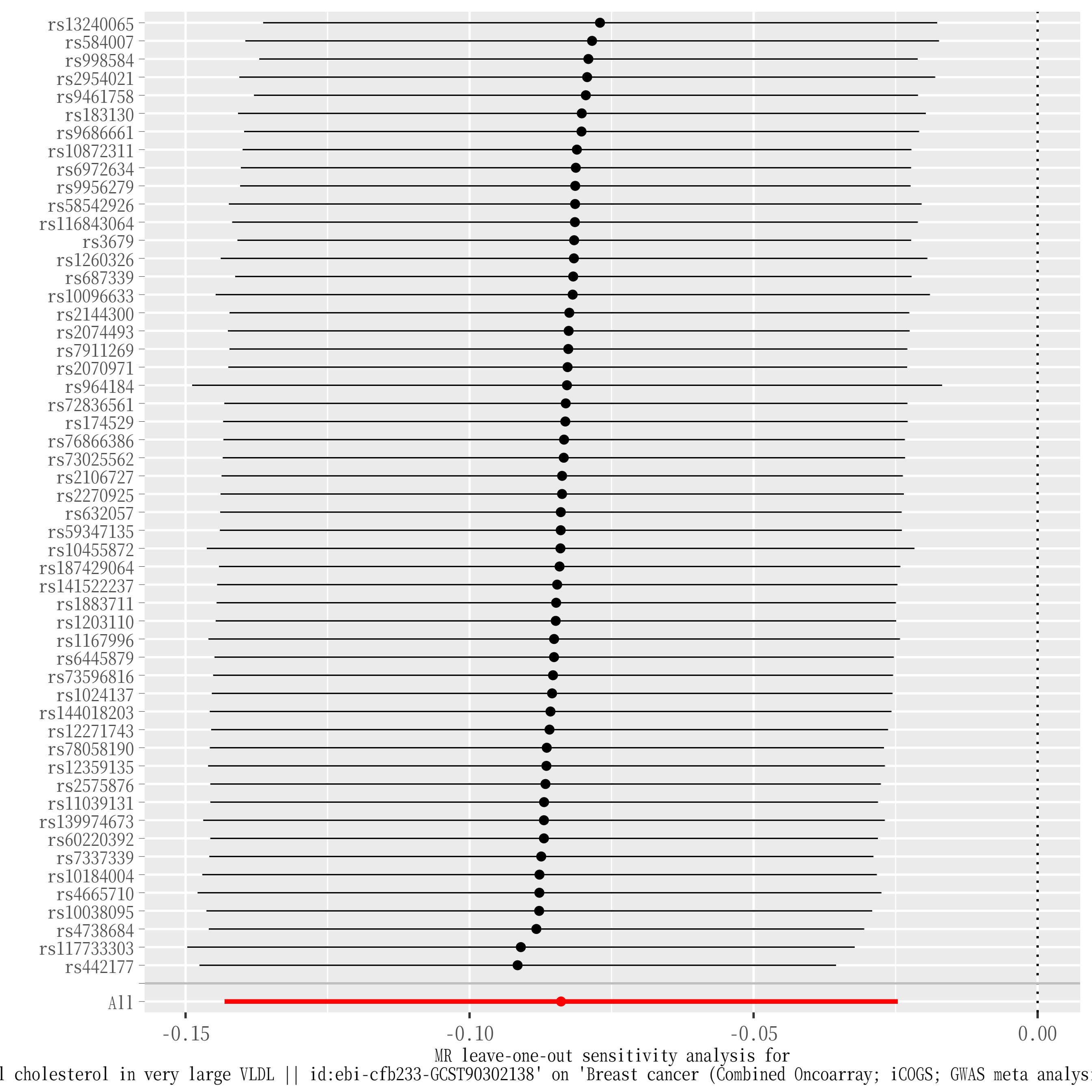

rs13240065
rs584007
rs998584
rs2954021
rs9461758
rs183130
rs9686661
rs10872311
rs6972634
rs9956279
rs58542926
rs116843064
rs3679
rs1260326
rs687339
rs10096633
rs2144300
rs2074493
rs7911269
rs2070971
rs964184
rs72836561
rs174529
rs76866386
rs73025562
rs2106727
rs2270925
rs632057
rs59347135
rs10455872
rs187429064
rs141522237
rs1883711
rs1203110
rs1167996
rs6445879
rs73596816
rs1024137
rs144018203
rs12271743
rs78058190
rs12359135
rs2575876
rs11039131
rs139974673
rs60220392
rs7337339
rs10184004
rs4665710
rs10038095
rs4738684
rs117733303
rs442177
All
-0.15
-0.10
-0.05
0.00
MR leave-one-out sensitivity analysis for
l cholesterol in very large VLDL || id:ebi-cfb233-GCST90302138' on 'Breast cancer (Combined Oncoarray; iCOGS; GWAS meta analys

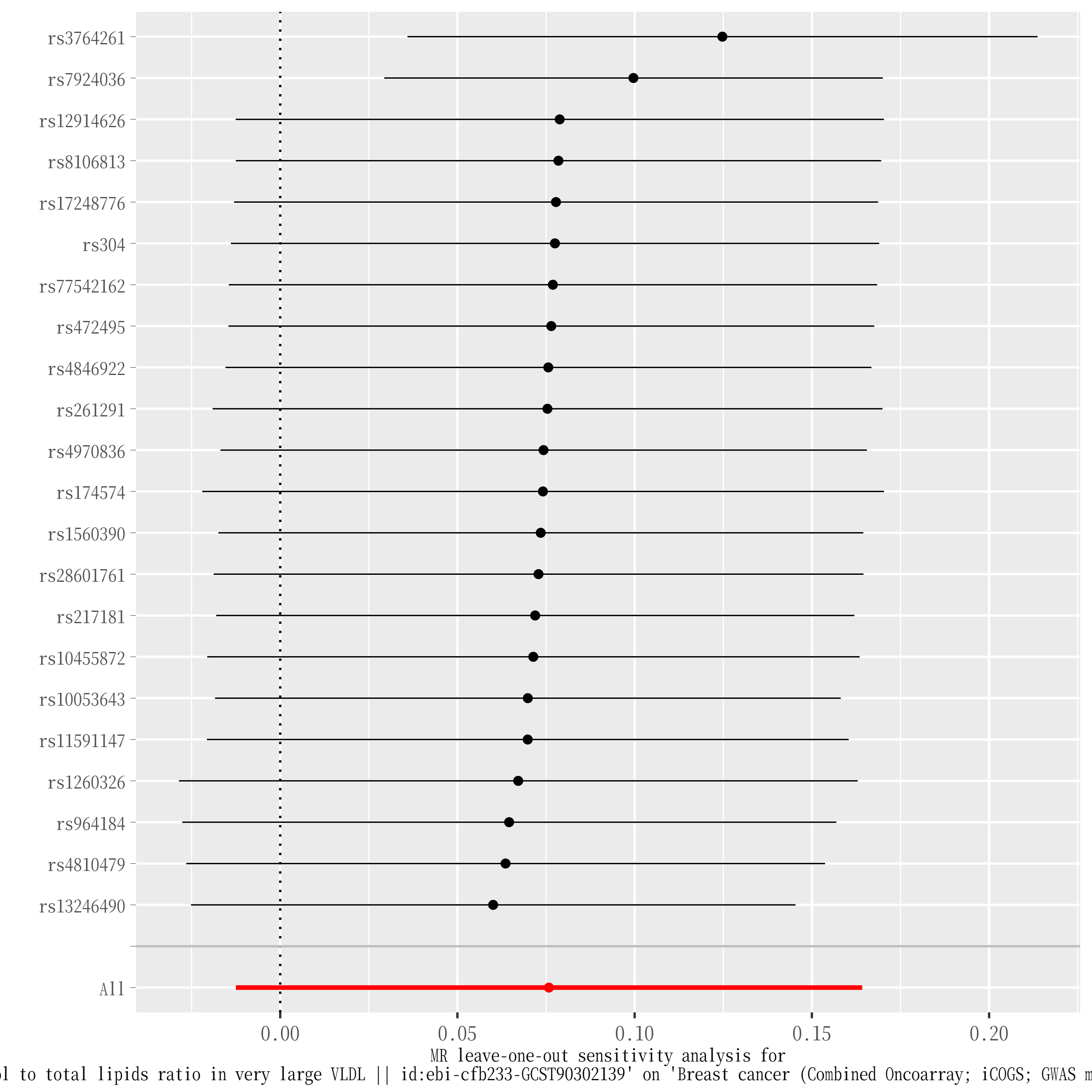
rs3764261
rs7924036
rs12914626
rs8106813
rs17248776
rs304
rs77542162
rs472495
rs4846922
rs261291
rs4970836
rs174574
rs1560390
rs28601761
rs217181
rs10455872
rs10053643
rs11591147
rs1260326
rs964184
rs4810479
rs13246490
All
0.00
0.05
0.10
0.15
0.20
MR leave-one-out sensitivity analysis for
l to total lipids ratio in very large VLDL || id:ebi-cfb233-GCST90302139' on 'Breast cancer (Combined Oncoarray; iCOGS; GWAS m

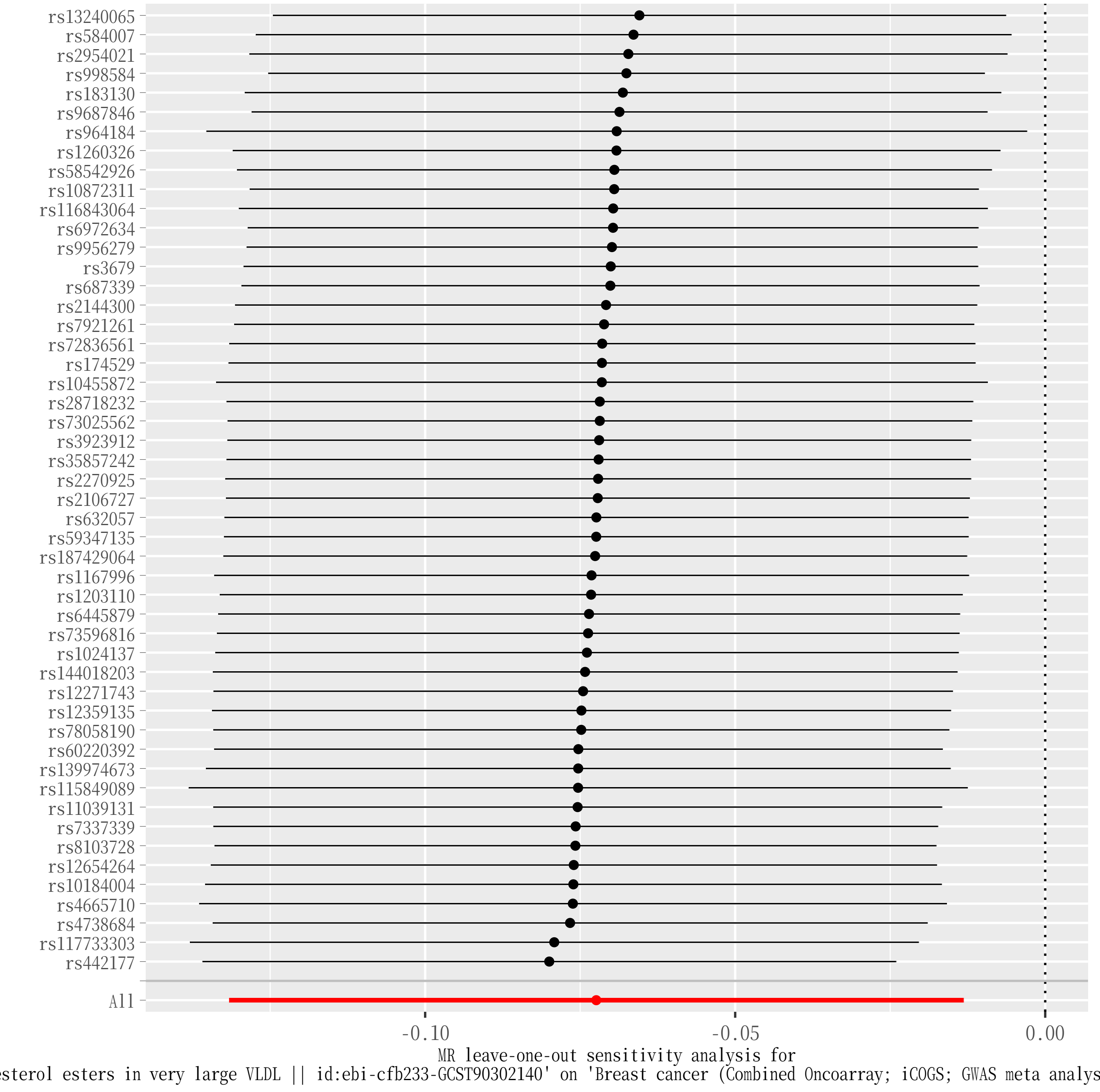

rs13240065
rs584007
rs2954021
rs998584
rs183130
rs9687846
rs964184
rs1260326
rs58542926
rs10872311
rs116843064
rs6972634
rs9956279
rs3679
rs687339
rs2144300
rs7921261
rs72836561
rs174529
rs10455872
rs28718232
rs73025562
rs3923912
rs35857242
rs2270925
rs2106727
rs632057
rs59347135
rs187429064
rs1167996
rs1203110
rs6445879
rs73596816
rs1024137
rs144018203
rs12271743
rs12359135
rs78058190
rs60220392
rs139974673
rs115849089
rs11039131
rs7337339
rs8103728
rs12654264
rs10184004
rs4665710
rs4738684
rs117733303
rs442177
All
-0.10
-0.05
0.00
MR leave-one-out sensitivity analysis for
esterol esters in very large VLDL || id:ebi-cfb233-GCST90302140' on 'Breast cancer (Combined Oncoarray; iCOGS; GWAS meta analys

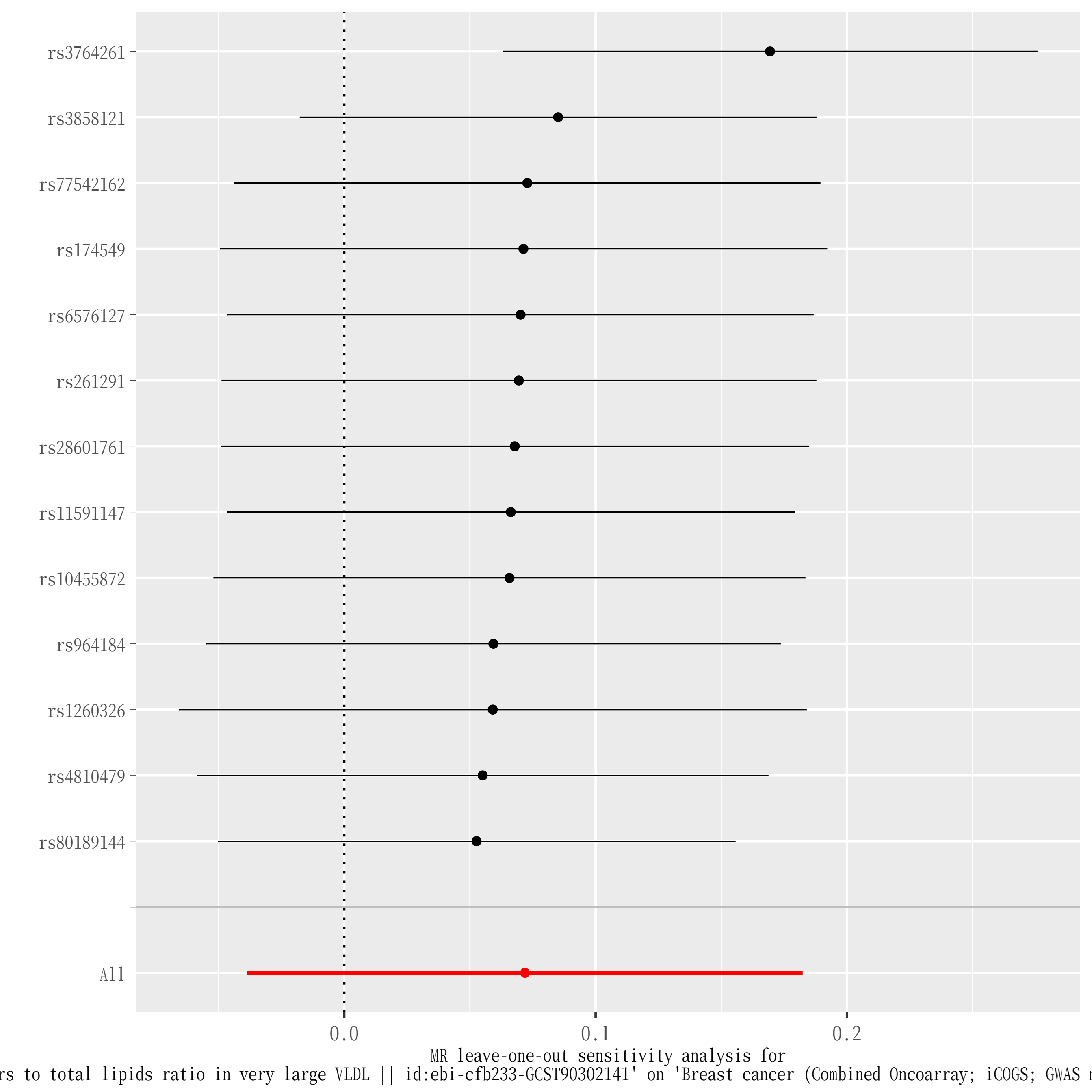
rs3764261
rs3858121
rs77542162
rs174549
rs6576127
rs261291
rs28601761
rs11591147
rs10455872
rs964184
rs1260326
rs4810479
rs80189144
All
0.0
0.1
0.2
MR leave-one-out sensitivity analysis for
rs to total lipids ratio in very large VLDL || id:ebi-cfb233-GCST90302141' on 'Breast cancer (Combined Oncoarray; iCOGS; GWAS m

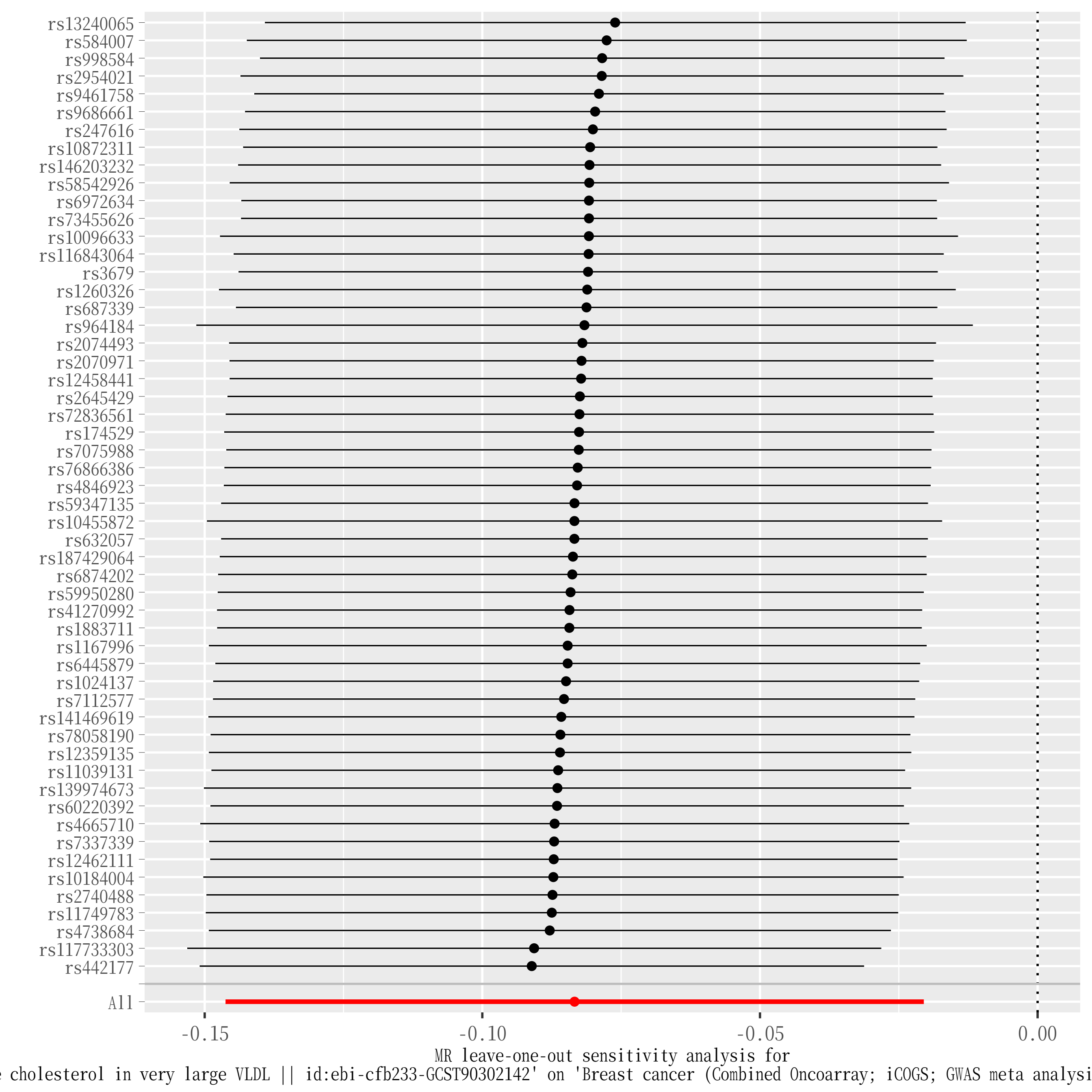
rs13240065
rs584007
rs998584
rs2954021
rs9461758
rs9686661
rs247616
rs10872311
rs146203232
rs58542926
rs6972634
rs73455626
rs10096633
rs116843064
rs3679
rs1260326
rs687339
rs964184
rs2074493
rs2070971
rs12458441
rs2645429
rs72836561
rs174529
rs7075988
rs76866386
rs4846923
rs59347135
rs10455872
rs632057
rs187429064
rs6874202
rs59950280
rs41270992
rs1883711
rs1167996
rs6445879
rs1024137
rs7112577
rs141469619
rs78058190
rs12359135
rs11039131
rs139974673
rs60220392
rs4665710
rs7337339
rs12462111
rs10184004
rs2740488
rs11749783
rs4738684
rs117733303
rs442177
All
-0.15
-0.10
-0.05
0.00
MR leave-one-out sensitivity analysis for
e cholesterol in very large VLDL || id:ebi-cfb233-GCST90302142' on 'Breast cancer (Combined Oncoarray; iCOGS; GWAS meta analysi

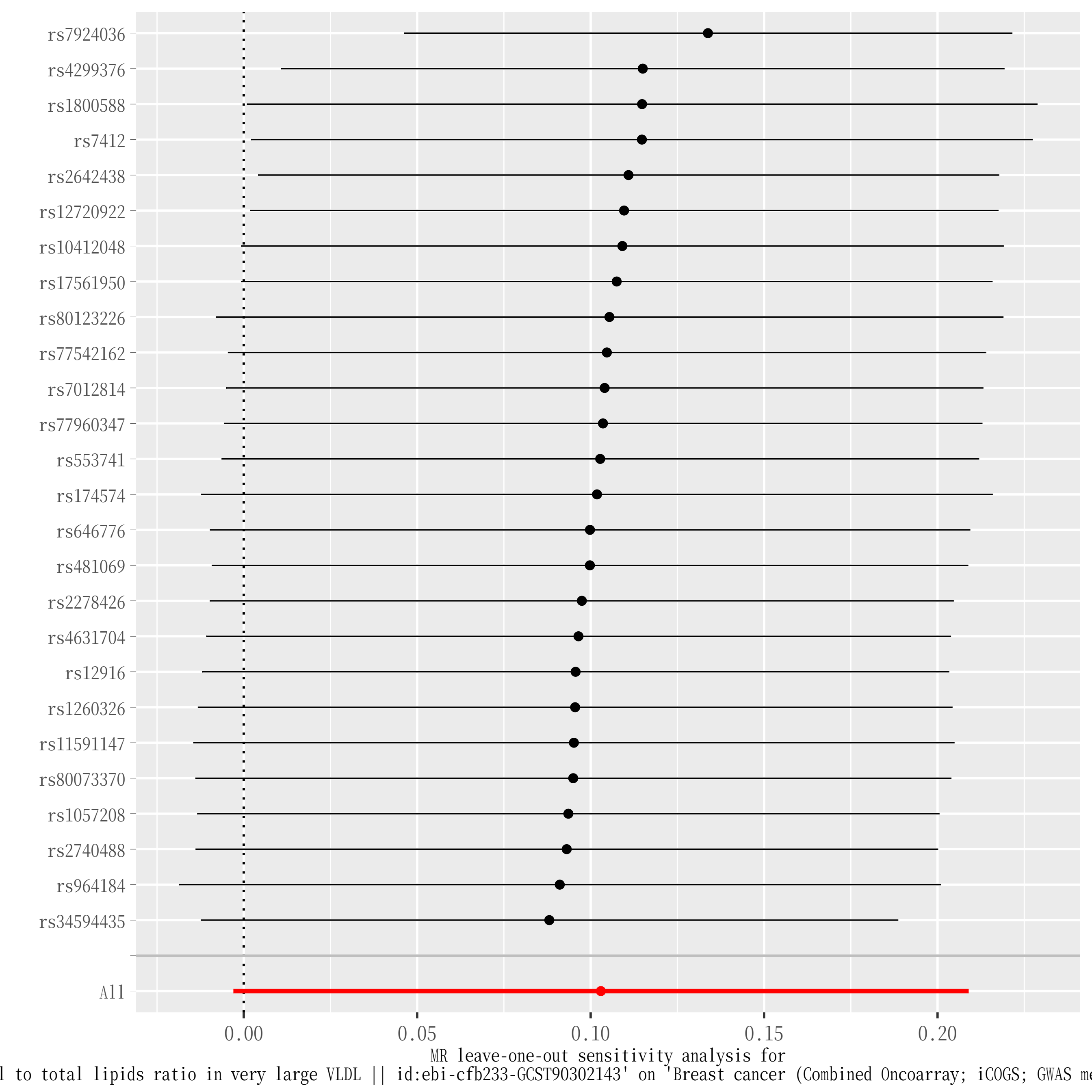
rs7924036
rs4299376
rs1800588
rs7412
rs2642438
rs12720922
rs10412048
rs17561950
rs80123226
rs77542162
rs7012814
rs77960347
rs553741
rs174574
rs646776
rs481069
rs2278426
rs4631704
rs12916
rs1260326
rs11591147
rs80073370
rs1057208
rs2740488
rs964184
rs34594435
All
0.00
0.05
0.10
0.15
0.20
MR leave-one-out sensitivity analysis for
l to total lipids ratio in very large VLDL || id:ebi-cfb233-GCST90302143' on 'Breast cancer (Combined Oncoarray; iCOGS; GWAS me

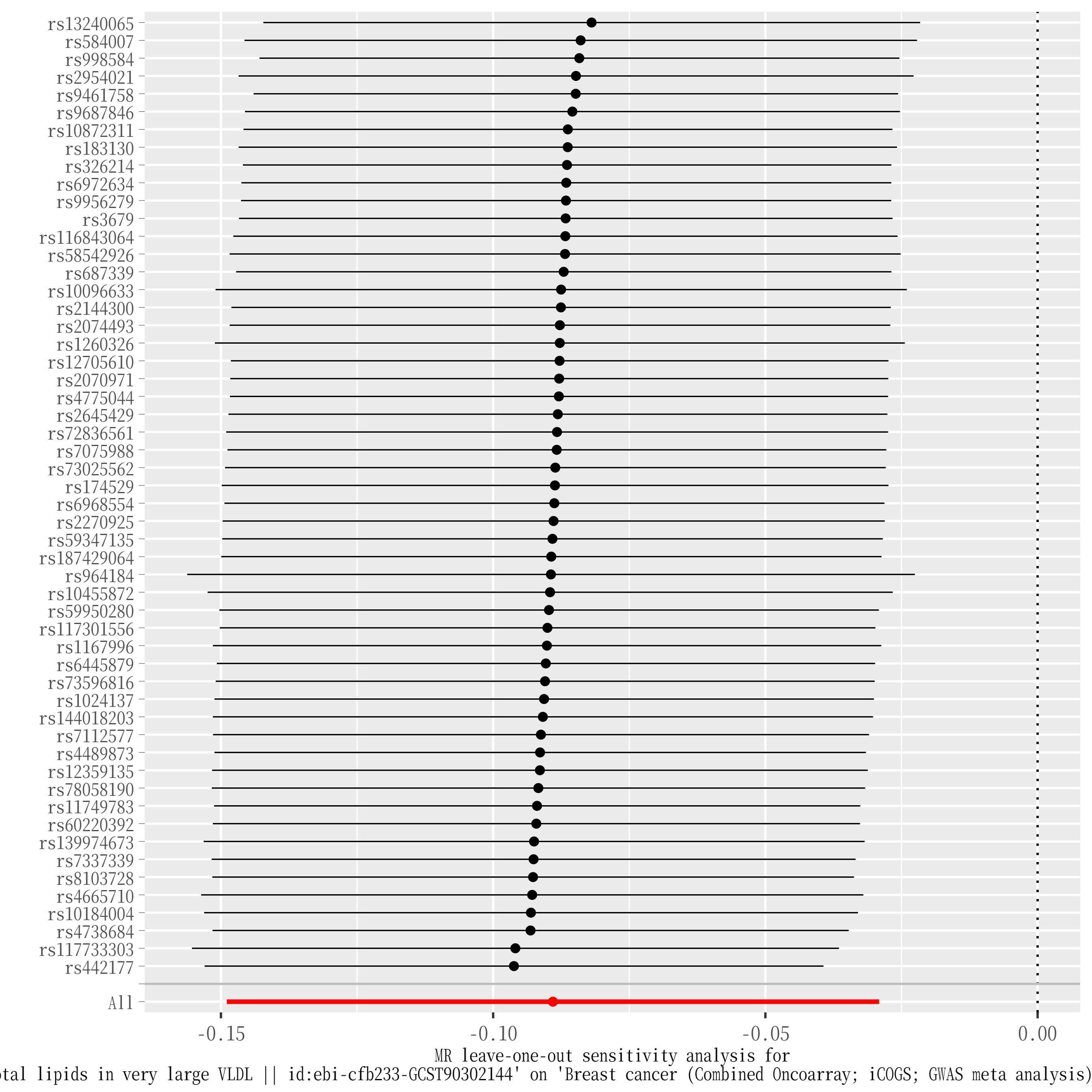

rs13240065
rs584007
rs998584
rs2954021
rs9461758
rs9687846
rs10872311
rs183130
rs326214
rs6972634
rs9956279
rs3679
rs116843064
rs58542926
rs687339
rs10096633
rs2144300
rs2074493
rs1260326
rs12705610
rs2070971
rs4775044
rs2645429
rs72836561
rs7075988
rs73025562
rs174529
rs6968554
rs2270925
rs59347135
rs187429064
rs964184
rs10455872
rs59950280
rs117301556
rs1167996
rs6445879
rs73596816
rs1024137
rs144018203
rs7112577
rs4489873
rs12359135
rs78058190
rs11749783
rs60220392
rs139974673
rs7337339
rs8103728
rs4665710
rs10184004
rs4738684
rs117733303
rs442177
All
-0.15
-0.10
-0.05
0.00
MR leave-one-out sensitivity analysis for
otal lipids in very large VLDL || id:ebi-cfb233-GCST90302144' on 'Breast cancer (Combined Oncoarray; iCOGS; GWAS meta analysis)

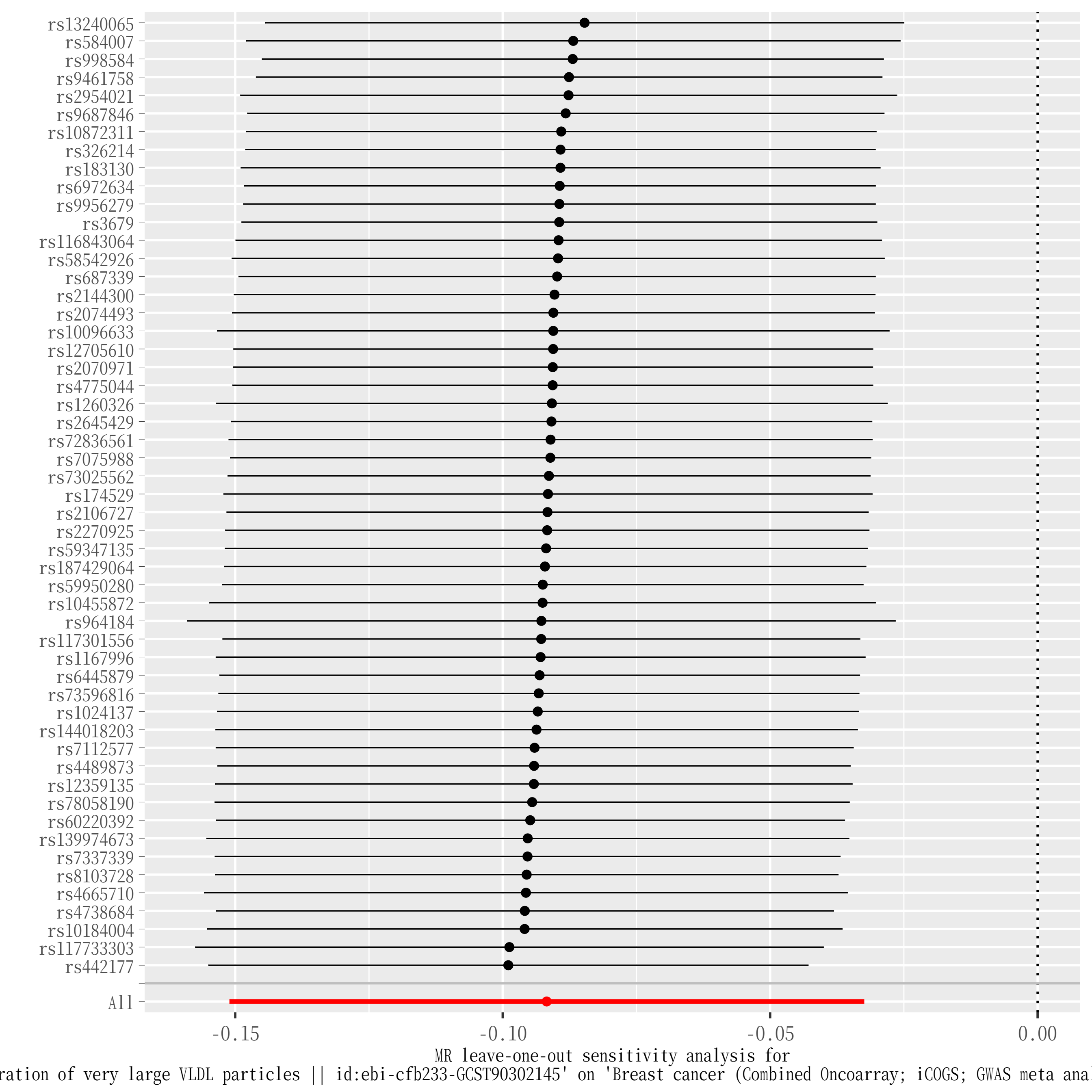
rs13240065
rs584007
rs998584
rs9461758
rs2954021
rs9687846
rs10872311
rs326214
rs183130
rs6972634
rs9956279
rs3679
rs116843064
rs58542926
rs687339
rs2144300
rs2074493
rs10096633
rs12705610
rs2070971
rs4775044
rs1260326
rs2645429
rs72836561
rs7075988
rs73025562
rs174529
rs2106727
rs2270925
rs59347135
rs187429064
rs59950280
rs10455872
rs964184
rs117301556
rs1167996
rs6445879
rs73596816
rs1024137
rs144018203
rs7112577
rs4489873
rs12359135
rs78058190
rs60220392
rs139974673
rs7337339
rs8103728
rs4665710
rs4738684
rs10184004
rs117733303
rs442177
All
-0.15
-0.10
-0.05
0.00
MR leave-one-out sensitivity analysis for
ration of very large VLDL particles || id:ebi-cfb233-GCST90302145' on 'Breast cancer (Combined Oncoarray; iCOGS; GWAS meta anal

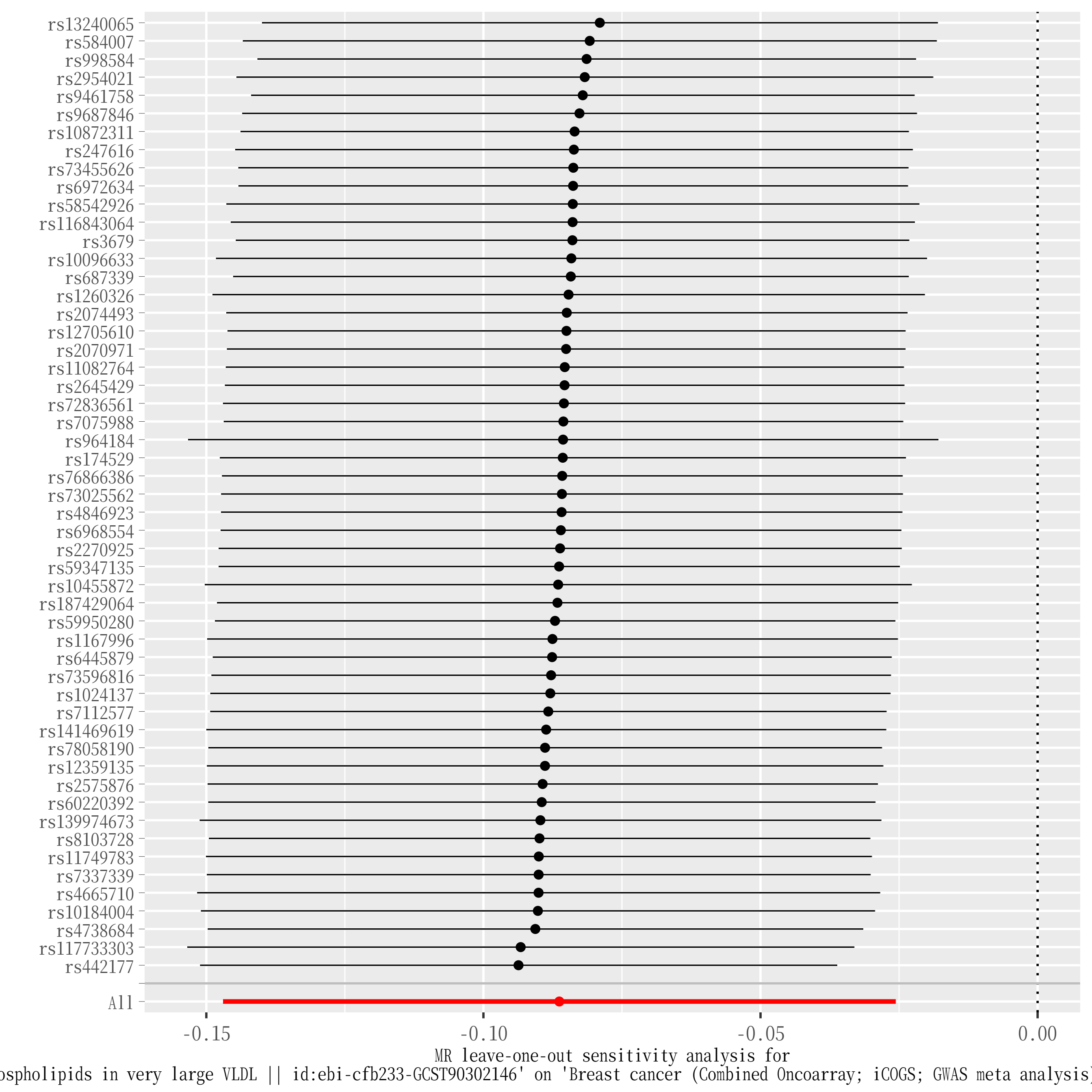

rs13240065
rs584007
rs998584
rs2954021
rs9461758
rs9687846
rs10872311
rs247616
rs73455626
rs6972634
rs58542926
rs116843064
rs3679
rs10096633
rs687339
rs1260326
rs2074493
rs12705610
rs2070971
rs11082764
rs2645429
rs72836561
rs7075988
rs964184
rs174529
rs76866386
rs73025562
rs4846923
rs6968554
rs2270925
rs59347135
rs10455872
rs187429064
rs59950280
rs1167996
rs6445879
rs73596816
rs1024137
rs7112577
rs141469619
rs78058190
rs12359135
rs2575876
rs60220392
rs139974673
rs8103728
rs11749783
rs7337339
rs4665710
rs10184004
rs4738684
rs117733303
rs442177
All
-0.15
-0.10
-0.05
0.00
MR leave-one-out sensitivity analysis for
ospholipids in very large VLDL || id:ebi-cfb233-GCST90302146' on 'Breast cancer (Combined Oncoarray; iCOGS; GWAS meta analysis)

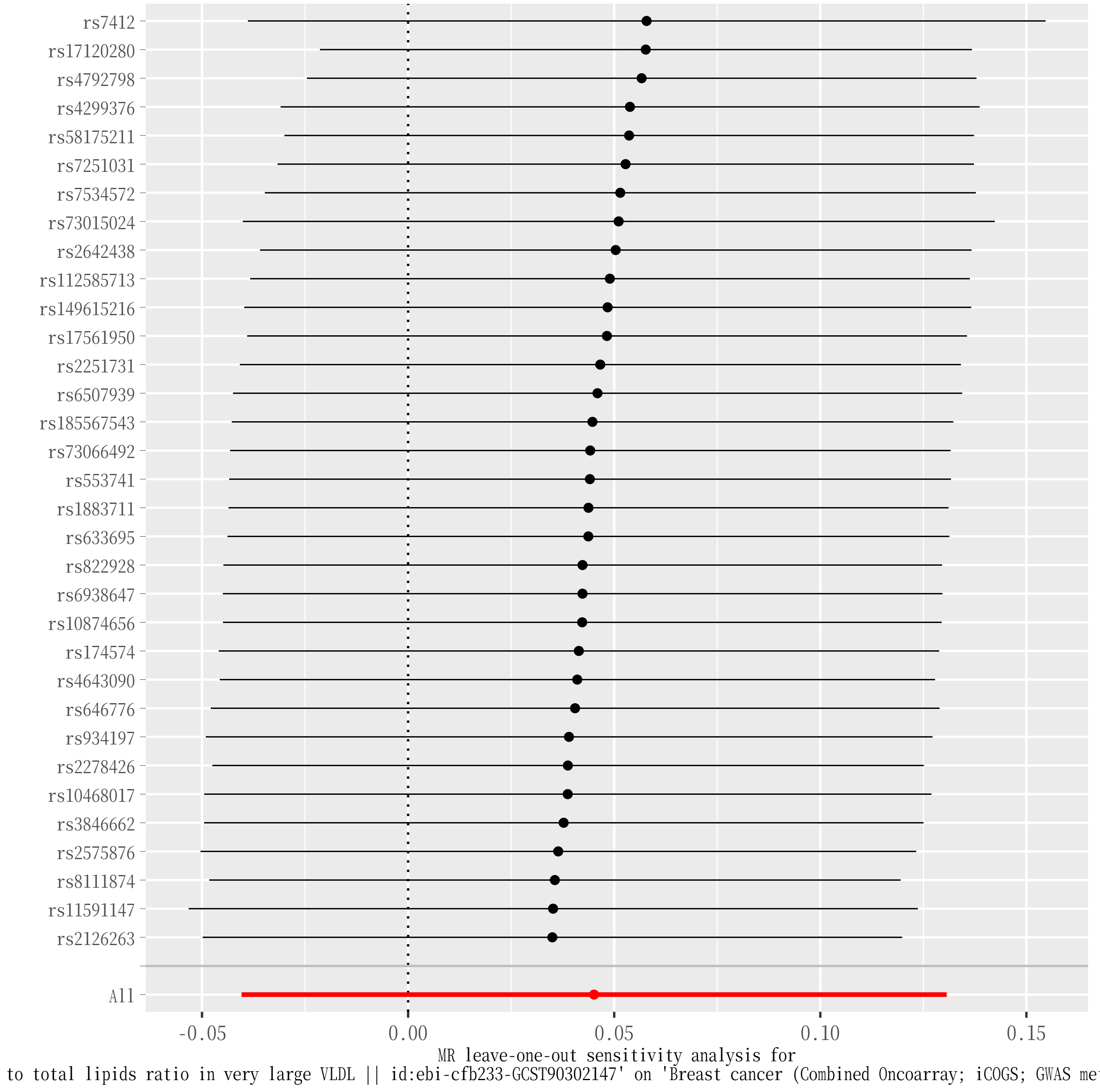

rs7412
rs17120280
rs4792798
rs4299376
rs58175211
rs7251031
rs7534572
rs73015024
rs2642438
rs112585713
rs149615216
rs17561950
rs2251731
rs6507939
rs185567543
rs73066492
rs553741
rs1883711
rs633695
rs822928
rs6938647
rs10874656
rs174574
rs4643090
rs646776
rs934197
rs2278426
rs10468017
rs3846662
rs2575876
rs8111874
rs11591147
rs2126263
All
-0.05
0.00
0.05
0.10
0.15
MR leave-one-out sensitivity analysis for
to total lipids ratio in very large VLDL || id:ebi-cfb233-GCST90302147' on 'Breast cancer (Combined Oncoarray; iCOGS; GWAS me

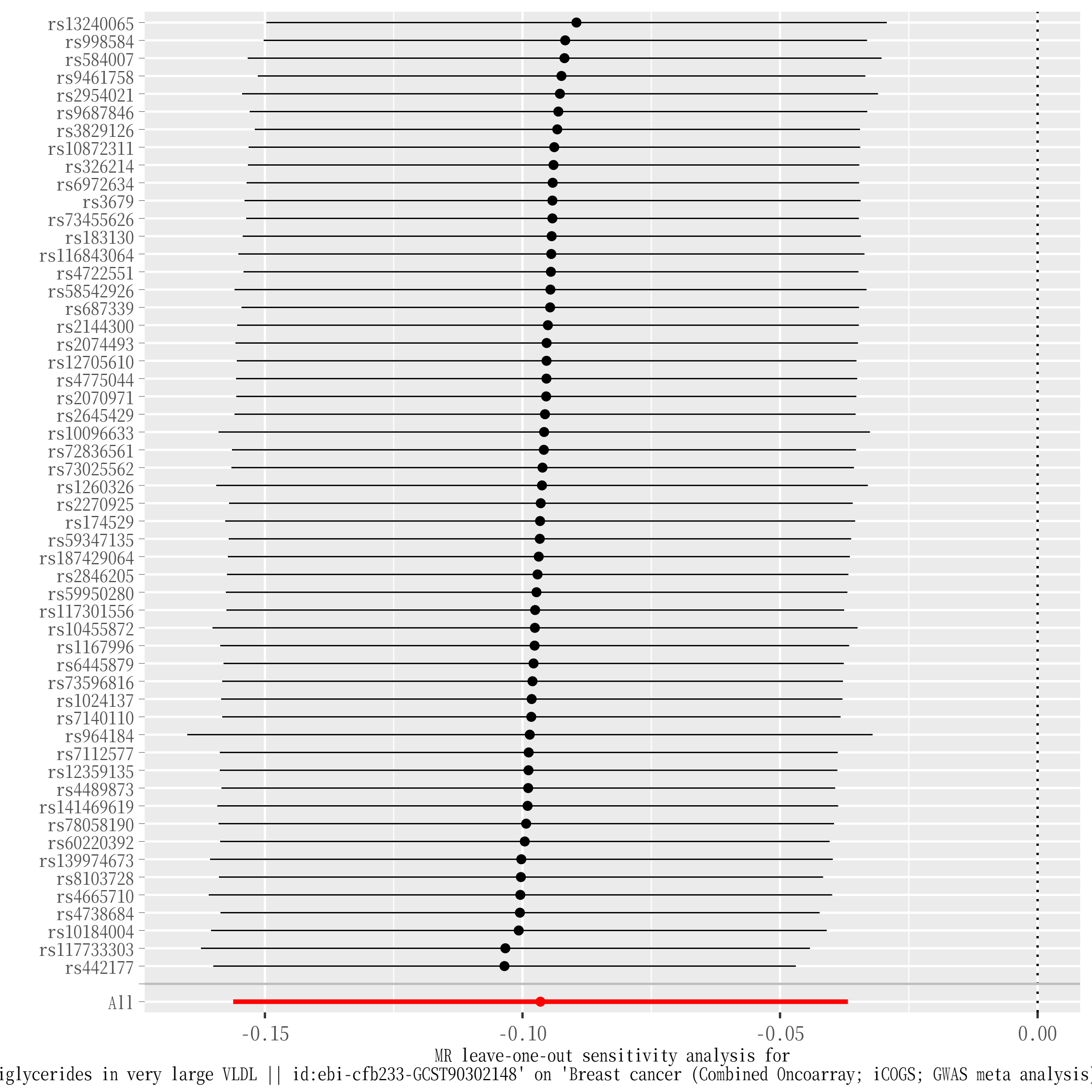
rs13240065
rs998584
rs584007
rs9461758
rs2954021
rs9687846
rs3829126
rs10872311
rs326214
rs6972634
rs3679
rs73455626
rs183130
rs116843064
rs4722551
rs58542926
rs687339
rs2144300
rs2074493
rs12705610
rs4775044
rs2070971
rs2645429
rs10096633
rs72836561
rs73025562
rs1260326
rs2270925
rs174529
rs59347135
rs187429064
rs2846205
rs59950280
rs117301556
rs10455872
rs1167996
rs6445879
rs73596816
rs1024137
rs7140110
rs964184
rs7112577
rs12359135
rs4489873
rs141469619
rs78058190
rs60220392
rs139974673
rs8103728
rs4665710
rs4738684
rs10184004
rs117733303
rs442177
All
-0.15
-0.10
-0.05
0.00
MR leave-one-out sensitivity analysis for
iglycerides in very large VLDL || id:ebi-cfb233-GCST90302148' on 'Breast cancer (Combined Oncoarray; iCOGS; GWAS meta analysis)

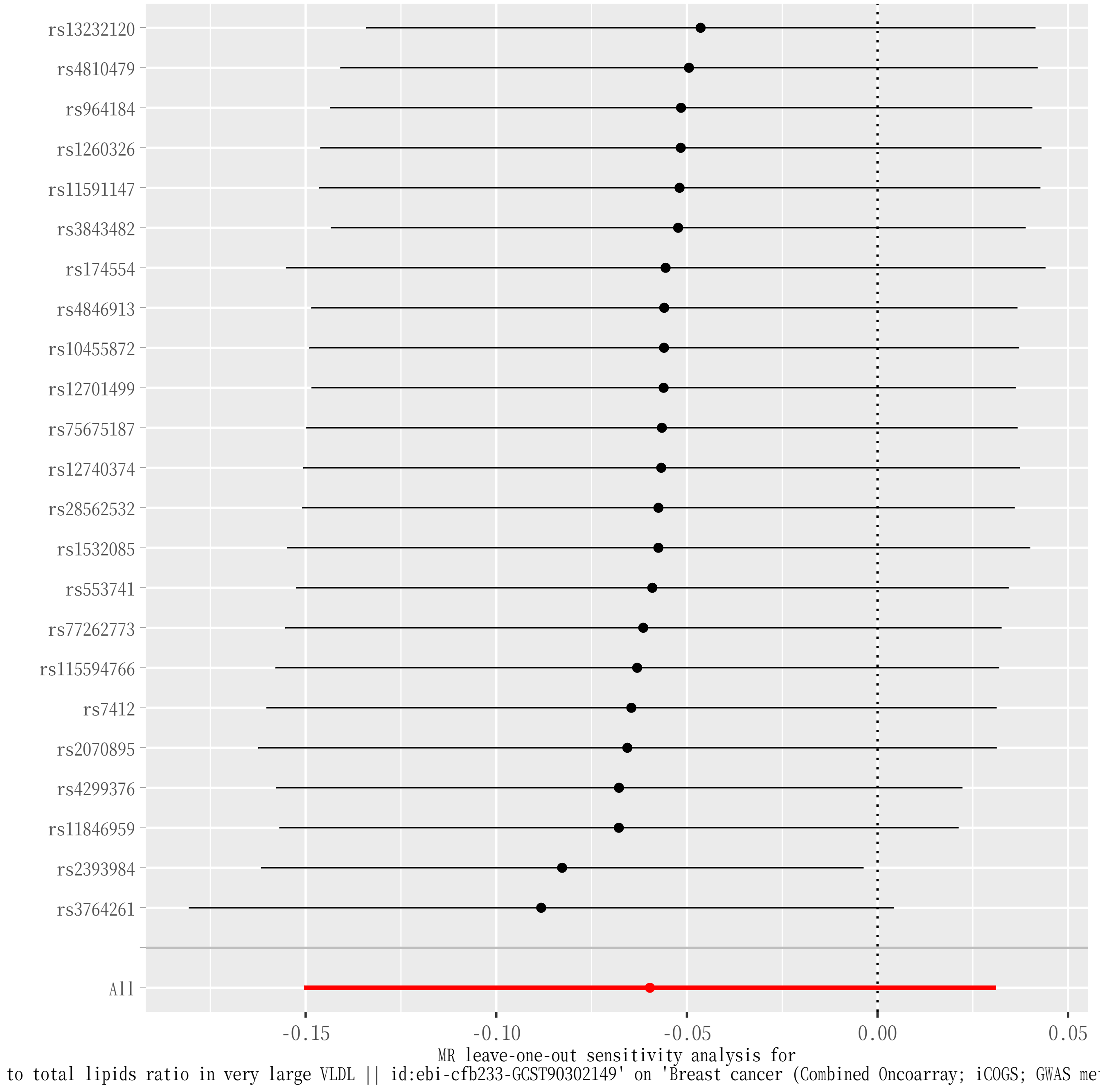

rs13232120
rs4810479
rs964184
rs1260326
rs11591147
rs3843482
rs174554
rs4846913
rs10455872
rs12701499
rs75675187
rs12740374
rs28562532
rs1532085
rs553741
rs77262773
rs115594766
rs7412
rs2070895
rs4299376
rs11846959
rs2393984
rs3764261
All
-0.15
-0.10
-0.05
0.00
0.05
MR leave-one-out sensitivity analysis for
to total lipids ratio in very large VLDL || id:ebi-cfb233-GCST90302149' on 'Breast cancer (Combined Oncoarray; iCOGS; GWAS me

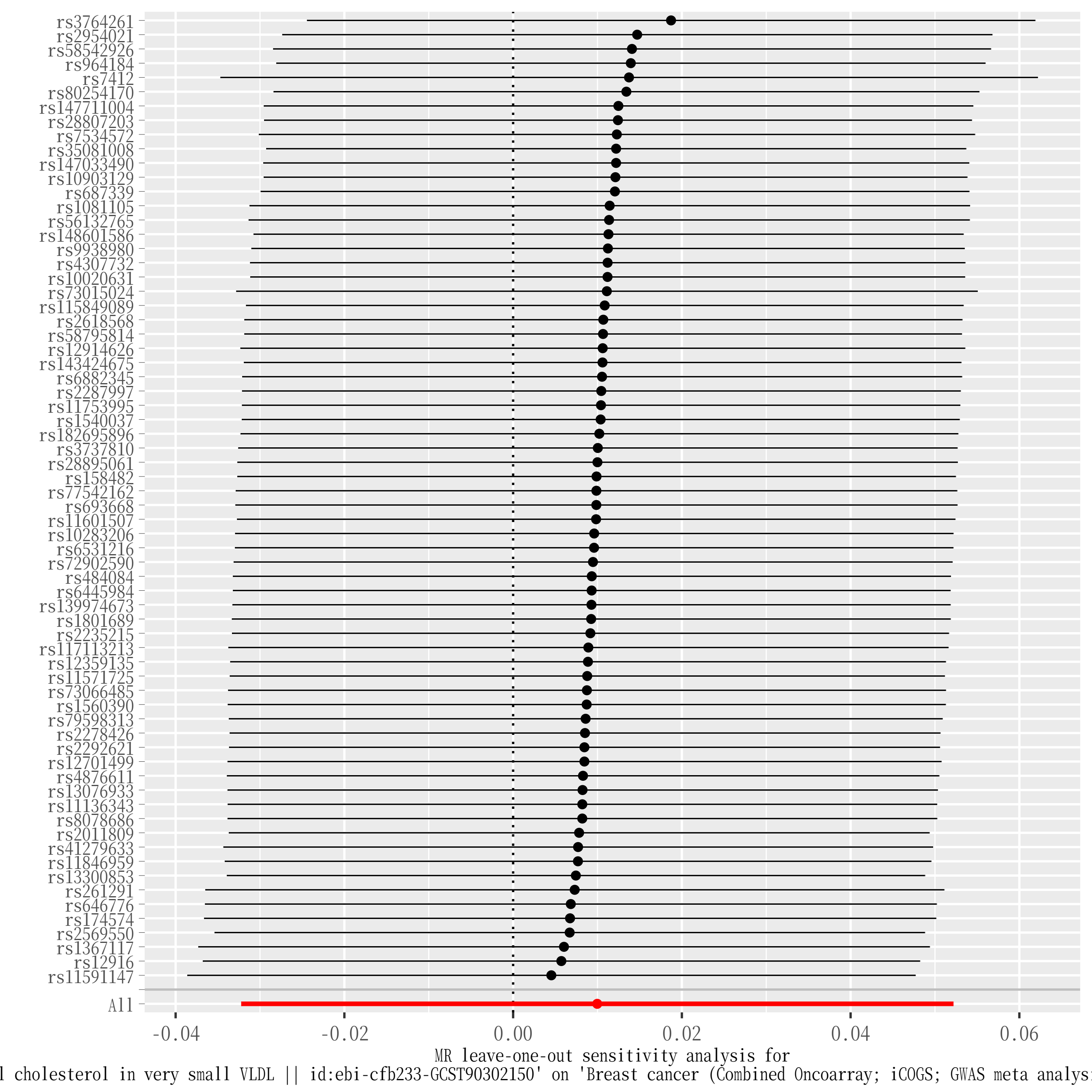
rs3764261
rs2954021
rs58542926
rs964184
rs7412
rs80254170
rs147711004
rs28807203
rs7534572
rs35081008
rs147033490
rs10903129
rs687339
rs1081105
rs56132765
rs148601586
rs9938980
rs4307732
rs10020631
rs73015024
rs115849089
rs2618568
rs58795814
rs12914626
rs143424675
rs6882345
rs2287997
rs11753995
rs1540037
rs182695896
rs3737810
rs28895061
rs158482
rs77542162
rs693668
rs11601507
rs10283206
rs6531216
rs72902590
rs484084
rs6445984
rs139974673
rs1801689
rs2235215
rs117113213
rs12359135
rs11571725
rs73066485
rs1560390
rs79598313
rs2278426
rs2292621
rs12701499
rs4876611
rs13076933
rs11136343
rs8078686
rs2011809
rs41279633
rs11846959
rs13300853
rs261291
rs646776
rs174574
rs2569550
rs1367117
rs12916
rs11591147
All
-0.04
-0.02
0.00
0.02
0.04
0.06
MR leave-one-out sensitivity analysis for
l cholesterol in very small VLDL || id:ebi-cfb233-GCST90302150' on 'Breast cancer (Combined Oncoarray; iCOGS; GWAS meta analys

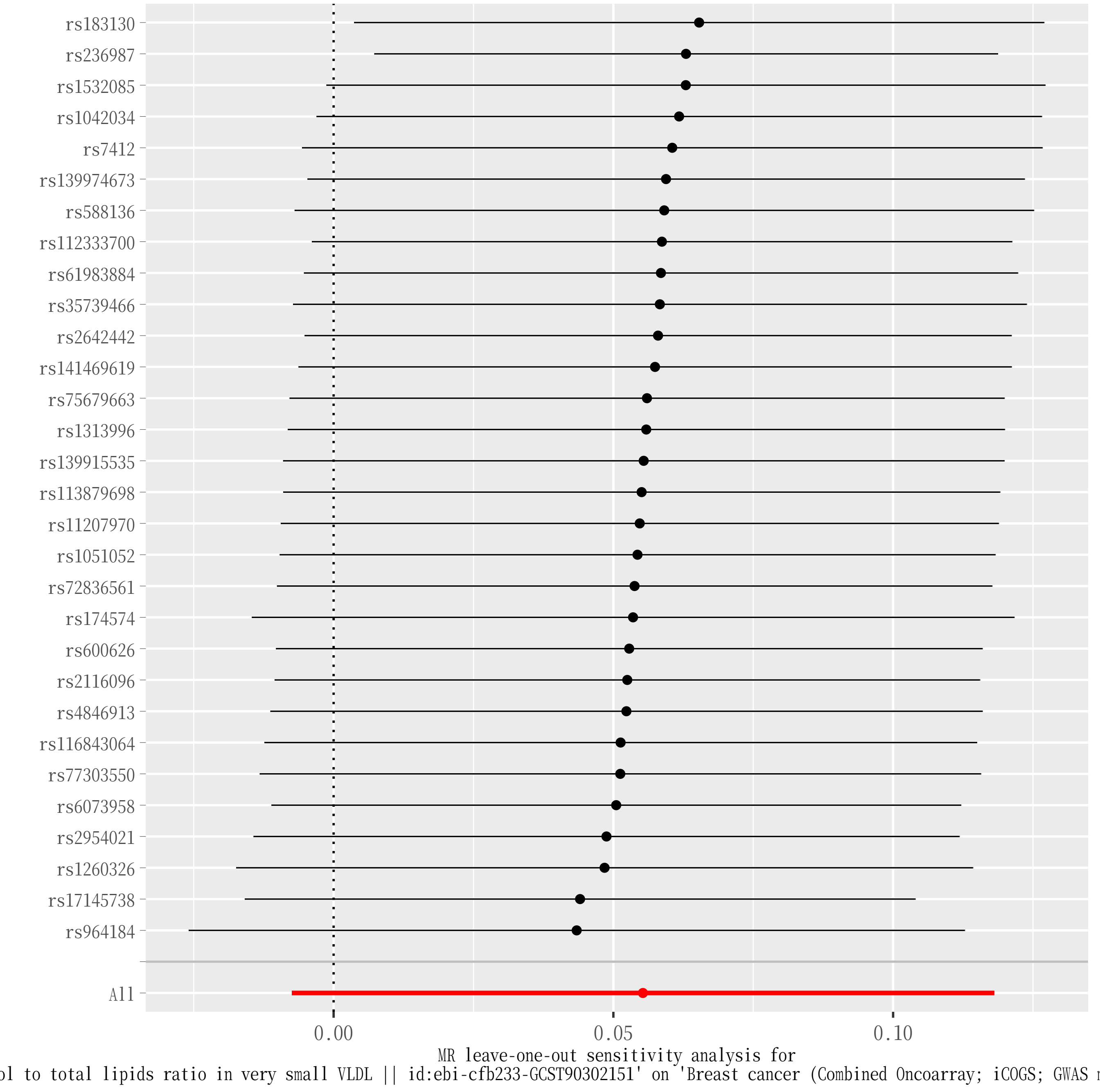

rs183130
rs236987
rs1532085
rs1042034
rs7412
rs139974673
rs588136
rs112333700
rs61983884
rs35739466
rs2642442
rs141469619
rs75679663
rs1313996
rs139915535
rs113879698
rs11207970
rs1051052
rs72836561
rs174574
rs600626
rs2116096
rs4846913
rs116843064
rs77303550
rs6073958
rs2954021
rs1260326
rs17145738
rs964184
All
0.00
0.05
0.10
MR leave-one-out sensitivity analysis for
ol to total lipids ratio in very small VLDL || id:ebi-cfb233-GCST90302151' on 'Breast cancer (Combined Oncoarray; iCOGS; GWAS m

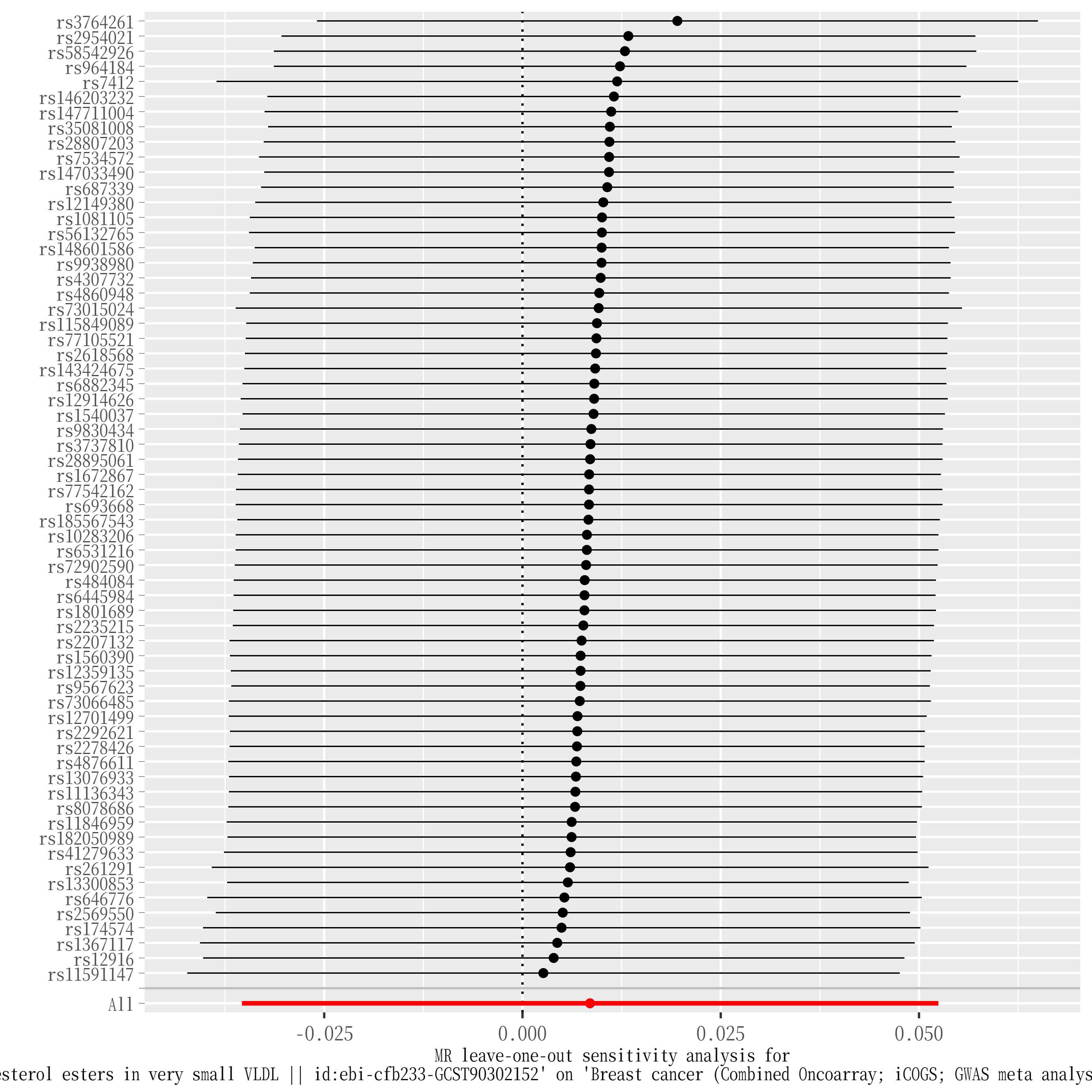

rs3764261
rs2954021
rs58542926
rs964184
rs7412
rs146203232
rs147711004
rs35081008
rs28807203
rs7534572
rs147033490
rs687339
rs12149380
rs1081105
rs56132765
rs148601586
rs9938980
rs4307732
rs4860948
rs73015024
rs115849089
rs77105521
rs2618568
rs143424675
rs6882345
rs12914626
rs1540037
rs9830434
rs3737810
rs28895061
rs1672867
rs77542162
rs693668
rs185567543
rs10283206
rs6531216
rs72902590
rs484084
rs6445984
rs1801689
rs2235215
rs2207132
rs1560390
rs12359135
rs9567623
rs73066485
rs12701499
rs2292621
rs2278426
rs4876611
rs13076933
rs11136343
rs8078686
rs11846959
rs182050989
rs41279633
rs261291
rs13300853
rs646776
rs2569550
rs174574
rs1367117
rs12916
rs11591147
All
-0.025
0.000
0.025
0.050
MR leave-one-out sensitivity analysis for
esterol esters in very small VLDL || id:ebi-cfb233-GCST90302152' on 'Breast cancer (Combined Oncoarray; iCOGS; GWAS meta analys

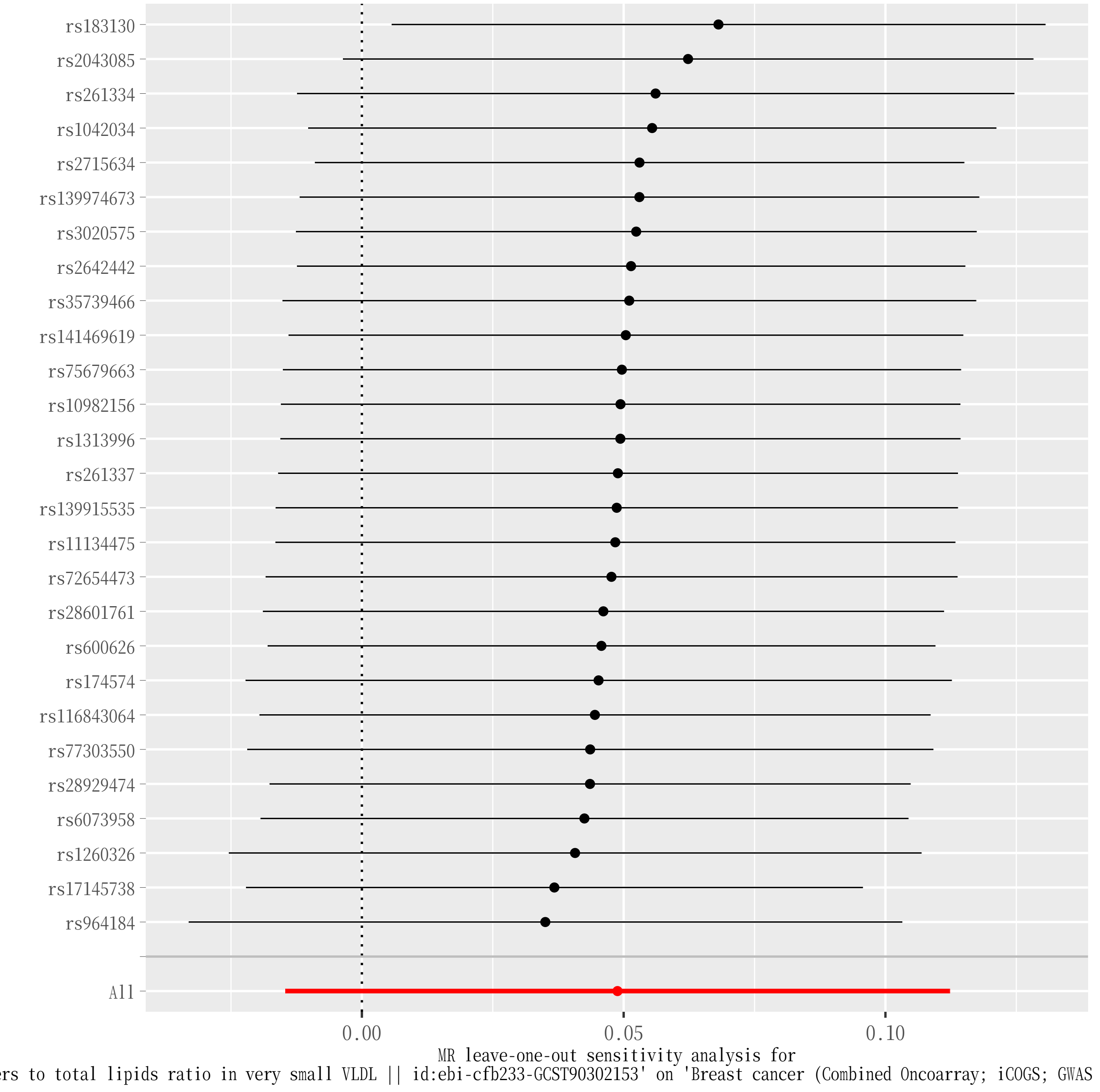
rs183130
rs2043085
rs261334
rs1042034
rs2715634
rs139974673
rs3020575
rs2642442
rs35739466
rs141469619
rs75679663
rs10982156
rs1313996
rs261337
rs139915535
rs11134475
rs72654473
rs28601761
rs600626
rs174574
rs116843064
rs77303550
rs28929474
rs6073958
rs1260326
rs17145738
rs964184
All
0.00
0.05
0.10
MR leave-one-out sensitivity analysis for
ers to total lipids ratio in very small VLDL || id:ebi-cfb233-GCST90302153' on 'Breast cancer (Combined Oncoarray; iCOGS; GWAS

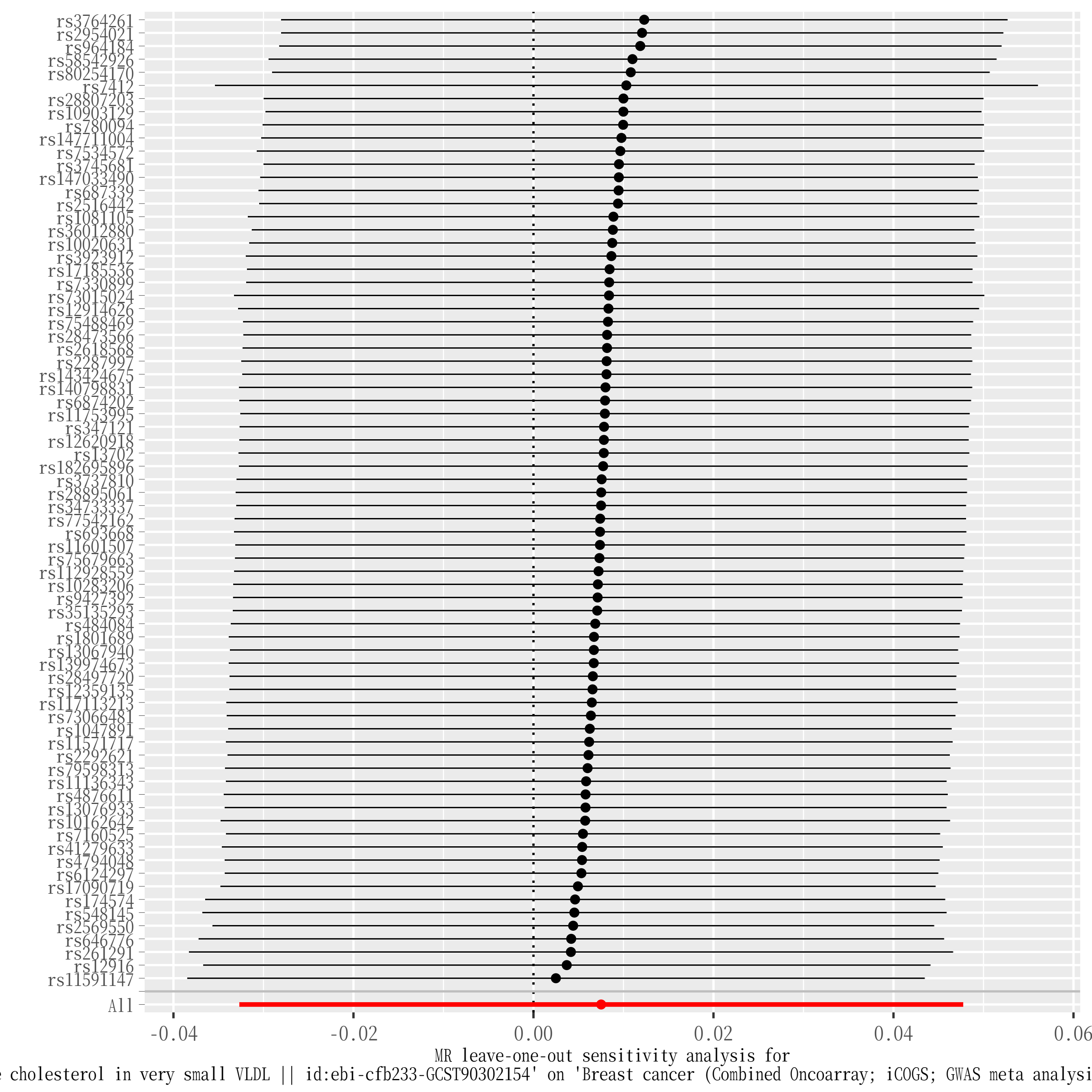

rs3764261
rs2954021
rs964184
rs58542926
rs80254170
rs7412
rs28807203
rs10903129
rs780094
rs147711004
rs7534572
rs3745681
rs147033490
rs687339
rs2516442
rs1081105
rs36012880
rs10020631
rs3923912
rs17185536
rs7330899
rs73015024
rs12914626
rs75488469
rs28473566
rs2618568
rs2287997
rs143424675
rs140798831
rs6874202
rs11753995
rs347121
rs12620918
rs13702
rs182695896
rs3737810
rs28895061
rs34733337
rs77542162
rs693668
rs11601507
rs75679663
rs112928559
rs10283206
rs9427392
rs35135293
rs484084
rs1801689
rs13067940
rs139974673
rs28497720
rs12359135
rs117113213
rs73066481
rs1047891
rs11571717
rs2292621
rs79598313
rs11136343
rs4876611
rs13076933
rs10162642
rs7160525
rs41279633
rs4794048
rs6124297
rs17090719
rs174574
rs548145
rs2569550
rs646776
rs261291
rs12916
rs11591147
All
-0.04
-0.02
0.00
0.02
0.04
0.06
MR leave-one-out sensitivity analysis for
e cholesterol in very small VLDL || id:ebi-cfb233-GCST90302154' on 'Breast cancer (Combined Oncoarray; iCOGS; GWAS meta analysi

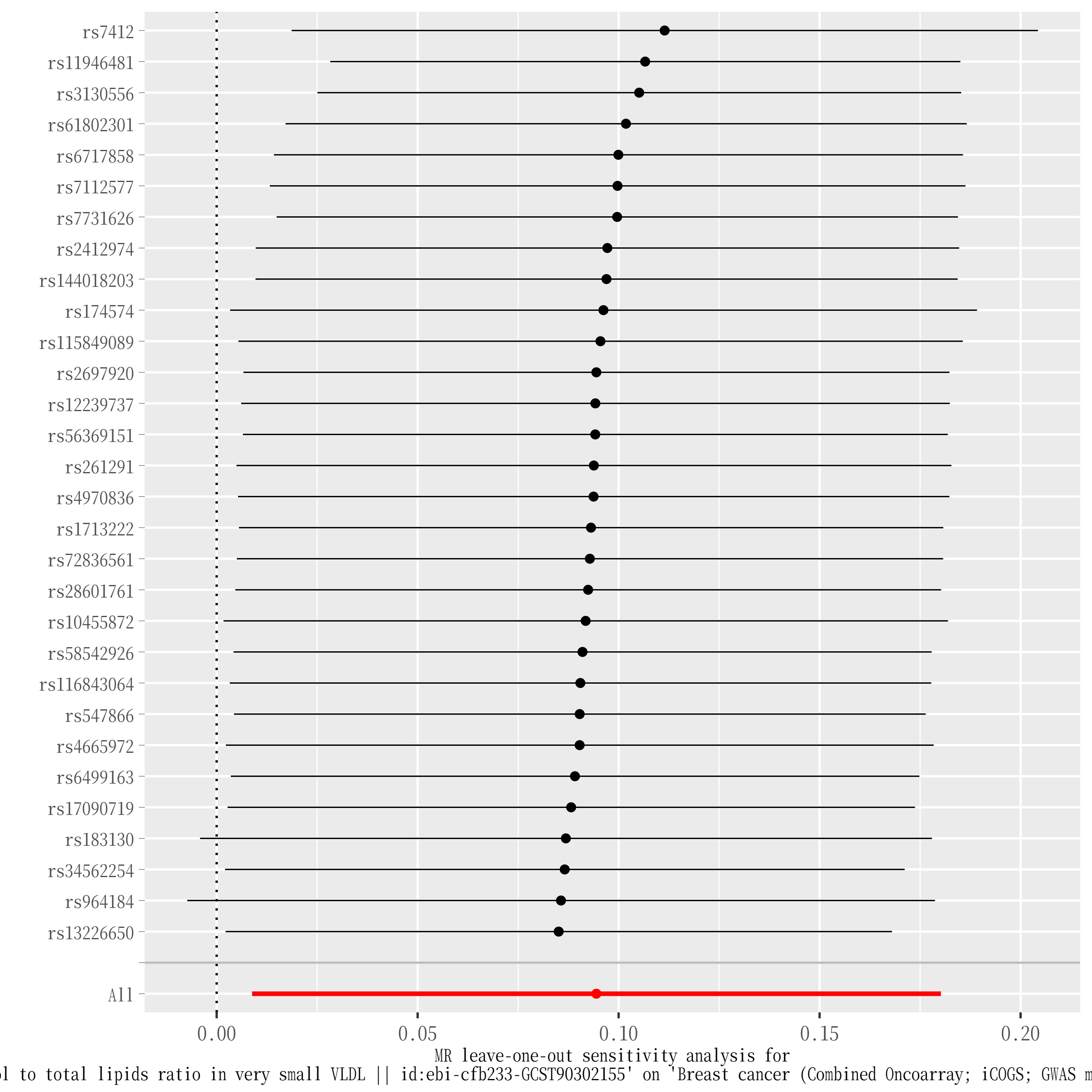

rs7412
rs11946481
rs3130556
rs61802301
rs6717858
rs7112577
rs7731626
rs2412974
rs144018203
rs174574
rs115849089
rs2697920
rs12239737
rs56369151
rs261291
rs4970836
rs1713222
rs72836561
rs28601761
rs10455872
rs58542926
rs116843064
rs547866
rs4665972
rs6499163
rs17090719
rs183130
rs34562254
rs964184
rs13226650
All
0.00
0.05
0.10
0.15
0.20
MR leave-one-out sensitivity analysis for
l to total lipids ratio in very small VLDL || id:ebi-cfb233-GCST90302155' on 'Breast cancer (Combined Oncoarray; iCOGS; GWAS m

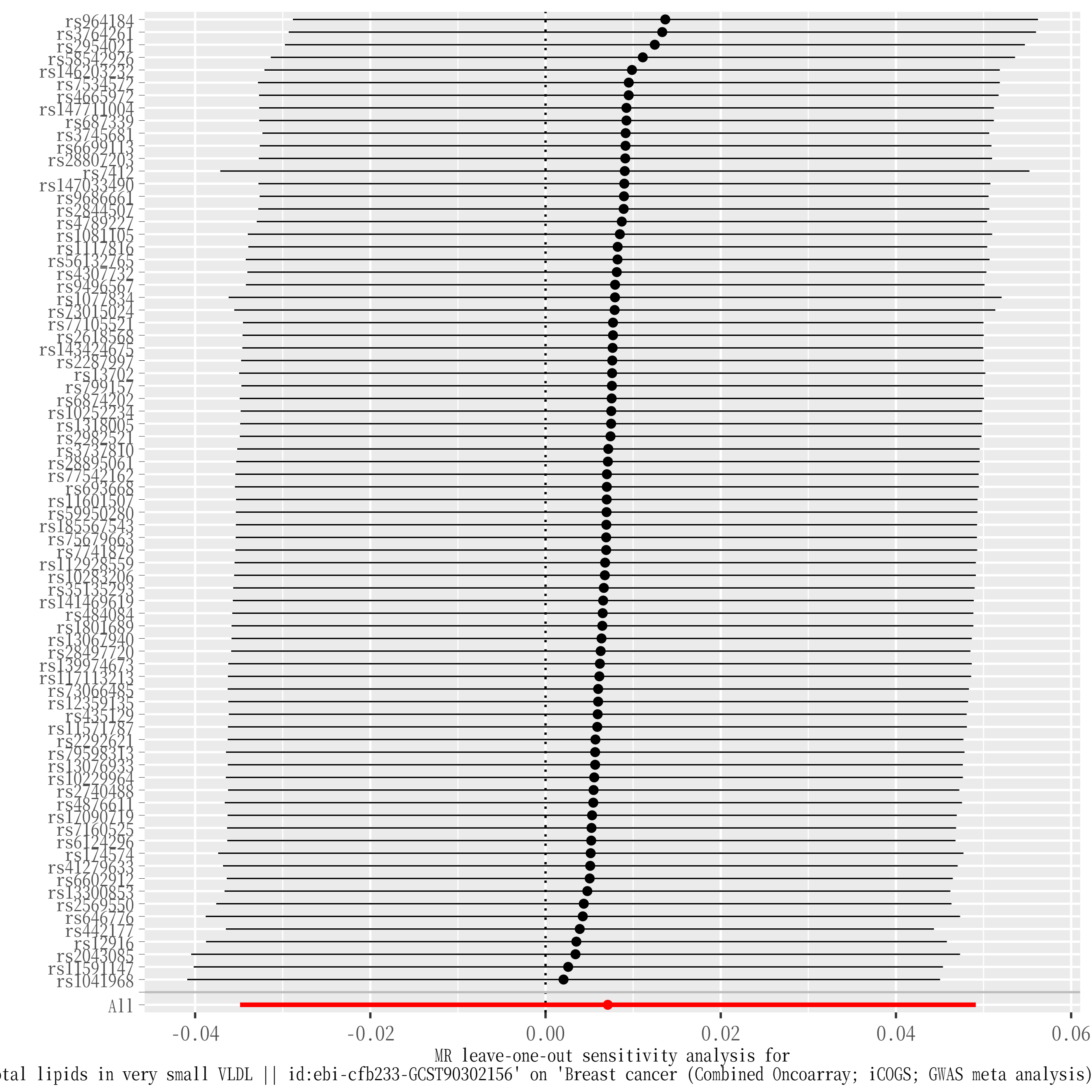
rs964184
rs3764261
rs2954021
rs58542926
rs146203232
rs7534572
rs4665972
rs147711004
rs687339
rs3745681
rs6699113
rs28807203
rs7412
rs147033490
rs9686661
rs2844507
rs4789227
rs1081105
rs1117816
rs56132765
rs4307732
rs9496567
rs1077834
rs73015024
rs77105521
rs2618568
rs143424675
rs2287997
rs13702
rs799157
rs6874202
rs10252234
rs1318005
rs2982521
rs3737810
rs28895061
rs77542162
rs693668
rs11601507
rs59950280
rs185567543
rs75679663
rs7741879
rs112928559
rs10283206
rs35135293
rs141469619
rs484084
rs1801689
rs13067940
rs28497720
rs139974673
rs117113213
rs73066485
rs12359135
rs435129
rs11571787
rs2292621
rs79598313
rs13076933
rs10229964
rs2740488
rs4876611
rs17090719
rs7160525
rs6124296
rs174574
rs41279633
rs6602912
rs13300853
rs2569550
rs646776
rs442177
rs12916
rs2043085
rs11591147
rs1041968
All
-0.04
-0.02
0.00
0.02
0.04
0.06
MR leave-one-out sensitivity analysis for
otal lipids in very small VLDL || id:ebi-cfb233-GCST90302156' on 'Breast cancer (Combined Oncoarray; iCOGS; GWAS meta analysis)

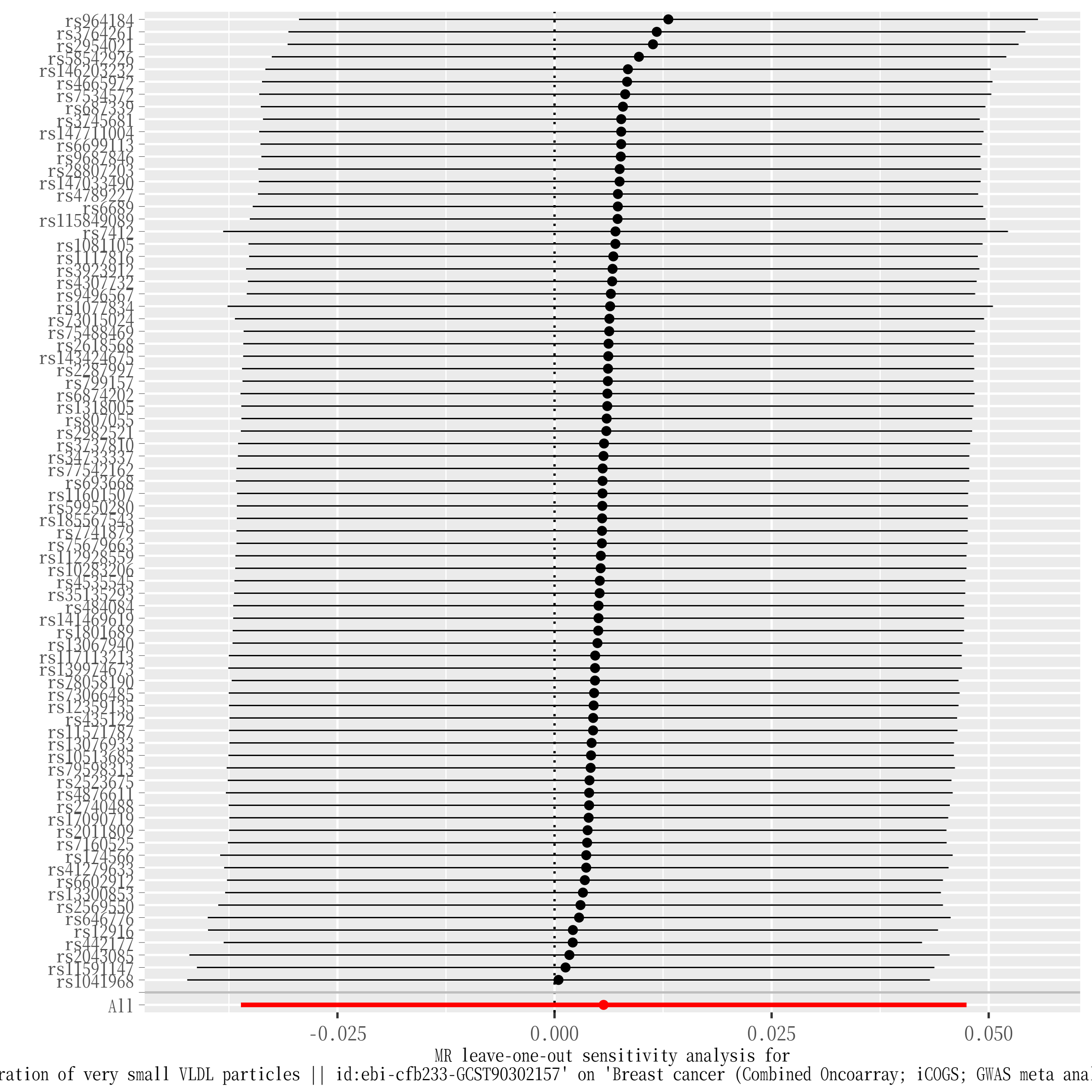
rs964184
rs3764261
rs2954021
rs58542926
rs146203232
rs4665972
rs7534572
rs687339
rs3745681
rs147711004
rs6699113
rs9687846
rs28807203
rs147033490
rs4789227
rs6689
rs115849089
rs7412
rs1081105
rs1117816
rs3923912
rs4307732
rs9496567
rs1077834
rs73015024
rs75488469
rs2618568
rs143424675
rs2287997
rs799157
rs6874202
rs1318005
rs807055
rs2982521
rs3737810
rs34733337
rs77542162
rs693668
rs11601507
rs59950280
rs185567543
rs7741879
rs75679663
rs112928559
rs10283206
rs4535545
rs35135293
rs484084
rs141469619
rs1801689
rs13067940
rs117113213
rs139974673
rs78058190
rs73066485
rs12359135
rs435129
rs11571787
rs13076933
rs10513685
rs79598313
rs2523675
rs4876611
rs2740488
rs17090719
rs2011809
rs7160525
rs174566
rs41279633
rs6602912
rs13300853
rs2569550
rs646776
rs12916
rs442177
rs2043085
rs11591147
rs1041968
All
-0.025
0.000
0.025
0.050
MR leave-one-out sensitivity analysis for
ration of very small VLDL particles || id:ebi-cfb233-GCST90302157' on 'Breast cancer (Combined Oncoarray; iCOGS; GWAS meta anal

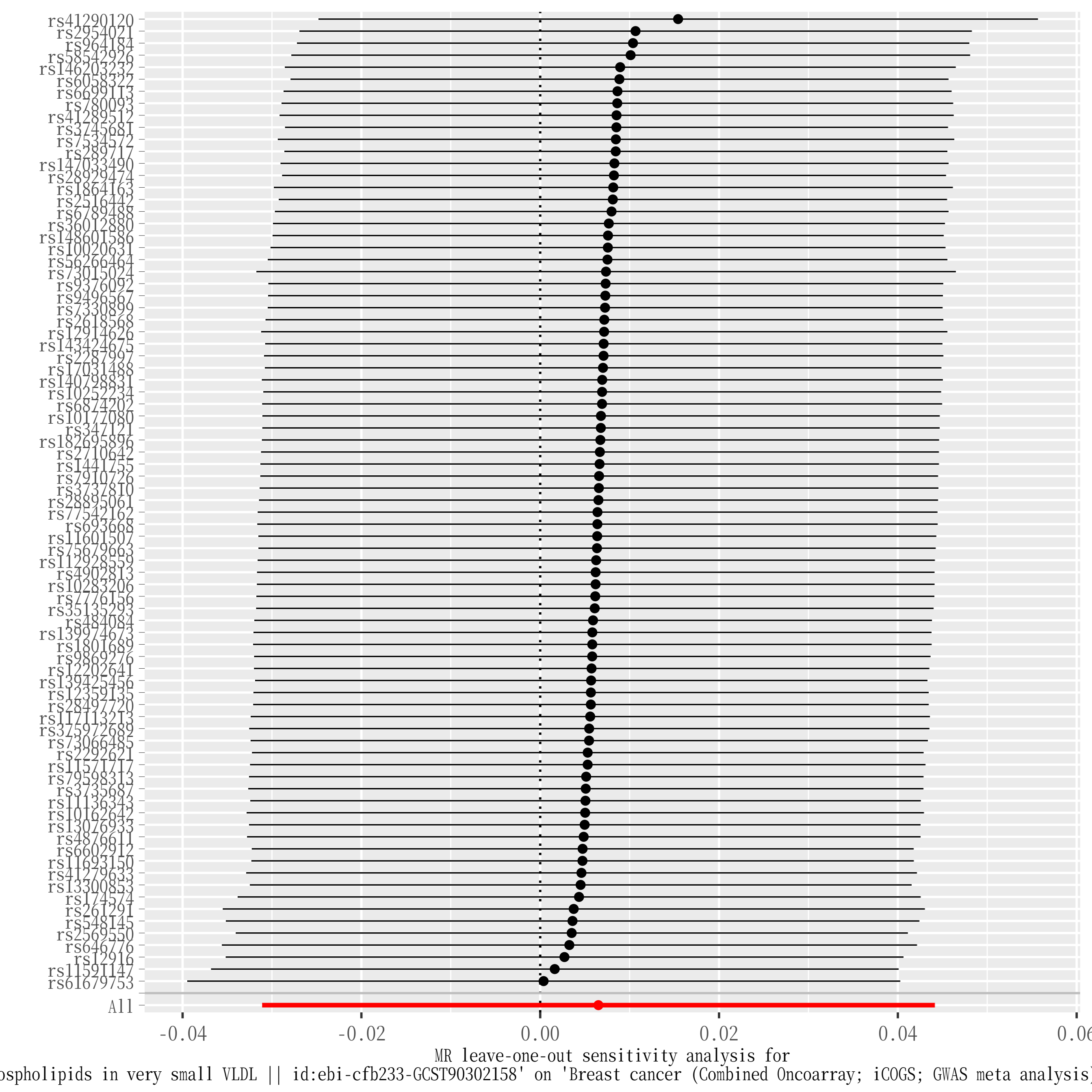
rs41290120
rs2954021
rs964184
rs58542926
rs146203232
rs6058322
rs6699113
rs780093
rs41289512
rs3745681
rs7534572
rs289717
rs147033490
rs28929474
rs1864163
rs2516442
rs6789488
rs36012880
rs148601586
rs10020631
rs56266464
rs73015024
rs9376092
rs9496567
rs7330899
rs2618568
rs12914626
rs143424675
rs2287997
rs17031488
rs140798831
rs10252234
rs6874202
rs10177080
rs347121
rs182695896
rs2710642
rs1441755
rs7910726
rs3737810
rs28895061
rs77542162
rs693668
rs11601507
rs75679663
rs112928559
rs4902813
rs10283206
rs7776156
rs35135293
rs484084
rs139974673
rs1801689
rs9869276
rs12202641
rs139425456
rs12359135
rs28497720
rs117113213
rs375972689
rs73066485
rs2292621
rs11571717
rs79598313
rs3735687
rs11136343
rs10162642
rs13076933
rs4876611
rs6602912
rs11693150
rs41279633
rs13300853
rs174574
rs261291
rs548145
rs2569550
rs646776
rs12916
rs11591147
rs61679753
All
-0.04
-0.02
0.00
0.02
0.04
0.06
MR leave-one-out sensitivity analysis for
ospholipids in very small VLDL || id:ebi-cfb233-GCST90302158' on 'Breast cancer (Combined Oncoarray; iCOGS; GWAS meta analysis)

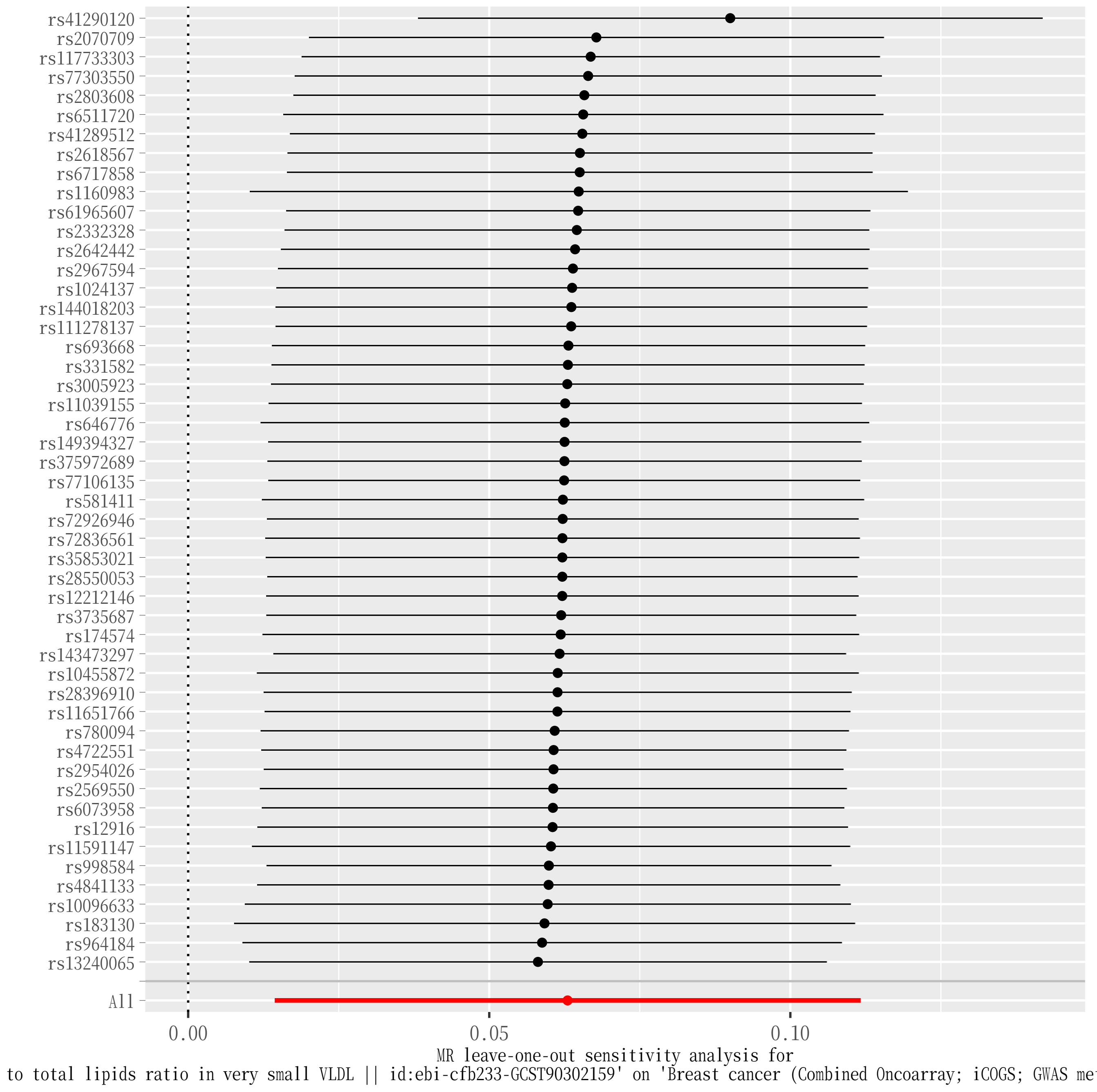

rs41290120
rs2070709
rs117733303
rs77303550
rs2803608
rs6511720
rs41289512
rs2618567
rs6717858
rs1160983
rs61965607
rs2332328
rs2642442
rs2967594
rs1024137
rs144018203
rs111278137
rs693668
rs331582
rs3005923
rs11039155
rs646776
rs149394327
rs375972689
rs77106135
rs581411
rs72926946
rs72836561
rs35853021
rs28550053
rs12212146
rs3735687
rs174574
rs143473297
rs10455872
rs28396910
rs11651766
rs780094
rs4722551
rs2954026
rs2569550
rs6073958
rs12916
rs11591147
rs998584
rs4841133
rs10096633
rs183130
rs964184
rs13240065
All
0.00
0.05
0.10
MR leave-one-out sensitivity analysis for
to total lipids ratio in very small VLDL || id:ebi-cfb233-GCST90302159' on 'Breast cancer (Combined Oncoarray; iCOGS; GWAS me

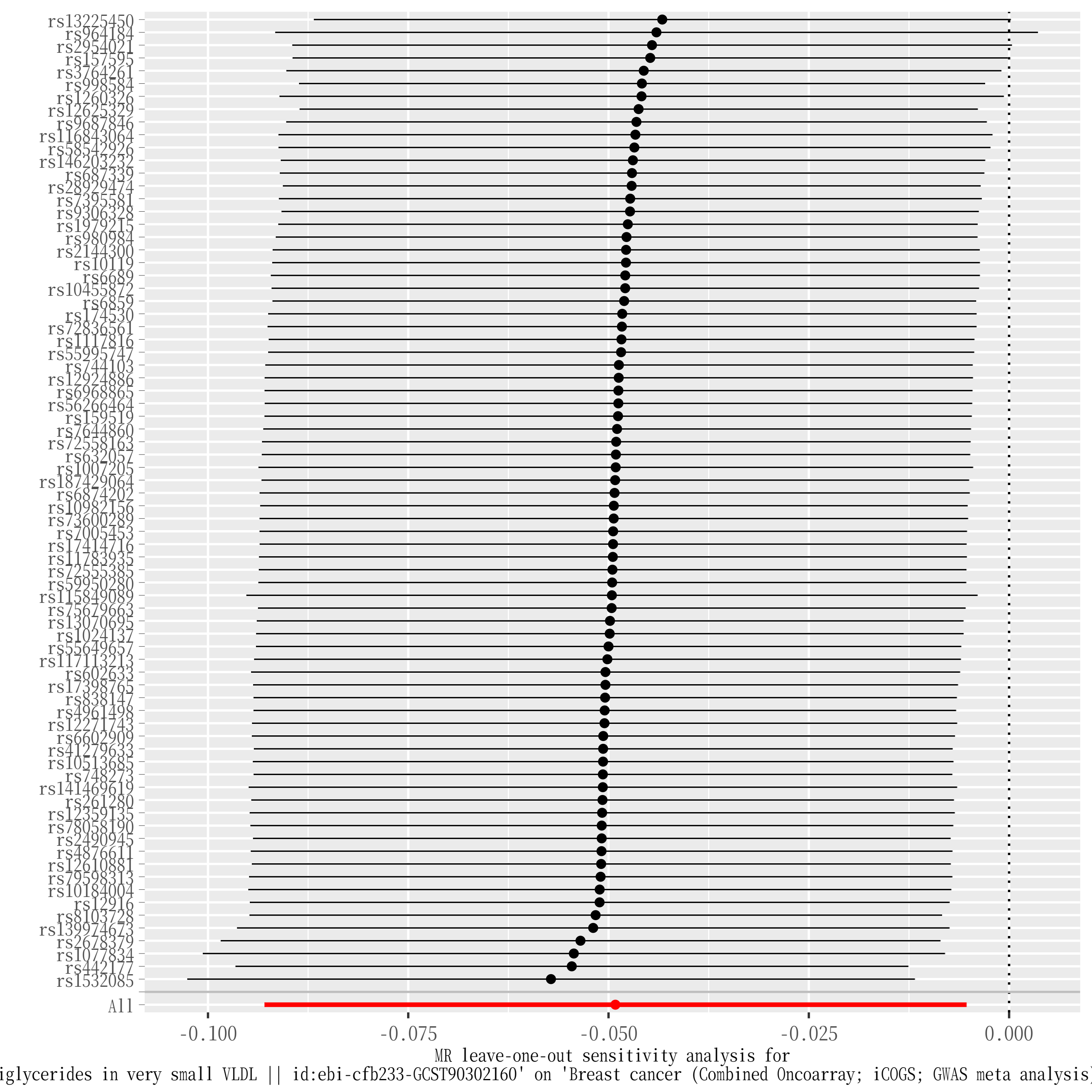
rs13225450
rs964184
rs2954021
rs157595
rs3764261
rs998584
rs1260326
rs12625329
rs9687846
rs116843064
rs58542926
rs146203232
rs687339
rs28929474
rs7395581
rs9306328
rs1979215
rs980984
rs2144300
rs10119
rs6689
rs10455872
rs6859
rs174530
rs72836561
rs1117816
rs55995747
rs744103
rs12924886
rs6968865
rs56266464
rs159519
rs7644860
rs72558163
rs632057
rs1007205
rs187429064
rs6874202
rs10982156
rs73600289
rs7005453
rs17414716
rs11783935
rs72555385
rs59950280
rs115849089
rs75679663
rs13070695
rs1024137
rs55649657
rs117113213
rs602633
rs17398765
rs838147
rs4961498
rs12271743
rs6602909
rs41279633
rs10513685
rs748273
rs141469619
rs261280
rs12359135
rs78058190
rs2490945
rs4876611
rs12610881
rs79598313
rs10184004
rs12916
rs8103728
rs139974673
rs2678379
rs1077834
rs442177
rs1532085
All
-0.100
-0.075
-0.050
-0.025
0.000
MR leave-one-out sensitivity analysis for
iglycerides in very small VLDL || id:ebi-cfb233-GCST90302160' on 'Breast cancer (Combined Oncoarray; iCOGS; GWAS meta analysis)

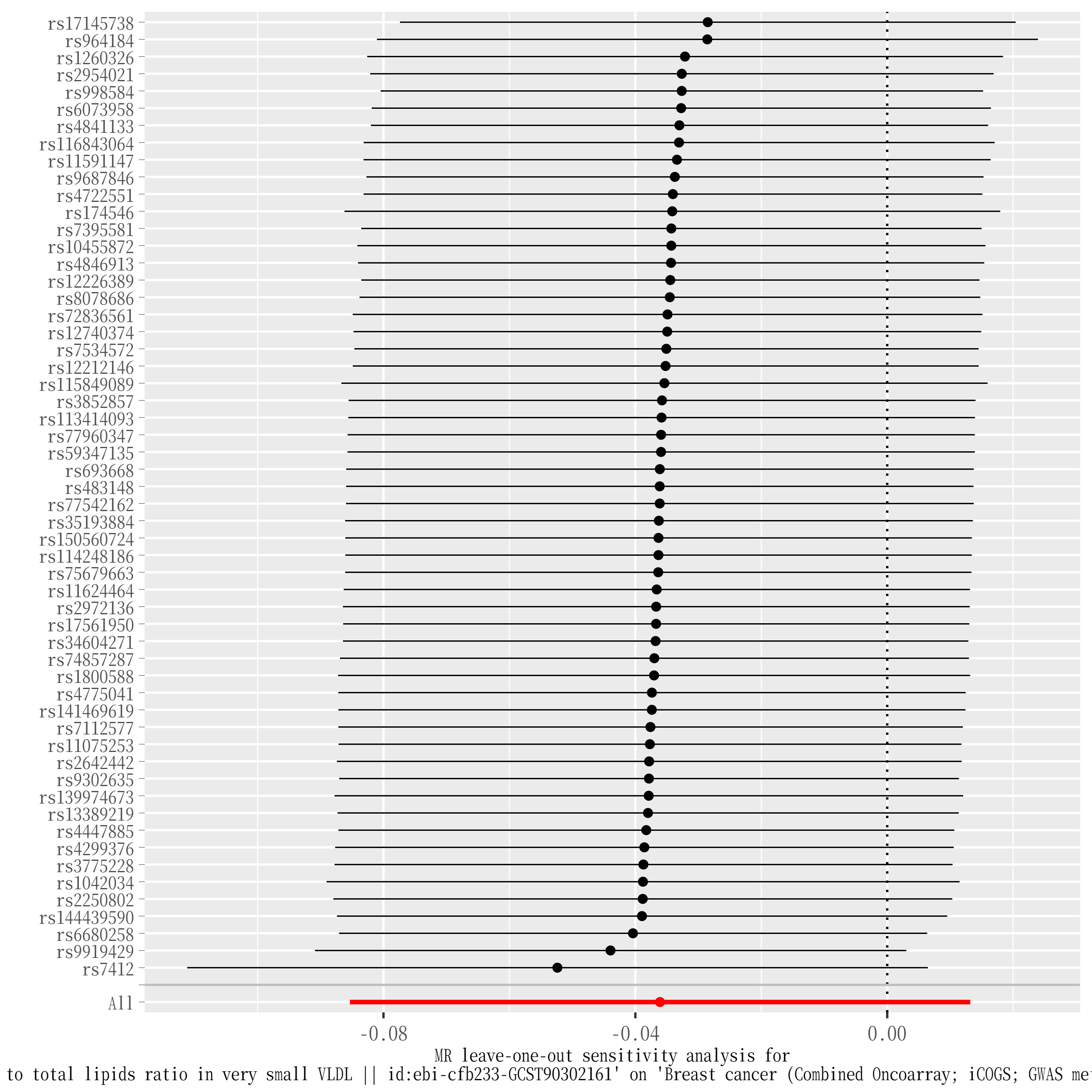

rs17145738
rs964184
rs1260326
rs2954021
rs998584
rs6073958
rs4841133
rs116843064
rs11591147
rs9687846
rs4722551
rs174546
rs7395581
rs10455872
rs4846913
rs12226389
rs8078686
rs72836561
rs12740374
rs7534572
rs12212146
rs115849089
rs3852857
rs113414093
rs77960347
rs59347135
rs693668
rs483148
rs77542162
rs35193884
rs150560724
rs114248186
rs75679663
rs11624464
rs2972136
rs17561950
rs34604271
rs74857287
rs1800588
rs4775041
rs141469619
rs7112577
rs11075253
rs2642442
rs9302635
rs139974673
rs13389219
rs4447885
rs4299376
rs3775228
rs1042034
rs2250802
rs144439590
rs6680258
rs9919429
rs7412
All
-0.08
-0.04
0.00
MR leave-one-out sensitivity analysis for
to total lipids ratio in very small VLDL || id:ebi-cfb233-GCST90302161' on 'Breast cancer (Combined Oncoarray; iCOGS; GWAS me

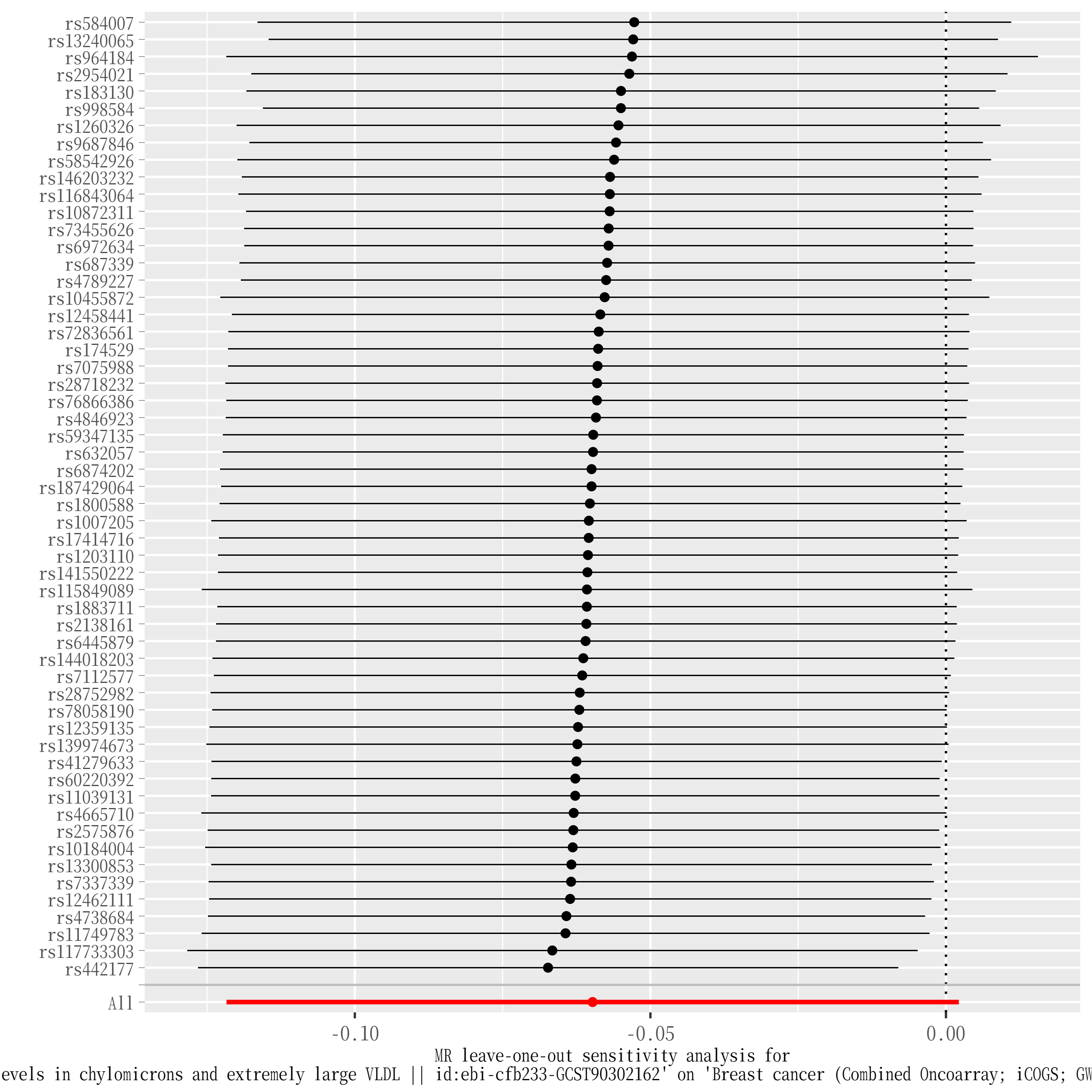

rs584007
rs13240065
rs964184
rs2954021
rs183130
rs998584
rs1260326
rs9687846
rs58542926
rs146203232
rs116843064
rs10872311
rs73455626
rs6972634
rs687339
rs4789227
rs10455872
rs12458441
rs72836561
rs174529
rs7075988
rs28718232
rs76866386
rs4846923
rs59347135
rs632057
rs6874202
rs187429064
rs1800588
rs1007205
rs17414716
rs1203110
rs141550222
rs115849089
rs1883711
rs2138161
rs6445879
rs144018203
rs7112577
rs28752982
rs78058190
rs12359135
rs139974673
rs41279633
rs60220392
rs11039131
rs4665710
rs2575876
rs10184004
rs13300853
rs7337339
rs12462111
rs4738684
rs11749783
rs117733303
rs442177
All
-0.10
-0.05
0.00
MR leave-one-out sensitivity analysis for
evels in chylomicrons and extremely large VLDL || id:ebi-cfb233-GCST90302162' on 'Breast cancer (Combined Oncoarray; iCOGS; GW

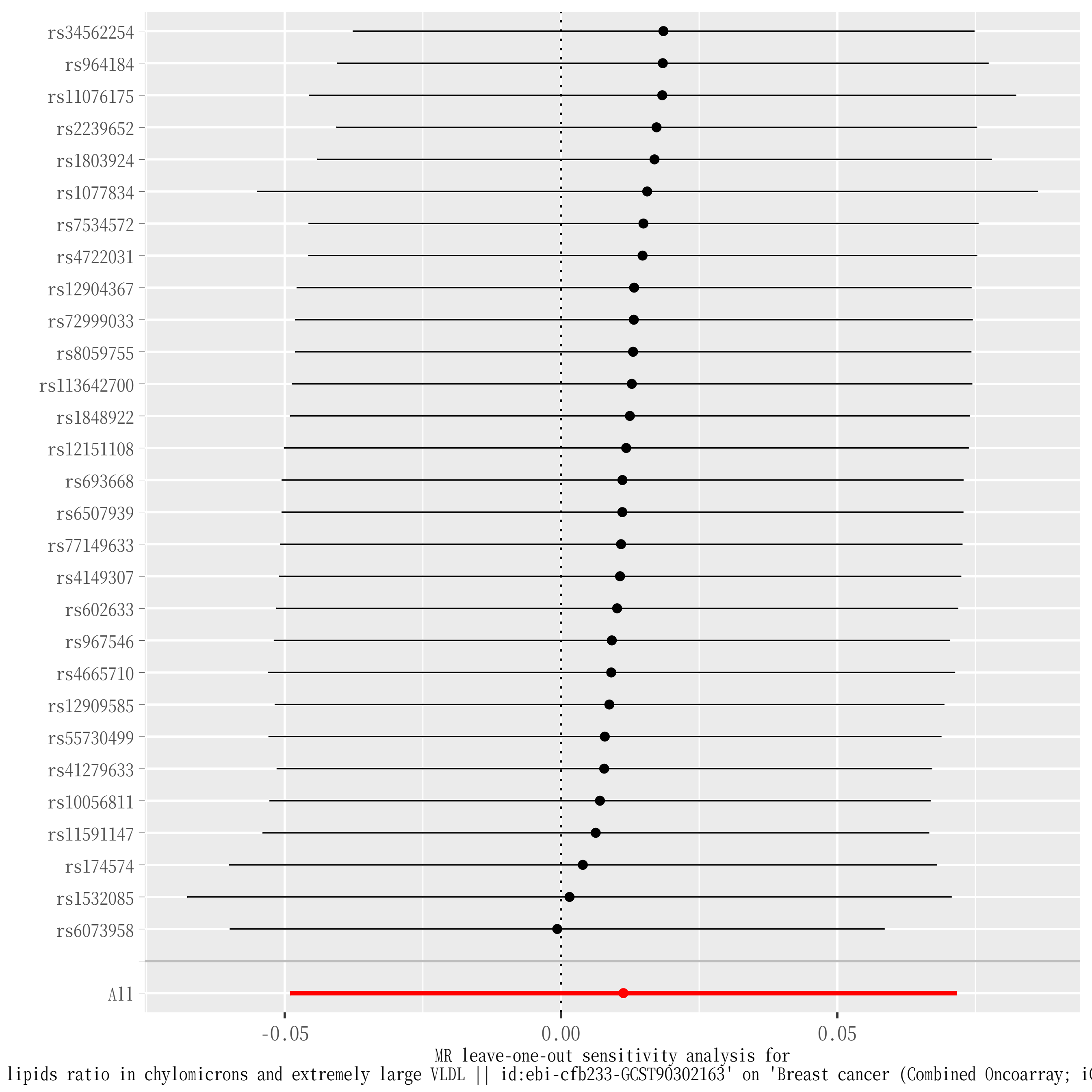

rs34562254
rs964184
rs11076175
rs2239652
rs1803924
rs1077834
rs7534572
rs4722031
rs12904367
rs72999033
rs8059755
rs113642700
rs1848922
rs12151108
rs693668
rs6507939
rs77149633
rs4149307
rs602633
rs967546
rs4665710
rs12909585
rs55730499
rs41279633
rs10056811
rs11591147
rs174574
rs1532085
rs6073958
All
-0.05
0.00
0.05
MR leave-one-out sensitivity analysis for
lipids ratio in chylomicrons and extremely large VLDL || id:ebi-cfb233-GCST90302163' on 'Breast cancer (Combined Oncoarray; i

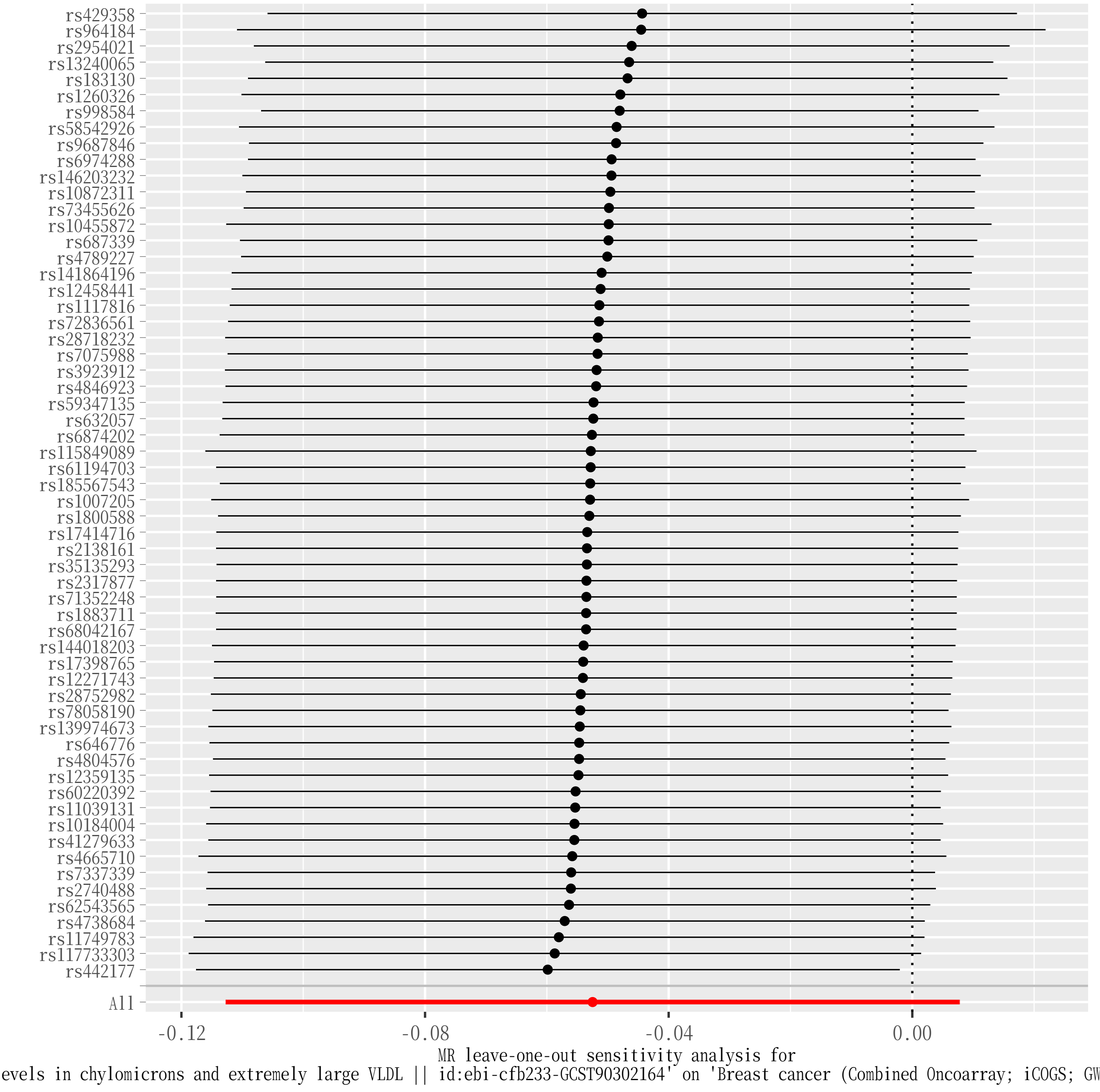
rs429358
rs964184
rs2954021
rs13240065
rs183130
rs1260326
rs998584
rs58542926
rs9687846
rs6974288
rs146203232
rs10872311
rs73455626
rs10455872
rs687339
rs4789227
rs141864196
rs12458441
rs1117816
rs72836561
rs28718232
rs7075988
rs3923912
rs4846923
rs59347135
rs632057
rs6874202
rs115849089
rs61194703
rs185567543
rs1007205
rs1800588
rs17414716
rs2138161
rs35135293
rs2317877
rs71352248
rs1883711
rs68042167
rs144018203
rs17398765
rs12271743
rs28752982
rs78058190
rs139974673
rs646776
rs4804576
rs12359135
rs60220392
rs11039131
rs10184004
rs41279633
rs4665710
rs7337339
rs2740488
rs62543565
rs4738684
rs11749783
rs117733303
rs442177
All
-0.12
-0.08
-0.04
0.00
MR leave-one-out sensitivity analysis for
evels in chylomicrons and extremely large VLDL || id:ebi-cfb233-GCST90302164' on 'Breast cancer (Combined Oncoarray; iCOGS; GW

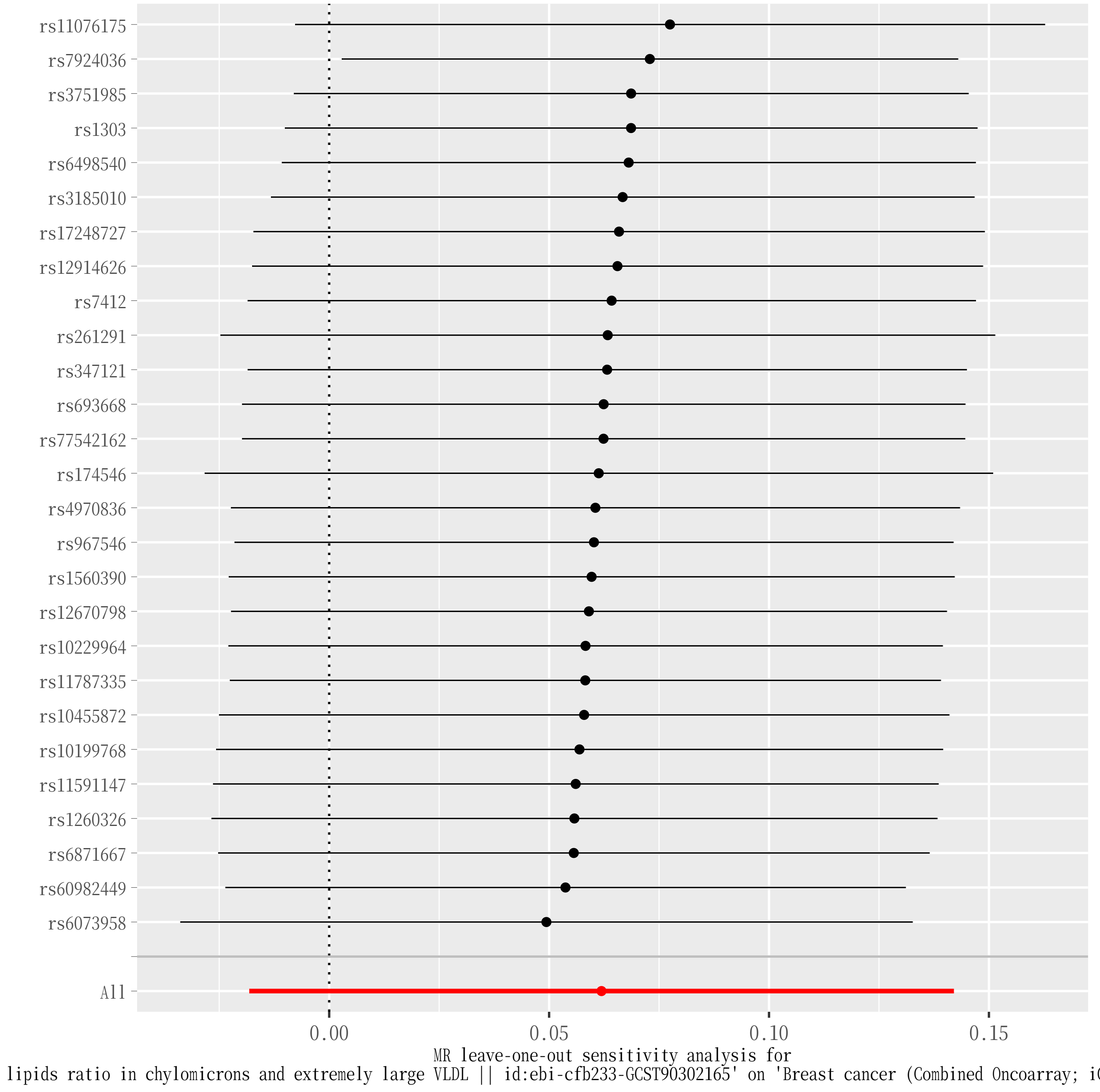

rs11076175
rs7924036
rs3751985
rs1303
rs6498540
rs3185010
rs17248727
rs12914626
rs7412
rs261291
rs347121
rs693668
rs77542162
rs174546
rs4970836
rs967546
rs1560390
rs12670798
rs10229964
rs11787335
rs10455872
rs10199768
rs11591147
rs1260326
rs6871667
rs60982449
rs6073958
All
0.00
0.05
0.10
0.15
MR leave-one-out sensitivity analysis for
lipids ratio in chylomicrons and extremely large VLDL || id:ebi-cfb233-GCST90302165' on 'Breast cancer (Combined Oncoarray; iC

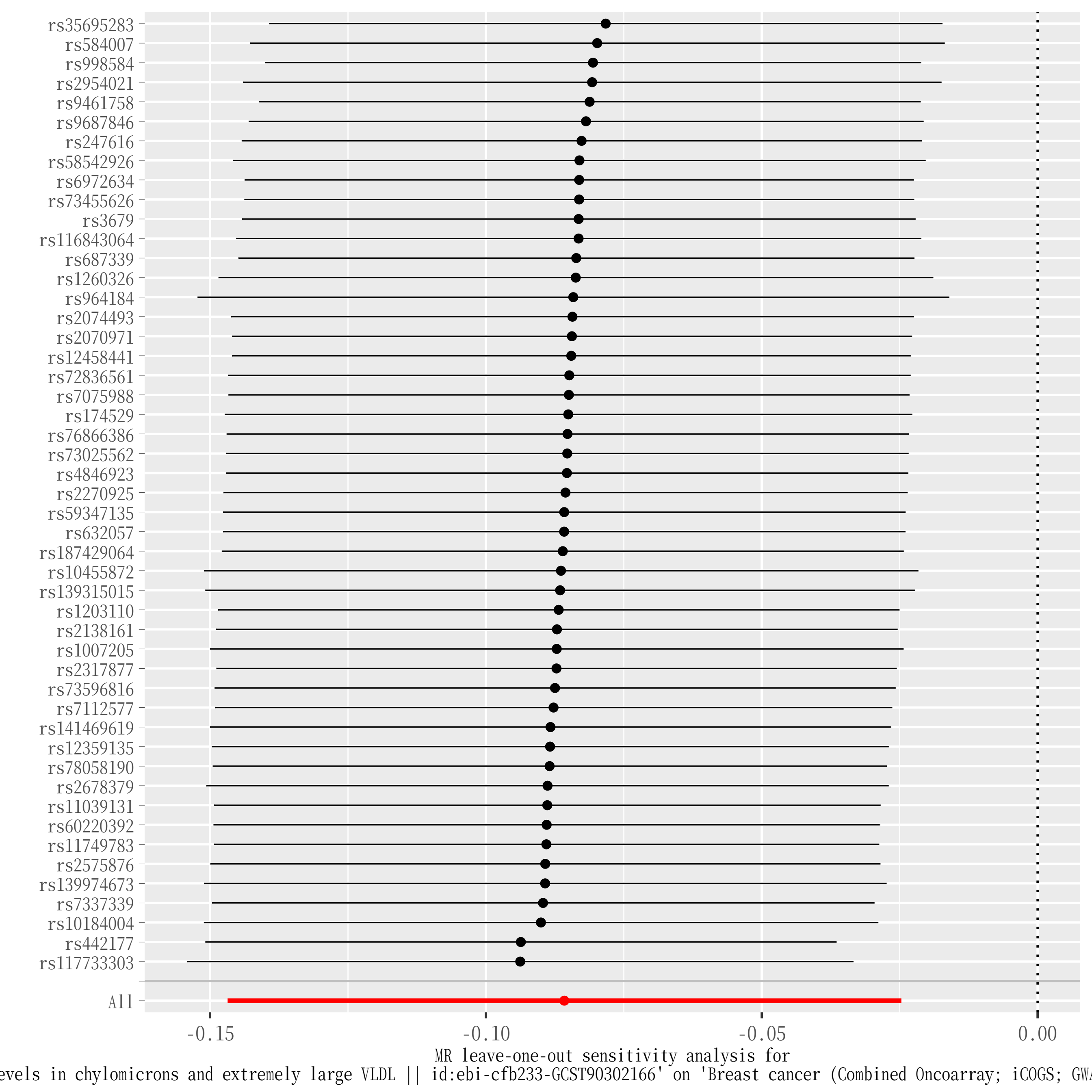

rs35695283
rs584007
rs998584
rs2954021
rs9461758
rs9687846
rs247616
rs58542926
rs6972634
rs73455626
rs3679
rs116843064
rs687339
rs1260326
rs964184
rs2074493
rs2070971
rs12458441
rs72836561
rs7075988
rs174529
rs76866386
rs73025562
rs4846923
rs2270925
rs59347135
rs632057
rs187429064
rs10455872
rs139315015
rs1203110
rs2138161
rs1007205
rs2317877
rs73596816
rs7112577
rs141469619
rs12359135
rs78058190
rs2678379
rs11039131
rs60220392
rs11749783
rs2575876
rs139974673
rs7337339
rs10184004
rs442177
rs117733303
All
-0.15
-0.10
-0.05
0.00
MR leave-one-out sensitivity analysis for
evels in chylomicrons and extremely large VLDL || id:ebi-cfb233-GCST90302166' on 'Breast cancer (Combined Oncoarray; iCOGS; GW

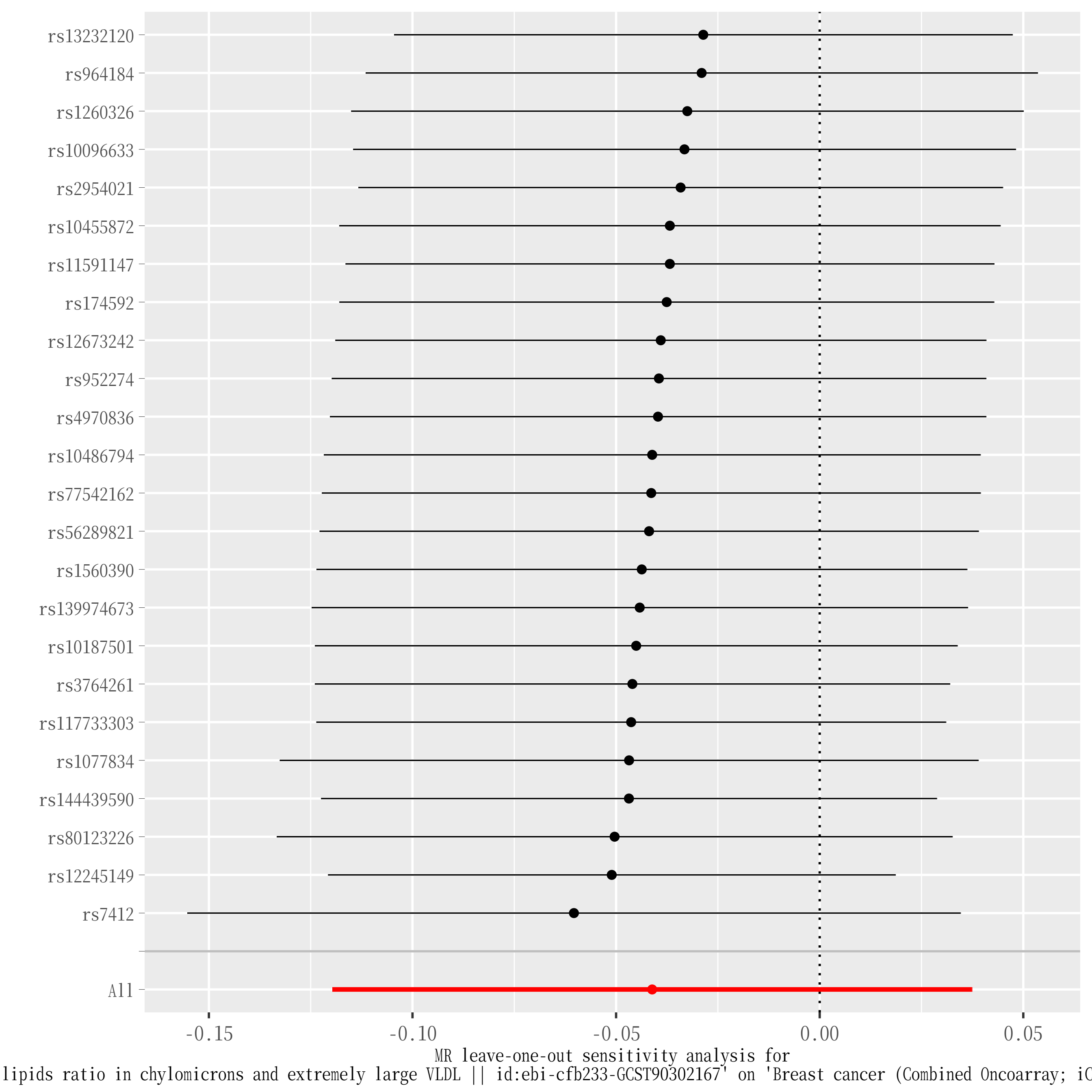
rs13232120
rs964184
rs1260326
rs10096633
rs2954021
rs10455872
rs11591147
rs174592
rs12673242
rs952274
rs4970836
rs10486794
rs77542162
rs56289821
rs1560390
rs139974673
rs10187501
rs3764261
rs117733303
rs1077834
rs144439590
rs80123226
rs12245149
rs7412
All
-0.15
-0.10
-0.05
0.00
0.05
MR leave-one-out sensitivity analysis for
lipids ratio in chylomicrons and extremely large VLDL || id:ebi-cfb233-GCST90302167' on 'Breast cancer (Combined Oncoarray; iC

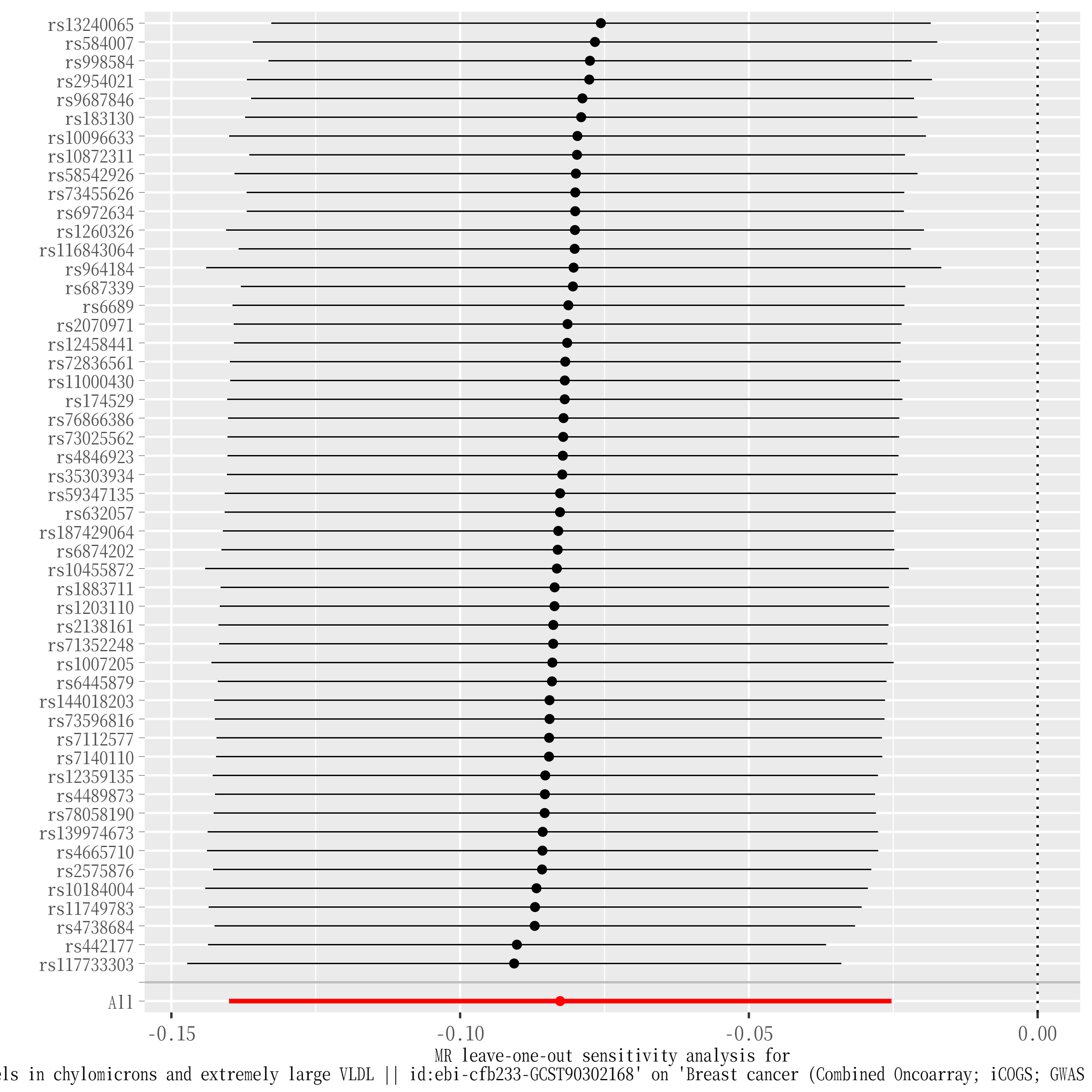

rs13240065
rs584007
rs998584
rs2954021
rs9687846
rs183130
rs10096633
rs10872311
rs58542926
rs73455626
rs6972634
rs1260326
rs116843064
rs964184
rs687339
rs6689
rs2070971
rs12458441
rs72836561
rs11000430
rs174529
rs76866386
rs73025562
rs4846923
rs35303934
rs59347135
rs632057
rs187429064
rs6874202
rs10455872
rs1883711
rs1203110
rs2138161
rs71352248
rs1007205
rs6445879
rs144018203
rs73596816
rs7112577
rs7140110
rs12359135
rs4489873
rs78058190
rs139974673
rs4665710
rs2575876
rs10184004
rs11749783
rs4738684
rs442177
rs117733303
All
-0.15
-0.10
-0.05
0.00
MR leave-one-out sensitivity analysis for
els in chylomicrons and extremely large VLDL || id:ebi-cfb233-GCST90302168' on 'Breast cancer (Combined Oncoarray; iCOGS; GWAS

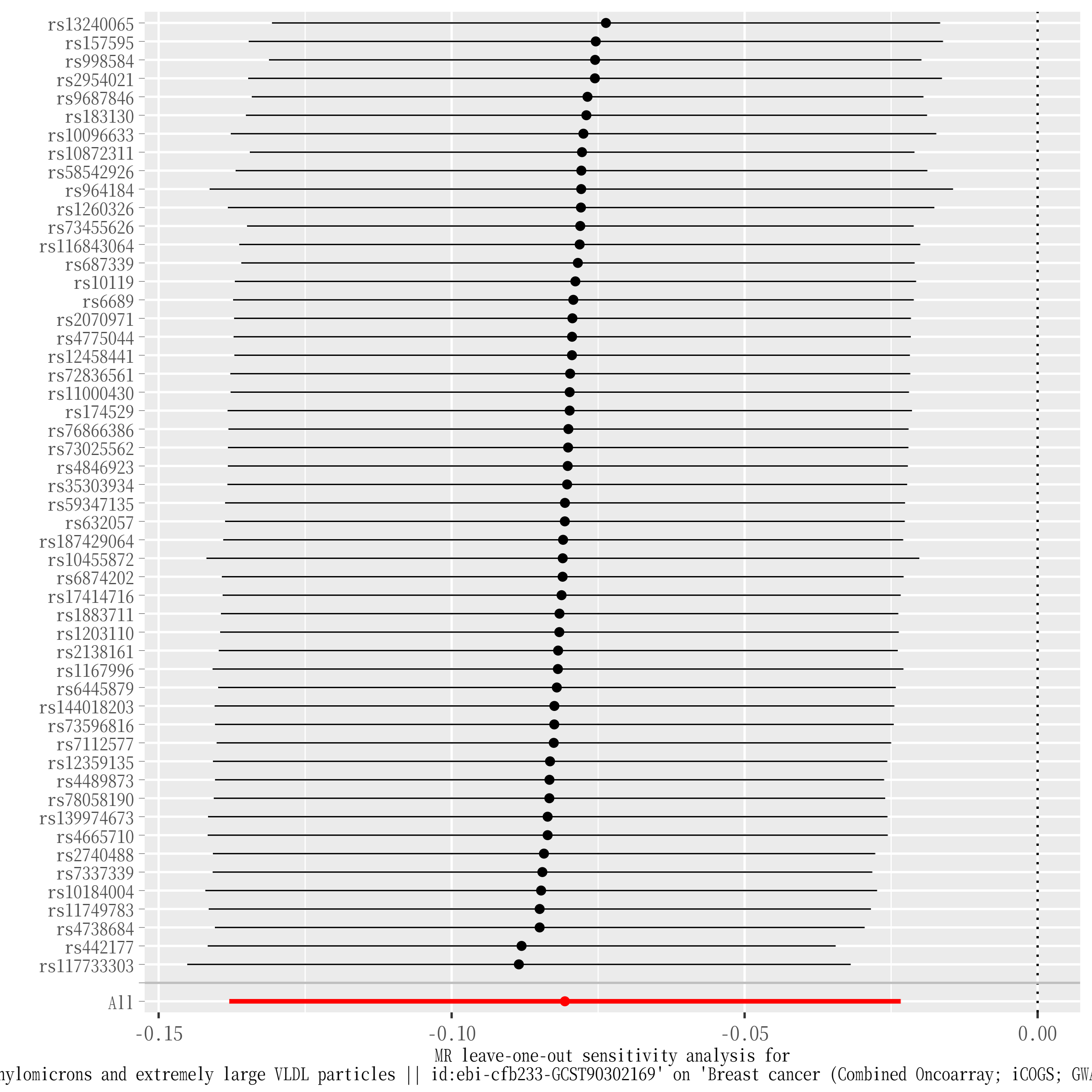
rs13240065
rs157595
rs998584
rs2954021
rs9687846
rs183130
rs10096633
rs10872311
rs58542926
rs964184
rs1260326
rs73455626
rs116843064
rs687339
rs10119
rs6689
rs2070971
rs4775044
rs12458441
rs72836561
rs11000430
rs174529
rs76866386
rs73025562
rs4846923
rs35303934
rs59347135
rs632057
rs187429064
rs10455872
rs6874202
rs17414716
rs1883711
rs1203110
rs2138161
rs1167996
rs6445879
rs144018203
rs73596816
rs7112577
rs12359135
rs4489873
rs78058190
rs139974673
rs4665710
rs2740488
rs7337339
rs10184004
rs11749783
rs4738684
rs442177
rs117733303
All
-0.15
-0.10
-0.05
0.00
MR leave-one-out sensitivity analysis for
hylomicrons and extremely large VLDL particles || id:ebi-cfb233-GCST90302169' on 'Breast cancer (Combined Oncoarray; iCOGS; GW

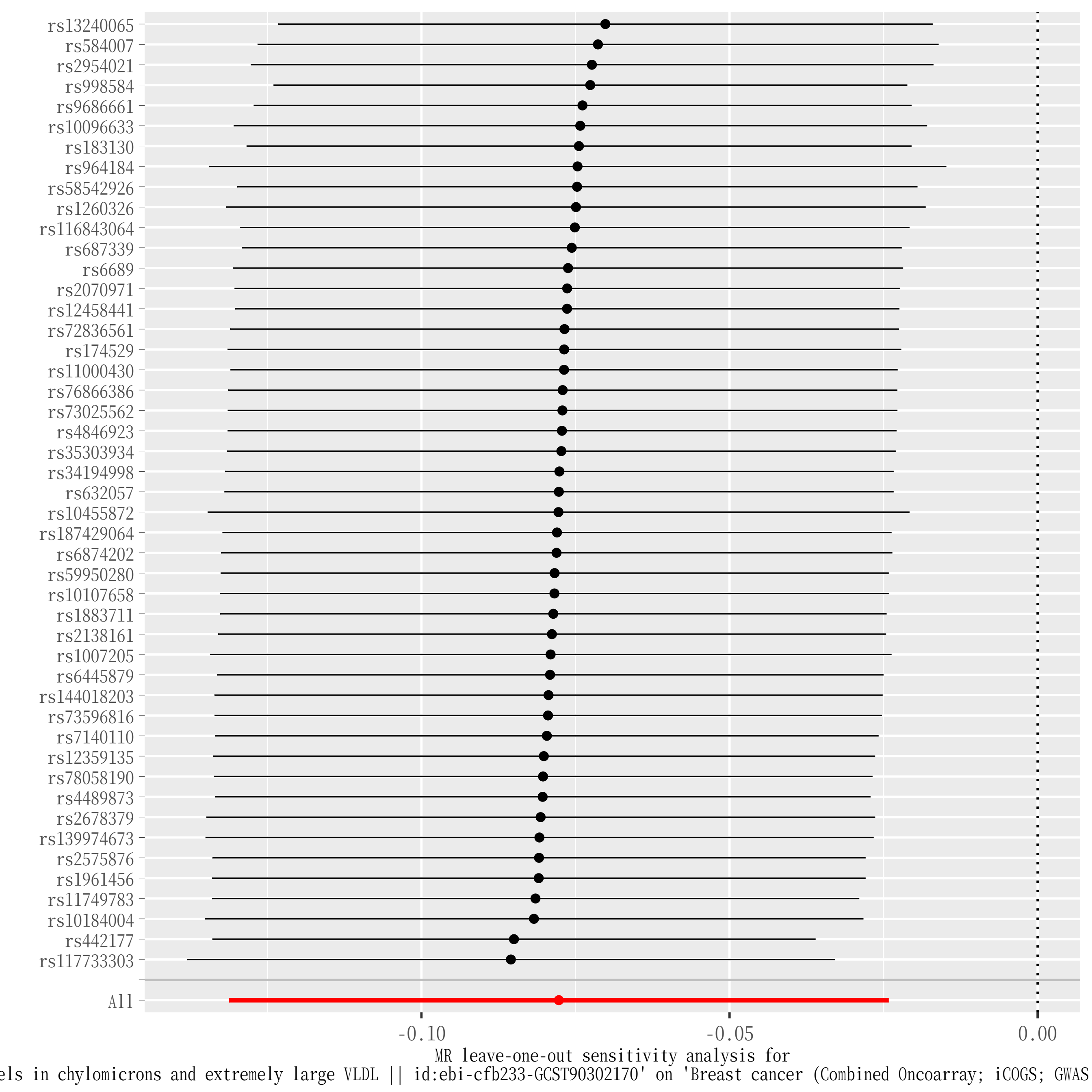

rs13240065
rs584007
rs2954021
rs998584
rs9686661
rs10096633
rs183130
rs964184
rs58542926
rs1260326
rs116843064
rs687339
rs6689
rs2070971
rs12458441
rs72836561
rs174529
rs11000430
rs76866386
rs73025562
rs4846923
rs35303934
rs34194998
rs632057
rs10455872
rs187429064
rs6874202
rs59950280
rs10107658
rs1883711
rs2138161
rs1007205
rs6445879
rs144018203
rs73596816
rs7140110
rs12359135
rs78058190
rs4489873
rs2678379
rs139974673
rs2575876
rs1961456
rs11749783
rs10184004
rs442177
rs117733303
All
-0.10
-0.05
0.00
MR leave-one-out sensitivity analysis for
els in chylomicrons and extremely large VLDL || id:ebi-cfb233-GCST90302170' on 'Breast cancer (Combined Oncoarray; iCOGS; GWAS

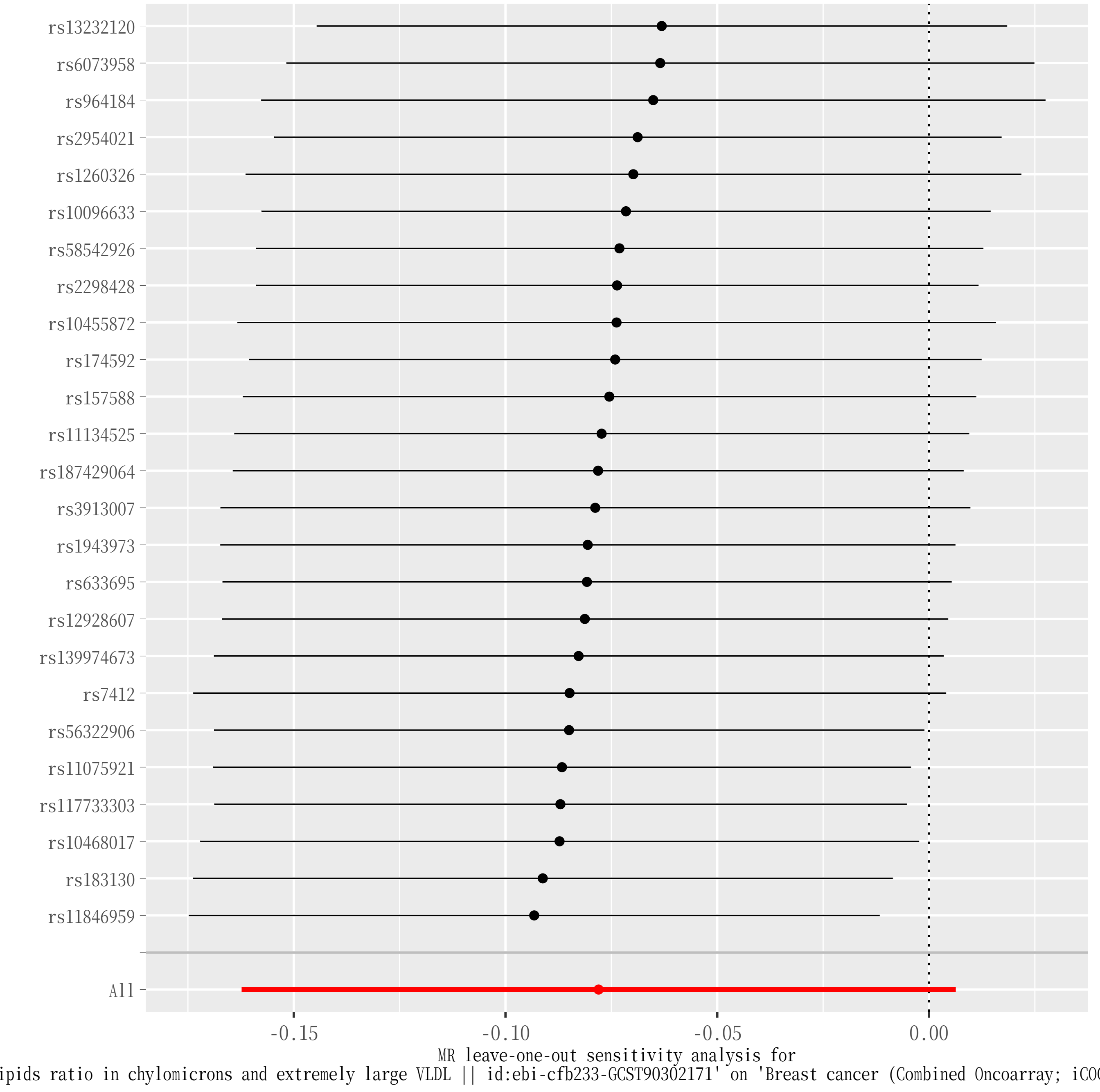
rs13232120
rs6073958
rs964184
rs2954021
rs1260326
rs10096633
rs58542926
rs2298428
rs10455872
rs174592
rs157588
rs11134525
rs187429064
rs3913007
rs1943973
rs633695
rs12928607
rs139974673
rs7412
rs56322906
rs11075921
rs117733303
rs10468017
rs183130
rs11846959
All
-0.15
-0.10
-0.05
0.00
MR leave-one-out sensitivity analysis for
ipids ratio in chylomicrons and extremely large VLDL || id:ebi-cfb233-GCST90302171' on 'Breast cancer (Combined Oncoarray; iCO

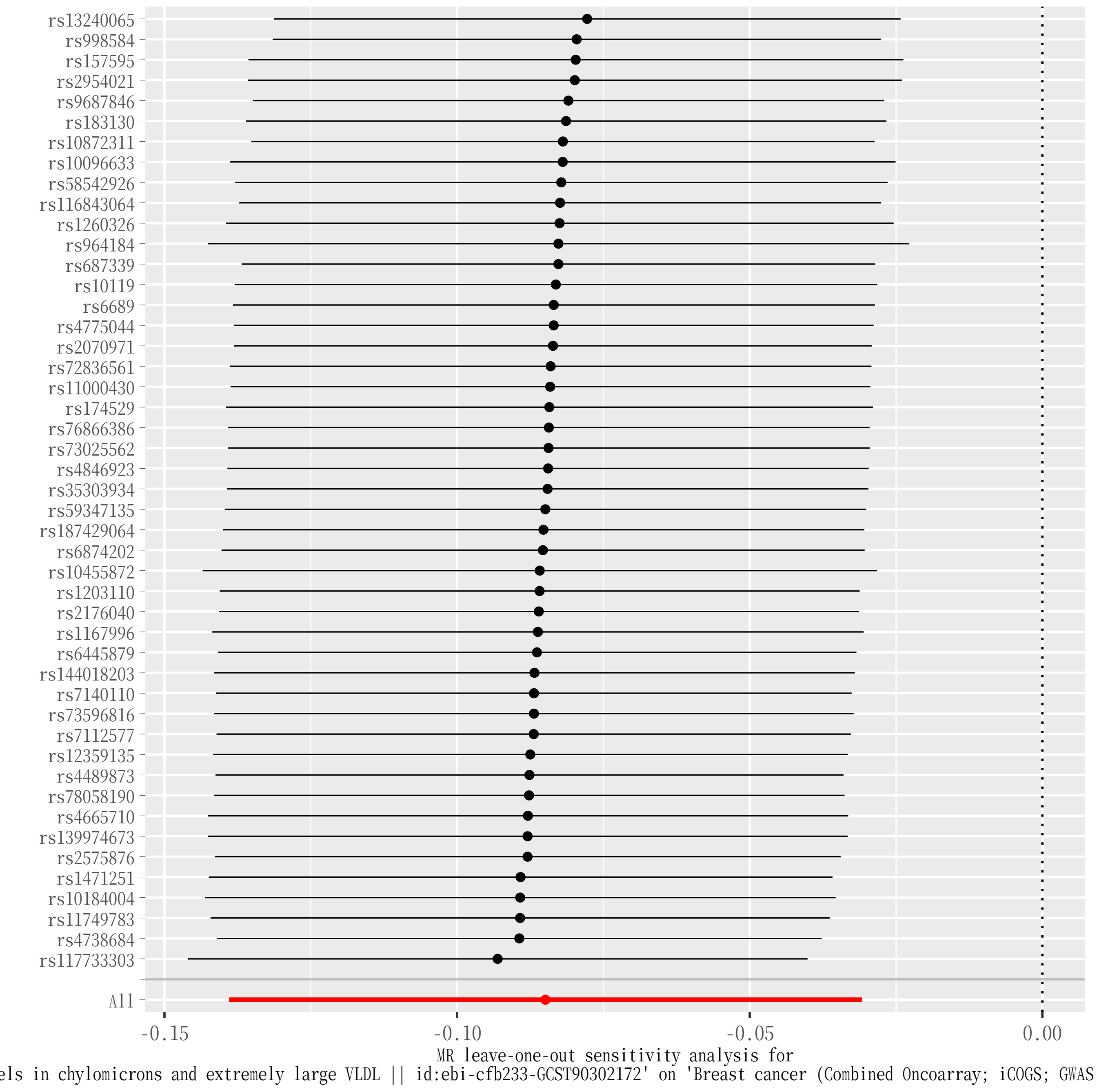
rs13240065
rs998584
rs157595
rs2954021
rs9687846
rs183130
rs10872311
rs10096633
rs58542926
rs116843064
rs1260326
rs964184
rs687339
rs10119
rs6689
rs4775044
rs2070971
rs72836561
rs11000430
rs174529
rs76866386
rs73025562
rs4846923
rs35303934
rs59347135
rs187429064
rs6874202
rs10455872
rs1203110
rs2176040
rs1167996
rs6445879
rs144018203
rs7140110
rs73596816
rs7112577
rs12359135
rs4489873
rs78058190
rs4665710
rs139974673
rs2575876
rs1471251
rs10184004
rs11749783
rs4738684
rs117733303
All
-0.15
-0.10
-0.05
0.00
MR leave-one-out sensitivity analysis for
els in chylomicrons and extremely large VLDL || id:ebi-cfb233-GCST90302172' on 'Breast cancer (Combined Oncoarray; iCOGS; GWAS

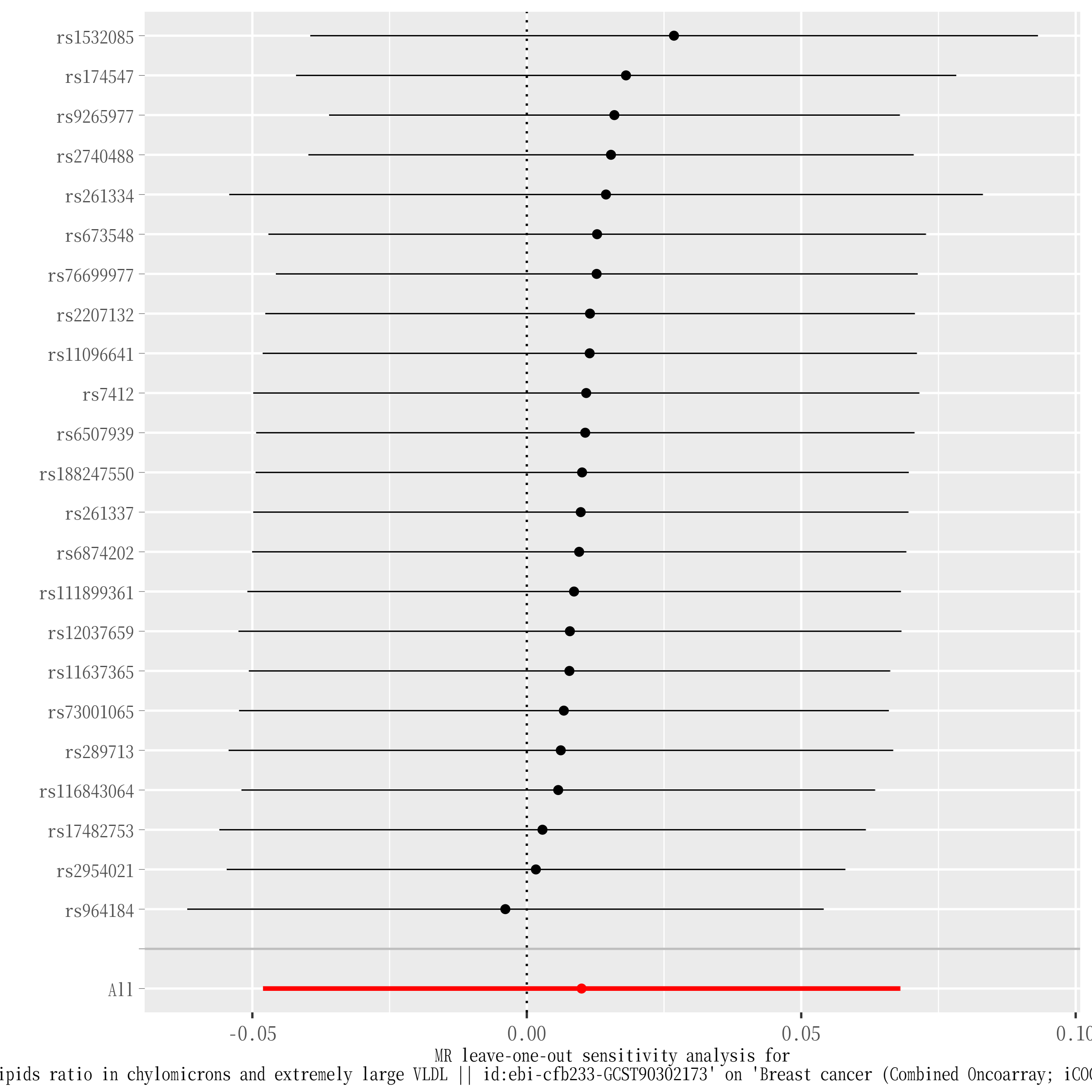
rs1532085
rs174547
rs9265977
rs2740488
rs261334
rs673548
rs76699977
rs2207132
rs11096641
rs7412
rs6507939
rs188247550
rs261337
rs6874202
rs111899361
rs12037659
rs11637365
rs73001065
rs289713
rs116843064
rs17482753
rs2954021
rs964184
All
-0.05
0.00
0.05
0.10
MR leave-one-out sensitivity analysis for
ipids ratio in chylomicrons and extremely large VLDL || id:ebi-cfb233-GCST90302173' on 'Breast cancer (Combined Oncoarray; iCO
