# Supplementary material for: Assessing the causal relationships between circulating metabolic biomarkers and breast cancer by using mendelian randomization
Source: Front Genet. 2024 Dec 18;15:1448748. doi: 10.3389/fgene.2024.1448748 (PMC11688392; doi:10.3389/fgene.2024.1448748)
Supplement: Supplementary file 2 [file DataSheet13.pdf]

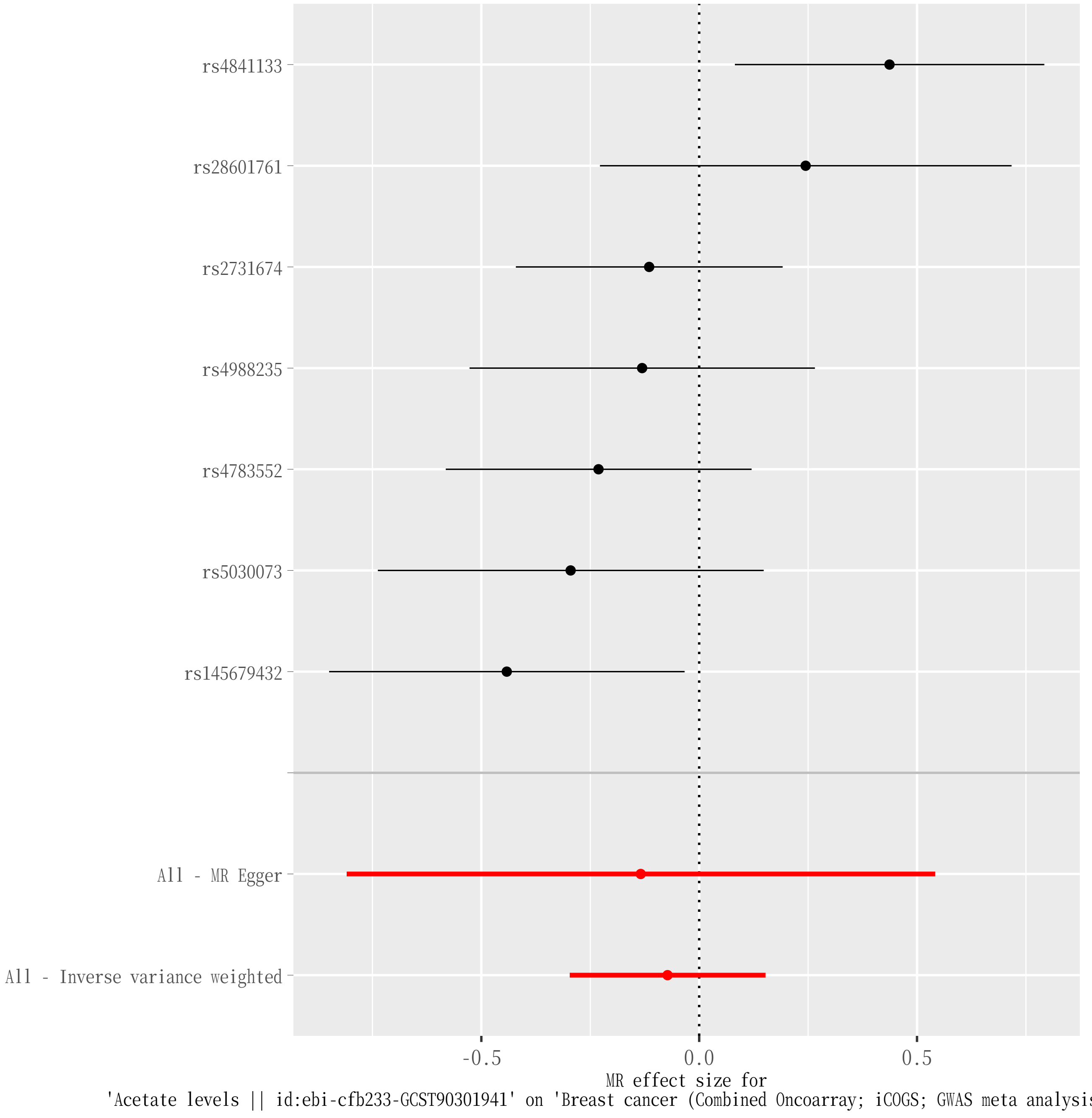

rs4841133
rs28601761
rs2731674
rs4988235
rs4783552
rs5030073
rs145679432
All - MR Egger
All - Inverse variance weighted
-0.5
0.0
0.5
MR effect size for
'Acetate levels || id:ebi-cfb233-GCST90301941' on 'Breast cancer (Combined Oncoarray; iCOGS; GWAS meta analysis

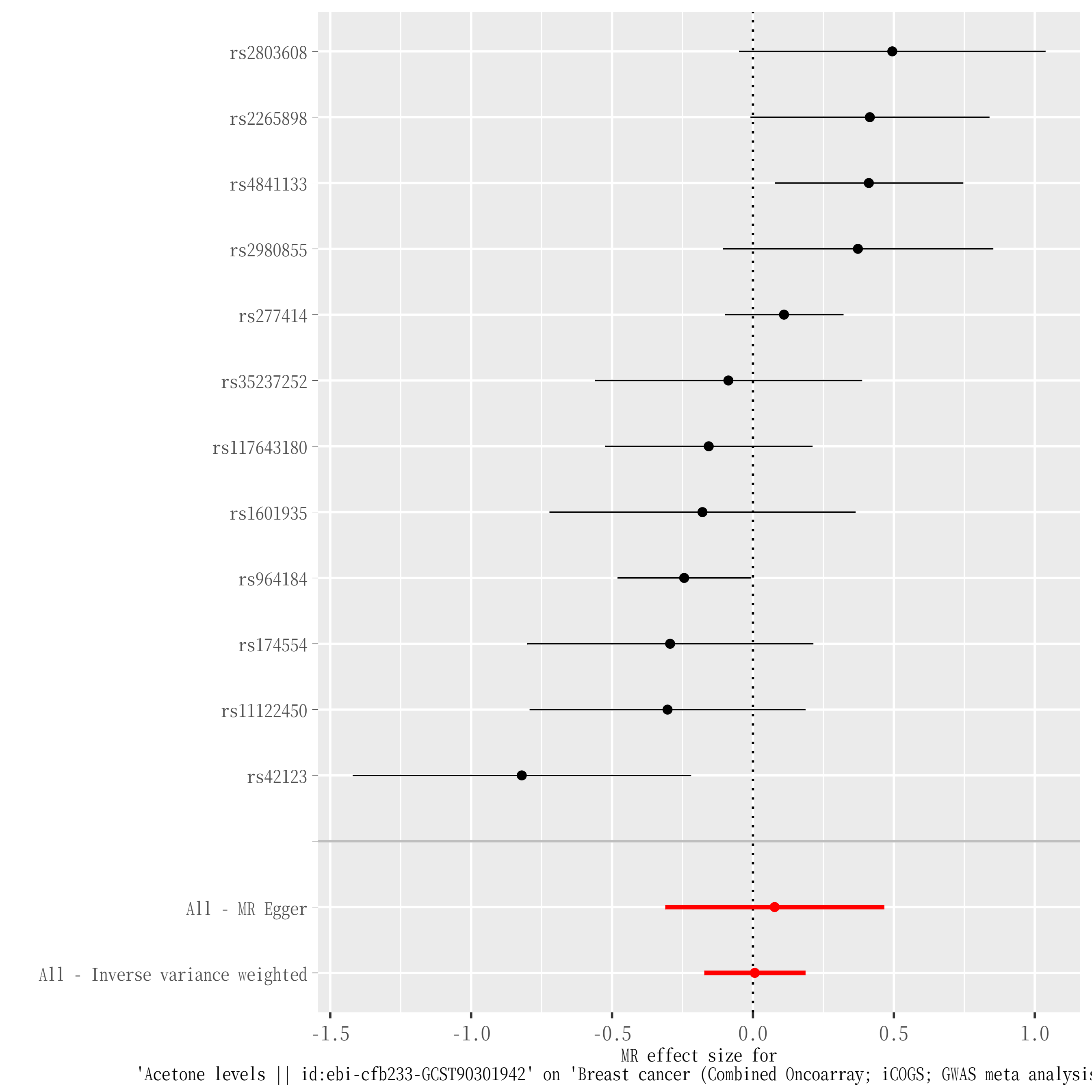
rs2803608
rs2265898
rs4841133
rs2980855
rs277414
rs35237252
rs117643180
rs1601935
rs964184
rs174554
rs11122450
rs42123
All - MR Egger
All - Inverse variance weighted
-1.5
-1.0
-0.5
0.0
0.5
1.0
MR effect size for
'Acetone levels || id:ebi-cfb233-GCST90301942' on 'Breast cancer (Combined Oncoarray; iCOGS; GWAS meta analysis

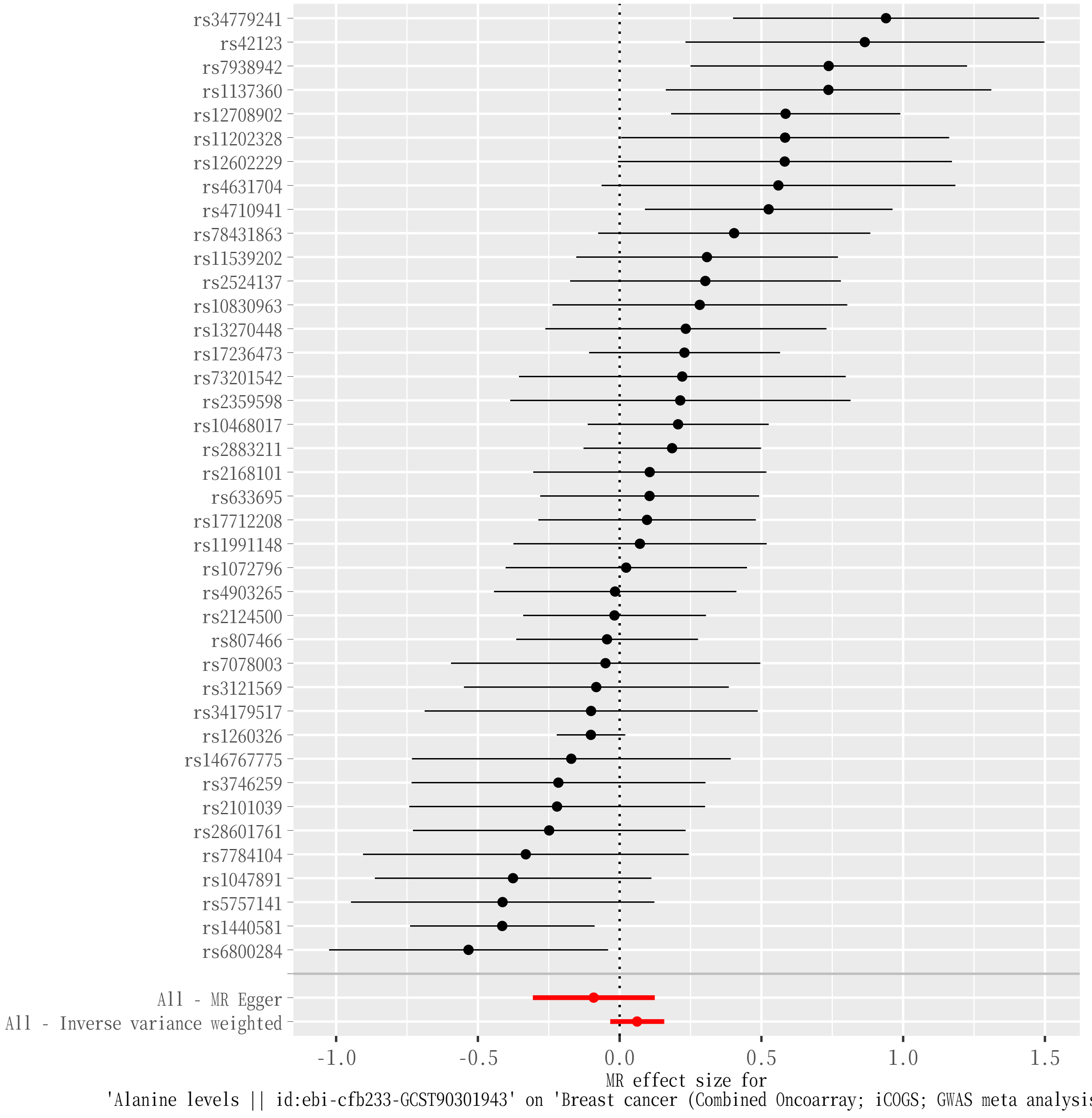

rs34779241
rs42123
rs7938942
rs1137360
rs12708902
rs11202328
rs12602229
rs4631704
rs4710941
rs78431863
rs11539202
rs2524137
rs10830963
rs13270448
rs17236473
rs73201542
rs2359598
rs10468017
rs2883211
rs2168101
rs633695
rs17712208
rs11991148
rs1072796
rs4903265
rs2124500
rs807466
rs7078003
rs3121569
rs34179517
rs1260326
rs146767775
rs3746259
rs2101039
rs28601761
rs7784104
rs1047891
rs5757141
rs1440581
rs6800284
All - MR Egger
All - Inverse variance weighted
-1.0
-0.5
0.0
0.5
1.0
1.5
MR effect size for
'Alanine levels || id:ebi-cfb233-GCST90301943' on 'Breast cancer (Combined Oncoarray; iCOGS; GWAS meta analysis

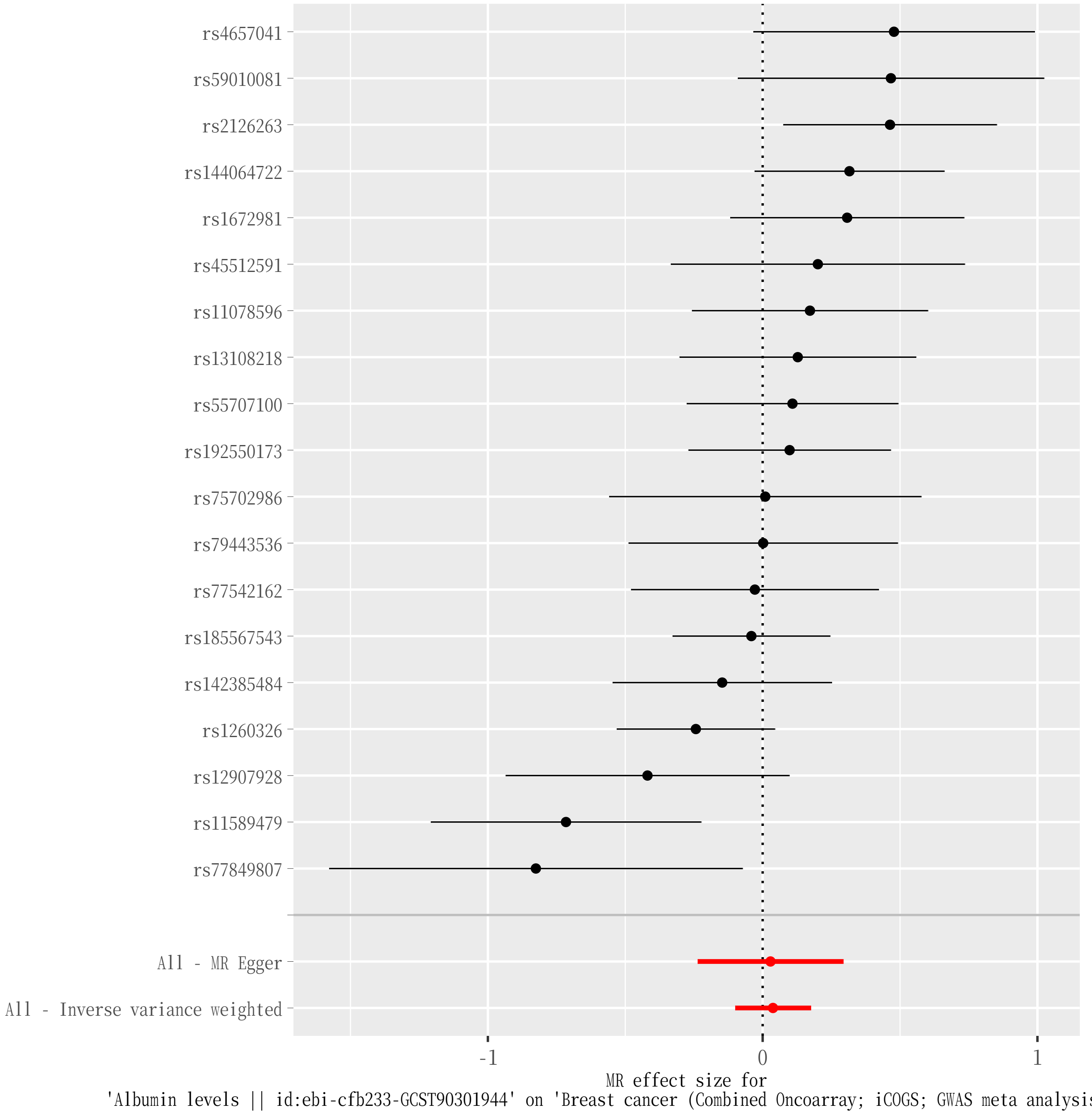
rs4657041
rs59010081
rs2126263
rs144064722
rs1672981
rs45512591
rs11078596
rs13108218
rs55707100
rs192550173
rs75702986
rs79443536
rs77542162
rs185567543
rs142385484
rs1260326
rs12907928
rs11589479
rs77849807
All - MR Egger
All - Inverse variance weighted
-1
0
1
MR effect size for
'Albumin levels || id:ebi-cfb233-GCST90301944' on 'Breast cancer (Combined Oncoarray; iCOGS; GWAS meta analysis

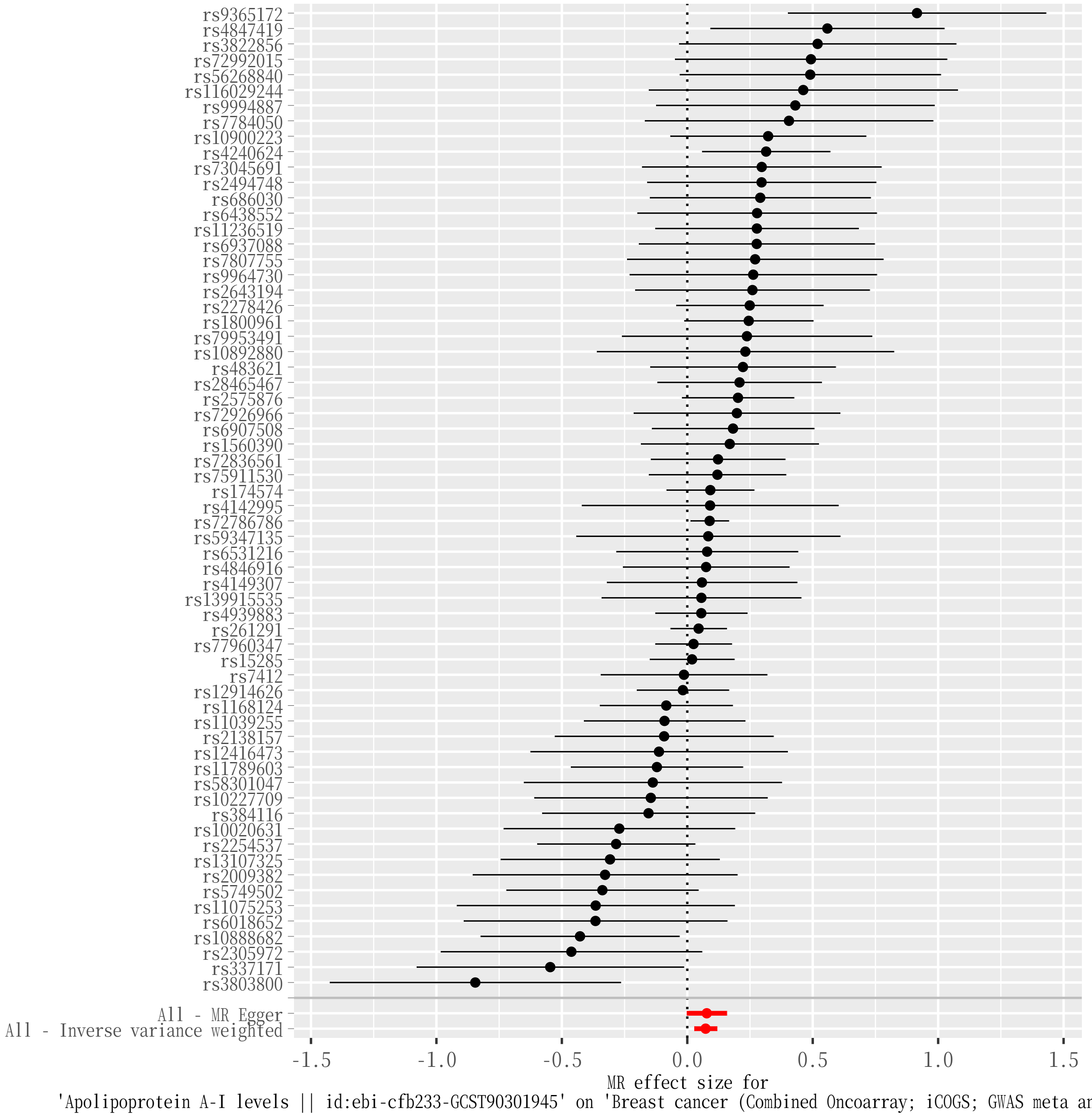
rs9365172
rs4847419
rs3822856
rs72992015
rs56268840
rs116029244
rs9994887
rs7784050
rs10900223
rs4240624
rs73045691
rs2494748
rs686030
rs6438552
rs11236519
rs6937088
rs7807755
rs9964730
rs2643194
rs2278426
rs1800961
rs79953491
rs10892880
rs483621
rs28465467
rs2575876
rs72926966
rs6907508
rs1560390
rs72836561
rs75911530
rs174574
rs4142995
rs72786786
rs59347135
rs6531216
rs4846916
rs4149307
rs139915535
rs4939883
rs261291
rs77960347
rs15285
rs7412
rs12914626
rs1168124
rs11039255
rs2138157
rs12416473
rs11789603
rs58301047
rs10227709
rs384116
rs10020631
rs2254537
rs13107325
rs2009382
rs5749502
rs11075253
rs6018652
rs10888682
rs2305972
rs337171
rs3803800
All - MR Egger
All - Inverse variance weighted
-1.5
-1.0
-0.5
0.0
0.5
1.0
1.5
MR effect size for
'Apolipoprotein A-I levels || id:ebi-cfb233-GCST90301945' on 'Breast cancer (Combined Oncoarray; iCOGS; GWAS meta an

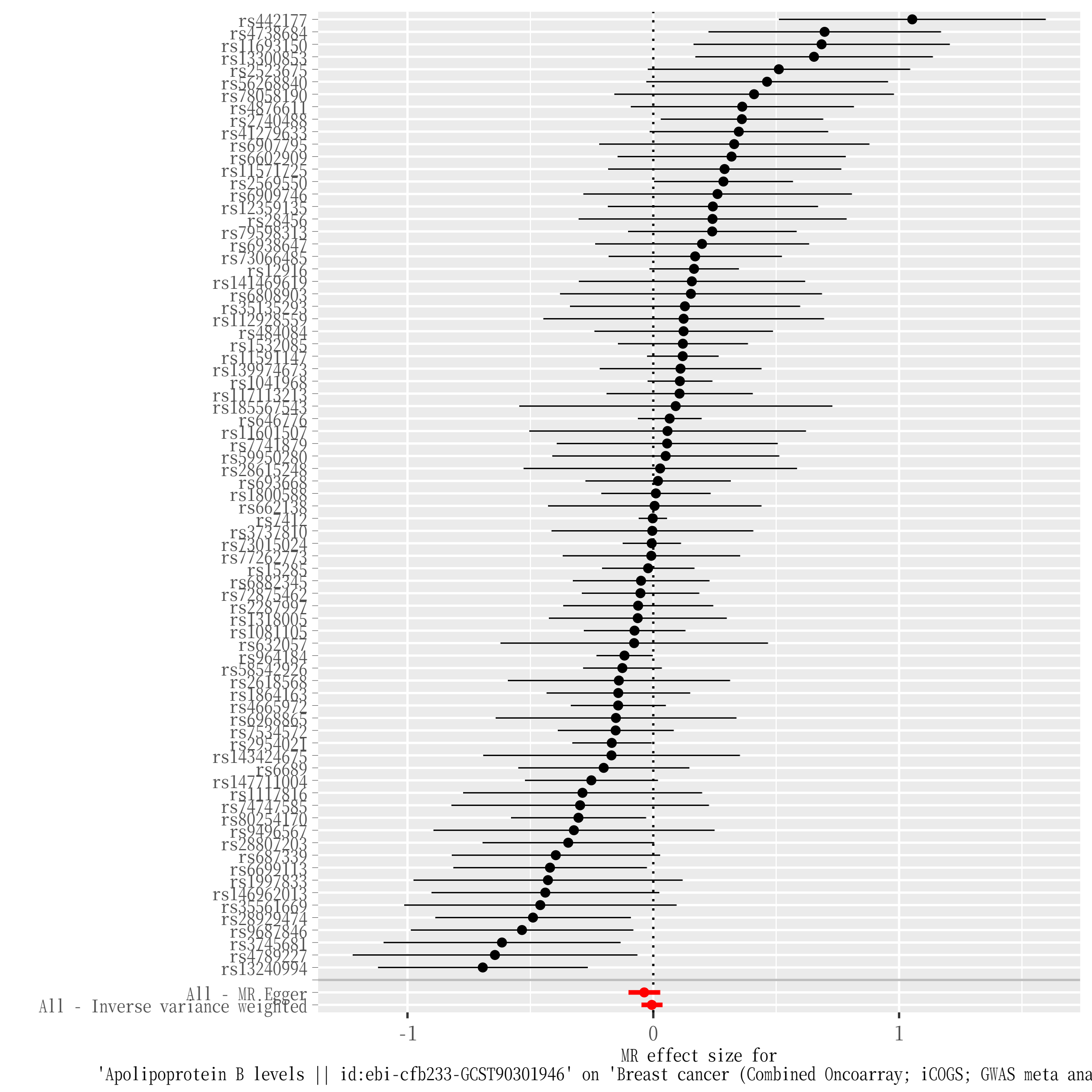
rs442177
rs4738684
rs11693150
rs13300853
rs2523675
rs56268840
rs78058190
rs4876611
rs2740488
rs41279633
rs6907795
rs6602909
rs11571725
rs2569550
rs6909746
rs12359135
rs28456
rs79598313
rs6938647
rs73066485
rs12916
rs141469619
rs6808903
rs35135293
rs112928559
rs484084
rs1532085
rs11591147
rs139974673
rs1041968
rs117113213
rs185567543
rs646776
rs11601507
rs7741879
rs59950280
rs28615248
rs693668
rs1800588
rs662138
rs7412
rs3737810
rs73015024
rs77262773
rs15285
rs6882345
rs72875462
rs2287997
rs1318005
rs1081105
rs632057
rs964184
rs58542926
rs2618568
rs1864163
rs4665972
rs6968865
rs7534572
rs2954021
rs143424675
rs6689
rs147711004
rs1117816
rs74747585
rs80254170
rs9496567
rs28807203
rs687339
rs6699113
rs1997833
rs146962013
rs35561669
rs28929474
rs9687846
rs3745681
rs4789227
rs13240994
All - MR Egger
All - Inverse variance weighted
-1
0
1
MR effect size for
'Apolipoprotein B levels || id:ebi-cfb233-GCST90301946' on 'Breast cancer (Combined Oncoarray; iCOGS; GWAS meta ana

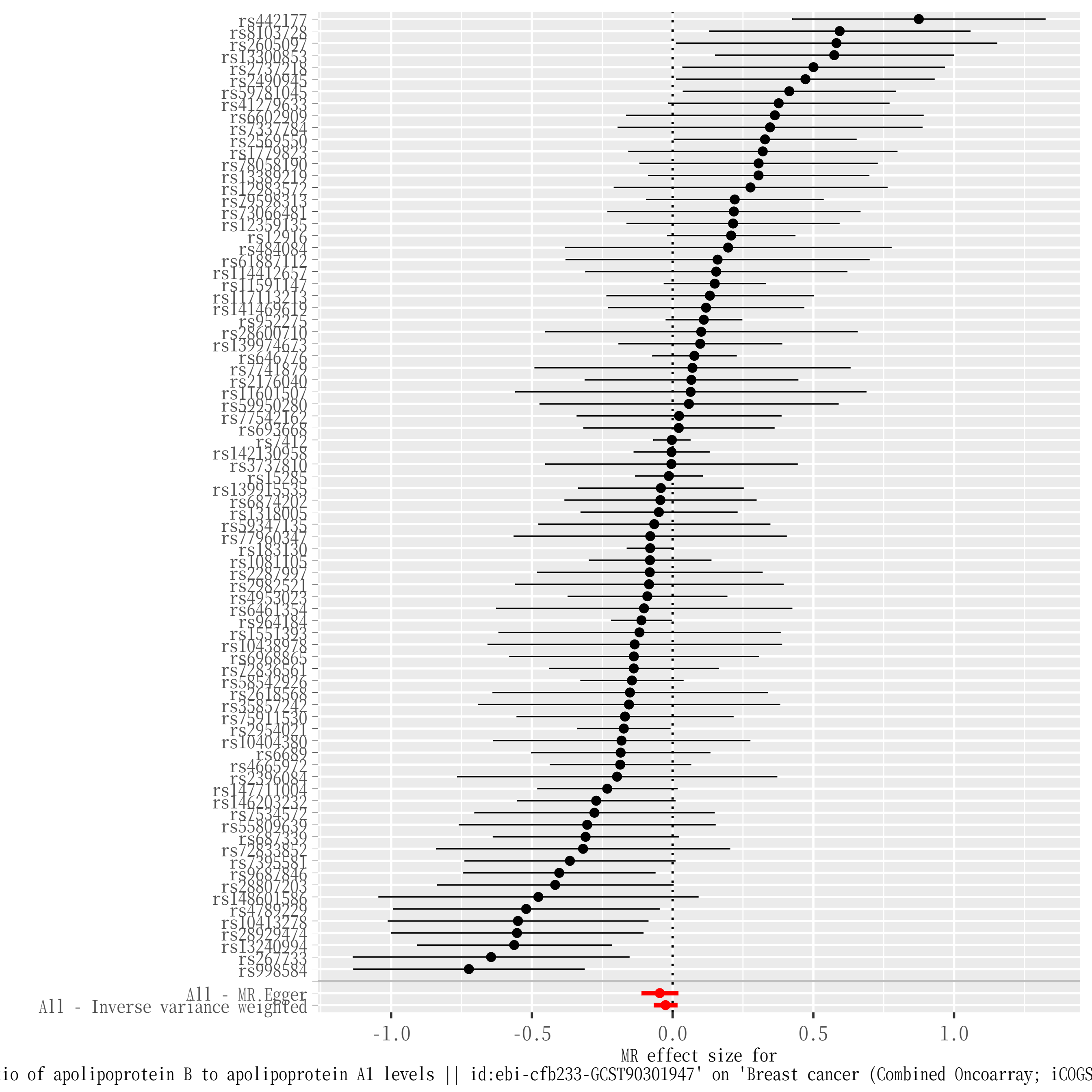
rs442177
rs8103728
rs2605097
rs13300853
rs2737218
rs2490945
rs59781045
rs41279633
rs6602909
rs7337784
rs2569550
rs1779823
rs78058190
rs13389219
rs12983572
rs79598313
rs73066481
rs12359135
rs12916
rs484084
rs61887112
rs114412657
rs11591147
rs117113213
rs141469619
rs952275
rs28600710
rs139974673
rs646776
rs7741879
rs2176040
rs11601507
rs59950280
rs77542162
rs693668
rs7412
rs142130958
rs3737810
rs15285
rs139915535
rs6874202
rs1318005
rs59347135
rs77960347
rs183130
rs1081105
rs2287997
rs2982521
rs4953023
rs6461354
rs964184
rs1551393
rs10438978
rs6968865
rs72836561
rs58542926
rs2618568
rs35857242
rs75911530
rs2954021
rs10404380
rs6689
rs4665972
rs2396084
rs147711004
rs146203232
rs7534572
rs55809639
rs687339
rs72833852
rs7395581
rs9687846
rs28807203
rs148601586
rs4789229
rs10413278
rs28929474
rs13240994
rs267733
rs998584
All - MR Egger
All - Inverse variance weighted
-1.0
-0.5
0.0
0.5
1.0
MR effect size for
io of apolipoprotein B to apolipoprotein A1 levels || id:ebi-cfb233-GCST90301947' on 'Breast cancer (Combined Oncoarray; iCOGS

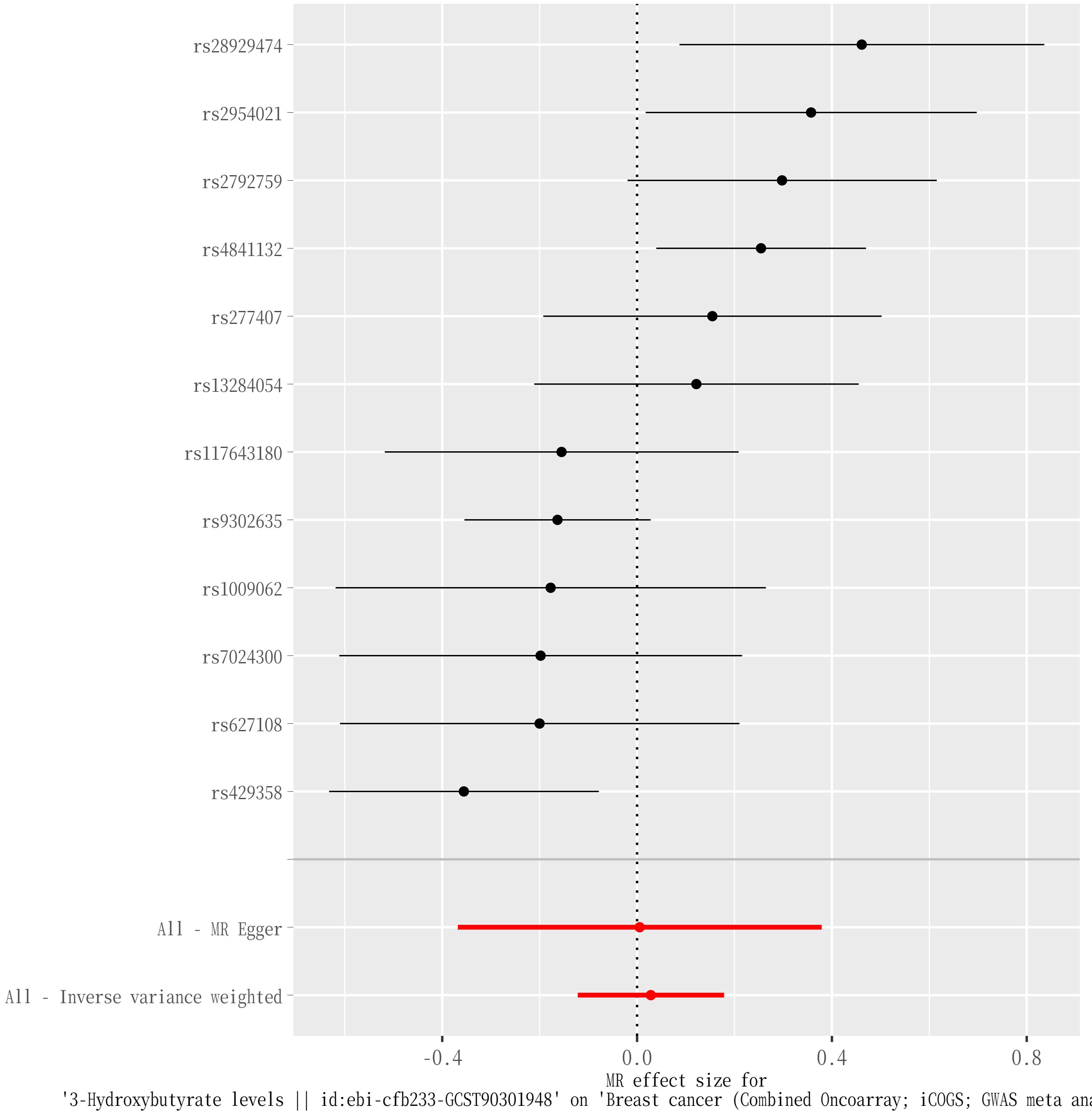
rs28929474
rs2954021
rs2792759
rs4841132
rs277407
rs13284054
rs117643180
rs9302635
rs1009062
rs7024300
rs627108
rs429358
All - MR Egger
All - Inverse variance weighted
-0.4
0.0
0.4
0.8
MR effect size for
'3-Hydroxybutyrate levels || id:ebi-cfb233-GCST90301948' on 'Breast cancer (Combined Oncoarray; iCOGS; GWAS meta an

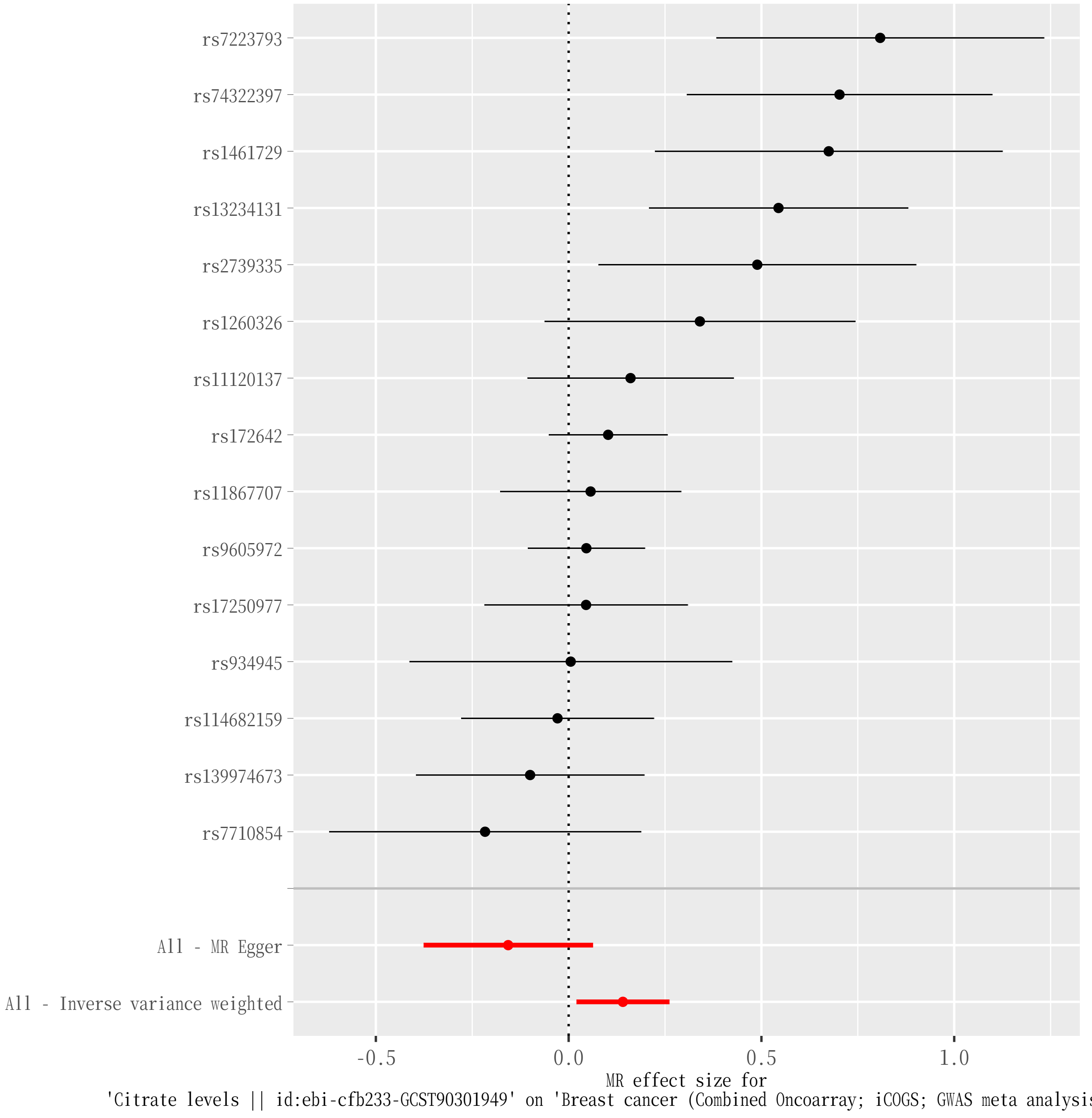
rs7223793
rs74322397
rs1461729
rs13234131
rs2739335
rs1260326
rs11120137
rs172642
rs11867707
rs9605972
rs17250977
rs934945
rs114682159
rs139974673
rs7710854
All - MR Egger
All - Inverse variance weighted
-0.5
0.0
0.5
1.0
MR effect size for
'Citrate levels || id:ebi-cfb233-GCST90301949' on 'Breast cancer (Combined Oncoarray; iCOGS; GWAS meta analysis

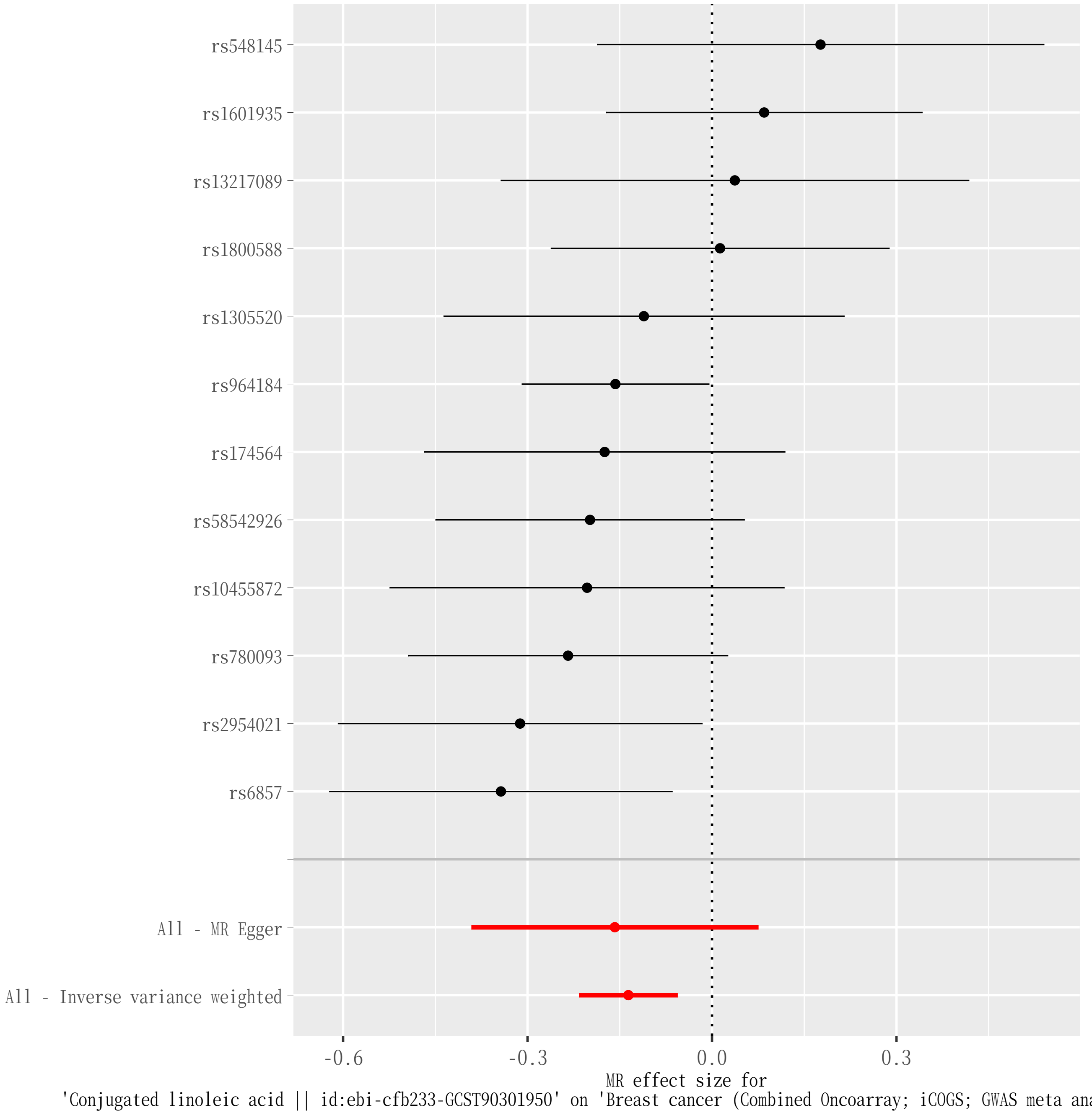
rs548145
rs1601935
rs13217089
rs1800588
rs1305520
rs964184
rs174564
rs58542926
rs10455872
rs780093
rs2954021
rs6857
All - MR Egger
All - Inverse variance weighted
-0.6
-0.3
0.0
0.3
MR effect size for
'Conjugated linoleic acid || id:ebi-cfb233-GCST90301950' on 'Breast cancer (Combined Oncoarray; iCOGS; GWAS meta ana

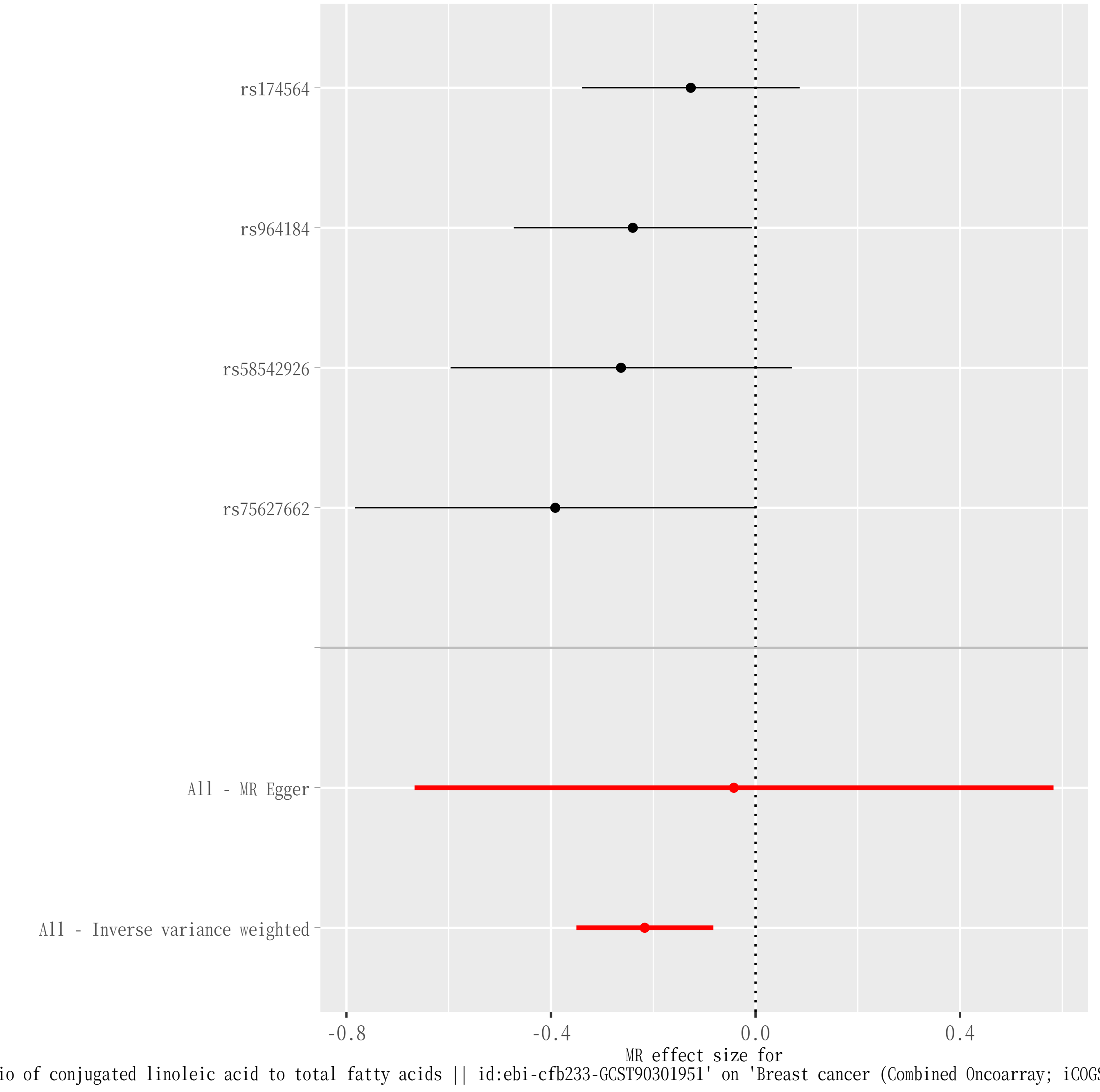
rs174564
rs964184
rs58542926
rs75627662
All - MR Egger
All - Inverse variance weighted
-0.8
-0.4
0.0
0.4
MR effect size for
io of conjugated linoleic acid to total fatty acids || id:ebi-cfb233-GCST90301951' on 'Breast cancer (Combined Oncoarray; iCOG

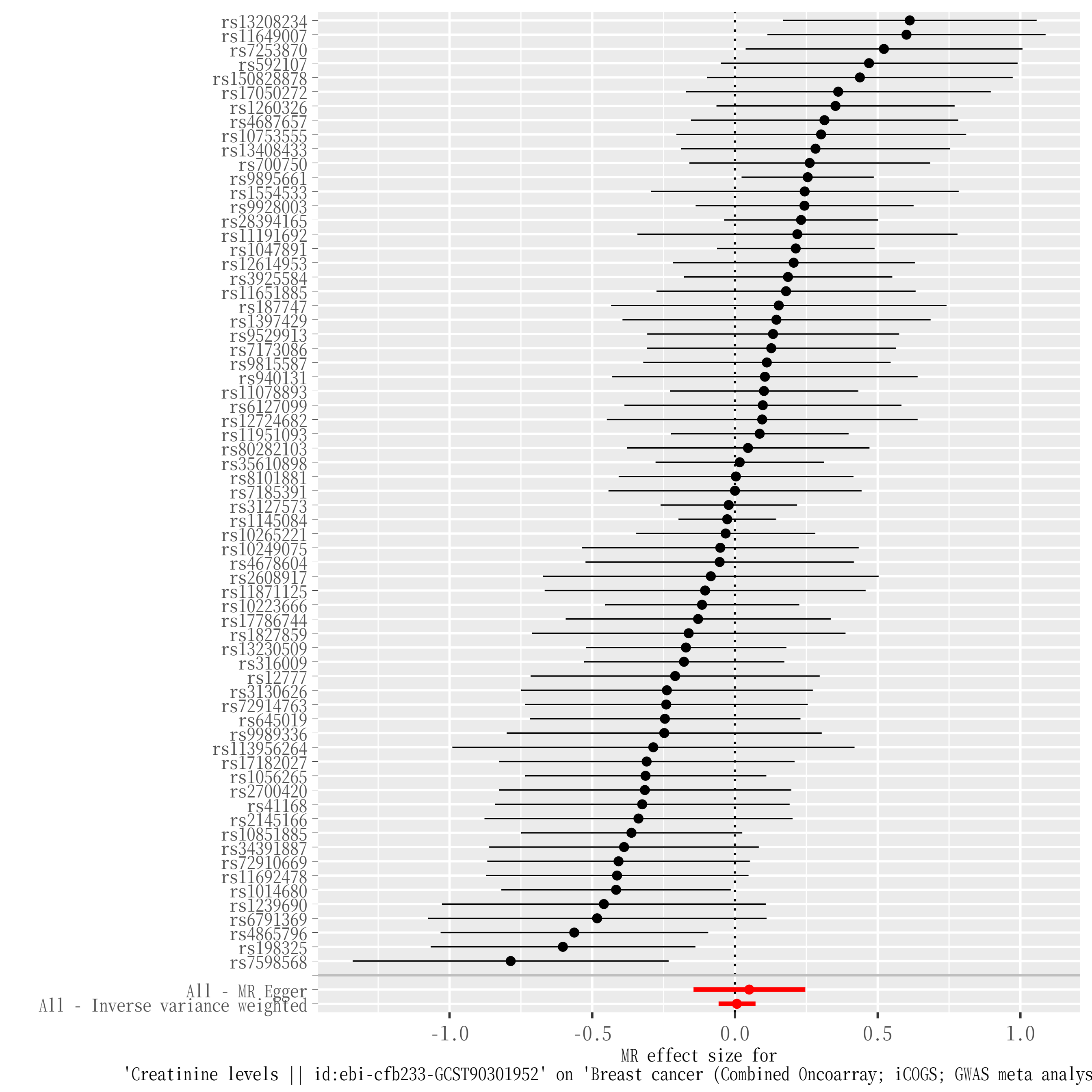

rs13208234
rs11649007
rs7253870
rs592107
rs150828878
rs17050272
rs1260326
rs4687657
rs10753555
rs13408433
rs700750
rs9895661
rs1554533
rs9928003
rs28394165
rs11191692
rs1047891
rs12614953
rs3925584
rs11651885
rs187747
rs1397429
rs9529913
rs7173086
rs9815587
rs940131
rs11078893
rs6127099
rs12724682
rs11951093
rs80282103
rs35610898
rs8101881
rs7185391
rs3127573
rs1145084
rs10265221
rs10249075
rs4678604
rs2608917
rs11871125
rs10223666
rs17786744
rs1827859
rs13230509
rs316009
rs12777
rs3130626
rs72914763
rs645019
rs9989336
rs113956264
rs17182027
rs1056265
rs2700420
rs41168
rs2145166
rs10851885
rs34391887
rs72910669
rs11692478
rs1014680
rs1239690
rs6791369
rs4865796
rs198325
rs7598568
All - MR Egger
All - Inverse variance weighted
-1.0
-0.5
0.0
0.5
1.0
MR effect size for
'Creatinine levels || id:ebi-cfb233-GCST90301952' on 'Breast cancer (Combined Oncoarray; iCOGS; GWAS meta analys

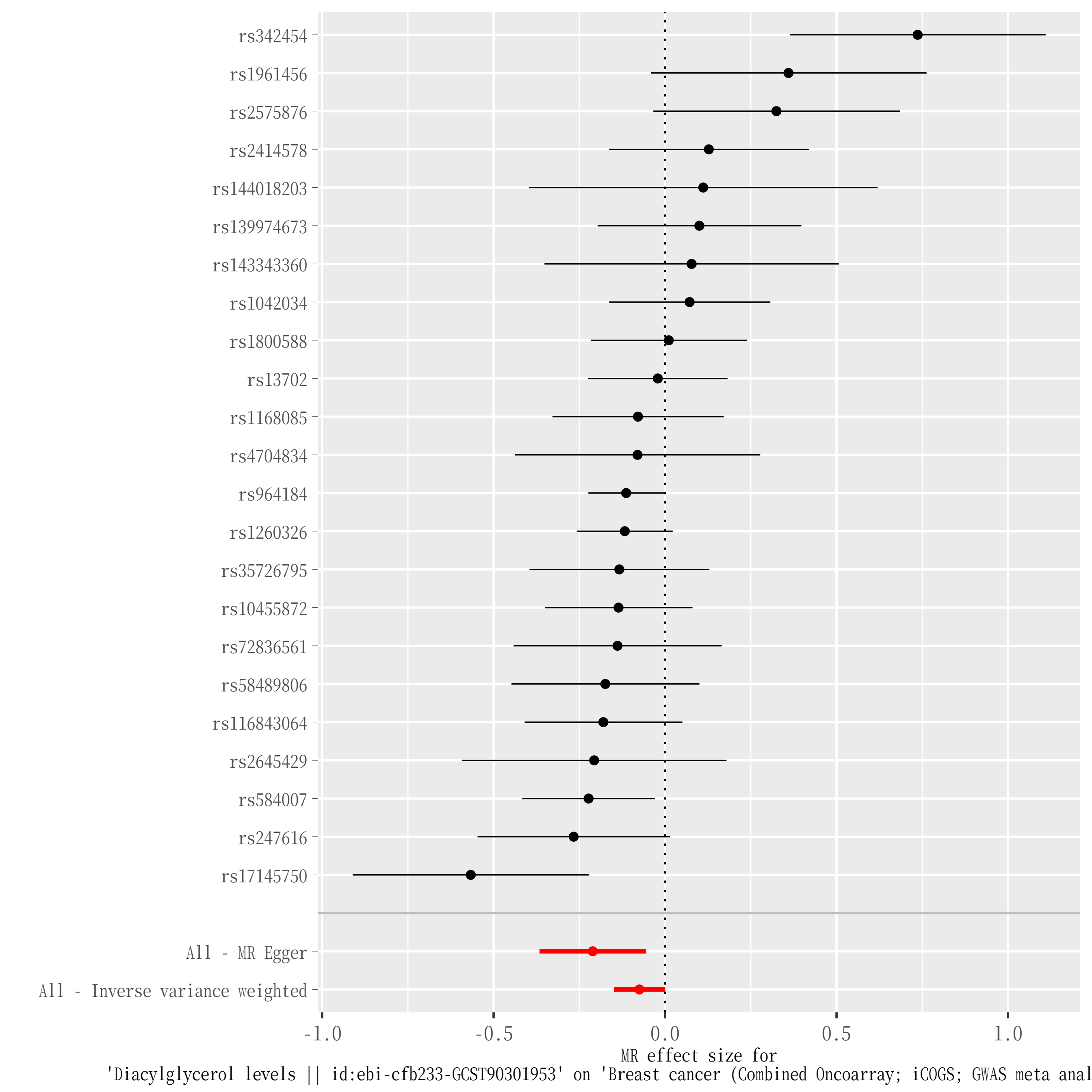

rs342454
rs1961456
rs2575876
rs2414578
rs144018203
rs139974673
rs143343360
rs1042034
rs1800588
rs13702
rs1168085
rs4704834
rs964184
rs1260326
rs35726795
rs10455872
rs72836561
rs58489806
rs116843064
rs2645429
rs584007
rs247616
rs17145750
All - MR Egger
All - Inverse variance weighted
-1.0
-0.5
0.0
0.5
1.0
MR effect size for
'Diacylglycerol levels || id:ebi-cfb233-GCST90301953' on 'Breast cancer (Combined Oncoarray; iCOGS; GWAS meta anal

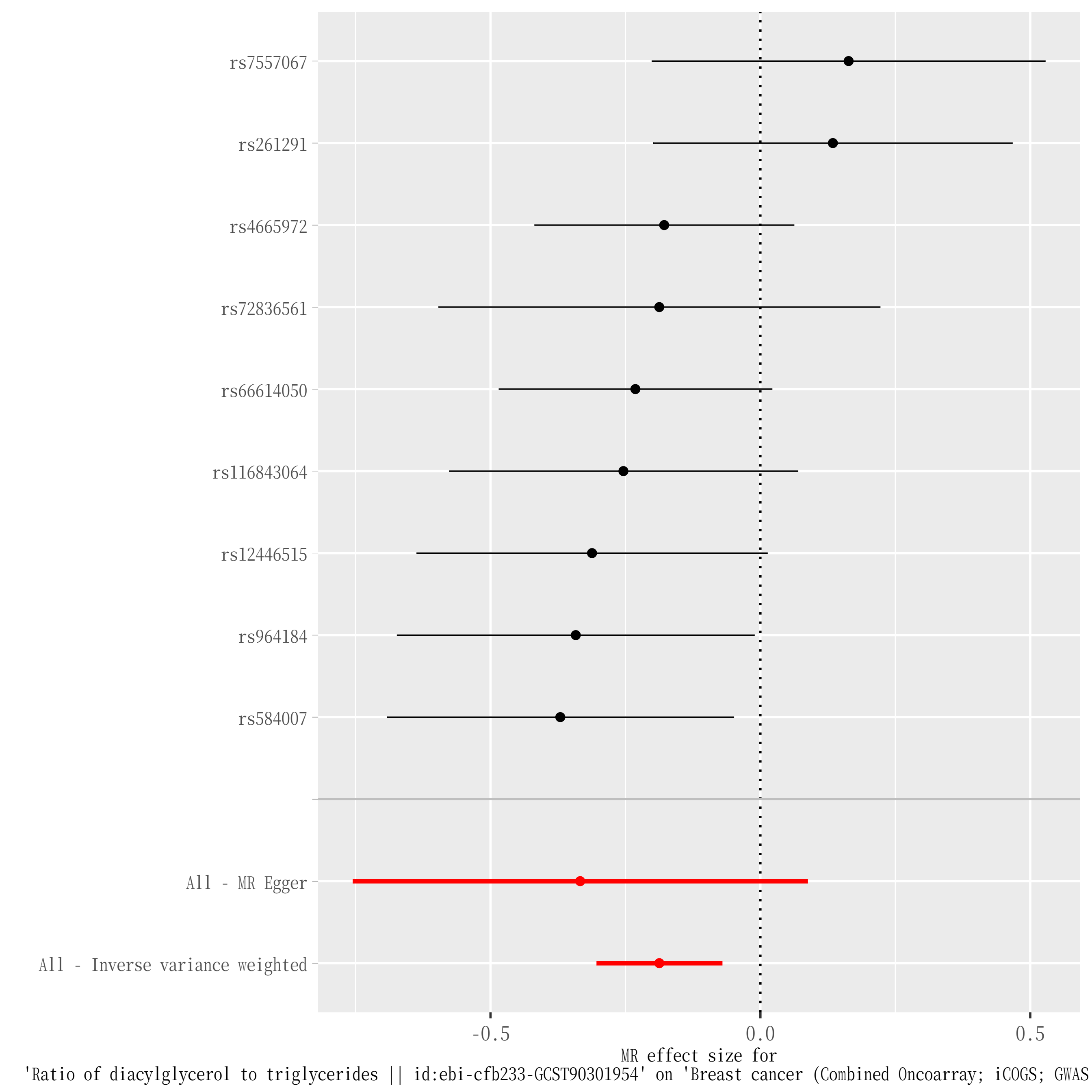
rs7557067
rs261291
rs4665972
rs72836561
rs66614050
rs116843064
rs12446515
rs964184
rs584007
All - MR Egger
All - Inverse variance weighted
-0.5
0.0
0.5
MR effect size for
'Ratio of diacylglycerol to triglycerides || id:ebi-cfb233-GCST90301954' on 'Breast cancer (Combined Oncoarray; iCOGS; GWAS

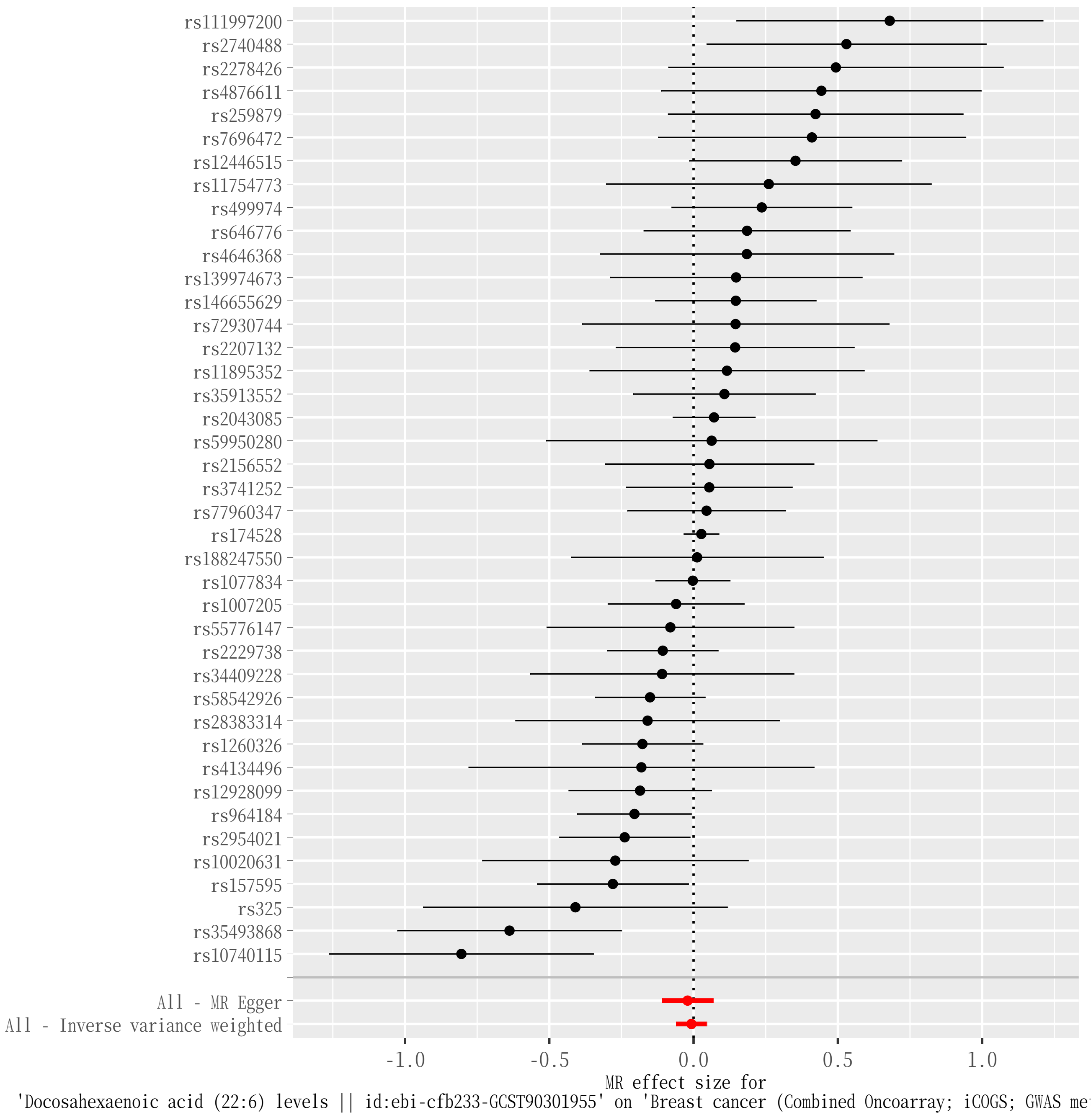

rs111997200
rs2740488
rs2278426
rs4876611
rs259879
rs7696472
rs12446515
rs11754773
rs499974
rs646776
rs4646368
rs139974673
rs146655629
rs72930744
rs2207132
rs11895352
rs35913552
rs2043085
rs59950280
rs2156552
rs3741252
rs77960347
rs174528
rs188247550
rs1077834
rs1007205
rs55776147
rs2229738
rs34409228
rs58542926
rs28383314
rs1260326
rs4134496
rs12928099
rs964184
rs2954021
rs10020631
rs157595
rs325
rs35493868
rs10740115
All - MR Egger
All - Inverse variance weighted
-1.0
-0.5
0.0
0.5
1.0
MR effect size for
'Docosahexaenoic acid (22:6) levels || id:ebi-cfb233-GCST90301955' on 'Breast cancer (Combined Oncoarray; iCOGS; GWAS me

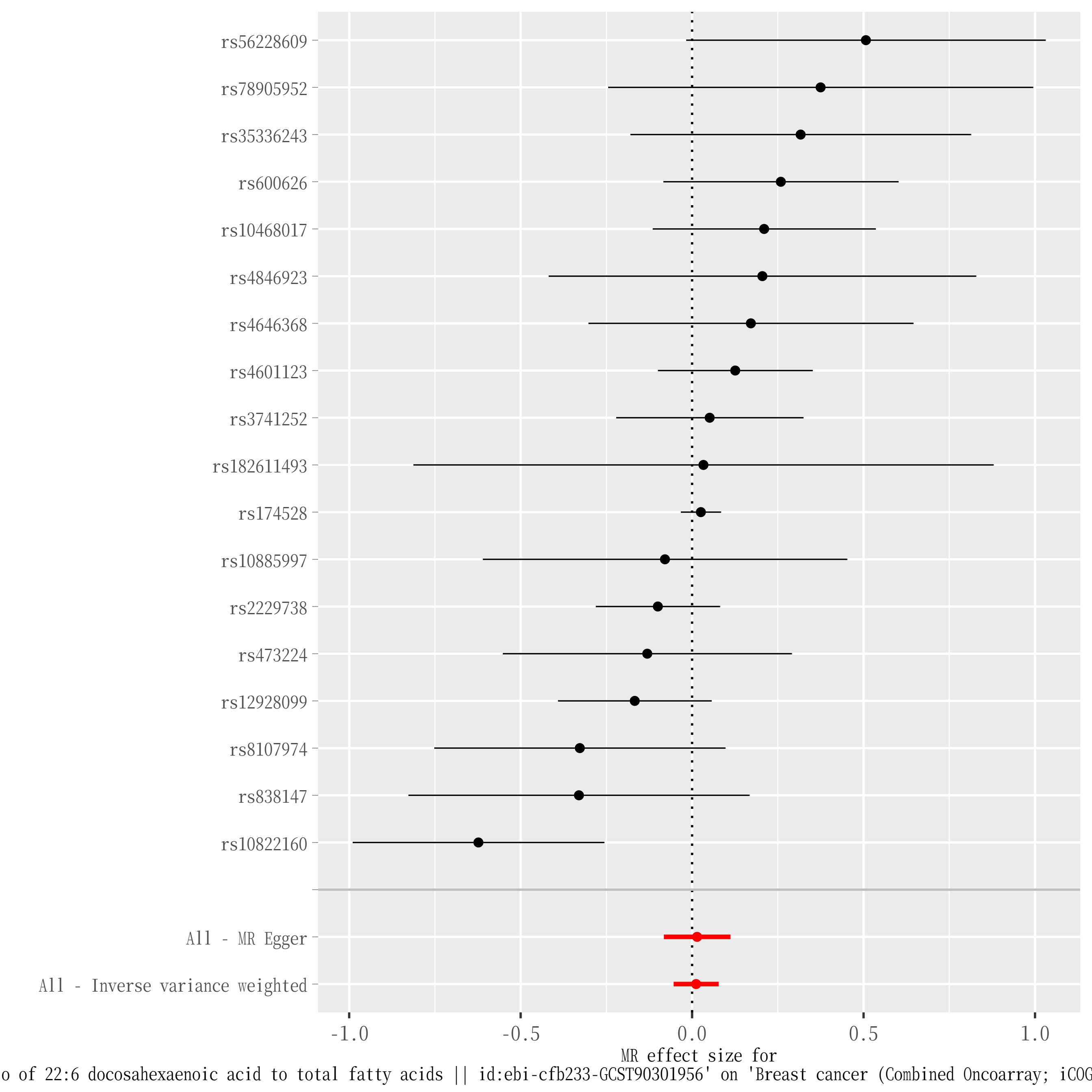

rs56228609
rs78905952
rs35336243
rs600626
rs10468017
rs4846923
rs4646368
rs4601123
rs3741252
rs182611493
rs174528
rs10885997
rs2229738
rs473224
rs12928099
rs8107974
rs838147
rs10822160
All - MR Egger
All - Inverse variance weighted
-1.0
-0.5
0.0
0.5
1.0
MR effect size for
o of 22:6 docosahexaenoic acid to total fatty acids || id:ebi-cfb233-GCST90301956' on 'Breast cancer (Combined Oncoarray; iCOG

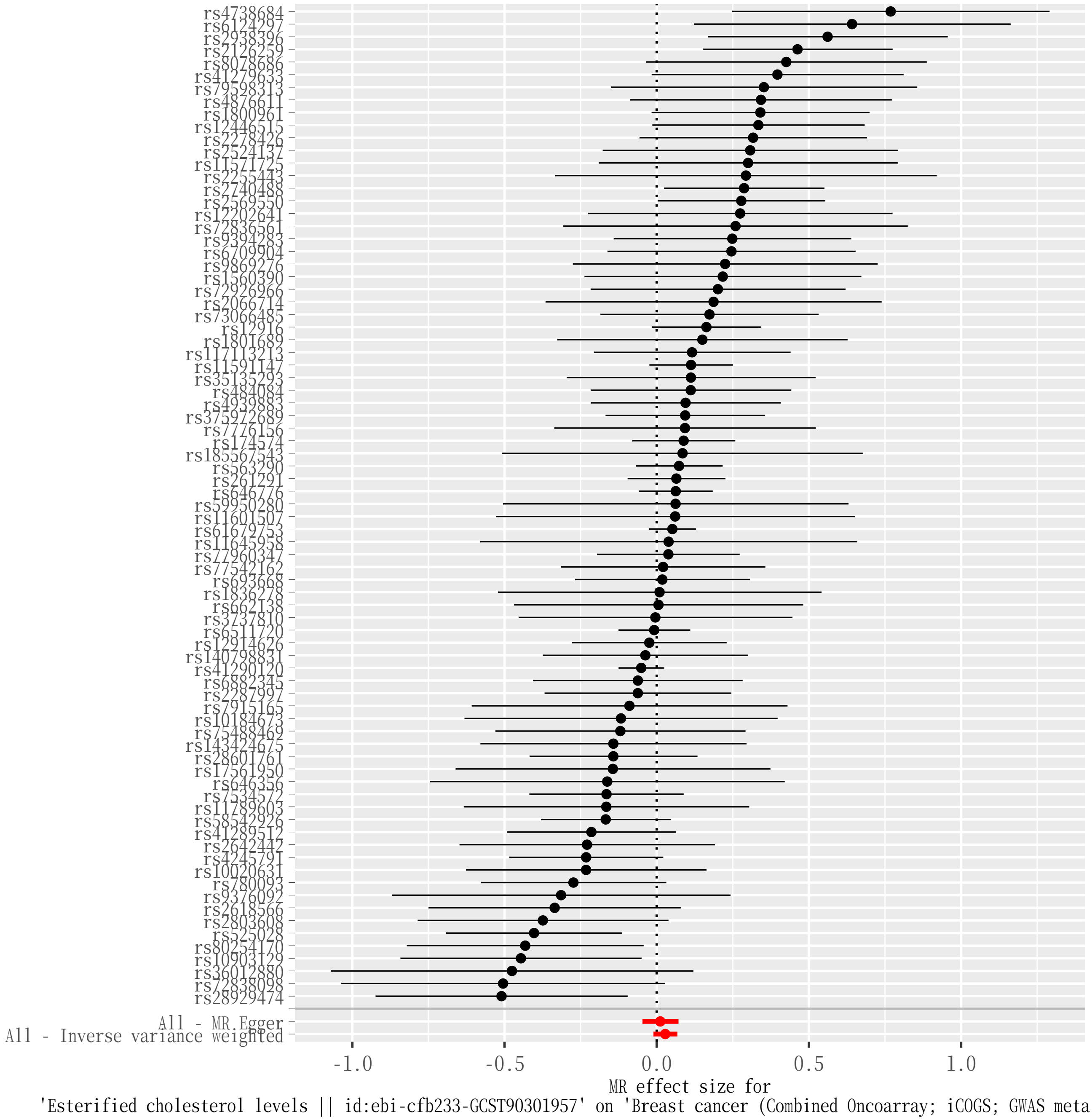
rs4738684
rs6124297
rs2938396
rs2126259
rs8078686
rs41279633
rs79598313
rs4876611
rs1800961
rs12446515
rs2278426
rs2524137
rs11571725
rs2255443
rs2740488
rs2569550
rs1220261
rs72836561
rs9394283
rs6709904
rs9869276
rs1560390
rs72926966
rs2066714
rs73066485
rs12916
rs1801689
rs117113213
rs11591147
rs35135293
rs484084
rs4939883
rs375972689
rs7776156
rs174574
rs185567543
rs563290
rs261291
rs646776
rs59950280
rs11601507
rs61679753
rs11645938
rs72960347
rs77542162
rs693668
rs1836278
rs662138
rs3737810
rs6511720
rs12914626
rs140798831
rs41290120
rs6882345
rs2287997
rs915165
rs10184673
rs75488469
rs143424675
rs28601761
rs17561950
rs646356
rs7534572
rs11789603
rs58542926
rs41289512
rs2642442
rs4245791
rs1002631
rs780093
rs9376092
rs2618566
rs2803608
rs525028
rs80254170
rs10903129
rs36012880
rs72838028
rs28929474
All - MR Egger
All - Inverse variance weighted
-1.0
-0.5
0.0
0.5
1.0
MR effect size for
'Esterified cholesterol levels || id:ebi-cfb233-GCST90301957' on 'Breast cancer (Combined Oncoarray; iCOGS; GWAS meta

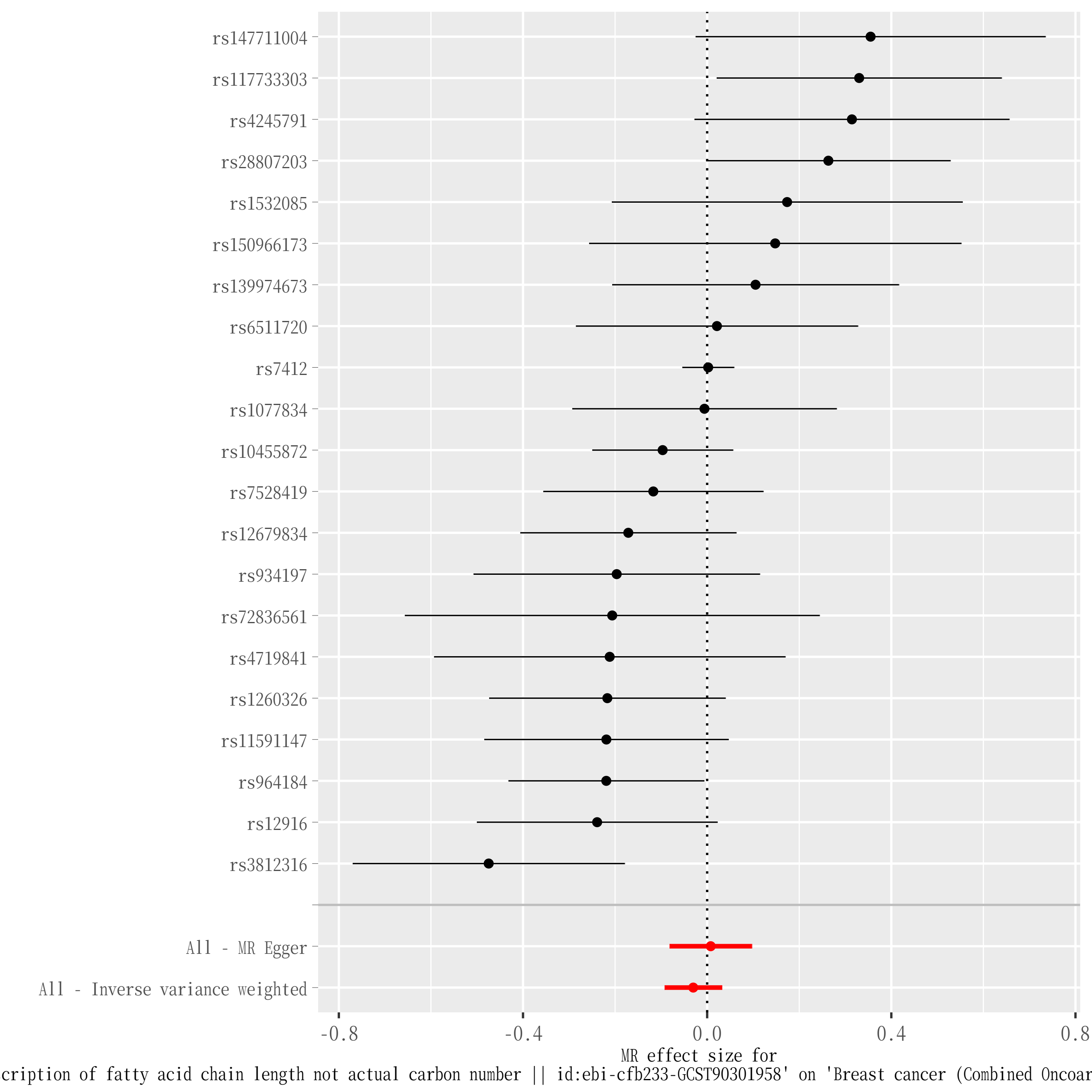
rs147711004
rs117733303
rs4245791
rs28807203
rs1532085
rs150966173
rs139974673
rs6511720
rs7412
rs1077834
rs10455872
rs7528419
rs12679834
rs934197
rs72836561
rs4719841
rs1260326
rs11591147
rs964184
rs12916
rs3812316
All - MR Egger
All - Inverse variance weighted
-0.8
-0.4
0.0
0.4
0.8
MR effect size for
cription of fatty acid chain length not actual carbon number || id:ebi-cfb233-GCST90301958' on 'Breast cancer (Combined Oncoar

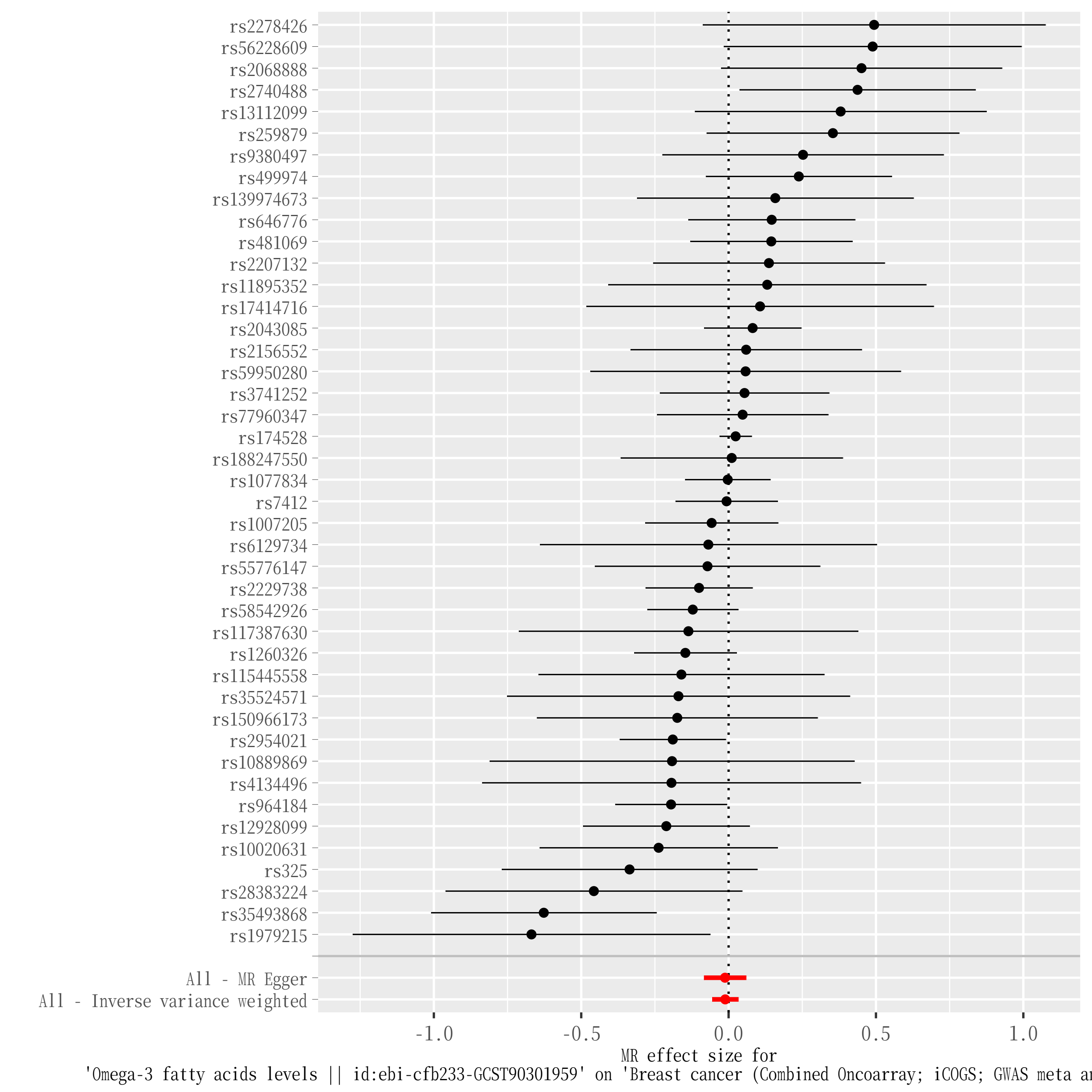
rs2278426
rs56228609
rs2068888
rs2740488
rs13112099
rs259879
rs9380497
rs499974
rs139974673
rs646776
rs481069
rs2207132
rs11895352
rs17414716
rs2043085
rs2156552
rs59950280
rs3741252
rs77960347
rs174528
rs188247550
rs1077834
rs7412
rs1007205
rs6129734
rs55776147
rs2229738
rs58542926
rs117387630
rs1260326
rs115445558
rs35524571
rs150966173
rs2954021
rs10889869
rs4134496
rs964184
rs12928099
rs10020631
rs325
rs28383224
rs35493868
rs1979215
All - MR Egger
All - Inverse variance weighted
-1.0
-0.5
0.0
0.5
1.0
MR effect size for
'Omega-3 fatty acids levels || id:ebi-cfb233-GCST90301959' on 'Breast cancer (Combined Oncoarray; iCOGS; GWAS meta an

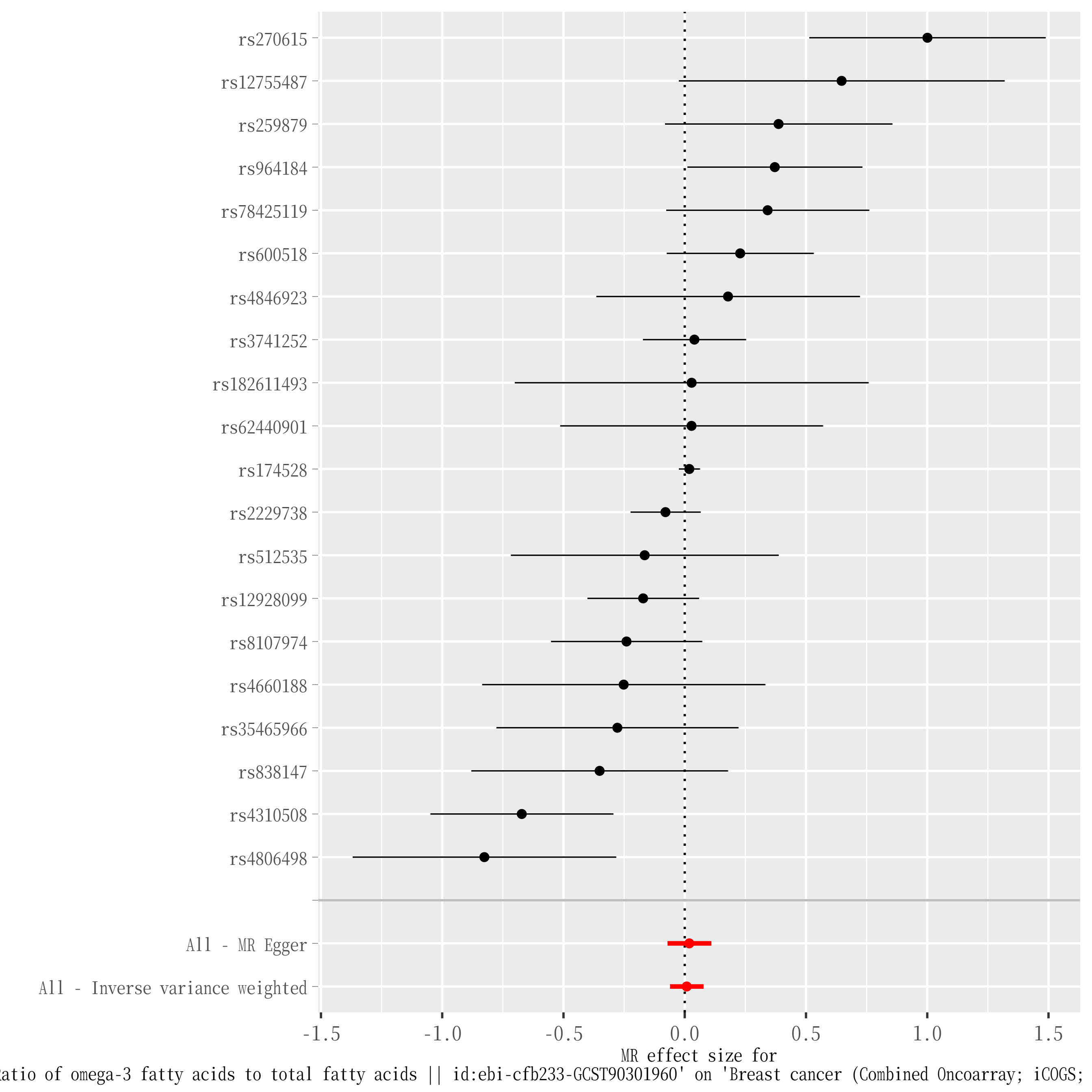

rs270615
rs12755487
rs259879
rs964184
rs78425119
rs600518
rs4846923
rs3741252
rs182611493
rs62440901
rs174528
rs2229738
rs512535
rs12928099
rs8107974
rs4660188
rs35465966
rs838147
rs4310508
rs4806498
All - MR Egger
All - Inverse variance weighted
-1.5
-1.0
-0.5
0.0
0.5
1.0
1.5
MR effect size for
Ratio of omega-3 fatty acids to total fatty acids || id:ebi-cfb233-GCST90301960' on 'Breast cancer (Combined Oncoarray; iCOGS;

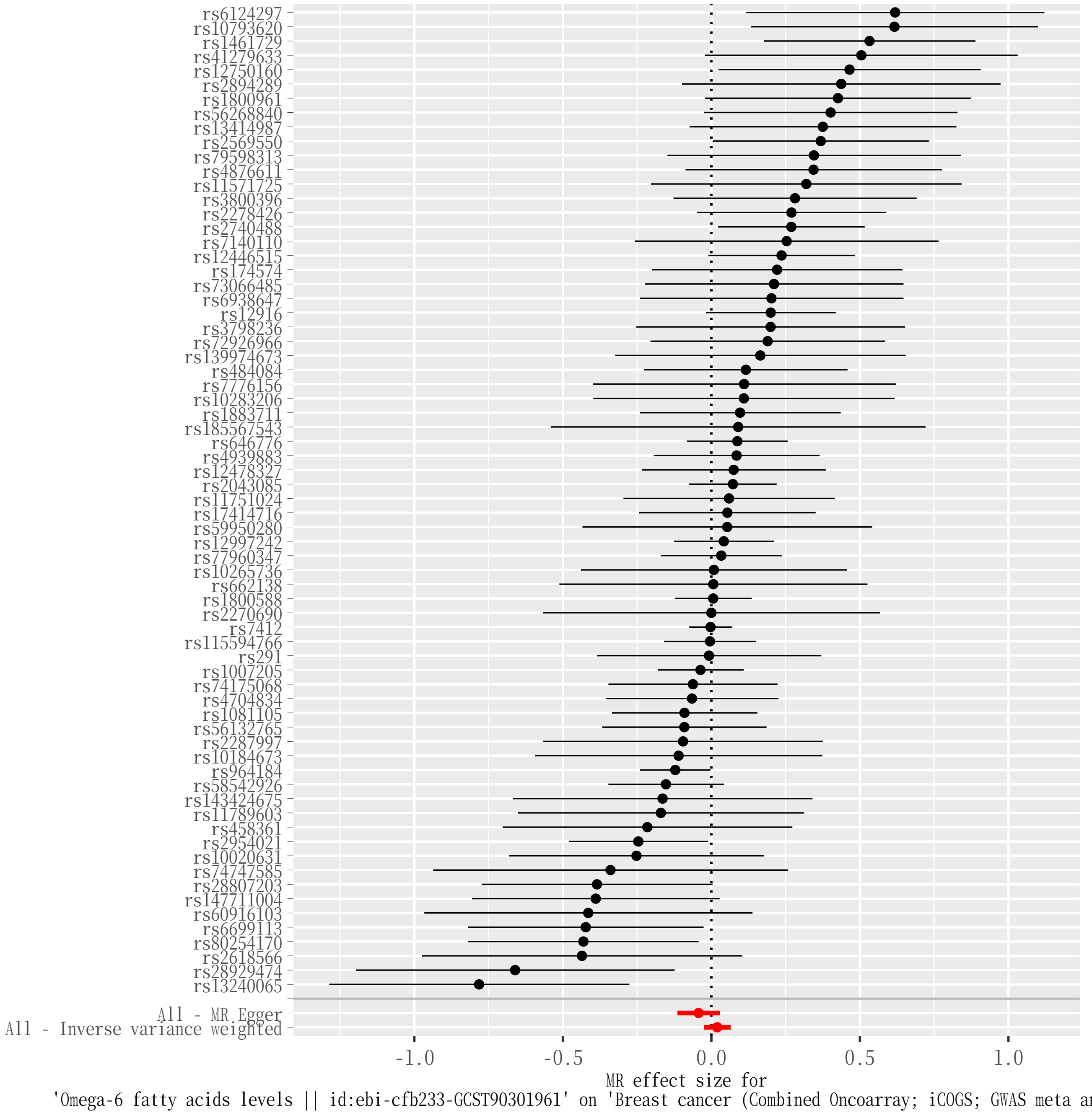
rs6124297
rs10793620
rs1461729
rs41279633
rs12750160
rs2894289
rs1800961
rs56268840
rs13414987
rs2569550
rs79598313
rs4876611
rs11571725
rs3800396
rs2278426
rs2740488
rs7140110
rs12446515
rs174574
rs73066485
rs6938647
rs12916
rs3798236
rs72926966
rs139974673
rs484084
rs7776156
rs10283206
rs1883711
rs185567543
rs646776
rs4939883
rs12478327
rs2043085
rs11751024
rs17414716
rs59950280
rs12997242
rs77960347
rs10265736
rs662138
rs1800588
rs2270690
rs7412
rs115594766
rs291
rs1007205
rs74175068
rs4704834
rs1081105
rs56132765
rs2287997
rs10184673
rs964184
rs58542926
rs143424675
rs11789603
rs458361
rs2954021
rs10020631
rs74747585
rs28807203
rs147711004
rs60916103
rs6699113
rs80254170
rs2618566
rs28929474
rs13240065
All - MR Egger
All - Inverse variance weighted
-1.0
-0.5
0.0
0.5
1.0
MR effect size for
'Omega-6 fatty acids levels || id:ebi-cfb233-GCST90301961' on 'Breast cancer (Combined Oncoarray; iCOGS; GWAS meta ar

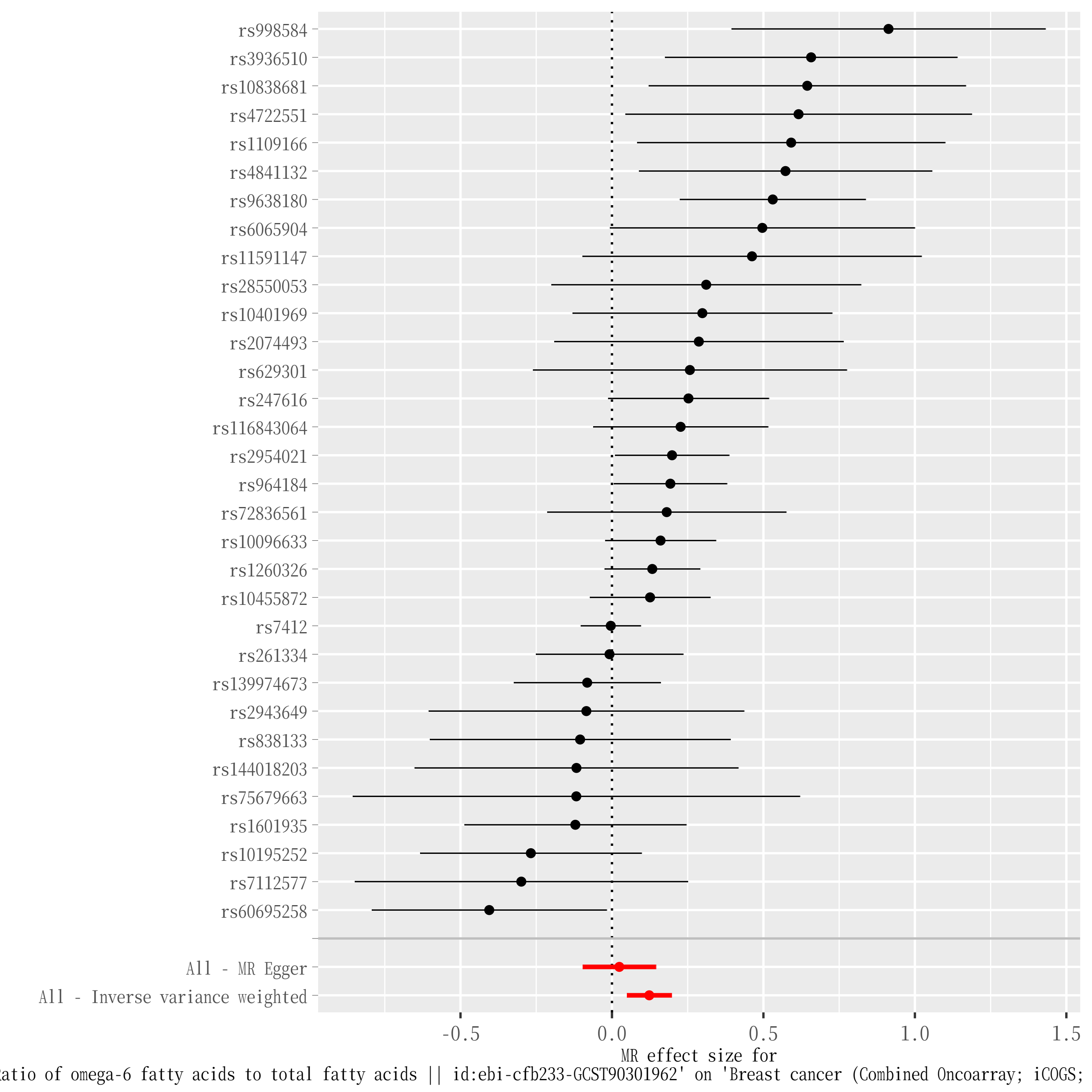
rs998584
rs3936510
rs10838681
rs4722551
rs1109166
rs4841132
rs9638180
rs6065904
rs11591147
rs28550053
rs10401969
rs2074493
rs629301
rs247616
rs116843064
rs2954021
rs964184
rs72836561
rs10096633
rs1260326
rs10455872
rs7412
rs261334
rs139974673
rs2943649
rs838133
rs144018203
rs75679663
rs1601935
rs10195252
rs7112577
rs60695258
All - MR Egger
All - Inverse variance weighted
-0.5
0.0
0.5
1.0
1.5
MR effect size for
atio of omega-6 fatty acids to total fatty acids || id:ebi-cfb233-GCST90301962' on 'Breast cancer (Combined Oncoarray; iCOGS;

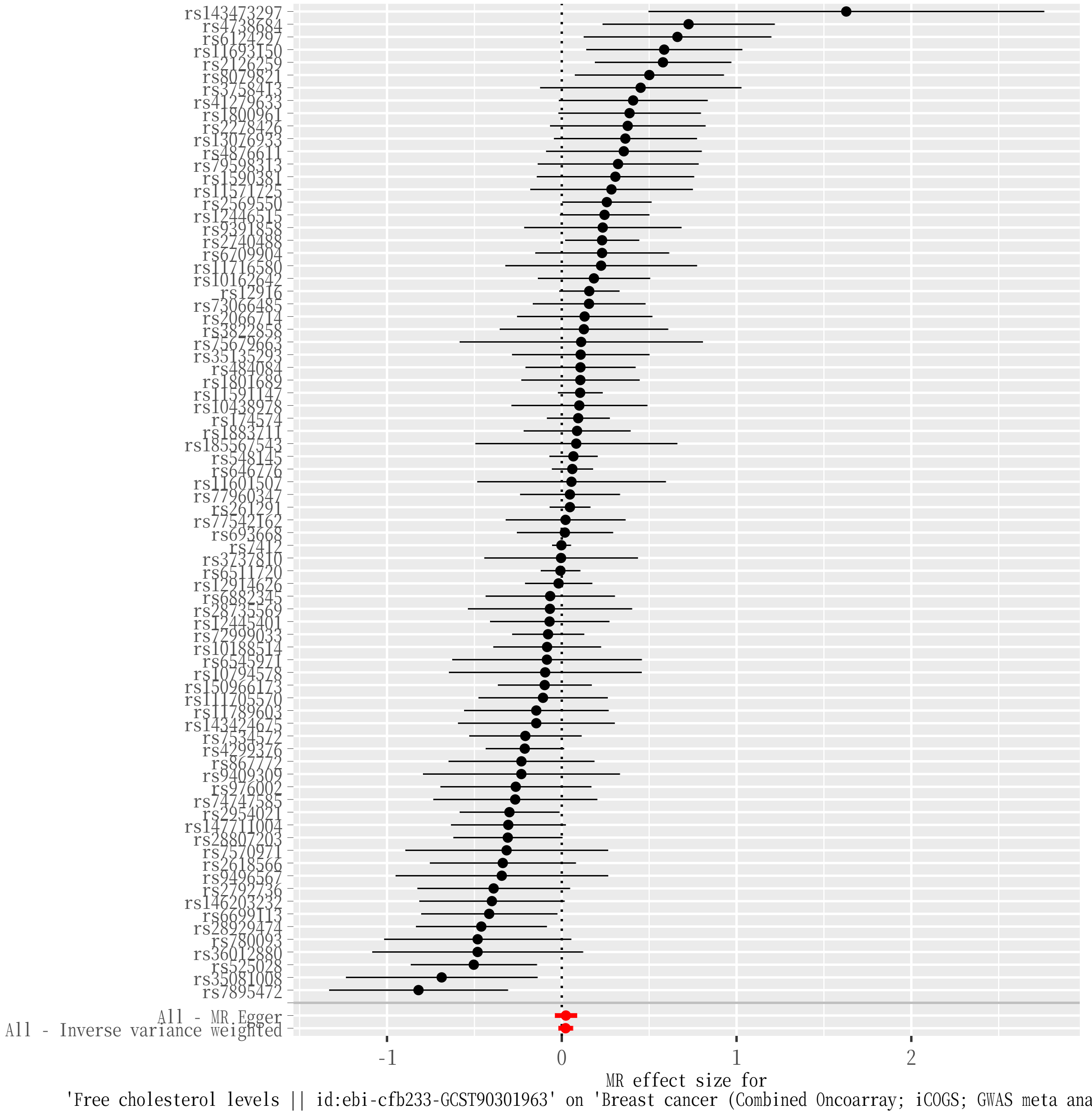

rs143473297
rs4738684
rs6124297
rs11693150
rs2126259
rs8079821
rs3758413
rs41279633
rs1800961
rs2278426
rs13076933
rs4876611
rs79598313
rs1590381
rs11571725
rs2569550
rs12446515
rs9391858
rs2740488
rs6709904
rs11716580
rs10162642
rs12916
rs73066485
rs2066714
rs3822858
rs75672663
rs35135293
rs484084
rs1801689
rs11591147
rs10438978
rs174574
rs1883711
rs185567543
rs548145
rs646776
rs11601507
rs77960347
rs261291
rs77542162
rs693668
rs7412
rs3737810
rs6511720
rs12914626
rs6882345
rs28735569
rs12445401
rs72999033
rs10188514
rs6545971
rs10794578
rs150266173
rs111705570
rs11789603
rs143424675
rs7534572
rs4299376
rs867772
rs9409309
rs976002
rs74747585
rs2954021
rs147711004
rs28807203
rs7570971
rs2618566
rs9496567
rs2792736
rs146203232
rs6699113
rs28929474
rs780093
rs36012880
rs525028
rs35081008
rs7895472
All - MR Egger
All - Inverse variance weighted
-1
0
1
2
MR effect size for
'Free cholesterol levels || id:ebi-cfb233-GCST90301963' on 'Breast cancer (Combined Oncoarray; iCOGS; GWAS meta ana

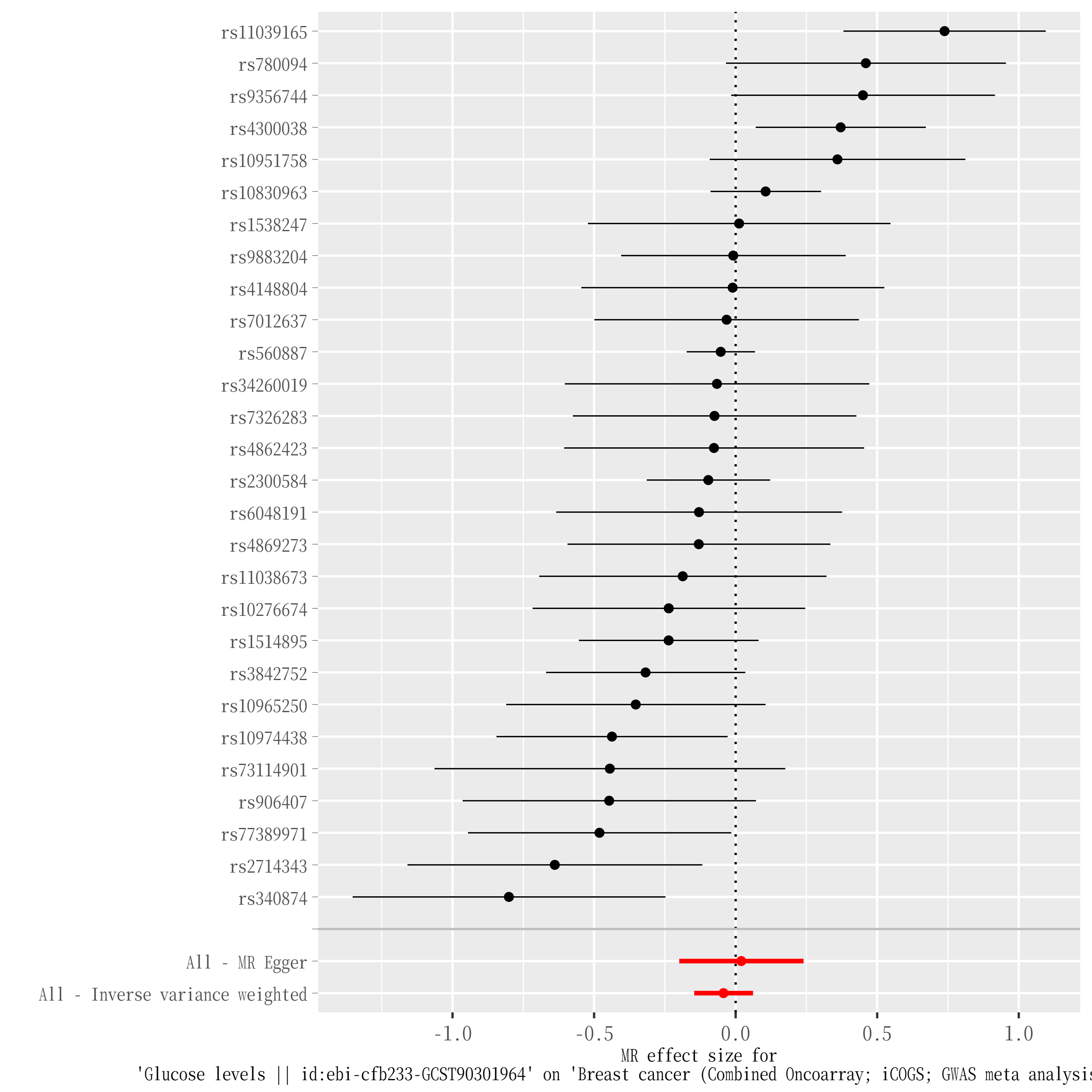
rs11039165
rs780094
rs9356744
rs4300038
rs10951758
rs10830963
rs1538247
rs9883204
rs4148804
rs7012637
rs560887
rs34260019
rs7326283
rs4862423
rs2300584
rs6048191
rs4869273
rs11038673
rs10276674
rs1514895
rs3842752
rs10965250
rs10974438
rs73114901
rs906407
rs77389971
rs2714343
rs340874
All - MR Egger
All - Inverse variance weighted
-1.0
-0.5
0.0
0.5
1.0
MR effect size for
'Glucose levels || id:ebi-cfb233-GCST90301964' on 'Breast cancer (Combined Oncoarray; iCOGS; GWAS meta analysis

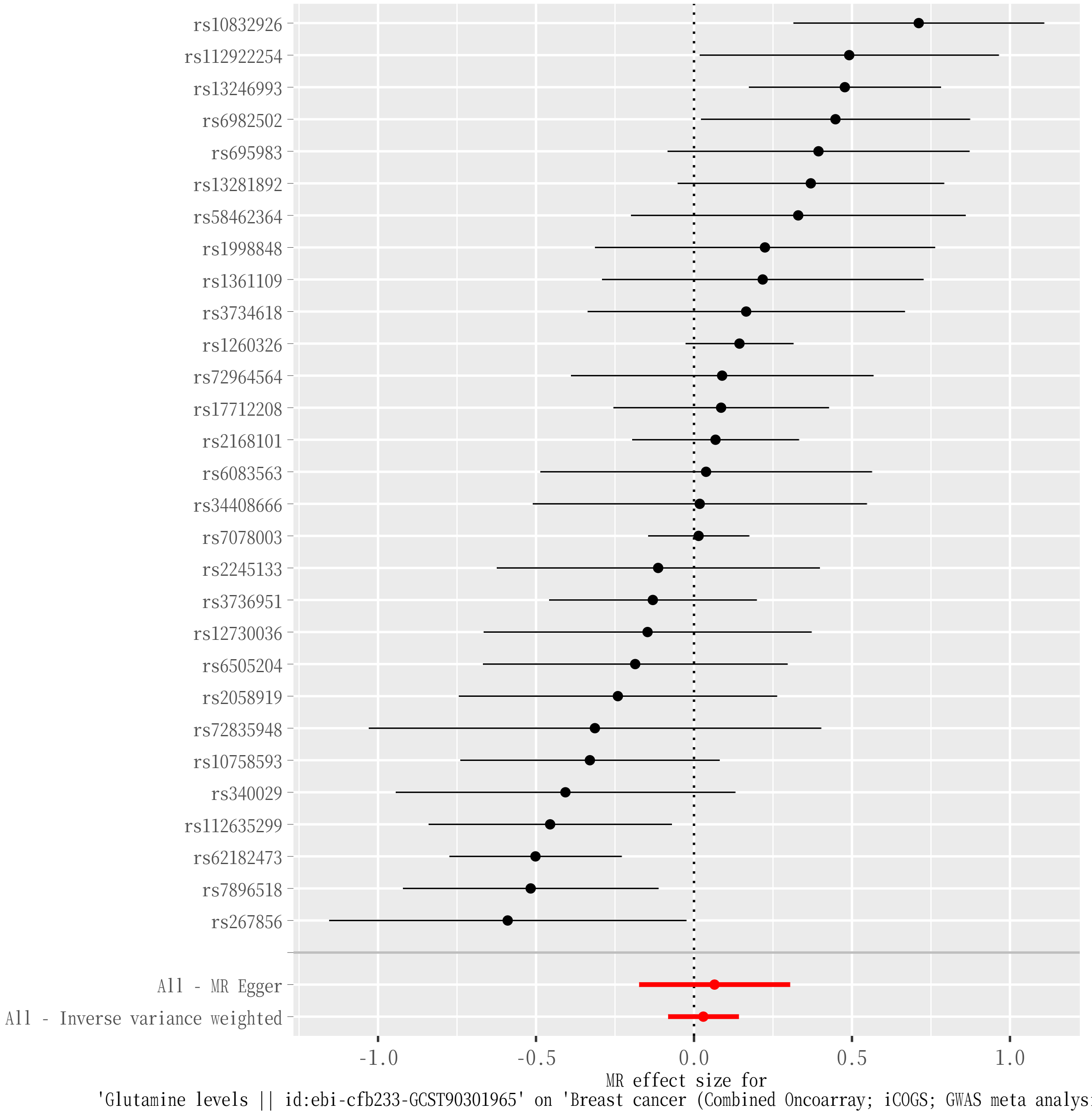

rs10832926
rs112922254
rs13246993
rs6982502
rs695983
rs13281892
rs58462364
rs1998848
rs1361109
rs3734618
rs1260326
rs72964564
rs17712208
rs2168101
rs6083563
rs34408666
rs7078003
rs2245133
rs3736951
rs12730036
rs6505204
rs2058919
rs72835948
rs10758593
rs340029
rs112635299
rs62182473
rs7896518
rs267856
All - MR Egger
All - Inverse variance weighted
-1.0
-0.5
0.0
0.5
1.0
MR effect size for
'Glutamine levels || id:ebi-cfb233-GCST90301965' on 'Breast cancer (Combined Oncoarray; iCOGS; GWAS meta analys

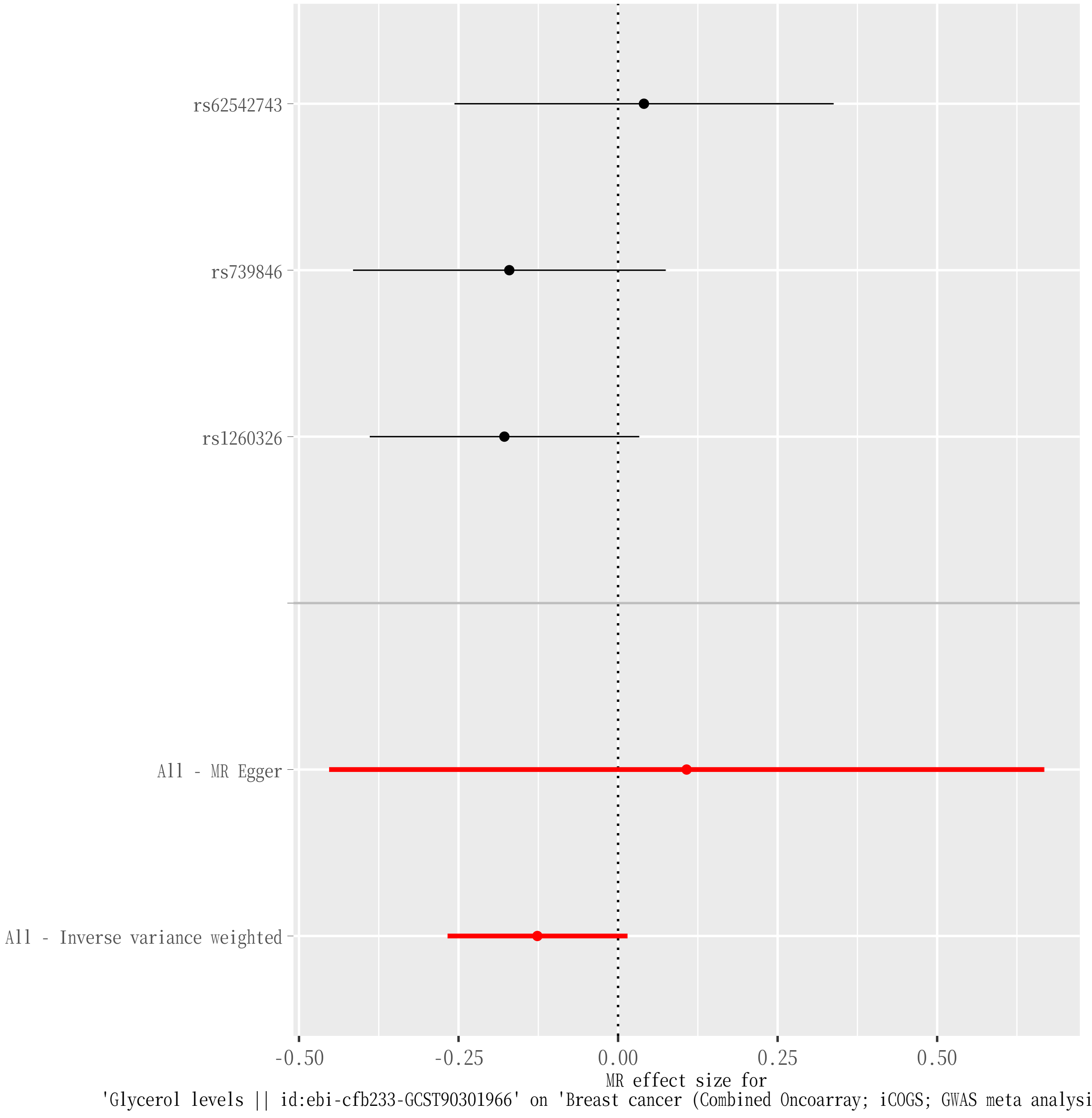

rs62542743
rs739846
rs1260326
All - MR Egger
All - Inverse variance weighted
-0.50
-0.25
0.00
0.25
0.50
MR effect size for
'Glycerol levels || id:ebi-cfb233-GCST90301966' on 'Breast cancer (Combined Oncoarray; iCOGS; GWAS meta analysi

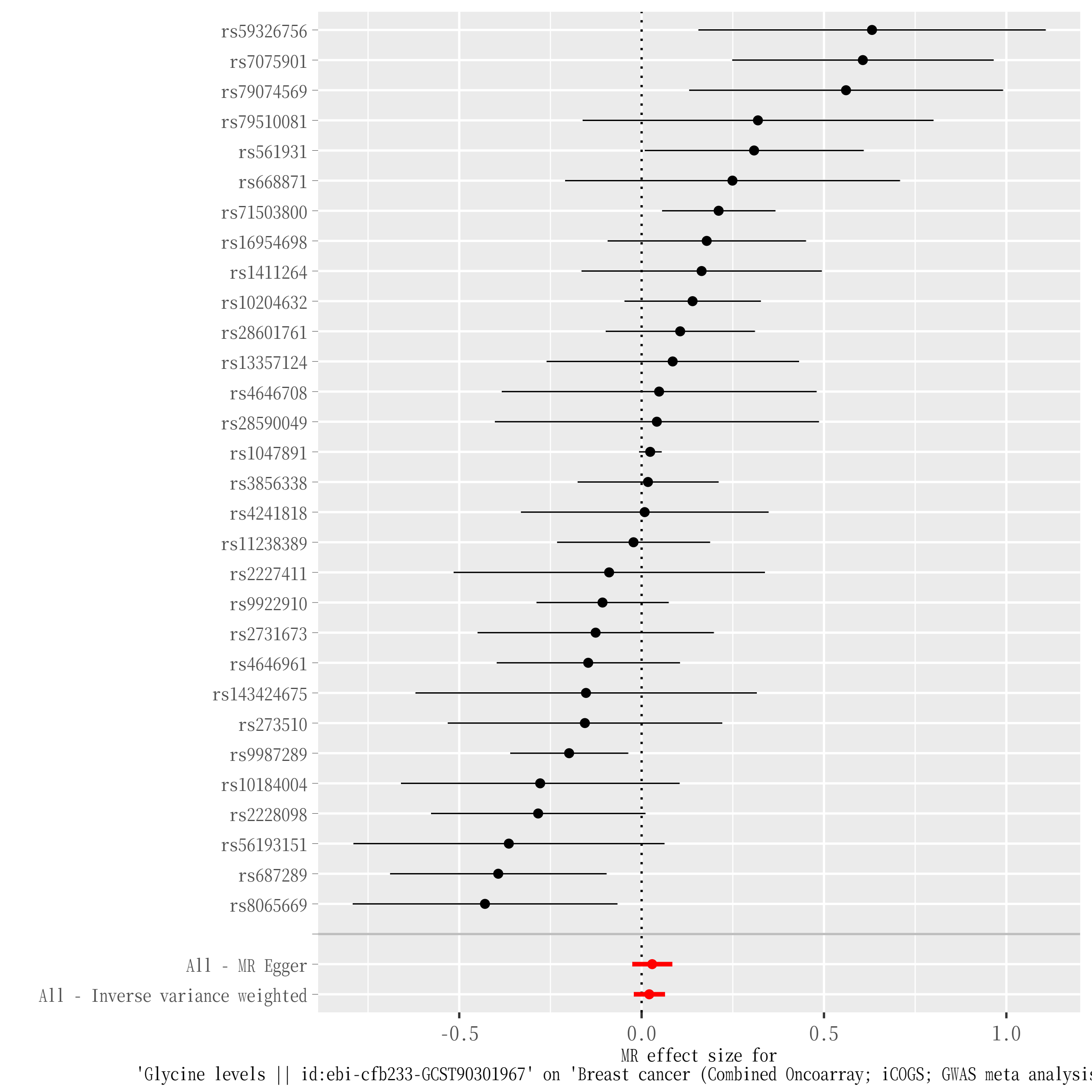
rs59326756
rs7075901
rs79074569
rs79510081
rs561931
rs668871
rs71503800
rs16954698
rs1411264
rs10204632
rs28601761
rs13357124
rs4646708
rs28590049
rs1047891
rs3856338
rs4241818
rs11238389
rs2227411
rs9922910
rs2731673
rs4646961
rs143424675
rs273510
rs9987289
rs10184004
rs2228098
rs56193151
rs687289
rs8065669
All - MR Egger
All - Inverse variance weighted
-0.5
0.0
0.5
1.0
MR effect size for
'Glycine levels || id:ebi-cfb233-GCST90301967' on 'Breast cancer (Combined Oncoarray; iCOGS; GWAS meta analysis

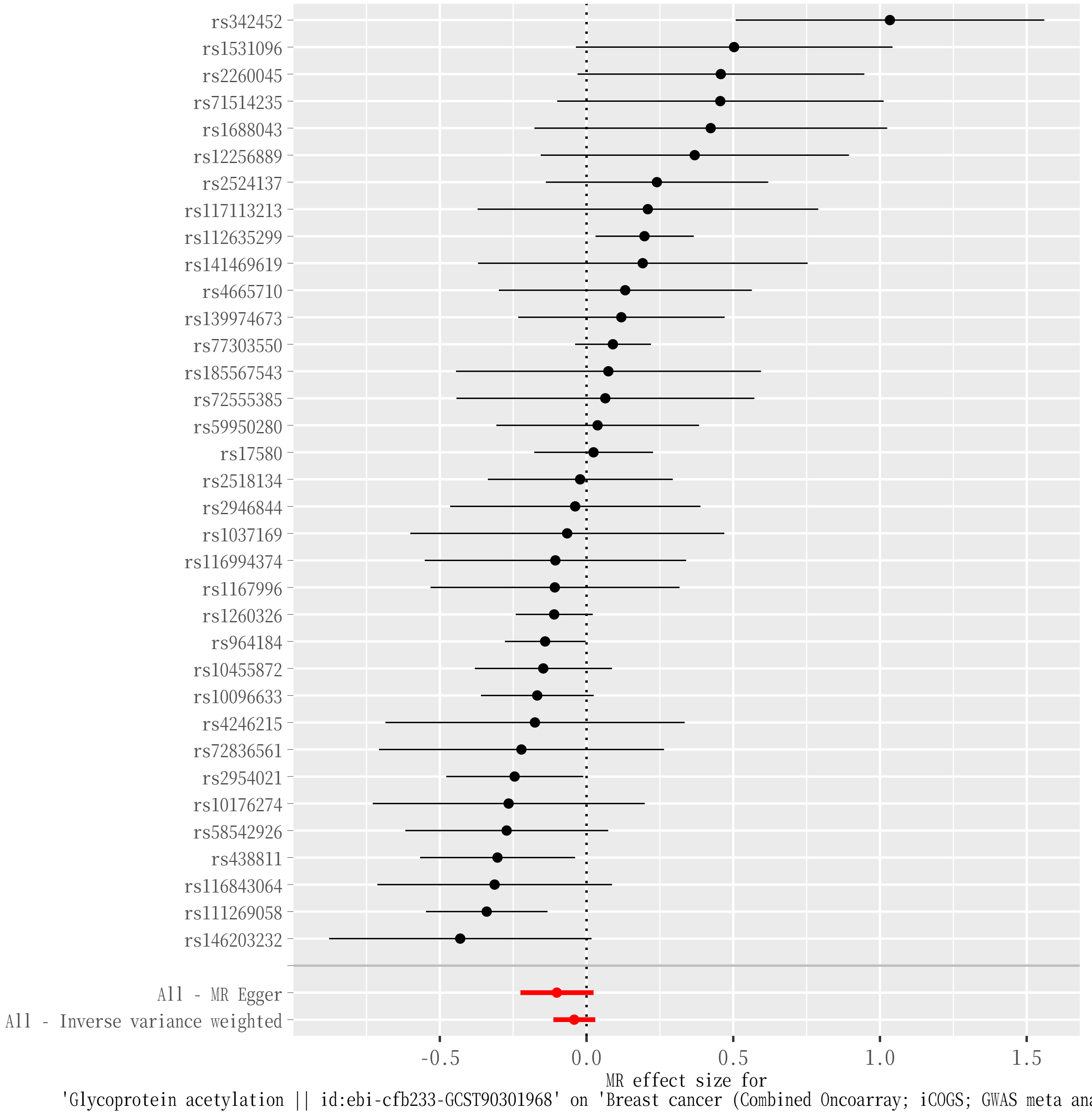
rs342452
rs1531096
rs2260045
rs71514235
rs1688043
rs12256889
rs2524137
rs117113213
rs112635299
rs141469619
rs4665710
rs139974673
rs77303550
rs185567543
rs72555385
rs59950280
rs17580
rs2518134
rs2946844
rs1037169
rs116994374
rs1167996
rs1260326
rs964184
rs10455872
rs10096633
rs4246215
rs72836561
rs2954021
rs10176274
rs58542926
rs438811
rs116843064
rs111269058
rs146203232
All - MR Egger
All - Inverse variance weighted
-0.5
0.0
0.5
1.0
1.5
MR effect size for
'Glycoprotein acetylation || id:ebi-cfb233-GCST90301968' on 'Breast cancer (Combined Oncoarray; iCOGS; GWAS meta ana

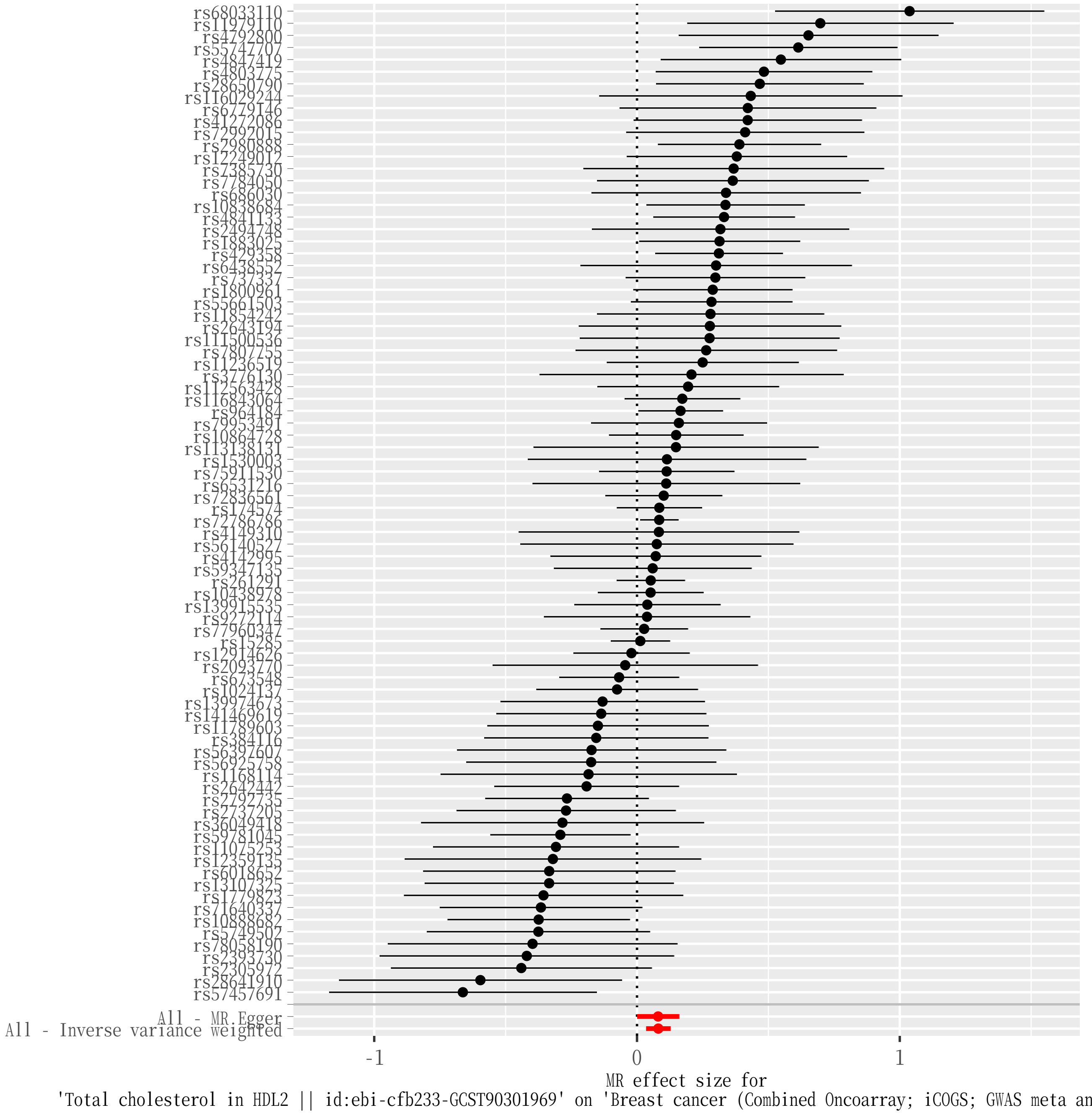
rs68033110
rs11979110
rs4792800
rs55747707
rs4847419
rs4803775
rs28650790
rs116029244
rs6779146
rs41272086
rs72992015
rs2980888
rs12249012
rs7385730
rs7784050
rs686030
rs10838684
rs4841133
rs2494748
rs1883025
rs429358
rs6438552
rs737337
rs1800961
rs55661503
rs11854242
rs2643194
rs111500536
rs7807755
rs11236519
rs3776130
rs112563428
rs116843064
rs964184
rs79953491
rs10864728
rs113138131
rs1530003
rs75911530
rs6531216
rs72836561
rs174574
rs72786786
rs4149310
rs56140527
rs4142995
rs59347135
rs261291
rs10438978
rs139915535
rs9272114
rs77960347
rs15285
rs12914626
rs2093770
rs673548
rs1024137
rs139974673
rs141469619
rs11789603
rs384116
rs56397607
rs56925758
rs1168114
rs2642442
rs2292735
rs2737205
rs36049418
rs59781045
rs11075253
rs12359135
rs6018652
rs13107325
rs1779823
rs71640337
rs10888682
rs5749502
rs78058190
rs2393730
rs2305972
rs28641910
rs57457691
All - MR Egger
All - Inverse variance weighted
-1
0
1
MR effect size for
'Total cholesterol in HDL2 || id:ebi-cfb233-GCST90301969' on 'Breast cancer (Combined Oncoarray; iCOGS; GWAS meta an

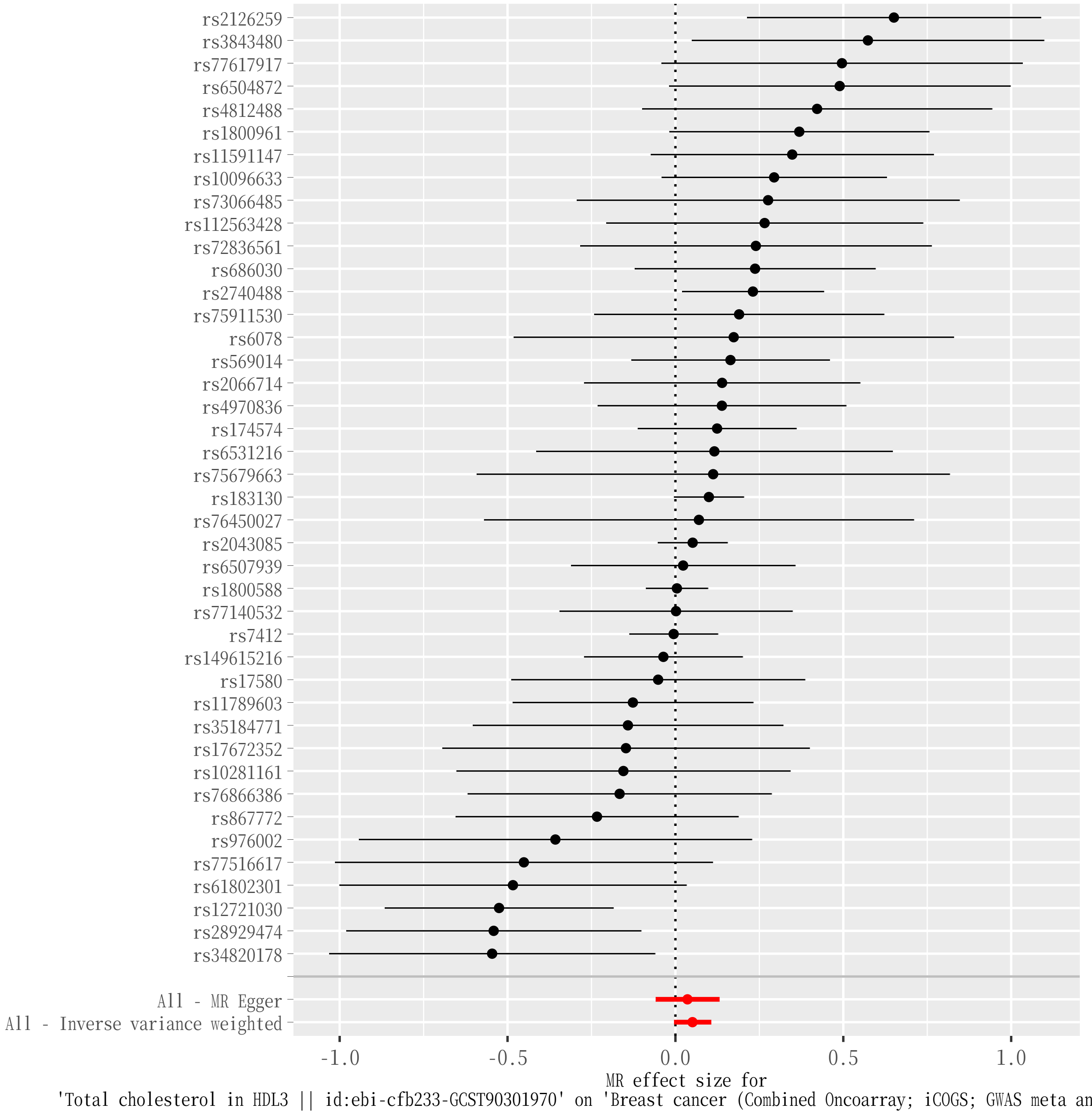
rs2126259
rs3843480
rs77617917
rs6504872
rs4812488
rs1800961
rs11591147
rs10096633
rs73066485
rs112563428
rs72836561
rs686030
rs2740488
rs75911530
rs6078
rs569014
rs2066714
rs4970836
rs174574
rs6531216
rs75679663
rs183130
rs76450027
rs2043085
rs6507939
rs1800588
rs77140532
rs7412
rs149615216
rs17580
rs11789603
rs35184771
rs17672352
rs10281161
rs76866386
rs867772
rs976002
rs77516617
rs61802301
rs12721030
rs28929474
rs34820178
All - MR Egger
All - Inverse variance weighted
-1.0
-0.5
0.0
0.5
1.0
MR effect size for
'Total cholesterol in HDL3 || id:ebi-cfb233-GCST90301970' on 'Breast cancer (Combined Oncoarray; iCOGS; GWAS meta an

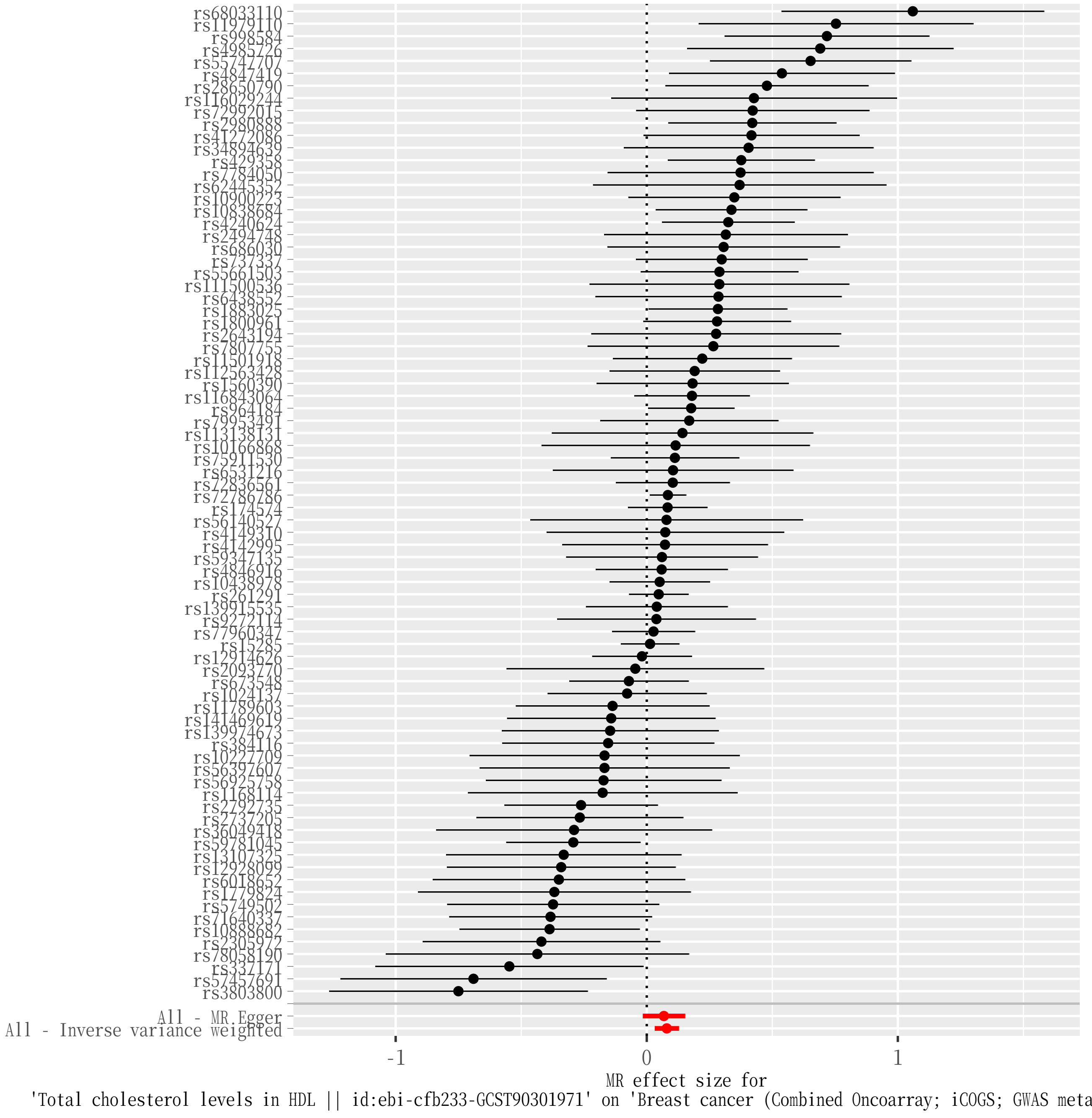
rs68033110
rs11979110
rs998584
rs4285726
rs55747707
rs4847419
rs28650790
rs116029244
rs72992015
rs2980888
rs41272086
rs34894639
rs429358
rs7784050
rs62445352
rs10900223
rs10838684
rs4240624
rs2494748
rs686030
rs737337
rs55661503
rs111500536
rs6438552
rs1883025
rs1800961
rs2643194
rs7807755
rs11501918
rs112563428
rs1560390
rs116843064
rs964184
rs79953491
rs113138131
rs10166868
rs75911530
rs6531216
rs72836561
rs72786786
rs174574
rs56140527
rs4149310
rs4142995
rs59347135
rs4846916
rs10438978
rs261291
rs139915535
rs9272114
rs77960347
rs15285
rs12914626
rs2023770
rs673548
rs1024137
rs11789603
rs141469619
rs139974673
rs384116
rs10227709
rs56397607
rs56925758
rs1168114
rs2792735
rs2737205
rs36049418
rs59781045
rs13107325
rs12928099
rs6018652
rs1779824
rs5749502
rs71640337
rs10888682
rs2305972
rs78058190
rs337171
rs57457691
rs3803800
All - MR Egger
All - Inverse variance weighted
-1
0
1
MR effect size for
'Total cholesterol levels in HDL || id:ebi-cfb233-GCST90301971' on 'Breast cancer (Combined Oncoarray; iCOGS; GWAS meta

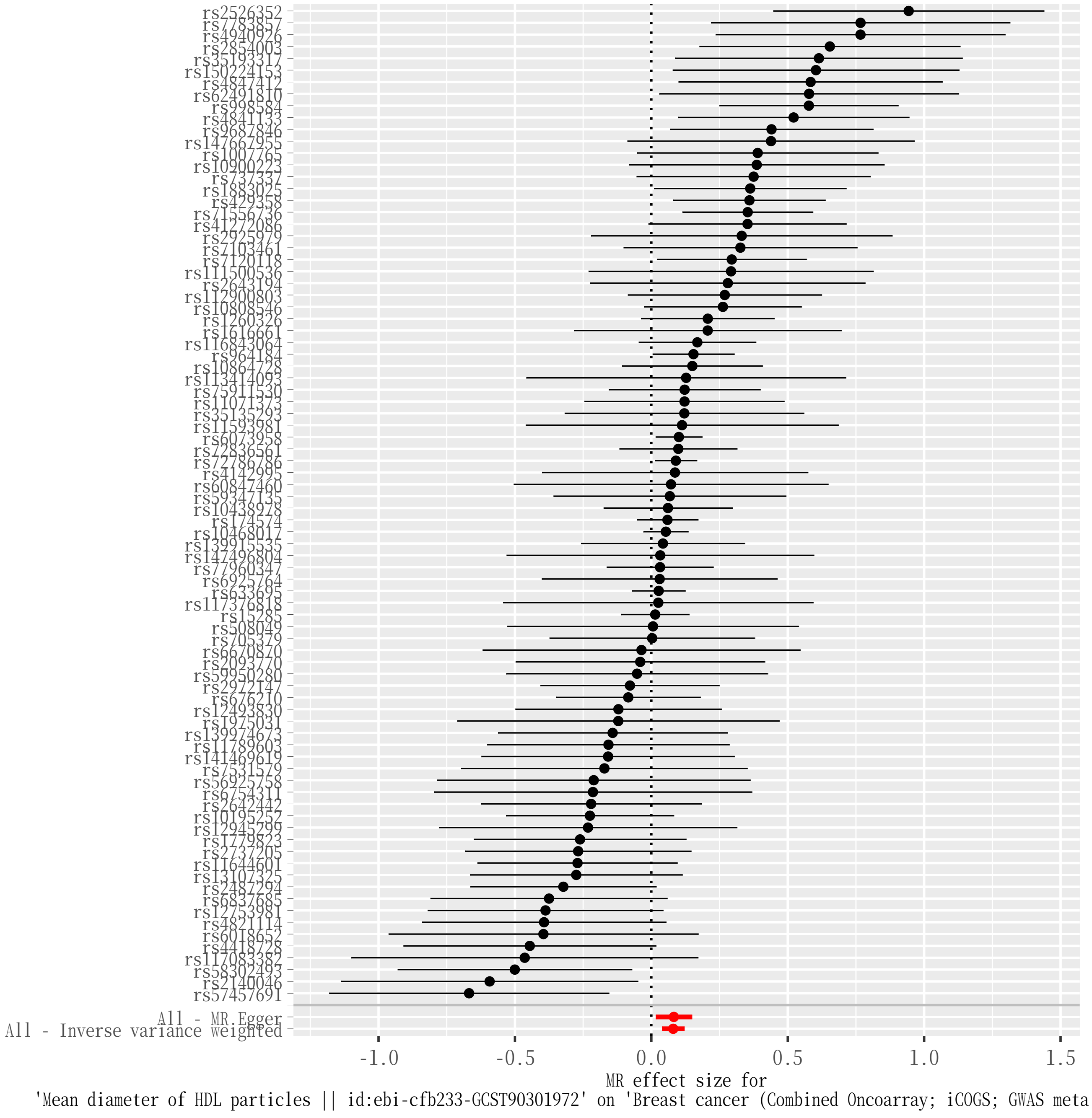

rs2526352
rs7783857
rs4940926
rs2854003
rs35193317
rs150224153
rs4847412
rs62491810
rs998584
rs4841133
rs9687846
rs147667955
rs1007765
rs10900223
rs737337
rs1883025
rs429358
rs71556736
rs41272086
rs2925979
rs7103461
rs7120118
rs111500536
rs2643194
rs112900803
rs10808546
rs1260326
rs1616661
rs116843064
rs964184
rs10864728
rs113414093
rs75911530
rs11071373
rs35135293
rs11593981
rs6073958
rs72836561
rs72786786
rs4142995
rs60847460
rs59347135
rs10438978
rs174574
rs10468017
rs139915535
rs147496804
rs77960347
rs6925764
rs633695
rs117376818
rs15285
rs508049
rs705379
rs6670870
rs2093770
rs59950280
rs2972147
rs676210
rs12493830
rs1975031
rs139974673
rs11789603
rs141469619
rs7531579
rs56925758
rs6754311
rs2642442
rs10195252
rs12945299
rs1779823
rs2737205
rs11644601
rs13107325
rs2487294
rs6837685
rs12753981
rs4821114
rs6018652
rs4418728
rs117083382
rs58302493
rs2140046
rs57457691
All - MR Egger
All - Inverse variance weighted
-1.0
-0.5
0.0
0.5
1.0
1.5
MR effect size for
'Mean diameter of HDL particles || id:ebi-cfb233-GCST90301972' on 'Breast cancer (Combined Oncoarray; iCOGS; GWAS meta

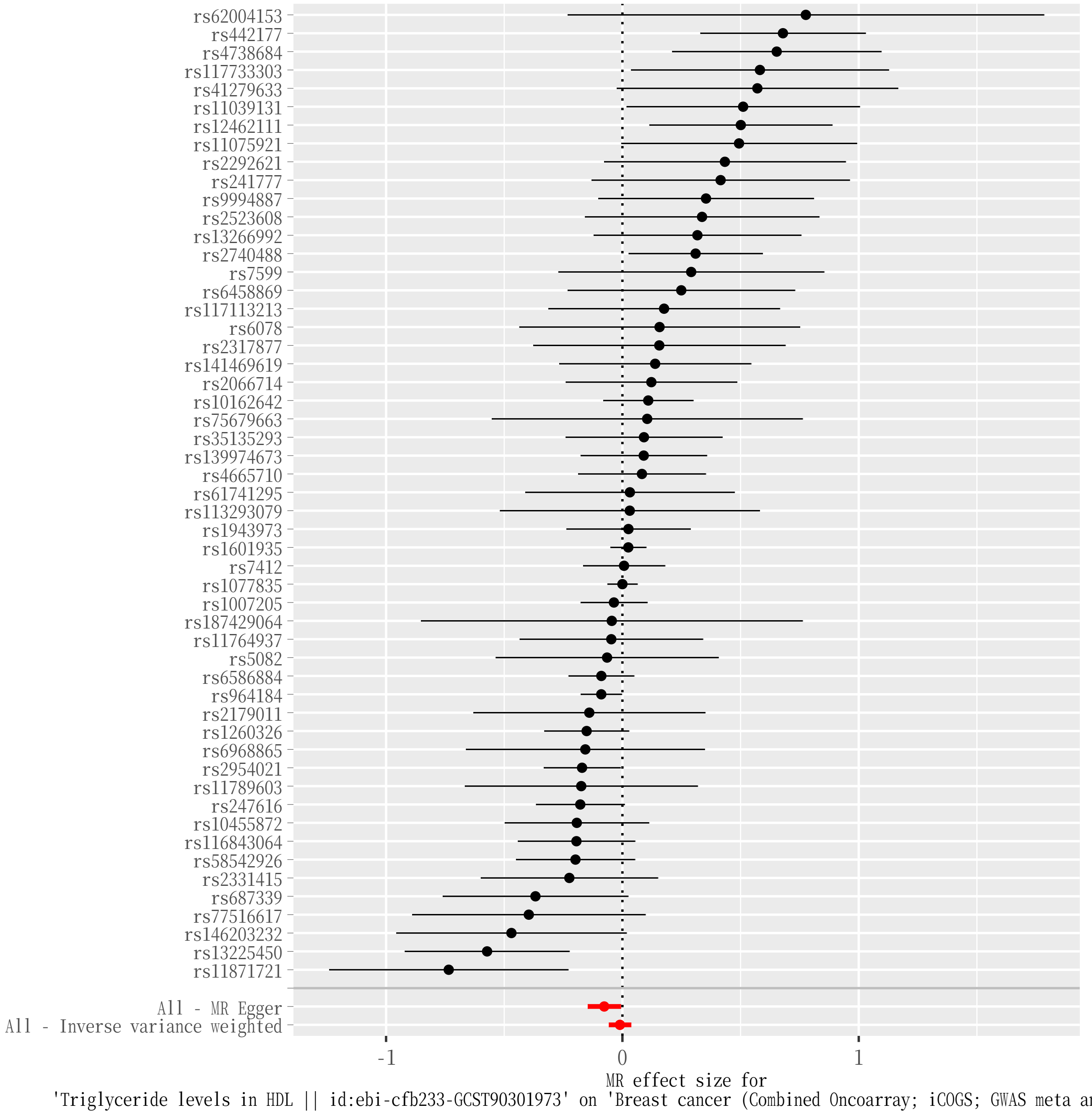
rs62004153
rs442177
rs4738684
rs117733303
rs41279633
rs11039131
rs12462111
rs11075921
rs2292621
rs241777
rs9994887
rs2523608
rs13266992
rs2740488
rs7599
rs6458869
rs117113213
rs6078
rs2317877
rs141469619
rs2066714
rs10162642
rs75679663
rs35135293
rs139974673
rs4665710
rs61741295
rs113293079
rs1943973
rs1601935
rs7412
rs1077835
rs1007205
rs187429064
rs11764937
rs5082
rs6586884
rs964184
rs2179011
rs1260326
rs6968865
rs2954021
rs11789603
rs247616
rs10455872
rs116843064
rs58542926
rs2331415
rs687339
rs77516617
rs146203232
rs13225450
rs11871721
All - MR Egger
All - Inverse variance weighted
-1
0
1
MR effect size for
'Triglyceride levels in HDL || id:ebi-cfb233-GCST90301973' on 'Breast cancer (Combined Oncoarray; iCOGS; GWAS meta ar

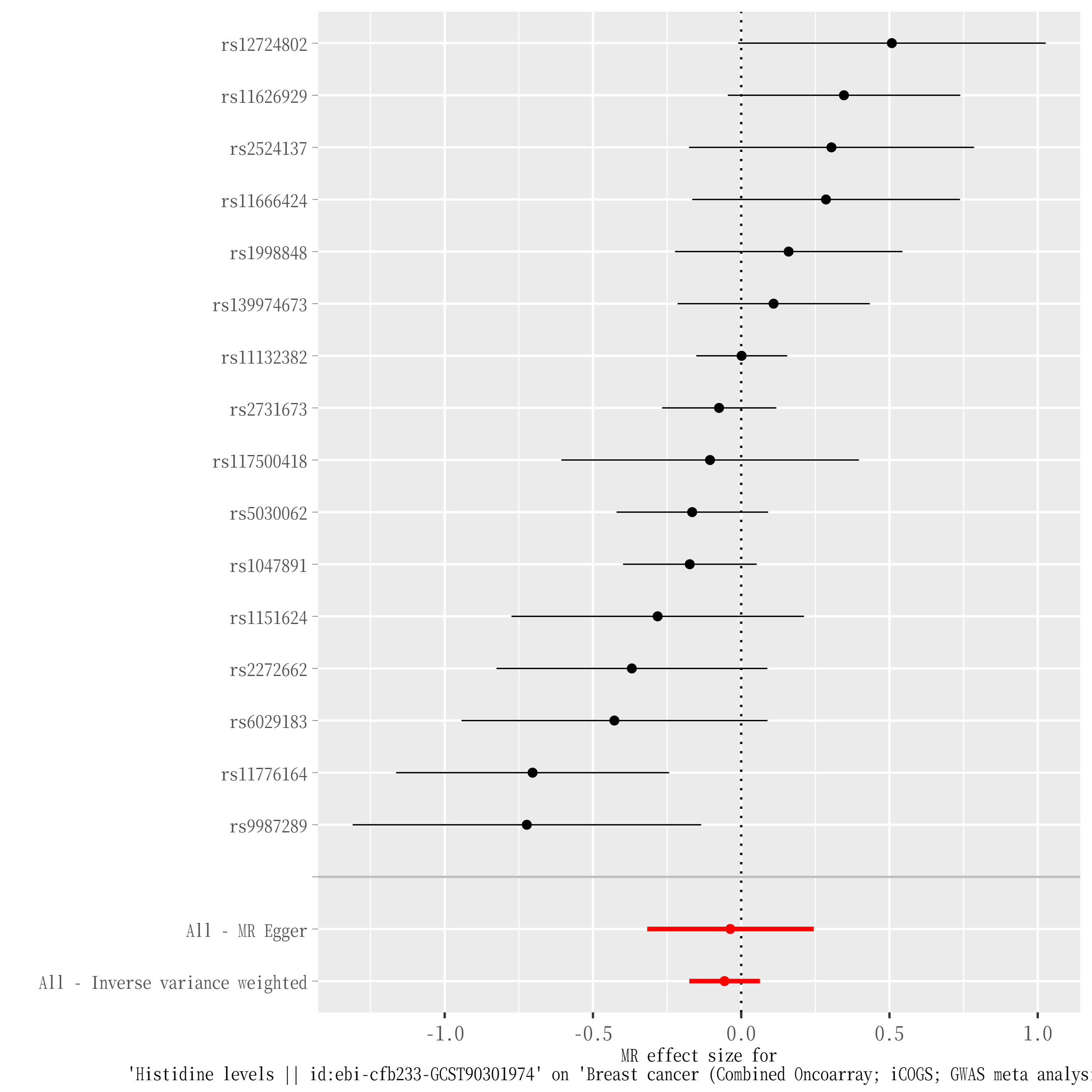
rs12724802
rs11626929
rs2524137
rs11666424
rs1998848
rs139974673
rs11132382
rs2731673
rs117500418
rs5030062
rs1047891
rs1151624
rs2272662
rs6029183
rs11776164
rs9987289
All - MR Egger
All - Inverse variance weighted
-1.0
-0.5
0.0
0.5
1.0
MR effect size for
'Histidine levels || id:ebi-cfb233-GCST90301974' on 'Breast cancer (Combined Oncoarray; iCOGS; GWAS meta analys

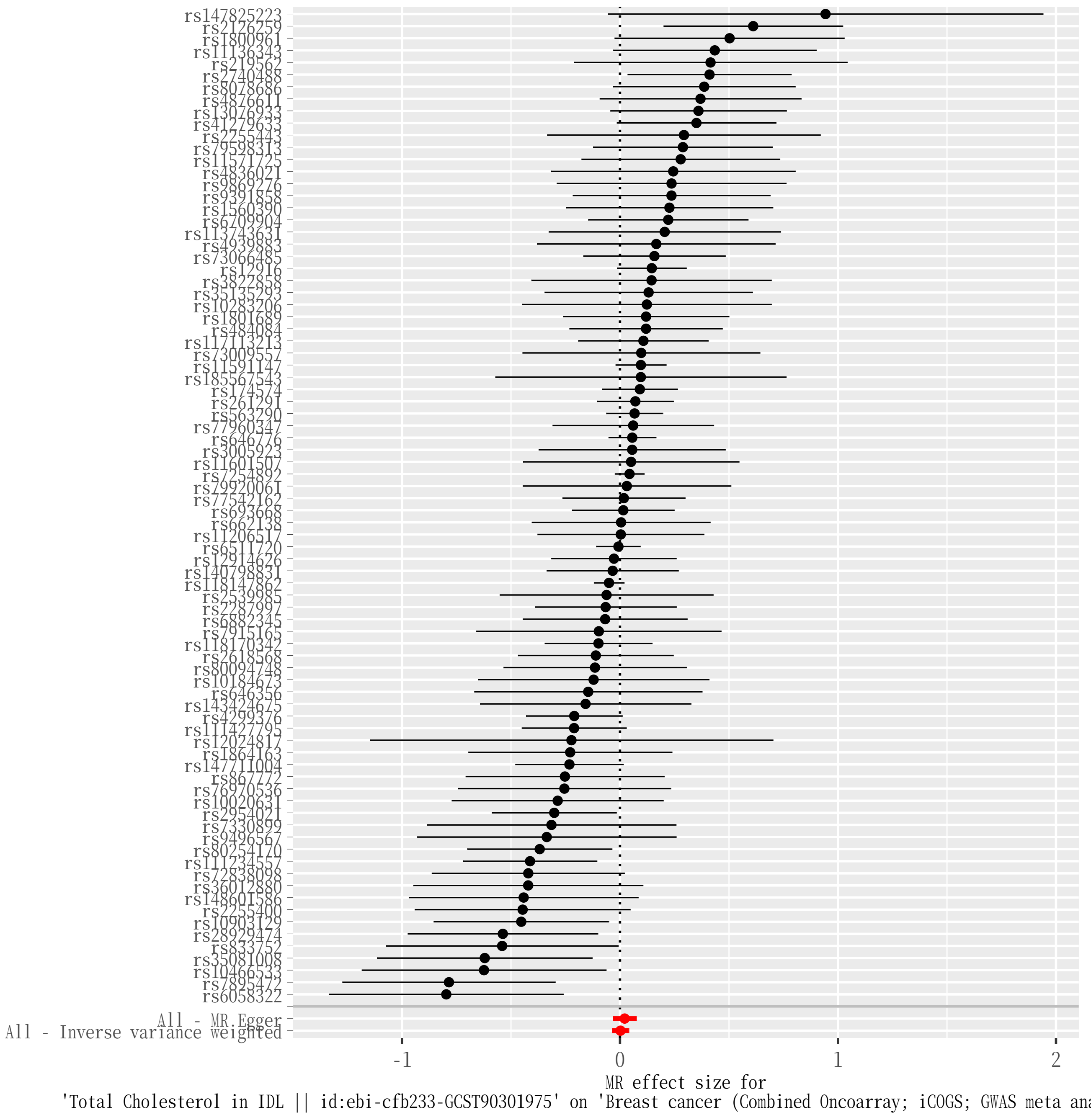
rs147825223
rs2126259
rs1800961
rs11136343
rs219562
rs2740488
rs8078686
rs4876611
rs13076933
rs41279633
rs2255443
rs79598313
rs11571725
rs4836021
rs9869276
rs9391858
rs1560390
rs6709904
rs113743631
rs4939883
rs73066485
rs12916
rs3822858
rs35135293
rs10283206
rs1801689
rs484084
rs117113213
rs73009557
rs11591147
rs185567543
rs174574
rs261291
rs563290
rs7796034?
rs646776
rs3005923
rs11601507
rs7254892
rs79920061
rs77542162
rs693668
rs662138
rs11206517
rs6511720
rs12914626
rs140798831
rs118147862
rs2539985
rs2287997
rs6882345
rs7915165
rs118170342
rs2618568
rs80094748
rs10184673
rs646356
rs143424675
rs4299376
rs111427795
rs12024817
rs1864163
rs147711004
rs867772
rs76970536
rs10020631
rs2954021
rs7330899
rs9496567
rs80254170
rs111234557
rs72838098
rs36012880
rs148601586
rs2255400
rs10903129
rs28929474
rs833752
rs35081008
rs10466533
rs7895472
rs6058322
All - MR Egger
All - Inverse variance weighted
-1
0
1
2
MR effect size for
'Total Cholesterol in IDL || id:ebi-cfb233-GCST90301975' on 'Breast cancer (Combined Oncoarray; iCOGS; GWAS meta ana

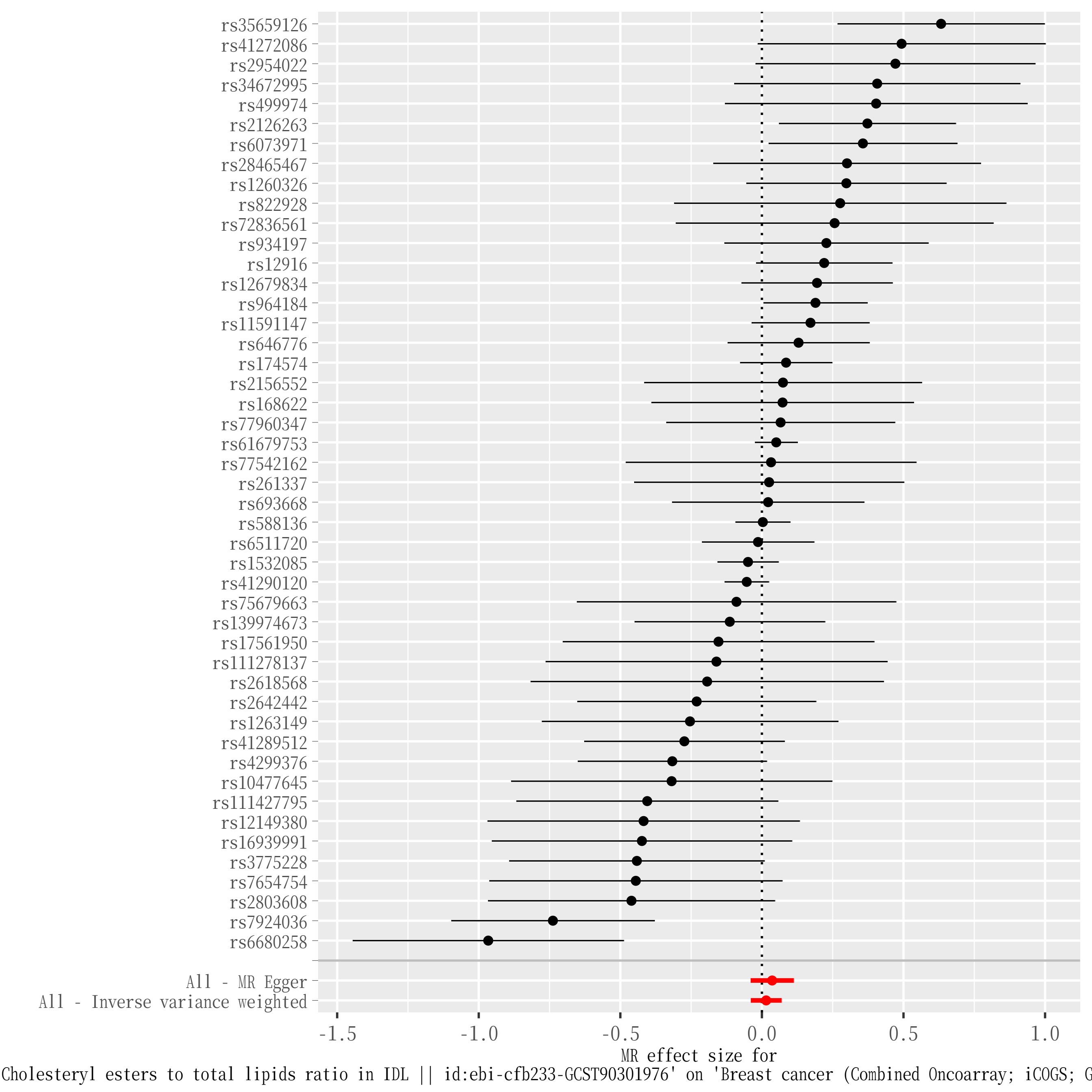
rs35659126
rs41272086
rs2954022
rs34672995
rs499974
rs2126263
rs6073971
rs28465467
rs1260326
rs822928
rs72836561
rs934197
rs12916
rs12679834
rs964184
rs11591147
rs646776
rs174574
rs2156552
rs168622
rs77960347
rs61679753
rs77542162
rs261337
rs693668
rs588136
rs6511720
rs1532085
rs41290120
rs75679663
rs139974673
rs17561950
rs111278137
rs2618568
rs2642442
rs1263149
rs41289512
rs4299376
rs10477645
rs111427795
rs12149380
rs16939991
rs3775228
rs7654754
rs2803608
rs7924036
rs6680258
All - MR Egger
All - Inverse variance weighted
-1.5
-1.0
-0.5
0.0
0.5
1.0
MR effect size for
Cholesteryl esters to total lipids ratio in IDL || id:ebi-cfb233-GCST90301976' on 'Breast cancer (Combined Oncoarray; iCOGS; G

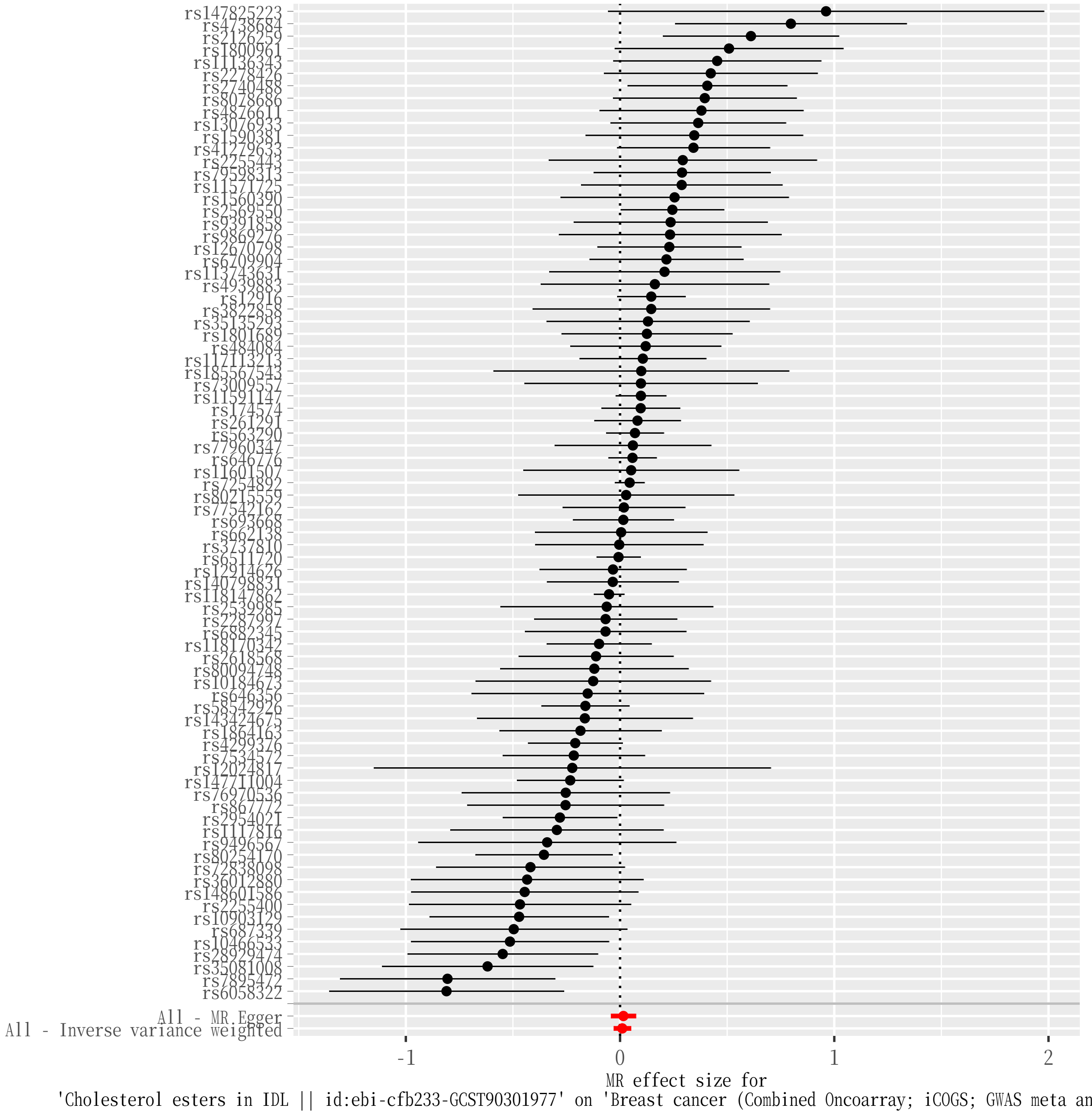
rs147825223
rs4738684
rs2126259
rs1800961
rs11136343
rs2278426
rs2740488
rs8078686
rs4876611
rs13076933
rs1590381
rs41272633
rs2255443
rs79598313
rs11571725
rs1560390
rs2569550
rs9391858
rs9869276
rs12670798
rs6709904
rs113743631
rs4939883
rs12916
rs3822858
rs35135293
rs1801689
rs484084
rs117113213
rs185567543
rs73009552
rs11591147
rs174574
rs261291
rs563290
rs77960347
rs646776
rs11601507
rs7254892
rs80215559
rs77542162
rs693668
rs662138
rs3737810
rs6511720
rs12914626
rs140798831
rs118147862
rs2539985
rs2287977
rs6882345
rs118170342
rs2618568
rs80094748
rs10184673
rs646356
rs58542926
rs143424675
rs1864163
rs4299376
rs7534572
rs12024817
rs147711004
rs76970536
rs867772
rs2954021
rs1117816
rs9496567
rs80254170
rs72838098
rs36012880
rs148601586
rs2255400
rs10903129
rs687339
rs10466533
rs28929474
rs35081008
rs7825472
rs6058322
All - MR Egger
All - Inverse variance weighted
-1
0
1
2
MR effect size for
'Cholesterol esters in IDL || id:ebi-cfb233-GCST90301977' on 'Breast cancer (Combined Oncoarray; iCOGS; GWAS meta an

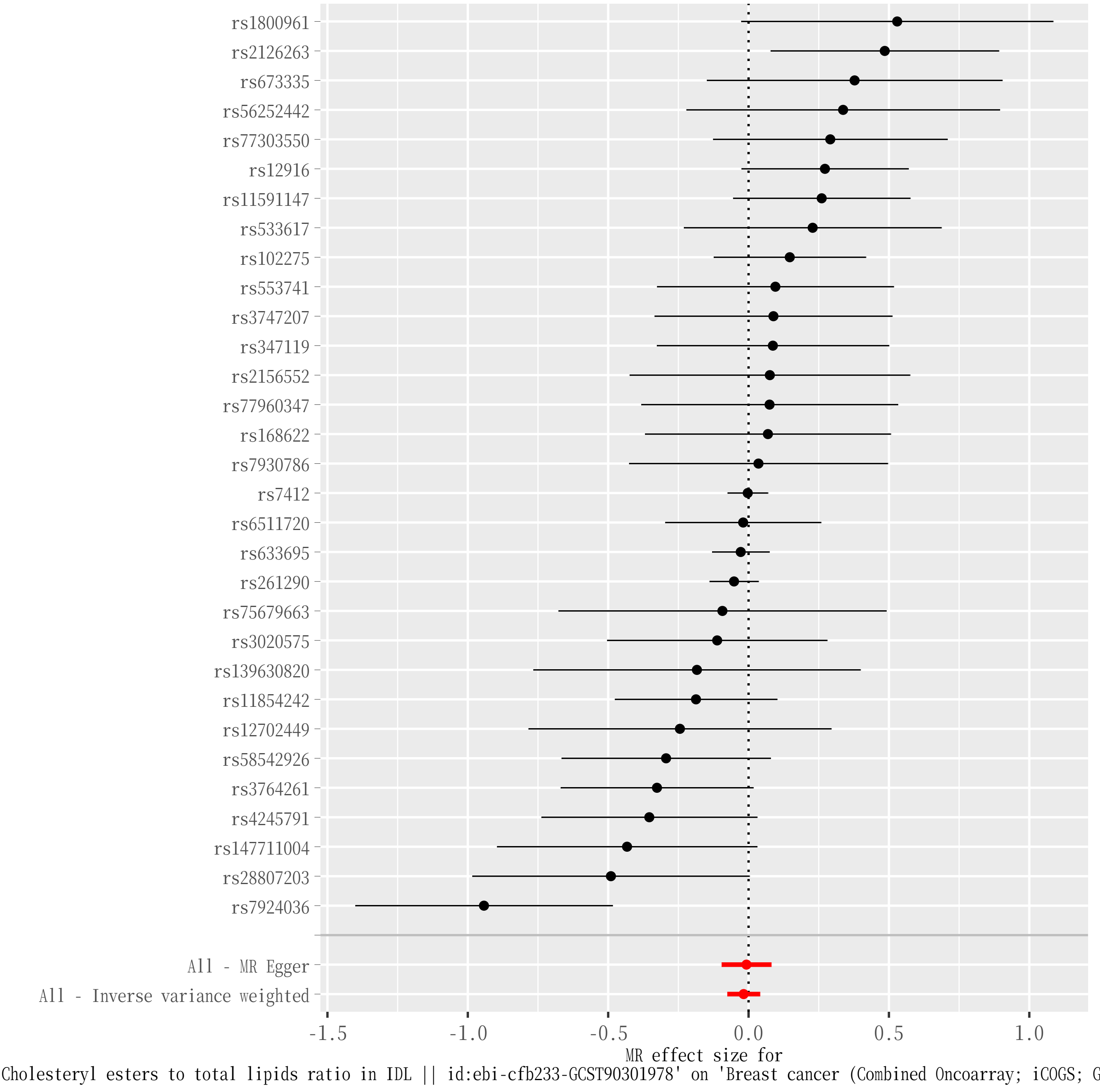

rs1800961
rs2126263
rs673335
rs56252442
rs77303550
rs12916
rs11591147
rs533617
rs102275
rs553741
rs3747207
rs347119
rs2156552
rs77960347
rs168622
rs7930786
rs7412
rs6511720
rs633695
rs261290
rs75679663
rs3020575
rs139630820
rs11854242
rs12702449
rs58542926
rs3764261
rs4245791
rs147711004
rs28807203
rs7924036
All - MR Egger
All - Inverse variance weighted
-1.5
-1.0
-0.5
0.0
0.5
1.0
MR effect size for
Cholesteryl esters to total lipids ratio in IDL || id:ebi-cfb233-GCST90301978' on 'Breast cancer (Combined Oncoarray; iCOGS; G

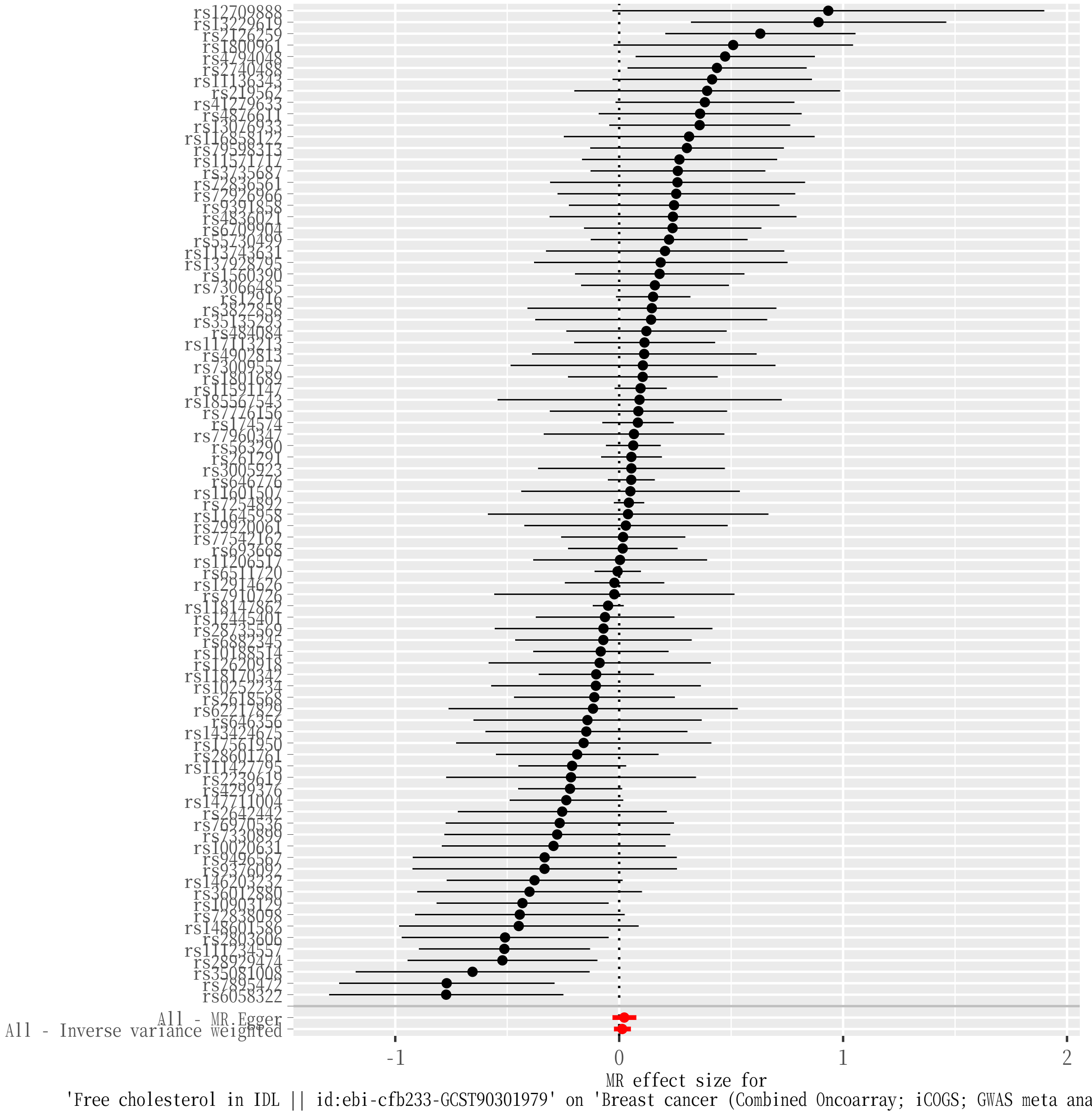
rs12709888
rs13229619
rs2126259
rs1800961
rs4794048
rs2740488
rs11136343
rs219562
rs41279633
rs4876611
rs13076933
rs116858122
rs79598313
rs11571717
rs3735687
rs72836561
rs72926966
rs9391858
rs4836021
rs6709904
rs55730499
rs113743631
rs137928795
rs1560390
rs73066485
rs12916
rs3822858
rs35135293
rs484084
rs117113213
rs4902813
rs73009557
rs1801689
rs11591147
rs185567543
rs7776156
rs174574
rs77960347
rs563290
rs261291
rs3005923
rs646776
rs11601507
rs7254892
rs11645958
rs79920061
rs77542162
rs693668
rs11206517
rs6511720
rs12914626
rs7910726
rs118147862
rs12445401
rs28735569
rs6882345
rs10188514
rs12620918
rs118170342
rs10252234
rs2618568
rs6217829
rs646356
rs143424675
rs17561950
rs28601761
rs111427795
rs2239619
rs4299376
rs147711004
rs2642442
rs76970536
rs7330899
rs1002063
rs9496567
rs9376092
rs146203232
rs36012880
rs10903129
rs72838098
rs148601586
rs2803606
rs111234557
rs2929474
rs35081008
rs7895472
rs6058322
All - MR Egger
All - Inverse variance weighted
-1
0
1
2
MR effect size for
'Free cholesterol in IDL || id:ebi-cfb233-GCST90301979' on 'Breast cancer (Combined Oncoarray; iCOGS; GWAS meta ana

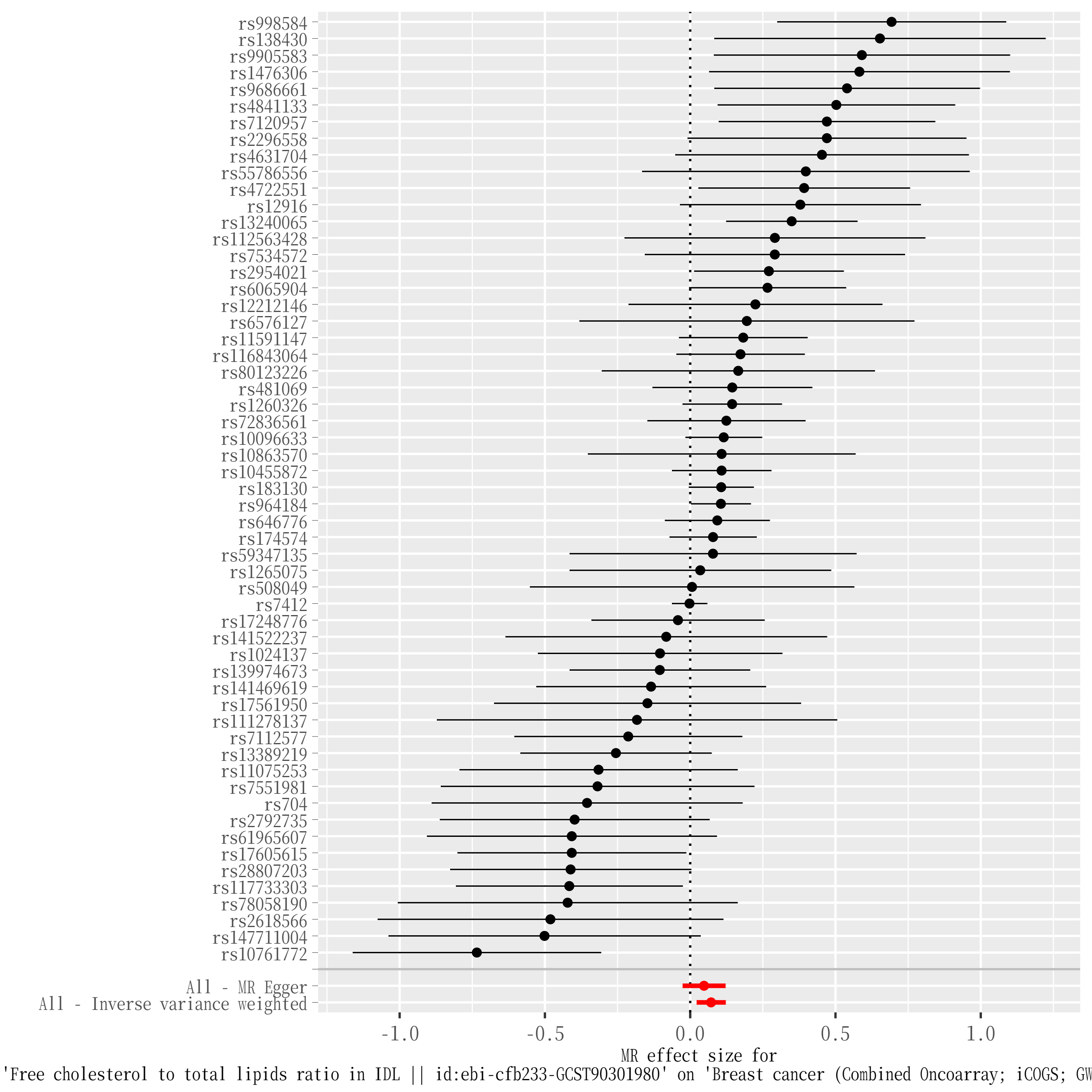
rs998584
rs138430
rs9905583
rs1476306
rs9686661
rs4841133
rs7120957
rs2296558
rs4631704
rs55786556
rs4722551
rs12916
rs13240065
rs112563428
rs7534572
rs2954021
rs6065904
rs12212146
rs6576127
rs11591147
rs116843064
rs80123226
rs481069
rs1260326
rs72836561
rs10096633
rs10863570
rs10455872
rs183130
rs964184
rs646776
rs174574
rs59347135
rs1265075
rs508049
rs7412
rs17248776
rs141522237
rs1024137
rs139974673
rs141469619
rs17561950
rs111278137
rs7112577
rs13389219
rs11075253
rs7551981
rs704
rs2792735
rs61965607
rs17605615
rs28807203
rs117733303
rs78058190
rs2618566
rs147711004
rs10761772
All - MR Egger
All - Inverse variance weighted
-1.0
-0.5
0.0
0.5
1.0
MR effect size for
'Free cholesterol to total lipids ratio in IDL || id:ebi-cfb233-GCST90301980' on 'Breast cancer (Combined Oncoarray; iCOGS; GW

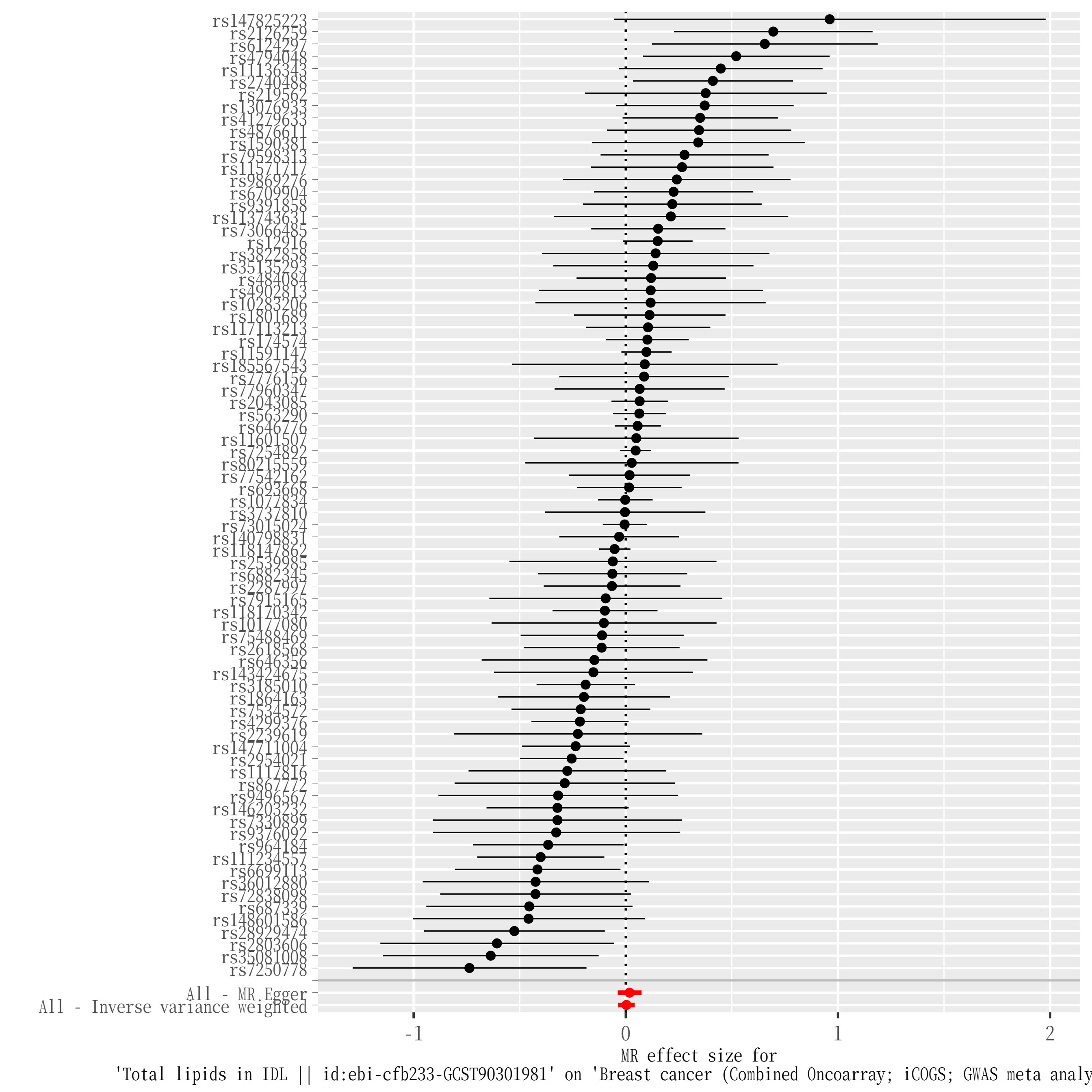
rs147825223
rs2126259
rs6124297
rs4794048
rs11136343
rs2740488
rs219562
rs13076933
rs41279633
rs4876611
rs1590381
rs79598313
rs11571717
rs9869276
rs6709904
rs9391858
rs113743631
rs73066485
rs12916
rs3822858
rs35135293
rs484084
rs4902813
rs10283206
rs1801689
rs117113213
rs174574
rs11591147
rs185567543
rs7776156
rs77960347
rs2043085
rs563290
rs646776
rs11601507
rs7254892
rs80215559
rs77542162
rs693668
rs1077834
rs3737810
rs73015024
rs140798831
rs118147862
rs2539985
rs6882345
rs2287997
rs7915165
rs118170342
rs10177080
rs75488469
rs2618568
rs646356
rs143424675
rs3185010
rs1864163
rs7534572
rs4299376
rs2239619
rs147711004
rs2954021
rs1117816
rs867772
rs9496567
rs146203232
rs7330899
rs9376092
rs964184
rs111234557
rs6699113
rs36012880
rs72838098
rs687339
rs148601586
rs28929474
rs2803606
rs35081008
rs7250778
All - MR Egger
All - Inverse variance weighted
-1
0
1
2
MR effect size for
'Total lipids in IDL || id:ebi-cfb233-GCST90301981' on 'Breast cancer (Combined Oncoarray; iCOGS; GWAS meta analy

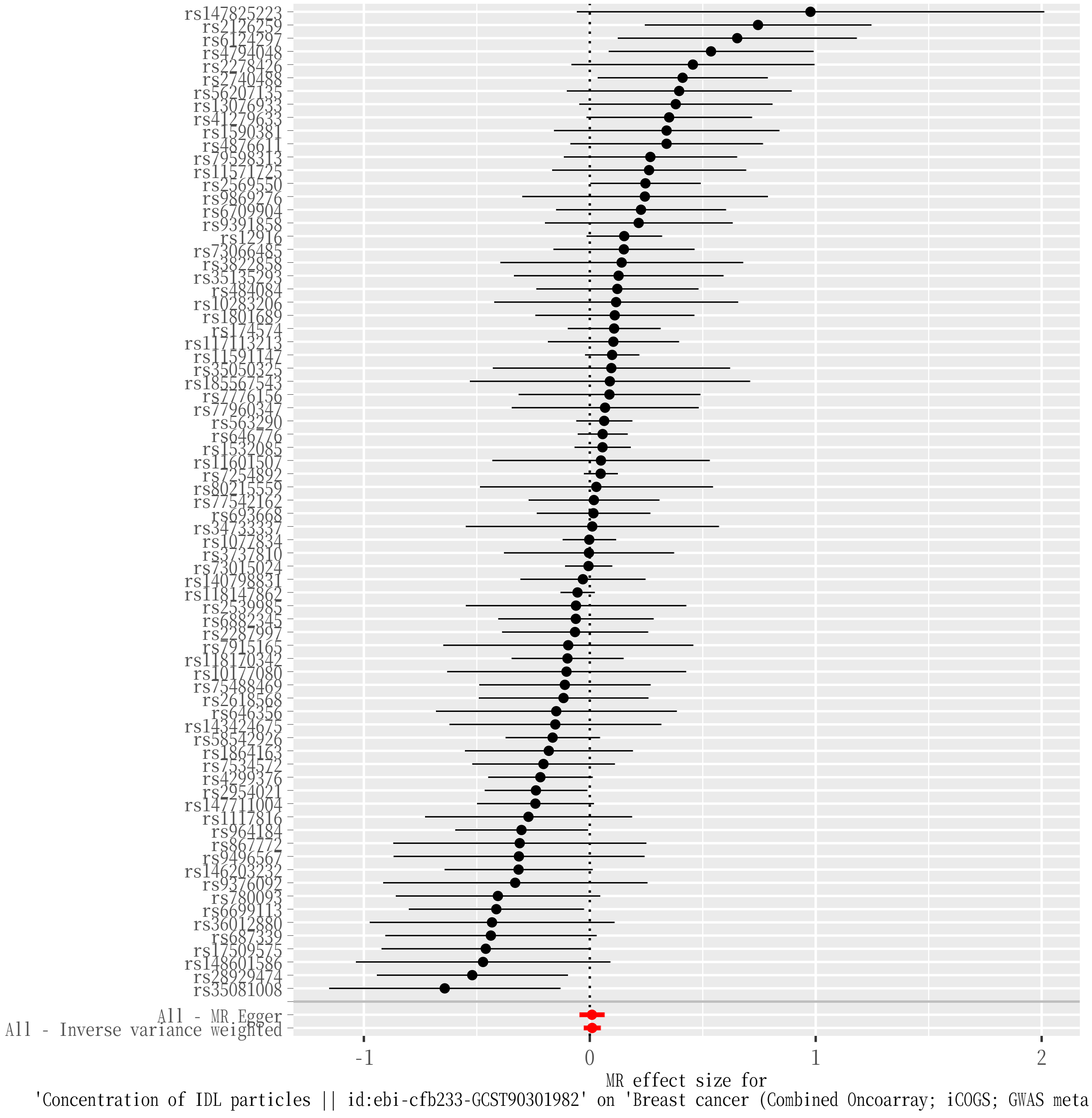
rs147825223
rs2126259
rs6124297
rs4794048
rs2278426
rs2740488
rs56207135
rs13076933
rs41279633
rs1590381
rs4876611
rs79598313
rs11571725
rs2569550
rs9869276
rs6709904
rs9391858
rs12916
rs73066485
rs3822858
rs35135293
rs484084
rs10283206
rs1801689
rs174574
rs117113213
rs11591147
rs35050325
rs185567543
rs7776156
rs77960347
rs563290
rs646776
rs1532085
rs11601507
rs7254892
rs80215559
rs77542162
rs693668
rs34733337
rs1077834
rs3737810
rs73015024
rs140798831
rs118147862
rs2539985
rs6882345
rs2287997
rs7915165
rs118170342
rs10177080
rs75488469
rs2618568
rs646356
rs143424675
rs58542926
rs1864163
rs7534572
rs4299376
rs2954021
rs147711004
rs1117816
rs964184
rs867772
rs9496567
rs146203232
rs9376092
rs780093
rs6699113
rs36012880
rs687339
rs17509575
rs148601586
rs28929474
rs35081008
All - MR Egger
All - Inverse variance weighted
-1
0
1
2
MR effect size for
'Concentration of IDL particles || id:ebi-cfb233-GCST90301982' on 'Breast cancer (Combined Oncoarray; iCOGS; GWAS meta

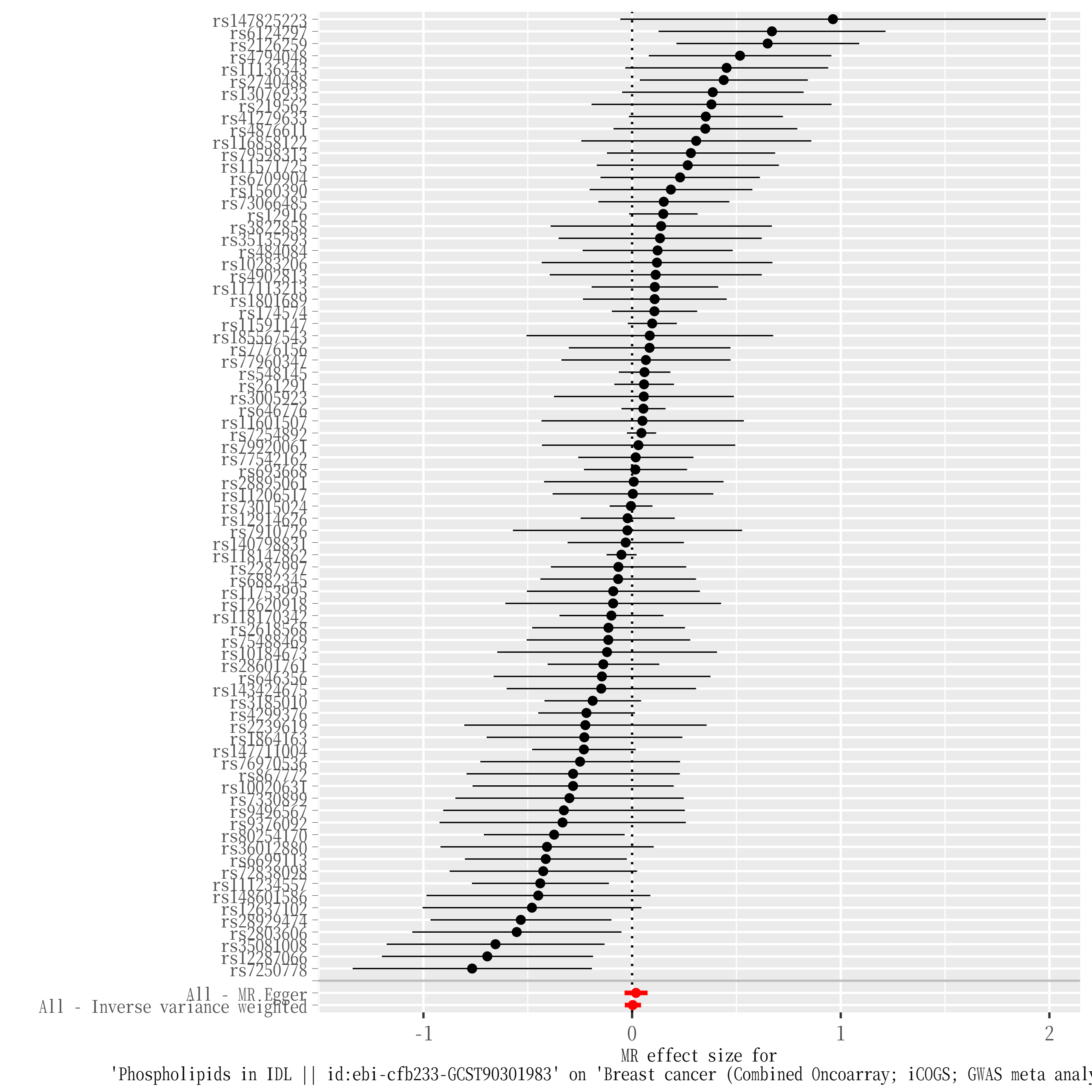
rs147825223
rs6124297
rs2126259
rs4794048
rs11136343
rs2740488
rs13076933
rs219562
rs41279633
rs4876611
rs116858122
rs79598313
rs11571725
rs6709904
rs1560390
rs73066485
rs12916
rs3822858
rs35135293
rs484084
rs10283206
rs4902813
rs117113213
rs1801689
rs174574
rs11591147
rs185567543
rs7776156
rs77960347
rs548145
rs261291
rs3005923
rs646776
rs11601507
rs7254892
rs79920061
rs77542162
rs693668
rs28895061
rs11206517
rs73015024
rs12914626
rs7910726
rs140798831
rs118147862
rs2287997
rs6882345
rs11753995
rs12620918
rs118170342
rs2618568
rs75488469
rs10184673
rs28601761
rs646356
rs143424675
rs3185010
rs4299376
rs2239619
rs1864163
rs147711004
rs76970536
rs867772
rs10020631
rs7330899
rs9496567
rs9376092
rs80254170
rs36012880
rs6699113
rs72838098
rs111234557
rs148601586
rs12637102
rs28929474
rs2803606
rs35081008
rs12287066
rs7250778
All - MR Egger
All - Inverse variance weighted
-1
0
1
2
MR effect size for
'Phospholipids in IDL || id:ebi-cfb233-GCST90301983' on 'Breast cancer (Combined Oncoarray; iCOGS; GWAS meta analy

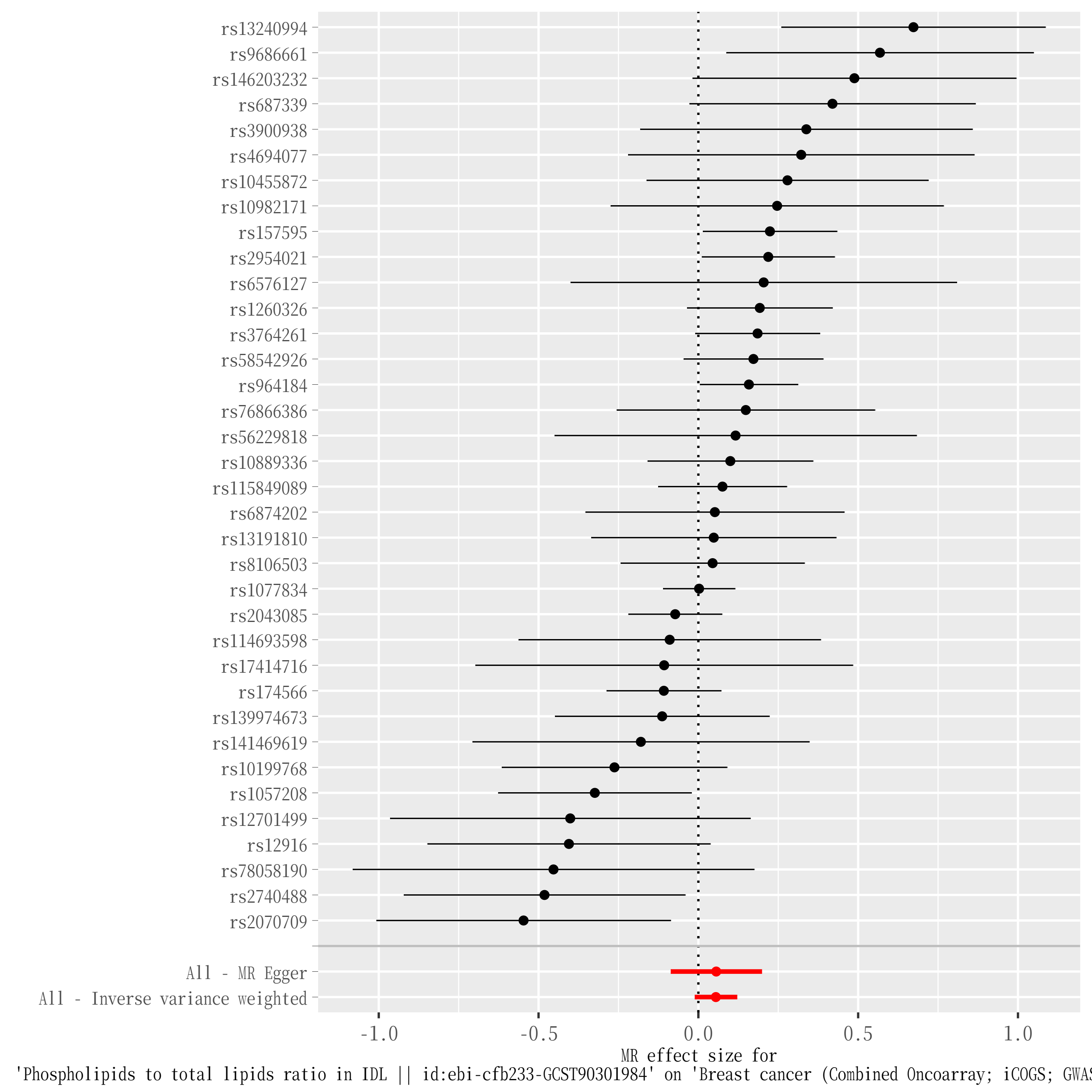

rs13240994
rs9686661
rs146203232
rs687339
rs3900938
rs4694077
rs10455872
rs10982171
rs157595
rs2954021
rs6576127
rs1260326
rs3764261
rs58542926
rs964184
rs76866386
rs56229818
rs10889336
rs115849089
rs6874202
rs13191810
rs8106503
rs1077834
rs2043085
rs114693598
rs17414716
rs174566
rs139974673
rs141469619
rs10199768
rs1057208
rs12701499
rs12916
rs78058190
rs2740488
rs2070709
All - MR Egger
All - Inverse variance weighted
-1.0
-0.5
0.0
0.5
1.0
MR effect size for
'Phospholipids to total lipids ratio in IDL || id:ebi-cfb233-GCST90301984' on 'Breast cancer (Combined Oncoarray; iCOGS; GWAS

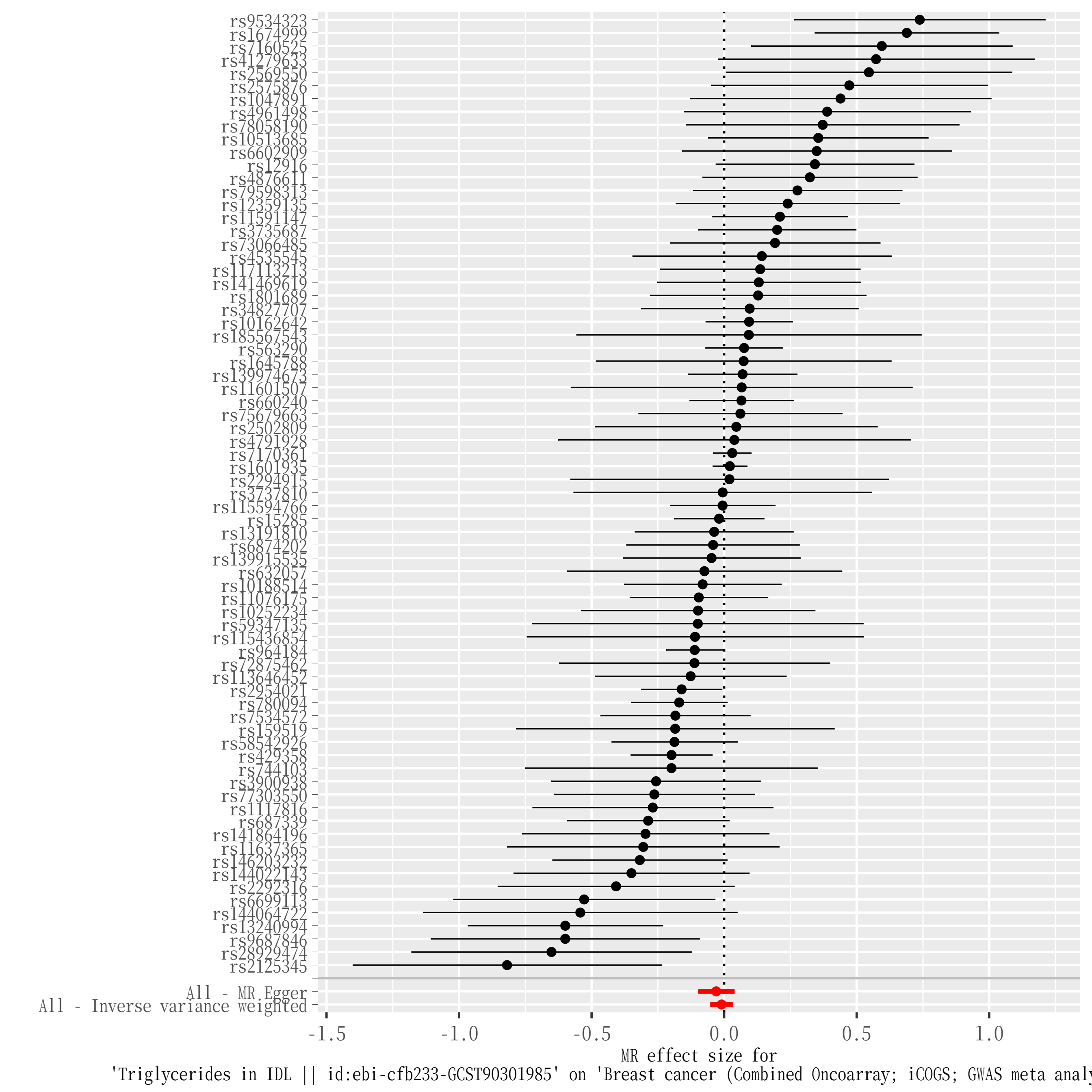
rs9534323
rs1674999
rs7160525
rs41279633
rs2569550
rs2575876
rs1047891
rs4961498
rs78058190
rs10513685
rs6602909
rs12916
rs4876611
rs79598313
rs12359135
rs11591147
rs3735687
rs73066485
rs4535545
rs117113213
rs141469619
rs1801689
rs34827707
rs10162642
rs185567543
rs563290
rs1645788
rs139974673
rs11601507
rs660240
rs75679663
rs2502809
rs4791928
rs7170361
rs1601935
rs2294915
rs3737810
rs115594766
rs15285
rs13191810
rs6874202
rs139915535
rs632057
rs10188514
rs11076175
rs10252234
rs59347135
rs115436854
rs964184
rs72875462
rs113646452
rs2954021
rs780094
rs7534572
rs159519
rs58542926
rs429358
rs744103
rs3900938
rs77303550
rs1117816
rs687339
rs141864196
rs11637365
rs146203232
rs144022143
rs2292316
rs6699113
rs144064722
rs13240994
rs9687846
rs28929474
rs2125345
All - MR Egger
All - Inverse variance weighted
-1.5
-1.0
-0.5
0.0
0.5
1.0
MR effect size for
'Triglycerides in IDL || id:ebi-cfb233-GCST90301985' on 'Breast cancer (Combined Oncoarray; iCOGS; GWAS meta analy

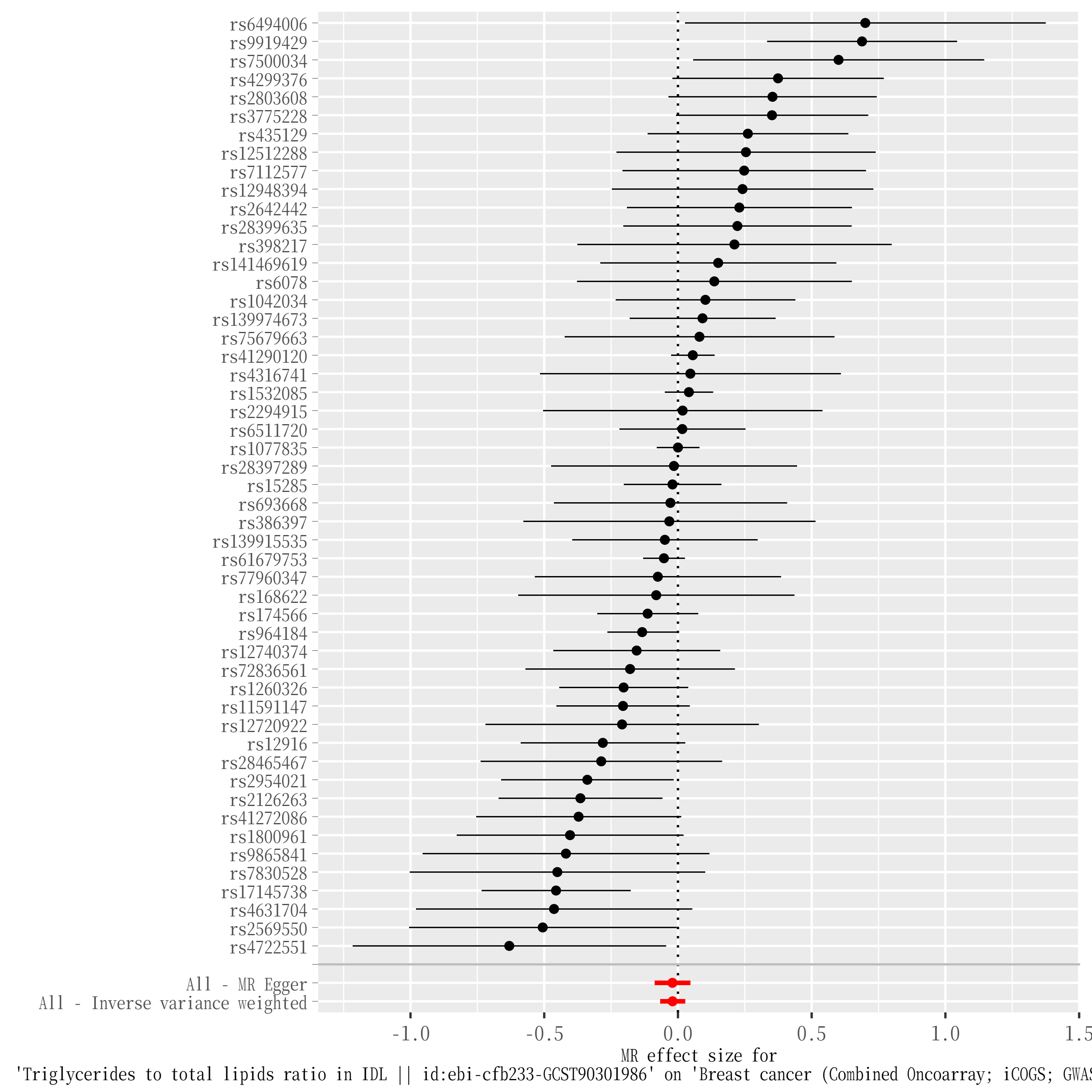
rs6494006
rs9919429
rs7500034
rs4299376
rs2803608
rs3775228
rs435129
rs12512288
rs7112577
rs12948394
rs2642442
rs28399635
rs398217
rs141469619
rs6078
rs1042034
rs139974673
rs75679663
rs41290120
rs4316741
rs1532085
rs2294915
rs6511720
rs1077835
rs28397289
rs15285
rs693668
rs386397
rs139915535
rs61679753
rs77960347
rs168622
rs174566
rs964184
rs12740374
rs72836561
rs1260326
rs11591147
rs12720922
rs12916
rs28465467
rs2954021
rs2126263
rs41272086
rs1800961
rs9865841
rs7830528
rs17145738
rs4631704
rs2569550
rs4722551
All - MR Egger
All - Inverse variance weighted
-1.0
-0.5
0.0
0.5
1.0
1.5
MR effect size for
'Triglycerides to total lipids ratio in IDL || id:ebi-cfb233-GCST90301986' on 'Breast cancer (Combined Oncoarray; iCOGS; GWAS

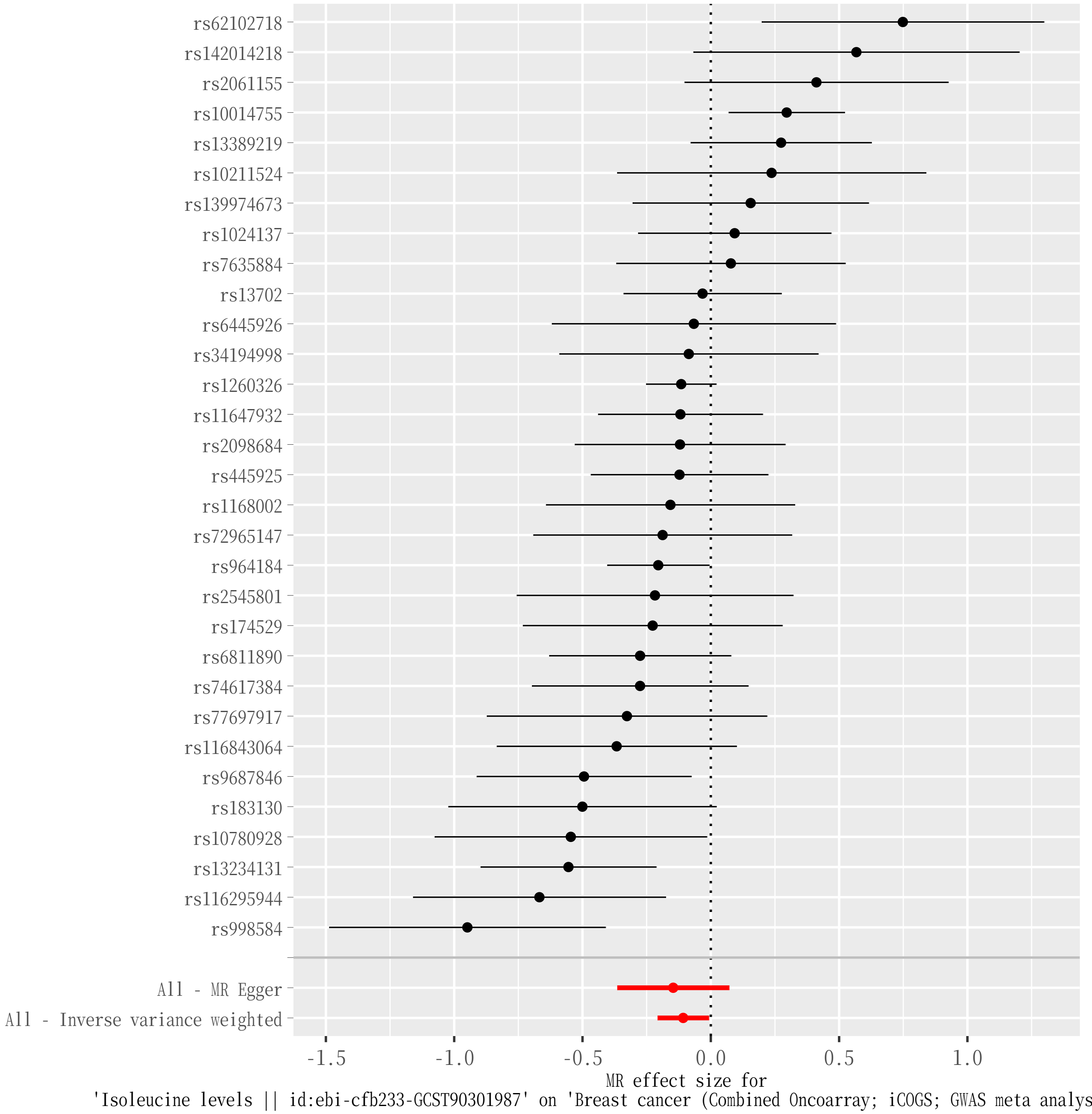
rs62102718
rs142014218
rs2061155
rs10014755
rs13389219
rs10211524
rs139974673
rs1024137
rs7635884
rs13702
rs6445926
rs34194998
rs1260326
rs11647932
rs2098684
rs445925
rs1168002
rs72965147
rs964184
rs2545801
rs174529
rs6811890
rs74617384
rs77697917
rs116843064
rs9687846
rs183130
rs10780928
rs13234131
rs116295944
rs998584
All - MR Egger
All - Inverse variance weighted
-1.5
-1.0
-0.5
0.0
0.5
1.0
MR effect size for
'Isoleucine levels || id:ebi-cfb233-GCST90301987' on 'Breast cancer (Combined Oncoarray; iCOGS; GWAS meta analys

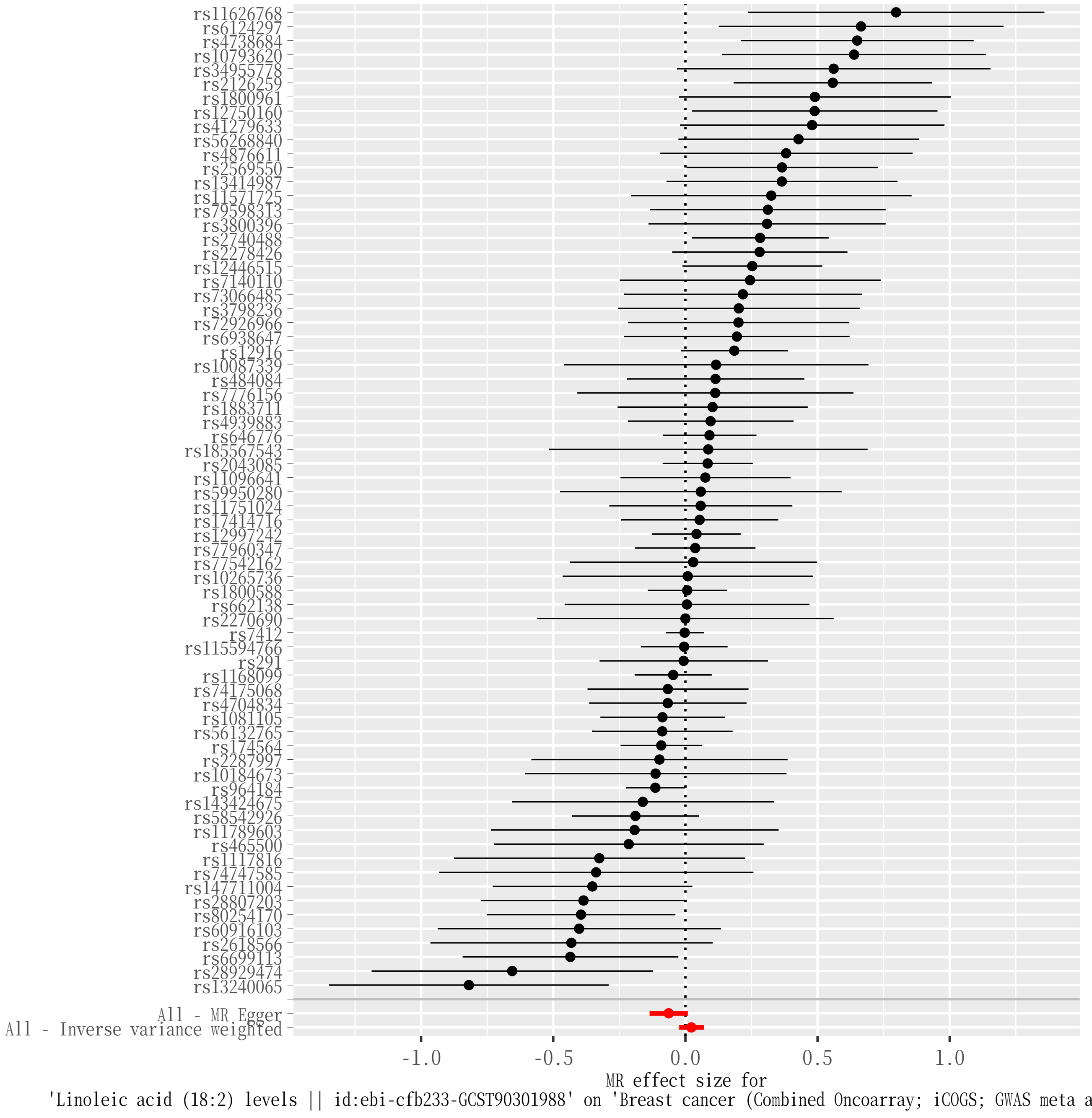

rs11626768
rs6124297
rs4738684
rs10793620
rs34955778
rs2126259
rs1800961
rs12750160
rs41279633
rs56268840
rs4876611
rs2569550
rs13414987
rs11571725
rs79598313
rs3800396
rs2740488
rs2278426
rs12446515
rs7140110
rs73066485
rs3798236
rs72926966
rs6938647
rs12916
rs10087339
rs484084
rs7776156
rs1883711
rs4939883
rs646776
rs185567543
rs2043085
rs11096641
rs59950280
rs11751024
rs17414716
rs12997242
rs77960347
rs77542162
rs10265736
rs1800588
rs662138
rs2270690
rs7412
rs115594766
rs291
rs1168099
rs74175068
rs4704834
rs1081105
rs56132765
rs174564
rs2287997
rs10184673
rs964184
rs143424675
rs58542926
rs11789603
rs465500
rs1117816
rs74747585
rs147711004
rs28807203
rs80254170
rs60916103
rs2618566
rs6699113
rs28929474
rs13240065
All - MR Egger
All - Inverse variance weighted
-1.0
-0.5
0.0
0.5
1.0
MR effect size for
'Linoleic acid (18:2) levels || id:ebi-cfb233-GCST90301988' on 'Breast cancer (Combined Oncoarray; iCOGS; GWAS meta a

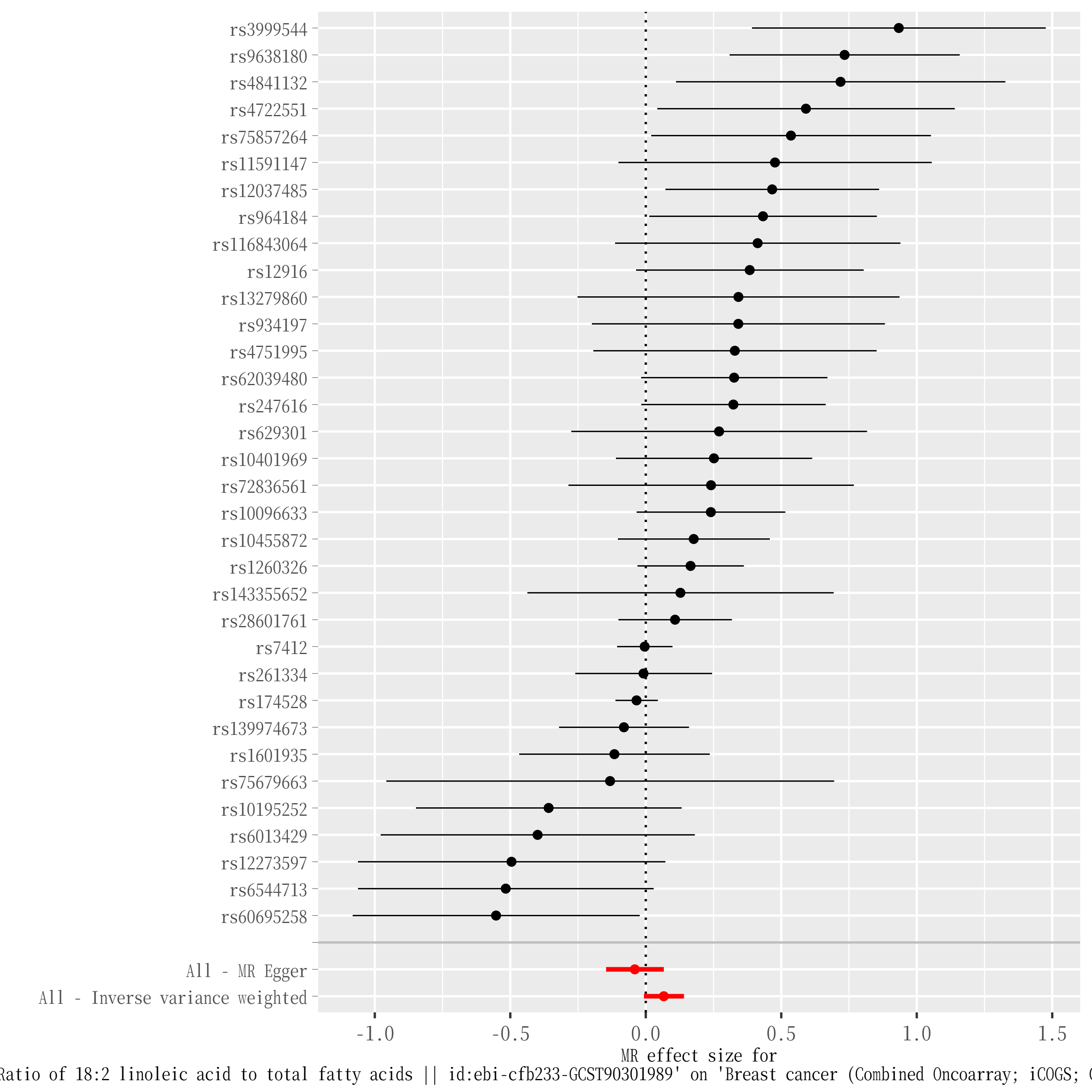
rs3999544
rs9638180
rs4841132
rs4722551
rs75857264
rs11591147
rs12037485
rs964184
rs116843064
rs12916
rs13279860
rs934197
rs4751995
rs62039480
rs247616
rs629301
rs10401969
rs72836561
rs10096633
rs10455872
rs1260326
rs143355652
rs28601761
rs7412
rs261334
rs174528
rs139974673
rs1601935
rs75679663
rs10195252
rs6013429
rs12273597
rs6544713
rs60695258
All - MR Egger
All - Inverse variance weighted
-1.0
-0.5
0.0
0.5
1.0
1.5
MR effect size for
Ratio of 18:2 linoleic acid to total fatty acids || id:ebi-cfb233-GCST90301989' on 'Breast cancer (Combined Oncoarray; iCOGS; G

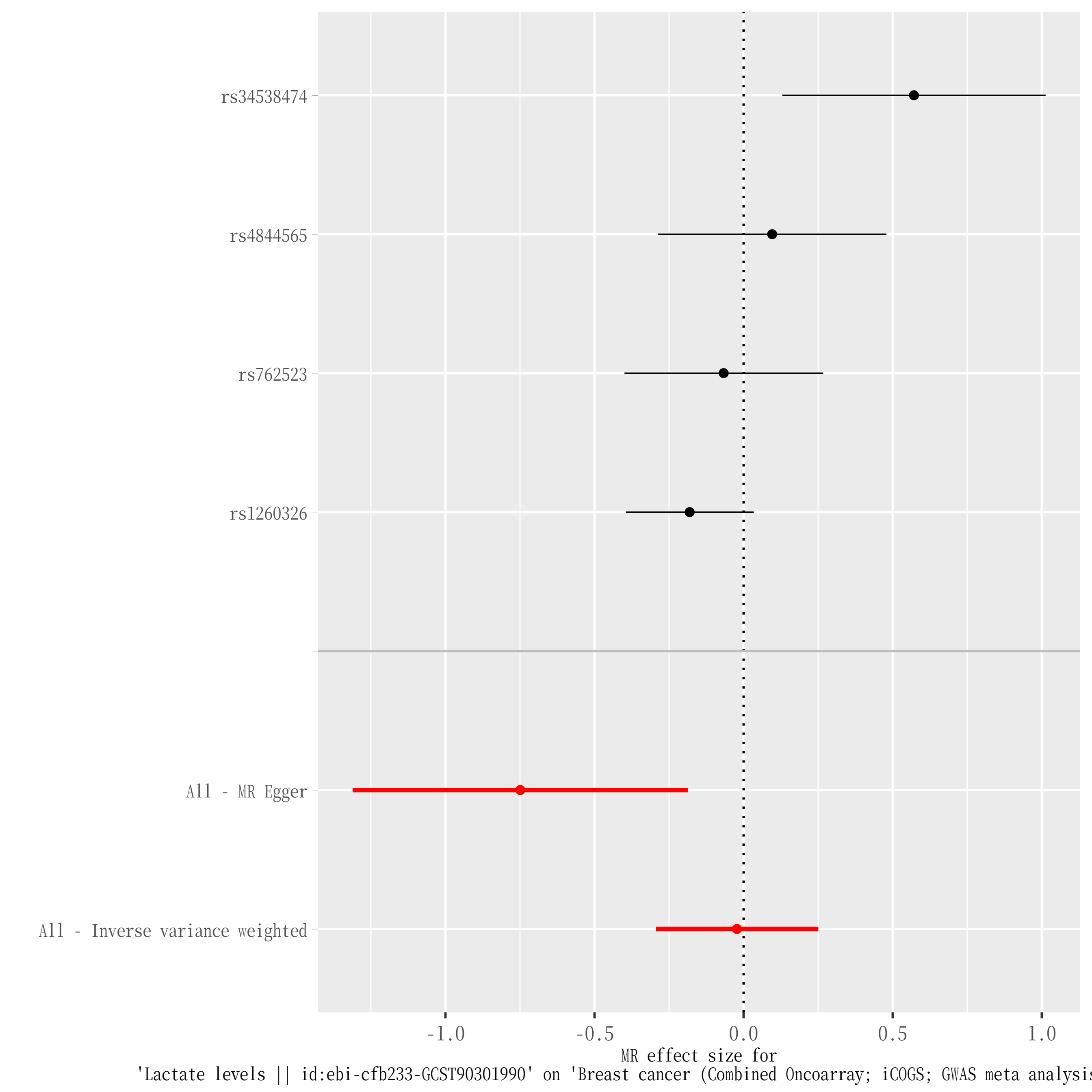

rs34538474
rs4844565
rs762523
rs1260326
All - MR Egger
All - Inverse variance weighted
-1.0
-0.5
0.0
0.5
1.0
MR effect size for
'Lactate levels || id:ebi-cfb233-GCST90301990' on 'Breast cancer (Combined Oncoarray; iCOGS; GWAS meta analysis

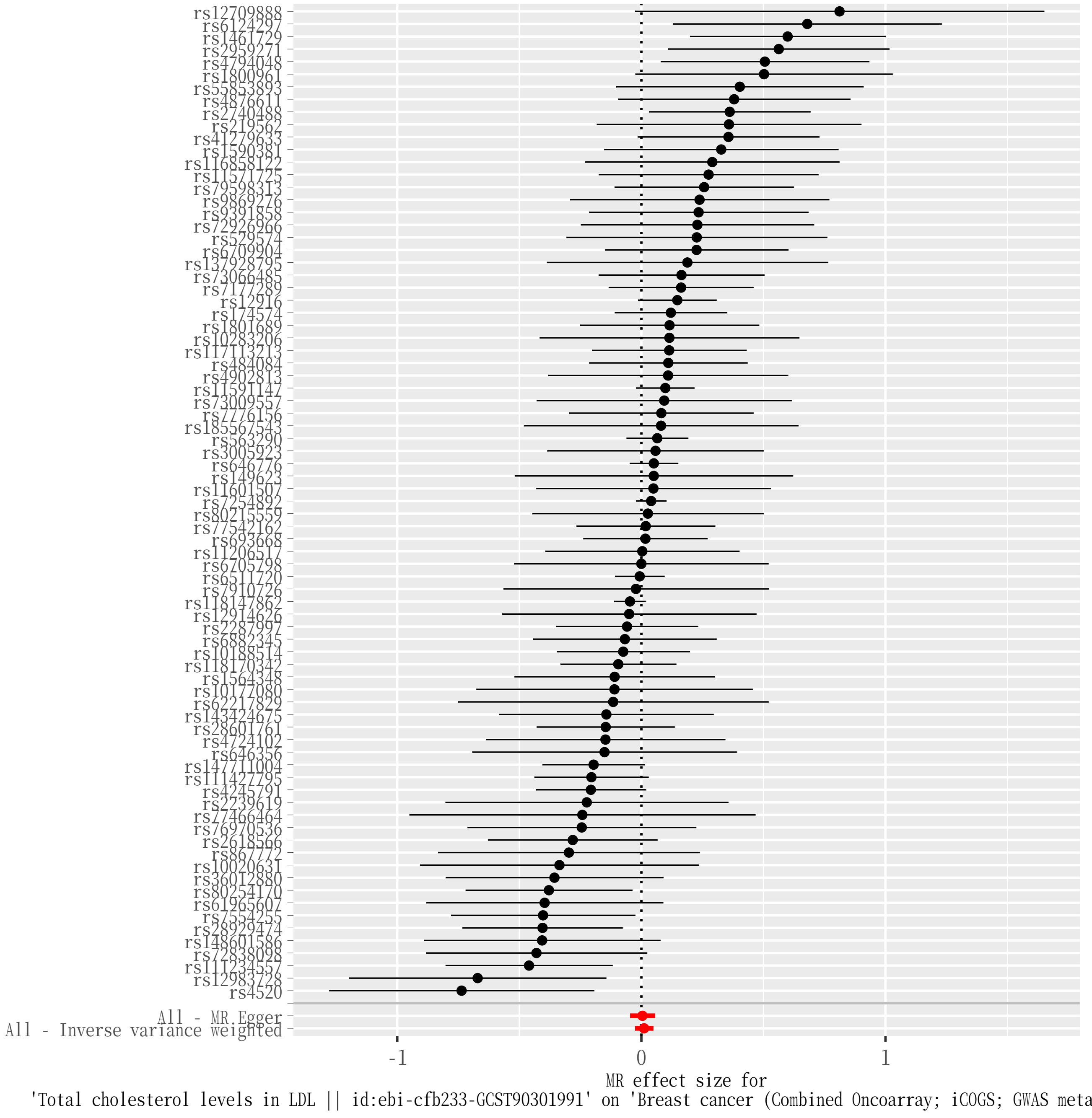
rs12709888
rs6124297
rs1461729
rs2959271
rs4794048
rs1800961
rs55853893
rs4876611
rs2740488
rs219562
rs41279633
rs1590381
rs116858122
rs11571725
rs79598313
rs9869276
rs9391858
rs72926966
rs529574
rs6709904
rs137928795
rs73066485
rs7177289
rs12916
rs174574
rs1801689
rs10283206
rs117113213
rs484084
rs4902813
rs11591147
rs73009557
rs7776156
rs185567543
rs563290
rs3005923
rs646776
rs149623
rs11601507
rs7254892
rs80215559
rs77542162
rs693668
rs11206517
rs6705798
rs6511720
rs7910726
rs118147862
rs12914626
rs2287997
rs6882345
rs10188514
rs118170342
rs1564348
rs10177080
rs62217829
rs143424675
rs28601761
rs4724102
rs646356
rs147711004
rs111427795
rs4245791
rs2239619
rs77466464
rs76970536
rs2618566
rs867772
rs10020631
rs36012880
rs80254170
rs61965607
rs7554255
rs28929474
rs148601586
rs72838028
rs111234557
rs12983728
rs4520
All - MR Egger
All - Inverse variance weighted
-1
0
1
MR effect size for
'Total cholesterol levels in LDL || id:ebi-cfb233-GCST90301991' on 'Breast cancer (Combined Oncoarray; iCOGS; GWAS meta

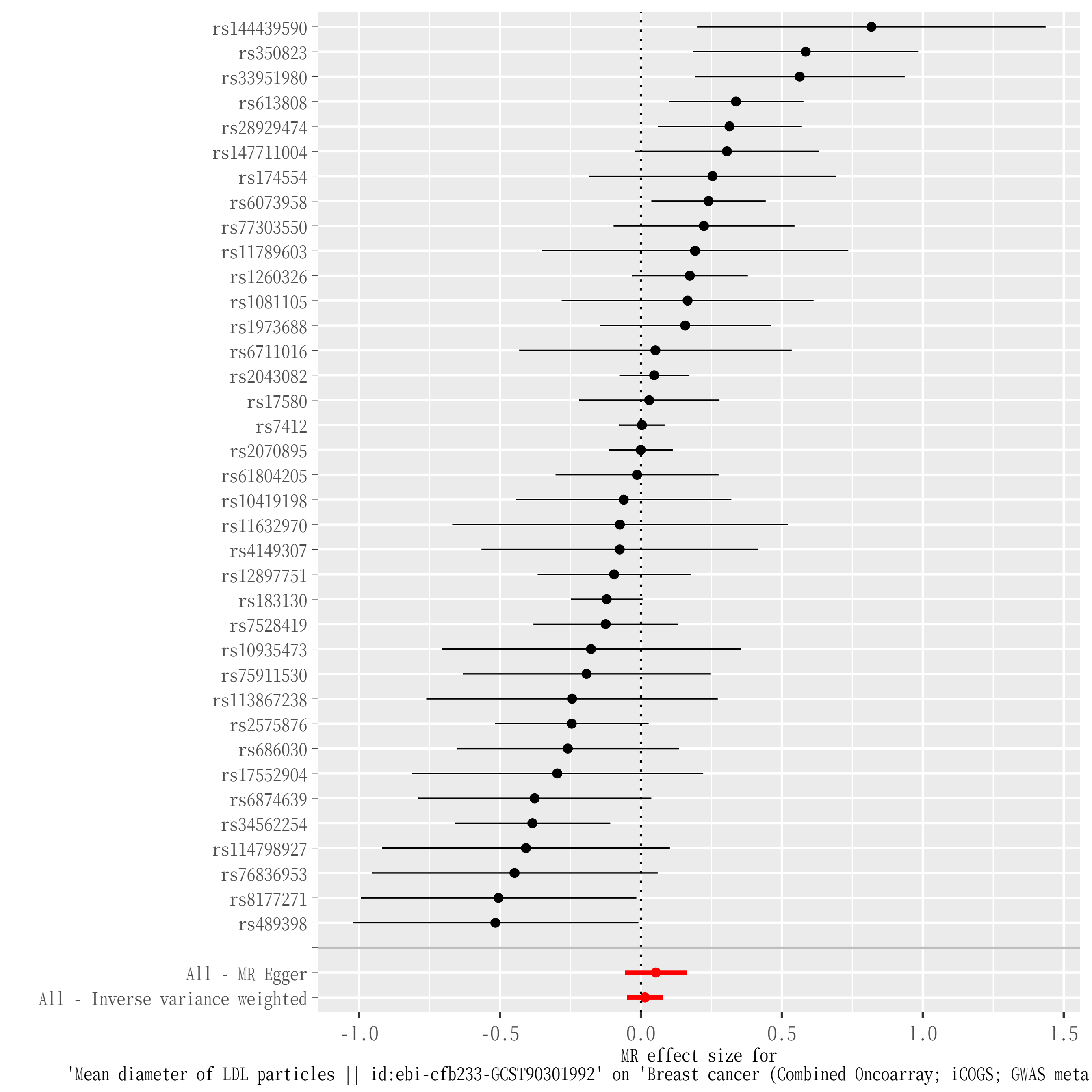

rs144439590
rs350823
rs33951980
rs613808
rs28929474
rs147711004
rs174554
rs6073958
rs77303550
rs11789603
rs1260326
rs1081105
rs1973688
rs6711016
rs2043082
rs17580
rs7412
rs2070895
rs61804205
rs10419198
rs11632970
rs4149307
rs12897751
rs183130
rs7528419
rs10935473
rs75911530
rs113867238
rs2575876
rs686030
rs17552904
rs6874639
rs34562254
rs114798927
rs76836953
rs8177271
rs489398
All - MR Egger
All - Inverse variance weighted
-1.0
-0.5
0.0
0.5
1.0
1.5
MR effect size for
'Mean diameter of LDL particles || id:ebi-cfb233-GCST90301992' on 'Breast cancer (Combined Oncoarray; iCOGS; GWAS meta

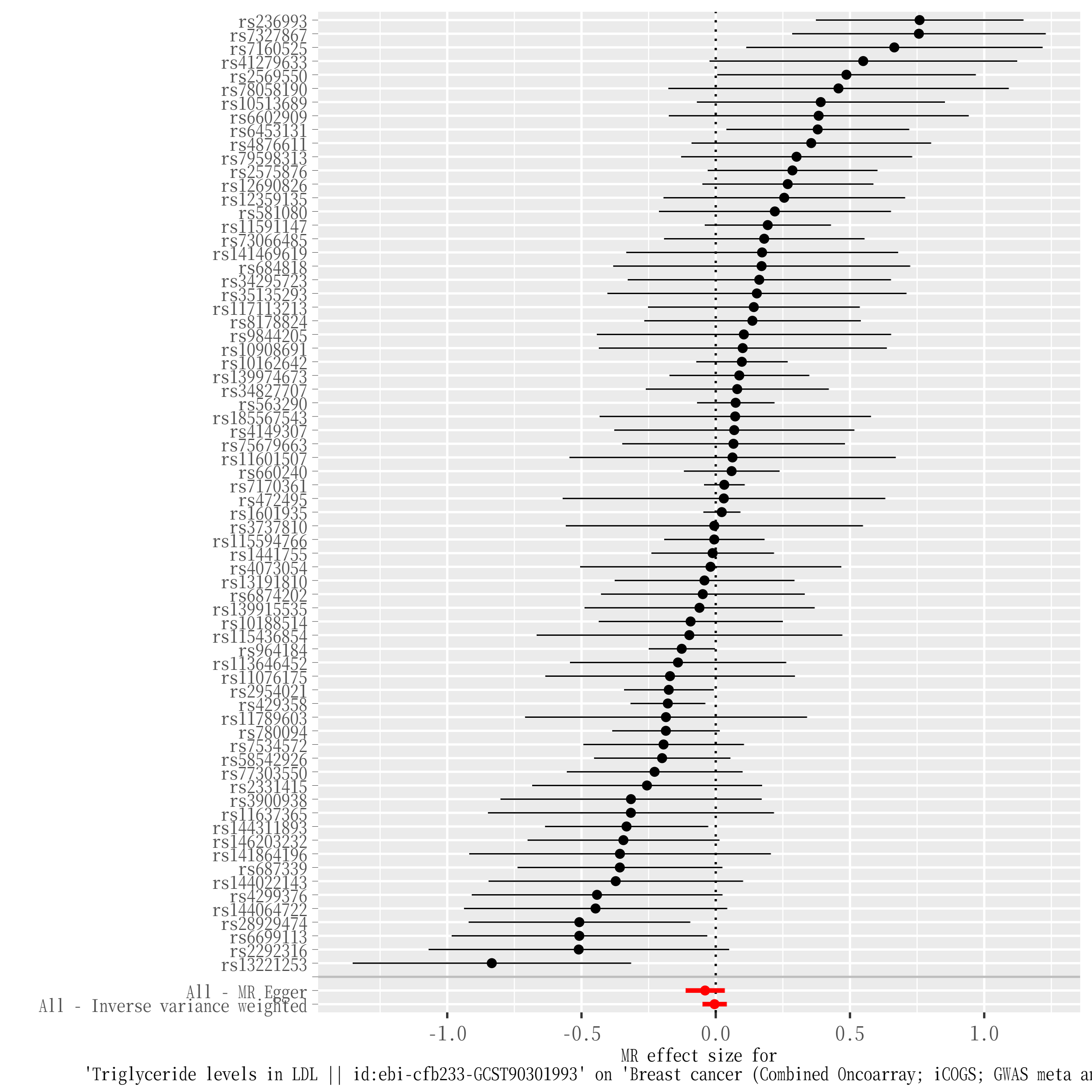
rs236993
rs7327867
rs7160525
rs41279633
rs2569550
rs78058190
rs10513689
rs6602909
rs6453131
rs4876611
rs79598313
rs2575876
rs12690826
rs12359135
rs581080
rs11591147
rs73066485
rs141469619
rs684818
rs34295723
rs35135293
rs117113213
rs8178824
rs9844205
rs10908691
rs10162642
rs139974673
rs34827707
rs563290
rs185567543
rs4149307
rs75679663
rs11601507
rs660240
rs7170361
rs472495
rs1601935
rs3737810
rs115594766
rs1441755
rs4073054
rs13191810
rs6874202
rs139915535
rs10188514
rs115436854
rs964184
rs113646452
rs11076175
rs2954021
rs429358
rs11789603
rs780094
rs7534572
rs58542926
rs77303550
rs2331415
rs3900938
rs11637365
rs144311893
rs146203232
rs141864196
rs687339
rs144022143
rs4299376
rs144064722
rs28929474
rs6699113
rs2292316
rs13221253
All - MR Egger
All - Inverse variance weighted
-1.0
-0.5
0.0
0.5
1.0
MR effect size for
'Triglyceride levels in LDL || id:ebi-cfb233-GCST90301993' on 'Breast cancer (Combined Oncoarray; iCOGS; GWAS meta an

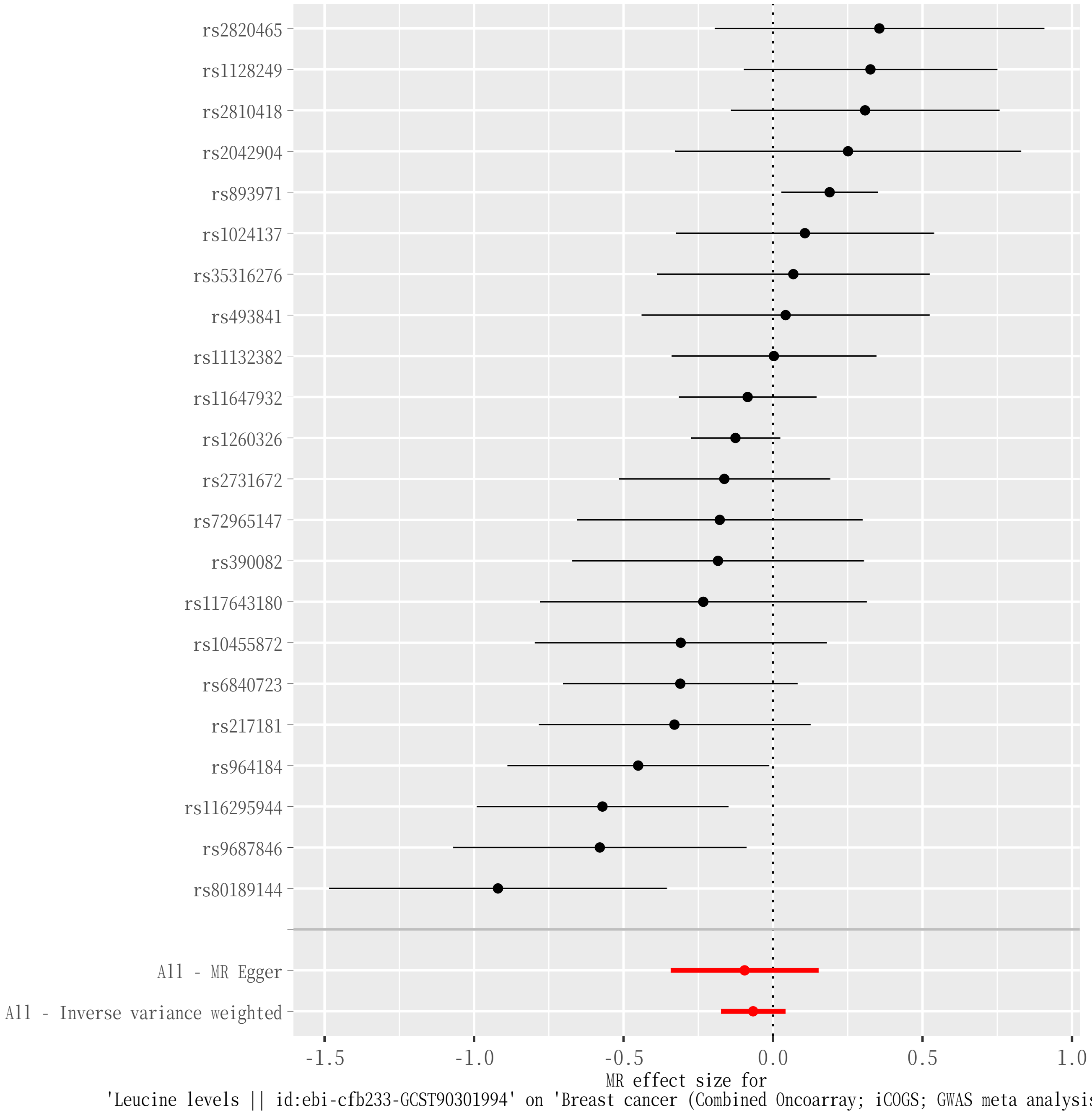
rs2820465
rs1128249
rs2810418
rs2042904
rs893971
rs1024137
rs35316276
rs493841
rs11132382
rs11647932
rs1260326
rs2731672
rs72965147
rs390082
rs117643180
rs10455872
rs6840723
rs217181
rs964184
rs116295944
rs9687846
rs80189144
All - MR Egger
All - Inverse variance weighted
-1.5
-1.0
-0.5
0.0
0.5
1.0
MR effect size for
'Leucine levels || id:ebi-cfb233-GCST90301994' on 'Breast cancer (Combined Oncoarray; iCOGS; GWAS meta analysis

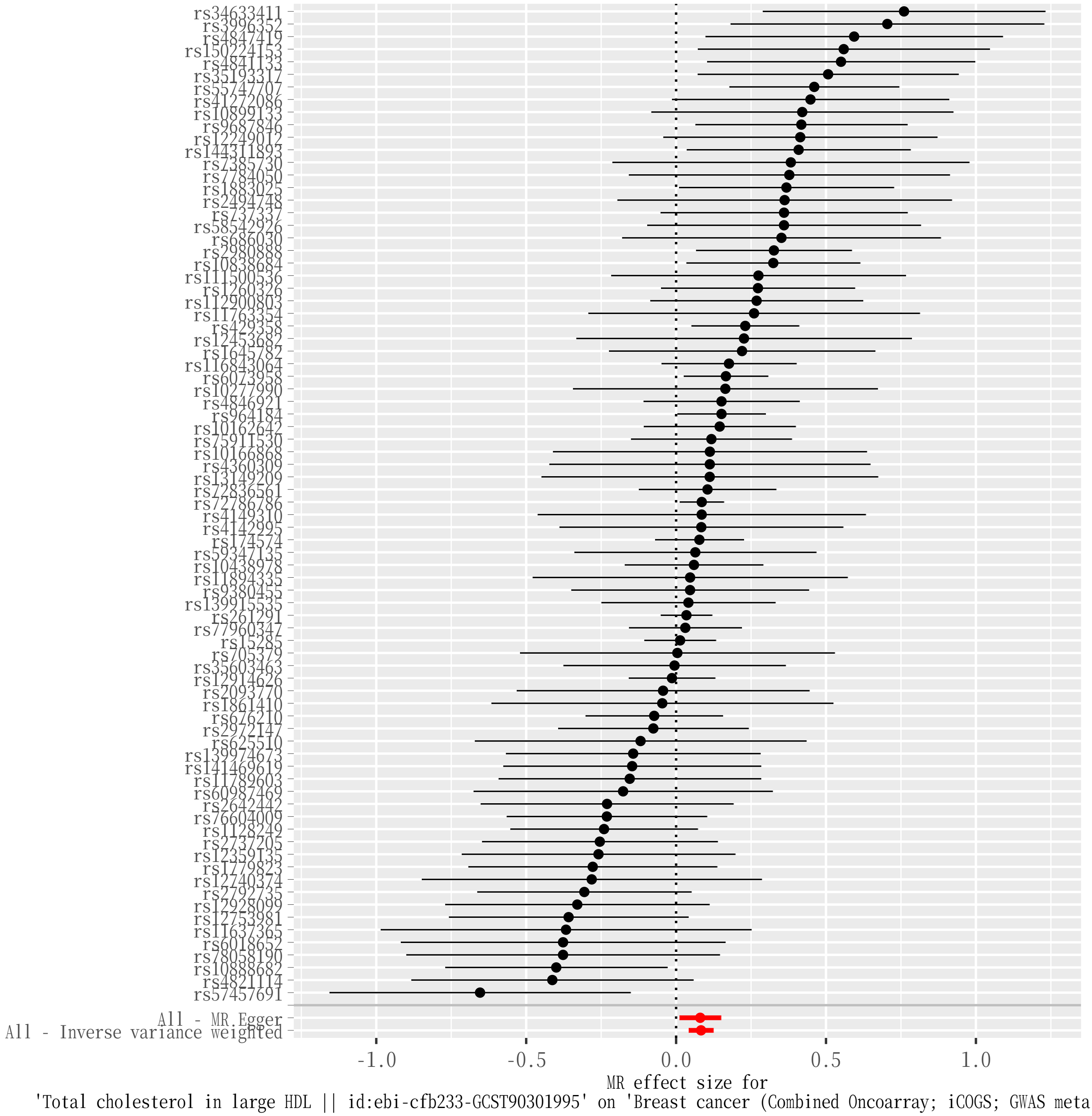

rs34633411
rs3996352
rs4847419
rs150224153
rs4841133
rs35193317
rs55747707
rs41272086
rs10899133
rs9687846
rs12249012
rs144311893
rs7385730
rs7784050
rs1883025
rs2494748
rs737337
rs58542926
rs686030
rs2980888
rs10838684
rs111500536
rs1260326
rs112900803
rs11763354
rs429358
rs12453682
rs1645782
rs116843064
rs6073958
rs10277990
rs4846921
rs964184
rs10162642
rs75911530
rs10166868
rs4360309
rs13149209
rs72836561
rs72786786
rs4149310
rs4142995
rs174574
rs59347135
rs10438978
rs11894335
rs9380455
rs139915535
rs261291
rs77960347
rs15285
rs705379
rs35603463
rs12914626
rs2093770
rs1861410
rs676210
rs2972147
rs625510
rs139974673
rs141469619
rs11789603
rs60987469
rs2642442
rs76604009
rs1128249
rs2737205
rs12359135
rs1779823
rs12740374
rs2792735
rs12928099
rs12753981
rs11637365
rs6018652
rs78058190
rs10888682
rs4821114
rs57457691
All - MR Egger
All - Inverse variance weighted
-1.0
-0.5
0.0
0.5
1.0
MR effect size for
'Total cholesterol in large HDL || id:ebi-cfb233-GCST90301995' on 'Breast cancer (Combined Oncoarray; iCOGS; GWAS meta

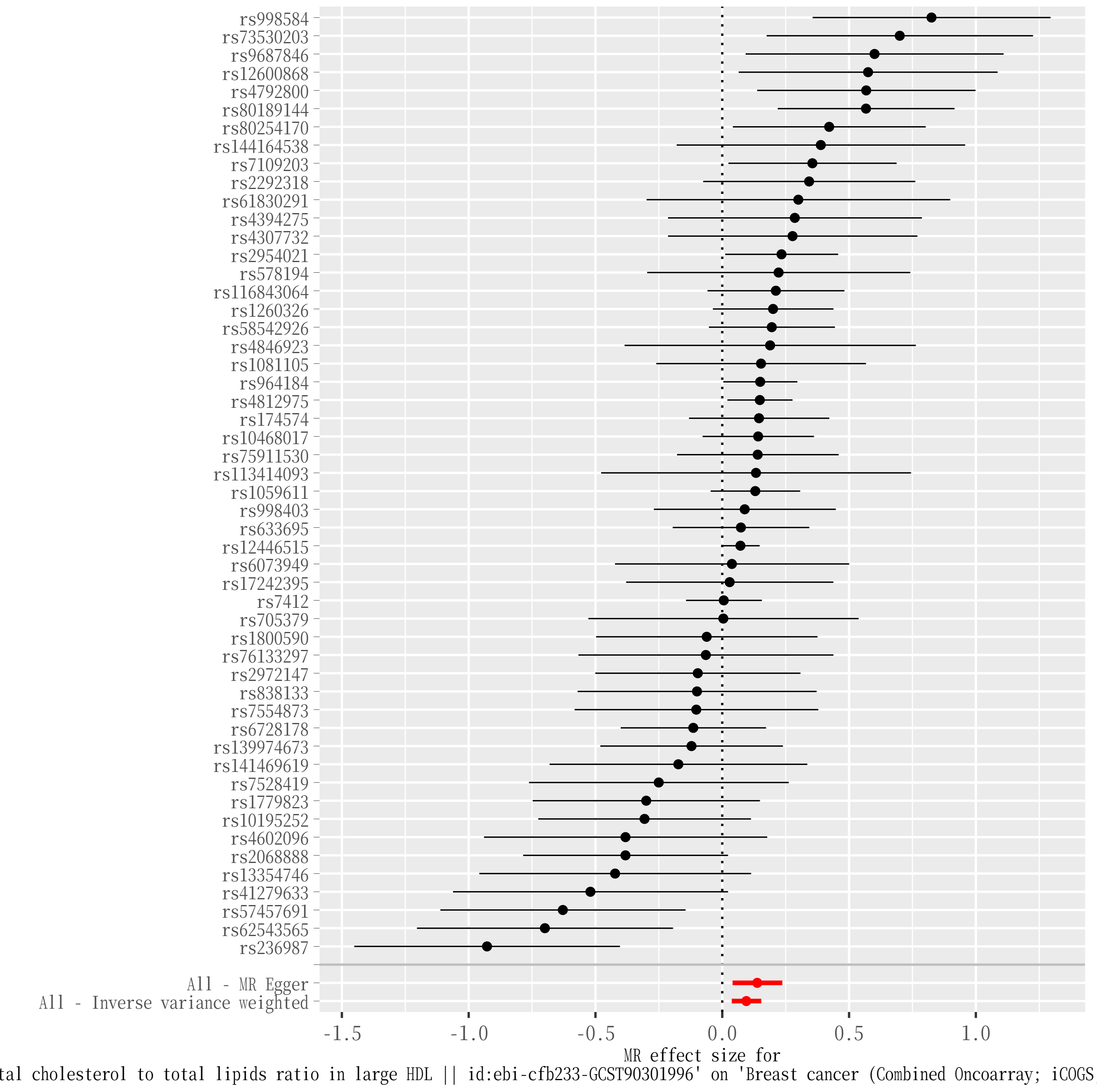
rs998584
rs73530203
rs9687846
rs12600868
rs4792800
rs80189144
rs80254170
rs144164538
rs7109203
rs2292318
rs61830291
rs4394275
rs4307732
rs2954021
rs578194
rs116843064
rs1260326
rs58542926
rs4846923
rs1081105
rs964184
rs4812975
rs174574
rs10468017
rs75911530
rs113414093
rs1059611
rs998403
rs633695
rs12446515
rs6073949
rs17242395
rs7412
rs705379
rs1800590
rs76133297
rs2972147
rs838133
rs7554873
rs6728178
rs139974673
rs141469619
rs7528419
rs1779823
rs10195252
rs4602096
rs2068888
rs13354746
rs41279633
rs57457691
rs62543565
rs236987
All - MR Egger
All - Inverse variance weighted
-1.5
-1.0
-0.5
0.0
0.5
1.0
MR effect size for
tal cholesterol to total lipids ratio in large HDL || id:ebi-cfb233-GCST90301996' on 'Breast cancer (Combined Oncoarray; iCOGS

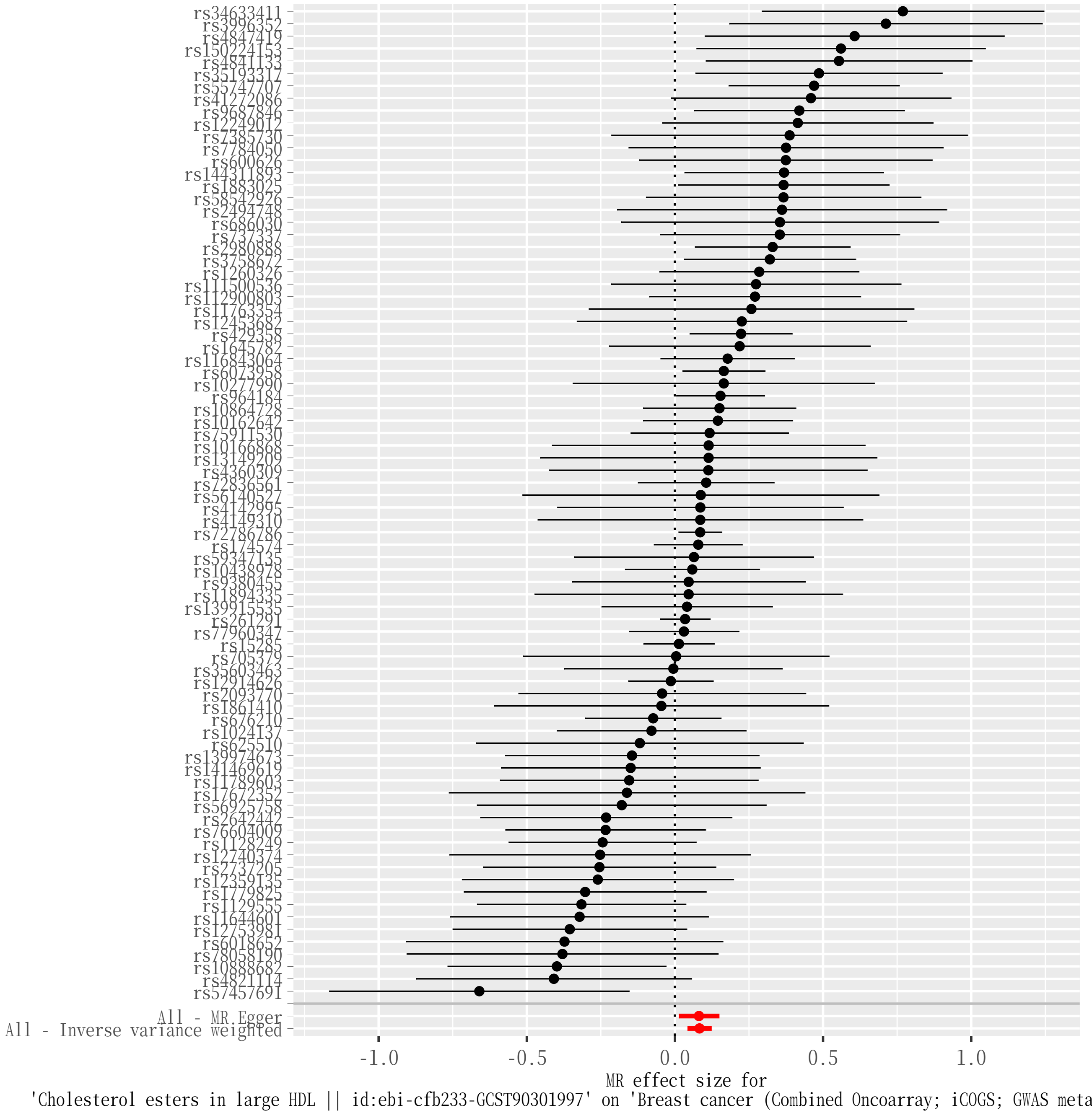
rs34633411
rs3996352
rs4847419
rs150224153
rs4841133
rs35193317
rs55747707
rs41272086
rs9687846
rs12249012
rs7385730
rs7784050
rs600626
rs144311893
rs1883025
rs58542926
rs2494748
rs686030
rs737337
rs2980888
rs3758672
rs1260326
rs111500536
rs112900803
rs11763354
rs12453682
rs429358
rs1645782
rs116843064
rs6073958
rs10277990
rs964184
rs10864728
rs10162642
rs75911530
rs10166868
rs13149209
rs4360309
rs72836561
rs56140527
rs4142995
rs4149310
rs72786786
rs174574
rs59347135
rs10438978
rs9380455
rs11894335
rs139915535
rs261291
rs77960347
rs15285
rs705379
rs35603463
rs12914626
rs2093770
rs1861410
rs676210
rs1024137
rs625510
rs139974673
rs141469619
rs11789603
rs17672352
rs56925758
rs2642442
rs76604009
rs1128249
rs12740374
rs2737205
rs12359135
rs1779825
rs1129555
rs11644601
rs12753981
rs6018652
rs78058190
rs10888682
rs4821114
rs57457691
All - MR Egger
All - Inverse variance weighted
-1.0
-0.5
0.0
0.5
1.0
MR effect size for
'Cholesterol esters in large HDL || id:ebi-cfb233-GCST90301997' on 'Breast cancer (Combined Oncoarray; iCOGS; GWAS meta

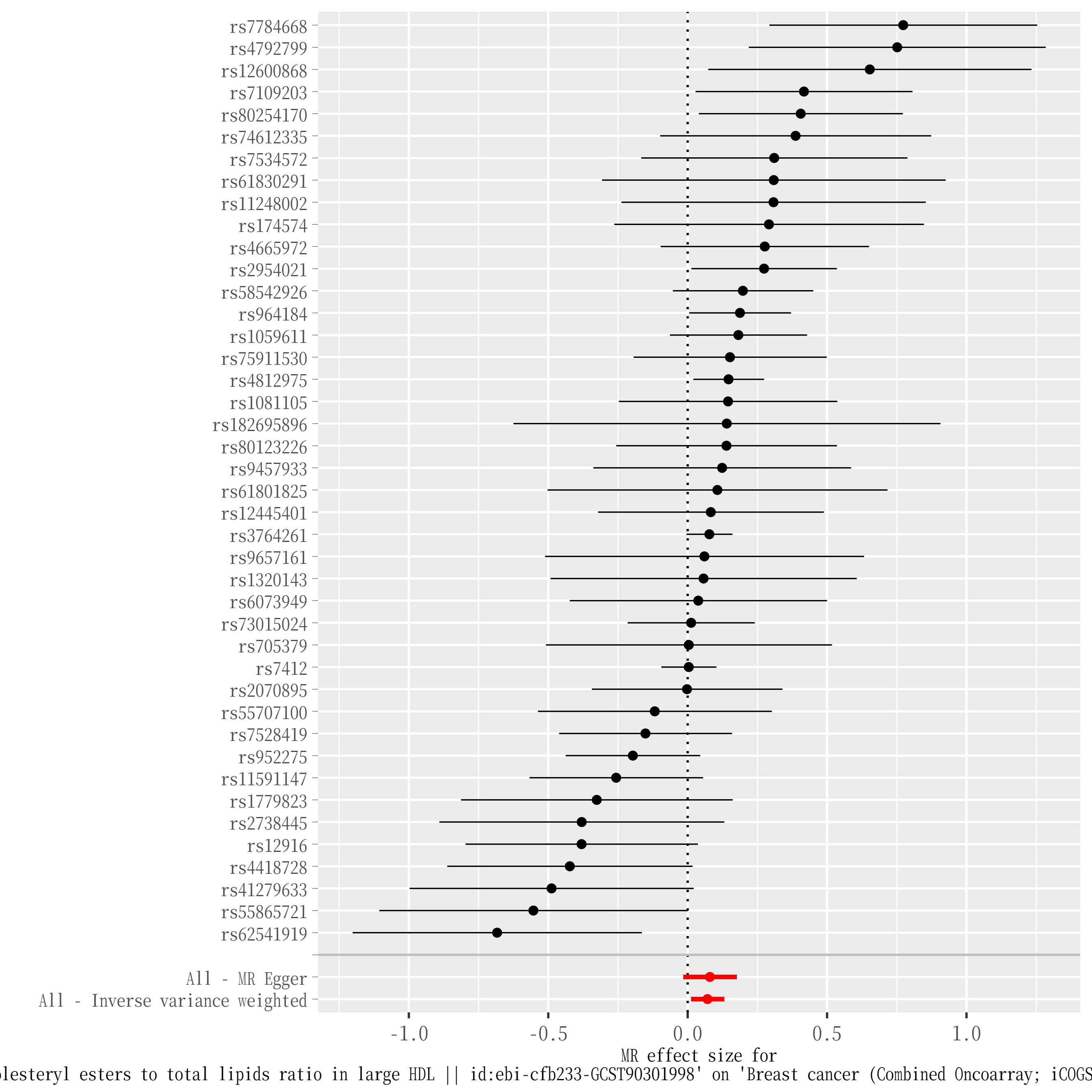

rs7784668
rs4792799
rs12600868
rs7109203
rs80254170
rs74612335
rs7534572
rs61830291
rs11248002
rs174574
rs4665972
rs2954021
rs58542926
rs964184
rs1059611
rs75911530
rs4812975
rs1081105
rs182695896
rs80123226
rs9457933
rs61801825
rs12445401
rs3764261
rs9657161
rs1320143
rs6073949
rs73015024
rs705379
rs7412
rs2070895
rs55707100
rs7528419
rs952275
rs11591147
rs1779823
rs2738445
rs12916
rs4418728
rs41279633
rs55865721
rs62541919
All - MR Egger
All - Inverse variance weighted
-1.0
-0.5
0.0
0.5
1.0
MR effect size for
lesteryl esters to total lipids ratio in large HDL || id:ebi-cfb233-GCST90301998' on 'Breast cancer (Combined Oncoarray; iCOGS

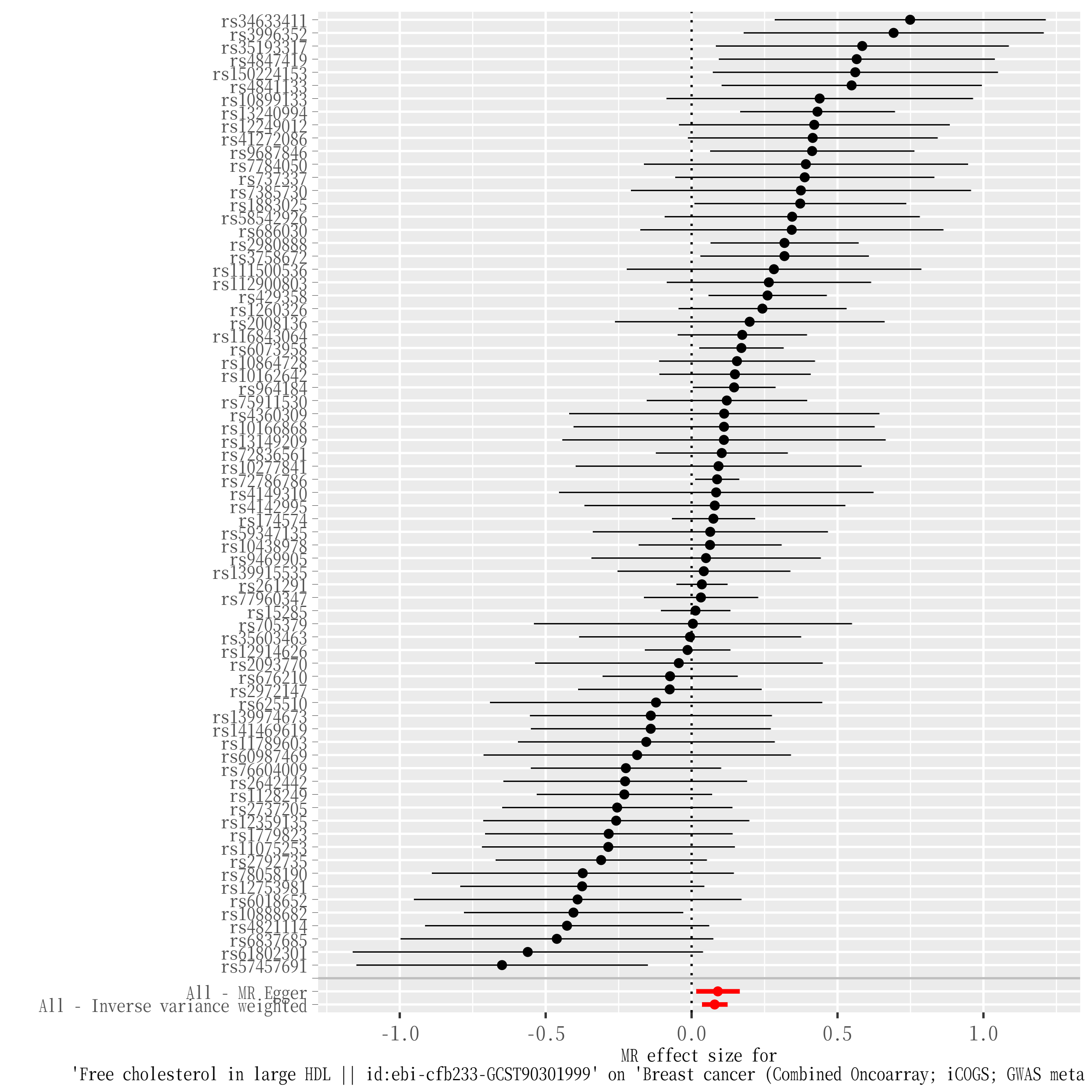

rs34633411
rs3996352
rs35193317
rs4847419
rs150224153
rs4841133
rs10899133
rs13240994
rs12249012
rs41272086
rs9687846
rs7784050
rs737337
rs7385730
rs1883025
rs58542926
rs686030
rs2980888
rs3758672
rs111500536
rs112900803
rs429358
rs1260326
rs2008136
rs116843064
rs6073958
rs10864728
rs10162642
rs964184
rs75911530
rs4360309
rs10166868
rs13149209
rs72836561
rs10277841
rs72786786
rs4149310
rs4142995
rs174574
rs59347135
rs10438978
rs9469905
rs139915535
rs261291
rs77960347
rs15285
rs705379
rs35603463
rs12914626
rs2093770
rs676210
rs2972147
rs625510
rs139974673
rs141469619
rs11789603
rs60987469
rs76604009
rs2642442
rs1128249
rs2737205
rs12359135
rs1779823
rs11075253
rs2792735
rs78058190
rs12753981
rs6018652
rs10888682
rs4821114
rs6837685
rs61802301
rs57457691
All - MR Egger
All - Inverse variance weighted
-1.0
-0.5
0.0
0.5
1.0
MR effect size for
'Free cholesterol in large HDL || id:ebi-cfb233-GCST90301999' on 'Breast cancer (Combined Oncoarray; iCOGS; GWAS meta

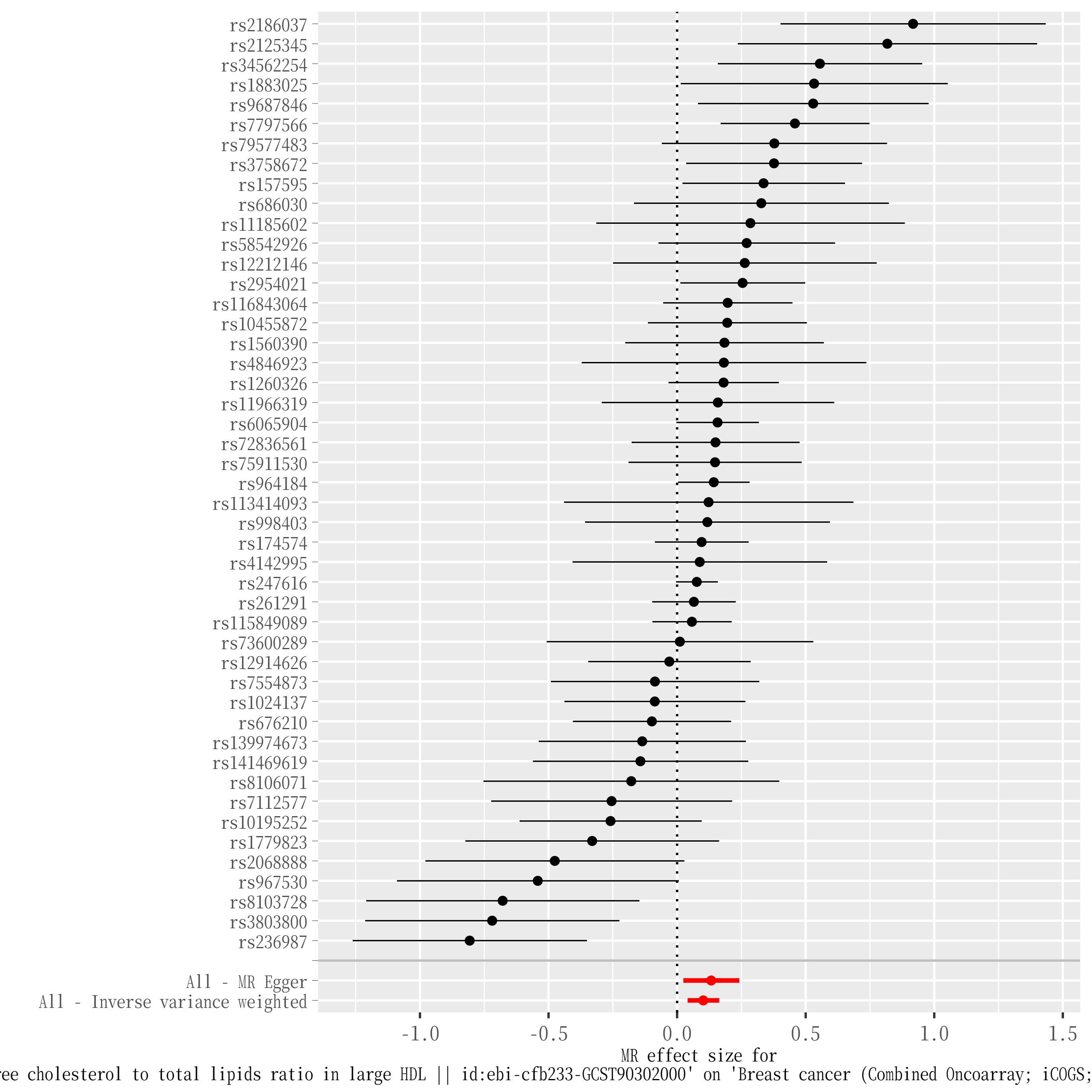
rs2186037
rs2125345
rs34562254
rs1883025
rs9687846
rs7797566
rs79577483
rs3758672
rs157595
rs686030
rs11185602
rs58542926
rs12212146
rs2954021
rs116843064
rs10455872
rs1560390
rs4846923
rs1260326
rs11966319
rs6065904
rs72836561
rs75911530
rs964184
rs113414093
rs998403
rs174574
rs4142995
rs247616
rs261291
rs115849089
rs73600289
rs12914626
rs7554873
rs1024137
rs676210
rs139974673
rs141469619
rs8106071
rs7112577
rs10195252
rs1779823
rs2068888
rs967530
rs8103728
rs3803800
rs236987
All - MR Egger
All - Inverse variance weighted
-1.0
-0.5
0.0
0.5
1.0
1.5
MR effect size for
ree cholesterol to total lipids ratio in large HDL || id:ebi-cfb233-GCST90302000' on 'Breast cancer (Combined Oncoarray; iCOGS;

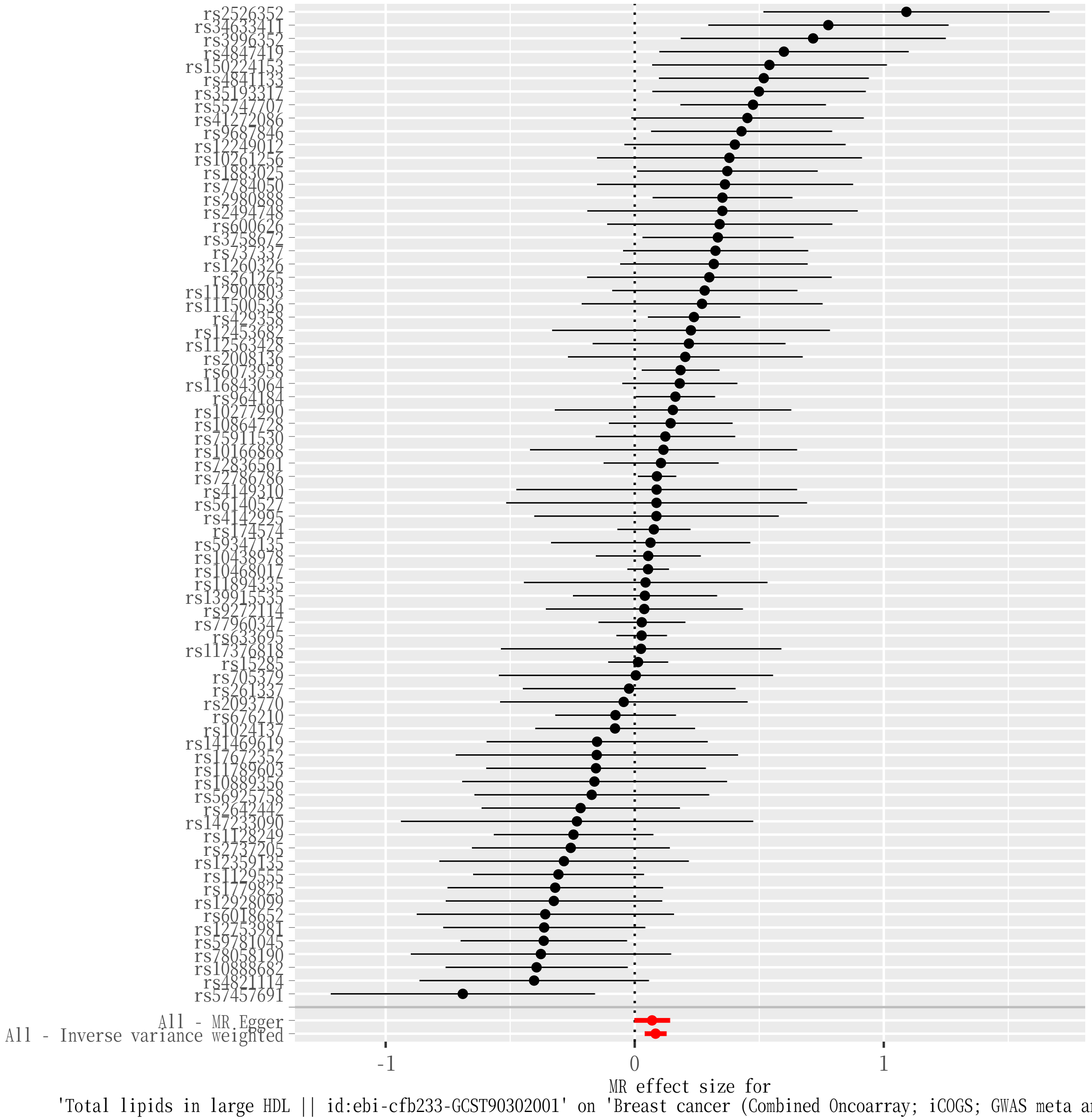
rs2526352
rs34633411
rs3996352
rs4847419
rs150224153
rs4841133
rs35193317
rs55747707
rs41272086
rs9687846
rs12249012
rs10261256
rs1883025
rs7784050
rs2980888
rs2494748
rs600626
rs3758672
rs737337
rs1260326
rs261265
rs112900803
rs111500536
rs429358
rs12453682
rs112563428
rs2008136
rs6073958
rs116843064
rs964184
rs10277990
rs10864728
rs75911530
rs10166868
rs72836561
rs72786786
rs4149310
rs56140527
rs4142995
rs174574
rs59347135
rs10438978
rs10468017
rs11894335
rs139915535
rs9272114
rs77960347
rs633695
rs117376818
rs15285
rs705379
rs261337
rs2093770
rs676210
rs1024137
rs141469619
rs17672352
rs11789603
rs10889356
rs56925758
rs2642442
rs147233090
rs1128249
rs2737205
rs12359135
rs1129555
rs1779825
rs12928099
rs6018652
rs12753981
rs59781045
rs78058190
rs10888682
rs4821114
rs57457691
All - MR Egger
All - Inverse variance weighted
-1
0
1
MR effect size for
'Total lipids in large HDL || id:ebi-cfb233-GCST90302001' on 'Breast cancer (Combined Oncoarray; iCOGS; GWAS meta an

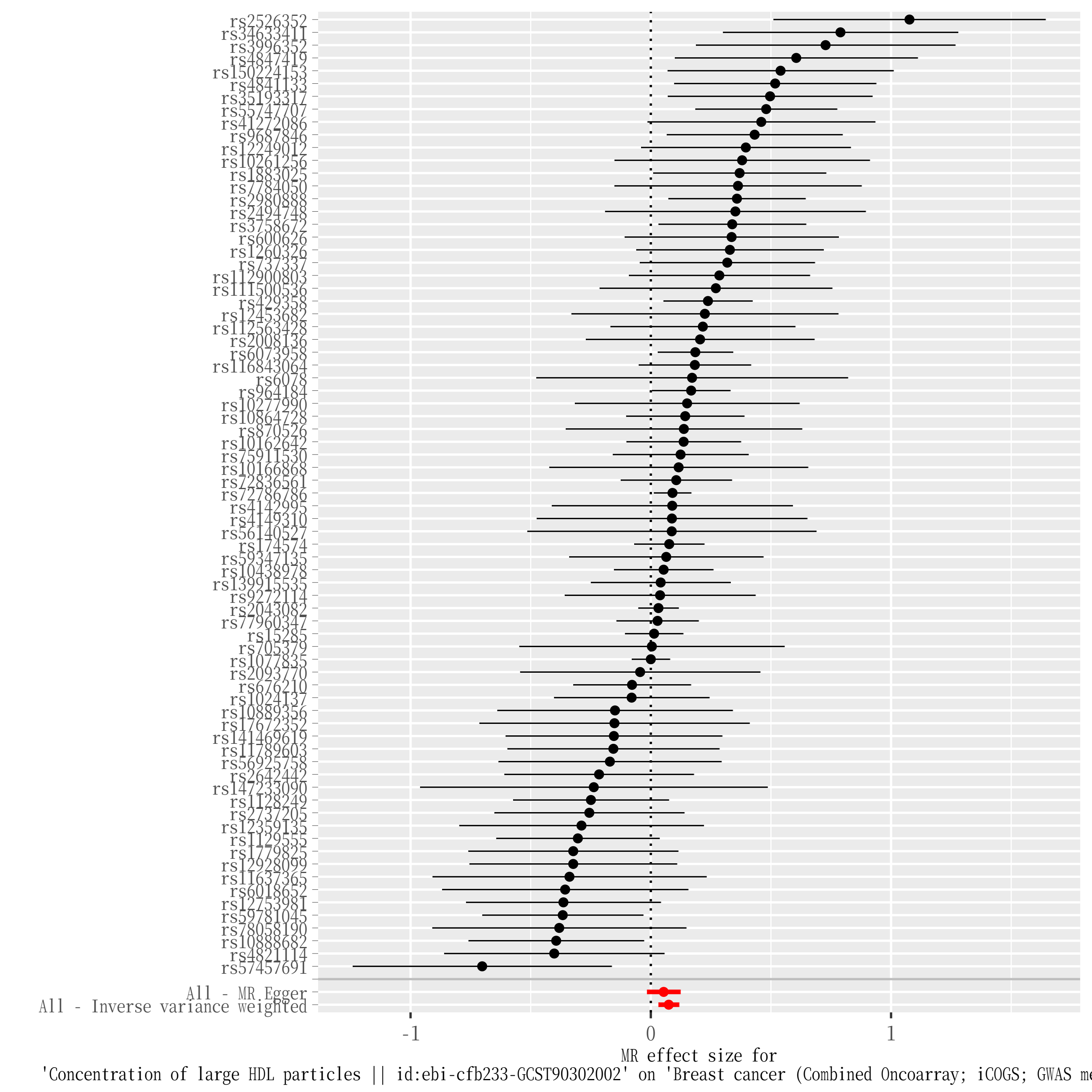
rs2526352
rs34633411
rs3996352
rs4847419
rs150224153
rs4841133
rs35193317
rs55747707
rs41272086
rs9687846
rs12249012
rs10261256
rs1883025
rs7784050
rs2980888
rs2494748
rs3758672
rs600626
rs1260326
rs737337
rs112900803
rs111500536
rs429358
rs12453682
rs112563428
rs2008136
rs6073958
rs116843064
rs6078
rs964184
rs10277990
rs10864728
rs870526
rs1016262
rs75911530
rs10166868
rs72836561
rs72786786
rs4142995
rs4149310
rs56140527
rs174574
rs59347135
rs10438978
rs139915535
rs9272114
rs2043082
rs77960347
rs15285
rs705379
rs1077835
rs2093770
rs676210
rs1024137
rs10889356
rs17672352
rs141469619
rs11789603
rs56925758
rs264242
rs147233090
rs1128249
rs2737205
rs12359135
rs1129555
rs1779825
rs12928099
rs11637365
rs6018652
rs12753981
rs59781045
rs78058190
rs10888682
rs4821114
rs57457691
All - MR Egger
All - Inverse variance weighted
-1
0
1
MR effect size for
'Concentration of large HDL particles || id:ebi-cfb233-GCST90302002' on 'Breast cancer (Combined Oncoarray; iCOGS; GWAS me

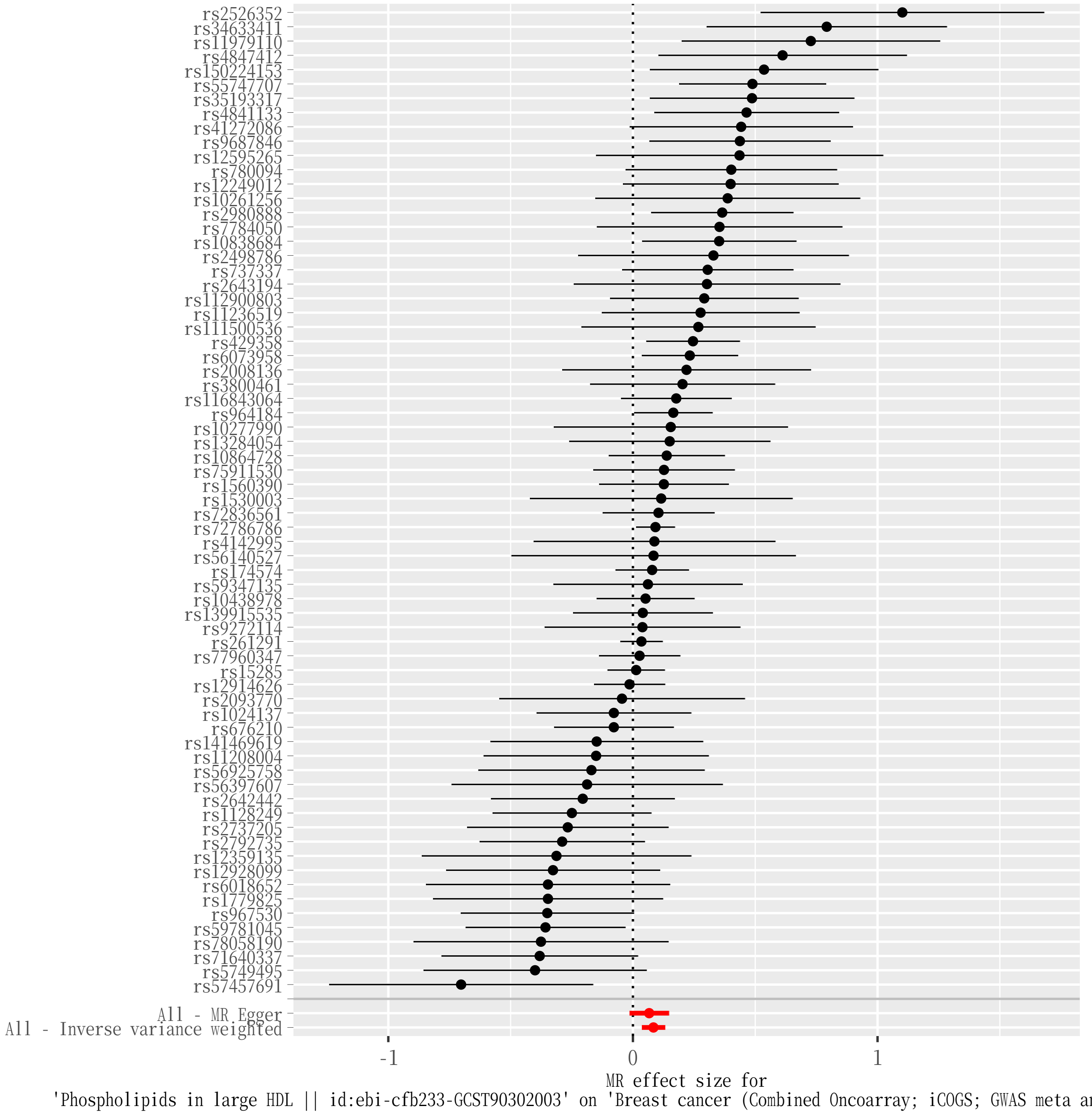

rs2526352
rs34633411
rs11979110
rs4847412
rs150224153
rs55747707
rs35193317
rs4841133
rs41272086
rs9687846
rs12595265
rs780094
rs12249012
rs10261256
rs2980888
rs7784050
rs10838684
rs2498786
rs737337
rs2643194
rs112900803
rs11236519
rs111500536
rs429358
rs6073958
rs2008136
rs3800461
rs116843064
rs964184
rs10277990
rs13284054
rs10864728
rs75911530
rs1560390
rs1530003
rs72836561
rs72786786
rs4142995
rs56140527
rs174574
rs59347135
rs10438978
rs139915535
rs9272114
rs261291
rs77960347
rs15285
rs12914626
rs2093770
rs1024137
rs676210
rs141469619
rs11208004
rs56925758
rs56397607
rs2642442
rs1128249
rs2737205
rs2792735
rs12359135
rs12928099
rs6018652
rs1779825
rs967530
rs59781045
rs78058190
rs71640337
rs5749495
rs57457691
All - MR Egger
All - Inverse variance weighted
-1
0
1
MR effect size for
'Phospholipids in large HDL || id:ebi-cfb233-GCST90302003' on 'Breast cancer (Combined Oncoarray; iCOGS; GWAS meta an

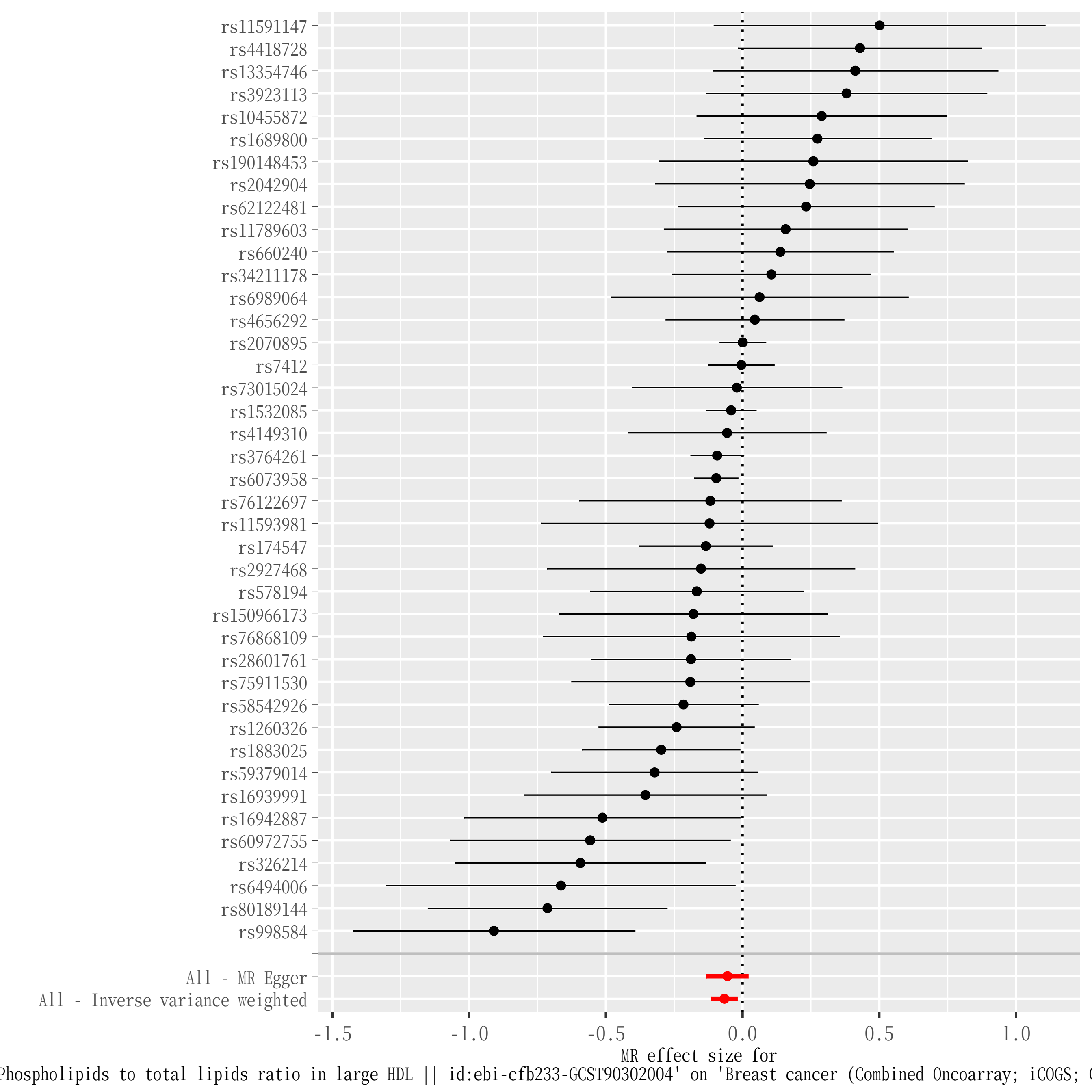
rs11591147
rs4418728
rs13354746
rs3923113
rs10455872
rs1689800
rs190148453
rs2042904
rs62122481
rs11789603
rs660240
rs34211178
rs6989064
rs4656292
rs2070895
rs7412
rs73015024
rs1532085
rs4149310
rs3764261
rs6073958
rs76122697
rs11593981
rs174547
rs2927468
rs578194
rs150966173
rs76868109
rs28601761
rs75911530
rs58542926
rs1260326
rs1883025
rs59379014
rs16939991
rs16942887
rs60972755
rs326214
rs6494006
rs80189144
rs998584
All - MR Egger
All - Inverse variance weighted
-1.5
-1.0
-0.5
0.0
0.5
1.0
MR effect size for
Phospholipids to total lipids ratio in large HDL || id:ebi-cfb233-GCST90302004' on 'Breast cancer (Combined Oncoarray; iCOGS; G

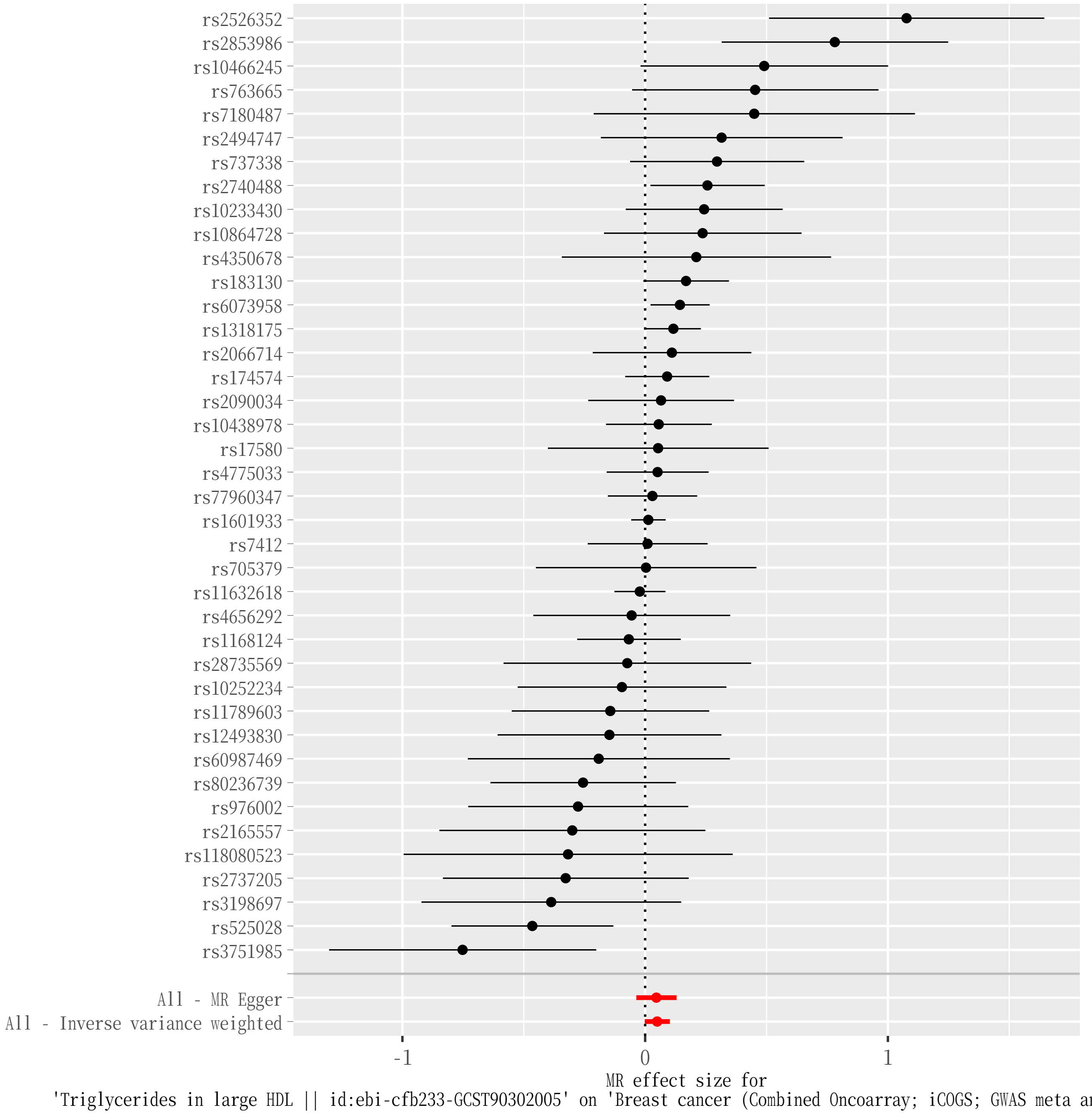
rs2526352
rs2853986
rs10466245
rs763665
rs7180487
rs2494747
rs737338
rs2740488
rs10233430
rs10864728
rs4350678
rs183130
rs6073958
rs1318175
rs2066714
rs174574
rs2090034
rs10438978
rs17580
rs4775033
rs77960347
rs1601933
rs7412
rs705379
rs11632618
rs4656292
rs1168124
rs28735569
rs10252234
rs11789603
rs12493830
rs60987469
rs80236739
rs976002
rs2165557
rs118080523
rs2737205
rs3198697
rs525028
rs3751985
All - MR Egger
All - Inverse variance weighted
-1
0
1
MR effect size for
'Triglycerides in large HDL || id:ebi-cfb233-GCST90302005' on 'Breast cancer (Combined Oncoarray; iCOGS; GWAS meta ar

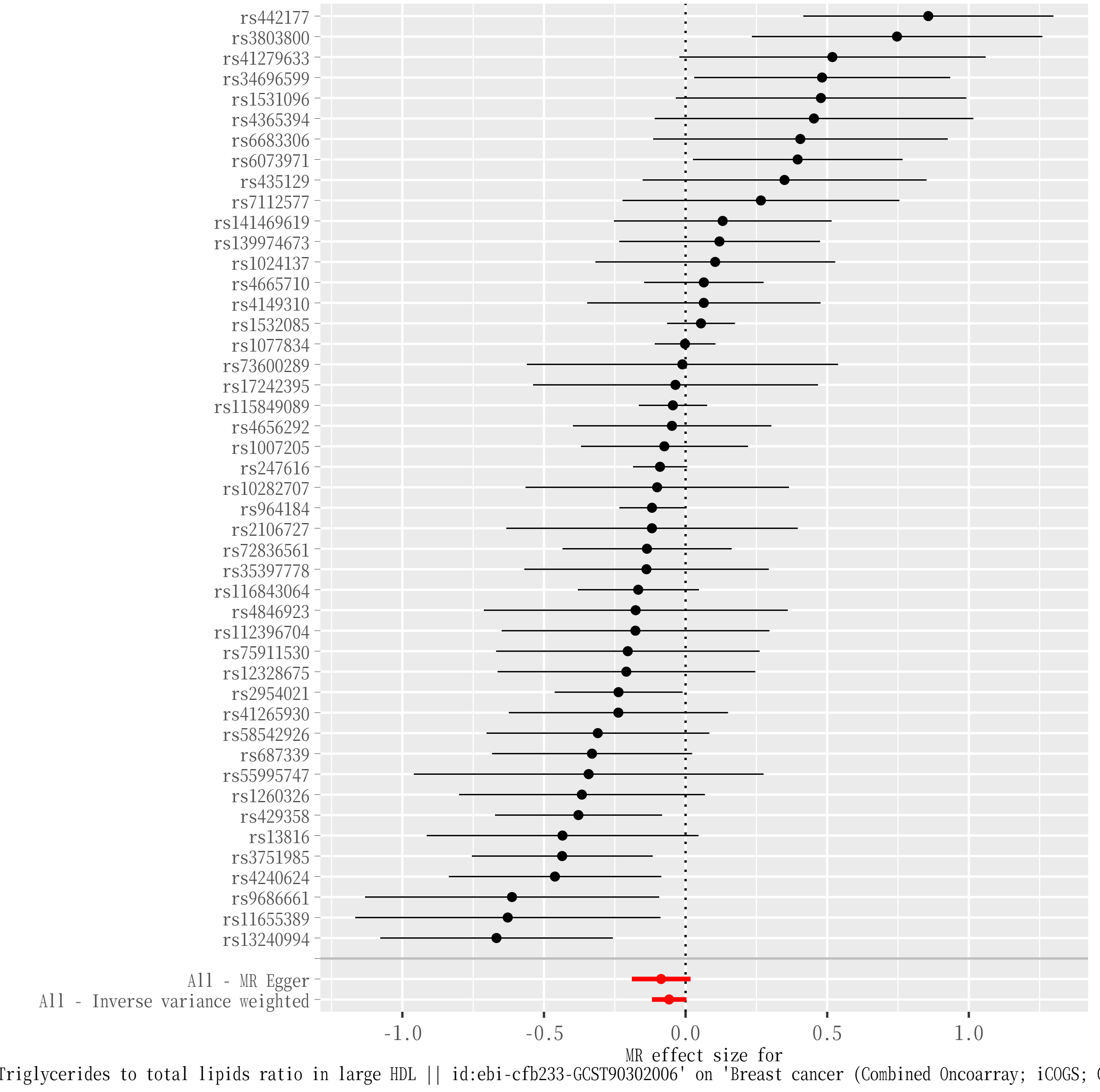
rs442177
rs3803800
rs41279633
rs34696599
rs1531096
rs4365394
rs6683306
rs6073971
rs435129
rs7112577
rs141469619
rs139974673
rs1024137
rs4665710
rs4149310
rs1532085
rs1077834
rs73600289
rs17242395
rs115849089
rs4656292
rs1007205
rs247616
rs10282707
rs964184
rs2106727
rs72836561
rs35397778
rs116843064
rs4846923
rs112396704
rs75911530
rs12328675
rs2954021
rs41265930
rs58542926
rs687339
rs55995747
rs1260326
rs429358
rs13816
rs3751985
rs4240624
rs9686661
rs11655389
rs13240994
All - MR Egger
All - Inverse variance weighted
-1.0
-0.5
0.0
0.5
1.0
MR effect size for
Triglycerides to total lipids ratio in large HDL || id:ebi-cfb233-GCST90302006' on 'Breast cancer (Combined Oncoarray; iCOGS; (

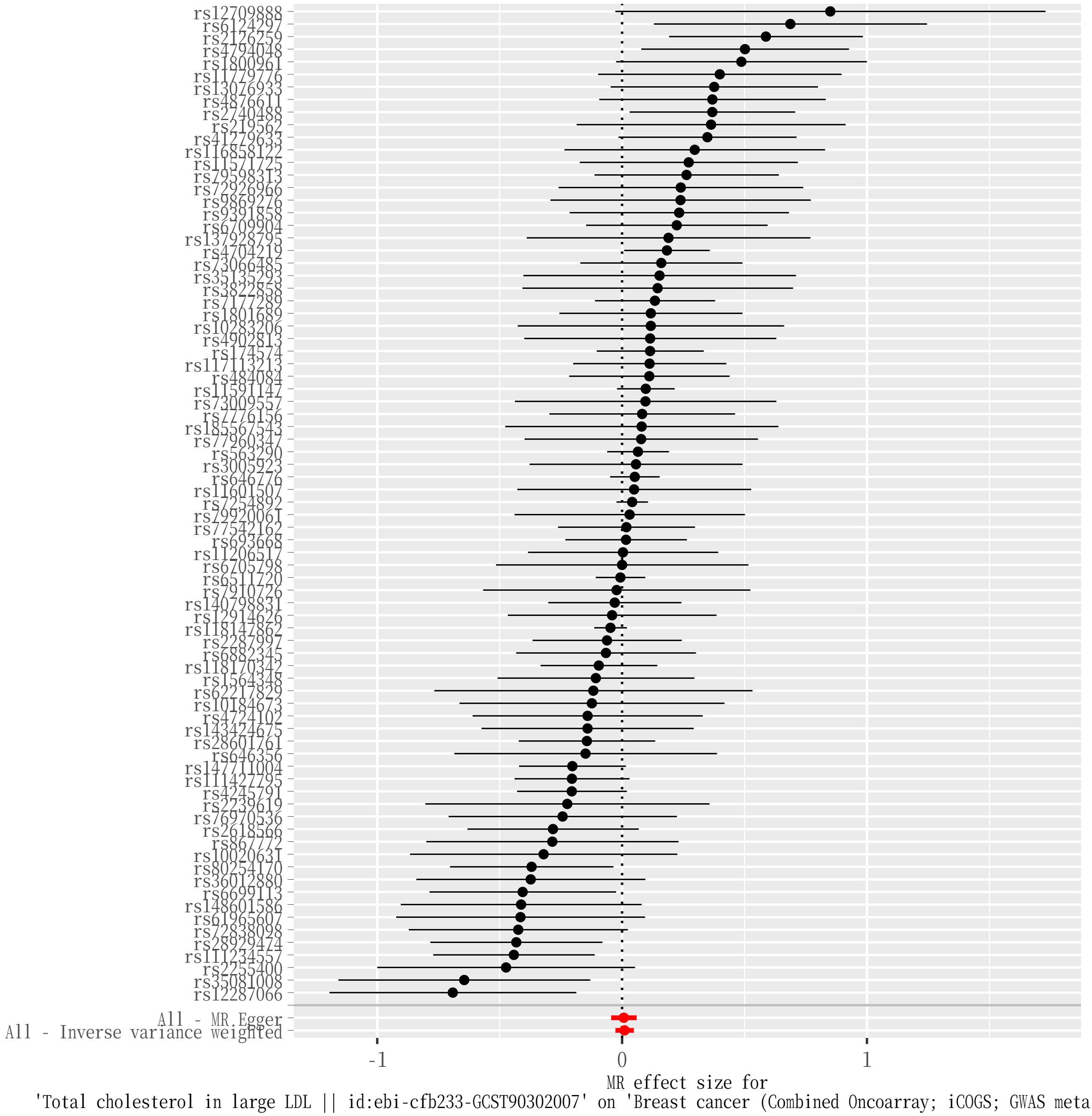
rs12709888
rs6124227
rs2126259
rs4794048
rs1800961
rs11779776
rs13076933
rs4876611
rs2740488
rs219562
rs41279633
rs116858122
rs11571725
rs79598313
rs72926966
rs9869276
rs9391858
rs6709904
rs137928795
rs4704219
rs73066485
rs35135293
rs3822858
rs7177289
rs1801689
rs10283206
rs4902813
rs174574
rs117113213
rs484084
rs11591147
rs73009557
rs7776156
rs185567543
rs7960347
rs563290
rs3005923
rs646776
rs11601507
rs7254892
rs79220061
rs77542162
rs693668
rs11206517
rs6705798
rs6511720
rs7910726
rs140798831
rs12914626
rs118147862
rs2287997
rs6882345
rs118170342
rs1564348
rs62217829
rs10184673
rs4724102
rs143424675
rs28601761
rs646356
rs147711004
rs111427795
rs4245791
rs2239619
rs76970536
rs2618566
rs867772
rs10020631
rs80254170
rs36012880
rs6699113
rs148601586
rs61965607
rs72838098
rs28929474
rs111234557
rs2255400
rs35081008
rs12287066
All - MR Egger
All - Inverse variance weighted
-1
0
1
MR effect size for
'Total cholesterol in large LDL || id:ebi-cfb233-GCST90302007' on 'Breast cancer (Combined Oncoarray; iCOGS; GWAS meta

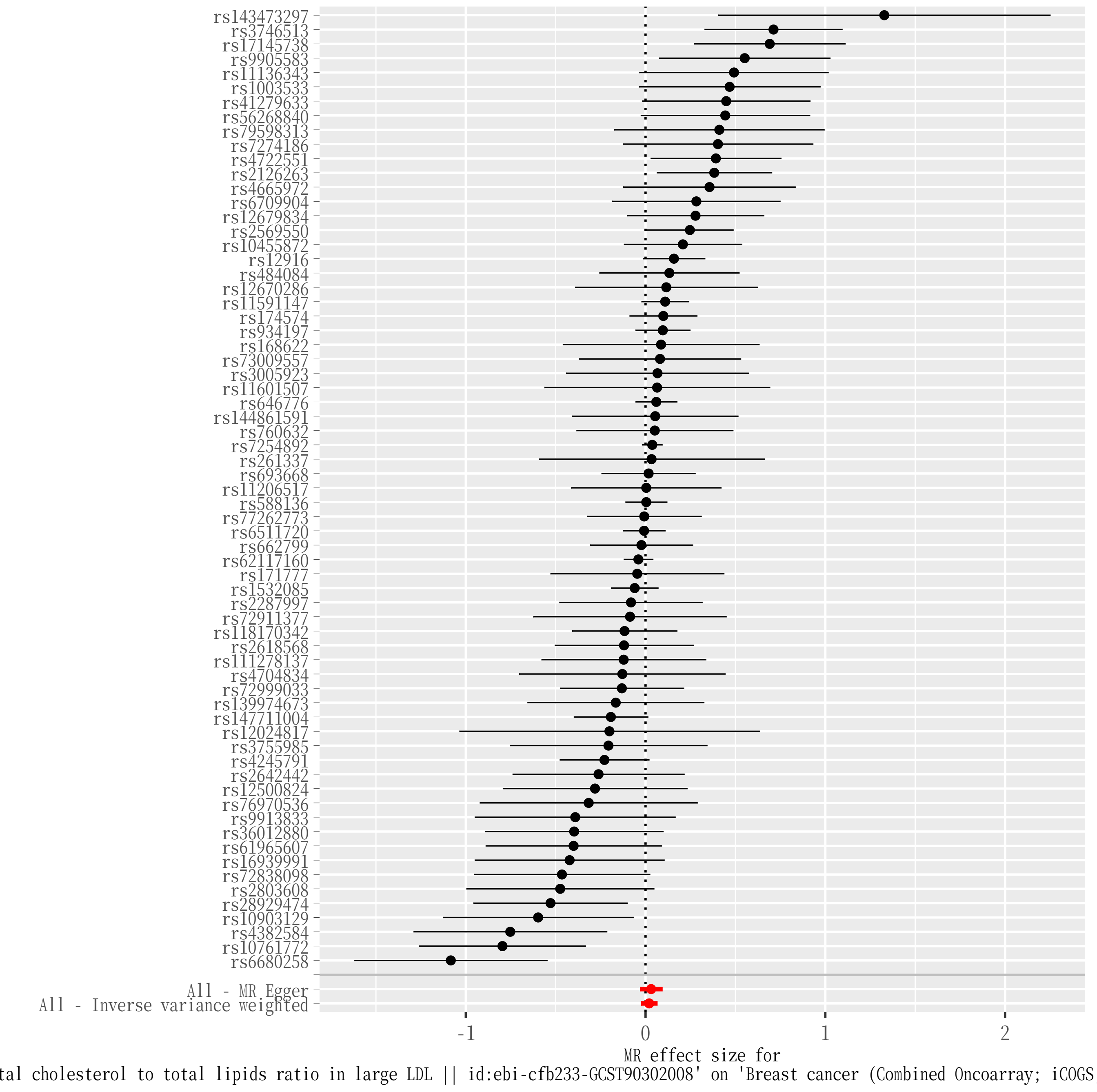
rs143473297
rs3746513
rs17145738
rs9905583
rs11136343
rs1003533
rs41279633
rs56268840
rs79598313
rs7274186
rs4722551
rs2126263
rs4665972
rs6709904
rs12679834
rs2569550
rs10455872
rs12916
rs484084
rs12670286
rs11591147
rs174574
rs934197
rs168622
rs73009557
rs3005923
rs11601507
rs646776
rs144861591
rs760632
rs7254892
rs261337
rs693668
rs11206517
rs588136
rs77262773
rs6511720
rs662799
rs62117160
rs171777
rs1532085
rs2287997
rs72911377
rs118170342
rs2618568
rs111278137
rs4704834
rs72999033
rs139974673
rs147711004
rs12024817
rs3755985
rs4245791
rs2642442
rs12500824
rs76970536
rs9913833
rs36012880
rs61965607
rs16939991
rs72838098
rs2803608
rs28929474
rs10903129
rs4382584
rs10761772
rs6680258
All - MR Egger
All - Inverse variance weighted
-1
0
1
2
MR effect size for
tal cholesterol to total lipids ratio in large LDL || id:ebi-cfb233-GCST90302008' on 'Breast cancer (Combined Oncoarray; iCOGS

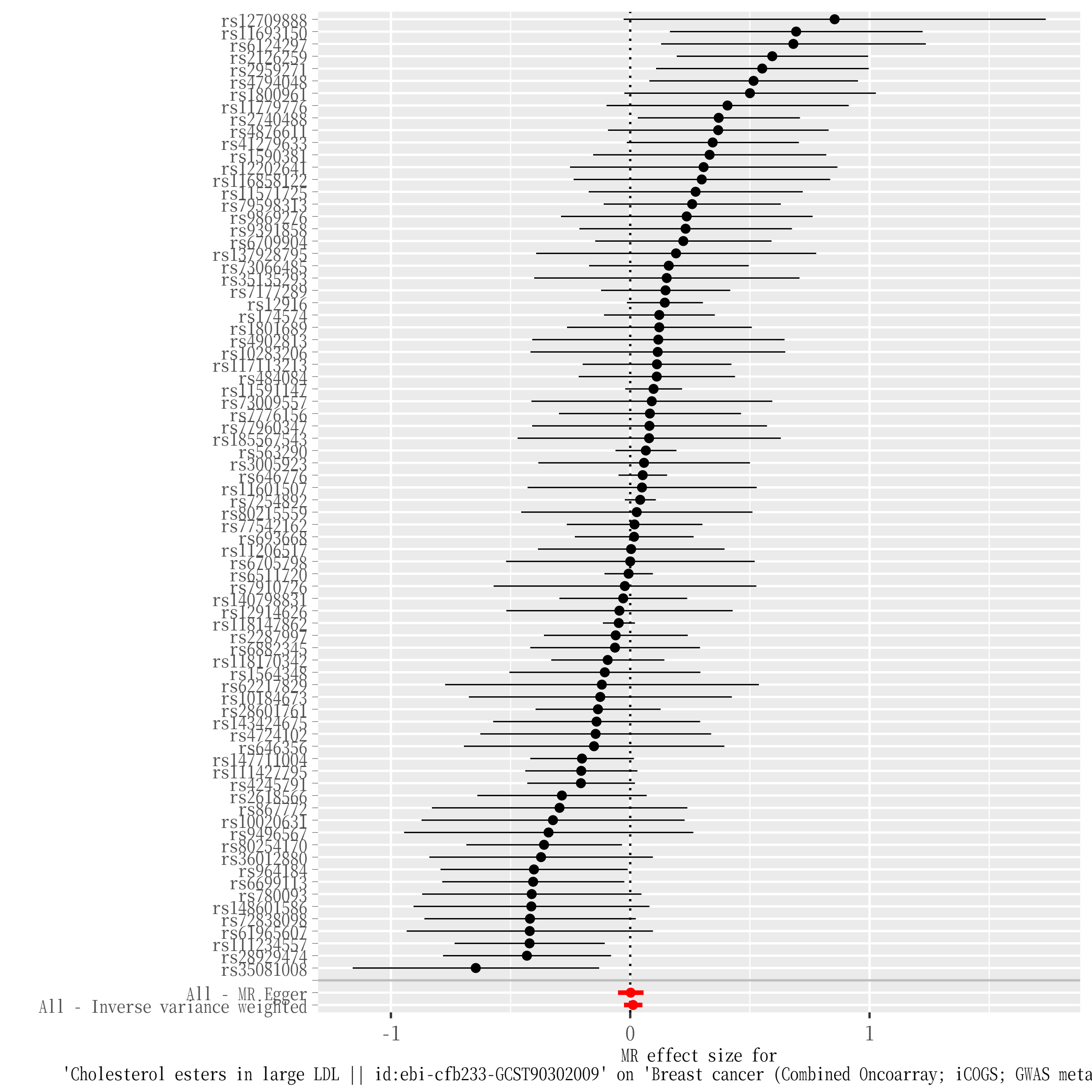
rs12709888
rs11693150
rs6124297
rs2126259
rs2959271
rs4794048
rs1800961
rs11779776
rs2740488
rs4876611
rs41279633
rs1590381
rs12202641
rs116858122
rs11571725
rs79598313
rs9869276
rs9391858
rs6709904
rs137928795
rs73066485
rs35135293
rs7177289
rs12916
rs174574
rs1801689
rs4902813
rs10283206
rs117113213
rs484084
rs11591147
rs73009557
rs7776156
rs77960347
rs185567543
rs563290
rs3005923
rs646776
rs11601507
rs7254892
rs80215559
rs77542162
rs693668
rs11206517
rs6705798
rs6511720
rs7910726
rs140798831
rs12914626
rs118147862
rs2287997
rs6882345
rs118170342
rs1564348
rs62217829
rs10184673
rs28601761
rs143424675
rs4724102
rs646356
rs147711004
rs111427795
rs4245791
rs2618566
rs867772
rs1002631
rs9426567
rs80254170
rs36012880
rs964184
rs6699113
rs780093
rs148601586
rs72838098
rs61965607
rs111234557
rs28929474
rs35081008
All - MR Egger
All - Inverse variance weighted
-1
0
1
MR effect size for
'Cholesterol esters in large LDL || id:ebi-cfb233-GCST90302009' on 'Breast cancer (Combined Oncoarray; iCOGS; GWAS meta

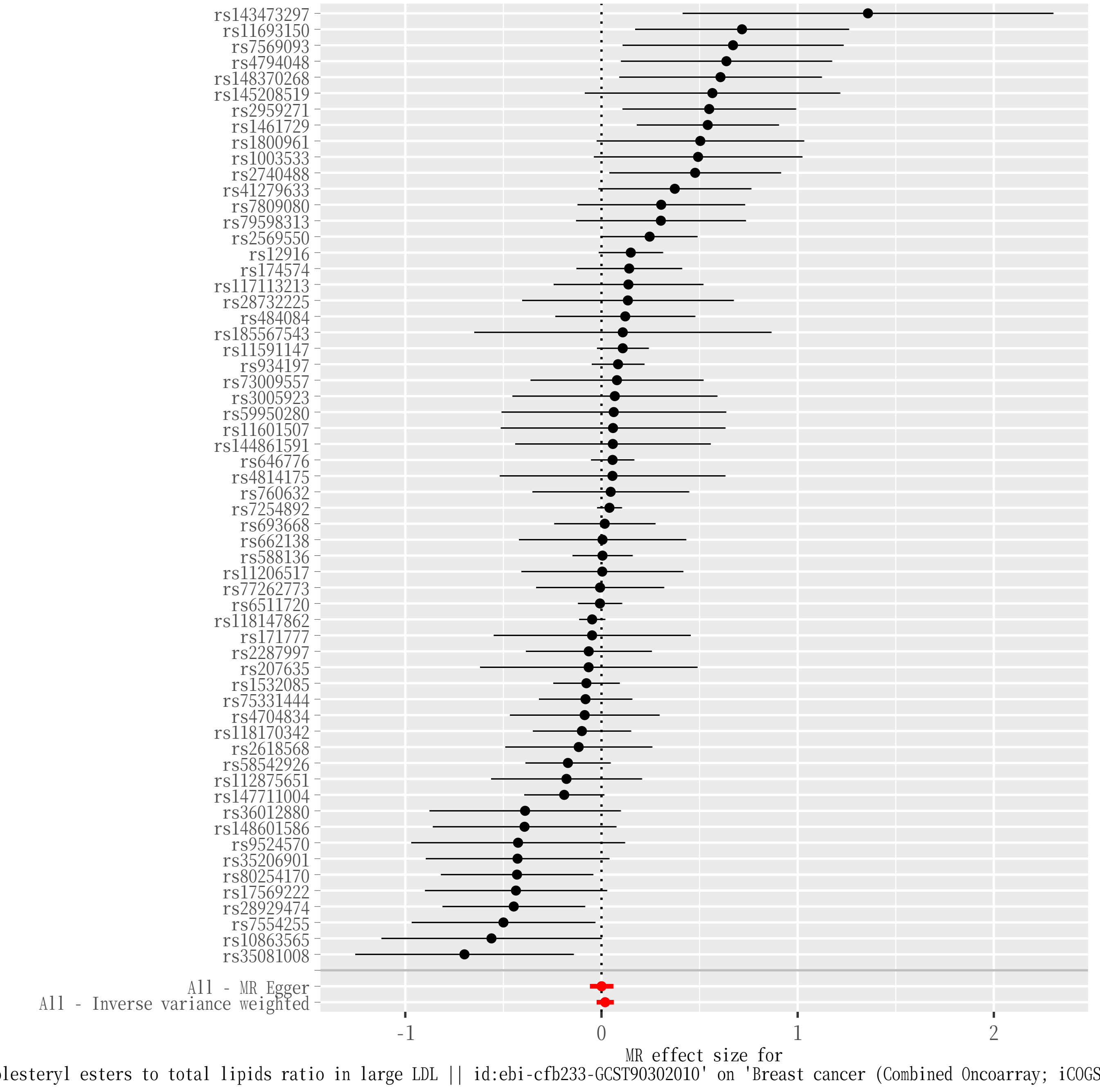
rs143473297
rs11693150
rs7569093
rs4794048
rs148370268
rs145208519
rs2959271
rs1461729
rs1800961
rs1003533
rs2740488
rs41279633
rs7809080
rs79598313
rs2569550
rs12916
rs174574
rs117113213
rs28732225
rs484084
rs185567543
rs11591147
rs934197
rs73009557
rs3005923
rs59950280
rs11601507
rs144861591
rs646776
rs4814175
rs760632
rs7254892
rs693668
rs662138
rs588136
rs11206517
rs77262773
rs6511720
rs118147862
rs171777
rs2287997
rs207635
rs1532085
rs75331444
rs4704834
rs118170342
rs2618568
rs58542926
rs112875651
rs147711004
rs36012880
rs148601586
rs9524570
rs35206901
rs80254170
rs17569222
rs28929474
rs7554255
rs10863565
rs35081008
All - MR Egger
All - Inverse variance weighted
-1
0
1
2
MR effect size for
lesteryl esters to total lipids ratio in large LDL || id:ebi-cfb233-GCST90302010' on 'Breast cancer (Combined Oncoarray; iCOGS

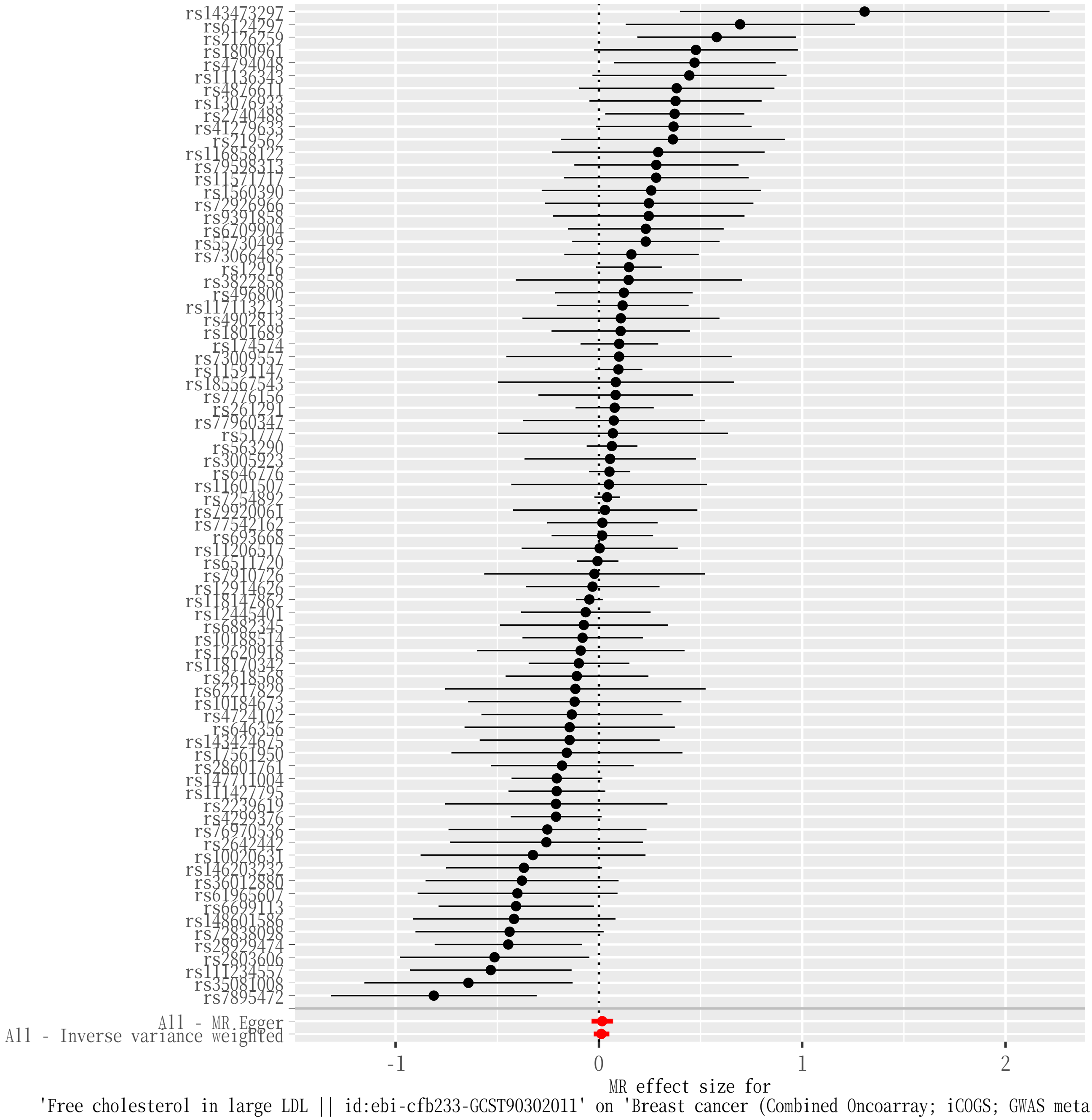
rs143473297
rs6124297
rs2126259
rs1800961
rs4794048
rs11136343
rs4876611
rs13076933
rs2740488
rs41279633
rs219562
rs116858122
rs79598313
rs11571717
rs1560390
rs72926966
rs9391858
rs6709904
rs55730499
rs73066485
rs12916
rs3822858
rs496800
rs117113213
rs4902813
rs1801689
rs174574
rs73009557
rs11591147
rs185567543
rs7776156
rs261291
rs77960347
rs51777
rs563290
rs3005923
rs646776
rs11601507
rs7254892
rs79920061
rs77542162
rs693668
rs11206517
rs6511720
rs7910726
rs12914626
rs118147862
rs12445401
rs6882345
rs10188514
rs12620918
rs118170342
rs2618568
rs62217829
rs10184673
rs4724102
rs646356
rs143424675
rs17561950
rs28601761
rs147711004
rs111427795
rs2239619
rs4299376
rs76970536
rs2642442
rs10020631
rs146203232
rs36012880
rs61965607
rs6699113
rs148601586
rs72838098
rs28929474
rs2803606
rs111234557
rs35081008
rs7895472
All - MR Egger
All - Inverse variance weighted
-1
0
1
2
MR effect size for
'Free cholesterol in large LDL || id:ebi-cfb233-GCST90302011' on 'Breast cancer (Combined Oncoarray; iCOGS; GWAS meta

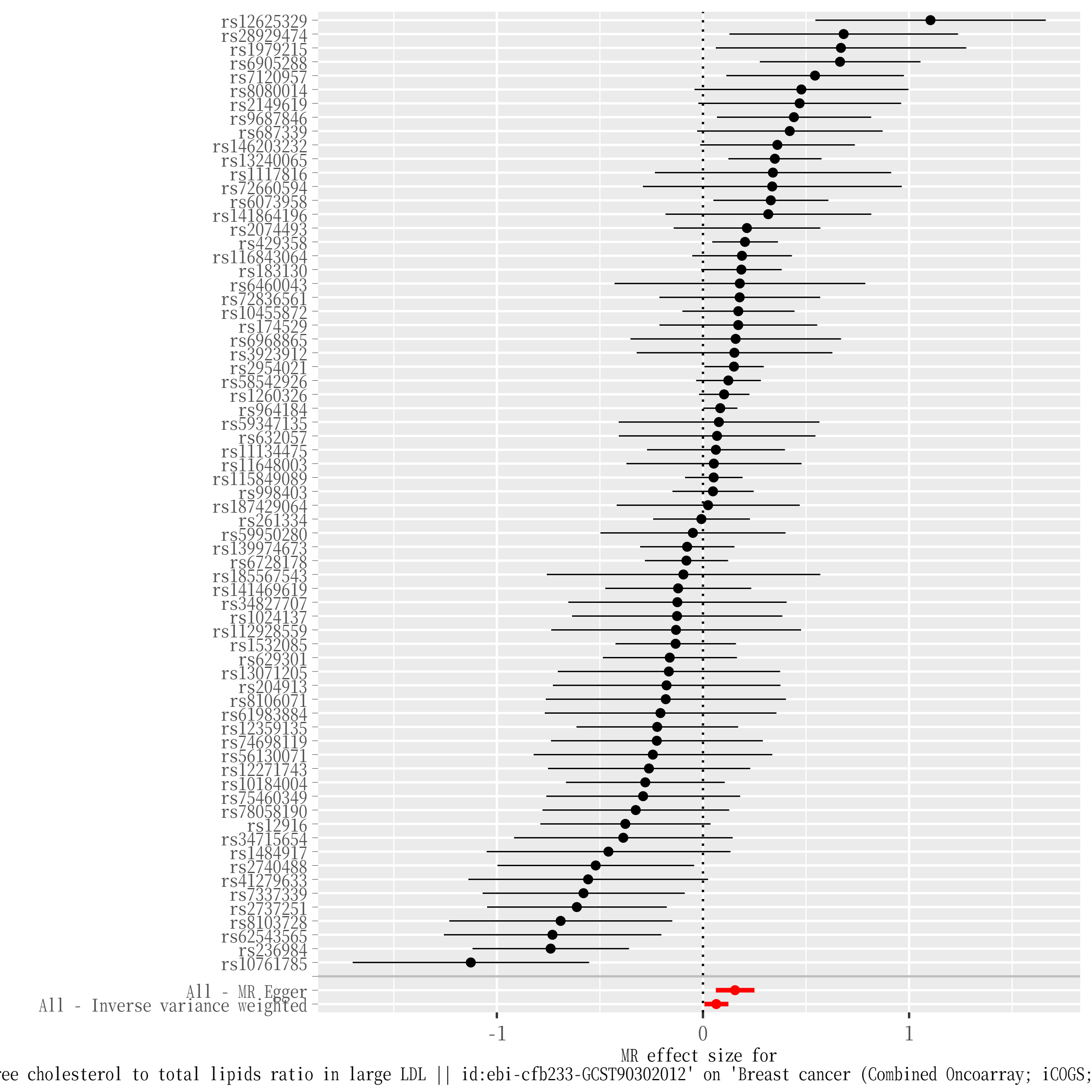
rs12625329
rs28929474
rs1979215
rs6905288
rs7120957
rs8080014
rs2149619
rs9687846
rs687339
rs146203232
rs13240065
rs1117816
rs72660594
rs6073958
rs141864196
rs2074493
rs429358
rs116843064
rs183130
rs6460043
rs72836561
rs10455872
rs174529
rs6968865
rs3923912
rs2954021
rs58542926
rs1260326
rs964184
rs59347135
rs632057
rs11134475
rs11648003
rs115849089
rs998403
rs187429064
rs261334
rs59950280
rs139974673
rs6728178
rs185567543
rs141469619
rs34827707
rs1024137
rs112928559
rs1532085
rs629301
rs13071205
rs204913
rs8106071
rs61983884
rs12359135
rs74698119
rs56130071
rs12271743
rs10184004
rs75460349
rs78058190
rs12916
rs34715654
rs1484917
rs2740488
rs41279633
rs7337339
rs2737251
rs8103728
rs62543565
rs236984
rs10761785
All - MR Egger
All - Inverse variance weighted
-1
0
1
MR effect size for
ree cholesterol to total lipids ratio in large LDL || id:ebi-cfb233-GCST90302012' on 'Breast cancer (Combined Oncoarray; iCOGS;

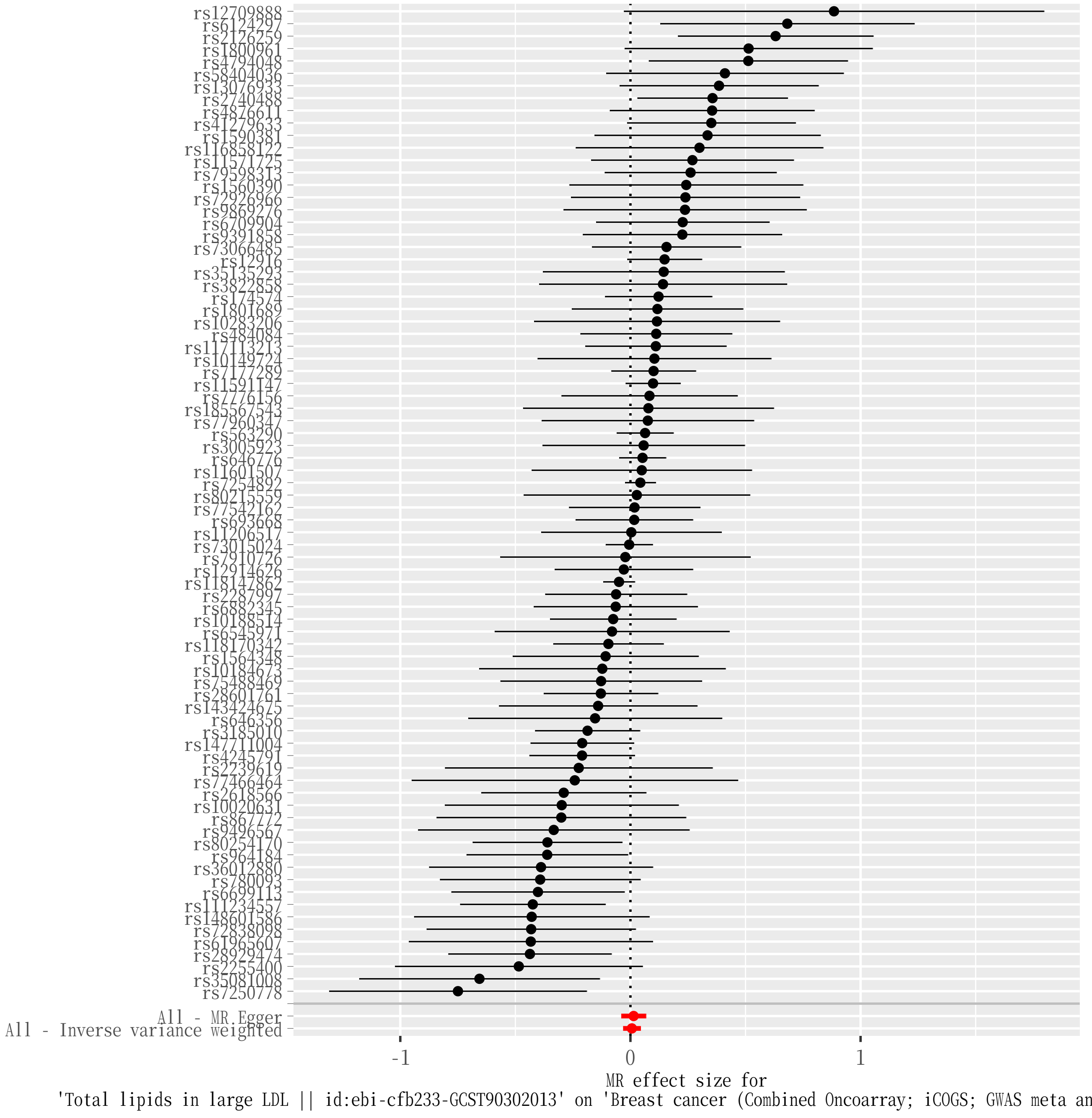

rs12709888
rs6124297
rs2126259
rs1800961
rs4794048
rs58404036
rs13076933
rs2740488
rs4876611
rs41279633
rs1590381
rs116858122
rs11571725
rs79598313
rs1560390
rs72926966
rs9869276
rs6709904
rs9391858
rs73066485
rs12916
rs35135293
rs3822858
rs174574
rs1801689
rs10283206
rs484084
rs117113213
rs10149724
rs7177289
rs11521147
rs7776156
rs185567543
rs77960347
rs563290
rs3005923
rs646776
rs11601507
rs7254892
rs80215559
rs77542162
rs693668
rs11206517
rs73015024
rs7910726
rs12914626
rs118147862
rs2287997
rs6882345
rs10188514
rs6545971
rs118170342
rs1564348
rs10184673
rs75488469
rs28601761
rs143424675
rs646356
rs3185010
rs147711004
rs4245791
rs2239619
rs77466464
rs2618566
rs10020631
rs867772
rs9496567
rs80254170
rs964184
rs36012880
rs780093
rs6699113
rs111234557
rs148601586
rs72838098
rs61965607
rs28929474
rs2255400
rs35081008
rs7250778
All - MR Egger
All - Inverse variance weighted
-1
0
1
MR effect size for
'Total lipids in large LDL || id:ebi-cfb233-GCST90302013' on 'Breast cancer (Combined Oncoarray; iCOGS; GWAS meta an

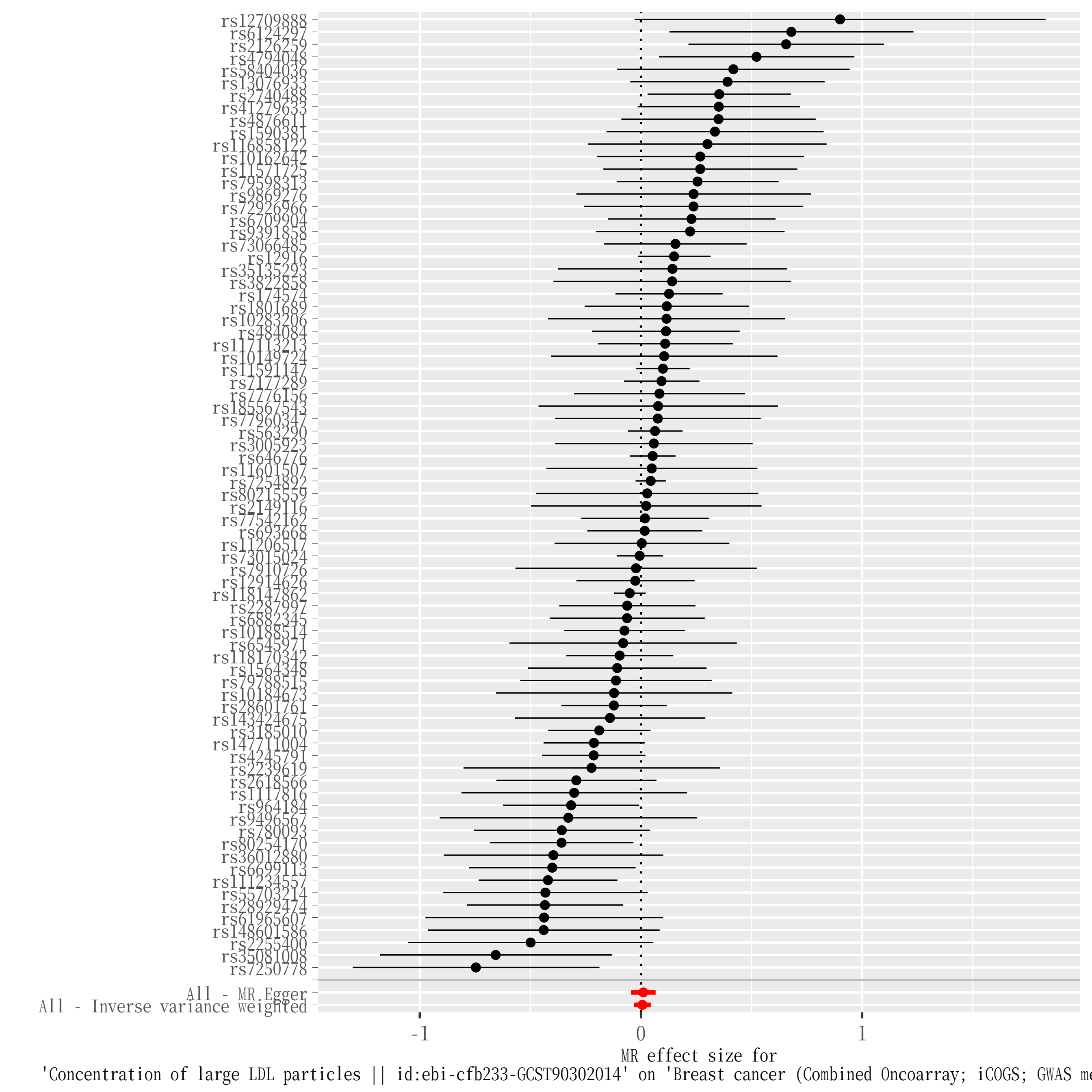
rs12709888
rs6124297
rs2126259
rs4794048
rs58404036
rs13076933
rs2740488
rs41279633
rs4876611
rs1590381
rs116858122
rs10162642
rs11571725
rs79598313
rs9869276
rs72926966
rs6709904
rs9391858
rs73066485
rs12916
rs35135293
rs3822858
rs174574
rs1801689
rs10283206
rs484084
rs117113213
rs10149724
rs11591147
rs7177289
rs7776156
rs185567543
rs77960347
rs563290
rs3005923
rs646776
rs11601507
rs7254892
rs80215559
rs2149116
rs77542162
rs693668
rs11206517
rs73015024
rs7910776
rs12914626
rs118147862
rs2287997
rs6882345
rs10188514
rs6545971
rs118170342
rs1564348
rs79788515
rs10184673
rs28601761
rs143424675
rs3185010
rs147711004
rs4245791
rs2239619
rs2618566
rs1117816
rs964184
rs9496567
rs780093
rs80254170
rs36012880
rs6699113
rs111234557
rs55703214
rs28929474
rs61965607
rs148601586
rs2255400
rs35081008
rs7250778
All - MR Egger
All - Inverse variance weighted
-1
0
1
MR effect size for
'Concentration of large LDL particles || id:ebi-cfb233-GCST90302014' on 'Breast cancer (Combined Oncoarray; iCOGS; GWAS me

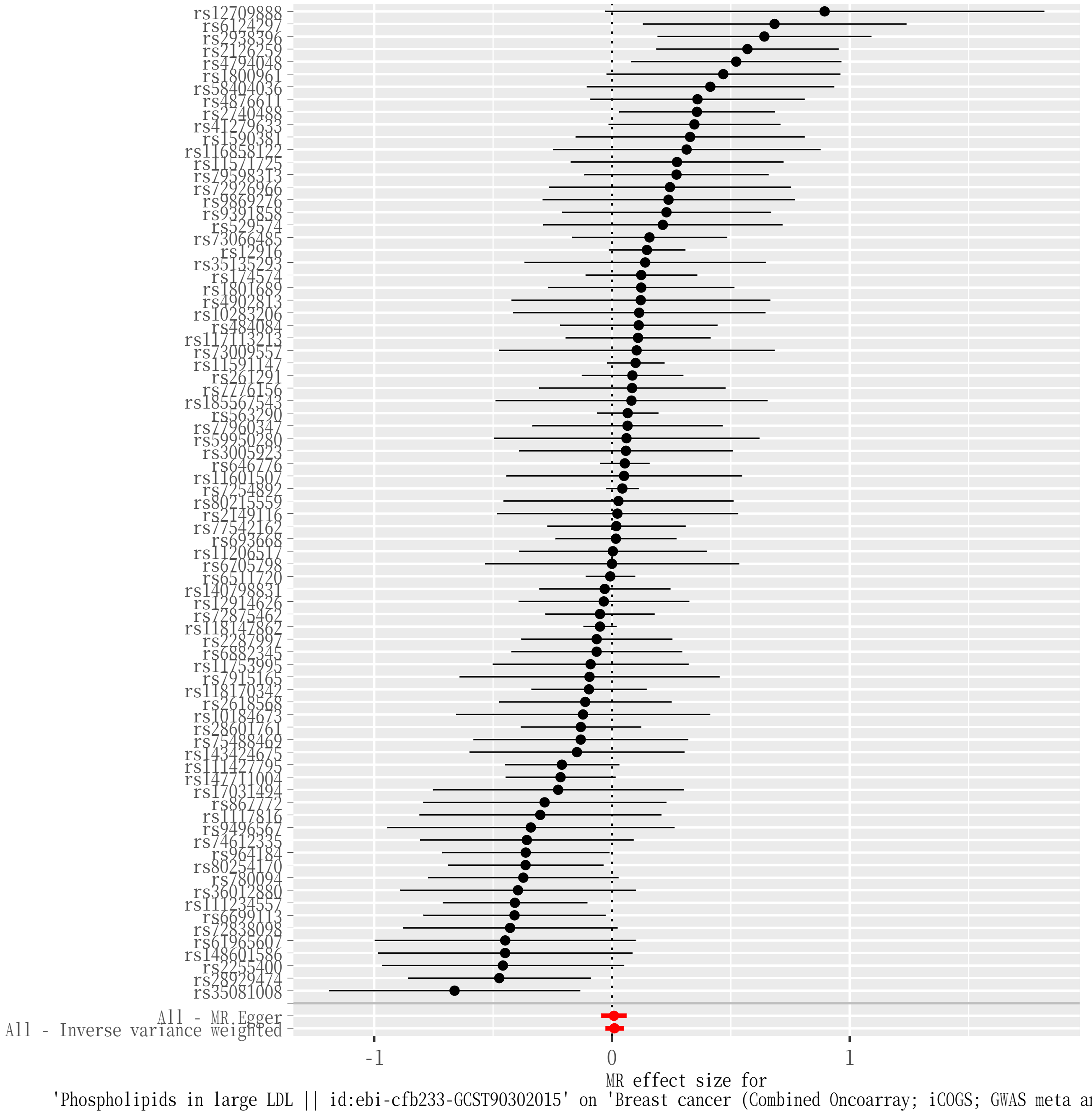
rs12709888
rs6124297
rs2938396
rs2126259
rs4794048
rs1800961
rs58404036
rs4876611
rs2740488
rs41279633
rs1590381
rs116858122
rs11571725
rs79598313
rs72926966
rs9869276
rs9391858
rs529574
rs73066485
rs12916
rs35135293
rs174574
rs1801689
rs4902813
rs10283206
rs484084
rs117113213
rs73009557
rs11591147
rs261291
rs7776156
rs185567543
rs563290
rs77960347
rs59950280
rs3005923
rs646776
rs11601507
rs7254892
rs80215559
rs2149116
rs77542162
rs693668
rs11206517
rs6705798
rs6511720
rs140798831
rs12914626
rs72875462
rs118147862
rs2287997
rs6882345
rs11753995
rs7915165
rs118170342
rs2618568
rs10184673
rs28601761
rs75488469
rs143424675
rs111427795
rs147711004
rs17031494
rs867772
rs1117816
rs9496567
rs74612335
rs964184
rs80254170
rs780094
rs36012880
rs111234557
rs6699113
rs72838098
rs61965607
rs148601586
rs2255400
rs28929474
rs35081008
All - MR.Egger
All - Inverse variance weighted
-1
0
1
MR effect size for
'Phospholipids in large LDL || id:ebi-cfb233-GCST90302015' on 'Breast cancer (Combined Oncoarray; iCOGS; GWAS meta an

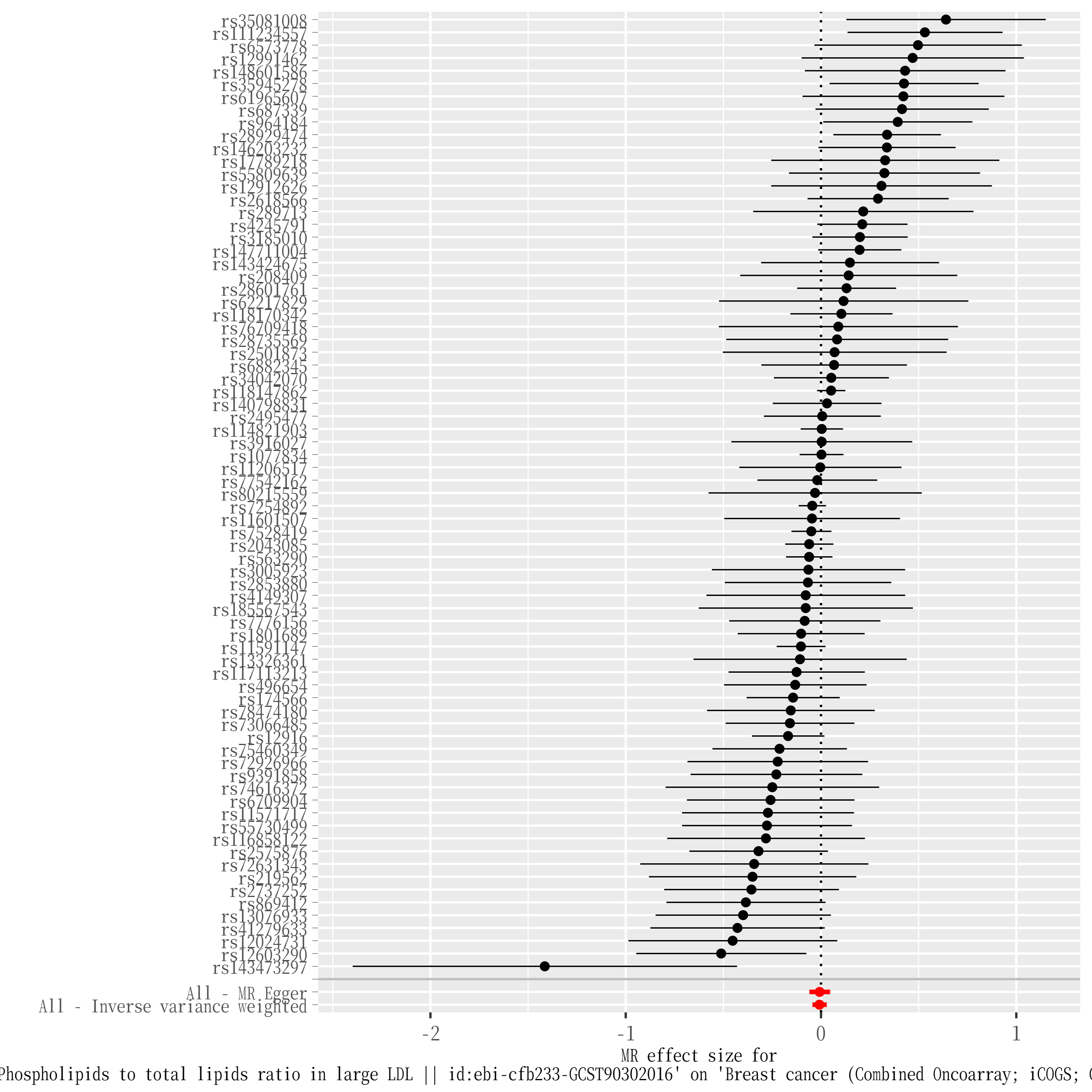
rs35081008
rs111234557
rs6573778
rs12991462
rs148601586
rs35945278
rs61965607
rs687339
rs964184
rs28929474
rs146203232
rs17789218
rs55809639
rs12912626
rs2618566
rs289713
rs4245791
rs3185010
rs147711004
rs143424675
rs208409
rs28601761
rs62217829
rs118170342
rs76709418
rs28735569
rs2501873
rs6882345
rs34042070
rs118147862
rs140798831
rs2495477
rs114821903
rs3916027
rs1077834
rs11206517
rs77542162
rs80215559
rs7254892
rs11601507
rs7528419
rs2043085
rs563290
rs3005923
rs2853880
rs4149307
rs185567543
rs7776156
rs1801689
rs11591147
rs13326361
rs117113213
rs496654
rs174566
rs78474180
rs73066485
rs12916
rs75460349
rs72926966
rs9391858
rs74616372
rs6709904
rs11571717
rs55730499
rs116858122
rs2575876
rs72631343
rs219562
rs2737252
rs869412
rs13076933
rs41279633
rs12024731
rs12603290
rs143473297
All - MR Egger
All - Inverse variance weighted
-2
-1
0
1
MR effect size for
'Phospholipids to total lipids ratio in large LDL || id:ebi-cfb233-GCST90302016' on 'Breast cancer (Combined Oncoarray; iCOGS; G

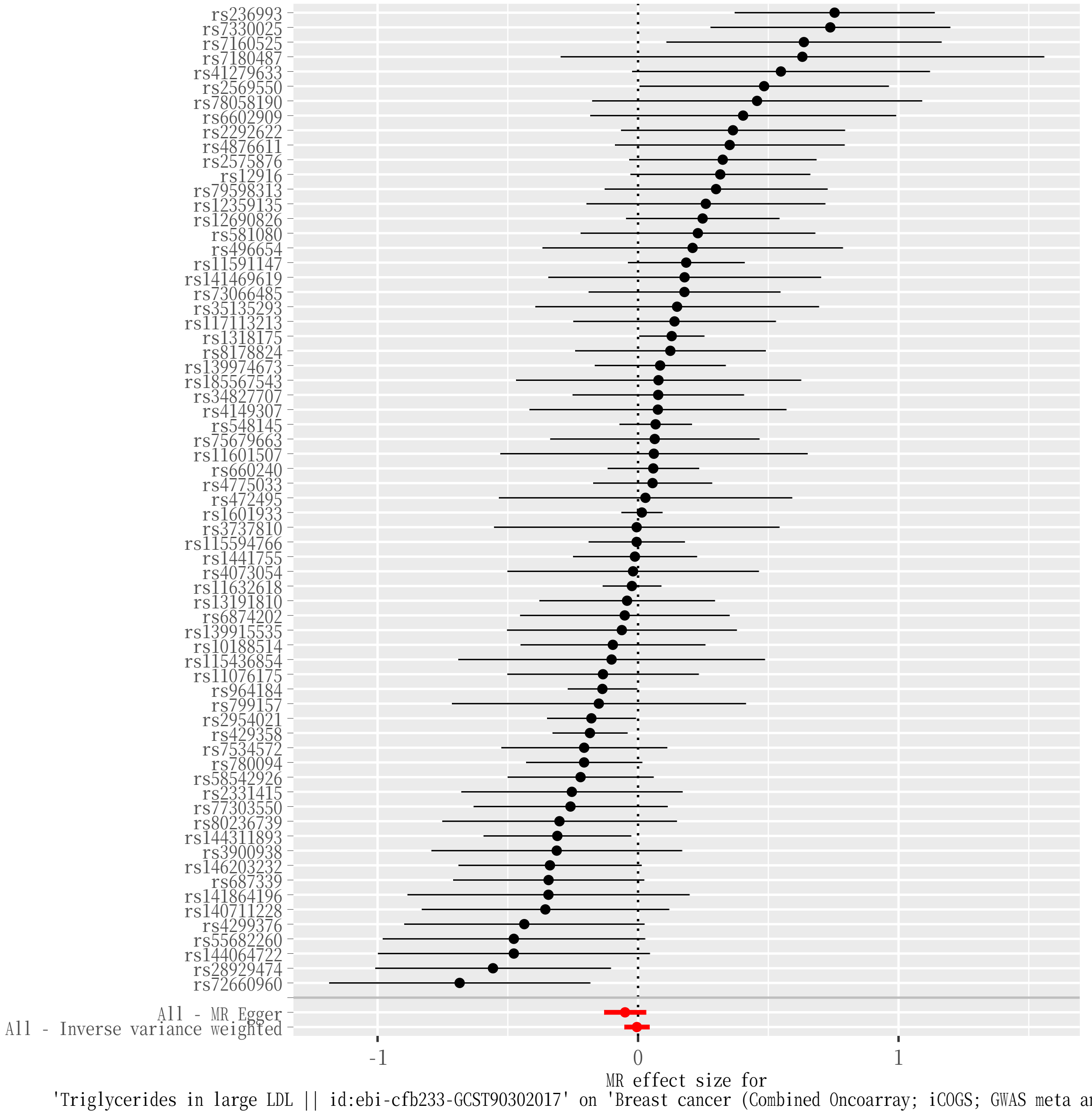

rs236993
rs7330025
rs7160525
rs7180487
rs41279633
rs2569550
rs78058190
rs6602909
rs2292622
rs4876611
rs2575876
rs12916
rs79598313
rs12359135
rs12690826
rs581080
rs496654
rs11591147
rs141469619
rs73066485
rs35135293
rs117113213
rs1318175
rs8178824
rs139974673
rs185567543
rs34827707
rs4149307
rs548145
rs75679663
rs11601507
rs660240
rs4775033
rs472495
rs1601933
rs3737810
rs115594766
rs1441755
rs4073054
rs11632618
rs13191810
rs6874202
rs139915535
rs10188514
rs115436854
rs11076175
rs964184
rs799157
rs2954021
rs429358
rs7534572
rs780094
rs58542926
rs2331415
rs77303550
rs80236739
rs144311893
rs3900938
rs146203232
rs687339
rs141864196
rs140711228
rs4299376
rs55682260
rs144064722
rs28929474
rs72660960
All - MR Egger
All - Inverse variance weighted
-1
0
1
MR effect size for
'Triglycerides in large LDL || id:ebi-cfb233-GCST90302017' on 'Breast cancer (Combined Oncoarray; iCOGS; GWAS meta ar

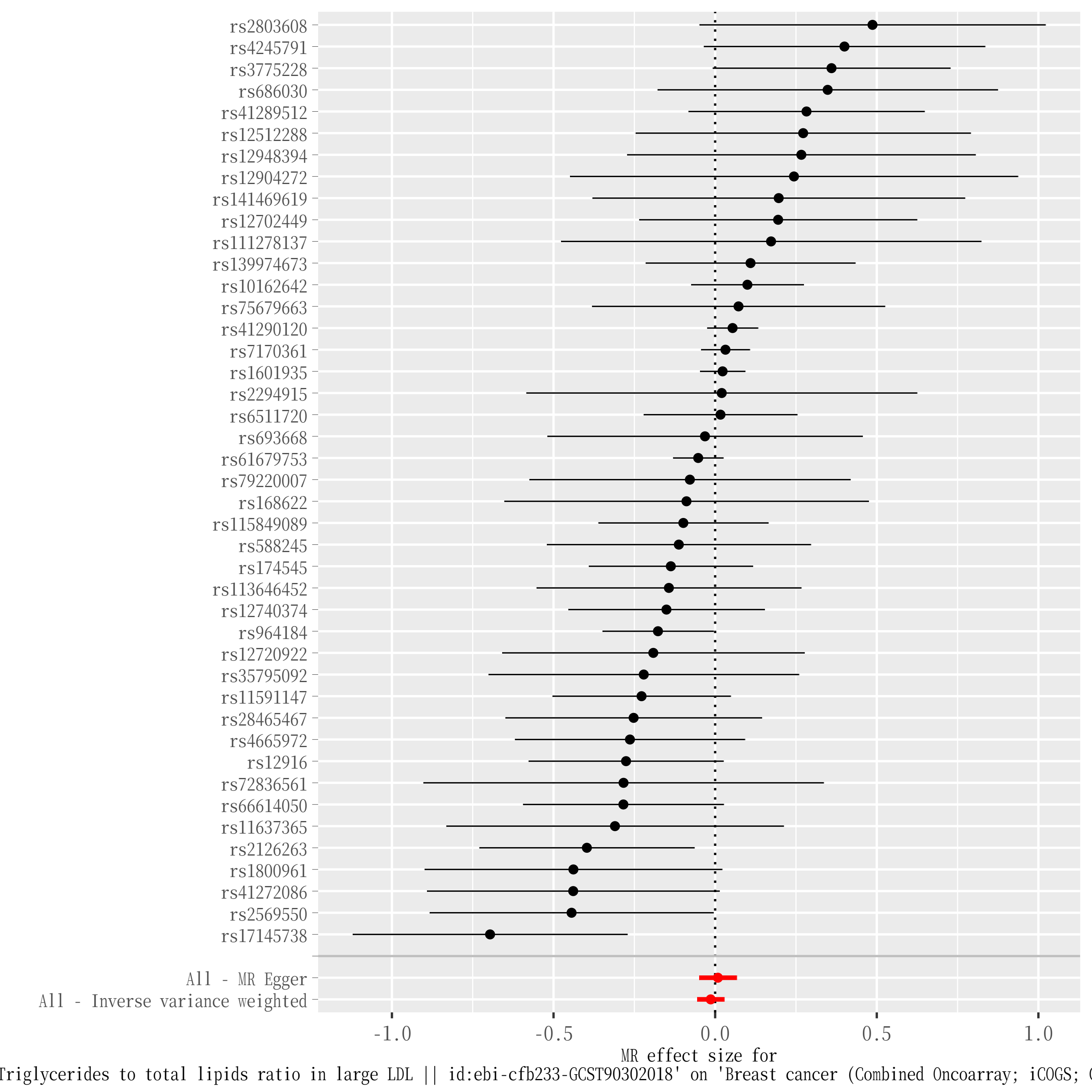

rs2803608
rs4245791
rs3775228
rs686030
rs41289512
rs12512288
rs12948394
rs12904272
rs141469619
rs12702449
rs111278137
rs139974673
rs10162642
rs75679663
rs41290120
rs7170361
rs1601935
rs2294915
rs6511720
rs693668
rs61679753
rs79220007
rs168622
rs115849089
rs588245
rs174545
rs113646452
rs12740374
rs964184
rs12720922
rs35795092
rs11591147
rs28465467
rs4665972
rs12916
rs72836561
rs66614050
rs11637365
rs2126263
rs1800961
rs41272086
rs2569550
rs17145738
All - MR Egger
All - Inverse variance weighted
-1.0
-0.5
0.0
0.5
1.0
MR effect size for
Triglycerides to total lipids ratio in large LDL || id:ebi-cfb233-GCST90302018' on 'Breast cancer (Combined Oncoarray; iCOGS; G

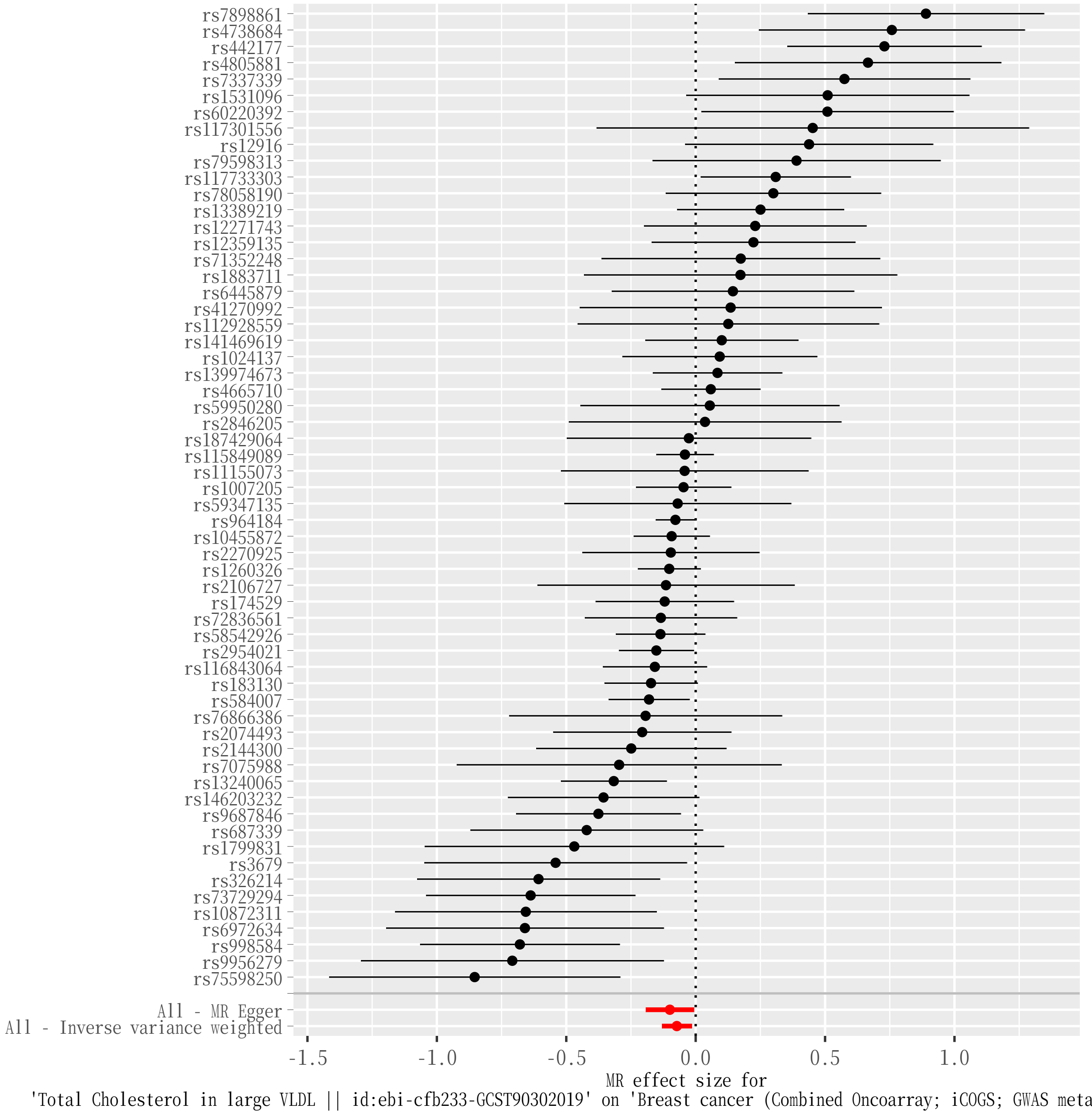
rs7898861
rs4738684
rs442177
rs4805881
rs7337339
rs1531096
rs60220392
rs117301556
rs12916
rs79598313
rs117733303
rs78058190
rs13389219
rs12271743
rs12359135
rs71352248
rs1883711
rs6445879
rs41270992
rs112928559
rs141469619
rs1024137
rs139974673
rs4665710
rs59950280
rs2846205
rs187429064
rs115849089
rs11155073
rs1007205
rs59347135
rs964184
rs10455872
rs2270925
rs1260326
rs2106727
rs174529
rs72836561
rs58542926
rs2954021
rs116843064
rs183130
rs584007
rs76866386
rs2074493
rs2144300
rs7075988
rs13240065
rs146203232
rs9687846
rs687339
rs1799831
rs3679
rs326214
rs73729294
rs10872311
rs6972634
rs998584
rs9956279
rs75598250
All - MR Egger
All - Inverse variance weighted
-1.5
-1.0
-0.5
0.0
0.5
1.0
MR effect size for
'Total Cholesterol in large VLDL || id:ebi-cfb233-GCST90302019' on 'Breast cancer (Combined Oncoarray; iCOGS; GWAS meta

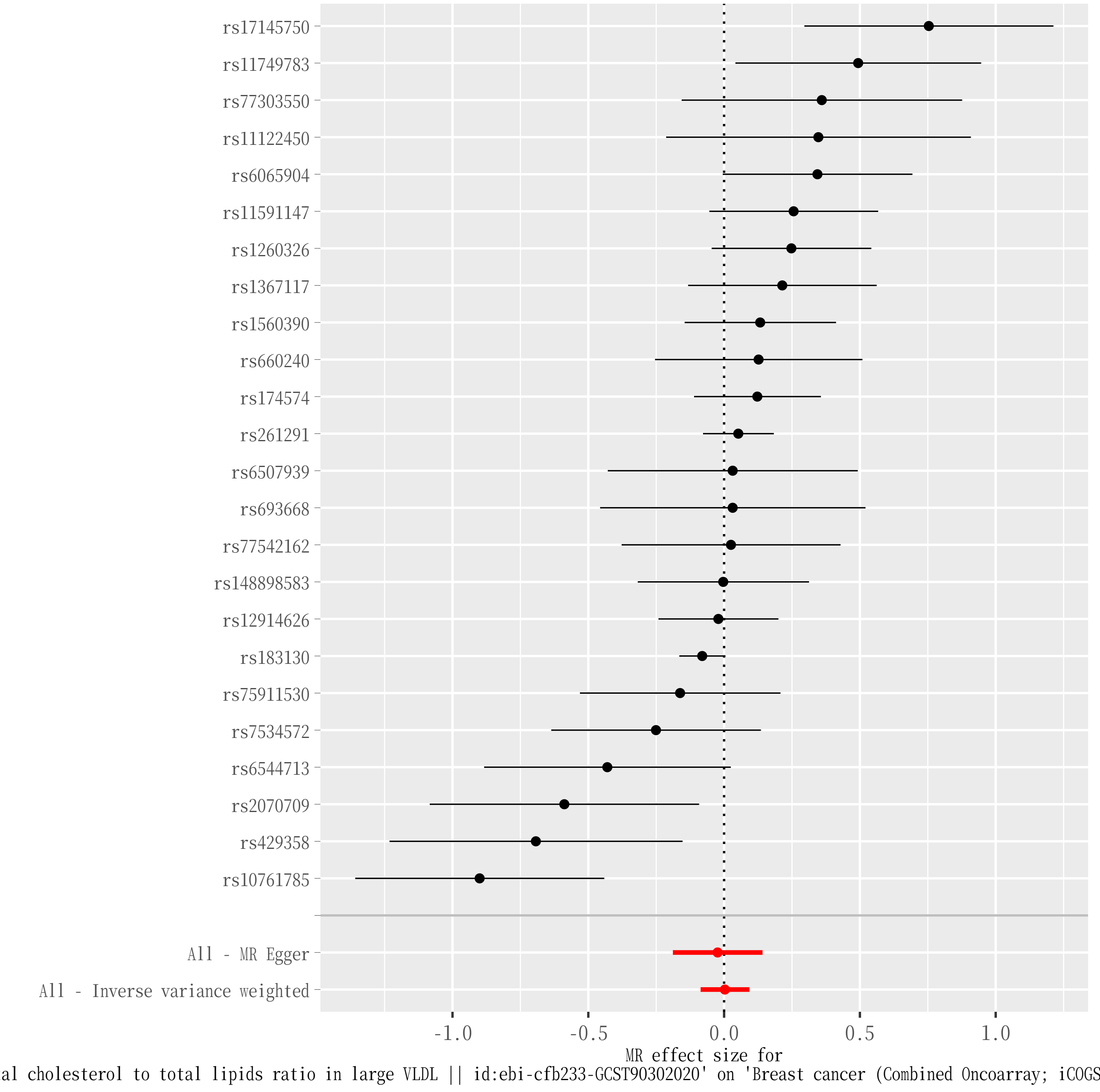
rs17145750
rs11749783
rs77303550
rs11122450
rs6065904
rs11591147
rs1260326
rs1367117
rs1560390
rs660240
rs174574
rs261291
rs6507939
rs693668
rs77542162
rs148898583
rs12914626
rs183130
rs75911530
rs7534572
rs6544713
rs2070709
rs429358
rs10761785
All - MR Egger
All - Inverse variance weighted
-1.0
-0.5
0.0
0.5
1.0
MR effect size for
al cholesterol to total lipids ratio in large VLDL || id:ebi-cfb233-GCST90302020' on 'Breast cancer (Combined Oncoarray; iCOGS

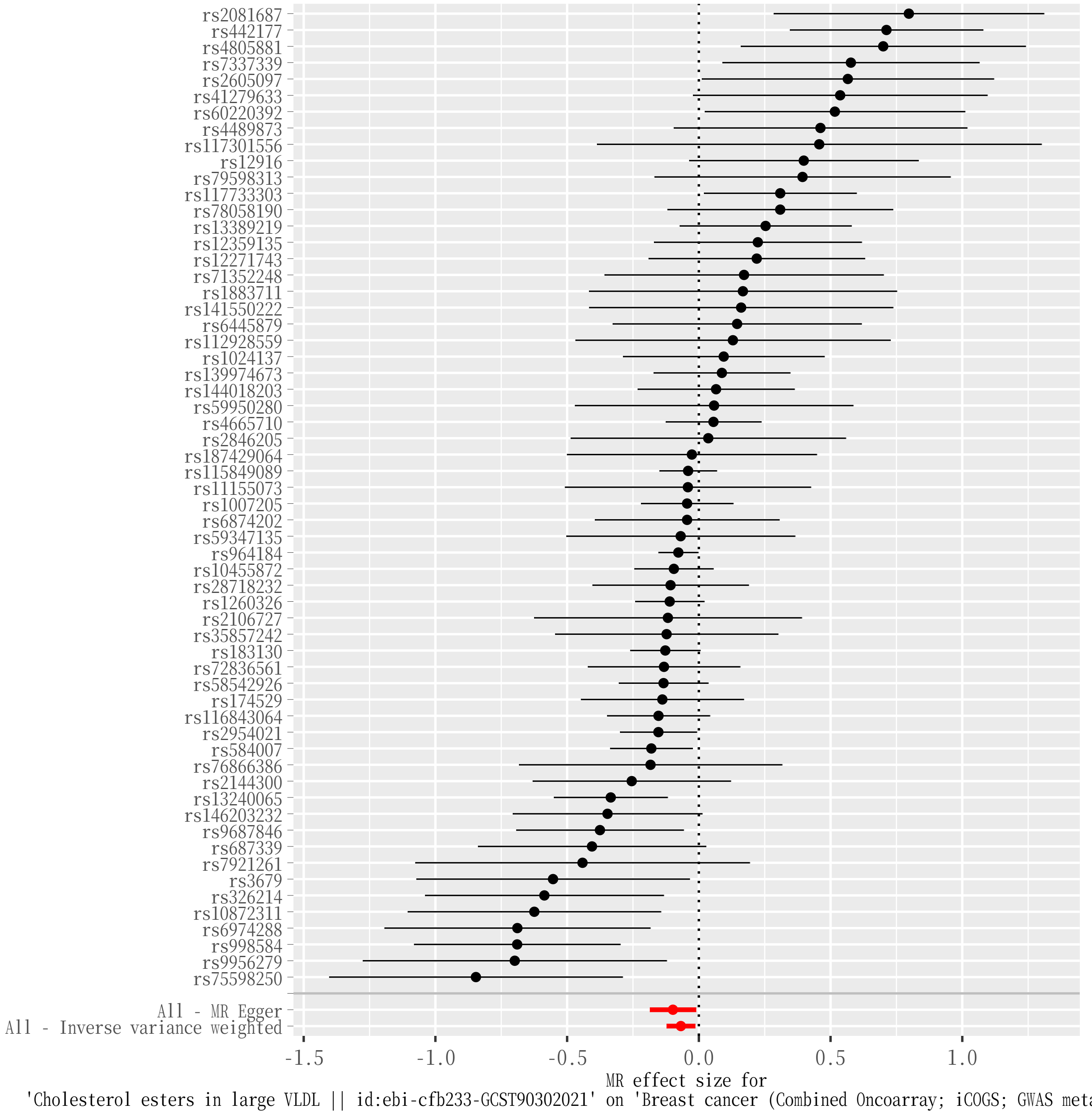
rs2081687
rs442177
rs4805881
rs7337339
rs2605097
rs41279633
rs60220392
rs4489873
rs117301556
rs12916
rs79598313
rs117733303
rs78058190
rs13389219
rs12359135
rs12271743
rs71352248
rs1883711
rs141550222
rs6445879
rs112928559
rs1024137
rs139974673
rs144018203
rs59950280
rs4665710
rs2846205
rs187429064
rs115849089
rs11155073
rs1007205
rs6874202
rs59347135
rs964184
rs10455872
rs28718232
rs1260326
rs2106727
rs35857242
rs183130
rs72836561
rs58542926
rs174529
rs116843064
rs2954021
rs584007
rs76866386
rs2144300
rs13240065
rs146203232
rs9687846
rs687339
rs7921261
rs3679
rs326214
rs10872311
rs6974288
rs998584
rs9956279
rs75598250
All - MR Egger
All - Inverse variance weighted
-1.5
-1.0
-0.5
0.0
0.5
1.0
MR effect size for
'Cholesterol esters in large VLDL || id:ebi-cfb233-GCST90302021' on 'Breast cancer (Combined Oncoarray; iCOGS; GWAS meta

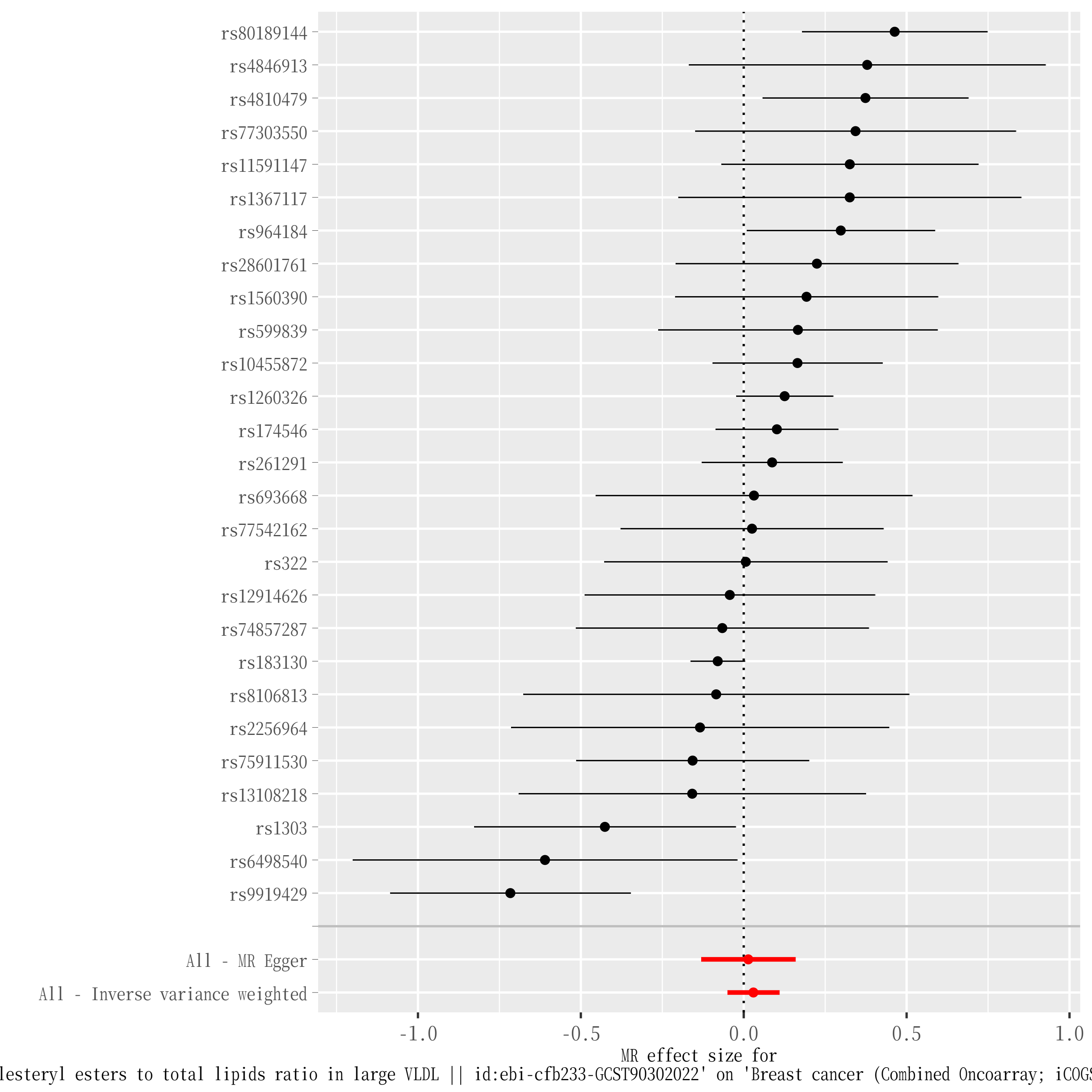
rs80189144
rs4846913
rs4810479
rs77303550
rs11591147
rs1367117
rs964184
rs28601761
rs1560390
rs599839
rs10455872
rs1260326
rs174546
rs261291
rs693668
rs77542162
rs322
rs12914626
rs74857287
rs183130
rs8106813
rs2256964
rs75911530
rs13108218
rs1303
rs6498540
rs9919429
All - MR Egger
All - Inverse variance weighted
-1.0
-0.5
0.0
0.5
1.0
MR effect size for
lesteryl esters to total lipids ratio in large VLDL || id:ebi-cfb233-GCST90302022' on 'Breast cancer (Combined Oncoarray; iCOGS

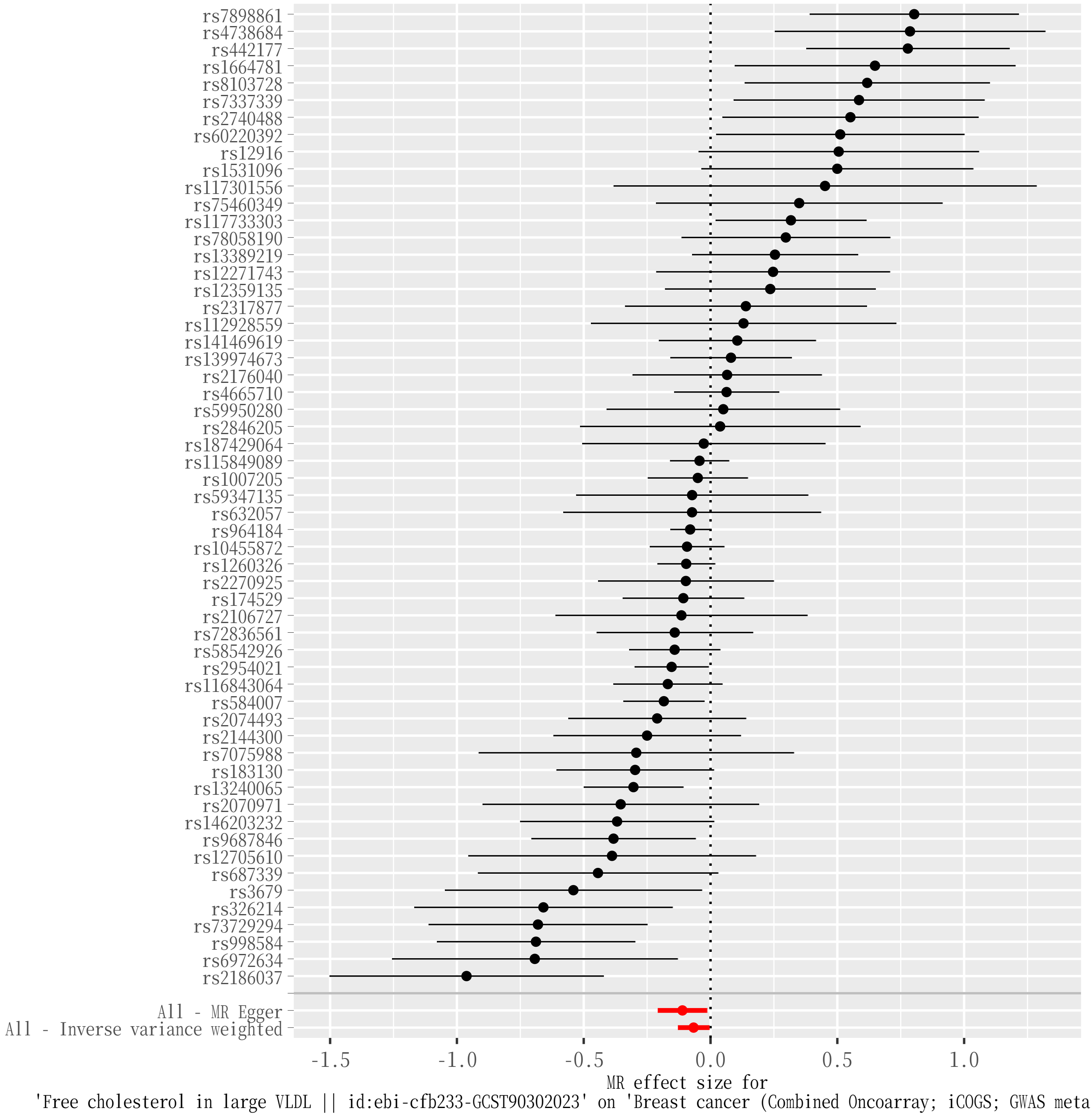

rs7898861
rs4738684
rs442177
rs1664781
rs8103728
rs7337339
rs2740488
rs60220392
rs12916
rs1531096
rs117301556
rs75460349
rs117733303
rs78058190
rs13389219
rs12271743
rs12359135
rs2317877
rs112928559
rs141469619
rs139974673
rs2176040
rs4665710
rs59950280
rs2846205
rs187429064
rs115849089
rs1007205
rs59347135
rs632057
rs964184
rs10455872
rs1260326
rs2270925
rs174529
rs2106727
rs72836561
rs58542926
rs2954021
rs116843064
rs584007
rs2074493
rs2144300
rs7075988
rs183130
rs13240065
rs2070971
rs146203232
rs9687846
rs12705610
rs687339
rs3679
rs326214
rs73729294
rs998584
rs6972634
rs2186037
All - MR Egger
All - Inverse variance weighted
-1.5
-1.0
-0.5
0.0
0.5
1.0
MR effect size for
'Free cholesterol in large VLDL || id:ebi-cfb233-GCST90302023' on 'Breast cancer (Combined Oncoarray; iCOGS; GWAS meta

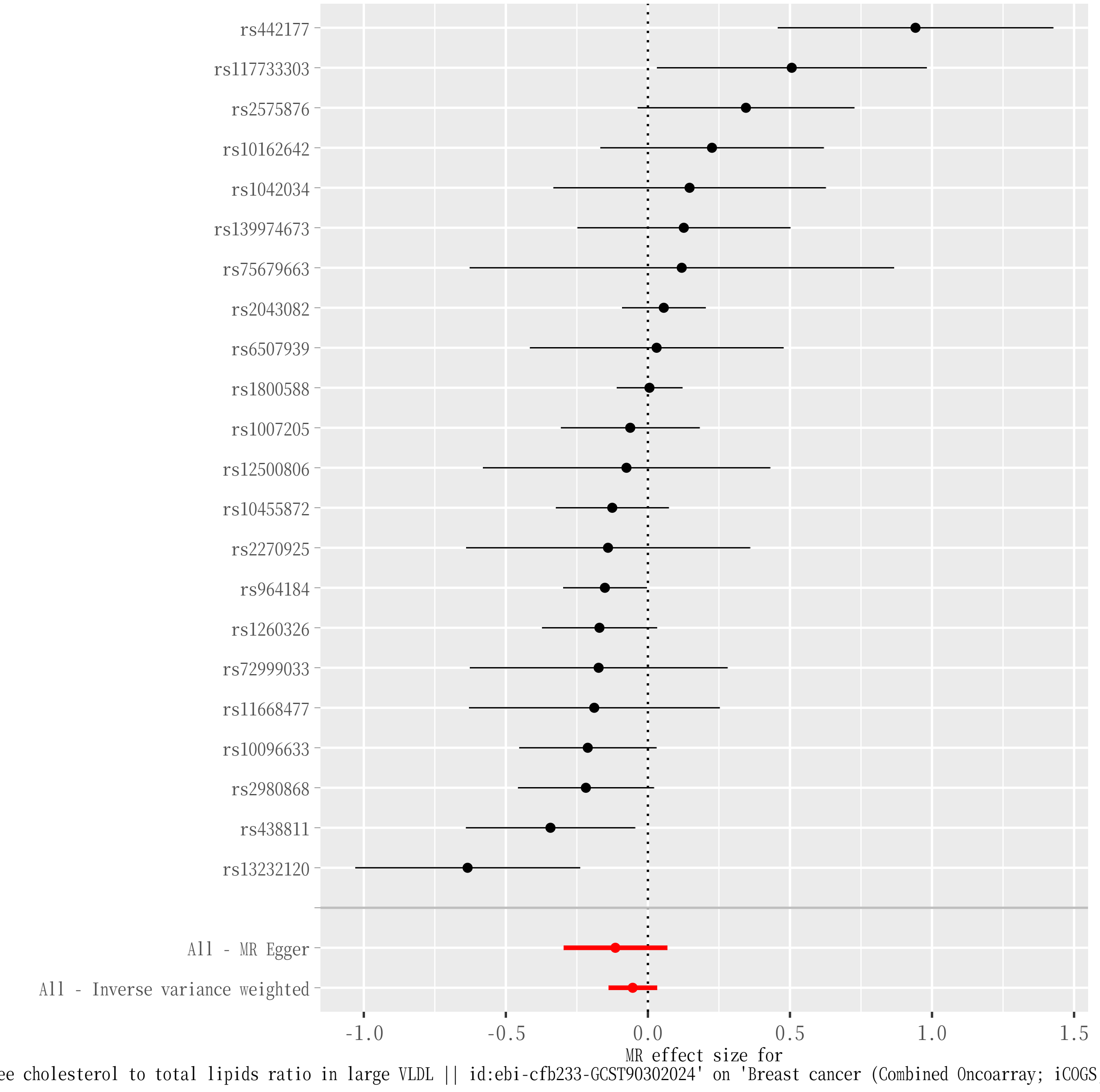
rs442177
rs117733303
rs2575876
rs10162642
rs1042034
rs139974673
rs75679663
rs2043082
rs6507939
rs1800588
rs1007205
rs12500806
rs10455872
rs2270925
rs964184
rs1260326
rs72999033
rs11668477
rs10096633
rs2980868
rs438811
rs13232120
All - MR Egger
All - Inverse variance weighted
-1.0
-0.5
0.0
0.5
1.0
1.5
MR effect size for
ee cholesterol to total lipids ratio in large VLDL || id:ebi-cfb233-GCST90302024' on 'Breast cancer (Combined Oncoarray; iCOGS

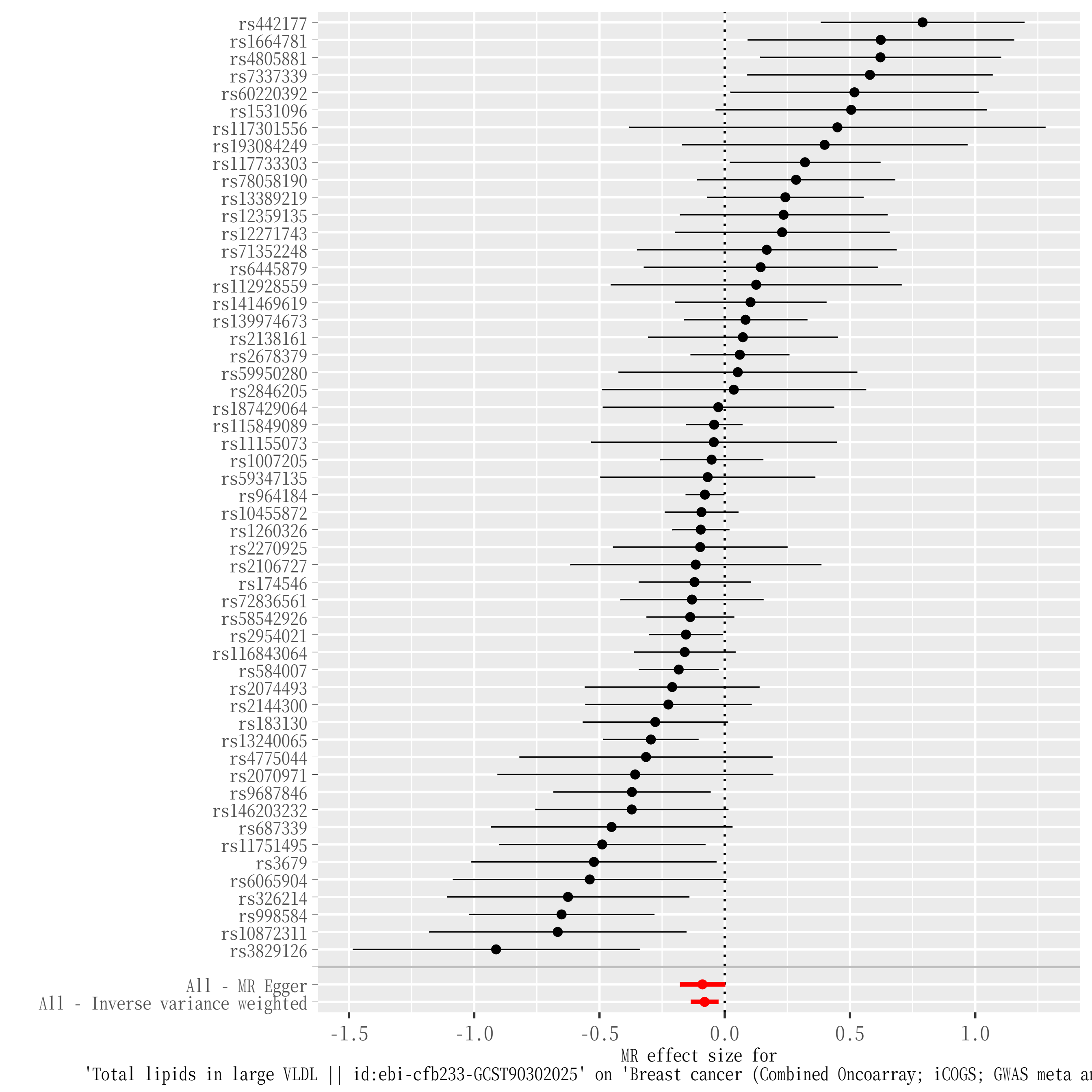
rs442177
rs1664781
rs4805881
rs7337339
rs60220392
rs1531096
rs117301556
rs193084249
rs117733303
rs78058190
rs13389219
rs12359135
rs12271743
rs71352248
rs6445879
rs112928559
rs141469619
rs139974673
rs2138161
rs2678379
rs59950280
rs2846205
rs187429064
rs115849089
rs11155073
rs1007205
rs59347135
rs964184
rs10455872
rs1260326
rs2270925
rs2106727
rs174546
rs72836561
rs58542926
rs2954021
rs116843064
rs584007
rs2074493
rs2144300
rs183130
rs13240065
rs4775044
rs2070971
rs9687846
rs146203232
rs687339
rs11751495
rs3679
rs6065904
rs326214
rs998584
rs10872311
rs3829126
All - MR Egger
All - Inverse variance weighted
-1.5
-1.0
-0.5
0.0
0.5
1.0
MR effect size for
'Total lipids in large VLDL || id:ebi-cfb233-GCST90302025' on 'Breast cancer (Combined Oncoarray; iCOGS; GWAS meta ar

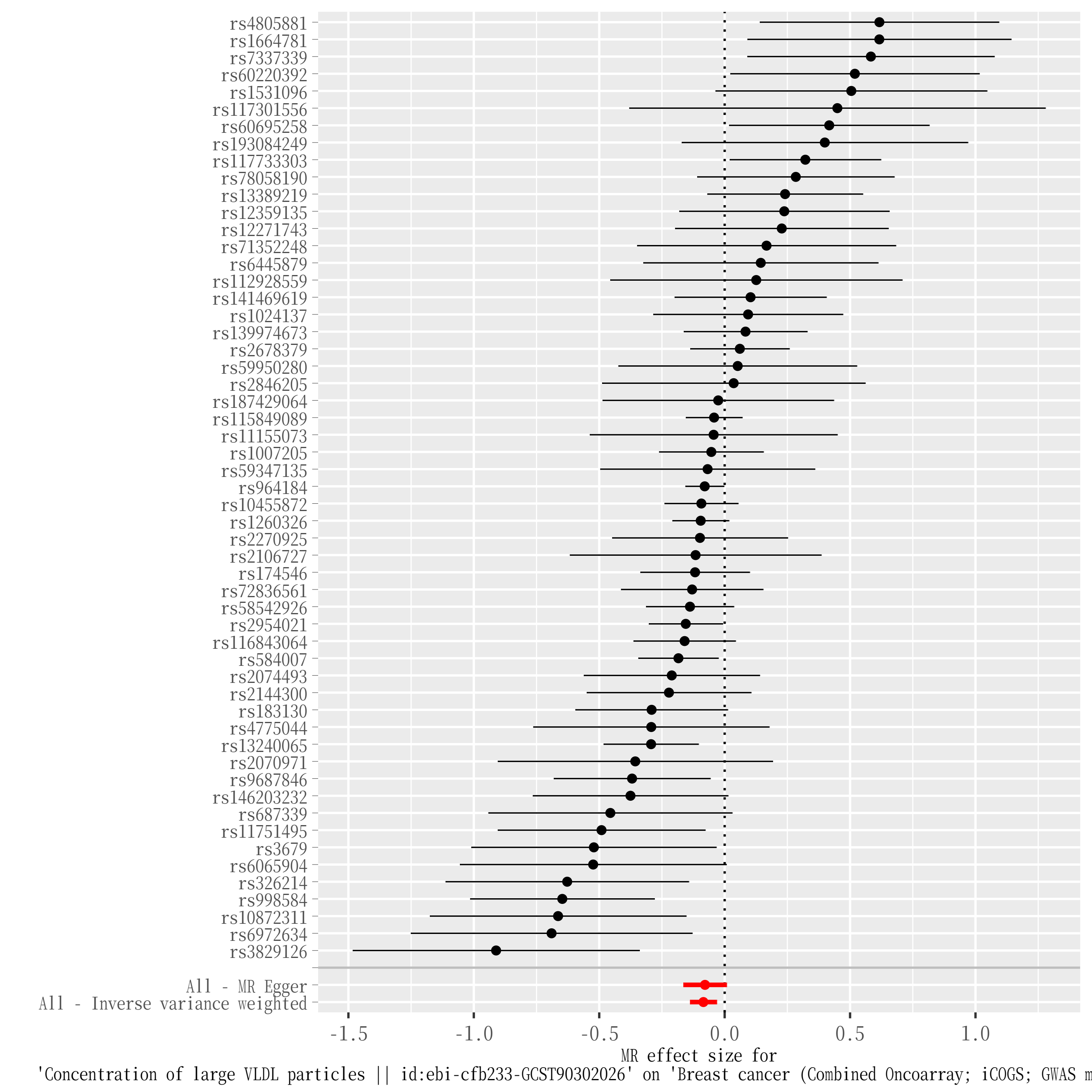

rs4805881
rs1664781
rs7337339
rs60220392
rs1531096
rs117301556
rs60695258
rs193084249
rs117733303
rs78058190
rs13389219
rs12359135
rs12271743
rs71352248
rs6445879
rs112928559
rs141469619
rs1024137
rs139974673
rs2678379
rs59950280
rs2846205
rs187429064
rs115849089
rs11155073
rs1007205
rs59347135
rs964184
rs10455872
rs1260326
rs2270925
rs2106727
rs174546
rs72836561
rs58542926
rs2954021
rs116843064
rs584007
rs2074493
rs2144300
rs183130
rs4775044
rs13240065
rs2070971
rs9687846
rs146203232
rs687339
rs11751495
rs3679
rs6065904
rs326214
rs998584
rs10872311
rs6972634
rs3829126
All - MR Egger
All - Inverse variance weighted
-1.5
-1.0
-0.5
0.0
0.5
1.0
MR effect size for
'Concentration of large VLDL particles || id:ebi-cfb233-GCST90302026' on 'Breast cancer (Combined Oncoarray; iCOGS; GWAS m

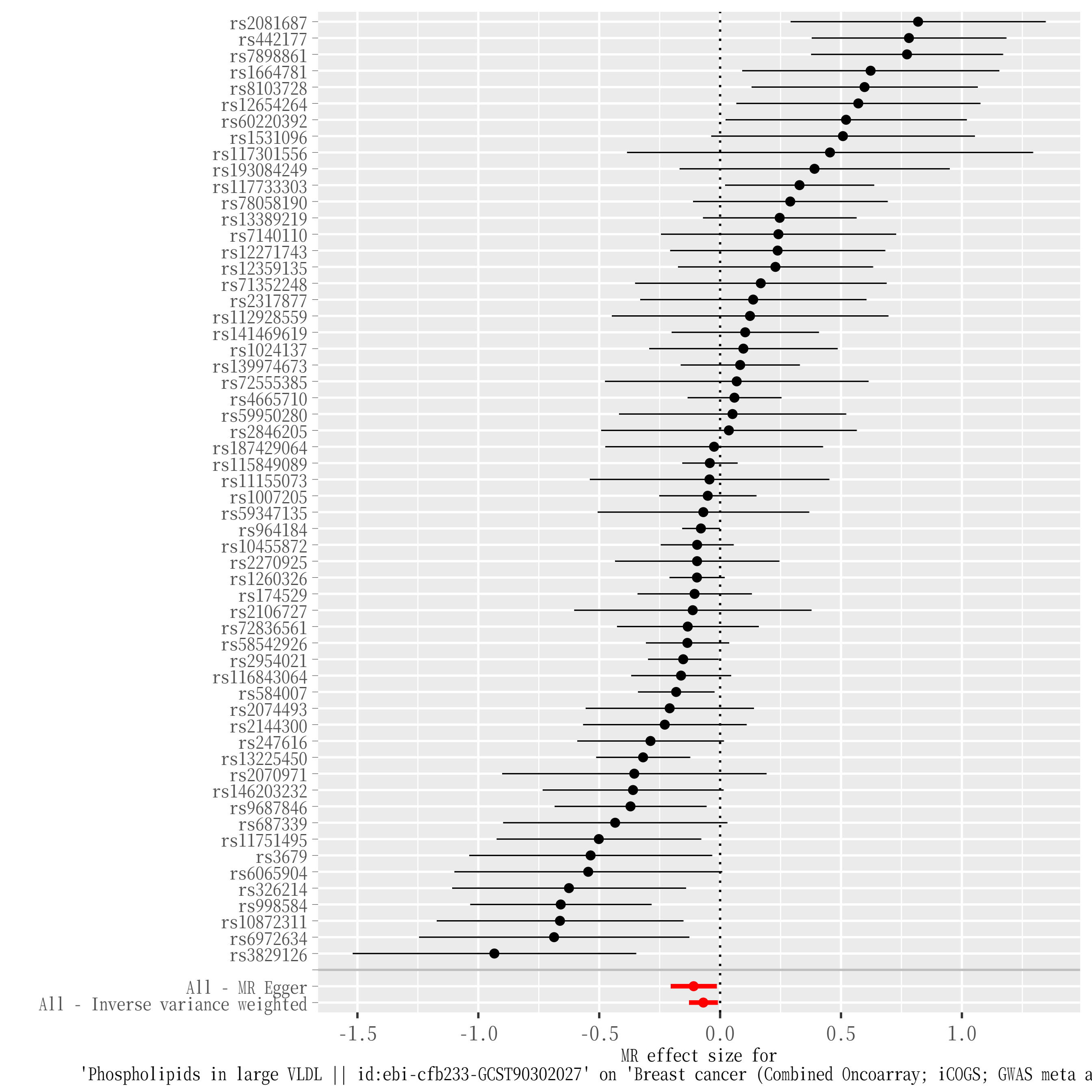

rs2081687
rs442177
rs7898861
rs1664781
rs8103728
rs12654264
rs60220392
rs1531096
rs117301556
rs193084249
rs117733303
rs78058190
rs13389219
rs7140110
rs12271743
rs12359135
rs71352248
rs2317877
rs112928559
rs141469619
rs1024137
rs139974673
rs72555385
rs4665710
rs59950280
rs2846205
rs187429064
rs115849089
rs11155073
rs1007205
rs59347135
rs964184
rs10455872
rs2270925
rs1260326
rs174529
rs2106727
rs72836561
rs58542926
rs2954021
rs116843064
rs584007
rs2074493
rs2144300
rs247616
rs13225450
rs2070971
rs146203232
rs9687846
rs687339
rs11751495
rs3679
rs6065904
rs326214
rs998584
rs10872311
rs6972634
rs3829126
All - MR Egger
All - Inverse variance weighted
-1.5
-1.0
-0.5
0.0
0.5
1.0
MR effect size for
'Phospholipids in large VLDL || id:ebi-cfb233-GCST90302027' on 'Breast cancer (Combined Oncoarray; iCOGS; GWAS meta a

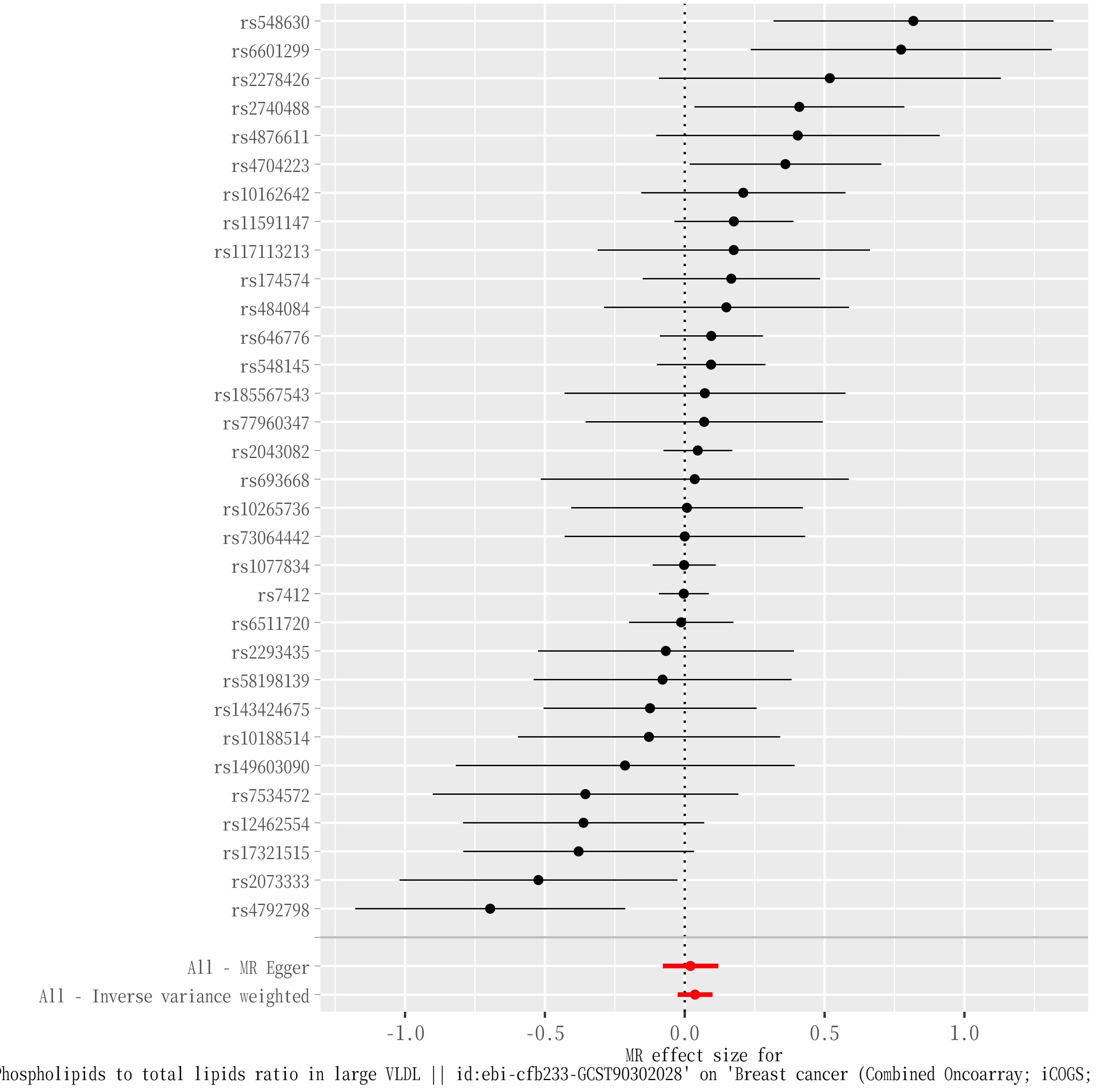
rs548630
rs6601299
rs2278426
rs2740488
rs4876611
rs4704223
rs10162642
rs11591147
rs117113213
rs174574
rs484084
rs646776
rs548145
rs185567543
rs77960347
rs2043082
rs693668
rs10265736
rs73064442
rs1077834
rs7412
rs6511720
rs2293435
rs58198139
rs143424675
rs10188514
rs149603090
rs7534572
rs12462554
rs17321515
rs2073333
rs4792798
All - MR Egger
All - Inverse variance weighted
-1.0
-0.5
0.0
0.5
1.0
MR effect size for
Phospholipids to total lipids ratio in large VLDL || id:ebi-cfb233-GCST90302028' on 'Breast cancer (Combined Oncoarray; iCOGS;

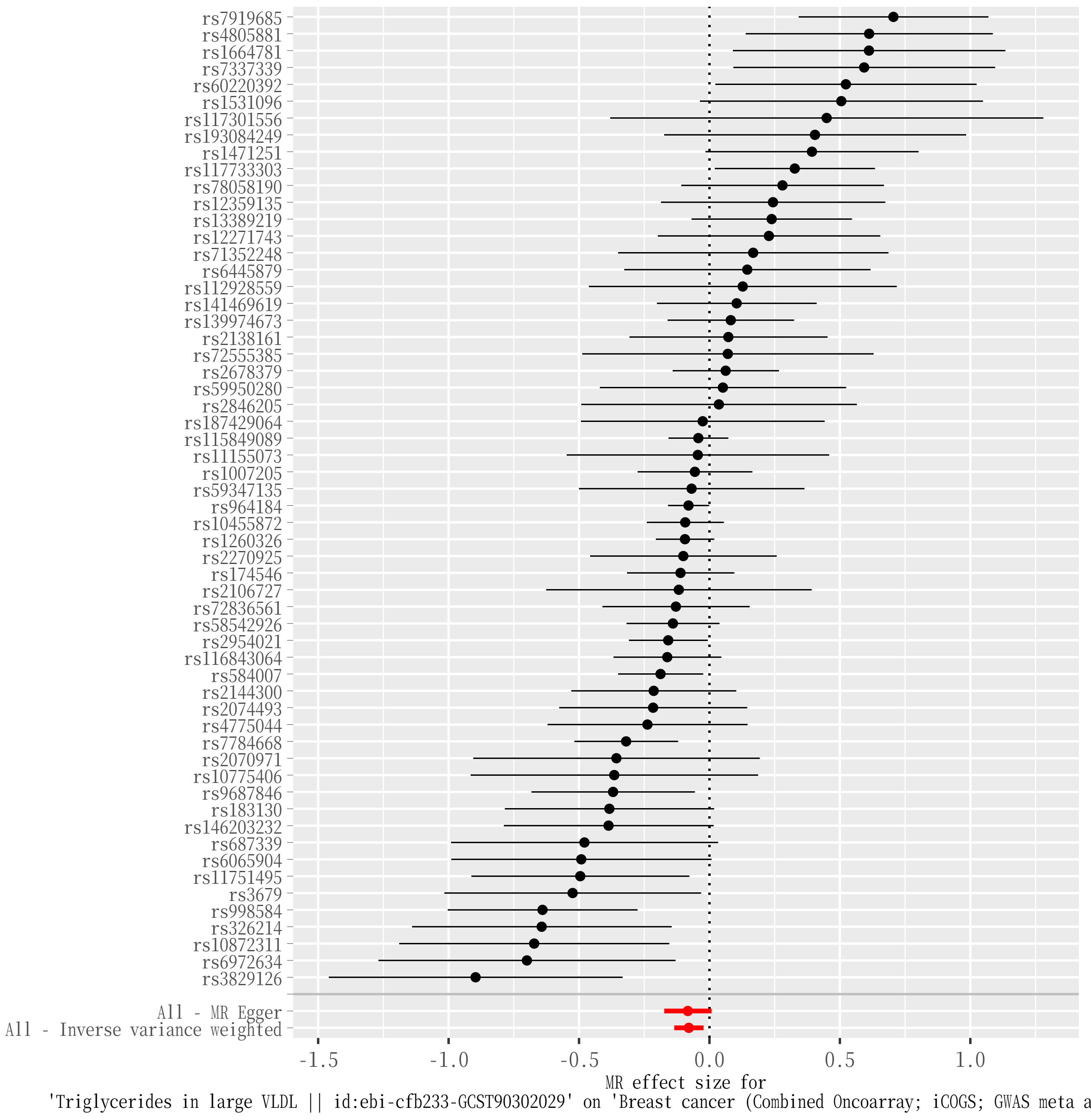

rs7919685
rs4805881
rs1664781
rs7337339
rs60220392
rs1531096
rs117301556
rs193084249
rs1471251
rs117733303
rs78058190
rs12359135
rs13389219
rs12271743
rs71352248
rs6445879
rs112928559
rs141469619
rs139974673
rs2138161
rs72555385
rs2678379
rs59950280
rs2846205
rs187429064
rs115849089
rs11155073
rs1007205
rs59347135
rs964184
rs10455872
rs1260326
rs2270925
rs174546
rs2106727
rs72836561
rs58542926
rs2954021
rs116843064
rs584007
rs2144300
rs2074493
rs4775044
rs7784668
rs2070971
rs10775406
rs9687846
rs183130
rs146203232
rs687339
rs6065904
rs11751495
rs3679
rs998584
rs326214
rs10872311
rs6972634
rs3829126
All - MR Egger
All - Inverse variance weighted
-1.5
-1.0
-0.5
0.0
0.5
1.0
MR effect size for
'Triglycerides in large VLDL || id:ebi-cfb233-GCST90302029' on 'Breast cancer (Combined Oncoarray; iCOGS; GWAS meta a

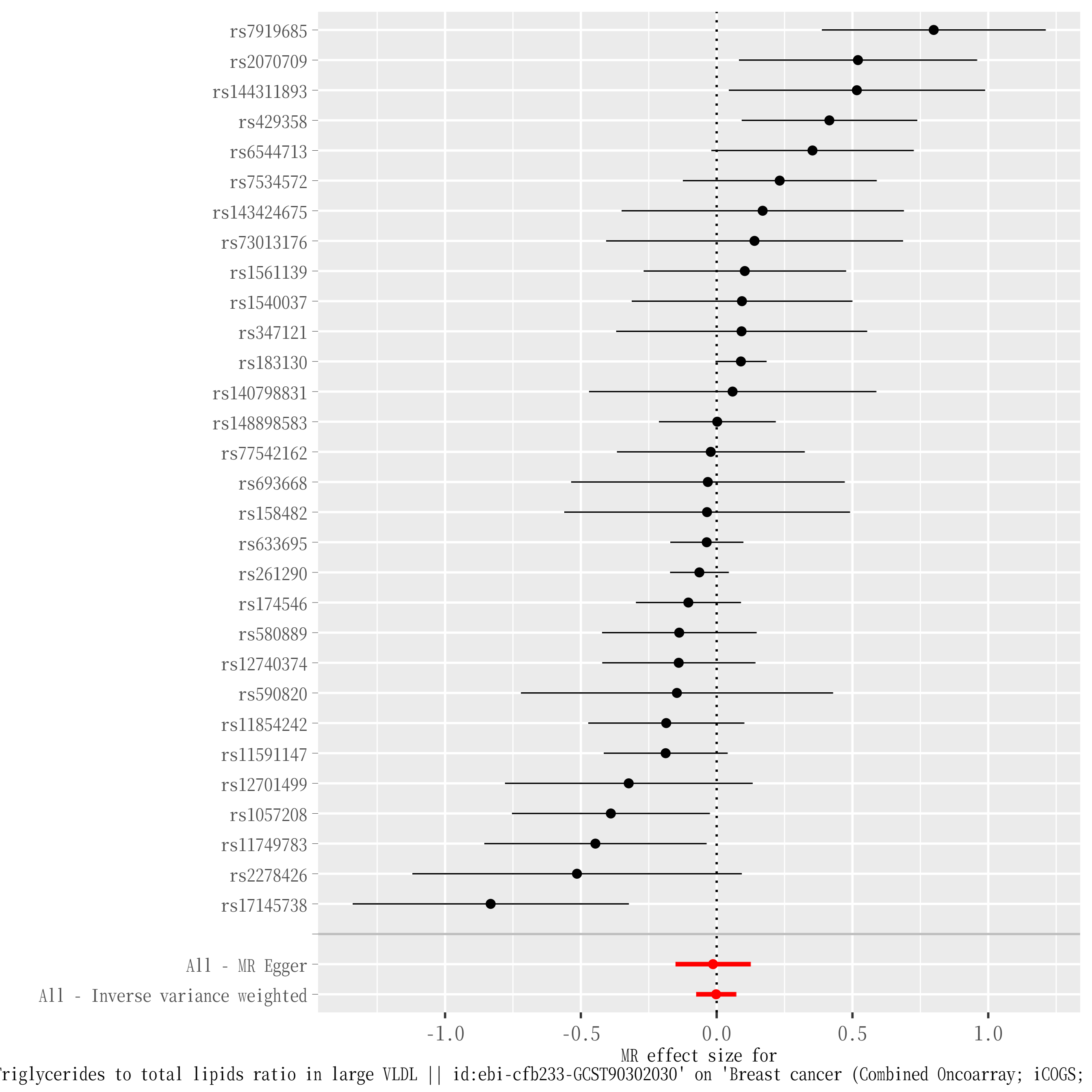
rs7919685
rs2070709
rs144311893
rs429358
rs6544713
rs7534572
rs143424675
rs73013176
rs1561139
rs1540037
rs347121
rs183130
rs140798831
rs148898583
rs77542162
rs693668
rs158482
rs633695
rs261290
rs174546
rs580889
rs12740374
rs590820
rs11854242
rs11591147
rs12701499
rs1057208
rs11749783
rs2278426
rs17145738
All - MR Egger
All - Inverse variance weighted
-1.0
-0.5
0.0
0.5
1.0
MR effect size for
'Triglycerides to total lipids ratio in large VLDL || id:ebi-cfb233-GCST90302030' on 'Breast cancer (Combined Oncoarray; iCOGS;

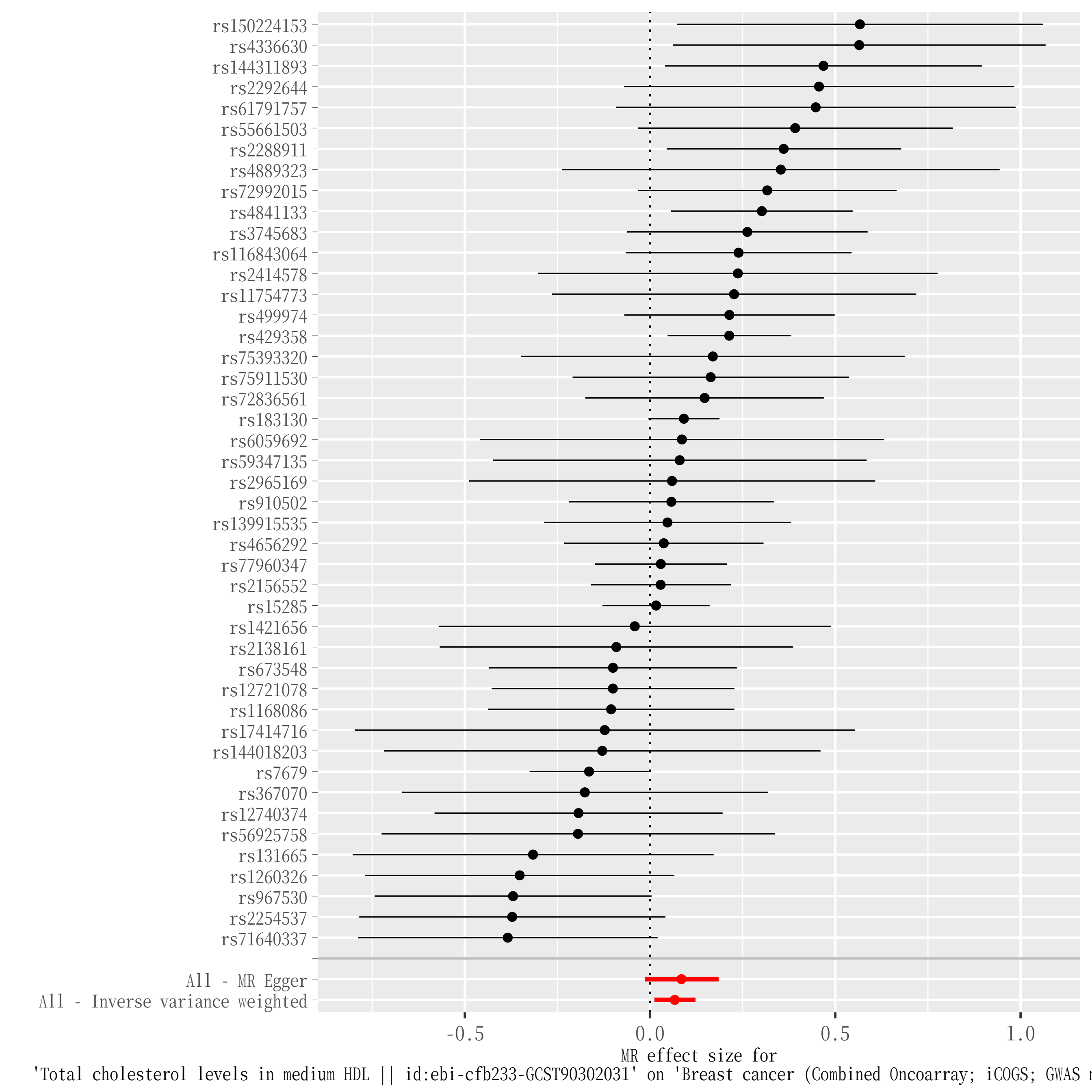

rs150224153
rs4336630
rs144311893
rs2292644
rs61791757
rs55661503
rs2288911
rs4889323
rs72992015
rs4841133
rs3745683
rs116843064
rs2414578
rs11754773
rs499974
rs429358
rs75393320
rs75911530
rs72836561
rs183130
rs6059692
rs59347135
rs2965169
rs910502
rs139915535
rs4656292
rs77960347
rs2156552
rs15285
rs1421656
rs2138161
rs673548
rs12721078
rs1168086
rs17414716
rs144018203
rs7679
rs367070
rs12740374
rs56925758
rs131665
rs1260326
rs967530
rs2254537
rs71640337
All - MR Egger
All - Inverse variance weighted
-0.5
0.0
0.5
1.0
MR effect size for
'Total cholesterol levels in medium HDL || id:ebi-cfb233-GCST90302031' on 'Breast cancer (Combined Oncoarray; iCOGS; GWAS m

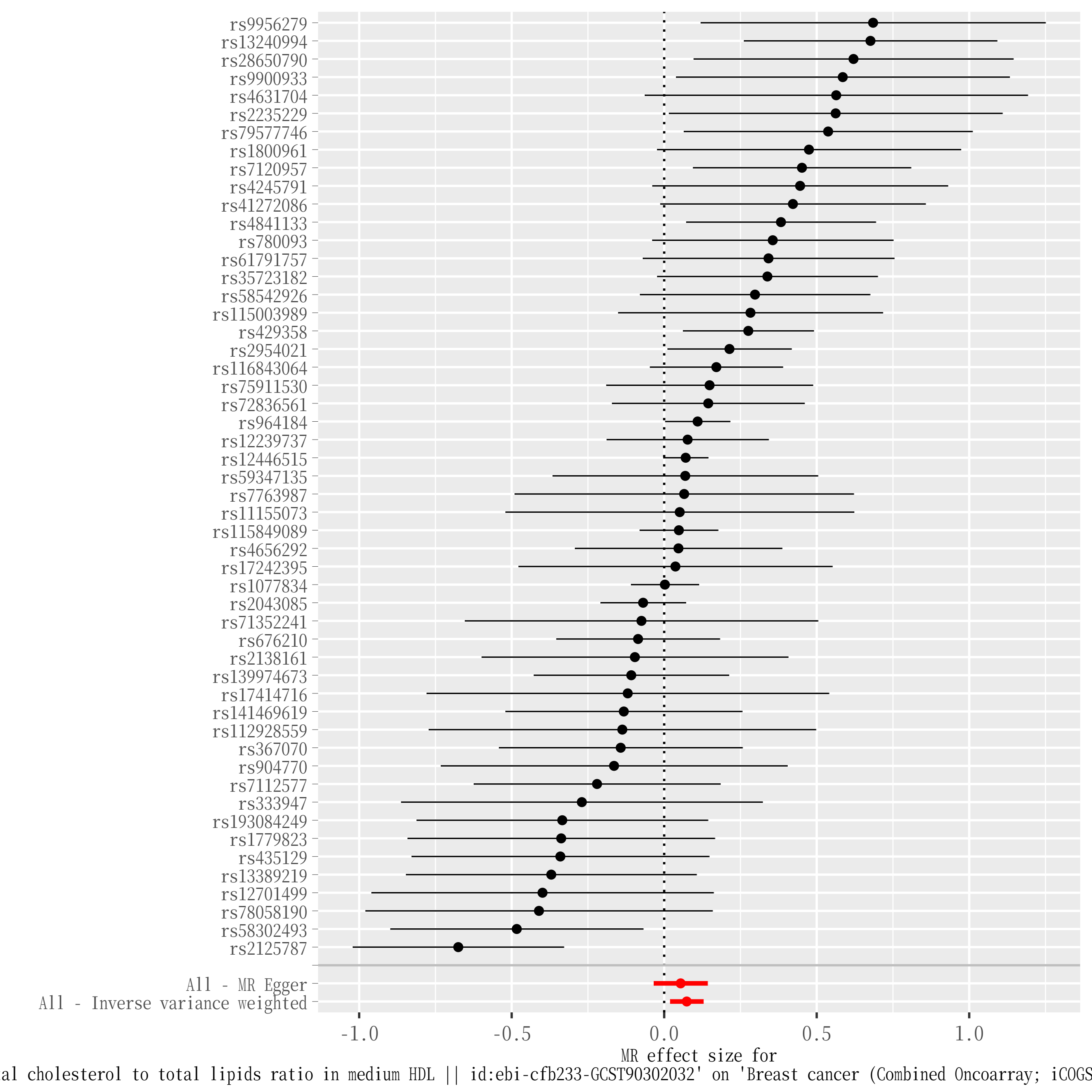
rs9956279
rs13240994
rs28650790
rs9900933
rs4631704
rs2235229
rs79577746
rs1800961
rs7120957
rs4245791
rs41272086
rs4841133
rs780093
rs61791757
rs35723182
rs58542926
rs115003989
rs429358
rs2954021
rs116843064
rs75911530
rs72836561
rs964184
rs12239737
rs12446515
rs59347135
rs7763987
rs11155073
rs115849089
rs4656292
rs17242395
rs1077834
rs2043085
rs71352241
rs676210
rs2138161
rs139974673
rs17414716
rs141469619
rs112928559
rs367070
rs904770
rs7112577
rs333947
rs193084249
rs1779823
rs435129
rs13389219
rs12701499
rs78058190
rs58302493
rs2125787
All - MR Egger
All - Inverse variance weighted
-1.0
-0.5
0.0
0.5
1.0
MR effect size for
al cholesterol to total lipids ratio in medium HDL || id:ebi-cfb233-GCST90302032' on 'Breast cancer (Combined Oncoarray; iCOGS

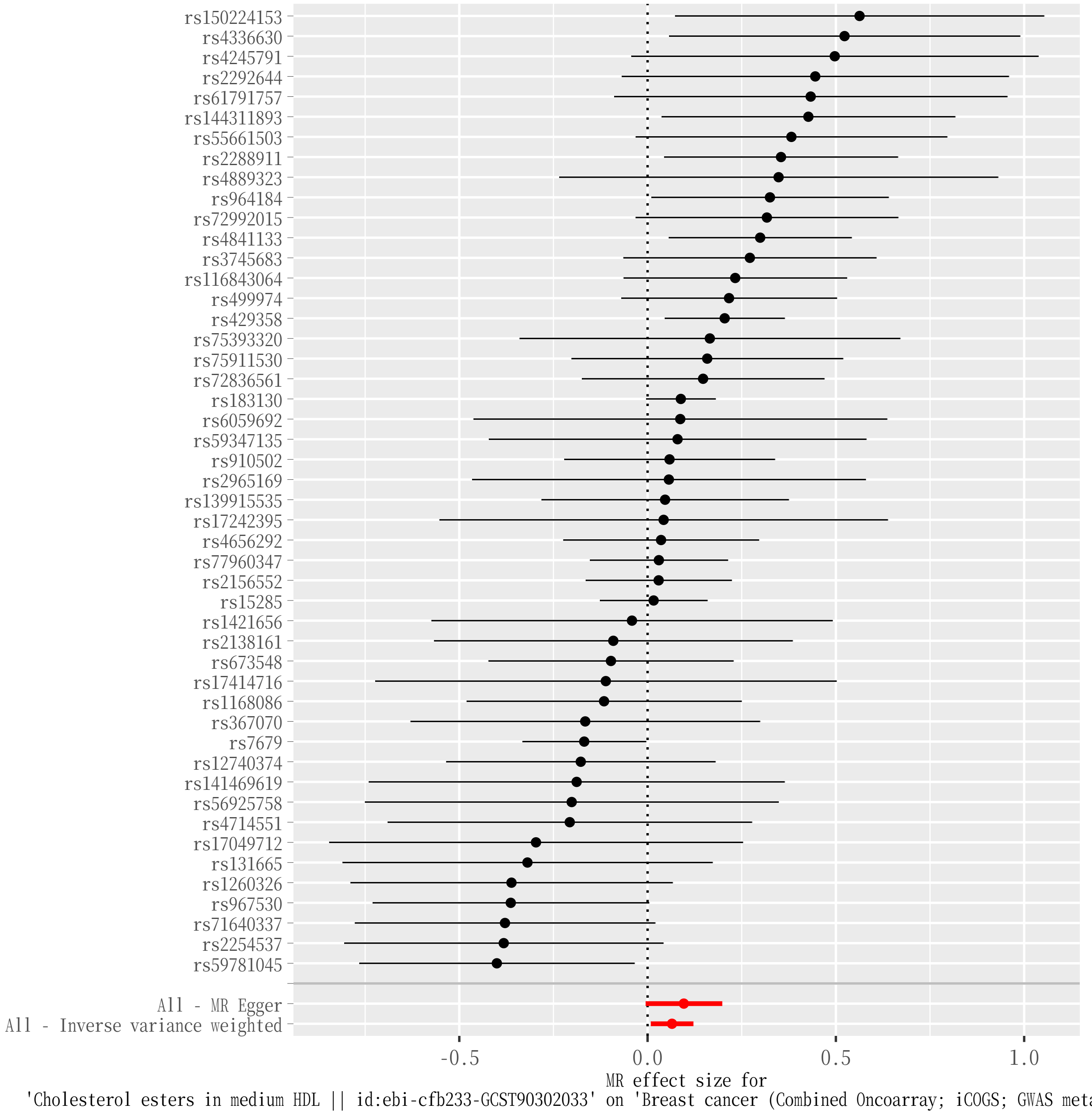

rs150224153
rs4336630
rs4245791
rs2292644
rs61791757
rs144311893
rs55661503
rs2288911
rs4889323
rs964184
rs72992015
rs4841133
rs3745683
rs116843064
rs499974
rs429358
rs75393320
rs75911530
rs72836561
rs183130
rs6059692
rs59347135
rs910502
rs2965169
rs139915535
rs17242395
rs4656292
rs77960347
rs2156552
rs15285
rs1421656
rs2138161
rs673548
rs17414716
rs1168086
rs367070
rs7679
rs12740374
rs141469619
rs56925758
rs4714551
rs17049712
rs131665
rs1260326
rs967530
rs71640337
rs2254537
rs59781045
All - MR Egger
All - Inverse variance weighted
-0.5
0.0
0.5
1.0
MR effect size for
'Cholesterol esters in medium HDL || id:ebi-cfb233-GCST90302033' on 'Breast cancer (Combined Oncoarray; iCOGS; GWAS meta

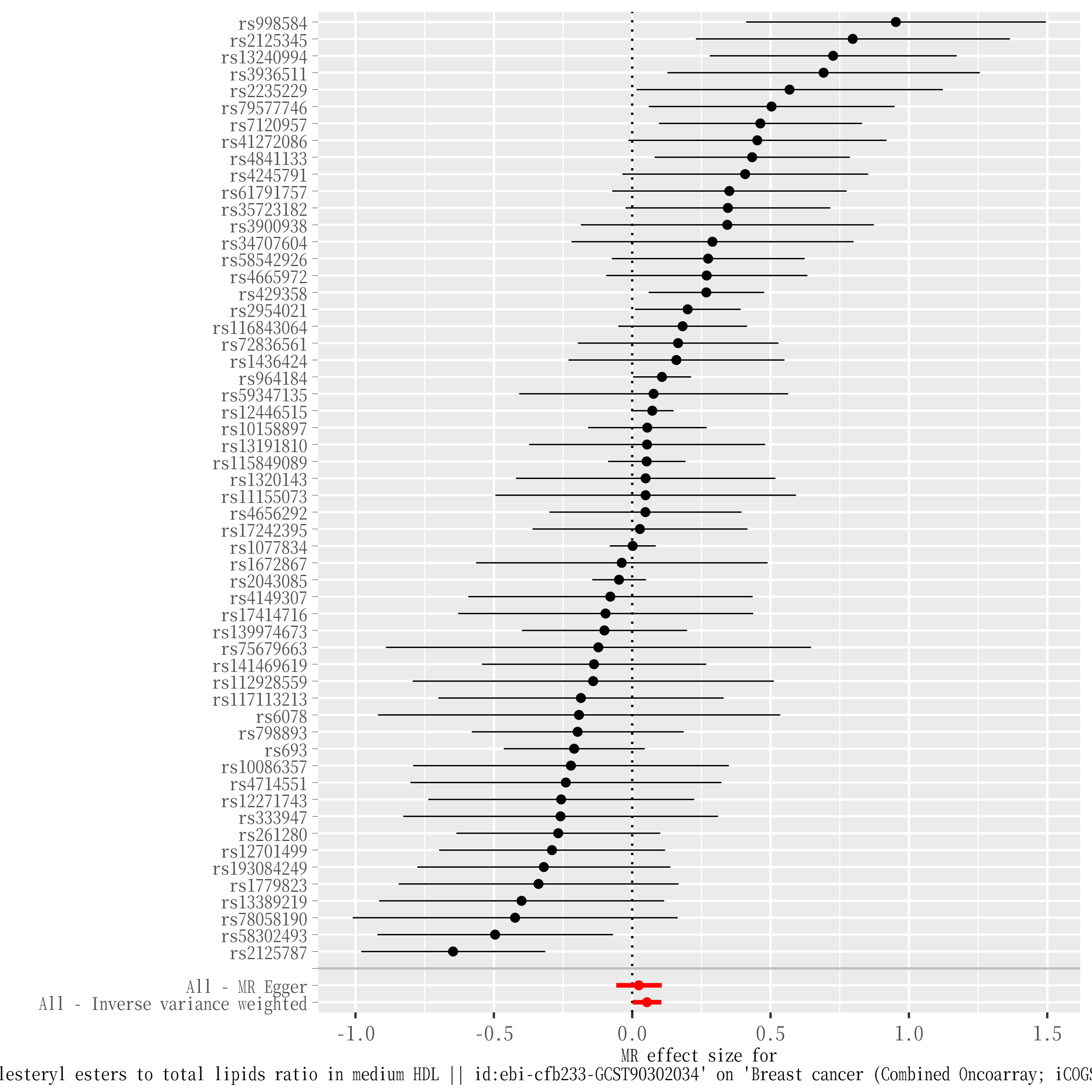

rs998584
rs2125345
rs13240994
rs3936511
rs2235229
rs79577746
rs7120957
rs41272086
rs4841133
rs4245791
rs61791757
rs35723182
rs3900938
rs34707604
rs58542926
rs4665972
rs429358
rs2954021
rs116843064
rs72836561
rs1436424
rs964184
rs59347135
rs12446515
rs10158897
rs13191810
rs115849089
rs1320143
rs11155073
rs4656292
rs17242395
rs1077834
rs1672867
rs2043085
rs4149307
rs17414716
rs139974673
rs75679663
rs141469619
rs112928559
rs117113213
rs6078
rs798893
rs693
rs10086357
rs4714551
rs12271743
rs333947
rs261280
rs12701499
rs193084249
rs1779823
rs13389219
rs78058190
rs58302493
rs2125787
All - MR Egger
All - Inverse variance weighted
-1.0
-0.5
0.0
0.5
1.0
1.5
MR effect size for
lesteryl esters to total lipids ratio in medium HDL || id:ebi-cfb233-GCST90302034' on 'Breast cancer (Combined Oncoarray; iCOGS

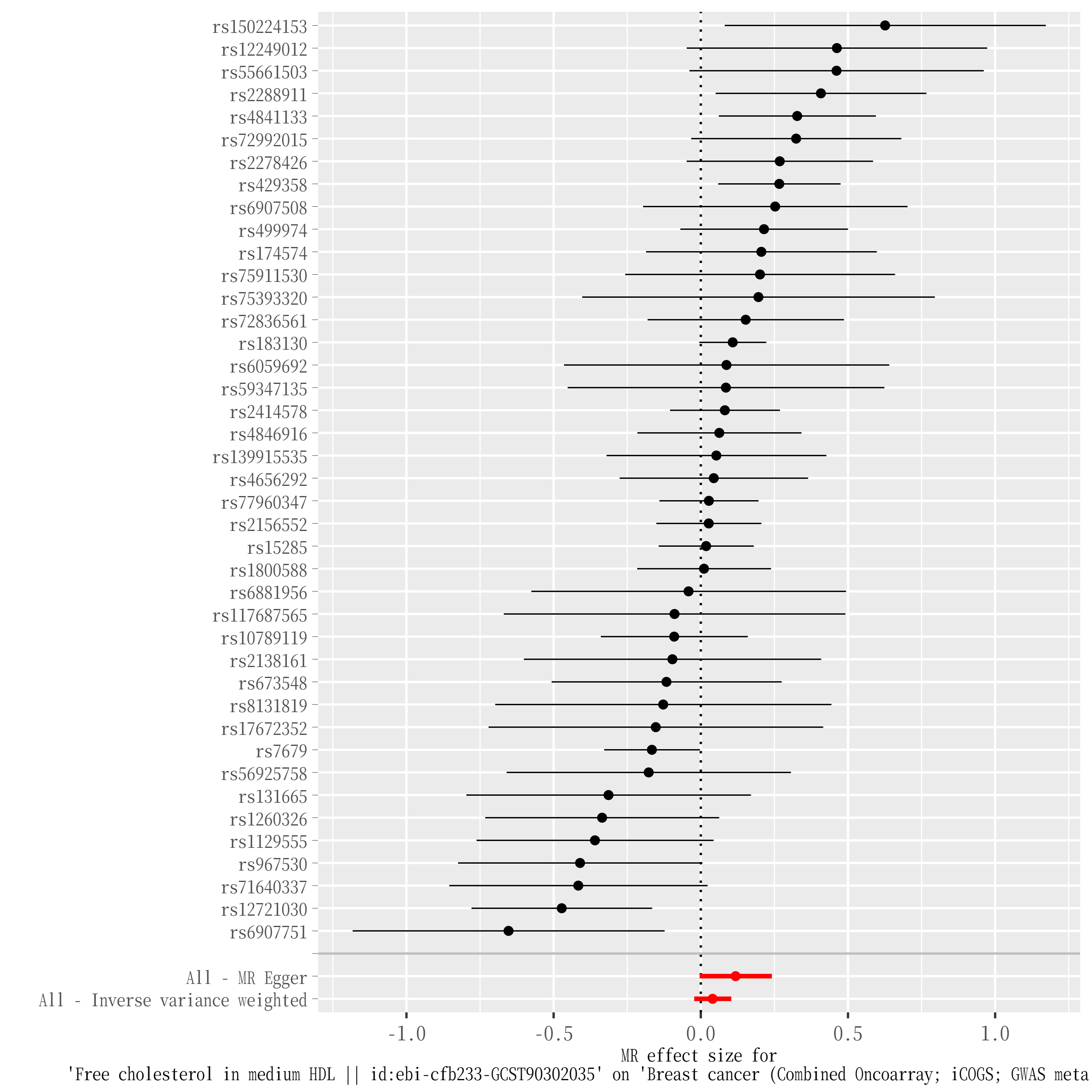
rs150224153
rs12249012
rs55661503
rs2288911
rs4841133
rs72992015
rs2278426
rs429358
rs6907508
rs499974
rs174574
rs75911530
rs75393320
rs72836561
rs183130
rs6059692
rs59347135
rs2414578
rs4846916
rs139915535
rs4656292
rs77960347
rs2156552
rs15285
rs1800588
rs6881956
rs117687565
rs10789119
rs2138161
rs673548
rs8131819
rs17672352
rs7679
rs56925758
rs131665
rs1260326
rs1129555
rs967530
rs71640337
rs12721030
rs6907751
All - MR Egger
All - Inverse variance weighted
-1.0
-0.5
0.0
0.5
1.0
MR effect size for
'Free cholesterol in medium HDL || id:ebi-cfb233-GCST90302035' on 'Breast cancer (Combined Oncoarray; iCOGS; GWAS meta

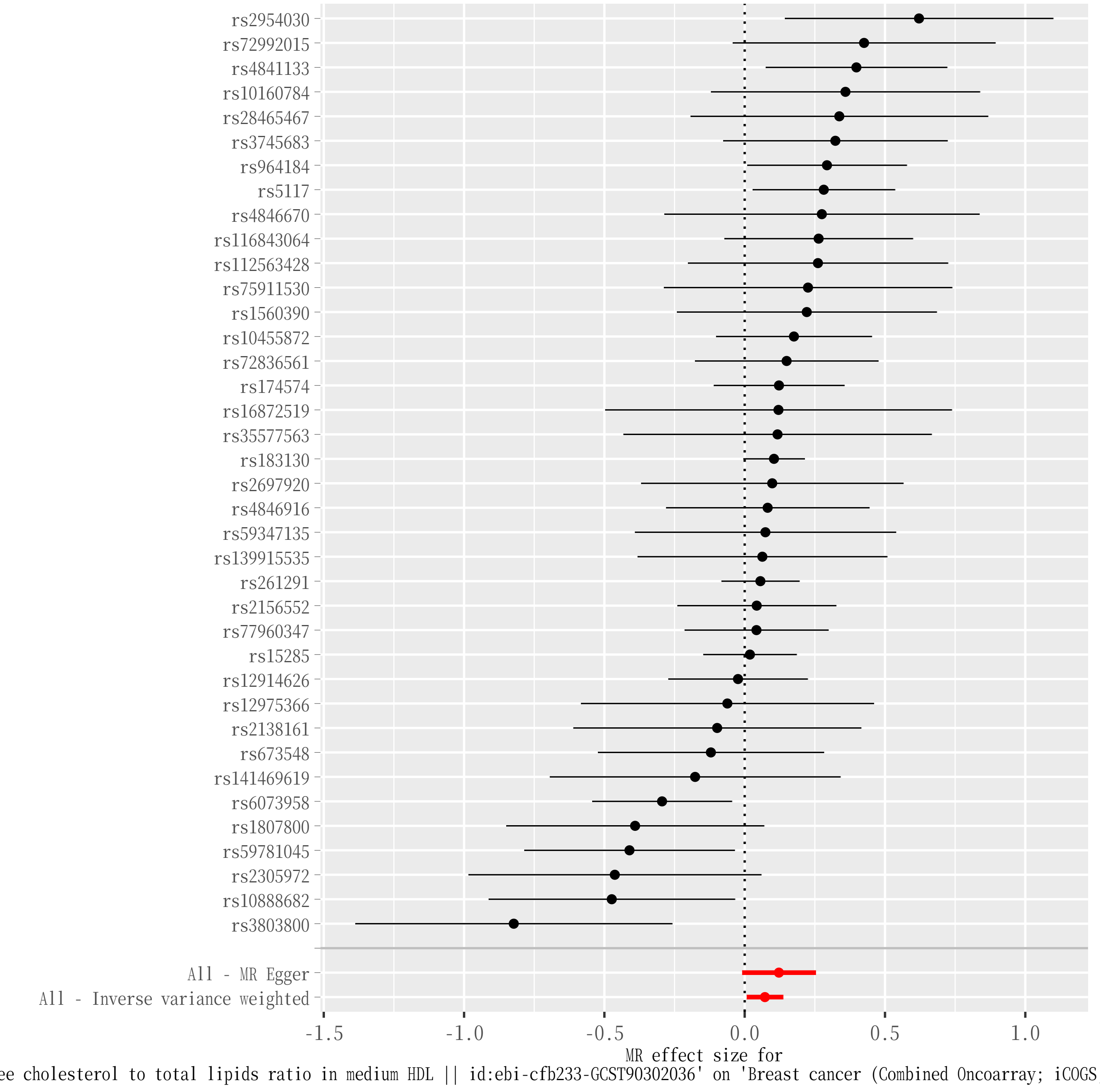
rs2954030
rs72992015
rs4841133
rs10160784
rs28465467
rs3745683
rs964184
rs5117
rs4846670
rs116843064
rs112563428
rs75911530
rs1560390
rs10455872
rs72836561
rs174574
rs16872519
rs35577563
rs183130
rs2697920
rs4846916
rs59347135
rs139915535
rs261291
rs2156552
rs77960347
rs15285
rs12914626
rs12975366
rs2138161
rs673548
rs141469619
rs6073958
rs1807800
rs59781045
rs2305972
rs10888682
rs3803800
All - MR Egger
All - Inverse variance weighted
-1.5
-1.0
-0.5
0.0
0.5
1.0
MR effect size for
ee cholesterol to total lipids ratio in medium HDL || id:ebi-cfb233-GCST90302036' on 'Breast cancer (Combined Oncoarray; iCOGS

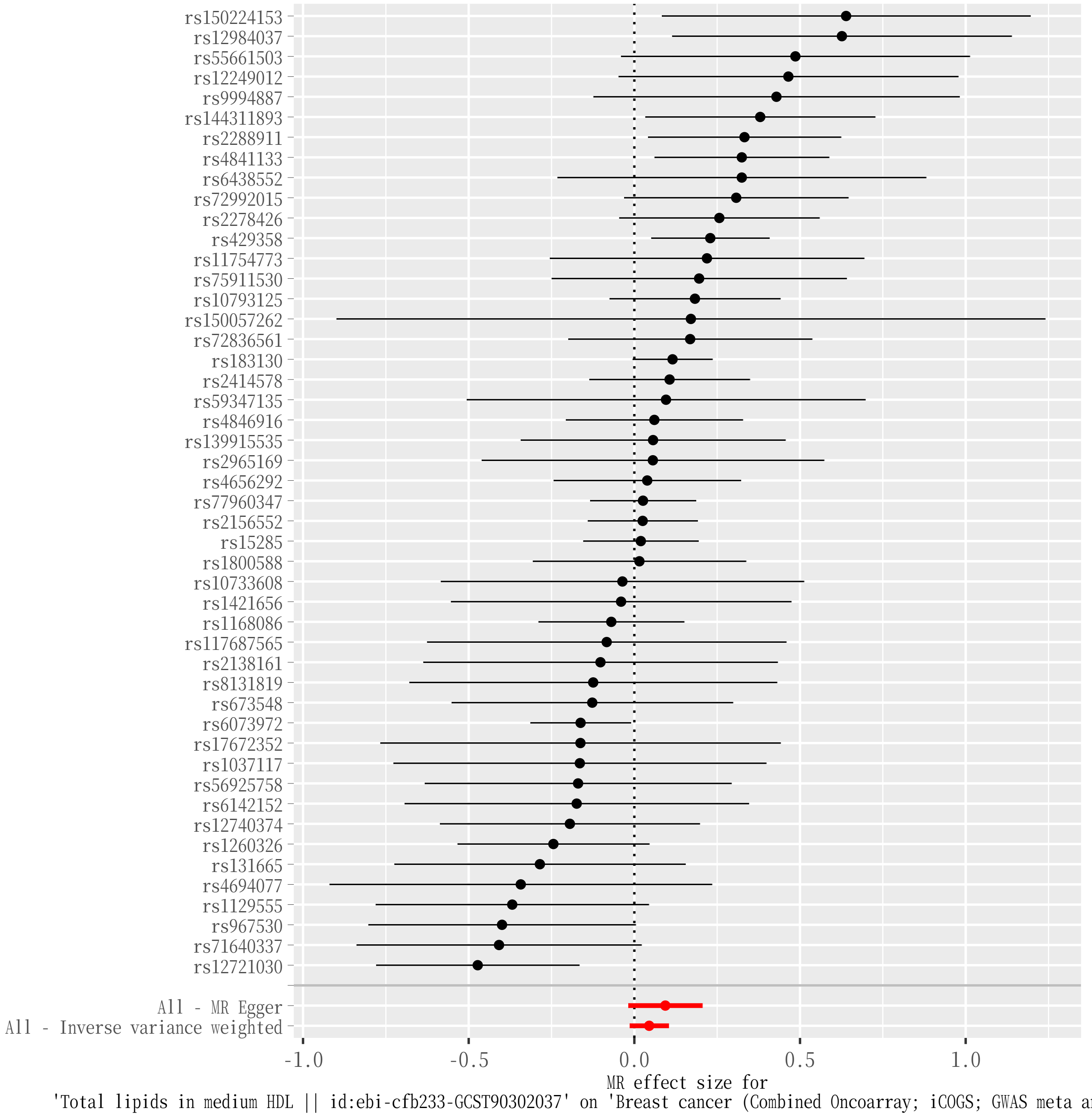
rs150224153
rs12984037
rs55661503
rs12249012
rs9994887
rs144311893
rs2288911
rs4841133
rs6438552
rs72992015
rs2278426
rs429358
rs11754773
rs75911530
rs10793125
rs150057262
rs72836561
rs183130
rs2414578
rs59347135
rs4846916
rs139915535
rs2965169
rs4656292
rs77960347
rs2156552
rs15285
rs1800588
rs10733608
rs1421656
rs1168086
rs117687565
rs2138161
rs8131819
rs673548
rs6073972
rs17672352
rs1037117
rs56925758
rs6142152
rs12740374
rs1260326
rs131665
rs4694077
rs1129555
rs967530
rs71640337
rs12721030
All - MR Egger
All - Inverse variance weighted
-1.0
-0.5
0.0
0.5
1.0
MR effect size for
'Total lipids in medium HDL || id:ebi-cfb233-GCST90302037' on 'Breast cancer (Combined Oncoarray; iCOGS; GWAS meta ar

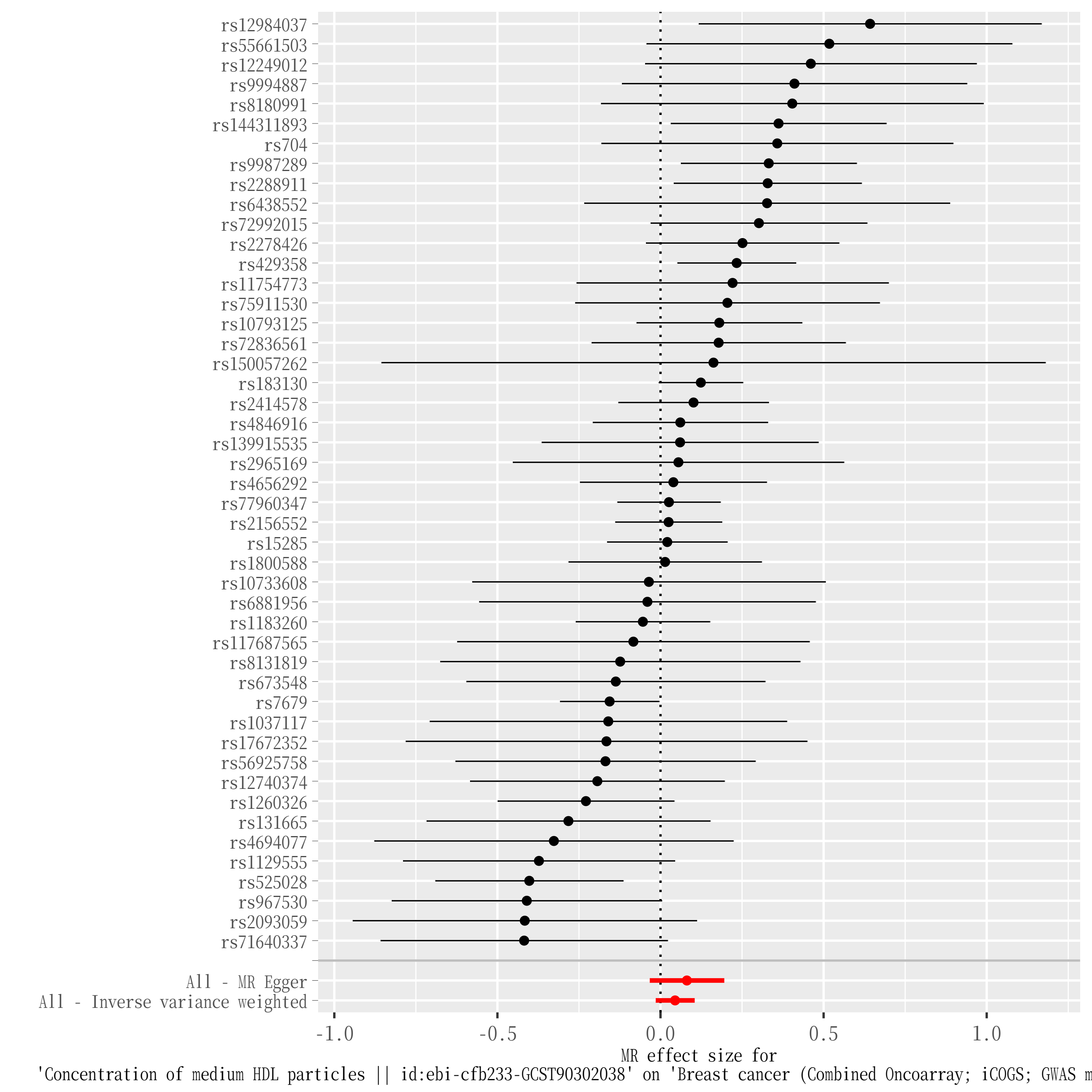
rs12984037
rs55661503
rs12249012
rs9994887
rs8180991
rs144311893
rs704
rs9987289
rs2288911
rs6438552
rs72992015
rs2278426
rs429358
rs11754773
rs75911530
rs10793125
rs72836561
rs150057262
rs183130
rs2414578
rs4846916
rs139915535
rs2965169
rs4656292
rs77960347
rs2156552
rs15285
rs1800588
rs10733608
rs6881956
rs1183260
rs117687565
rs8131819
rs673548
rs7679
rs1037117
rs17672352
rs56925758
rs12740374
rs1260326
rs131665
rs4694077
rs1129555
rs525028
rs967530
rs2093059
rs71640337
All - MR Egger
All - Inverse variance weighted
-1.0
-0.5
0.0
0.5
1.0
MR effect size for
'Concentration of medium HDL particles || id:ebi-cfb233-GCST90302038' on 'Breast cancer (Combined Oncoarray; iCOGS; GWAS m

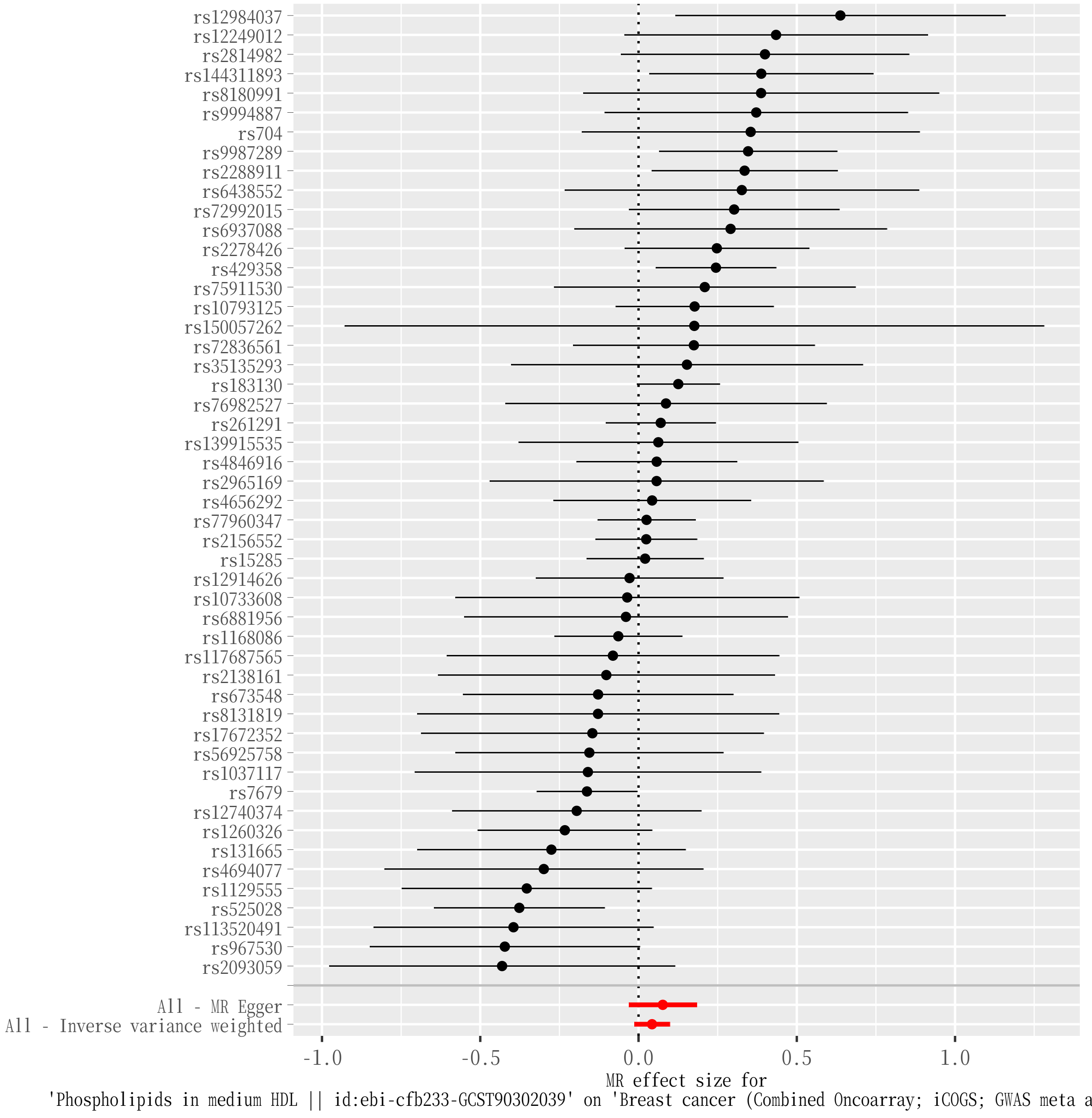

rs12984037
rs12249012
rs2814982
rs144311893
rs8180991
rs9994887
rs704
rs9987289
rs2288911
rs6438552
rs72992015
rs6937088
rs2278426
rs429358
rs75911530
rs10793125
rs150057262
rs72836561
rs35135293
rs183130
rs76982527
rs261291
rs139915535
rs4846916
rs2965169
rs4656292
rs77960347
rs2156552
rs15285
rs12914626
rs10733608
rs6881956
rs1168086
rs117687565
rs2138161
rs673548
rs8131819
rs17672352
rs56925758
rs1037117
rs7679
rs12740374
rs1260326
rs131665
rs4694077
rs1129555
rs525028
rs113520491
rs967530
rs2093059
All - MR Egger
All - Inverse variance weighted
-1.0
-0.5
0.0
0.5
1.0
MR effect size for
'Phospholipids in medium HDL || id:ebi-cfb233-GCST90302039' on 'Breast cancer (Combined Oncoarray; iCOGS; GWAS meta a

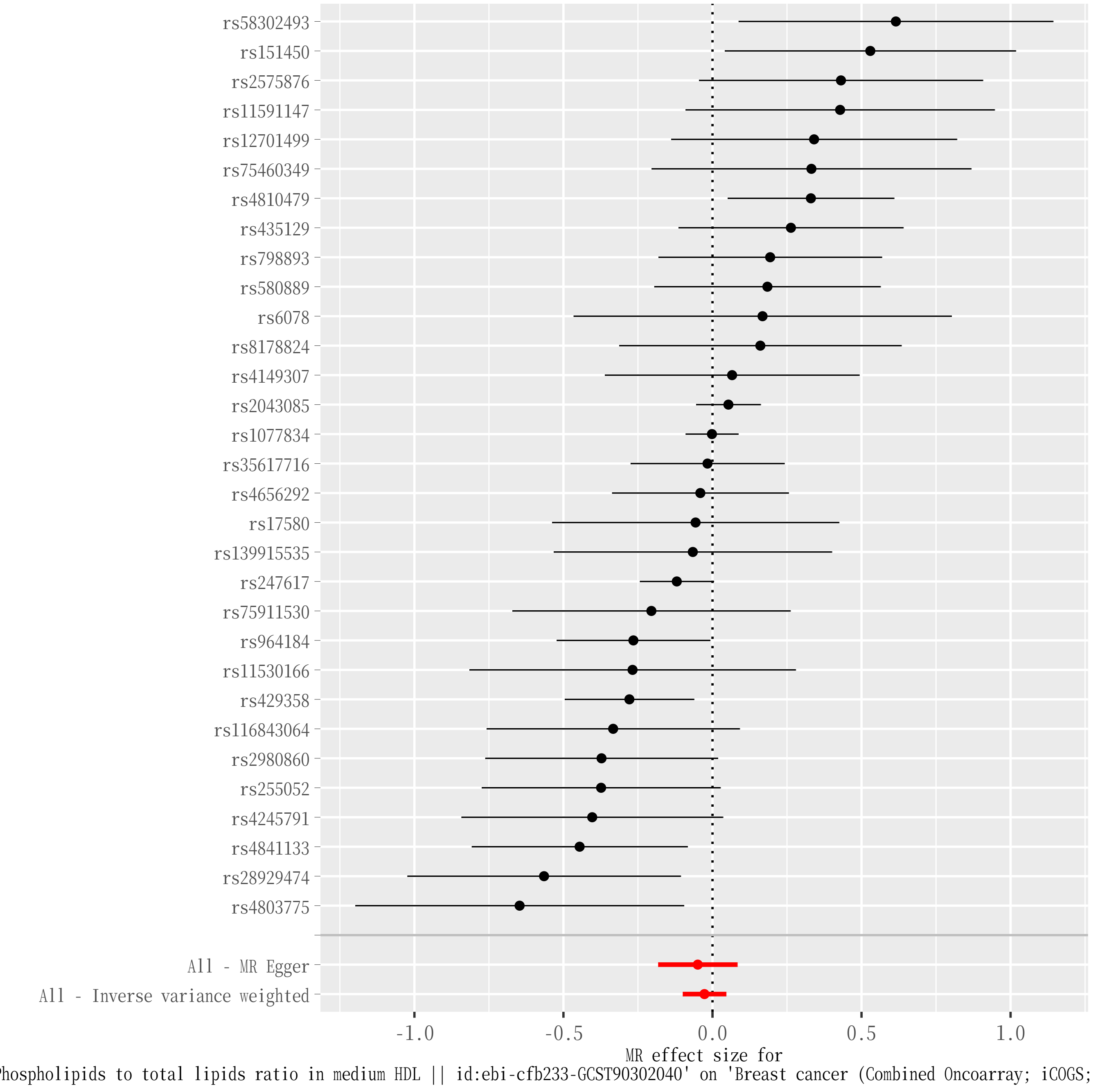
rs58302493
rs151450
rs2575876
rs11591147
rs12701499
rs75460349
rs4810479
rs435129
rs798893
rs580889
rs6078
rs8178824
rs4149307
rs2043085
rs1077834
rs35617716
rs4656292
rs17580
rs139915535
rs247617
rs75911530
rs964184
rs11530166
rs429358
rs116843064
rs2980860
rs255052
rs4245791
rs4841133
rs28929474
rs4803775
All - MR Egger
All - Inverse variance weighted
-1.0
-0.5
0.0
0.5
1.0
MR effect size for
Phospholipids to total lipids ratio in medium HDL || id:ebi-cfb233-GCST90302040' on 'Breast cancer (Combined Oncoarray; iCOGS;

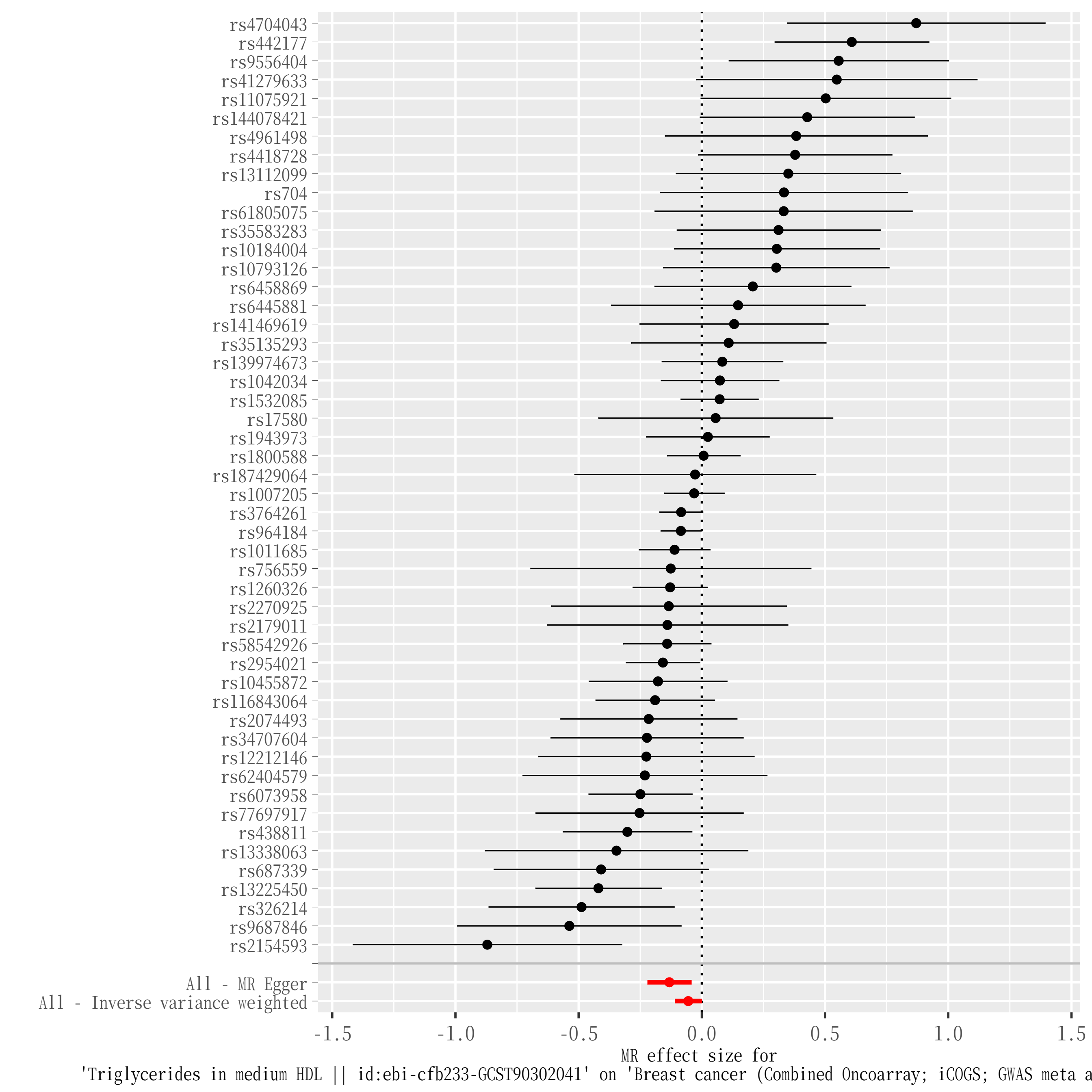
rs4704043
rs442177
rs9556404
rs41279633
rs11075921
rs144078421
rs4961498
rs4418728
rs13112099
rs704
rs61805075
rs35583283
rs10184004
rs10793126
rs6458869
rs6445881
rs141469619
rs35135293
rs139974673
rs1042034
rs1532085
rs17580
rs1943973
rs1800588
rs187429064
rs1007205
rs3764261
rs964184
rs1011685
rs756559
rs1260326
rs2270925
rs2179011
rs58542926
rs2954021
rs10455872
rs116843064
rs2074493
rs34707604
rs12212146
rs62404579
rs6073958
rs77697917
rs438811
rs13338063
rs687339
rs13225450
rs326214
rs9687846
rs2154593
All - MR Egger
All - Inverse variance weighted
-1.5
-1.0
-0.5
0.0
0.5
1.0
1.5
MR effect size for
'Triglycerides in medium HDL || id:ebi-cfb233-GCST90302041' on 'Breast cancer (Combined Oncoarray; iCOGS; GWAS meta a

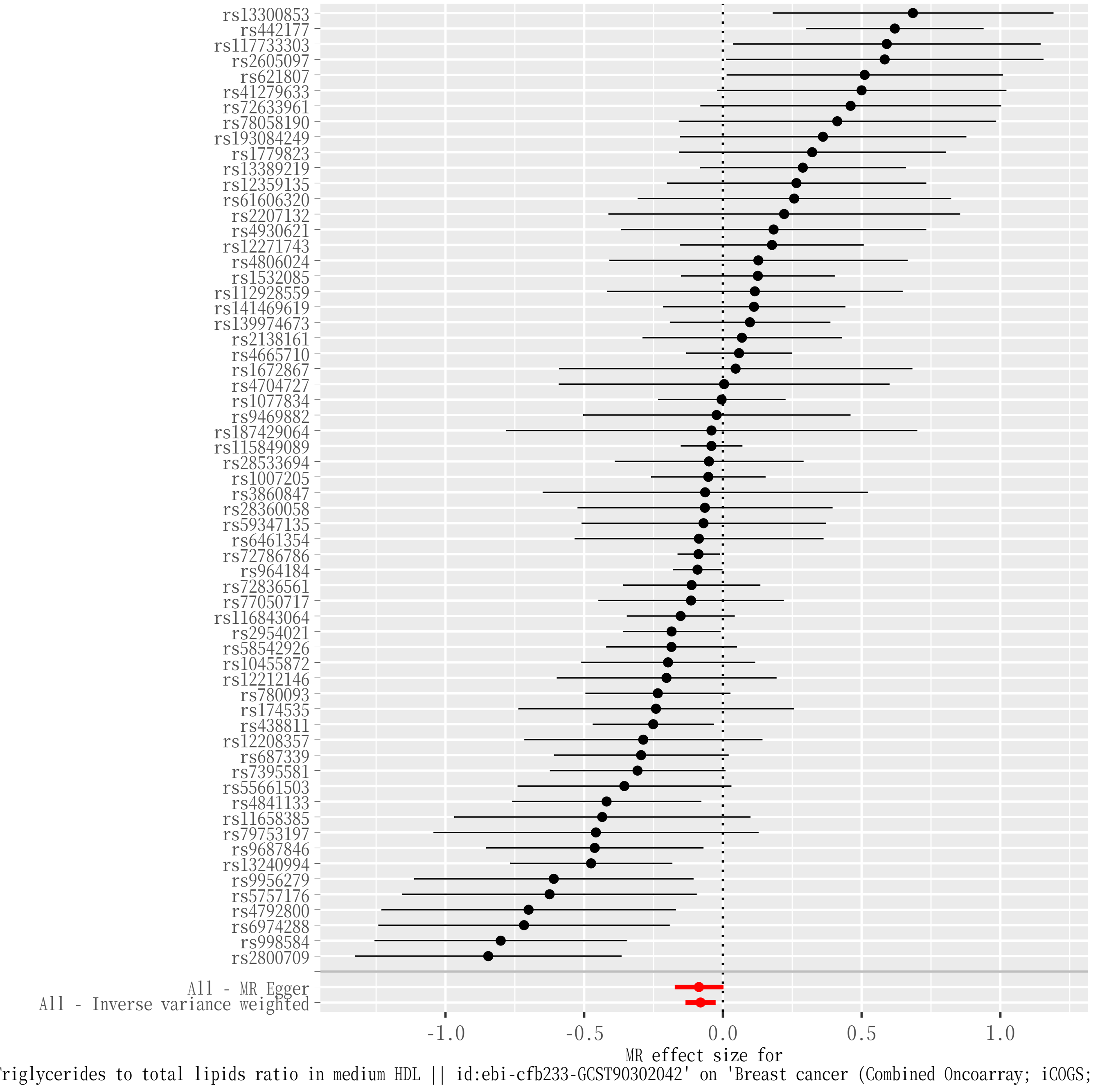
rs13300853
rs442177
rs117733303
rs2605097
rs621807
rs41279633
rs72633961
rs78058190
rs193084249
rs1779823
rs13389219
rs12359135
rs61606320
rs2207132
rs4930621
rs12271743
rs4806024
rs1532085
rs112928559
rs141469619
rs139974673
rs2138161
rs4665710
rs1672867
rs4704727
rs1077834
rs9469882
rs187429064
rs115849089
rs28533694
rs1007205
rs3860847
rs28360058
rs59347135
rs6461354
rs72786786
rs964184
rs72836561
rs77050717
rs116843064
rs2954021
rs58542926
rs10455872
rs12212146
rs780093
rs174535
rs438811
rs12208357
rs687339
rs7395581
rs55661503
rs4841133
rs11658385
rs79753197
rs9687846
rs13240994
rs9956279
rs5757176
rs4792800
rs6974288
rs998584
rs2800709
All - MR Egger
All - Inverse variance weighted
-1.0
-0.5
0.0
0.5
1.0
MR effect size for
'Triglycerides to total lipids ratio in medium HDL || id:ebi-cfb233-GCST90302042' on 'Breast cancer (Combined Oncoarray; iCOGS;

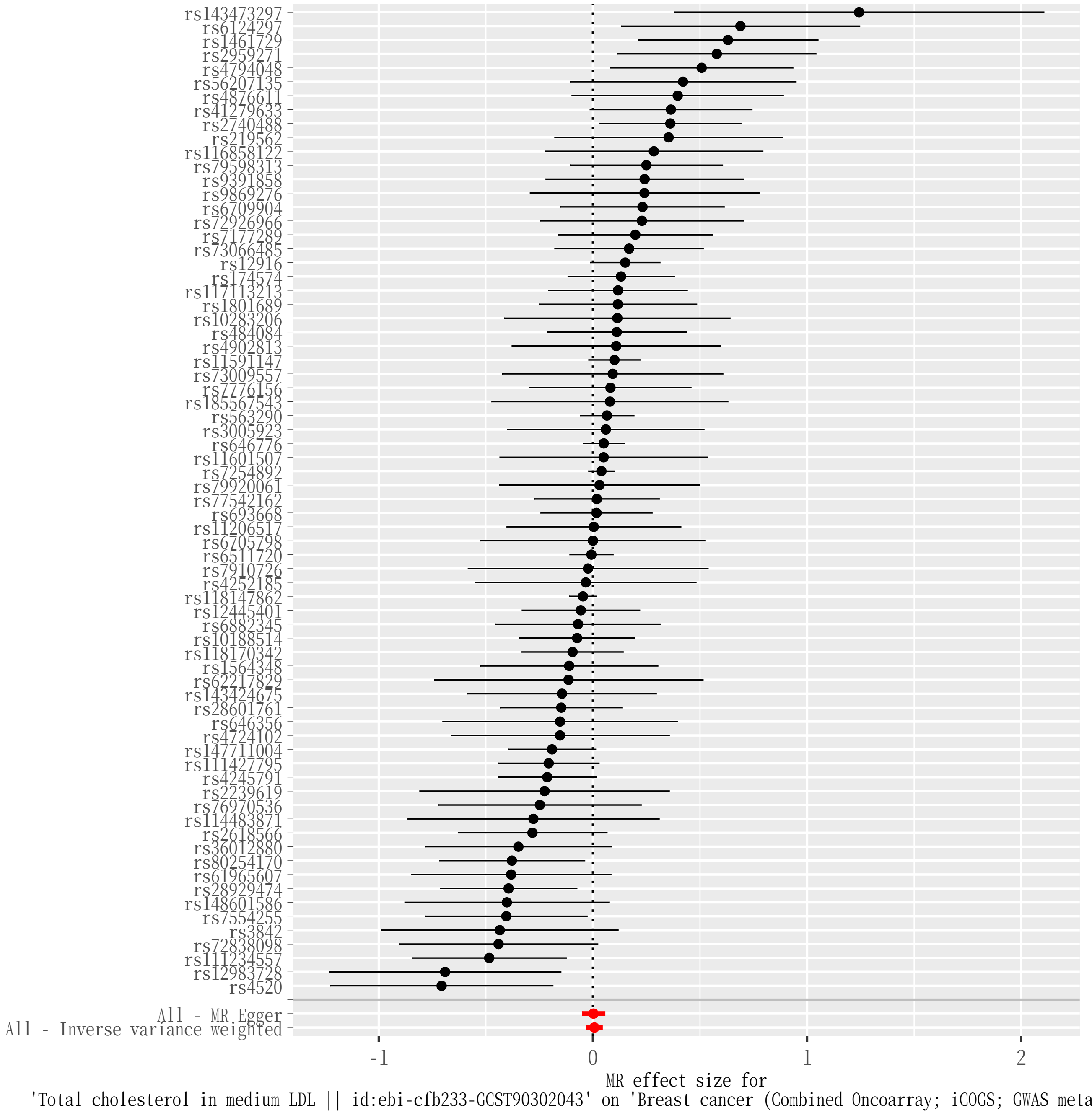
rs143473297
rs6124297
rs1461729
rs2959271
rs4794048
rs56207135
rs4876611
rs41279633
rs2740488
rs219562
rs116858122
rs79598313
rs9391858
rs9869276
rs6709904
rs72926966
rs7177289
rs73066485
rs12916
rs174574
rs117113213
rs1801689
rs10283206
rs484084
rs4902813
rs11591147
rs73009557
rs7776156
rs185567543
rs563290
rs3005923
rs646776
rs11601507
rs7254892
rs79920061
rs77542162
rs693668
rs11206517
rs6705798
rs6511720
rs7910726
rs4252185
rs118147862
rs12445401
rs6882345
rs10188514
rs118170342
rs1564348
rs62217829
rs143424675
rs28601761
rs646356
rs4724102
rs147711004
rs111427795
rs4245791
rs2239619
rs76970536
rs114483871
rs2618566
rs36012880
rs80254170
rs61965607
rs28929474
rs148601586
rs7554255
rs3842
rs72838098
rs111234557
rs12983728
rs4520
All - MR Egger
All - Inverse variance weighted
-1
0
1
2
MR effect size for
'Total cholesterol in medium LDL || id:ebi-cfb233-GCST90302043' on 'Breast cancer (Combined Oncoarray; iCOGS; GWAS meta

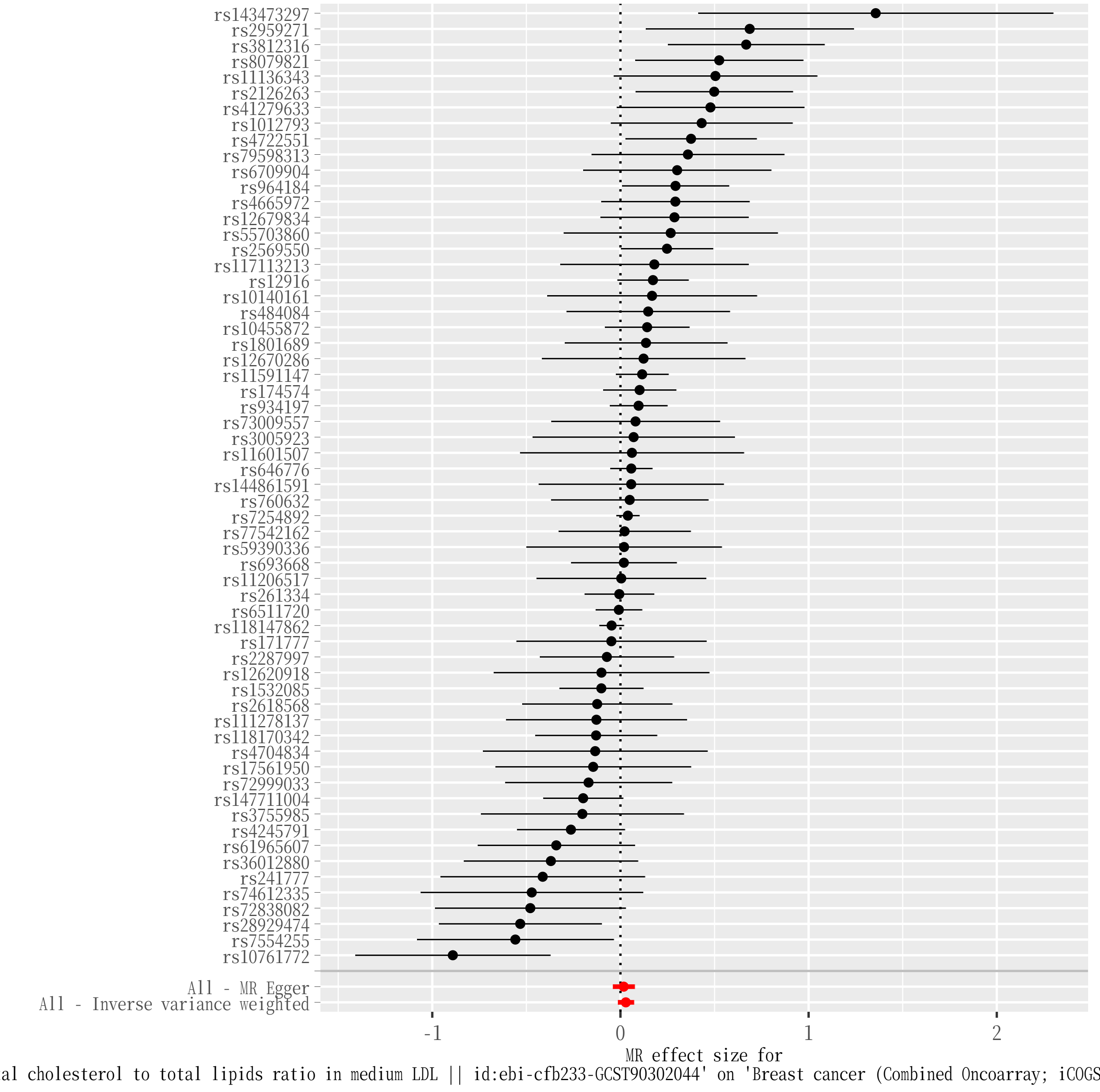
rs143473297
rs2959271
rs3812316
rs8079821
rs11136343
rs2126263
rs41279633
rs1012793
rs4722551
rs79598313
rs6709904
rs964184
rs4665972
rs12679834
rs55703860
rs2569550
rs117113213
rs12916
rs10140161
rs484084
rs10455872
rs1801689
rs12670286
rs11591147
rs174574
rs934197
rs73009557
rs3005923
rs11601507
rs646776
rs144861591
rs760632
rs7254892
rs77542162
rs59390336
rs693668
rs11206517
rs261334
rs6511720
rs118147862
rs171777
rs2287997
rs12620918
rs1532085
rs2618568
rs111278137
rs118170342
rs4704834
rs17561950
rs72999033
rs147711004
rs3755985
rs4245791
rs61965607
rs36012880
rs241777
rs74612335
rs72838082
rs28929474
rs7554255
rs10761772
All - MR Egger
All - Inverse variance weighted
-1
0
1
2
MR effect size for
al cholesterol to total lipids ratio in medium LDL || id:ebi-cfb233-GCST90302044' on 'Breast cancer (Combined Oncoarray; iCOGS

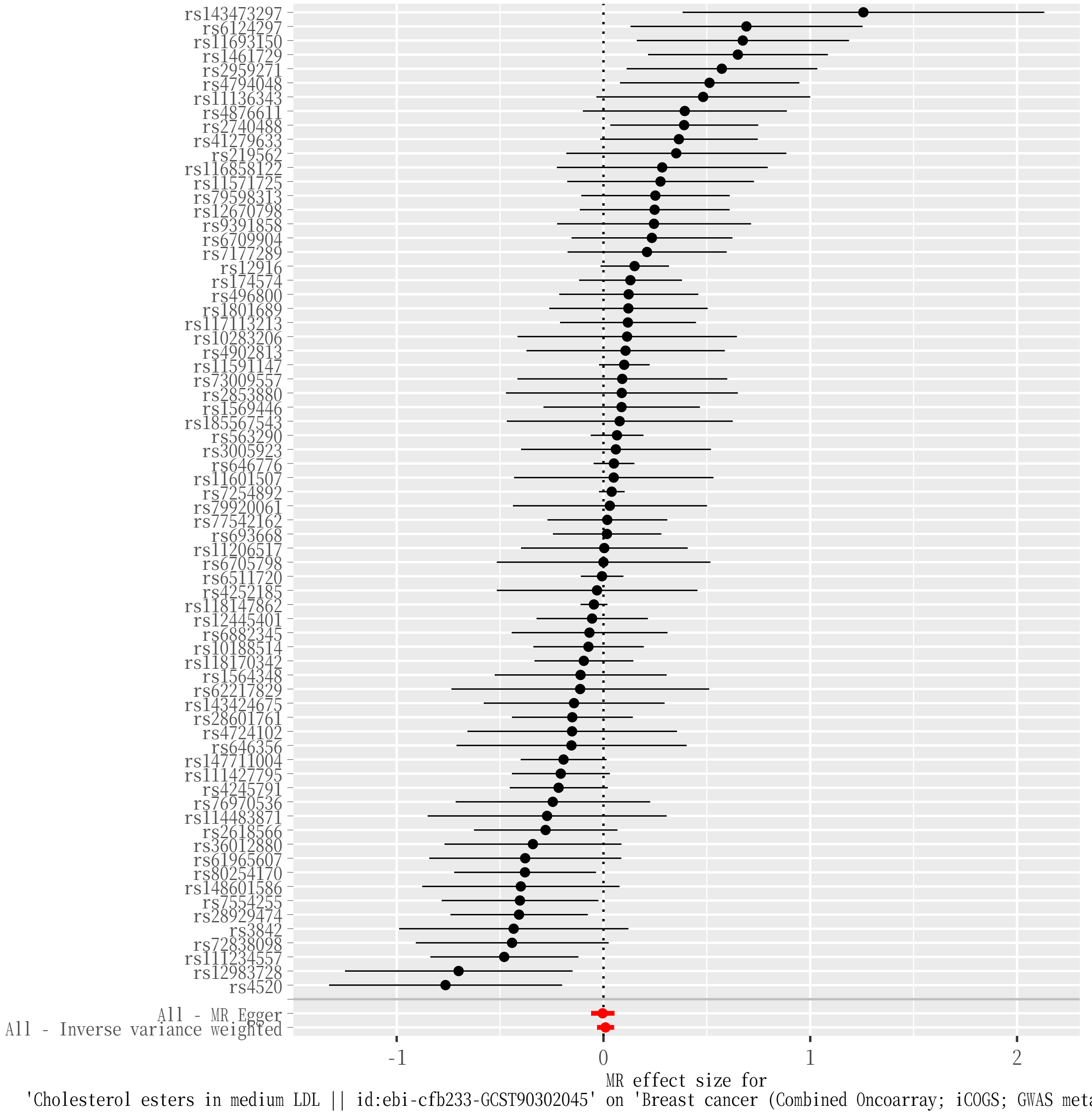
rs143473297
rs6124297
rs11693150
rs1461729
rs2959271
rs4794048
rs11136343
rs4876611
rs2740488
rs41279633
rs219562
rs116858122
rs11571725
rs79598313
rs12670798
rs9391858
rs6709904
rs7177289
rs12916
rs174574
rs496800
rs1801689
rs117113213
rs10283206
rs4902813
rs11591147
rs73009557
rs2853880
rs1569446
rs185567543
rs563290
rs3005923
rs646776
rs11601507
rs7254892
rs79920061
rs77542162
rs693668
rs11206517
rs6705798
rs6511720
rs4252185
rs118147862
rs12445401
rs6882345
rs10188514
rs118170342
rs1564348
rs62217829
rs143424675
rs28601761
rs4724102
rs646356
rs147711004
rs111427795
rs4245791
rs76970536
rs114483871
rs2618566
rs36012880
rs61965607
rs80254170
rs148601586
rs7554255
rs28929474
rs3842
rs72838098
rs111234557
rs12983728
rs4520
All - MR Egger
All - Inverse variance weighted
-1
0
1
2
MR effect size for
'Cholesterol esters in medium LDL || id:ebi-cfb233-GCST90302045' on 'Breast cancer (Combined Oncoarray; iCOGS; GWAS meta

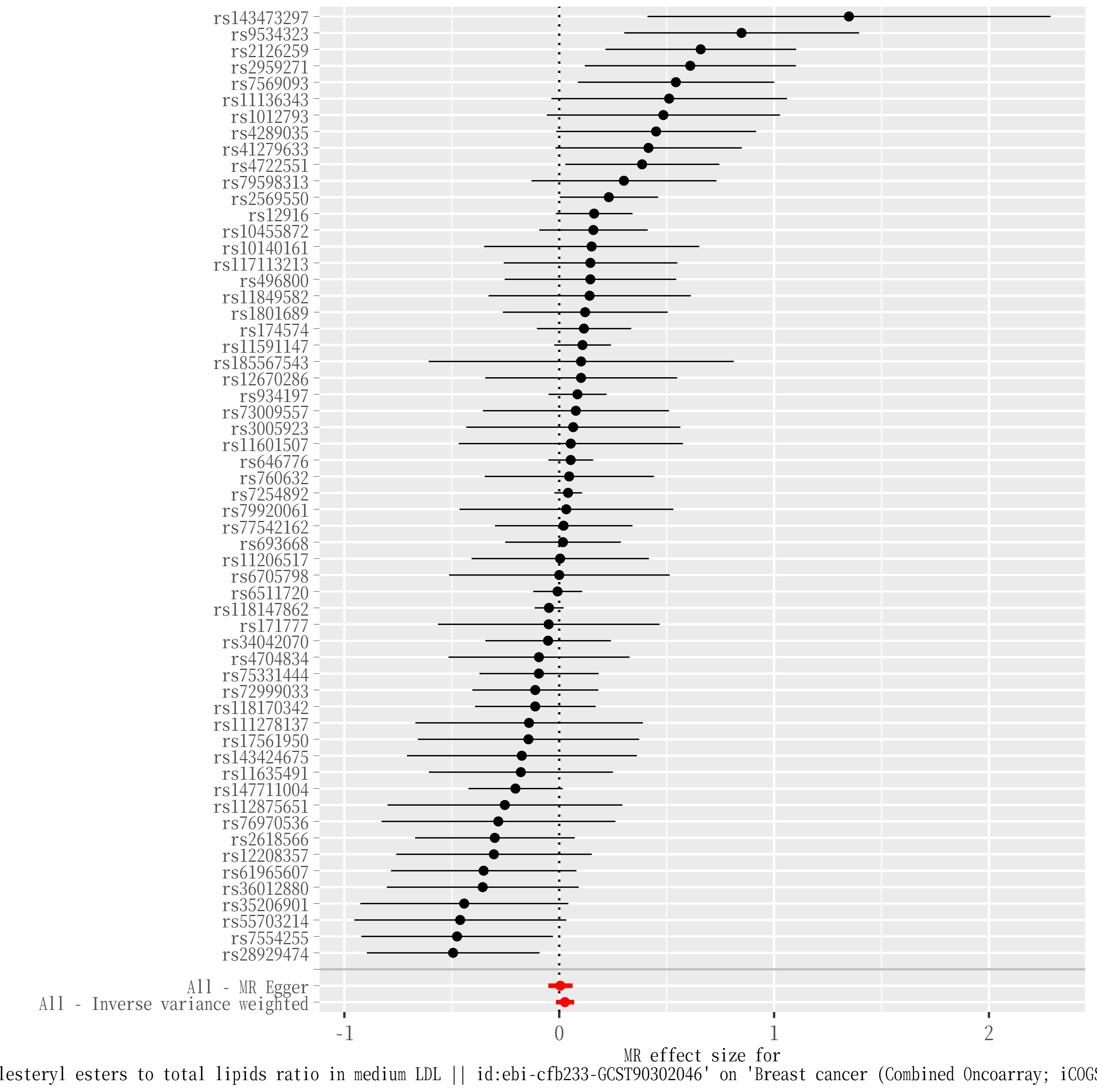

rs143473297
rs9534323
rs2126259
rs2959271
rs7569093
rs11136343
rs1012793
rs4289035
rs41279633
rs4722551
rs79598313
rs2569550
rs12916
rs10455872
rs10140161
rs117113213
rs496800
rs11849582
rs1801689
rs174574
rs11591147
rs185567543
rs12670286
rs934197
rs73009557
rs3005923
rs11601507
rs646776
rs760632
rs7254892
rs79920061
rs77542162
rs693668
rs11206517
rs6705798
rs6511720
rs118147862
rs171777
rs34042070
rs4704834
rs75331444
rs72999033
rs118170342
rs111278137
rs17561950
rs143424675
rs11635491
rs147711004
rs112875651
rs76970536
rs2618566
rs12208357
rs61965607
rs36012880
rs35206901
rs55703214
rs7554255
rs28929474
All - MR Egger
All - Inverse variance weighted
-1
0
1
2
MR effect size for
lesteryl esters to total lipids ratio in medium LDL || id:ebi-cfb233-GCST90302046' on 'Breast cancer (Combined Oncoarray; iCOGS

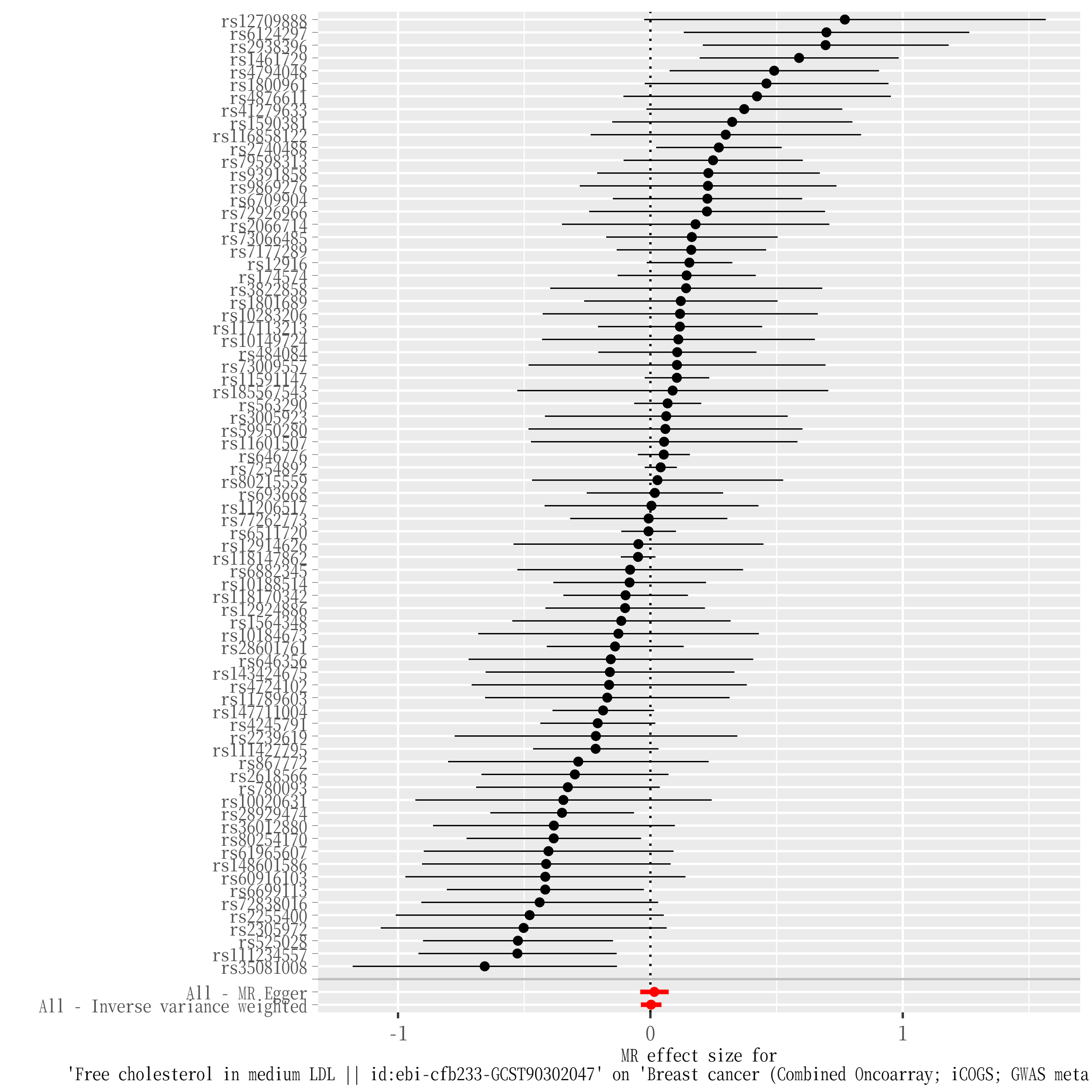

rs12709888
rs6124297
rs2938396
rs1461729
rs4794048
rs1800961
rs4876611
rs41279633
rs1590381
rs116858122
rs2740488
rs79598313
rs9391858
rs9869276
rs6709904
rs72926966
rs2066714
rs73066485
rs7177289
rs12916
rs174574
rs3822858
rs1801689
rs10283206
rs117113213
rs10149724
rs484084
rs73009557
rs11591147
rs185567543
rs563290
rs3005923
rs59950280
rs11601507
rs646776
rs7254892
rs80215559
rs693668
rs11206517
rs77262773
rs6511720
rs12914626
rs118147862
rs6882345
rs10188514
rs118170342
rs12924886
rs1564348
rs10184673
rs28601761
rs646356
rs143424675
rs4724102
rs11789603
rs147711004
rs4245791
rs2239619
rs111427725
rs867772
rs2618566
rs780093
rs10020631
rs28929474
rs36012880
rs80254170
rs61965607
rs148601586
rs60916103
rs6699113
rs72838016
rs2255400
rs2305972
rs525028
rs111234557
rs35081008
All - MR Egger
All - Inverse variance weighted
-1
0
1
MR effect size for
'Free cholesterol in medium LDL || id:ebi-cfb233-GCST90302047' on 'Breast cancer (Combined Oncoarray; iCOGS; GWAS meta

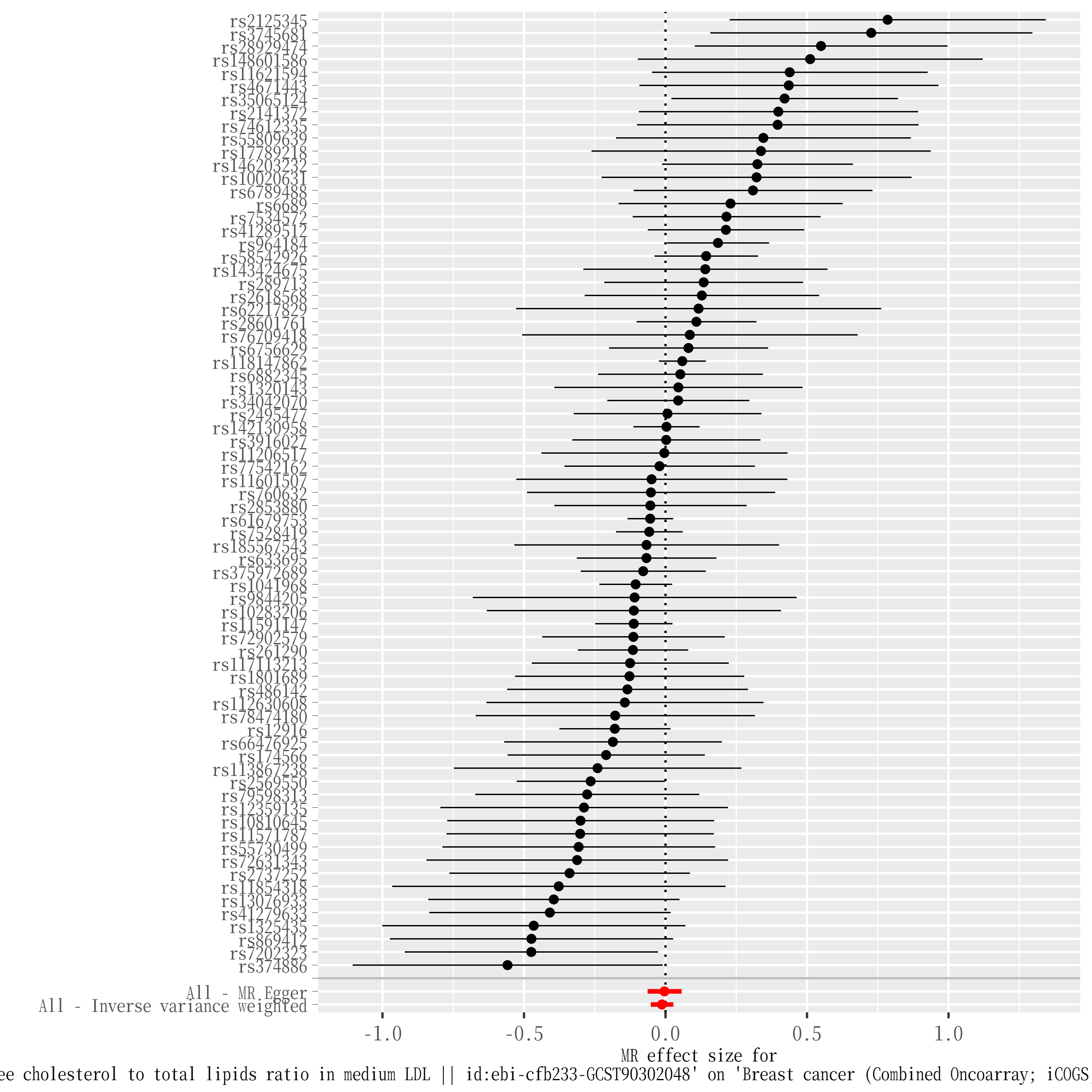
rs2125345
rs3745681
rs28929474
rs148601586
rs11621594
rs4671443
rs35065124
rs2141372
rs74612335
rs55809639
rs17789218
rs146203232
rs10020631
rs6789488
rs6689
rs7534572
rs41289512
rs964184
rs58542926
rs143424675
rs289713
rs2618568
rs62217829
rs28601761
rs76709418
rs6756629
rs118147862
rs6882345
rs1320143
rs34042070
rs2495477
rs142130958
rs3916027
rs11206517
rs77542162
rs11601507
rs760632
rs2853880
rs61679753
rs7528419
rs185567543
rs633695
rs375972689
rs1041968
rs9844205
rs10283206
rs11591147
rs72902579
rs261290
rs117113213
rs1801689
rs486142
rs112630608
rs78474180
rs12916
rs66476925
rs174566
rs113867238
rs2569550
rs79598313
rs12359135
rs10810645
rs11571787
rs55730499
rs72631343
rs2737252
rs11854318
rs13076933
rs41279633
rs1325435
rs869412
rs7202323
rs374886
All - MR Egger
All - Inverse variance weighted
-1.0
-0.5
0.0
0.5
1.0
MR effect size for
ee cholesterol to total lipids ratio in medium LDL || id:ebi-cfb233-GCST90302048' on 'Breast cancer (Combined Oncoarray; iCOGS

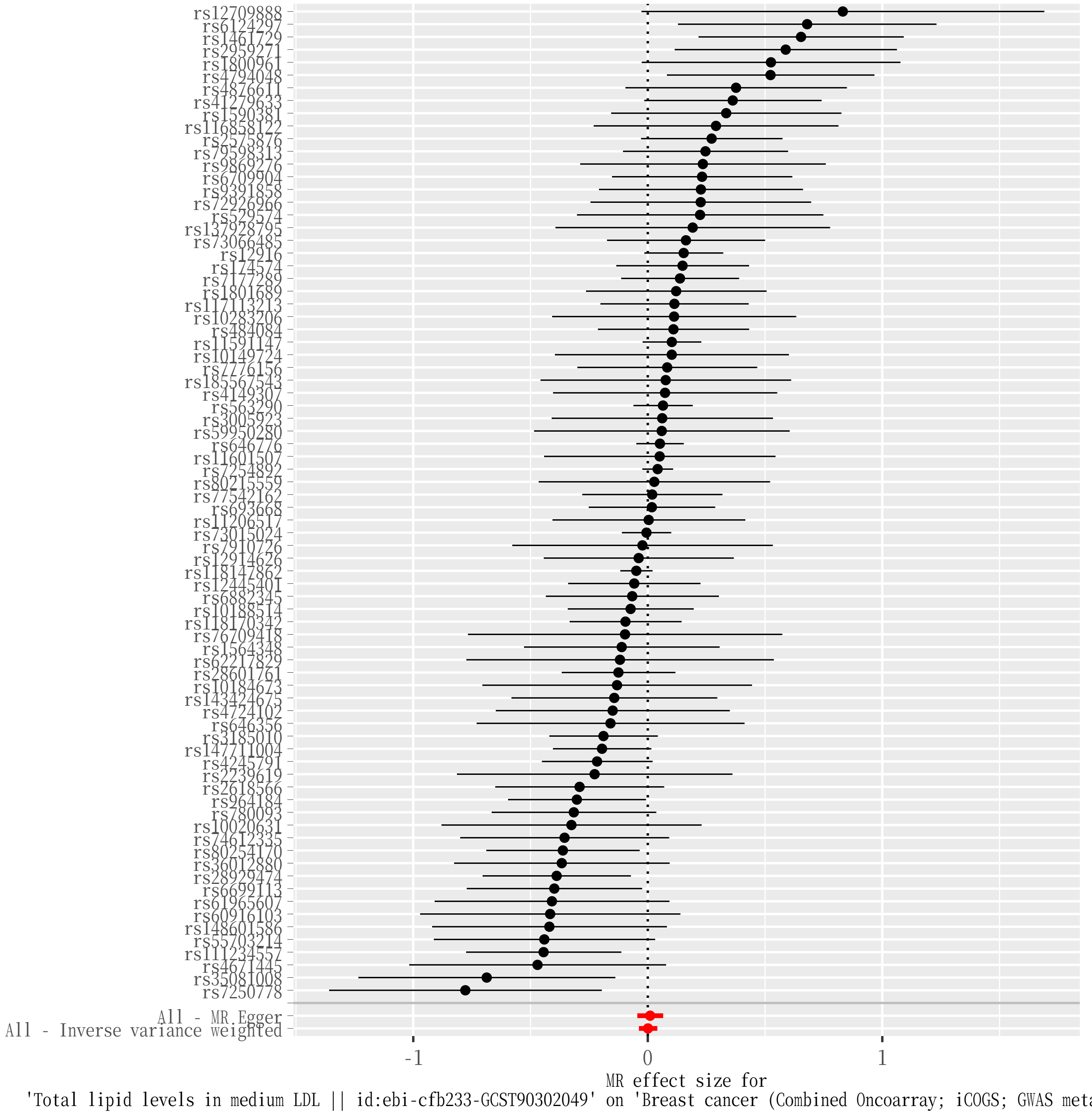
rs12709888
rs6124297
rs1461729
rs2959271
rs1800961
rs4794048
rs4876611
rs41279633
rs1590381
rs116858122
rs2575876
rs79598313
rs9869276
rs6709904
rs9391858
rs72926966
rs529574
rs137928795
rs73066485
rs12916
rs174574
rs7177289
rs1801689
rs117113213
rs10283206
rs484084
rs11591147
rs10149724
rs7776156
rs185567543
rs4149307
rs563290
rs3005923
rs59950280
rs646776
rs11601507
rs7254892
rs80215559
rs77542162
rs693668
rs11206517
rs73015024
rs7910776
rs12914626
rs118147862
rs12445401
rs6882345
rs10188514
rs118170342
rs76709418
rs1564348
rs62217829
rs28601761
rs10184673
rs143424675
rs4724102
rs646356
rs3185010
rs147711004
rs4245791
rs2239619
rs2618566
rs264184
rs780093
rs10020631
rs74612335
rs80254170
rs36012880
rs28929474
rs6699113
rs61965607
rs60916103
rs148601586
rs55703214
rs111234557
rs4671445
rs35081008
rs7250778
All - MR Egger
All - Inverse variance weighted
-1
0
1
MR effect size for
'Total lipid levels in medium LDL || id:ebi-cfb233-GCST90302049' on 'Breast cancer (Combined Oncoarray; iCOGS; GWAS meta

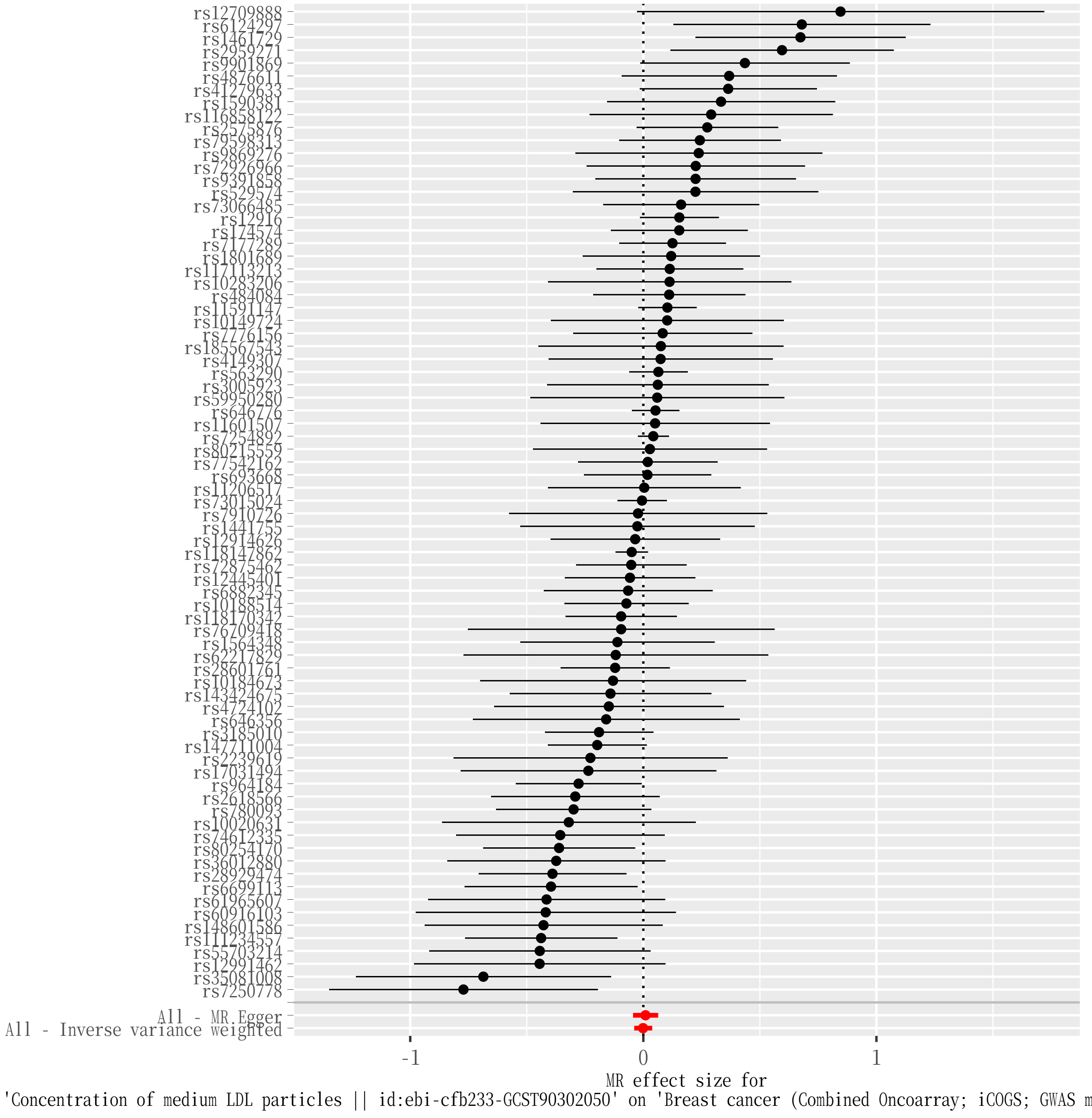
rs12709888
rs6124297
rs1461729
rs2959271
rs9901869
rs4876611
rs41279633
rs1590381
rs116858122
rs2575876
rs79598313
rs9869276
rs72926966
rs9391858
rs529574
rs73066485
rs12916
rs174574
rs7177289
rs1801689
rs117113213
rs10283206
rs484084
rs11591147
rs10149724
rs7776156
rs185567543
rs4149307
rs563290
rs3005923
rs59950280
rs646776
rs11601507
rs7254892
rs80215559
rs77542162
rs693668
rs11206517
rs73015024
rs7910726
rs1441755
rs12914626
rs118147862
rs72875462
rs12445401
rs6882345
rs10188514
rs118170342
rs76709418
rs1564348
rs62217829
rs28601761
rs10184673
rs143424675
rs4724102
rs646356
rs3185010
rs147711004
rs2239619
rs17031494
rs964184
rs2618566
rs780093
rs10020631
rs74612335
rs80254170
rs36012880
rs28929474
rs6699113
rs61965607
rs60916103
rs148601586
rs111234557
rs55703214
rs12991462
rs35081008
rs7250778
All - MR Egger
All - Inverse variance weighted
-1
0
1
MR effect size for
'Concentration of medium LDL particles || id:ebi-cfb233-GCST90302050' on 'Breast cancer (Combined Oncoarray; iCOGS; GWAS m

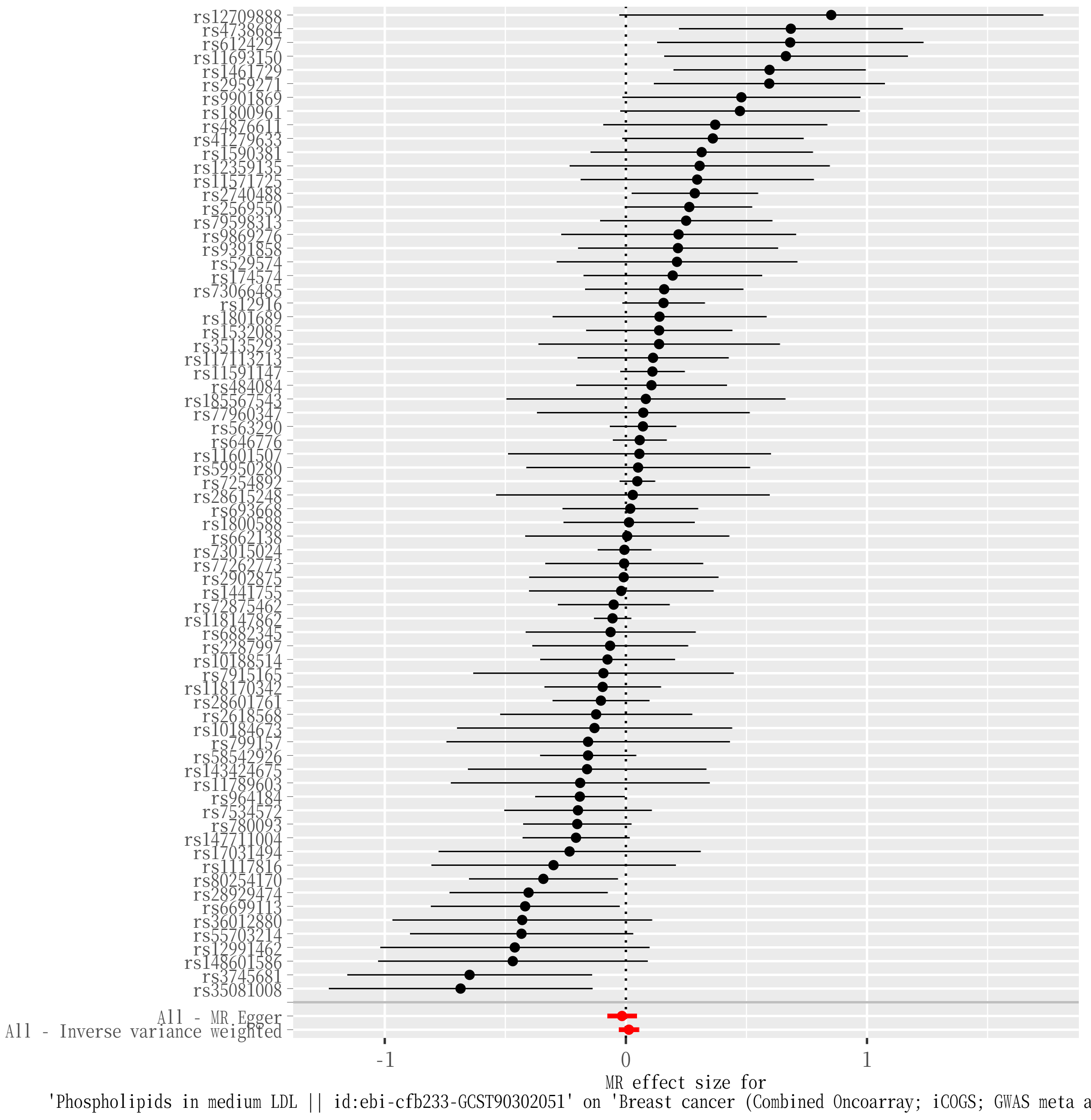
rs12709888
rs4738684
rs6124297
rs11693150
rs1461729
rs2959271
rs9901869
rs1800961
rs4876611
rs41279633
rs1590381
rs12359135
rs11571725
rs2740488
rs2569550
rs79598313
rs9869276
rs9391858
rs529574
rs174574
rs73066485
rs12916
rs1801689
rs1532085
rs35135293
rs117113213
rs11591147
rs484084
rs185567543
rs77960347
rs563290
rs646776
rs11601507
rs59950280
rs7254892
rs28615248
rs693668
rs1800588
rs662138
rs73015024
rs77262773
rs2902875
rs1441755
rs72875462
rs118147862
rs6882345
rs2287997
rs10188514
rs7915165
rs118170342
rs28601761
rs2618568
rs10184673
rs799157
rs58542926
rs143424675
rs11789603
rs964184
rs7534572
rs780093
rs147711004
rs17031494
rs1117816
rs80254170
rs28929474
rs6699113
rs36012880
rs55703214
rs12991462
rs148601586
rs3745681
rs35081008
All - MR Egger
All - Inverse variance weighted
-1
0
1
MR effect size for
'Phospholipids in medium LDL || id:ebi-cfb233-GCST90302051' on 'Breast cancer (Combined Oncoarray; iCOGS; GWAS meta a

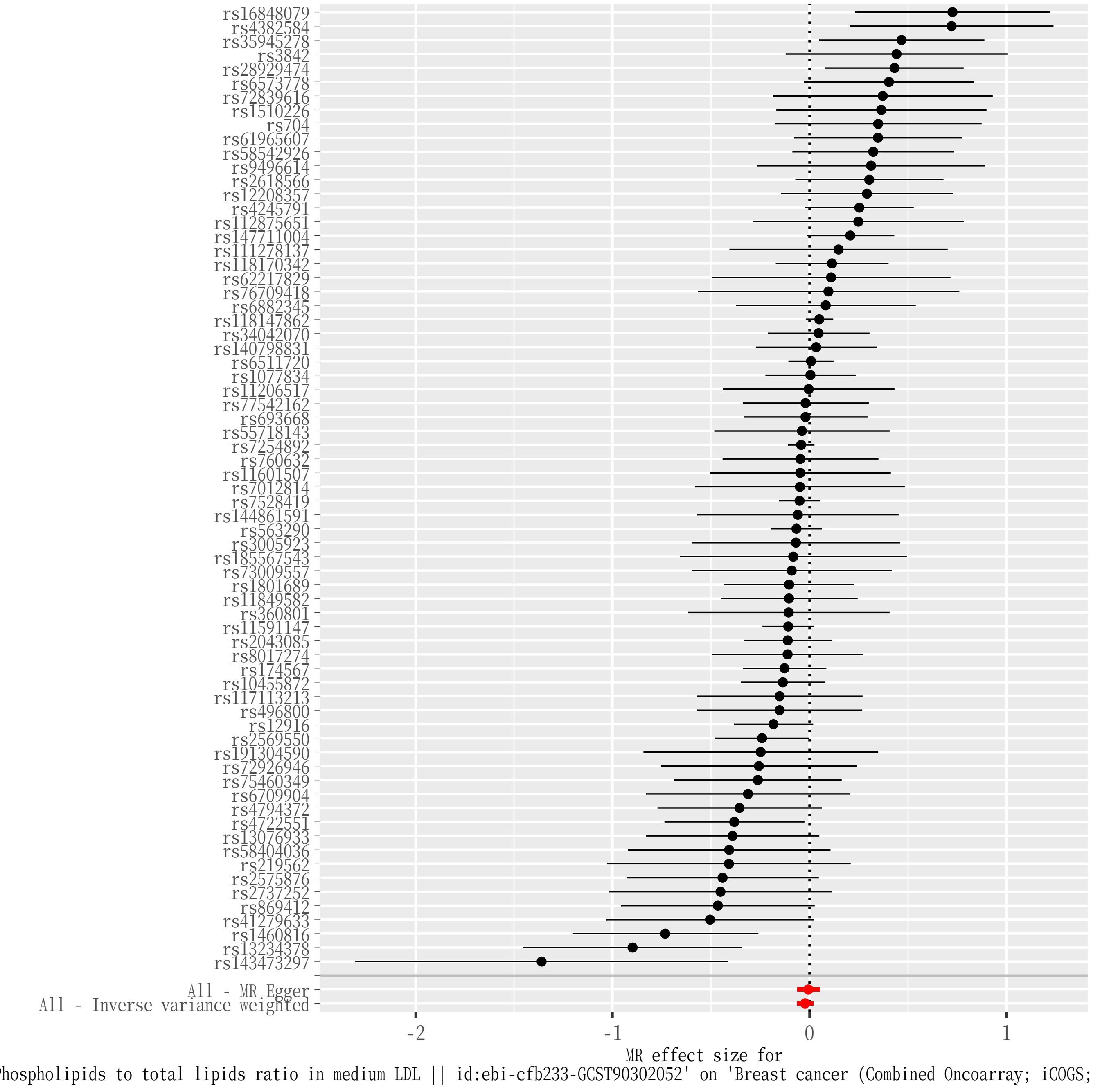
rs16848079
rs4382584
rs35945278
rs3842
rs28929474
rs6573778
rs72839616
rs1510226
rs704
rs61965607
rs58542926
rs9496614
rs2618566
rs12208357
rs4245791
rs112875651
rs147711004
rs111278137
rs118170342
rs62217829
rs76709418
rs6882345
rs118147862
rs34042070
rs140798831
rs6511720
rs1077834
rs11206517
rs77542162
rs693668
rs55718143
rs7254892
rs760632
rs11601507
rs7012814
rs7528419
rs144861591
rs563290
rs3005923
rs185567543
rs73009557
rs1801689
rs11849582
rs360801
rs11591147
rs2043085
rs8017274
rs174567
rs10455872
rs117113213
rs496800
rs12916
rs2569550
rs191304590
rs72926946
rs75460349
rs6709904
rs4794372
rs4722551
rs13076933
rs58404036
rs219562
rs2575876
rs2737252
rs869412
rs41279633
rs1460816
rs13234378
rs143473297
All - MR Egger
All - Inverse variance weighted
-2
-1
0
1
MR effect size for
Phospholipids to total lipids ratio in medium LDL || id:ebi-cfb233-GCST90302052' on 'Breast cancer (Combined Oncoarray; iCOGS;

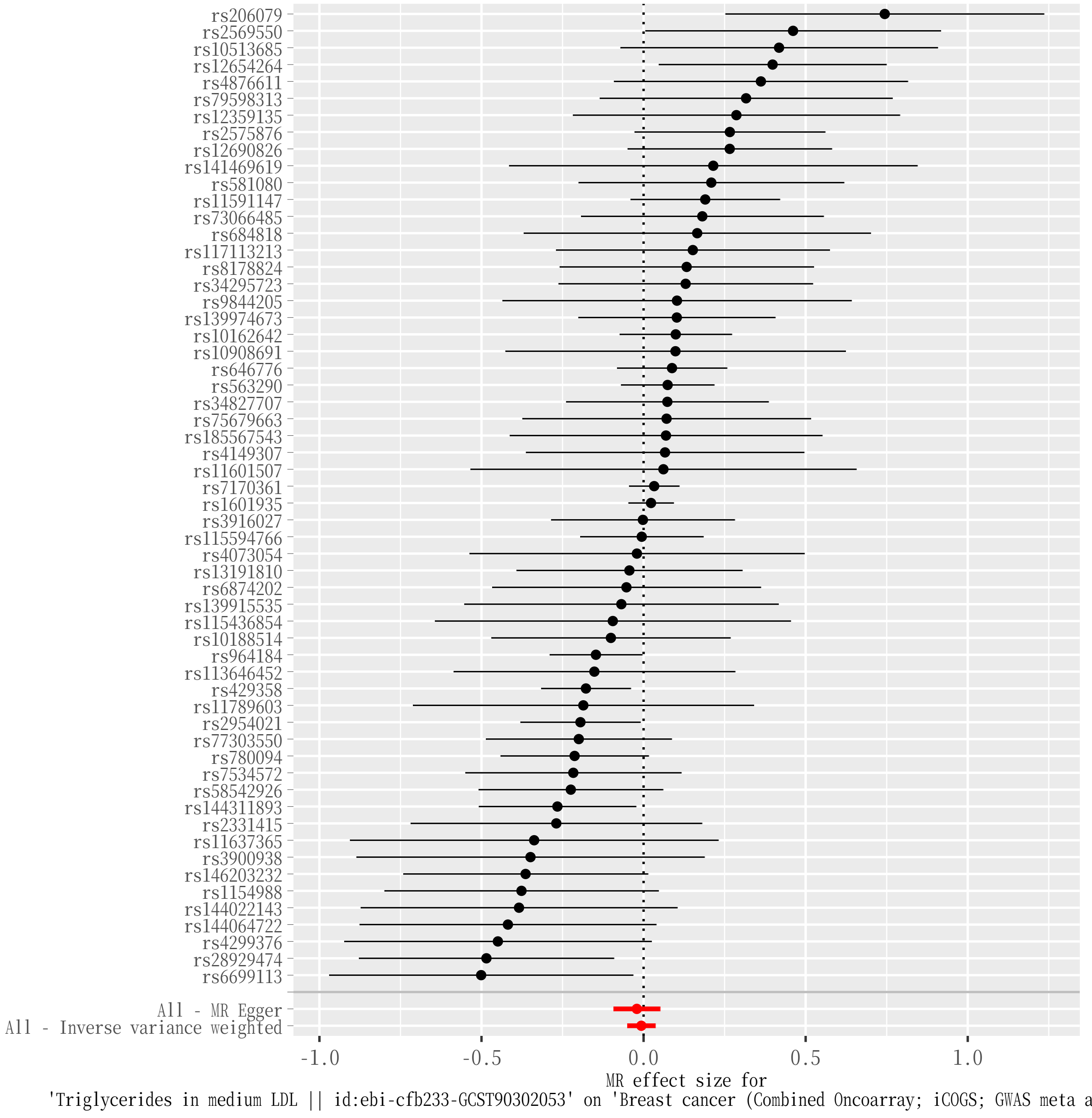

rs206079
rs2569550
rs10513685
rs12654264
rs4876611
rs79598313
rs12359135
rs2575876
rs12690826
rs141469619
rs581080
rs11591147
rs73066485
rs684818
rs117113213
rs8178824
rs34295723
rs9844205
rs139974673
rs10162642
rs10908691
rs646776
rs563290
rs34827707
rs75679663
rs185567543
rs4149307
rs11601507
rs7170361
rs1601935
rs3916027
rs115594766
rs4073054
rs13191810
rs6874202
rs139915535
rs115436854
rs10188514
rs964184
rs113646452
rs429358
rs11789603
rs2954021
rs77303550
rs780094
rs7534572
rs58542926
rs144311893
rs2331415
rs11637365
rs3900938
rs146203232
rs1154988
rs144022143
rs144064722
rs4299376
rs28929474
rs6699113
All - MR Egger
All - Inverse variance weighted
-1.0
-0.5
0.0
0.5
1.0
MR effect size for
'Triglycerides in medium LDL || id:ebi-cfb233-GCST90302053' on 'Breast cancer (Combined Oncoarray; iCOGS; GWAS meta a

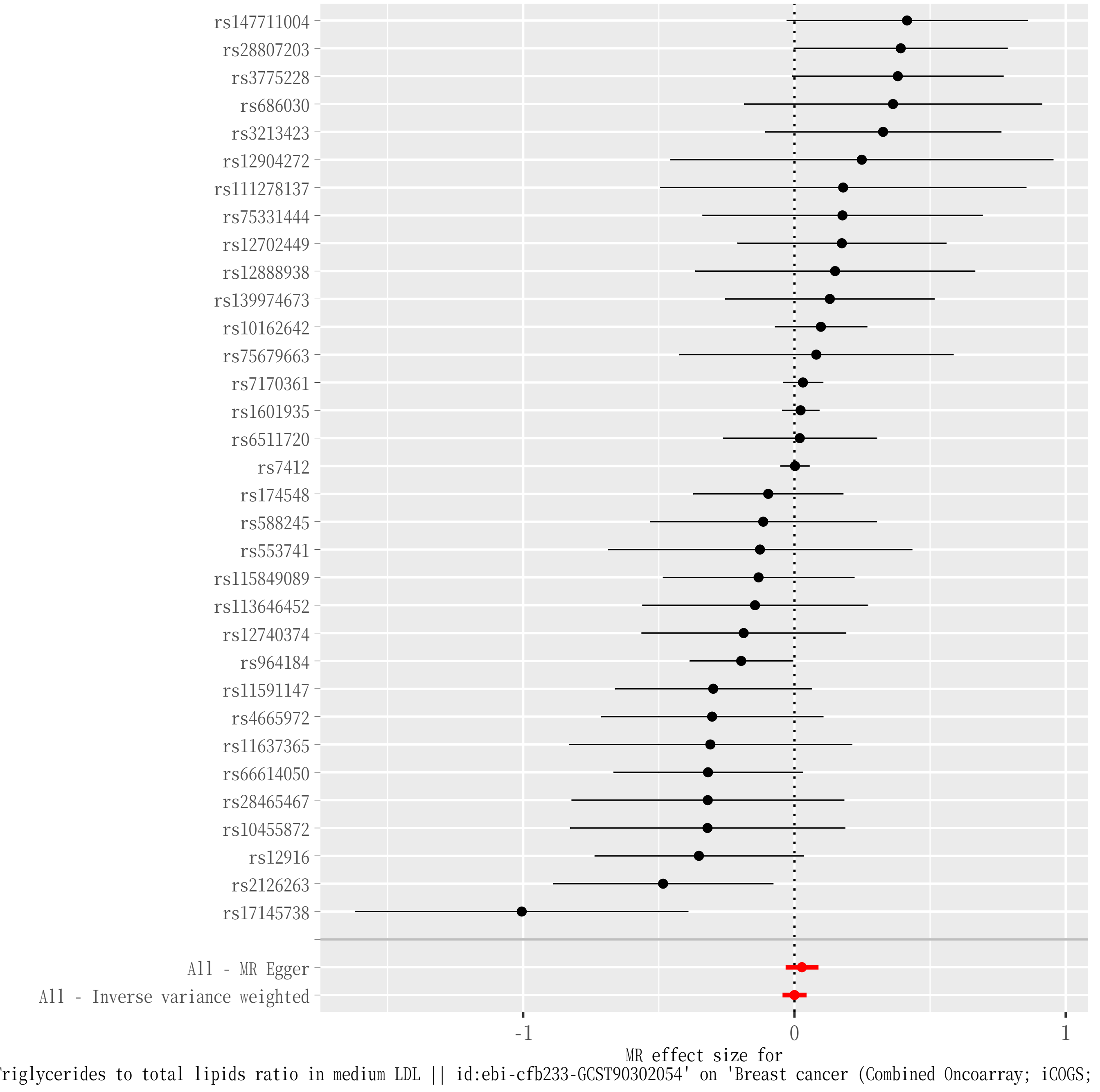
rs147711004
rs28807203
rs3775228
rs686030
rs3213423
rs12904272
rs111278137
rs75331444
rs12702449
rs12888938
rs139974673
rs10162642
rs75679663
rs7170361
rs1601935
rs6511720
rs7412
rs174548
rs588245
rs553741
rs115849089
rs113646452
rs12740374
rs964184
rs11591147
rs4665972
rs11637365
rs66614050
rs28465467
rs10455872
rs12916
rs2126263
rs17145738
All - MR Egger
All - Inverse variance weighted
-1
0
1
MR effect size for
'riglycerides to total lipids ratio in medium LDL || id:ebi-cfb233-GCST90302054' on 'Breast cancer (Combined Oncoarray; iCOGS;

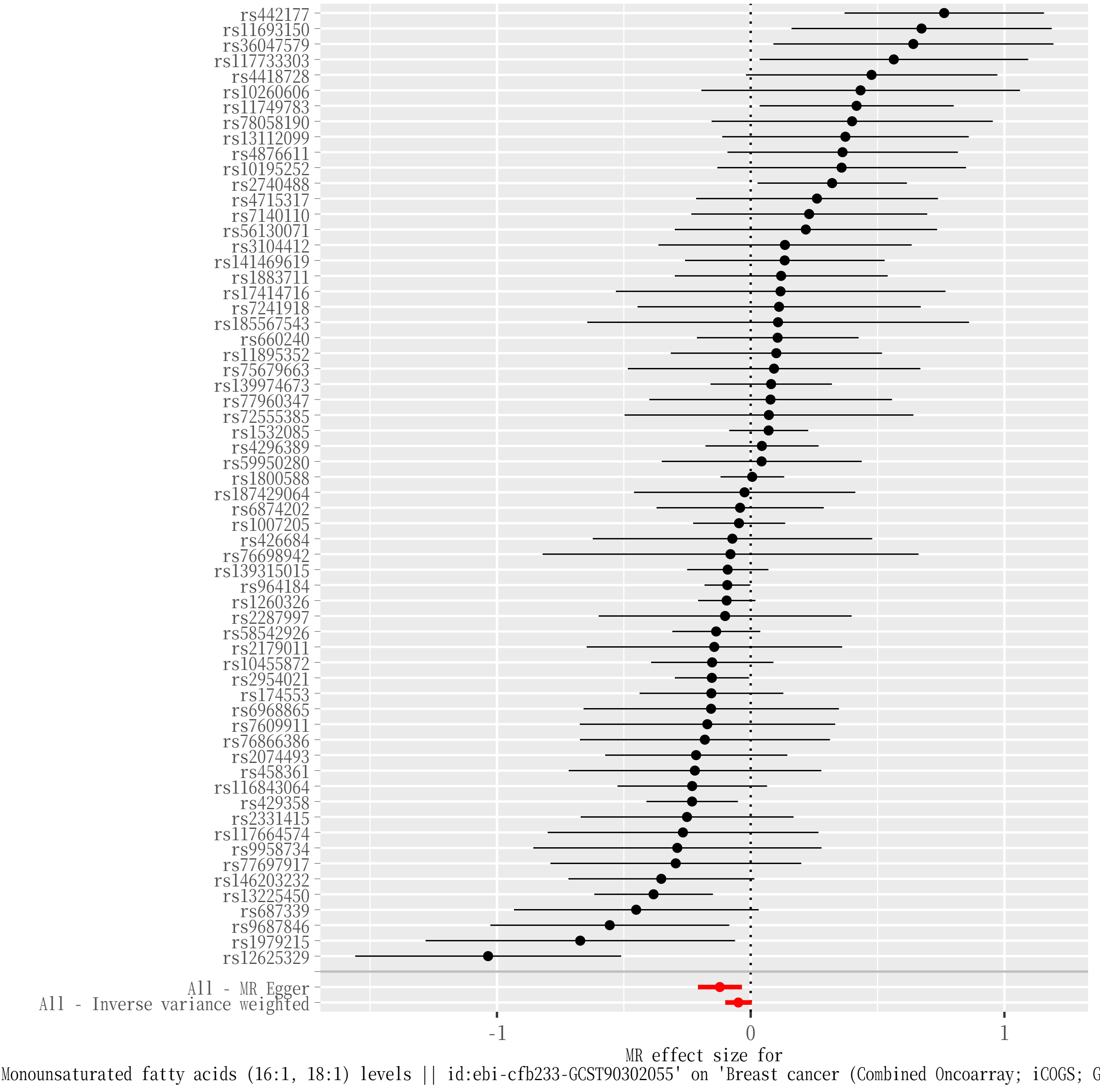
rs442177
rs11693150
rs36047579
rs117733303
rs4418728
rs10260606
rs11749783
rs78058190
rs13112099
rs4876611
rs10195252
rs2740488
rs4715317
rs7140110
rs56130071
rs3104412
rs141469619
rs1883711
rs17414716
rs7241918
rs185567543
rs660240
rs11895352
rs75679663
rs139974673
rs77960347
rs72555385
rs1532085
rs4296389
rs59950280
rs1800588
rs187429064
rs6874202
rs1007205
rs426684
rs76698942
rs139315015
rs964184
rs1260326
rs2287997
rs58542926
rs2179011
rs10455872
rs2954021
rs174553
rs6968865
rs7609911
rs76866386
rs2074493
rs458361
rs116843064
rs429358
rs2331415
rs117664574
rs9958734
rs77697917
rs146203232
rs13225450
rs687339
rs9687846
rs1979215
rs12625329
All - MR Egger
All - Inverse variance weighted
-1
0
1
MR effect size for
'Monounsaturated fatty acids (16:1, 18:1) levels || id:ebi-cfb233-GCST90302055' on 'Breast cancer (Combined Oncoarray; iCOGS; G

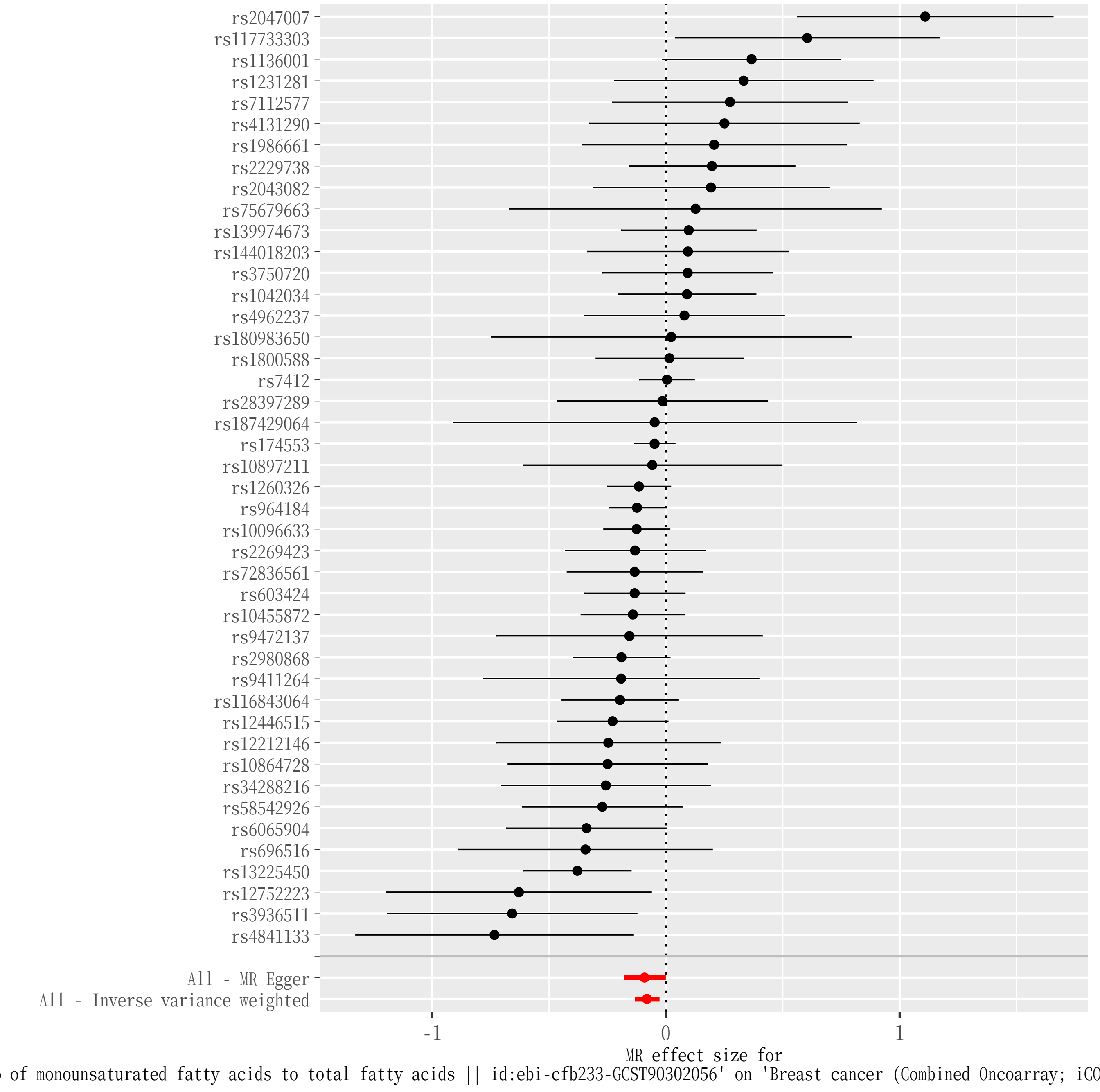
rs2047007
rs117733303
rs1136001
rs1231281
rs7112577
rs4131290
rs1986661
rs2229738
rs2043082
rs75679663
rs139974673
rs144018203
rs3750720
rs1042034
rs4962237
rs180983650
rs1800588
rs7412
rs28397289
rs187429064
rs174553
rs10897211
rs1260326
rs964184
rs10096633
rs2269423
rs72836561
rs603424
rs10455872
rs9472137
rs2980868
rs9411264
rs116843064
rs12446515
rs12212146
rs10864728
rs34288216
rs58542926
rs6065904
rs696516
rs13225450
rs12752223
rs3936511
rs4841133
All - MR Egger
All - Inverse variance weighted
-1
0
1
MR effect size for
o of monounsaturated fatty acids to total fatty acids || id:ebi-cfb233-GCST90302056' on 'Breast cancer (Combined Oncoarray; iCO

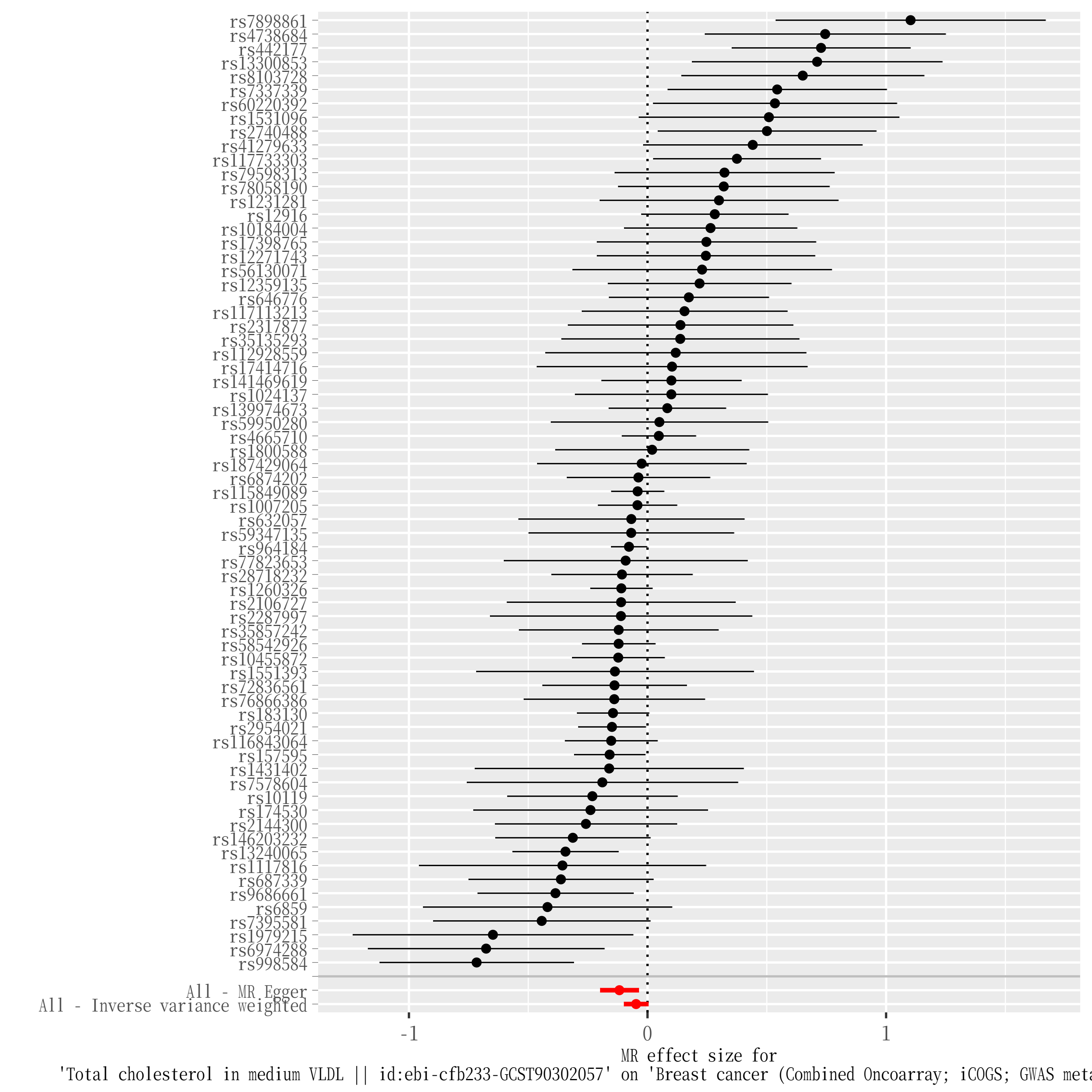
rs7898861
rs4738684
rs442177
rs13300853
rs8103728
rs7337339
rs60220392
rs1531096
rs2740488
rs41279633
rs117733303
rs79598313
rs78058190
rs1231281
rs12916
rs10184004
rs17398765
rs12271743
rs56130071
rs12359135
rs646776
rs117113213
rs2317877
rs35135293
rs112928559
rs17414716
rs141469619
rs1024137
rs139974673
rs59950280
rs4665710
rs1800588
rs187429064
rs6874202
rs115849089
rs1007205
rs632057
rs59347135
rs964184
rs77823653
rs28718232
rs1260326
rs2106727
rs2287997
rs35857242
rs58542926
rs10455872
rs1551393
rs72836561
rs76866386
rs183130
rs2954021
rs116843064
rs157595
rs1431402
rs7578604
rs10119
rs174530
rs2144300
rs146203232
rs13240065
rs1117816
rs687339
rs9686661
rs6859
rs7395581
rs1979215
rs6974288
rs998584
All - MR Egger
All - Inverse variance weighted
-1
0
1
MR effect size for
'Total cholesterol in medium VLDL || id:ebi-cfb233-GCST90302057' on 'Breast cancer (Combined Oncoarray; iCOGS; GWAS meta

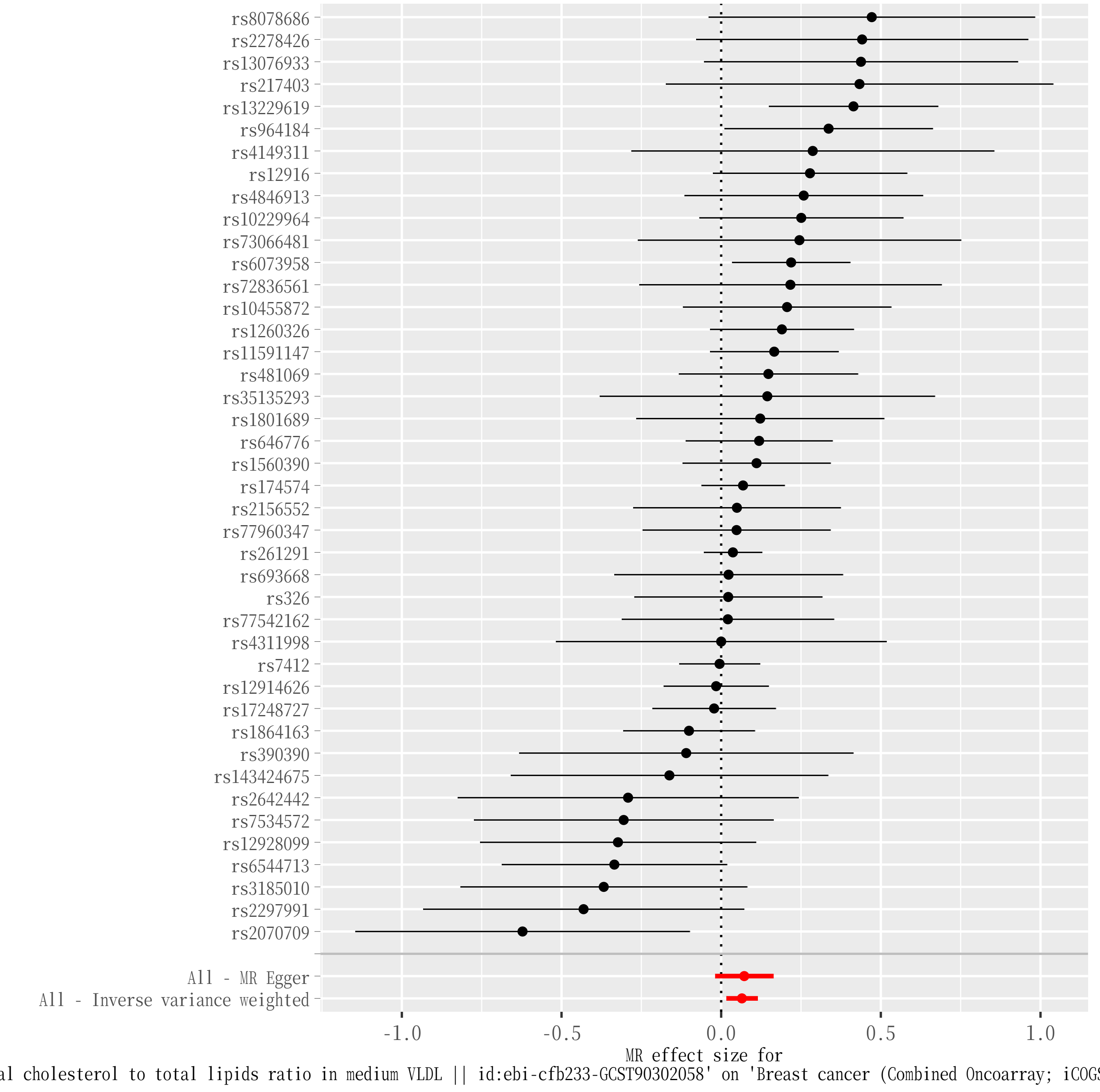
rs8078686
rs2278426
rs13076933
rs217403
rs13229619
rs964184
rs4149311
rs12916
rs4846913
rs10229964
rs73066481
rs6073958
rs72836561
rs10455872
rs1260326
rs11591147
rs481069
rs35135293
rs1801689
rs646776
rs1560390
rs174574
rs2156552
rs77960347
rs261291
rs693668
rs326
rs77542162
rs4311998
rs7412
rs12914626
rs17248727
rs1864163
rs390390
rs143424675
rs2642442
rs7534572
rs12928099
rs6544713
rs3185010
rs2297991
rs2070709
All - MR Egger
All - Inverse variance weighted
-1.0
-0.5
0.0
0.5
1.0
MR effect size for
al cholesterol to total lipids ratio in medium VLDL || id:ebi-cfb233-GCST90302058' on 'Breast cancer (Combined Oncoarray; iCOGS

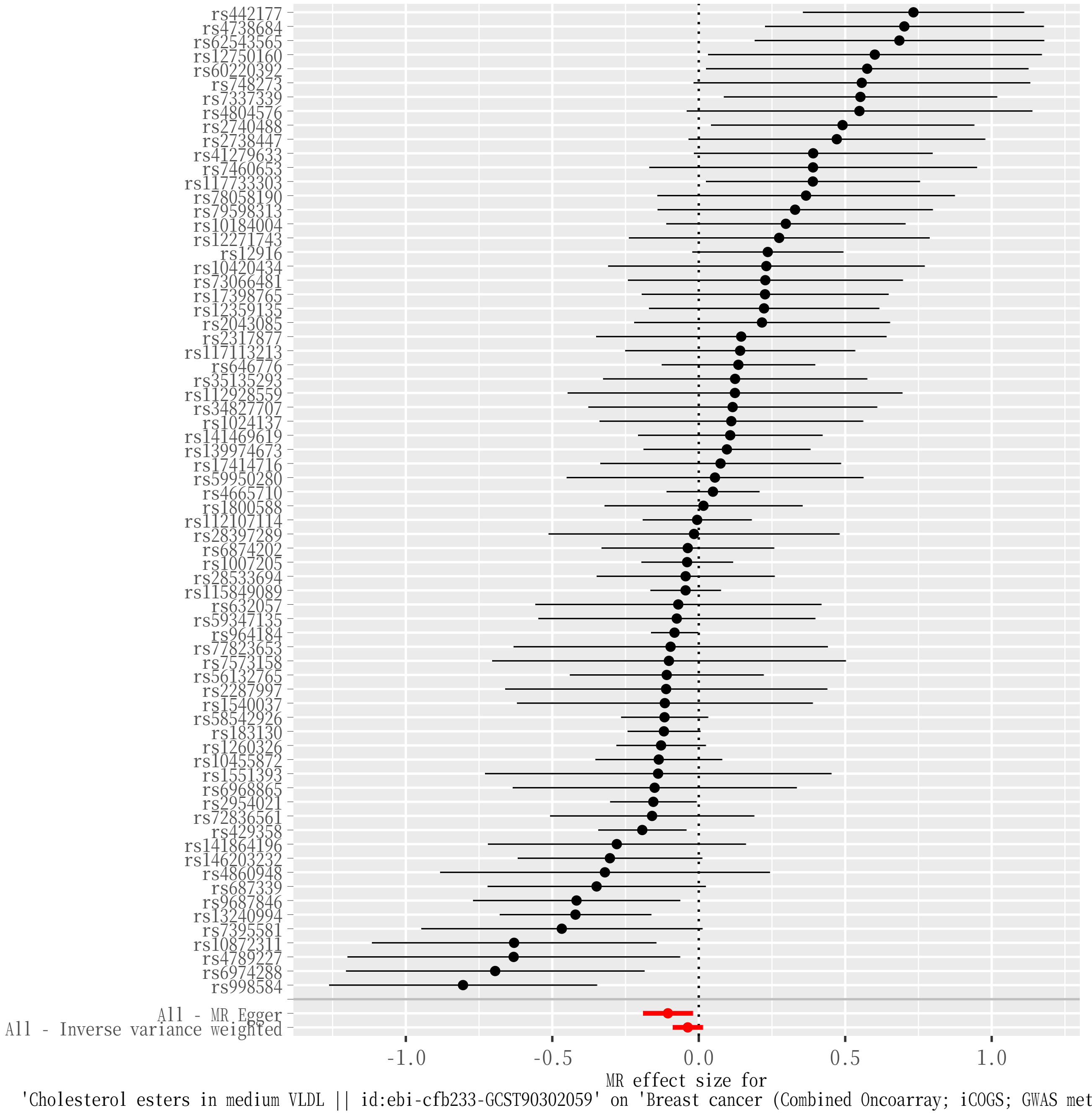

rs442177
rs4738684
rs62543565
rs12750160
rs60220392
rs748273
rs7337339
rs4804576
rs2740488
rs2738447
rs41279633
rs7460653
rs117733303
rs78058190
rs79598313
rs10184004
rs12271743
rs12916
rs10420434
rs73066481
rs17398765
rs12359135
rs2043085
rs2317877
rs117113213
rs646776
rs35135293
rs112928559
rs34827707
rs1024137
rs141469619
rs139974673
rs17414716
rs59950280
rs4665710
rs1800588
rs112107114
rs28397289
rs6874202
rs1007205
rs28533694
rs115849089
rs632057
rs59347135
rs964184
rs77823653
rs7573158
rs56132765
rs2287997
rs1540037
rs58542926
rs183130
rs1260326
rs10455872
rs1551393
rs6968865
rs2954021
rs72836561
rs429358
rs141864196
rs146203232
rs4860948
rs687339
rs9687846
rs13240994
rs7395581
rs10872311
rs4789227
rs6974288
rs998584
All - MR Egger
All - Inverse variance weighted
-1.0
-0.5
0.0
0.5
1.0
MR effect size for
'Cholesterol esters in medium VLDL || id:ebi-cfb233-GCST90302059' on 'Breast cancer (Combined Oncoarray; iCOGS; GWAS met

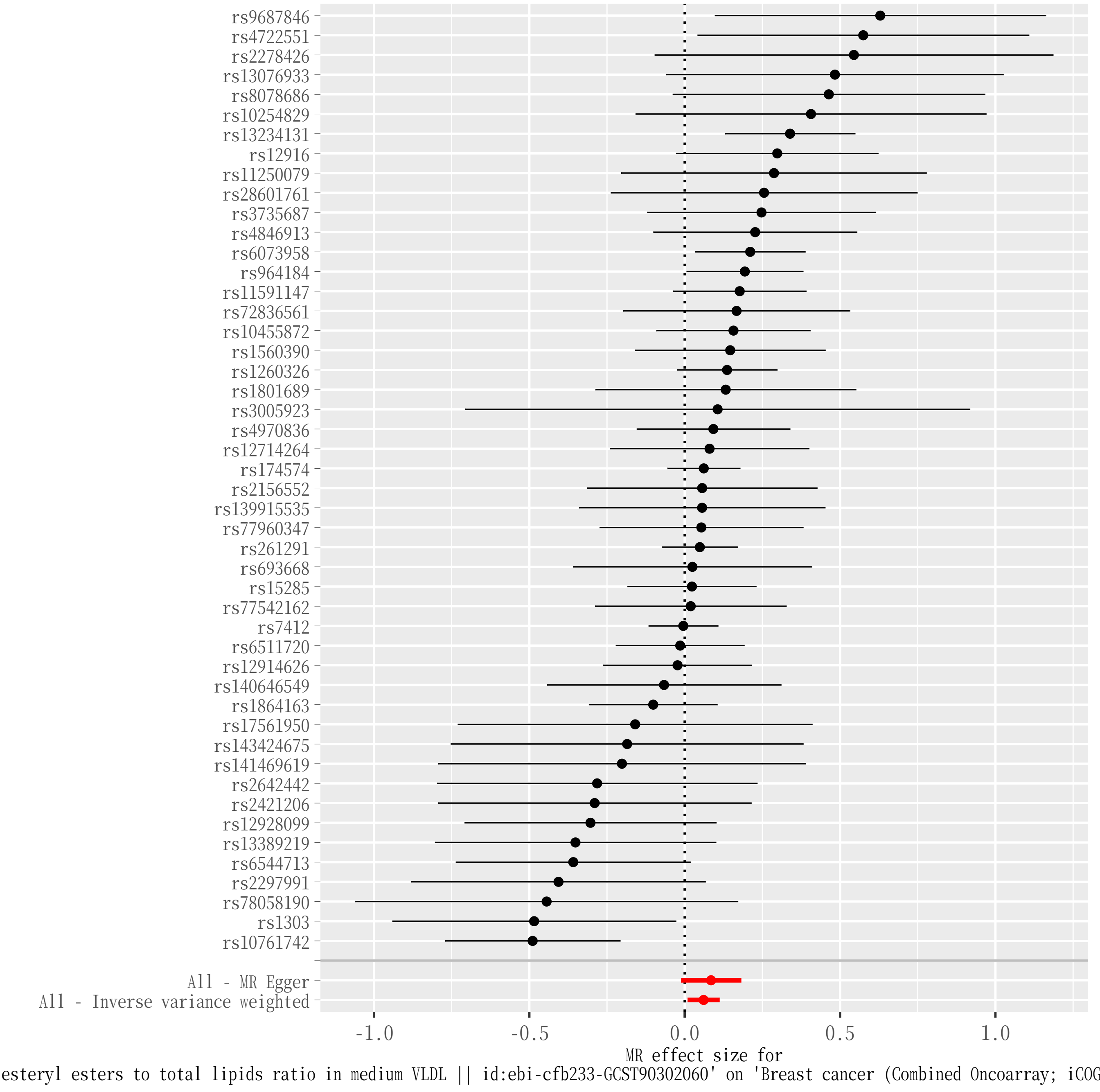
rs9687846
rs4722551
rs2278426
rs13076933
rs8078686
rs10254829
rs13234131
rs12916
rs11250079
rs28601761
rs3735687
rs4846913
rs6073958
rs964184
rs11591147
rs72836561
rs10455872
rs1560390
rs1260326
rs1801689
rs3005923
rs4970836
rs12714264
rs174574
rs2156552
rs139915535
rs77960347
rs261291
rs693668
rs15285
rs77542162
rs7412
rs6511720
rs12914626
rs140646549
rs1864163
rs17561950
rs143424675
rs141469619
rs2642442
rs2421206
rs12928099
rs13389219
rs6544713
rs2297991
rs78058190
rs1303
rs10761742
All - MR Egger
All - Inverse variance weighted
-1.0
-0.5
0.0
0.5
1.0
MR effect size for
esteryl esters to total lipids ratio in medium VLDL || id:ebi-cfb233-GCST90302060' on 'Breast cancer (Combined Oncoarray; iCOG

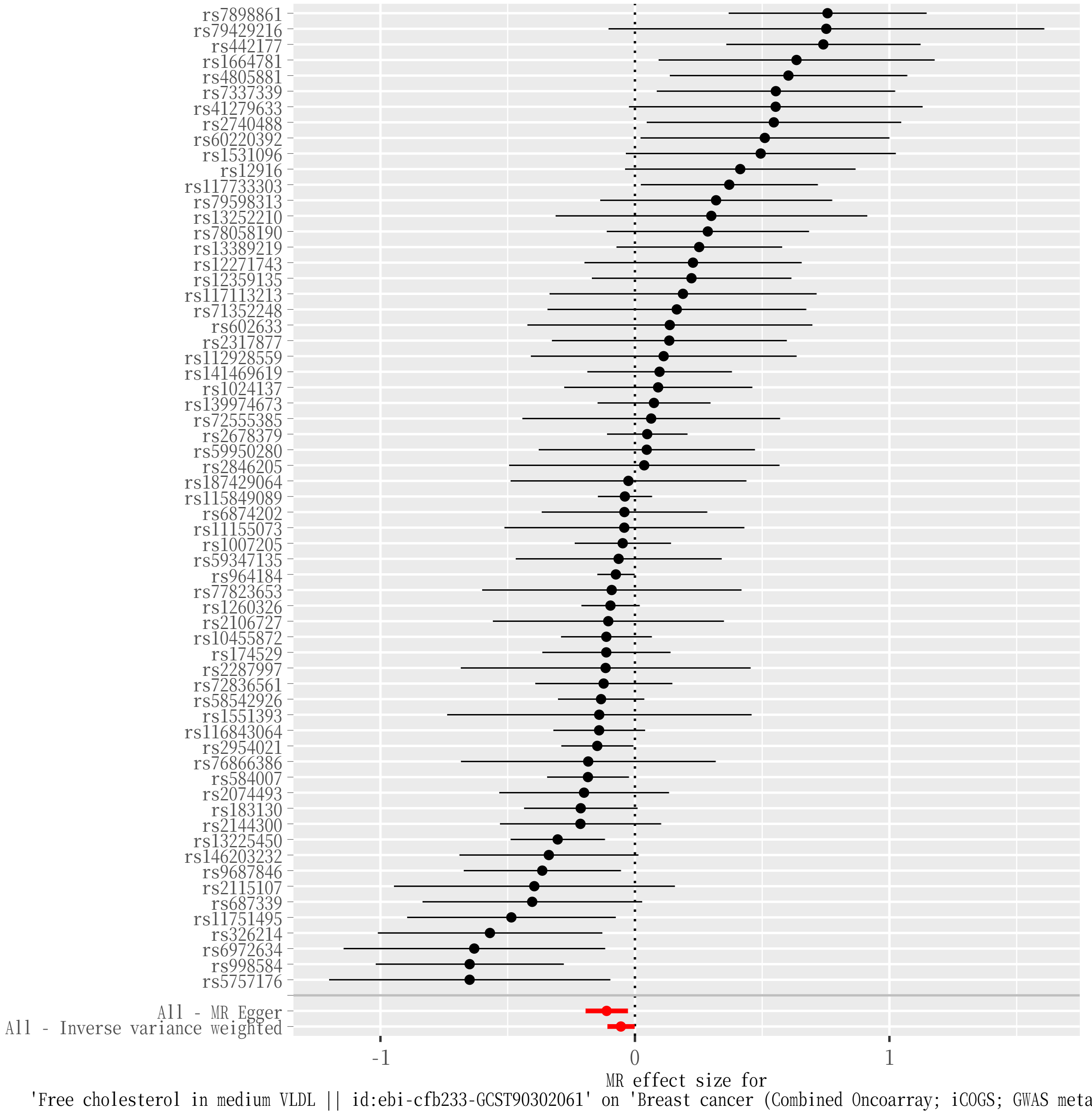

rs7898861
rs79429216
rs442177
rs1664781
rs4805881
rs7337339
rs41279633
rs2740488
rs60220392
rs1531096
rs12916
rs117733303
rs79598313
rs13252210
rs78058190
rs13389219
rs12271743
rs12359135
rs117113213
rs71352248
rs602633
rs2317877
rs112928559
rs141469619
rs1024137
rs139974673
rs72555385
rs2678379
rs59950280
rs2846205
rs187429064
rs115849089
rs6874202
rs11155073
rs1007205
rs59347135
rs964184
rs77823653
rs1260326
rs2106727
rs10455872
rs174529
rs2287997
rs72836561
rs58542926
rs1551393
rs116843064
rs2954021
rs76866386
rs584007
rs2074493
rs183130
rs2144300
rs13225450
rs146203232
rs9687846
rs2115107
rs687339
rs11751495
rs326214
rs6972634
rs998584
rs5757176
All - MR Egger
All - Inverse variance weighted
-1
0
1
MR effect size for
'Free cholesterol in medium VLDL || id:ebi-cfb233-GCST90302061' on 'Breast cancer (Combined Oncoarray; iCOGS; GWAS meta

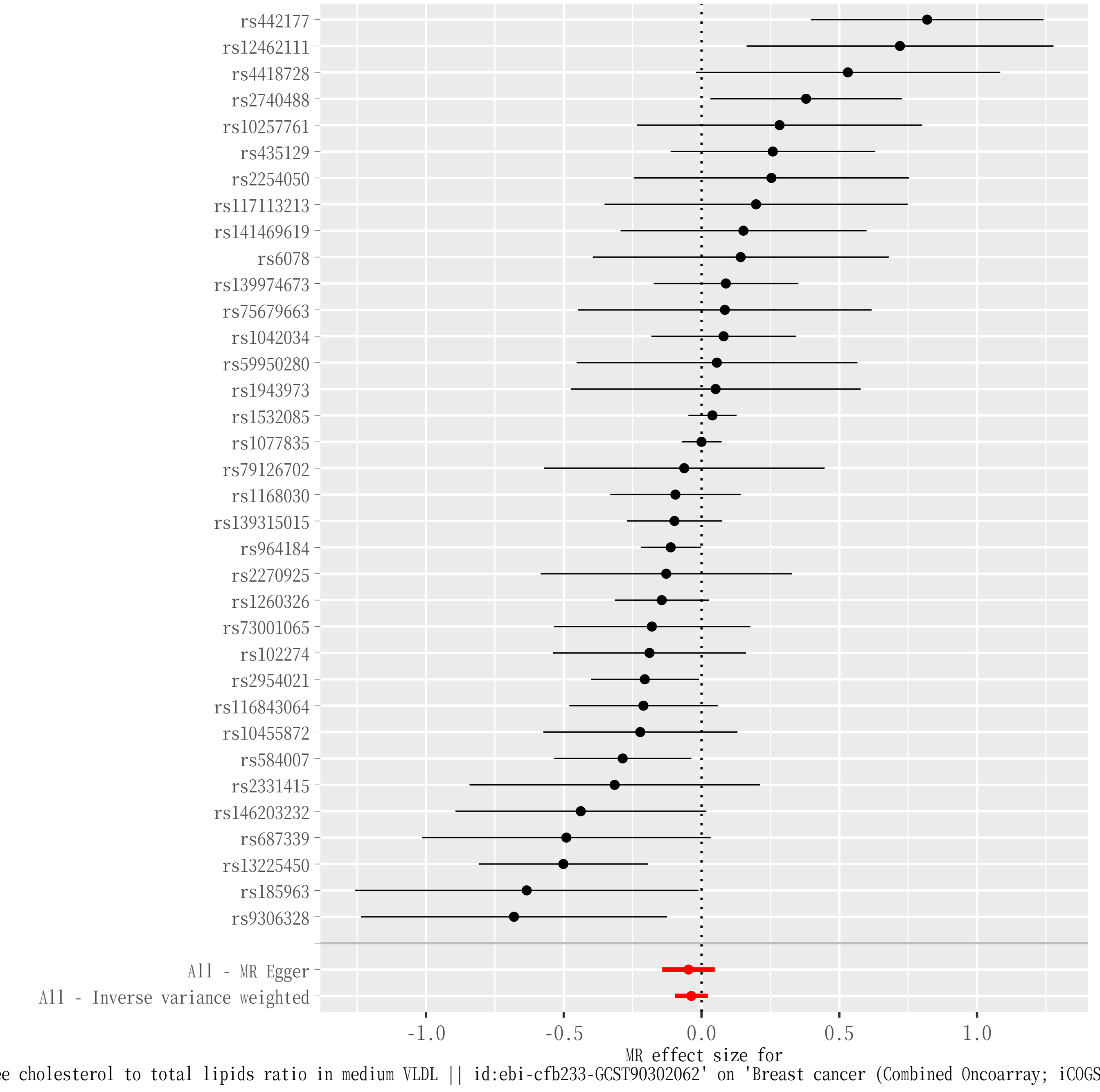
rs442177
rs12462111
rs4418728
rs2740488
rs10257761
rs435129
rs2254050
rs117113213
rs141469619
rs6078
rs139974673
rs75679663
rs1042034
rs59950280
rs1943973
rs1532085
rs1077835
rs79126702
rs1168030
rs139315015
rs964184
rs2270925
rs1260326
rs73001065
rs102274
rs2954021
rs116843064
rs10455872
rs584007
rs2331415
rs146203232
rs687339
rs13225450
rs185963
rs9306328
All - MR Egger
All - Inverse variance weighted
-1.0
-0.5
0.0
0.5
1.0
MR effect size for
e cholesterol to total lipids ratio in medium VLDL || id:ebi-cfb233-GCST90302062' on 'Breast cancer (Combined Oncoarray; iCOGS

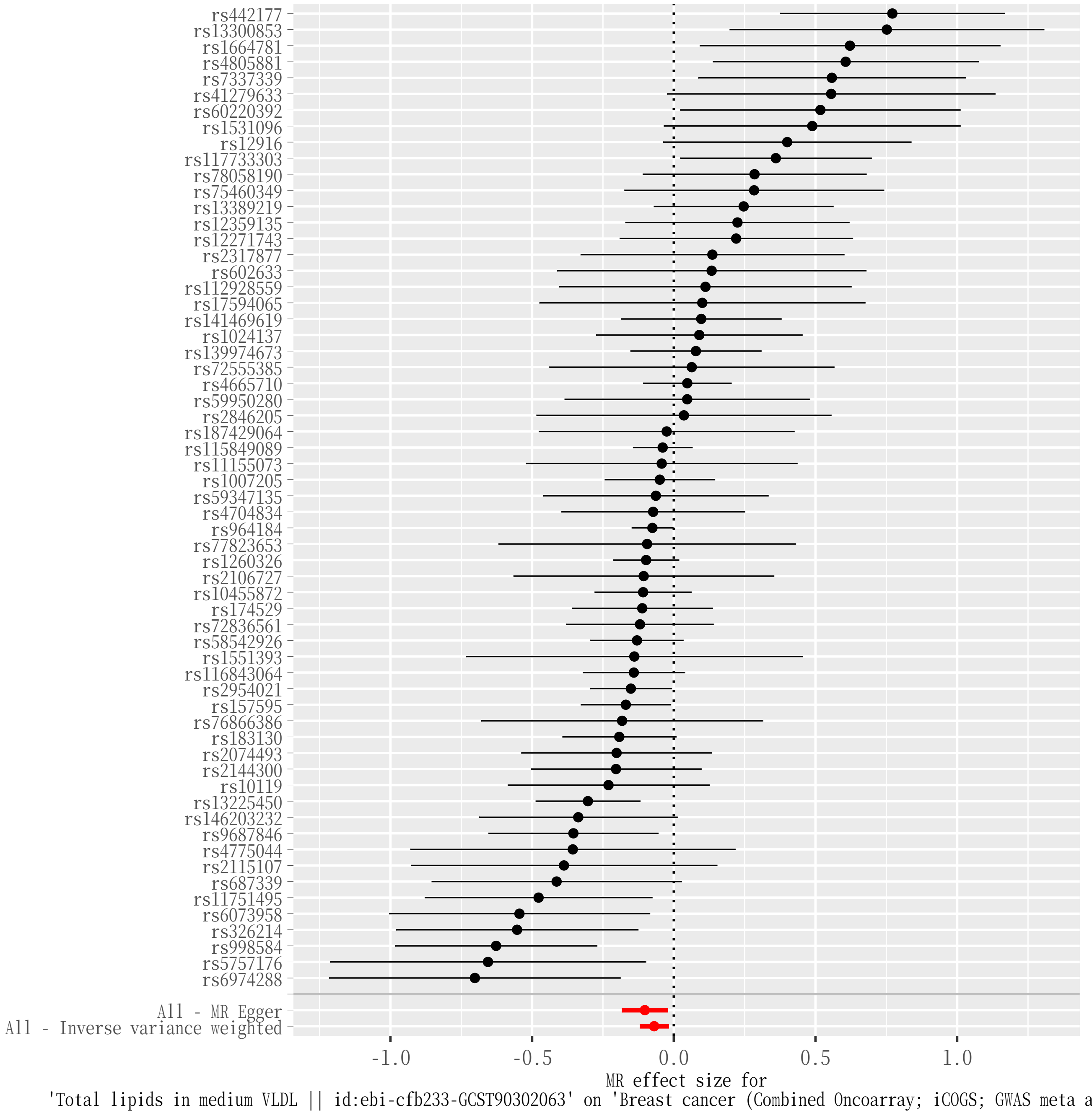
rs442177
rs13300853
rs1664781
rs4805881
rs7337339
rs41279633
rs60220392
rs1531096
rs12916
rs117733303
rs78058190
rs75460349
rs13389219
rs12359135
rs12271743
rs2317877
rs602633
rs112928559
rs17594065
rs141469619
rs1024137
rs139974673
rs72555385
rs4665710
rs59950280
rs2846205
rs187429064
rs115849089
rs11155073
rs1007205
rs59347135
rs4704834
rs964184
rs77823653
rs1260326
rs2106727
rs10455872
rs174529
rs72836561
rs58542926
rs1551393
rs116843064
rs2954021
rs157595
rs76866386
rs183130
rs2074493
rs2144300
rs10119
rs13225450
rs146203232
rs9687846
rs4775044
rs2115107
rs687339
rs11751495
rs6073958
rs326214
rs998584
rs5757176
rs6974288
All - MR Egger
All - Inverse variance weighted
-1.0
-0.5
0.0
0.5
1.0
MR effect size for
'Total lipids in medium VLDL || id:ebi-cfb233-GCST90302063' on 'Breast cancer (Combined Oncoarray; iCOGS; GWAS meta a

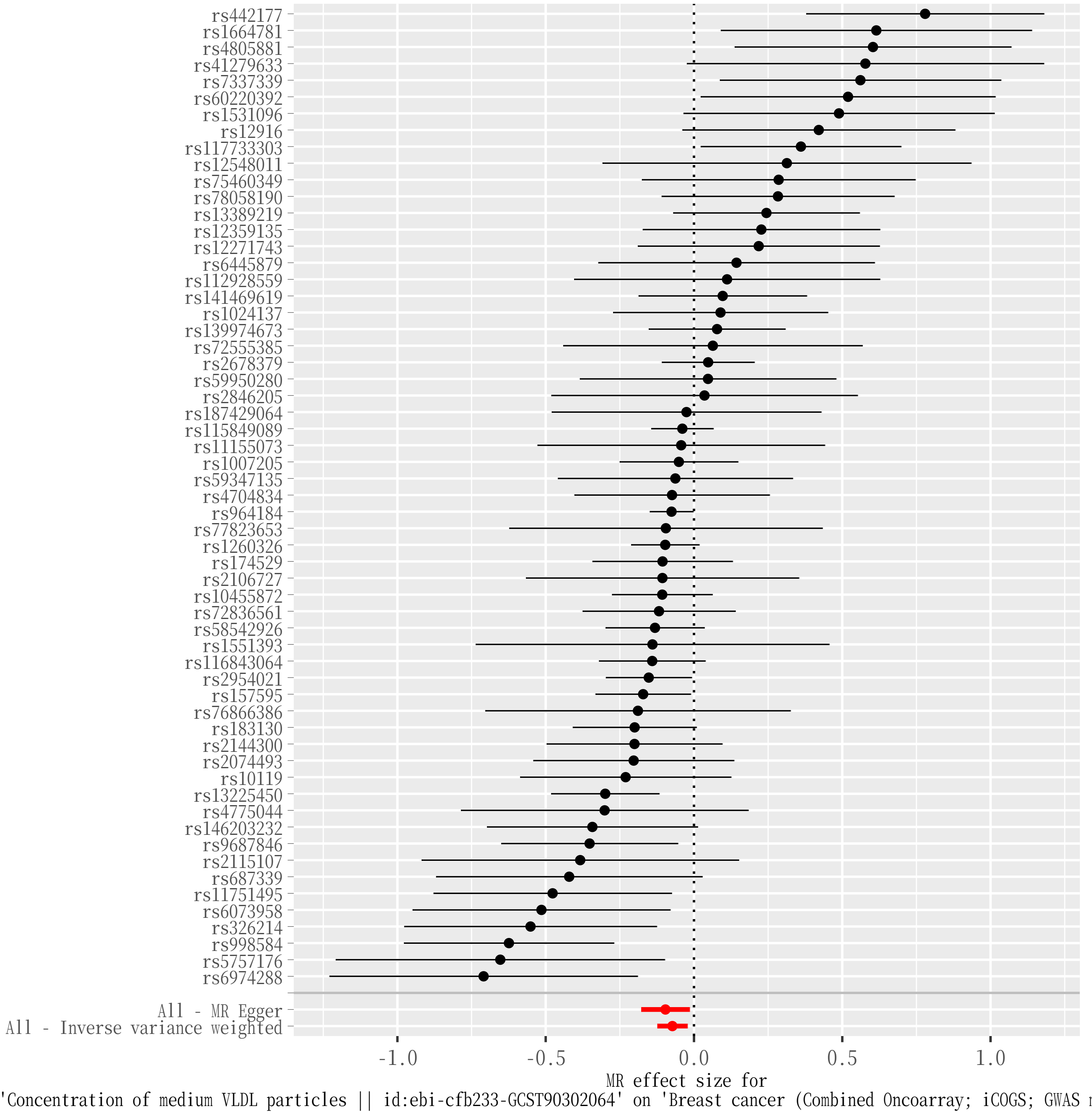
rs442177
rs1664781
rs4805881
rs41279633
rs7337339
rs60220392
rs1531096
rs12916
rs117733303
rs12548011
rs75460349
rs78058190
rs13389219
rs12359135
rs12271743
rs6445879
rs112928559
rs141469619
rs1024137
rs139974673
rs72555385
rs2678379
rs59950280
rs2846205
rs187429064
rs115849089
rs11155073
rs1007205
rs59347135
rs4704834
rs964184
rs77823653
rs1260326
rs174529
rs2106727
rs10455872
rs72836561
rs58542926
rs1551393
rs116843064
rs2954021
rs157595
rs76866386
rs183130
rs2144300
rs2074493
rs10119
rs13225450
rs4775044
rs146203232
rs9687846
rs2115107
rs687339
rs11751495
rs6073958
rs326214
rs998584
rs5757176
rs6974288
All - MR Egger
All - Inverse variance weighted
-1.0
-0.5
0.0
0.5
1.0
MR effect size for
'Concentration of medium VLDL particles || id:ebi-cfb233-GCST90302064' on 'Breast cancer (Combined Oncoarray; iCOGS; GWAS m

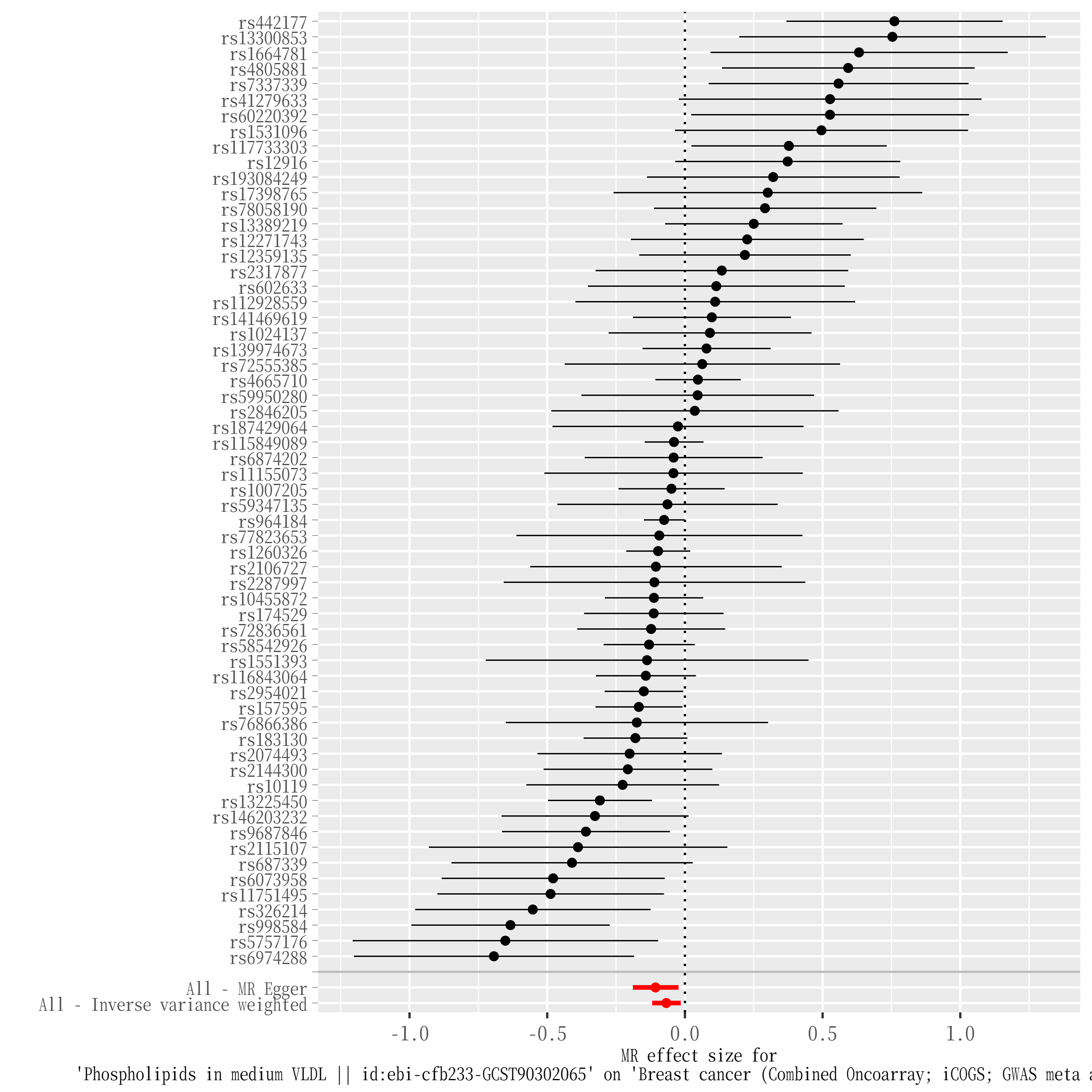
rs442177
rs13300853
rs1664781
rs4805881
rs7337339
rs41279633
rs60220392
rs1531096
rs117733303
rs12916
rs193084249
rs17398765
rs78058190
rs13389219
rs12271743
rs12359135
rs2317877
rs602633
rs112928559
rs141469619
rs1024137
rs139974673
rs72555385
rs4665710
rs59950280
rs2846205
rs187429064
rs115849089
rs6874202
rs11155073
rs1007205
rs59347135
rs964184
rs77823653
rs1260326
rs2106727
rs2287997
rs10455872
rs174529
rs72836561
rs58542926
rs1551393
rs116843064
rs2954021
rs157595
rs76866386
rs183130
rs2074493
rs2144300
rs10119
rs13225450
rs146203232
rs9687846
rs2115107
rs687339
rs6073958
rs11751495
rs326214
rs998584
rs5757176
rs6974288
All - MR Egger
All - Inverse variance weighted
-1.0
-0.5
0.0
0.5
1.0
MR effect size for
'Phospholipids in medium VLDL || id:ebi-cfb233-GCST90302065' on 'Breast cancer (Combined Oncoarray; iCOGS; GWAS meta a

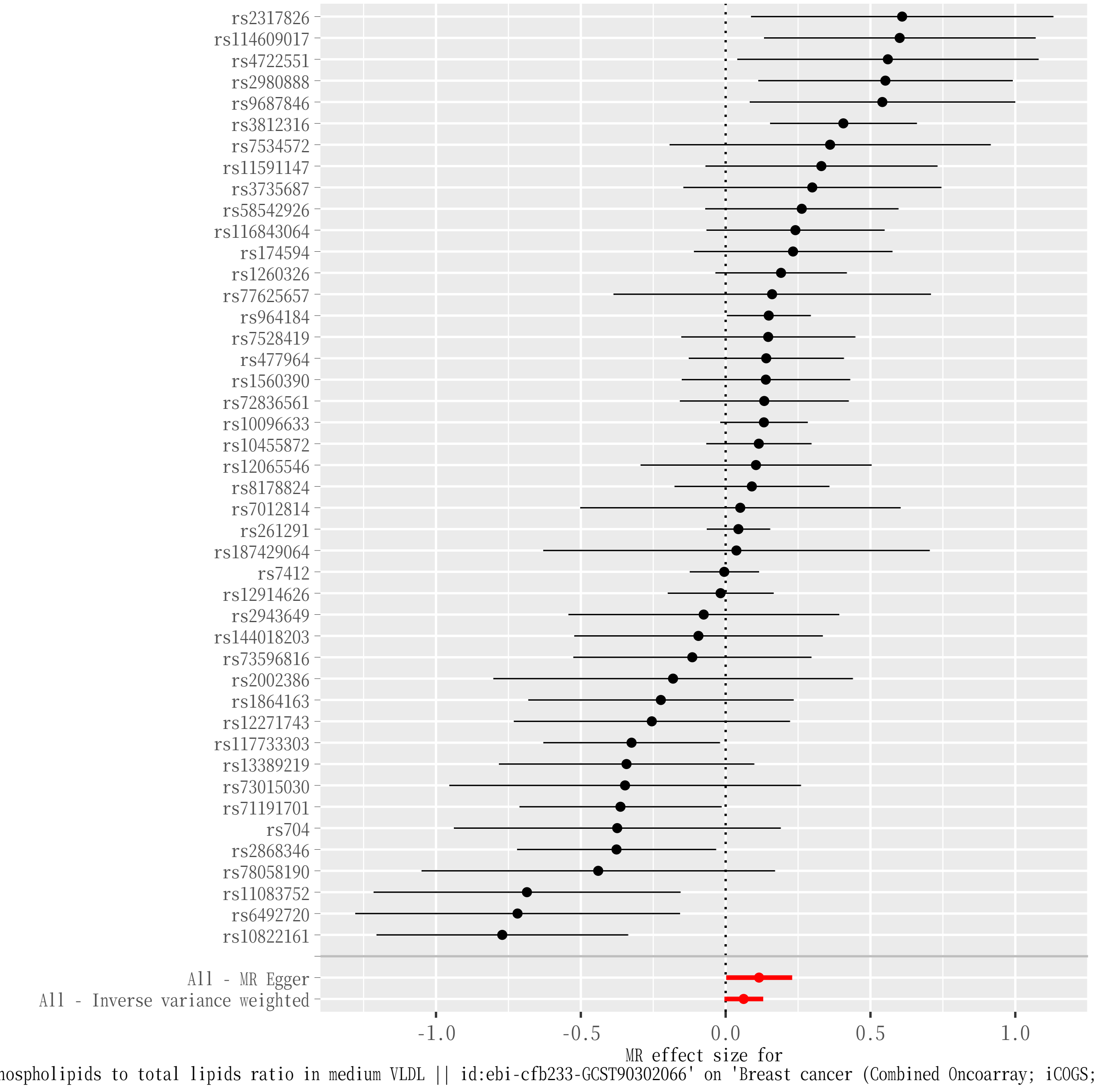

rs2317826
rs114609017
rs4722551
rs2980888
rs9687846
rs3812316
rs7534572
rs11591147
rs3735687
rs58542926
rs116843064
rs174594
rs1260326
rs77625657
rs964184
rs7528419
rs477964
rs1560390
rs72836561
rs10096633
rs10455872
rs12065546
rs8178824
rs7012814
rs261291
rs187429064
rs7412
rs12914626
rs2943649
rs144018203
rs73596816
rs2002386
rs1864163
rs12271743
rs117733303
rs13389219
rs73015030
rs71191701
rs704
rs2868346
rs78058190
rs11083752
rs6492720
rs10822161
All - MR Egger
All - Inverse variance weighted
-1.0
-0.5
0.0
0.5
1.0
MR effect size for
hospholipids to total lipids ratio in medium VLDL || id:ebi-cfb233-GCST90302066' on 'Breast cancer (Combined Oncoarray; iCOGS;

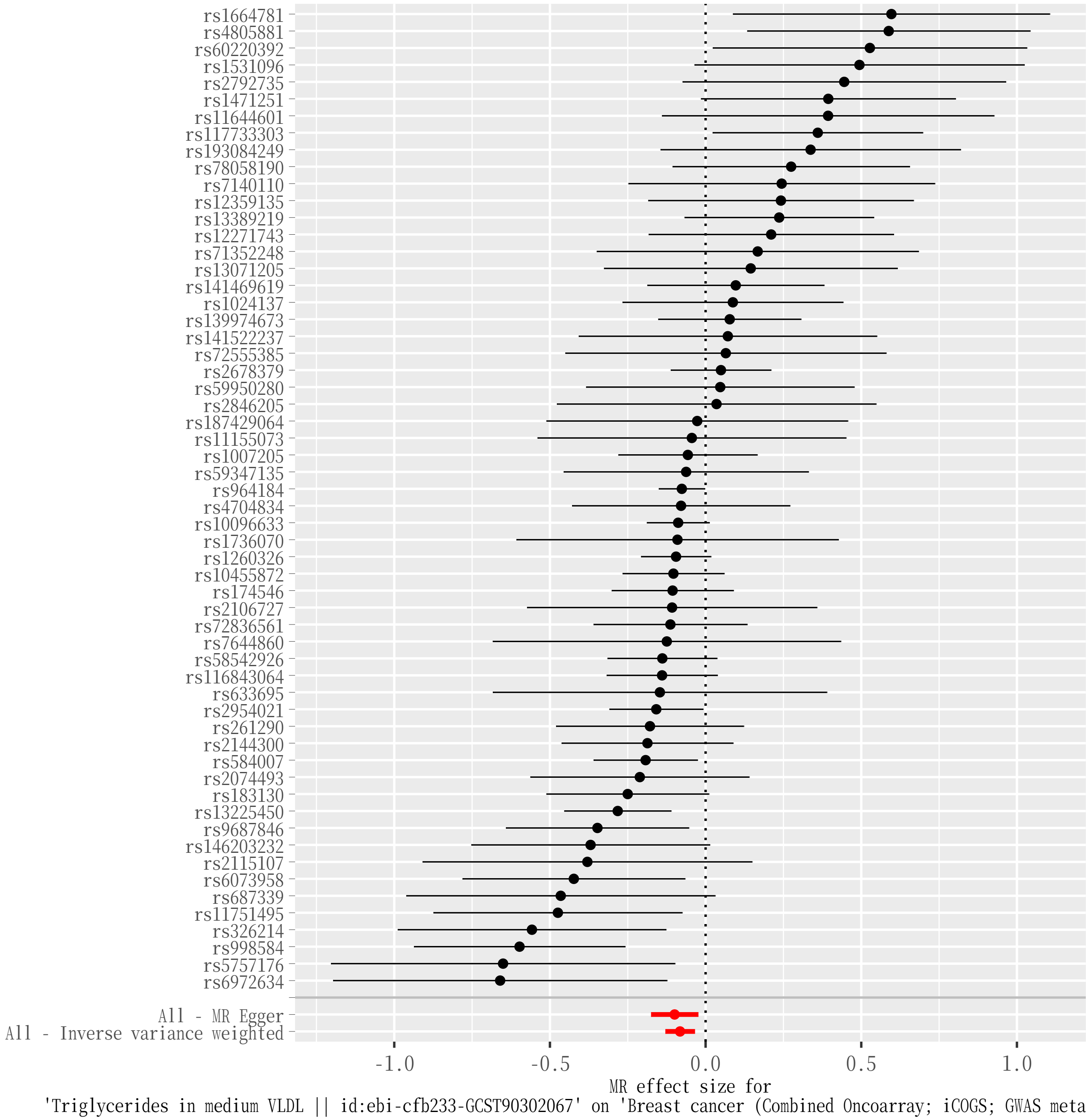
rs1664781
rs4805881
rs60220392
rs1531096
rs2792735
rs1471251
rs11644601
rs117733303
rs193084249
rs78058190
rs7140110
rs12359135
rs13389219
rs12271743
rs71352248
rs13071205
rs141469619
rs1024137
rs139974673
rs141522237
rs72555385
rs2678379
rs59950280
rs2846205
rs187429064
rs11155073
rs1007205
rs59347135
rs964184
rs4704834
rs10096633
rs1736070
rs1260326
rs10455872
rs174546
rs2106727
rs72836561
rs7644860
rs58542926
rs116843064
rs633695
rs2954021
rs261290
rs2144300
rs584007
rs2074493
rs183130
rs13225450
rs9687846
rs146203232
rs2115107
rs6073958
rs687339
rs11751495
rs326214
rs998584
rs5757176
rs6972634
All - MR Egger
All - Inverse variance weighted
-1.0
-0.5
0.0
0.5
1.0
MR effect size for
'Triglycerides in medium VLDL || id:ebi-cfb233-GCST90302067' on 'Breast cancer (Combined Oncoarray; iCOGS; GWAS meta a

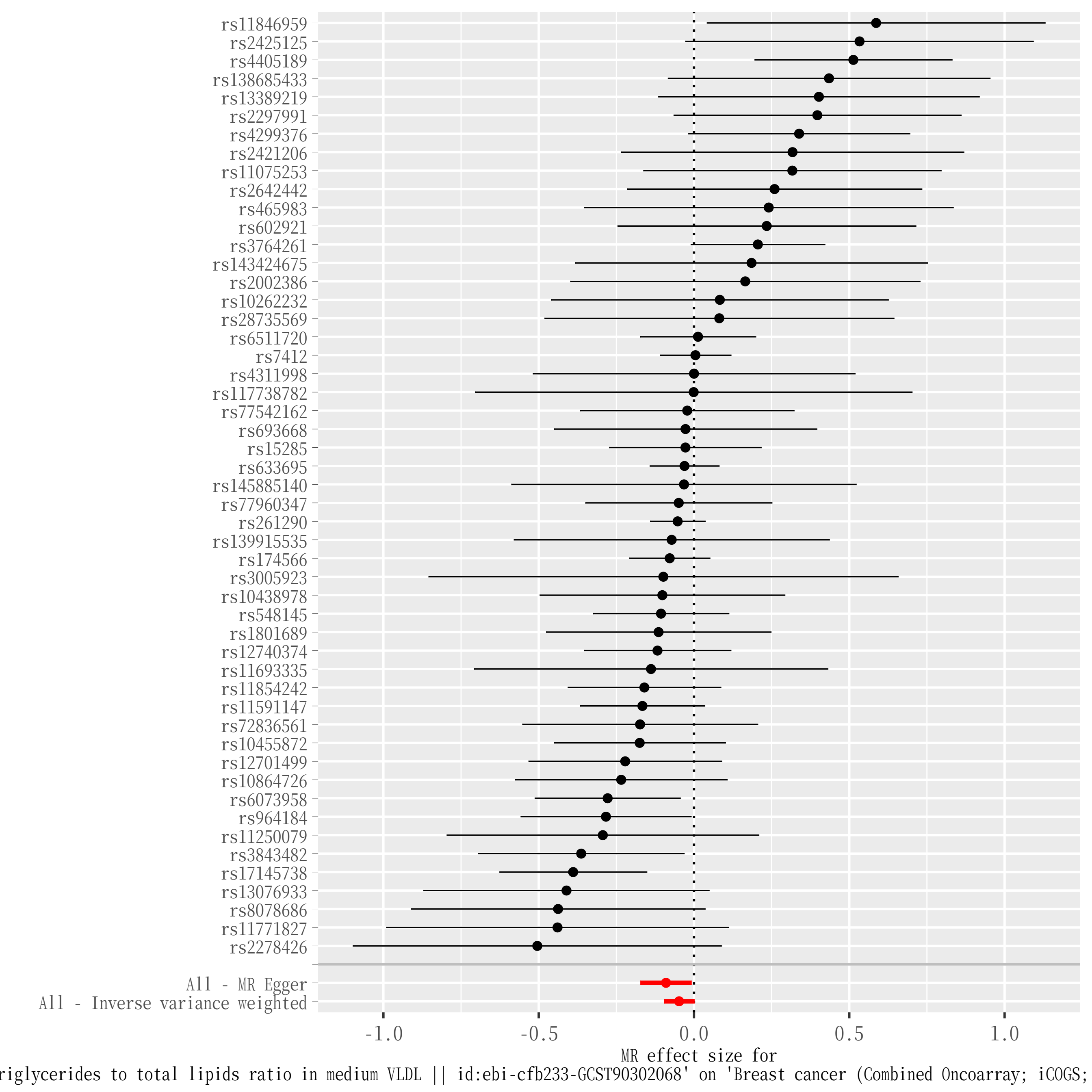
rs11846959
rs2425125
rs4405189
rs138685433
rs13389219
rs2297991
rs4299376
rs2421206
rs11075253
rs2642442
rs465983
rs602921
rs3764261
rs143424675
rs2002386
rs10262232
rs28735569
rs6511720
rs7412
rs4311998
rs117738782
rs77542162
rs693668
rs15285
rs633695
rs145885140
rs77960347
rs261290
rs139915535
rs174566
rs3005923
rs10438978
rs548145
rs1801689
rs12740374
rs11693335
rs11854242
rs11591147
rs72836561
rs10455872
rs12701499
rs10864726
rs6073958
rs964184
rs11250079
rs3843482
rs17145738
rs13076933
rs8078686
rs11771827
rs2278426
All - MR Egger
All - Inverse variance weighted
-1.0
-0.5
0.0
0.5
1.0
MR effect size for
riglycerides to total lipids ratio in medium VLDL || id:ebi-cfb233-GCST90302068' on 'Breast cancer (Combined Oncoarray; iCOGS;

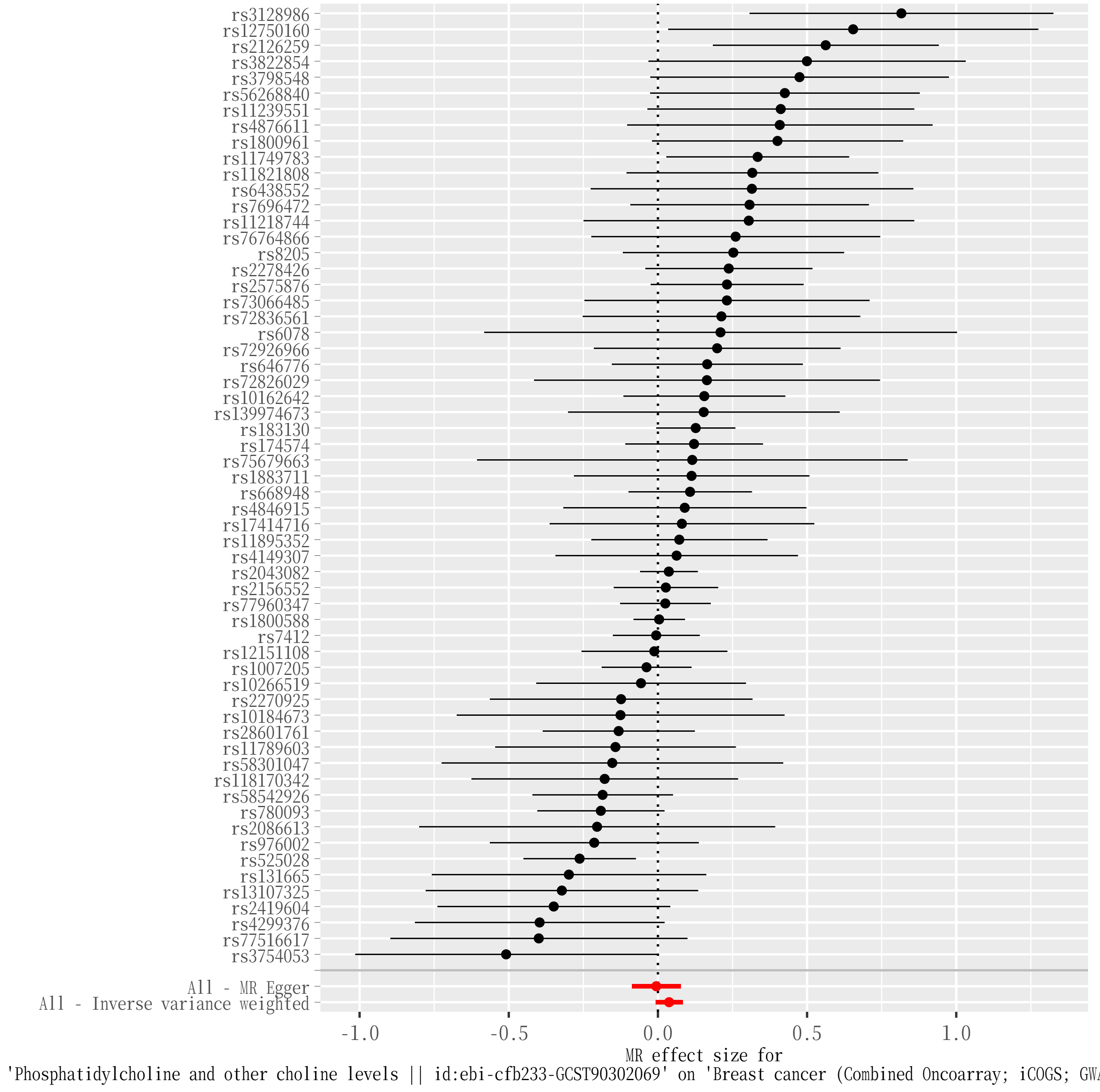

rs3128986
rs12750160
rs2126259
rs3822854
rs3798548
rs56268840
rs11239551
rs4876611
rs1800961
rs11749783
rs11821808
rs6438552
rs7696472
rs11218744
rs76764866
rs8205
rs2278426
rs2575876
rs73066485
rs72836561
rs6078
rs72926966
rs646776
rs72826029
rs10162642
rs139974673
rs183130
rs174574
rs75679663
rs1883711
rs668948
rs4846915
rs17414716
rs11895352
rs4149307
rs2043082
rs2156552
rs77960347
rs1800588
rs7412
rs12151108
rs1007205
rs10266519
rs2270925
rs10184673
rs28601761
rs11789603
rs58301047
rs118170342
rs58542926
rs780093
rs2086613
rs976002
rs525028
rs131665
rs13107325
rs2419604
rs4299376
rs77516617
rs3754053
All - MR Egger
All - Inverse variance weighted
-1.0
-0.5
0.0
0.5
1.0
MR effect size for
'Phosphatidylcholine and other choline levels || id:ebi-cfb233-GCST90302069' on 'Breast cancer (Combined Oncoarray; iCOGS; GWA

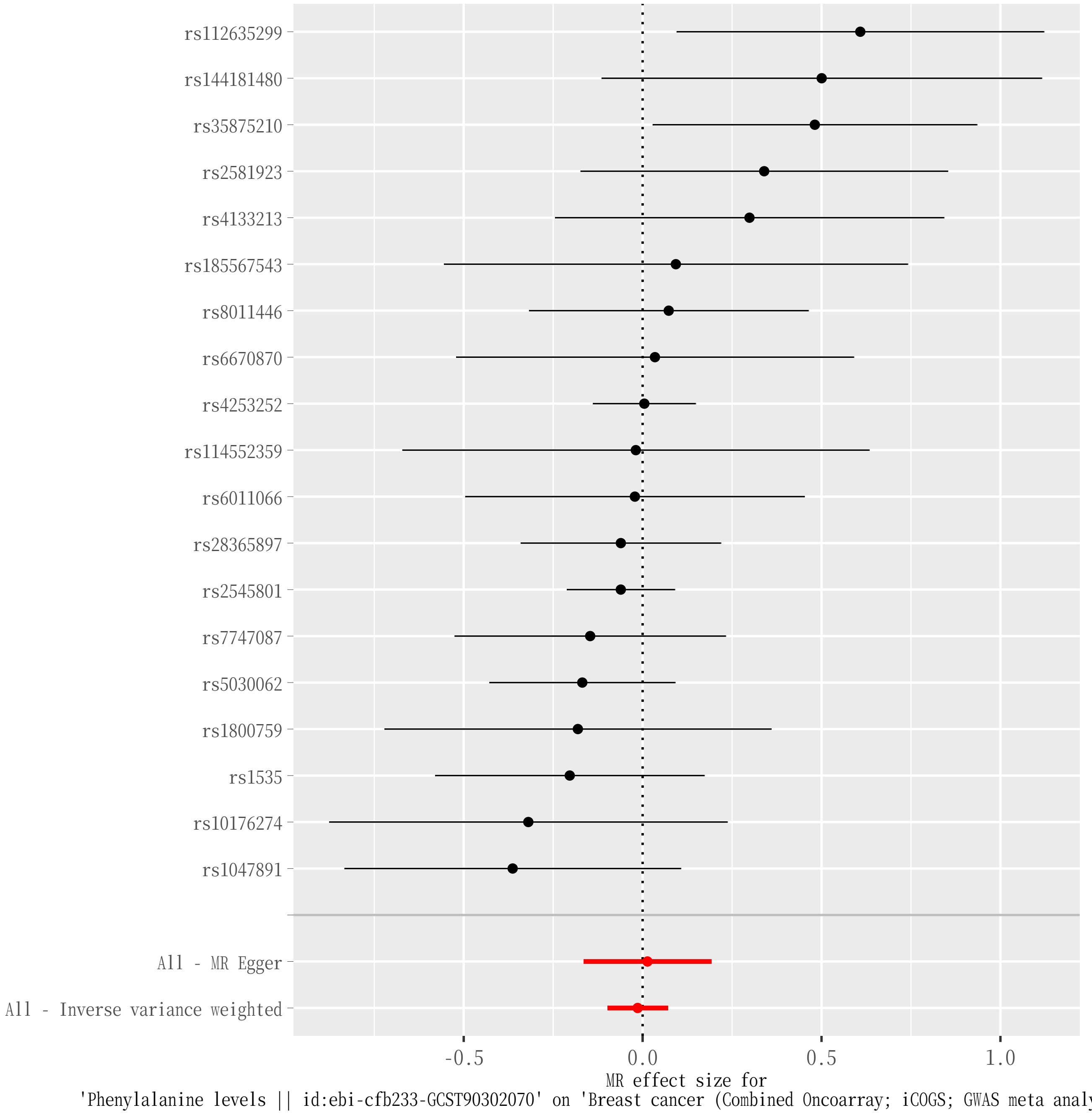

rs112635299
rs144181480
rs35875210
rs2581923
rs4133213
rs185567543
rs8011446
rs6670870
rs4253252
rs114552359
rs6011066
rs28365897
rs2545801
rs7747087
rs5030062
rs1800759
rs1535
rs10176274
rs1047891
All - MR Egger
All - Inverse variance weighted
-0.5
0.0
0.5
1.0
MR effect size for
'Phenylalanine levels || id:ebi-cfb233-GCST90302070' on 'Breast cancer (Combined Oncoarray; iCOGS; GWAS meta analy

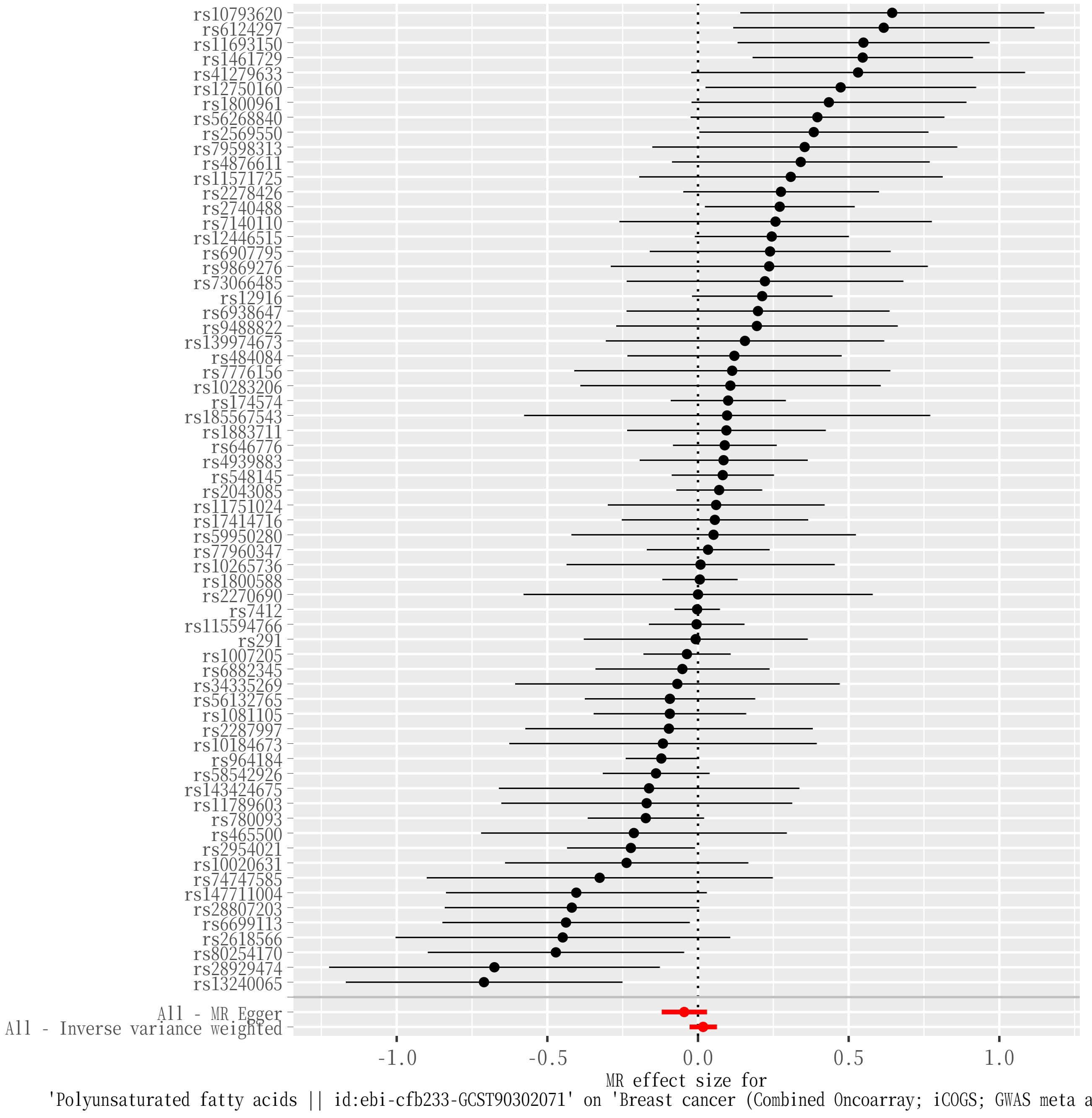
rs10793620
rs6124297
rs11693150
rs1461729
rs41279633
rs12750160
rs1800961
rs56268840
rs2569550
rs79598313
rs4876611
rs11571725
rs2278426
rs2740488
rs7140110
rs12446515
rs6907795
rs9869276
rs73066485
rs12916
rs6938647
rs9488822
rs139974673
rs484084
rs7776156
rs10283206
rs174574
rs185567543
rs1883711
rs646776
rs4939883
rs548145
rs2043085
rs11751024
rs17414716
rs59950280
rs77960347
rs10265736
rs1800588
rs2270690
rs7412
rs115594766
rs291
rs1007205
rs6882345
rs34335269
rs56132765
rs1081105
rs2287997
rs10184673
rs964184
rs58542926
rs143424675
rs11789603
rs780093
rs465500
rs2954021
rs10020631
rs74747585
rs147711004
rs28807203
rs6699113
rs2618566
rs80254170
rs28929474
rs13240065
All - MR Egger
All - Inverse variance weighted
-1.0
-0.5
0.0
0.5
1.0
MR effect size for
'Polyunsaturated fatty acids || id:ebi-cfb233-GCST90302071' on 'Breast cancer (Combined Oncoarray; iCOGS; GWAS meta a

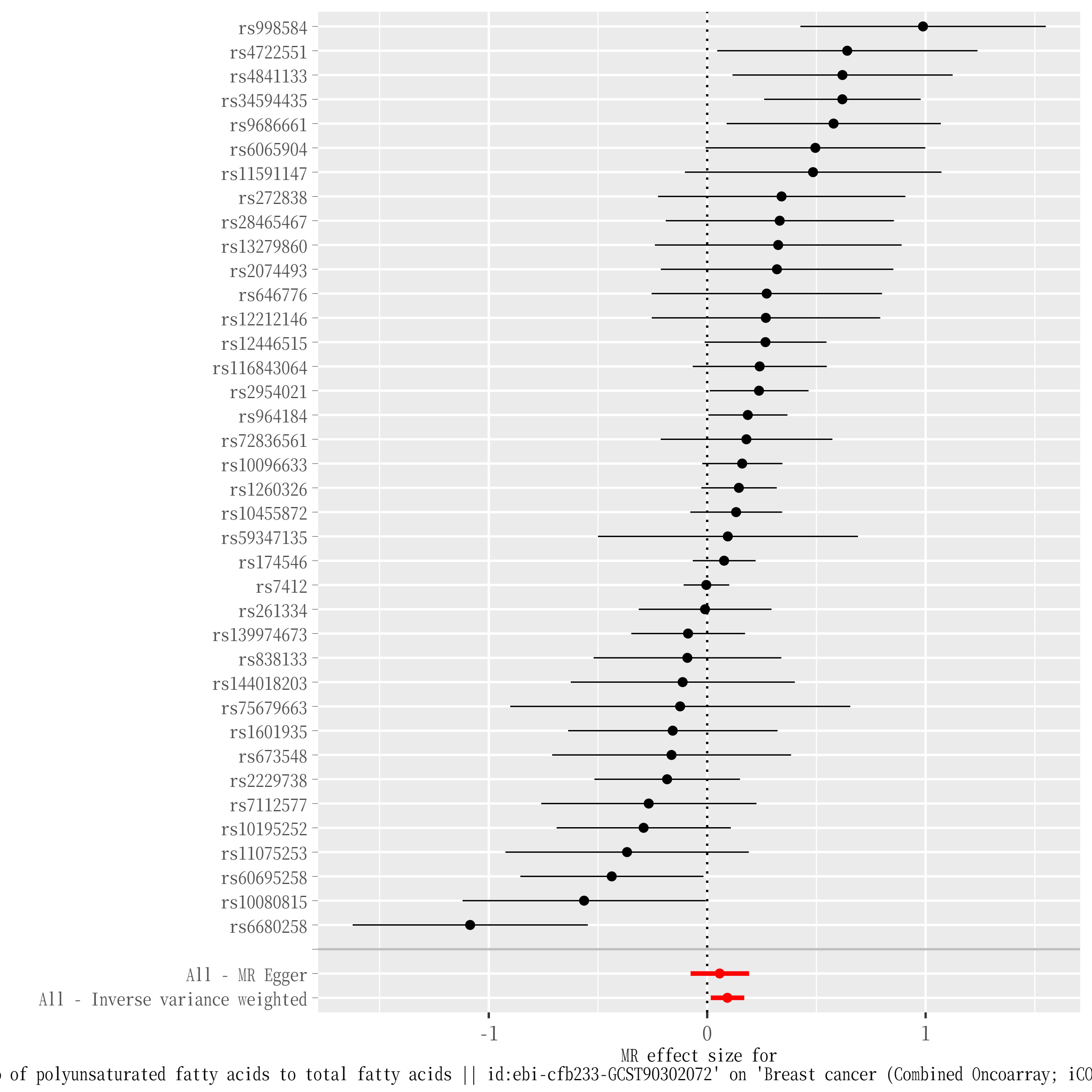
rs998584
rs4722551
rs4841133
rs34594435
rs9686661
rs6065904
rs11591147
rs272838
rs28465467
rs13279860
rs2074493
rs646776
rs12212146
rs12446515
rs116843064
rs2954021
rs964184
rs72836561
rs10096633
rs1260326
rs10455872
rs59347135
rs174546
rs7412
rs261334
rs139974673
rs838133
rs144018203
rs75679663
rs1601935
rs673548
rs2229738
rs7112577
rs10195252
rs11075253
rs60695258
rs10080815
rs6680258
All - MR Egger
All - Inverse variance weighted
-1
0
1
MR effect size for
o of polyunsaturated fatty acids to total fatty acids || id:ebi-cfb233-GCST90302072' on 'Breast cancer (Combined Oncoarray; iCO

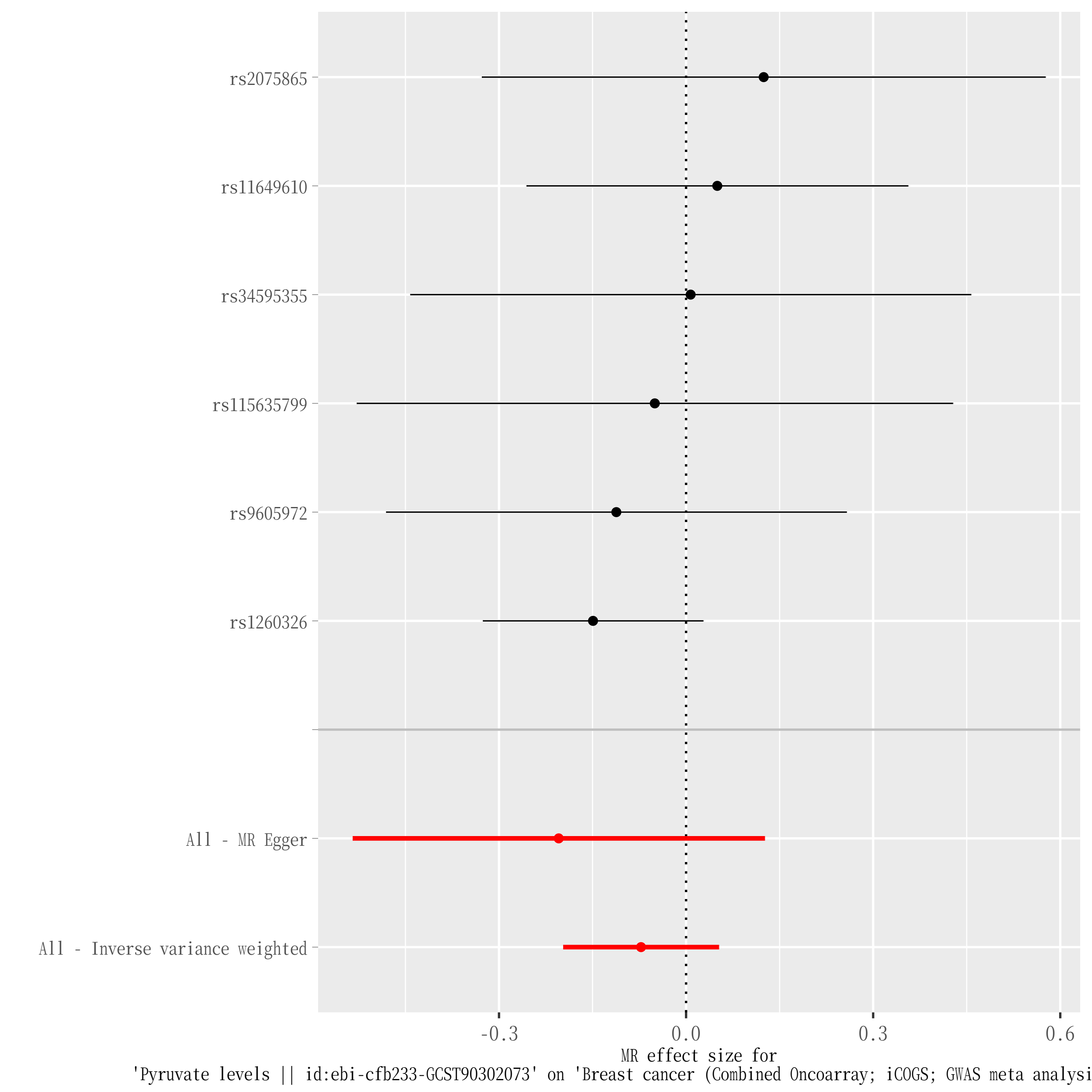
rs2075865
rs11649610
rs34595355
rs115635799
rs9605972
rs1260326
All - MR Egger
All - Inverse variance weighted
-0.3
0.0
0.3
0.6
MR effect size for
'Pyruvate levels || id:ebi-cfb233-GCST90302073' on 'Breast cancer (Combined Oncoarray; iCOGS; GWAS meta analysi

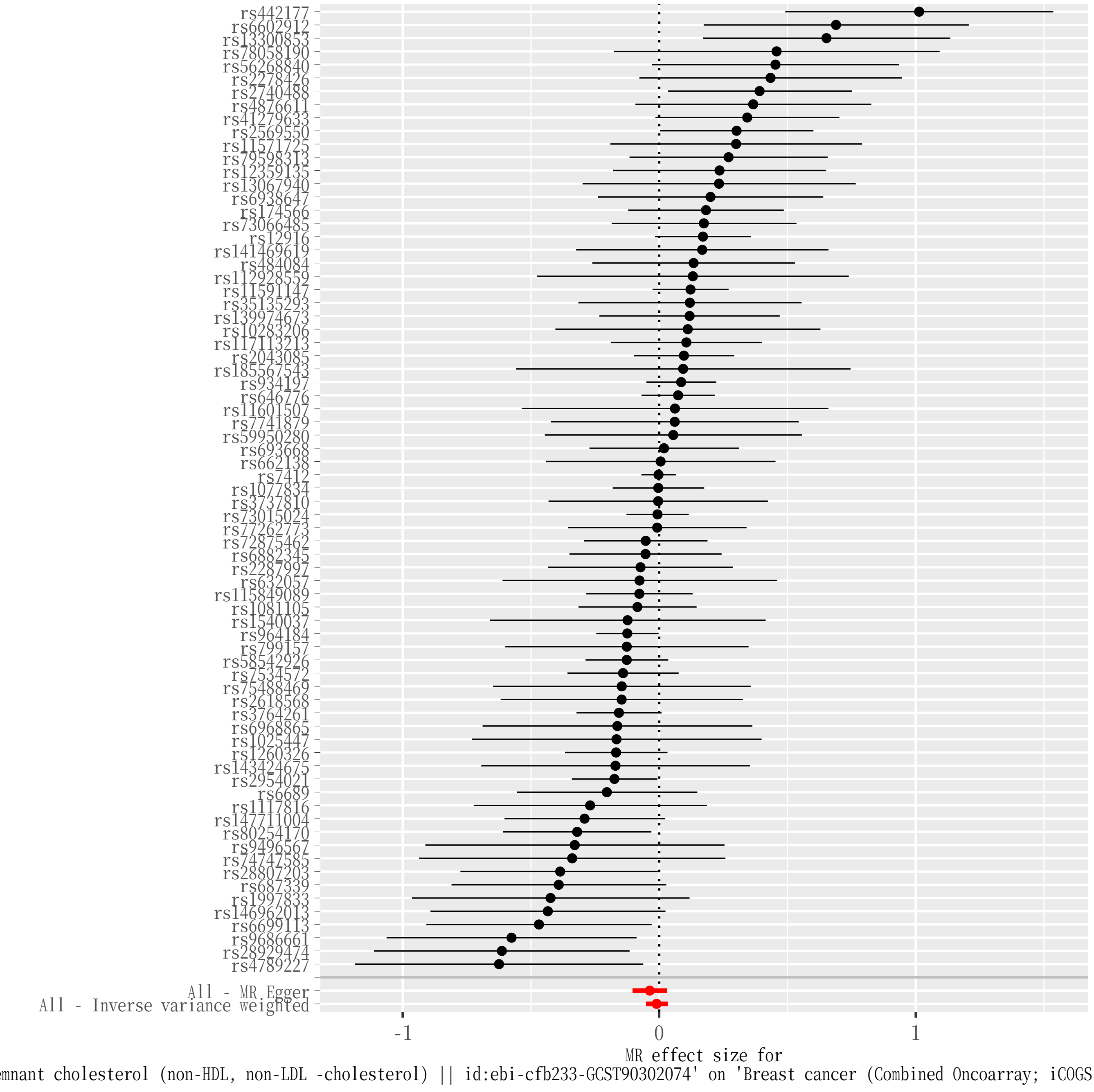
rs442177
rs6602912
rs13300853
rs78058190
rs56268840
rs2278426
rs2740488
rs4876611
rs41279633
rs2569550
rs11571725
rs79598313
rs12359135
rs13067940
rs6938647
rs174566
rs73066485
rs12916
rs141469619
rs484084
rs112928559
rs11591147
rs35135293
rs139974673
rs10283206
rs117113213
rs2043085
rs185567543
rs934197
rs646776
rs11601507
rs7741879
rs59950280
rs693668
rs662138
rs7412
rs1077834
rs3737810
rs73015024
rs77262773
rs72875462
rs6882345
rs2287997
rs632057
rs115849089
rs1081105
rs1540037
rs964184
rs799157
rs58542926
rs7534572
rs75488469
rs2618568
rs3764261
rs6968865
rs1025447
rs1260326
rs143424675
rs2954021
rs6689
rs1117816
rs147711004
rs80254170
rs9496567
rs74747585
rs28807203
rs687339
rs1997833
rs146962013
rs6699113
rs9686661
rs28929474
rs4789227
All - MR Egger
All - Inverse variance weighted
-1
0
1
MR effect size for
emnant cholesterol (non-HDL, non-LDL -cholesterol) || id:ebi-cfb233-GCST90302074' on 'Breast cancer (Combined Oncoarray; iCOGS;

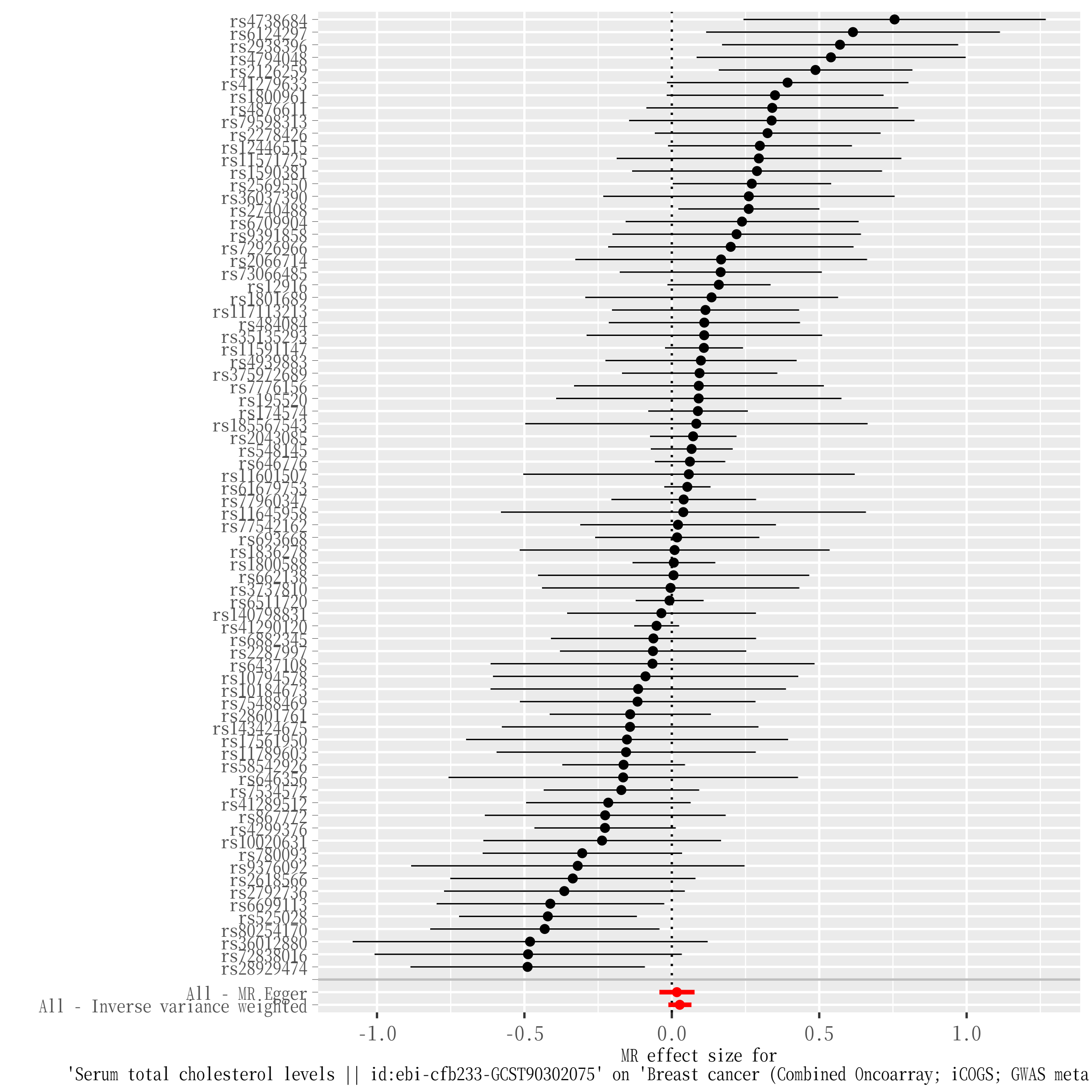
rs4738684
rs6124297
rs2938396
rs4794048
rs2126259
rs41279633
rs1800961
rs4876611
rs79598313
rs2278426
rs12446515
rs11571725
rs1590381
rs2569550
rs36037390
rs2740488
rs6709904
rs9391858
rs72926966
rs2066714
rs73066485
rs12916
rs1801689
rs117113213
rs484084
rs35135293
rs11591147
rs4939883
rs375972689
rs7776156
rs195520
rs174574
rs185567543
rs2043085
rs548145
rs646776
rs11601507
rs61679753
rs77960347
rs11645958
rs77542162
rs693668
rs1836278
rs1800588
rs662138
rs3737810
rs6511720
rs140798831
rs41290120
rs6882345
rs2287997
rs6437108
rs10794578
rs10184673
rs75488469
rs28601761
rs143424675
rs17561950
rs11789603
rs58542926
rs646356
rs7534572
rs41289512
rs867772
rs4299376
rs10020631
rs780093
rs9376092
rs2618566
rs2792736
rs6699113
rs525028
rs80254170
rs36012880
rs72838016
rs28929474
All - MR Egger
All - Inverse variance weighted
-1.0
-0.5
0.0
0.5
1.0
MR effect size for
'Serum total cholesterol levels || id:ebi-cfb233-GCST90302075' on 'Breast cancer (Combined Oncoarray; iCOGS; GWAS meta

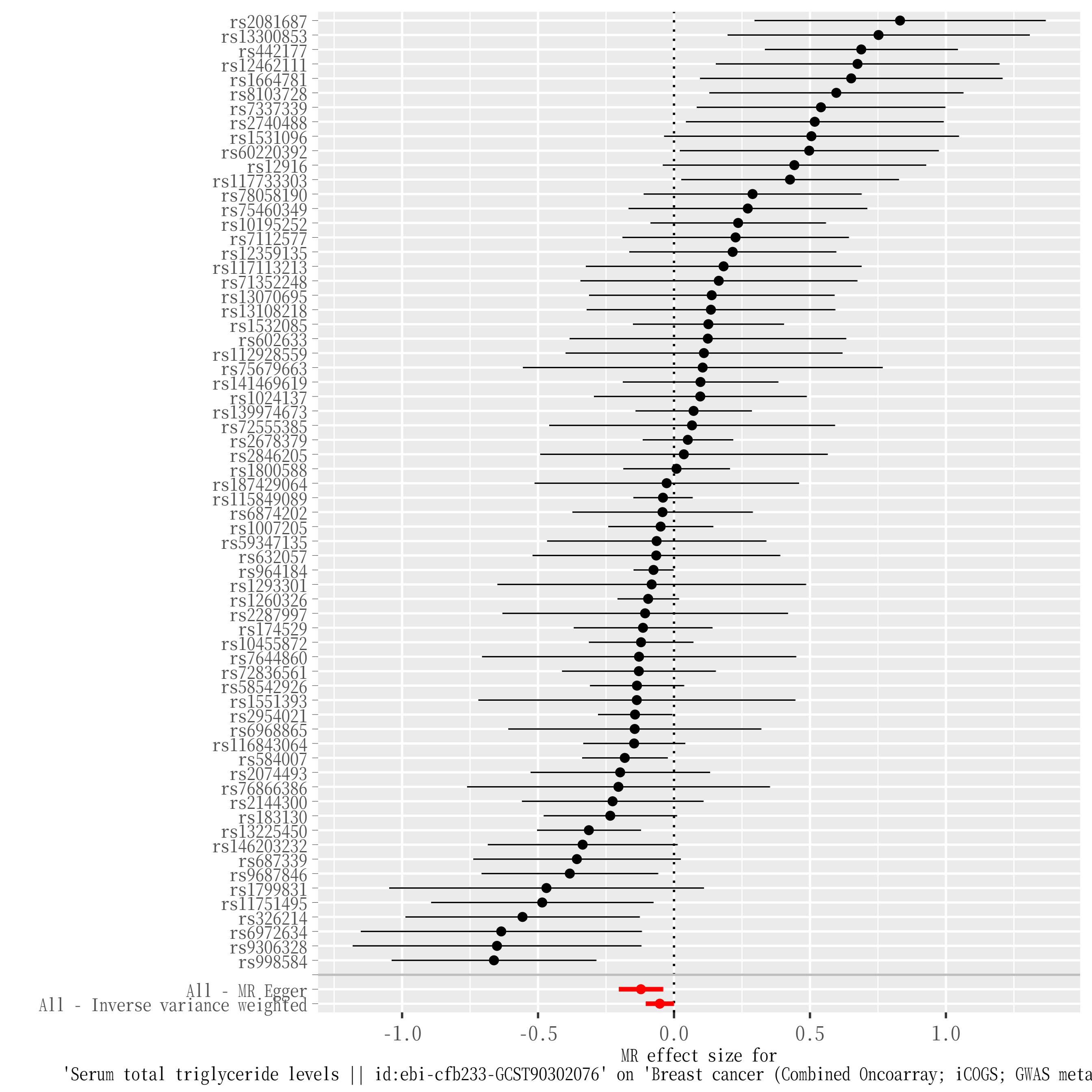
rs2081687
rs13300853
rs442177
rs12462111
rs1664781
rs8103728
rs7337339
rs2740488
rs1531096
rs60220392
rs12916
rs117733303
rs78058190
rs75460349
rs10195252
rs7112577
rs12359135
rs117113213
rs71352248
rs13070695
rs13108218
rs1532085
rs602633
rs112928559
rs75679663
rs141469619
rs1024137
rs139974673
rs72555385
rs2678379
rs2846205
rs1800588
rs187429064
rs115849089
rs6874202
rs1007205
rs59347135
rs632057
rs964184
rs1293301
rs1260326
rs2287997
rs174529
rs10455872
rs7644860
rs72836561
rs58542926
rs1551393
rs2954021
rs6968865
rs116843064
rs584007
rs2074493
rs76866386
rs2144300
rs183130
rs13225450
rs146203232
rs687339
rs9687846
rs1799831
rs11751495
rs326214
rs6972634
rs9306328
rs998584
All - MR Egger
All - Inverse variance weighted
-1.0
-0.5
0.0
0.5
1.0
MR effect size for
'Serum total triglyceride levels || id:ebi-cfb233-GCST90302076' on 'Breast cancer (Combined Oncoarray; iCOGS; GWAS meta

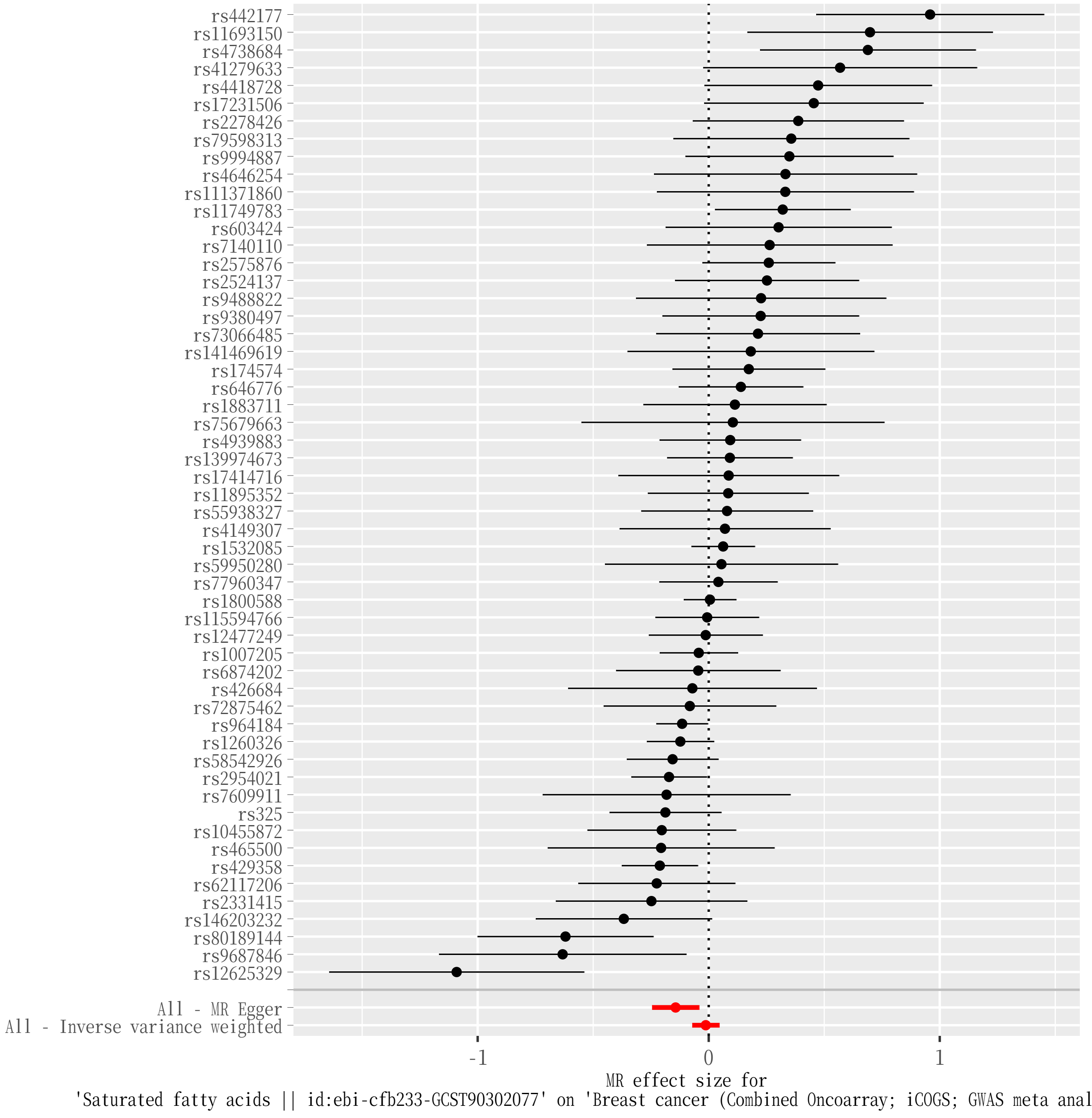

rs442177
rs11693150
rs4738684
rs41279633
rs4418728
rs17231506
rs2278426
rs79598313
rs9994887
rs4646254
rs111371860
rs11749783
rs603424
rs7140110
rs2575876
rs2524137
rs9488822
rs9380497
rs73066485
rs141469619
rs174574
rs646776
rs1883711
rs75679663
rs4939883
rs139974673
rs17414716
rs11895352
rs55938327
rs4149307
rs1532085
rs59950280
rs77960347
rs1800588
rs115594766
rs12477249
rs1007205
rs6874202
rs426684
rs72875462
rs964184
rs1260326
rs58542926
rs2954021
rs7609911
rs325
rs10455872
rs465500
rs429358
rs62117206
rs2331415
rs146203232
rs80189144
rs9687846
rs12625329
All - MR Egger
All - Inverse variance weighted
-1
0
1
MR effect size for
'Saturated fatty acids || id:ebi-cfb233-GCST90302077' on 'Breast cancer (Combined Oncoarray; iCOGS; GWAS meta anal

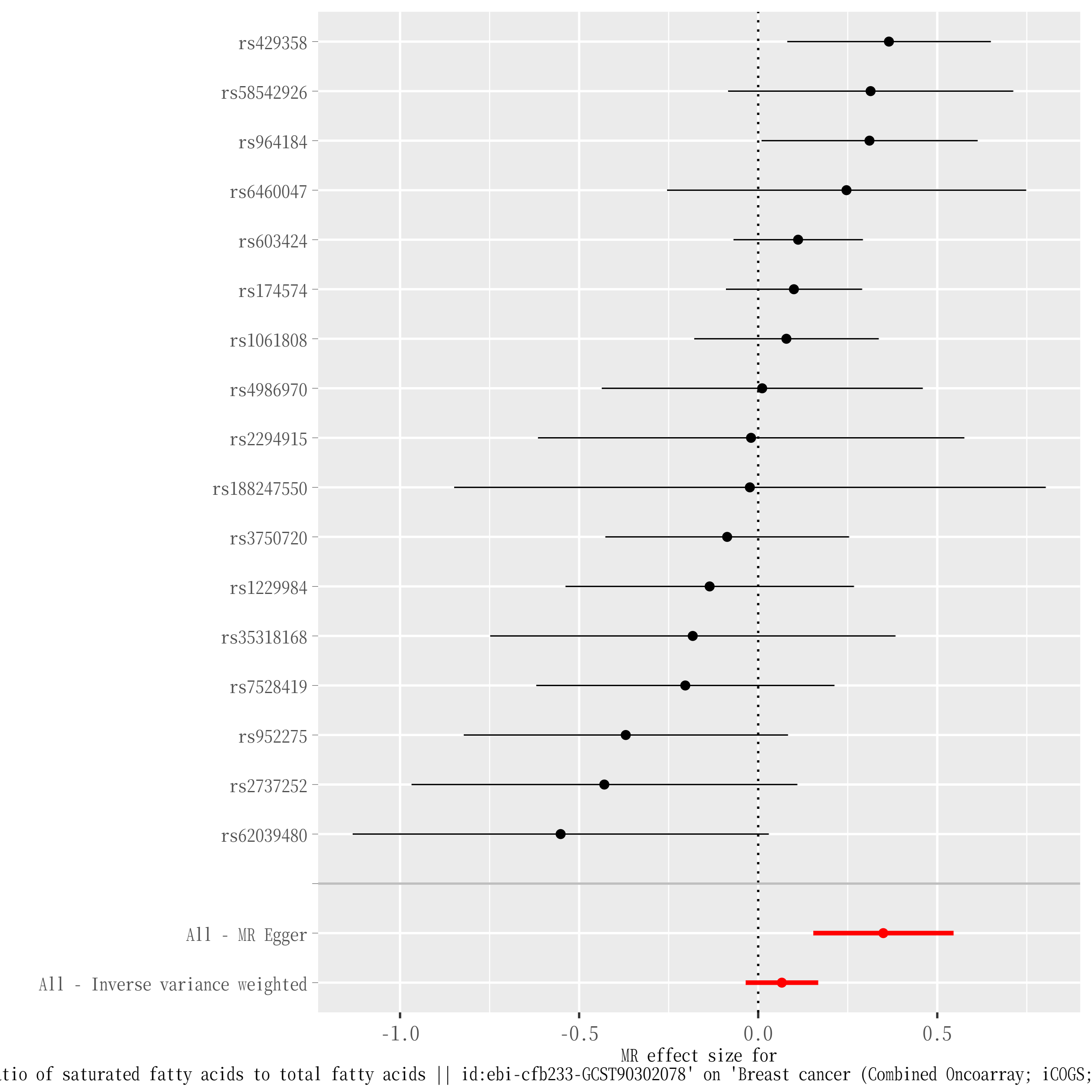

rs429358
rs58542926
rs964184
rs6460047
rs603424
rs174574
rs1061808
rs4986970
rs2294915
rs188247550
rs3750720
rs1229984
rs35318168
rs7528419
rs952275
rs2737252
rs62039480
All - MR Egger
All - Inverse variance weighted
-1.0
-0.5
0.0
0.5
MR effect size for
tio of saturated fatty acids to total fatty acids || id:ebi-cfb233-GCST90302078' on 'Breast cancer (Combined Oncoarray; iCOGS;

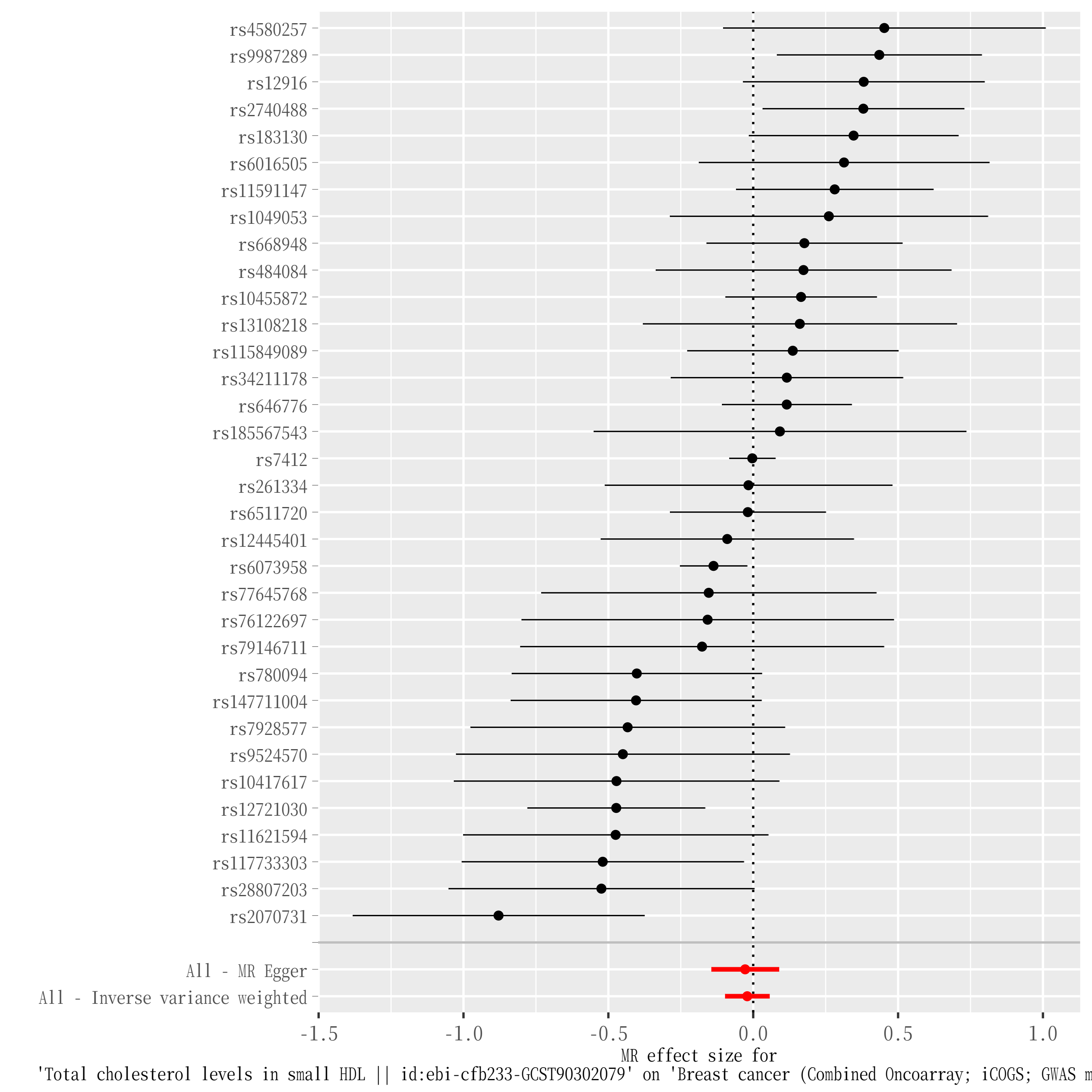
rs4580257
rs9987289
rs12916
rs2740488
rs183130
rs6016505
rs11591147
rs1049053
rs668948
rs484084
rs10455872
rs13108218
rs115849089
rs34211178
rs646776
rs185567543
rs7412
rs261334
rs6511720
rs12445401
rs6073958
rs77645768
rs76122697
rs79146711
rs780094
rs147711004
rs7928577
rs9524570
rs10417617
rs12721030
rs11621594
rs117733303
rs28807203
rs2070731
All - MR Egger
All - Inverse variance weighted
-1.5
-1.0
-0.5
0.0
0.5
1.0
MR effect size for
'Total cholesterol levels in small HDL || id:ebi-cfb233-GCST90302079' on 'Breast cancer (Combined Oncoarray; iCOGS; GWAS m

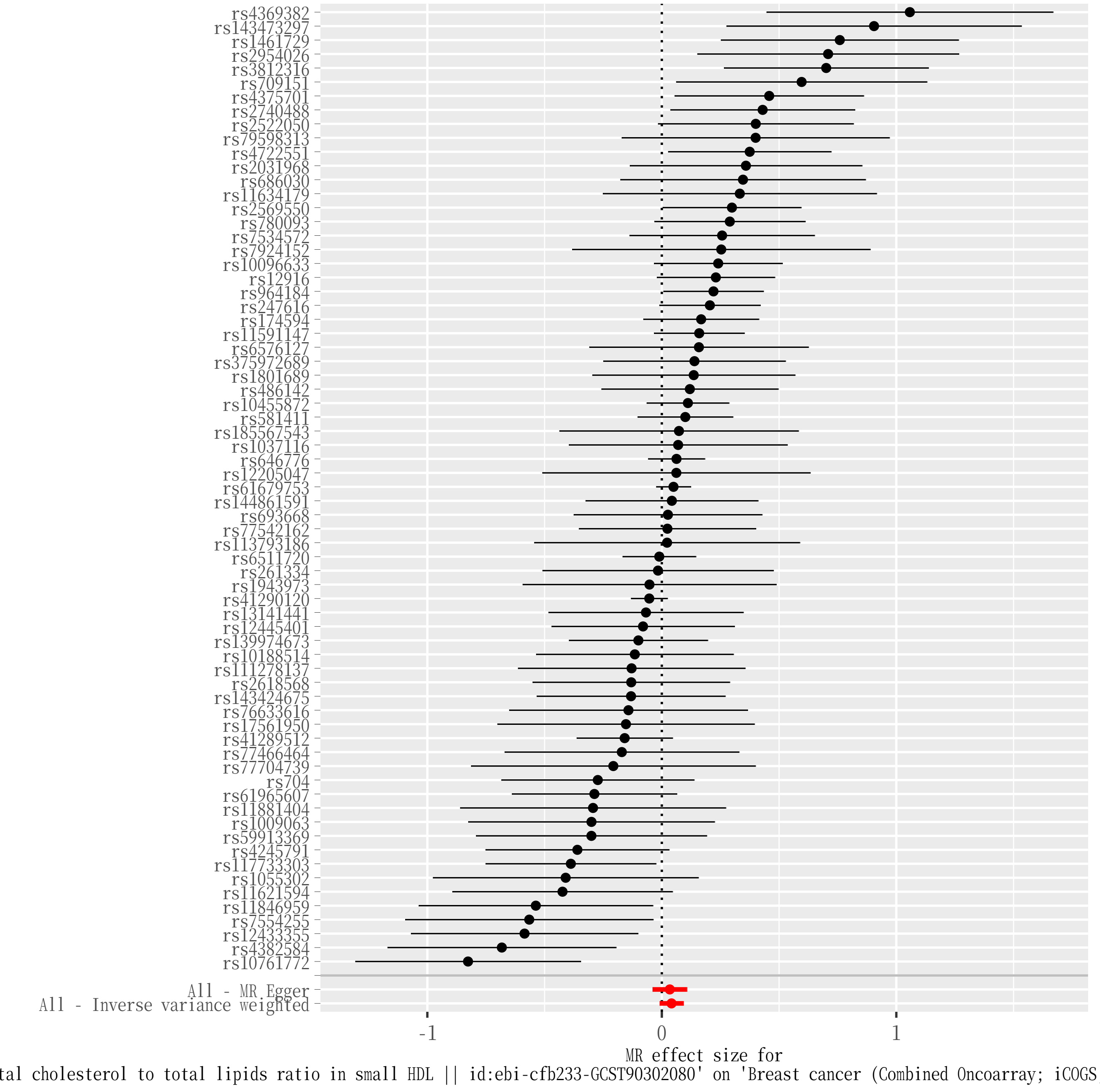

rs4369382
rs143473297
rs1461729
rs2954026
rs3812316
rs709151
rs4375701
rs2740488
rs2522050
rs79598313
rs4722551
rs2031968
rs686030
rs11634179
rs2569550
rs780093
rs7534572
rs7924152
rs10096633
rs12916
rs964184
rs247616
rs174594
rs11591147
rs6576127
rs375972689
rs1801689
rs486142
rs10455872
rs581411
rs185567543
rs1037116
rs646776
rs12205047
rs61679753
rs144861591
rs693668
rs77542162
rs113793186
rs6511720
rs261334
rs1943973
rs41290120
rs13141441
rs12445401
rs139974673
rs10188514
rs111278137
rs2618568
rs143424675
rs76633616
rs17561950
rs41289512
rs77466464
rs77704739
rs704
rs61965607
rs11881404
rs1009063
rs59913369
rs4245791
rs117733303
rs1055302
rs11621594
rs11846959
rs7554255
rs12433355
rs4382584
rs10761772
All - MR Egger
All - Inverse variance weighted
-1
0
1
MR effect size for
tal cholesterol to total lipids ratio in small HDL || id:ebi-cfb233-GCST90302080' on 'Breast cancer (Combined Oncoarray; iCOGS

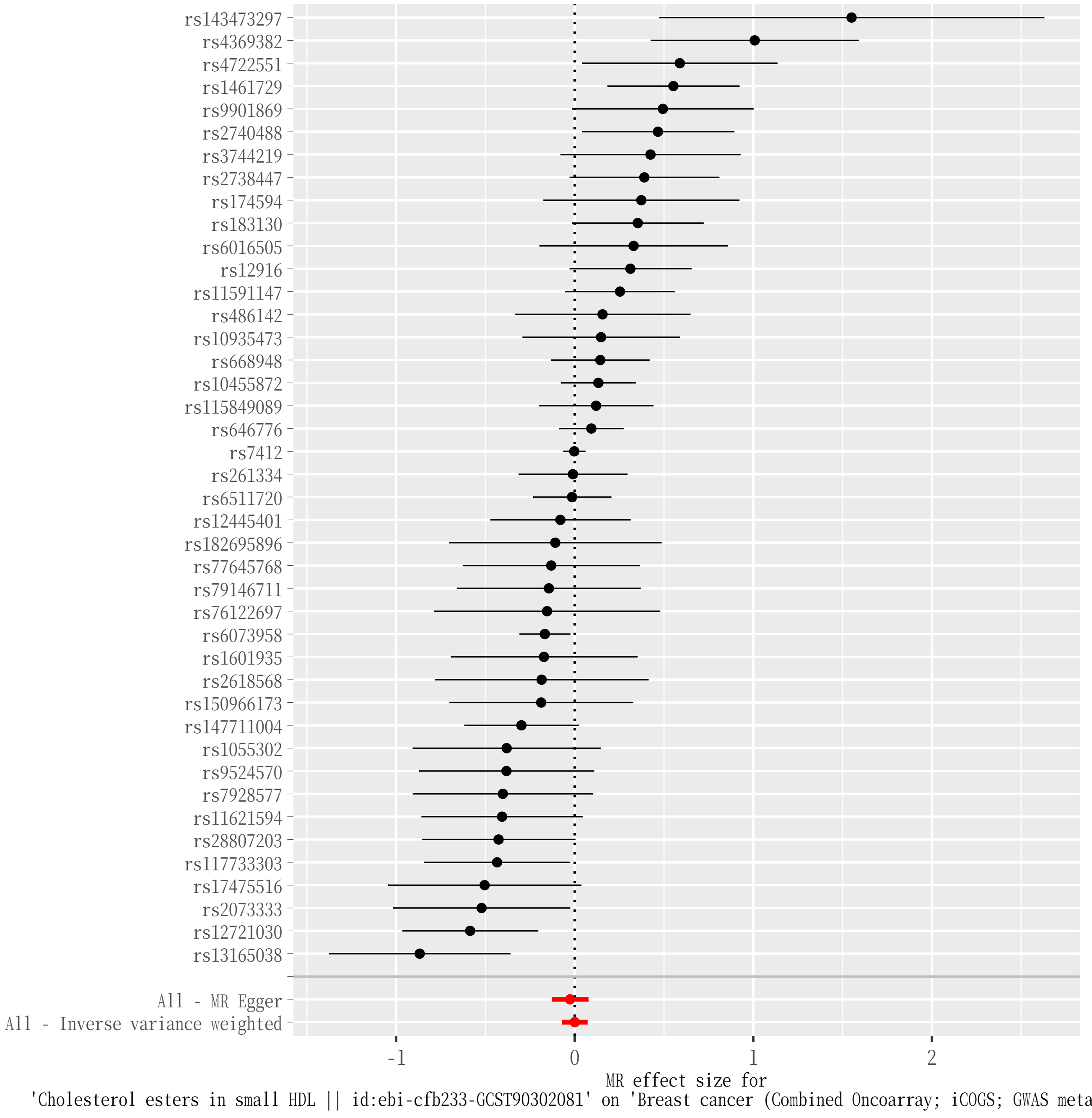

rs143473297
rs4369382
rs4722551
rs1461729
rs9901869
rs2740488
rs3744219
rs2738447
rs174594
rs183130
rs6016505
rs12916
rs11591147
rs486142
rs10935473
rs668948
rs10455872
rs115849089
rs646776
rs7412
rs261334
rs6511720
rs12445401
rs182695896
rs77645768
rs79146711
rs76122697
rs6073958
rs1601935
rs2618568
rs150966173
rs147711004
rs1055302
rs9524570
rs7928577
rs11621594
rs28807203
rs117733303
rs17475516
rs2073333
rs12721030
rs13165038
All - MR Egger
All - Inverse variance weighted
-1
0
1
2
MR effect size for
'Cholesterol esters in small HDL || id:ebi-cfb233-GCST90302081' on 'Breast cancer (Combined Oncoarray; iCOGS; GWAS meta

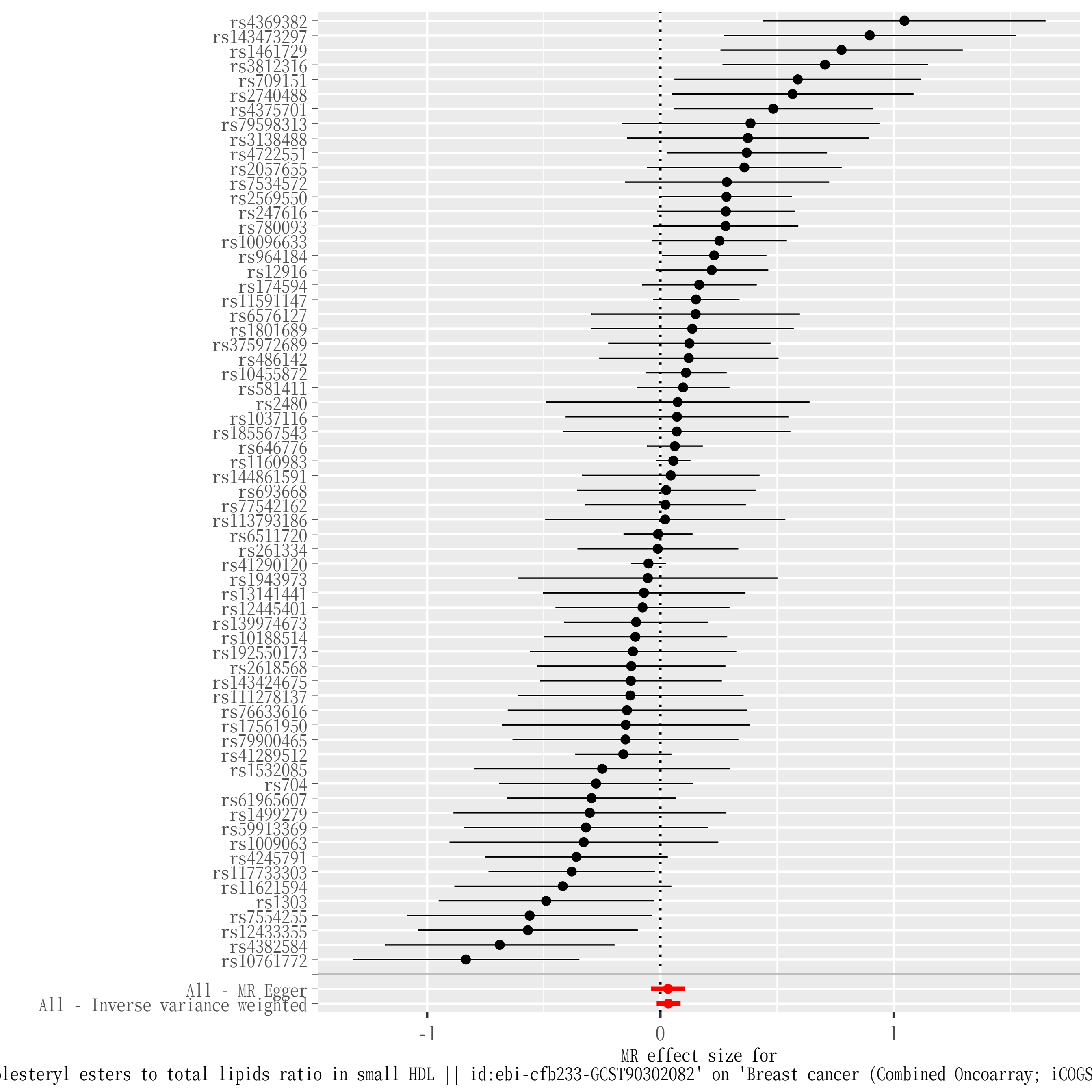
rs4369382
rs143473297
rs1461729
rs3812316
rs709151
rs2740488
rs4375701
rs79598313
rs3138488
rs4722551
rs2057655
rs7534572
rs2569550
rs247616
rs780093
rs10096633
rs964184
rs12916
rs174594
rs11591147
rs6576127
rs1801689
rs375972689
rs486142
rs10455872
rs581411
rs2480
rs1037116
rs185567543
rs646776
rs1160983
rs144861591
rs693668
rs77542162
rs113793186
rs6511720
rs261334
rs41290120
rs1943973
rs13141441
rs12445401
rs139974673
rs10188514
rs192550173
rs2618568
rs143424675
rs111278137
rs76633616
rs17561950
rs79900465
rs41289512
rs1532085
rs704
rs61965607
rs1499279
rs59913369
rs1009063
rs4245791
rs117733303
rs11621594
rs1303
rs7554255
rs12433355
rs4382584
rs10761772
All - MR Egger
All - Inverse variance weighted
-1
0
1
MR effect size for
olesteryl esters to total lipids ratio in small HDL || id:ebi-cfb233-GCST90302082' on 'Breast cancer (Combined Oncoarray; iCOGS

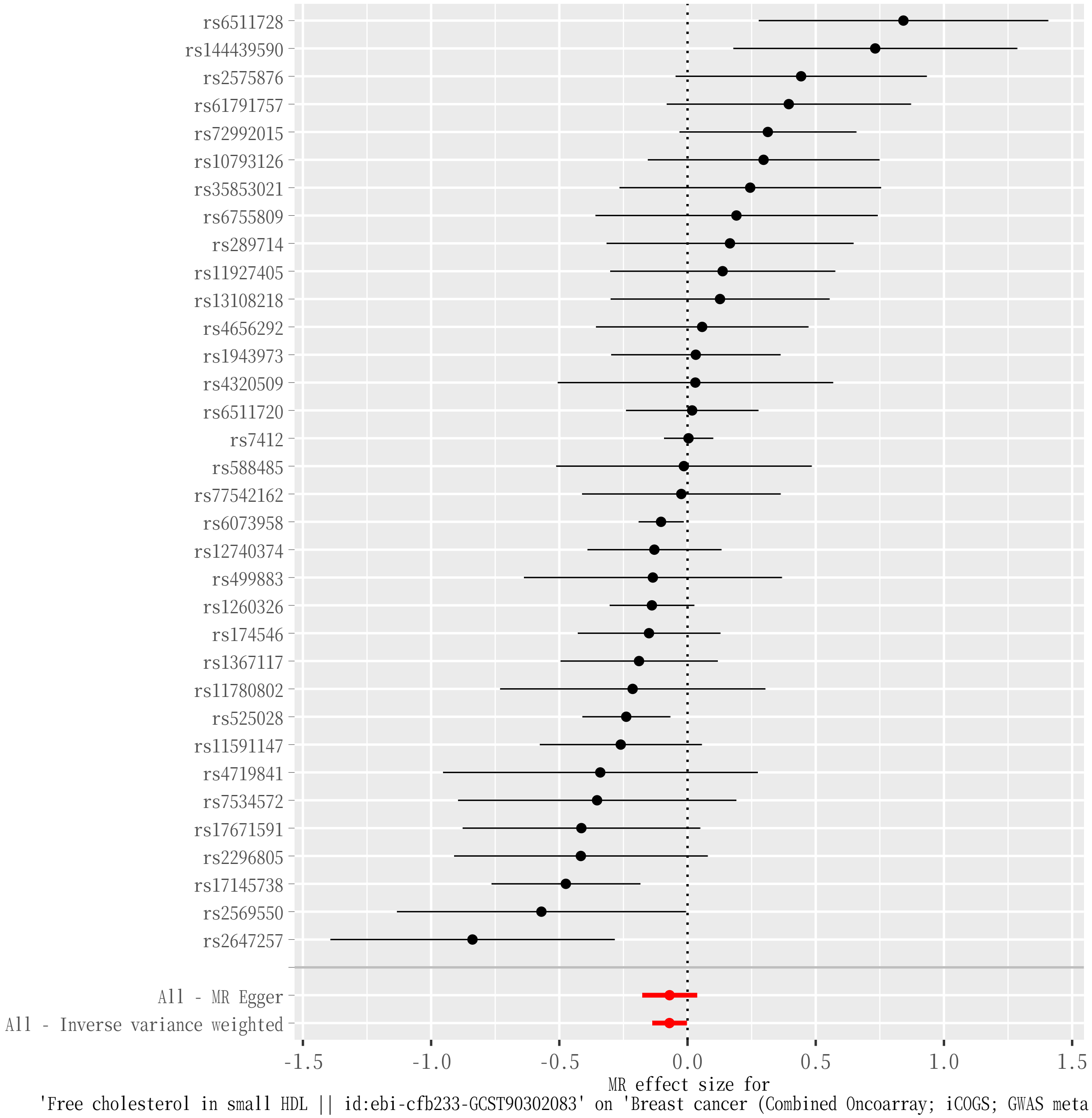
rs6511728
rs144439590
rs2575876
rs61791757
rs72992015
rs10793126
rs35853021
rs6755809
rs289714
rs11927405
rs13108218
rs4656292
rs1943973
rs4320509
rs6511720
rs7412
rs588485
rs77542162
rs6073958
rs12740374
rs499883
rs1260326
rs174546
rs1367117
rs11780802
rs525028
rs11591147
rs4719841
rs7534572
rs17671591
rs2296805
rs17145738
rs2569550
rs2647257
All - MR Egger
All - Inverse variance weighted
-1.5
-1.0
-0.5
0.0
0.5
1.0
1.5
MR effect size for
'Free cholesterol in small HDL || id:ebi-cfb233-GCST90302083' on 'Breast cancer (Combined Oncoarray; iCOGS; GWAS meta

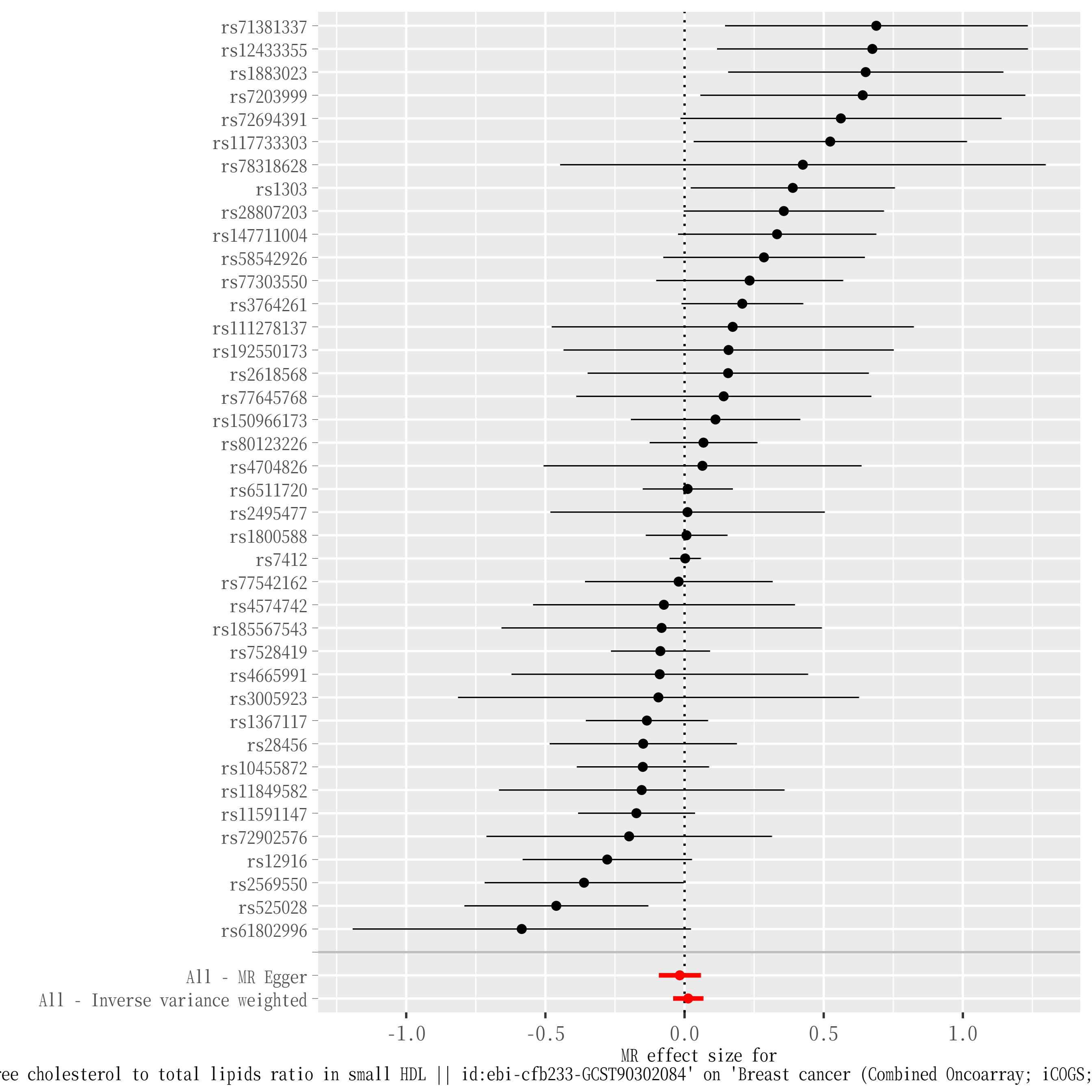
rs71381337
rs12433355
rs1883023
rs7203999
rs72694391
rs117733303
rs78318628
rs1303
rs28807203
rs147711004
rs58542926
rs77303550
rs3764261
rs111278137
rs192550173
rs2618568
rs77645768
rs150966173
rs80123226
rs4704826
rs6511720
rs2495477
rs1800588
rs7412
rs77542162
rs4574742
rs185567543
rs7528419
rs4665991
rs3005923
rs1367117
rs28456
rs10455872
rs11849582
rs11591147
rs72902576
rs12916
rs2569550
rs525028
rs61802996
All - MR Egger
All - Inverse variance weighted
-1.0
-0.5
0.0
0.5
1.0
MR effect size for
ree cholesterol to total lipids ratio in small HDL || id:ebi-cfb233-GCST90302084' on 'Breast cancer (Combined Oncoarray; iCOGS;

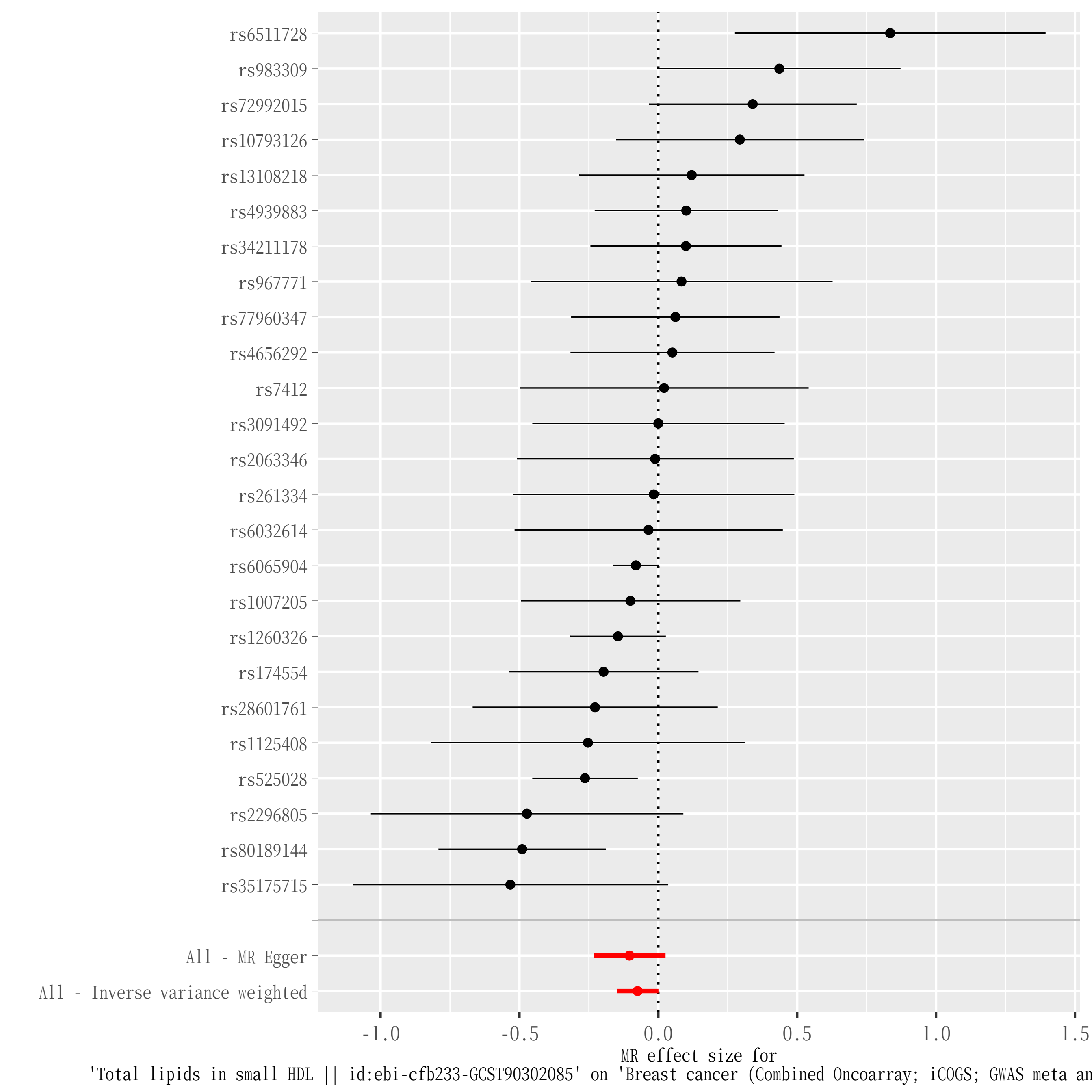

rs6511728
rs983309
rs72992015
rs10793126
rs13108218
rs4939883
rs34211178
rs967771
rs77960347
rs4656292
rs7412
rs3091492
rs2063346
rs261334
rs6032614
rs6065904
rs1007205
rs1260326
rs174554
rs28601761
rs1125408
rs525028
rs2296805
rs80189144
rs35175715
All - MR Egger
All - Inverse variance weighted
-1.0
-0.5
0.0
0.5
1.0
1.5
MR effect size for
'Total lipids in small HDL || id:ebi-cfb233-GCST90302085' on 'Breast cancer (Combined Oncoarray; iCOGS; GWAS meta an

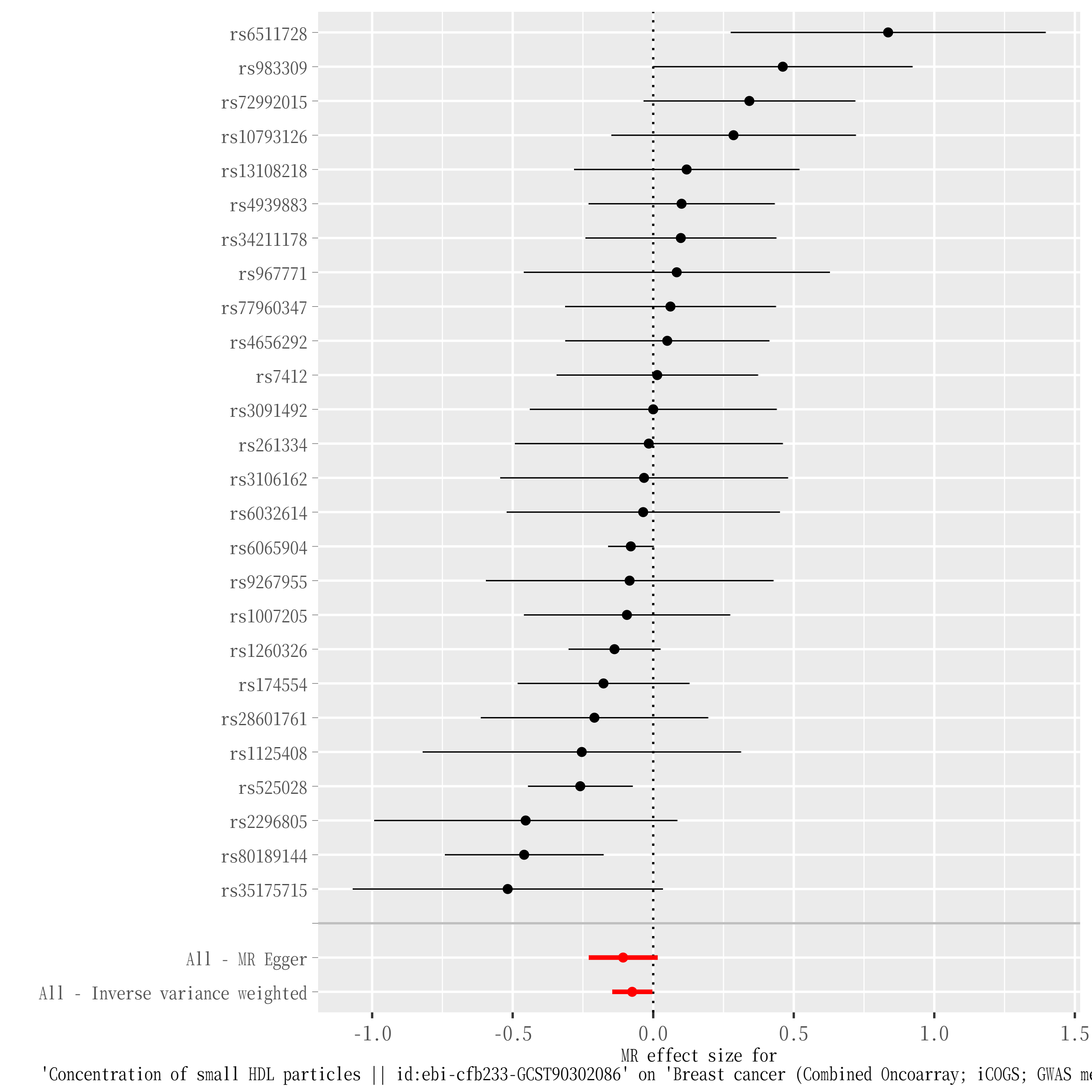
rs6511728
rs983309
rs72992015
rs10793126
rs13108218
rs4939883
rs34211178
rs967771
rs77960347
rs4656292
rs7412
rs3091492
rs261334
rs3106162
rs6032614
rs6065904
rs9267955
rs1007205
rs1260326
rs174554
rs28601761
rs1125408
rs525028
rs2296805
rs80189144
rs35175715
All - MR Egger
All - Inverse variance weighted
-1.0
-0.5
0.0
0.5
1.0
1.5
MR effect size for
'Concentration of small HDL particles || id:ebi-cfb233-GCST90302086' on 'Breast cancer (Combined Oncoarray; iCOGS; GWAS me

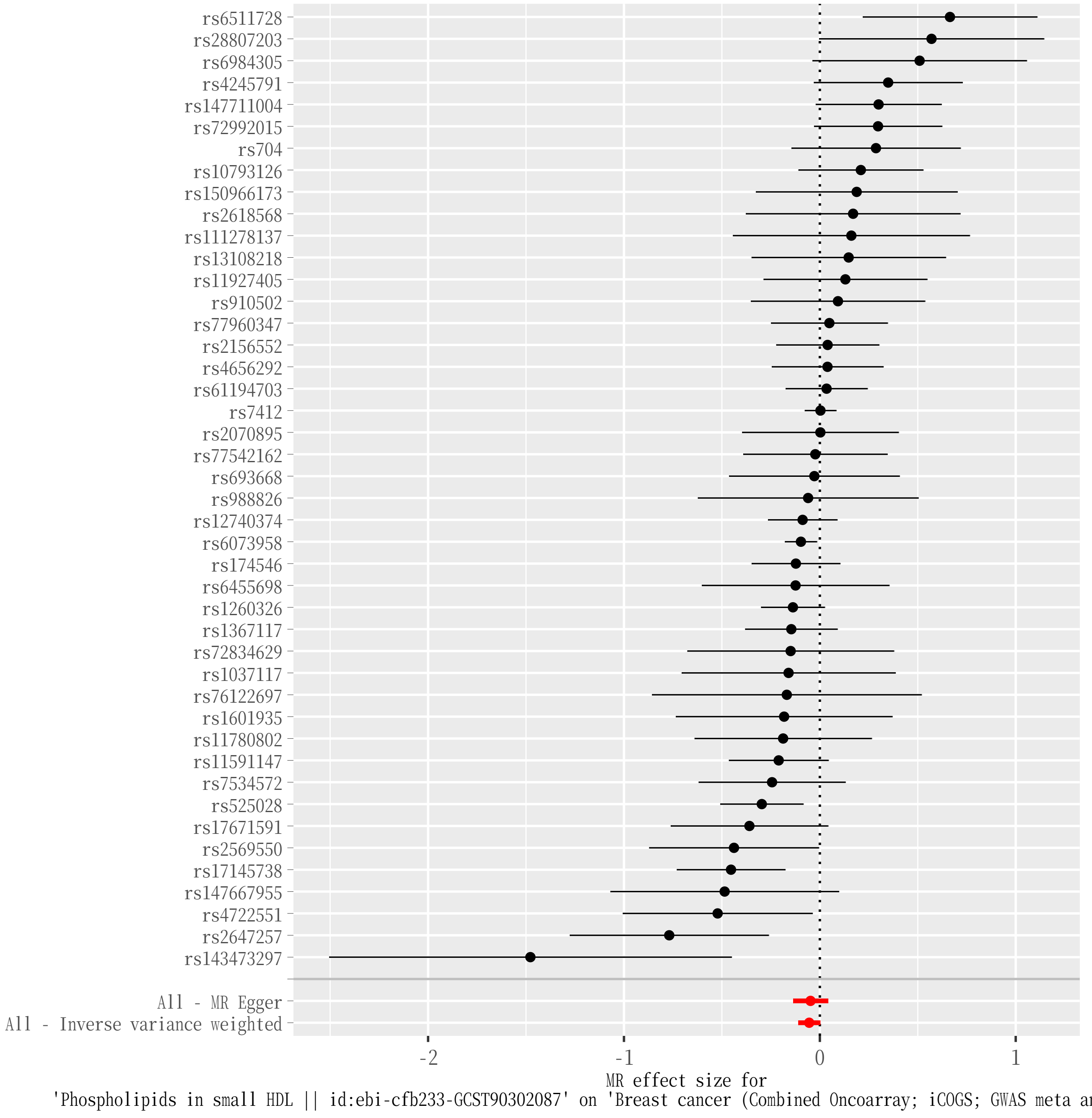
rs6511728
rs28807203
rs6984305
rs4245791
rs147711004
rs72992015
rs704
rs10793126
rs150966173
rs2618568
rs111278137
rs13108218
rs11927405
rs910502
rs77960347
rs2156552
rs4656292
rs61194703
rs7412
rs2070895
rs77542162
rs693668
rs988826
rs12740374
rs6073958
rs174546
rs6455698
rs1260326
rs1367117
rs72834629
rs1037117
rs76122697
rs1601935
rs11780802
rs11591147
rs7534572
rs525028
rs17671591
rs2569550
rs17145738
rs147667955
rs4722551
rs2647257
rs143473297
All - MR Egger
All - Inverse variance weighted
-2
-1
0
1
MR effect size for
'Phospholipids in small HDL || id:ebi-cfb233-GCST90302087' on 'Breast cancer (Combined Oncoarray; iCOGS; GWAS meta ar

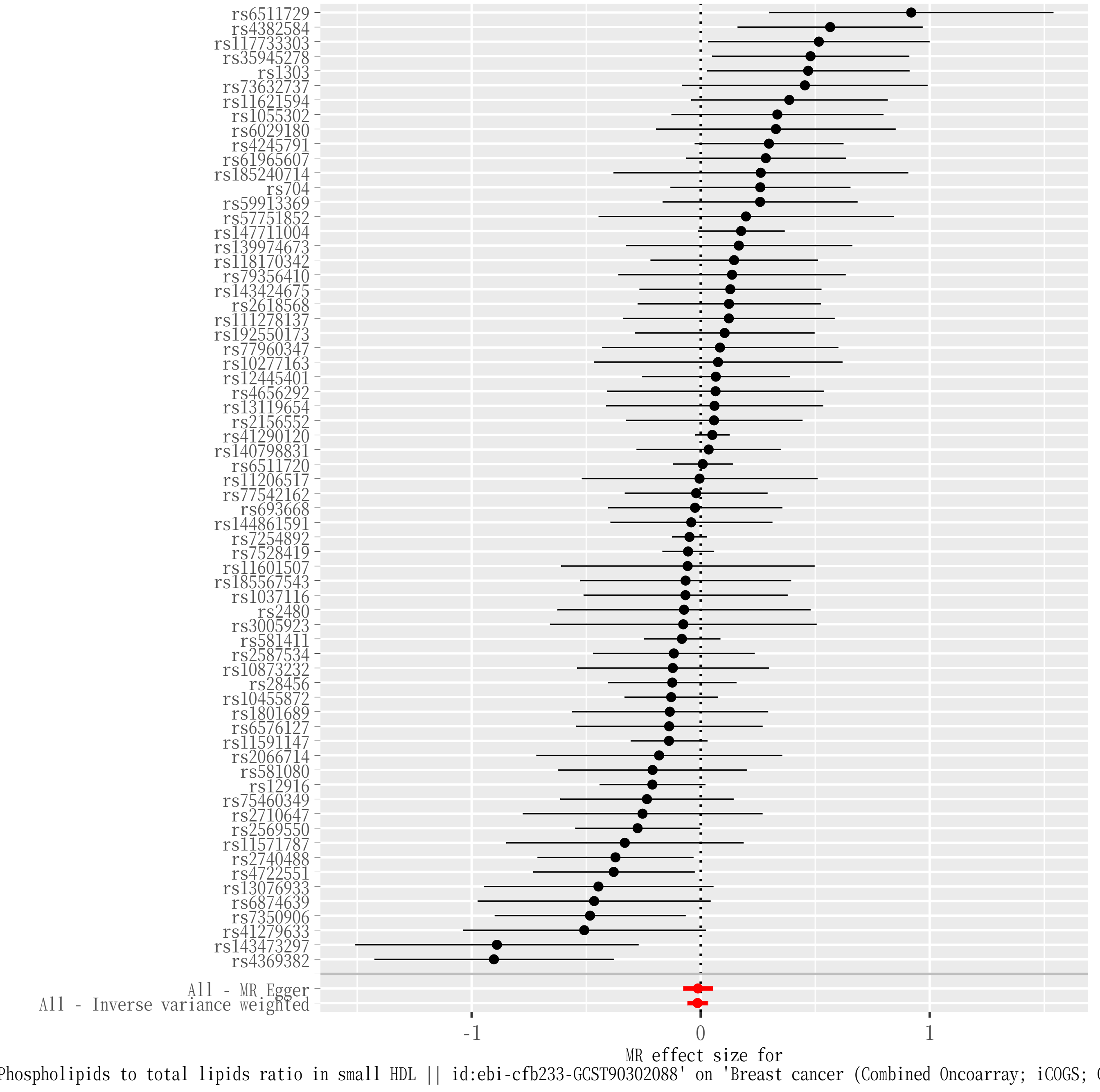
rs6511729
rs4382584
rs117733303
rs35945278
rs1303
rs73632737
rs11621594
rs1055302
rs6029180
rs4245791
rs61965607
rs185240714
rs704
rs59913369
rs57751852
rs147711004
rs139974673
rs118170342
rs79356410
rs143424675
rs2618568
rs111278137
rs192550173
rs77960347
rs10277163
rs12445401
rs4656292
rs13119654
rs2156552
rs41290120
rs140798831
rs6511720
rs11206517
rs77542162
rs693668
rs144861591
rs7254892
rs7528419
rs11601507
rs185567543
rs1037116
rs2480
rs3005923
rs581411
rs2587534
rs10873232
rs28456
rs10455872
rs1801689
rs6576127
rs11591147
rs2066714
rs581080
rs12916
rs75460349
rs2710647
rs2569550
rs11571787
rs2740488
rs4722551
rs13076933
rs6874639
rs7350906
rs41279633
rs143473297
rs4369382
All - MR Egger
All - Inverse variance weighted
-1
0
1
MR effect size for
'Phospholipids to total lipids ratio in small HDL || id:ebi-cfb233-GCST90302088' on 'Breast cancer (Combined Oncoarray; iCOGS; G

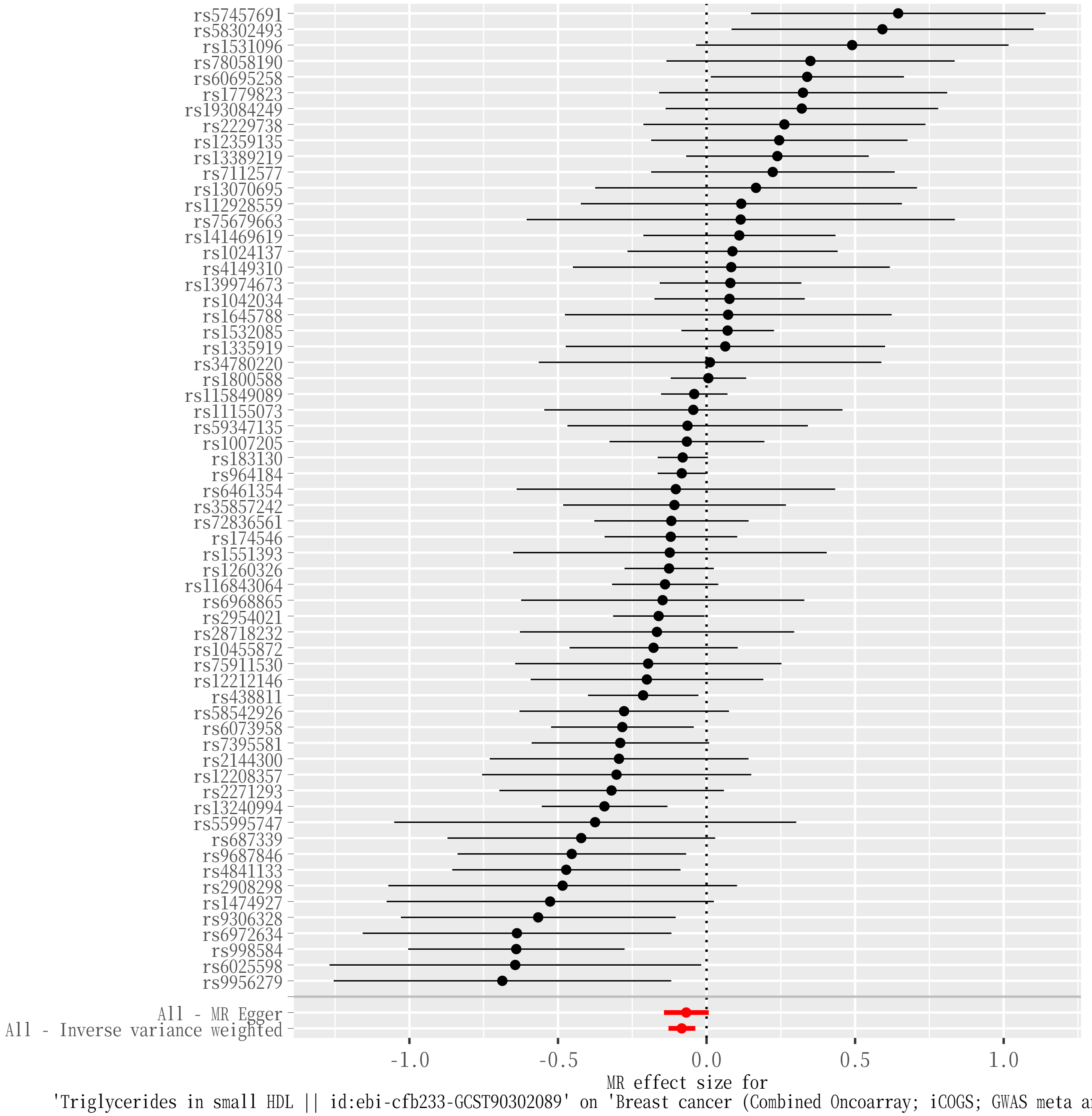
rs57457691
rs58302493
rs1531096
rs78058190
rs60695258
rs1779823
rs193084249
rs2229738
rs12359135
rs13389219
rs7112577
rs13070695
rs112928559
rs75679663
rs141469619
rs1024137
rs4149310
rs139974673
rs1042034
rs1645788
rs1532085
rs1335919
rs34780220
rs1800588
rs115849089
rs11155073
rs59347135
rs1007205
rs183130
rs964184
rs6461354
rs35857242
rs72836561
rs174546
rs1551393
rs1260326
rs116843064
rs6968865
rs2954021
rs28718232
rs10455872
rs75911530
rs12212146
rs438811
rs58542926
rs6073958
rs7395581
rs2144300
rs12208357
rs2271293
rs13240994
rs55995747
rs687339
rs9687846
rs4841133
rs2908298
rs1474927
rs9306328
rs6972634
rs998584
rs6025598
rs9956279
All - MR Egger
All - Inverse variance weighted
-1.0
-0.5
0.0
0.5
1.0
MR effect size for
'Triglycerides in small HDL || id:ebi-cfb233-GCST90302089' on 'Breast cancer (Combined Oncoarray; iCOGS; GWAS meta an

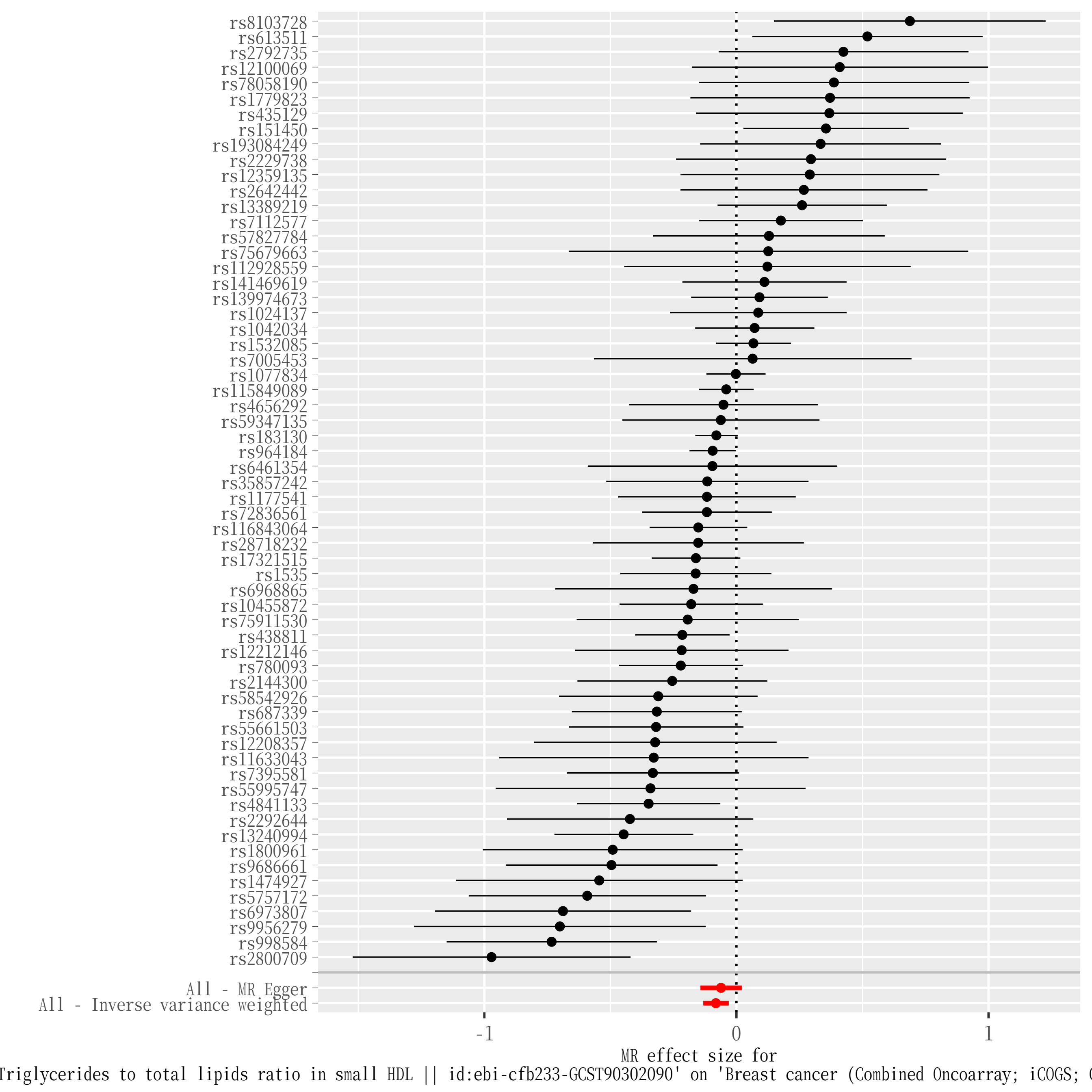

rs8103728
rs613511
rs2792735
rs12100069
rs78058190
rs1779823
rs435129
rs151450
rs193084249
rs2229738
rs12359135
rs2642442
rs13389219
rs7112577
rs57827784
rs75679663
rs112928559
rs141469619
rs139974673
rs1024137
rs1042034
rs1532085
rs7005453
rs1077834
rs115849089
rs4656292
rs59347135
rs183130
rs964184
rs6461354
rs35857242
rs1177541
rs72836561
rs116843064
rs28718232
rs17321515
rs1535
rs6968865
rs10455872
rs75911530
rs438811
rs12212146
rs780093
rs2144300
rs58542926
rs687339
rs55661503
rs12208357
rs11633043
rs7395581
rs55995747
rs4841133
rs2292644
rs13240994
rs1800961
rs9686661
rs1474927
rs5757172
rs6973807
rs9956279
rs998584
rs2800709
All - MR Egger
All - Inverse variance weighted
-1
0
1
MR effect size for
Triglycerides to total lipids ratio in small HDL || id:ebi-cfb233-GCST90302090' on 'Breast cancer (Combined Oncoarray; iCOGS; (

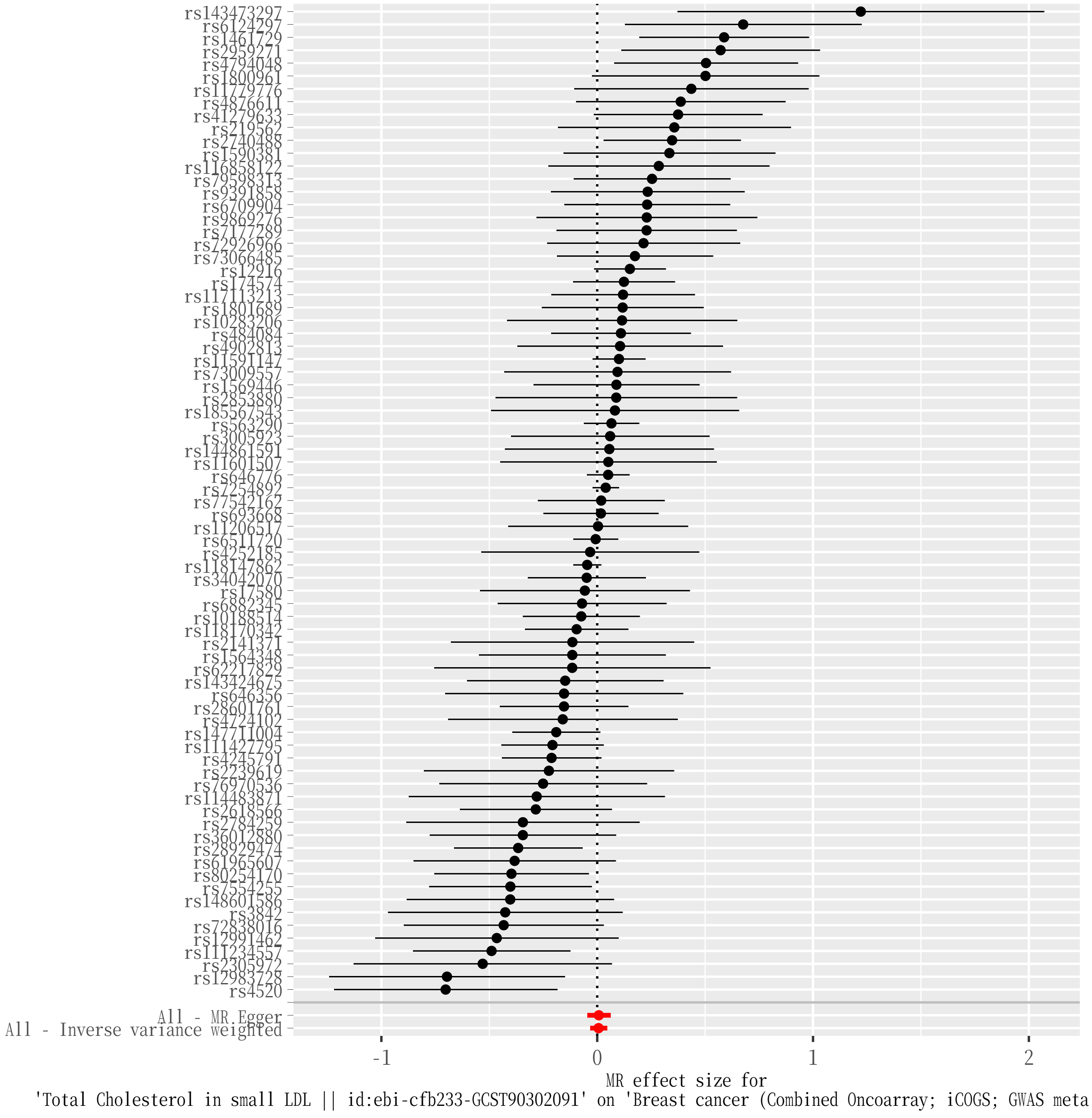
rs143473297
rs6124297
rs1461729
rs2959271
rs4794048
rs1800961
rs11779776
rs4876611
rs41279633
rs219562
rs2740488
rs1590381
rs116858122
rs79598313
rs9391858
rs6709904
rs9869276
rs7177289
rs72926966
rs73066485
rs12916
rs174574
rs117113213
rs1801689
rs10283206
rs484084
rs4902813
rs11591147
rs73009557
rs1569446
rs2853880
rs185567543
rs563290
rs3005923
rs144861591
rs11601507
rs646776
rs7254892
rs77542162
rs693668
rs11206517
rs6511720
rs4252185
rs118147862
rs34042070
rs17580
rs6882345
rs10188514
rs118170342
rs2141371
rs1564348
rs62217829
rs143424675
rs646356
rs28601761
rs4724102
rs147711004
rs111427795
rs4245791
rs2239619
rs76970536
rs114483871
rs2618566
rs2784259
rs36012880
rs28929474
rs61965607
rs80254170
rs7554255
rs148601586
rs3842
rs72838016
rs12991462
rs111234557
rs2305972
rs12983728
rs4520
All - MR Egger
All - Inverse variance weighted
-1
0
1
2
MR effect size for
'Total Cholesterol in small LDL || id:ebi-cfb233-GCST90302091' on 'Breast cancer (Combined Oncoarray; iCOGS; GWAS meta

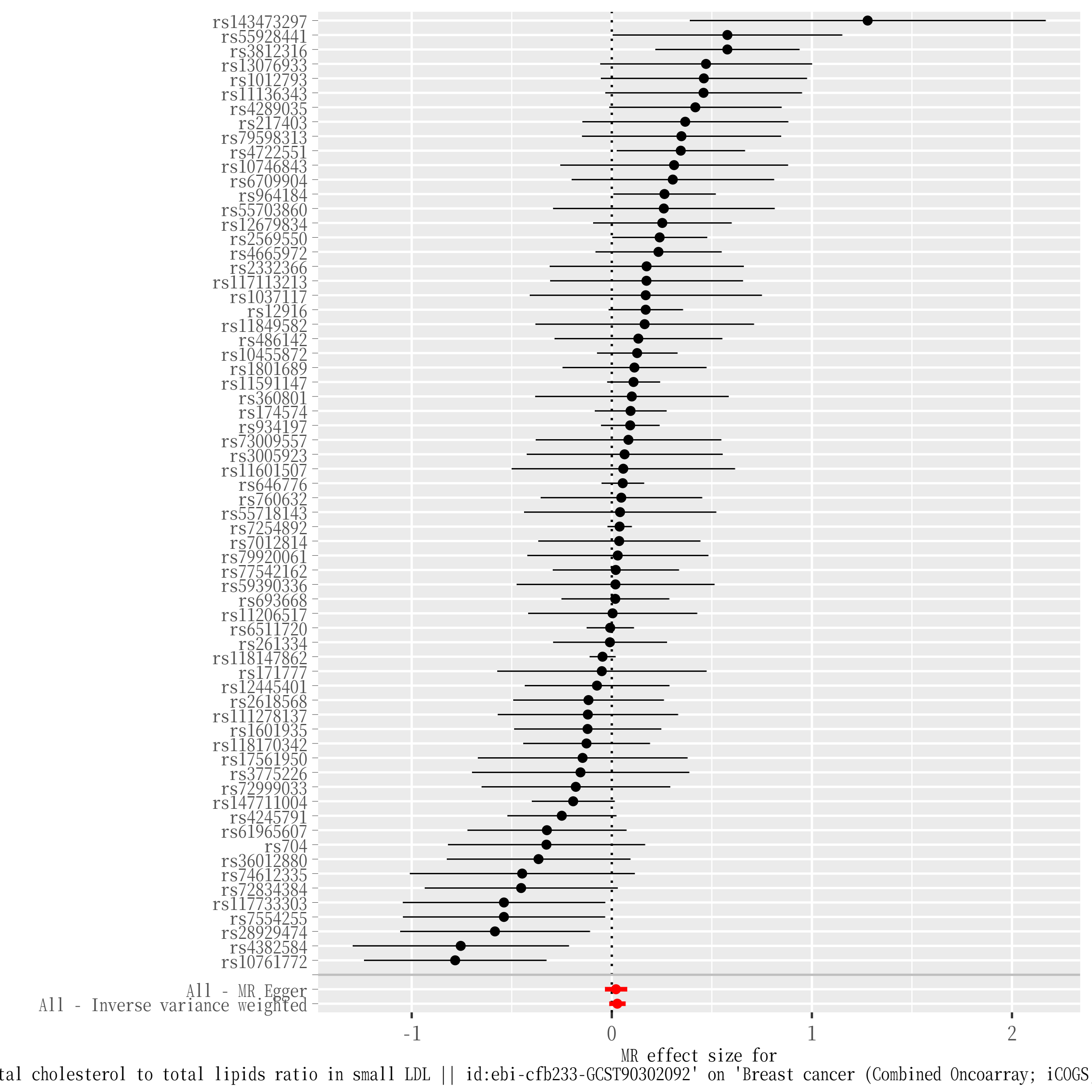
rs143473297
rs55928441
rs3812316
rs13076933
rs1012793
rs11136343
rs4289035
rs217403
rs79598313
rs4722551
rs10746843
rs6709904
rs964184
rs55703860
rs12679834
rs2569550
rs4665972
rs2332366
rs117113213
rs1037117
rs12916
rs11849582
rs486142
rs10455872
rs1801689
rs11591147
rs360801
rs174574
rs934197
rs73009557
rs3005923
rs11601507
rs646776
rs760632
rs55718143
rs7254892
rs7012814
rs79920061
rs77542162
rs59390336
rs693668
rs11206517
rs6511720
rs261334
rs118147862
rs171777
rs12445401
rs2618568
rs111278137
rs1601935
rs118170342
rs17561950
rs3775226
rs72999033
rs147711004
rs4245791
rs61965607
rs704
rs36012880
rs74612335
rs72834384
rs117733303
rs7554255
rs28929474
rs4382584
rs10761772
All - MR Egger
All - Inverse variance weighted
-1
0
1
2
MR effect size for
tal cholesterol to total lipids ratio in small LDL || id:ebi-cfb233-GCST90302092' on 'Breast cancer (Combined Oncoarray; iCOGS

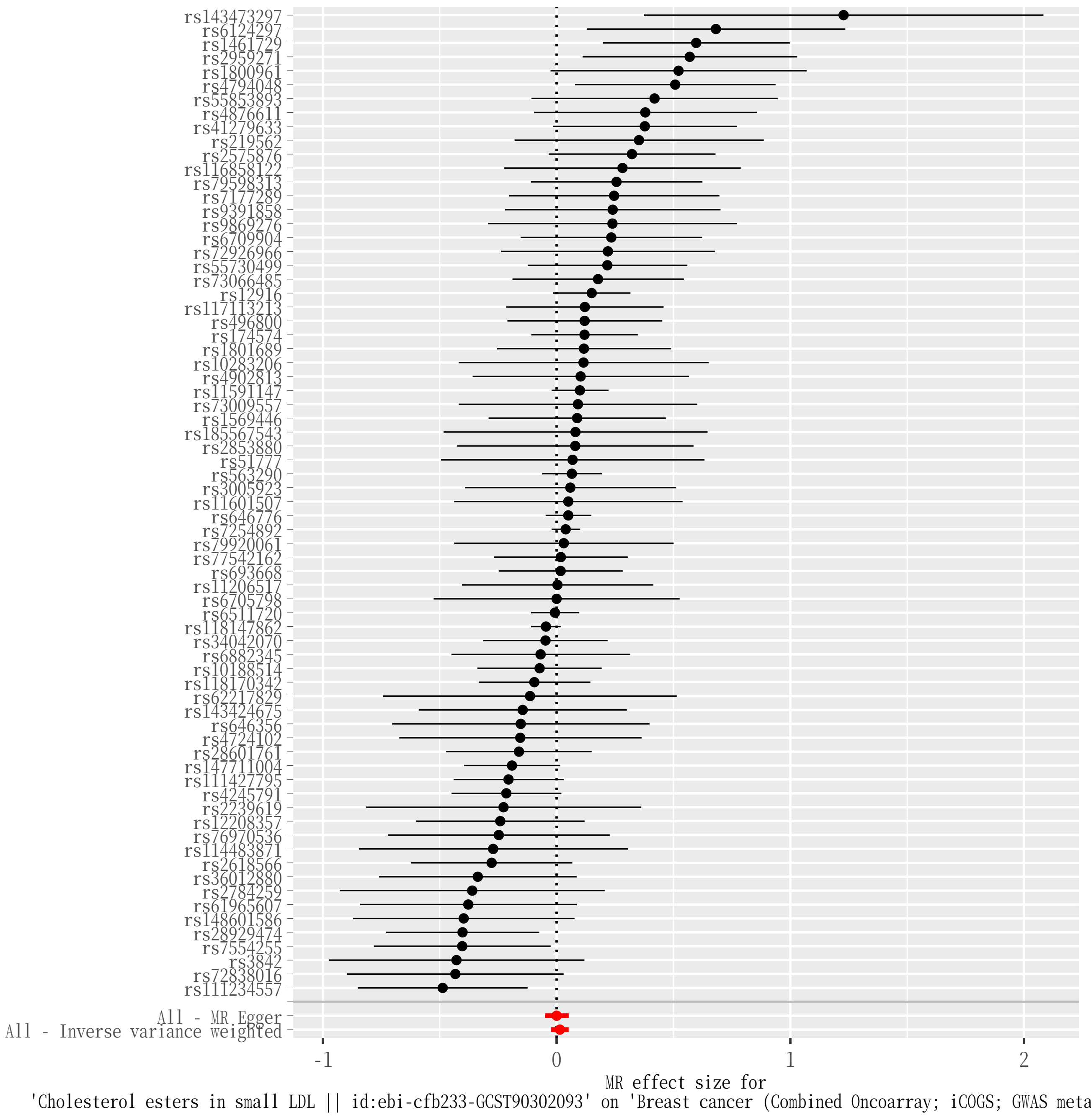

rs143473297
rs6124297
rs1461729
rs2959271
rs1800961
rs4794048
rs55853893
rs4876611
rs41279633
rs219562
rs2575876
rs116858122
rs79598313
rs7177289
rs9391858
rs9869276
rs6709904
rs72926966
rs55730499
rs73066485
rs12916
rs117113213
rs496800
rs174574
rs1801689
rs10283206
rs4902813
rs11591147
rs73009557
rs1569446
rs185567543
rs2853880
rs51777
rs563290
rs3005923
rs11601507
rs646776
rs7254892
rs79920061
rs77542162
rs693668
rs11206517
rs6705798
rs6511720
rs118147862
rs34042070
rs6882345
rs10188514
rs118170342
rs62217829
rs143424675
rs646356
rs4724102
rs28601761
rs147711004
rs111427795
rs4245791
rs2239619
rs12208357
rs76970536
rs114483871
rs2618566
rs36012880
rs2784259
rs61965607
rs148601586
rs28929474
rs7554255
rs3842
rs72838016
rs111234557
All - MR Egger
All - Inverse variance weighted
-1
0
1
2
MR effect size for
'Cholesterol esters in small LDL || id:ebi-cfb233-GCST90302093' on 'Breast cancer (Combined Oncoarray; iCOGS; GWAS meta

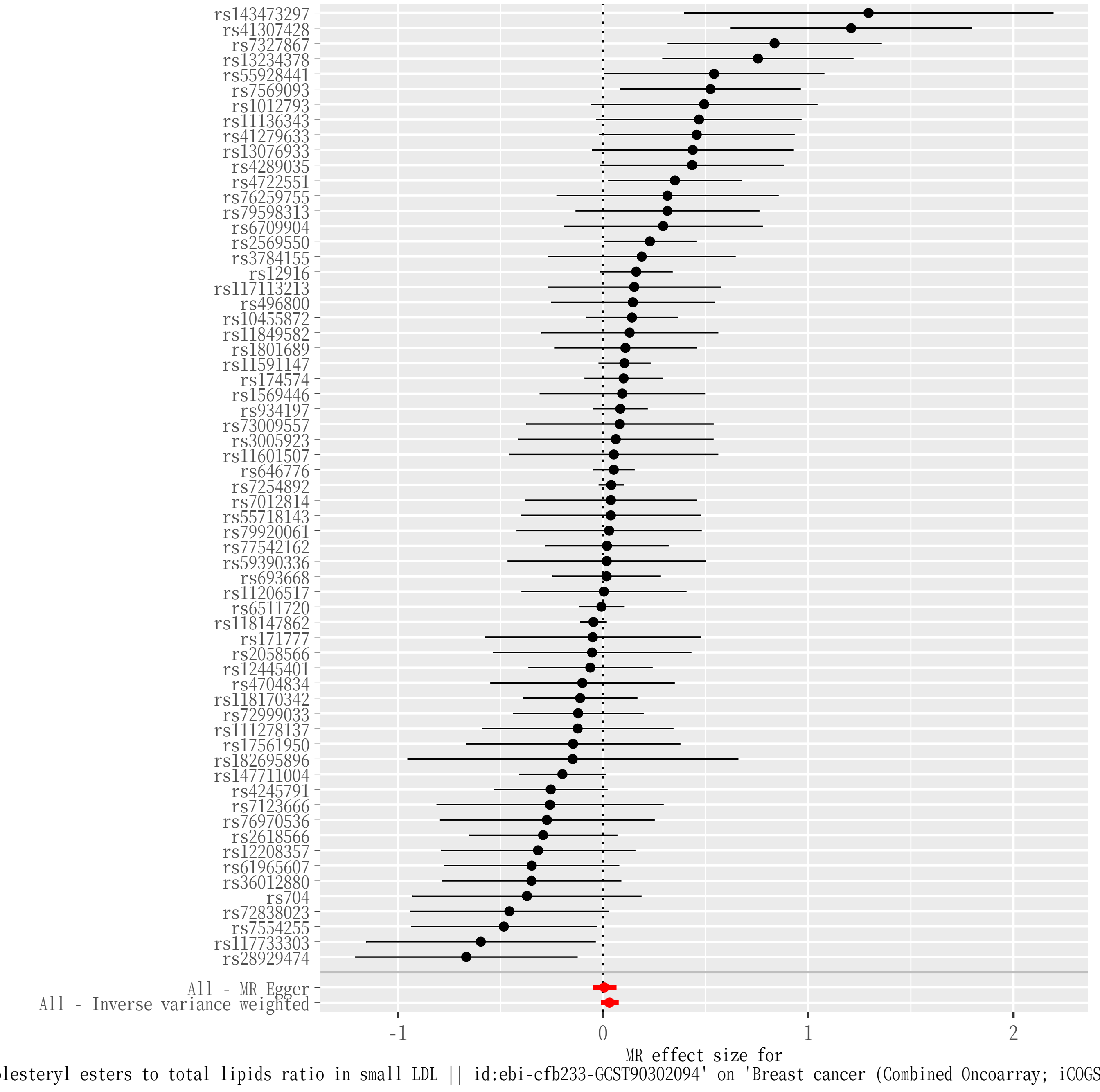

rs143473297
rs41307428
rs7327867
rs13234378
rs55928441
rs7569093
rs1012793
rs11136343
rs41279633
rs13076933
rs4289035
rs4722551
rs76259755
rs79598313
rs6709904
rs2569550
rs3784155
rs12916
rs117113213
rs496800
rs10455872
rs11849582
rs1801689
rs11591147
rs174574
rs1569446
rs934197
rs73009557
rs3005923
rs11601507
rs646776
rs7254892
rs7012814
rs55718143
rs79920061
rs77542162
rs59390336
rs693668
rs11206517
rs6511720
rs118147862
rs171777
rs2058566
rs12445401
rs4704834
rs118170342
rs72999033
rs111278137
rs17561950
rs182695896
rs147711004
rs4245791
rs7123666
rs76970536
rs2618566
rs12208357
rs61965607
rs36012880
rs704
rs72838023
rs7554255
rs117733303
rs28929474
All - MR Egger
All - Inverse variance weighted
-1
0
1
2
MR effect size for
olesteryl esters to total lipids ratio in small LDL || id:ebi-cfb233-GCST90302094' on 'Breast cancer (Combined Oncoarray; iCOGS

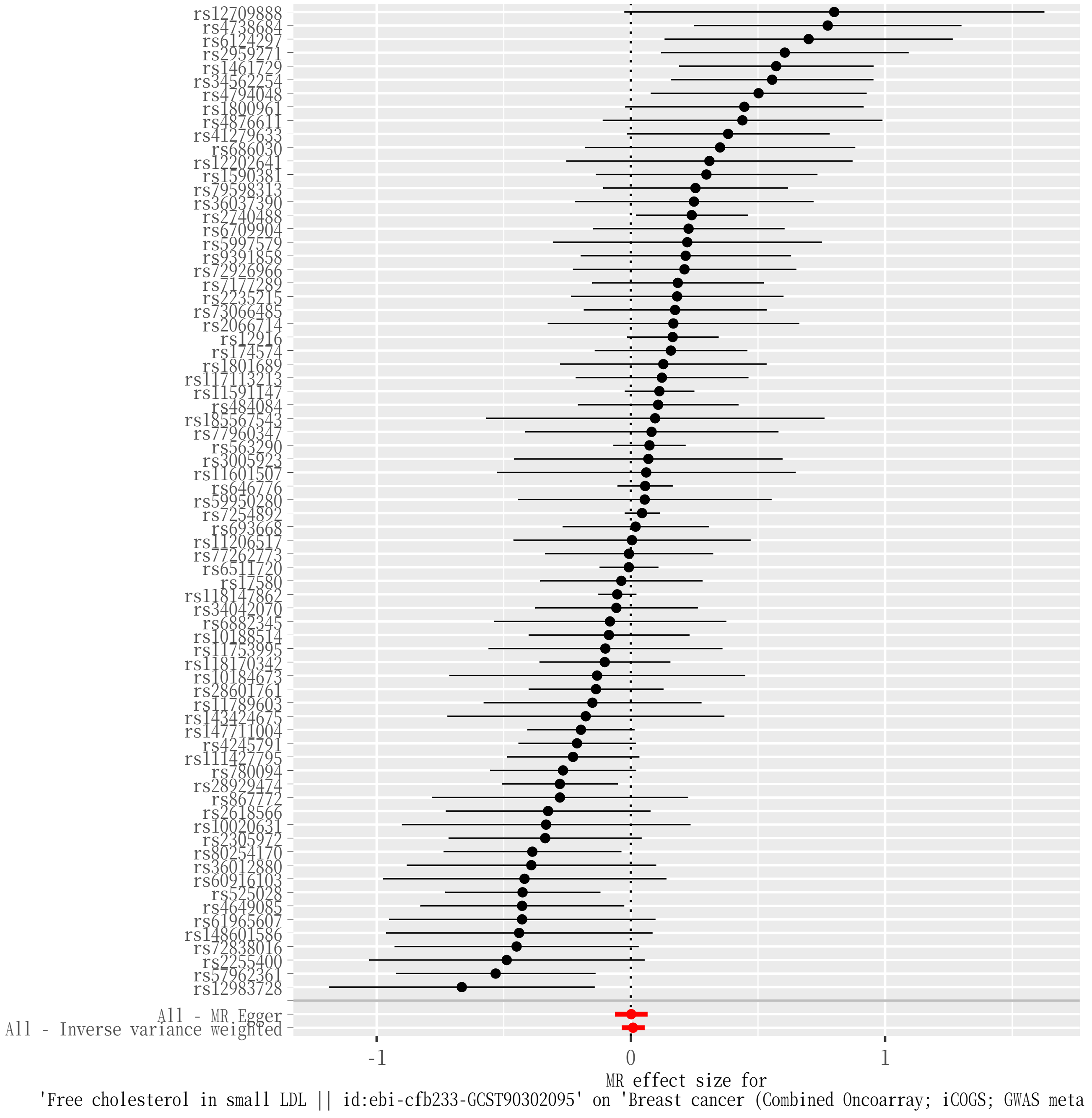
rs12709888
rs4738684
rs6124297
rs2959271
rs1461729
rs34562254
rs4794048
rs1800961
rs4876611
rs41279633
rs686030
rs12202641
rs1590381
rs79598313
rs36037390
rs2740488
rs6709904
rs5997579
rs9391858
rs72926966
rs7177289
rs2235215
rs73066485
rs2066714
rs12916
rs174574
rs1801689
rs117113213
rs11591147
rs484084
rs185567543
rs77960347
rs563290
rs3005923
rs11601507
rs646776
rs59950280
rs7254892
rs693668
rs11206517
rs77262773
rs6511720
rs17580
rs118147862
rs34042070
rs6882345
rs10188514
rs11753995
rs118170342
rs10184673
rs28601761
rs11789603
rs143424675
rs147711004
rs4245791
rs111427795
rs780094
rs28929474
rs867772
rs2618566
rs10020631
rs2305972
rs80254170
rs36012880
rs60916103
rs525028
rs4649085
rs61965607
rs148601586
rs72838016
rs2255400
rs57962361
rs12983728
All - MR Egger
All - Inverse variance weighted
-1
0
1
MR effect size for
'Free cholesterol in small LDL || id:ebi-cfb233-GCST90302095' on 'Breast cancer (Combined Oncoarray; iCOGS; GWAS meta

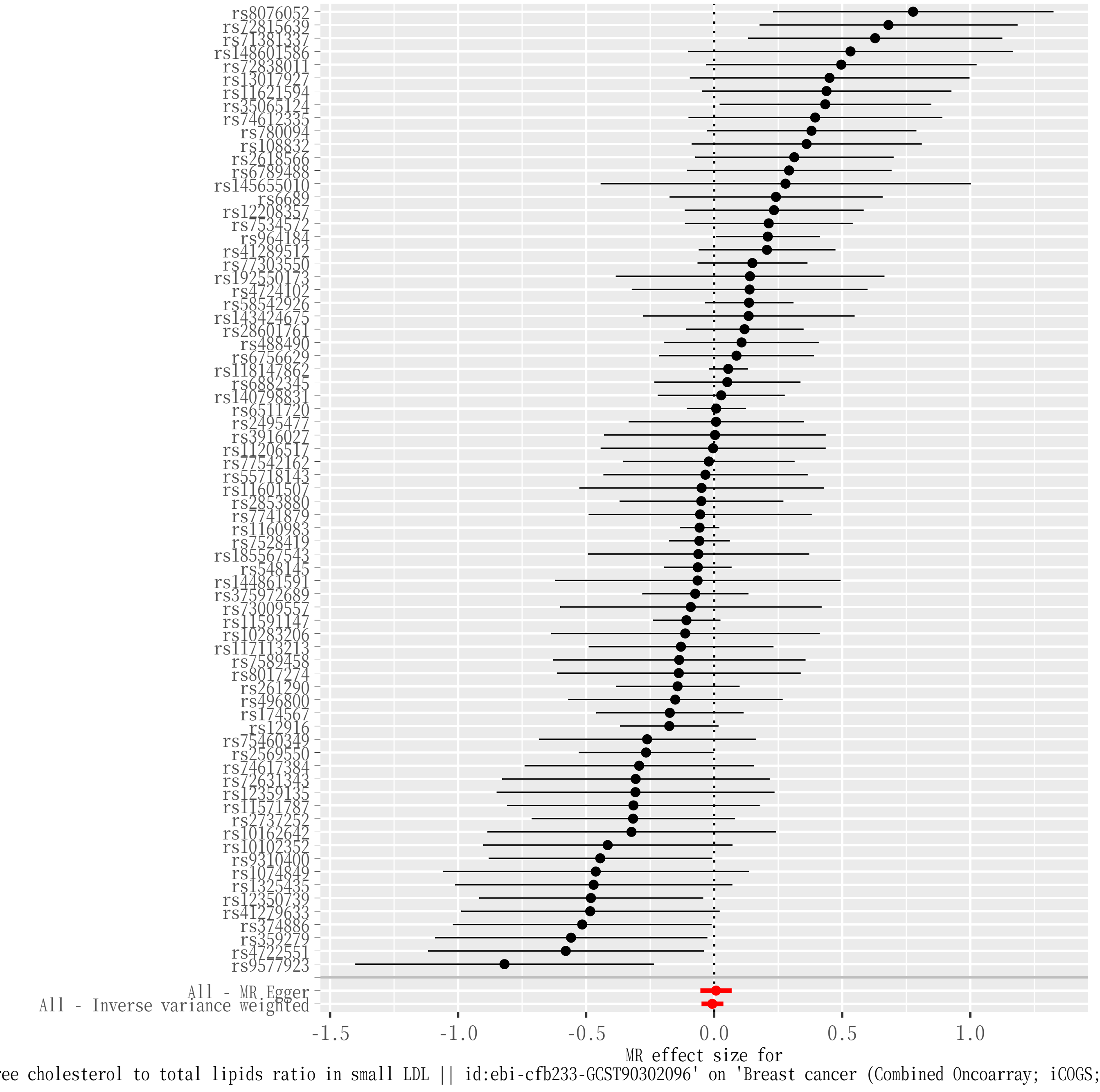
rs8076052
rs72815639
rs71381337
rs148601586
rs72838011
rs13017927
rs11621594
rs35065124
rs74612335
rs780094
rs108832
rs2618566
rs6789488
rs145655010
rs6689
rs12208357
rs7534572
rs964184
rs41289512
rs77303550
rs192550173
rs4724102
rs58542926
rs143424675
rs28601761
rs488490
rs6756629
rs118147862
rs6882345
rs140798831
rs6511720
rs2495477
rs3916027
rs11206517
rs77542162
rs55718143
rs11601507
rs2853880
rs7741879
rs1160983
rs7528419
rs185567543
rs548145
rs144861591
rs375972689
rs73009557
rs11591147
rs10283206
rs117113213
rs7589458
rs8017274
rs261290
rs496800
rs174567
rs12916
rs75460349
rs2569550
rs74617384
rs72631343
rs12359135
rs11571787
rs2737252
rs10162642
rs10102352
rs9310400
rs1074849
rs1325435
rs12350739
rs41279633
rs374886
rs359279
rs4722551
rs9577923
All - MR Egger
All - Inverse variance weighted
-1.5
-1.0
-0.5
0.0
0.5
1.0
MR effect size for
ree cholesterol to total lipids ratio in small LDL || id:ebi-cfb233-GCST90302096' on 'Breast cancer (Combined Oncoarray; iCOGS;

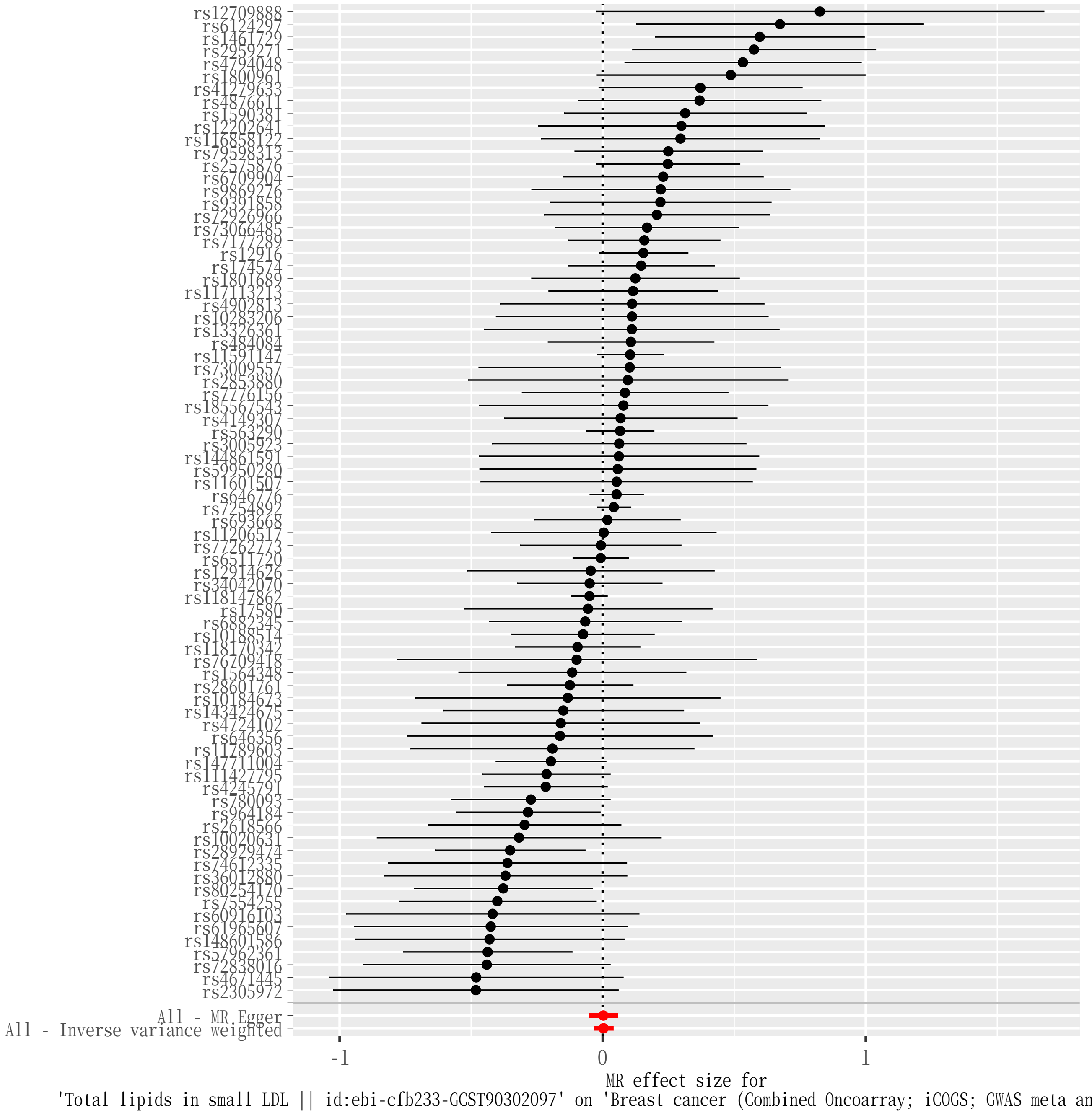
rs12709888
rs6124297
rs1461729
rs2959271
rs4794048
rs1800961
rs41279633
rs4876611
rs1590381
rs12202641
rs116858122
rs79598313
rs2575876
rs6709904
rs9869276
rs9391858
rs72926966
rs73066485
rs7177289
rs12916
rs174574
rs1801689
rs117113213
rs4902813
rs10283206
rs13326361
rs484084
rs11591147
rs73009557
rs2853880
rs7276156
rs185567543
rs4149307
rs563290
rs3005923
rs144861591
rs59950280
rs11601507
rs646776
rs7254892
rs693668
rs11206517
rs77262773
rs6511720
rs12914626
rs34042070
rs118147862
rs17580
rs6882345
rs10188514
rs118170342
rs76709418
rs1564348
rs28601761
rs10184673
rs143424675
rs4724102
rs646356
rs11789603
rs147711004
rs111427795
rs4245791
rs780093
rs964184
rs2618566
rs10020631
rs28929474
rs74612335
rs36012880
rs80254170
rs7554255
rs60916103
rs61965607
rs148601586
rs57962361
rs72838016
rs4671445
rs2305972
All - MR Egger
All - Inverse variance weighted
-1
0
1
MR effect size for
'Total lipids in small LDL || id:ebi-cfb233-GCST90302097' on 'Breast cancer (Combined Oncoarray; iCOGS; GWAS meta an

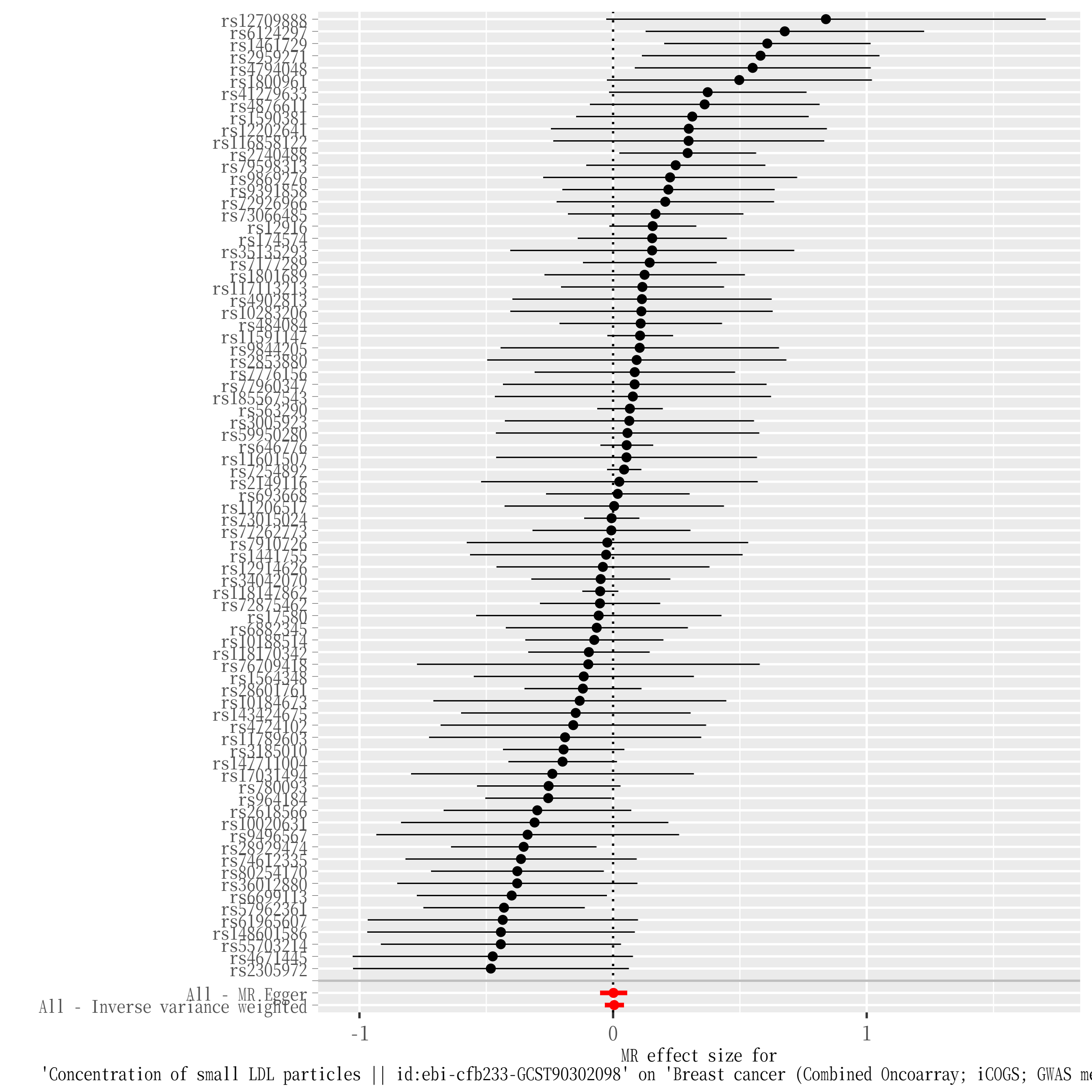

rs12709888
rs6124297
rs1461729
rs2959271
rs4794048
rs1800961
rs41279633
rs4876611
rs1590381
rs12202641
rs116858122
rs2740488
rs79598313
rs9869276
rs9391858
rs72926966
rs73066485
rs12916
rs174574
rs35135293
rs7177289
rs1801689
rs117113213
rs4902813
rs10283206
rs484084
rs11591147
rs9844205
rs2853880
rs7776156
rs77960347
rs185567543
rs563290
rs3005923
rs59950280
rs646776
rs11601507
rs7254892
rs2149116
rs693668
rs11206517
rs73015024
rs77262773
rs7910726
rs1441755
rs12914626
rs34042070
rs118147862
rs72875462
rs17580
rs6882345
rs10188514
rs118170342
rs76709418
rs1564348
rs28601761
rs10184673
rs143424675
rs4724102
rs11789603
rs3185010
rs147711004
rs17031494
rs780093
rs964184
rs2618566
rs10020631
rs9496567
rs28929474
rs74612335
rs80254170
rs36012880
rs6699113
rs57962361
rs61965607
rs148601586
rs55703214
rs4671445
rs2305972
All - MR Egger
All - Inverse variance weighted
-1
0
1
MR effect size for
'Concentration of small LDL particles || id:ebi-cfb233-GCST90302098' on 'Breast cancer (Combined Oncoarray; iCOGS; GWAS me

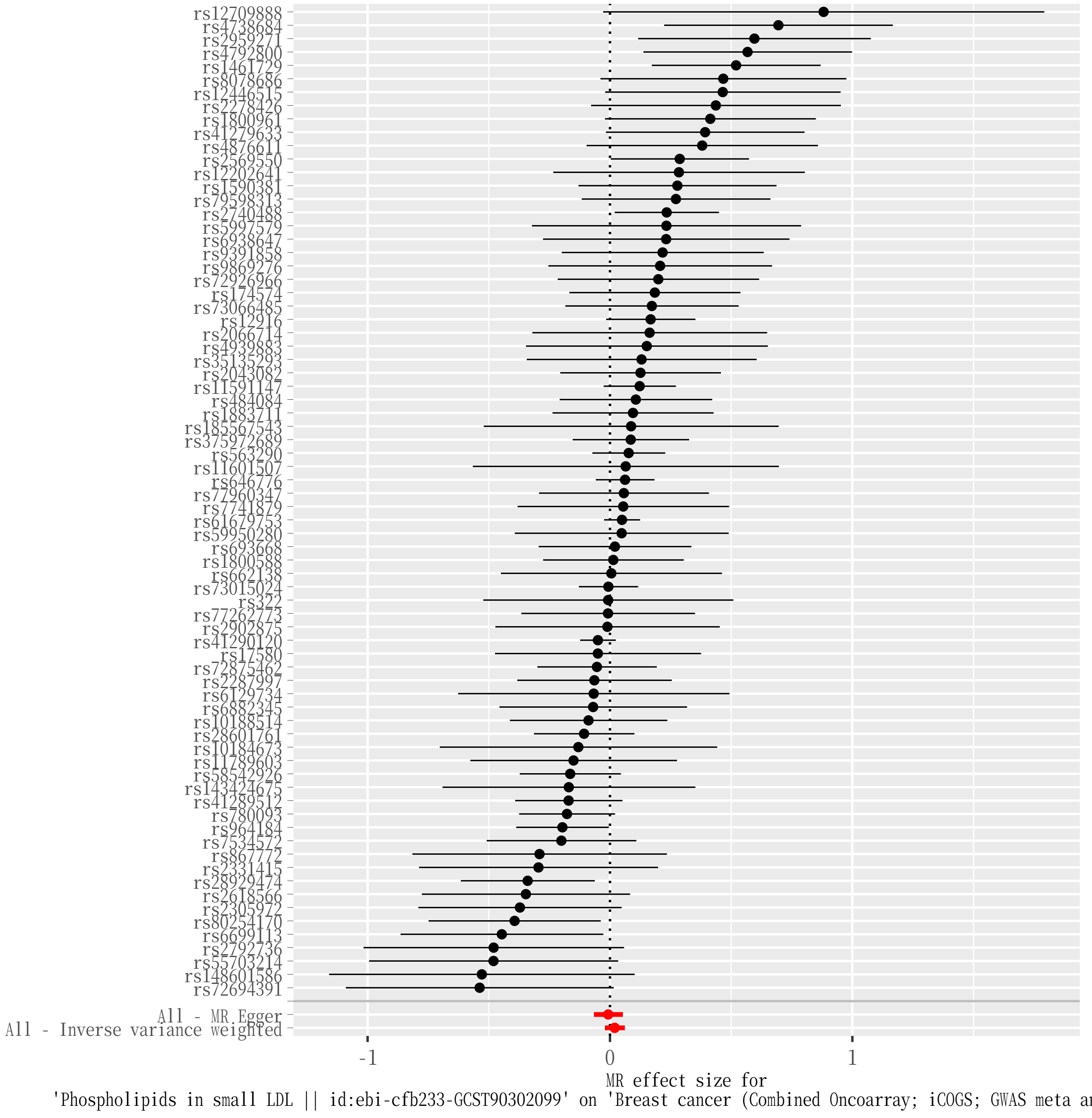
rs12709888
rs4738684
rs2959271
rs4792800
rs1461729
rs8078686
rs12446515
rs2278426
rs1800961
rs41279633
rs4876611
rs2569550
rs12202641
rs1590381
rs79598313
rs2740488
rs5997579
rs6938647
rs9391858
rs9869276
rs72926966
rs174574
rs73066485
rs12916
rs2066714
rs4939883
rs35135293
rs2043082
rs11591147
rs484084
rs1883711
rs185567543
rs375972689
rs563290
rs11601507
rs646776
rs72960347
rs7741879
rs61679753
rs59950280
rs693668
rs1800588
rs662138
rs73015024
rs322
rs77262773
rs2902875
rs41290120
rs17580
rs72875462
rs2287997
rs6129734
rs6882345
rs10188514
rs28601761
rs10184673
rs11789603
rs58542926
rs143424675
rs41289512
rs780093
rs964184
rs7534572
rs867772
rs2331415
rs28929474
rs2618566
rs2305972
rs80254170
rs6699113
rs2792736
rs55703214
rs148601586
rs72694391
All - MR Egger
All - Inverse variance weighted
-1
0
1
MR effect size for
'Phospholipids in small LDL || id:ebi-cfb233-GCST90302099' on 'Breast cancer (Combined Oncoarray; iCOGS; GWAS meta an

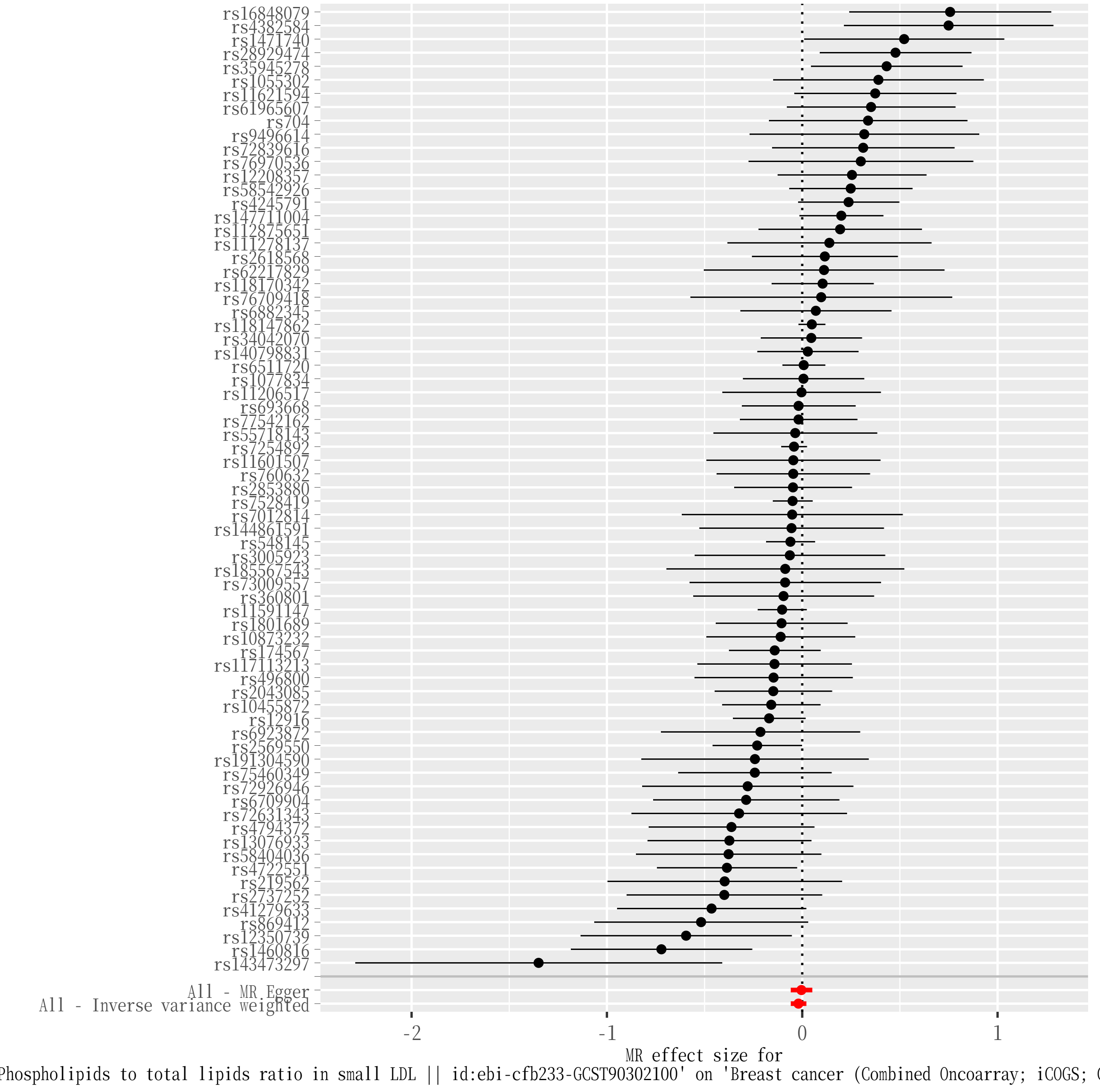
rs16848079
rs4382584
rs1471740
rs28929474
rs35945278
rs1055302
rs11621594
rs61965607
rs704
rs9496614
rs72839616
rs76970536
rs12208357
rs58542926
rs4245791
rs147711004
rs112875651
rs111278137
rs2618568
rs62217829
rs118170342
rs76709418
rs6882345
rs118147862
rs34042070
rs140798831
rs6511720
rs1077834
rs11206517
rs693668
rs77542162
rs55718143
rs7254892
rs11601507
rs760632
rs2853880
rs7528419
rs7012814
rs144861591
rs548145
rs3005923
rs185567543
rs73009557
rs360801
rs11591147
rs1801689
rs10873232
rs174567
rs117113213
rs496800
rs2043085
rs10455872
rs12916
rs6923872
rs2569550
rs191304590
rs75460349
rs72926946
rs6709904
rs72631343
rs4794372
rs13076933
rs58404036
rs4722551
rs219562
rs2737252
rs41279633
rs869412
rs12350739
rs1460816
rs143473297
All - MR Egger
All - Inverse variance weighted
-2
-1
0
1
MR effect size for
Phospholipids to total lipids ratio in small LDL || id:ebi-cfb233-GCST90302100' on 'Breast cancer (Combined Oncoarray; iCOGS; G

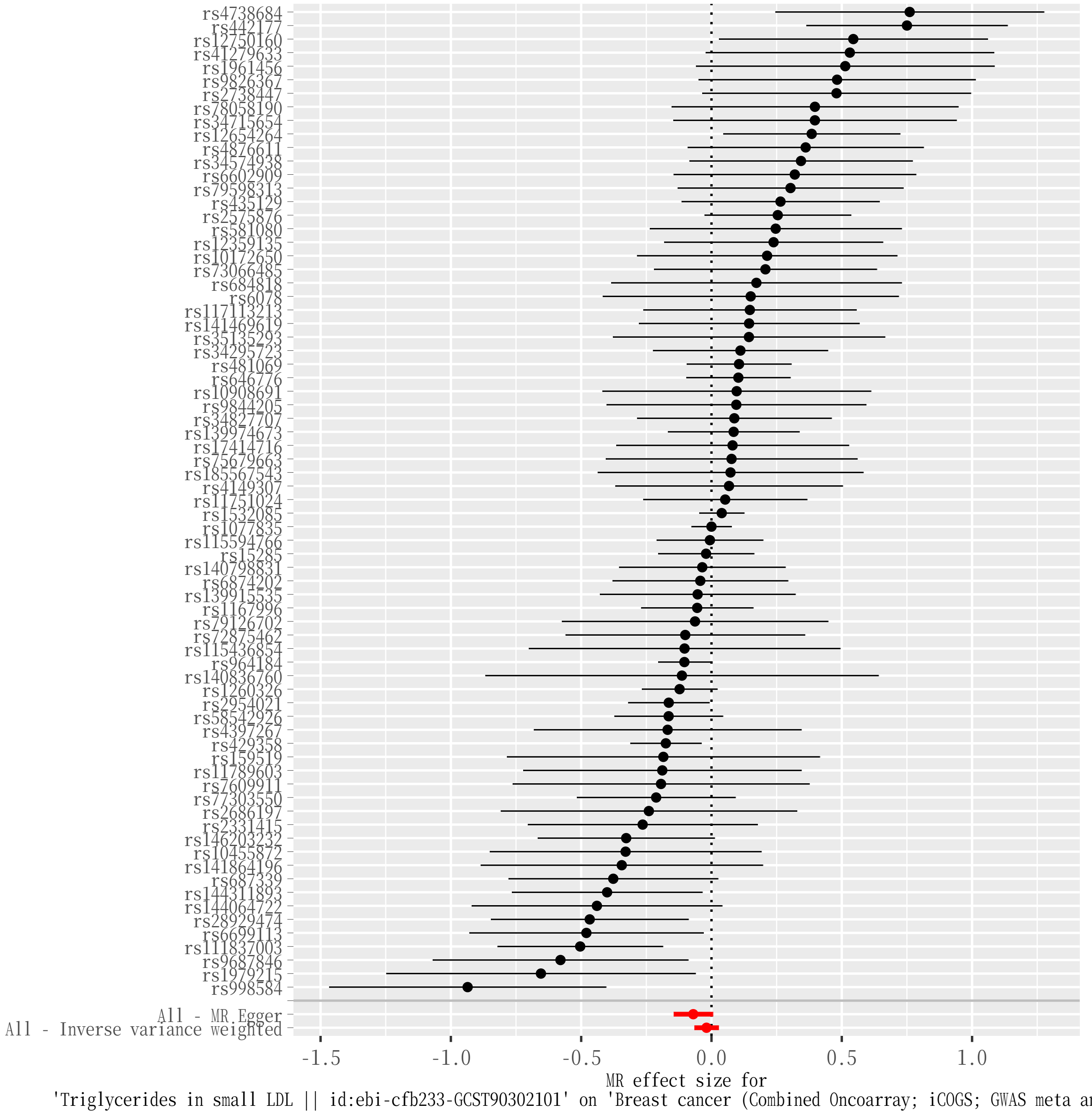
rs4738684
rs442177
rs12750160
rs41279633
rs1961456
rs9826367
rs2738447
rs78058190
rs34715654
rs12654264
rs4876611
rs34574938
rs6602909
rs79598313
rs435129
rs2575876
rs581080
rs12359135
rs10172650
rs73066485
rs684818
rs6078
rs117113213
rs141469619
rs35135293
rs34295723
rs481069
rs646776
rs10908691
rs9844205
rs34827707
rs139974673
rs17414716
rs75679663
rs185567543
rs4149307
rs11751024
rs1532085
rs1077835
rs115594766
rs15285
rs140798831
rs6874202
rs139915535
rs1167996
rs79126702
rs72875462
rs115436854
rs964184
rs140836760
rs1260326
rs2954021
rs58542926
rs4397267
rs429358
rs159519
rs11789603
rs7609911
rs77303550
rs2686197
rs2331415
rs146203232
rs10455872
rs141864196
rs687339
rs144311893
rs144064722
rs28929474
rs6699113
rs111837003
rs9687846
rs1979215
rs998584
All - MR Egger
All - Inverse variance weighted
-1.5
-1.0
-0.5
0.0
0.5
1.0
MR effect size for
'Triglycerides in small LDL || id:ebi-cfb233-GCST90302101' on 'Breast cancer (Combined Oncoarray; iCOGS; GWAS meta an

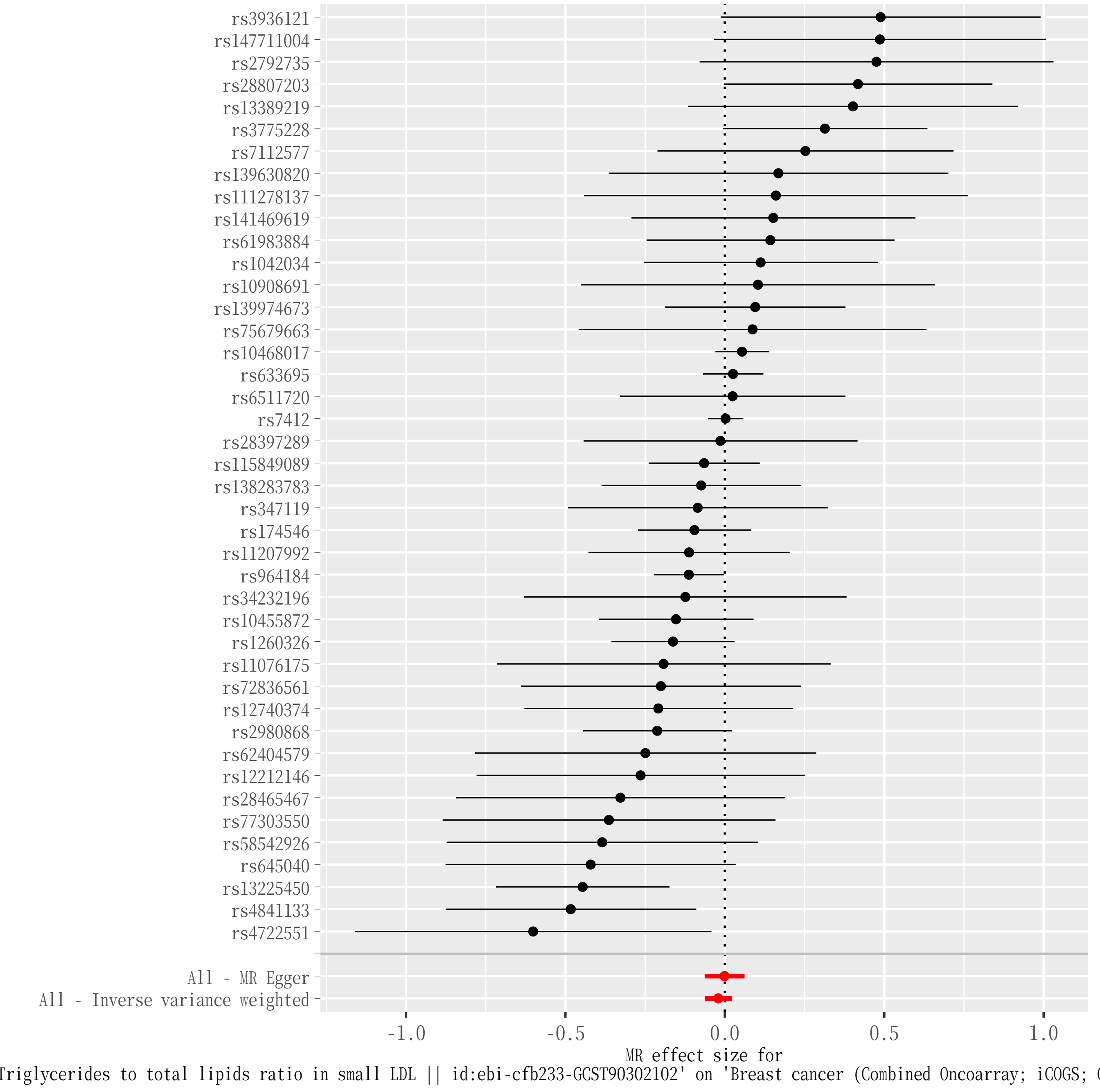
rs3936121
rs147711004
rs2792735
rs28807203
rs13389219
rs3775228
rs7112577
rs139630820
rs111278137
rs141469619
rs61983884
rs1042034
rs10908691
rs139974673
rs75679663
rs10468017
rs633695
rs6511720
rs7412
rs28397289
rs115849089
rs138283783
rs347119
rs174546
rs11207992
rs964184
rs34232196
rs10455872
rs1260326
rs11076175
rs72836561
rs12740374
rs2980868
rs62404579
rs12212146
rs28465467
rs77303550
rs58542926
rs645040
rs13225450
rs4841133
rs4722551
All - MR Egger
All - Inverse variance weighted
-1.0
-0.5
0.0
0.5
1.0
MR effect size for
'Triglycerides to total lipids ratio in small LDL || id:ebi-cfb233-GCST90302102' on 'Breast cancer (Combined Oncoarray; iCOGS; (

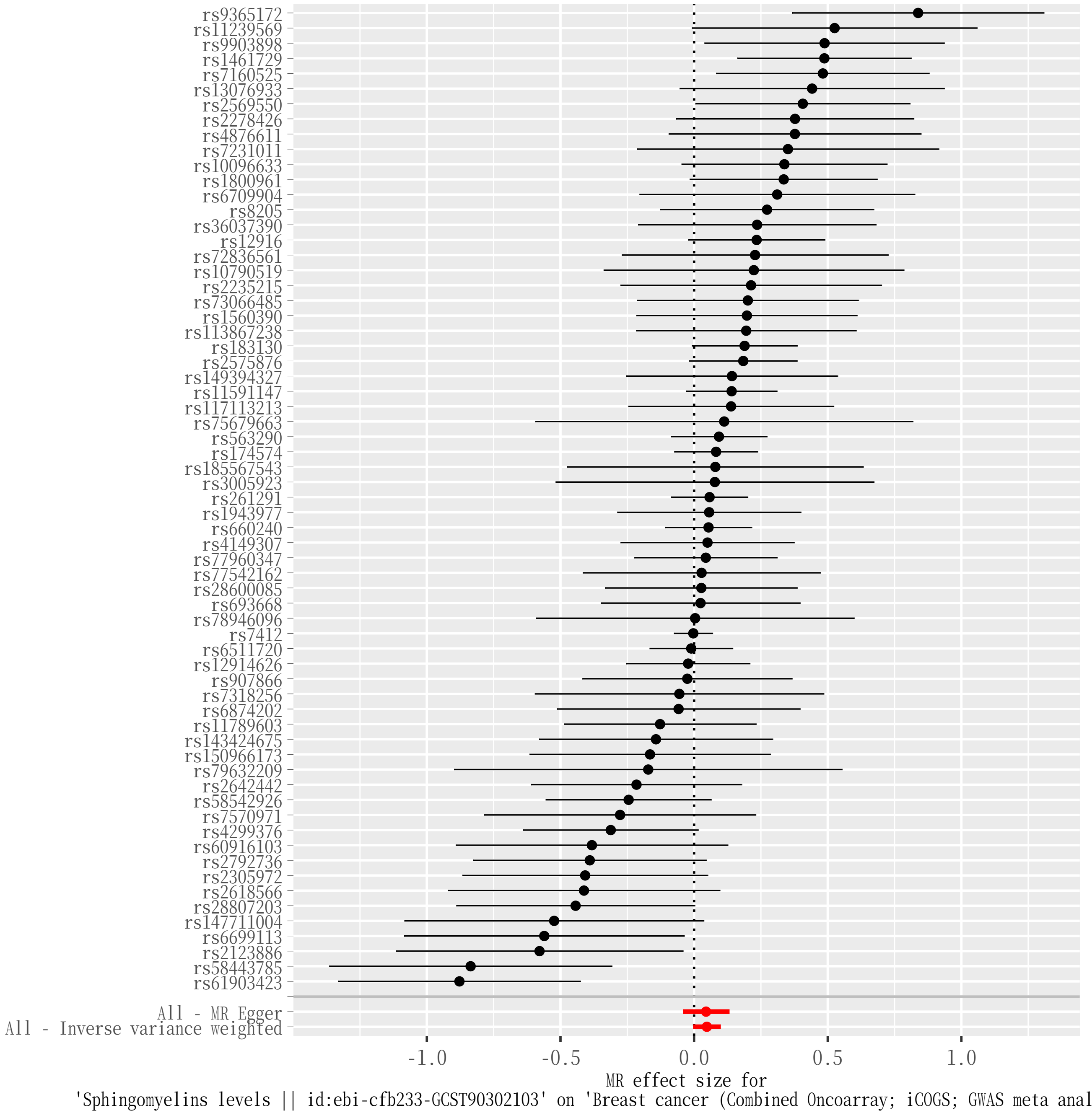
rs9365172
rs11239569
rs9903898
rs1461729
rs7160525
rs13076933
rs2569550
rs2278426
rs4876611
rs7231011
rs10096633
rs1800961
rs6709904
rs8205
rs36037390
rs12916
rs72836561
rs10790519
rs2235215
rs73066485
rs1560390
rs113867238
rs183130
rs2575876
rs149394327
rs11591147
rs117113213
rs75679663
rs563290
rs174574
rs185567543
rs3005923
rs261291
rs1943977
rs660240
rs4149307
rs77960347
rs77542162
rs28600085
rs693668
rs78946096
rs7412
rs6511720
rs12914626
rs907866
rs7318256
rs6874202
rs11789603
rs143424675
rs150966173
rs79632209
rs2642442
rs58542926
rs7570971
rs4299376
rs60916103
rs2792736
rs2305972
rs2618566
rs28807203
rs147711004
rs6699113
rs2123886
rs58443785
rs61903423
All - MR Egger
All - Inverse variance weighted
-1.0
-0.5
0.0
0.5
1.0
MR effect size for
'Sphingomyelins levels || id:ebi-cfb233-GCST90302103' on 'Breast cancer (Combined Oncoarray; iCOGS; GWAS meta anal

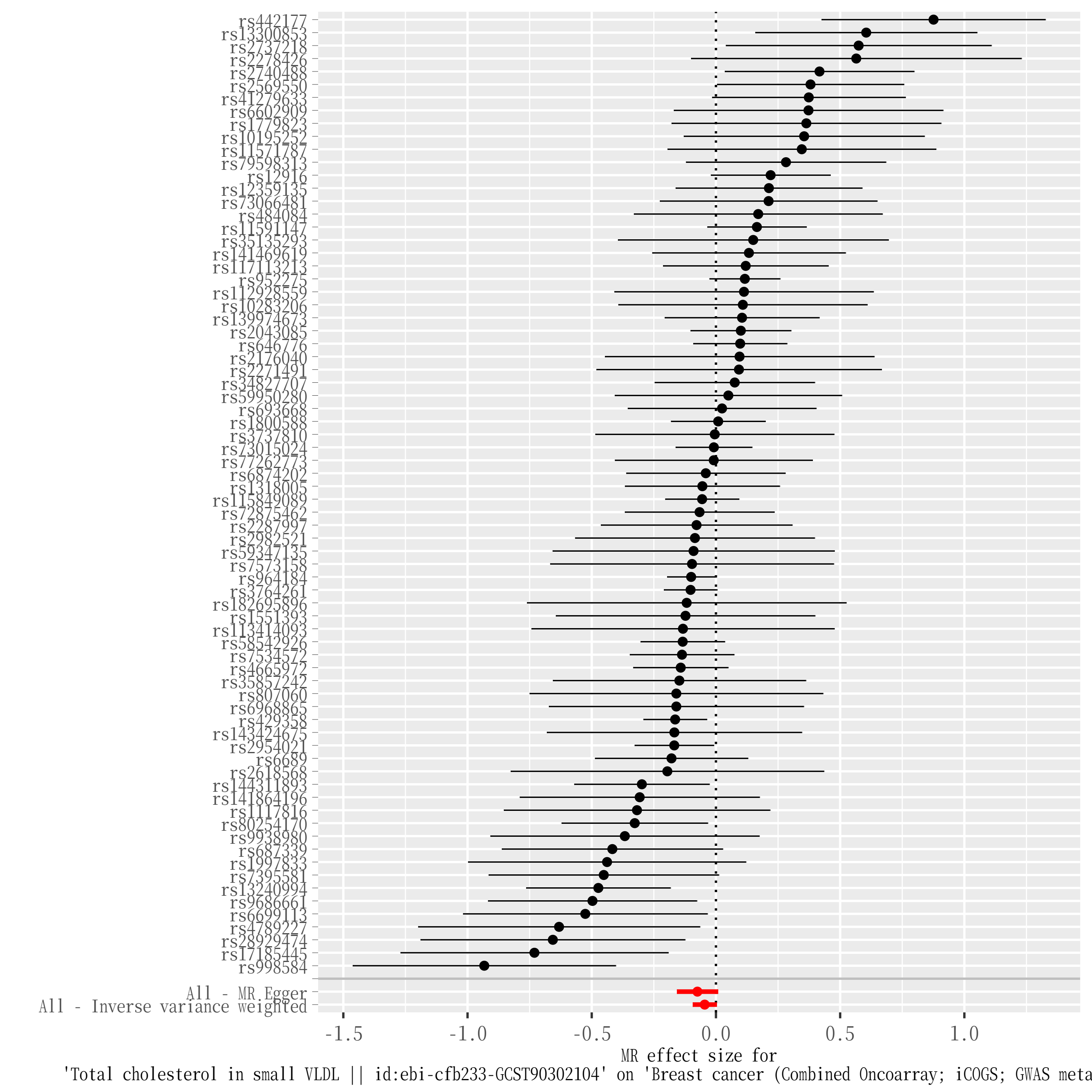
rs442177
rs13300853
rs2737218
rs2278426
rs2740488
rs2569550
rs41279633
rs6602909
rs1779823
rs10195252
rs11571787
rs79598313
rs12916
rs12359135
rs73066481
rs484084
rs11591147
rs35135293
rs141469619
rs117113213
rs952275
rs112928559
rs10283206
rs139974673
rs2043085
rs646776
rs2176040
rs2271491
rs34827707
rs59950280
rs693668
rs1800588
rs3737810
rs73015024
rs77262773
rs6874202
rs1318005
rs115849089
rs72875462
rs2287997
rs2982521
rs59347135
rs7573158
rs964184
rs3764261
rs182695896
rs1551393
rs113414093
rs58542926
rs7534572
rs4665972
rs35857242
rs807060
rs6968865
rs429358
rs143424675
rs2954021
rs6689
rs2618568
rs144311893
rs141864196
rs1117816
rs80254170
rs9938980
rs687339
rs1997833
rs7395581
rs13240994
rs9686661
rs6699113
rs4789227
rs28929474
rs17185445
rs998584
All - MR Egger
All - Inverse variance weighted
-1.5
-1.0
-0.5
0.0
0.5
1.0
MR effect size for
'Total cholesterol in small VLDL || id:ebi-cfb233-GCST90302104' on 'Breast cancer (Combined Oncoarray; iCOGS; GWAS meta

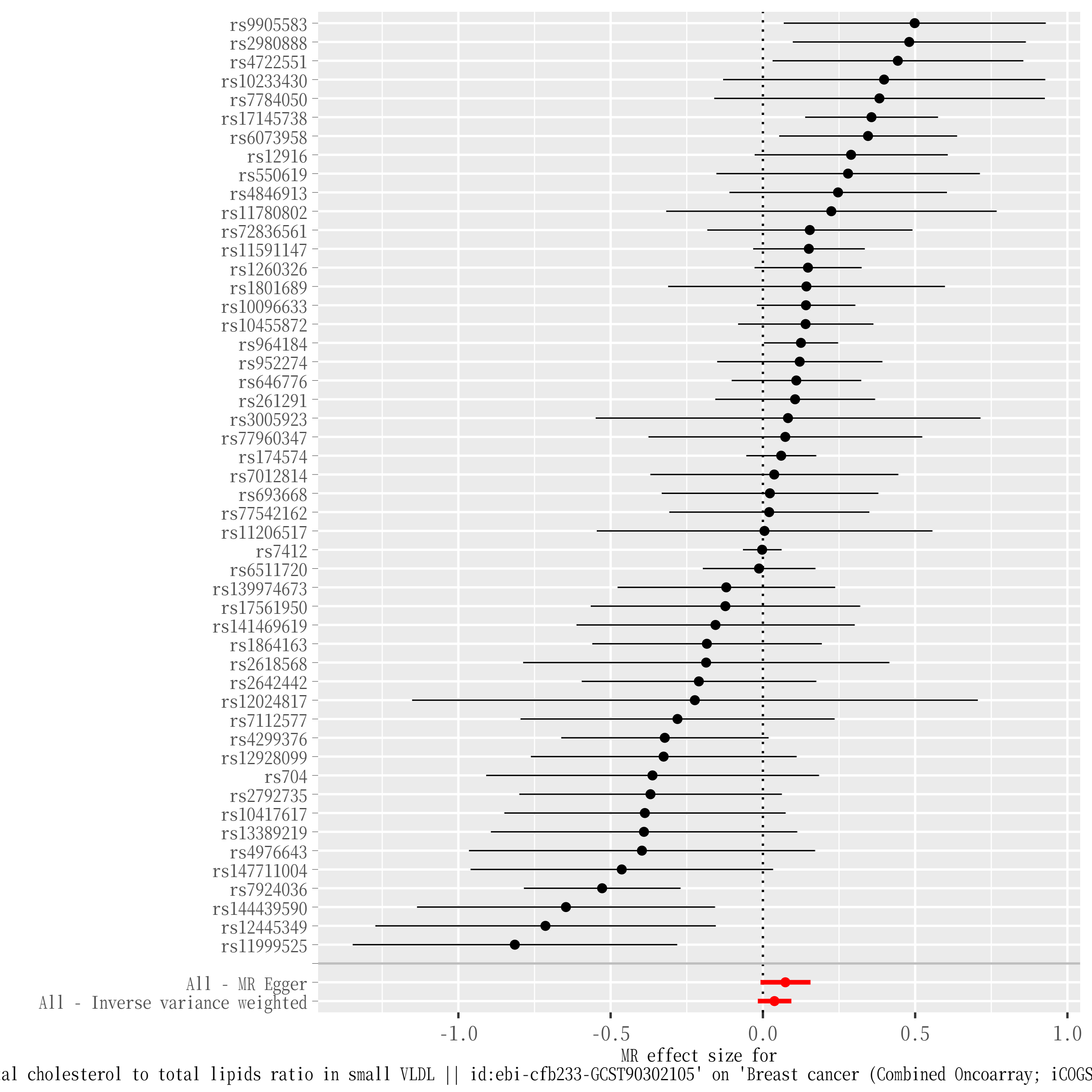

rs9905583
rs2980888
rs4722551
rs10233430
rs7784050
rs17145738
rs6073958
rs12916
rs550619
rs4846913
rs11780802
rs72836561
rs11591147
rs1260326
rs1801689
rs10096633
rs10455872
rs964184
rs952274
rs646776
rs261291
rs3005923
rs77960347
rs174574
rs7012814
rs693668
rs77542162
rs11206517
rs7412
rs6511720
rs139974673
rs17561950
rs141469619
rs1864163
rs2618568
rs2642442
rs12024817
rs7112577
rs4299376
rs12928099
rs704
rs2792735
rs10417617
rs13389219
rs4976643
rs147711004
rs7924036
rs144439590
rs12445349
rs11999525
All - MR Egger
All - Inverse variance weighted
-1.0
-0.5
0.0
0.5
1.0
MR effect size for
al cholesterol to total lipids ratio in small VLDL || id:ebi-cfb233-GCST90302105' on 'Breast cancer (Combined Oncoarray; iCOGS

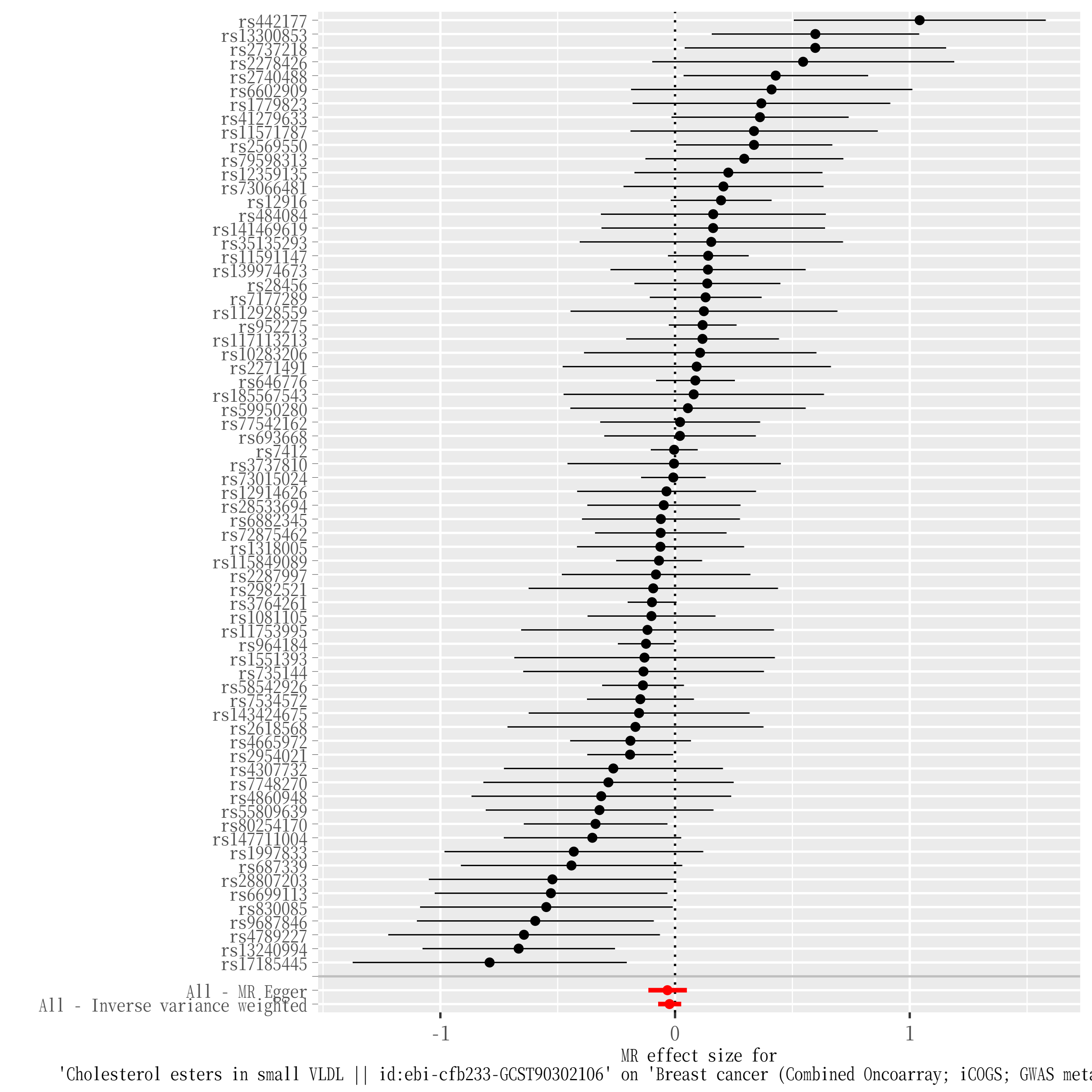
rs442177
rs13300853
rs2737218
rs2278426
rs2740488
rs6602909
rs1779823
rs41279633
rs11571787
rs2569550
rs79598313
rs12359135
rs73066481
rs12916
rs484084
rs141469619
rs35135293
rs11591147
rs139974673
rs28456
rs7177289
rs112928559
rs952275
rs117113213
rs10283206
rs2271491
rs646776
rs185567543
rs59950280
rs77542162
rs693668
rs7412
rs3737810
rs73015024
rs12914626
rs28533694
rs6882345
rs72875462
rs1318005
rs115849089
rs2287997
rs2982521
rs3764261
rs1081105
rs11753995
rs964184
rs1551393
rs735144
rs58542926
rs7534572
rs143424675
rs2618568
rs4665972
rs2954021
rs4307732
rs7748270
rs4860948
rs55809639
rs80254170
rs147711004
rs1997833
rs687339
rs28807203
rs6699113
rs830085
rs9687846
rs4789227
rs13240994
rs17185445
All - MR Egger
All - Inverse variance weighted
-1
0
1
MR effect size for
'Cholesterol esters in small VLDL || id:ebi-cfb233-GCST90302106' on 'Breast cancer (Combined Oncoarray; iCOGS; GWAS meta

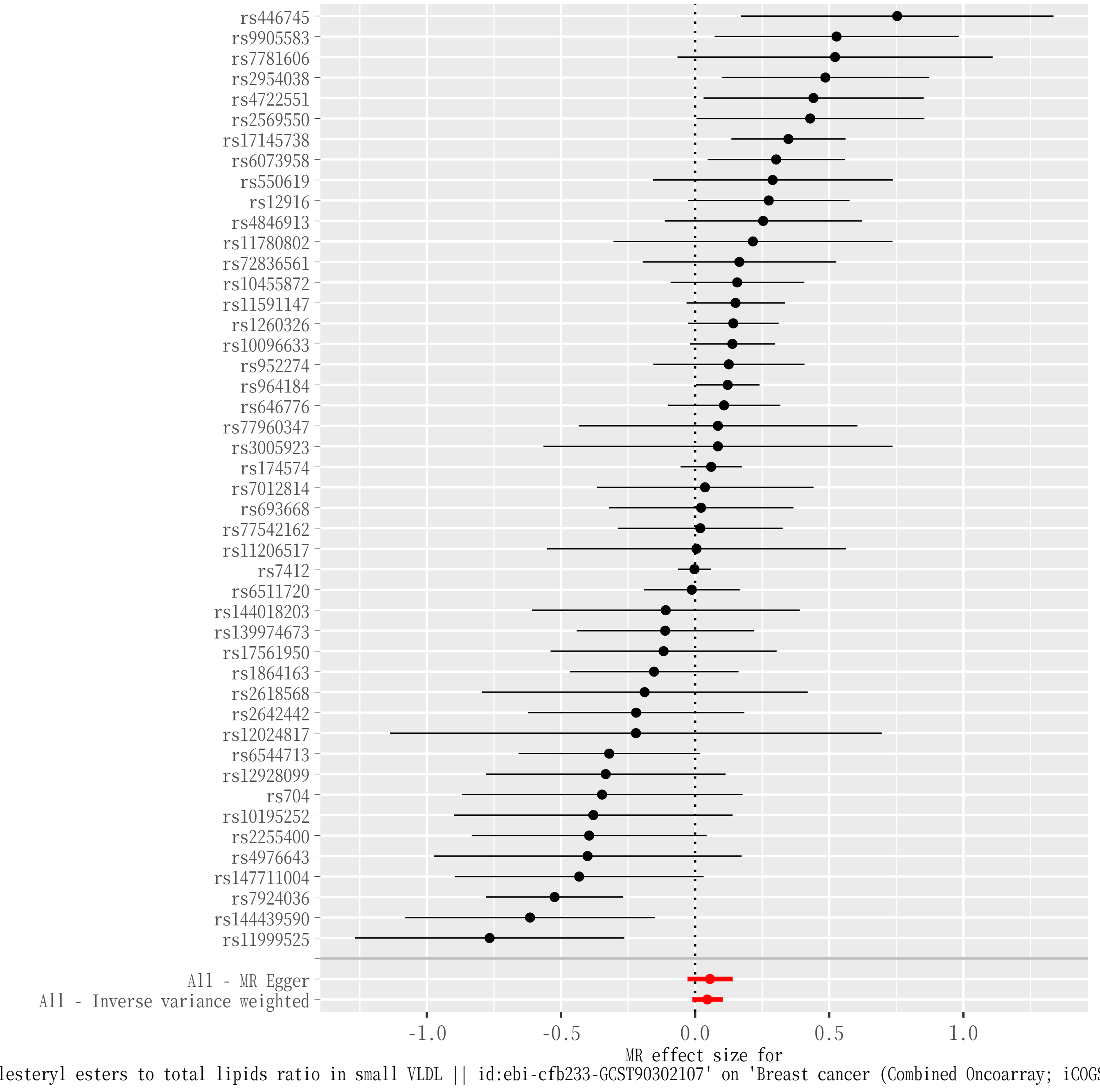
rs446745
rs9905583
rs7781606
rs2954038
rs4722551
rs2569550
rs17145738
rs6073958
rs550619
rs12916
rs4846913
rs11780802
rs72836561
rs10455872
rs11591147
rs1260326
rs10096633
rs952274
rs964184
rs646776
rs77960347
rs3005923
rs174574
rs7012814
rs693668
rs77542162
rs11206517
rs7412
rs6511720
rs144018203
rs139974673
rs17561950
rs1864163
rs2618568
rs2642442
rs12024817
rs6544713
rs12928099
rs704
rs10195252
rs2255400
rs4976643
rs147711004
rs7924036
rs144439590
rs11999525
All - MR Egger
All - Inverse variance weighted
-1.0
-0.5
0.0
0.5
1.0
MR effect size for
lesteryl esters to total lipids ratio in small VLDL || id:ebi-cfb233-GCST90302107' on 'Breast cancer (Combined Oncoarray; iCOGS

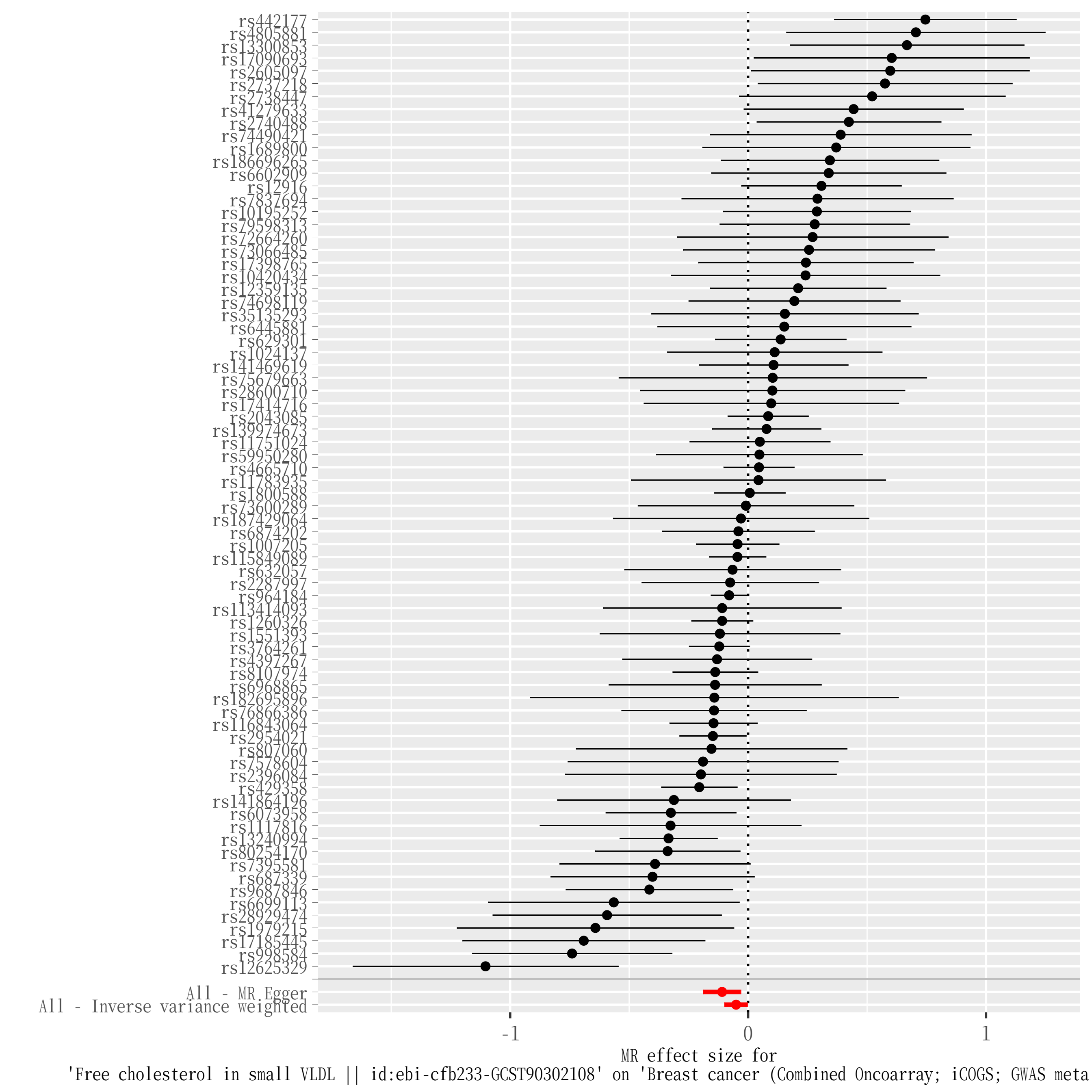

rs442177
rs4805881
rs13300853
rs17090693
rs2605097
rs2737218
rs2738447
rs41279633
rs2740488
rs74490421
rs1689800
rs186696265
rs6602909
rs12916
rs7837694
rs10195252
rs79598313
rs72664260
rs73066485
rs17398765
rs10420434
rs12359135
rs74698119
rs35135293
rs6445881
rs629301
rs1024137
rs141469619
rs75679663
rs28600710
rs17414716
rs2043085
rs139974673
rs11751024
rs59950280
rs4665710
rs11783935
rs1800588
rs73600289
rs187429064
rs6874202
rs1007205
rs115849089
rs632057
rs2287997
rs964184
rs113414093
rs1260326
rs1551393
rs3764261
rs4397267
rs8107974
rs6968865
rs182695896
rs76866386
rs116843064
rs2954021
rs807060
rs7578604
rs2396084
rs429358
rs141864196
rs6073958
rs1117816
rs13240994
rs80254170
rs7395581
rs687339
rs9687846
rs6699113
rs28929474
rs1979215
rs17185445
rs998584
rs12625329
All - MR Egger
All - Inverse variance weighted
-1
0
1
MR effect size for
'Free cholesterol in small VLDL || id:ebi-cfb233-GCST90302108' on 'Breast cancer (Combined Oncoarray; iCOGS; GWAS meta

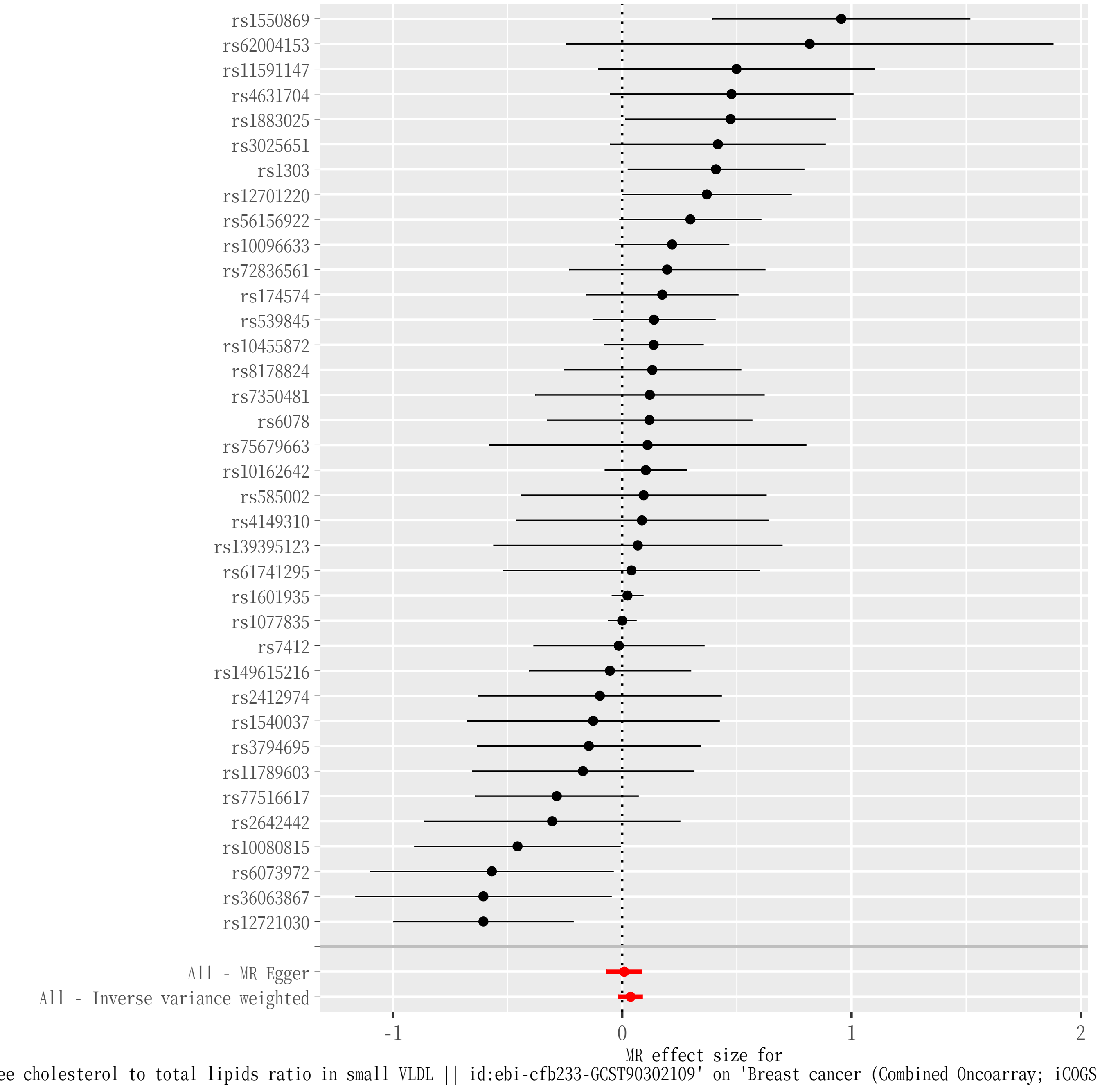
rs1550869
rs62004153
rs11591147
rs4631704
rs1883025
rs3025651
rs1303
rs12701220
rs56156922
rs10096633
rs72836561
rs174574
rs539845
rs10455872
rs8178824
rs7350481
rs6078
rs75679663
rs10162642
rs585002
rs4149310
rs139395123
rs61741295
rs1601935
rs1077835
rs7412
rs149615216
rs2412974
rs1540037
rs3794695
rs11789603
rs77516617
rs2642442
rs10080815
rs6073972
rs36063867
rs12721030
All - MR Egger
All - Inverse variance weighted
-1
0
1
2
MR effect size for
ee cholesterol to total lipids ratio in small VLDL || id:ebi-cfb233-GCST90302109' on 'Breast cancer (Combined Oncoarray; iCOGS

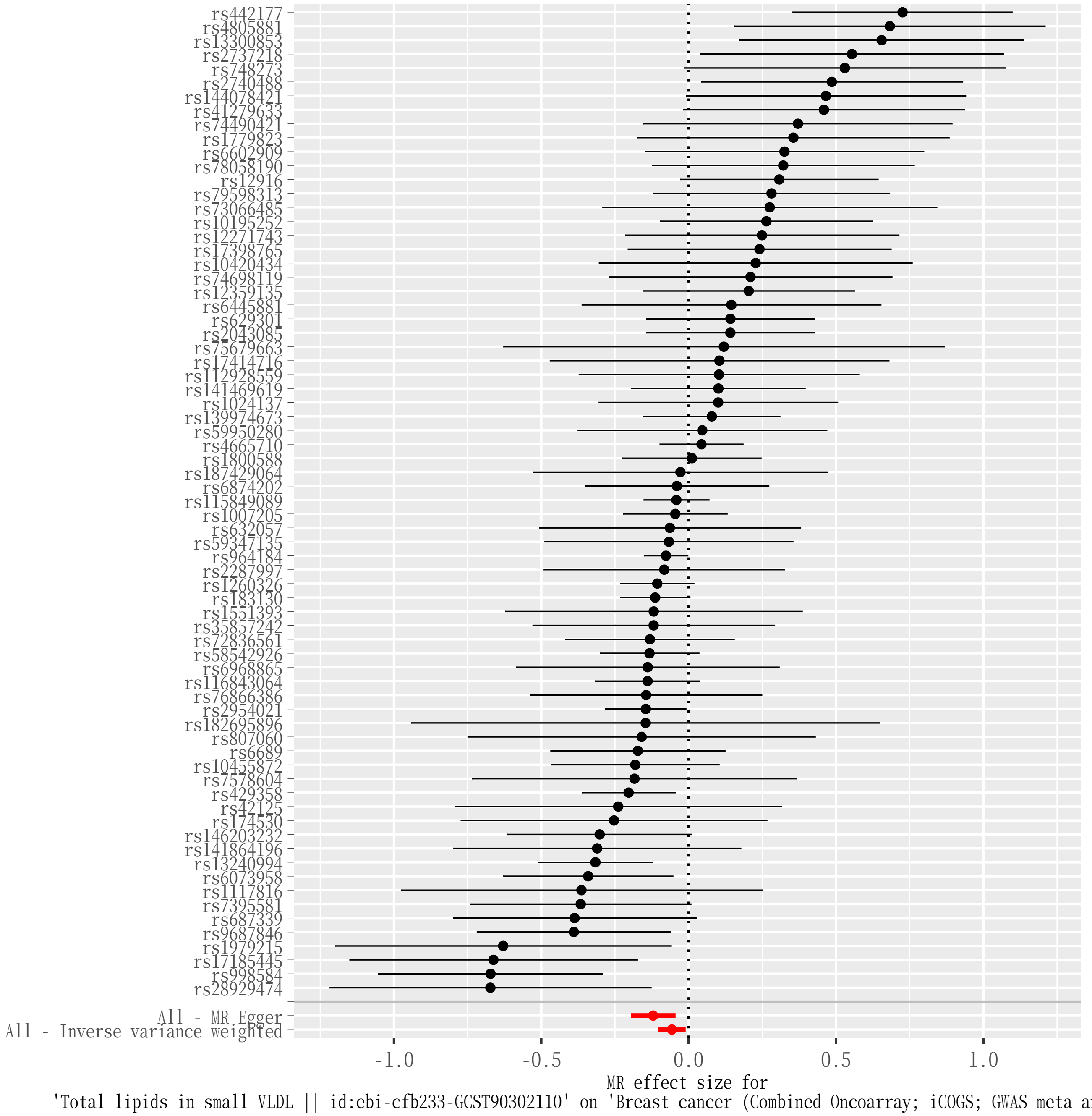

rs442177
rs4805881
rs13300853
rs2737218
rs748273
rs2740488
rs144078421
rs41279633
rs74490421
rs1779823
rs6602909
rs78058190
rs12916
rs79598313
rs73066485
rs10195252
rs12271743
rs17398765
rs10420434
rs74698119
rs12359135
rs6445881
rs629301
rs2043085
rs75679663
rs17414716
rs112928559
rs141469619
rs1024137
rs139974673
rs59950280
rs4665710
rs1800588
rs187429064
rs6874202
rs115849089
rs1007205
rs632057
rs59347135
rs964184
rs2287997
rs1260326
rs183130
rs1551393
rs35857242
rs72836561
rs58542926
rs6968865
rs116843064
rs76866386
rs2954021
rs182695896
rs807060
rs6689
rs10455872
rs7578604
rs429358
rs42125
rs174530
rs146203232
rs141864196
rs13240994
rs6073958
rs1117816
rs7395581
rs687339
rs9687846
rs1979215
rs17185445
rs998584
rs28929474
All - MR Egger
All - Inverse variance weighted
-1.0
-0.5
0.0
0.5
1.0
MR effect size for
'Total lipids in small VLDL || id:ebi-cfb233-GCST90302110' on 'Breast cancer (Combined Oncoarray; iCOGS; GWAS meta an

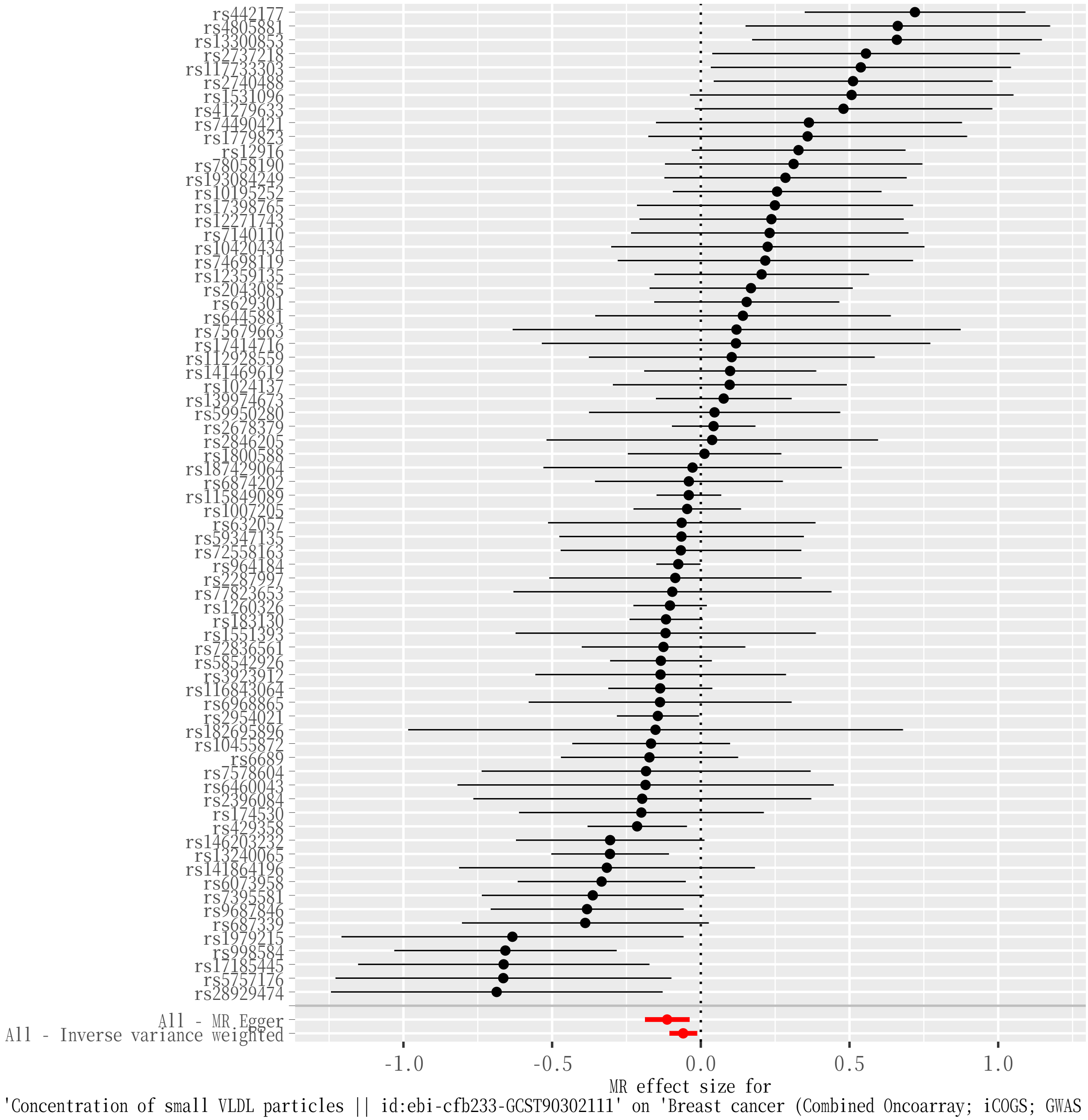
rs442177
rs4805881
rs13300853
rs2737218
rs117733303
rs2740488
rs1531096
rs41279633
rs74490421
rs1779823
rs12916
rs78058190
rs193084249
rs10195252
rs17398765
rs12271743
rs7140110
rs10420434
rs74698119
rs12359135
rs2043085
rs629301
rs6445881
rs75679663
rs17414716
rs112928559
rs141469619
rs1024137
rs139974673
rs59950280
rs2678379
rs2846205
rs1800588
rs187429064
rs6874202
rs115849089
rs1007205
rs632057
rs59347135
rs72558163
rs964184
rs2287997
rs77823653
rs1260326
rs183130
rs1551393
rs72836561
rs58542926
rs3923912
rs116843064
rs6968865
rs2954021
rs182695826
rs10455872
rs6689
rs7578604
rs6460043
rs2396084
rs174530
rs429358
rs146203232
rs13240065
rs141864196
rs6073958
rs7395581
rs9687846
rs687339
rs1979215
rs998584
rs17185445
rs5757176
rs28929474
All - MR Egger
All - Inverse variance weighted
-1.0
-0.5
0.0
0.5
1.0
MR effect size for
'Concentration of small VLDL particles || id:ebi-cfb233-GCST90302111' on 'Breast cancer (Combined Oncoarray; iCOGS; GWAS m

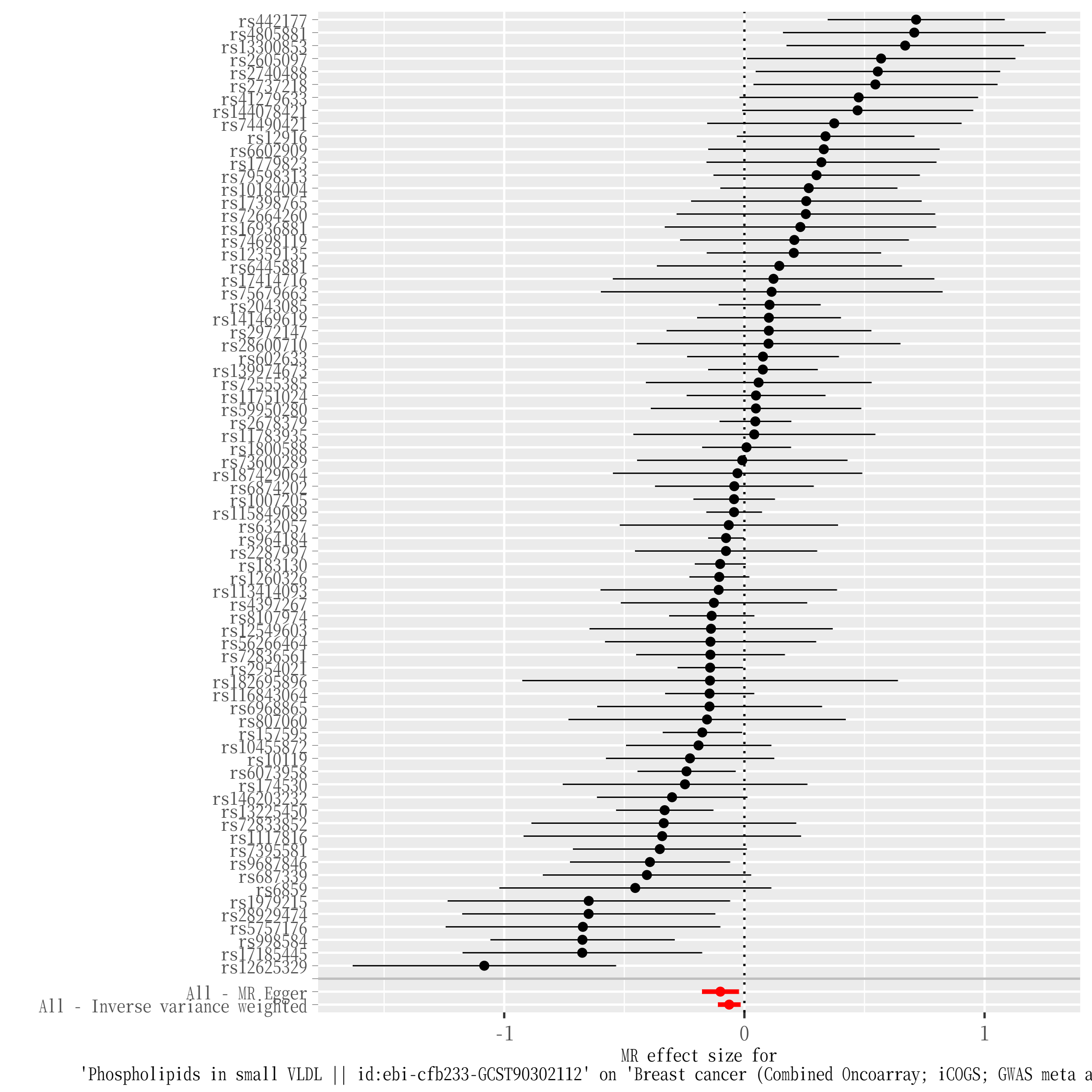
rs442177
rs4805881
rs13300853
rs2605097
rs2740488
rs2737218
rs41279633
rs144078421
rs74490421
rs12916
rs6602909
rs1779823
rs79598313
rs10184004
rs17398765
rs72664260
rs16936881
rs74698119
rs12359135
rs6445881
rs17414716
rs75679663
rs2043085
rs141469619
rs2972147
rs28600710
rs602633
rs139974673
rs72555385
rs11751024
rs59950280
rs2678379
rs11783935
rs1800588
rs73600289
rs187429064
rs6874202
rs1007205
rs115849089
rs632057
rs964184
rs2287997
rs183130
rs1260326
rs113414093
rs4397267
rs8107974
rs12549603
rs56266464
rs72836561
rs2954021
rs182695896
rs116843064
rs6968865
rs807060
rs157595
rs10455872
rs10119
rs6073958
rs174530
rs146203232
rs13225450
rs72833852
rs1117816
rs7395581
rs9687846
rs687339
rs6859
rs1979215
rs28929474
rs5757176
rs998584
rs17185445
rs12625329
All - MR Egger
All - Inverse variance weighted
-1
0
1
MR effect size for
'Phospholipids in small VLDL || id:ebi-cfb233-GCST90302112' on 'Breast cancer (Combined Oncoarray; iCOGS; GWAS meta a

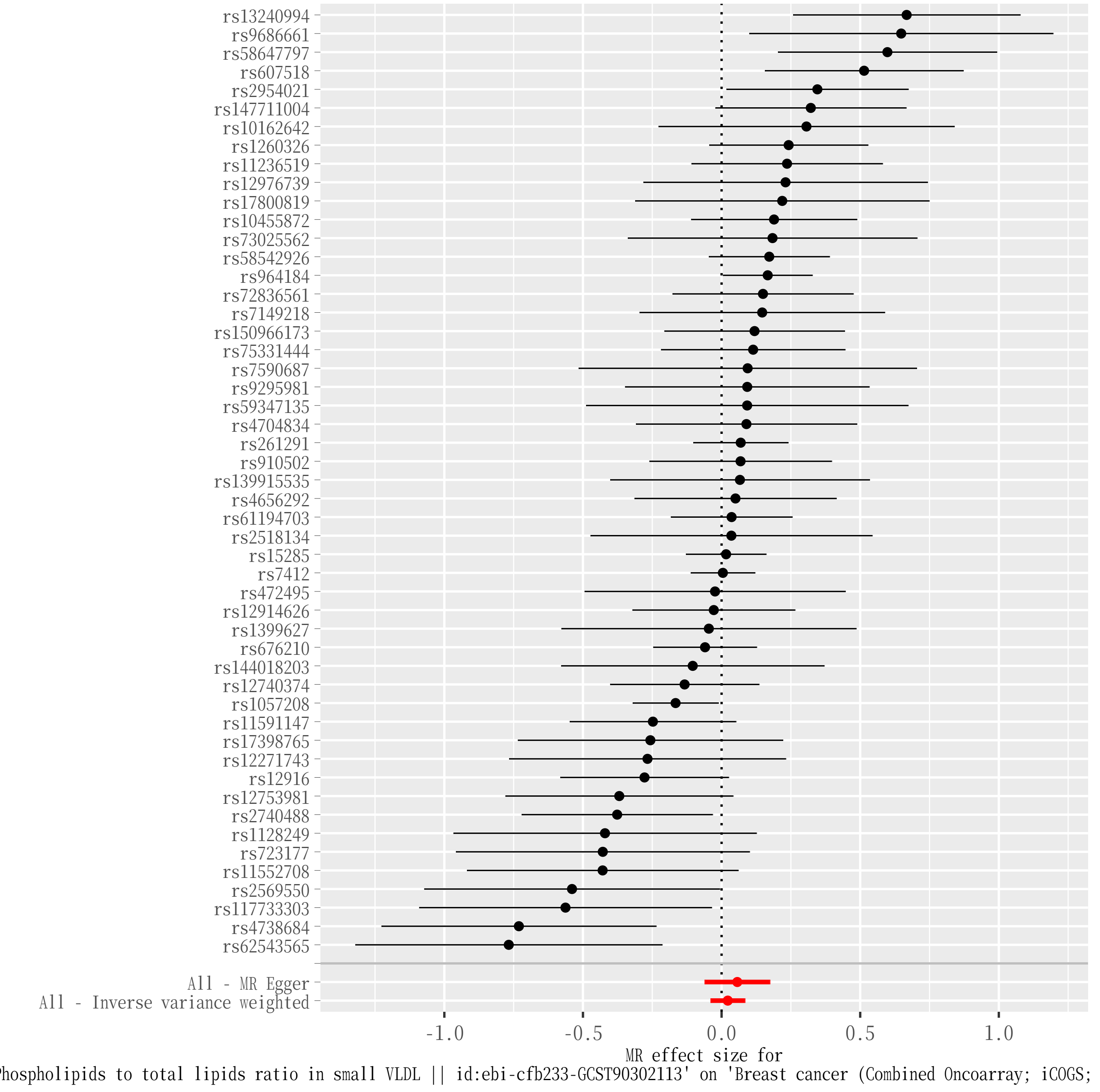
rs13240994
rs9686661
rs58647797
rs607518
rs2954021
rs147711004
rs10162642
rs1260326
rs11236519
rs12976739
rs17800819
rs10455872
rs73025562
rs58542926
rs964184
rs72836561
rs7149218
rs150966173
rs75331444
rs7590687
rs9295981
rs59347135
rs4704834
rs261291
rs910502
rs139915535
rs4656292
rs61194703
rs2518134
rs15285
rs7412
rs472495
rs12914626
rs1399627
rs676210
rs144018203
rs12740374
rs1057208
rs11591147
rs17398765
rs12271743
rs12916
rs12753981
rs2740488
rs1128249
rs723177
rs11552708
rs2569550
rs117733303
rs4738684
rs62543565
All - MR Egger
All - Inverse variance weighted
-1.0
-0.5
0.0
0.5
1.0
MR effect size for
Phospholipids to total lipids ratio in small VLDL || id:ebi-cfb233-GCST90302113' on 'Breast cancer (Combined Oncoarray; iCOGS;

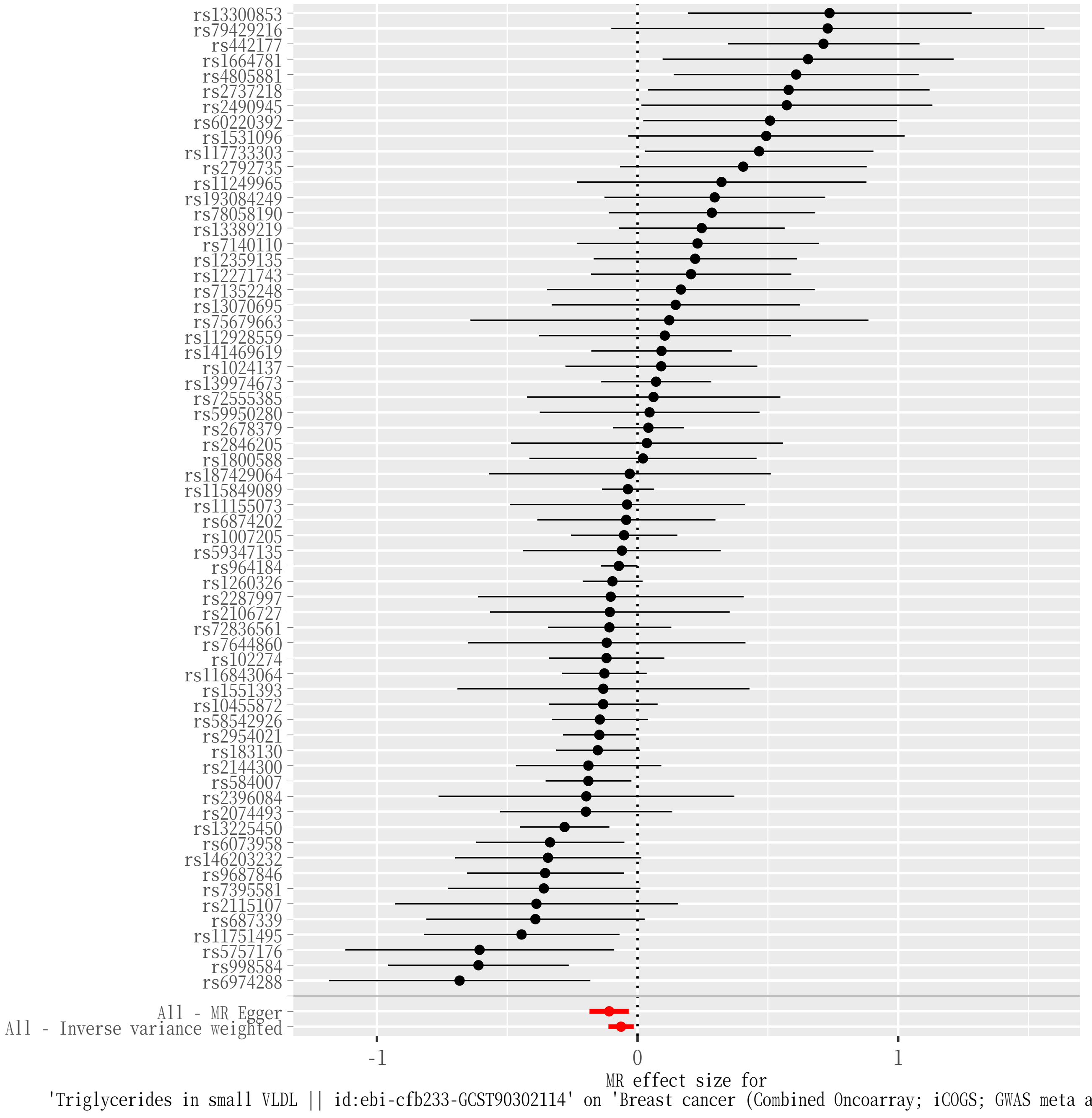
rs13300853
rs79429216
rs442177
rs1664781
rs4805881
rs2737218
rs2490945
rs60220392
rs1531096
rs117733303
rs2792735
rs11249965
rs193084249
rs78058190
rs13389219
rs7140110
rs12359135
rs12271743
rs71352248
rs13070695
rs75679663
rs112928559
rs141469619
rs1024137
rs139974673
rs72555385
rs59950280
rs2678379
rs2846205
rs1800588
rs187429064
rs115849089
rs11155073
rs6874202
rs1007205
rs59347135
rs964184
rs1260326
rs2287997
rs2106727
rs72836561
rs7644860
rs102274
rs116843064
rs1551393
rs10455872
rs58542926
rs2954021
rs183130
rs2144300
rs584007
rs2396084
rs2074493
rs13225450
rs6073958
rs146203232
rs9687846
rs7395581
rs2115107
rs687339
rs11751495
rs5757176
rs998584
rs6974288
All - MR Egger
All - Inverse variance weighted
-1
0
1
MR effect size for
'Triglycerides in small VLDL || id:ebi-cfb233-GCST90302114' on 'Breast cancer (Combined Oncoarray; iCOGS; GWAS meta a

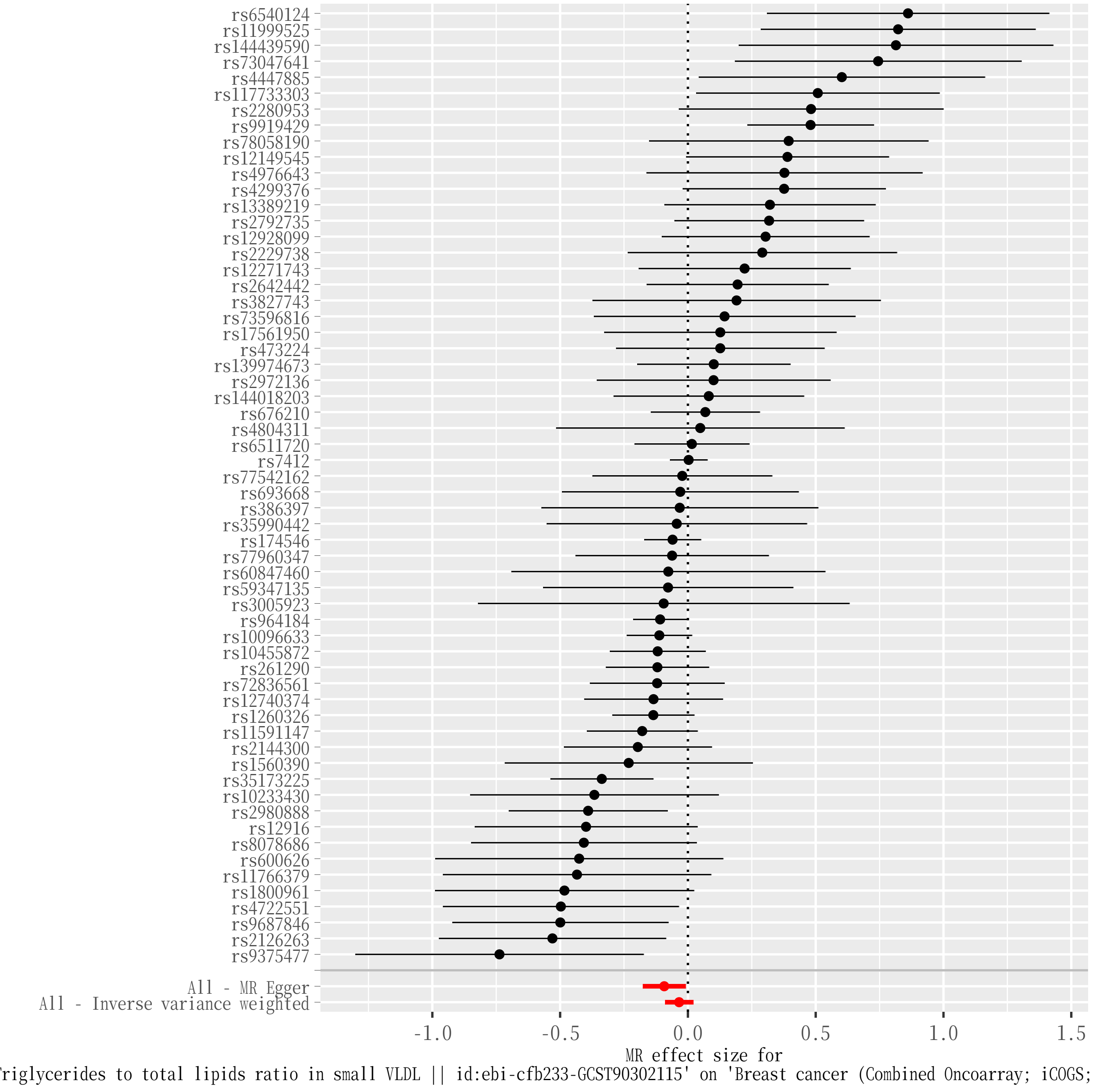
rs6540124
rs11999525
rs144439590
rs73047641
rs4447885
rs117733303
rs2280953
rs9919429
rs78058190
rs12149545
rs4976643
rs4299376
rs13389219
rs2792735
rs12928099
rs2229738
rs12271743
rs2642442
rs3827743
rs73596816
rs17561950
rs473224
rs139974673
rs2972136
rs144018203
rs676210
rs4804311
rs6511720
rs7412
rs77542162
rs693668
rs386397
rs35990442
rs174546
rs77960347
rs60847460
rs59347135
rs3005923
rs964184
rs10096633
rs10455872
rs261290
rs72836561
rs12740374
rs1260326
rs11591147
rs2144300
rs1560390
rs35173225
rs10233430
rs2980888
rs12916
rs8078686
rs600626
rs11766379
rs1800961
rs4722551
rs9687846
rs2126263
rs9375477
All - MR Egger
All - Inverse variance weighted
-1.0
-0.5
0.0
0.5
1.0
1.5
MR effect size for
'Triglycerides to total lipids ratio in small VLDL || id:ebi-cfb233-GCST90302115' on 'Breast cancer (Combined Oncoarray; iCOGS;

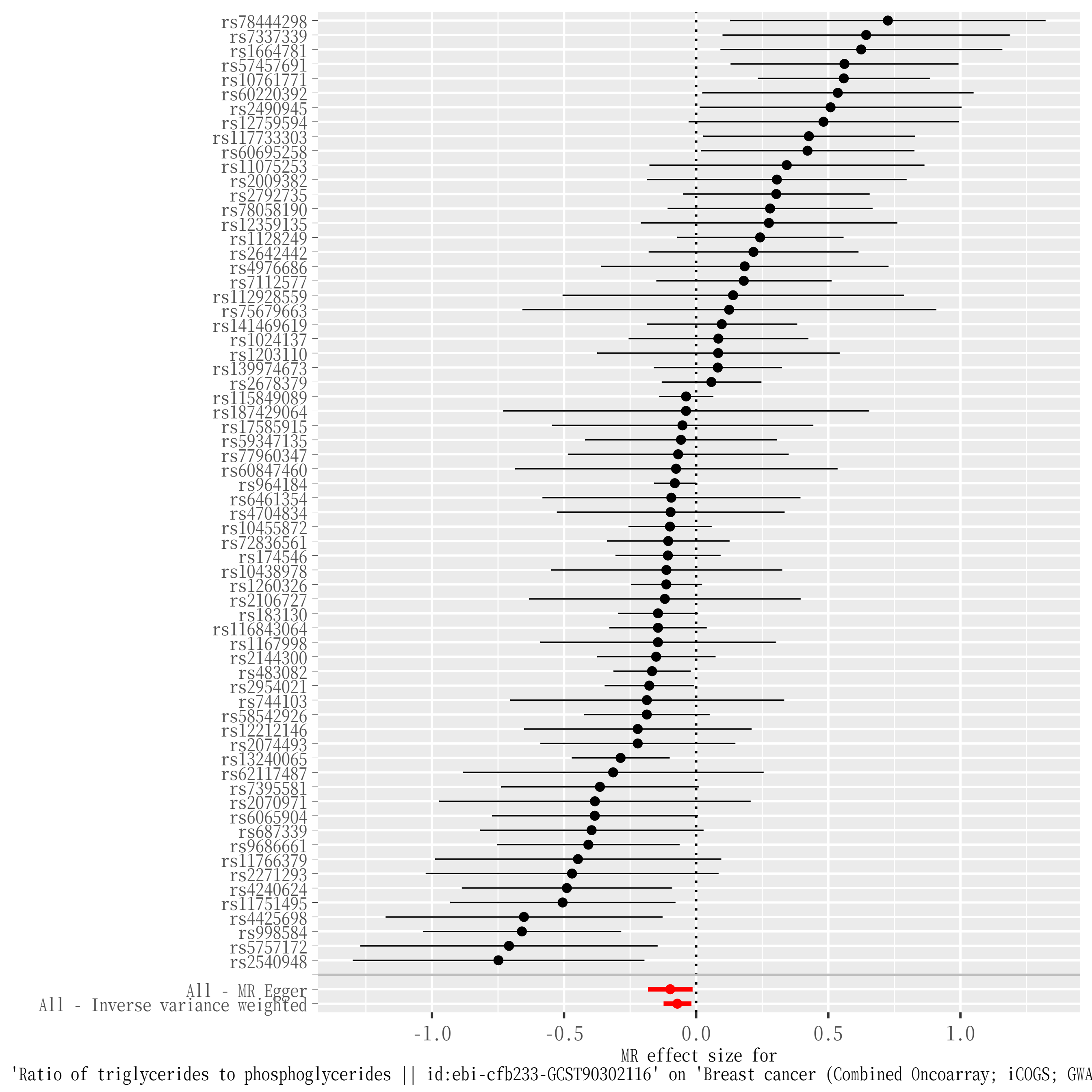
rs78444298
rs7337339
rs1664781
rs57457691
rs10761771
rs60220392
rs2490945
rs12759594
rs117733303
rs60695258
rs11075253
rs2009382
rs2792735
rs78058190
rs12359135
rs1128249
rs2642442
rs4976686
rs7112577
rs112928559
rs75679663
rs141469619
rs1024137
rs1203110
rs139974673
rs2678379
rs115849089
rs187429064
rs17585915
rs59347135
rs77960347
rs60847460
rs964184
rs6461354
rs4704834
rs10455872
rs72836561
rs174546
rs10438978
rs1260326
rs2106727
rs183130
rs116843064
rs1167998
rs2144300
rs483082
rs2954021
rs744103
rs58542926
rs12212146
rs2074493
rs13240065
rs62117487
rs7395581
rs2070971
rs6065904
rs687339
rs9686661
rs11766379
rs2271293
rs4240624
rs11751495
rs4425698
rs998584
rs5757172
rs2540948
All - MR Egger
All - Inverse variance weighted
-1.0
-0.5
0.0
0.5
1.0
MR effect size for
'Ratio of triglycerides to phosphoglycerides || id:ebi-cfb233-GCST90302116' on 'Breast cancer (Combined Oncoarray; iCOGS; GWA

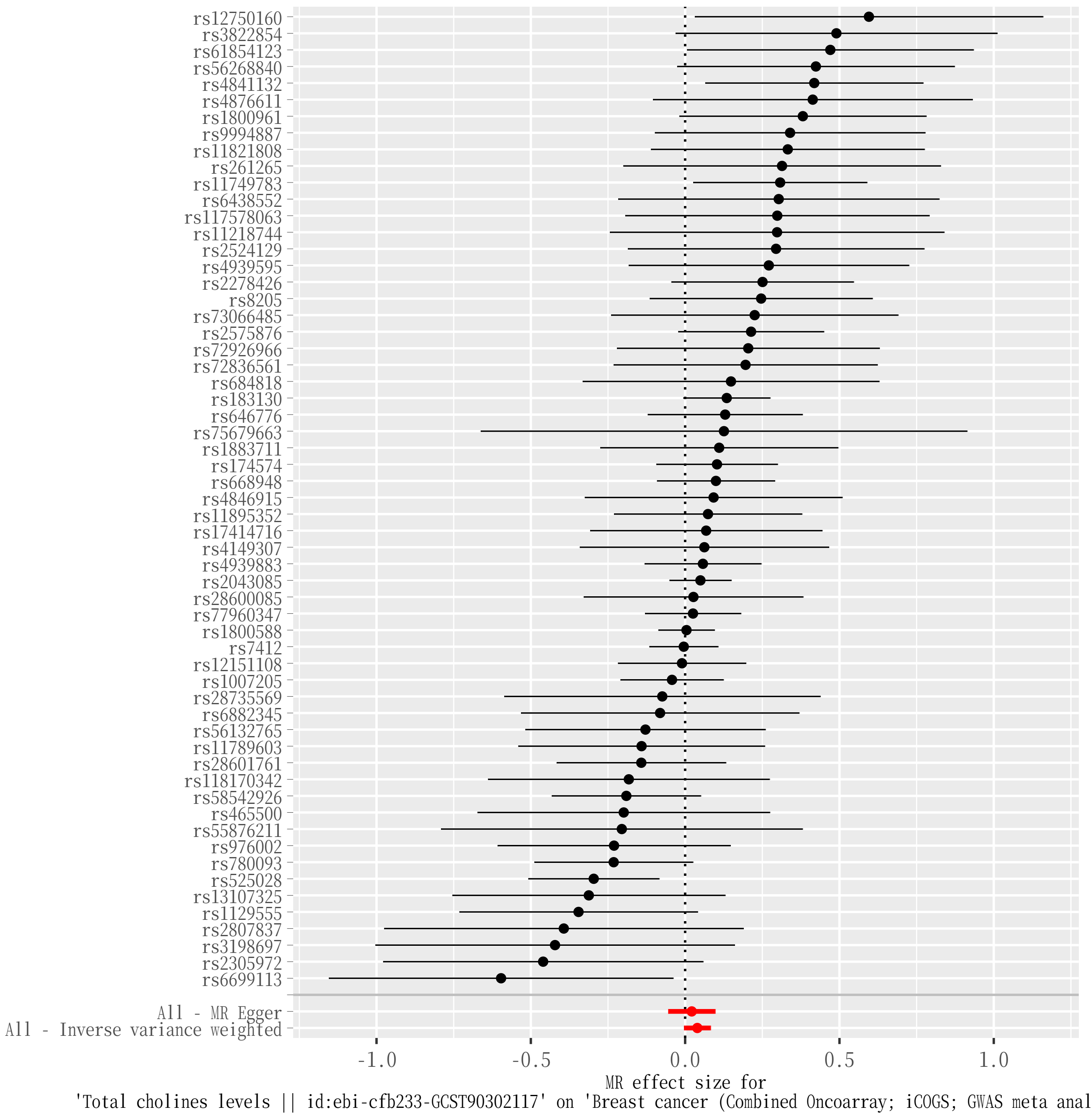

rs12750160
rs3822854
rs61854123
rs56268840
rs4841132
rs4876611
rs1800961
rs9994887
rs11821808
rs261265
rs11749783
rs6438552
rs117578063
rs11218744
rs2524129
rs4939595
rs2278426
rs8205
rs73066485
rs2575876
rs72926966
rs72836561
rs684818
rs183130
rs646776
rs75679663
rs1883711
rs174574
rs668948
rs4846915
rs11895352
rs17414716
rs4149307
rs4939883
rs2043085
rs28600085
rs77960347
rs1800588
rs7412
rs12151108
rs1007205
rs28735569
rs6882345
rs56132765
rs11789603
rs28601761
rs118170342
rs58542926
rs465500
rs55876211
rs976002
rs780093
rs525028
rs13107325
rs1129555
rs2807837
rs3198697
rs2305972
rs6699113
All - MR Egger
All - Inverse variance weighted
-1.0
-0.5
0.0
0.5
1.0
MR effect size for
'Total cholines levels || id:ebi-cfb233-GCST90302117' on 'Breast cancer (Combined Oncoarray; iCOGS; GWAS meta anal

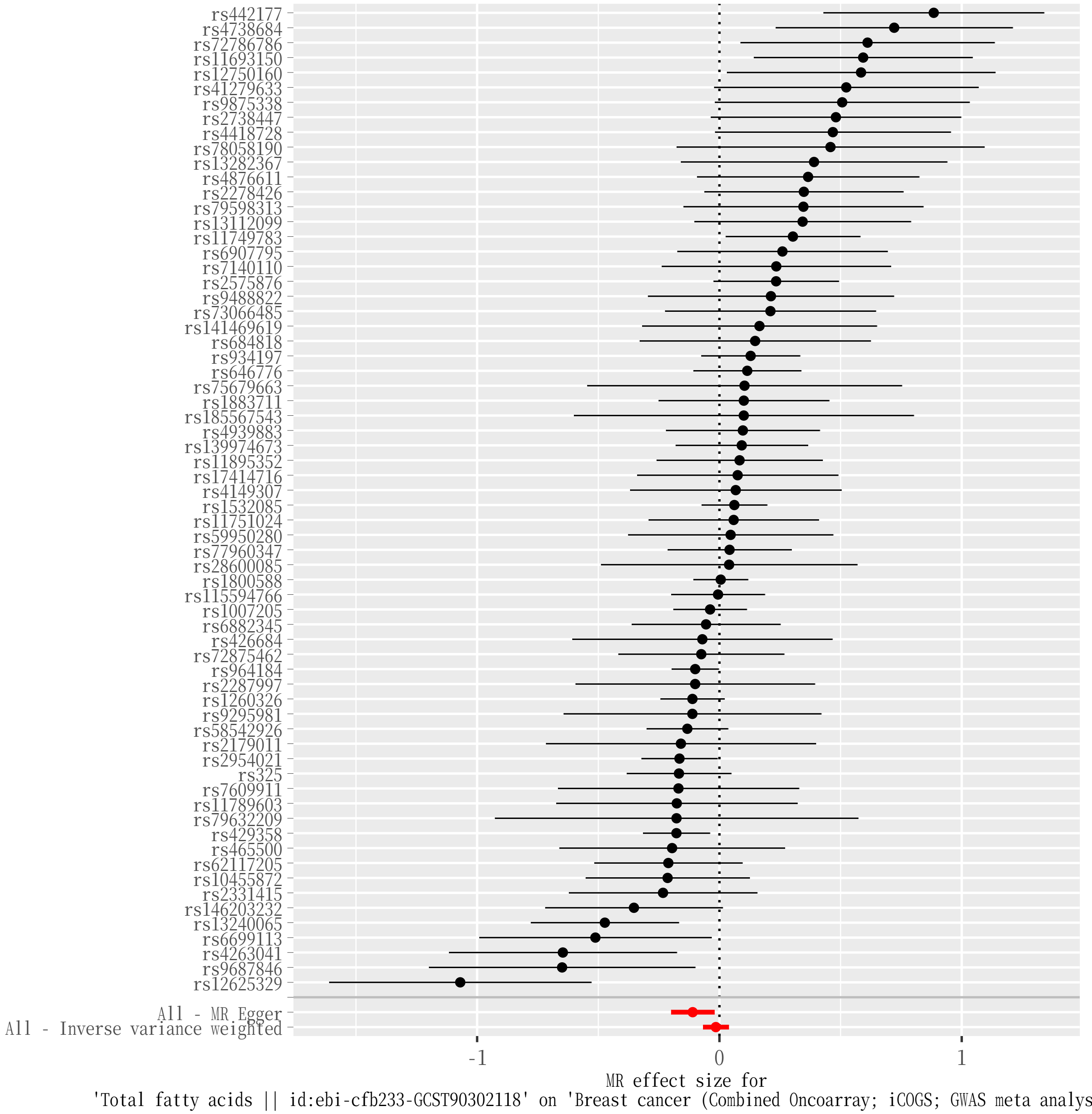

rs442177
rs4738684
rs72786786
rs11693150
rs12750160
rs41279633
rs9875338
rs2738447
rs4418728
rs78058190
rs13282367
rs4876611
rs2278426
rs79598313
rs13112099
rs11749783
rs6907795
rs7140110
rs2575876
rs9488822
rs73066485
rs141469619
rs684818
rs934197
rs646776
rs75679663
rs1883711
rs185567543
rs4939883
rs139974673
rs11895352
rs17414716
rs4149307
rs1532085
rs11751024
rs59950280
rs77960347
rs28600085
rs1800588
rs115594766
rs1007205
rs6882345
rs426684
rs72875462
rs964184
rs2287997
rs1260326
rs9295981
rs58542926
rs2179011
rs2954021
rs325
rs7609911
rs11789603
rs79632209
rs429358
rs465500
rs62117205
rs10455872
rs2331415
rs146203232
rs13240065
rs6699113
rs4263041
rs9687846
rs12625329
All - MR Egger
All - Inverse variance weighted
-1
0
1
MR effect size for
'Total fatty acids || id:ebi-cfb233-GCST90302118' on 'Breast cancer (Combined Oncoarray; iCOGS; GWAS meta analys

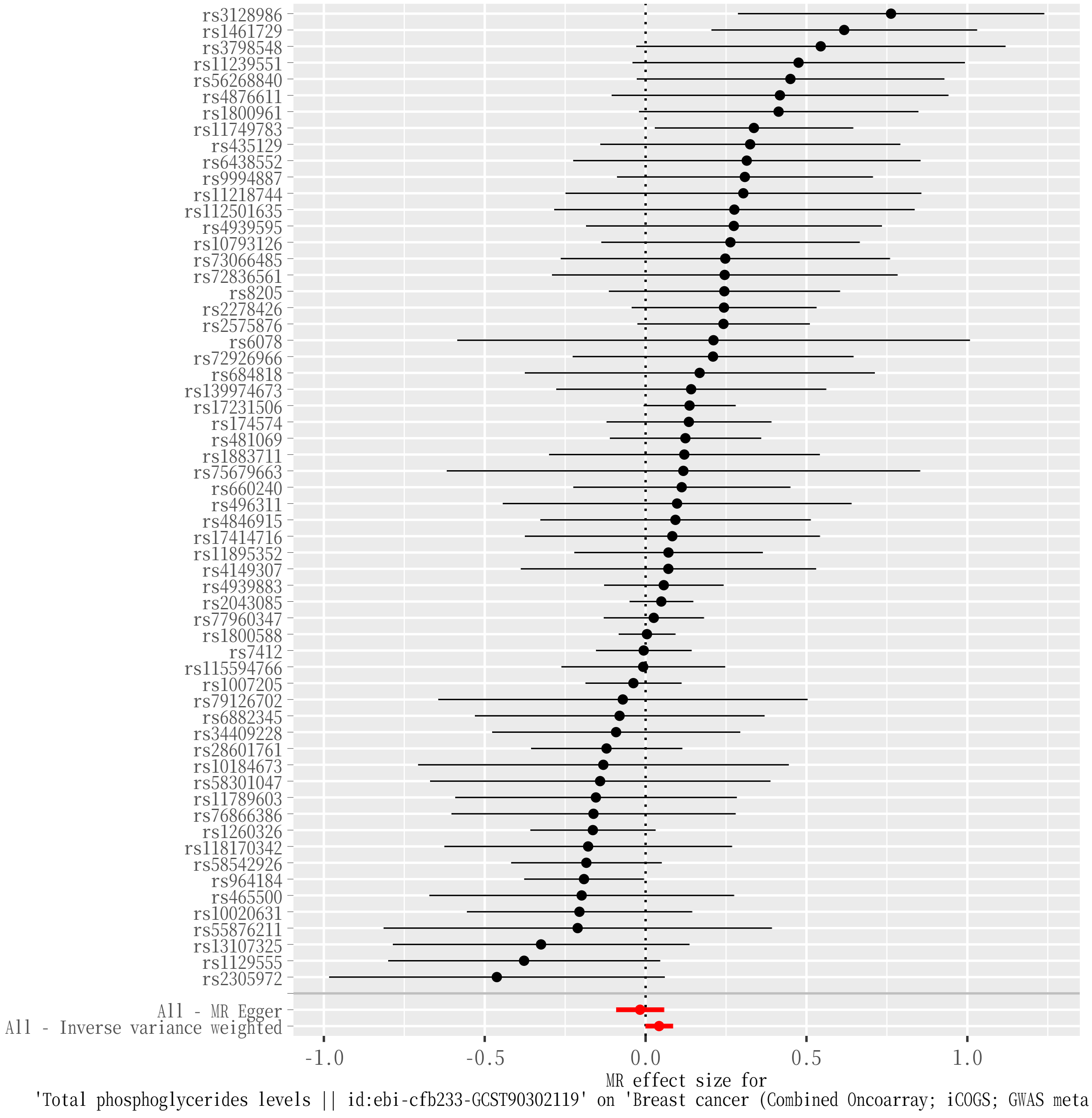

rs3128986
rs1461729
rs3798548
rs11239551
rs56268840
rs4876611
rs1800961
rs11749783
rs435129
rs6438552
rs9994887
rs11218744
rs112501635
rs4939595
rs10793126
rs73066485
rs72836561
rs8205
rs2278426
rs2575876
rs6078
rs72926966
rs684818
rs139974673
rs17231506
rs174574
rs481069
rs1883711
rs75679663
rs660240
rs496311
rs4846915
rs17414716
rs11895352
rs4149307
rs4939883
rs2043085
rs77960347
rs1800588
rs7412
rs115594766
rs1007205
rs79126702
rs6882345
rs34409228
rs28601761
rs10184673
rs58301047
rs11789603
rs76866386
rs1260326
rs118170342
rs58542926
rs964184
rs465500
rs10020631
rs55876211
rs13107325
rs1129555
rs2305972
All - MR Egger
All - Inverse variance weighted
-1.0
-0.5
0.0
0.5
1.0
MR effect size for
'Total phosphoglycerides levels || id:ebi-cfb233-GCST90302119' on 'Breast cancer (Combined Oncoarray; iCOGS; GWAS meta

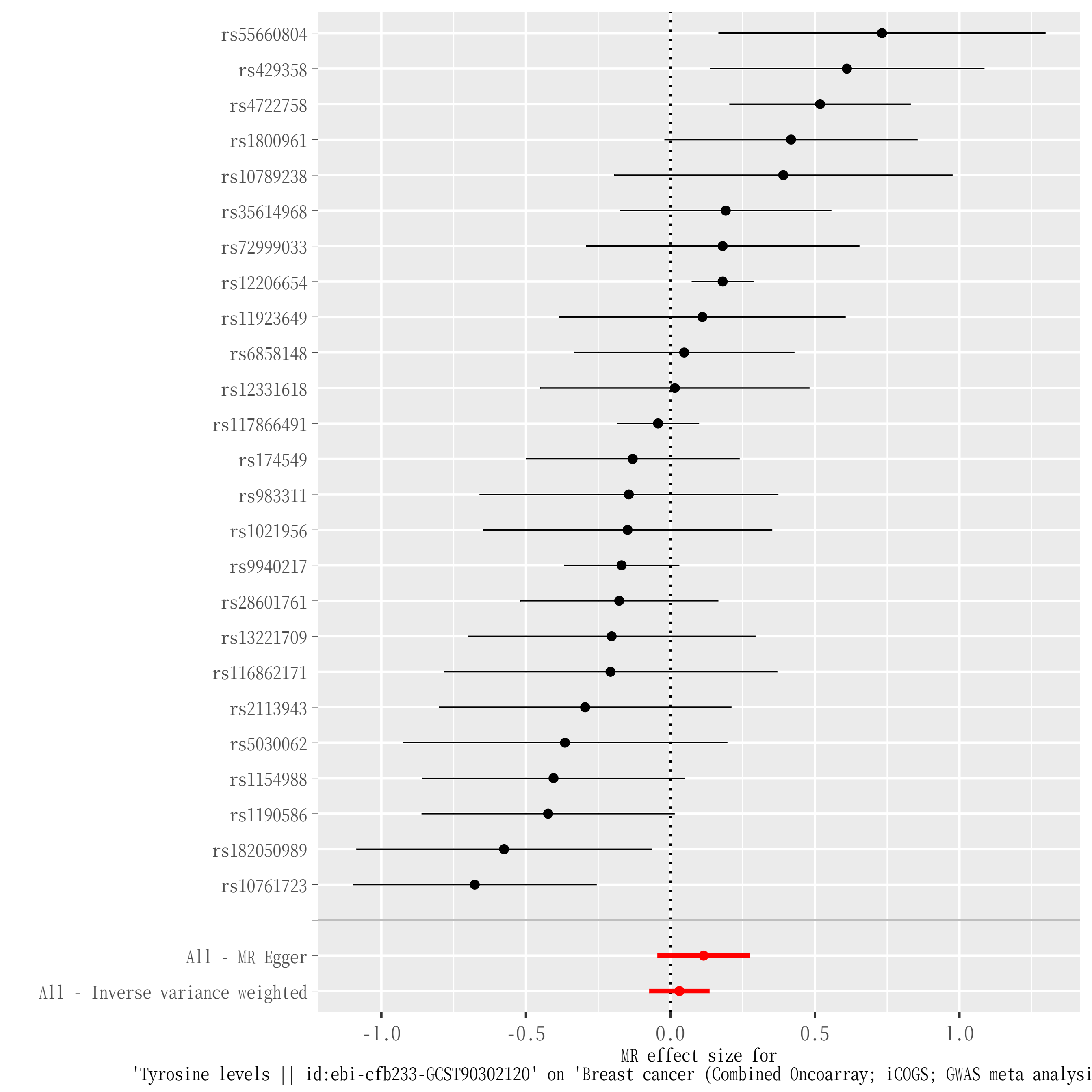
rs55660804
rs429358
rs4722758
rs1800961
rs10789238
rs35614968
rs72999033
rs12206654
rs11923649
rs6858148
rs12331618
rs117866491
rs174549
rs983311
rs1021956
rs9940217
rs28601761
rs13221709
rs116862171
rs2113943
rs5030062
rs1154988
rs1190586
rs182050989
rs10761723
All - MR Egger
All - Inverse variance weighted
-1.0
-0.5
0.0
0.5
1.0
MR effect size for
'Tyrosine levels || id:ebi-cfb233-GCST90302120' on 'Breast cancer (Combined Oncoarray; iCOGS; GWAS meta analysi

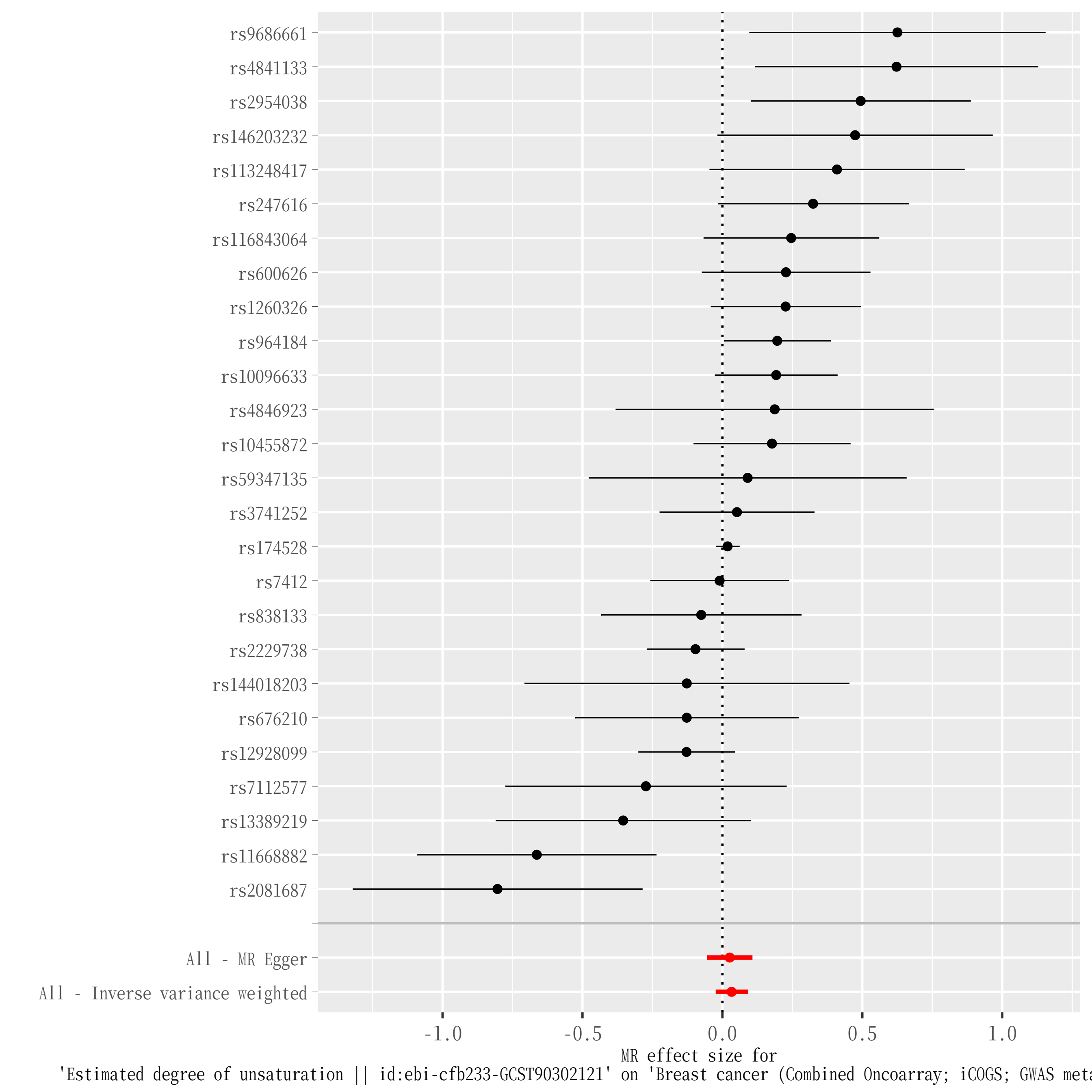

rs9686661
rs4841133
rs2954038
rs146203232
rs113248417
rs247616
rs116843064
rs600626
rs1260326
rs964184
rs10096633
rs4846923
rs10455872
rs59347135
rs3741252
rs174528
rs7412
rs838133
rs2229738
rs144018203
rs676210
rs12928099
rs7112577
rs13389219
rs11668882
rs2081687
All - MR Egger
All - Inverse variance weighted
-1.0
-0.5
0.0
0.5
1.0
MR effect size for
'Estimated degree of unsaturation || id:ebi-cfb233-GCST90302121' on 'Breast cancer (Combined Oncoarray; iCOGS; GWAS meta

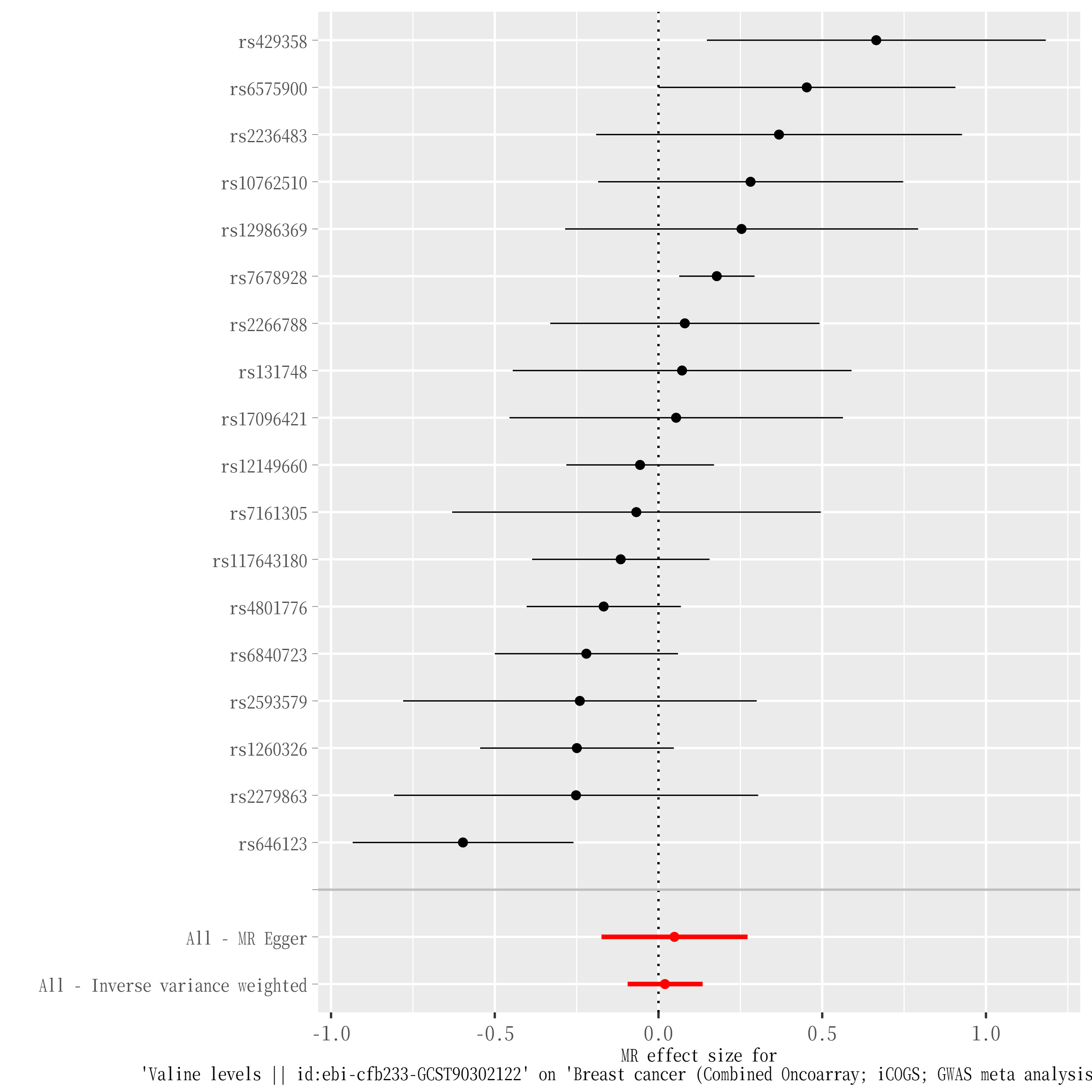
rs429358
rs6575900
rs2236483
rs10762510
rs12986369
rs7678928
rs2266788
rs131748
rs17096421
rs12149660
rs7161305
rs117643180
rs4801776
rs6840723
rs2593579
rs1260326
rs2279863
rs646123
All - MR Egger
All - Inverse variance weighted
-1.0
-0.5
0.0
0.5
1.0
MR effect size for
'Valine levels || id:ebi-cfb233-GCST90302122' on 'Breast cancer (Combined Oncoarray; iCOGS; GWAS meta analysis

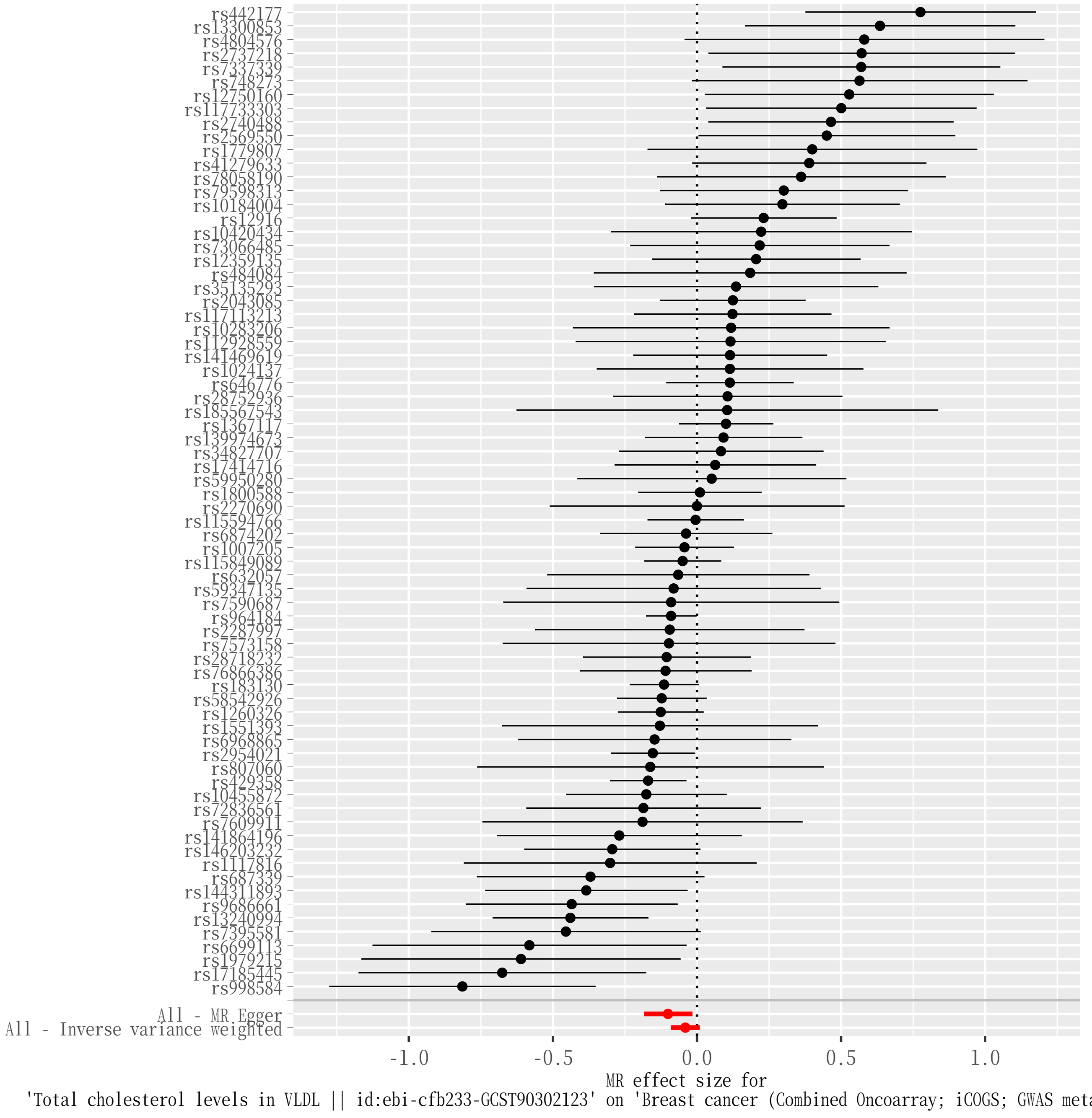
rs442177
rs13300853
rs4804576
rs2737218
rs7337339
rs748273
rs12750160
rs117733303
rs2740488
rs2569550
rs1779807
rs41279633
rs78058190
rs79598313
rs10184004
rs12916
rs10420434
rs73066485
rs12359135
rs484084
rs35135293
rs2043085
rs117113213
rs10283206
rs112928559
rs141469619
rs1024137
rs646776
rs28752936
rs185567543
rs1367117
rs139974673
rs34827707
rs17414716
rs59950280
rs1800588
rs2270690
rs115594766
rs6874202
rs1007205
rs115849089
rs632057
rs59347135
rs7590687
rs964184
rs2287997
rs7573158
rs28718232
rs76866386
rs183130
rs58542926
rs1260326
rs1551393
rs6968865
rs2954021
rs807060
rs429358
rs10455872
rs72836561
rs7609911
rs141864196
rs146203232
rs1117816
rs687339
rs144311893
rs9686661
rs13240994
rs7395581
rs6699113
rs1979215
rs17185445
rs998584
All - MR Egger
All - Inverse variance weighted
-1.0
-0.5
0.0
0.5
1.0
MR effect size for
'Total cholesterol levels in VLDL || id:ebi-cfb233-GCST90302123' on 'Breast cancer (Combined Oncoarray; iCOGS; GWAS meta

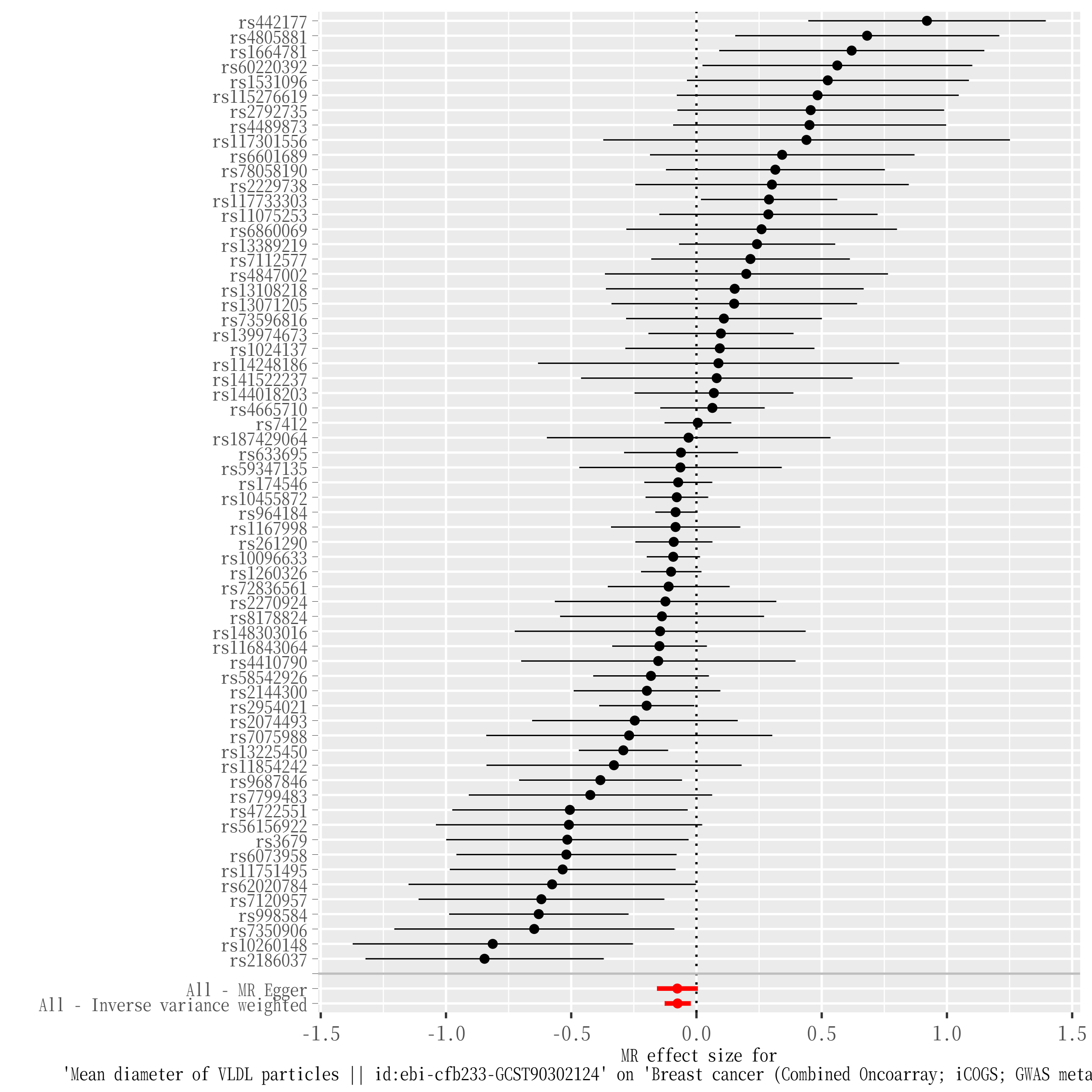
rs442177
rs4805881
rs1664781
rs60220392
rs1531096
rs115276619
rs2792735
rs4489873
rs117301556
rs6601689
rs78058190
rs2229738
rs117733303
rs11075253
rs6860069
rs13389219
rs7112577
rs4847002
rs13108218
rs13071205
rs73596816
rs139974673
rs1024137
rs114248186
rs141522237
rs144018203
rs4665710
rs7412
rs187429064
rs633695
rs59347135
rs174546
rs10455872
rs964184
rs1167998
rs261290
rs10096633
rs1260326
rs72836561
rs2270924
rs8178824
rs148303016
rs116843064
rs4410790
rs58542926
rs2144300
rs2954021
rs2074493
rs7075988
rs13225450
rs11854242
rs9687846
rs7799483
rs4722551
rs56156922
rs3679
rs6073958
rs11751495
rs62020784
rs7120957
rs998584
rs7350906
rs10260148
rs2186037
All - MR Egger
All - Inverse variance weighted
-1.5
-1.0
-0.5
0.0
0.5
1.0
1.5
MR effect size for
'Mean diameter of VLDL particles || id:ebi-cfb233-GCST90302124' on 'Breast cancer (Combined Oncoarray; iCOGS; GWAS meta

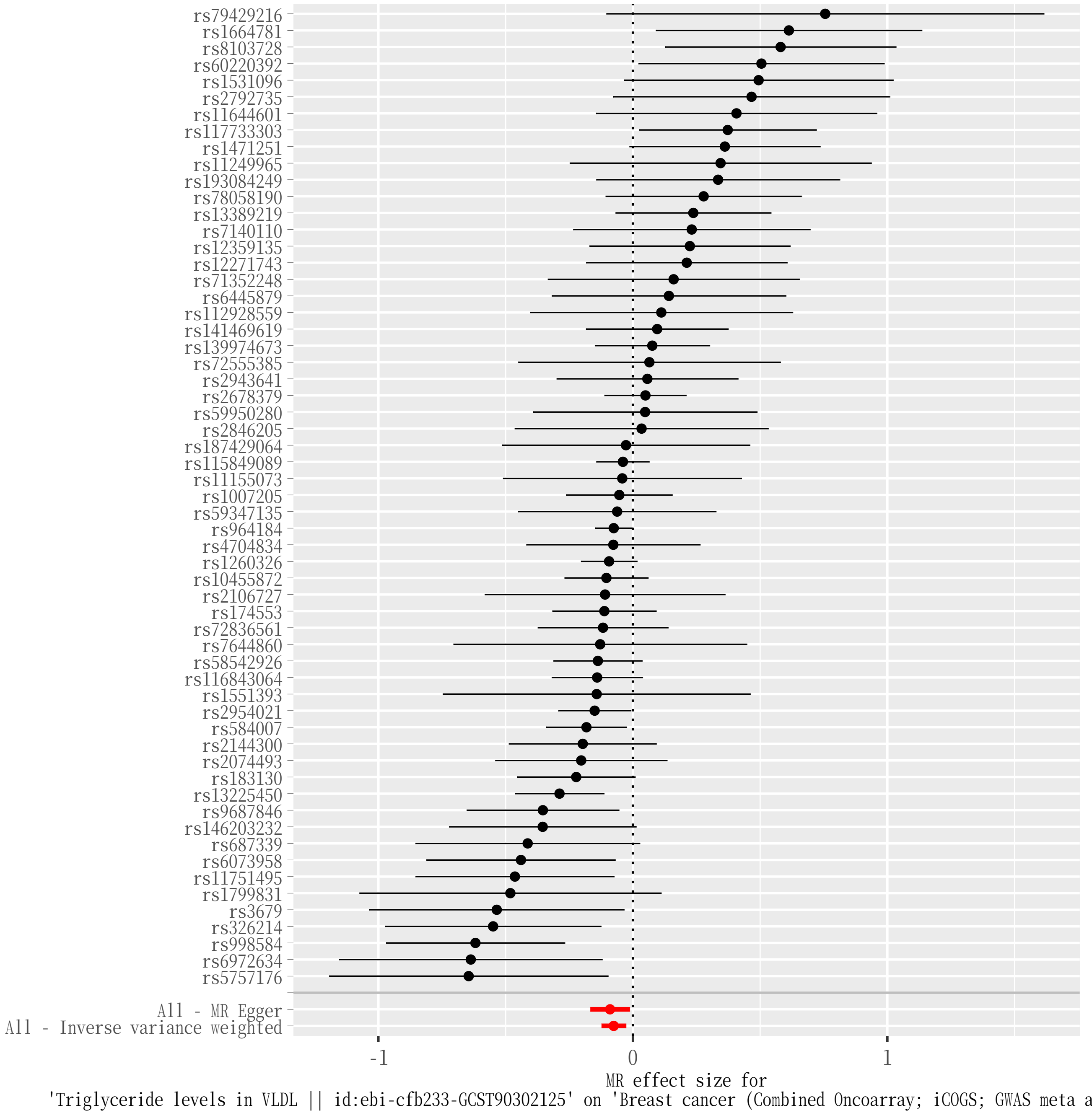
rs79429216
rs1664781
rs8103728
rs60220392
rs1531096
rs2792735
rs11644601
rs117733303
rs1471251
rs11249965
rs193084249
rs78058190
rs13389219
rs7140110
rs12359135
rs12271743
rs71352248
rs6445879
rs112928559
rs141469619
rs139974673
rs72555385
rs2943641
rs2678379
rs59950280
rs2846205
rs187429064
rs115849089
rs11155073
rs1007205
rs59347135
rs964184
rs4704834
rs1260326
rs10455872
rs2106727
rs174553
rs72836561
rs7644860
rs58542926
rs116843064
rs1551393
rs2954021
rs584007
rs2144300
rs2074493
rs183130
rs13225450
rs9687846
rs146203232
rs687339
rs6073958
rs11751495
rs1799831
rs3679
rs326214
rs998584
rs6972634
rs5757176
All - MR Egger
All - Inverse variance weighted
-1
0
1
MR effect size for
'Triglyceride levels in VLDL || id:ebi-cfb233-GCST90302125' on 'Breast cancer (Combined Oncoarray; iCOGS; GWAS meta a

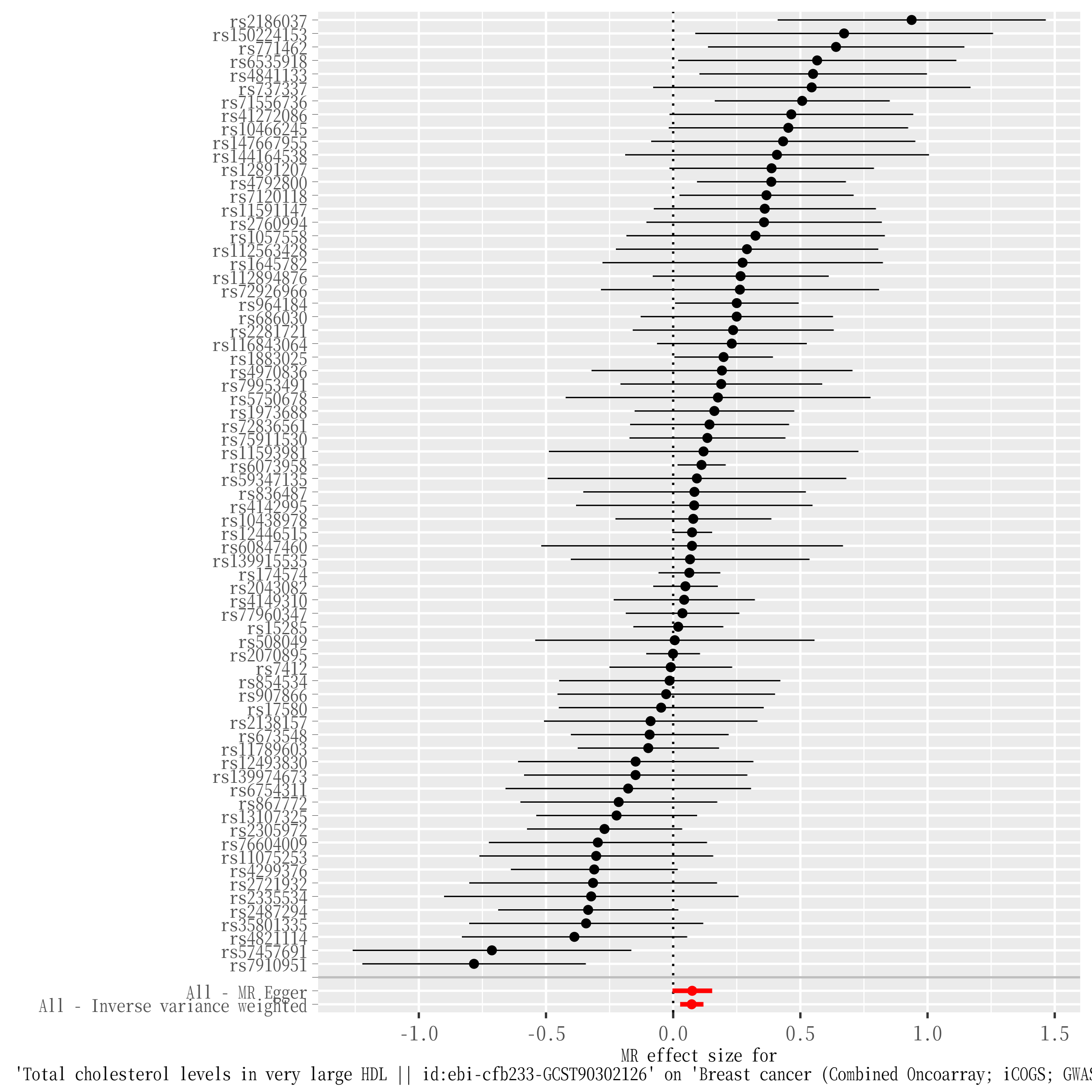
rs2186037
rs150224153
rs771462
rs6535918
rs4841133
rs737337
rs71556736
rs41272086
rs10466245
rs147667955
rs144164538
rs12891207
rs4792800
rs7120118
rs11591147
rs2760994
rs1057558
rs112563428
rs1645782
rs112894876
rs72926966
rs964184
rs686030
rs2281721
rs116843064
rs1883025
rs4970836
rs79953491
rs5750678
rs1973688
rs72836561
rs75911530
rs11593981
rs6073958
rs59347135
rs836487
rs4142995
rs10438978
rs12446515
rs60847460
rs139915535
rs174574
rs2043082
rs4149310
rs77960347
rs15285
rs508049
rs2070895
rs7412
rs854534
rs907866
rs17580
rs2138157
rs673548
rs11789603
rs12493830
rs139974673
rs6754311
rs867772
rs13107325
rs2305972
rs76604009
rs11075253
rs4299376
rs2721932
rs2335534
rs2487294
rs35801335
rs4821114
rs57457691
rs7910951
All - MR Egger
All - Inverse variance weighted
-1.0
-0.5
0.0
0.5
1.0
1.5
MR effect size for
'Total cholesterol levels in very large HDL || id:ebi-cfb233-GCST90302126' on 'Breast cancer (Combined Oncoarray; iCOGS; GWAS

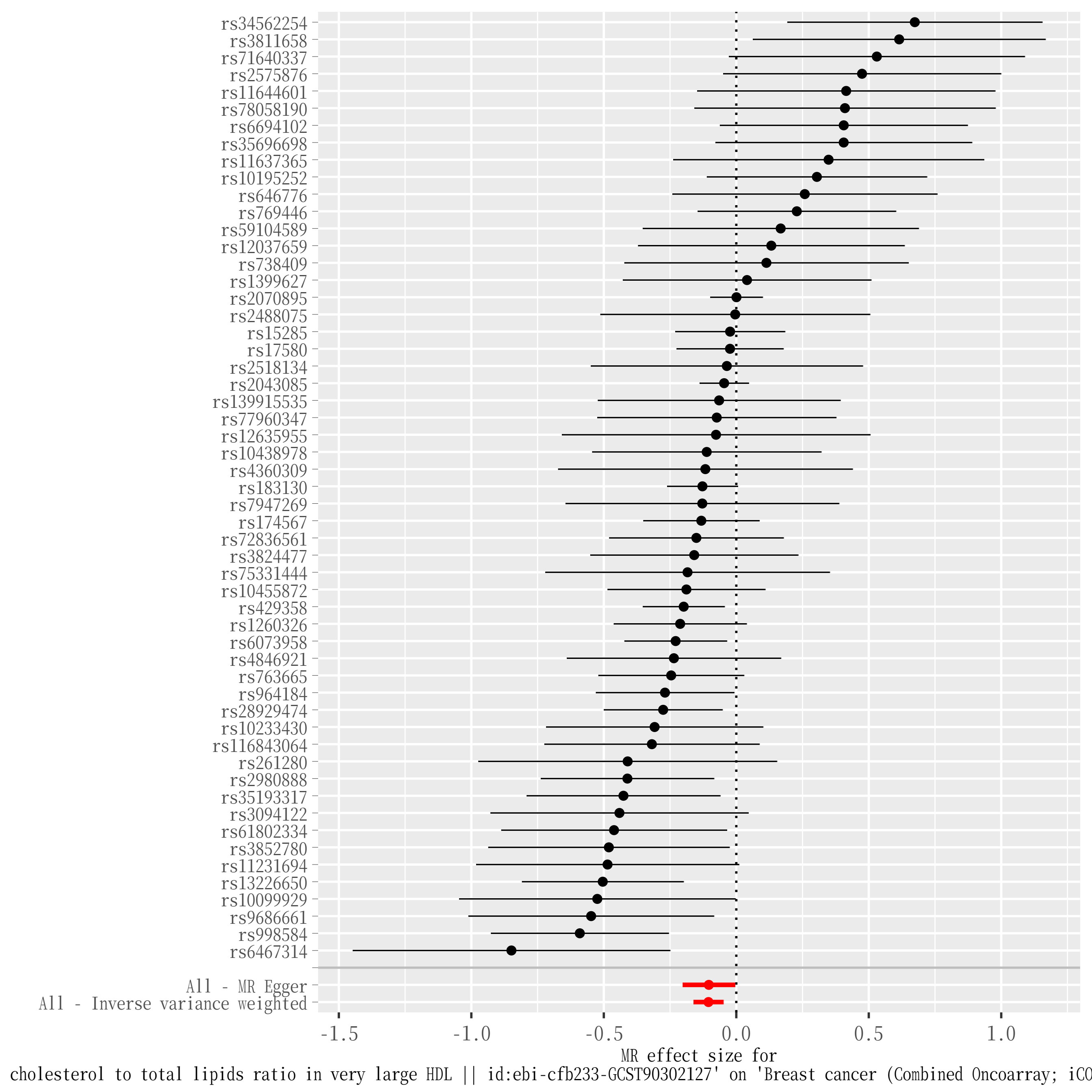

rs34562254
rs3811658
rs71640337
rs2575876
rs11644601
rs78058190
rs6694102
rs35696698
rs11637365
rs10195252
rs646776
rs769446
rs59104589
rs12037659
rs738409
rs1399627
rs2070895
rs2488075
rs15285
rs17580
rs2518134
rs2043085
rs139915535
rs77960347
rs12635955
rs10438978
rs4360309
rs183130
rs7947269
rs174567
rs72836561
rs3824477
rs75331444
rs10455872
rs429358
rs1260326
rs6073958
rs4846921
rs763665
rs964184
rs28929474
rs10233430
rs116843064
rs261280
rs2980888
rs35193317
rs3094122
rs61802334
rs3852780
rs11231694
rs13226650
rs10099929
rs9686661
rs998584
rs6467314
All - MR Egger
All - Inverse variance weighted
-1.5
-1.0
-0.5
0.0
0.5
1.0
MR effect size for
cholesterol to total lipids ratio in very large HDL || id:ebi-cfb233-GCST90302127' on 'Breast cancer (Combined Oncoarray; iCO

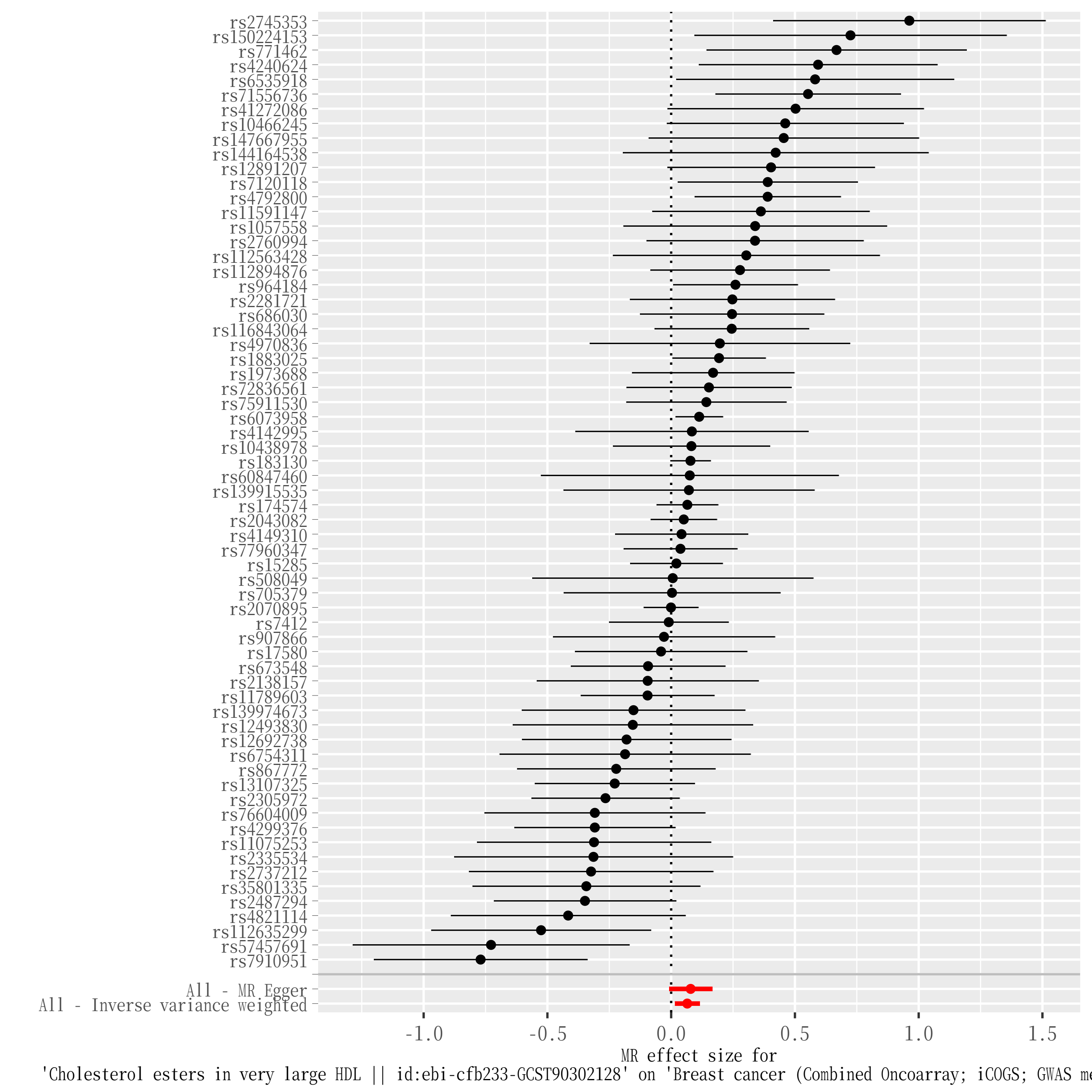

rs2745353
rs150224153
rs771462
rs4240624
rs6535918
rs71556736
rs41272086
rs10466245
rs147667955
rs144164538
rs12891207
rs7120118
rs4792800
rs11591147
rs1057558
rs2760994
rs112563428
rs112894876
rs964184
rs2281721
rs686030
rs116843064
rs4970836
rs1883025
rs1973688
rs72836561
rs75911530
rs6073958
rs4142995
rs10438978
rs183130
rs60847460
rs139915535
rs174574
rs2043082
rs4149310
rs77960347
rs15285
rs508049
rs705379
rs2070895
rs7412
rs907866
rs17580
rs673548
rs2138157
rs11789603
rs139974673
rs12493830
rs12692738
rs6754311
rs867772
rs13107325
rs2305972
rs76604009
rs4299376
rs11075253
rs2335534
rs2737212
rs35801335
rs2487294
rs4821114
rs112635299
rs57457691
rs7910951
All - MR Egger
All - Inverse variance weighted
-1.0
-0.5
0.0
0.5
1.0
1.5
MR effect size for
'Cholesterol esters in very large HDL || id:ebi-cfb233-GCST90302128' on 'Breast cancer (Combined Oncoarray; iCOGS; GWAS me

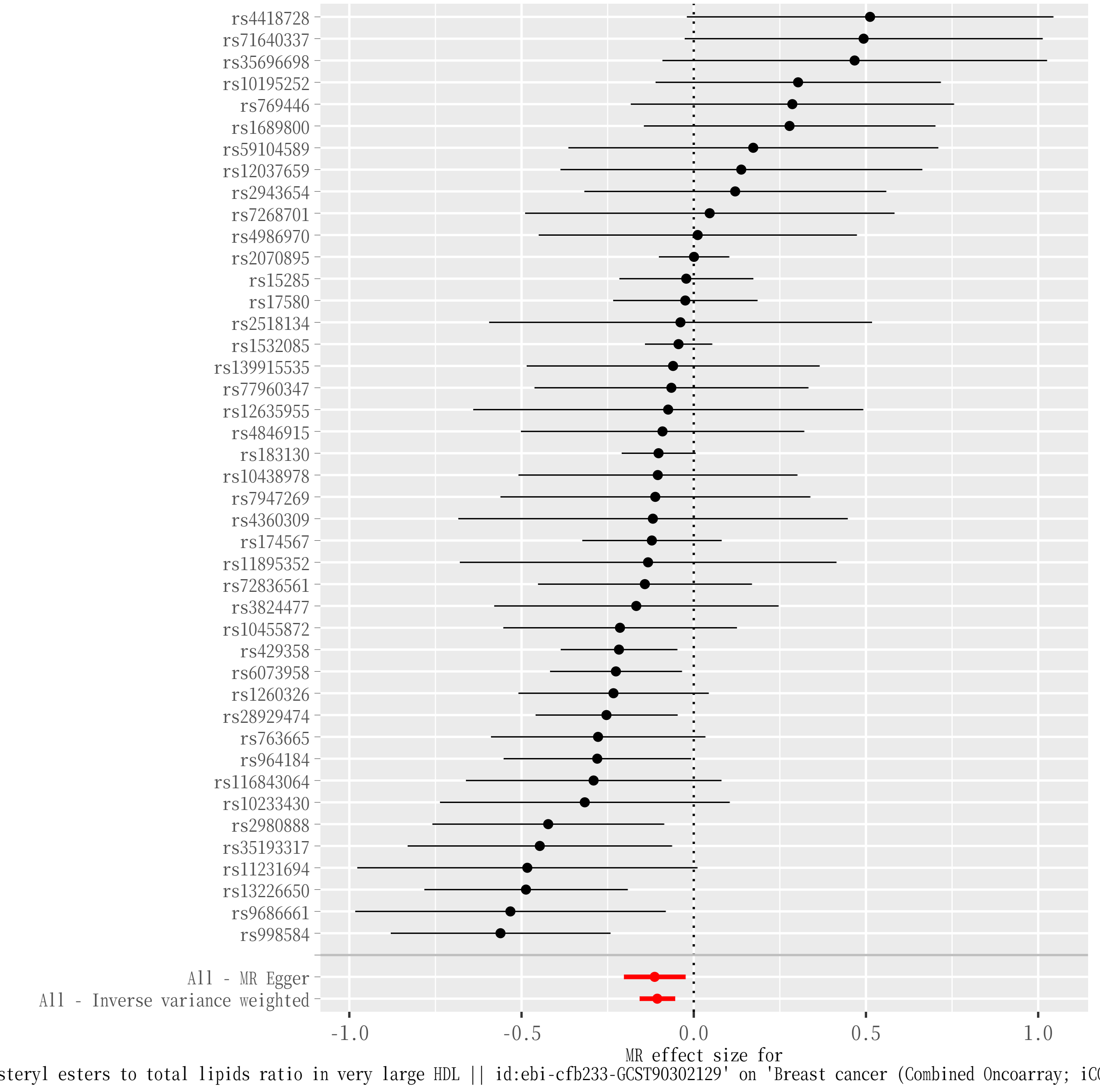
rs4418728
rs71640337
rs35696698
rs10195252
rs769446
rs1689800
rs59104589
rs12037659
rs2943654
rs7268701
rs4986970
rs2070895
rs15285
rs17580
rs2518134
rs1532085
rs139915535
rs77960347
rs12635955
rs4846915
rs183130
rs10438978
rs7947269
rs4360309
rs174567
rs11895352
rs72836561
rs3824477
rs10455872
rs429358
rs6073958
rs1260326
rs28929474
rs763665
rs964184
rs116843064
rs10233430
rs2980888
rs35193317
rs11231694
rs13226650
rs9686661
rs998584
All - MR Egger
All - Inverse variance weighted
-1.0
-0.5
0.0
0.5
1.0
MR effect size for
steryl esters to total lipids ratio in very large HDL || id:ebi-cfb233-GCST90302129' on 'Breast cancer (Combined Oncoarray; iC

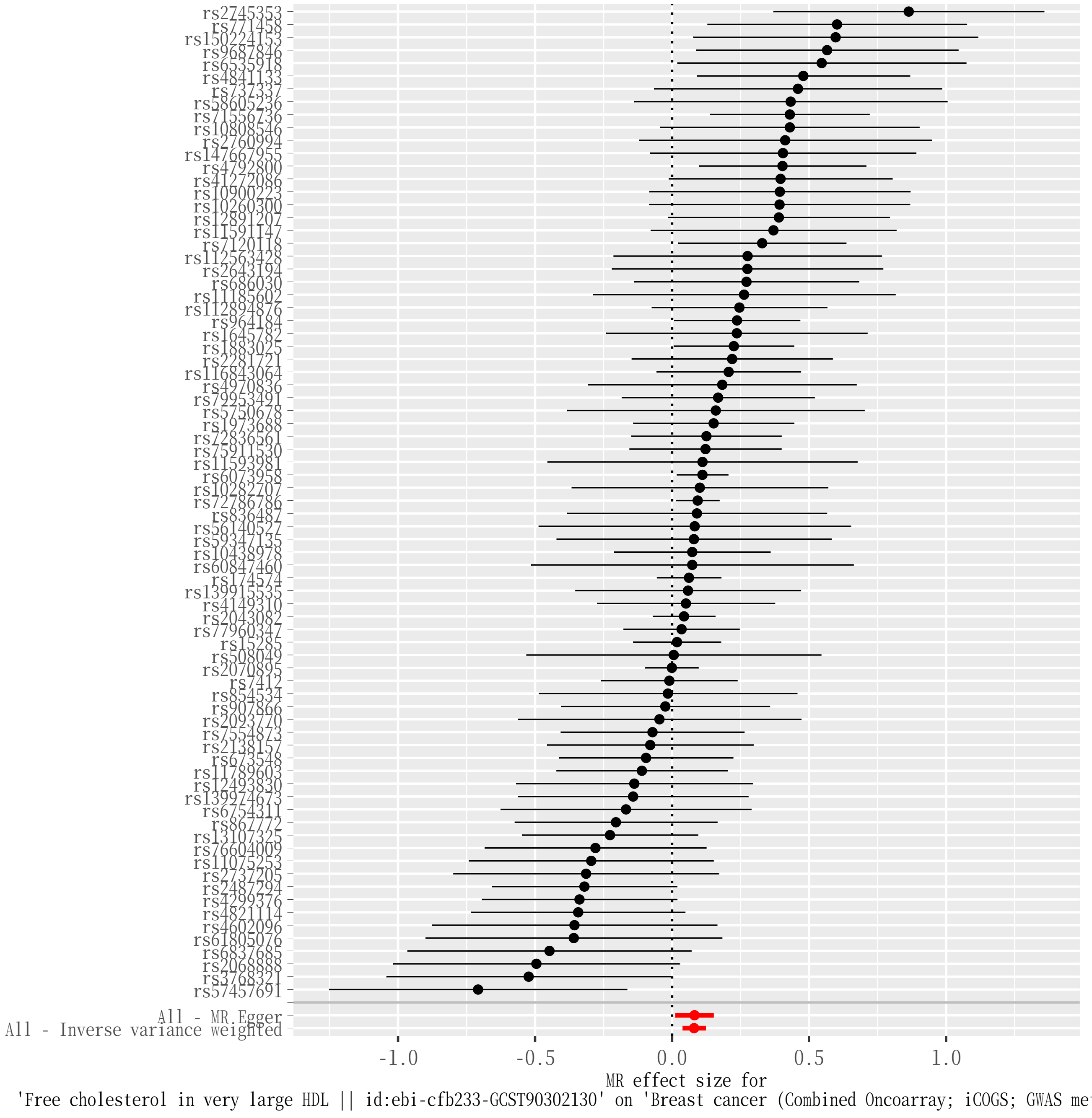
rs2745353
rs771458
rs150224153
rs9687846
rs6535918
rs4841133
rs737337
rs58605236
rs71556736
rs10808546
rs2760994
rs147667955
rs4792800
rs41272086
rs10900223
rs10260300
rs12891207
rs11591147
rs7120118
rs112563428
rs2643194
rs686030
rs11185602
rs112894876
rs964184
rs1645782
rs1883025
rs2281721
rs116843064
rs4970836
rs7995491
rs5750678
rs1973688
rs72836561
rs75911530
rs11593981
rs6073958
rs10282707
rs72786786
rs836487
rs56140527
rs59347135
rs10438978
rs60847460
rs174574
rs139915535
rs4149310
rs2043082
rs77960347
rs15285
rs508049
rs2070895
rs7412
rs854534
rs907866
rs2093770
rs7554873
rs2138157
rs673548
rs11789603
rs12493830
rs139974673
rs6754311
rs862772
rs13107325
rs76604009
rs11075253
rs2737205
rs2487294
rs4299376
rs4821114
rs4602096
rs61805076
rs6837685
rs2068888
rs3768321
rs57457691
All - MR Egger
All - Inverse variance weighted
-1.0
-0.5
0.0
0.5
1.0
MR effect size for
'Free cholesterol in very large HDL || id:ebi-cfb233-GCST90302130' on 'Breast cancer (Combined Oncoarray; iCOGS; GWAS me

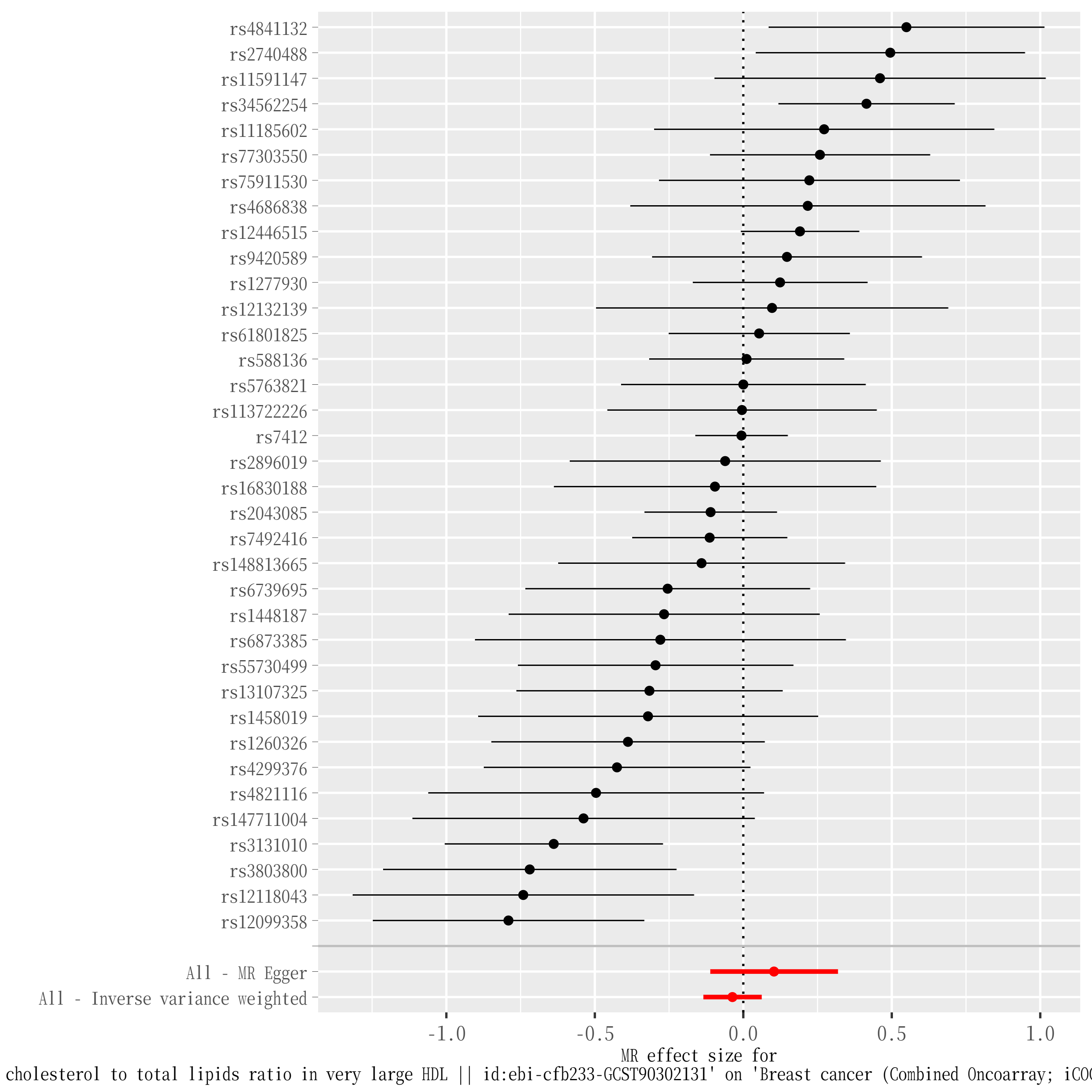
rs4841132
rs2740488
rs11591147
rs34562254
rs11185602
rs77303550
rs75911530
rs4686838
rs12446515
rs9420589
rs1277930
rs12132139
rs61801825
rs588136
rs5763821
rs113722226
rs7412
rs2896019
rs16830188
rs2043085
rs7492416
rs148813665
rs6739695
rs1448187
rs6873385
rs55730499
rs13107325
rs1458019
rs1260326
rs4299376
rs4821116
rs147711004
rs3131010
rs3803800
rs12118043
rs12099358
All - MR Egger
All - Inverse variance weighted
-1.0
-0.5
0.0
0.5
1.0
MR effect size for
cholesterol to total lipids ratio in very large HDL || id:ebi-cfb233-GCST90302131' on 'Breast cancer (Combined Oncoarray; iCO

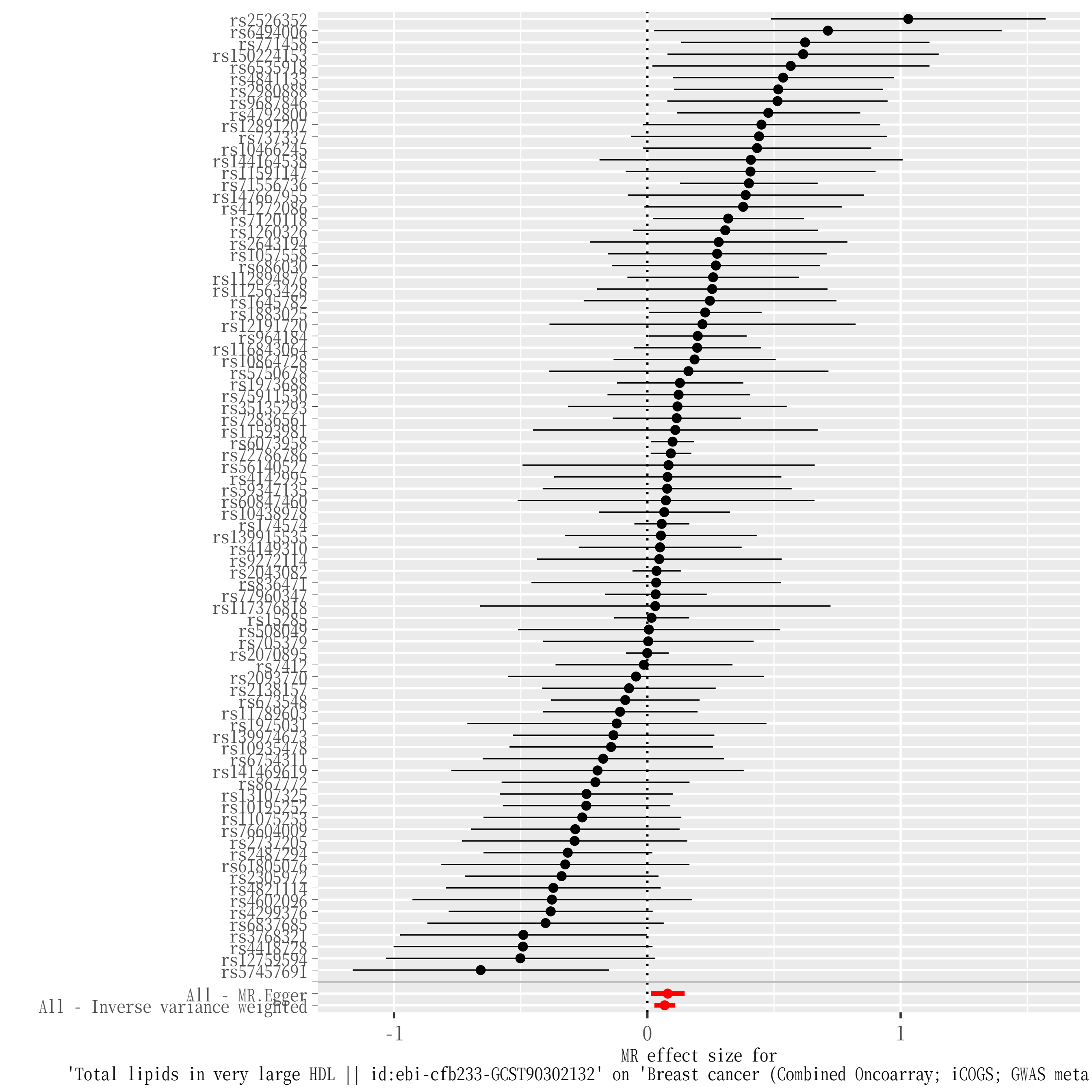
rs2526352
rs6424006
rs771458
rs150224153
rs6535918
rs4841133
rs2980888
rs9687846
rs4792800
rs12891207
rs737337
rs10466245
rs144164538
rs11591147
rs71556736
rs147667955
rs41272086
rs7120118
rs1260326
rs2643194
rs1057558
rs686030
rs112894876
rs112563428
rs1645782
rs1883025
rs12191720
rs964184
rs116843064
rs10864728
rs5750678
rs1973688
rs75911530
rs35135293
rs72836561
rs11593981
rs6073958
rs72786786
rs56140527
rs4142995
rs59347135
rs60847460
rs10438978
rs174574
rs139915535
rs4149310
rs9272114
rs2043082
rs836471
rs77960347
rs117376818
rs15285
rs508049
rs705379
rs2070895
rs7412
rs2093770
rs2138157
rs673548
rs11789603
rs1975031
rs139974673
rs10935478
rs6754311
rs141469619
rs862772
rs13107325
rs10195252
rs11075253
rs76604009
rs2737205
rs2487794
rs61805076
rs2305972
rs4821114
rs4602096
rs4299376
rs6837685
rs3768321
rs4418728
rs12759594
rs57457691
All - MR Egger
All - Inverse variance weighted
-1
0
1
MR effect size for
'Total lipids in very large HDL || id:ebi-cfb233-GCST90302132' on 'Breast cancer (Combined Oncoarray; iCOGS; GWAS meta

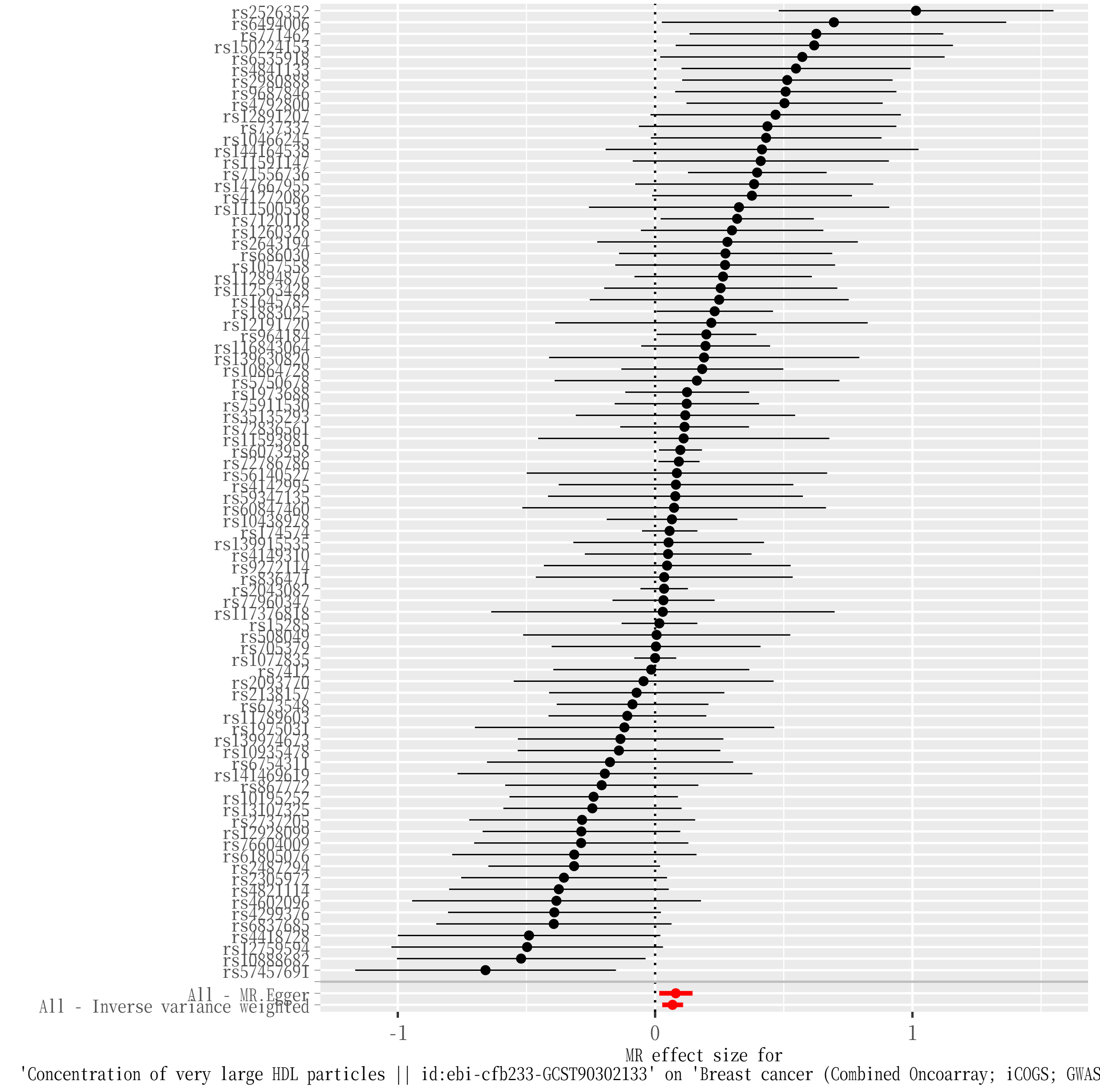
rs2526352
rs6424006
rs771462
rs150224153
rs6535918
rs4841133
rs2980888
rs9687846
rs4792800
rs12891207
rs737337
rs10466245
rs144164538
rs11591147
rs71556736
rs147667955
rs41272086
rs111500536
rs7120118
rs1260326
rs2643194
rs686030
rs1057558
rs112894876
rs112563428
rs1645782
rs1883025
rs12191720
rs964184
rs116843064
rs139630820
rs10864728
rs5750678
rs1973688
rs75911530
rs35135293
rs72836561
rs11593981
rs6073958
rs72786786
rs56140527
rs4142995
rs59347135
rs60847460
rs10438978
rs174574
rs139915535
rs4149310
rs9272114
rs836471
rs2043082
rs77960347
rs117376818
rs15285
rs508049
rs705379
rs1077835
rs7412
rs2093770
rs2138157
rs673548
rs11789603
rs1975031
rs139974673
rs10935478
rs6754311
rs141469619
rs867772
rs10195252
rs13107325
rs2737205
rs12928099
rs76604009
rs61805076
rs2487294
rs2305972
rs4821114
rs4602096
rs4299376
rs6837285
rs4418728
rs12759594
rs10888682
rs57457691
All - MR Egger
All - Inverse variance weighted
-1
0
1
MR effect size for
'Concentration of very large HDL particles || id:ebi-cfb233-GCST90302133' on 'Breast cancer (Combined Oncoarray; iCOGS; GWAS

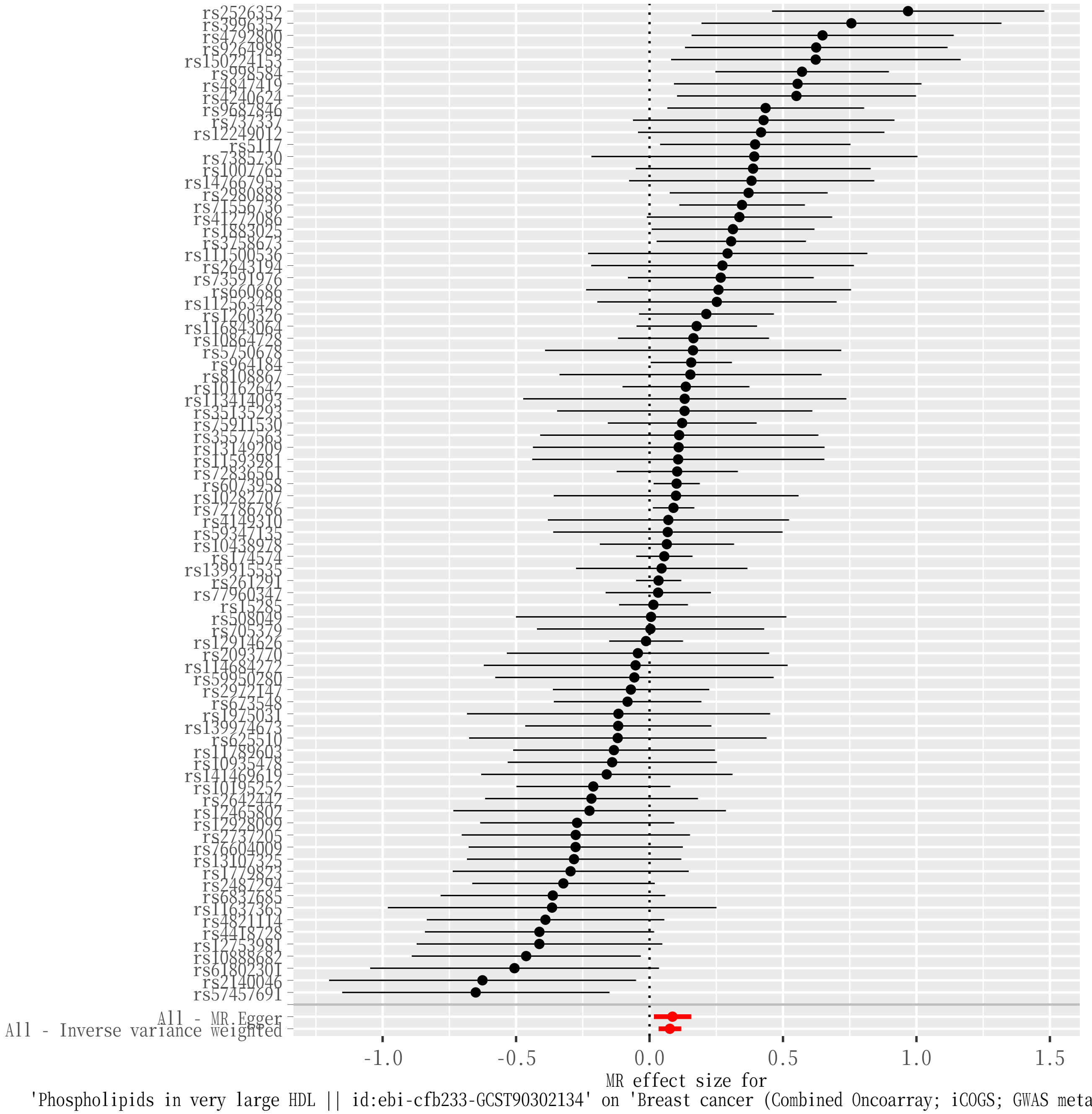
rs2526352
rs3992352
rs4792800
rs9264988
rs150224153
rs998584
rs4847419
rs4240624
rs9687846
rs737337
rs12249012
rs5117
rs7385730
rs1007765
rs147667955
rs2980888
rs71556736
rs41272086
rs1883025
rs3758673
rs111500536
rs2643194
rs73591976
rs660686
rs112563428
rs1260326
rs116843064
rs10864728
rs5750678
rs964184
rs8108867
rs10162642
rs113414093
rs35135293
rs75211530
rs35577563
rs13149209
rs11593981
rs72836561
rs6073958
rs10282707
rs72786786
rs4149310
rs59347135
rs10438978
rs174574
rs139915535
rs261291
rs77960347
rs15285
rs508049
rs705379
rs12914626
rs2093770
rs114684272
rs59950280
rs2972147
rs673548
rs1975031
rs139974673
rs625510
rs11789603
rs10935478
rs141469619
rs10195252
rs2642442
rs12465802
rs12928099
rs2737205
rs76604009
rs13107325
rs1779873
rs2487294
rs6837685
rs11637365
rs4821114
rs4418728
rs12753981
rs10888682
rs61802301
rs2140046
rs57457691
All - MR Egger
All - Inverse variance weighted
-1.0
-0.5
0.0
0.5
1.0
1.5
MR effect size for
'Phospholipids in very large HDL || id:ebi-cfb233-GCST90302134' on 'Breast cancer (Combined Oncoarray; iCOGS; GWAS meta

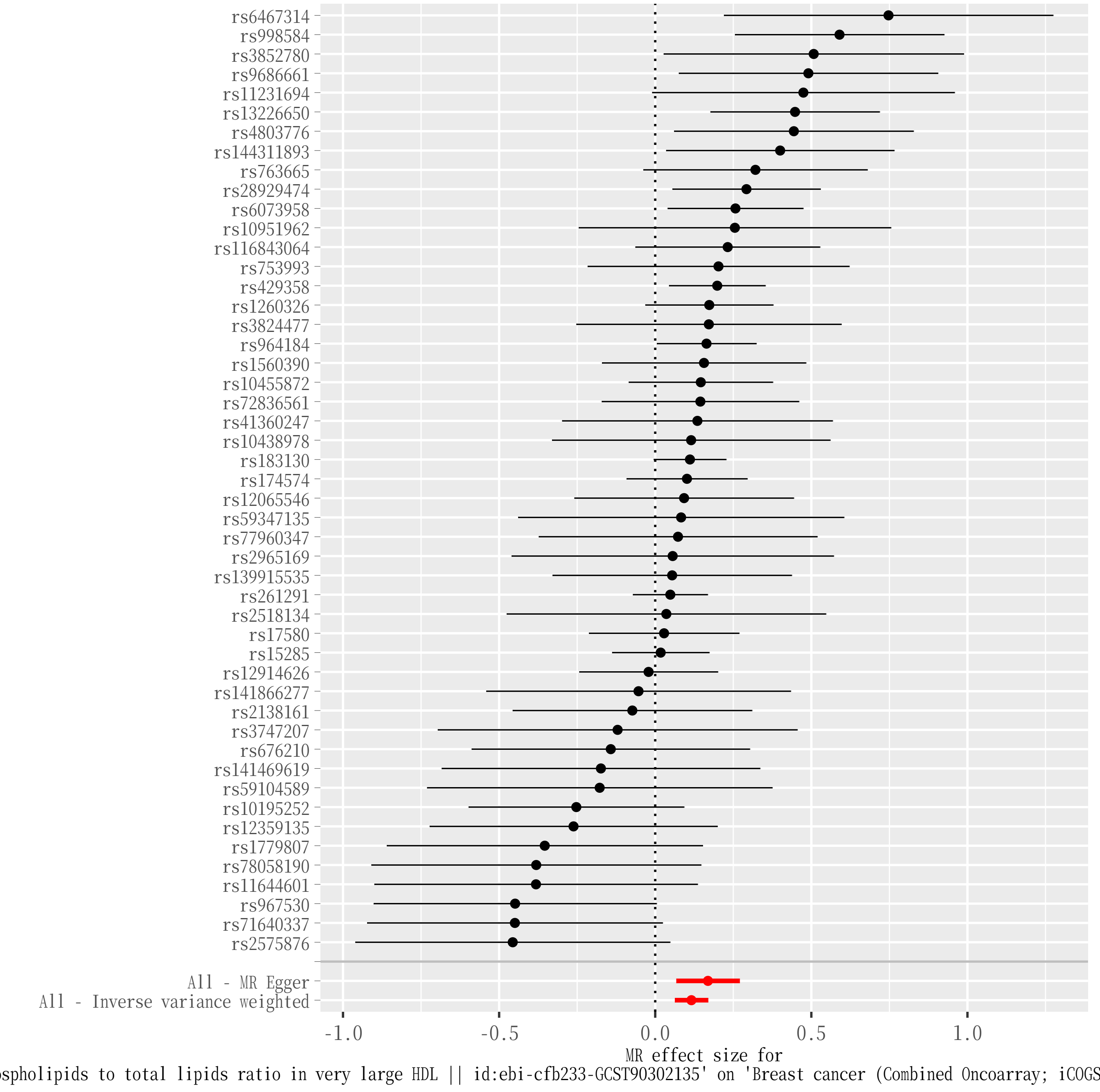
rs6467314
rs998584
rs3852780
rs9686661
rs11231694
rs13226650
rs4803776
rs144311893
rs763665
rs28929474
rs6073958
rs10951962
rs116843064
rs753993
rs429358
rs1260326
rs3824477
rs964184
rs1560390
rs10455872
rs72836561
rs41360247
rs10438978
rs183130
rs174574
rs12065546
rs59347135
rs77960347
rs2965169
rs139915535
rs261291
rs2518134
rs17580
rs15285
rs12914626
rs141866277
rs2138161
rs3747207
rs676210
rs141469619
rs59104589
rs10195252
rs12359135
rs1779807
rs78058190
rs11644601
rs967530
rs71640337
rs2575876
All - MR Egger
All - Inverse variance weighted
-1.0
-0.5
0.0
0.5
1.0
MR effect size for
ospholipids to total lipids ratio in very large HDL || id:ebi-cfb233-GCST90302135' on 'Breast cancer (Combined Oncoarray; iCOGS

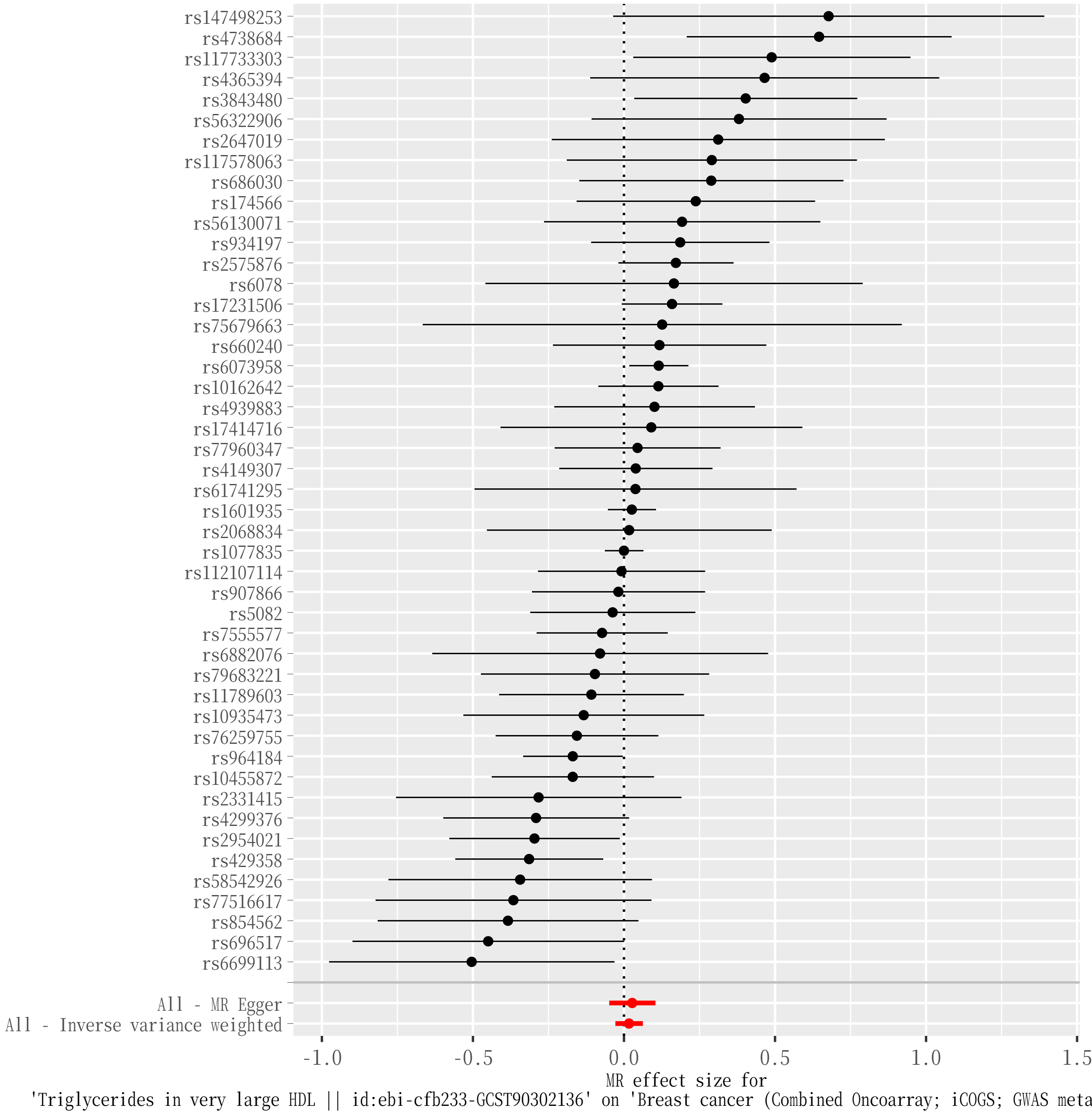
rs147498253
rs4738684
rs117733303
rs4365394
rs3843480
rs56322906
rs2647019
rs117578063
rs686030
rs174566
rs56130071
rs934197
rs2575876
rs6078
rs17231506
rs75679663
rs660240
rs6073958
rs10162642
rs4939883
rs17414716
rs77960347
rs4149307
rs61741295
rs1601935
rs2068834
rs1077835
rs112107114
rs907866
rs5082
rs7555577
rs6882076
rs79683221
rs11789603
rs10935473
rs76259755
rs964184
rs10455872
rs2331415
rs4299376
rs2954021
rs429358
rs58542926
rs77516617
rs854562
rs696517
rs6699113
All - MR Egger
All - Inverse variance weighted
-1.0
-0.5
0.0
0.5
1.0
1.5
MR effect size for
'Triglycerides in very large HDL || id:ebi-cfb233-GCST90302136' on 'Breast cancer (Combined Oncoarray; iCOGS; GWAS meta

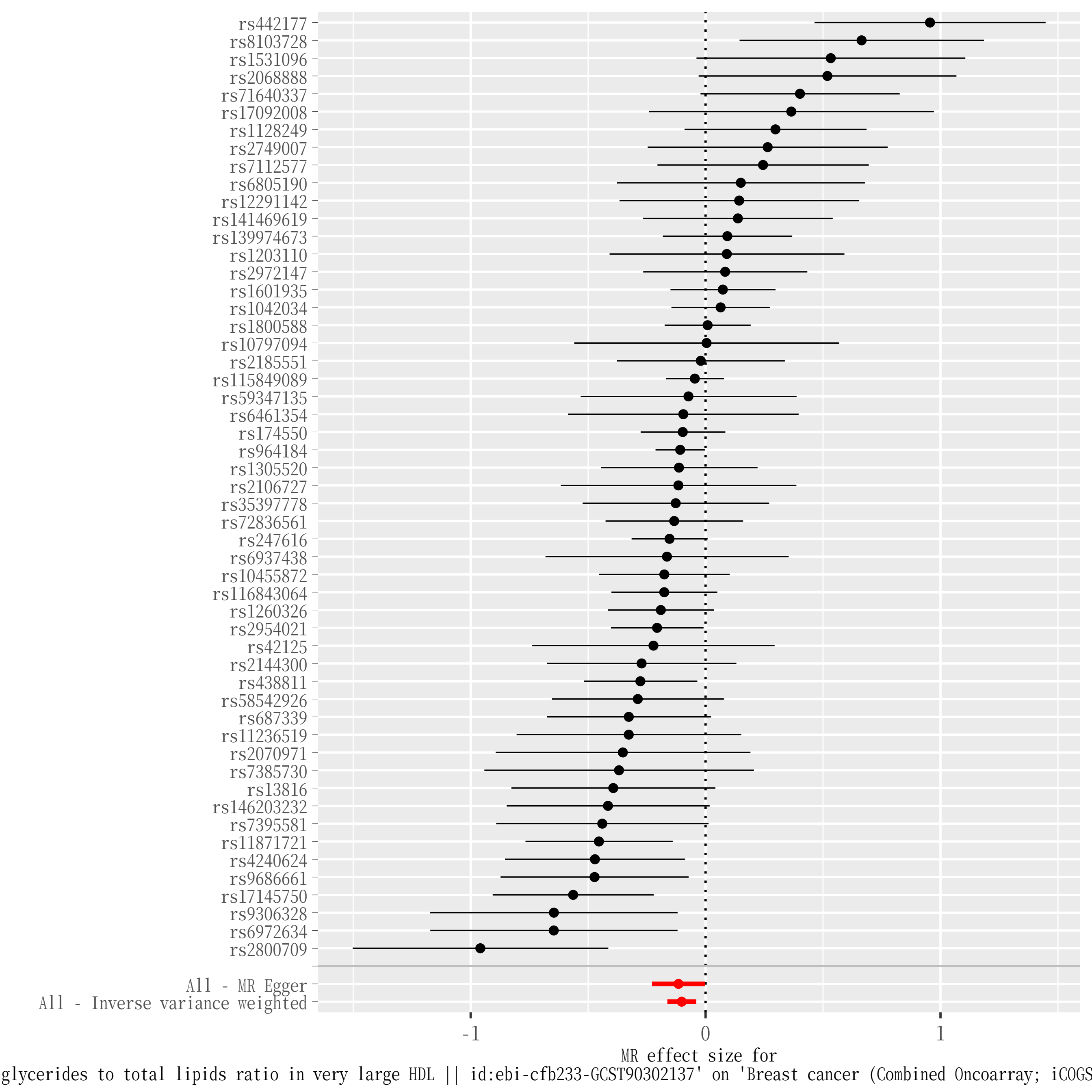

rs442177
rs8103728
rs1531096
rs2068888
rs71640337
rs17092008
rs1128249
rs2749007
rs7112577
rs6805190
rs12291142
rs141469619
rs139974673
rs1203110
rs2972147
rs1601935
rs1042034
rs1800588
rs10797094
rs2185551
rs115849089
rs59347135
rs6461354
rs174550
rs964184
rs1305520
rs2106727
rs35397778
rs72836561
rs247616
rs6937438
rs10455872
rs116843064
rs1260326
rs2954021
rs42125
rs2144300
rs438811
rs58542926
rs687339
rs11236519
rs2070971
rs7385730
rs13816
rs146203232
rs7395581
rs11871721
rs4240624
rs9686661
rs17145750
rs9306328
rs6972634
rs2800709
All - MR Egger
All - Inverse variance weighted
-1
0
1
MR effect size for
glycerides to total lipids ratio in very large HDL || id:ebi-cfb233-GCST90302137' on 'Breast cancer (Combined Oncoarray; iCOGS

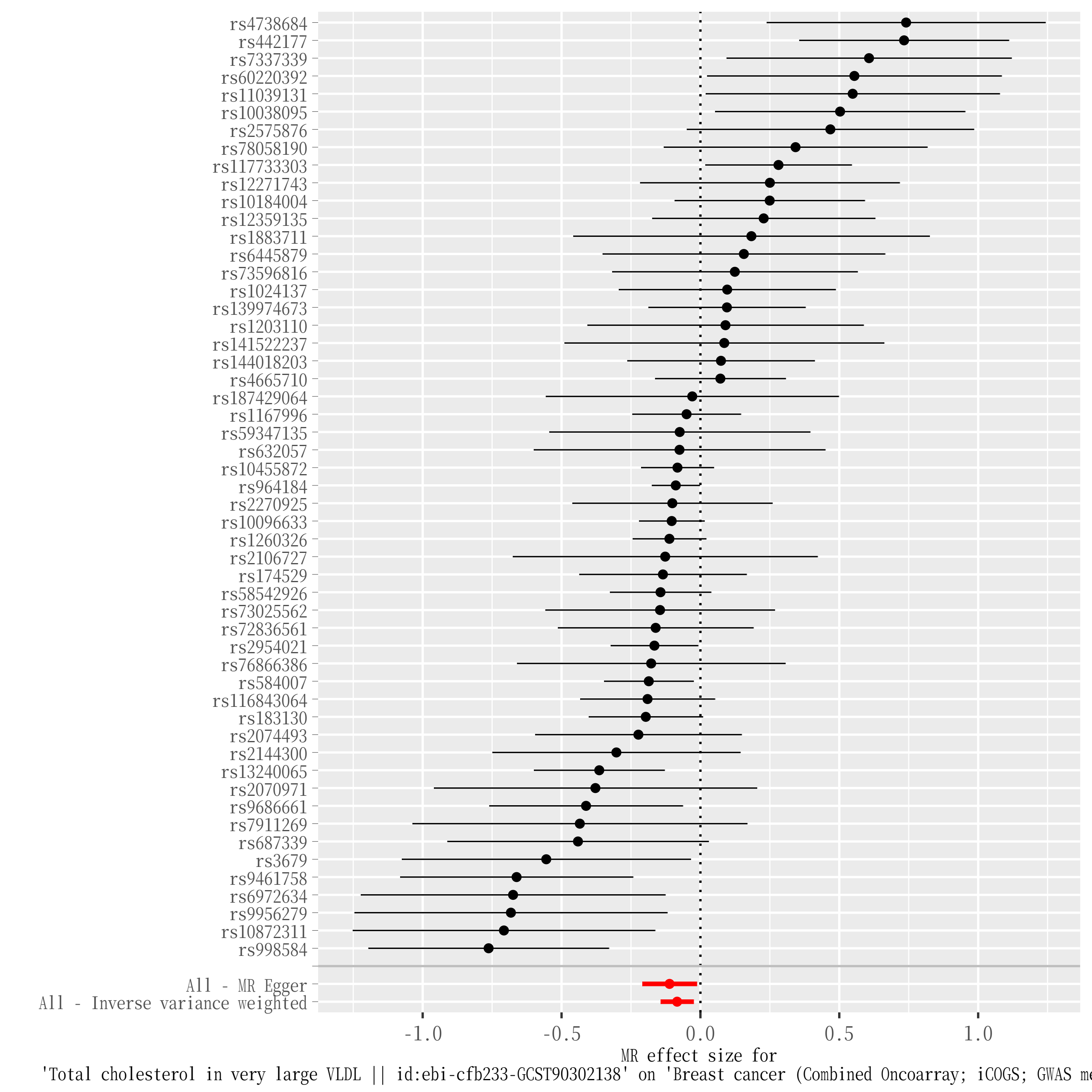
rs4738684
rs442177
rs7337339
rs60220392
rs11039131
rs10038095
rs2575876
rs78058190
rs117733303
rs12271743
rs10184004
rs12359135
rs1883711
rs6445879
rs73596816
rs1024137
rs139974673
rs1203110
rs141522237
rs144018203
rs4665710
rs187429064
rs1167996
rs59347135
rs632057
rs10455872
rs964184
rs2270925
rs10096633
rs1260326
rs2106727
rs174529
rs58542926
rs73025562
rs72836561
rs2954021
rs76866386
rs584007
rs116843064
rs183130
rs2074493
rs2144300
rs13240065
rs2070971
rs9686661
rs7911269
rs687339
rs3679
rs9461758
rs6972634
rs9956279
rs10872311
rs998584
All - MR Egger
All - Inverse variance weighted
-1.0
-0.5
0.0
0.5
1.0
MR effect size for
'Total cholesterol in very large VLDL || id:ebi-cfb233-GCST90302138' on 'Breast cancer (Combined Oncoarray; iCOGS; GWAS me

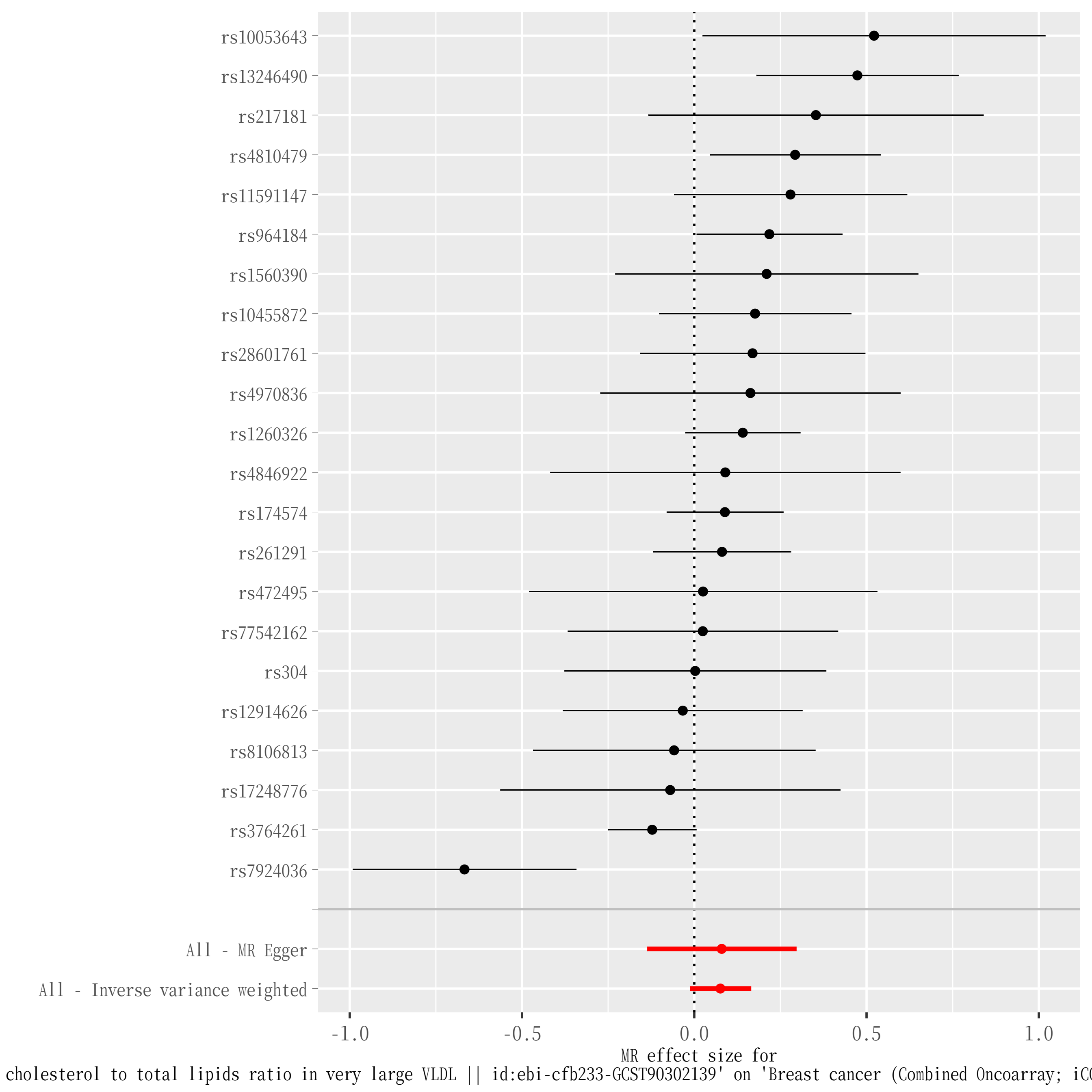
rs10053643
rs13246490
rs217181
rs4810479
rs11591147
rs964184
rs1560390
rs10455872
rs28601761
rs4970836
rs1260326
rs4846922
rs174574
rs261291
rs472495
rs77542162
rs304
rs12914626
rs8106813
rs17248776
rs3764261
rs7924036
All - MR Egger
All - Inverse variance weighted
-1.0
-0.5
0.0
0.5
1.0
MR effect size for
cholesterol to total lipids ratio in very large VLDL || id:ebi-cfb233-GCST90302139' on 'Breast cancer (Combined Oncoarray; iC

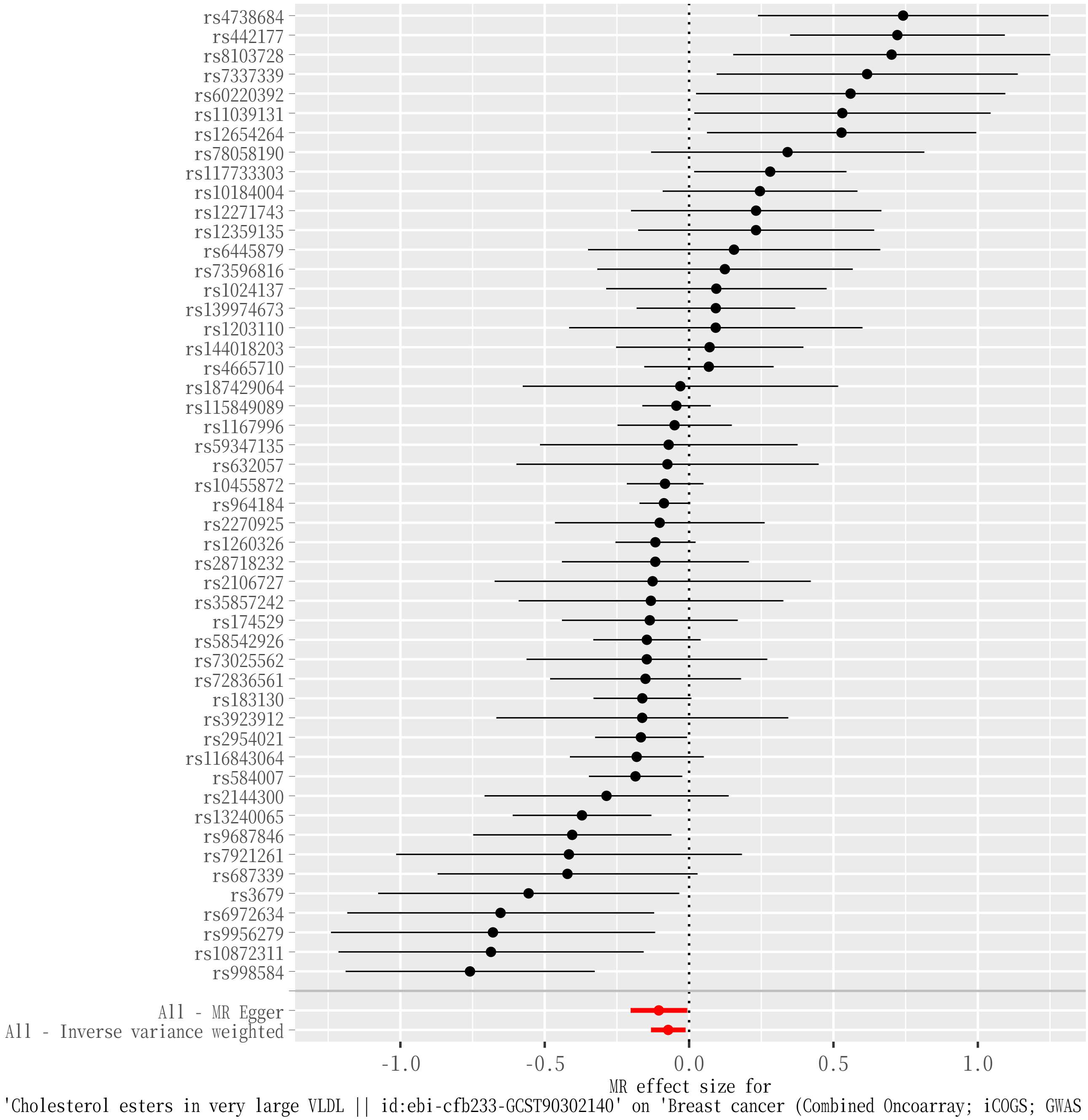
rs4738684
rs442177
rs8103728
rs7337339
rs60220392
rs11039131
rs12654264
rs78058190
rs117733303
rs10184004
rs12271743
rs12359135
rs6445879
rs73596816
rs1024137
rs139974673
rs1203110
rs144018203
rs4665710
rs187429064
rs115849089
rs1167996
rs59347135
rs632057
rs10455872
rs964184
rs2270925
rs1260326
rs28718232
rs2106727
rs35857242
rs174529
rs58542926
rs73025562
rs72836561
rs183130
rs3923912
rs2954021
rs116843064
rs584007
rs2144300
rs13240065
rs9687846
rs7921261
rs687339
rs3679
rs6972634
rs9956279
rs10872311
rs998584
All - MR Egger
All - Inverse variance weighted
-1.0
-0.5
0.0
0.5
1.0
MR effect size for
'Cholesterol esters in very large VLDL || id:ebi-cfb233-GCST90302140' on 'Breast cancer (Combined Oncoarray; iCOGS; GWAS m

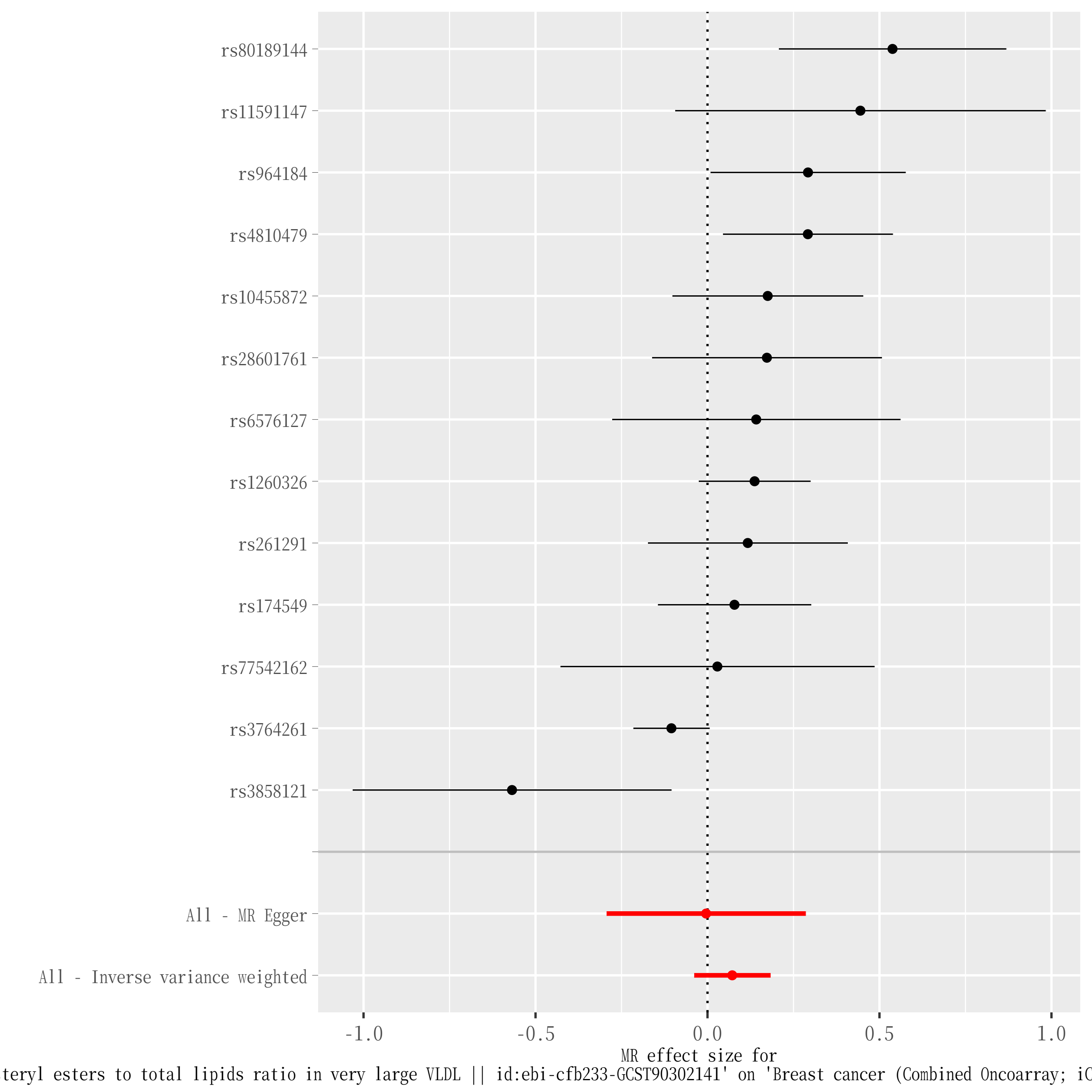

rs80189144
rs11591147
rs964184
rs4810479
rs10455872
rs28601761
rs6576127
rs1260326
rs261291
rs174549
rs77542162
rs3764261
rs3858121
All - MR Egger
All - Inverse variance weighted
-1.0
-0.5
0.0
0.5
1.0
MR effect size for
teryl esters to total lipids ratio in very large VLDL || id:ebi-cfb233-GCST90302141' on 'Breast cancer (Combined Oncoarray; iC

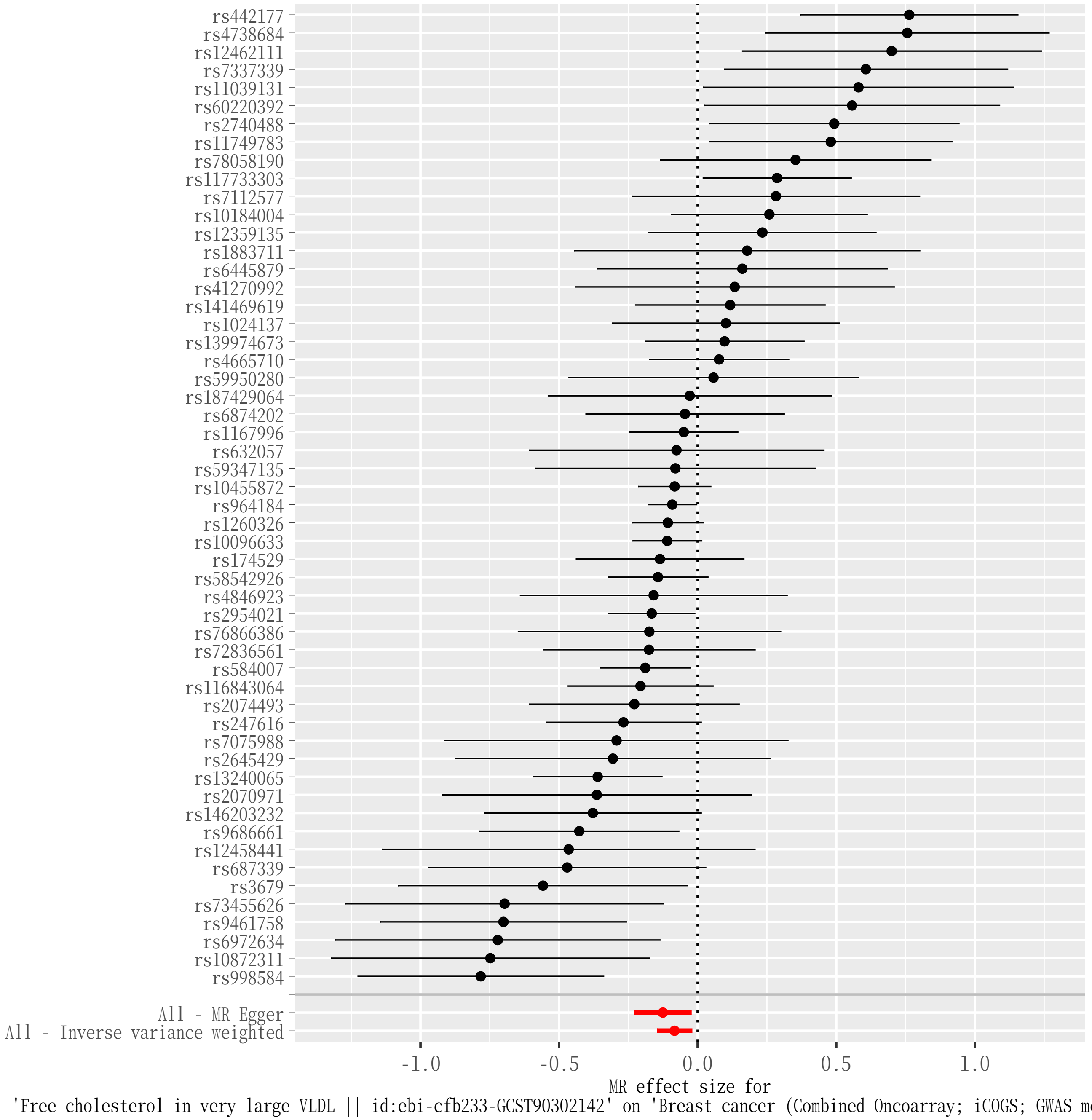
rs442177
rs4738684
rs12462111
rs7337339
rs11039131
rs60220392
rs2740488
rs11749783
rs78058190
rs117733303
rs7112577
rs10184004
rs12359135
rs1883711
rs6445879
rs41270992
rs141469619
rs1024137
rs139974673
rs4665710
rs59950280
rs187429064
rs6874202
rs1167996
rs632057
rs59347135
rs10455872
rs964184
rs1260326
rs10096633
rs174529
rs58542926
rs4846923
rs2954021
rs76866386
rs72836561
rs584007
rs116843064
rs2074493
rs247616
rs7075988
rs2645429
rs13240065
rs2070971
rs146203232
rs9686661
rs12458441
rs687339
rs3679
rs73455626
rs9461758
rs6972634
rs10872311
rs998584
All - MR Egger
All - Inverse variance weighted
-1.0
-0.5
0.0
0.5
1.0
MR effect size for
'Free cholesterol in very large VLDL || id:ebi-cfb233-GCST90302142' on 'Breast cancer (Combined Oncoarray; iCOGS; GWAS me

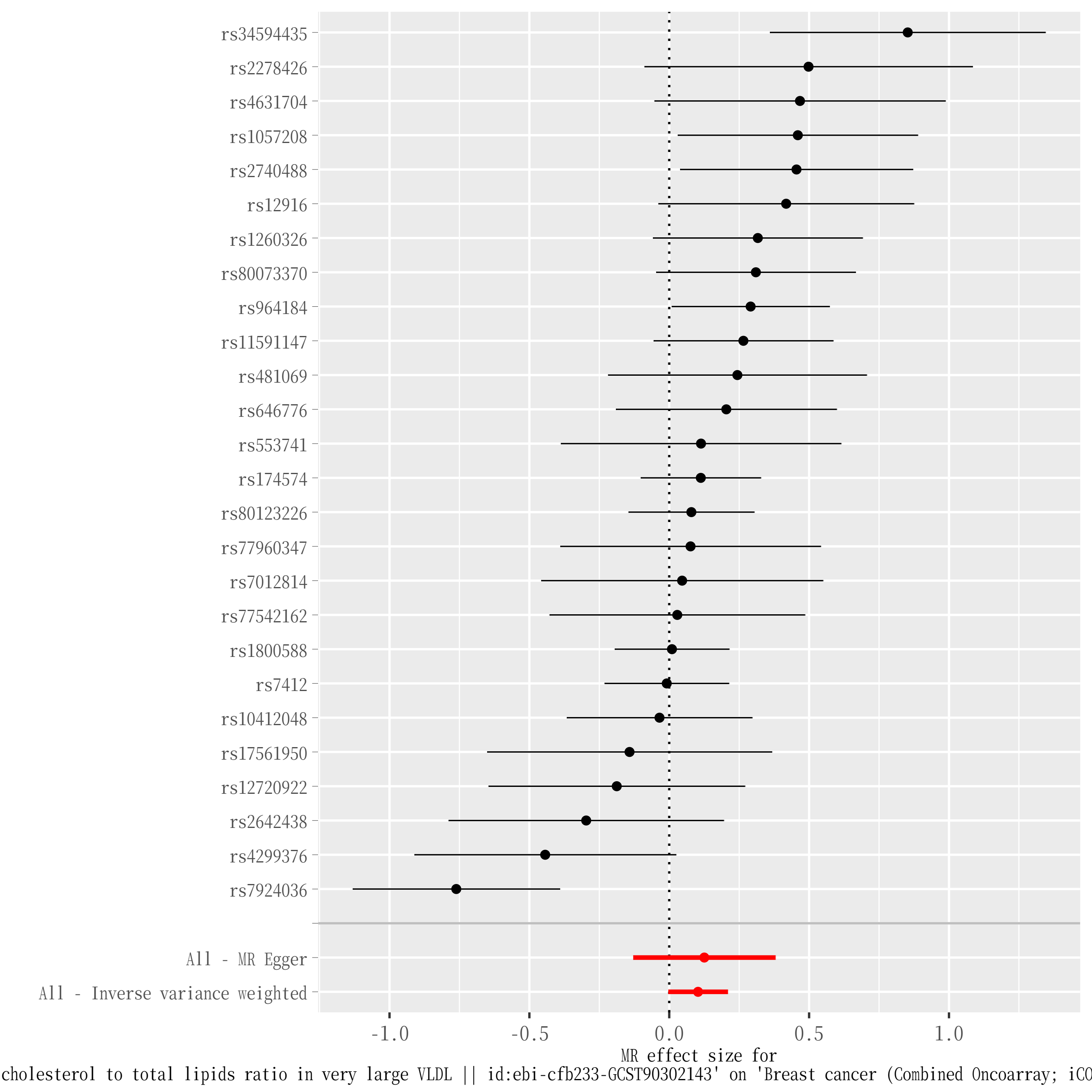
rs34594435
rs2278426
rs4631704
rs1057208
rs2740488
rs12916
rs1260326
rs80073370
rs964184
rs11591147
rs481069
rs646776
rs553741
rs174574
rs80123226
rs77960347
rs7012814
rs77542162
rs1800588
rs7412
rs10412048
rs17561950
rs12720922
rs2642438
rs4299376
rs7924036
All - MR Egger
All - Inverse variance weighted
-1.0
-0.5
0.0
0.5
1.0
MR effect size for
cholesterol to total lipids ratio in very large VLDL || id:ebi-cfb233-GCST90302143' on 'Breast cancer (Combined Oncoarray; iCO

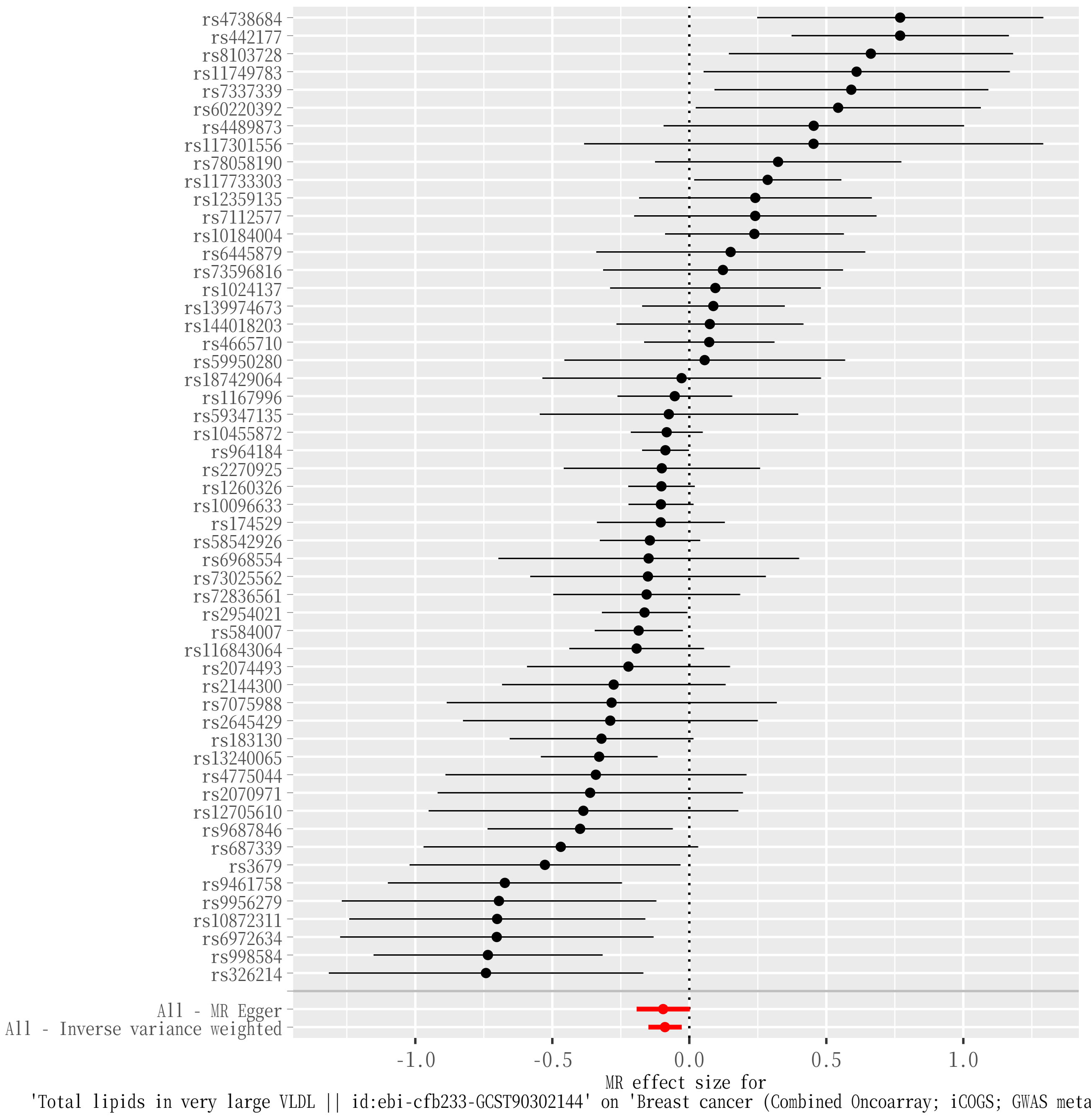
rs4738684
rs442177
rs8103728
rs11749783
rs7337339
rs60220392
rs4489873
rs117301556
rs78058190
rs117733303
rs12359135
rs7112577
rs10184004
rs6445879
rs73596816
rs1024137
rs139974673
rs144018203
rs4665710
rs59950280
rs187429064
rs1167996
rs59347135
rs10455872
rs964184
rs2270925
rs1260326
rs10096633
rs174529
rs58542926
rs6968554
rs73025562
rs72836561
rs2954021
rs584007
rs116843064
rs2074493
rs2144300
rs7075988
rs2645429
rs183130
rs13240065
rs4775044
rs2070971
rs12705610
rs9687846
rs687339
rs3679
rs9461758
rs9956279
rs10872311
rs6972634
rs998584
rs326214
All - MR Egger
All - Inverse variance weighted
-1.0
-0.5
0.0
0.5
1.0
MR effect size for
'Total lipids in very large VLDL || id:ebi-cfb233-GCST90302144' on 'Breast cancer (Combined Oncoarray; iCOGS; GWAS meta

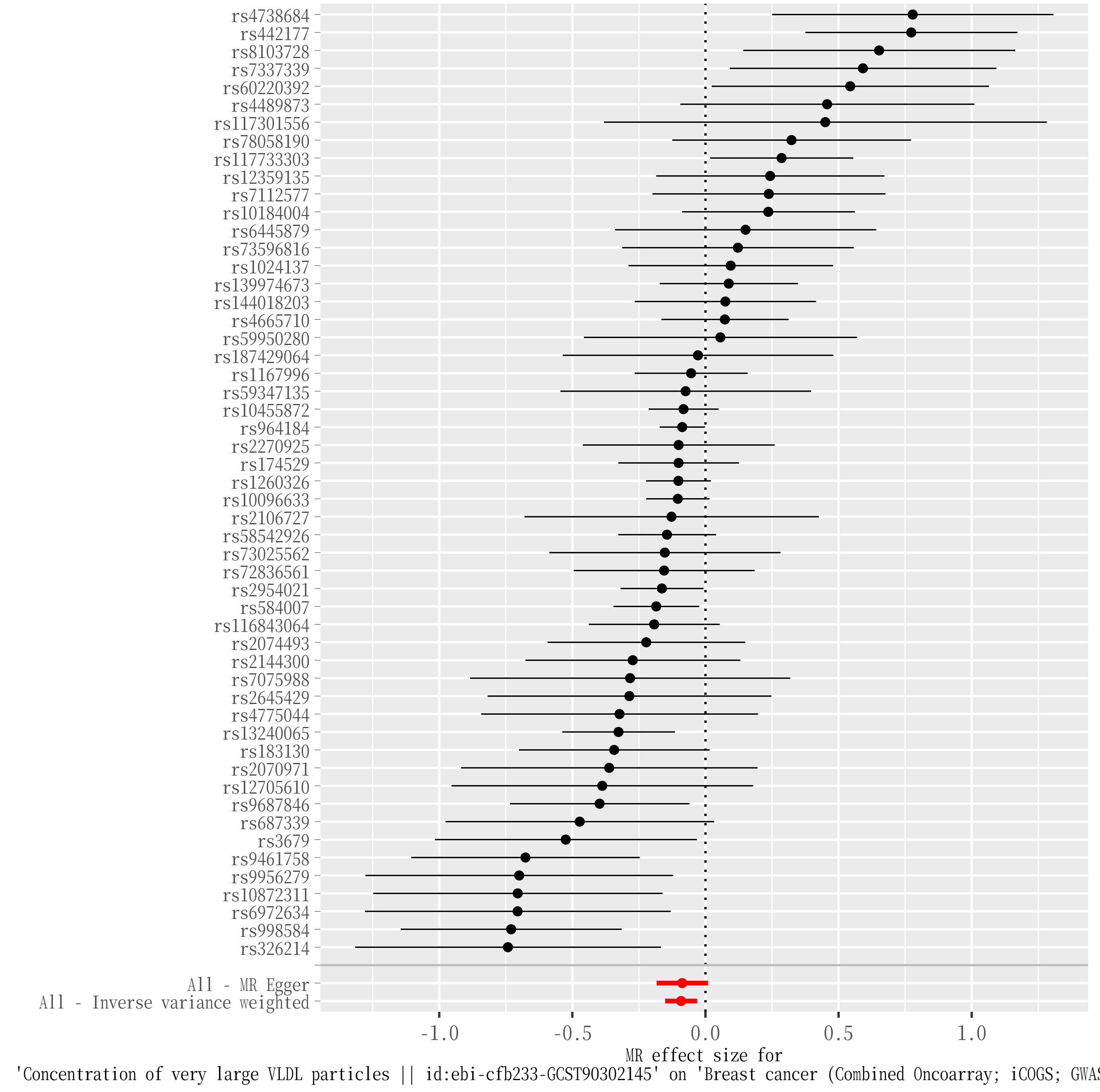
rs4738684
rs442177
rs8103728
rs7337339
rs60220392
rs4489873
rs117301556
rs78058190
rs117733303
rs12359135
rs7112577
rs10184004
rs6445879
rs73596816
rs1024137
rs139974673
rs144018203
rs4665710
rs59950280
rs187429064
rs1167996
rs59347135
rs10455872
rs964184
rs2270925
rs174529
rs1260326
rs10096633
rs2106727
rs58542926
rs73025562
rs72836561
rs2954021
rs584007
rs116843064
rs2074493
rs2144300
rs7075988
rs2645429
rs4775044
rs13240065
rs183130
rs2070971
rs12705610
rs9687846
rs687339
rs3679
rs9461758
rs9956279
rs10872311
rs6972634
rs998584
rs326214
All - MR Egger
All - Inverse variance weighted
-1.0
-0.5
0.0
0.5
1.0
MR effect size for
'Concentration of very large VLDL particles || id:ebi-cfb233-GCST90302145' on 'Breast cancer (Combined Oncoarray; iCOGS; GWAS

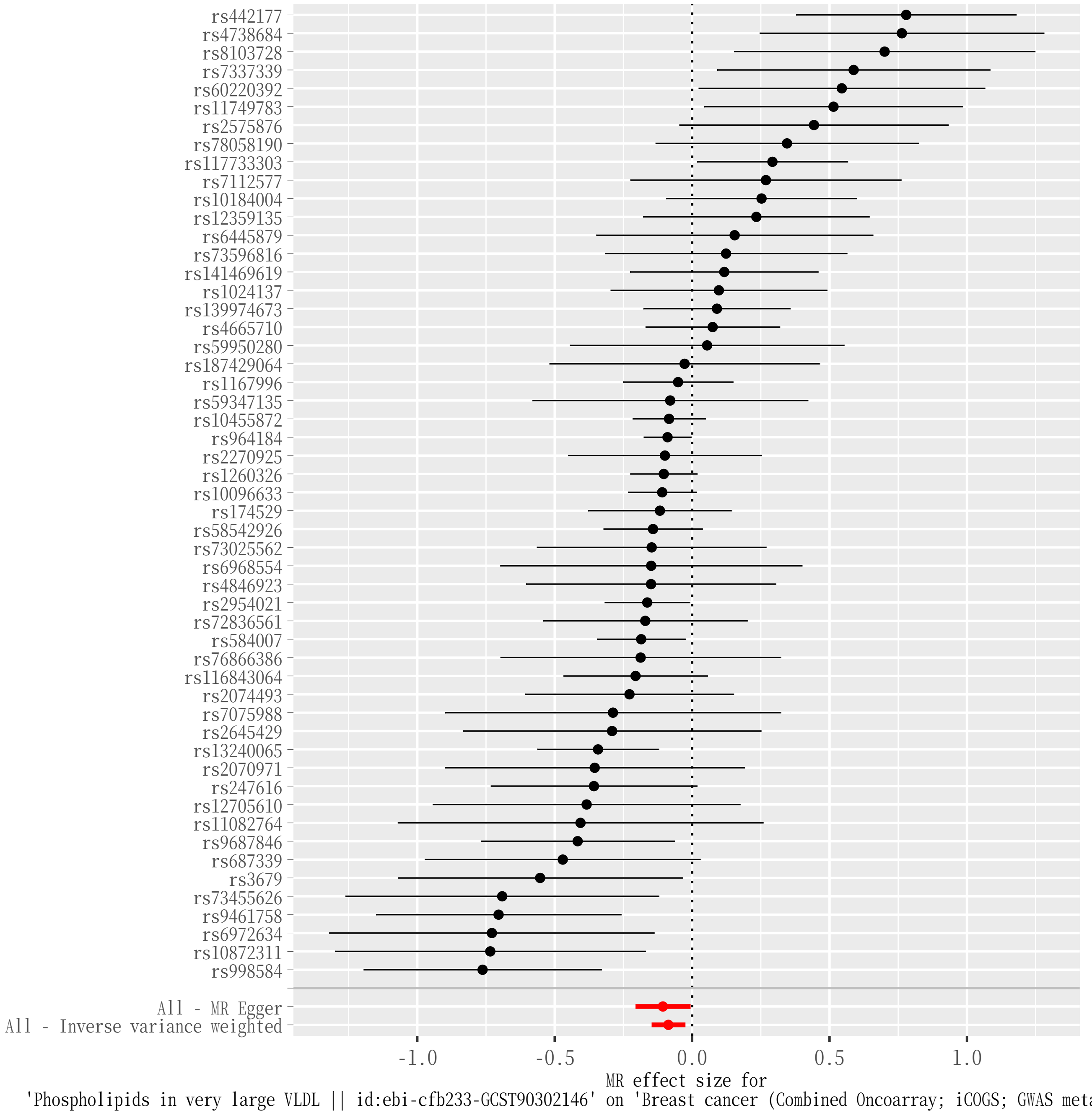
rs442177
rs4738684
rs8103728
rs7337339
rs60220392
rs11749783
rs2575876
rs78058190
rs117733303
rs7112577
rs10184004
rs12359135
rs6445879
rs73596816
rs141469619
rs1024137
rs139974673
rs4665710
rs59950280
rs187429064
rs1167996
rs59347135
rs10455872
rs964184
rs2270925
rs1260326
rs10096633
rs174529
rs58542926
rs73025562
rs6968554
rs4846923
rs2954021
rs72836561
rs584007
rs76866386
rs116843064
rs2074493
rs7075988
rs2645429
rs13240065
rs2070971
rs247616
rs12705610
rs11082764
rs9687846
rs687339
rs3679
rs73455626
rs9461758
rs6972634
rs10872311
rs998584
All - MR Egger
All - Inverse variance weighted
-1.0
-0.5
0.0
0.5
1.0
MR effect size for
'Phospholipids in very large VLDL || id:ebi-cfb233-GCST90302146' on 'Breast cancer (Combined Oncoarray; iCOGS; GWAS meta

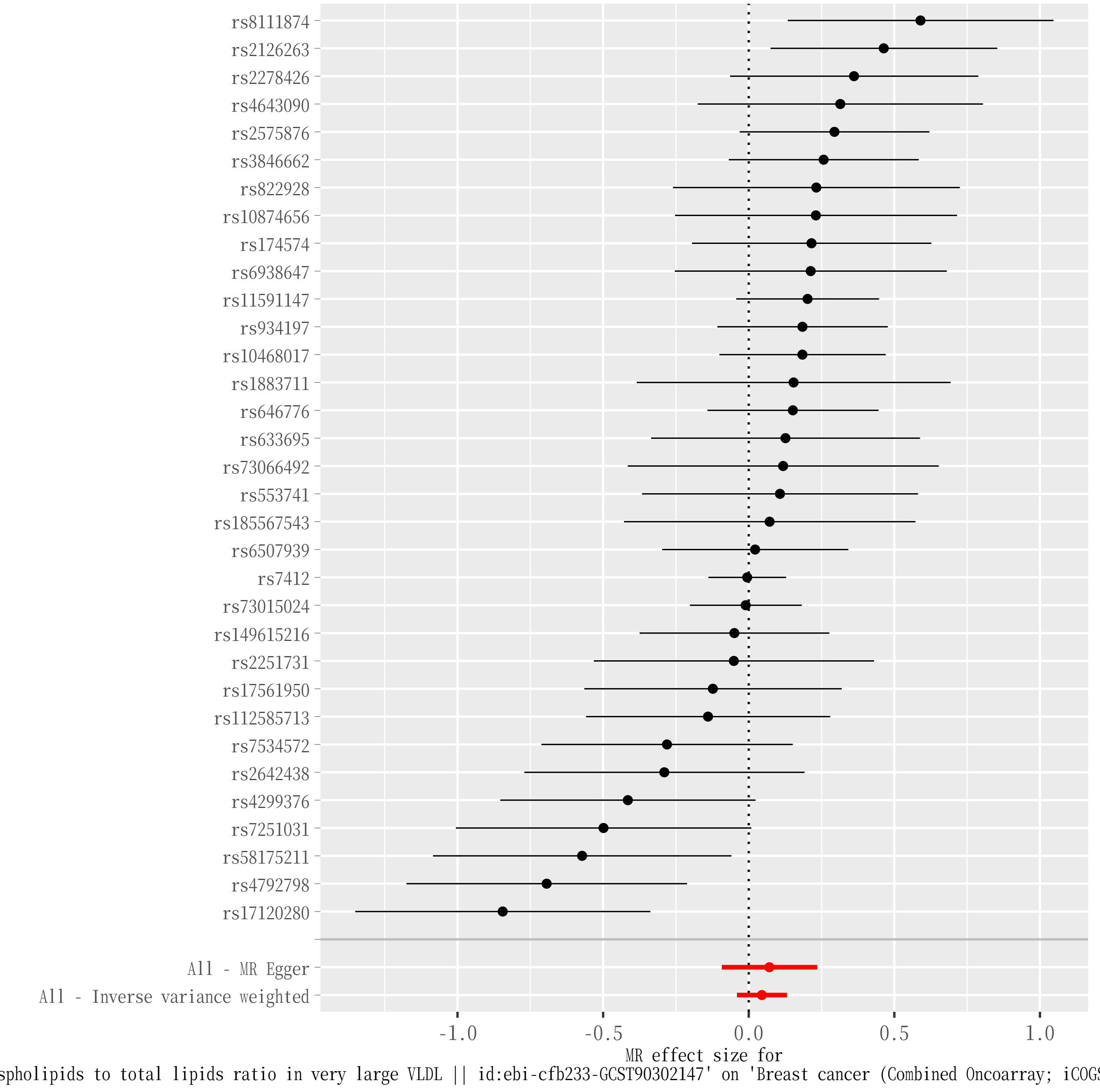

rs8111874
rs2126263
rs2278426
rs4643090
rs2575876
rs3846662
rs822928
rs10874656
rs174574
rs6938647
rs11591147
rs934197
rs10468017
rs1883711
rs646776
rs633695
rs73066492
rs553741
rs185567543
rs6507939
rs7412
rs73015024
rs149615216
rs2251731
rs17561950
rs112585713
rs7534572
rs2642438
rs4299376
rs7251031
rs58175211
rs4792798
rs17120280
All - MR Egger
All - Inverse variance weighted
-1.0
-0.5
0.0
0.5
1.0
MR effect size for
spholipids to total lipids ratio in very large VLDL || id:ebi-cfb233-GCST90302147' on 'Breast cancer (Combined Oncoarray; iCOGS

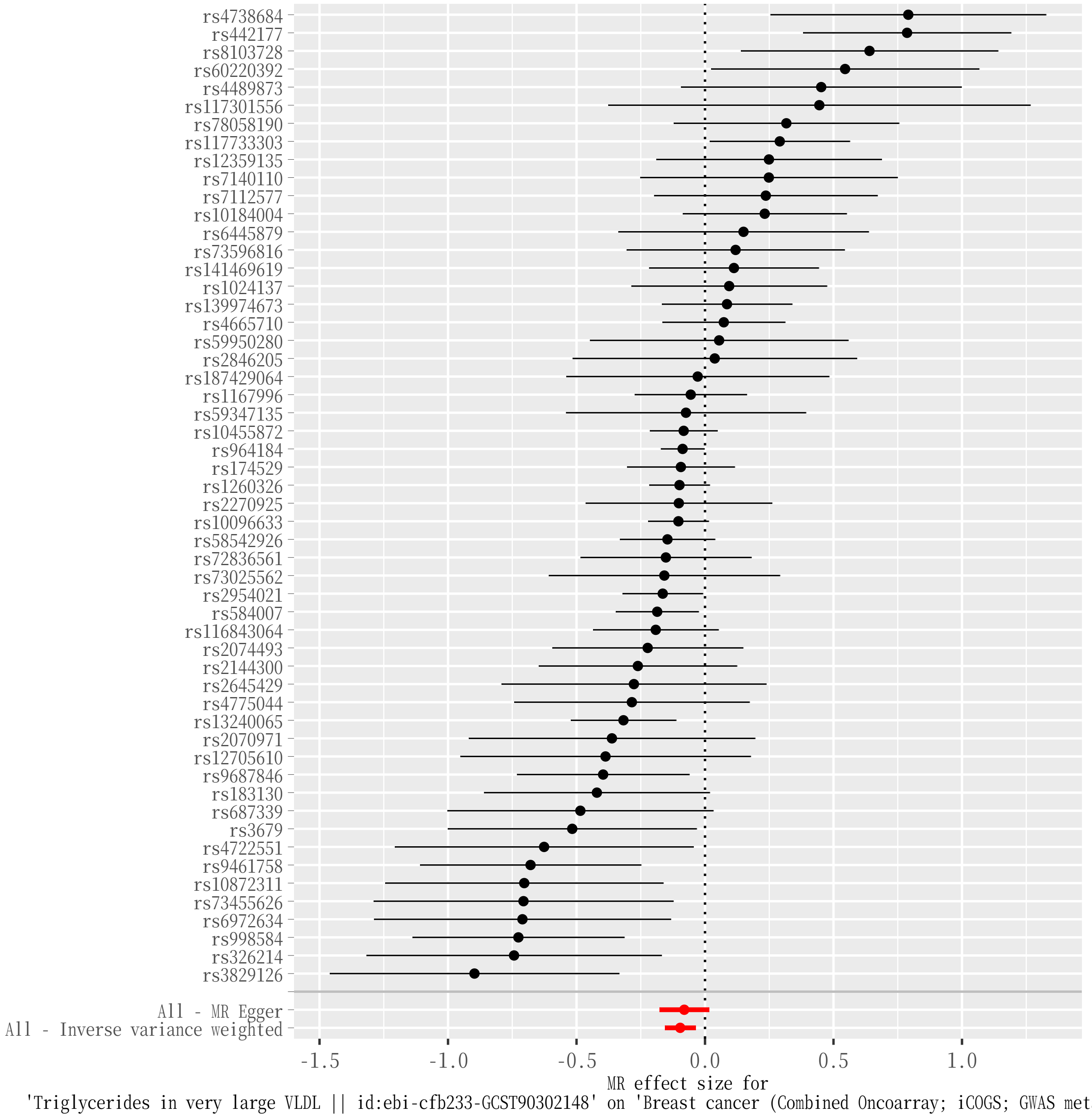
rs4738684
rs442177
rs8103728
rs60220392
rs4489873
rs117301556
rs78058190
rs117733303
rs12359135
rs7140110
rs7112577
rs10184004
rs6445879
rs73596816
rs141469619
rs1024137
rs139974673
rs4665710
rs59950280
rs2846205
rs187429064
rs1167996
rs59347135
rs10455872
rs964184
rs174529
rs1260326
rs2270925
rs10096633
rs58542926
rs72836561
rs73025562
rs2954021
rs584007
rs116843064
rs2074493
rs2144300
rs2645429
rs4775044
rs13240065
rs2070971
rs12705610
rs9687846
rs183130
rs687339
rs3679
rs4722551
rs9461758
rs10872311
rs73455626
rs6972634
rs998584
rs326214
rs3829126
All - MR Egger
All - Inverse variance weighted
-1.5
-1.0
-0.5
0.0
0.5
1.0
MR effect size for
'Triglycerides in very large VLDL || id:ebi-cfb233-GCST90302148' on 'Breast cancer (Combined Oncoarray; iCOGS; GWAS meta

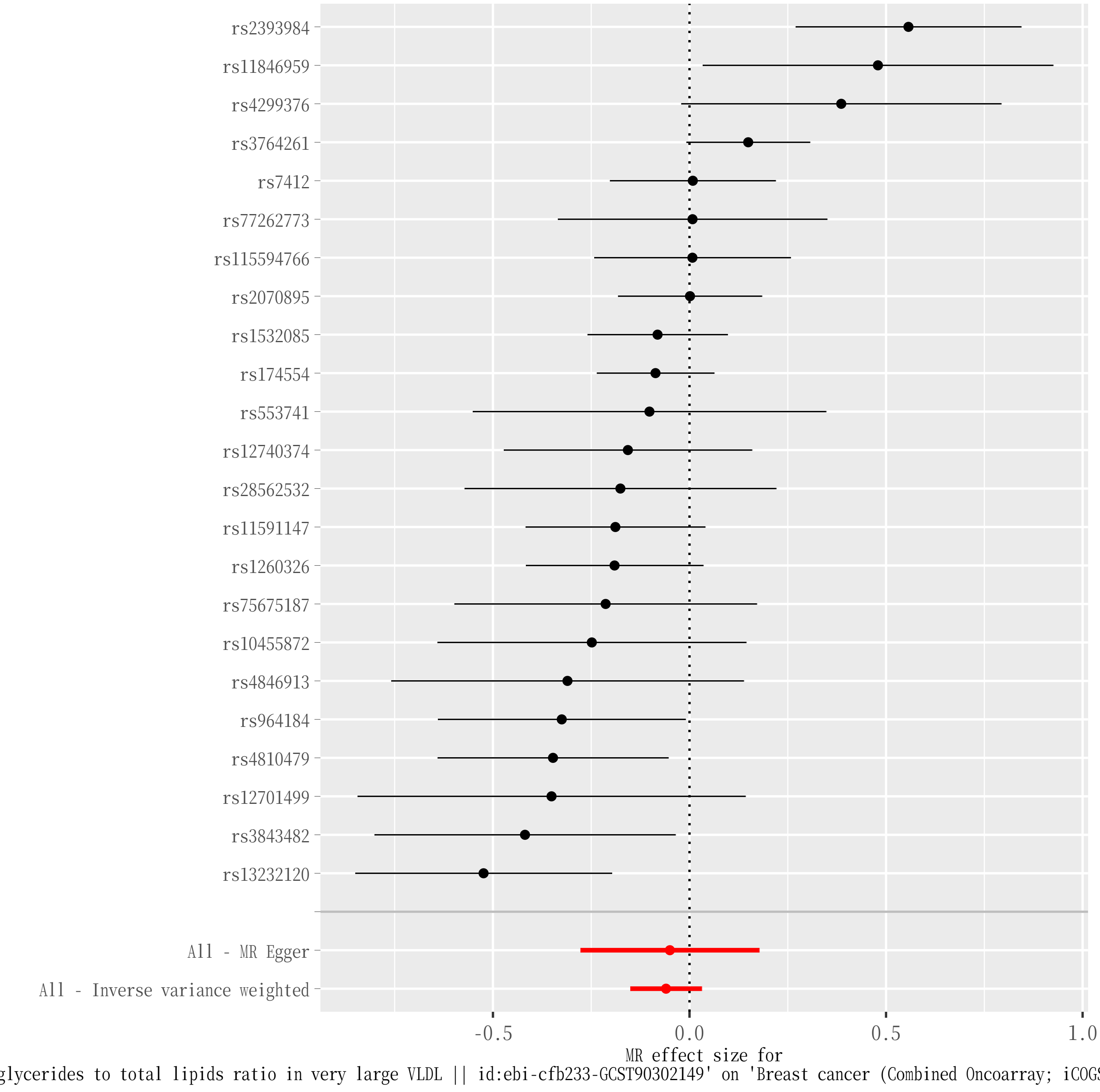

rs2393984
rs11846959
rs4299376
rs3764261
rs7412
rs77262773
rs115594766
rs2070895
rs1532085
rs174554
rs553741
rs12740374
rs28562532
rs11591147
rs1260326
rs75675187
rs10455872
rs4846913
rs964184
rs4810479
rs12701499
rs3843482
rs13232120
All - MR Egger
All - Inverse variance weighted
-0.5
0.0
0.5
1.0
MR effect size for
glycerides to total lipids ratio in very large VLDL || id:ebi-cfb233-GCST90302149' on 'Breast cancer (Combined Oncoarray; iCOGS

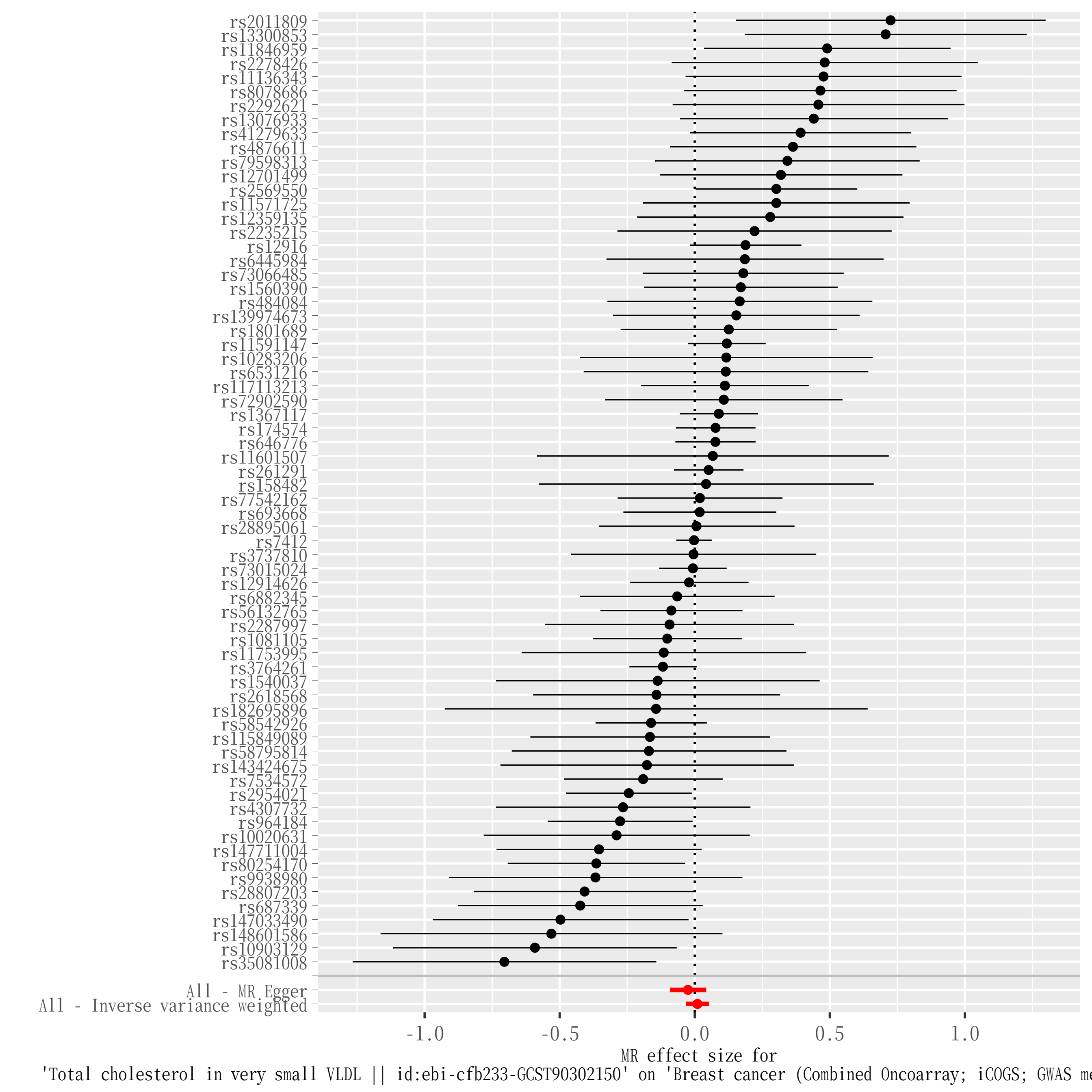

rs2011809
rs13300853
rs11846959
rs2278426
rs11136343
rs8078686
rs2292621
rs13076933
rs41279633
rs4876611
rs79598313
rs12701499
rs2569550
rs11571725
rs12359135
rs2235215
rs12916
rs6445984
rs73066485
rs1560390
rs484084
rs139974673
rs1801689
rs11591147
rs10283206
rs6531216
rs117113213
rs72902590
rs1367117
rs174574
rs646776
rs11601507
rs261291
rs158482
rs77542162
rs693668
rs28895061
rs7412
rs3737810
rs73015024
rs12914626
rs6882345
rs56132765
rs2287997
rs1081105
rs11753995
rs3764261
rs1540037
rs2618568
rs182695896
rs58542926
rs115849089
rs58795814
rs143424675
rs7534572
rs2954021
rs4307732
rs964184
rs10020631
rs147711004
rs80254170
rs9938980
rs28807203
rs687339
rs147033490
rs148601586
rs10903129
rs35081008
All - MR Egger
All - Inverse variance weighted
-1.0
-0.5
0.0
0.5
1.0
MR effect size for
'Total cholesterol in very small VLDL || id:ebi-cfb233-GCST90302150' on 'Breast cancer (Combined Oncoarray; iCOGS; GWAS me

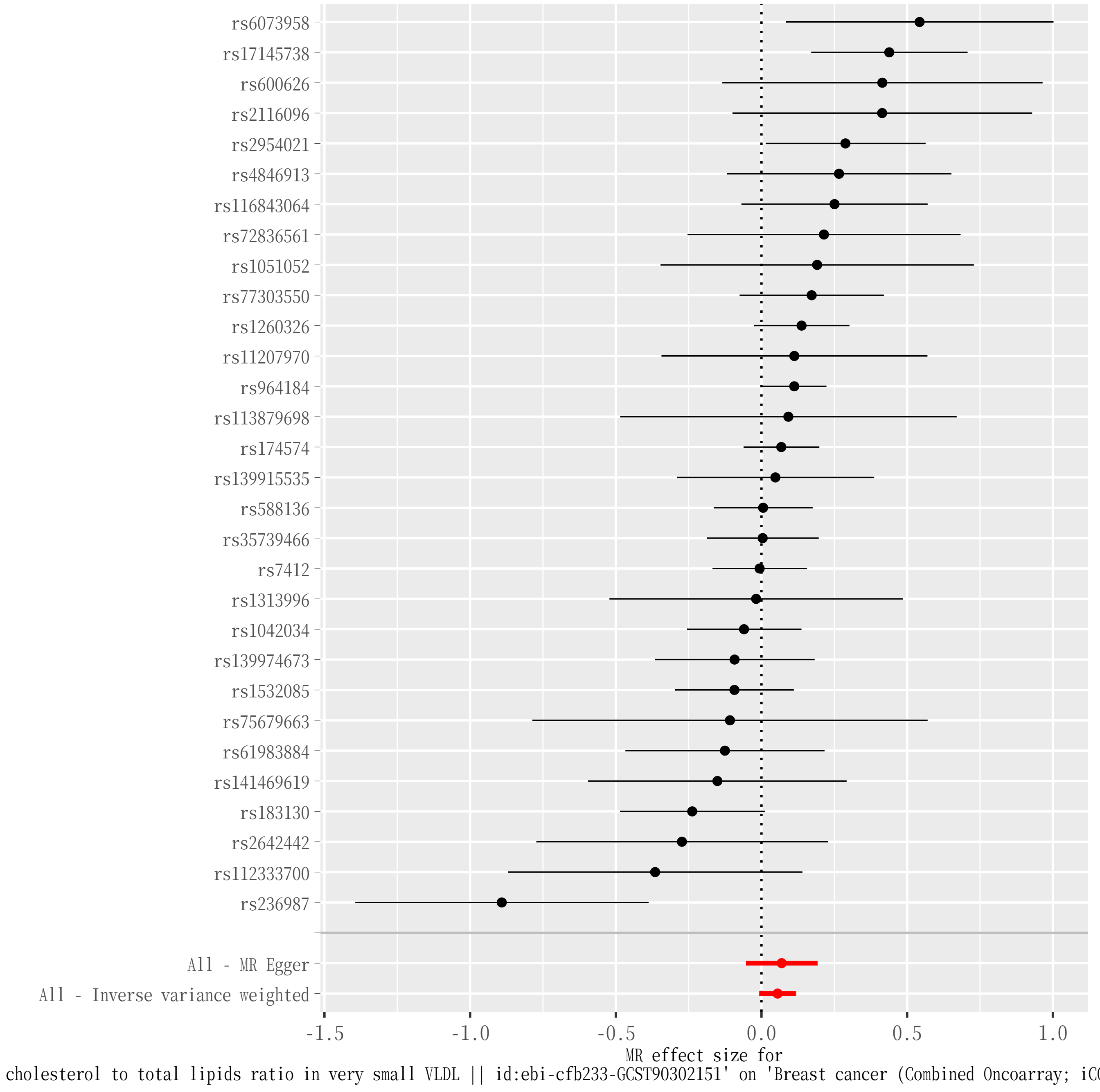
rs6073958
rs17145738
rs600626
rs2116096
rs2954021
rs4846913
rs116843064
rs72836561
rs1051052
rs77303550
rs1260326
rs11207970
rs964184
rs113879698
rs174574
rs139915535
rs588136
rs35739466
rs7412
rs1313996
rs1042034
rs139974673
rs1532085
rs75679663
rs61983884
rs141469619
rs183130
rs2642442
rs112333700
rs236987
All - MR Egger
All - Inverse variance weighted
-1.5
-1.0
-0.5
0.0
0.5
1.0
MR effect size for
cholesterol to total lipids ratio in very small VLDL || id:ebi-cfb233-GCST90302151' on 'Breast cancer (Combined Oncoarray; iC

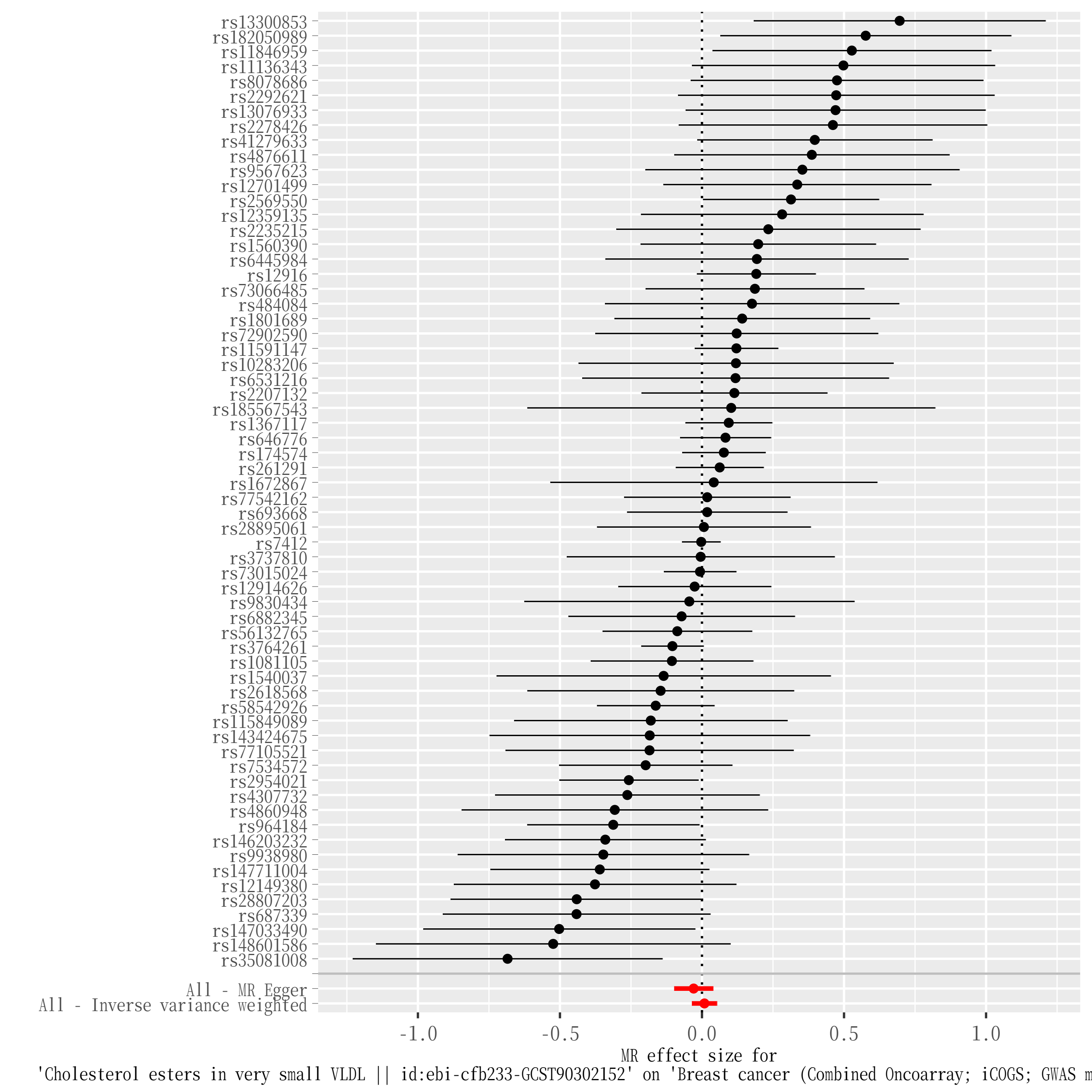

rs13300853
rs182050989
rs11846959
rs11136343
rs8078686
rs2292621
rs13076933
rs2278426
rs41279633
rs4876611
rs9567623
rs12701499
rs2569550
rs12359135
rs2235215
rs1560390
rs6445984
rs12916
rs73066485
rs484084
rs1801689
rs72902590
rs11591147
rs10283206
rs6531216
rs2207132
rs185567543
rs1367117
rs646776
rs174574
rs261291
rs1672867
rs77542162
rs693668
rs28895061
rs7412
rs3737810
rs73015024
rs12914626
rs9830434
rs6882345
rs56132765
rs3764261
rs1081105
rs1540037
rs2618568
rs58542926
rs115849089
rs143424675
rs77105521
rs7534572
rs2954021
rs4307732
rs4860948
rs964184
rs146203232
rs9938980
rs147711004
rs12149380
rs28807203
rs687339
rs147033490
rs148601586
rs35081008
All - MR Egger
All - Inverse variance weighted
-1.0
-0.5
0.0
0.5
1.0
MR effect size for
'Cholesterol esters in very small VLDL || id:ebi-cfb233-GCST90302152' on 'Breast cancer (Combined Oncoarray; iCOGS; GWAS m

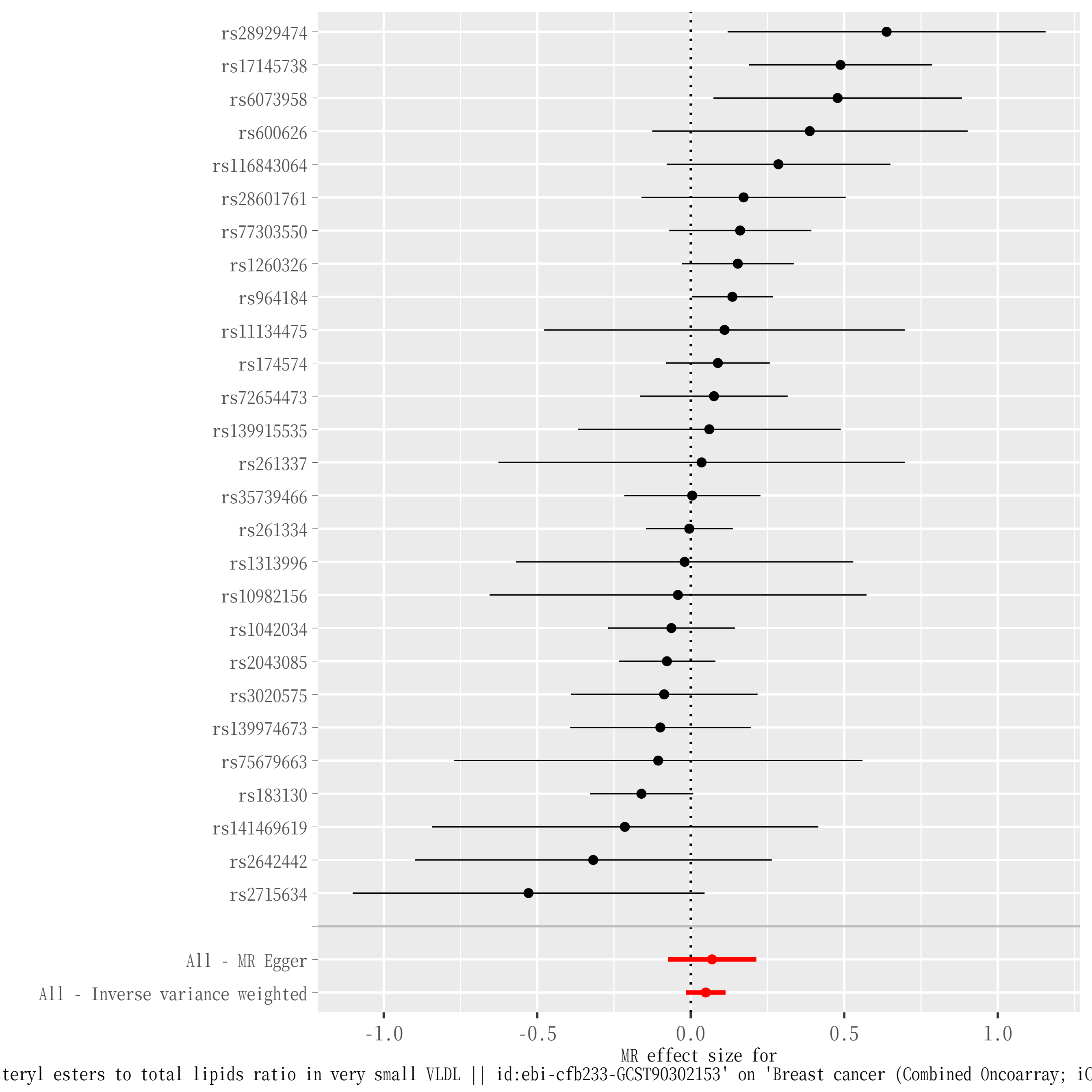

rs28929474
rs17145738
rs6073958
rs600626
rs116843064
rs28601761
rs77303550
rs1260326
rs964184
rs11134475
rs174574
rs72654473
rs139915535
rs261337
rs35739466
rs261334
rs1313996
rs10982156
rs1042034
rs2043085
rs3020575
rs139974673
rs75679663
rs183130
rs141469619
rs2642442
rs2715634
All - MR Egger
All - Inverse variance weighted
-1.0
-0.5
0.0
0.5
1.0
MR effect size for
teryl esters to total lipids ratio in very small VLDL || id:ebi-cfb233-GCST90302153' on 'Breast cancer (Combined Oncoarray; iC

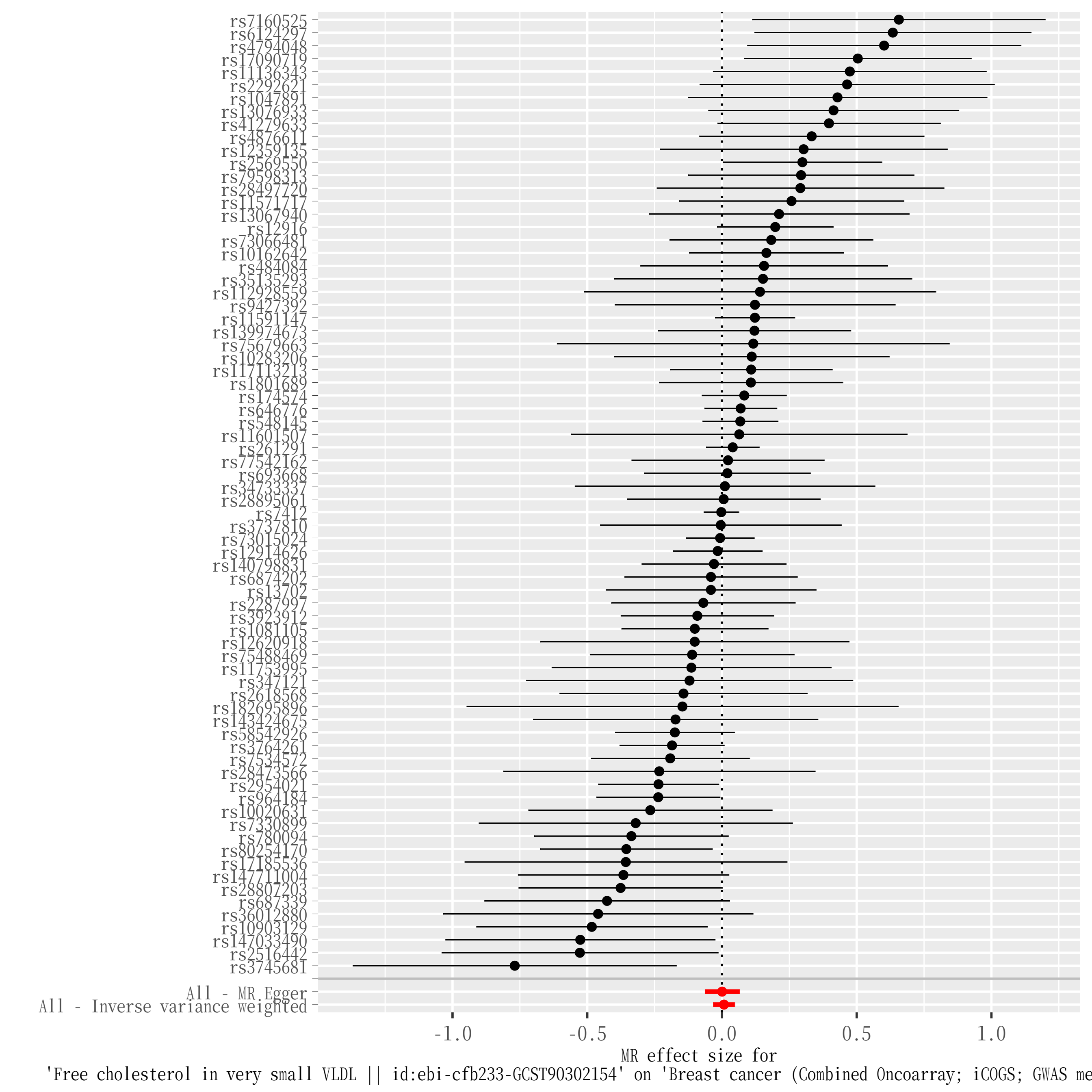
rs7160525
rs6124297
rs4794048
rs17090719
rs11136343
rs2292621
rs1047891
rs13076933
rs41279633
rs4876611
rs12359135
rs2569550
rs79598313
rs28497720
rs11571717
rs13067940
rs12916
rs73066481
rs10162642
rs484084
rs35135293
rs112928559
rs9427392
rs11591147
rs139974673
rs75679663
rs10283206
rs117113213
rs1801689
rs174574
rs646776
rs548145
rs11601507
rs261291
rs77542162
rs693668
rs34733337
rs28895061
rs7412
rs3737810
rs73015024
rs12914626
rs140798831
rs6874202
rs13702
rs2287997
rs3923912
rs1081105
rs12620918
rs75488469
rs11753995
rs347121
rs2618568
rs182695896
rs143424675
rs58542926
rs3764261
rs7534572
rs28473566
rs2954021
rs964184
rs10020631
rs7330899
rs780094
rs80254170
rs17185536
rs147711004
rs28807203
rs687339
rs36012880
rs10903129
rs147033490
rs2516442
rs3745681
All - MR Egger
All - Inverse variance weighted
-1.0
-0.5
0.0
0.5
1.0
MR effect size for
'Free cholesterol in very small VLDL || id:ebi-cfb233-GCST90302154' on 'Breast cancer (Combined Oncoarray; iCOGS; GWAS me

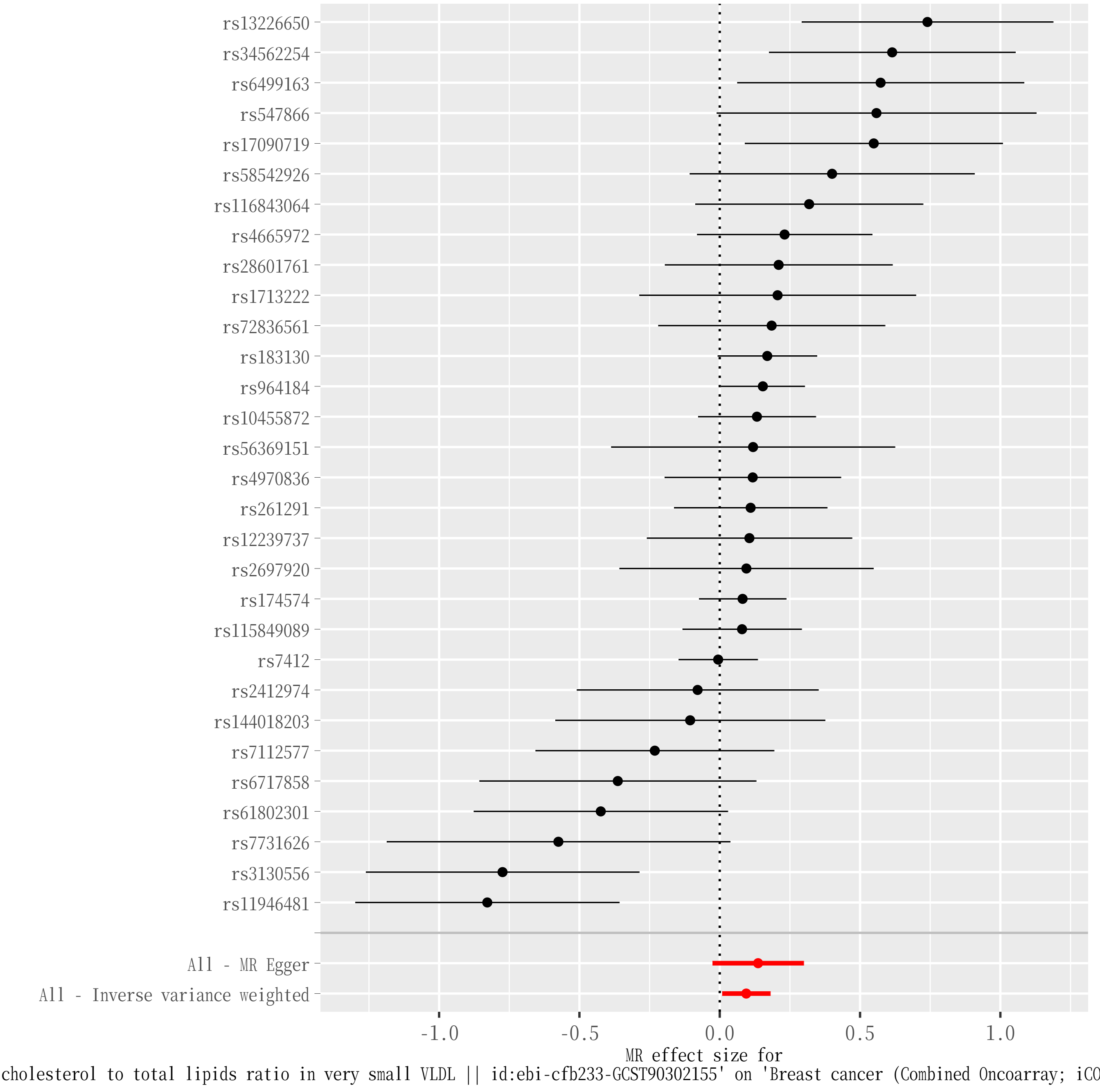

rs13226650
rs34562254
rs6499163
rs547866
rs17090719
rs58542926
rs116843064
rs4665972
rs28601761
rs1713222
rs72836561
rs183130
rs964184
rs10455872
rs56369151
rs4970836
rs261291
rs12239737
rs2697920
rs174574
rs115849089
rs7412
rs2412974
rs144018203
rs7112577
rs6717858
rs61802301
rs7731626
rs3130556
rs11946481
All - MR Egger
All - Inverse variance weighted
-1.0
-0.5
0.0
0.5
1.0
MR effect size for
cholesterol to total lipids ratio in very small VLDL || id:ebi-cfb233-GCST90302155' on 'Breast cancer (Combined Oncoarray; iCO

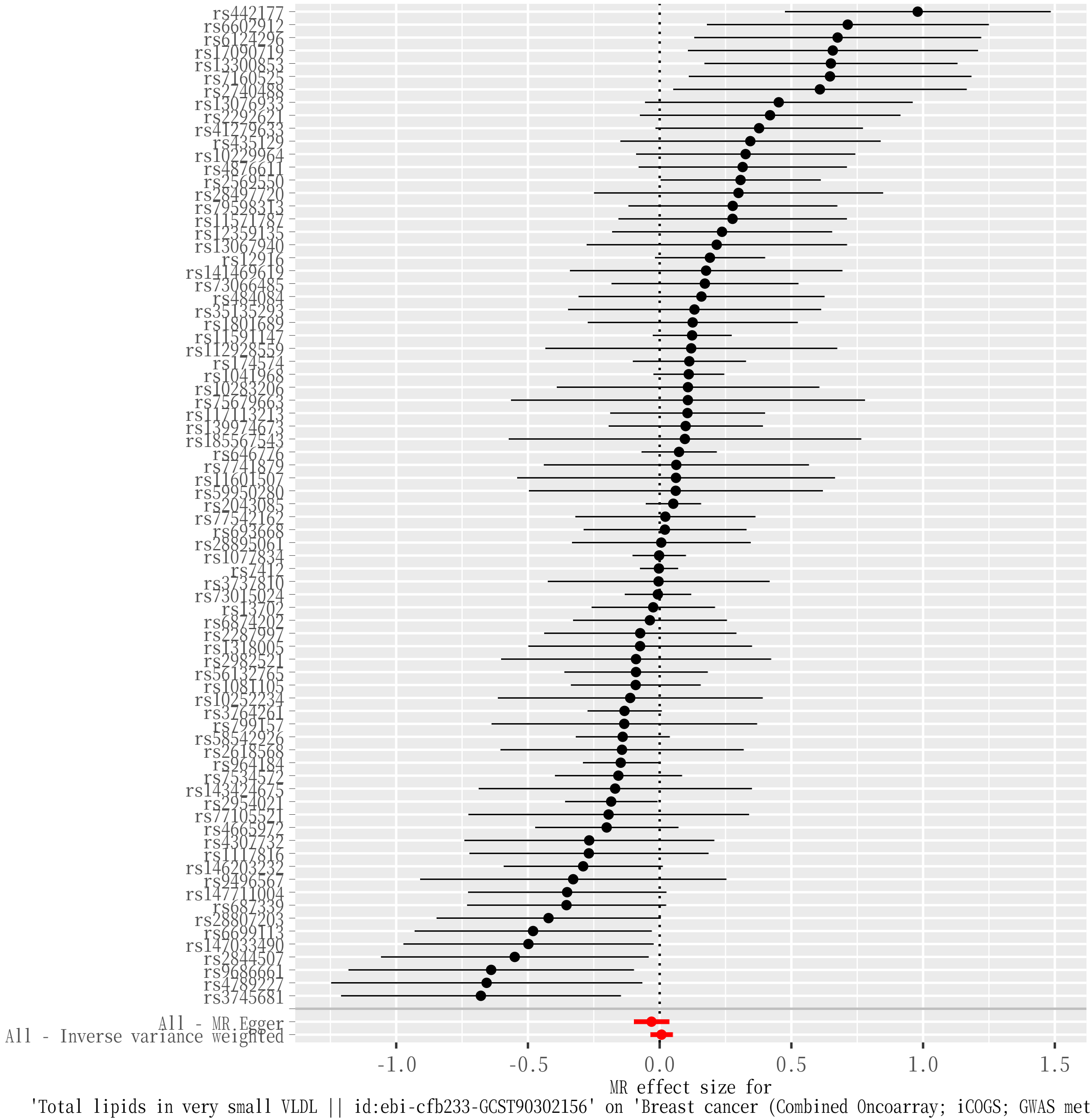
rs442177
rs6602912
rs6124296
rs17090719
rs13300853
rs7160525
rs2740488
rs13076933
rs2292621
rs41279633
rs435129
rs10229964
rs4876611
rs2562550
rs28497720
rs79598313
rs11571787
rs12359135
rs13067940
rs12916
rs141469619
rs73066485
rs484084
rs35135293
rs1801689
rs11591147
rs112928559
rs174574
rs1041968
rs10283206
rs75679663
rs117113213
rs139974673
rs185567543
rs646776
rs7741879
rs11601507
rs59950280
rs2043085
rs77542162
rs693668
rs28895061
rs1077834
rs7412
rs3737810
rs73015024
rs13702
rs6874202
rs2287997
rs1318005
rs2982521
rs56132765
rs1081105
rs10252234
rs3764261
rs799157
rs58542926
rs2618568
rs964184
rs7534572
rs143424675
rs2954021
rs77105521
rs4665972
rs4307732
rs1117816
rs146203232
rs9496567
rs147711004
rs687339
rs28807203
rs6699113
rs147033490
rs2844507
rs9686661
rs4789227
rs3745681
All - MR Egger
All - Inverse variance weighted
-1.0
-0.5
0.0
0.5
1.0
1.5
MR effect size for
'Total lipids in very small VLDL || id:ebi-cfb233-GCST90302156' on 'Breast cancer (Combined Oncoarray; iCOGS; GWAS meta

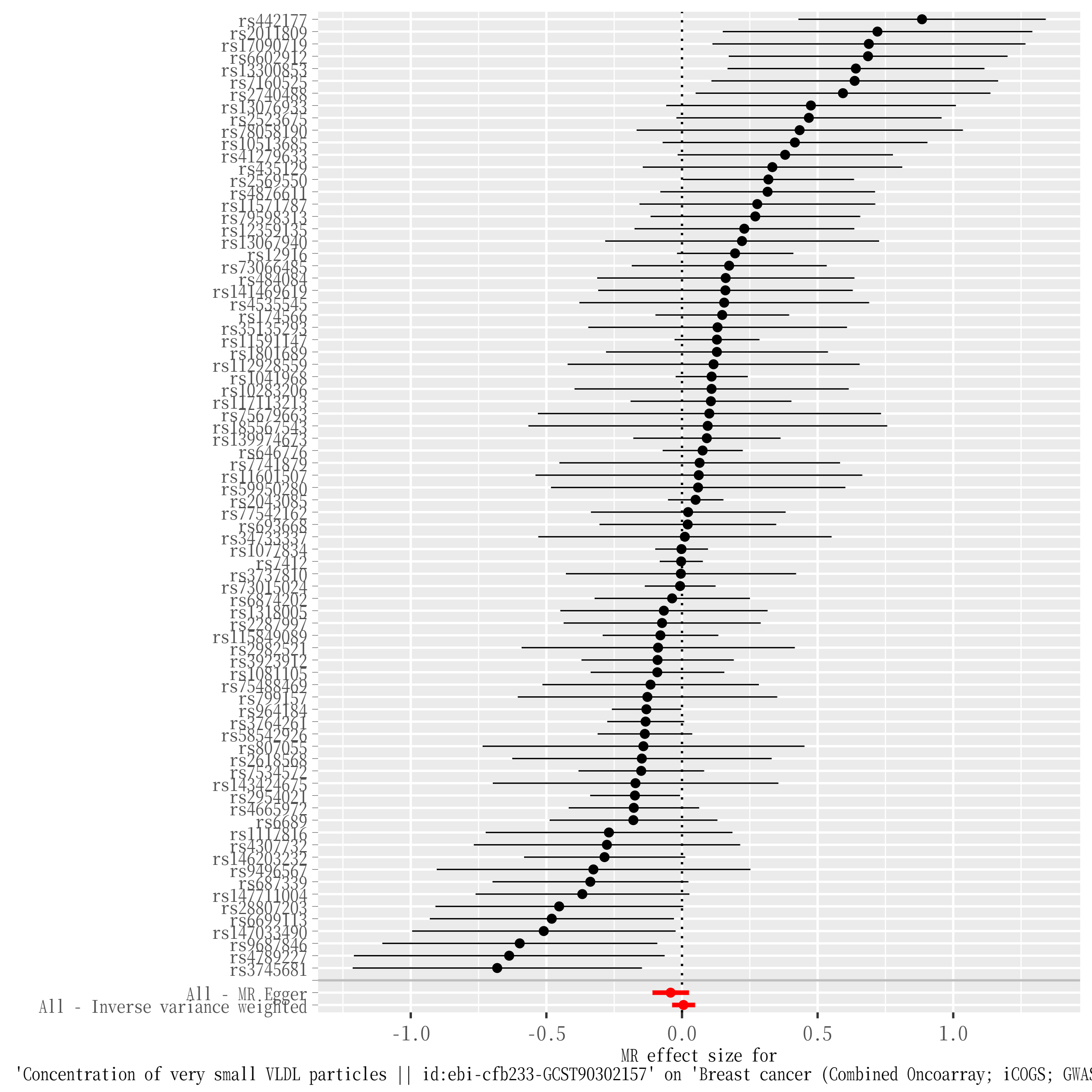

rs442177
rs2011809
rs17090719
rs6602912
rs13300853
rs7160525
rs2740488
rs13076933
rs2523675
rs78058190
rs10513685
rs41279633
rs435129
rs2569550
rs4876611
rs11571787
rs79598313
rs12359135
rs13067940
rs12916
rs73066485
rs484084
rs141469619
rs4535545
rs174566
rs35135293
rs11591147
rs1801689
rs112928559
rs1041968
rs10283206
rs117113213
rs75679663
rs185567543
rs139974673
rs646776
rs7741879
rs11601507
rs59950280
rs2043085
rs77542162
rs693668
rs34733337
rs1077834
rs7412
rs3737810
rs73015024
rs6874202
rs1318005
rs2287997
rs115849089
rs2982521
rs3923912
rs1081105
rs75488469
rs799157
rs964184
rs3764261
rs58542926
rs807055
rs2618568
rs7534572
rs143424675
rs2954021
rs4665972
rs6689
rs1117816
rs4307732
rs146203232
rs9496567
rs687339
rs147711004
rs28807203
rs6699113
rs147033490
rs9687846
rs4789227
rs3745681
All - MR Egger
All - Inverse variance weighted
-1.0
-0.5
0.0
0.5
1.0
MR effect size for
'Concentration of very small VLDL particles || id:ebi-cfb233-GCST90302157' on 'Breast cancer (Combined Oncoarray; iCOGS; GWAS

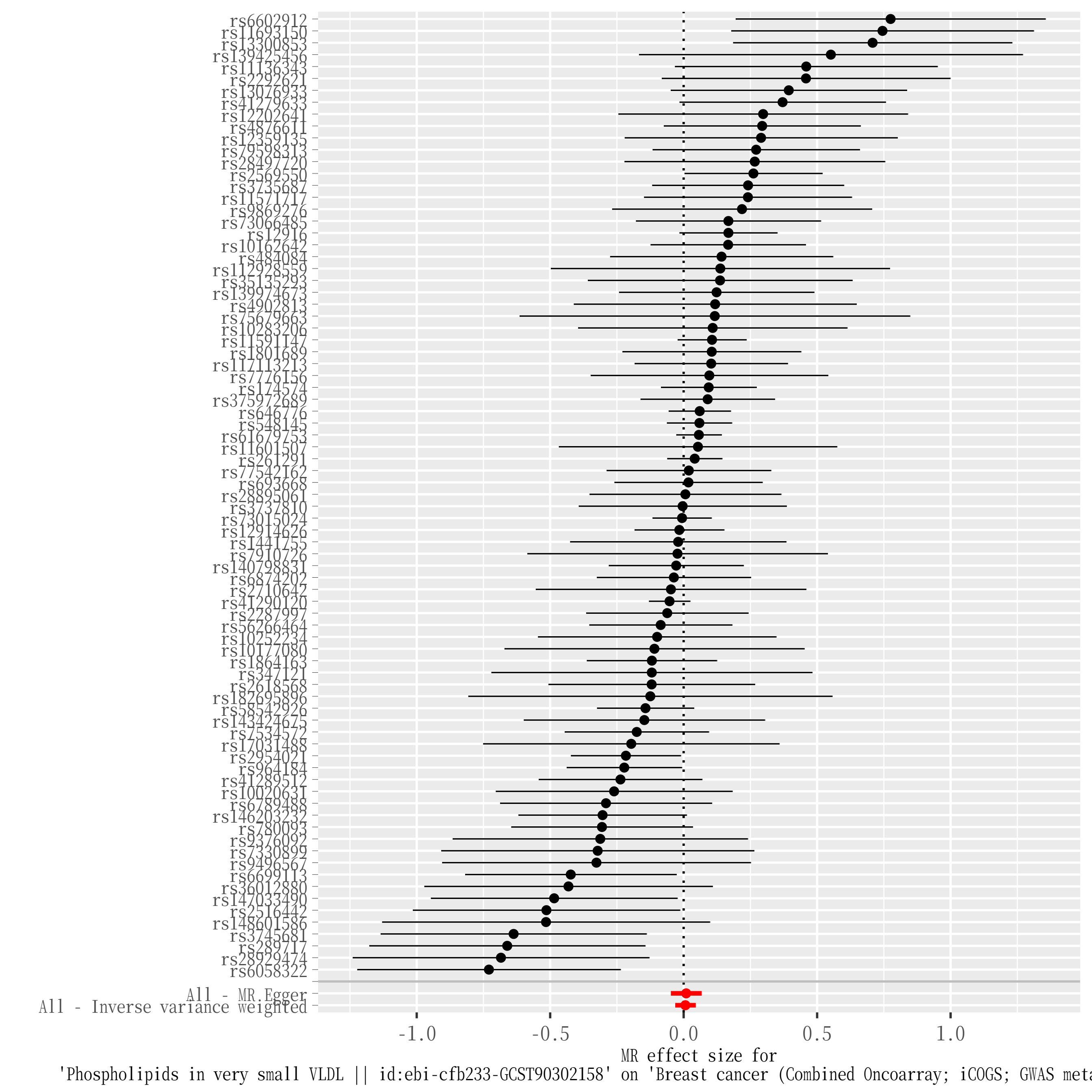
rs6602912
rs11693150
rs13300853
rs139425456
rs11136343
rs2292621
rs13076933
rs41279633
rs12202641
rs4876611
rs12359135
rs79598313
rs28497720
rs2569550
rs3735687
rs11571717
rs9869276
rs73066485
rs12916
rs10162642
rs484084
rs112928559
rs35135293
rs139974673
rs4902813
rs75679863
rs10283206
rs11591147
rs1801689
rs112113213
rs7776156
rs174574
rs375972689
rs646776
rs548145
rs61679753
rs11601507
rs261291
rs77542162
rs693668
rs28895061
rs3737810
rs73015024
rs12914626
rs1441755
rs7910726
rs140798831
rs6874202
rs2710642
rs41290120
rs2287997
rs56266464
rs10252234
rs10177080
rs1864163
rs347121
rs2618568
rs182695896
rs58542926
rs143424675
rs7534572
rs17031488
rs2954021
rs964184
rs41289512
rs10020631
rs6789488
rs146203232
rs780093
rs2376092
rs7330899
rs9496567
rs6699113
rs36012880
rs147033490
rs2516442
rs148601586
rs3745681
rs289717
rs28929474
rs6058322
All - MR Egger
All - Inverse variance weighted
-1.0
-0.5
0.0
0.5
1.0
MR effect size for
'Phospholipids in very small VLDL || id:ebi-cfb233-GCST90302158' on 'Breast cancer (Combined Oncoarray; iCOGS; GWAS meta

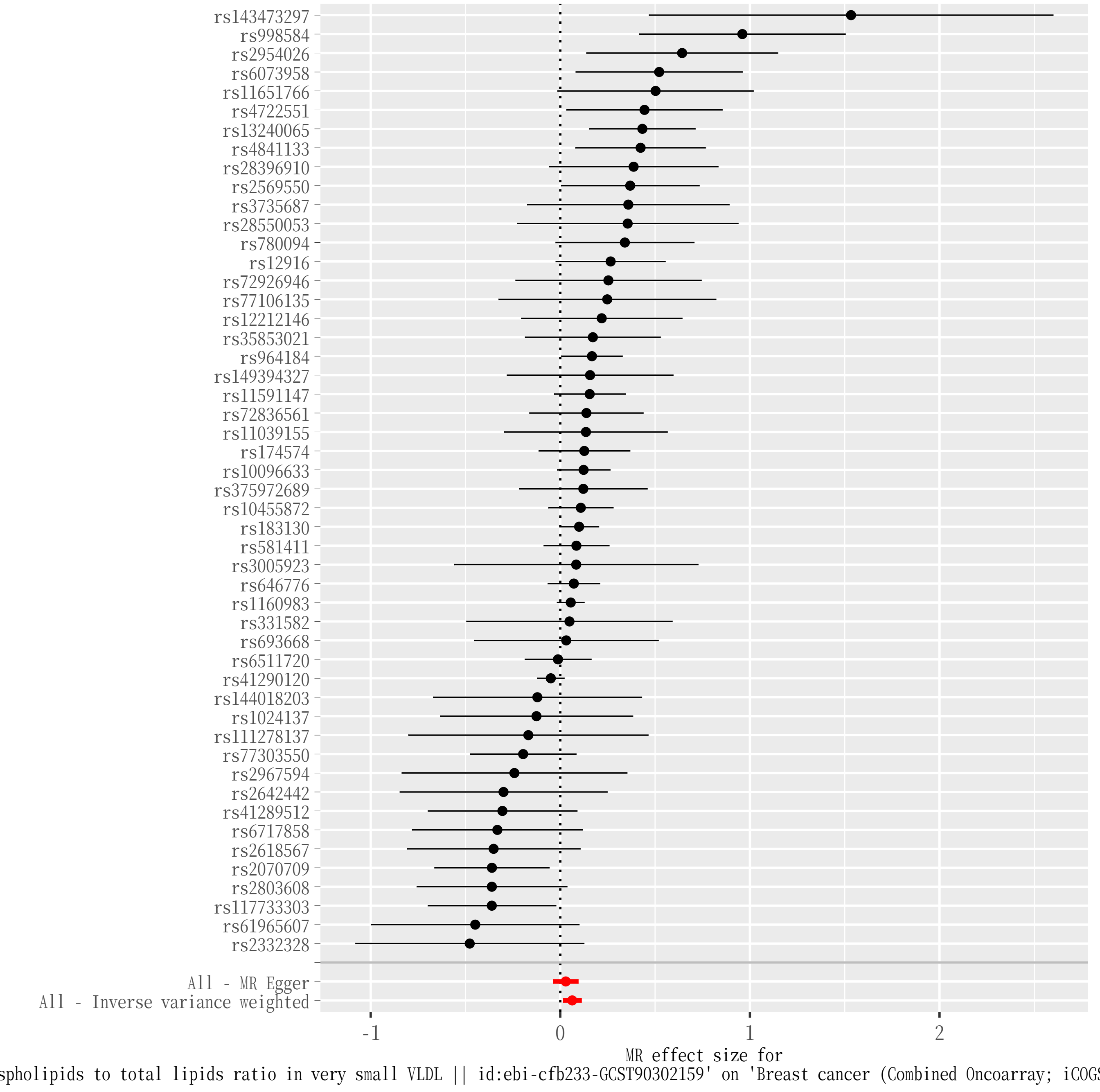
rs143473297
rs998584
rs2954026
rs6073958
rs11651766
rs4722551
rs13240065
rs4841133
rs28396910
rs2569550
rs3735687
rs28550053
rs780094
rs12916
rs72926946
rs77106135
rs12212146
rs35853021
rs964184
rs149394327
rs11591147
rs72836561
rs11039155
rs174574
rs10096633
rs375972689
rs10455872
rs183130
rs581411
rs3005923
rs646776
rs1160983
rs331582
rs693668
rs6511720
rs41290120
rs144018203
rs1024137
rs111278137
rs77303550
rs2967594
rs2642442
rs41289512
rs6717858
rs2618567
rs2070709
rs2803608
rs117733303
rs61965607
rs2332328
All - MR Egger
All - Inverse variance weighted
-1
0
1
2
MR effect size for
spholipids to total lipids ratio in very small VLDL || id:ebi-cfb233-GCST90302159' on 'Breast cancer (Combined Oncoarray; iCOGS

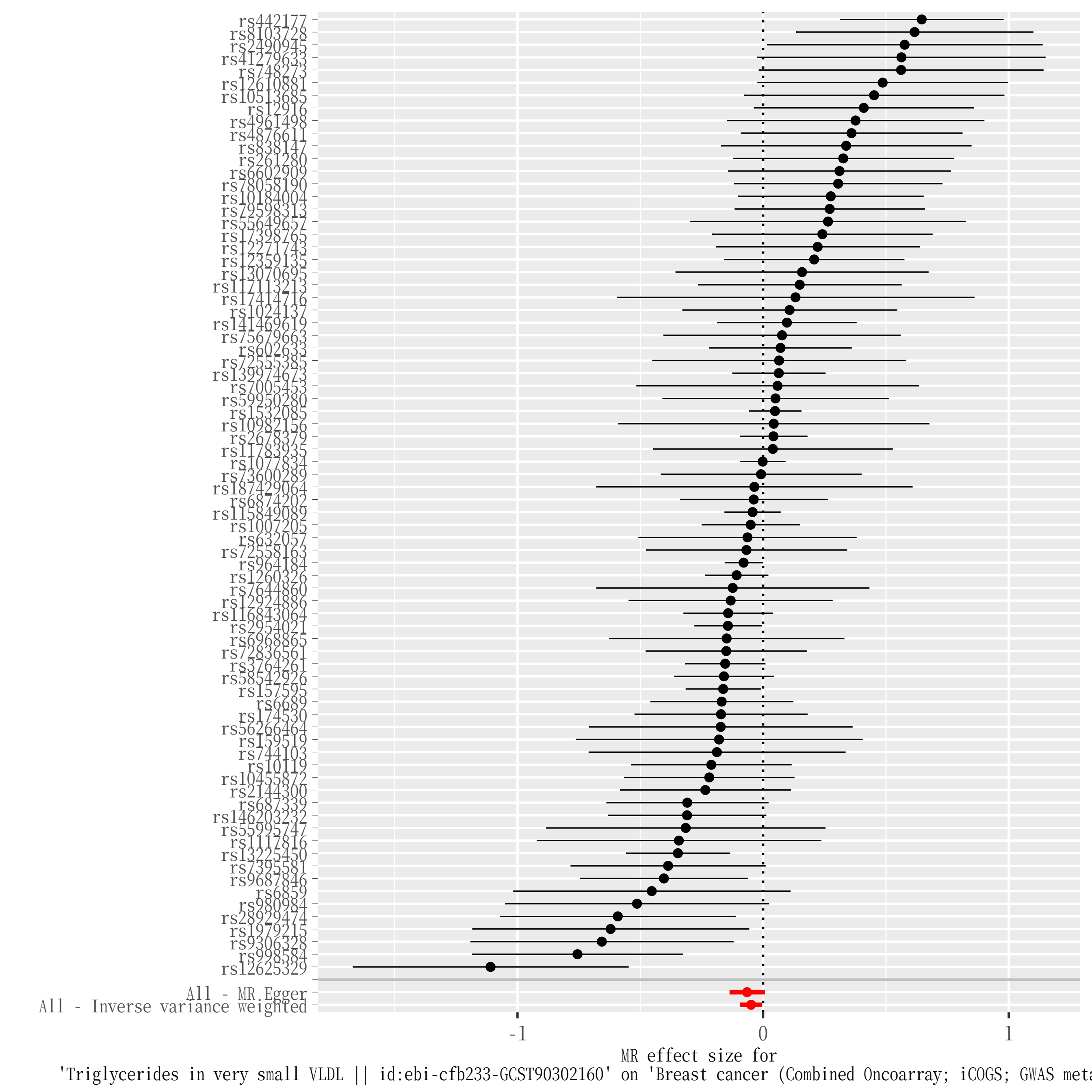
rs442177
rs8103728
rs2490945
rs41279633
rs748273
rs12610881
rs10513685
rs12916
rs4961498
rs4876611
rs838147
rs261280
rs6602909
rs78058190
rs10184004
rs79598313
rs55649657
rs17398765
rs12271743
rs12359135
rs13070695
rs117113213
rs17414716
rs1024137
rs141469619
rs75679663
rs602633
rs72555385
rs139974673
rs7005453
rs59950280
rs1532085
rs10982156
rs2678379
rs11783935
rs1077834
rs73600289
rs187429064
rs6874202
rs115849089
rs1007205
rs632057
rs72558163
rs964184
rs1260326
rs7644860
rs12924886
rs116843064
rs2954021
rs6968865
rs72836561
rs3764261
rs58542926
rs157595
rs6689
rs174530
rs56266464
rs159519
rs744103
rs10119
rs10455872
rs2144300
rs687339
rs146203232
rs55995747
rs1117816
rs13225450
rs7395581
rs9687846
rs6859
rs980984
rs28929474
rs1979215
rs9306328
rs998584
rs12625329
All - MR Egger
All - Inverse variance weighted
-1
0
1
MR effect size for
'Triglycerides in very small VLDL || id:ebi-cfb233-GCST90302160' on 'Breast cancer (Combined Oncoarray; iCOGS; GWAS meta

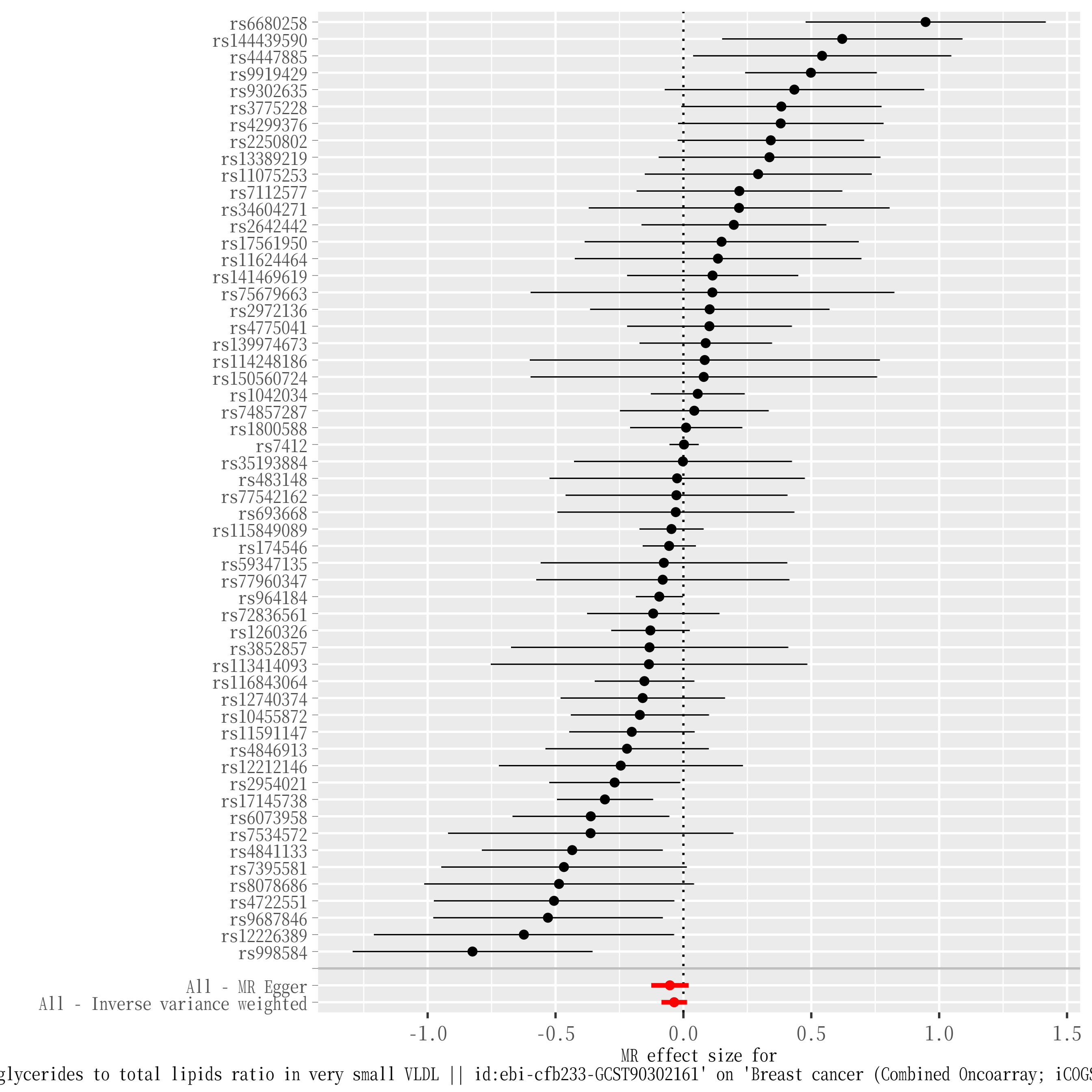
rs6680258
rs144439590
rs4447885
rs9919429
rs9302635
rs3775228
rs4299376
rs2250802
rs13389219
rs11075253
rs7112577
rs34604271
rs2642442
rs17561950
rs11624464
rs141469619
rs75679663
rs2972136
rs4775041
rs139974673
rs114248186
rs150560724
rs1042034
rs74857287
rs1800588
rs7412
rs35193884
rs483148
rs77542162
rs693668
rs115849089
rs174546
rs59347135
rs77960347
rs964184
rs72836561
rs1260326
rs3852857
rs113414093
rs116843064
rs12740374
rs10455872
rs11591147
rs4846913
rs12212146
rs2954021
rs17145738
rs6073958
rs7534572
rs4841133
rs7395581
rs8078686
rs4722551
rs9687846
rs12226389
rs998584
All - MR Egger
All - Inverse variance weighted
-1.0
-0.5
0.0
0.5
1.0
1.5
MR effect size for
glycerides to total lipids ratio in very small VLDL || id:ebi-cfb233-GCST90302161' on 'Breast cancer (Combined Oncoarray; iCOGS

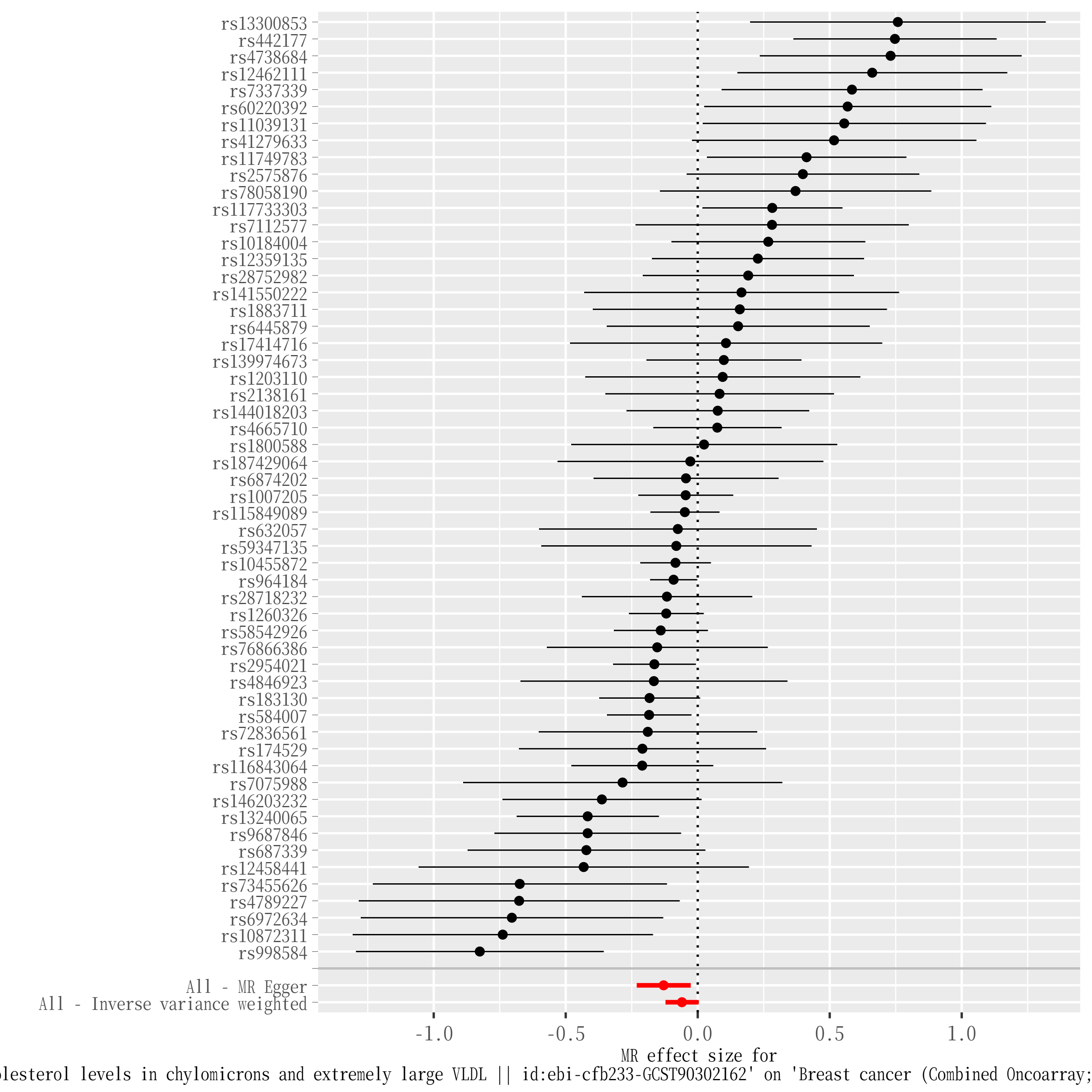
rs13300853
rs442177
rs4738684
rs12462111
rs7337339
rs60220392
rs11039131
rs41279633
rs11749783
rs2575876
rs78058190
rs117733303
rs7112577
rs10184004
rs12359135
rs28752982
rs141550222
rs1883711
rs6445879
rs17414716
rs139974673
rs1203110
rs2138161
rs144018203
rs4665710
rs1800588
rs187429064
rs6874202
rs1007205
rs115849089
rs632057
rs59347135
rs10455872
rs964184
rs28718232
rs1260326
rs58542926
rs76866386
rs2954021
rs4846923
rs183130
rs584007
rs72836561
rs174529
rs116843064
rs7075988
rs146203232
rs13240065
rs9687846
rs687339
rs12458441
rs73455626
rs4789227
rs6972634
rs10872311
rs998584
All - MR Egger
All - Inverse variance weighted
-1.0
-0.5
0.0
0.5
1.0
MR effect size for
olesterol levels in chylomicrons and extremely large VLDL || id:ebi-cfb233-GCST90302162' on 'Breast cancer (Combined Oncoarray;

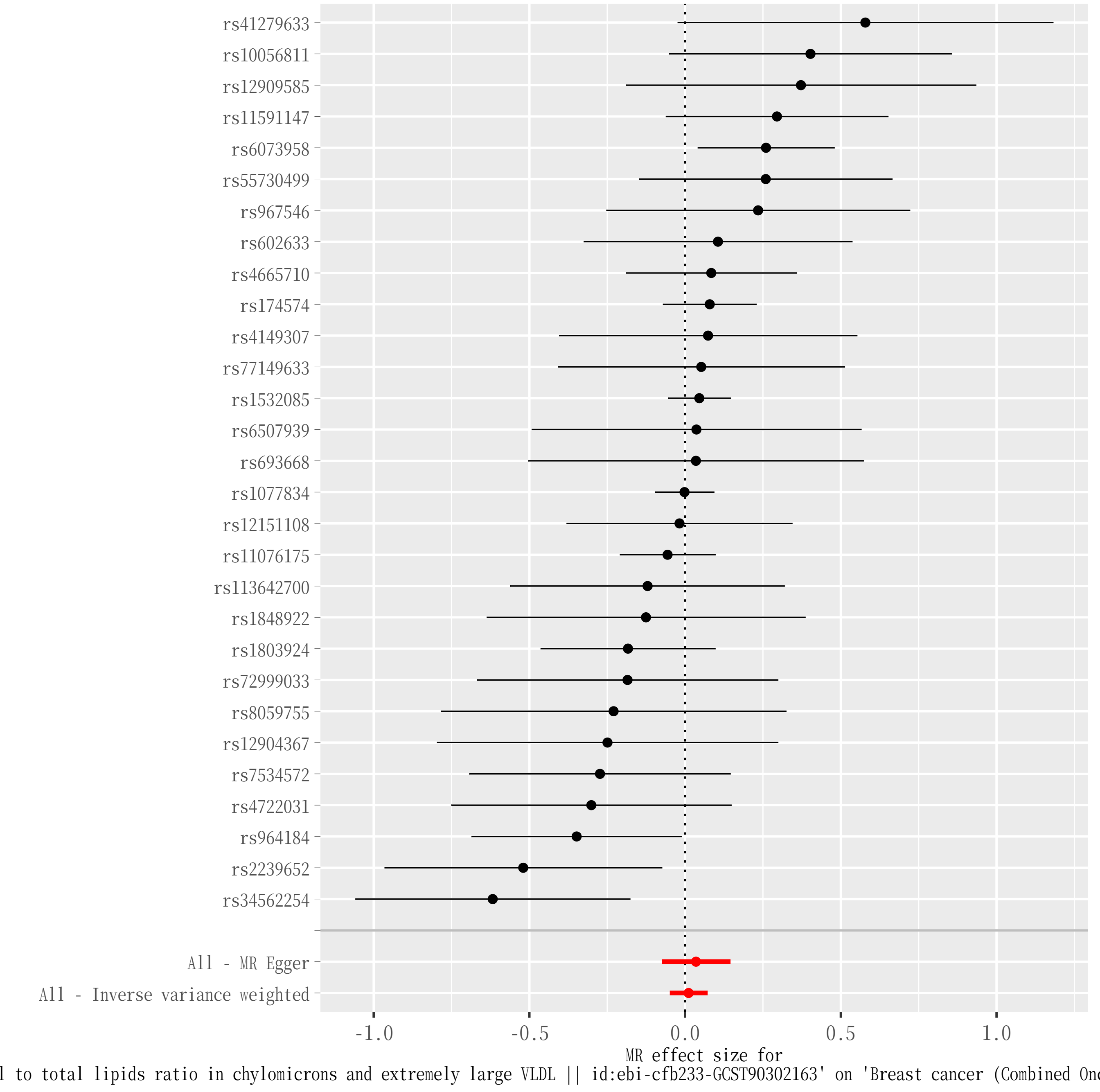

rs41279633
rs10056811
rs12909585
rs11591147
rs6073958
rs55730499
rs967546
rs602633
rs4665710
rs174574
rs4149307
rs77149633
rs1532085
rs6507939
rs693668
rs1077834
rs12151108
rs11076175
rs113642700
rs1848922
rs1803924
rs72999033
rs8059755
rs12904367
rs7534572
rs4722031
rs964184
rs2239652
rs34562254
All - MR Egger
All - Inverse variance weighted
-1.0
-0.5
0.0
0.5
1.0
MR effect size for
l to total lipids ratio in chylomicrons and extremely large VLDL || id:ebi-cfb233-GCST90302163' on 'Breast cancer (Combined Onc

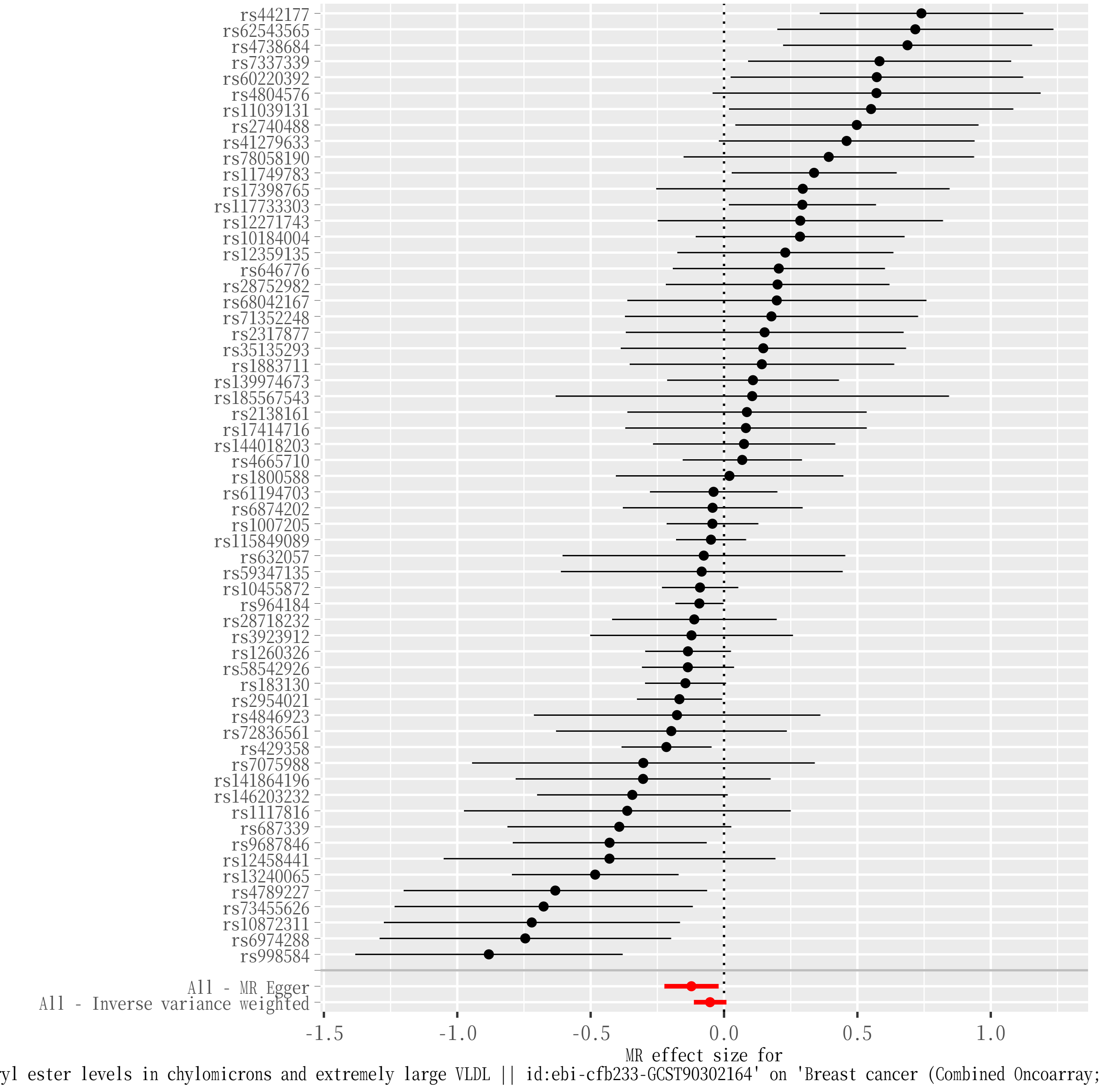

rs442177
rs62543565
rs4738684
rs7337339
rs60220392
rs4804576
rs11039131
rs2740488
rs41279633
rs78058190
rs11749783
rs17398765
rs117733303
rs12271743
rs10184004
rs12359135
rs646776
rs28752982
rs68042167
rs71352248
rs2317877
rs35135293
rs1883711
rs139974673
rs185567543
rs2138161
rs17414716
rs144018203
rs4665710
rs1800588
rs61194703
rs6874202
rs1007205
rs115849089
rs632057
rs59347135
rs10455872
rs964184
rs28718232
rs3923912
rs1260326
rs58542926
rs183130
rs2954021
rs4846923
rs72836561
rs429358
rs7075988
rs141864196
rs146203232
rs1117816
rs687339
rs9687846
rs12458441
rs13240065
rs4789227
rs73455626
rs10872311
rs6974288
rs998584
All - MR Egger
All - Inverse variance weighted
-1.5
-1.0
-0.5
0.0
0.5
1.0
MR effect size for
yl ester levels in chylomicrons and extremely large VLDL || id:ebi-cfb233-GCST90302164' on 'Breast cancer (Combined Oncoarray;

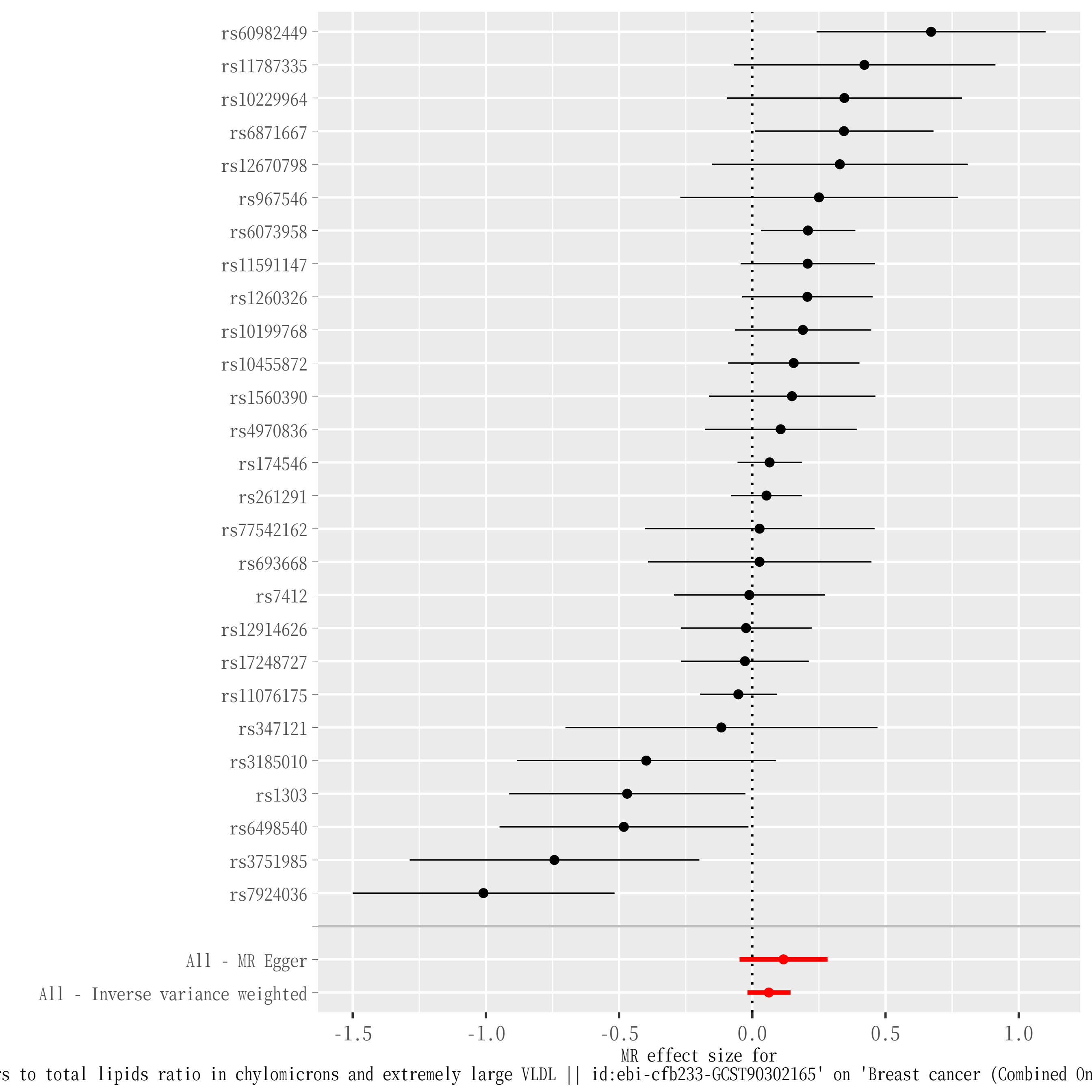

rs60982449
rs11787335
rs10229964
rs6871667
rs12670798
rs967546
rs6073958
rs11591147
rs1260326
rs10199768
rs10455872
rs1560390
rs4970836
rs174546
rs261291
rs77542162
rs693668
rs7412
rs12914626
rs17248727
rs11076175
rs347121
rs3185010
rs1303
rs6498540
rs3751985
rs7924036
All - MR Egger
All - Inverse variance weighted
-1.5
-1.0
-0.5
0.0
0.5
1.0
MR effect size for
rs to total lipids ratio in chylomicrons and extremely large VLDL || id:ebi-cfb233-GCST90302165' on 'Breast cancer (Combined On

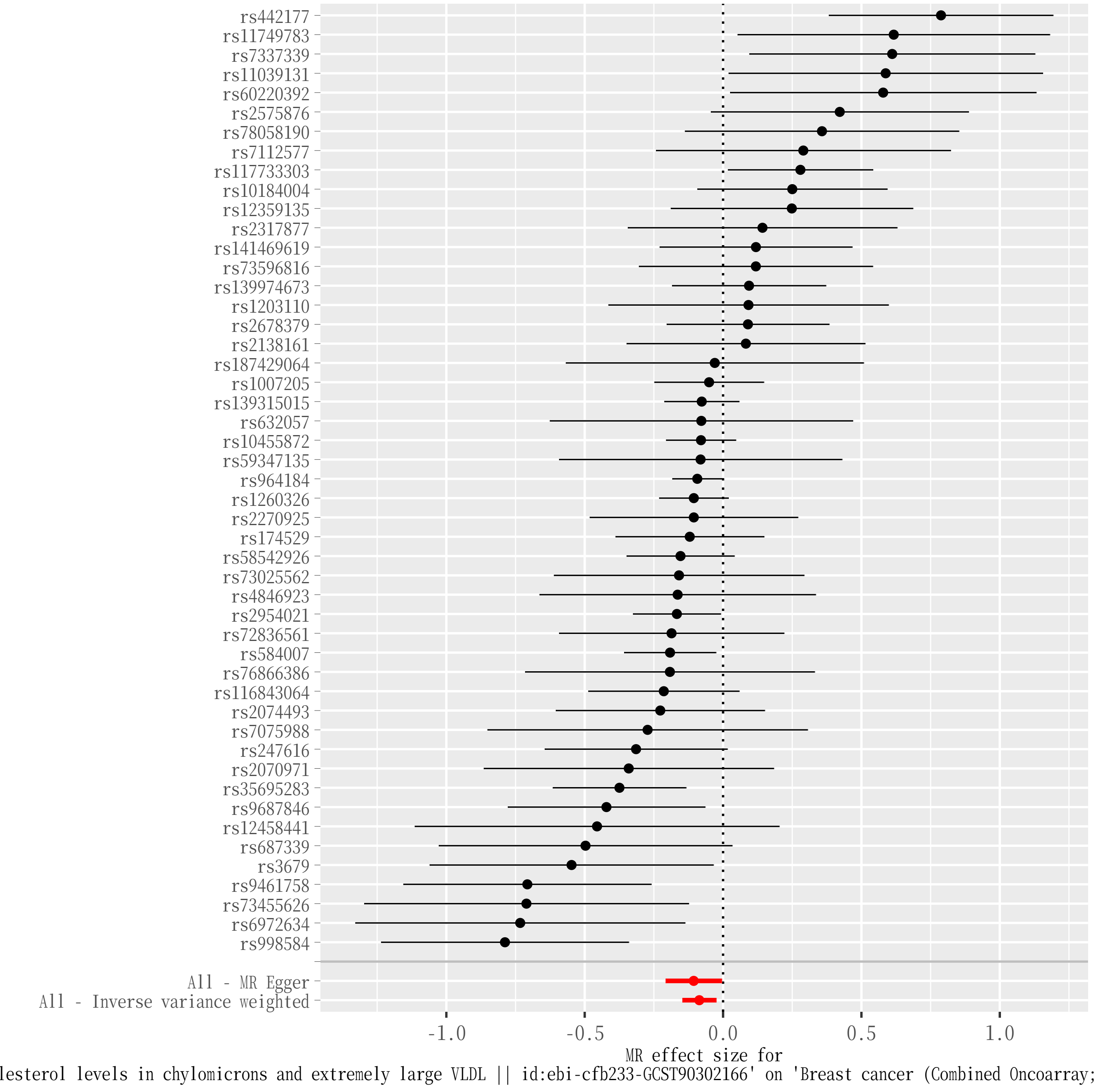
rs442177
rs11749783
rs7337339
rs11039131
rs60220392
rs2575876
rs78058190
rs7112577
rs117733303
rs10184004
rs12359135
rs2317877
rs141469619
rs73596816
rs139974673
rs1203110
rs2678379
rs2138161
rs187429064
rs1007205
rs139315015
rs632057
rs10455872
rs59347135
rs964184
rs1260326
rs2270925
rs174529
rs58542926
rs73025562
rs4846923
rs2954021
rs72836561
rs584007
rs76866386
rs116843064
rs2074493
rs7075988
rs247616
rs2070971
rs35695283
rs9687846
rs12458441
rs687339
rs3679
rs9461758
rs73455626
rs6972634
rs998584
All - MR Egger
All - Inverse variance weighted
-1.0
-0.5
0.0
0.5
1.0
MR effect size for
lesterol levels in chylomicrons and extremely large VLDL || id:ebi-cfb233-GCST90302166' on 'Breast cancer (Combined Oncoarray;

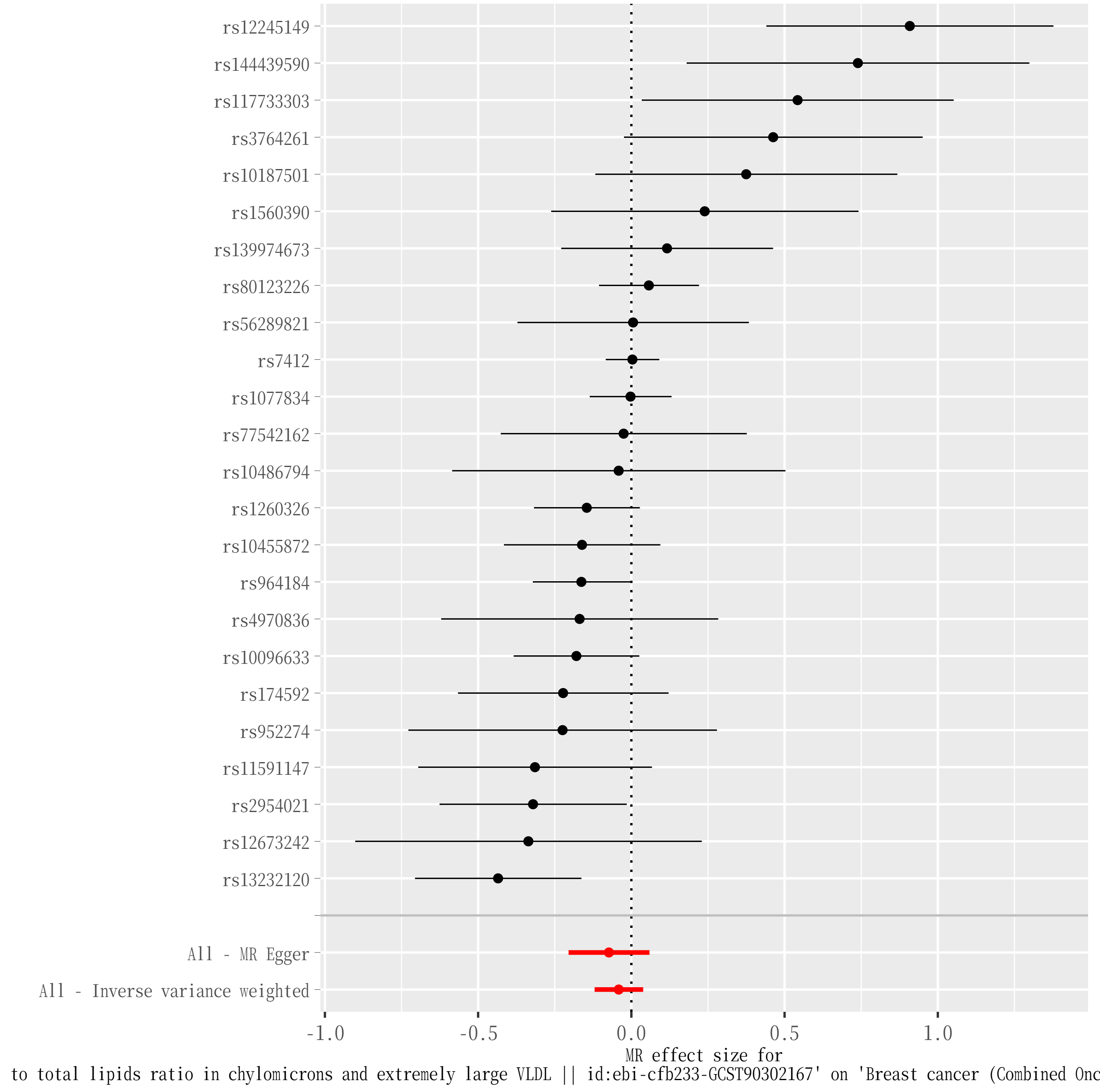
rs12245149
rs144439590
rs117733303
rs3764261
rs10187501
rs1560390
rs139974673
rs80123226
rs56289821
rs7412
rs1077834
rs77542162
rs10486794
rs1260326
rs10455872
rs964184
rs4970836
rs10096633
rs174592
rs952274
rs11591147
rs2954021
rs12673242
rs13232120
All - MR Egger
All - Inverse variance weighted
-1.0
-0.5
0.0
0.5
1.0
MR effect size for
to total lipids ratio in chylomicrons and extremely large VLDL || id:ebi-cfb233-GCST90302167' on 'Breast cancer (Combined Onc

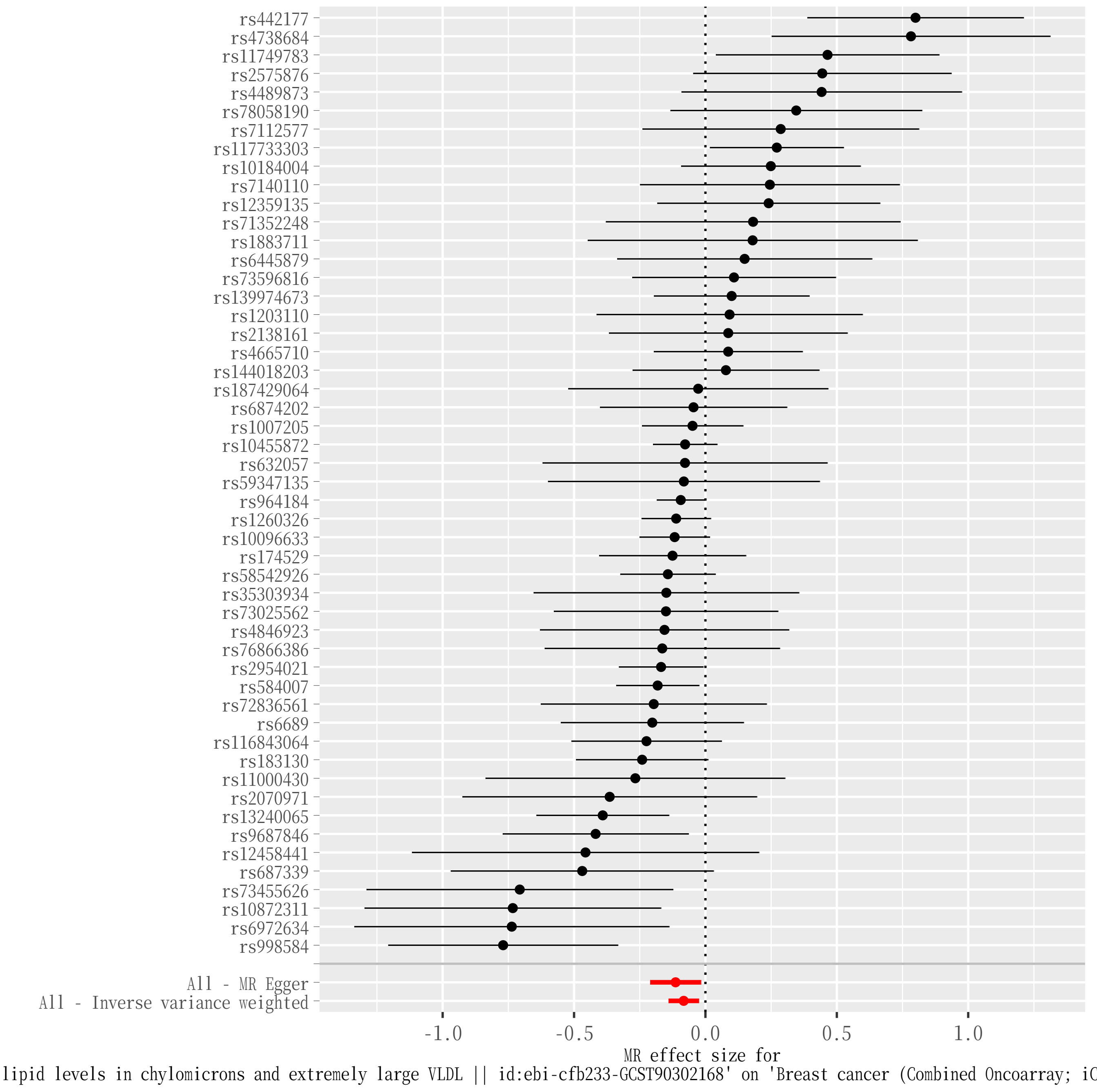
rs442177
rs4738684
rs11749783
rs2575876
rs4489873
rs78058190
rs7112577
rs117733303
rs10184004
rs7140110
rs12359135
rs71352248
rs1883711
rs6445879
rs73596816
rs139974673
rs1203110
rs2138161
rs4665710
rs144018203
rs187429064
rs6874202
rs1007205
rs10455872
rs632057
rs59347135
rs964184
rs1260326
rs10096633
rs174529
rs58542926
rs35303934
rs73025562
rs4846923
rs76866386
rs2954021
rs584007
rs72836561
rs6689
rs116843064
rs183130
rs11000430
rs2070971
rs13240065
rs9687846
rs12458441
rs687339
rs73455626
rs10872311
rs6972634
rs998584
All - MR Egger
All - Inverse variance weighted
-1.0
-0.5
0.0
0.5
1.0
MR effect size for
lipid levels in chylomicrons and extremely large VLDL || id:ebi-cfb233-GCST90302168' on 'Breast cancer (Combined Oncoarray; iC

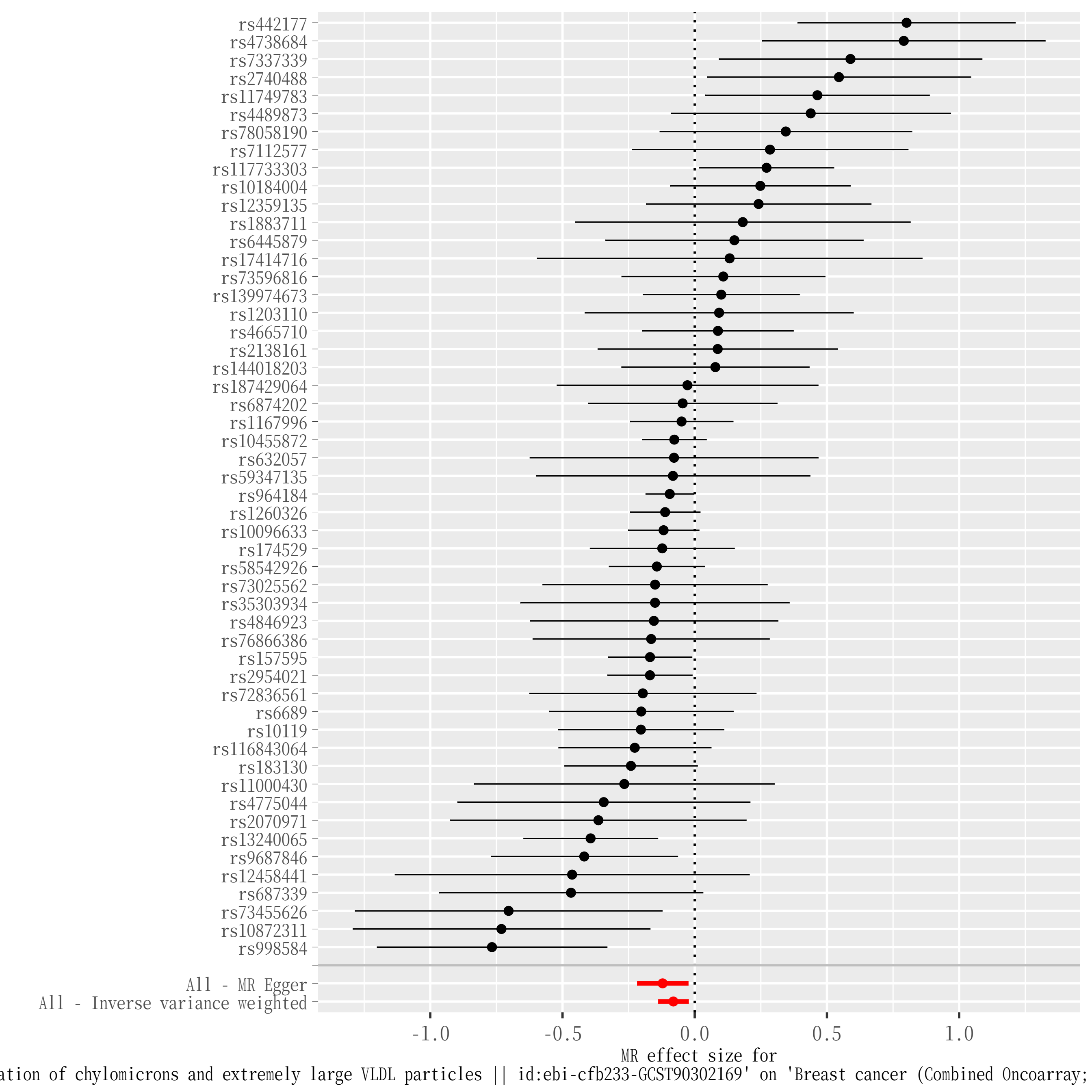

rs442177
rs4738684
rs7337339
rs2740488
rs11749783
rs4489873
rs78058190
rs7112577
rs117733303
rs10184004
rs12359135
rs1883711
rs6445879
rs17414716
rs73596816
rs139974673
rs1203110
rs4665710
rs2138161
rs144018203
rs187429064
rs6874202
rs1167996
rs10455872
rs632057
rs59347135
rs964184
rs1260326
rs10096633
rs174529
rs58542926
rs73025562
rs35303934
rs4846923
rs76866386
rs157595
rs2954021
rs72836561
rs6689
rs10119
rs116843064
rs183130
rs11000430
rs4775044
rs2070971
rs13240065
rs9687846
rs12458441
rs687339
rs73455626
rs10872311
rs998584
All - MR Egger
All - Inverse variance weighted
-1.0
-0.5
0.0
0.5
1.0
MR effect size for
ation of chylomicrons and extremely large VLDL particles || id:ebi-cfb233-GCST90302169' on 'Breast cancer (Combined Oncoarray;

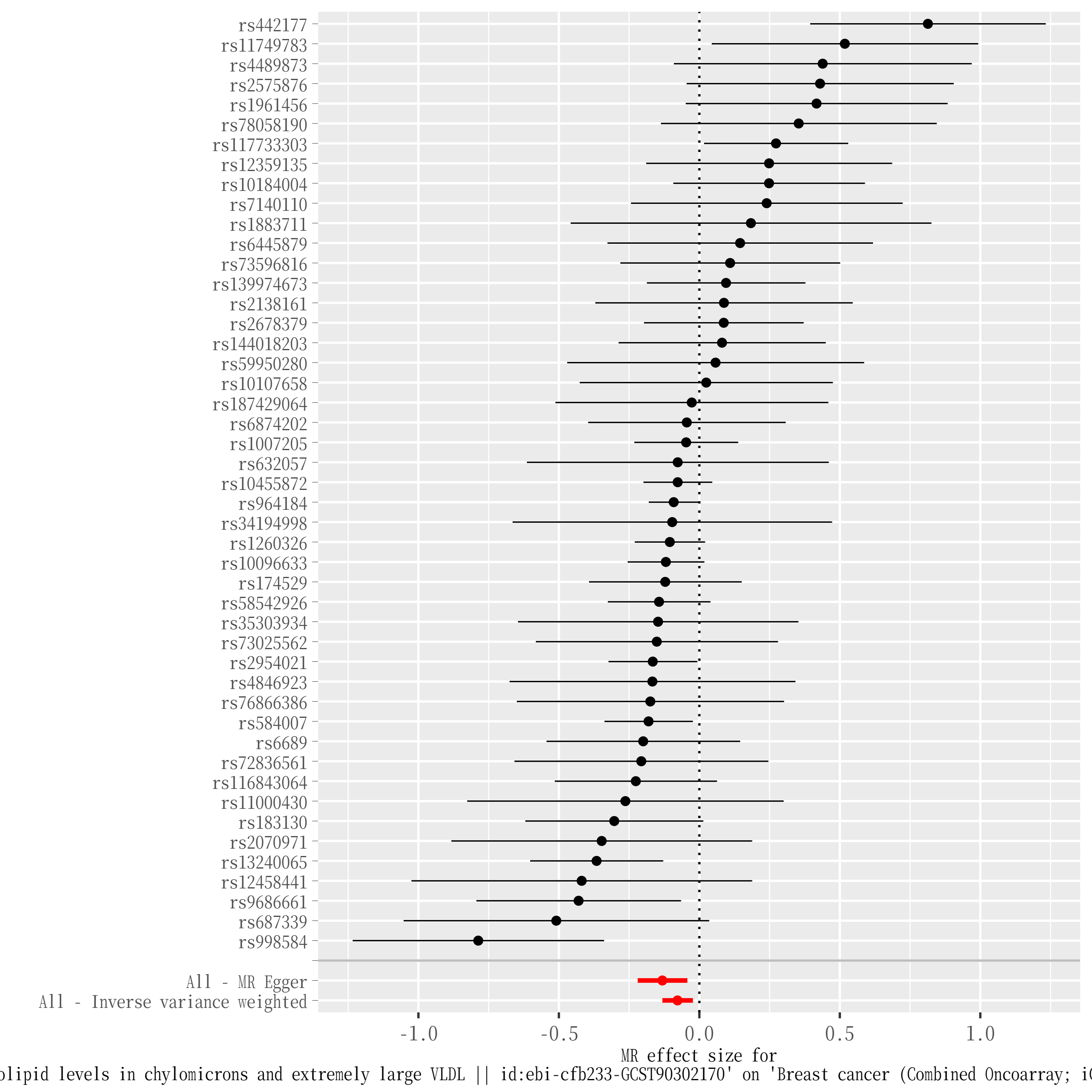
rs442177
rs11749783
rs4489873
rs2575876
rs1961456
rs78058190
rs117733303
rs12359135
rs10184004
rs7140110
rs1883711
rs6445879
rs73596816
rs139974673
rs2138161
rs2678379
rs144018203
rs59950280
rs10107658
rs187429064
rs6874202
rs1007205
rs632057
rs10455872
rs964184
rs34194998
rs1260326
rs10096633
rs174529
rs58542926
rs35303934
rs73025562
rs2954021
rs4846923
rs76866386
rs584007
rs6689
rs72836561
rs116843064
rs11000430
rs183130
rs2070971
rs13240065
rs12458441
rs9686661
rs687339
rs998584
All - MR Egger
All - Inverse variance weighted
-1.0
-0.5
0.0
0.5
1.0
MR effect size for
olipid levels in chylomicrons and extremely large VLDL || id:ebi-cfb233-GCST90302170' on 'Breast cancer (Combined Oncoarray; i

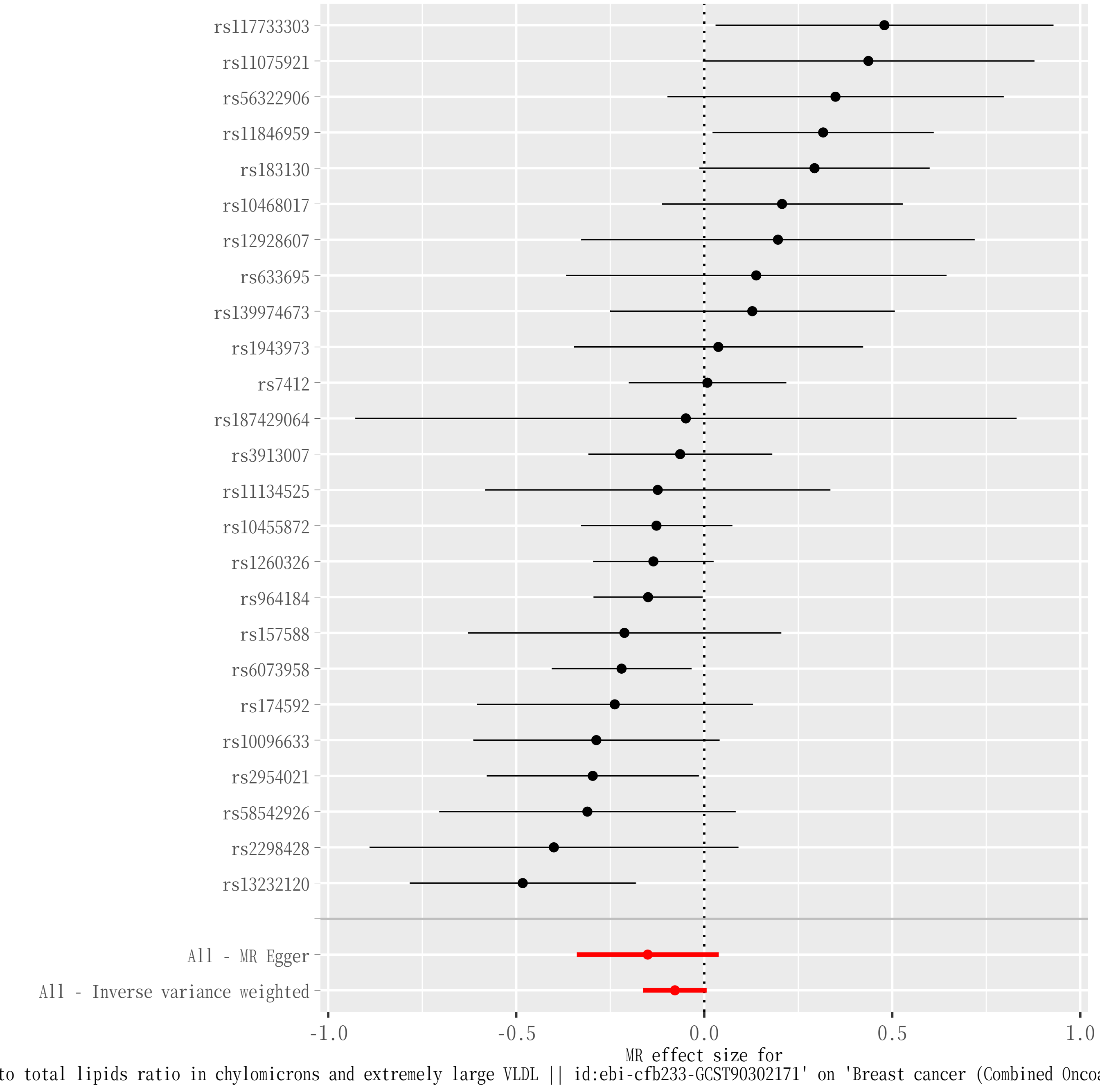

rs117733303
rs11075921
rs56322906
rs11846959
rs183130
rs10468017
rs12928607
rs633695
rs139974673
rs1943973
rs7412
rs187429064
rs3913007
rs11134525
rs10455872
rs1260326
rs964184
rs157588
rs6073958
rs174592
rs10096633
rs2954021
rs58542926
rs2298428
rs13232120
All - MR Egger
All - Inverse variance weighted
-1.0
-0.5
0.0
0.5
1.0
MR effect size for
to total lipids ratio in chylomicrons and extremely large VLDL || id:ebi-cfb233-GCST90302171' on 'Breast cancer (Combined Onco

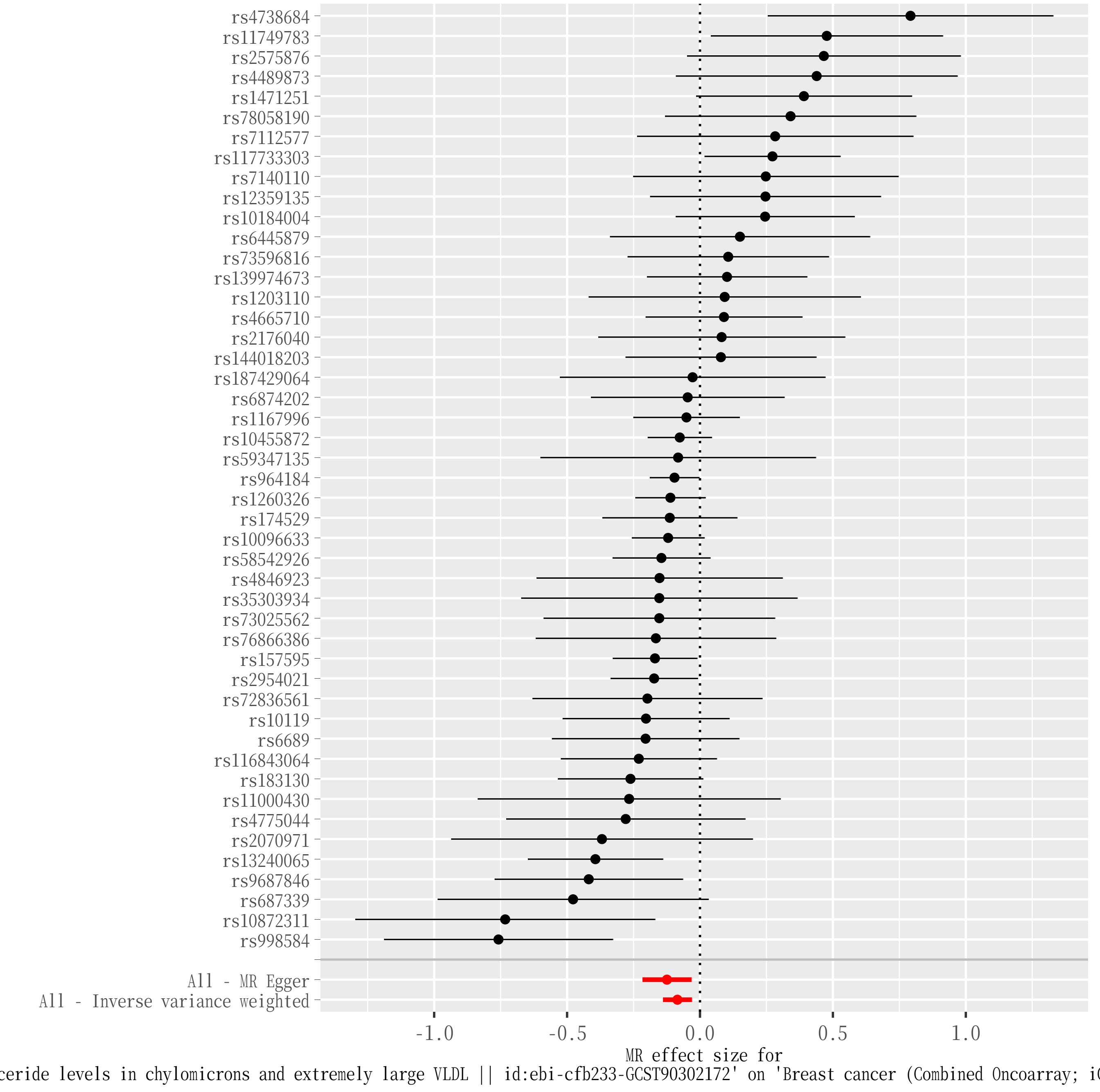
rs4738684
rs11749783
rs2575876
rs4489873
rs1471251
rs78058190
rs7112577
rs117733303
rs7140110
rs12359135
rs10184004
rs6445879
rs73596816
rs139974673
rs1203110
rs4665710
rs2176040
rs144018203
rs187429064
rs6874202
rs1167996
rs10455872
rs59347135
rs964184
rs1260326
rs174529
rs10096633
rs58542926
rs4846923
rs35303934
rs73025562
rs76866386
rs157595
rs2954021
rs72836561
rs10119
rs6689
rs116843064
rs183130
rs11000430
rs4775044
rs2070971
rs13240065
rs9687846
rs687339
rs10872311
rs998584
All - MR Egger
All - Inverse variance weighted
-1.0
-0.5
0.0
0.5
1.0
MR effect size for
ceride levels in chylomicrons and extremely large VLDL || id:ebi-cfb233-GCST90302172' on 'Breast cancer (Combined Oncoarray; iC

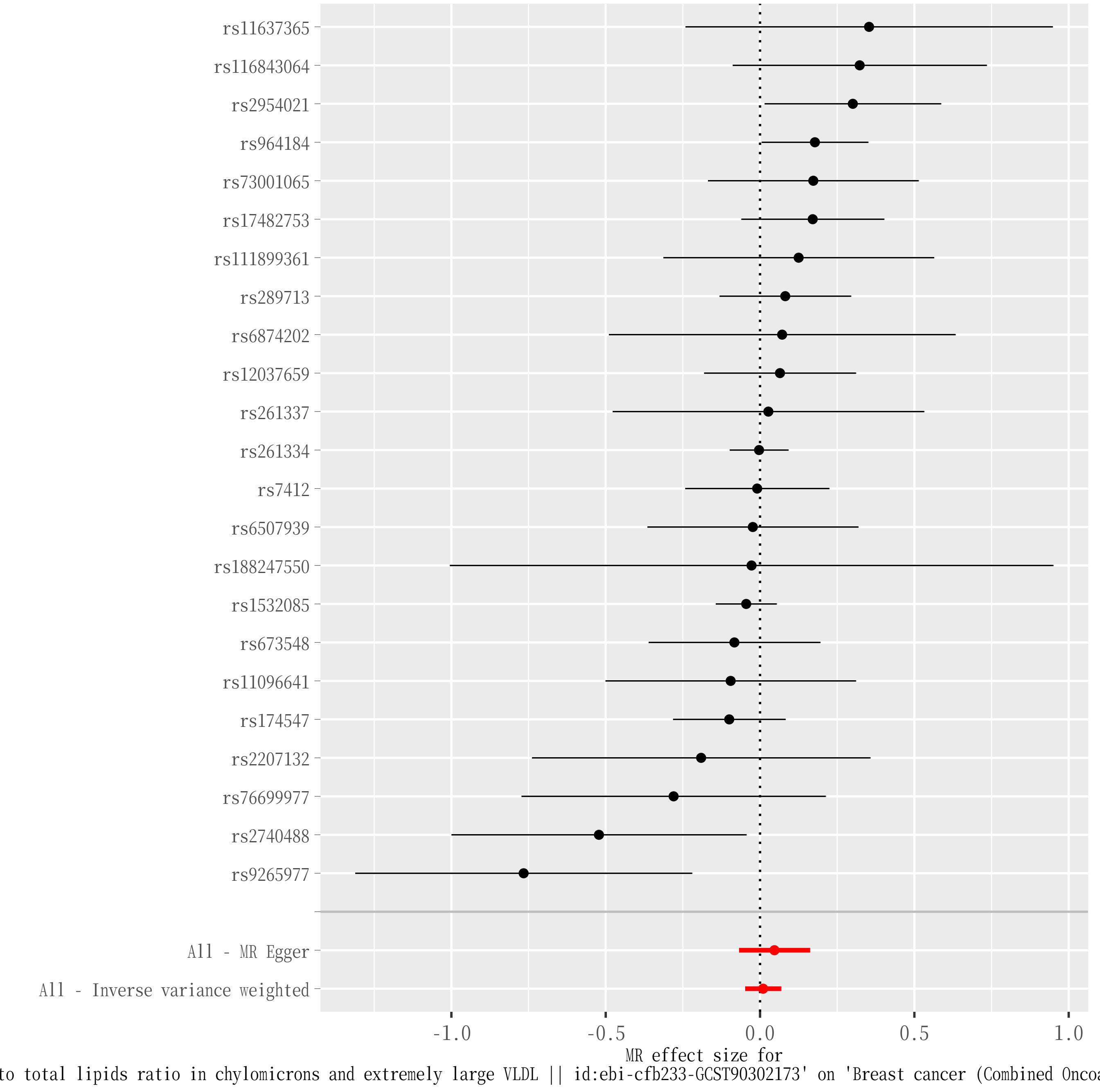
rs11637365
rs116843064
rs2954021
rs964184
rs73001065
rs17482753
rs111899361
rs289713
rs6874202
rs12037659
rs261337
rs261334
rs7412
rs6507939
rs188247550
rs1532085
rs673548
rs11096641
rs174547
rs2207132
rs76699977
rs2740488
rs9265977
All - MR Egger
All - Inverse variance weighted
-1.0
-0.5
0.0
0.5
1.0
MR effect size for
to total lipids ratio in chylomicrons and extremely large VLDL || id:ebi-cfb233-GCST90302173' on 'Breast cancer (Combined Onco
