# Supplementary material for: Assessing the causal relationships between circulating metabolic biomarkers and breast cancer by using mendelian randomization
Source: Front Genet. 2024 Dec 18;15:1448748. doi: 10.3389/fgene.2024.1448748 (PMC11688392; doi:10.3389/fgene.2024.1448748)
Supplement: Supplementary file 4 [file DataSheet4.pdf]

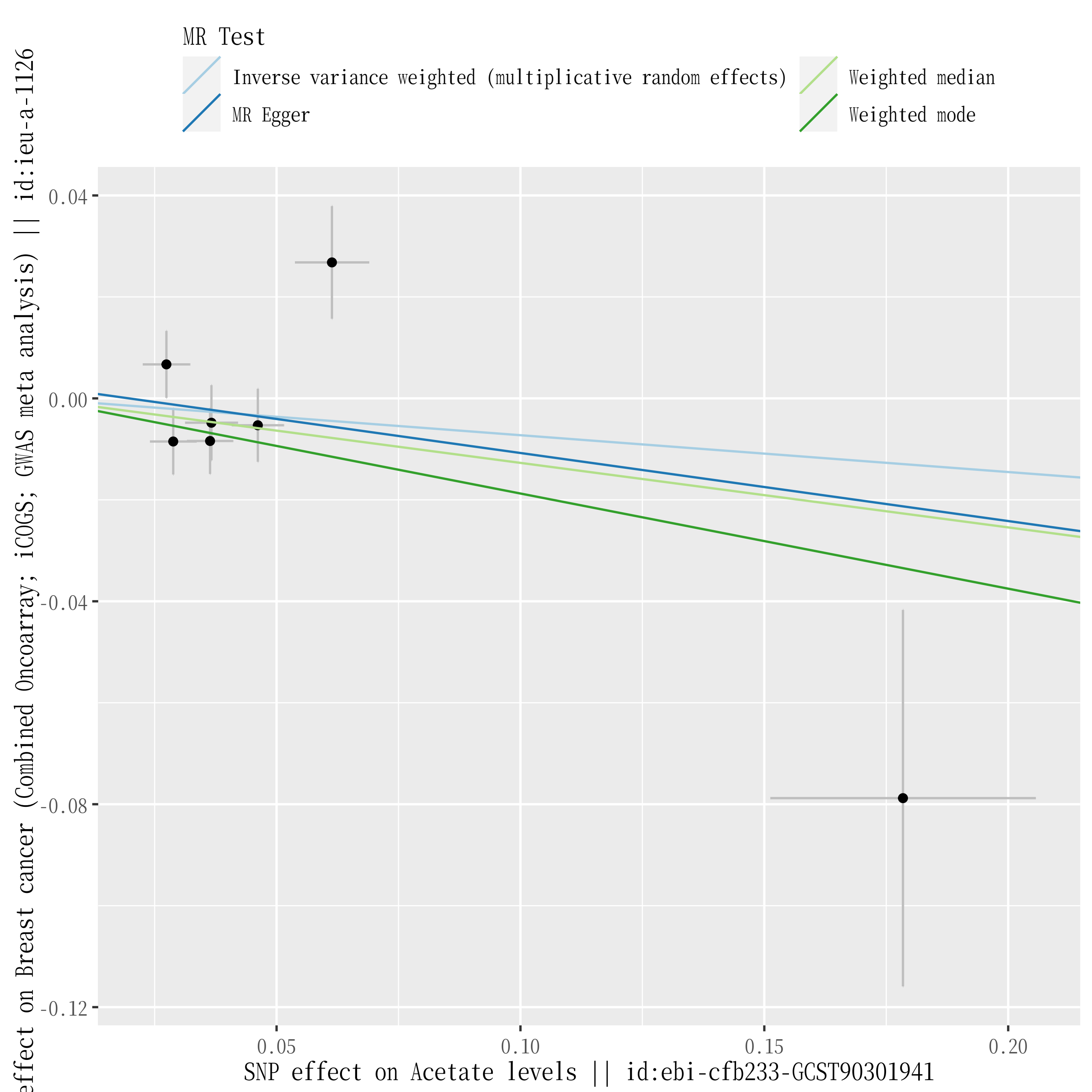
MR Test
Inverse variance weighted (multiplicative random effects)
MR Egger
Weighted median
Weighted mode
0.04
0.00
-0.04
-0.08
-0.12
0.05
0.10
0.15
0.20
SNP effect on Acetate levels || id:ebi-cfb233-GCST90301941
effect on Breast cancer (Combined Oncoarray; iCOGS; GWAS meta analysis) || id:ieu-a-1126

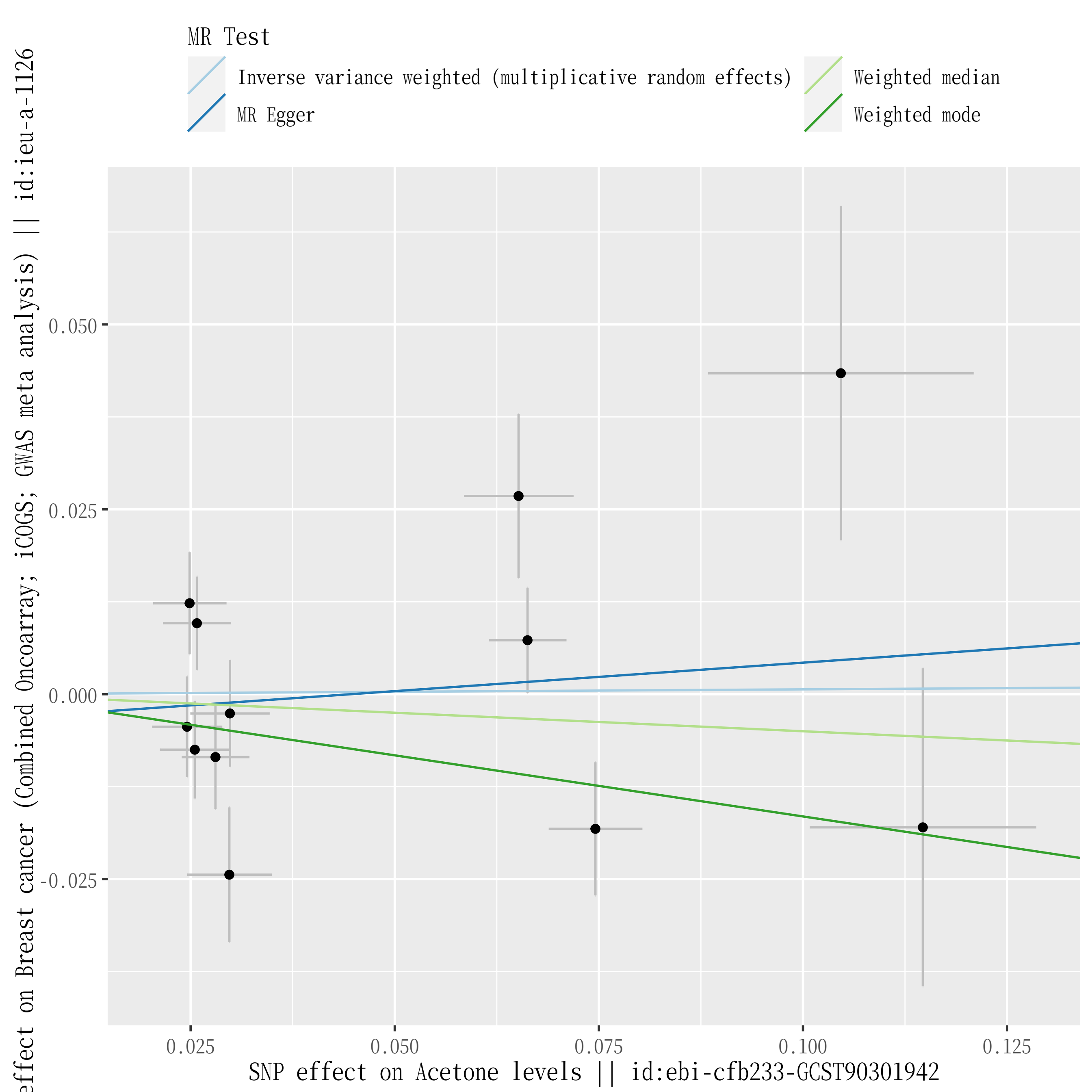
MR Test
Inverse variance weighted (multiplicative random effects)
MR Egger
Weighted median
Weighted mode
effect on Breast cancer (Combined Oncoarray; iCOGS; GWAS meta analysis) || id:ieu-a-1126
0.050
0.025
0.000
-0.025
0.025
0.050
0.075
0.100
0.125
SNP effect on Acetone levels || id:ebi-cfb233-GCST90301942

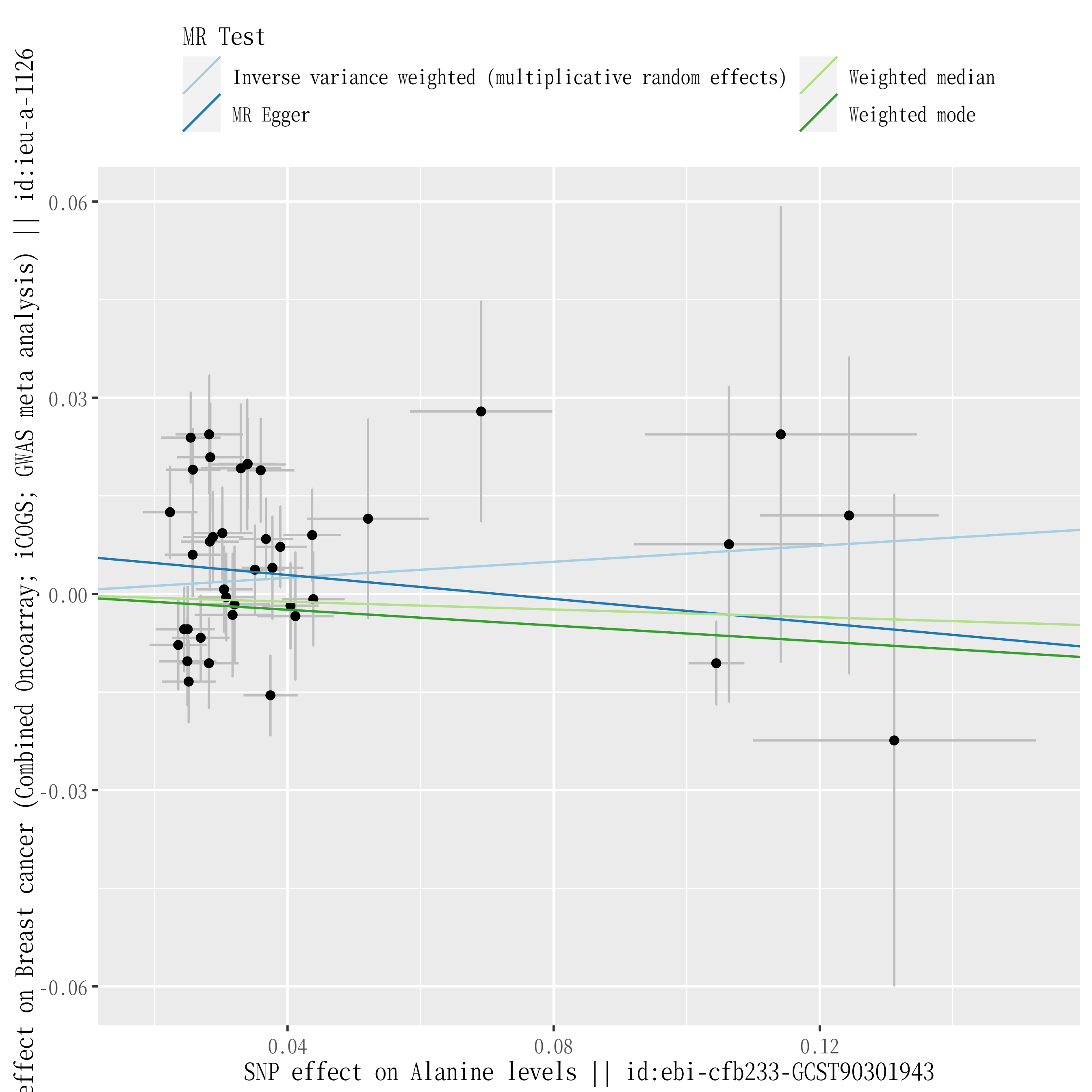

MR Test
Inverse variance weighted (multiplicative random effects)
MR Egger
Weighted median
Weighted mode
0.06
0.03
0.00
-0.03
-0.06
0.04
0.08
0.12
SNP effect on Alanine levels || id:ebi-cfb233-GCST90301943
effect on Breast cancer (Combined Oncoarray; iCOGS; GWAS meta analysis) || id:ieu-a-1126

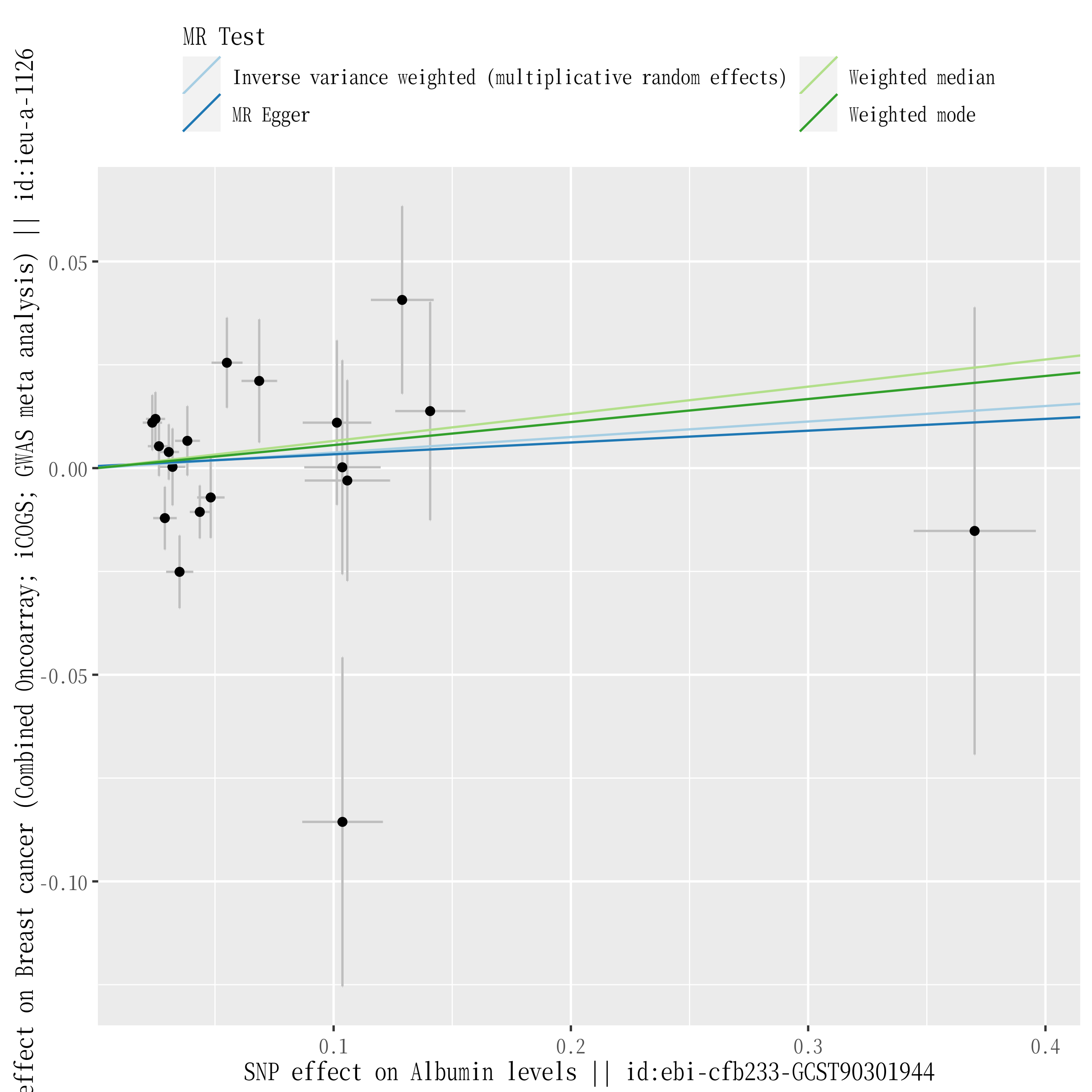

MR Test
Inverse variance weighted (multiplicative random effects)
MR Egger
Weighted median
Weighted mode
0.05
0.00
-0.05
-0.10
0.1
0.2
0.3
0.4
SNP effect on Albumin levels || id:ebi-cfb233-GCST90301944
effect on Breast cancer (Combined Oncoarray; iCOGS; GWAS meta analysis) || id:ieu-a-1126

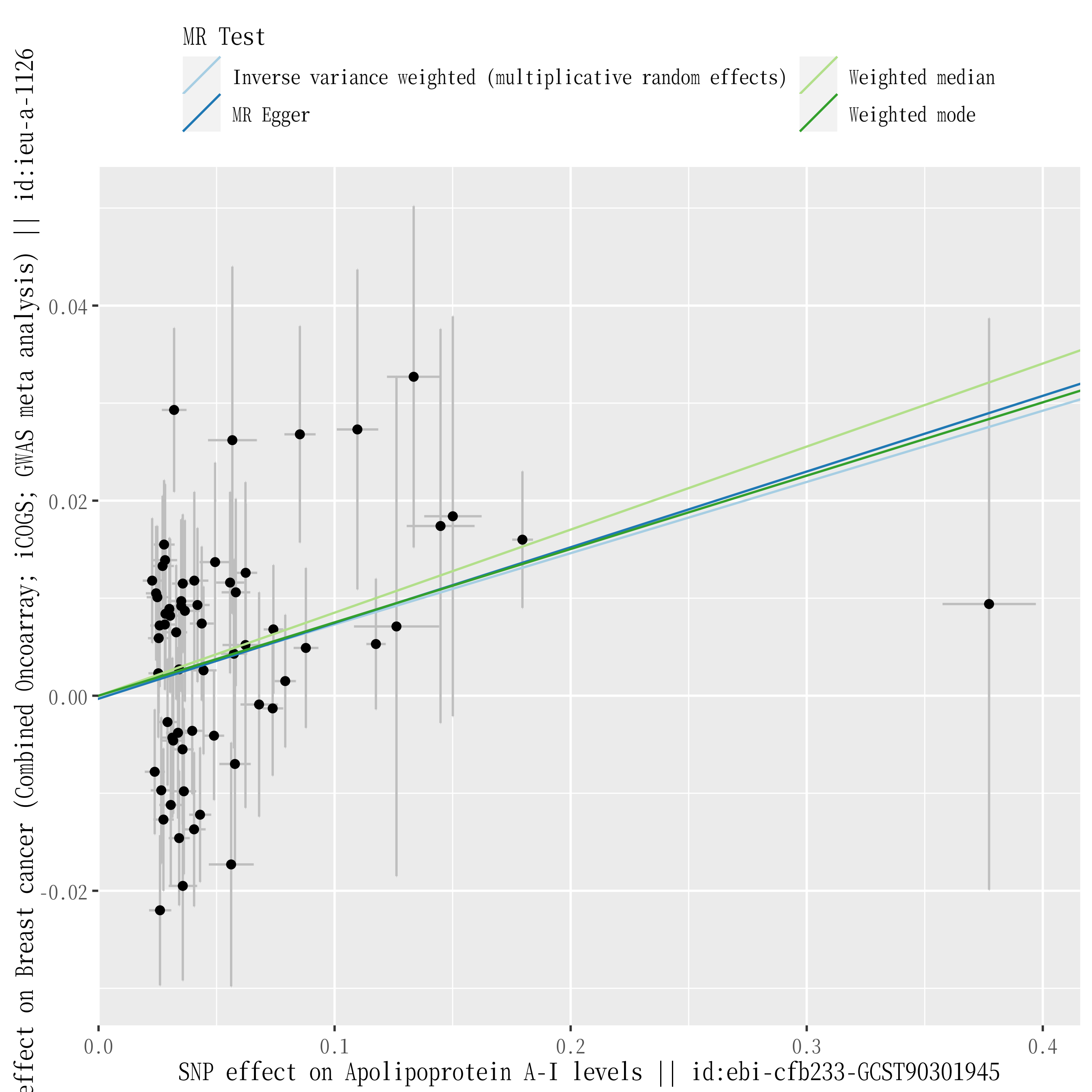

MR Test
Inverse variance weighted (multiplicative random effects)
MR Egger
Weighted median
Weighted mode
0.04
0.02
0.00
-0.02
0.0
0.1
0.2
0.3
0.4
SNP effect on Apolipoprotein A-I levels || id:ebi-cfb233-GCST90301945
effect on Breast cancer (Combined Oncoarray; iCOGS; GWAS meta analysis) || id:ieu-a-1126

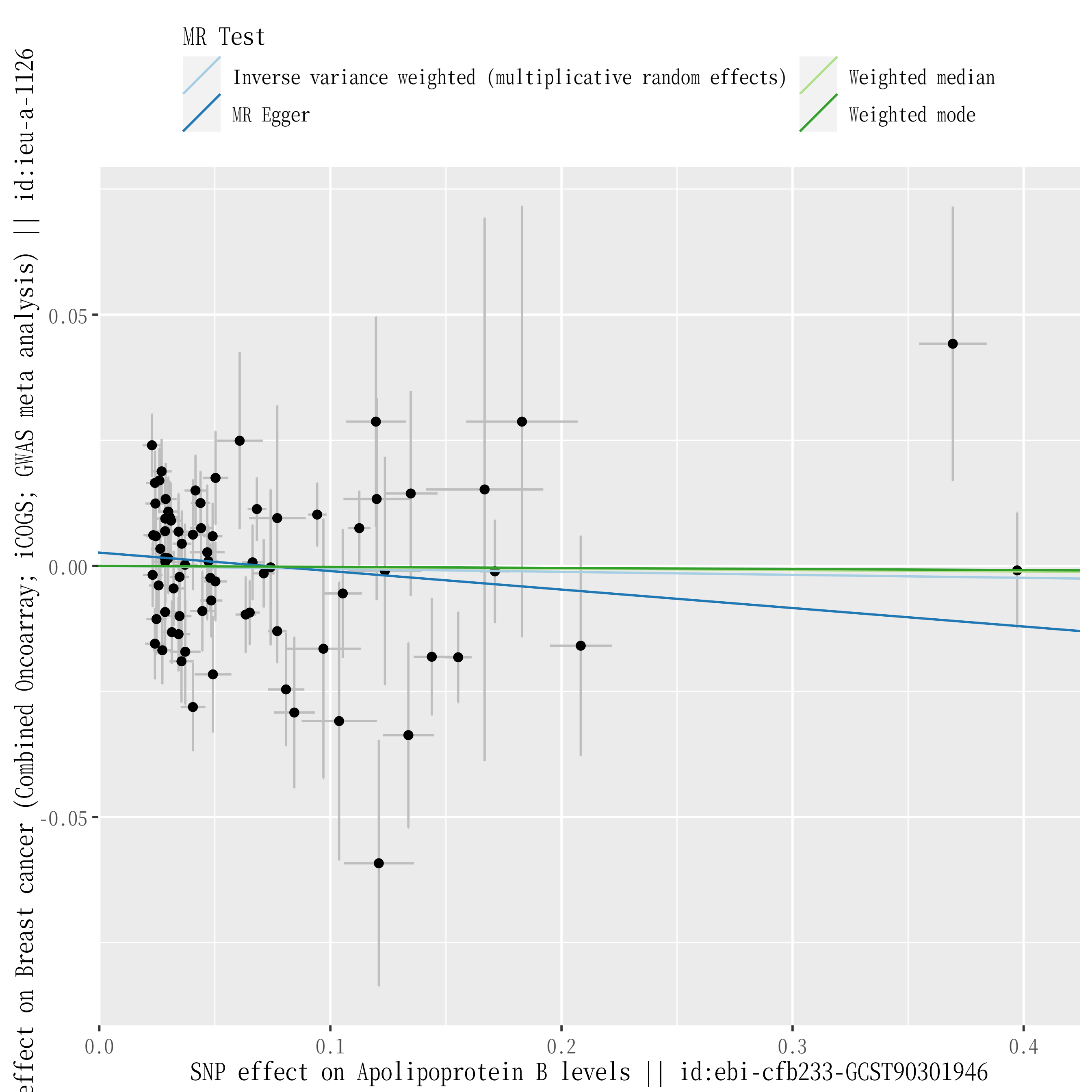
MR Test
Inverse variance weighted (multiplicative random effects)
MR Egger
Weighted median
Weighted mode
0.05
0.00
-0.05
0.0
0.1
0.2
0.3
0.4
SNP effect on Apolipoprotein B levels || id:ebi-cfb233-GCST90301946
effect on Breast cancer (Combined Oncoarray; iCOGS; GWAS meta analysis) || id:ieu-a-1126

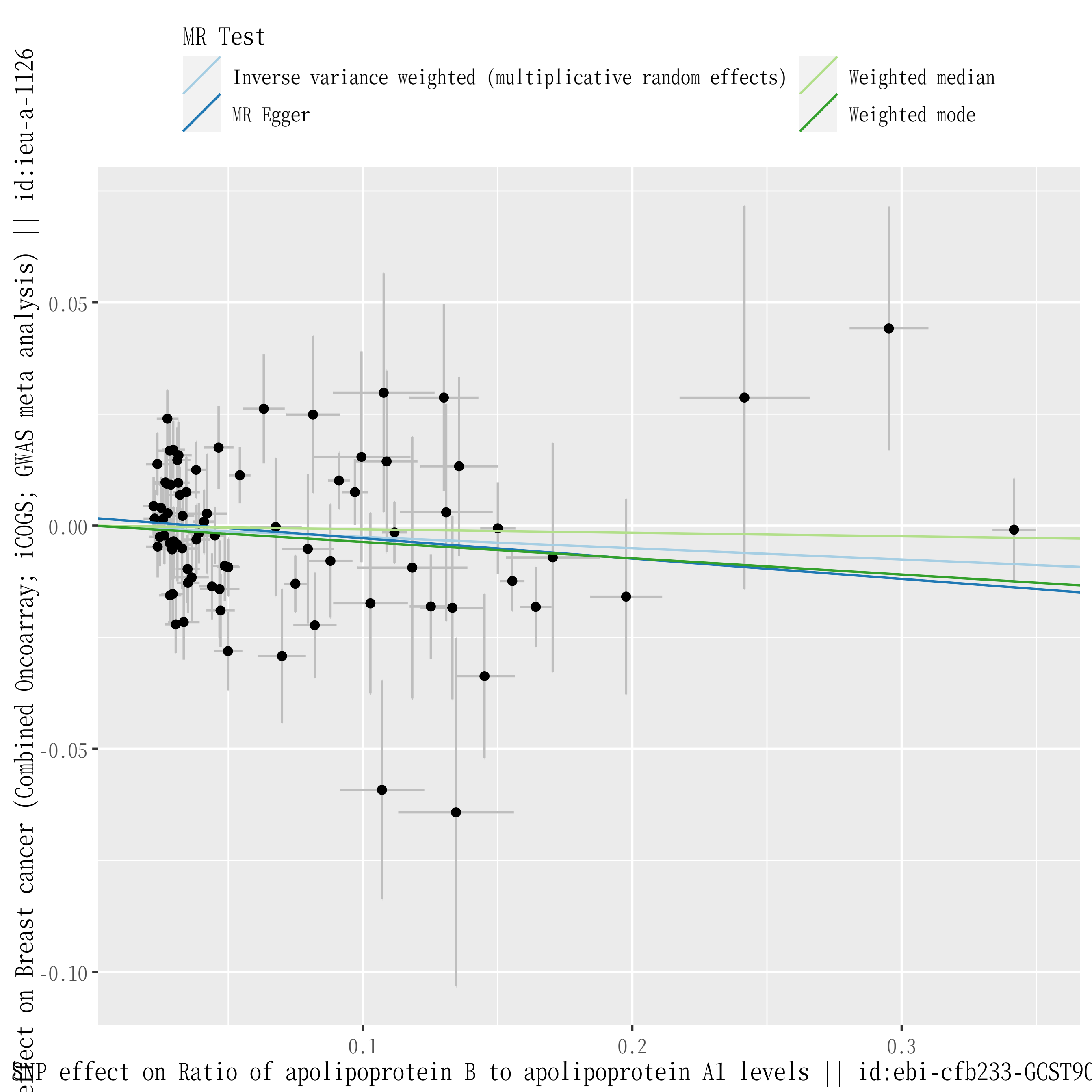

MR Test
Inverse variance weighted (multiplicative random effects)
MR Egger
Weighted median
Weighted mode
0.05
0.00
-0.05
-0.10
0.1
0.2
0.3
SNP effect on Ratio of apolipoprotein B to apolipoprotein A1 levels || id:ebi-cfb233-GCST90
effect on Breast cancer (Combined Oncoarray; iCOGS; GWAS meta analysis) || id:ieu-a-1126

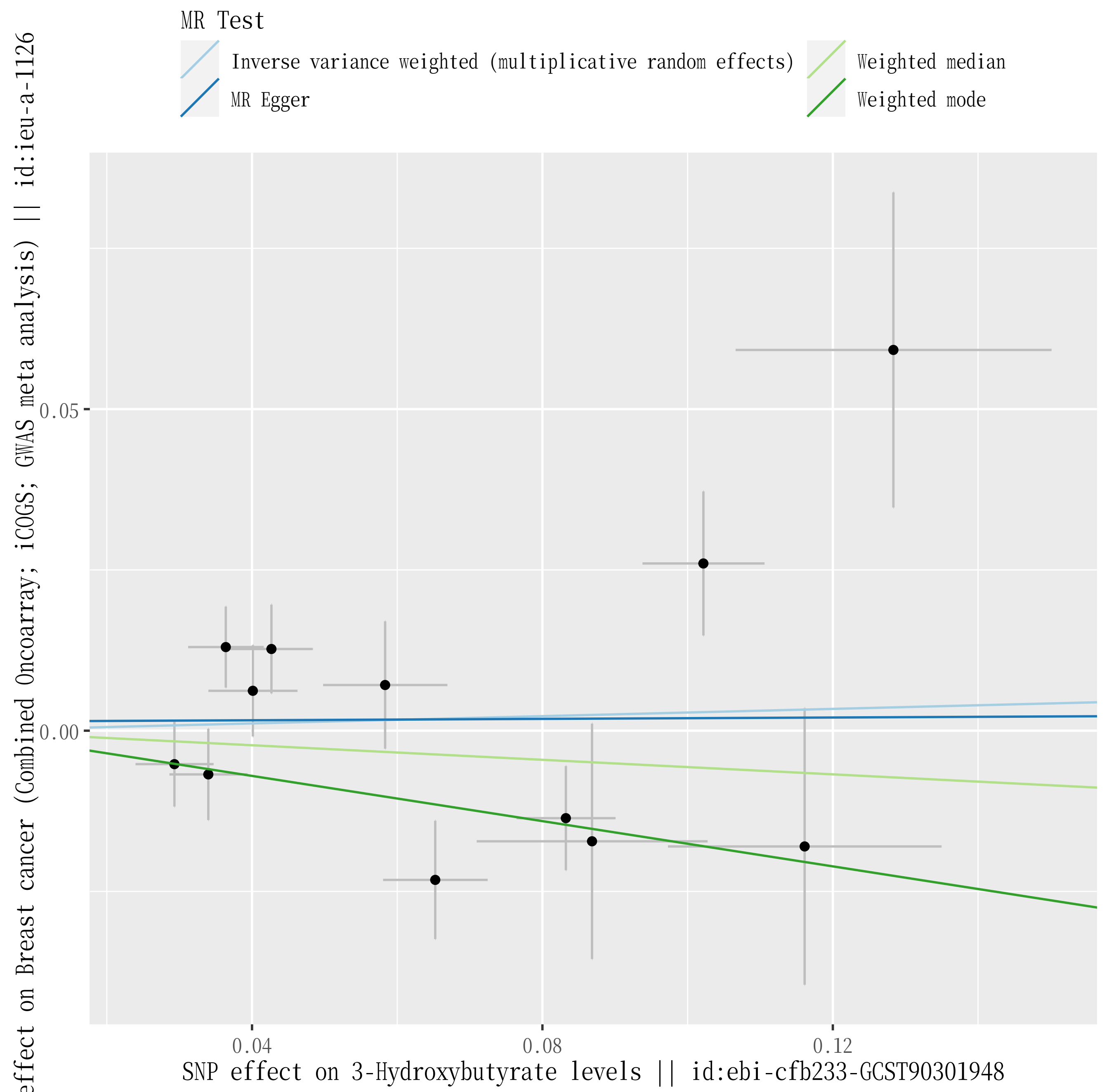

MR Test
Inverse variance weighted (multiplicative random effects)
MR Egger
Weighted median
Weighted mode
0.05
0.00
0.04
0.08
0.12
SNP effect on 3-Hydroxybutyrate levels || id:ebi-cfb233-GCST90301948
effect on Breast cancer (Combined Oncoarray; iCOGS; GWAS meta analysis) || id:ieu-a-1126

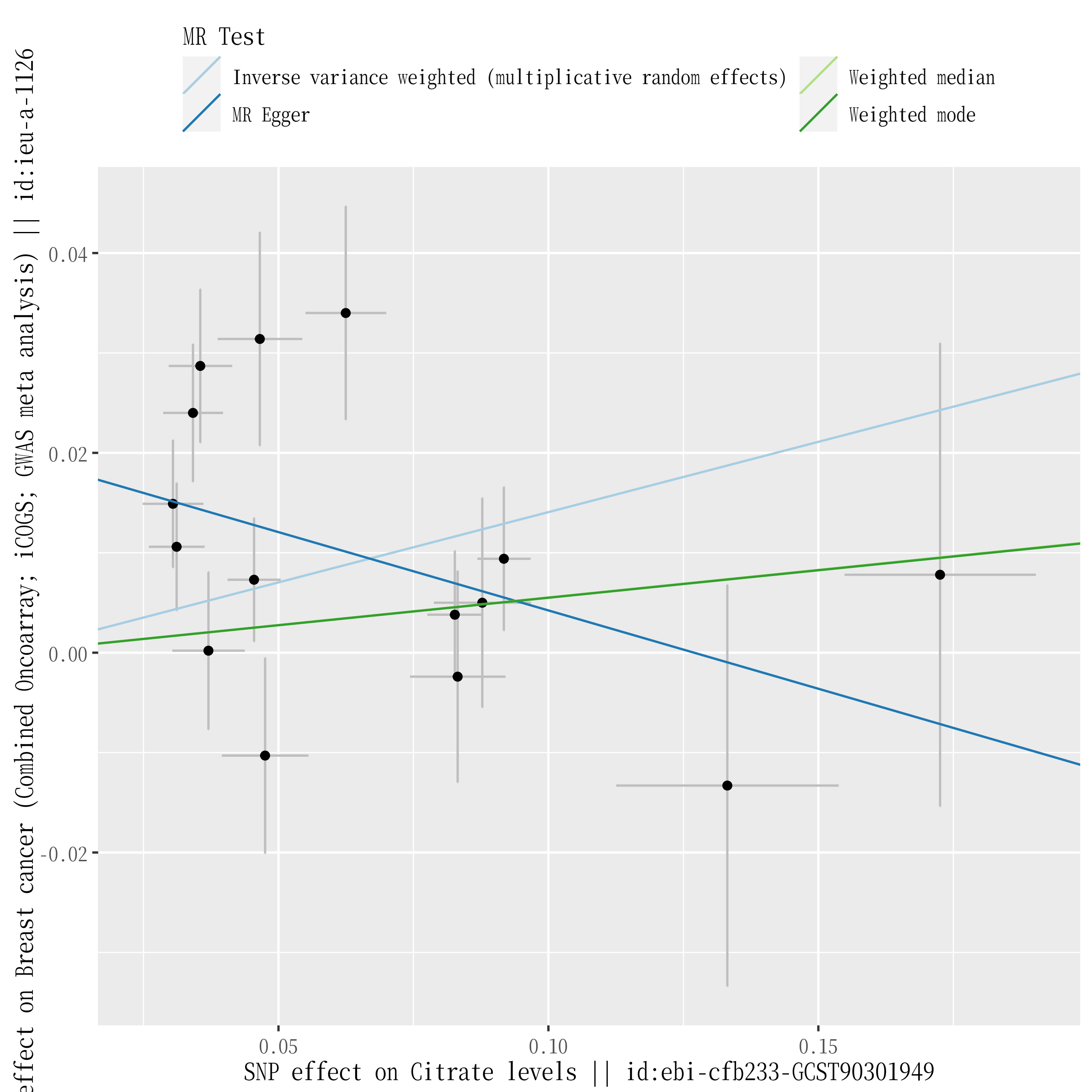
MR Test
Inverse variance weighted (multiplicative random effects)
MR Egger
Weighted median
Weighted mode
0.04
0.02
0.00
-0.02
0.05
0.10
0.15
SNP effect on Citrate levels || id:ebi-cfb233-GCST90301949
effect on Breast cancer (Combined Oncoarray; iCOGS; GWAS meta analysis) || id:ieu-a-1126

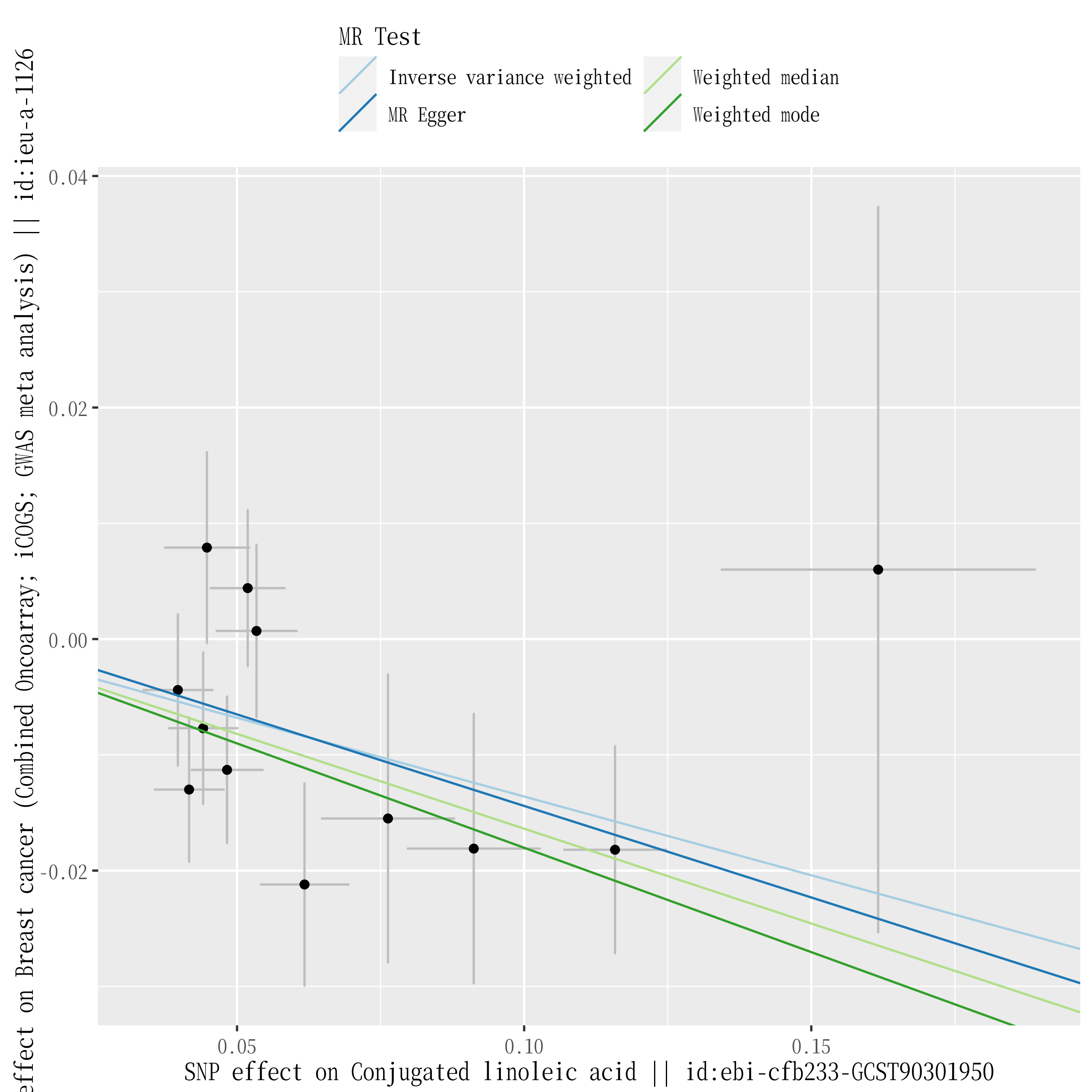
MR Test
Inverse variance weighted
MR Egger
Weighted median
Weighted mode
0.04
0.02
0.00
-0.02
0.05
0.10
0.15
SNP effect on Conjugated linoleic acid || id:ebi-cfb233-GCST90301950
effect on Breast cancer (Combined Oncoarray; iCOGS; GWAS meta analysis) || id:ieu-a-1126

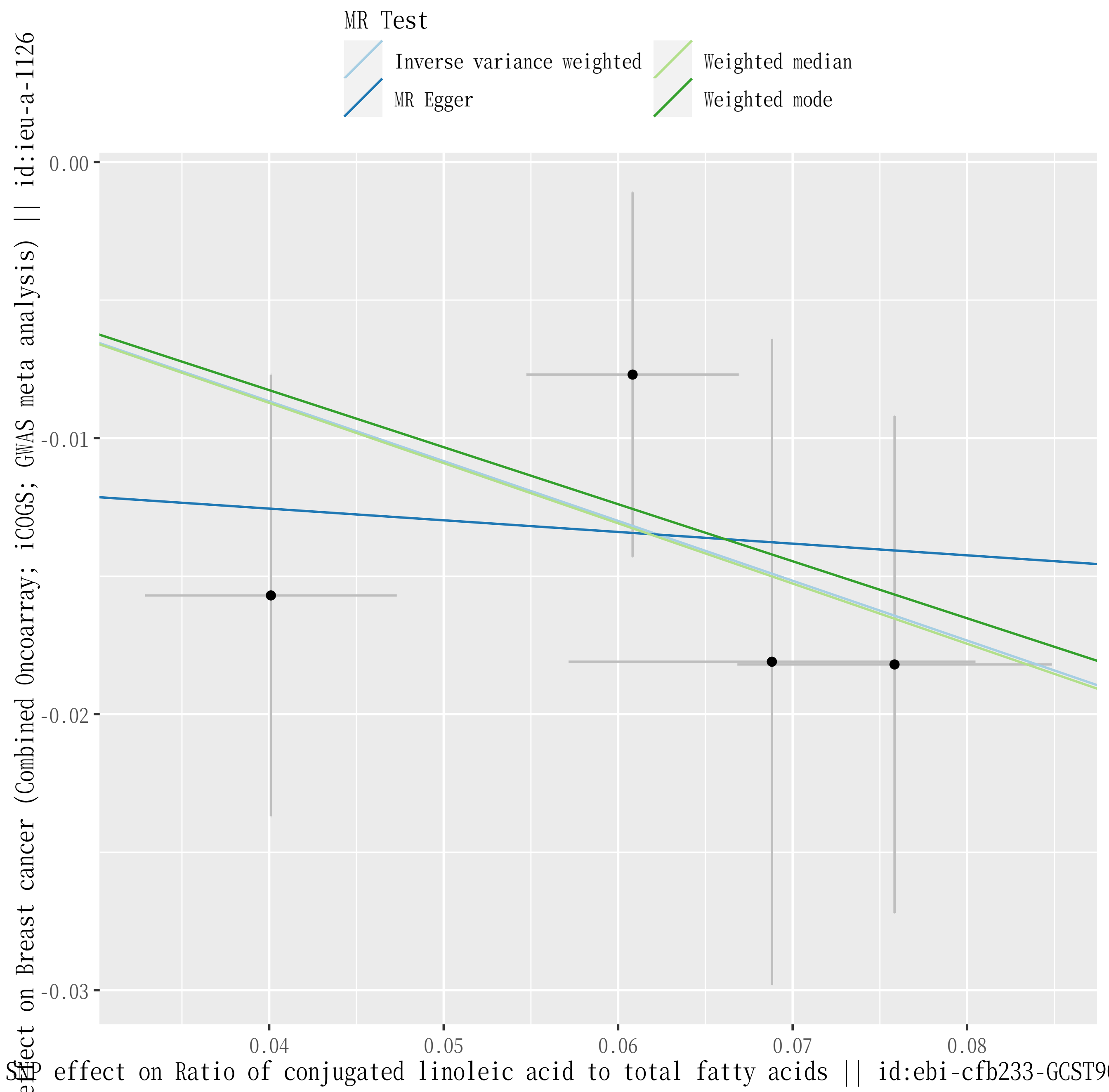
MR Test
Inverse variance weighted
MR Egger
Weighted median
Weighted mode
0.00
-0.01
-0.02
-0.03
0.04
0.05
0.06
0.07
0.08
SNP effect on Ratio of conjugated linoleic acid to total fatty acids || id:ebi-cfb233-GCST90
effect on Breast cancer (Combined Oncoarray; iCOGS; GWAS meta analysis) || id:ieu-a-1126

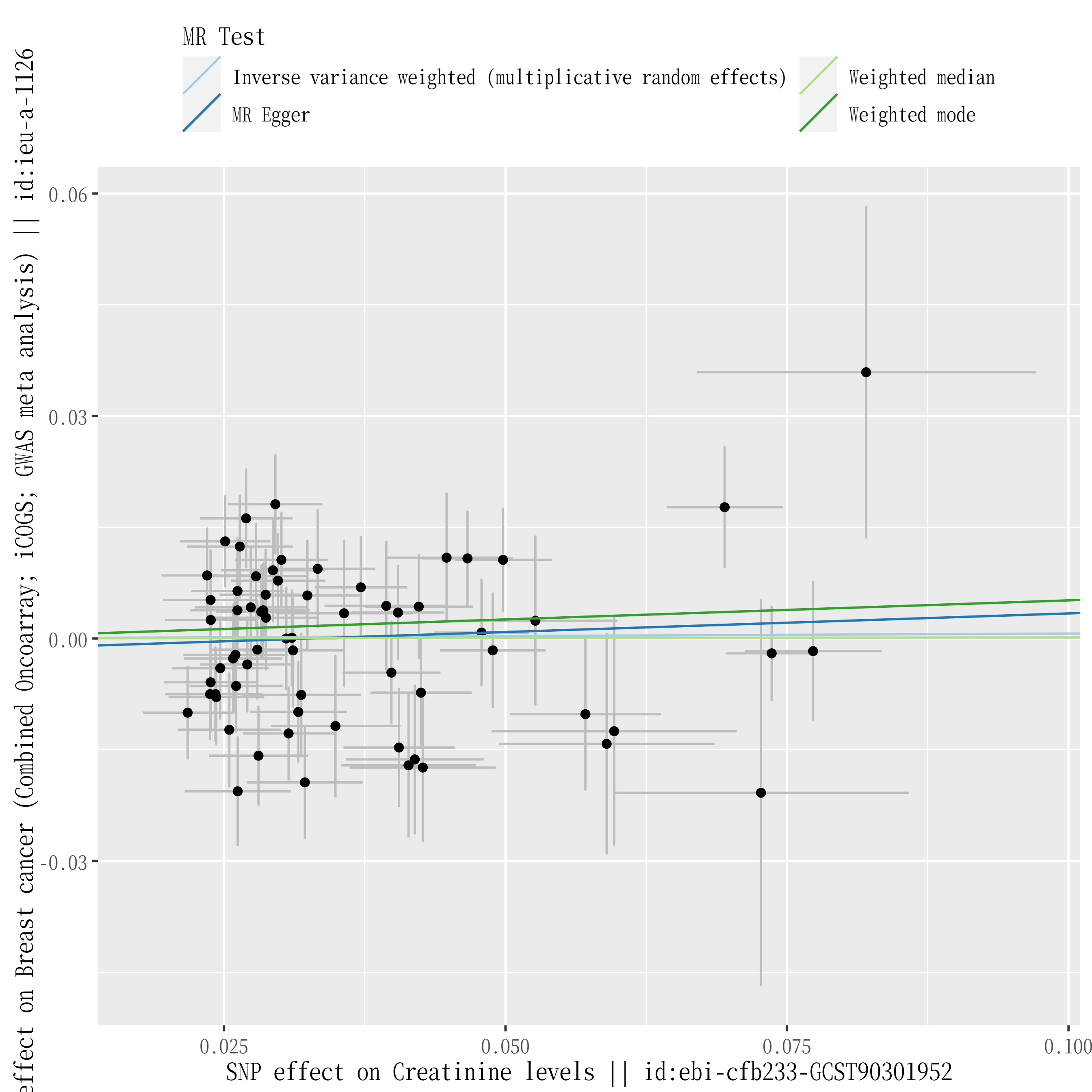

MR Test
Inverse variance weighted (multiplicative random effects)
MR Egger
Weighted median
Weighted mode
0.06
0.03
0.00
-0.03
0.025
0.050
0.075
0.100
SNP effect on Creatinine levels || id:ebi-cfb233-GCST90301952
effect on Breast cancer (Combined Oncoarray; iCOGS; GWAS meta analysis) || id:ieu-a-1126

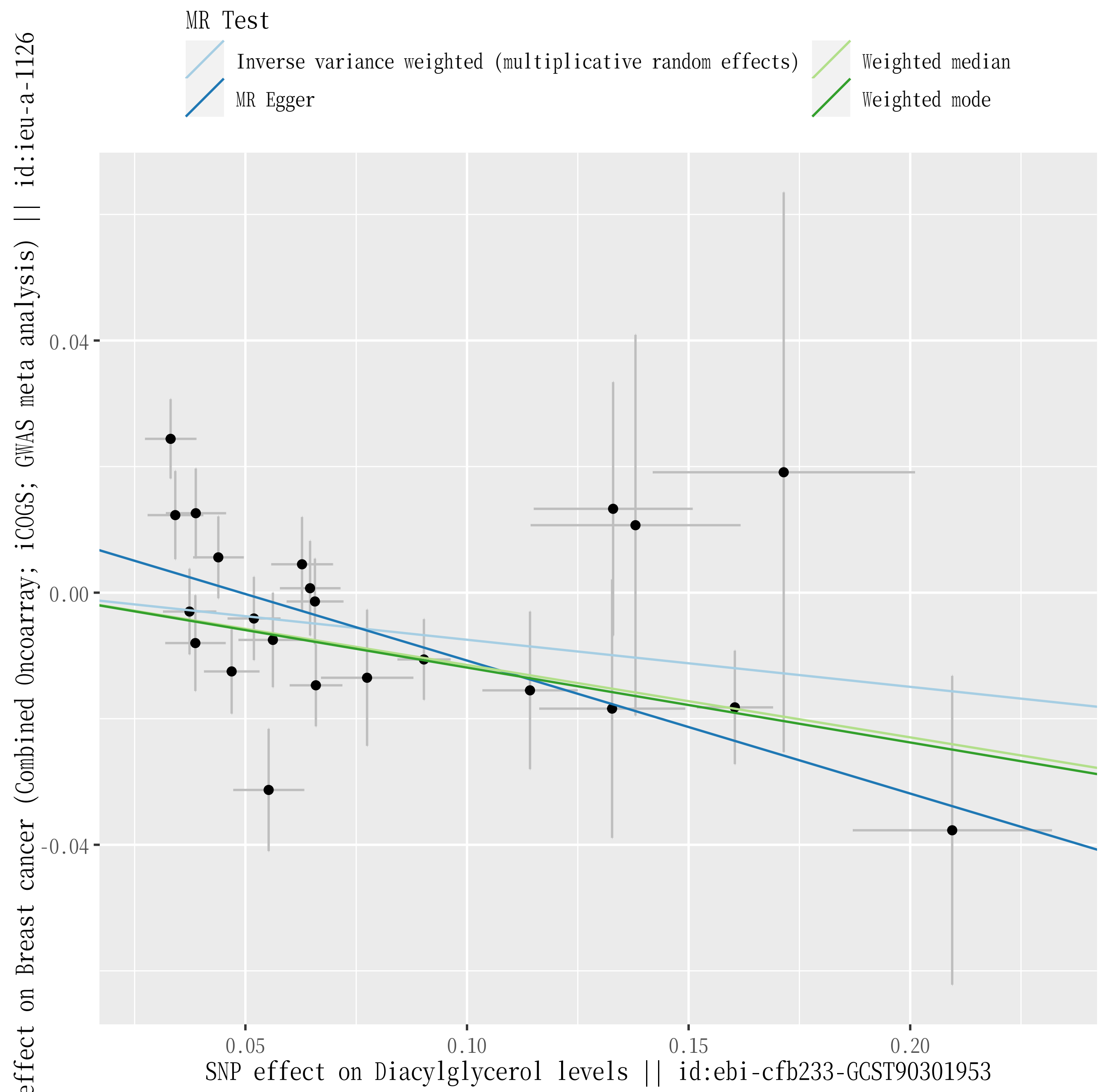

MR Test
Inverse variance weighted (multiplicative random effects)
MR Egger
Weighted median
Weighted mode
0.04
0.00
-0.04
0.05
0.10
0.15
0.20
SNP effect on Diacylglycerol levels || id:ebi-cfb233-GCST90301953
effect on Breast cancer (Combined Oncoarray; iCOGS; GWAS meta analysis) || id:ieu-a-1126

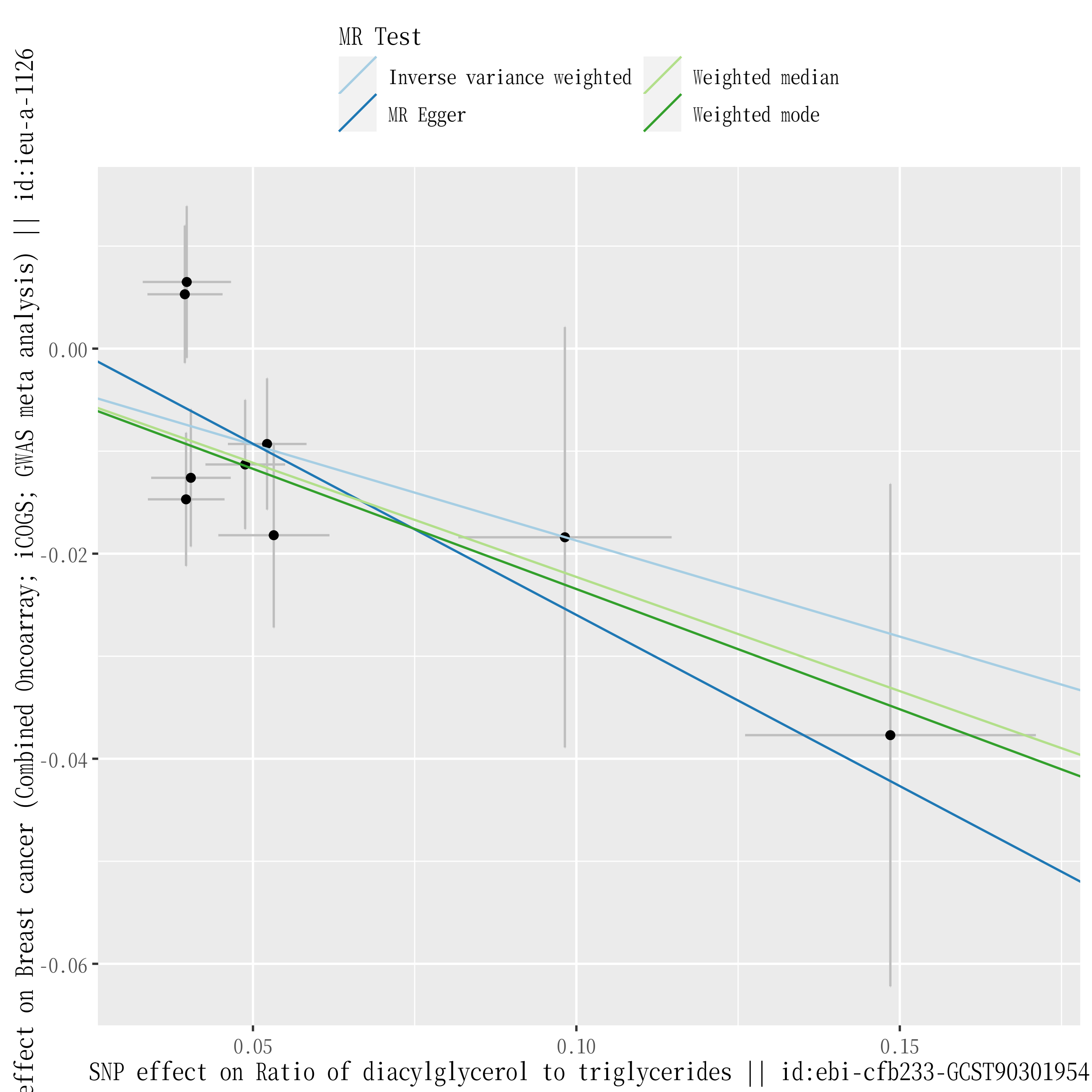

MR Test
Inverse variance weighted
MR Egger
Weighted median
Weighted mode
0.00
-0.02
-0.04
-0.06
0.05
0.10
0.15
SNP effect on Ratio of diacylglycerol to triglycerides || id:ebi-cfb233-GCST90301954
effect on Breast cancer (Combined Oncoarray; iCOGS; GWAS meta analysis) || id:ieu-a-1126

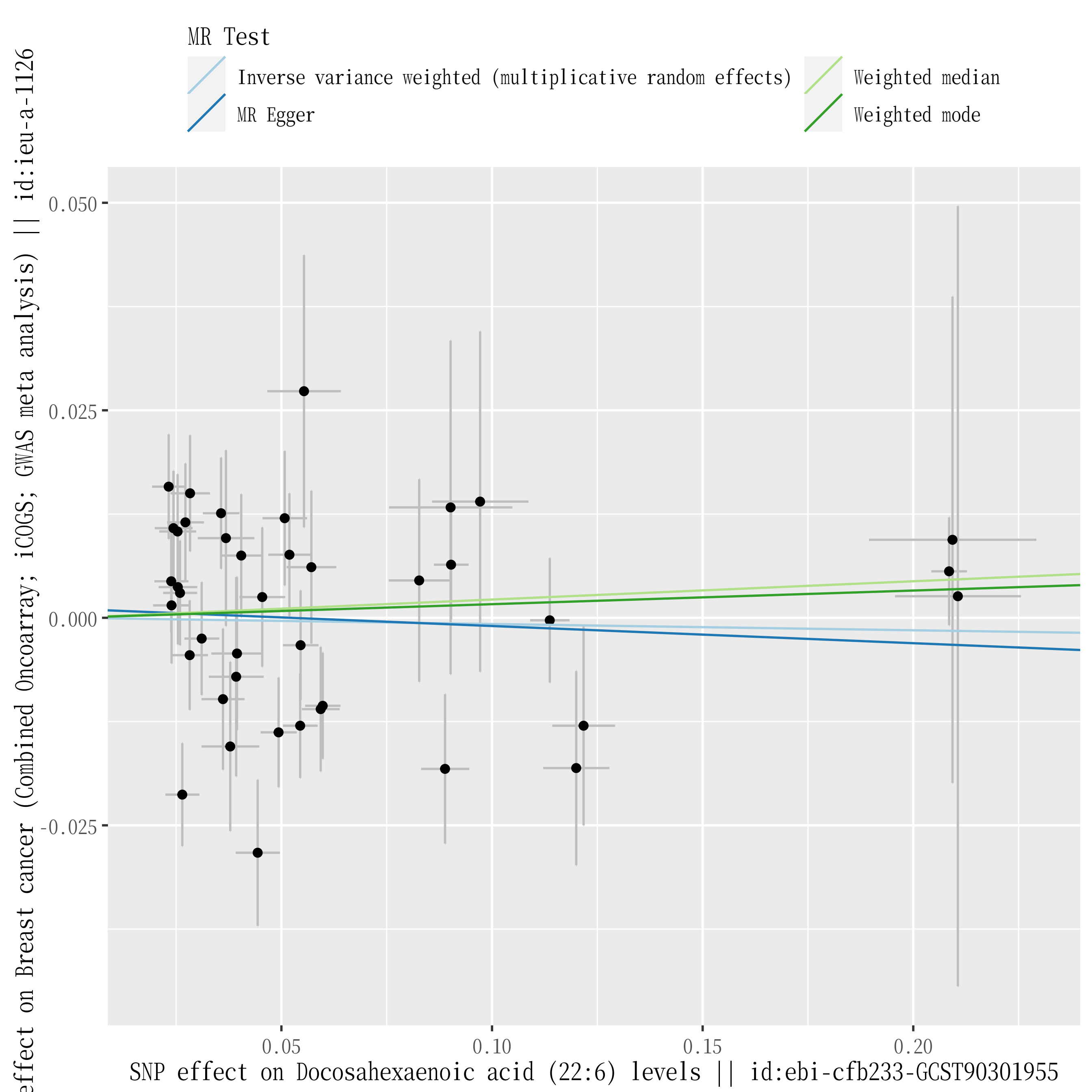
MR Test
Inverse variance weighted (multiplicative random effects)
MR Egger
Weighted median
Weighted mode
0.050
0.025
0.000
-0.025
0.05
0.10
0.15
0.20
SNP effect on Docosahexaenoic acid (22:6) levels || id:ebi-cfb233-GCST90301955
effect on Breast cancer (Combined Oncoarray; iCOGS; GWAS meta analysis) || id:ieu-a-1126

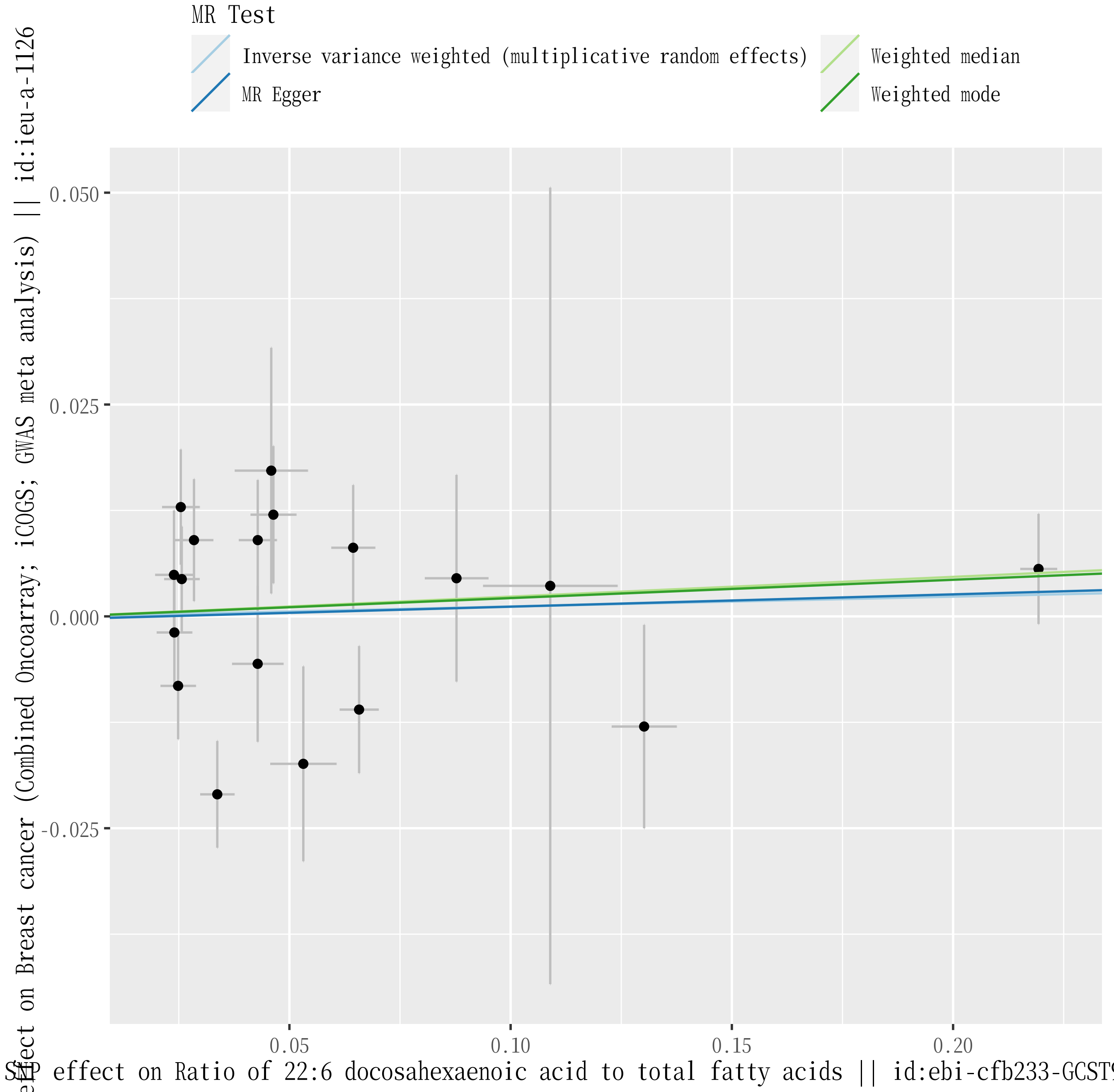

MR Test
Inverse variance weighted (multiplicative random effects)
MR Egger
Weighted median
Weighted mode
0.050
0.025
0.000
-0.025
0.05
0.10
0.15
0.20
SNP effect on Ratio of 22:6 docosahexaenoic acid to total fatty acids || id:ebi-cfb233-GCST
effect on Breast cancer (Combined Oncoarray; iCOGS; GWAS meta analysis) || id:ieu-a-1126

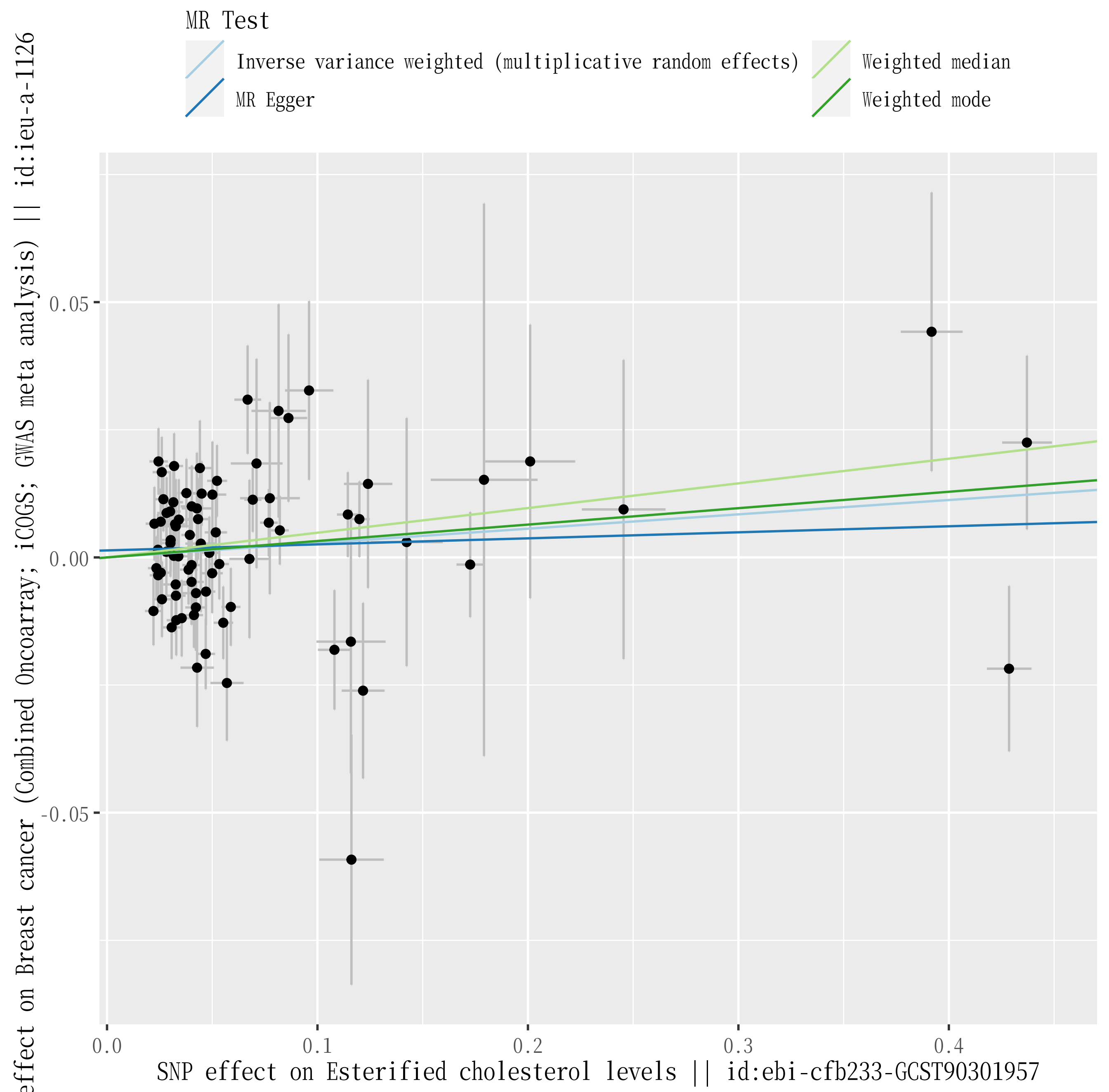
MR Test
Inverse variance weighted (multiplicative random effects)
MR Egger
Weighted median
Weighted mode
0.05
0.00
-0.05
0.0
0.1
0.2
0.3
0.4
SNP effect on Esterified cholesterol levels || id:ebi-cfb233-GCST90301957
effect on Breast cancer (Combined Oncoarray; iCOGS; GWAS meta analysis) || id:ieu-a-1126

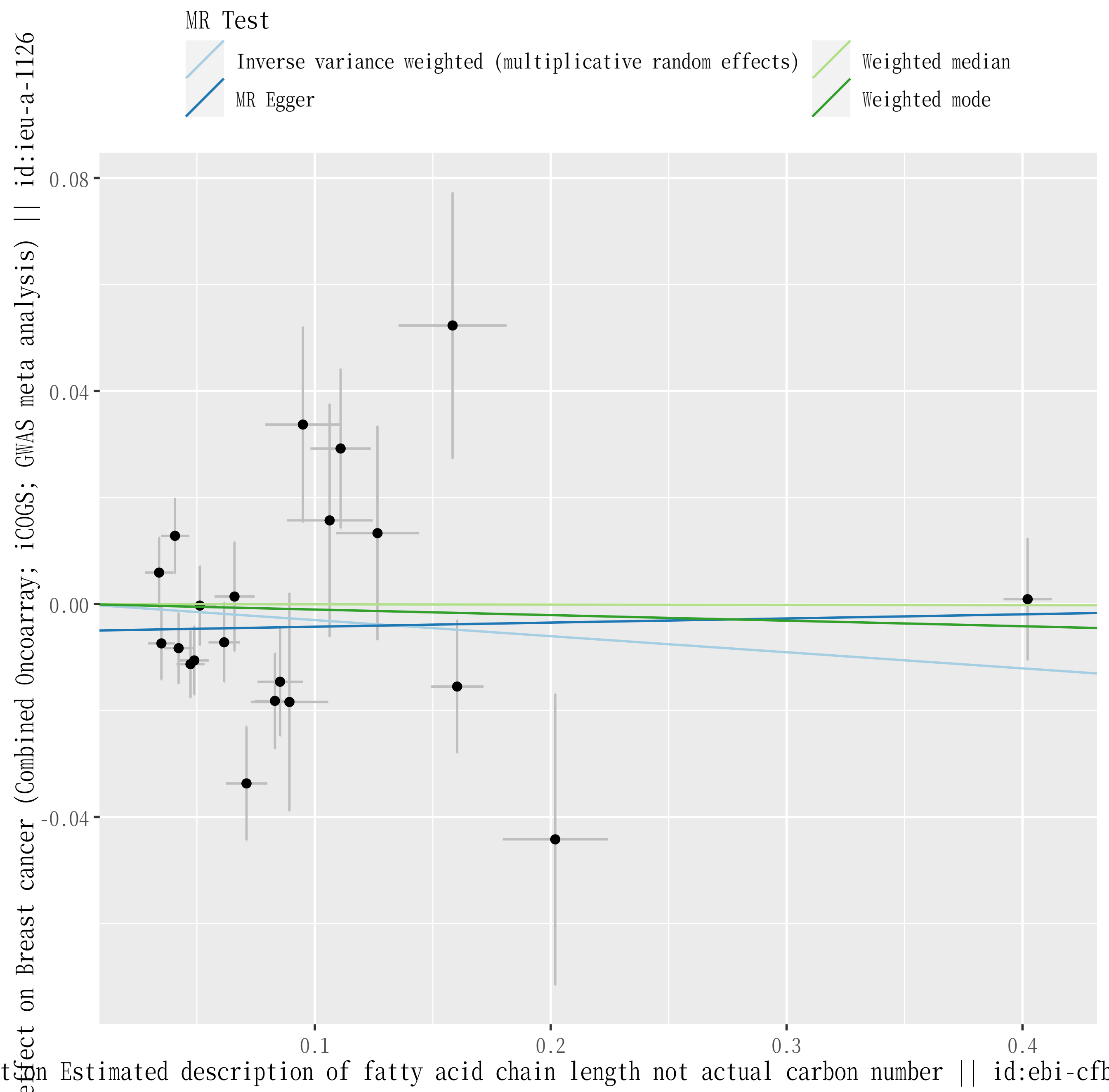

MR Test
Inverse variance weighted (multiplicative random effects)
MR Egger
Weighted median
Weighted mode
0.08
0.04
0.00
-0.04
0.1
0.2
0.3
0.4
effect on Breast cancer (Combined Oncoarray; iCOGS; GWAS meta analysis) || id:ieu-a-1126
t effect on Estimated description of fatty acid chain length not actual carbon number || id:ebi-cfb

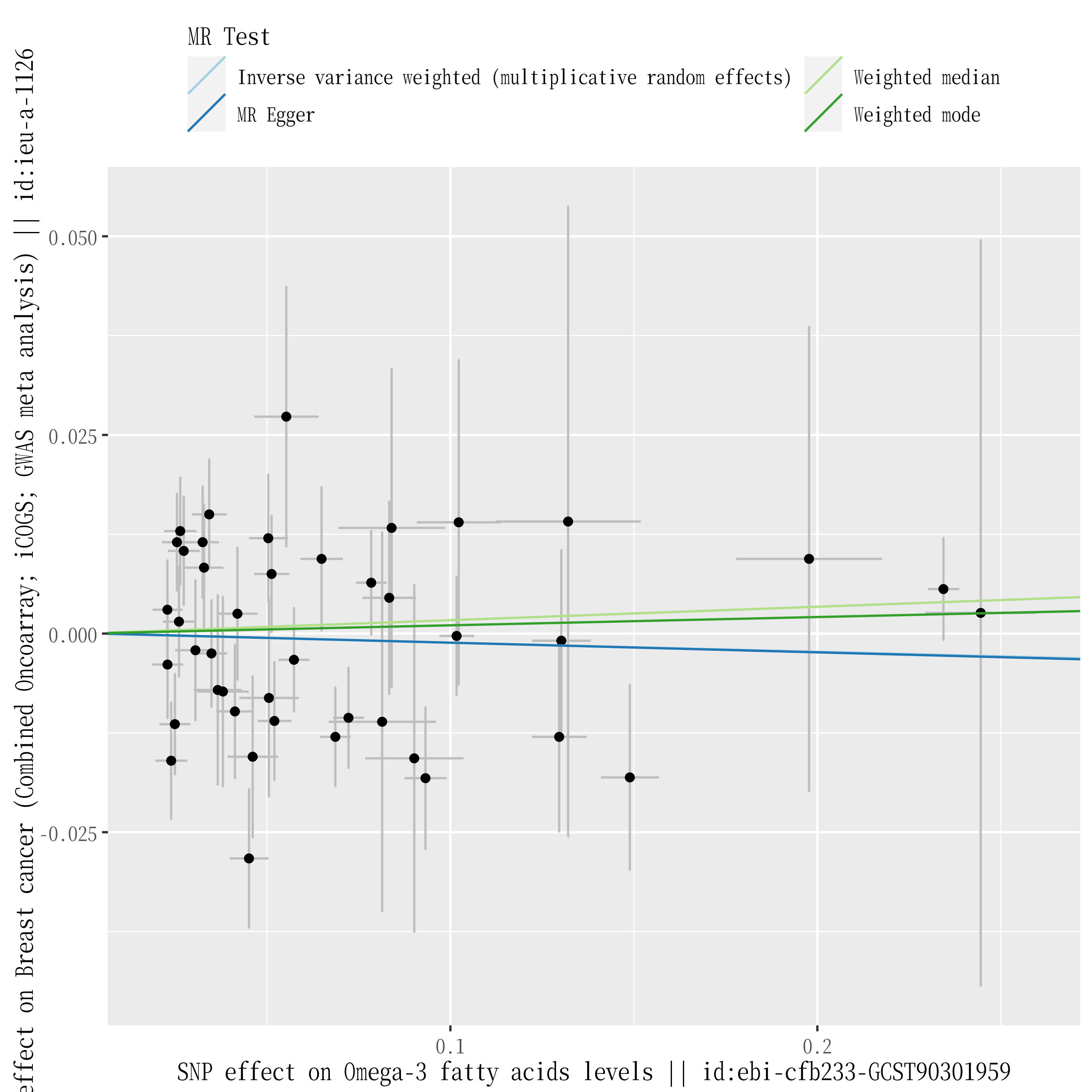
MR Test
Inverse variance weighted (multiplicative random effects)
MR Egger
Weighted median
Weighted mode
SNP effect on Breast cancer (Combined Oncoarray; iCOGS; GWAS meta analysis) || id:ieu-a-1126
0.050
0.025
0.000
-0.025
0.1
0.2
SNP effect on Omega-3 fatty acids levels || id:ebi-cfb233-GCST90301959

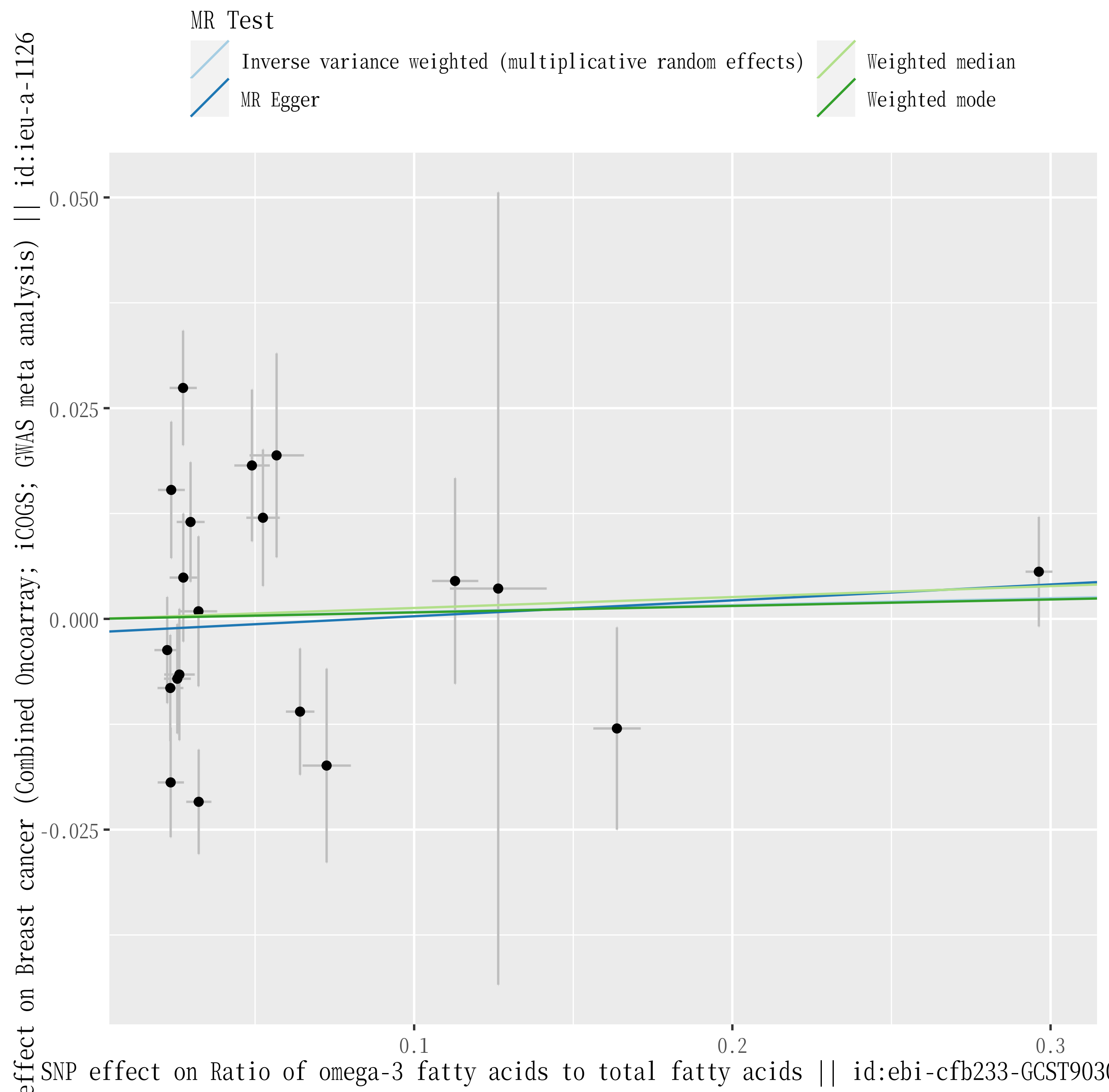

MR Test
Inverse variance weighted (multiplicative random effects)
MR Egger
Weighted median
Weighted mode
0.050
0.025
0.000
-0.025
0.1
0.2
0.3
SNP effect on Breast cancer (Combined Oncoarray; iCOGS; GWAS meta analysis) || id:ieu-a-1126
SNP effect on Ratio of omega-3 fatty acids to total fatty acids || id:ebi-cfb233-GCST903

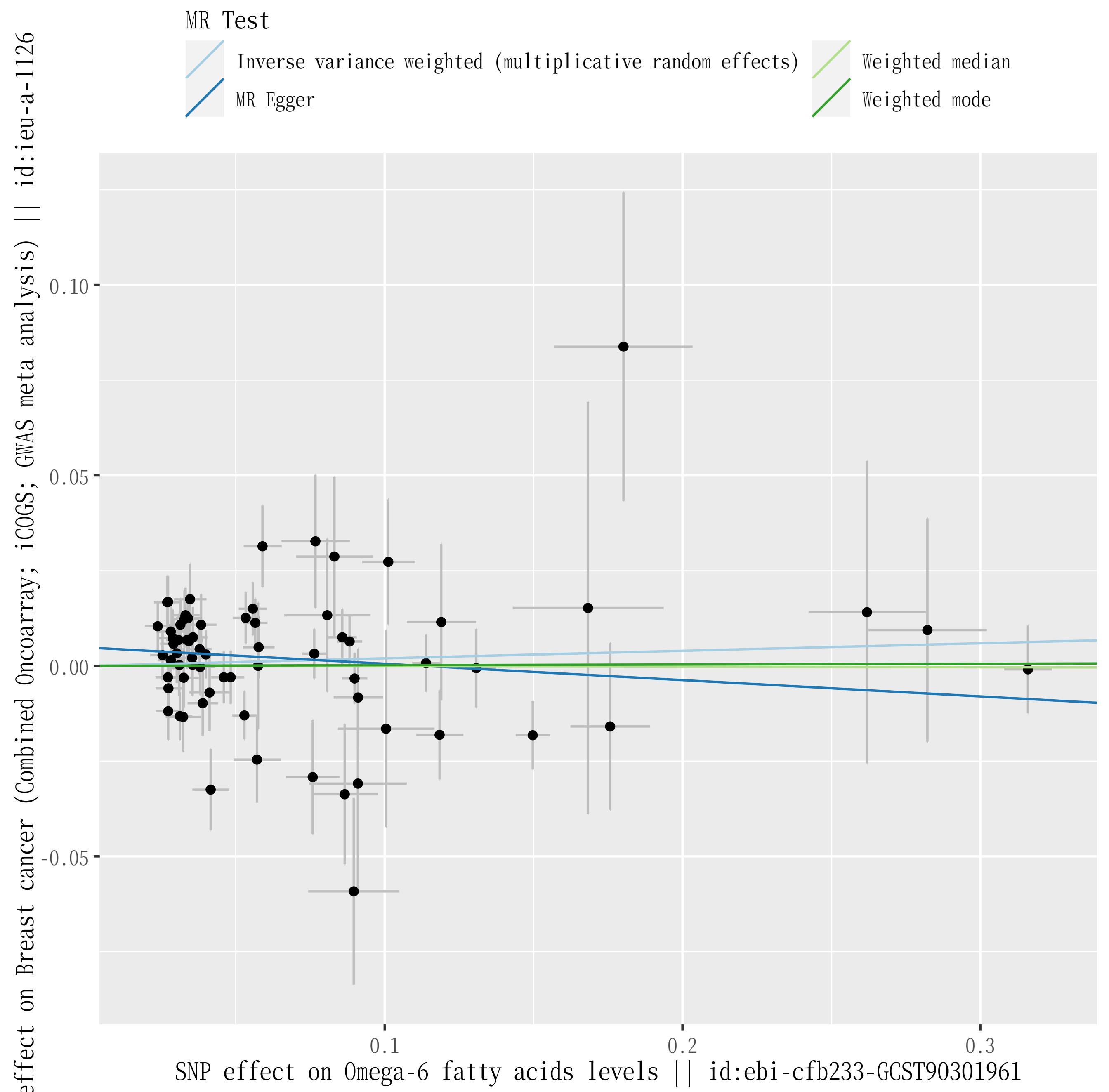
MR Test
Inverse variance weighted (multiplicative random effects)
MR Egger
Weighted median
Weighted mode
SNP effect on Breast cancer (Combined Oncoarray; iCOGS; GWAS meta analysis) || id:ieu-a-1126
0.10
0.05
0.00
-0.05
0.1
0.2
0.3
SNP effect on Omega-6 fatty acids levels || id:ebi-cfb233-GCST90301961

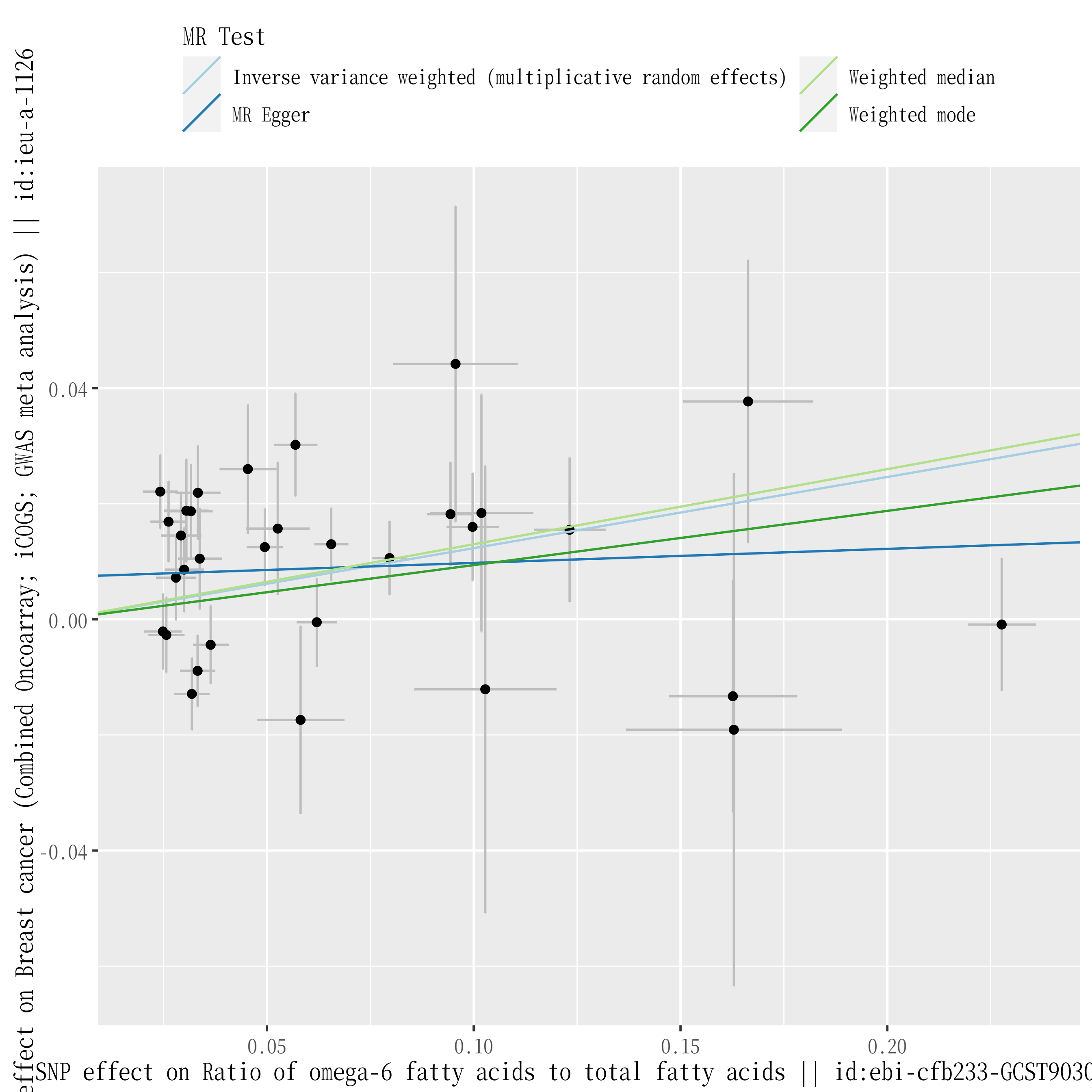

MR Test
Inverse variance weighted (multiplicative random effects)
MR Egger
Weighted median
Weighted mode
0.04
0.00
-0.04
0.05
0.10
0.15
0.20
SNP effect on Ratio of omega-6 fatty acids to total fatty acids || id:ebi-cfb233-GCST9030
effect on Breast cancer (Combined Oncoarray; iCOGS; GWAS meta analysis) || id:ieu-a-1126

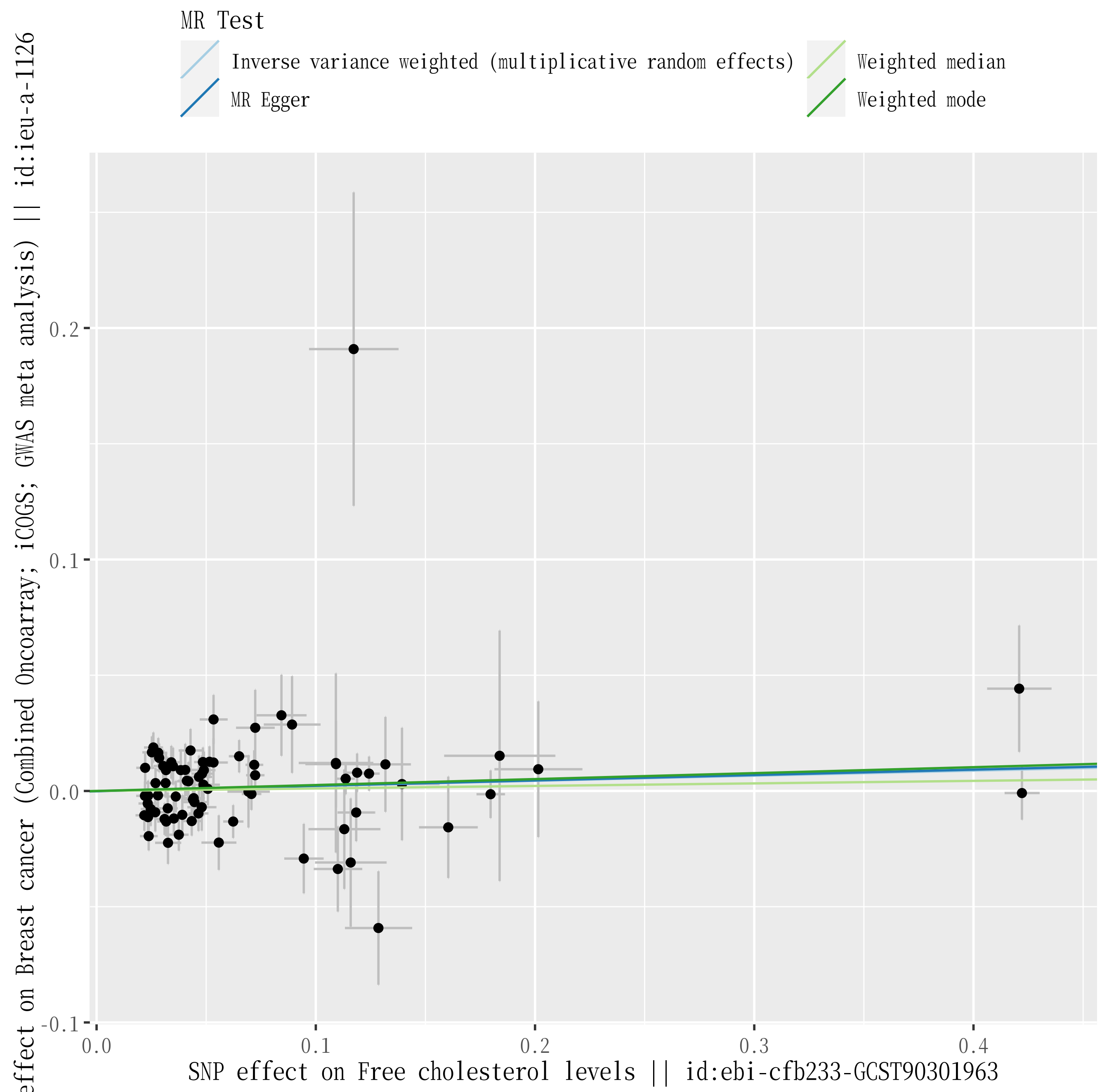
MR Test
Inverse variance weighted (multiplicative random effects)
MR Egger
Weighted median
Weighted mode
SNP effect on Breast cancer (Combined Oncoarray; iCOGS; GWAS meta analysis) || id:ieu-a-1126
0.2
0.1
0.0
-0.1
0.0
0.1
0.2
0.3
0.4
SNP effect on Free cholesterol levels || id:ebi-cfb233-GCST90301963

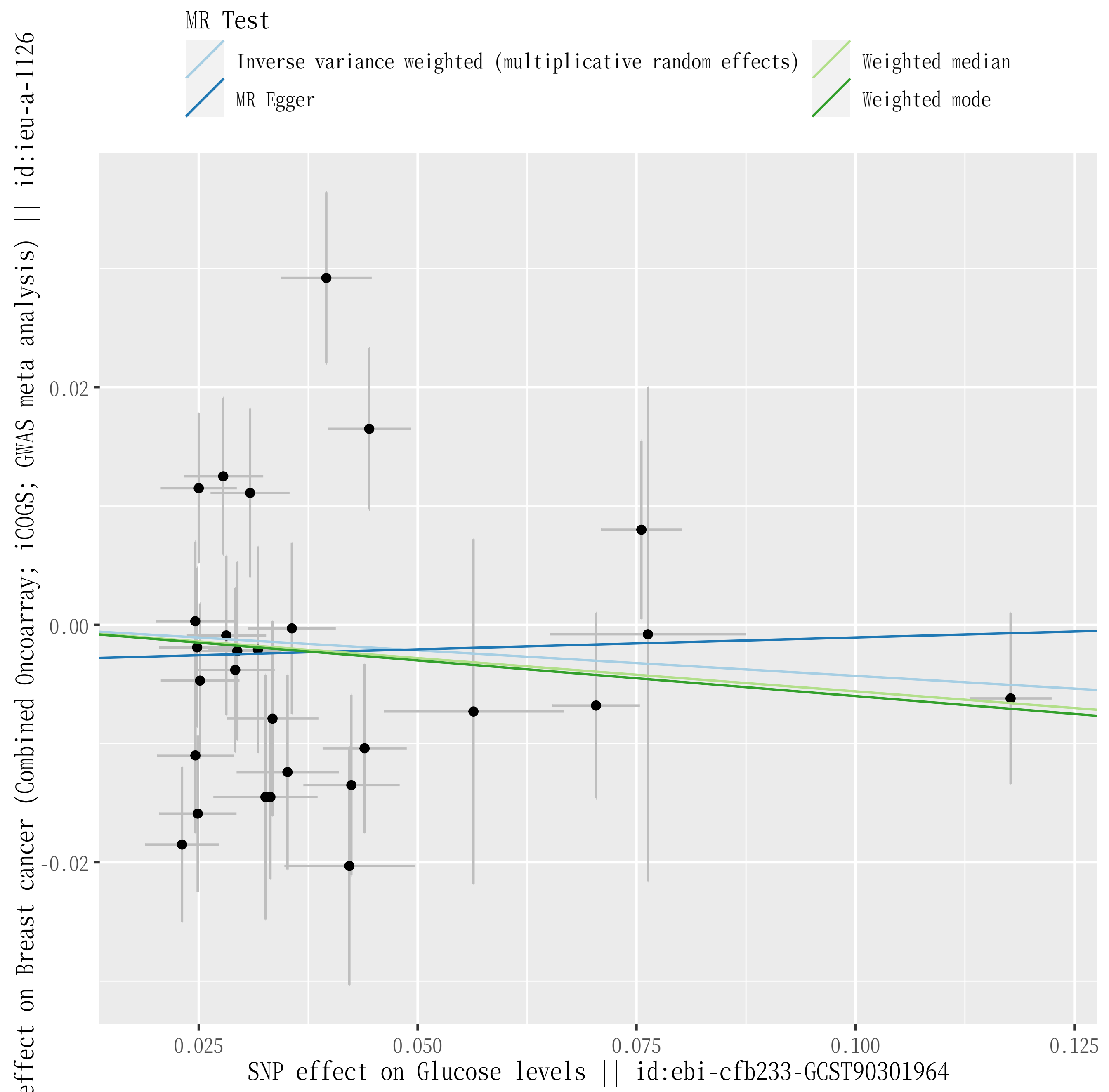
MR Test
Inverse variance weighted (multiplicative random effects)
MR Egger
Weighted median
Weighted mode
0.02
0.00
-0.02
0.025
0.050
0.075
0.100
0.125
SNP effect on Glucose levels || id:ebi-cfb233-GCST90301964
effect on Breast cancer (Combined Oncoarray; iCOGS; GWAS meta analysis) || id:ieu-a-1126

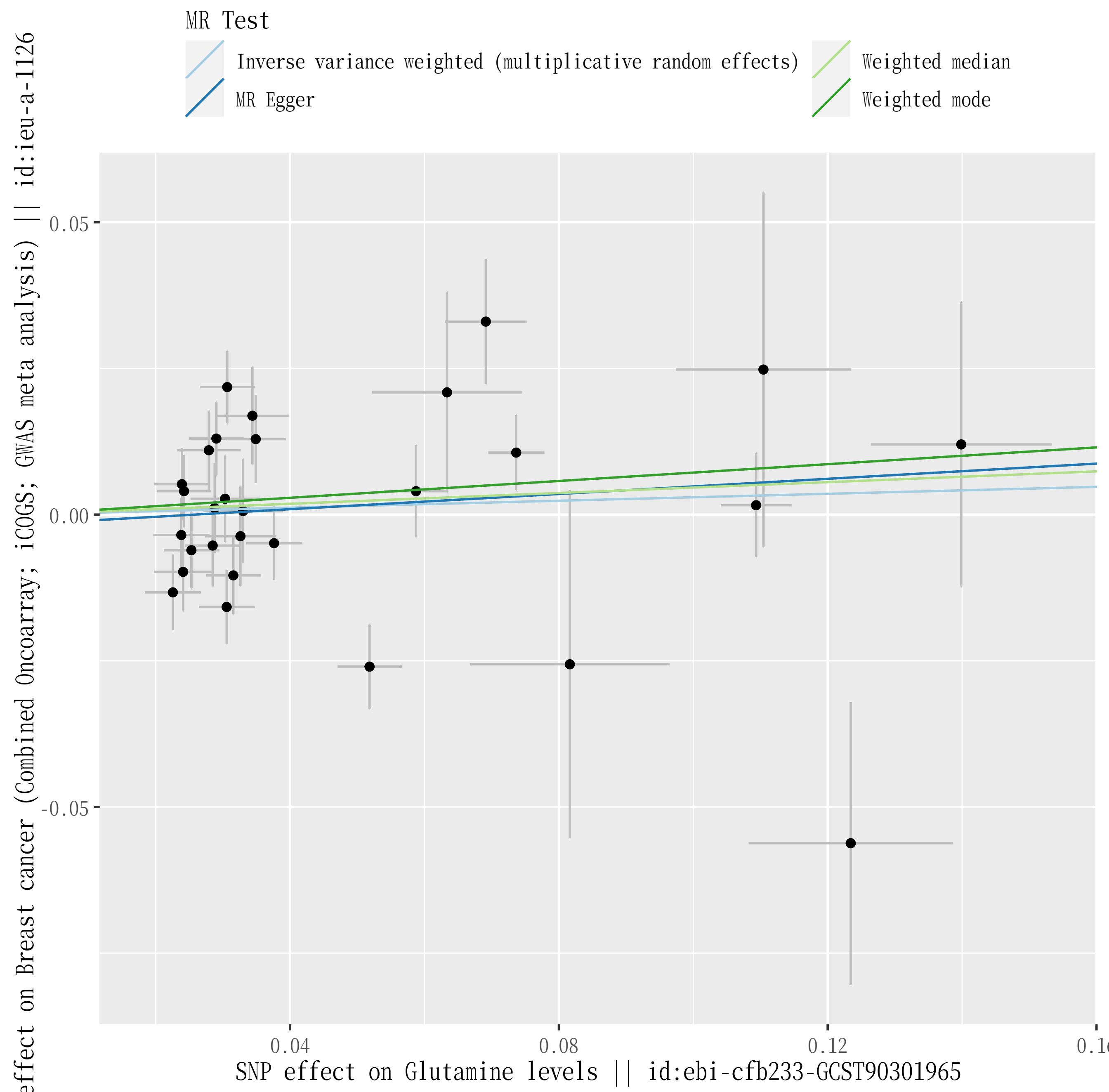
MR Test
Inverse variance weighted (multiplicative random effects)
MR Egger
Weighted median
Weighted mode
0.05
0.00
-0.05
0.04
0.08
0.12
SNP effect on Glutamine levels || id:ebi-cfb233-GCST90301965
effect on Breast cancer (Combined Oncoarray; iCOGS; GWAS meta analysis) || id:ieu-a-1126

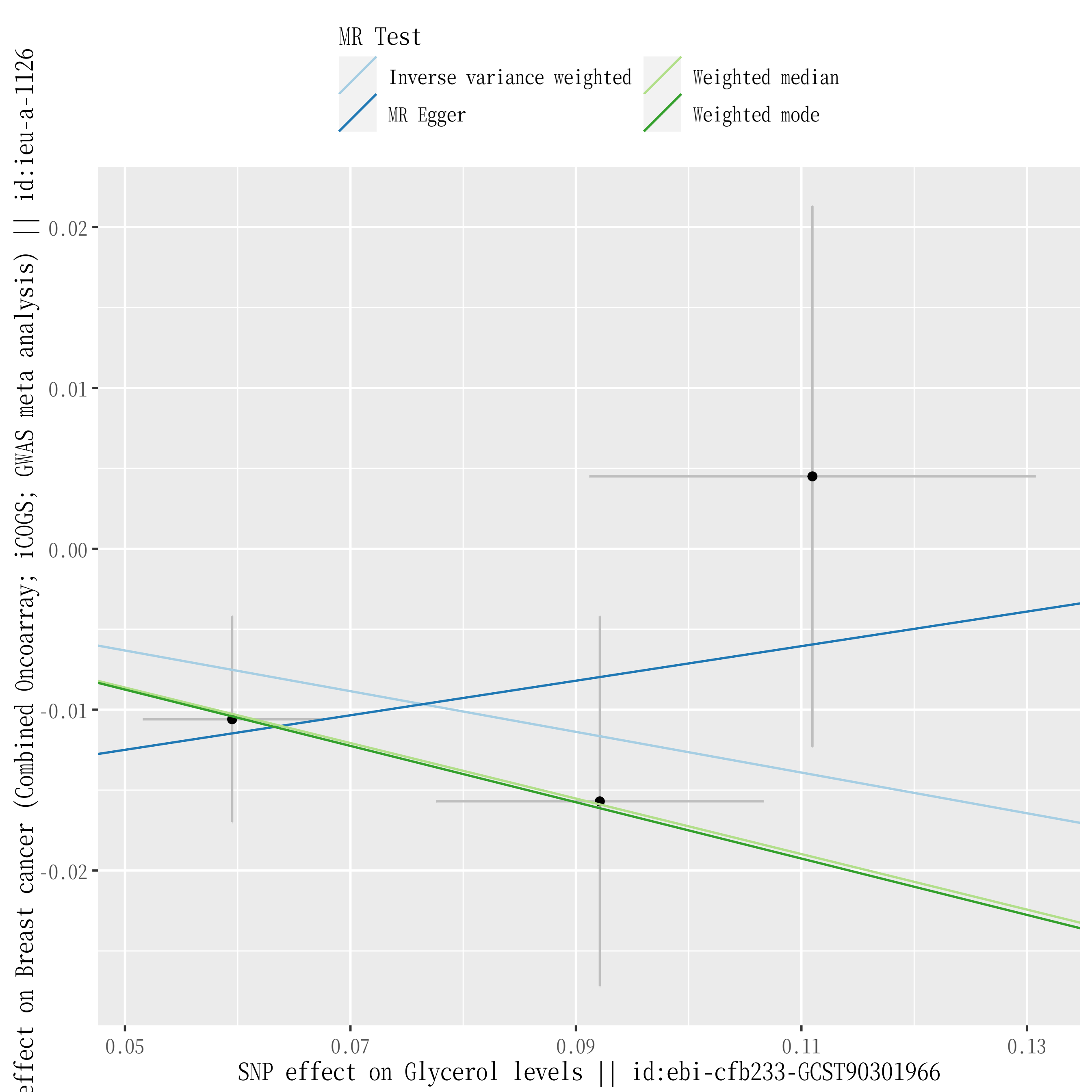
MR Test
Inverse variance weighted
MR Egger
Weighted median
Weighted mode
SNP effect on Breast cancer (Combined Oncoarray; iCOGS; GWAS meta analysis) || id:ieu-a-1126
0.02
0.01
0.00
-0.01
-0.02
0.05
0.07
0.09
0.11
0.13
SNP effect on Glycerol levels || id:ebi-cfb233-GCST90301966

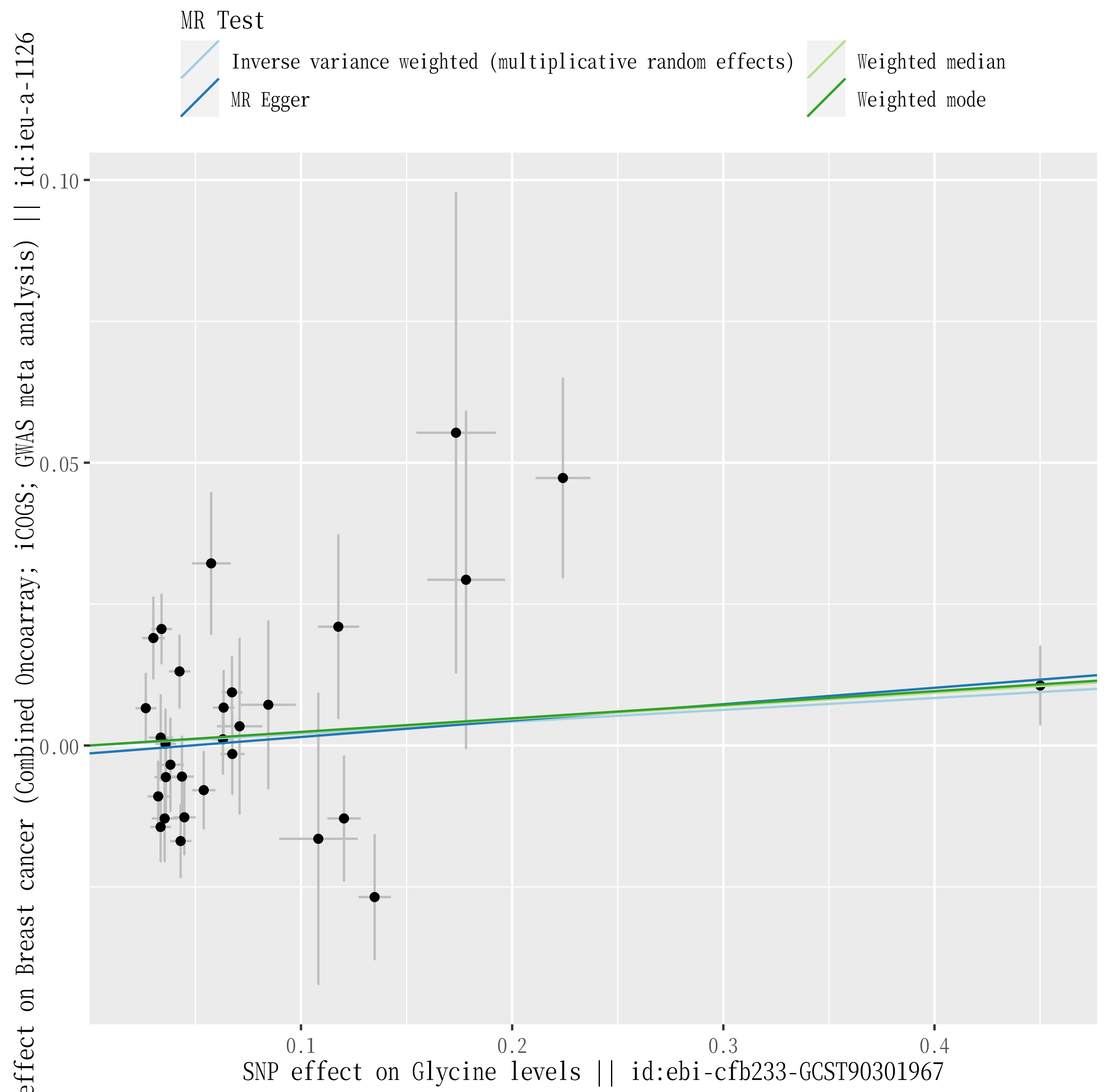
MR Test
Inverse variance weighted (multiplicative random effects)
MR Egger
Weighted median
Weighted mode
0.10
0.05
0.00
0.1
0.2
0.3
0.4
SNP effect on Glycine levels || id:ebi-cfb233-GCST90301967
effect on Breast cancer (Combined Oncoarray; iCOGS; GWAS meta analysis) || id:ieu-a-1126

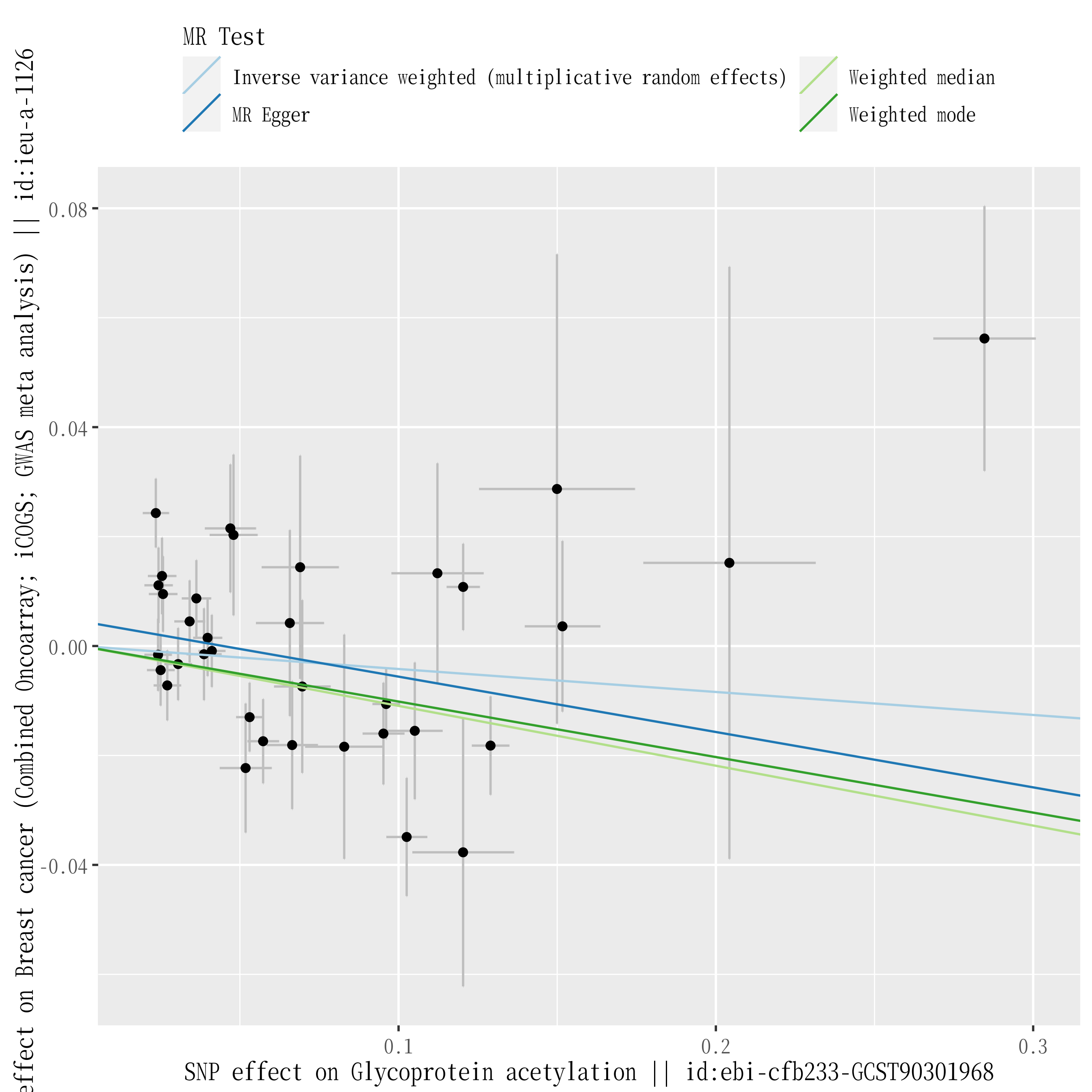

MR Test
Inverse variance weighted (multiplicative random effects)
MR Egger
Weighted median
Weighted mode
0.08
0.04
0.00
-0.04
0.1
0.2
0.3
SNP effect on Glycoprotein acetylation || id:ebi-cfb233-GCST90301968
effect on Breast cancer (Combined Oncoarray; iCOGS; GWAS meta analysis) || id:ieu-a-1126

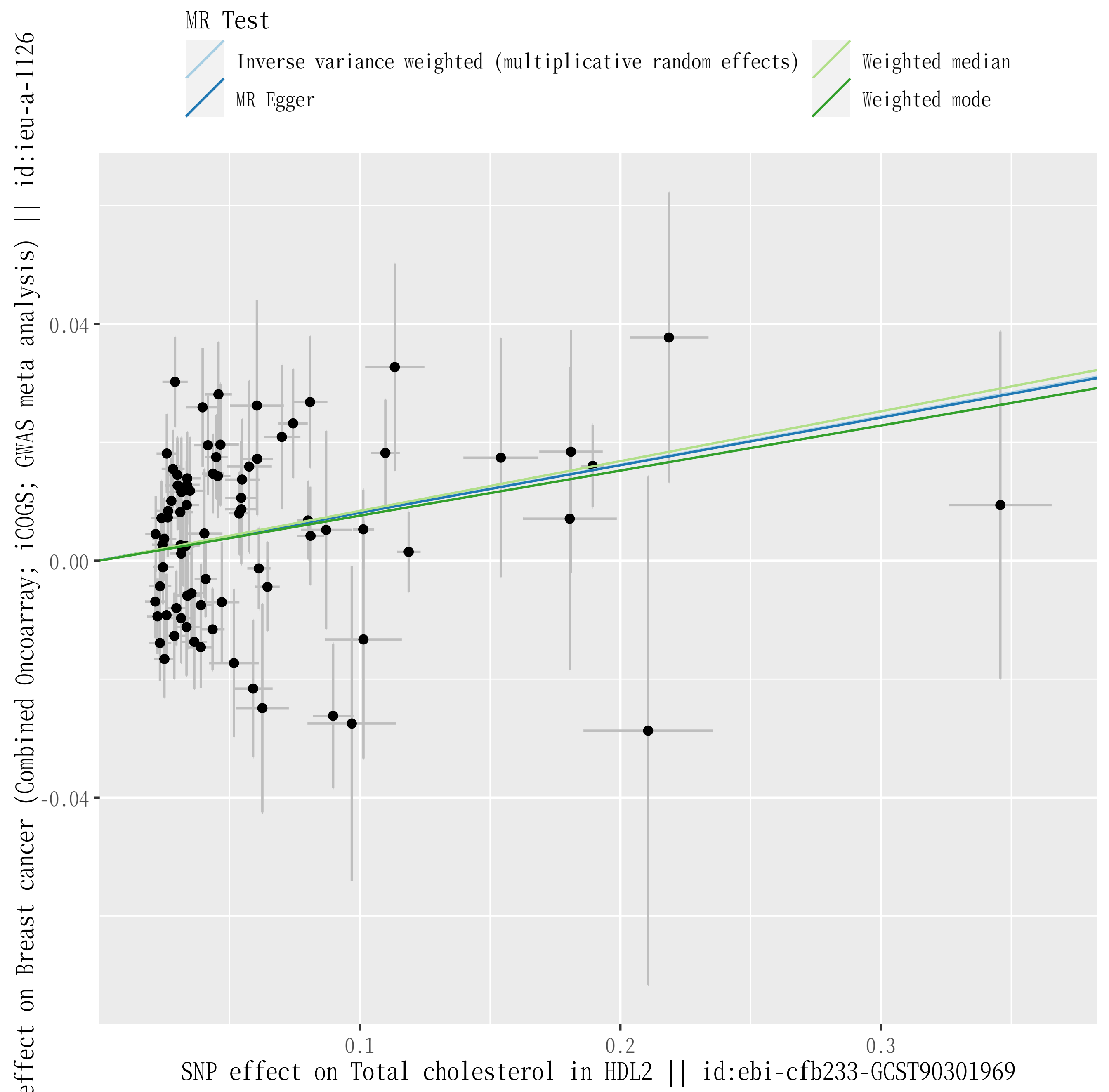
MR Test
Inverse variance weighted (multiplicative random effects)
MR Egger
Weighted median
Weighted mode
0.04
0.00
-0.04
0.1
0.2
0.3
SNP effect on Total cholesterol in HDL2 || id:ebi-cfb233-GCST90301969
effect on Breast cancer (Combined Oncoarray; iCOGS; GWAS meta analysis) || id:ieu-a-1126

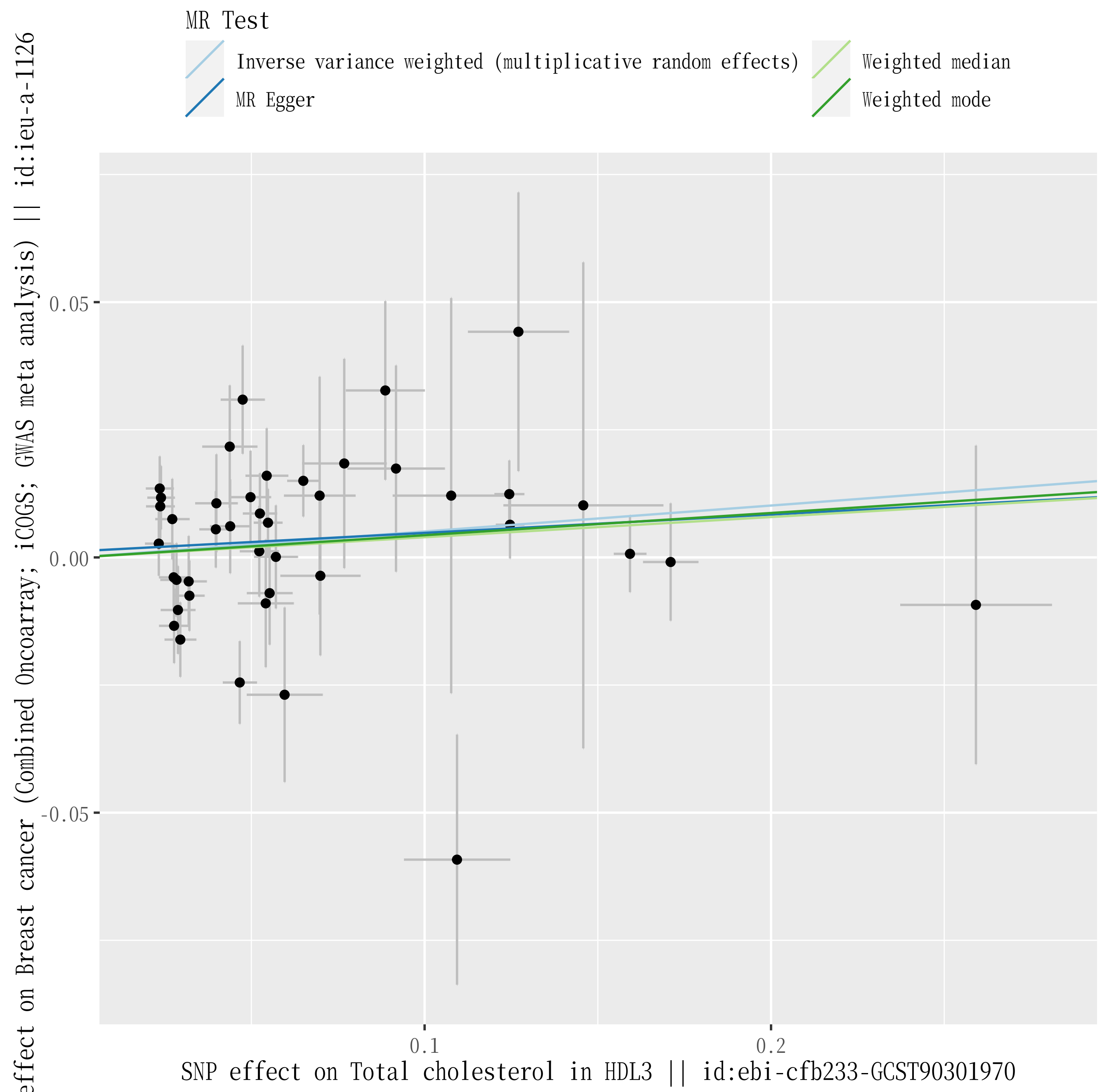

MR Test
Inverse variance weighted (multiplicative random effects)
MR Egger
Weighted median
Weighted mode
0.05
0.00
-0.05
0.1
0.2
SNP effect on Total cholesterol in HDL3 || id:ebi-cfb233-GCST90301970
effect on Breast cancer (Combined Oncoarray; iCOGS; GWAS meta analysis) || id:ieu-a-1126

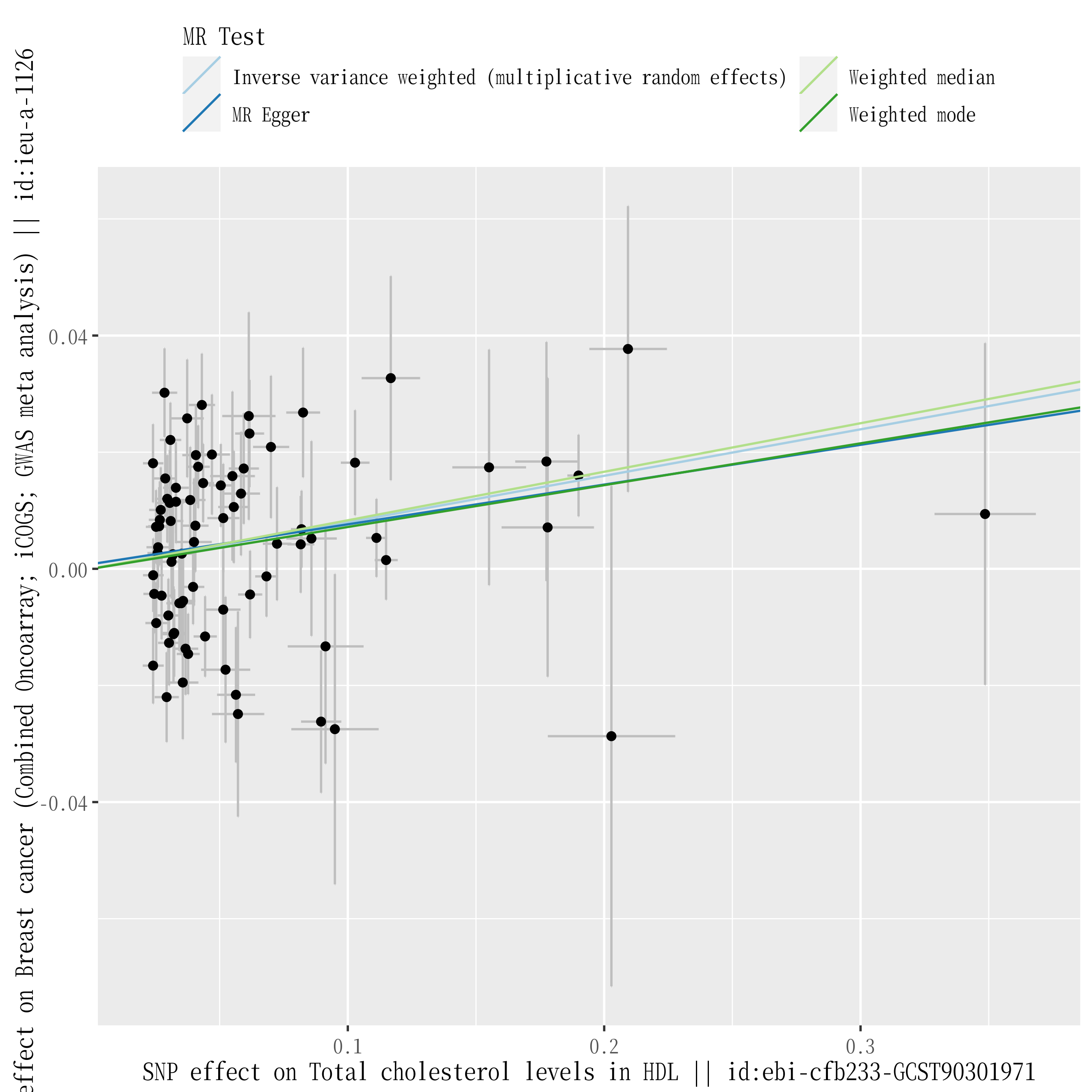
MR Test
Inverse variance weighted (multiplicative random effects)
MR Egger
Weighted median
Weighted mode
0.04
0.00
-0.04
0.1
0.2
0.3
effect on Breast cancer (Combined Oncoarray; iCOGS; GWAS meta analysis) || id:ieu-a-1126
SNP effect on Total cholesterol levels in HDL || id:ebi-cfb233-GCST90301971

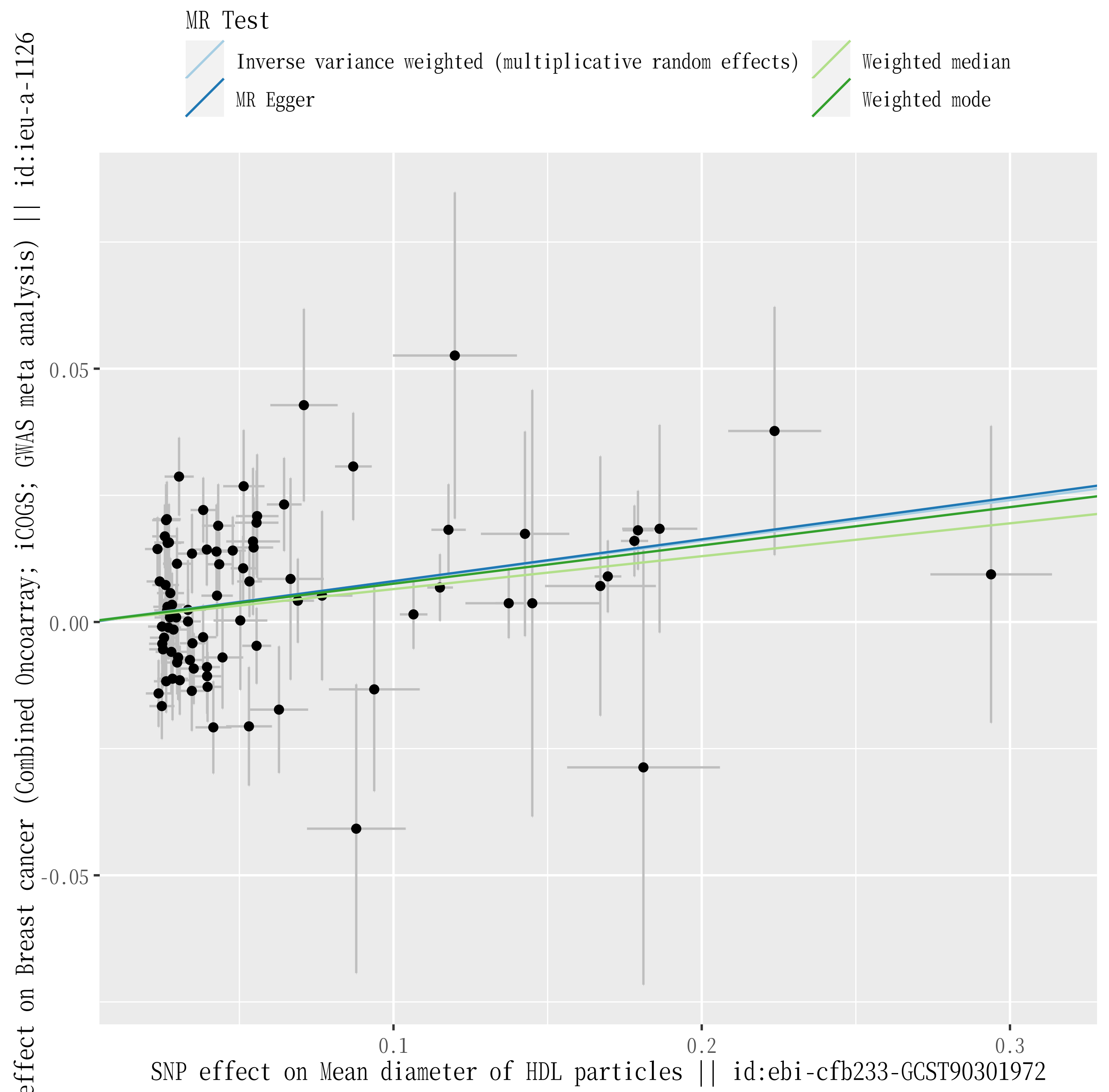

MR Test
Inverse variance weighted (multiplicative random effects)
MR Egger
Weighted median
Weighted mode
0.05
0.00
-0.05
0.1
0.2
0.3
SNP effect on Mean diameter of HDL particles || id:ebi-cfb233-GCST90301972
effect on Breast cancer (Combined Oncoarray; iCOGS; GWAS meta analysis) || id:ieu-a-1126

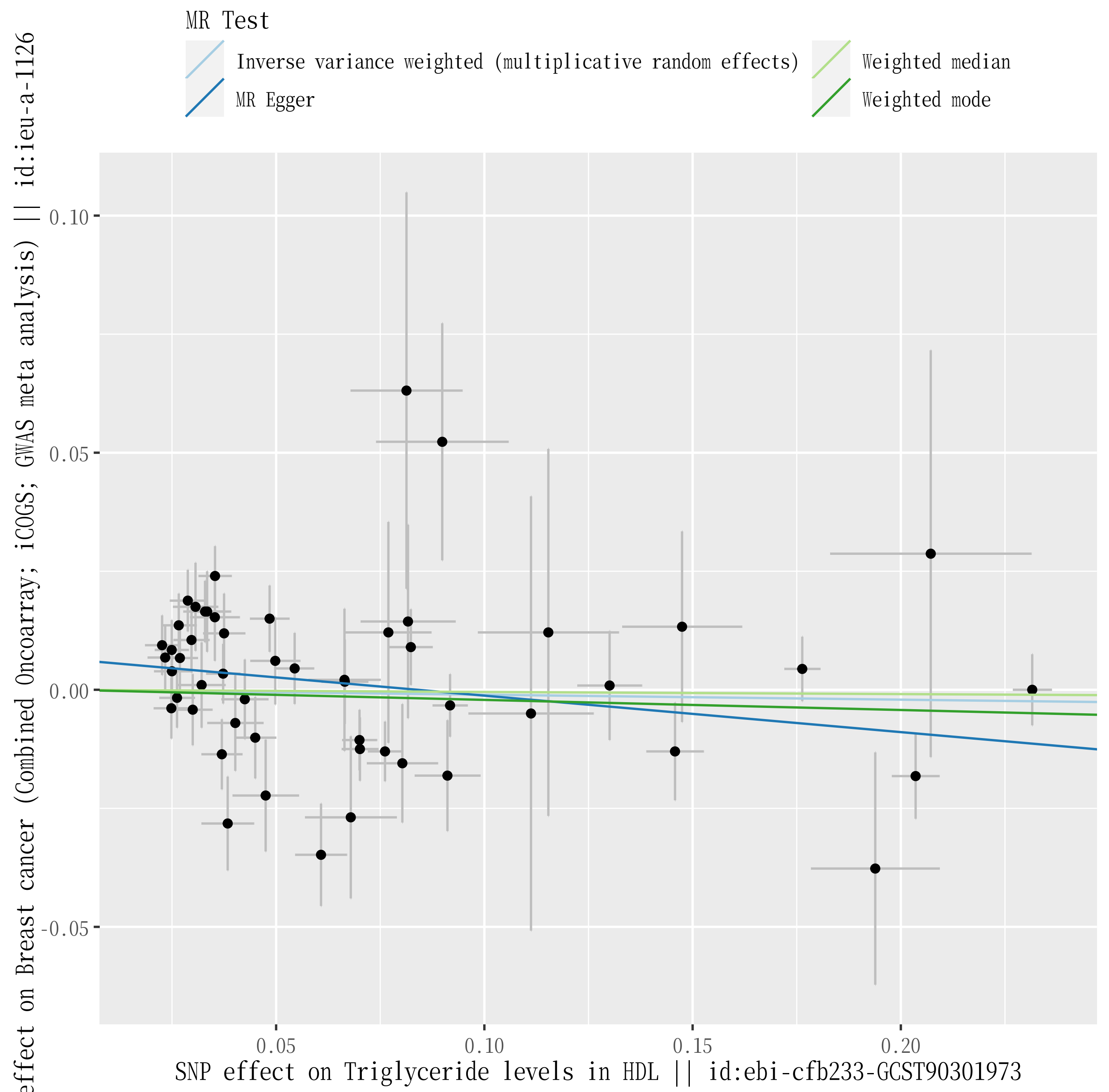

MR Test
Inverse variance weighted (multiplicative random effects)
MR Egger
Weighted median
Weighted mode
0.10
0.05
0.00
-0.05
0.05
0.10
0.15
0.20
SNP effect on Triglyceride levels in HDL || id:ebi-cfb233-GCST90301973
effect on Breast cancer (Combined Oncoarray; iCOGS; GWAS meta analysis) || id:ieu-a-1126

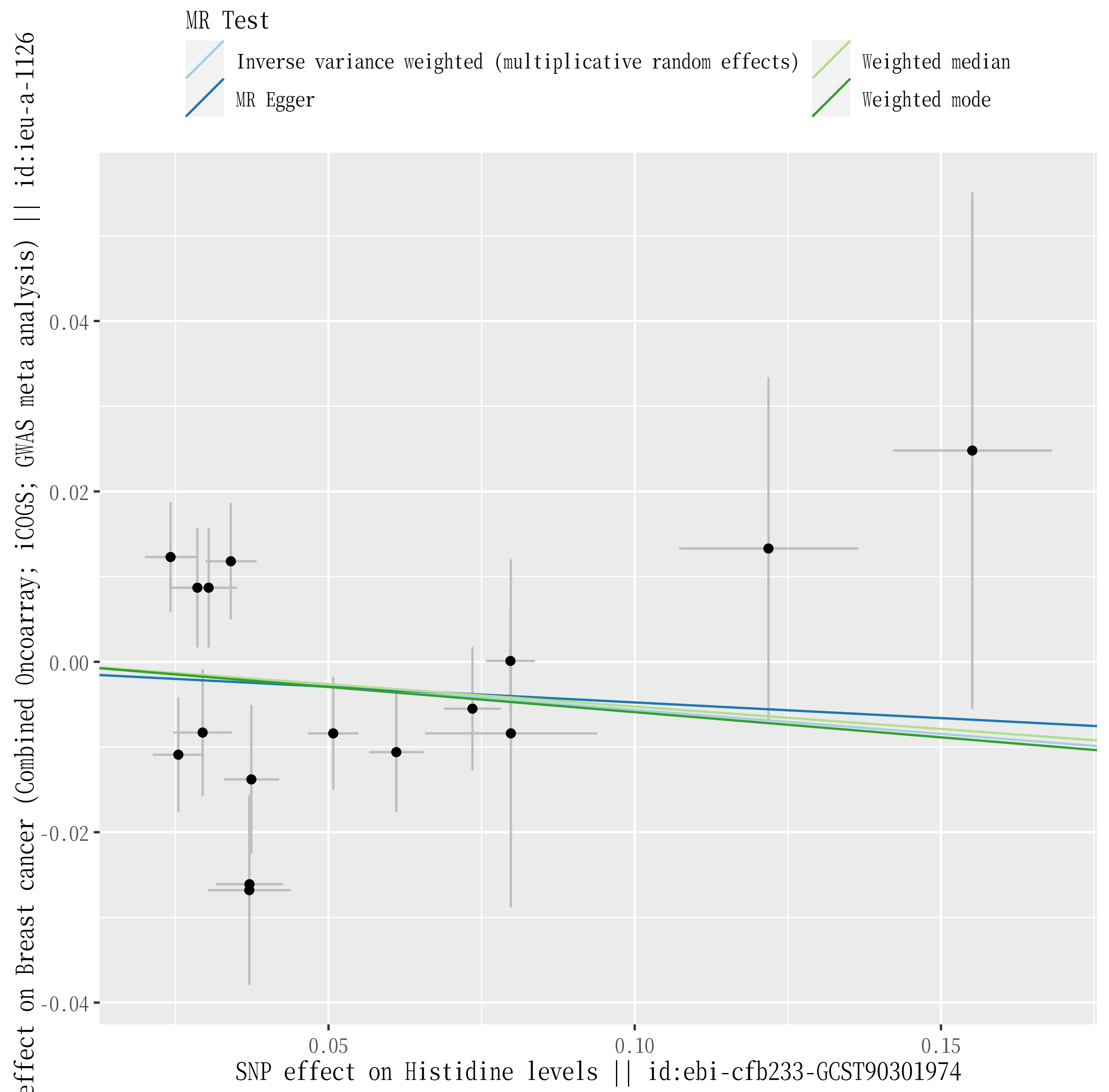
MR Test
Inverse variance weighted (multiplicative random effects)
MR Egger
Weighted median
Weighted mode
0.04
0.02
0.00
-0.02
-0.04
0.05
0.10
0.15
SNP effect on Histidine levels || id:ebi-cfb233-GCST90301974
effect on Breast cancer (Combined Oncoarray; iCOGS; GWAS meta analysis) || id:ieu-a-1126

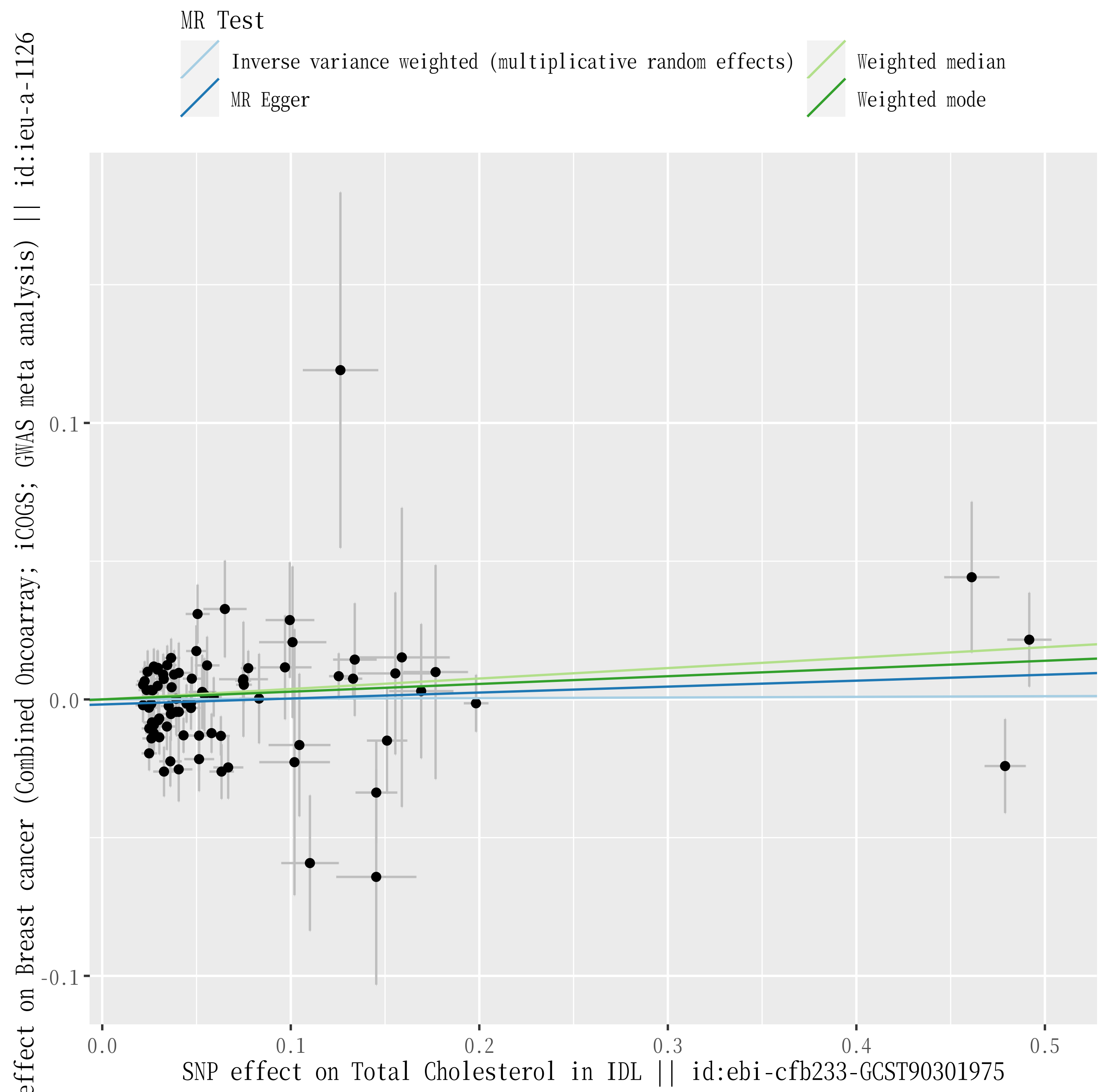

MR Test
Inverse variance weighted (multiplicative random effects)
MR Egger
Weighted median
Weighted mode
0.1
0.0
-0.1
0.0
0.1
0.2
0.3
0.4
0.5
SNP effect on Total Cholesterol in IDL || id:ebi-cfb233-GCST90301975
effect on Breast cancer (Combined Oncoarray; iCOGS; GWAS meta analysis) || id:ieu-a-1126

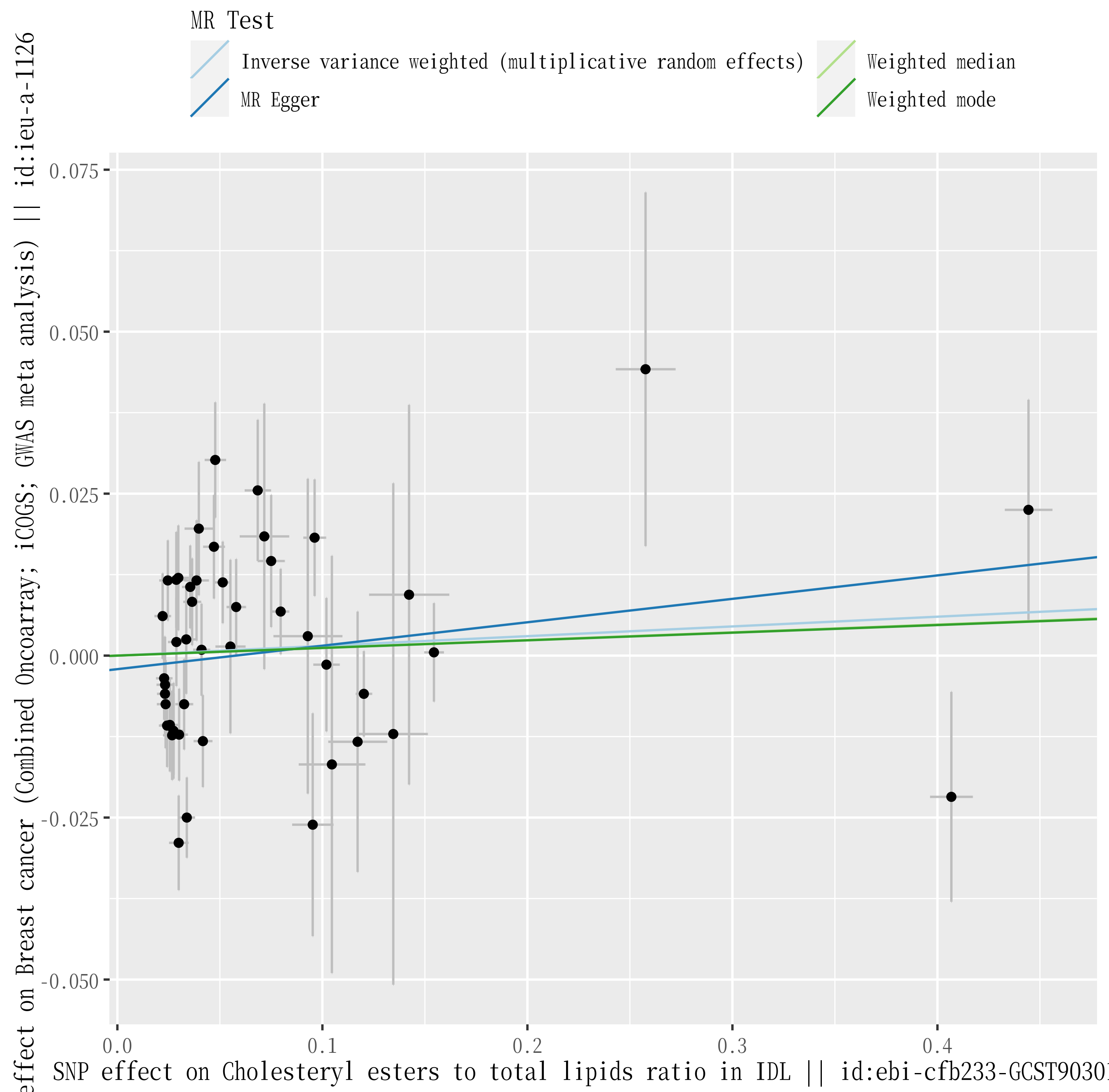
MR Test
Inverse variance weighted (multiplicative random effects)
MR Egger
Weighted median
Weighted mode
0.075
0.050
0.025
0.000
-0.025
-0.050
0.0
0.1
0.2
0.3
0.4
SNP effect on Cholesteryl esters to total lipids ratio in IDL || id:ebi-cfb233-GCST9030
effect on Breast cancer (Combined Oncoarray; iCOGS; GWAS meta analysis) || id:ieu-a-1126

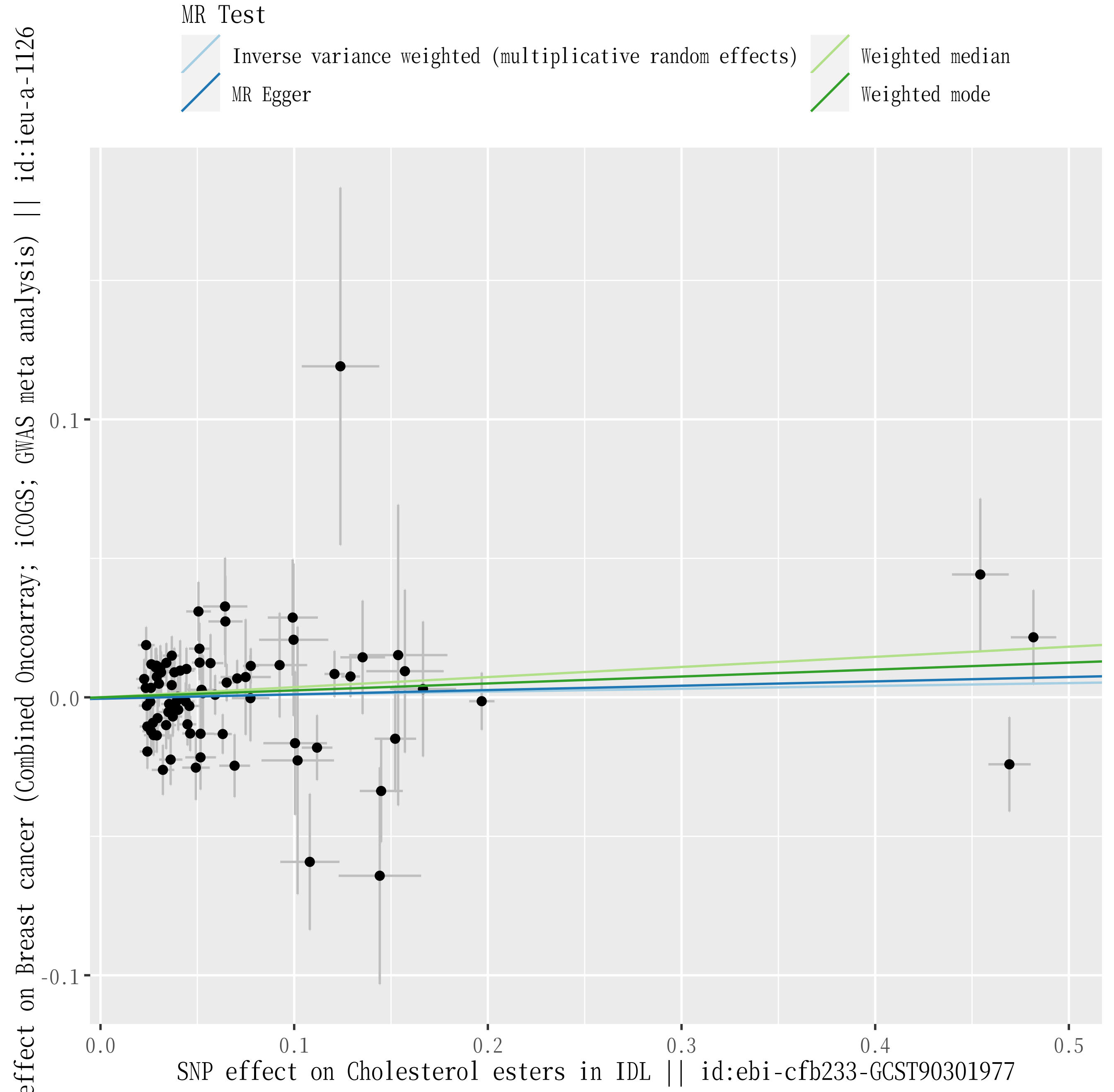
MR Test
Inverse variance weighted (multiplicative random effects)
MR Egger
Weighted median
Weighted mode
SNP effect on Breast cancer (Combined Oncoarray; iCOGS; GWAS meta analysis) || id:ieu-a-1126
0.1
0.0
-0.1
0.0
0.1
0.2
0.3
0.4
0.5
SNP effect on Cholesterol esters in IDL || id:ebi-cfb233-GCST90301977

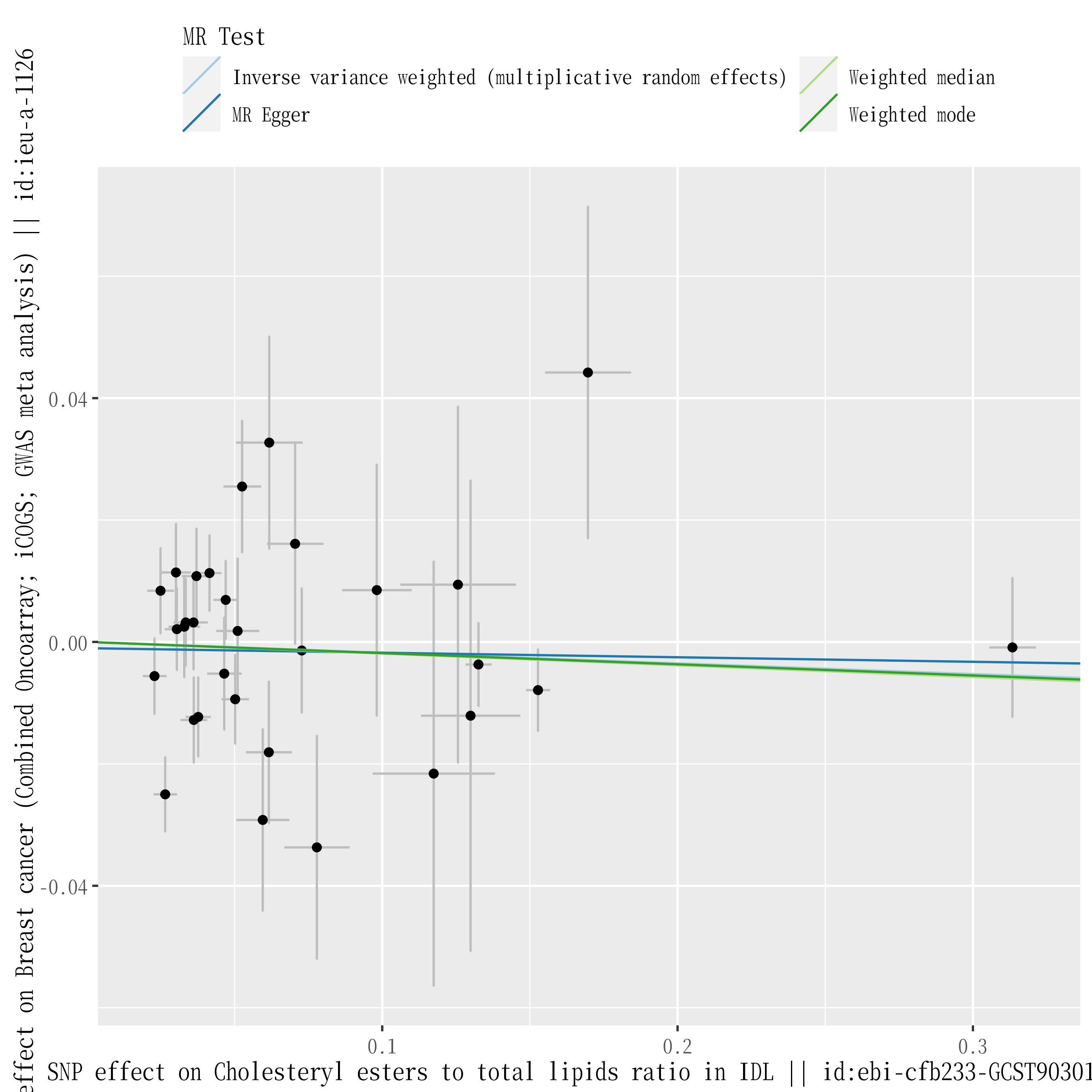
MR Test
Inverse variance weighted (multiplicative random effects)
MR Egger
Weighted median
Weighted mode
0.04
0.00
-0.04
0.1
0.2
0.3
SNP effect on Cholesteryl esters to total lipids ratio in IDL || id:ebi-cfb233-GCST90301
effect on Breast cancer (Combined Oncoarray; iCOGS; GWAS meta analysis) || id:ieu-a-1126

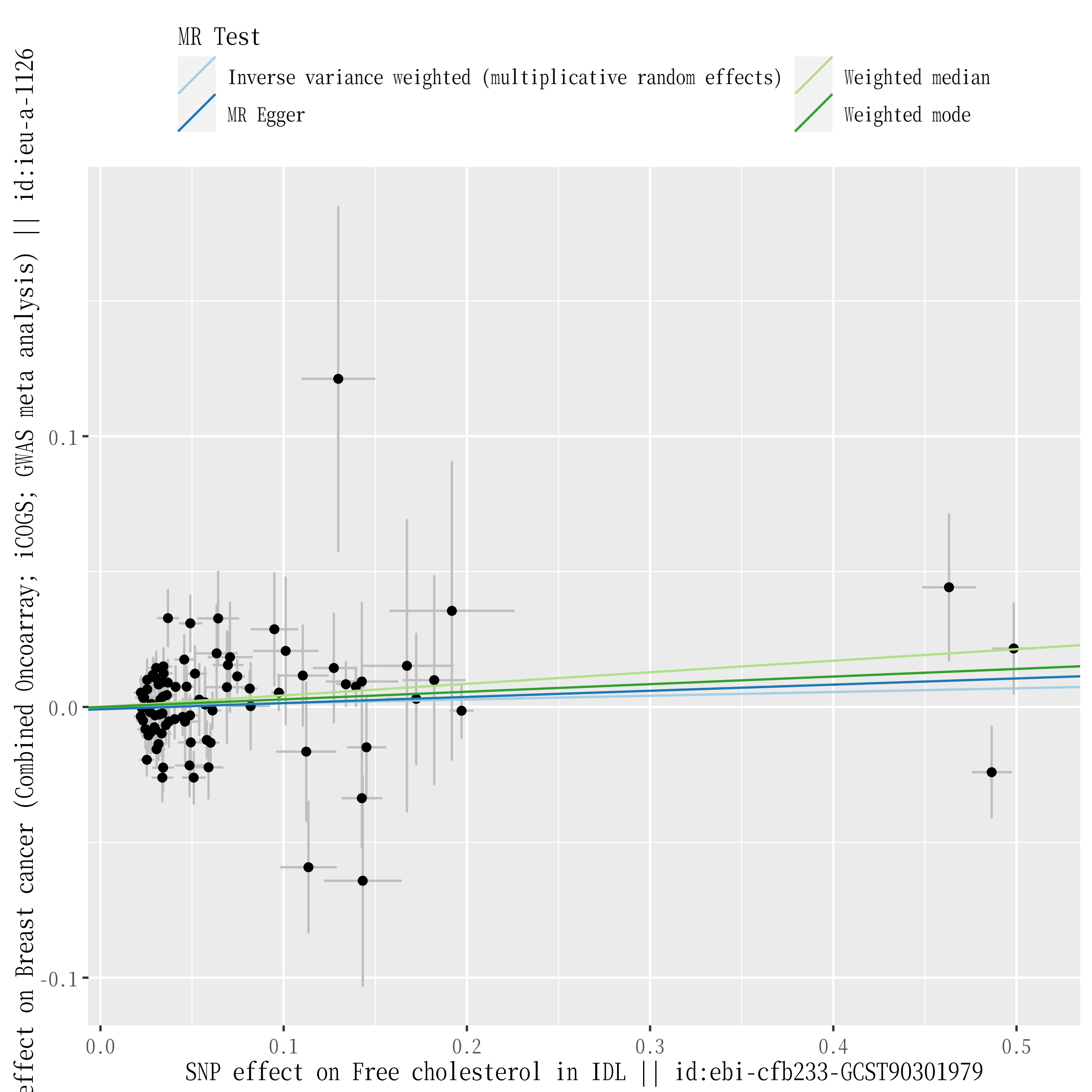

MR Test
Inverse variance weighted (multiplicative random effects)
MR Egger
Weighted median
Weighted mode
SNP effect on Breast cancer (Combined Oncoarray; iCOGS; GWAS meta analysis) || id:ieu-a-1126
0.1
0.0
-0.1
0.0
0.1
0.2
0.3
0.4
0.5
SNP effect on Free cholesterol in IDL || id:ebi-cfb233-GCST90301979

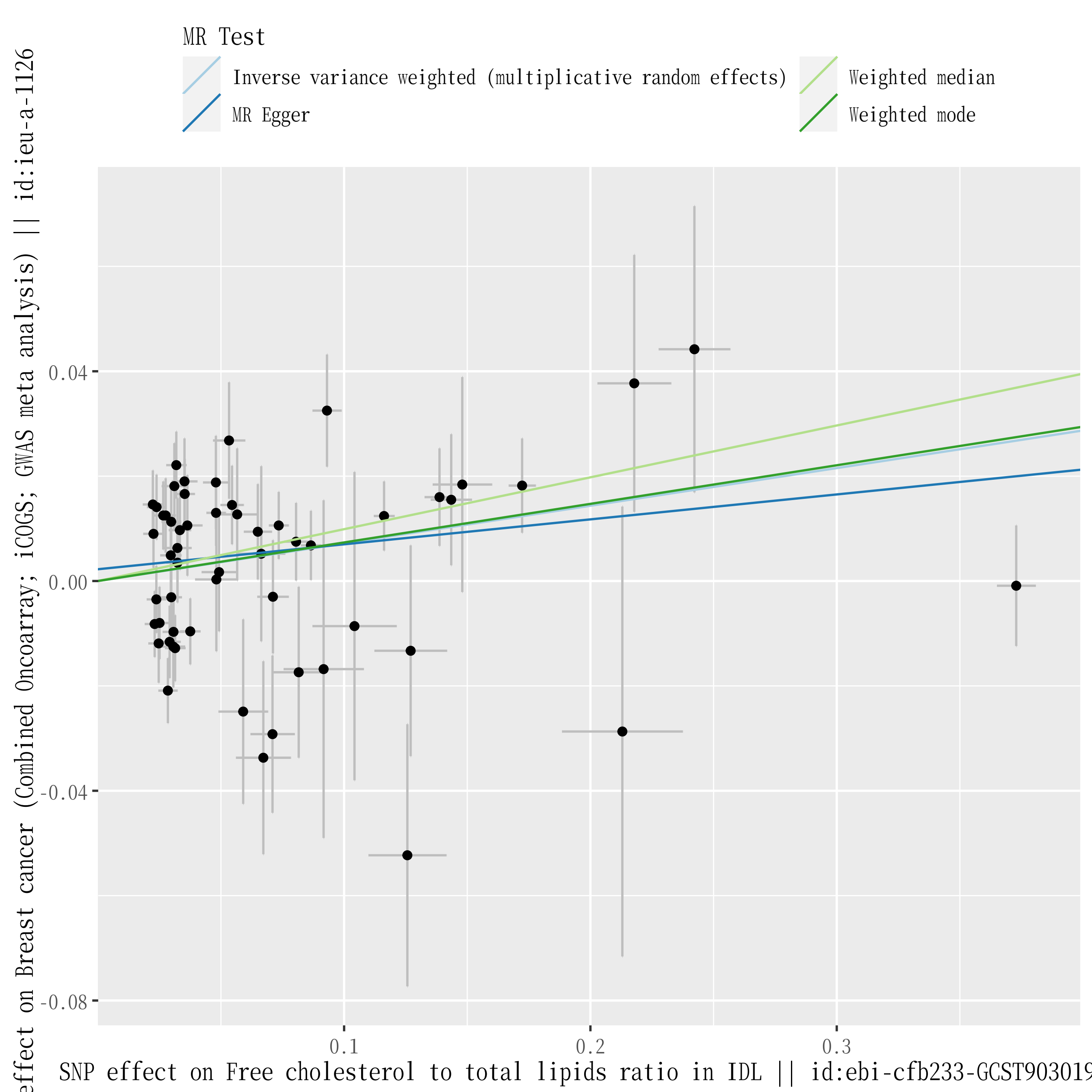

MR Test
Inverse variance weighted (multiplicative random effects)
MR Egger
Weighted median
Weighted mode
0.04
0.00
-0.04
-0.08
0.1
0.2
0.3
SNP effect on Free cholesterol to total lipids ratio in IDL || id:ebi-cfb233-GCST903019
effect on Breast cancer (Combined Oncoarray; iCOGS; GWAS meta analysis) || id:ieu-a-1126

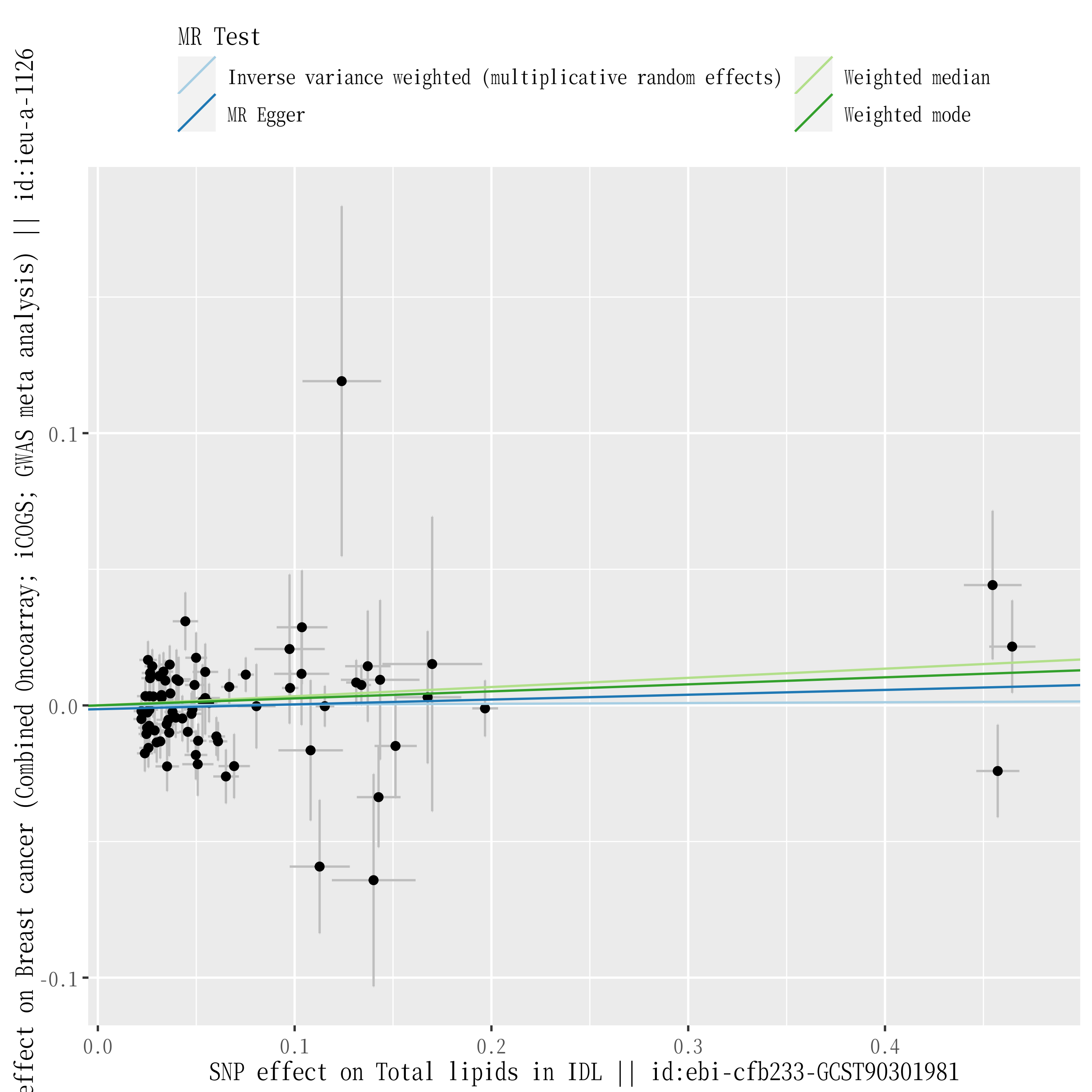

MR Test
Inverse variance weighted (multiplicative random effects)
MR Egger
Weighted median
Weighted mode
0.1
0.0
-0.1
0.0
0.1
0.2
0.3
0.4
SNP effect on Total lipids in IDL || id:ebi-cfb233-GCST90301981
effect on Breast cancer (Combined Oncoarray; iCOGS; GWAS meta analysis) || id:ieu-a-1126

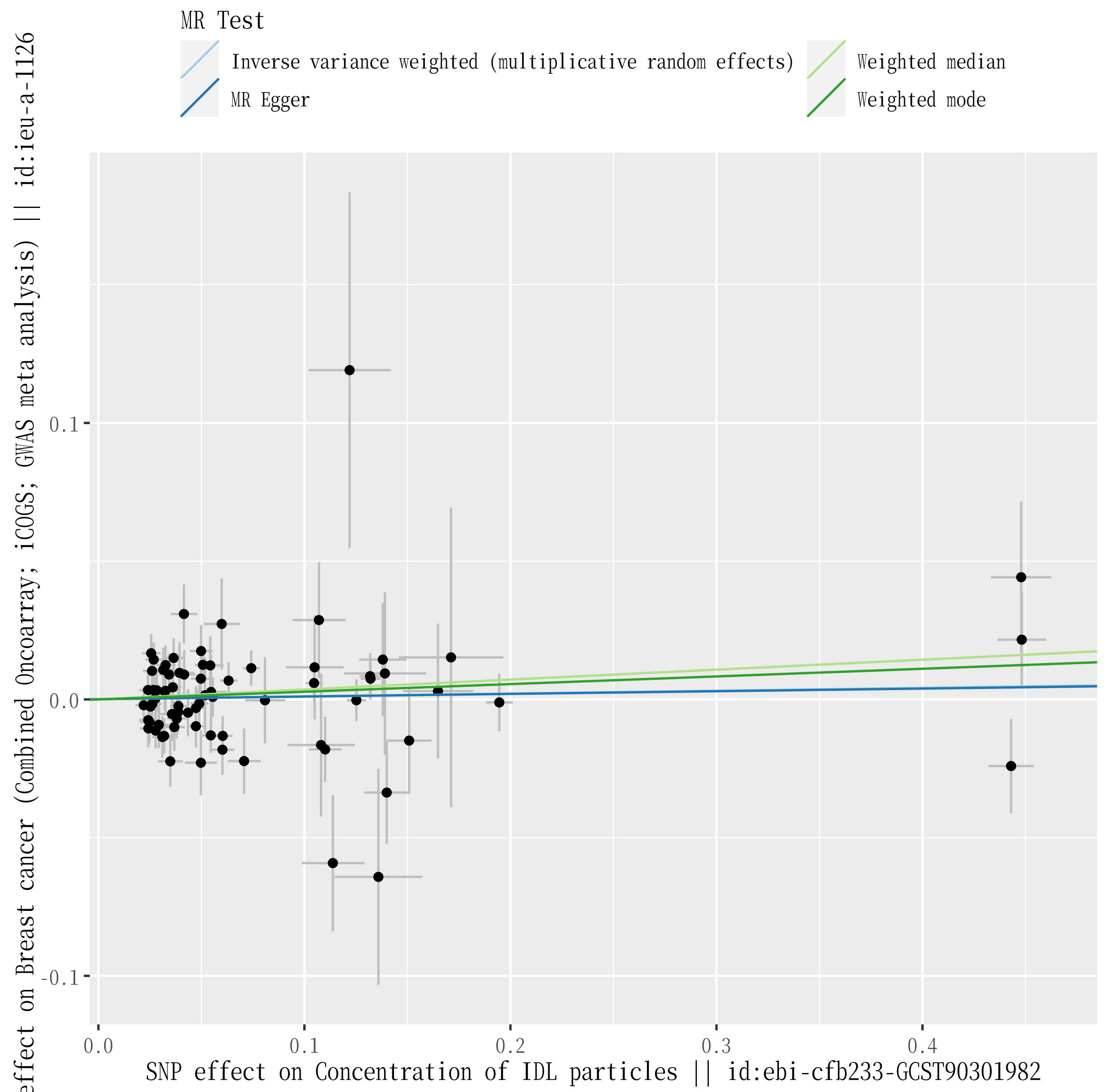
MR Test
Inverse variance weighted (multiplicative random effects)
MR Egger
Weighted median
Weighted mode
SNP effect on Breast cancer (Combined Oncoarray; iCOGS; GWAS meta analysis) || id:ieu-a-1126
0.1
0.0
-0.1
0.0
0.1
0.2
0.3
0.4
SNP effect on Concentration of IDL particles || id:ebi-cfb233-GCST90301982

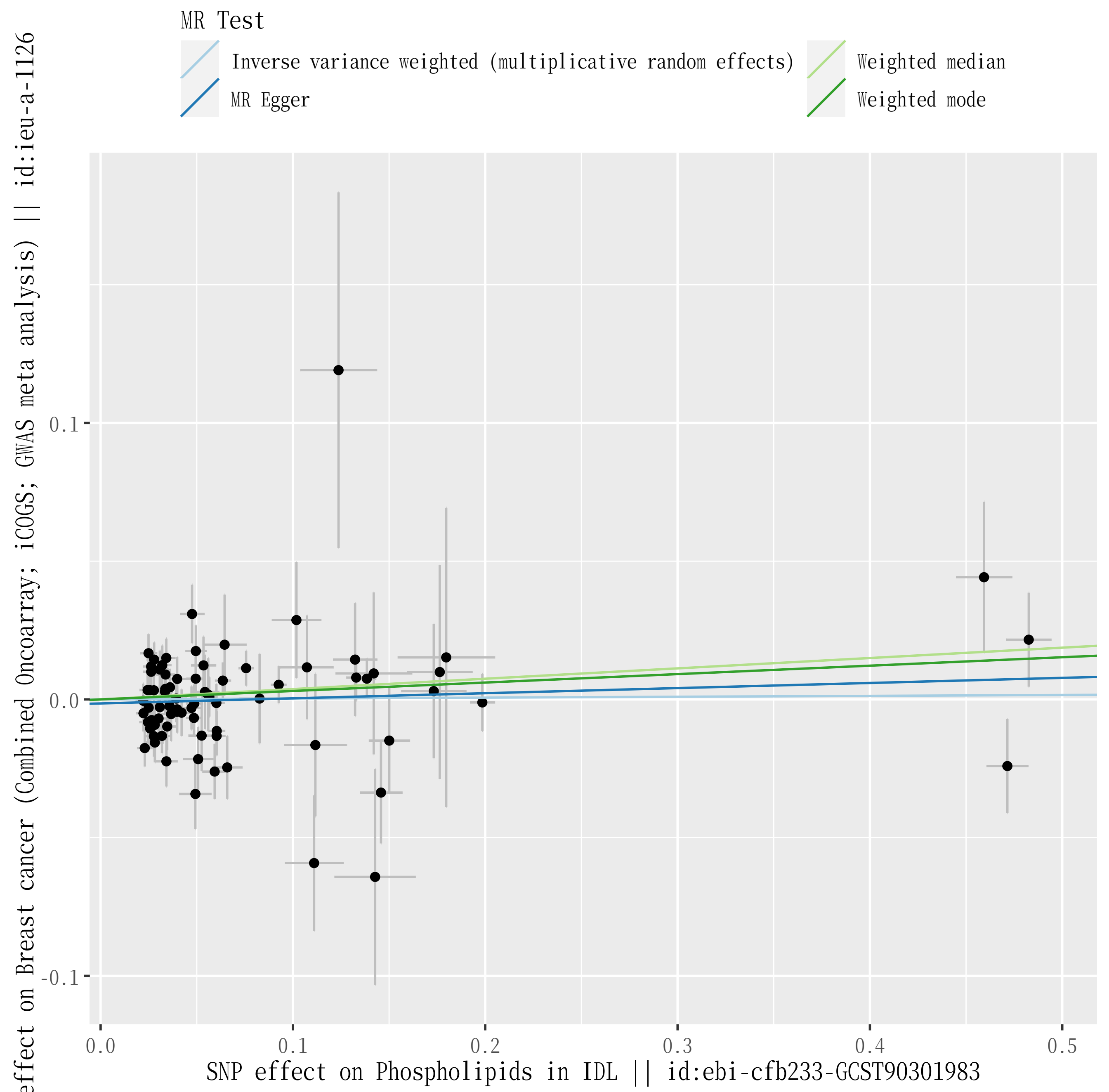
MR Test
Inverse variance weighted (multiplicative random effects)
MR Egger
Weighted median
Weighted mode
0.1
0.0
-0.1
0.0
0.1
0.2
0.3
0.4
0.5
SNP effect on Phospholipids in IDL || id:ebi-cfb233-GCST90301983
effect on Breast cancer (Combined Oncoarray; iCOGS; GWAS meta analysis) || id:ieu-a-1126

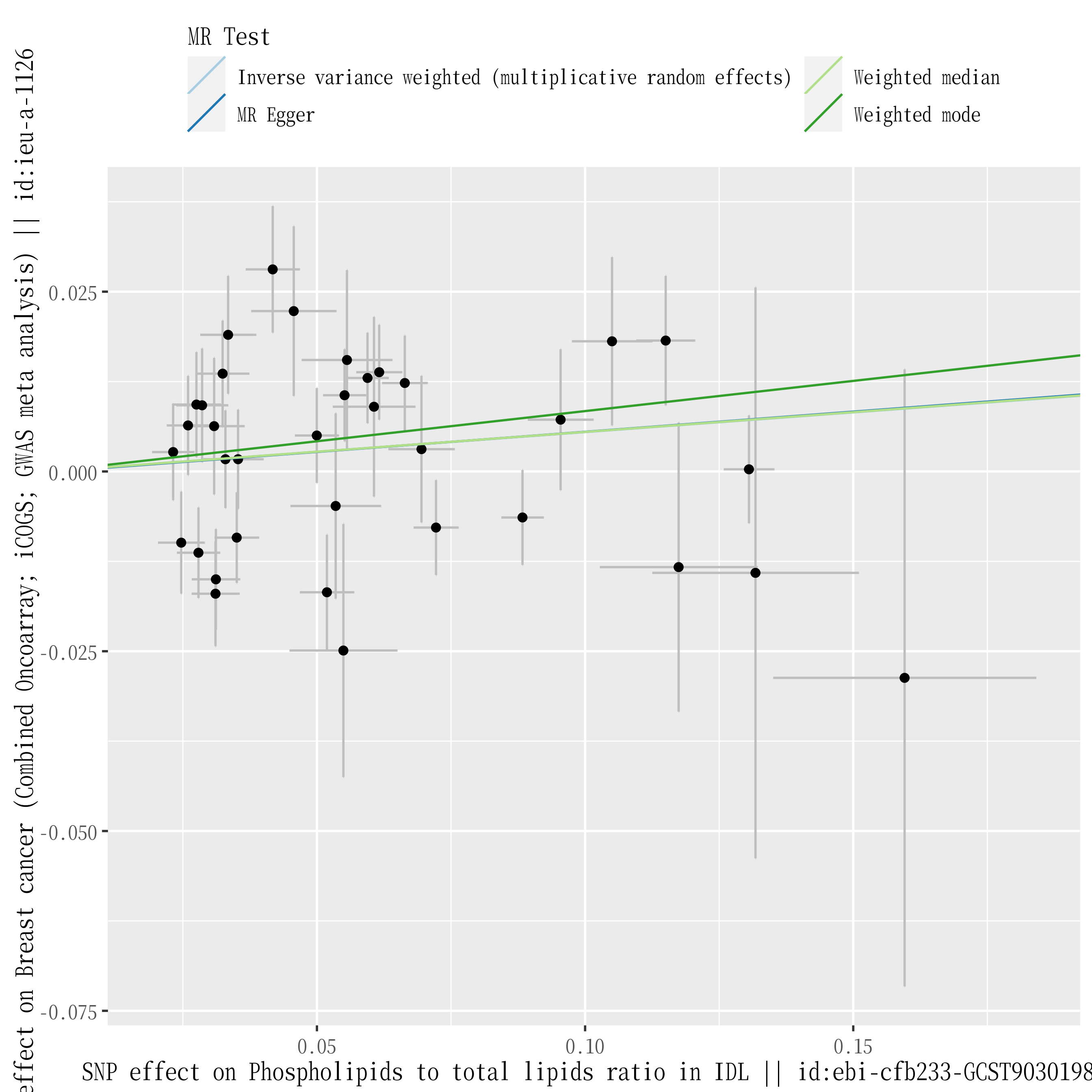
MR Test
Inverse variance weighted (multiplicative random effects)
MR Egger
Weighted median
Weighted mode
0.025
0.000
-0.025
-0.050
-0.075
0.05
0.10
0.15
SNP effect on Phospholipids to total lipids ratio in IDL || id:ebi-cfb233-GCST9030198
effect on Breast cancer (Combined Oncoarray; iCOGS; GWAS meta analysis) || id:ieu-a-1126

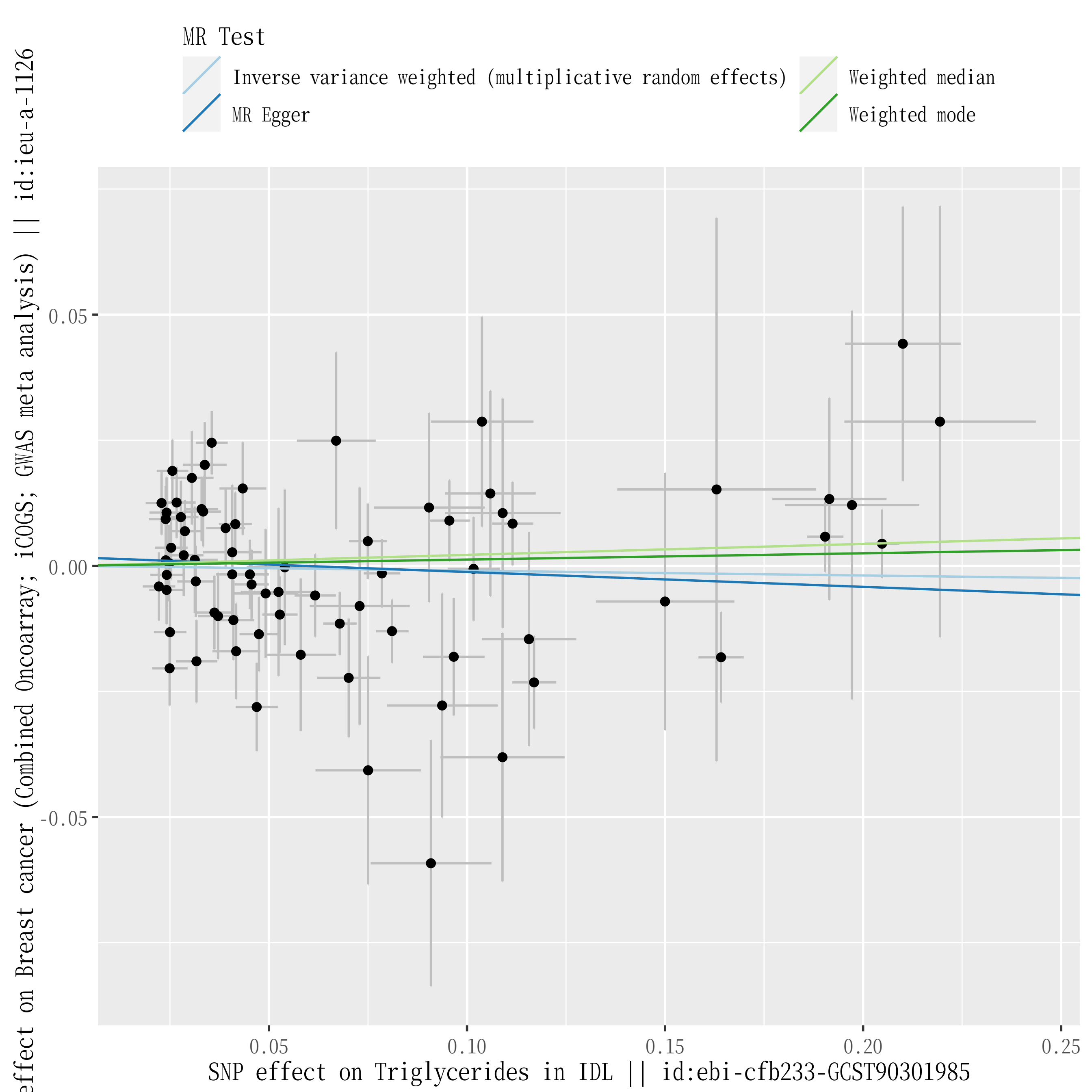

MR Test
Inverse variance weighted (multiplicative random effects)
MR Egger
Weighted median
Weighted mode
0.05
0.00
-0.05
0.05
0.10
0.15
0.20
0.25
SNP effect on Triglycerides in IDL || id:ebi-cfb233-GCST90301985
effect on Breast cancer (Combined Oncoarray; iCOGS; GWAS meta analysis) || id:ieu-a-1126

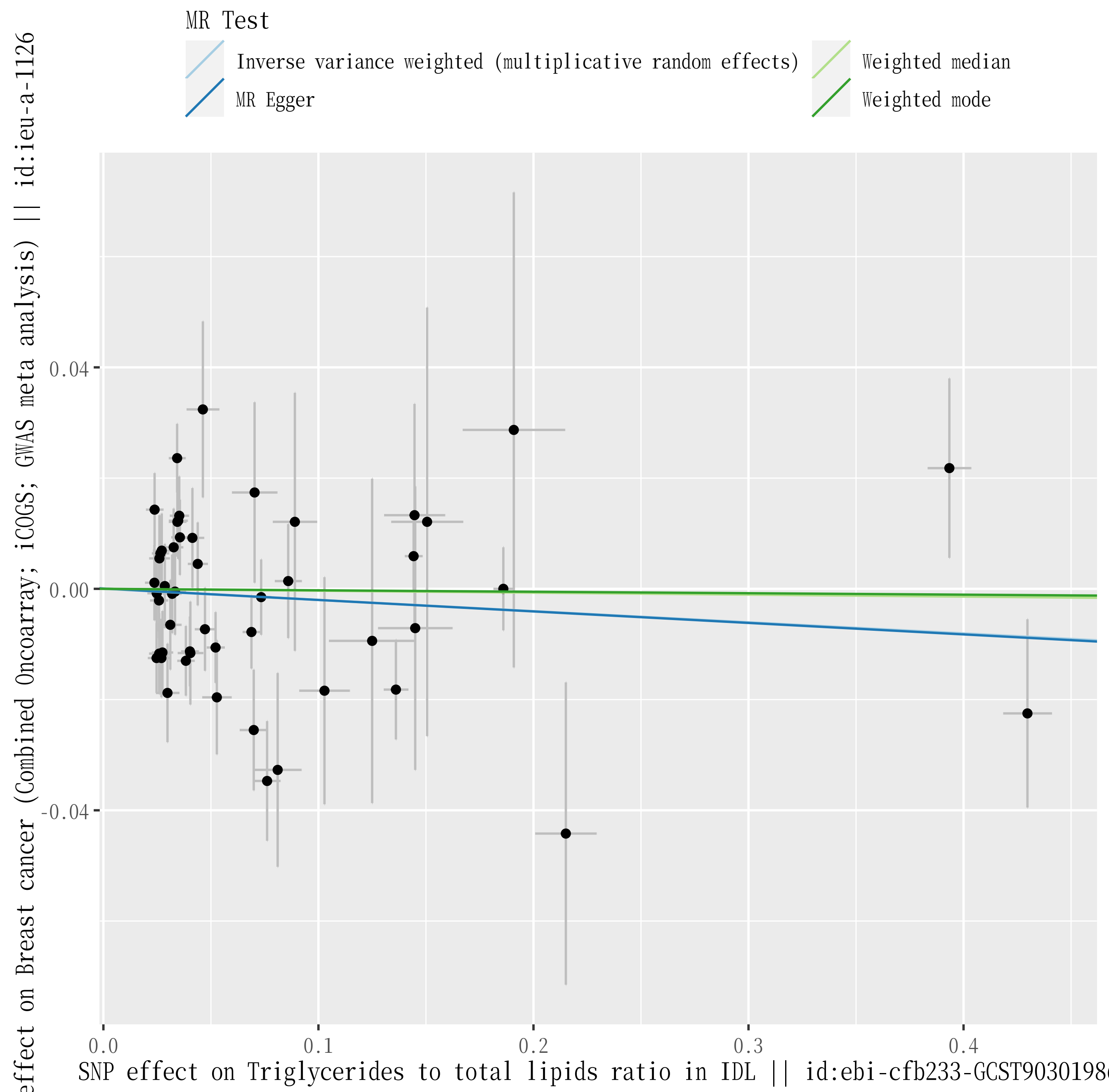
MR Test
Inverse variance weighted (multiplicative random effects)
MR Egger
Weighted median
Weighted mode
0.04
0.00
-0.04
0.0
0.1
0.2
0.3
0.4
SNP effect on Triglycerides to total lipids ratio in IDL || id:ebi-cfb233-GCST9030198
effect on Breast cancer (Combined Oncoarray; iCOGS; GWAS meta analysis) || id:ieu-a-1126

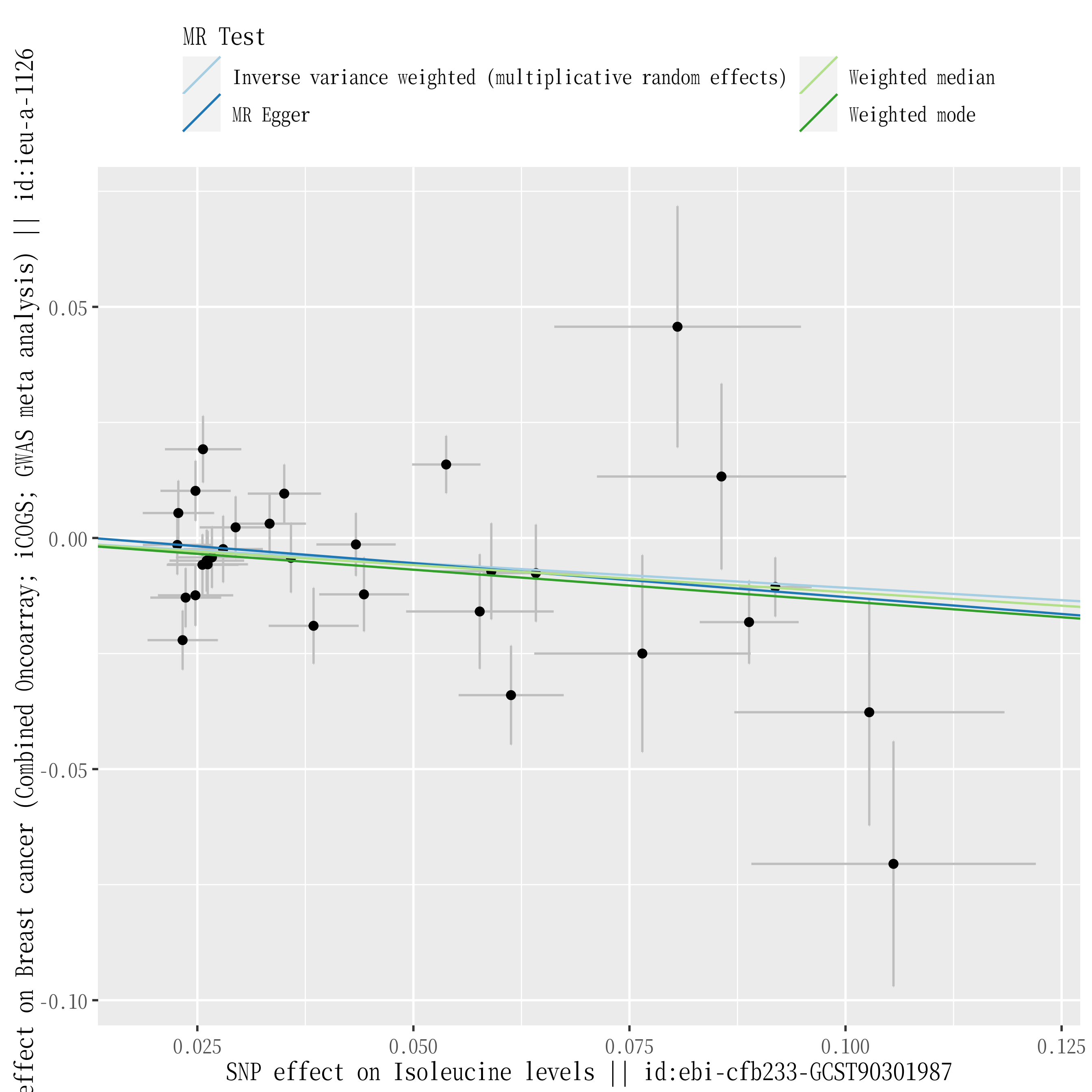
MR Test
Inverse variance weighted (multiplicative random effects)
MR Egger
Weighted median
Weighted mode
0.05
0.00
-0.05
-0.10
0.025
0.050
0.075
0.100
0.125
SNP effect on Isoleucine levels || id:ebi-cfb233-GCST90301987
effect on Breast cancer (Combined Oncoarray; iCOGS; GWAS meta analysis) || id:ieu-a-1126

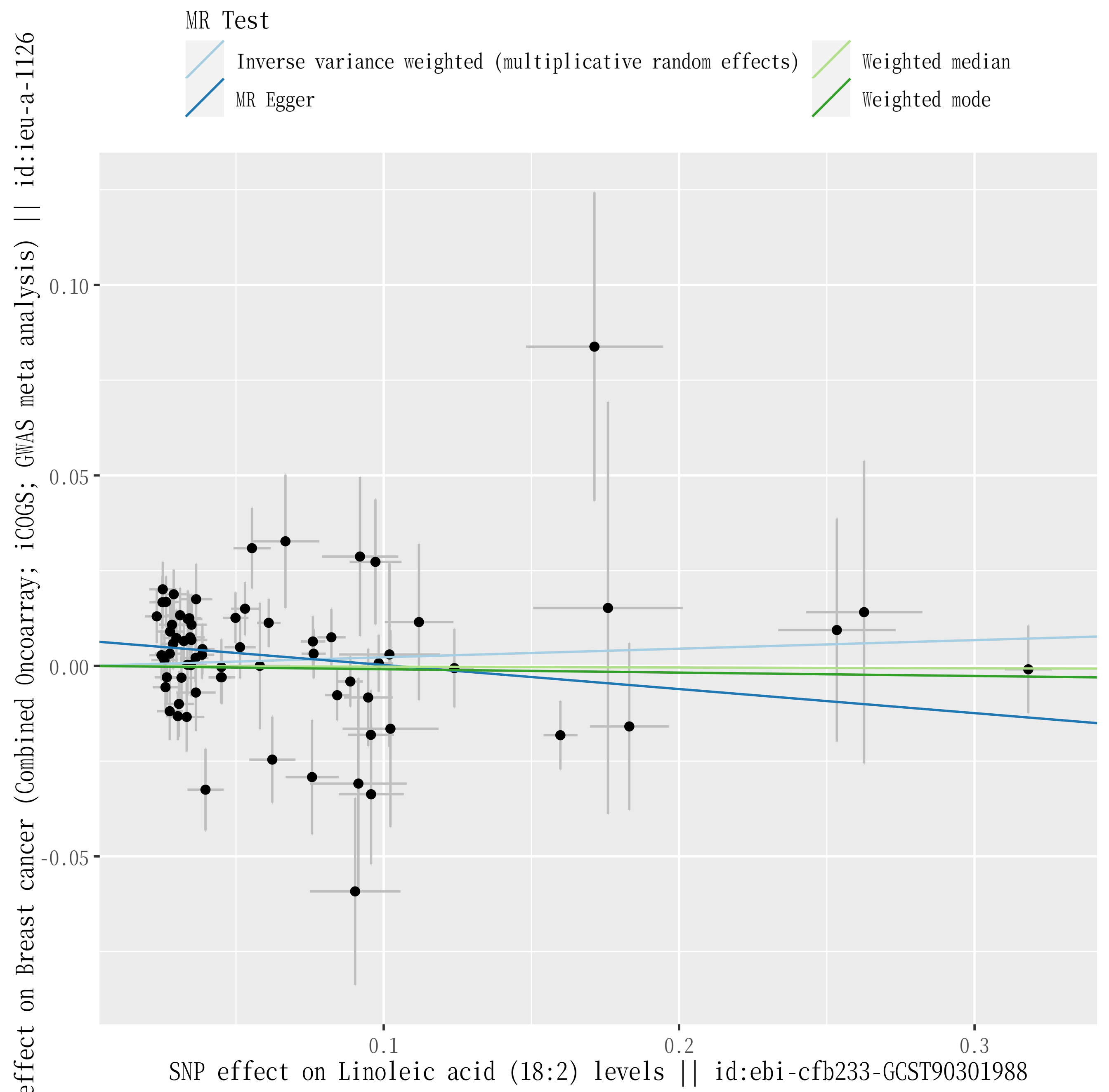

MR Test
Inverse variance weighted (multiplicative random effects)
MR Egger
Weighted median
Weighted mode
0.10
0.05
0.00
-0.05
0.1
0.2
0.3
SNP effect on Linoleic acid (18:2) levels || id:ebi-cfb233-GCST90301988
effect on Breast cancer (Combined Oncoarray; iCOGS; GWAS meta analysis) || id:ieu-a-1126

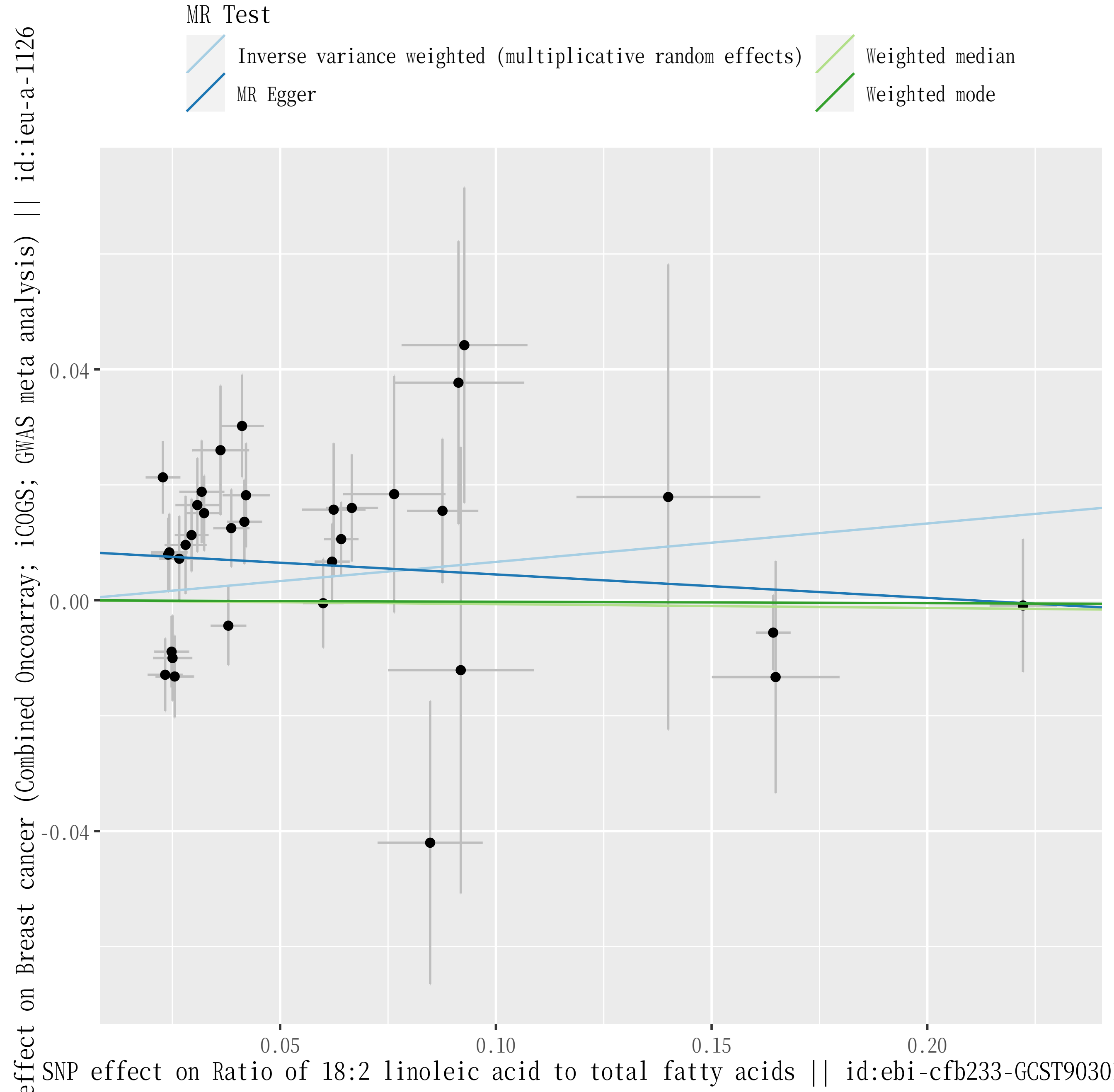

MR Test
Inverse variance weighted (multiplicative random effects)
MR Egger
Weighted median
Weighted mode
0.04
0.00
-0.04
0.05
0.10
0.15
0.20
SNP effect on Ratio of 18:2 linoleic acid to total fatty acids || id:ebi-cfb233-GCST9030
effect on Breast cancer (Combined Oncoarray; iCOGS; GWAS meta analysis) || id:ieu-a-1126

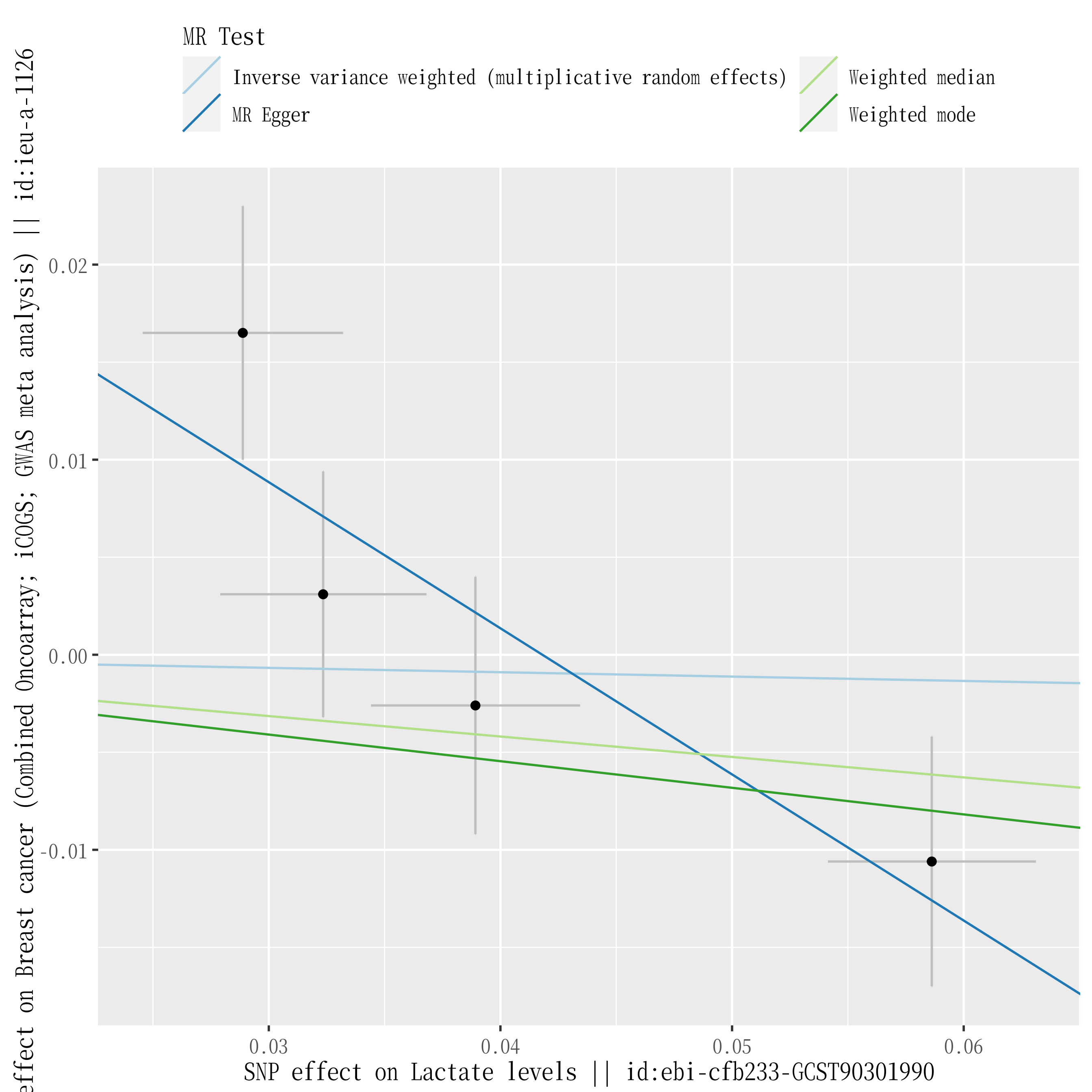

MR Test
Inverse variance weighted (multiplicative random effects)
MR Egger
Weighted median
Weighted mode
0.02
0.01
0.00
-0.01
0.03
0.04
0.05
0.06
SNP effect on Lactate levels || id:ebi-cfb233-GCST90301990
effect on Breast cancer (Combined Oncoarray; iCOGS; GWAS meta analysis) || id:ieu-a-1126

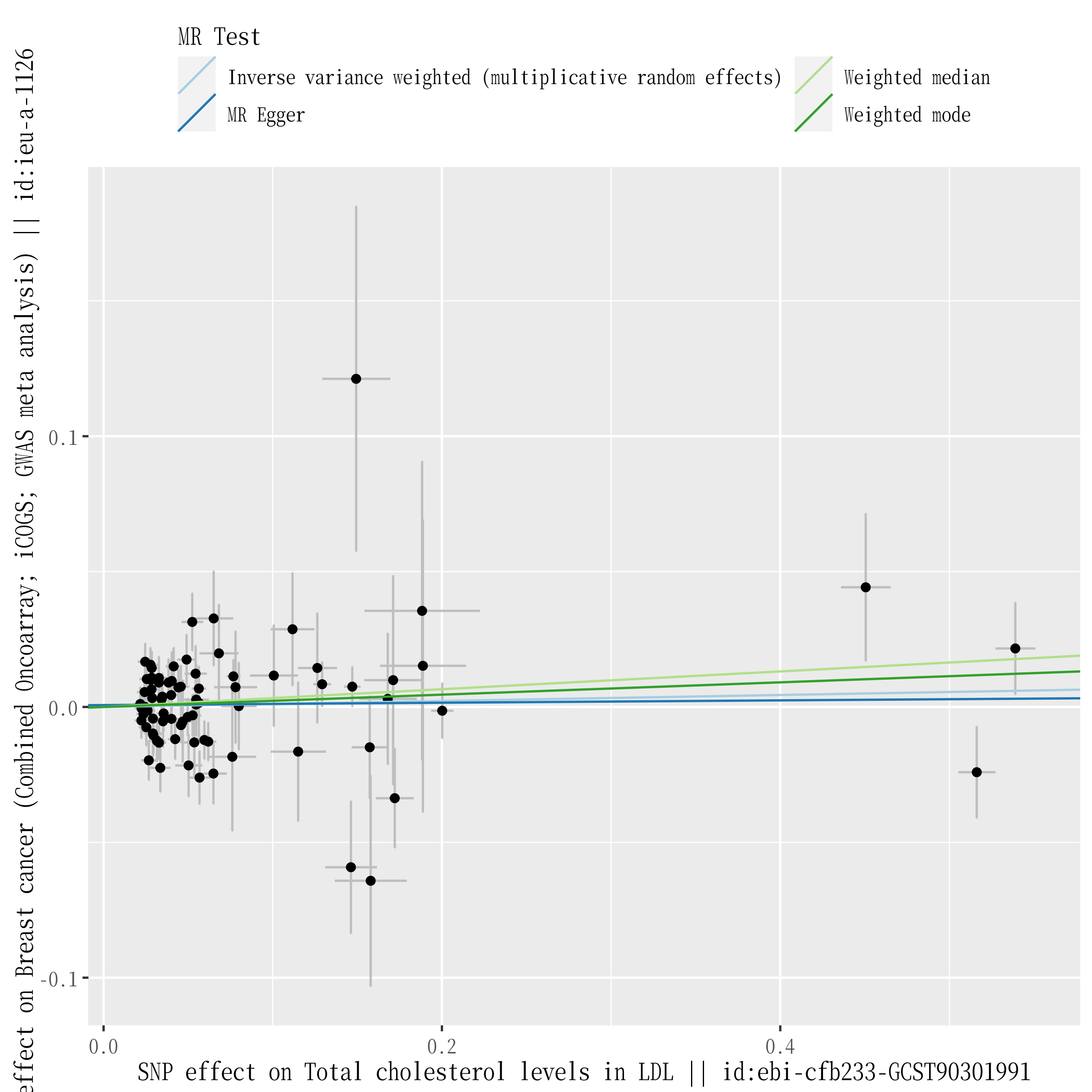
MR Test
Inverse variance weighted (multiplicative random effects)
MR Egger
Weighted median
Weighted mode
0.1
0.0
-0.1
0.0
0.2
0.4
SNP effect on Total cholesterol levels in LDL || id:ebi-cfb233-GCST90301991
effect on Breast cancer (Combined Oncoarray; iCOGS; GWAS meta analysis) || id:ieu-a-1126

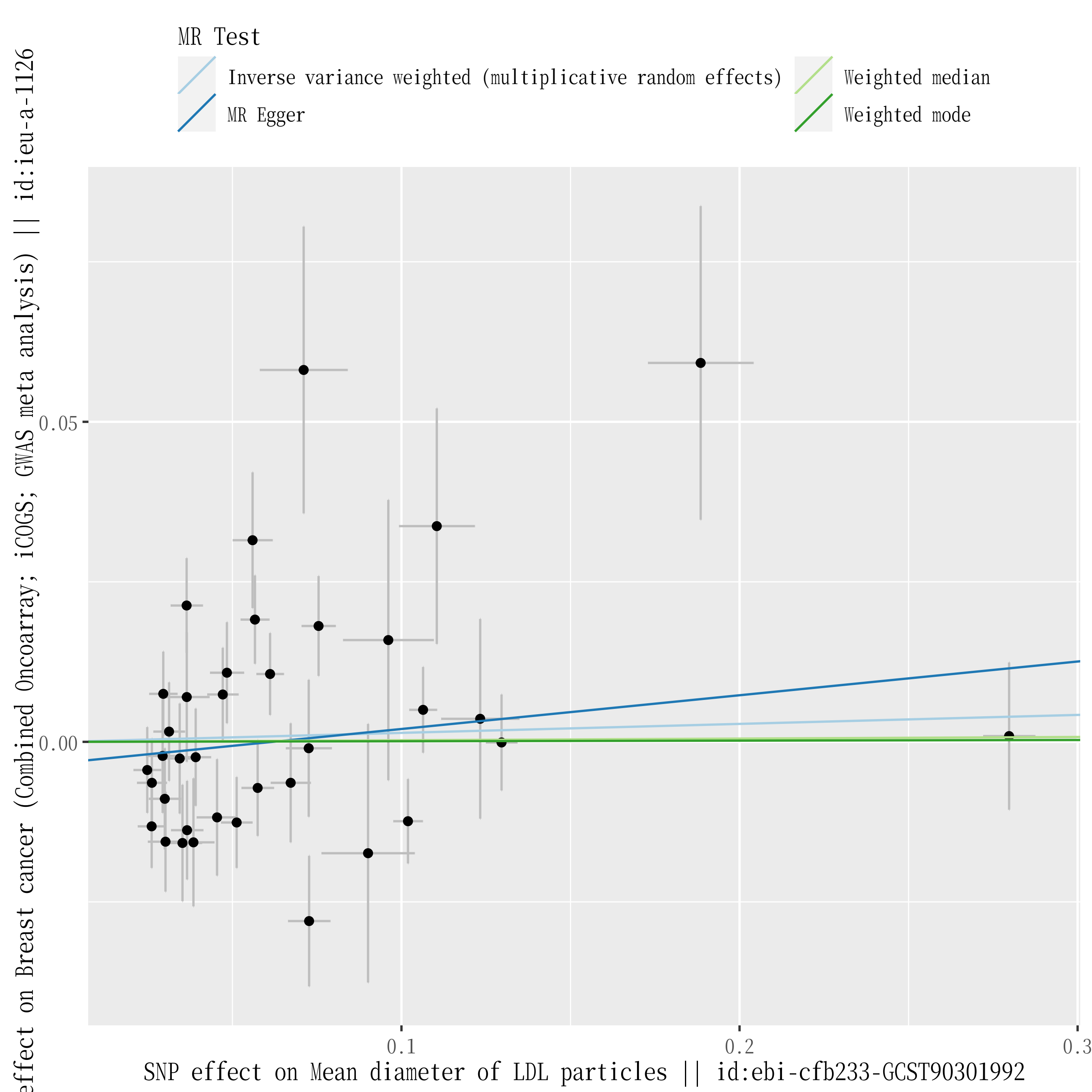
MR Test
Inverse variance weighted (multiplicative random effects)
MR Egger
Weighted median
Weighted mode
0.05
0.00
0.1
0.2
0.3
SNP effect on Mean diameter of LDL particles || id:ebi-cfb233-GCST90301992
effect on Breast cancer (Combined Oncoarray; iCOGS; GWAS meta analysis) || id:ieu-a-1126

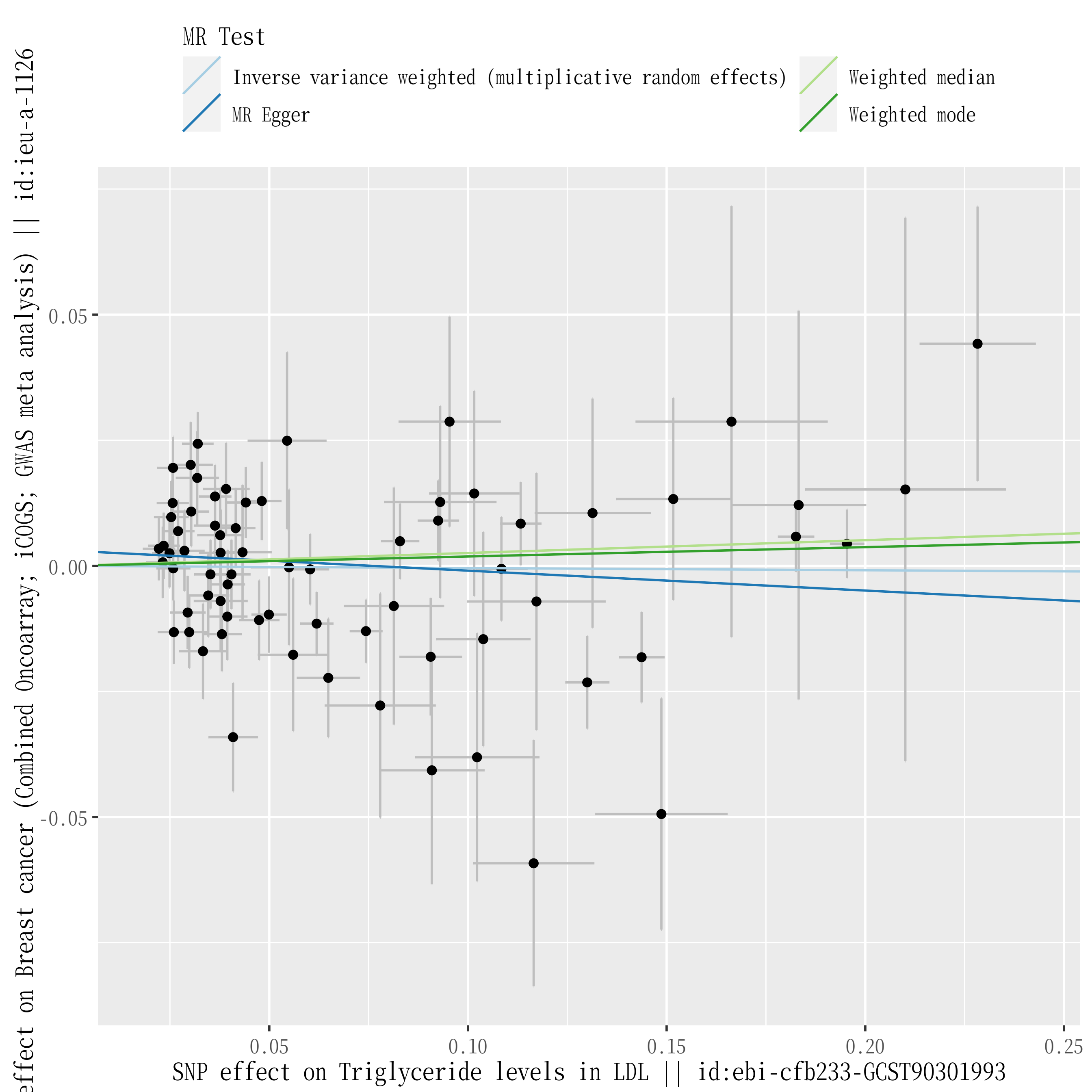

MR Test
Inverse variance weighted (multiplicative random effects)
MR Egger
Weighted median
Weighted mode
0.05
0.00
-0.05
0.05
0.10
0.15
0.20
0.25
SNP effect on Triglyceride levels in LDL || id:ebi-cfb233-GCST90301993
effect on Breast cancer (Combined Oncoarray; iCOGS; GWAS meta analysis) || id:ieu-a-1126

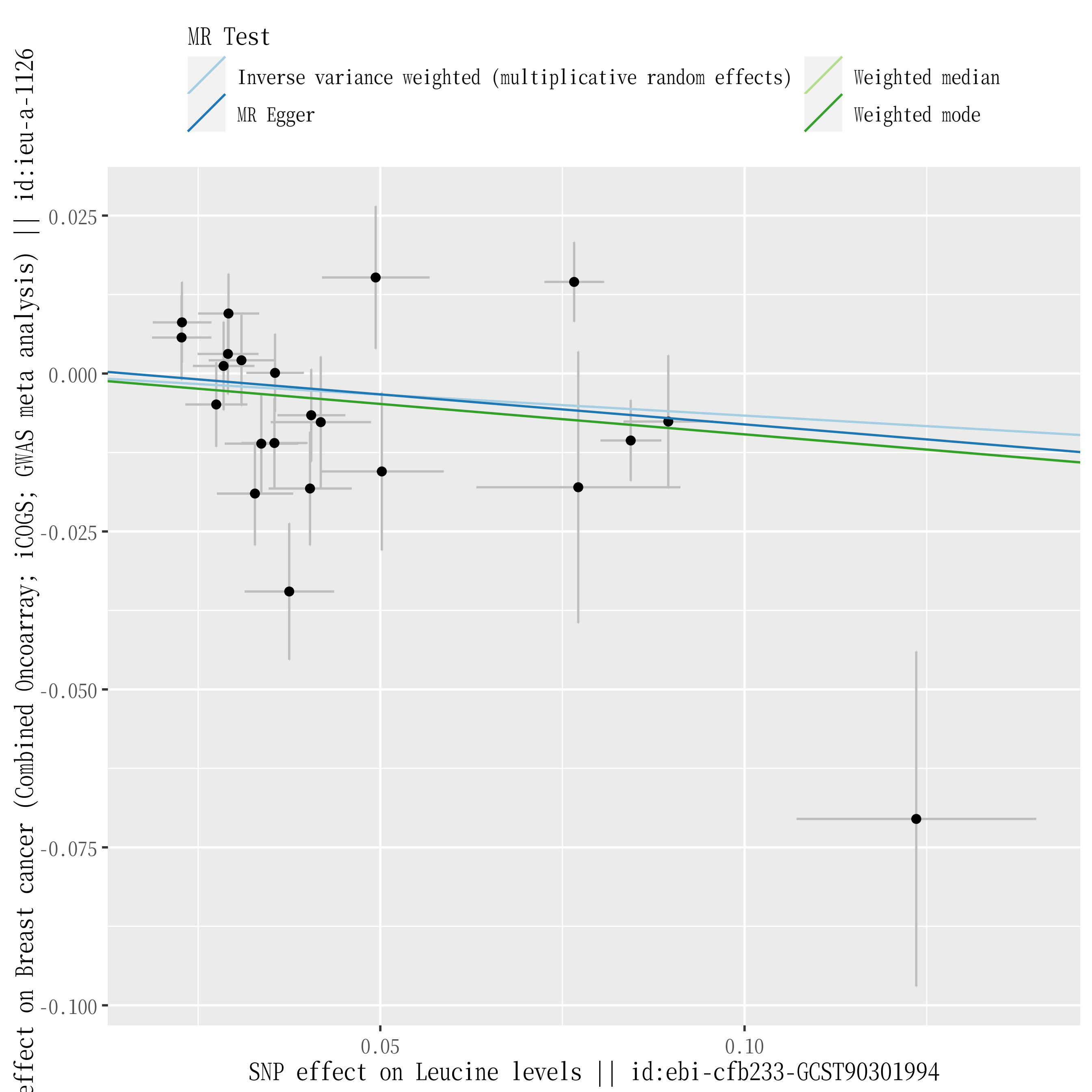

MR Test
Inverse variance weighted (multiplicative random effects)
MR Egger
Weighted median
Weighted mode
0.025
0.000
-0.025
-0.050
-0.075
-0.100
0.05
0.10
SNP effect on Leucine levels || id:ebi-cfb233-GCST90301994
effect on Breast cancer (Combined Oncoarray; iCOGS; GWAS meta analysis) || id:ieu-a-1126

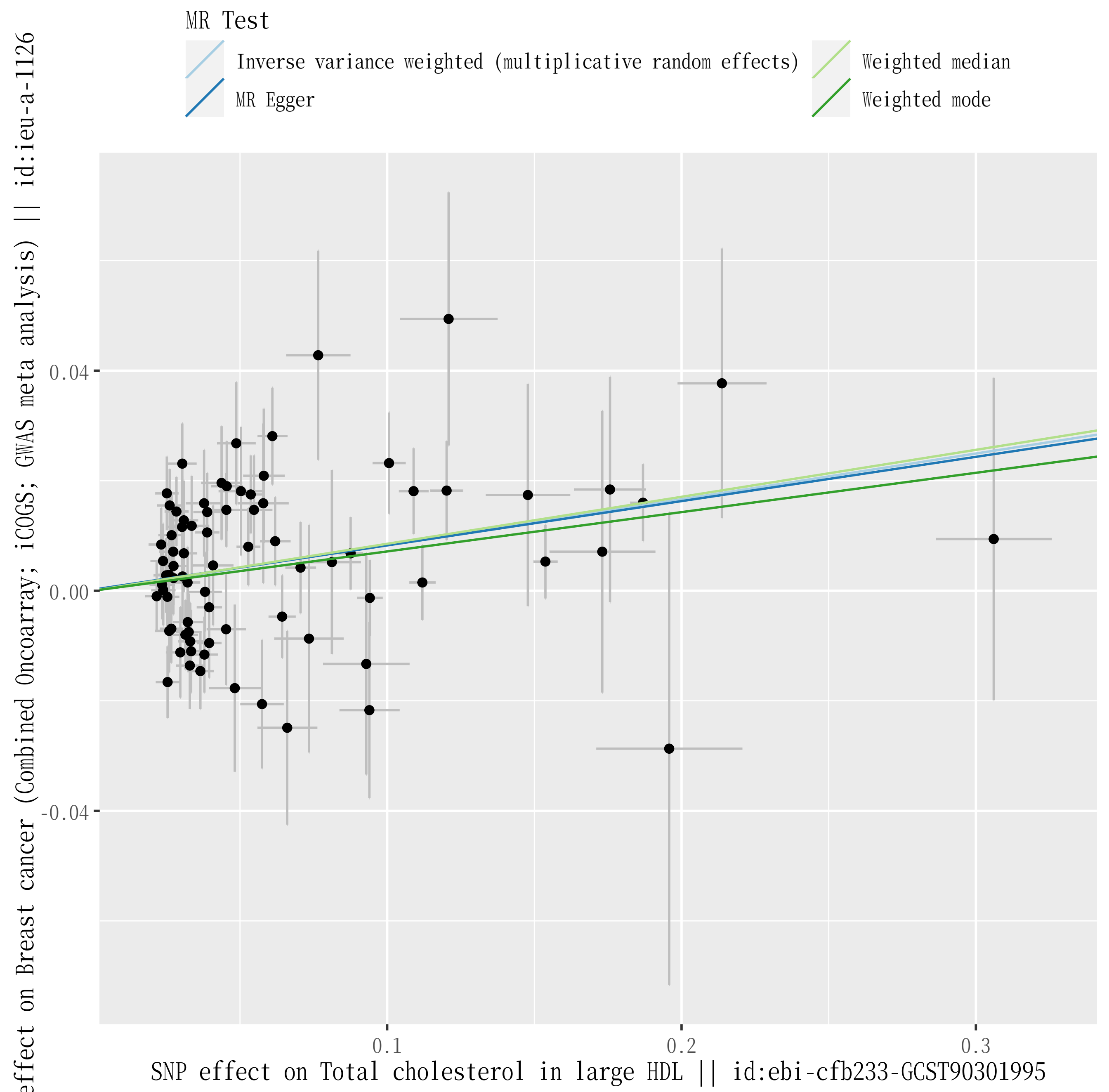
MR Test
Inverse variance weighted (multiplicative random effects)
MR Egger
Weighted median
Weighted mode
0.04
0.00
-0.04
0.1
0.2
0.3
SNP effect on Total cholesterol in large HDL || id:ebi-cfb233-GCST90301995
effect on Breast cancer (Combined Oncoarray; iCOGS; GWAS meta analysis) || id:ieu-a-1126

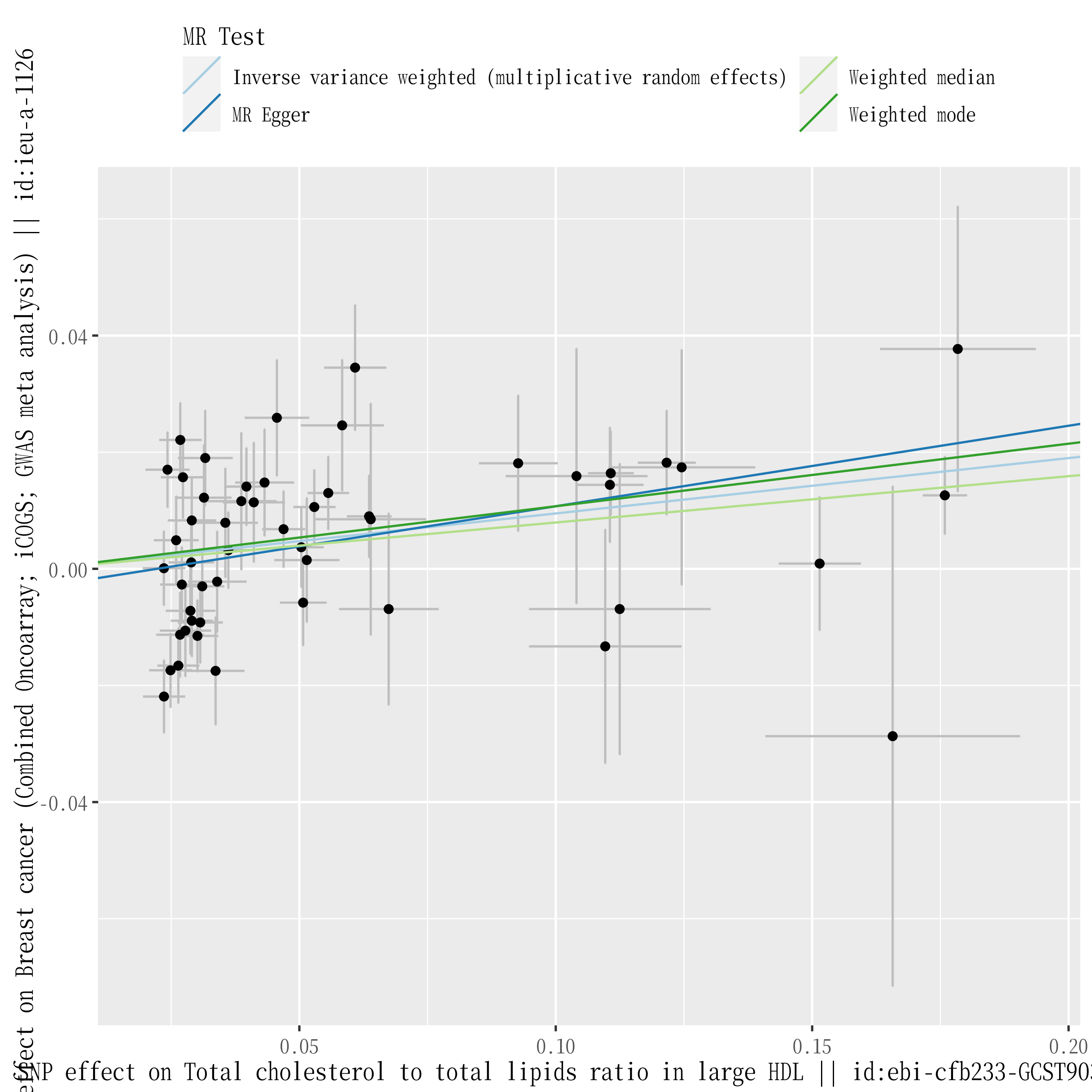

MR Test
Inverse variance weighted (multiplicative random effects)
MR Egger
Weighted median
Weighted mode
0.04
0.00
-0.04
0.05
0.10
0.15
0.20
SNP effect on Total cholesterol to total lipids ratio in large HDL || id:ebi-cfb233-GCST90
effect on Breast cancer (Combined Oncoarray; iCOGS; GWAS meta analysis) || id:ieu-a-1126

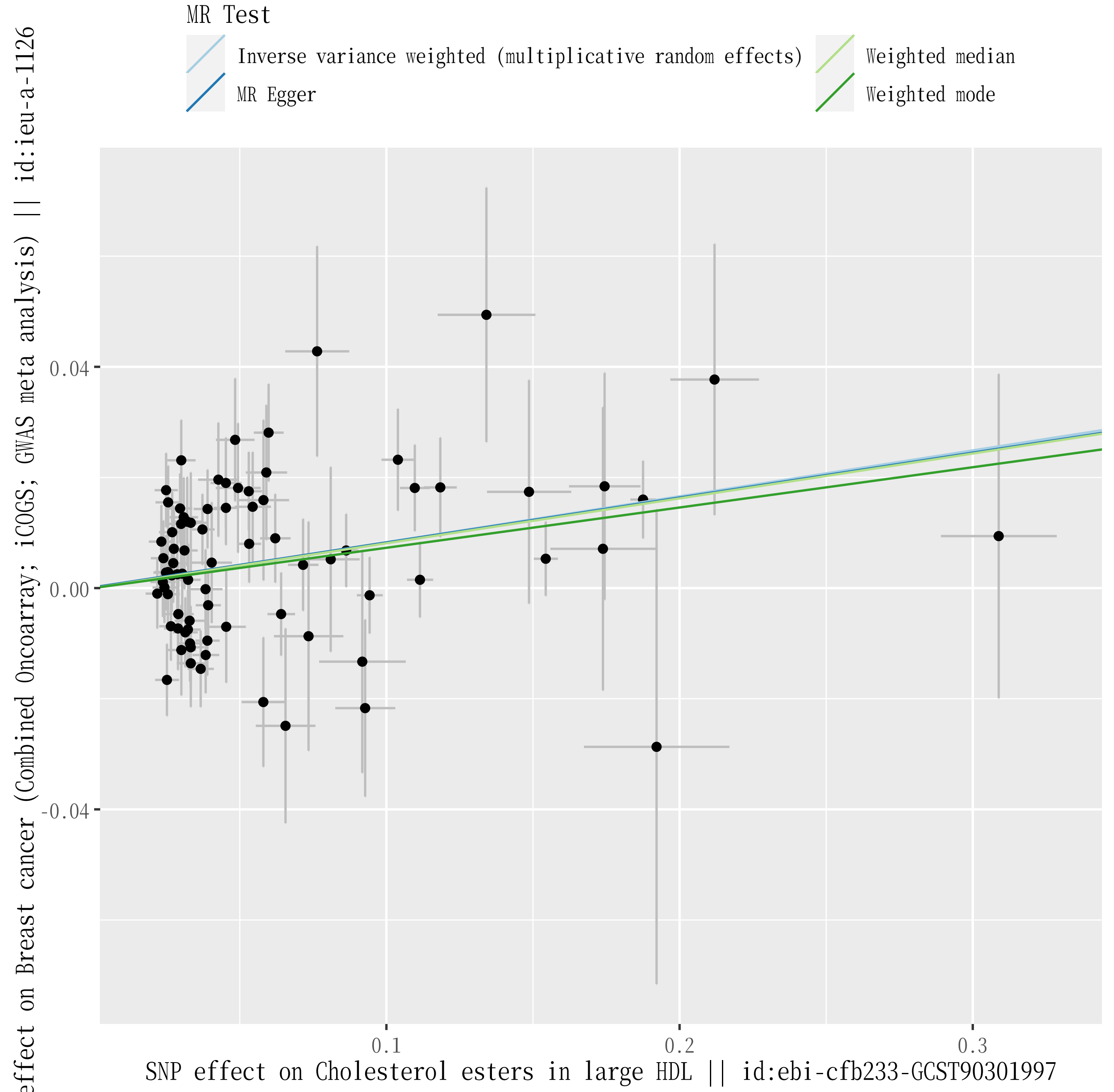

MR Test
Inverse variance weighted (multiplicative random effects)
MR Egger
Weighted median
Weighted mode
0.04
0.00
-0.04
0.1
0.2
0.3
SNP effect on Cholesterol esters in large HDL || id:ebi-cfb233-GCST90301997
effect on Breast cancer (Combined Oncoarray; iCOGS; GWAS meta analysis) || id:ieu-a-1126

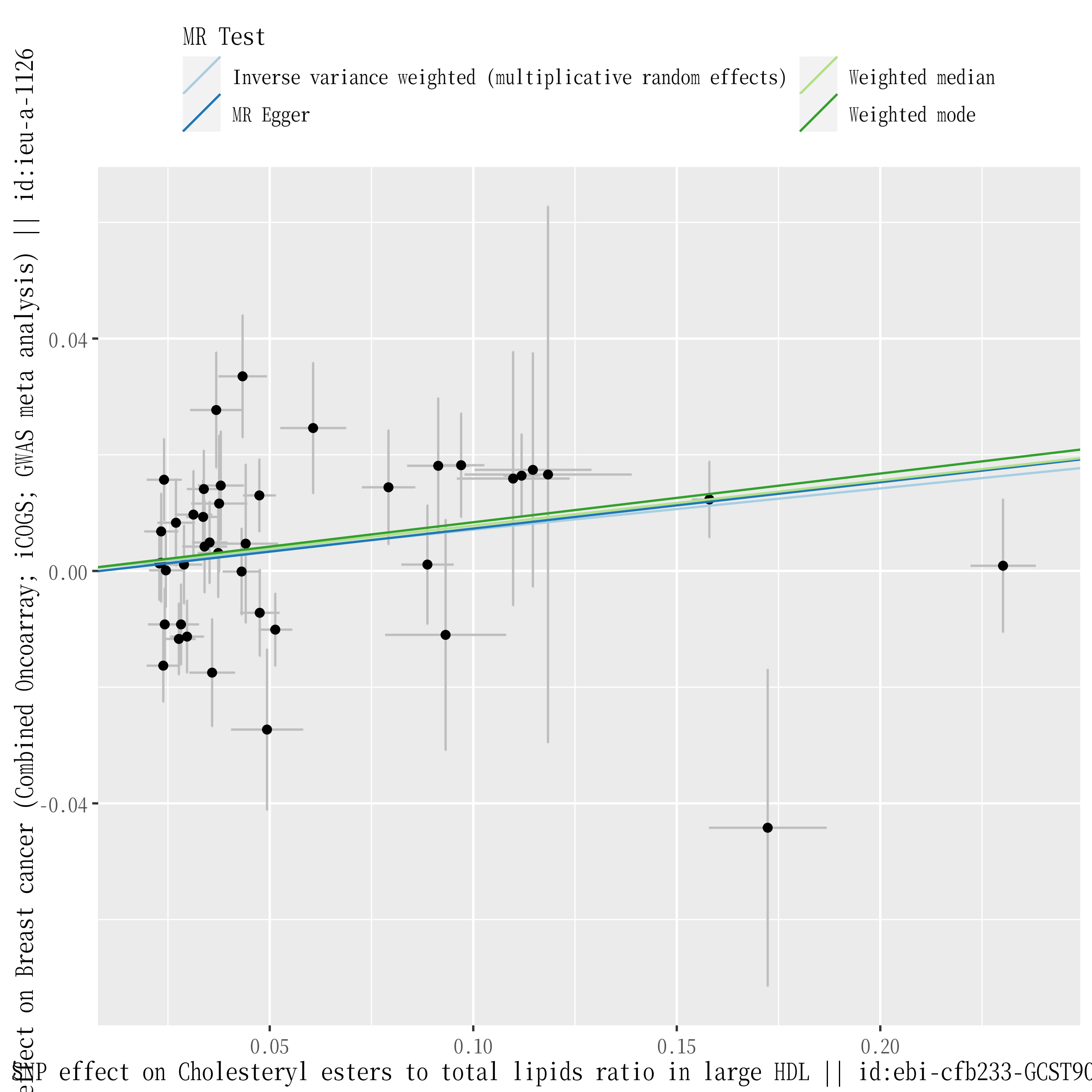

MR Test
Inverse variance weighted (multiplicative random effects)
MR Egger
Weighted median
Weighted mode
0.04
0.00
-0.04
0.05
0.10
0.15
0.20
SNP effect on Cholesteryl esters to total lipids ratio in large HDL || id:ebi-cfb233-GCST90
effect on Breast cancer (Combined Oncoarray; iCOGS; GWAS meta analysis) || id:ieu-a-1126

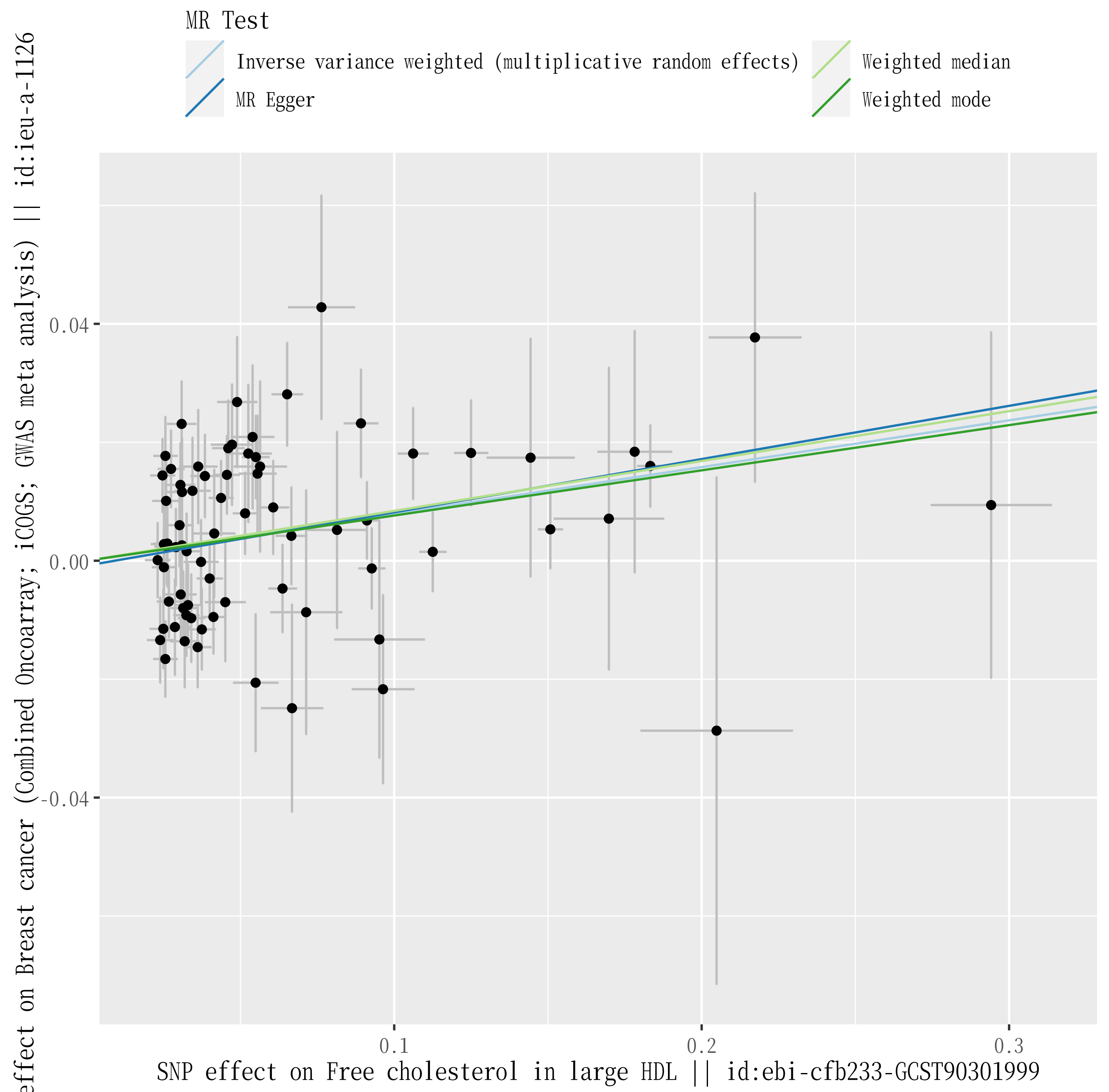
MR Test
Inverse variance weighted (multiplicative random effects)
MR Egger
Weighted median
Weighted mode
SNP effect on Breast cancer (Combined Oncoarray; iCOGS; GWAS meta analysis) || id:ieu-a-1126
0.04
0.00
-0.04
0.1
0.2
0.3
SNP effect on Free cholesterol in large HDL || id:ebi-cfb233-GCST90301999

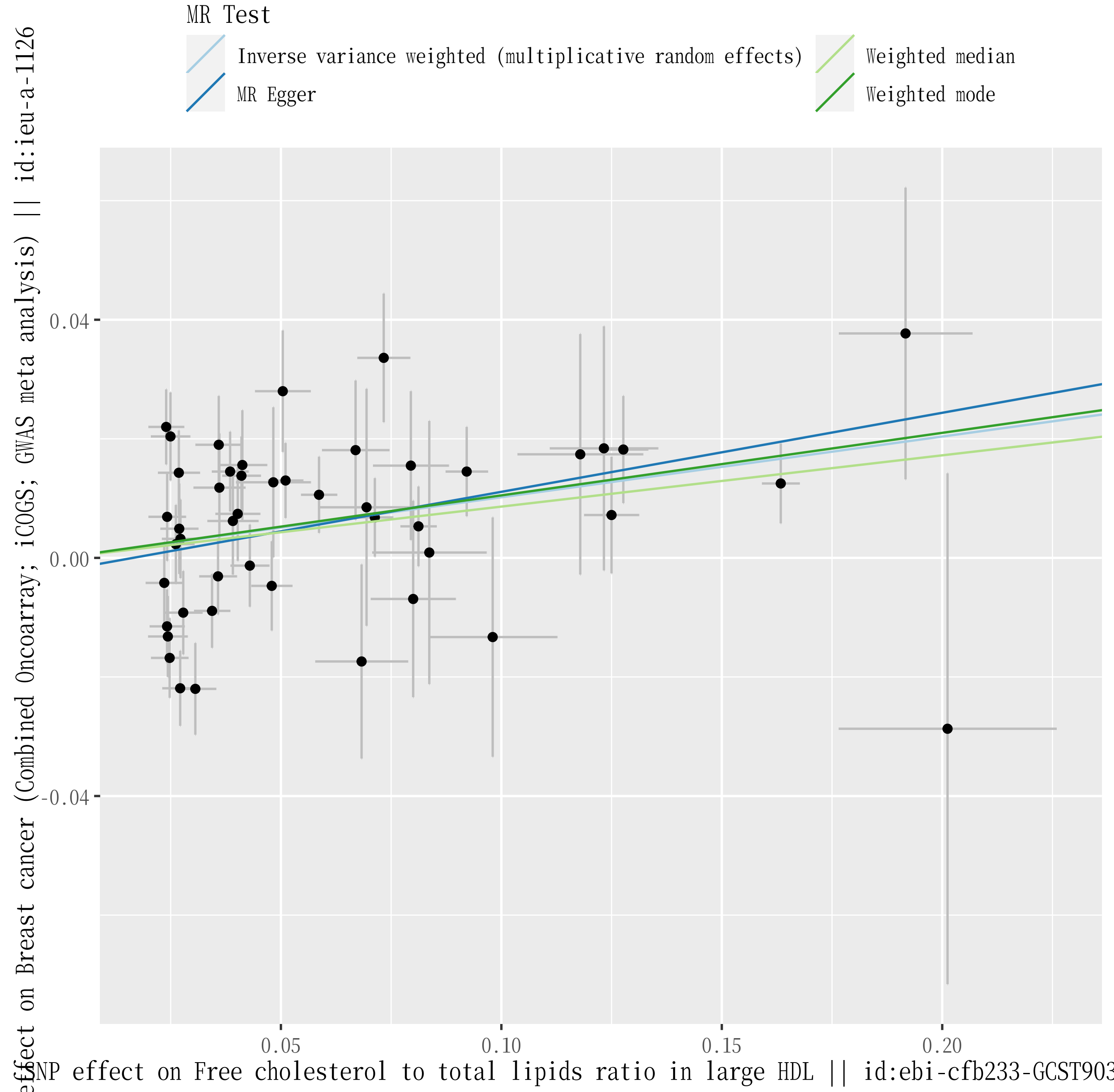
MR Test
Inverse variance weighted (multiplicative random effects)
MR Egger
Weighted median
Weighted mode
0.04
0.00
-0.04
0.05
0.10
0.15
0.20
SNP effect on Free cholesterol to total lipids ratio in large HDL || id:ebi-cfb233-GCST903
effect on Breast cancer (Combined Oncoarray; iCOGS; GWAS meta analysis) || id:ieu-a-1126

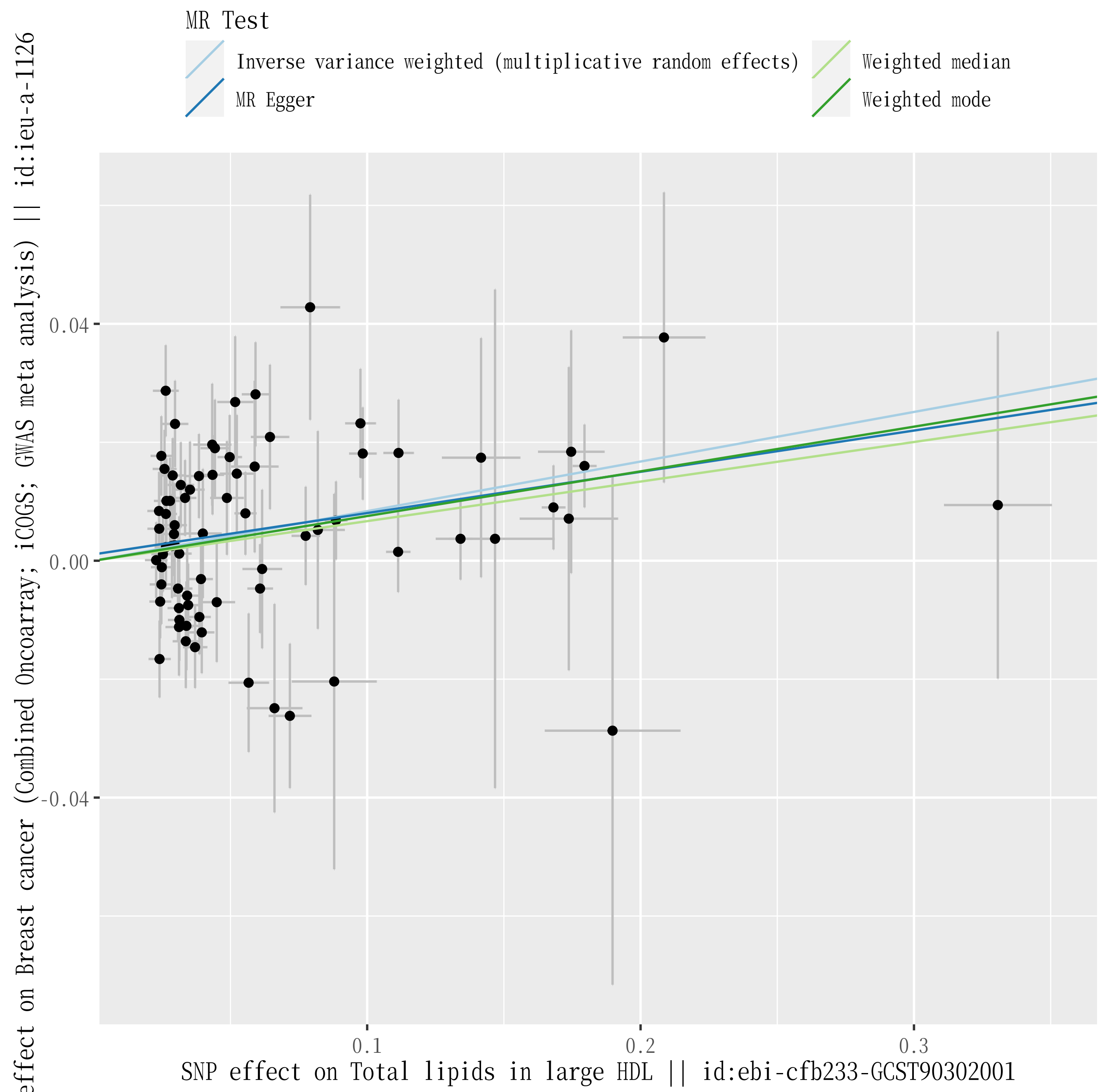

MR Test
Inverse variance weighted (multiplicative random effects)
MR Egger
Weighted median
Weighted mode
0.04
0.00
-0.04
0.1
0.2
0.3
SNP effect on Total lipids in large HDL || id:ebi-cfb233-GCST90302001
effect on Breast cancer (Combined Oncoarray; iCOGS; GWAS meta analysis) || id:ieu-a-1126

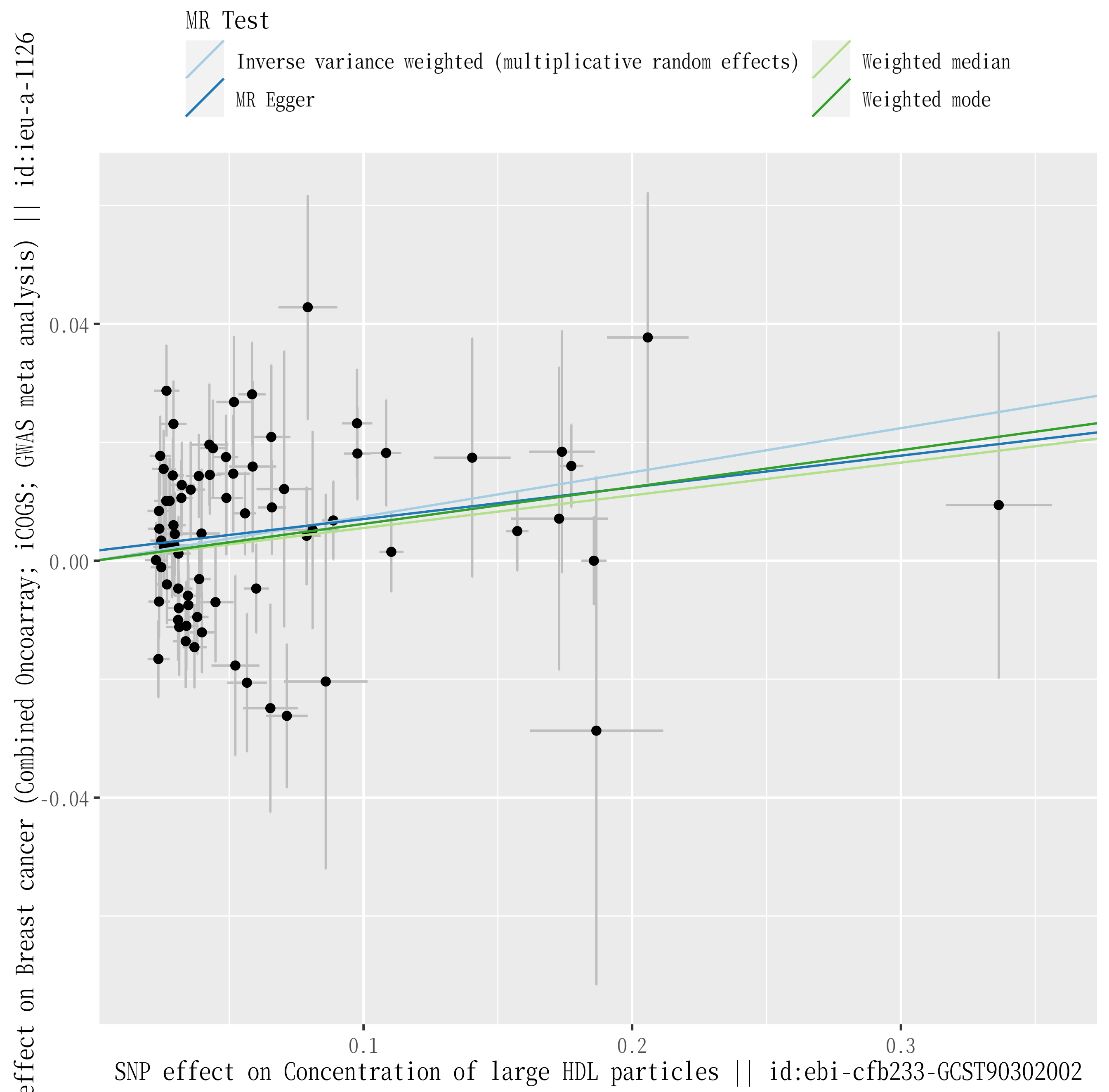

MR Test
Inverse variance weighted (multiplicative random effects)
MR Egger
Weighted median
Weighted mode
0.04
0.00
-0.04
0.1
0.2
0.3
SNP effect on Concentration of large HDL particles || id:ebi-cfb233-GCST90302002
effect on Breast cancer (Combined Oncoarray; iCOGS; GWAS meta analysis) || id:ieu-a-1126

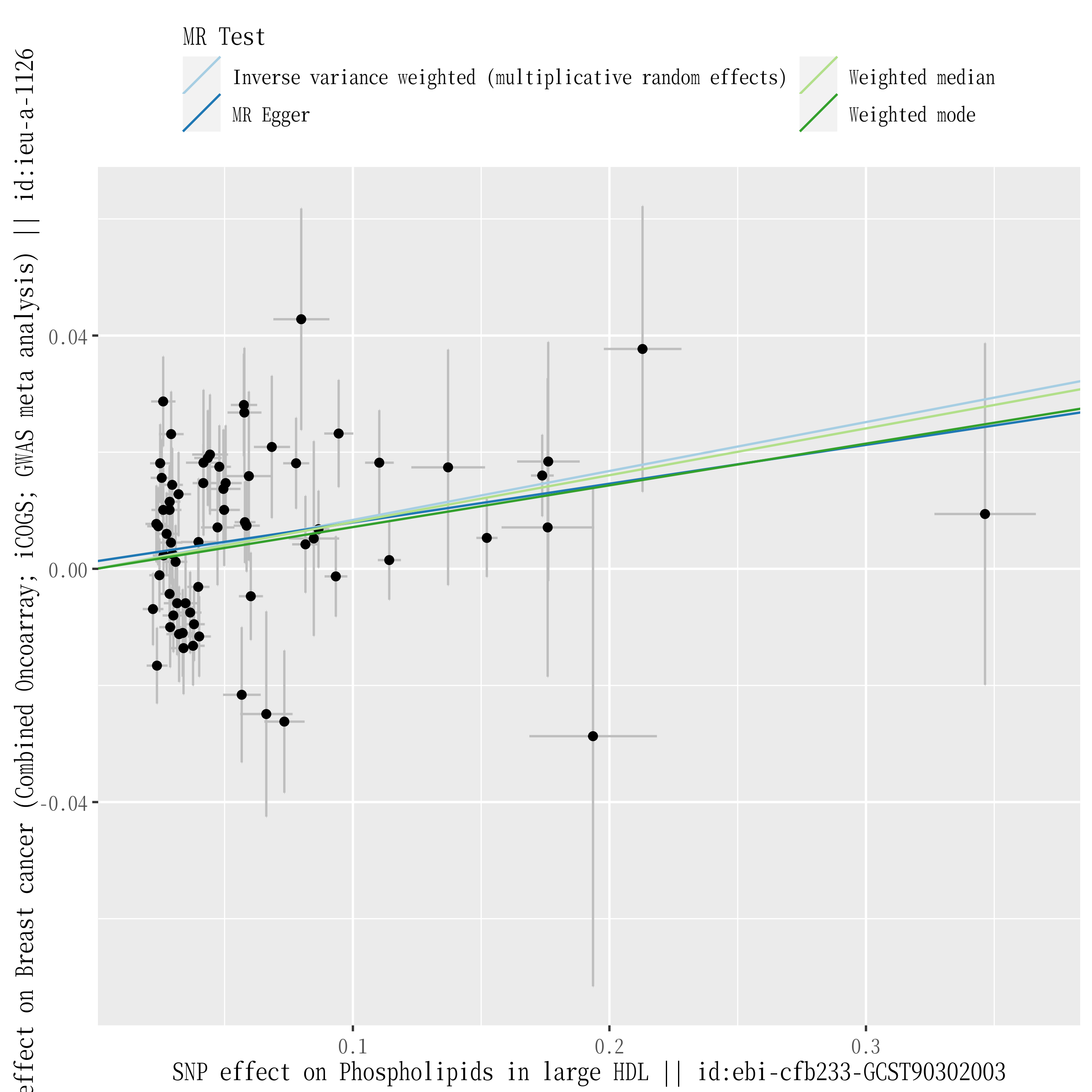

MR Test
Inverse variance weighted (multiplicative random effects)
MR Egger
Weighted median
Weighted mode
0.04
0.00
-0.04
0.1
0.2
0.3
SNP effect on Phospholipids in large HDL || id:ebi-cfb233-GCST90302003
effect on Breast cancer (Combined Oncoarray; iCOGS; GWAS meta analysis) || id:ieu-a-1126

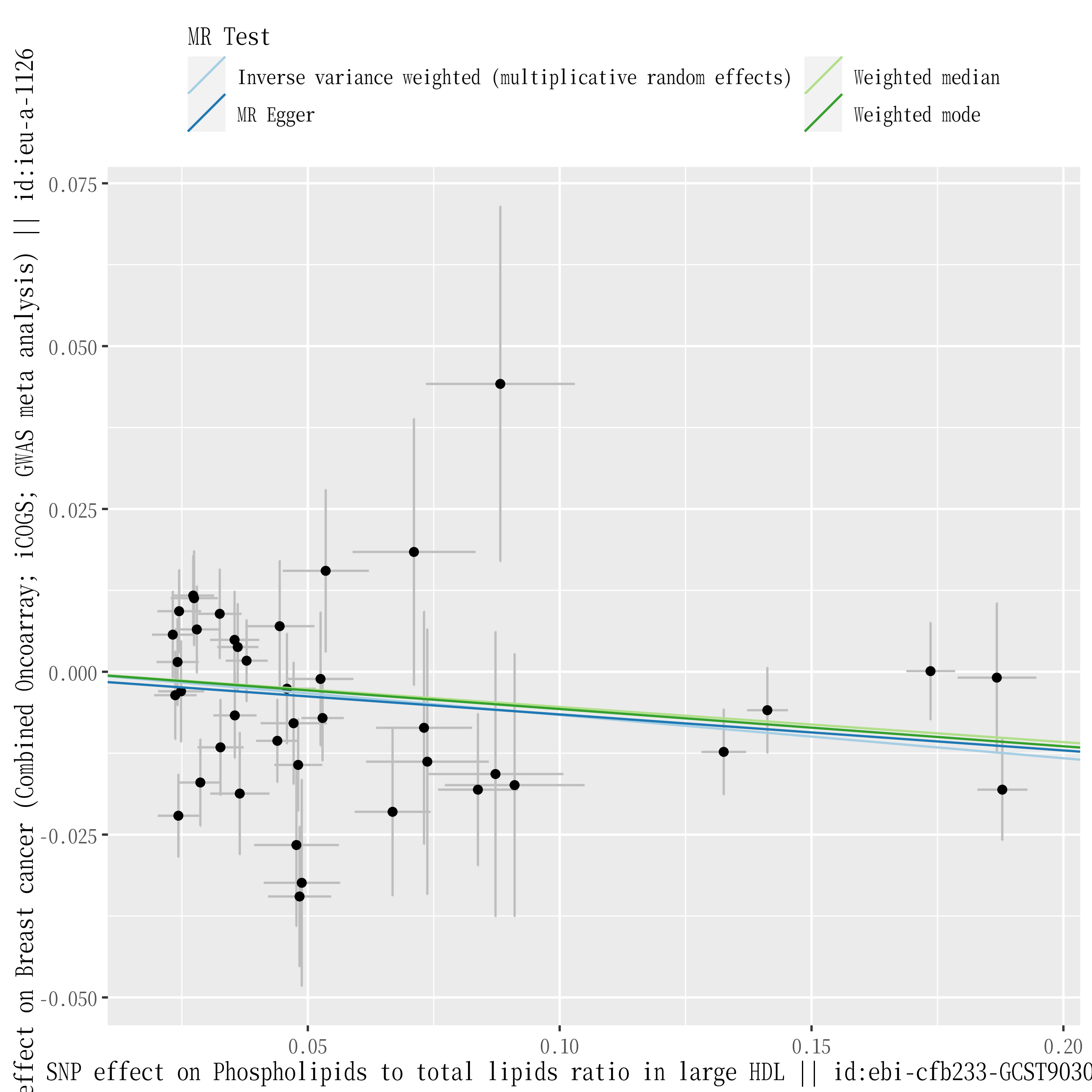
MR Test
Inverse variance weighted (multiplicative random effects)
MR Egger
Weighted median
Weighted mode
0.075
0.050
0.025
0.000
-0.025
-0.050
0.05
0.10
0.15
0.20
SNP effect on Phospholipids to total lipids ratio in large HDL || id:ebi-cfb233-GCST9030
effect on Breast cancer (Combined Oncoarray; iCOGS; GWAS meta analysis) || id:ieu-a-1126

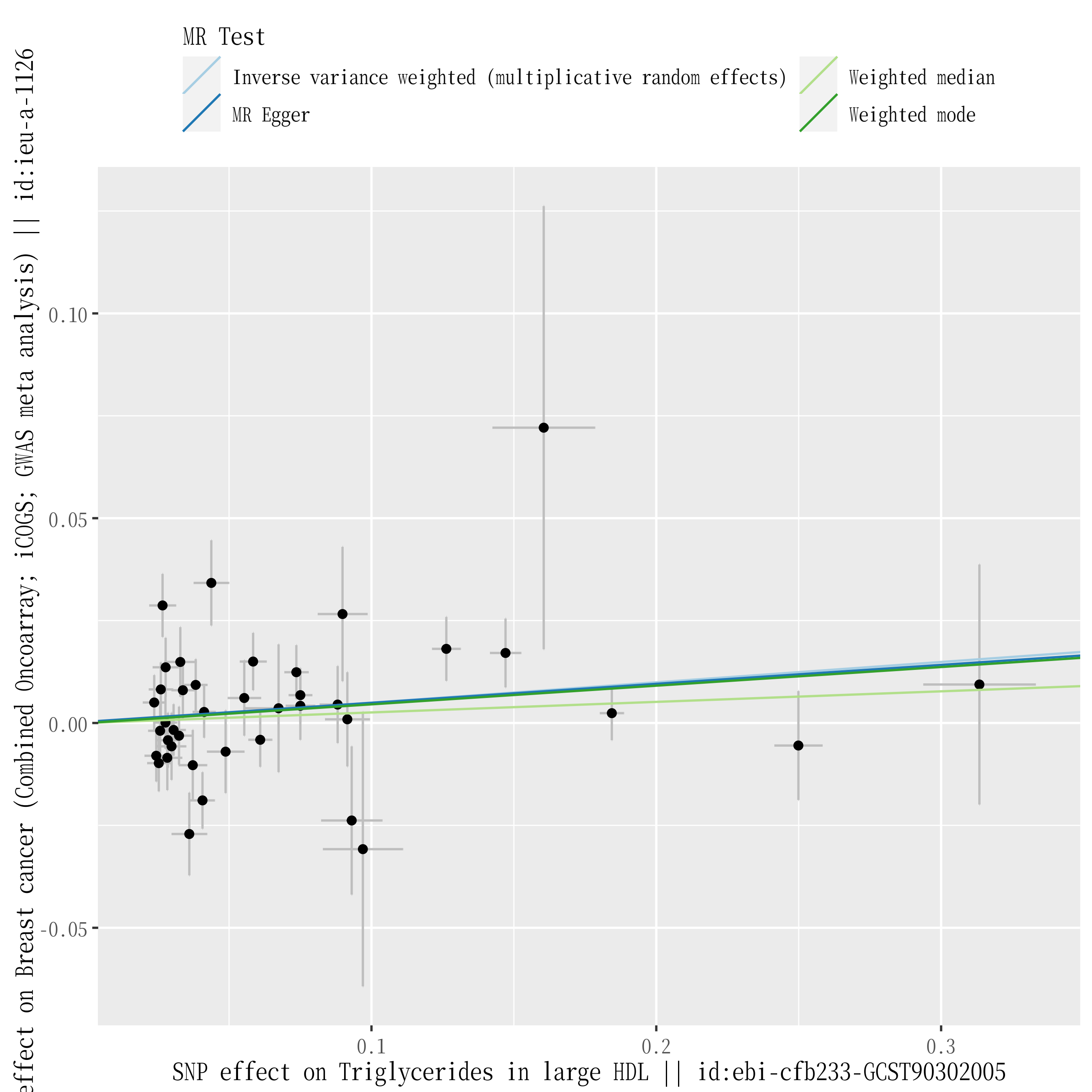
MR Test
Inverse variance weighted (multiplicative random effects)
MR Egger
Weighted median
Weighted mode
SNP effect on Breast cancer (Combined Oncoarray; iCOGS; GWAS meta analysis) || id:ieu-a-1126
0.10
0.05
0.00
-0.05
0.1
0.2
0.3
SNP effect on Triglycerides in large HDL || id:ebi-cfb233-GCST90302005

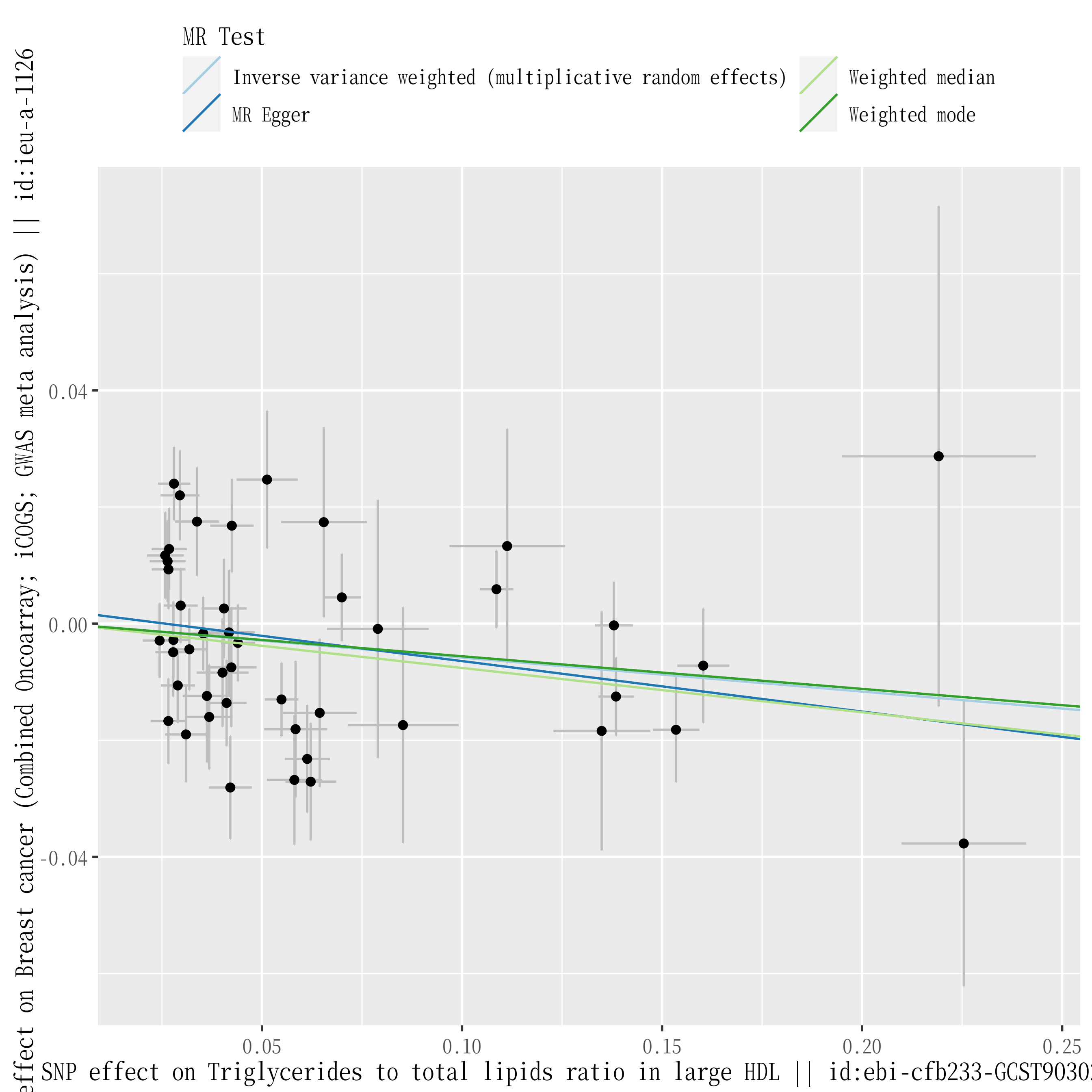

MR Test
Inverse variance weighted (multiplicative random effects)
MR Egger
Weighted median
Weighted mode
0.04
0.00
-0.04
0.05
0.10
0.15
0.20
0.25
SNP effect on Triglycerides to total lipids ratio in large HDL || id:ebi-a-GCST9030
effect on Breast cancer (Combined Oncoarray; iCOGS; GWAS meta analysis) || id:ieu-a-1126

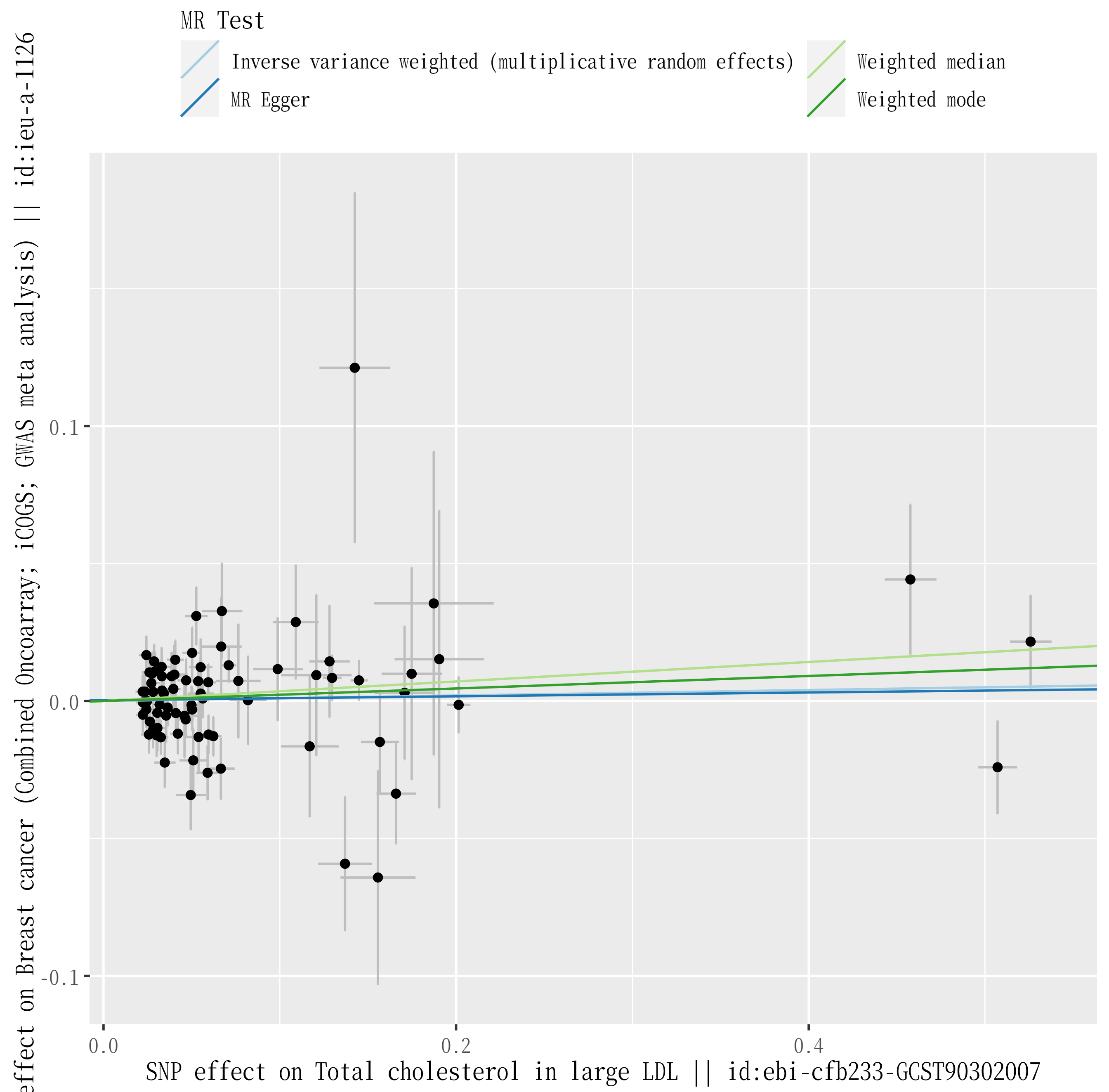

MR Test
Inverse variance weighted (multiplicative random effects)
MR Egger
Weighted median
Weighted mode
0.1
0.0
-0.1
0.0
0.2
0.4
SNP effect on Total cholesterol in large LDL || id:ebi-cfb233-GCST90302007
effect on Breast cancer (Combined Oncoarray; iCOGS; GWAS meta analysis) || id:ieu-a-1126

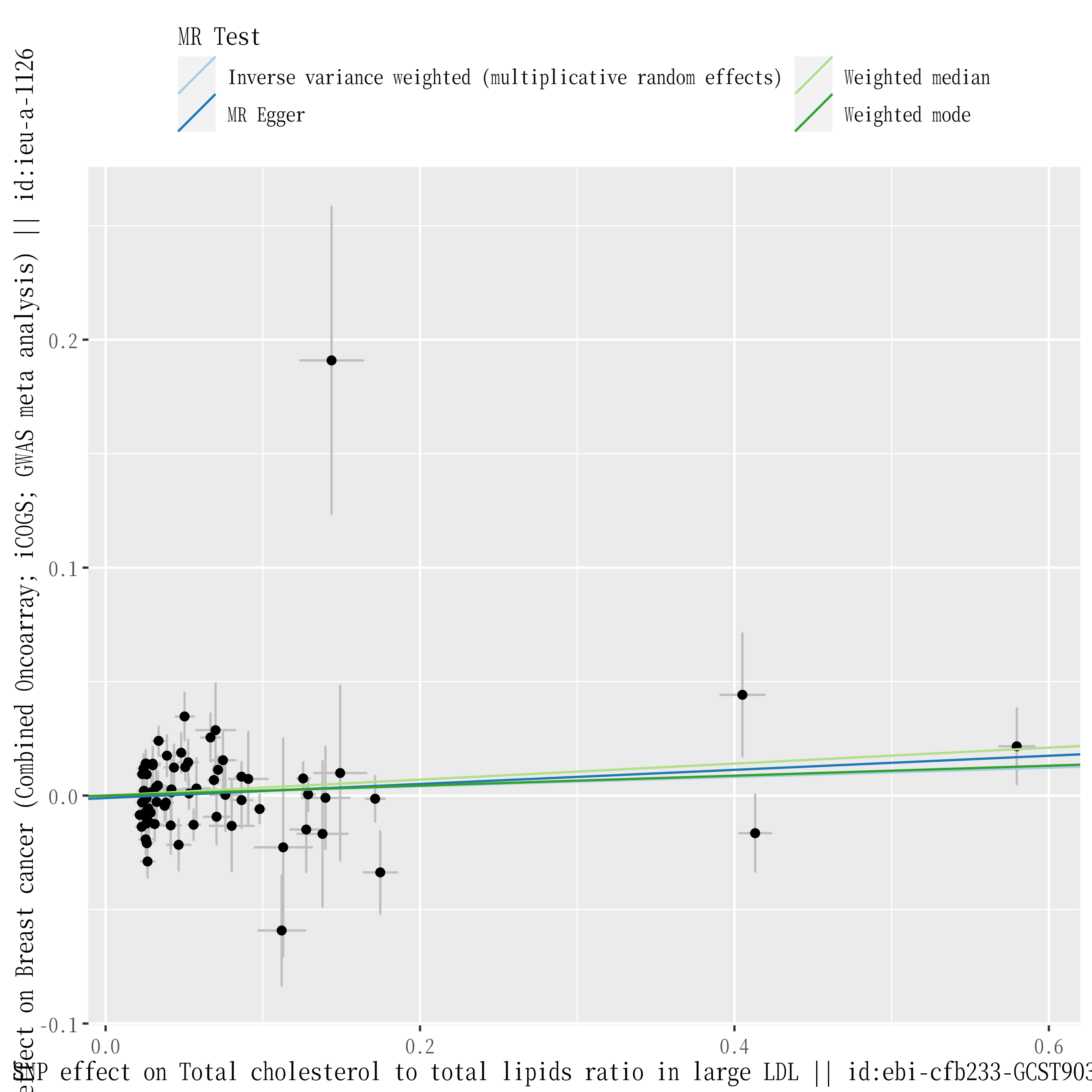
MR Test
Inverse variance weighted (multiplicative random effects)
MR Egger
Weighted median
Weighted mode
0.2
0.1
0.0
-0.1
0.0
0.2
0.4
0.6
SNP effect on Total cholesterol to total lipids ratio in large LDL || id:ebi-cfb233-GCST903
effect on Breast cancer (Combined Oncoarray; iCOGS; GWAS meta analysis) || id:ieu-a-1126

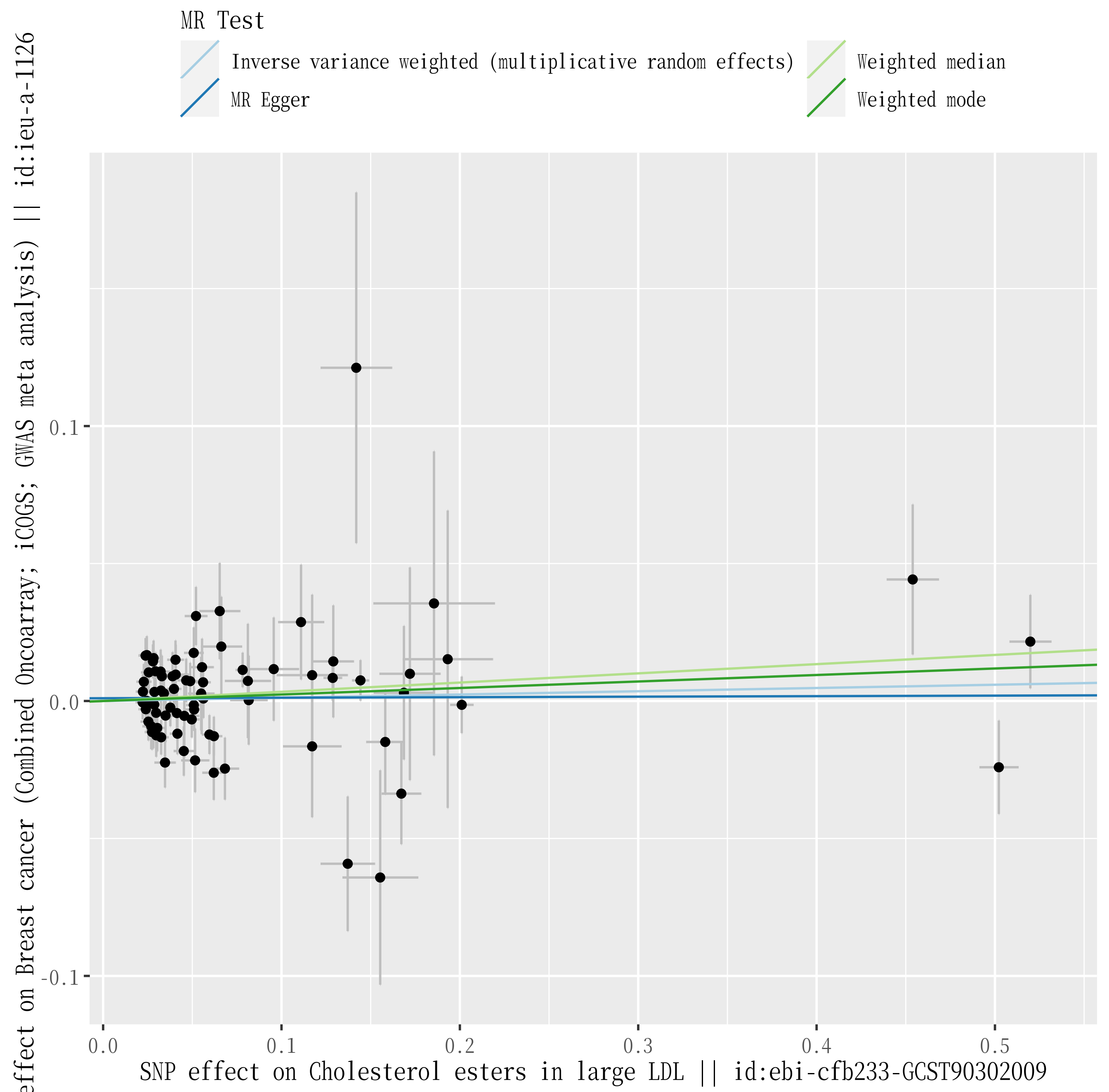

MR Test
Inverse variance weighted (multiplicative random effects)
MR Egger
Weighted median
Weighted mode
0.1
0.0
-0.1
0.0
0.1
0.2
0.3
0.4
0.5
SNP effect on Cholesterol esters in large LDL || id:ebi-cfb233-GCST90302009
SNP effect on Breast cancer (Combined Oncoarray; iCOGS; GWAS meta analysis) || id:ieu-a-1126

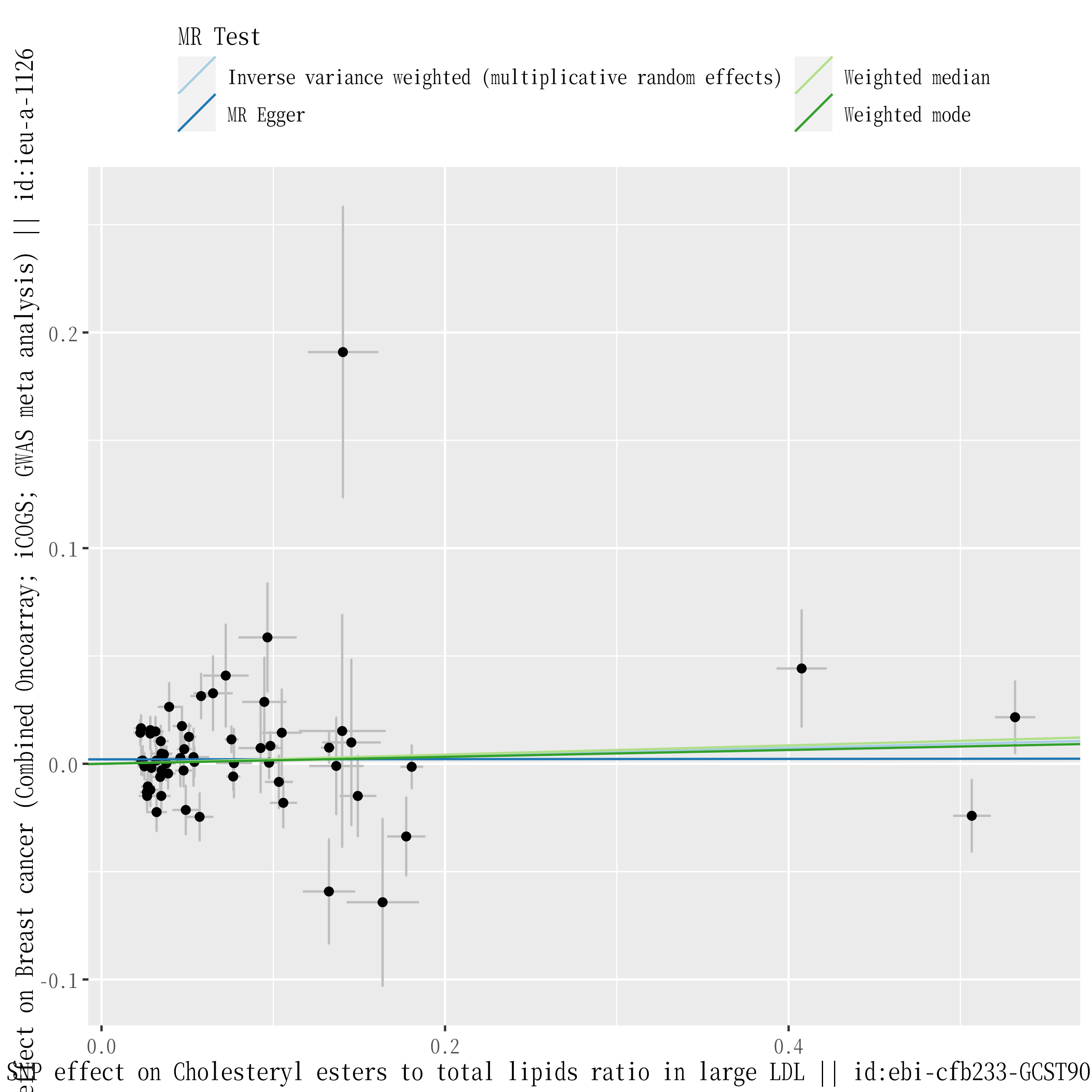

MR Test
Inverse variance weighted (multiplicative random effects)
MR Egger
Weighted median
Weighted mode
0.2
0.1
0.0
-0.1
0.0
0.2
0.4
SNP effect on Cholesteryl esters to total lipids ratio in large LDL || id:ebi-cfb233-GCST90
effect on Breast cancer (Combined Oncoarray; iCOGS; GWAS meta analysis) || id:ieu-a-1126

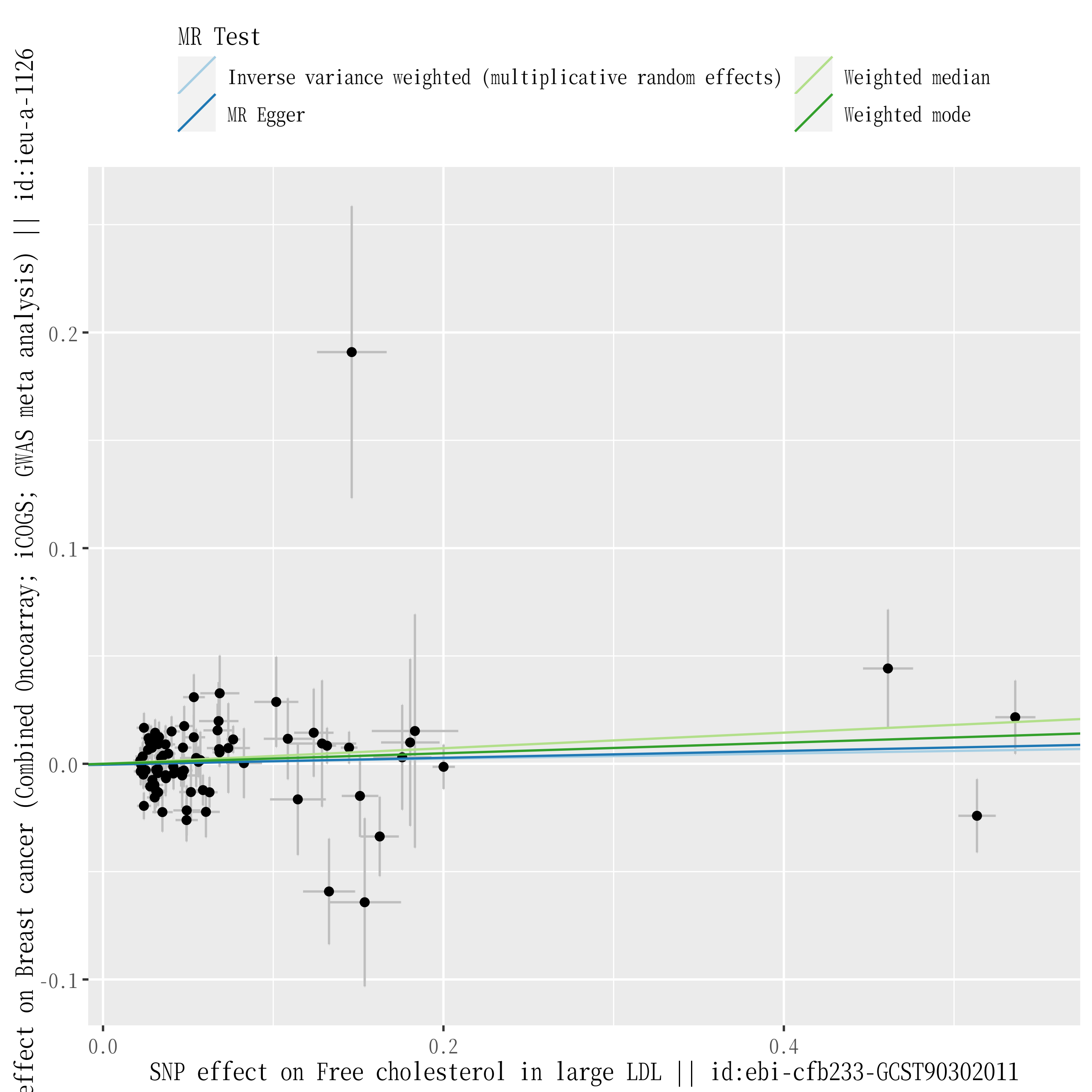
MR Test
Inverse variance weighted (multiplicative random effects)
MR Egger
Weighted median
Weighted mode
effect on Breast cancer (Combined Oncoarray; iCOGS; GWAS meta analysis) || id:ieu-a-1126
0.2
0.1
0.0
-0.1
0.0
0.2
0.4
SNP effect on Free cholesterol in large LDL || id:ebi-cfb233-GCST90302011

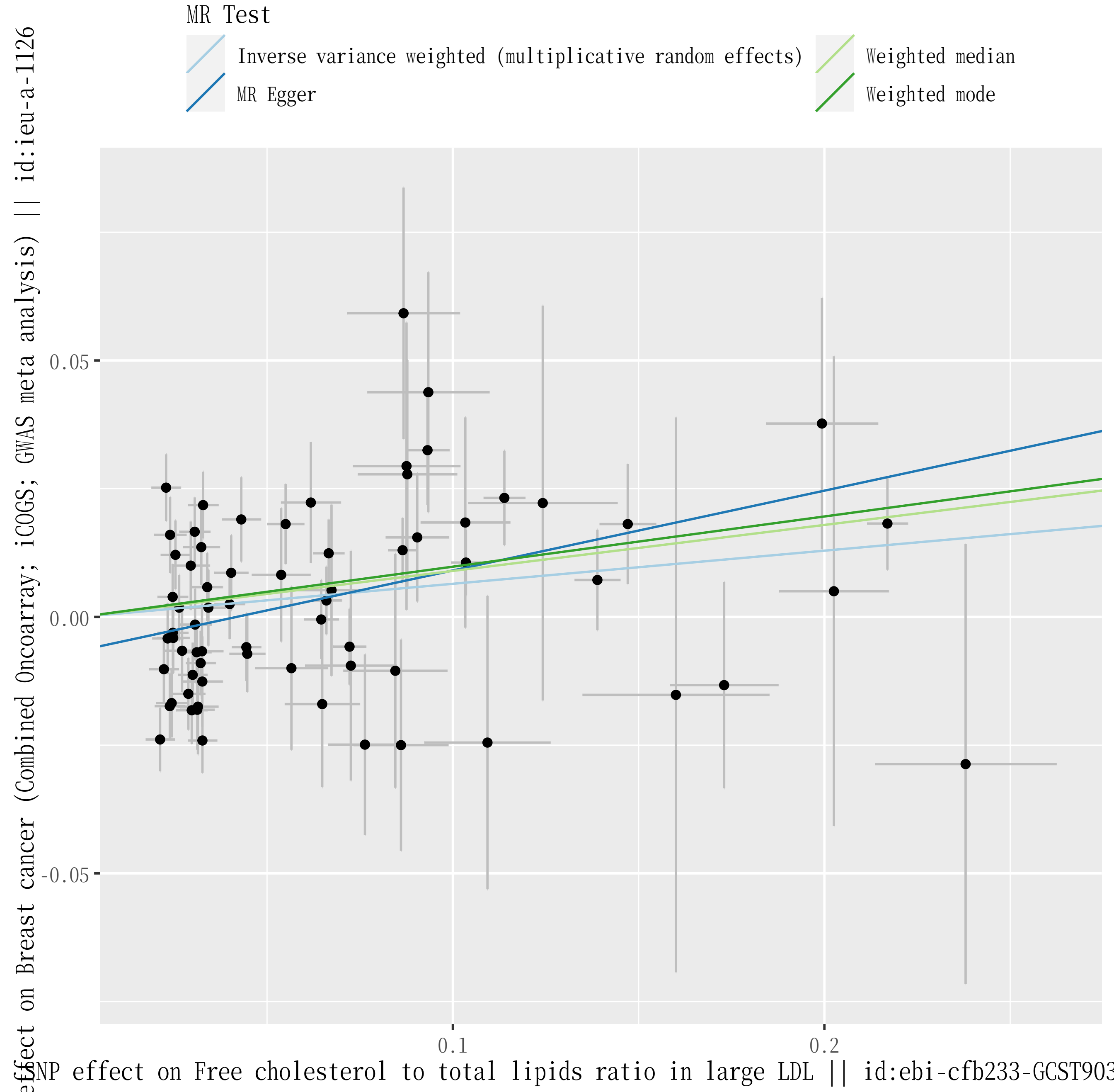

MR Test
Inverse variance weighted (multiplicative random effects)
MR Egger
Weighted median
Weighted mode
0.05
0.00
-0.05
0.1
0.2
SNP effect on Free cholesterol to total lipids ratio in large LDL || id:ebi-cfb233-GCST903
effect on Breast cancer (Combined Oncoarray; iCOGS; GWAS meta analysis) || id:ieu-a-1126

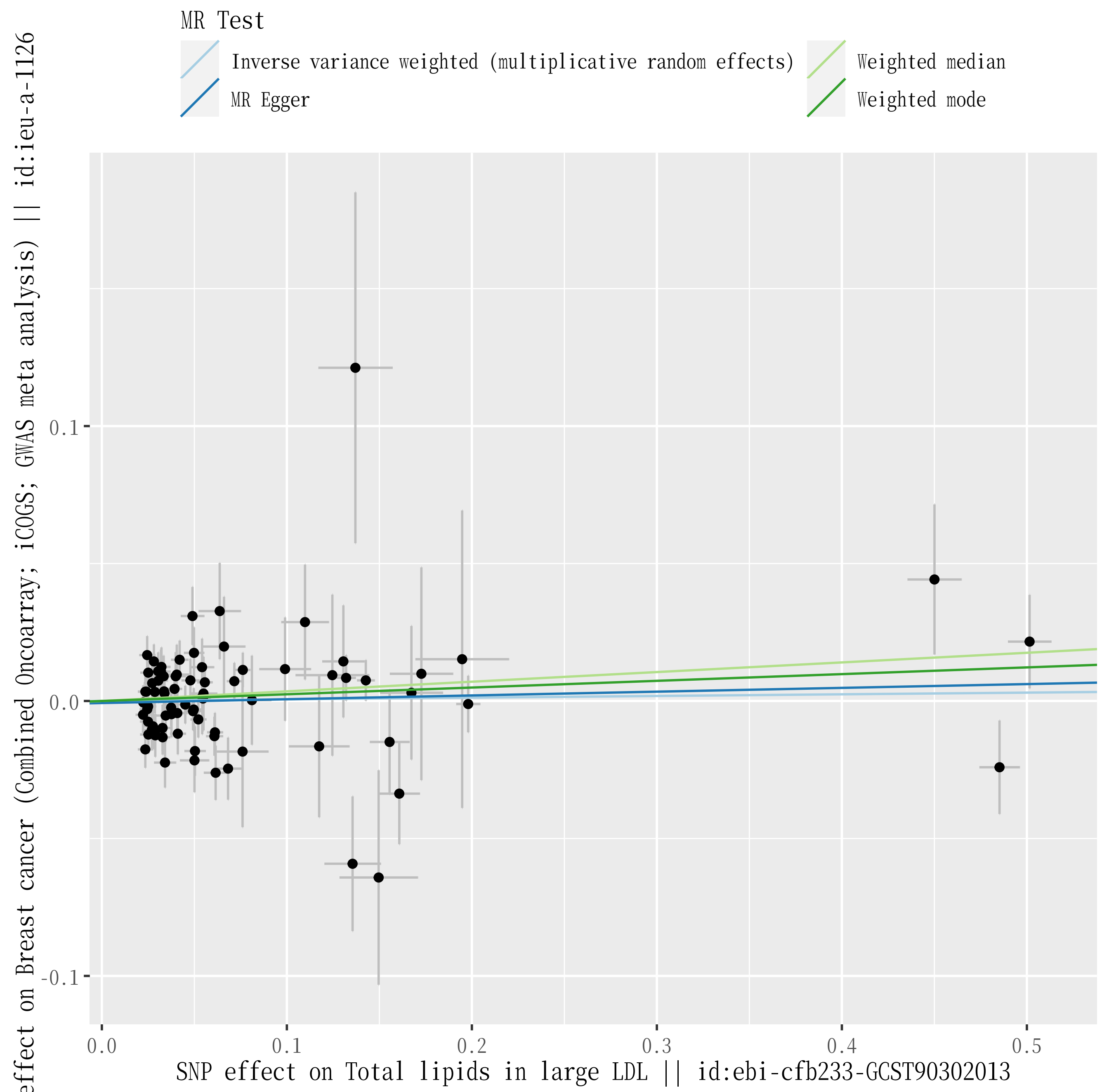

MR Test
Inverse variance weighted (multiplicative random effects)
MR Egger
Weighted median
Weighted mode
0.1
0.0
-0.1
0.0
0.1
0.2
0.3
0.4
0.5
SNP effect on Total lipids in large LDL || id:ebi-cfb233-GCST90302013
effect on Breast cancer (Combined Oncoarray; iCOGS; GWAS meta analysis) || id:ieu-a-1126

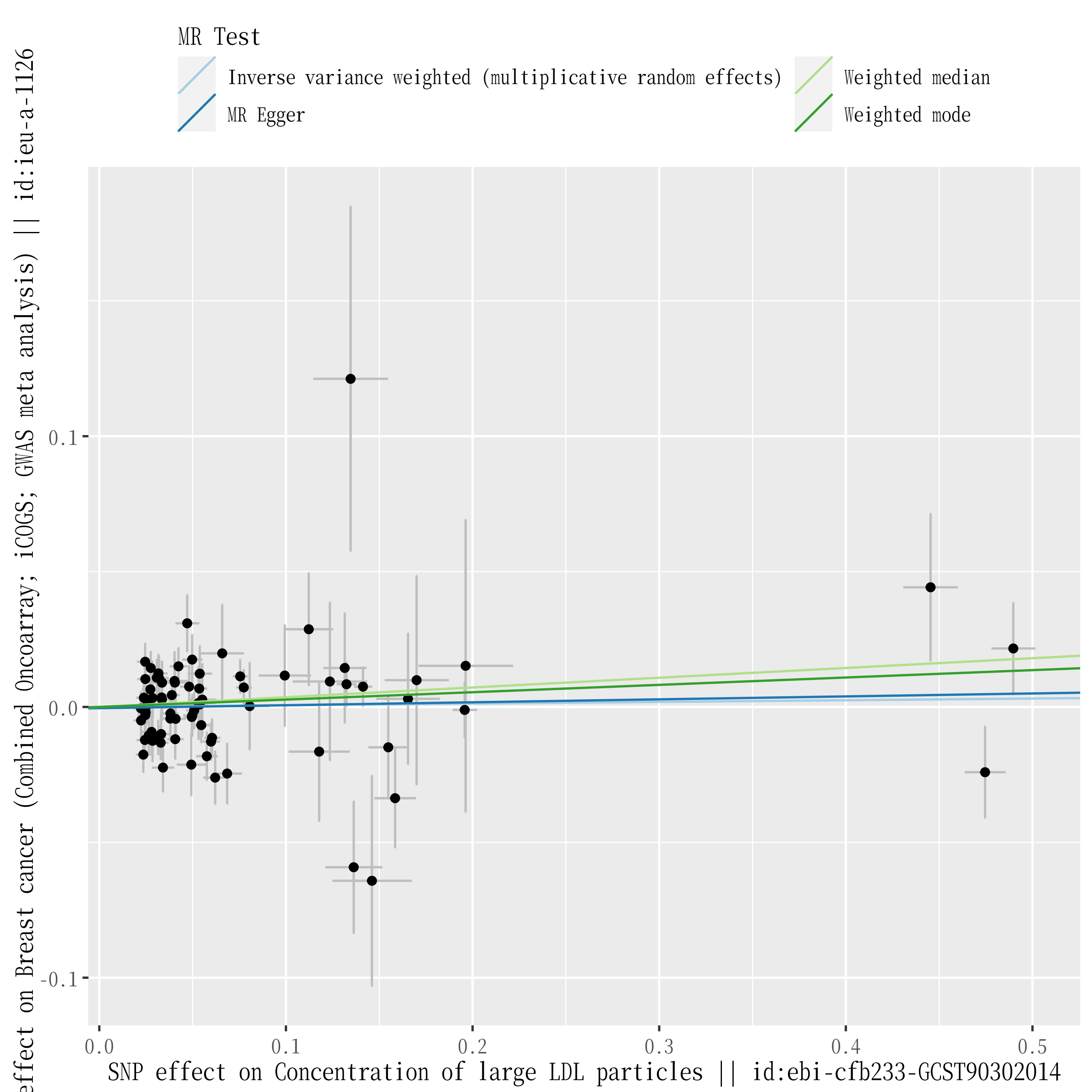

MR Test
Inverse variance weighted (multiplicative random effects)
MR Egger
Weighted median
Weighted mode
SNP effect on Breast cancer (Combined Oncoarray; iCOGS; GWAS meta analysis) || id:ieu-a-1126
0.1
0.0
-0.1
0.0
0.1
0.2
0.3
0.4
0.5
SNP effect on Concentration of large LDL particles || id:ebi-cfb233-GCST90302014

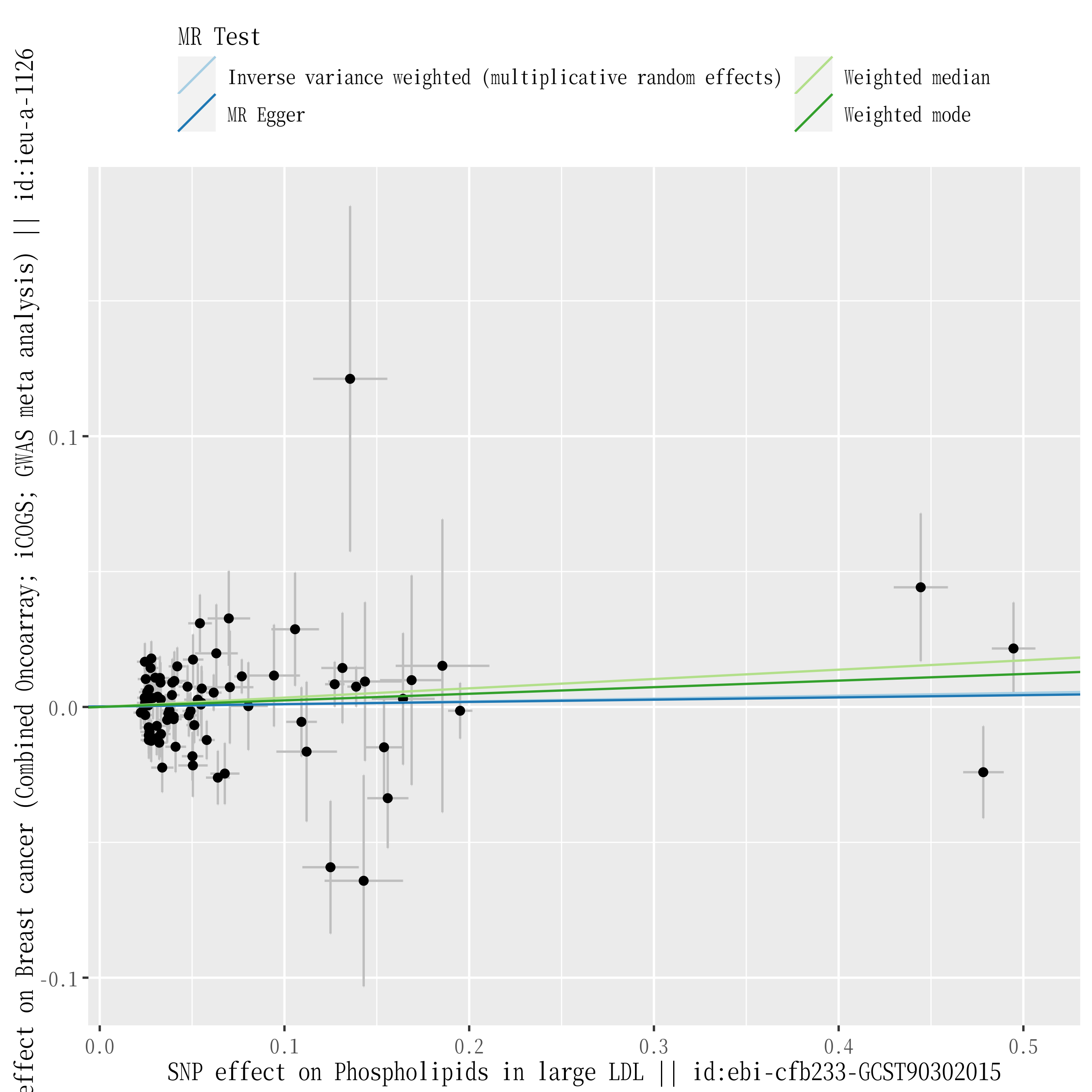

MR Test
Inverse variance weighted (multiplicative random effects)
MR Egger
Weighted median
Weighted mode
0.1
0.0
-0.1
0.0
0.1
0.2
0.3
0.4
0.5
SNP effect on Phospholipids in large LDL || id:ebi-cfb233-GCST90302015
effect on Breast cancer (Combined Oncoarray; iCOGS; GWAS meta analysis) || id:ieu-a-1126

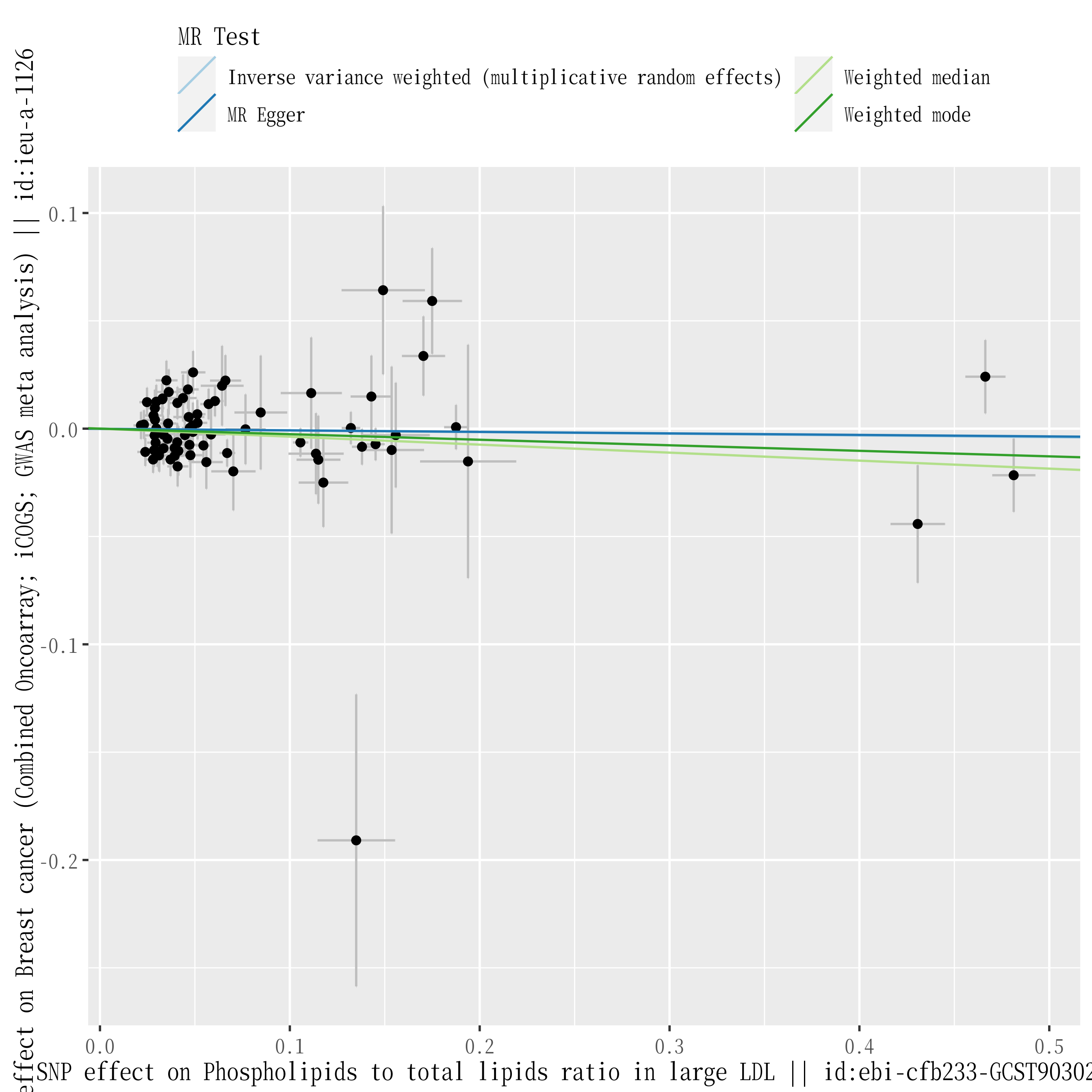
MR Test
Inverse variance weighted (multiplicative random effects)
MR Egger
Weighted median
Weighted mode
0.1
0.0
-0.1
-0.2
0.0
0.1
0.2
0.3
0.4
0.5
effect on Breast cancer (Combined Oncoarray; iCOGS; GWAS meta analysis) || id:ieu-a-1126
SNP effect on Phospholipids to total lipids ratio in large LDL || id:ebi-cfb233-GCST90302

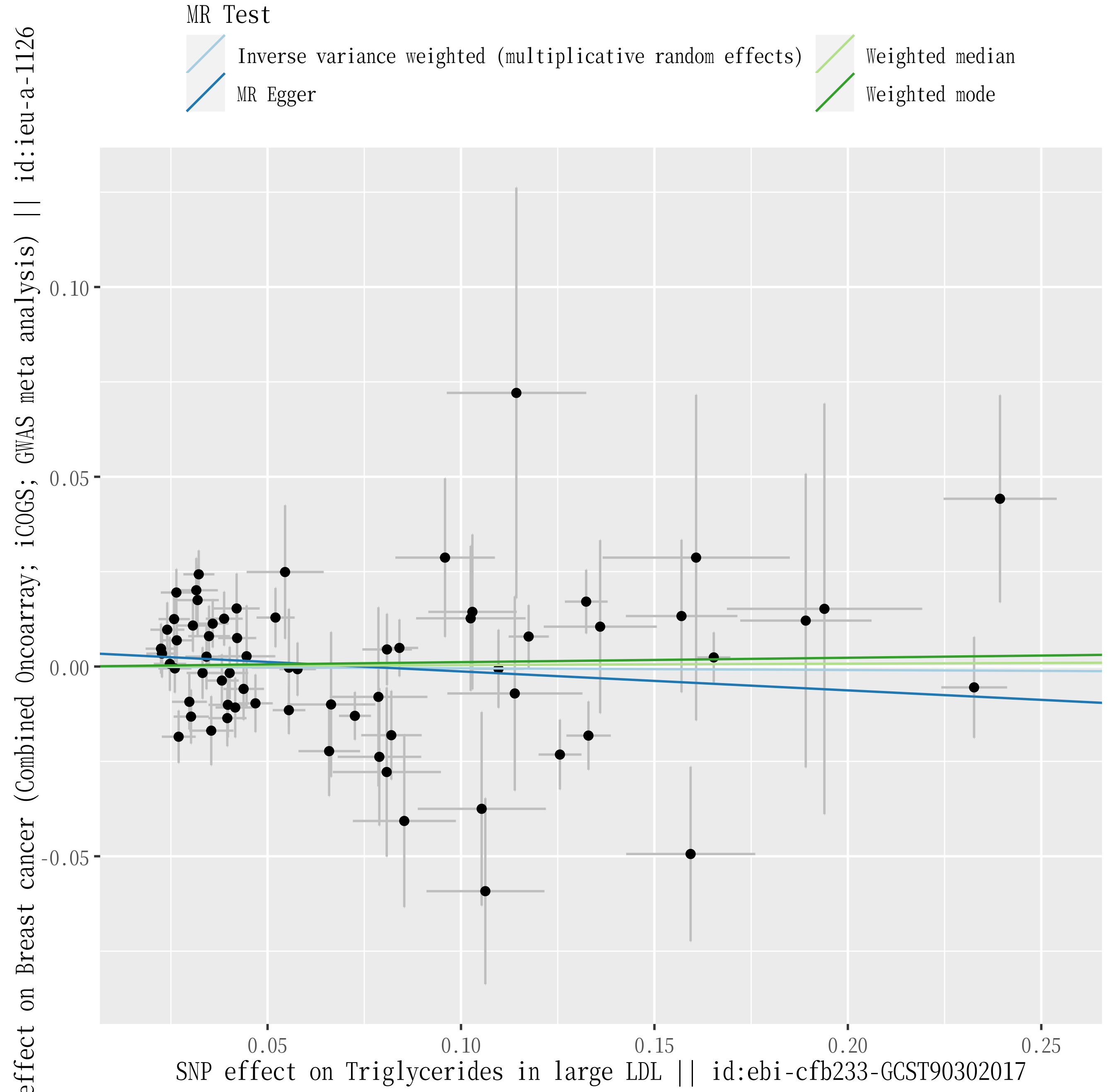

MR Test
Inverse variance weighted (multiplicative random effects)
MR Egger
Weighted median
Weighted mode
SNP effect on Breast cancer (Combined Oncoarray; iCOGS; GWAS meta analysis) || id:ieu-a-1126
0.10
0.05
0.00
-0.05
0.05
0.10
0.15
0.20
0.25
SNP effect on Triglycerides in large LDL || id:ebi-cfb233-GCST90302017

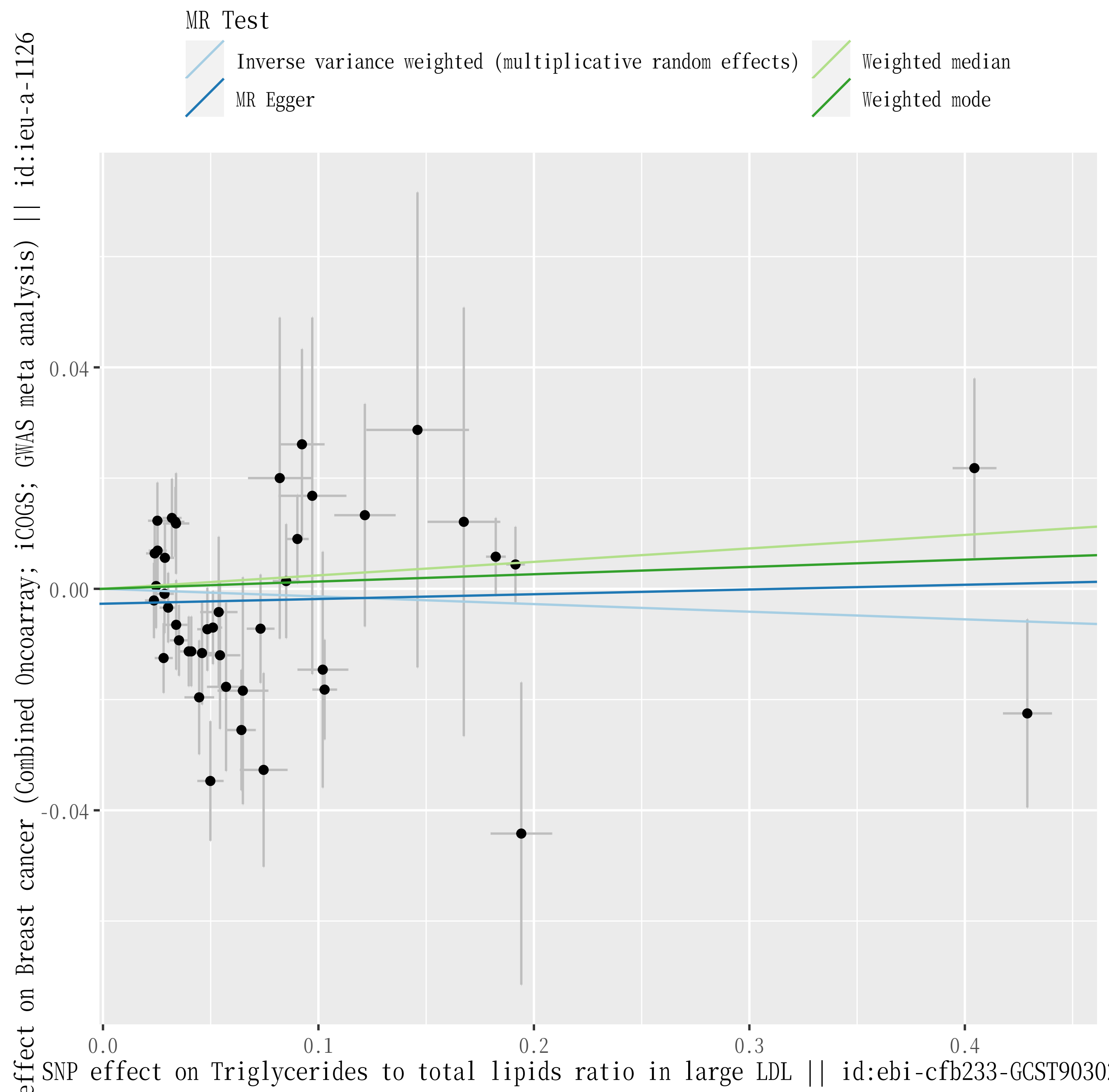
MR Test
Inverse variance weighted (multiplicative random effects)
MR Egger
Weighted median
Weighted mode
0.04
0.00
-0.04
0.0
0.1
0.2
0.3
0.4
SNP effect on Triglycerides to total lipids ratio in large LDL || id:ebi-cfb233-GCST9030
effect on Breast cancer (Combined Oncoarray; iCOGS; GWAS meta analysis) || id:ieu-a-1126

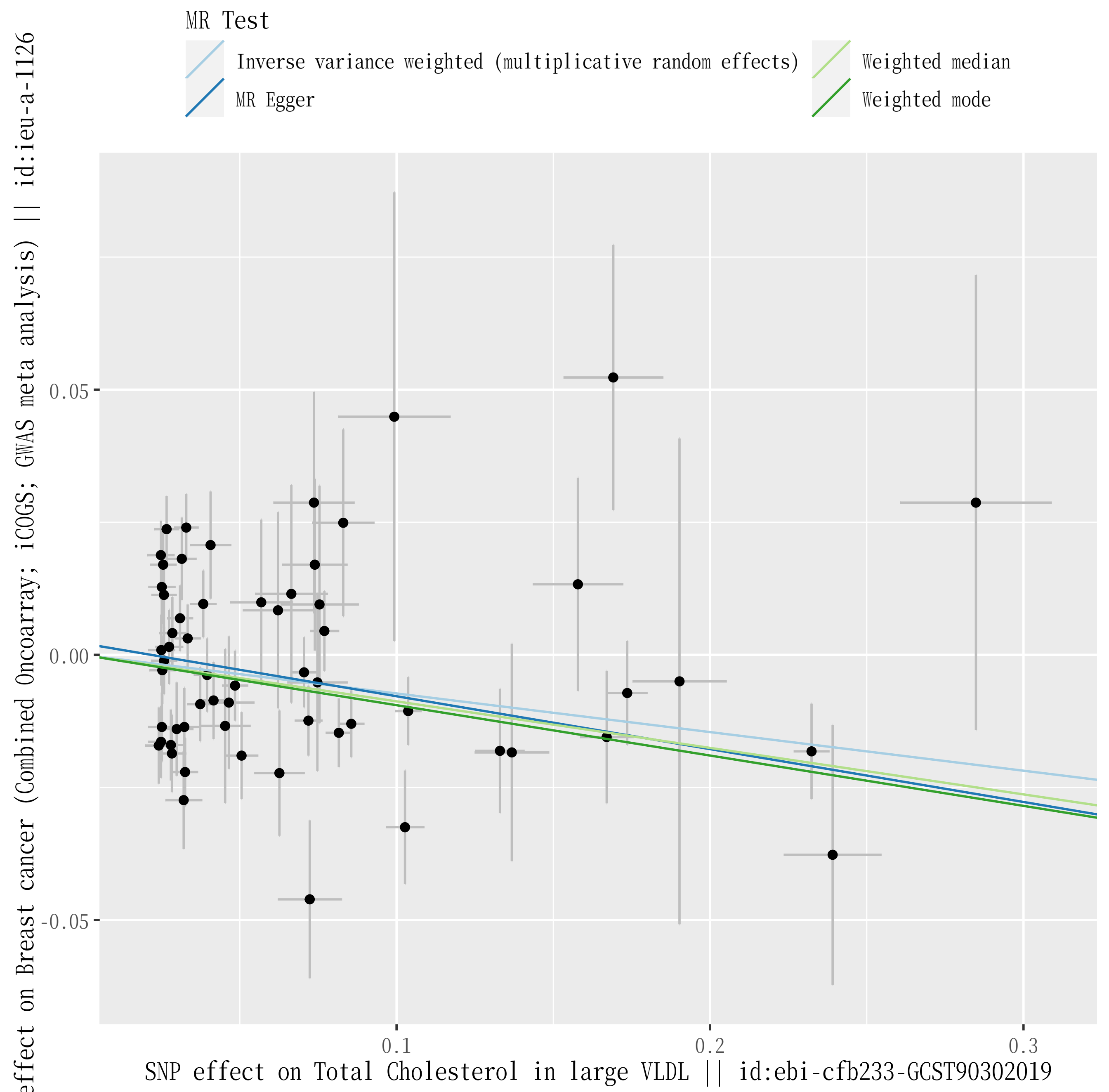
MR Test
Inverse variance weighted (multiplicative random effects)
MR Egger
Weighted median
Weighted mode
0.05
0.00
-0.05
0.1
0.2
0.3
SNP effect on Total Cholesterol in large VLDL || id:ebi-cfb233-GCST90302019
effect on Breast cancer (Combined Oncoarray; iCOGS; GWAS meta analysis) || id:ieu-a-1126

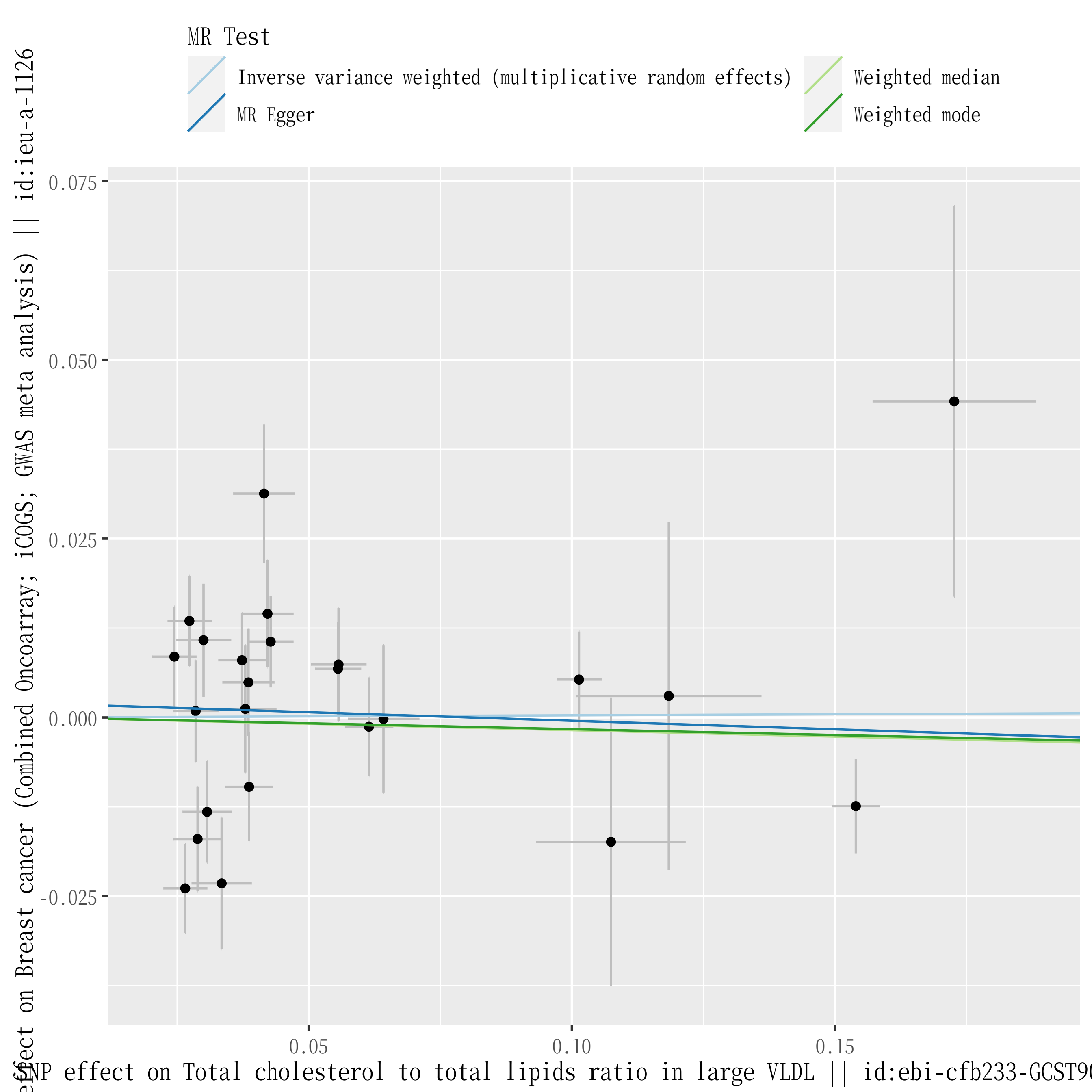

MR Test
Inverse variance weighted (multiplicative random effects)
MR Egger
Weighted median
Weighted mode
0.075
0.050
0.025
0.000
-0.025
0.05
0.10
0.15
SNP effect on Total cholesterol to total lipids ratio in large VLDL || id:ebi-cfb233-GCST90
effect on Breast cancer (Combined Oncoarray; iCOGS; GWAS meta analysis) || id:ieu-a-1126

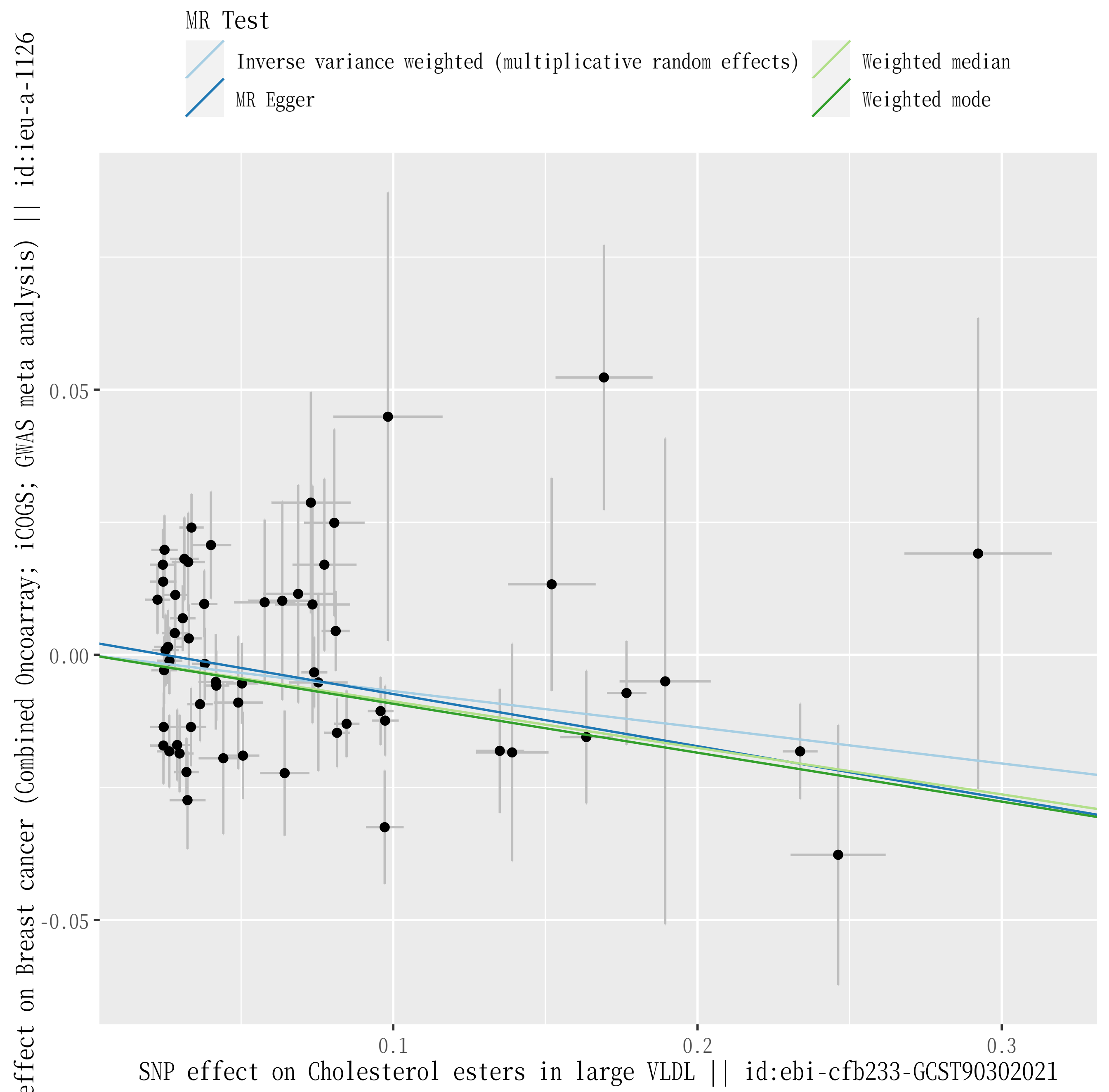
MR Test
Inverse variance weighted (multiplicative random effects)
MR Egger
Weighted median
Weighted mode
0.05
0.00
-0.05
0.1
0.2
0.3
SNP effect on Cholesterol esters in large VLDL || id:ebi-cfb233-GCST90302021
effect on Breast cancer (Combined Oncoarray; iCOGS; GWAS meta analysis) || id:ieu-a-1126

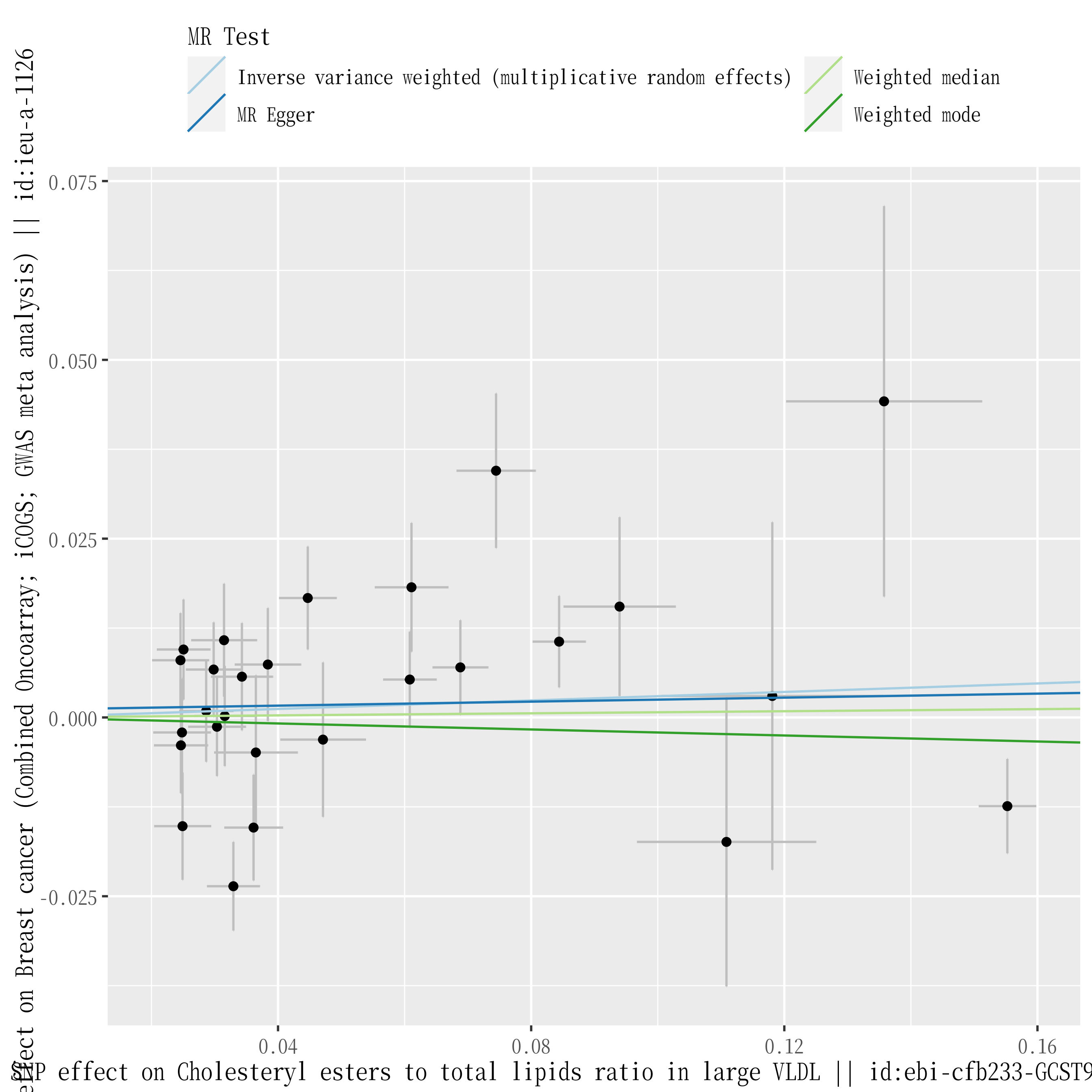

MR Test
Inverse variance weighted (multiplicative random effects)
MR Egger
Weighted median
Weighted mode
0.075
0.050
0.025
0.000
-0.025
0.04
0.08
0.12
0.16
SNP effect on Cholesteryl esters to total lipids ratio in large VLDL || id:ebi-cfb233-GCST9
effect on Breast cancer (Combined Oncoarray; iCOGS; GWAS meta analysis) || id:ieu-a-1126

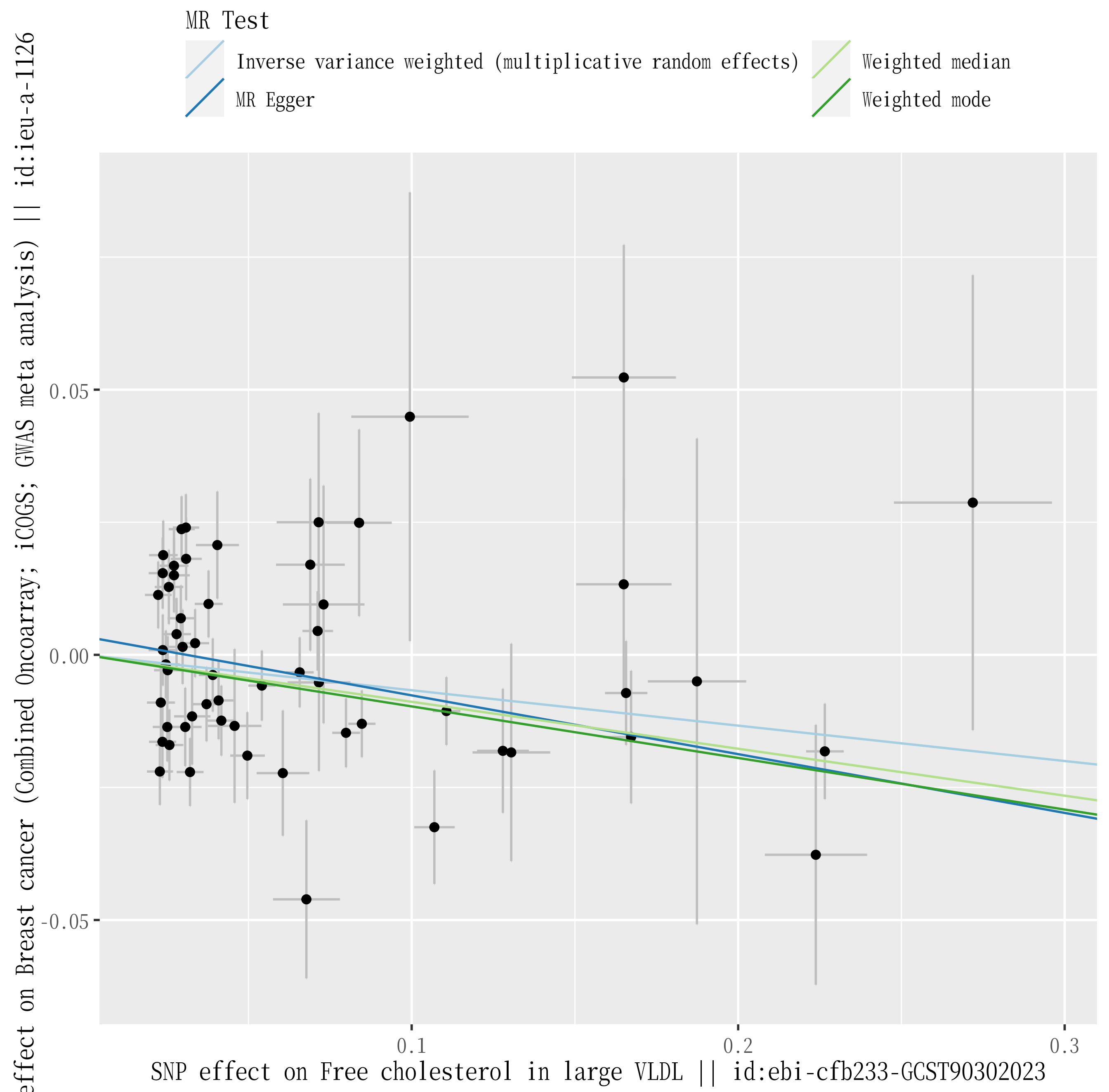
MR Test
Inverse variance weighted (multiplicative random effects)
MR Egger
Weighted median
Weighted mode
0.05
0.00
-0.05
0.1
0.2
0.3
SNP effect on Free cholesterol in large VLDL || id:ebi-cfb233-GCST90302023
effect on Breast cancer (Combined Oncoarray; iCOGS; GWAS meta analysis) || id:ieu-a-1126

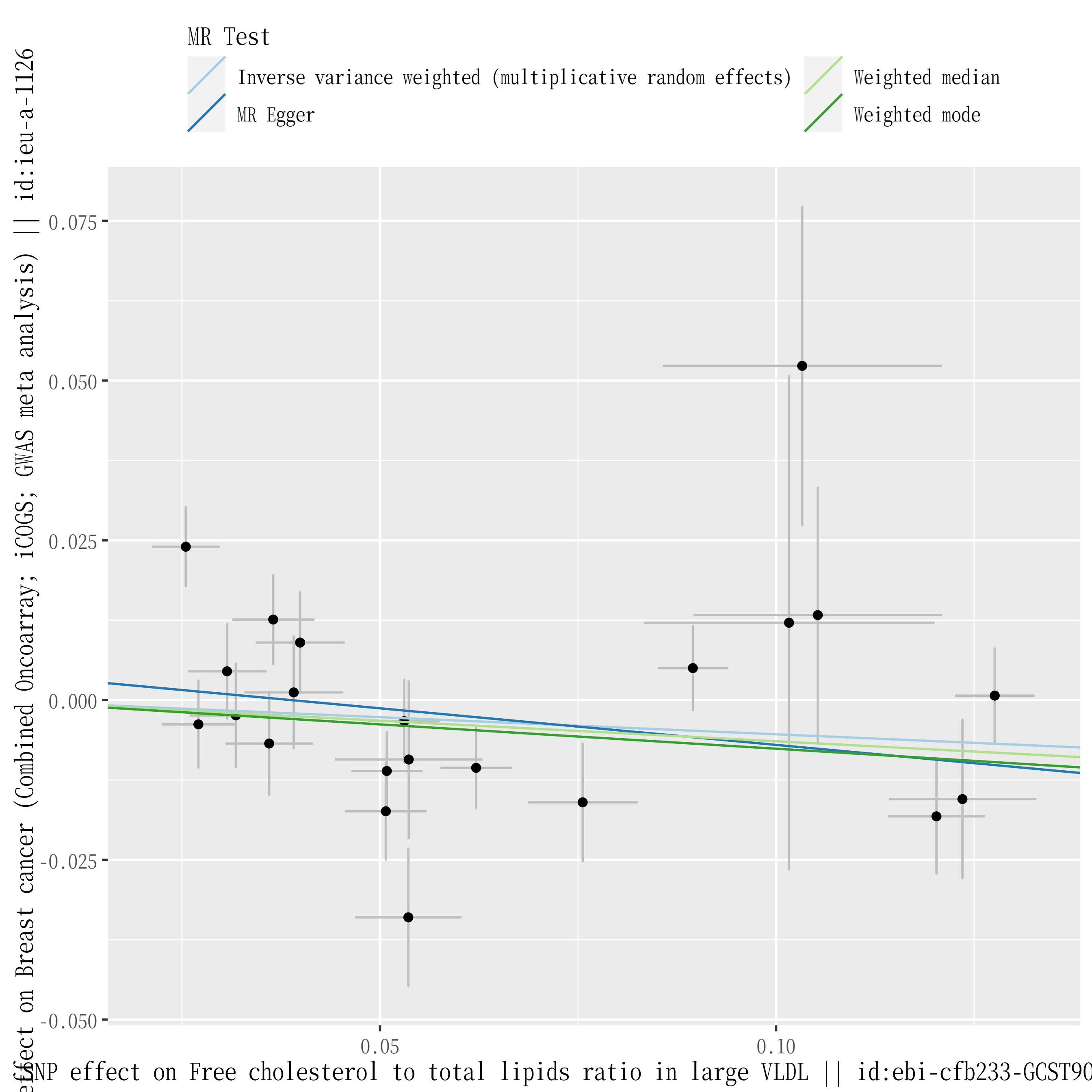
MR Test
Inverse variance weighted (multiplicative random effects)
MR Egger
Weighted median
Weighted mode
0.075
0.050
0.025
0.000
-0.025
-0.050
0.05
0.10
SNP effect on Free cholesterol to total lipids ratio in large VLDL || id:ebi-cfb233-GCST90
SNP effect on Breast cancer (Combined Oncoarray; iCOGS; GWAS meta analysis) || id:ieu-a-1126

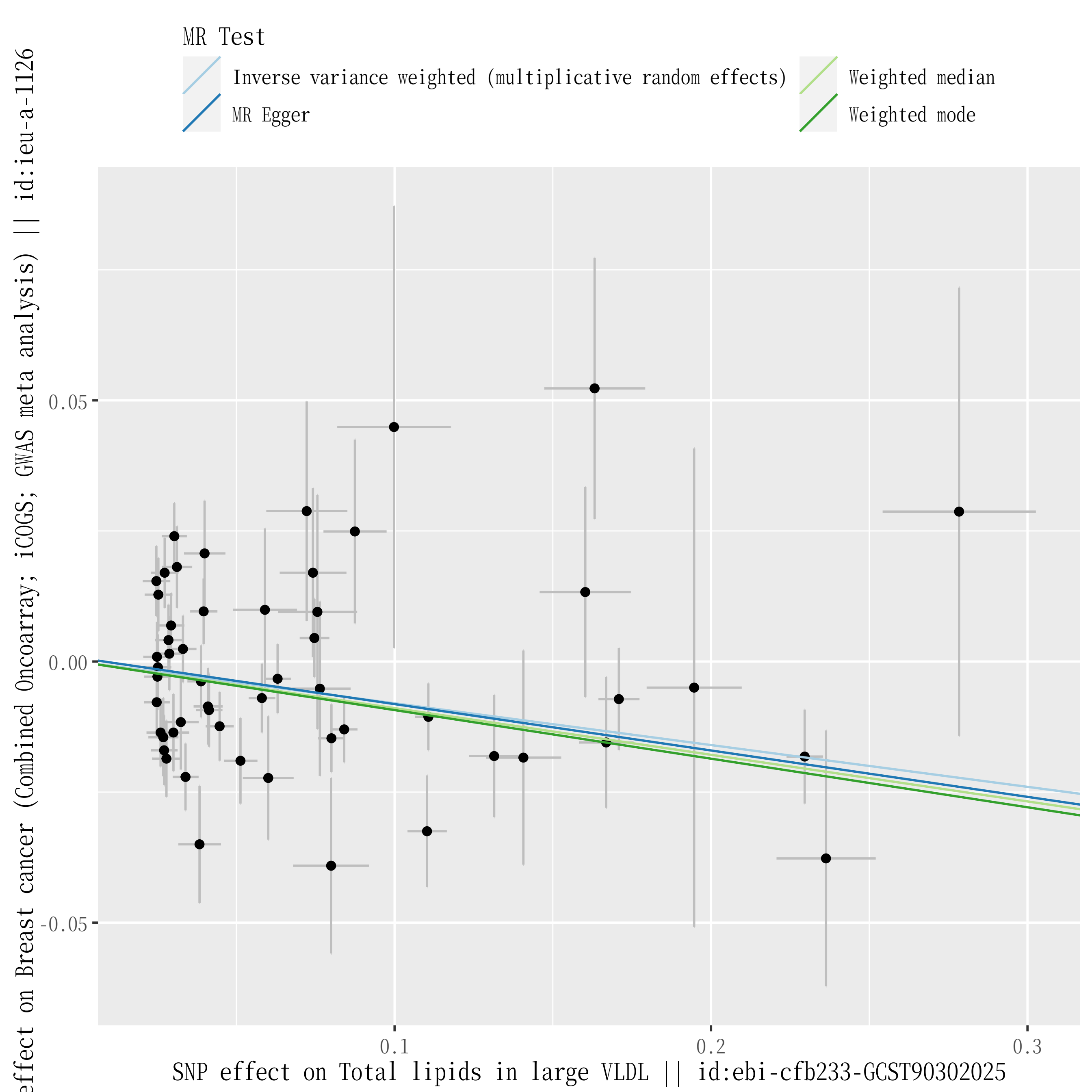

MR Test
Inverse variance weighted (multiplicative random effects)
MR Egger
Weighted median
Weighted mode
0.05
0.00
-0.05
0.1
0.2
0.3
SNP effect on Total lipids in large VLDL || id:ebi-cfb233-GCST90302025
effect on Breast cancer (Combined Oncoarray; iCOGS; GWAS meta analysis) || id:ieu-a-1126

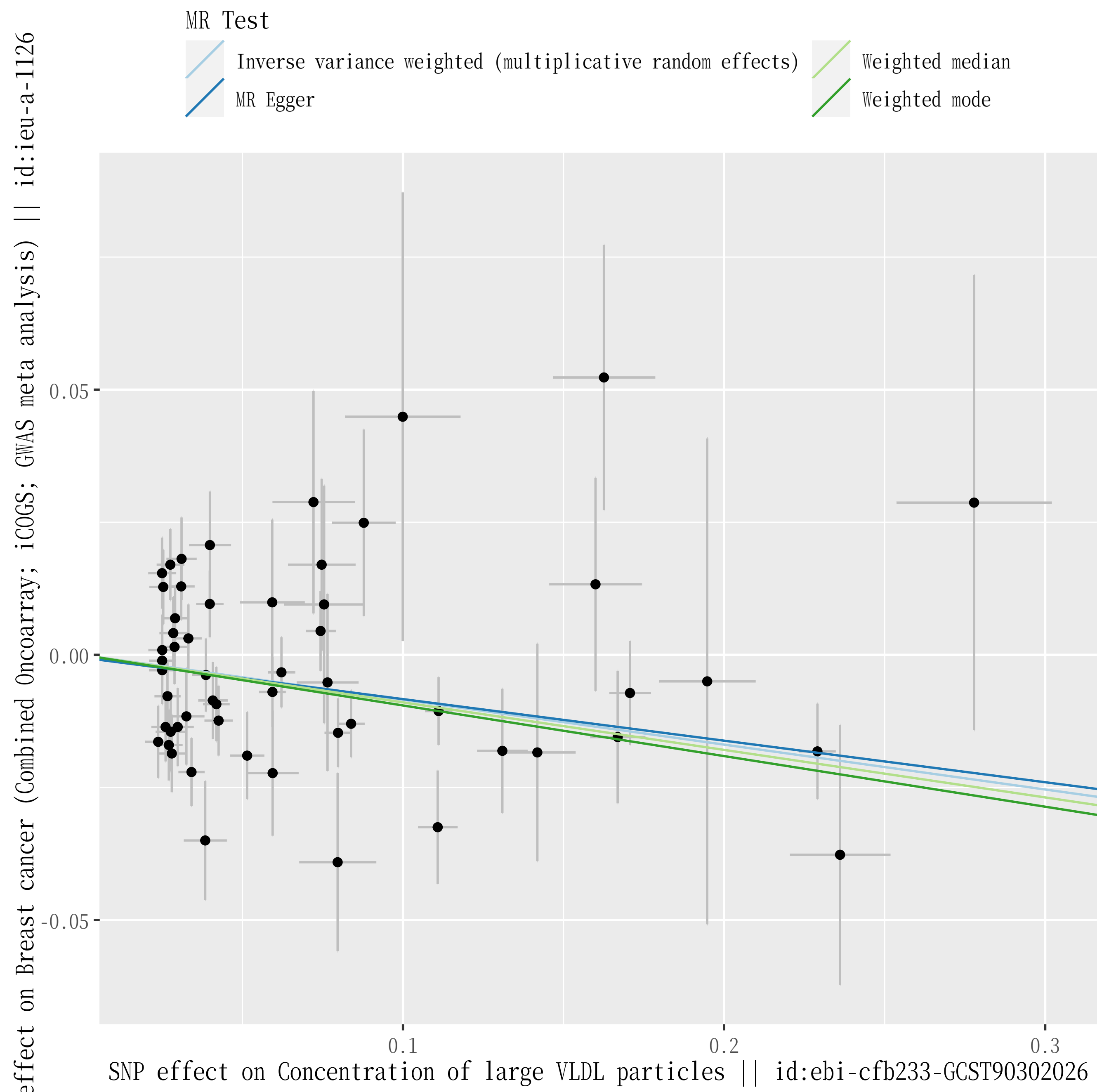
MR Test
Inverse variance weighted (multiplicative random effects)
MR Egger
Weighted median
Weighted mode
0.05
0.00
-0.05
0.1
0.2
0.3
SNP effect on Concentration of large VLDL particles || id:ebi-cfb233-GCST90302026
effect on Breast cancer (Combined Oncoarray; iCOGS; GWAS meta analysis) || id:ieu-a-1126

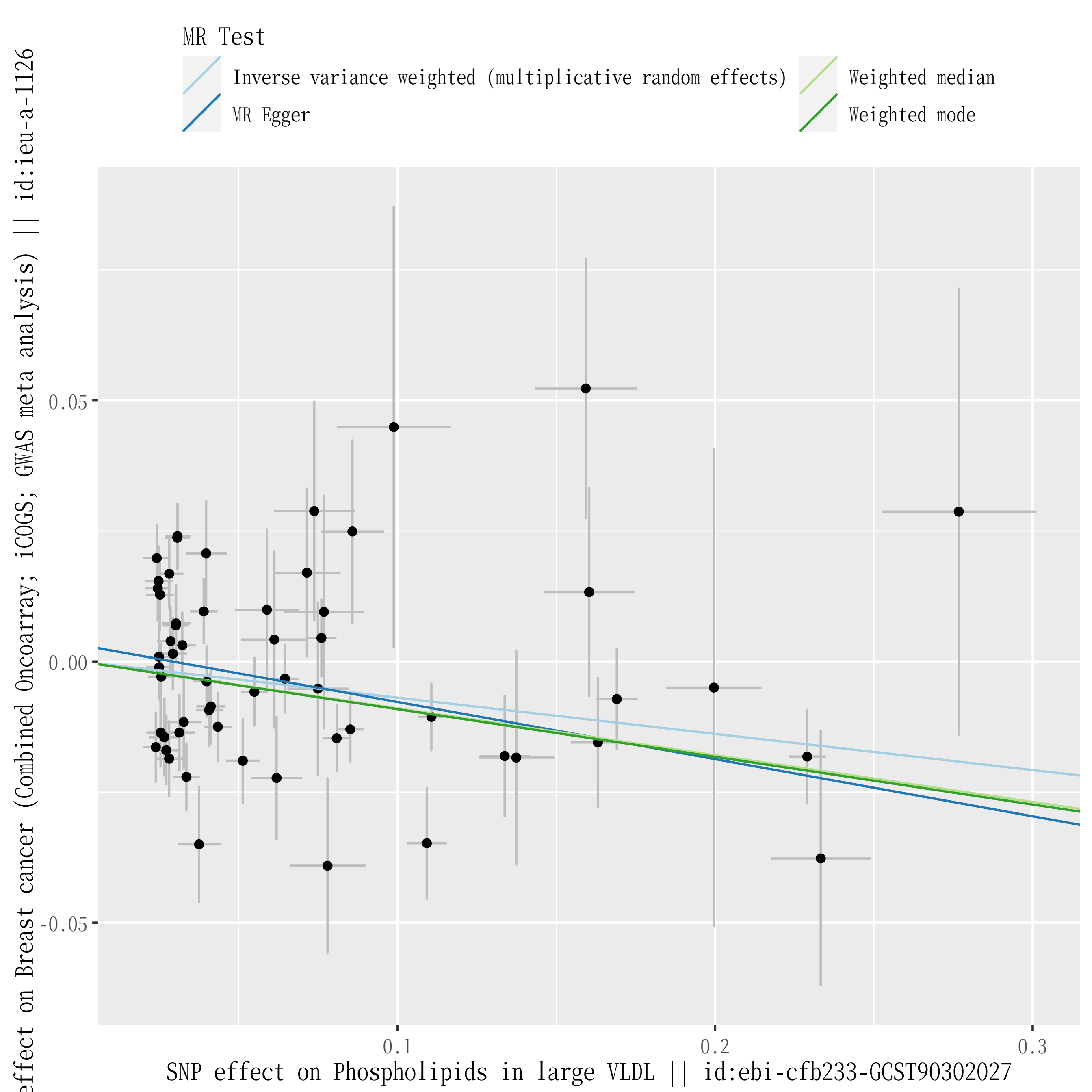
MR Test
Inverse variance weighted (multiplicative random effects)
MR Egger
Weighted median
Weighted mode
0.05
0.00
-0.05
0.1
0.2
0.3
SNP effect on Phospholipids in large VLDL || id:ebi-cfb233-GCST90302027
effect on Breast cancer (Combined Oncoarray; iCOGS; GWAS meta analysis) || id:ieu-a-1126

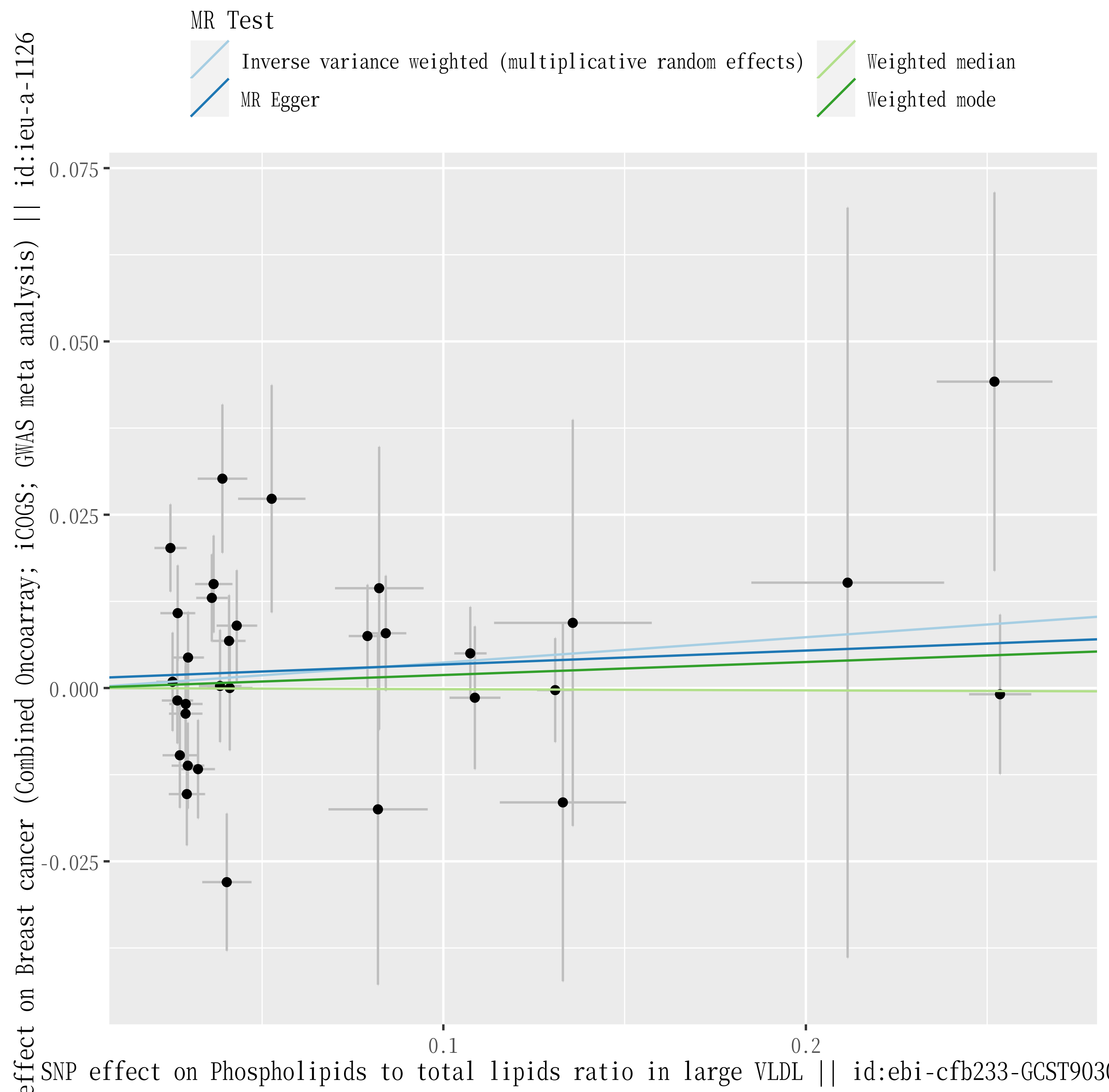

MR Test
Inverse variance weighted (multiplicative random effects)
MR Egger
Weighted median
Weighted mode
0.075
0.050
0.025
0.000
-0.025
0.1
0.2
SNP effect on Phospholipids to total lipids ratio in large VLDL || id:ebi-cfb233-GCST903
effect on Breast cancer (Combined Oncoarray; iCOGS; GWAS meta analysis) || id:ieu-a-1126

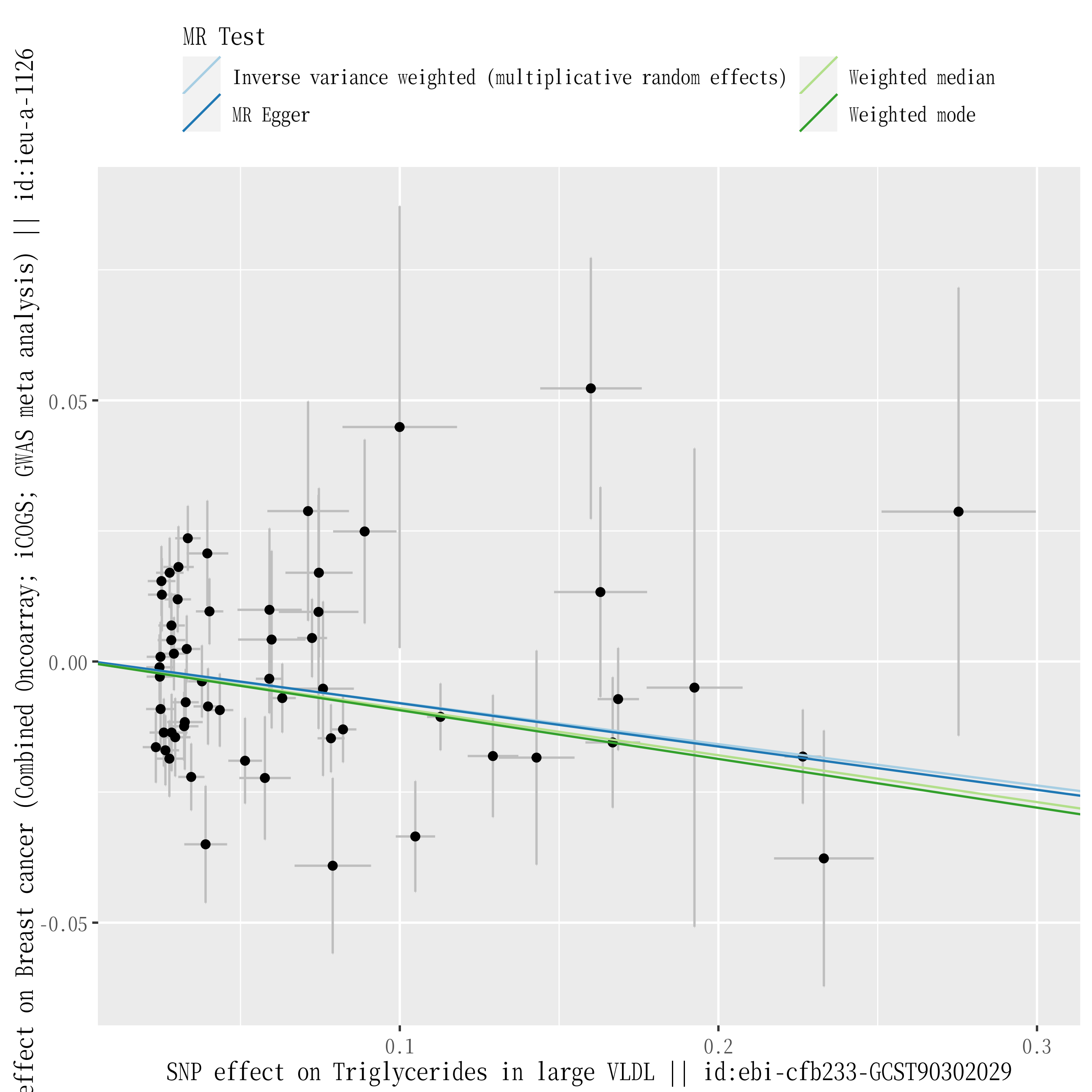
MR Test
Inverse variance weighted (multiplicative random effects)
MR Egger
Weighted median
Weighted mode
0.05
0.00
-0.05
0.1
0.2
0.3
SNP effect on Triglycerides in large VLDL || id:ebi-cfb233-GCST90302029
effect on Breast cancer (Combined Oncoarray; iCOGS; GWAS meta analysis) || id:ieu-a-1126

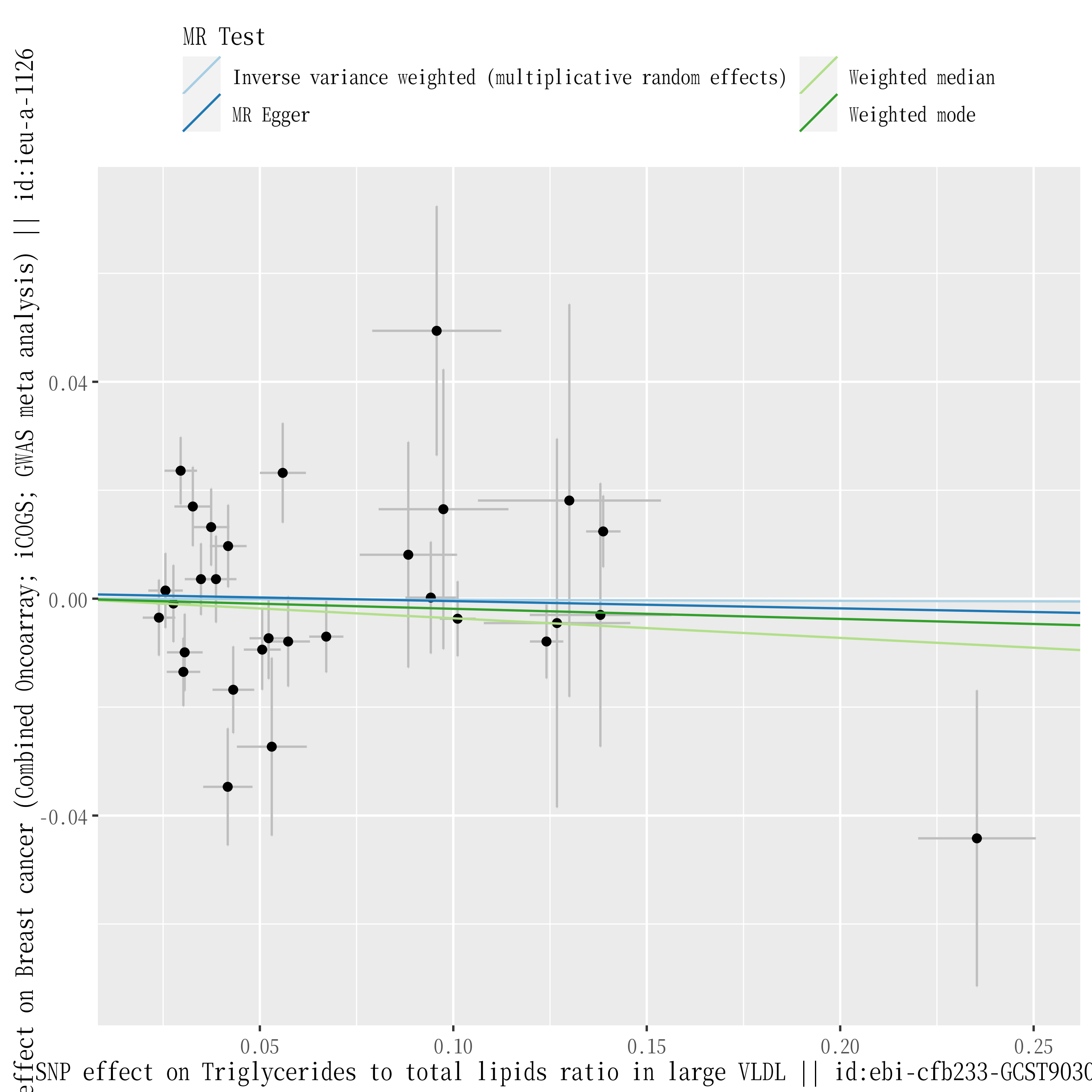

MR Test
Inverse variance weighted (multiplicative random effects)
MR Egger
Weighted median
Weighted mode
0.04
0.00
-0.04
0.05
0.10
0.15
0.20
0.25
SNP effect on Triglycerides to total lipids ratio in large VLDL || id:ebi-cfb233-GCST9030
effect on Breast cancer (Combined Oncoarray; iCOGS; GWAS meta analysis) || id:ieu-a-1126

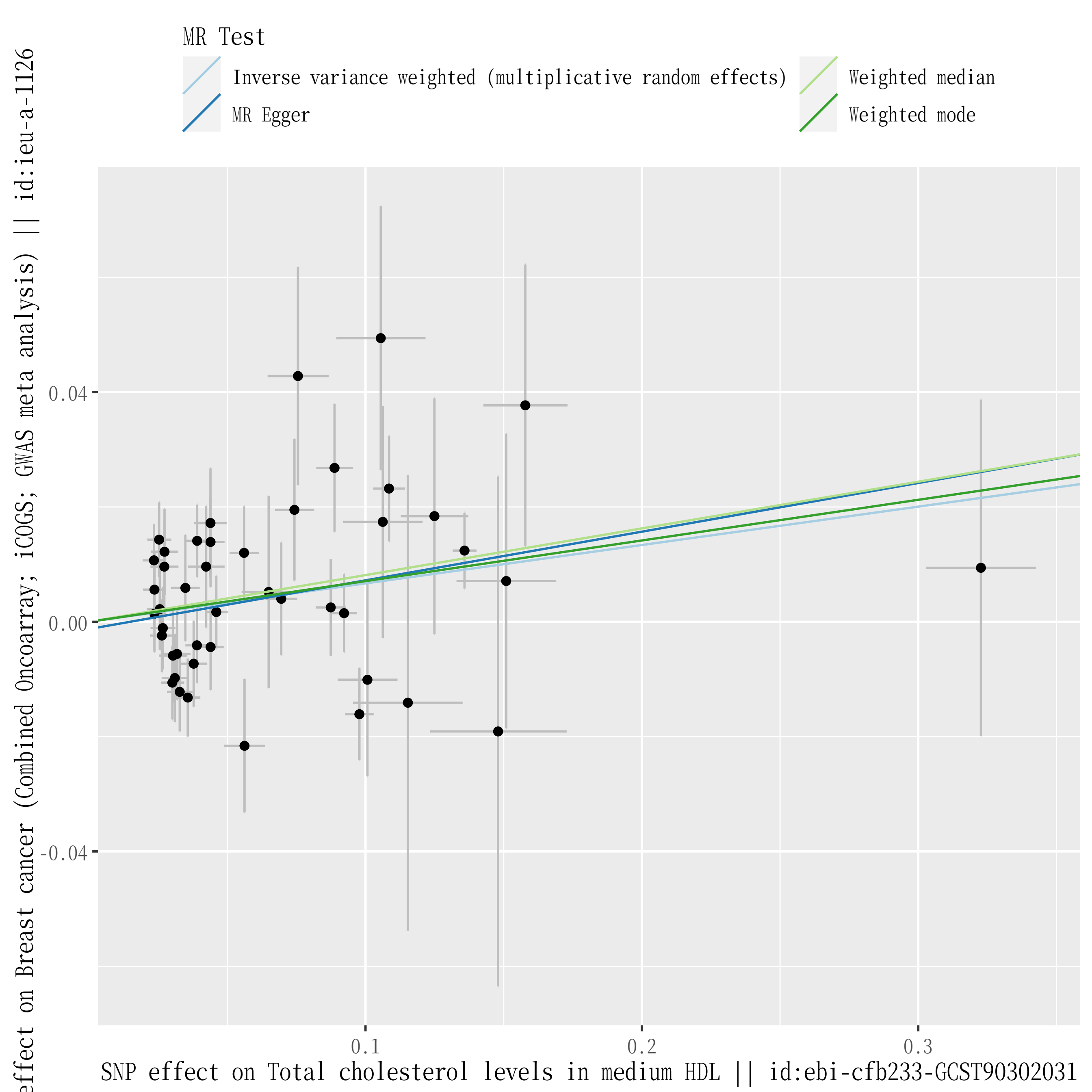

MR Test
Inverse variance weighted (multiplicative random effects)
MR Egger
Weighted median
Weighted mode
0.04
0.00
-0.04
0.1
0.2
0.3
SNP effect on Total cholesterol levels in medium HDL || id:ebi-cfb233-GCST90302031
effect on Breast cancer (Combined Oncoarray; iCOGS; GWAS meta analysis) || id:ieu-a-1126

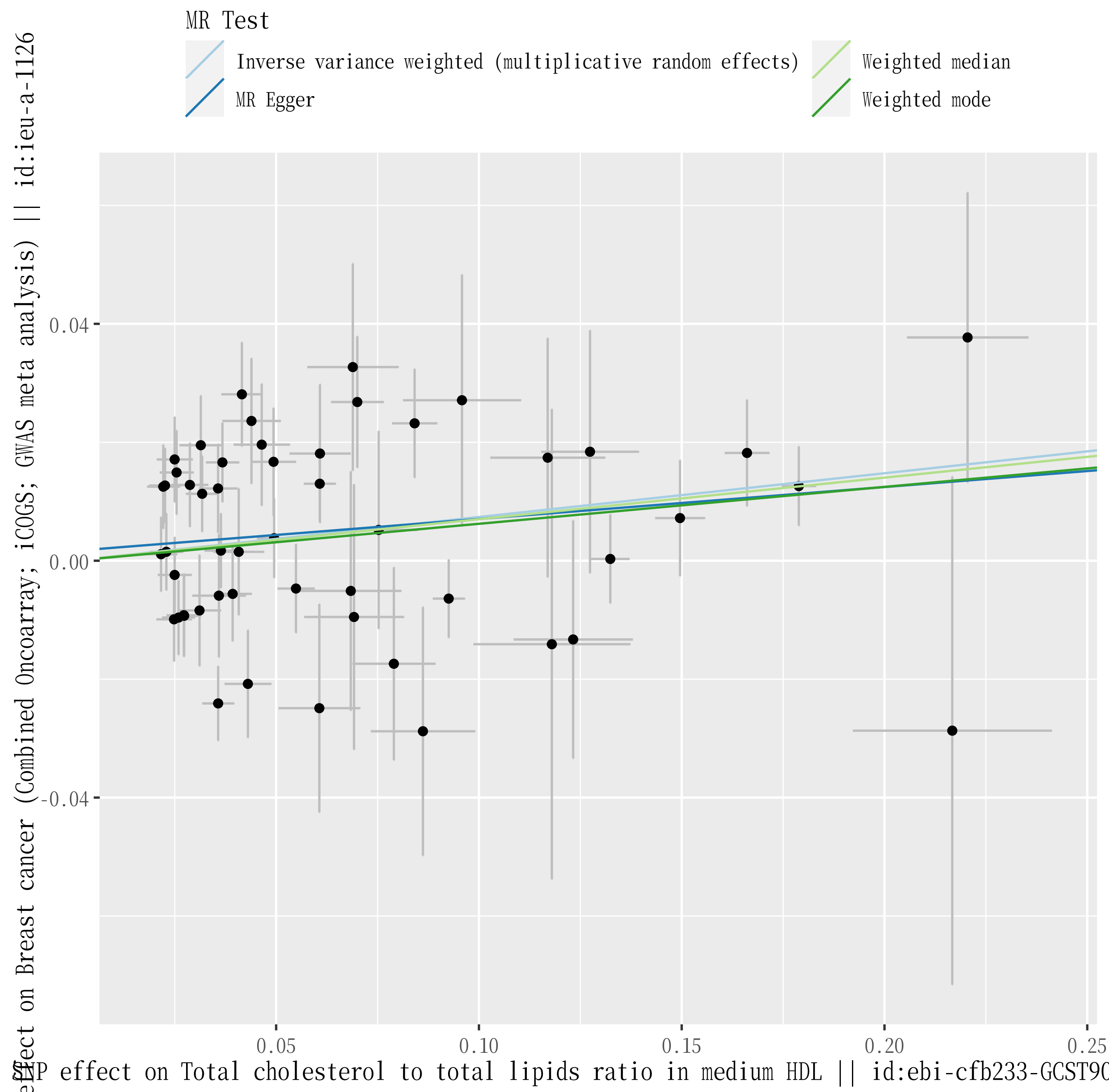

MR Test
Inverse variance weighted (multiplicative random effects)
MR Egger
Weighted median
Weighted mode
0.04
0.00
-0.04
0.05
0.10
0.15
0.20
0.25
SNP effect on Total cholesterol to total lipids ratio in medium HDL || id:ebi-cfb233-GCST90
SNP effect on Breast cancer (Combined Oncoarray; iCOGS; GWAS meta analysis) || id:ieu-a-1126

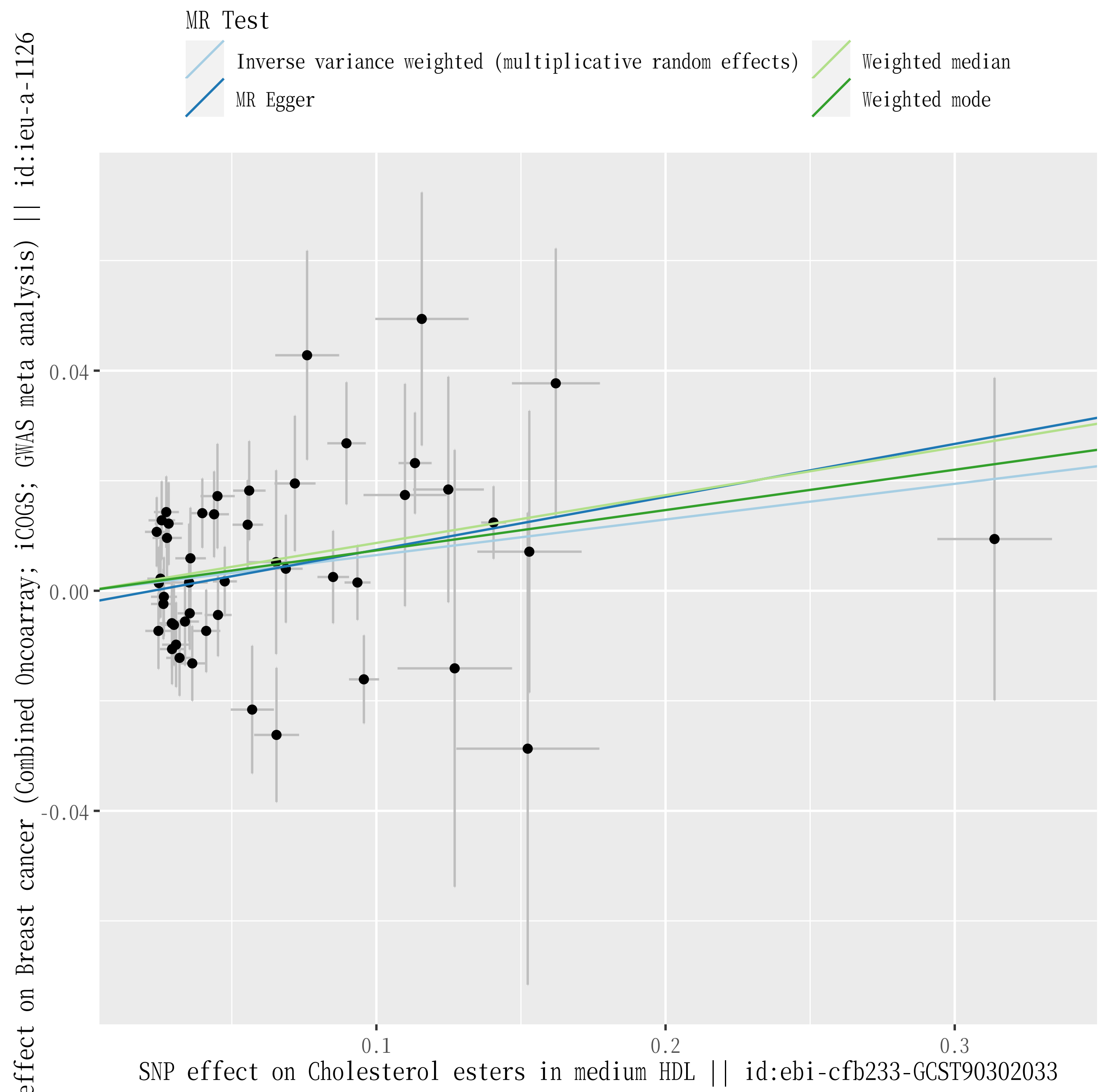

MR Test
Inverse variance weighted (multiplicative random effects)
MR Egger
Weighted median
Weighted mode
0.04
0.00
-0.04
0.1
0.2
0.3
SNP effect on Cholesterol esters in medium HDL || id:ebi-cfb233-GCST90302033
effect on Breast cancer (Combined Oncoarray; iCOGS; GWAS meta analysis) || id:ieu-a-1126

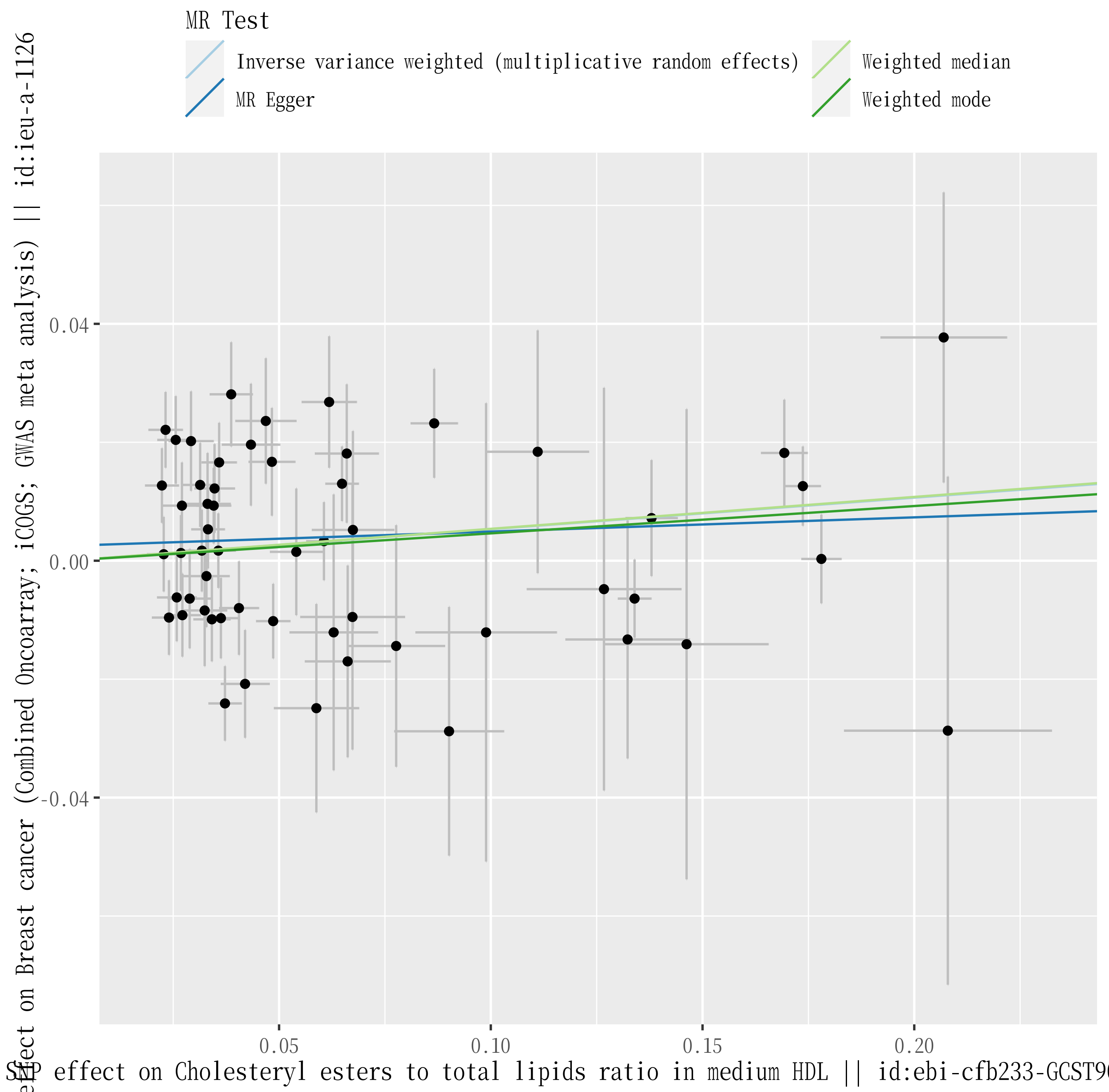
MR Test
Inverse variance weighted (multiplicative random effects)
MR Egger
Weighted median
Weighted mode
0.04
0.00
-0.04
0.05
0.10
0.15
0.20
SNP effect on Cholesteryl esters to total lipids ratio in medium HDL || id:ebi-cfb233-GCST90
SNP effect on Breast cancer (Combined Oncoarray; iCOGS; GWAS meta analysis) || id:ieu-a-1126

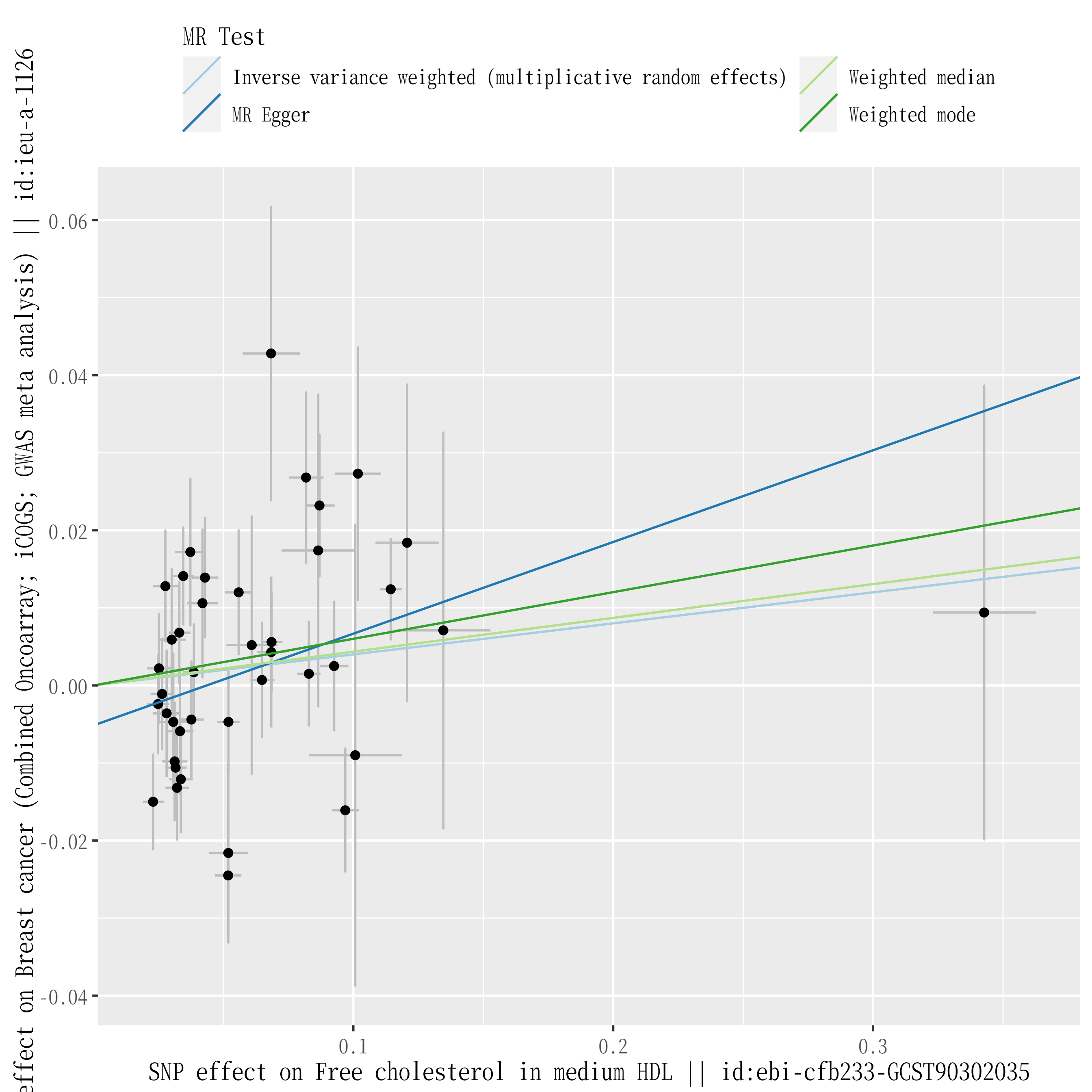

MR Test
Inverse variance weighted (multiplicative random effects)
MR Egger
Weighted median
Weighted mode
0.06
0.04
0.02
0.00
-0.02
-0.04
0.1
0.2
0.3
SNP effect on Free cholesterol in medium HDL || id:ebi-cfb233-GCST90302035
effect on Breast cancer (Combined Oncoarray; iCOGS; GWAS meta analysis) || id:ieu-a-1126

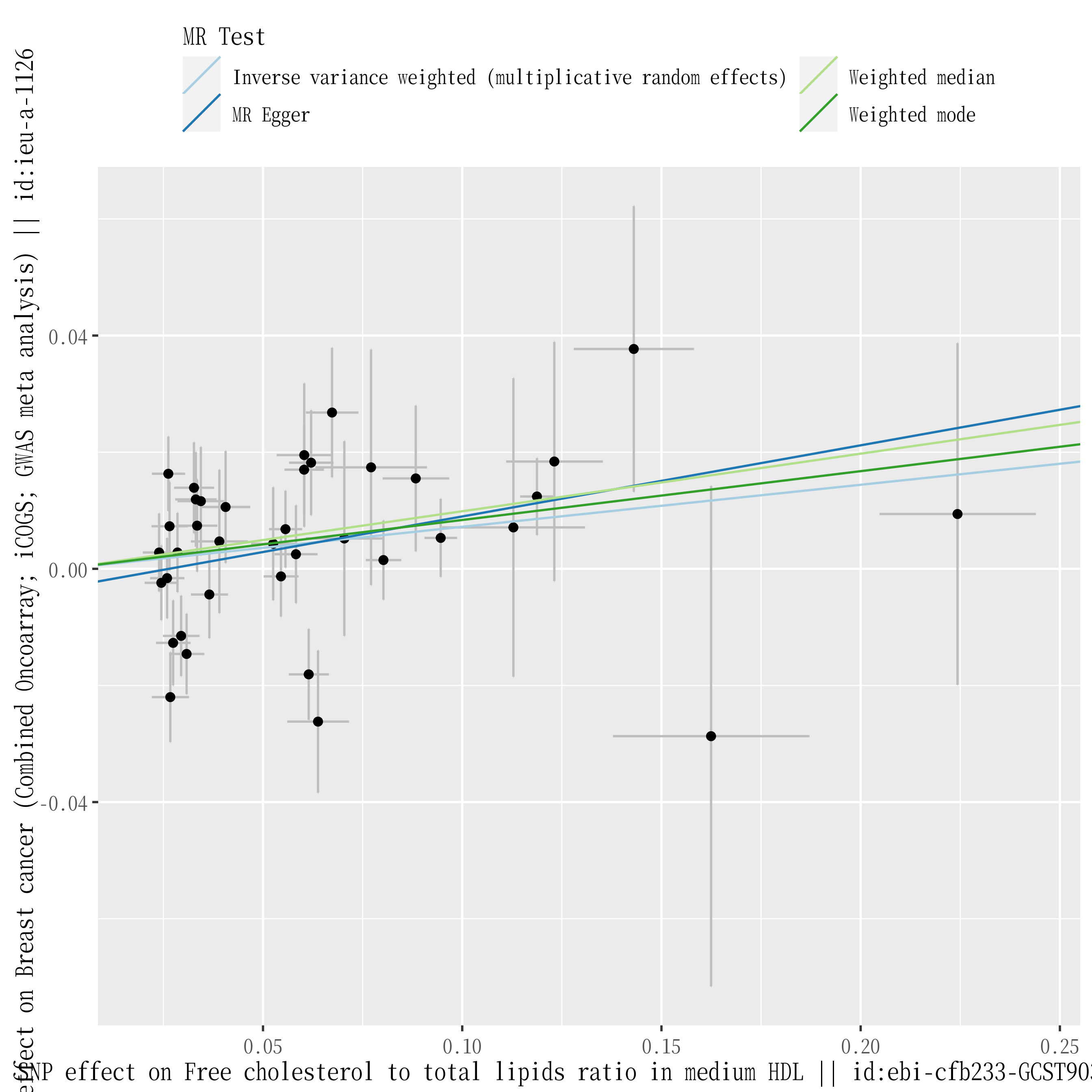
MR Test
Inverse variance weighted (multiplicative random effects)
MR Egger
Weighted median
Weighted mode
0.04
0.00
-0.04
0.05
0.10
0.15
0.20
0.25
SNP effect on Free cholesterol to total lipids ratio in medium HDL || id:ebi-cfb233-GCST90
effect on Breast cancer (Combined Oncoarray; iCOGS; GWAS meta analysis) || id:ieu-a-1126

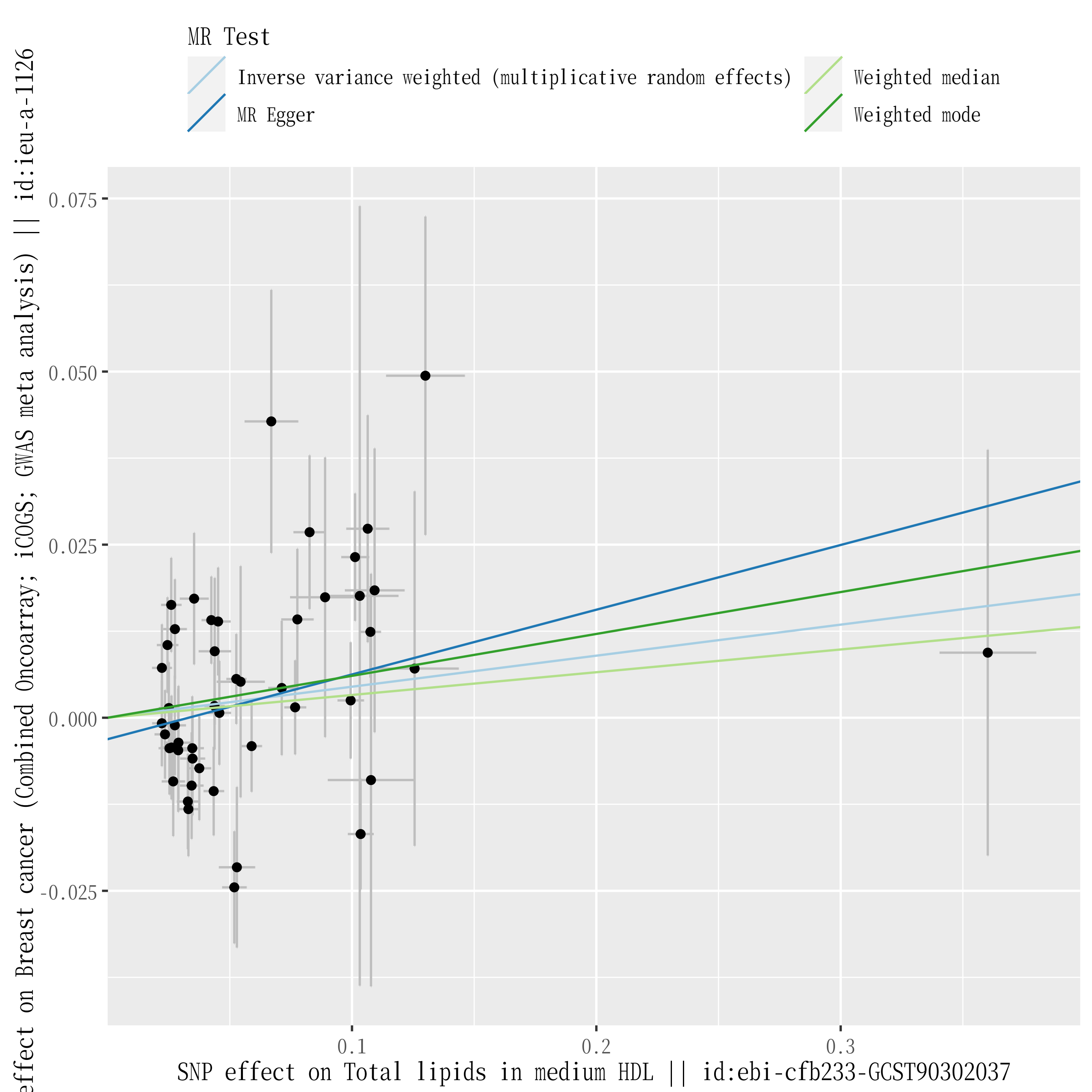
MR Test
Inverse variance weighted (multiplicative random effects)
MR Egger
Weighted median
Weighted mode
0.075
0.050
0.025
0.000
-0.025
0.1
0.2
0.3
SNP effect on Total lipids in medium HDL || id:ebi-cfb233-GCST90302037
effect on Breast cancer (Combined Oncoarray; iCOGS; GWAS meta analysis) || id:ieu-a-1126

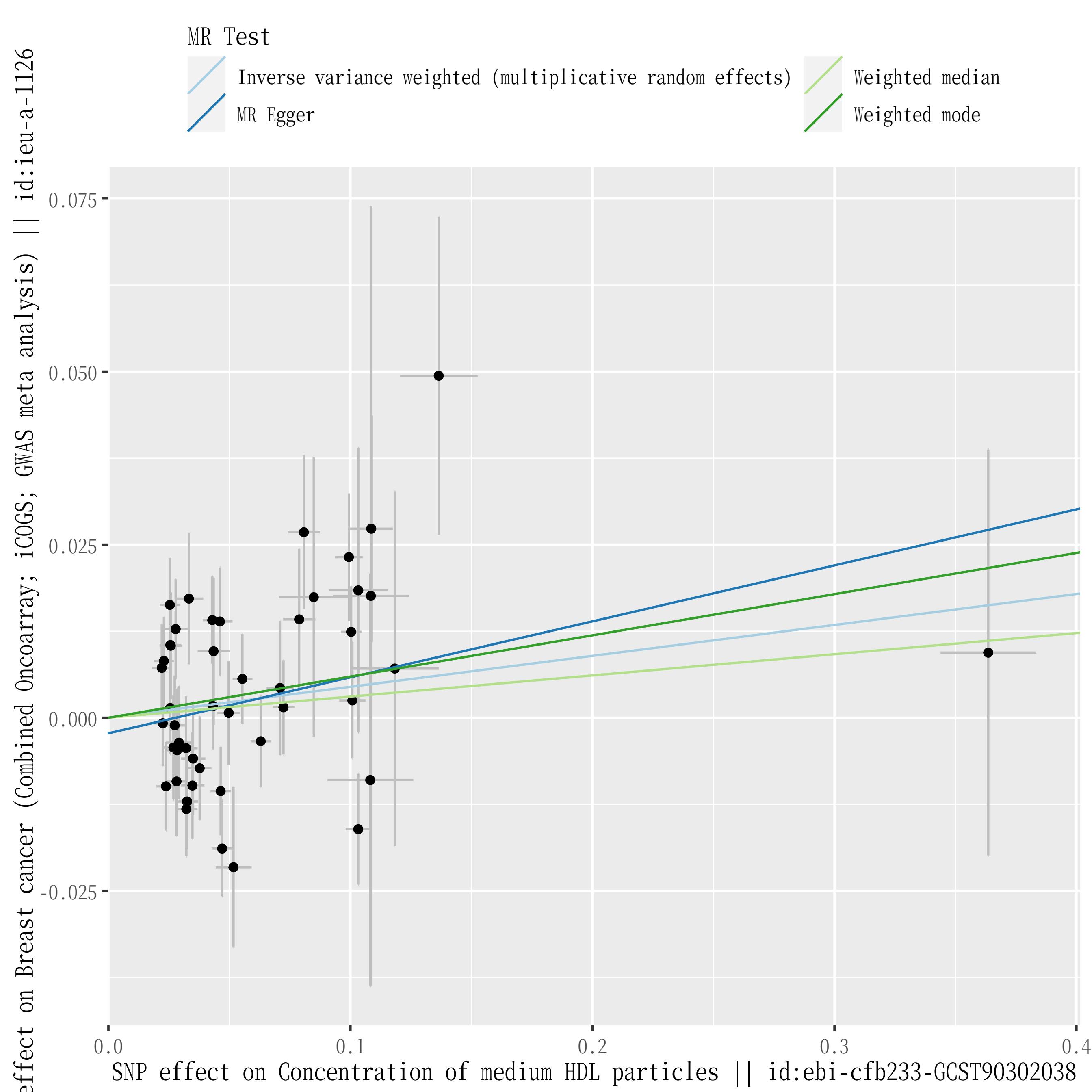
MR Test
Inverse variance weighted (multiplicative random effects)
MR Egger
Weighted median
Weighted mode
0.075
0.050
0.025
0.000
-0.025
0.0
0.1
0.2
0.3
0.4
SNP effect on Concentration of medium HDL particles || id:ebi-cfb233-GCST90302038
effect on Breast cancer (Combined Oncoarray; iCOGS; GWAS meta analysis) || id:ieu-a-1126

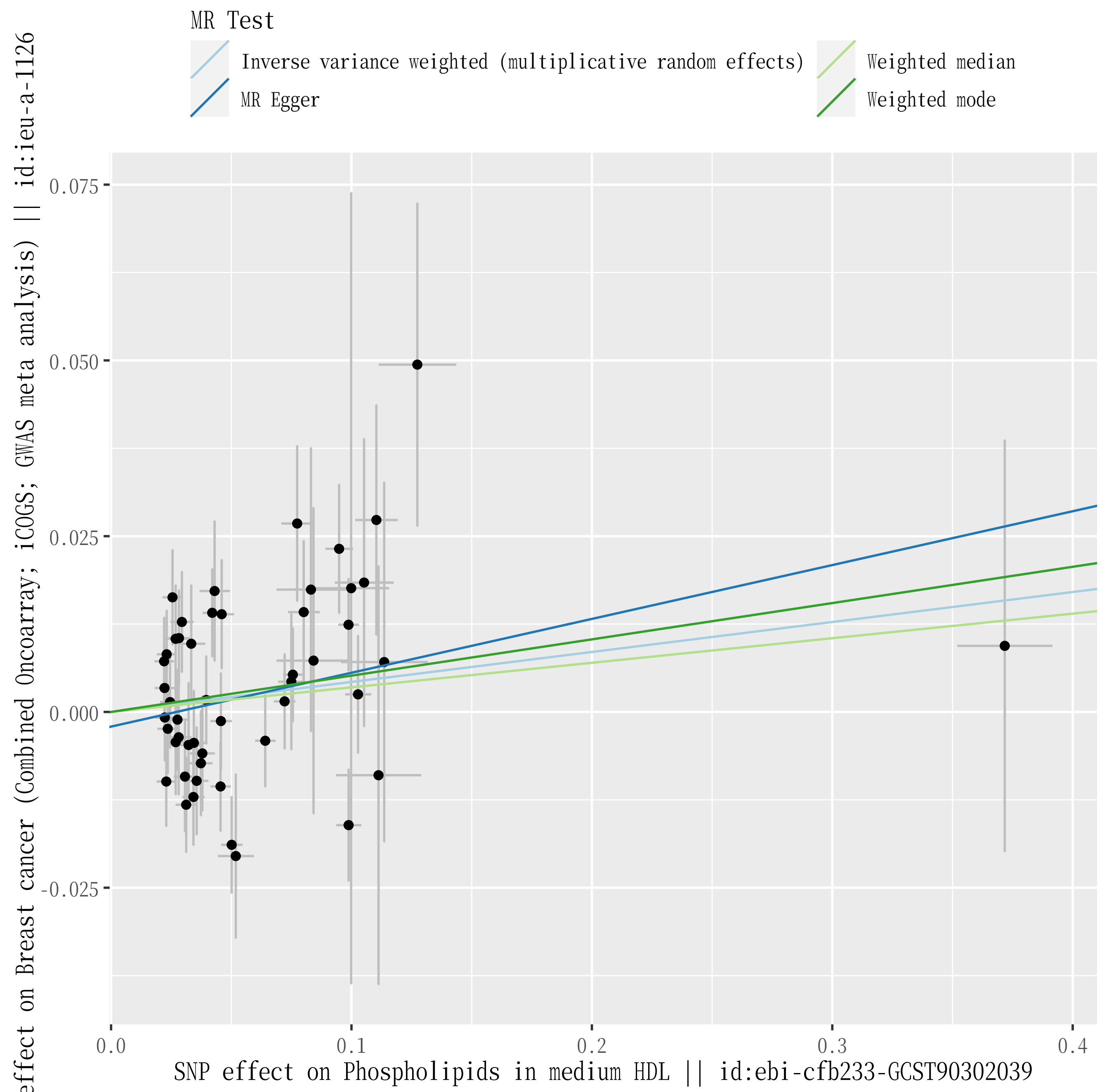

MR Test
Inverse variance weighted (multiplicative random effects)
MR Egger
Weighted median
Weighted mode
0.075
0.050
0.025
0.000
-0.025
0.0
0.1
0.2
0.3
0.4
SNP effect on Phospholipids in medium HDL || id:ebi-cfb233-GCST90302039
effect on Breast cancer (Combined Oncoarray; iCOGS; GWAS meta analysis) || id:ieu-a-1126

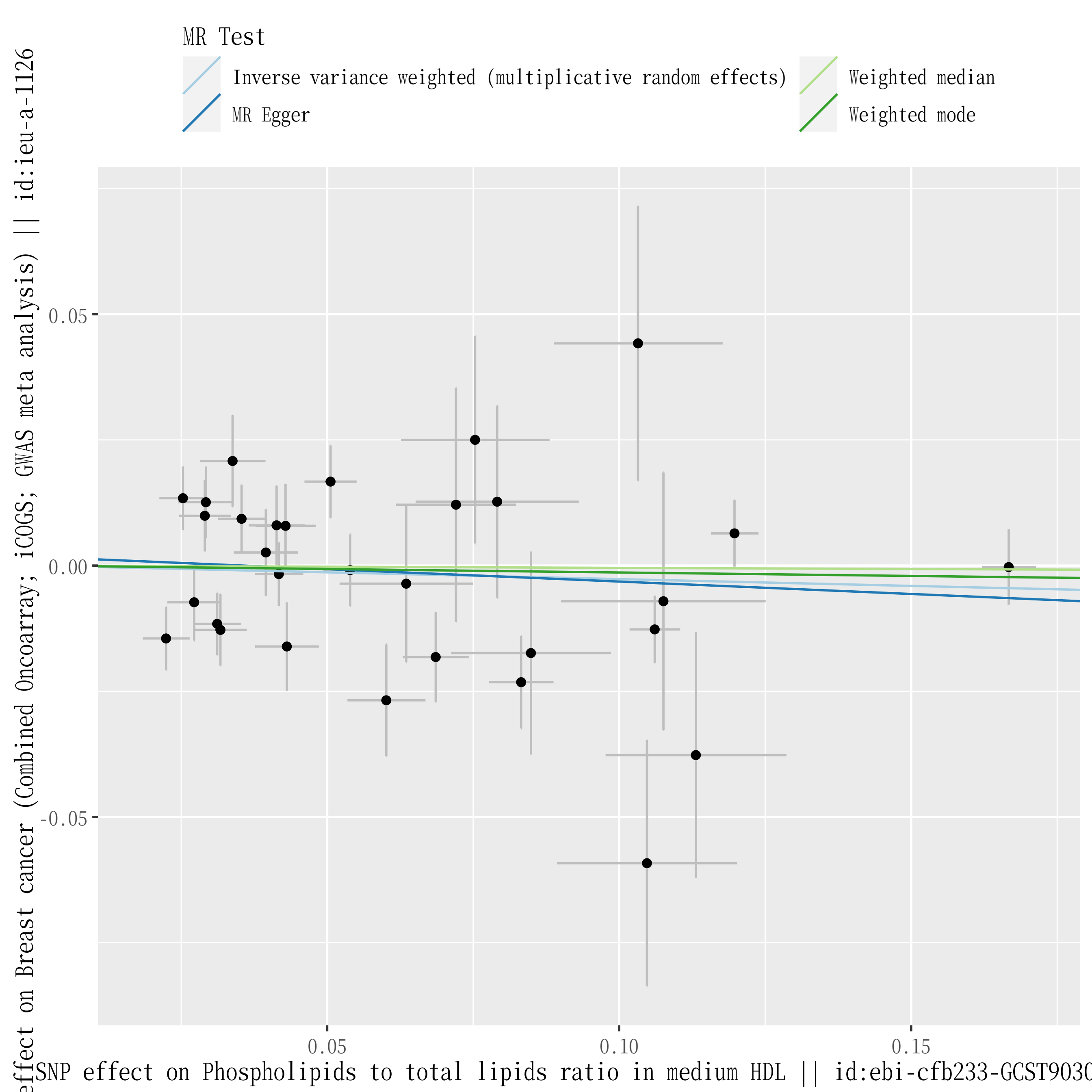

MR Test
Inverse variance weighted (multiplicative random effects)
MR Egger
Weighted median
Weighted mode
0.05
0.00
-0.05
0.05
0.10
0.15
SNP effect on Phospholipids to total lipids ratio in medium HDL || id:ebi-cfb233-GCST9030
effect on Breast cancer (Combined Oncoarray; iCOGS; GWAS meta analysis) || id:ieu-a-1126

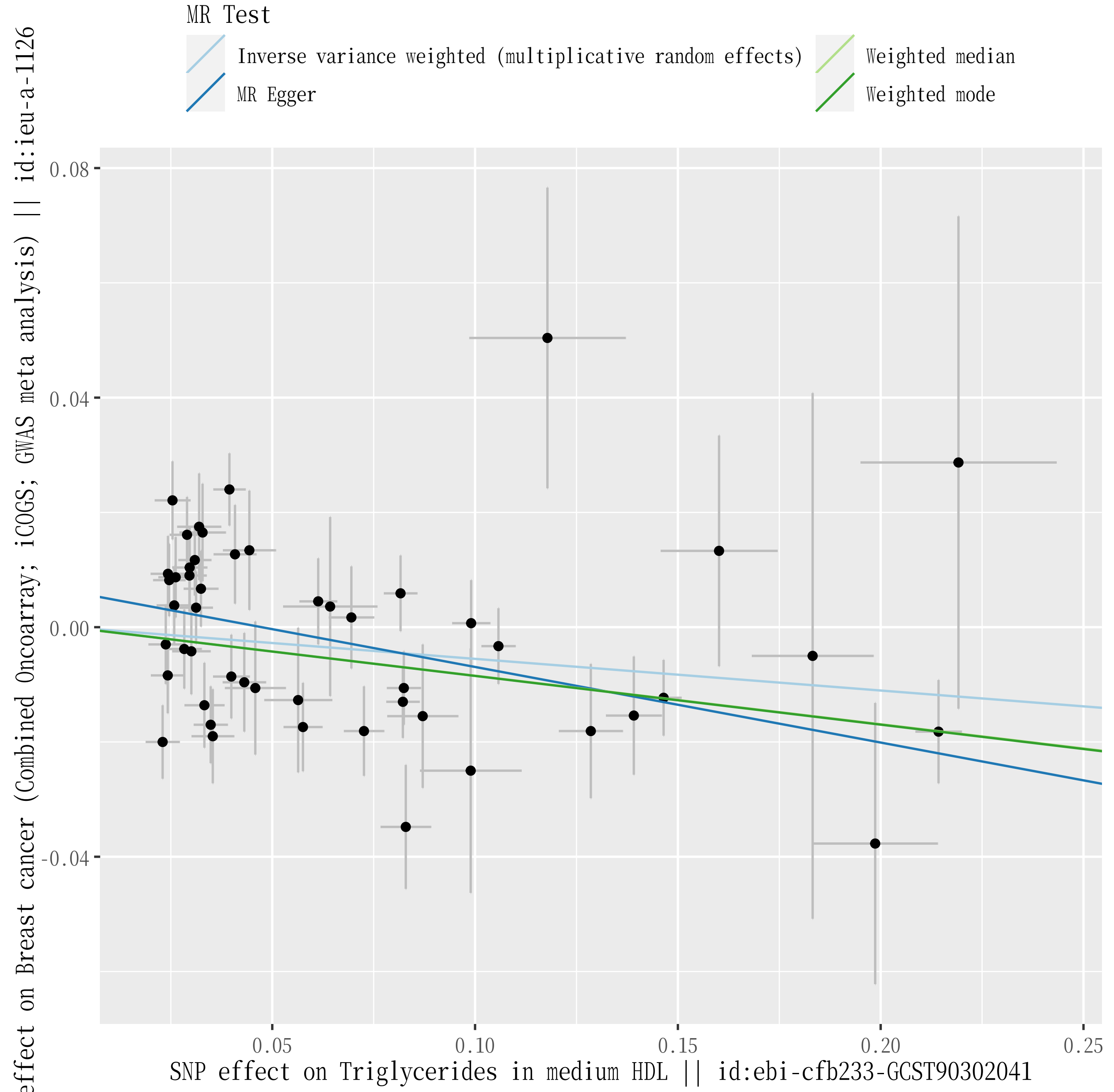

MR Test
Inverse variance weighted (multiplicative random effects)
MR Egger
Weighted median
Weighted mode
0.08
0.04
0.00
-0.04
0.05
0.10
0.15
0.20
0.25
SNP effect on Triglycerides in medium HDL || id:ebi-cfb233-GCST90302041
effect on Breast cancer (Combined Oncoarray; iCOGS; GWAS meta analysis) || id:ieu-a-1126

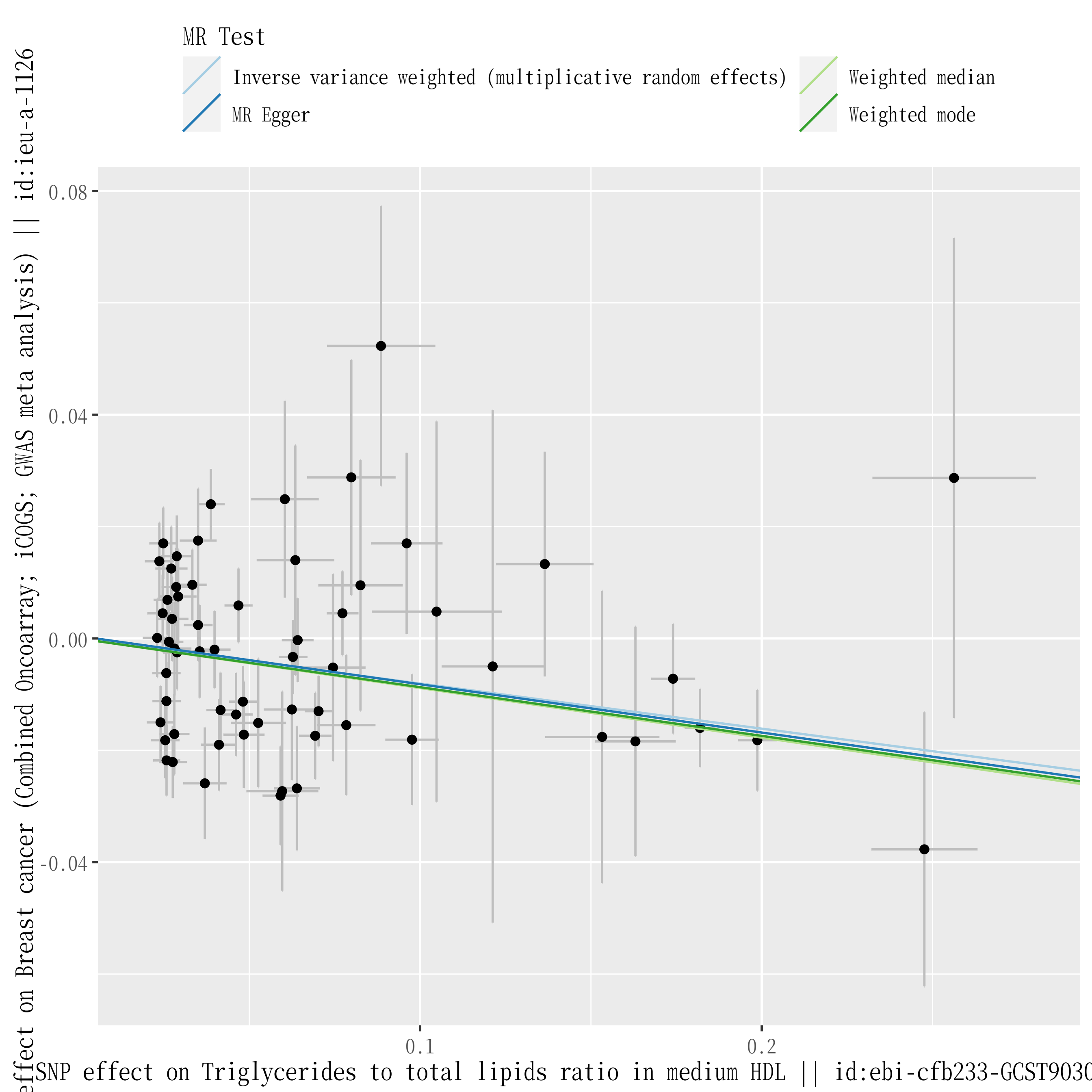

MR Test
Inverse variance weighted (multiplicative random effects)
MR Egger
Weighted median
Weighted mode
0.08
0.04
0.00
-0.04
0.1
0.2
SNP effect on Triglycerides to total lipids ratio in medium HDL || id:ebi-cfb233-GCST9030
effect on Breast cancer (Combined Oncoarray; iCOGS; GWAS meta analysis) || id:ieu-a-1126

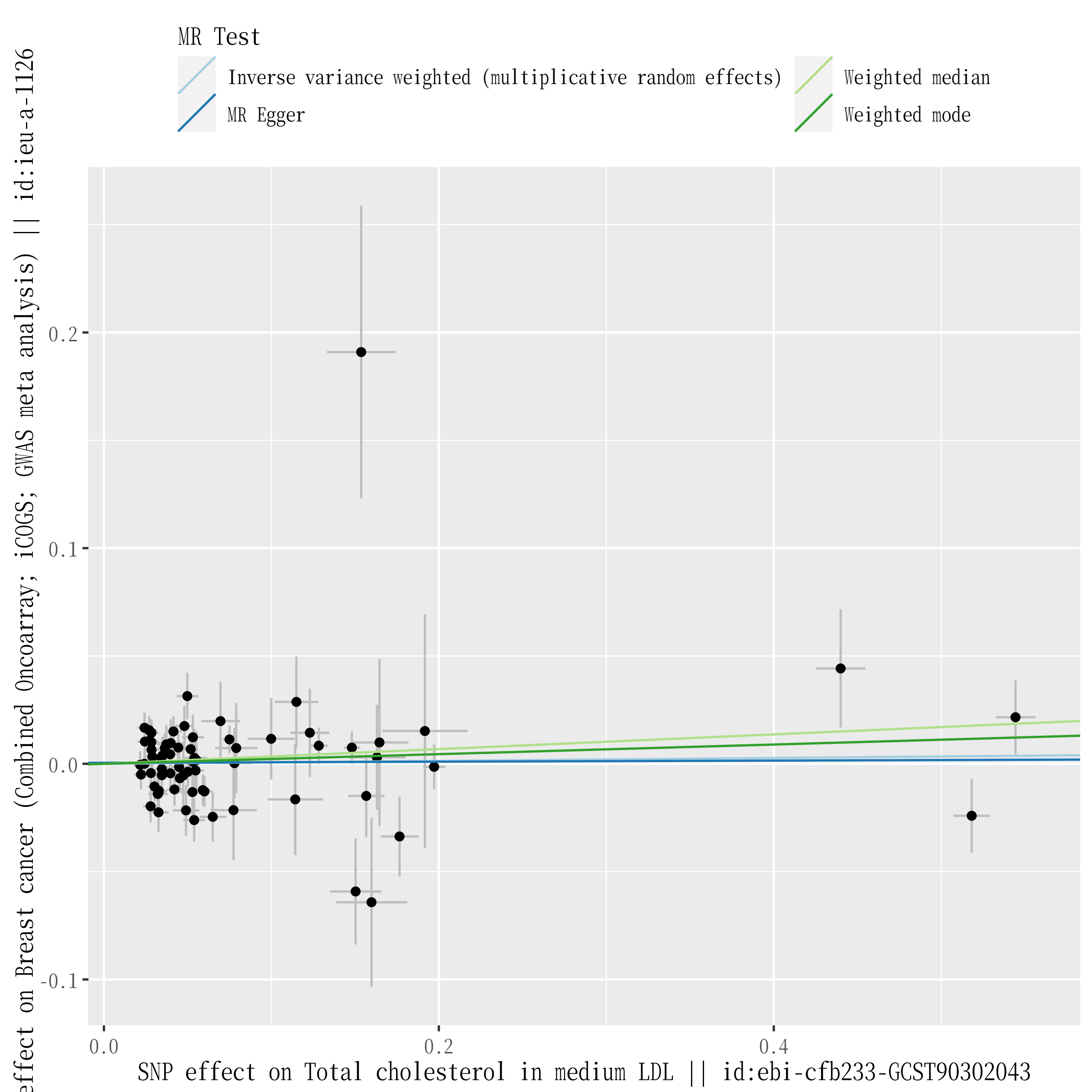

MR Test
Inverse variance weighted (multiplicative random effects)
MR Egger
Weighted median
Weighted mode
0.2
0.1
0.0
-0.1
0.0
0.2
0.4
SNP effect on Total cholesterol in medium LDL || id:ebi-cfb233-GCST90302043
effect on Breast cancer (Combined Oncoarray; iCOGS; GWAS meta analysis) || id:ieu-a-1126

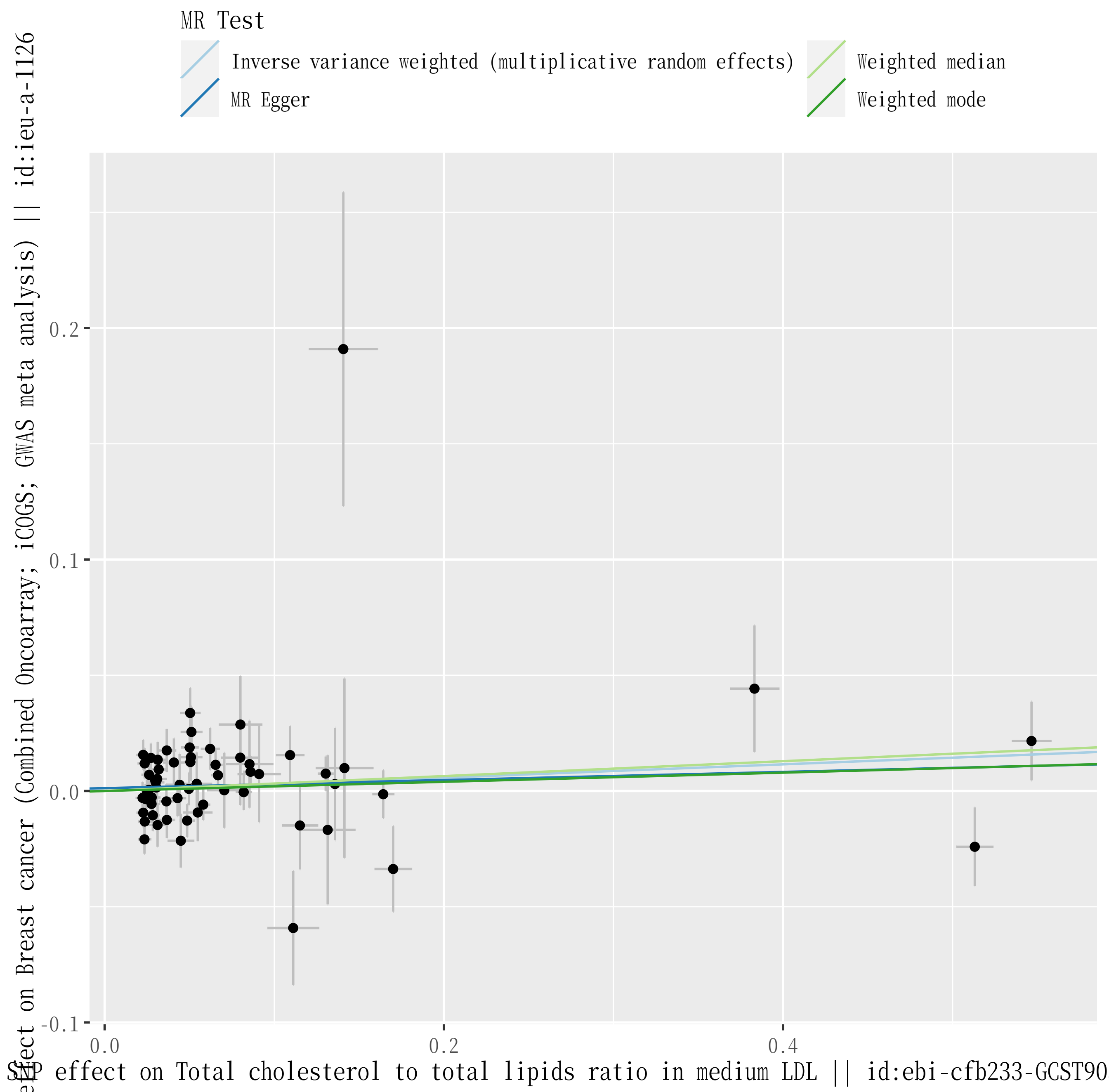
MR Test
Inverse variance weighted (multiplicative random effects)
MR Egger
Weighted median
Weighted mode
0.2
0.1
0.0
-0.1
0.0
0.2
0.4
SNP effect on Total cholesterol to total lipids ratio in medium LDL || id:ebi-cfb233-GCST90
effect on Breast cancer (Combined Oncoarray; iCOGS; GWAS meta analysis) || id:ieu-a-1126

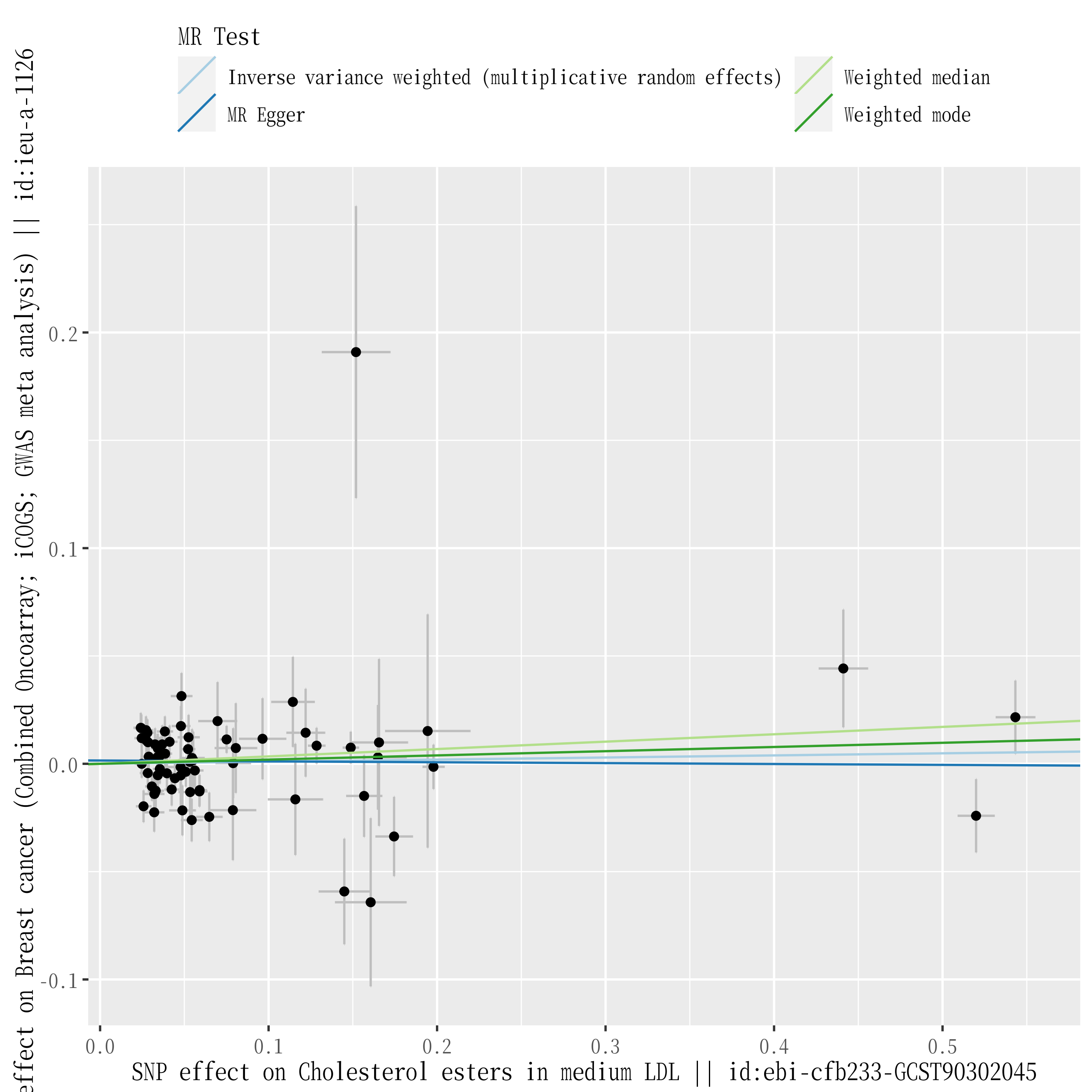
MR Test
Inverse variance weighted (multiplicative random effects)
MR Egger
Weighted median
Weighted mode
SNP effect on Breast cancer (Combined Oncoarray; iCOGS; GWAS meta analysis) || id:ieu-a-1126
SNP effect on Cholesterol esters in medium LDL || id:ebi-cfb233-GCST90302045
0.2
0.1
0.0
-0.1
0.0
0.1
0.2
0.3
0.4
0.5

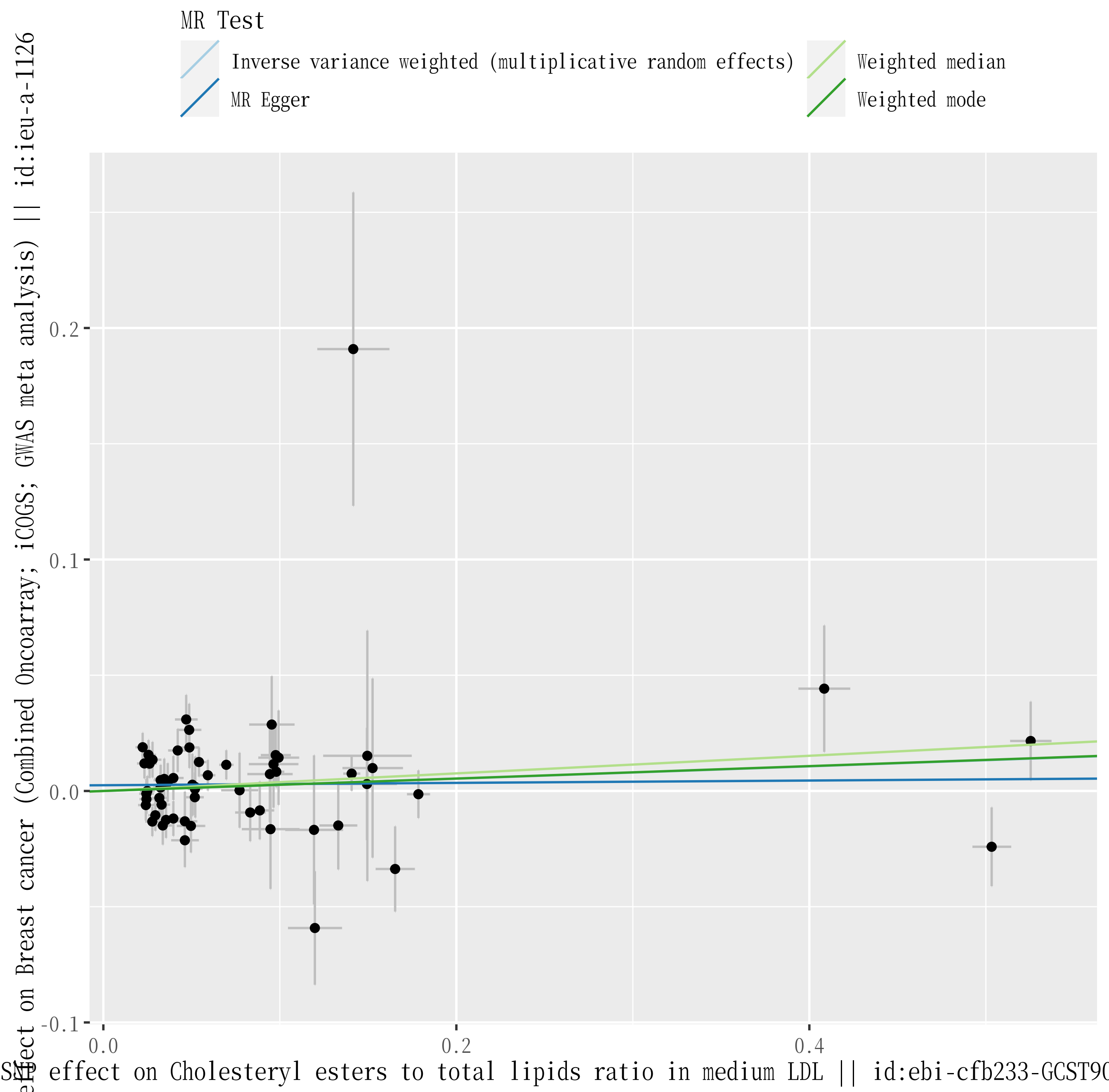

MR Test
Inverse variance weighted (multiplicative random effects)
MR Egger
Weighted median
Weighted mode
0.2
0.1
0.0
-0.1
0.0
0.2
0.4
SNP effect on Cholesteryl esters to total lipids ratio in medium LDL || id:ebi-cfb233-GCST90
SNP effect on Breast cancer (Combined Oncoarray; iCOGS; GWAS meta analysis) || id:ieu-a-1126

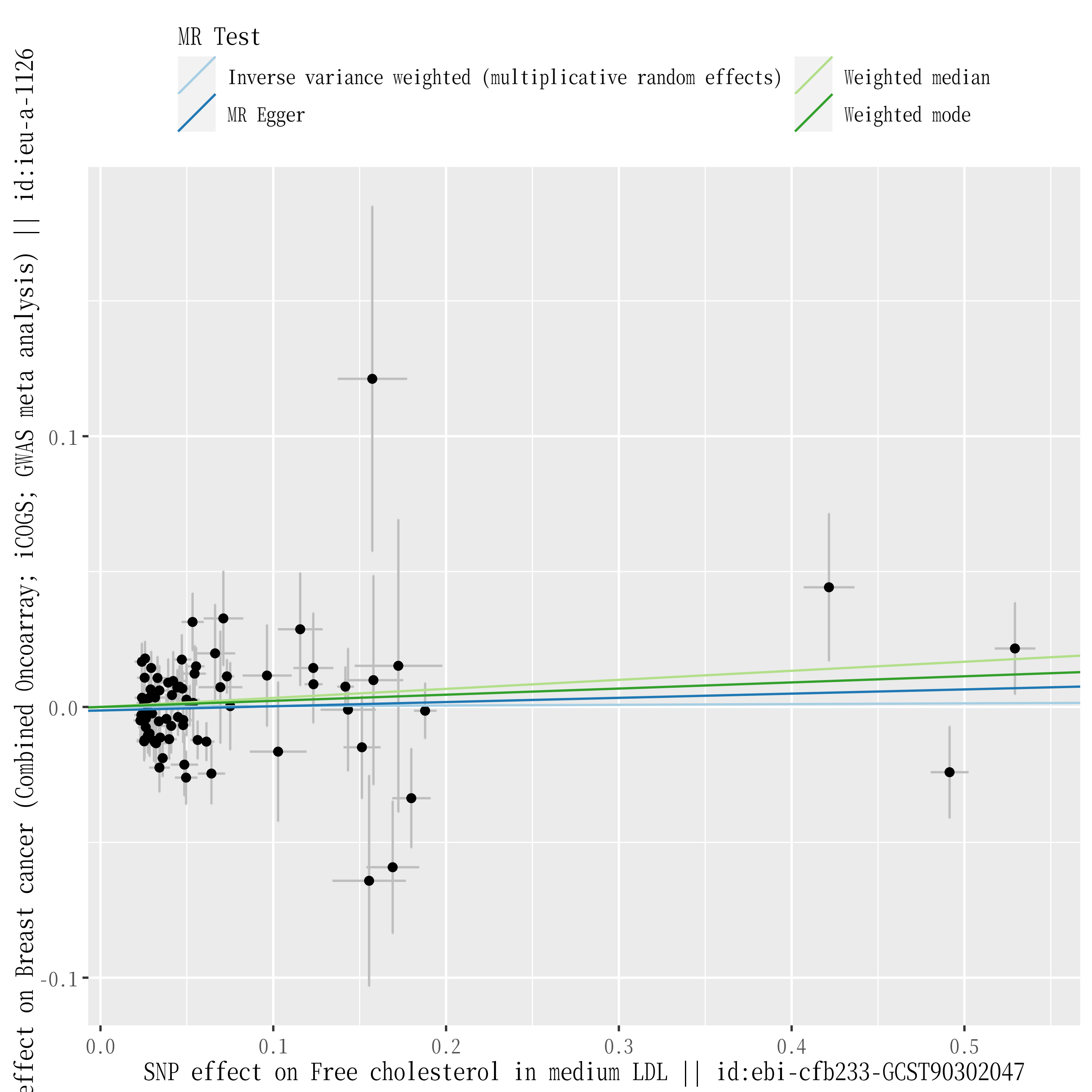

MR Test
Inverse variance weighted (multiplicative random effects)
MR Egger
Weighted median
Weighted mode
0.1
0.0
-0.1
0.0
0.1
0.2
0.3
0.4
0.5
SNP effect on Free cholesterol in medium LDL || id:ebi-cfb233-GCST90302047
effect on Breast cancer (Combined Oncoarray; iCOGS; GWAS meta analysis) || id:ieu-a-1126

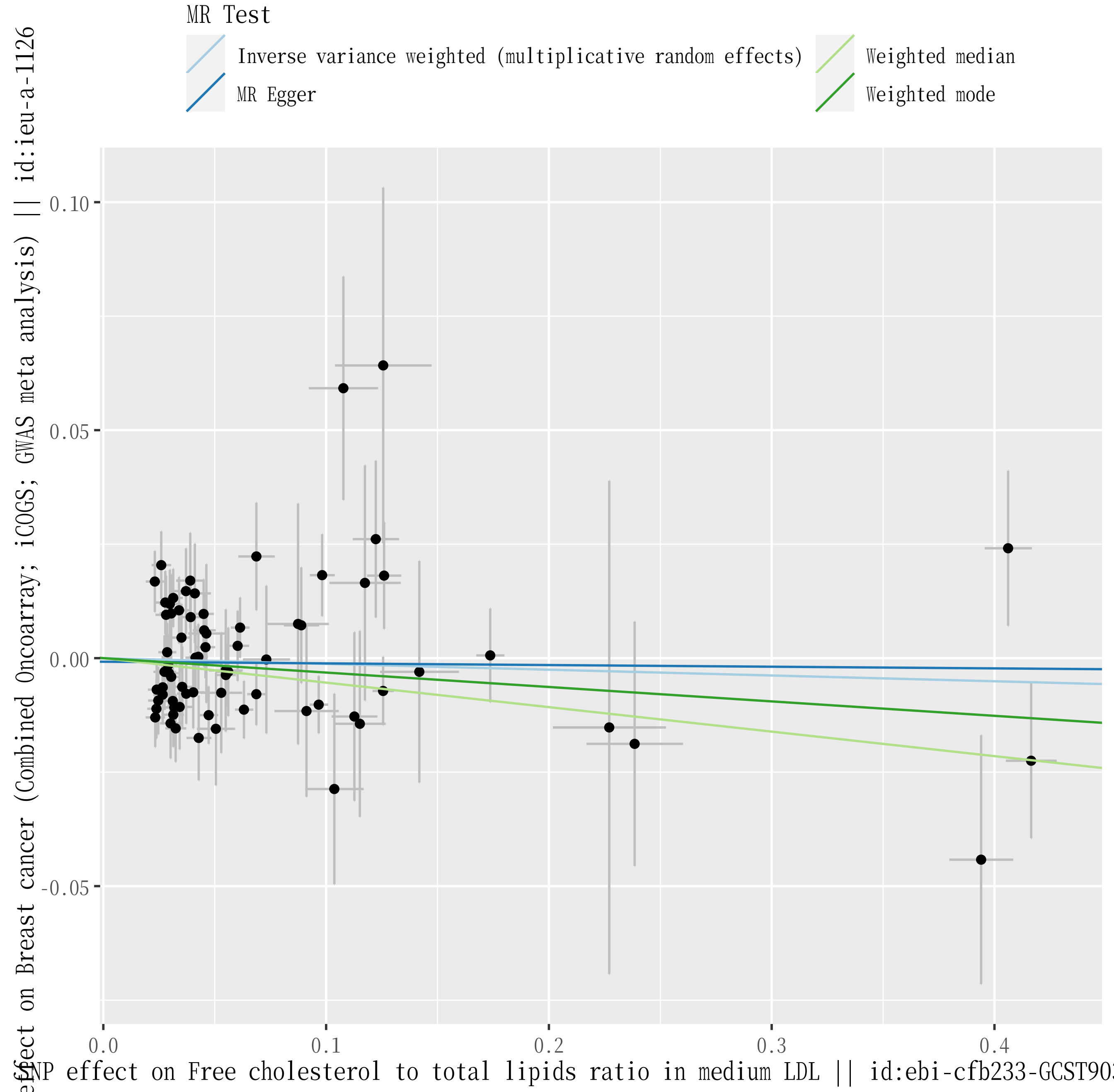

MR Test
Inverse variance weighted (multiplicative random effects)
MR Egger
Weighted median
Weighted mode
0.10
0.05
0.00
-0.05
0.0
0.1
0.2
0.3
0.4
SNP effect on Free cholesterol to total lipids ratio in medium LDL || id:ebi-cfb233-GCST90
SNP effect on Breast cancer (Combined Oncoarray; iCOGS; GWAS meta analysis) || id:ieu-a-1126

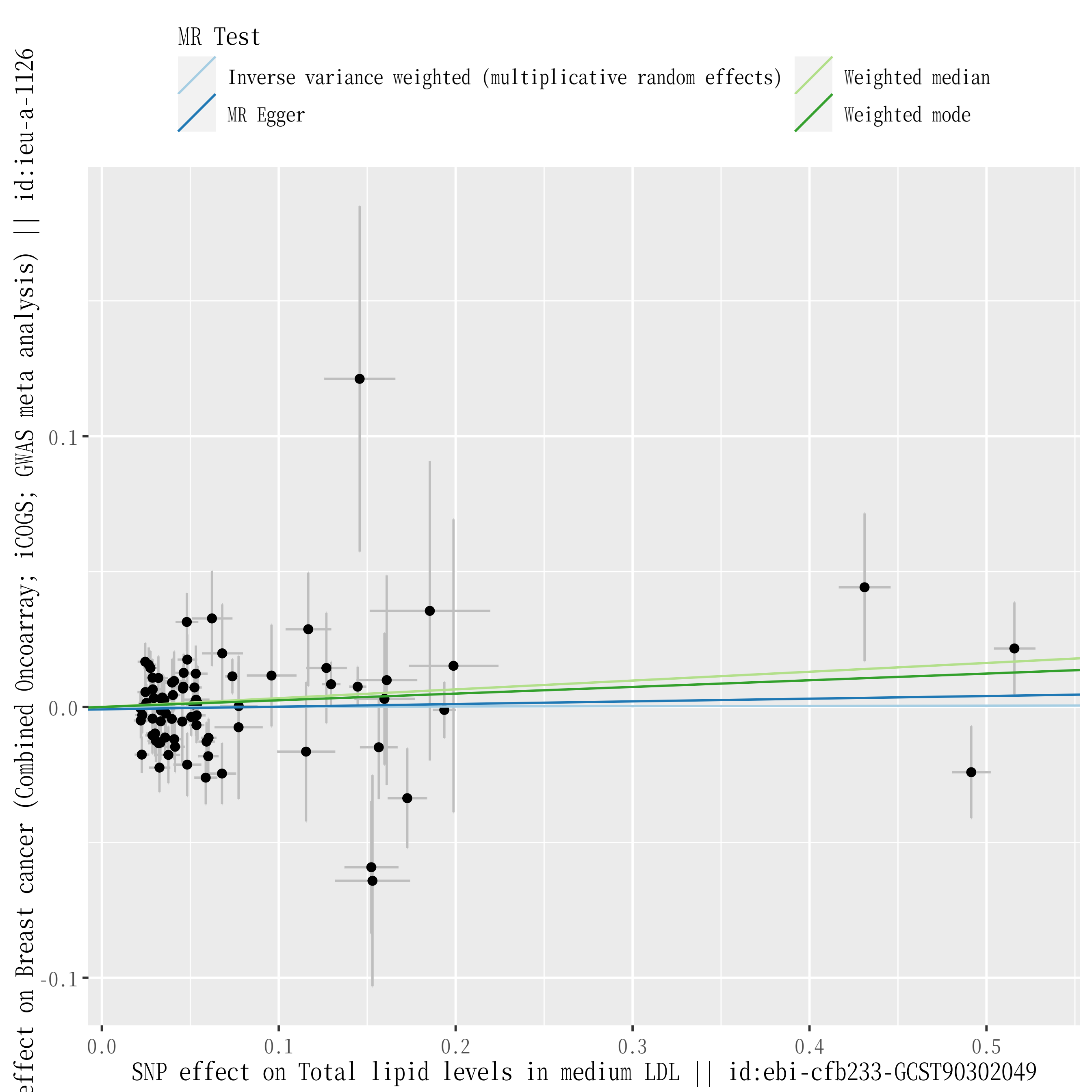
MR Test
Inverse variance weighted (multiplicative random effects)
MR Egger
Weighted median
Weighted mode
0.1
0.0
-0.1
0.0
0.1
0.2
0.3
0.4
0.5
SNP effect on Total lipid levels in medium LDL || id:ebi-cfb233-GCST90302049
effect on Breast cancer (Combined Oncoarray; iCOGS; GWAS meta analysis) || id:ieu-a-1126

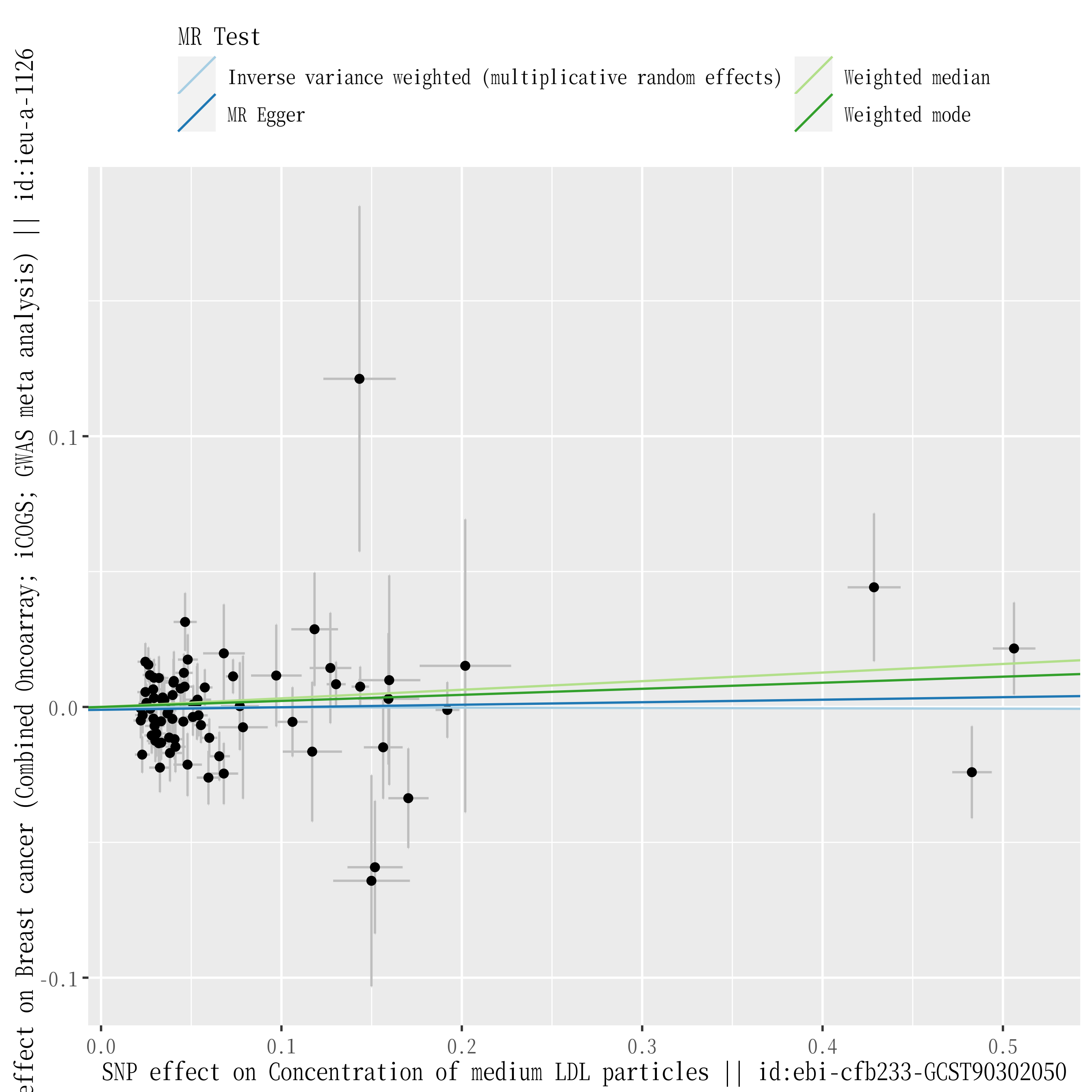

MR Test
Inverse variance weighted (multiplicative random effects)
MR Egger
Weighted median
Weighted mode
0.1
0.0
-0.1
0.0
0.1
0.2
0.3
0.4
0.5
SNP effect on Concentration of medium LDL particles || id:ebi-cfb233-GCST90302050
SNP effect on Breast cancer (Combined Oncoarray; iCOGS; GWAS meta analysis) || id:ieu-a-1126

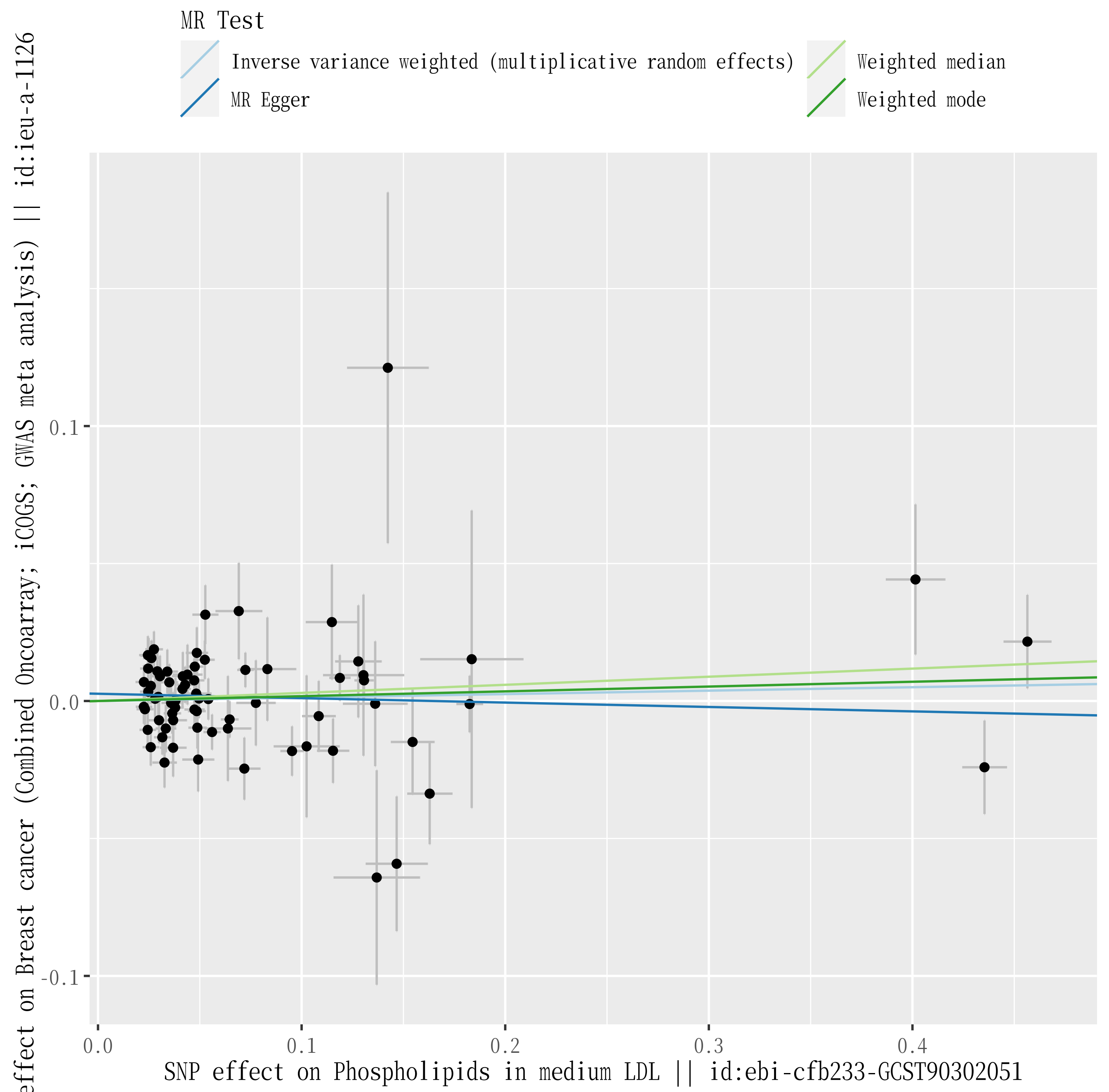

MR Test
Inverse variance weighted (multiplicative random effects)
MR Egger
Weighted median
Weighted mode
0.1
0.0
-0.1
0.0
0.1
0.2
0.3
0.4
SNP effect on Phospholipids in medium LDL || id:ebi-cfb233-GCST90302051
effect on Breast cancer (Combined Oncoarray; iCOGS; GWAS meta analysis) || id:ieu-a-1126

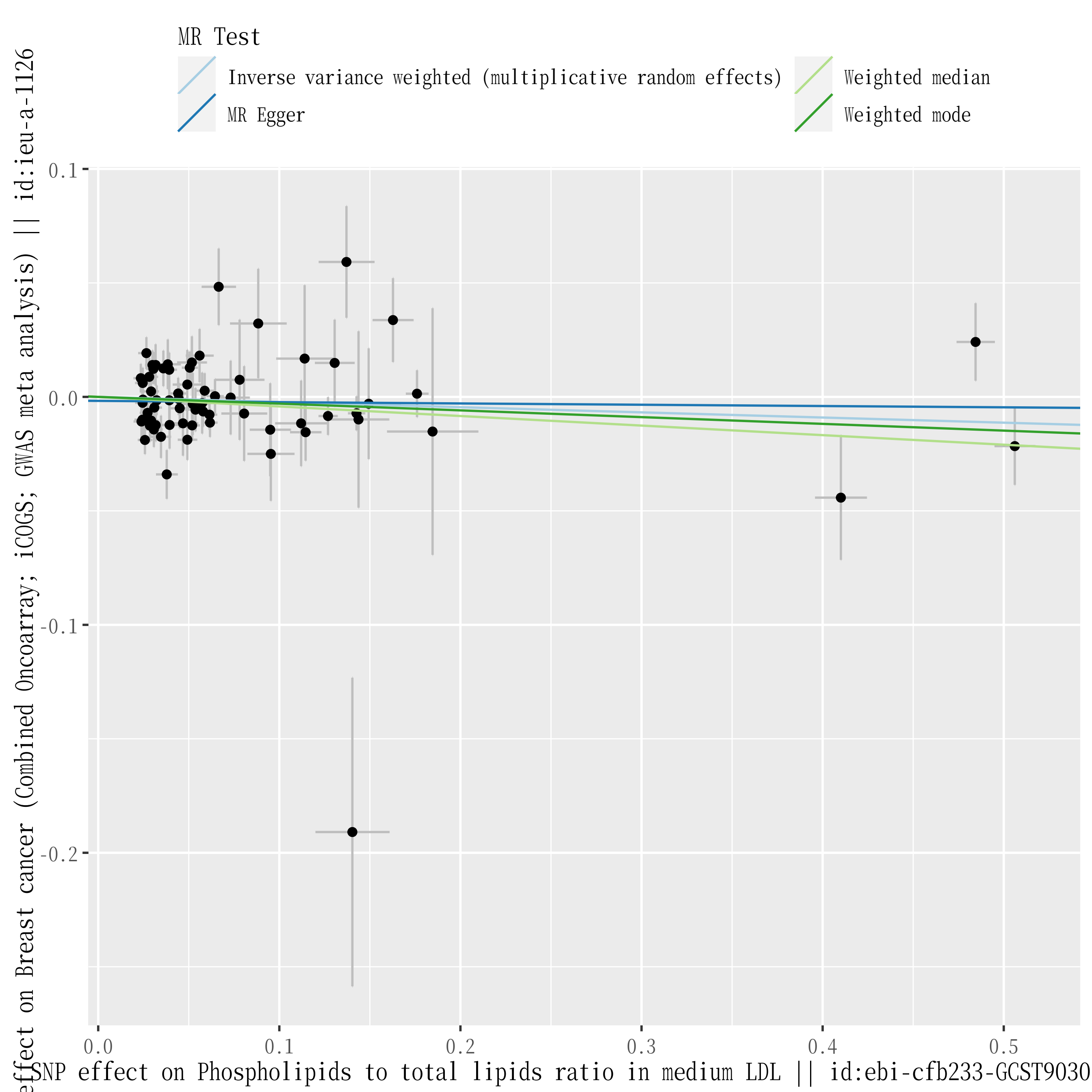

MR Test
Inverse variance weighted (multiplicative random effects)
MR Egger
Weighted median
Weighted mode
0.1
0.0
-0.1
-0.2
0.0
0.1
0.2
0.3
0.4
0.5
SNP effect on Phospholipids to total lipids ratio in medium LDL || id:ebi-cfb233-GCST9030
effect on Breast cancer (Combined Oncoarray; iCOGS; GWAS meta analysis) || id:ieu-a-1126

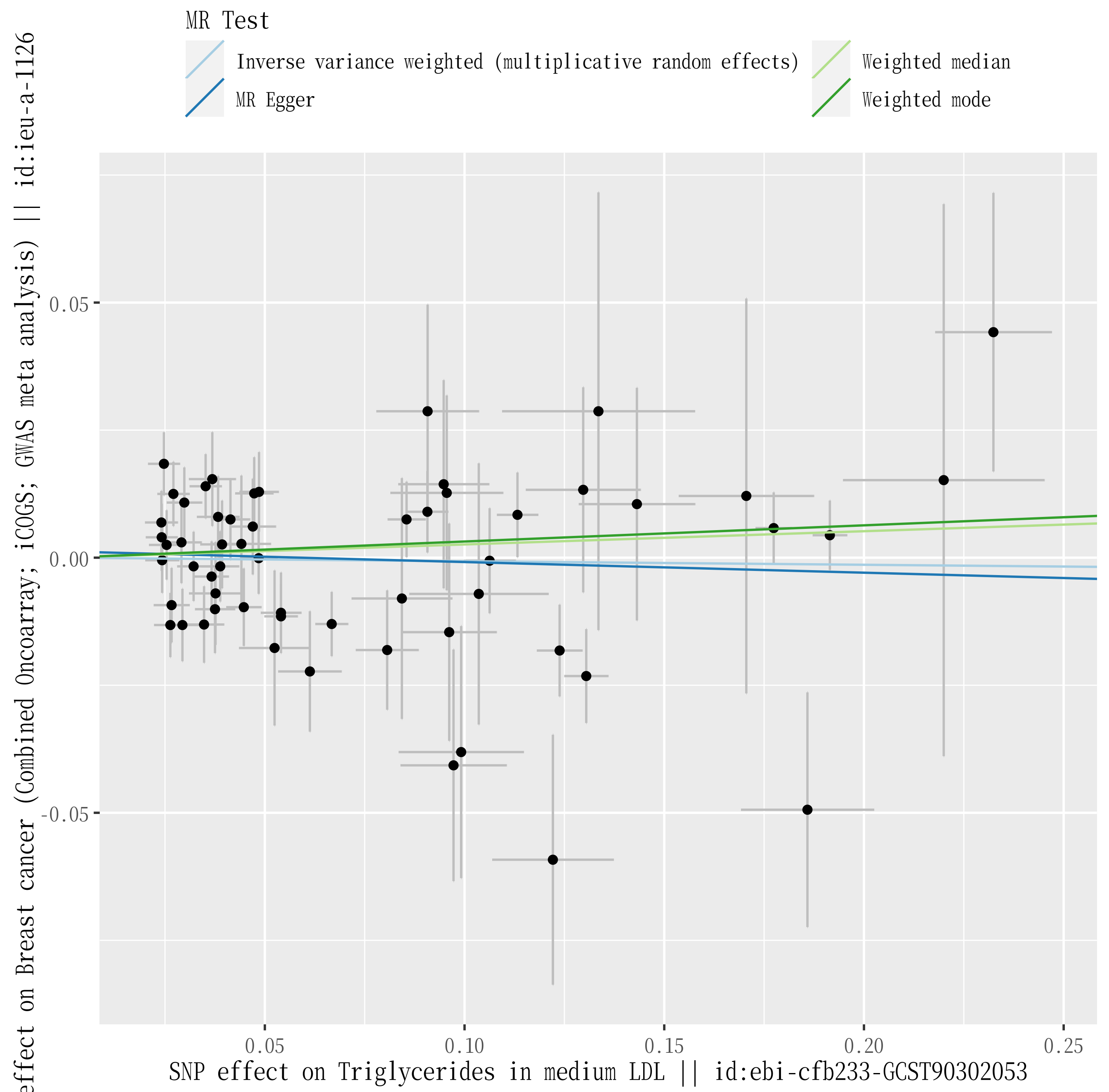

MR Test
Inverse variance weighted (multiplicative random effects)
MR Egger
Weighted median
Weighted mode
0.05
0.00
-0.05
0.05
0.10
0.15
0.20
0.25
SNP effect on Triglycerides in medium LDL || id:ebi-cfb233-GCST90302053
effect on Breast cancer (Combined Oncoarray; iCOGS; GWAS meta analysis) || id:ieu-a-1126

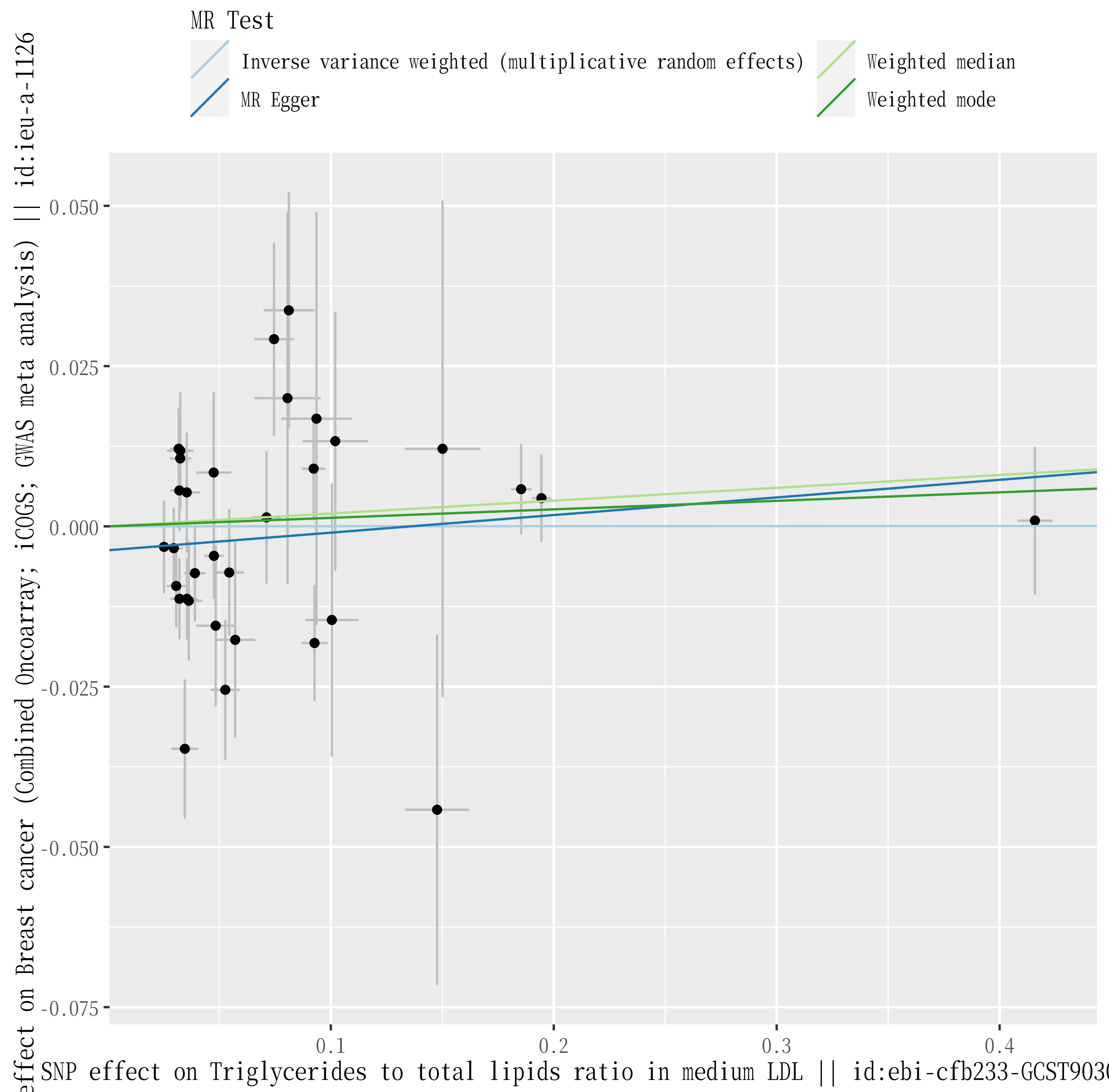
MR Test
Inverse variance weighted (multiplicative random effects)
MR Egger
Weighted median
Weighted mode
0.050
0.025
0.000
-0.025
-0.050
-0.075
0.1
0.2
0.3
0.4
SNP effect on Breast cancer (Combined Oncoarray; iCOGS; GWAS meta analysis) || id:ieu-a-1126
SNP effect on Triglycerides to total lipids ratio in medium LDL || id:ebi-cfb233-GCST903

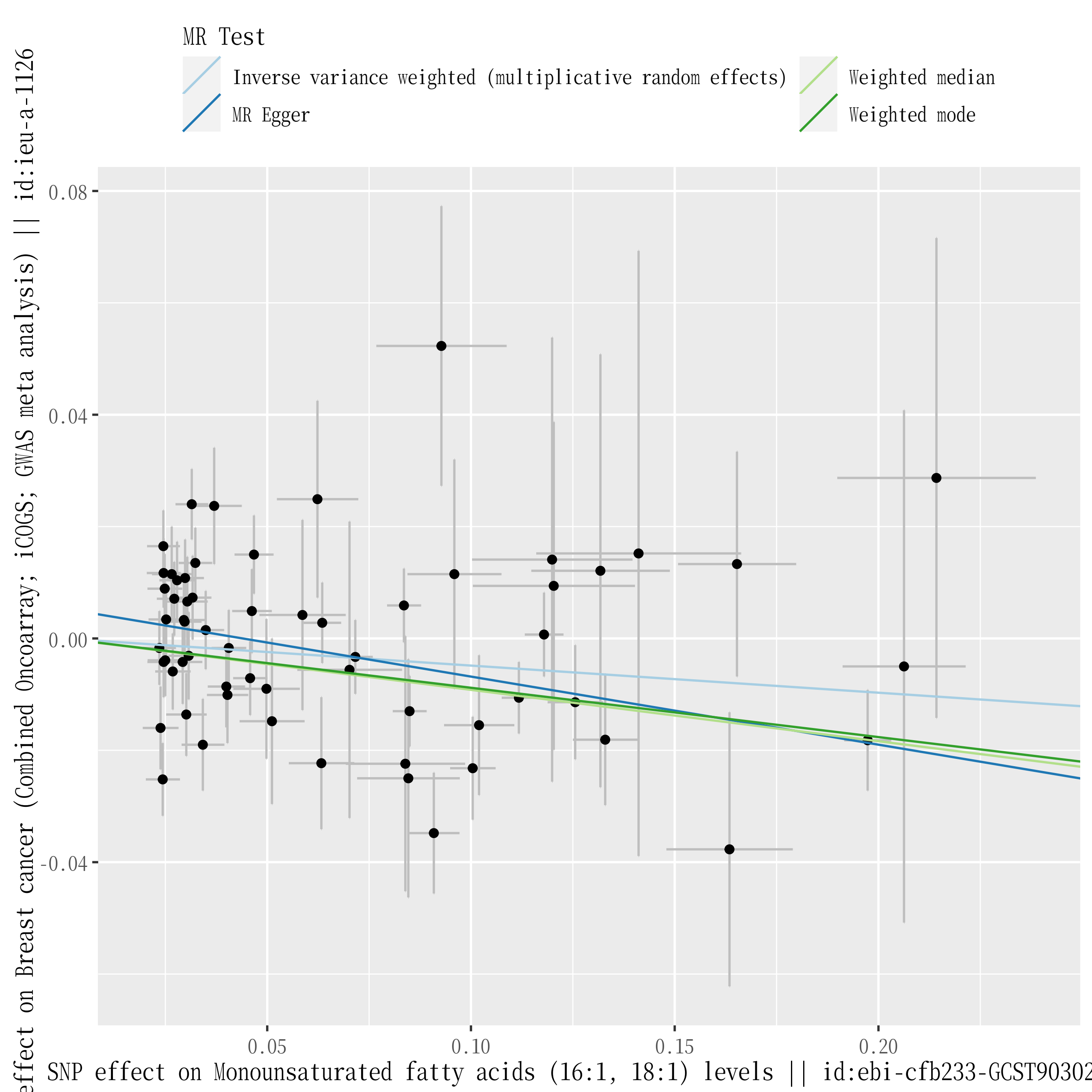

MR Test
Inverse variance weighted (multiplicative random effects)
MR Egger
Weighted median
Weighted mode
0.08
0.04
0.00
-0.04
0.05
0.10
0.15
0.20
SNP effect on Monounsaturated fatty acids (16:1, 18:1) levels || id:ebi-cfb233-GCST90302
effect on Breast cancer (Combined Oncoarray; iCOGS; GWAS meta analysis) || id:ieu-a-1126

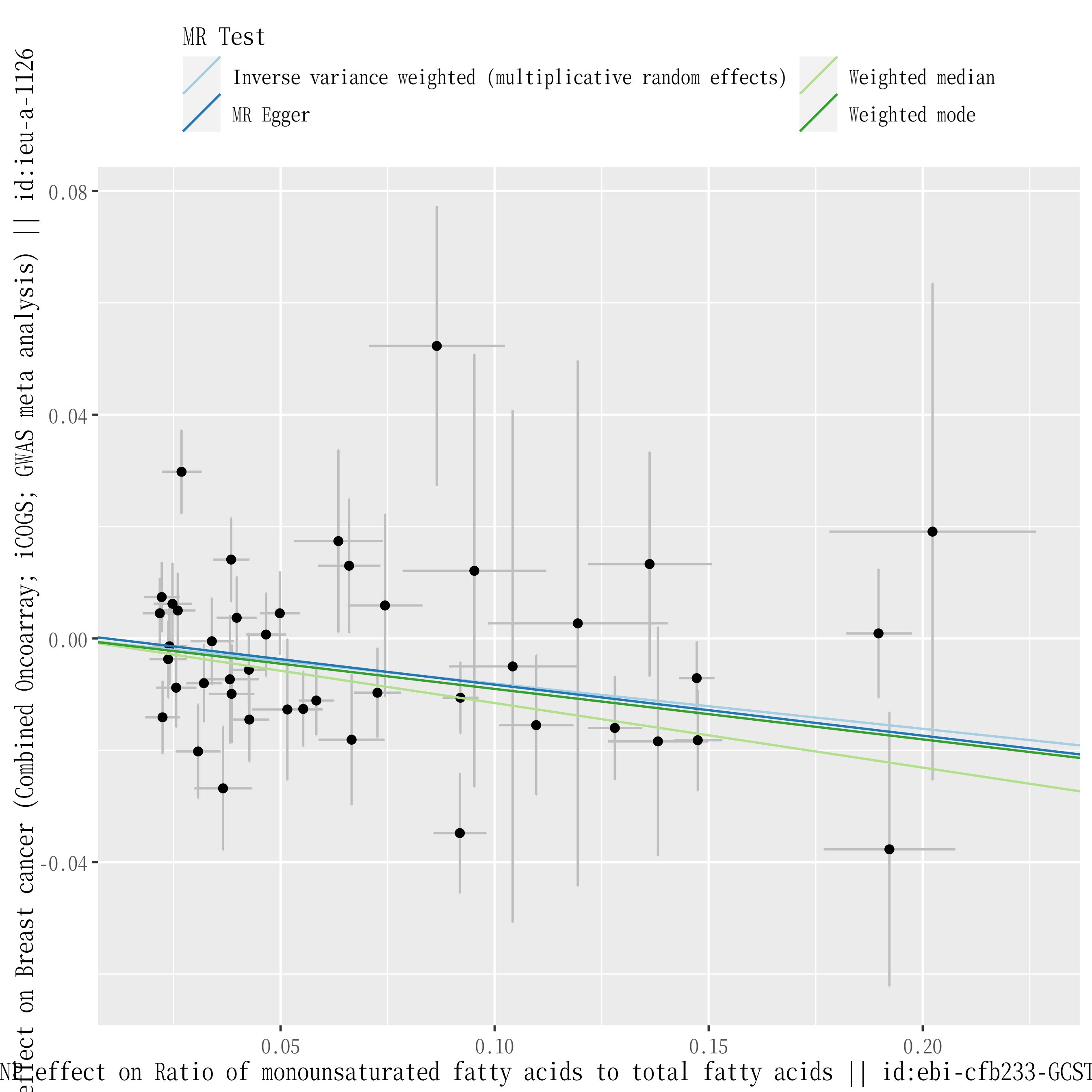

MR Test
Inverse variance weighted (multiplicative random effects)
MR Egger
Weighted median
Weighted mode
0.08
0.04
0.00
-0.04
0.05
0.10
0.15
0.20
SNP effect on Ratio of monounsaturated fatty acids to total fatty acids || id:ebi-cfb233-GCST
SNP effect on Breast cancer (Combined Oncoarray; iCOGS; GWAS meta analysis) || id:ieu-a-1126

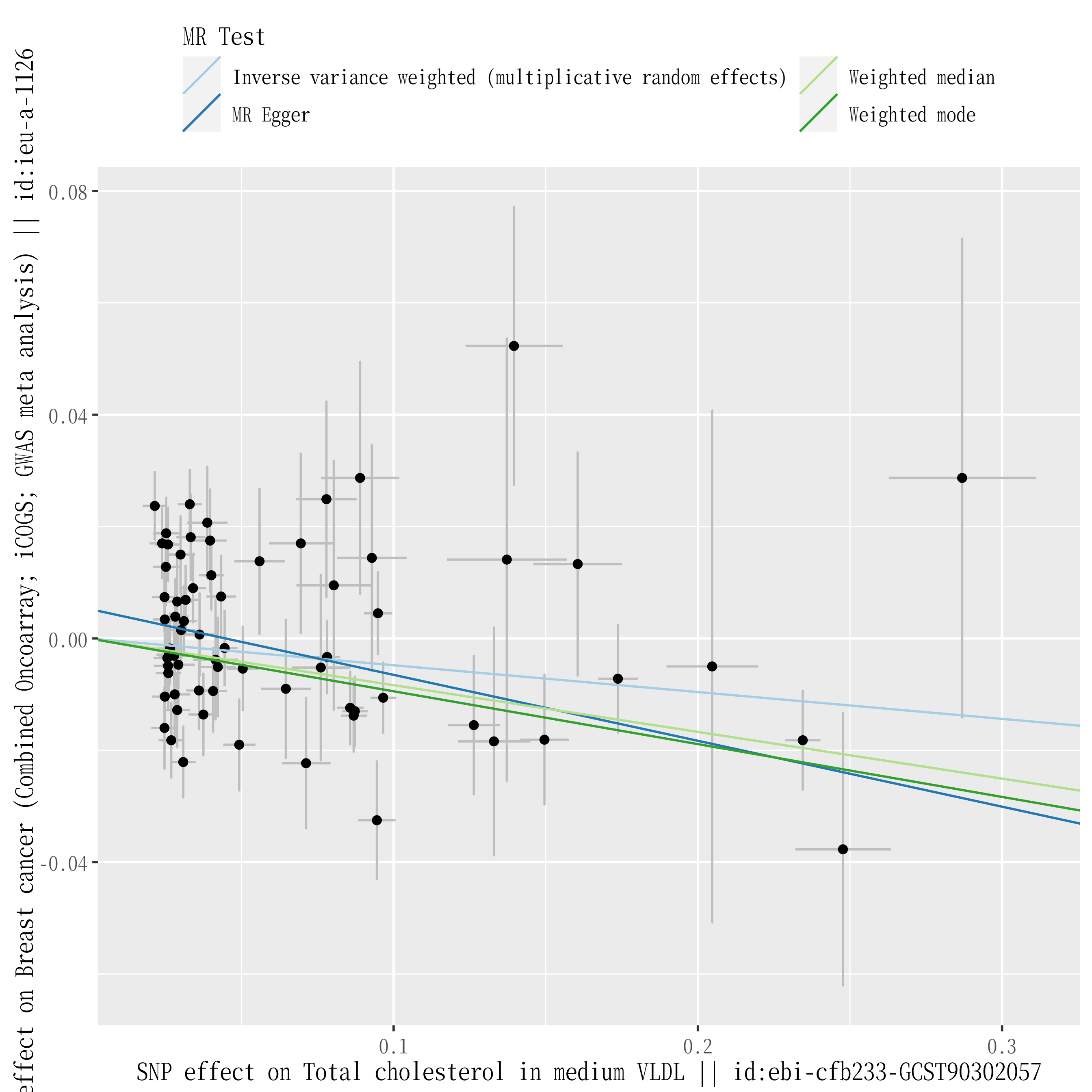
MR Test
Inverse variance weighted (multiplicative random effects)
MR Egger
Weighted median
Weighted mode
effect on Breast cancer (Combined Oncoarray; iCOGS; GWAS meta analysis) || id:ieu-a-1126
0.08
0.04
0.00
-0.04
0.1
0.2
0.3
SNP effect on Total cholesterol in medium VLDL || id:ebi-cfb233-GCST90302057

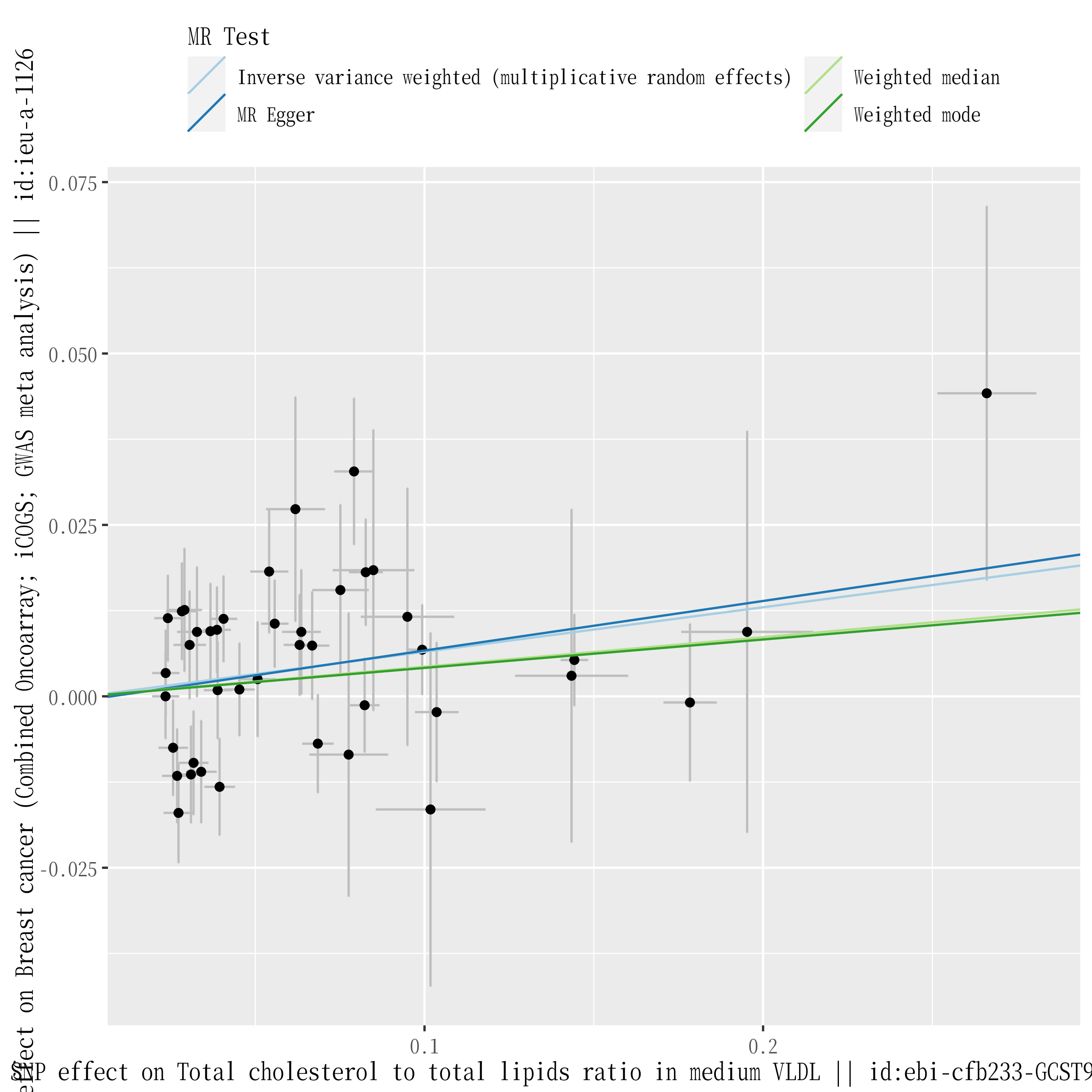

MR Test
Inverse variance weighted (multiplicative random effects)
MR Egger
Weighted median
Weighted mode
0.075
0.050
0.025
0.000
-0.025
0.1
0.2
SNP effect on Total cholesterol to total lipids ratio in medium VLDL || id:ebi-cfb233-GCST9
effect on Breast cancer (Combined Oncoarray; iCOGS; GWAS meta analysis) || id:ieu-a-1126

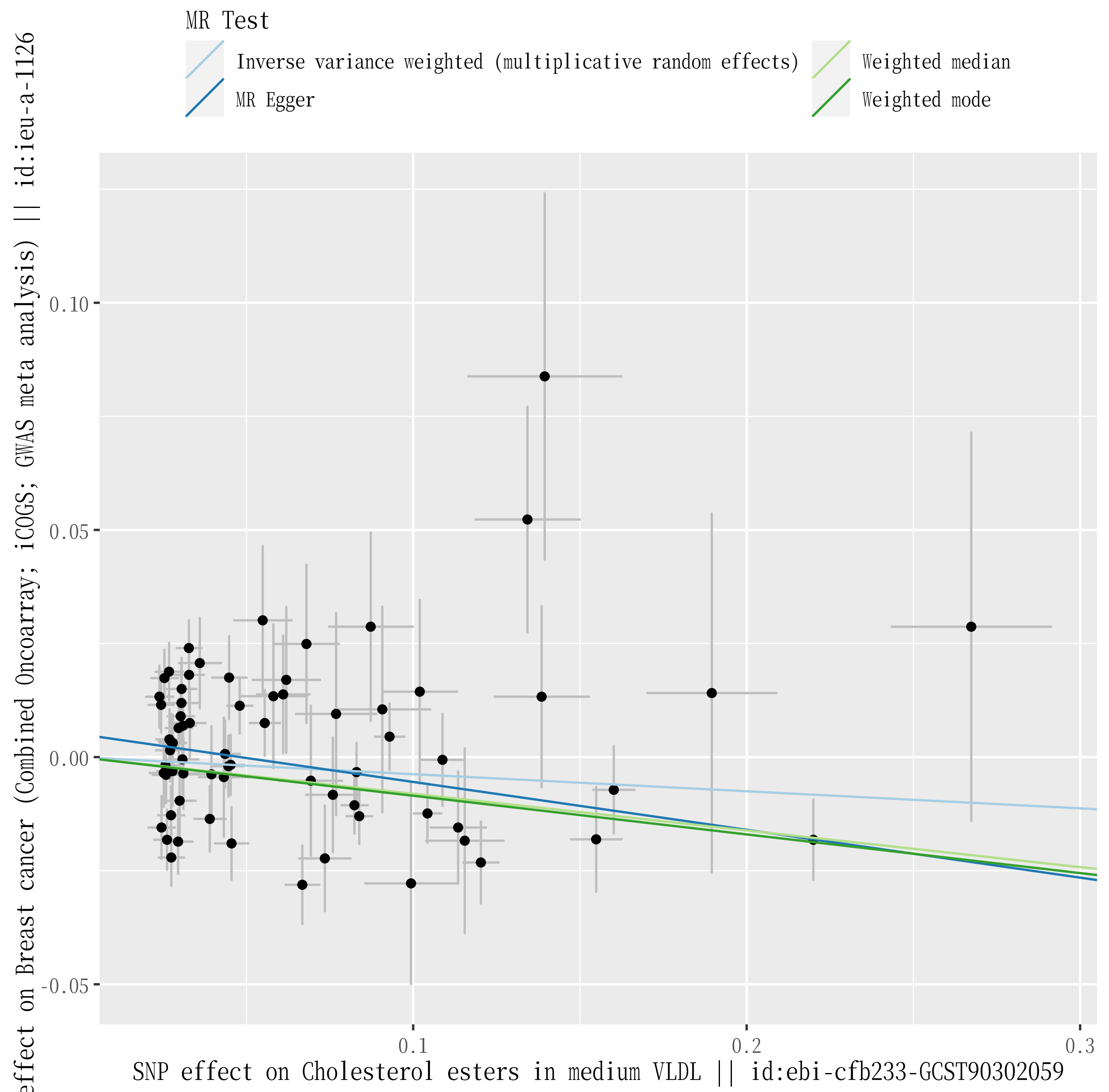
MR Test
Inverse variance weighted (multiplicative random effects)
MR Egger
Weighted median
Weighted mode
0.10
0.05
0.00
-0.05
0.1
0.2
0.3
SNP effect on Cholesterol esters in medium VLDL || id:ebi-cfb233-GCST90302059
effect on Breast cancer (Combined Oncoarray; iCOGS; GWAS meta analysis) || id:ieu-a-1126

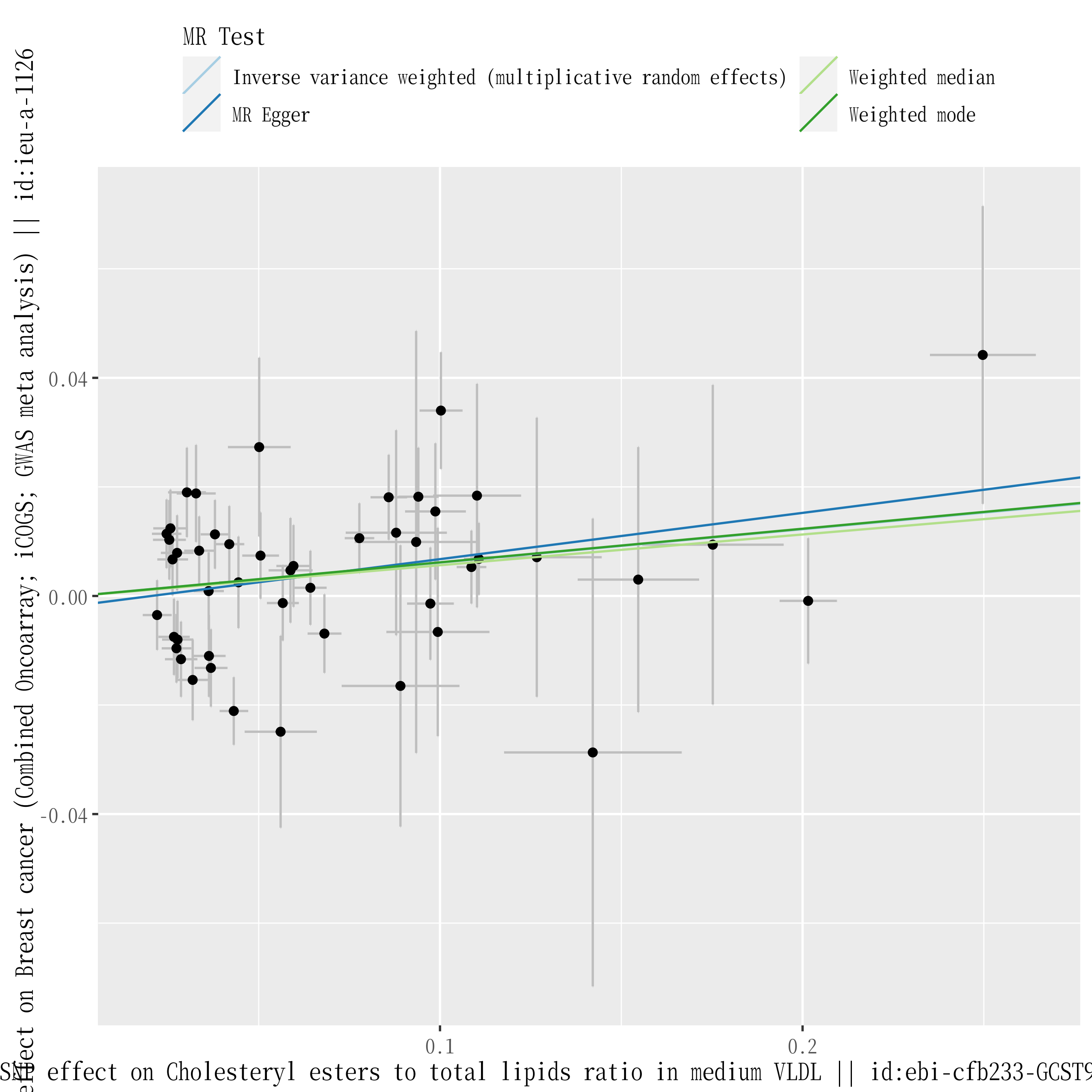

MR Test
Inverse variance weighted (multiplicative random effects)
MR Egger
Weighted median
Weighted mode
0.04
0.00
-0.04
0.1
0.2
SNP effect on Cholesteryl esters to total lipids ratio in medium VLDL || id:ebi-cfb233-GCST9
effect on Breast cancer (Combined Oncoarray; iCOGS; GWAS meta analysis) || id:ieu-a-1126

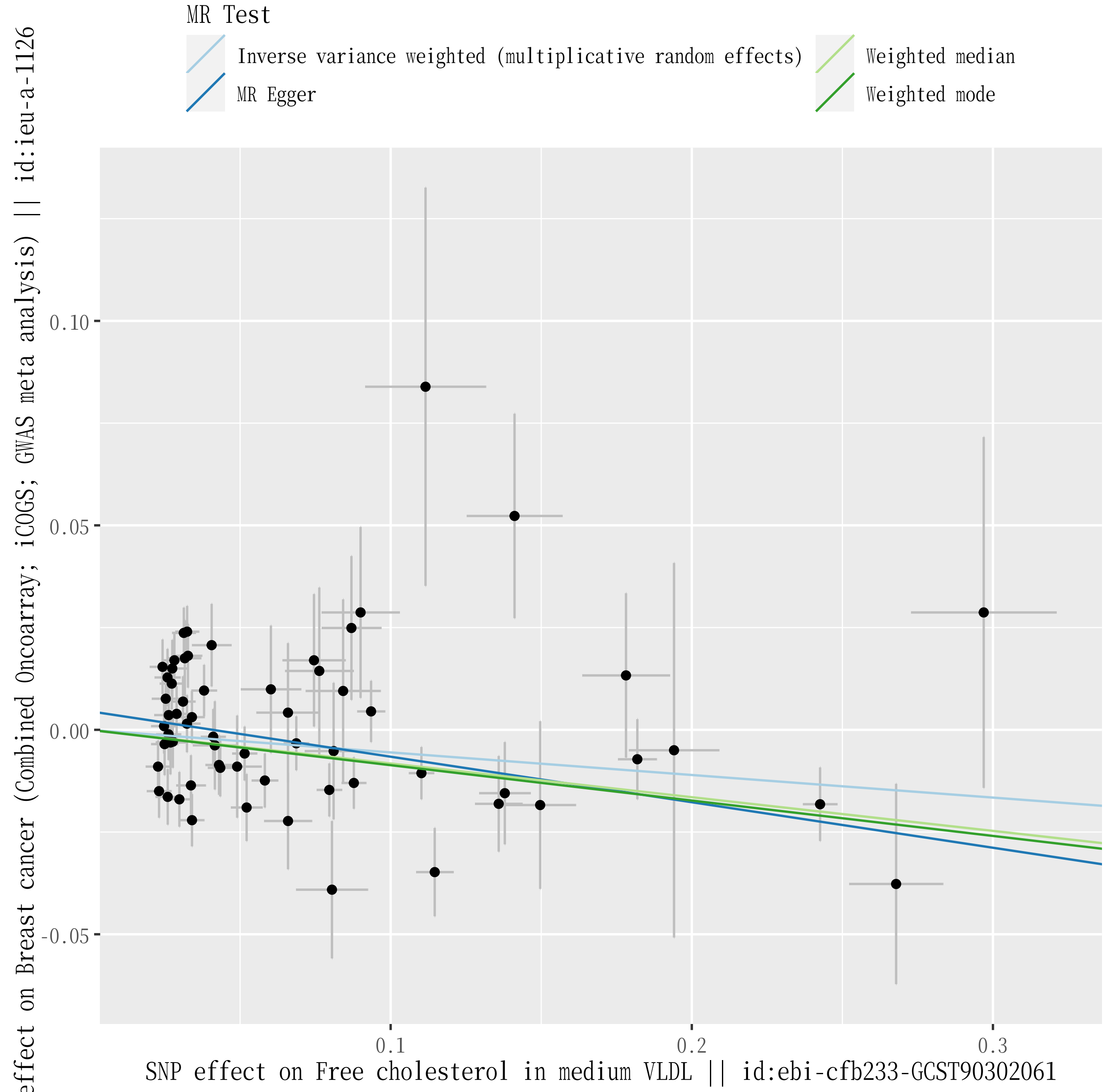
MR Test
Inverse variance weighted (multiplicative random effects)
MR Egger
Weighted median
Weighted mode
0.10
0.05
0.00
-0.05
0.1
0.2
0.3
SNP effect on Free cholesterol in medium VLDL || id:ebi-cfb233-GCST90302061
effect on Breast cancer (Combined Oncoarray; iCOGS; GWAS meta analysis) || id:ieu-a-1126

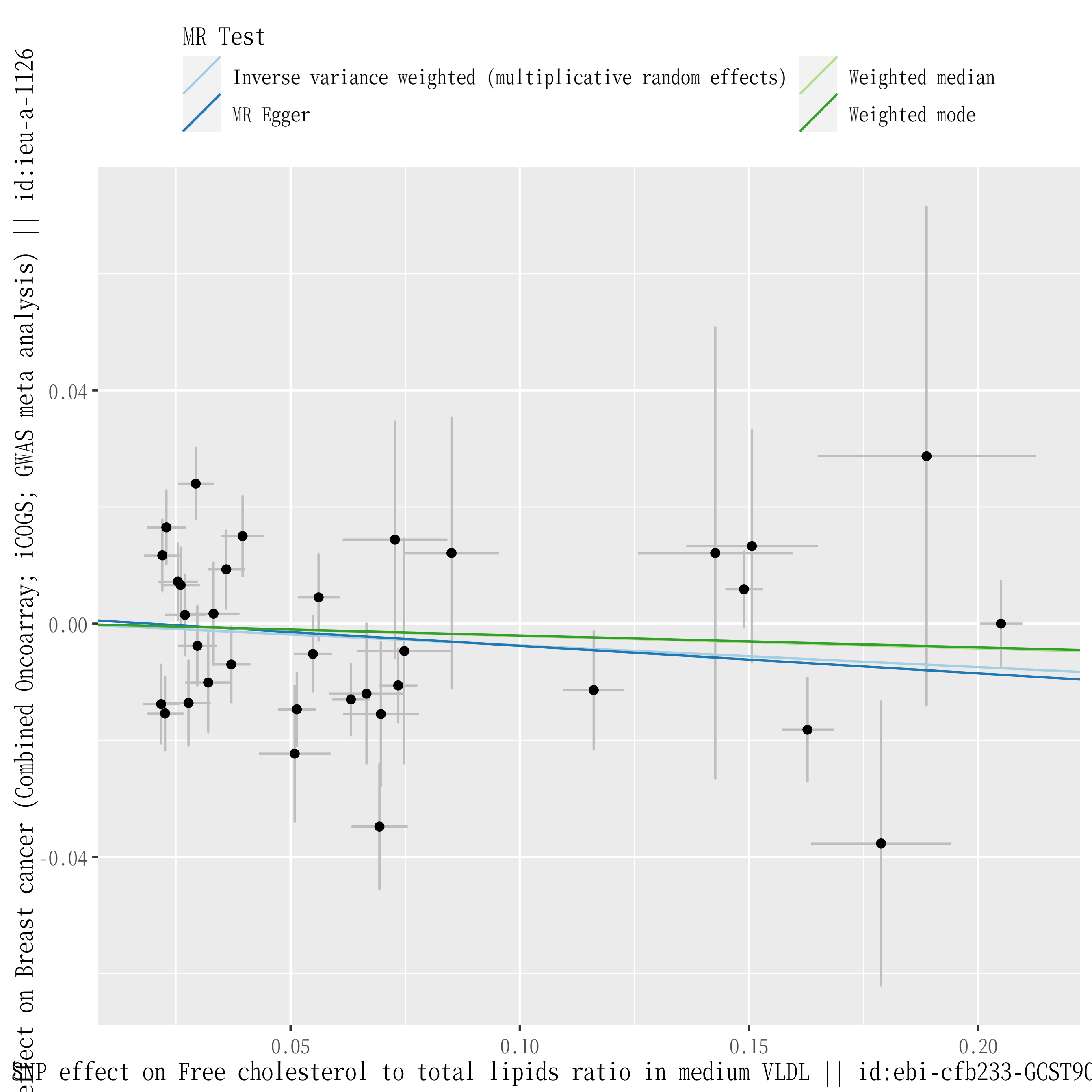

MR Test
Inverse variance weighted (multiplicative random effects)
MR Egger
Weighted median
Weighted mode
0.04
0.00
-0.04
0.05
0.10
0.15
0.20
SNP effect on Free cholesterol to total lipids ratio in medium VLDL || id:ebi-cfb233-GCST90
effect on Breast cancer (Combined Oncoarray; iCOGS; GWAS meta analysis) || id:ieu-a-1126

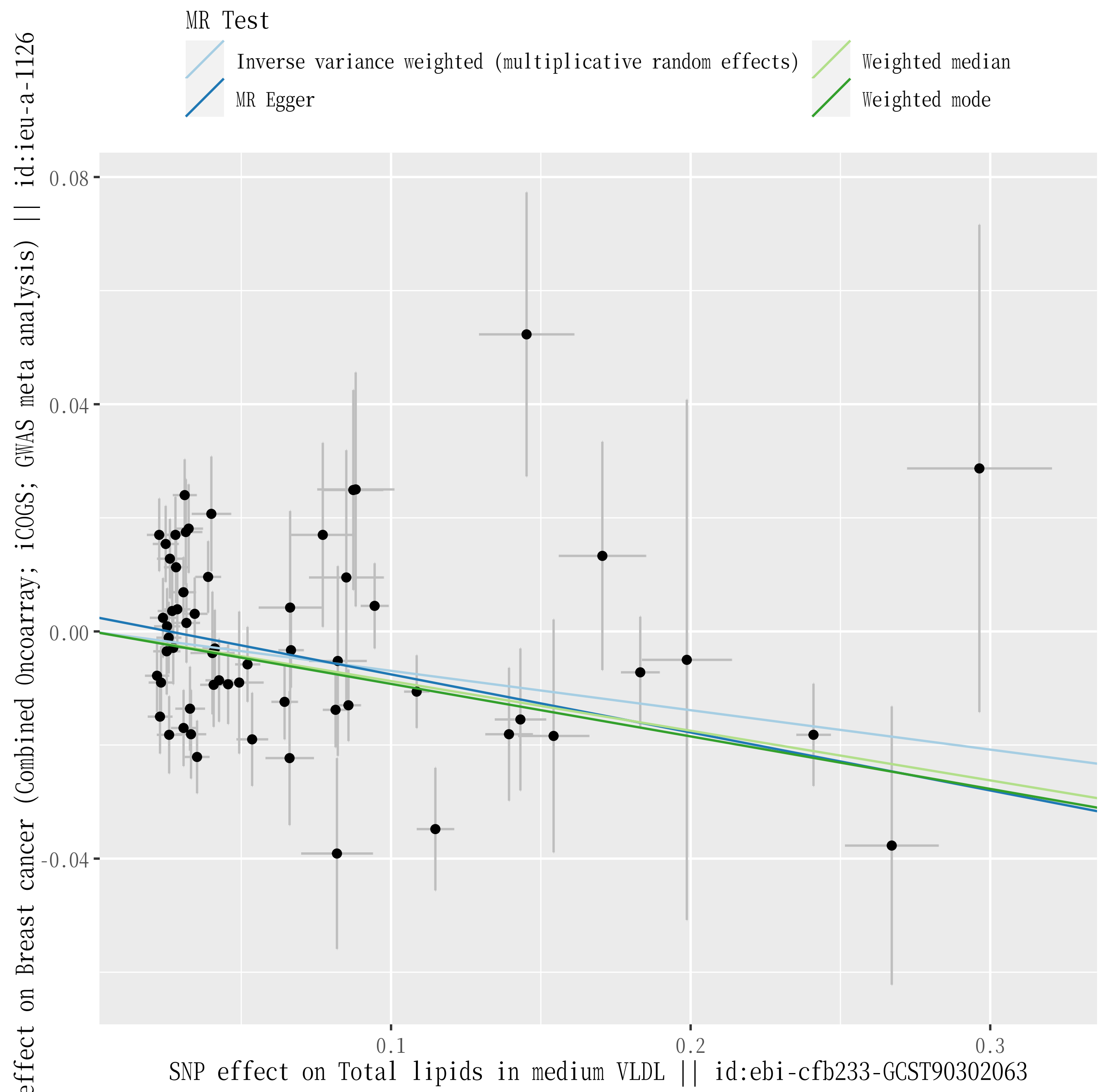

MR Test
Inverse variance weighted (multiplicative random effects)
MR Egger
Weighted median
Weighted mode
0.08
0.04
0.00
-0.04
0.1
0.2
0.3
SNP effect on Total lipids in medium VLDL || id:ebi-cfb233-GCST90302063
effect on Breast cancer (Combined Oncoarray; iCOGS; GWAS meta analysis) || id:ieu-a-1126

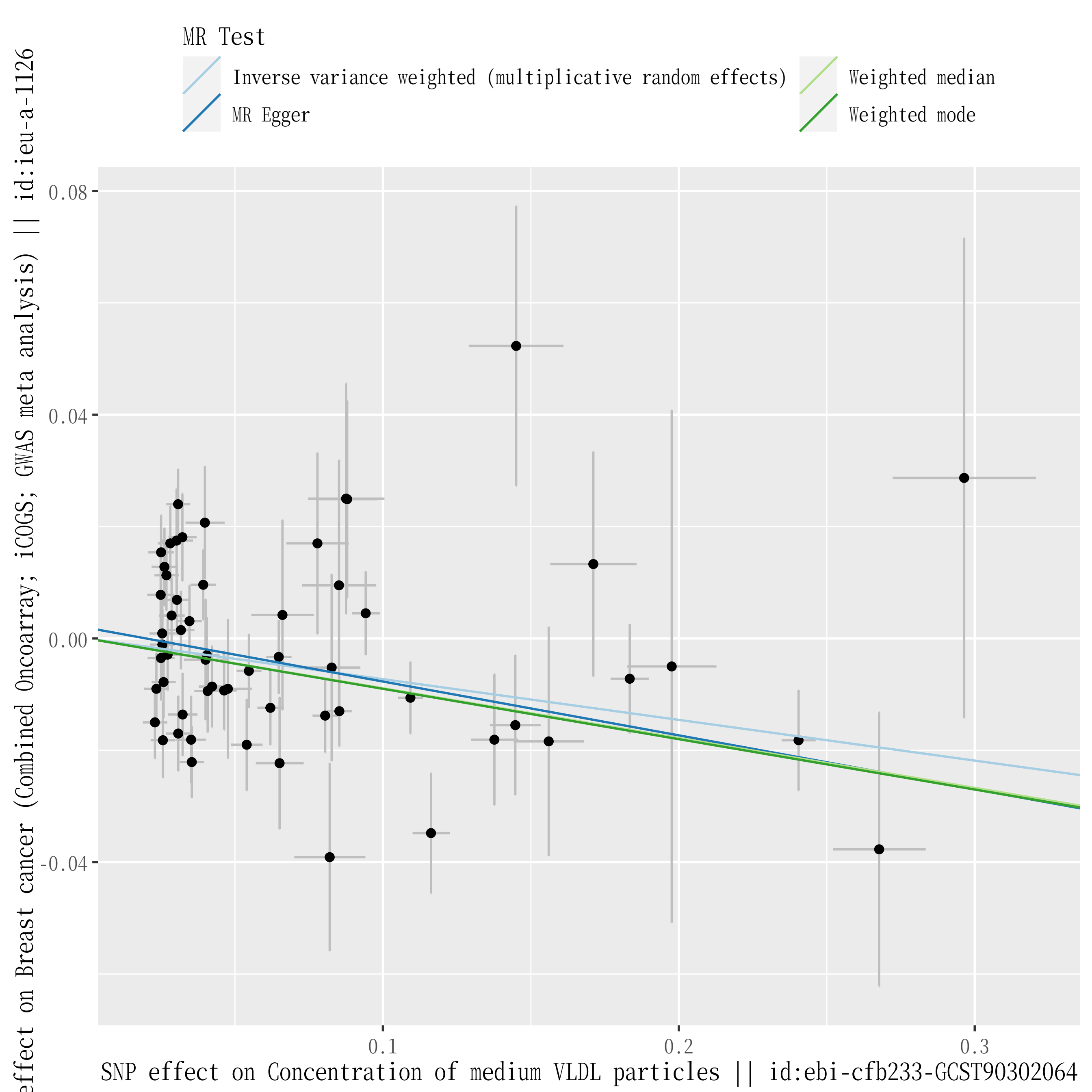

MR Test
Inverse variance weighted (multiplicative random effects)
MR Egger
Weighted median
Weighted mode
0.08
0.04
0.00
-0.04
0.1
0.2
0.3
SNP effect on Concentration of medium VLDL particles || id:ebi-cfb233-GCST90302064
effect on Breast cancer (Combined Oncoarray; iCOGS; GWAS meta analysis) || id:ieu-a-1126

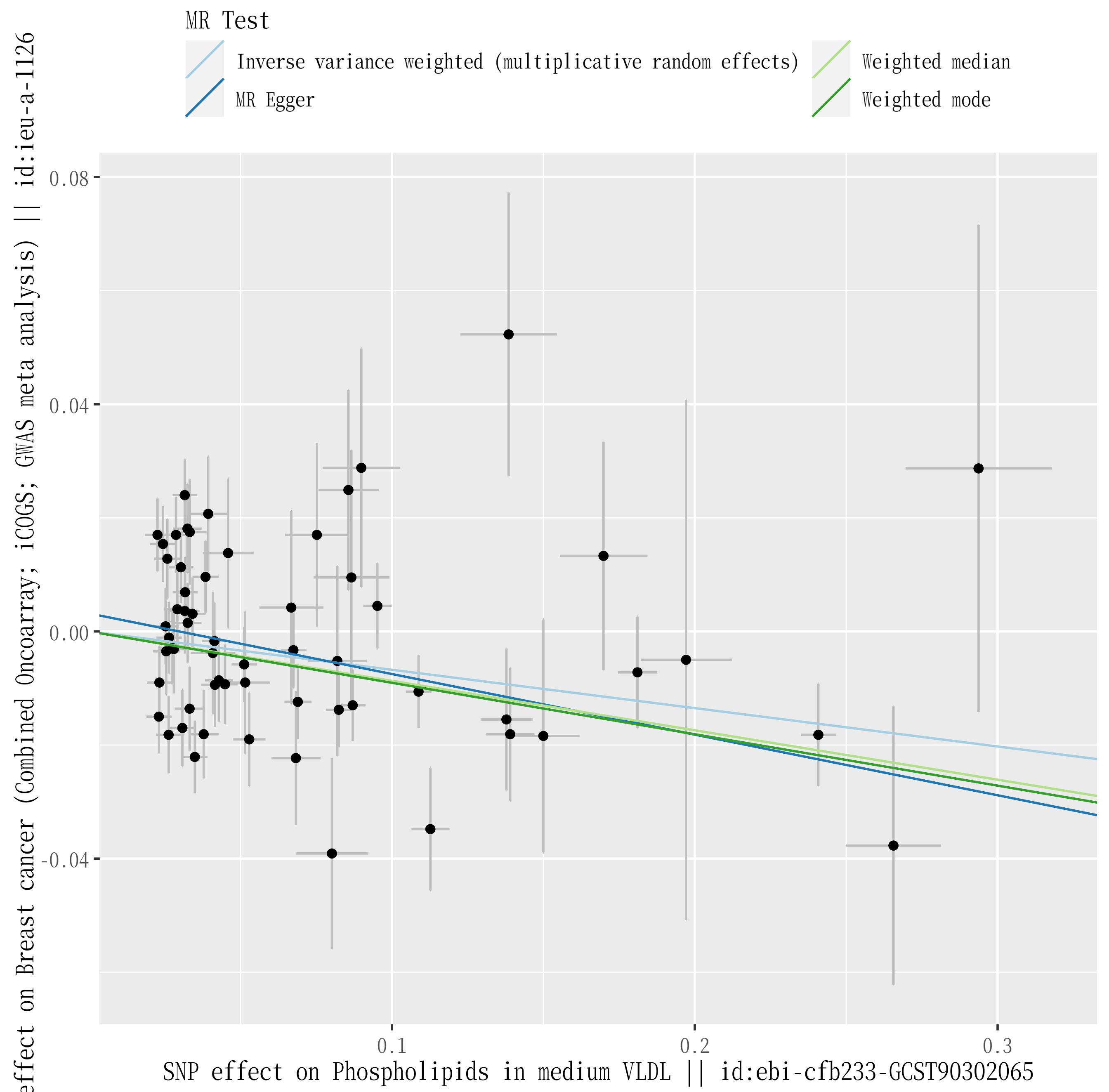

MR Test
Inverse variance weighted (multiplicative random effects)
MR Egger
Weighted median
Weighted mode
0.08
0.04
0.00
-0.04
0.1
0.2
0.3
SNP effect on Phospholipids in medium VLDL || id:ebi-cfb233-GCST90302065
effect on Breast cancer (Combined Oncoarray; iCOGS; GWAS meta analysis) || id:ieu-a-1126

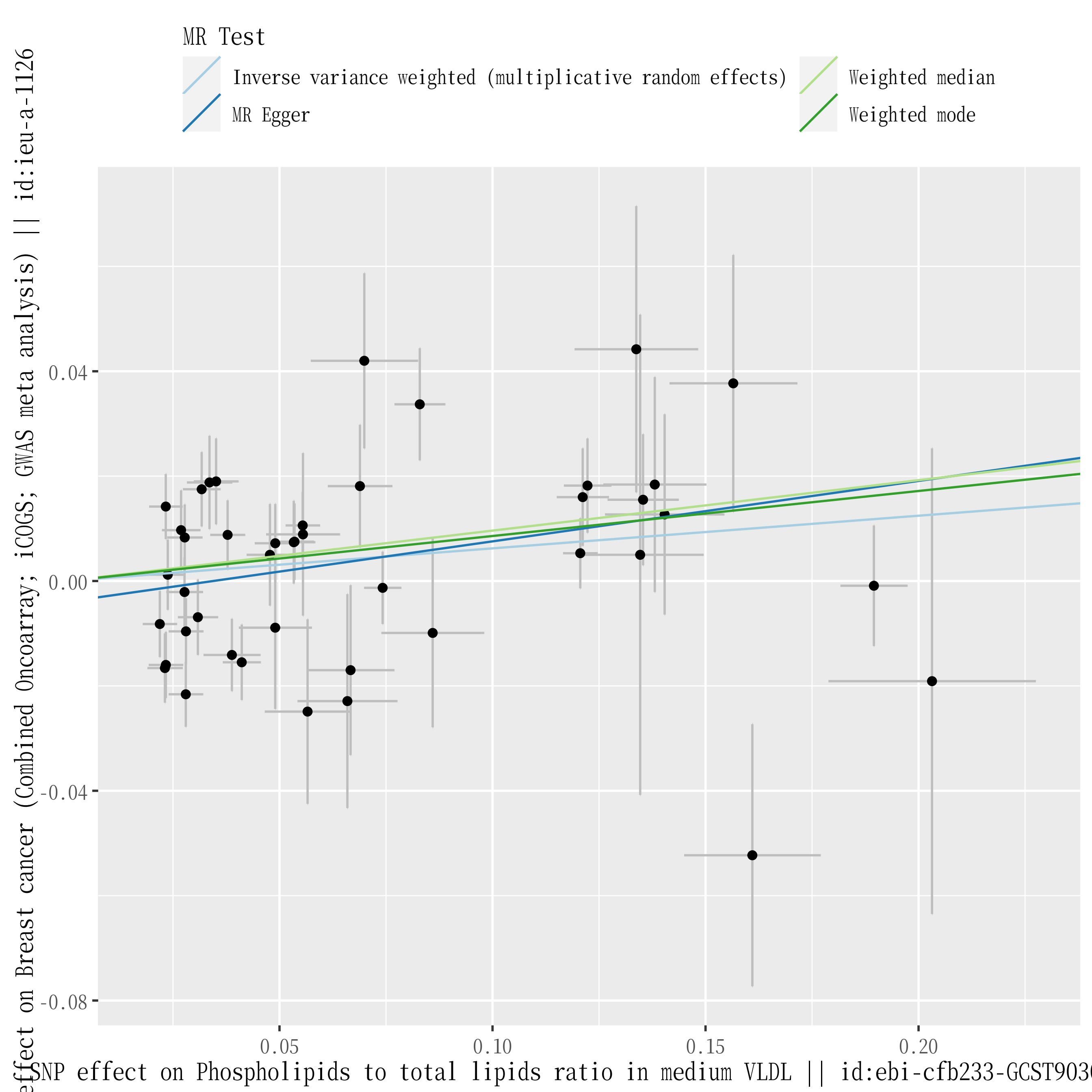
MR Test
Inverse variance weighted (multiplicative random effects)
MR Egger
Weighted median
Weighted mode
0.04
0.00
-0.04
-0.08
0.05
0.10
0.15
0.20
SNP effect on Phospholipids to total lipids ratio in medium VLDL || id:ebi-cfb233-GCST903
effect on Breast cancer (Combined Oncoarray; iCOGS; GWAS meta analysis) || id:ieu-a-1126

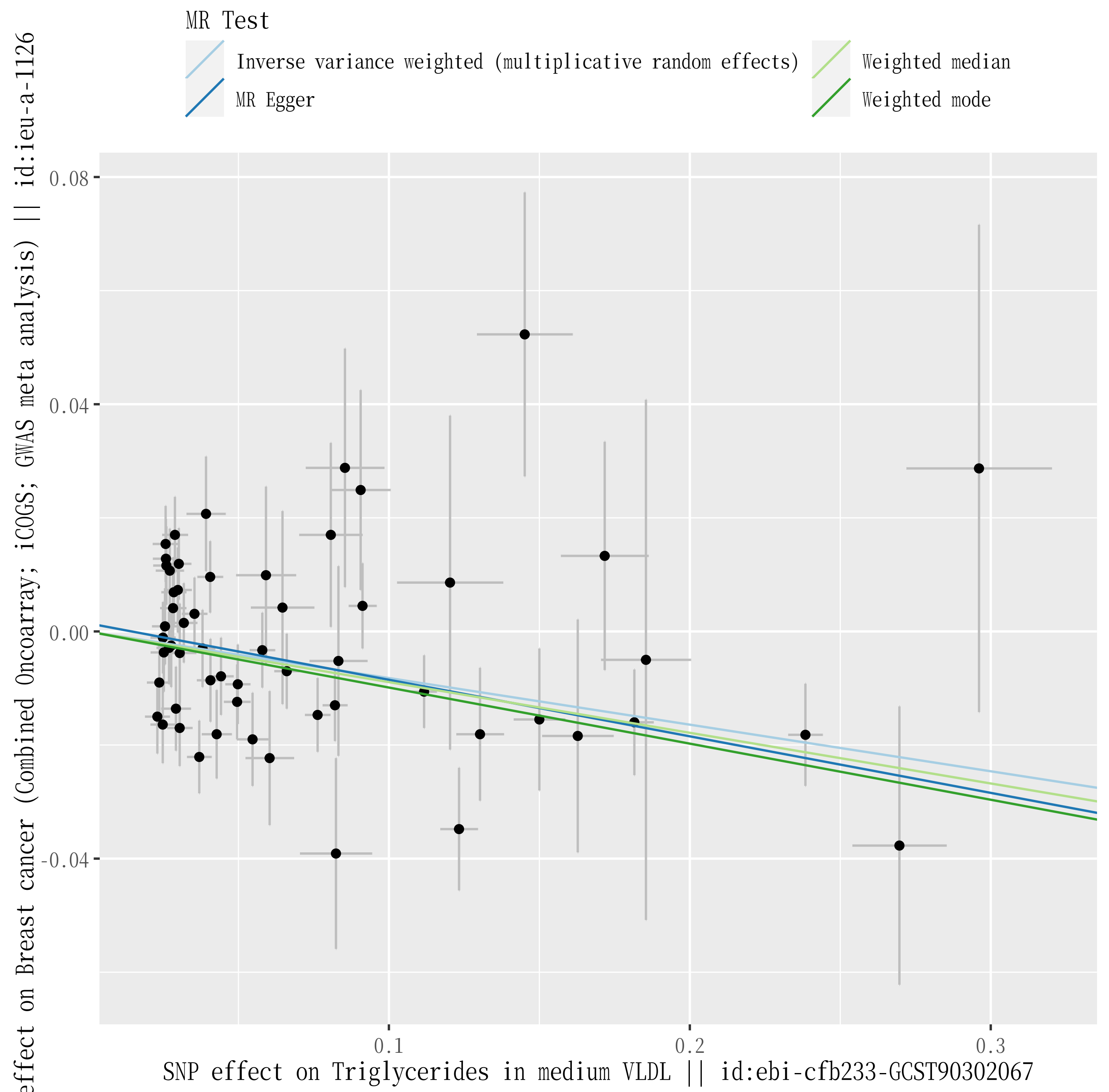

MR Test
Inverse variance weighted (multiplicative random effects)
MR Egger
Weighted median
Weighted mode
0.08
0.04
0.00
-0.04
0.1
0.2
0.3
SNP effect on Triglycerides in medium VLDL || id:ebi-cfb233-GCST90302067
effect on Breast cancer (Combined Oncoarray; iCOGS; GWAS meta analysis) || id:ieu-a-1126

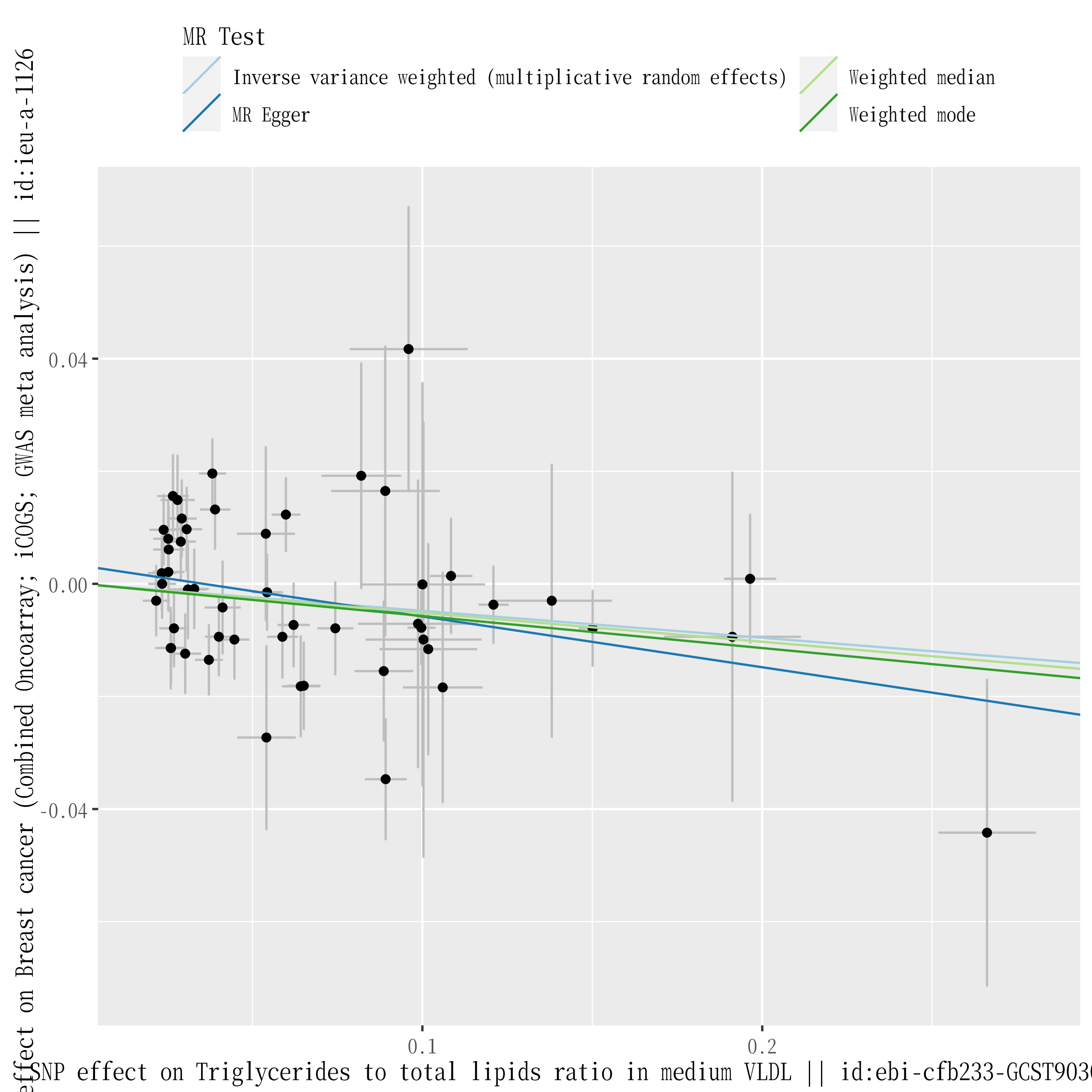
MR Test
Inverse variance weighted (multiplicative random effects)
MR Egger
Weighted median
Weighted mode
0.04
0.00
-0.04
0.1
0.2
SNP effect on Triglycerides to total lipids ratio in medium VLDL || id:ebi-cfb233-GCST903
effect on Breast cancer (Combined Oncoarray; iCOGS; GWAS meta analysis) || id:ieu-a-1126

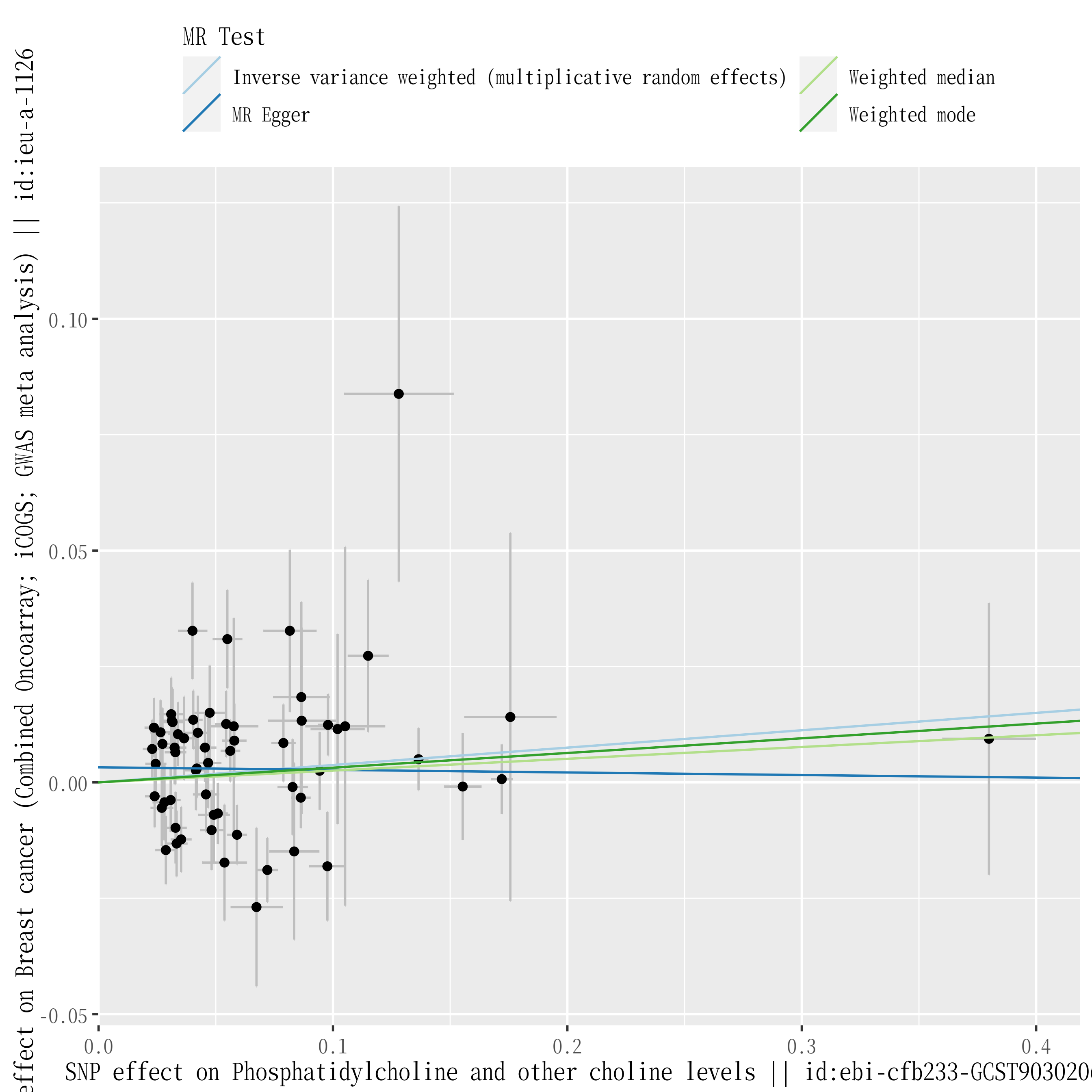
MR Test
Inverse variance weighted (multiplicative random effects)
MR Egger
Weighted median
Weighted mode
SNP effect on Breast cancer (Combined Oncoarray; iCOGS; GWAS meta analysis) || id:ieu-a-1126
0.10
0.05
0.00
-0.05
0.0
0.1
0.2
0.3
0.4
SNP effect on Phosphatidylcholine and other choline levels || id:ebi-cfb233-GCST90302

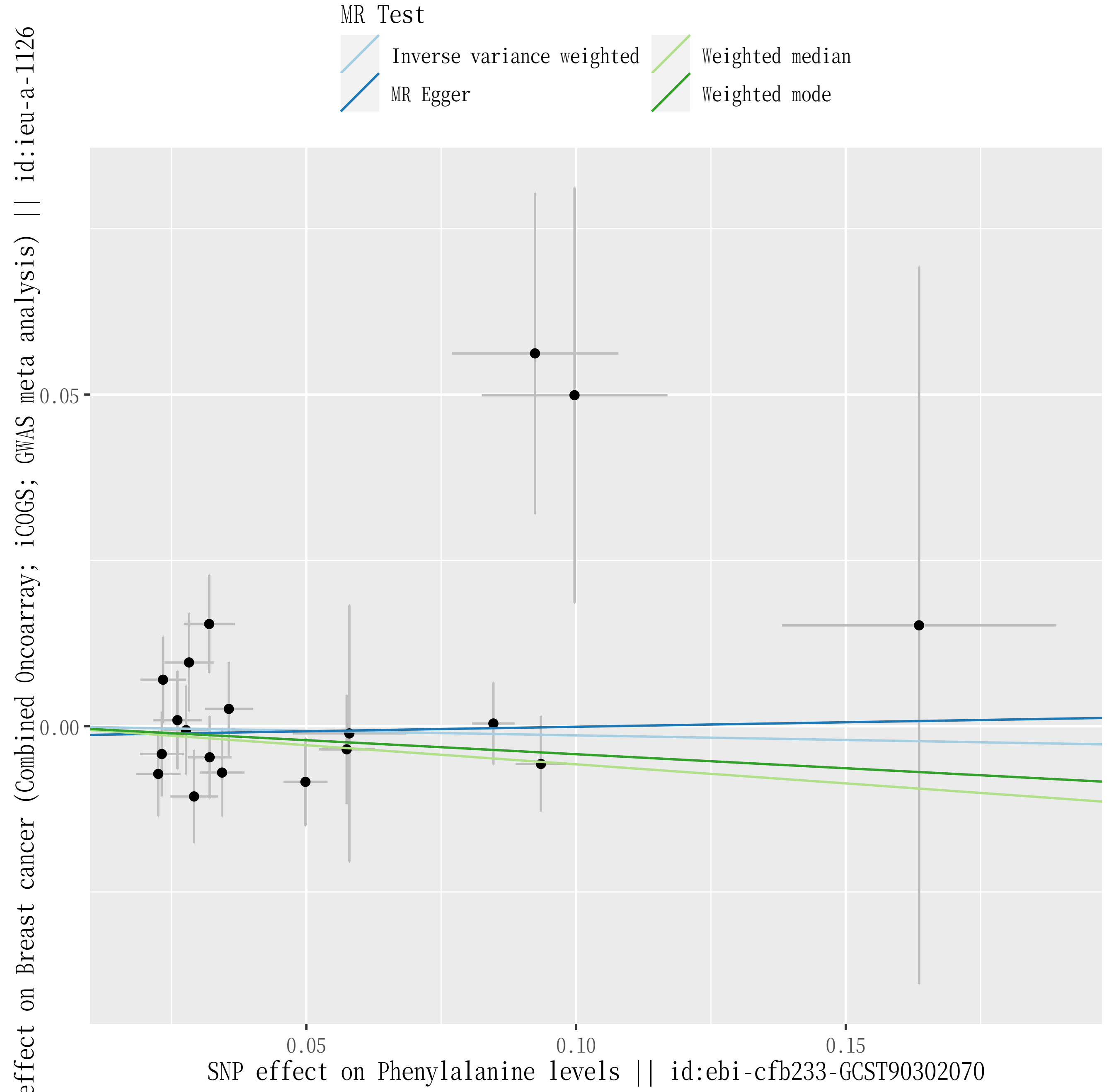
MR Test
Inverse variance weighted
MR Egger
Weighted median
Weighted mode
0.05
0.00
0.05
0.10
0.15
SNP effect on Phenylalanine levels || id:ebi-cfb233-GCST90302070
effect on Breast cancer (Combined Oncoarray; iCOGS; GWAS meta analysis) || id:ieu-a-1126

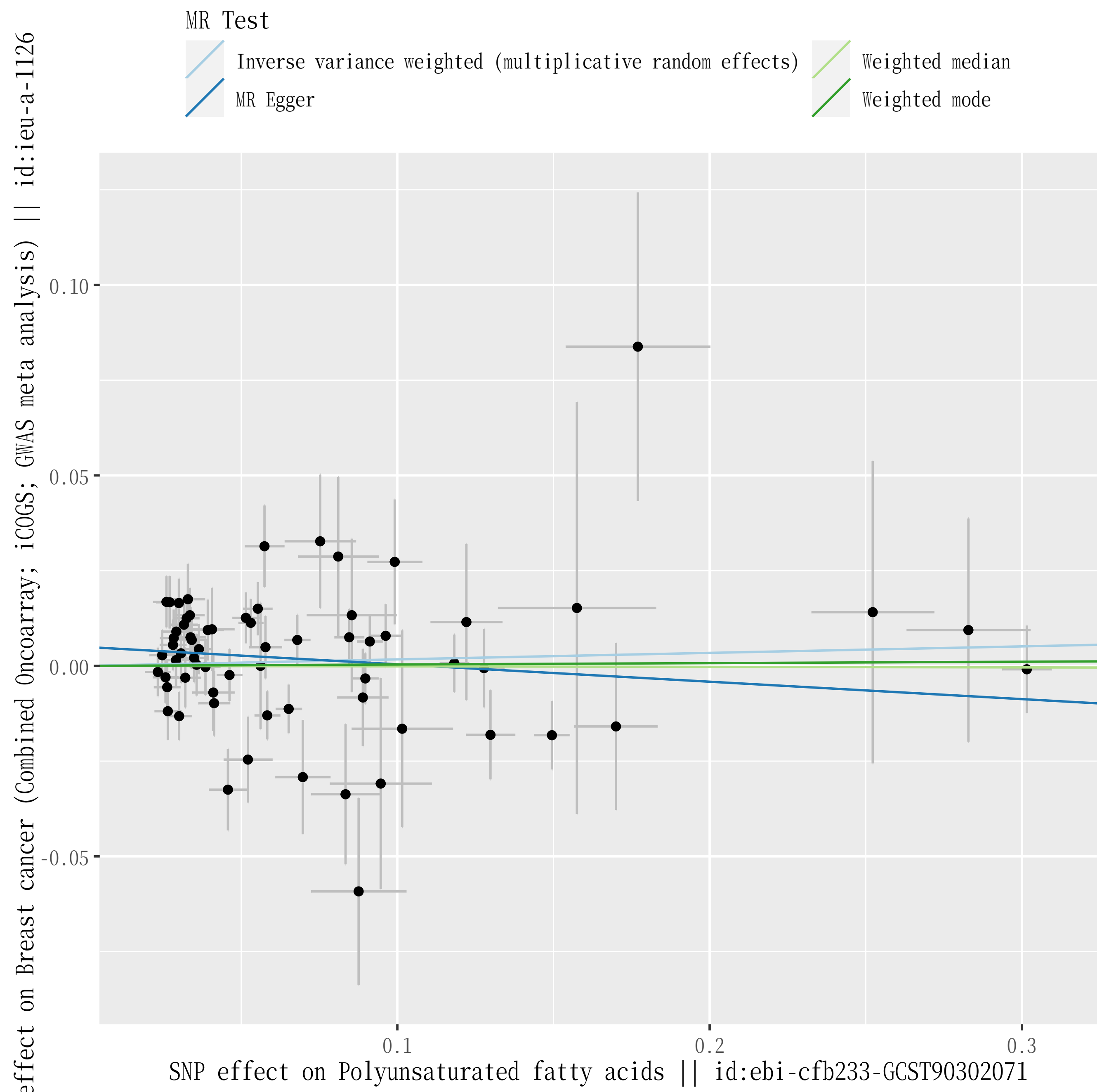
MR Test
Inverse variance weighted (multiplicative random effects)
MR Egger
Weighted median
Weighted mode
0.10
0.05
0.00
-0.05
0.1
0.2
0.3
SNP effect on Polyunsaturated fatty acids || id:ebi-cfb233-GCST90302071
effect on Breast cancer (Combined Oncoarray; iCOGS; GWAS meta analysis) || id:ieu-a-1126

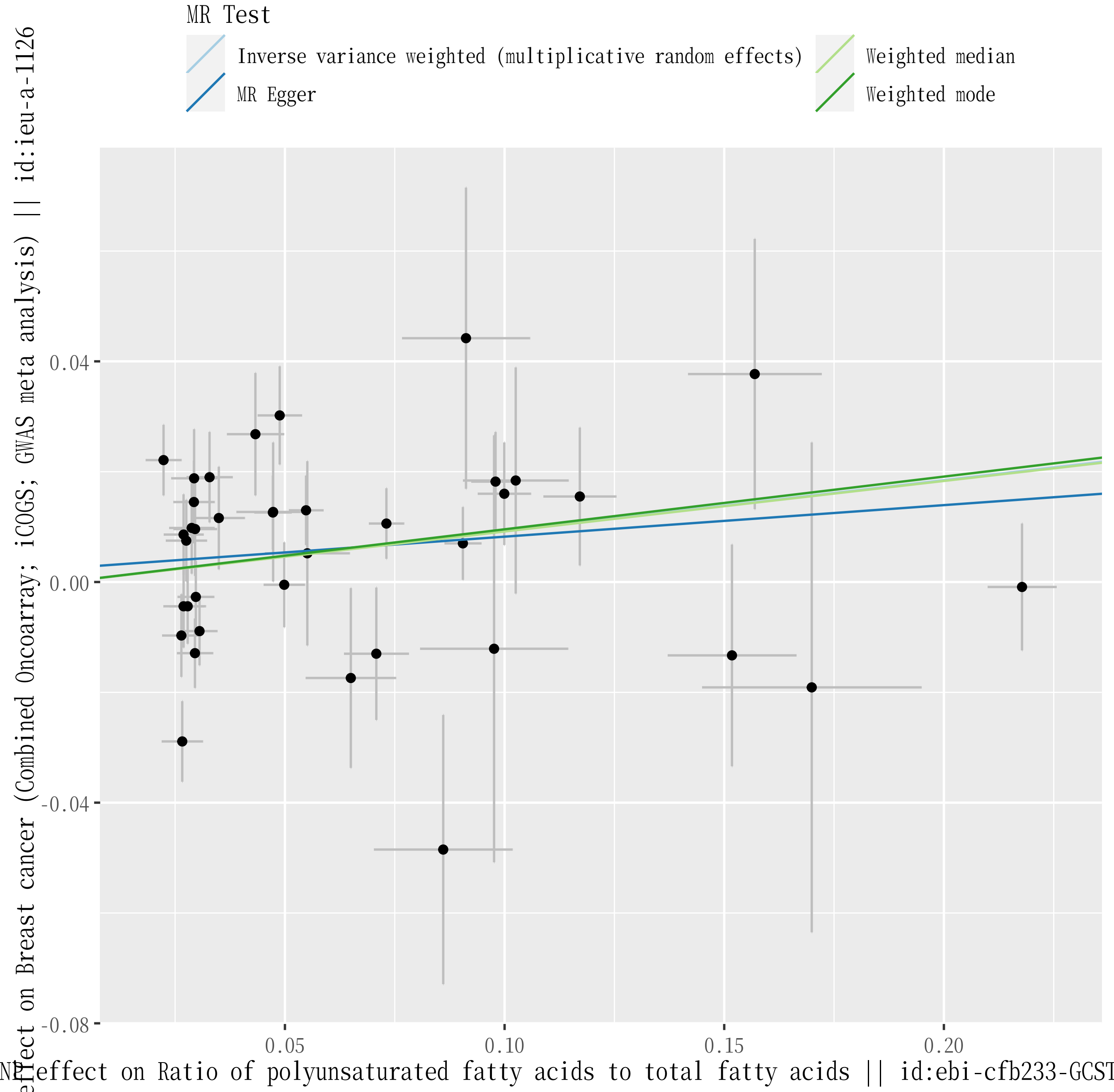

MR Test
Inverse variance weighted (multiplicative random effects)
MR Egger
Weighted median
Weighted mode
0.04
0.00
-0.04
-0.08
0.05
0.10
0.15
0.20
SNP effect on Ratio of polyunsaturated fatty acids to total fatty acids || id:ebi-cfb233-GCST
SNP effect on Breast cancer (Combined Oncoarray; iCOGS; GWAS meta analysis) || id:ieu-a-1126

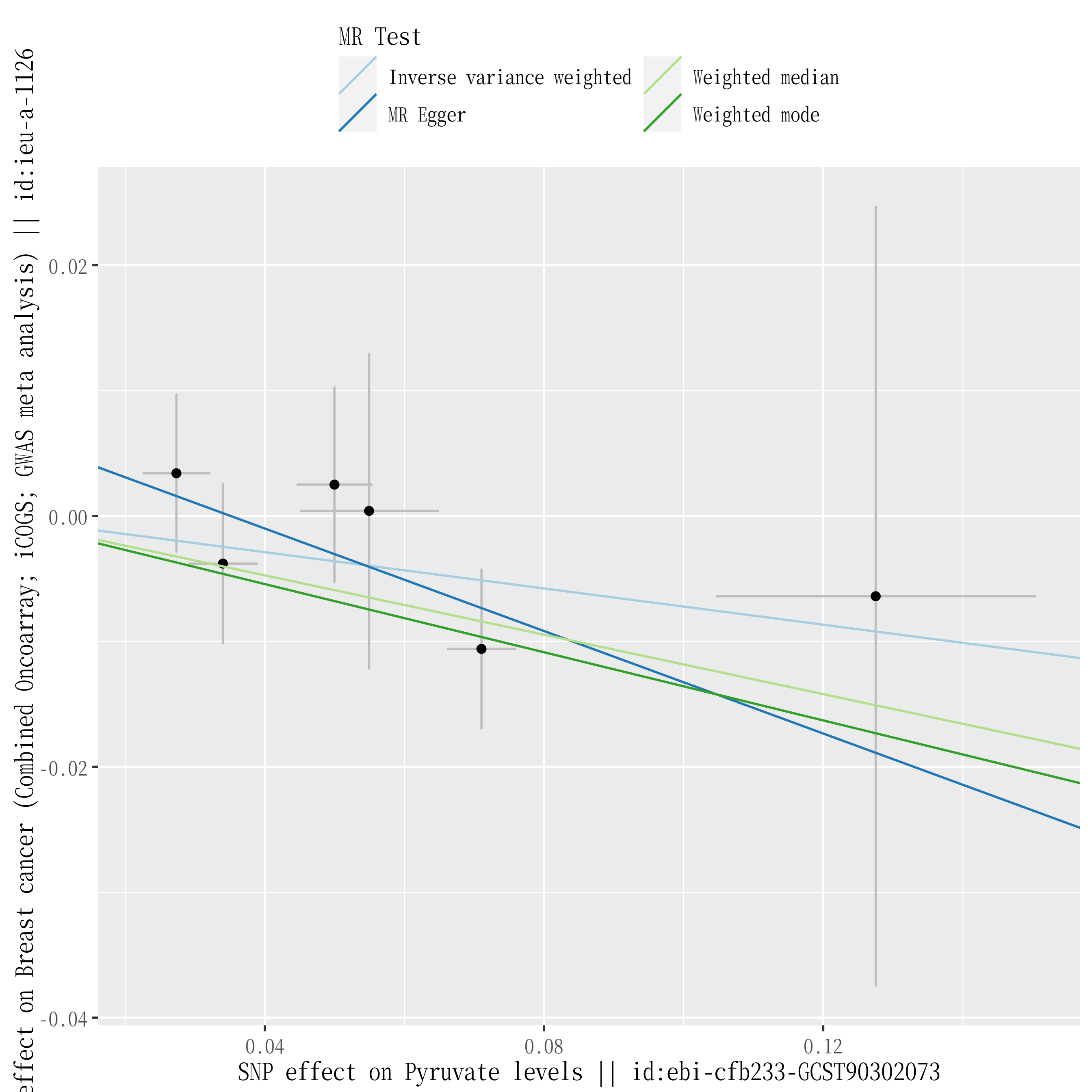

MR Test
Inverse variance weighted
MR Egger
Weighted median
Weighted mode
0.02
0.00
-0.02
-0.04
0.04
0.08
0.12
SNP effect on Pyruvate levels || id:ebi-cfb233-GCST90302073
effect on Breast cancer (Combined Oncoarray; iCOGS; GWAS meta analysis) || id:ieu-a-1126

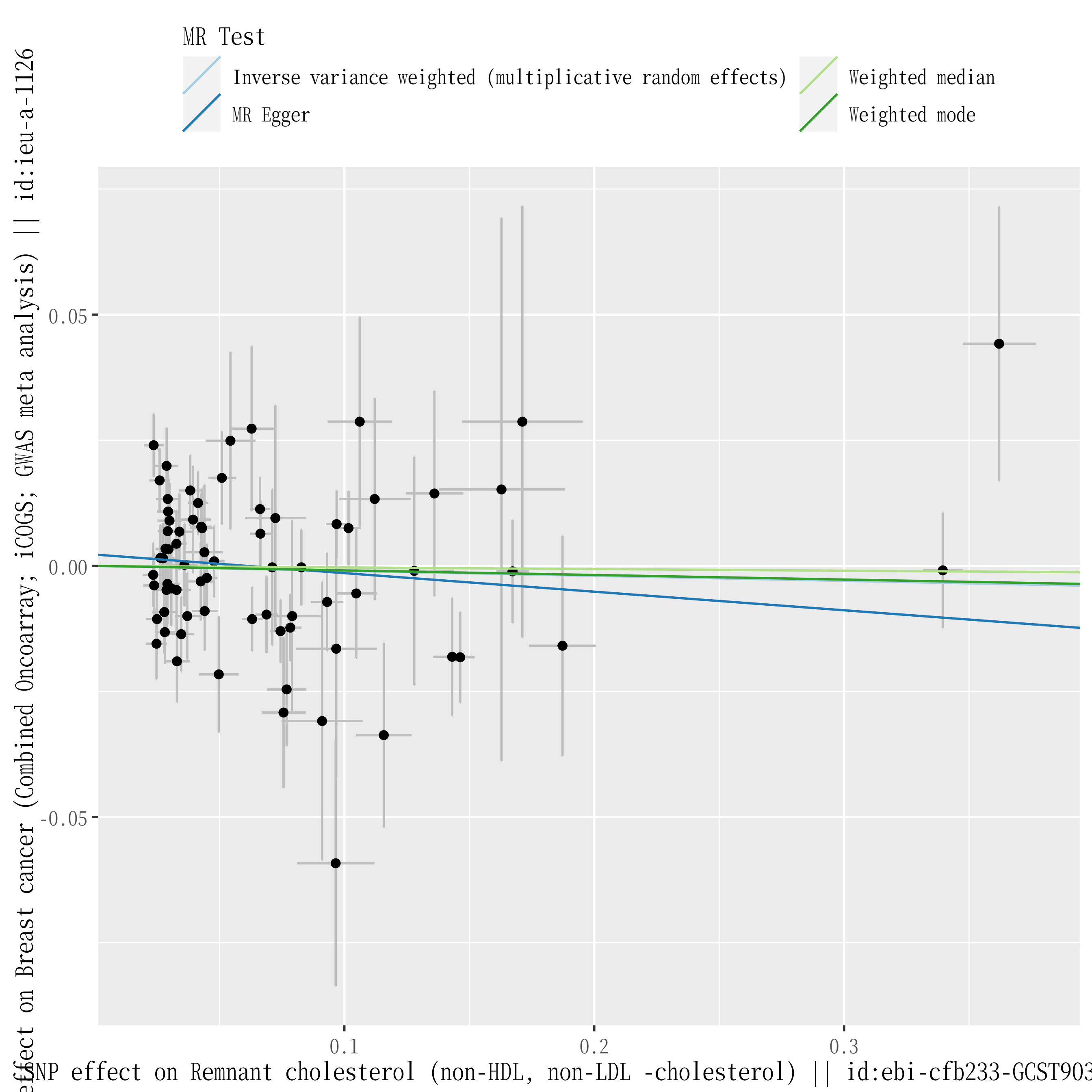

MR Test
Inverse variance weighted (multiplicative random effects)
MR Egger
Weighted median
Weighted mode
0.05
0.00
-0.05
0.1
0.2
0.3
SNP effect on Breast cancer (Combined Oncoarray; iCOGS; GWAS meta analysis) || id:ieu-a-1126
SNP effect on Remnant cholesterol (non-HDL, non-LDL -cholesterol) || id:ebi-cfb233-GCST903

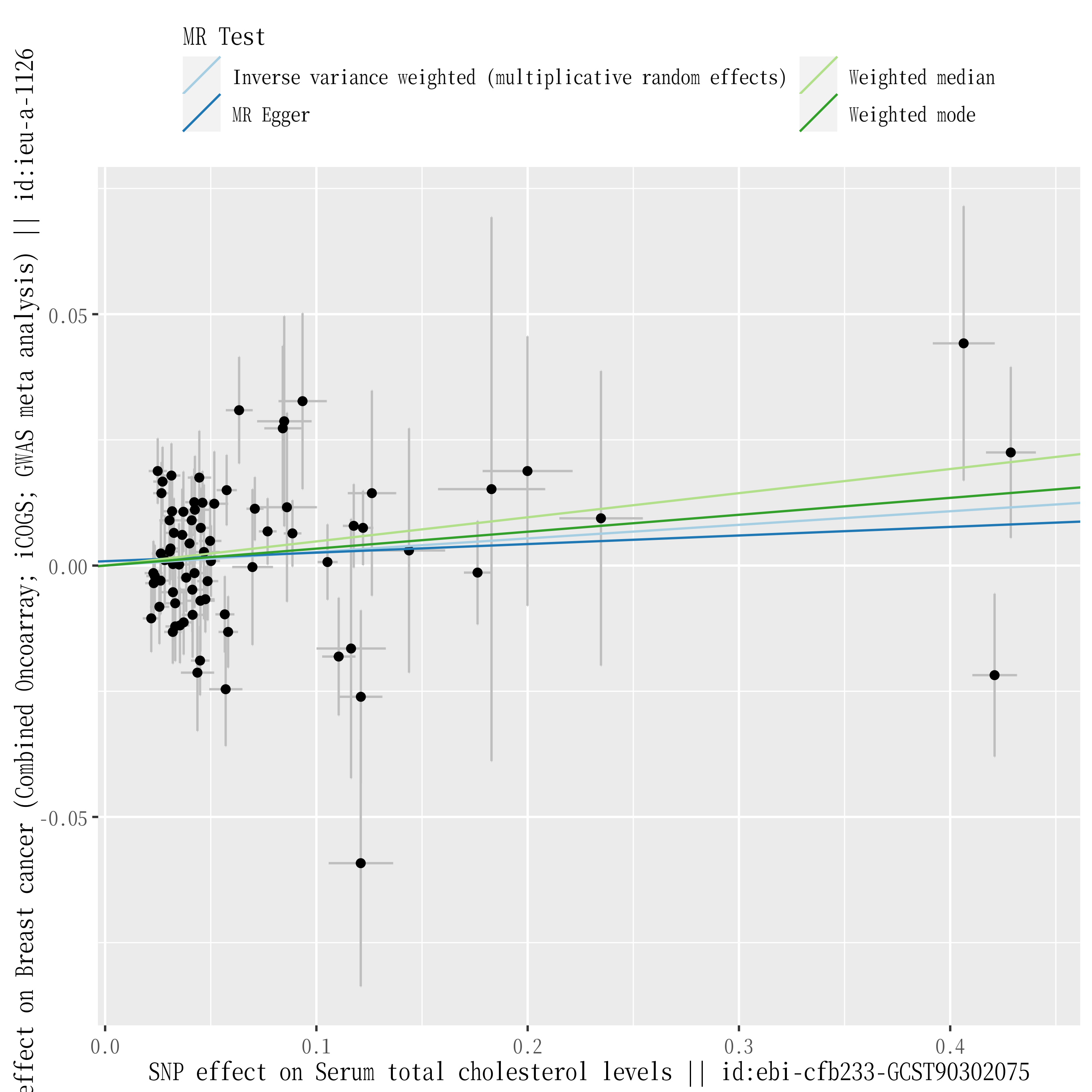

MR Test
Inverse variance weighted (multiplicative random effects)
MR Egger
Weighted median
Weighted mode
0.05
0.00
-0.05
0.0
0.1
0.2
0.3
0.4
SNP effect on Serum total cholesterol levels || id:ebi-cfb233-GCST90302075
effect on Breast cancer (Combined Oncoarray; iCOGS; GWAS meta analysis) || id:ieu-a-1126

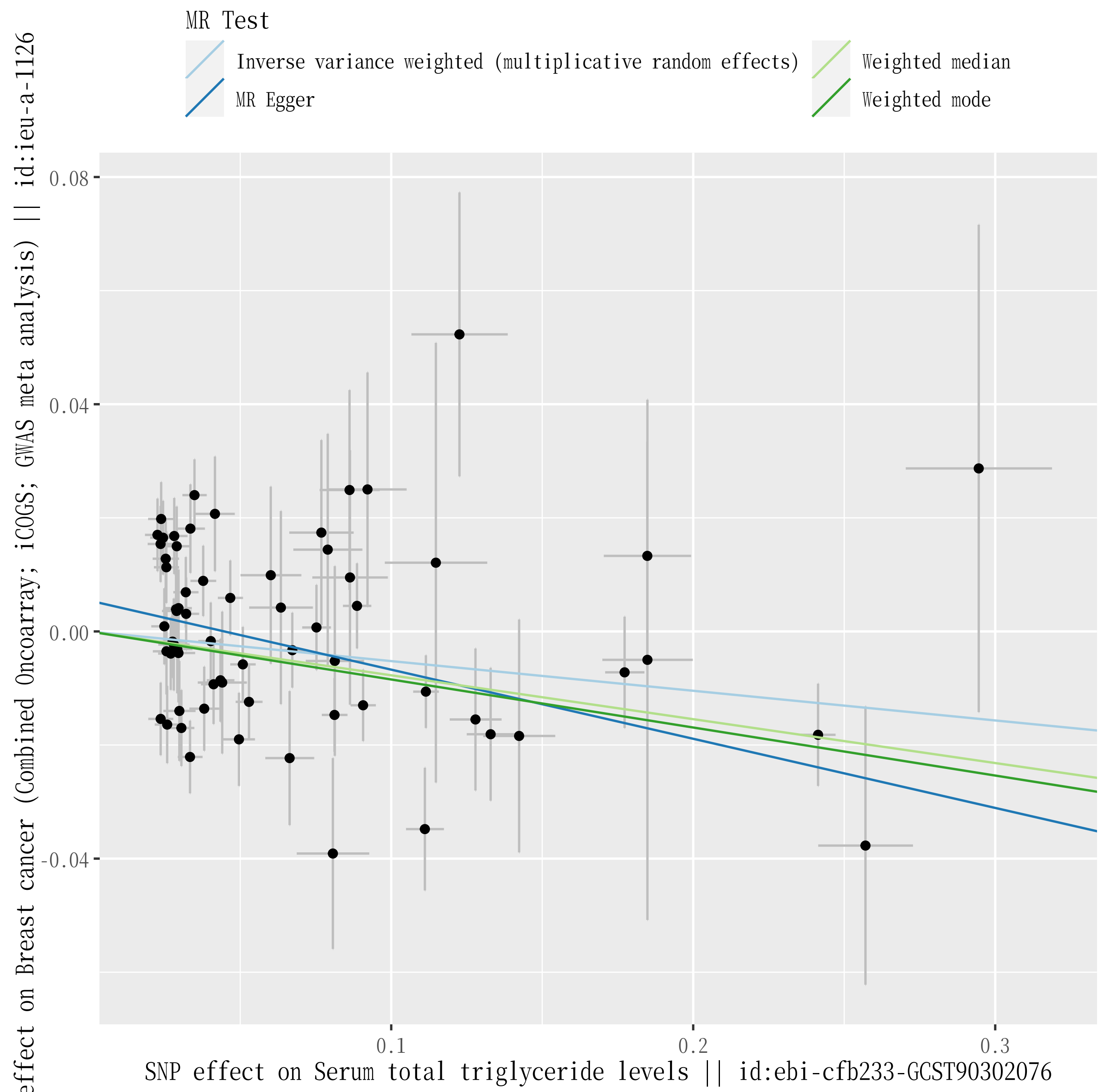

MR Test
Inverse variance weighted (multiplicative random effects)
MR Egger
Weighted median
Weighted mode
0.08
0.04
0.00
-0.04
0.1
0.2
0.3
SNP effect on Serum total triglyceride levels || id:ebi-cfb233-GCST90302076
effect on Breast cancer (Combined Oncoarray; iCOGS; GWAS meta analysis) || id:ieu-a-1126

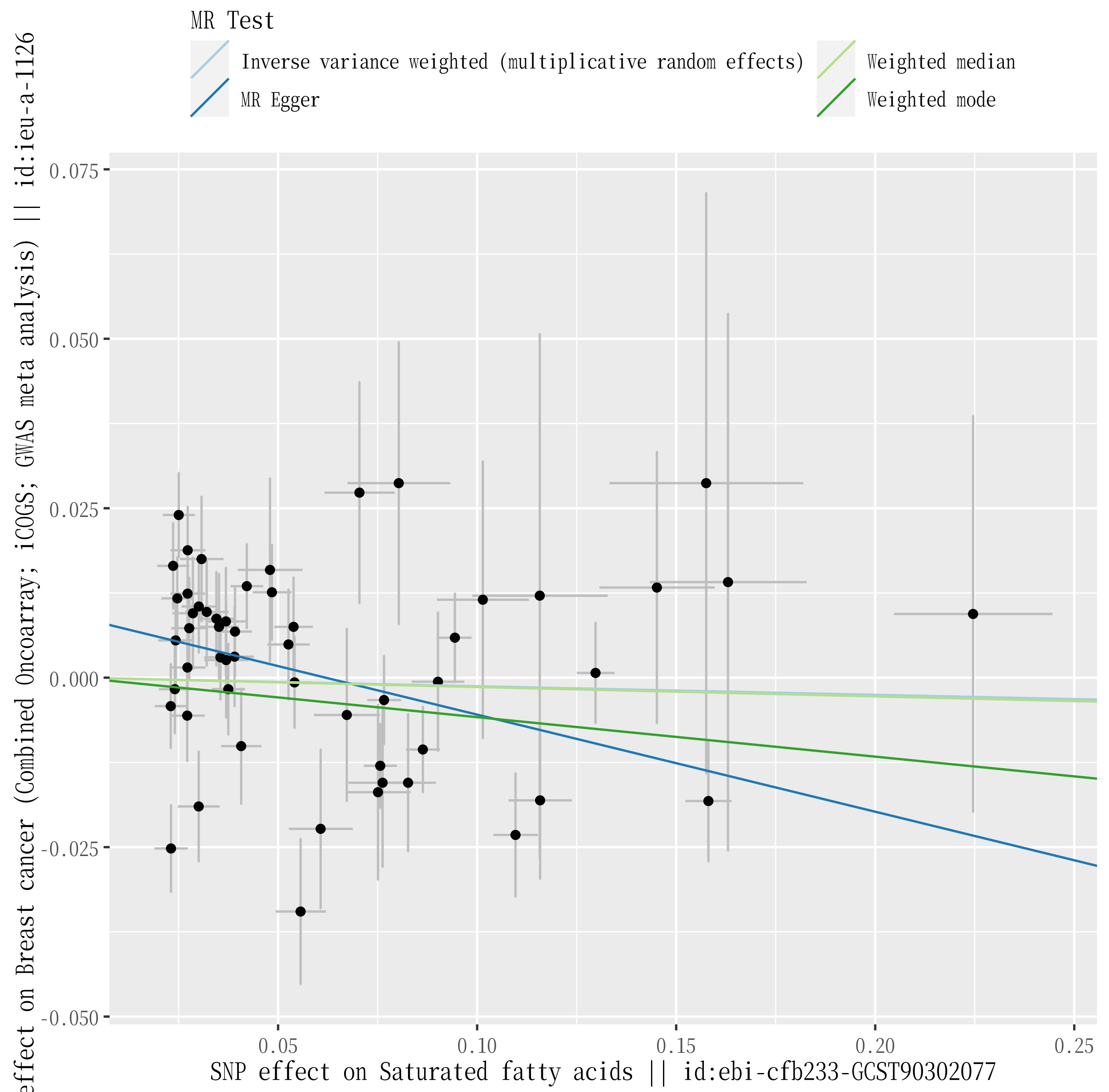
MR Test
Inverse variance weighted (multiplicative random effects)
MR Egger
Weighted median
Weighted mode
0.075
0.050
0.025
0.000
-0.025
-0.050
0.05
0.10
0.15
0.20
0.25
SNP effect on Saturated fatty acids || id:ebi-cfb233-GCST90302077
effect on Breast cancer (Combined Oncoarray; iCOGS; GWAS meta analysis) || id:ieu-a-1126

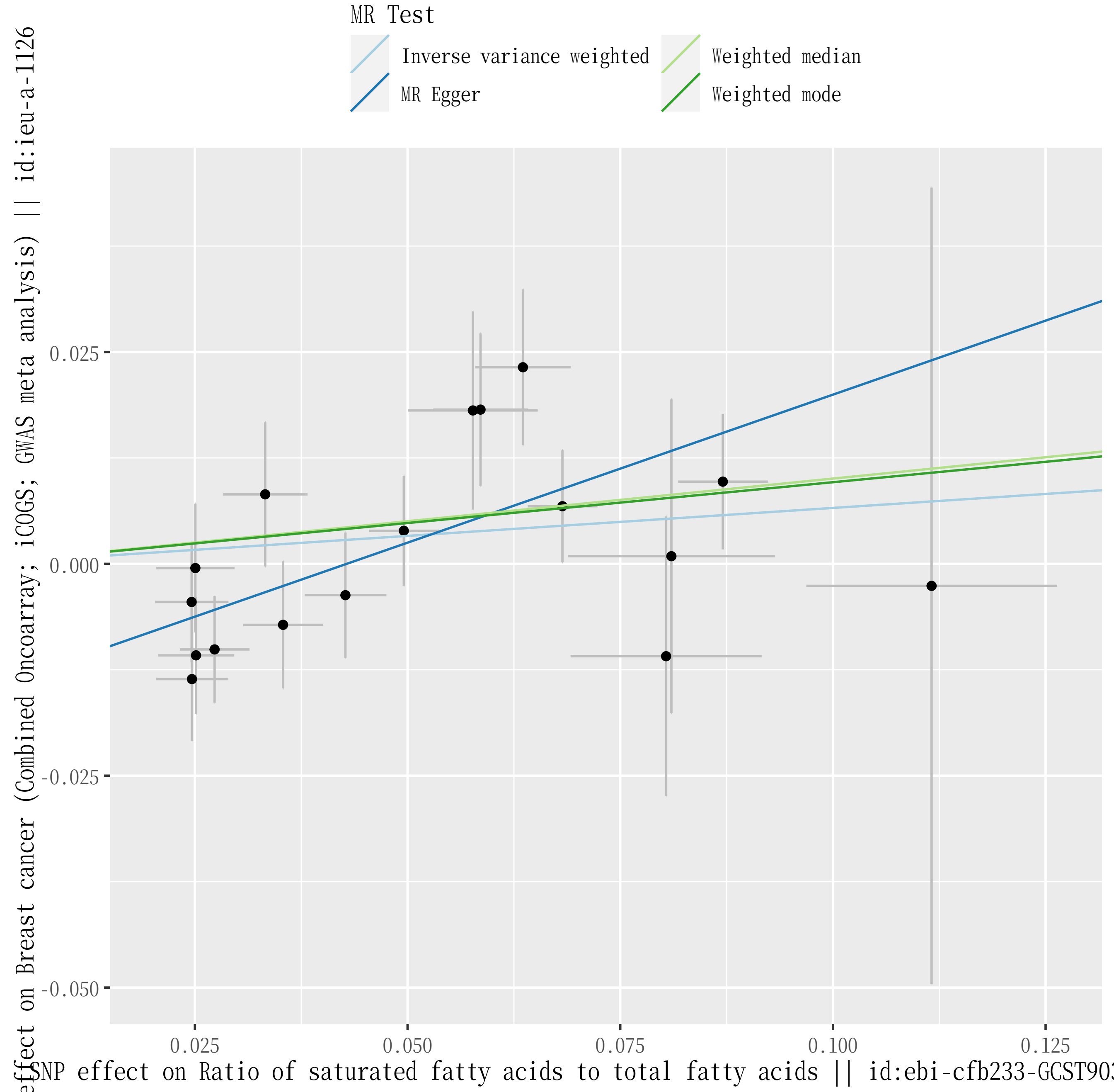
MR Test
Inverse variance weighted
MR Egger
Weighted median
Weighted mode
0.025
0.000
-0.025
-0.050
0.025
0.050
0.075
0.100
0.125
SNP effect on Breast cancer (Combined Oncoarray; iCOGS; GWAS meta analysis) || id:ieu-a-1126
SNP effect on Ratio of saturated fatty acids to total fatty acids || id:ebi-cfb233-GCST90

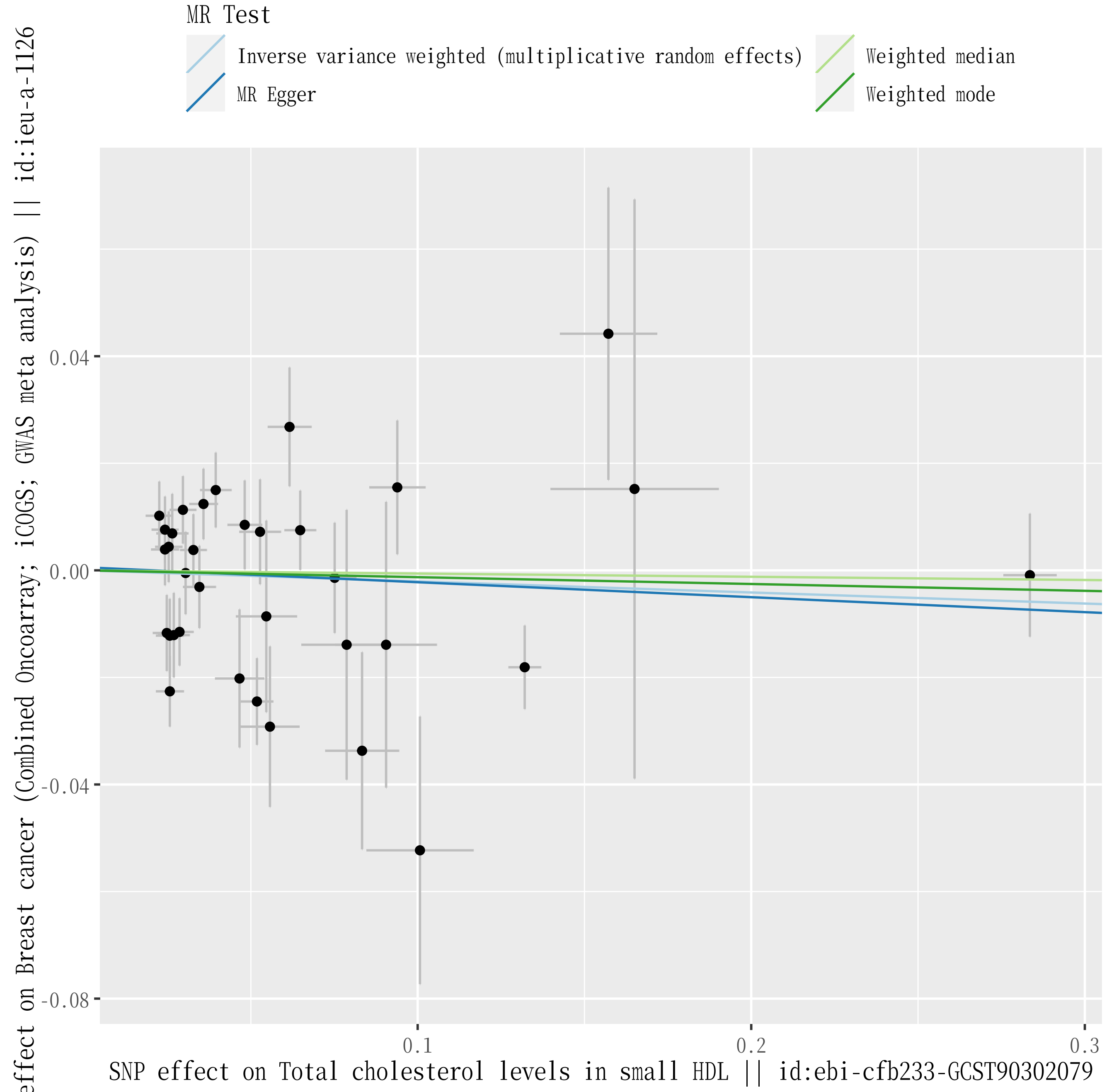

MR Test
Inverse variance weighted (multiplicative random effects)
MR Egger
Weighted median
Weighted mode
0.04
0.00
-0.04
-0.08
0.1
0.2
0.3
SNP effect on Total cholesterol levels in small HDL || id:ebi-cfb233-GCST90302079
effect on Breast cancer (Combined Oncoarray; iCOGS; GWAS meta analysis) || id:ieu-a-1126

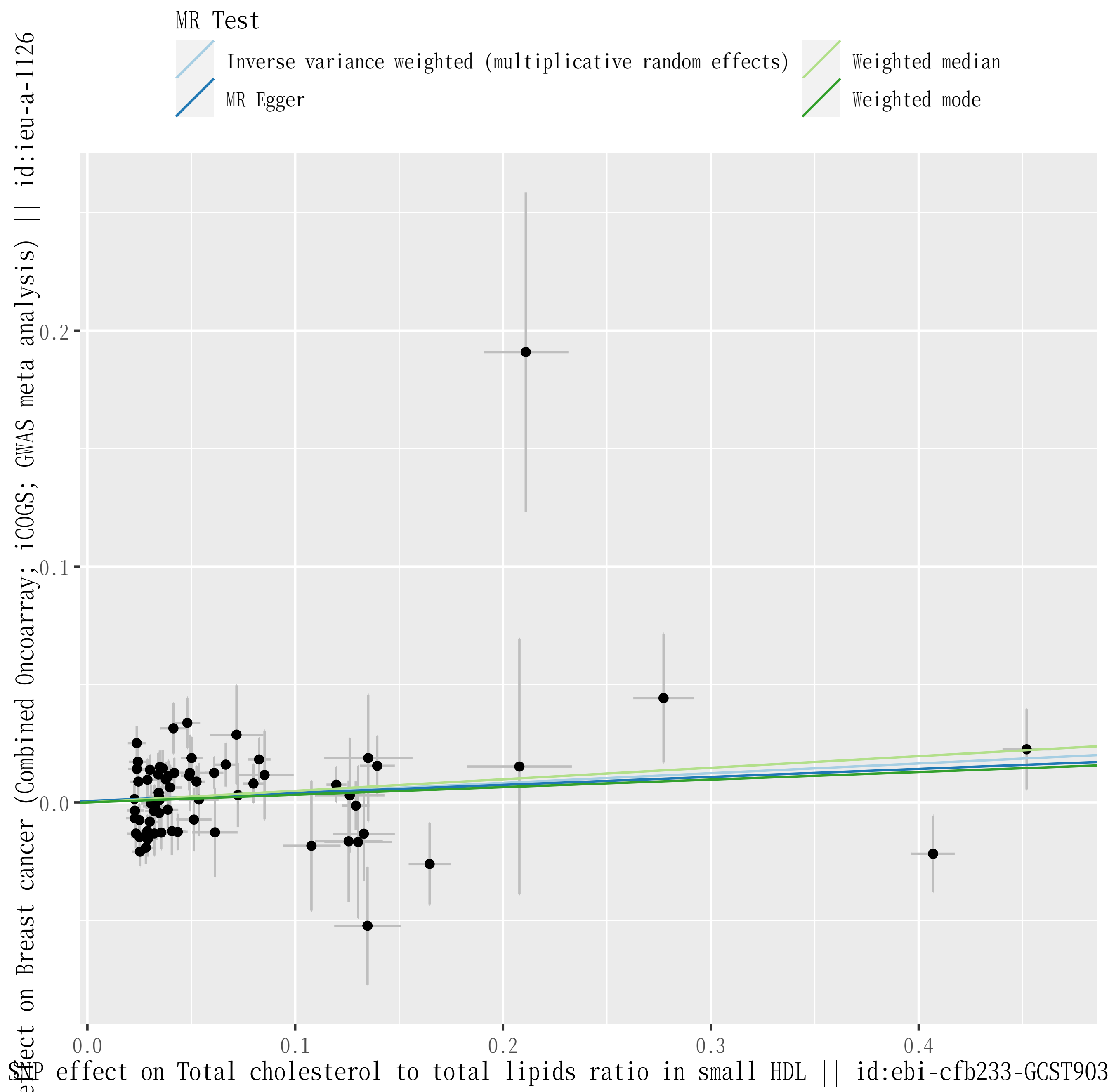

MR Test
Inverse variance weighted (multiplicative random effects)
MR Egger
Weighted median
Weighted mode
0.0
0.1
0.2
0.3
0.4
0.0
0.1
0.2
SNP effect on Total cholesterol to total lipids ratio in small HDL || id:ebi-cfb233-GCST903
effect on Breast cancer (Combined Oncoarray; iCOGS; GWAS meta analysis) || id:ieu-a-1126

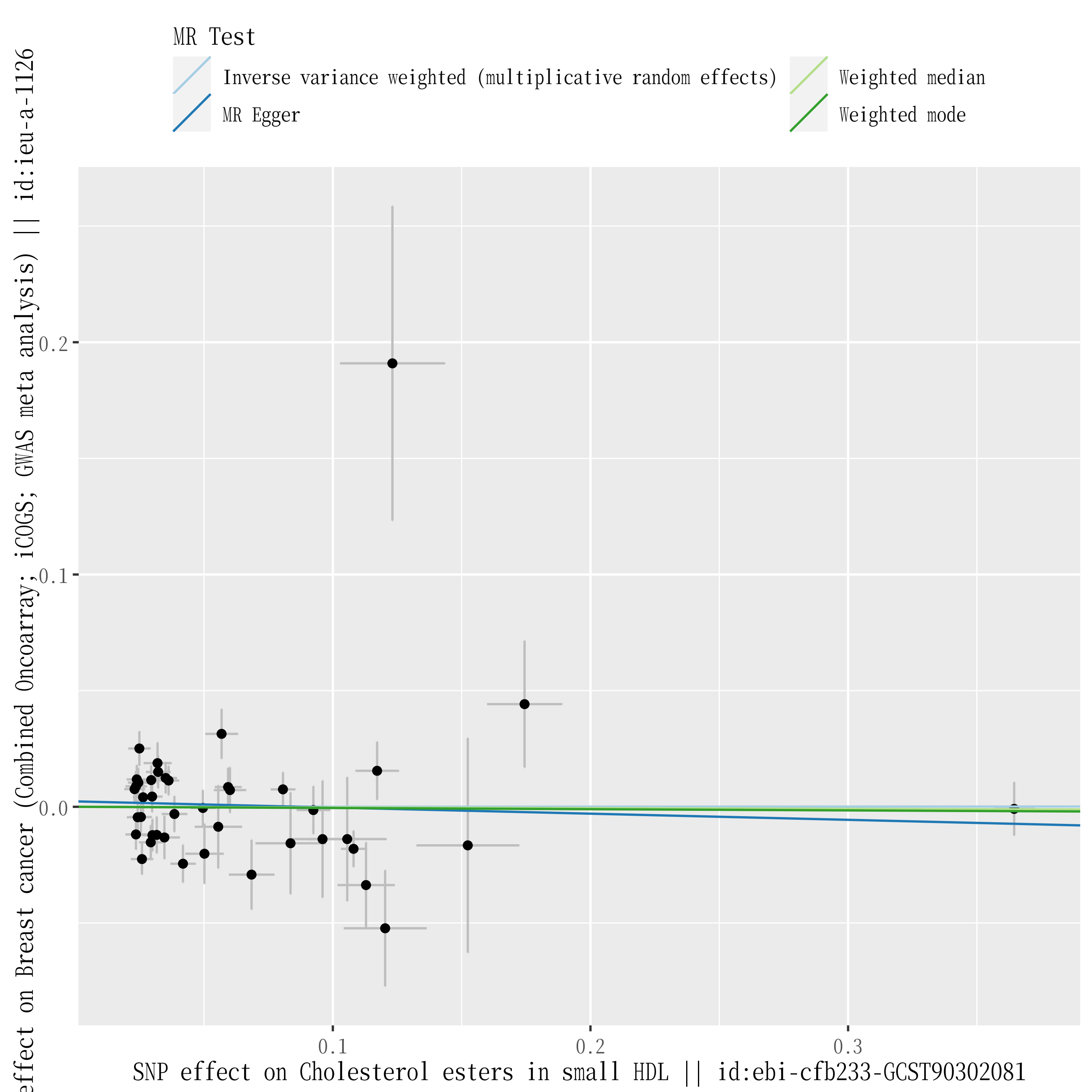

MR Test
Inverse variance weighted (multiplicative random effects)
MR Egger
Weighted median
Weighted mode
0.2
0.1
0.0
0.1
0.2
0.3
SNP effect on Cholesterol esters in small HDL || id:ebi-cfb233-GCST90302081
effect on Breast cancer (Combined Oncoarray; iCOGS; GWAS meta analysis) || id:ieu-a-1126

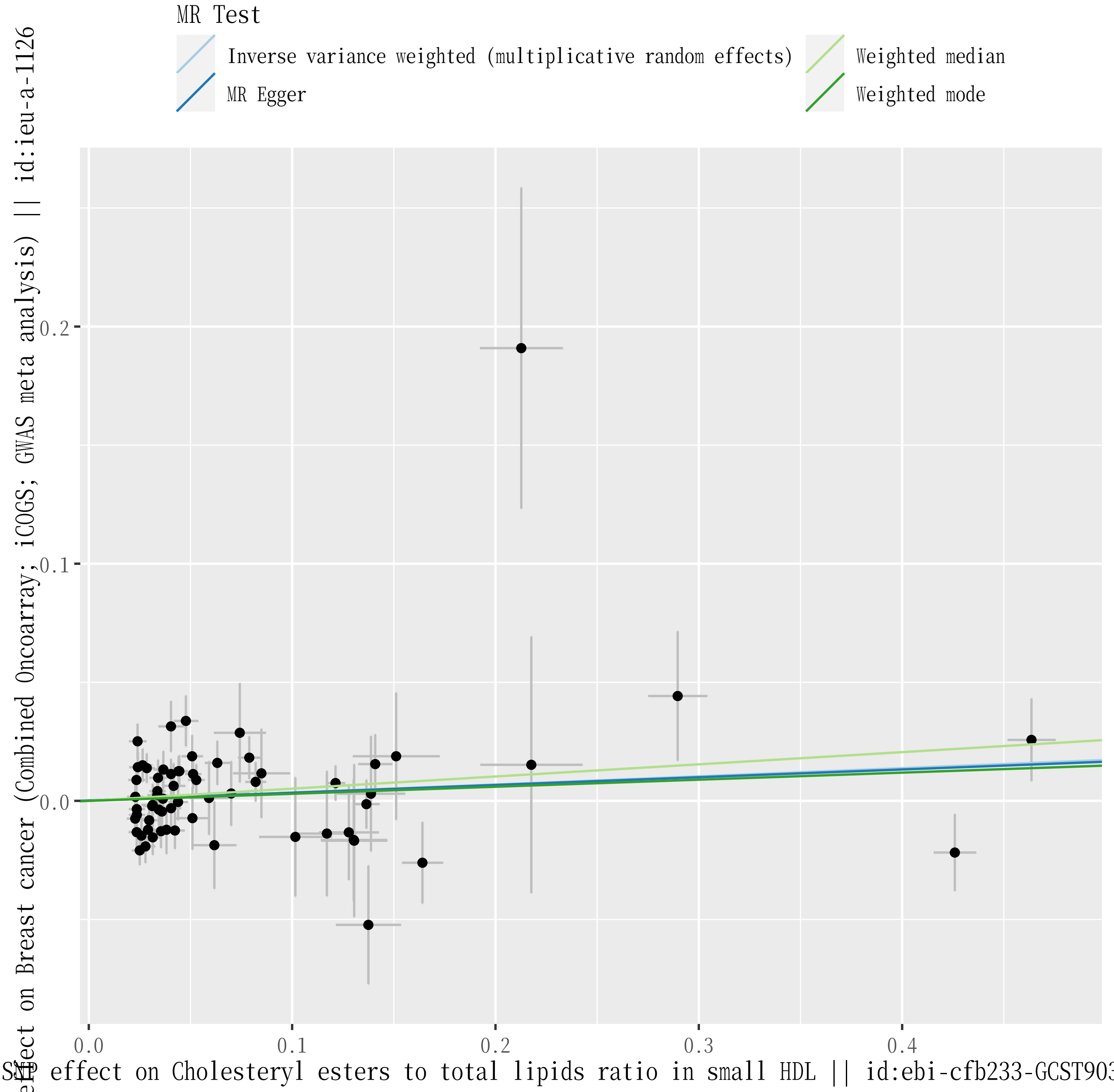
MR Test
Inverse variance weighted (multiplicative random effects)
MR Egger
Weighted median
Weighted mode
0.0
0.1
0.2
0.3
0.4
SNP effect on Cholesteryl esters to total lipids ratio in small HDL || id:ebi-cfb233-GCST903
effect on Breast cancer (Combined Oncoarray; iCOGS; GWAS meta analysis) || id:ieu-a-1126

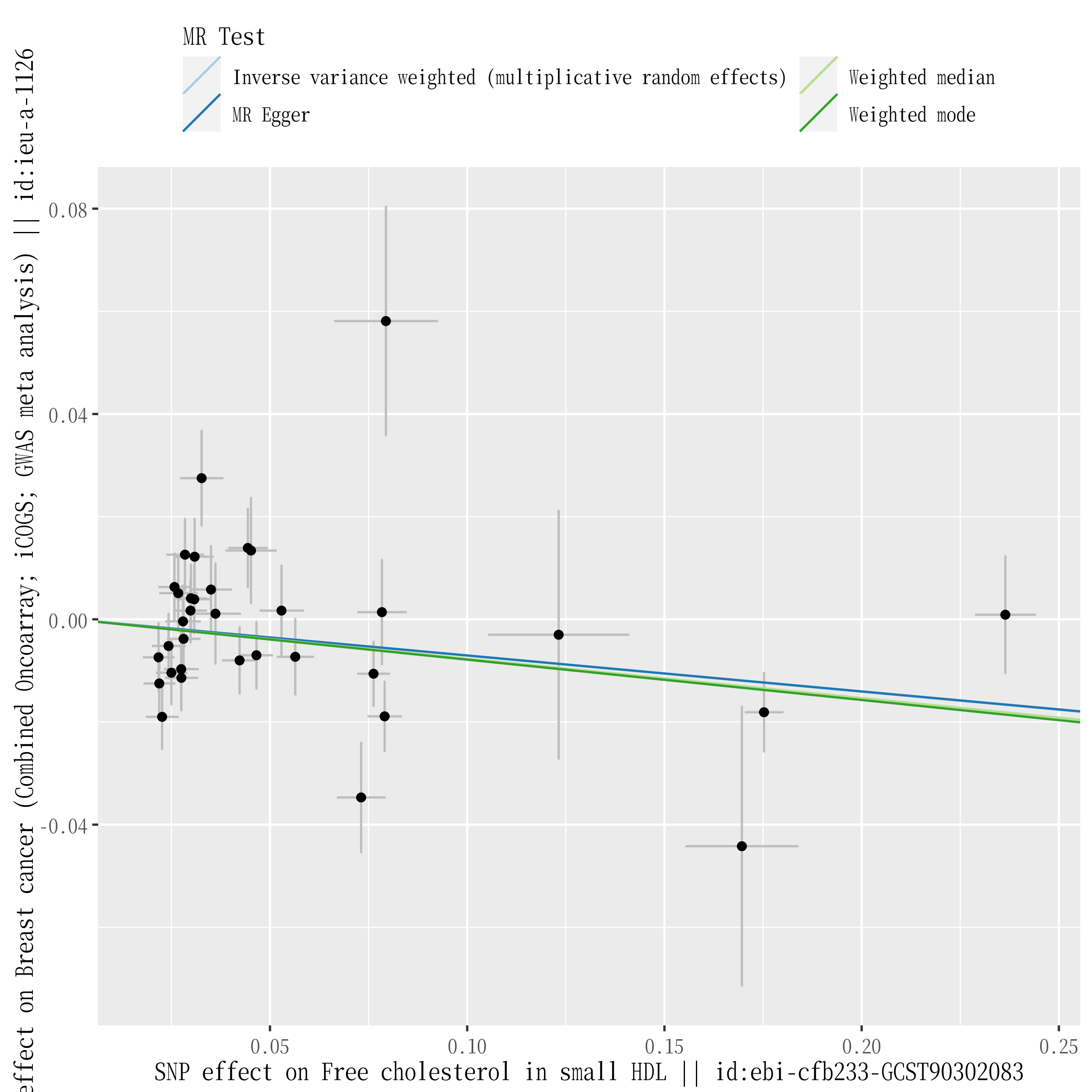

MR Test
Inverse variance weighted (multiplicative random effects)
MR Egger
Weighted median
Weighted mode
0.08
0.04
0.00
-0.04
0.05
0.10
0.15
0.20
0.25
SNP effect on Free cholesterol in small HDL || id:ebi-cfb233-GCST90302083
effect on Breast cancer (Combined Oncoarray; iCOGS; GWAS meta analysis) || id:ieu-a-1126

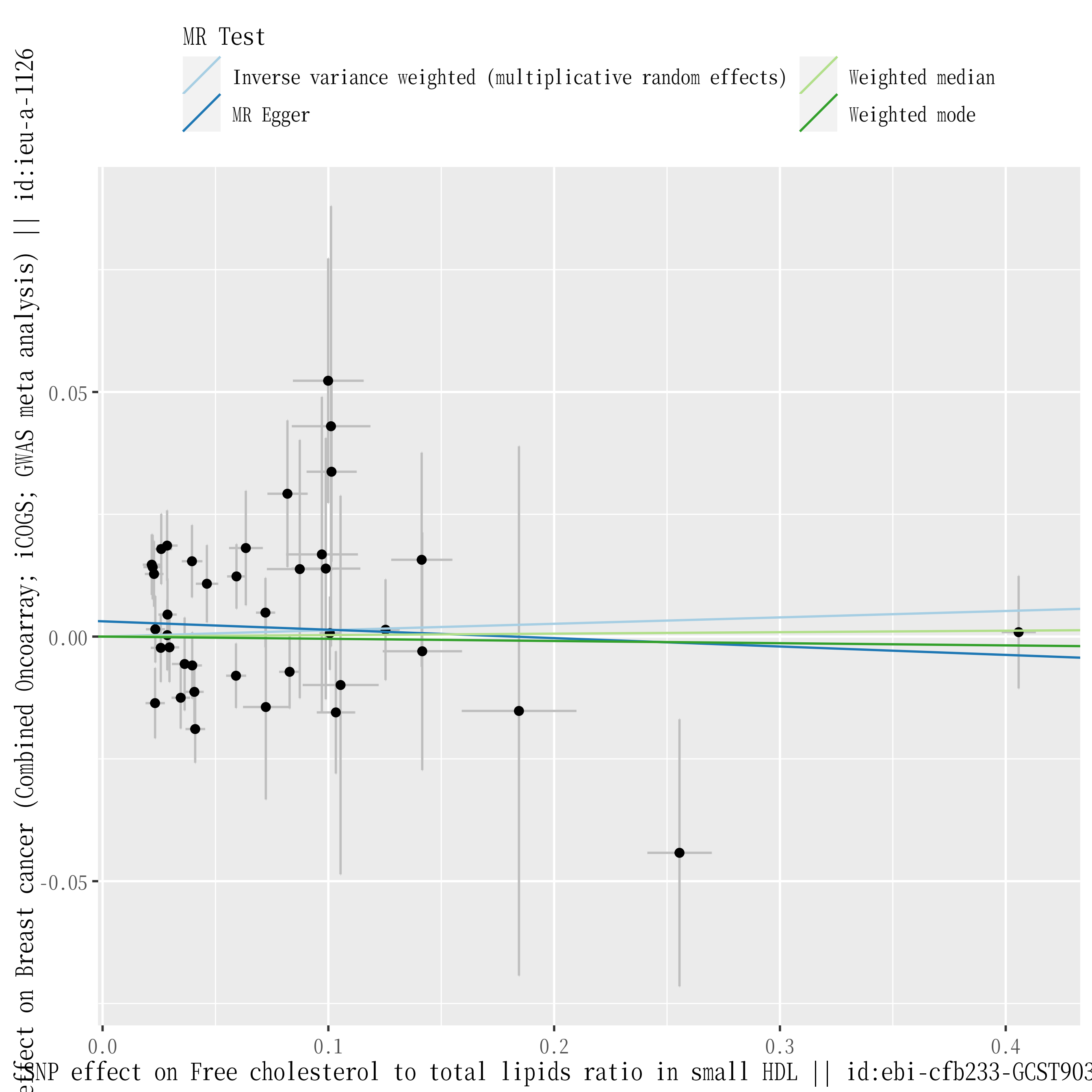

MR Test
Inverse variance weighted (multiplicative random effects)
MR Egger
Weighted median
Weighted mode
0.05
0.00
-0.05
0.0
0.1
0.2
0.3
0.4
SNP effect on Free cholesterol to total lipids ratio in small HDL || id:ebi-cfb233-GCST903
effect on Breast cancer (Combined Oncoarray; iCOGS; GWAS meta analysis) || id:ieu-a-1126

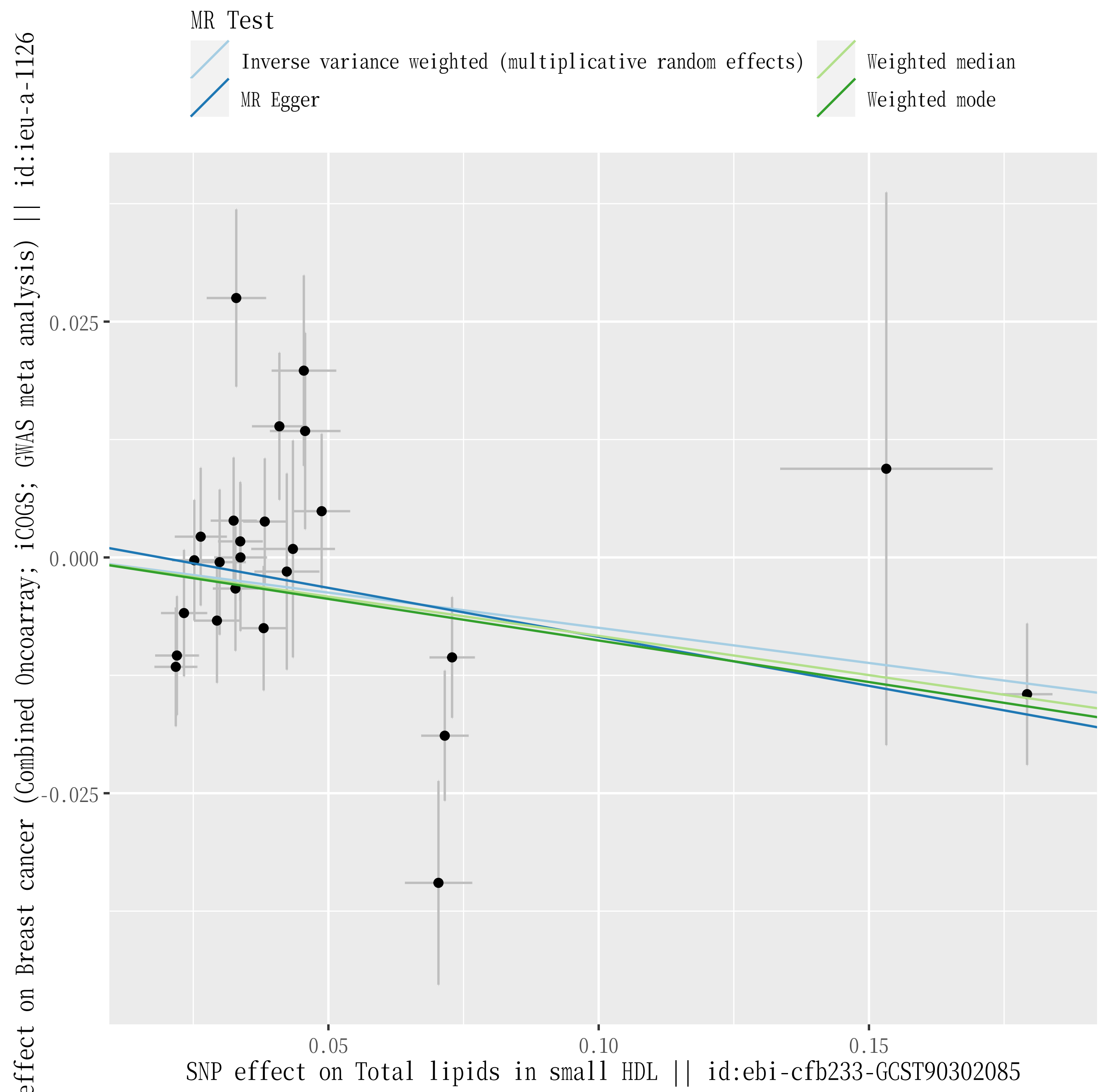

MR Test
Inverse variance weighted (multiplicative random effects)
MR Egger
Weighted median
Weighted mode
0.025
0.000
-0.025
0.05
0.10
0.15
SNP effect on Total lipids in small HDL || id:ebi-cfb233-GCST90302085
effect on Breast cancer (Combined Oncoarray; iCOGS; GWAS meta analysis) || id:ieu-a-1126

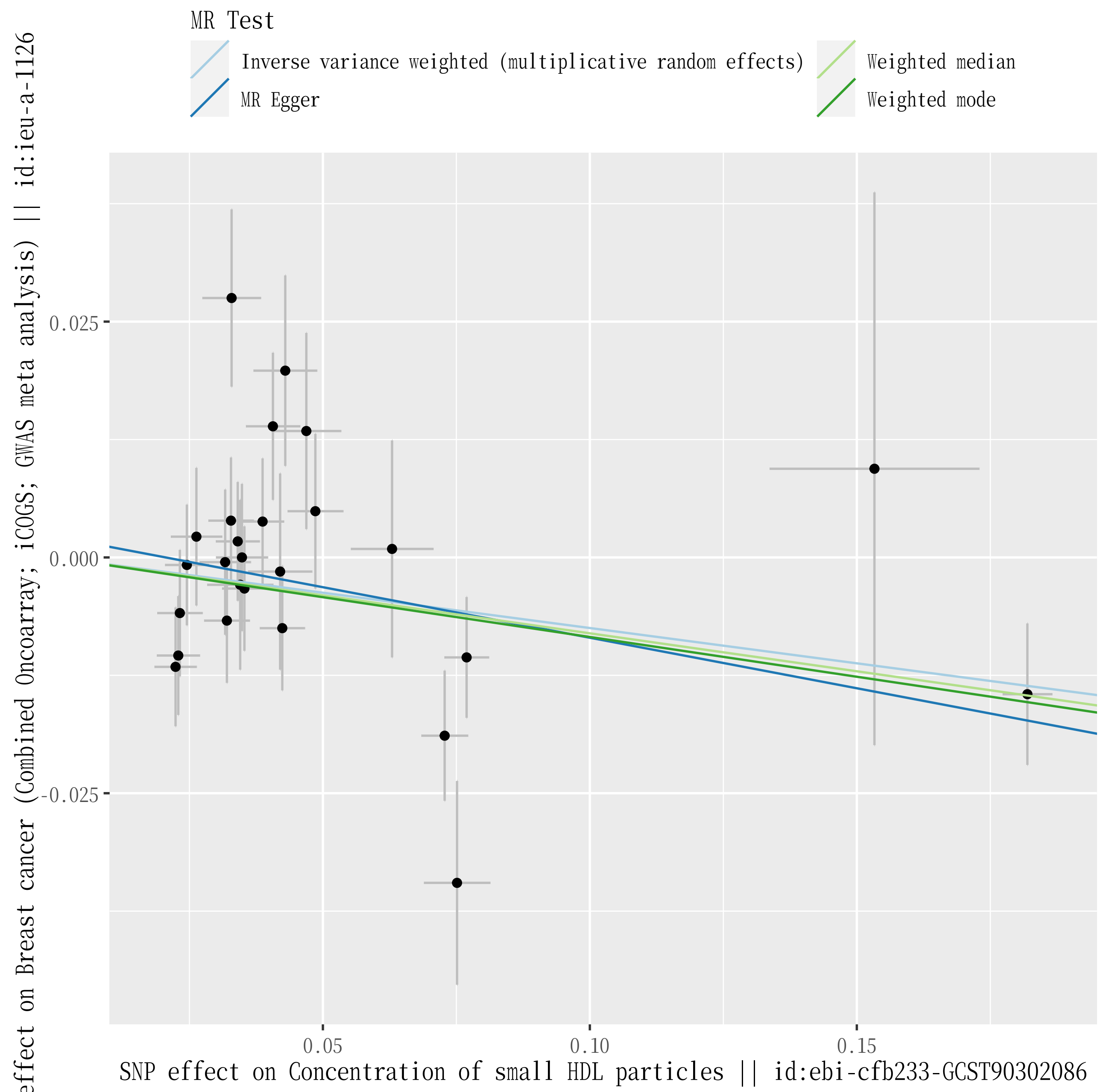
MR Test
Inverse variance weighted (multiplicative random effects)
MR Egger
Weighted median
Weighted mode
0.025
0.000
-0.025
0.05
0.10
0.15
SNP effect on Concentration of small HDL particles || id:ebi-cfb233-GCST90302086
effect on Breast cancer (Combined Oncoarray; iCOGS; GWAS meta analysis) || id:ieu-a-1126

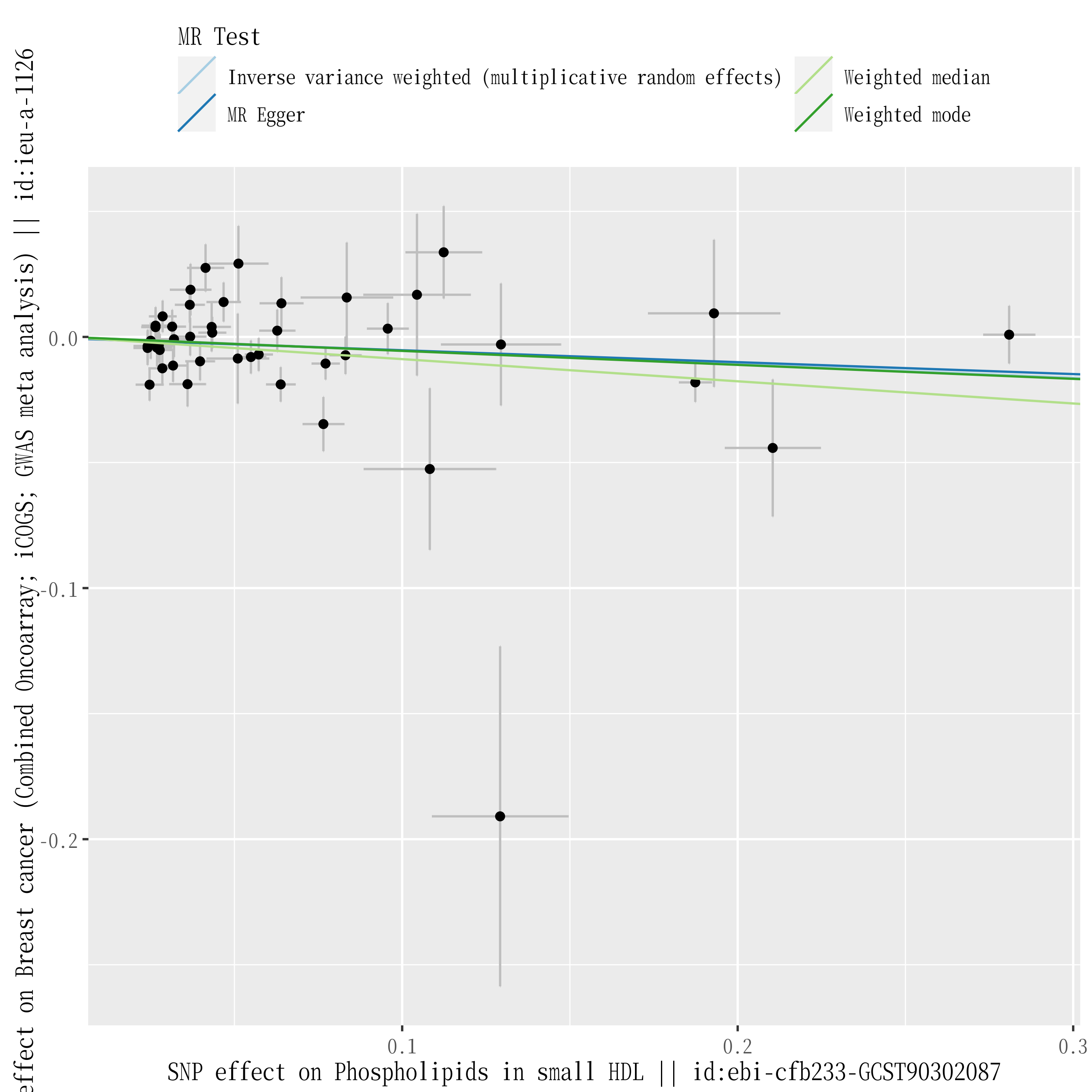
MR Test
Inverse variance weighted (multiplicative random effects)
MR Egger
Weighted median
Weighted mode
0.0
-0.1
-0.2
0.1
0.2
0.3
SNP effect on Phospholipids in small HDL || id:ebi-cfb233-GCST90302087
effect on Breast cancer (Combined Oncoarray; iCOGS; GWAS meta analysis) || id:ieu-a-1126

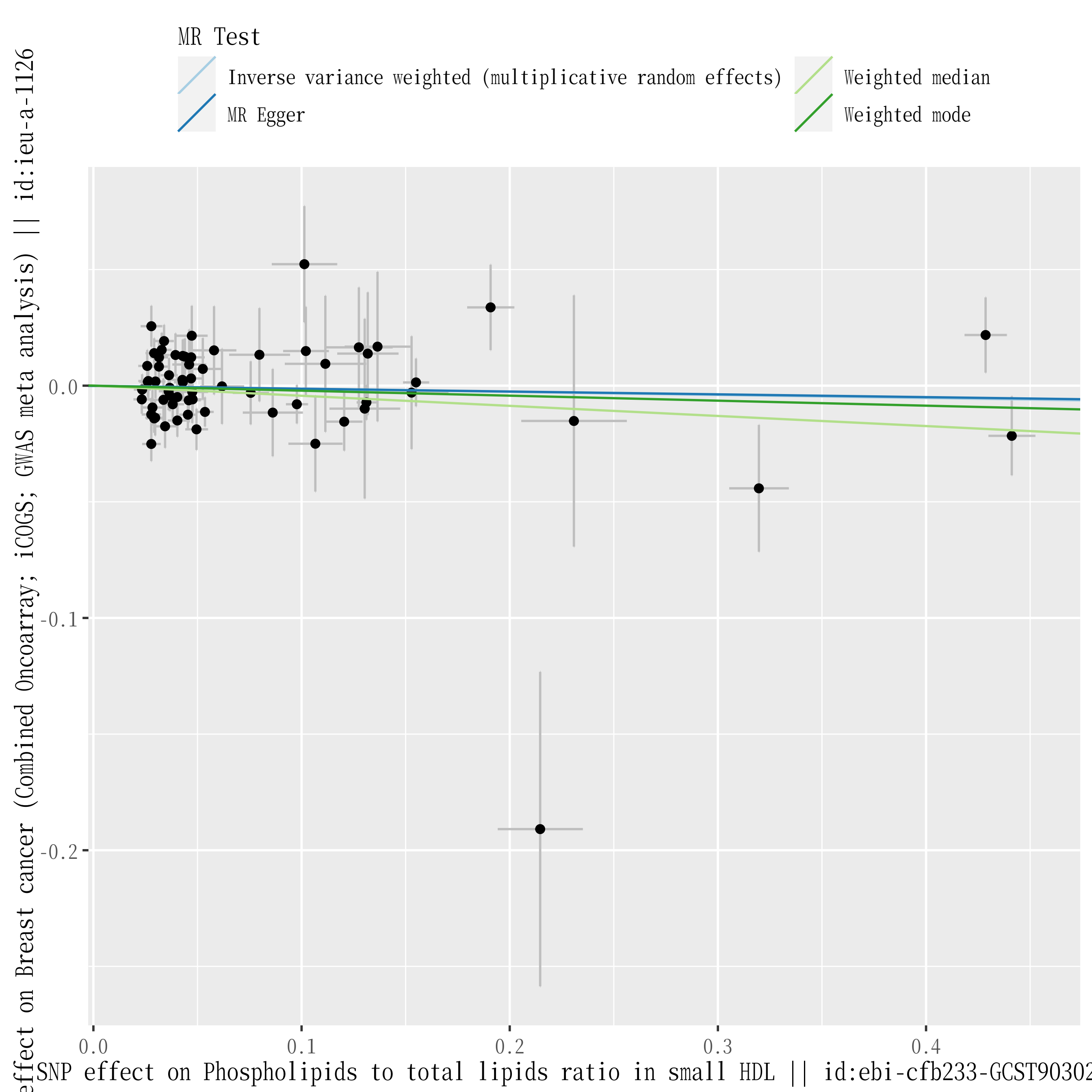
MR Test
Inverse variance weighted (multiplicative random effects)
MR Egger
Weighted median
Weighted mode
0.0
-0.1
-0.2
0.0
0.1
0.2
0.3
0.4
SNP effect on Phospholipids to total lipids ratio in small HDL || id:ebi-cfb233-GCST90302
effect on Breast cancer (Combined Oncoarray; iCOGS; GWAS meta analysis) || id:ieu-a-1126

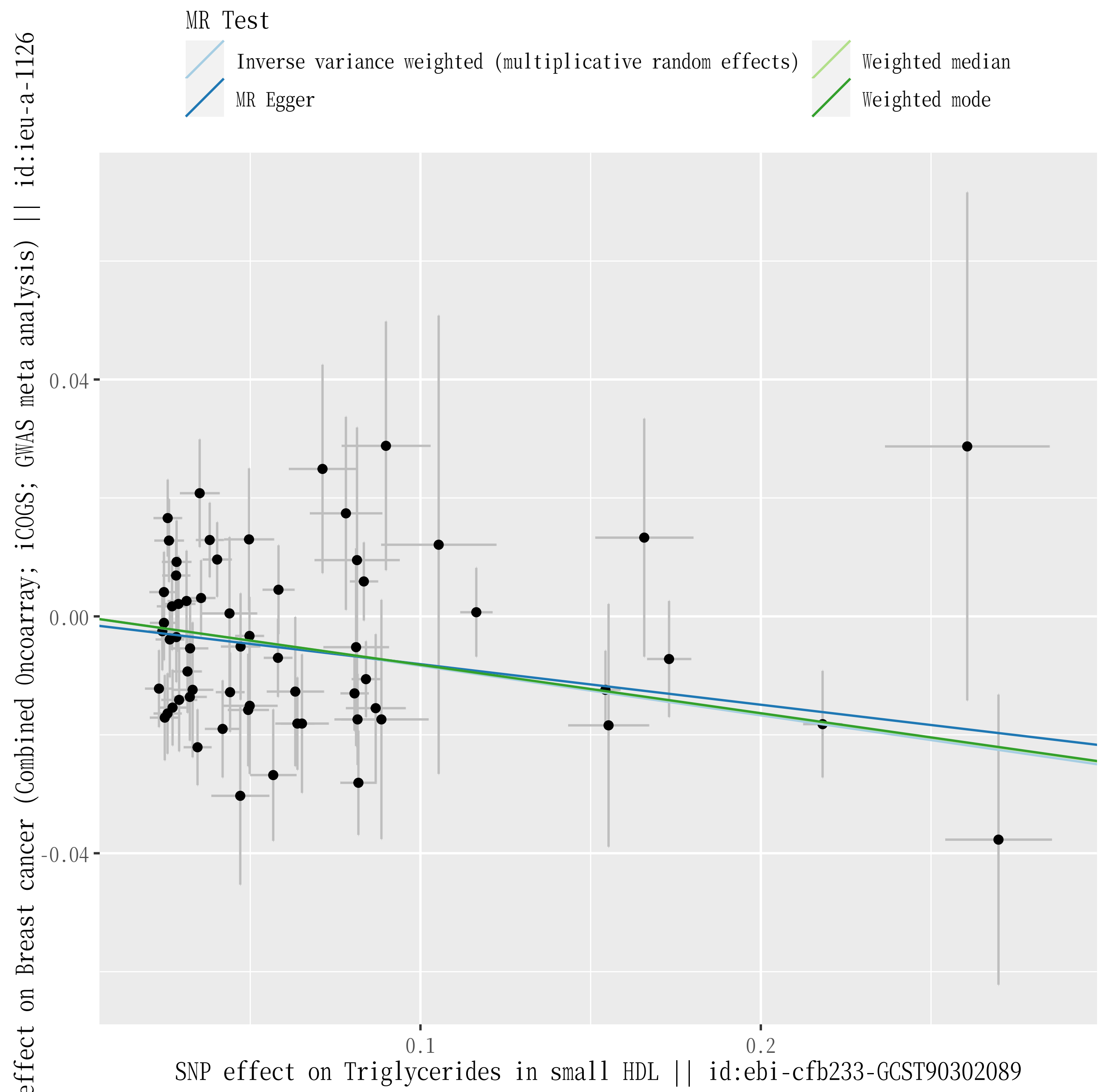

MR Test
Inverse variance weighted (multiplicative random effects)
MR Egger
Weighted median
Weighted mode
0.04
0.00
-0.04
0.1
0.2
SNP effect on Triglycerides in small HDL || id:ebi-cfb233-GCST90302089
effect on Breast cancer (Combined Oncoarray; iCOGS; GWAS meta analysis) || id:ieu-a-1126

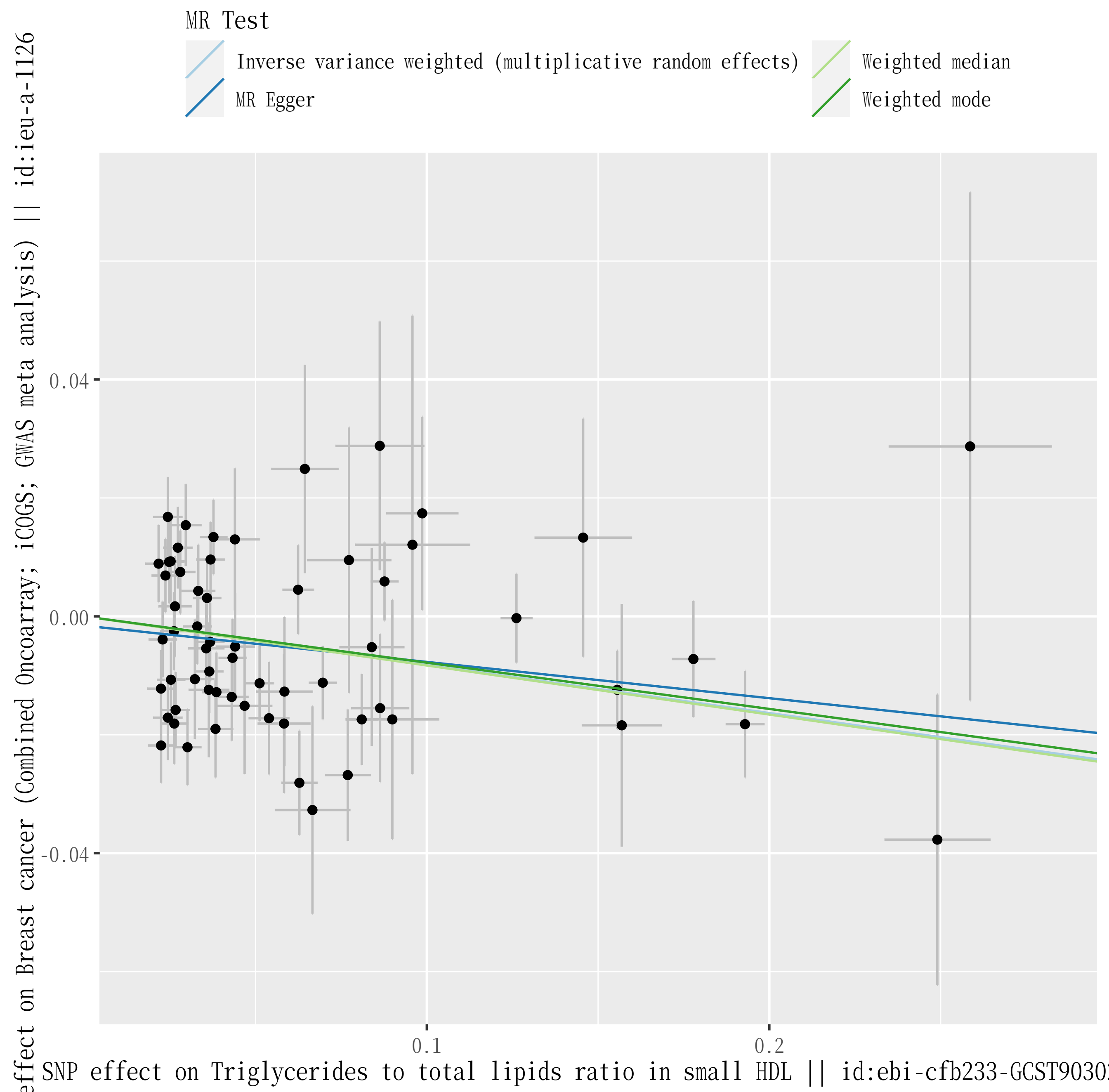
MR Test
Inverse variance weighted (multiplicative random effects)
MR Egger
Weighted median
Weighted mode
0.04
0.00
-0.04
0.1
0.2
SNP effect on Triglycerides to total lipids ratio in small HDL || id:ebi-cfb233-GCST9030
effect on Breast cancer (Combined Oncoarray; iCOGS; GWAS meta analysis) || id:ieu-a-1126

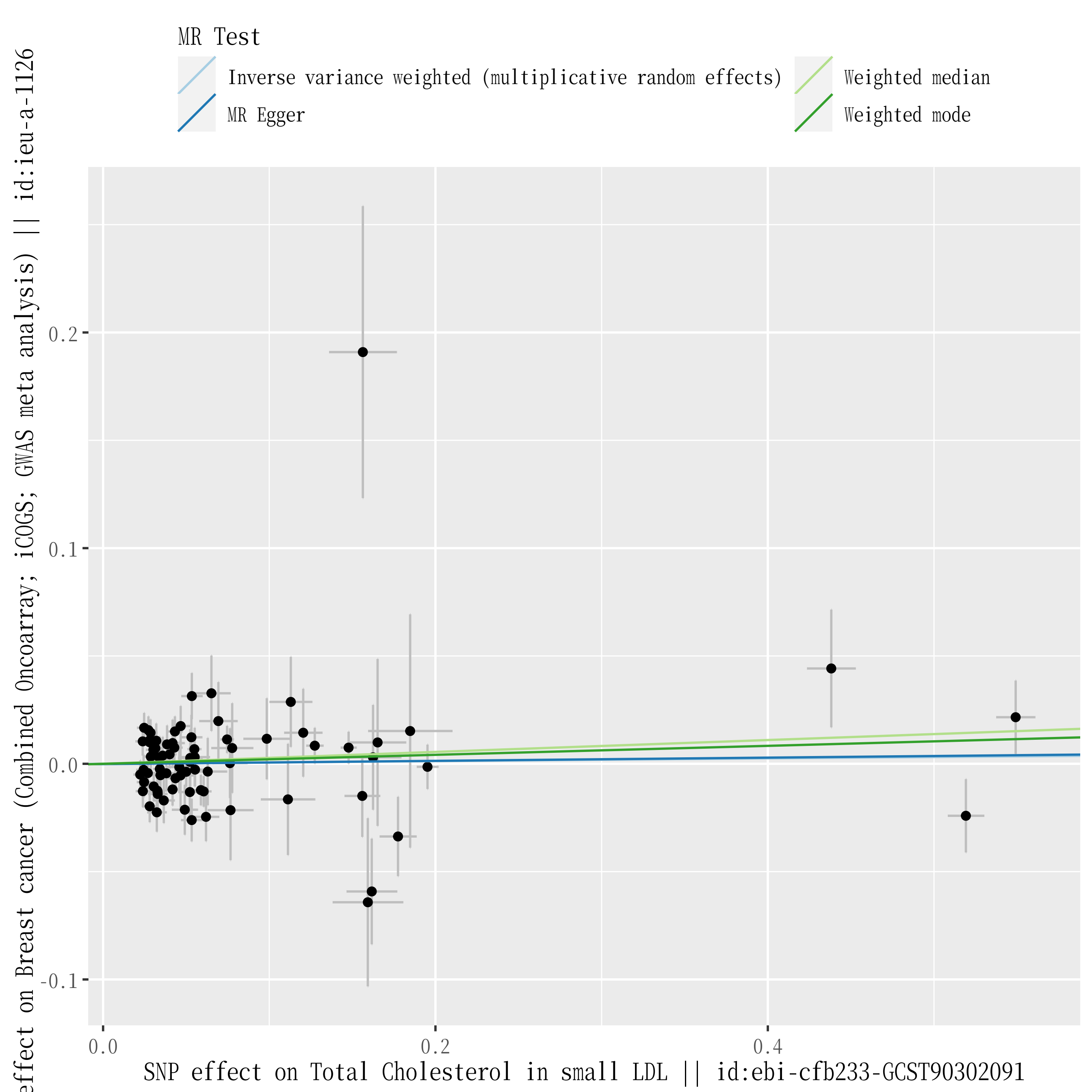
MR Test
Inverse variance weighted (multiplicative random effects)
MR Egger
Weighted median
Weighted mode
effect on Breast cancer (Combined Oncoarray; iCOGS; GWAS meta analysis) || id:ieu-a-1126
0.2
0.1
0.0
-0.1
0.0
0.2
0.4
SNP effect on Total Cholesterol in small LDL || id:ebi-cfb233-GCST90302091

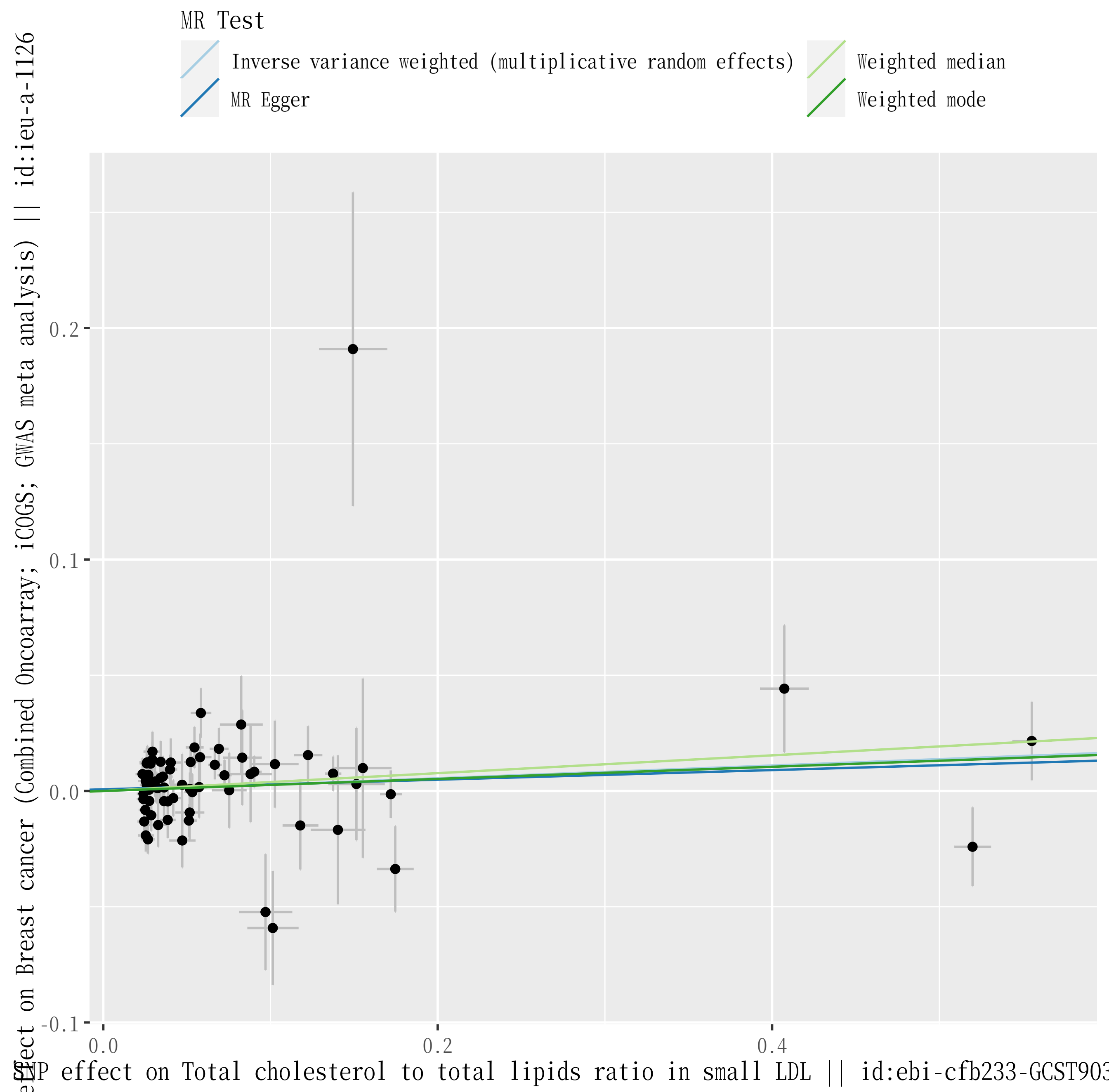
MR Test
Inverse variance weighted (multiplicative random effects)
MR Egger
Weighted median
Weighted mode
0.2
0.1
0.0
-0.1
0.0
0.2
0.4
SNP effect on Total cholesterol to total lipids ratio in small LDL || id:ebi-cfb233-GCST903
effect on Breast cancer (Combined Oncoarray; iCOGS; GWAS meta analysis) || id:ieu-a-1126

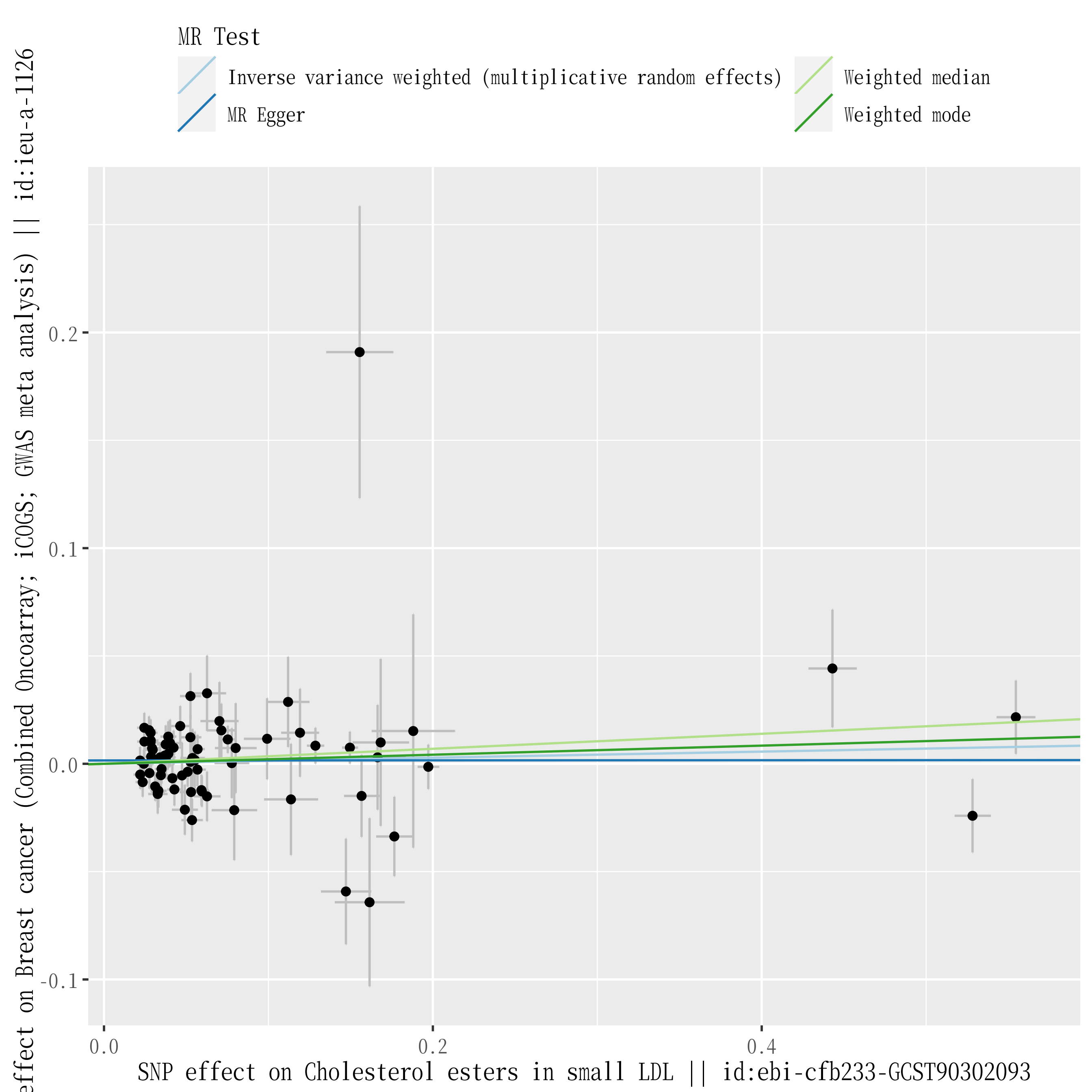

MR Test
Inverse variance weighted (multiplicative random effects)
MR Egger
Weighted median
Weighted mode
effect on Breast cancer (Combined Oncoarray; iCOGS; GWAS meta analysis) || id:ieu-a-1126
0.2
0.1
0.0
-0.1
0.0
0.2
0.4
SNP effect on Cholesterol esters in small LDL || id:ebi-cfb233-GCST90302093

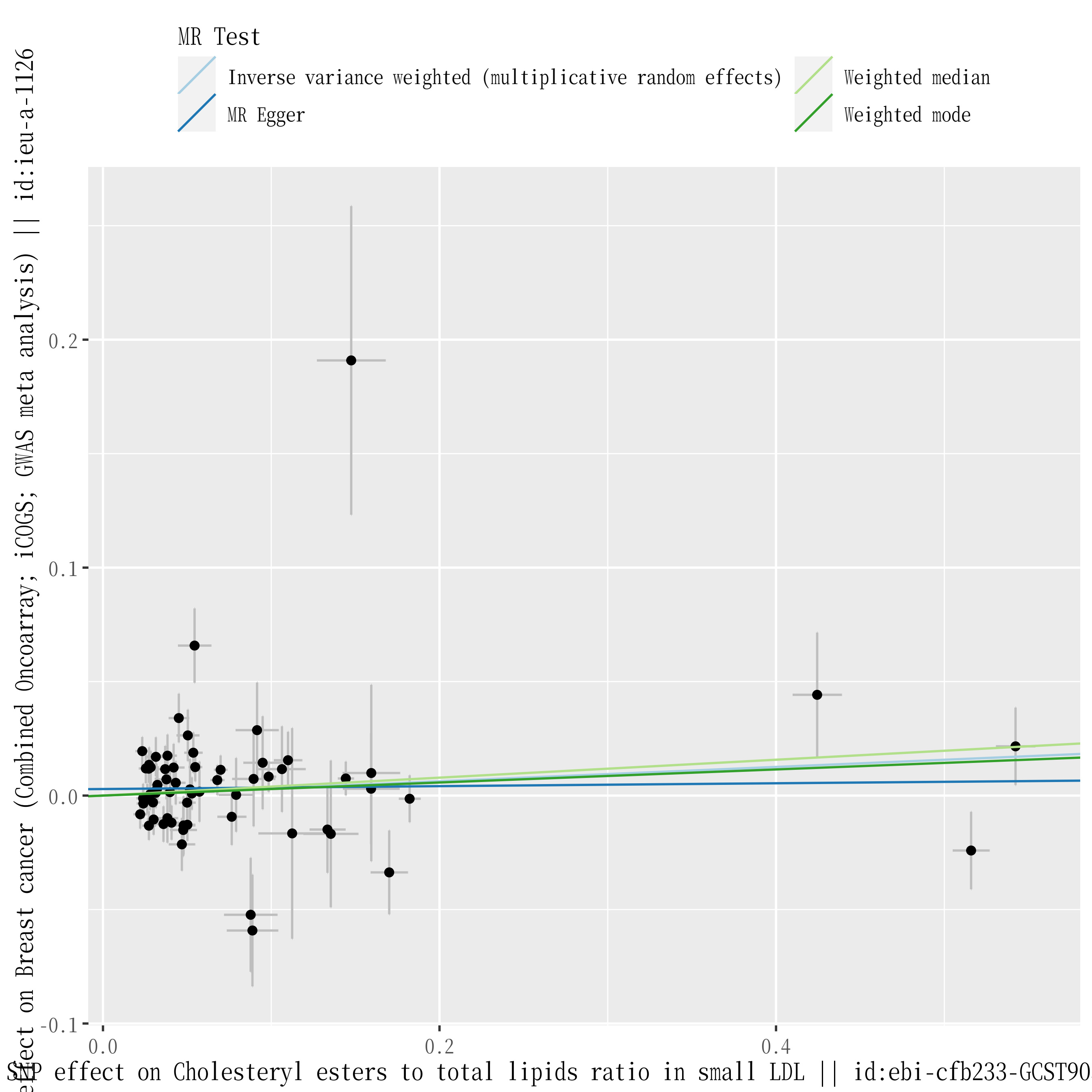

MR Test
Inverse variance weighted (multiplicative random effects)
MR Egger
Weighted median
Weighted mode
0.2
0.1
0.0
-0.1
0.0
0.2
0.4
SNP effect on Cholesteryl esters to total lipids ratio in small LDL || id:ebi-cfb233-GCST90
effect on Breast cancer (Combined Oncoarray; iCOGS; GWAS meta analysis) || id:ieu-a-1126

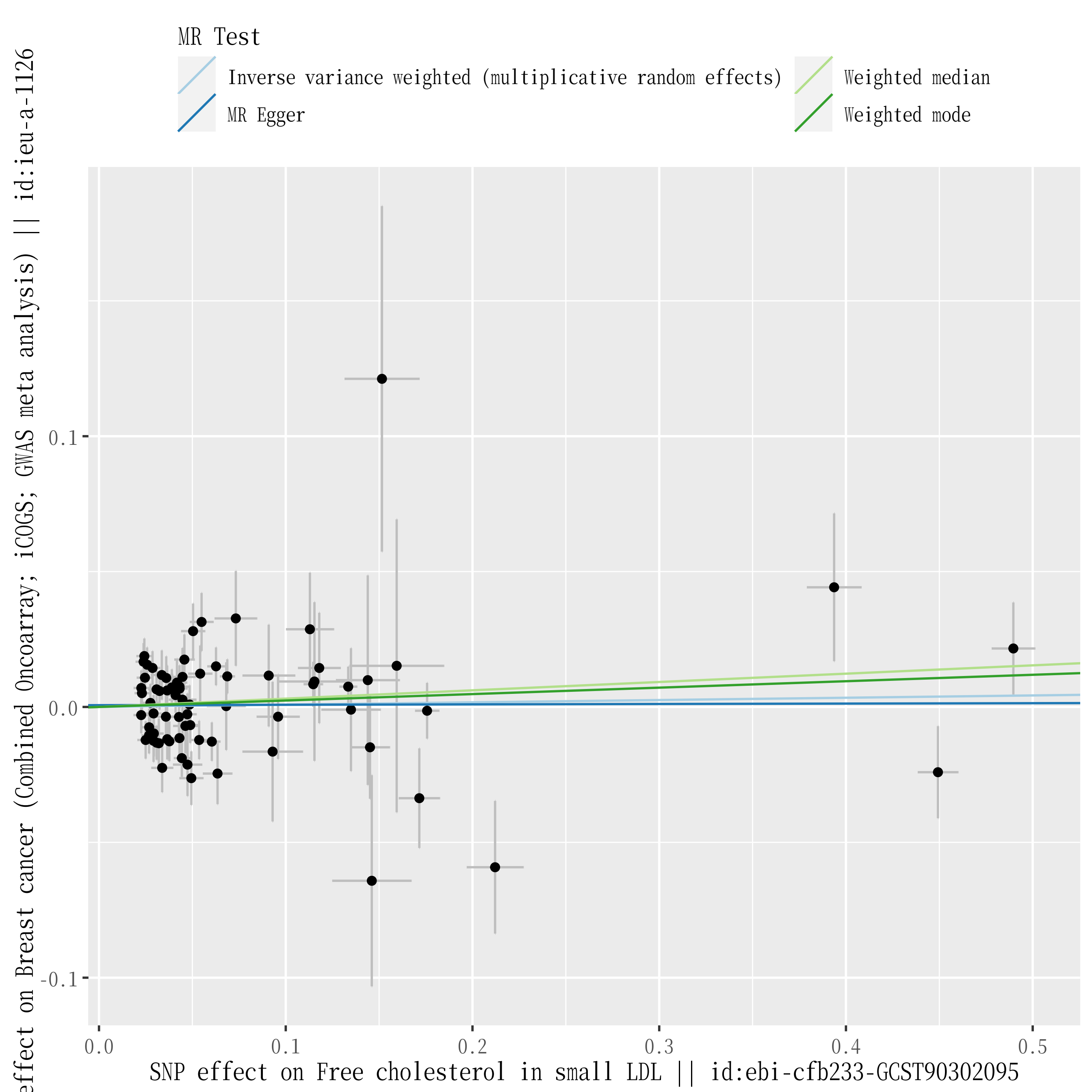

MR Test
Inverse variance weighted (multiplicative random effects)
MR Egger
Weighted median
Weighted mode
SNP effect on Breast cancer (Combined Oncoarray; iCOGS; GWAS meta analysis) || id:ieu-a-1126
0.1
0.0
-0.1
0.0
0.1
0.2
0.3
0.4
0.5
SNP effect on Free cholesterol in small LDL || id:ebi-cfb233-GCST90302095

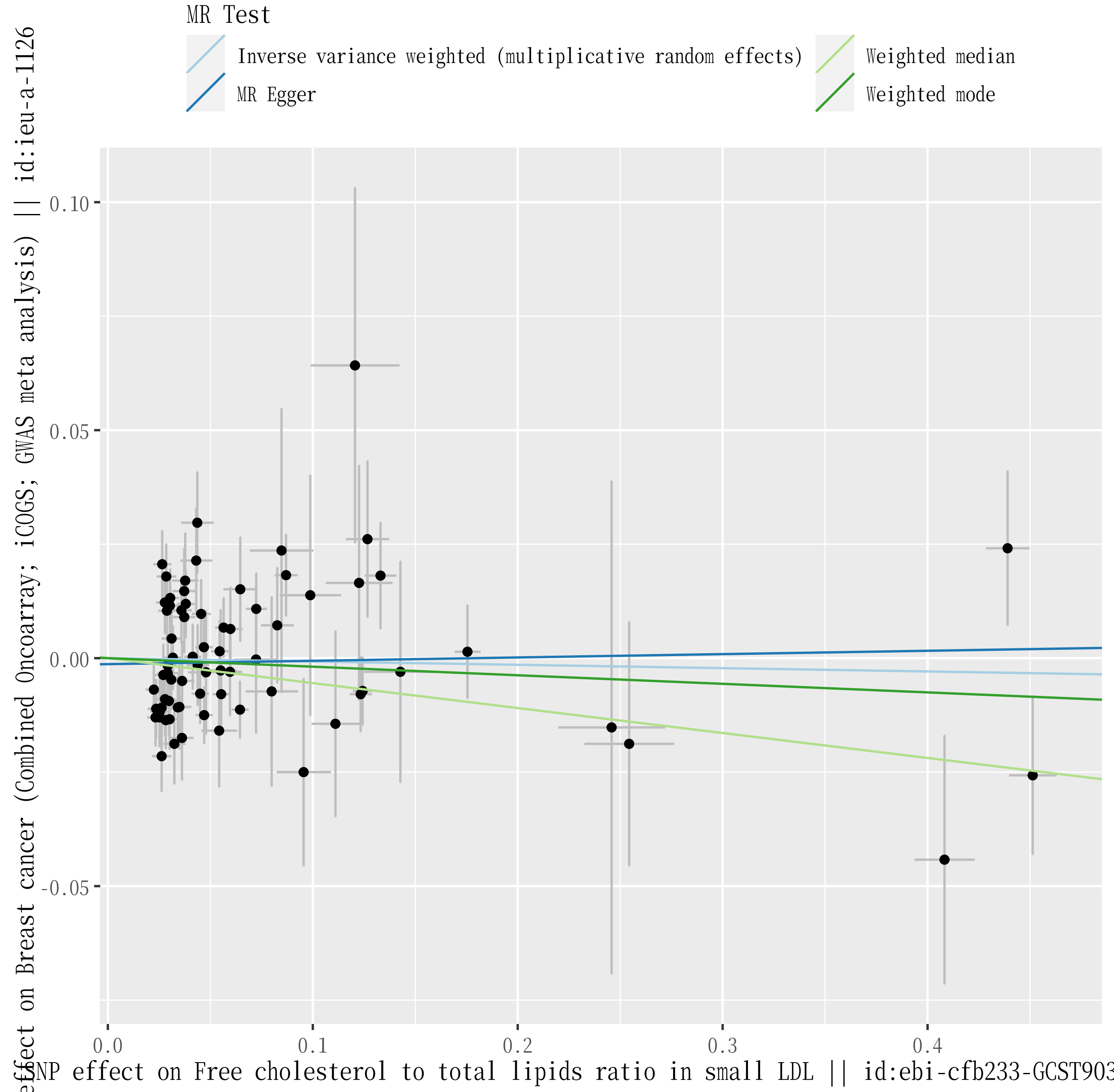

MR Test
Inverse variance weighted (multiplicative random effects)
MR Egger
Weighted median
Weighted mode
0.10
0.05
0.00
-0.05
0.0
0.1
0.2
0.3
0.4
SNP effect on Free cholesterol to total lipids ratio in small LDL || id:ebi-cfb233-GCST903
effect on Breast cancer (Combined Oncoarray; iCOGS; GWAS meta analysis) || id:ieu-a-1126

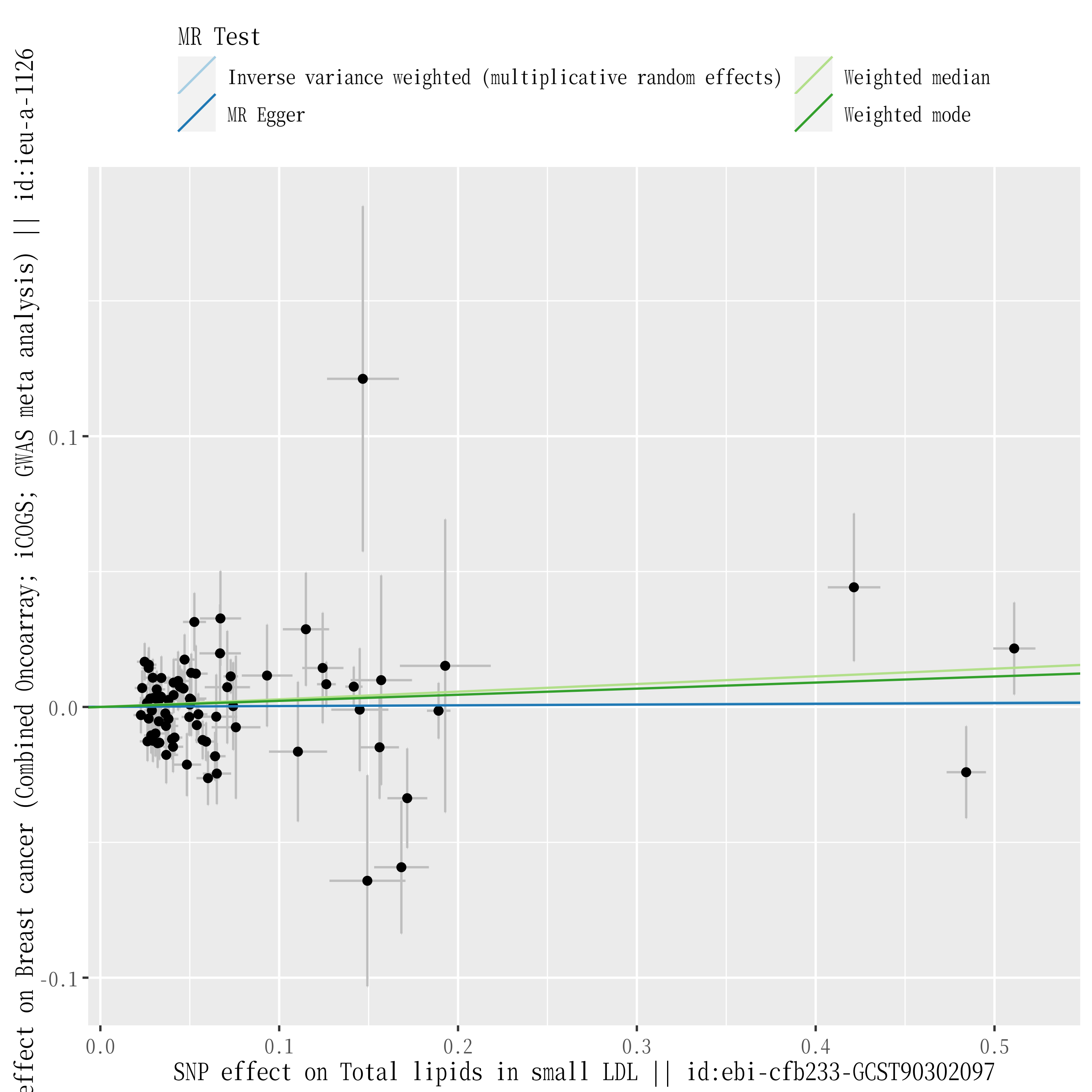
MR Test
Inverse variance weighted (multiplicative random effects)
MR Egger
Weighted median
Weighted mode
SNP effect on Breast cancer (Combined Oncoarray; iCOGS; GWAS meta analysis) || id:ieu-a-1126
0.1
0.0
-0.1
0.0
0.1
0.2
0.3
0.4
0.5
SNP effect on Total lipids in small LDL || id:ebi-cfb233-GCST90302097

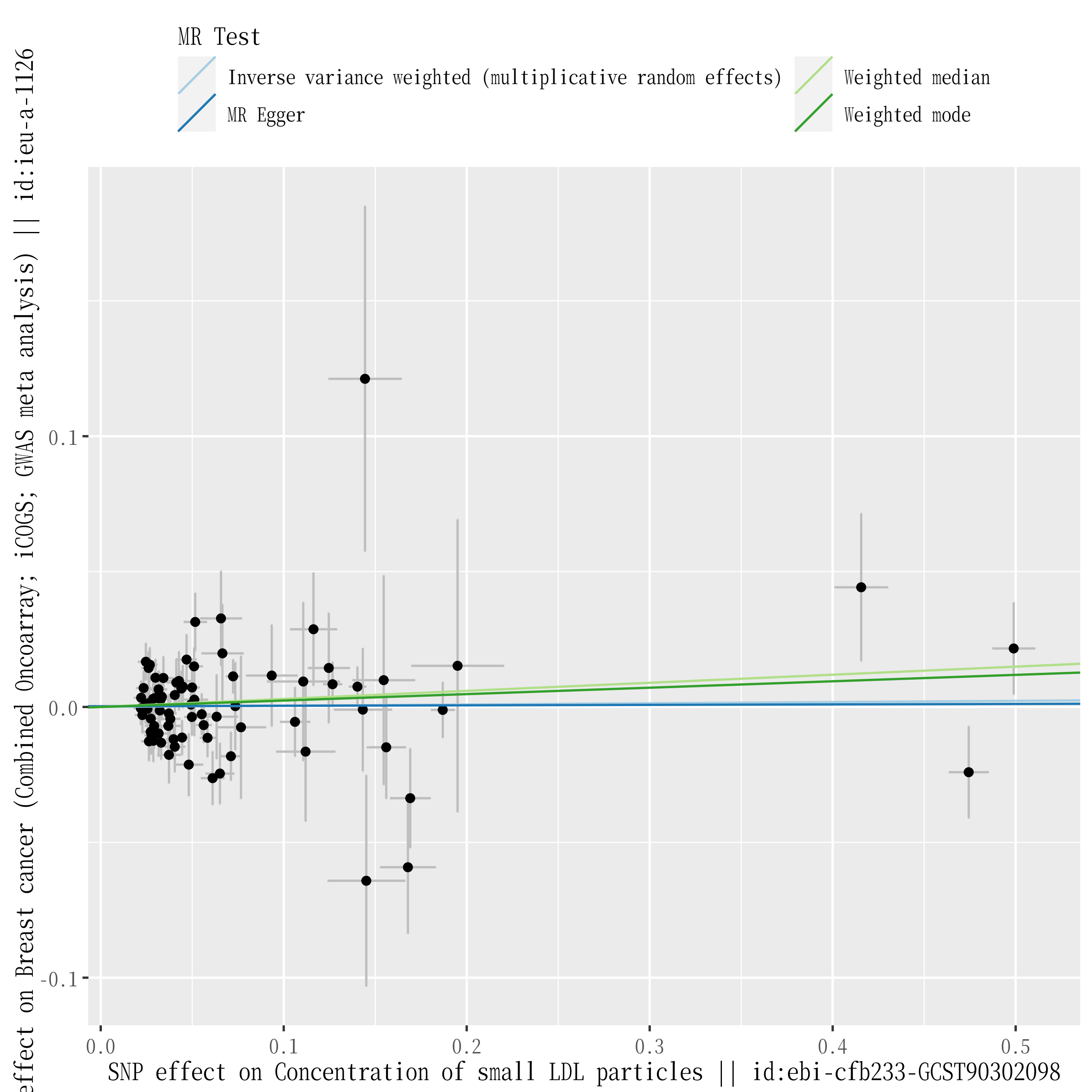
MR Test
Inverse variance weighted (multiplicative random effects)
MR Egger
Weighted median
Weighted mode
SNP effect on Breast cancer (Combined Oncoarray; iCOGS; GWAS meta analysis) || id:ieu-a-1126
0.1
0.0
-0.1
0.0
0.1
0.2
0.3
0.4
0.5
SNP effect on Concentration of small LDL particles || id:ebi-cfb233-GCST90302098

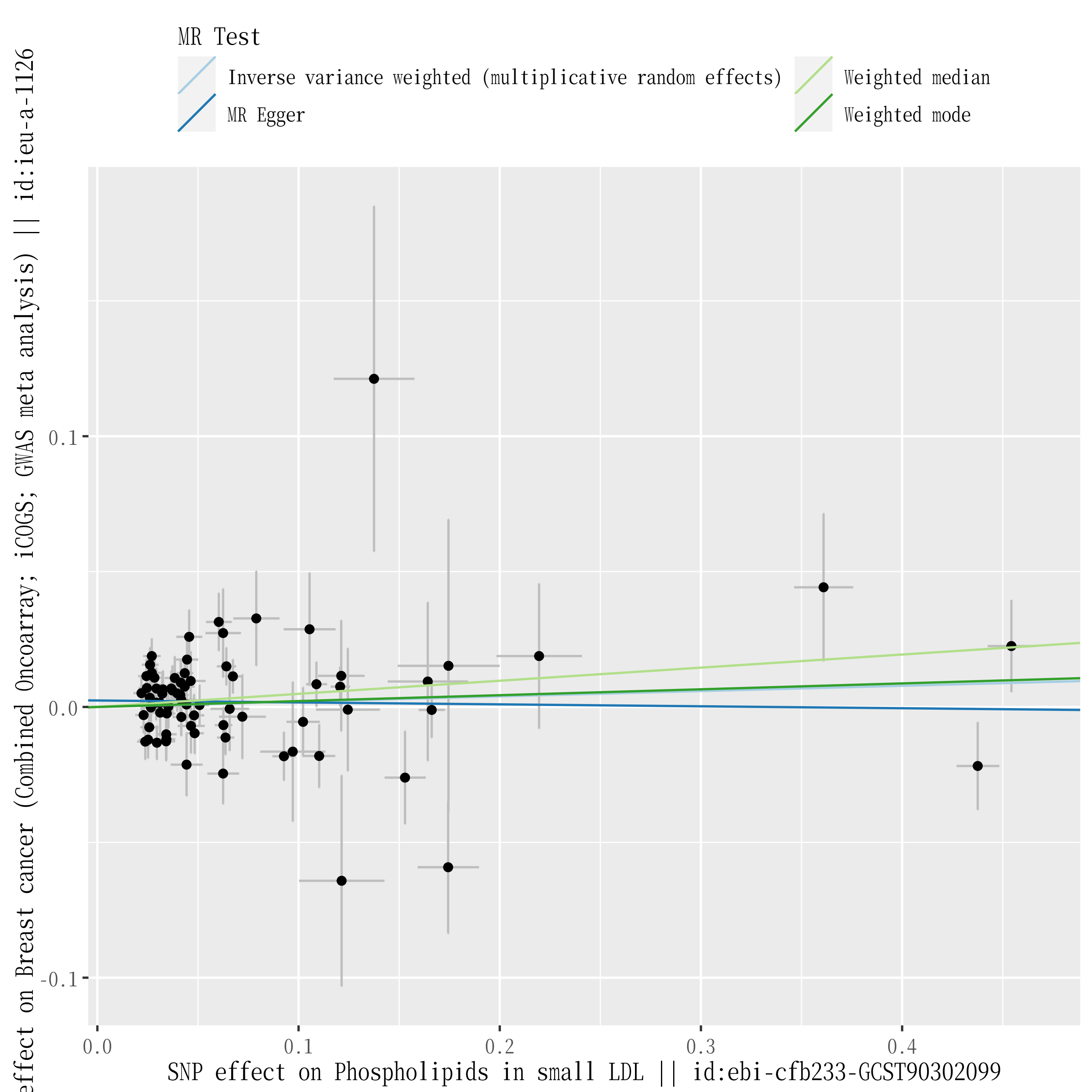
MR Test
Inverse variance weighted (multiplicative random effects)
MR Egger
Weighted median
Weighted mode
SNP effect on Breast cancer (Combined Oncoarray; iCOGS; GWAS meta analysis) || id:ieu-a-1126
0.1
0.0
-0.1
0.0
0.1
0.2
0.3
0.4
SNP effect on Phospholipids in small LDL || id:ebi-cfb233-GCST90302099

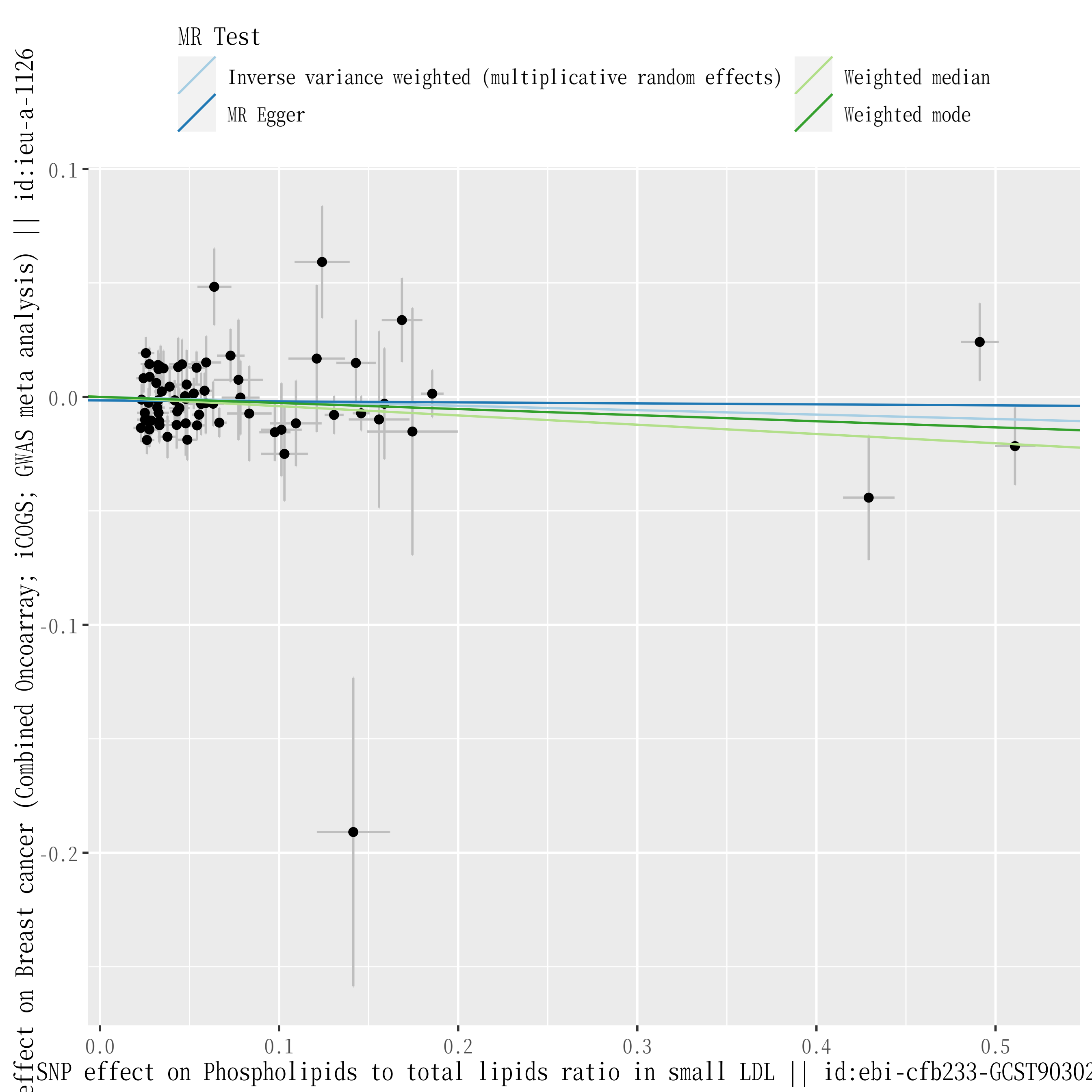

MR Test
Inverse variance weighted (multiplicative random effects)
MR Egger
Weighted median
Weighted mode
0.1
0.0
-0.1
-0.2
0.0
0.1
0.2
0.3
0.4
0.5
SNP effect on Phospholipids to total lipids ratio in small LDL || id:ebi-cfb233-GCST90302
effect on Breast cancer (Combined Oncoarray; iCOGS; GWAS meta analysis) || id:ieu-a-1126

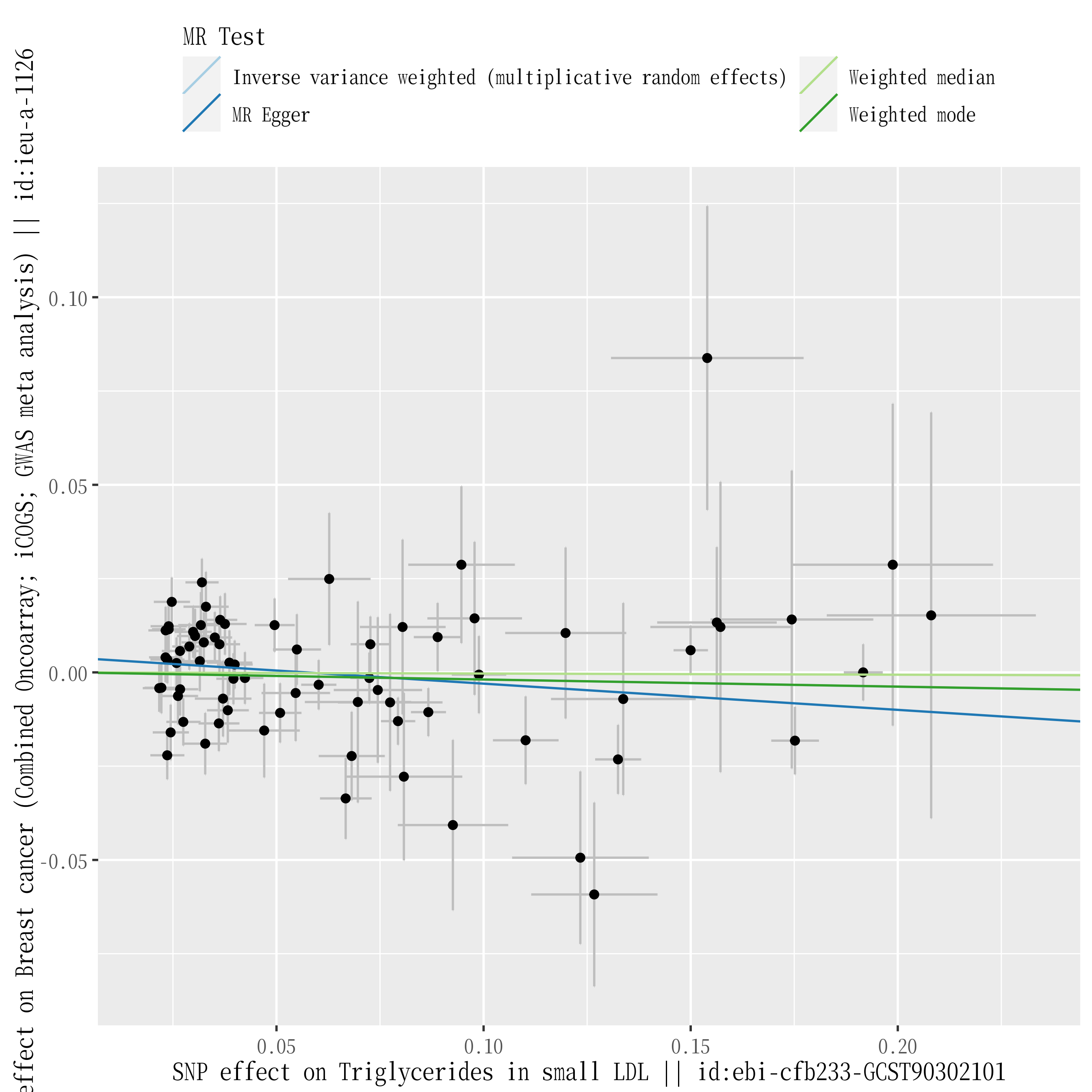

MR Test
Inverse variance weighted (multiplicative random effects)
MR Egger
Weighted median
Weighted mode
effect on Breast cancer (Combined Oncoarray; iCOGS; GWAS meta analysis) || id:ieu-a-1126
0.10
0.05
0.00
-0.05
0.05
0.10
0.15
0.20
SNP effect on Triglycerides in small LDL || id:ebi-cfb233-GCST90302101

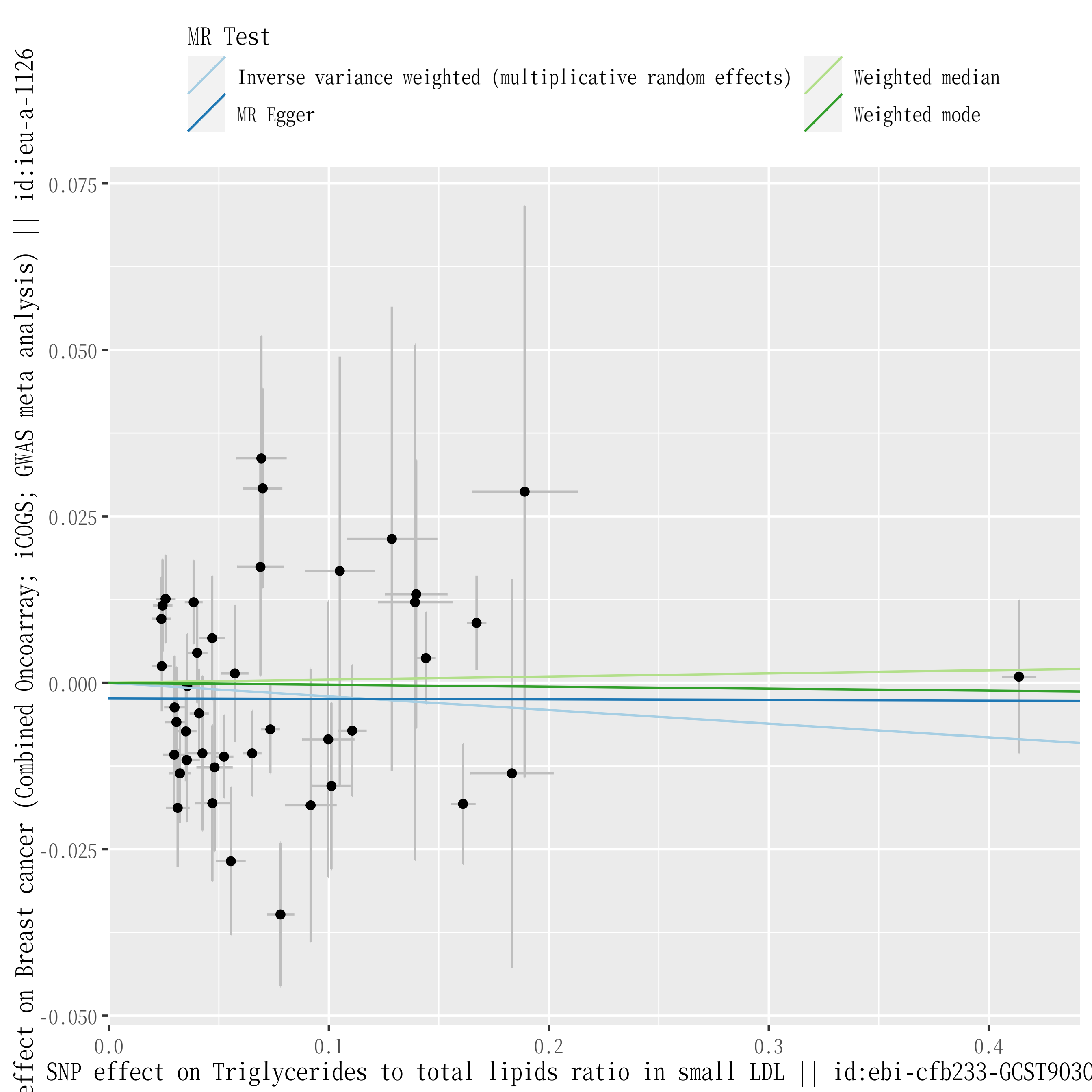
MR Test
Inverse variance weighted (multiplicative random effects)
MR Egger
Weighted median
Weighted mode
0.075
0.050
0.025
0.000
-0.025
-0.050
0.0
0.1
0.2
0.3
0.4
SNP effect on Triglycerides to total lipids ratio in small LDL || id:ebi-cfb233-GCST9030
effect on Breast cancer (Combined Oncoarray; iCOGS; GWAS meta analysis) || id:ieu-a-1126

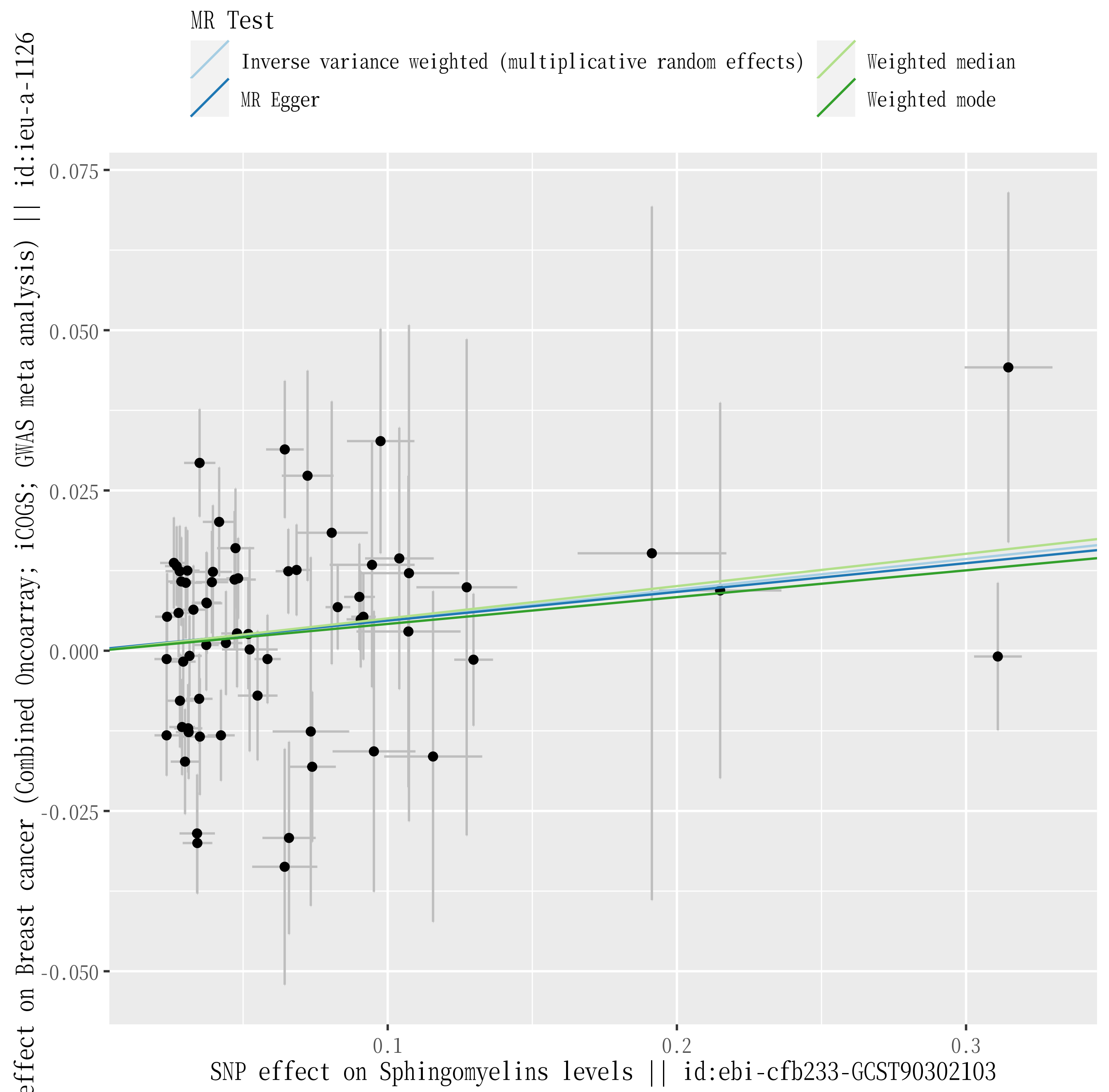
MR Test
Inverse variance weighted (multiplicative random effects)
MR Egger
Weighted median
Weighted mode
0.075
0.050
0.025
0.000
-0.025
-0.050
0.1
0.2
0.3
SNP effect on Sphingomyelins levels || id:ebi-cfb233-GCST90302103
effect on Breast cancer (Combined Oncoarray; iCOGS; GWAS meta analysis) || id:ieu-a-1126

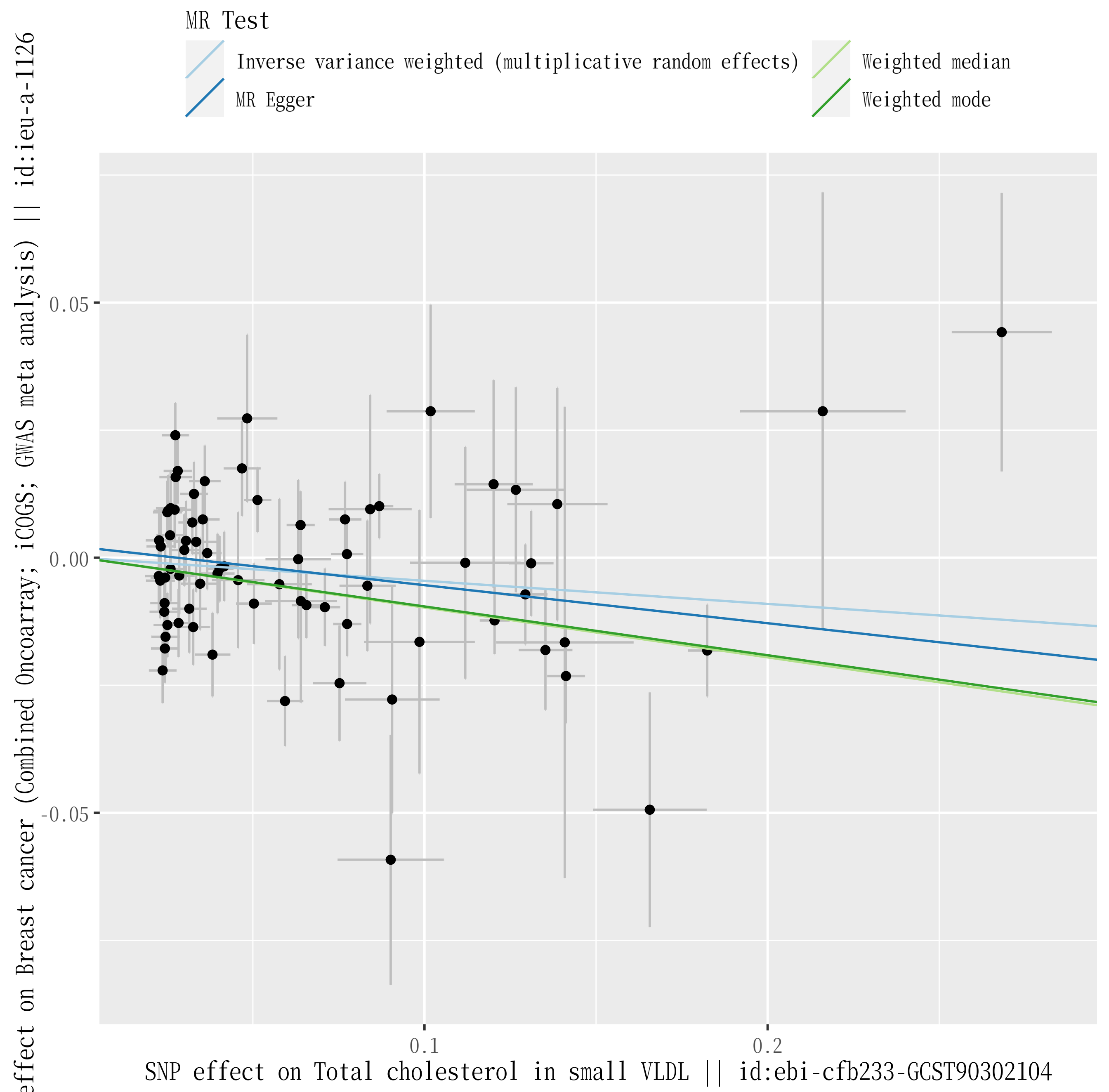

MR Test
Inverse variance weighted (multiplicative random effects)
MR Egger
Weighted median
Weighted mode
0.05
0.00
-0.05
0.1
0.2
SNP effect on Total cholesterol in small VLDL || id:ebi-cfb233-GCST90302104
effect on Breast cancer (Combined Oncoarray; iCOGS; GWAS meta analysis) || id:ieu-a-1126

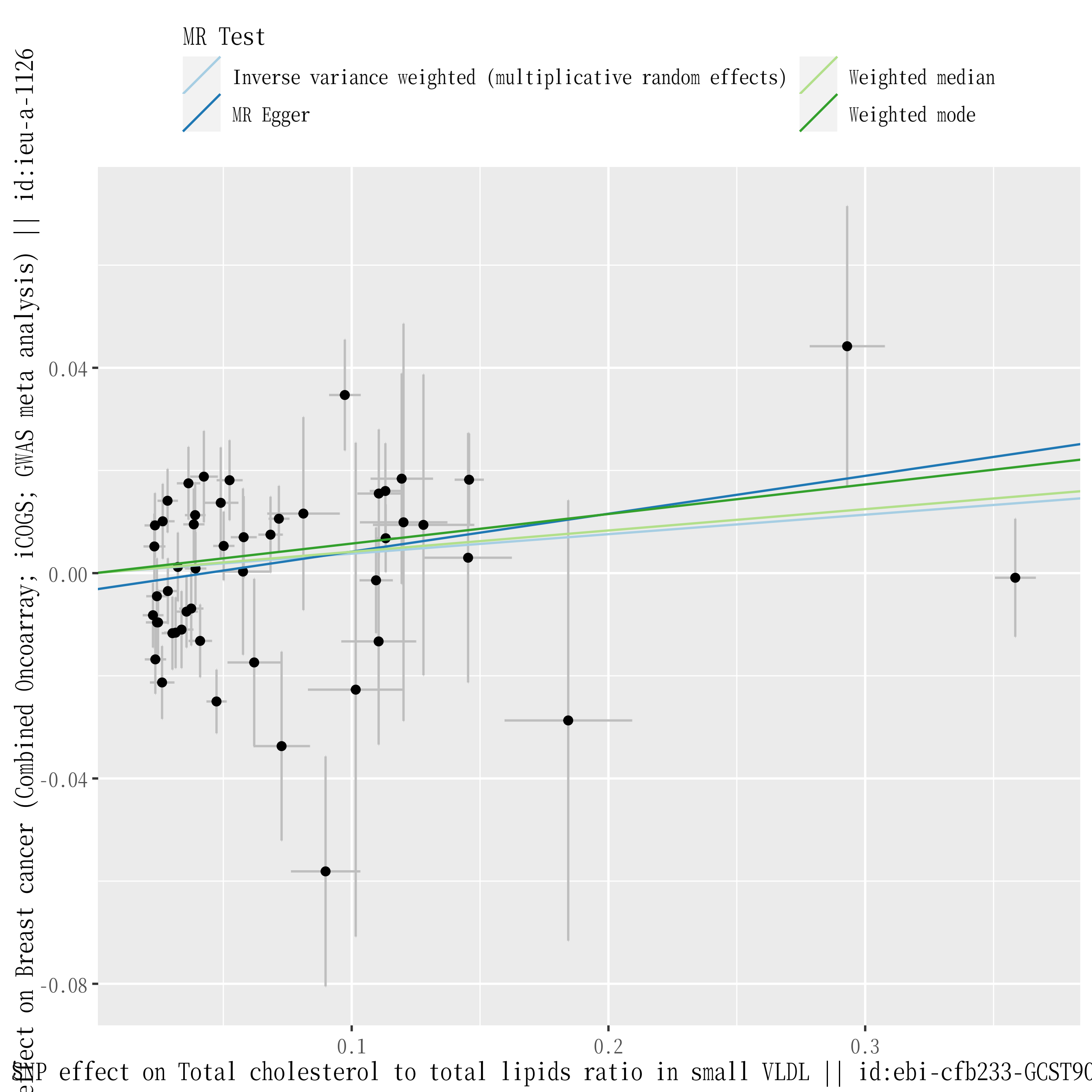

MR Test
Inverse variance weighted (multiplicative random effects)
MR Egger
Weighted median
Weighted mode
0.04
0.00
-0.04
-0.08
0.1
0.2
0.3
SNP effect on Total cholesterol to total lipids ratio in small VLDL || id:ebi-a-GCST90
SNP effect on Breast cancer (Combined Oncoarray; iCOGS; GWAS meta analysis) || id:ieu-a-1126

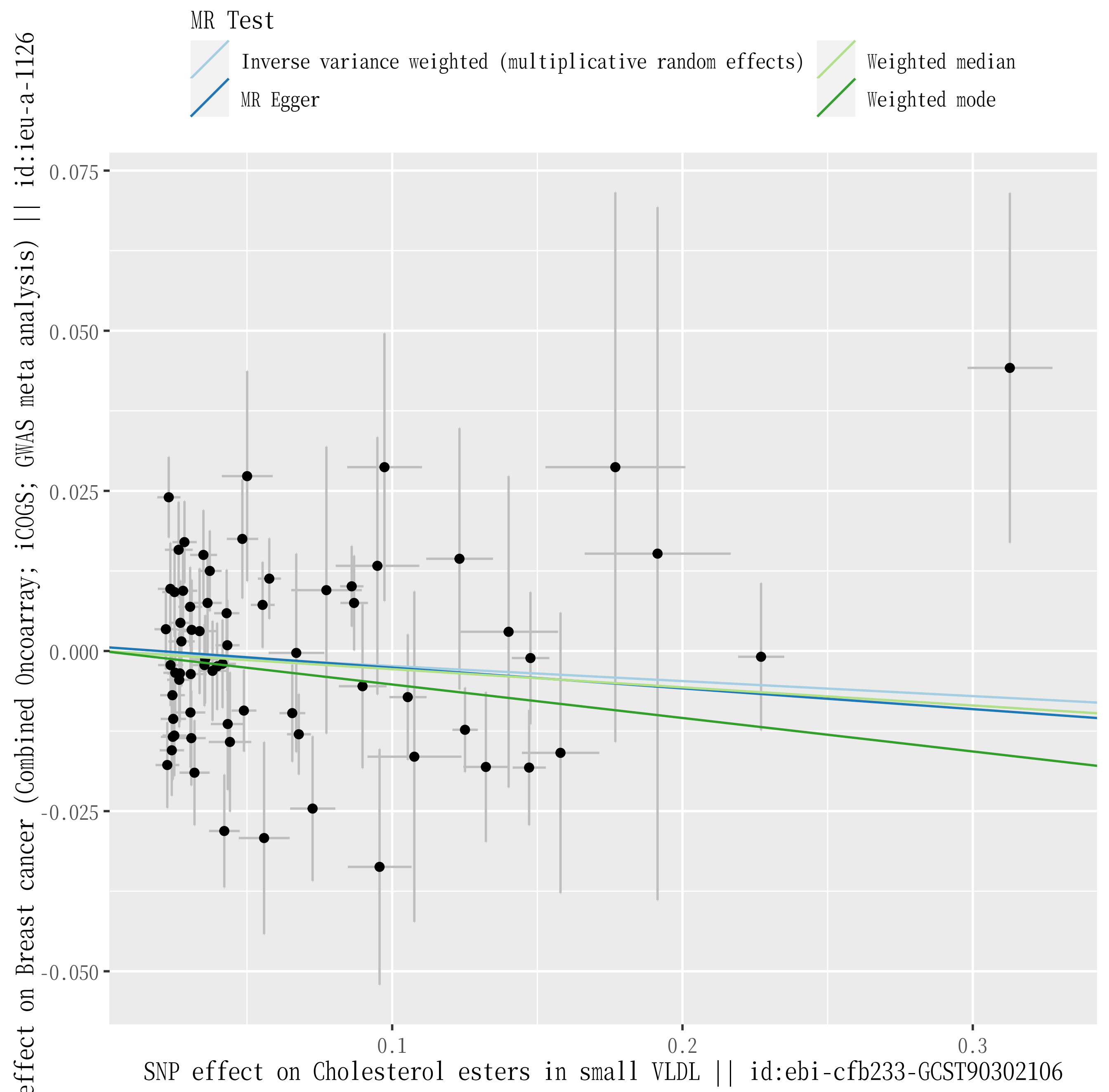

MR Test
Inverse variance weighted (multiplicative random effects)
MR Egger
Weighted median
Weighted mode
0.075
0.050
0.025
0.000
-0.025
-0.050
0.1
0.2
0.3
SNP effect on Cholesterol esters in small VLDL || id:ebi-cfb233-GCST90302106
effect on Breast cancer (Combined Oncoarray; iCOGS; GWAS meta analysis) || id:ieu-a-1126

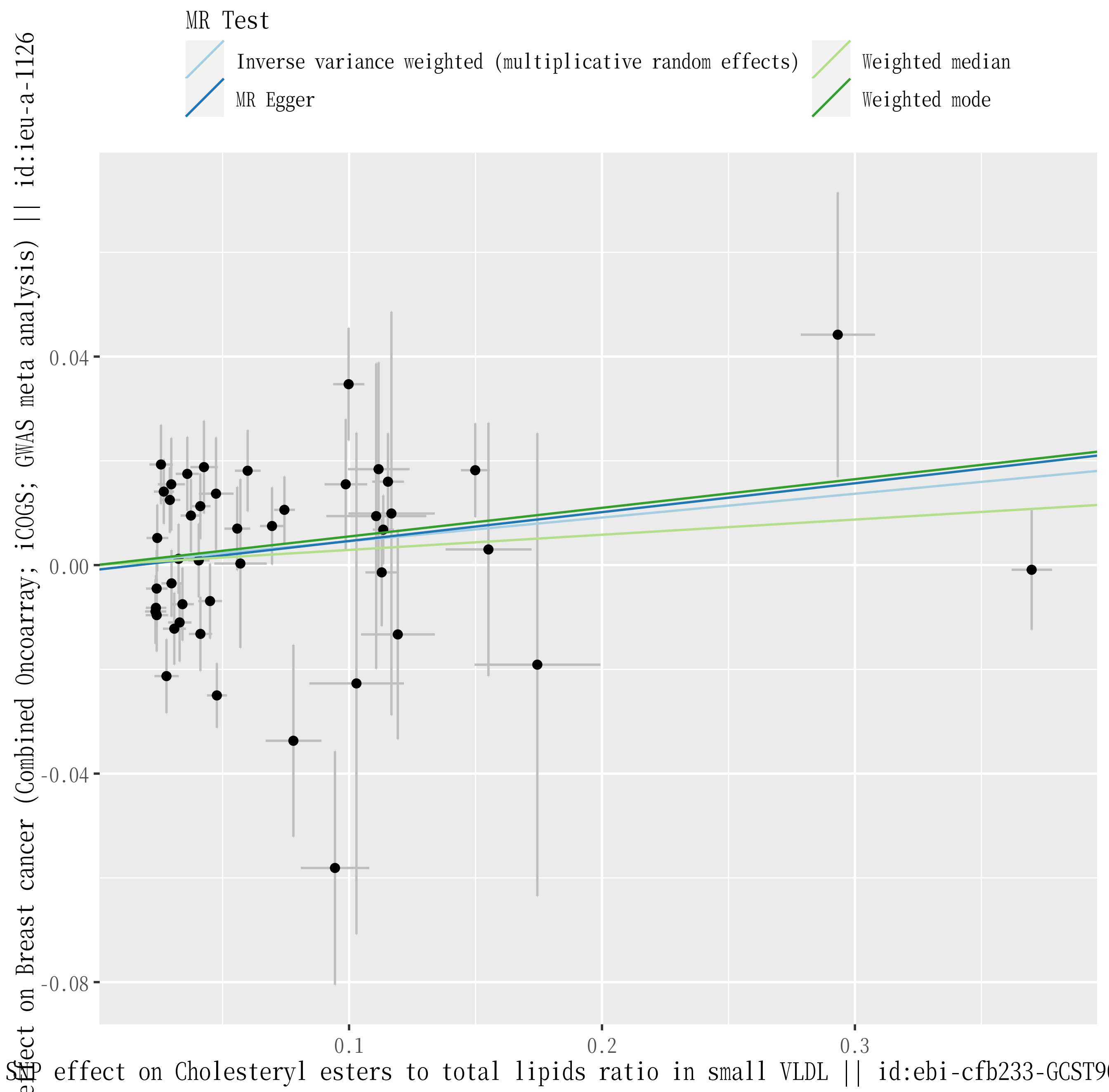
MR Test
Inverse variance weighted (multiplicative random effects)
MR Egger
Weighted median
Weighted mode
0.04
0.00
-0.04
-0.08
0.1
0.2
0.3
SNP effect on Cholesteryl esters to total lipids ratio in small VLDL || id:ebi-cfb233-GCST90
effect on Breast cancer (Combined Oncoarray; iCOGS; GWAS meta analysis) || id:ieu-a-1126

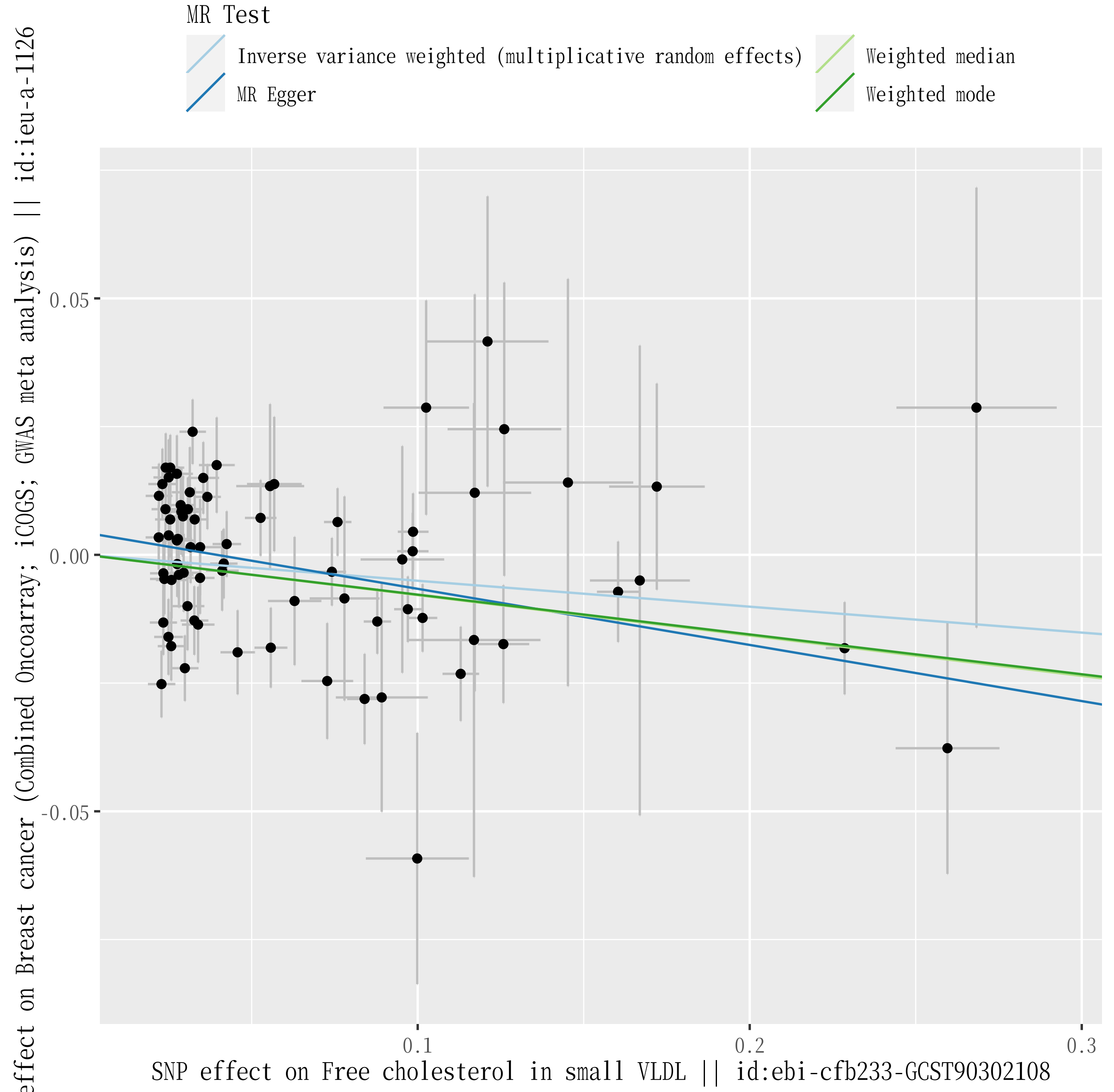
MR Test
Inverse variance weighted (multiplicative random effects)
MR Egger
Weighted median
Weighted mode
0.05
0.00
-0.05
0.1
0.2
0.3
SNP effect on Free cholesterol in small VLDL || id:ebi-cfb233-GCST90302108
effect on Breast cancer (Combined Oncoarray; iCOGS; GWAS meta analysis) || id:ieu-a-1126

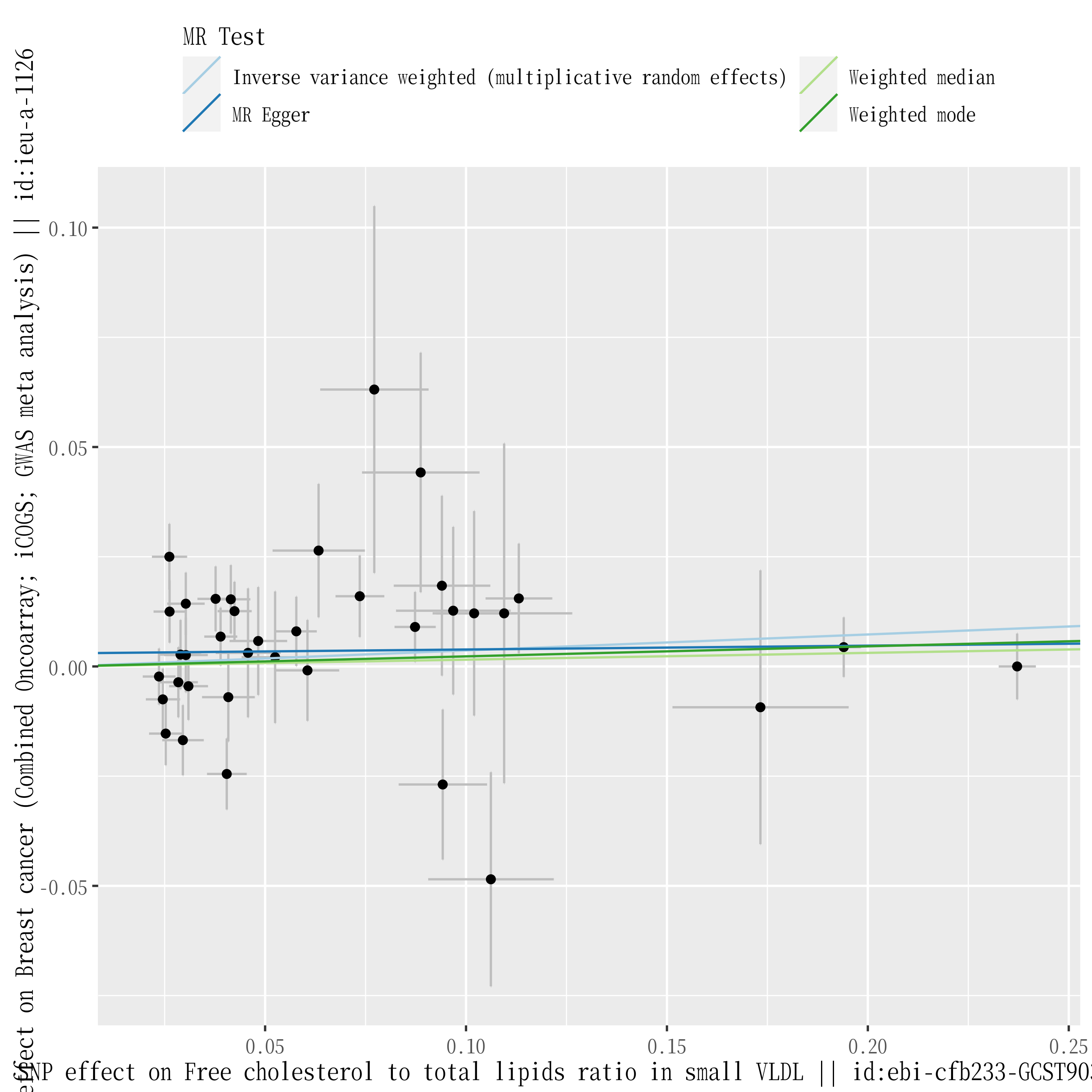
MR Test
Inverse variance weighted (multiplicative random effects)
MR Egger
Weighted median
Weighted mode
0.10
0.05
0.00
-0.05
0.05
0.10
0.15
0.20
SNP effect on Free cholesterol to total lipids ratio in small VLDL || id:ebi-cfb233-GCST90
SNP effect on Breast cancer (Combined Oncoarray; iCOGS; GWAS meta analysis) || id:ieu-a-1126

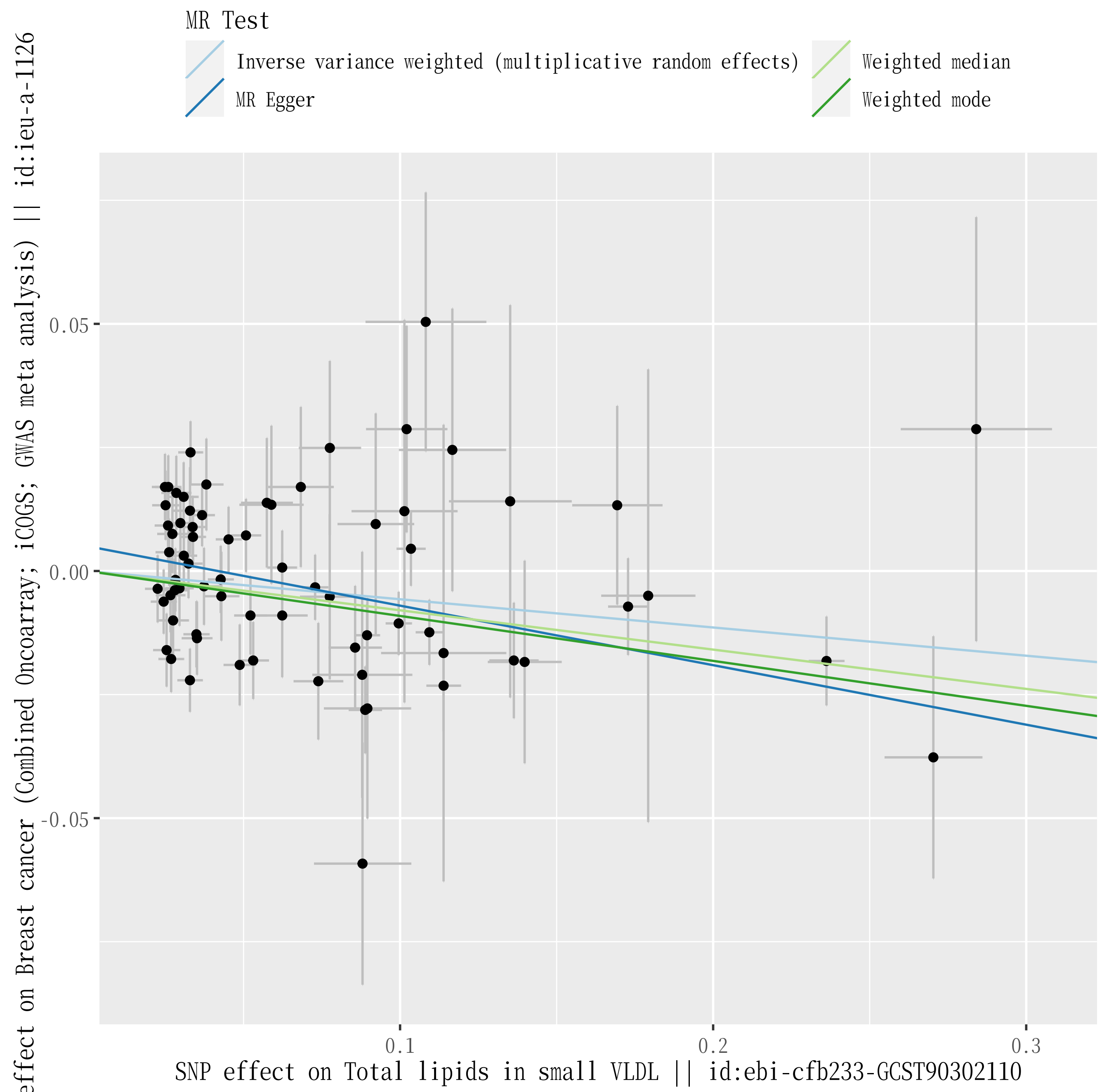

MR Test
Inverse variance weighted (multiplicative random effects)
MR Egger
Weighted median
Weighted mode
SNP effect on Breast cancer (Combined Oncoarray; iCOGS; GWAS meta analysis) || id:ieu-a-1126
0.05
0.00
-0.05
0.1
0.2
0.3
SNP effect on Total lipids in small VLDL || id:ebi-cfb233-GCST90302110

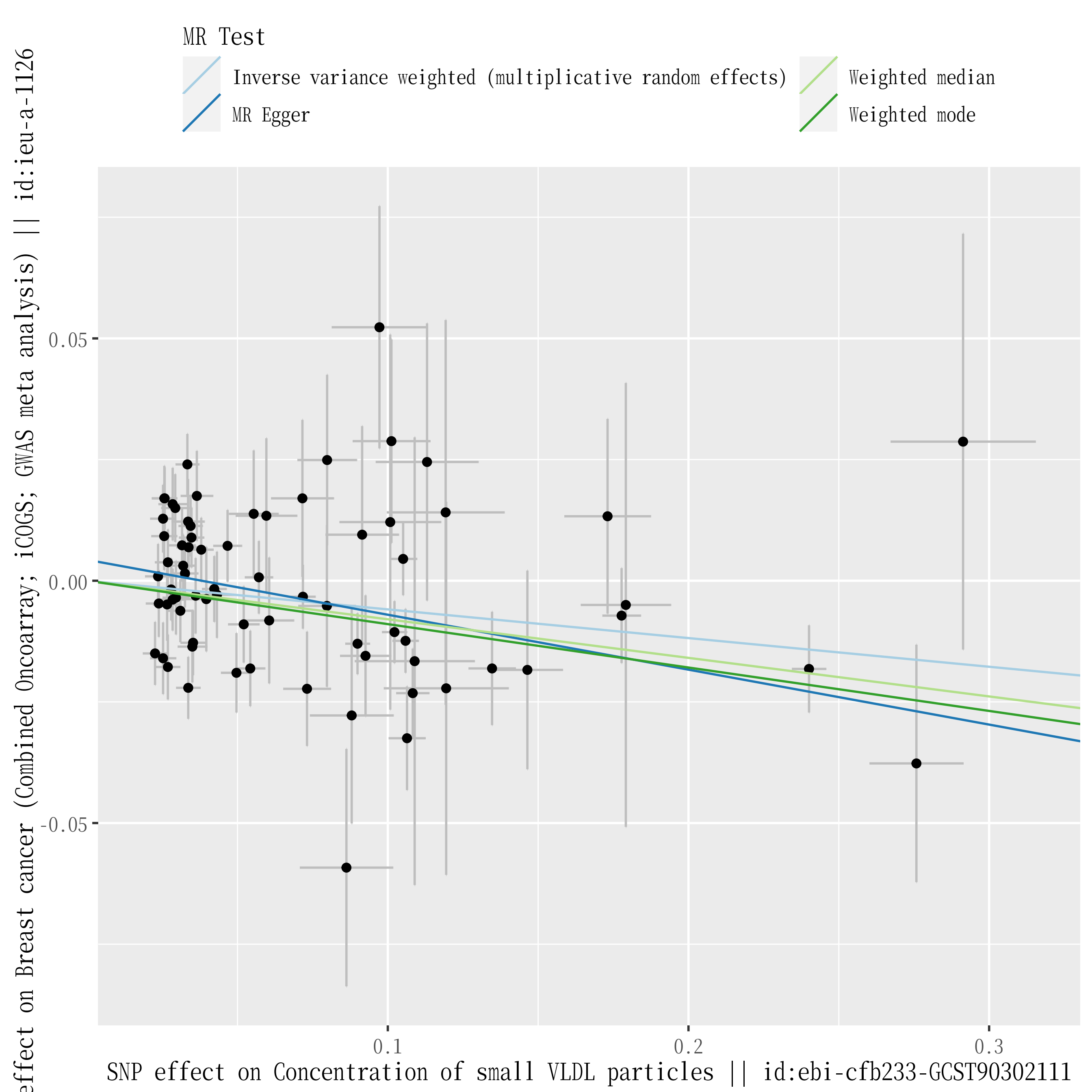
MR Test
Inverse variance weighted (multiplicative random effects)
MR Egger
Weighted median
Weighted mode
0.05
0.00
-0.05
0.1
0.2
0.3
SNP effect on Concentration of small VLDL particles || id:ebi-cfb233-GCST90302111
effect on Breast cancer (Combined Oncoarray; iCOGS; GWAS meta analysis) || id:ieu-a-1126

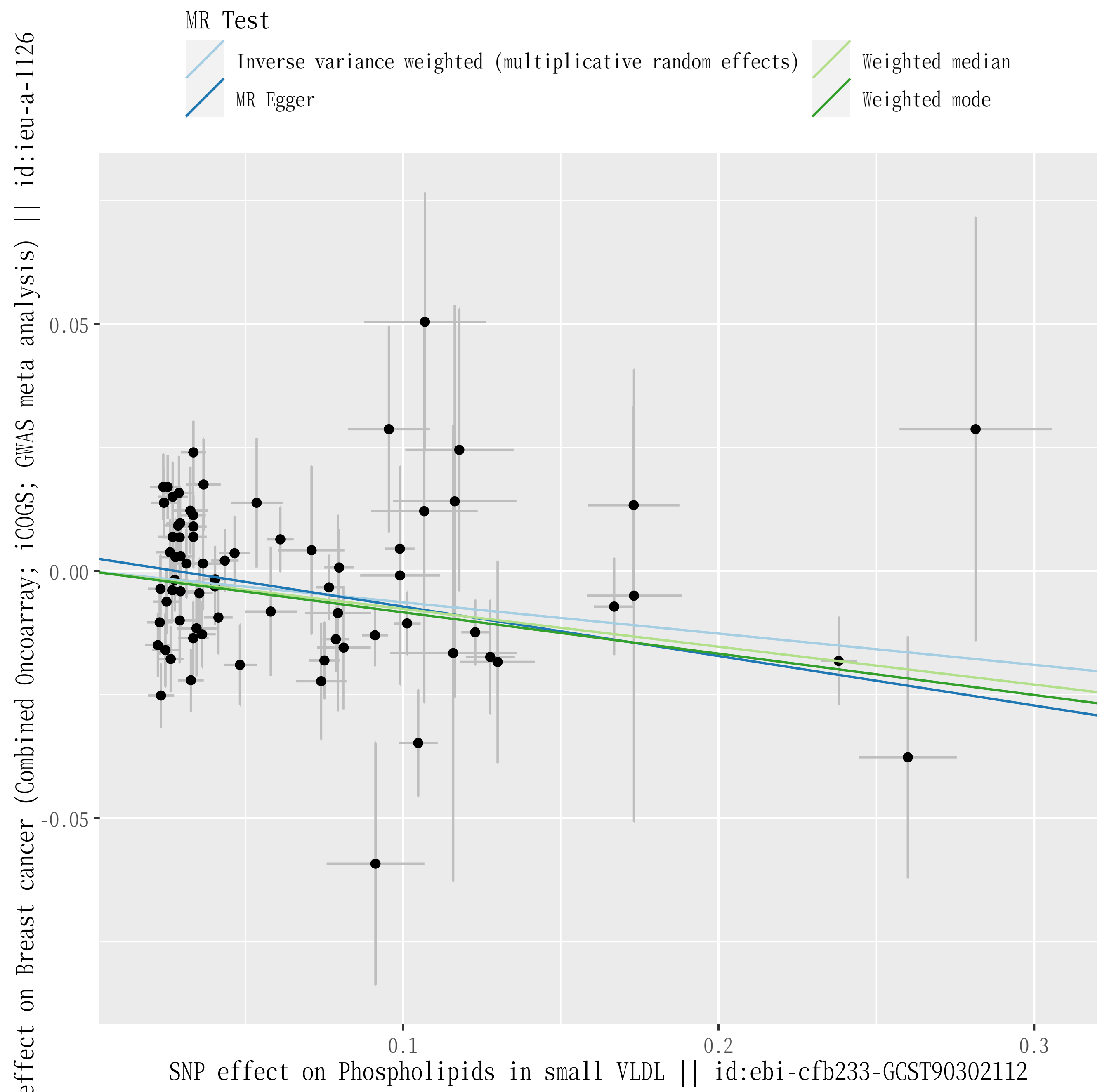
MR Test
Inverse variance weighted (multiplicative random effects)
MR Egger
Weighted median
Weighted mode
0.05
0.00
-0.05
0.1
0.2
0.3
SNP effect on Phospholipids in small VLDL || id:ebi-cfb233-GCST90302112
effect on Breast cancer (Combined Oncoarray; iCOGS; GWAS meta analysis) || id:ieu-a-1126

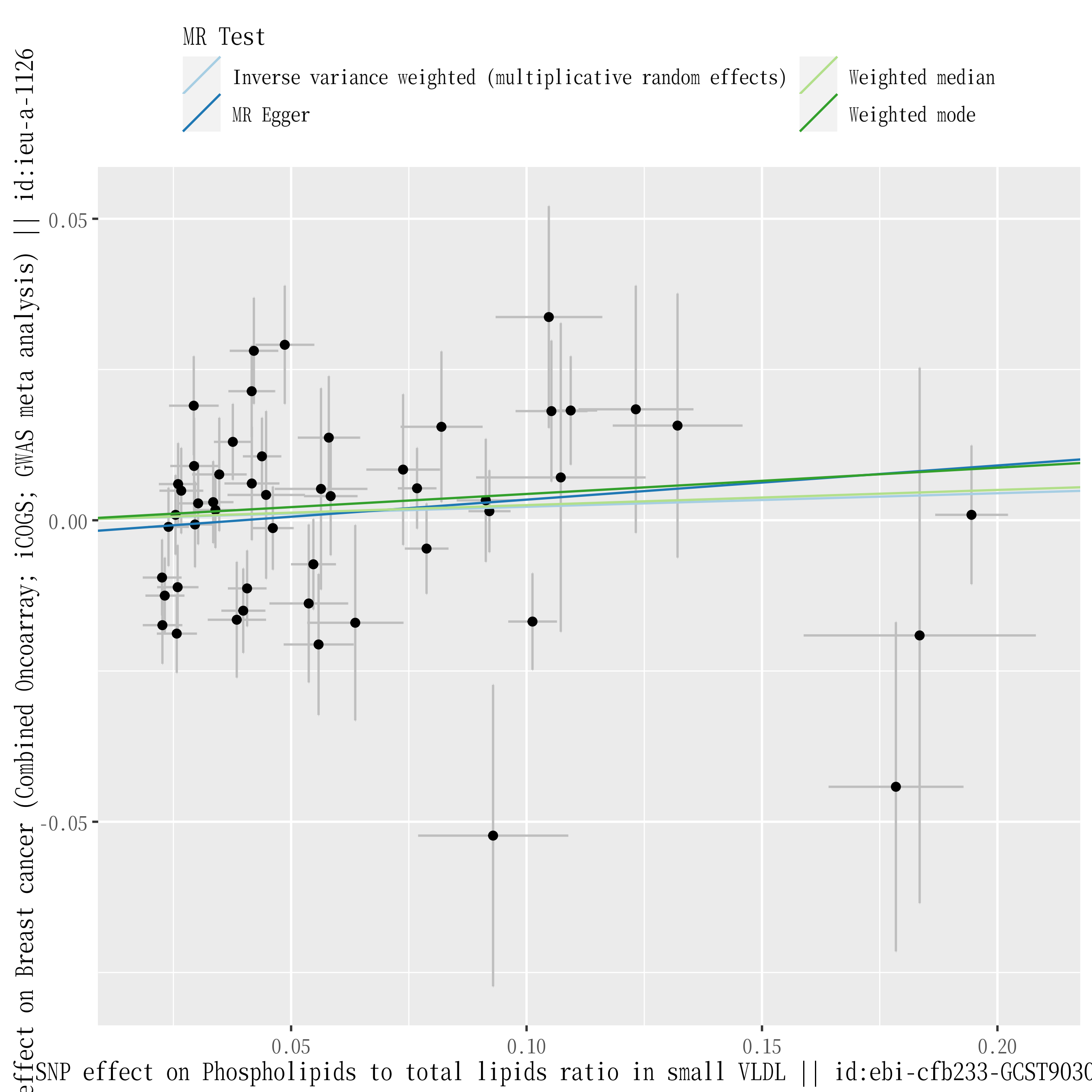

MR Test
Inverse variance weighted (multiplicative random effects)
MR Egger
Weighted median
Weighted mode
0.05
0.00
-0.05
0.05
0.10
0.15
0.20
SNP effect on Phospholipids to total lipids ratio in small VLDL || id:ebi-cfb233-GCST9030
effect on Breast cancer (Combined Oncoarray; iCOGS; GWAS meta analysis) || id:ieu-a-1126

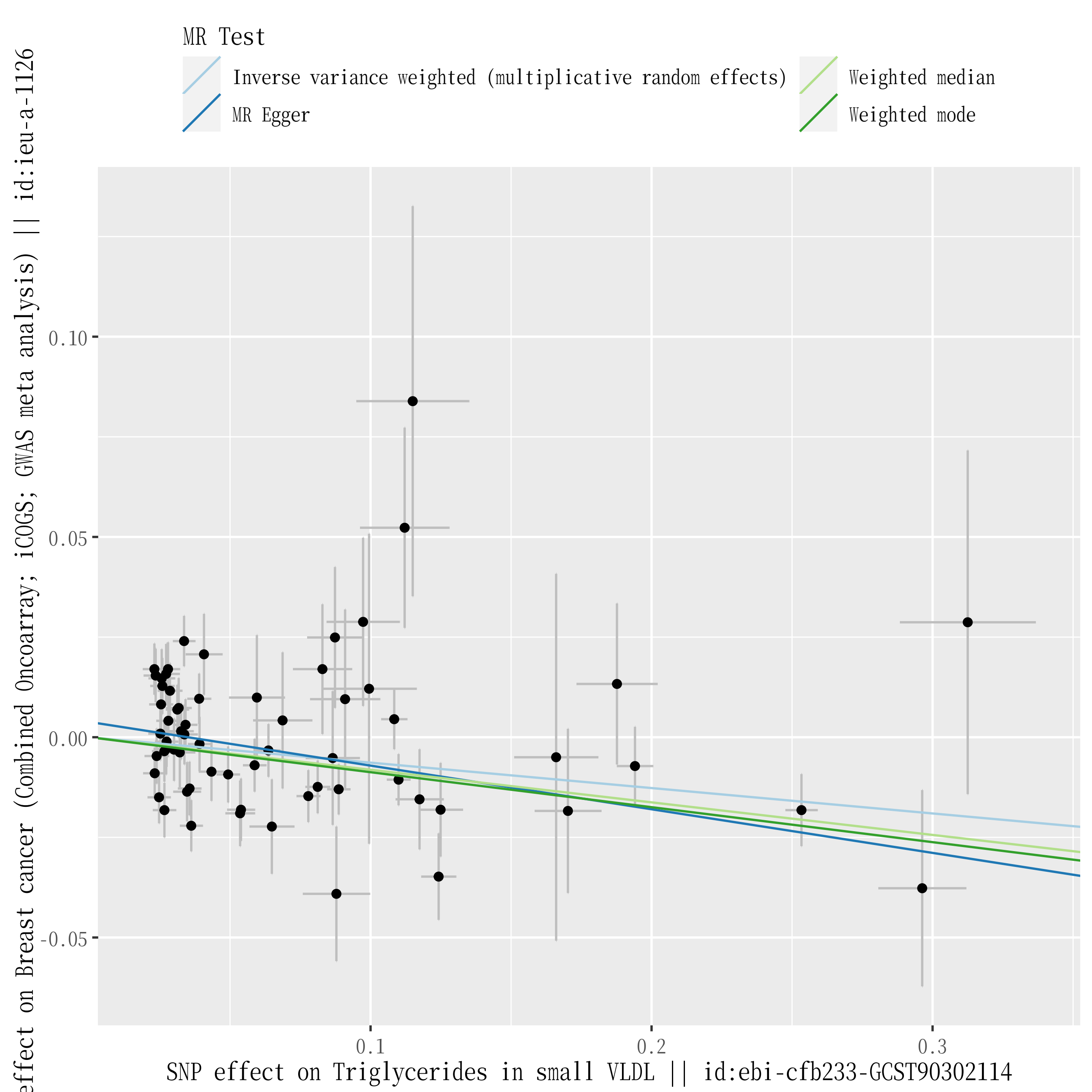
MR Test
Inverse variance weighted (multiplicative random effects)
MR Egger
Weighted median
Weighted mode
0.10
0.05
0.00
-0.05
0.1
0.2
0.3
SNP effect on Triglycerides in small VLDL || id:ebi-cfb233-GCST90302114
effect on Breast cancer (Combined Oncoarray; iCOGS; GWAS meta analysis) || id:ieu-a-1126

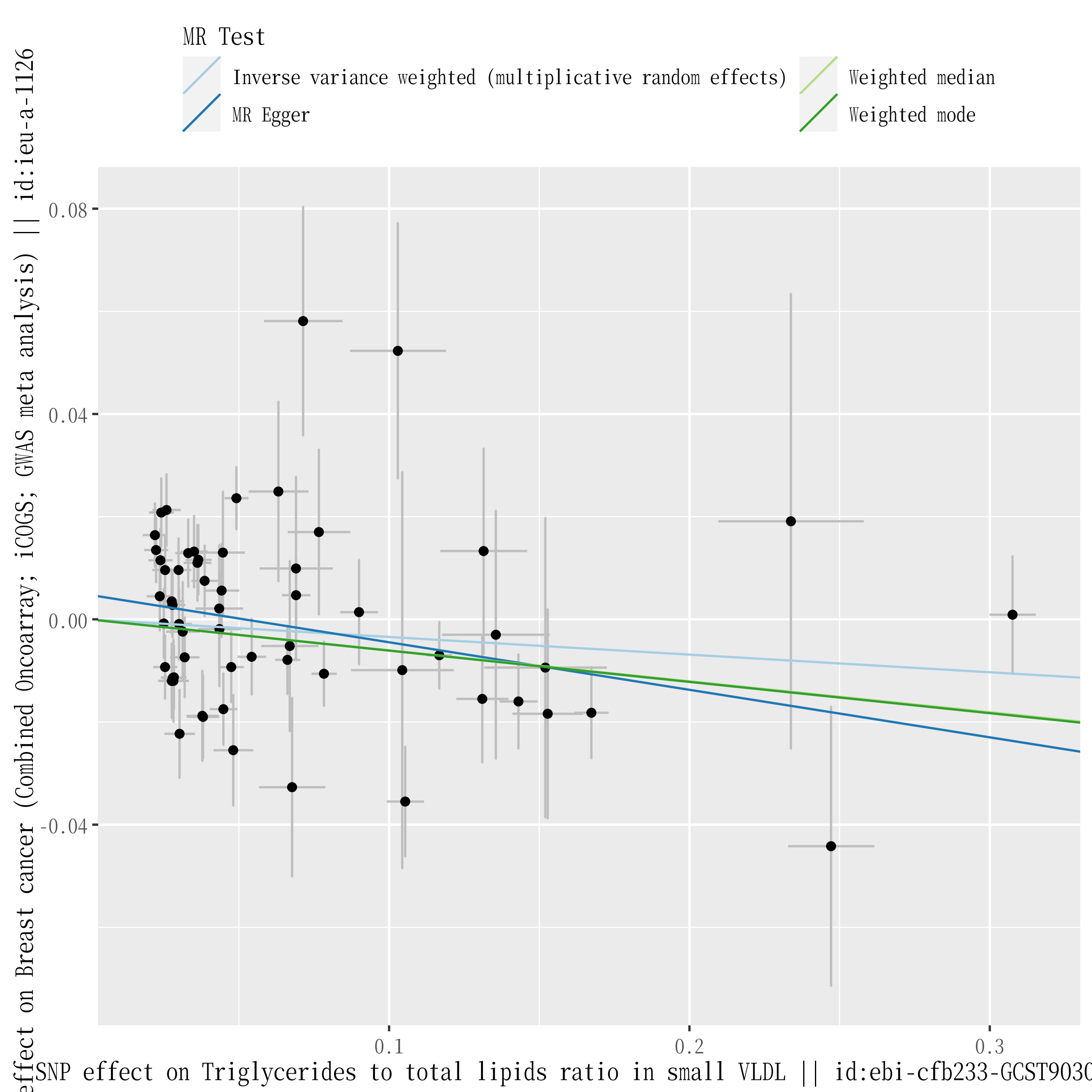
MR Test
Inverse variance weighted (multiplicative random effects)
MR Egger
Weighted median
Weighted mode
0.08
0.04
0.00
-0.04
0.1
0.2
0.3
SNP effect on Triglycerides to total lipids ratio in small VLDL || id:ebi-cfb233-GCST9030
effect on Breast cancer (Combined Oncoarray; iCOGS; GWAS meta analysis) || id:ieu-a-1126

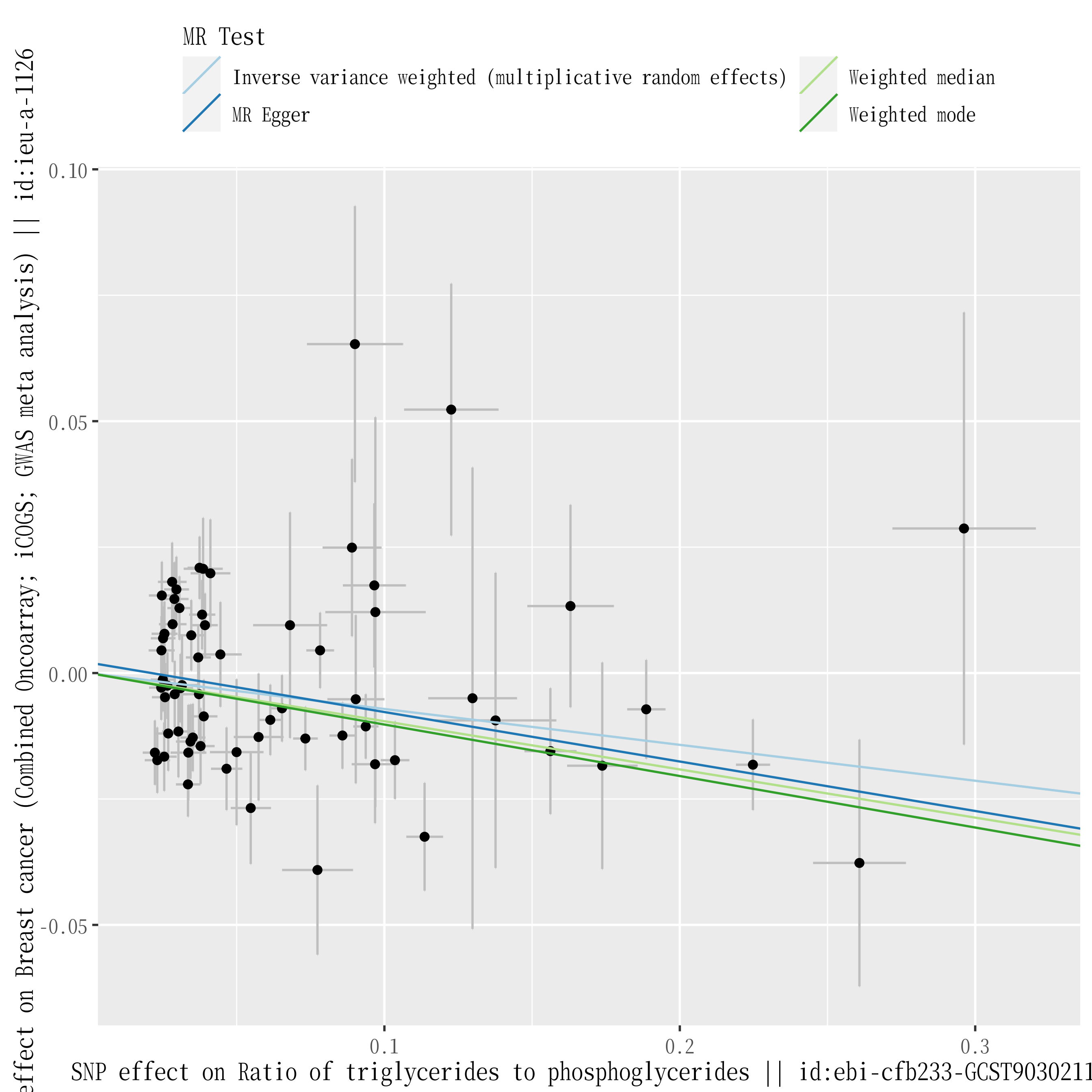
MR Test
Inverse variance weighted (multiplicative random effects)
MR Egger
Weighted median
Weighted mode
0.10
0.05
0.00
-0.05
0.1
0.2
0.3
SNP effect on Ratio of triglycerides to phosphoglycerides || id:ebi-cfb233-GCST9030211
effect on Breast cancer (Combined Oncoarray; iCOGS; GWAS meta analysis) || id:ieu-a-1126

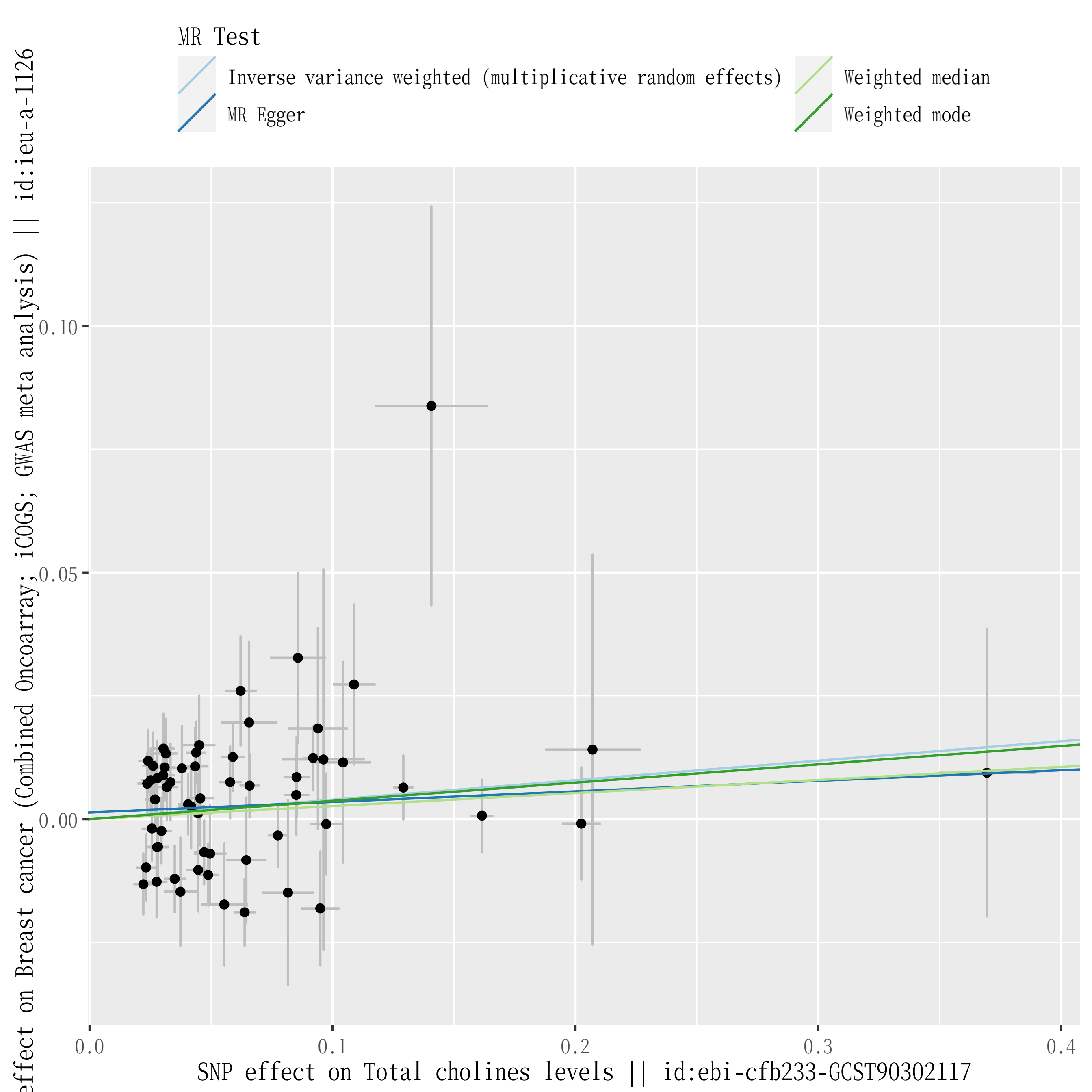
MR Test
Inverse variance weighted (multiplicative random effects)
MR Egger
Weighted median
Weighted mode
0.10
0.05
0.00
0.0
0.1
0.2
0.3
0.4
SNP effect on Total cholines levels || id:ebi-cfb233-GCST90302117
effect on Breast cancer (Combined Oncoarray; iCOGS; GWAS meta analysis) || id:ieu-a-1126

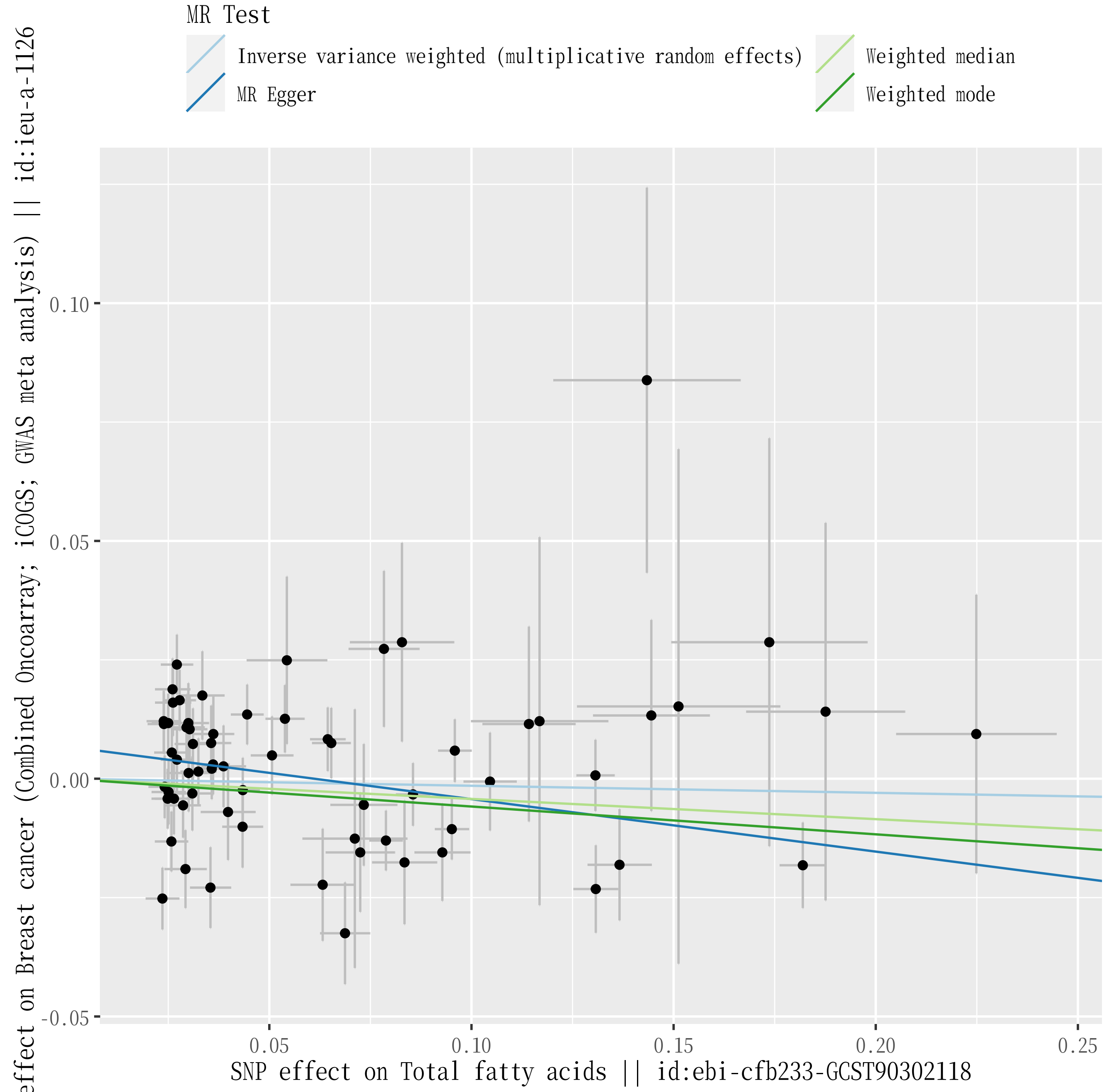

MR Test
Inverse variance weighted (multiplicative random effects)
MR Egger
Weighted median
Weighted mode
0.10
0.05
0.00
-0.05
0.05
0.10
0.15
0.20
0.25
SNP effect on Total fatty acids || id:ebi-cfb233-GCST90302118
effect on Breast cancer (Combined Oncoarray; iCOGS; GWAS meta analysis) || id:ieu-a-1126

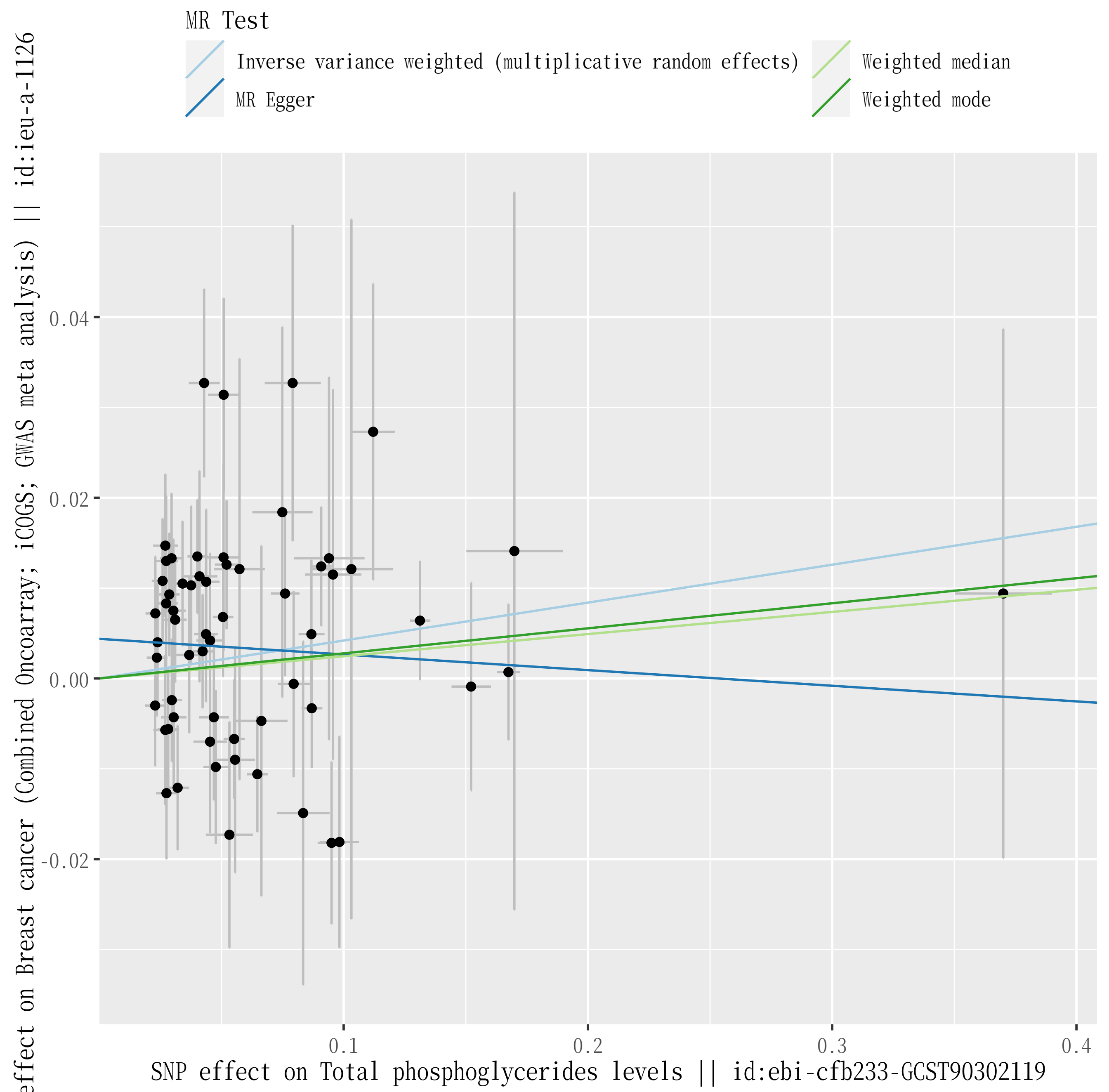

MR Test
Inverse variance weighted (multiplicative random effects)
MR Egger
Weighted median
Weighted mode
0.04
0.02
0.00
-0.02
0.1
0.2
0.3
0.4
SNP effect on Total phosphoglycerides levels || id:ebi-cfb233-GCST90302119
effect on Breast cancer (Combined Oncoarray; iCOGS; GWAS meta analysis) || id:ieu-a-1126

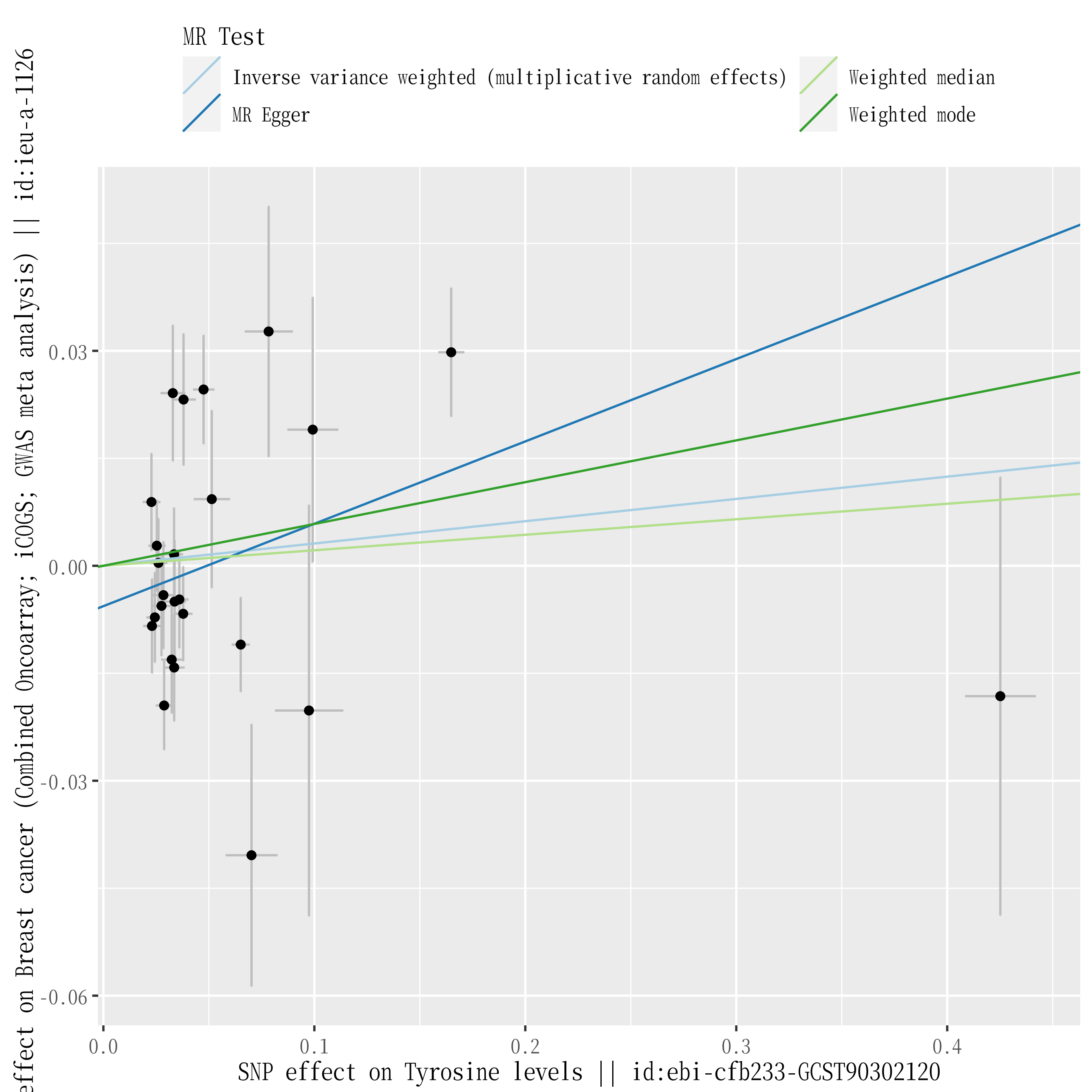

MR Test
Inverse variance weighted (multiplicative random effects)
MR Egger
Weighted median
Weighted mode
0.03
0.00
-0.03
-0.06
0.0
0.1
0.2
0.3
0.4
SNP effect on Tyrosine levels || id:ebi-cfb233-GCST90302120
effect on Breast cancer (Combined Oncoarray; iCOGS; GWAS meta analysis) || id:ieu-a-1126

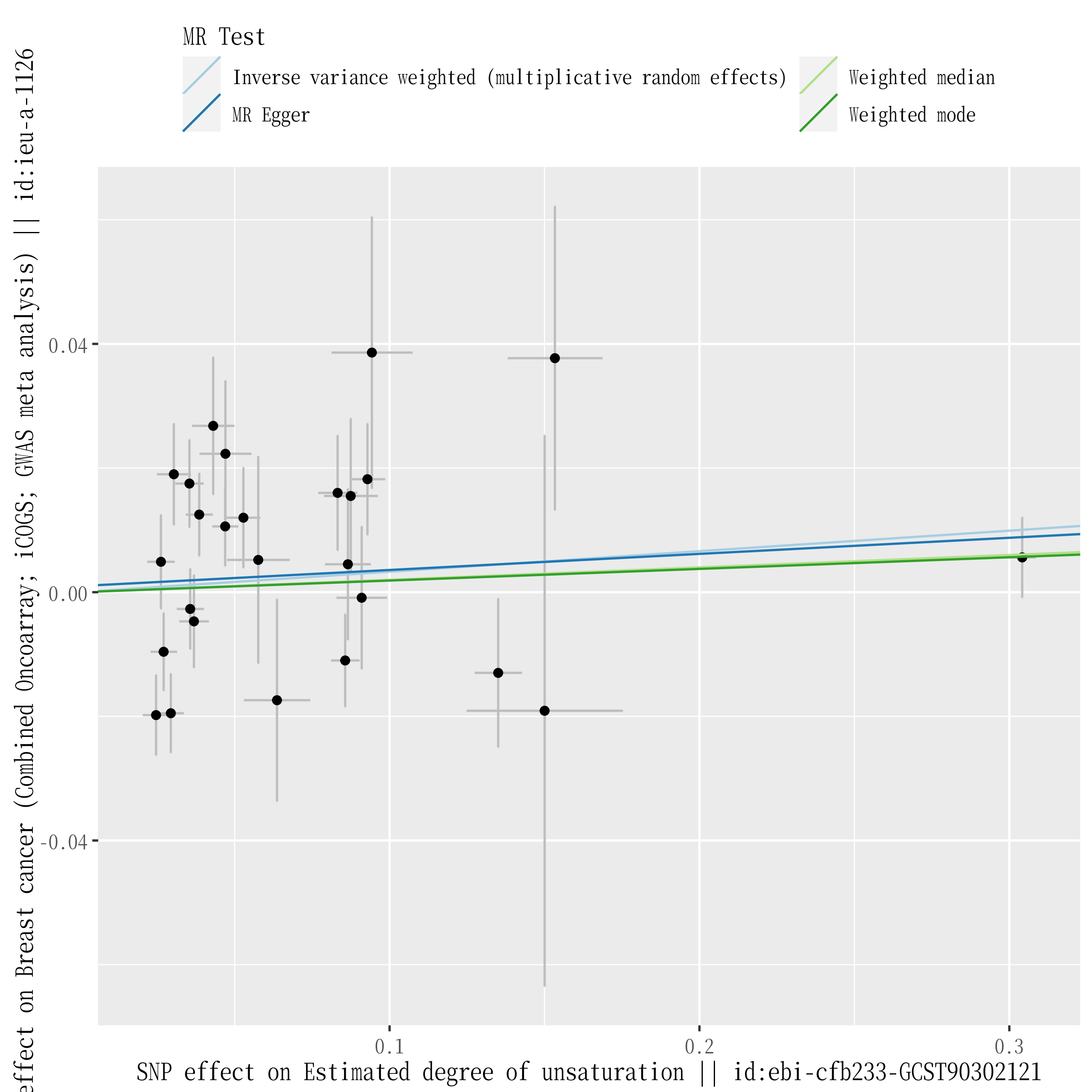

MR Test
Inverse variance weighted (multiplicative random effects)
MR Egger
Weighted median
Weighted mode
0.04
0.00
-0.04
0.1
0.2
0.3
SNP effect on Estimated degree of unsaturation || id:ebi-cfb233-GCST90302121
effect on Breast cancer (Combined Oncoarray; iCOGS; GWAS meta analysis) || id:ieu-a-1126

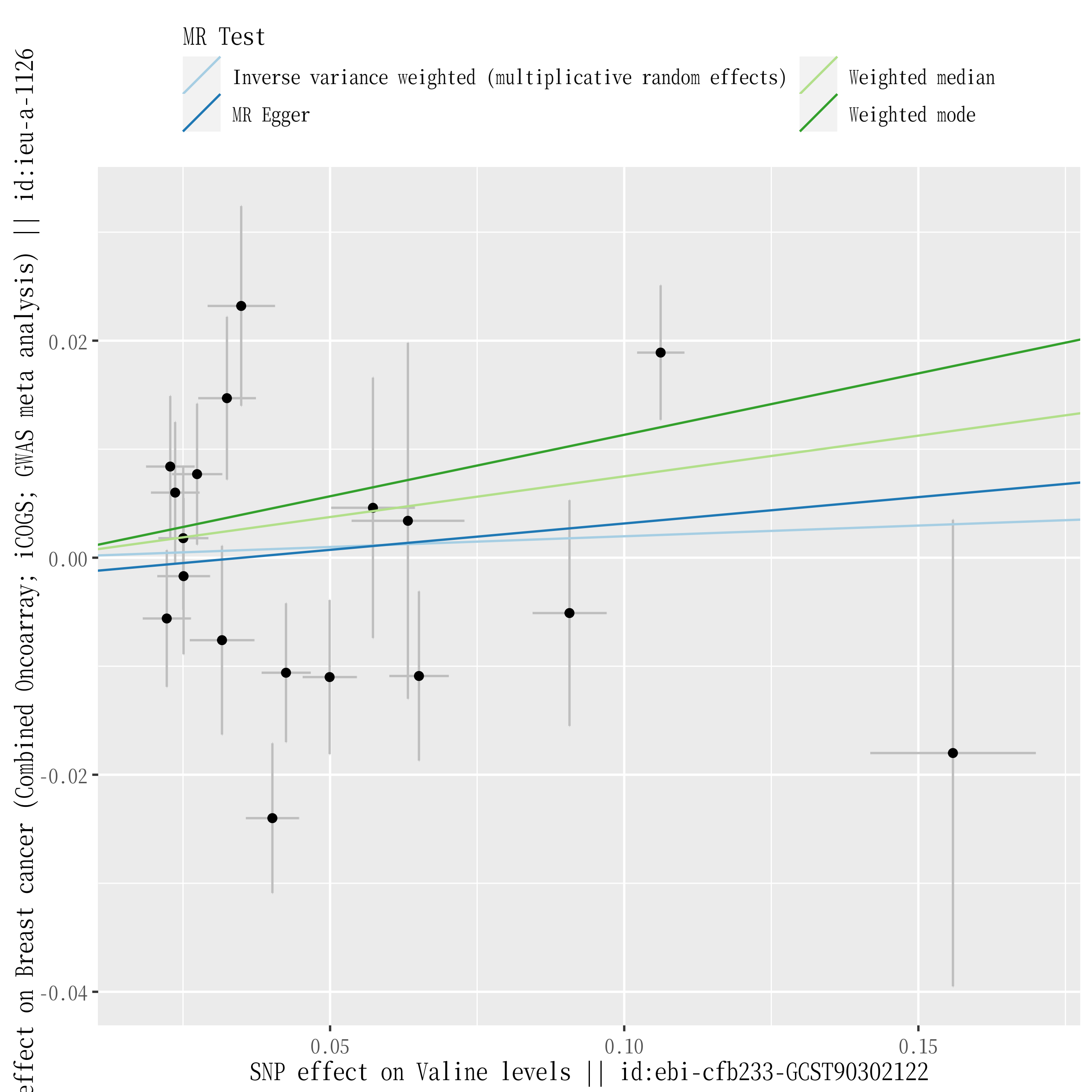

MR Test
Inverse variance weighted (multiplicative random effects)
MR Egger
Weighted median
Weighted mode
0.02
0.00
-0.02
-0.04
0.05
0.10
0.15
SNP effect on Valine levels || id:ebi-cfb233-GCST90302122
effect on Breast cancer (Combined Oncoarray; iCOGS; GWAS meta analysis) || id:ieu-a-1126

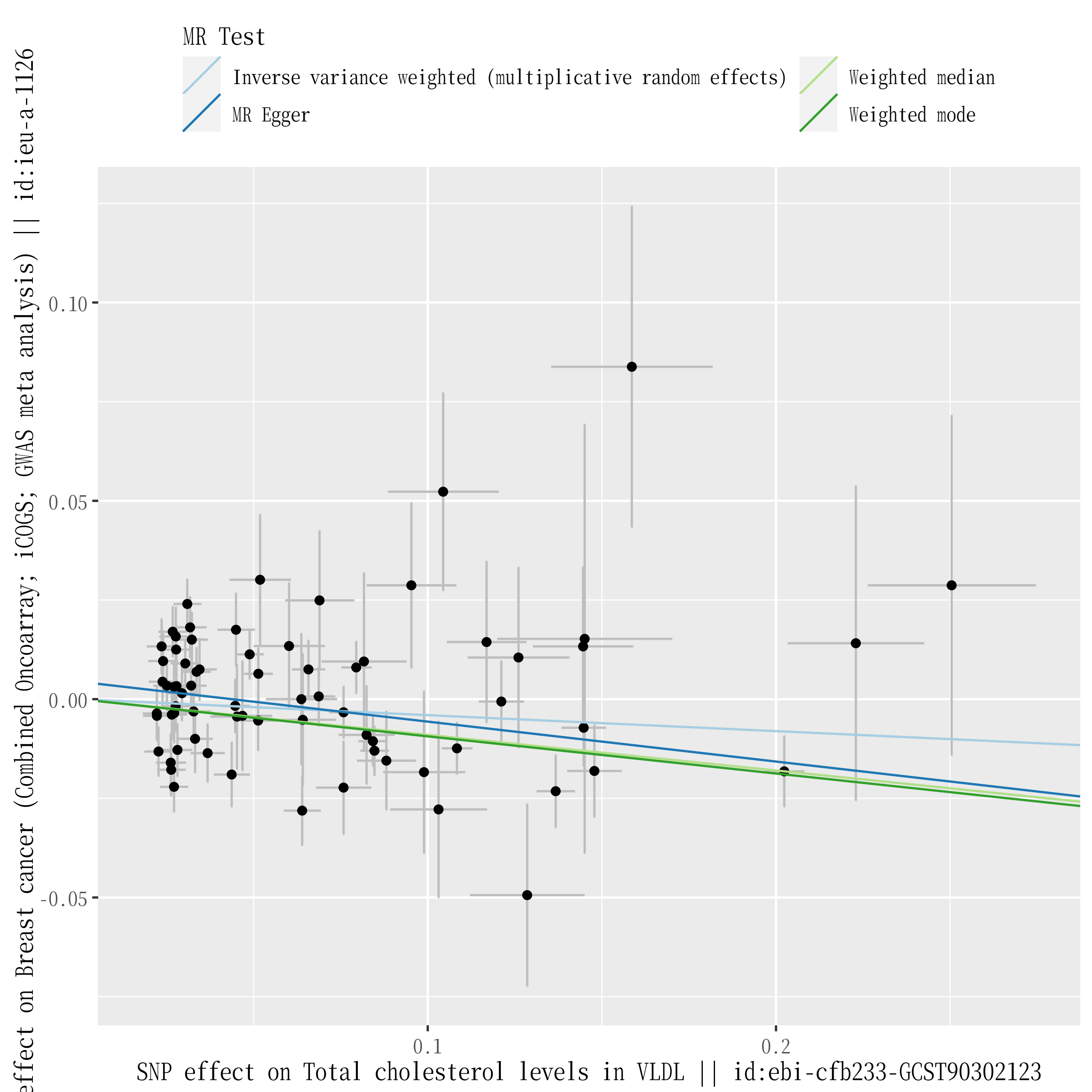
MR Test
Inverse variance weighted (multiplicative random effects)
MR Egger
Weighted median
Weighted mode
0.10
0.05
0.00
-0.05
0.1
0.2
SNP effect on Total cholesterol levels in VLDL || id:ebi-cfb233-GCST90302123
effect on Breast cancer (Combined Oncoarray; iCOGS; GWAS meta analysis) || id:ieu-a-1126

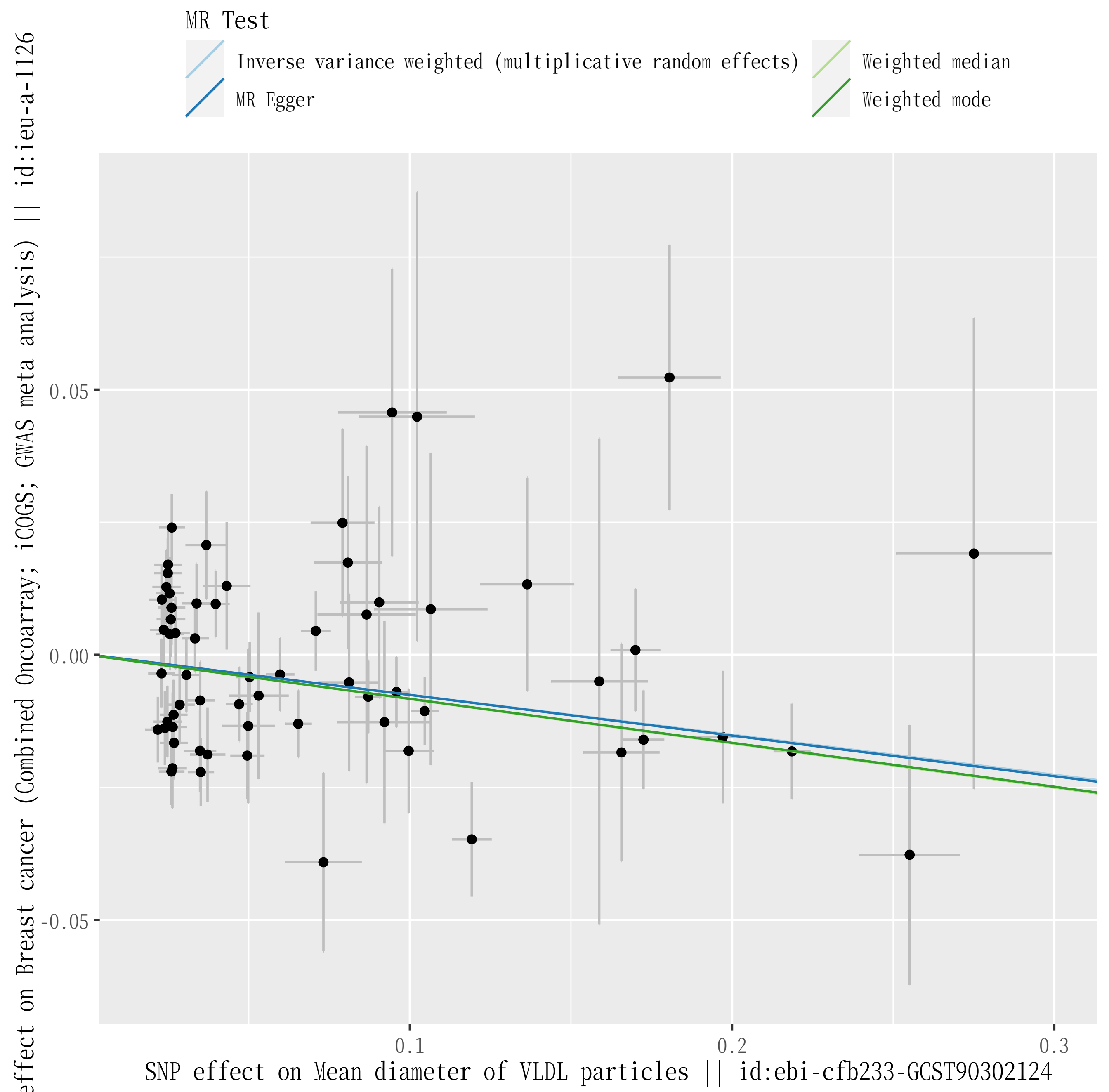

MR Test
Inverse variance weighted (multiplicative random effects)
MR Egger
Weighted median
Weighted mode
0.05
0.00
-0.05
0.1
0.2
0.3
SNP effect on Mean diameter of VLDL particles || id:ebi-cfb233-GCST90302124
effect on Breast cancer (Combined Oncoarray; iCOGS; GWAS meta analysis) || id:ieu-a-1126

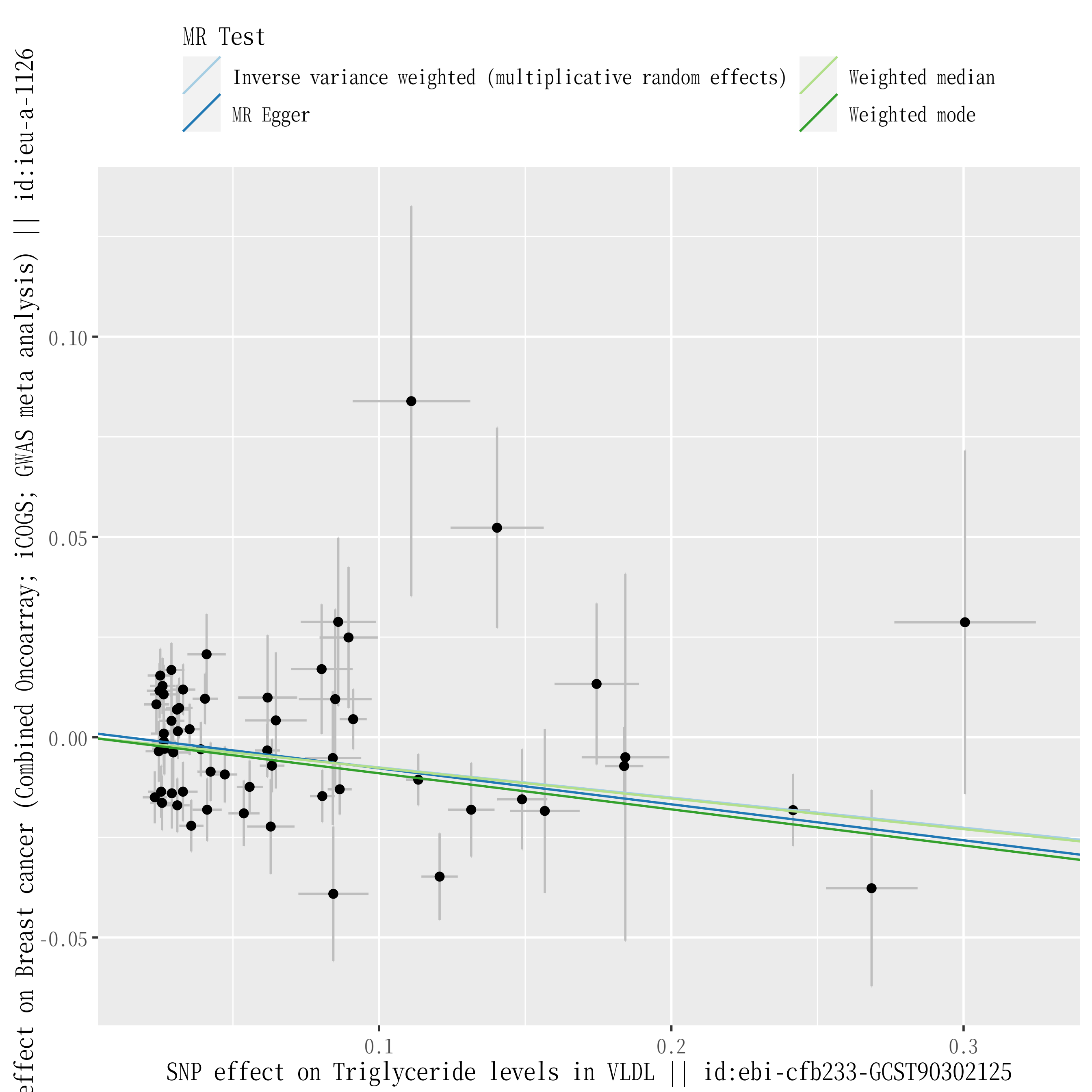

MR Test
Inverse variance weighted (multiplicative random effects)
MR Egger
Weighted median
Weighted mode
0.10
0.05
0.00
-0.05
0.1
0.2
0.3
SNP effect on Triglyceride levels in VLDL || id:ebi-cfb233-GCST90302125
effect on Breast cancer (Combined Oncoarray; iCOGS; GWAS meta analysis) || id:ieu-a-1126

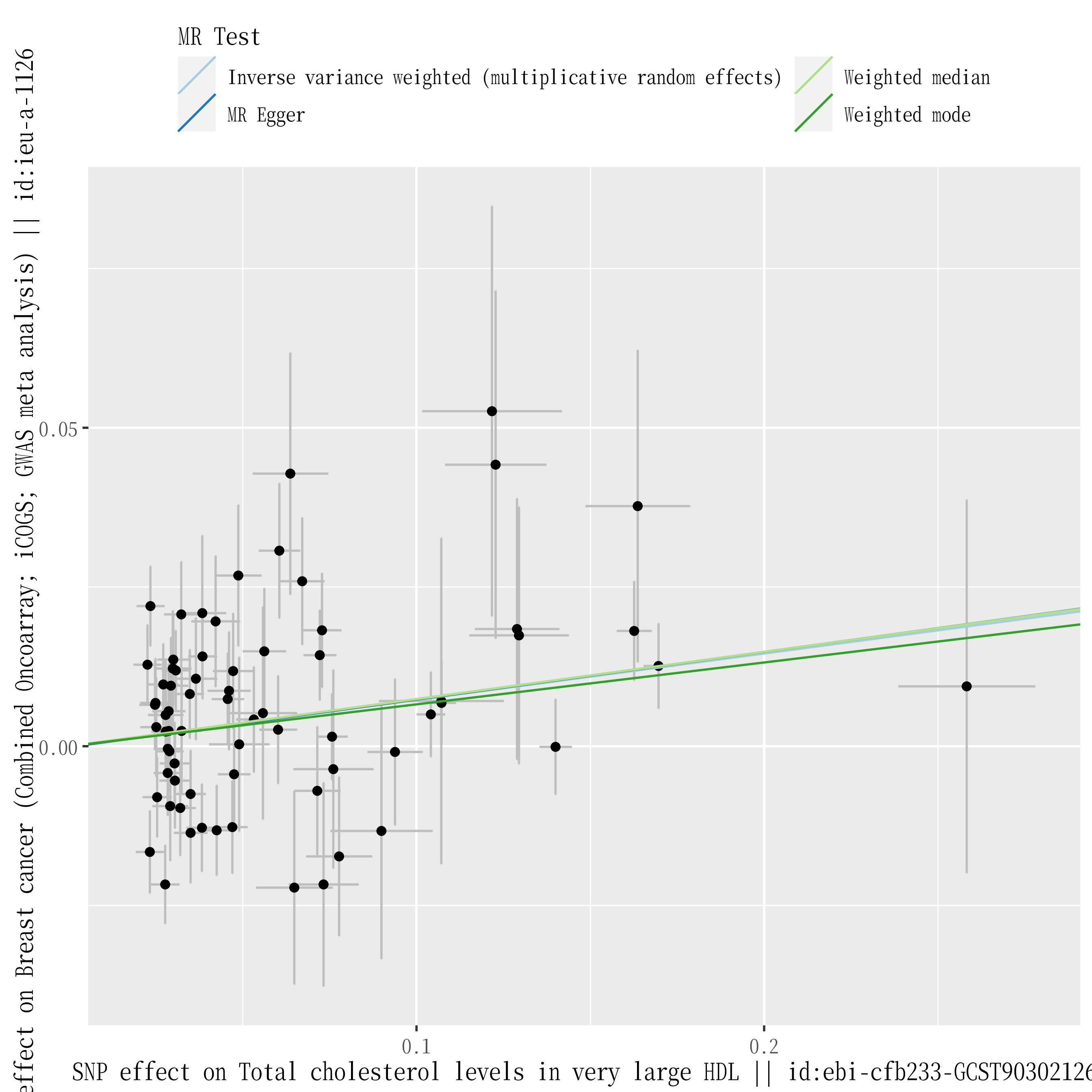
MR Test
Inverse variance weighted (multiplicative random effects)
MR Egger
Weighted median
Weighted mode
0.05
0.00
0.1
0.2
SNP effect on Total cholesterol levels in very large HDL || id:ebi-cfb233-GCST90302126
effect on Breast cancer (Combined Oncoarray; iCOGS; GWAS meta analysis) || id:ieu-a-1126

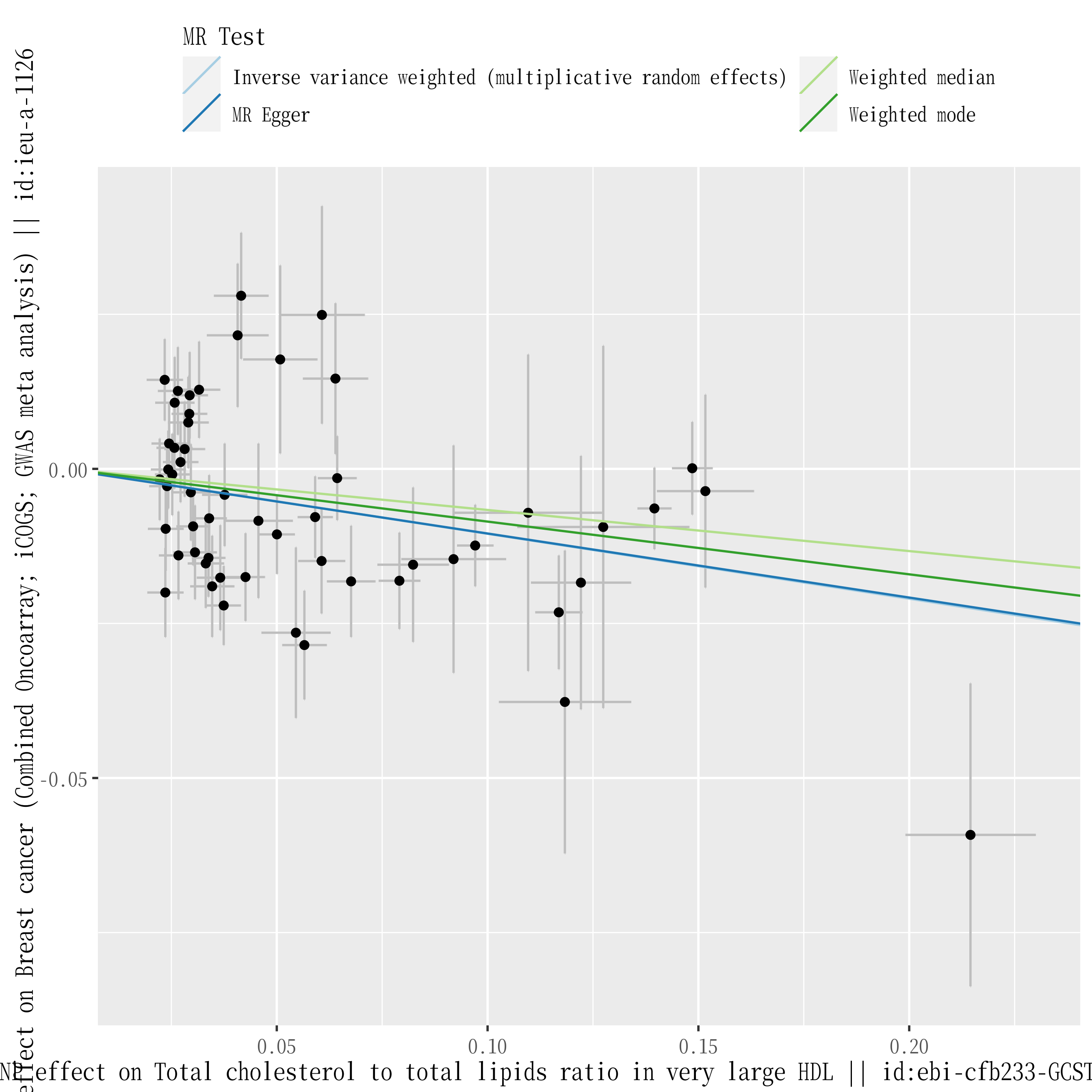

MR Test
Inverse variance weighted (multiplicative random effects)
MR Egger
Weighted median
Weighted mode
0.00
-0.05
0.05
0.10
0.15
0.20
SNP effect on Total cholesterol to total lipids ratio in very large HDL || id:ebi-cfb233-GCST
SNP effect on Breast cancer (Combined Oncoarray; iCOGS; GWAS meta analysis) || id:ieu-a-1126

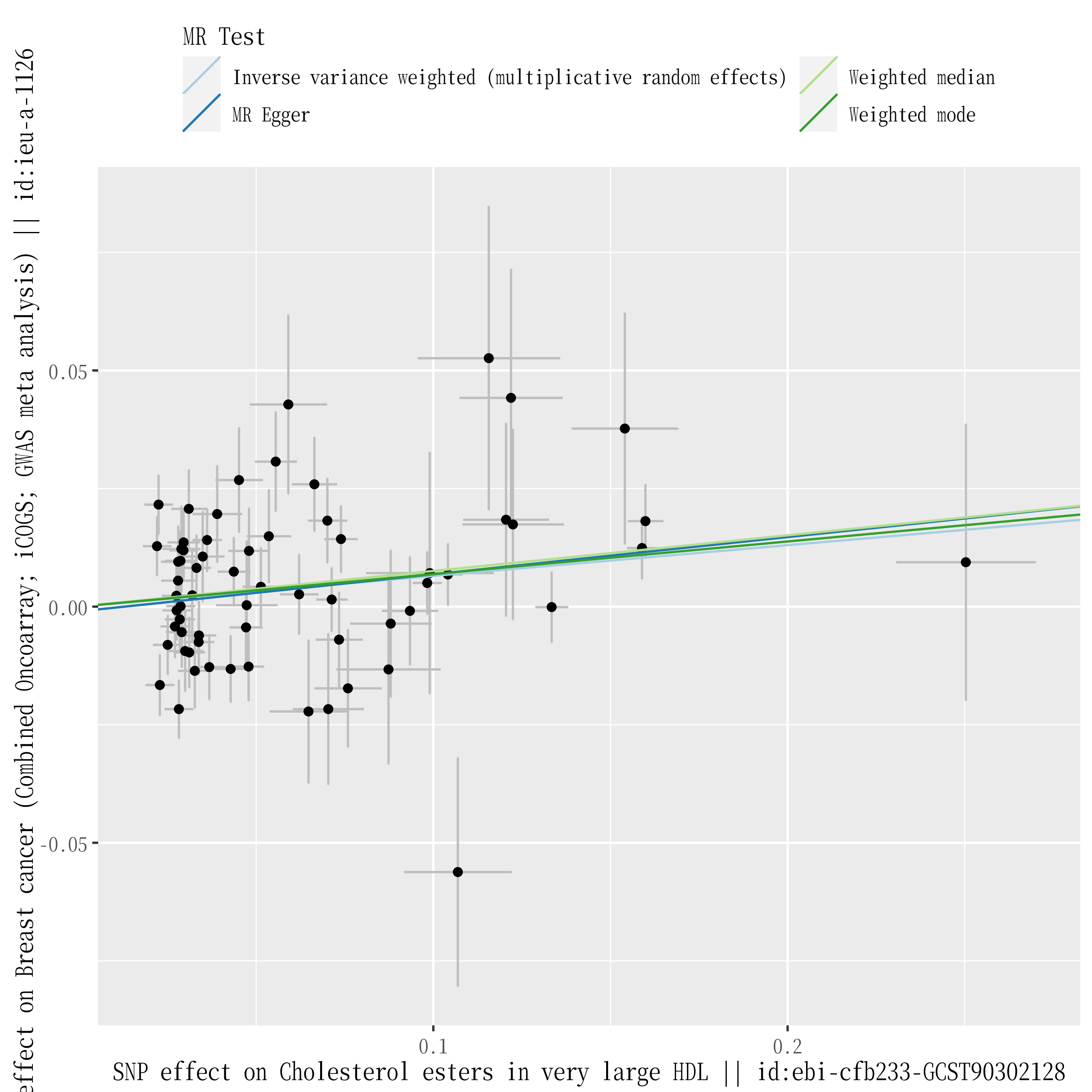
MR Test
Inverse variance weighted (multiplicative random effects)
MR Egger
Weighted median
Weighted mode
0.05
0.00
-0.05
0.1
0.2
SNP effect on Cholesterol esters in very large HDL || id:ebi-cfb233-GCST90302128
effect on Breast cancer (Combined Oncoarray; iCOGS; GWAS meta analysis) || id:ieu-a-1126

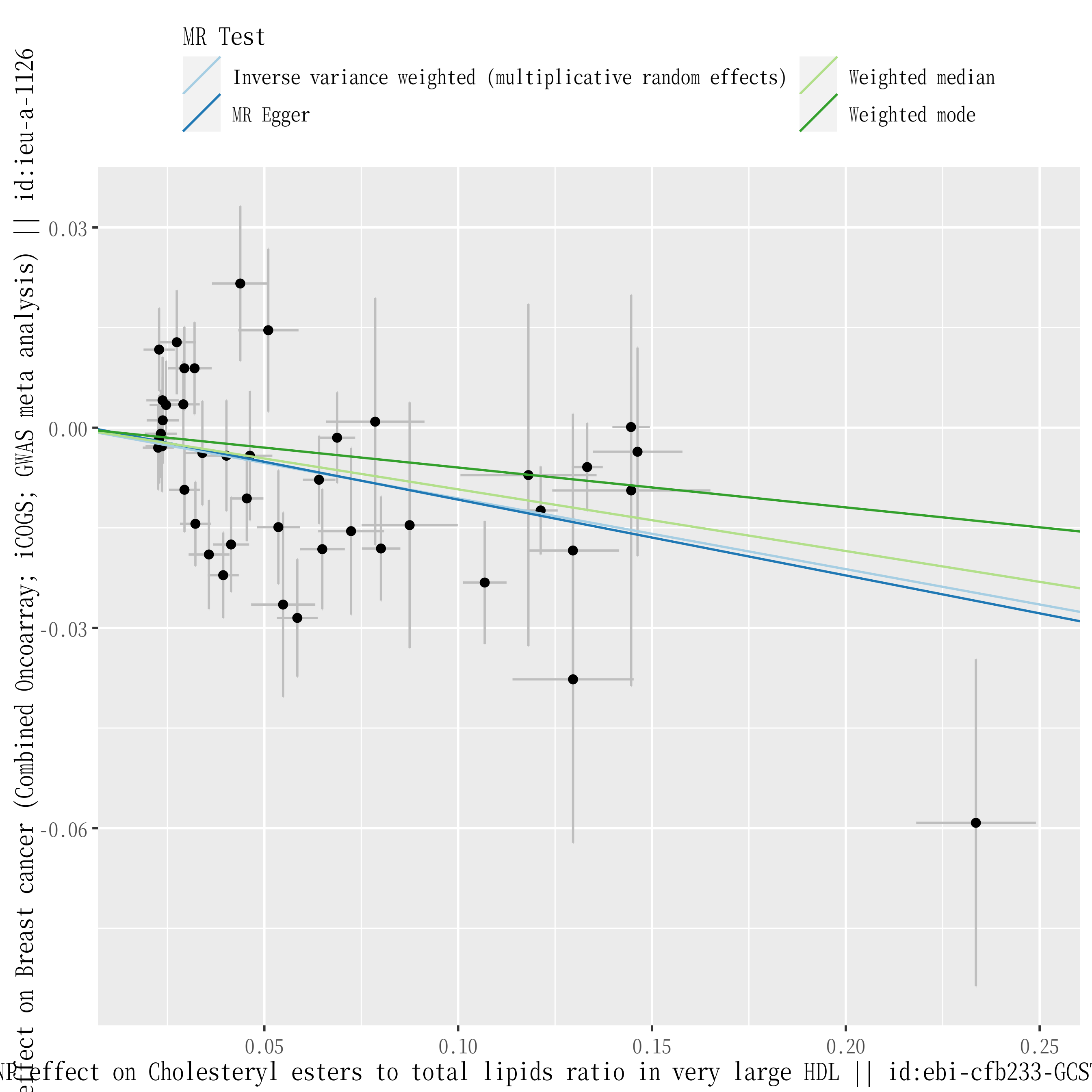
MR Test
Inverse variance weighted (multiplicative random effects)
MR Egger
Weighted median
Weighted mode
0.03
0.00
-0.03
-0.06
0.05
0.10
0.15
0.20
0.25
SNP effect on Cholesteryl esters to total lipids ratio in very large HDL || id:ebi-cfb233-GCST
SNP effect on Breast cancer (Combined Oncoarray; iCOGS; GWAS meta analysis) || id:ieu-a-1126

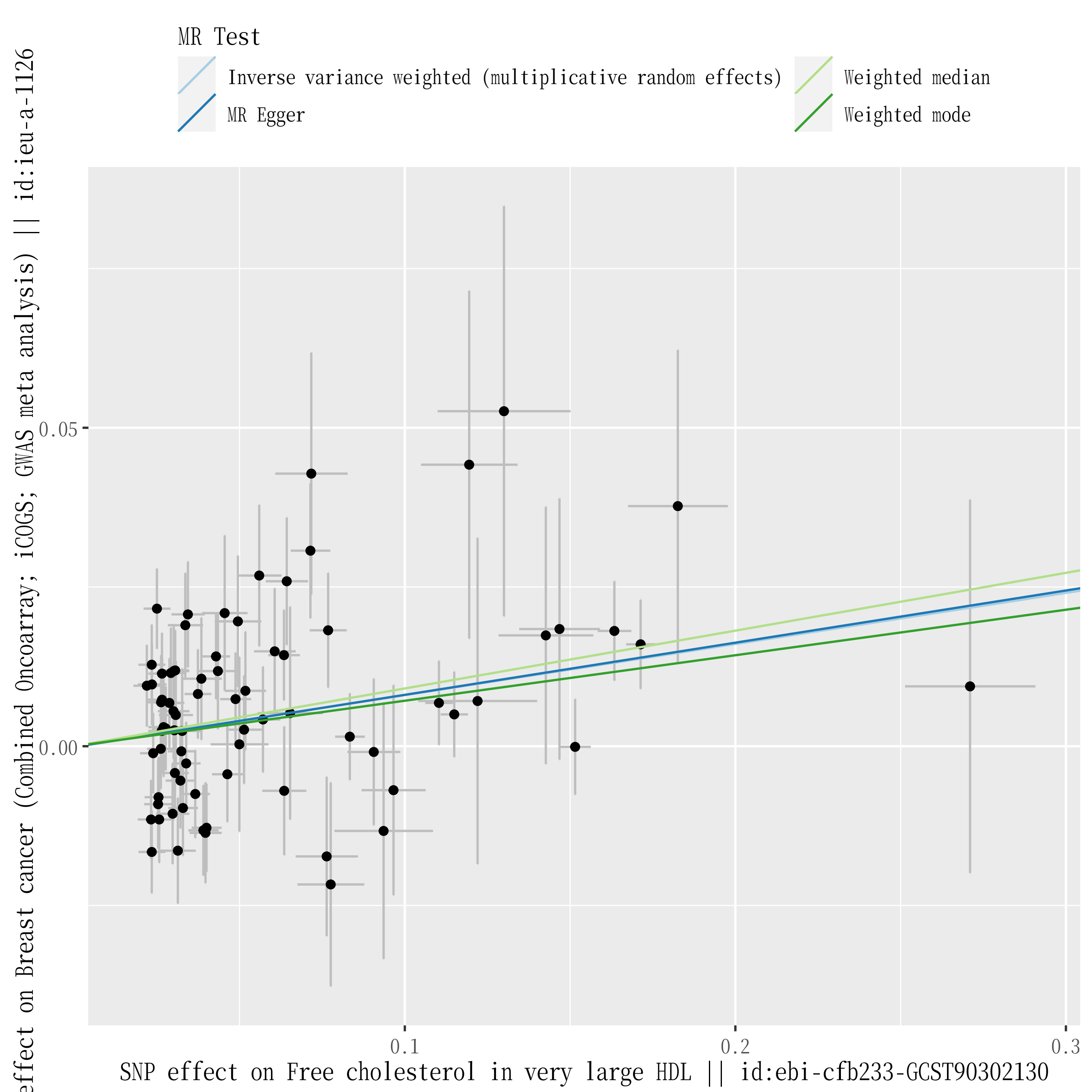

MR Test
Inverse variance weighted (multiplicative random effects)
MR Egger
Weighted median
Weighted mode
0.05
0.00
0.1
0.2
0.3
SNP effect on Free cholesterol in very large HDL || id:ebi-cfb233-GCST90302130
effect on Breast cancer (Combined Oncoarray; iCOGS; GWAS meta analysis) || id:ieu-a-1126

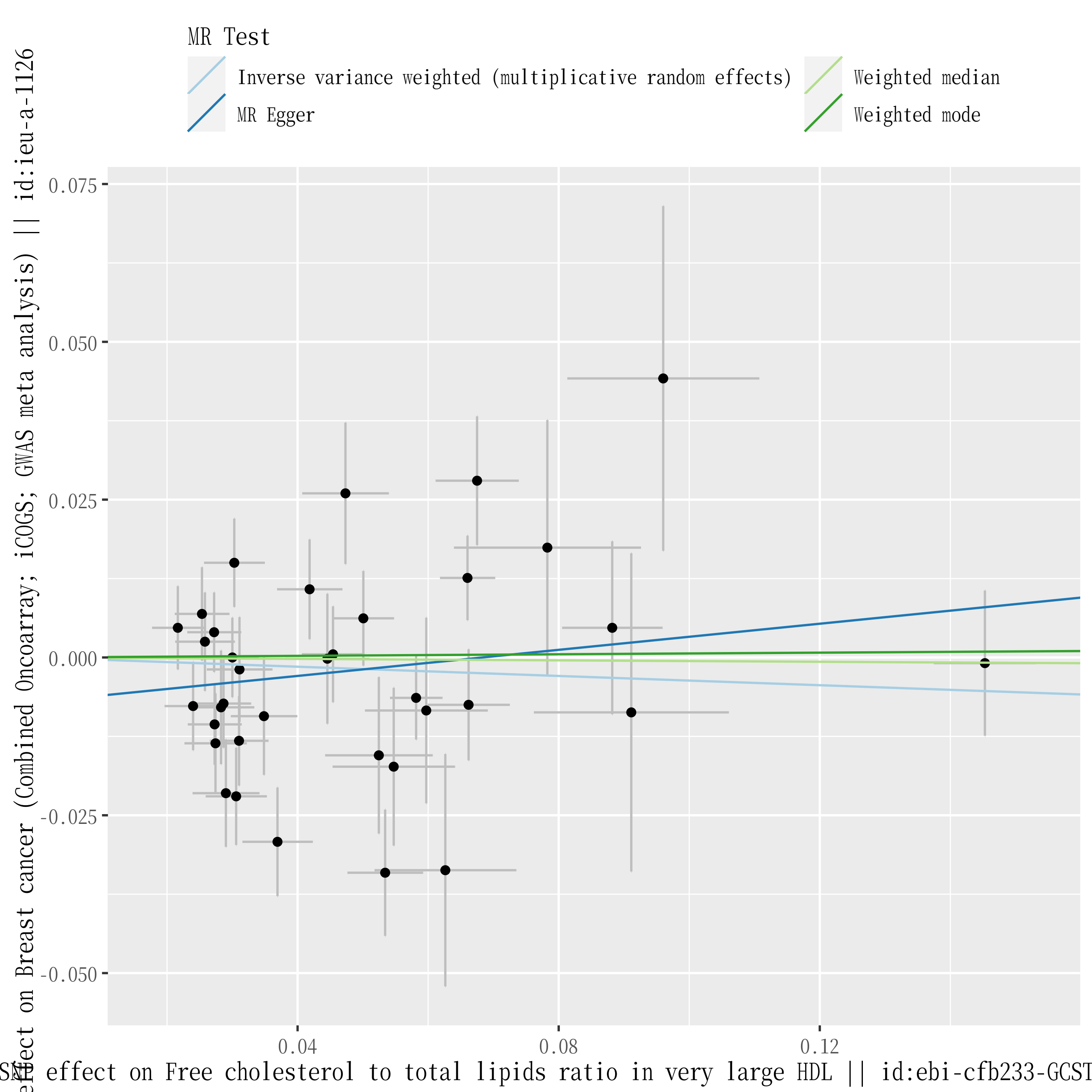
MR Test
Inverse variance weighted (multiplicative random effects)
MR Egger
Weighted median
Weighted mode
0.075
0.050
0.025
0.000
-0.025
-0.050
0.04
0.08
0.12
SNP effect on Free cholesterol to total lipids ratio in very large HDL || id:ebi-cfb233-GCST
SNP effect on Breast cancer (Combined Oncoarray; iCOGS; GWAS meta analysis) || id:ieu-a-1126

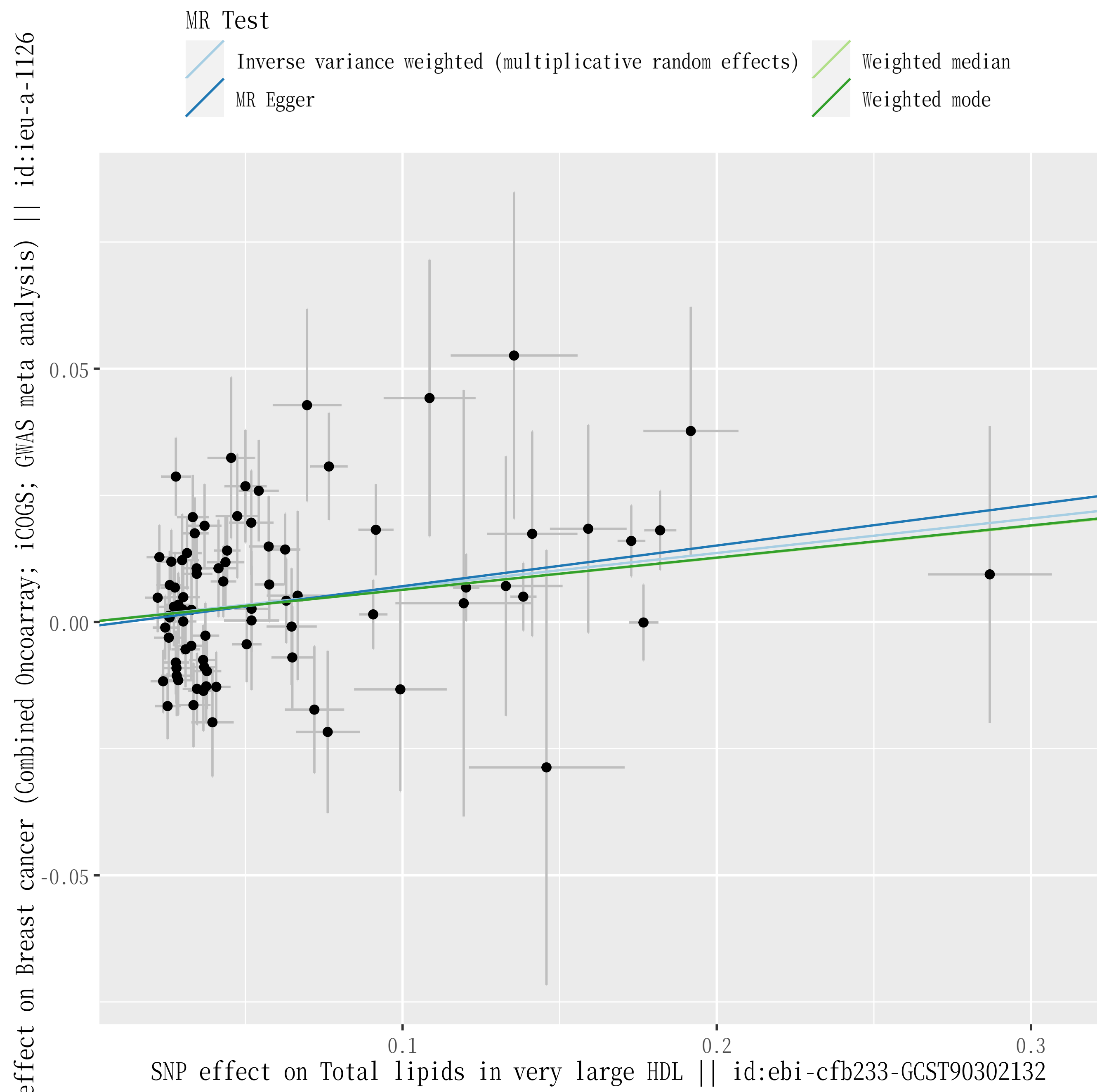

MR Test
Inverse variance weighted (multiplicative random effects)
MR Egger
Weighted median
Weighted mode
0.05
0.00
-0.05
0.1
0.2
0.3
SNP effect on Total lipids in very large HDL || id:ebi-cfb233-GCST90302132
effect on Breast cancer (Combined Oncoarray; iCOGS; GWAS meta analysis) || id:ieu-a-1126

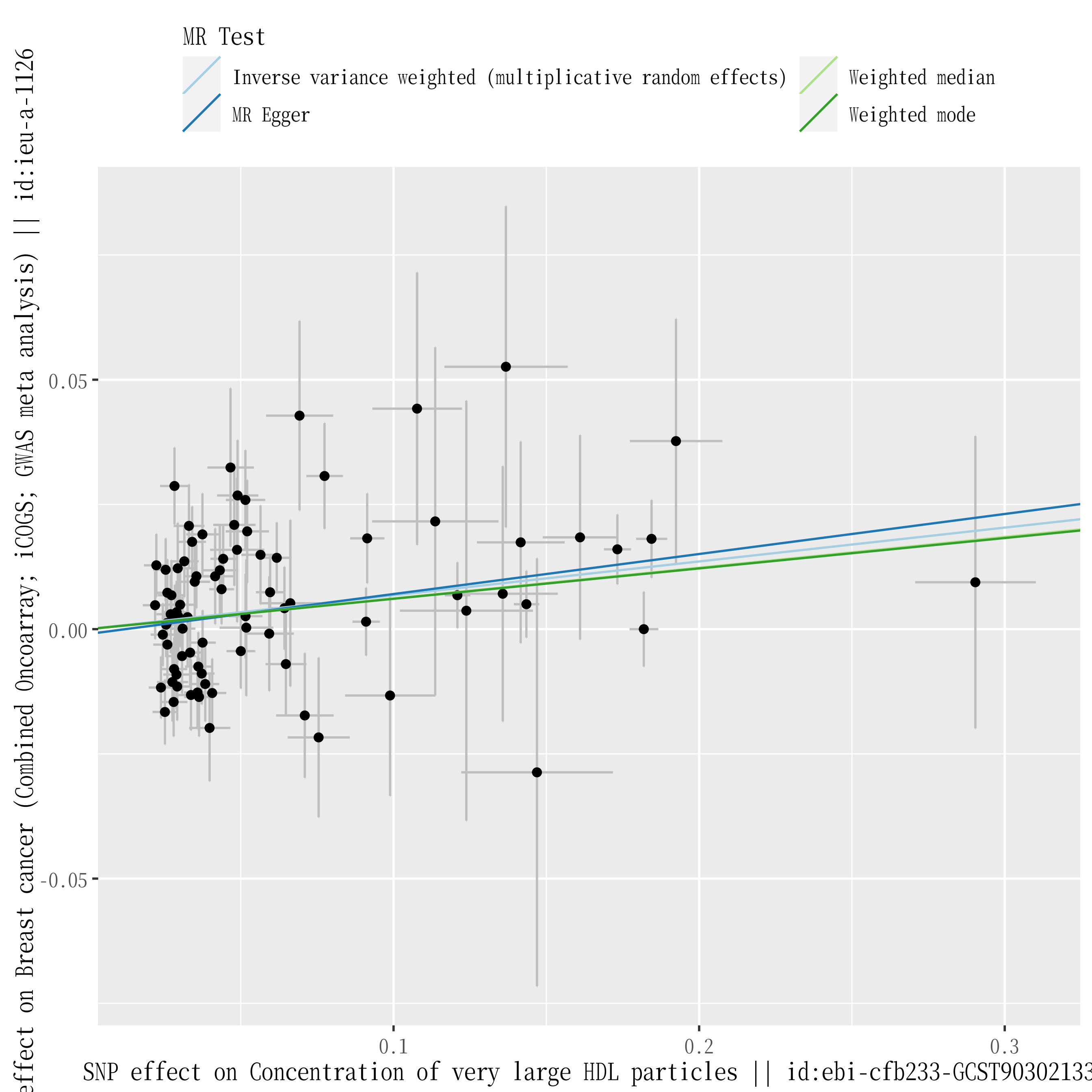

MR Test
Inverse variance weighted (multiplicative random effects)
MR Egger
Weighted median
Weighted mode
0.05
0.00
-0.05
0.1
0.2
0.3
SNP effect on Concentration of very large HDL particles || id:ebi-cfb233-GCST90302133
effect on Breast cancer (Combined Oncoarray; iCOGS; GWAS meta analysis) || id:ieu-a-1126

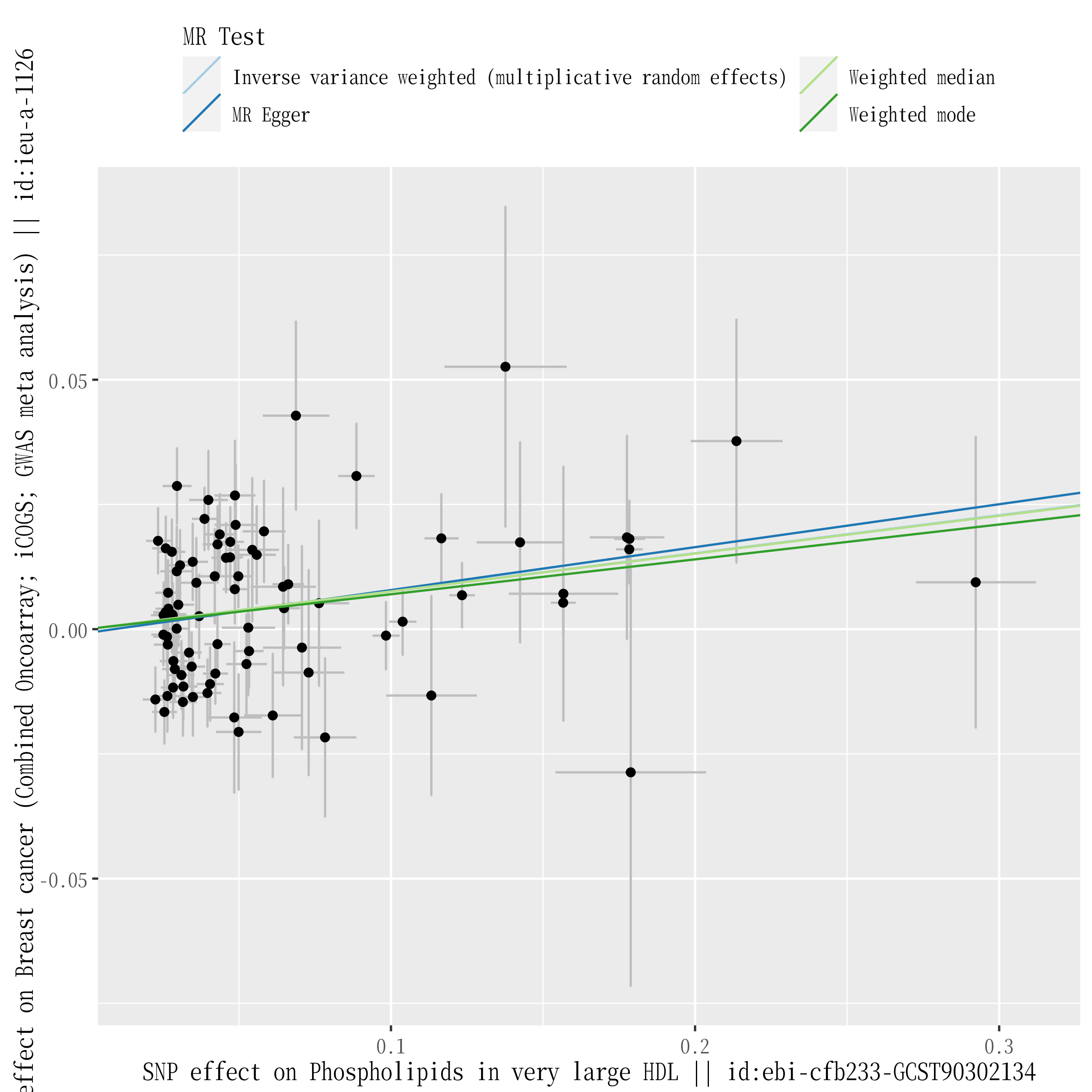
MR Test
Inverse variance weighted (multiplicative random effects)
MR Egger
Weighted median
Weighted mode
0.05
0.00
-0.05
0.1
0.2
0.3
SNP effect on Phospholipids in very large HDL || id:ebi-cfb233-GCST90302134
effect on Breast cancer (Combined Oncoarray; iCOGS; GWAS meta analysis) || id:ieu-a-1126

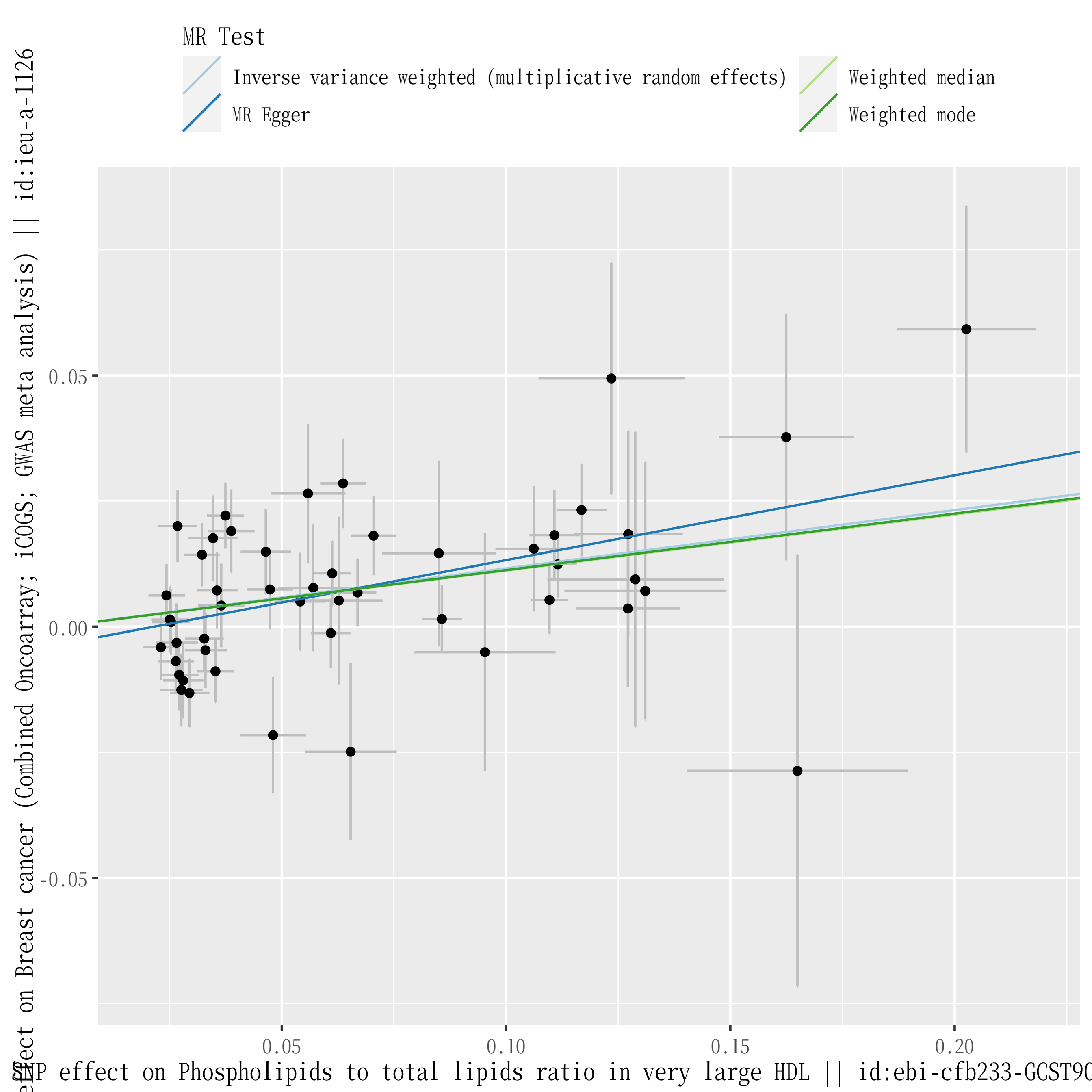
MR Test
Inverse variance weighted (multiplicative random effects)
MR Egger
Weighted median
Weighted mode
0.05
0.00
-0.05
0.05
0.10
0.15
0.20
SNP effect on Phospholipids to total lipids ratio in very large HDL || id:ebi-cfb233-GCST90
effect on Breast cancer (Combined Oncoarray; iCOGS; GWAS meta analysis) || id:ieu-a-1126

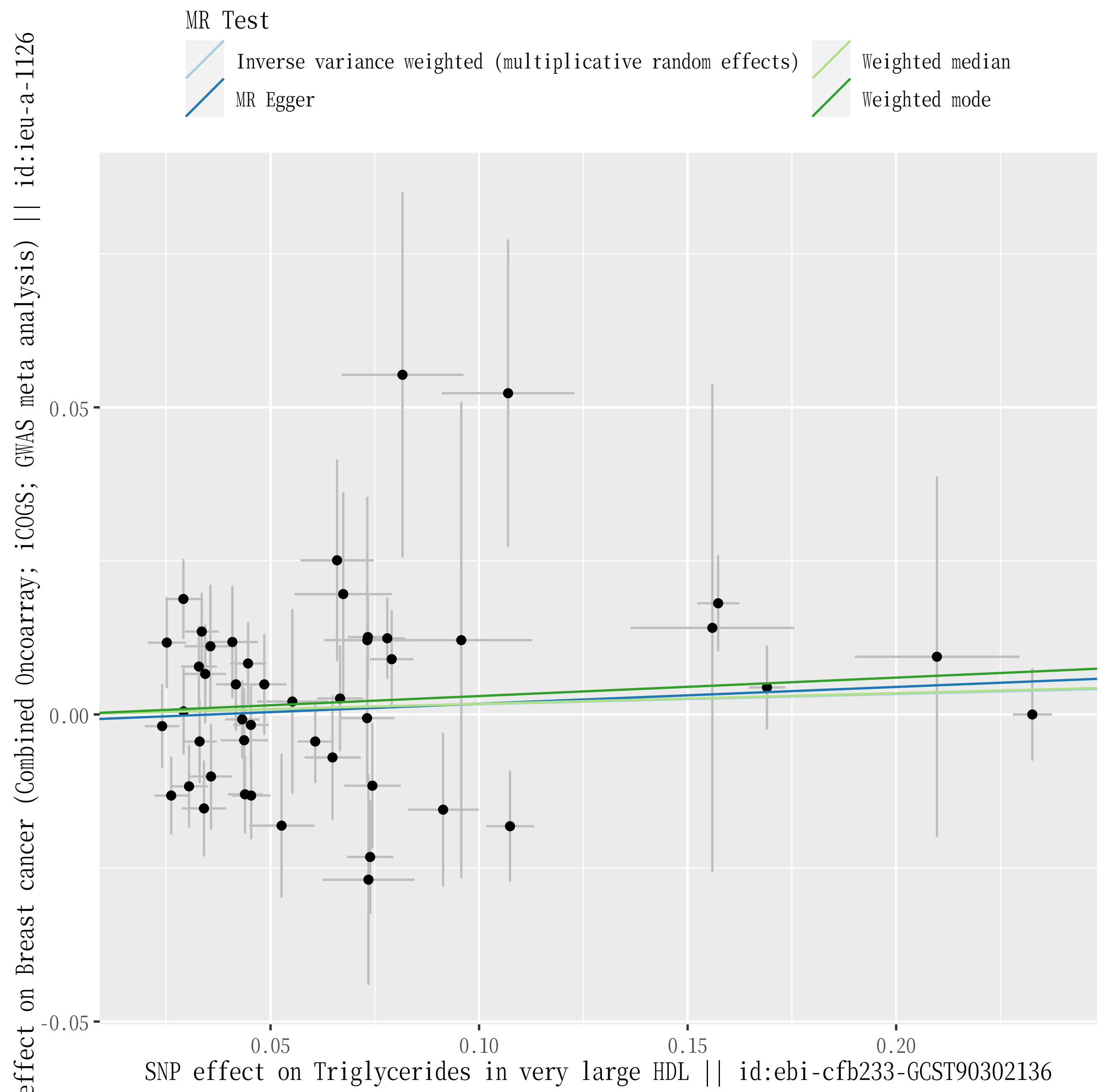

MR Test
Inverse variance weighted (multiplicative random effects)
MR Egger
Weighted median
Weighted mode
0.05
0.00
-0.05
0.05
0.10
0.15
0.20
SNP effect on Triglycerides in very large HDL || id:ebi-cfb233-GCST90302136
effect on Breast cancer (Combined Oncoarray; iCOGS; GWAS meta analysis) || id:ieu-a-1126

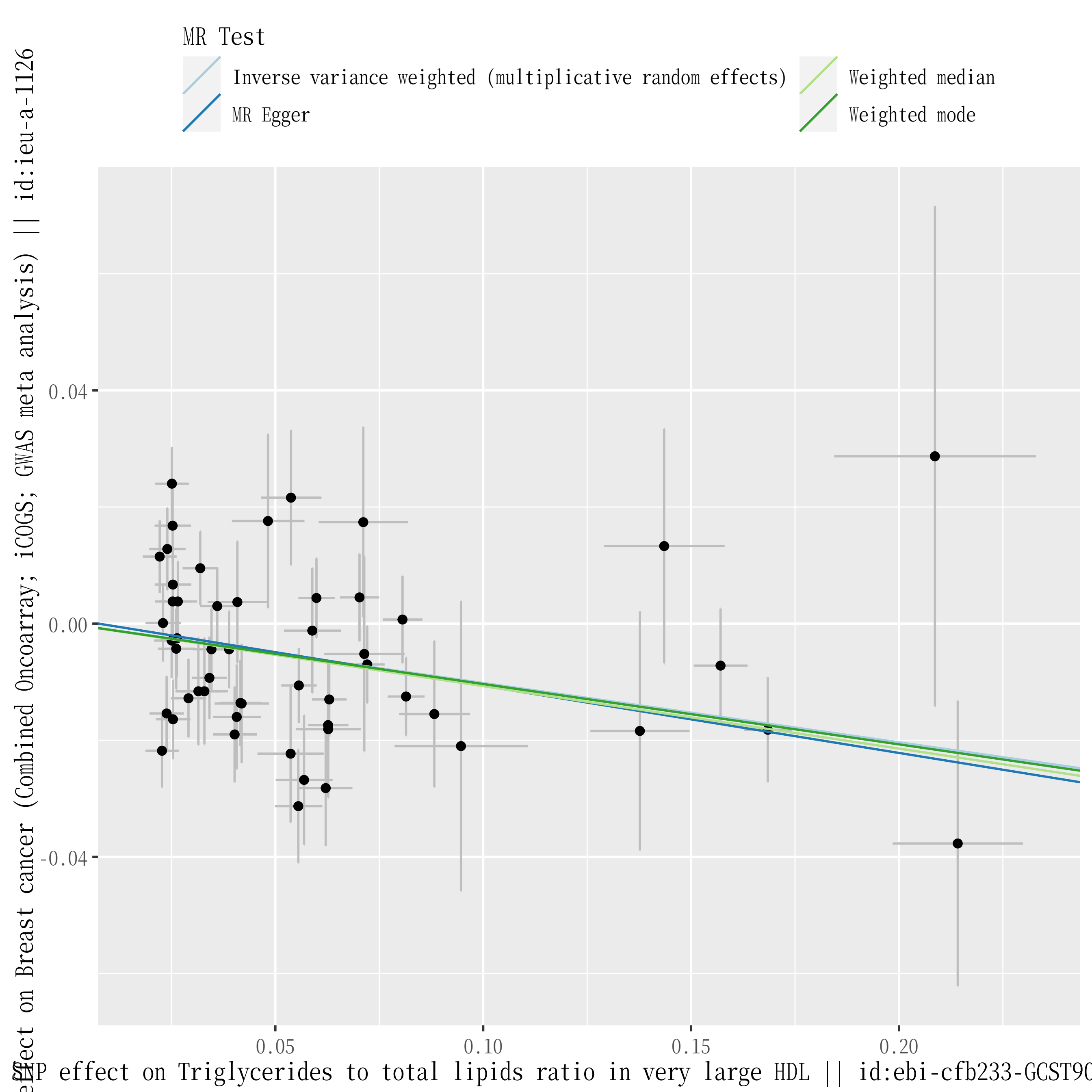

MR Test
Inverse variance weighted (multiplicative random effects)
MR Egger
Weighted median
Weighted mode
0.04
0.00
-0.04
0.05
0.10
0.15
0.20
SNP effect on Triglycerides to total lipids ratio in very large HDL || id:ebi-cfb233-GCST90
effect on Breast cancer (Combined Oncoarray; iCOGS; GWAS meta analysis) || id:ieu-a-1126

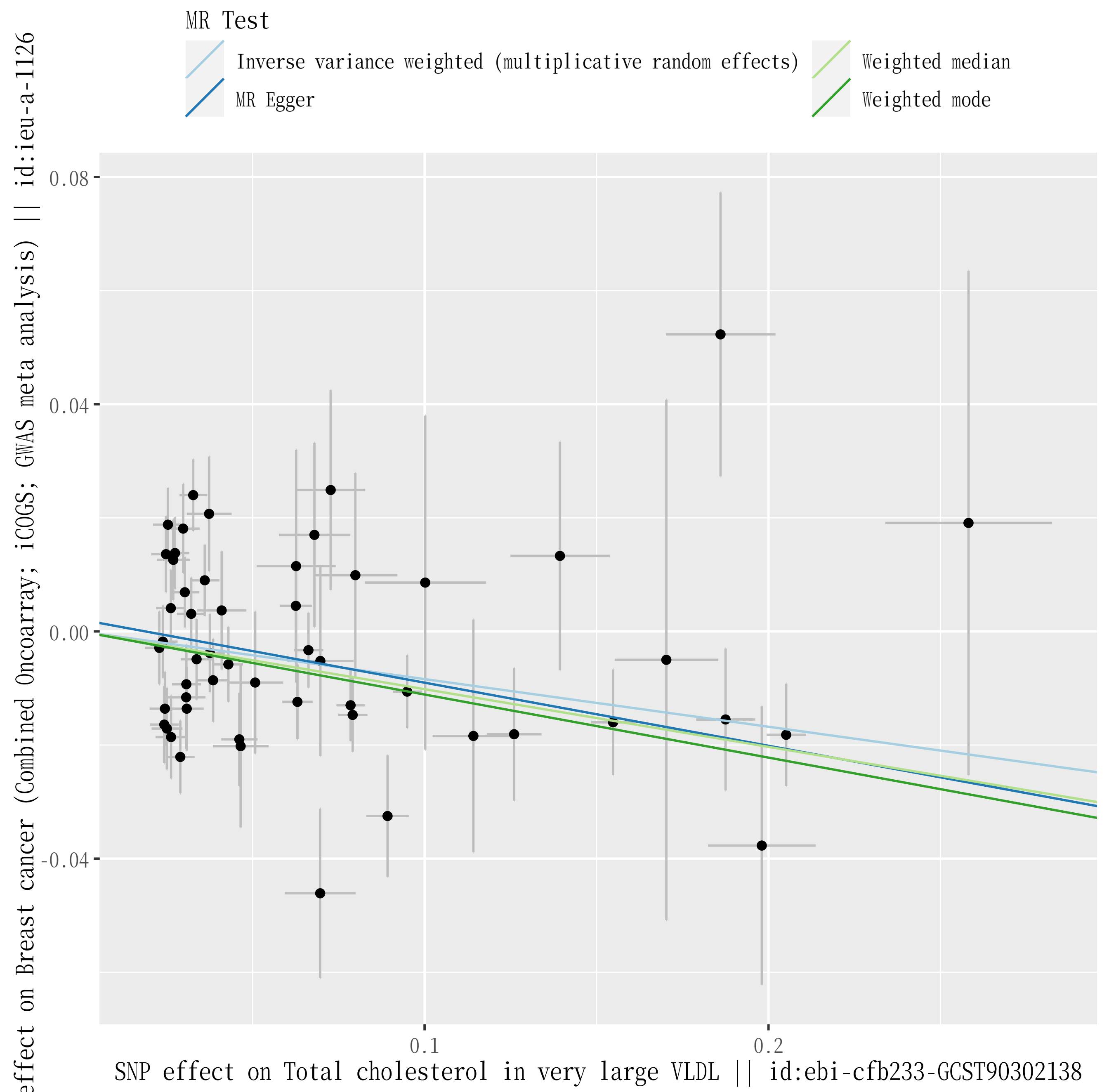

MR Test
Inverse variance weighted (multiplicative random effects)
MR Egger
Weighted median
Weighted mode
0.08
0.04
0.00
-0.04
0.1
0.2
SNP effect on Total cholesterol in very large VLDL || id:ebi-cfb233-GCST90302138
effect on Breast cancer (Combined Oncoarray; iCOGS; GWAS meta analysis) || id:ieu-a-1126

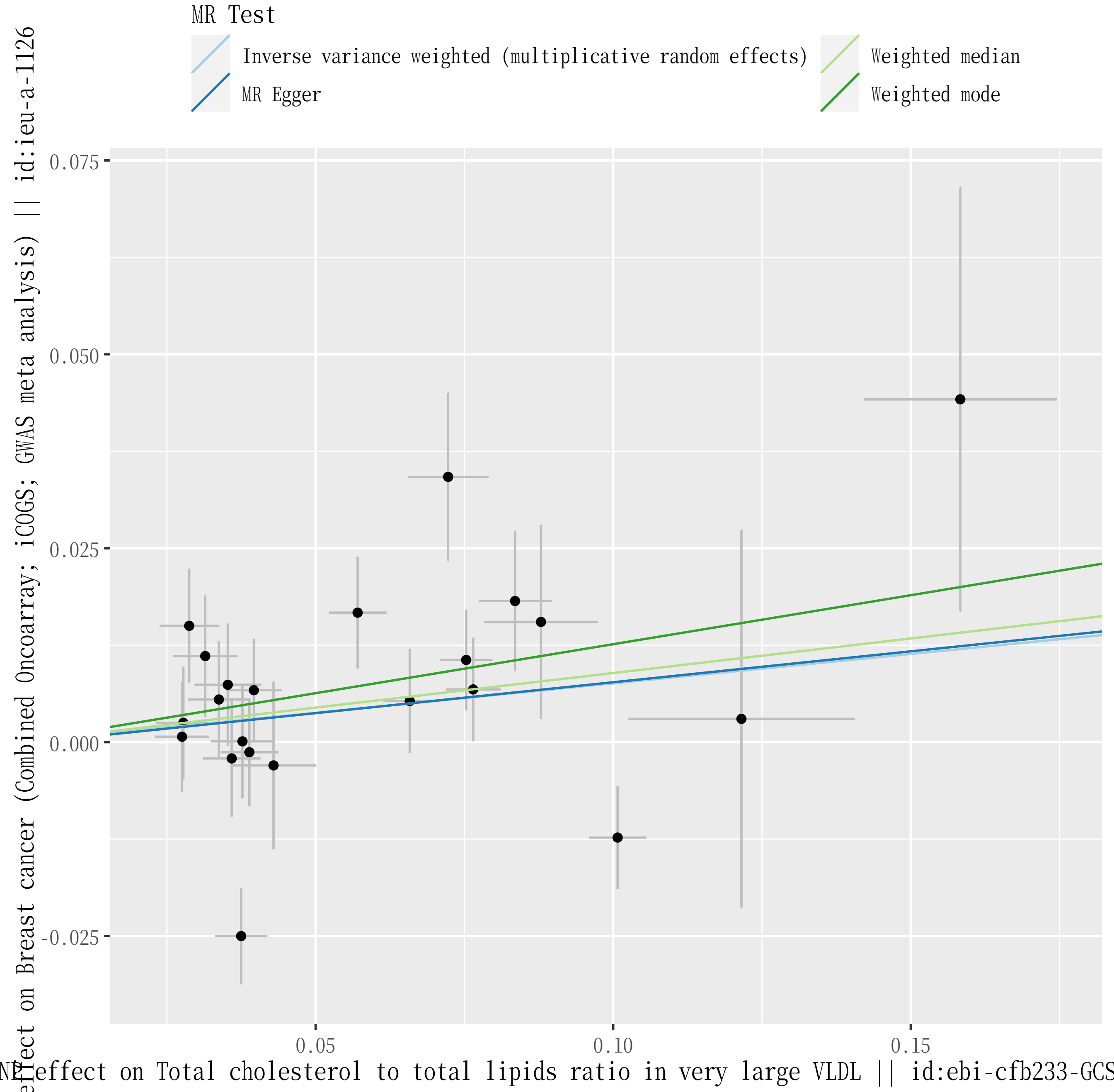
MR Test
Inverse variance weighted (multiplicative random effects)
MR Egger
Weighted median
Weighted mode
0.075
0.050
0.025
0.000
-0.025
0.05
0.10
0.15
SNP effect on Total cholesterol to total lipids ratio in very large VLDL || id:ebi-cfb233-GCS
SNP effect on Breast cancer (Combined Oncoarray; iCOGS; GWAS meta analysis) || id:ieu-a-1126

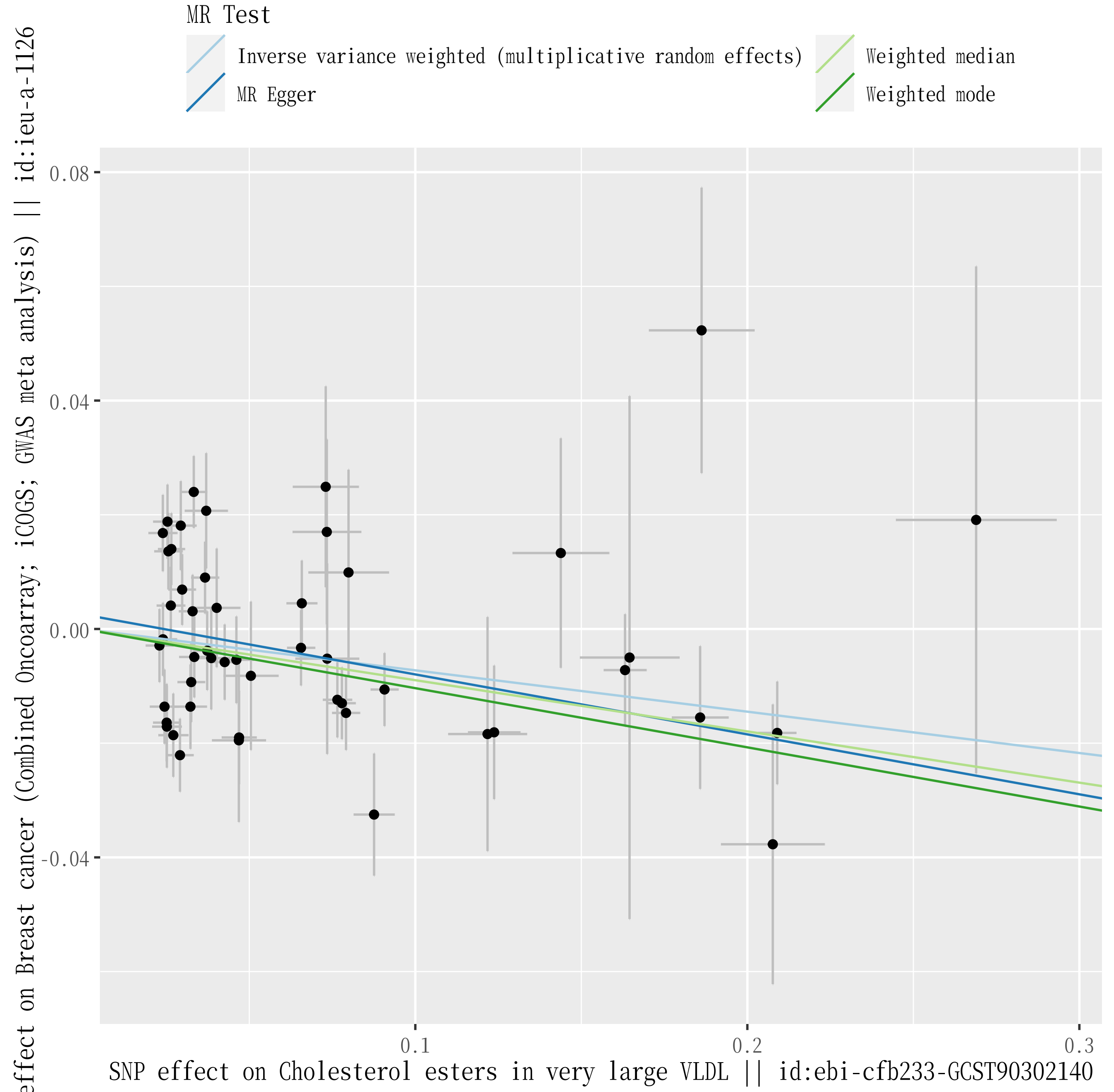
MR Test
Inverse variance weighted (multiplicative random effects)
MR Egger
Weighted median
Weighted mode
0.08
0.04
0.00
-0.04
0.1
0.2
0.3
SNP effect on Cholesterol esters in very large VLDL || id:ebi-cfb233-GCST90302140
effect on Breast cancer (Combined Oncoarray; iCOGS; GWAS meta analysis) || id:ieu-a-1126

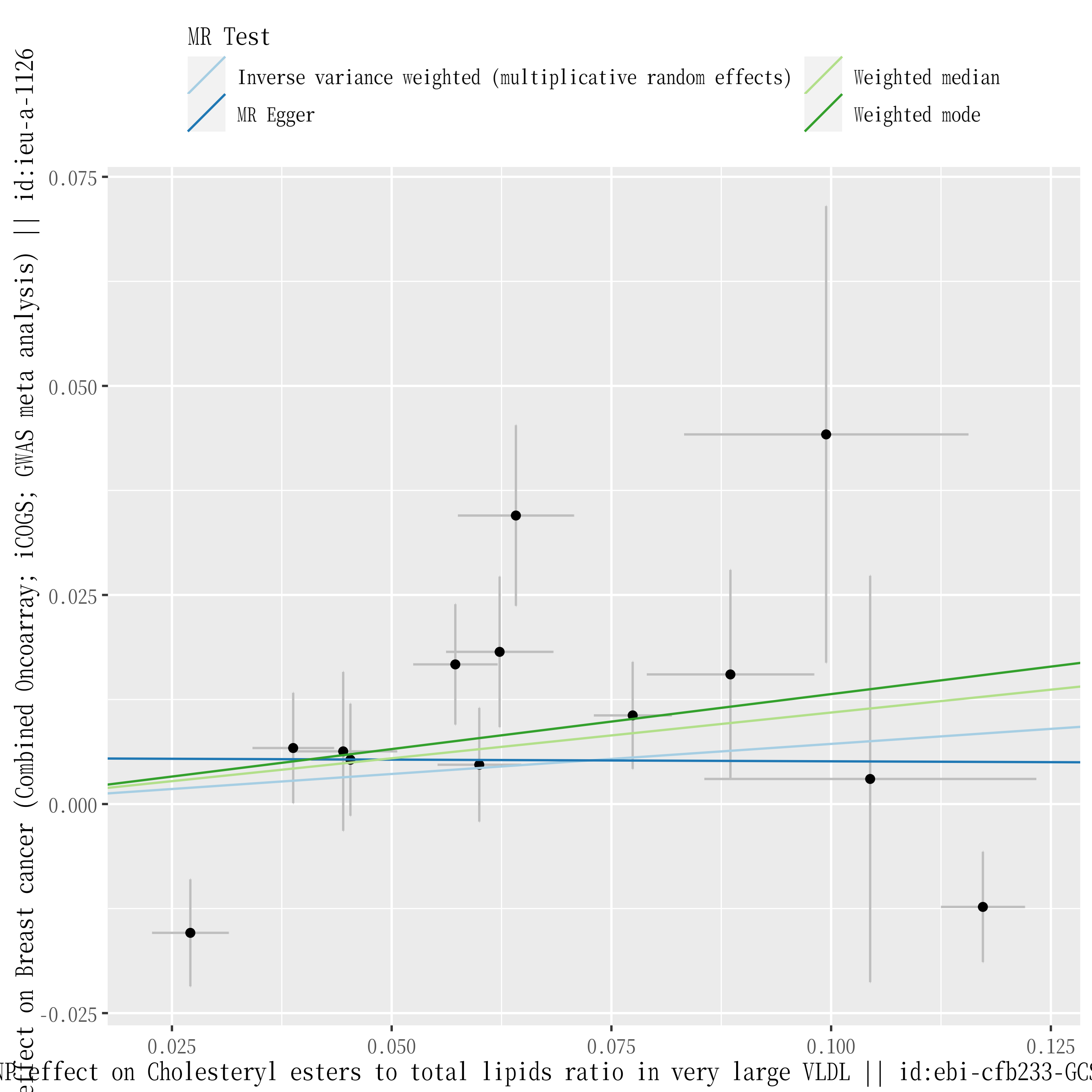

MR Test
Inverse variance weighted (multiplicative random effects)
MR Egger
Weighted median
Weighted mode
0.075
0.050
0.025
0.000
-0.025
0.025
0.050
0.075
0.100
0.125
SNP effect on Cholesteryl esters to total lipids ratio in very large VLDL || id:ebi-cfb233-GCS
effect on Breast cancer (Combined Oncoarray; iCOGS; GWAS meta analysis) || id:ieu-a-1126

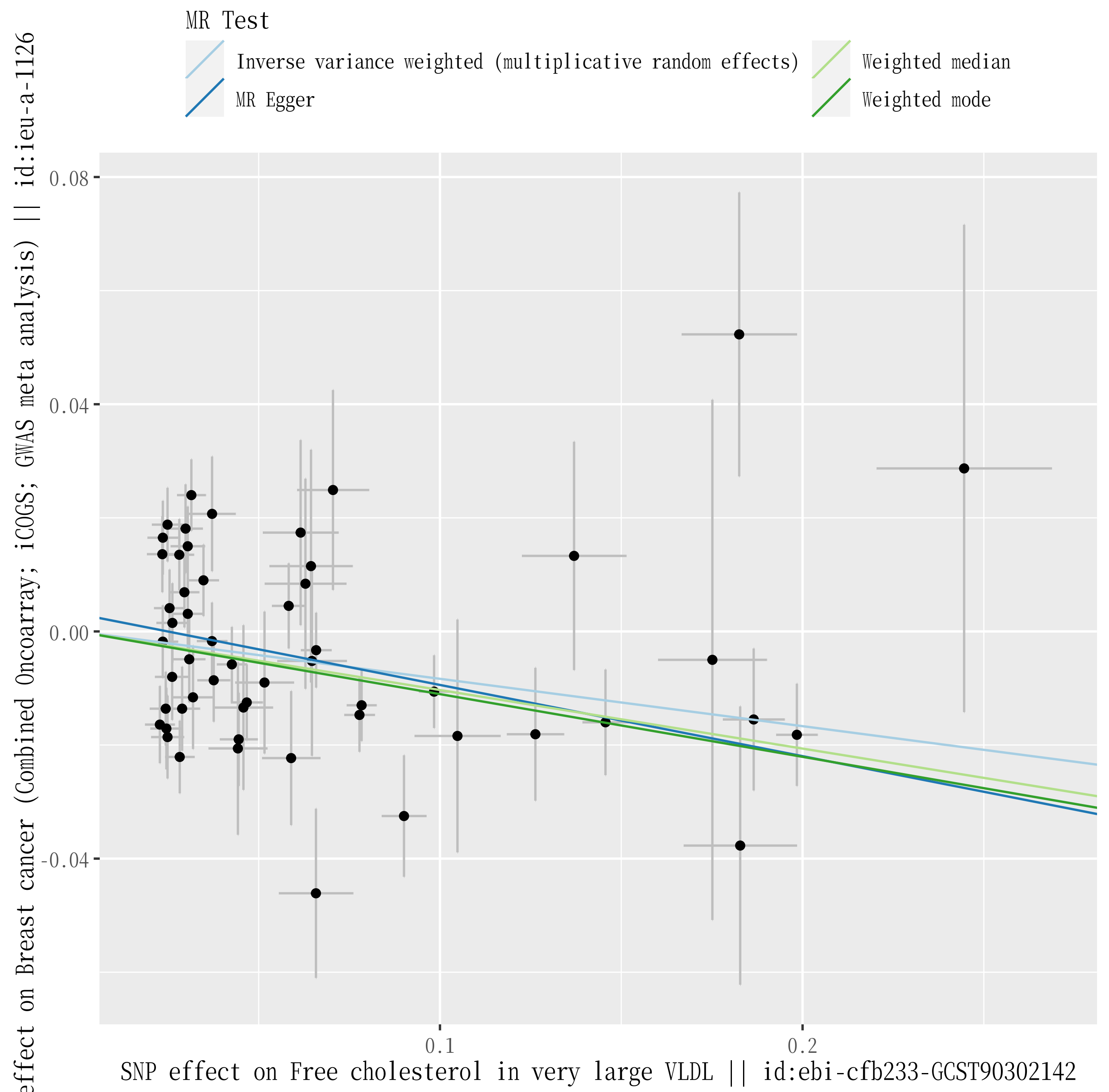

MR Test
Inverse variance weighted (multiplicative random effects)
MR Egger
Weighted median
Weighted mode
0.08
0.04
0.00
-0.04
0.1
0.2
SNP effect on Free cholesterol in very large VLDL || id:ebi-cfb233-GCST90302142
effect on Breast cancer (Combined Oncoarray; iCOGS; GWAS meta analysis) || id:ieu-a-1126

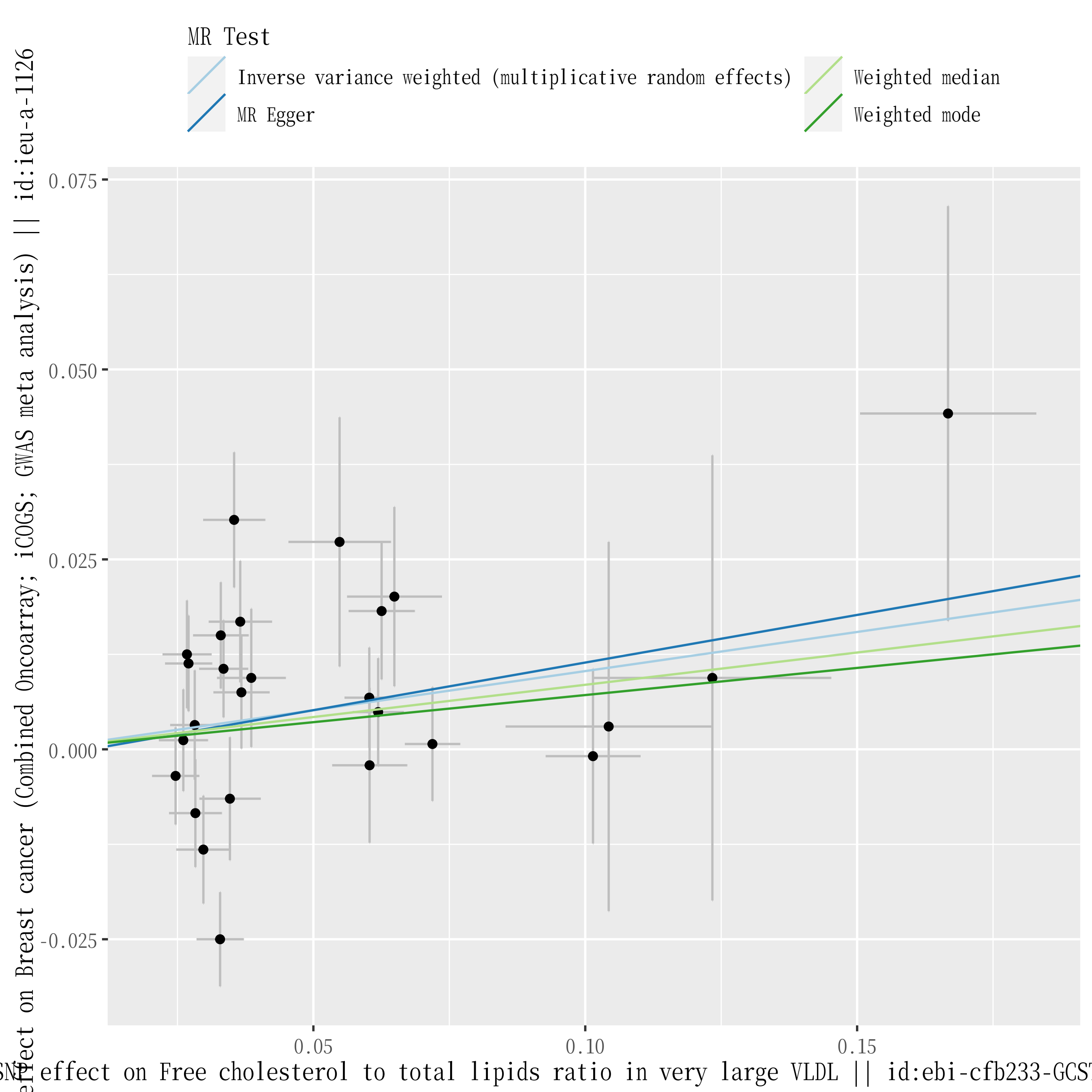

MR Test
Inverse variance weighted (multiplicative random effects)
MR Egger
Weighted median
Weighted mode
0.075
0.050
0.025
0.000
-0.025
0.05
0.10
0.15
SNP effect on Free cholesterol to total lipids ratio in very large VLDL || id:ebi-cfb233-GCST
SNP effect on Breast cancer (Combined Oncoarray; iCOGS; GWAS meta analysis) || id:ieu-a-1126

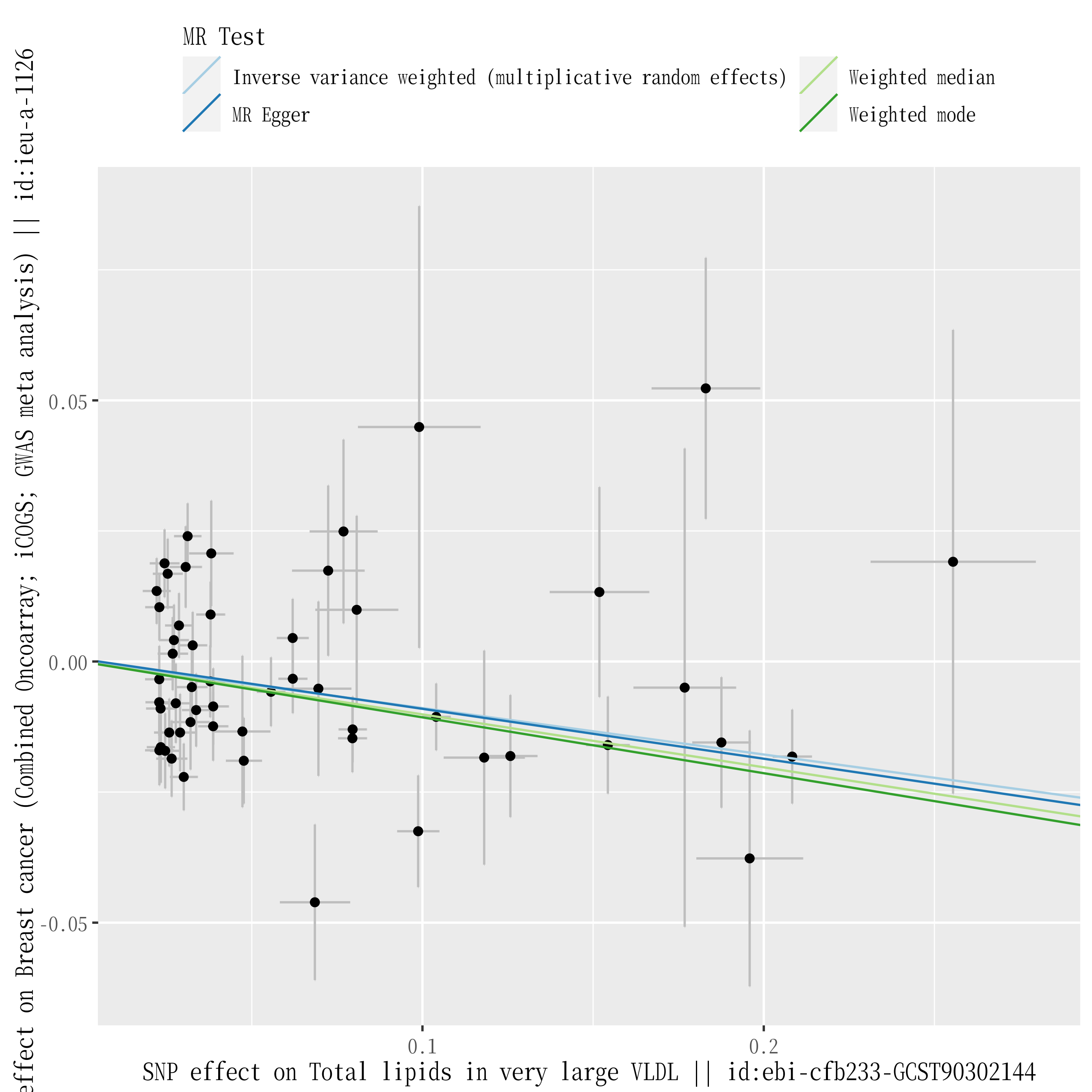

MR Test
Inverse variance weighted (multiplicative random effects)
MR Egger
Weighted median
Weighted mode
0.05
0.00
-0.05
0.1
0.2
SNP effect on Total lipids in very large VLDL || id:ebi-cfb233-GCST90302144
effect on Breast cancer (Combined Oncoarray; iCOGS; GWAS meta analysis) || id:ieu-a-1126

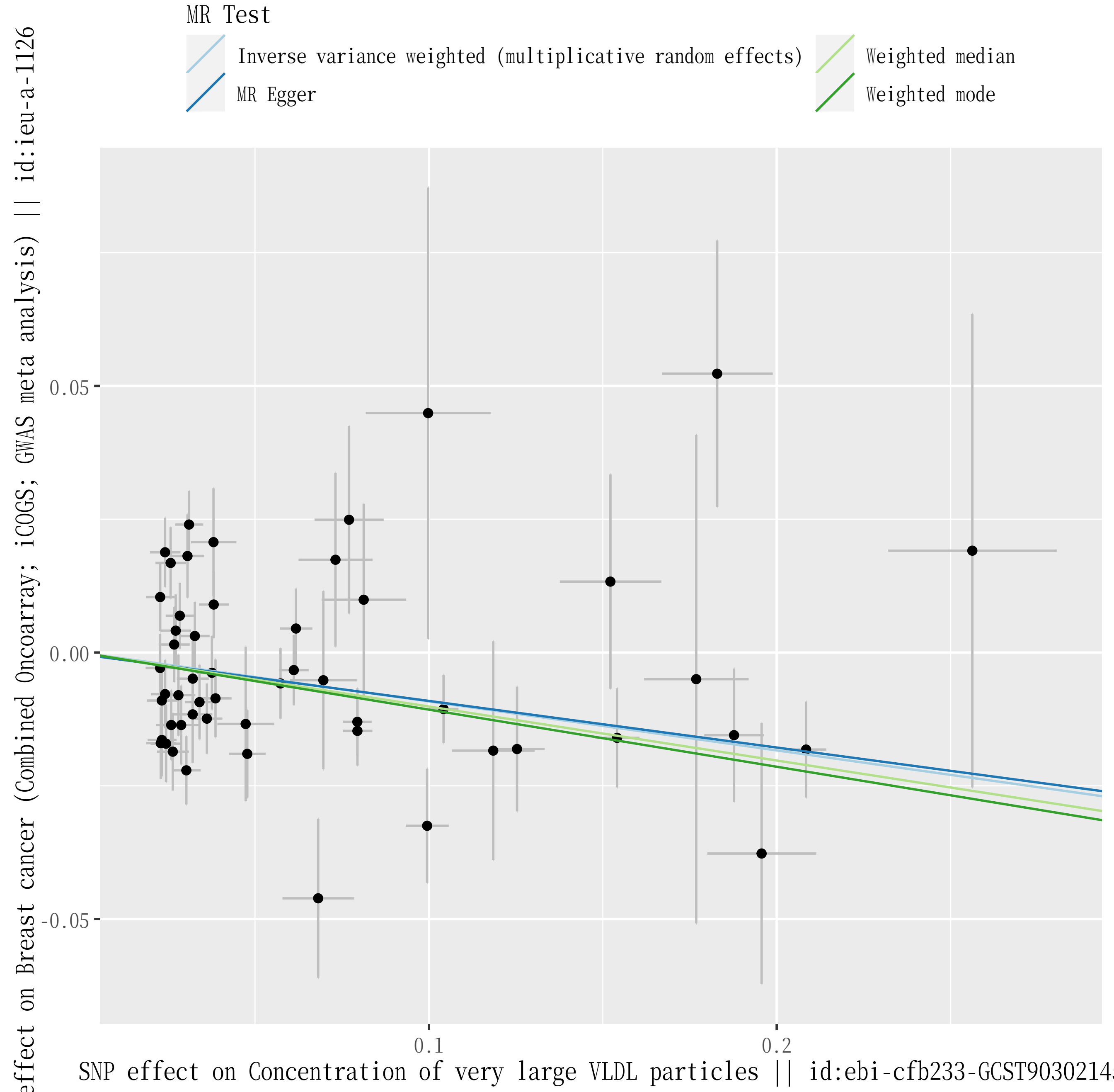

MR Test
Inverse variance weighted (multiplicative random effects)
MR Egger
Weighted median
Weighted mode
0.05
0.00
-0.05
0.1
0.2
SNP effect on Concentration of very large VLDL particles || id:ebi-cfb233-GCST9030214
effect on Breast cancer (Combined Oncoarray; iCOGS; GWAS meta analysis) || id:ieu-a-1126

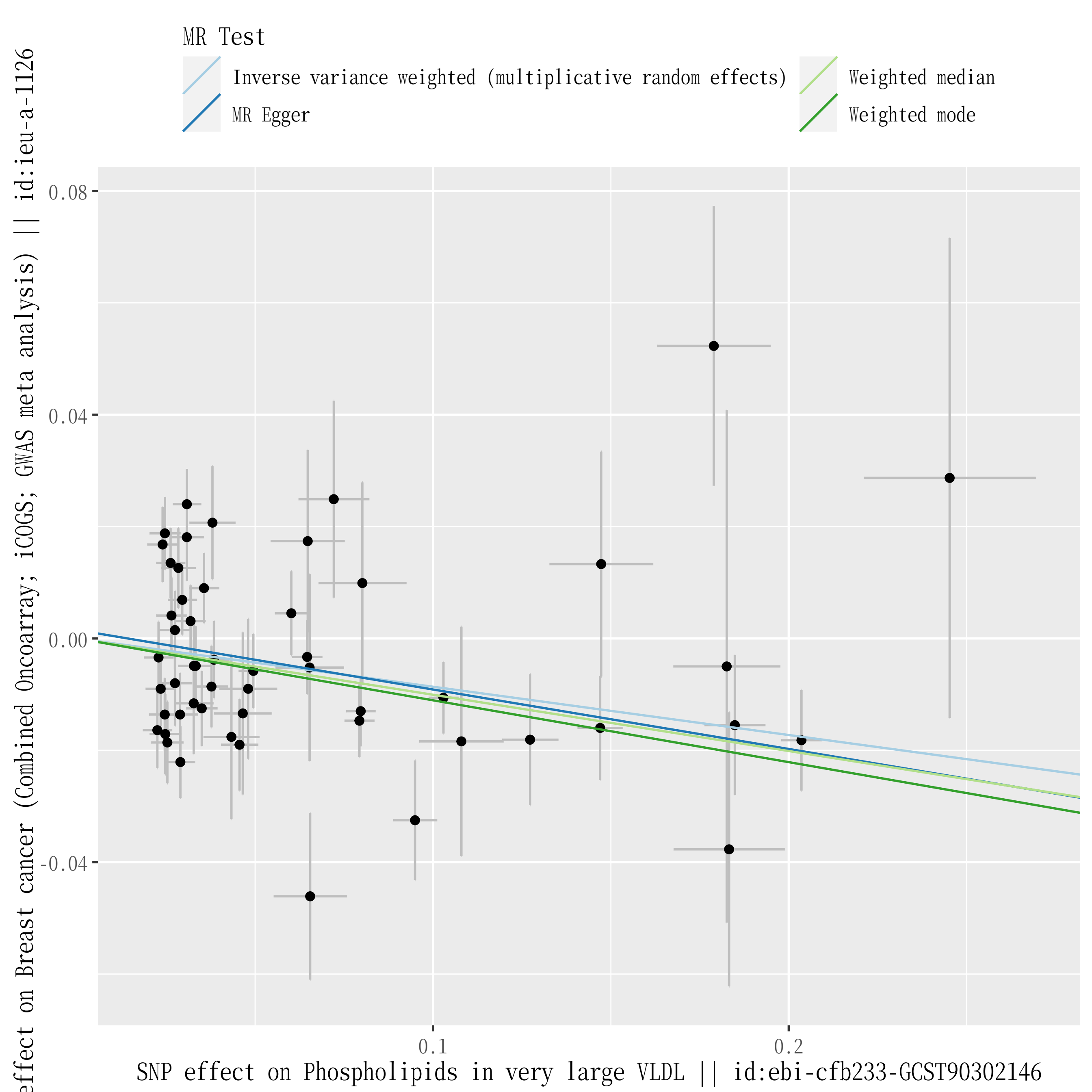
MR Test
Inverse variance weighted (multiplicative random effects)
MR Egger
Weighted median
Weighted mode
0.08
0.04
0.00
-0.04
0.1
0.2
SNP effect on Phospholipids in very large VLDL || id:ebi-cfb233-GCST90302146
effect on Breast cancer (Combined Oncoarray; iCOGS; GWAS meta analysis) || id:ieu-a-1126

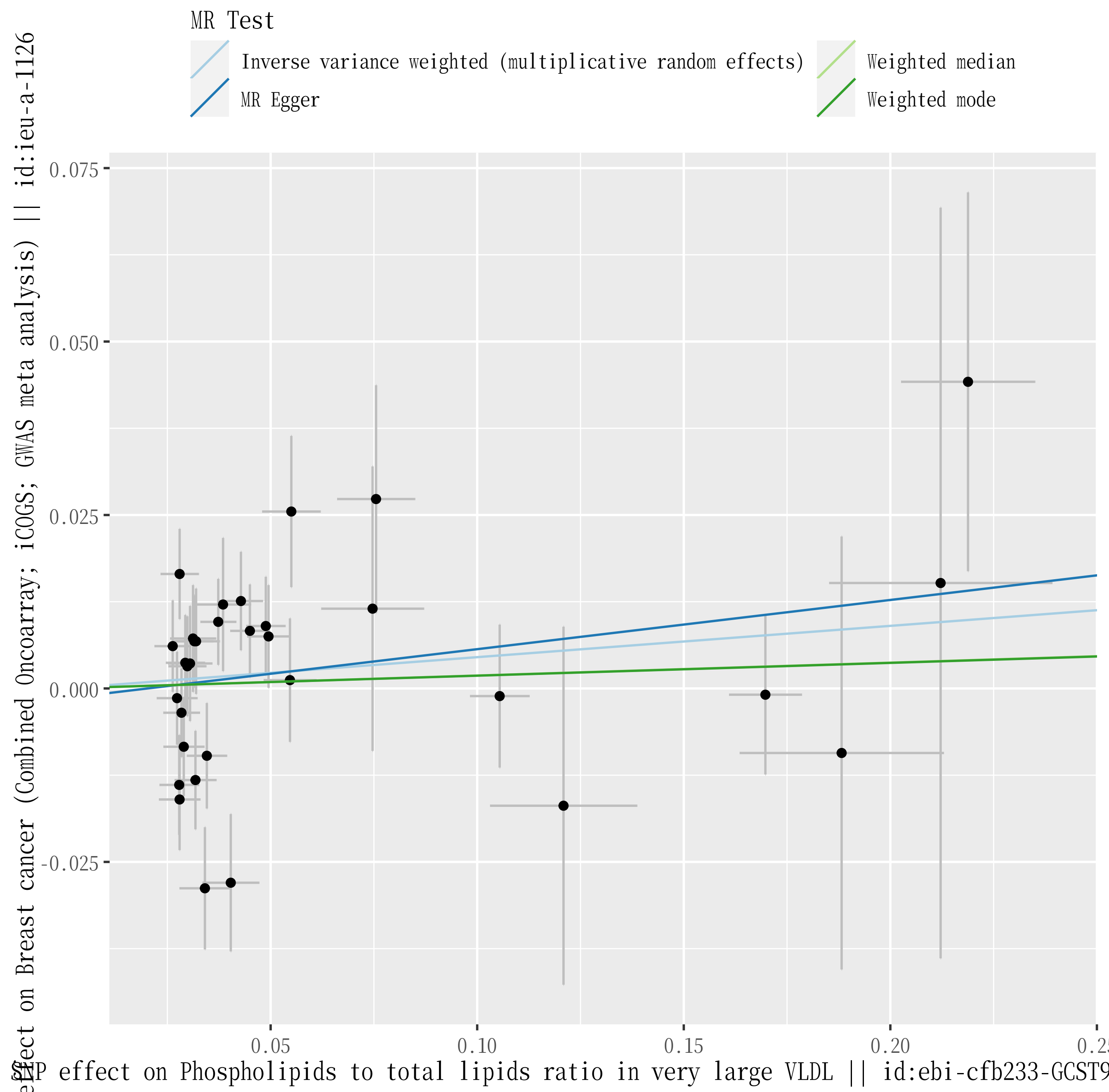
MR Test
Inverse variance weighted (multiplicative random effects)
MR Egger
Weighted median
Weighted mode
0.075
0.050
0.025
0.000
-0.025
0.05
0.10
0.15
0.20
SNP effect on Phospholipids to total lipids ratio in very large VLDL || id:ebi-cfb233-GCST9
effect on Breast cancer (Combined Oncoarray; iCOGS; GWAS meta analysis) || id:ieu-a-1126

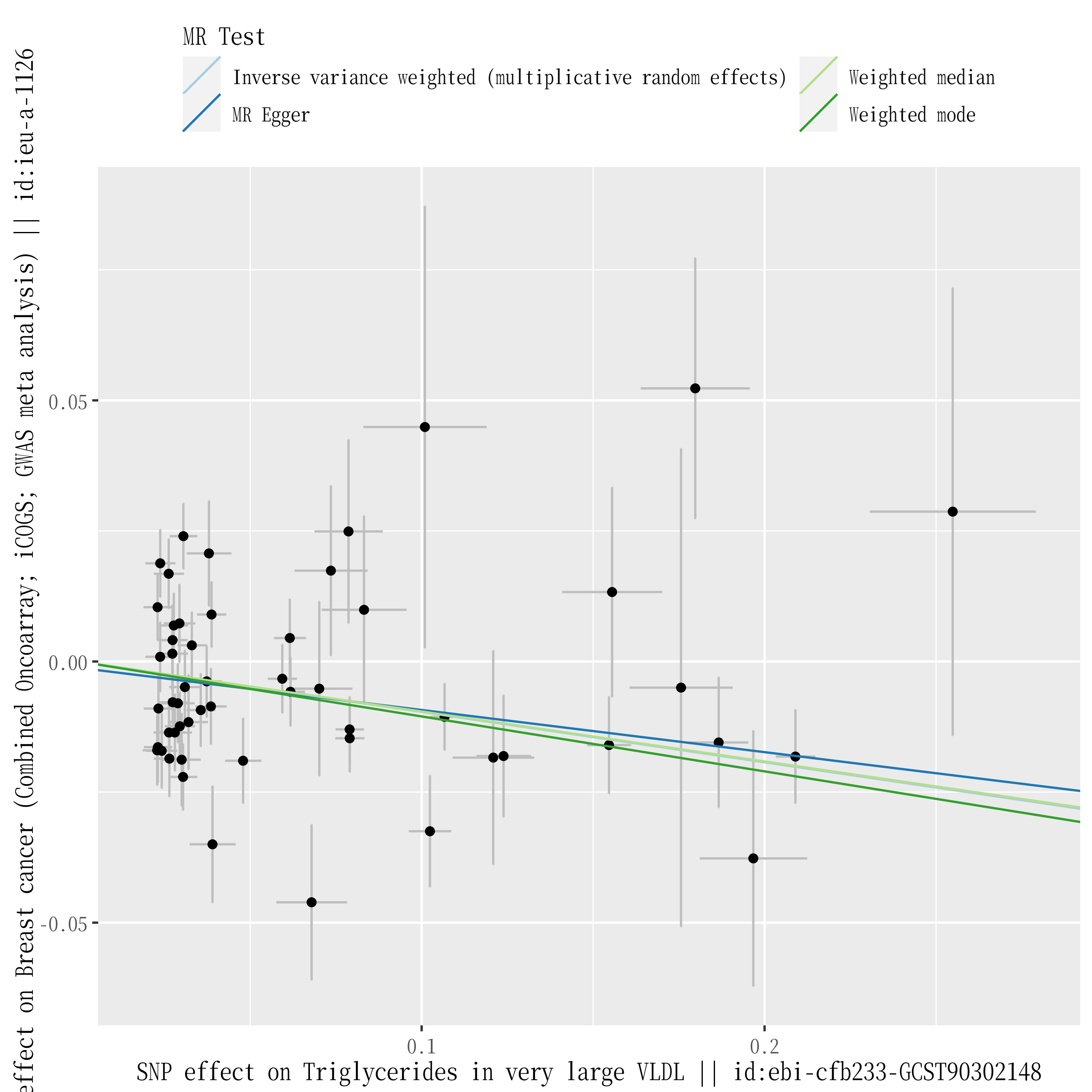
MR Test
Inverse variance weighted (multiplicative random effects)
MR Egger
Weighted median
Weighted mode
0.05
0.00
-0.05
0.1
0.2
SNP effect on Triglycerides in very large VLDL || id:ebi-cfb233-GCST90302148
effect on Breast cancer (Combined Oncoarray; iCOGS; GWAS meta analysis) || id:ieu-a-1126

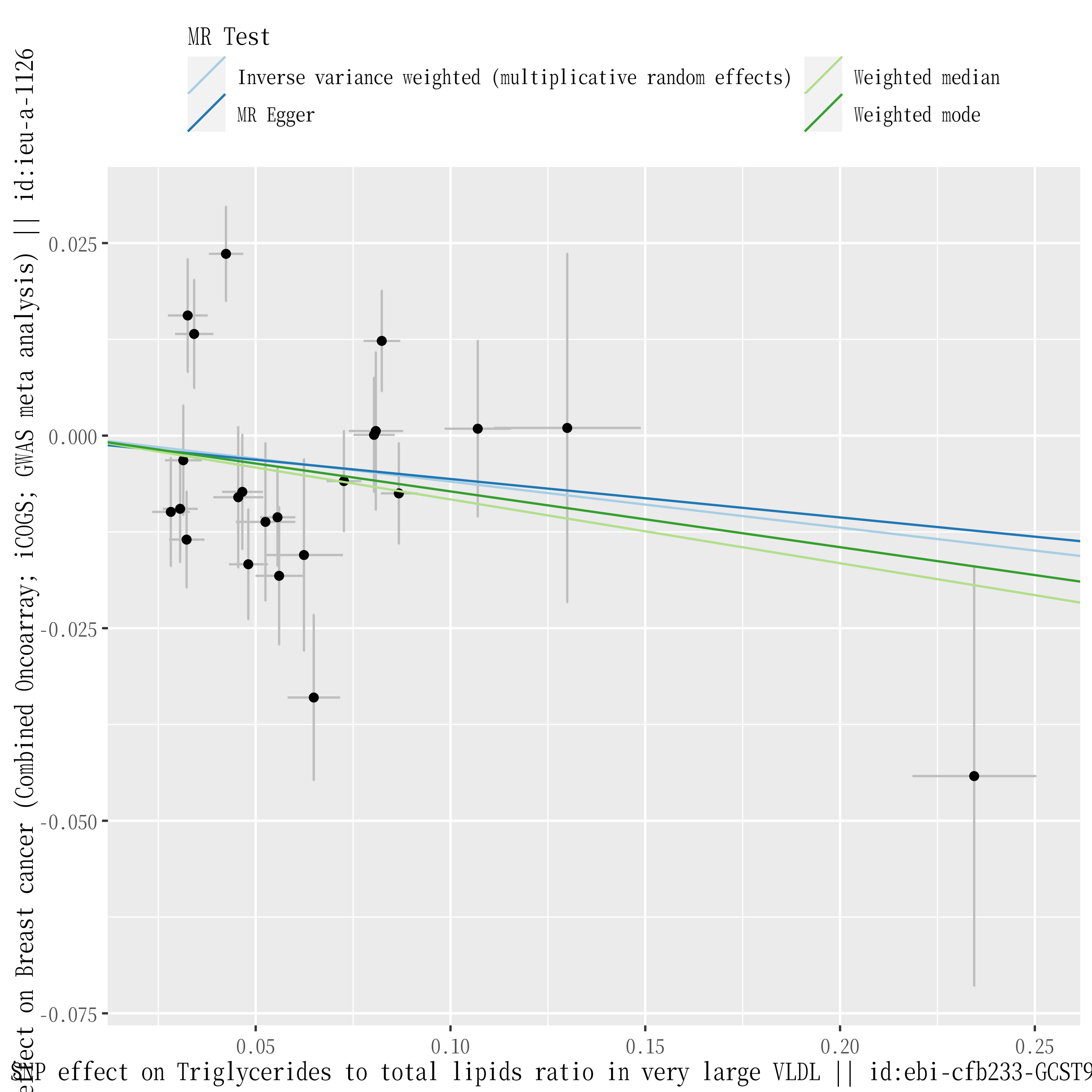
MR Test
Inverse variance weighted (multiplicative random effects)
MR Egger
Weighted median
Weighted mode
0.025
0.000
-0.025
-0.050
-0.075
0.05
0.10
0.15
0.20
0.25
SNP effect on Triglycerides to total lipids ratio in very large VLDL || id:ebi-cfb233-GCST9
effect on Breast cancer (Combined Oncoarray; iCOGS; GWAS meta analysis) || id:ieu-a-1126

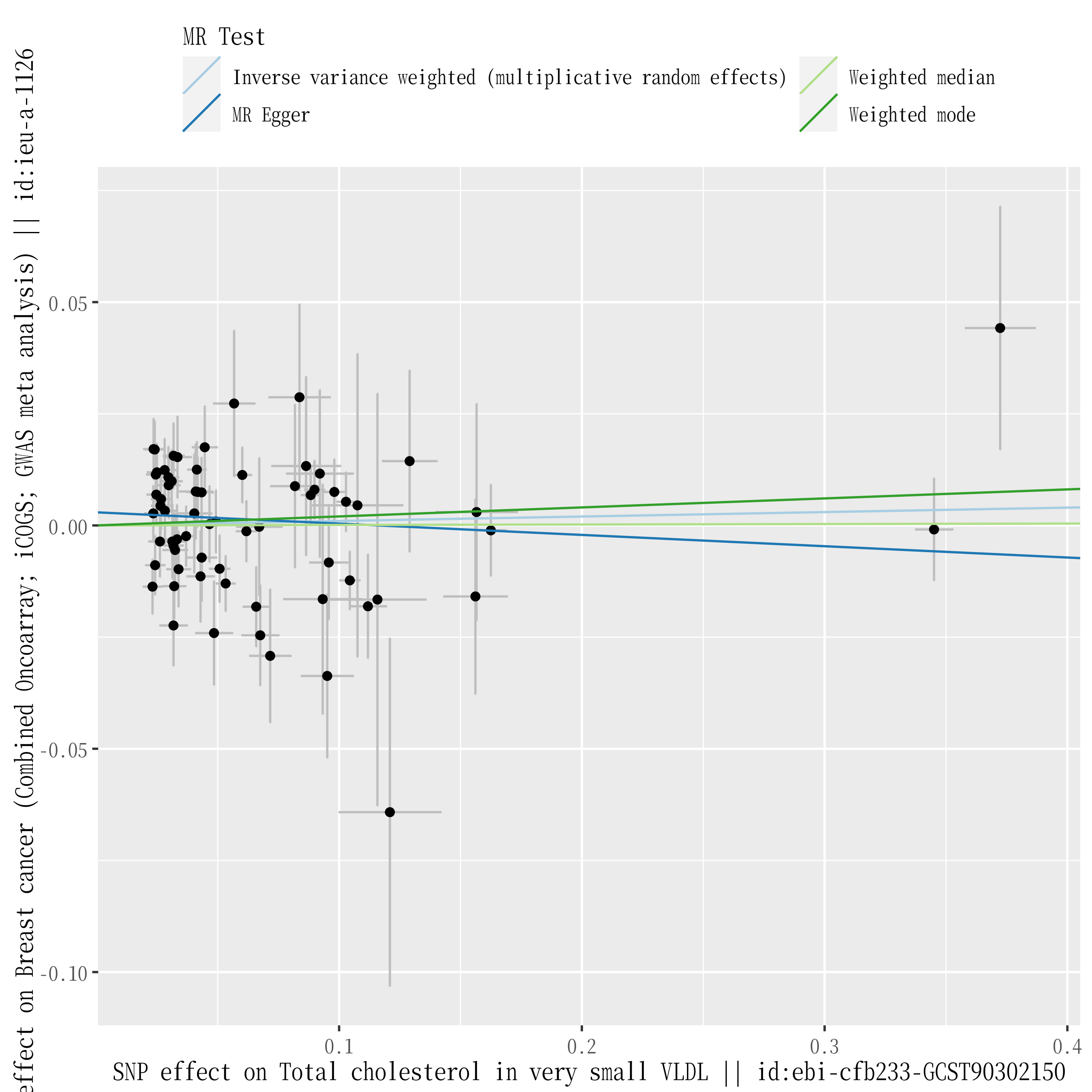

MR Test
Inverse variance weighted (multiplicative random effects)
MR Egger
Weighted median
Weighted mode
0.05
0.00
-0.05
-0.10
0.1
0.2
0.3
0.4
SNP effect on Total cholesterol in very small VLDL || id:ebi-cfb233-GCST90302150
effect on Breast cancer (Combined Oncoarray; iCOGS; GWAS meta analysis) || id:ieu-a-1126

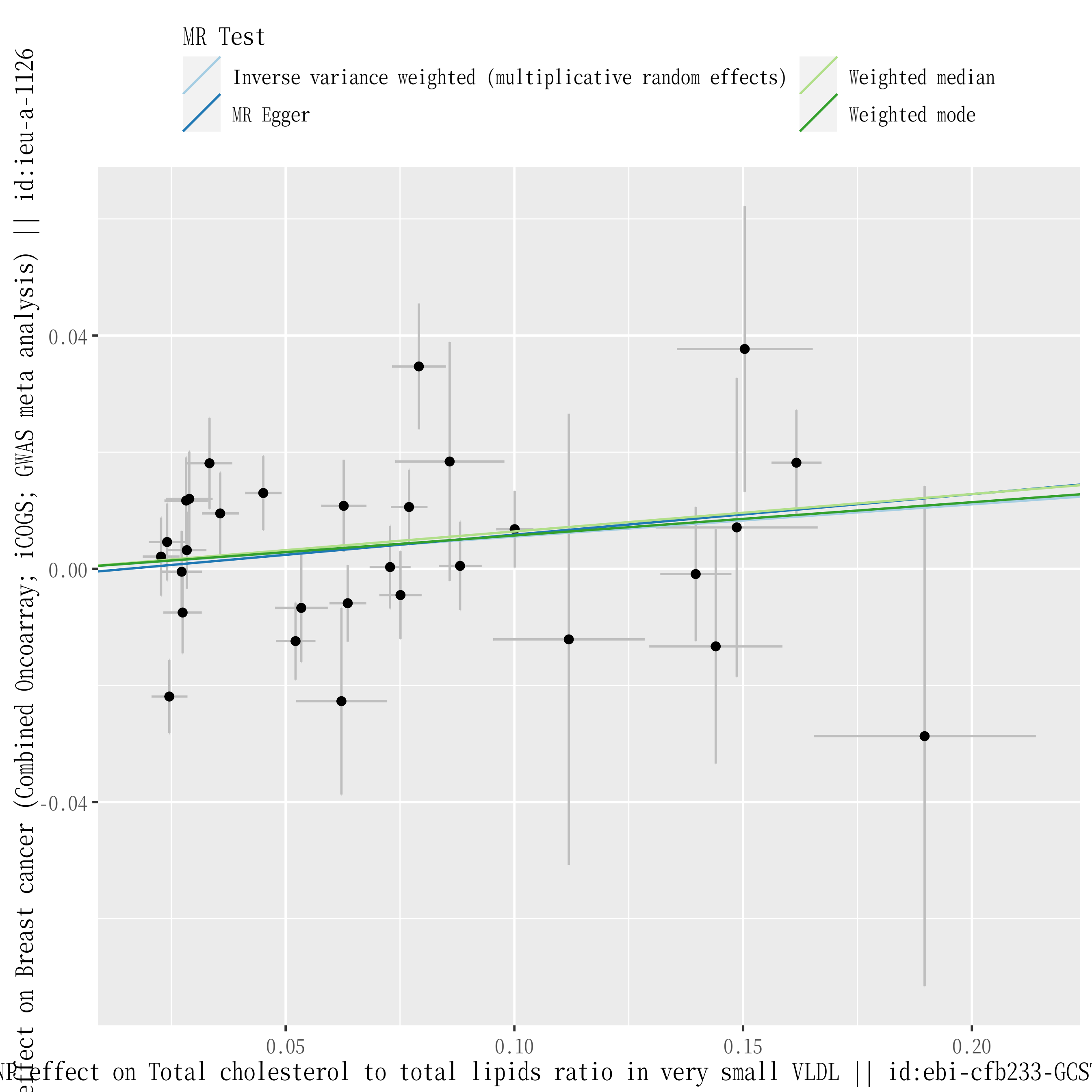

MR Test
Inverse variance weighted (multiplicative random effects)
MR Egger
Weighted median
Weighted mode
0.04
0.00
-0.04
0.05
0.10
0.15
0.20
SNP effect on Breast cancer (Combined Oncoarray; iCOGS; GWAS meta analysis) || id:ieu-a-1126
SNP effect on Total cholesterol to total lipids ratio in very small VLDL || id:ebi-cfb233-GCST

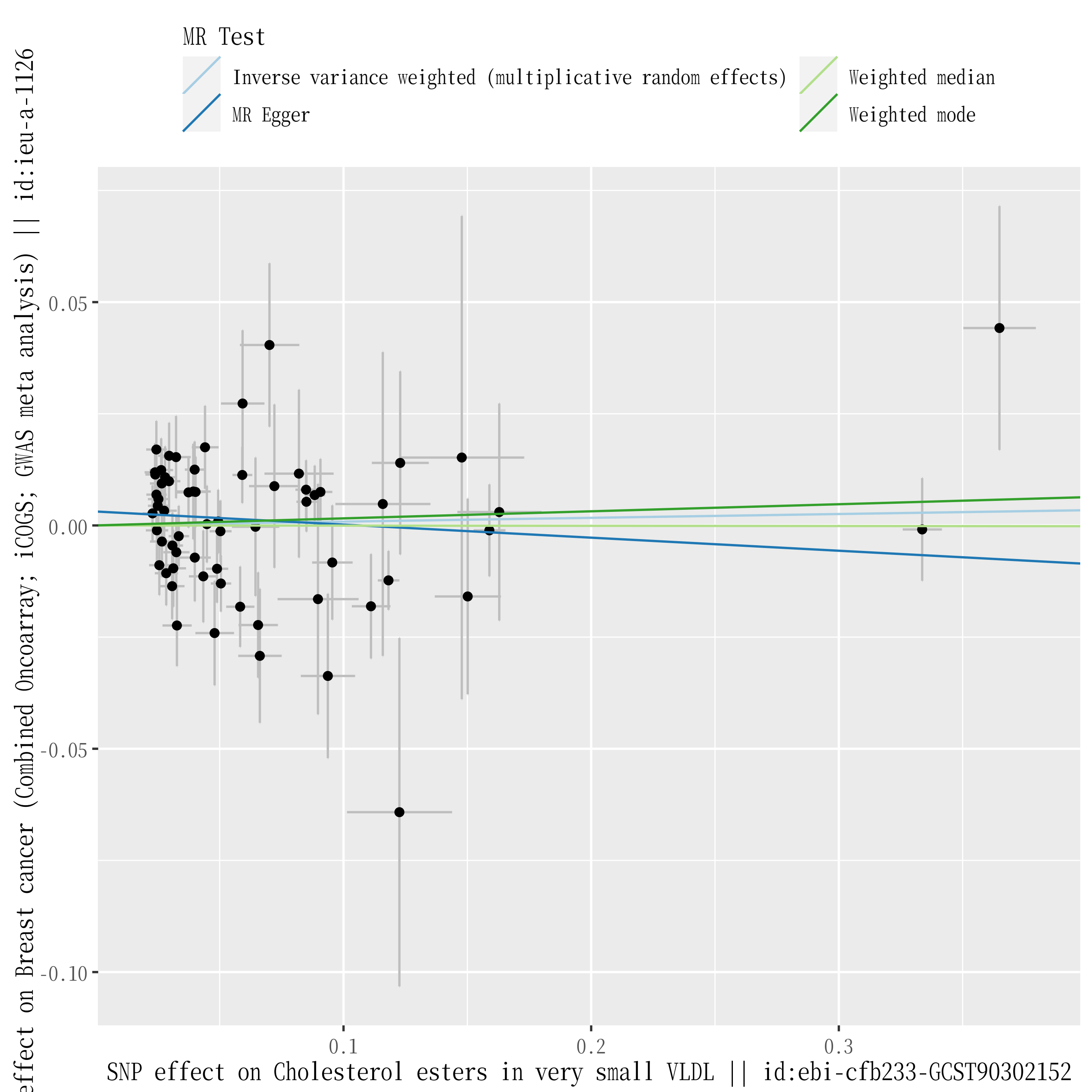
MR Test
Inverse variance weighted (multiplicative random effects)
MR Egger
Weighted median
Weighted mode
0.05
0.00
-0.05
-0.10
0.1
0.2
0.3
SNP effect on Cholesterol esters in very small VLDL || id:ebi-cfb233-GCST90302152
effect on Breast cancer (Combined Oncoarray; iCOGS; GWAS meta analysis) || id:ieu-a-1126

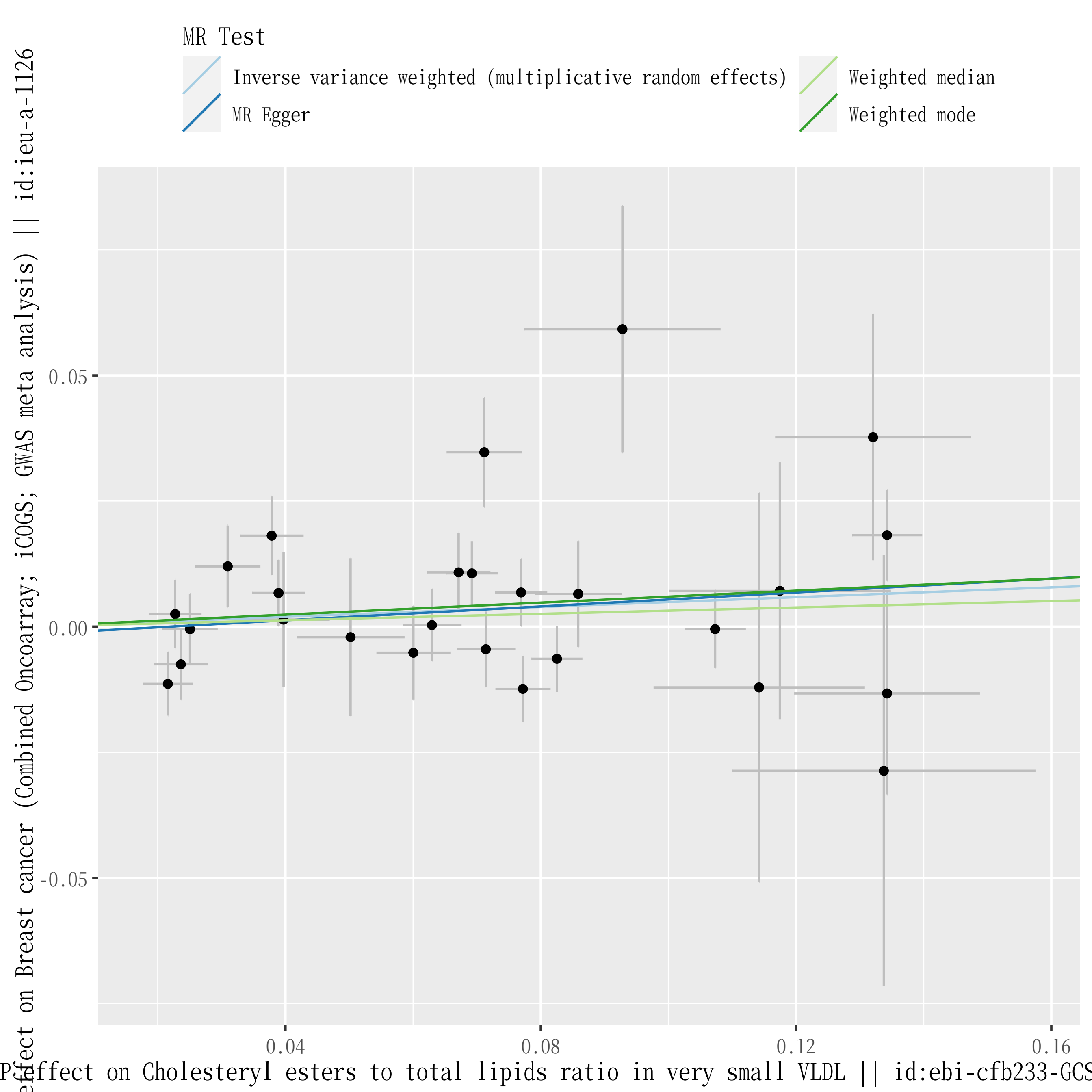
MR Test
Inverse variance weighted (multiplicative random effects)
MR Egger
Weighted median
Weighted mode
0.05
0.00
-0.05
0.04
0.08
0.12
0.16
SNP effect on Cholesteryl esters to total lipids ratio in very small VLDL || id:ebi-cfb233-GCS
SNP effect on Breast cancer (Combined Oncoarray; iCOGS; GWAS meta analysis) || id:ieu-a-1126

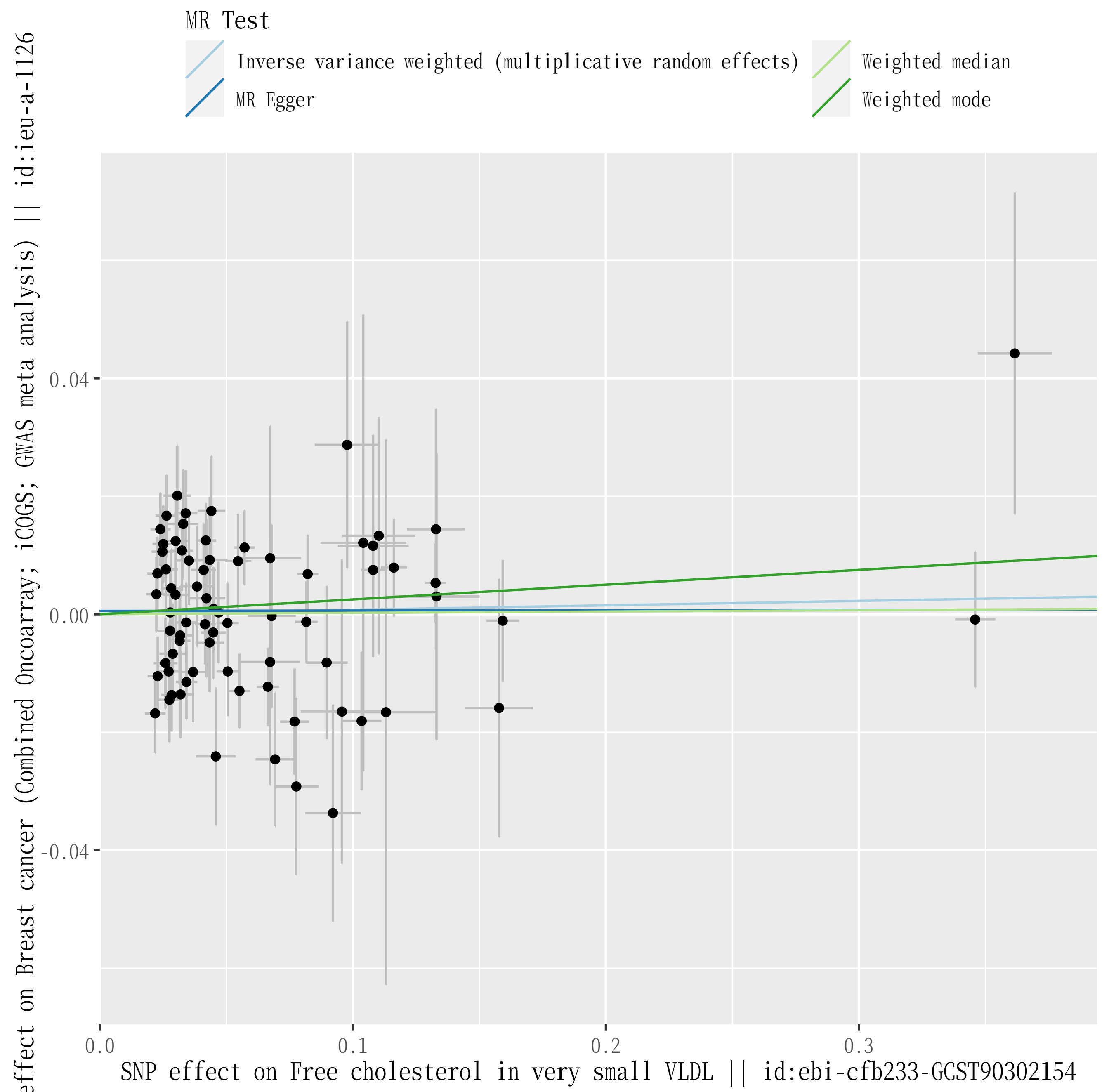

MR Test
Inverse variance weighted (multiplicative random effects)
MR Egger
Weighted median
Weighted mode
0.04
0.00
-0.04
0.0
0.1
0.2
0.3
SNP effect on Free cholesterol in very small VLDL || id:ebi-cfb233-GCST90302154
effect on Breast cancer (Combined Oncoarray; iCOGS; GWAS meta analysis) || id:ieu-a-1126

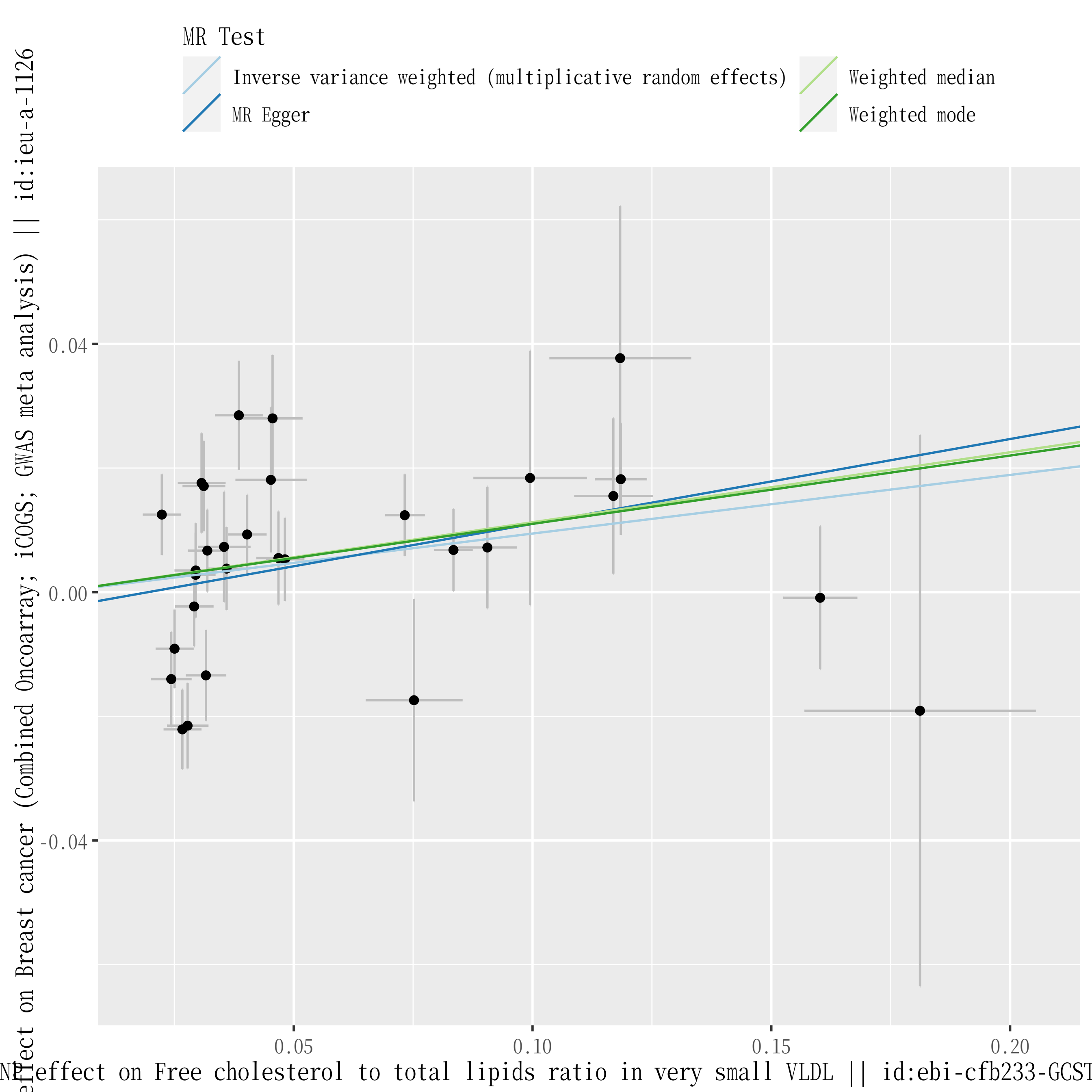
MR Test
Inverse variance weighted (multiplicative random effects)
MR Egger
Weighted median
Weighted mode
0.04
0.00
-0.04
0.05
0.10
0.15
0.20
effect on Breast cancer (Combined Oncoarray; iCOGS; GWAS meta analysis) || id:ieu-a-1126
effect on Free cholesterol to total lipids ratio in very small VLDL || id:ebi-cfb233-GCST

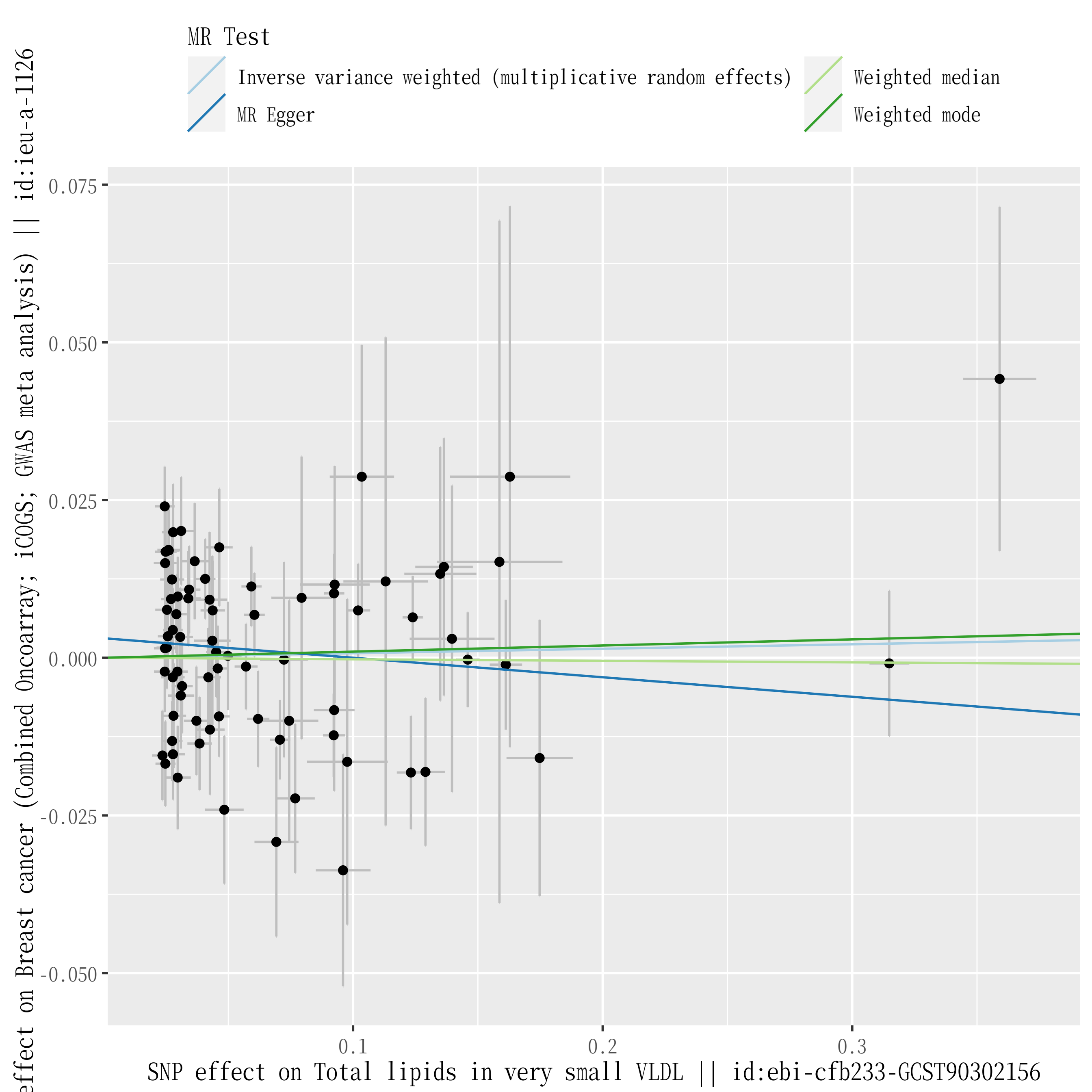
MR Test
Inverse variance weighted (multiplicative random effects)
MR Egger
Weighted median
Weighted mode
0.075
0.050
0.025
0.000
-0.025
-0.050
0.1
0.2
0.3
SNP effect on Breast cancer (Combined Oncoarray; iCOGS; GWAS meta analysis) || id:ieu-a-1126
SNP effect on Total lipids in very small VLDL || id:ebi-cfb233-GCST90302156

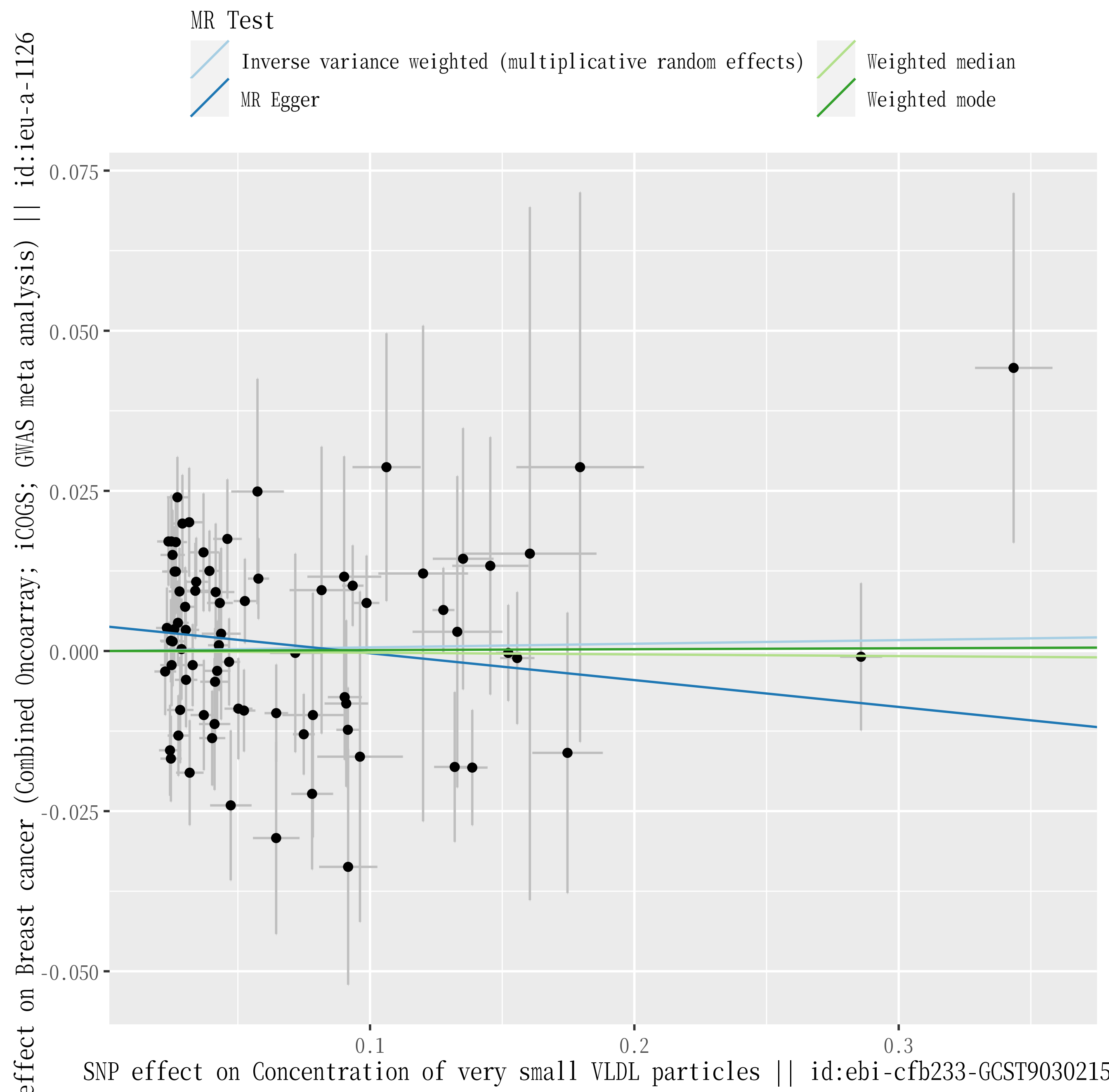

MR Test
Inverse variance weighted (multiplicative random effects)
MR Egger
Weighted median
Weighted mode
0.075
0.050
0.025
0.000
-0.025
-0.050
0.1
0.2
0.3
SNP effect on Concentration of very small VLDL particles || id:ebi-cfb233-GCST9030215
effect on Breast cancer (Combined Oncoarray; iCOGS; GWAS meta analysis) || id:ieu-a-1126

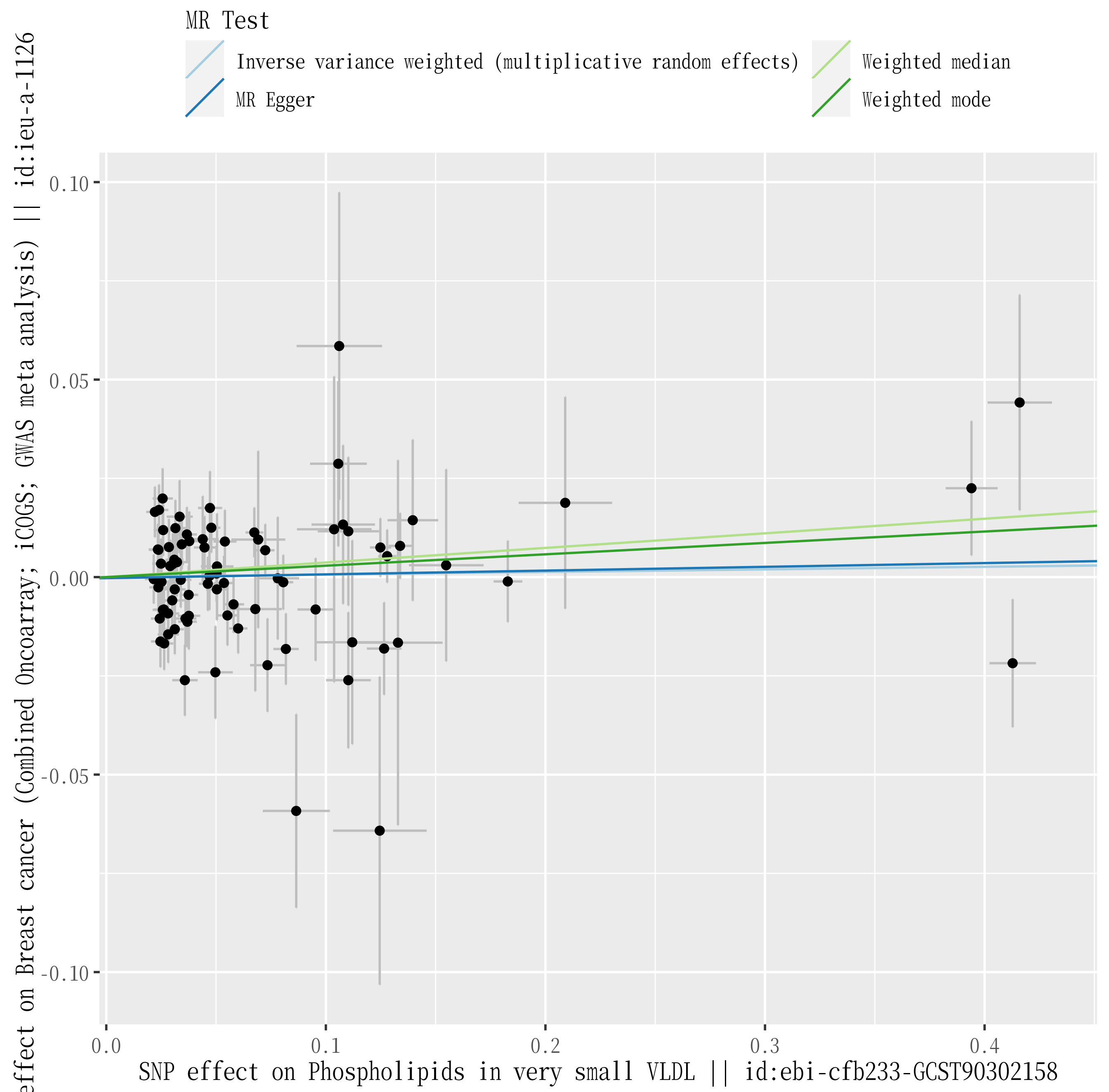
MR Test
Inverse variance weighted (multiplicative random effects)
MR Egger
Weighted median
Weighted mode
SNP effect on Breast cancer (Combined Oncoarray; iCOGS; GWAS meta analysis) || id:ieu-a-1126
0.10
0.05
0.00
-0.05
-0.10
0.0
0.1
0.2
0.3
0.4
SNP effect on Phospholipids in very small VLDL || id:ebi-cfb233-GCST90302158

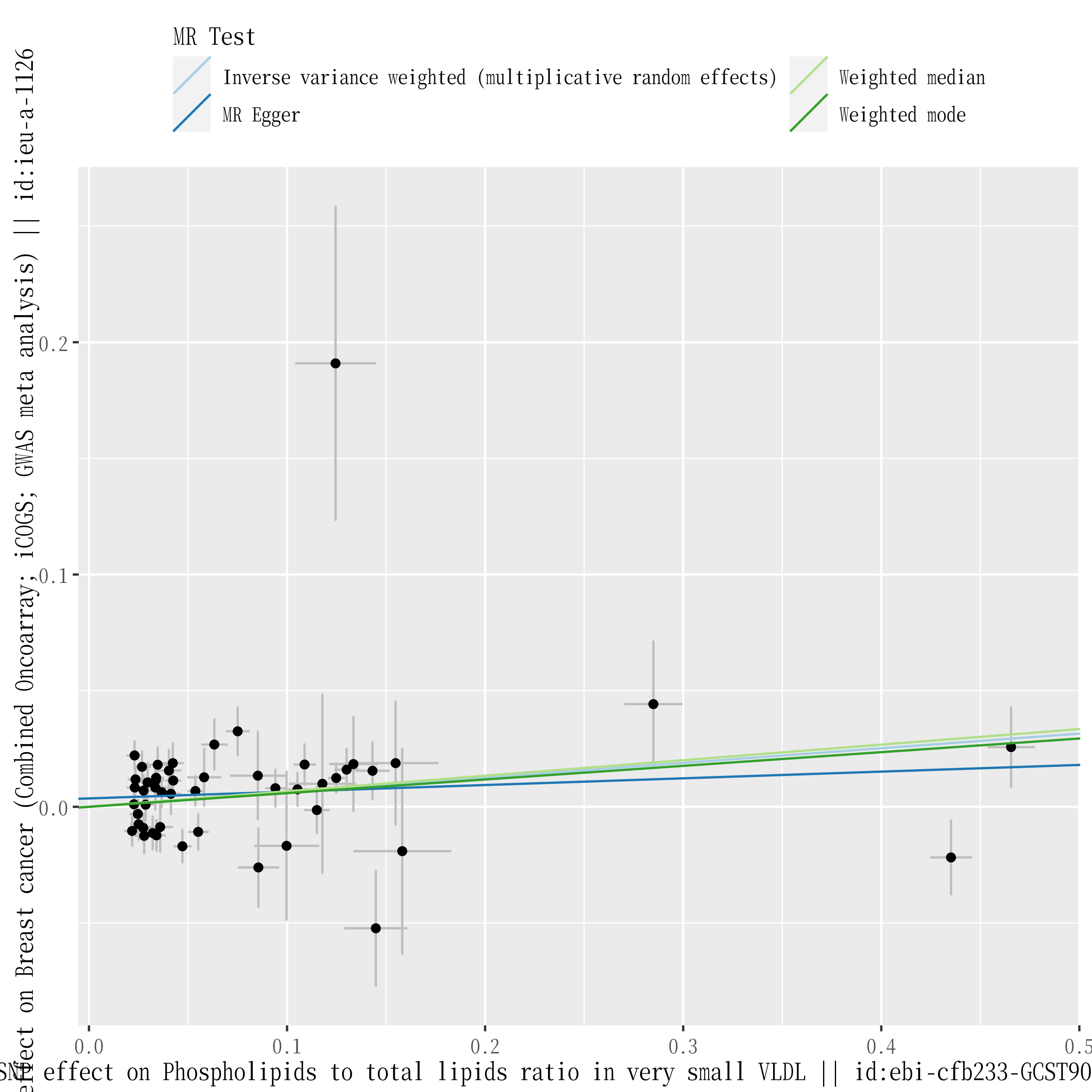

MR Test
Inverse variance weighted (multiplicative random effects)
MR Egger
Weighted median
Weighted mode
0.0
0.1
0.2
0.3
0.4
0.5
SNP effect on Phospholipids to total lipids ratio in very small VLDL || id:ebi-cfb233-GCST90
effect on Breast cancer (Combined Oncoarray; iCOGS; GWAS meta analysis) || id:ieu-a-1126

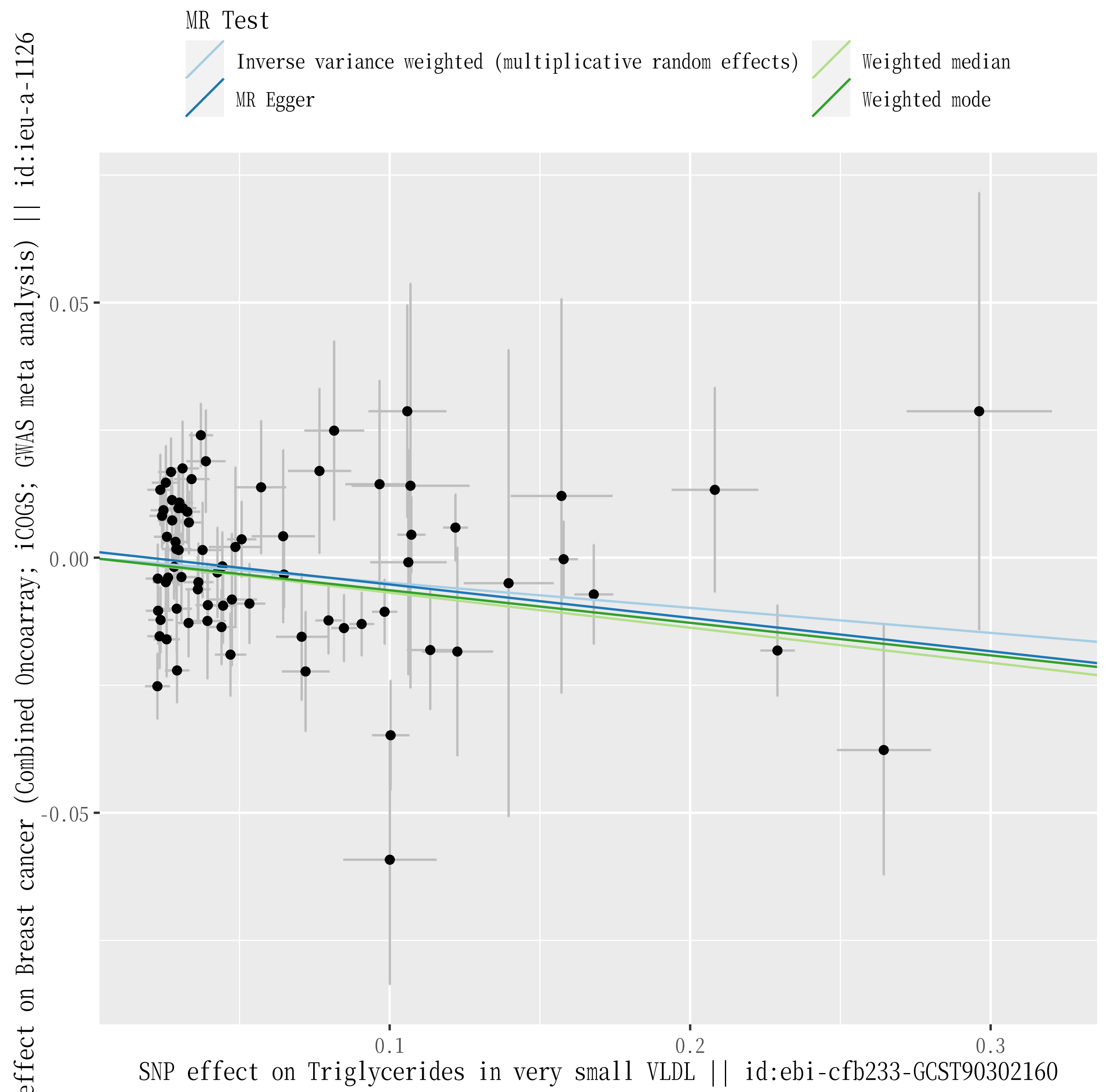
MR Test
Inverse variance weighted (multiplicative random effects)
MR Egger
Weighted median
Weighted mode
0.05
0.00
-0.05
0.1
0.2
0.3
SNP effect on Triglycerides in very small VLDL || id:ebi-cfb233-GCST90302160
effect on Breast cancer (Combined Oncoarray; iCOGS; GWAS meta analysis) || id:ieu-a-1126

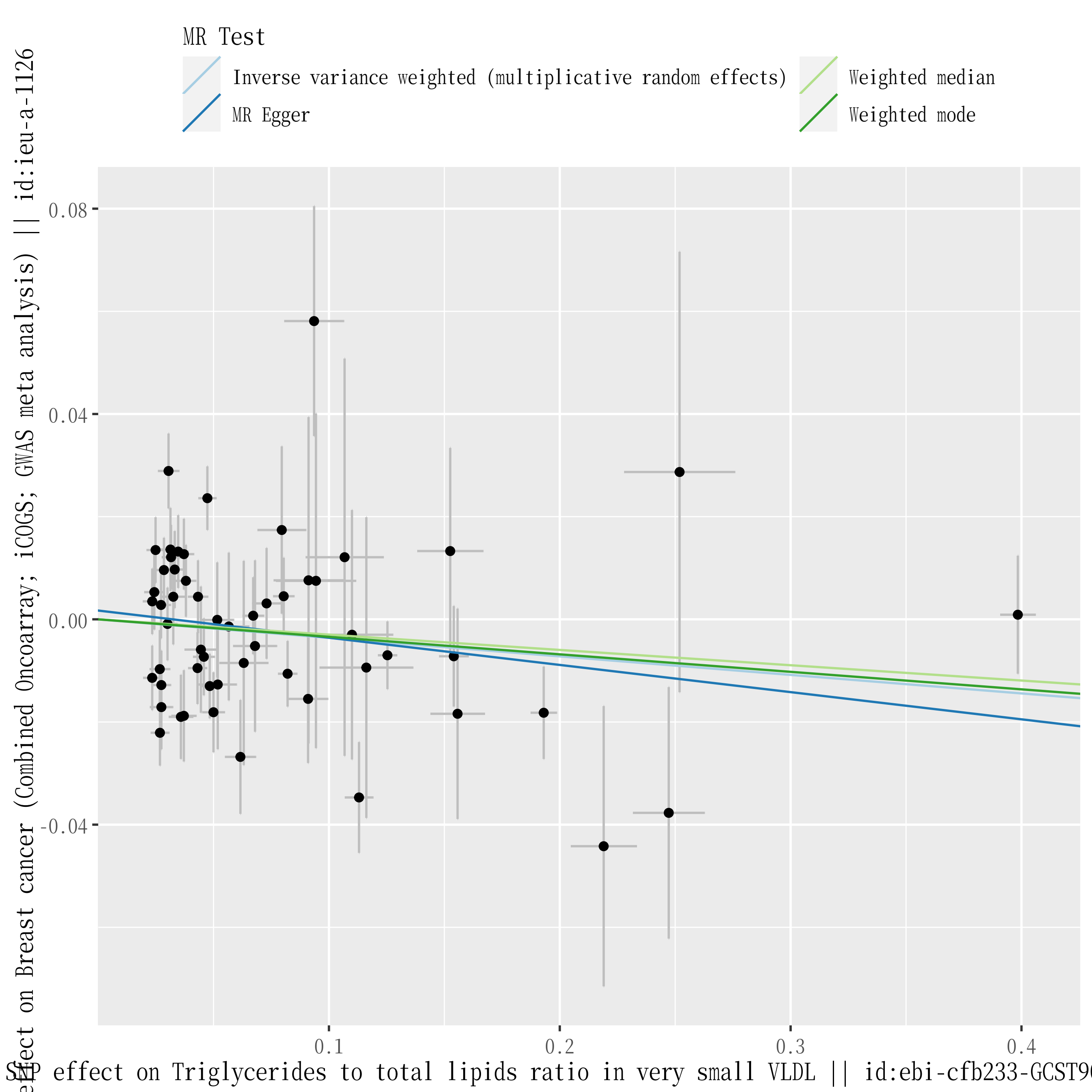

MR Test
Inverse variance weighted (multiplicative random effects)
MR Egger
Weighted median
Weighted mode
0.08
0.04
0.00
-0.04
0.1
0.2
0.3
0.4
SNP effect on Triglycerides to total lipids ratio in very small VLDL || id:ebi-cfb233-GCST90
effect on Breast cancer (Combined Oncoarray; iCOGS; GWAS meta analysis) || id:ieu-a-1126

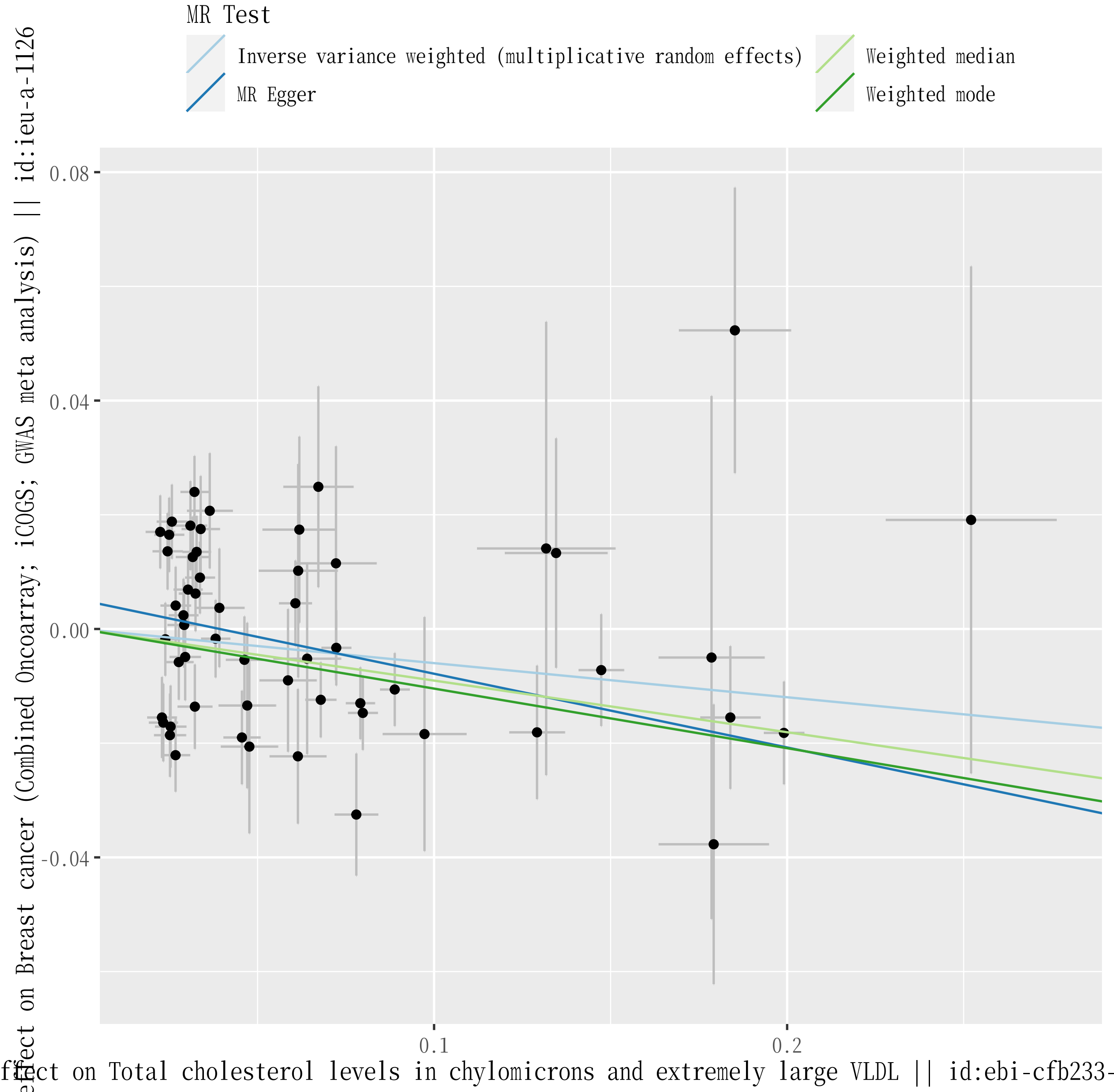

MR Test
Inverse variance weighted (multiplicative random effects)
MR Egger
Weighted median
Weighted mode
0.08
0.04
0.00
-0.04
0.1
0.2
effect on Breast cancer (Combined Oncoarray; iCOGS; GWAS meta analysis) || id:ieu-a-1126
ffect on Total cholesterol levels in chylomicrons and extremely large VLDL || id:ebi-cfb233-

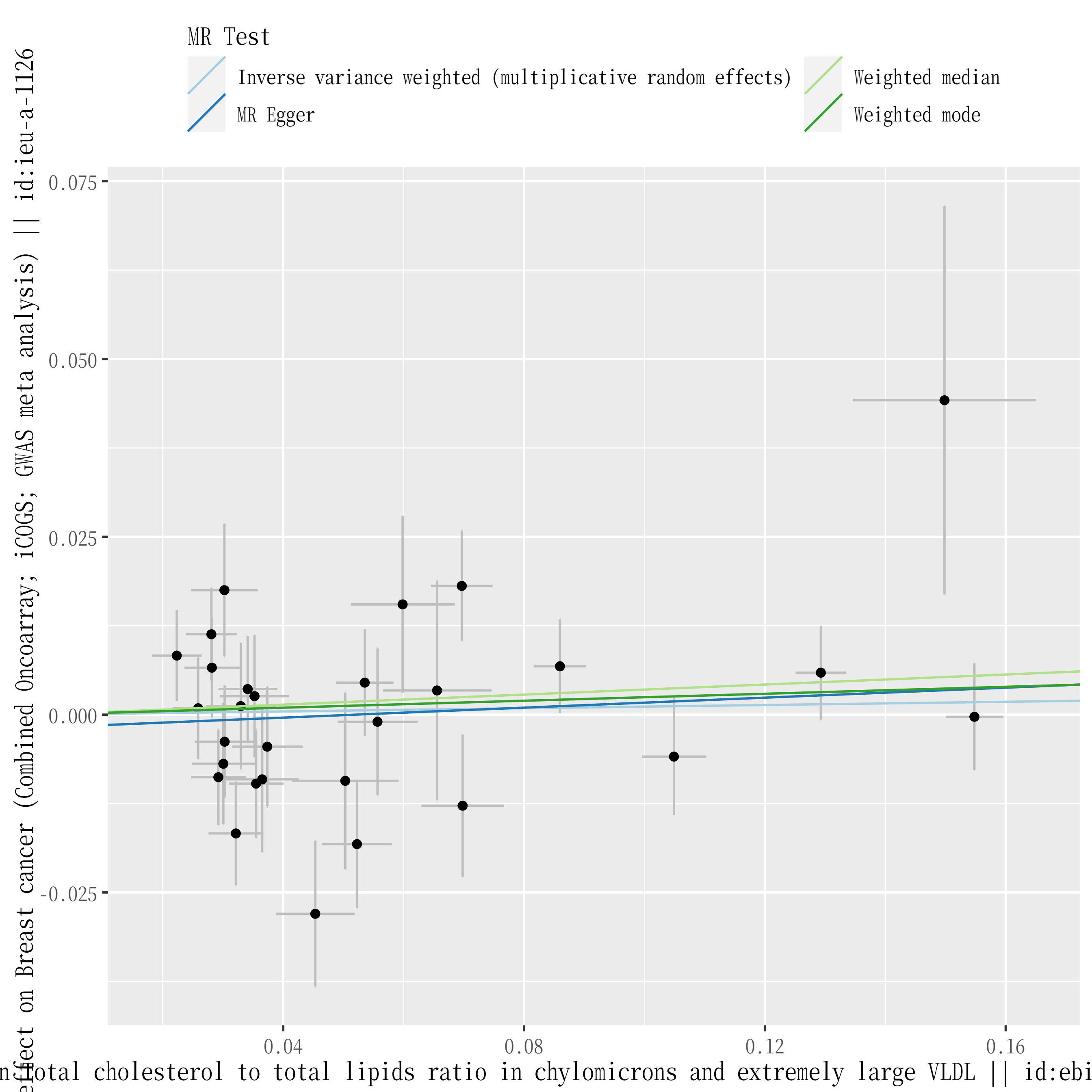
MR Test
Inverse variance weighted (multiplicative random effects)
MR Egger
Weighted median
Weighted mode
0.075
0.050
0.025
0.000
-0.025
0.04
0.08
0.12
0.16
n Total cholesterol to total lipids ratio in chylomicrons and extremely large VLDL || id:ebi
effect on Breast cancer (Combined Oncoarray; iCOGS; GWAS meta analysis) || id:ieu-a-1126

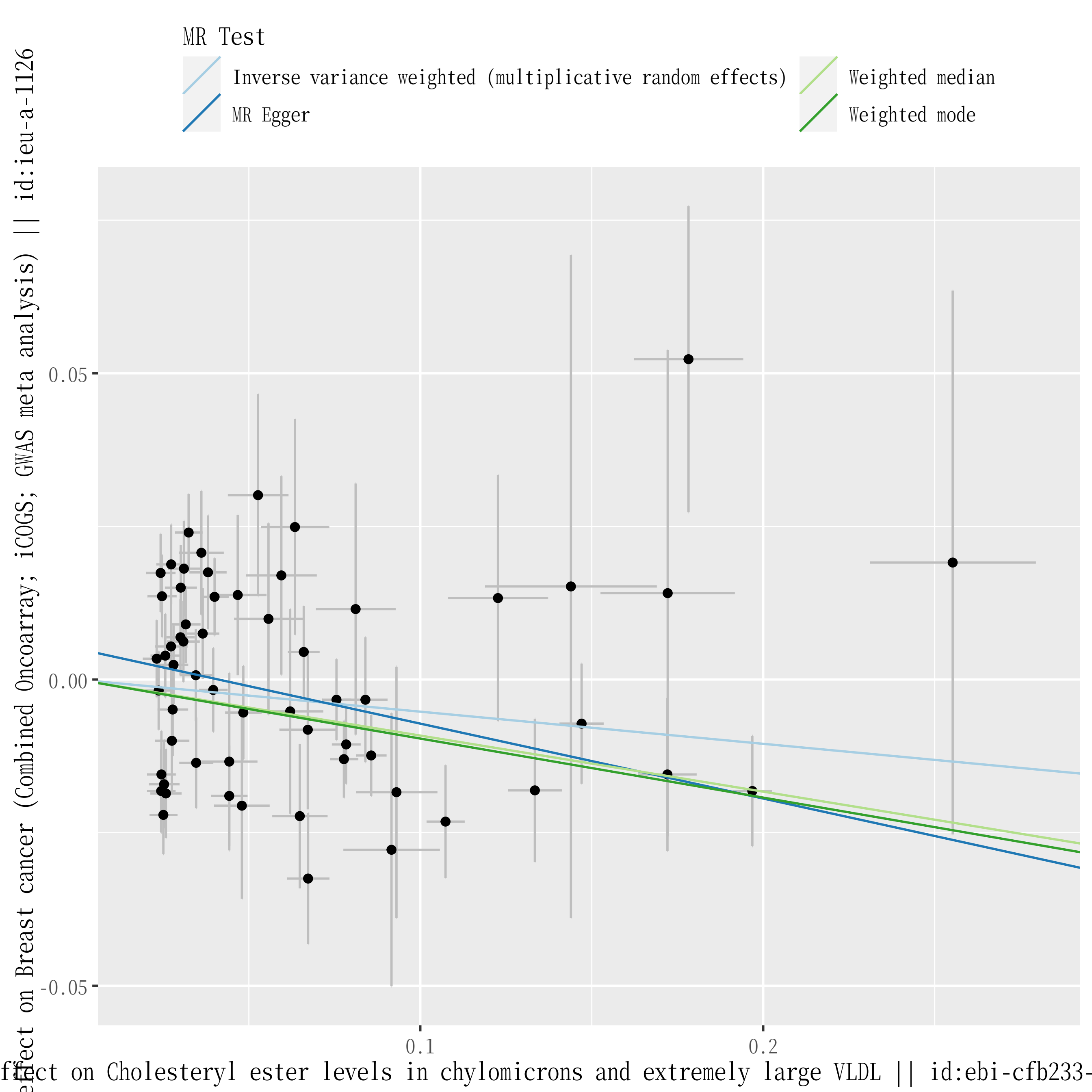

MR Test
Inverse variance weighted (multiplicative random effects)
MR Egger
Weighted median
Weighted mode
0.05
0.00
-0.05
0.1
0.2
SNP effect on Breast cancer (Combined Oncoarray; iCOGS; GWAS meta analysis) || id:ieu-a-1126
SNP effect on Cholesteryl ester levels in chylomicrons and extremely large VLDL || id:ebi-cfb233-

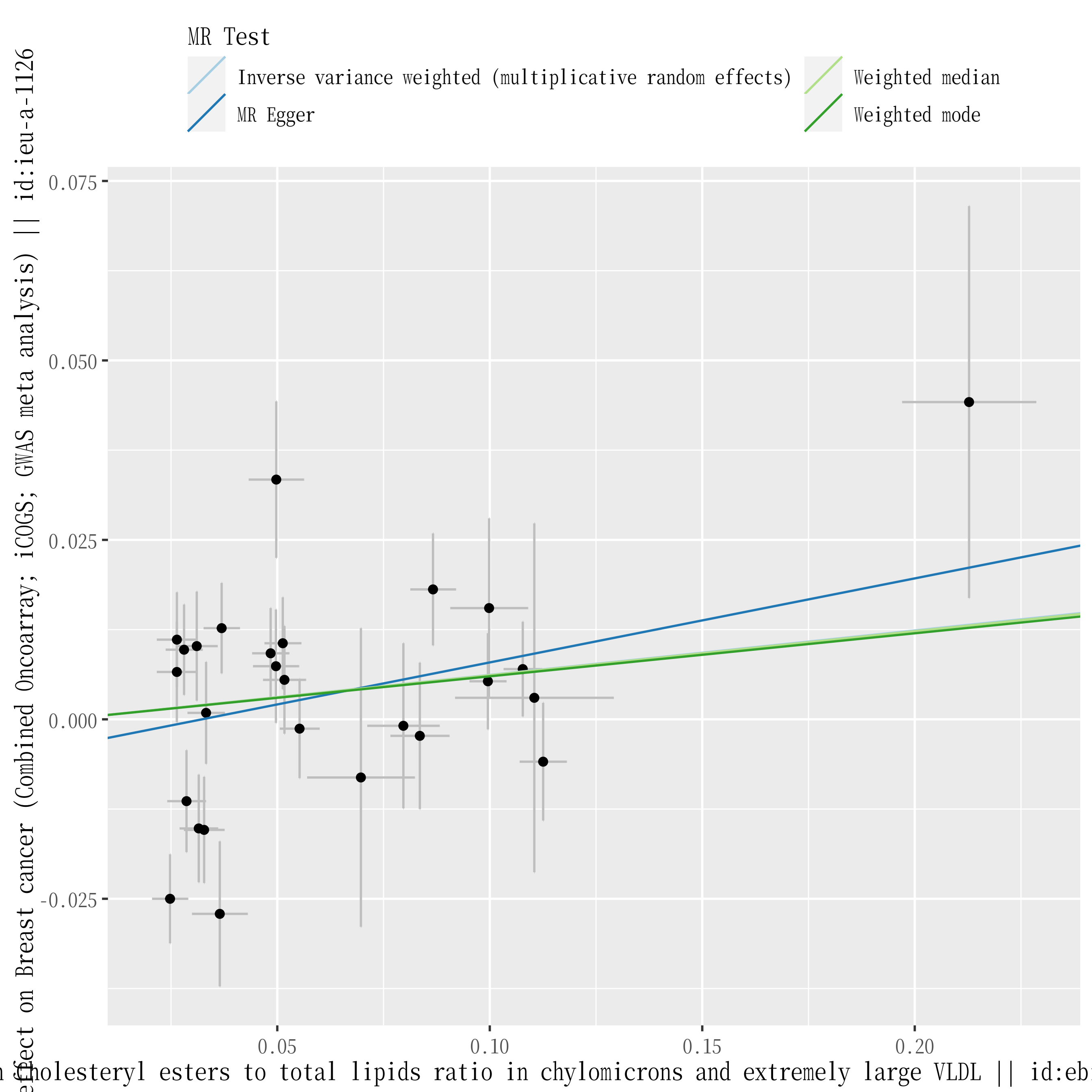

MR Test
Inverse variance weighted (multiplicative random effects)
MR Egger
Weighted median
Weighted mode
0.075
0.050
0.025
0.000
-0.025
0.05
0.10
0.15
0.20
effect on Breast cancer (Combined Oncoarray; iCOGS; GWAS meta analysis) || id:ieu-a-1126
cholesteryl esters to total lipids ratio in chylomicrons and extremely large VLDL || id:eb

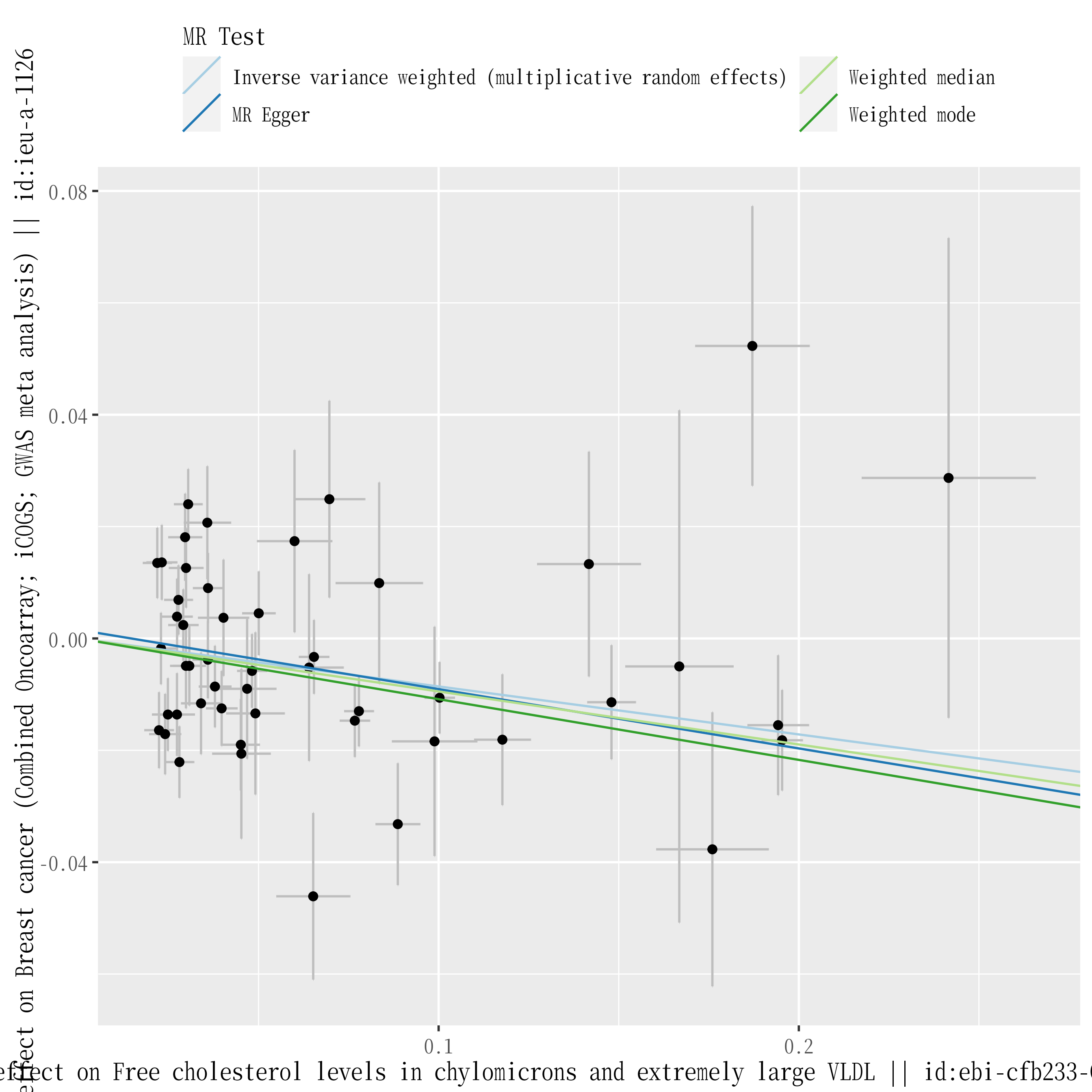

MR Test
Inverse variance weighted (multiplicative random effects)
MR Egger
Weighted median
Weighted mode
0.08
0.04
0.00
-0.04
0.1
0.2
effect on Breast cancer (Combined Oncoarray; iCOGS; GWAS meta analysis) || id:ieu-a-1126
effect on Free cholesterol levels in chylomicrons and extremely large VLDL || id:ebi-cfb233-

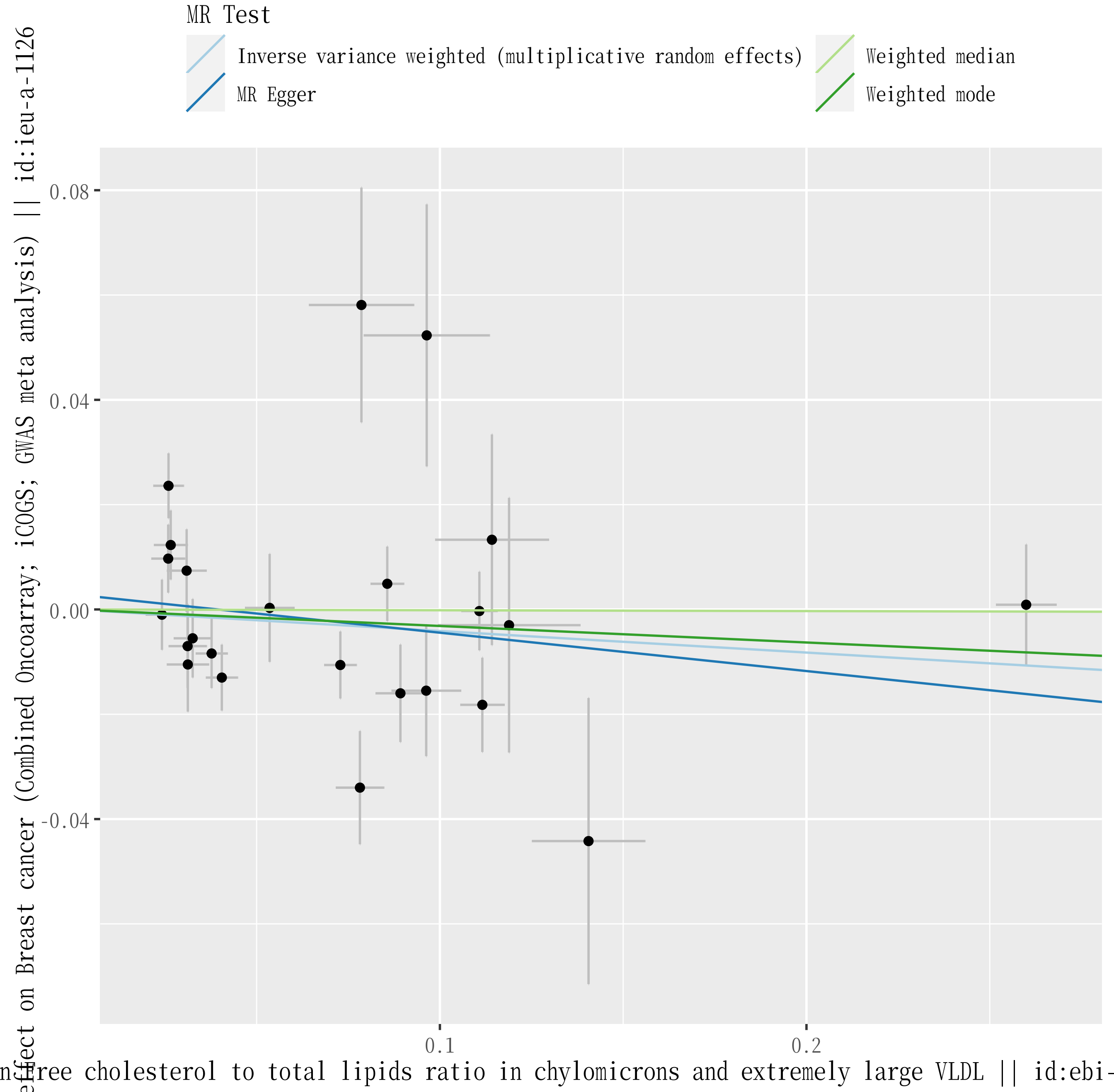

MR Test
Inverse variance weighted (multiplicative random effects)
MR Egger
Weighted median
Weighted mode
0.08
0.04
0.00
-0.04
0.1
0.2
n-free cholesterol to total lipids ratio in chylomicrons and extremely large VLDL || id:ebi-
effect on Breast cancer (Combined Oncoarray; iCOGS; GWAS meta analysis) || id:ieu-a-1126

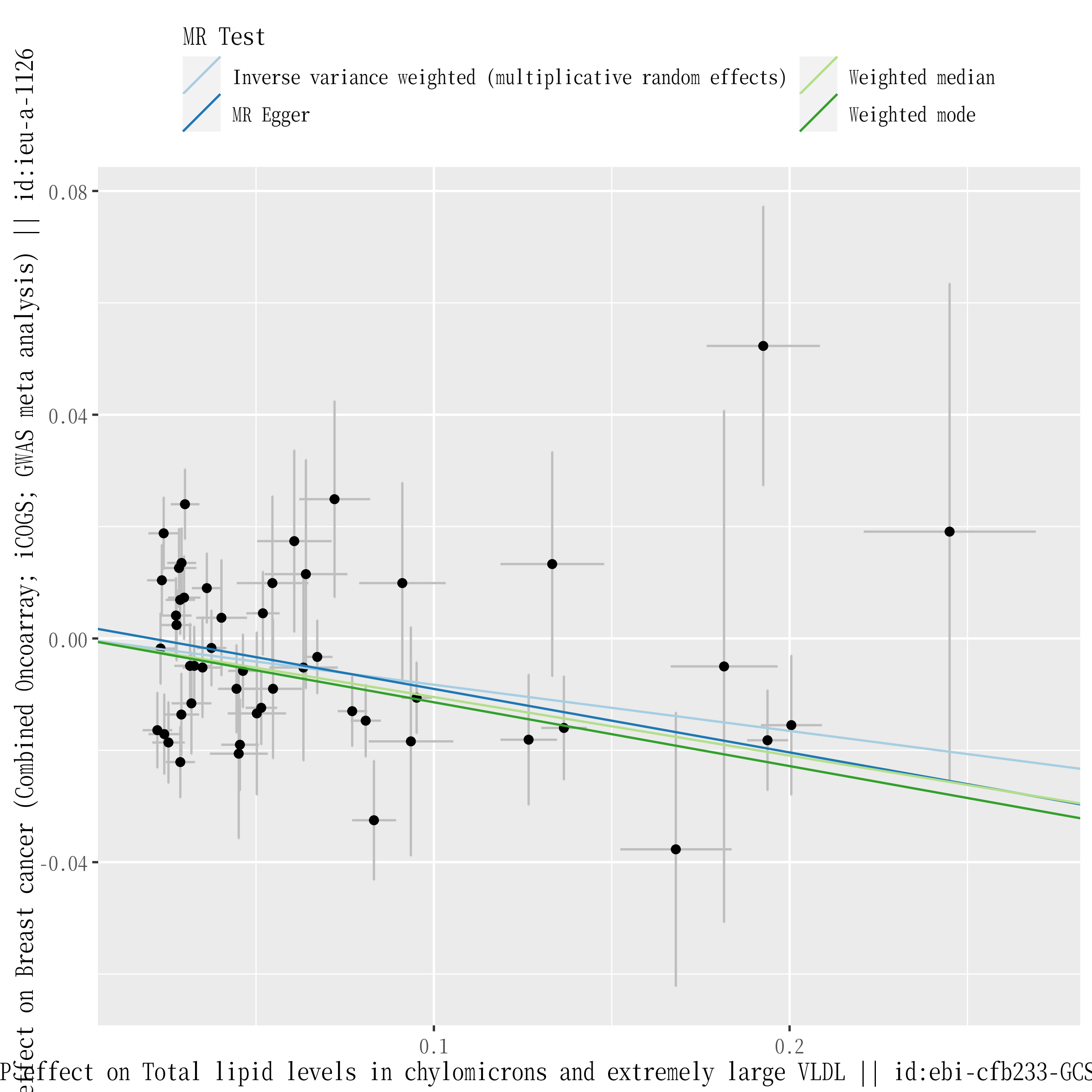
MR Test
Inverse variance weighted (multiplicative random effects)
MR Egger
Weighted median
Weighted mode
0.08
0.04
0.00
-0.04
0.1
0.2
SNP effect on Total lipid levels in chylomicrons and extremely large VLDL || id:ebi-cfb233-GCS
SNP effect on Breast cancer (Combined Oncoarray; iCOGS; GWAS meta analysis) || id:ieu-a-1126

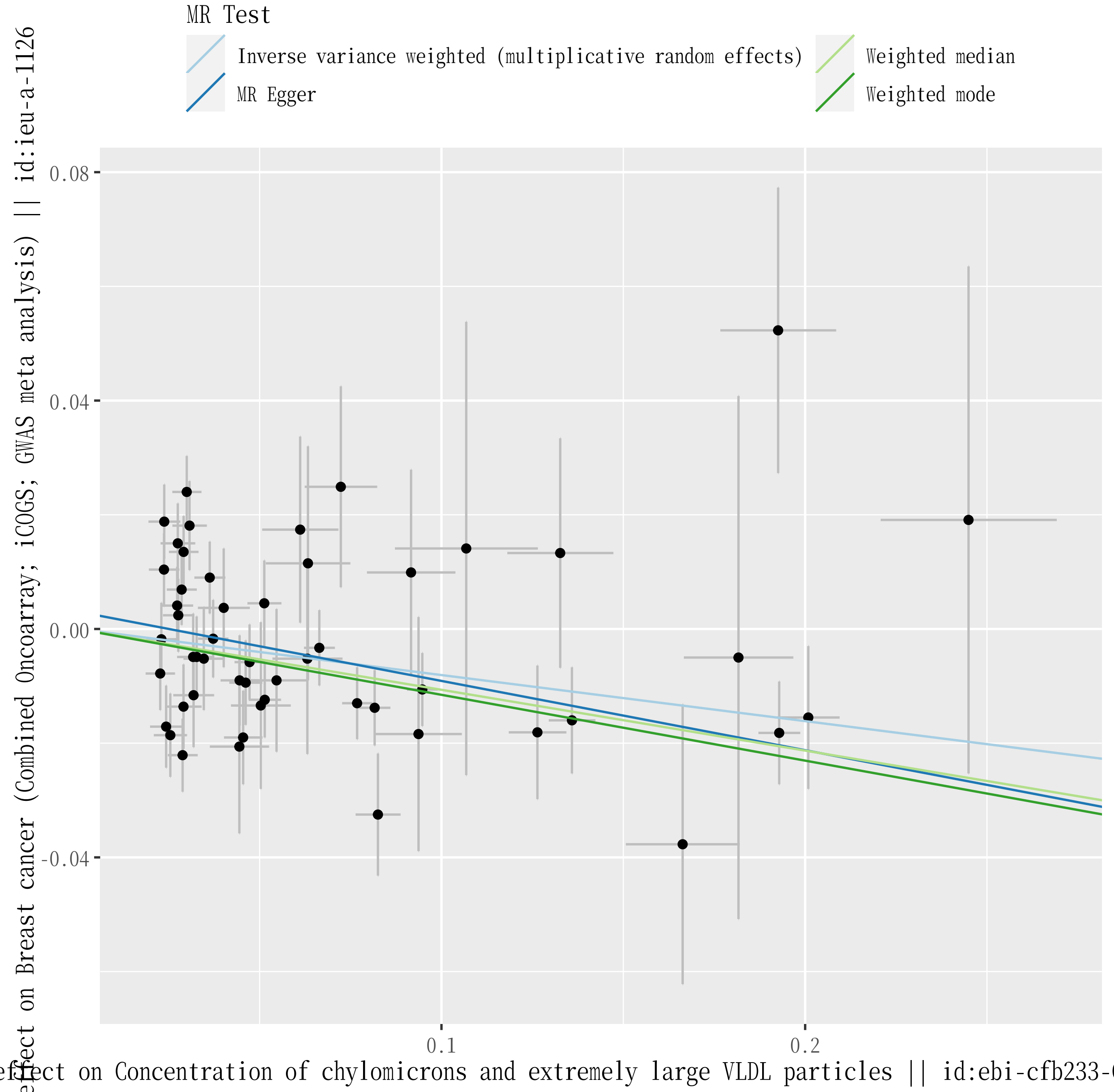
MR Test
Inverse variance weighted (multiplicative random effects)
MR Egger
Weighted median
Weighted mode
0.08
0.04
0.00
-0.04
0.1
0.2
effect on Concentration of chylomicrons and extremely large VLDL particles || id:ebi-cfb233-
effect on Breast cancer (Combined Oncoarray; iCOGS; GWAS meta analysis) || id:ieu-a-1126

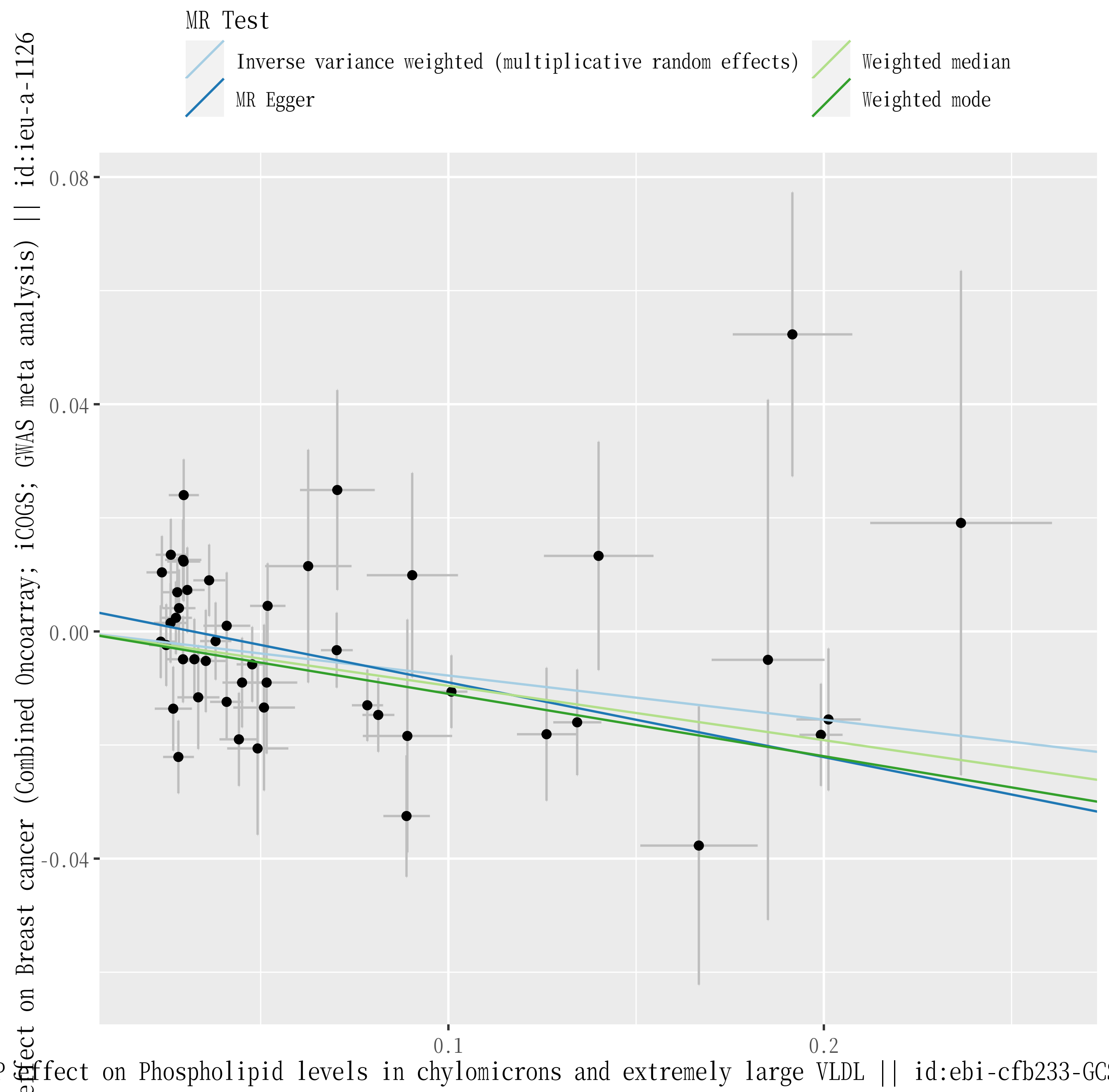

MR Test
Inverse variance weighted (multiplicative random effects)
MR Egger
Weighted median
Weighted mode
0.08
0.04
0.00
-0.04
0.1
0.2
effect on Breast cancer (Combined Oncoarray; iCOGS; GWAS meta analysis) || id:ieu-a-1126
effect on Phospholipid levels in chylomicrons and extremely large VLDL || id:ebi-a-GCST

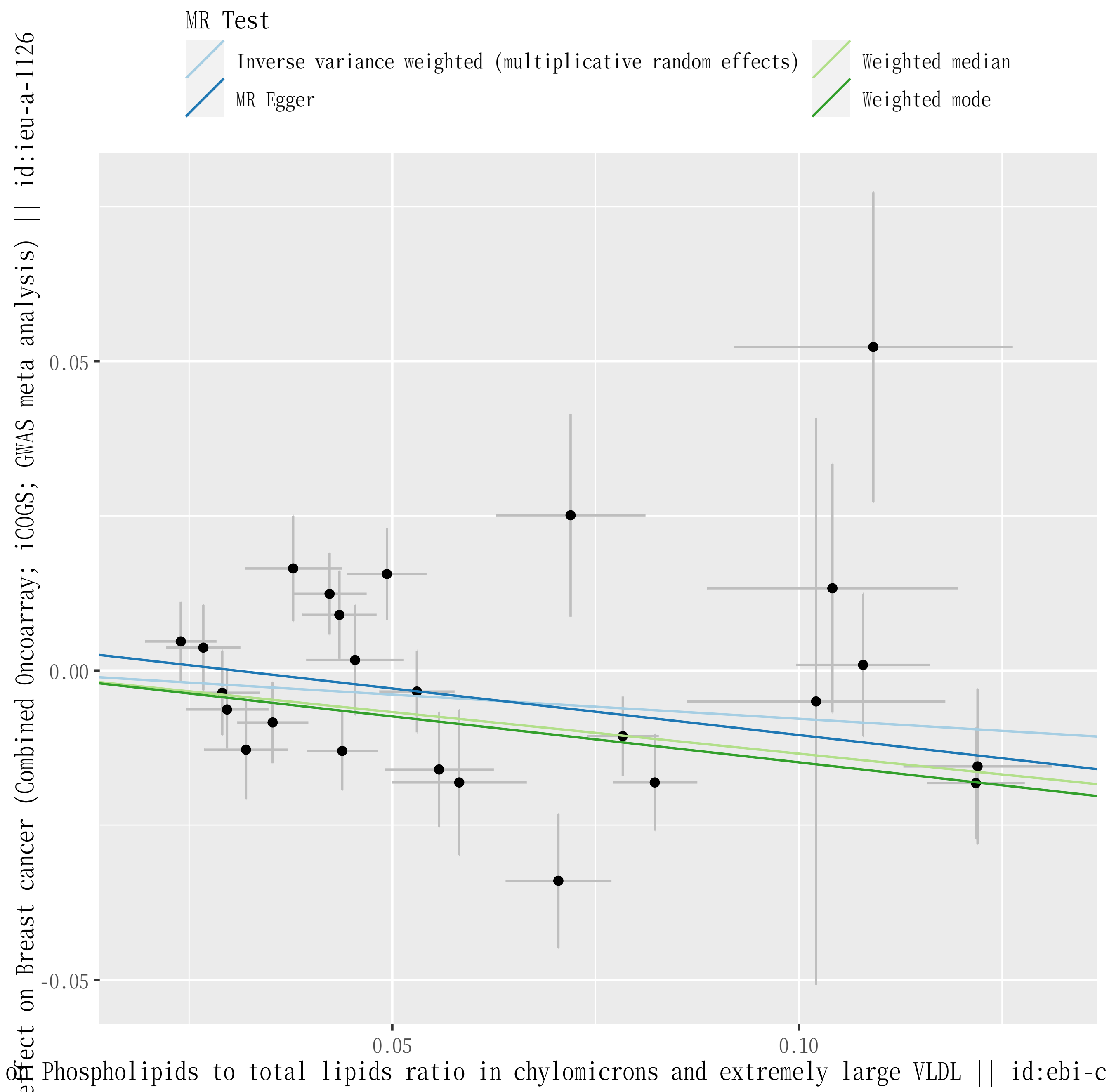

MR Test
Inverse variance weighted (multiplicative random effects)
MR Egger
Weighted median
Weighted mode
0.05
0.00
-0.05
0.05
0.10
SNP effect on Breast cancer (Combined Oncoarray; iCOGS; GWAS meta analysis) || id:ieu-a-1126
SNP effect on Phospholipids to total lipids ratio in chylomicrons and extremely large VLDL || id:ebi-a

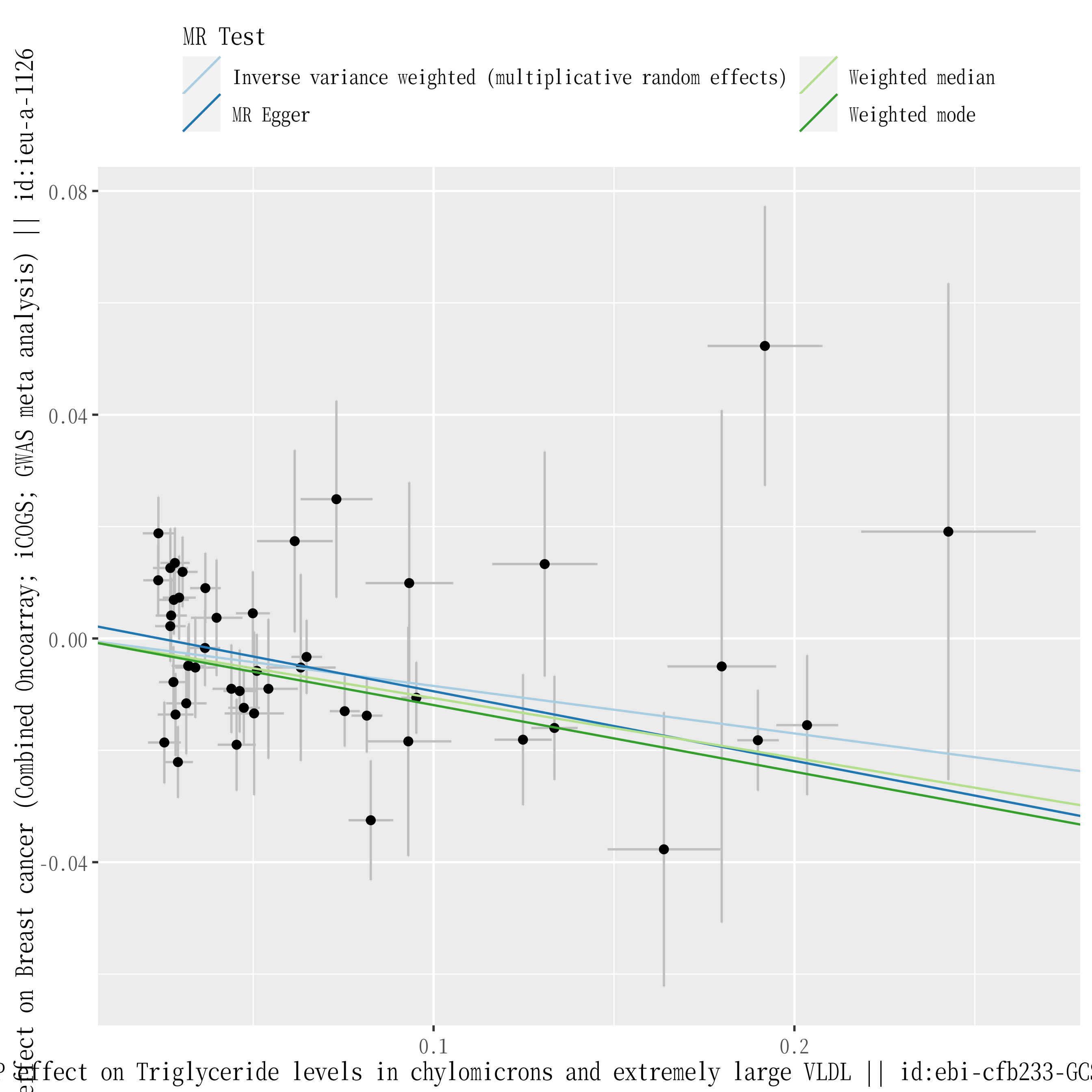

MR Test
Inverse variance weighted (multiplicative random effects)
MR Egger
Weighted median
Weighted mode
0.08
0.04
0.00
-0.04
0.1
0.2
SNP effect on Triglyceride levels in chylomicrons and extremely large VLDL || id:ebi-cfb233-GCS
SNP effect on Breast cancer (Combined Oncoarray; iCOGS; GWAS meta analysis) || id:ieu-a-1126

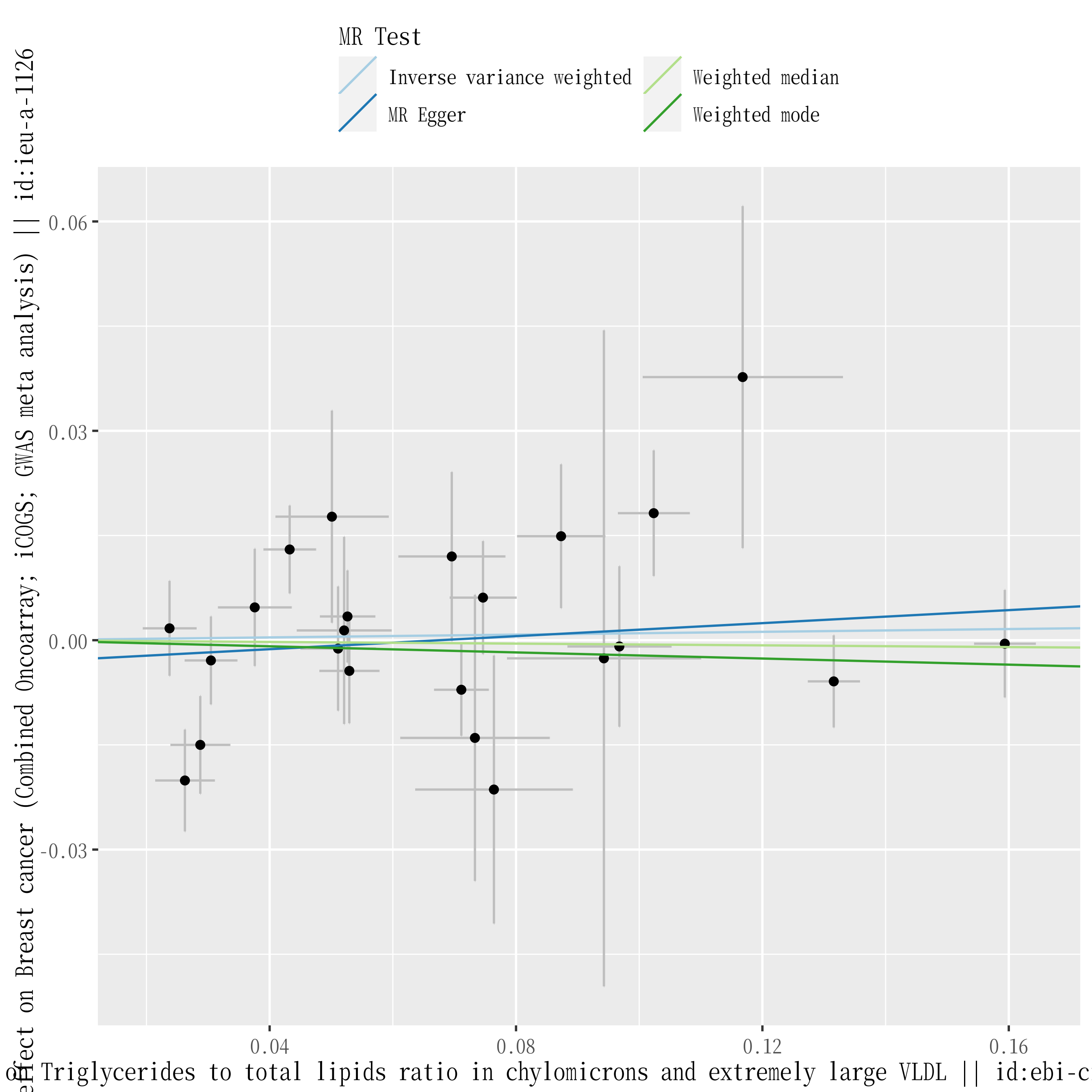
MR Test
Inverse variance weighted
MR Egger
Weighted median
Weighted mode
0.06
0.03
0.00
-0.03
0.04
0.08
0.12
0.16
effect on Breast cancer (Combined Oncoarray; iCOGS; GWAS meta analysis) || id:ieu-a-1126
effect on Triglycerides to total lipids ratio in chylomicrons and extremely large VLDL || id:ebi-c
