# Supplementary material for: Assessing the causal relationships between circulating metabolic biomarkers and breast cancer by using mendelian randomization
Source: Front Genet. 2024 Dec 18;15:1448748. doi: 10.3389/fgene.2024.1448748 (PMC11688392; doi:10.3389/fgene.2024.1448748)
Supplement: Supplementary file 5 [file DataSheet6.pdf]

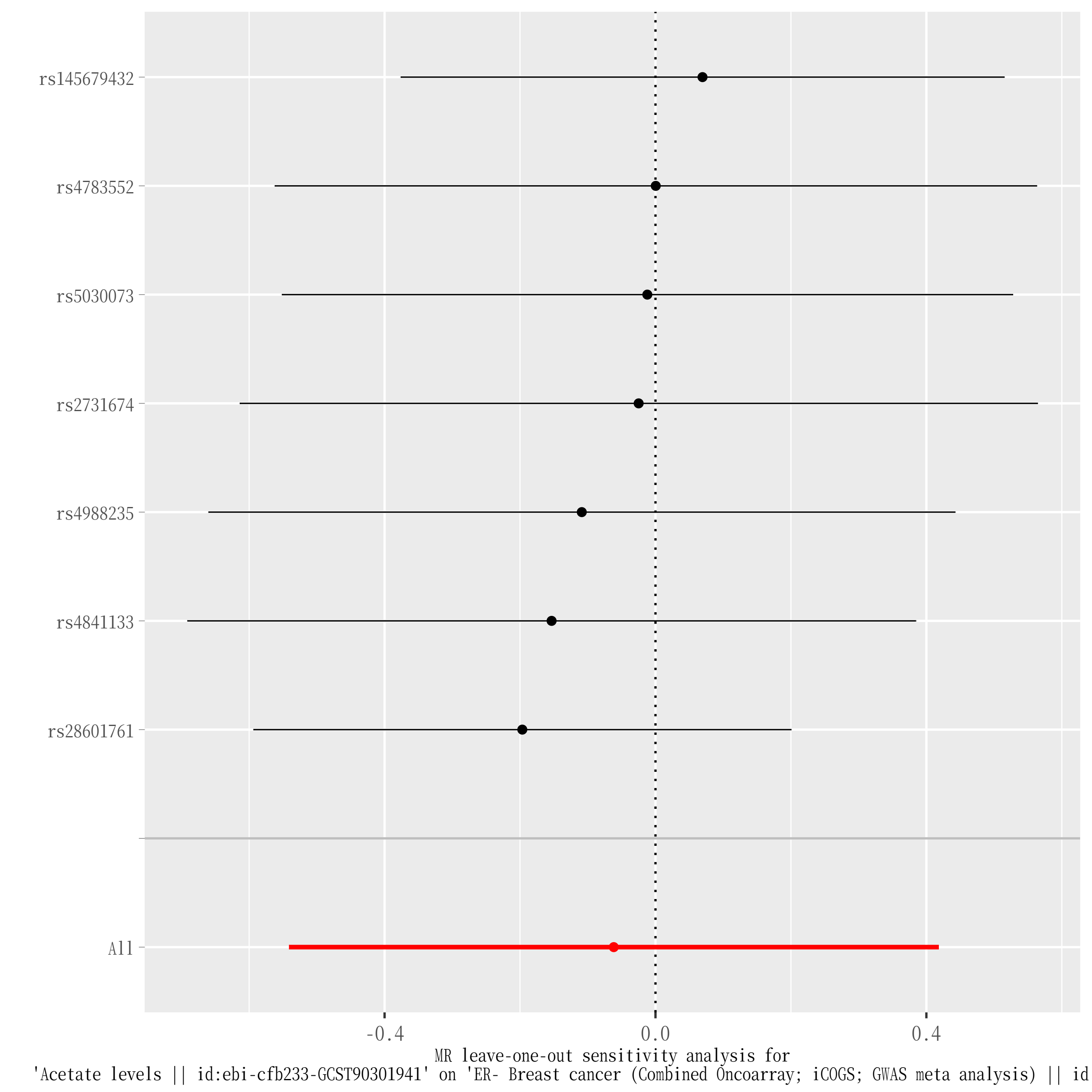

rs145679432
rs4783552
rs5030073
rs2731674
rs4988235
rs4841133
rs28601761
All
-0.4
0.0
0.4
MR leave-one-out sensitivity analysis for
'Acetate levels || id:ebi-cfb233-GCST90301941' on 'ER- Breast cancer (Combined Oncoarray; iCOGS; GWAS meta analysis) || id

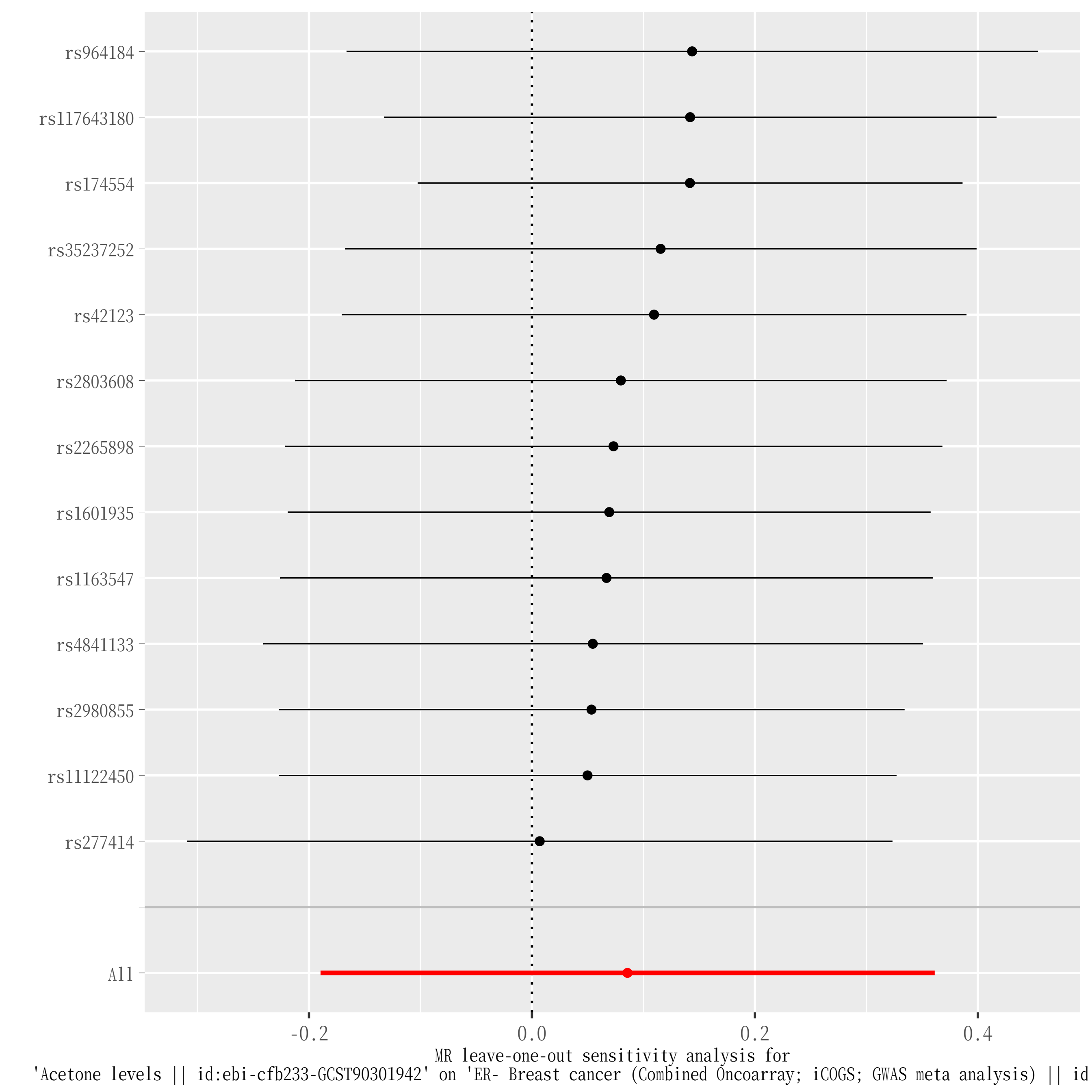

rs964184
rs117643180
rs174554
rs35237252
rs42123
rs2803608
rs2265898
rs1601935
rs1163547
rs4841133
rs2980855
rs11122450
rs277414
All
-0.2
0.0
0.2
0.4
MR leave-one-out sensitivity analysis for
'Acetone levels || id:ebi-cfb233-GCST90301942' on 'ER- Breast cancer (Combined Oncoarray; iCOGS; GWAS meta analysis) || id

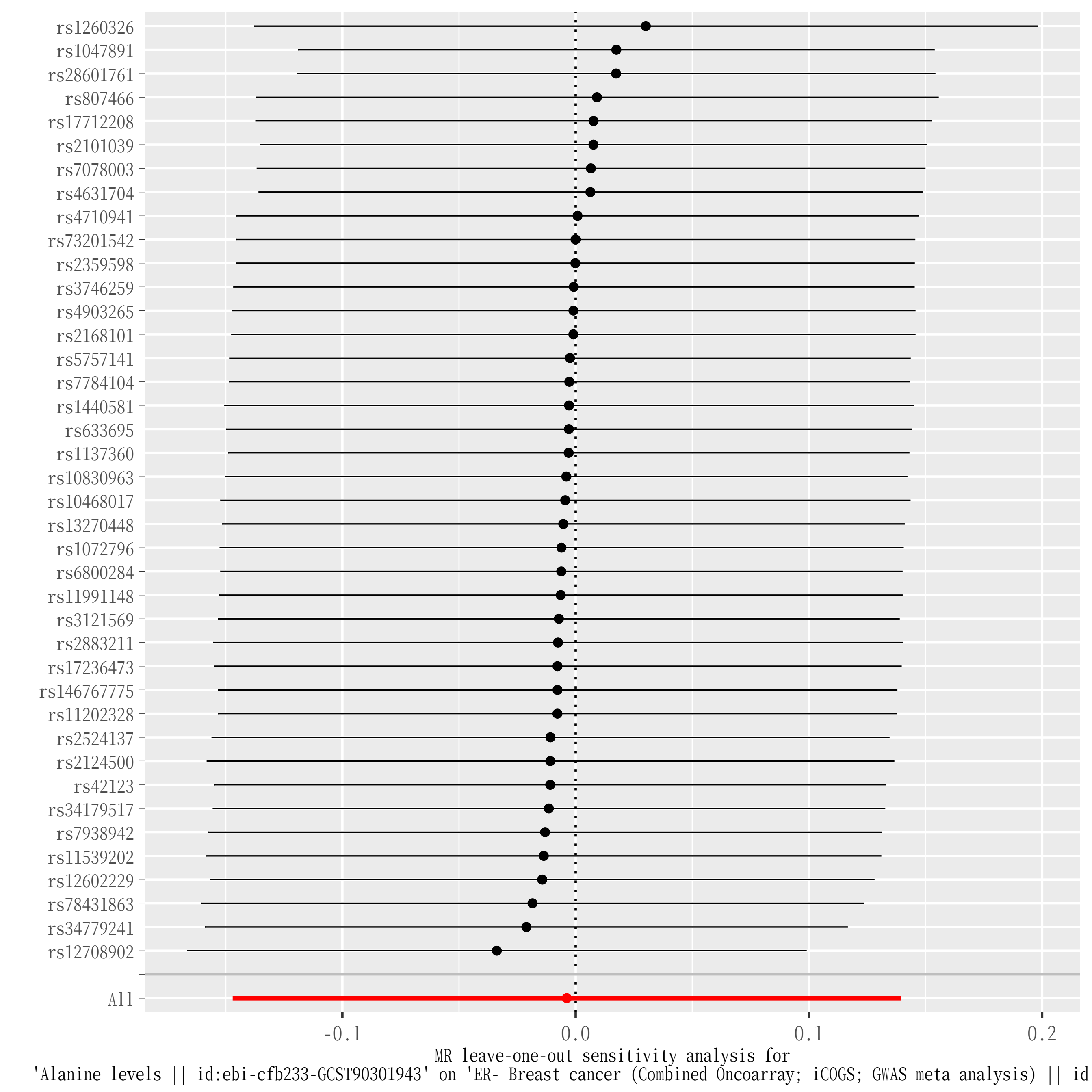
rs1260326
rs1047891
rs28601761
rs807466
rs17712208
rs2101039
rs7078003
rs4631704
rs4710941
rs73201542
rs2359598
rs3746259
rs4903265
rs2168101
rs5757141
rs7784104
rs1440581
rs633695
rs1137360
rs10830963
rs10468017
rs13270448
rs1072796
rs6800284
rs11991148
rs3121569
rs2883211
rs17236473
rs146767775
rs11202328
rs2524137
rs2124500
rs42123
rs34179517
rs7938942
rs11539202
rs12602229
rs78431863
rs34779241
rs12708902
All
-0.1
0.0
0.1
0.2
MR leave-one-out sensitivity analysis for
'Alanine levels || id:ebi-cfb233-GCST90301943' on 'ER- Breast cancer (Combined Oncoarray; iCOGS; GWAS meta analysis) || id

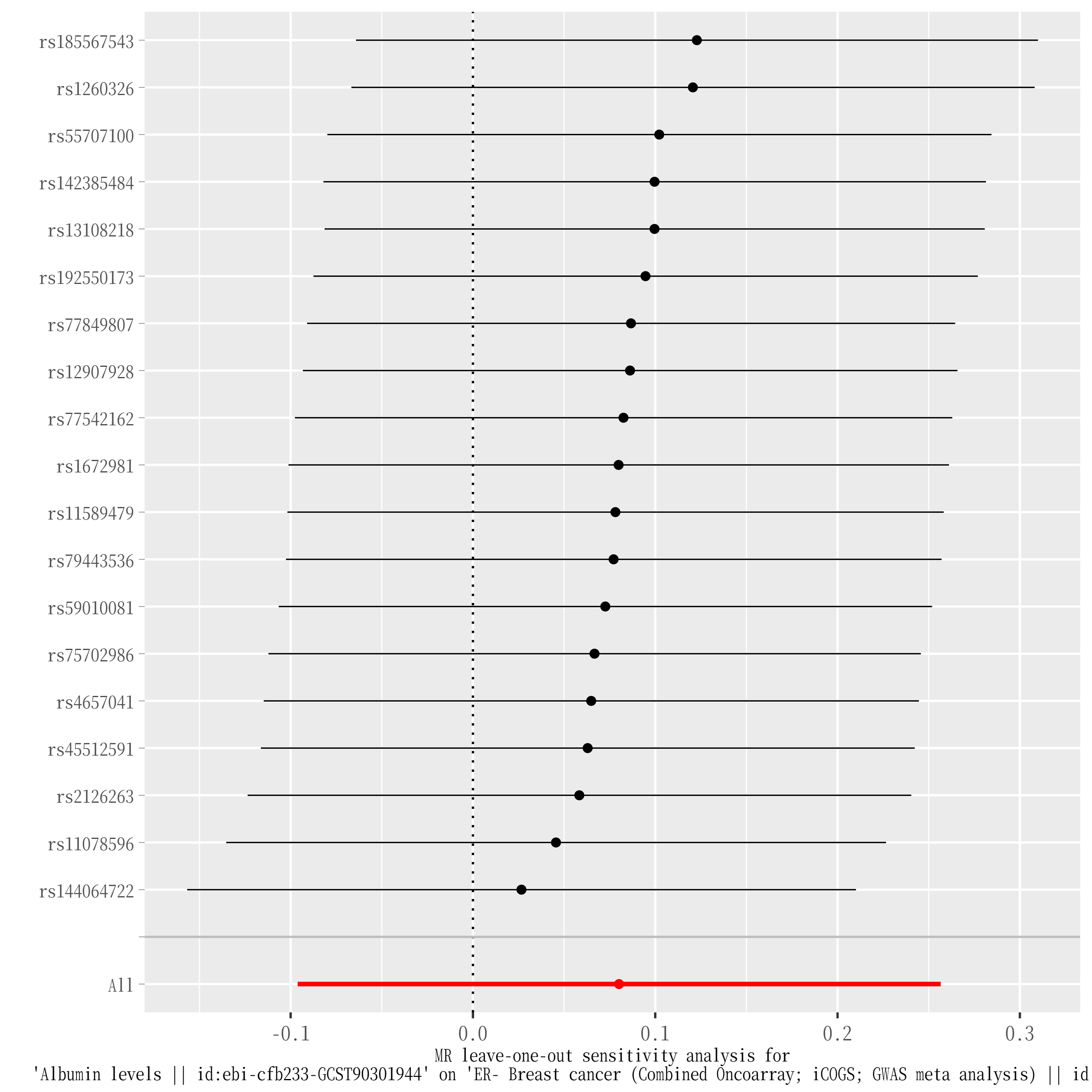
rs185567543
rs1260326
rs55707100
rs142385484
rs13108218
rs192550173
rs77849807
rs12907928
rs77542162
rs1672981
rs11589479
rs79443536
rs59010081
rs75702986
rs4657041
rs45512591
rs2126263
rs11078596
rs144064722
All
-0.1
0.0
0.1
0.2
0.3
MR leave-one-out sensitivity analysis for
'Albumin levels || id:ebi-cfb233-GCST90301944' on 'ER- Breast cancer (Combined Oncoarray; iCOGS; GWAS meta analysis) || id

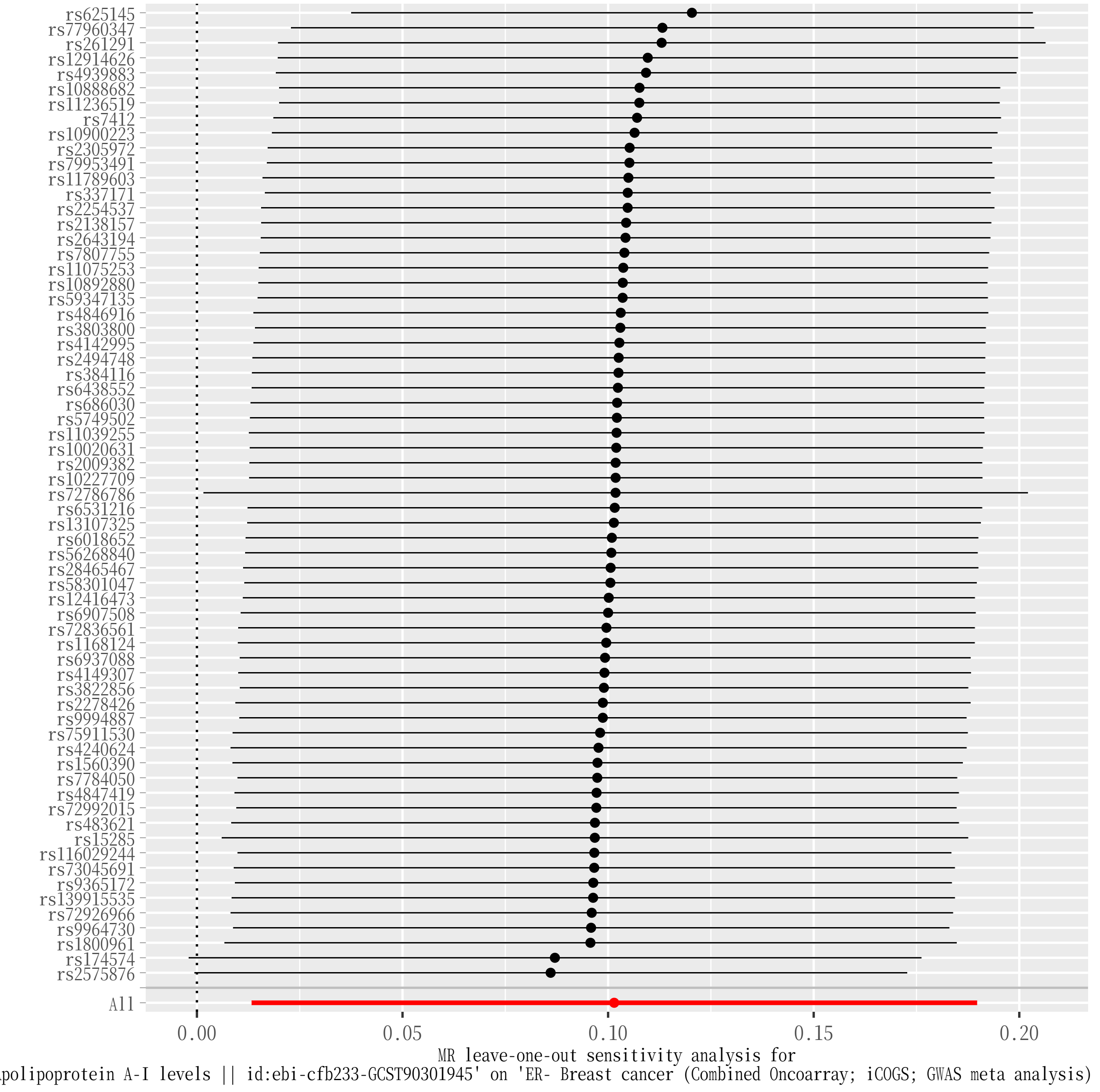

rs625145
rs77960347
rs261291
rs12914626
rs4939883
rs10888682
rs11236519
rs7412
rs10900223
rs2305972
rs79953491
rs11789603
rs337171
rs2254537
rs2138157
rs2643194
rs7807755
rs11075253
rs10892880
rs59347135
rs4846916
rs3803800
rs4142995
rs2494748
rs384116
rs6438552
rs686030
rs5749502
rs11039255
rs10020631
rs2009382
rs10227709
rs72786786
rs6531216
rs13107325
rs6018652
rs56268840
rs28465467
rs58301047
rs12416473
rs6907508
rs72836561
rs1168124
rs6937088
rs4149307
rs3822856
rs2278426
rs9994887
rs75911530
rs4240624
rs1560390
rs7784050
rs4847419
rs72992015
rs483621
rs15285
rs116029244
rs73045691
rs9365172
rs139915535
rs72926966
rs9964730
rs1800961
rs174574
rs2575876
All
0.00
0.05
0.10
0.15
0.20
MR leave-one-out sensitivity analysis for
polipoprotein A-I levels || id:ebi-cfb233-GCST90301945' on 'ER- Breast cancer (Combined Oncoarray; iCOGS; GWAS meta analysis)

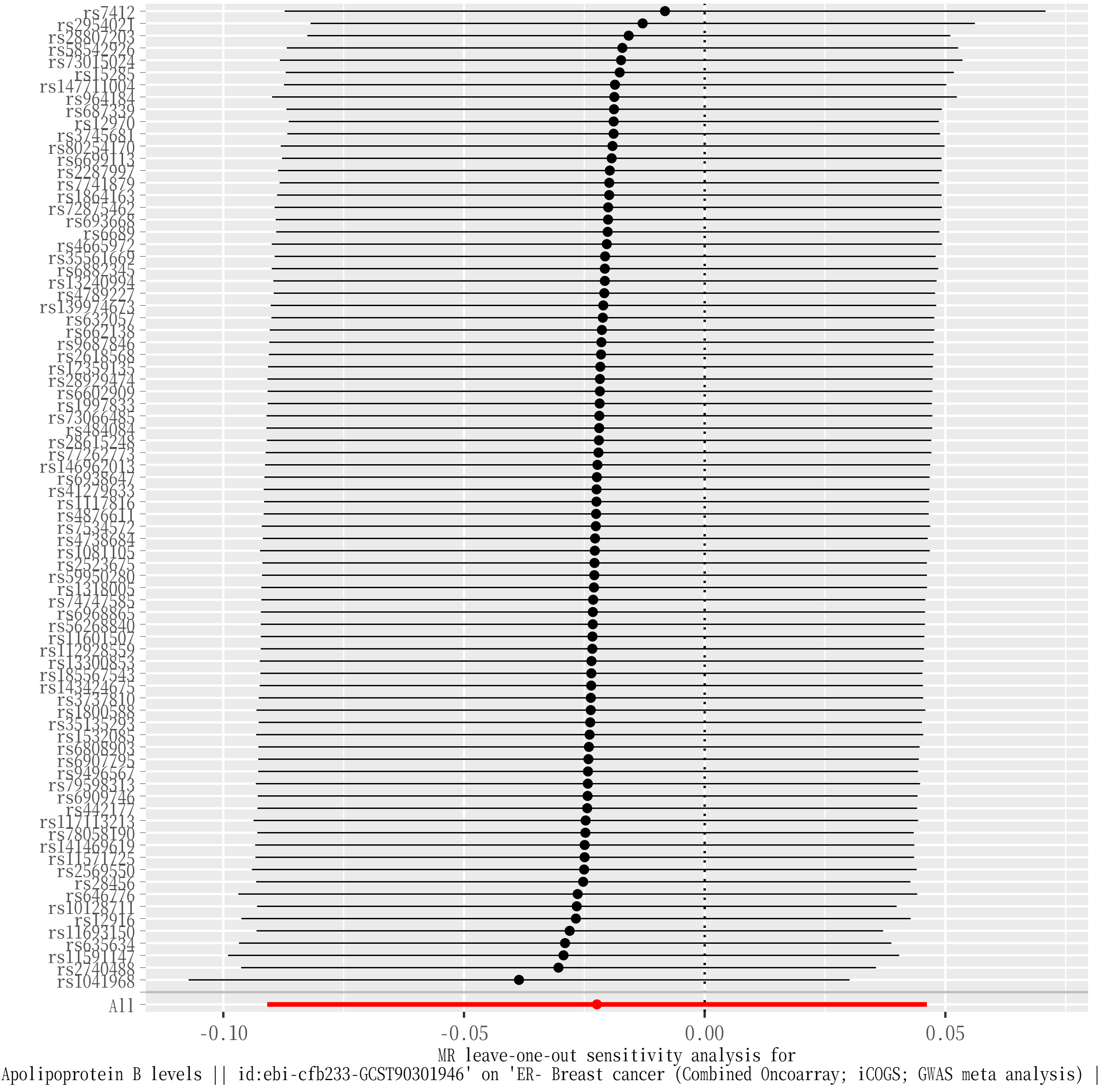
rs7412
rs2954021
rs28807203
rs58542926
rs73015024
rs15285
rs147711004
rs964184
rs687339
rs12970
rs3745681
rs80254170
rs6699113
rs2287997
rs7741879
rs1864163
rs72875462
rs693668
rs6689
rs4665972
rs35561669
rs6882345
rs13240994
rs4789227
rs139974673
rs632057
rs662138
rs9687846
rs2618568
rs12359135
rs28929474
rs6602909
rs1997833
rs73066485
rs484084
rs28615248
rs77262773
rs146962013
rs6938647
rs41279633
rs1117816
rs4876611
rs7534572
rs4738684
rs1081105
rs2523675
rs59950280
rs1318005
rs74747585
rs6968865
rs56268840
rs11601507
rs112928559
rs13300853
rs185567543
rs143424675
rs3737810
rs1800588
rs35135293
rs1532085
rs6808903
rs6907795
rs9496567
rs79598313
rs6909746
rs442177
rs117113213
rs78058190
rs141469619
rs11571725
rs2569550
rs28456
rs646776
rs10128711
rs12916
rs11693150
rs635634
rs11591147
rs2740488
rs1041968
All
-0.10
-0.05
0.00
0.05
MR leave-one-out sensitivity analysis for
Apolipoprotein B levels || id:ebi-cfb233-GCST90301946' on 'ER- Breast cancer (Combined Oncoarray; iCOGS; GWAS meta analysis) |

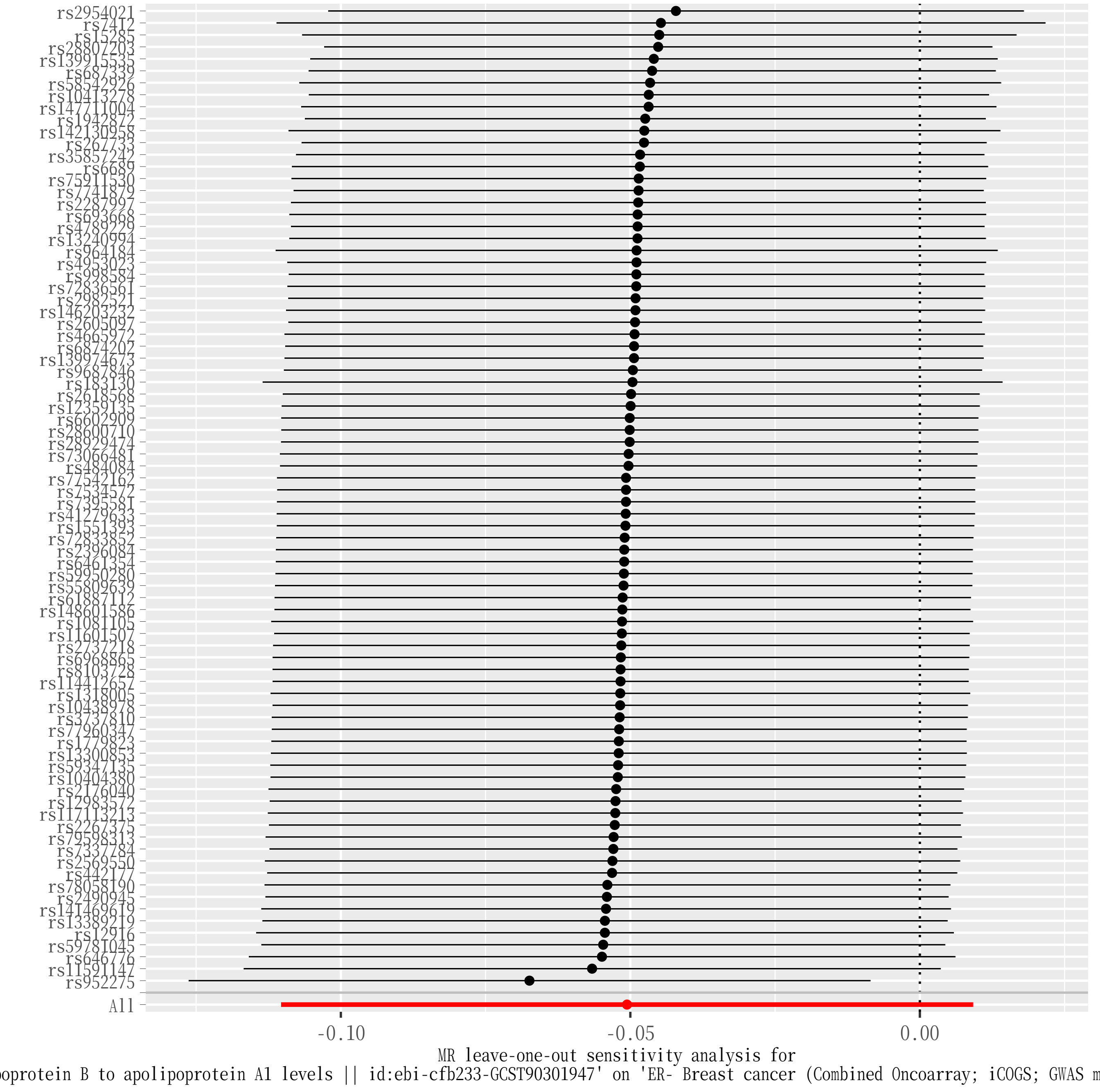
rs2954021
rs7412
rs15285
rs28807203
rs139915535
rs687339
rs58542926
rs10413278
rs147711004
rs1942872
rs142130958
rs267733
rs35857242
rs6689
rs75911530
rs7741879
rs2287997
rs693668
rs4789229
rs13240994
rs964184
rs4953023
rs998584
rs72836561
rs2982521
rs146203232
rs2605097
rs4665972
rs6874202
rs139974673
rs9687846
rs183130
rs2618568
rs12359135
rs6602909
rs28600710
rs28929474
rs73066481
rs484084
rs77542162
rs7534572
rs7395581
rs41279633
rs1551393
rs72833852
rs2396084
rs6461354
rs59950280
rs55809639
rs61887112
rs148601586
rs1081105
rs11601507
rs2737218
rs6968865
rs8103728
rs114412657
rs1318005
rs10438978
rs3737810
rs77960347
rs1779823
rs13300853
rs59347135
rs10404380
rs2176040
rs12983572
rs117113213
rs2267375
rs79598313
rs7337784
rs2569550
rs442177
rs78058190
rs2490945
rs141469619
rs13389219
rs12916
rs59781045
rs646776
rs11591147
rs952275
All
-0.10
-0.05
0.00
MR leave-one-out sensitivity analysis for
oprotein B to apolipoprotein A1 levels || id:ebi-cfb233-GCST90301947' on 'ER- Breast cancer (Combined Oncoarray; iCOGS; GWAS m

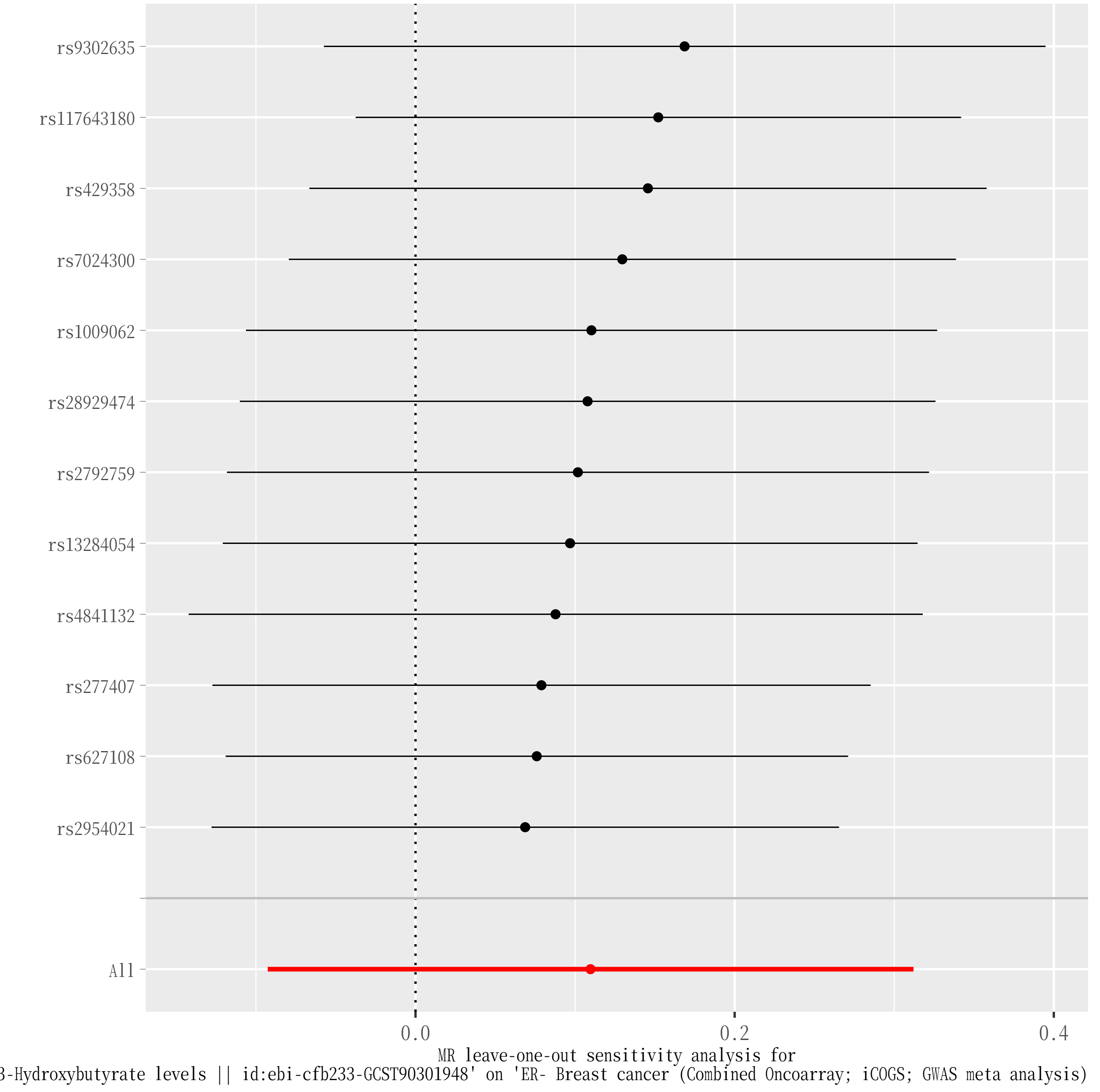

rs9302635
rs117643180
rs429358
rs7024300
rs1009062
rs28929474
rs2792759
rs13284054
rs4841132
rs277407
rs627108
rs2954021
All
0.0
0.2
0.4
MR leave-one-out sensitivity analysis for
3-Hydroxybutyrate levels || id:ebi-cfb233-GCST90301948' on 'ER- Breast cancer (Combined Oncoarray; iCOGS; GWAS meta analysis)

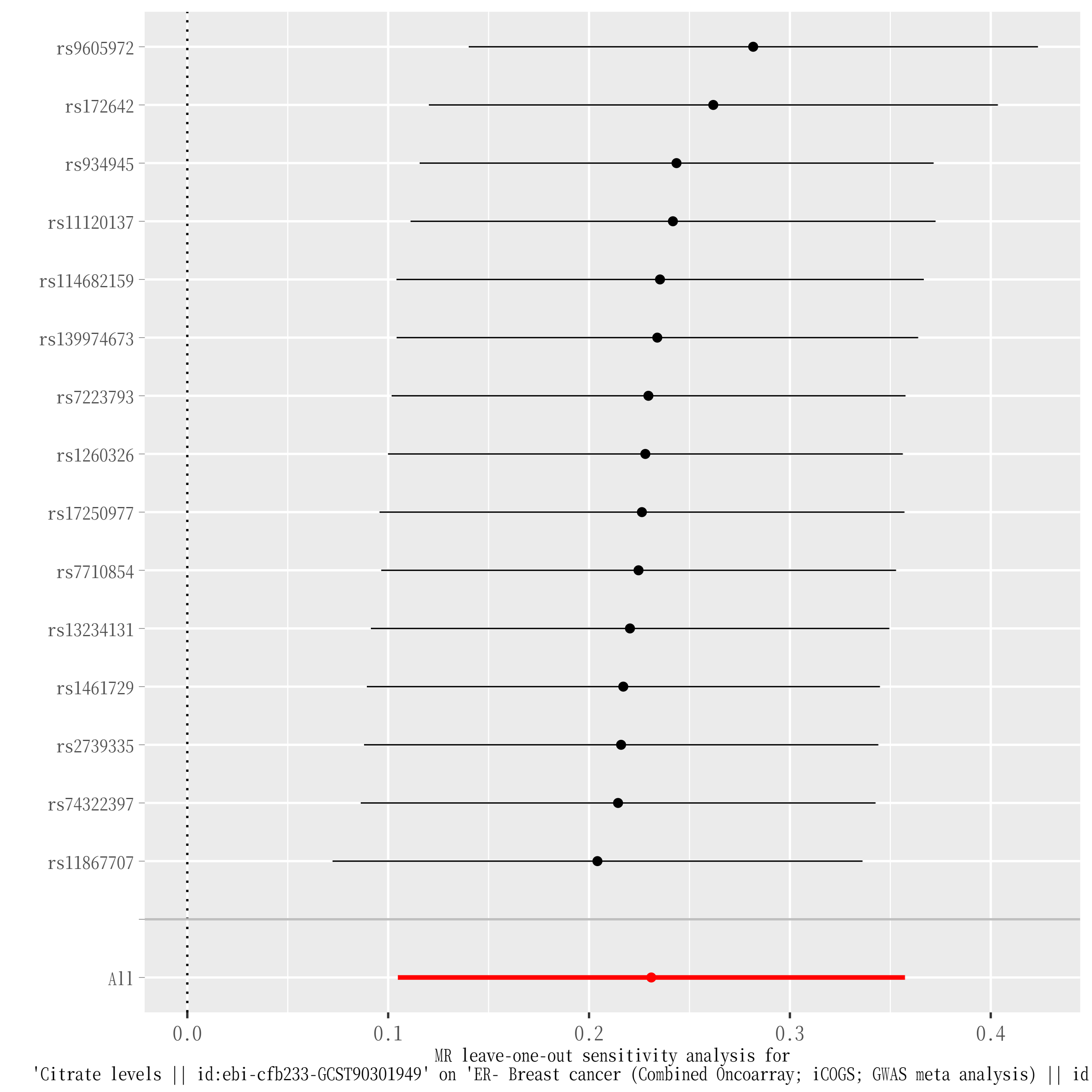

rs9605972
rs172642
rs934945
rs11120137
rs114682159
rs139974673
rs7223793
rs1260326
rs17250977
rs7710854
rs13234131
rs1461729
rs2739335
rs74322397
rs11867707
All
0.0
0.1
0.2
0.3
0.4
MR leave-one-out sensitivity analysis for
'Citrate levels || id:ebi-cfb233-GCST90301949' on 'ER- Breast cancer (Combined Oncoarray; iCOGS; GWAS meta analysis) || id

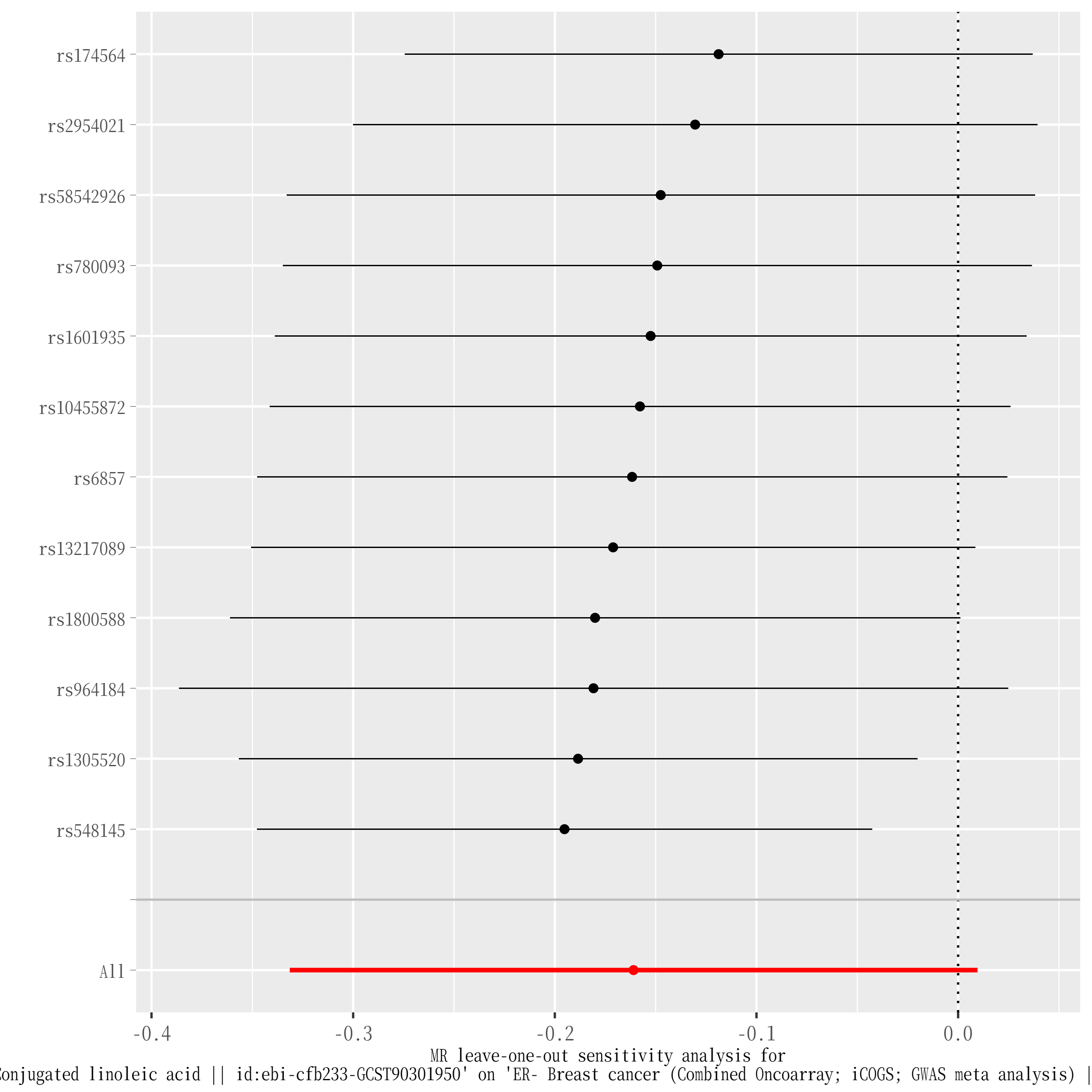
rs174564
rs2954021
rs58542926
rs780093
rs1601935
rs10455872
rs6857
rs13217089
rs1800588
rs964184
rs1305520
rs548145
All
-0.4
-0.3
-0.2
-0.1
0.0
MR leave-one-out sensitivity analysis for
Conjugated linoleic acid || id:ebi-cfb233-GCST90301950' on 'ER- Breast cancer (Combined Oncoarray; iCOGS; GWAS meta analysis) |

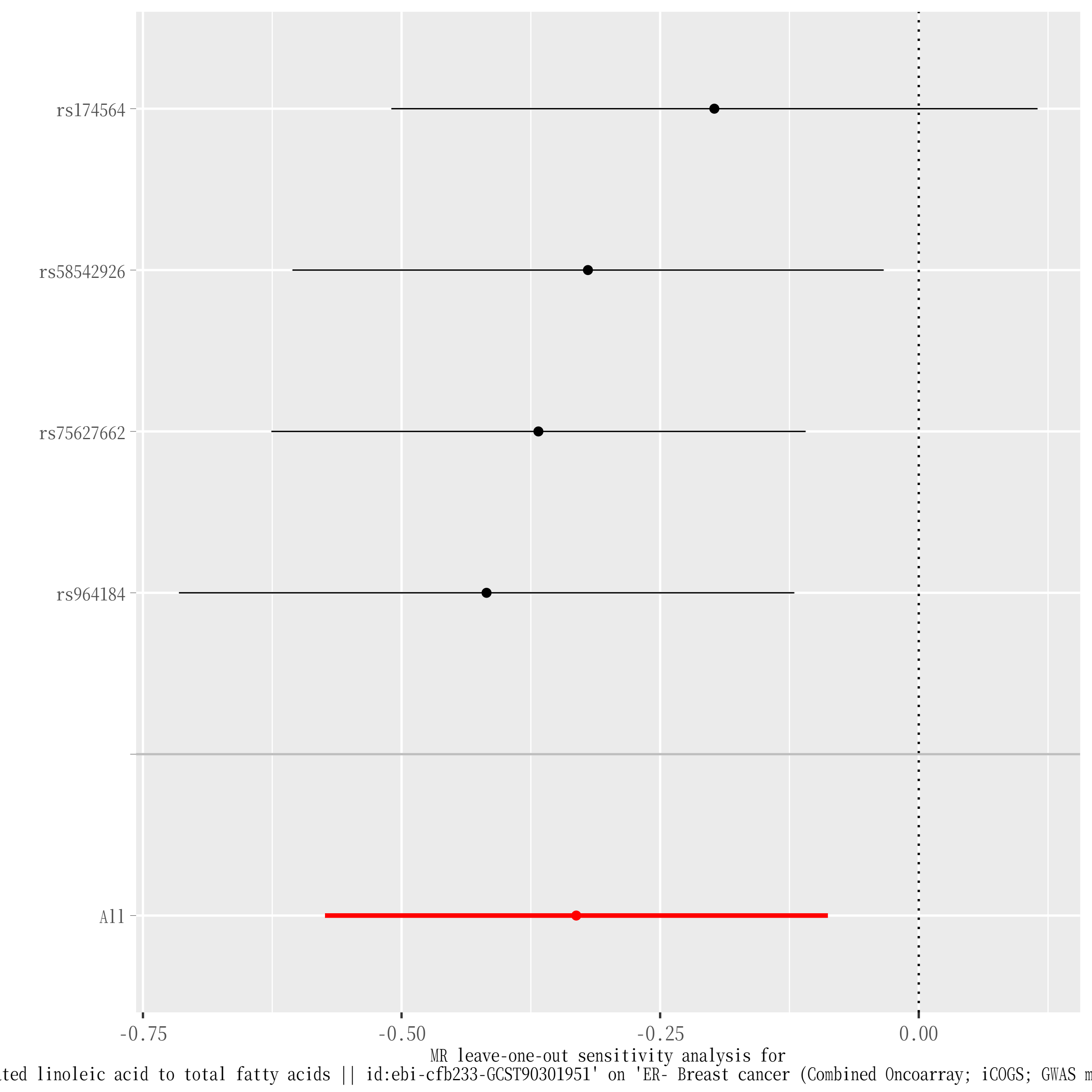
rs174564
rs58542926
rs75627662
rs964184
All
-0.75
-0.50
-0.25
0.00
MR leave-one-out sensitivity analysis for
ted linoleic acid to total fatty acids || id:ebi-cfb233-GCST90301951' on 'ER- Breast cancer (Combined Oncoarray; iCOGS; GWAS m

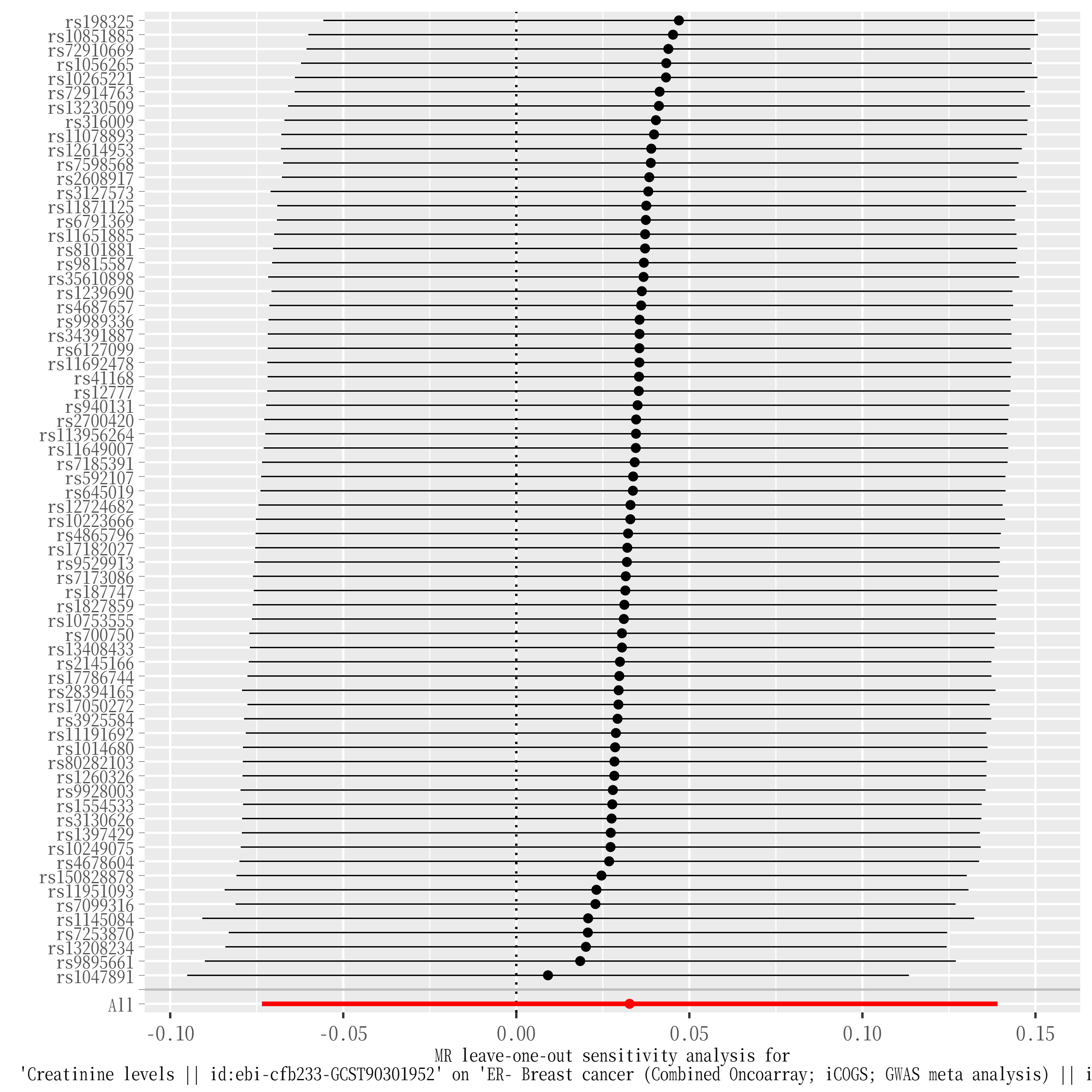

rs198325
rs10851885
rs72910669
rs1056265
rs10265221
rs72914763
rs13230509
rs316009
rs11078893
rs12614953
rs7598568
rs2608917
rs3127573
rs11871125
rs6791369
rs11651885
rs8101881
rs9815587
rs35610898
rs1239690
rs4687657
rs9989336
rs34391887
rs6127099
rs11692478
rs41168
rs12777
rs940131
rs2700420
rs113956264
rs11649007
rs7185391
rs592107
rs645019
rs12724682
rs10223666
rs4865796
rs17182027
rs9529913
rs7173086
rs187747
rs1827859
rs10753555
rs700750
rs13408433
rs2145166
rs17786744
rs28394165
rs17050272
rs3925584
rs11191692
rs1014680
rs80282103
rs1260326
rs9928003
rs1554533
rs3130626
rs1397429
rs10249075
rs4678604
rs150828878
rs11951093
rs7099316
rs1145084
rs7253870
rs13208234
rs9895661
rs1047891
All
-0.10
-0.05
0.00
0.05
0.10
0.15
MR leave-one-out sensitivity analysis for
'Creatinine levels || id:ebi-cfb233-GCST90301952' on 'ER- Breast cancer (Combined Oncoarray; iCOGS; GWAS meta analysis) || i

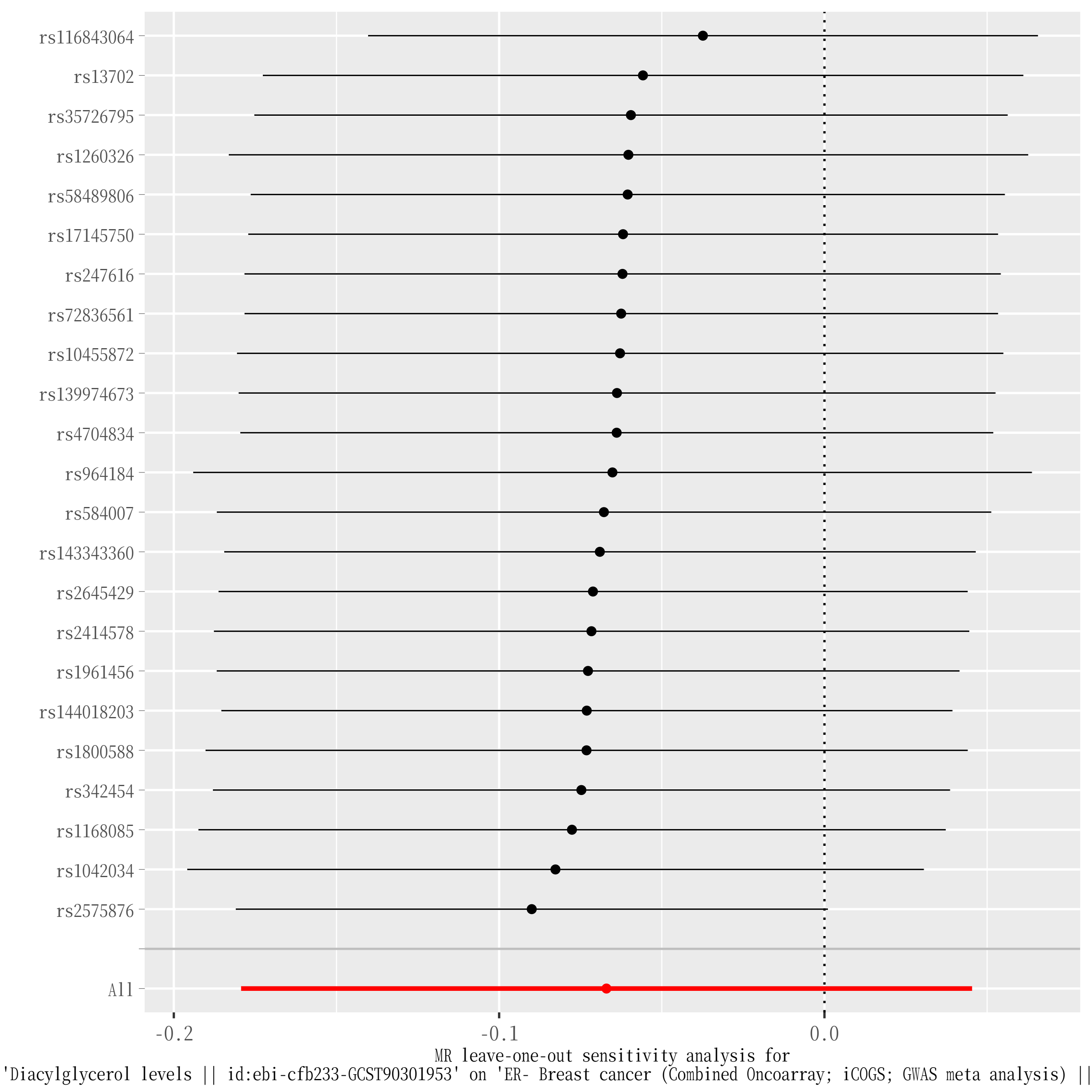

rs116843064
rs13702
rs35726795
rs1260326
rs58489806
rs17145750
rs247616
rs72836561
rs10455872
rs139974673
rs4704834
rs964184
rs584007
rs143343360
rs2645429
rs2414578
rs1961456
rs144018203
rs1800588
rs342454
rs1168085
rs1042034
rs2575876
All
-0.2
-0.1
0.0
MR leave-one-out sensitivity analysis for
'Diacylglycerol levels || id:ebi-cfb233-GCST90301953' on 'ER- Breast cancer (Combined Oncoarray; iCOGS; GWAS meta analysis) ||

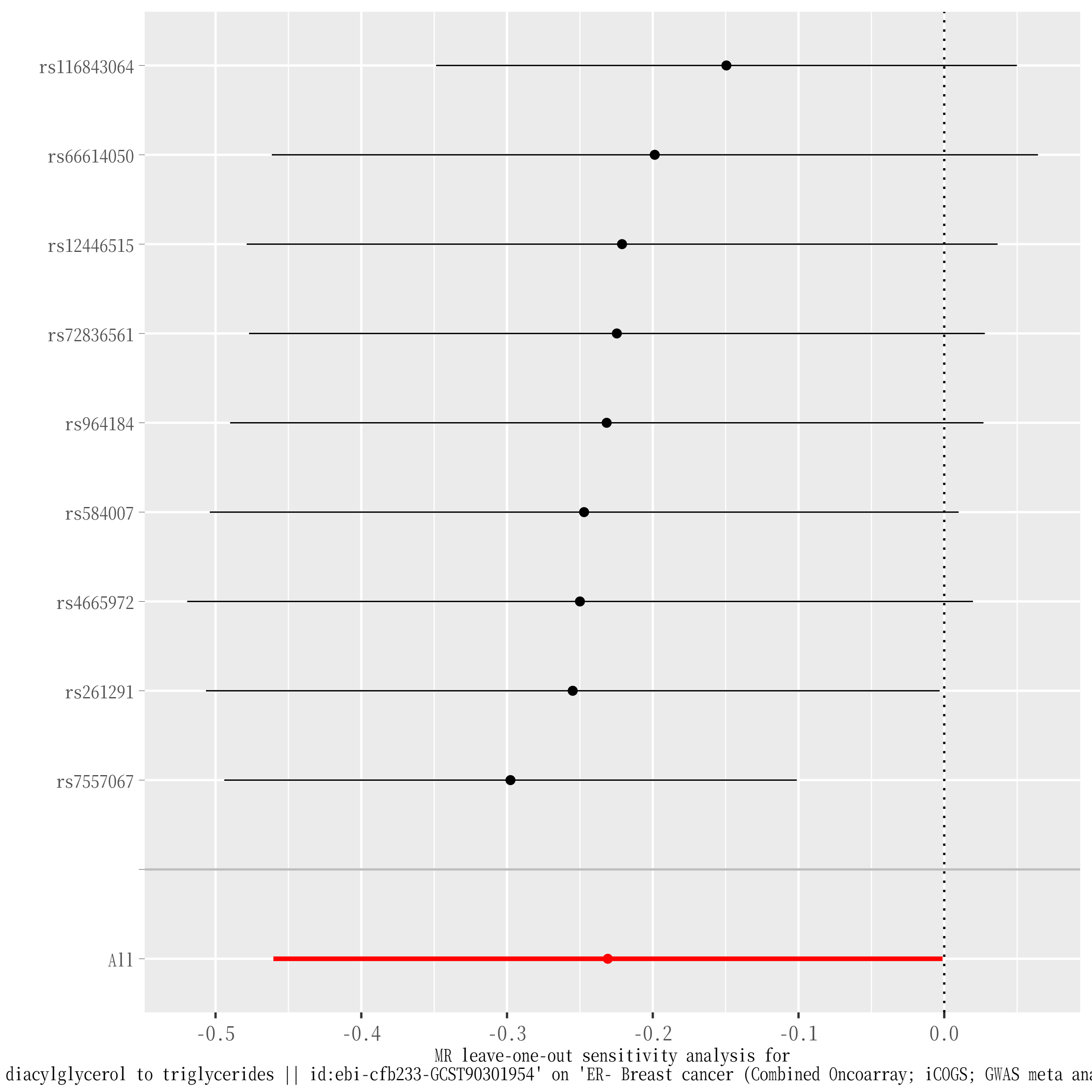
rs116843064
rs66614050
rs12446515
rs72836561
rs964184
rs584007
rs4665972
rs261291
rs7557067
All
-0.5
-0.4
-0.3
-0.2
-0.1
0.0
MR leave-one-out sensitivity analysis for
diacylglycerol to triglycerides || id:ebi-cfb233-GCST90301954' on 'ER- Breast cancer (Combined Oncoarray; iCOGS; GWAS meta ana

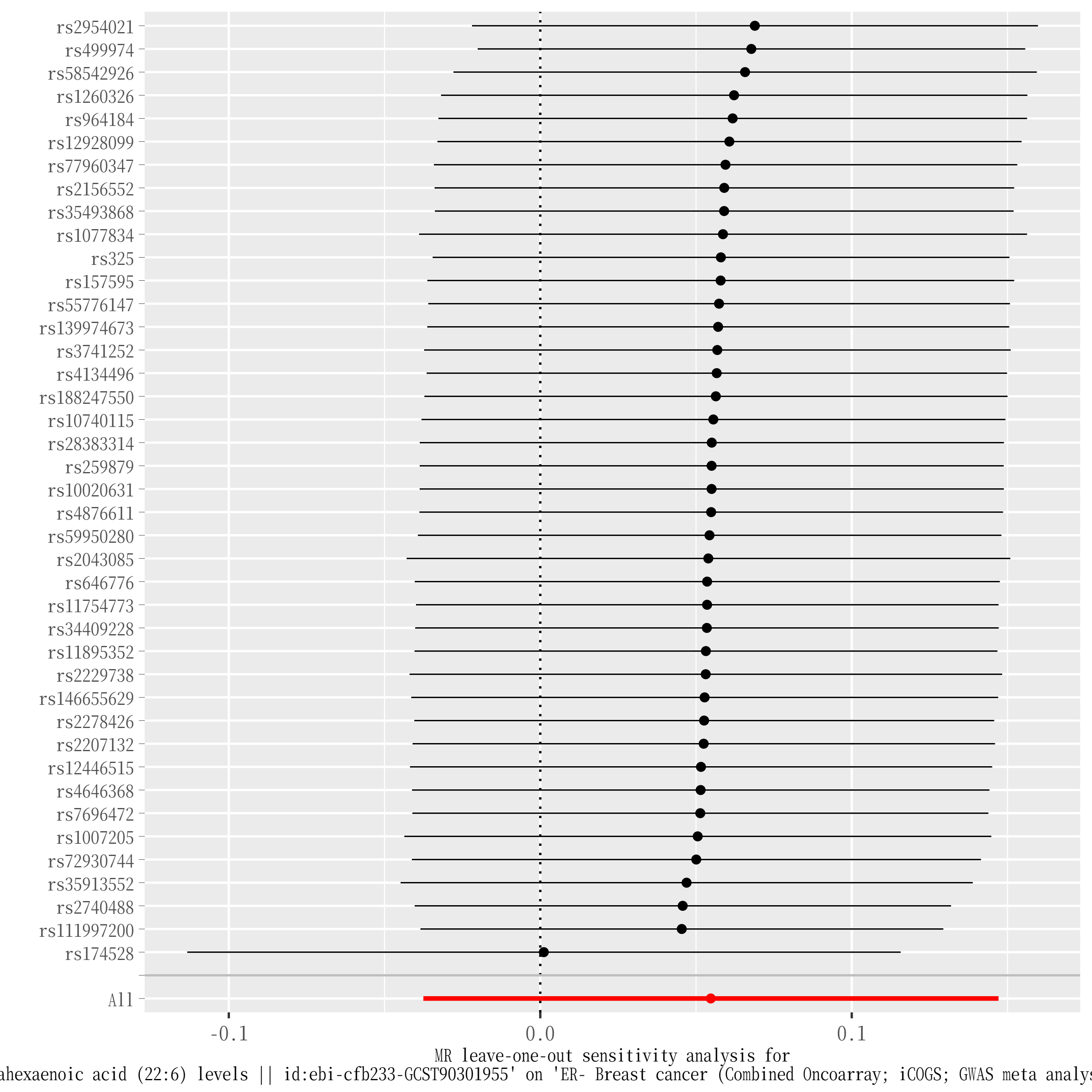

rs2954021
rs499974
rs58542926
rs1260326
rs964184
rs12928099
rs77960347
rs2156552
rs35493868
rs1077834
rs325
rs157595
rs55776147
rs139974673
rs3741252
rs4134496
rs188247550
rs10740115
rs28383314
rs259879
rs10020631
rs4876611
rs59950280
rs2043085
rs646776
rs11754773
rs34409228
rs11895352
rs2229738
rs146655629
rs2278426
rs2207132
rs12446515
rs4646368
rs7696472
rs1007205
rs72930744
rs35913552
rs2740488
rs111997200
rs174528
All
-0.1
0.0
0.1
MR leave-one-out sensitivity analysis for
ahexaenoic acid (22:6) levels || id:ebi-cfb233-GCST90301955' on 'ER- Breast cancer (Combined Oncoarray; iCOGS; GWAS meta analys

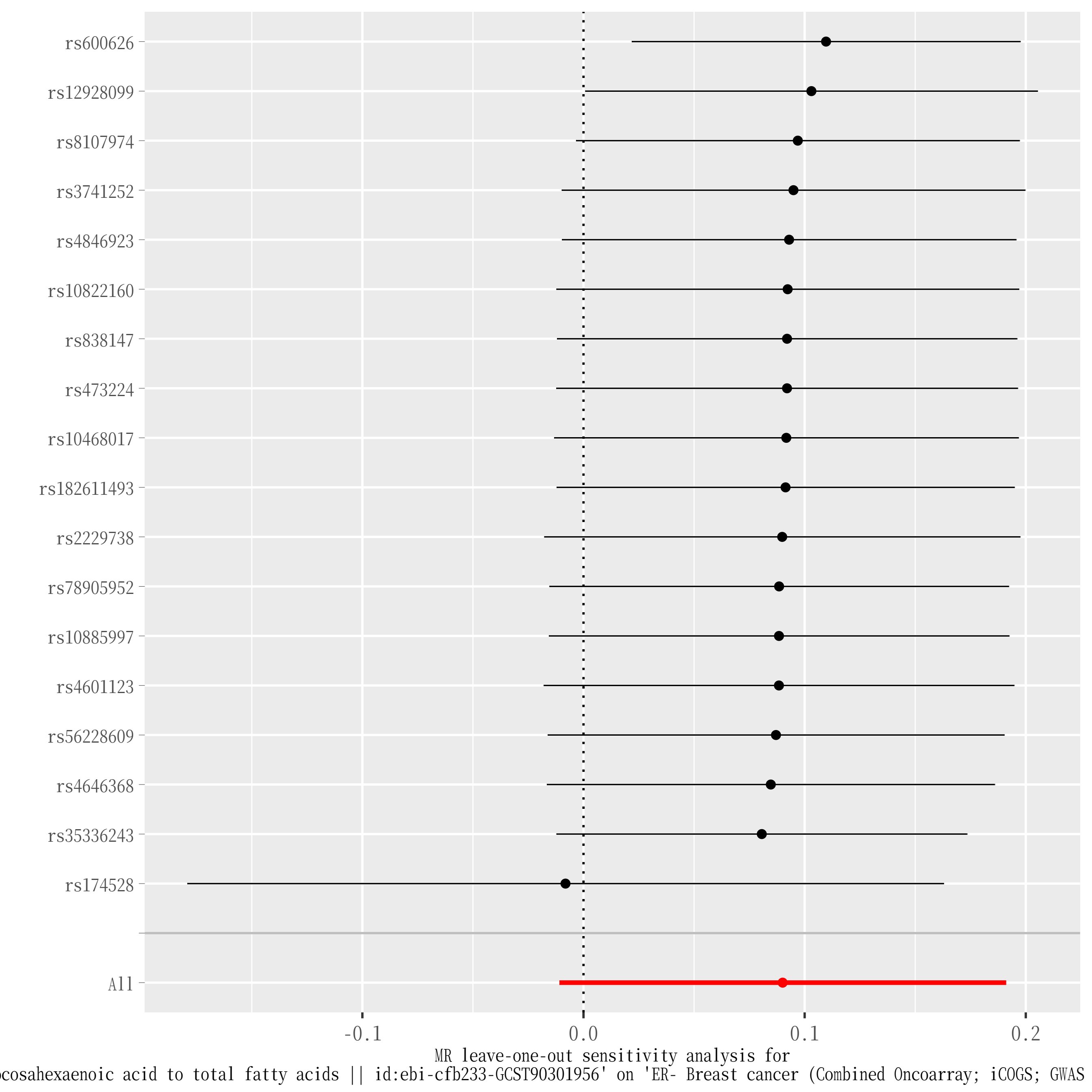
rs600626
rs12928099
rs8107974
rs3741252
rs4846923
rs10822160
rs838147
rs473224
rs10468017
rs182611493
rs2229738
rs78905952
rs10885997
rs4601123
rs56228609
rs4646368
rs35336243
rs174528
All
-0.1
0.0
0.1
0.2
MR leave-one-out sensitivity analysis for
ocosahexaenoic acid to total fatty acids || id:ebi-cfb233-GCST90301956' on 'ER- Breast cancer (Combined Oncoarray; iCOGS; GWAS

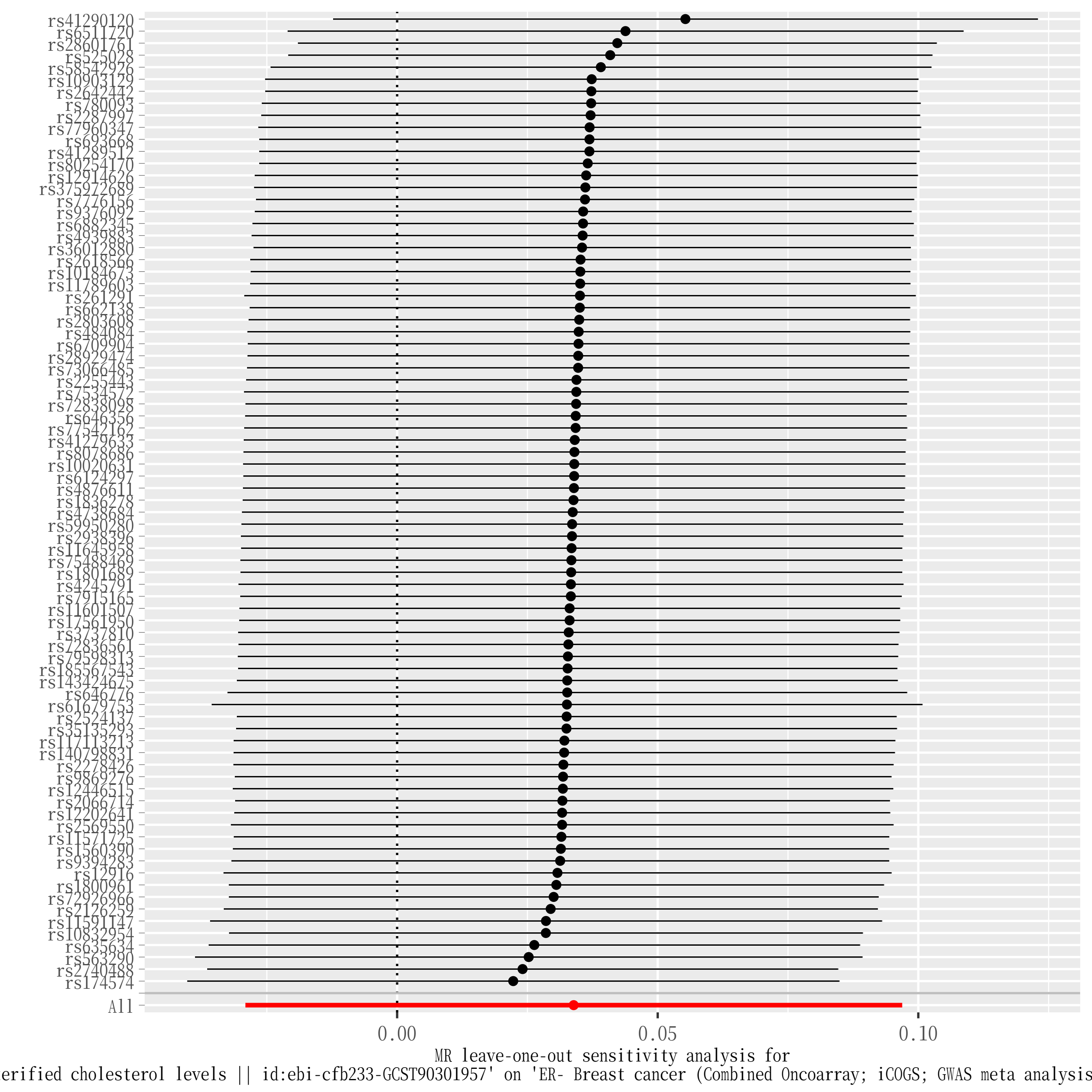

rs41290120
rs6511720
rs28601761
rs525028
rs58542926
rs10903129
rs2642442
rs780093
rs2287997
rs77960347
rs693668
rs41289512
rs80254170
rs12914626
rs375272689
rs7776156
rs9376092
rs6882345
rs4939883
rs36012880
rs2618566
rs10184673
rs11789603
rs261291
rs662138
rs2803608
rs484084
rs6709904
rs28929474
rs73066485
rs2255443
rs7534572
rs72838028
rs646356
rs77542162
rs41279633
rs8078686
rs10020631
rs6124297
rs4876611
rs1836278
rs4738684
rs59950280
rs2938396
rs11645958
rs75488469
rs1801689
rs4245791
rs7915165
rs11601507
rs17561950
rs3737810
rs72836561
rs79598313
rs185567543
rs143424675
rs646776
rs61679753
rs2524137
rs35135293
rs117113213
rs140798831
rs2278426
rs9869276
rs12446515
rs2066714
rs12202641
rs2569550
rs11571725
rs1560390
rs9394283
rs12916
rs1800961
rs72926966
rs2126259
rs11591147
rs10832954
rs635634
rs563290
rs2740488
rs174574
All
0.00
0.05
0.10
MR leave-one-out sensitivity analysis for
erified cholesterol levels || id:ebi-cfb233-GCST90301957' on 'ER- Breast cancer (Combined Oncoarray; iCOGS; GWAS meta analysis

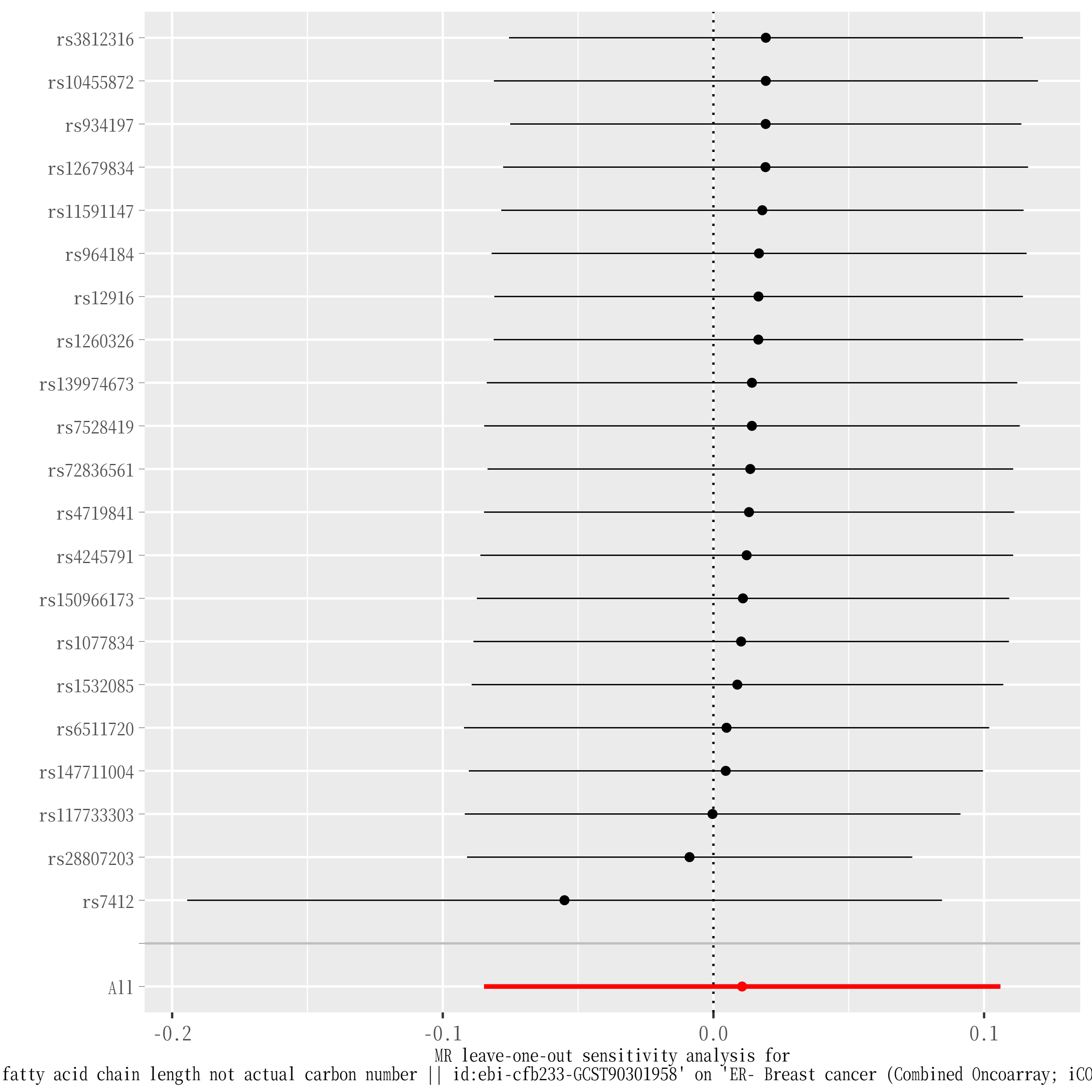

rs3812316
rs10455872
rs934197
rs12679834
rs11591147
rs964184
rs12916
rs1260326
rs139974673
rs7528419
rs72836561
rs4719841
rs4245791
rs150966173
rs1077834
rs1532085
rs6511720
rs147711004
rs117733303
rs28807203
rs7412
All
-0.2
-0.1
0.0
0.1
MR leave-one-out sensitivity analysis for
fatty acid chain length not actual carbon number || id:ebi-cfb233-GCST90301958' on 'ER- Breast cancer (Combined Oncoarray; iC0

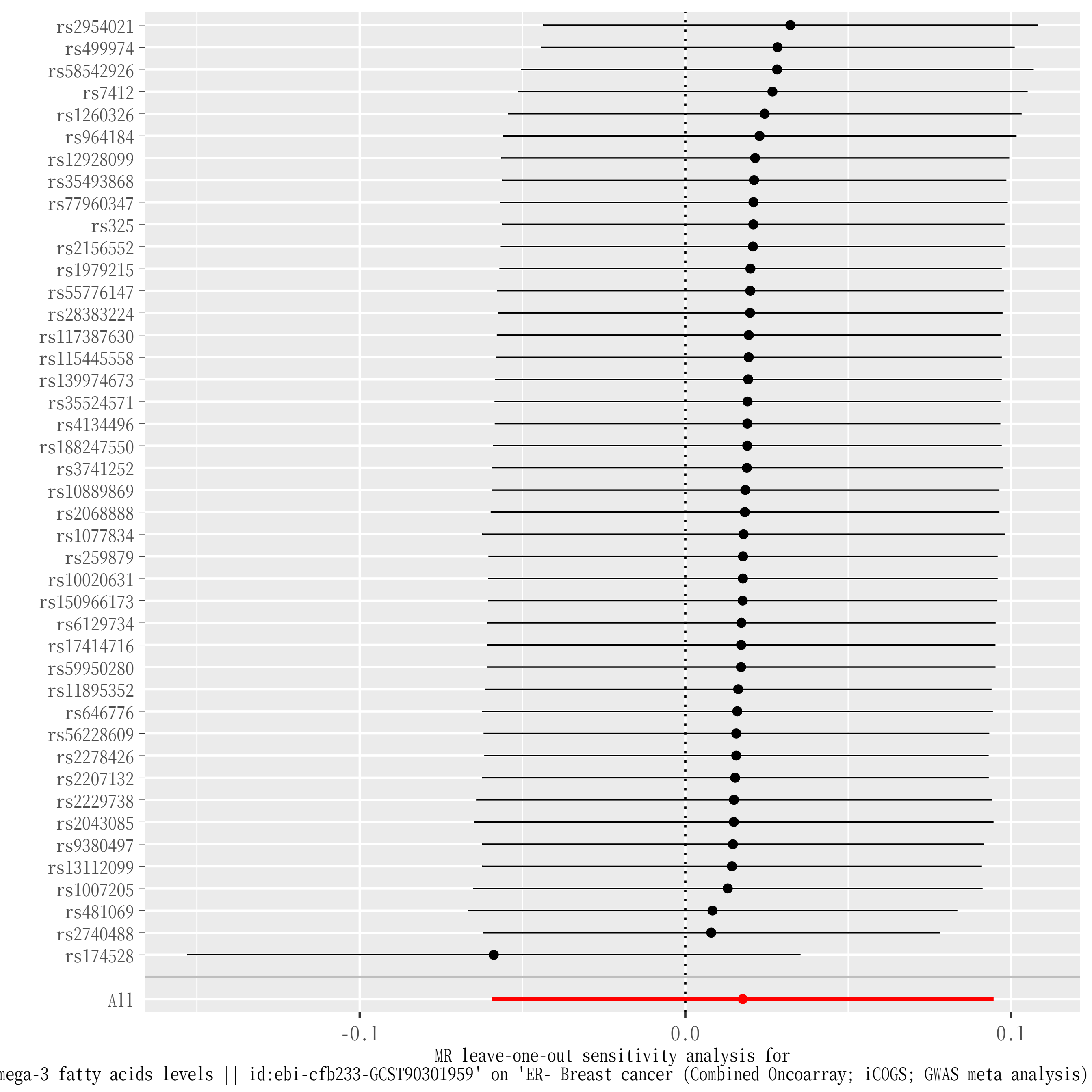
rs2954021
rs499974
rs58542926
rs7412
rs1260326
rs964184
rs12928099
rs35493868
rs77960347
rs325
rs2156552
rs1979215
rs55776147
rs28383224
rs117387630
rs115445558
rs139974673
rs35524571
rs4134496
rs188247550
rs3741252
rs10889869
rs2068888
rs1077834
rs259879
rs10020631
rs150966173
rs6129734
rs17414716
rs59950280
rs11895352
rs646776
rs56228609
rs2278426
rs2207132
rs2229738
rs2043085
rs9380497
rs13112099
rs1007205
rs481069
rs2740488
rs174528
All
-0.1
0.0
0.1
MR leave-one-out sensitivity analysis for
mega-3 fatty acids levels || id:ebi-cfb233-GCST90301959' on 'ER- Breast cancer (Combined Oncoarray; iCOGS; GWAS meta analysis)

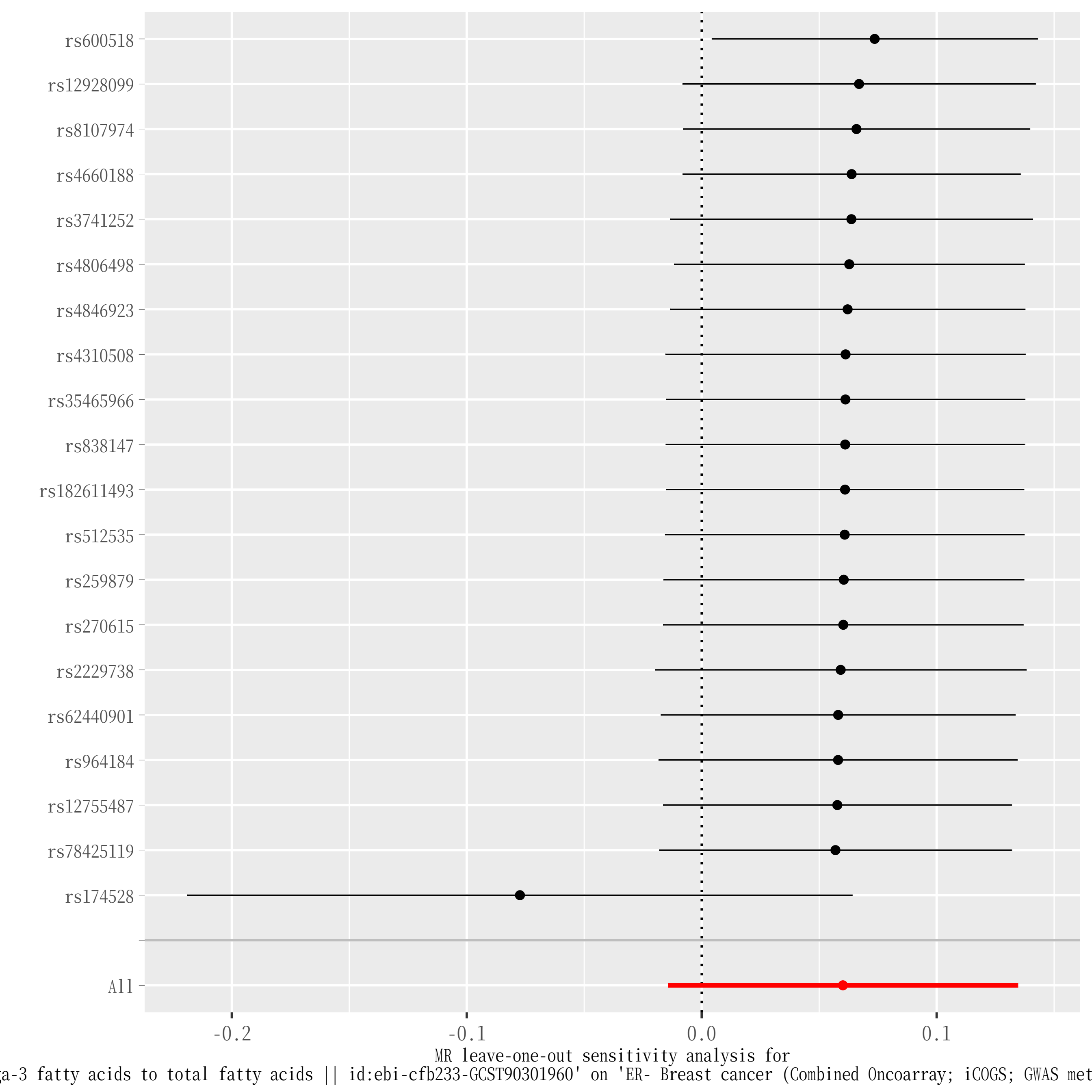
rs600518
rs12928099
rs8107974
rs4660188
rs3741252
rs4806498
rs4846923
rs4310508
rs35465966
rs838147
rs182611493
rs512535
rs259879
rs270615
rs2229738
rs62440901
rs964184
rs12755487
rs78425119
rs174528
All
-0.2
-0.1
0.0
0.1
MR leave-one-out sensitivity analysis for
a-3 fatty acids to total fatty acids || id:ebi-cfb233-GCST90301960' on 'ER- Breast cancer (Combined Oncoarray; iCOGS; GWAS met

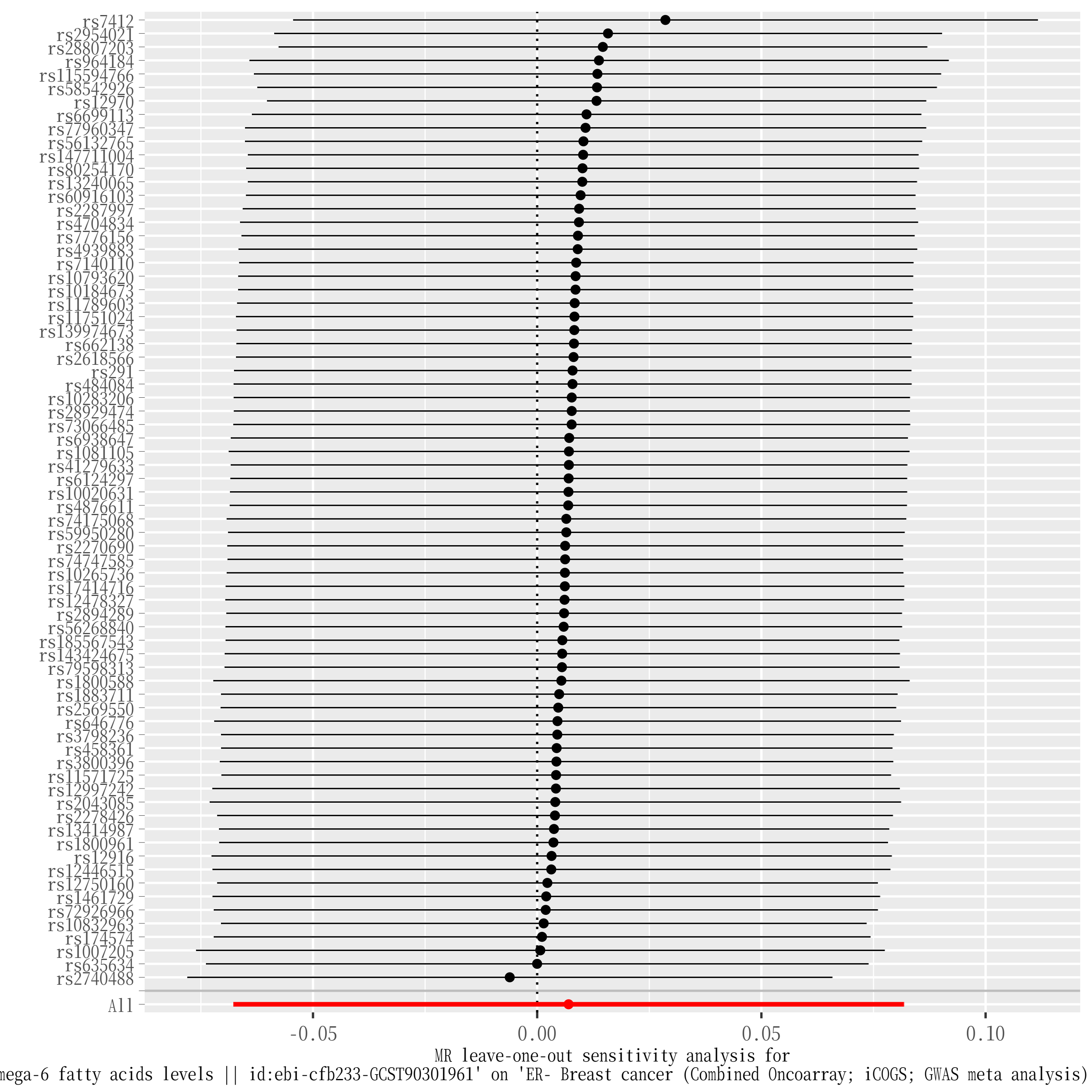
rs7412
rs2954021
rs28807203
rs964184
rs115594766
rs58542926
rs12970
rs6699113
rs77960347
rs56132765
rs147711004
rs80254170
rs13240065
rs60916103
rs2287997
rs4704834
rs7776156
rs4939883
rs7140110
rs10793620
rs10184673
rs11789603
rs11751024
rs139974673
rs662138
rs2618566
rs291
rs484084
rs10283206
rs28929474
rs73066485
rs6938647
rs1081105
rs41279633
rs6124297
rs10020631
rs4876611
rs74175068
rs59950280
rs2270690
rs74747585
rs10265736
rs17414716
rs12478327
rs2894289
rs56268840
rs185567543
rs143424675
rs79598313
rs1800588
rs1883711
rs2569550
rs646776
rs3798236
rs458361
rs3800396
rs11571725
rs12997242
rs2043085
rs2278426
rs13414987
rs1800961
rs12916
rs12446515
rs12750160
rs1461729
rs72926966
rs10832963
rs174574
rs1007205
rs635634
rs2740488
All
-0.05
0.00
0.05
0.10
MR leave-one-out sensitivity analysis for
mega-6 fatty acids levels || id:ebi-cfb233-GCST90301961' on 'ER- Breast cancer (Combined Oncoarray; iCOGS; GWAS meta analysis)

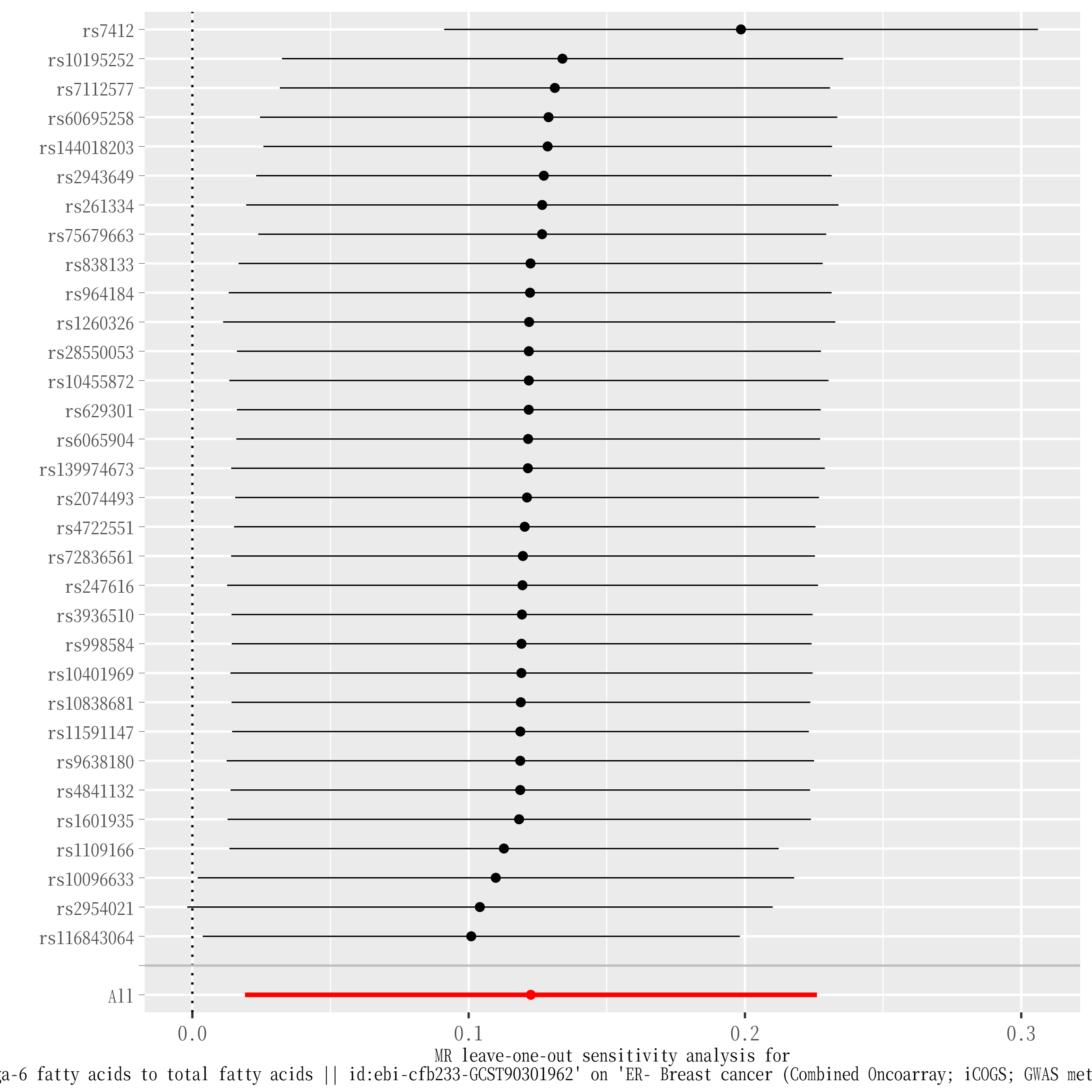
rs7412
rs10195252
rs7112577
rs60695258
rs144018203
rs2943649
rs261334
rs75679663
rs838133
rs964184
rs1260326
rs28550053
rs10455872
rs629301
rs6065904
rs139974673
rs2074493
rs4722551
rs72836561
rs247616
rs3936510
rs998584
rs10401969
rs10838681
rs11591147
rs9638180
rs4841132
rs1601935
rs1109166
rs10096633
rs2954021
rs116843064
All
0.0
0.1
0.2
0.3
MR leave-one-out sensitivity analysis for
a-6 fatty acids to total fatty acids || id:ebi-cfb233-GCST90301962' on 'ER- Breast cancer (Combined Oncoarray; iCOGS; GWAS met

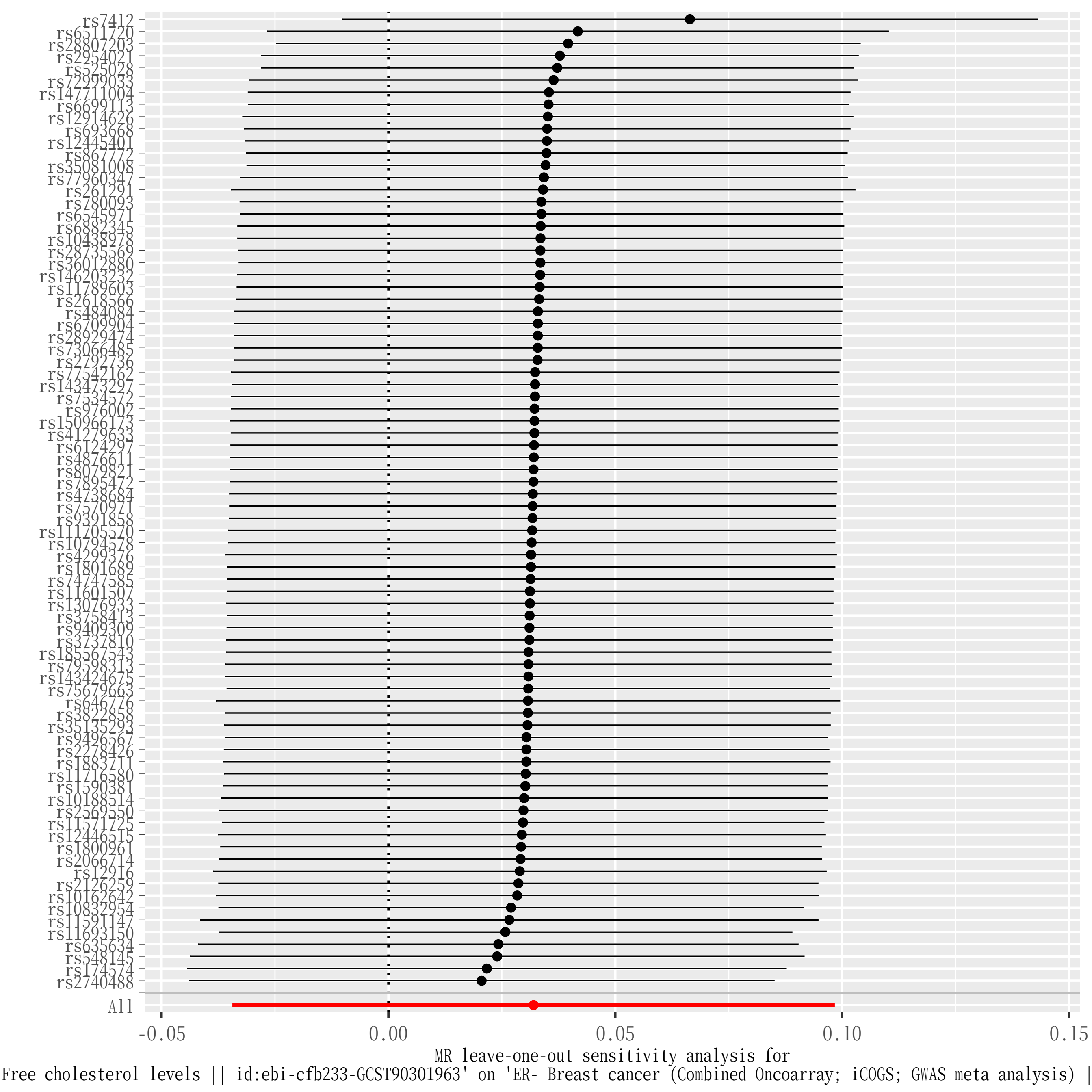
rs7412
rs6511720
rs28807203
rs2954021
rs525028
rs72999033
rs147711004
rs6699113
rs12914626
rs693668
rs12445401
rs867772
rs35081008
rs77960347
rs261291
rs780093
rs6545971
rs6882345
rs10438978
rs28735569
rs36012880
rs146203232
rs11789603
rs2618566
rs484084
rs6709904
rs28929474
rs73066485
rs2792736
rs77542162
rs143473297
rs7534572
rs976002
rs150966173
rs41279633
rs6124297
rs4876611
rs8079821
rs7895472
rs4738684
rs7570971
rs9391858
rs111705570
rs10794578
rs4299376
rs1801689
rs74747585
rs11601507
rs13076933
rs3758413
rs9409309
rs3737810
rs185567543
rs79598313
rs143424675
rs75679663
rs646776
rs3822858
rs35135293
rs9496567
rs2278426
rs1883711
rs11716580
rs1590381
rs10188514
rs2569550
rs11571725
rs12446515
rs1800961
rs2066714
rs12916
rs2126259
rs10162642
rs10832954
rs11591147
rs11693150
rs635634
rs548145
rs174574
rs2740488
All
-0.05
0.00
0.05
0.10
0.15
MR leave-one-out sensitivity analysis for
Free cholesterol levels || id:ebi-cfb233-GCST90301963' on 'ER- Breast cancer (Combined Oncoarray; iCOGS; GWAS meta analysis) |

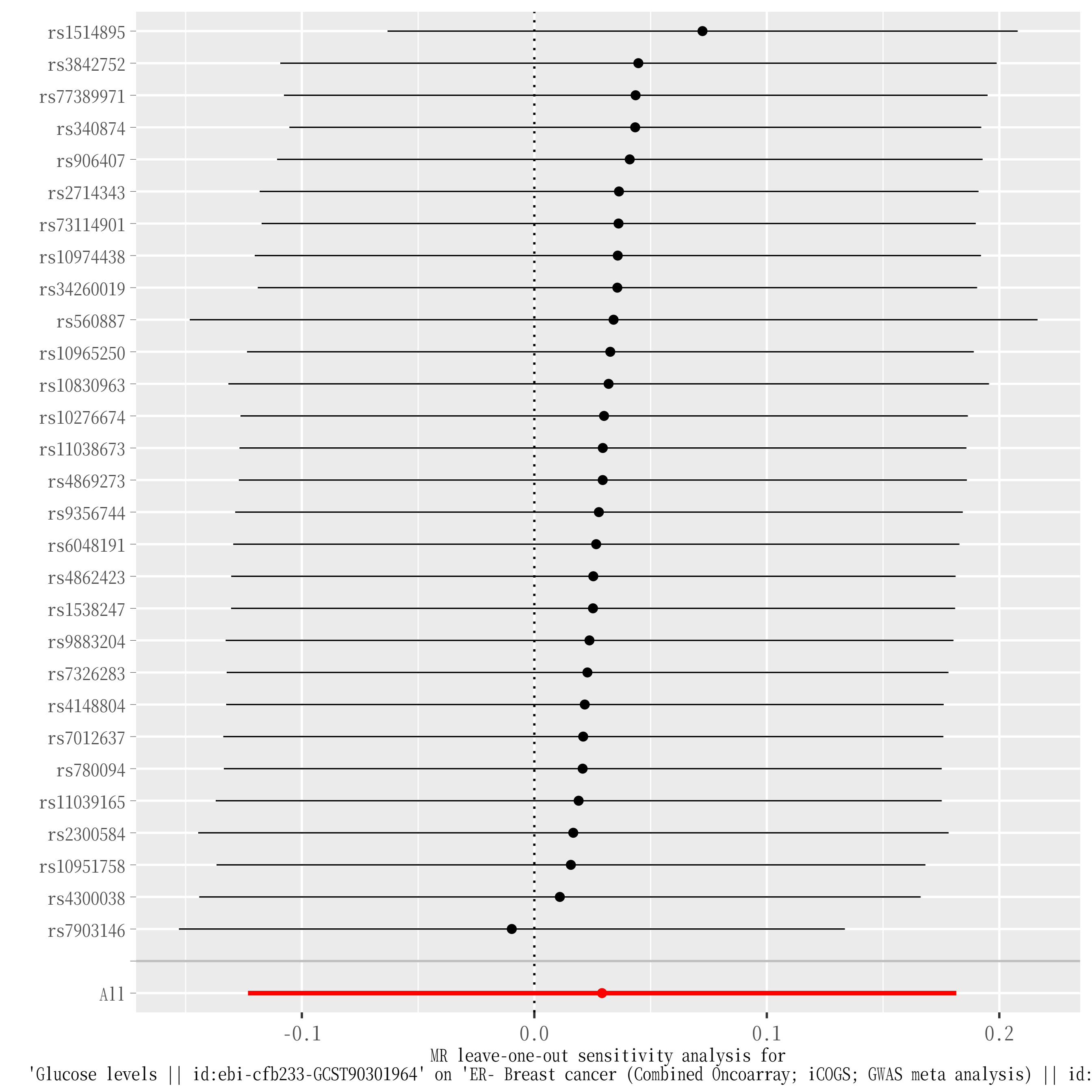
rs1514895
rs3842752
rs77389971
rs340874
rs906407
rs2714343
rs73114901
rs10974438
rs34260019
rs560887
rs10965250
rs10830963
rs10276674
rs11038673
rs4869273
rs9356744
rs6048191
rs4862423
rs1538247
rs9883204
rs7326283
rs4148804
rs7012637
rs780094
rs11039165
rs2300584
rs10951758
rs4300038
rs7903146
All
-0.1
0.0
0.1
0.2
MR leave-one-out sensitivity analysis for
'Glucose levels || id:ebi-cfb233-GCST90301964' on 'ER- Breast cancer (Combined Oncoarray; iCOGS; GWAS meta analysis) || id:

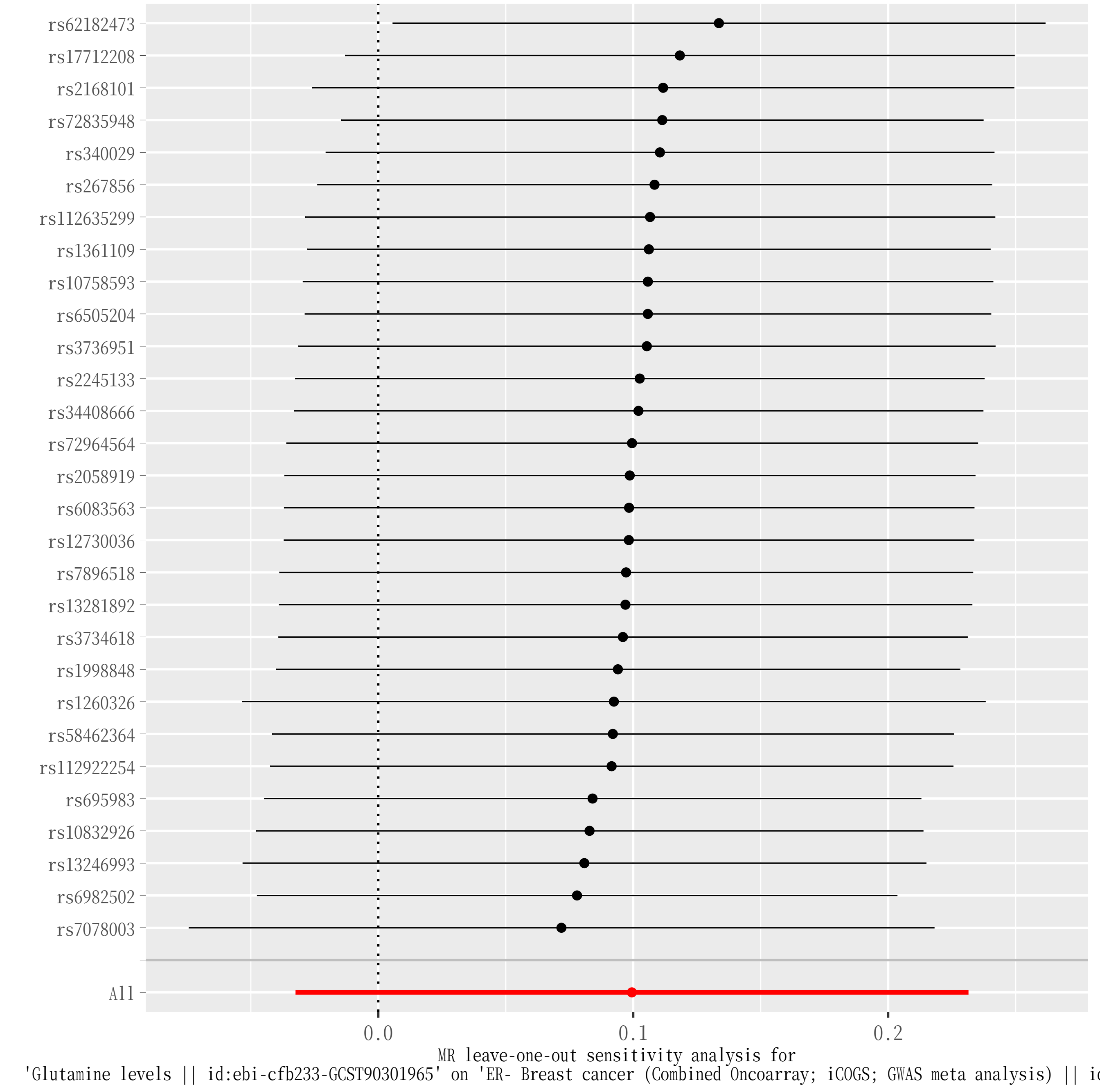
rs62182473
rs17712208
rs2168101
rs72835948
rs340029
rs267856
rs112635299
rs1361109
rs10758593
rs6505204
rs3736951
rs2245133
rs34408666
rs72964564
rs2058919
rs6083563
rs12730036
rs7896518
rs13281892
rs3734618
rs1998848
rs1260326
rs58462364
rs112922254
rs695983
rs10832926
rs13246993
rs6982502
rs7078003
All
0.0
0.1
0.2
MR leave-one-out sensitivity analysis for
'Glutamine levels || id:ebi-cfb233-GCST90301965' on 'ER- Breast cancer (Combined Oncoarray; iCOGS; GWAS meta analysis) || i

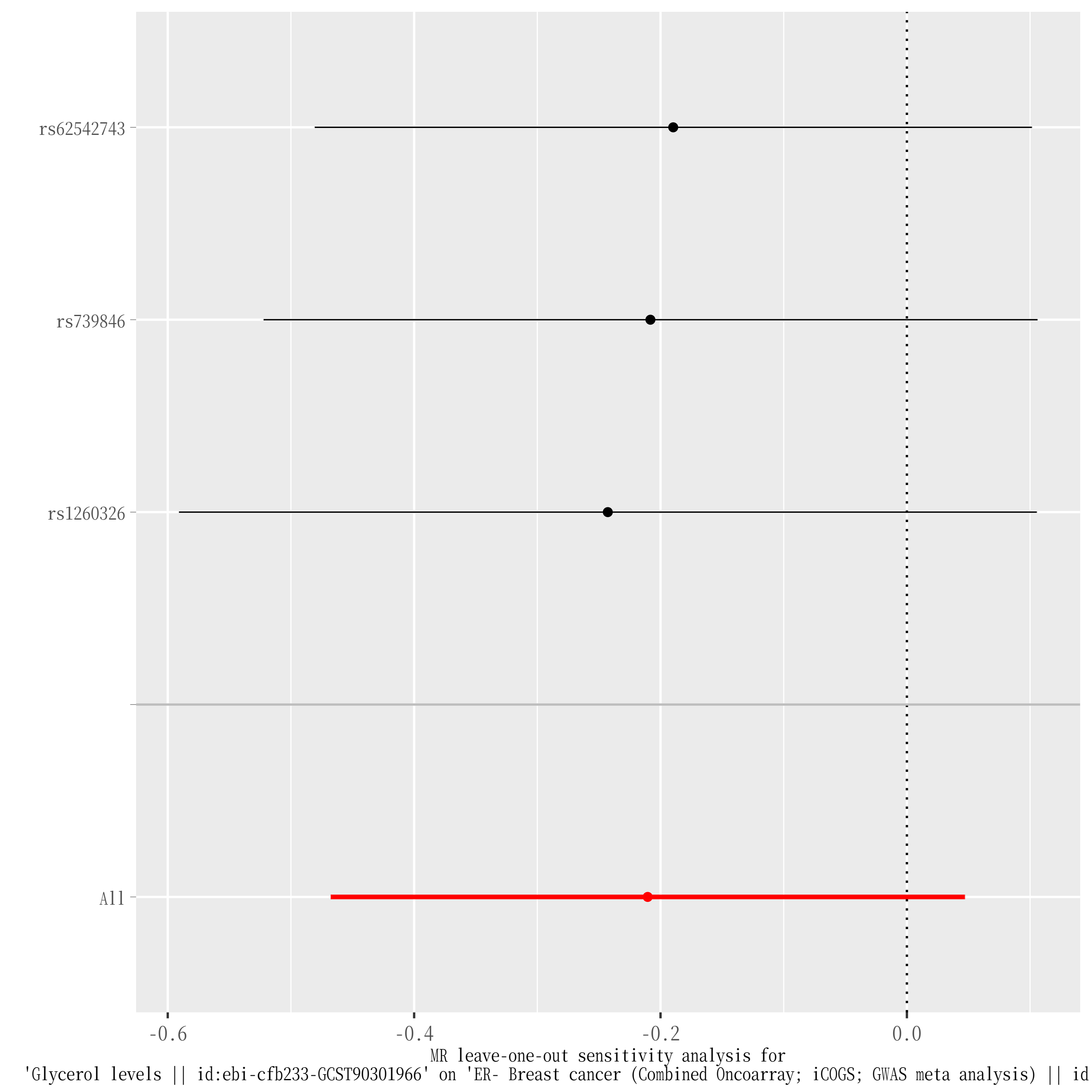

rs62542743
rs739846
rs1260326
All
-0.6
-0.4
-0.2
0.0
MR leave-one-out sensitivity analysis for
'Glycerol levels || id:ebi-cfb233-GCST90301966' on 'ER- Breast cancer (Combined Oncoarray; iCOGS; GWAS meta analysis) || id

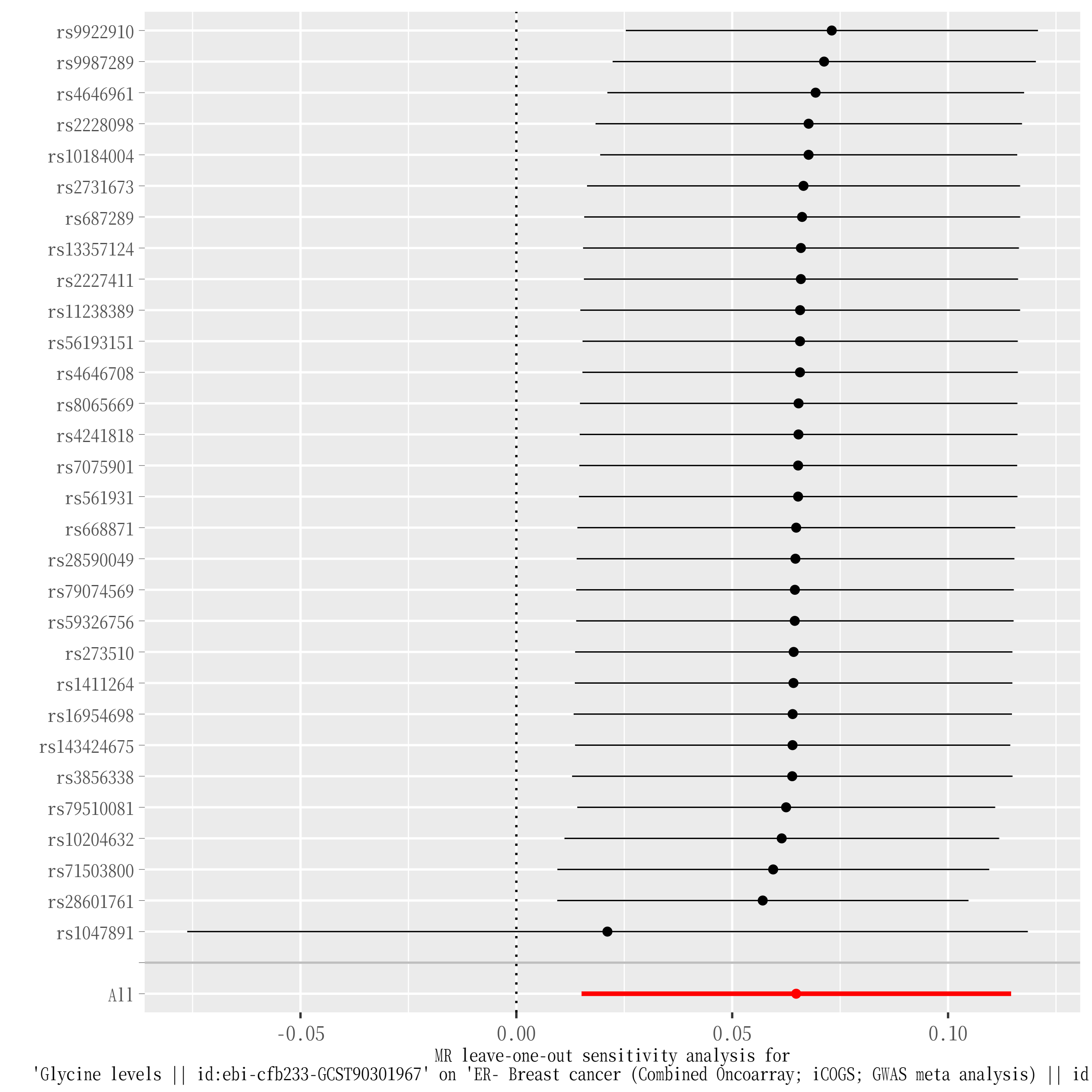
rs9922910
rs9987289
rs4646961
rs2228098
rs10184004
rs2731673
rs687289
rs13357124
rs2227411
rs11238389
rs56193151
rs4646708
rs8065669
rs4241818
rs7075901
rs561931
rs668871
rs28590049
rs79074569
rs59326756
rs273510
rs1411264
rs16954698
rs143424675
rs3856338
rs79510081
rs10204632
rs71503800
rs28601761
rs1047891
All
-0.05
0.00
0.05
0.10
MR leave-one-out sensitivity analysis for
'Glycine levels || id:ebi-cfb233-GCST90301967' on 'ER- Breast cancer (Combined Oncoarray; iCOGS; GWAS meta analysis) || id

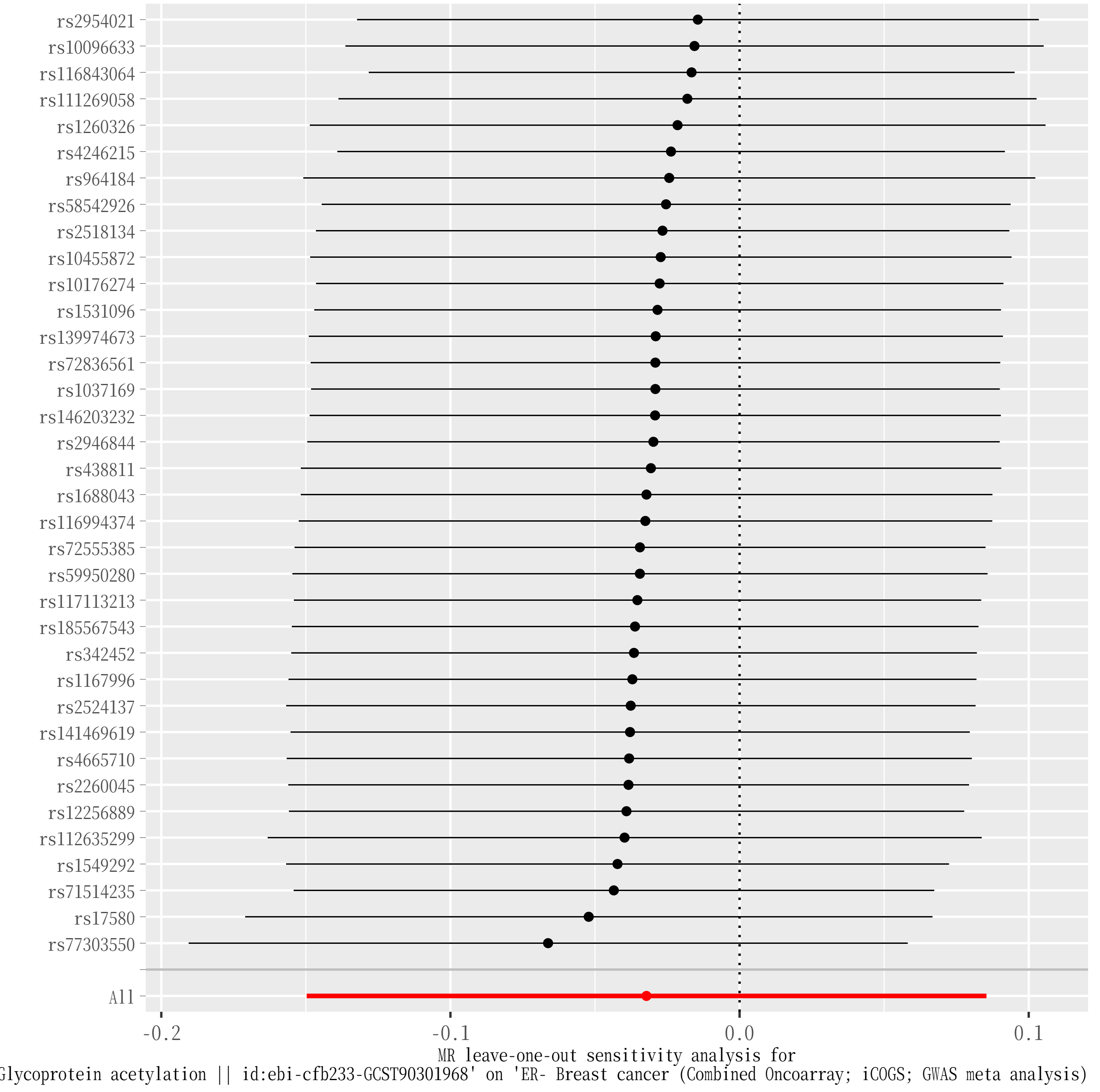
rs2954021
rs10096633
rs116843064
rs111269058
rs1260326
rs4246215
rs964184
rs58542926
rs2518134
rs10455872
rs10176274
rs1531096
rs139974673
rs72836561
rs1037169
rs146203232
rs2946844
rs438811
rs1688043
rs116994374
rs72555385
rs59950280
rs117113213
rs185567543
rs342452
rs1167996
rs2524137
rs141469619
rs4665710
rs2260045
rs12256889
rs112635299
rs1549292
rs71514235
rs17580
rs77303550
All
-0.2
-0.1
0.0
0.1
MR leave-one-out sensitivity analysis for
'Glycoprotein acetylation || id:ebi-cfb233-GCST90301968' on 'ER- Breast cancer (Combined Oncoarray; iCOGS; GWAS meta analysis)'

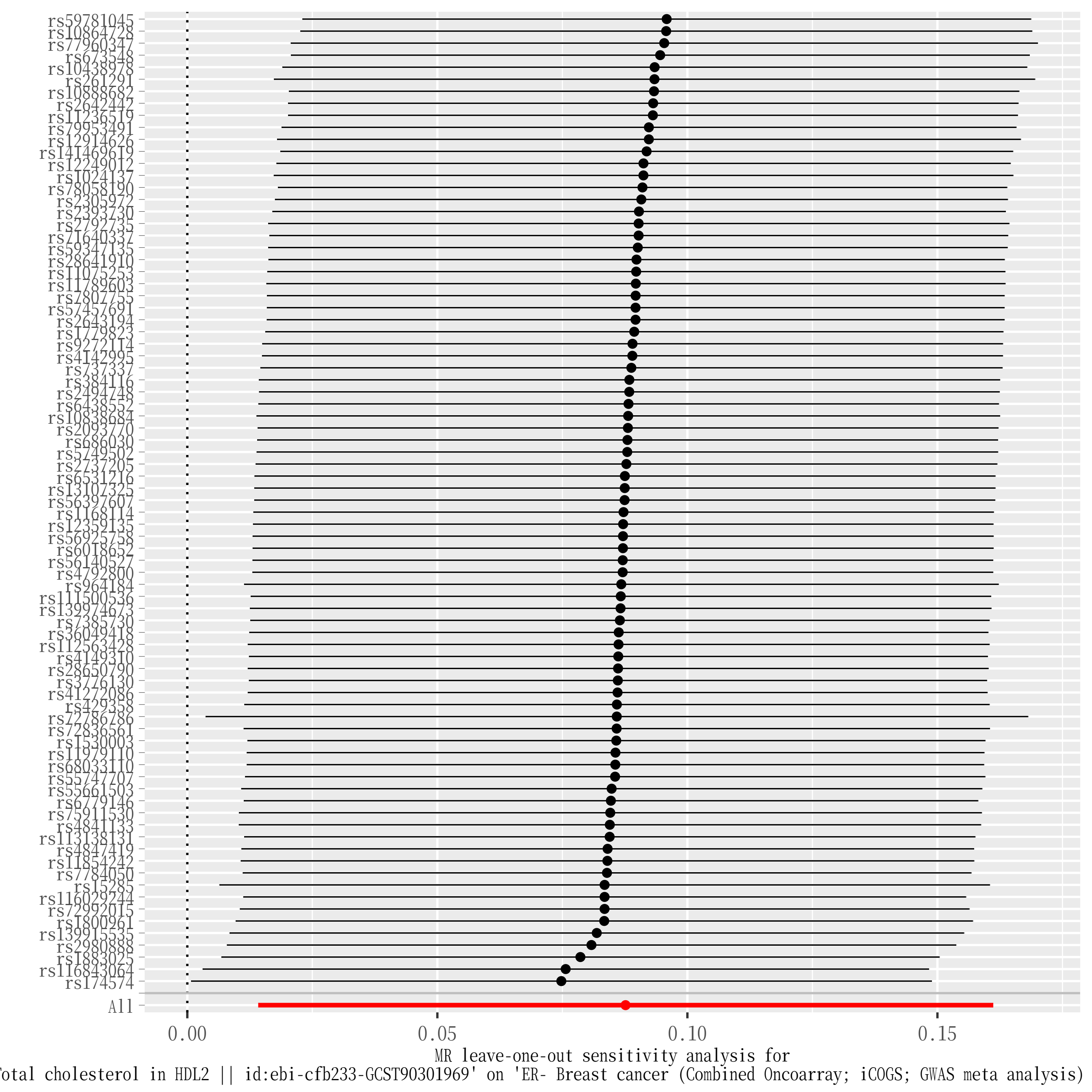
rs59781045
rs10864728
rs77960347
rs673548
rs10438978
rs261291
rs10888682
rs2642442
rs11236519
rs79953491
rs12914626
rs141469619
rs12249012
rs1024137
rs78058190
rs2305972
rs2393730
rs2792735
rs71640337
rs59347135
rs28641910
rs11075253
rs11789603
rs7807755
rs57457691
rs2643194
rs1779823
rs9272114
rs4142995
rs737337
rs384116
rs2494748
rs6438552
rs10838684
rs2093770
rs686030
rs5749502
rs2737205
rs6531216
rs13107325
rs56397607
rs1168114
rs12359135
rs56925758
rs6018652
rs56140527
rs4792800
rs964184
rs111500536
rs139974673
rs7385730
rs36049418
rs112563428
rs4149310
rs28650790
rs3776130
rs41272086
rs429358
rs72786786
rs72836561
rs1530003
rs11979110
rs68033110
rs55747707
rs55661503
rs6779146
rs75911530
rs4841133
rs113138131
rs4847419
rs11854242
rs7784050
rs15285
rs116029244
rs72992015
rs1800961
rs139915535
rs2980888
rs1883025
rs116843064
rs174574
All
0.00
0.05
0.10
0.15
MR leave-one-out sensitivity analysis for
otal cholesterol in HDL2 || id:ebi-cfb233-GCST90301969' on 'ER- Breast cancer (Combined Oncoarray; iCOGS; GWAS meta analysis)

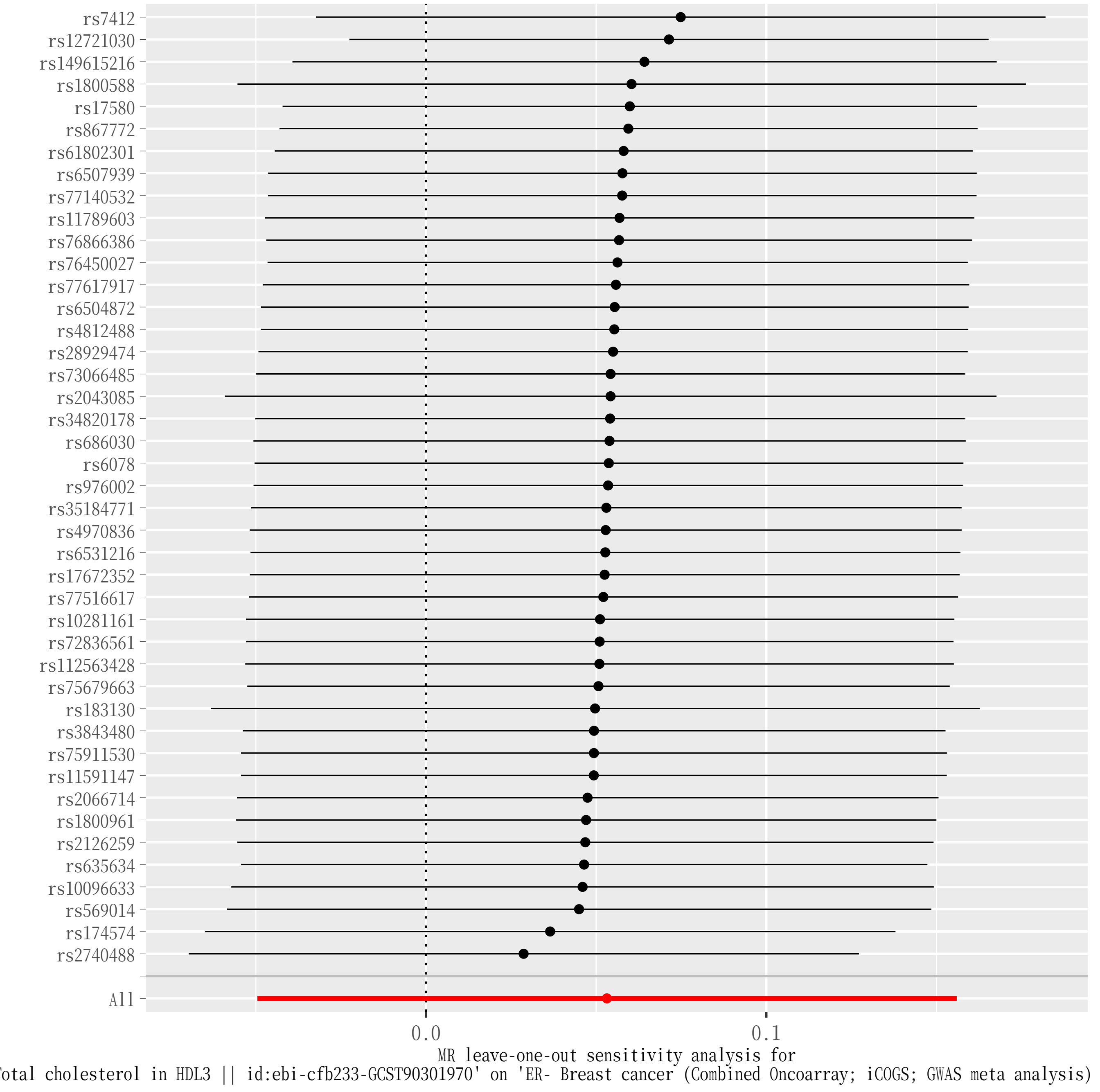

rs7412
rs12721030
rs149615216
rs1800588
rs17580
rs867772
rs61802301
rs6507939
rs77140532
rs11789603
rs76866386
rs76450027
rs77617917
rs6504872
rs4812488
rs28929474
rs73066485
rs2043085
rs34820178
rs686030
rs6078
rs976002
rs35184771
rs4970836
rs6531216
rs17672352
rs77516617
rs10281161
rs72836561
rs112563428
rs75679663
rs183130
rs3843480
rs75911530
rs11591147
rs2066714
rs1800961
rs2126259
rs635634
rs10096633
rs569014
rs174574
rs2740488
All
0.0
0.1
MR leave-one-out sensitivity analysis for
'Total cholesterol in HDL3 || id:ebi-cfb233-GCST90301970' on 'ER- Breast cancer (Combined Oncoarray; iCOGS; GWAS meta analysis)'

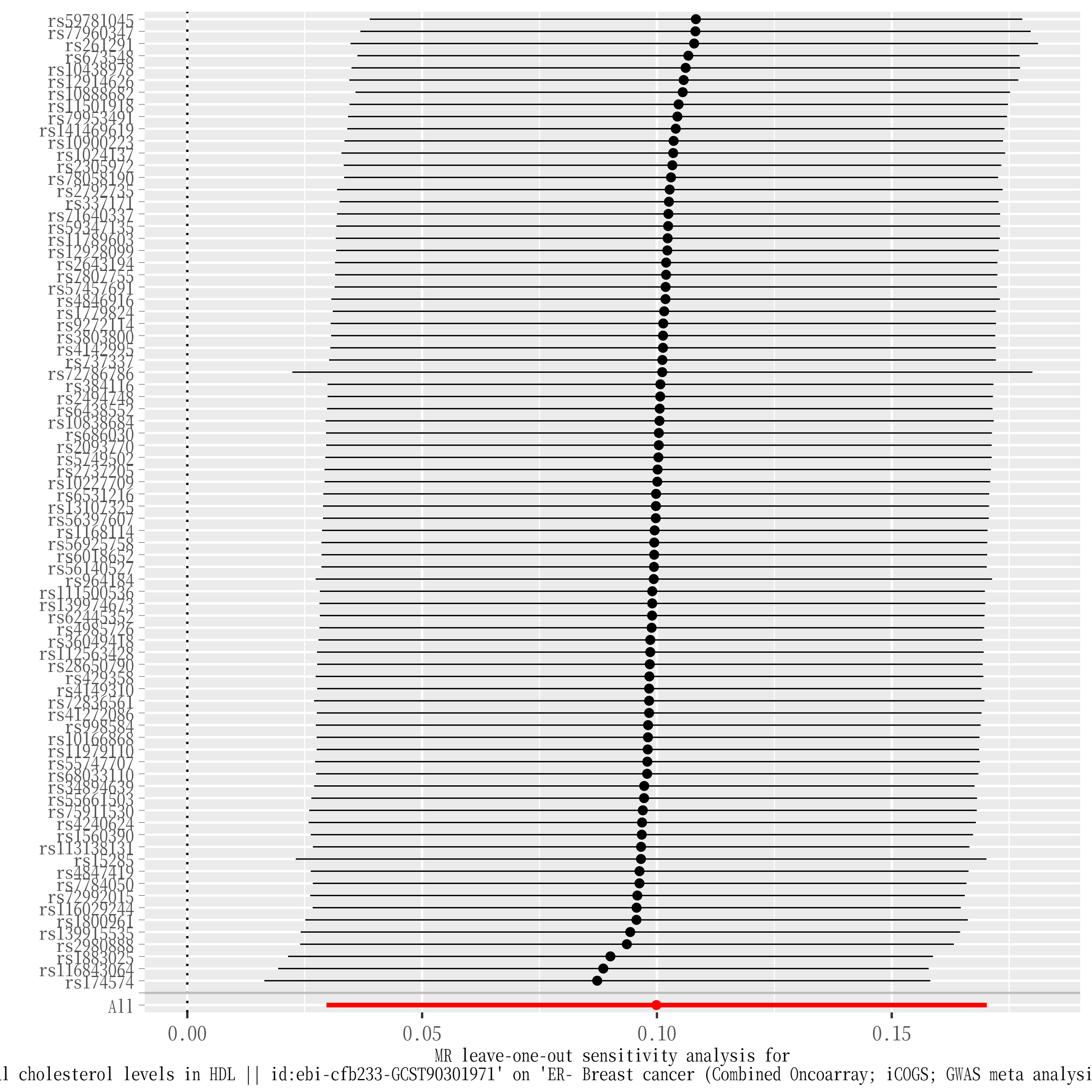
rs59781045
rs77960347
rs261291
rs673548
rs10438978
rs12914626
rs10888682
rs11501918
rs79953491
rs141469619
rs10900223
rs1024137
rs2305972
rs78058190
rs2792735
rs337171
rs71640337
rs59347135
rs11789603
rs12928099
rs2643194
rs7807755
rs57457691
rs4846916
rs1779824
rs9272114
rs3803800
rs4142995
rs737337
rs72786786
rs384116
rs2494748
rs6438552
rs10838684
rs686030
rs2093770
rs5749502
rs2737205
rs10227709
rs6531216
rs13107325
rs56397607
rs1168114
rs56925758
rs6018652
rs56140527
rs964184
rs111500536
rs139974673
rs62445352
rs4985726
rs36049418
rs112563428
rs28650790
rs429358
rs4149310
rs72836561
rs41272086
rs998584
rs10166868
rs11979110
rs55747707
rs68033110
rs34894639
rs55661503
rs75911530
rs4240624
rs1560390
rs113138131
rs15285
rs4847419
rs7784050
rs72992015
rs116029244
rs1800961
rs139915535
rs2980888
rs1883025
rs116843064
rs174574
All
0.00
0.05
0.10
0.15
MR leave-one-out sensitivity analysis for
l cholesterol levels in HDL || id:ebi-cfb233-GCST90301971' on 'ER- Breast cancer (Combined Oncoarray; iCOGS; GWAS meta analysi

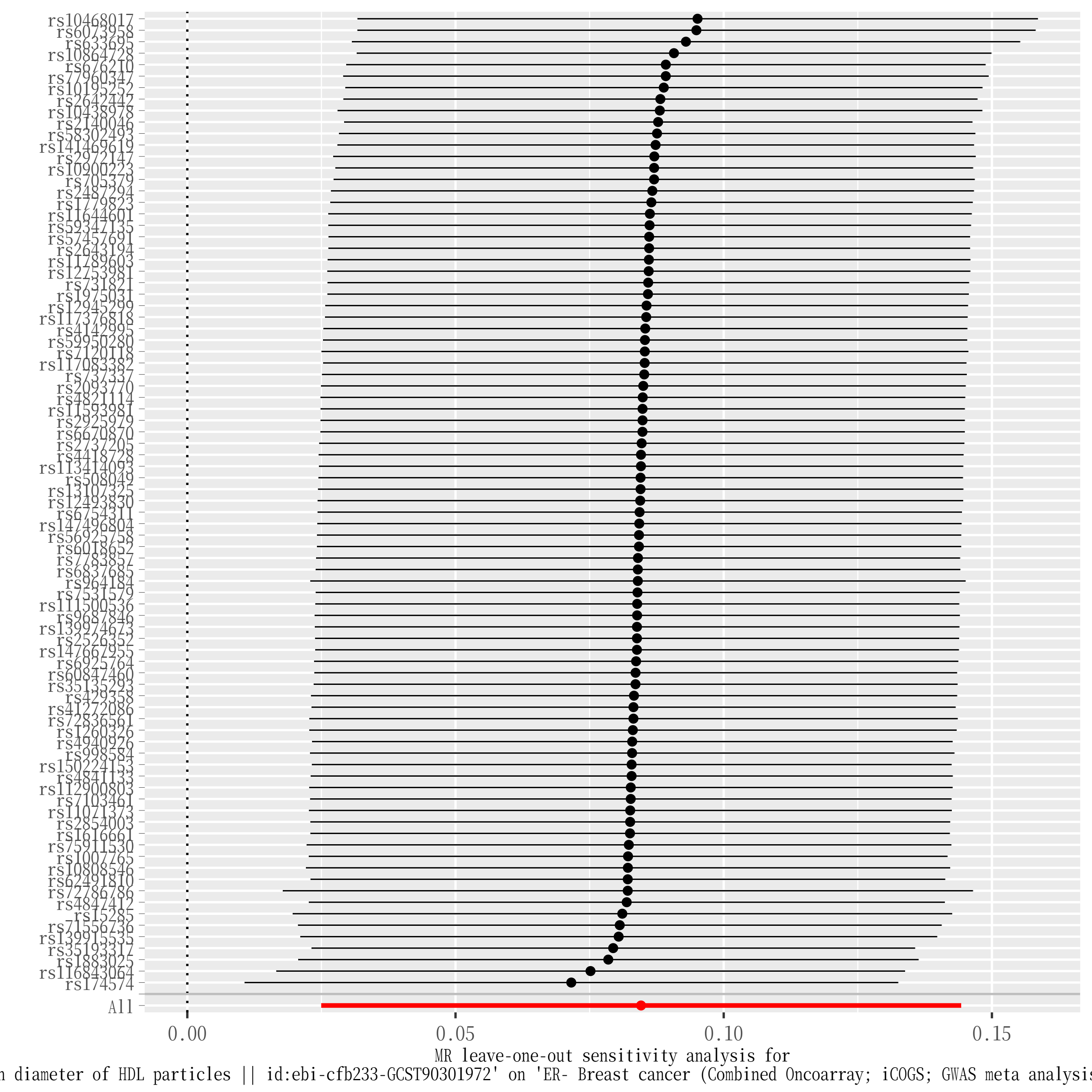
rs10468017
rs6073958
rs633695
rs10864728
rs676210
rs77960347
rs10195252
rs2642442
rs10438978
rs2140046
rs58302493
rs141469619
rs2972147
rs10900223
rs705379
rs2487294
rs1779823
rs11644601
rs59347135
rs57457691
rs2643194
rs11789603
rs12753981
rs731821
rs1975031
rs12945299
rs117376818
rs4142995
rs59950280
rs7120118
rs117083382
rs737337
rs2093770
rs4821114
rs11593981
rs2925979
rs6670870
rs2737205
rs4418728
rs113414093
rs508049
rs13107325
rs12493830
rs6754311
rs147496804
rs56925758
rs6018652
rs7783857
rs6837685
rs964184
rs7531579
rs111500536
rs9687846
rs139974673
rs2526352
rs147667955
rs6925764
rs60847460
rs35135293
rs429358
rs41272086
rs72836561
rs1260326
rs4940926
rs998584
rs150224153
rs4841133
rs112900803
rs7103461
rs11071373
rs2854003
rs1616661
rs75911530
rs1007765
rs10808546
rs62491810
rs72786786
rs4847412
rs15285
rs71556736
rs139915535
rs35193317
rs1883025
rs116843064
rs174574
All
0.00
0.05
0.10
0.15
MR leave-one-out sensitivity analysis for
n diameter of HDL particles || id:ebi-cfb233-GCST90301972' on 'ER- Breast cancer (Combined Oncoarray; iCOGS; GWAS meta analysis

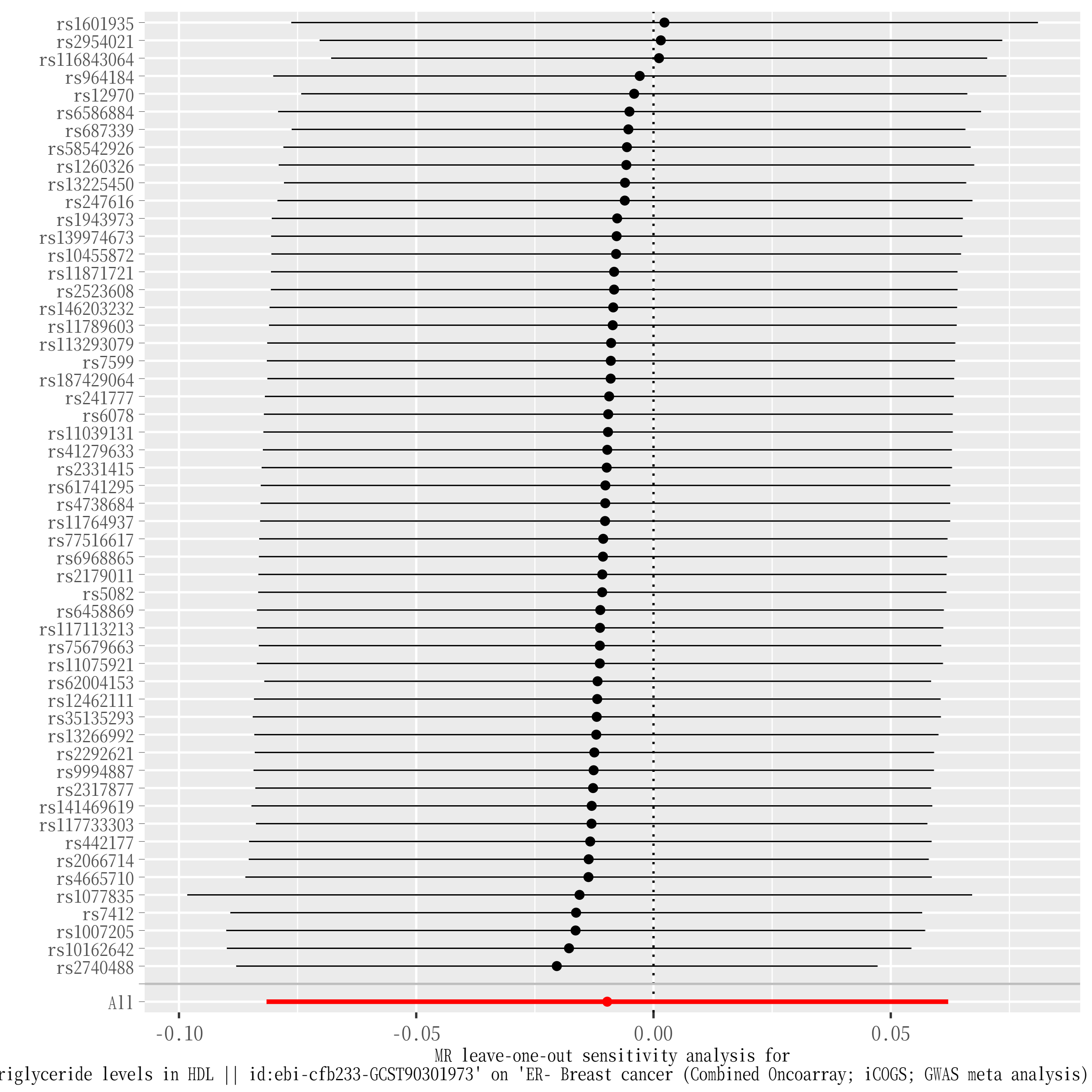
rs1601935
rs2954021
rs116843064
rs964184
rs12970
rs6586884
rs687339
rs58542926
rs1260326
rs13225450
rs247616
rs1943973
rs139974673
rs10455872
rs11871721
rs2523608
rs146203232
rs11789603
rs113293079
rs7599
rs187429064
rs241777
rs6078
rs11039131
rs41279633
rs2331415
rs61741295
rs4738684
rs11764937
rs77516617
rs6968865
rs2179011
rs5082
rs6458869
rs117113213
rs75679663
rs11075921
rs62004153
rs12462111
rs35135293
rs13266992
rs2292621
rs9994887
rs2317877
rs141469619
rs117733303
rs442177
rs2066714
rs4665710
rs1077835
rs7412
rs1007205
rs10162642
rs2740488
All
-0.10
-0.05
0.00
0.05
MR leave-one-out sensitivity analysis for
riglyceride levels in HDL || id:ebi-cfb233-GCST90301973' on 'ER- Breast cancer (Combined Oncoarray; iCOGS; GWAS meta analysis)

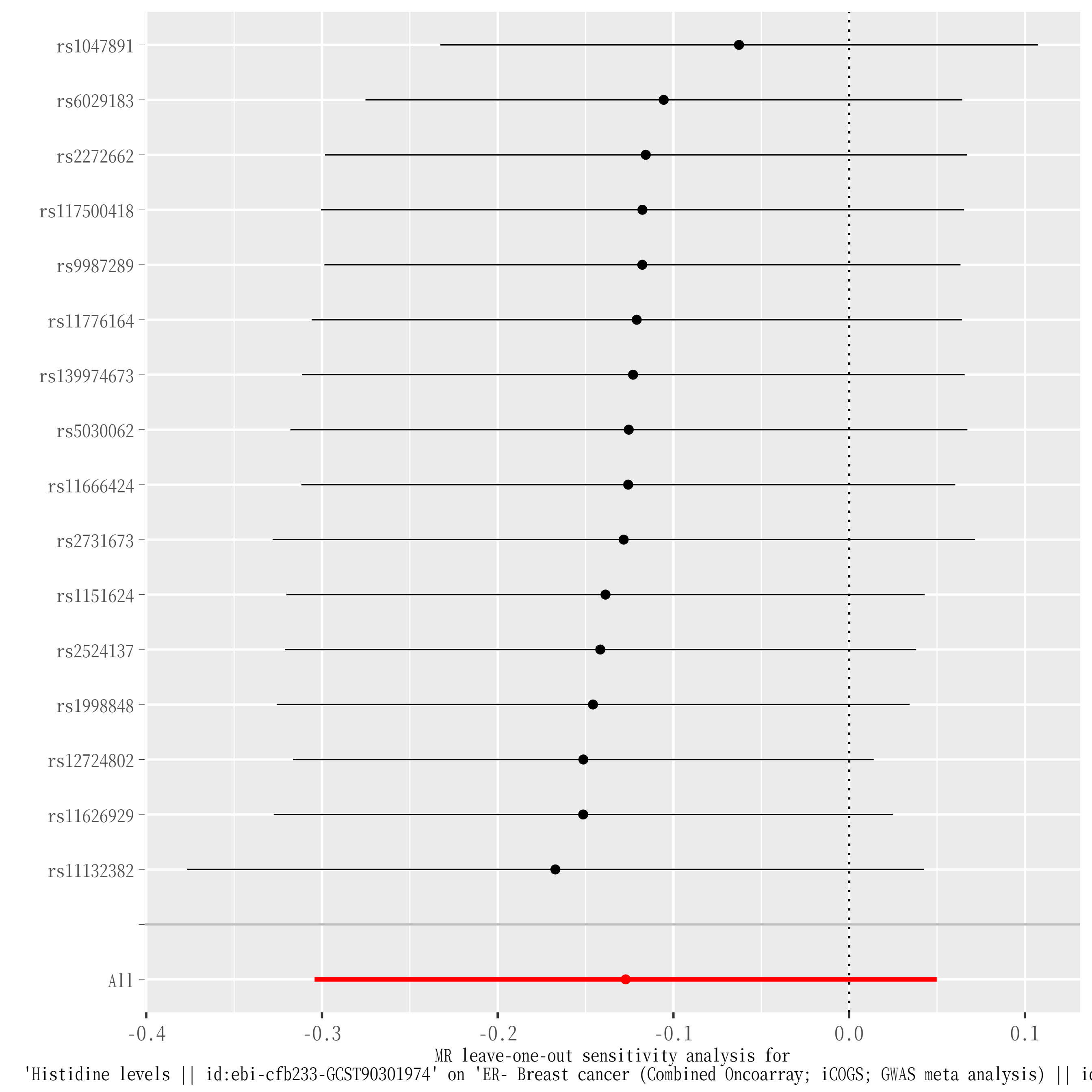
rs1047891
rs6029183
rs2272662
rs117500418
rs9987289
rs11776164
rs139974673
rs5030062
rs11666424
rs2731673
rs1151624
rs2524137
rs1998848
rs12724802
rs11626929
rs11132382
All
-0.4
-0.3
-0.2
-0.1
0.0
0.1
MR leave-one-out sensitivity analysis for
'Histidine levels || id:ebi-cfb233-GCST90301974' on 'ER- Breast cancer (Combined Oncoarray; iCOGS; GWAS meta analysis) || id

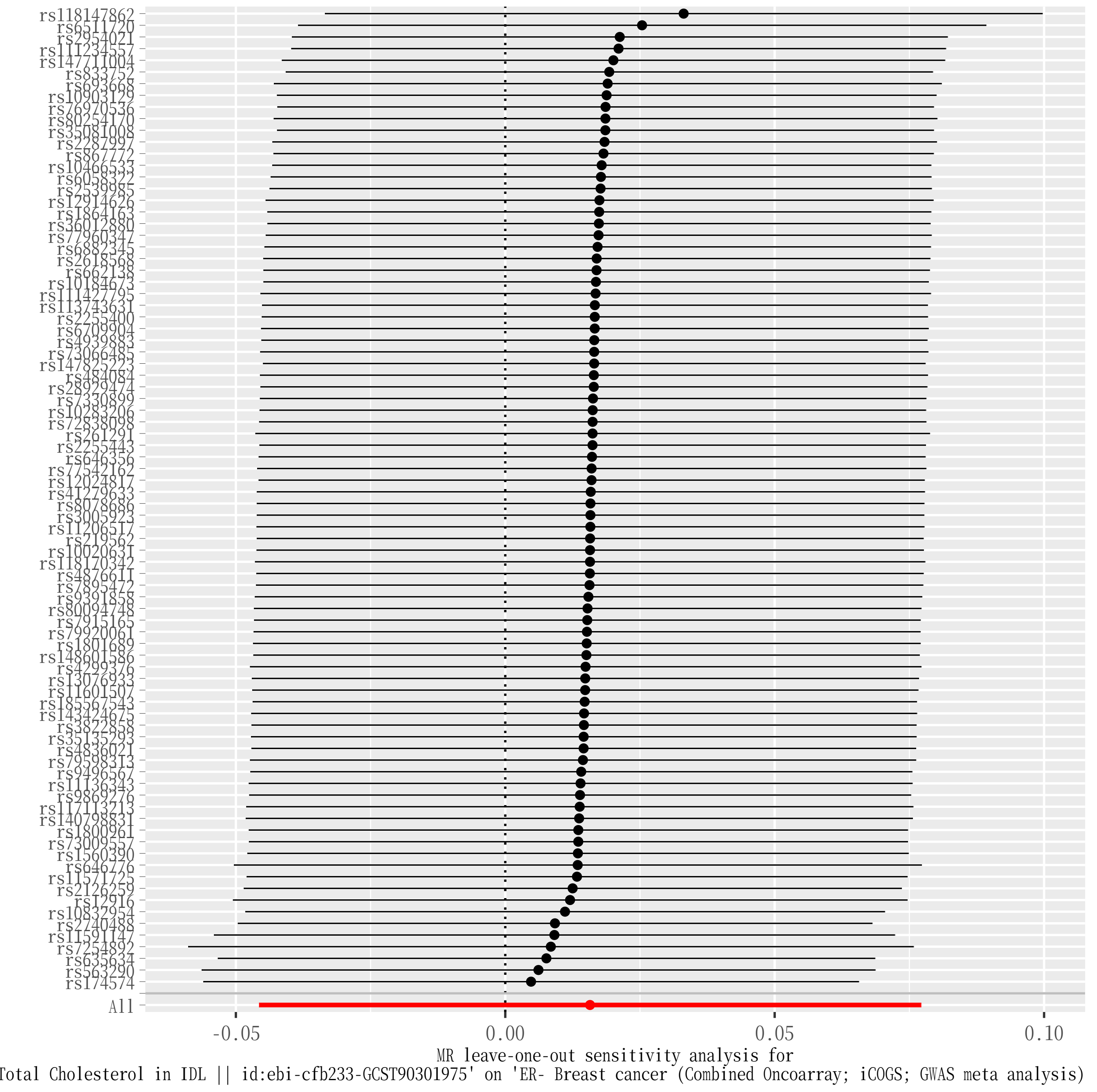
rs118147862
rs6511720
rs2954021
rs111234557
rs147711004
rs833752
rs693668
rs10903129
rs76970536
rs80254170
rs35081008
rs2287997
rs867772
rs10466533
rs6058322
rs2539985
rs12914626
rs1864163
rs36012880
rs77960347
rs6882345
rs2618568
rs662138
rs10184673
rs111427795
rs113743631
rs2255400
rs6709904
rs4939883
rs73066485
rs147825223
rs484084
rs28929474
rs7330899
rs10283206
rs72838098
rs261291
rs2255443
rs646356
rs77542162
rs12024817
rs41279633
rs8078686
rs3005923
rs11206517
rs219562
rs10020631
rs118170342
rs4876611
rs7895472
rs9391858
rs80094748
rs7915165
rs79920061
rs1801689
rs148601586
rs4299376
rs13076933
rs11601507
rs185567543
rs143424675
rs3822858
rs35135293
rs4836021
rs79598313
rs9496567
rs11136343
rs9869276
rs117113213
rs140798831
rs1800961
rs73009557
rs1560320
rs646776
rs11571725
rs2126259
rs12916
rs10832954
rs2740488
rs11591147
rs7254892
rs635634
rs563290
rs174574
All
-0.05
0.00
0.05
0.10
MR leave-one-out sensitivity analysis for
'Total Cholesterol in IDL || id:ebi-cfb233-GCST90301975' on 'ER- Breast cancer (Combined Oncoarray; iCOGS; GWAS meta analysis)'

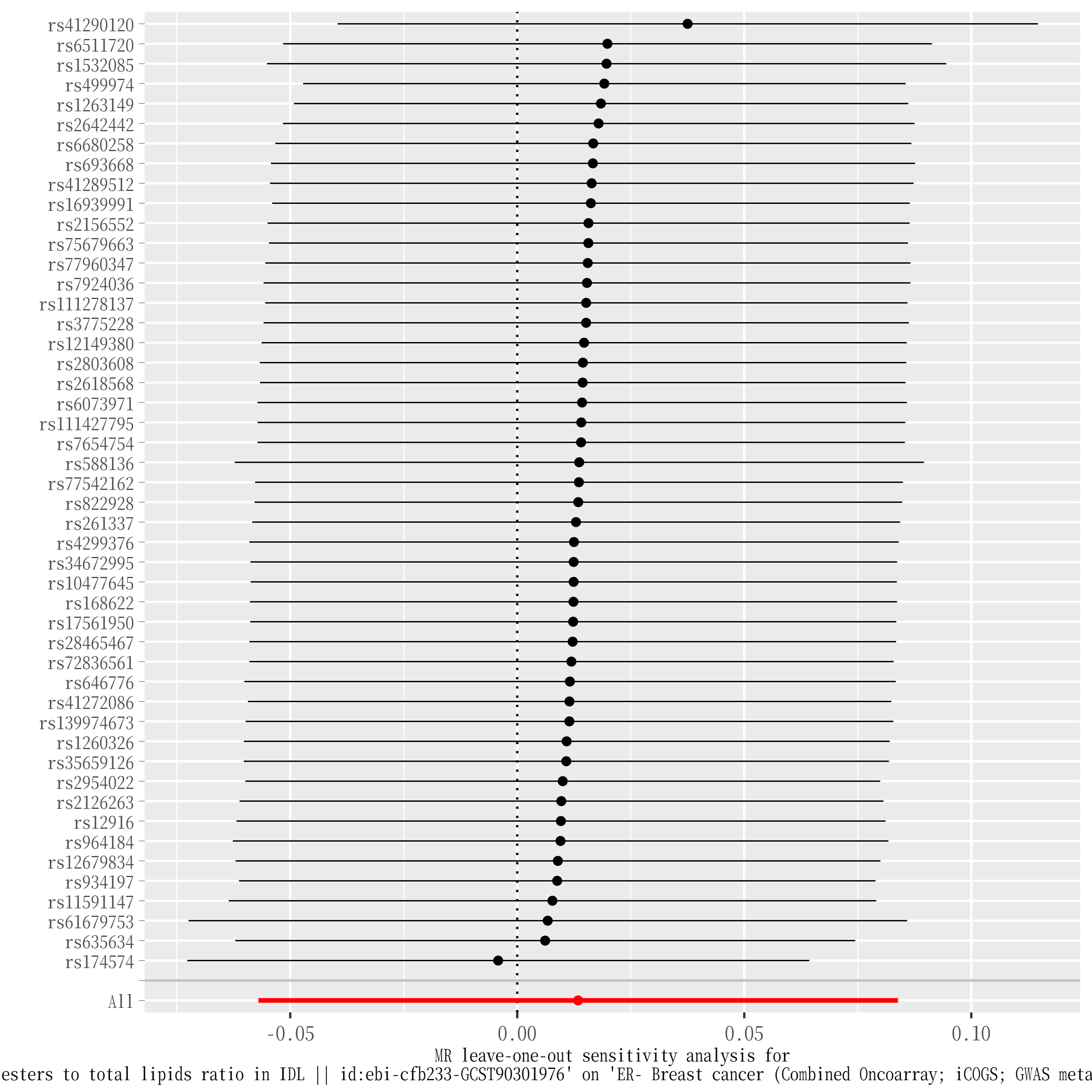

rs41290120
rs6511720
rs1532085
rs499974
rs1263149
rs2642442
rs6680258
rs693668
rs41289512
rs16939991
rs2156552
rs75679663
rs77960347
rs7924036
rs111278137
rs3775228
rs12149380
rs2803608
rs2618568
rs6073971
rs111427795
rs7654754
rs588136
rs77542162
rs822928
rs261337
rs4299376
rs34672995
rs10477645
rs168622
rs17561950
rs28465467
rs72836561
rs646776
rs41272086
rs139974673
rs1260326
rs35659126
rs2954022
rs2126263
rs12916
rs964184
rs12679834
rs934197
rs11591147
rs61679753
rs635634
rs174574
All
-0.05
0.00
0.05
0.10
MR leave-one-out sensitivity analysis for
esters to total lipids ratio in IDL || id:ebi-cfb233-GCST90301976' on 'ER- Breast cancer (Combined Oncoarray; iCOGS; GWAS meta

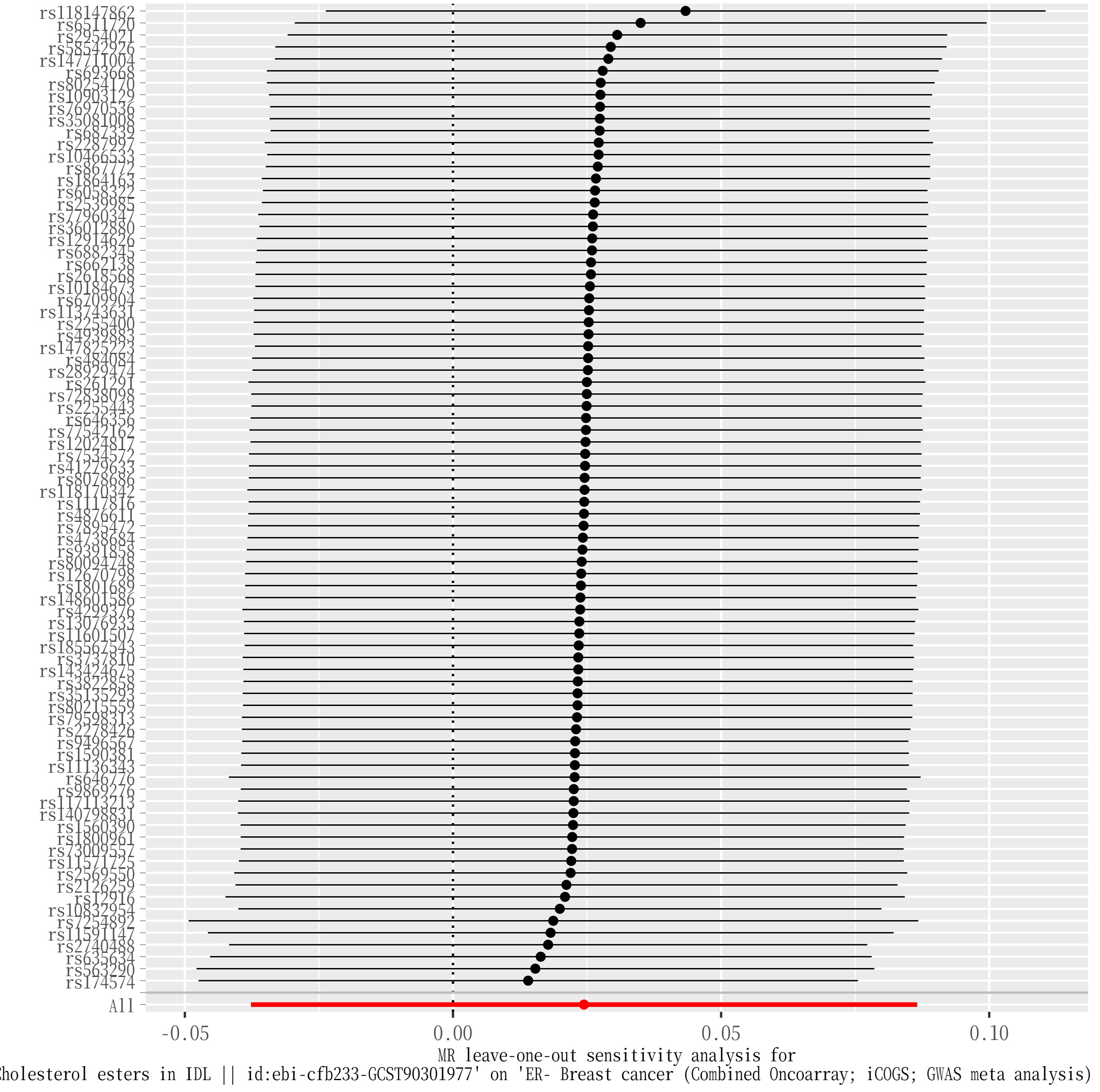

rs118147862
rs6511720
rs2954021
rs58542926
rs147711004
rs693668
rs80254170
rs10903129
rs76970536
rs35081008
rs687339
rs2287887
rs10466533
rs867772
rs1864163
rs6058322
rs2539985
rs77960347
rs36012880
rs12914626
rs6882345
rs662138
rs2618568
rs10184673
rs6709904
rs113743631
rs2255400
rs4939883
rs147825223
rs484084
rs28929474
rs261291
rs72838098
rs2255443
rs646356
rs77542162
rs12024817
rs7534572
rs41279633
rs8078686
rs118170342
rs1117816
rs4876611
rs7895472
rs4738684
rs9391858
rs80094748
rs12670798
rs1801689
rs148601586
rs4299376
rs13076933
rs11601507
rs185567543
rs3737810
rs143424675
rs3822858
rs35135293
rs80215559
rs79598313
rs2278426
rs9496567
rs1590381
rs11136343
rs646776
rs9869276
rs117113213
rs140798831
rs1560390
rs1800961
rs73009557
rs11571725
rs2569550
rs2126259
rs12916
rs10832954
rs7254892
rs115911147
rs2740488
rs635634
rs563290
rs174574
All
-0.05
0.00
0.05
0.10
MR leave-one-out sensitivity analysis for
'Cholesterol esters in IDL || id:ebi-cfb233-GCST90301977' on 'ER- Breast cancer (Combined Oncoarray; iCOGS; GWAS meta analysis)'

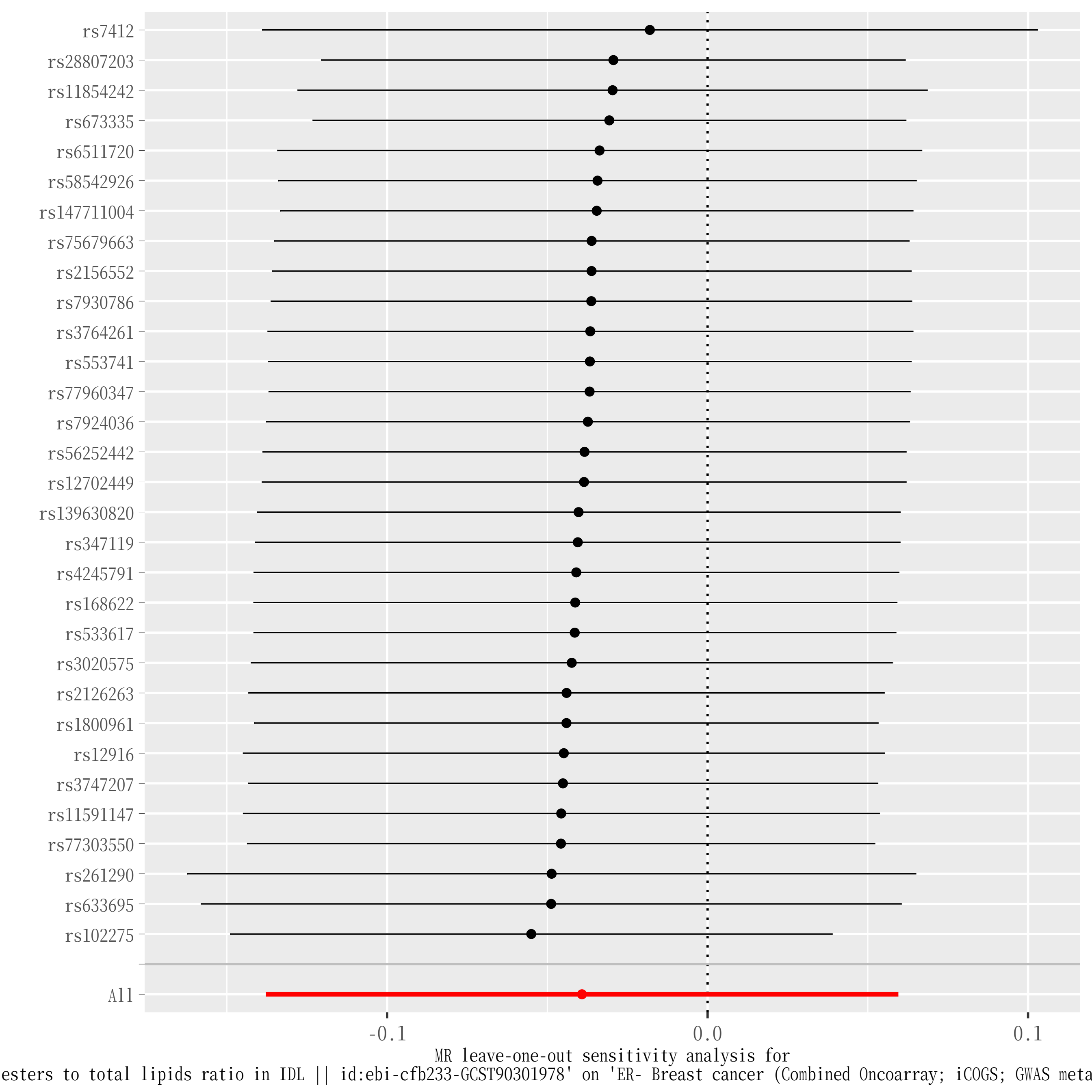
rs7412
rs28807203
rs11854242
rs673335
rs6511720
rs58542926
rs147711004
rs75679663
rs2156552
rs7930786
rs3764261
rs553741
rs77960347
rs7924036
rs56252442
rs12702449
rs139630820
rs347119
rs4245791
rs168622
rs533617
rs3020575
rs2126263
rs1800961
rs12916
rs3747207
rs11591147
rs77303550
rs261290
rs633695
rs102275
All
-0.1
0.0
0.1
MR leave-one-out sensitivity analysis for
esters to total lipids ratio in IDL || id:ebi-cfb233-GCST90301978' on 'ER- Breast cancer (Combined Oncoarray; iCOGS; GWAS meta

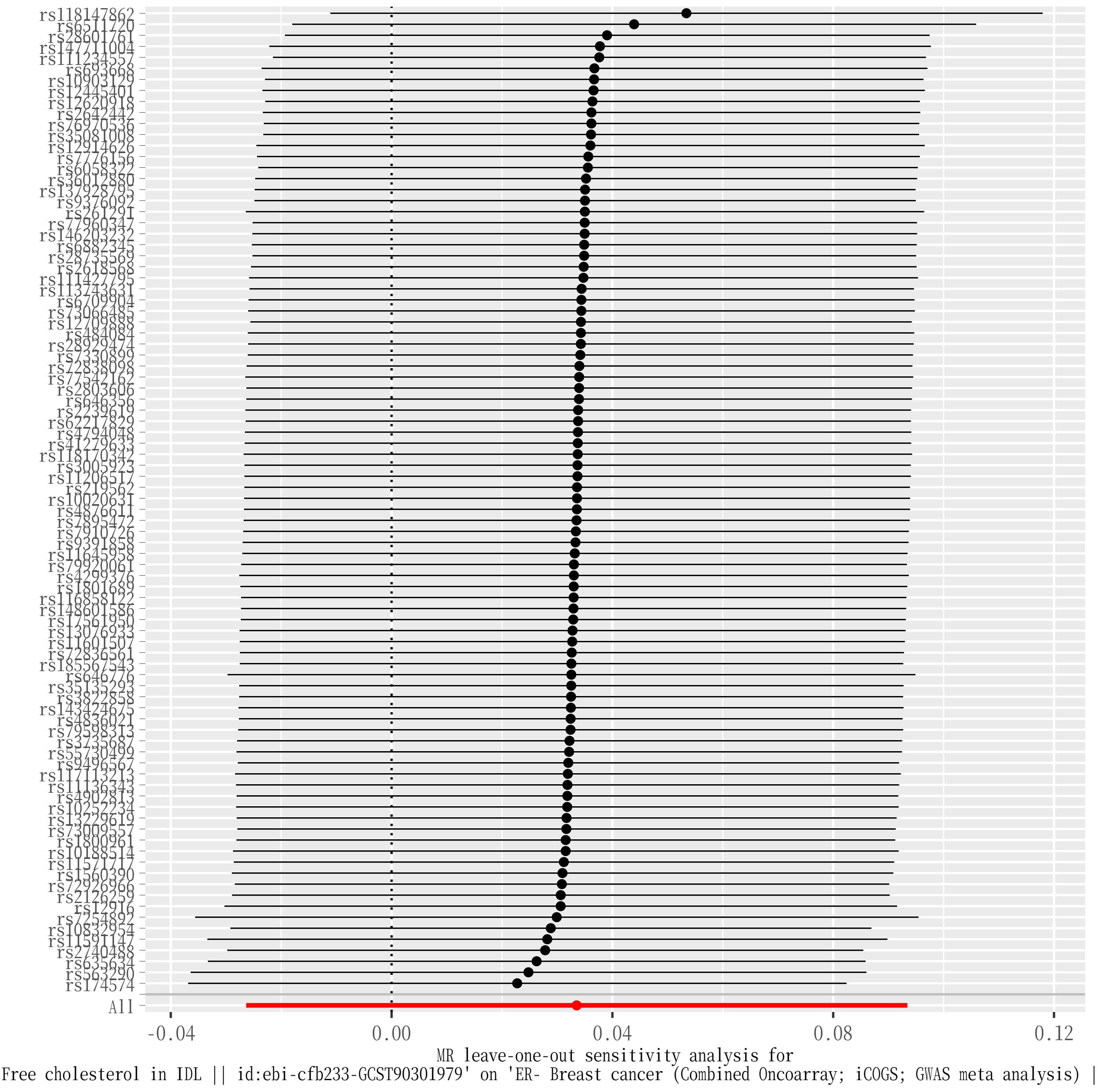
All
-0.04
0.00
0.04
0.08
0.12
MR leave-one-out sensitivity analysis for
Free cholesterol in IDL || id:ebi-cfb233-GCST90301979' on 'ER- Breast cancer (Combined Oncoarray; iCOGS; GWAS meta analysis) |

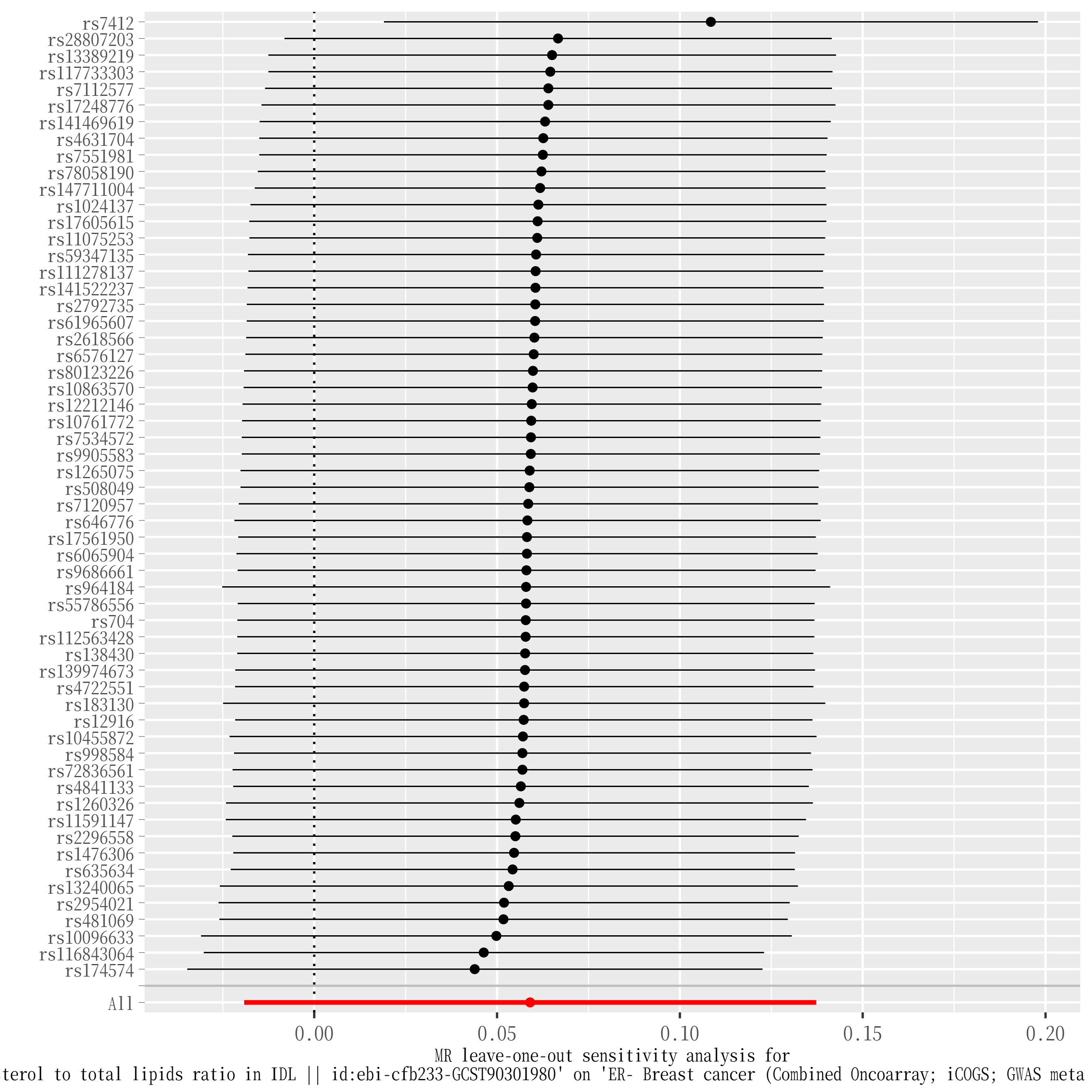
rs7412
rs28807203
rs13389219
rs117733303
rs7112577
rs17248776
rs141469619
rs4631704
rs7551981
rs78058190
rs147711004
rs1024137
rs17605615
rs11075253
rs59347135
rs111278137
rs141522237
rs2792735
rs61965607
rs2618566
rs6576127
rs80123226
rs10863570
rs12212146
rs10761772
rs7534572
rs9905583
rs1265075
rs508049
rs7120957
rs646776
rs17561950
rs6065904
rs9686661
rs964184
rs55786556
rs704
rs112563428
rs138430
rs139974673
rs4722551
rs183130
rs12916
rs10455872
rs998584
rs72836561
rs4841133
rs1260326
rs11591147
rs2296558
rs1476306
rs635634
rs13240065
rs2954021
rs481069
rs10096633
rs116843064
rs174574
All
0.00
0.05
0.10
0.15
0.20
MR leave-one-out sensitivity analysis for
terol to total lipids ratio in IDL || id:ebi-cfb233-GCST90301980' on 'ER- Breast cancer (Combined Oncoarray; iCOGS; GWAS meta

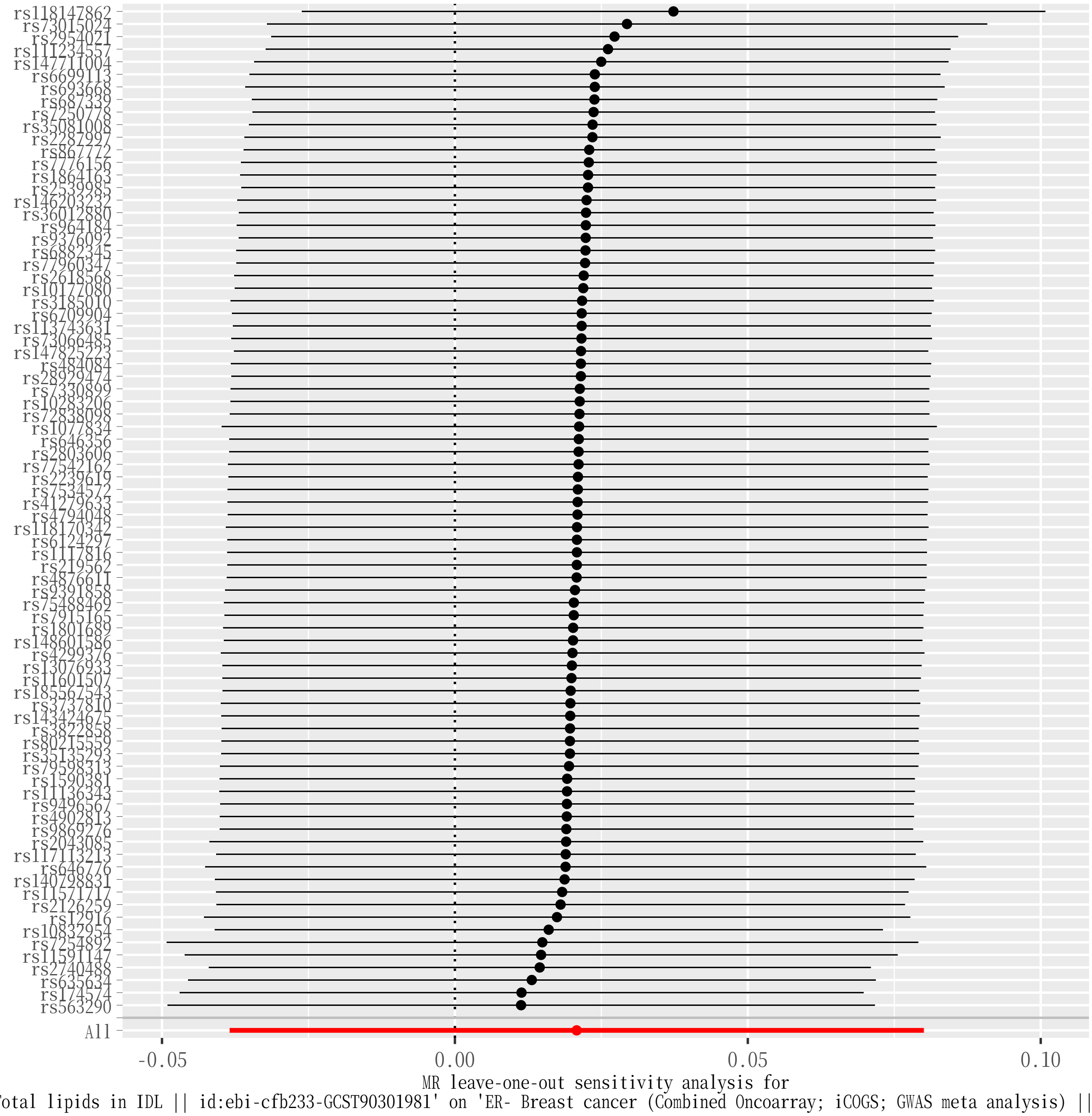
rs118147862
rs73015024
rs2954021
rs111234557
rs147711004
rs6699113
rs693668
rs687339
rs7250778
rs35081008
rs2287997
rs867772
rs7776156
rs1864163
rs2539985
rs146203232
rs36012880
rs964184
rs9376092
rs6882345
rs77960347
rs2618568
rs10177080
rs3185010
rs6709904
rs113743631
rs73066485
rs147825223
rs484084
rs28929474
rs7330899
rs10283206
rs72838098
rs1077834
rs646356
rs2803606
rs77542162
rs2239619
rs7534572
rs41279633
rs4794048
rs118170342
rs6124297
rs1117816
rs219562
rs4876611
rs9391858
rs75488469
rs7915165
rs1801689
rs148601586
rs4299376
rs13076933
rs11601507
rs185567543
rs3737810
rs143424675
rs3822858
rs80215559
rs35135293
rs79598313
rs1590381
rs11136343
rs9496567
rs4902813
rs9869276
rs2043085
rs117113213
rs646776
rs140798831
rs11571717
rs2126259
rs12916
rs10832954
rs7254892
rs11591147
rs2740488
rs635634
rs174574
rs563290
All
-0.05
0.00
0.05
0.10
MR leave-one-out sensitivity analysis for
'Total lipids in IDL || id:ebi-cfb233-GCST90301981' on 'ER- Breast cancer (Combined Oncoarray; iCOGS; GWAS meta analysis) ||

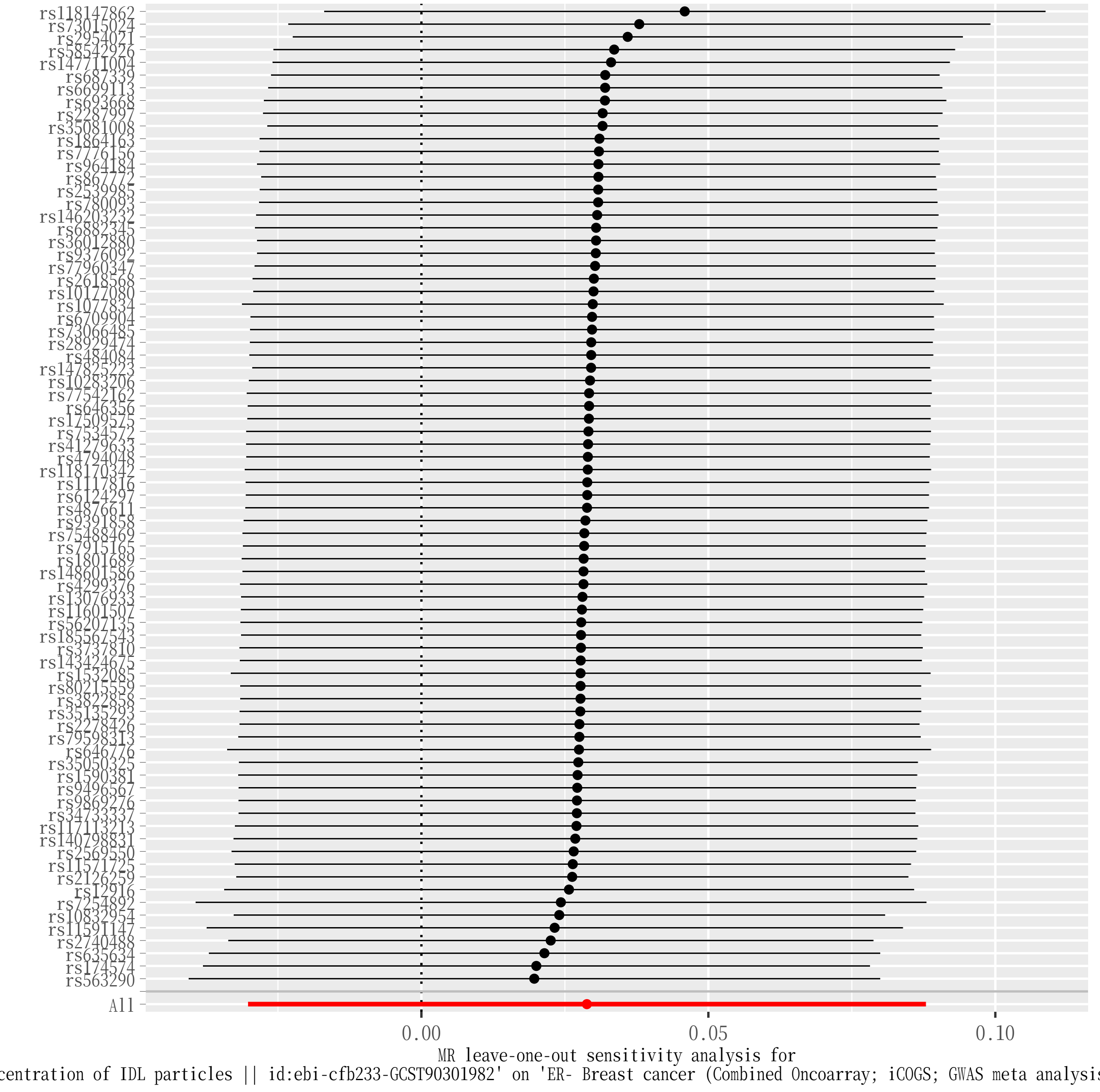
rs118147862
rs73015024
rs2954021
rs58542926
rs147711004
rs687339
rs6699113
rs693668
rs2287997
rs35081008
rs1864163
rs7776156
rs964184
rs867772
rs2539985
rs780093
rs146203232
rs6882345
rs36012880
rs9376092
rs77960347
rs2618568
rs10177080
rs1077834
rs6709904
rs73066485
rs28929474
rs484084
rs147825223
rs10283206
rs77542162
rs646356
rs17509575
rs7534572
rs41279633
rs4794048
rs118170342
rs1117816
rs6124297
rs4876611
rs9391858
rs75488469
rs7915165
rs1801689
rs148601586
rs4299376
rs13076933
rs11601507
rs56207135
rs185567543
rs3737810
rs143424675
rs1532085
rs80215559
rs3822858
rs35135293
rs2278426
rs79598313
rs646776
rs35050325
rs1590381
rs9496567
rs9869276
rs34733337
rs117113213
rs140798831
rs2569550
rs11571725
rs2126259
rs12916
rs7254892
rs10832954
rs11591147
rs2740488
rs635634
rs174574
rs563290
All
0.00
0.05
0.10
MR leave-one-out sensitivity analysis for
centration of IDL particles || id:ebi-cfb233-GCST90301982' on 'ER- Breast cancer (Combined Oncoarray; iCOGS; GWAS meta analysis

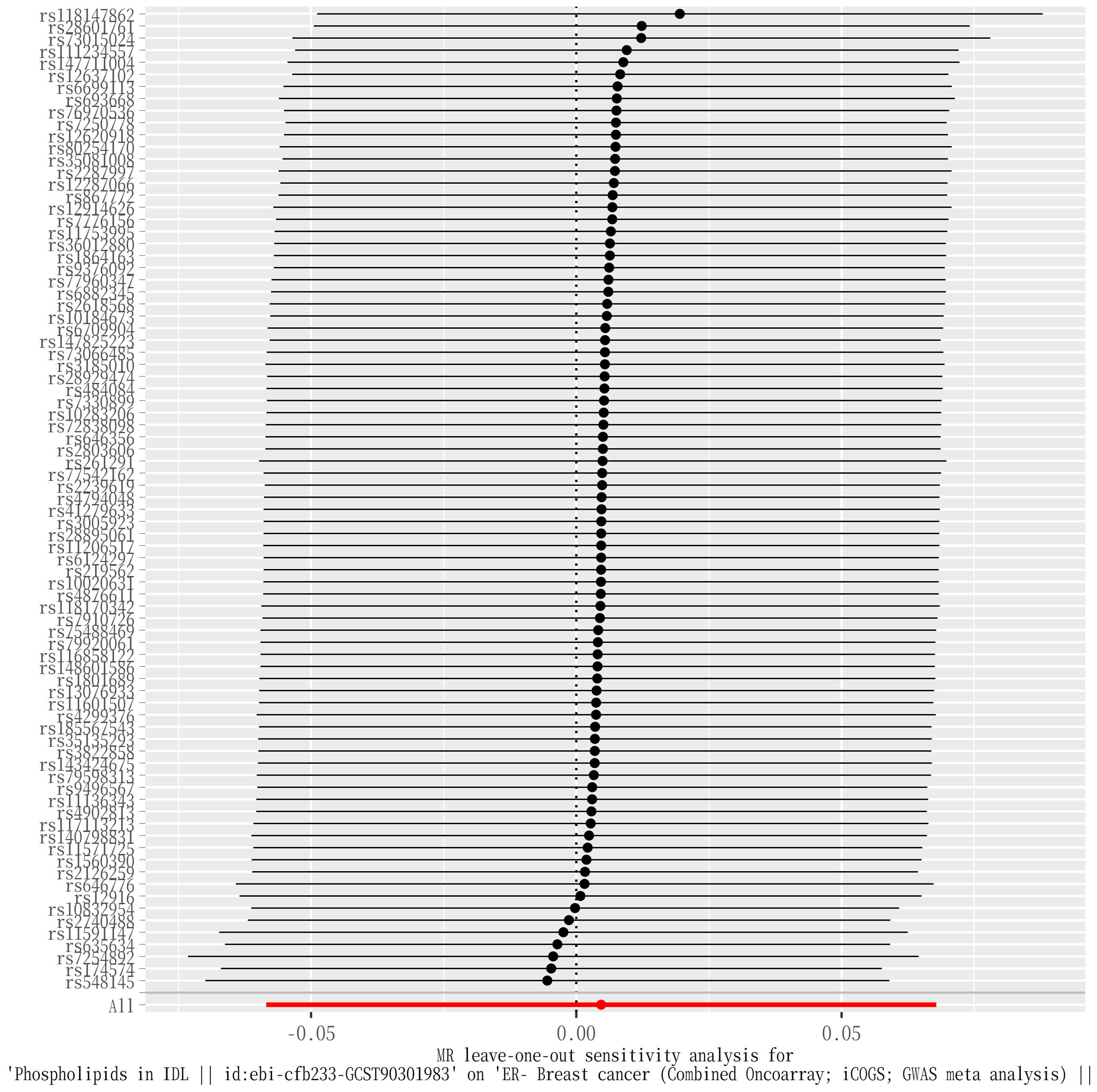
rs118147862
rs28601761
rs73015024
rs111234557
rs147711004
rs12637102
rs6699113
rs693668
rs76970536
rs7250778
rs12620918
rs80254170
rs35081008
rs2287997
rs12287066
rs867772
rs12914626
rs7776156
rs11753995
rs36012880
rs1864163
rs9376092
rs77960347
rs6882345
rs2618568
rs10184673
rs6709904
rs147825223
rs73066485
rs3185010
rs28929474
rs484084
rs7330899
rs10283206
rs72838028
rs646356
rs2803606
rs261291
rs77542162
rs2239619
rs4794048
rs41279633
rs3005923
rs28895061
rs11206517
rs6124297
rs219562
rs10020631
rs4876611
rs118170342
rs7910726
rs75488469
rs79920061
rs116858122
rs148601586
rs1801689
rs13076933
rs11601507
rs4299376
rs185567543
rs35135293
rs3822858
rs143474675
rs79598313
rs9496567
rs11136343
rs4902813
rs117113213
rs140798831
rs11571725
rs1560320
rs2126259
rs646776
rs12916
rs10832954
rs2740488
rs11591147
rs635634
rs7254892
rs174574
rs548145
All
-0.05
0.00
0.05
MR leave-one-out sensitivity analysis for
'Phospholipids in IDL || id:ebi-cfb233-GCST90301983' on 'ER- Breast cancer (Combined Oncoarray; iCOGS; GWAS meta analysis) ||

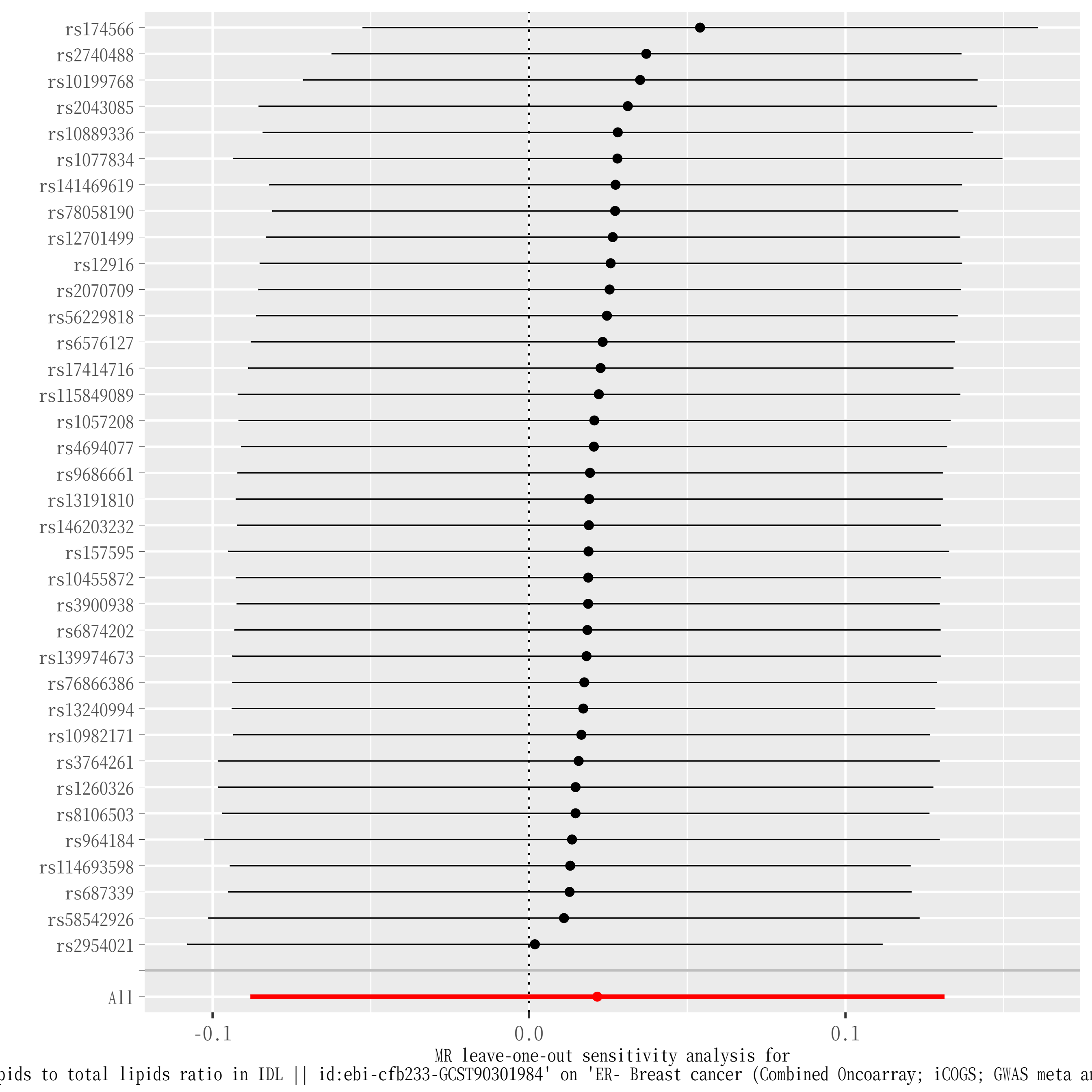

rs174566
rs2740488
rs10199768
rs2043085
rs10889336
rs1077834
rs141469619
rs78058190
rs12701499
rs12916
rs2070709
rs56229818
rs6576127
rs17414716
rs115849089
rs1057208
rs4694077
rs9686661
rs13191810
rs146203232
rs157595
rs10455872
rs3900938
rs6874202
rs139974673
rs76866386
rs13240994
rs10982171
rs3764261
rs1260326
rs8106503
rs964184
rs114693598
rs687339
rs58542926
rs2954021
All
-0.1
0.0
0.1
MR leave-one-out sensitivity analysis for
pids to total lipids ratio in IDL || id:ebi-cfb233-GCST90301984' on 'ER- Breast cancer (Combined Oncoarray; iCOGS; GWAS meta an

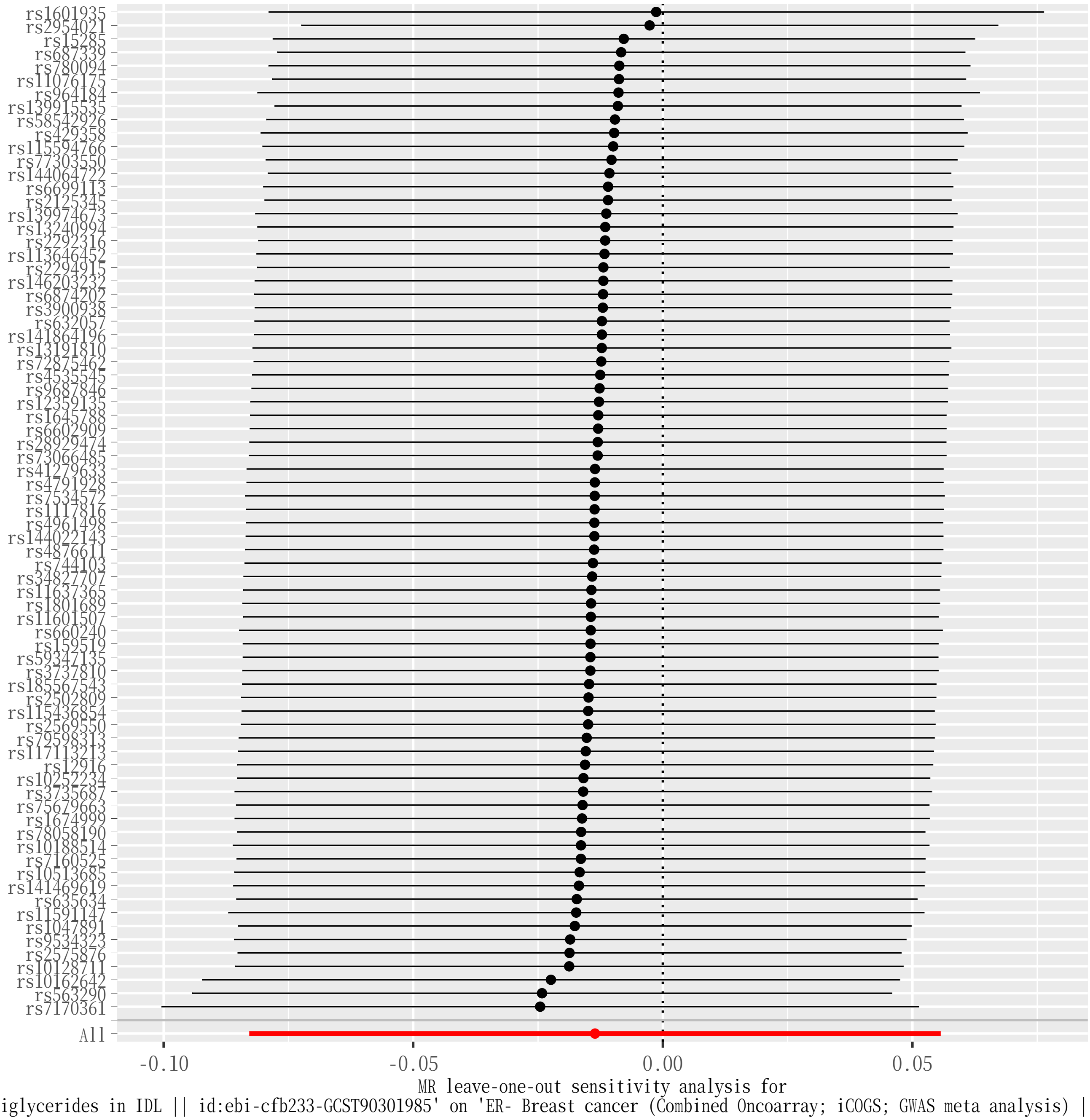
rs1601935
rs2954021
rs15285
rs687339
rs780094
rs11076175
rs964184
rs139215535
rs58542926
rs429358
rs115594766
rs77303550
rs144064722
rs6699113
rs2125345
rs139974673
rs13240994
rs2292316
rs113646452
rs2294915
rs146203232
rs6874202
rs3900938
rs632057
rs141864196
rs13191810
rs72875462
rs4535545
rs9687846
rs12359135
rs1645788
rs6602909
rs28929474
rs73066485
rs41279633
rs4791928
rs7534572
rs1117816
rs4961498
rs144022143
rs4876611
rs744103
rs34827707
rs11637365
rs1801689
rs11601507
rs660240
rs159519
rs59347135
rs3737810
rs185567543
rs2502809
rs115436854
rs2569550
rs79598313
rs117113213
rs12916
rs10252234
rs3735687
rs75679663
rs1674999
rs78058190
rs10188514
rs7160525
rs10513685
rs141469619
rs635634
rs11591147
rs1047891
rs9534323
rs2575876
rs10128711
rs10162642
rs563290
rs7170361
All
-0.10
-0.05
0.00
0.05
MR leave-one-out sensitivity analysis for
'Triglycerides in IDL || id:ebi-cfb233-GCST90301985' on 'ER- Breast cancer (Combined Oncoarray; iCOGS; GWAS meta analysis) ||

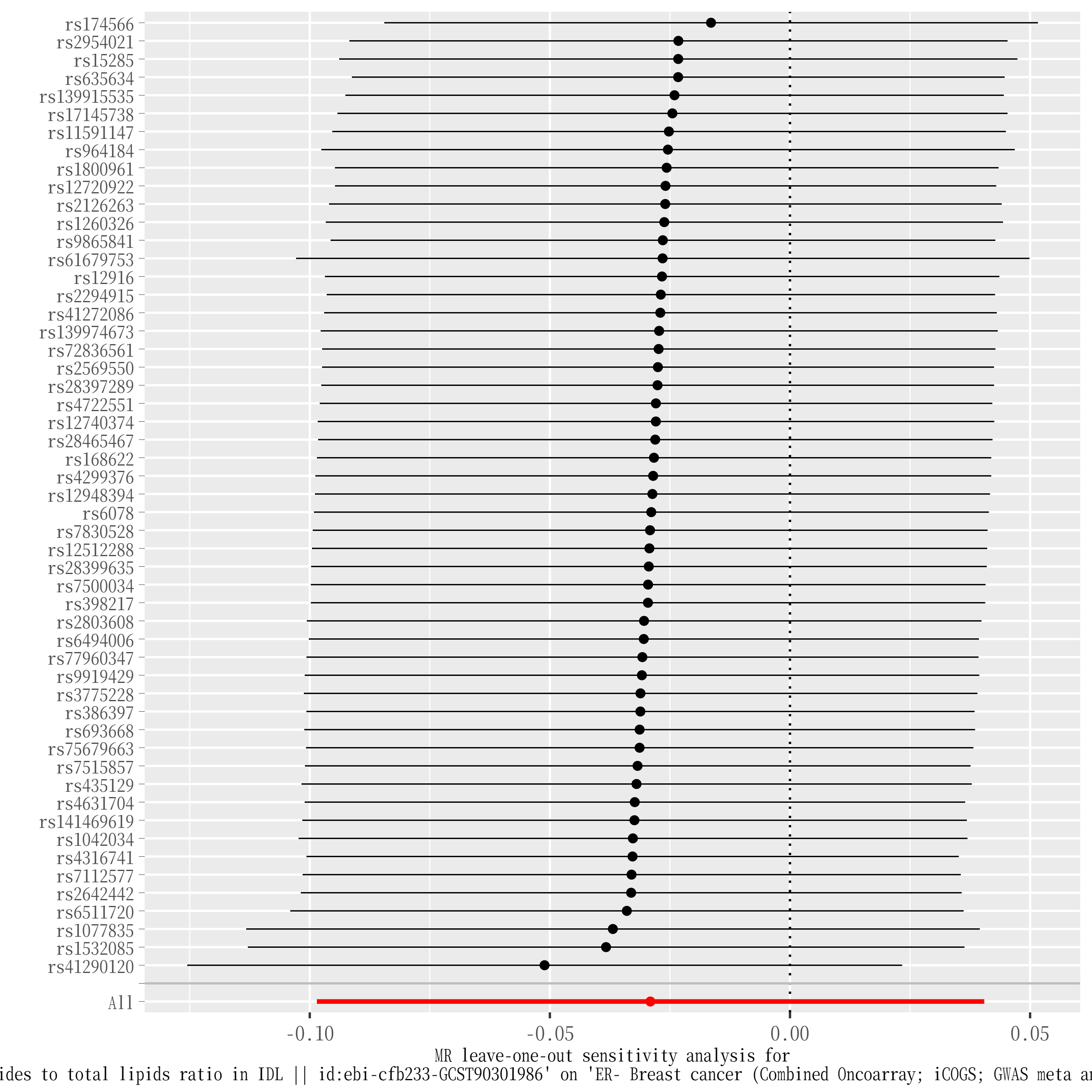

rs174566
rs2954021
rs15285
rs635634
rs139915535
rs17145738
rs11591147
rs964184
rs1800961
rs12720922
rs2126263
rs1260326
rs9865841
rs61679753
rs12916
rs2294915
rs41272086
rs139974673
rs72836561
rs2569550
rs28397289
rs4722551
rs12740374
rs28465467
rs168622
rs4299376
rs12948394
rs6078
rs7830528
rs12512288
rs28399635
rs7500034
rs398217
rs2803608
rs6494006
rs77960347
rs9919429
rs3775228
rs386397
rs693668
rs75679663
rs7515857
rs435129
rs4631704
rs141469619
rs1042034
rs4316741
rs7112577
rs2642442
rs6511720
rs1077835
rs1532085
rs41290120
All
-0.10
-0.05
0.00
0.05
MR leave-one-out sensitivity analysis for
ides to total lipids ratio in IDL || id:ebi-cfb233-GCST90301986' on 'ER- Breast cancer (Combined Oncoarray; iCOGS; GWAS meta an

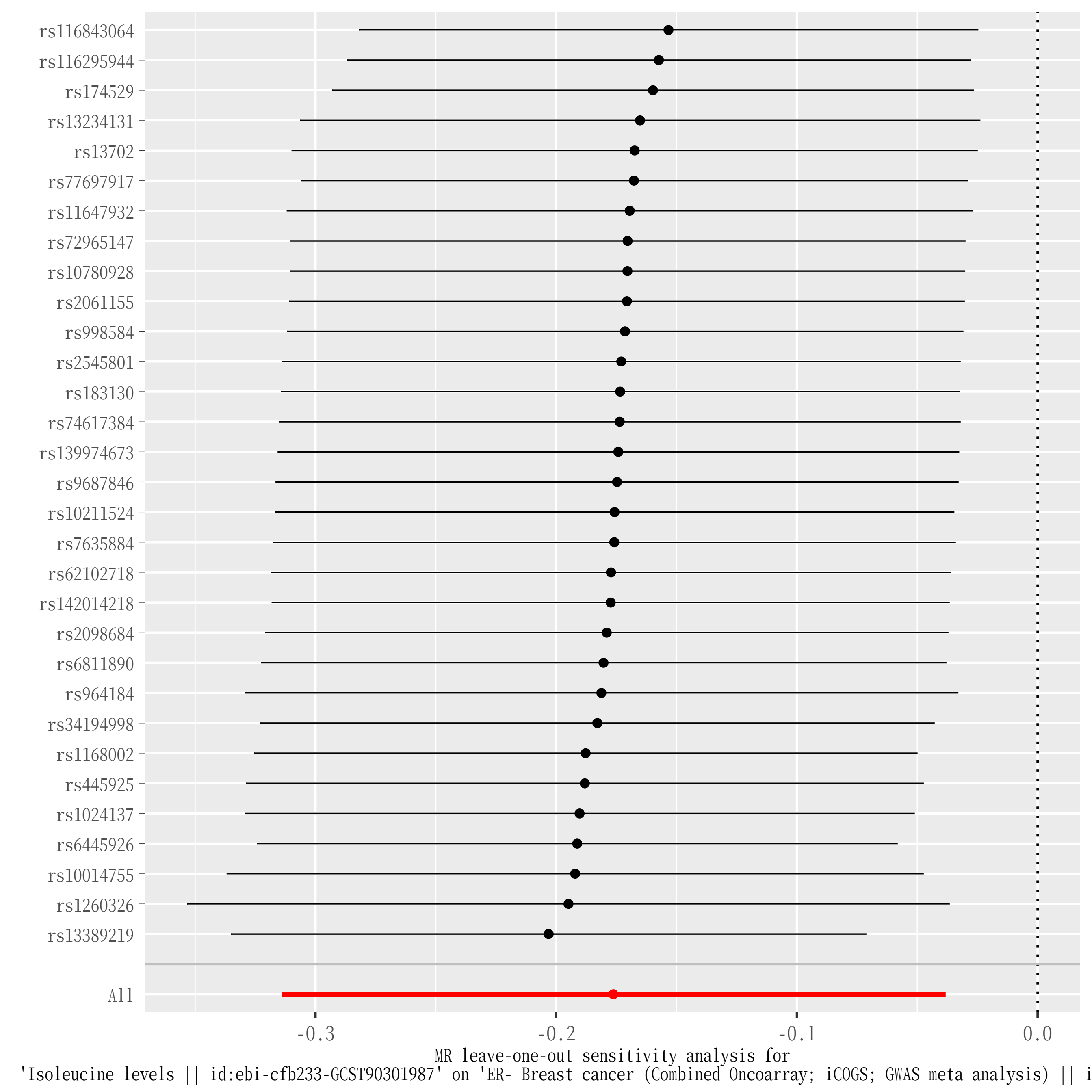

rs116843064
rs116295944
rs174529
rs13234131
rs13702
rs77697917
rs11647932
rs72965147
rs10780928
rs2061155
rs998584
rs2545801
rs183130
rs74617384
rs139974673
rs9687846
rs10211524
rs7635884
rs62102718
rs142014218
rs2098684
rs6811890
rs964184
rs34194998
rs1168002
rs445925
rs1024137
rs6445926
rs10014755
rs1260326
rs13389219
All
-0.3
-0.2
-0.1
0.0
MR leave-one-out sensitivity analysis for
'Isoleucine levels || id:ebi-cfb233-GCST90301987' on 'ER- Breast cancer (Combined Oncoarray; iCOGS; GWAS meta analysis) || i

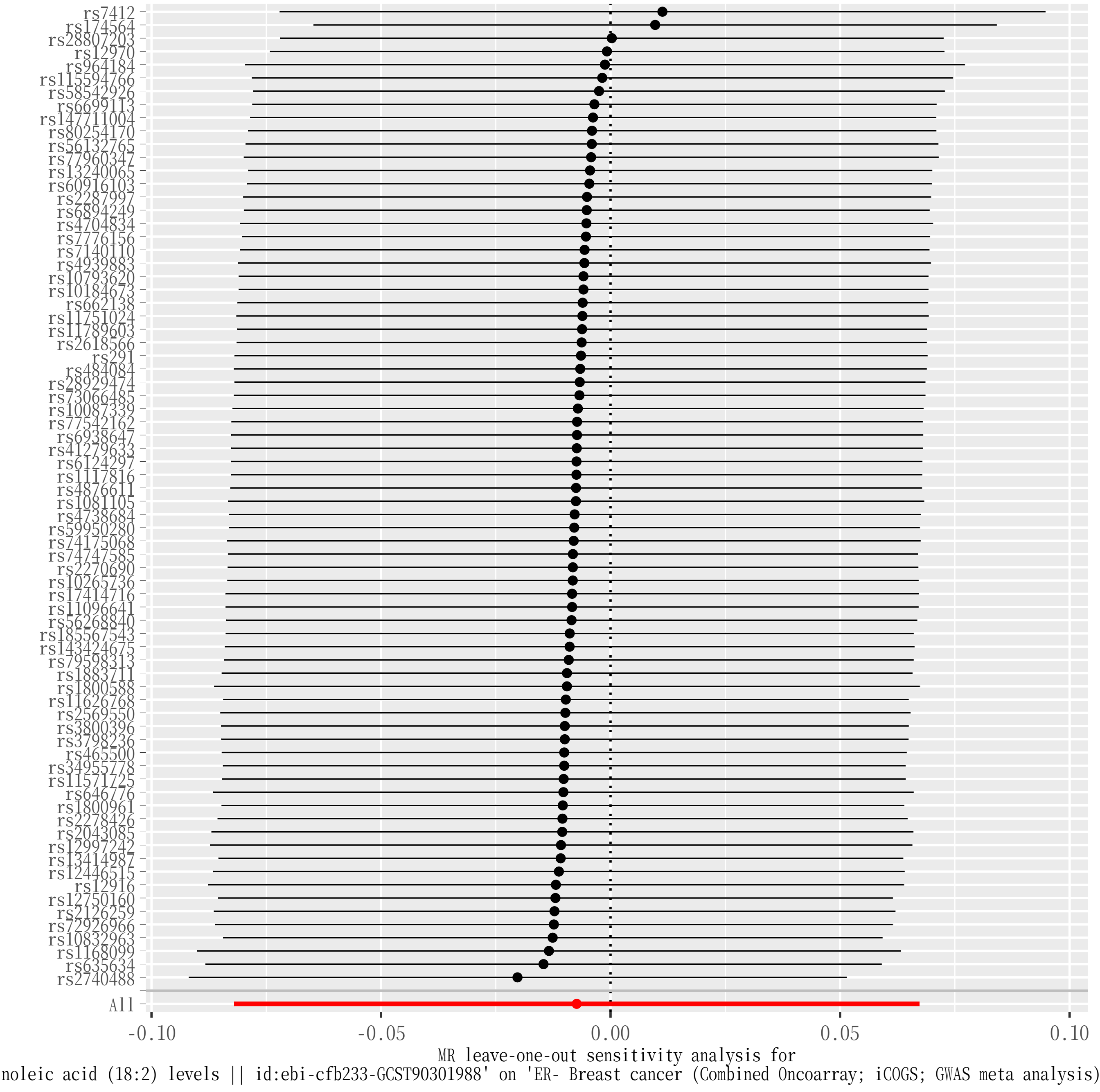
rs7412
rs174564
rs28807203
rs12970
rs964184
rs115594766
rs58542926
rs6699113
rs147711004
rs80254170
rs56132765
rs77960347
rs13240065
rs60916103
rs2287997
rs6894249
rs4704834
rs7776156
rs7140110
rs4939883
rs10793620
rs10184673
rs662138
rs11751024
rs11789603
rs2618566
rs291
rs484084
rs28929474
rs73066485
rs10087339
rs77542162
rs6938647
rs41279633
rs6124297
rs1117816
rs4876611
rs1081105
rs4738684
rs59950280
rs74175068
rs74747585
rs2270690
rs10265736
rs17414716
rs11096641
rs56268840
rs185567543
rs143424675
rs79598313
rs1883711
rs1800588
rs11626768
rs2569550
rs3800396
rs3798236
rs465500
rs34955778
rs11571725
rs646776
rs1800961
rs2278426
rs2043085
rs12997242
rs13414987
rs12446515
rs12916
rs12750160
rs2126259
rs72926966
rs10832963
rs1168099
rs635634
rs2740488
All
-0.10
-0.05
0.00
0.05
0.10
MR leave-one-out sensitivity analysis for
noleic acid (18:2) levels || id:ebi-cfb233-GCST90301988' on 'ER- Breast cancer (Combined Oncoarray; iCOGS; GWAS meta analysis)

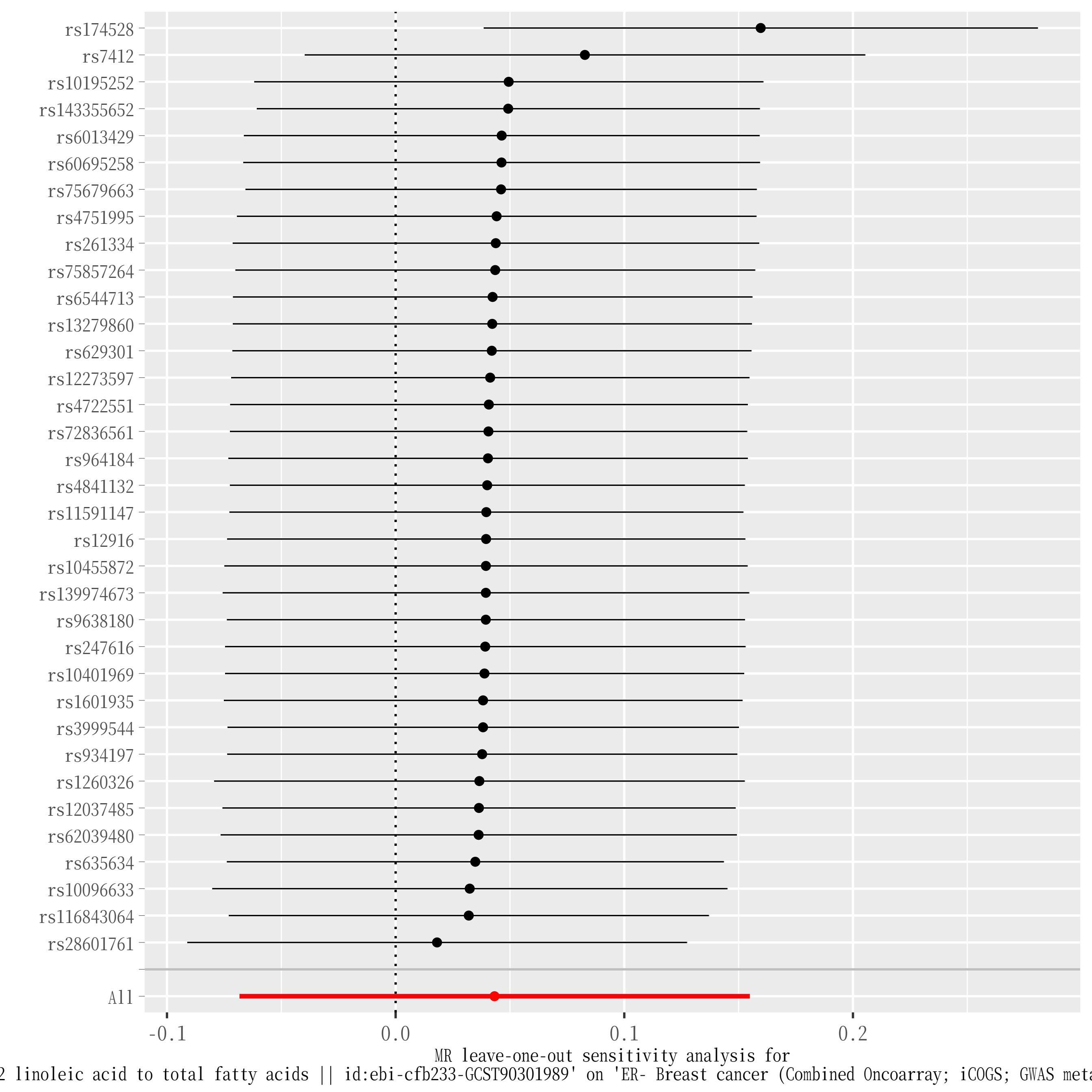
rs174528
rs7412
rs10195252
rs143355652
rs6013429
rs60695258
rs75679663
rs4751995
rs261334
rs75857264
rs6544713
rs13279860
rs629301
rs12273597
rs4722551
rs72836561
rs964184
rs4841132
rs11591147
rs12916
rs10455872
rs139974673
rs9638180
rs247616
rs10401969
rs1601935
rs3999544
rs934197
rs1260326
rs12037485
rs62039480
rs635634
rs10096633
rs116843064
rs28601761
All
-0.1
0.0
0.1
0.2
MR leave-one-out sensitivity analysis for
2 linoleic acid to total fatty acids || id:ebi-cfb233-GCST90301989' on 'ER- Breast cancer (Combined Oncoarray; iCOGS; GWAS meta

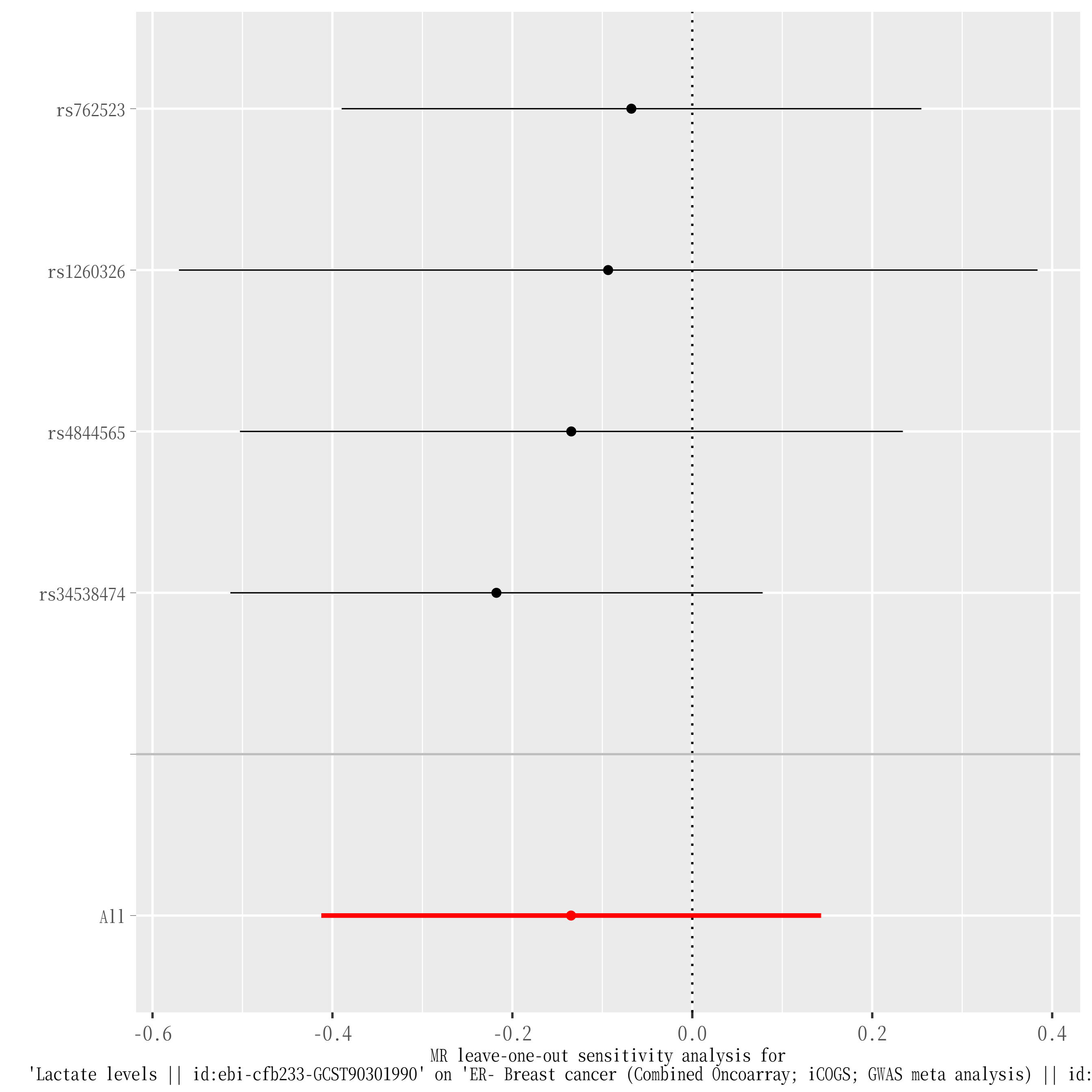
rs762523
rs1260326
rs4844565
rs34538474
All
-0.6
-0.4
-0.2
0.0
0.2
0.4
MR leave-one-out sensitivity analysis for
'Lactate levels || id:ebi-cfb233-GCST90301990' on 'ER- Breast cancer (Combined Oncoarray; iCOGS; GWAS meta analysis) || id:

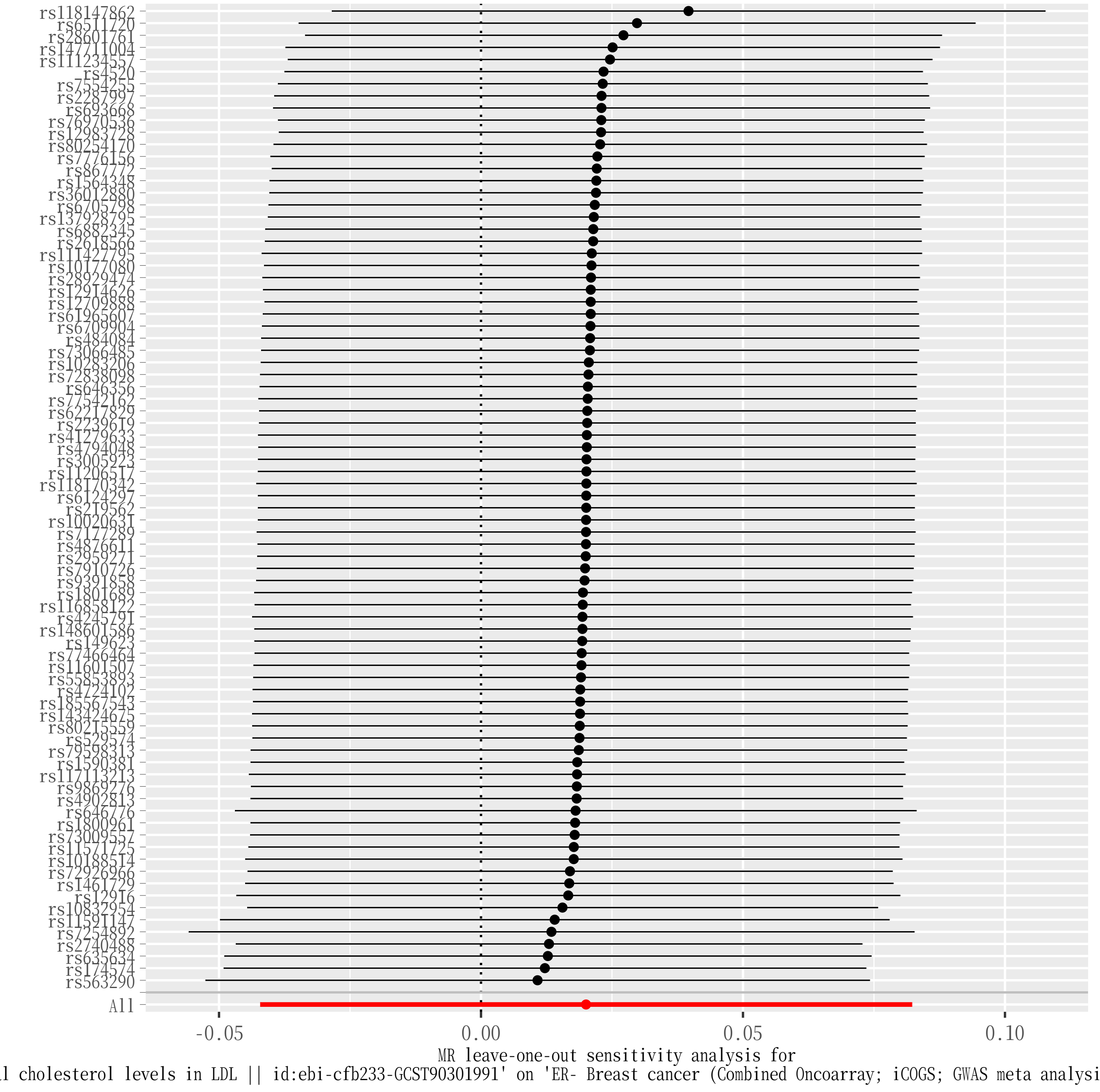
rs118147862
rs6511720
rs28601761
rs147711004
rs111234557
rs4520
rs7554255
rs2287997
rs693668
rs76970536
rs12983728
rs80254170
rs7776156
rs867772
rs1564348
rs36012880
rs6705798
rs137928795
rs6882345
rs2618566
rs111427795
rs10177080
rs28929474
rs12914626
rs12709888
rs61965607
rs6709904
rs484084
rs73066485
rs10283206
rs72838028
rs646356
rs77542162
rs62217829
rs2239619
rs41279633
rs4794048
rs3005923
rs11206517
rs118170342
rs6124297
rs219562
rs10020631
rs7177289
rs4876611
rs2959271
rs7910726
rs9391858
rs1801689
rs116858122
rs4245791
rs148601586
rs149623
rs77466464
rs11601507
rs55853893
rs4724102
rs185567543
rs143424675
rs80215559
rs529574
rs79598313
rs1590381
rs117113213
rs9869276
rs4902813
rs646776
rs1800961
rs73009557
rs11571725
rs10188514
rs72926966
rs1461729
rs12916
rs10832954
rs11591147
rs7254892
rs2740488
rs635634
rs174574
rs563290
All
-0.05
0.00
0.05
0.10
MR leave-one-out sensitivity analysis for
l cholesterol levels in LDL || id:ebi-cfb233-GCST90301991' on 'ER- Breast cancer (Combined Oncoarray; iCOGS; GWAS meta analysi

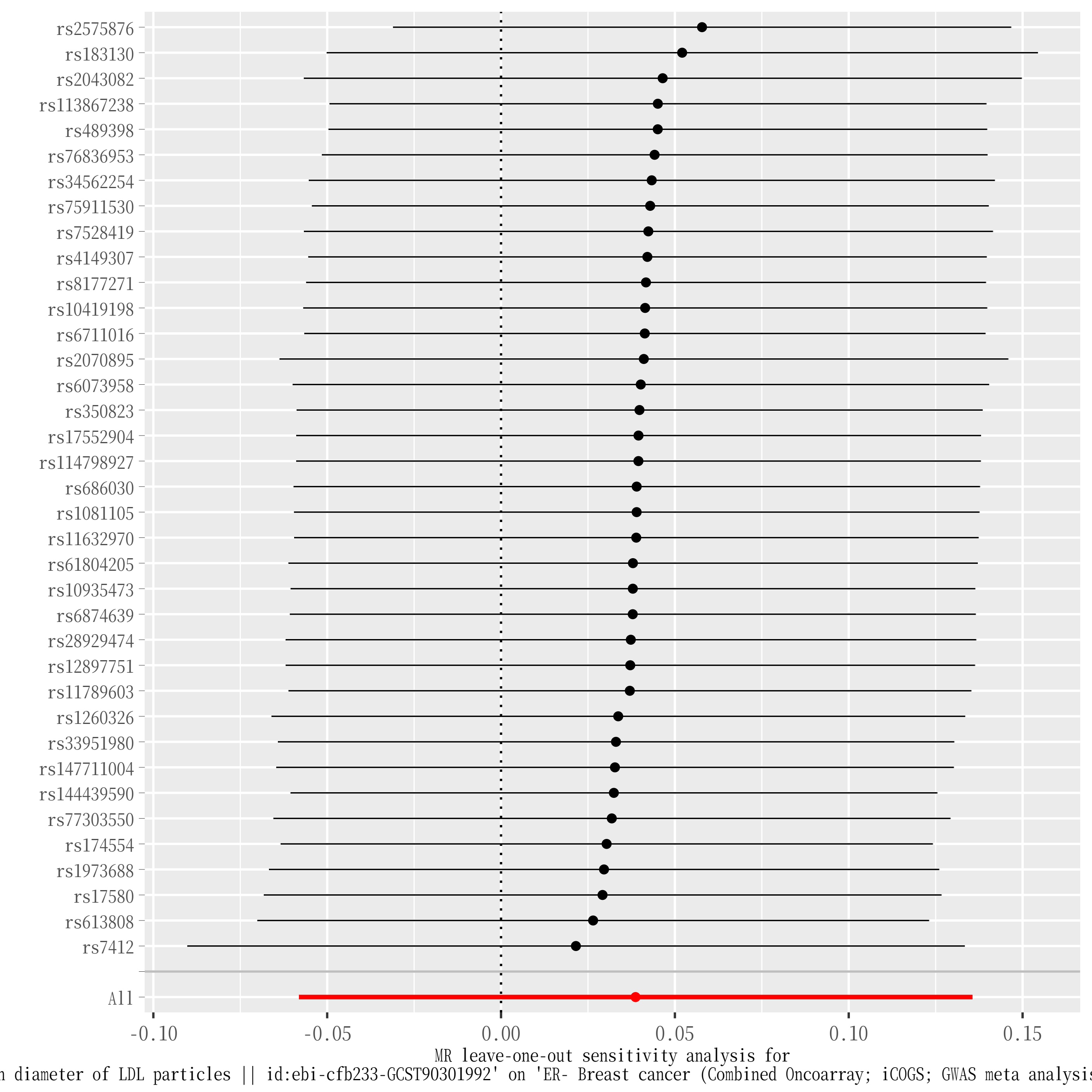
rs2575876
rs183130
rs2043082
rs113867238
rs489398
rs76836953
rs34562254
rs75911530
rs7528419
rs4149307
rs8177271
rs10419198
rs6711016
rs2070895
rs6073958
rs350823
rs17552904
rs114798927
rs686030
rs1081105
rs11632970
rs61804205
rs10935473
rs6874639
rs28929474
rs12897751
rs11789603
rs1260326
rs33951980
rs147711004
rs144439590
rs77303550
rs174554
rs1973688
rs17580
rs613808
rs7412
All
-0.10
-0.05
0.00
0.05
0.10
0.15
MR leave-one-out sensitivity analysis for
n diameter of LDL particles || id:ebi-cfb233-GCST90301992' on 'ER- Breast cancer (Combined Oncoarray; iCOGS; GWAS meta analysis

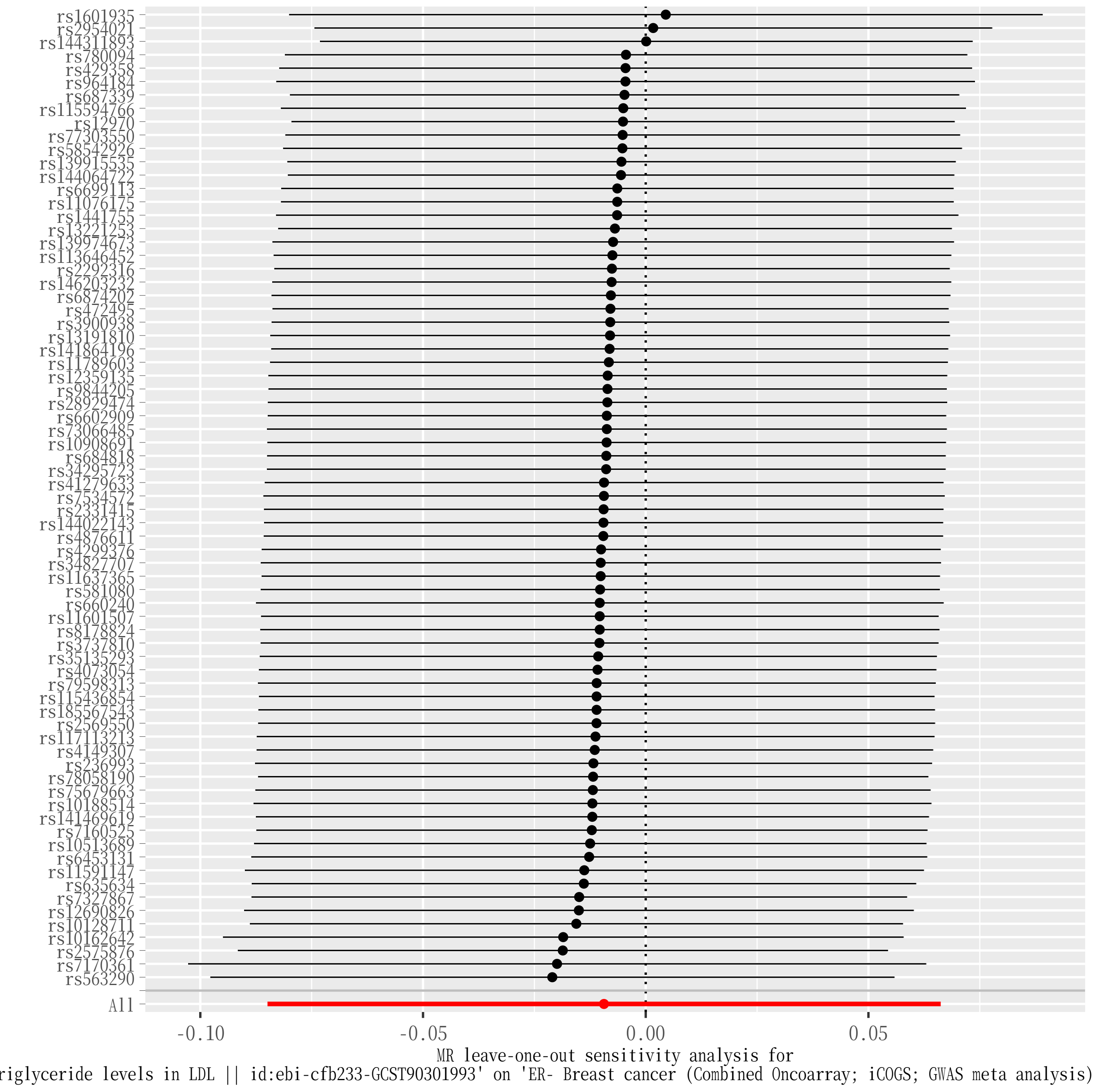
rs1601935
rs2954021
rs144311893
rs780094
rs429358
rs964184
rs687339
rs115594766
rs12970
rs77303550
rs58542926
rs139915535
rs144064722
rs6699113
rs11076175
rs1441755
rs13221253
rs139974673
rs113646452
rs2292316
rs146203232
rs6874202
rs472495
rs3900938
rs13191810
rs141864196
rs11789603
rs12359135
rs9844205
rs28929474
rs6602909
rs73066485
rs10908691
rs684818
rs34295723
rs41279633
rs7534572
rs2331415
rs144022143
rs4876611
rs4299376
rs34827707
rs11637365
rs581080
rs660240
rs11601507
rs8178824
rs3737810
rs35135293
rs4073054
rs79598313
rs115436854
rs185567543
rs2569550
rs117113213
rs4149307
rs236993
rs78058190
rs75679663
rs10188514
rs141469619
rs7160525
rs10513689
rs6453131
rs11591147
rs635634
rs7327867
rs12690826
rs10128711
rs10162642
rs2575876
rs7170361
rs563290
All
-0.10
-0.05
0.00
0.05
MR leave-one-out sensitivity analysis for
riglyceride levels in LDL || id:ebi-cfb233-GCST90301993' on 'ER- Breast cancer (Combined Oncoarray; iCOGS; GWAS meta analysis)

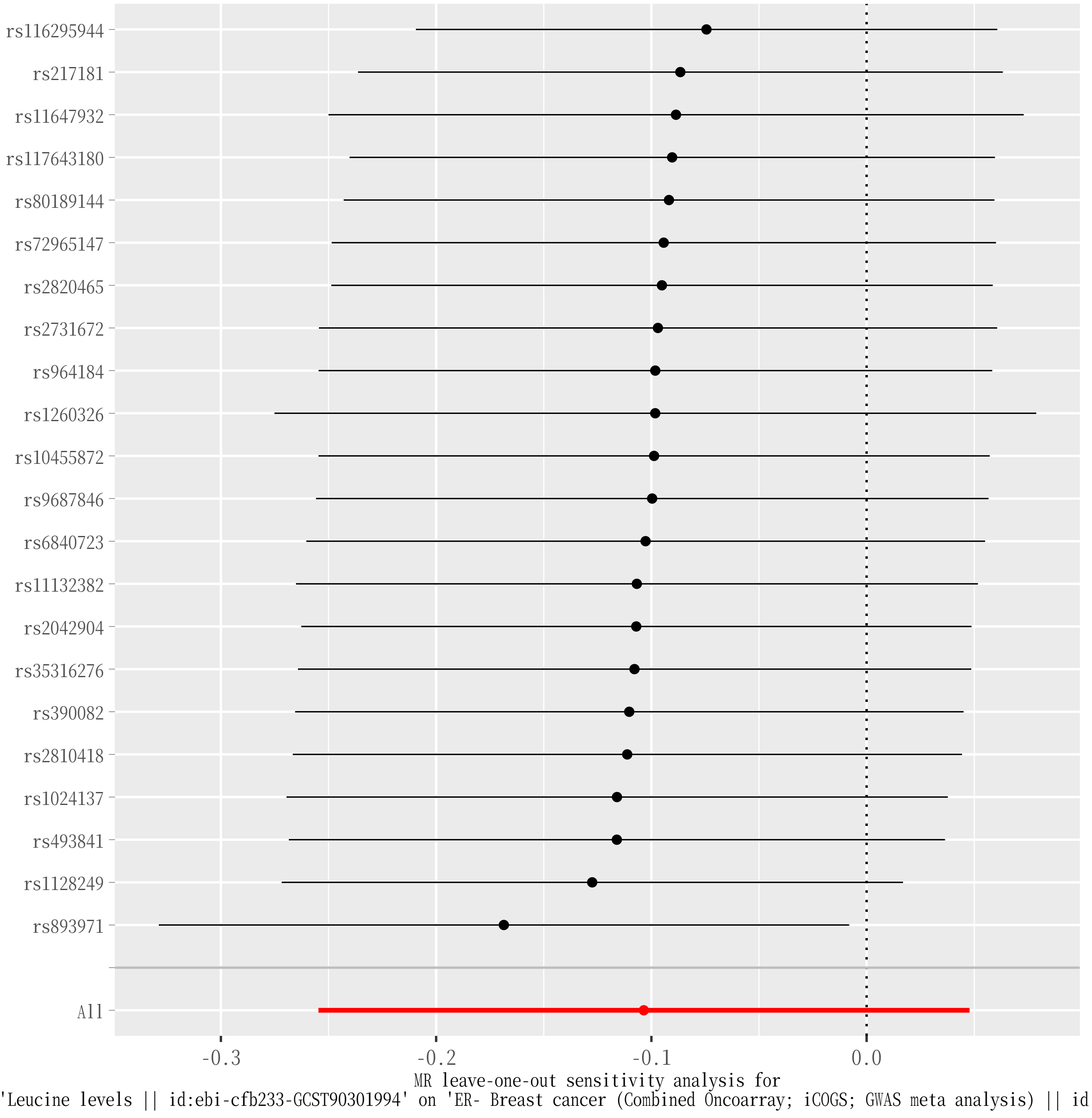
rs116295944
rs217181
rs11647932
rs117643180
rs80189144
rs72965147
rs2820465
rs2731672
rs964184
rs1260326
rs10455872
rs9687846
rs6840723
rs11132382
rs2042904
rs35316276
rs390082
rs2810418
rs1024137
rs493841
rs1128249
rs893971
All
-0.3
-0.2
-0.1
0.0
MR leave-one-out sensitivity analysis for
'Leucine levels || id:ebi-cfb233-GCST90301994' on 'ER- Breast cancer (Combined Oncoarray; iCOGS; GWAS meta analysis) || id

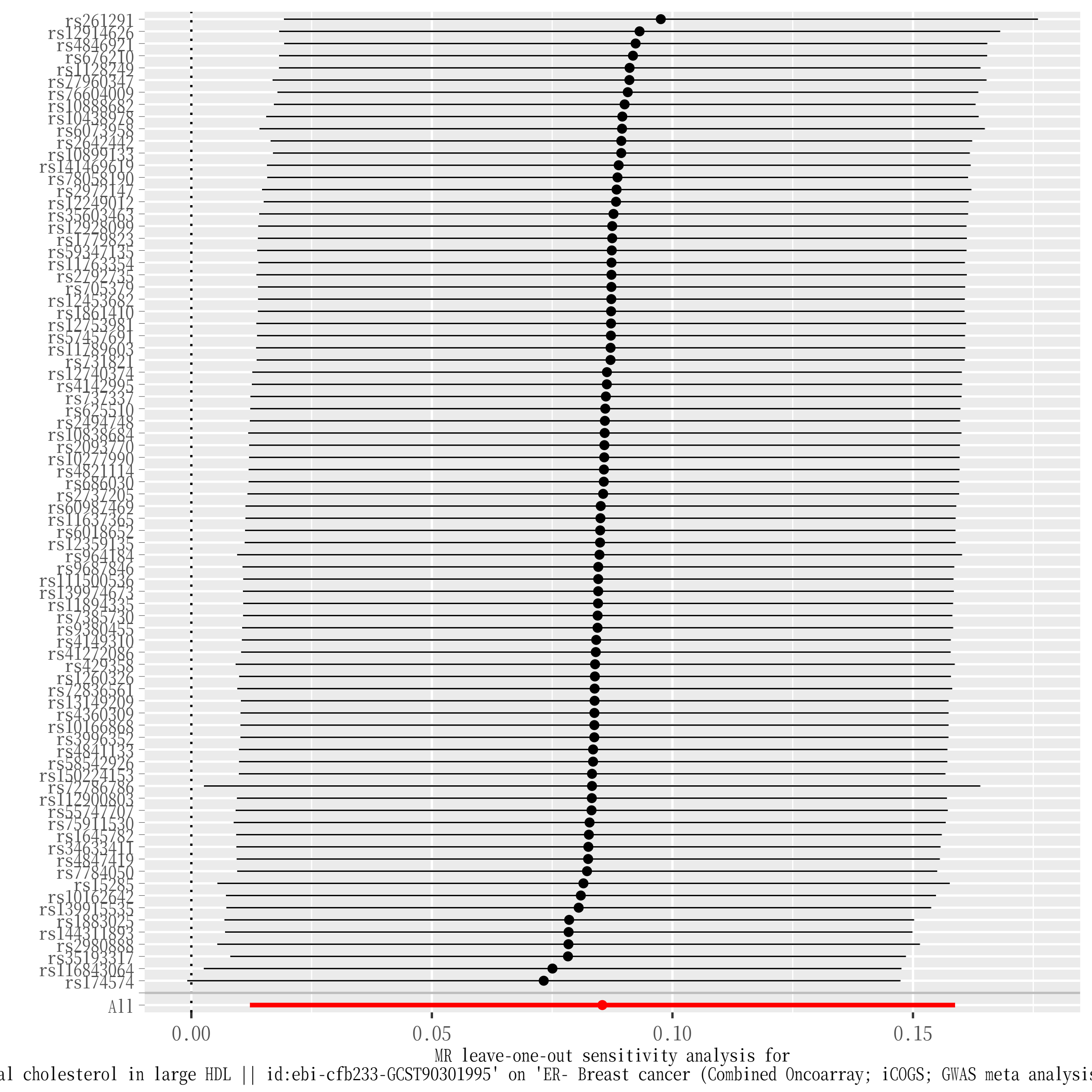

rs261291
rs12914626
rs4846921
rs676210
rs1128249
rs77960347
rs76604009
rs10888682
rs10438978
rs6073958
rs2642442
rs10899133
rs141469619
rs78058190
rs2972147
rs12249012
rs35603463
rs12928099
rs1779823
rs59347135
rs11763354
rs2792735
rs705379
rs12453682
rs1861410
rs12753981
rs57457691
rs11789603
rs731821
rs12740374
rs4142995
rs737337
rs625510
rs2494748
rs10838684
rs2093770
rs10277990
rs4821114
rs686030
rs2737205
rs60987469
rs11637365
rs6018652
rs12359135
rs964184
rs9687846
rs111500536
rs139974673
rs11894335
rs7385730
rs9380455
rs4149310
rs41272086
rs429358
rs1260326
rs72836561
rs13149209
rs4360309
rs10166868
rs3996352
rs4841133
rs58542926
rs150224153
rs72786786
rs112900803
rs55747707
rs75911530
rs1645782
rs34633411
rs4847419
rs7784050
rs15285
rs10162642
rs139915535
rs1883025
rs144311893
rs2980888
rs35193317
rs116843064
rs174574
All
0.00
0.05
0.10
0.15
MR leave-one-out sensitivity analysis for
al cholesterol in large HDL || id:ebi-cfb233-GCST90301995' on 'ER- Breast cancer (Combined Oncoarray; iCOGS; GWAS meta analysis

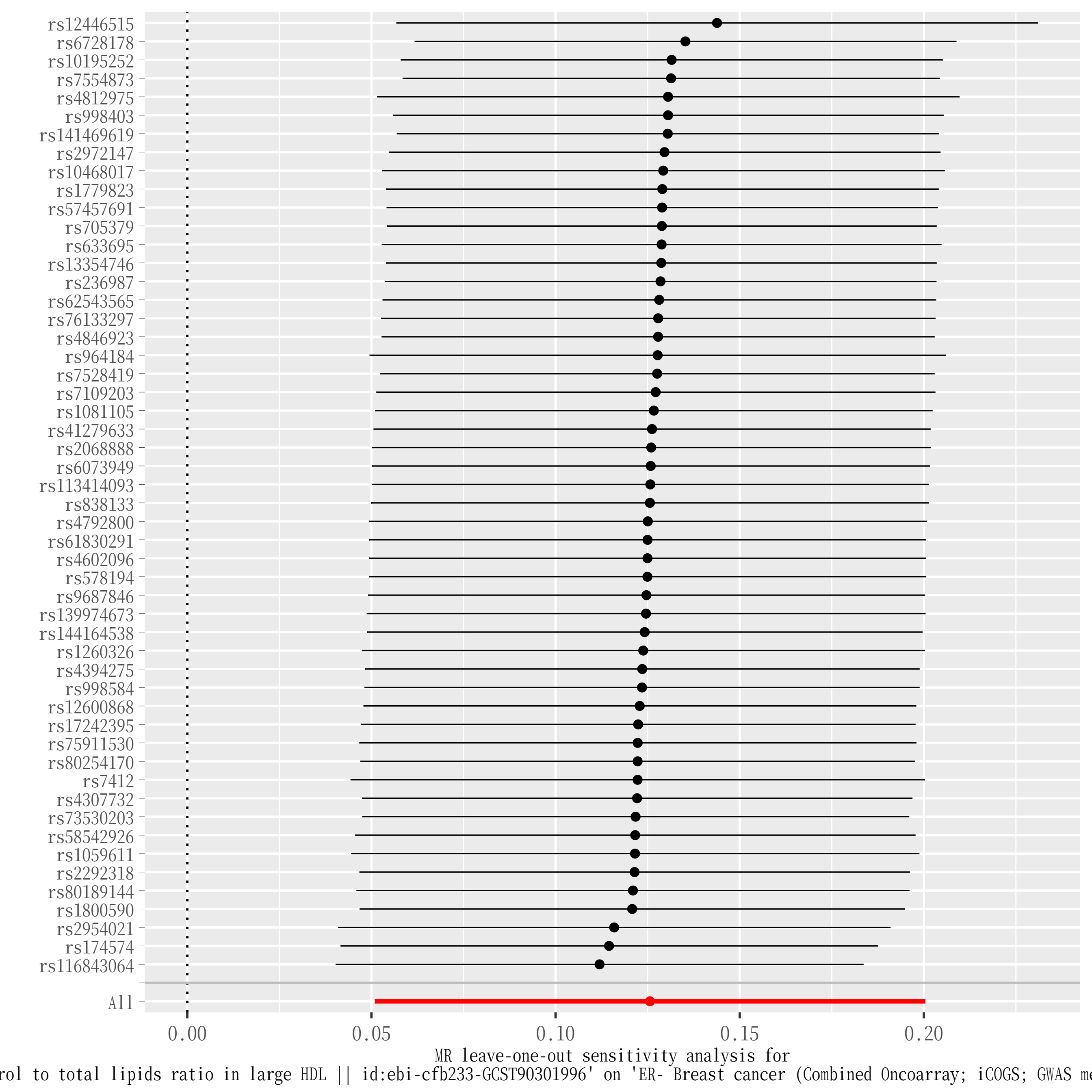

rs12446515
rs6728178
rs10195252
rs7554873
rs4812975
rs998403
rs141469619
rs2972147
rs10468017
rs1779823
rs57457691
rs705379
rs633695
rs13354746
rs236987
rs62543565
rs76133297
rs4846923
rs964184
rs7528419
rs7109203
rs1081105
rs41279633
rs2068888
rs6073949
rs113414093
rs838133
rs4792800
rs61830291
rs4602096
rs578194
rs9687846
rs139974673
rs144164538
rs1260326
rs4394275
rs998584
rs12600868
rs17242395
rs75911530
rs80254170
rs7412
rs4307732
rs73530203
rs58542926
rs1059611
rs2292318
rs80189144
rs1800590
rs2954021
rs174574
rs116843064
All
0.00
0.05
0.10
0.15
0.20
MR leave-one-out sensitivity analysis for
rol to total lipids ratio in large HDL || id:ebi-cfb233-GCST90301996' on 'ER- Breast cancer (Combined Oncoarray; iCOGS; GWAS me

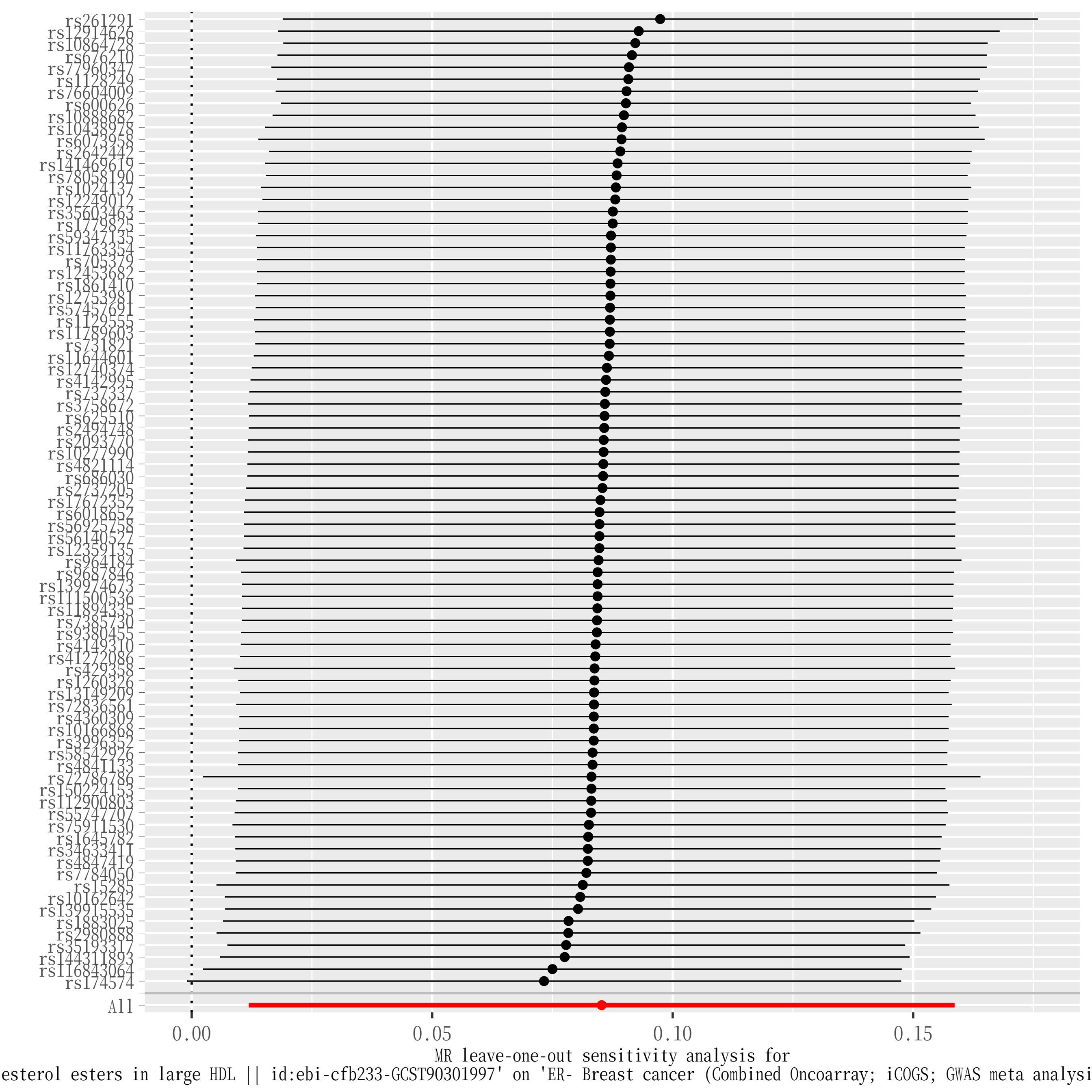

rs261291
rs12914626
rs10864728
rs676210
rs77960347
rs1128249
rs76604009
rs600626
rs10888682
rs10438978
rs6073958
rs2642442
rs141469619
rs78058190
rs1024137
rs12249012
rs35603463
rs1779825
rs59347135
rs11763354
rs705379
rs12453682
rs1861410
rs12753981
rs57457621
rs1129555
rs11789603
rs731821
rs11644601
rs12740374
rs4142995
rs737337
rs3758672
rs625510
rs2494748
rs2093770
rs10277990
rs4821114
rs686030
rs2737205
rs17672352
rs6018652
rs56925758
rs56140527
rs12359135
rs964184
rs9687846
rs139974673
rs111500536
rs11894335
rs7385730
rs9380455
rs4149310
rs41272086
rs429358
rs1260326
rs13149209
rs72836561
rs4360309
rs10166868
rs3996352
rs58542926
rs4841133
rs72786786
rs150224153
rs112900803
rs55747707
rs75911530
rs1645782
rs34633411
rs4847419
rs7784050
rs15285
rs10162642
rs139915535
rs1883025
rs2980888
rs35193317
rs144311893
rs116843064
rs174574
All
0.00
0.05
0.10
0.15
MR leave-one-out sensitivity analysis for
esterol esters in large HDL || id:ebi-cfb233-GCST90301997' on 'ER- Breast cancer (Combined Oncoarray; iCOGS; GWAS meta analysi

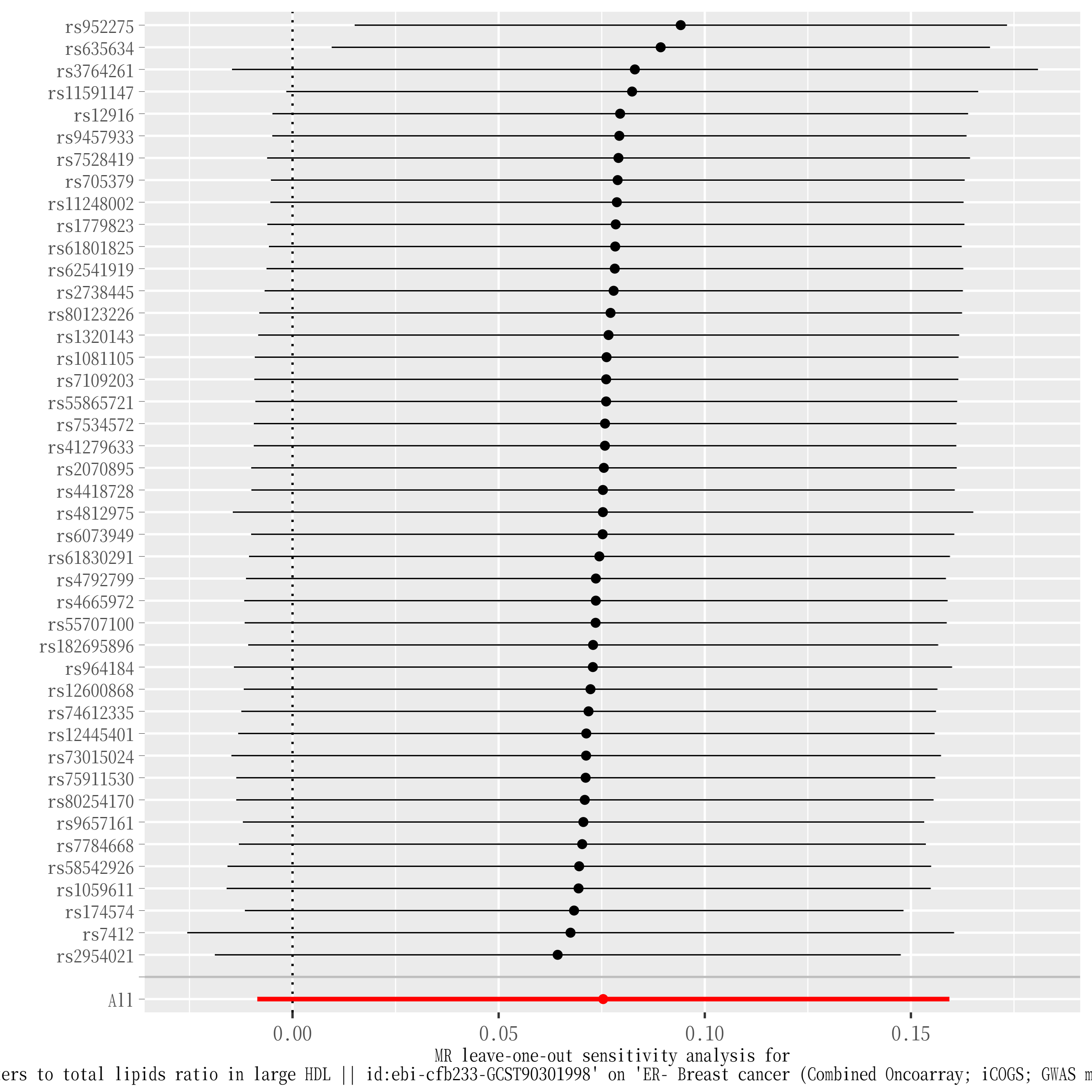

rs952275
rs635634
rs3764261
rs11591147
rs12916
rs9457933
rs7528419
rs705379
rs11248002
rs1779823
rs61801825
rs62541919
rs2738445
rs80123226
rs1320143
rs1081105
rs7109203
rs55865721
rs7534572
rs41279633
rs2070895
rs4418728
rs4812975
rs6073949
rs61830291
rs4792799
rs4665972
rs55707100
rs182695896
rs964184
rs12600868
rs74612335
rs12445401
rs73015024
rs75911530
rs80254170
rs9657161
rs7784668
rs58542926
rs1059611
rs174574
rs7412
rs2954021
All
0.00
0.05
0.10
0.15
MR leave-one-out sensitivity analysis for
ers to total lipids ratio in large HDL || id:ebi-cfb233-GCST90301998' on 'ER- Breast cancer (Combined Oncoarray; iCOGS; GWAS m

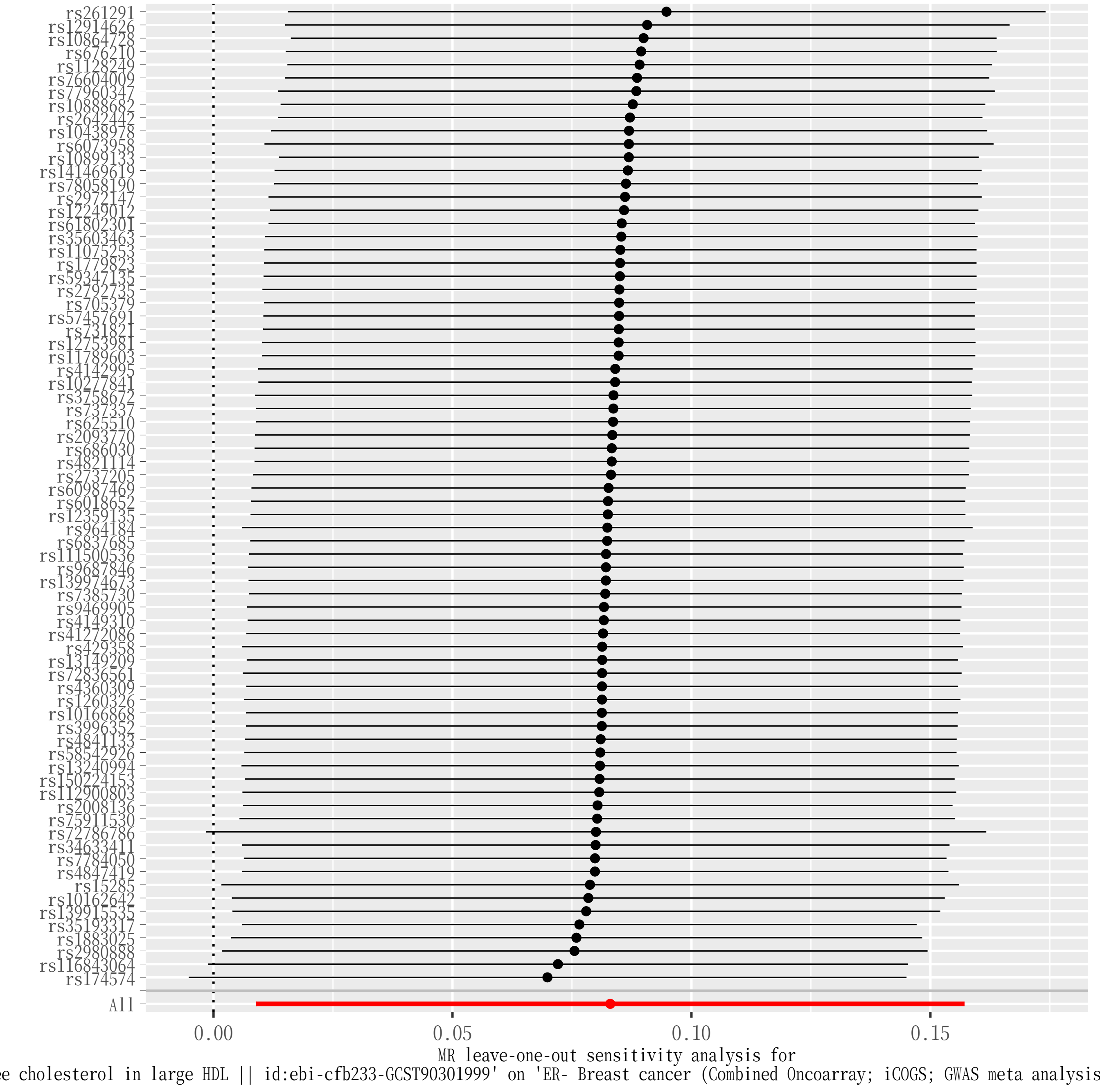
rs261291
rs12914626
rs10864728
rs676210
rs1128249
rs76604009
rs77960347
rs10888682
rs2642442
rs10438978
rs6073958
rs10899133
rs141469619
rs78058190
rs2972147
rs12249012
rs61802301
rs35603463
rs11075253
rs1779823
rs59347135
rs2792735
rs705379
rs57457691
rs731821
rs12753981
rs11789603
rs4142995
rs10277841
rs3758672
rs737337
rs625510
rs2093770
rs686030
rs4821114
rs2737205
rs60987469
rs6018652
rs12359135
rs964184
rs6837685
rs111500536
rs9687846
rs139974673
rs7385730
rs9469905
rs4149310
rs41272086
rs429358
rs13149209
rs72836561
rs4360309
rs1260326
rs10166868
rs3996352
rs4841133
rs58542926
rs13240994
rs150224153
rs112900803
rs2008136
rs75911530
rs72786786
rs34633411
rs7784050
rs4847419
rs15285
rs10162642
rs139915535
rs35193317
rs1883025
rs2980888
rs116843064
rs174574
All
0.00
0.05
0.10
0.15
MR leave-one-out sensitivity analysis for
ee cholesterol in large HDL || id:ebi-cfb233-GCST90301999' on 'ER- Breast cancer (Combined Oncoarray; iCOGS; GWAS meta analysis

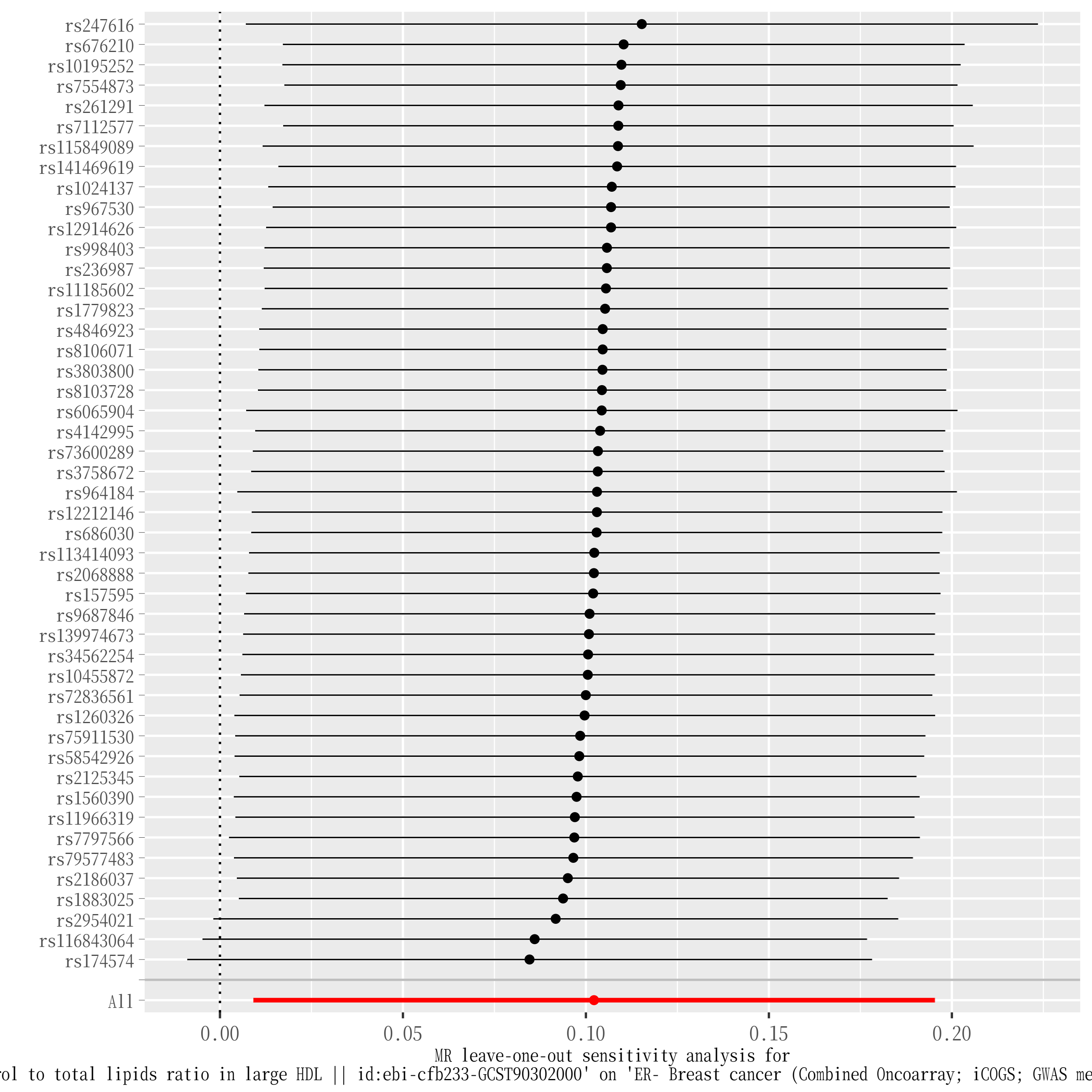

rs247616
rs676210
rs10195252
rs7554873
rs261291
rs7112577
rs115849089
rs141469619
rs1024137
rs967530
rs12914626
rs998403
rs236987
rs11185602
rs1779823
rs4846923
rs8106071
rs3803800
rs8103728
rs6065904
rs4142995
rs73600289
rs3758672
rs964184
rs12212146
rs686030
rs113414093
rs2068888
rs157595
rs9687846
rs139974673
rs34562254
rs10455872
rs72836561
rs1260326
rs75911530
rs58542926
rs2125345
rs1560390
rs11966319
rs7797566
rs79577483
rs2186037
rs1883025
rs2954021
rs116843064
rs174574
All
0.00
0.05
0.10
0.15
0.20
MR leave-one-out sensitivity analysis for
rol to total lipids ratio in large HDL || id:ebi-cfb233-GCST90302000' on 'ER- Breast cancer (Combined Oncoarray; iCOGS; GWAS me

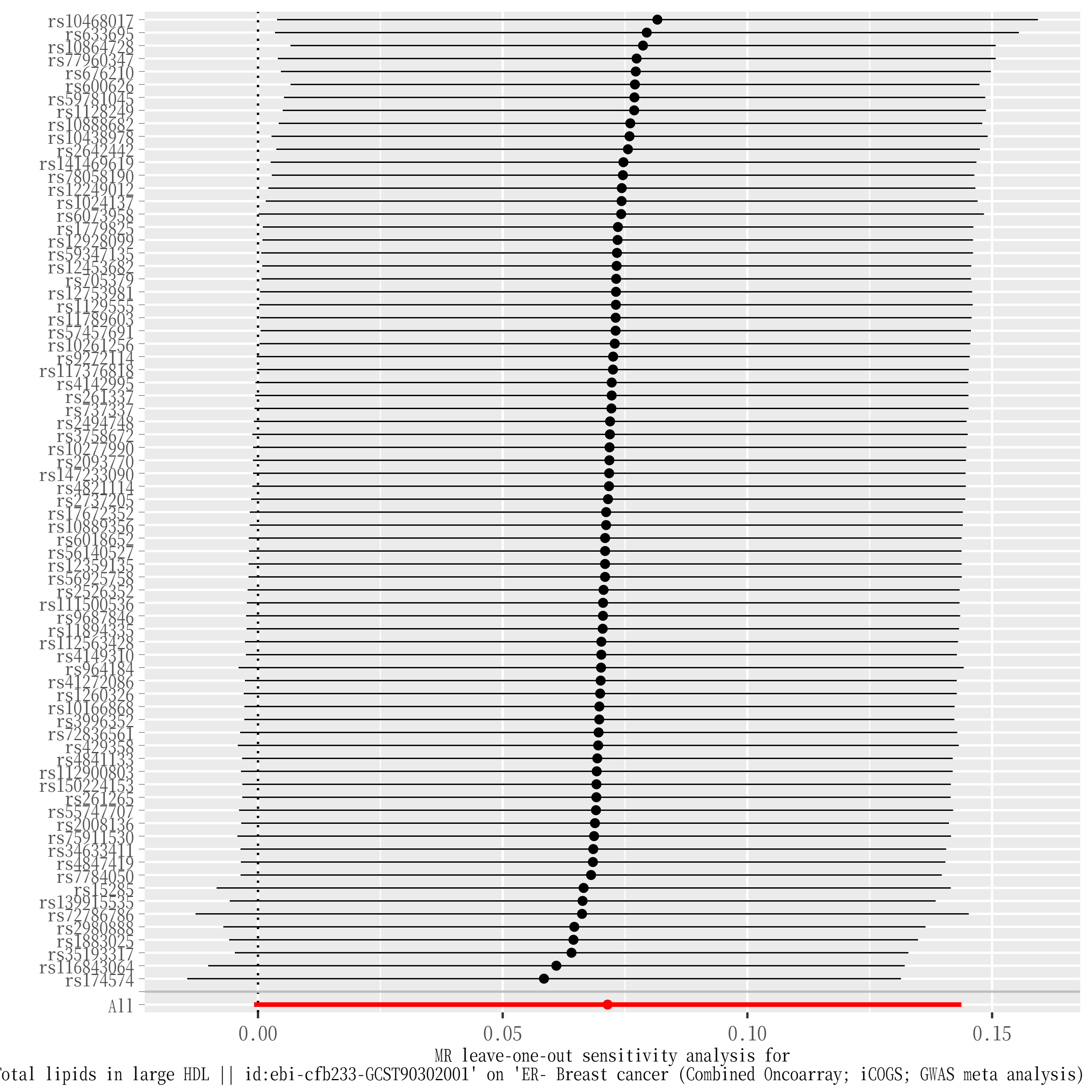

rs10468017
rs633695
rs10864728
rs77960347
rs676210
rs600626
rs59781045
rs1128249
rs10888682
rs10438978
rs2642442
rs141469619
rs78058190
rs12249012
rs1024137
rs6073958
rs1779825
rs12928099
rs59347135
rs12453682
rs705379
rs12753981
rs1129555
rs11789603
rs57457691
rs10261256
rs9272114
rs117376818
rs4142995
rs261337
rs737337
rs2494748
rs3758672
rs10277990
rs2093770
rs147233090
rs4821114
rs2737205
rs17672352
rs10889356
rs6018652
rs56140527
rs12359135
rs56925758
rs2526352
rs111500536
rs9687846
rs11894335
rs112563428
rs4149310
rs964184
rs41272086
rs1260326
rs10166868
rs3996352
rs72836561
rs429358
rs4841133
rs112900803
rs150224153
rs261265
rs55747707
rs2008136
rs75911530
rs34633411
rs4847419
rs7784050
rs15285
rs139915535
rs72786786
rs2980888
rs1883025
rs35193317
rs116843064
rs174574
All
0.00
0.05
0.10
0.15
MR leave-one-out sensitivity analysis for
Total lipids in large HDL || id:ebi-cfb233-GCST90302001' on 'ER- Breast cancer (Combined Oncoarray; iCOGS; GWAS meta analysis)'

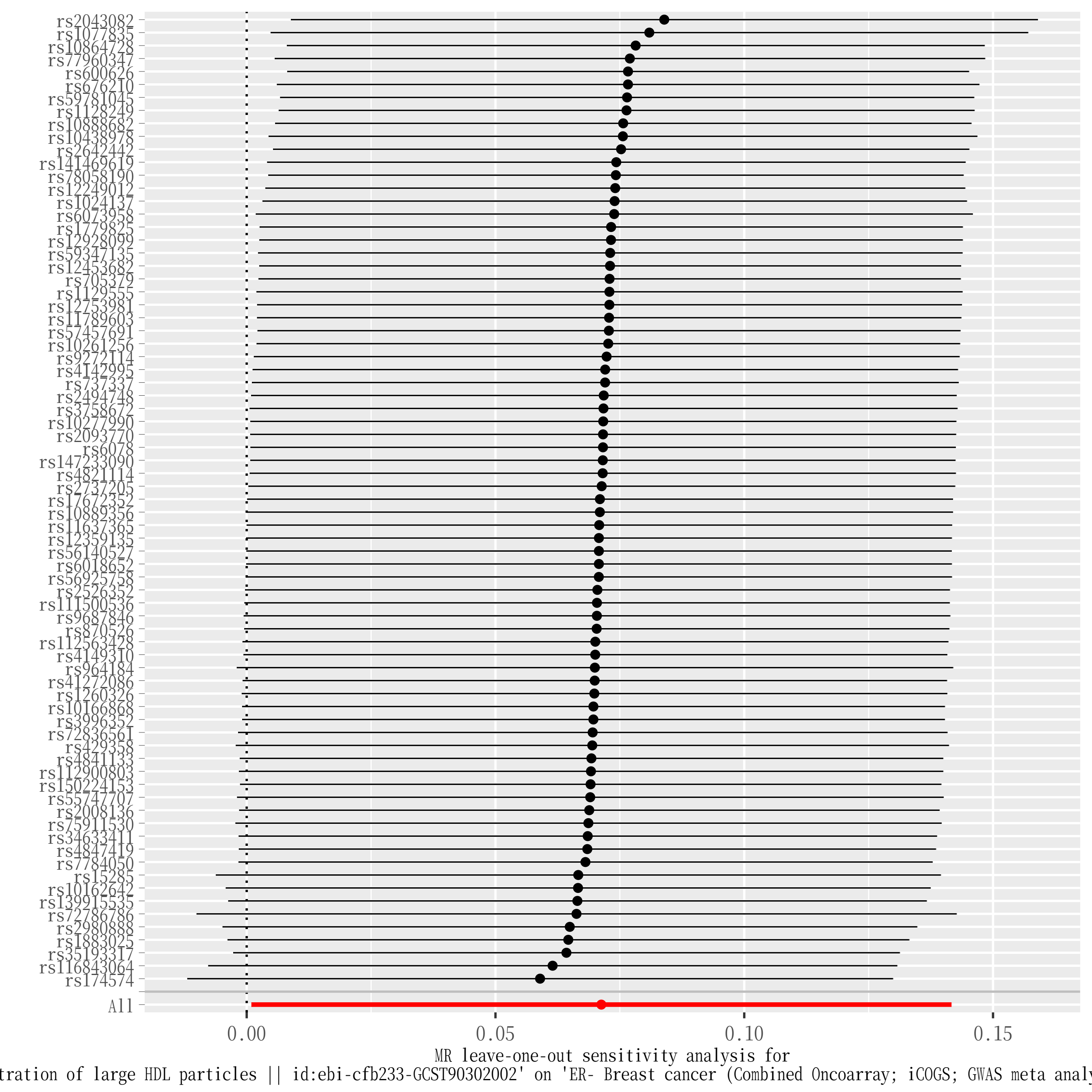

rs2043082
rs1077835
rs10864728
rs77960347
rs600626
rs676210
rs59781045
rs1128249
rs10888682
rs10438978
rs2642442
rs141469619
rs78058190
rs12249012
rs1024137
rs6073958
rs1779825
rs12928099
rs59347135
rs12453682
rs705379
rs1129555
rs12753981
rs11789603
rs57457691
rs10261256
rs9272114
rs4142995
rs737337
rs2494748
rs3758672
rs10277990
rs2093770
rs6078
rs147233090
rs4821114
rs2737205
rs17672352
rs10889356
rs11637365
rs12359135
rs56140527
rs6018652
rs56925758
rs2526352
rs111500536
rs9687846
rs870526
rs112563428
rs4149310
rs964184
rs41272086
rs1260326
rs10166868
rs3996352
rs72836561
rs429358
rs4841133
rs112900803
rs150224153
rs55747707
rs2008136
rs75911530
rs34633411
rs4847419
rs7784050
rs15285
rs10162642
rs139915535
rs72786786
rs2980888
rs1883025
rs35193317
rs116843064
rs174574
All
0.00
0.05
0.10
0.15
MR leave-one-out sensitivity analysis for
tration of large HDL particles || id:ebi-cfb233-GCST90302002' on 'ER- Breast cancer (Combined Oncoarray; iCOGS; GWAS meta analy

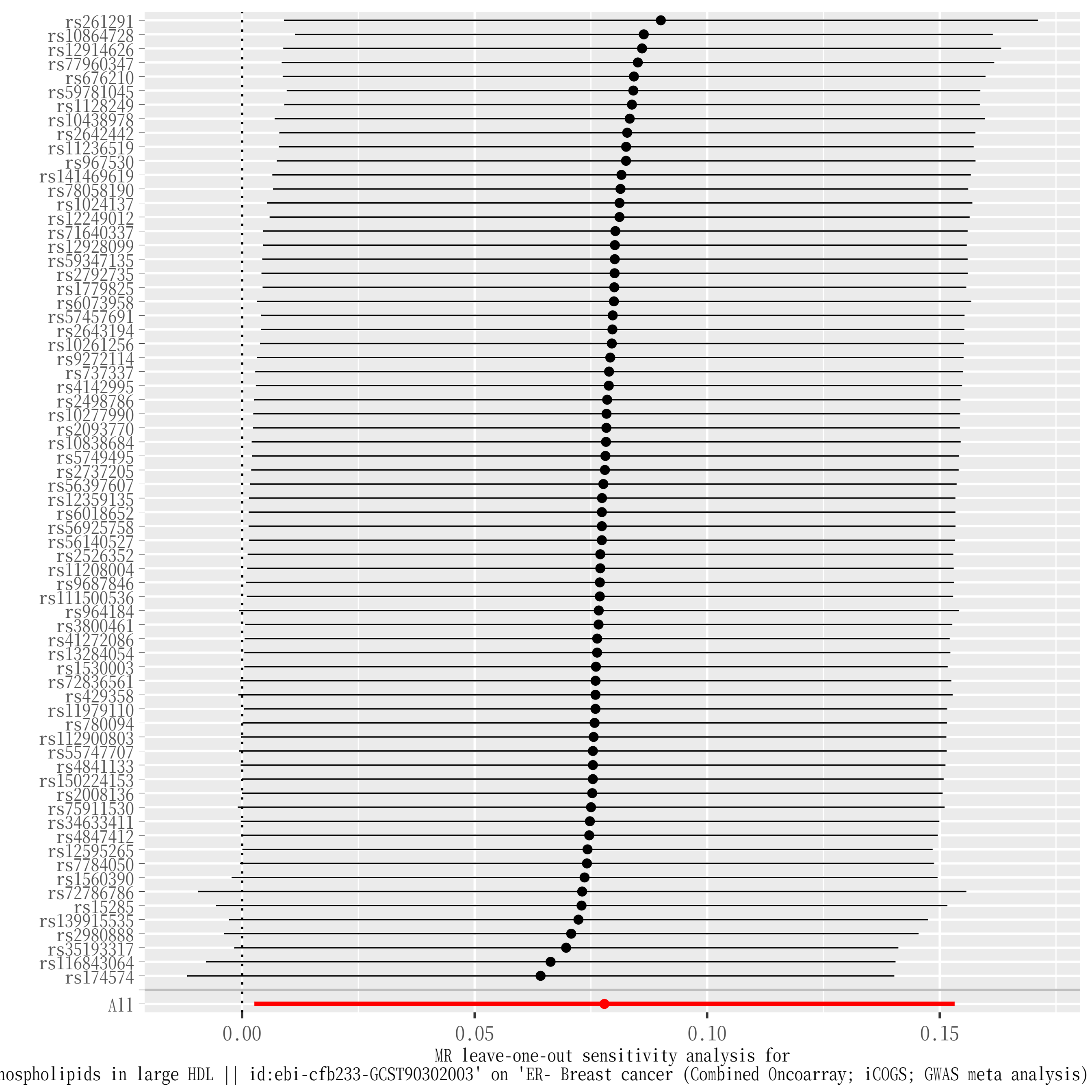

rs261291
rs10864728
rs12914626
rs77960347
rs676210
rs59781045
rs1128249
rs10438978
rs2642442
rs11236519
rs967530
rs141469619
rs78058190
rs1024137
rs12249012
rs71640337
rs12928099
rs59347135
rs2792735
rs1779825
rs6073958
rs57457691
rs2643194
rs10261256
rs9272114
rs737337
rs4142995
rs2498786
rs10277990
rs2093770
rs10838684
rs5749495
rs2737205
rs56397607
rs12359135
rs6018652
rs56925758
rs56140527
rs2526352
rs11208004
rs9687846
rs111500536
rs964184
rs3800461
rs41272086
rs13284054
rs1530003
rs72836561
rs429358
rs11979110
rs780094
rs112900803
rs55747707
rs4841133
rs150224153
rs2008136
rs75911530
rs34633411
rs4847412
rs12595265
rs7784050
rs1560390
rs72786786
rs15285
rs139915535
rs2980888
rs35193317
rs116843064
rs174574
All
0.00
0.05
0.10
0.15
MR leave-one-out sensitivity analysis for
hospholipids in large HDL || id:ebi-cfb233-GCST90302003' on 'ER- Breast cancer (Combined Oncoarray; iCOGS; GWAS meta analysis)

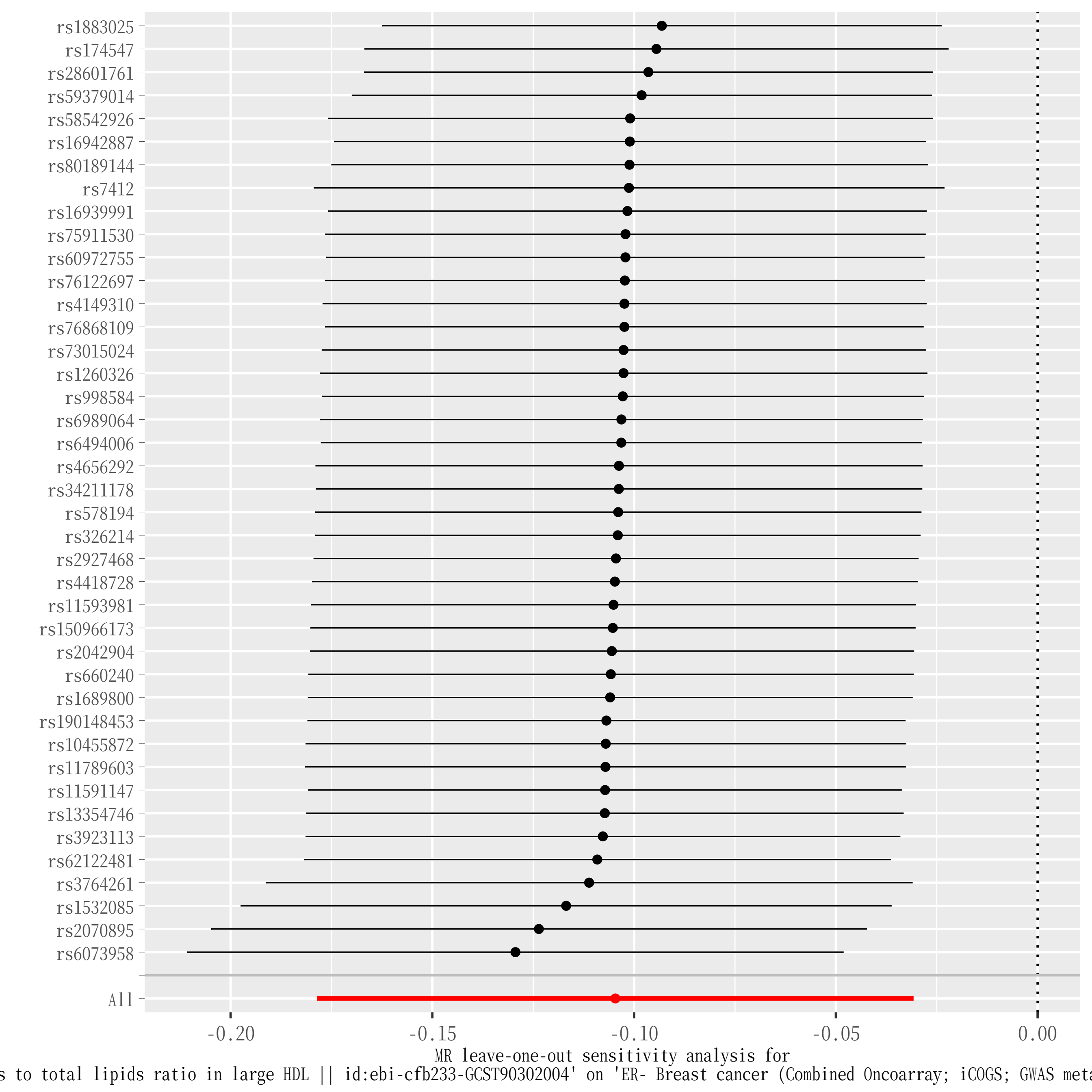

rs1883025
rs174547
rs28601761
rs59379014
rs58542926
rs16942887
rs80189144
rs7412
rs16939991
rs75911530
rs60972755
rs76122697
rs4149310
rs76868109
rs73015024
rs1260326
rs998584
rs6989064
rs6494006
rs4656292
rs34211178
rs578194
rs326214
rs2927468
rs4418728
rs11593981
rs150966173
rs2042904
rs660240
rs1689800
rs190148453
rs10455872
rs11789603
rs11591147
rs13354746
rs3923113
rs62122481
rs3764261
rs1532085
rs2070895
rs6073958
All
-0.20
-0.15
-0.10
-0.05
0.00
MR leave-one-out sensitivity analysis for
s to total lipids ratio in large HDL || id:ebi-cfb233-GCST90302004' on 'ER- Breast cancer (Combined Oncoarray; iCOGS; GWAS meta

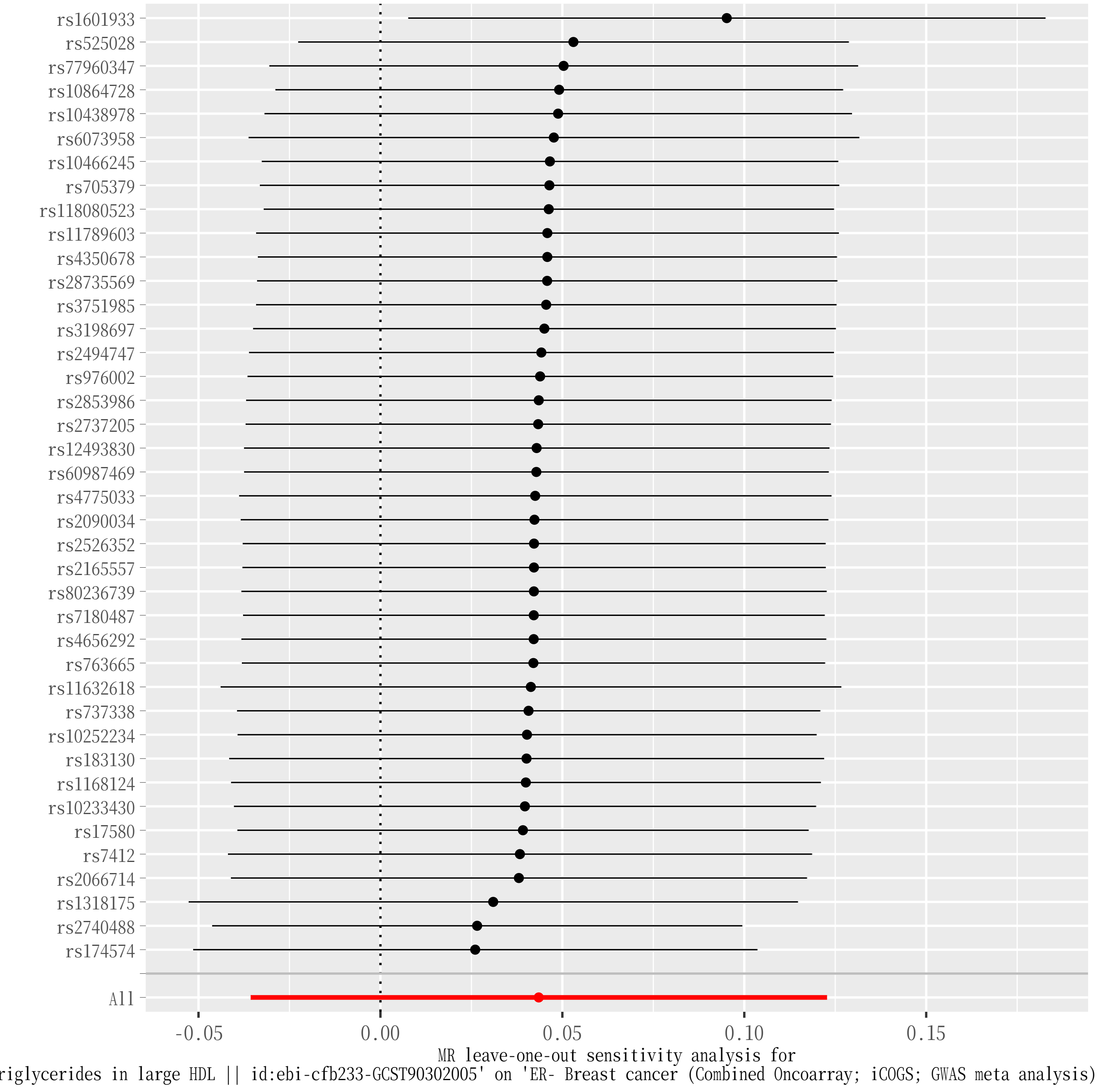

rs1601933
rs525028
rs77960347
rs10864728
rs10438978
rs6073958
rs10466245
rs705379
rs118080523
rs11789603
rs4350678
rs28735569
rs3751985
rs3198697
rs2494747
rs976002
rs2853986
rs2737205
rs12493830
rs60987469
rs4775033
rs2090034
rs2526352
rs2165557
rs80236739
rs7180487
rs4656292
rs763665
rs11632618
rs737338
rs10252234
rs183130
rs1168124
rs10233430
rs17580
rs7412
rs2066714
rs1318175
rs2740488
rs174574
All
-0.05
0.00
0.05
0.10
0.15
MR leave-one-out sensitivity analysis for
riglycerides in large HDL || id:ebi-cfb233-GCST90302005' on 'ER- Breast cancer (Combined Oncoarray; iCOGS; GWAS meta analysis)

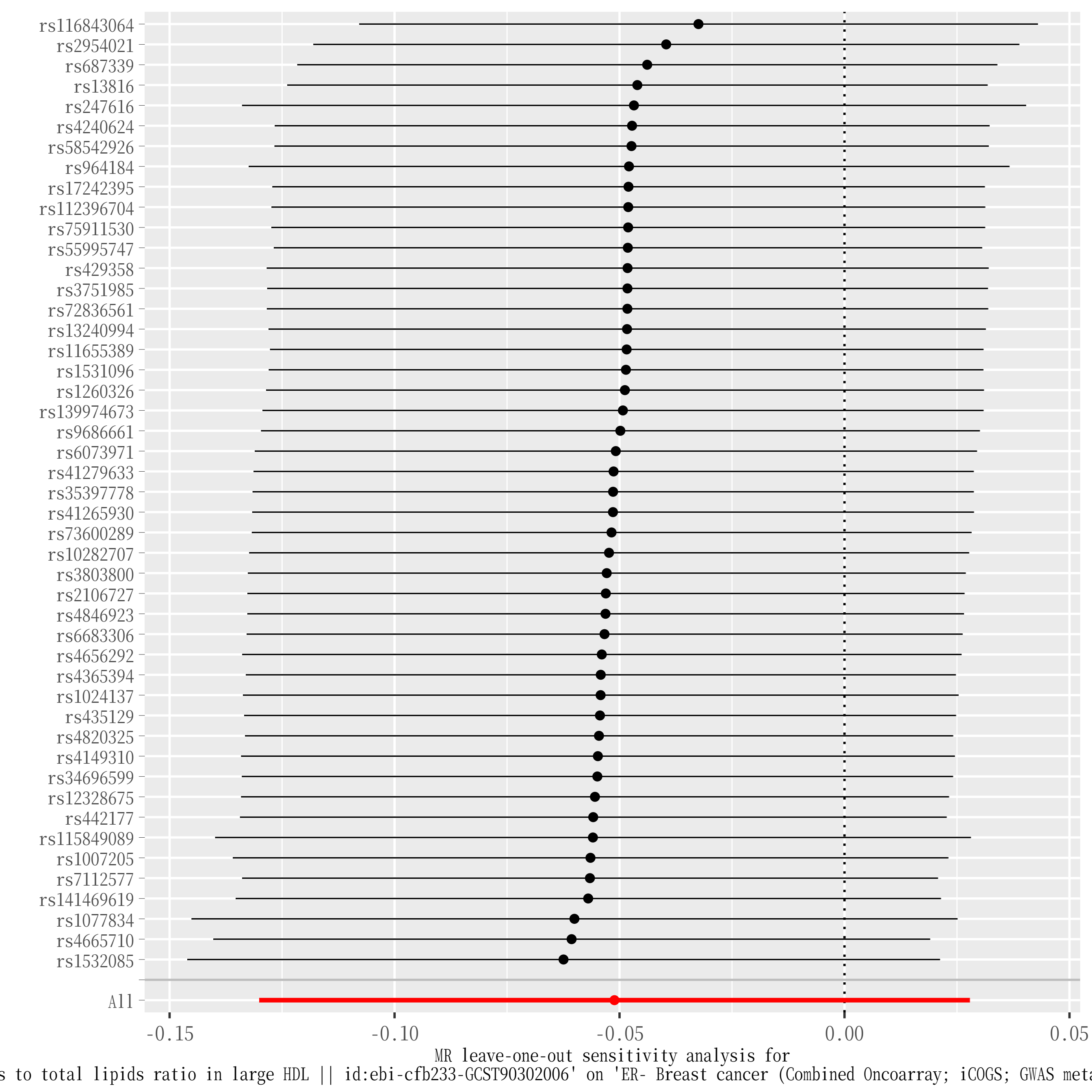

rs116843064
rs2954021
rs687339
rs13816
rs247616
rs4240624
rs58542926
rs964184
rs17242395
rs112396704
rs75911530
rs55995747
rs429358
rs3751985
rs72836561
rs13240994
rs11655389
rs1531096
rs1260326
rs139974673
rs9686661
rs6073971
rs41279633
rs35397778
rs41265930
rs73600289
rs10282707
rs3803800
rs2106727
rs4846923
rs6683306
rs4656292
rs4365394
rs1024137
rs435129
rs4820325
rs4149310
rs34696599
rs12328675
rs442177
rs115849089
rs1007205
rs7112577
rs141469619
rs1077834
rs4665710
rs1532085
All
-0.15
-0.10
-0.05
0.00
0.05
MR leave-one-out sensitivity analysis for
s to total lipids ratio in large HDL || id:ebi-cfb233-GCST90302006' on 'ER- Breast cancer (Combined Oncoarray; iCOGS; GWAS meta

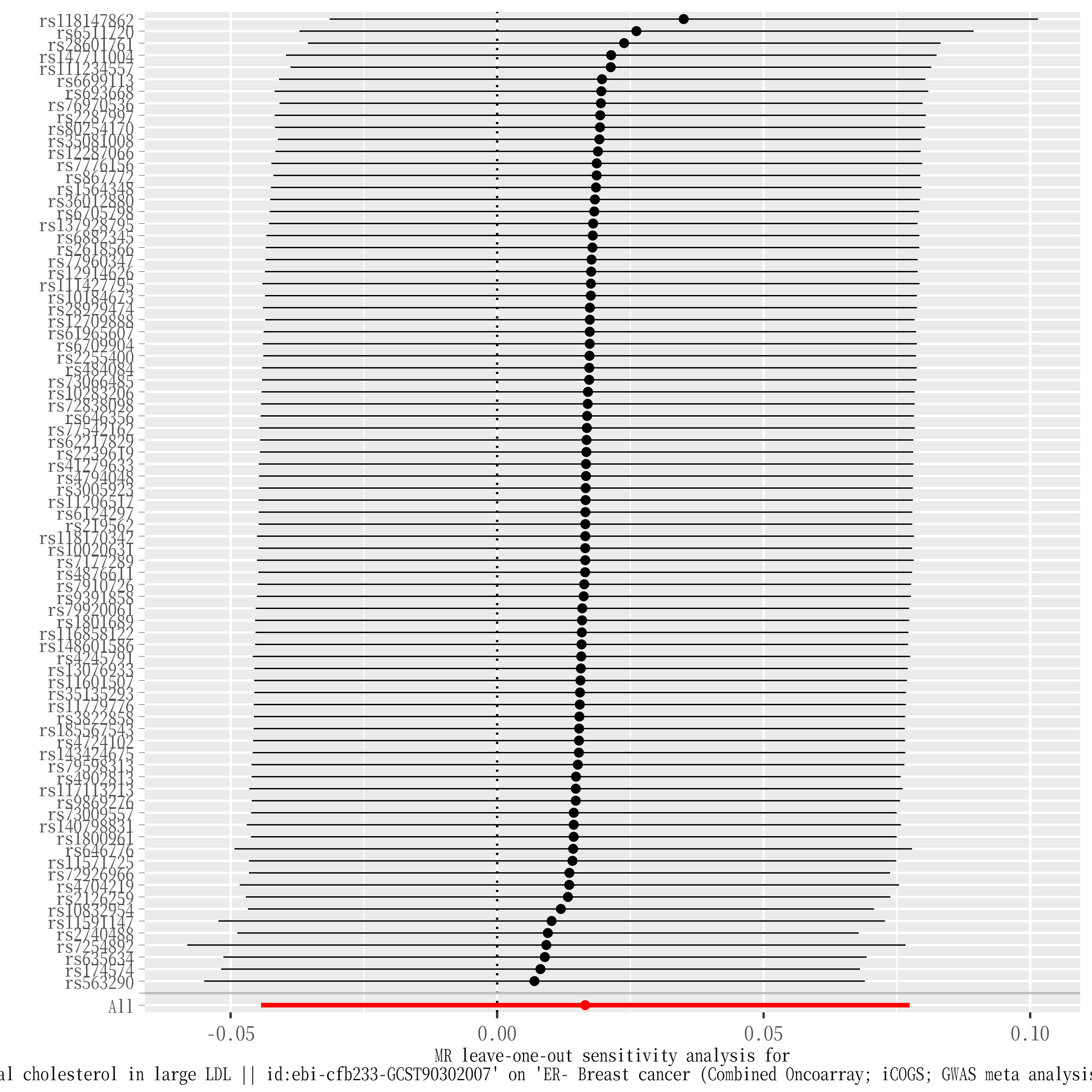
rs118147862
rs6511720
rs28601761
rs147711004
rs111234557
rs6699113
rs693668
rs76970536
rs2287997
rs80254170
rs35081008
rs12287066
rs7776156
rs867772
rs1564348
rs36012880
rs6705798
rs137928795
rs6882345
rs2618566
rs77960347
rs12914626
rs111427795
rs10184673
rs28929474
rs12709888
rs61265607
rs6709904
rs2255400
rs484084
rs73066485
rs10283206
rs72838098
rs646356
rs77542162
rs62217829
rs2239619
rs41279633
rs4794048
rs3005923
rs11206517
rs6124297
rs219562
rs118170342
rs10020631
rs7127289
rs4876611
rs7910726
rs9391858
rs79920061
rs1801689
rs116858122
rs148601586
rs4245791
rs13076933
rs11601507
rs35135293
rs11779776
rs3822858
rs185567543
rs4724102
rs143424675
rs79598313
rs4902813
rs117113213
rs9869276
rs73009557
rs140798831
rs1800961
rs646776
rs11571725
rs72926966
rs4704219
rs2126259
rs10832954
rs11591147
rs2740488
rs7254892
rs635634
rs174574
rs563290
All
-0.05
0.00
0.05
0.10
MR leave-one-out sensitivity analysis for
al cholesterol in large LDL || id:ebi-cfb233-GCST90302007' on 'ER- Breast cancer (Combined Oncoarray; iCOGS; GWAS meta analysis

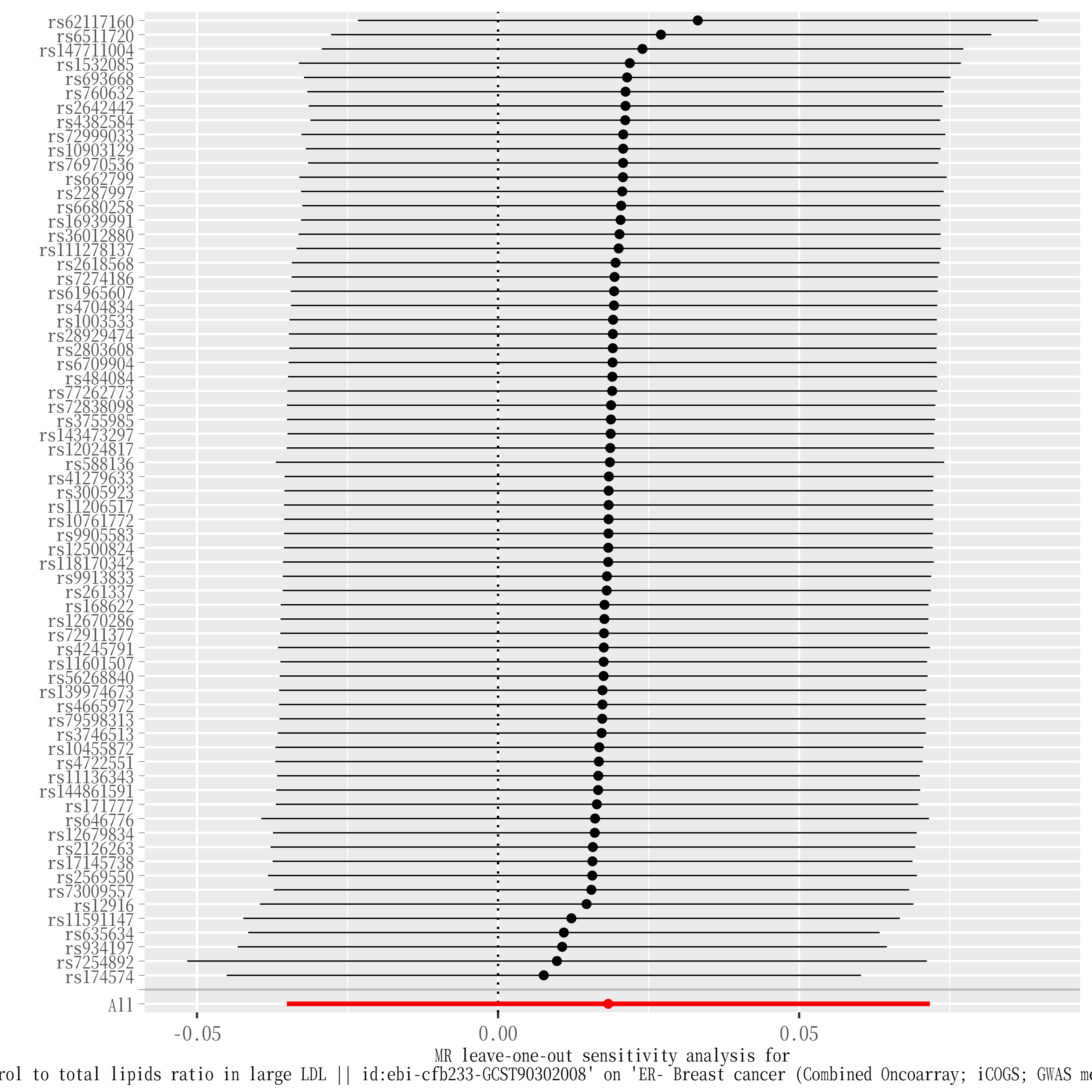

rs62117160
rs6511720
rs147711004
rs1532085
rs693668
rs760632
rs2642442
rs4382584
rs72999033
rs10903129
rs76970536
rs662799
rs2287997
rs6680258
rs16939991
rs36012880
rs111278137
rs2618568
rs7274186
rs61965607
rs4704834
rs1003533
rs28929474
rs2803608
rs6709904
rs484084
rs77262773
rs72838098
rs3755985
rs143473297
rs12024817
rs588136
rs41279633
rs3005923
rs11206517
rs10761772
rs9905583
rs12500824
rs118170342
rs9913833
rs261337
rs168622
rs12670286
rs72911377
rs4245791
rs11601507
rs56268840
rs139974673
rs4665972
rs79598313
rs3746513
rs10455872
rs4722551
rs11136343
rs144861591
rs171777
rs646776
rs12679834
rs2126263
rs17145738
rs2569550
rs73009557
rs12916
rs11591147
rs635634
rs934197
rs7254892
rs174574
All
-0.05
0.00
0.05
MR leave-one-out sensitivity analysis for
rol to total lipids ratio in large LDL || id:ebi-cfb233-GCST90302008' on 'ER- Breast cancer (Combined Oncoarray; iCOGS; GWAS me

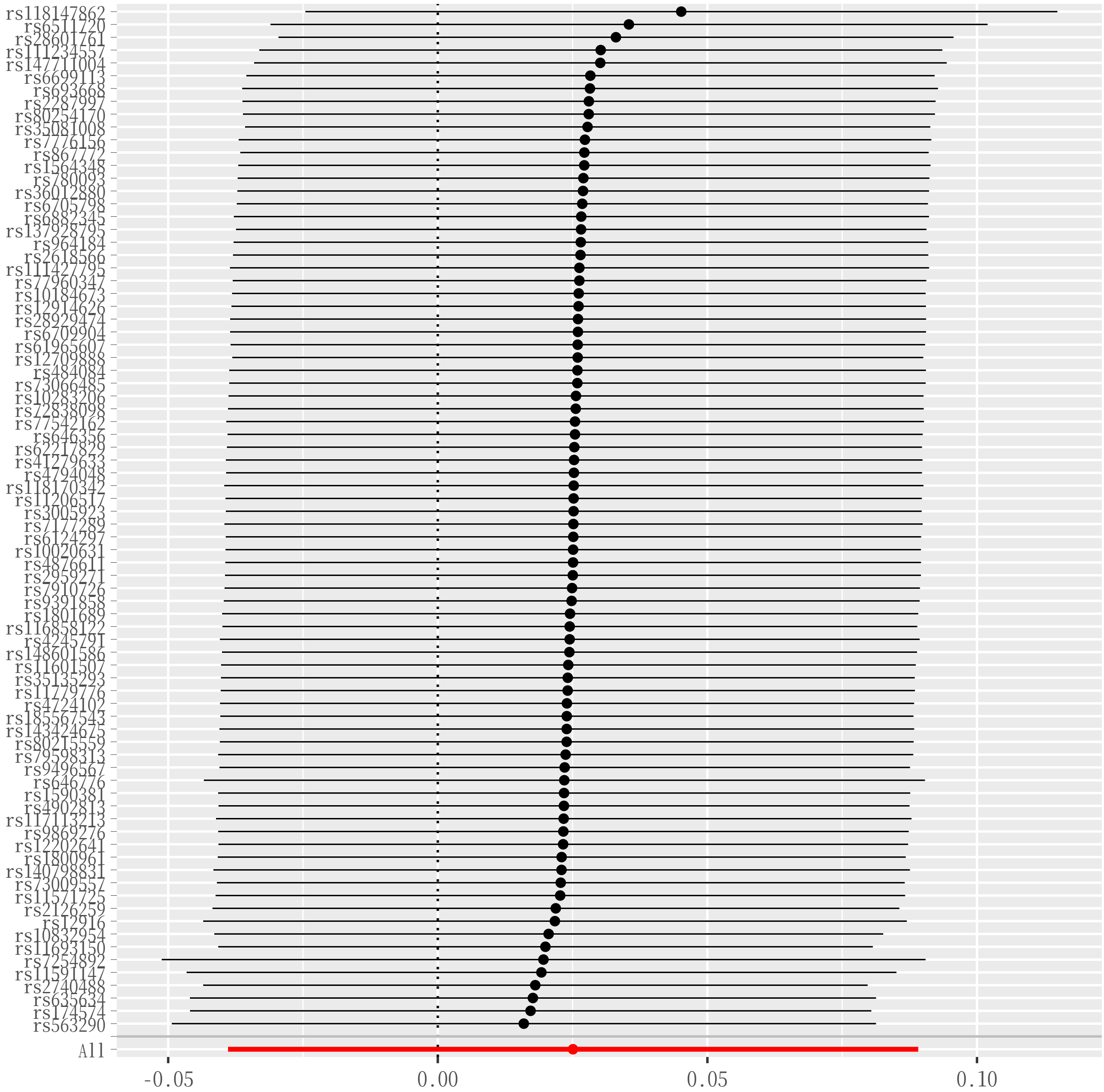

MR leave-one-out sensitivity analysis for
esterol esters in large LDL || id:ebi-cfb233-GCST90302009' on 'ER- Breast cancer (Combined Oncoarray; iCOGS; GWAS meta analysi

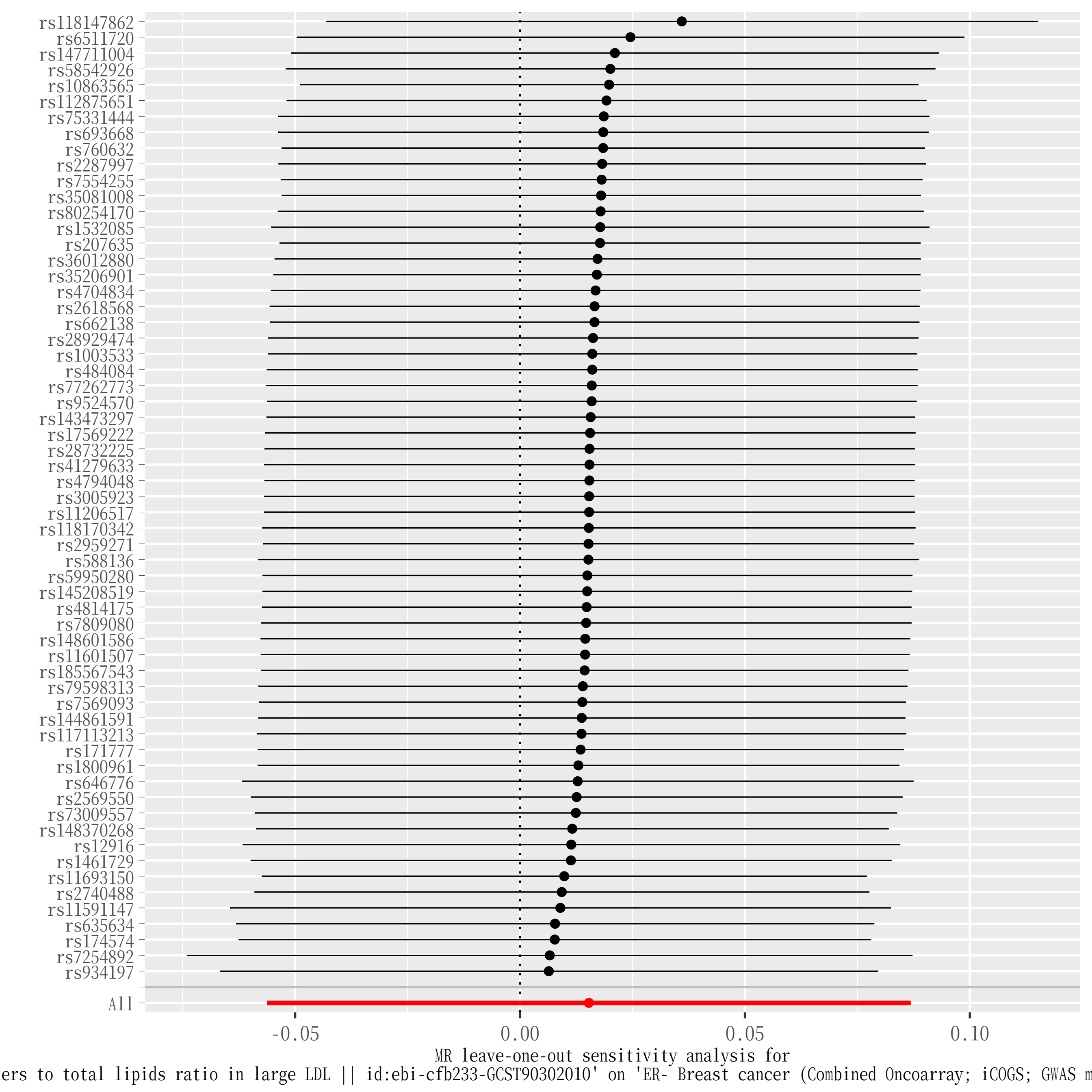

rs118147862
rs6511720
rs147711004
rs58542926
rs10863565
rs112875651
rs75331444
rs693668
rs760632
rs2287997
rs7554255
rs35081008
rs80254170
rs1532085
rs207635
rs36012880
rs35206901
rs4704834
rs2618568
rs662138
rs28929474
rs1003533
rs484084
rs77262773
rs9524570
rs143473297
rs17569222
rs28732225
rs41279633
rs4794048
rs3005923
rs11206517
rs118170342
rs2959271
rs588136
rs59950280
rs145208519
rs4814175
rs7809080
rs148601586
rs11601507
rs185567543
rs79598313
rs7569093
rs144861591
rs117113213
rs171777
rs1800961
rs646776
rs2569550
rs73009557
rs148370268
rs12916
rs1461729
rs11693150
rs2740488
rs11591147
rs635634
rs174574
rs7254892
rs934197
All
-0.05
0.00
0.05
0.10
MR leave-one-out sensitivity analysis for
ers to total lipids ratio in large LDL || id:ebi-cfb233-GCST90302010' on 'ER- Breast cancer (Combined Oncoarray; iCOGS; GWAS m

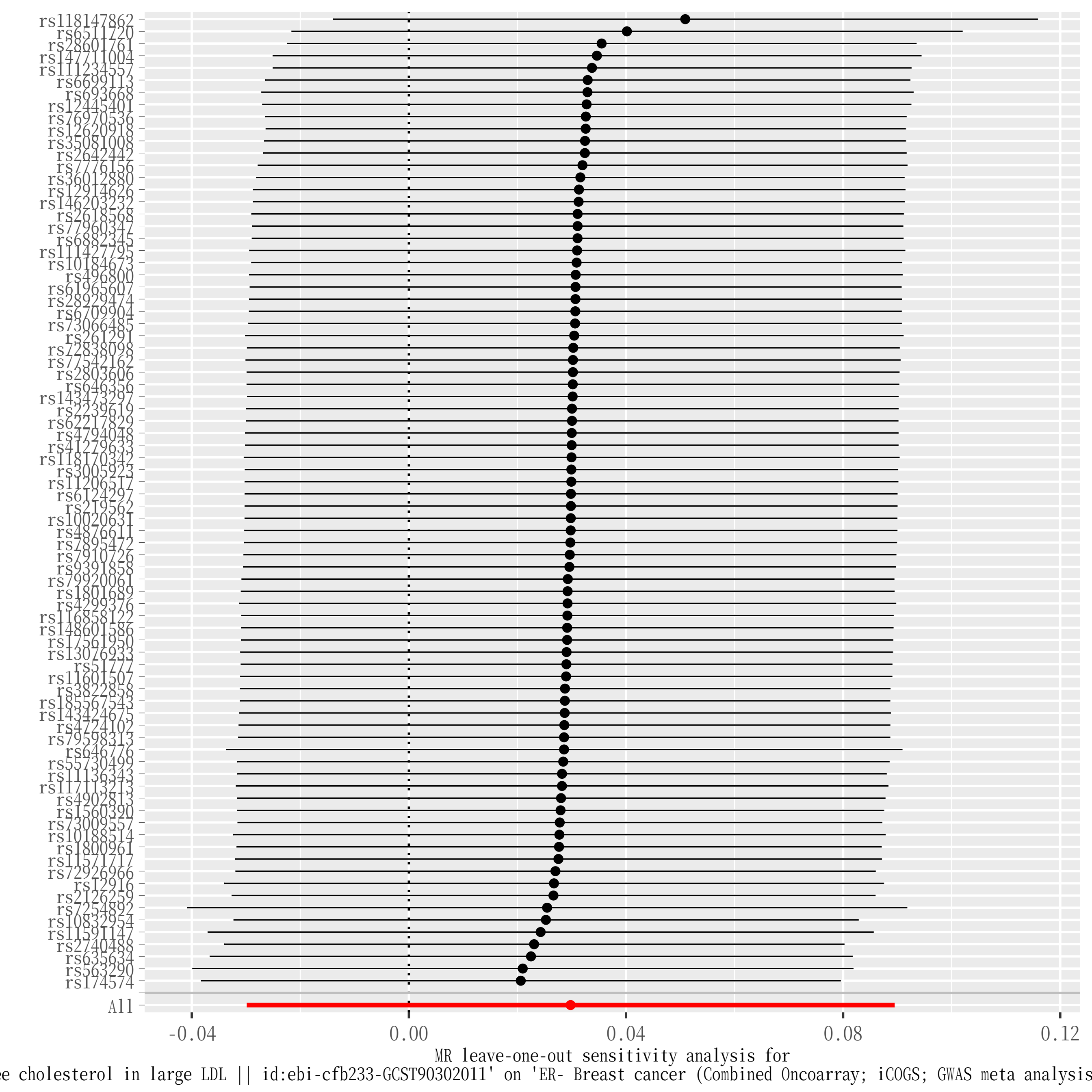
rs118147862
rs6511720
rs28601761
rs147711004
rs111234557
rs6699113
rs693668
rs12445401
rs76970536
rs12620918
rs35081008
rs2642442
rs7776156
rs36012880
rs12914626
rs146203232
rs2618568
rs77960347
rs6882345
rs111427795
rs10184673
rs496800
rs61965607
rs28929474
rs6709904
rs73066485
rs261291
rs72838098
rs77542162
rs2803606
rs646356
rs143473297
rs2239619
rs62217829
rs4794048
rs41279633
rs118170342
rs3005923
rs11206517
rs6124297
rs219562
rs10020631
rs4876611
rs7895472
rs7910726
rs9391858
rs79920061
rs1801689
rs4299376
rs116858122
rs148601586
rs17561950
rs13076933
rs51777
rs11601507
rs3822858
rs185567543
rs143424675
rs4724102
rs79598313
rs646776
rs55730499
rs11136343
rs117113213
rs4902813
rs1560390
rs73009557
rs10188514
rs1800961
rs11571717
rs72926966
rs12916
rs2126259
rs7254892
rs10832954
rs11591147
rs2740488
rs635634
rs563290
rs174574
All
-0.04
0.00
0.04
0.08
0.12
MR leave-one-out sensitivity analysis for
ee cholesterol in large LDL || id:ebi-cfb233-GCST90302011' on 'ER- Breast cancer (Combined Oncoarray; iCOGS; GWAS meta analysis

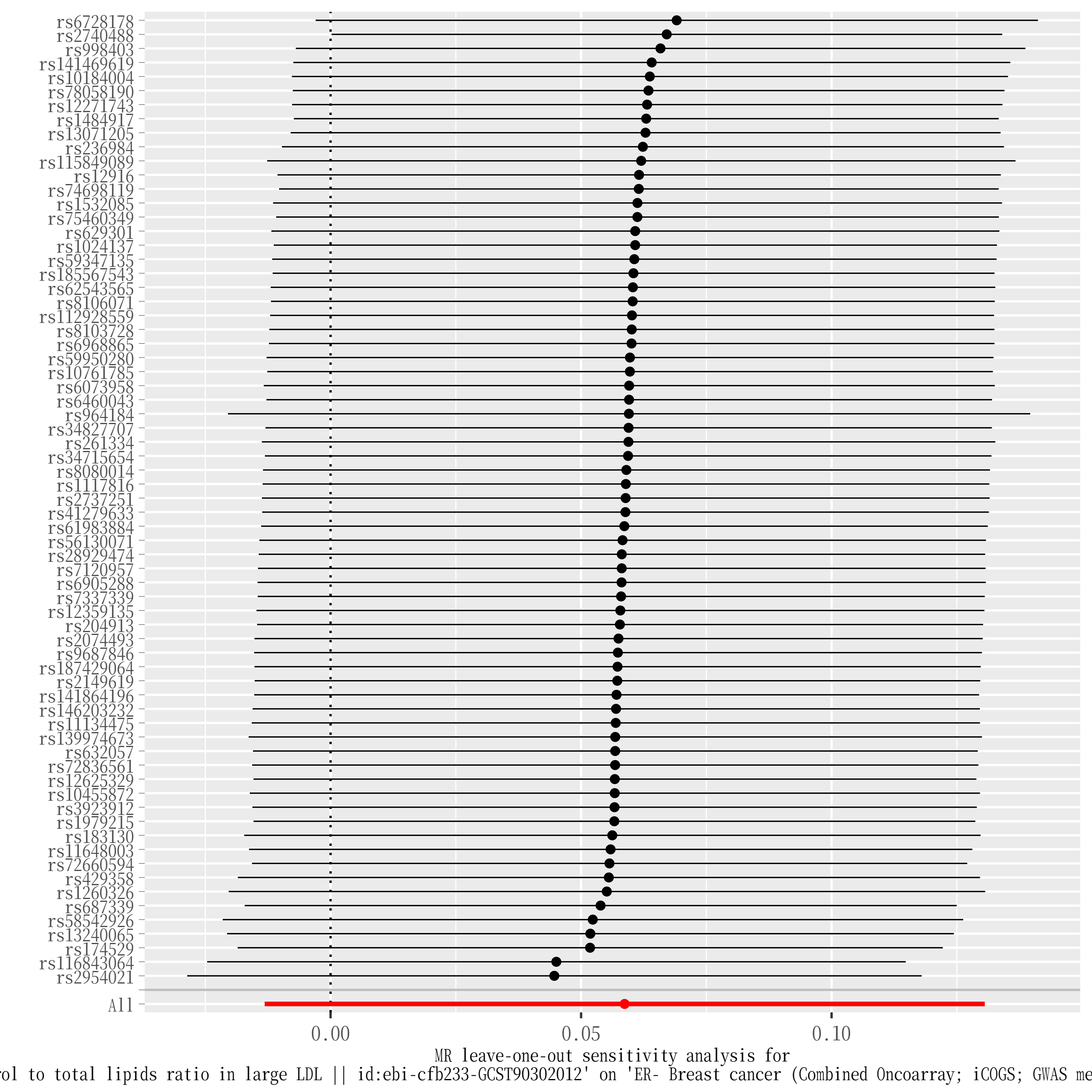

rs6728178
rs2740488
rs998403
rs141469619
rs10184004
rs78058190
rs12271743
rs1484917
rs13071205
rs236984
rs115849089
rs12916
rs74698119
rs1532085
rs75460349
rs629301
rs1024137
rs59347135
rs185567543
rs62543565
rs8106071
rs112928559
rs8103728
rs6968865
rs59950280
rs10761785
rs6073958
rs6460043
rs964184
rs34827707
rs261334
rs34715654
rs8080014
rs1117816
rs2737251
rs41279633
rs61983884
rs56130071
rs28929474
rs7120957
rs6905288
rs7337339
rs12359135
rs204913
rs2074493
rs9687846
rs187429064
rs2149619
rs141864196
rs146203232
rs11134475
rs139974673
rs632057
rs72836561
rs12625329
rs10455872
rs3923912
rs1979215
rs183130
rs11648003
rs72660594
rs429358
rs1260326
rs687339
rs58542926
rs13240065
rs174529
rs116843064
rs2954021
All
0.00
0.05
0.10
MR leave-one-out sensitivity analysis for
rol to total lipids ratio in large LDL || id:ebi-cfb233-GCST90302012' on 'ER- Breast cancer (Combined Oncoarray; iCOGS; GWAS me

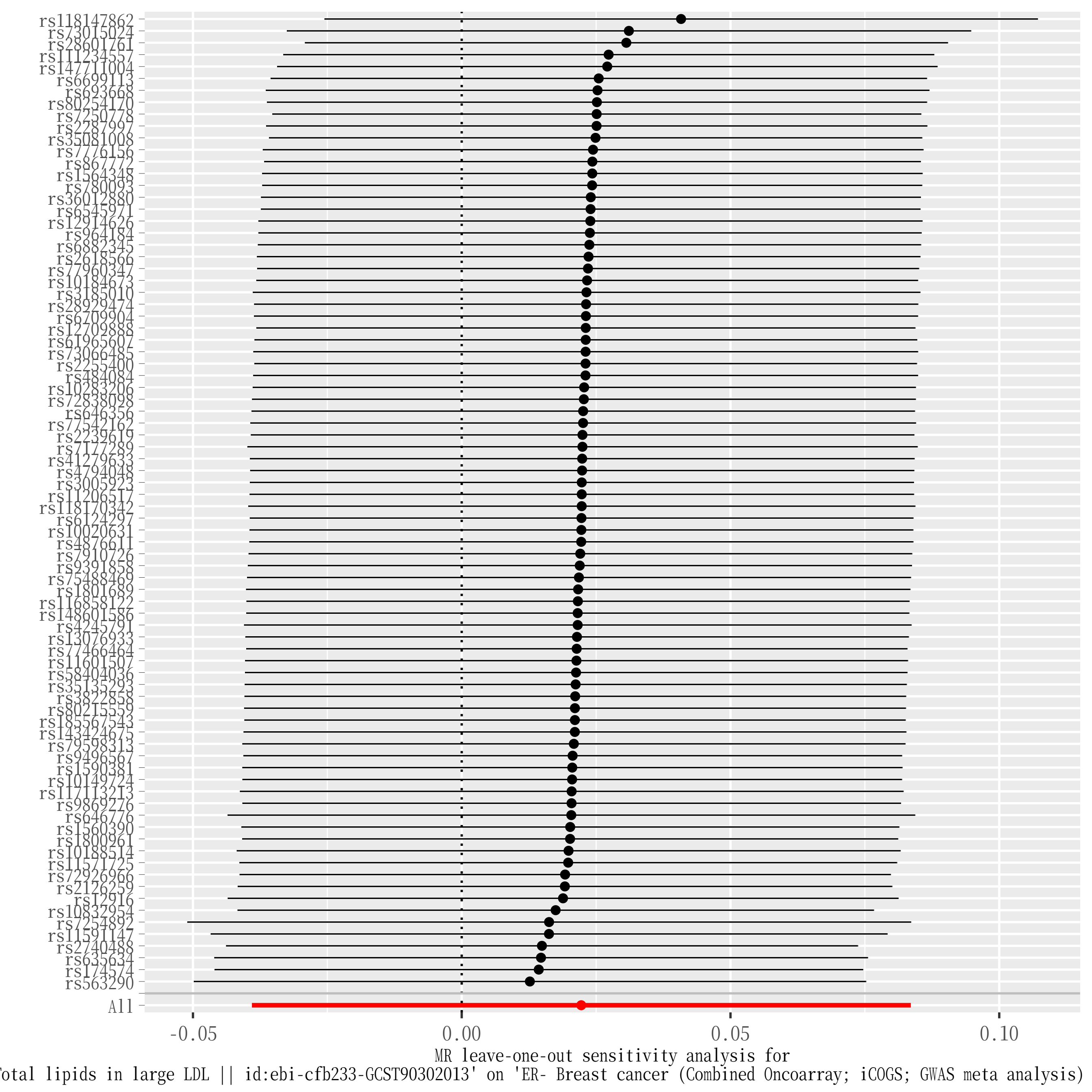

rs118147862
rs73015024
rs28601761
rs111234557
rs147711004
rs6699113
rs693668
rs80254170
rs7250778
rs2287997
rs35081008
rs7776156
rs867772
rs1564348
rs780093
rs36012880
rs6545971
rs12914626
rs964184
rs6882345
rs2618566
rs77960347
rs10184673
rs3185010
rs28929474
rs6709904
rs12709888
rs61965607
rs73066485
rs2255400
rs484084
rs10283206
rs72838098
rs646356
rs77542162
rs2232619
rs7177289
rs41279633
rs4794048
rs3005923
rs11206517
rs118170342
rs6124297
rs10020631
rs4876611
rs7910726
rs9391858
rs75488469
rs1801689
rs116858122
rs148601586
rs4245791
rs13076933
rs77466464
rs11601507
rs58404036
rs35135293
rs3822858
rs80215559
rs185567543
rs143424675
rs79598313
rs9496567
rs1590381
rs10149724
rs117113213
rs9869276
rs646776
rs1560390
rs1800961
rs10188514
rs11571725
rs72926966
rs2126259
rs12916
rs10832954
rs7254892
rs11591147
rs2740488
rs635634
rs174574
rs563290
All
-0.05
0.00
0.05
0.10
MR leave-one-out sensitivity analysis for
Total lipids in large LDL || id:ebi-cfb233-GCST90302013' on 'ER- Breast cancer (Combined Oncoarray; iCOGS; GWAS meta analysis)

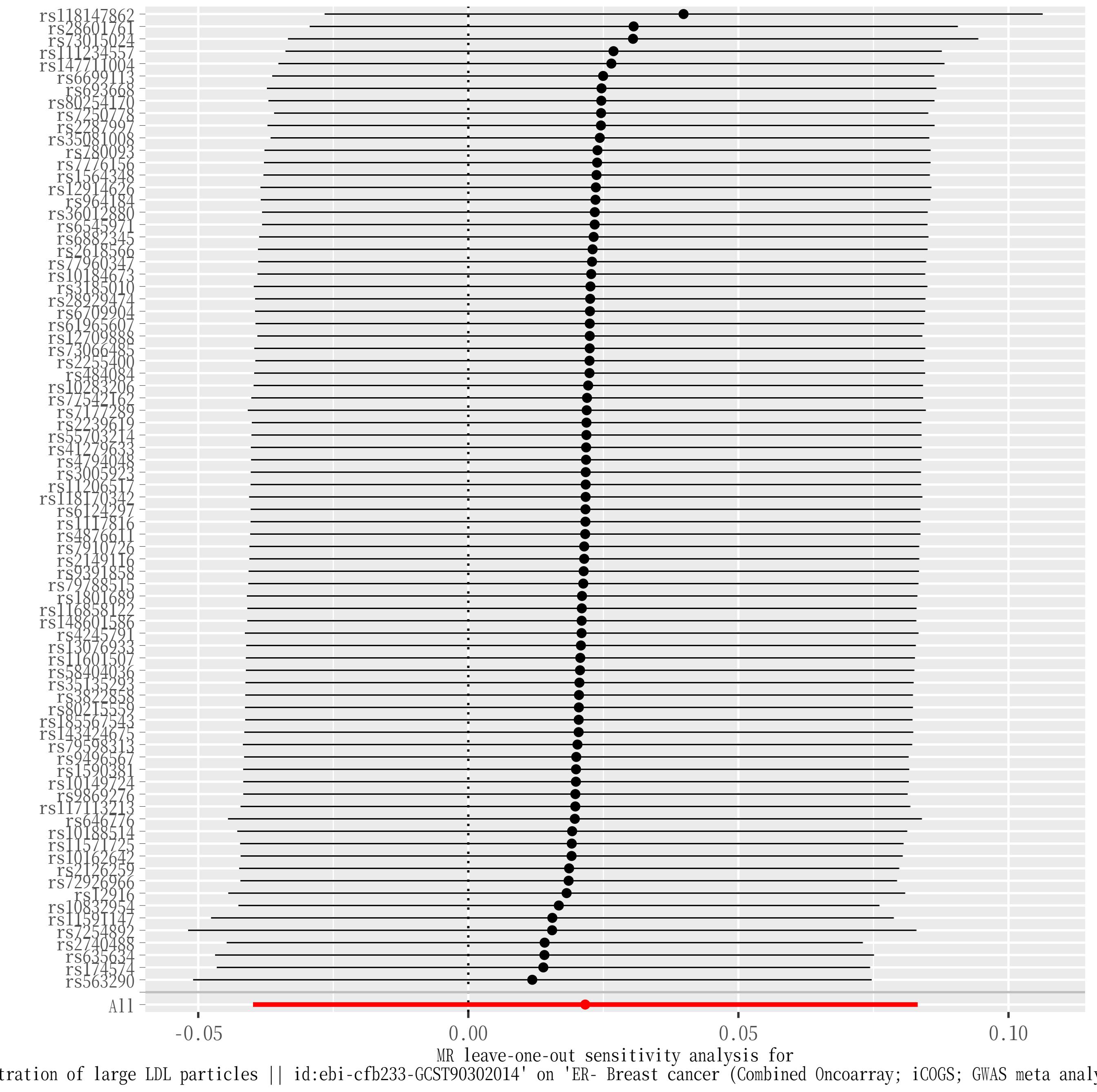
rs118147862
rs28601761
rs73015024
rs111234557
rs147711004
rs6699113
rs693668
rs80254170
rs7250778
rs2287997
rs35081008
rs780093
rs7776156
rs1564348
rs12914626
rs964184
rs36012880
rs6545971
rs6882345
rs2618566
rs77960347
rs10184673
rs3185010
rs28929474
rs6709904
rs61965607
rs12709888
rs73066485
rs2255400
rs484084
rs10283206
rs77542162
rs7177289
rs2239619
rs55703214
rs41279633
rs4794048
rs3005923
rs11206517
rs118170342
rs6124297
rs1117816
rs4876611
rs7910726
rs2149116
rs9391858
rs79788515
rs1801689
rs116858122
rs148601586
rs4245791
rs13076933
rs11601507
rs58404036
rs35135293
rs3822858
rs80215559
rs185567543
rs143424675
rs79598313
rs9496567
rs1590381
rs10149724
rs9869276
rs117113213
rs646776
rs10188514
rs11571725
rs10162642
rs2126259
rs72926966
rs12916
rs10832954
rs11591147
rs7254892
rs2740488
rs635634
rs174574
rs563290
All
-0.05
0.00
0.05
0.10
MR leave-one-out sensitivity analysis for
tration of large LDL particles || id:ebi-cfb233-GCST90302014' on 'ER- Breast cancer (Combined Oncoarray; iCOGS; GWAS meta analy

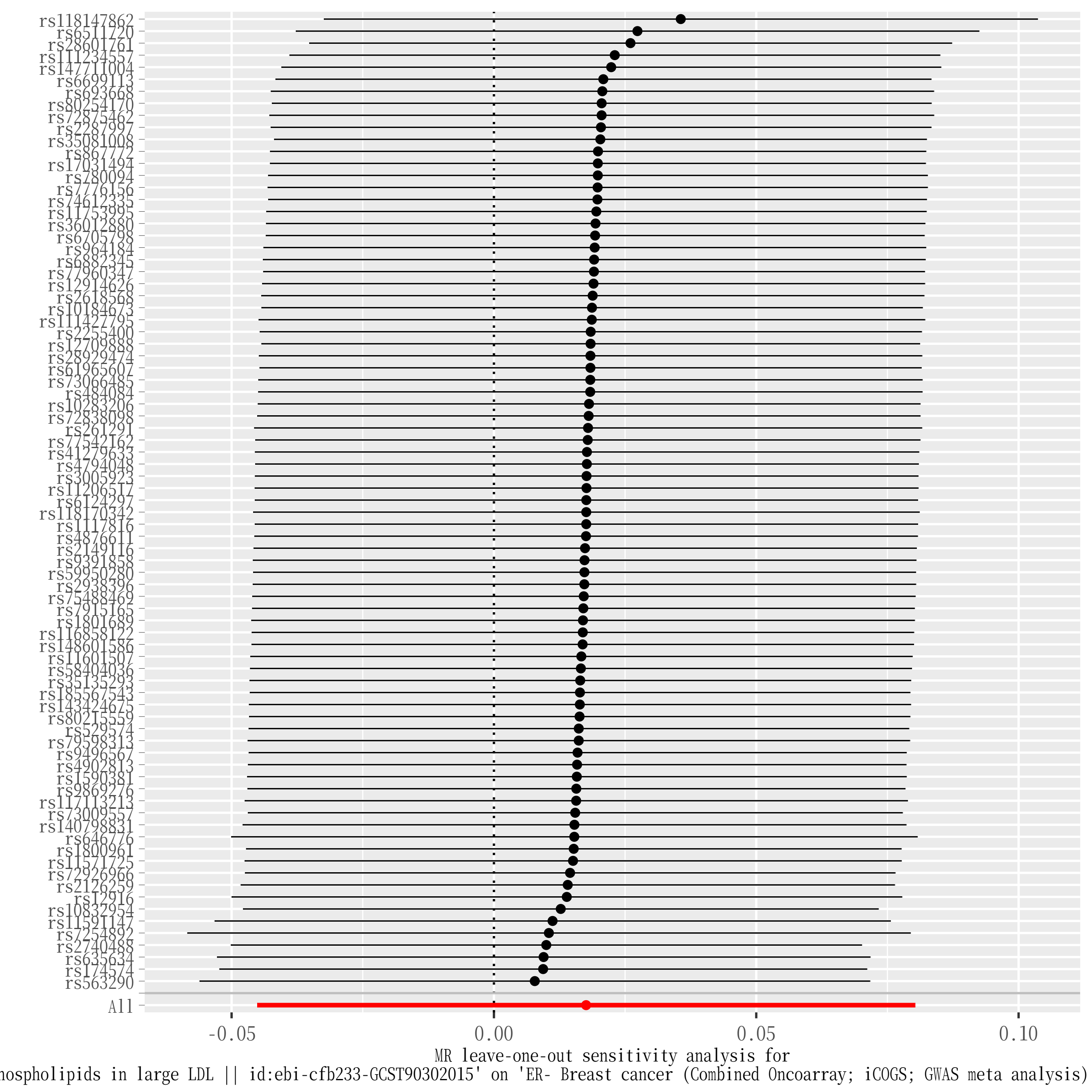
rs118147862
rs6511720
rs28601761
rs111234557
rs147711004
rs6699113
rs693668
rs80254170
rs72875462
rs2287997
rs35081008
rs867772
rs17031494
rs280094
rs7776156
rs74612335
rs11753995
rs36012880
rs6705798
rs964184
rs6882345
rs77960347
rs12914626
rs2618568
rs10184673
rs111427795
rs2255400
rs12709888
rs28929474
rs61965607
rs73066485
rs484084
rs10283206
rs72838098
rs261291
rs77542162
rs41279633
rs4794048
rs3005923
rs11206517
rs6124297
rs118170342
rs1117816
rs4876611
rs2149116
rs9391858
rs59950280
rs2938396
rs75488469
rs7915165
rs1801689
rs116858122
rs148601586
rs11601507
rs58404036
rs35135293
rs185567543
rs143424675
rs80215559
rs529574
rs79598313
rs9496567
rs4902813
rs1590381
rs9869276
rs117113213
rs73009557
rs140798831
rs646776
rs1800961
rs11571725
rs72926966
rs2126259
rs12916
rs10832954
rs11591147
rs7254892
rs2740488
rs635634
rs174574
rs563290
All
-0.05
0.00
0.05
0.10
MR leave-one-out sensitivity analysis for
hospholipids in large LDL || id:ebi-cfb233-GCST90302015' on 'ER- Breast cancer (Combined Oncoarray; iCOGS; GWAS meta analysis)

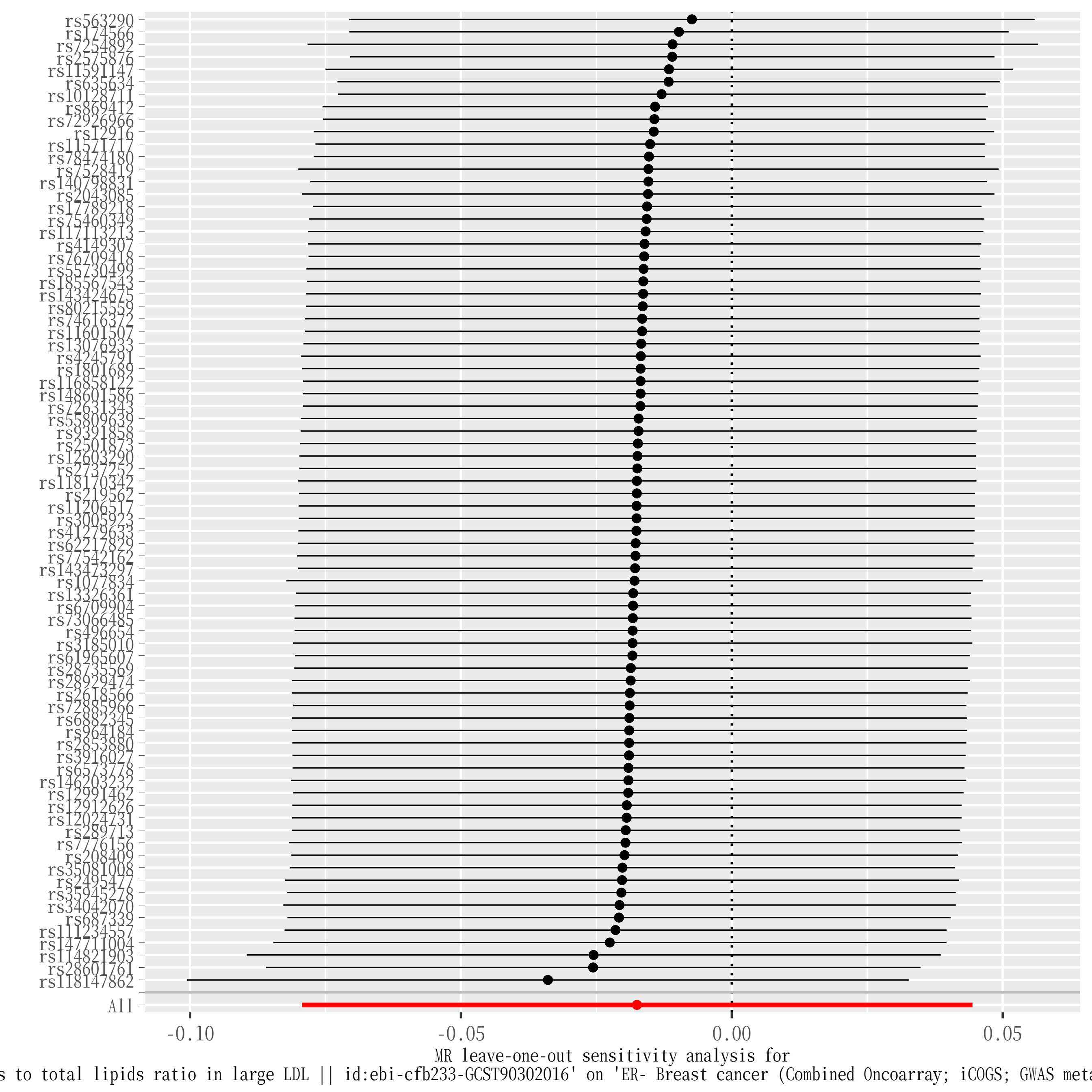

rs563290
rs174566
rs7254892
rs2575876
rs11591147
rs635634
rs10128711
rs869412
rs72926966
rs12916
rs11571717
rs78474180
rs7528419
rs140798831
rs2043085
rs17789218
rs75460349
rs117113213
rs4149307
rs76209418
rs55730499
rs185567543
rs143424675
rs80215559
rs74616372
rs11601507
rs13076933
rs4245791
rs1801689
rs116858122
rs148601586
rs72631343
rs55809639
rs9391858
rs2501873
rs12603290
rs2737252
rs118170342
rs219562
rs11206517
rs3005923
rs41279633
rs62217829
rs77542162
rs143473297
rs1077834
rs13326361
rs6709904
rs73066485
rs496654
rs3185010
rs61965607
rs28735569
rs28929474
rs2618566
rs72885966
rs6882345
rs964184
rs2853880
rs3916027
rs6573778
rs146203232
rs12991462
rs12912626
rs12024731
rs289713
rs7776156
rs208409
rs35081008
rs2495477
rs35945278
rs34042070
rs687339
rs111234557
rs147711004
rs114821903
rs28601761
rs118147862
All
-0.10
-0.05
0.00
0.05
MR leave-one-out sensitivity analysis for
s to total lipids ratio in large LDL || id:ebi-cfb233-GCST90302016' on 'ER- Breast cancer (Combined Oncoarray; iCOGS; GWAS meta

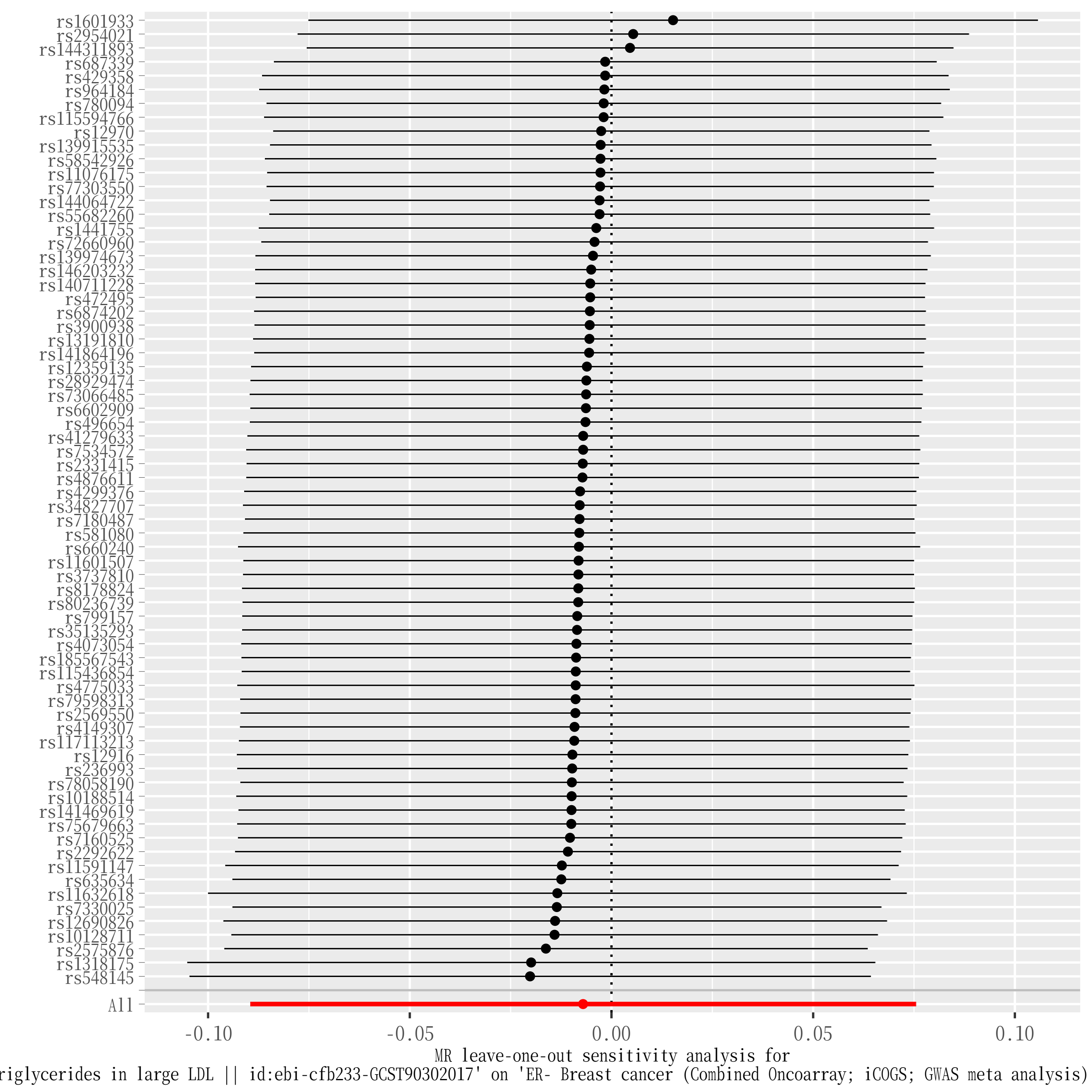

rs1601933
rs2954021
rs144311893
rs687339
rs429358
rs964184
rs780094
rs115594766
rs12970
rs139915535
rs58542926
rs11076175
rs77303550
rs144064722
rs55682260
rs1441755
rs72660960
rs139974673
rs146203232
rs140711228
rs472495
rs6874202
rs3900938
rs13191810
rs141864196
rs12359135
rs28929474
rs73066485
rs6602909
rs496654
rs41279633
rs7534572
rs2331415
rs4876611
rs4299376
rs34827707
rs7180487
rs581080
rs660240
rs11601507
rs3737810
rs8178824
rs80236739
rs799157
rs35135293
rs4073054
rs185567543
rs115436854
rs4775033
rs79598313
rs2569550
rs4149307
rs117113213
rs12916
rs236993
rs78058190
rs10188514
rs141469619
rs75679663
rs7160525
rs2292622
rs11591147
rs635634
rs11632618
rs7330025
rs12690826
rs10128711
rs2575876
rs1318175
rs548145
All
-0.10
-0.05
0.00
0.05
0.10
MR leave-one-out sensitivity analysis for
riglycerides in large LDL || id:ebi-cfb233-GCST90302017' on 'ER- Breast cancer (Combined Oncoarray; iCOGS; GWAS meta analysis)

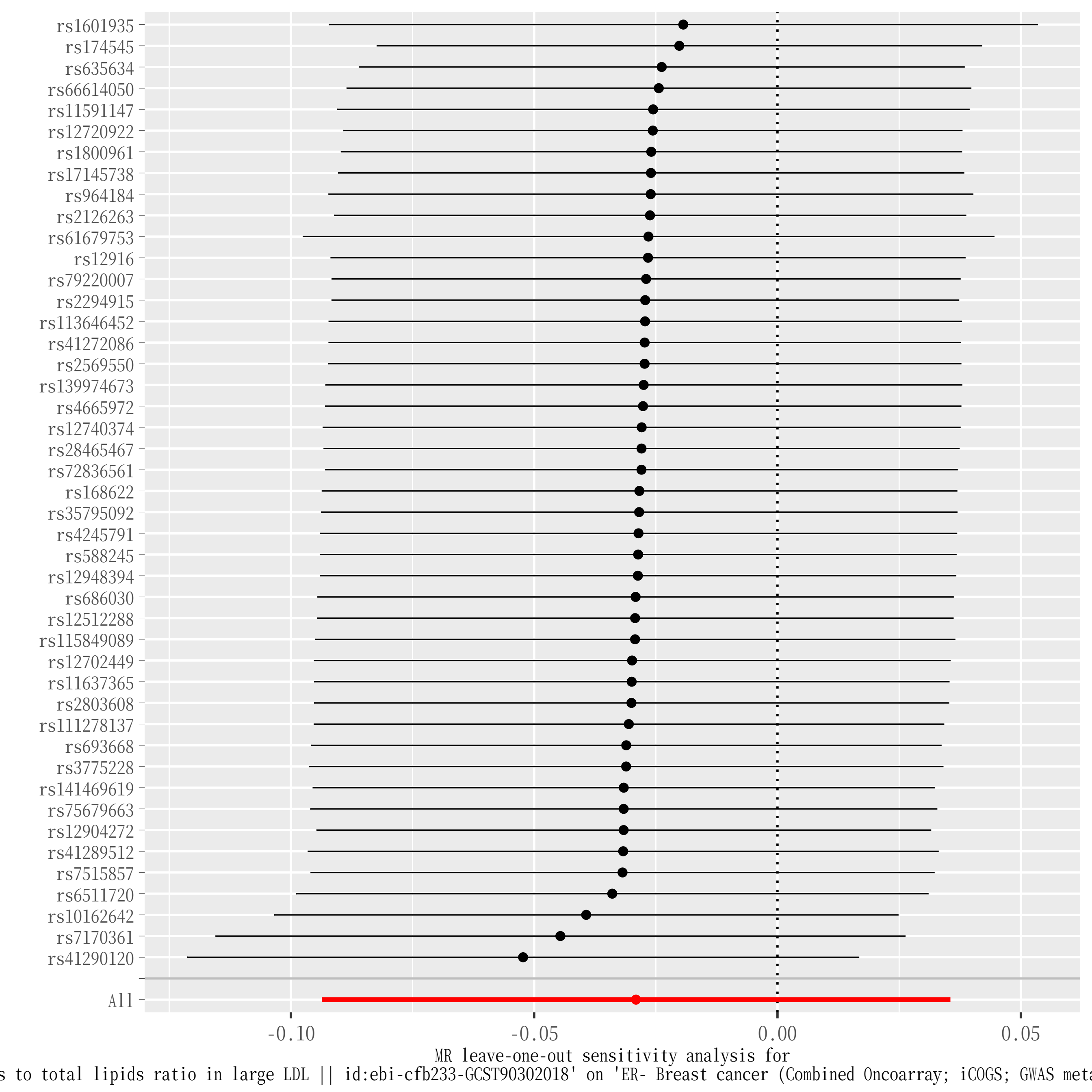
rs1601935
rs174545
rs635634
rs66614050
rs11591147
rs12720922
rs1800961
rs17145738
rs964184
rs2126263
rs61679753
rs12916
rs79220007
rs2294915
rs113646452
rs41272086
rs2569550
rs139974673
rs4665972
rs12740374
rs28465467
rs72836561
rs168622
rs35795092
rs4245791
rs588245
rs12948394
rs686030
rs12512288
rs115849089
rs12702449
rs11637365
rs2803608
rs111278137
rs693668
rs3775228
rs141469619
rs75679663
rs12904272
rs41289512
rs7515857
rs6511720
rs10162642
rs7170361
rs41290120
All
-0.10
-0.05
0.00
0.05
MR leave-one-out sensitivity analysis for
s to total lipids ratio in large LDL || id:ebi-cfb233-GCST90302018' on 'ER- Breast cancer (Combined Oncoarray; iCOGS; GWAS meta

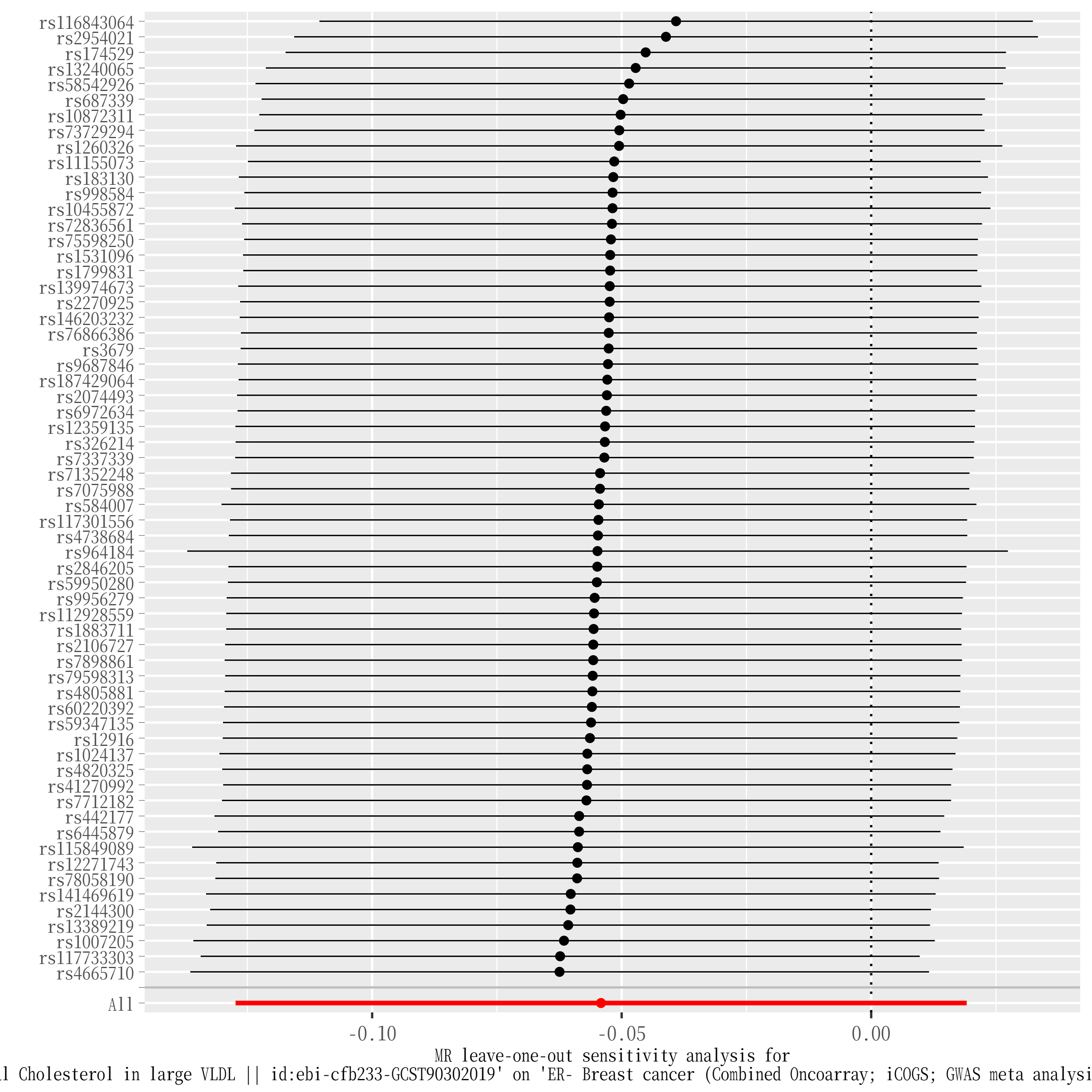

rs116843064
rs2954021
rs174529
rs13240065
rs58542926
rs687339
rs10872311
rs73729294
rs1260326
rs11155073
rs183130
rs998584
rs10455872
rs72836561
rs75598250
rs1531096
rs1799831
rs139974673
rs2270925
rs146203232
rs76866386
rs3679
rs9687846
rs187429064
rs2074493
rs6972634
rs12359135
rs326214
rs7337339
rs71352248
rs7075988
rs584007
rs117301556
rs4738684
rs964184
rs2846205
rs59950280
rs9956279
rs112928559
rs1883711
rs2106727
rs7898861
rs79598313
rs4805881
rs60220392
rs59347135
rs12916
rs1024137
rs4820325
rs41270992
rs7712182
rs442177
rs6445879
rs115849089
rs12271743
rs78058190
rs141469619
rs2144300
rs13389219
rs1007205
rs117733303
rs4665710
All
-0.10
-0.05
0.00
MR leave-one-out sensitivity analysis for
l Cholesterol in large VLDL || id:ebi-cfb233-GCST90302019' on 'ER- Breast cancer (Combined Oncoarray; iCOGS; GWAS meta analysi

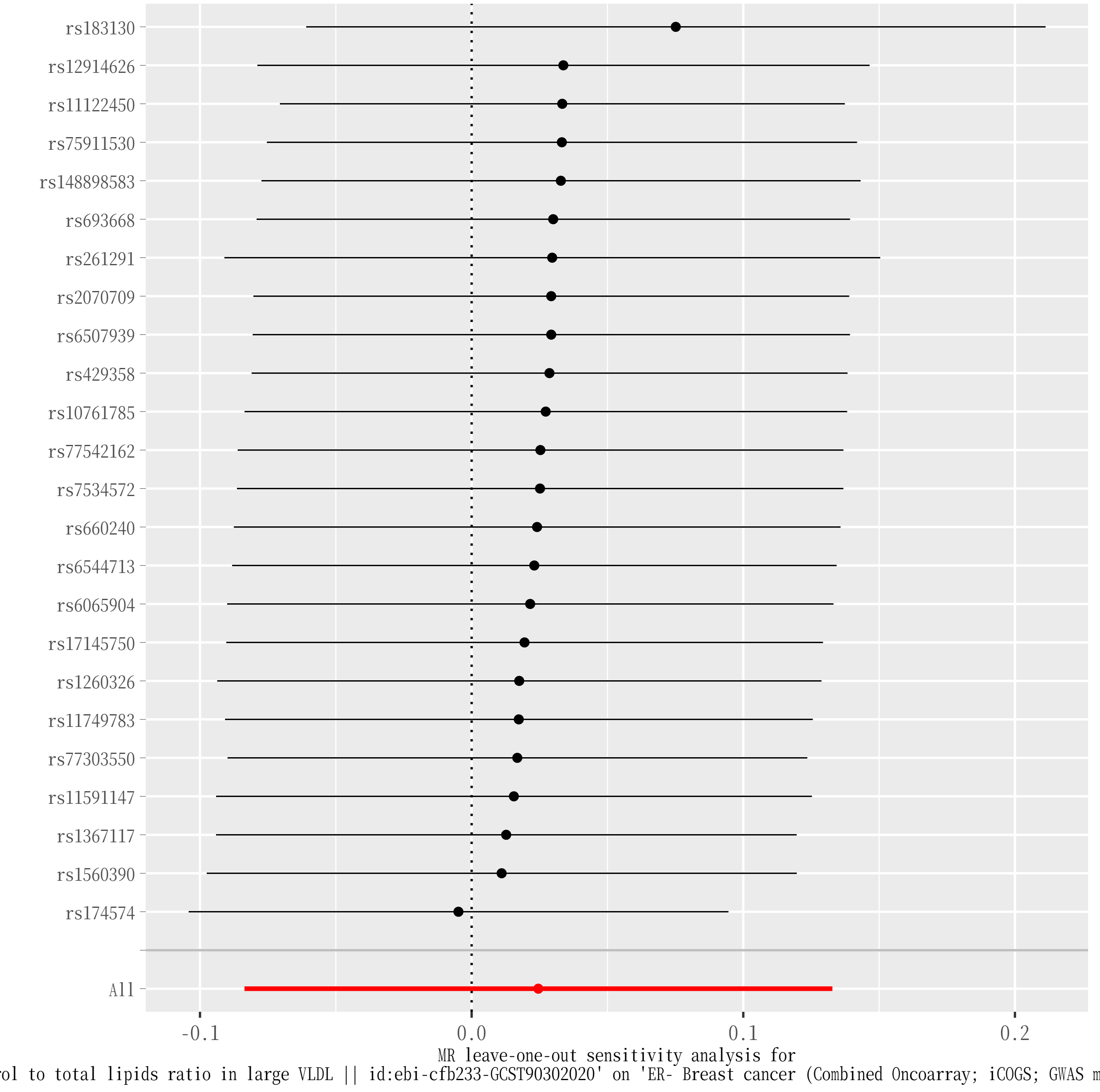
rs183130
rs12914626
rs11122450
rs75911530
rs148898583
rs693668
rs261291
rs2070709
rs6507939
rs429358
rs10761785
rs77542162
rs7534572
rs660240
rs6544713
rs6065904
rs17145750
rs1260326
rs11749783
rs77303550
rs11591147
rs1367117
rs1560390
rs174574
All
-0.1
0.0
0.1
0.2
MR leave-one-out sensitivity analysis for
rol to total lipids ratio in large VLDL || id:ebi-cfb233-GCST90302020' on 'ER- Breast cancer (Combined Oncoarray; iCOGS; GWAS m

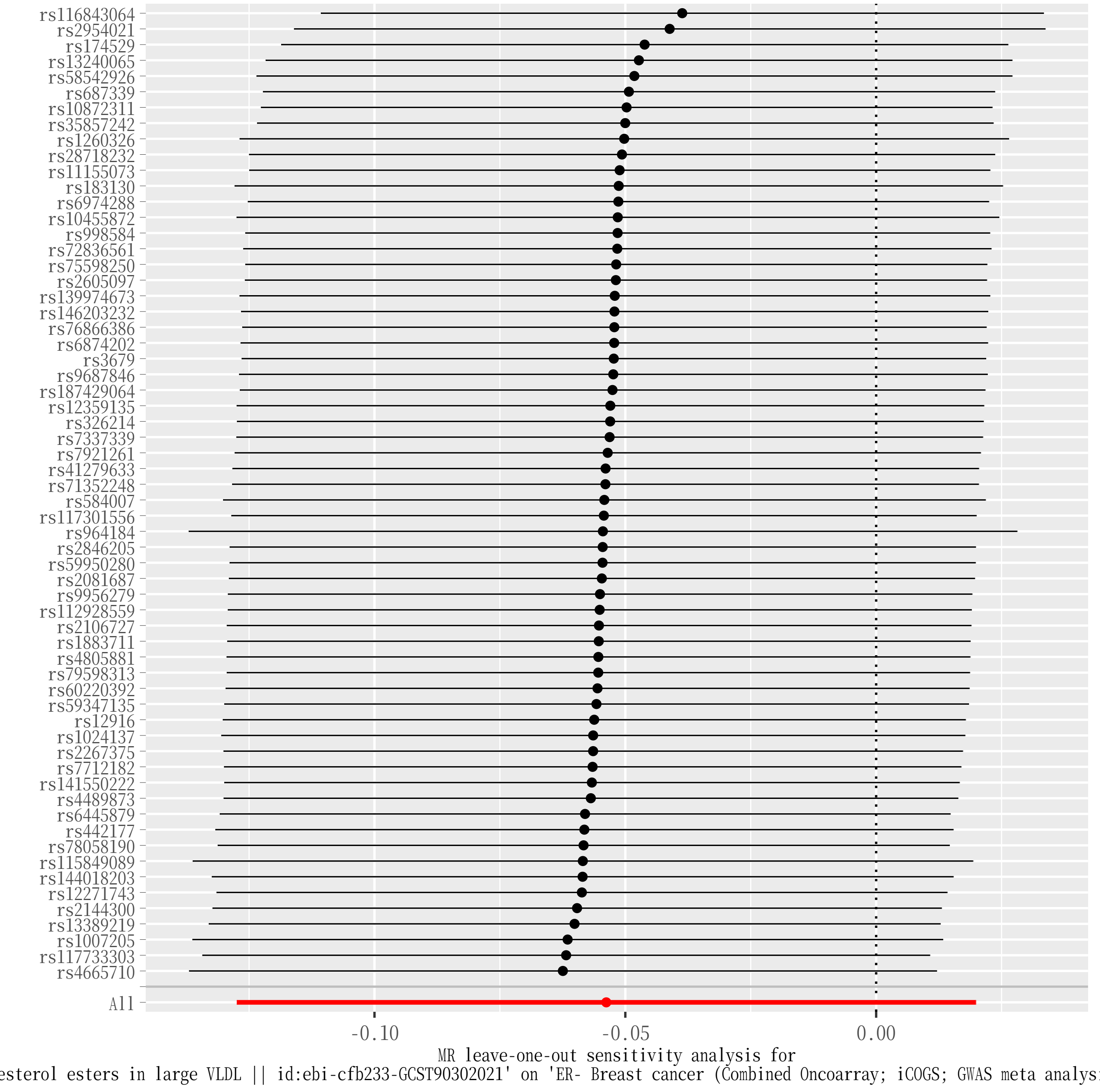
rs116843064
rs2954021
rs174529
rs13240065
rs58542926
rs687339
rs10872311
rs35857242
rs1260326
rs28718232
rs11155073
rs183130
rs6974288
rs10455872
rs998584
rs72836561
rs75598250
rs2605097
rs139974673
rs146203232
rs76866386
rs6874202
rs3679
rs9687846
rs187429064
rs12359135
rs326214
rs7337339
rs7921261
rs41279633
rs71352248
rs584007
rs117301556
rs964184
rs2846205
rs59950280
rs2081687
rs9956279
rs112928559
rs2106727
rs1883711
rs4805881
rs79598313
rs60220392
rs59347135
rs12916
rs1024137
rs2267375
rs7712182
rs141550222
rs4489873
rs6445879
rs442177
rs78058190
rs115849089
rs144018203
rs12271743
rs2144300
rs13389219
rs1007205
rs117733303
rs4665710
All
-0.10
-0.05
0.00
MR leave-one-out sensitivity analysis for
esterol esters in large VLDL || id:ebi-cfb233-GCST90302021' on 'ER- Breast cancer (Combined Oncoarray; iCOGS; GWAS meta analys

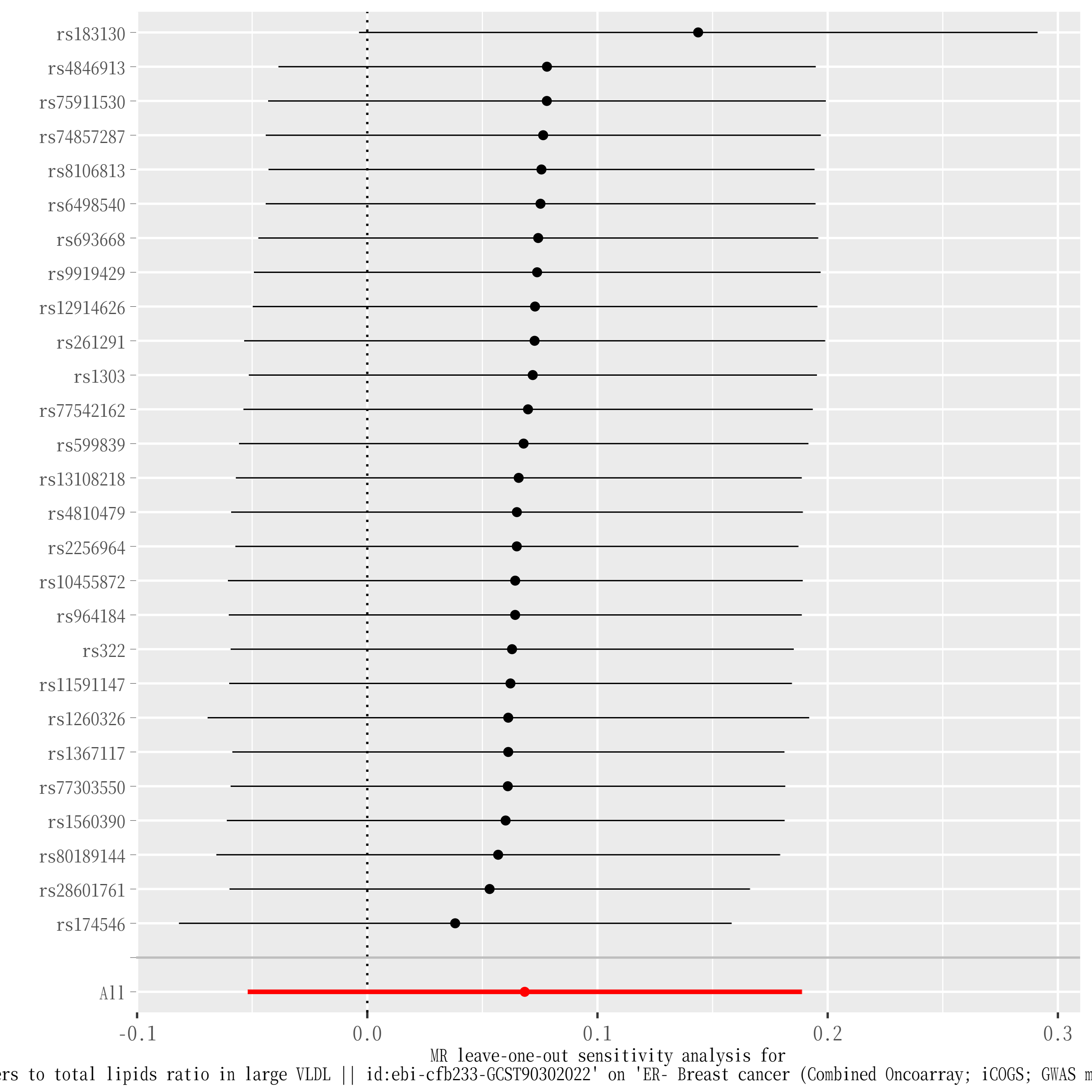
rs183130
rs4846913
rs75911530
rs74857287
rs8106813
rs6498540
rs693668
rs9919429
rs12914626
rs261291
rs1303
rs77542162
rs599839
rs13108218
rs4810479
rs2256964
rs10455872
rs964184
rs322
rs11591147
rs1260326
rs1367117
rs77303550
rs1560390
rs80189144
rs28601761
rs174546
All
-0.1
0.0
0.1
0.2
0.3
MR leave-one-out sensitivity analysis for
ers to total lipids ratio in large VLDL || id:ebi-cfb233-GCST90302022' on 'ER- Breast cancer (Combined Oncoarray; iCOGS; GWAS m

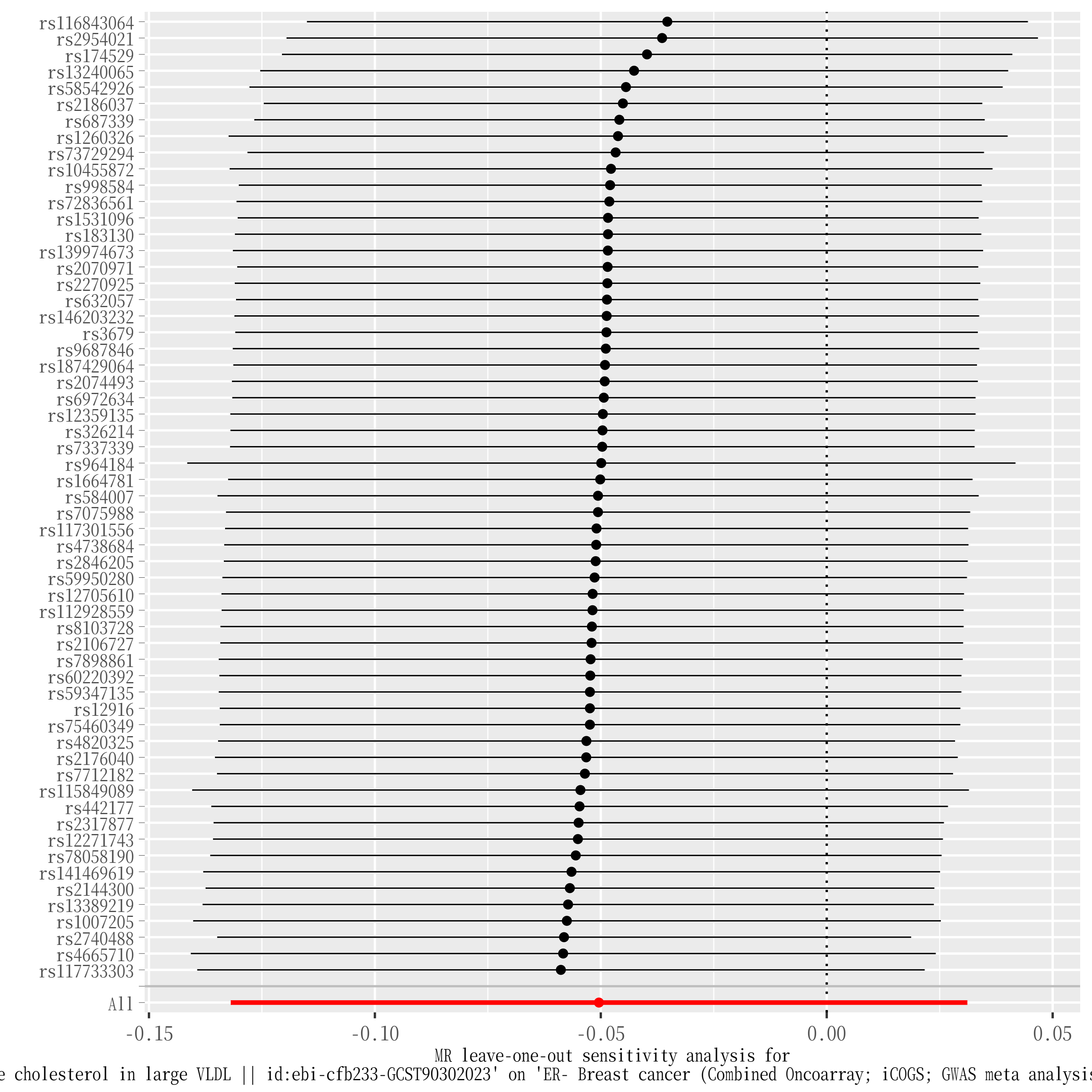
rs116843064
rs2954021
rs174529
rs13240065
rs58542926
rs2186037
rs687339
rs1260326
rs73729294
rs10455872
rs998584
rs72836561
rs1531096
rs183130
rs139974673
rs2070971
rs2270925
rs632057
rs146203232
rs3679
rs9687846
rs187429064
rs2074493
rs6972634
rs12359135
rs326214
rs7337339
rs964184
rs1664781
rs584007
rs7075988
rs117301556
rs4738684
rs2846205
rs59950280
rs12705610
rs112928559
rs8103728
rs2106727
rs7898861
rs60220392
rs59347135
rs12916
rs75460349
rs4820325
rs2176040
rs7712182
rs115849089
rs442177
rs2317877
rs12271743
rs78058190
rs141469619
rs2144300
rs13389219
rs1007205
rs2740488
rs4665710
rs117733303
All
-0.15
-0.10
-0.05
0.00
0.05
MR leave-one-out sensitivity analysis for
e cholesterol in large VLDL || id:ebi-cfb233-GCST90302023' on 'ER- Breast cancer (Combined Oncoarray; iCOGS; GWAS meta analysis

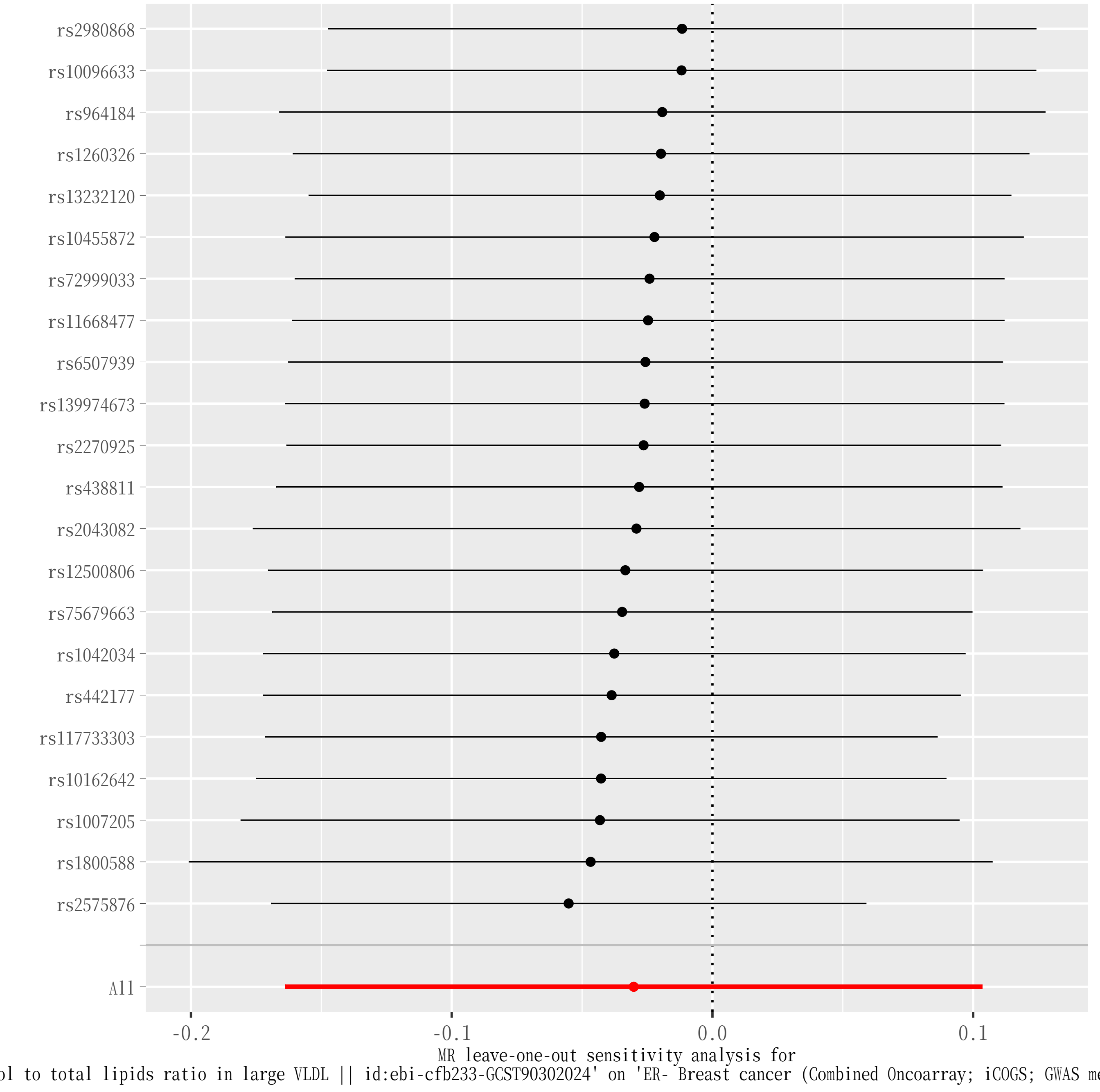

rs2980868
rs10096633
rs964184
rs1260326
rs13232120
rs10455872
rs72999033
rs11668477
rs6507939
rs139974673
rs2270925
rs438811
rs2043082
rs12500806
rs75679663
rs1042034
rs442177
rs117733303
rs10162642
rs1007205
rs1800588
rs2575876
All
-0.2
-0.1
0.0
0.1
MR leave-one-out sensitivity analysis for
ol to total lipids ratio in large VLDL || id:ebi-cfb233-GCST90302024' on 'ER- Breast cancer (Combined Oncoarray; iCOGS; GWAS me

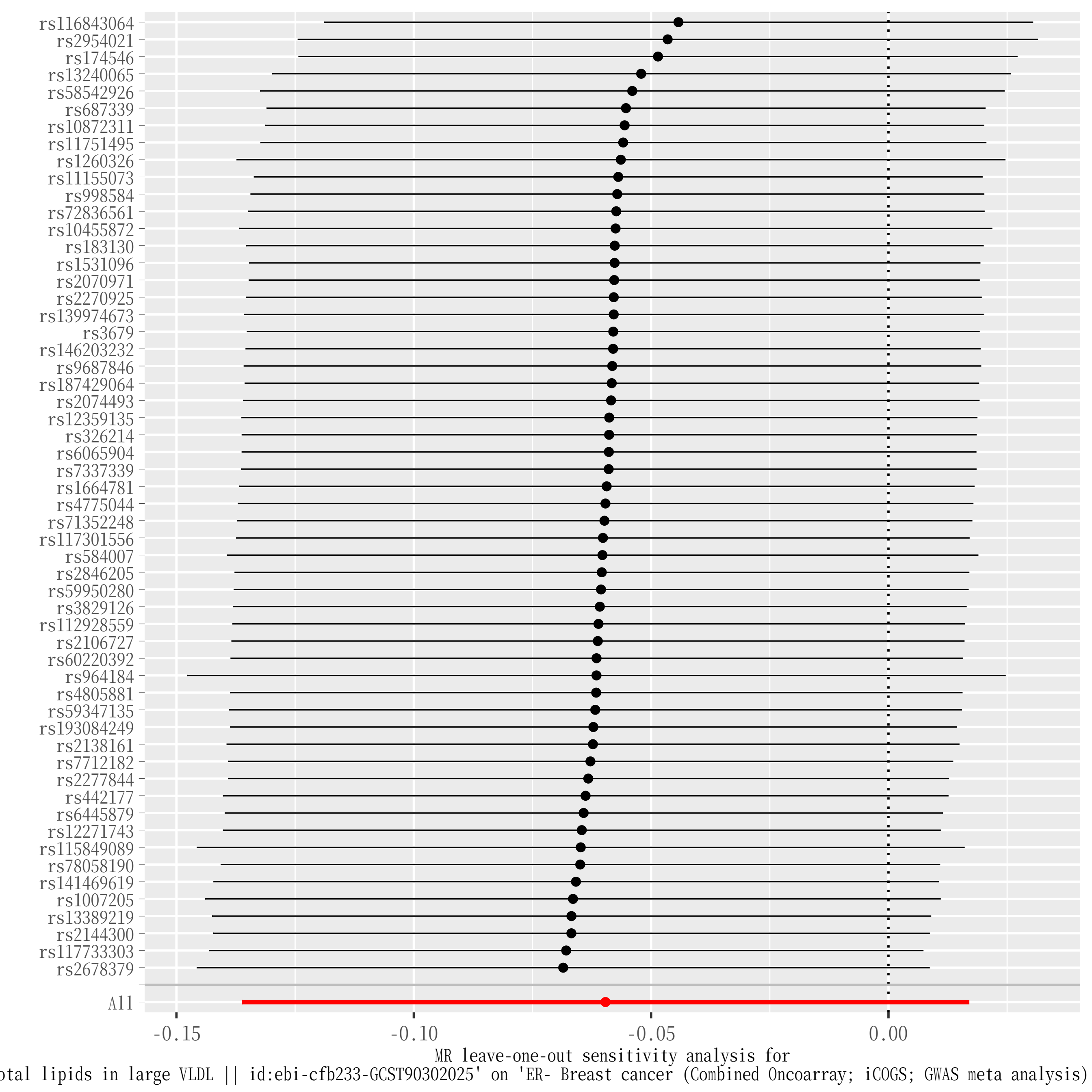

rs116843064
rs2954021
rs174546
rs13240065
rs58542926
rs687339
rs10872311
rs11751495
rs1260326
rs11155073
rs998584
rs72836561
rs10455872
rs183130
rs1531096
rs2070971
rs2270925
rs139974673
rs3679
rs146203232
rs9687846
rs187429064
rs2074493
rs12359135
rs326214
rs6065904
rs7337339
rs1664781
rs4775044
rs71352248
rs117301556
rs584007
rs2846205
rs59950280
rs3829126
rs112928559
rs2106727
rs60220392
rs964184
rs4805881
rs59347135
rs193084249
rs2138161
rs7712182
rs2277844
rs442177
rs6445879
rs12271743
rs115849089
rs78058190
rs141469619
rs1007205
rs13389219
rs2144300
rs117733303
rs2678379
All
-0.15
-0.10
-0.05
0.00
MR leave-one-out sensitivity analysis for
otal lipids in large VLDL || id:ebi-cfb233-GCST90302025' on 'ER- Breast cancer (Combined Oncoarray; iCOGS; GWAS meta analysis)

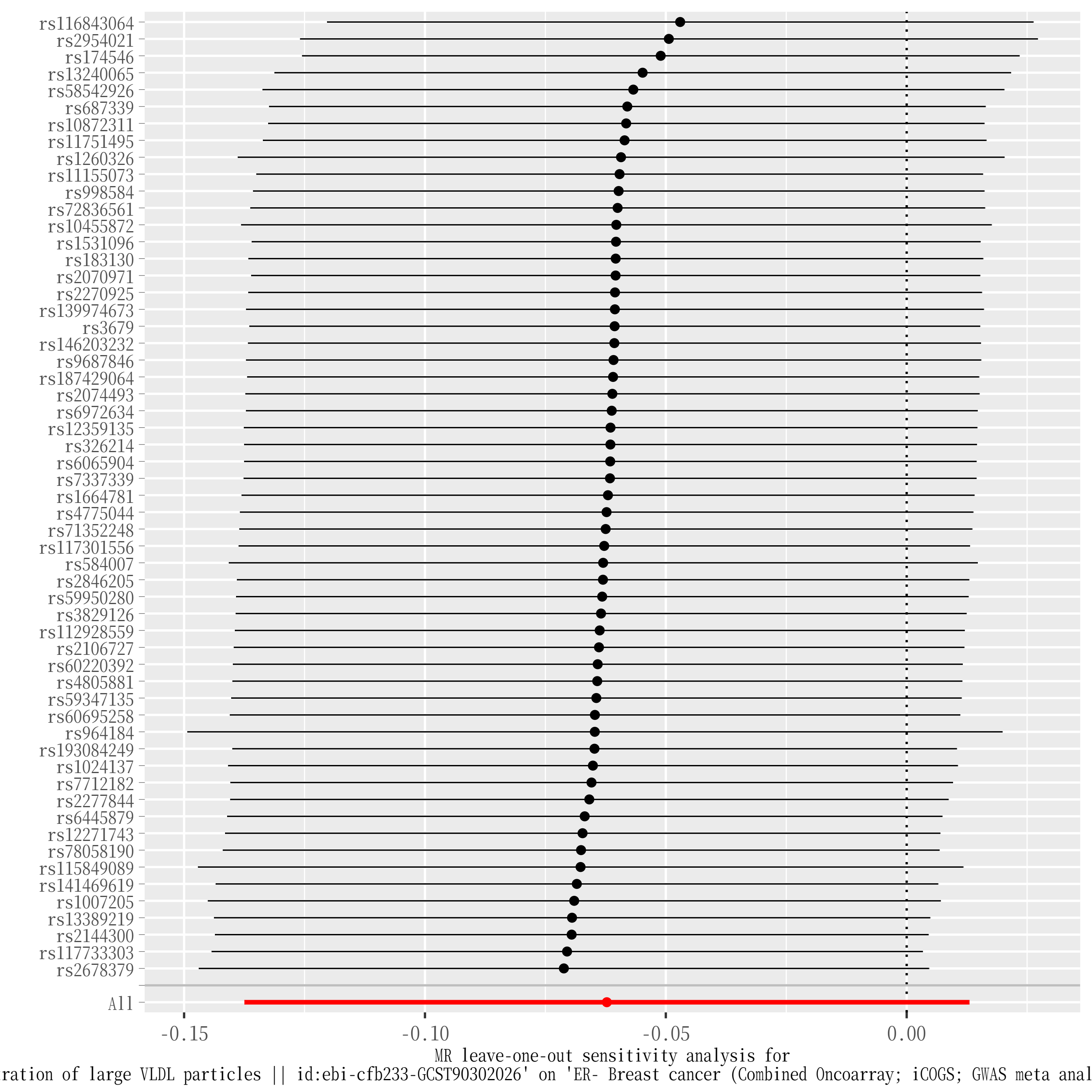
rs116843064
rs2954021
rs174546
rs13240065
rs58542926
rs687339
rs10872311
rs11751495
rs1260326
rs11155073
rs998584
rs72836561
rs10455872
rs1531096
rs183130
rs2070971
rs2270925
rs139974673
rs3679
rs146203232
rs9687846
rs187429064
rs2074493
rs6972634
rs12359135
rs326214
rs6065904
rs7337339
rs1664781
rs4775044
rs71352248
rs117301556
rs584007
rs2846205
rs59950280
rs3829126
rs112928559
rs2106727
rs60220392
rs4805881
rs59347135
rs60695258
rs964184
rs193084249
rs1024137
rs7712182
rs2277844
rs6445879
rs12271743
rs78058190
rs115849089
rs141469619
rs1007205
rs13389219
rs2144300
rs117733303
rs2678379
All
-0.15
-0.10
-0.05
0.00
MR leave-one-out sensitivity analysis for
ration of large VLDL particles || id:ebi-cfb233-GCST90302026' on 'ER- Breast cancer (Combined Oncoarray; iCOGS; GWAS meta anal

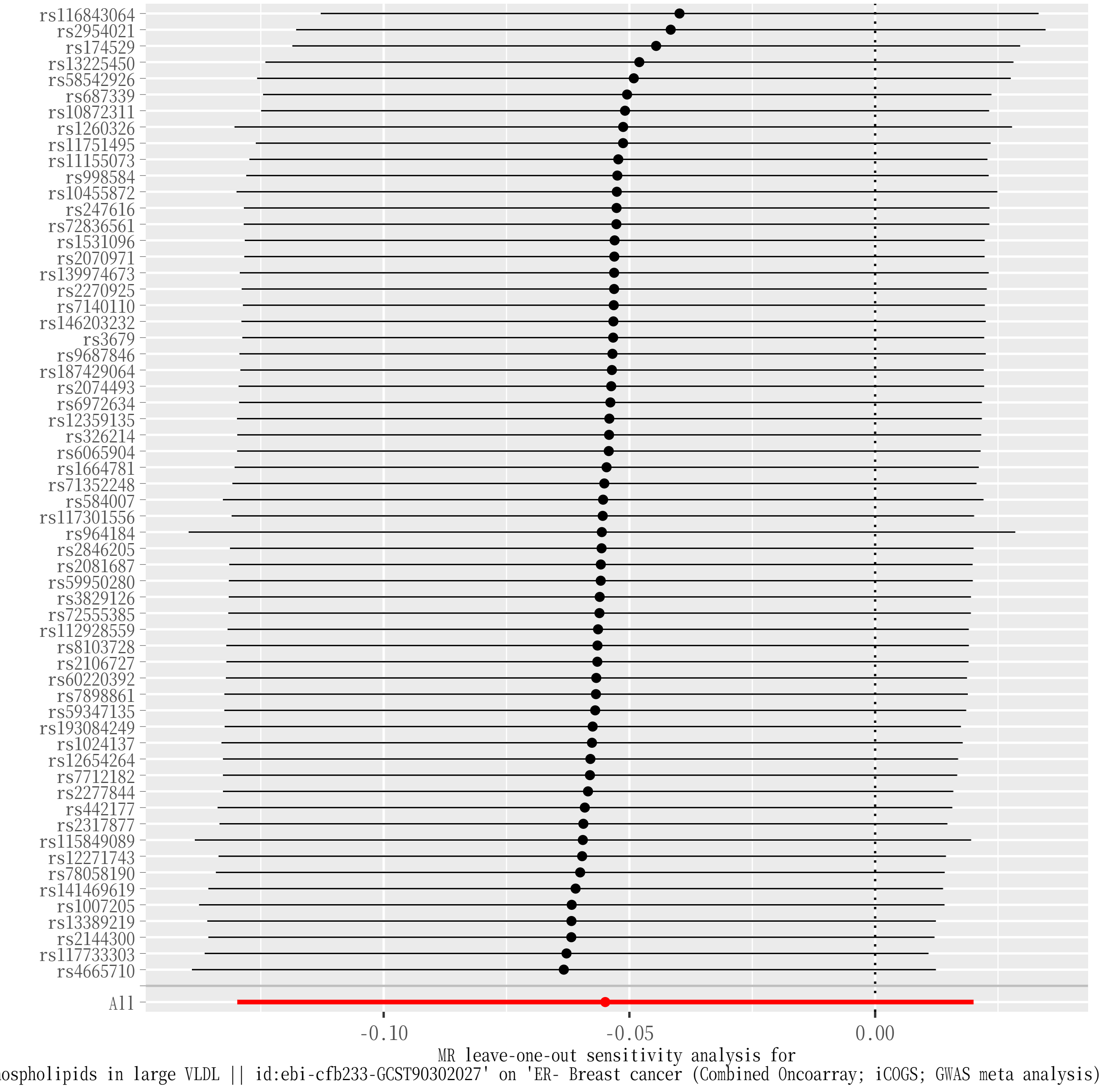
rs116843064
rs2954021
rs174529
rs13225450
rs58542926
rs687339
rs10872311
rs1260326
rs11751495
rs11155073
rs998584
rs10455872
rs247616
rs72836561
rs1531096
rs2070971
rs139974673
rs2270925
rs7140110
rs146203232
rs3679
rs9687846
rs187429064
rs2074493
rs6972634
rs12359135
rs326214
rs6065904
rs1664781
rs71352248
rs584007
rs117301556
rs964184
rs2846205
rs2081687
rs59950280
rs3829126
rs72555385
rs112928559
rs8103728
rs2106727
rs60220392
rs7898861
rs59347135
rs193084249
rs1024137
rs12654264
rs7712182
rs2277844
rs442177
rs2317877
rs115849089
rs12271743
rs78058190
rs141469619
rs1007205
rs13389219
rs2144300
rs117733303
rs4665710
All
-0.10
-0.05
0.00
MR leave-one-out sensitivity analysis for
ospholipids in large VLDL || id:ebi-cfb233-GCST90302027' on 'ER- Breast cancer (Combined Oncoarray; iCOGS; GWAS meta analysis)

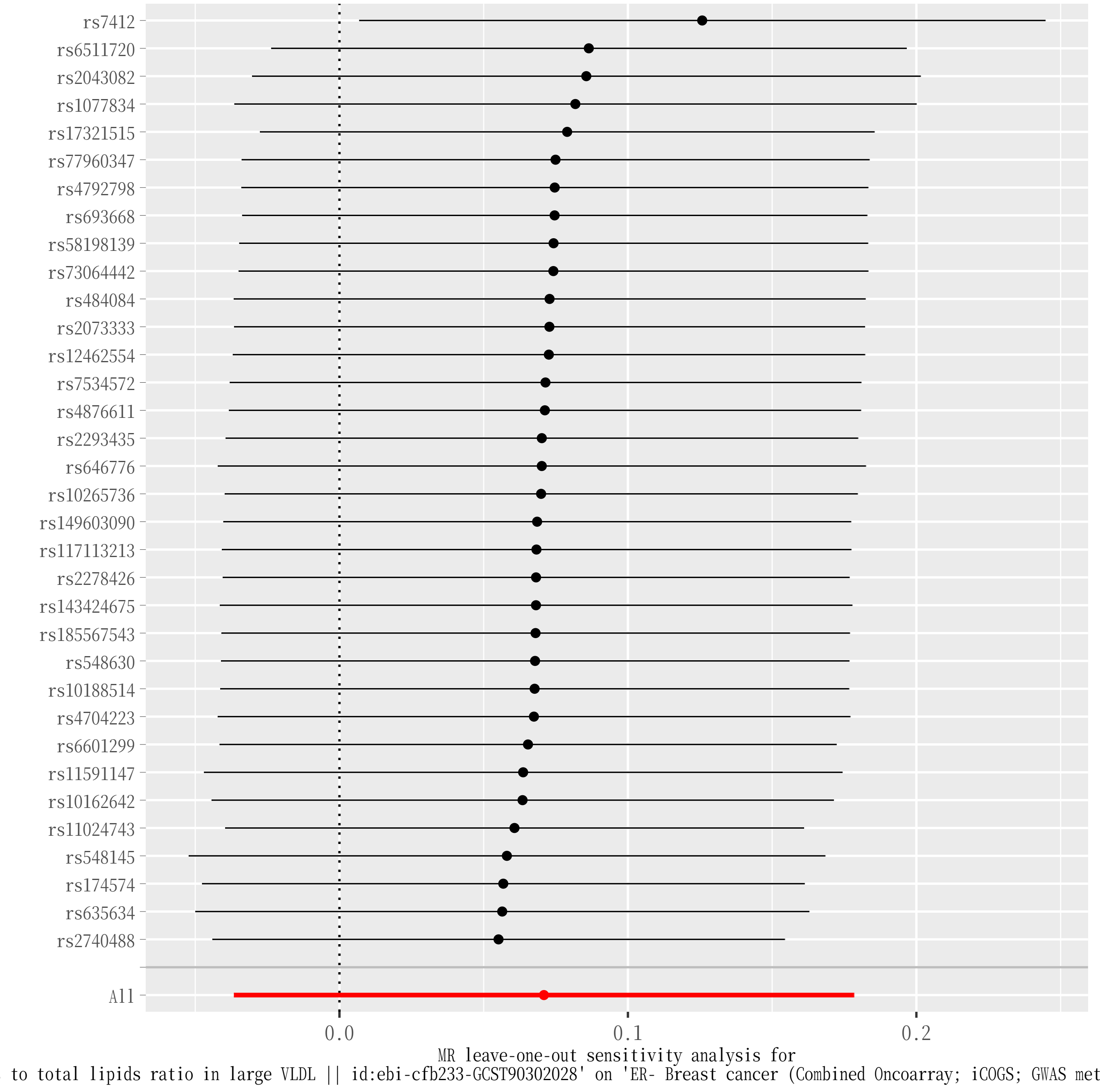

rs7412
rs6511720
rs2043082
rs1077834
rs17321515
rs77960347
rs4792798
rs693668
rs58198139
rs73064442
rs484084
rs2073333
rs12462554
rs7534572
rs4876611
rs2293435
rs646776
rs10265736
rs149603090
rs117113213
rs2278426
rs143424675
rs185567543
rs548630
rs10188514
rs4704223
rs6601299
rs11591147
rs10162642
rs11024743
rs548145
rs174574
rs635634
rs2740488
All
0.0
0.1
0.2
MR leave-one-out sensitivity analysis for
to total lipids ratio in large VLDL || id:ebi-cfb233-GCST90302028' on 'ER- Breast cancer (Combined Oncoarray; iCOGS; GWAS met

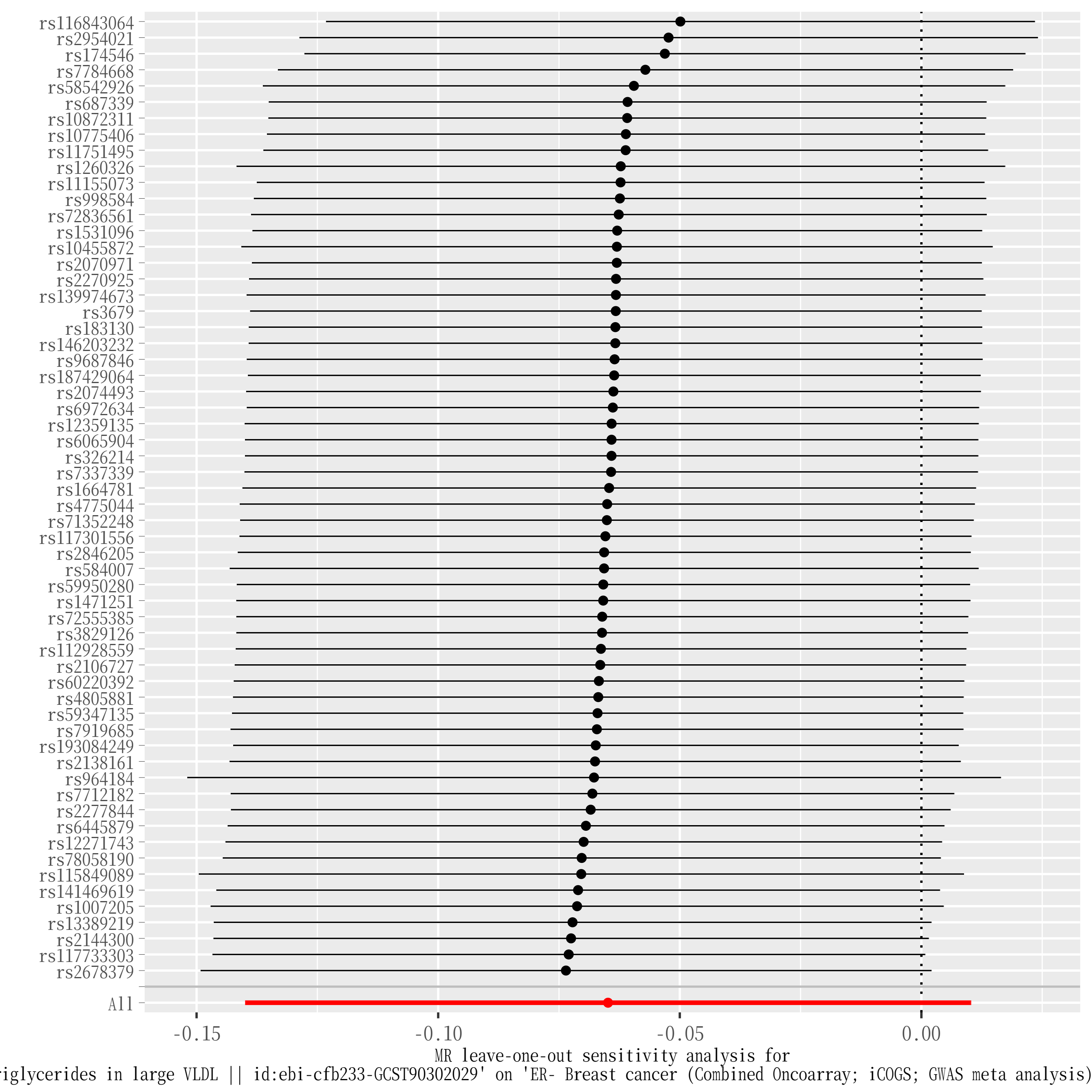
rs116843064
rs2954021
rs174546
rs7784668
rs58542926
rs687339
rs10872311
rs10775406
rs11751495
rs1260326
rs11155073
rs998584
rs72836561
rs1531096
rs10455872
rs2070971
rs2270925
rs139974673
rs3679
rs183130
rs146203232
rs9687846
rs187429064
rs2074493
rs6972634
rs12359135
rs6065904
rs326214
rs7337339
rs1664781
rs4775044
rs71352248
rs117301556
rs2846205
rs584007
rs59950280
rs1471251
rs72555385
rs3829126
rs112928559
rs2106727
rs60220392
rs4805881
rs59347135
rs7919685
rs193084249
rs2138161
rs964184
rs7712182
rs2277844
rs6445879
rs12271743
rs78058190
rs115849089
rs141469619
rs1007205
rs13389219
rs2144300
rs117733303
rs2678379
All
-0.15
-0.10
-0.05
0.00
MR leave-one-out sensitivity analysis for
riglycerides in large VLDL || id:ebi-cfb233-GCST90302029' on 'ER- Breast cancer (Combined Oncoarray; iCOGS; GWAS meta analysis)

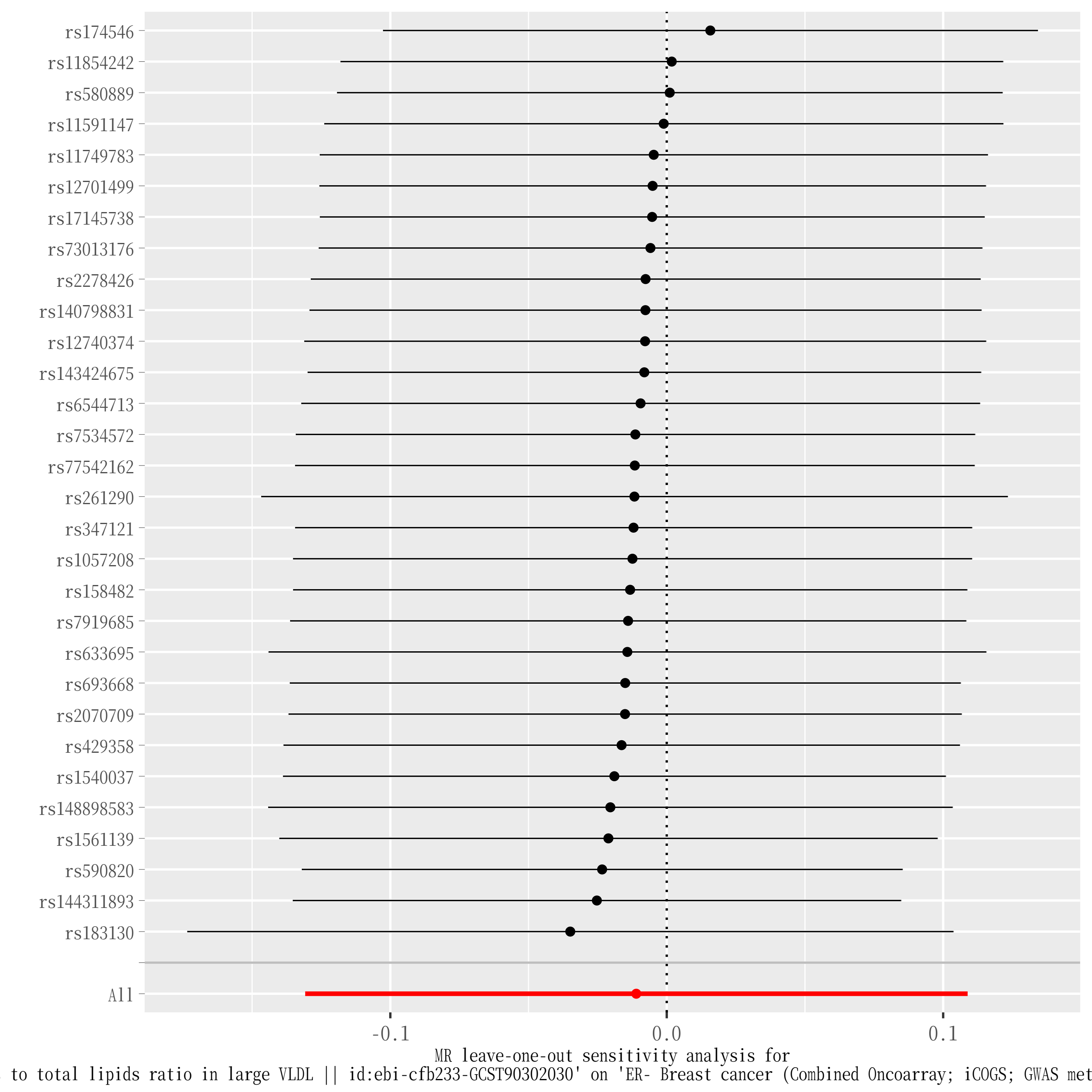
rs174546
rs11854242
rs580889
rs11591147
rs11749783
rs12701499
rs17145738
rs73013176
rs2278426
rs140798831
rs12740374
rs143424675
rs6544713
rs7534572
rs77542162
rs261290
rs347121
rs1057208
rs158482
rs7919685
rs633695
rs693668
rs2070709
rs429358
rs1540037
rs148898583
rs1561139
rs590820
rs144311893
rs183130
All
-0.1
0.0
0.1
MR leave-one-out sensitivity analysis for
to total lipids ratio in large VLDL || id:ebi-cfb233-GCST90302030' on 'ER- Breast cancer (Combined Oncoarray; iCOGS; GWAS met

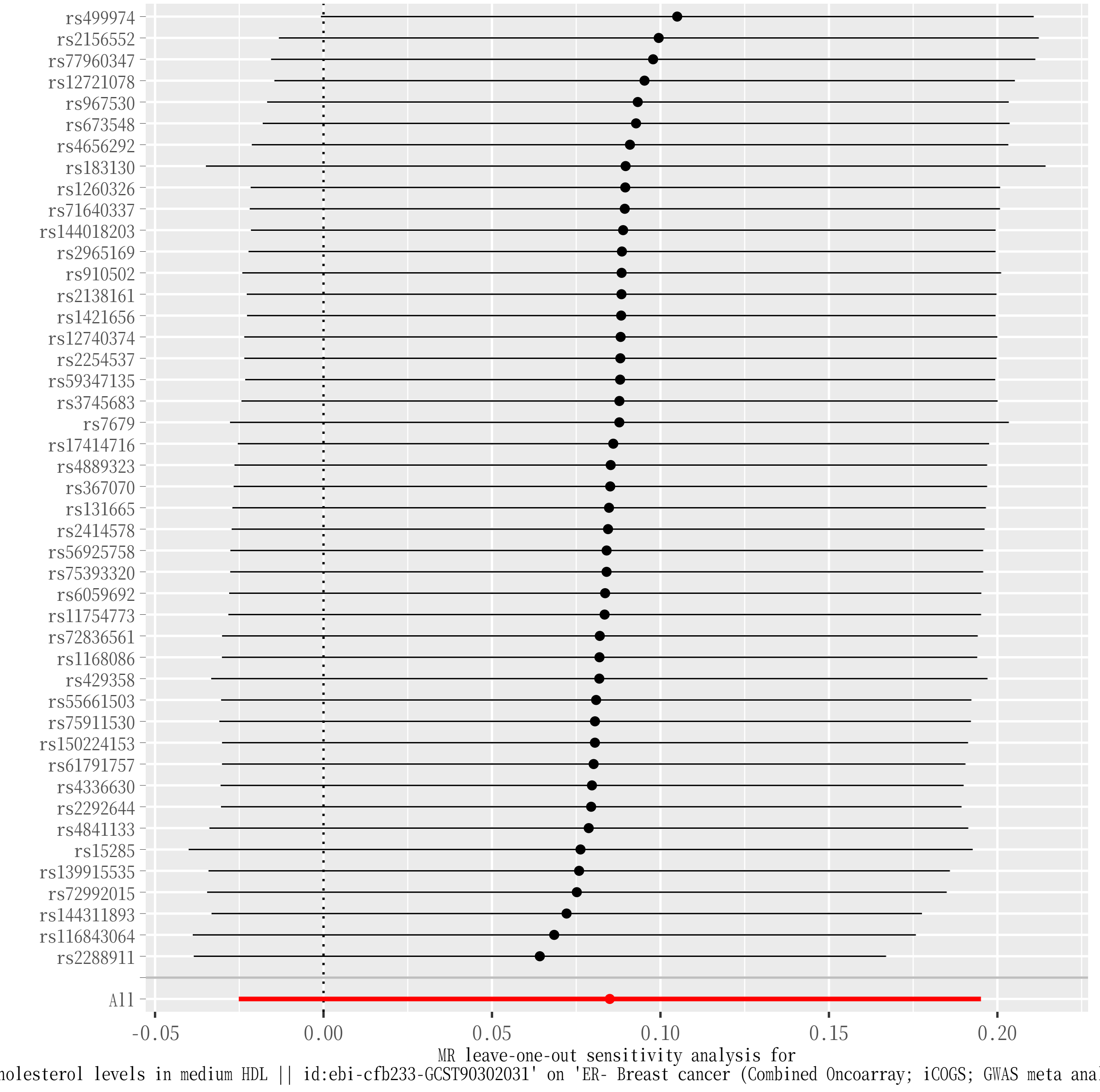

rs499974
rs2156552
rs77960347
rs12721078
rs967530
rs673548
rs4656292
rs183130
rs1260326
rs71640337
rs144018203
rs2965169
rs910502
rs2138161
rs1421656
rs12740374
rs2254537
rs59347135
rs3745683
rs7679
rs17414716
rs4889323
rs367070
rs131665
rs2414578
rs56925758
rs75393320
rs6059692
rs11754773
rs72836561
rs1168086
rs429358
rs55661503
rs75911530
rs150224153
rs61791757
rs4336630
rs2292644
rs4841133
rs15285
rs139915535
rs72992015
rs144311893
rs116843064
rs2288911
All
-0.05
0.00
0.05
0.10
0.15
0.20
MR leave-one-out sensitivity analysis for
holesterol levels in medium HDL || id:ebi-cfb233-GCST90302031' on 'ER- Breast cancer (Combined Oncoarray; iCOGS; GWAS meta anal

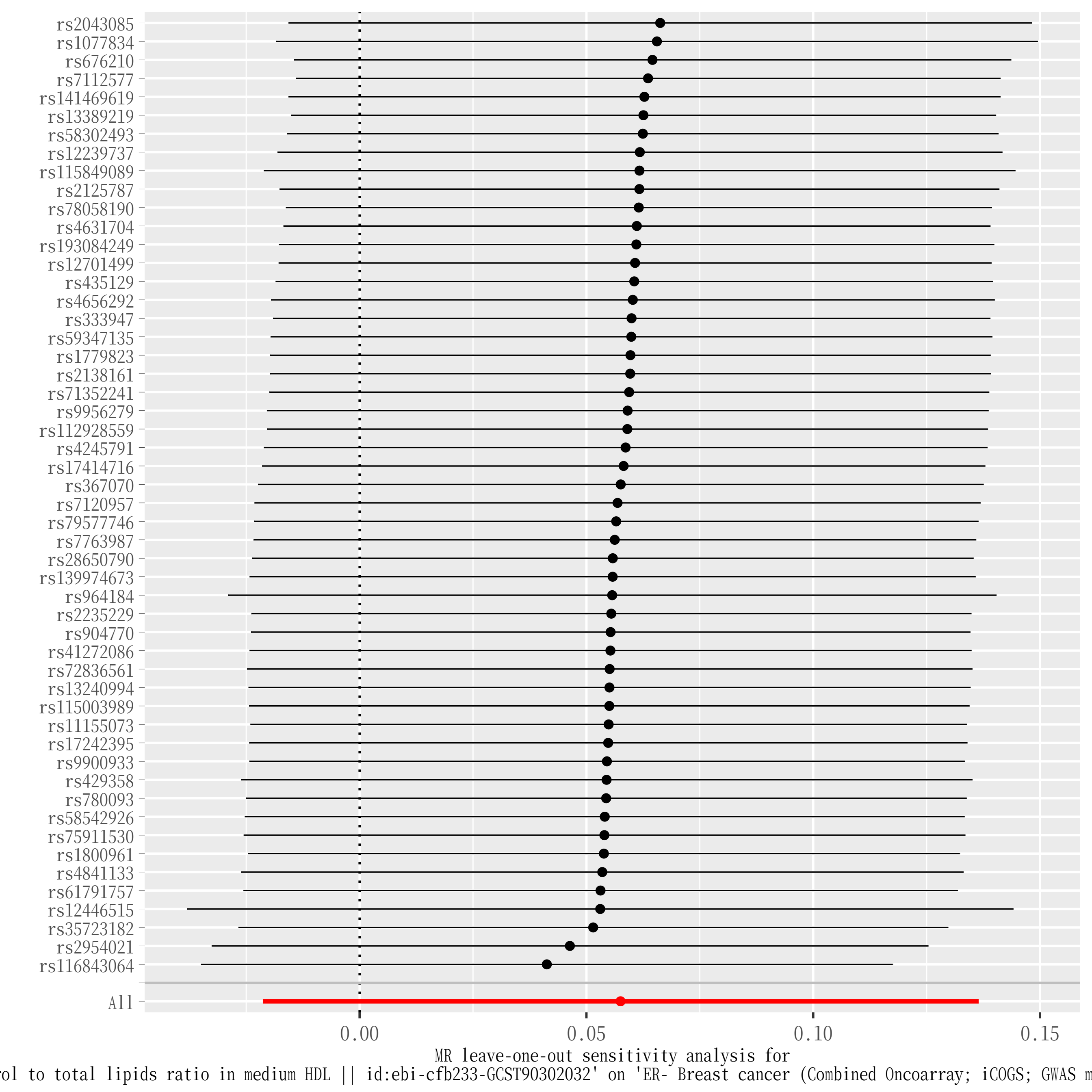

rs2043085
rs1077834
rs676210
rs7112577
rs141469619
rs13389219
rs58302493
rs12239737
rs115849089
rs2125787
rs78058190
rs4631704
rs193084249
rs12701499
rs435129
rs4656292
rs333947
rs59347135
rs1779823
rs2138161
rs71352241
rs9956279
rs112928559
rs4245791
rs17414716
rs367070
rs7120957
rs79577746
rs7763987
rs28650790
rs139974673
rs964184
rs2235229
rs904770
rs41272086
rs72836561
rs13240994
rs115003989
rs11155073
rs17242395
rs9900933
rs429358
rs780093
rs58542926
rs75911530
rs1800961
rs4841133
rs61791757
rs12446515
rs35723182
rs2954021
rs116843064
All
0.00
0.05
0.10
0.15
MR leave-one-out sensitivity analysis for
rol to total lipids ratio in medium HDL || id:ebi-cfb233-GCST90302032' on 'ER- Breast cancer (Combined Oncoarray; iCOGS; GWAS m

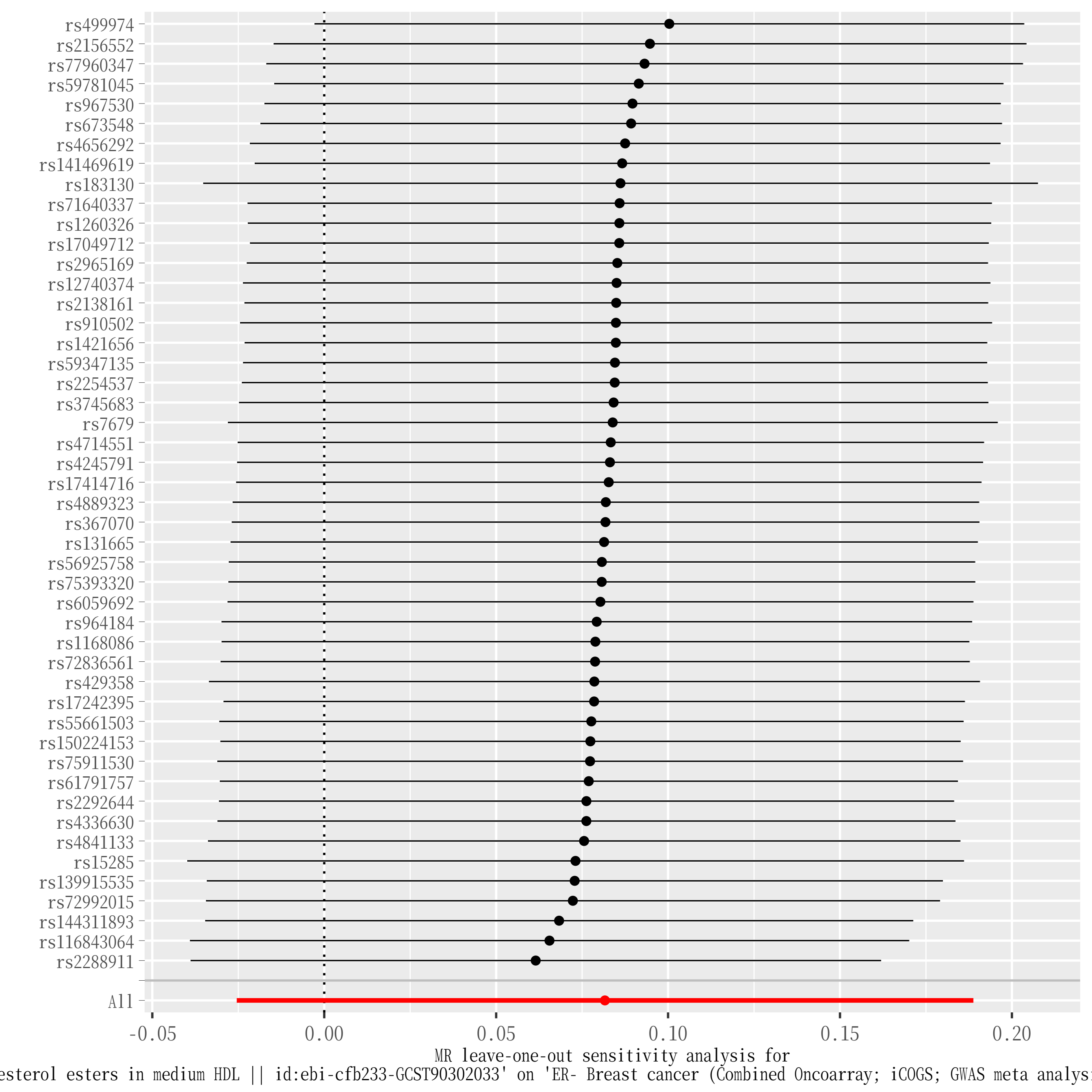
rs499974
rs2156552
rs77960347
rs59781045
rs967530
rs673548
rs4656292
rs141469619
rs183130
rs71640337
rs1260326
rs17049712
rs2965169
rs12740374
rs2138161
rs910502
rs1421656
rs59347135
rs2254537
rs3745683
rs7679
rs4714551
rs4245791
rs17414716
rs4889323
rs367070
rs131665
rs56925758
rs75393320
rs6059692
rs964184
rs1168086
rs72836561
rs429358
rs17242395
rs55661503
rs150224153
rs75911530
rs61791757
rs2292644
rs4336630
rs4841133
rs15285
rs139915535
rs72992015
rs144311893
rs116843064
rs2288911
All
-0.05
0.00
0.05
0.10
0.15
0.20
MR leave-one-out sensitivity analysis for
esterol esters in medium HDL || id:ebi-cfb233-GCST90302033' on 'ER- Breast cancer (Combined Oncoarray; iCOGS; GWAS meta analys

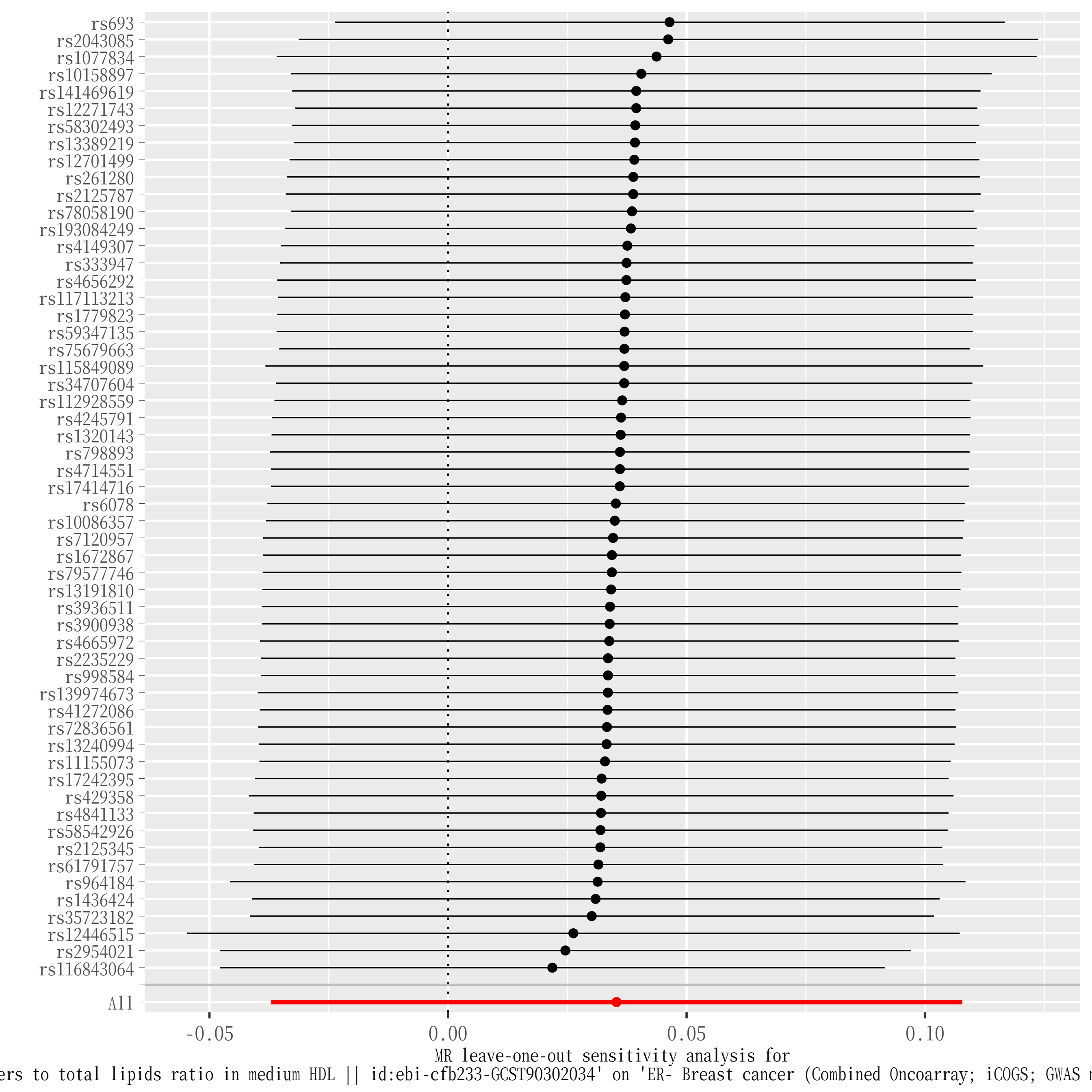

rs693
rs2043085
rs1077834
rs10158897
rs141469619
rs12271743
rs58302493
rs13389219
rs12701499
rs261280
rs2125787
rs78058190
rs193084249
rs4149307
rs333947
rs4656292
rs117113213
rs1779823
rs59347135
rs75679663
rs115849089
rs34707604
rs112928559
rs4245791
rs1320143
rs798893
rs4714551
rs17414716
rs6078
rs10086357
rs7120957
rs1672867
rs79577746
rs13191810
rs3936511
rs3900938
rs4665972
rs2235229
rs998584
rs139974673
rs41272086
rs72836561
rs13240994
rs11155073
rs17242395
rs429358
rs4841133
rs58542926
rs2125345
rs61791757
rs964184
rs1436424
rs35723182
rs12446515
rs2954021
rs116843064
All
-0.05
0.00
0.05
0.10
MR leave-one-out sensitivity analysis for
ers to total lipids ratio in medium HDL || id:ebi-cfb233-GCST90302034' on 'ER- Breast cancer (Combined Oncoarray; iCOGS; GWAS m

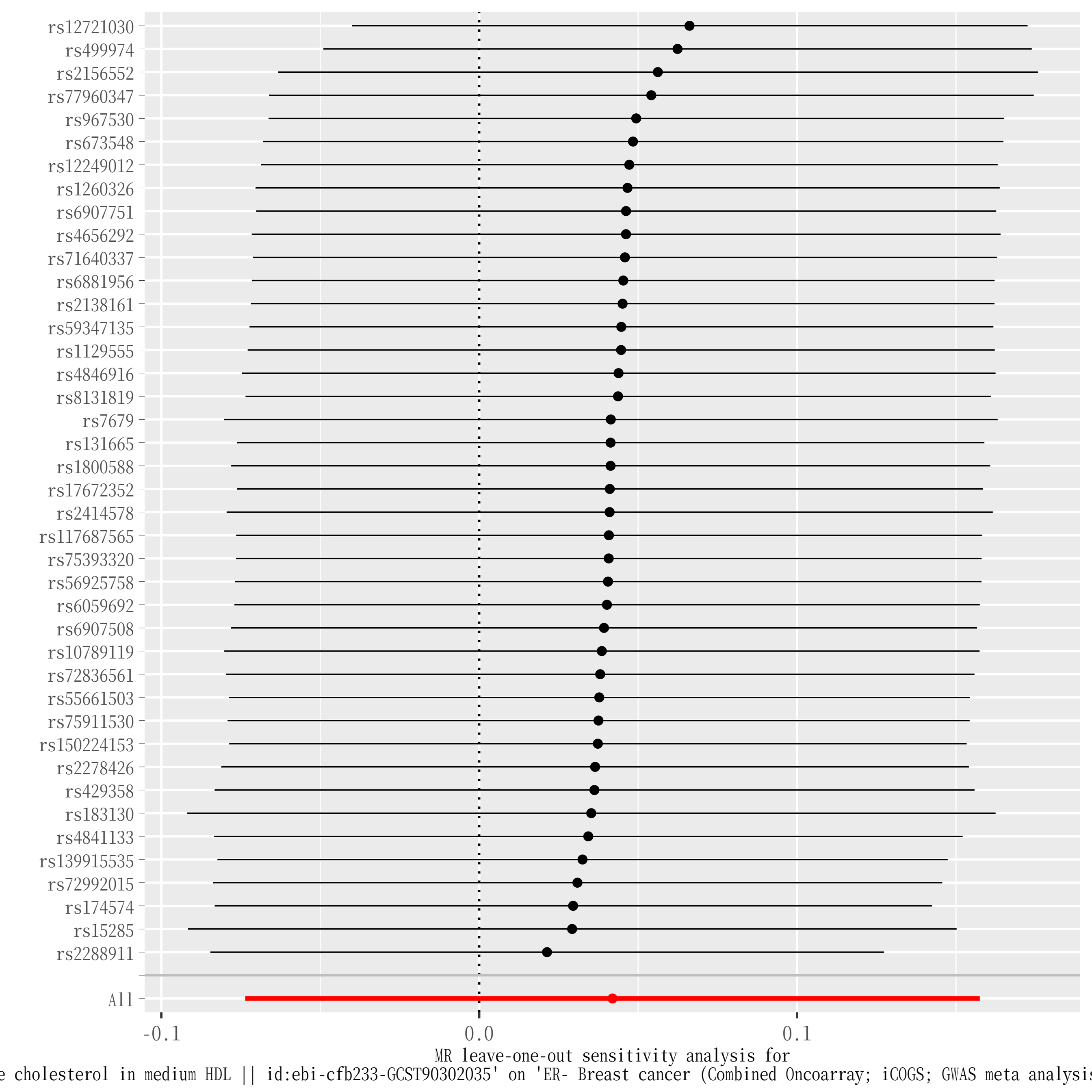
rs12721030
rs499974
rs2156552
rs77960347
rs967530
rs673548
rs12249012
rs1260326
rs6907751
rs4656292
rs71640337
rs6881956
rs2138161
rs59347135
rs1129555
rs4846916
rs8131819
rs7679
rs131665
rs1800588
rs17672352
rs2414578
rs117687565
rs75393320
rs56925758
rs6059692
rs6907508
rs10789119
rs72836561
rs55661503
rs75911530
rs150224153
rs2278426
rs429358
rs183130
rs4841133
rs139915535
rs72992015
rs174574
rs15285
rs2288911
All
-0.1
0.0
0.1
MR leave-one-out sensitivity analysis for
e cholesterol in medium HDL || id:ebi-cfb233-GCST90302035' on 'ER- Breast cancer (Combined Oncoarray; iCOGS; GWAS meta analysis

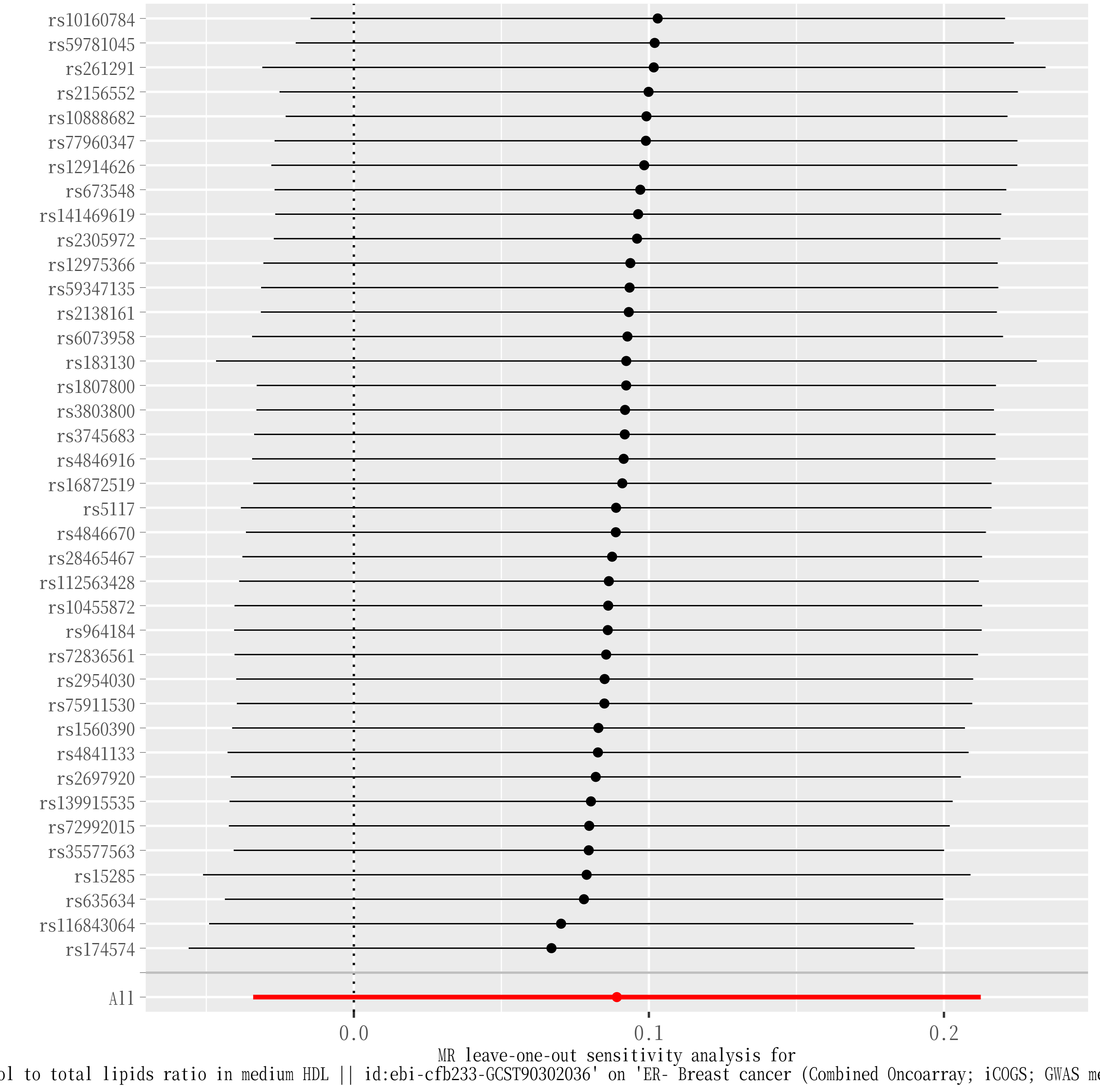

rs10160784
rs59781045
rs261291
rs2156552
rs10888682
rs77960347
rs12914626
rs673548
rs141469619
rs2305972
rs12975366
rs59347135
rs2138161
rs6073958
rs183130
rs1807800
rs3803800
rs3745683
rs4846916
rs16872519
rs5117
rs4846670
rs28465467
rs112563428
rs10455872
rs964184
rs72836561
rs2954030
rs75911530
rs1560390
rs4841133
rs2697920
rs139915535
rs72992015
rs35577563
rs15285
rs635634
rs116843064
rs174574
All
0.0
0.1
0.2
MR leave-one-out sensitivity analysis for
ol to total lipids ratio in medium HDL || id:ebi-cfb233-GCST90302036' on 'ER- Breast cancer (Combined Oncoarray; iCOGS; GWAS me

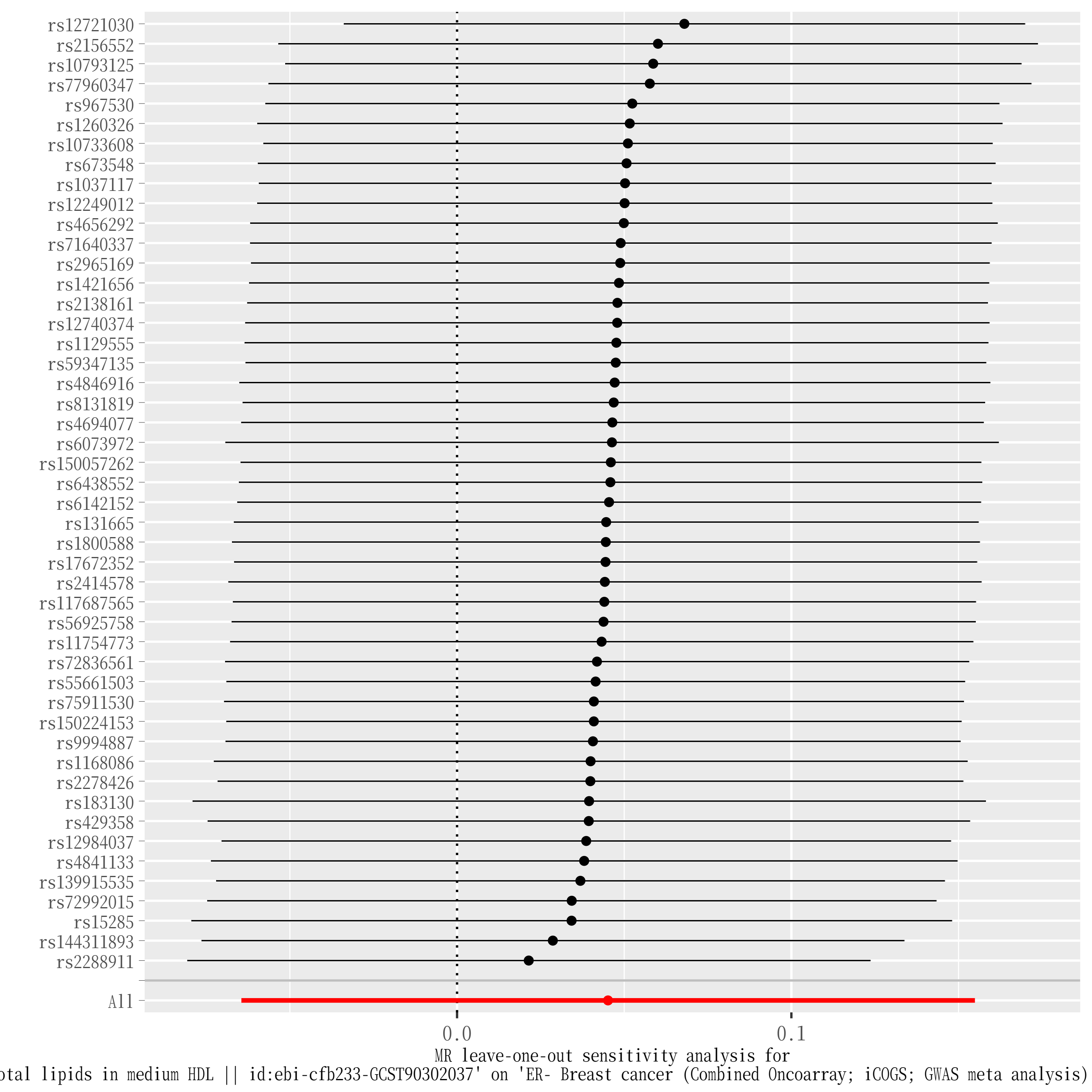
rs12721030
rs2156552
rs10793125
rs77960347
rs967530
rs1260326
rs10733608
rs673548
rs1037117
rs12249012
rs4656292
rs71640337
rs2965169
rs1421656
rs2138161
rs12740374
rs1129555
rs59347135
rs4846916
rs8131819
rs4694077
rs6073972
rs150057262
rs6438552
rs6142152
rs131665
rs1800588
rs17672352
rs2414578
rs117687565
rs56925758
rs11754773
rs72836561
rs55661503
rs75911530
rs150224153
rs9994887
rs1168086
rs2278426
rs183130
rs429358
rs12984037
rs4841133
rs139915535
rs72992015
rs15285
rs144311893
rs2288911
All
0.0
0.1
MR leave-one-out sensitivity analysis for
otal lipids in medium HDL || id:ebi-cfb233-GCST90302037' on 'ER- Breast cancer (Combined Oncoarray; iCOGS; GWAS meta analysis)

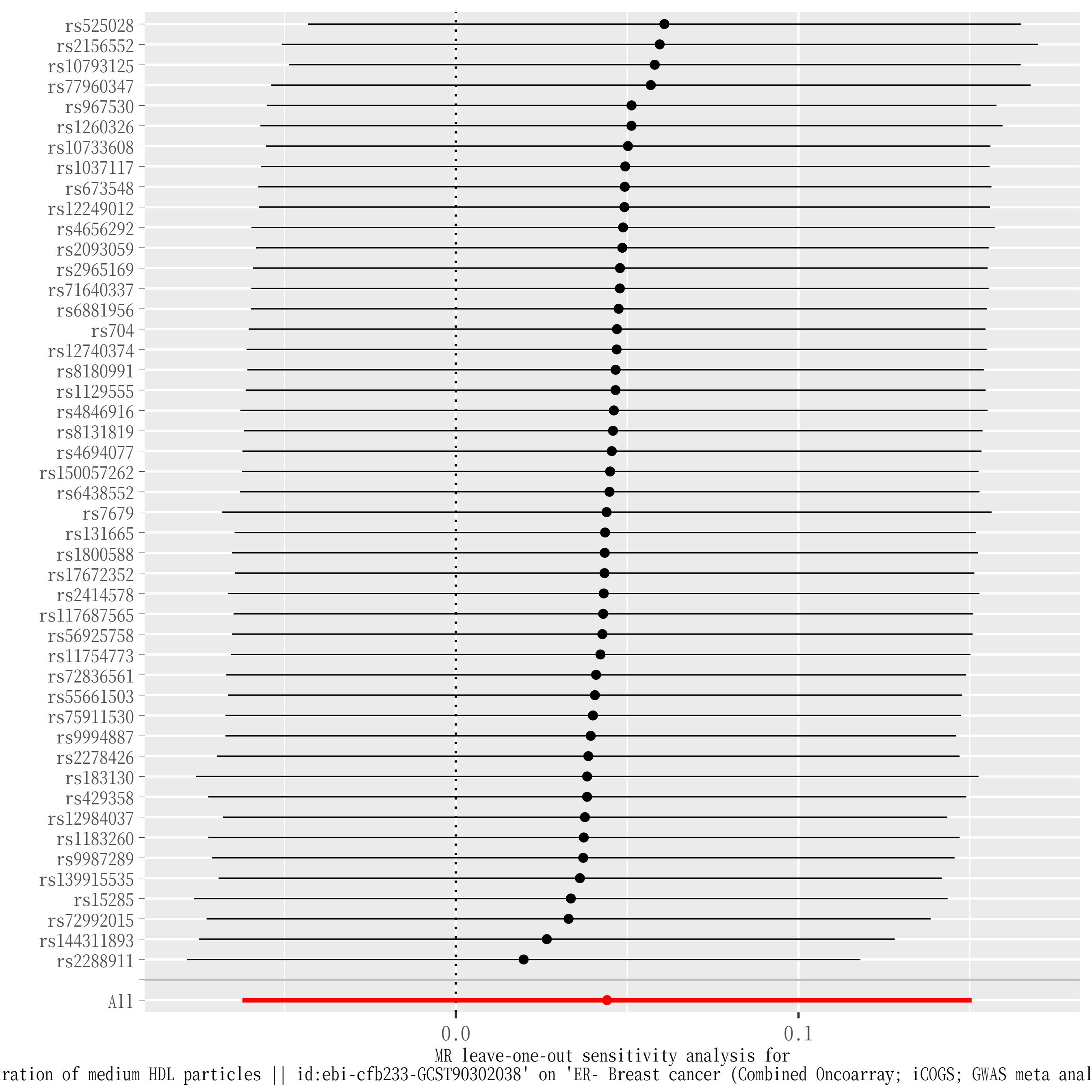
rs525028
rs2156552
rs10793125
rs77960347
rs967530
rs1260326
rs10733608
rs1037117
rs673548
rs12249012
rs4656292
rs2093059
rs2965169
rs71640337
rs6881956
rs704
rs12740374
rs8180991
rs1129555
rs4846916
rs8131819
rs4694077
rs150057262
rs6438552
rs7679
rs131665
rs1800588
rs17672352
rs2414578
rs117687565
rs56925758
rs11754773
rs72836561
rs55661503
rs75911530
rs9994887
rs2278426
rs183130
rs429358
rs12984037
rs1183260
rs9987289
rs139915535
rs15285
rs72992015
rs144311893
rs2288911
All
0.0
0.1
MR leave-one-out sensitivity analysis for
ration of medium HDL particles || id:ebi-cfb233-GCST90302038' on 'ER- Breast cancer (Combined Oncoarray; iCOGS; GWAS meta anal

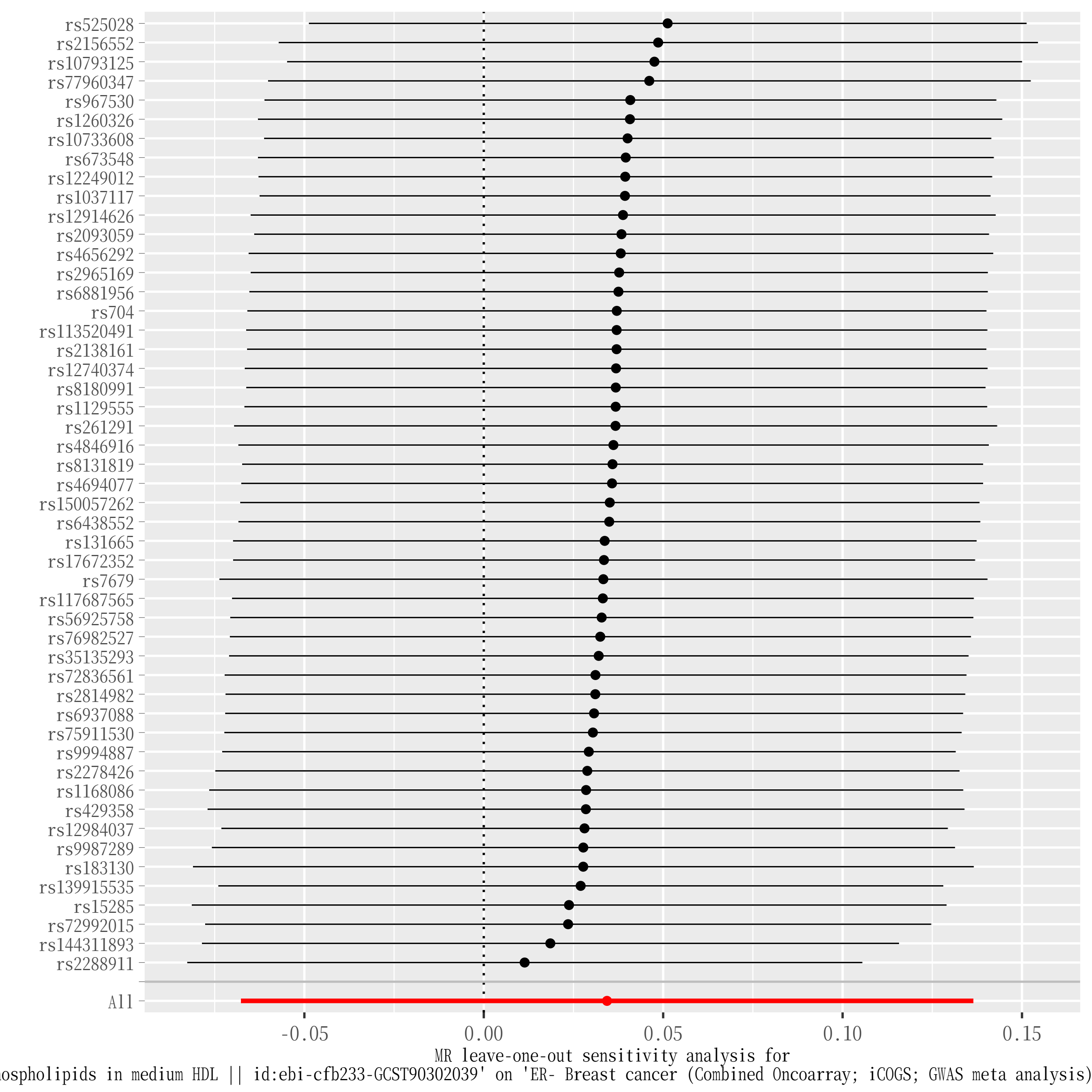
rs525028
rs2156552
rs10793125
rs77960347
rs967530
rs1260326
rs10733608
rs673548
rs12249012
rs1037117
rs12914626
rs2093059
rs4656292
rs2965169
rs6881956
rs704
rs113520491
rs2138161
rs12740374
rs8180991
rs1129555
rs261291
rs4846916
rs8131819
rs4694077
rs150057262
rs6438552
rs131665
rs17672352
rs7679
rs117687565
rs56925758
rs76982527
rs35135293
rs72836561
rs2814982
rs6937088
rs75911530
rs9994887
rs2278426
rs1168086
rs429358
rs12984037
rs9987289
rs183130
rs139915535
rs15285
rs72992015
rs144311893
rs2288911
All
-0.05
0.00
0.05
0.10
0.15
MR leave-one-out sensitivity analysis for
ospholipids in medium HDL || id:ebi-cfb233-GCST90302039' on 'ER- Breast cancer (Combined Oncoarray; iCOGS; GWAS meta analysis)

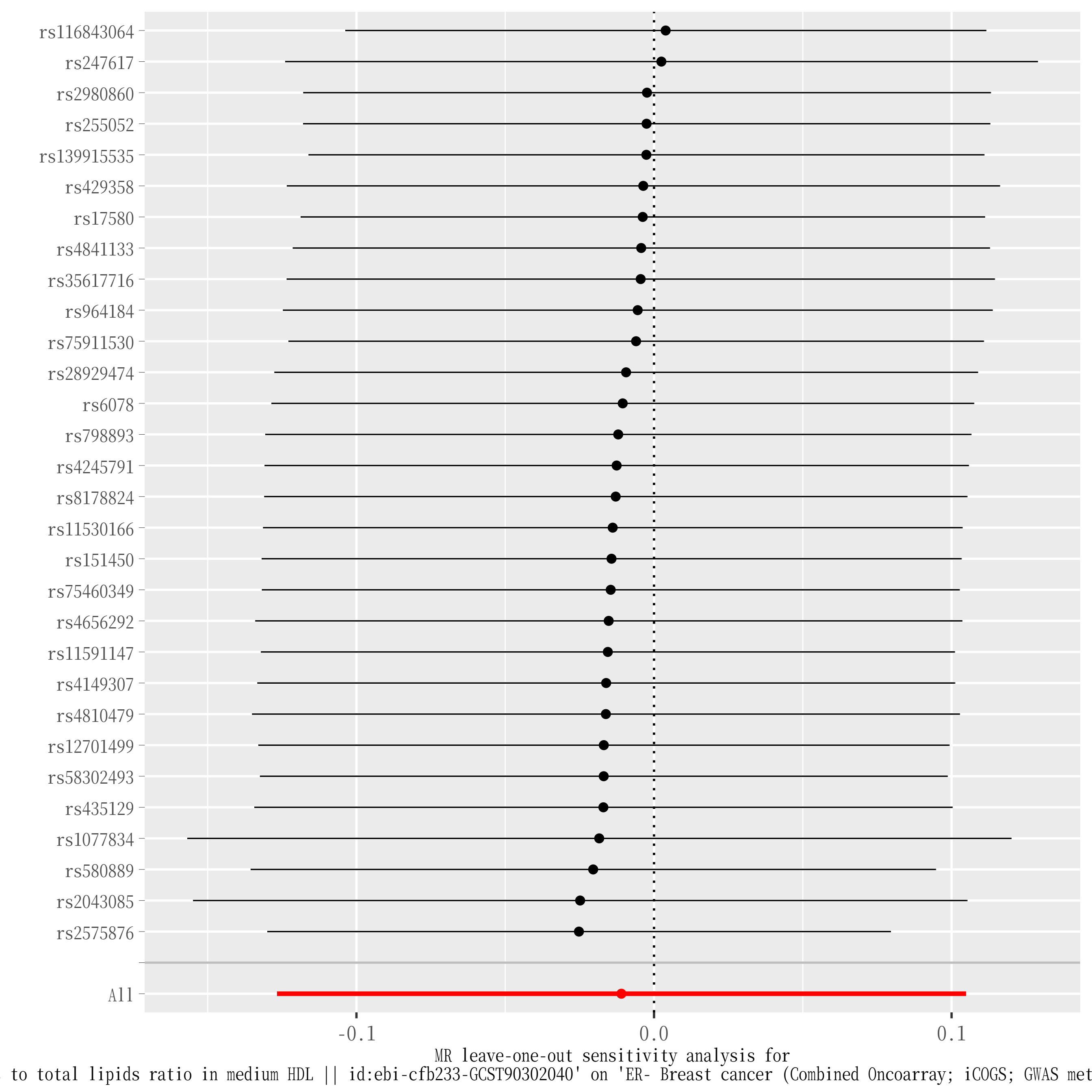
rs116843064
rs247617
rs2980860
rs255052
rs139915535
rs429358
rs17580
rs4841133
rs35617716
rs964184
rs75911530
rs28929474
rs6078
rs798893
rs4245791
rs8178824
rs11530166
rs151450
rs75460349
rs4656292
rs11591147
rs4149307
rs4810479
rs12701499
rs58302493
rs435129
rs1077834
rs580889
rs2043085
rs2575876
All
-0.1
0.0
0.1
MR leave-one-out sensitivity analysis for
to total lipids ratio in medium HDL || id:ebi-cfb233-GCST90302040' on 'ER- Breast cancer (Combined Oncoarray; iCOGS; GWAS met

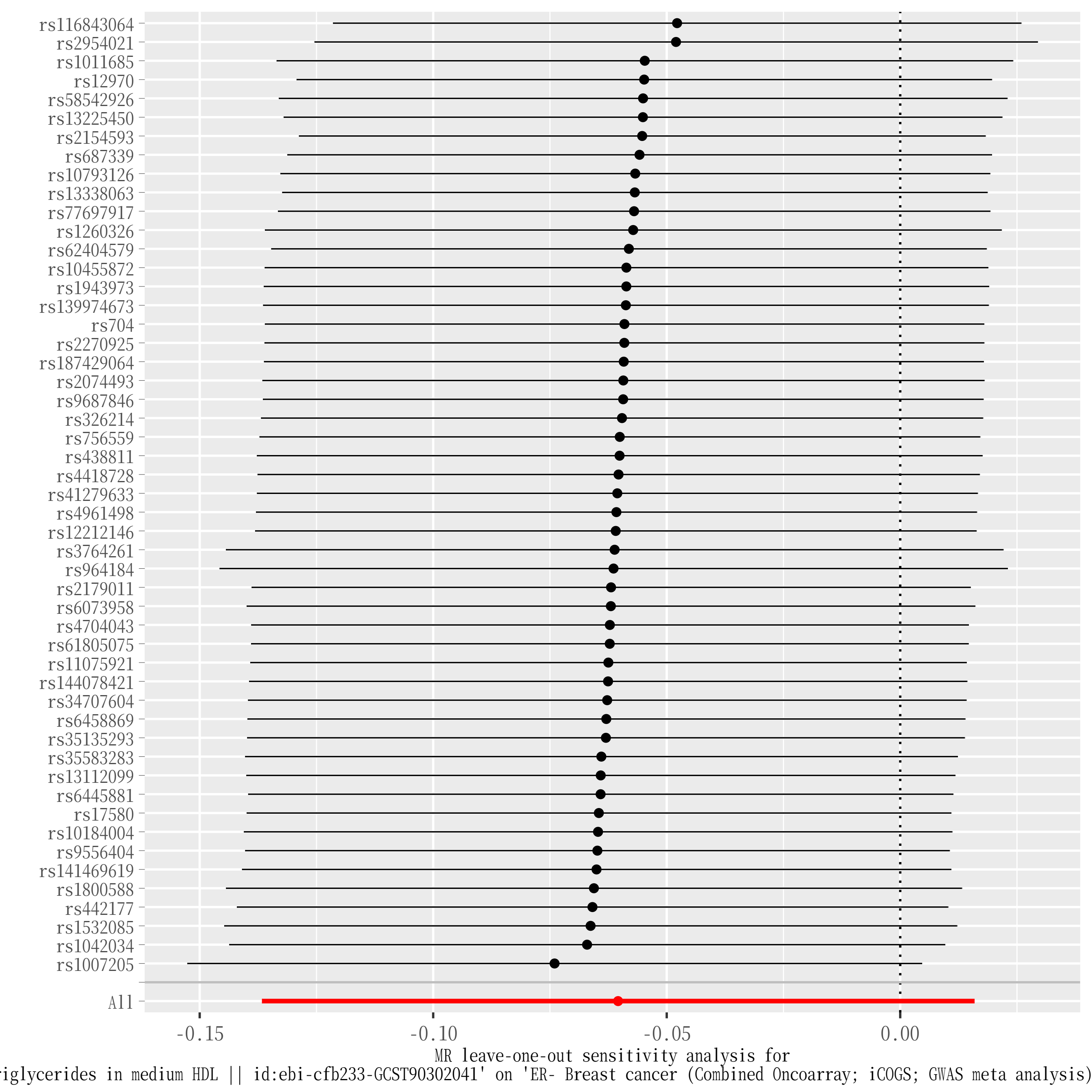
rs116843064
rs2954021
rs1011685
rs12970
rs58542926
rs13225450
rs2154593
rs687339
rs10793126
rs13338063
rs77697917
rs1260326
rs62404579
rs10455872
rs1943973
rs139974673
rs704
rs2270925
rs187429064
rs2074493
rs9687846
rs326214
rs756559
rs438811
rs4418728
rs41279633
rs4961498
rs12212146
rs3764261
rs964184
rs2179011
rs6073958
rs4704043
rs61805075
rs11075921
rs144078421
rs34707604
rs6458869
rs35135293
rs35583283
rs13112099
rs6445881
rs17580
rs10184004
rs9556404
rs141469619
rs1800588
rs442177
rs1532085
rs1042034
rs1007205
All
-0.15
-0.10
-0.05
0.00
MR leave-one-out sensitivity analysis for
riglycerides in medium HDL || id:ebi-cfb233-GCST90302041' on 'ER- Breast cancer (Combined Oncoarray; iCOGS; GWAS meta analysis)

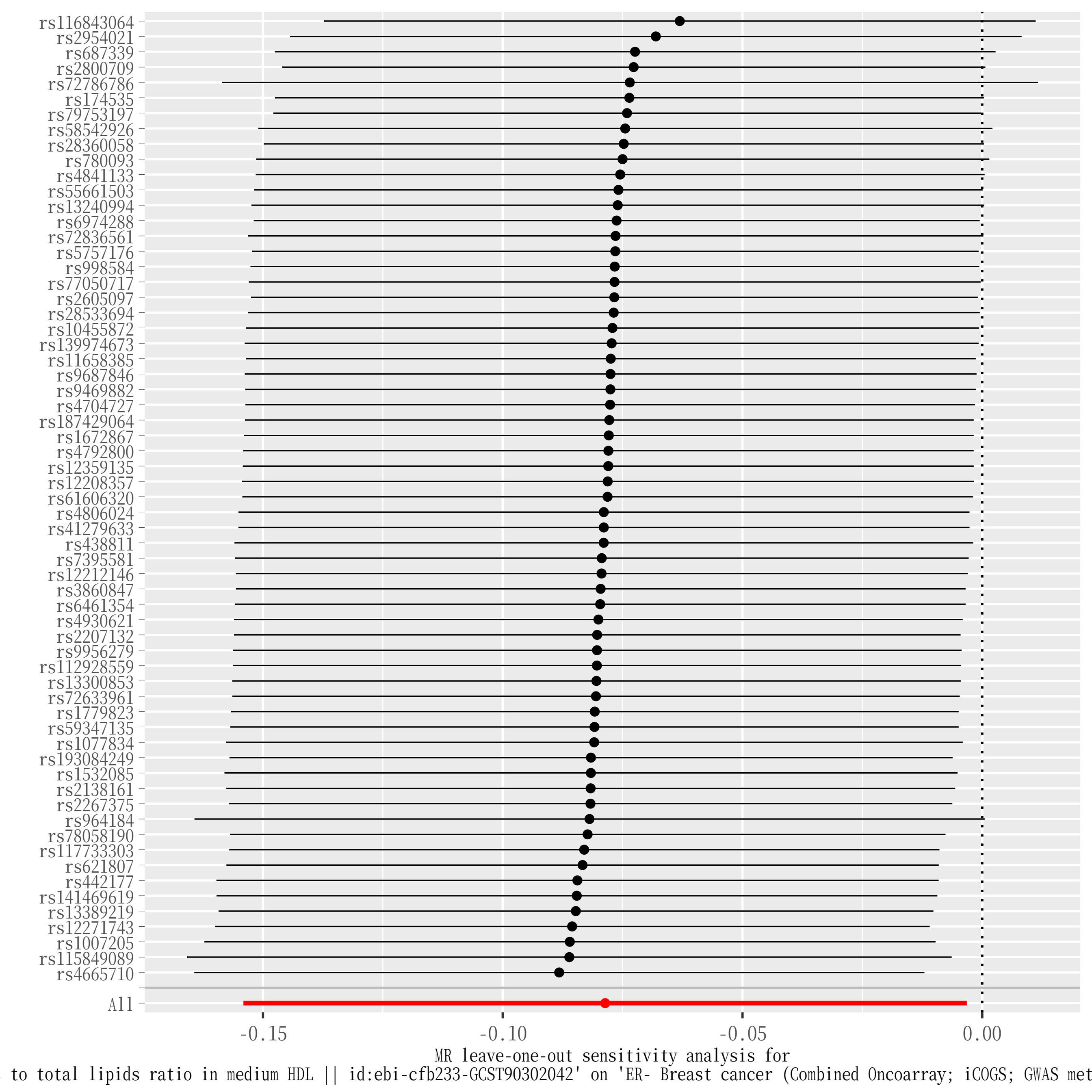

rs116843064
rs2954021
rs687339
rs2800709
rs72786786
rs174535
rs79753197
rs58542926
rs28360058
rs780093
rs4841133
rs55661503
rs13240994
rs6974288
rs72836561
rs5757176
rs998584
rs77050717
rs2605097
rs28533694
rs10455872
rs139974673
rs11658385
rs9687846
rs9469882
rs4704727
rs187429064
rs1672867
rs4792800
rs12359135
rs12208357
rs61606320
rs4806024
rs41279633
rs438811
rs7395581
rs12212146
rs3860847
rs6461354
rs4930621
rs2207132
rs9956279
rs112928559
rs13300853
rs72633961
rs1779823
rs59347135
rs1077834
rs193084249
rs1532085
rs2138161
rs2267375
rs964184
rs78058190
rs117733303
rs621807
rs442177
rs141469619
rs13389219
rs12271743
rs1007205
rs115849089
rs4665710
All
-0.15
-0.10
-0.05
0.00
MR leave-one-out sensitivity analysis for
to total lipids ratio in medium HDL || id:ebi-cfb233-GCST90302042' on 'ER- Breast cancer (Combined Oncoarray; iCOGS; GWAS met

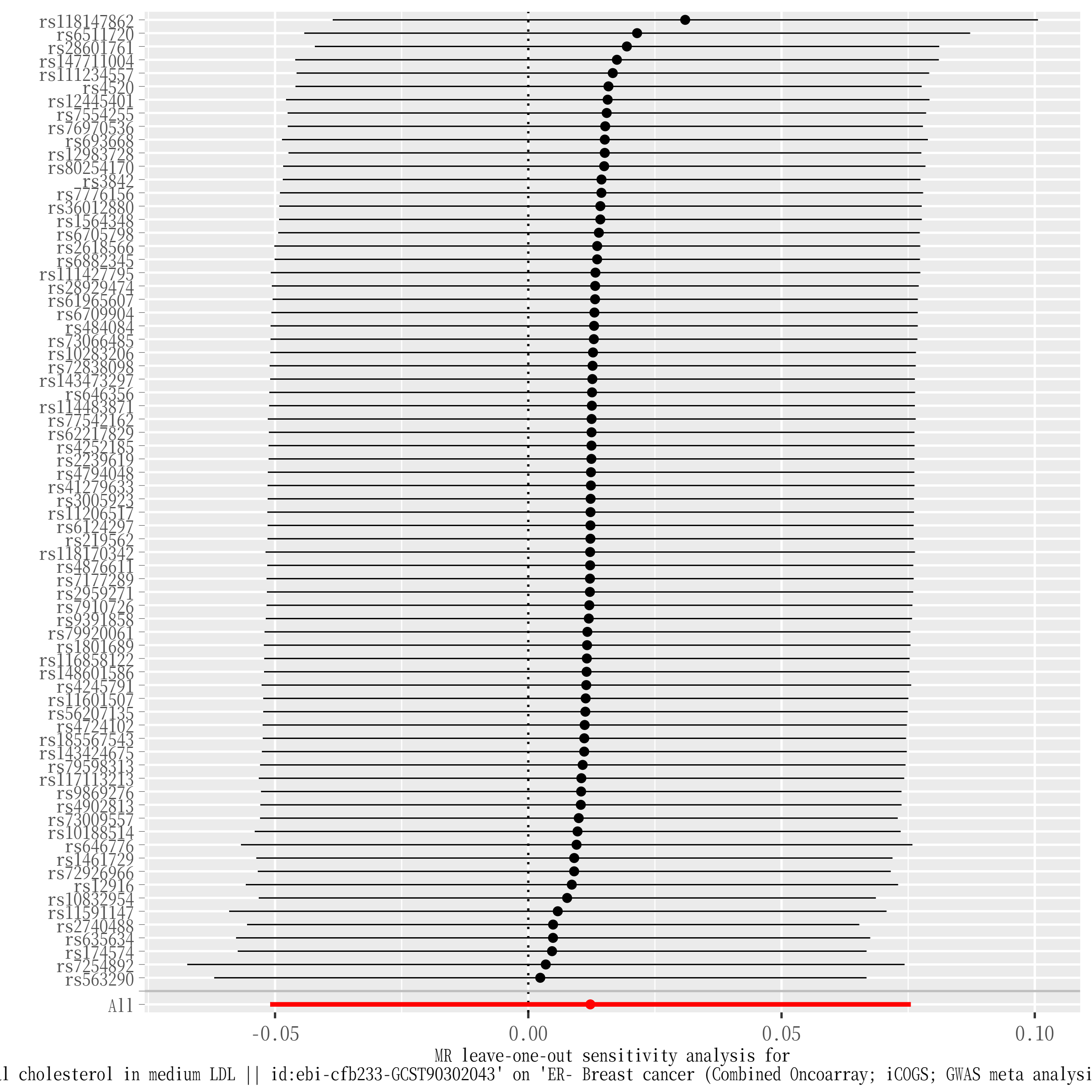

rs118147862
rs6511720
rs28601761
rs147711004
rs111234557
rs4520
rs12445401
rs7554255
rs76970536
rs693668
rs12983728
rs80254170
rs3842
rs7776156
rs36012880
rs1564348
rs6705798
rs2618566
rs6882345
rs111427795
rs28929474
rs61965607
rs6709904
rs484084
rs73066485
rs10283206
rs72838098
rs143473297
rs646356
rs114483871
rs77542162
rs62217829
rs4252185
rs2239619
rs4794048
rs41279633
rs3005923
rs11206517
rs6124297
rs219562
rs118170342
rs4876611
rs7177289
rs2959271
rs7910726
rs9391858
rs79920061
rs1801689
rs116858122
rs148601586
rs4245791
rs11601507
rs56207135
rs4724102
rs185567543
rs143424675
rs79598313
rs117113213
rs9869276
rs4902813
rs73009557
rs10188514
rs646776
rs1461729
rs72926966
rs12916
rs10832954
rs11591147
rs2740488
rs635634
rs174574
rs7254892
rs563290
All
-0.05
0.00
0.05
0.10
MR leave-one-out sensitivity analysis for
l cholesterol in medium LDL || id:ebi-cfb233-GCST90302043' on 'ER- Breast cancer (Combined Oncoarray; iCOGS; GWAS meta analysi

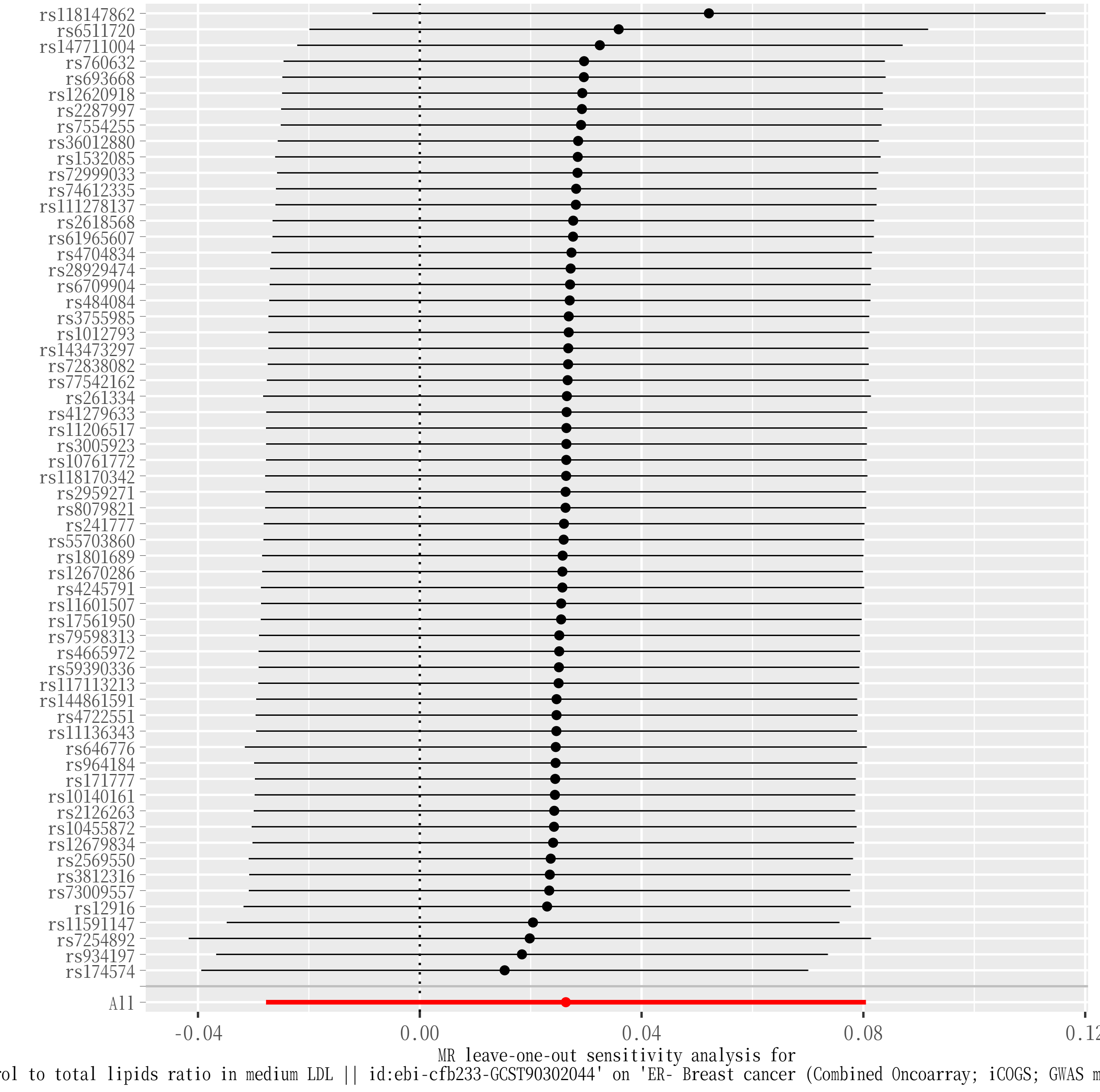
rs118147862
rs6511720
rs147711004
rs760632
rs693668
rs12620918
rs2287997
rs7554255
rs36012880
rs1532085
rs72999033
rs74612335
rs111278137
rs2618568
rs61965607
rs4704834
rs28929474
rs6709904
rs484084
rs3755985
rs1012793
rs143473297
rs72838082
rs77542162
rs261334
rs41279633
rs11206517
rs3005923
rs10761772
rs118170342
rs2959271
rs8079821
rs241777
rs55703860
rs1801689
rs12670286
rs4245791
rs11601507
rs17561950
rs79598313
rs4665972
rs59390336
rs117113213
rs144861591
rs4722551
rs11136343
rs646776
rs964184
rs171777
rs10140161
rs2126263
rs10455872
rs12679834
rs2569550
rs3812316
rs73009557
rs12916
rs11591147
rs7254892
rs934197
rs174574
All
-0.04
0.00
0.04
0.08
0.12
MR leave-one-out sensitivity analysis for
rol to total lipids ratio in medium LDL || id:ebi-cfb233-GCST90302044' on 'ER- Breast cancer (Combined Oncoarray; iCOGS; GWAS m

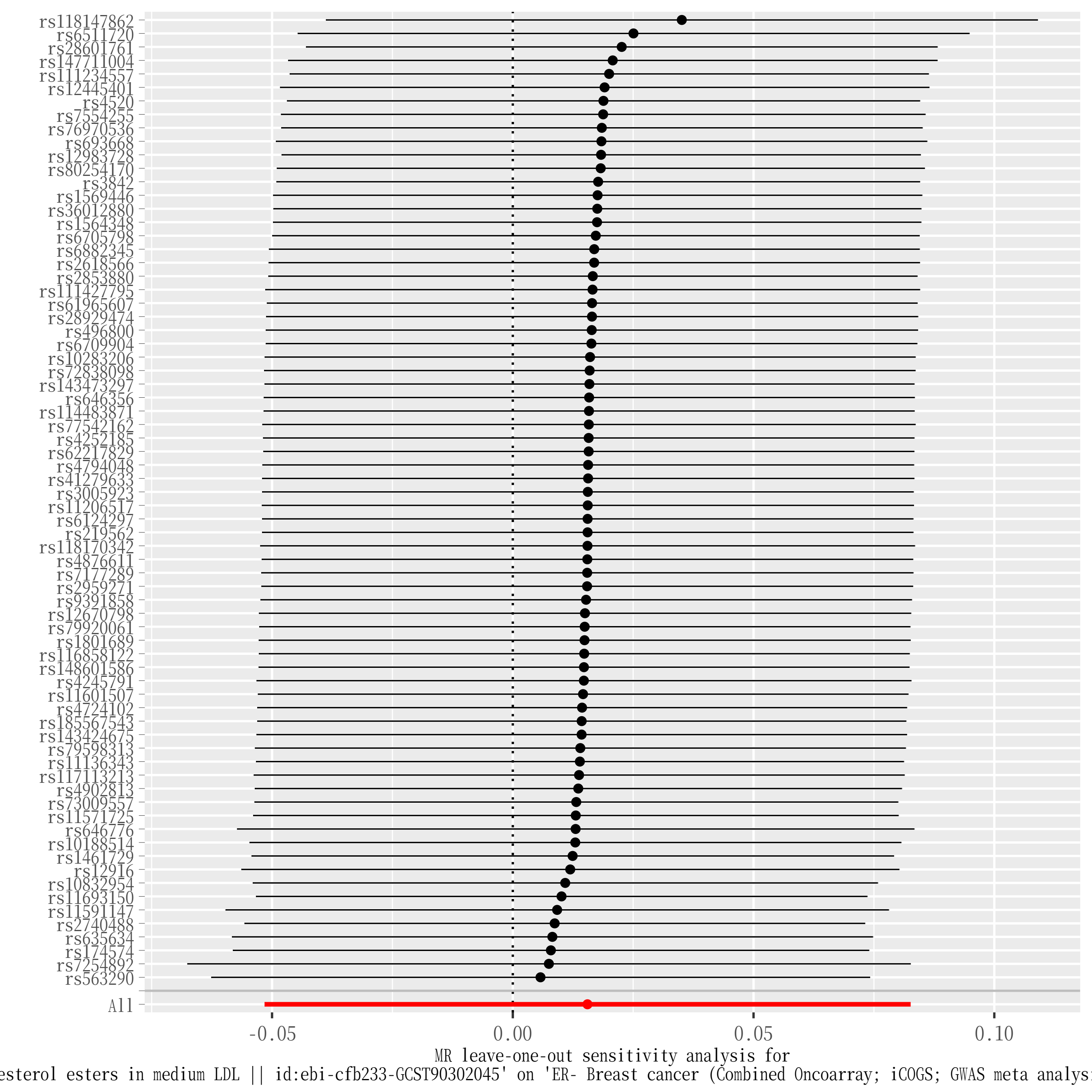
rs118147862
rs6511720
rs28601761
rs147711004
rs111234557
rs12445401
rs4520
rs7554255
rs76970536
rs693668
rs12983728
rs80254170
rs3842
rs1569446
rs36012880
rs1564348
rs6705798
rs6882345
rs2618566
rs2853880
rs111427795
rs61965607
rs28929474
rs496800
rs6709904
rs10283206
rs72838098
rs143473297
rs646356
rs114483871
rs77542162
rs4252185
rs62217829
rs4794048
rs41279633
rs3005923
rs11206517
rs6124297
rs219562
rs118170342
rs4876611
rs7177289
rs2959271
rs9391858
rs12670798
rs79920061
rs1801689
rs116858122
rs148601586
rs4245791
rs11601507
rs4724102
rs185567543
rs143424675
rs79598313
rs11136343
rs117113213
rs4902813
rs73009557
rs11571725
rs646776
rs10188514
rs1461729
rs12916
rs10832954
rs11693150
rs11591147
rs2740488
rs635634
rs174574
rs7254892
rs563290
All
-0.05
0.00
0.05
0.10
MR leave-one-out sensitivity analysis for
esterol esters in medium LDL || id:ebi-cfb233-GCST90302045' on 'ER- Breast cancer (Combined Oncoarray; iCOGS; GWAS meta analys

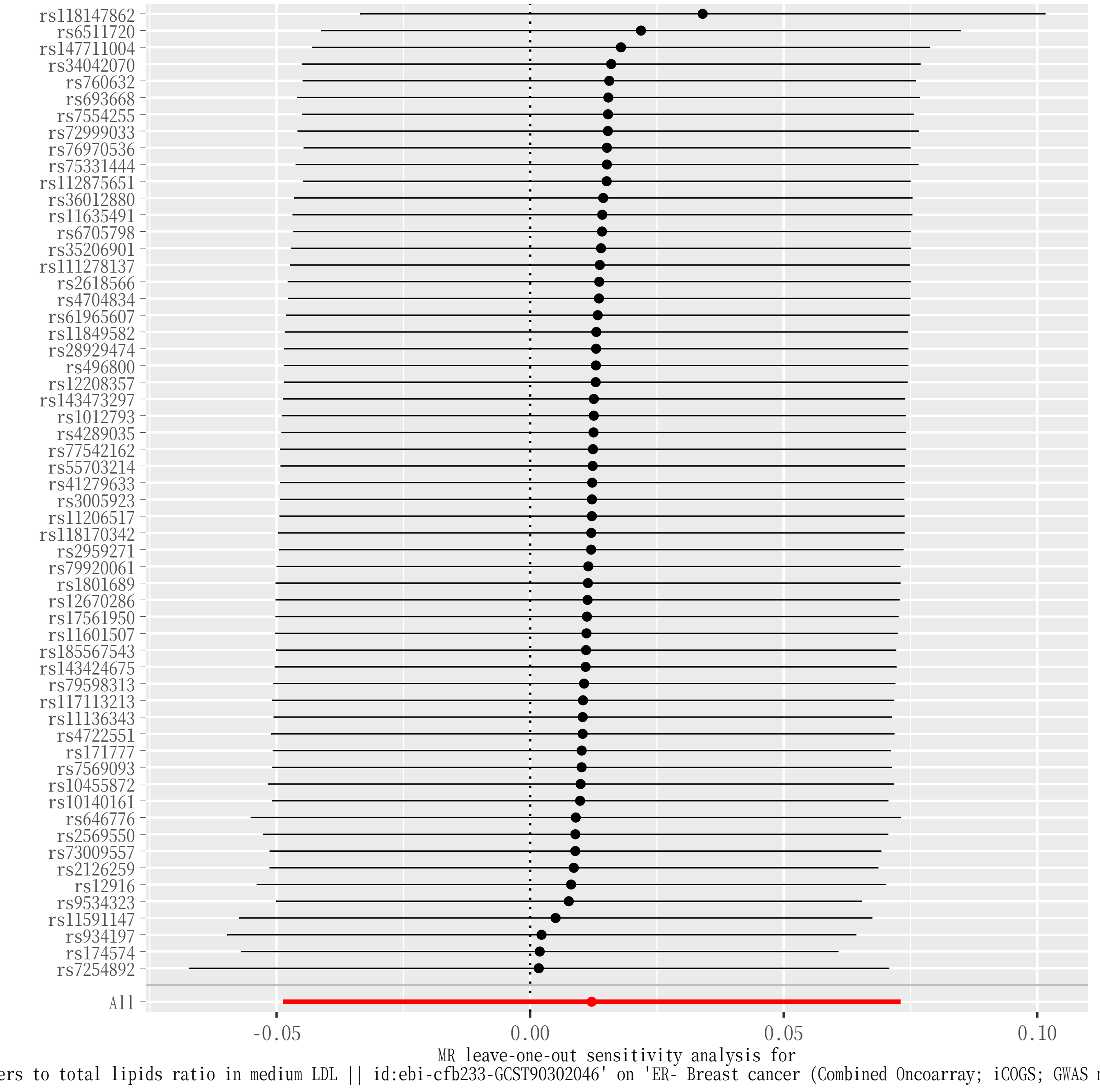
rs118147862
rs6511720
rs147711004
rs34042070
rs760632
rs693668
rs7554255
rs72999033
rs76970536
rs75331444
rs112875651
rs36012880
rs11635491
rs6705798
rs35206901
rs111278137
rs2618566
rs4704834
rs61965607
rs11849582
rs28929474
rs496800
rs12208357
rs143473297
rs1012793
rs4289035
rs77542162
rs55703214
rs41279633
rs3005923
rs11206517
rs118170342
rs2959271
rs79920061
rs1801689
rs12670286
rs17561950
rs11601507
rs185567543
rs143424675
rs79598313
rs117113213
rs11136343
rs4722551
rs171777
rs7569093
rs10455872
rs10140161
rs646776
rs2569550
rs73009557
rs2126259
rs12916
rs9534323
rs11591147
rs934197
rs174574
rs7254892
All
-0.05
0.00
0.05
0.10
MR leave-one-out sensitivity analysis for
ers to total lipids ratio in medium LDL || id:ebi-cfb233-GCST90302046' on 'ER- Breast cancer (Combined Oncoarray; iCOGS; GWAS m

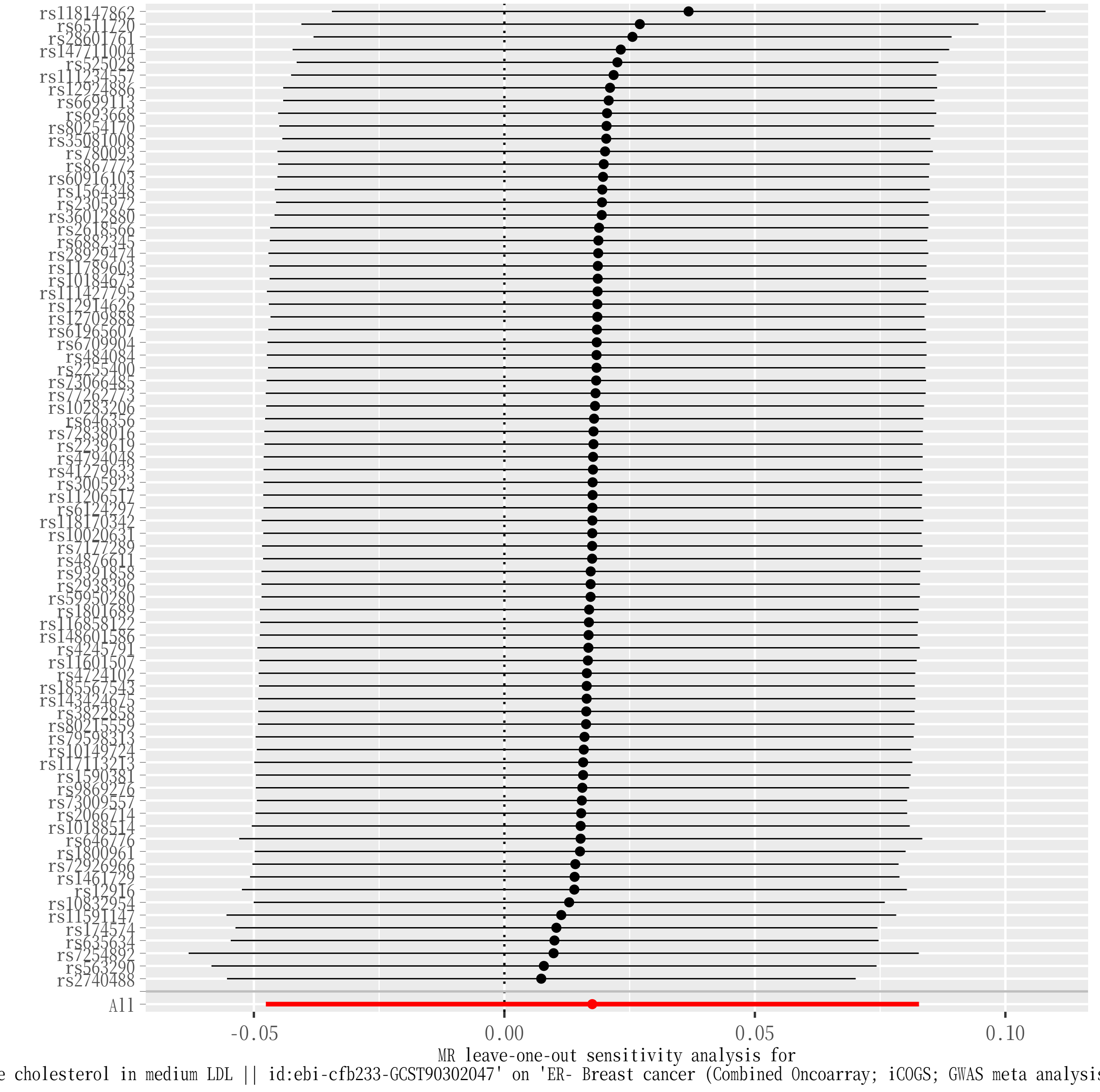
rs118147862
rs6511720
rs28601761
rs147711004
rs525028
rs111234557
rs12924886
rs6699113
rs693668
rs80254170
rs35081008
rs780093
rs867772
rs60916103
rs1564348
rs2305972
rs36012880
rs2618566
rs6882345
rs28929474
rs11789603
rs10184673
rs111427795
rs12914626
rs12709888
rs61965607
rs6709904
rs484084
rs2255400
rs73066485
rs77262773
rs10283206
rs646356
rs72838016
rs2239619
rs4794048
rs41279633
rs3005923
rs11206517
rs6124297
rs118170342
rs10020631
rs7177289
rs4876611
rs9391858
rs2938396
rs59950280
rs1801689
rs116858122
rs148601586
rs4245791
rs11601507
rs4724102
rs185567543
rs143424675
rs3822858
rs80215559
rs79598313
rs10149724
rs117113213
rs1590381
rs9869276
rs73009557
rs2066714
rs10188514
rs646776
rs1800961
rs72926966
rs1461729
rs12916
rs10832954
rs11591147
rs174574
rs635634
rs7254892
rs563290
rs2740488
All
-0.05
0.00
0.05
0.10
MR leave-one-out sensitivity analysis for
e cholesterol in medium LDL || id:ebi-cfb233-GCST90302047' on 'ER- Breast cancer (Combined Oncoarray; iCOGS; GWAS meta analysis

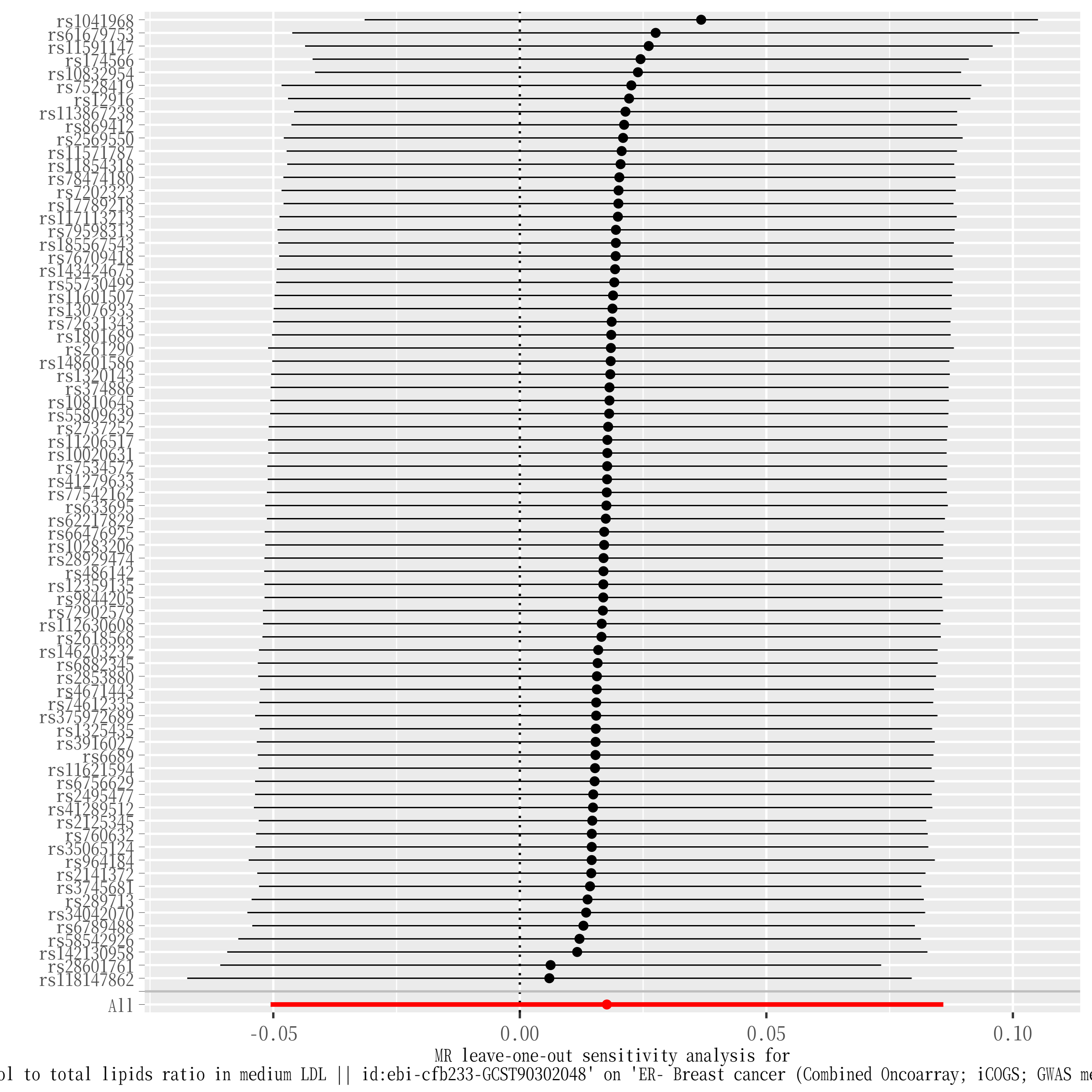
rs1041968
rs61679753
rs11591147
rs174566
rs10832954
rs7528419
rs12916
rs113867238
rs869412
rs2569550
rs11571787
rs11854318
rs78474180
rs7202323
rs17789218
rs117113213
rs79598313
rs185567543
rs76709418
rs143424675
rs55730499
rs11601507
rs13076933
rs72631343
rs1801689
rs261290
rs148601586
rs1320143
rs374886
rs10810645
rs55809639
rs2737252
rs11206517
rs10020631
rs7534572
rs41279633
rs77542162
rs633695
rs62217829
rs66476925
rs10283206
rs28929474
rs486142
rs12359135
rs9844205
rs72902579
rs112630608
rs2618568
rs146203232
rs6882345
rs2853880
rs4671443
rs74612335
rs375972689
rs1325435
rs3916027
rs6689
rs11621594
rs6756629
rs2495477
rs41289512
rs2125345
rs760632
rs35065124
rs964184
rs2141372
rs3745681
rs289713
rs34042070
rs6789488
rs58542926
rs142130958
rs28601761
rs118147862
All
-0.05
0.00
0.05
0.10
MR leave-one-out sensitivity analysis for
ol to total lipids ratio in medium LDL || id:ebi-cfb233-GCST90302048' on 'ER- Breast cancer (Combined Oncoarray; iCOGS; GWAS me

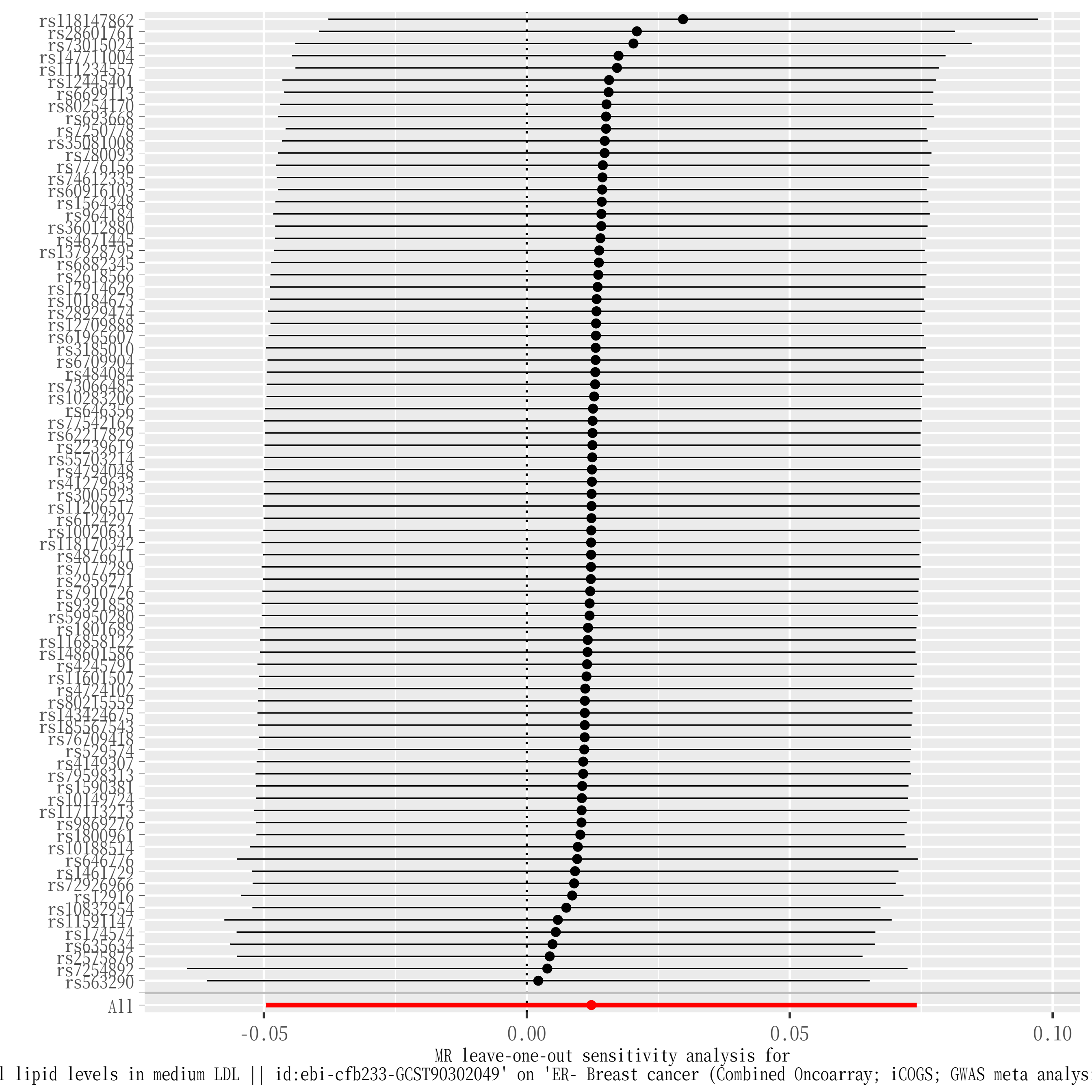

rs118147862
rs28601761
rs73015024
rs147711004
rs111234557
rs12445401
rs6699113
rs80254170
rs693668
rs7250778
rs35081008
rs780093
rs7776156
rs74612335
rs60916103
rs1564348
rs964184
rs36012880
rs4671445
rs137928795
rs6882345
rs2618566
rs12914626
rs10184673
rs28929474
rs12709888
rs61965607
rs3185010
rs6709904
rs484084
rs73066485
rs10283206
rs646356
rs77542162
rs62217829
rs2239619
rs55703214
rs4794048
rs41279633
rs3005923
rs11206517
rs6124297
rs10020631
rs118170342
rs4876611
rs7177289
rs2959271
rs7910726
rs9391858
rs59950280
rs1801689
rs116858122
rs148601586
rs4245791
rs11601507
rs4724102
rs80215559
rs143424675
rs185567543
rs76709418
rs529574
rs4149307
rs79598313
rs1590381
rs10149724
rs117113213
rs9869276
rs1800961
rs10188514
rs646776
rs1461729
rs72926966
rs12916
rs10832954
rs115911147
rs174574
rs635634
rs2575876
rs7254892
rs563290
All
-0.05
0.00
0.05
0.10
MR leave-one-out sensitivity analysis for
l lipid levels in medium LDL || id:ebi-cfb233-GCST90302049' on 'ER- Breast cancer (Combined Oncoarray; iCOGS; GWAS meta analys

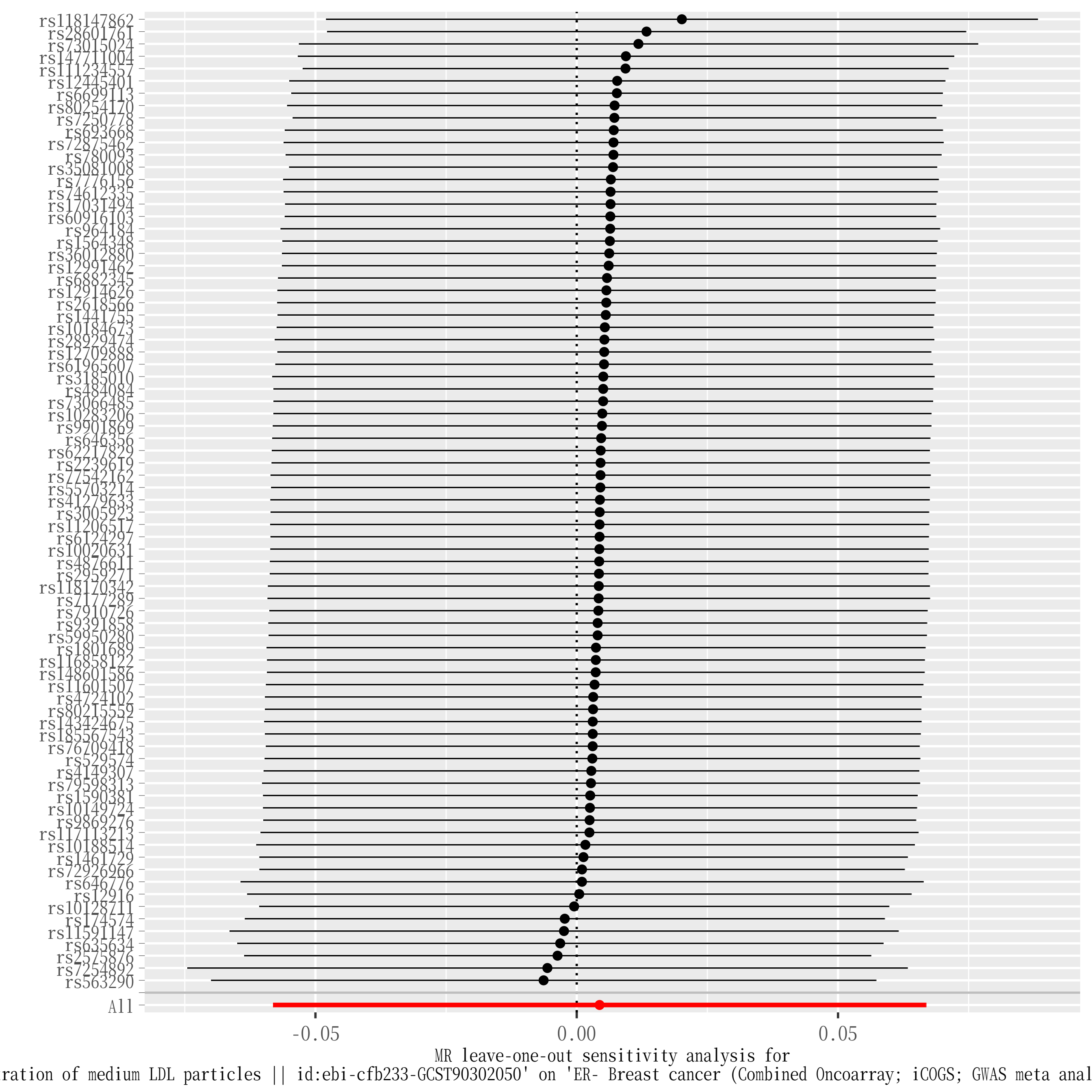
rs118147862
rs28601761
rs73015024
rs147711004
rs111234557
rs12445401
rs6699113
rs80254170
rs7250778
rs693668
rs72875462
rs780093
rs35081008
rs7776156
rs74612335
rs17031494
rs60916103
rs964184
rs1564348
rs36012880
rs12991462
rs6882345
rs12914626
rs2618566
rs1441755
rs10184673
rs28929474
rs12709888
rs61965607
rs3185010
rs484084
rs73066485
rs10283206
rs9901869
rs646356
rs62217829
rs2239619
rs77542162
rs55703214
rs41279633
rs3005923
rs11206517
rs6124297
rs10020631
rs4876611
rs2959271
rs118170342
rs2177289
rs7910726
rs9391858
rs59950280
rs1801689
rs116858122
rs148601586
rs11601507
rs4724102
rs80215559
rs143424675
rs185567543
rs76709418
rs529574
rs4149307
rs79598313
rs1590381
rs10149724
rs9869276
rs117113213
rs10188514
rs1461729
rs72926966
rs646776
rs12916
rs10128711
rs174574
rs11591147
rs635634
rs2575876
rs7254892
rs563290
All
-0.05
0.00
0.05
MR leave-one-out sensitivity analysis for
ration of medium LDL particles || id:ebi-cfb233-GCST90302050' on 'ER- Breast cancer (Combined Oncoarray; iCOGS; GWAS meta anal

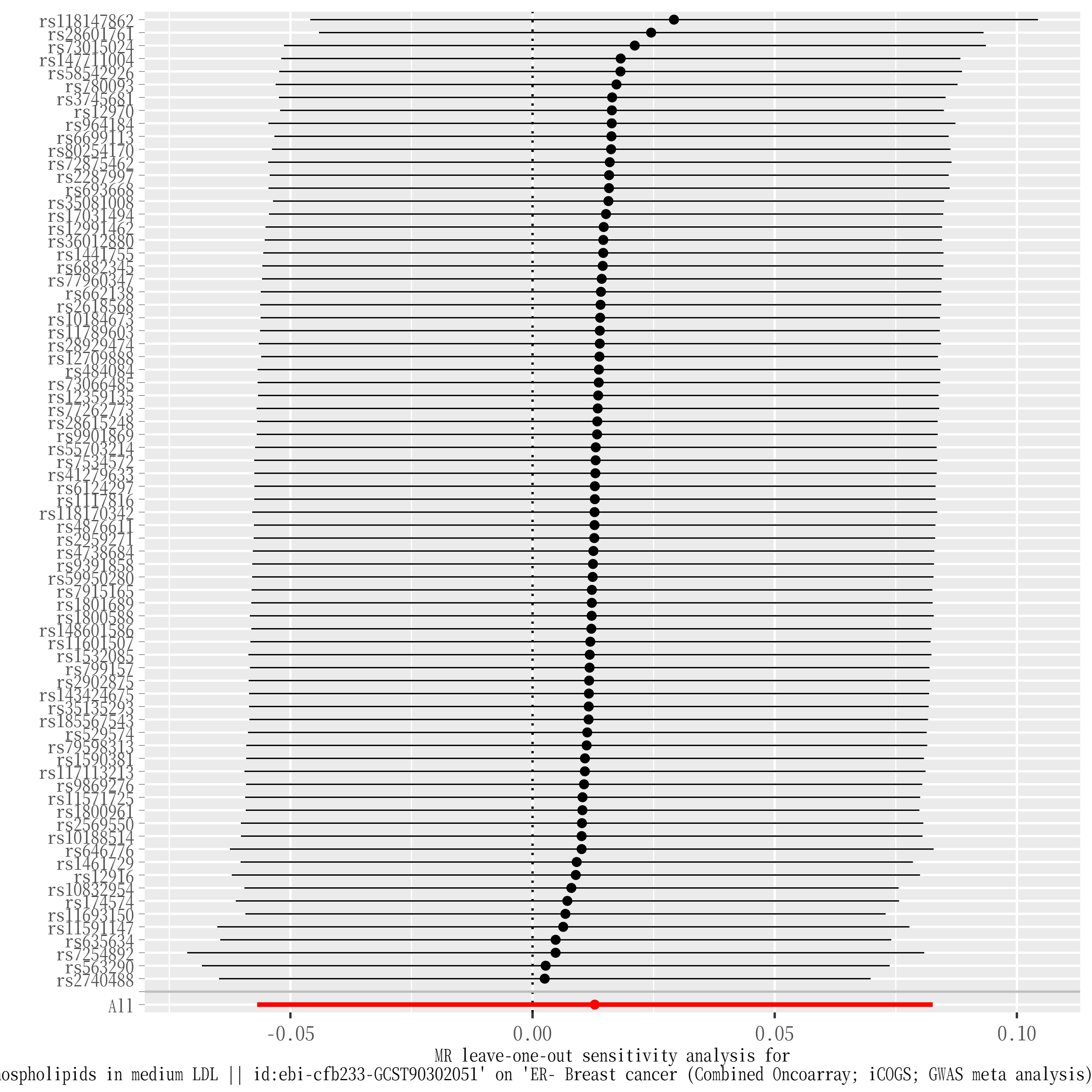

rs118147862
rs28601761
rs73015024
rs147711004
rs58542926
rs780093
rs3745681
rs12970
rs964184
rs6699113
rs80254170
rs72875462
rs2287997
rs693668
rs35081008
rs17031494
rs12991462
rs36012880
rs1441755
rs6882345
rs77960347
rs662138
rs2618568
rs10184673
rs11789603
rs28929474
rs12709888
rs484084
rs73066485
rs12359135
rs77262773
rs28615248
rs9901869
rs55703214
rs7534572
rs41279633
rs6124297
rs1117816
rs118170342
rs4876611
rs2959271
rs4738684
rs9391858
rs59950280
rs7915165
rs1801689
rs1800588
rs148601586
rs11601507
rs1532085
rs799157
rs2902875
rs143424675
rs35135293
rs185567543
rs529574
rs79598313
rs1590381
rs117113213
rs9869276
rs11571725
rs1800961
rs2569550
rs10188514
rs646776
rs1461729
rs12916
rs10832954
rs174574
rs11693150
rs11591147
rs635634
rs7254892
rs563290
rs2740488
All
-0.05
0.00
0.05
0.10
MR leave-one-out sensitivity analysis for
'Phospholipids in medium LDL || id:ebi-cfb233-GCST90302051' on 'ER- Breast cancer (Combined Oncoarray; iCOGS; GWAS meta analysis)'

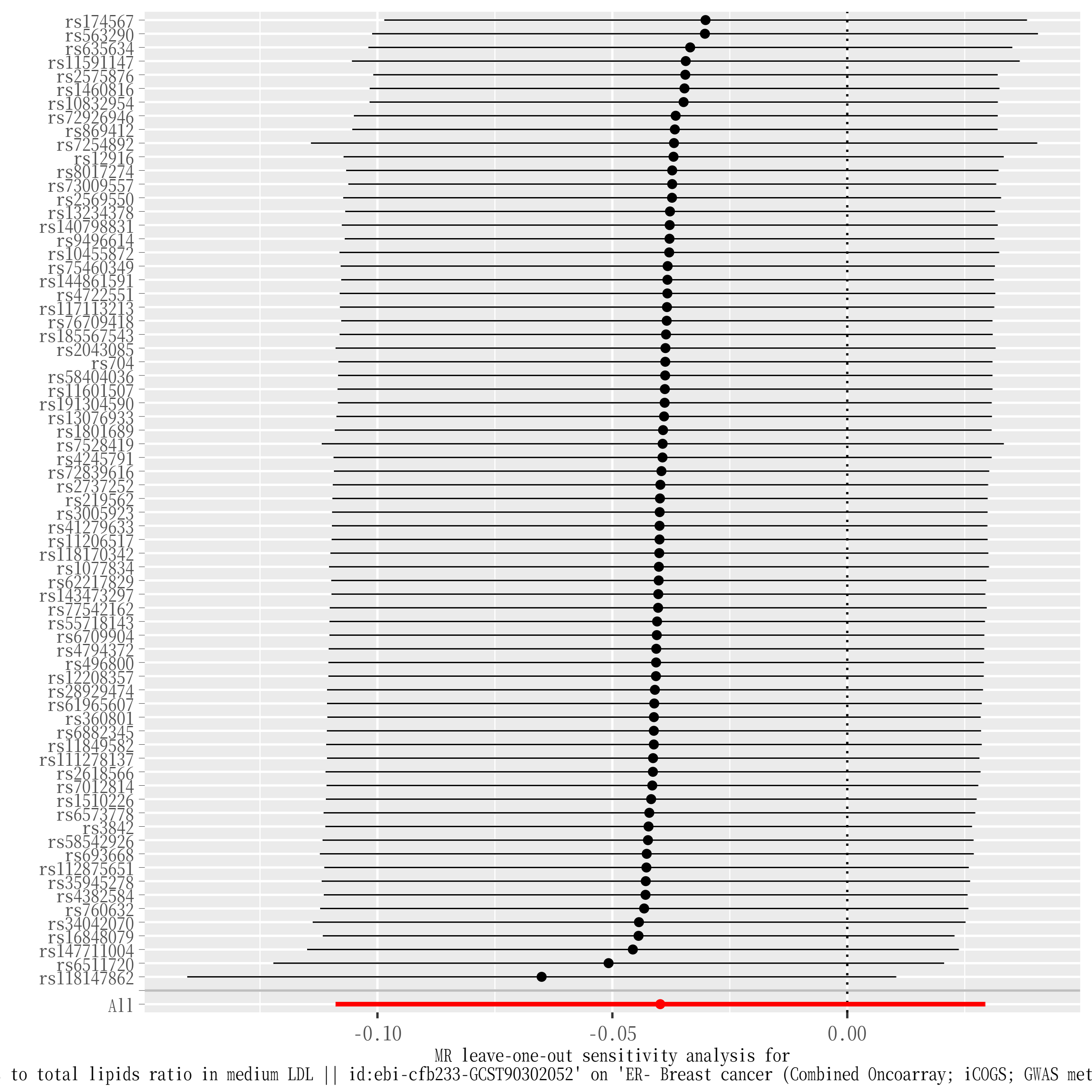
rs174567
rs563290
rs635634
rs11591147
rs2575876
rs1460816
rs10832954
rs72926946
rs869412
rs7254892
rs12916
rs8017274
rs73009557
rs2569550
rs13234378
rs140798831
rs9496614
rs10455872
rs75460349
rs144861591
rs4722551
rs117113213
rs76709418
rs185567543
rs2043085
rs704
rs58404036
rs11601507
rs191304590
rs13076933
rs1801689
rs7528419
rs4245791
rs72839616
rs2737252
rs219562
rs3005923
rs41279633
rs11206517
rs118170342
rs1077834
rs62217829
rs143473297
rs77542162
rs55718143
rs6709904
rs4794372
rs496800
rs12208357
rs28929474
rs61965607
rs360801
rs6882345
rs11849582
rs111278137
rs2618566
rs7012814
rs1510226
rs6573778
rs3842
rs58542926
rs693668
rs112875651
rs35945278
rs4382584
rs760632
rs34042070
rs16848079
rs147711004
rs6511720
rs118147862
All
-0.10
-0.05
0.00
MR leave-one-out sensitivity analysis for
to total lipids ratio in medium LDL || id:ebi-cfb233-GCST90302052' on 'ER- Breast cancer (Combined Oncoarray; iCOGS; GWAS met

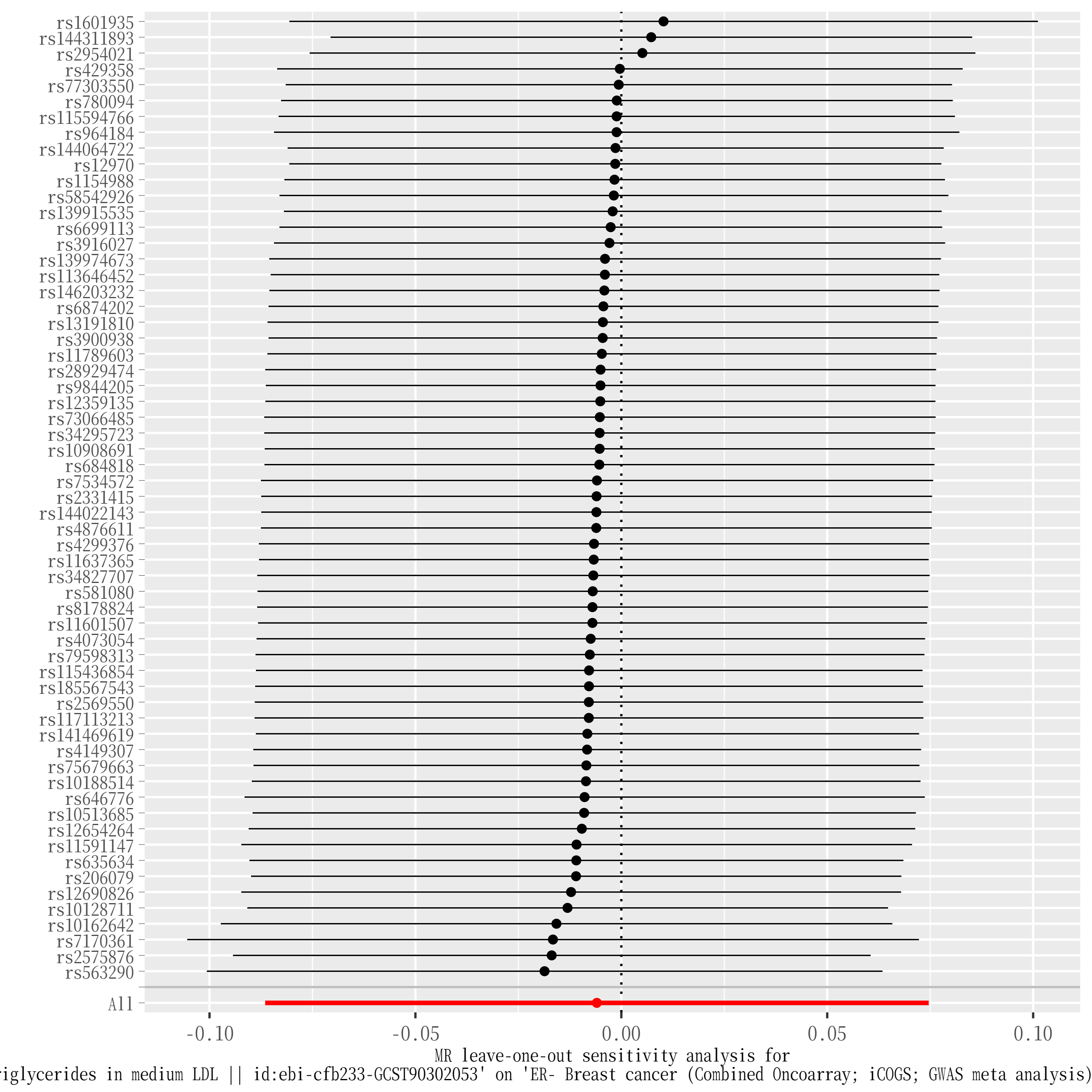

rs1601935
rs144311893
rs2954021
rs429358
rs77303550
rs780094
rs115594766
rs964184
rs144064722
rs12970
rs1154988
rs58542926
rs139915535
rs6699113
rs3916027
rs139974673
rs113646452
rs146203232
rs6874202
rs13191810
rs3900938
rs11789603
rs28929474
rs9844205
rs12359135
rs73066485
rs34295723
rs10908691
rs684818
rs7534572
rs2331415
rs144022143
rs4876611
rs4299376
rs11637365
rs34827707
rs581080
rs8178824
rs11601507
rs4073054
rs79598313
rs115436854
rs185567543
rs2569550
rs117113213
rs141469619
rs4149307
rs75679663
rs10188514
rs646776
rs10513685
rs12654264
rs11591147
rs635634
rs206079
rs12690826
rs10128711
rs10162642
rs7170361
rs2575876
rs563290
All
-0.10
-0.05
0.00
0.05
0.10
MR leave-one-out sensitivity analysis for
riglycerides in medium LDL || id:ebi-cfb233-GCST90302053' on 'ER- Breast cancer (Combined Oncoarray; iCOGS; GWAS meta analysis)

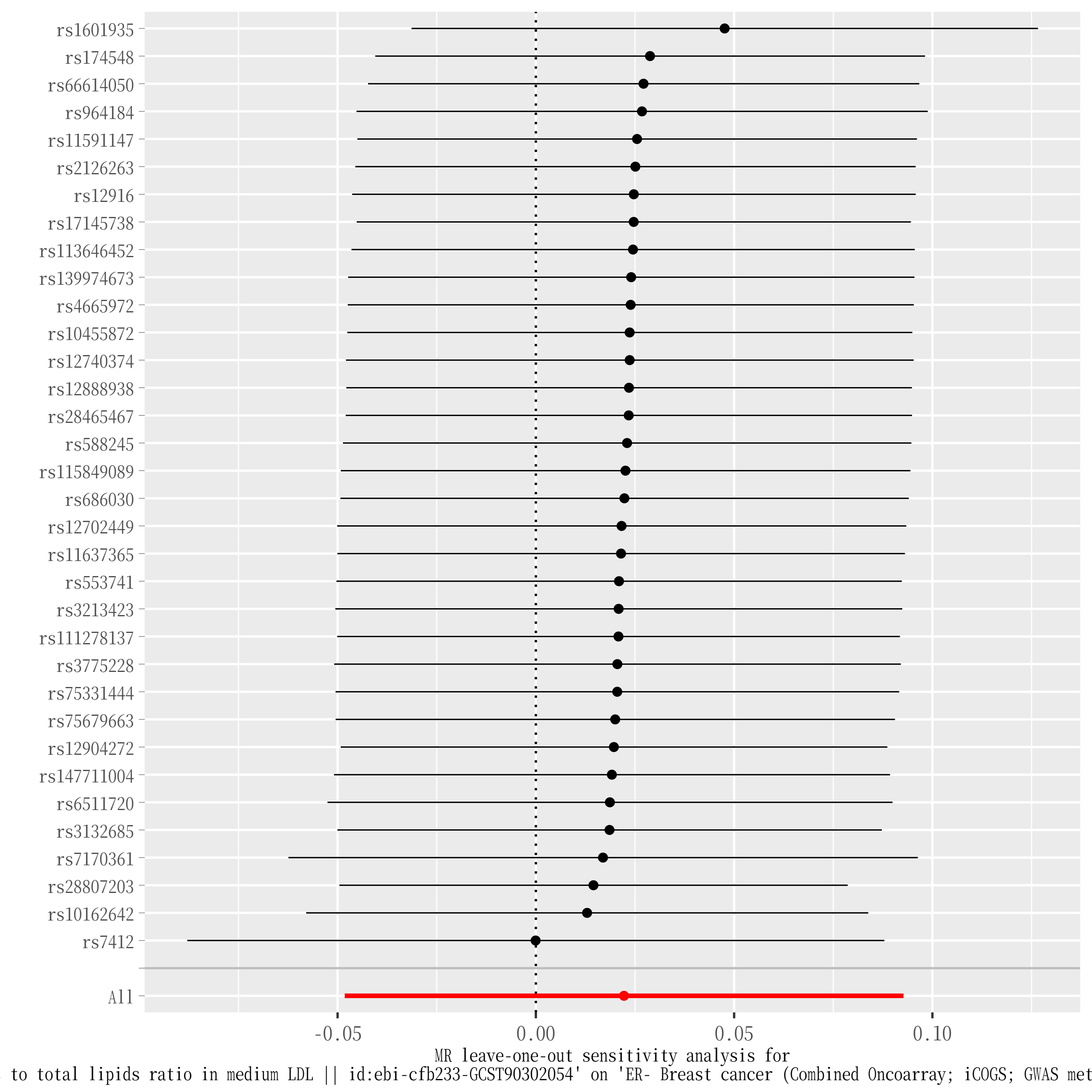

rs1601935
rs174548
rs66614050
rs964184
rs11591147
rs2126263
rs12916
rs17145738
rs113646452
rs139974673
rs4665972
rs10455872
rs12740374
rs12888938
rs28465467
rs588245
rs115849089
rs686030
rs12702449
rs11637365
rs553741
rs3213423
rs111278137
rs3775228
rs75331444
rs75679663
rs12904272
rs147711004
rs6511720
rs3132685
rs7170361
rs28807203
rs10162642
rs7412
All
-0.05
0.00
0.05
0.10
MR leave-one-out sensitivity analysis for
to total lipids ratio in medium LDL || id:ebi-cfb233-GCST90302054' on 'ER- Breast cancer (Combined Oncoarray; iCOGS; GWAS met

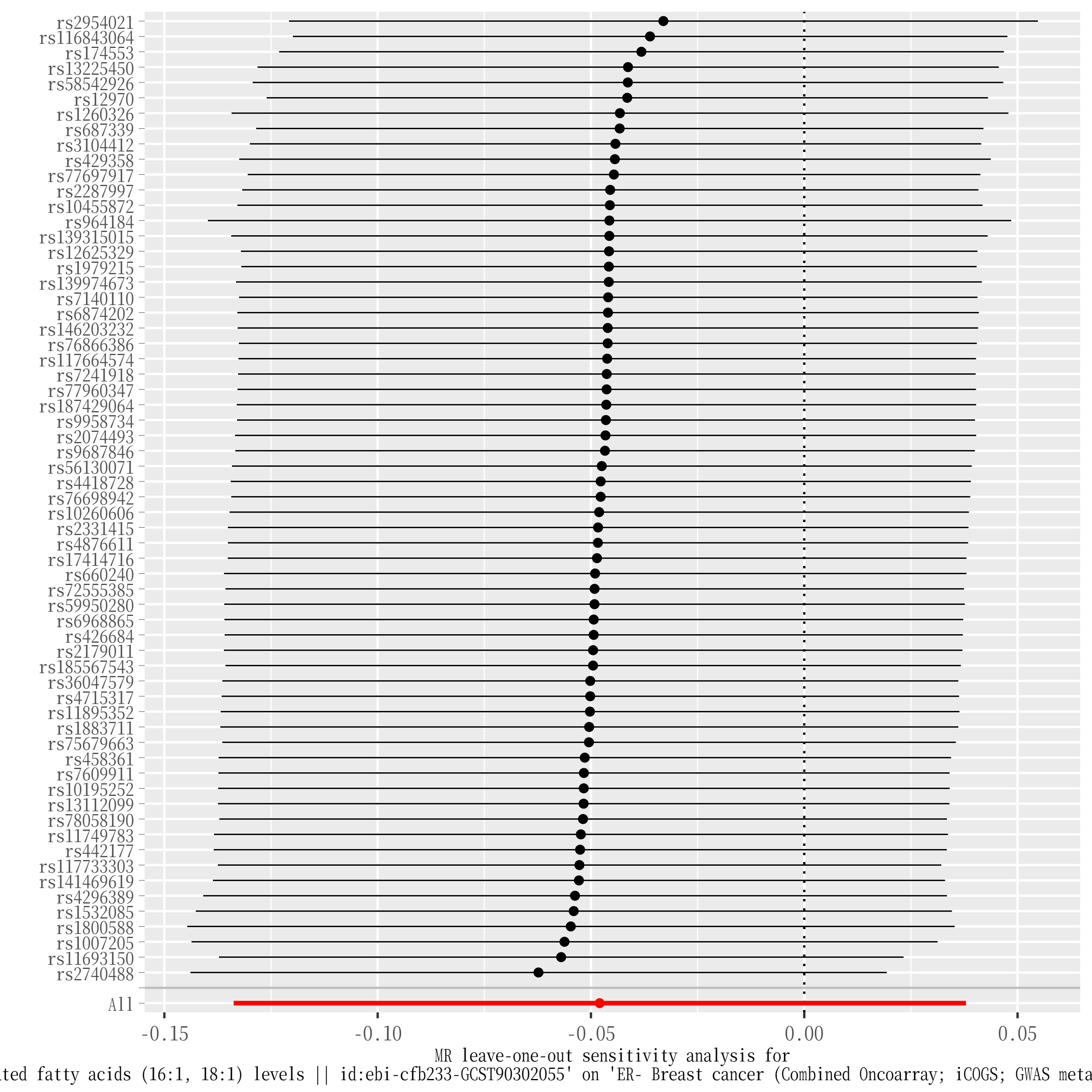

rs2954021
rs116843064
rs174553
rs13225450
rs58542926
rs12970
rs1260326
rs687339
rs3104412
rs429358
rs77697917
rs2287997
rs10455872
rs964184
rs139315015
rs12625329
rs1979215
rs139974673
rs7140110
rs6874202
rs146203232
rs76866386
rs117664574
rs7241918
rs77960347
rs187429064
rs9958734
rs2074493
rs9687846
rs56130071
rs4418728
rs76698942
rs10260606
rs2331415
rs4876611
rs17414716
rs660240
rs72555385
rs59950280
rs6968865
rs426684
rs2179011
rs185567543
rs36047579
rs4715317
rs11895352
rs1883711
rs75679663
rs458361
rs7609911
rs10195252
rs13112099
rs78058190
rs11749783
rs442177
rs117733303
rs141469619
rs4296389
rs1532085
rs1800588
rs1007205
rs11693150
rs2740488
All
-0.15
-0.10
-0.05
0.00
0.05
MR leave-one-out sensitivity analysis for
ted fatty acids (16:1, 18:1) levels || id:ebi-cfb233-GCST90302055' on 'ER- Breast cancer (Combined Oncoarray; iCOGS; GWAS meta

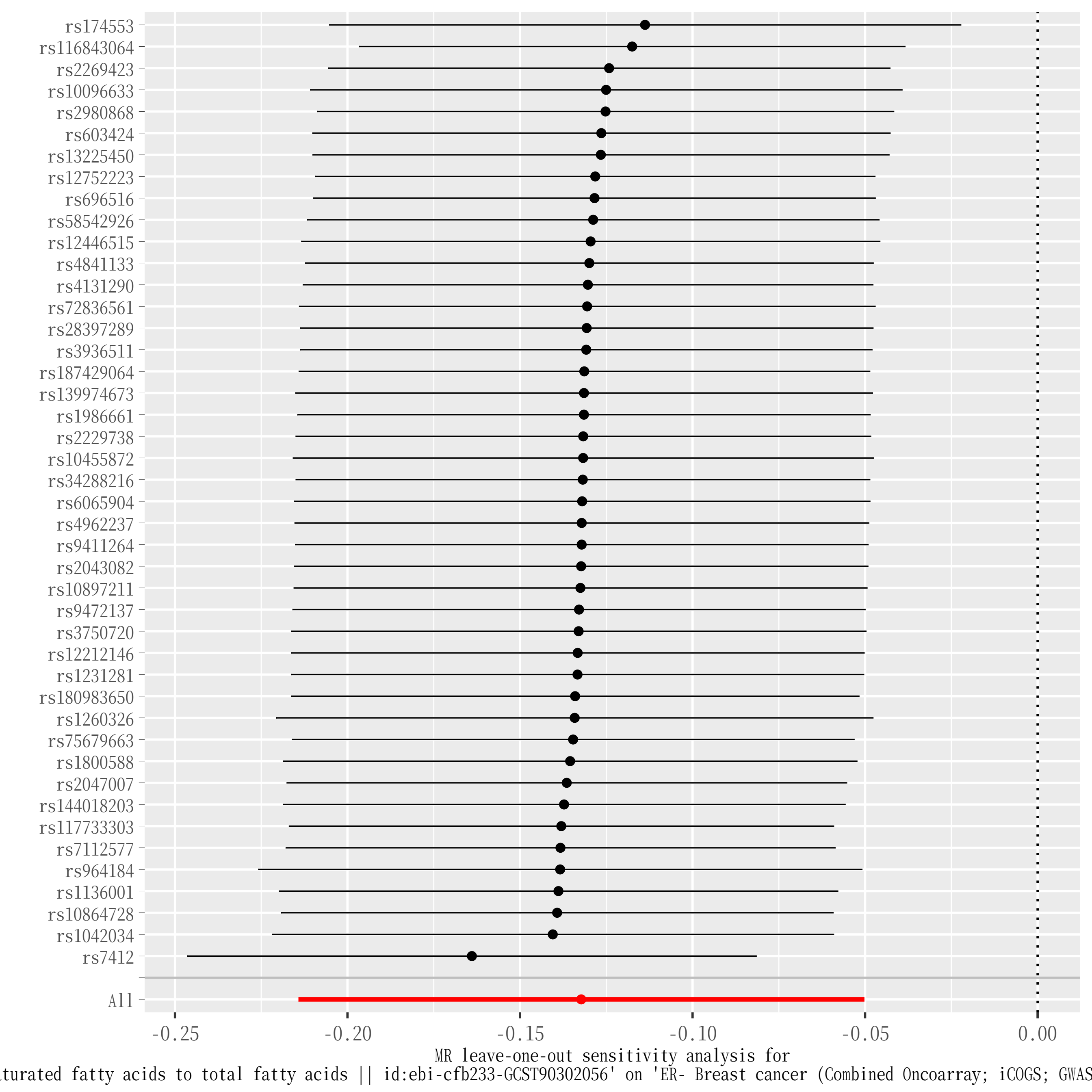

rs174553
rs116843064
rs2269423
rs10096633
rs2980868
rs603424
rs13225450
rs12752223
rs696516
rs58542926
rs12446515
rs4841133
rs4131290
rs72836561
rs28397289
rs3936511
rs187429064
rs139974673
rs1986661
rs2229738
rs10455872
rs34288216
rs6065904
rs4962237
rs9411264
rs2043082
rs10897211
rs9472137
rs3750720
rs12212146
rs1231281
rs180983650
rs1260326
rs75679663
rs1800588
rs2047007
rs144018203
rs117733303
rs7112577
rs964184
rs1136001
rs10864728
rs1042034
rs7412
All
-0.25
-0.20
-0.15
-0.10
-0.05
0.00
MR leave-one-out sensitivity analysis for
turated fatty acids to total fatty acids || id:ebi-cfb233-GCST90302056' on 'ER- Breast cancer (Combined Oncoarray; iCOGS; GWAS

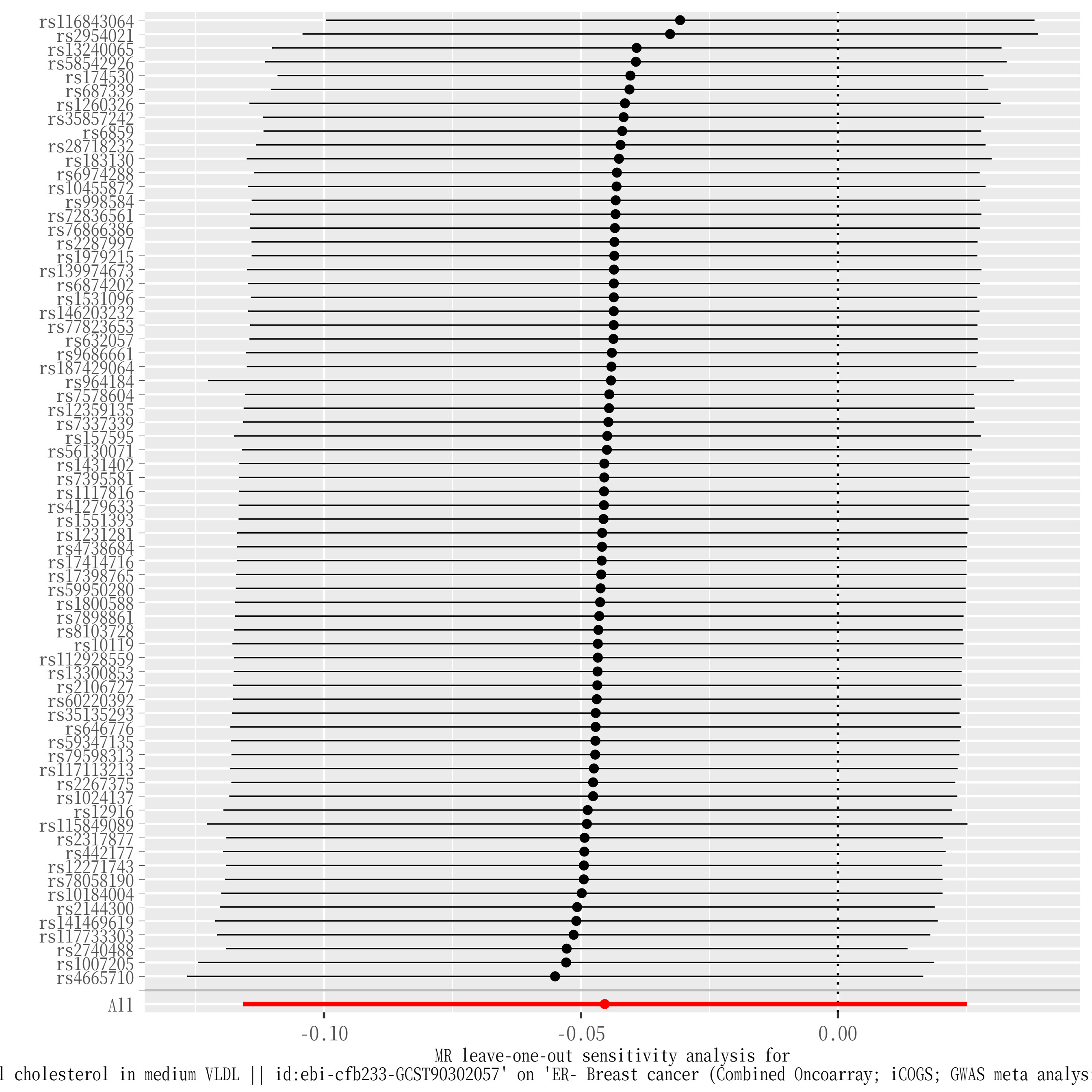

rs116843064
rs2954021
rs13240065
rs58542926
rs174530
rs687339
rs1260326
rs35857242
rs6859
rs28718232
rs183130
rs6974288
rs10455872
rs998584
rs72836561
rs76866386
rs2287997
rs1979215
rs139974673
rs6874202
rs1531096
rs146203232
rs77823653
rs632057
rs9686661
rs187429064
rs964184
rs7578604
rs12359135
rs7337339
rs157595
rs56130071
rs1431402
rs7395581
rs1117816
rs41279633
rs1551393
rs1231281
rs4738684
rs17414716
rs17398765
rs59950280
rs1800588
rs7898861
rs8103728
rs10119
rs112928559
rs13300853
rs2106727
rs60220392
rs35135293
rs646776
rs59347135
rs79598313
rs117113213
rs2267375
rs1024137
rs12916
rs115849089
rs2317877
rs442177
rs12271743
rs78058190
rs10184004
rs2144300
rs141469619
rs117733303
rs2740488
rs1007205
rs4665710
All
-0.10
-0.05
0.00
MR leave-one-out sensitivity analysis for
l cholesterol in medium VLDL || id:ebi-cfb233-GCST90302057' on 'ER- Breast cancer (Combined Oncoarray; iCOGS; GWAS meta analys

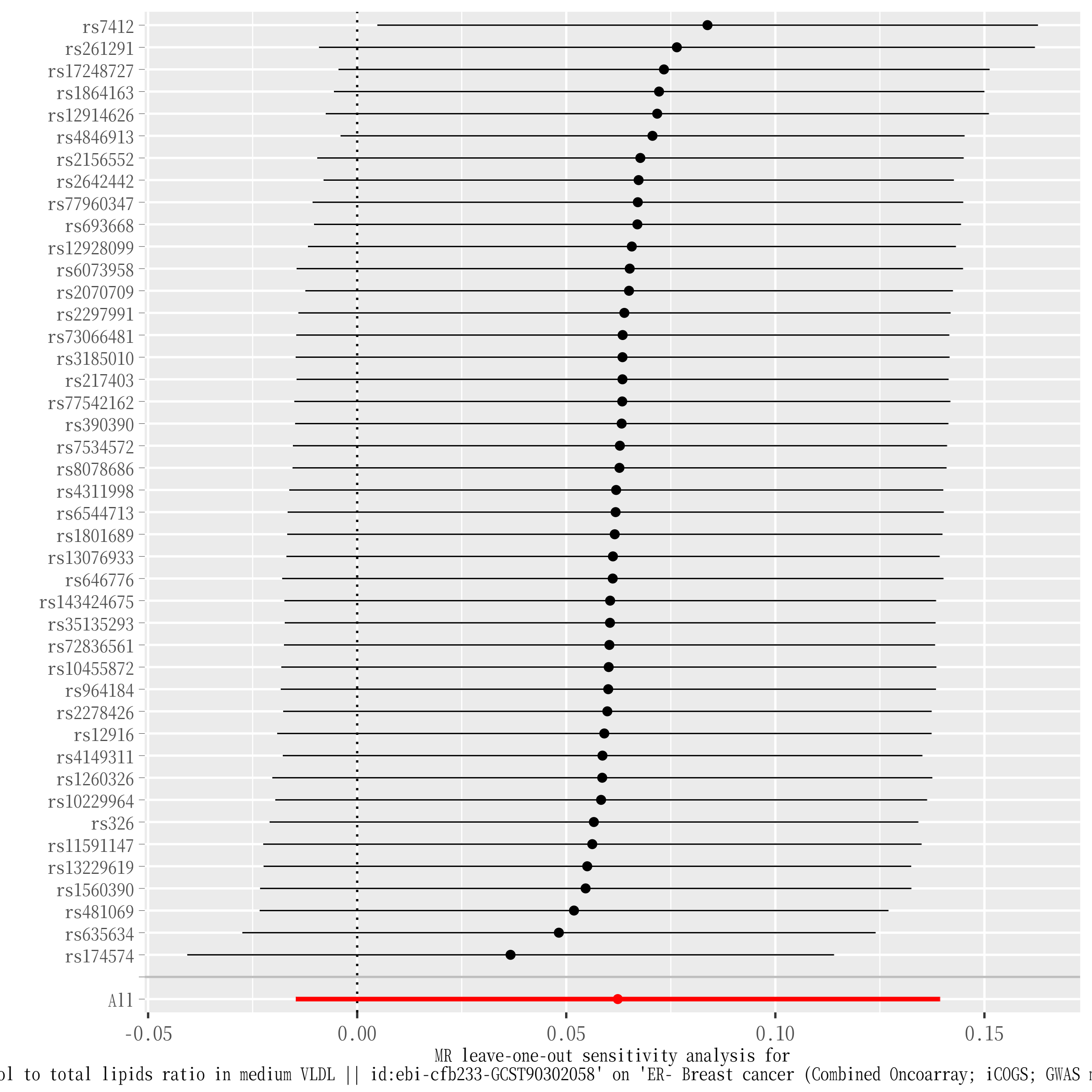
rs7412
rs261291
rs17248727
rs1864163
rs12914626
rs4846913
rs2156552
rs2642442
rs77960347
rs693668
rs12928099
rs6073958
rs2070709
rs2297991
rs73066481
rs3185010
rs217403
rs77542162
rs390390
rs7534572
rs8078686
rs4311998
rs6544713
rs1801689
rs13076933
rs646776
rs143424675
rs35135293
rs72836561
rs10455872
rs964184
rs2278426
rs12916
rs4149311
rs1260326
rs10229964
rs326
rs11591147
rs13229619
rs1560390
rs481069
rs635634
rs174574
All
-0.05
0.00
0.05
0.10
0.15
MR leave-one-out sensitivity analysis for
ol to total lipids ratio in medium VLDL || id:ebi-cfb233-GCST90302058' on 'ER- Breast cancer (Combined Oncoarray; iCOGS; GWAS r

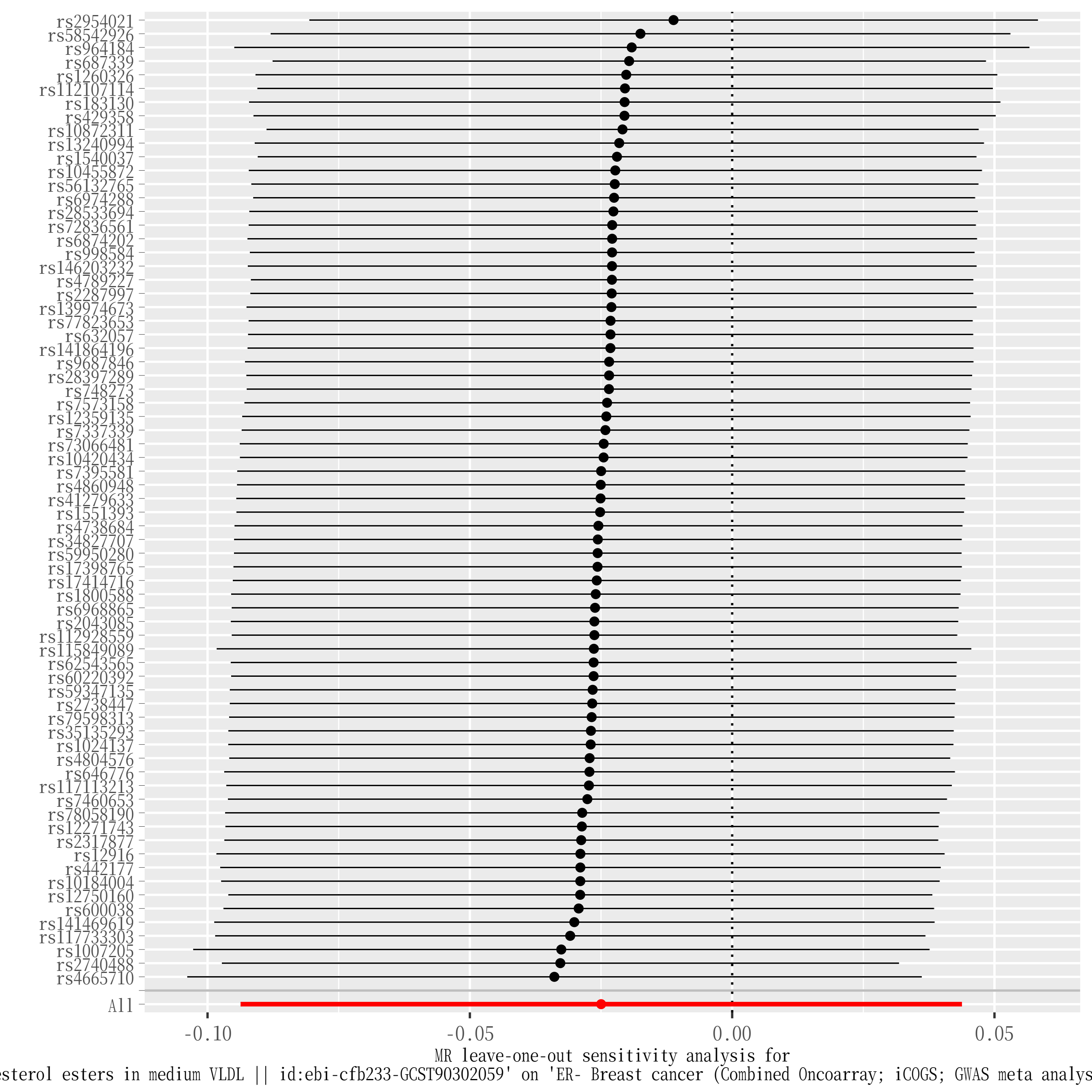
rs2954021
rs58542926
rs964184
rs687339
rs1260326
rs112107114
rs183130
rs429358
rs10872311
rs13240994
rs1540037
rs10455872
rs56132765
rs6974288
rs28533694
rs72836561
rs6874202
rs998584
rs146203232
rs4789227
rs2287997
rs139974673
rs77823653
rs632057
rs141864196
rs9687846
rs28397289
rs748273
rs7573158
rs12359135
rs7337339
rs73066481
rs10420434
rs7395581
rs4860948
rs41279633
rs1551393
rs4738684
rs34827707
rs59950280
rs17398765
rs17414716
rs1800588
rs6968865
rs2043085
rs112928559
rs115849089
rs62543565
rs60220392
rs59347135
rs2738447
rs79598313
rs35135293
rs1024137
rs4804576
rs646776
rs117113213
rs7460653
rs78058190
rs12271743
rs2317877
rs12916
rs442177
rs10184004
rs12750160
rs600038
rs141469619
rs117733303
rs1007205
rs2740488
rs4665710
All
-0.10
-0.05
0.00
0.05
MR leave-one-out sensitivity analysis for
esterol esters in medium VLDL || id:ebi-cfb233-GCST90302059' on 'ER- Breast cancer (Combined Oncoarray; iCOGS; GWAS meta analys

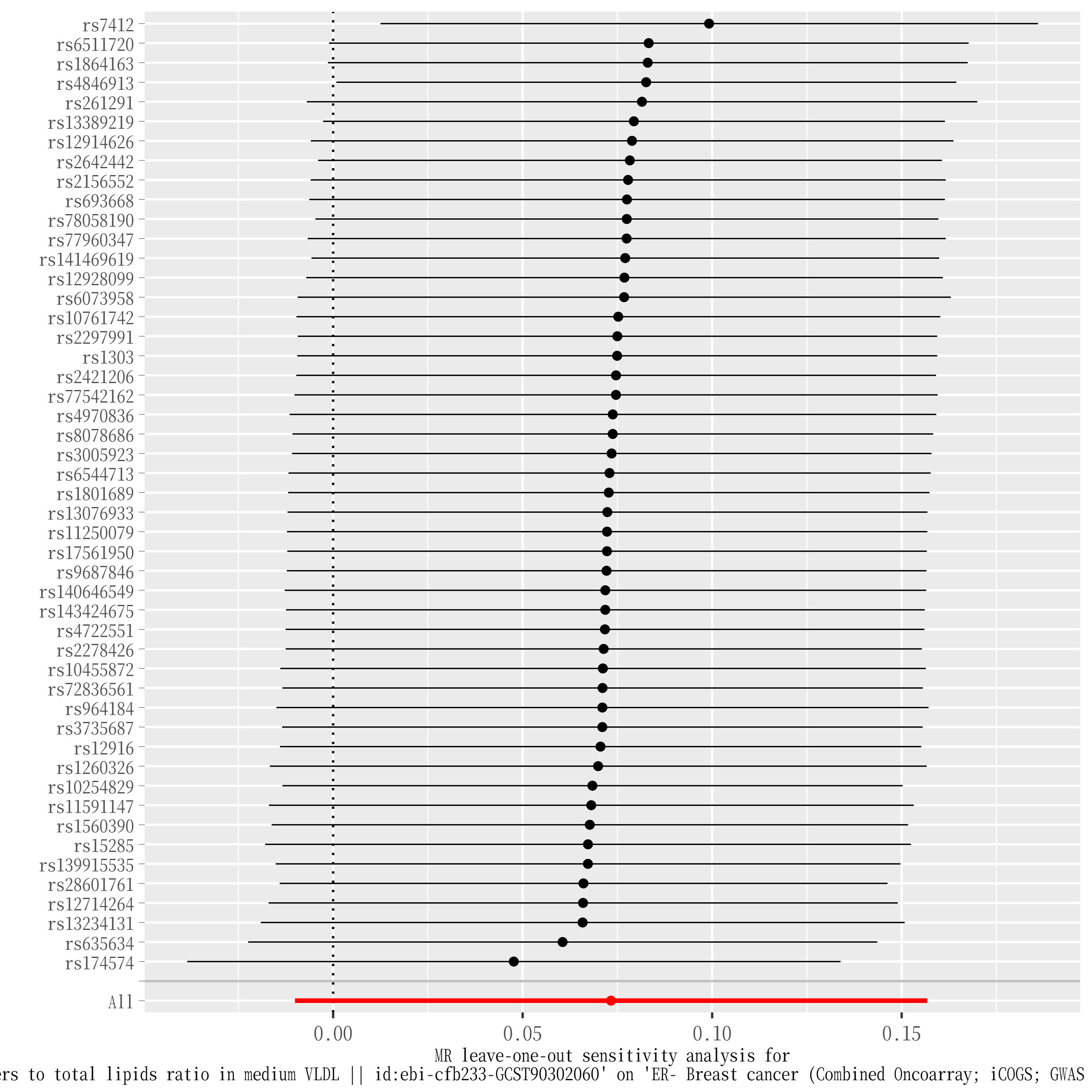

rs7412
rs6511720
rs1864163
rs4846913
rs261291
rs13389219
rs12914626
rs2642442
rs2156552
rs693668
rs78058190
rs77960347
rs141469619
rs12928099
rs6073958
rs10761742
rs2297991
rs1303
rs2421206
rs77542162
rs4970836
rs8078686
rs3005923
rs6544713
rs1801689
rs13076933
rs11250079
rs17561950
rs9687846
rs140646549
rs143424675
rs4722551
rs2278426
rs10455872
rs72836561
rs964184
rs3735687
rs12916
rs1260326
rs10254829
rs11591147
rs1560390
rs15285
rs139915535
rs28601761
rs12714264
rs13234131
rs635634
rs174574
All
0.00
0.05
0.10
0.15
MR leave-one-out sensitivity analysis for
ers to total lipids ratio in medium VLDL || id:ebi-cfb233-GCST90302060' on 'ER- Breast cancer (Combined Oncoarray; iCOGS; GWAS

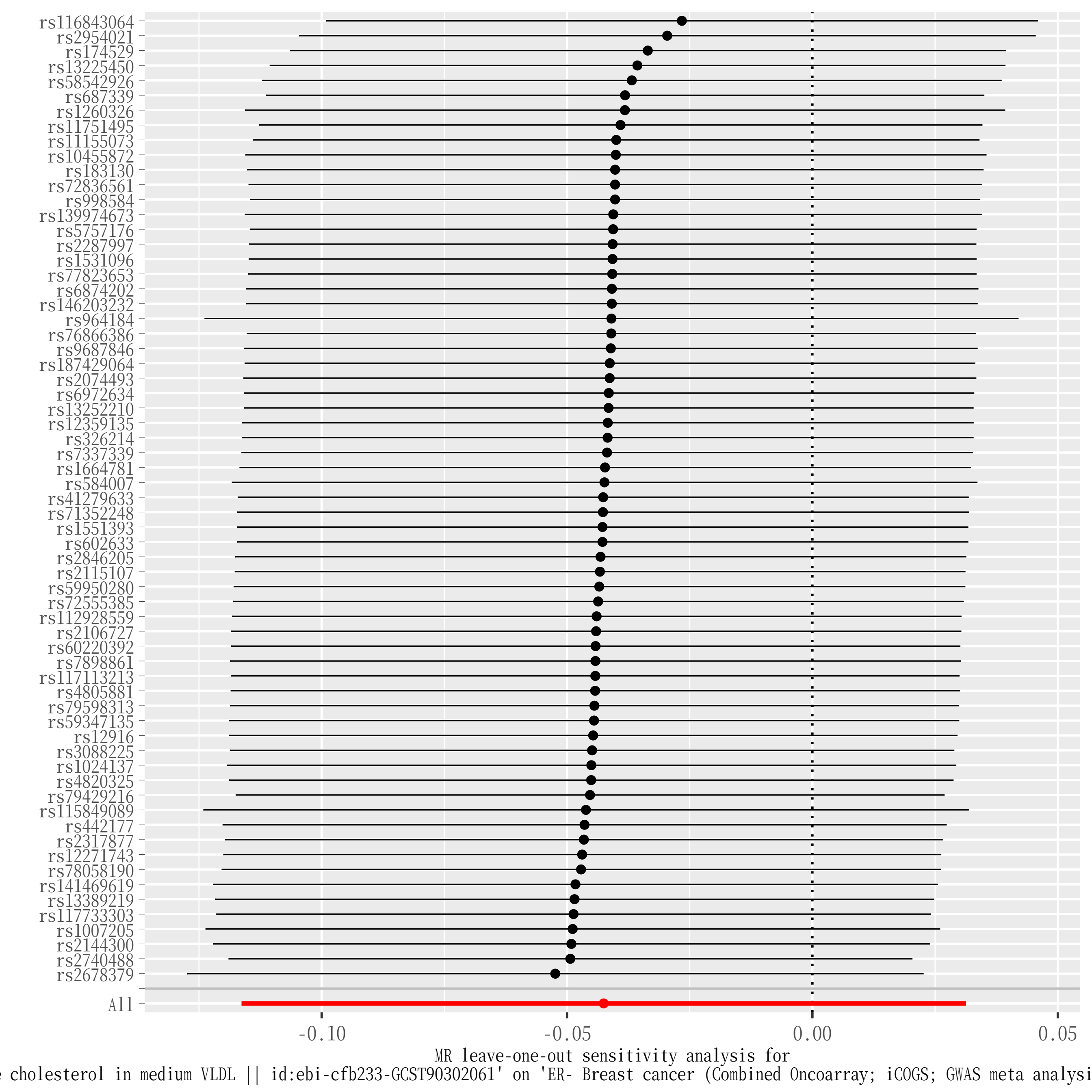
rs116843064
rs2954021
rs174529
rs13225450
rs58542926
rs687339
rs1260326
rs11751495
rs11155073
rs10455872
rs183130
rs72836561
rs998584
rs139974673
rs5757176
rs2287997
rs1531096
rs77823653
rs6874202
rs146203232
rs964184
rs76866386
rs9687846
rs187429064
rs2074493
rs6972634
rs13252210
rs12359135
rs326214
rs7337339
rs1664781
rs584007
rs41279633
rs71352248
rs1551393
rs602633
rs2846205
rs2115107
rs59950280
rs72555385
rs112928559
rs2106727
rs60220392
rs7898861
rs117113213
rs4805881
rs79598313
rs59347135
rs12916
rs3088225
rs1024137
rs4820325
rs79429216
rs115849089
rs442177
rs2317877
rs12271743
rs78058190
rs141469619
rs13389219
rs117733303
rs1007205
rs2144300
rs2740488
rs2678379
All
-0.10
-0.05
0.00
0.05
MR leave-one-out sensitivity analysis for
e cholesterol in medium VLDL || id:ebi-cfb233-GCST90302061' on 'ER- Breast cancer (Combined Oncoarray; iCOGS; GWAS meta analysi

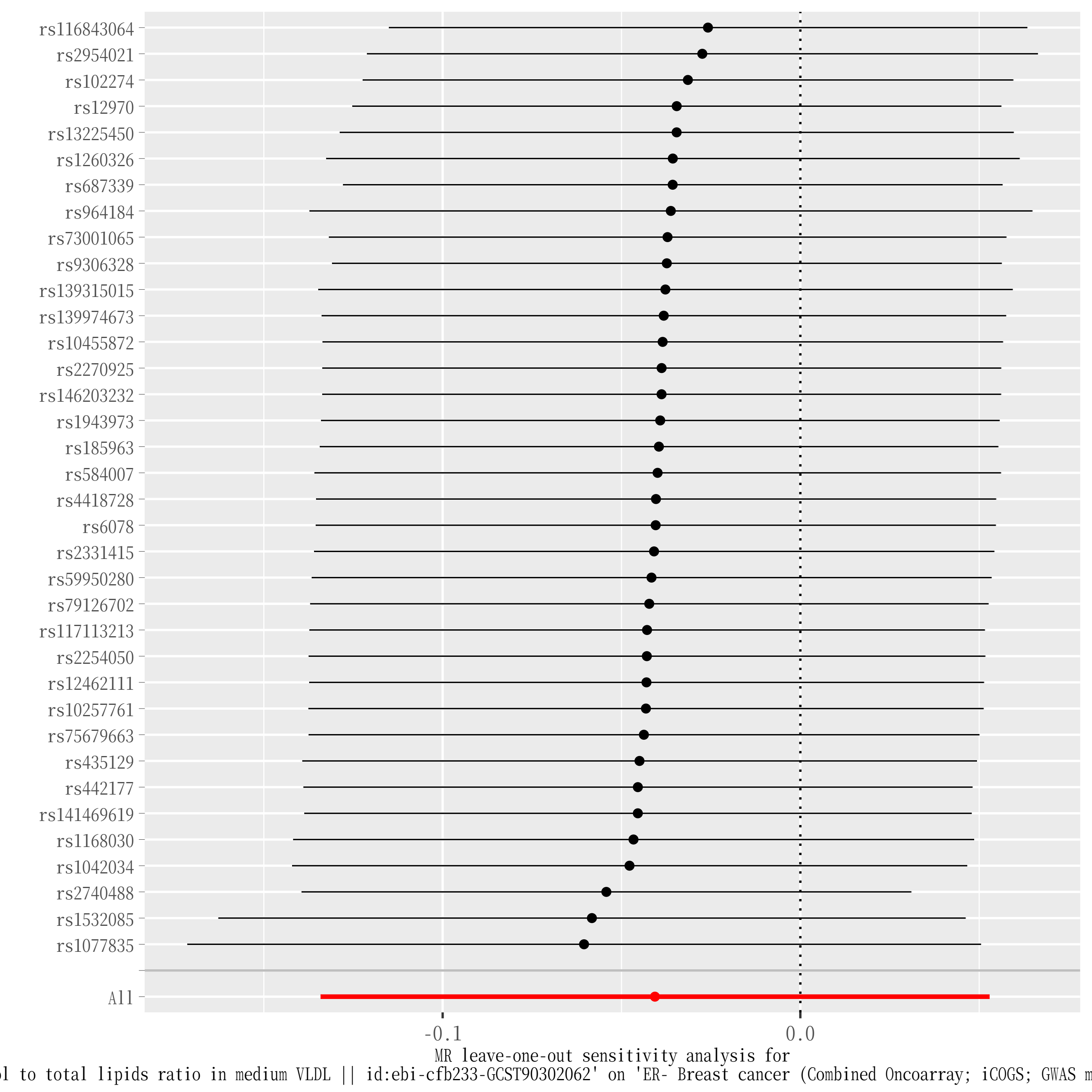

rs116843064
rs2954021
rs102274
rs12970
rs13225450
rs1260326
rs687339
rs964184
rs73001065
rs9306328
rs139315015
rs139974673
rs10455872
rs2270925
rs146203232
rs1943973
rs185963
rs584007
rs4418728
rs6078
rs2331415
rs59950280
rs79126702
rs117113213
rs2254050
rs12462111
rs10257761
rs75679663
rs435129
rs442177
rs141469619
rs1168030
rs1042034
rs2740488
rs1532085
rs1077835
All
-0.1
0.0
MR leave-one-out sensitivity analysis for
l to total lipids ratio in medium VLDL || id:ebi-cfb233-GCST90302062' on 'ER- Breast cancer (Combined Oncoarray; iCOGS; GWAS m

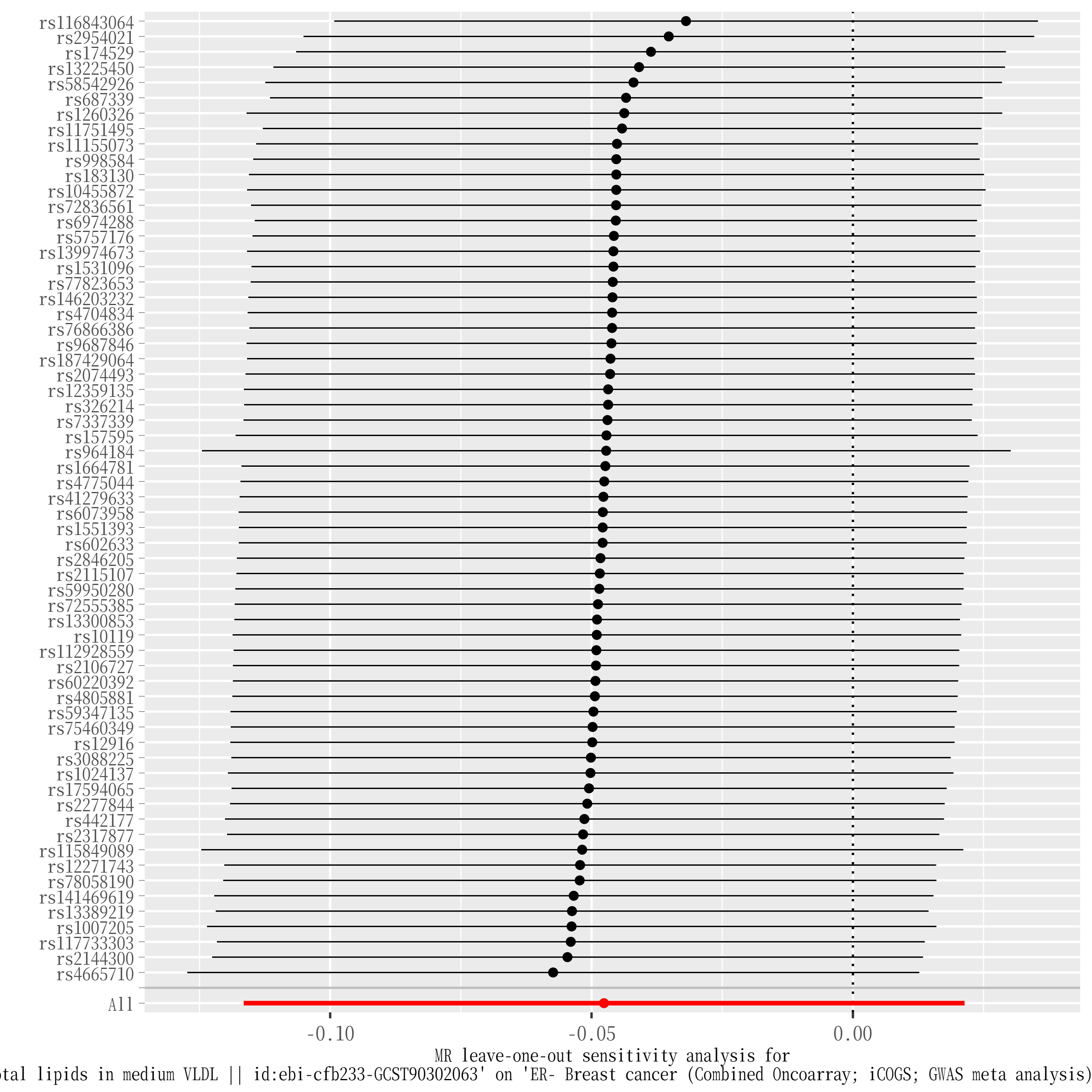
rs116843064
rs2954021
rs174529
rs13225450
rs58542926
rs687339
rs1260326
rs11751495
rs11155073
rs998584
rs183130
rs10455872
rs72836561
rs6974288
rs5757176
rs139974673
rs1531096
rs77823653
rs146203232
rs4704834
rs76866386
rs9687846
rs187429064
rs2074493
rs12359135
rs326214
rs7337339
rs157595
rs964184
rs1664781
rs4775044
rs41279633
rs6073958
rs1551393
rs602633
rs2846205
rs2115107
rs59950280
rs72555385
rs13300853
rs10119
rs112928559
rs2106727
rs60220392
rs4805881
rs59347135
rs75460349
rs12916
rs3088225
rs1024137
rs17594065
rs2277844
rs442177
rs2317877
rs115849089
rs12271743
rs78058190
rs141469619
rs13389219
rs1007205
rs117733303
rs2144300
rs4665710
All
-0.10
-0.05
0.00
MR leave-one-out sensitivity analysis for
otal lipids in medium VLDL || id:ebi-cfb233-GCST90302063' on 'ER- Breast cancer (Combined Oncoarray; iCOGS; GWAS meta analysis)

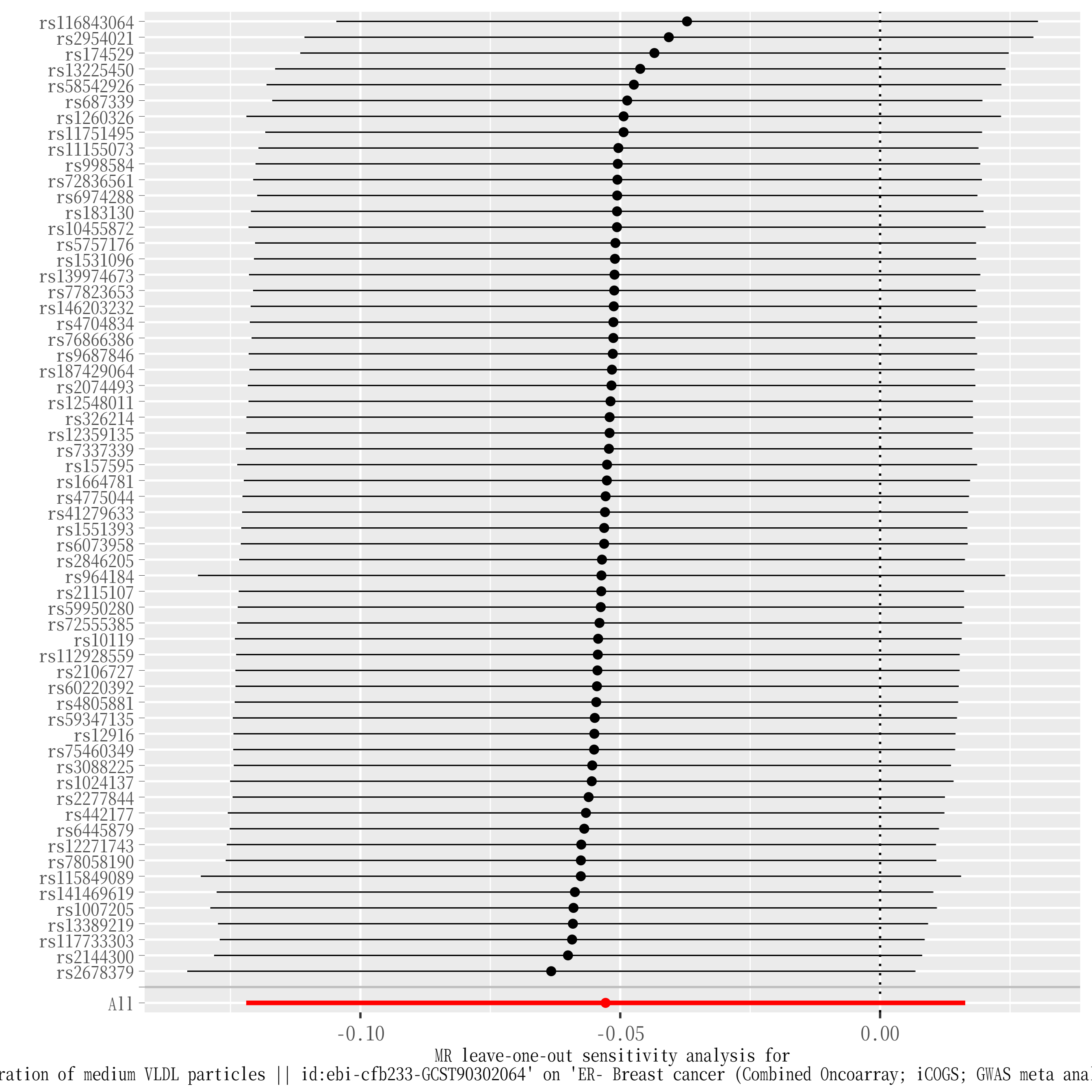

rs116843064
rs2954021
rs174529
rs13225450
rs58542926
rs687339
rs1260326
rs11751495
rs11155073
rs998584
rs72836561
rs6974288
rs183130
rs10455872
rs5757176
rs1531096
rs139974673
rs77823653
rs146203232
rs4704834
rs76866386
rs9687846
rs187429064
rs2074493
rs12548011
rs326214
rs12359135
rs7337339
rs157595
rs1664781
rs4775044
rs41279633
rs1551393
rs6073958
rs2846205
rs964184
rs2115107
rs59950280
rs72555385
rs10119
rs112928559
rs2106727
rs60220392
rs4805881
rs59347135
rs12916
rs75460349
rs3088225
rs1024137
rs2277844
rs442177
rs6445879
rs12271743
rs78058190
rs115849089
rs141469619
rs1007205
rs13389219
rs117733303
rs2144300
rs2678379
All
-0.10
-0.05
0.00
MR leave-one-out sensitivity analysis for
ration of medium VLDL particles || id:ebi-cfb233-GCST90302064' on 'ER- Breast cancer (Combined Oncoarray; iCOGS; GWAS meta anal

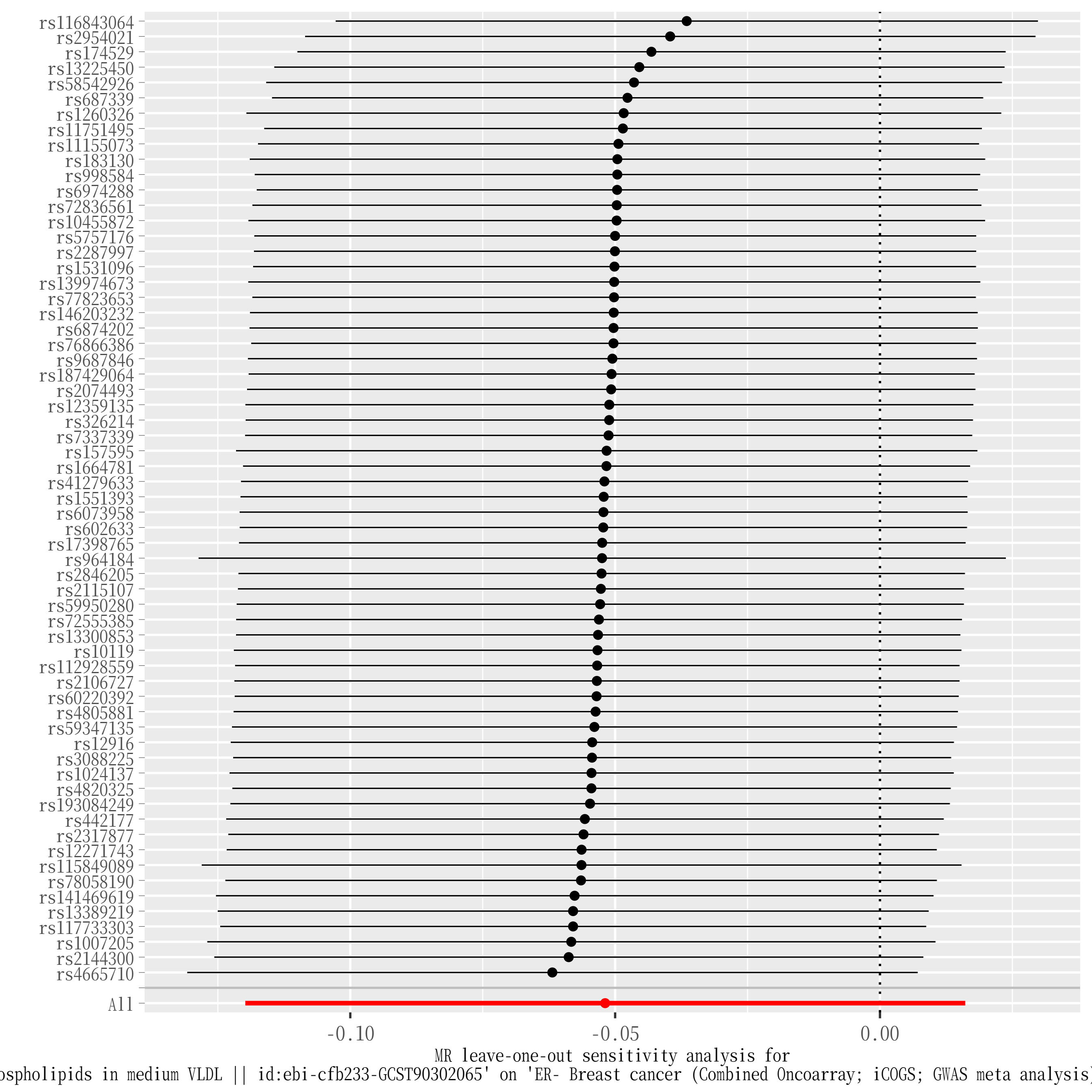

rs116843064
rs2954021
rs174529
rs13225450
rs58542926
rs687339
rs1260326
rs11751495
rs11155073
rs183130
rs998584
rs6974288
rs72836561
rs10455872
rs5757176
rs2287997
rs1531096
rs139974673
rs77823653
rs146203232
rs6874202
rs76866386
rs9687846
rs187429064
rs2074493
rs12359135
rs326214
rs7337339
rs157595
rs1664781
rs41279633
rs1551393
rs6073958
rs602633
rs17398765
rs964184
rs2846205
rs2115107
rs59950280
rs72555385
rs13300853
rs10119
rs112928559
rs2106727
rs60220392
rs4805881
rs59347135
rs12916
rs3088225
rs1024137
rs4820325
rs193084249
rs442177
rs2317877
rs12271743
rs115849089
rs78058190
rs141469619
rs13389219
rs117733303
rs1007205
rs2144300
rs4665710
All
-0.10
-0.05
0.00
MR leave-one-out sensitivity analysis for
ospholipids in medium VLDL || id:ebi-cfb233-GCST90302065' on 'ER- Breast cancer (Combined Oncoarray; iCOGS; GWAS meta analysis)

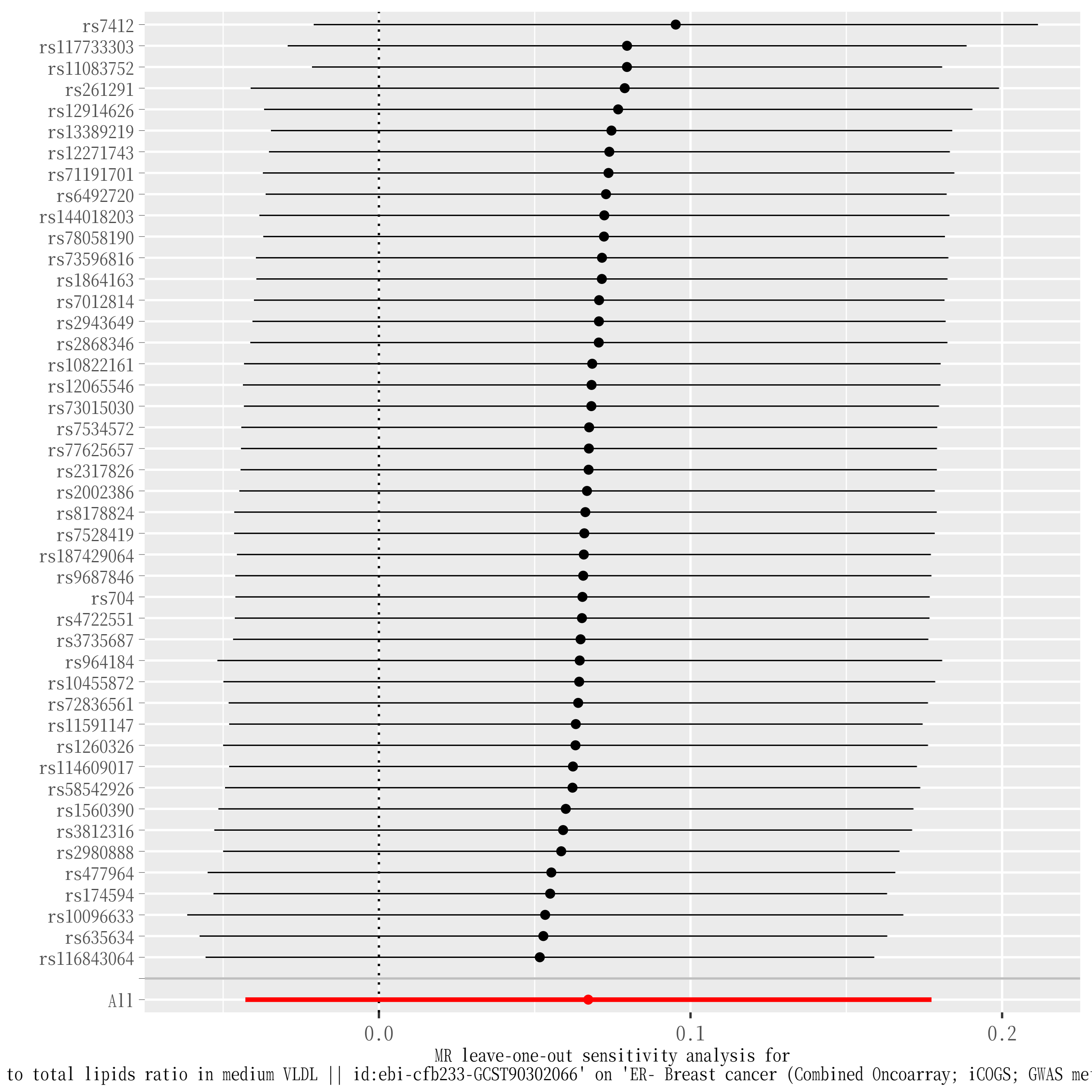
rs7412
rs117733303
rs11083752
rs261291
rs12914626
rs13389219
rs12271743
rs71191701
rs6492720
rs144018203
rs78058190
rs73596816
rs1864163
rs7012814
rs2943649
rs2868346
rs10822161
rs12065546
rs73015030
rs7534572
rs77625657
rs2317826
rs2002386
rs8178824
rs7528419
rs187429064
rs9687846
rs704
rs4722551
rs3735687
rs964184
rs10455872
rs72836561
rs11591147
rs1260326
rs114609017
rs58542926
rs1560390
rs3812316
rs2980888
rs477964
rs174594
rs10096633
rs635634
rs116843064
All
0.0
0.1
0.2
MR leave-one-out sensitivity analysis for
to total lipids ratio in medium VLDL || id:ebi-cfb233-GCST90302066' on 'ER- Breast cancer (Combined Oncoarray; iCOGS; GWAS me

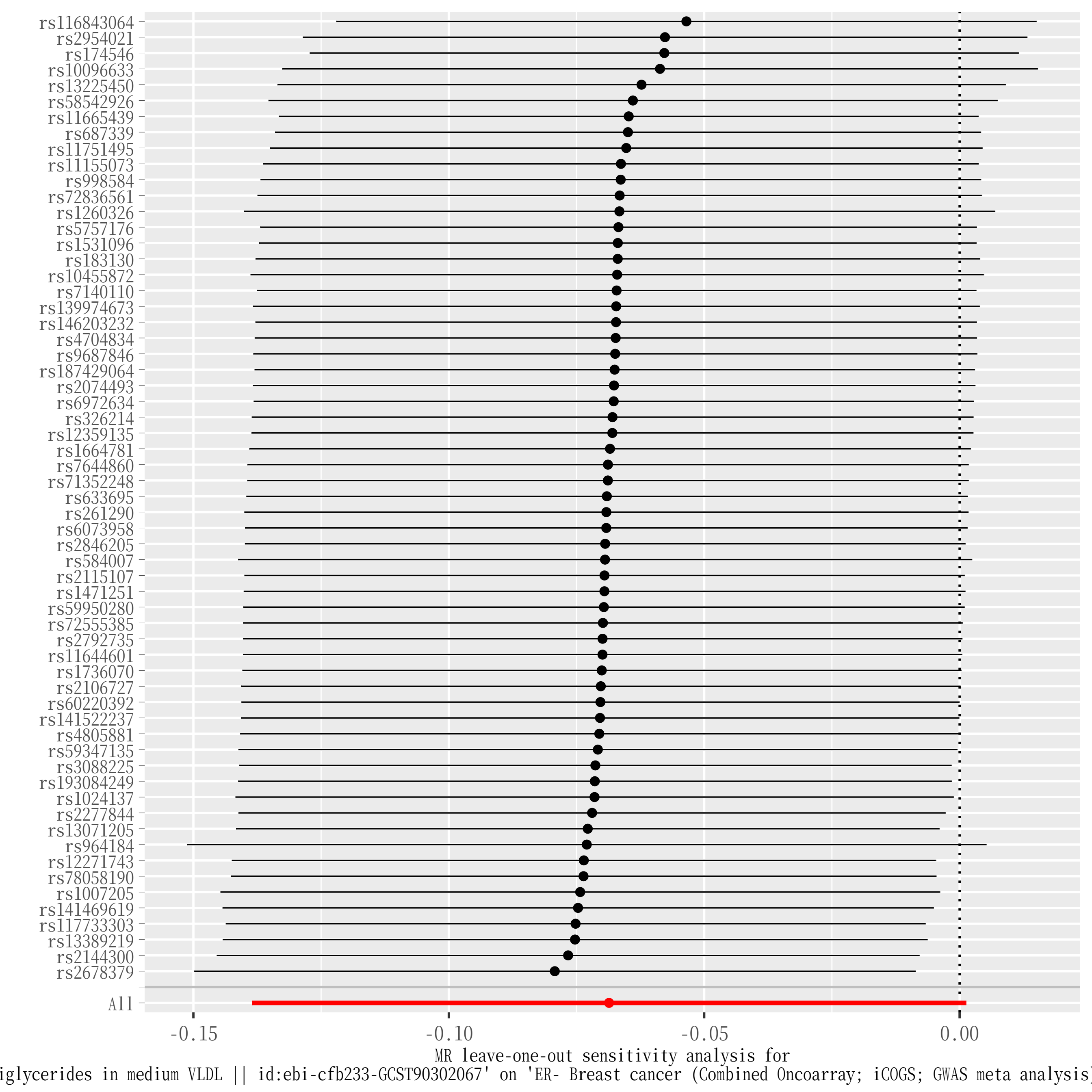

rs116843064
rs2954021
rs174546
rs10096633
rs13225450
rs58542926
rs11665439
rs687339
rs11751495
rs11155073
rs998584
rs72836561
rs1260326
rs5757176
rs1531096
rs183130
rs10455872
rs7140110
rs139974673
rs146203232
rs4704834
rs9687846
rs187429064
rs2074493
rs6972634
rs326214
rs12359135
rs1664781
rs7644860
rs71352248
rs633695
rs261290
rs6073958
rs2846205
rs584007
rs2115107
rs1471251
rs59950280
rs72555385
rs2792735
rs11644601
rs1736070
rs2106727
rs60220392
rs141522237
rs4805881
rs59347135
rs3088225
rs193084249
rs1024137
rs2277844
rs13071205
rs964184
rs12271743
rs78058190
rs1007205
rs141469619
rs117733303
rs13389219
rs2144300
rs2678379
All
-0.15
-0.10
-0.05
0.00
MR leave-one-out sensitivity analysis for
iglycerides in medium VLDL || id:ebi-cfb233-GCST90302067' on 'ER- Breast cancer (Combined Oncoarray; iCOGS; GWAS meta analysis)

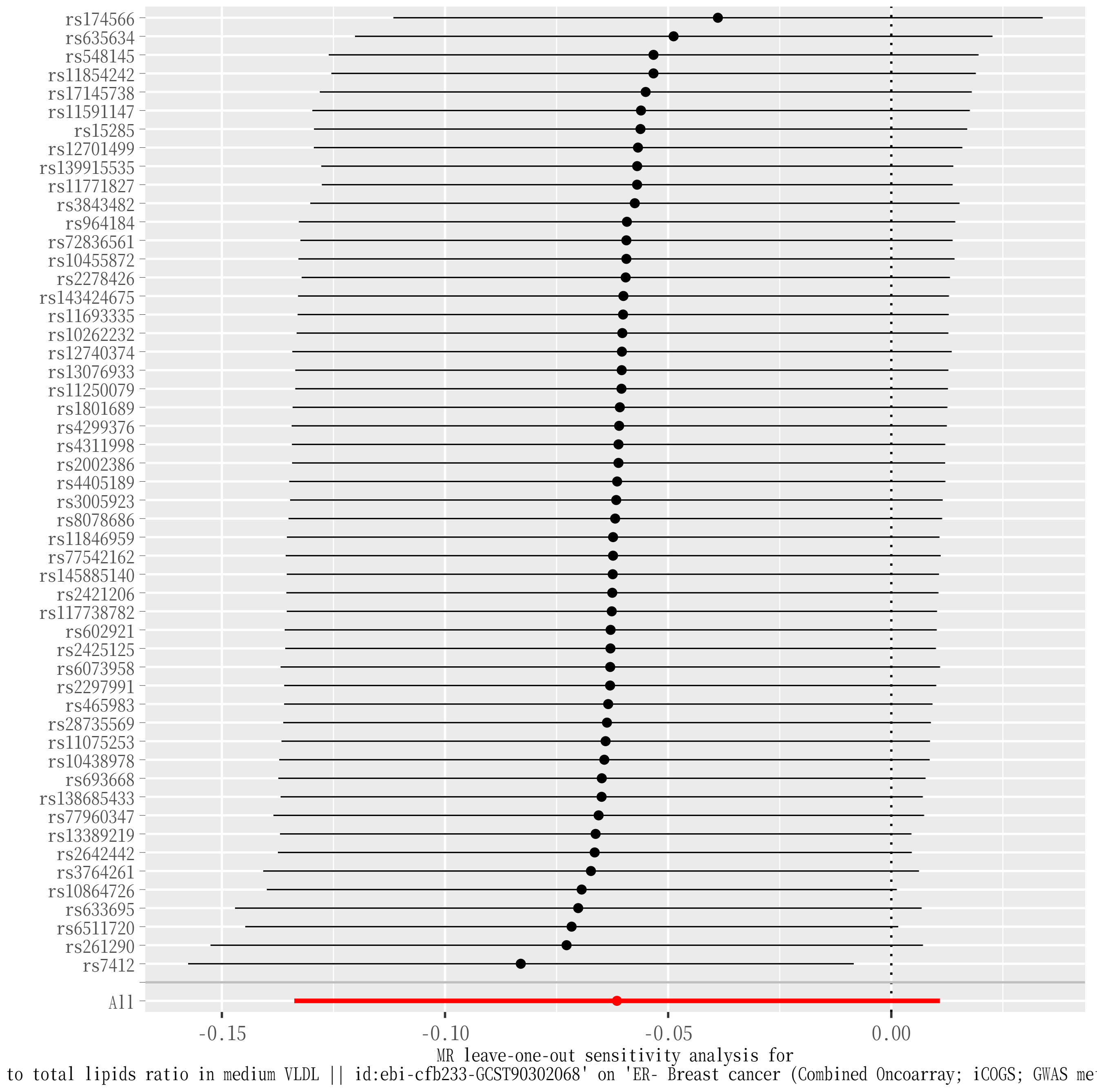
rs174566
rs635634
rs548145
rs11854242
rs17145738
rs11591147
rs15285
rs12701499
rs139915535
rs11771827
rs3843482
rs964184
rs72836561
rs10455872
rs2278426
rs143424675
rs11693335
rs10262232
rs12740374
rs13076933
rs11250079
rs1801689
rs4299376
rs4311998
rs2002386
rs4405189
rs3005923
rs8078686
rs11846959
rs77542162
rs145885140
rs2421206
rs117738782
rs602921
rs2425125
rs6073958
rs2297991
rs465983
rs28735569
rs11075253
rs10438978
rs693668
rs138685433
rs77960347
rs13389219
rs2642442
rs3764261
rs10864726
rs633695
rs6511720
rs261290
rs7412
All
-0.15
-0.10
-0.05
0.00
MR leave-one-out sensitivity analysis for
to total lipids ratio in medium VLDL || id:ebi-cfb233-GCST90302068' on 'ER- Breast cancer (Combined Oncoarray; iCOGS; GWAS me

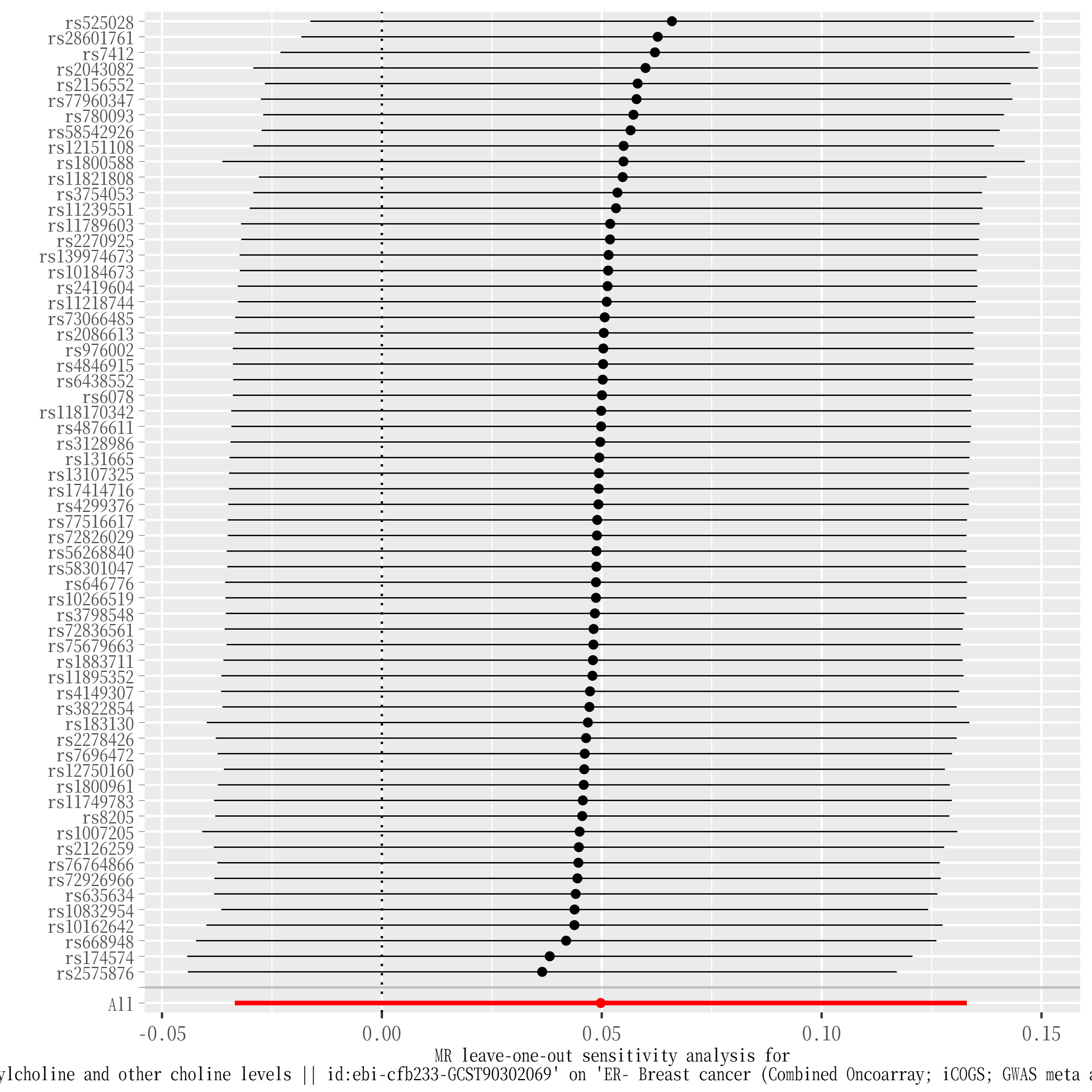
rs525028
rs28601761
rs7412
rs2043082
rs2156552
rs77960347
rs780093
rs58542926
rs12151108
rs1800588
rs11821808
rs3754053
rs11239551
rs11789603
rs2270925
rs139974673
rs10184673
rs2419604
rs11218744
rs73066485
rs2086613
rs976002
rs4846915
rs6438552
rs6078
rs118170342
rs4876611
rs3128986
rs131665
rs13107325
rs17414716
rs4299376
rs77516617
rs72826029
rs56268840
rs58301047
rs646776
rs10266519
rs3798548
rs72836561
rs75679663
rs1883711
rs11895352
rs4149307
rs3822854
rs183130
rs2278426
rs7696472
rs12750160
rs1800961
rs11749783
rs8205
rs1007205
rs2126259
rs76764866
rs72926966
rs635634
rs10832954
rs10162642
rs668948
rs174574
rs2575876
All
-0.05
0.00
0.05
0.10
0.15
MR leave-one-out sensitivity analysis for
ylcholine and other choline levels || id:ebi-cfb233-GCST90302069' on 'ER- Breast cancer (Combined Oncoarray; iCOGS; GWAS meta a

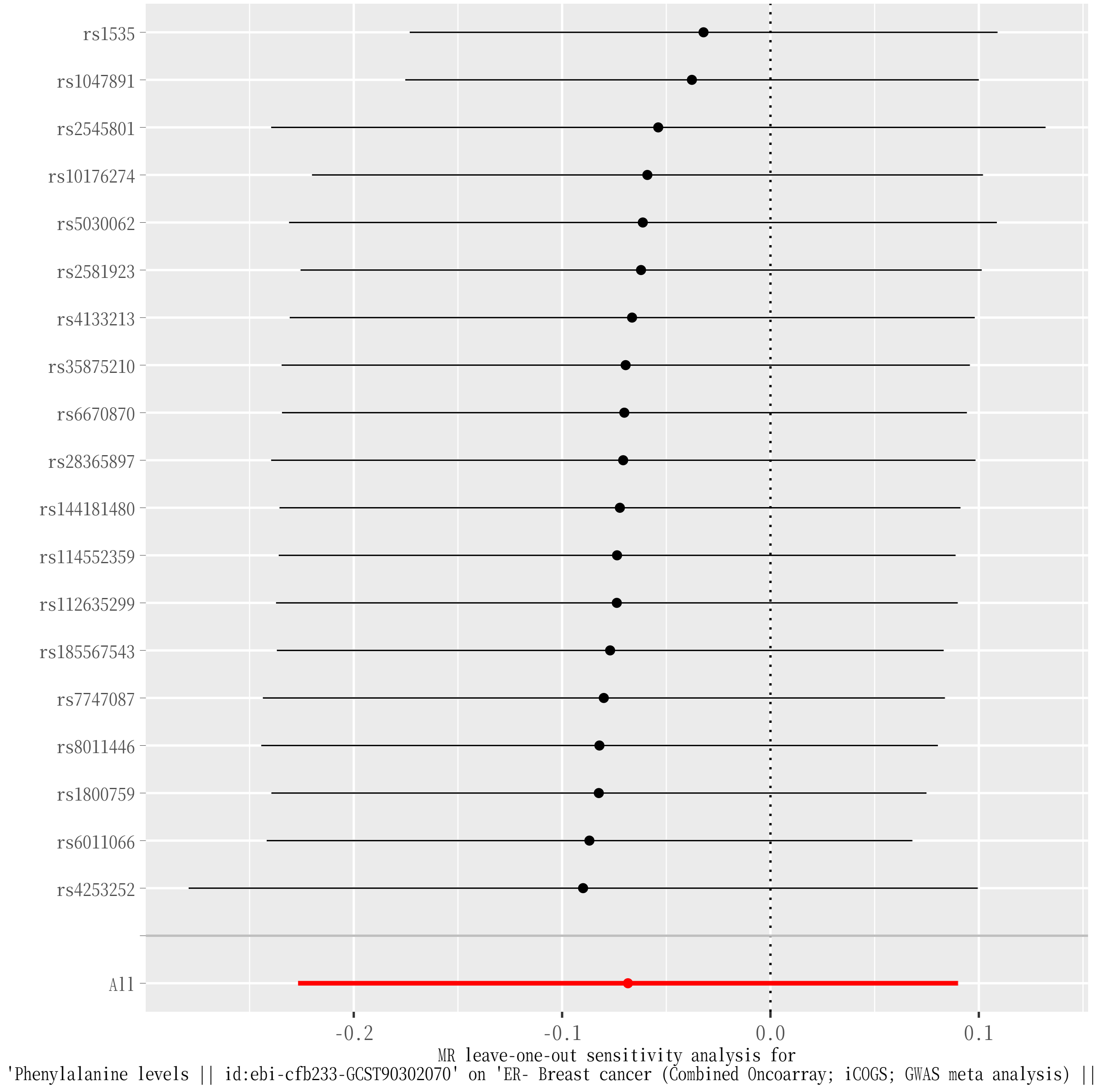
rs1535
rs1047891
rs2545801
rs10176274
rs5030062
rs2581923
rs4133213
rs35875210
rs6670870
rs28365897
rs144181480
rs114552359
rs112635299
rs185567543
rs7747087
rs8011446
rs1800759
rs6011066
rs4253252
All
-0.2
-0.1
0.0
0.1
MR leave-one-out sensitivity analysis for
'Phenylalanine levels || id:ebi-cfb233-GCST90302070' on 'ER- Breast cancer (Combined Oncoarray; iCOGS; GWAS meta analysis) ||

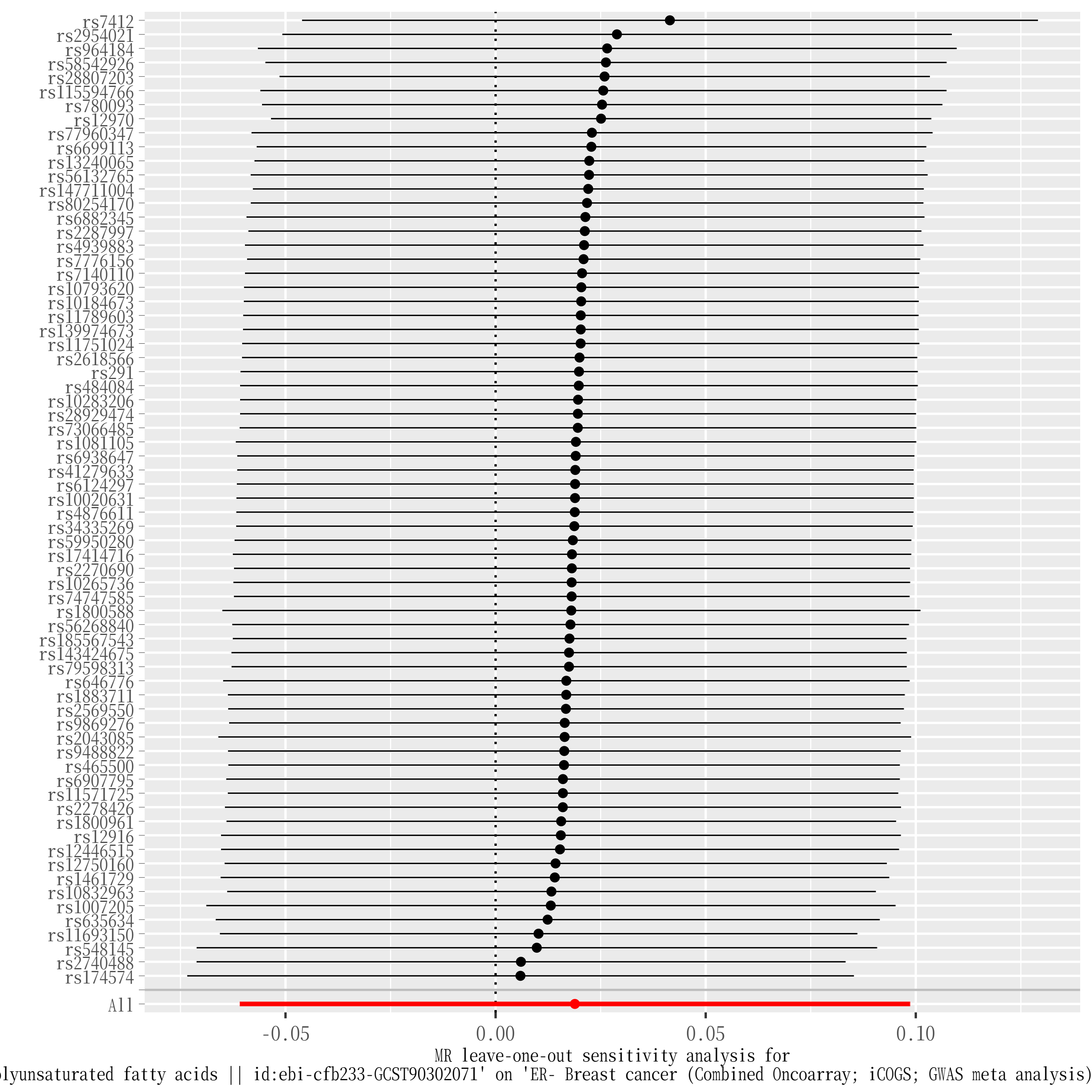

rs7412
rs2954021
rs964184
rs58542926
rs28807203
rs115594766
rs780093
rs12970
rs77960347
rs6699113
rs13240065
rs56132765
rs147711004
rs80254170
rs6882345
rs2287997
rs4939883
rs7776156
rs7140110
rs10793620
rs10184673
rs11789603
rs139974673
rs11751024
rs2618566
rs291
rs484084
rs10283206
rs28929474
rs73066485
rs1081105
rs6938647
rs41279633
rs6124297
rs10020631
rs4876611
rs34335269
rs59950280
rs17414716
rs2270690
rs10265736
rs74747585
rs1800588
rs56268840
rs185567543
rs143424675
rs79598313
rs646776
rs1883711
rs2569550
rs9869276
rs2043085
rs9488822
rs465500
rs6907795
rs11571725
rs2278426
rs1800961
rs12916
rs12446515
rs12750160
rs1461729
rs10832963
rs1007205
rs635634
rs11693150
rs548145
rs2740488
rs174574
All
-0.05
0.00
0.05
0.10
MR leave-one-out sensitivity analysis for
lyunsaturated fatty acids || id:ebi-cfb233-GCST90302071' on 'ER- Breast cancer (Combined Oncoarray; iCOGS; GWAS meta analysis)

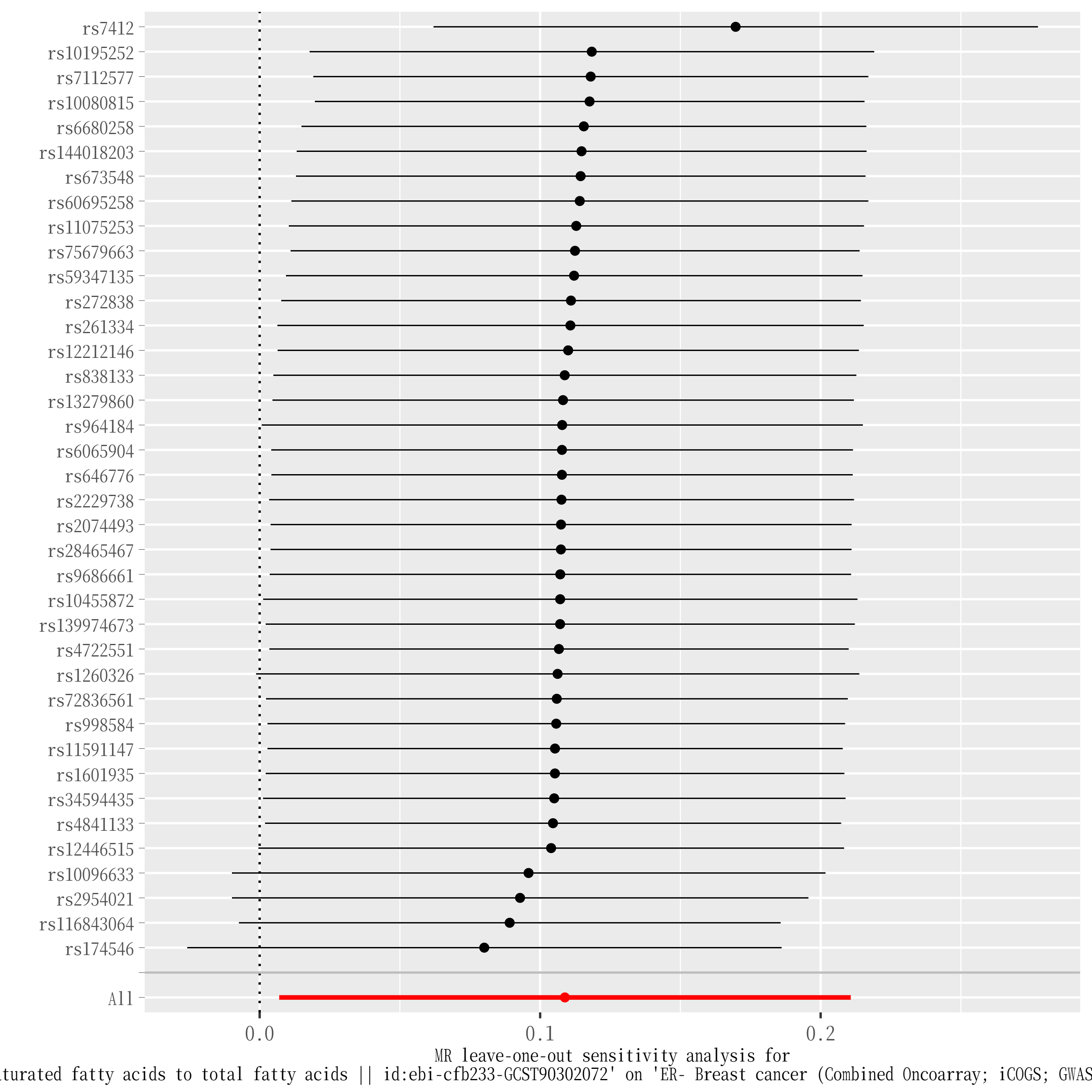

rs7412
rs10195252
rs7112577
rs10080815
rs6680258
rs144018203
rs673548
rs60695258
rs11075253
rs75679663
rs59347135
rs272838
rs261334
rs12212146
rs838133
rs13279860
rs964184
rs6065904
rs646776
rs2229738
rs2074493
rs28465467
rs9686661
rs10455872
rs139974673
rs4722551
rs1260326
rs72836561
rs998584
rs11591147
rs1601935
rs34594435
rs4841133
rs12446515
rs10096633
rs2954021
rs116843064
rs174546
All
0.0
0.1
0.2
MR leave-one-out sensitivity analysis for
turated fatty acids to total fatty acids || id:ebi-cfb233-GCST90302072' on 'ER- Breast cancer (Combined Oncoarray; iCOGS; GWAS

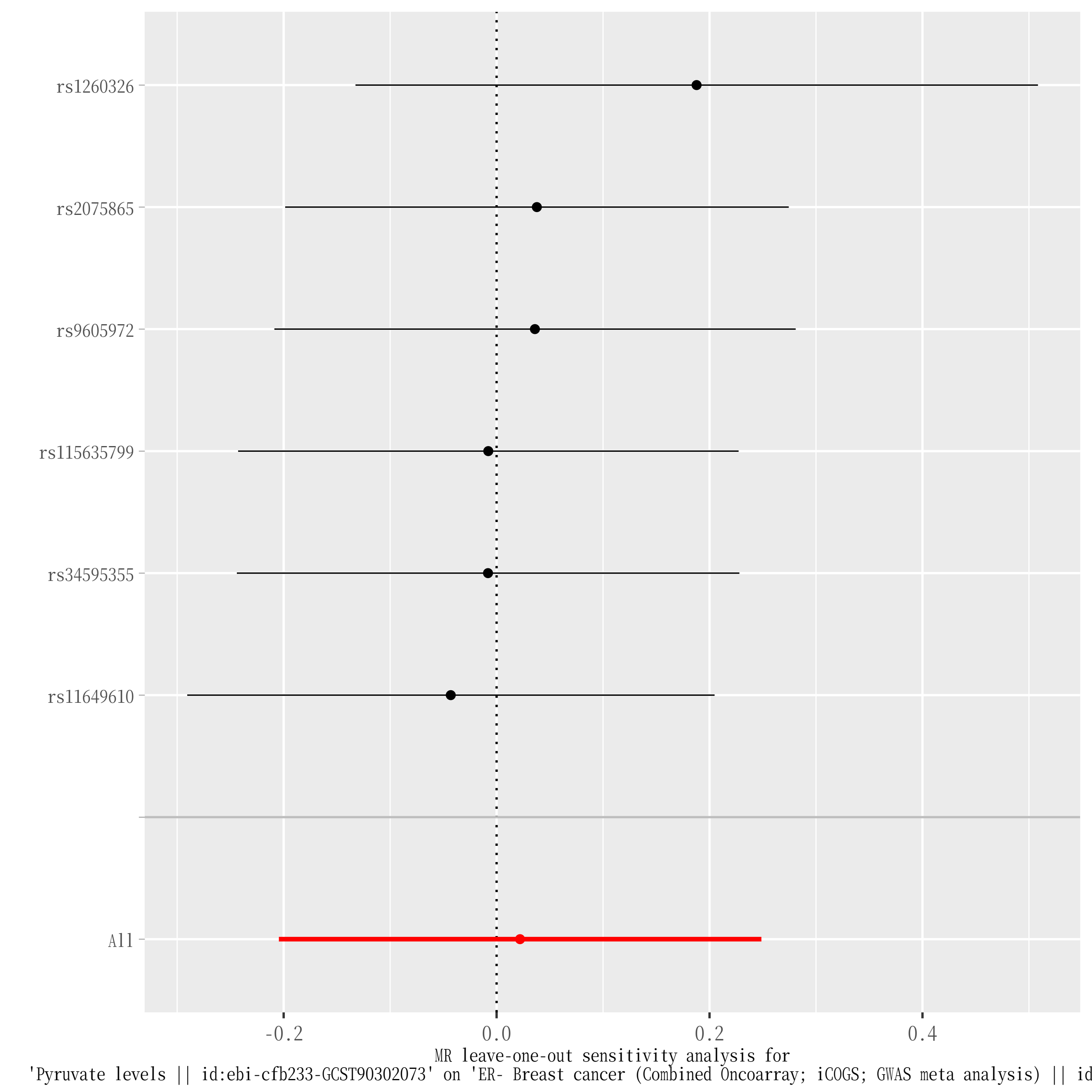
rs1260326
rs2075865
rs9605972
rs115635799
rs34595355
rs11649610
All
-0.2
0.0
0.2
0.4
MR leave-one-out sensitivity analysis for
'Pyruvate levels || id:ebi-cfb233-GCST90302073' on 'ER- Breast cancer (Combined Oncoarray; iCOGS; GWAS meta analysis) || id

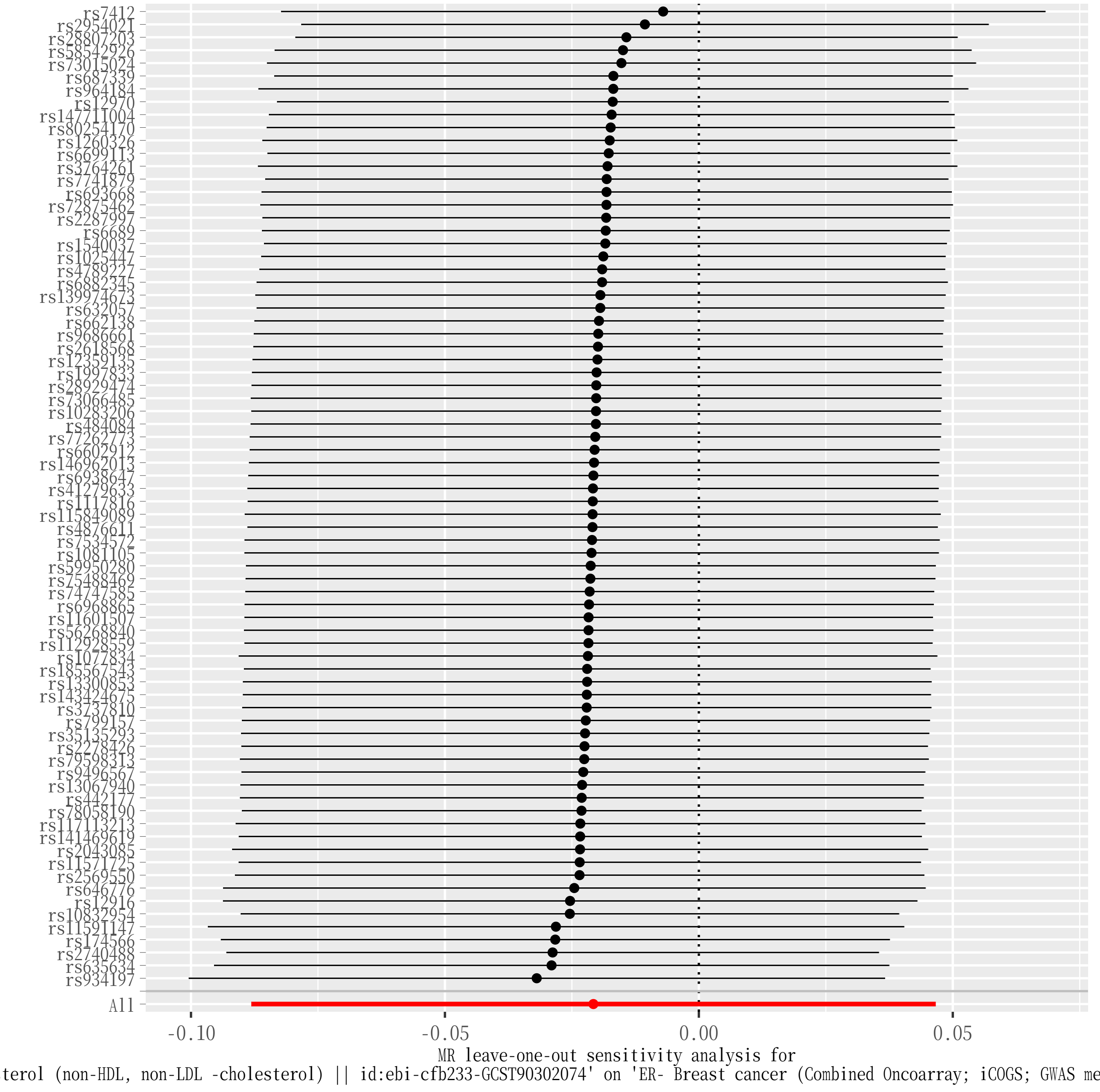
rs7412
rs2954021
rs28807203
rs58542926
rs73015024
rs687339
rs964184
rs12970
rs147711004
rs80254170
rs1260326
rs6699113
rs3764261
rs7741879
rs693668
rs72875462
rs2287997
rs6689
rs1540037
rs1025447
rs4789227
rs6882345
rs139974673
rs632057
rs662138
rs9686661
rs2618568
rs12359135
rs1997833
rs28929474
rs73066485
rs10283206
rs484084
rs77262773
rs6602912
rs146962013
rs6938647
rs41279633
rs1117816
rs115849089
rs4876611
rs7534572
rs1081105
rs59950280
rs75488469
rs74747585
rs6968865
rs11601507
rs56268840
rs112928559
rs1077834
rs185567543
rs13300853
rs143424675
rs3737810
rs799157
rs35135293
rs2278426
rs79598313
rs9496567
rs13067940
rs442177
rs78058190
rs117113213
rs141469619
rs2043085
rs11571725
rs2569550
rs646776
rs12916
rs10832954
rs11591147
rs174566
rs2740488
rs635634
rs934197
All
-0.10
-0.05
0.00
0.05
MR leave-one-out sensitivity analysis for
terol (non-HDL, non-LDL -cholesterol) || id:ebi-cfb233-GCST90302074' on 'ER- Breast cancer (Combined Oncoarray; iCOGS; GWAS me

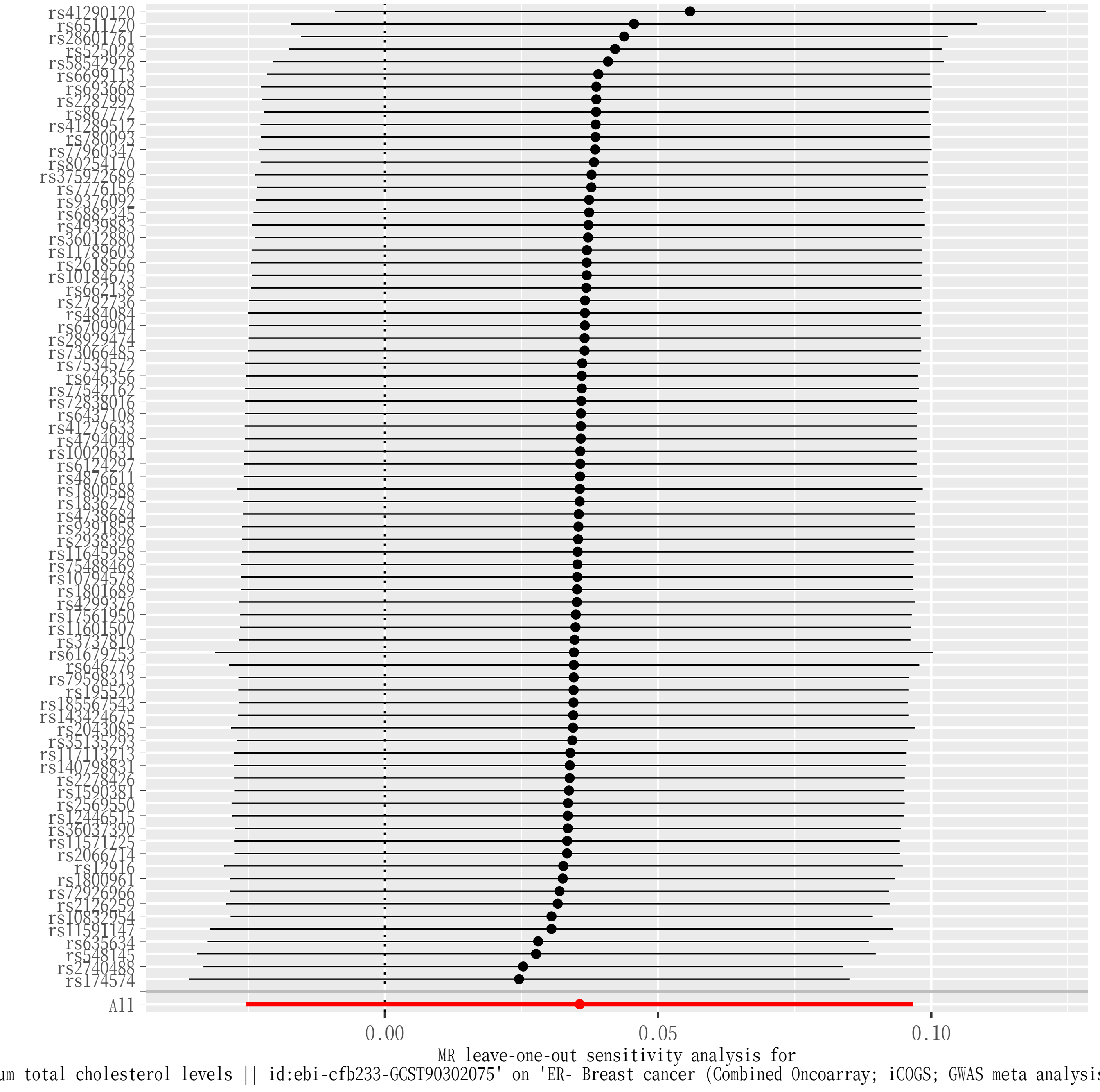
rs41290120
rs6511720
rs28601761
rs525028
rs58542926
rs6699113
rs693668
rs2287997
rs867772
rs41289512
rs780093
rs77960347
rs80254170
rs375272689
rs7776156
rs9376092
rs6882345
rs4939883
rs36012880
rs11789603
rs2618566
rs10184673
rs662138
rs2792736
rs484084
rs6709904
rs28929474
rs73066485
rs7534572
rs646356
rs77542162
rs72838016
rs6437108
rs41279633
rs4794048
rs10020631
rs6124297
rs4876611
rs1800588
rs1836278
rs4738684
rs9391858
rs2938396
rs11645958
rs75488469
rs10794578
rs1801689
rs4299376
rs17561950
rs11601507
rs3737810
rs61679753
rs646776
rs79598313
rs195520
rs185567543
rs143424675
rs2043085
rs35135293
rs117113213
rs140798831
rs2278426
rs1590381
rs2569550
rs12446515
rs36037390
rs11571725
rs2066714
rs12916
rs1800961
rs72926966
rs2126259
rs10832954
rs11591147
rs635634
rs548145
rs2740488
rs174574
All
0.00
0.05
0.10
MR leave-one-out sensitivity analysis for
um total cholesterol levels || id:ebi-cfb233-GCST90302075' on 'ER- Breast cancer (Combined Oncoarray; iCOGS; GWAS meta analysis

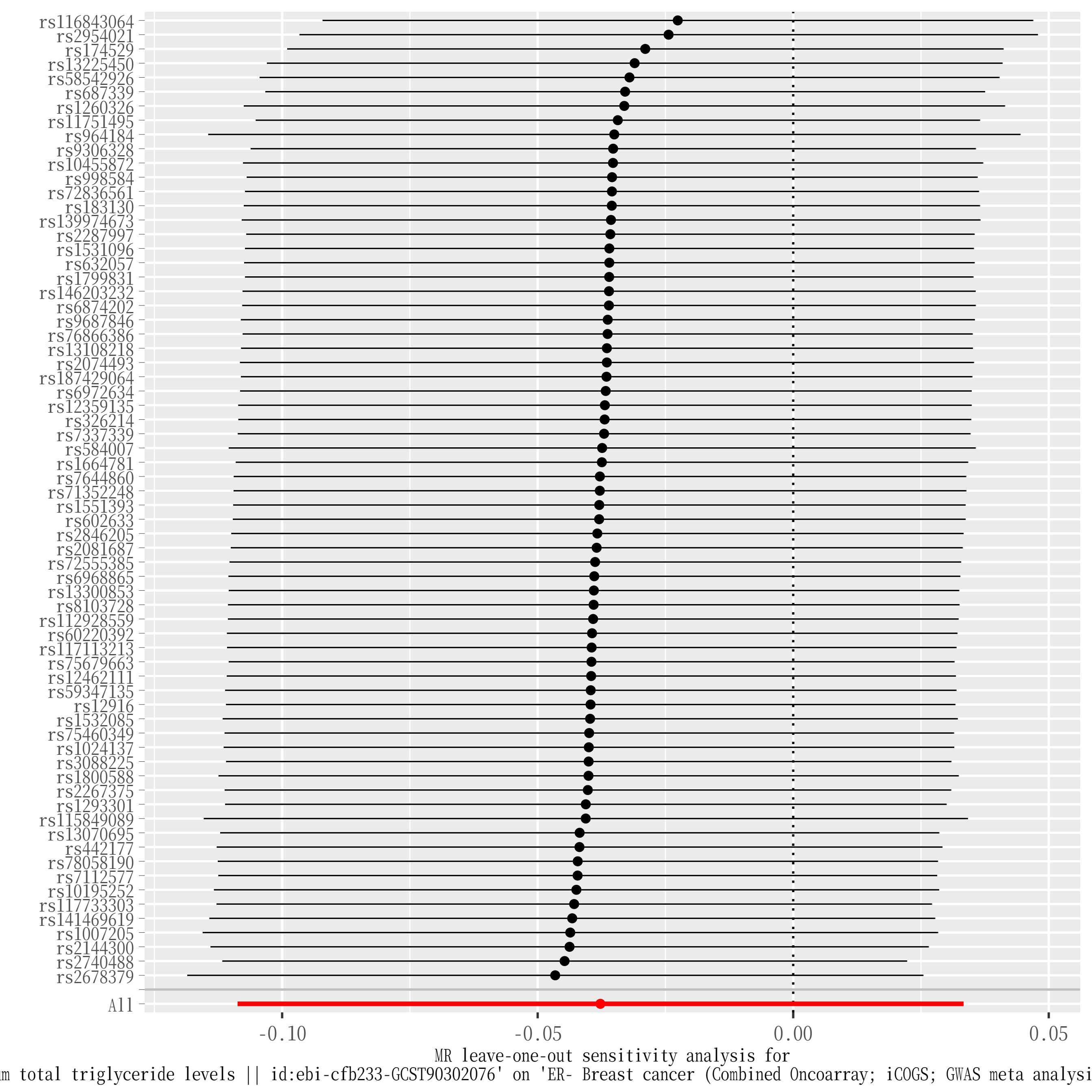

rs116843064
rs2954021
rs174529
rs13225450
rs58542926
rs687339
rs1260326
rs11751495
rs964184
rs9306328
rs10455872
rs998584
rs72836561
rs183130
rs139974673
rs2287997
rs1531096
rs632057
rs1799831
rs146203232
rs6874202
rs9687846
rs76866386
rs13108218
rs2074493
rs187429064
rs6972634
rs12359135
rs326214
rs7337339
rs584007
rs1664781
rs7644860
rs71352248
rs1551393
rs602633
rs2846205
rs2081687
rs72555385
rs6968865
rs13300853
rs8103728
rs112928559
rs60220392
rs117113213
rs75679663
rs12462111
rs59347135
rs12916
rs1532085
rs75460349
rs1024137
rs3088225
rs1800588
rs2267375
rs1293301
rs115849089
rs13070695
rs442177
rs78058190
rs7112577
rs10195252
rs117733303
rs141469619
rs1007205
rs2144300
rs2740488
rs2678379
All
-0.10
-0.05
0.00
0.05
MR leave-one-out sensitivity analysis for
m total triglyceride levels || id:ebi-cfb233-GCST90302076' on 'ER- Breast cancer (Combined Oncoarray; iCOGS; GWAS meta analysi

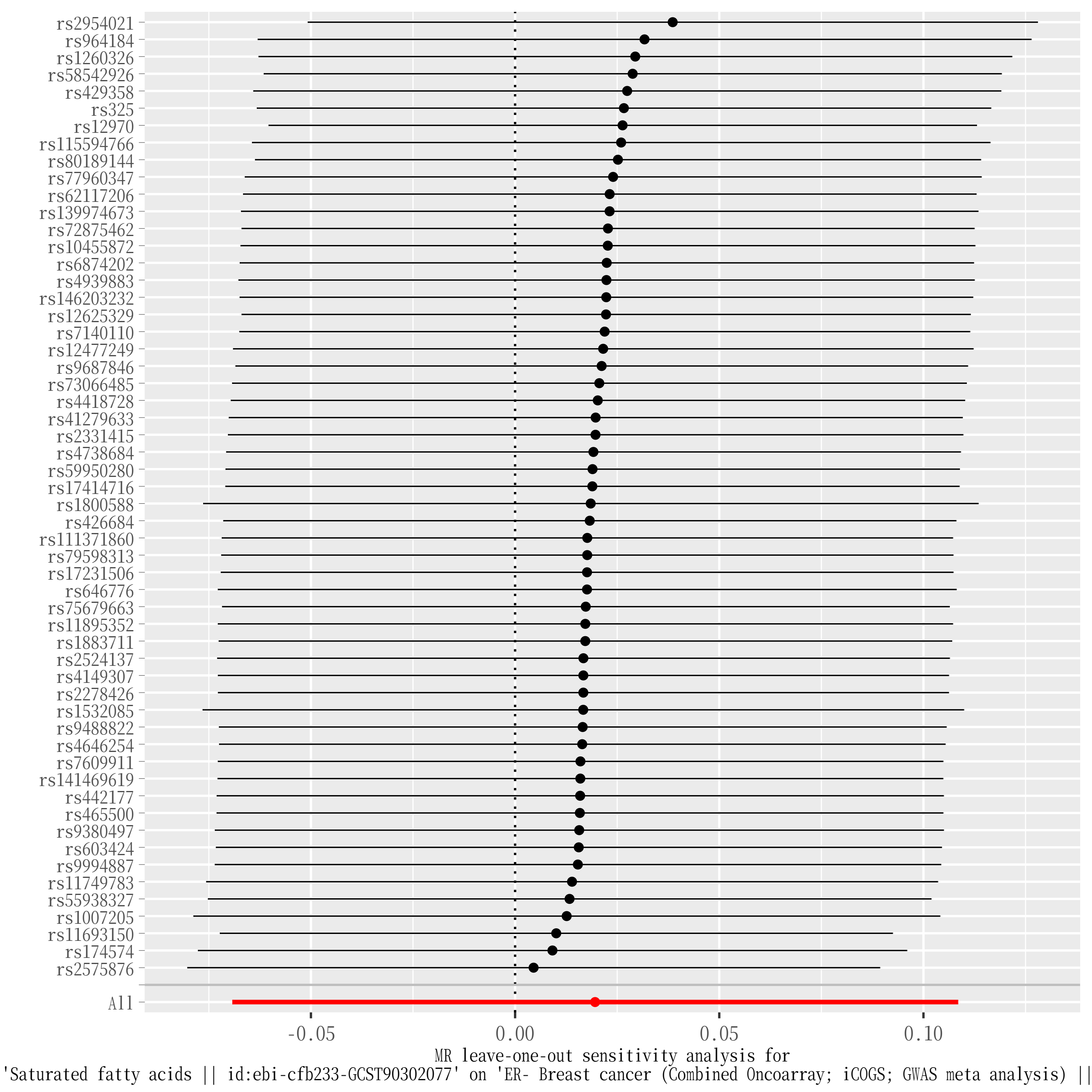

rs2954021
rs964184
rs1260326
rs58542926
rs429358
rs325
rs12970
rs115594766
rs80189144
rs77960347
rs62117206
rs139974673
rs72875462
rs10455872
rs6874202
rs4939883
rs146203232
rs12625329
rs7140110
rs12477249
rs9687846
rs73066485
rs4418728
rs41279633
rs2331415
rs4738684
rs59950280
rs17414716
rs1800588
rs426684
rs111371860
rs79598313
rs17231506
rs646776
rs75679663
rs11895352
rs1883711
rs2524137
rs4149307
rs2278426
rs1532085
rs9488822
rs4646254
rs7609911
rs141469619
rs442177
rs465500
rs9380497
rs603424
rs9994887
rs11749783
rs55938327
rs1007205
rs11693150
rs174574
rs2575876
All
-0.05
0.00
0.05
0.10
MR leave-one-out sensitivity analysis for
'Saturated fatty acids || id:ebi-cfb233-GCST90302077' on 'ER- Breast cancer (Combined Oncoarray; iCOGS; GWAS meta analysis) ||

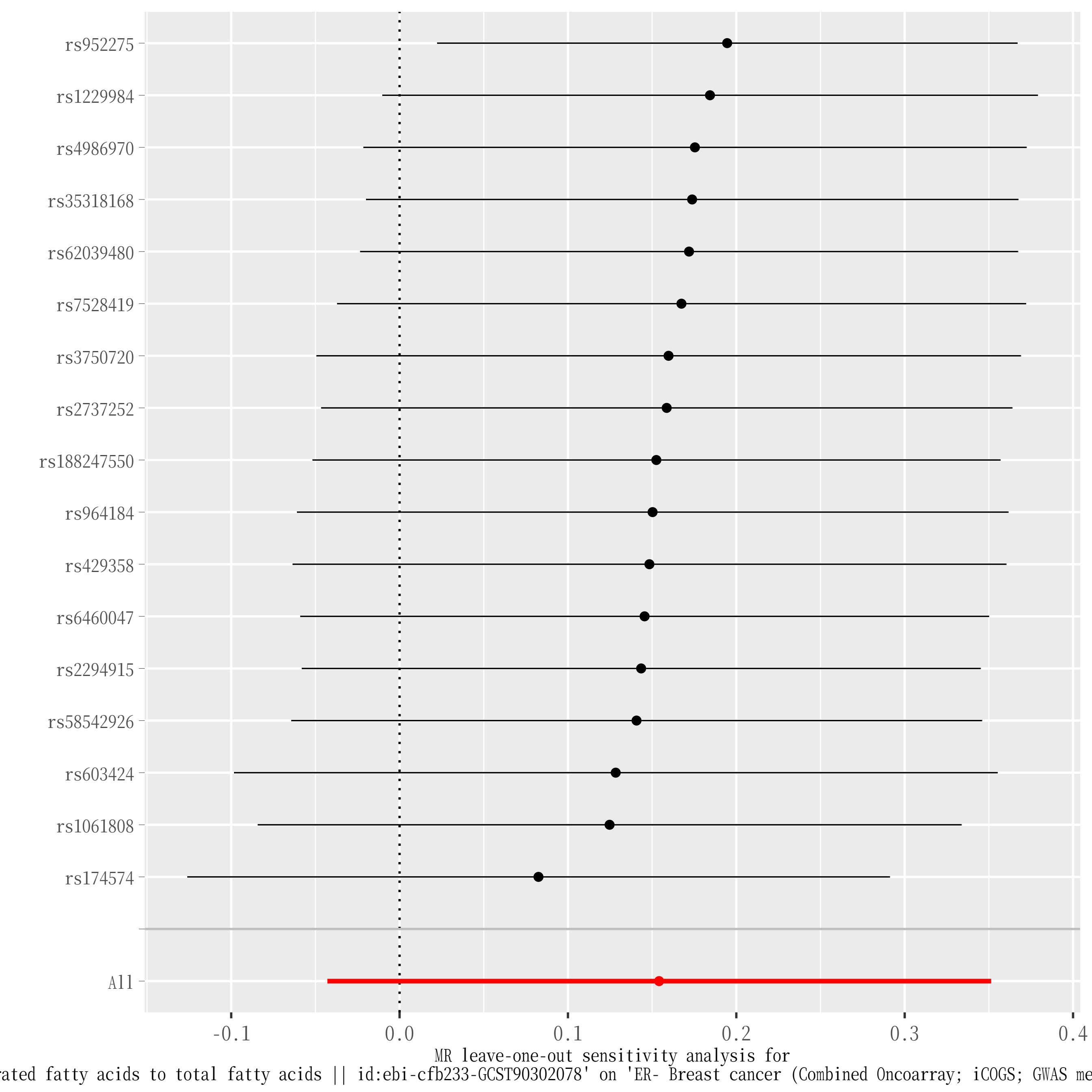
rs952275
rs1229984
rs4986970
rs35318168
rs62039480
rs7528419
rs3750720
rs2737252
rs188247550
rs964184
rs429358
rs6460047
rs2294915
rs58542926
rs603424
rs1061808
rs174574
All
-0.1
0.0
0.1
0.2
0.3
0.4
MR leave-one-out sensitivity analysis for
rated fatty acids to total fatty acids || id:ebi-cfb233-GCST90302078' on 'ER- Breast cancer (Combined Oncoarray; iCOGS; GWAS me

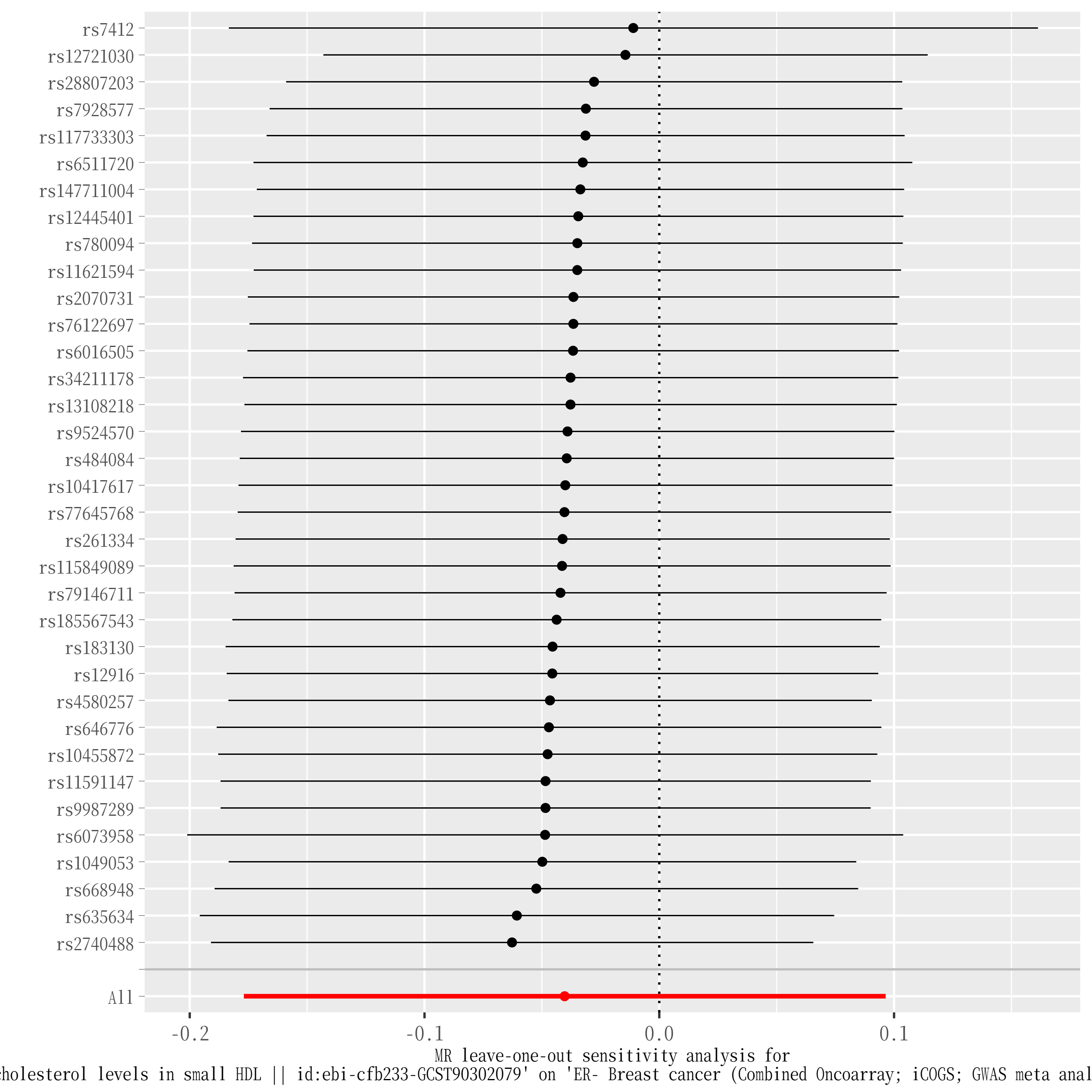
rs7412
rs12721030
rs28807203
rs7928577
rs117733303
rs6511720
rs147711004
rs12445401
rs780094
rs11621594
rs2070731
rs76122697
rs6016505
rs34211178
rs13108218
rs9524570
rs484084
rs10417617
rs77645768
rs261334
rs115849089
rs79146711
rs185567543
rs183130
rs12916
rs4580257
rs646776
rs10455872
rs11591147
rs9987289
rs6073958
rs1049053
rs668948
rs635634
rs2740488
All
-0.2
-0.1
0.0
0.1
MR leave-one-out sensitivity analysis for
holesterol levels in small HDL || id:ebi-cfb233-GCST90302079' on 'ER- Breast cancer (Combined Oncoarray; iCOGS; GWAS meta anal

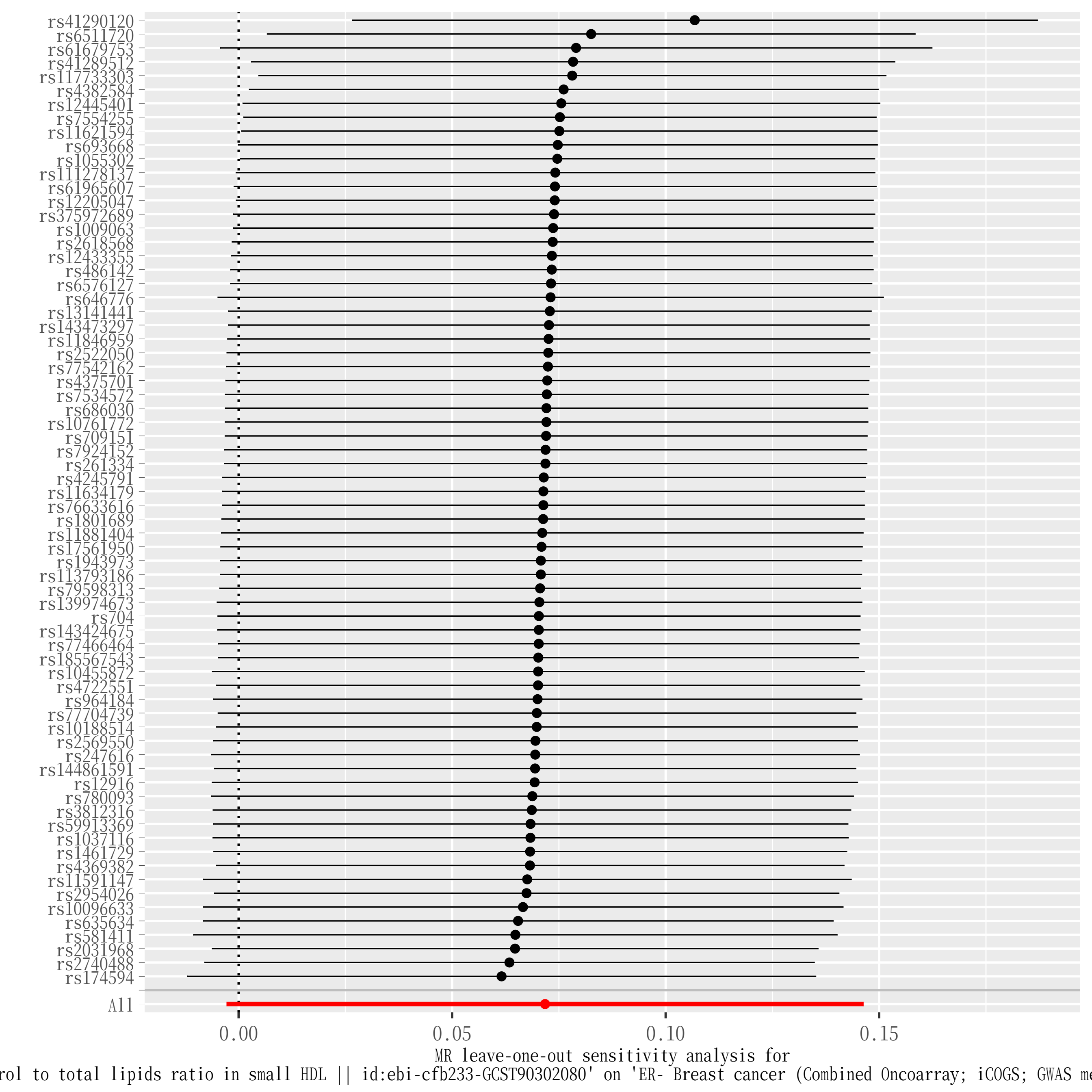

rs41290120
rs6511720
rs61679753
rs41289512
rs117733303
rs4382584
rs12445401
rs7554255
rs11621594
rs693668
rs1055302
rs111278137
rs61965607
rs12205047
rs375972689
rs1009063
rs2618568
rs12433355
rs486142
rs6576127
rs646776
rs13141441
rs143473297
rs11846959
rs2522050
rs77542162
rs4375701
rs7534572
rs686030
rs10761772
rs709151
rs7924152
rs261334
rs4245791
rs11634179
rs76633616
rs1801689
rs11881404
rs17561950
rs1943973
rs113793186
rs79598313
rs139974673
rs704
rs143424675
rs77466464
rs185567543
rs10455872
rs4722551
rs964184
rs77704739
rs10188514
rs2569550
rs247616
rs144861591
rs12916
rs780093
rs3812316
rs59913369
rs1037116
rs1461729
rs4369382
rs11591147
rs2954026
rs10096633
rs635634
rs581411
rs2031968
rs2740488
rs174594
All
0.00
0.05
0.10
0.15
MR leave-one-out sensitivity analysis for
rol to total lipids ratio in small HDL || id:ebi-cfb233-GCST90302080' on 'ER- Breast cancer (Combined Oncoarray; iCOGS; GWAS me

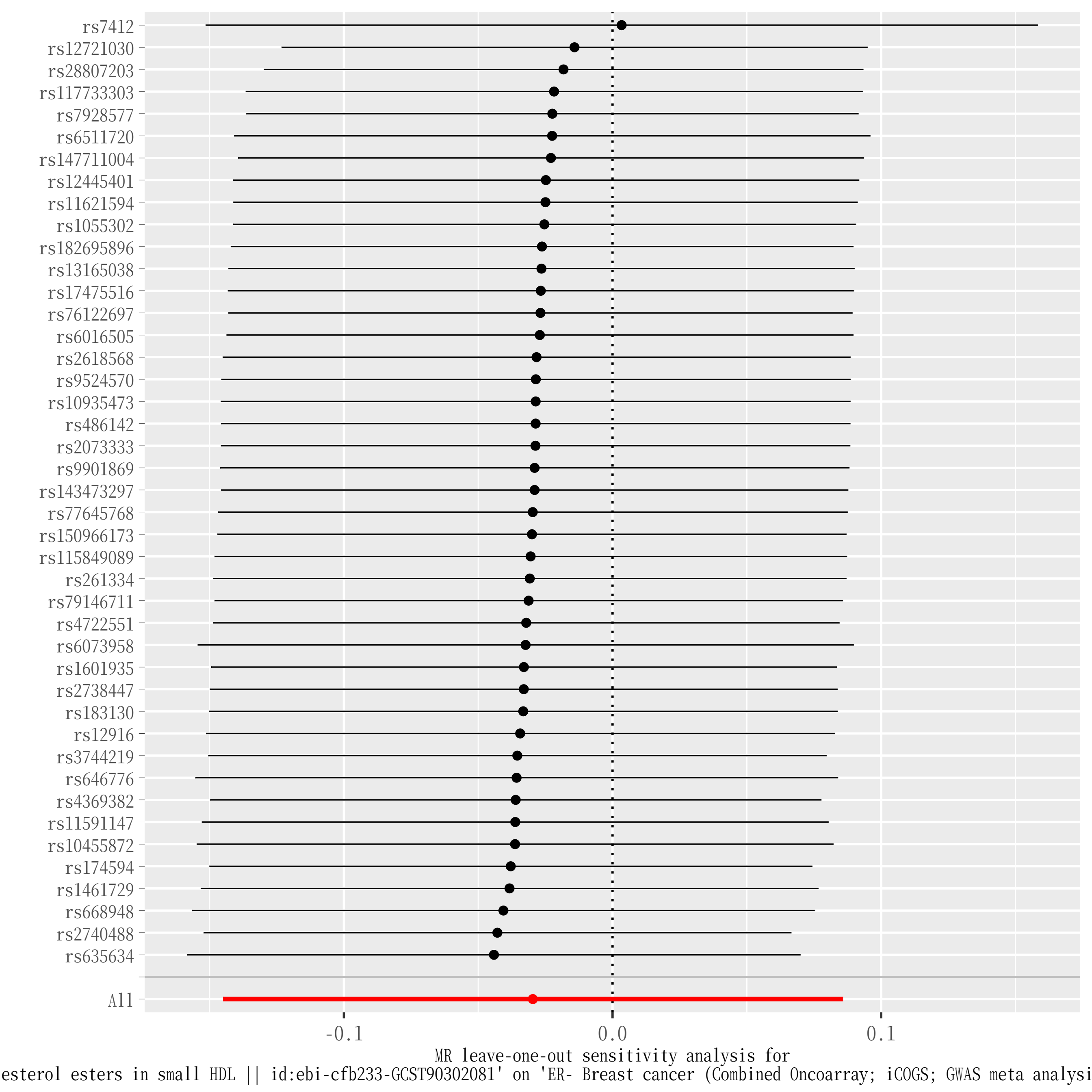
rs7412
rs12721030
rs28807203
rs117733303
rs7928577
rs6511720
rs147711004
rs12445401
rs11621594
rs1055302
rs182695896
rs13165038
rs17475516
rs76122697
rs6016505
rs2618568
rs9524570
rs10935473
rs486142
rs2073333
rs9901869
rs143473297
rs77645768
rs150966173
rs115849089
rs261334
rs79146711
rs4722551
rs6073958
rs1601935
rs2738447
rs183130
rs12916
rs3744219
rs646776
rs4369382
rs11591147
rs10455872
rs174594
rs1461729
rs668948
rs2740488
rs635634
All
-0.1
0.0
0.1
MR leave-one-out sensitivity analysis for
esterol esters in small HDL || id:ebi-cfb233-GCST90302081' on 'ER- Breast cancer (Combined Oncoarray; iCOGS; GWAS meta analysi

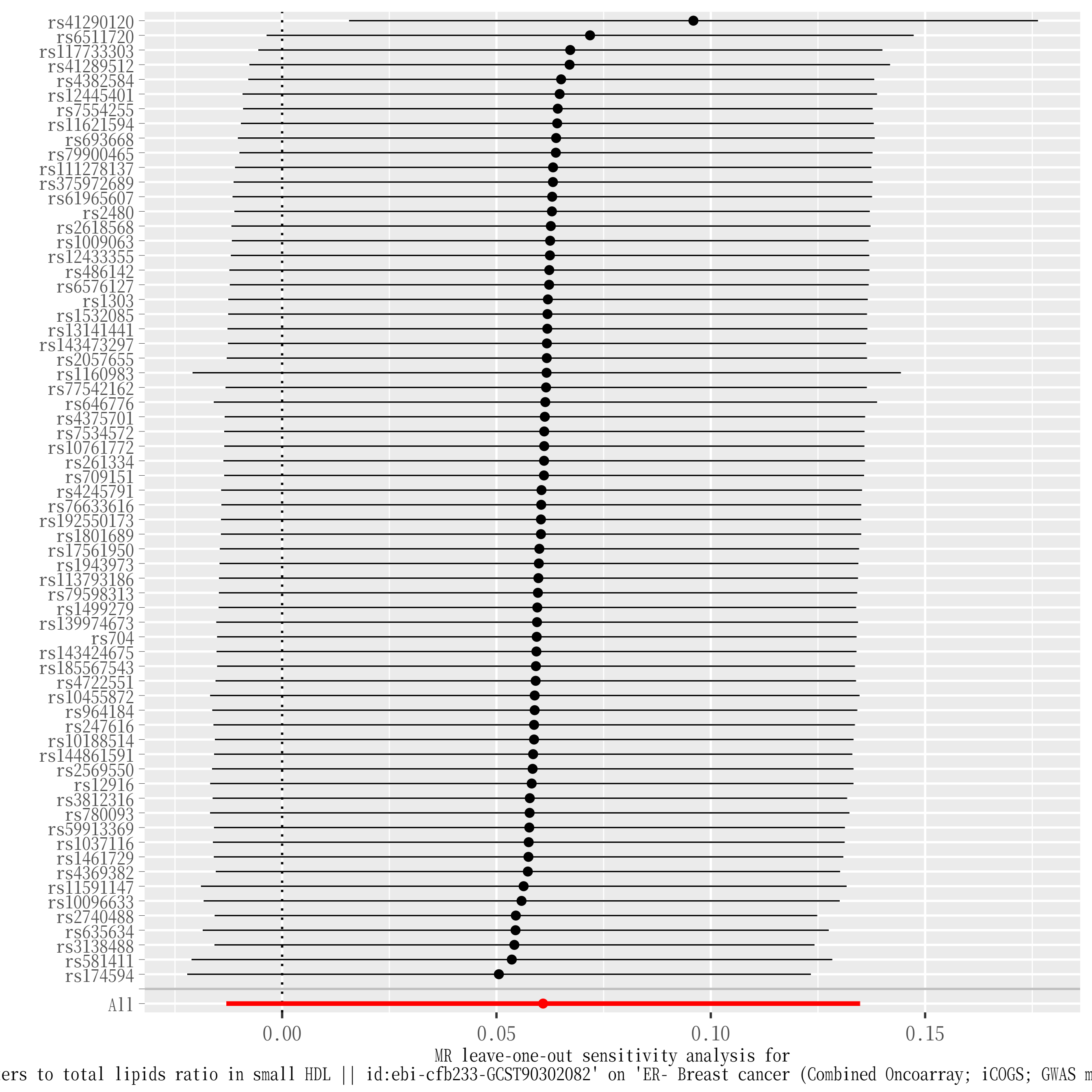
rs41290120
rs6511720
rs117733303
rs41289512
rs4382584
rs12445401
rs7554255
rs11621594
rs693668
rs79900465
rs111278137
rs375972689
rs61965607
rs2480
rs2618568
rs1009063
rs12433355
rs486142
rs6576127
rs1303
rs1532085
rs13141441
rs143473297
rs2057655
rs1160983
rs77542162
rs646776
rs4375701
rs7534572
rs10761772
rs261334
rs709151
rs4245791
rs76633616
rs192550173
rs1801689
rs17561950
rs1943973
rs113793186
rs79598313
rs1499279
rs139974673
rs704
rs143424675
rs185567543
rs4722551
rs10455872
rs964184
rs247616
rs10188514
rs144861591
rs2569550
rs12916
rs3812316
rs780093
rs59913369
rs1037116
rs1461729
rs4369382
rs11591147
rs10096633
rs2740488
rs635634
rs3138488
rs581411
rs174594
All
0.00
0.05
0.10
0.15
MR leave-one-out sensitivity analysis for
ers to total lipids ratio in small HDL || id:ebi-cfb233-GCST90302082' on 'ER- Breast cancer (Combined Oncoarray; iCOGS; GWAS m

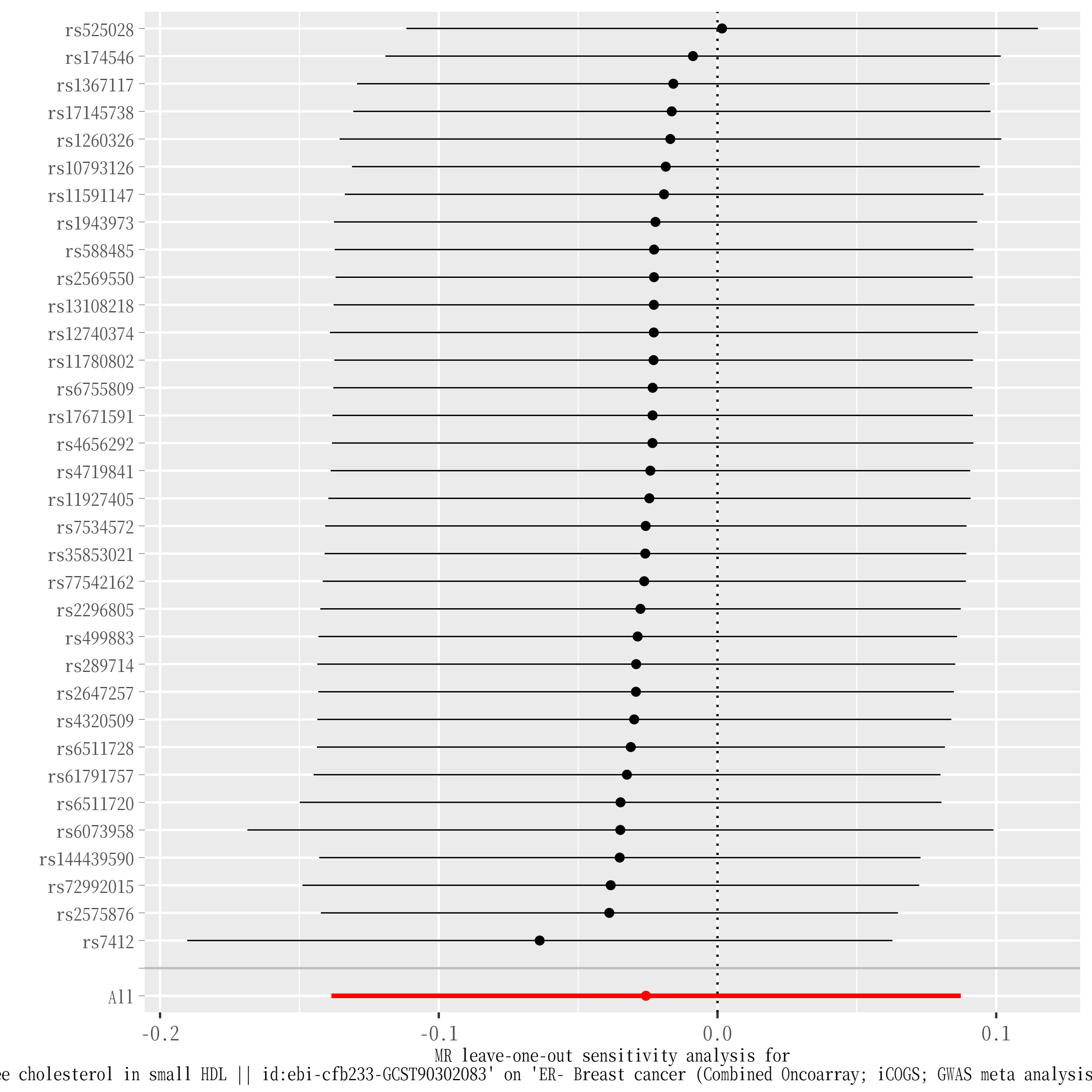
rs525028
rs174546
rs1367117
rs17145738
rs1260326
rs10793126
rs11591147
rs1943973
rs588485
rs2569550
rs13108218
rs12740374
rs11780802
rs6755809
rs17671591
rs4656292
rs4719841
rs11927405
rs7534572
rs35853021
rs77542162
rs2296805
rs499883
rs289714
rs2647257
rs4320509
rs6511728
rs61791757
rs6511720
rs6073958
rs144439590
rs72992015
rs2575876
rs7412
All
-0.2
-0.1
0.0
0.1
MR leave-one-out sensitivity analysis for
ee cholesterol in small HDL || id:ebi-cfb233-GCST90302083' on 'ER- Breast cancer (Combined Oncoarray; iCOGS; GWAS meta analysis

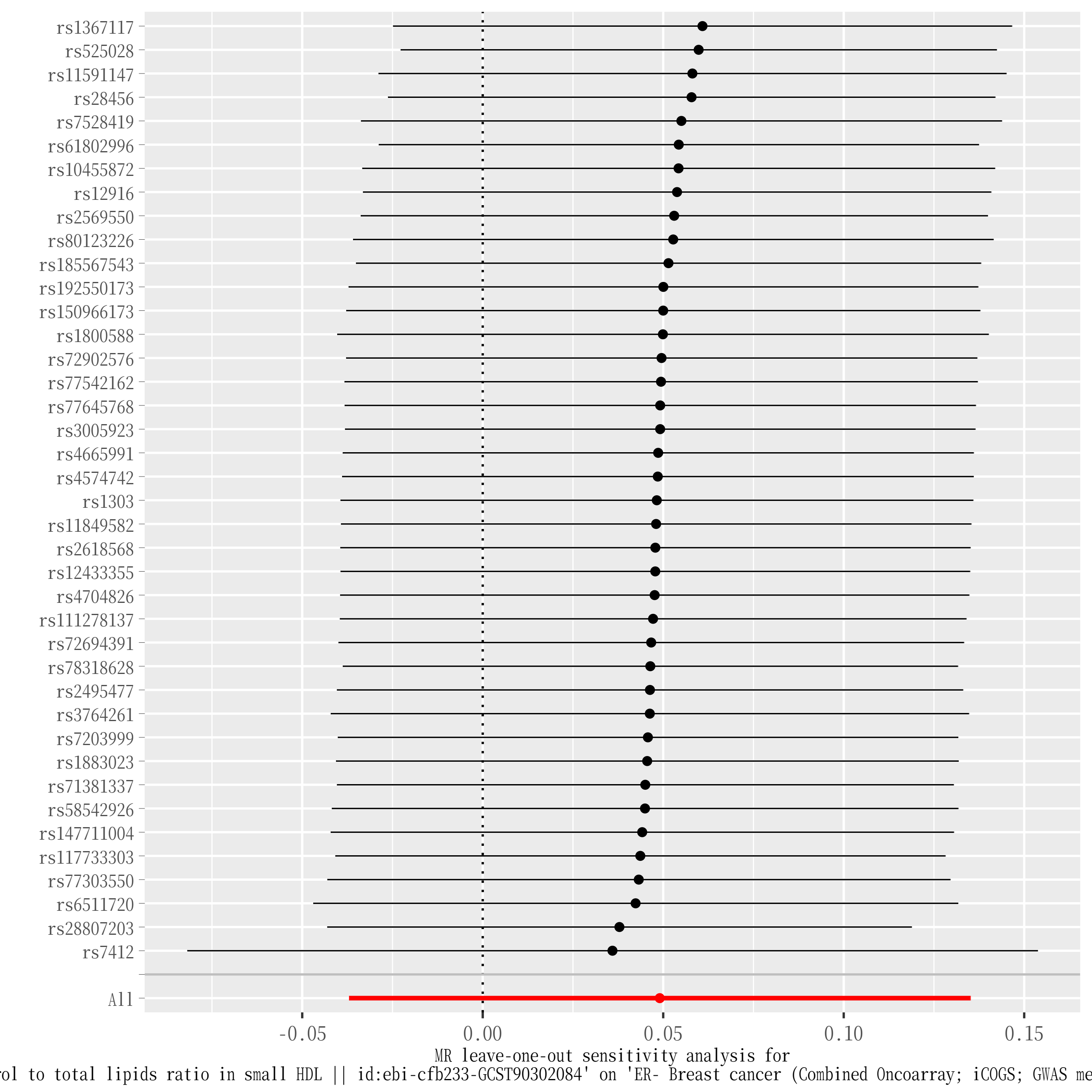

rs1367117
rs525028
rs11591147
rs28456
rs7528419
rs61802996
rs10455872
rs12916
rs2569550
rs80123226
rs185567543
rs192550173
rs150966173
rs1800588
rs72902576
rs77542162
rs77645768
rs3005923
rs4665991
rs4574742
rs1303
rs11849582
rs2618568
rs12433355
rs4704826
rs111278137
rs72694391
rs78318628
rs2495477
rs3764261
rs7203999
rs1883023
rs71381337
rs58542926
rs147711004
rs117733303
rs77303550
rs6511720
rs28807203
rs7412
All
-0.05
0.00
0.05
0.10
0.15
MR leave-one-out sensitivity analysis for
rol to total lipids ratio in small HDL || id:ebi-cfb233-GCST90302084' on 'ER- Breast cancer (Combined Oncoarray; iCOGS; GWAS me

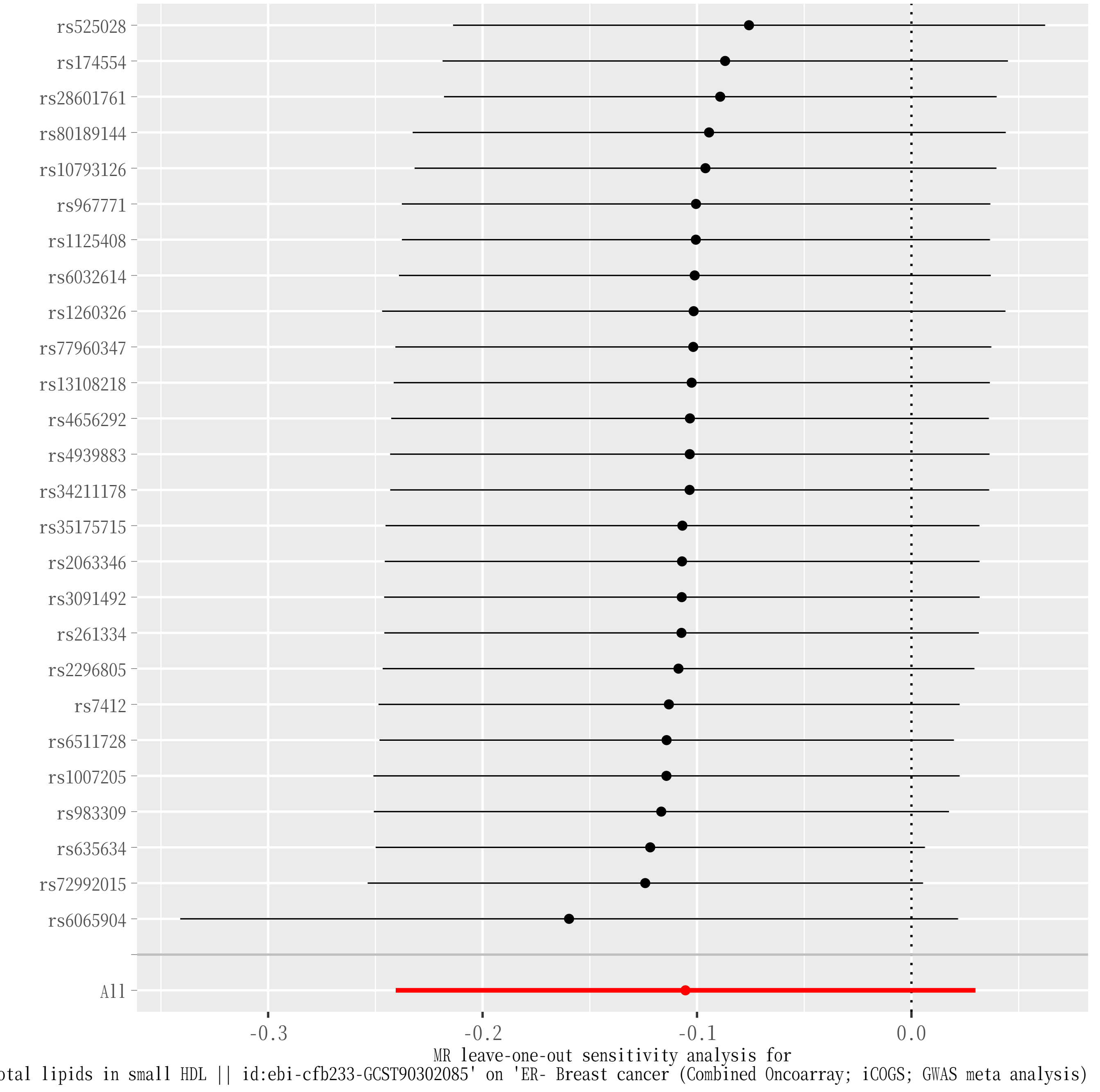

rs525028
rs174554
rs28601761
rs80189144
rs10793126
rs967771
rs1125408
rs6032614
rs1260326
rs77960347
rs13108218
rs4656292
rs4939883
rs34211178
rs35175715
rs2063346
rs3091492
rs261334
rs2296805
rs7412
rs6511728
rs1007205
rs983309
rs635634
rs72992015
rs6065904
All
-0.3
-0.2
-0.1
0.0
MR leave-one-out sensitivity analysis for
otal lipids in small HDL || id:ebi-cfb233-GCST90302085' on 'ER- Breast cancer (Combined Oncoarray; iCOGS; GWAS meta analysis)

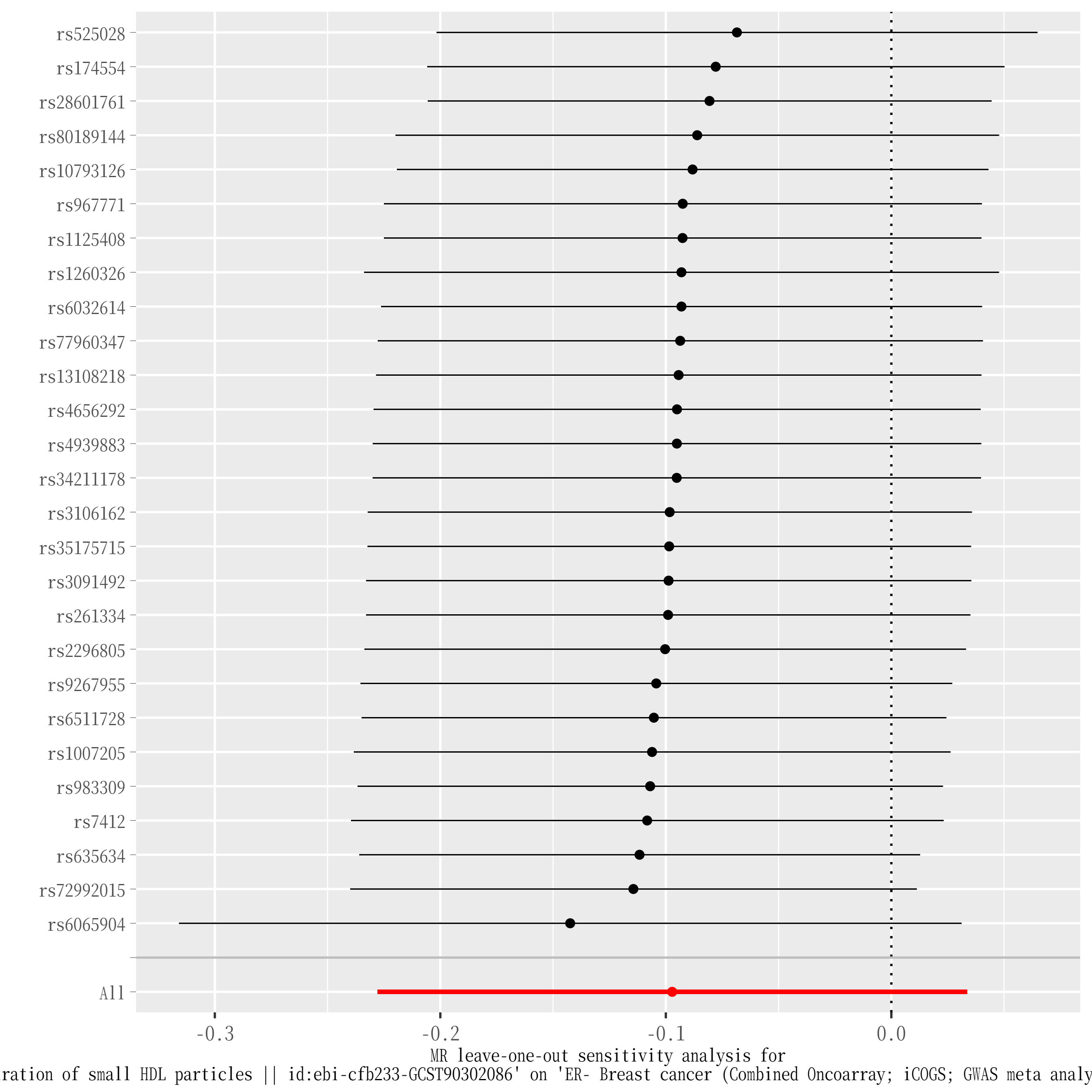

rs525028
rs174554
rs28601761
rs80189144
rs10793126
rs967771
rs1125408
rs1260326
rs6032614
rs77960347
rs13108218
rs4656292
rs4939883
rs34211178
rs3106162
rs35175715
rs3091492
rs261334
rs2296805
rs9267955
rs6511728
rs1007205
rs983309
rs7412
rs635634
rs72992015
rs6065904
All
-0.3
-0.2
-0.1
0.0
MR leave-one-out sensitivity analysis for
ration of small HDL particles || id:ebi-cfb233-GCST90302086' on 'ER- Breast cancer (Combined Oncoarray; iCOGS; GWAS meta analy

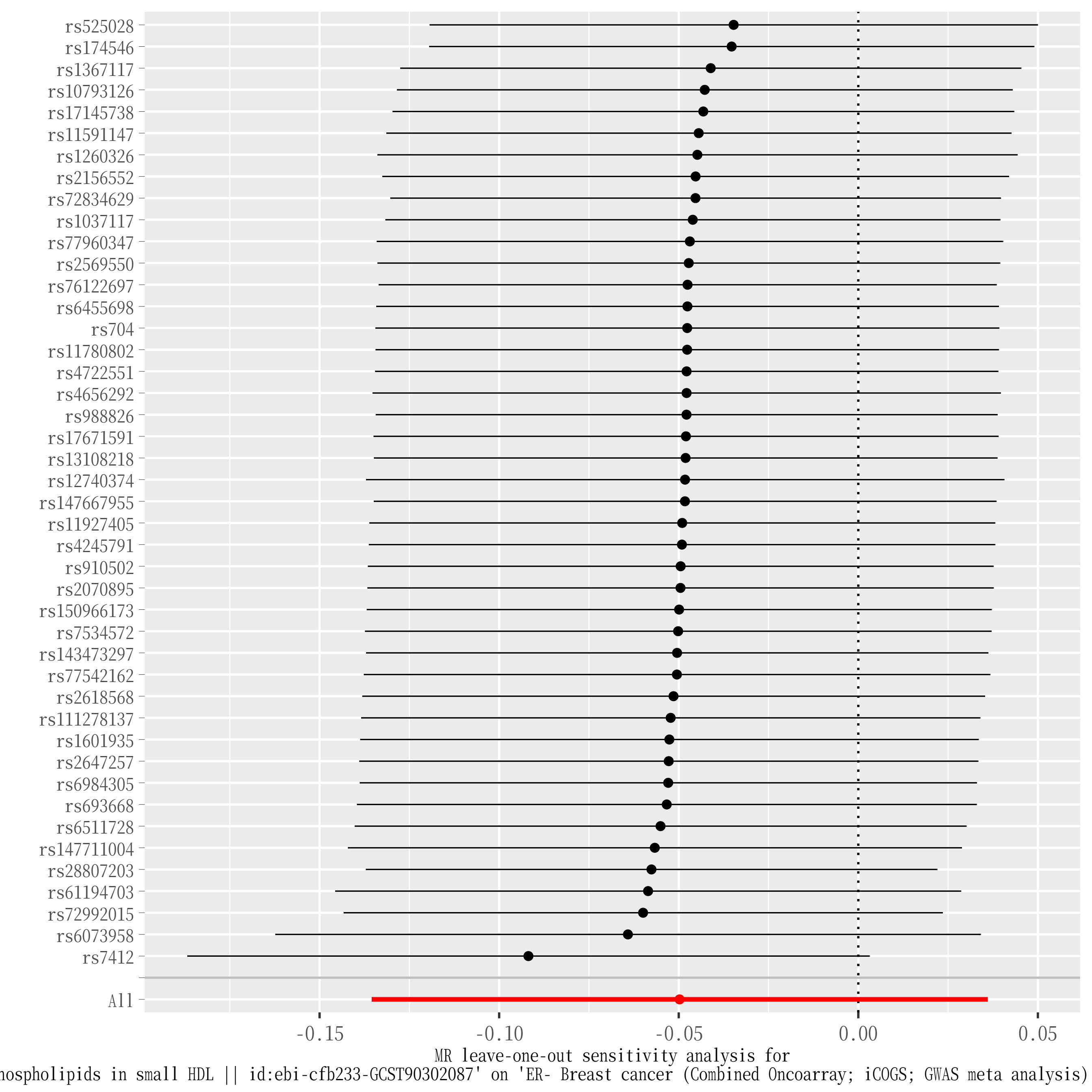

rs525028
rs174546
rs1367117
rs10793126
rs17145738
rs11591147
rs1260326
rs2156552
rs72834629
rs1037117
rs77960347
rs2569550
rs76122697
rs6455698
rs704
rs11780802
rs4722551
rs4656292
rs988826
rs17671591
rs13108218
rs12740374
rs147667955
rs11927405
rs4245791
rs910502
rs2070895
rs150966173
rs7534572
rs143473297
rs77542162
rs2618568
rs111278137
rs1601935
rs2647257
rs6984305
rs693668
rs6511728
rs147711004
rs28807203
rs61194703
rs72992015
rs6073958
rs7412
All
-0.15
-0.10
-0.05
0.00
0.05
MR leave-one-out sensitivity analysis for
hospholipids in small HDL || id:ebi-cfb233-GCST90302087' on 'ER- Breast cancer (Combined Oncoarray; iCOGS; GWAS meta analysis)

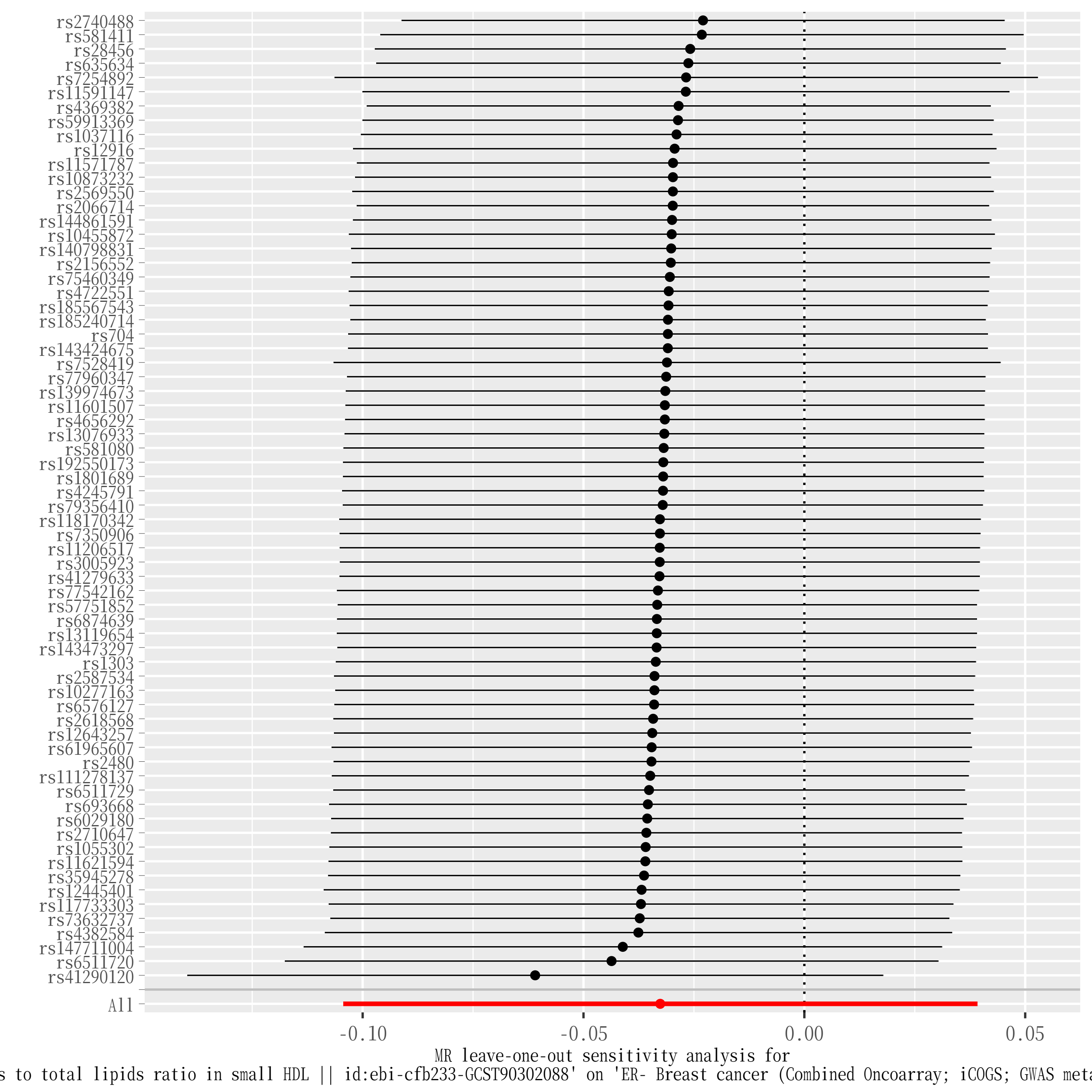

rs2740488
rs581411
rs28456
rs635634
rs7254892
rs11591147
rs4369382
rs59913369
rs1037116
rs12916
rs11571787
rs10873232
rs2569550
rs2066714
rs144861591
rs10455872
rs140798831
rs2156552
rs75460349
rs4722551
rs185567543
rs185240714
rs704
rs143424675
rs7528419
rs77960347
rs139974673
rs11601507
rs4656292
rs13076933
rs581080
rs192550173
rs1801689
rs4245791
rs79356410
rs118170342
rs7350906
rs11206517
rs3005923
rs41279633
rs77542162
rs57751852
rs6874639
rs13119654
rs143473297
rs1303
rs2587534
rs10277163
rs6576127
rs2618568
rs12643257
rs61965607
rs2480
rs111278137
rs6511729
rs693668
rs6029180
rs2710647
rs1055302
rs11621594
rs35945278
rs12445401
rs117733303
rs73632737
rs4382584
rs147711004
rs6511720
rs41290120
All
-0.10
-0.05
0.00
0.05
MR leave-one-out sensitivity analysis for
s to total lipids ratio in small HDL || id:ebi-cfb233-GCST90302088' on 'ER- Breast cancer (Combined Oncoarray; iCOGS; GWAS meta

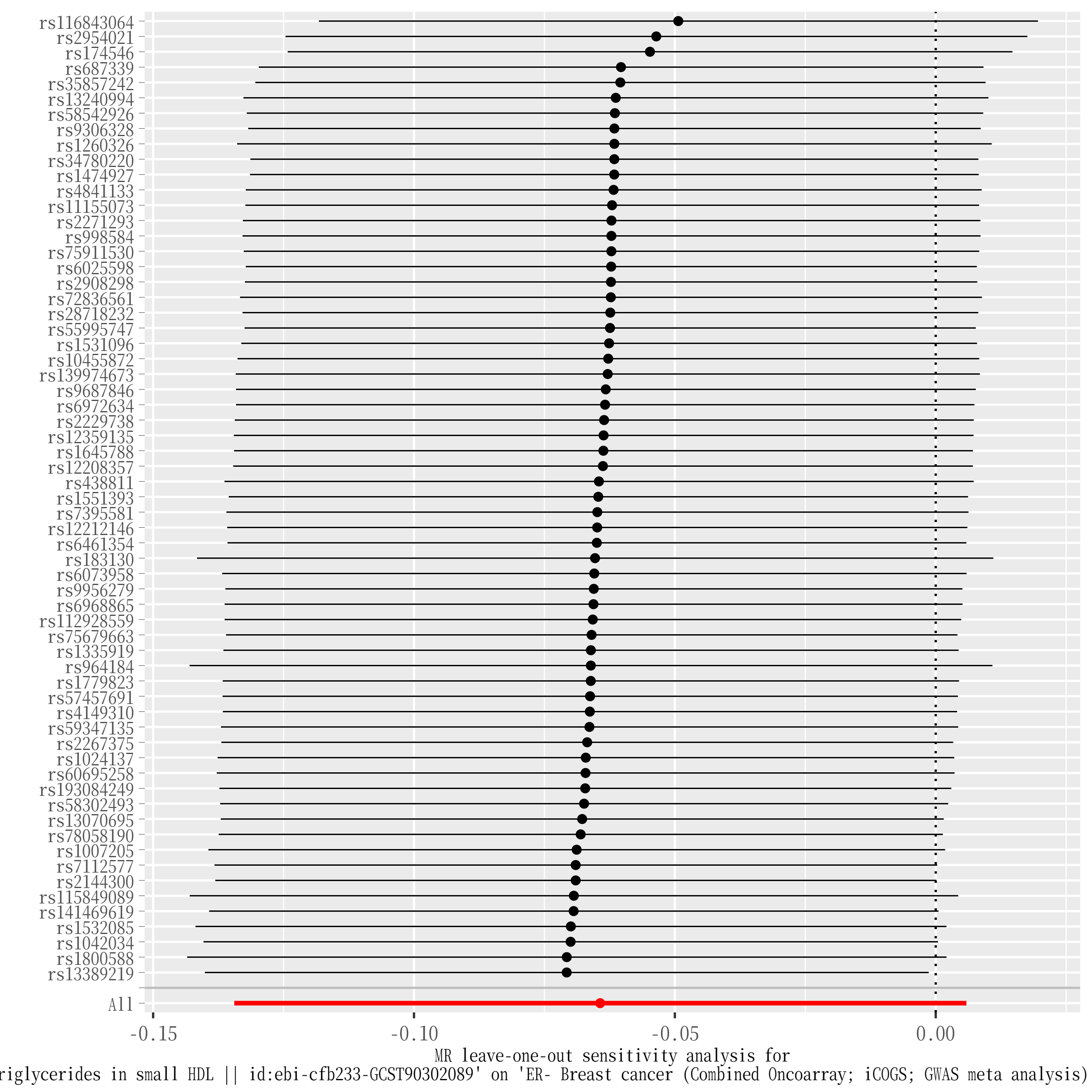

rs116843064
rs2954021
rs174546
rs687339
rs35857242
rs13240994
rs58542926
rs9306328
rs1260326
rs34780220
rs1474927
rs4841133
rs11155073
rs2271293
rs998584
rs75911530
rs6025598
rs2908298
rs72836561
rs28718232
rs55995747
rs1531096
rs10455872
rs139974673
rs9687846
rs6972634
rs2229738
rs12359135
rs1645788
rs12208357
rs438811
rs1551393
rs7395581
rs12212146
rs6461354
rs183130
rs6073958
rs9956279
rs6968865
rs112928559
rs75679663
rs1335919
rs964184
rs1779823
rs57457691
rs4149310
rs59347135
rs2267375
rs1024137
rs60695258
rs193084249
rs58302493
rs13070695
rs78058190
rs1007205
rs7112577
rs2144300
rs115849089
rs141469619
rs1532085
rs1042034
rs1800588
rs13389219
All
-0.15
-0.10
-0.05
0.00
MR leave-one-out sensitivity analysis for
riglycerides in small HDL || id:ebi-cfb233-GCST90302089' on 'ER- Breast cancer (Combined Oncoarray; iCOGS; GWAS meta analysis)

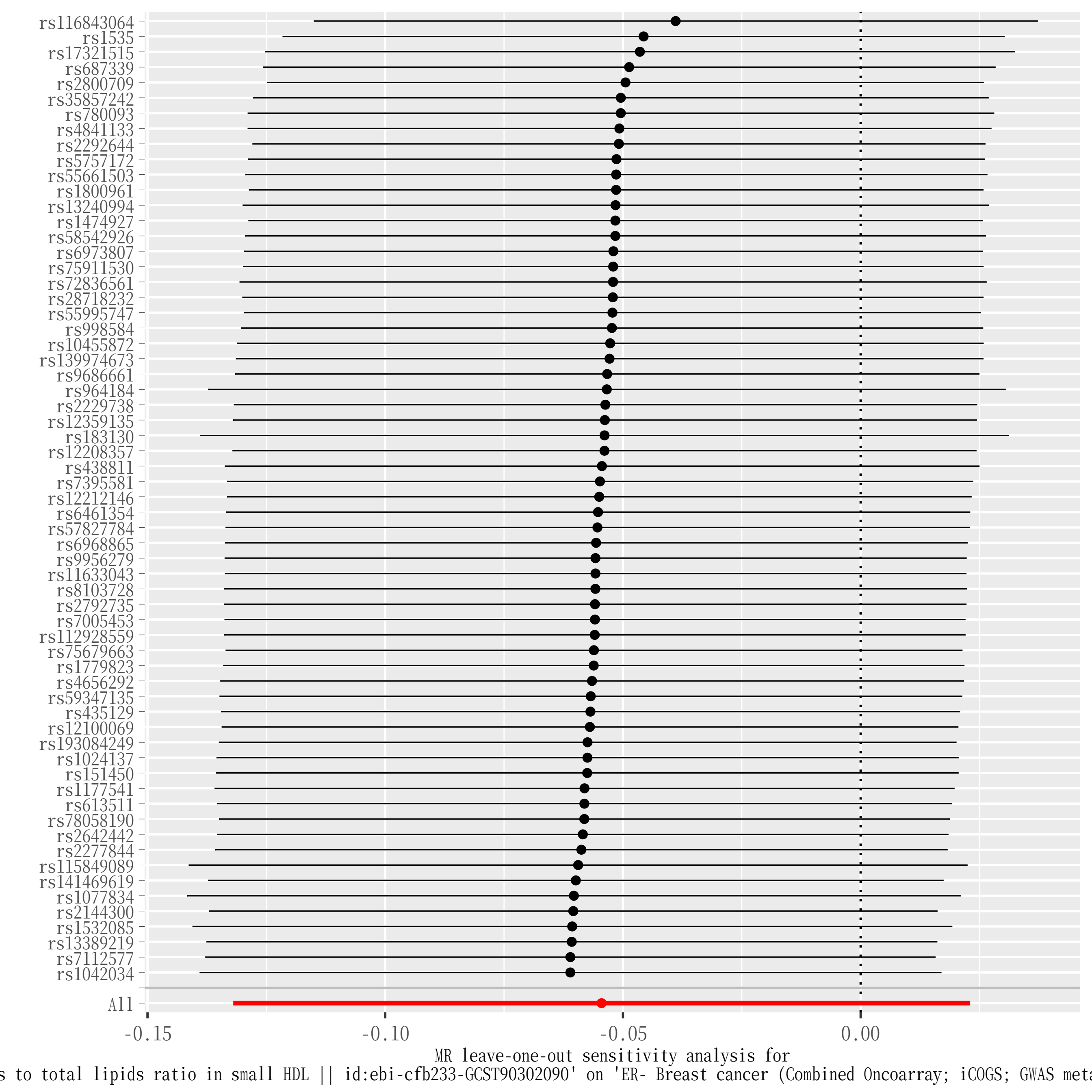

rs116843064
rs1535
rs17321515
rs687339
rs2800709
rs35857242
rs780093
rs4841133
rs2292644
rs5757172
rs55661503
rs1800961
rs13240994
rs1474927
rs58542926
rs6973807
rs75911530
rs72836561
rs28718232
rs55995747
rs998584
rs10455872
rs139974673
rs9686661
rs964184
rs2229738
rs12359135
rs183130
rs12208357
rs438811
rs7395581
rs12212146
rs6461354
rs57827784
rs6968865
rs9956279
rs11633043
rs8103728
rs2792735
rs7005453
rs112928559
rs75679663
rs1779823
rs4656292
rs59347135
rs435129
rs12100069
rs193084249
rs1024137
rs151450
rs1177541
rs613511
rs78058190
rs2642442
rs2277844
rs115849089
rs141469619
rs1077834
rs2144300
rs1532085
rs13389219
rs7112577
rs1042034
All
-0.15
-0.10
-0.05
0.00
MR leave-one-out sensitivity analysis for
s to total lipids ratio in small HDL || id:ebi-cfb233-GCST90302090' on 'ER- Breast cancer (Combined Oncoarray; iCOGS; GWAS meta

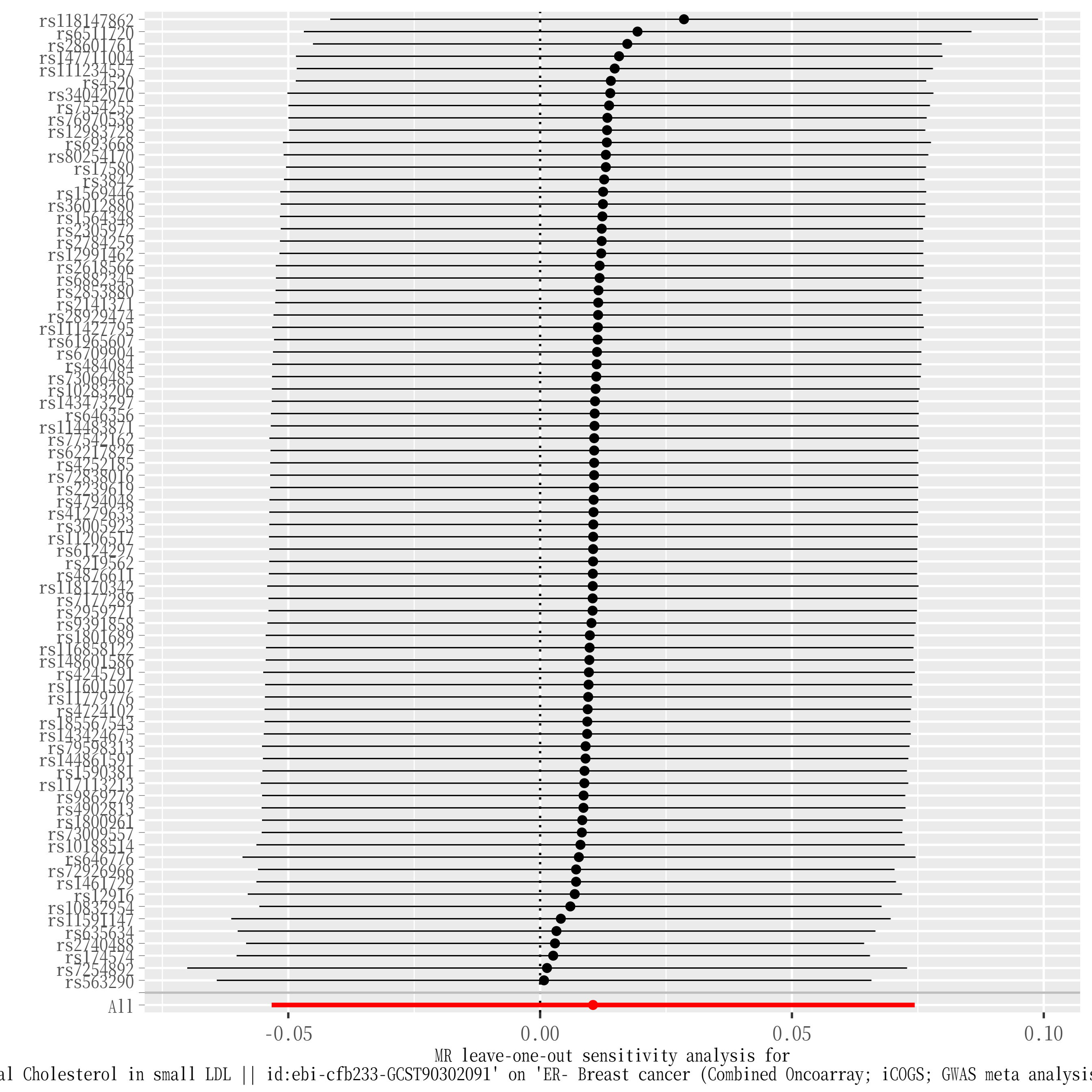

rs118147862
rs6511720
rs28601761
rs147711004
rs111234557
rs4520
rs34042070
rs7554255
rs76970536
rs12983728
rs623668
rs80254170
rs17580
rs3842
rs1569446
rs36012880
rs1564348
rs2305972
rs2784259
rs12991462
rs2618566
rs6882345
rs2853880
rs2141371
rs28929474
rs111427795
rs61265607
rs6709904
rs484084
rs73066485
rs10283206
rs143473297
rs646356
rs114483871
rs77542162
rs62217829
rs4252185
rs72838016
rs2239619
rs4794048
rs41279633
rs3005923
rs11206517
rs6124297
rs219562
rs4876611
rs118170342
rs7177289
rs2959271
rs9391858
rs1801689
rs116858122
rs148601586
rs4245791
rs11601507
rs11779776
rs4724102
rs185567543
rs143424675
rs79598313
rs144861591
rs1590381
rs117113213
rs9869276
rs4902813
rs1800961
rs73009557
rs10188514
rs646776
rs72926966
rs1461729
rs12916
rs10832954
rs115911147
rs635634
rs2740488
rs174574
rs7254892
rs563290
All
-0.05
0.00
0.05
0.10
MR leave-one-out sensitivity analysis for
al Cholesterol in small LDL || id:ebi-cfb233-GCST90302091' on 'ER- Breast cancer (Combined Oncoarray; iCOGS; GWAS meta analysis

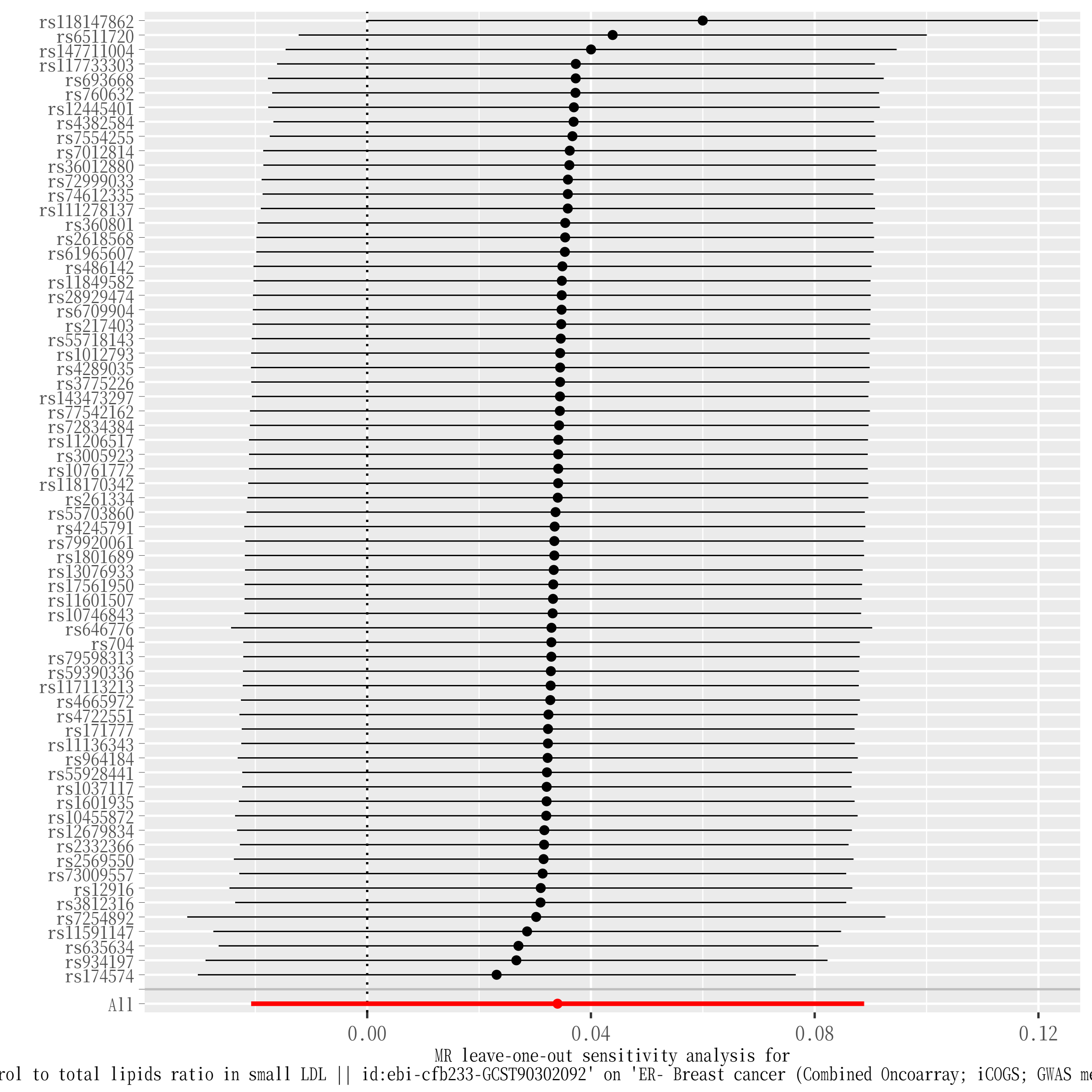

rs118147862
rs6511720
rs147711004
rs117733303
rs693668
rs760632
rs12445401
rs4382584
rs7554255
rs7012814
rs36012880
rs72999033
rs74612335
rs111278137
rs360801
rs2618568
rs61965607
rs486142
rs11849582
rs28929474
rs6709904
rs217403
rs55718143
rs1012793
rs4289035
rs3775226
rs143473297
rs77542162
rs72834384
rs11206517
rs3005923
rs10761772
rs118170342
rs261334
rs55703860
rs4245791
rs79920061
rs1801689
rs13076933
rs17561950
rs11601507
rs10746843
rs646776
rs704
rs79598313
rs59390336
rs117113213
rs4665972
rs4722551
rs171777
rs11136343
rs964184
rs55928441
rs1037117
rs1601935
rs10455872
rs12679834
rs2332366
rs2569550
rs73009557
rs12916
rs3812316
rs7254892
rs11591147
rs635634
rs934197
rs174574
All
0.00
0.04
0.08
0.12
MR leave-one-out sensitivity analysis for
rol to total lipids ratio in small LDL || id:ebi-cfb233-GCST90302092' on 'ER- Breast cancer (Combined Oncoarray; iCOGS; GWAS me

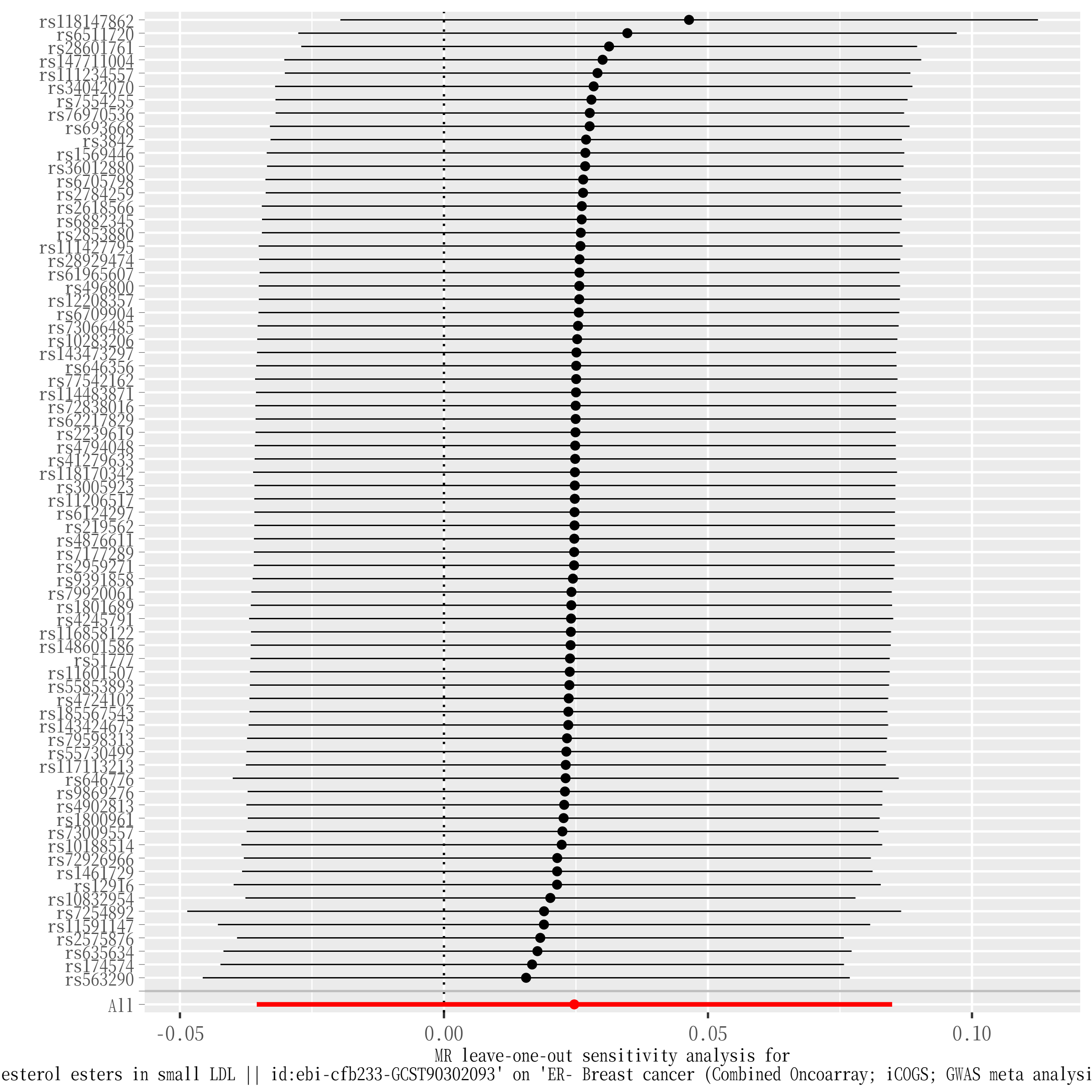

rs118147862
rs6511720
rs28601761
rs147711004
rs111234557
rs34042070
rs7554255
rs76970536
rs693668
rs3842
rs1569446
rs36012880
rs6705798
rs2784259
rs2618566
rs6882345
rs2853880
rs111427795
rs28929474
rs61965607
rs496800
rs12208357
rs6709904
rs73066485
rs10283206
rs143473297
rs646356
rs77542162
rs114483871
rs72838016
rs62217829
rs2239619
rs4794048
rs41279633
rs118170342
rs3005923
rs11206517
rs6124297
rs219562
rs4876611
rs7177289
rs2959271
rs9391858
rs79920061
rs1801689
rs4245791
rs116858122
rs148601586
rs51777
rs11601507
rs55853893
rs4724102
rs185567543
rs143424675
rs79598313
rs55730499
rs117113213
rs646776
rs9869276
rs4902813
rs1800961
rs73009557
rs10188514
rs72926966
rs1461729
rs12916
rs10832954
rs7254892
rs11591147
rs2575876
rs635634
rs174574
rs563290
All
-0.05
0.00
0.05
0.10
MR leave-one-out sensitivity analysis for
esterol esters in small LDL || id:ebi-cfb233-GCST90302093' on 'ER- Breast cancer (Combined Oncoarray; iCOGS; GWAS meta analysi

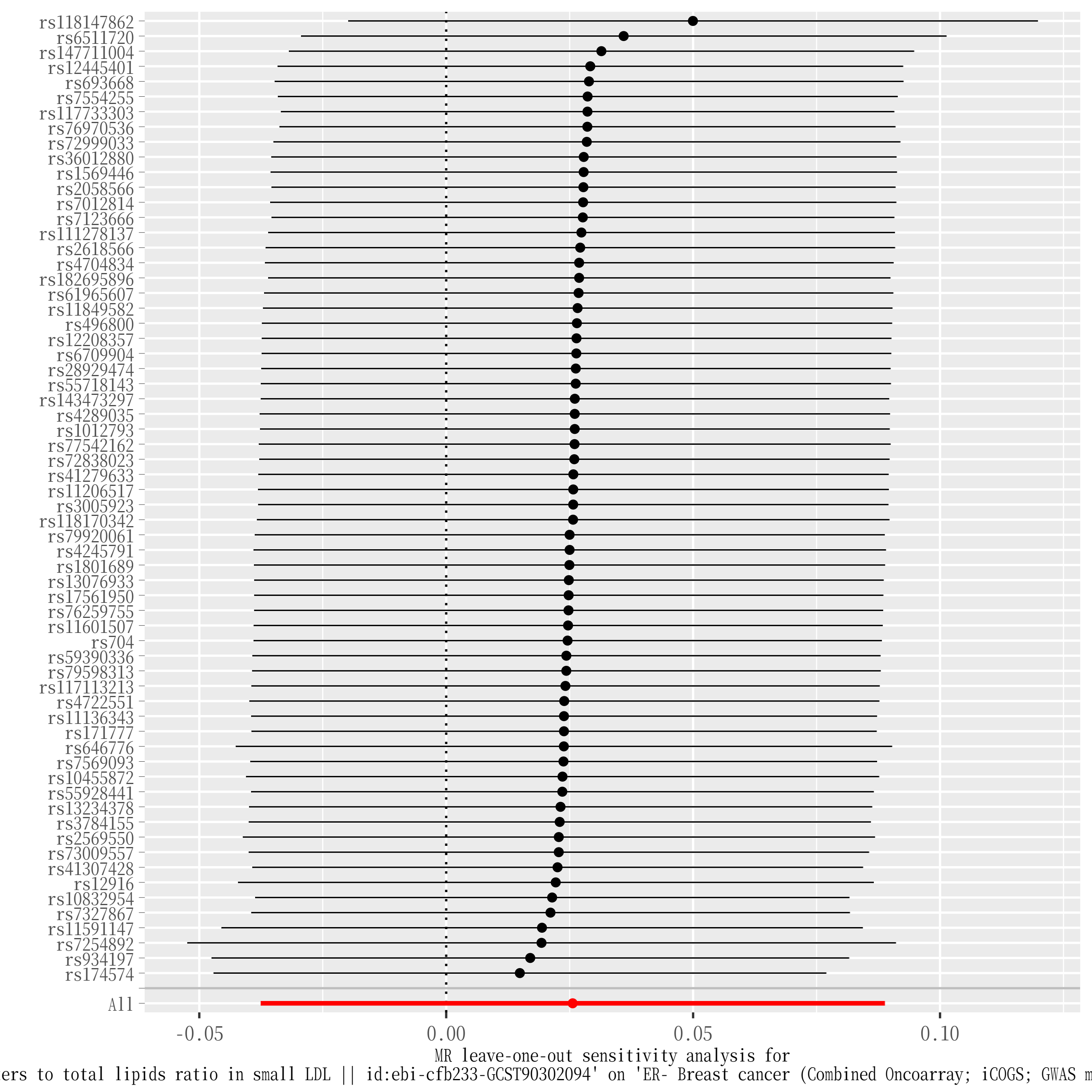

rs118147862
rs6511720
rs147711004
rs12445401
rs693668
rs7554255
rs117733303
rs76970536
rs72999033
rs36012880
rs1569446
rs2058566
rs7012814
rs7123666
rs111278137
rs2618566
rs4704834
rs182695896
rs61965607
rs11849582
rs496800
rs12208357
rs6709904
rs28929474
rs55718143
rs143473297
rs4289035
rs1012793
rs77542162
rs72838023
rs41279633
rs11206517
rs3005923
rs118170342
rs79920061
rs4245791
rs1801689
rs13076933
rs17561950
rs76259755
rs11601507
rs704
rs59390336
rs79598313
rs117113213
rs4722551
rs11136343
rs171777
rs646776
rs7569093
rs10455872
rs55928441
rs13234378
rs3784155
rs2569550
rs73009557
rs41307428
rs12916
rs10832954
rs7327867
rs11591147
rs7254892
rs934197
rs174574
All
-0.05
0.00
0.05
0.10
MR leave-one-out sensitivity analysis for
ers to total lipids ratio in small LDL || id:ebi-cfb233-GCST90302094' on 'ER- Breast cancer (Combined Oncoarray; iCOGS; GWAS m

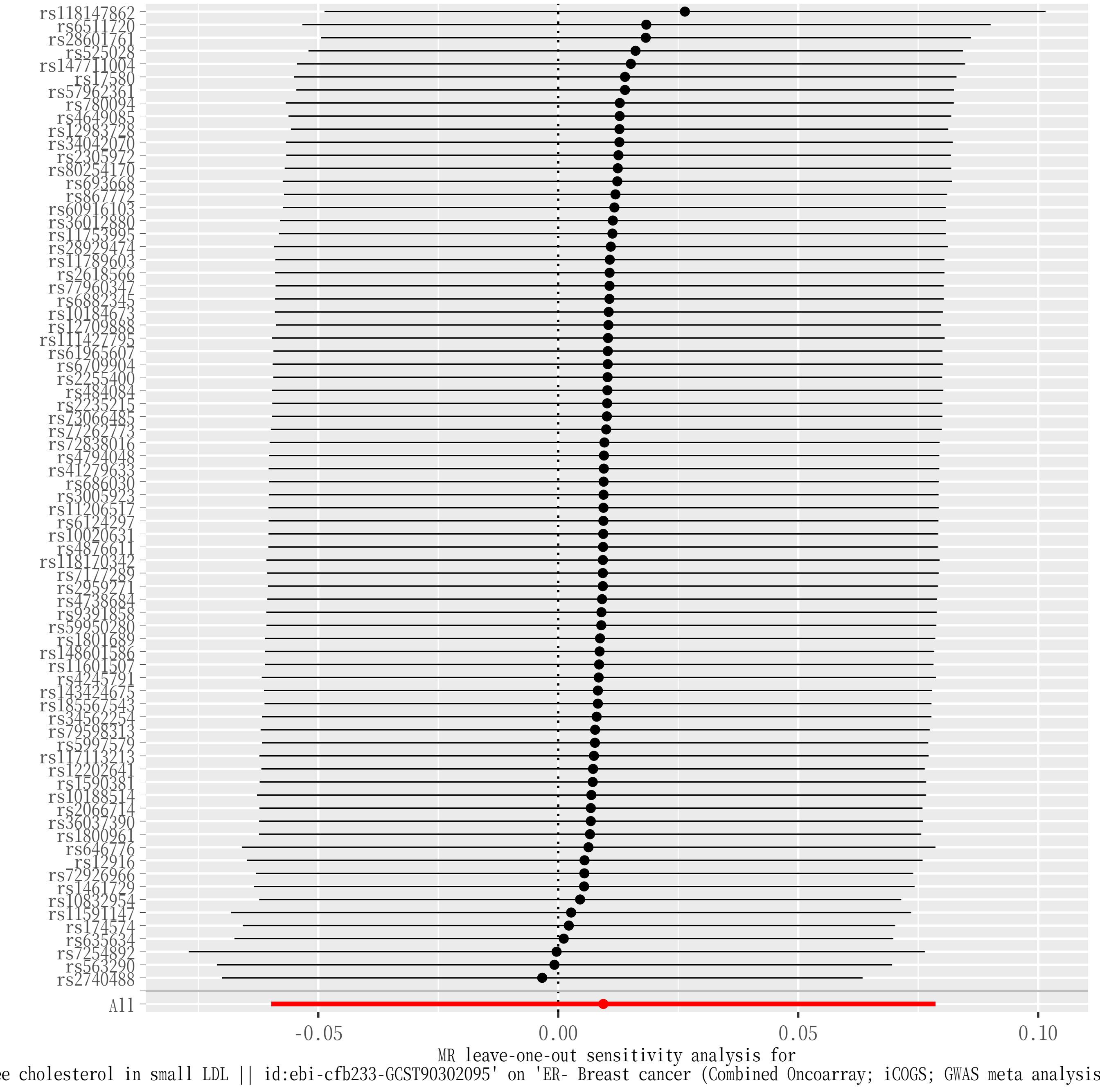

rs118147862
rs6511720
rs28601761
rs525028
rs147711004
rs17580
rs57962361
rs780094
rs4649085
rs12983728
rs34042070
rs2305972
rs80254170
rs693668
rs867772
rs60916103
rs36012880
rs11753995
rs28929474
rs11789603
rs2618566
rs77960347
rs6882345
rs10184673
rs12709888
rs111427795
rs61965607
rs6709904
rs2255400
rs484084
rs2235215
rs73066485
rs77262773
rs72838016
rs4794048
rs41279633
rs686030
rs3005923
rs11206517
rs6124297
rs10020631
rs4876611
rs118170342
rs7177289
rs2959271
rs4738684
rs9321858
rs59950280
rs1801689
rs148601586
rs11601507
rs4245791
rs143424675
rs185567543
rs34562254
rs79598313
rs5997579
rs117113213
rs12202641
rs1590381
rs10188514
rs2066714
rs36037390
rs1800961
rs646776
rs12916
rs72926966
rs1461729
rs10832954
rs11591147
rs174574
rs635634
rs7254892
rs563290
rs2740488
All
-0.05
0.00
0.05
0.10
MR leave-one-out sensitivity analysis for
e cholesterol in small LDL || id:ebi-cfb233-GCST90302095' on 'ER- Breast cancer (Combined Oncoarray; iCOGS; GWAS meta analysis

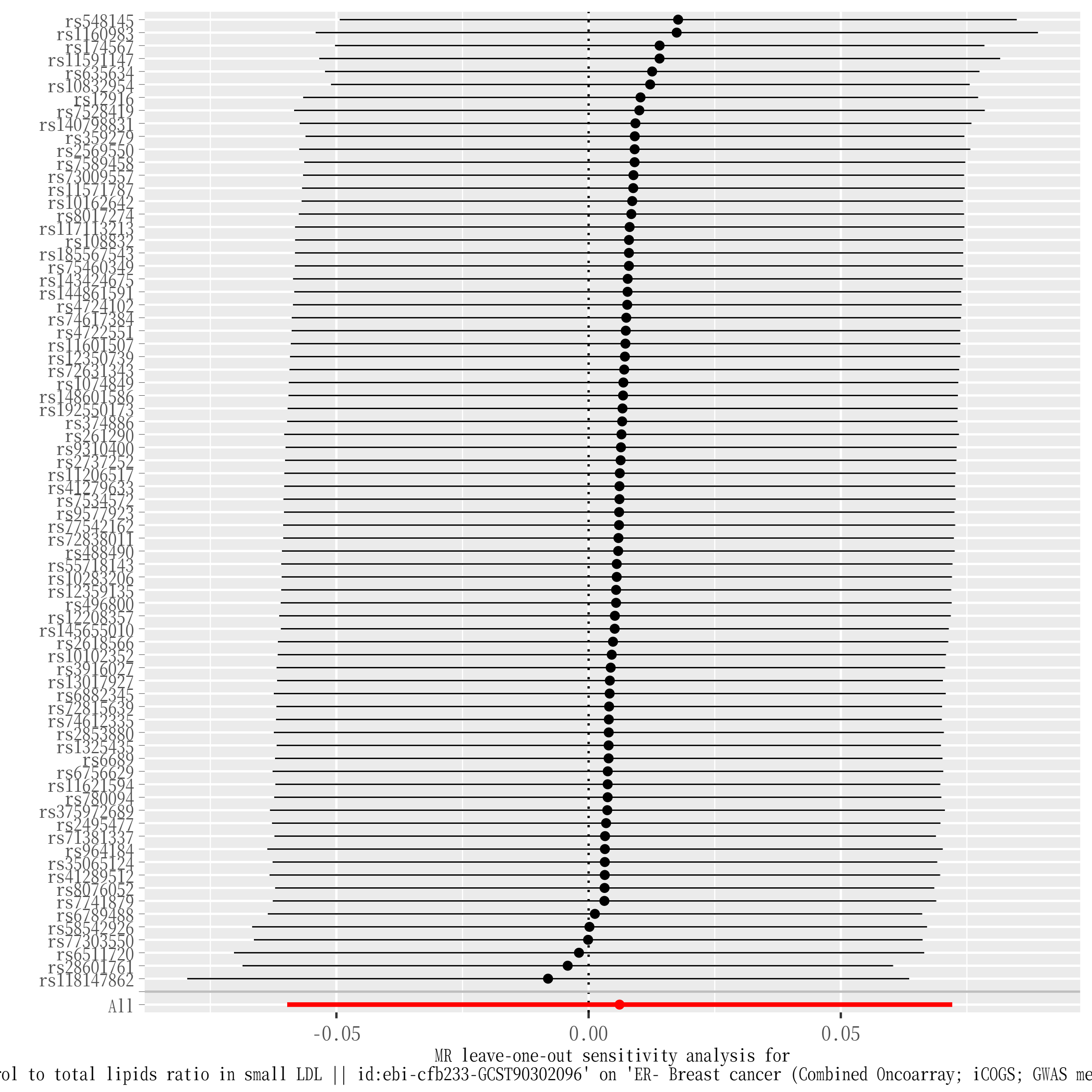

rs548145
rs1160983
rs174567
rs11591147
rs635634
rs10832954
rs12916
rs7528419
rs140798831
rs359279
rs2569550
rs7589458
rs73009557
rs11571787
rs10162642
rs8017274
rs117113213
rs108832
rs185567543
rs75460349
rs143424675
rs144861591
rs4724102
rs74617384
rs4722551
rs11601507
rs12350739
rs72631343
rs1074849
rs148601586
rs192550173
rs374886
rs261290
rs9310400
rs2737252
rs11206517
rs41279633
rs7534572
rs9577923
rs77542162
rs72838011
rs488490
rs55718143
rs10283206
rs12359135
rs496800
rs12208357
rs145655010
rs2618566
rs10102352
rs3916027
rs13017927
rs6882345
rs72815639
rs74612335
rs2853880
rs1325435
rs6689
rs6756629
rs11621594
rs780094
rs375972689
rs2495477
rs71381337
rs964184
rs35065124
rs41289512
rs8076052
rs7741879
rs6789488
rs58542926
rs77303550
rs6511720
rs28601761
rs118147862
All
-0.05
0.00
0.05
MR leave-one-out sensitivity analysis for
rol to total lipids ratio in small LDL || id:ebi-cfb233-GCST90302096' on 'ER- Breast cancer (Combined Oncoarray; iCOGS; GWAS me

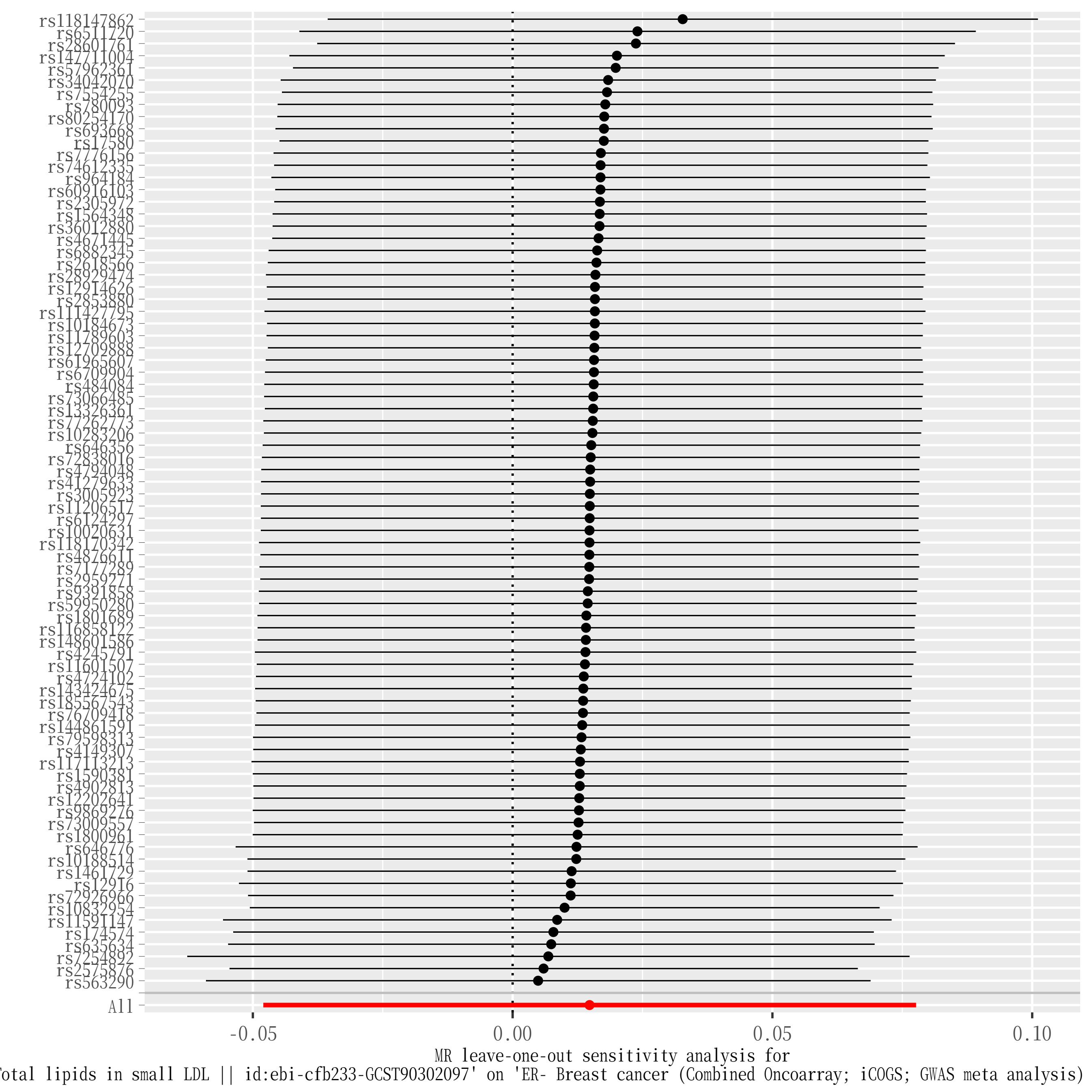
rs118147862
rs6511720
rs28601761
rs147711004
rs57962361
rs34042070
rs7554255
rs780093
rs80254170
rs693668
rs17580
rs7776156
rs74612335
rs964184
rs60916103
rs2305972
rs1564348
rs36012880
rs4671445
rs6882345
rs2618566
rs28929474
rs12914626
rs2853880
rs111427795
rs10184673
rs11789603
rs12709888
rs61965607
rs6709904
rs484084
rs73066485
rs13326361
rs77262773
rs10283206
rs646356
rs72838016
rs4794048
rs41279633
rs3005923
rs11206517
rs6124297
rs10020631
rs118170342
rs4876611
rs7177289
rs2959271
rs9391858
rs59950280
rs1801689
rs116858122
rs148601586
rs4245791
rs11601507
rs4724102
rs143424675
rs185567543
rs76709418
rs144861591
rs79598313
rs4149307
rs117113213
rs1590381
rs4902813
rs12207641
rs9869276
rs73009557
rs1800961
rs646776
rs10188514
rs1461729
rs12916
rs72926966
rs10832954
rs11591147
rs174574
rs635634
rs7254892
rs2575876
rs563290
All
-0.05
0.00
0.05
0.10
MR leave-one-out sensitivity analysis for
'Total lipids in small LDL || id:ebi-cfb233-GCST90302097' on 'ER- Breast cancer (Combined Oncoarray; iCOGS; GWAS meta analysis)'

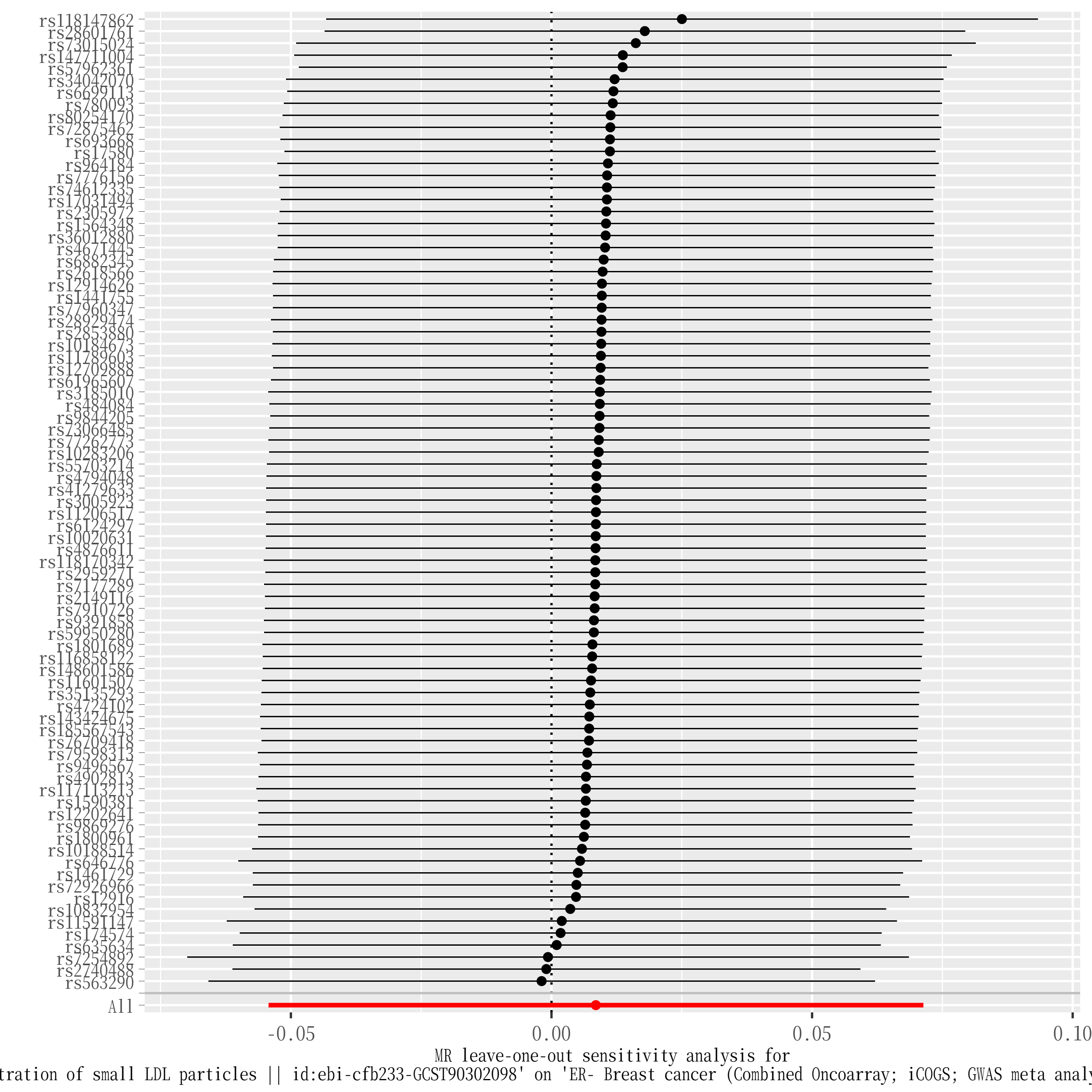

rs118147862
rs28601761
rs73015024
rs147711004
rs57962361
rs34042070
rs6699113
rs780093
rs80254170
rs72875462
rs693668
rs17580
rs964184
rs7776156
rs74612335
rs17031494
rs2305972
rs1564348
rs36012880
rs4671445
rs6882345
rs2618566
rs12914626
rs1441755
rs77960347
rs28929474
rs2853880
rs10184673
rs11789603
rs12709888
rs61965607
rs3185010
rs484084
rs9844205
rs73066485
rs77262773
rs10283206
rs55703214
rs4794048
rs41279633
rs3005923
rs11206517
rs6124297
rs10020631
rs4876611
rs118170342
rs2959271
rs7177289
rs2149116
rs7910726
rs9391858
rs59950280
rs1801689
rs116858122
rs148601586
rs11601507
rs35135293
rs4724102
rs143424675
rs185567543
rs76709418
rs79598313
rs9496567
rs4902813
rs117113213
rs1590381
rs12202641
rs9869276
rs1800961
rs10188514
rs646776
rs1461729
rs72926966
rs12916
rs10832954
rs11591147
rs174574
rs635634
rs7254892
rs2740488
rs563290
All
-0.05
0.00
0.05
0.10
MR leave-one-out sensitivity analysis for
tration of small LDL particles || id:ebi-cfb233-GCST90302098' on 'ER- Breast cancer (Combined Oncoarray; iCOGS; GWAS meta analy

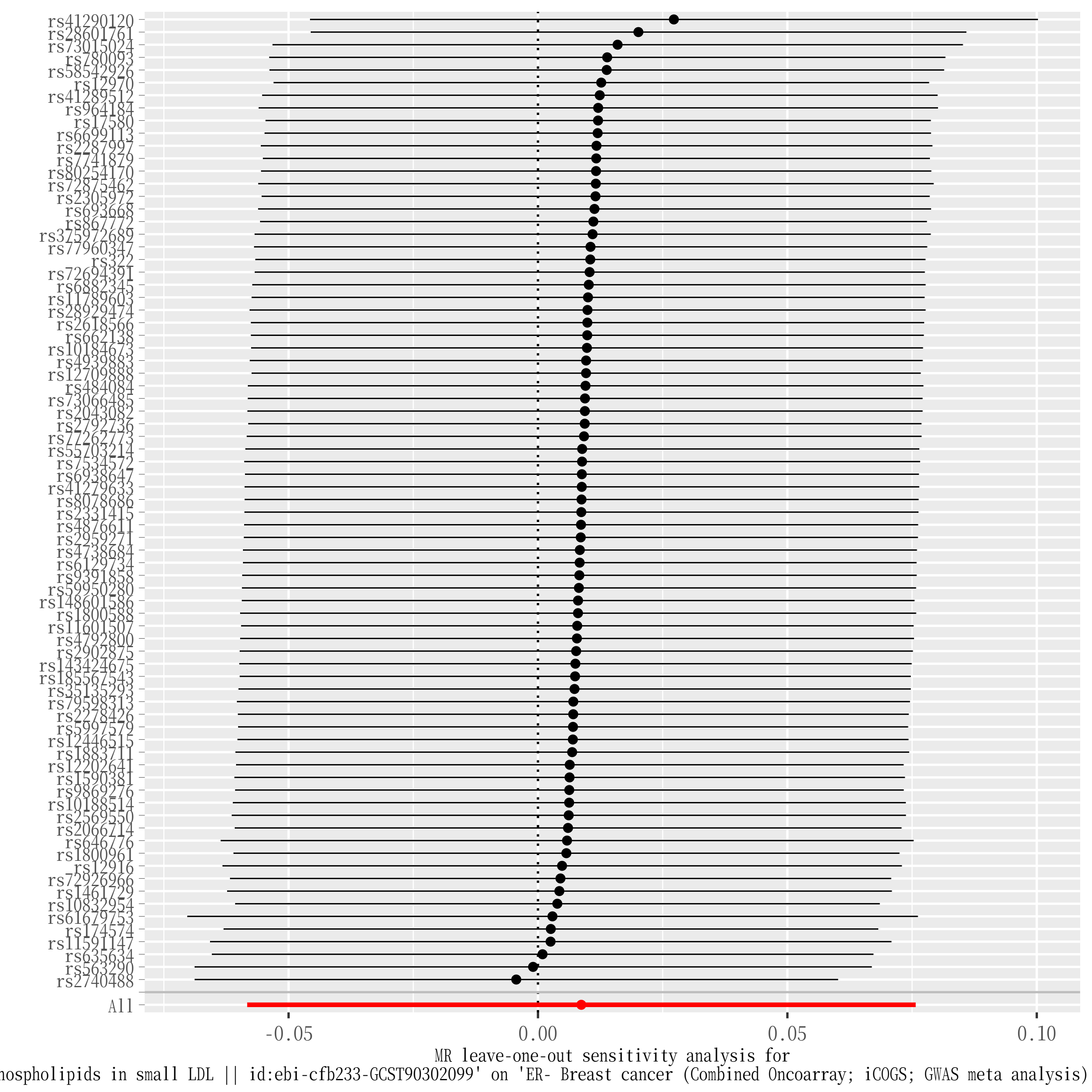

rs41290120
rs28601761
rs73015024
rs780093
rs58542926
rs12970
rs41289512
rs964184
rs17580
rs6699113
rs2287997
rs7741879
rs80254170
rs72875462
rs2305972
rs693668
rs867772
rs375972689
rs77960347
rs322
rs72694391
rs6882345
rs11789603
rs28929474
rs2618566
rs662138
rs10184673
rs4939883
rs12709888
rs484084
rs73066485
rs2043082
rs2792736
rs77262773
rs55703214
rs7534572
rs6938647
rs41279633
rs8078686
rs2331415
rs4876611
rs2959271
rs4738684
rs6129734
rs9391858
rs59950280
rs148601586
rs1800588
rs11601507
rs4792800
rs2902875
rs143424675
rs185567543
rs35135293
rs79598313
rs2278426
rs5997579
rs12446515
rs1883711
rs1220264
rs1590381
rs9869276
rs10188514
rs2569550
rs2066714
rs646776
rs1800961
rs12916
rs72926966
rs1461729
rs10832954
rs61679753
rs174574
rs11591147
rs635634
rs563290
rs2740488
All
-0.05
0.00
0.05
0.10
MR leave-one-out sensitivity analysis for
hospholipids in small LDL || id:ebi-cfb233-GCST90302099' on 'ER- Breast cancer (Combined Oncoarray; iCOGS; GWAS meta analysis)

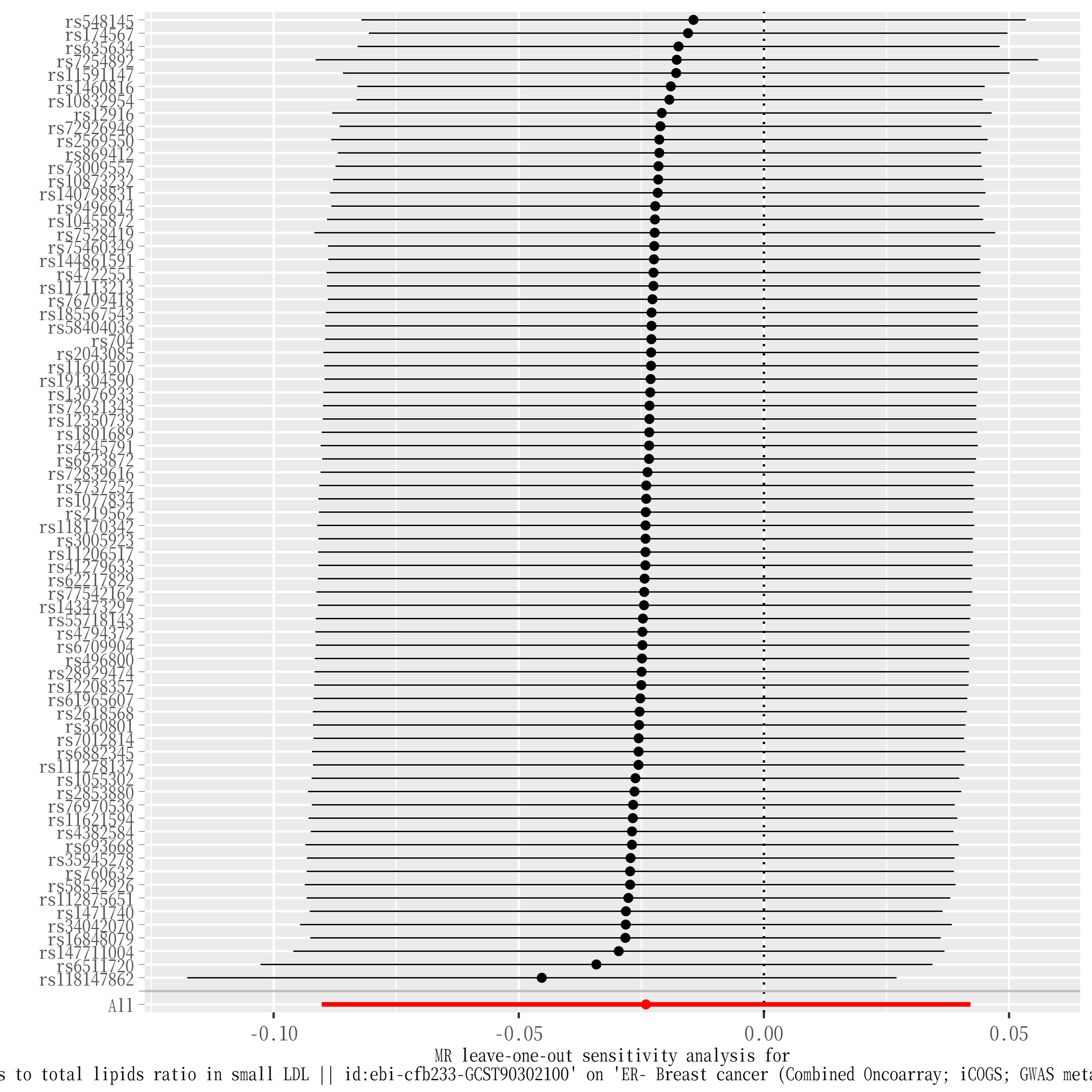
rs548145
rs174567
rs635634
rs7254892
rs11591147
rs1460816
rs10832954
rs12916
rs72926946
rs2569550
rs869412
rs73009557
rs10873232
rs140798831
rs9496614
rs10455872
rs7528419
rs75460349
rs144861591
rs4722551
rs117113213
rs76709418
rs185567543
rs58404036
rs704
rs2043085
rs11601507
rs191304590
rs13076933
rs72631343
rs12350739
rs1801689
rs4245791
rs6923872
rs72839616
rs2737252
rs1077834
rs219562
rs118170342
rs3005923
rs11206517
rs41279633
rs62217829
rs77542162
rs143473297
rs55718143
rs4794372
rs6709904
rs496800
rs28929474
rs12208357
rs61965607
rs2618568
rs360801
rs7012814
rs6882345
rs111278137
rs1055302
rs2853880
rs76970536
rs11621594
rs4382584
rs693668
rs35945278
rs760632
rs58542926
rs112875651
rs1471740
rs34042070
rs16848079
rs147711004
rs6511720
rs118147862
All
-0.10
-0.05
0.00
0.05
MR leave-one-out sensitivity analysis for
s to total lipids ratio in small LDL || id:ebi-cfb233-GCST90302100' on 'ER- Breast cancer (Combined Oncoarray; iCOGS; GWAS meta

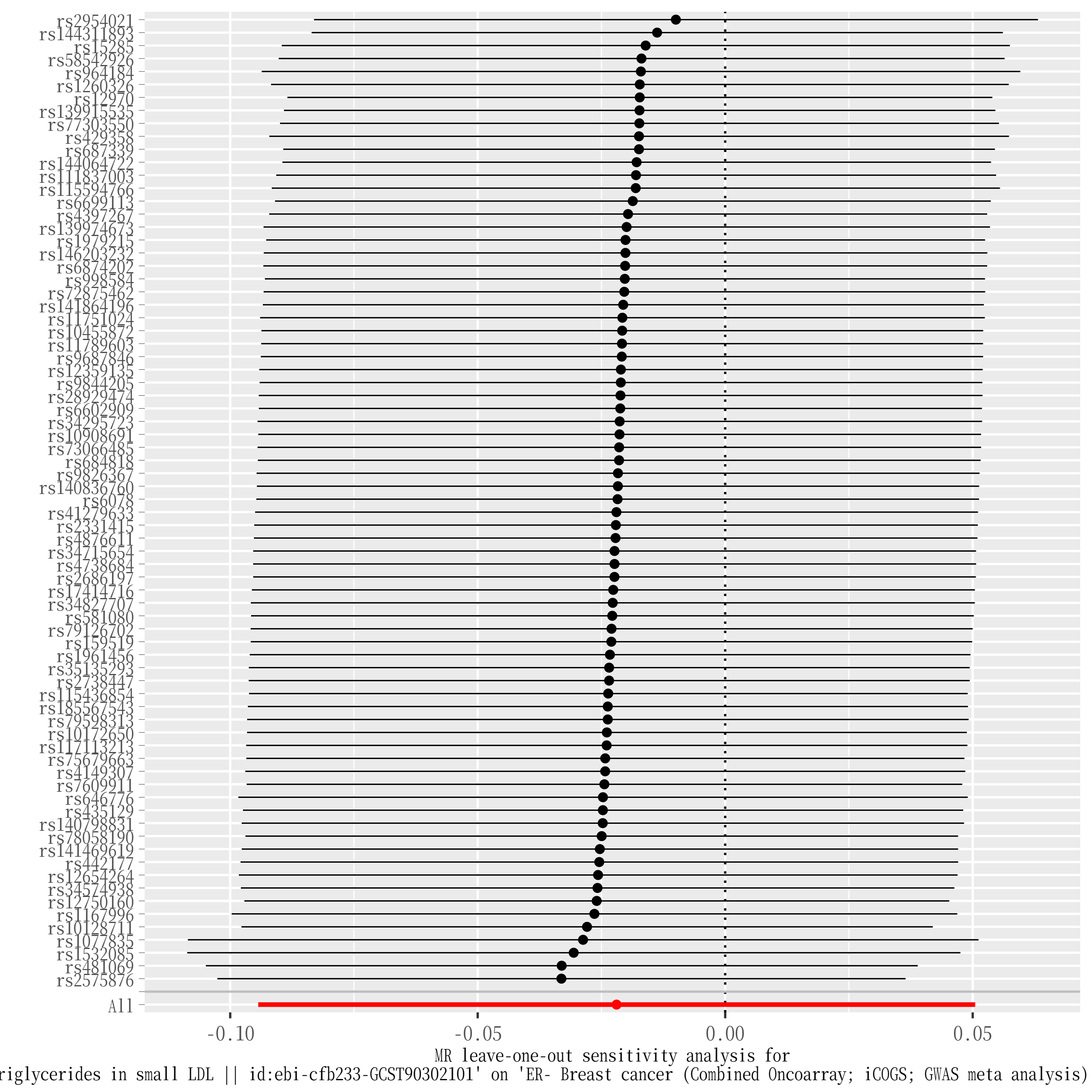
rs2954021
rs144311893
rs15285
rs58542926
rs964184
rs1260326
rs12970
rs139915535
rs77303550
rs429358
rs687339
rs144064722
rs111837003
rs115594766
rs6699113
rs4397267
rs139974673
rs1979215
rs146203232
rs6874202
rs998584
rs72875462
rs141864196
rs11751024
rs10455872
rs11789603
rs9687846
rs12359135
rs9844205
rs28929474
rs6602909
rs34295723
rs10908691
rs73066485
rs684818
rs9826367
rs140836760
rs6078
rs41279633
rs2331415
rs4876611
rs34715654
rs4738684
rs2686197
rs17414716
rs34827707
rs581080
rs79126702
rs159519
rs1961456
rs35135293
rs2738447
rs115436854
rs185567543
rs79528313
rs10172650
rs117113213
rs75679663
rs4149307
rs7609911
rs646776
rs435129
rs140798831
rs78058190
rs141469619
rs442177
rs12654264
rs34574938
rs12750160
rs1167996
rs10128711
rs1077835
rs1532085
rs481069
rs2575876
All
-0.10
-0.05
0.00
0.05
MR leave-one-out sensitivity analysis for
riglycerides in small LDL || id:ebi-cfb233-GCST90302101' on 'ER- Breast cancer (Combined Oncoarray; iCOGS; GWAS meta analysis)

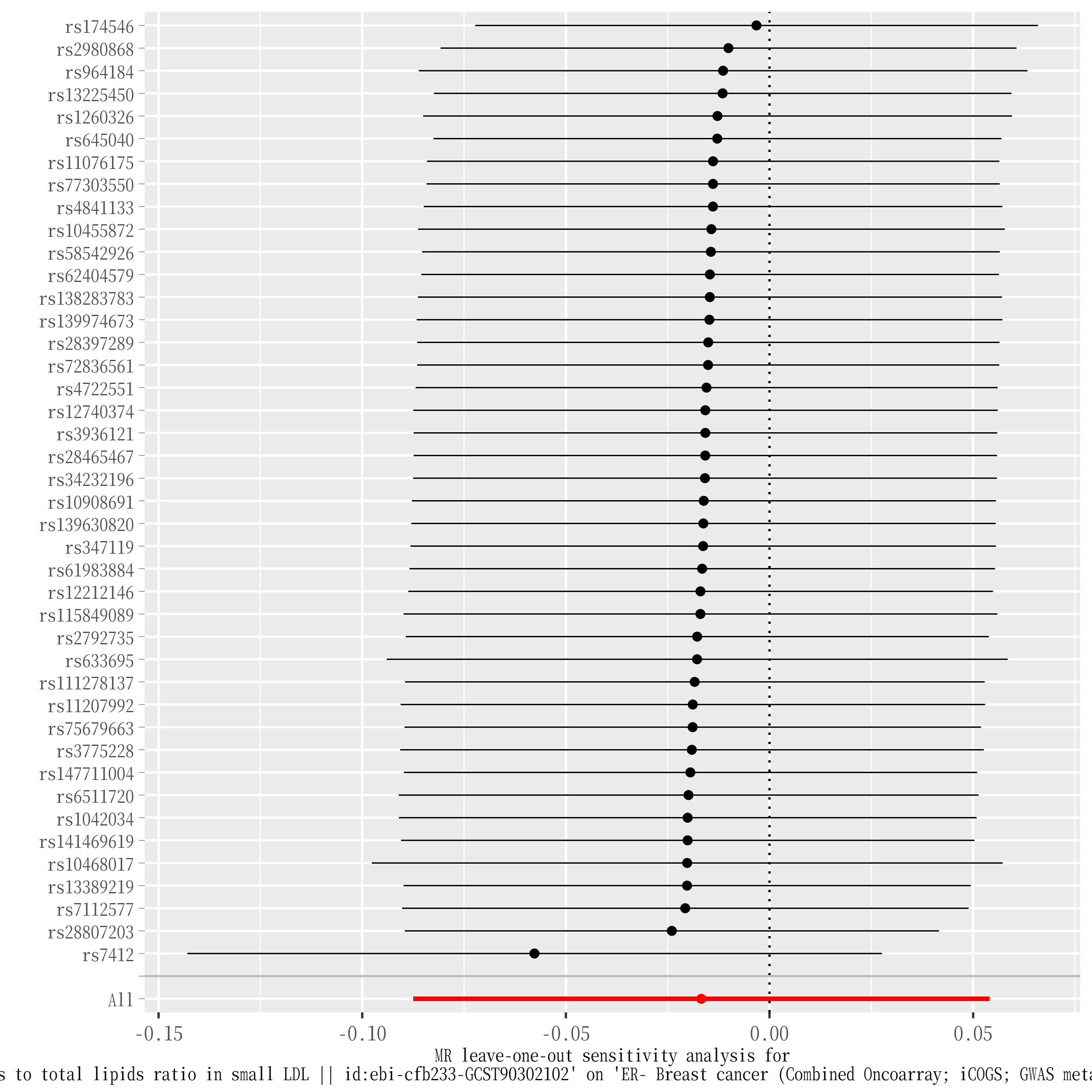

rs174546
rs2980868
rs964184
rs13225450
rs1260326
rs645040
rs11076175
rs77303550
rs4841133
rs10455872
rs58542926
rs62404579
rs138283783
rs139974673
rs28397289
rs72836561
rs4722551
rs12740374
rs3936121
rs28465467
rs34232196
rs10908691
rs139630820
rs347119
rs61983884
rs12212146
rs115849089
rs2792735
rs633695
rs111278137
rs11207992
rs75679663
rs3775228
rs147711004
rs6511720
rs1042034
rs141469619
rs10468017
rs13389219
rs7112577
rs28807203
rs7412
All
-0.15
-0.10
-0.05
0.00
0.05
MR leave-one-out sensitivity analysis for
s to total lipids ratio in small LDL || id:ebi-cfb233-GCST90302102' on 'ER- Breast cancer (Combined Oncoarray; iCOGS; GWAS meta

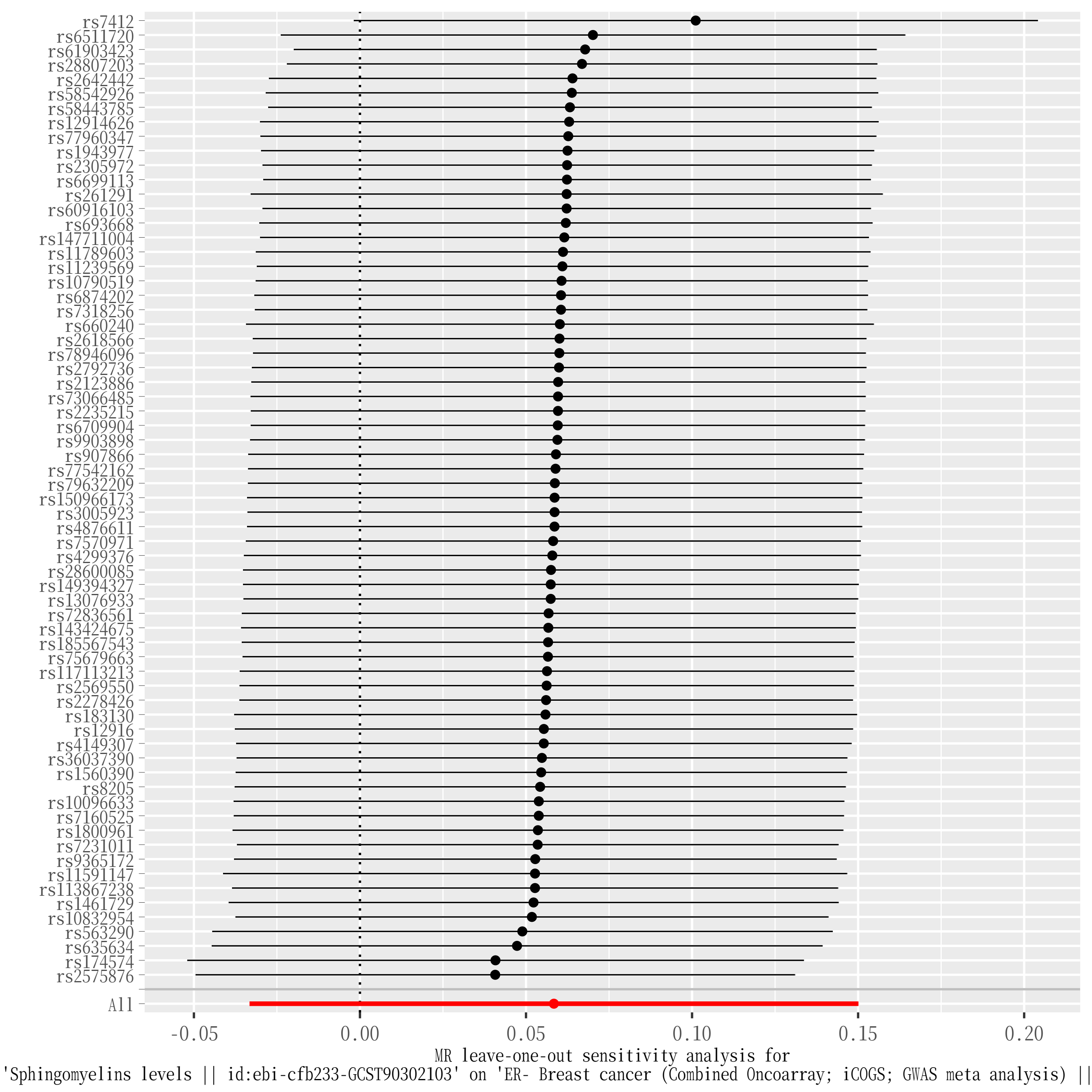

rs7412
rs6511720
rs61903423
rs28807203
rs2642442
rs58542926
rs58443785
rs12914626
rs77960347
rs1943977
rs2305972
rs6699113
rs261291
rs60916103
rs693668
rs147711004
rs11789603
rs11239569
rs10790519
rs6874202
rs7318256
rs660240
rs2618566
rs78946096
rs2792736
rs2123886
rs73066485
rs2235215
rs6709904
rs9903898
rs907866
rs77542162
rs79632209
rs150966173
rs3005923
rs4876611
rs7570971
rs4299376
rs28600085
rs149394327
rs13076933
rs72836561
rs143424675
rs185567543
rs75679663
rs117113213
rs2569550
rs2278426
rs183130
rs12916
rs4149307
rs36037390
rs1560390
rs8205
rs10096633
rs7160525
rs1800961
rs7231011
rs9365172
rs11591147
rs113867238
rs1461729
rs10832954
rs563290
rs635634
rs174574
rs2575876
All
-0.05
0.00
0.05
0.10
0.15
0.20
MR leave-one-out sensitivity analysis for
'Sphingomyelins levels || id:ebi-cfb233-GCST90302103' on 'ER- Breast cancer (Combined Oncoarray; iCOGS; GWAS meta analysis) ||

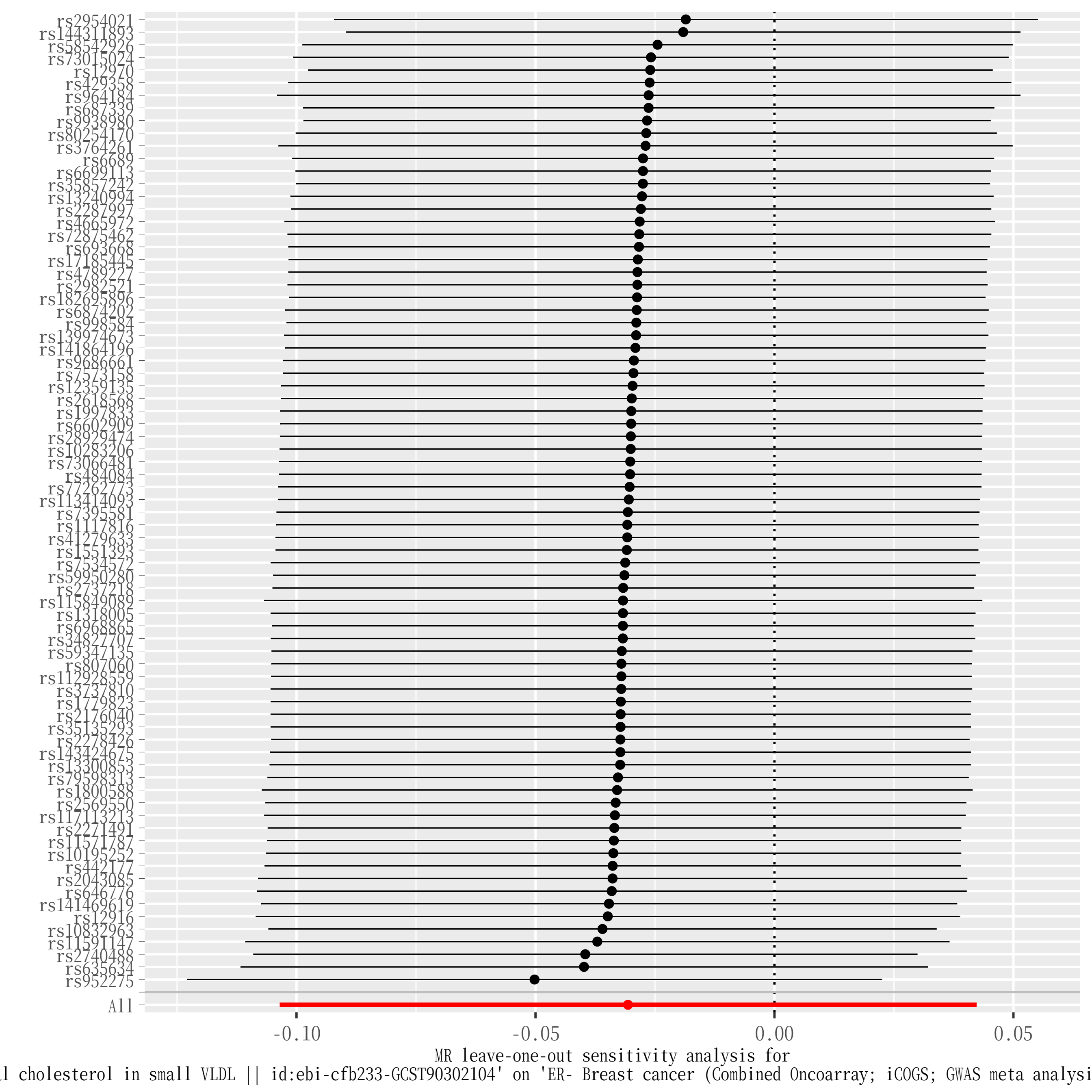
rs2954021
rs144311893
rs58542926
rs73015024
rs12970
rs429358
rs964184
rs687339
rs9938980
rs80254170
rs3764261
rs6689
rs6699113
rs35857242
rs13240994
rs2287997
rs4665972
rs72875462
rs693668
rs17185445
rs4789227
rs2982521
rs182695896
rs6874202
rs998584
rs139974673
rs141864196
rs9686661
rs7573158
rs12359135
rs2618568
rs1997833
rs6602909
rs28929474
rs10283206
rs73066481
rs484084
rs77262773
rs113414093
rs7395581
rs1117816
rs41279633
rs1551393
rs7534572
rs59950280
rs2737218
rs115849089
rs1318005
rs6968865
rs34827707
rs59347135
rs807060
rs112928559
rs3737810
rs1779823
rs2176040
rs35135293
rs2278426
rs143424675
rs13300853
rs79598313
rs1800588
rs2569550
rs117113213
rs2271491
rs11571787
rs10195252
rs442177
rs2043085
rs646776
rs141469619
rs12916
rs10832963
rs11591147
rs2740488
rs635634
rs952275
All
-0.10
-0.05
0.00
0.05
MR leave-one-out sensitivity analysis for
l cholesterol in small VLDL || id:ebi-cfb233-GCST90302104' on 'ER- Breast cancer (Combined Oncoarray; iCOGS; GWAS meta analysi

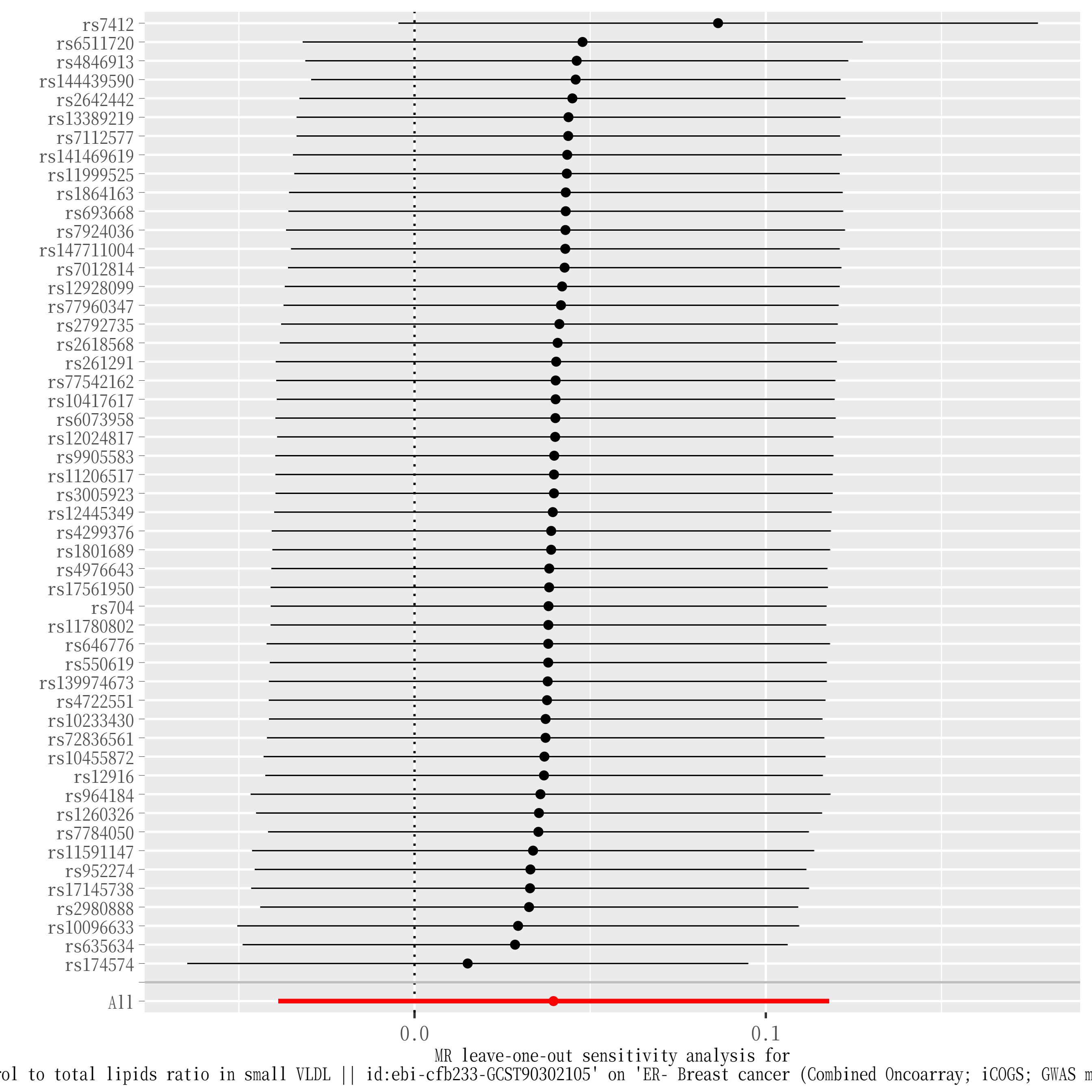
rs7412
rs6511720
rs4846913
rs144439590
rs2642442
rs13389219
rs7112577
rs141469619
rs11999525
rs1864163
rs693668
rs7924036
rs147711004
rs7012814
rs12928099
rs77960347
rs2792735
rs2618568
rs261291
rs77542162
rs10417617
rs6073958
rs12024817
rs9905583
rs11206517
rs3005923
rs12445349
rs4299376
rs1801689
rs4976643
rs17561950
rs704
rs11780802
rs646776
rs550619
rs139974673
rs4722551
rs10233430
rs72836561
rs10455872
rs12916
rs964184
rs1260326
rs7784050
rs11591147
rs952274
rs17145738
rs2980888
rs10096633
rs635634
rs174574
All
0.0
0.1
MR leave-one-out sensitivity analysis for
rol to total lipids ratio in small VLDL || id:ebi-cfb233-GCST90302105' on 'ER- Breast cancer (Combined Oncoarray; iCOGS; GWAS m

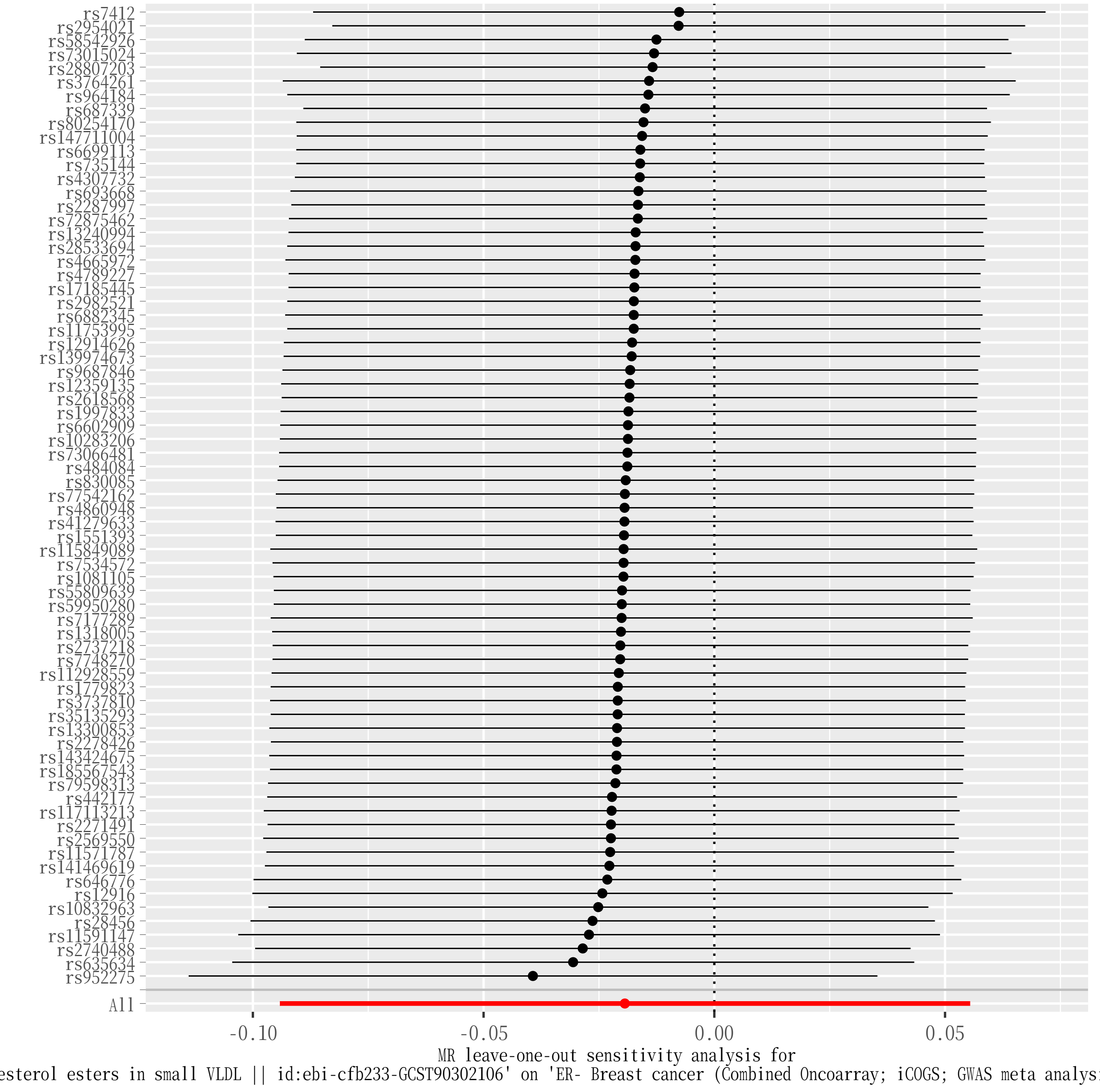
rs7412
rs2954021
rs58542926
rs73015024
rs28807203
rs3764261
rs964184
rs687339
rs80254170
rs147711004
rs6699113
rs735144
rs4307732
rs693668
rs2287997
rs72875462
rs13240994
rs28533694
rs4665972
rs4789227
rs17185445
rs2982521
rs6882345
rs11753995
rs12914626
rs139974673
rs9687846
rs12359135
rs2618568
rs1997833
rs6602909
rs10283206
rs73066481
rs484084
rs830085
rs77542162
rs4860948
rs41279633
rs1551393
rs115849089
rs7534572
rs1081105
rs55809639
rs59950280
rs7177289
rs1318005
rs2737218
rs7748270
rs112928559
rs1779823
rs3737810
rs35135293
rs13300853
rs2278426
rs143424675
rs185567543
rs79598313
rs442177
rs117113213
rs2271491
rs2569550
rs11571787
rs141469619
rs646776
rs12916
rs10832963
rs28456
rs11591147
rs2740488
rs635634
rs952275
All
-0.10
-0.05
0.00
0.05
MR leave-one-out sensitivity analysis for
esterol esters in small VLDL || id:ebi-cfb233-GCST90302106' on 'ER- Breast cancer (Combined Oncoarray; iCOGS; GWAS meta analys

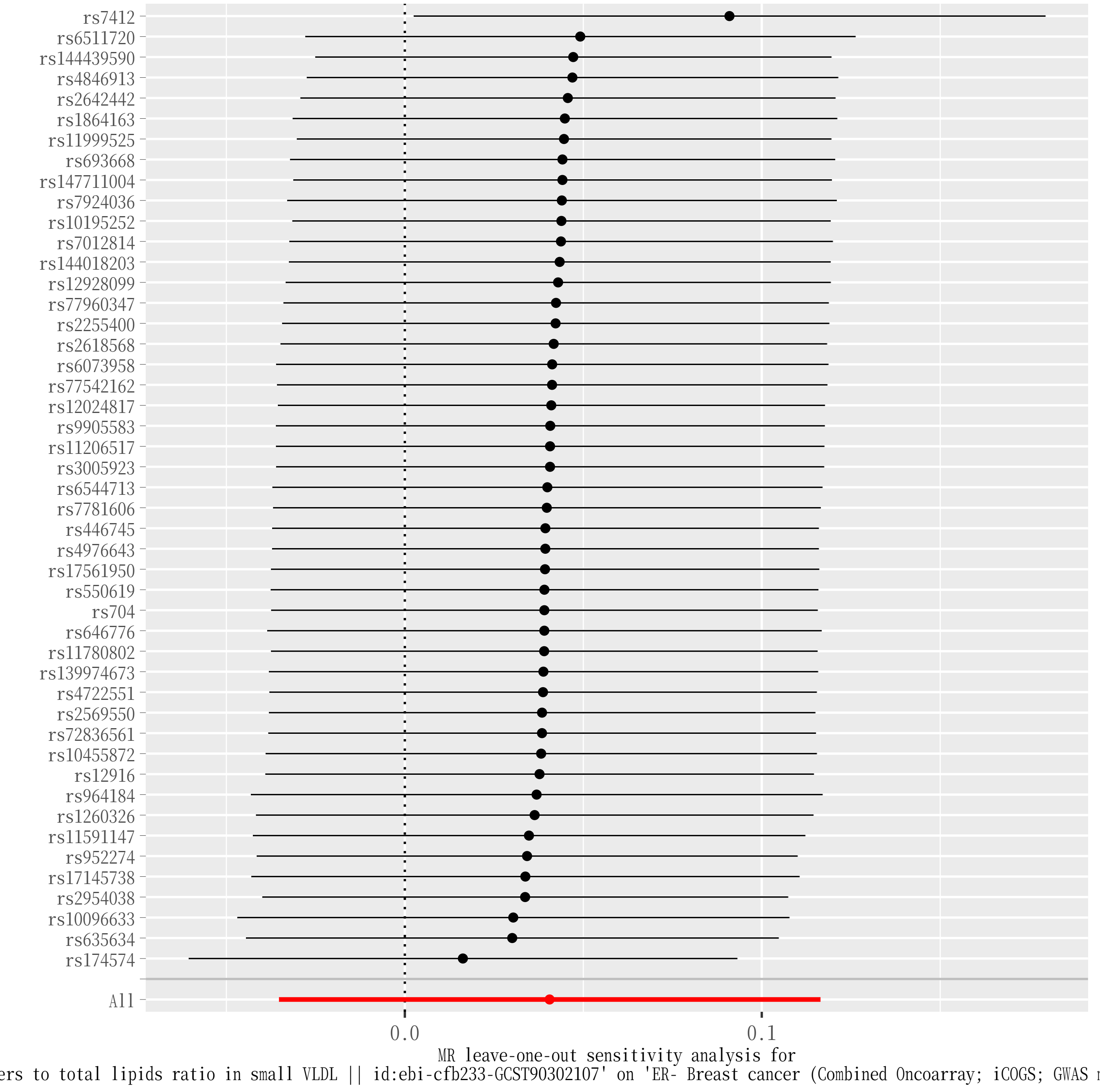
rs7412
rs6511720
rs144439590
rs4846913
rs2642442
rs1864163
rs11999525
rs693668
rs147711004
rs7924036
rs10195252
rs7012814
rs144018203
rs12928099
rs77960347
rs2255400
rs2618568
rs6073958
rs77542162
rs12024817
rs9905583
rs11206517
rs3005923
rs6544713
rs7781606
rs446745
rs4976643
rs17561950
rs550619
rs704
rs646776
rs11780802
rs139974673
rs4722551
rs2569550
rs72836561
rs10455872
rs12916
rs964184
rs1260326
rs11591147
rs952274
rs17145738
rs2954038
rs10096633
rs635634
rs174574
All
0.0
0.1
MR leave-one-out sensitivity analysis for
ers to total lipids ratio in small VLDL || id:ebi-cfb233-GCST90302107' on 'ER- Breast cancer (Combined Oncoarray; iCOGS; GWAS m

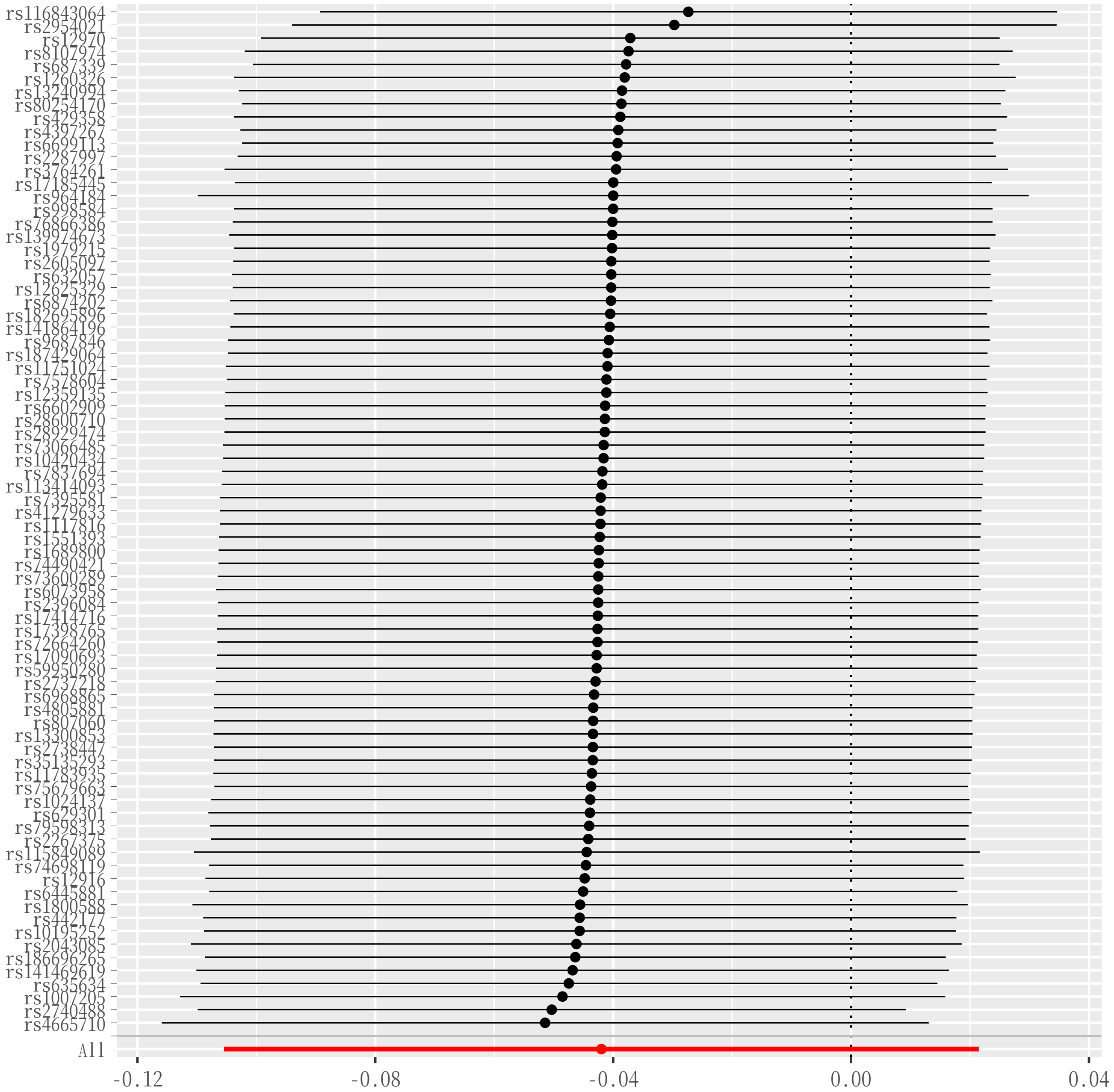

MR leave-one-out sensitivity analysis for
e cholesterol in small VLDL || id:ebi-cfb233-GCST90302108' on 'ER- Breast cancer (Combined Oncoarray; iCOGS; GWAS meta analysis

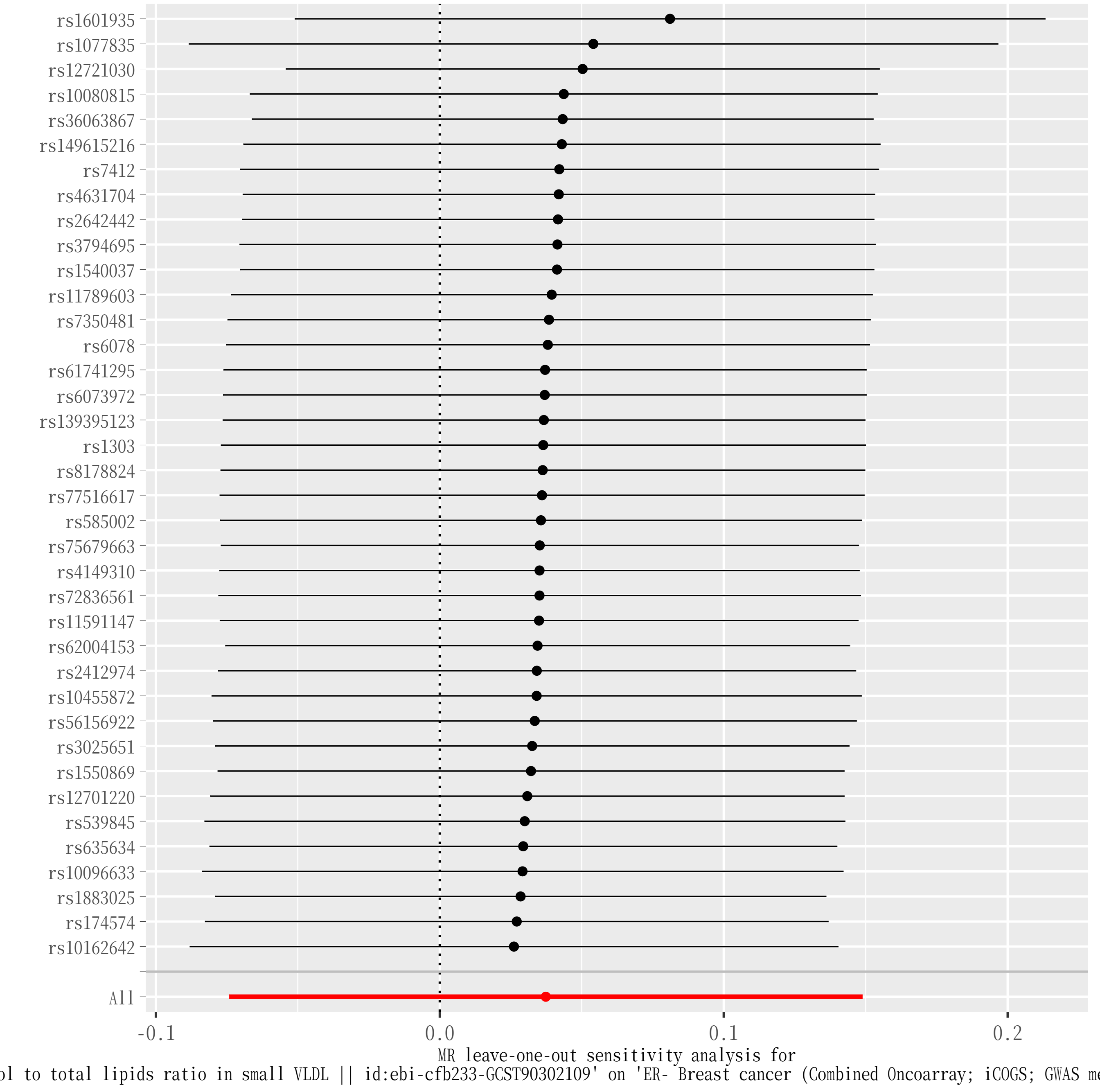
rs1601935
rs1077835
rs12721030
rs10080815
rs36063867
rs149615216
rs7412
rs4631704
rs2642442
rs3794695
rs1540037
rs11789603
rs7350481
rs6078
rs61741295
rs6073972
rs139395123
rs1303
rs8178824
rs77516617
rs585002
rs75679663
rs4149310
rs72836561
rs11591147
rs62004153
rs2412974
rs10455872
rs56156922
rs3025651
rs1550869
rs12701220
rs539845
rs635634
rs10096633
rs1883025
rs174574
rs10162642
All
-0.1
0.0
0.1
0.2
MR leave-one-out sensitivity analysis for
ol to total lipids ratio in small VLDL || id:ebi-cfb233-GCST90302109' on 'ER- Breast cancer (Combined Oncoarray; iCOGS; GWAS me

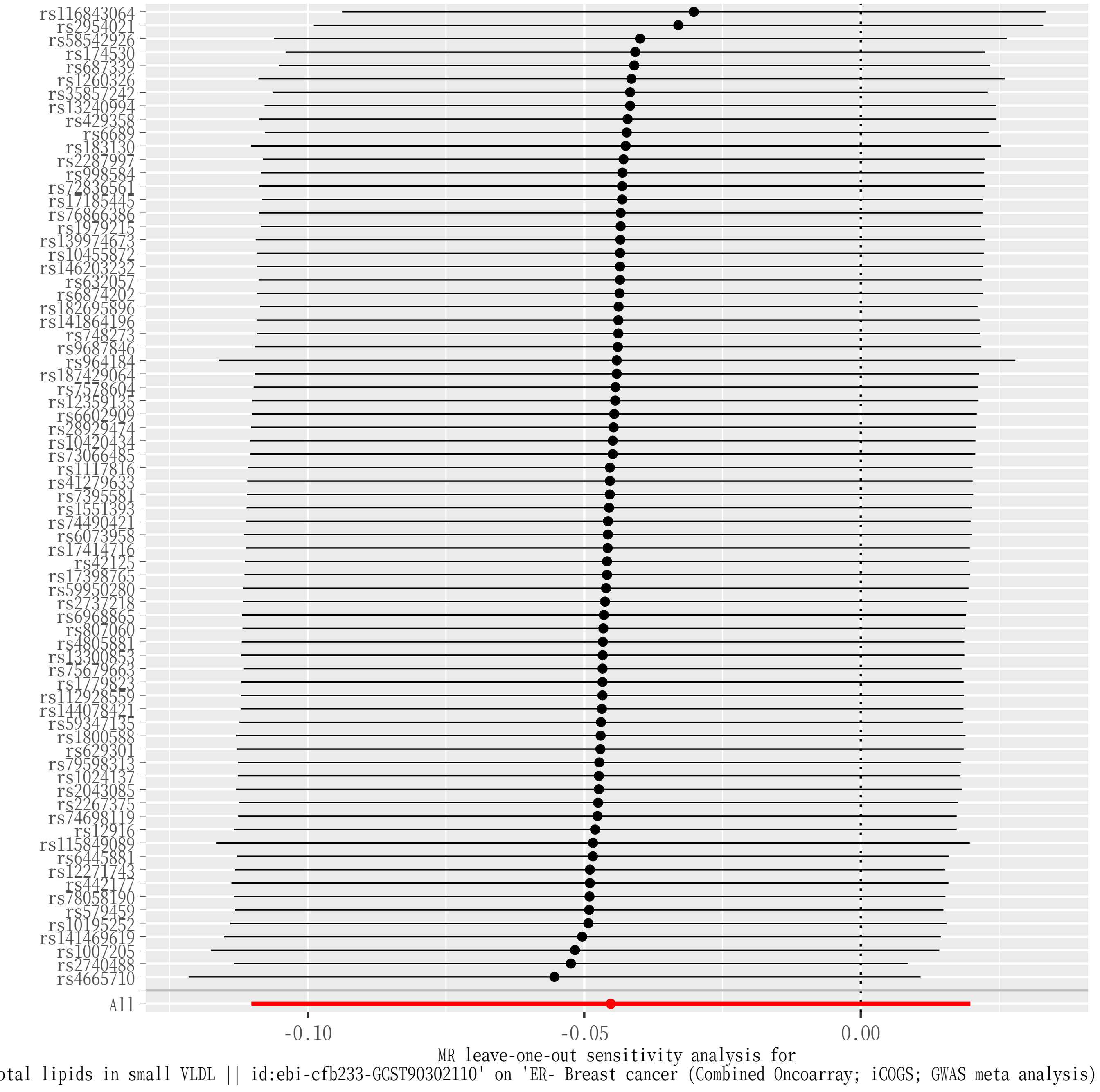
rs116843064
rs2954021
rs58542926
rs174530
rs687339
rs1260326
rs35857242
rs13240994
rs429358
rs6689
rs183130
rs2287997
rs998584
rs72836561
rs17185445
rs76866386
rs1979215
rs139974673
rs10455872
rs146203232
rs632057
rs6874202
rs182695896
rs141864196
rs748273
rs9687846
rs964184
rs187429064
rs7578604
rs12359135
rs6602909
rs28929474
rs10420434
rs73066485
rs1117816
rs41279633
rs7395581
rs1551393
rs74490421
rs6073958
rs17414716
rs42125
rs17398765
rs59950280
rs2737218
rs6968865
rs807060
rs4805881
rs13300853
rs75679663
rs1779823
rs112928559
rs144078421
rs59347135
rs1800588
rs629301
rs79598313
rs1024137
rs2043085
rs2267375
rs74698119
rs12916
rs115849089
rs6445881
rs12271743
rs442177
rs78058190
rs579459
rs10195252
rs141469619
rs1007205
rs2740488
rs4665710
All
-0.10
-0.05
0.00
MR leave-one-out sensitivity analysis for
otal lipids in small VLDL || id:ebi-cfb233-GCST90302110' on 'ER- Breast cancer (Combined Oncoarray; iCOGS; GWAS meta analysis)

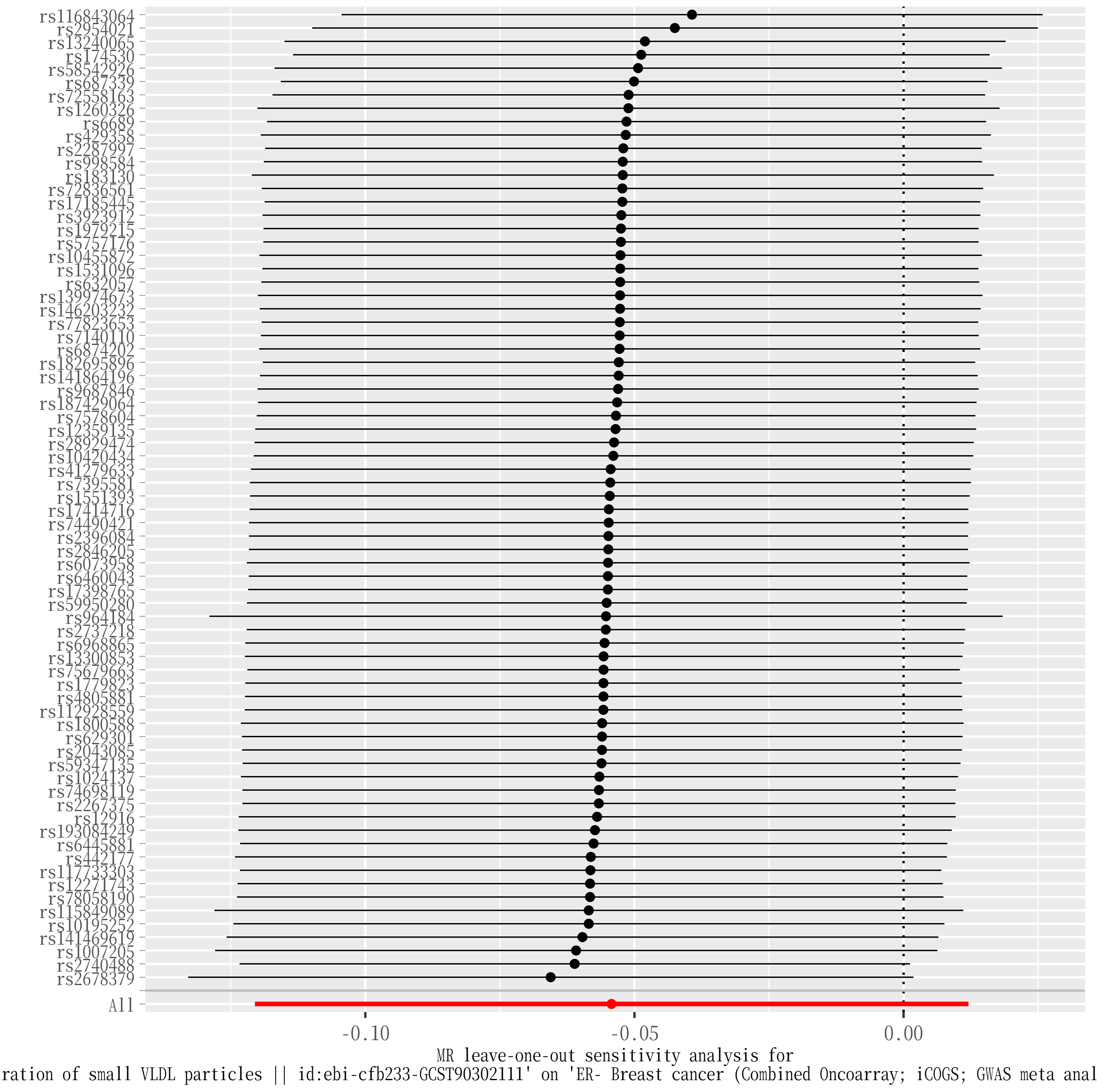

rs116843064
rs2954021
rs13240065
rs174530
rs58542926
rs687339
rs72558163
rs1260326
rs6689
rs429358
rs2287997
rs998584
rs183130
rs72836561
rs17185445
rs3923912
rs1979215
rs5757176
rs10455872
rs1531096
rs632057
rs139974673
rs146203232
rs77823653
rs7140110
rs6874202
rs182695896
rs141864196
rs9687846
rs187429064
rs7578604
rs12359135
rs28929474
rs10420434
rs41279633
rs7395581
rs1551393
rs17414716
rs74490421
rs2396084
rs2846205
rs6073958
rs6460043
rs17398765
rs59950280
rs964184
rs2737218
rs6968865
rs13300853
rs75679663
rs1779823
rs4805881
rs112928559
rs1800588
rs629301
rs2043085
rs59347135
rs1024137
rs74698119
rs2267375
rs12916
rs193084249
rs6445881
rs442177
rs117733303
rs12271743
rs78058190
rs115849089
rs10195252
rs141469619
rs1007205
rs2740488
rs2678379
All
-0.10
-0.05
0.00
MR leave-one-out sensitivity analysis for
ration of small VLDL particles || id:ebi-cfb233-GCST90302111' on 'ER- Breast cancer (Combined Oncoarray; iCOGS; GWAS meta anal

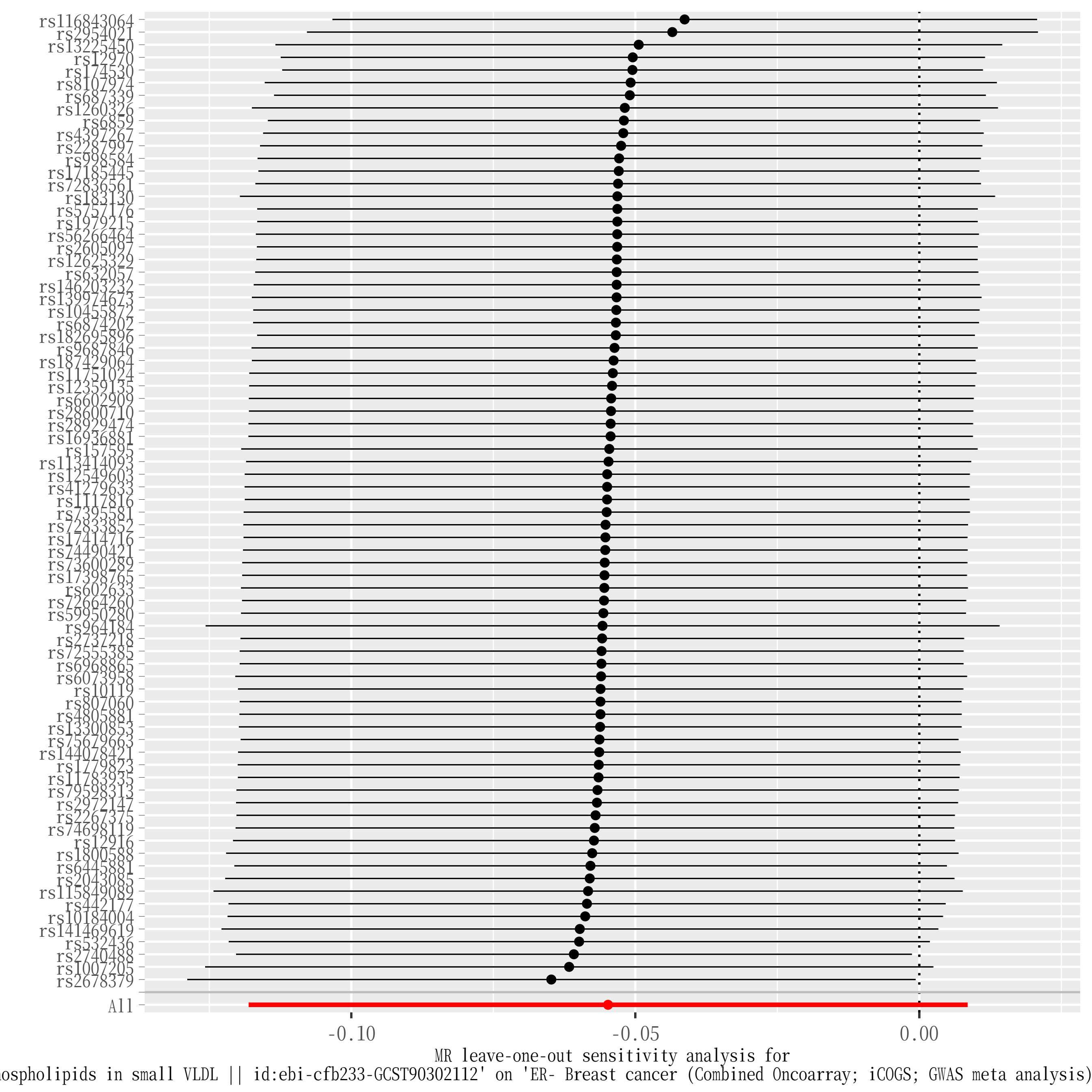

rs116843064
rs2954021
rs13225450
rs12970
rs174530
rs8107974
rs687339
rs1260326
rs6859
rs4397267
rs2287997
rs998584
rs17185445
rs72836561
rs183130
rs5757176
rs1979215
rs56266464
rs2605097
rs12625329
rs632057
rs146203232
rs139974673
rs10455872
rs6874202
rs182695896
rs9687846
rs187429064
rs11751024
rs12359135
rs6602909
rs28600710
rs28929474
rs1693681
rs157595
rs113414093
rs12549603
rs41279633
rs1117816
rs7395581
rs72833852
rs17414716
rs74490421
rs73600289
rs17398765
rs602633
rs72664260
rs59950280
rs964184
rs2737218
rs72555385
rs6968865
rs6073958
rs10119
rs807060
rs4805881
rs13300853
rs75679663
rs144078421
rs1779823
rs11783935
rs79598313
rs2972147
rs2267375
rs74698119
rs12916
rs1800588
rs6445881
rs2043085
rs115849089
rs442177
rs10184004
rs141469619
rs532436
rs2740488
rs1007205
rs2678379
All
-0.10
-0.05
0.00
MR leave-one-out sensitivity analysis for
ospholipids in small VLDL || id:ebi-cfb233-GCST90302112' on 'ER- Breast cancer (Combined Oncoarray; iCOGS; GWAS meta analysis)

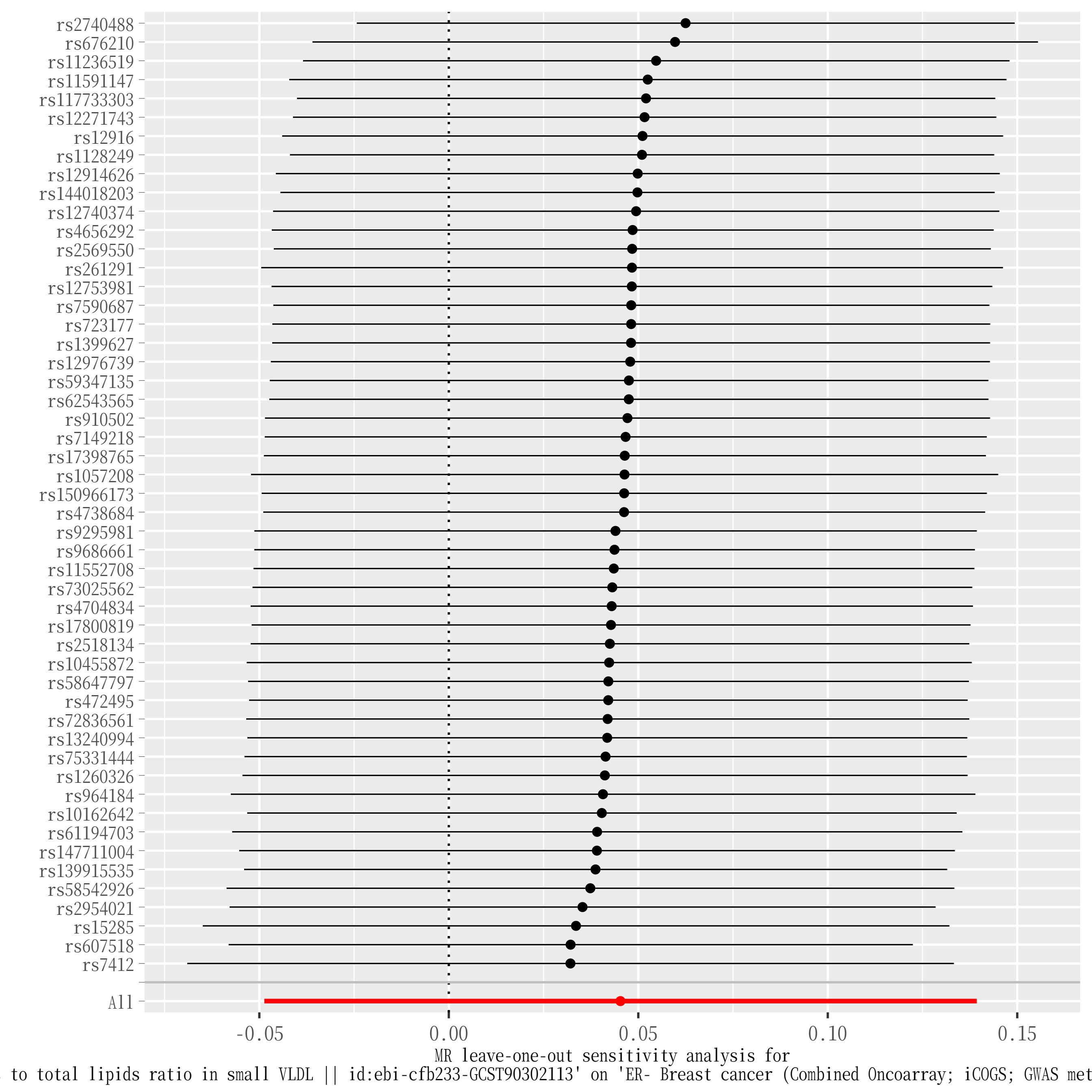

rs2740488
rs676210
rs11236519
rs11591147
rs117733303
rs12271743
rs12916
rs1128249
rs12914626
rs144018203
rs12740374
rs4656292
rs2569550
rs261291
rs12753981
rs7590687
rs723177
rs1399627
rs12976739
rs59347135
rs62543565
rs910502
rs7149218
rs17398765
rs1057208
rs150966173
rs4738684
rs9295981
rs9686661
rs11552708
rs73025562
rs4704834
rs17800819
rs2518134
rs10455872
rs58647797
rs472495
rs72836561
rs13240994
rs75331444
rs1260326
rs964184
rs10162642
rs61194703
rs147711004
rs139915535
rs58542926
rs2954021
rs15285
rs607518
rs7412
All
-0.05
0.00
0.05
0.10
0.15
MR leave-one-out sensitivity analysis for
to total lipids ratio in small VLDL || id:ebi-cfb233-GCST90302113' on 'ER- Breast cancer (Combined Oncoarray; iCOGS; GWAS met

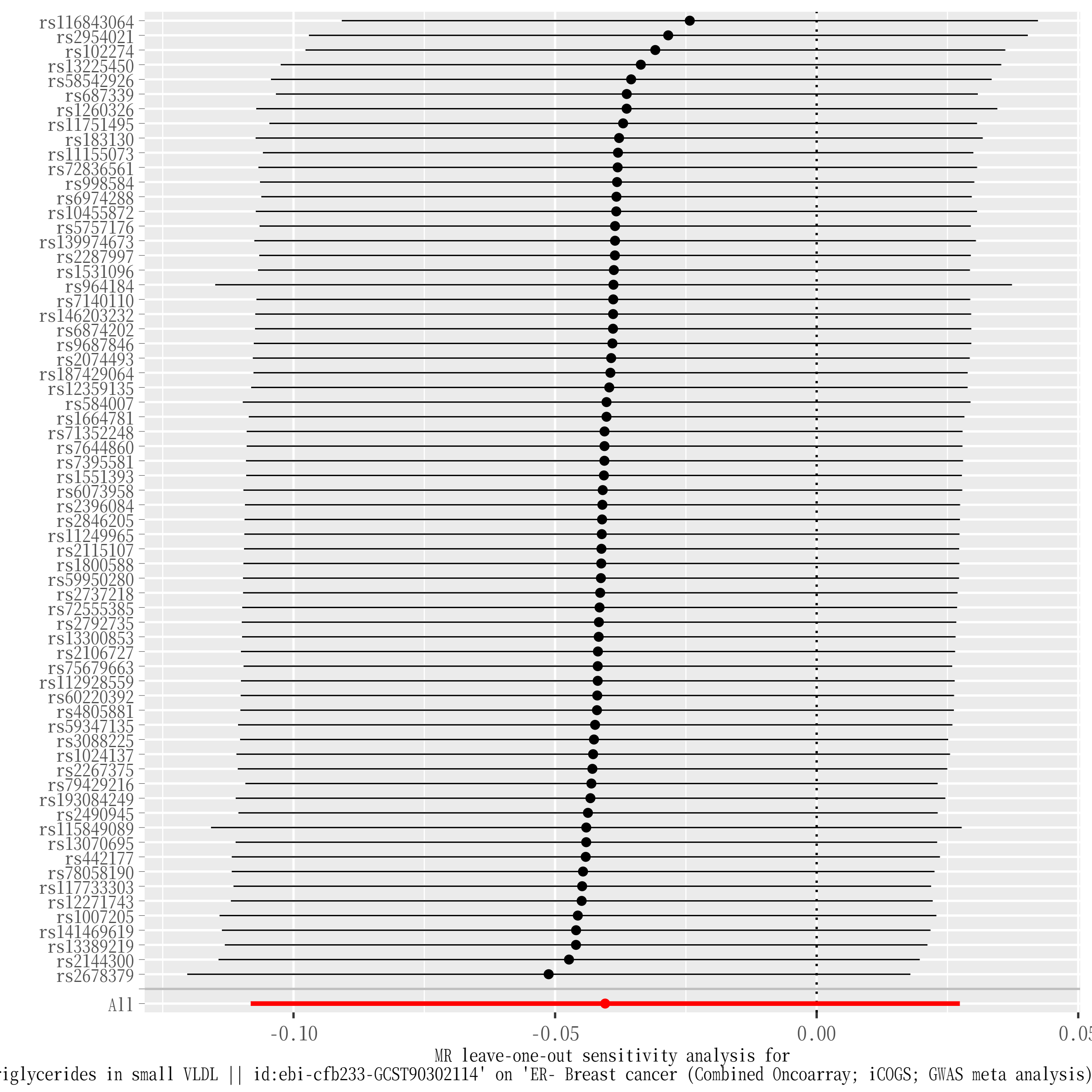
rs116843064
rs2954021
rs102274
rs13225450
rs58542926
rs687339
rs1260326
rs11751495
rs183130
rs11155073
rs72836561
rs998584
rs6974288
rs10455872
rs5757176
rs139974673
rs2287997
rs1531096
rs964184
rs7140110
rs146203232
rs6874202
rs9687846
rs2074493
rs187429064
rs12359135
rs584007
rs1664781
rs71352248
rs7644860
rs7395581
rs1551393
rs6073958
rs2396084
rs2846205
rs11249965
rs2115107
rs1800588
rs59950280
rs2737218
rs72555385
rs2792735
rs13300853
rs2106727
rs75679663
rs112928559
rs60220392
rs4805881
rs59347135
rs3088225
rs1024137
rs2267375
rs79429216
rs193084249
rs2490945
rs115849089
rs13070695
rs442177
rs78058190
rs117733303
rs12271743
rs1007205
rs141469619
rs13389219
rs2144300
rs2678379
All
-0.10
-0.05
0.00
0.05
MR leave-one-out sensitivity analysis for
'Triglycerides in small VLDL || id:ebi-cfb233-GCST90302114' on 'ER- Breast cancer (Combined Oncoarray; iCOGS; GWAS meta analysis)'

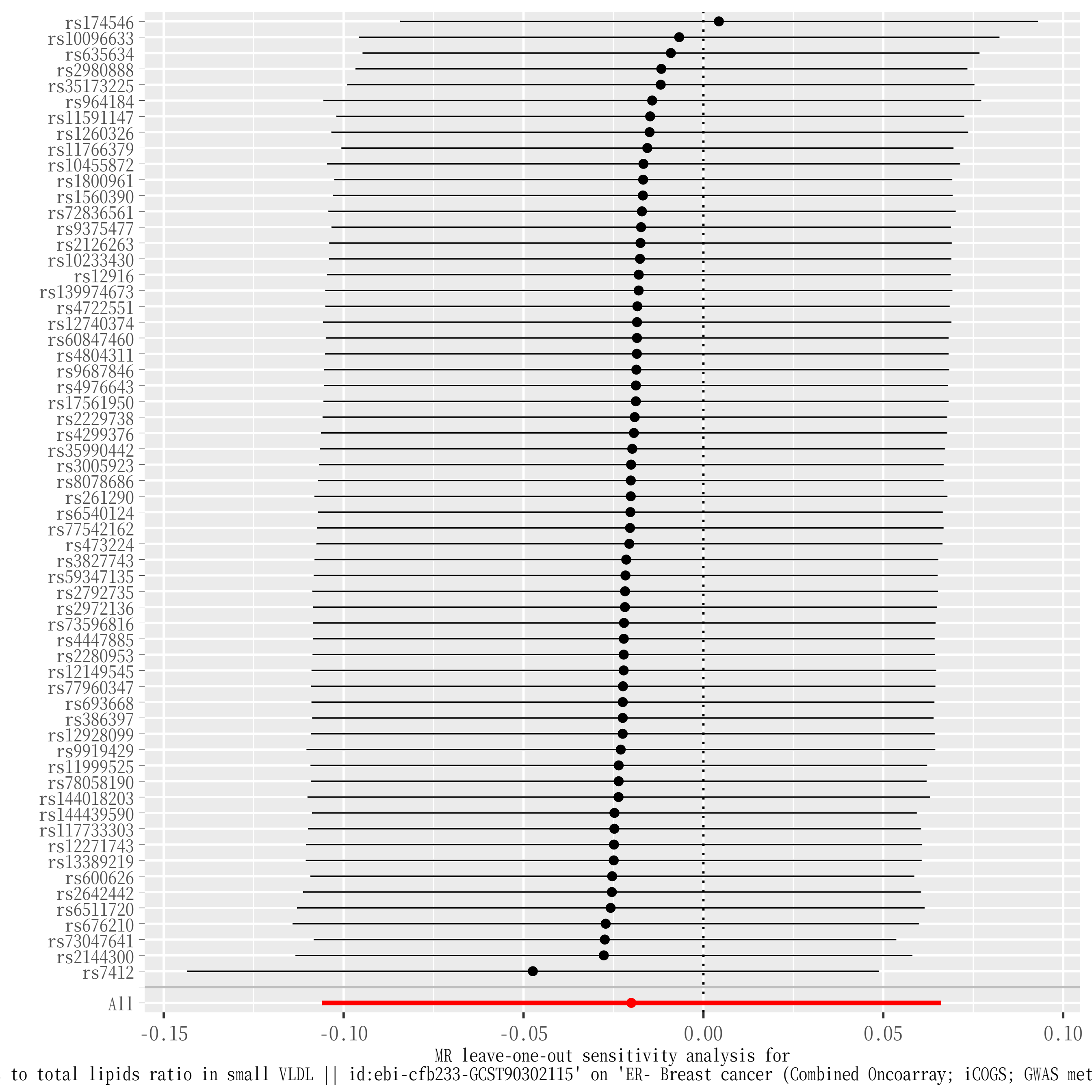
rs174546
rs10096633
rs635634
rs2980888
rs35173225
rs964184
rs11591147
rs1260326
rs11766379
rs10455872
rs1800961
rs1560390
rs72836561
rs9375477
rs2126263
rs10233430
rs12916
rs139974673
rs4722551
rs12740374
rs60847460
rs4804311
rs9687846
rs4976643
rs17561950
rs2229738
rs4299376
rs35990442
rs3005923
rs8078686
rs261290
rs6540124
rs77542162
rs473224
rs3827743
rs59347135
rs2792735
rs2972136
rs73596816
rs4447885
rs2280953
rs12149545
rs77960347
rs693668
rs386397
rs12928099
rs9919429
rs11999525
rs78058190
rs144018203
rs144439590
rs117733303
rs12271743
rs13389219
rs600626
rs2642442
rs6511720
rs676210
rs73047641
rs2144300
rs7412
All
-0.15
-0.10
-0.05
0.00
0.05
0.10
MR leave-one-out sensitivity analysis for
to total lipids ratio in small VLDL || id:ebi-cfb233-GCST90302115' on 'ER- Breast cancer (Combined Oncoarray; iCOGS; GWAS met

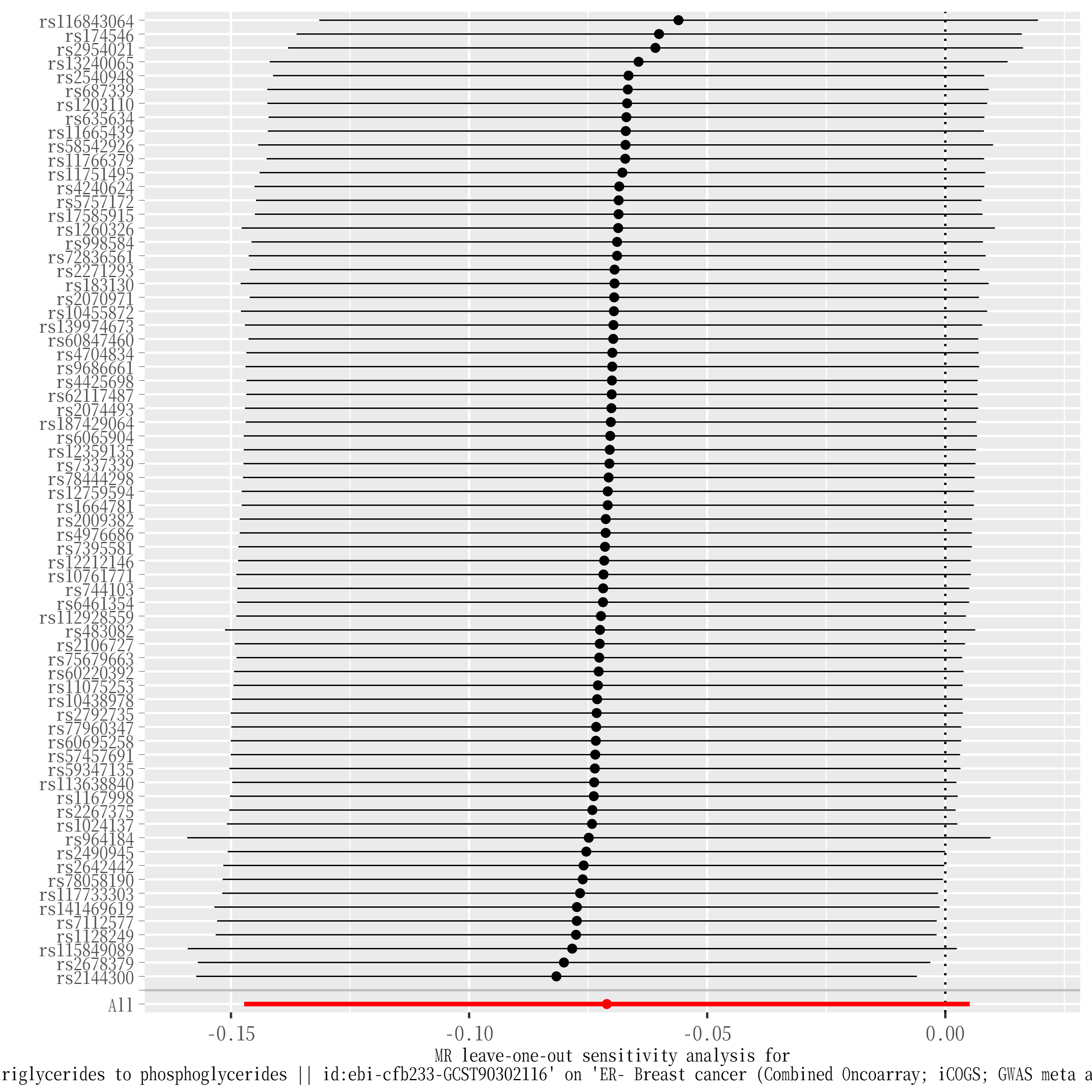
rs116843064
rs174546
rs2954021
rs13240065
rs2540948
rs687339
rs1203110
rs635634
rs11665439
rs58542926
rs11766379
rs11751495
rs4240624
rs5757172
rs17585915
rs1260326
rs998584
rs72836561
rs2271293
rs183130
rs2070971
rs10455872
rs139974673
rs60847460
rs4704834
rs9686661
rs4425698
rs62117487
rs2074493
rs187429064
rs6065904
rs12359135
rs7337339
rs78444298
rs12759594
rs1664781
rs2009382
rs4976686
rs7395581
rs12212146
rs10761771
rs744103
rs6461354
rs112928559
rs483082
rs2106727
rs75679663
rs60220392
rs11075253
rs10438978
rs2792735
rs77960347
rs60695258
rs57457691
rs59347135
rs113638840
rs1167998
rs2267375
rs1024137
rs964184
rs2490945
rs2642442
rs78058190
rs117733303
rs141469619
rs7112577
rs1128249
rs115849089
rs2678379
rs2144300
All
-0.15
-0.10
-0.05
0.00
MR leave-one-out sensitivity analysis for
riglycerides to phosphoglycerides || id:ebi-cfb233-GCST90302116' on 'ER- Breast cancer (Combined Oncoarray; iCOGS; GWAS meta a

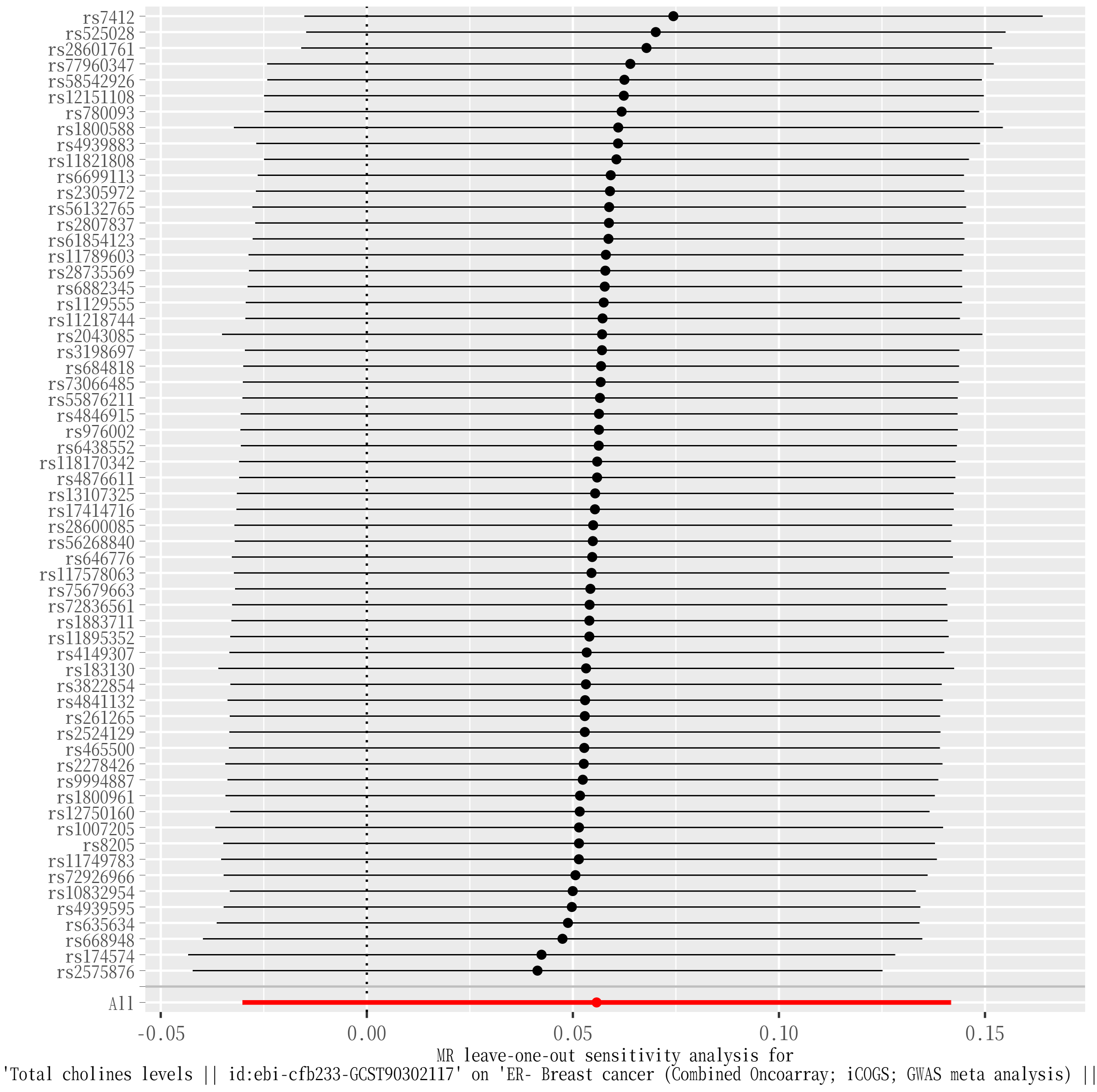
rs7412
rs525028
rs28601761
rs77960347
rs58542926
rs12151108
rs780093
rs1800588
rs4939883
rs11821808
rs6699113
rs2305972
rs56132765
rs2807837
rs61854123
rs11789603
rs28735569
rs6882345
rs1129555
rs11218744
rs2043085
rs3198697
rs684818
rs73066485
rs55876211
rs4846915
rs976002
rs6438552
rs118170342
rs4876611
rs13107325
rs17414716
rs28600085
rs56268840
rs646776
rs117578063
rs75679663
rs72836561
rs1883711
rs11895352
rs4149307
rs183130
rs3822854
rs4841132
rs261265
rs2524129
rs465500
rs2278426
rs9994887
rs1800961
rs12750160
rs1007205
rs8205
rs11749783
rs72926966
rs10832954
rs4939595
rs635634
rs668948
rs174574
rs2575876
All
-0.05
0.00
0.05
0.10
0.15
MR leave-one-out sensitivity analysis for
'Total cholines levels || id:ebi-cfb233-GCST90302117' on 'ER- Breast cancer (Combined Oncoarray; iCOGS; GWAS meta analysis) ||

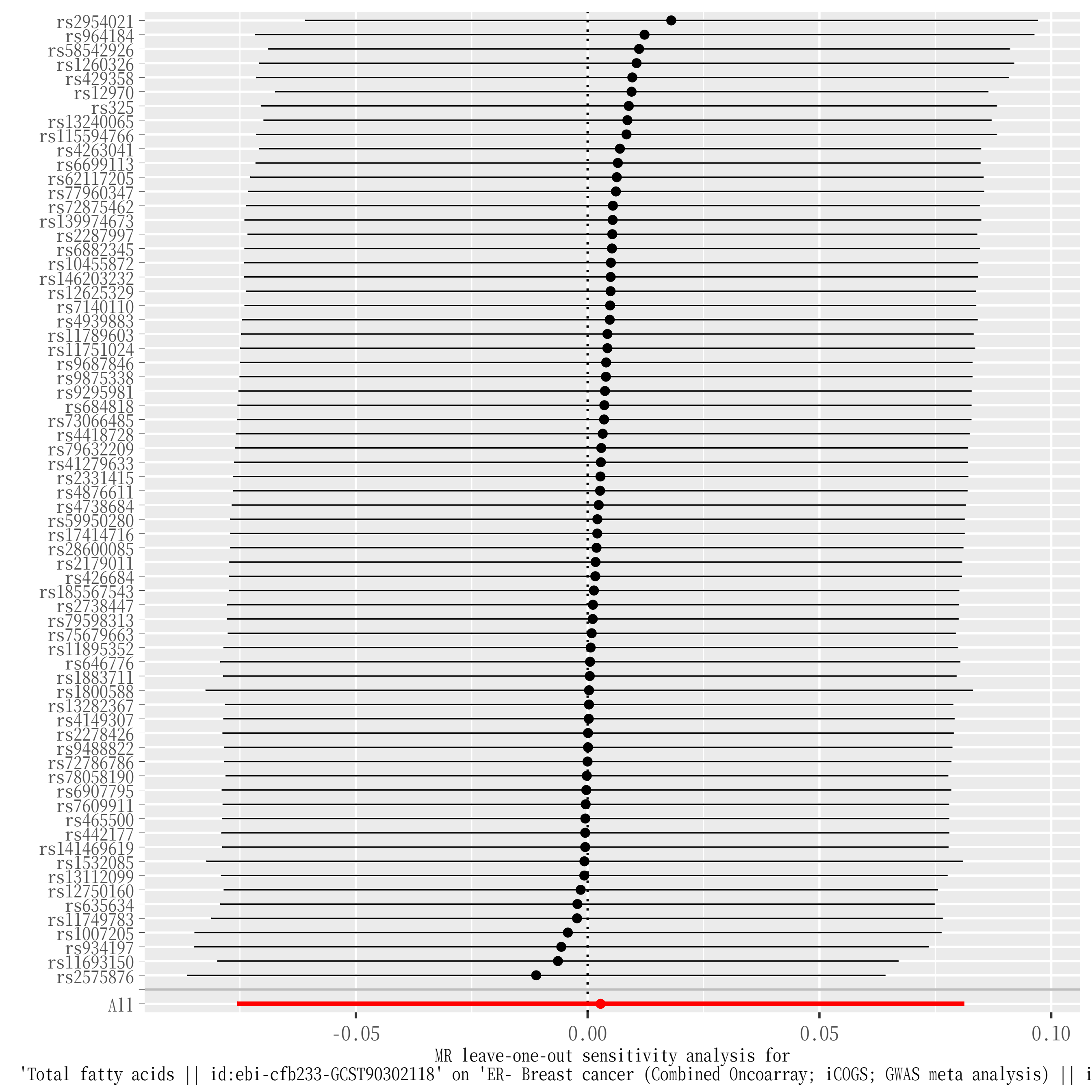

rs2954021
rs964184
rs58542926
rs1260326
rs429358
rs12970
rs325
rs13240065
rs115594766
rs4263041
rs6699113
rs62117205
rs77960347
rs72875462
rs139974673
rs2287997
rs6882345
rs10455872
rs146203232
rs12625329
rs7140110
rs4939883
rs11789603
rs11751024
rs9687846
rs9875338
rs9295981
rs684818
rs73066485
rs4418728
rs79632209
rs41279633
rs2331415
rs4876611
rs4738684
rs59950280
rs17414716
rs28600085
rs2179011
rs426684
rs185567543
rs2738447
rs79598313
rs75679663
rs11895352
rs646776
rs1883711
rs1800588
rs13282367
rs4149307
rs2278426
rs9488822
rs72786786
rs78058190
rs6907795
rs7609911
rs465500
rs442177
rs141469619
rs1532085
rs13112099
rs12750160
rs635634
rs11749783
rs1007205
rs934197
rs11693150
rs2575876
All
-0.05
0.00
0.05
0.10
MR leave-one-out sensitivity analysis for
'Total fatty acids || id:ebi-cfb233-GCST90302118' on 'ER- Breast cancer (Combined Oncoarray; iCOGS; GWAS meta analysis) || i

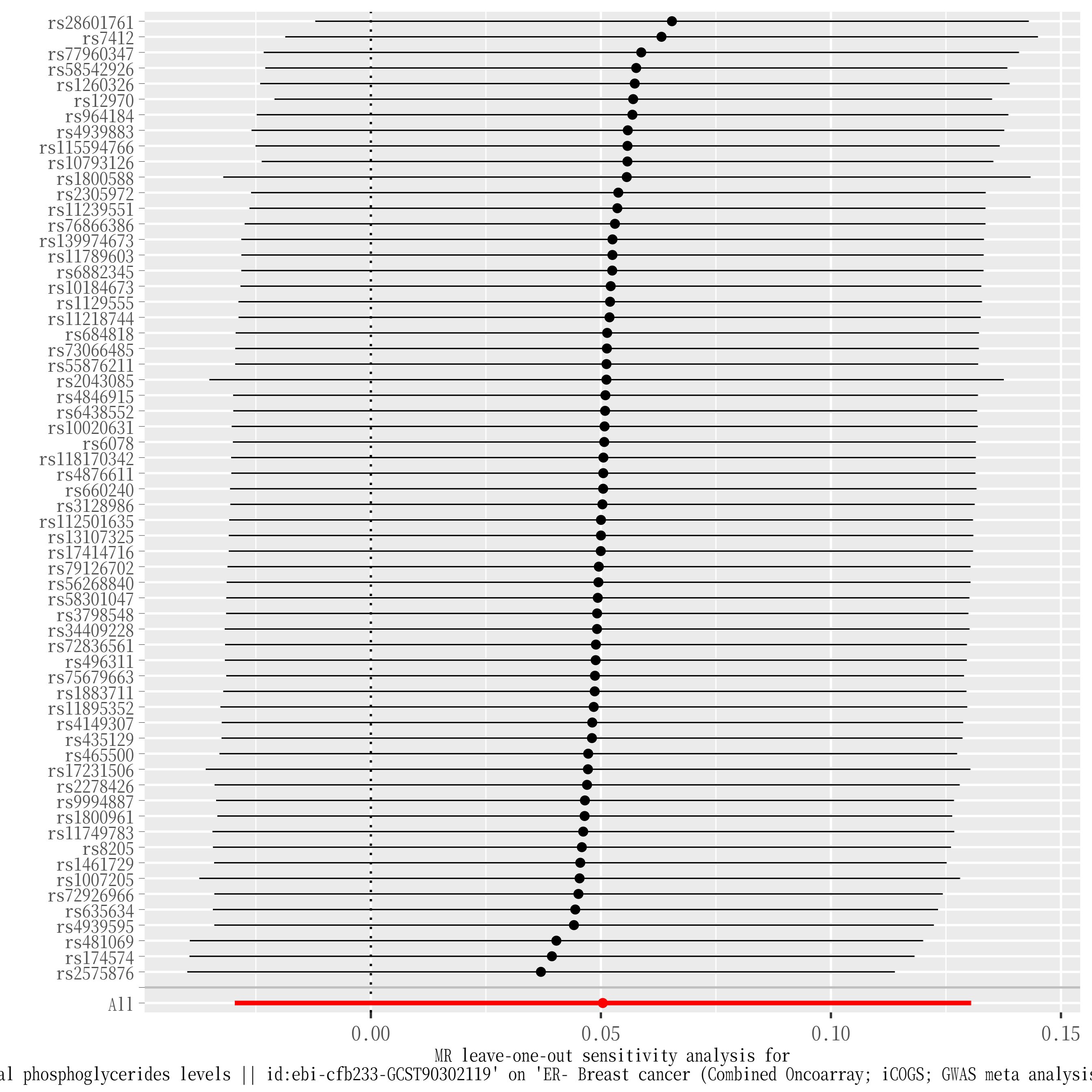
rs28601761
rs7412
rs77960347
rs58542926
rs1260326
rs12970
rs964184
rs4939883
rs115594766
rs10793126
rs1800588
rs2305972
rs11239551
rs76866386
rs139974673
rs11789603
rs6882345
rs10184673
rs1129555
rs11218744
rs684818
rs73066485
rs55876211
rs2043085
rs4846915
rs6438552
rs10020631
rs6078
rs118170342
rs4876611
rs660240
rs3128986
rs112501635
rs13107325
rs17414716
rs79126702
rs56268840
rs58301047
rs3798548
rs34409228
rs72836561
rs496311
rs75679663
rs1883711
rs11895352
rs4149307
rs435129
rs465500
rs17231506
rs2278426
rs9994887
rs1800961
rs11749783
rs8205
rs1461729
rs1007205
rs72926966
rs635634
rs4939595
rs481069
rs174574
rs2575876
All
0.00
0.05
0.10
0.15
MR leave-one-out sensitivity analysis for
al phosphoglycerides levels || id:ebi-cfb233-GCST90302119' on 'ER- Breast cancer (Combined Oncoarray; iCOGS; GWAS meta analysis

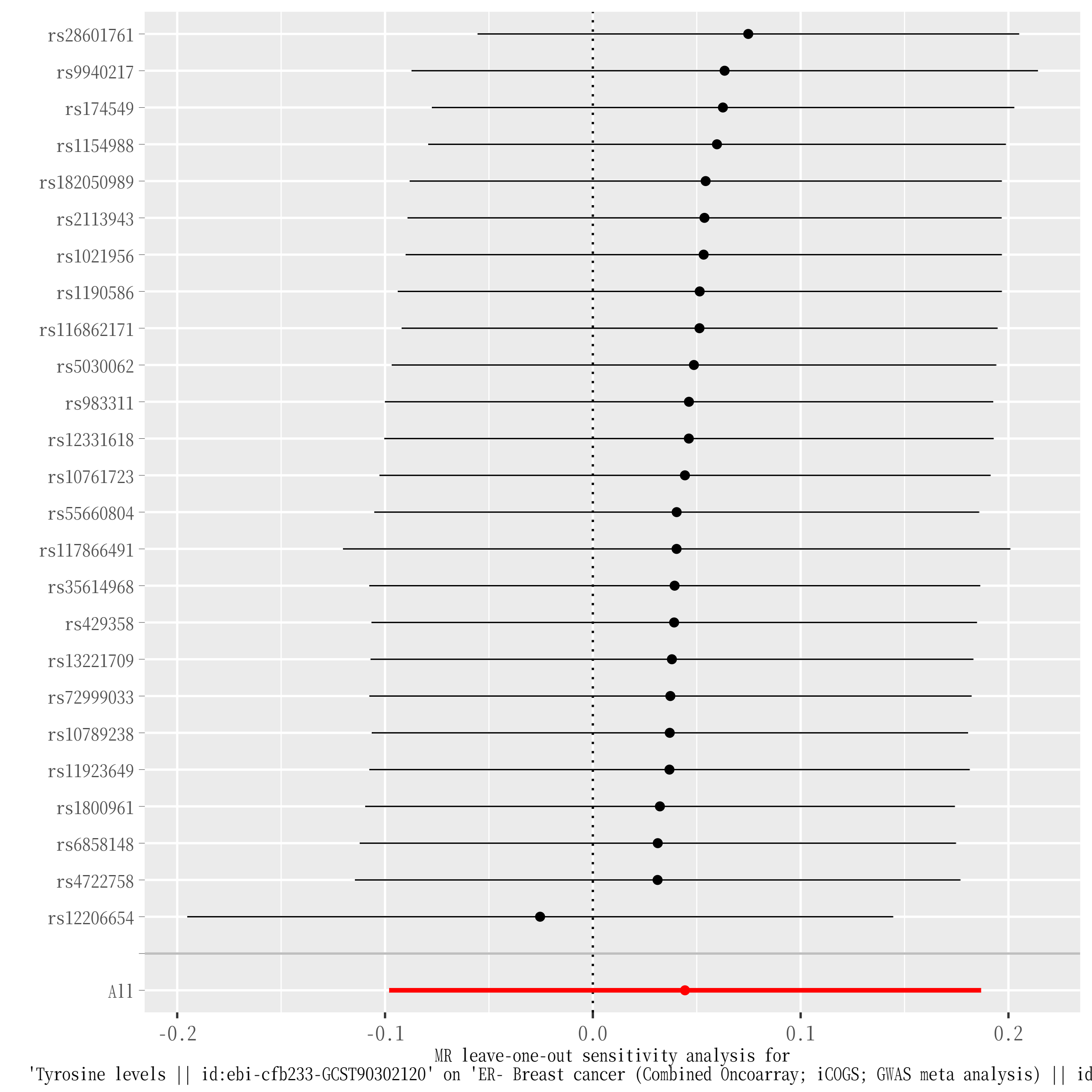
rs28601761
rs9940217
rs174549
rs1154988
rs182050989
rs2113943
rs1021956
rs1190586
rs116862171
rs5030062
rs983311
rs12331618
rs10761723
rs55660804
rs117866491
rs35614968
rs429358
rs13221709
rs72999033
rs10789238
rs11923649
rs1800961
rs6858148
rs4722758
rs12206654
All
-0.2
-0.1
0.0
0.1
0.2
MR leave-one-out sensitivity analysis for
'Tyrosine levels || id:ebi-cfb233-GCST90302120' on 'ER- Breast cancer (Combined Oncoarray; iCOGS; GWAS meta analysis) || id

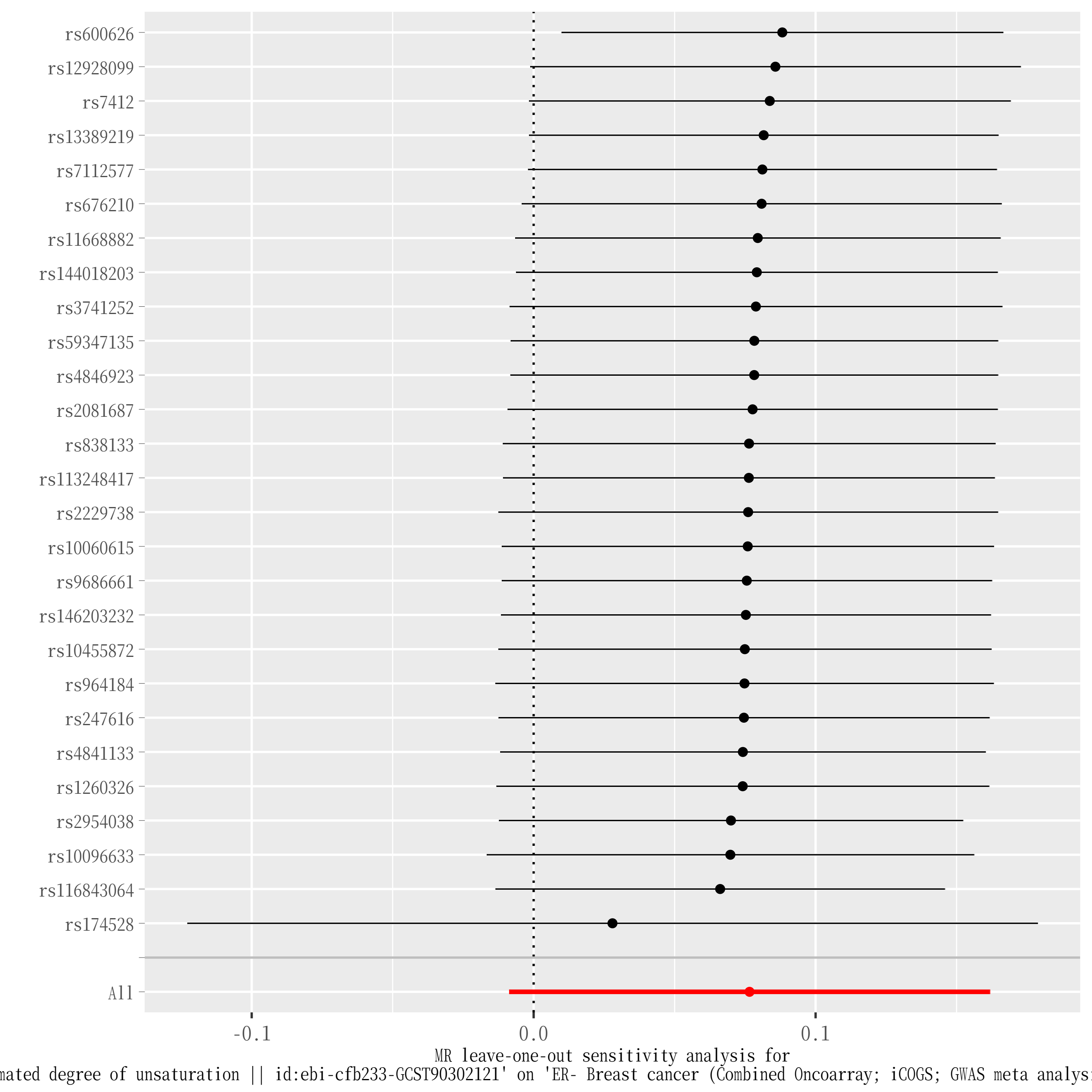
rs600626
rs12928099
rs7412
rs13389219
rs7112577
rs676210
rs11668882
rs144018203
rs3741252
rs59347135
rs4846923
rs2081687
rs838133
rs113248417
rs2229738
rs10060615
rs9686661
rs146203232
rs10455872
rs964184
rs247616
rs4841133
rs1260326
rs2954038
rs10096633
rs116843064
rs174528
All
-0.1
0.0
0.1
MR leave-one-out sensitivity analysis for
mated degree of unsaturation || id:ebi-cfb233-GCST90302121' on 'ER- Breast cancer (Combined Oncoarray; iCOGS; GWAS meta analys

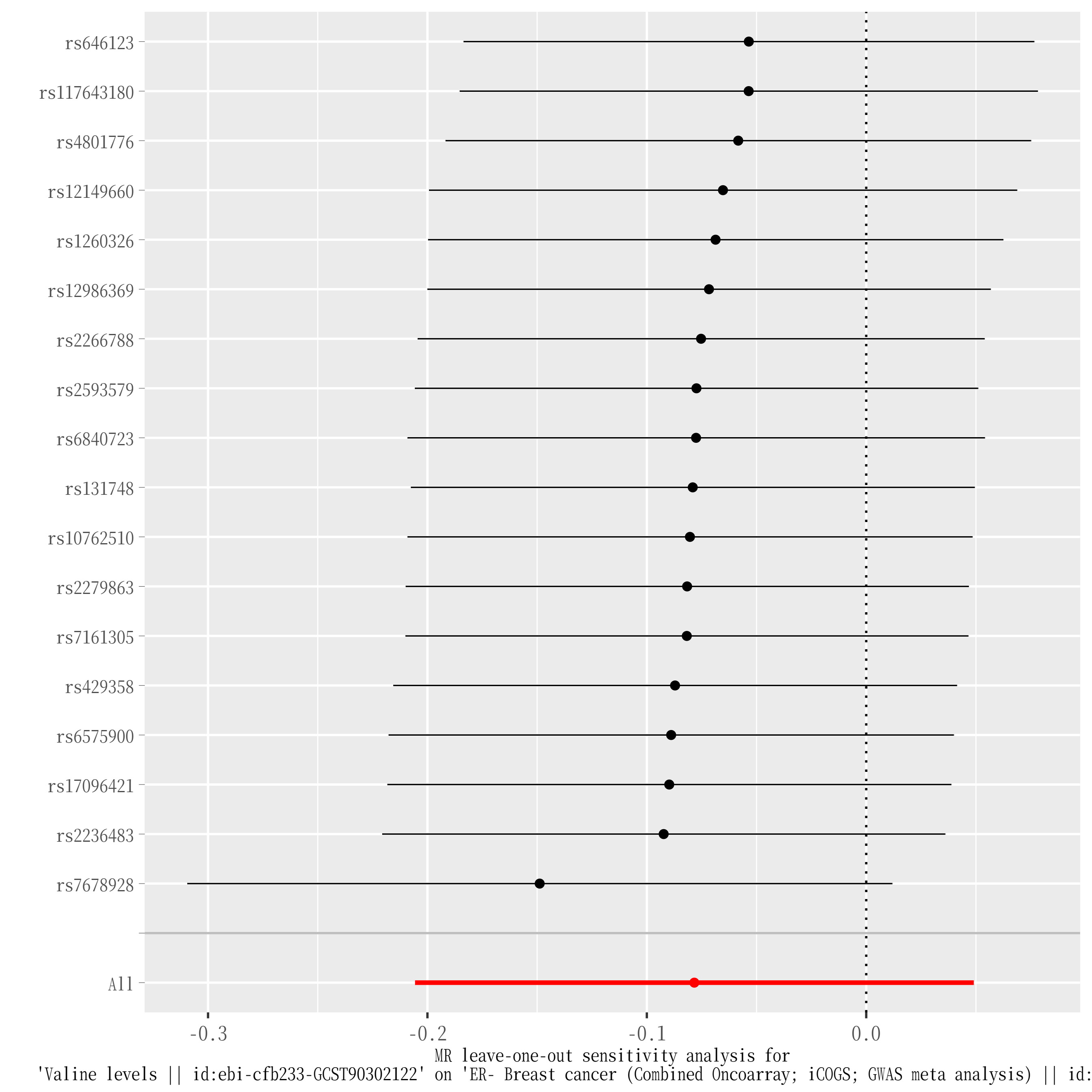
rs646123
rs117643180
rs4801776
rs12149660
rs1260326
rs12986369
rs2266788
rs2593579
rs6840723
rs131748
rs10762510
rs2279863
rs7161305
rs429358
rs6575900
rs17096421
rs2236483
rs7678928
All
-0.3
-0.2
-0.1
0.0
MR leave-one-out sensitivity analysis for
'Valine levels || id:ebi-cfb233-GCST90302122' on 'ER- Breast cancer (Combined Oncoarray; iCOGS; GWAS meta analysis) || id:

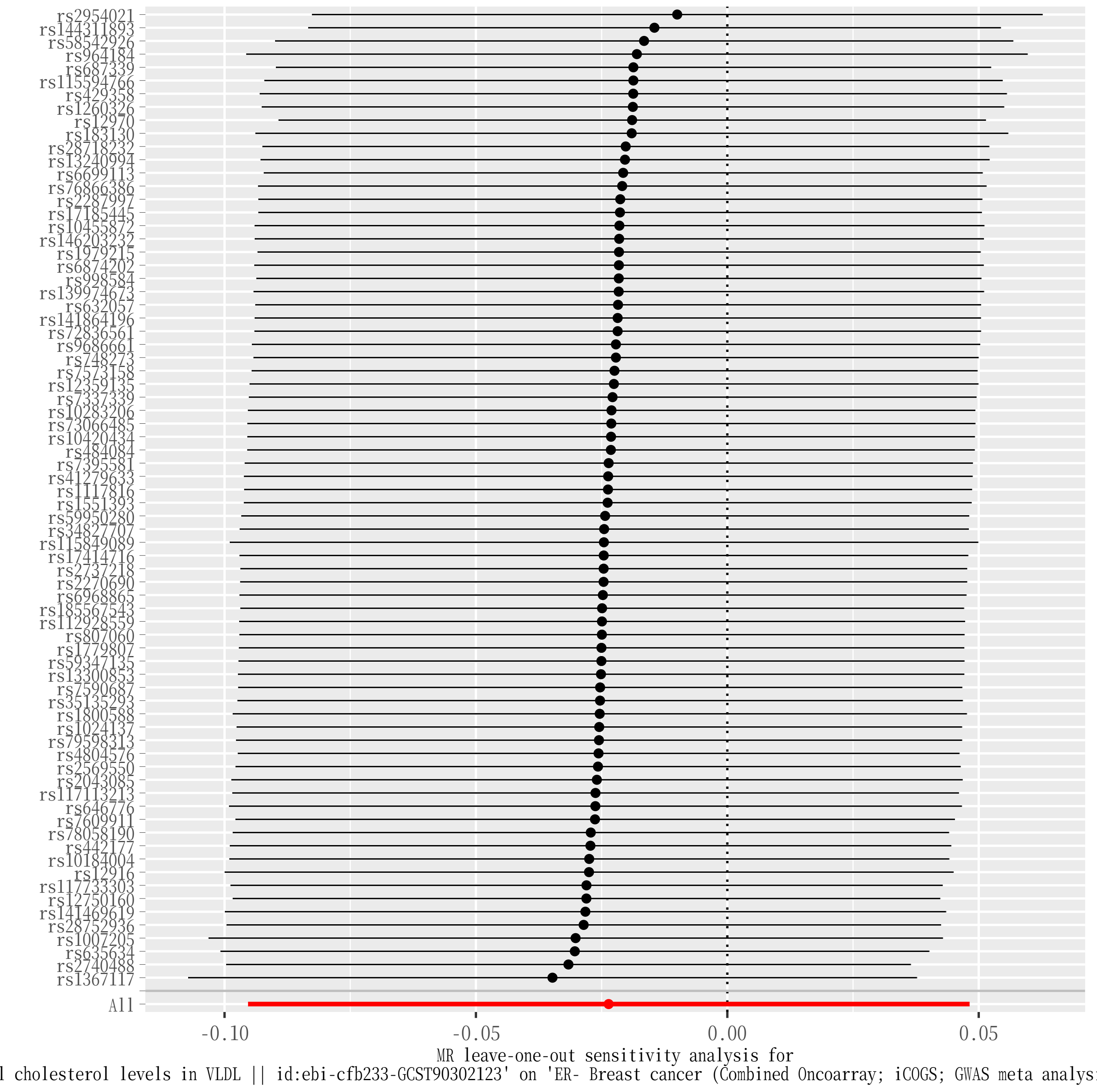

rs2954021
rs144311893
rs58542926
rs964184
rs687339
rs115594766
rs429358
rs1260326
rs12970
rs183130
rs28718232
rs13240994
rs6699113
rs76866386
rs2287997
rs17185445
rs10455872
rs146203232
rs1979215
rs6874202
rs998584
rs139974673
rs632057
rs141864196
rs72836561
rs9686661
rs748273
rs7573158
rs12359135
rs7337339
rs10283206
rs73066485
rs10420434
rs484084
rs7395581
rs41279633
rs1117816
rs1551393
rs59950280
rs34827707
rs115849089
rs17414716
rs2737218
rs2270690
rs6968865
rs185567543
rs112928559
rs807060
rs1779807
rs59347135
rs13300853
rs7590687
rs35135293
rs1800588
rs1024137
rs79598313
rs4804576
rs2569550
rs2043085
rs117113213
rs646776
rs7609911
rs78058190
rs442177
rs10184004
rs12916
rs117733303
rs12750160
rs141469619
rs28752936
rs1007205
rs635634
rs2740488
rs1367117
All
-0.10
-0.05
0.00
0.05
MR leave-one-out sensitivity analysis for
l cholesterol levels in VLDL || id:ebi-cfb233-GCST90302123' on 'ER- Breast cancer (Combined Oncoarray; iCOGS; GWAS meta analys

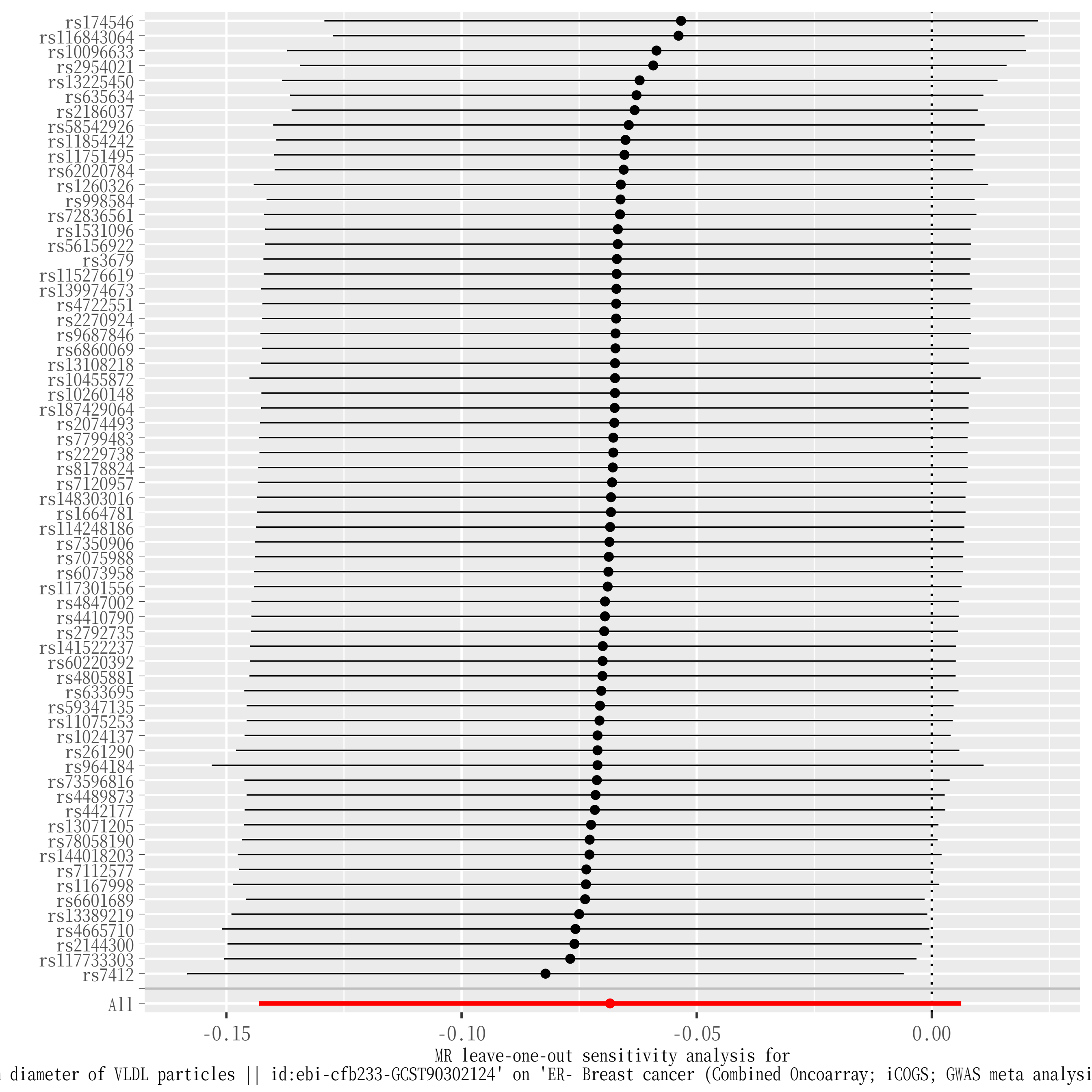
rs174546
rs116843064
rs10096633
rs2954021
rs13225450
rs635634
rs2186037
rs58542926
rs11854242
rs11751495
rs62020784
rs1260326
rs998584
rs72836561
rs1531096
rs56156922
rs3679
rs115276619
rs139974673
rs4722551
rs2270924
rs9687846
rs6860069
rs13108218
rs10455872
rs10260148
rs187429064
rs2074493
rs7799483
rs2229738
rs8178824
rs7120957
rs148303016
rs1664781
rs114248186
rs7350906
rs7075988
rs6073958
rs117301556
rs4847002
rs4410790
rs2792735
rs141522237
rs60220392
rs4805881
rs633695
rs59347135
rs11075253
rs1024137
rs261290
rs964184
rs73596816
rs4489873
rs442177
rs13071205
rs78058190
rs144018203
rs7112577
rs1167998
rs6601689
rs13389219
rs4665710
rs2144300
rs117733303
rs7412
All
-0.15
-0.10
-0.05
0.00
MR leave-one-out sensitivity analysis for
diameter of VLDL particles || id:ebi-cfb233-GCST90302124' on 'ER- Breast cancer (Combined Oncoarray; iCOGS; GWAS meta analysi

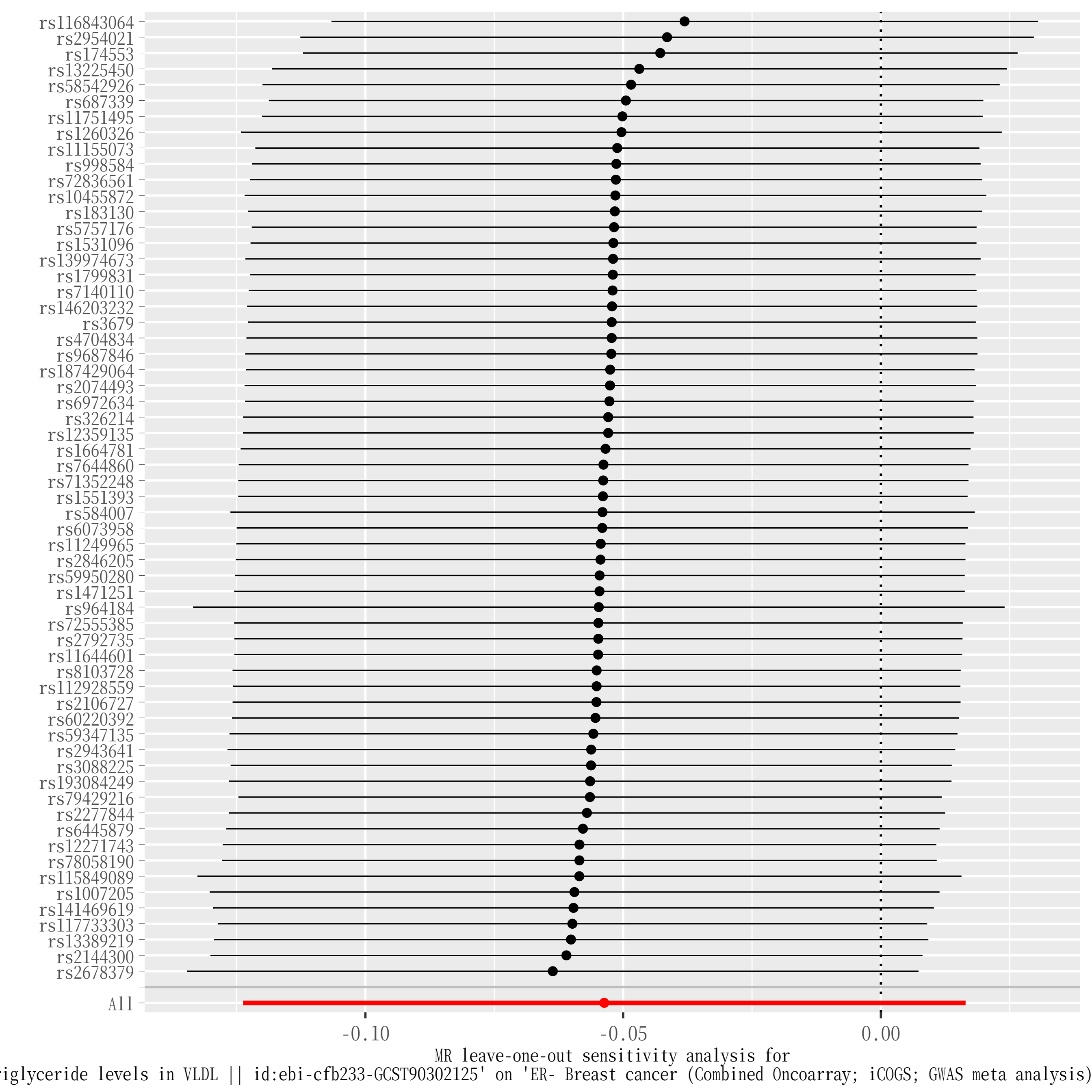
rs116843064
rs2954021
rs174553
rs13225450
rs58542926
rs687339
rs11751495
rs1260326
rs11155073
rs998584
rs72836561
rs10455872
rs183130
rs5757176
rs1531096
rs139974673
rs1799831
rs7140110
rs146203232
rs3679
rs4704834
rs9687846
rs187429064
rs2074493
rs6972634
rs326214
rs12359135
rs1664781
rs7644860
rs71352248
rs1551393
rs584007
rs6073958
rs11249965
rs2846205
rs59950280
rs1471251
rs964184
rs72555385
rs2792735
rs11644601
rs8103728
rs112928559
rs2106727
rs60220392
rs59347135
rs2943641
rs3088225
rs193084249
rs79429216
rs2277844
rs6445879
rs12271743
rs78058190
rs115849089
rs1007205
rs141469619
rs117733303
rs13389219
rs2144300
rs2678379
All
-0.10
-0.05
0.00
MR leave-one-out sensitivity analysis for
iglyceride levels in VLDL || id:ebi-cfb233-GCST90302125' on 'ER- Breast cancer (Combined Oncoarray; iCOGS; GWAS meta analysis)

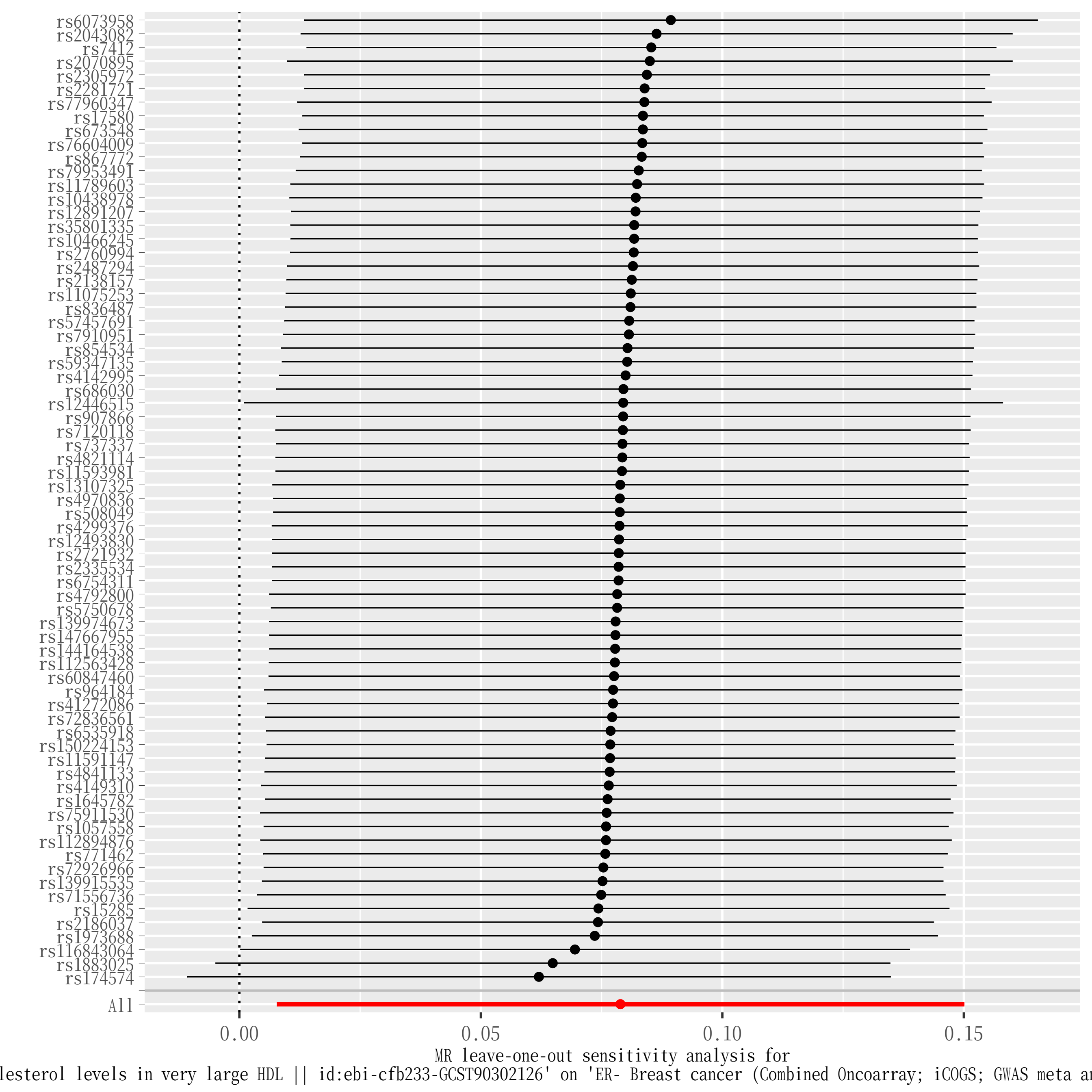

rs6073958
rs2043082
rs7412
rs2070895
rs2305972
rs2281721
rs77960347
rs17580
rs673548
rs76604009
rs867772
rs79953491
rs11789603
rs10438978
rs12891207
rs35801335
rs10466245
rs2760994
rs2487294
rs2138157
rs11075253
rs836487
rs57457691
rs7910951
rs854534
rs59347135
rs4142995
rs686030
rs12446515
rs907866
rs7120118
rs737337
rs4821114
rs11593981
rs13107325
rs4970836
rs508049
rs4299376
rs12493830
rs2721932
rs2335534
rs6754311
rs4792800
rs5750678
rs139974673
rs147667955
rs144164538
rs112563428
rs60847460
rs964184
rs41272086
rs72836561
rs6535918
rs150224153
rs11591147
rs4841133
rs4149310
rs1645782
rs75911530
rs1057558
rs112894876
rs771462
rs72926966
rs139915535
rs71556736
rs15285
rs2186037
rs1973688
rs116843064
rs1883025
rs174574
All
0.00
0.05
0.10
0.15
MR leave-one-out sensitivity analysis for
lesterol levels in very large HDL || id:ebi-cfb233-GCST90302126' on 'ER- Breast cancer (Combined Oncoarray; iCOGS; GWAS meta an

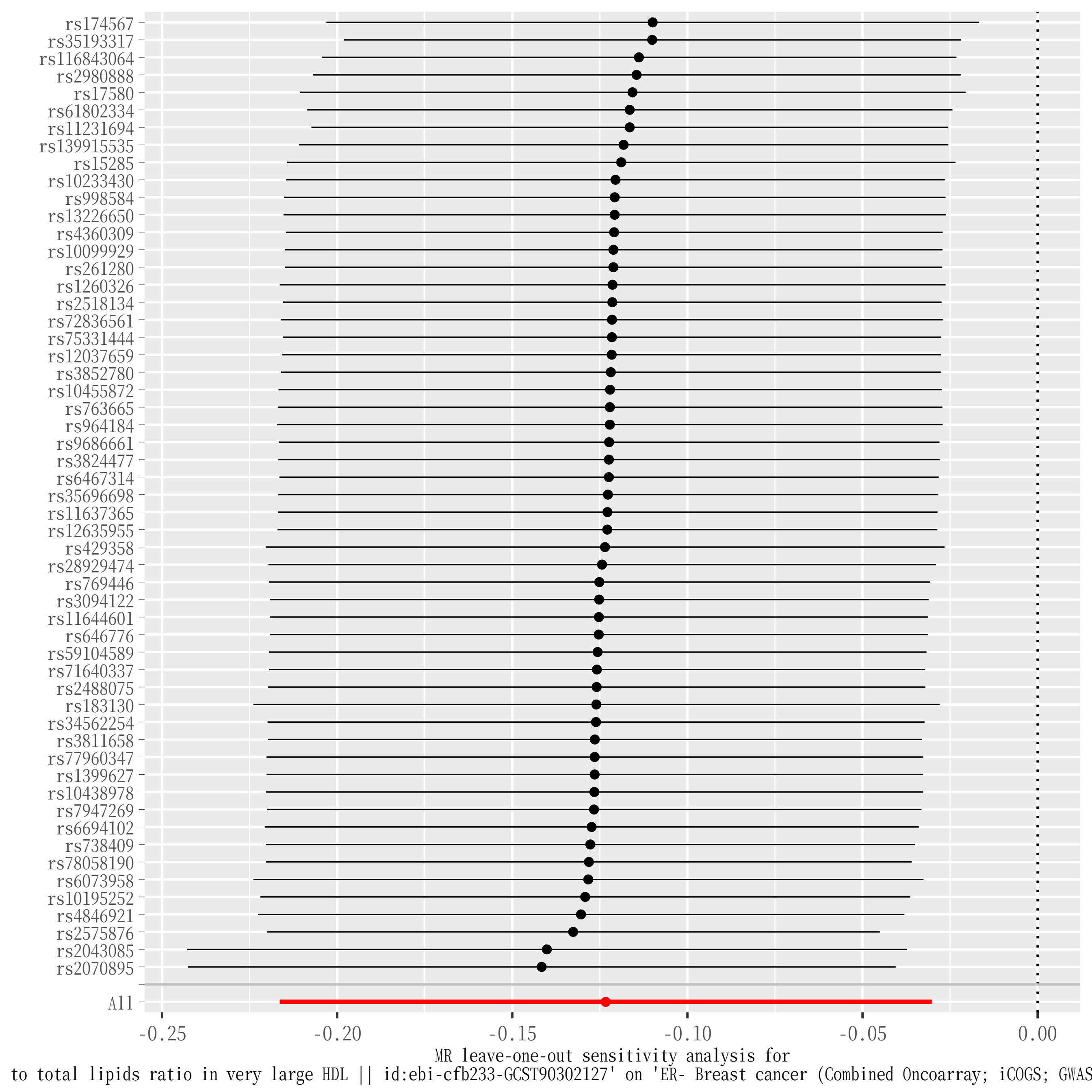
rs174567
rs35193317
rs116843064
rs2980888
rs17580
rs61802334
rs11231694
rs139915535
rs15285
rs10233430
rs998584
rs13226650
rs4360309
rs10099929
rs261280
rs1260326
rs2518134
rs72836561
rs75331444
rs12037659
rs3852780
rs10455872
rs763665
rs964184
rs9686661
rs3824477
rs6467314
rs35696698
rs11637365
rs12635955
rs429358
rs28929474
rs769446
rs3094122
rs11644601
rs646776
rs59104589
rs71640337
rs2488075
rs183130
rs34562254
rs3811658
rs77960347
rs1399627
rs10438978
rs7947269
rs6694102
rs738409
rs78058190
rs6073958
rs10195252
rs4846921
rs2575876
rs2043085
rs2070895
All
-0.25
-0.20
-0.15
-0.10
-0.05
0.00
MR leave-one-out sensitivity analysis for
to total lipids ratio in very large HDL || id:ebi-cfb233-GCST90302127' on 'ER- Breast cancer (Combined Oncoarray; iCOGS; GWAS

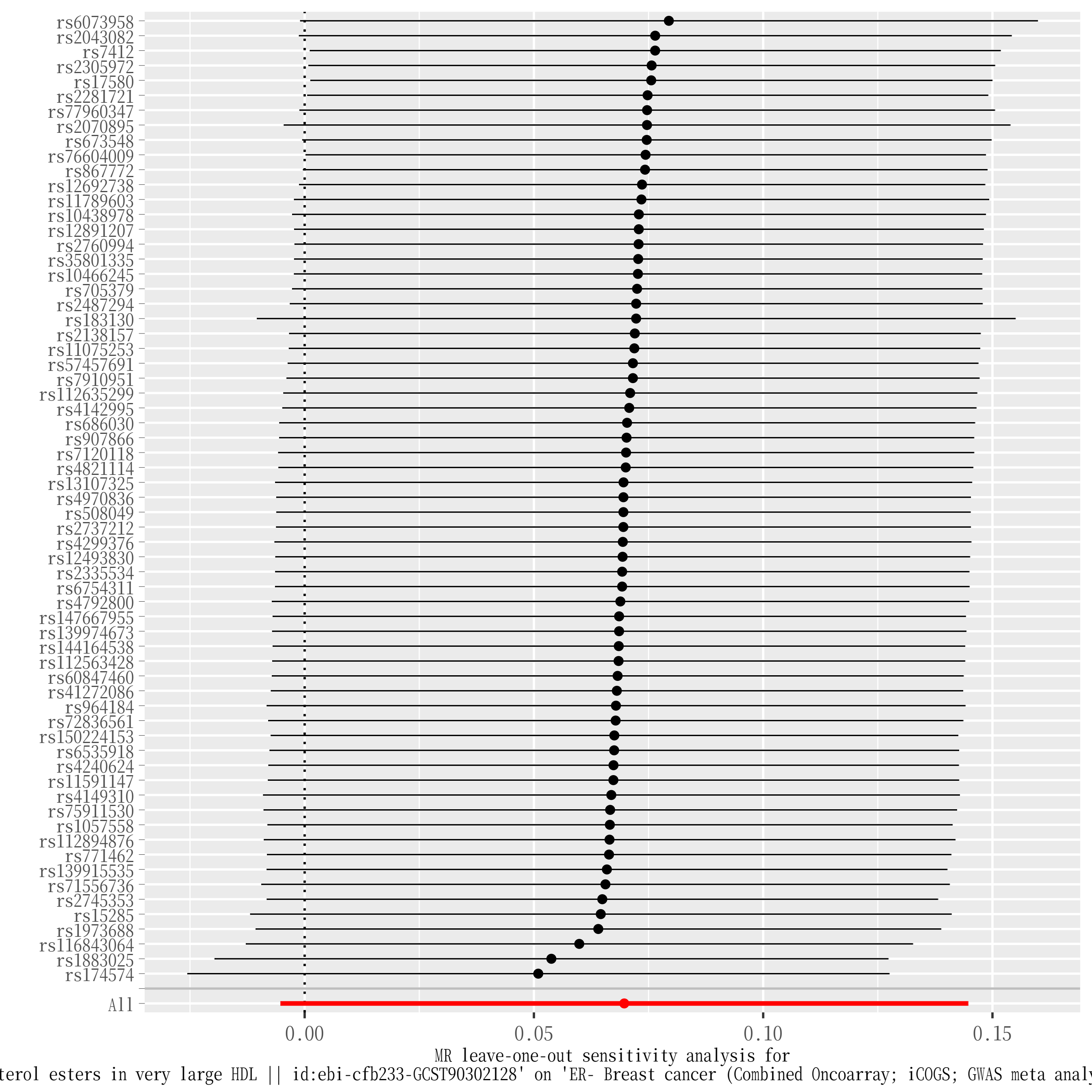

rs6073958
rs2043082
rs7412
rs2305972
rs17580
rs2281721
rs77960347
rs2070895
rs673548
rs76604009
rs867772
rs12692738
rs11789603
rs10438978
rs12891207
rs2760994
rs35801335
rs10466245
rs705379
rs2487294
rs183130
rs2138157
rs11075253
rs57457691
rs7910951
rs112635299
rs4142995
rs686030
rs907866
rs7120118
rs4821114
rs13107325
rs4970836
rs508049
rs2737212
rs4299376
rs12493830
rs2335534
rs6754311
rs4792800
rs147667955
rs139974673
rs144164538
rs112563428
rs60847460
rs41272086
rs964184
rs72836561
rs150224153
rs6535918
rs4240624
rs11591147
rs4149310
rs75911530
rs1057558
rs112894876
rs771462
rs139915535
rs71556736
rs2745353
rs15285
rs1973688
rs116843064
rs1883025
rs174574
All
0.00
0.05
0.10
0.15
MR leave-one-out sensitivity analysis for
terol esters in very large HDL || id:ebi-cfb233-GCST90302128' on 'ER- Breast cancer (Combined Oncoarray; iCOGS; GWAS meta analy

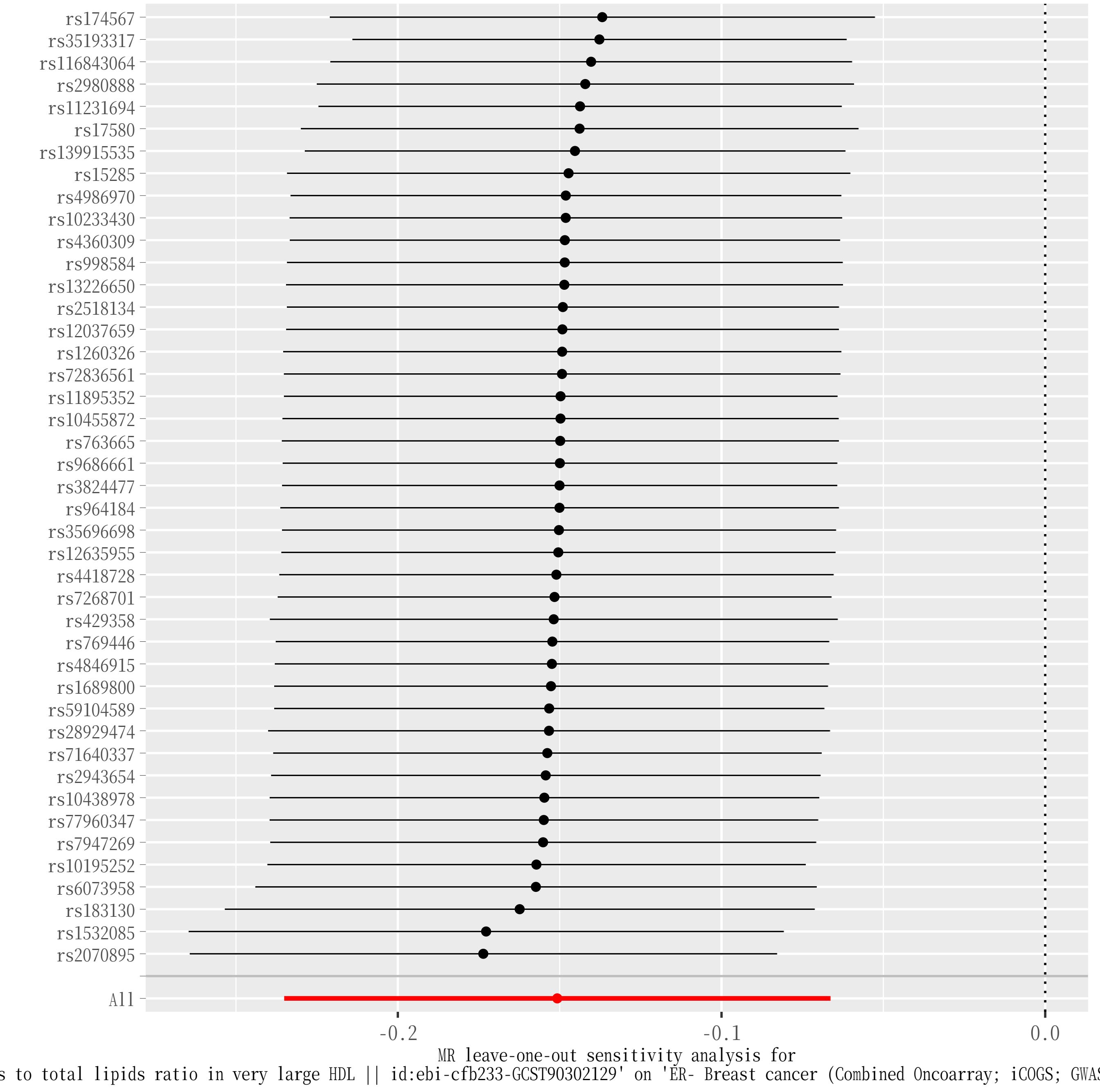

rs174567
rs35193317
rs116843064
rs2980888
rs11231694
rs17580
rs139915535
rs15285
rs4986970
rs10233430
rs4360309
rs998584
rs13226650
rs2518134
rs12037659
rs1260326
rs72836561
rs11895352
rs10455872
rs763665
rs9686661
rs3824477
rs964184
rs35696698
rs12635955
rs4418728
rs7268701
rs429358
rs769446
rs4846915
rs1689800
rs59104589
rs28929474
rs71640337
rs2943654
rs10438978
rs77960347
rs7947269
rs10195252
rs6073958
rs183130
rs1532085
rs2070895
All
-0.2
-0.1
0.0
MR leave-one-out sensitivity analysis for
s to total lipids ratio in very large HDL || id:ebi-cfb233-GCST90302129' on 'ER- Breast cancer (Combined Oncoarray; iCOGS; GWAS

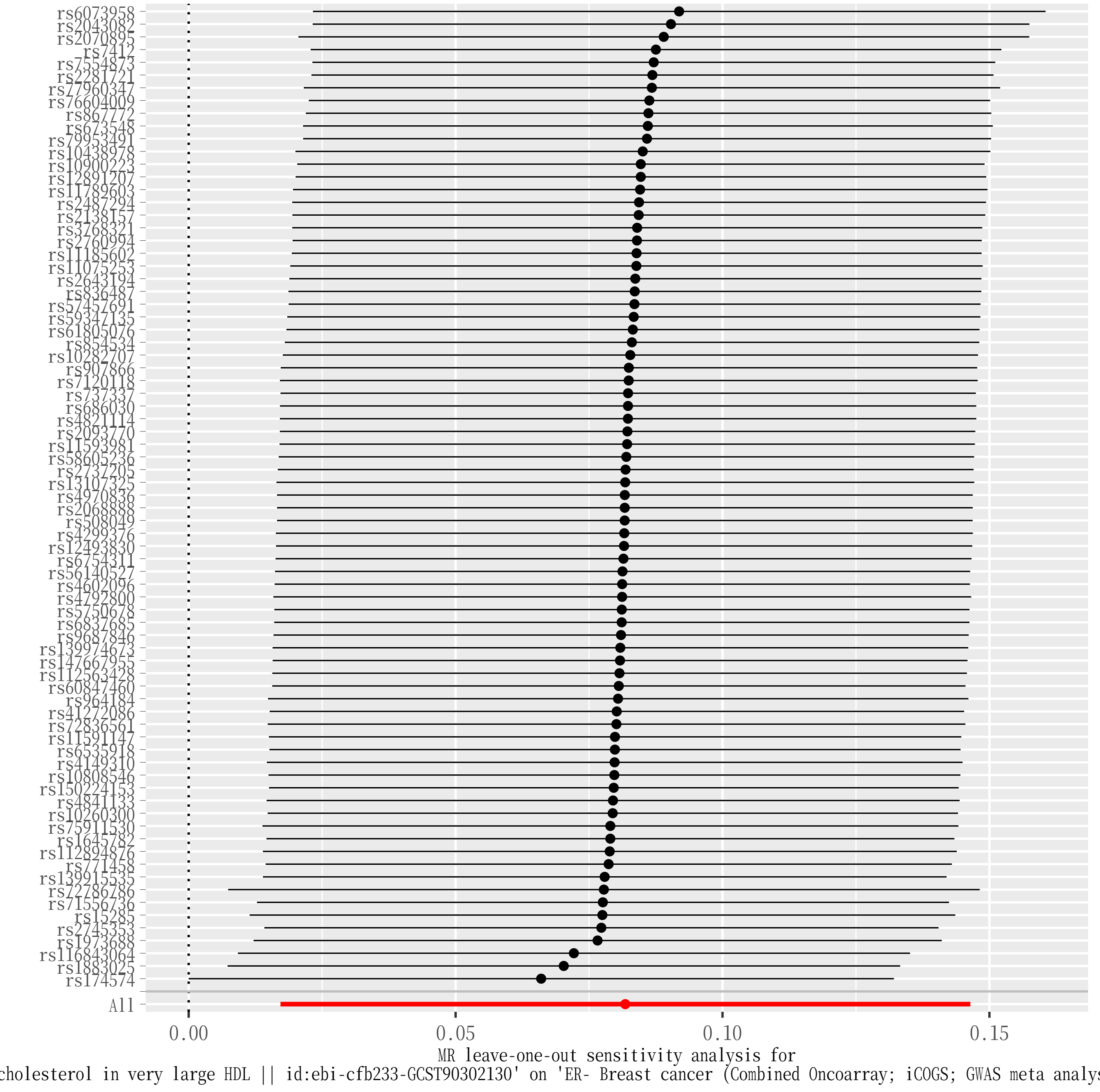
rs6073958
rs2043082
rs2070895
rs7412
rs7554873
rs2281721
rs77960347
rs76604009
rs867772
rs673548
rs79953491
rs10438978
rs10900223
rs12891207
rs11789603
rs2487294
rs2138157
rs3768321
rs2760994
rs11185602
rs11075253
rs2643194
rs836487
rs57457691
rs59347135
rs61805076
rs854534
rs10282707
rs907866
rs7120118
rs737337
rs686030
rs4821114
rs2093770
rs11593981
rs58605236
rs2737205
rs13107325
rs4970836
rs2068888
rs508049
rs4299376
rs12493830
rs6754311
rs56140527
rs4602096
rs4792800
rs5750678
rs6837685
rs9687846
rs139974673
rs147667955
rs112563428
rs60847460
rs964184
rs41272086
rs72836561
rs11591147
rs6535918
rs4149310
rs10808546
rs150224153
rs4841133
rs10260300
rs75911530
rs1645782
rs112894876
rs771458
rs139215535
rs72786786
rs71556736
rs15285
rs2745353
rs1973688
rs116843064
rs1883025
rs174574
All
0.00
0.05
0.10
0.15
MR leave-one-out sensitivity analysis for
cholesterol in very large HDL || id:ebi-cfb233-GCST90302130' on 'ER- Breast cancer (Combined Oncoarray; iCOGS; GWAS meta analys

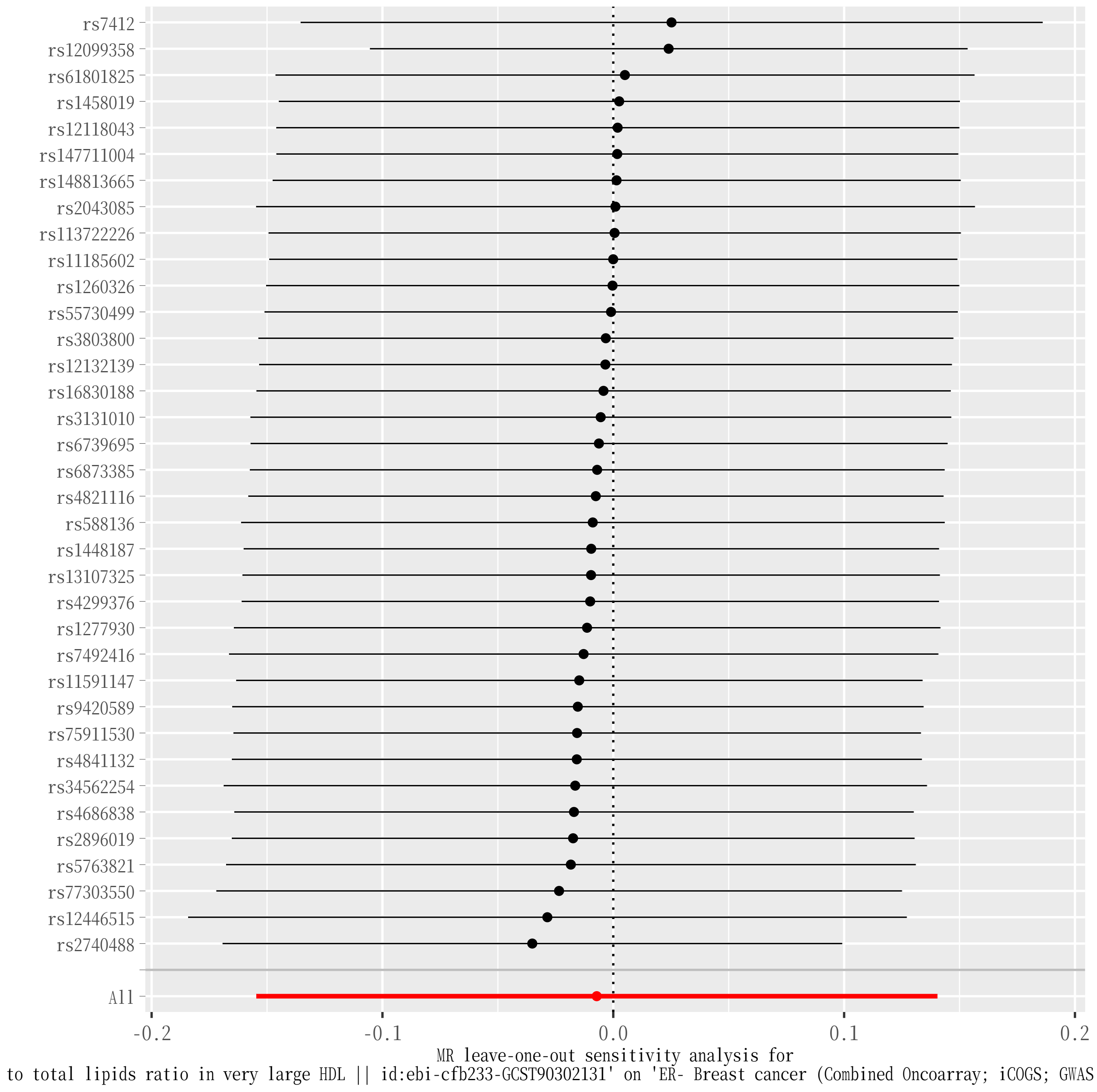
rs7412
rs12099358
rs61801825
rs1458019
rs12118043
rs147711004
rs148813665
rs2043085
rs113722226
rs11185602
rs1260326
rs55730499
rs3803800
rs12132139
rs16830188
rs3131010
rs6739695
rs6873385
rs4821116
rs588136
rs1448187
rs13107325
rs4299376
rs1277930
rs7492416
rs11591147
rs9420589
rs75911530
rs4841132
rs34562254
rs4686838
rs2896019
rs5763821
rs77303550
rs12446515
rs2740488
All
-0.2
-0.1
0.0
0.1
0.2
MR leave-one-out sensitivity analysis for
to total lipids ratio in very large HDL || id:ebi-cfb233-GCST90302131' on 'ER- Breast cancer (Combined Oncoarray; iCOGS; GWAS

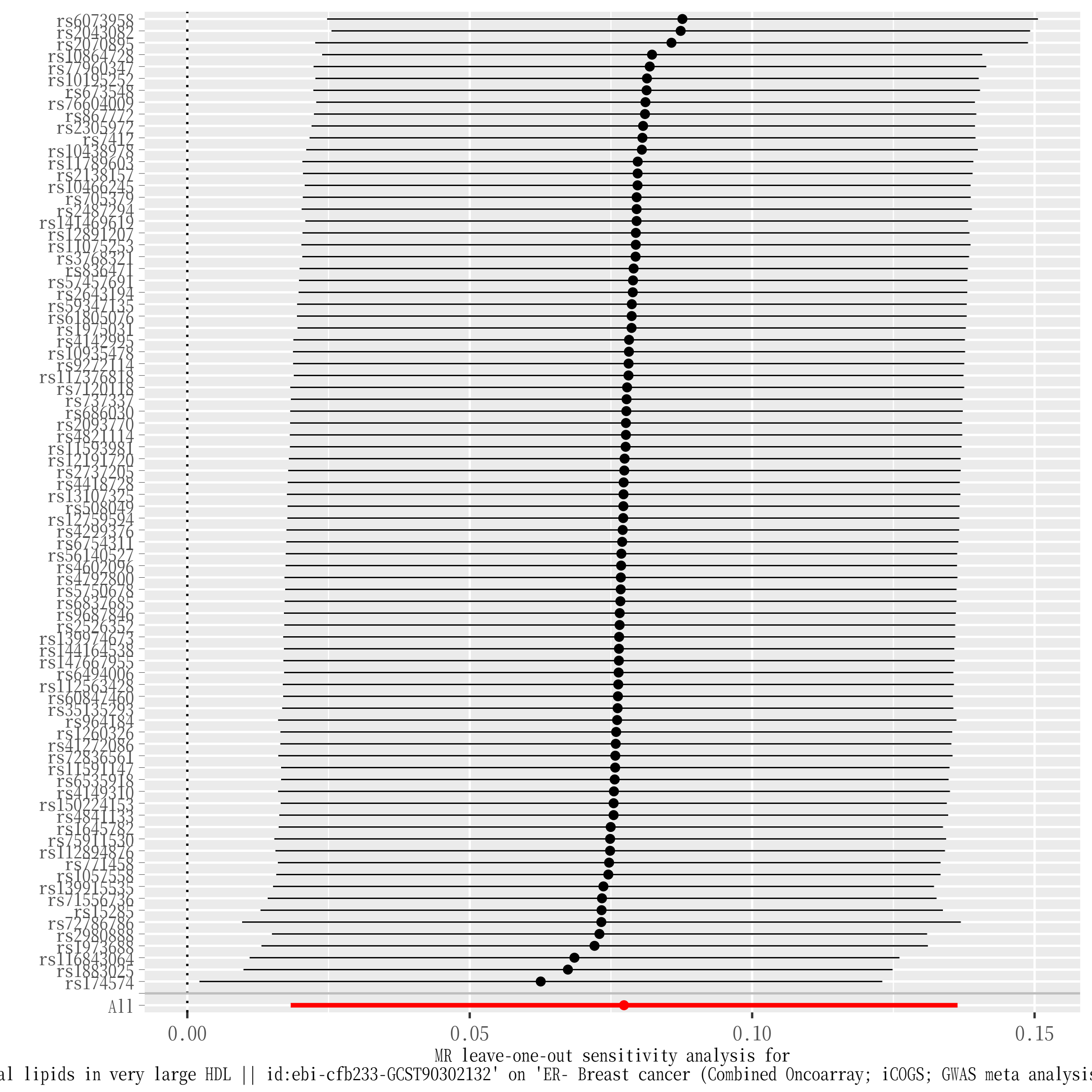
rs6073958
rs2043082
rs2070895
rs10864728
rs77960347
rs10195252
rs673548
rs76604009
rs867772
rs2305972
rs7412
rs10438978
rs11789603
rs2138157
rs10466245
rs705379
rs2487294
rs141469619
rs12891207
rs11075253
rs3768321
rs836471
rs57457691
rs2643194
rs59347135
rs61805076
rs1975031
rs4142995
rs10935478
rs9272114
rs117376818
rs7120118
rs737337
rs686030
rs2093770
rs4821114
rs11593981
rs12191720
rs2737205
rs4418728
rs13107325
rs508049
rs12759594
rs4299376
rs6754311
rs56140527
rs4602096
rs4792800
rs5750678
rs6837685
rs9687846
rs2526352
rs139974673
rs144164538
rs147667955
rs6494006
rs112563428
rs60847460
rs35135293
rs964184
rs1260326
rs41272086
rs72836561
rs11591147
rs6535918
rs4149310
rs150224153
rs4841133
rs1645782
rs75911530
rs112894876
rs771458
rs1057558
rs139915535
rs71556736
rs15285
rs72786786
rs2980888
rs1973688
rs116843064
rs1883025
rs174574
All
0.00
0.05
0.10
0.15
MR leave-one-out sensitivity analysis for
al lipids in very large HDL || id:ebi-cfb233-GCST90302132' on 'ER- Breast cancer (Combined Oncoarray; iCOGS; GWAS meta analysis

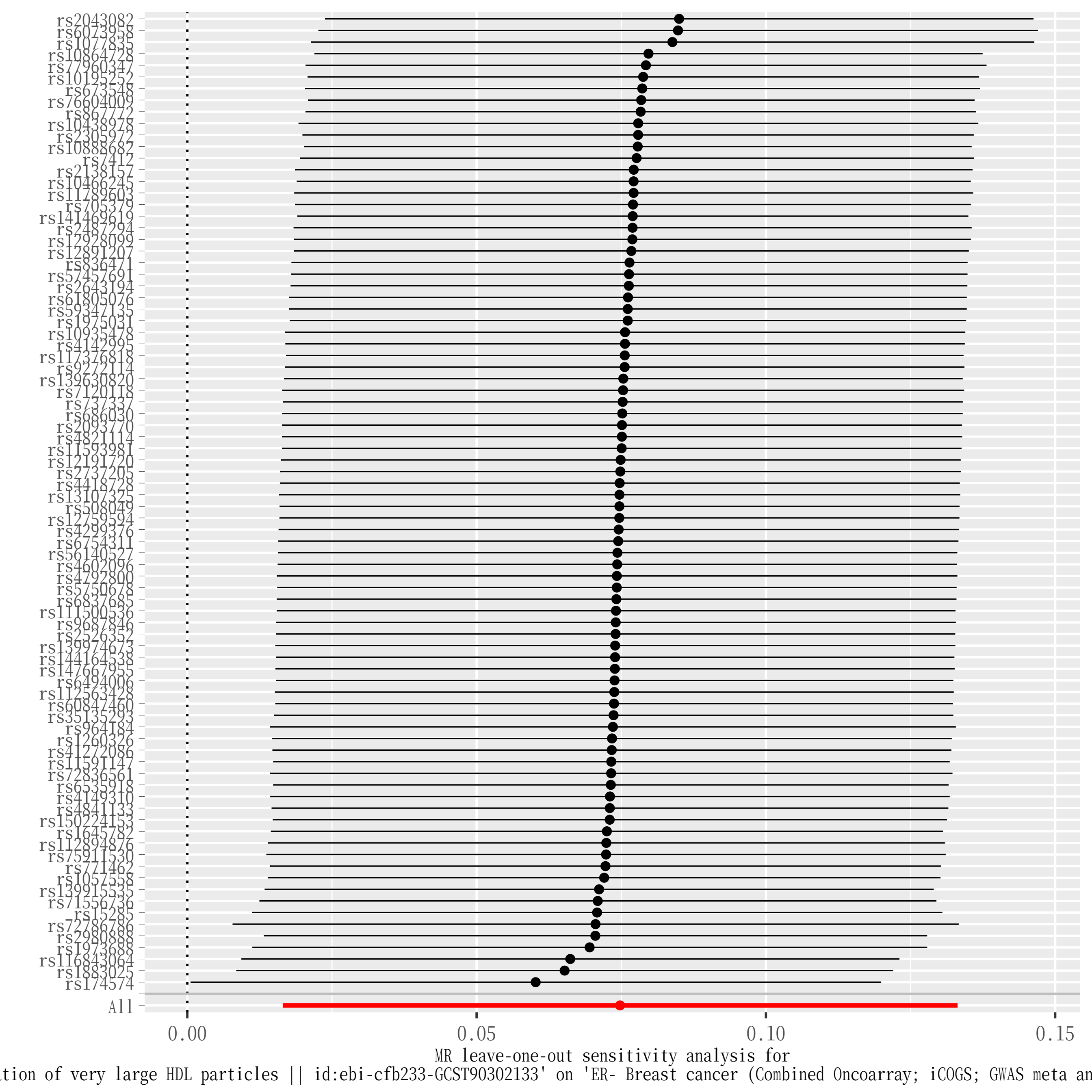
rs2043082
rs6073958
rs1077835
rs10864728
rs77960347
rs10195252
rs673548
rs76604009
rs867772
rs10438978
rs2305972
rs10888682
rs7412
rs2138157
rs10466245
rs11789603
rs705379
rs141469619
rs2487294
rs12928099
rs12891207
rs836471
rs57457691
rs2643194
rs61805076
rs59347135
rs1975031
rs10935478
rs4142995
rs117376818
rs9272114
rs139630820
rs7120118
rs737337
rs686030
rs2093770
rs4821114
rs11593981
rs12191720
rs2737205
rs4418728
rs13107325
rs508049
rs12759594
rs4299376
rs6754311
rs56140527
rs4602096
rs4792800
rs5750678
rs6837685
rs111500536
rs9687846
rs2526352
rs139974673
rs144164538
rs147667955
rs6494006
rs112563428
rs60847460
rs35135293
rs964184
rs1260326
rs41272086
rs11591147
rs72836561
rs6535918
rs4149310
rs4841133
rs150224153
rs1645782
rs112894876
rs75211530
rs771462
rs1057558
rs139915535
rs71556736
rs15285
rs72786786
rs2980888
rs1973688
rs116843064
rs1883025
rs174574
All
0.00
0.05
0.10
0.15
MR leave-one-out sensitivity analysis for
tion of very large HDL particles || id:ebi-cfb233-GCST90302133' on 'ER- Breast cancer (Combined Oncoarray; iCOGS; GWAS meta an

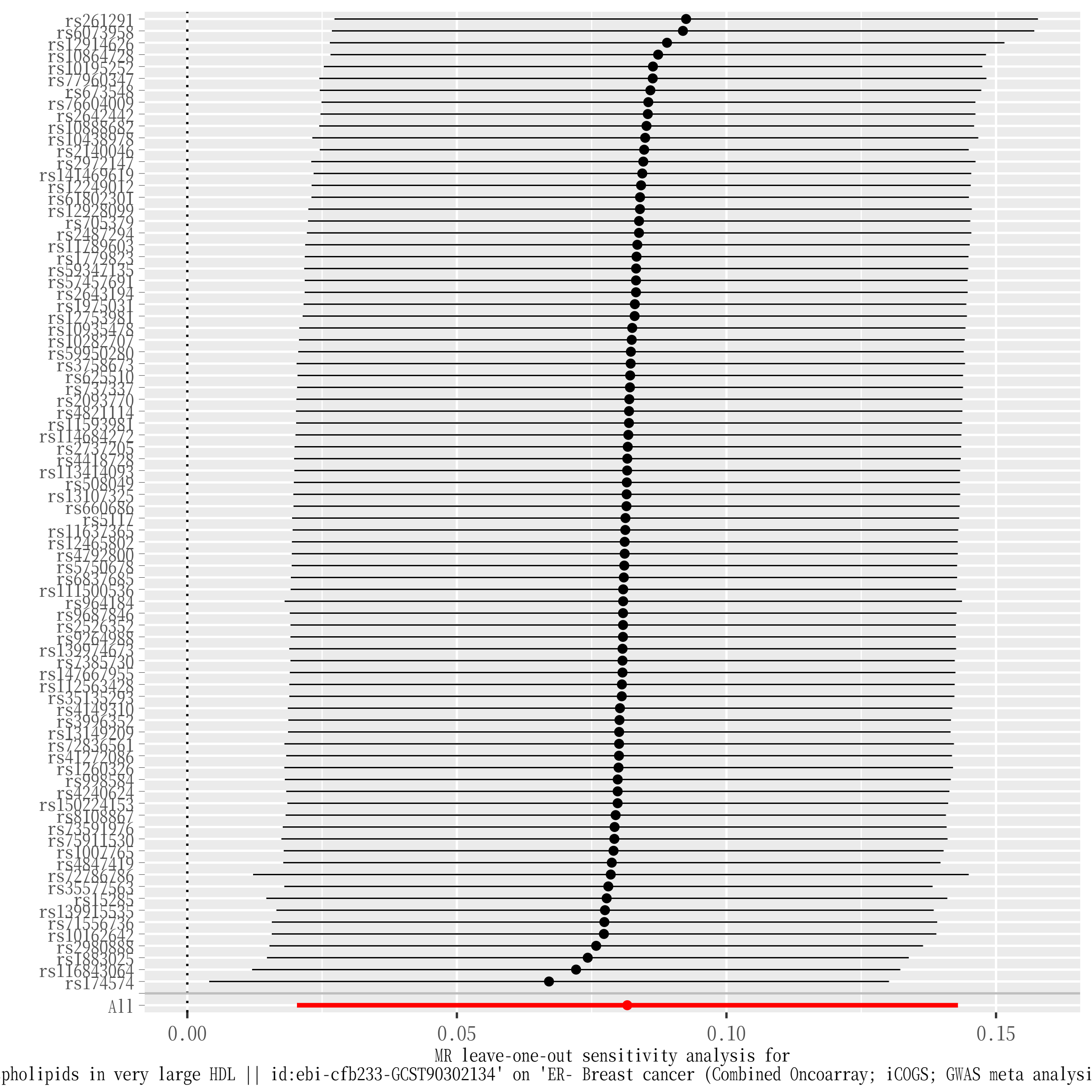
rs261291
rs6073958
rs12914626
rs10864728
rs10195252
rs77960347
rs673548
rs76604009
rs2642442
rs10888682
rs10438978
rs2140046
rs2972147
rs141469619
rs12249012
rs61802301
rs12928099
rs705379
rs2487294
rs11789603
rs1779823
rs59347135
rs57457691
rs2643194
rs1975031
rs12753981
rs10935478
rs10282707
rs59950280
rs3758673
rs625510
rs737337
rs2093770
rs4821114
rs11593981
rs114684272
rs2737205
rs4418728
rs113414093
rs508049
rs13107325
rs660686
rs5112
rs11637365
rs12465802
rs4792800
rs5750678
rs6837685
rs111500536
rs964184
rs9687846
rs2526352
rs9264988
rs139974673
rs7385730
rs147667955
rs112563428
rs35135293
rs4149310
rs3996352
rs13149209
rs72836561
rs41272086
rs1260326
rs998584
rs4240624
rs150224153
rs8108867
rs73591976
rs75911530
rs1007765
rs4847419
rs72786786
rs35577563
rs15285
rs139215535
rs71556736
rs10162642
rs2980888
rs1883025
rs116843064
rs174574
All
0.00
0.05
0.10
0.15
MR leave-one-out sensitivity analysis for
pholipids in very large HDL || id:ebi-cfb233-GCST90302134' on 'ER- Breast cancer (Combined Oncoarray; iCOGS; GWAS meta analysi

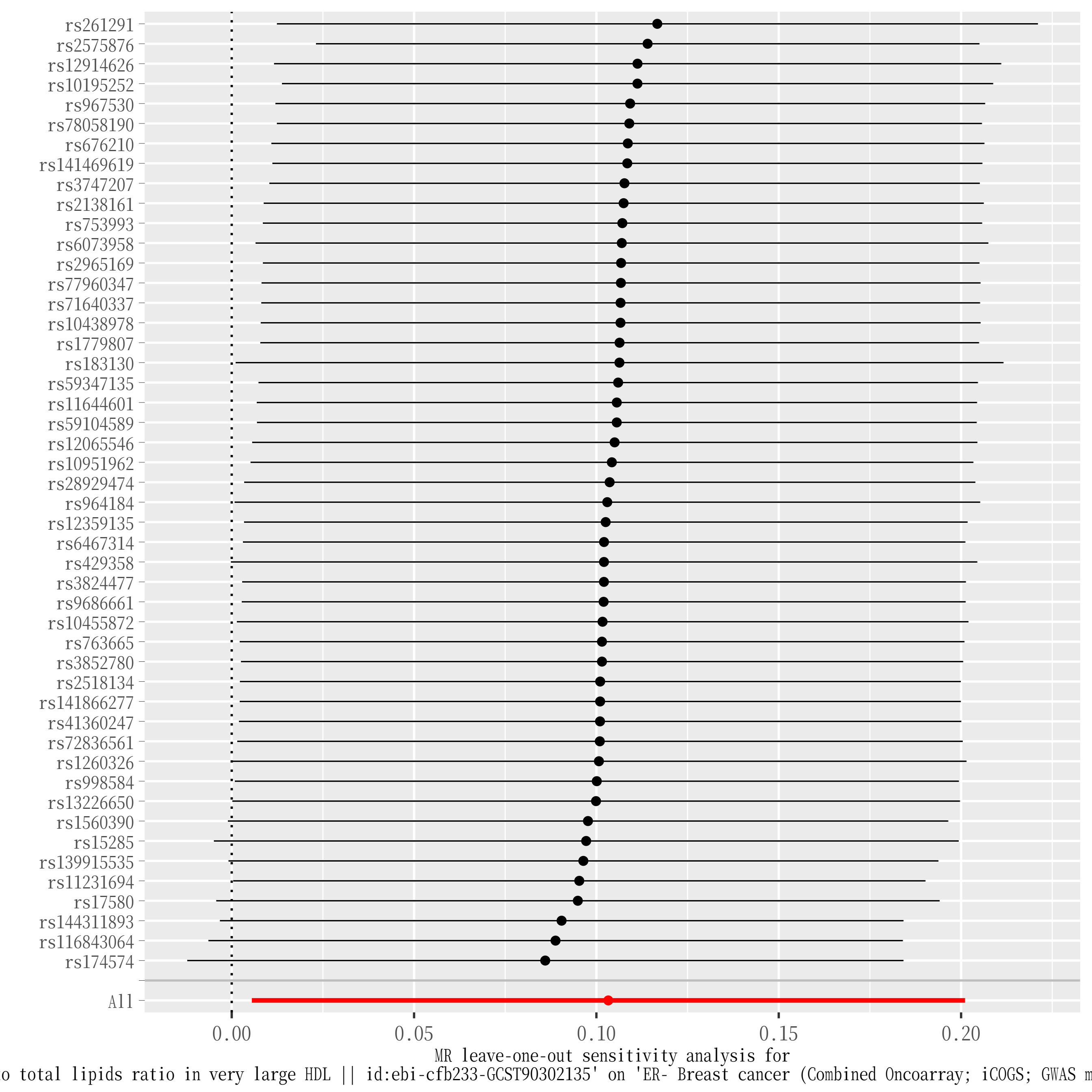

rs261291
rs2575876
rs12914626
rs10195252
rs967530
rs78058190
rs676210
rs141469619
rs3747207
rs2138161
rs753993
rs6073958
rs2965169
rs77960347
rs71640337
rs10438978
rs1779807
rs183130
rs59347135
rs11644601
rs59104589
rs12065546
rs10951962
rs28929474
rs964184
rs12359135
rs6467314
rs429358
rs3824477
rs9686661
rs10455872
rs763665
rs3852780
rs2518134
rs141866277
rs41360247
rs72836561
rs1260326
rs998584
rs13226650
rs1560390
rs15285
rs139915535
rs11231694
rs17580
rs144311893
rs116843064
rs174574
All
0.00
0.05
0.10
0.15
0.20
MR leave-one-out sensitivity analysis for
o total lipids ratio in very large HDL || id:ebi-cfb233-GCST90302135' on 'ER- Breast cancer (Combined Oncoarray; iCOGS; GWAS m

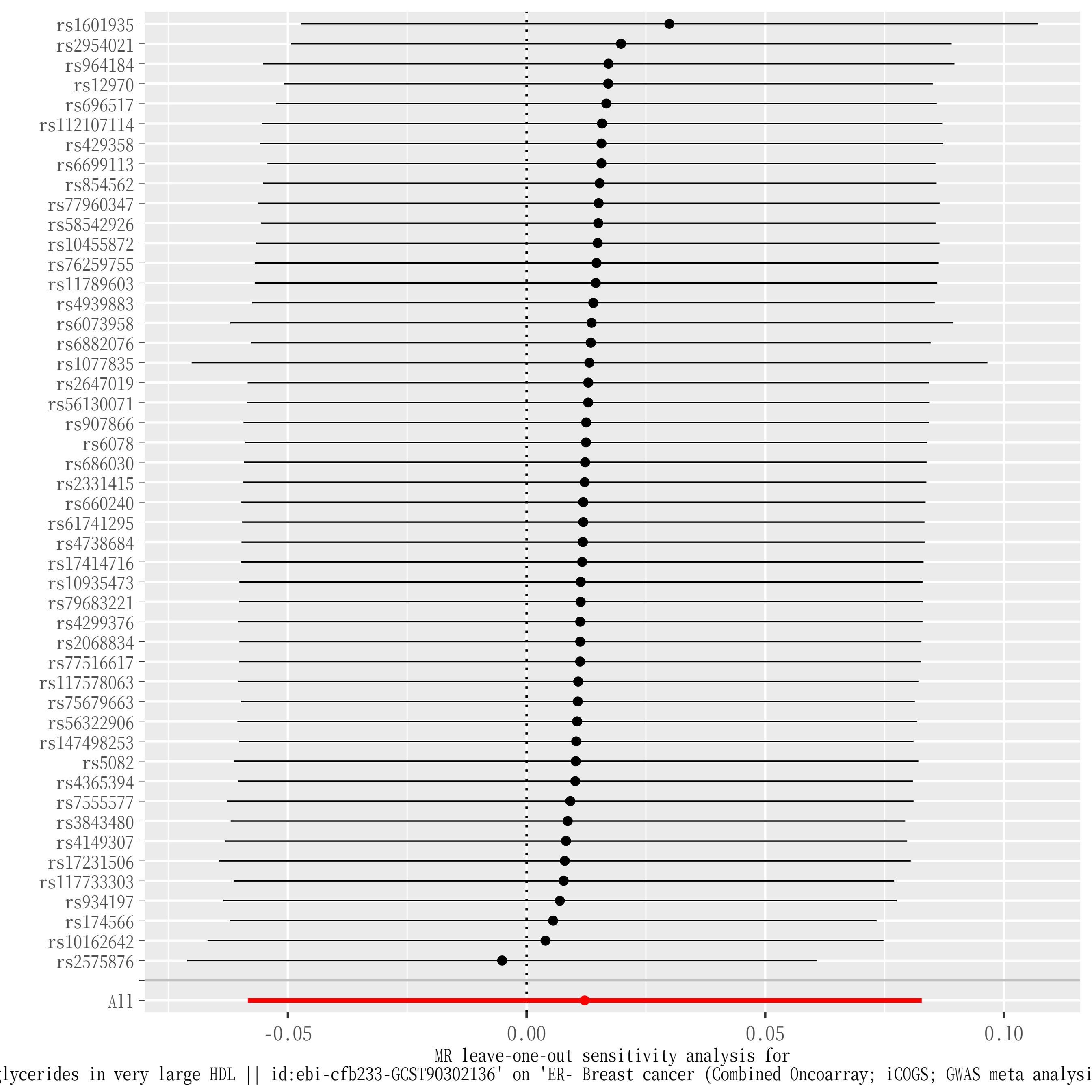
rs1601935
rs2954021
rs964184
rs12970
rs696517
rs112107114
rs429358
rs6699113
rs854562
rs77960347
rs58542926
rs10455872
rs76259755
rs11789603
rs4939883
rs6073958
rs6882076
rs1077835
rs2647019
rs56130071
rs907866
rs6078
rs686030
rs2331415
rs660240
rs61741295
rs4738684
rs17414716
rs10935473
rs79683221
rs4299376
rs2068834
rs77516617
rs117578063
rs75679663
rs56322906
rs147498253
rs5082
rs4365394
rs7555577
rs3843480
rs4149307
rs17231506
rs117733303
rs934197
rs174566
rs10162642
rs2575876
All
-0.05
0.00
0.05
0.10
MR leave-one-out sensitivity analysis for
lycerides in very large HDL || id:ebi-cfb233-GCST90302136' on 'ER- Breast cancer (Combined Oncoarray; iCOGS; GWAS meta analysi

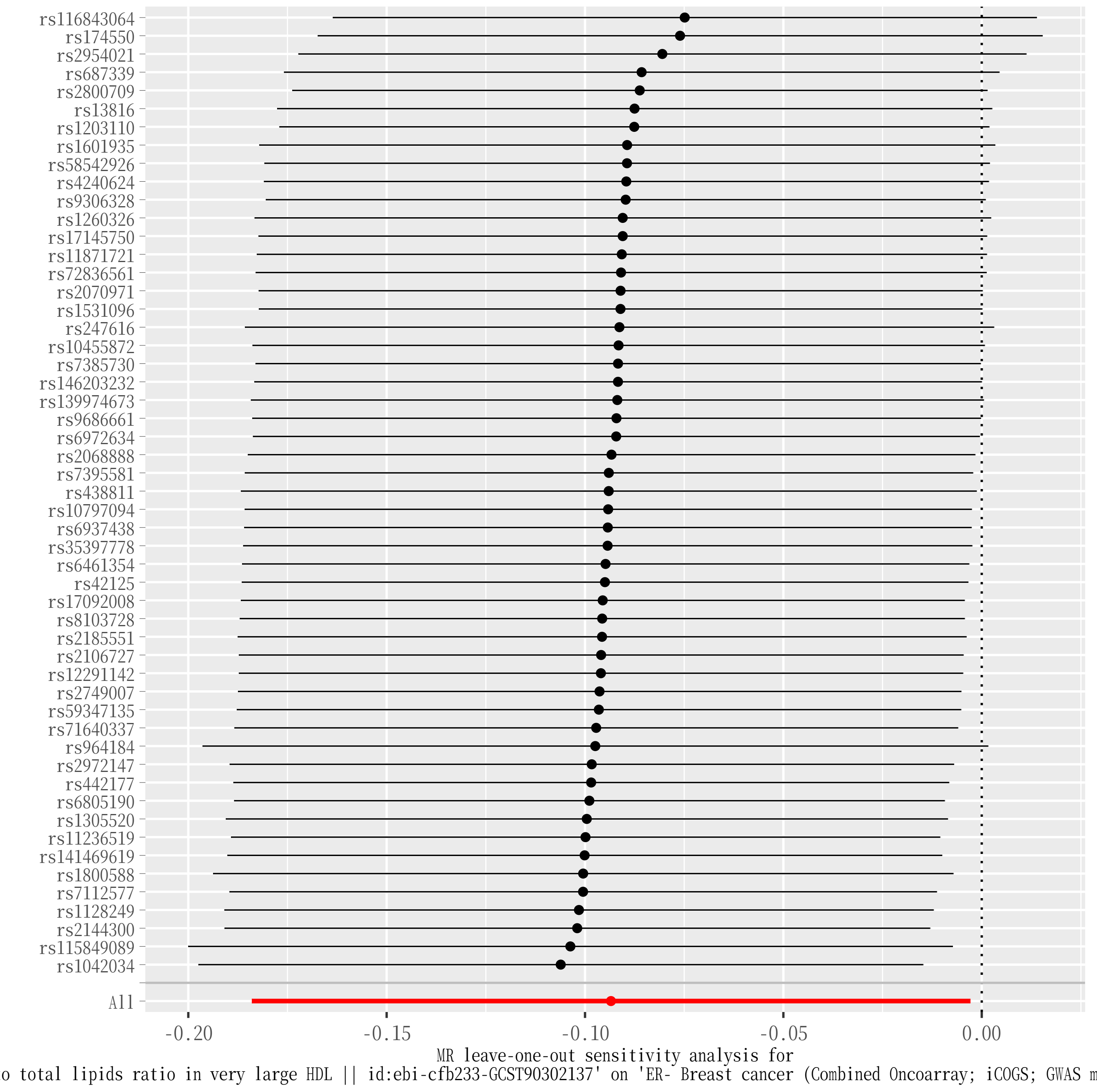
rs116843064
rs174550
rs2954021
rs687339
rs2800709
rs13816
rs1203110
rs1601935
rs58542926
rs4240624
rs9306328
rs1260326
rs17145750
rs11871721
rs72836561
rs2070971
rs1531096
rs247616
rs10455872
rs7385730
rs146203232
rs139974673
rs9686661
rs6972634
rs2068888
rs7395581
rs438811
rs10797094
rs6937438
rs35397778
rs6461354
rs42125
rs17092008
rs8103728
rs2185551
rs2106727
rs12291142
rs2749007
rs59347135
rs71640337
rs964184
rs2972147
rs442177
rs6805190
rs1305520
rs11236519
rs141469619
rs1800588
rs7112577
rs1128249
rs2144300
rs115849089
rs1042034
All
-0.20
-0.15
-0.10
-0.05
0.00
MR leave-one-out sensitivity analysis for
o total lipids ratio in very large HDL || id:ebi-cfb233-GCST90302137' on 'ER- Breast cancer (Combined Oncoarray; iCOGS; GWAS m

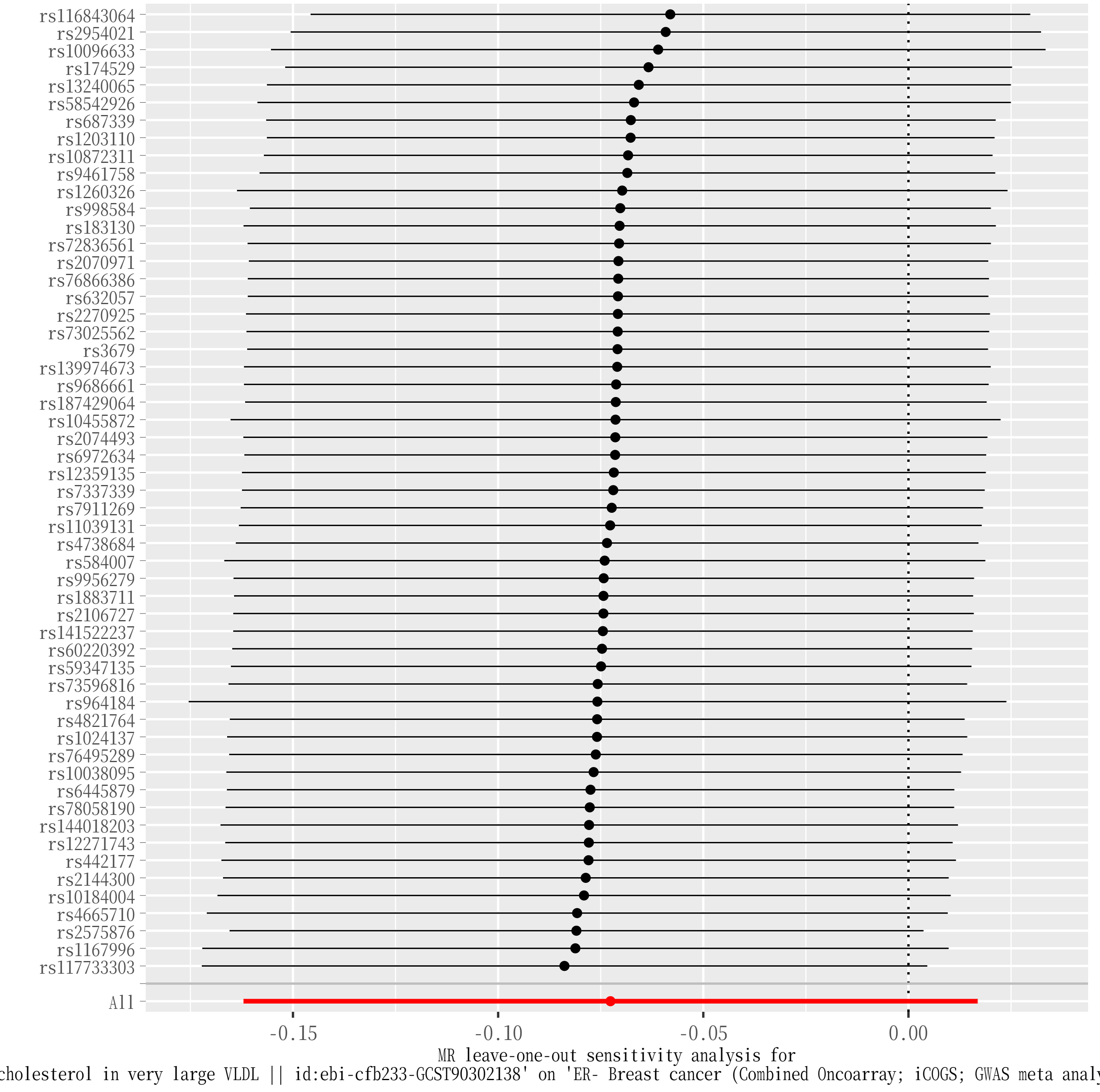

rs116843064
rs2954021
rs10096633
rs174529
rs13240065
rs58542926
rs687339
rs1203110
rs10872311
rs9461758
rs1260326
rs998584
rs183130
rs72836561
rs2070971
rs76866386
rs632057
rs2270925
rs73025562
rs3679
rs139974673
rs9686661
rs187429064
rs10455872
rs2074493
rs6972634
rs12359135
rs7337339
rs7911269
rs11039131
rs4738684
rs584007
rs9956279
rs1883711
rs2106727
rs141522237
rs60220392
rs59347135
rs73596816
rs964184
rs4821764
rs1024137
rs76495289
rs10038095
rs6445879
rs78058190
rs144018203
rs12271743
rs442177
rs2144300
rs10184004
rs4665710
rs2575876
rs1167996
rs117733303
All
-0.15
-0.10
-0.05
0.00
MR leave-one-out sensitivity analysis for
cholesterol in very large VLDL || id:ebi-cfb233-GCST90302138' on 'ER- Breast cancer (Combined Oncoarray; iCOGS; GWAS meta analy

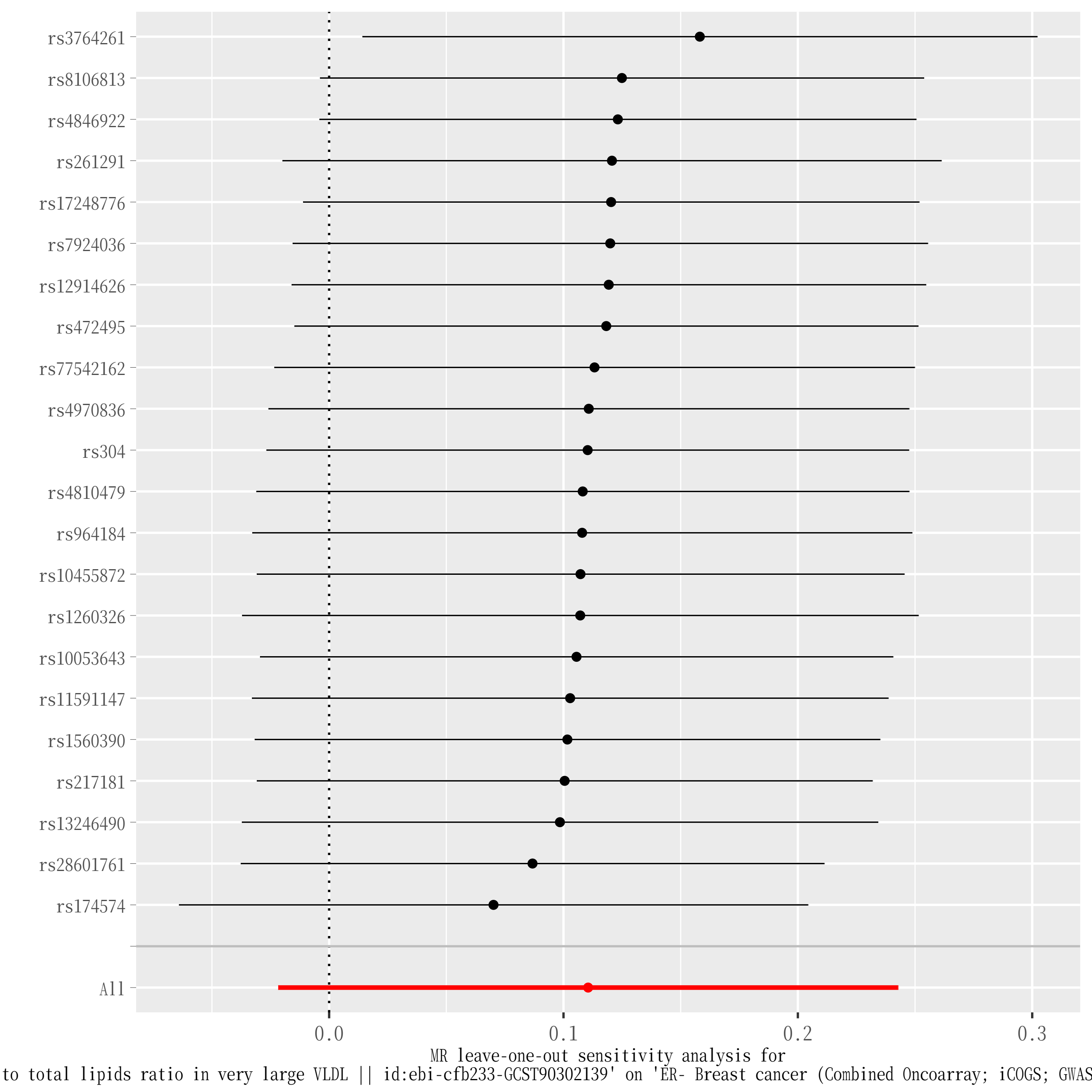

rs3764261
rs8106813
rs4846922
rs261291
rs17248776
rs7924036
rs12914626
rs472495
rs77542162
rs4970836
rs304
rs4810479
rs964184
rs10455872
rs1260326
rs10053643
rs11591147
rs1560390
rs217181
rs13246490
rs28601761
rs174574
All
0.0
0.1
0.2
0.3
MR leave-one-out sensitivity analysis for
to total lipids ratio in very large VLDL || id:ebi-cfb233-GCST90302139' on 'ER- Breast cancer (Combined Oncoarray; iCOGS; GWAS

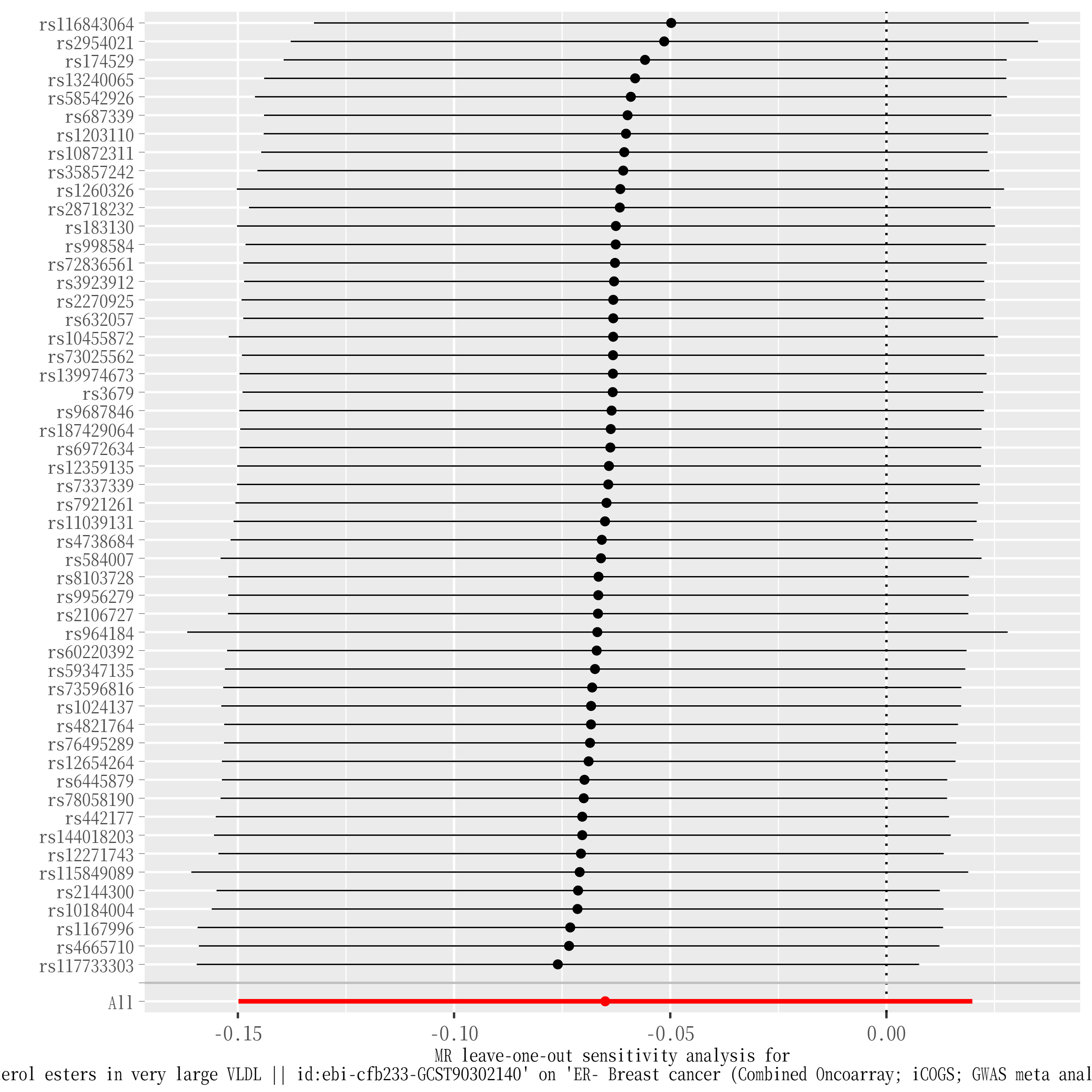
rs116843064
rs2954021
rs174529
rs13240065
rs58542926
rs687339
rs1203110
rs10872311
rs35857242
rs1260326
rs28718232
rs183130
rs998584
rs72836561
rs3923912
rs2270925
rs632057
rs10455872
rs73025562
rs139974673
rs3679
rs9687846
rs187429064
rs6972634
rs12359135
rs7337339
rs7921261
rs11039131
rs4738684
rs584007
rs8103728
rs9956279
rs2106727
rs964184
rs60220392
rs59347135
rs73596816
rs1024137
rs4821764
rs76495289
rs12654264
rs6445879
rs78058190
rs442177
rs144018203
rs12271743
rs115849089
rs2144300
rs10184004
rs1167996
rs4665710
rs117733303
All
-0.15
-0.10
-0.05
0.00
MR leave-one-out sensitivity analysis for
erol esters in very large VLDL || id:ebi-cfb233-GCST90302140' on 'ER- Breast cancer (Combined Oncoarray; iCOGS; GWAS meta anal

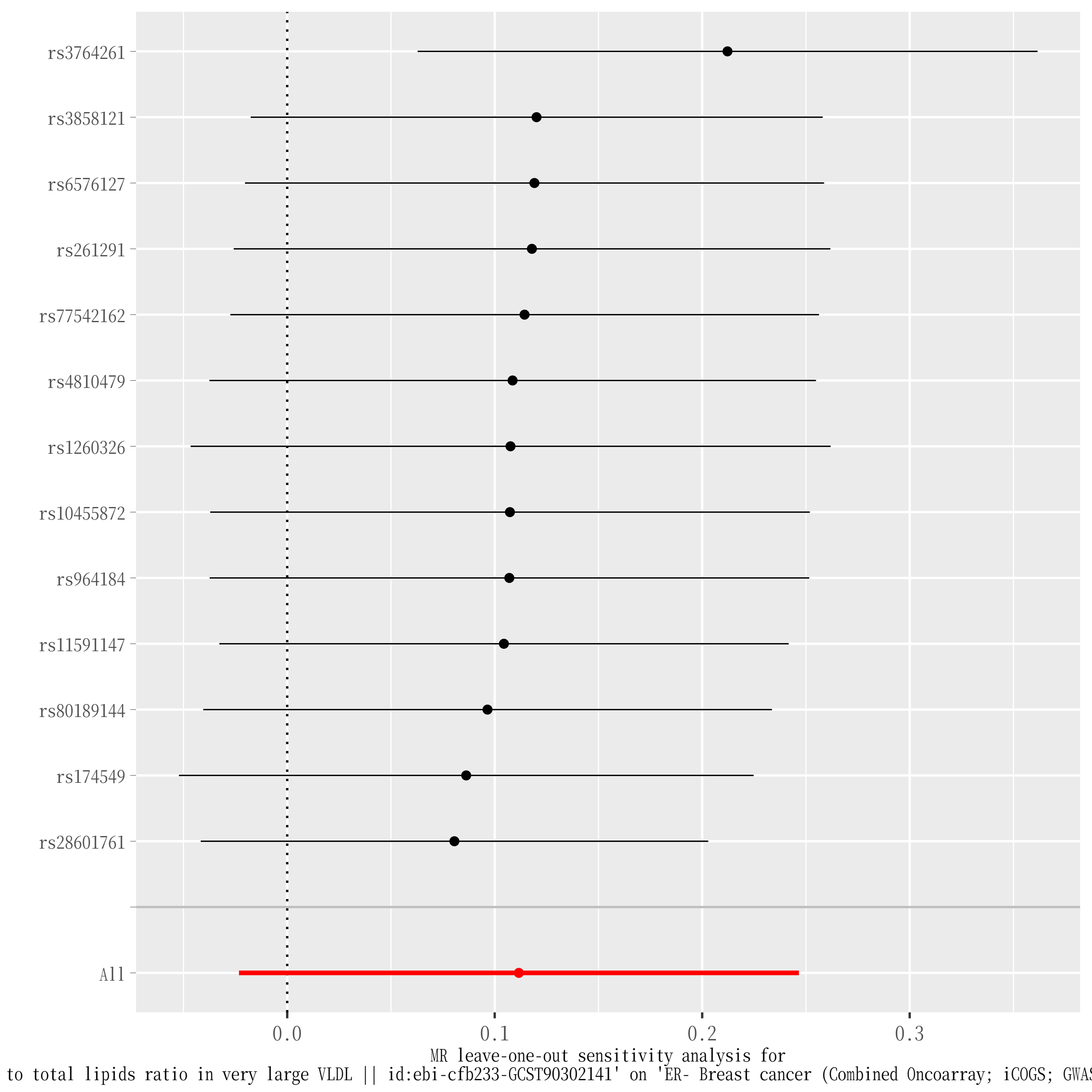

rs3764261
rs3858121
rs6576127
rs261291
rs77542162
rs4810479
rs1260326
rs10455872
rs964184
rs11591147
rs80189144
rs174549
rs28601761
All
0.0
0.1
0.2
0.3
MR leave-one-out sensitivity analysis for
to total lipids ratio in very large VLDL || id:ebi-cfb233-GCST90302141' on 'ER- Breast cancer (Combined Oncoarray; iCOGS; GWAS

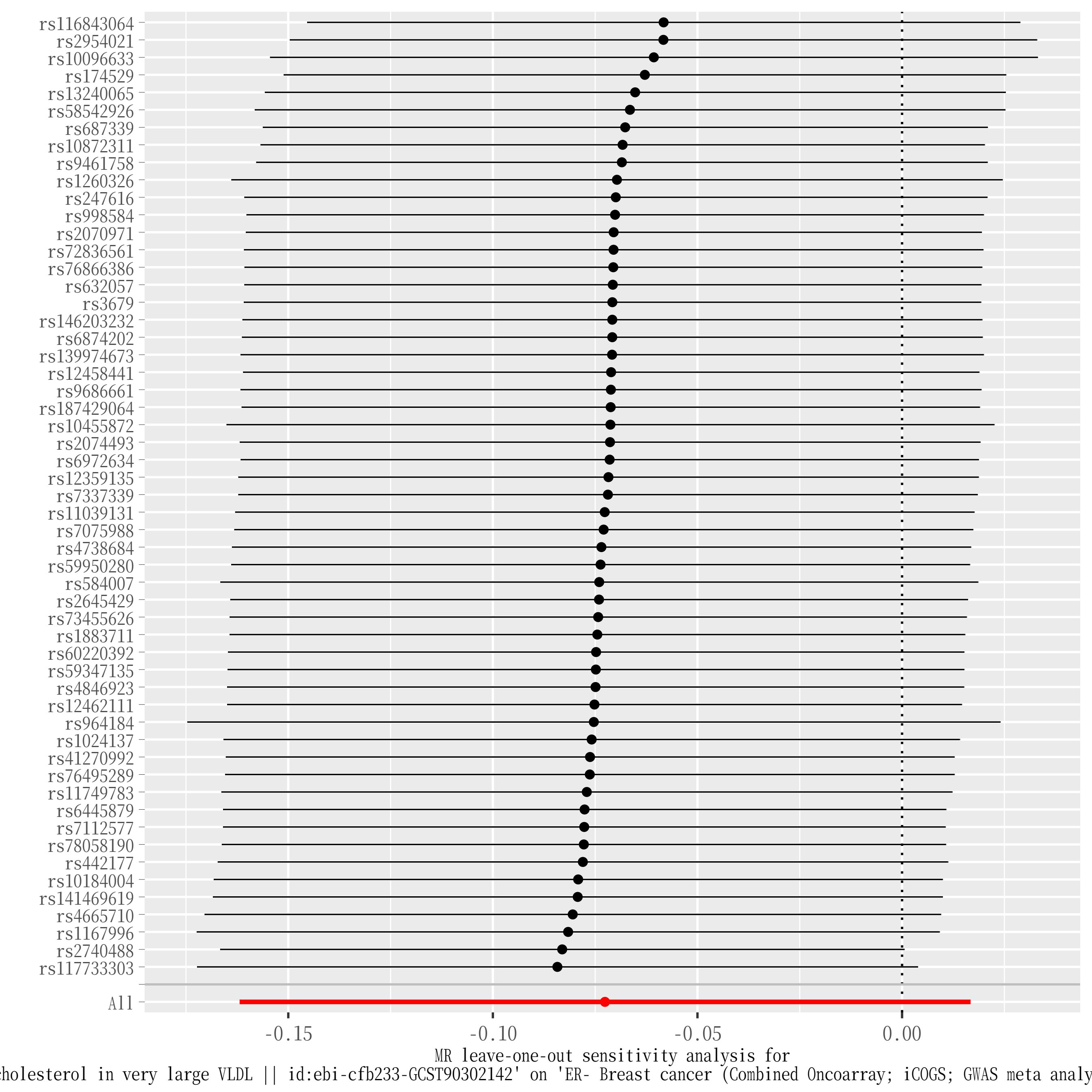

rs116843064
rs2954021
rs10096633
rs174529
rs13240065
rs58542926
rs687339
rs10872311
rs9461758
rs1260326
rs247616
rs998584
rs2070971
rs72836561
rs76866386
rs632057
rs3679
rs146203232
rs6874202
rs139974673
rs12458441
rs9686661
rs187429064
rs10455872
rs2074493
rs6972634
rs12359135
rs7337339
rs11039131
rs7075988
rs4738684
rs59950280
rs584007
rs2645429
rs73455626
rs1883711
rs60220392
rs59347135
rs4846923
rs12462111
rs964184
rs1024137
rs41270992
rs76495289
rs11749783
rs6445879
rs7112577
rs78058190
rs442177
rs10184004
rs141469619
rs4665710
rs1167996
rs2740488
rs117733303
All
-0.15
-0.10
-0.05
0.00
MR leave-one-out sensitivity analysis for
holesterol in very large VLDL || id:ebi-cfb233-GCST90302142' on 'ER- Breast cancer (Combined Oncoarray; iCOGS; GWAS meta analy

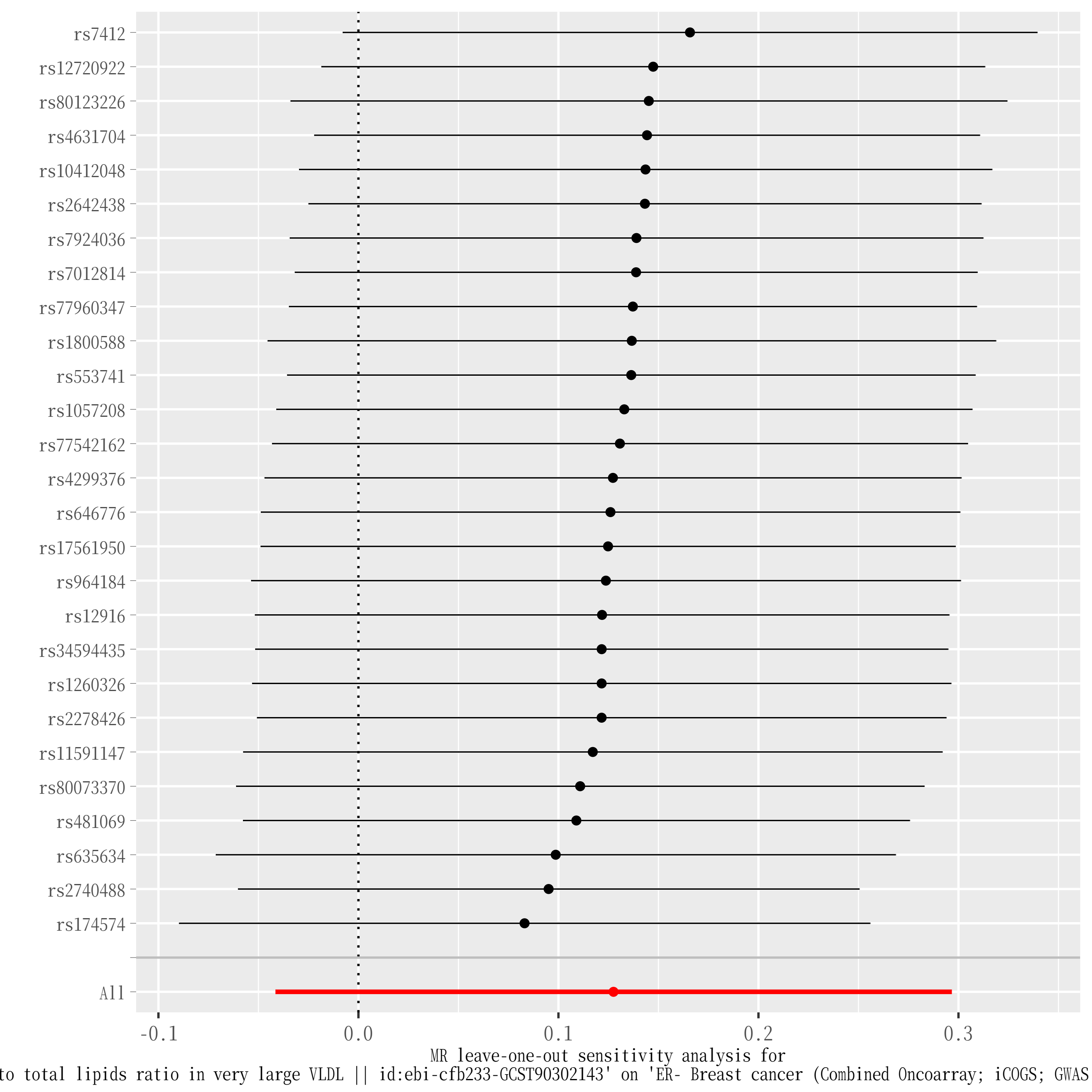
rs7412
rs12720922
rs80123226
rs4631704
rs10412048
rs2642438
rs7924036
rs7012814
rs77960347
rs1800588
rs553741
rs1057208
rs77542162
rs4299376
rs646776
rs17561950
rs964184
rs12916
rs34594435
rs1260326
rs2278426
rs11591147
rs80073370
rs481069
rs635634
rs2740488
rs174574
All
-0.1
0.0
0.1
0.2
0.3
MR leave-one-out sensitivity analysis for
to total lipids ratio in very large VLDL || id:ebi-cfb233-GCST90302143' on 'ER- Breast cancer (Combined Oncoarray; iCOGS; GWAS

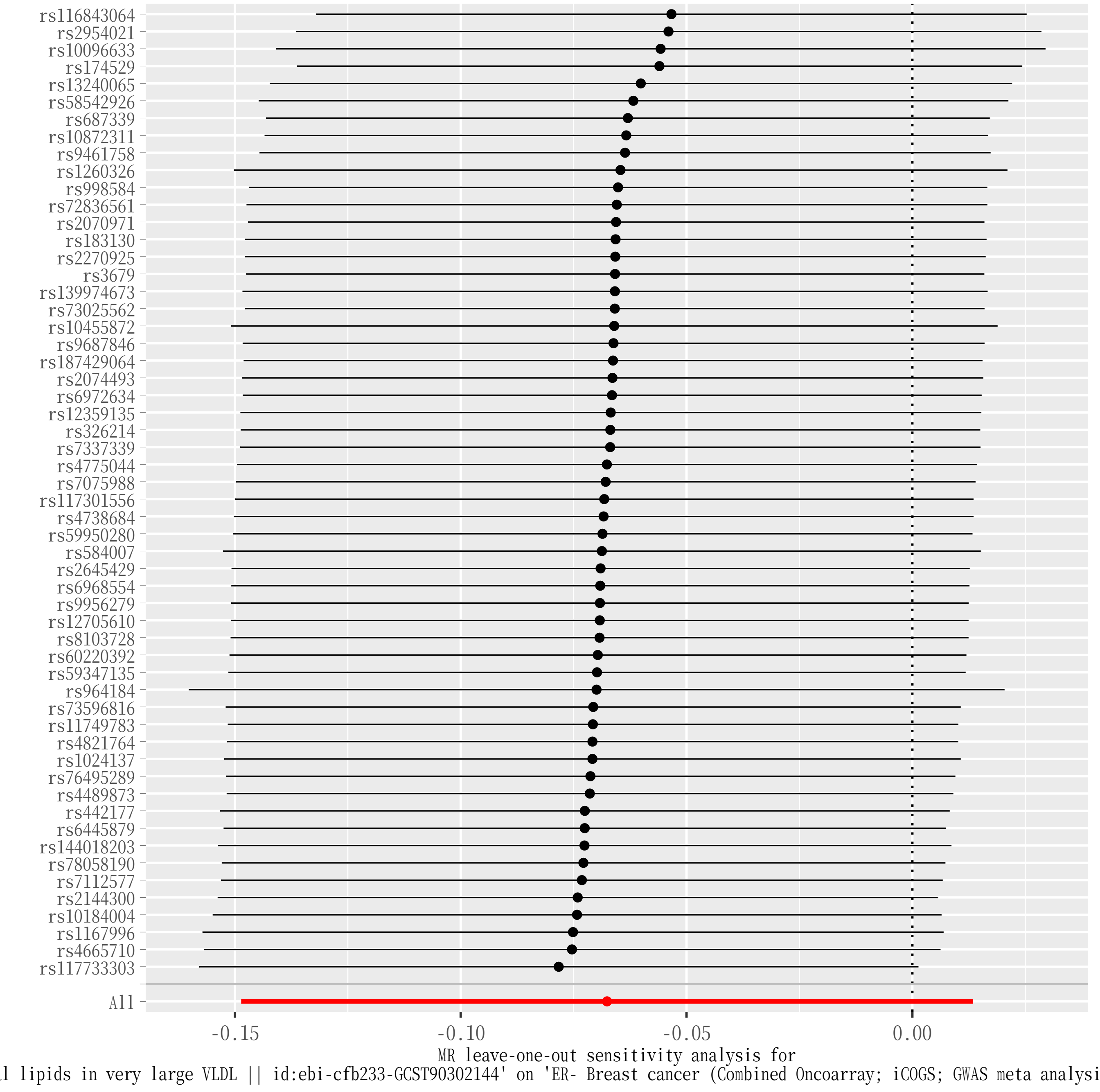

rs116843064
rs2954021
rs10096633
rs174529
rs13240065
rs58542926
rs687339
rs10872311
rs9461758
rs1260326
rs998584
rs72836561
rs2070971
rs183130
rs2270925
rs3679
rs139974673
rs73025562
rs10455872
rs9687846
rs187429064
rs2074493
rs6972634
rs12359135
rs326214
rs7337339
rs4775044
rs7075988
rs117301556
rs4738684
rs59950280
rs584007
rs2645429
rs6968554
rs9956279
rs12705610
rs8103728
rs60220392
rs59347135
rs964184
rs73596816
rs11749783
rs4821764
rs1024137
rs76495289
rs4489873
rs442177
rs6445879
rs144018203
rs78058190
rs7112577
rs2144300
rs10184004
rs1167996
rs4665710
rs117733303
All
-0.15
-0.10
-0.05
0.00
MR leave-one-out sensitivity analysis for
l lipids in very large VLDL || id:ebi-cfb233-GCST90302144' on 'ER- Breast cancer (Combined Oncoarray; iCOGS; GWAS meta analysi

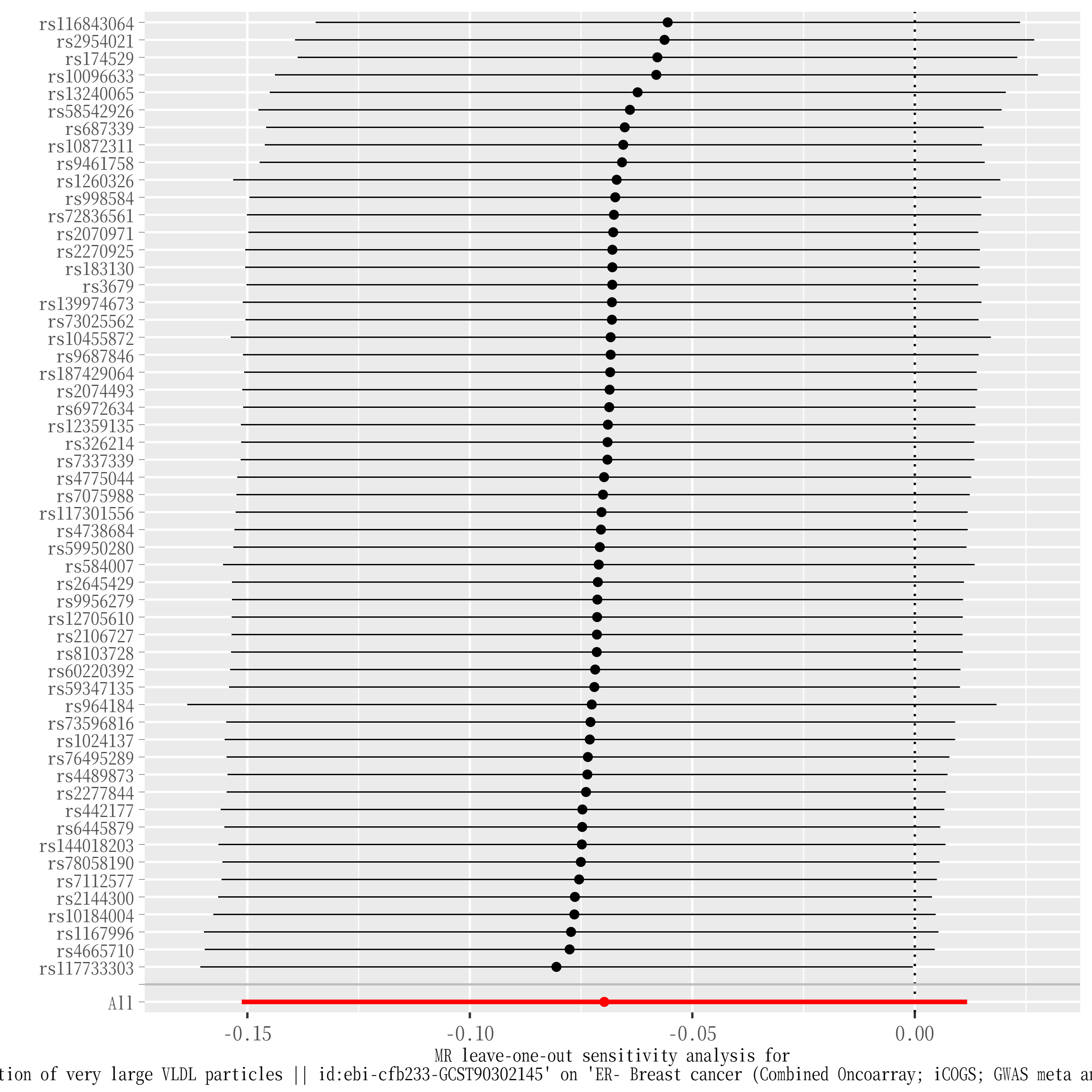
rs116843064
rs2954021
rs174529
rs10096633
rs13240065
rs58542926
rs687339
rs10872311
rs9461758
rs1260326
rs998584
rs72836561
rs2070971
rs2270925
rs183130
rs3679
rs139974673
rs73025562
rs10455872
rs9687846
rs187429064
rs2074493
rs6972634
rs12359135
rs326214
rs7337339
rs4775044
rs7075988
rs117301556
rs4738684
rs59950280
rs584007
rs2645429
rs9956279
rs12705610
rs2106727
rs8103728
rs60220392
rs59347135
rs964184
rs73596816
rs1024137
rs76495289
rs4489873
rs2277844
rs442177
rs6445879
rs144018203
rs78058190
rs7112577
rs2144300
rs10184004
rs1167996
rs4665710
rs117733303
All
-0.15
-0.10
-0.05
0.00
MR leave-one-out sensitivity analysis for
tion of very large VLDL particles || id:ebi-cfb233-GCST90302145' on 'ER- Breast cancer (Combined Oncoarray; iCOGS; GWAS meta ar

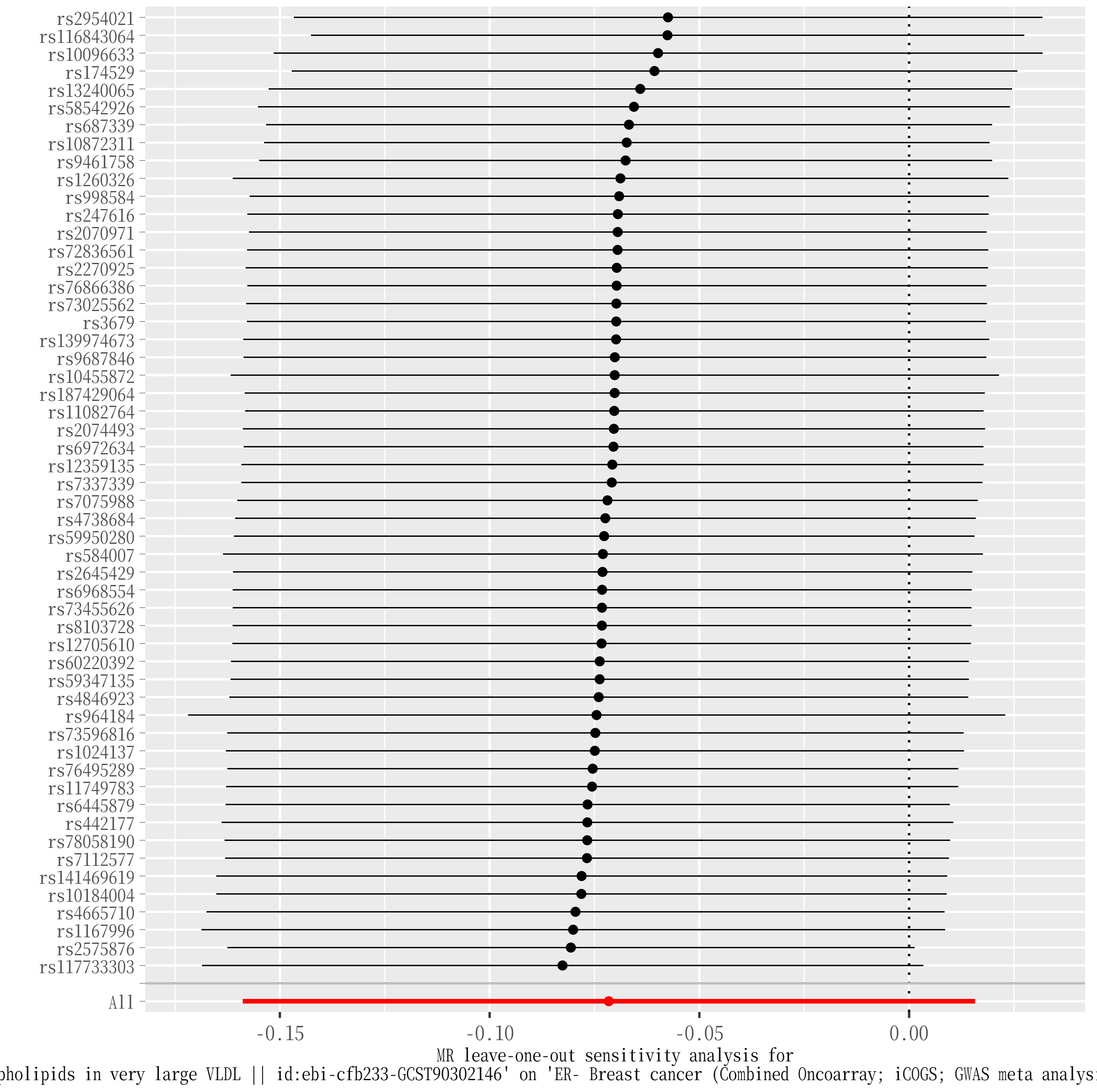

rs2954021
rs116843064
rs10096633
rs174529
rs13240065
rs58542926
rs687339
rs10872311
rs9461758
rs1260326
rs998584
rs247616
rs2070971
rs72836561
rs2270925
rs76866386
rs73025562
rs3679
rs139974673
rs9687846
rs10455872
rs187429064
rs11082764
rs2074493
rs6972634
rs12359135
rs7337339
rs7075988
rs4738684
rs59950280
rs584007
rs2645429
rs6968554
rs73455626
rs8103728
rs12705610
rs60220392
rs59347135
rs4846923
rs964184
rs73596816
rs1024137
rs76495289
rs11749783
rs6445879
rs442177
rs78058190
rs7112577
rs141469619
rs10184004
rs4665710
rs1167996
rs2575876
rs117733303
All
-0.15
-0.10
-0.05
0.00
MR leave-one-out sensitivity analysis for
pholipids in very large VLDL || id:ebi-cfb233-GCST90302146' on 'ER- Breast cancer (Combined Oncoarray; iCOGS; GWAS meta analys

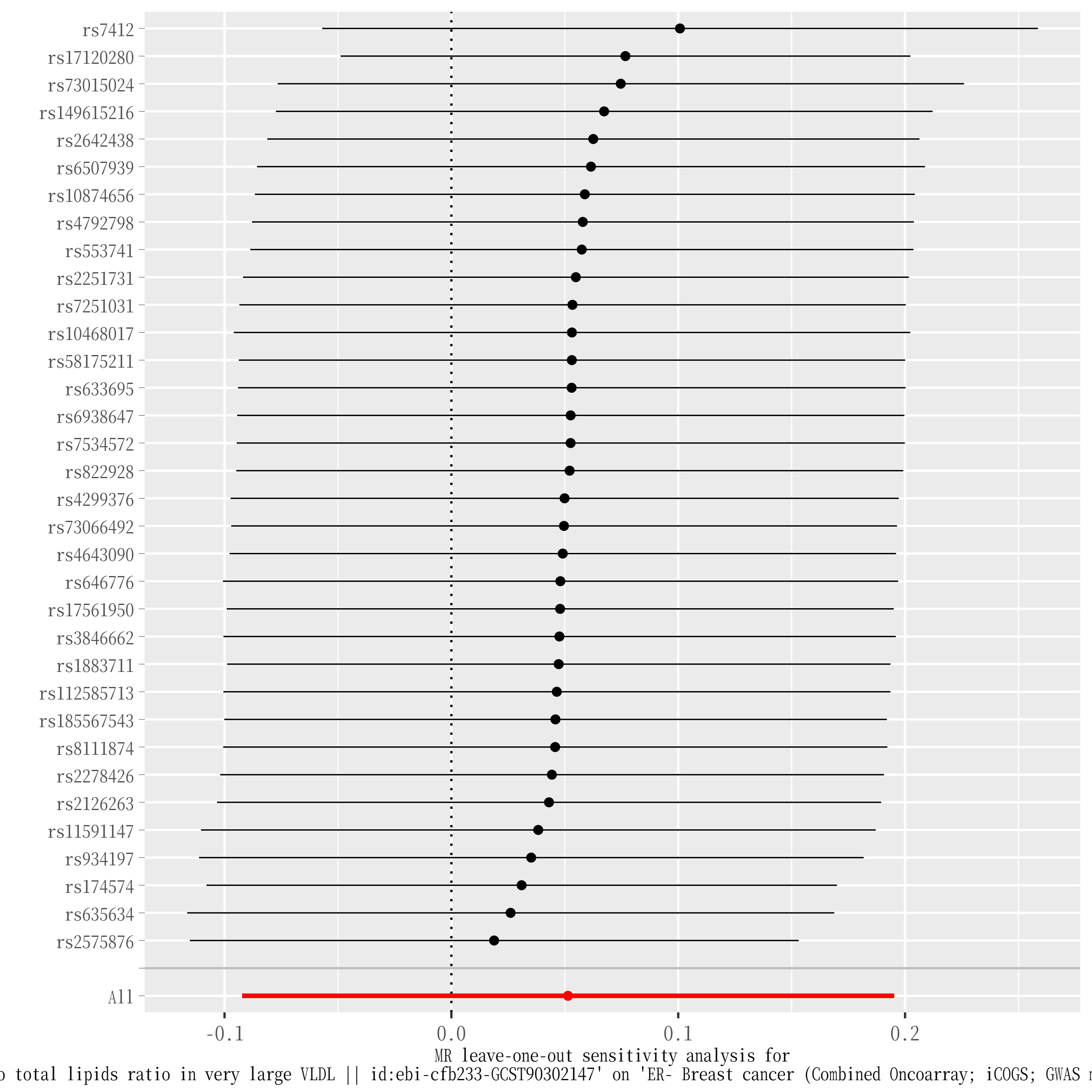

rs7412
rs17120280
rs73015024
rs149615216
rs2642438
rs6507939
rs10874656
rs4792798
rs553741
rs2251731
rs7251031
rs10468017
rs58175211
rs633695
rs6938647
rs7534572
rs822928
rs4299376
rs73066492
rs4643090
rs646776
rs17561950
rs3846662
rs1883711
rs112585713
rs185567543
rs8111874
rs2278426
rs2126263
rs11591147
rs934197
rs174574
rs635634
rs2575876
All
-0.1
0.0
0.1
0.2
MR leave-one-out sensitivity analysis for
o total lipids ratio in very large VLDL || id:ebi-cfb233-GCST90302147' on 'ER- Breast cancer (Combined Oncoarray; iCOGS; GWAS m

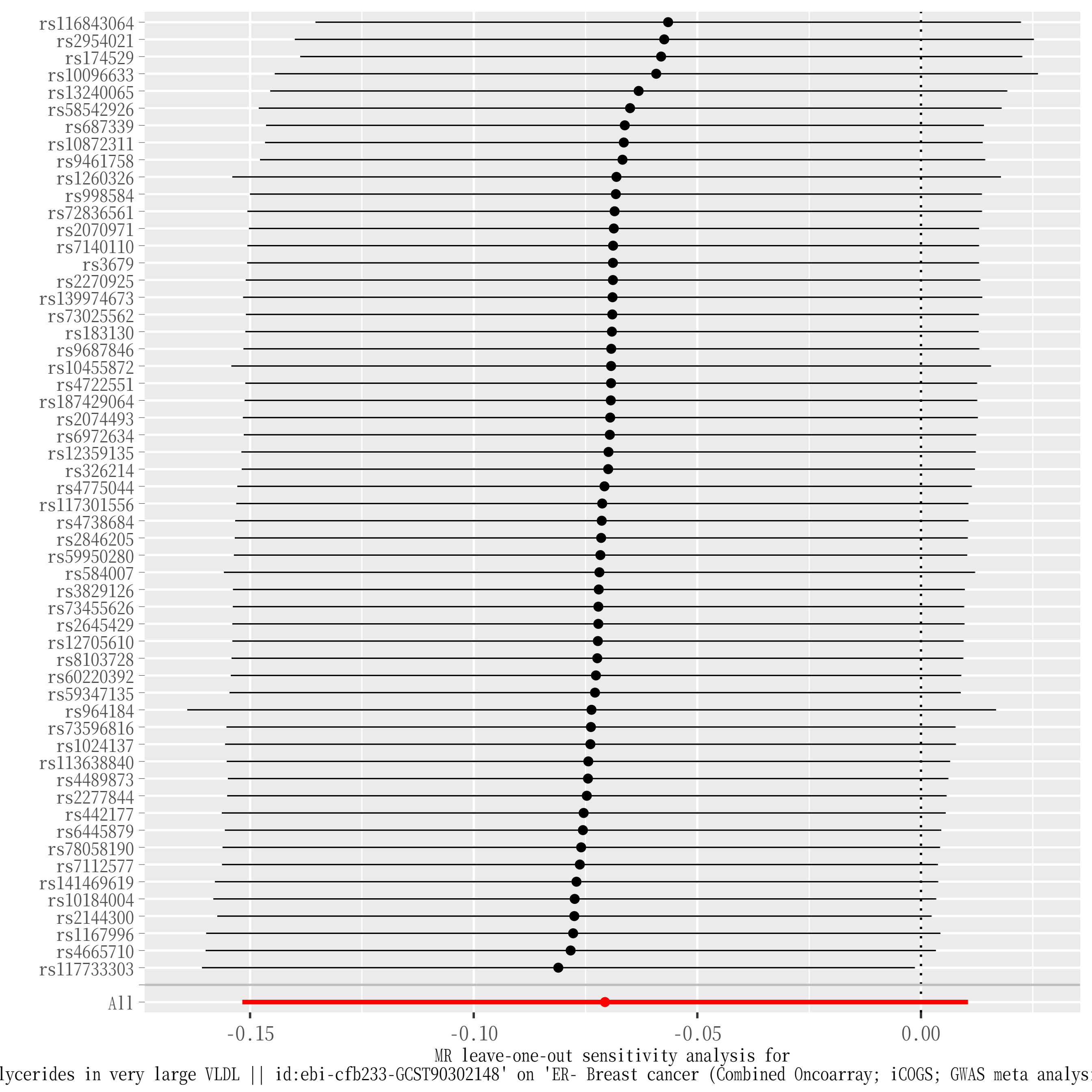
rs116843064
rs2954021
rs174529
rs10096633
rs13240065
rs58542926
rs687339
rs10872311
rs9461758
rs1260326
rs998584
rs72836561
rs2070971
rs7140110
rs3679
rs2270925
rs139974673
rs73025562
rs183130
rs9687846
rs10455872
rs4722551
rs187429064
rs2074493
rs6972634
rs12359135
rs326214
rs4775044
rs117301556
rs4738684
rs2846205
rs59950280
rs584007
rs3829126
rs73455626
rs2645429
rs12705610
rs8103728
rs60220392
rs59347135
rs964184
rs73596816
rs1024137
rs113638840
rs4489873
rs2277844
rs442177
rs6445879
rs78058190
rs7112577
rs141469619
rs10184004
rs2144300
rs1167996
rs4665710
rs117733303
All
-0.15
-0.10
-0.05
0.00
MR leave-one-out sensitivity analysis for
lycerides in very large VLDL || id:ebi-cfb233-GCST90302148' on 'ER- Breast cancer (Combined Oncoarray; iCOGS; GWAS meta analys

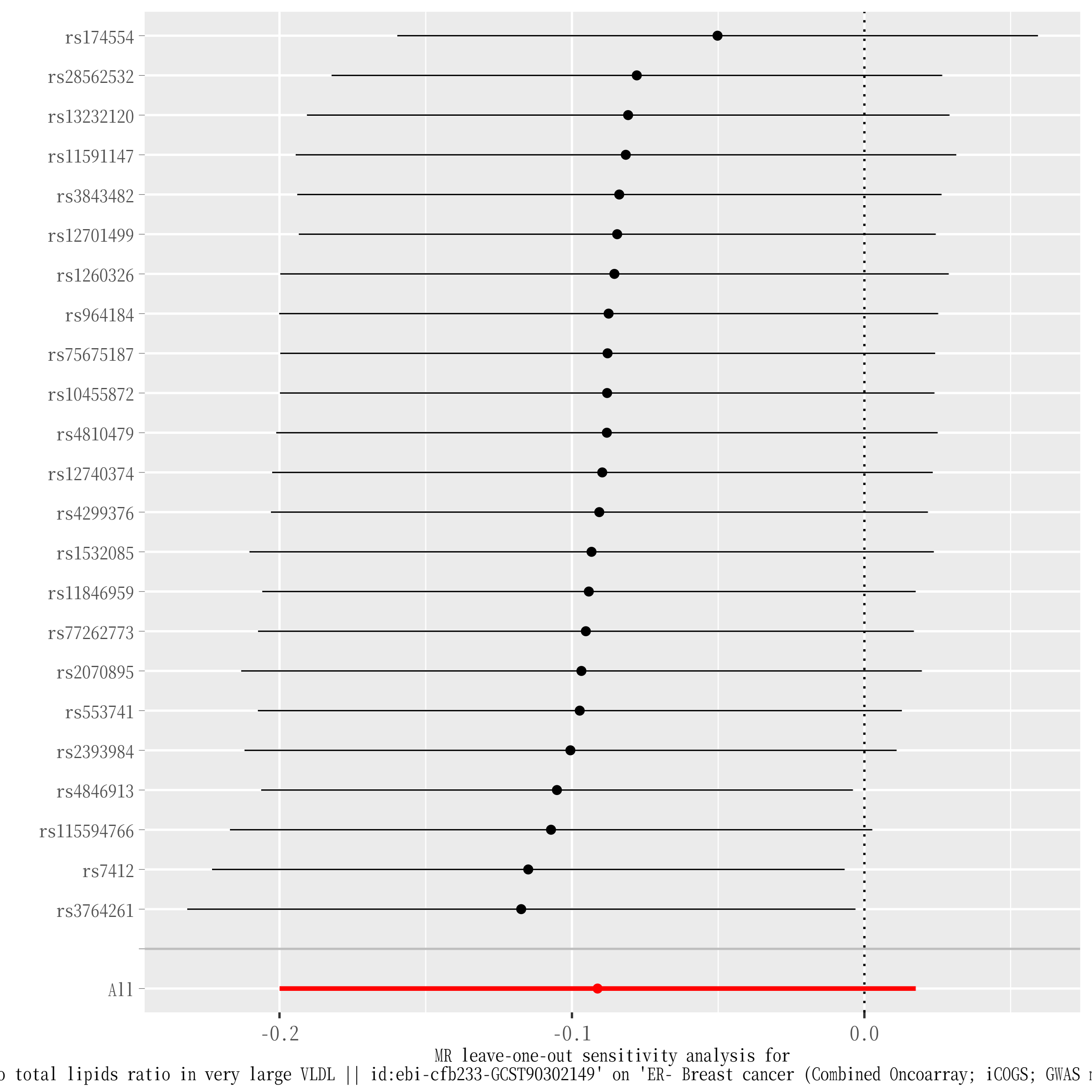
rs174554
rs28562532
rs13232120
rs11591147
rs3843482
rs12701499
rs1260326
rs964184
rs75675187
rs10455872
rs4810479
rs12740374
rs4299376
rs1532085
rs11846959
rs77262773
rs2070895
rs553741
rs2393984
rs4846913
rs115594766
rs7412
rs3764261
All
-0.2
-0.1
0.0
MR leave-one-out sensitivity analysis for
o total lipids ratio in very large VLDL || id:ebi-cfb233-GCST90302149' on 'ER- Breast cancer (Combined Oncoarray; iCOGS; GWAS m

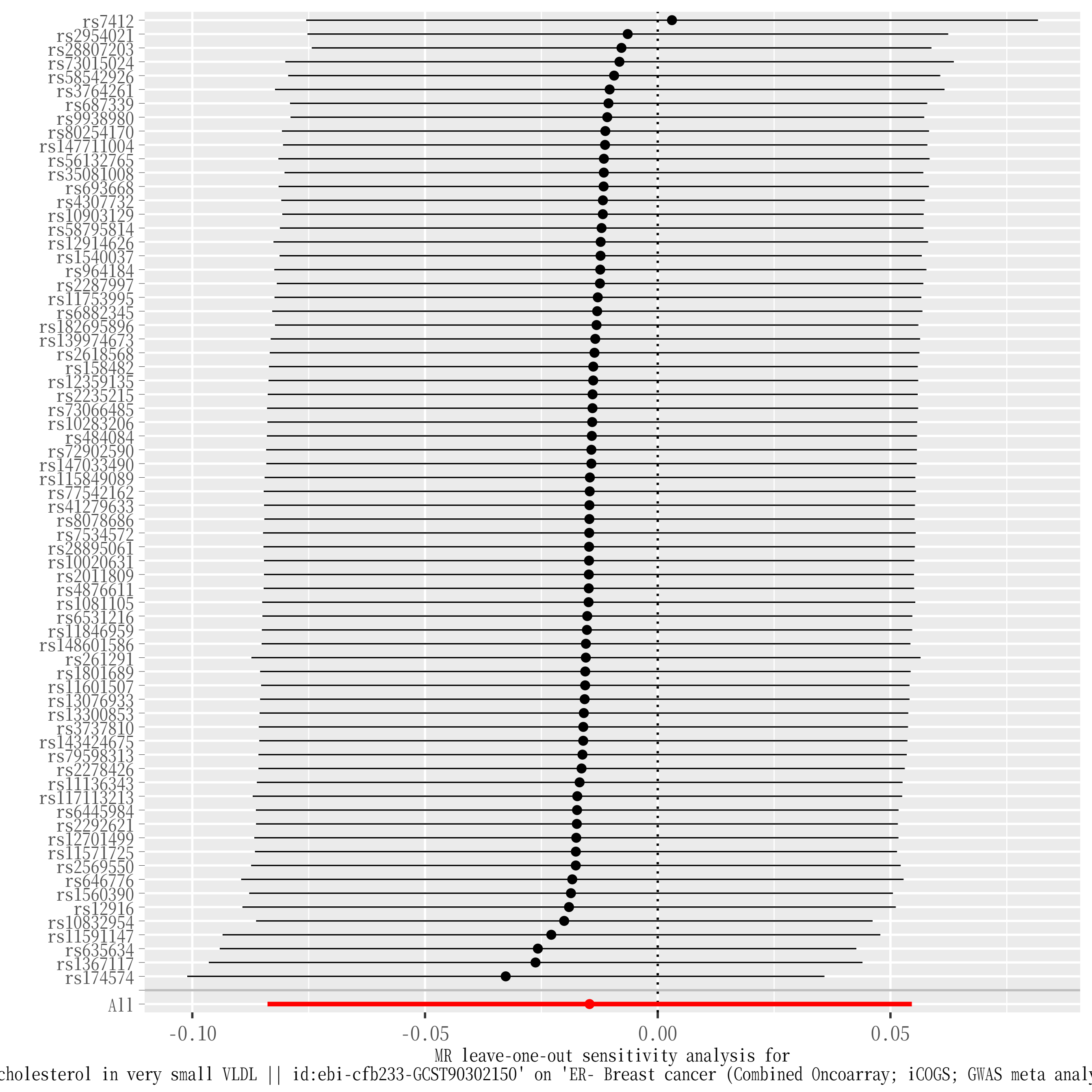

rs7412
rs2954021
rs28807203
rs73015024
rs58542926
rs3764261
rs687339
rs9938980
rs80254170
rs147711004
rs56132765
rs35081008
rs693668
rs4307732
rs10903129
rs58795814
rs12914626
rs1540037
rs964184
rs2287997
rs11753995
rs6882345
rs182695896
rs139974673
rs2618568
rs158482
rs12359135
rs2235215
rs73066485
rs10283206
rs484084
rs72902590
rs147033490
rs115849089
rs77542162
rs41279633
rs8078686
rs7534572
rs28895061
rs10020631
rs2011809
rs4876611
rs1081105
rs6531216
rs11846959
rs148601586
rs261291
rs1801689
rs11601507
rs13076933
rs13300853
rs3737810
rs143424675
rs79598313
rs2278426
rs11136343
rs117113213
rs6445984
rs2292621
rs12701499
rs11571725
rs2569550
rs646776
rs1560390
rs12916
rs10832954
rs11591147
rs635634
rs1367117
rs174574
All
-0.10
-0.05
0.00
0.05
MR leave-one-out sensitivity analysis for
cholesterol in very small VLDL || id:ebi-cfb233-GCST90302150' on 'ER- Breast cancer (Combined Oncoarray; iCOGS; GWAS meta analy

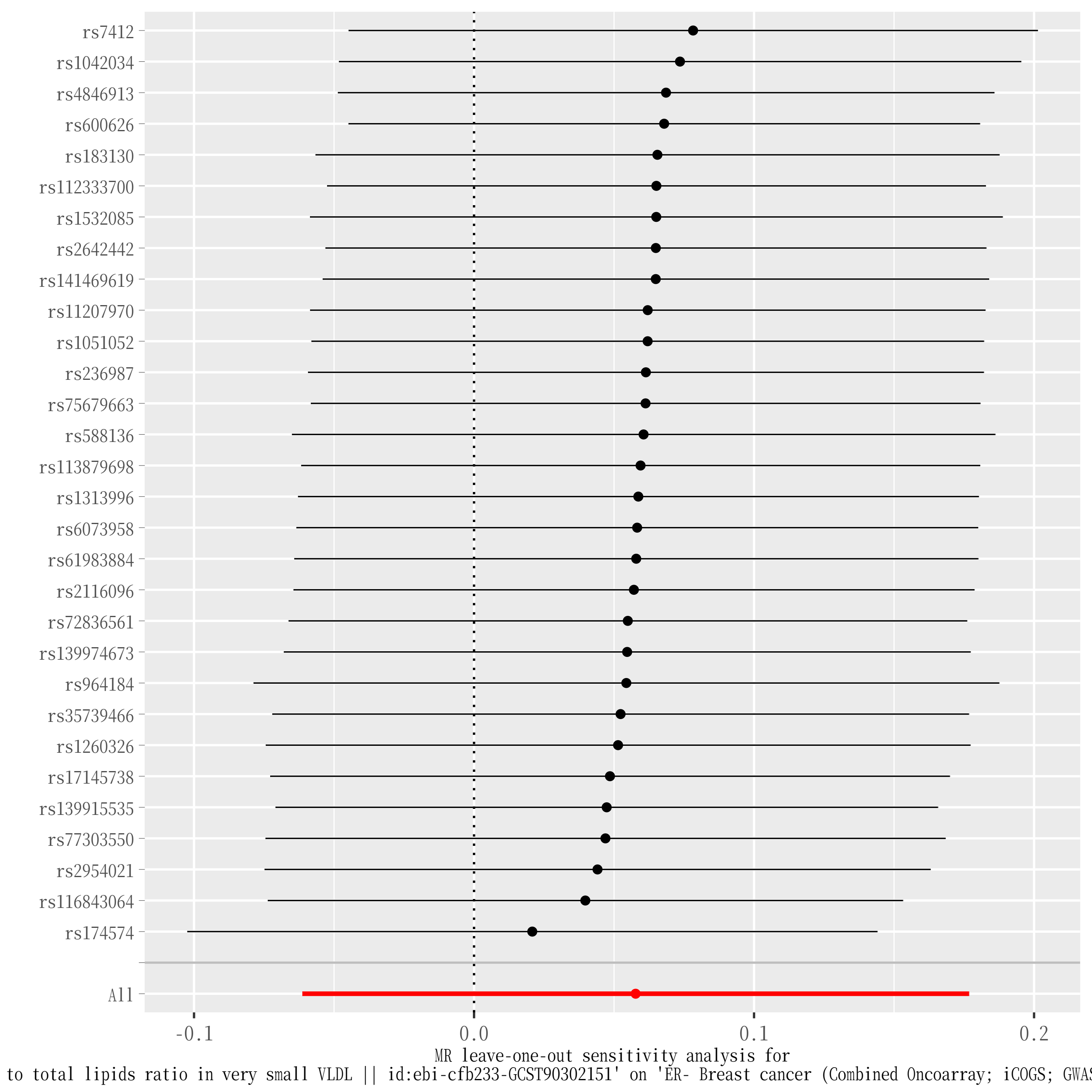

rs7412
rs1042034
rs4846913
rs600626
rs183130
rs112333700
rs1532085
rs2642442
rs141469619
rs11207970
rs1051052
rs236987
rs75679663
rs588136
rs113879698
rs1313996
rs6073958
rs61983884
rs2116096
rs72836561
rs139974673
rs964184
rs35739466
rs1260326
rs17145738
rs139915535
rs77303550
rs2954021
rs116843064
rs174574
All
-0.1
0.0
0.1
0.2
MR leave-one-out sensitivity analysis for
to total lipids ratio in very small VLDL || id:ebi-cfb233-GCST90302151' on 'ER- Breast cancer (Combined Oncoarray; iCOGS; GWAS

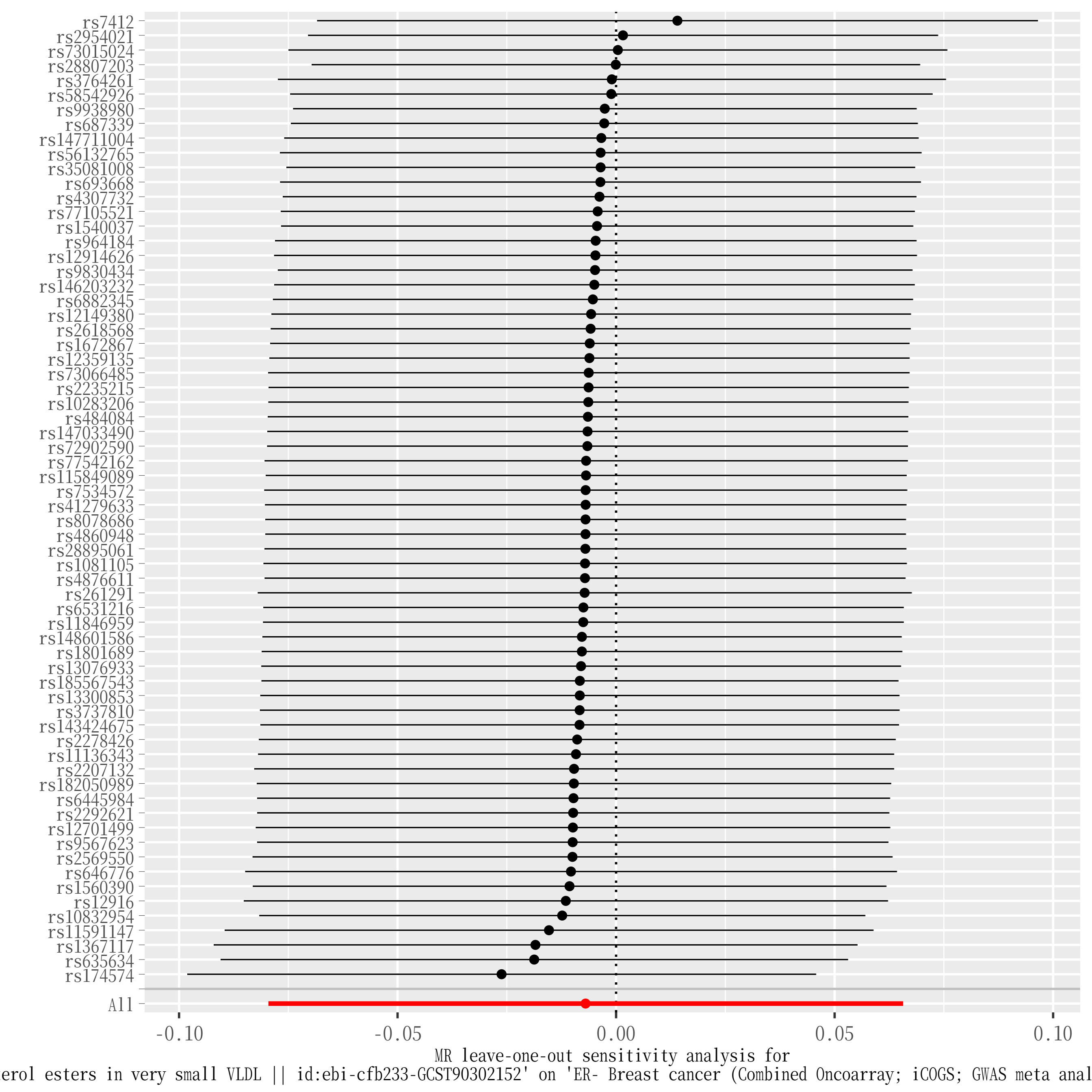

rs7412
rs2954021
rs73015024
rs28807203
rs3764261
rs58542926
rs9938980
rs687339
rs147711004
rs56132765
rs35081008
rs693668
rs4307732
rs77105521
rs1540037
rs964184
rs12914626
rs9830434
rs146203232
rs6882345
rs12149380
rs2618568
rs1672867
rs12359135
rs73066485
rs2235215
rs10283206
rs484084
rs147033490
rs72902590
rs77542162
rs115849089
rs7534572
rs41279633
rs8078686
rs4860948
rs28895061
rs1081105
rs4876611
rs261291
rs6531216
rs11846959
rs148601586
rs1801689
rs13076933
rs185567543
rs13300853
rs3737810
rs143424675
rs2278426
rs11136343
rs2207132
rs182050989
rs6445984
rs2292621
rs12701499
rs9567623
rs2569550
rs646776
rs1560390
rs12916
rs10832954
rs11591147
rs1367117
rs635634
rs174574
All
-0.10
-0.05
0.00
0.05
0.10
MR leave-one-out sensitivity analysis for
erol esters in very small VLDL || id:ebi-cfb233-GCST90302152' on 'ER- Breast cancer (Combined Oncoarray; iCOGS; GWAS meta anal

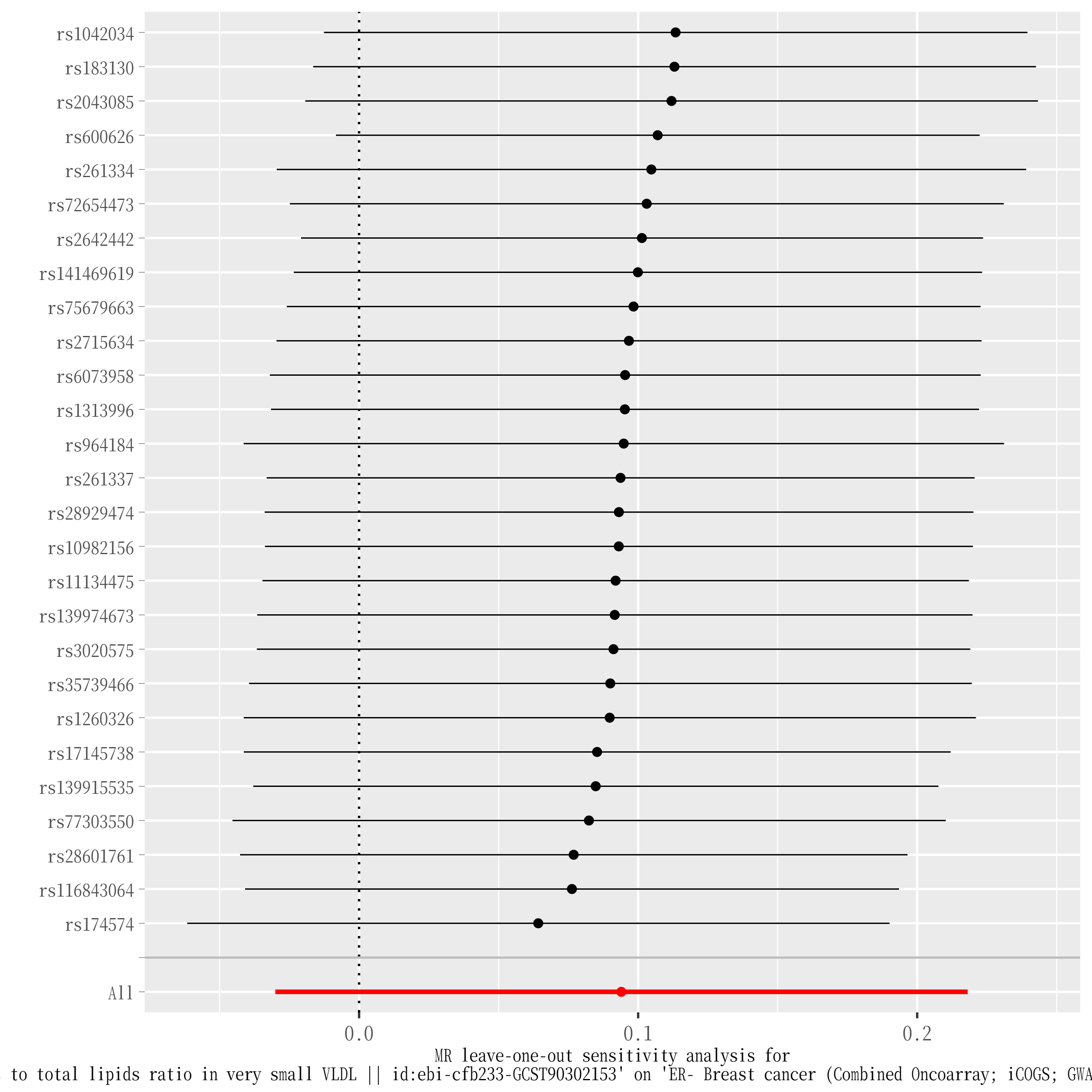

rs1042034
rs183130
rs2043085
rs600626
rs261334
rs72654473
rs2642442
rs141469619
rs75679663
rs2715634
rs6073958
rs1313996
rs964184
rs261337
rs28929474
rs10982156
rs11134475
rs139974673
rs3020575
rs35739466
rs1260326
rs17145738
rs139915535
rs77303550
rs28601761
rs116843064
rs174574
All
0.0
0.1
0.2
MR leave-one-out sensitivity analysis for
to total lipids ratio in very small VLDL || id:ebi-cfb233-GCST90302153' on 'ER- Breast cancer (Combined Oncoarray; iCOGS; GWA

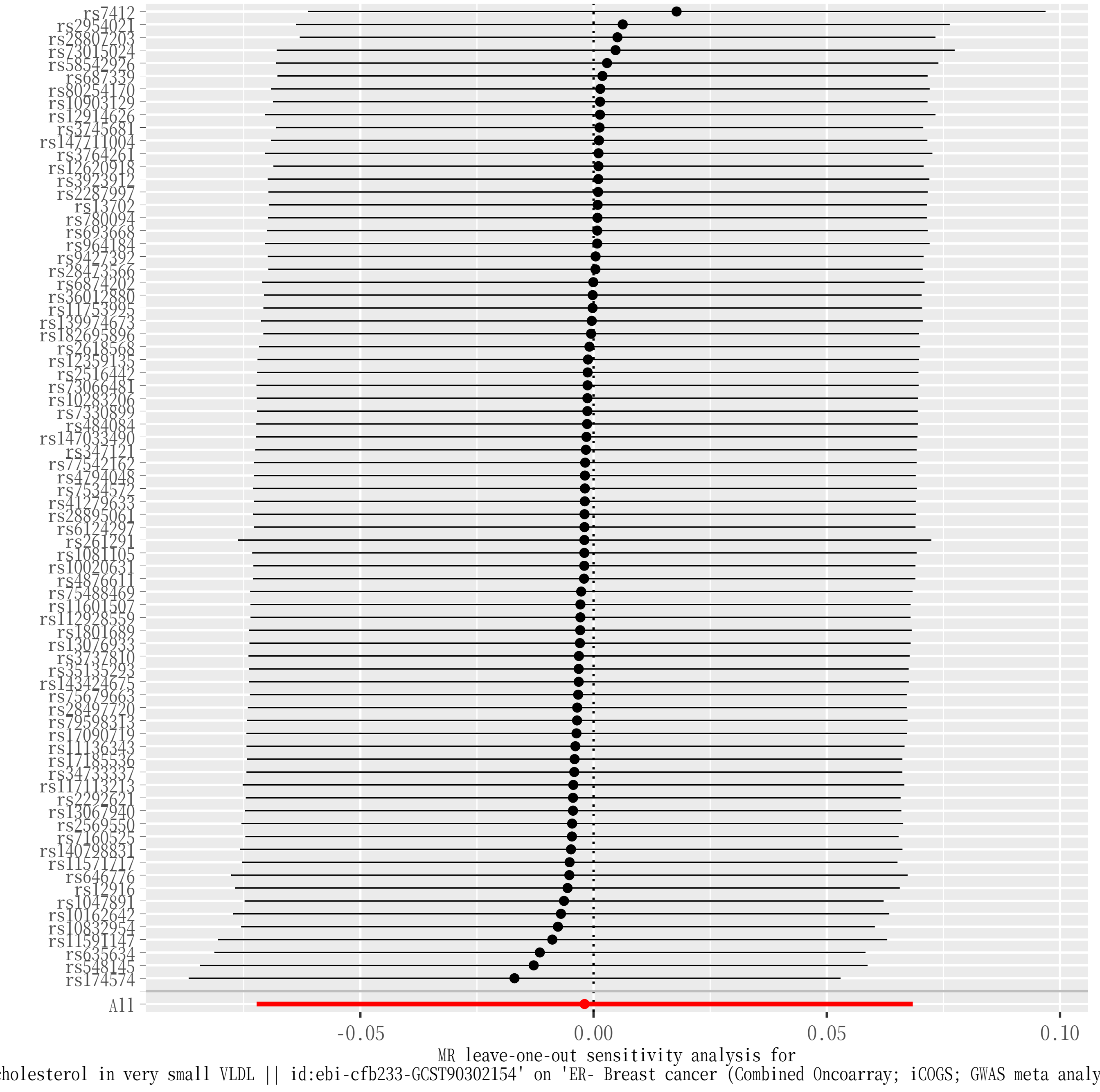

rs7412
rs2954021
rs28807203
rs73015024
rs58542926
rs687339
rs80254170
rs10903129
rs12914626
rs3745681
rs147711004
rs3764261
rs12620918
rs3923912
rs2287997
rs13702
rs780094
rs693668
rs964184
rs9427392
rs28473566
rs6874202
rs36012880
rs11753995
rs139974673
rs182695896
rs2618568
rs12359135
rs2516442
rs73066481
rs10283206
rs7330899
rs484084
rs147033490
rs347121
rs77542162
rs4794048
rs7534572
rs41279633
rs28895061
rs6124297
rs261291
rs1081105
rs10020631
rs4876611
rs75488469
rs11601507
rs112928559
rs1801689
rs13076933
rs3737810
rs35135293
rs143424675
rs75679663
rs28497720
rs79598313
rs17090719
rs11136343
rs17185536
rs34733337
rs117113213
rs2292621
rs13067940
rs2569550
rs7160525
rs140798831
rs11571717
rs646776
rs12916
rs1047891
rs10162642
rs10832954
rs11591147
rs635634
rs548145
rs174574
All
-0.05
0.00
0.05
0.10
MR leave-one-out sensitivity analysis for
cholesterol in very small VLDL || id:ebi-cfb233-GCST90302154' on 'ER- Breast cancer (Combined Oncoarray; iCOGS; GWAS meta analy

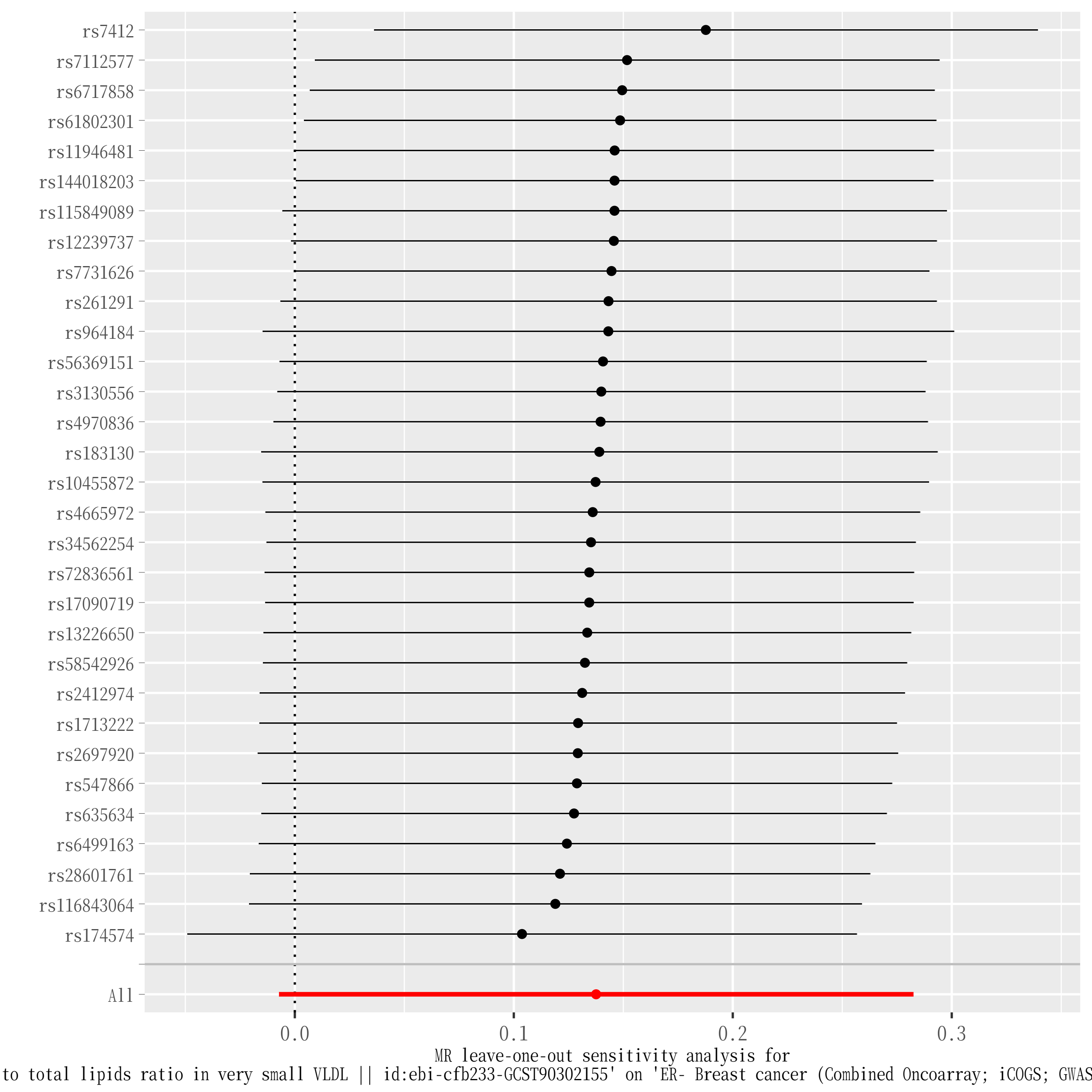
rs7412
rs7112577
rs6717858
rs61802301
rs11946481
rs144018203
rs115849089
rs12239737
rs7731626
rs261291
rs964184
rs56369151
rs3130556
rs4970836
rs183130
rs10455872
rs4665972
rs34562254
rs72836561
rs17090719
rs13226650
rs58542926
rs2412974
rs1713222
rs2697920
rs547866
rs635634
rs6499163
rs28601761
rs116843064
rs174574
All
0.0
0.1
0.2
0.3
MR leave-one-out sensitivity analysis for
to total lipids ratio in very small VLDL || id:ebi-cfb233-GCST90302155' on 'ER- Breast cancer (Combined Oncoarray; iCOGS; GWAS

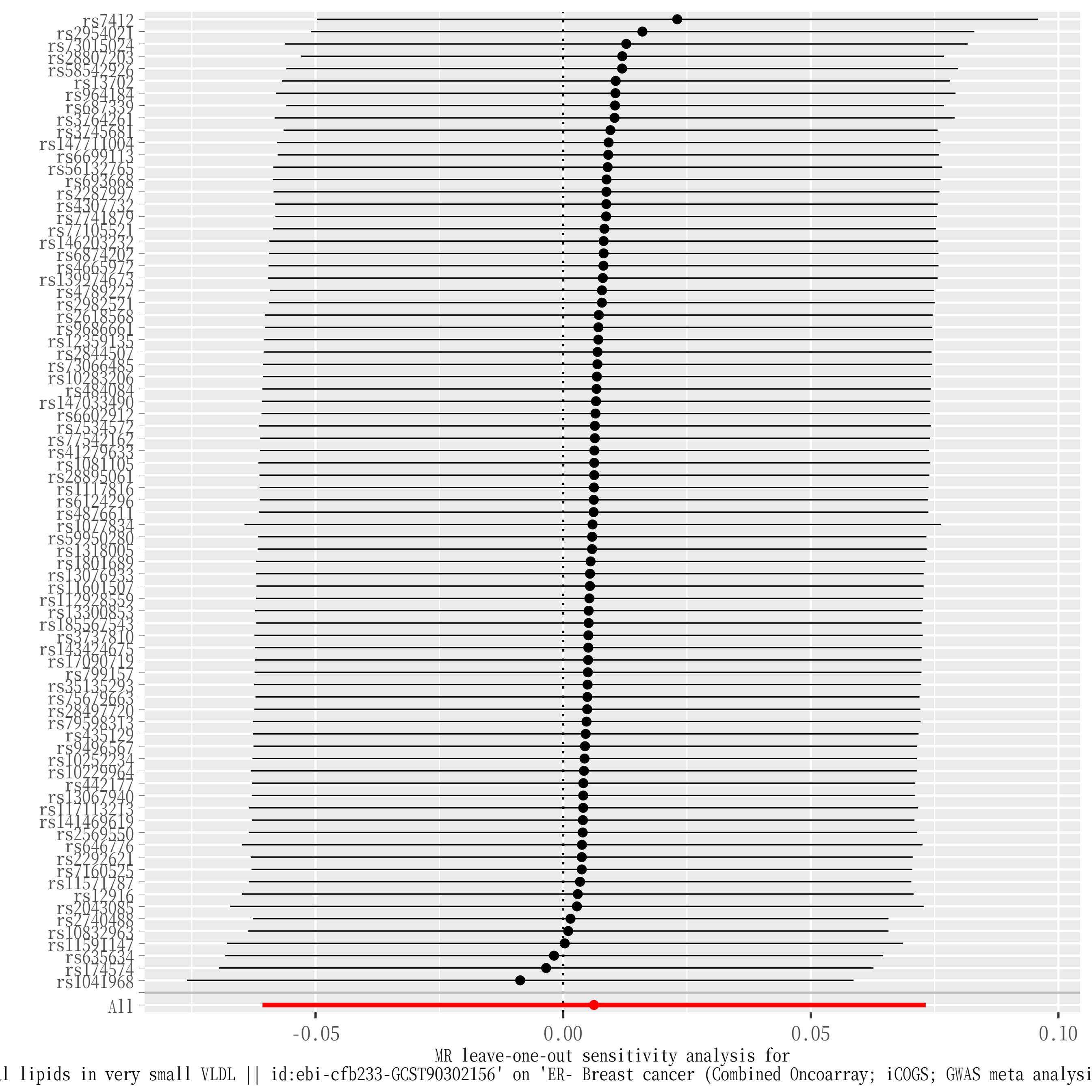
rs7412
rs2954021
rs73015024
rs28807203
rs58542926
rs13702
rs964184
rs687339
rs3764261
rs3745681
rs147711004
rs6699113
rs56132765
rs693668
rs2287997
rs4307732
rs7741879
rs77105521
rs146203232
rs6874202
rs4665972
rs139974673
rs4789227
rs2982521
rs2618568
rs9686661
rs12359135
rs2844507
rs73066485
rs10283206
rs484084
rs147033490
rs6602912
rs7534572
rs77542162
rs41279633
rs1081105
rs28895061
rs1117816
rs6124296
rs4876611
rs1077834
rs59950280
rs1318005
rs1801689
rs13076933
rs11601507
rs112928559
rs13300853
rs185567543
rs3737810
rs143424675
rs17090719
rs799157
rs35135293
rs75679663
rs28497720
rs79598313
rs435129
rs9496567
rs10252234
rs10229964
rs442177
rs13067940
rs117113213
rs141469619
rs2569550
rs646776
rs2292621
rs7160525
rs11571787
rs12916
rs2043085
rs2740488
rs10832963
rs115911147
rs635634
rs174574
rs1041968
All
-0.05
0.00
0.05
0.10
MR leave-one-out sensitivity analysis for
l lipids in very small VLDL || id:ebi-cfb233-GCST90302156' on 'ER- Breast cancer (Combined Oncoarray; iCOGS; GWAS meta analysi

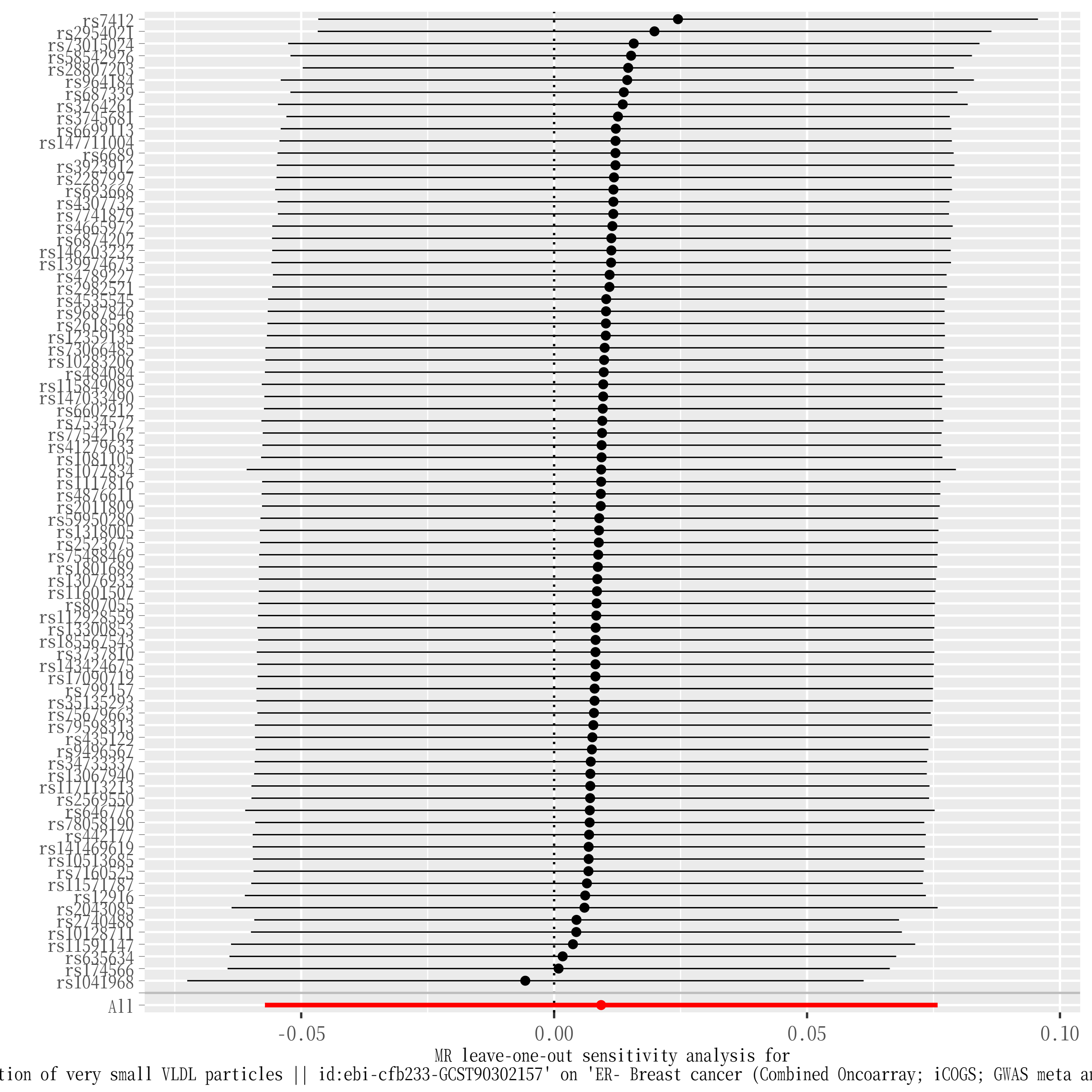
rs7412
rs2954021
rs73015024
rs58542926
rs28807203
rs964184
rs687339
rs3764261
rs3745681
rs6699113
rs147711004
rs6689
rs3923912
rs2287997
rs693668
rs4307732
rs7741879
rs4665972
rs6874202
rs146203232
rs139974673
rs4789227
rs2982521
rs4535545
rs9687846
rs2618568
rs12359135
rs73066485
rs10283206
rs484084
rs115849089
rs147033490
rs6602912
rs7534572
rs77542162
rs41279633
rs1081105
rs1077834
rs1117816
rs4876611
rs2011809
rs59950280
rs1318005
rs2523675
rs75488469
rs1801689
rs13076933
rs11601507
rs807055
rs112928559
rs13300853
rs185567543
rs3737810
rs143424675
rs17090719
rs792157
rs35135293
rs75679663
rs79598313
rs435129
rs9496567
rs34733337
rs13067940
rs117113213
rs2569550
rs646776
rs78058190
rs442177
rs141469619
rs10513685
rs7160525
rs11571787
rs12916
rs2043085
rs2740488
rs10128711
rs11591147
rs635634
rs174566
rs1041968
All
-0.05
0.00
0.05
0.10
MR leave-one-out sensitivity analysis for
tion of very small VLDL particles || id:ebi-cfb233-GCST90302157' on 'ER- Breast cancer (Combined Oncoarray; iCOGS; GWAS meta ar

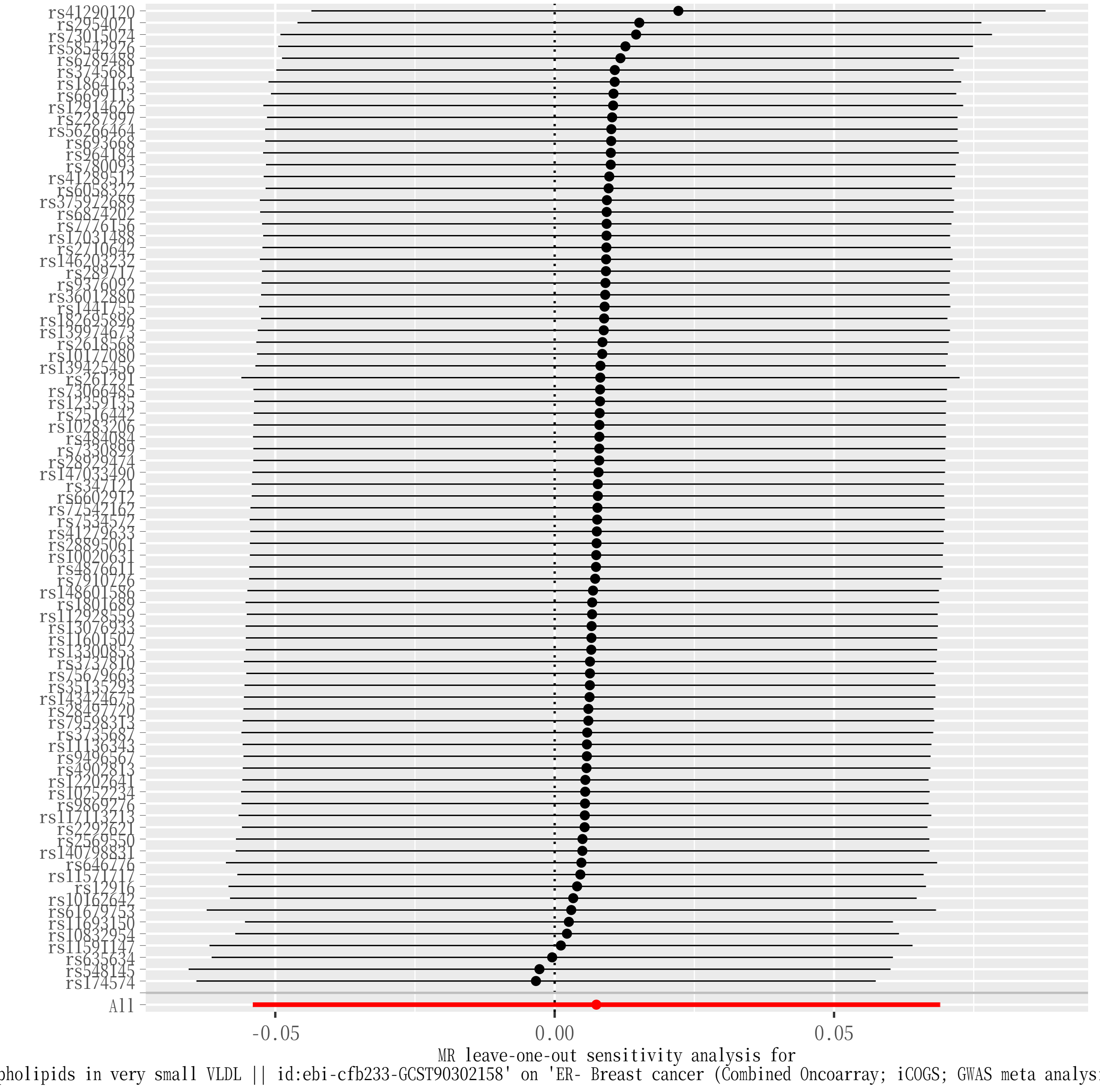
rs41290120
rs2954021
rs73015024
rs58542926
rs6789488
rs3745681
rs1864163
rs6699113
rs12914626
rs2287997
rs56266464
rs693668
rs964184
rs780093
rs41289512
rs6058327
rs375972689
rs6874202
rs7776156
rs17031488
rs2710642
rs146203232
rs289717
rs9376092
rs36012880
rs1441755
rs182695826
rs139974673
rs2618568
rs10177080
rs139425456
rs261291
rs73066485
rs12359135
rs2516442
rs10283206
rs484084
rs7330899
rs28929474
rs147033490
rs347121
rs6602912
rs72542162
rs7534572
rs41279633
rs28895061
rs10020631
rs4876611
rs7910726
rs148601586
rs1801689
rs112928559
rs13076933
rs11601507
rs13300853
rs3737810
rs75679663
rs35135223
rs143424675
rs28497720
rs79598313
rs3735687
rs11136343
rs9496567
rs4902813
rs12207641
rs10252234
rs9869276
rs117113213
rs229621
rs2569550
rs140798831
rs646776
rs11571717
rs12916
rs10162642
rs61679753
rs11693150
rs10832954
rs11591147
rs635634
rs548145
rs174574
All
-0.05
0.00
0.05
MR leave-one-out sensitivity analysis for
pholipids in very small VLDL || id:ebi-cfb233-GCST90302158' on 'ER- Breast cancer (Combined Oncoarray; iCOGS; GWAS meta analys

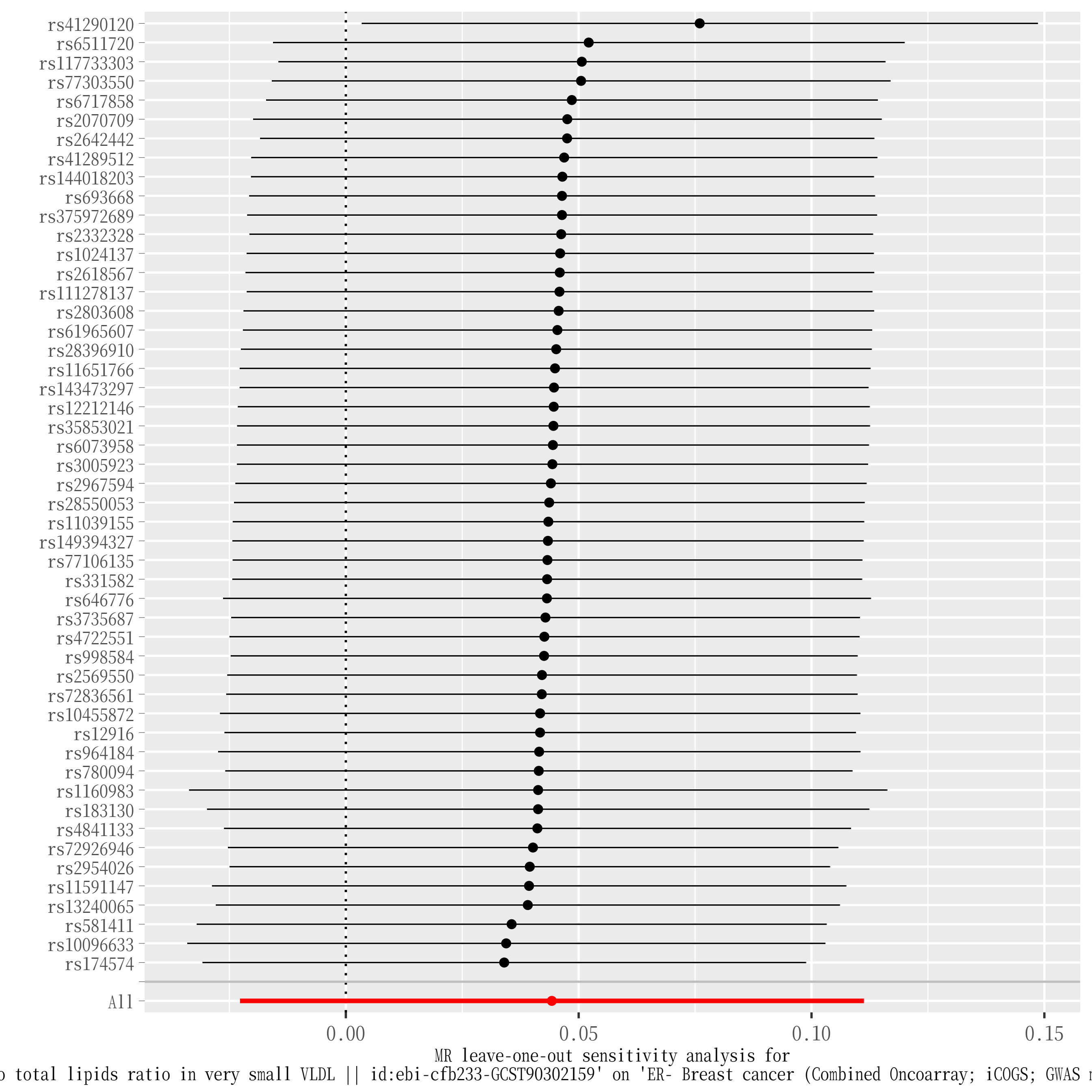

rs41290120
rs6511720
rs117733303
rs77303550
rs6717858
rs2070709
rs2642442
rs41289512
rs144018203
rs693668
rs375972689
rs2332328
rs1024137
rs2618567
rs111278137
rs2803608
rs61965607
rs28396910
rs11651766
rs143473297
rs12212146
rs35853021
rs6073958
rs3005923
rs2967594
rs28550053
rs11039155
rs149394327
rs77106135
rs331582
rs646776
rs3735687
rs4722551
rs998584
rs2569550
rs72836561
rs10455872
rs12916
rs964184
rs780094
rs1160983
rs183130
rs4841133
rs72926946
rs2954026
rs11591147
rs13240065
rs581411
rs10096633
rs174574
All
0.00
0.05
0.10
0.15
MR leave-one-out sensitivity analysis for
o total lipids ratio in very small VLDL || id:ebi-cfb233-GCST90302159' on 'ER- Breast cancer (Combined Oncoarray; iCOGS; GWAS m

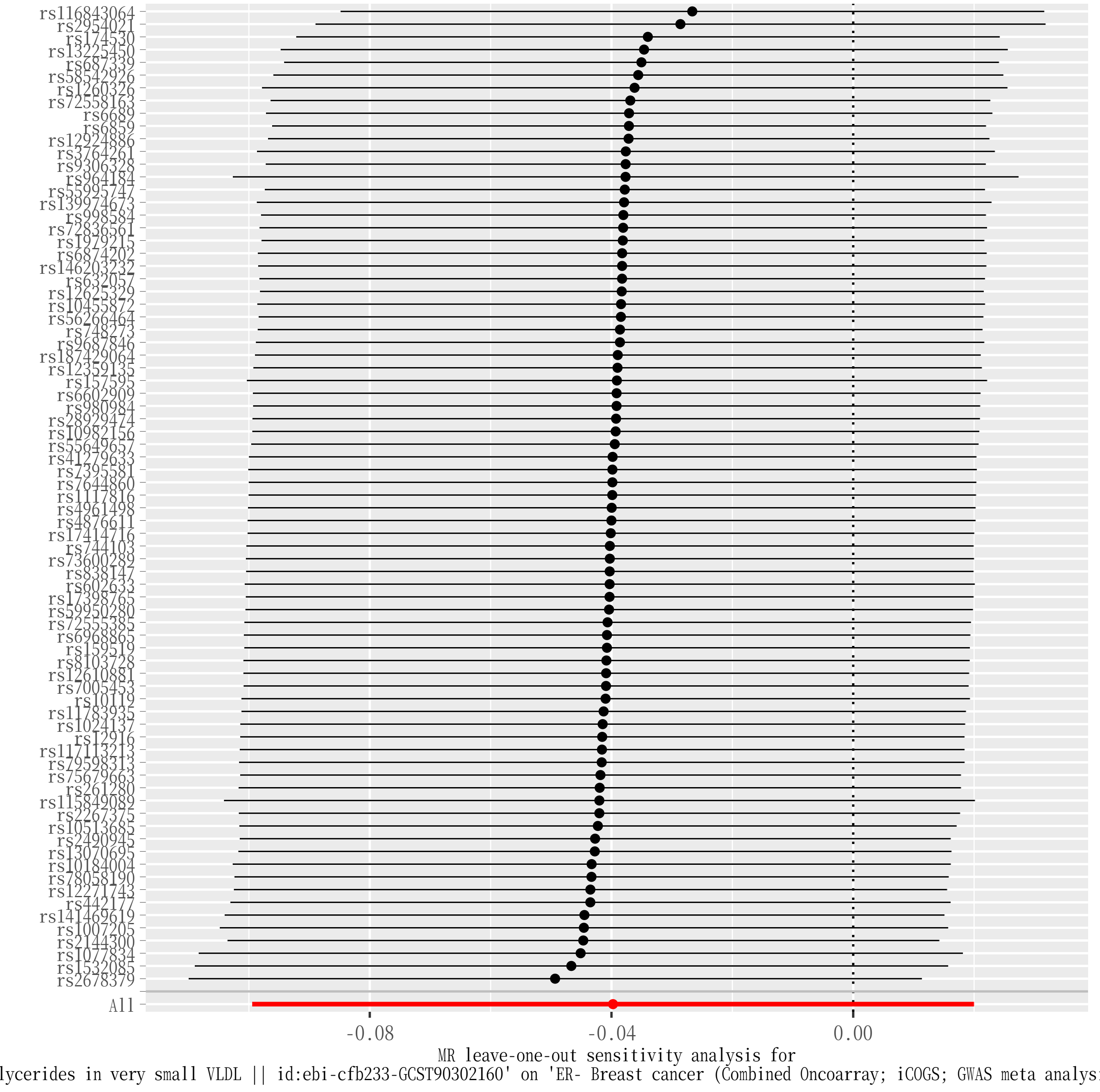
rs116843064
rs2954021
rs174530
rs13225450
rs687339
rs58542926
rs1260326
rs72558163
rs6689
rs6859
rs12924886
rs3764261
rs9306328
rs964184
rs55995747
rs139974673
rs998584
rs72836561
rs1979215
rs6874202
rs146203232
rs632057
rs12625329
rs10455872
rs56266464
rs748273
rs9687846
rs187429064
rs12359135
rs157595
rs6602909
rs980984
rs28929474
rs10982156
rs55649657
rs41279633
rs7395581
rs7644860
rs1117816
rs4961498
rs4876611
rs17414716
rs744103
rs73600289
rs838147
rs602633
rs17398765
rs59950280
rs72555385
rs6968865
rs159519
rs8103728
rs12610881
rs7005453
rs10119
rs11783935
rs1024137
rs12916
rs117113213
rs79598313
rs75679663
rs261280
rs115849089
rs2267375
rs10513685
rs2490945
rs13070695
rs10184004
rs78058190
rs12271743
rs442177
rs141469619
rs1007205
rs2144300
rs1077834
rs1532085
rs2678379
All
-0.08
-0.04
0.00
MR leave-one-out sensitivity analysis for
lycerides in very small VLDL || id:ebi-cfb233-GCST90302160' on 'ER- Breast cancer (Combined Oncoarray; iCOGS; GWAS meta analys

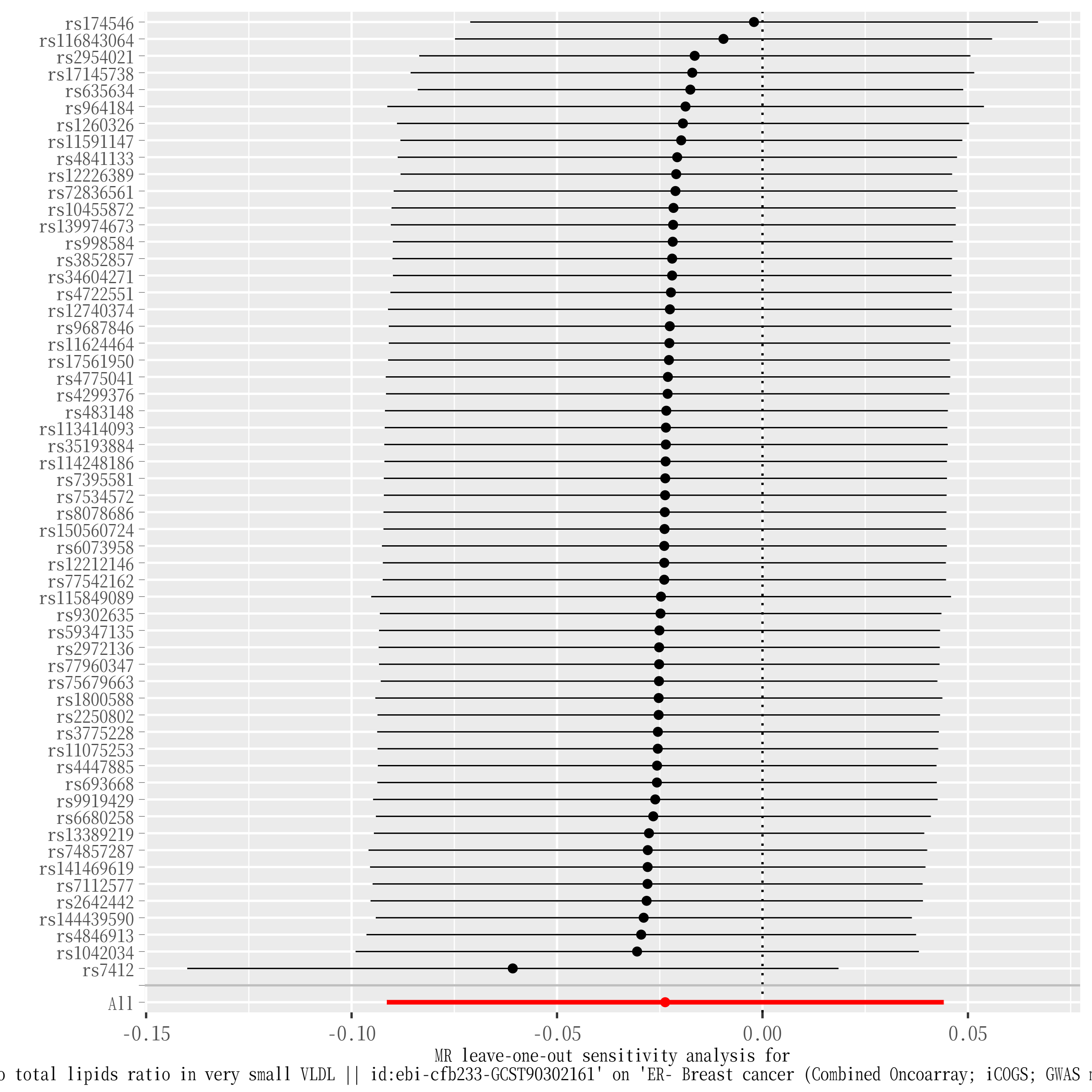

rs174546
rs116843064
rs2954021
rs17145738
rs635634
rs964184
rs1260326
rs11591147
rs4841133
rs12226389
rs72836561
rs10455872
rs139974673
rs998584
rs3852857
rs34604271
rs4722551
rs12740374
rs9687846
rs11624464
rs17561950
rs4775041
rs4299376
rs483148
rs113414093
rs35193884
rs114248186
rs7395581
rs7534572
rs8078686
rs150560724
rs6073958
rs12212146
rs77542162
rs115849089
rs9302635
rs59347135
rs2972136
rs77960347
rs75679663
rs1800588
rs2250802
rs3775228
rs11075253
rs4447885
rs693668
rs9919429
rs6680258
rs13389219
rs74857287
rs141469619
rs7112577
rs2642442
rs144439590
rs4846913
rs1042034
rs7412
All
-0.15
-0.10
-0.05
0.00
0.05
MR leave-one-out sensitivity analysis for
o total lipids ratio in very small VLDL || id:ebi-cfb233-GCST90302161' on 'ER- Breast cancer (Combined Oncoarray; iCOGS; GWAS m

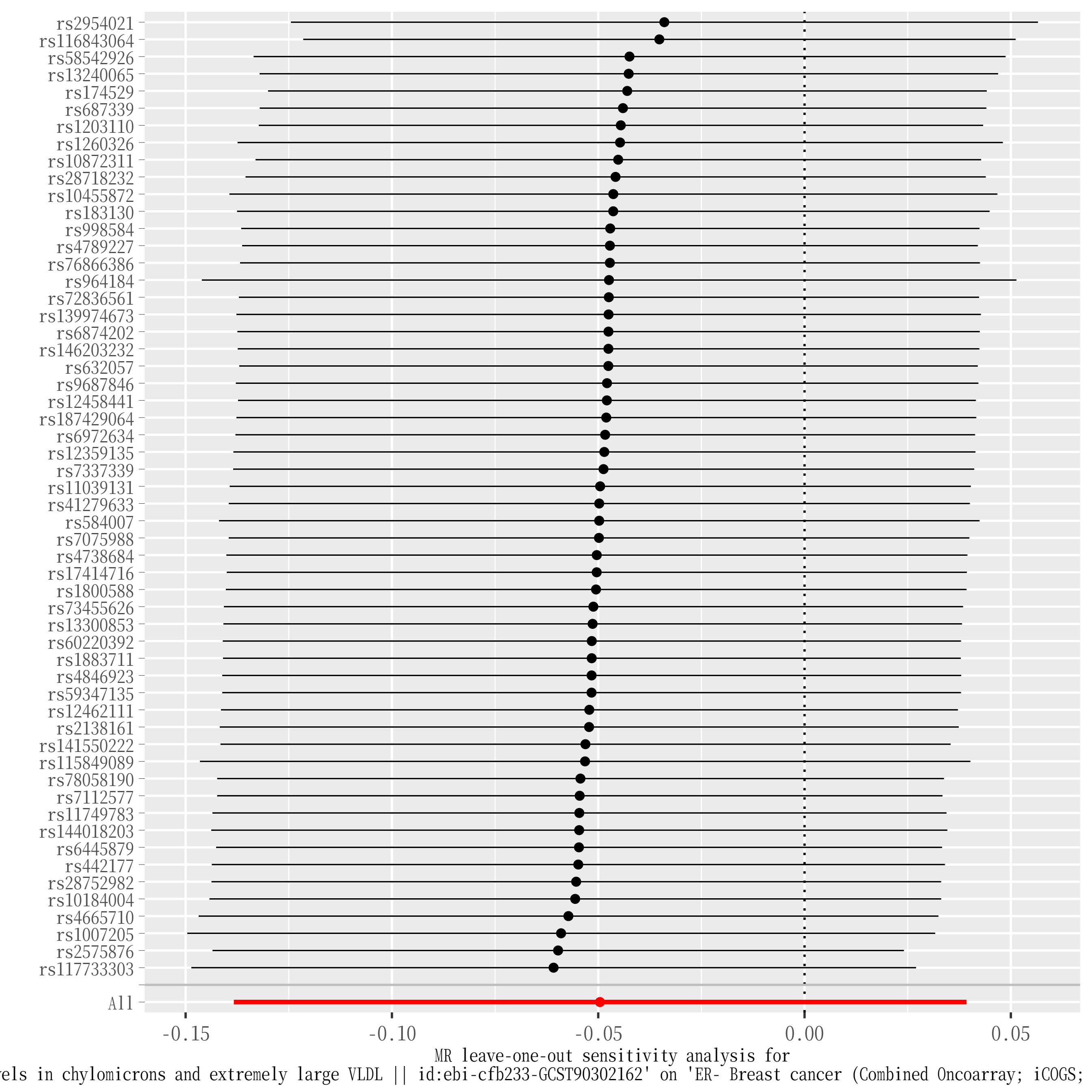
rs2954021
rs116843064
rs58542926
rs13240065
rs174529
rs687339
rs1203110
rs1260326
rs10872311
rs28718232
rs10455872
rs183130
rs998584
rs4789227
rs76866386
rs964184
rs72836561
rs139974673
rs6874202
rs146203232
rs632057
rs9687846
rs12458441
rs187429064
rs6972634
rs12359135
rs7337339
rs11039131
rs41279633
rs584007
rs7075988
rs4738684
rs17414716
rs1800588
rs73455626
rs13300853
rs60220392
rs1883711
rs4846923
rs59347135
rs12462111
rs2138161
rs141550222
rs115849089
rs78058190
rs7112577
rs11749783
rs144018203
rs6445879
rs442177
rs28752982
rs10184004
rs4665710
rs1007205
rs2575876
rs117733303
All
-0.15
-0.10
-0.05
0.00
0.05
MR leave-one-out sensitivity analysis for
rels in chylomicrons and extremely large VLDL || id:ebi-cfb233-GCST90302162' on 'ER- Breast cancer (Combined Oncoarray; iCOGS;

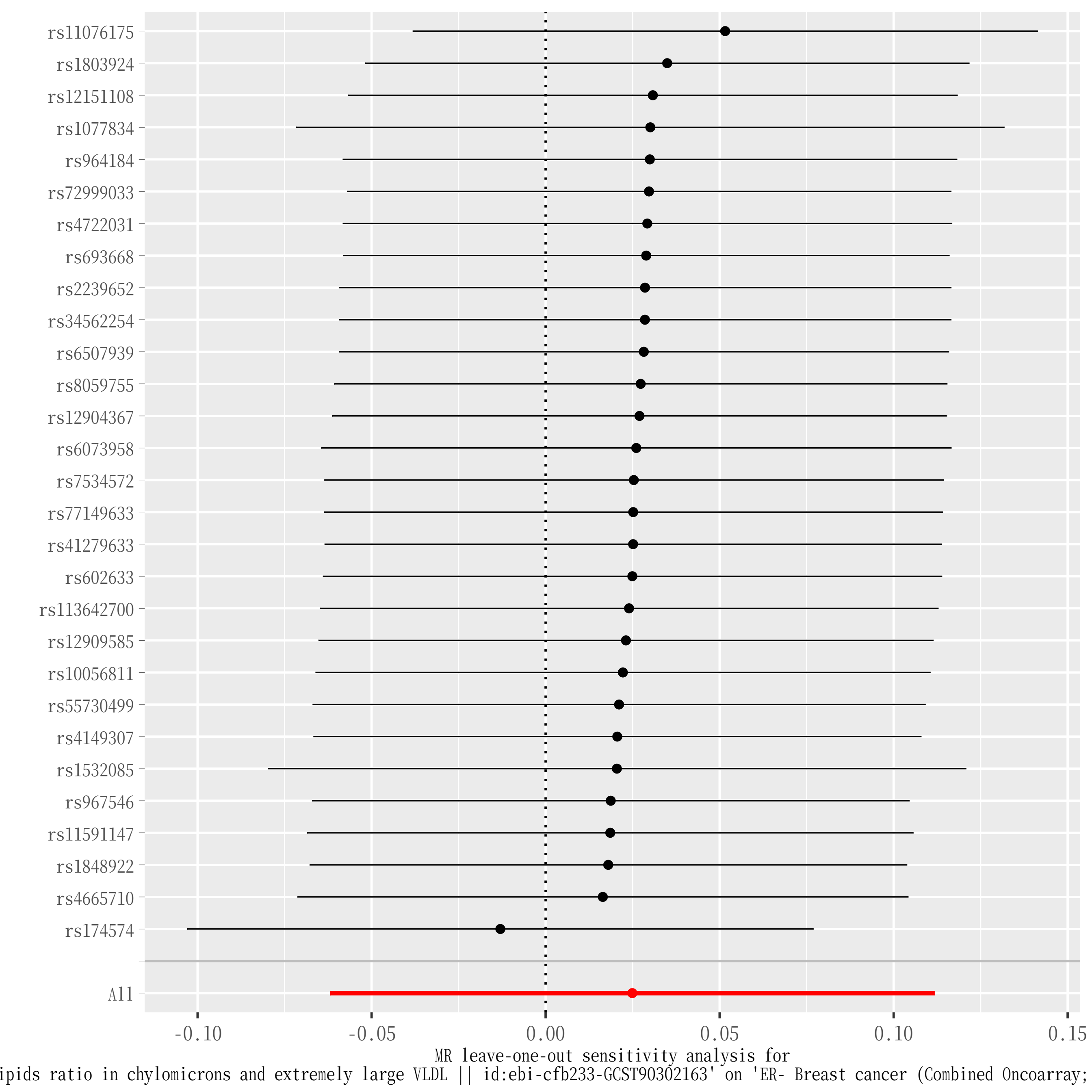
rs11076175
rs1803924
rs12151108
rs1077834
rs964184
rs72999033
rs4722031
rs693668
rs2239652
rs34562254
rs6507939
rs8059755
rs12904367
rs6073958
rs7534572
rs77149633
rs41279633
rs602633
rs113642700
rs12909585
rs10056811
rs55730499
rs4149307
rs1532085
rs967546
rs11591147
rs1848922
rs4665710
rs174574
All
-0.10
-0.05
0.00
0.05
0.10
0.15
MR leave-one-out sensitivity analysis for
ipids ratio in chylomicrons and extremely large VLDL || id:ebi-cfb233-GCST90302163' on 'ER- Breast cancer (Combined Oncoarray;

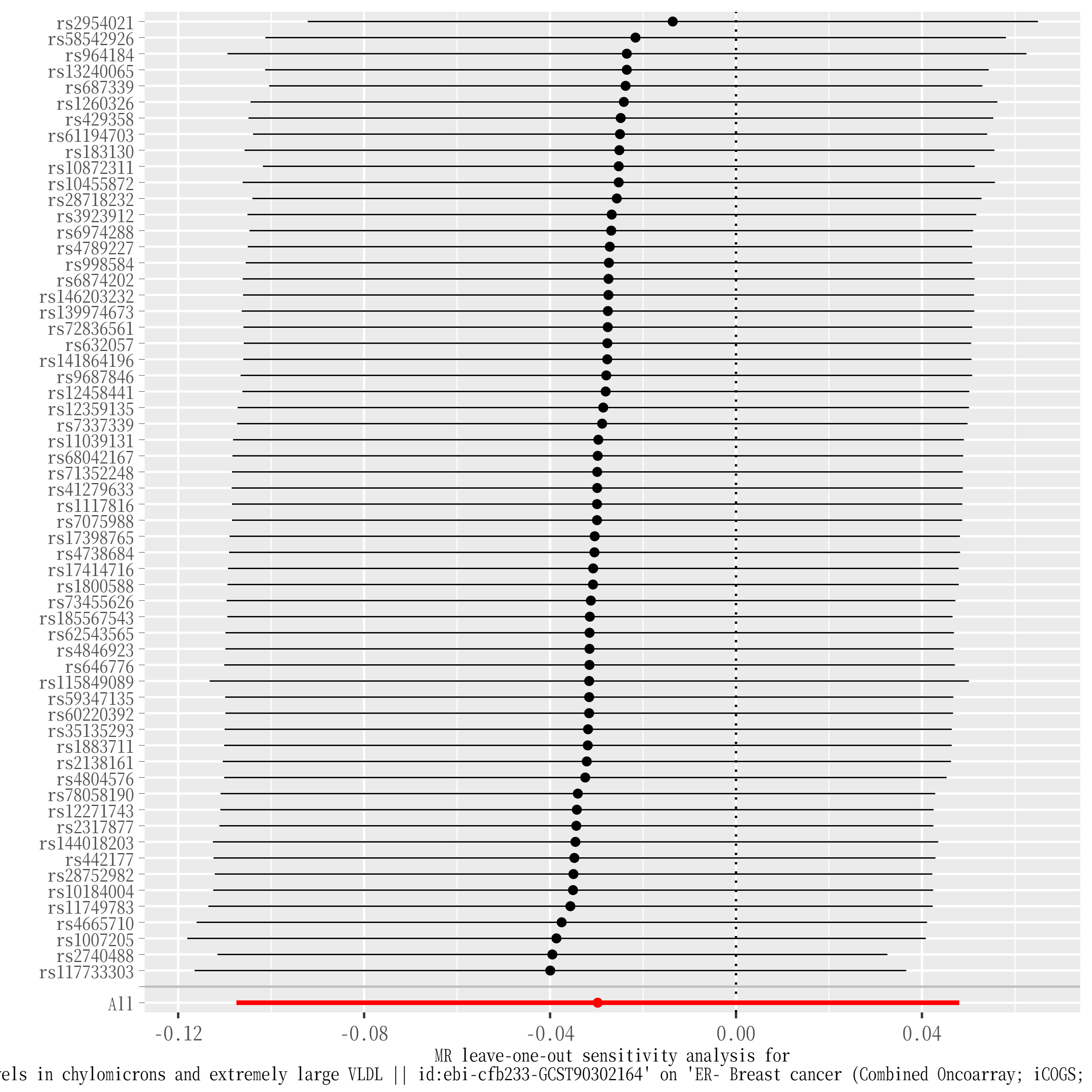
rs2954021
rs58542926
rs964184
rs13240065
rs687339
rs1260326
rs429358
rs61194703
rs183130
rs10872311
rs10455872
rs28718232
rs3923912
rs6974288
rs4789227
rs998584
rs6874202
rs146203232
rs139974673
rs72836561
rs632057
rs141864196
rs9687846
rs12458441
rs12359135
rs7337339
rs11039131
rs68042167
rs71352248
rs41279633
rs1117816
rs7075988
rs17398765
rs4738684
rs17414716
rs1800588
rs73455626
rs185567543
rs62543565
rs4846923
rs646776
rs115849089
rs59347135
rs60220392
rs35135293
rs1883711
rs2138161
rs4804576
rs78058190
rs12271743
rs2317877
rs144018203
rs442177
rs28752982
rs10184004
rs11749783
rs4665710
rs1007205
rs2740488
rs117733303
All
-0.12
-0.08
-0.04
0.00
0.04
MR leave-one-out sensitivity analysis for
rels in chylomicrons and extremely large VLDL || id:ebi-cfb233-GCST90302164' on 'ER- Breast cancer (Combined Oncoarray; iCOGS;

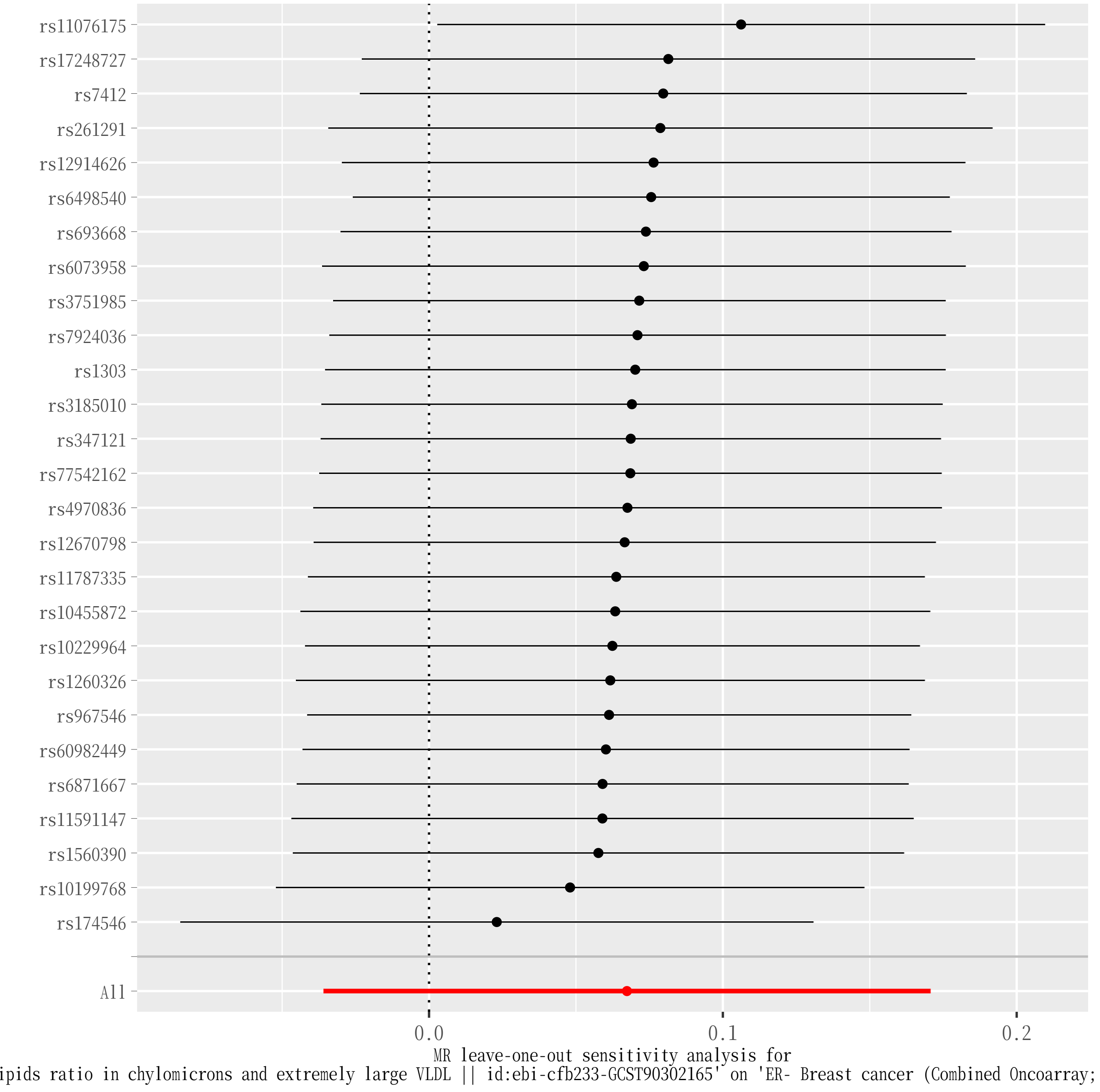
rs11076175
rs17248727
rs7412
rs261291
rs12914626
rs6498540
rs693668
rs6073958
rs3751985
rs7924036
rs1303
rs3185010
rs347121
rs77542162
rs4970836
rs12670798
rs11787335
rs10455872
rs10229964
rs1260326
rs967546
rs60982449
rs6871667
rs11591147
rs1560390
rs10199768
rs174546
All
0.0
0.1
0.2
MR leave-one-out sensitivity analysis for
ipids ratio in chylomicrons and extremely large VLDL || id:ebi-cfb233-GCST90302165' on 'ER- Breast cancer (Combined Oncoarray;

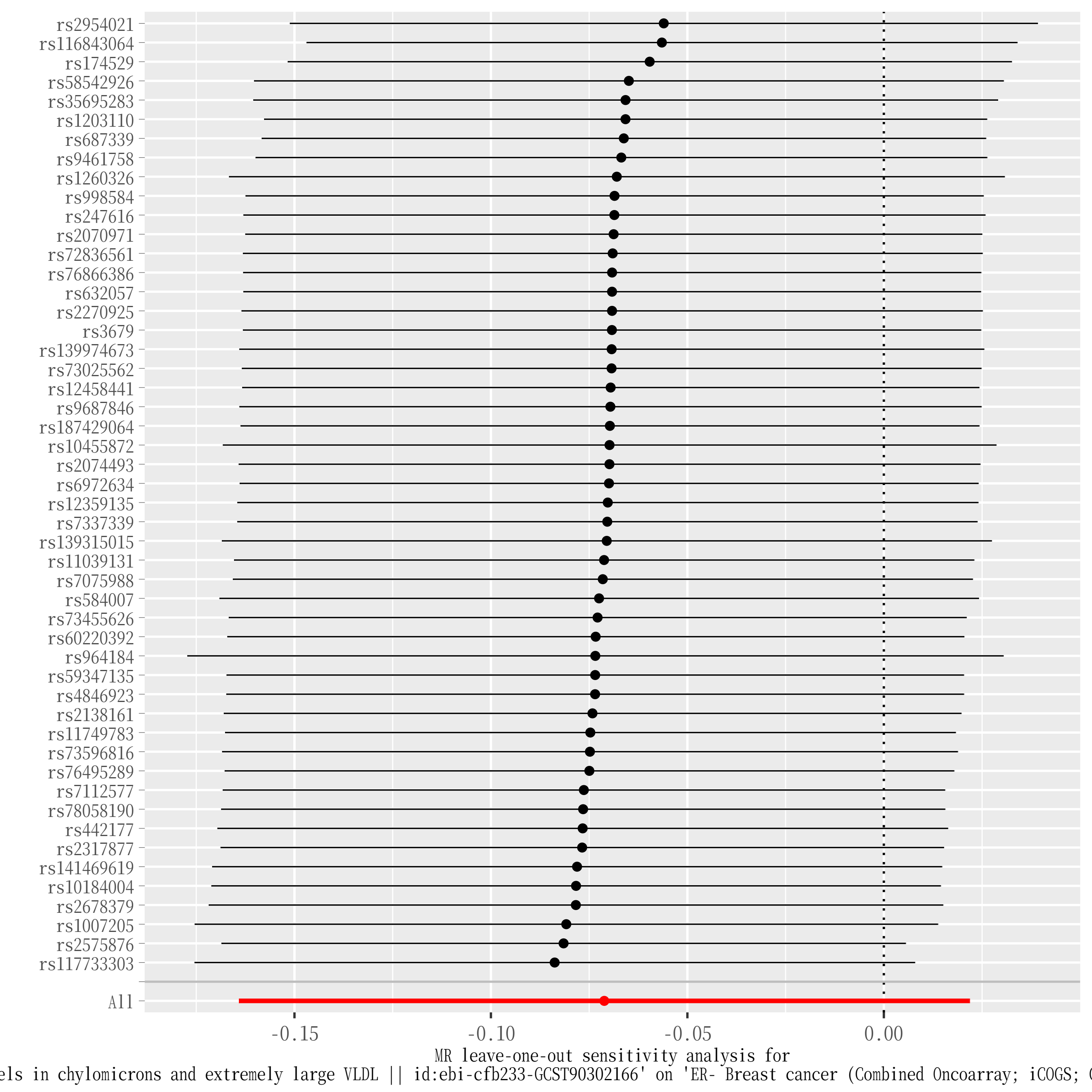
rs2954021
rs116843064
rs174529
rs58542926
rs35695283
rs1203110
rs687339
rs9461758
rs1260326
rs998584
rs247616
rs2070971
rs72836561
rs76866386
rs632057
rs2270925
rs3679
rs139974673
rs73025562
rs12458441
rs9687846
rs187429064
rs10455872
rs2074493
rs6972634
rs12359135
rs7337339
rs139315015
rs11039131
rs7075988
rs584007
rs73455626
rs60220392
rs964184
rs59347135
rs4846923
rs2138161
rs11749783
rs73596816
rs76495289
rs7112577
rs78058190
rs442177
rs2317877
rs141469619
rs10184004
rs2678379
rs1007205
rs2575876
rs117733303
All
-0.15
-0.10
-0.05
0.00
MR leave-one-out sensitivity analysis for
els in chylomicrons and extremely large VLDL || id:ebi-cfb233-GCST90302166' on 'ER- Breast cancer (Combined Oncoarray; iCOGS; (

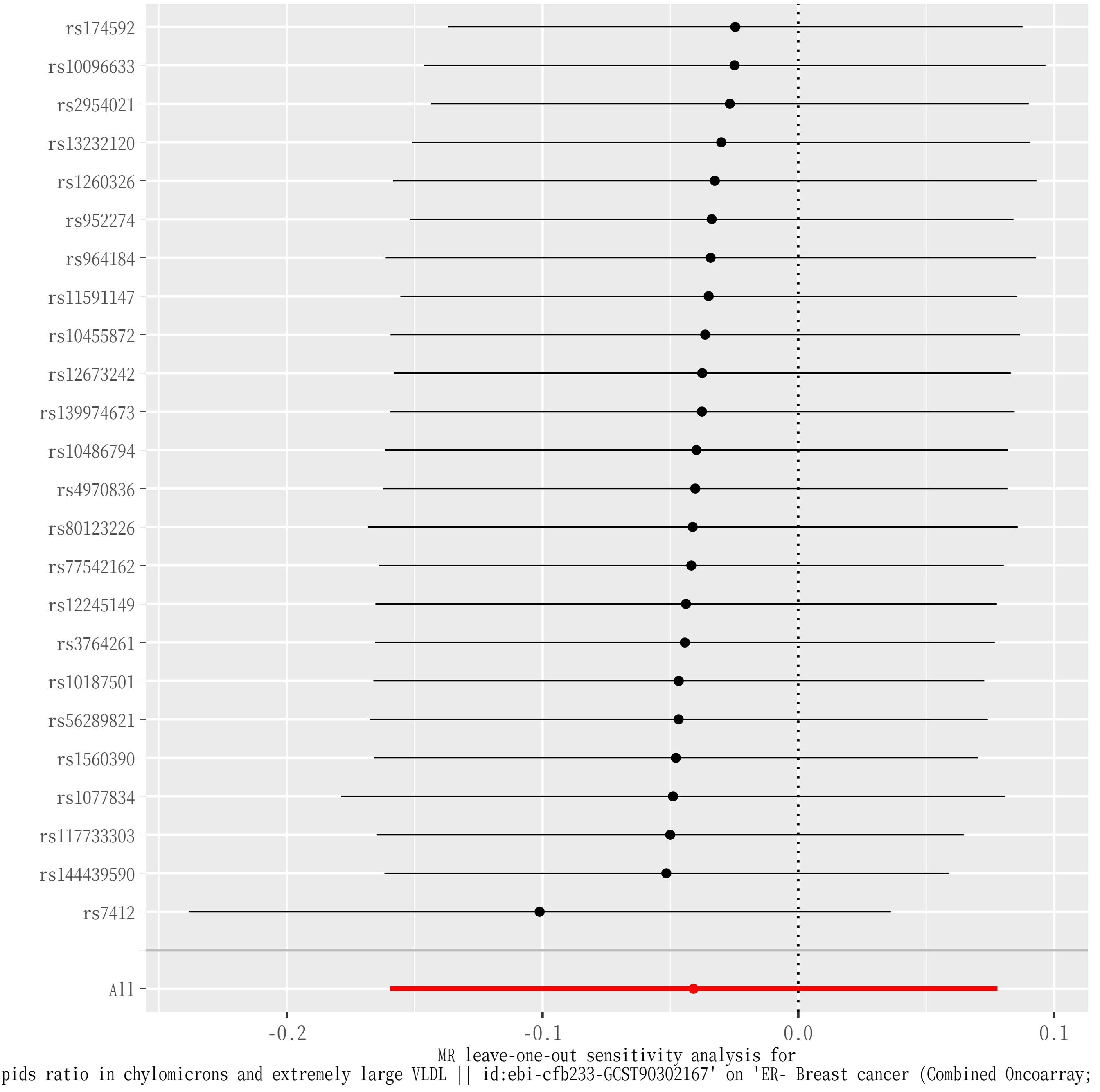

rs174592
rs10096633
rs2954021
rs13232120
rs1260326
rs952274
rs964184
rs11591147
rs10455872
rs12673242
rs139974673
rs10486794
rs4970836
rs80123226
rs77542162
rs12245149
rs3764261
rs10187501
rs56289821
rs1560390
rs1077834
rs117733303
rs144439590
rs7412
All
-0.2
-0.1
0.0
0.1
MR leave-one-out sensitivity analysis for
pids ratio in chylomicrons and extremely large VLDL || id:ebi-cfb233-GCST90302167' on 'ER- Breast cancer (Combined Oncoarray;

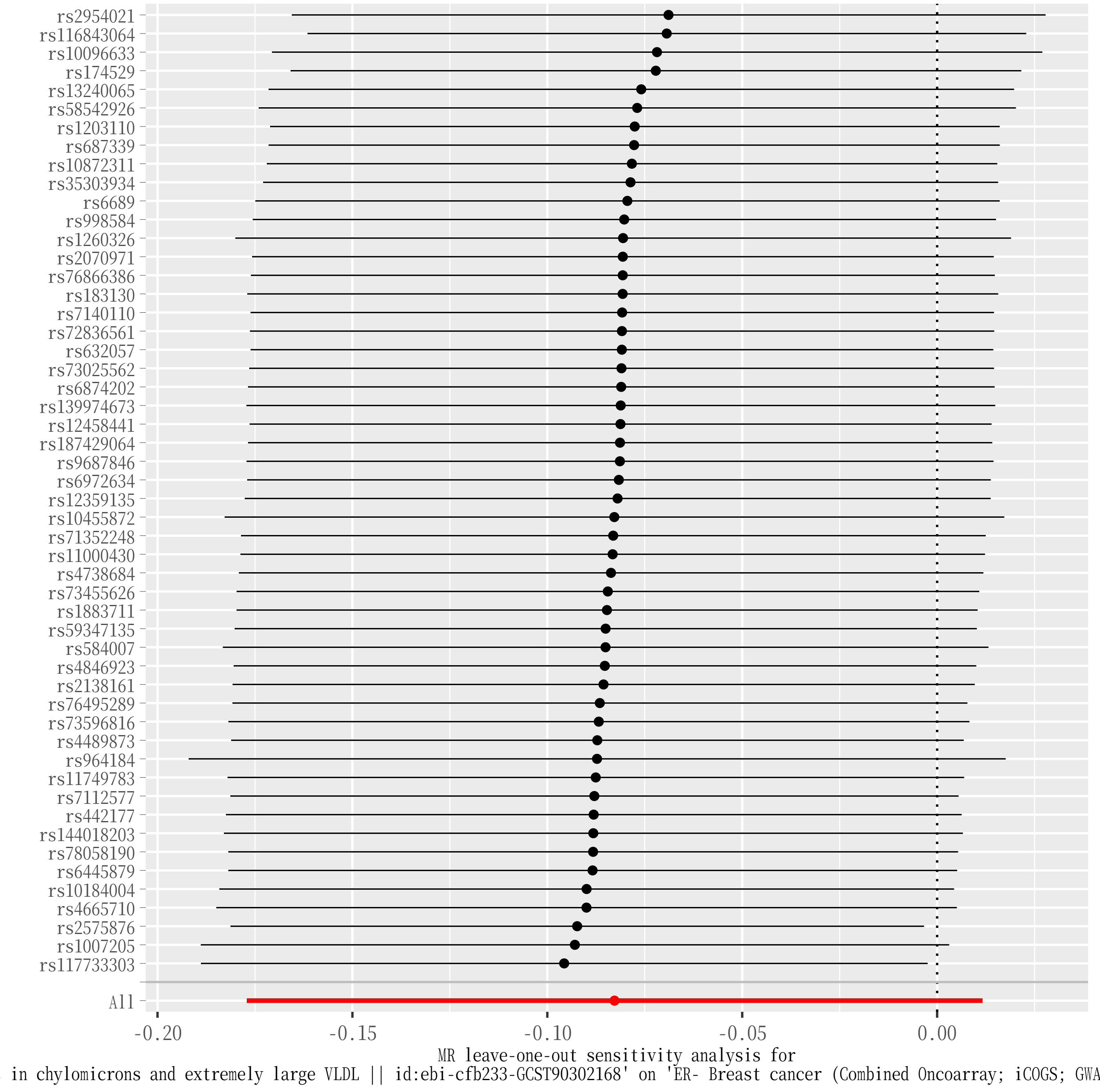

rs2954021
rs116843064
rs10096633
rs174529
rs13240065
rs58542926
rs1203110
rs687339
rs10872311
rs35303934
rs6689
rs998584
rs1260326
rs2070971
rs76866386
rs183130
rs7140110
rs72836561
rs632057
rs73025562
rs6874202
rs139974673
rs12458441
rs187429064
rs9687846
rs6972634
rs12359135
rs10455872
rs71352248
rs11000430
rs4738684
rs73455626
rs1883711
rs59347135
rs584007
rs4846923
rs2138161
rs76495289
rs73596816
rs4489873
rs964184
rs11749783
rs7112577
rs442177
rs144018203
rs78058190
rs6445879
rs10184004
rs4665710
rs2575876
rs1007205
rs117733303
All
-0.20
-0.15
-0.10
-0.05
0.00
MR leave-one-out sensitivity analysis for
in chylomicrons and extremely large VLDL || id:ebi-cfb233-GCST90302168' on 'ER- Breast cancer (Combined Oncoarray; iCOGS; GWA

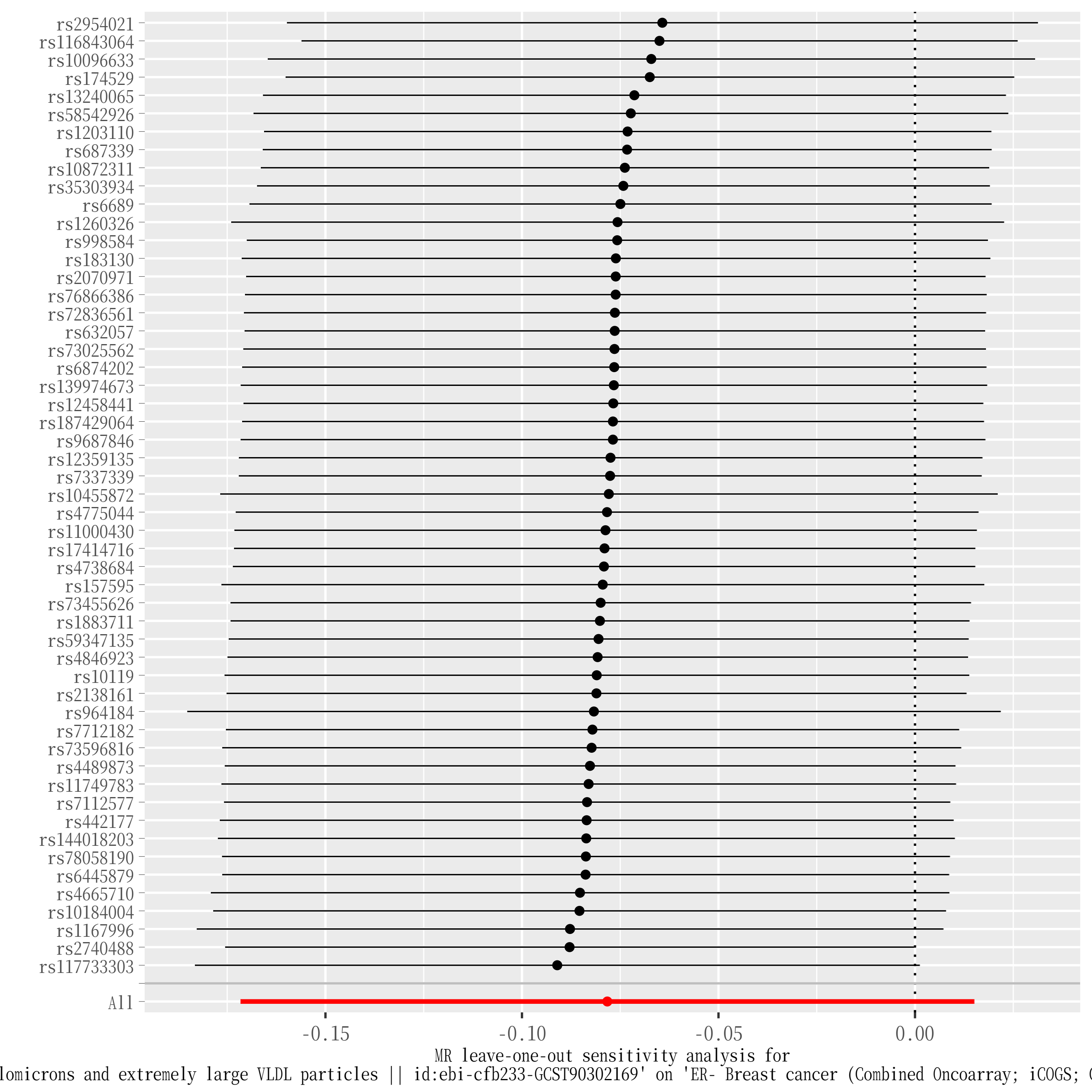

rs2954021
rs116843064
rs10096633
rs174529
rs13240065
rs58542926
rs1203110
rs687339
rs10872311
rs35303934
rs6689
rs1260326
rs998584
rs183130
rs2070971
rs76866386
rs72836561
rs632057
rs73025562
rs6874202
rs139974673
rs12458441
rs187429064
rs9687846
rs12359135
rs7337339
rs10455872
rs4775044
rs11000430
rs17414716
rs4738684
rs157595
rs73455626
rs1883711
rs59347135
rs4846923
rs10119
rs2138161
rs964184
rs7712182
rs73596816
rs4489873
rs11749783
rs7112577
rs442177
rs144018203
rs78058190
rs6445879
rs4665710
rs10184004
rs1167996
rs2740488
rs117733303
All
-0.15
-0.10
-0.05
0.00
MR leave-one-out sensitivity analysis for
lomicrons and extremely large VLDL particles || id:ebi-cfb233-GCST90302169' on 'ER- Breast cancer (Combined Oncoarray; iCOGS; (

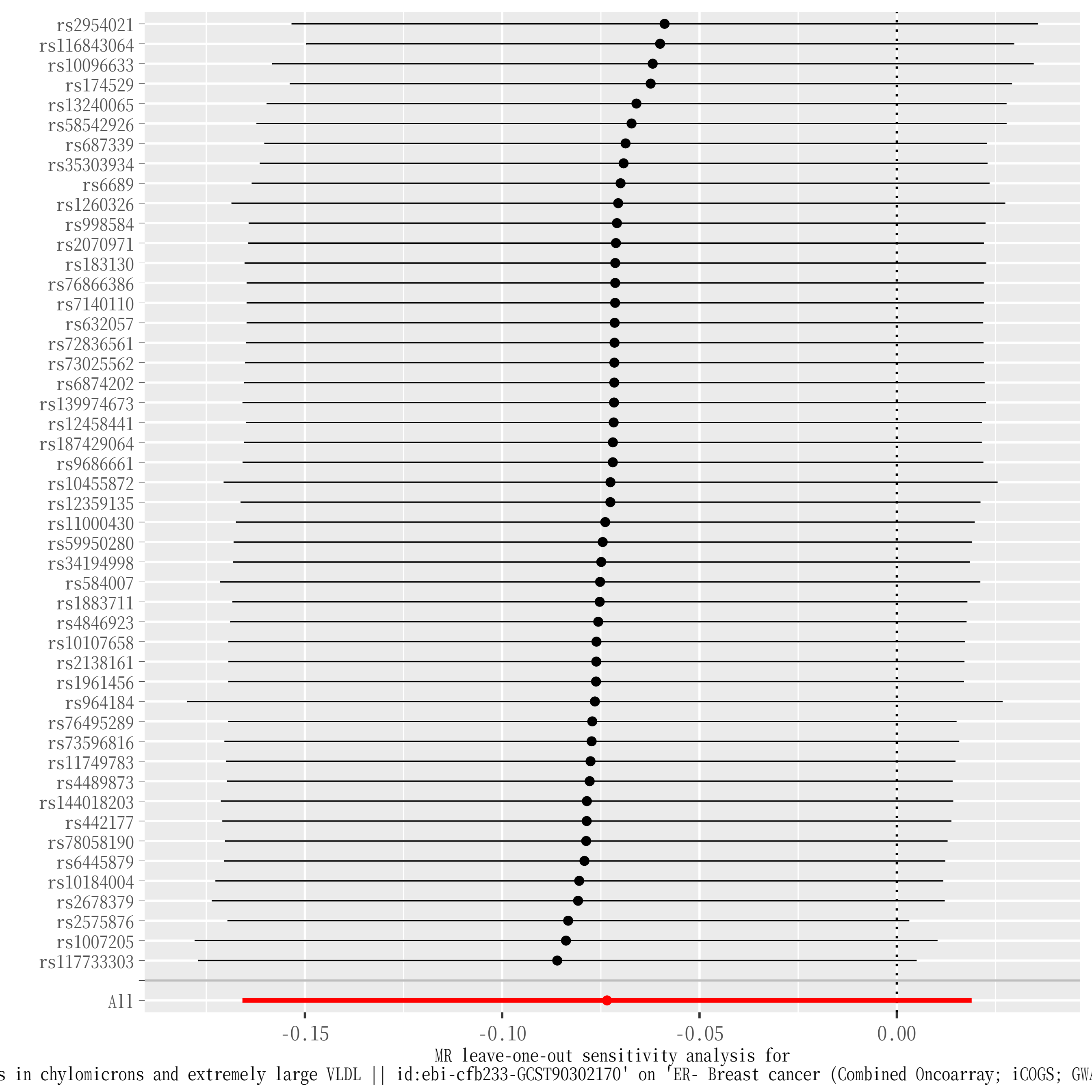

rs2954021
rs116843064
rs10096633
rs174529
rs13240065
rs58542926
rs687339
rs35303934
rs6689
rs1260326
rs998584
rs2070971
rs183130
rs76866386
rs7140110
rs632057
rs72836561
rs73025562
rs6874202
rs139974673
rs12458441
rs187429064
rs9686661
rs10455872
rs12359135
rs11000430
rs59950280
rs34194998
rs584007
rs1883711
rs4846923
rs10107658
rs2138161
rs1961456
rs964184
rs76495289
rs73596816
rs11749783
rs4489873
rs144018203
rs442177
rs78058190
rs6445879
rs10184004
rs2678379
rs2575876
rs1007205
rs117733303
All
-0.15
-0.10
-0.05
0.00
MR leave-one-out sensitivity analysis for
s in chylomicrons and extremely large VLDL || id:ebi-cfb233-GCST90302170' on 'ER- Breast cancer (Combined Oncoarray; iCOGS; GWA

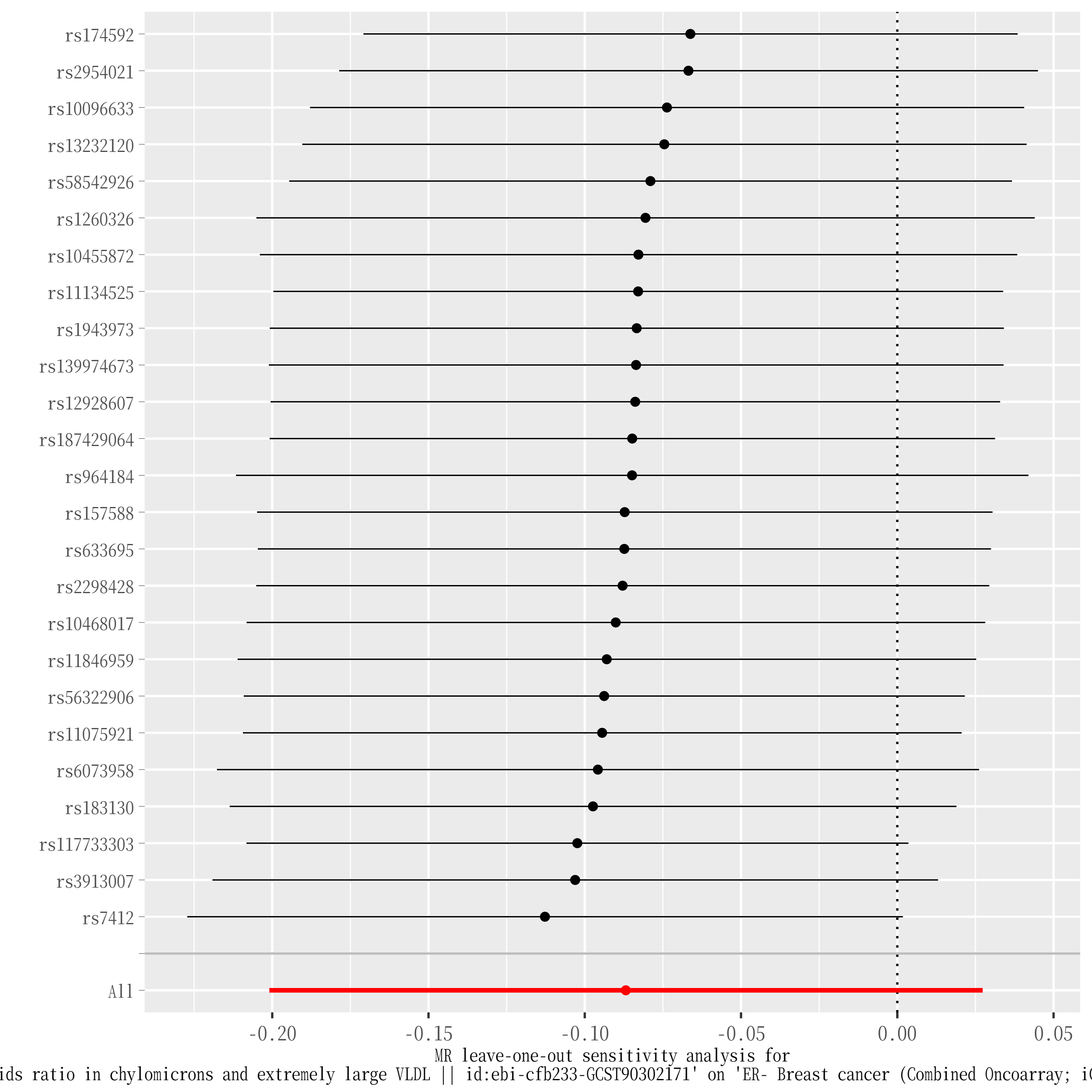
rs174592
rs2954021
rs10096633
rs13232120
rs58542926
rs1260326
rs10455872
rs11134525
rs1943973
rs139974673
rs12928607
rs187429064
rs964184
rs157588
rs633695
rs2298428
rs10468017
rs11846959
rs56322906
rs11075921
rs6073958
rs183130
rs117733303
rs3913007
rs7412
All
-0.20
-0.15
-0.10
-0.05
0.00
0.05
MR leave-one-out sensitivity analysis for
ids ratio in chylomicrons and extremely large VLDL || id:ebi-cfb233-GCST90302171' on 'ER- Breast cancer (Combined Oncoarray; i

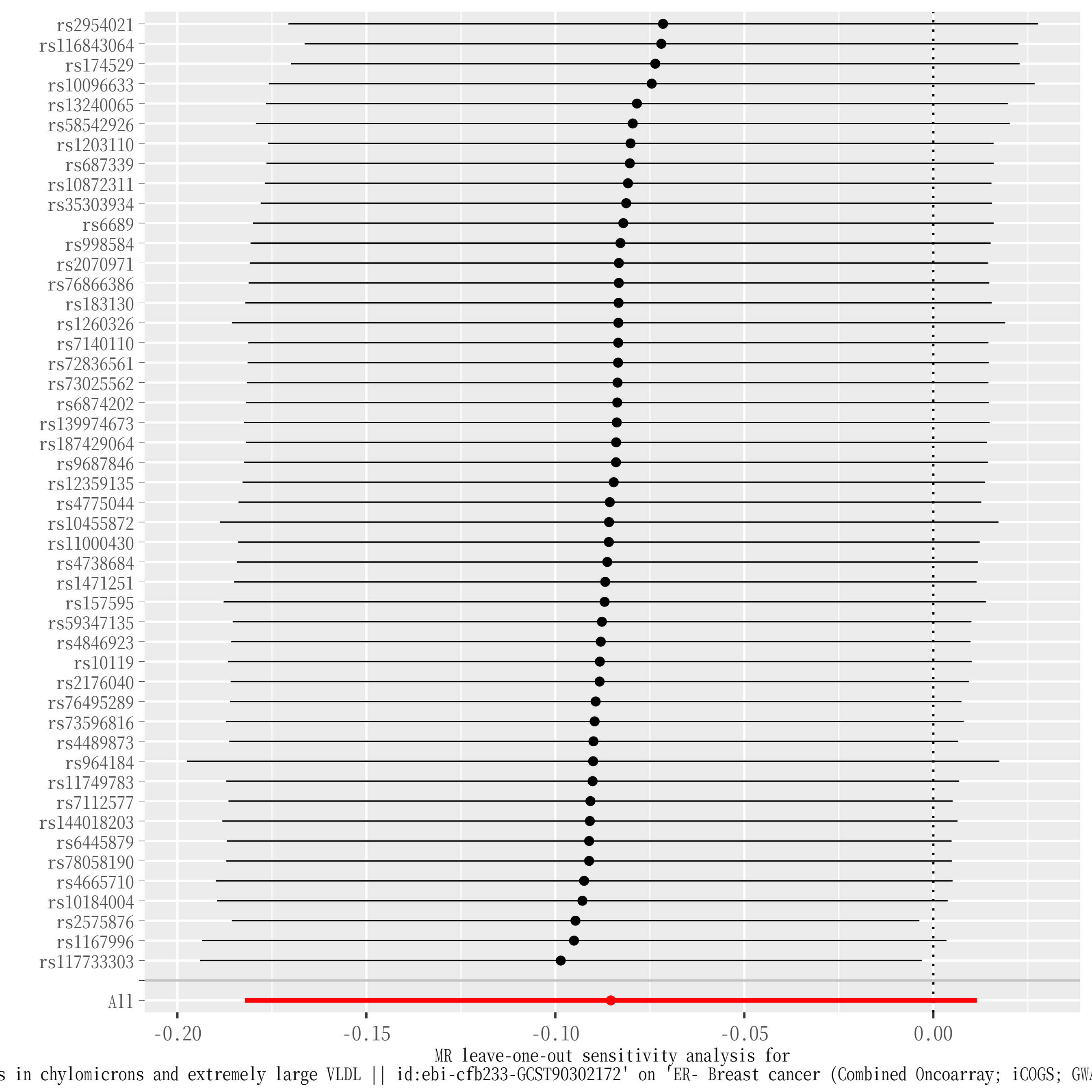
rs2954021
rs116843064
rs174529
rs10096633
rs13240065
rs58542926
rs1203110
rs687339
rs10872311
rs35303934
rs6689
rs998584
rs2070971
rs76866386
rs183130
rs1260326
rs7140110
rs72836561
rs73025562
rs6874202
rs139974673
rs187429064
rs9687846
rs12359135
rs4775044
rs10455872
rs11000430
rs4738684
rs1471251
rs157595
rs59347135
rs4846923
rs10119
rs2176040
rs76495289
rs73596816
rs4489873
rs964184
rs11749783
rs7112577
rs144018203
rs6445879
rs78058190
rs4665710
rs10184004
rs2575876
rs1167996
rs117733303
All
-0.20
-0.15
-0.10
-0.05
0.00
MR leave-one-out sensitivity analysis for
s in chylomicrons and extremely large VLDL || id:ebi-cfb233-GCST90302172' on 'ER- Breast cancer (Combined Oncoarray; iCOGS; GWA

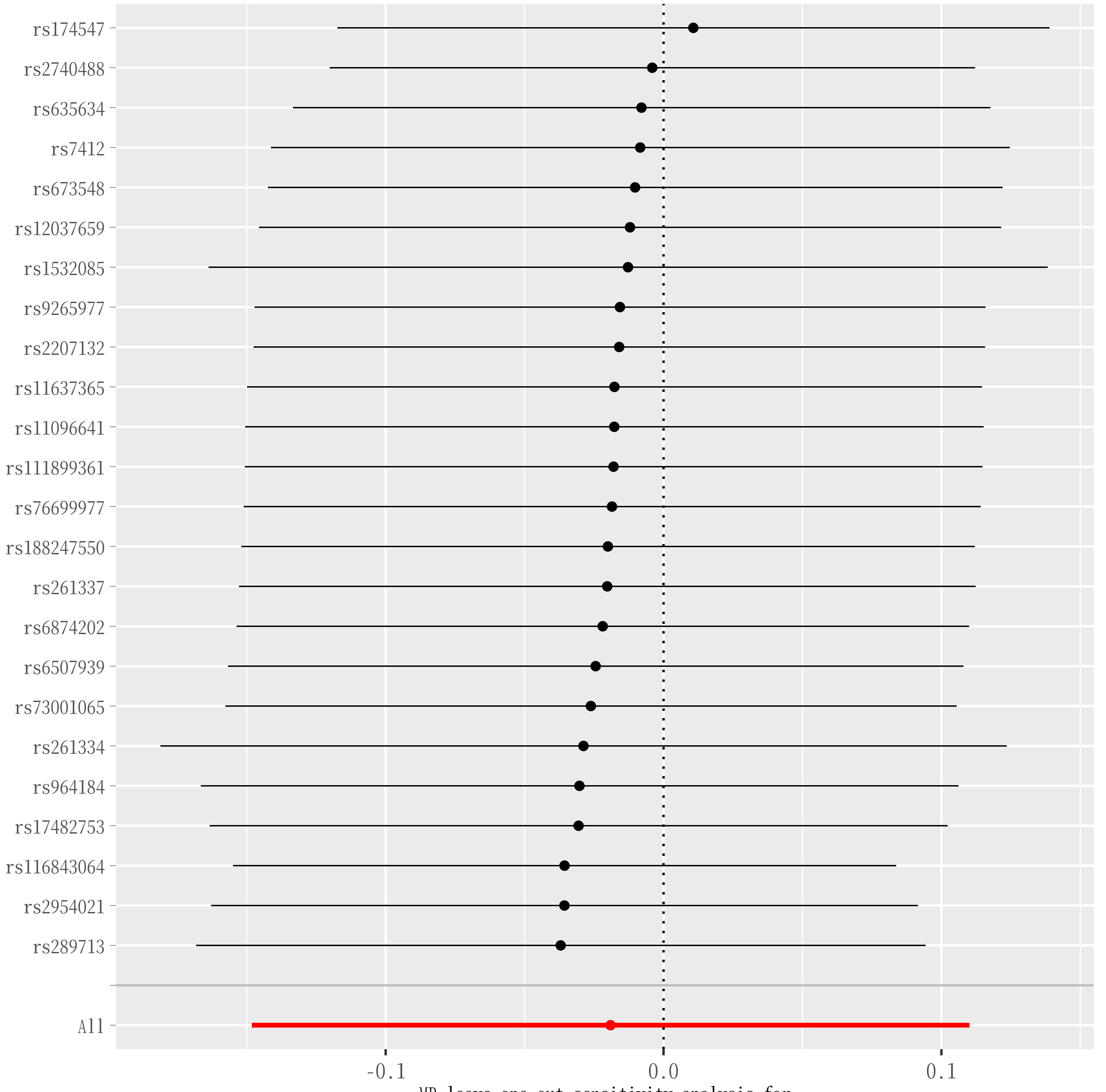

MR leave-one-out sensitivity analysis for
ids ratio in chylomicrons and extremely large VLDL || id:ebi-cfb233-GCST90302173' on 'ER- Breast cancer (Combined Oncoarray; i
