# Supplementary material for: Assessing the causal relationships between circulating metabolic biomarkers and breast cancer by using mendelian randomization
Source: Front Genet. 2024 Dec 18;15:1448748. doi: 10.3389/fgene.2024.1448748 (PMC11688392; doi:10.3389/fgene.2024.1448748)
Supplement: Supplementary file 6 [file DataSheet9.pdf]

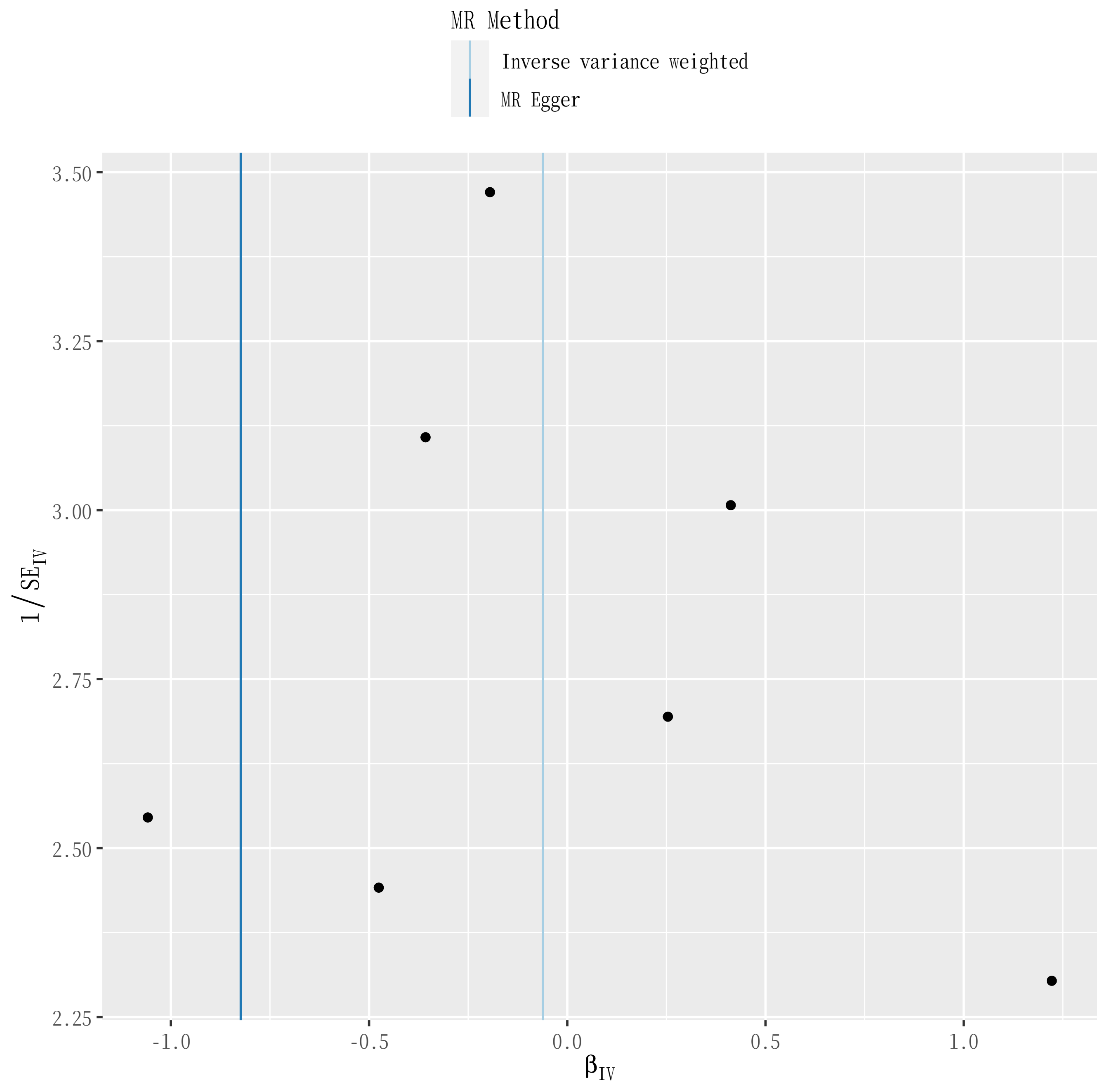

MR Method
Inverse variance weighted
MR Egger
3.50
3.25
3.00
2.75
2.50
2.25
1/SE_IV
-1.0
-0.5
0.0
0.5
1.0
β_IV

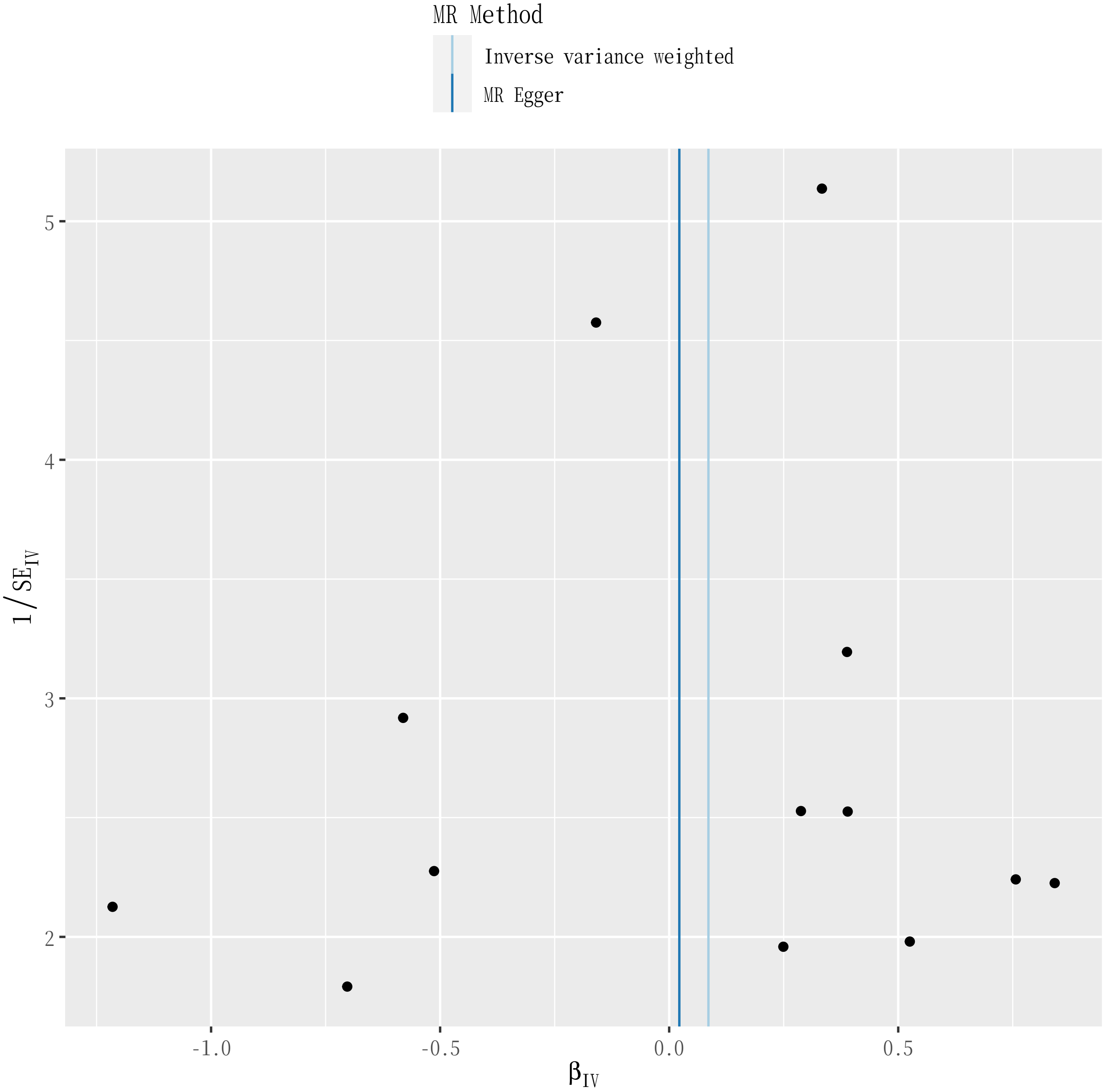
MR Method
Inverse variance weighted
MR Egger
5
4
3
2
-1.0
-0.5
0.0
0.5
1/SE_IV
β_IV

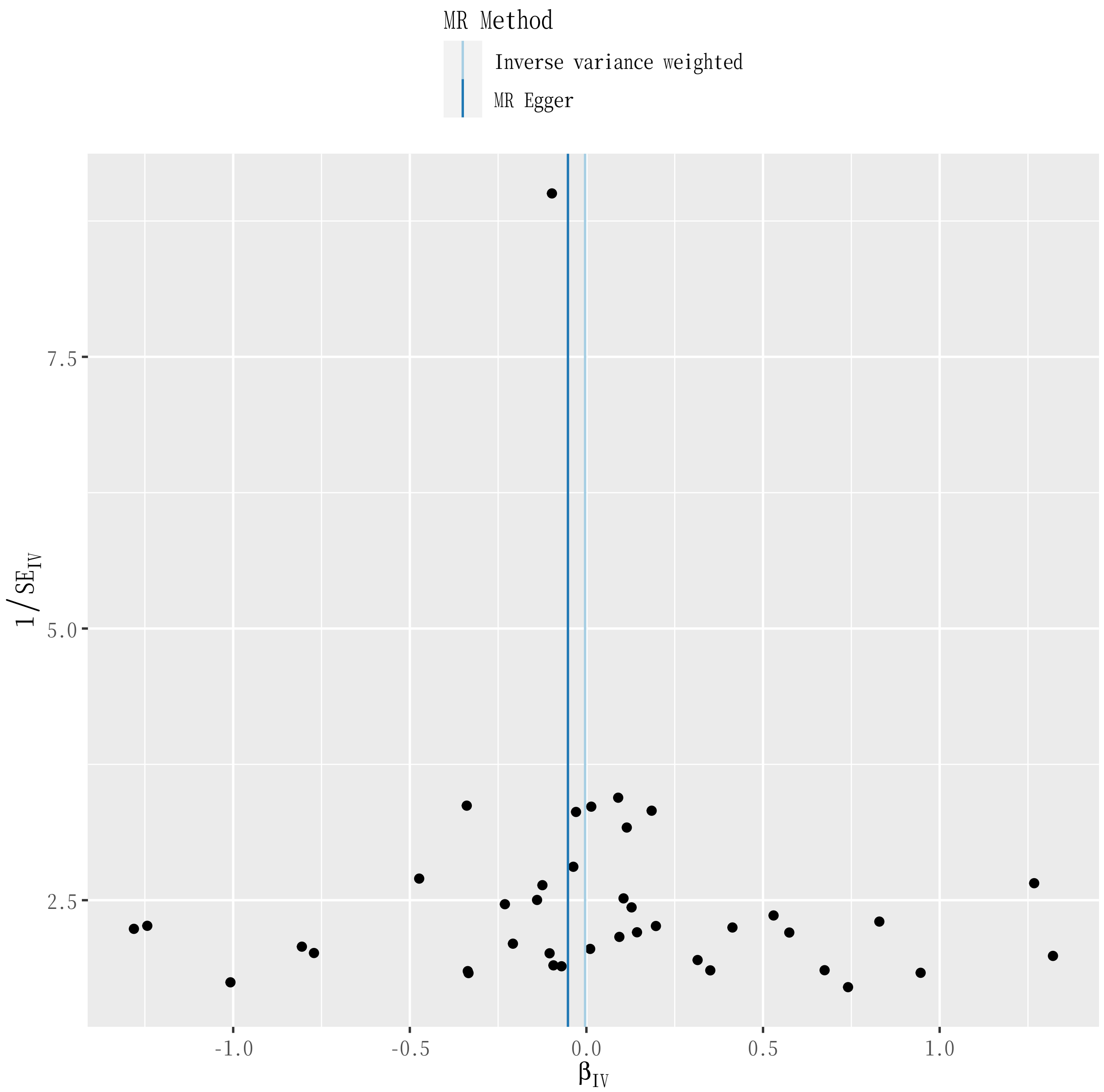

MR Method
Inverse variance weighted
MR Egger
7.5
5.0
2.5
-1.0
-0.5
0.0
0.5
1.0
1/SE_IV
β_IV

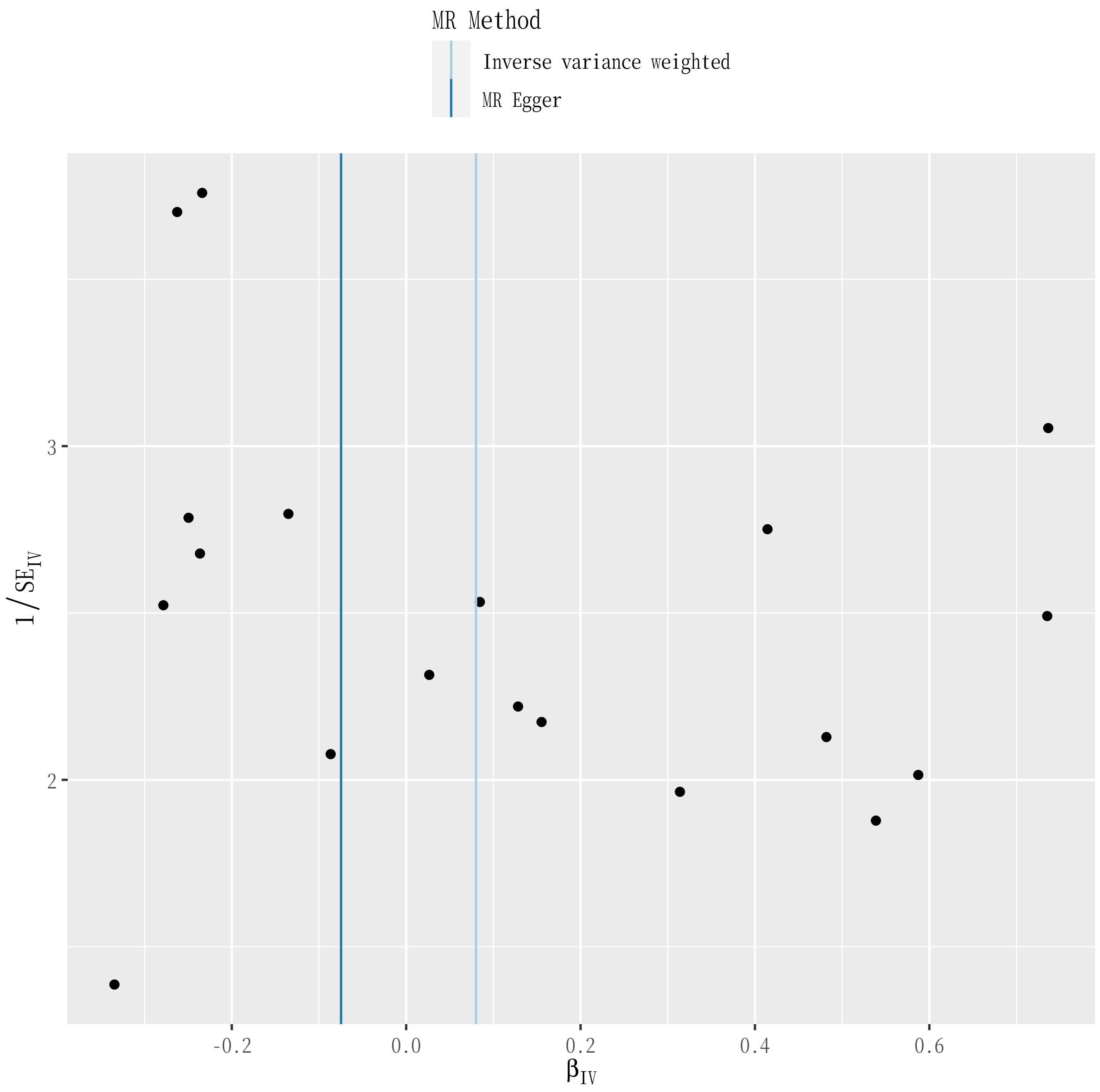

MR Method
Inverse variance weighted
MR Egger
3
2
1/SE_IV
-0.2
0.0
0.2
0.4
0.6
β_IV

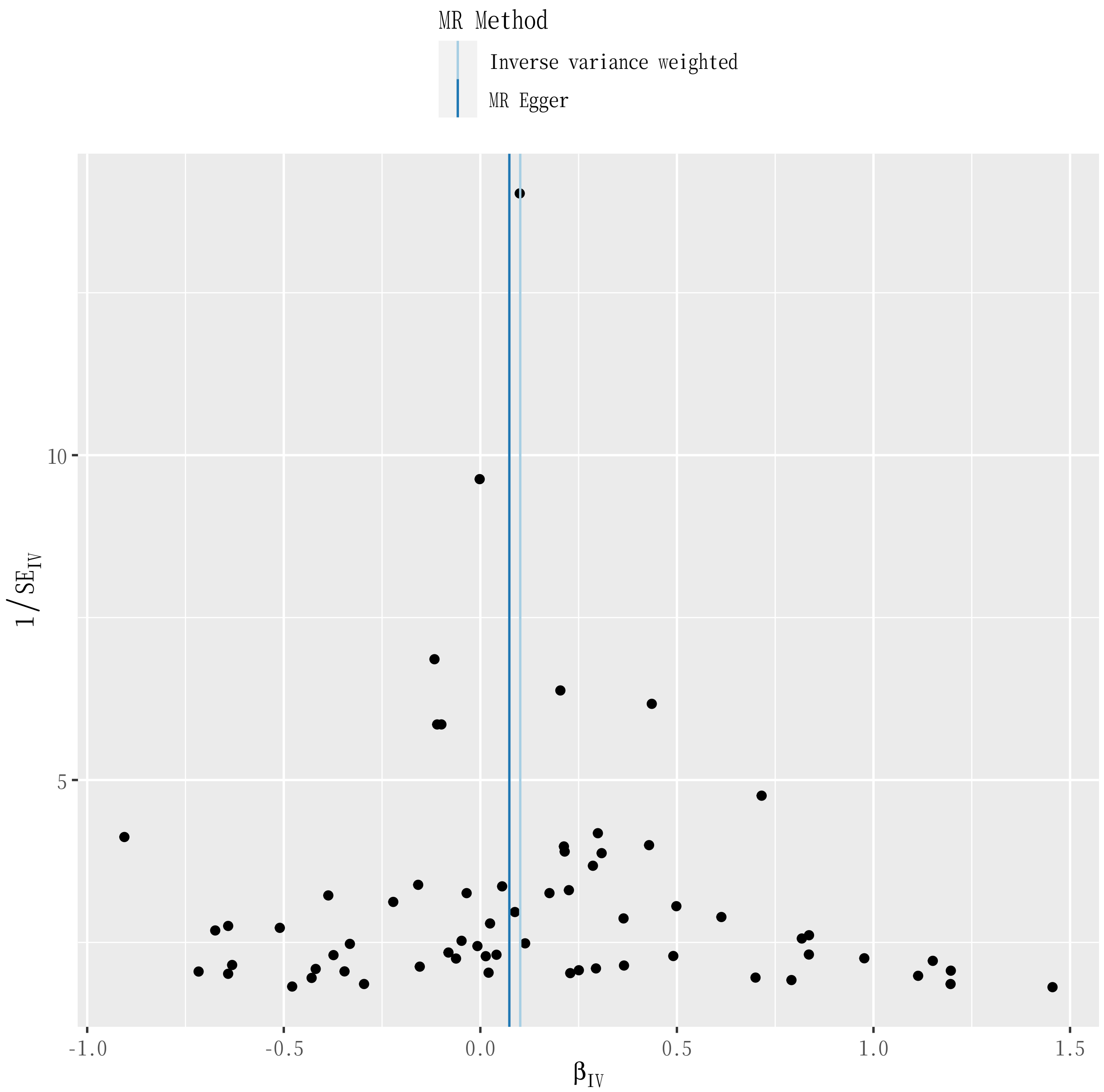
MR Method
Inverse variance weighted
MR Egger
10
5
$1/SE_{IV}$
-1.0
-0.5
0.0
0.5
1.0
1.5
β_{IV}

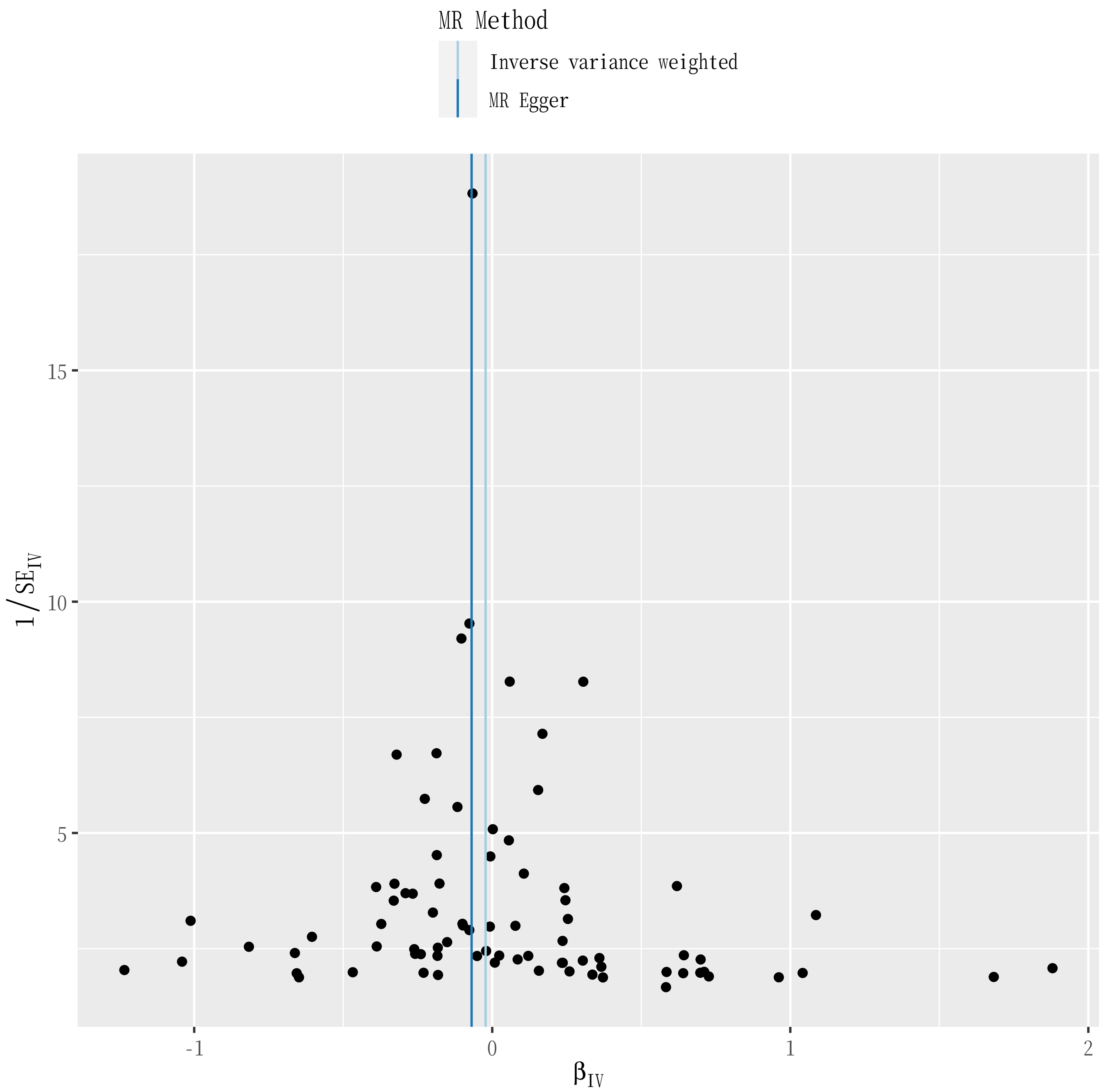
MR Method
Inverse variance weighted
MR Egger
15
10
5
-1
0
1
2
$1/SE_{IV}$
β_{IV}

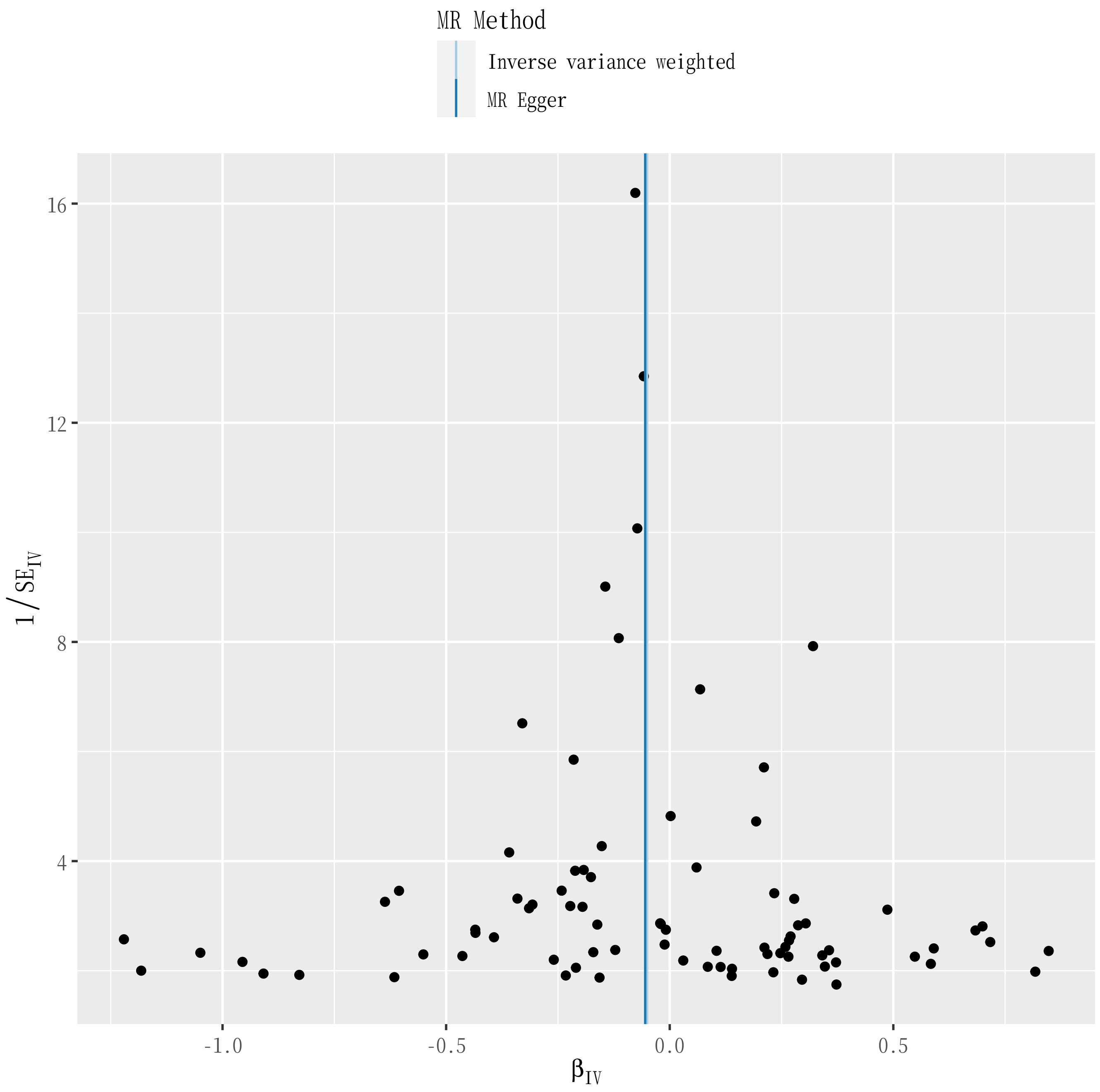
MR Method
Inverse variance weighted
MR Egger
16
12
8
4
1/SE$_{IV}$
-1.0
-0.5
0.0
0.5
β$_{IV}$

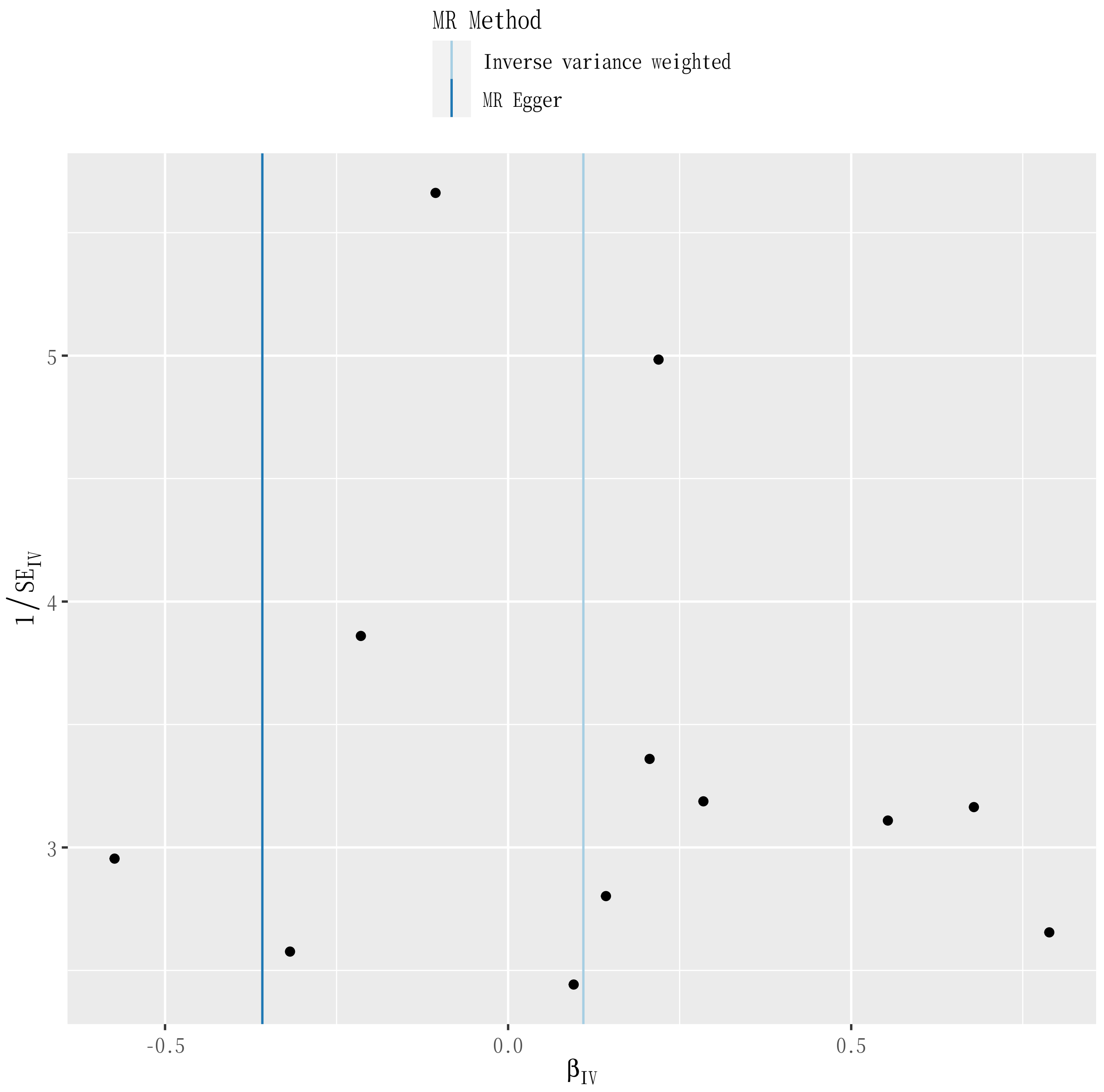

MR Method
Inverse variance weighted
MR Egger
5
4
3
$1/SE_{IV}$
-0.5
0.0
0.5
β_{IV}

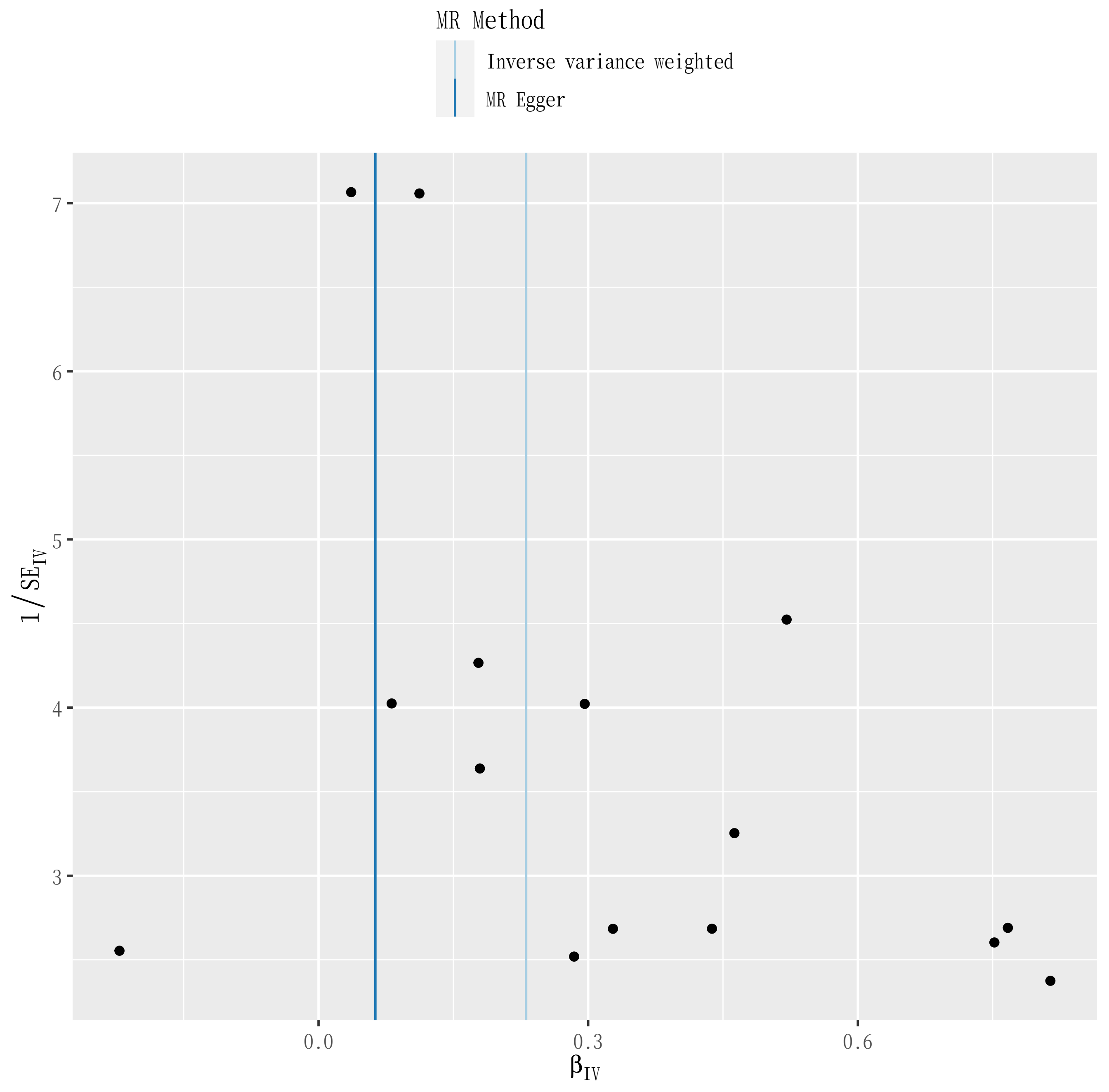
MR Method
Inverse variance weighted
MR Egger
7
6
5
4
3
$1/SE_{IV}$
0.0
0.3
0.6
β_{IV}

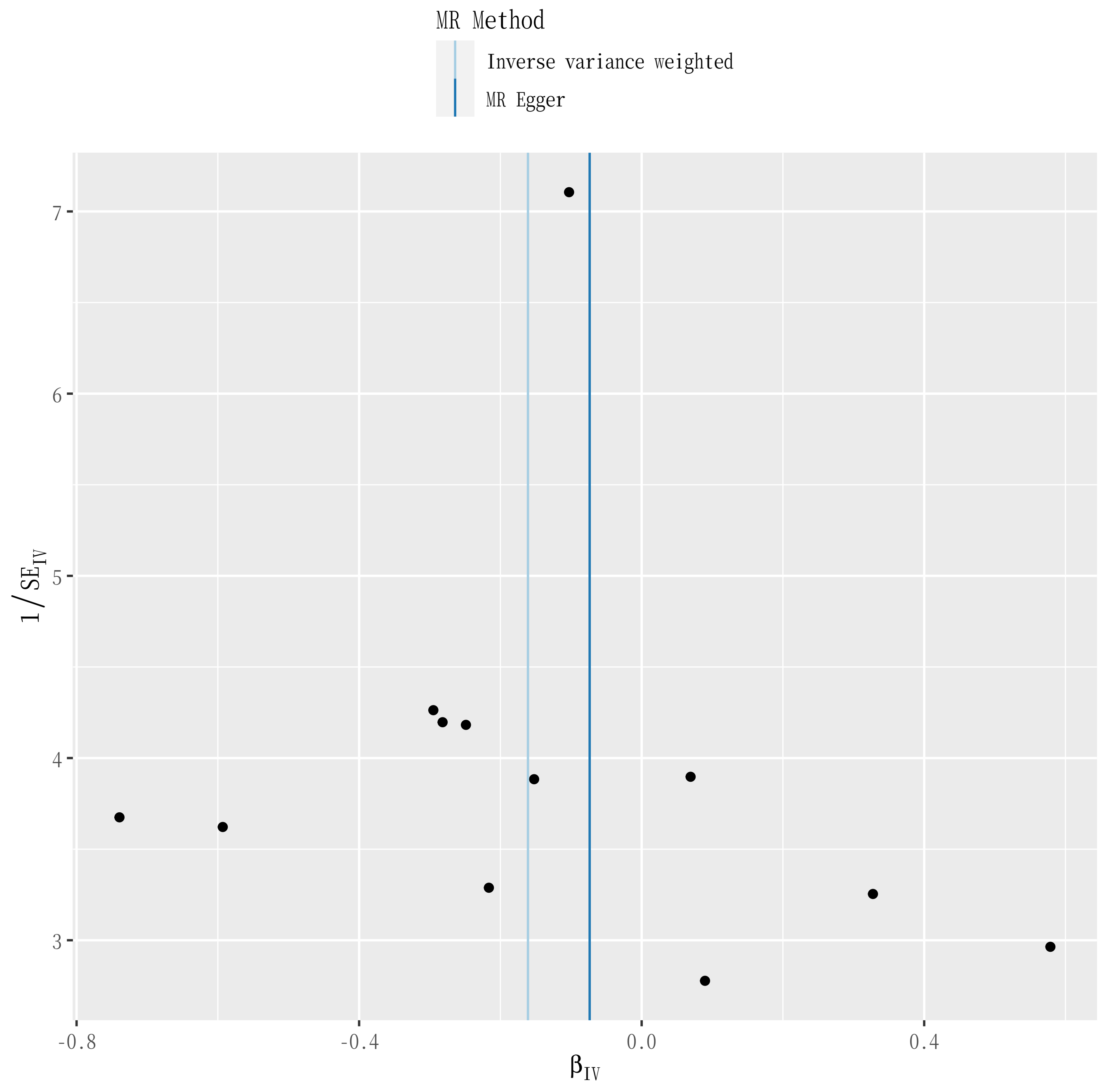
MR Method
Inverse variance weighted
MR Egger
$1/SE_{IV}$
β_{IV}
7
6
5
4
3
-0.8
-0.4
0.0
0.4

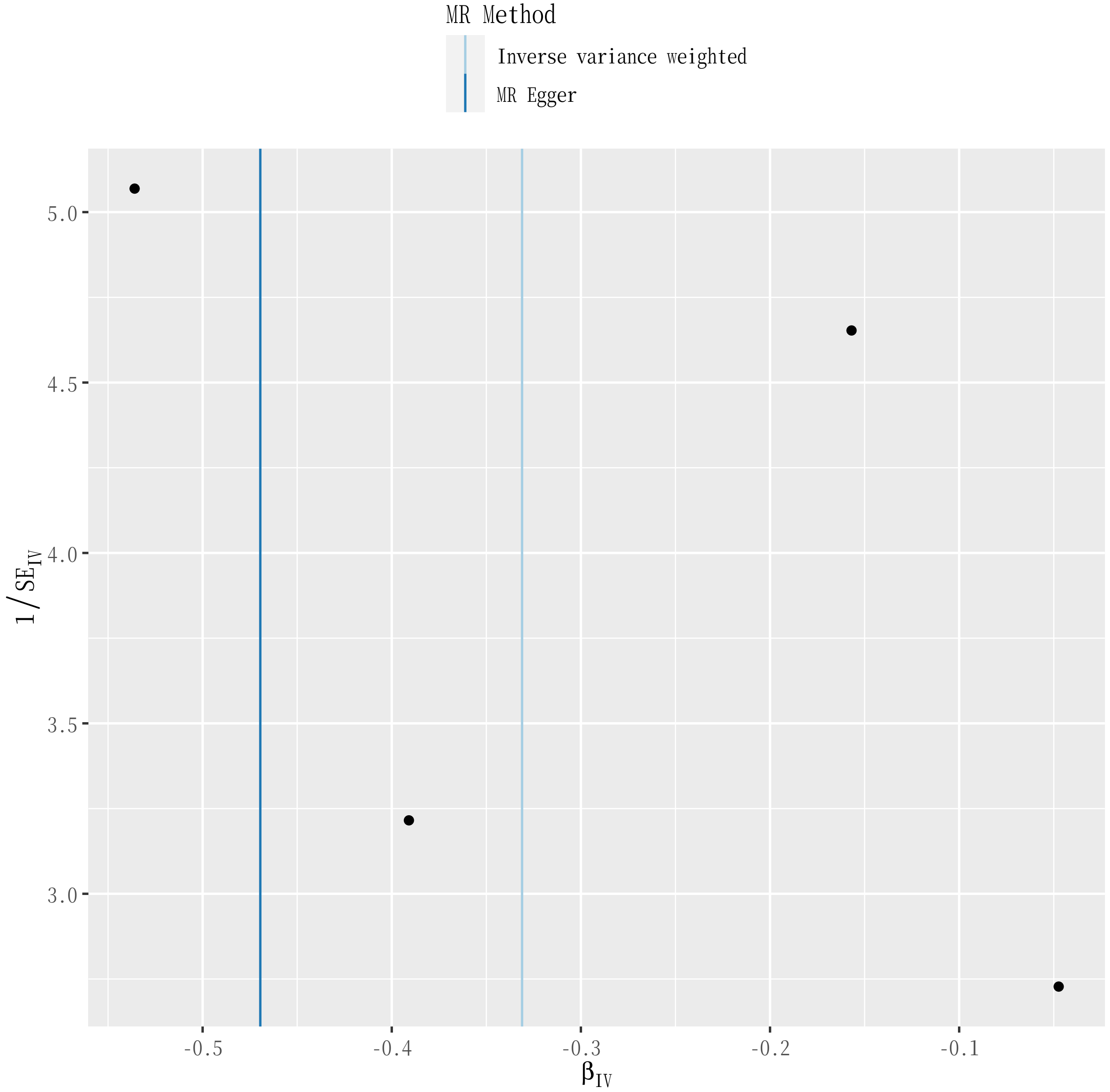

MR Method
Inverse variance weighted
MR Egger
5.0
4.5
4.0
3.5
3.0
1/SE$_{IV}$
-0.5
-0.4
-0.3
-0.2
-0.1
β$_{IV}$

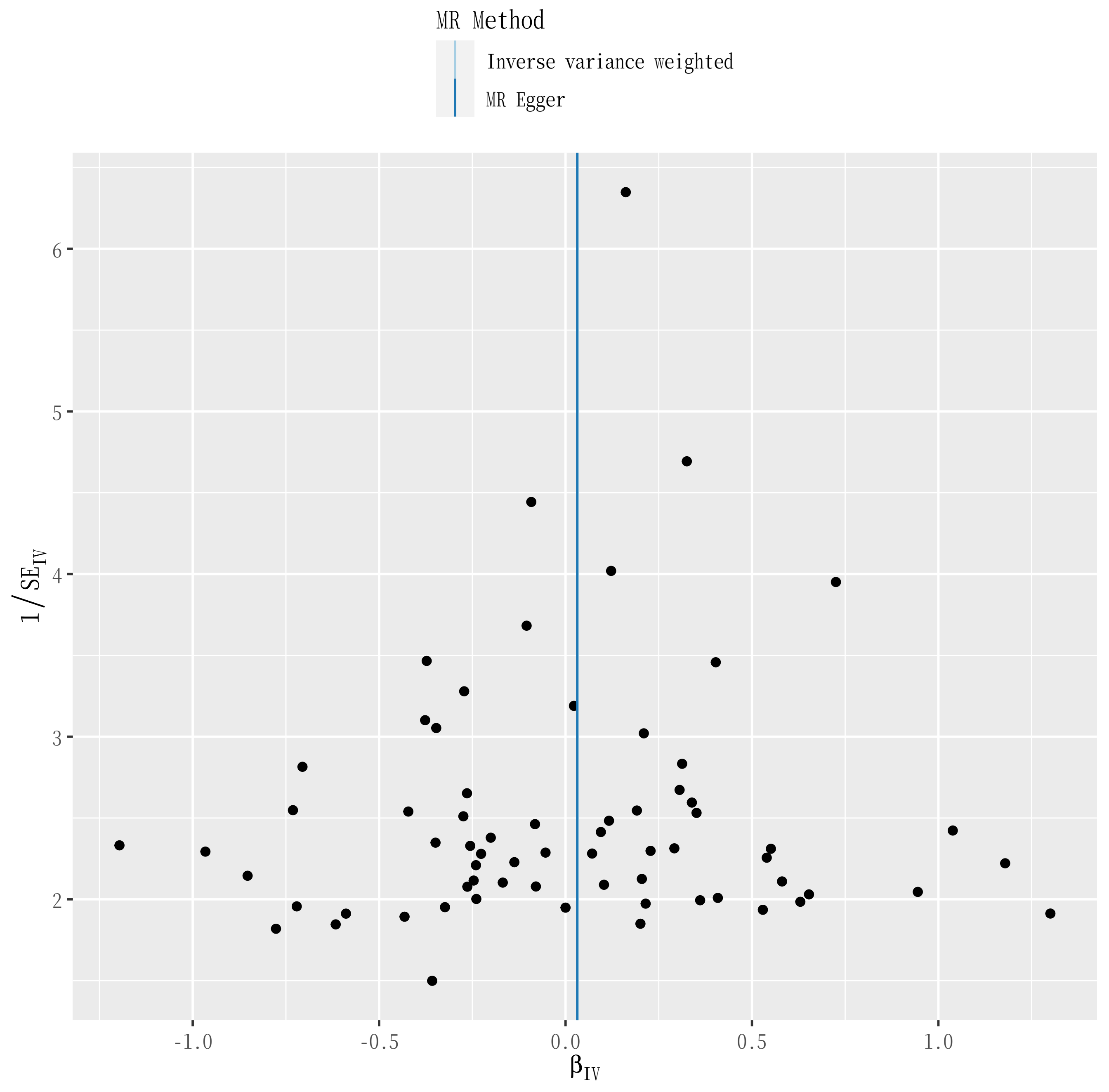

MR Method
Inverse variance weighted
MR Egger
6
5
4
3
2
$1/SE_{IV}$
-1.0
-0.5
0.0
0.5
1.0
β_{IV}

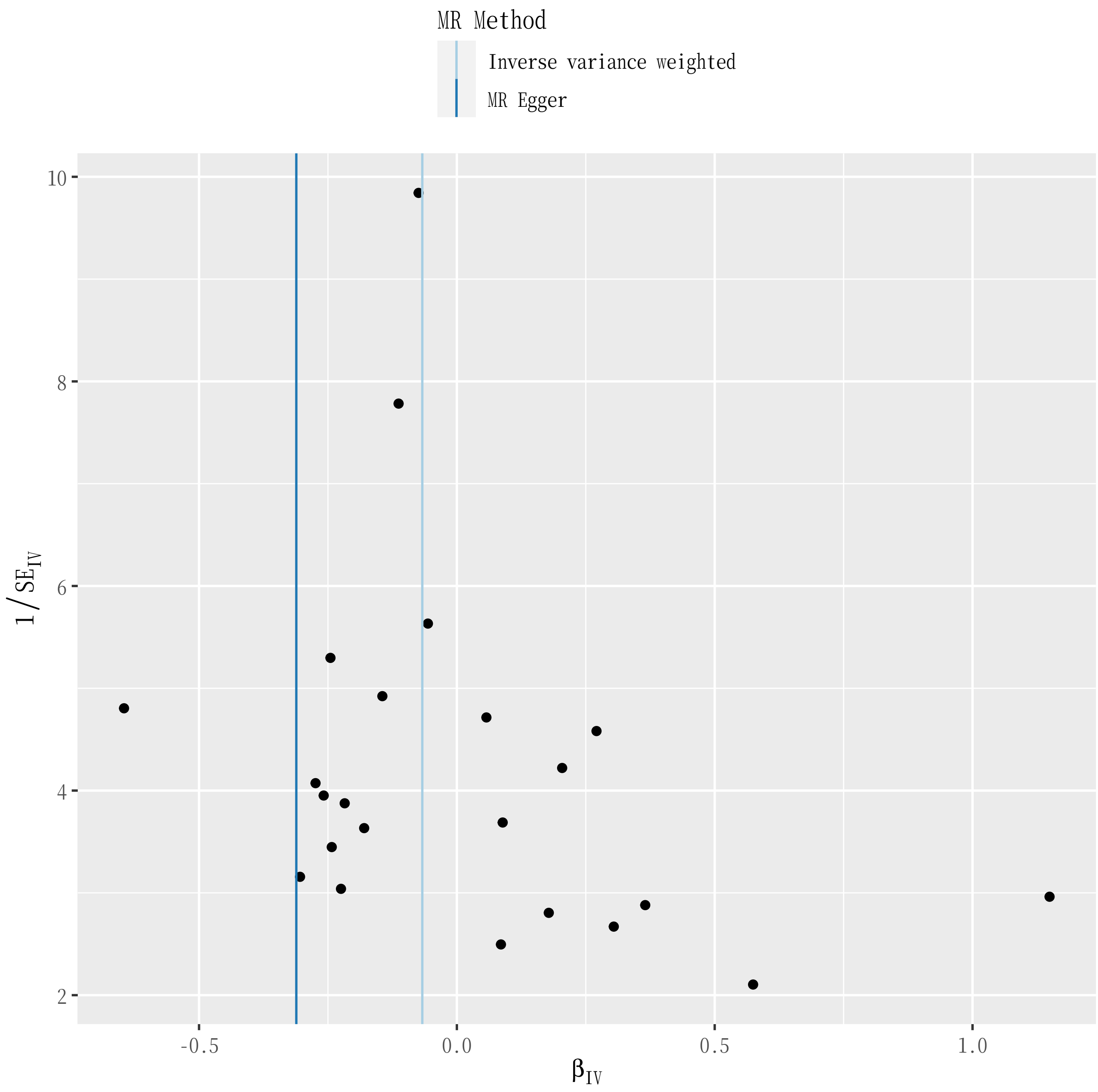

MR Method
Inverse variance weighted
MR Egger
10
8
6
4
2
1/SE_IV
-0.5
0.0
0.5
1.0
β_IV

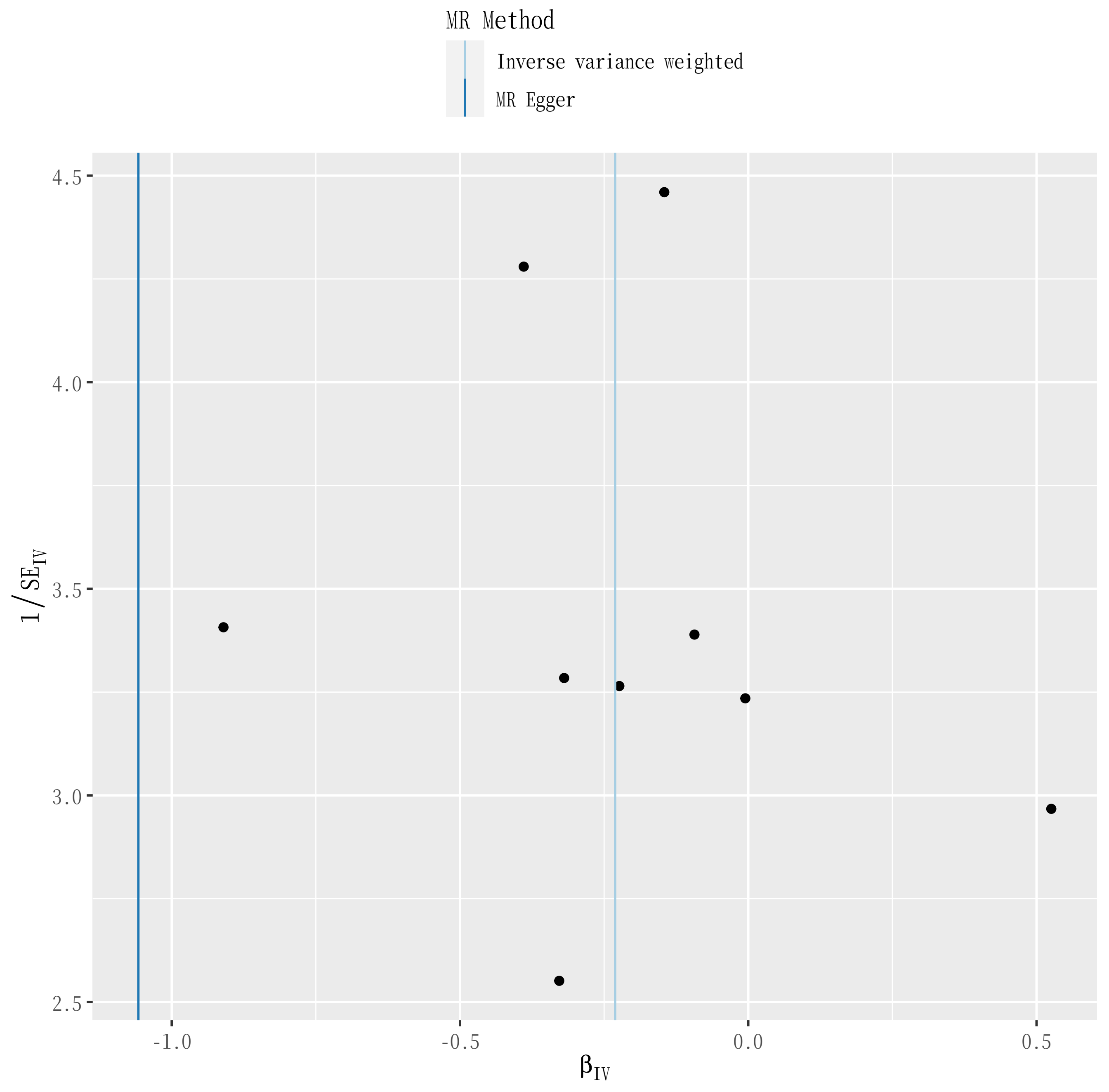
MR Method
Inverse variance weighted
MR Egger
4.5
4.0
3.5
3.0
2.5
1/SE_IV
-1.0
-0.5
0.0
0.5
β_IV

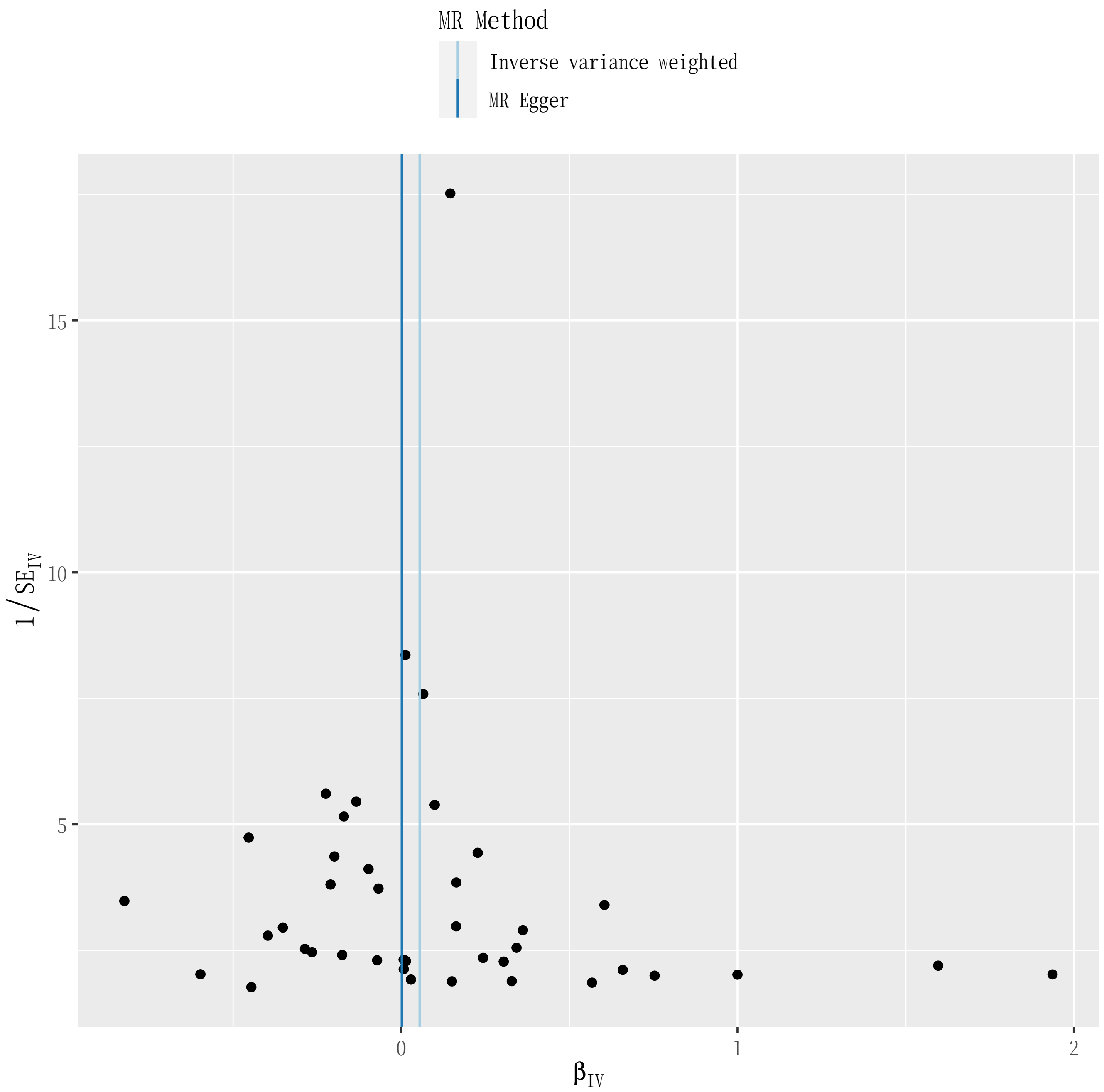
MR Method
Inverse variance weighted
MR Egger
15
10
5
1/SE_IV
0
1
2
β_IV

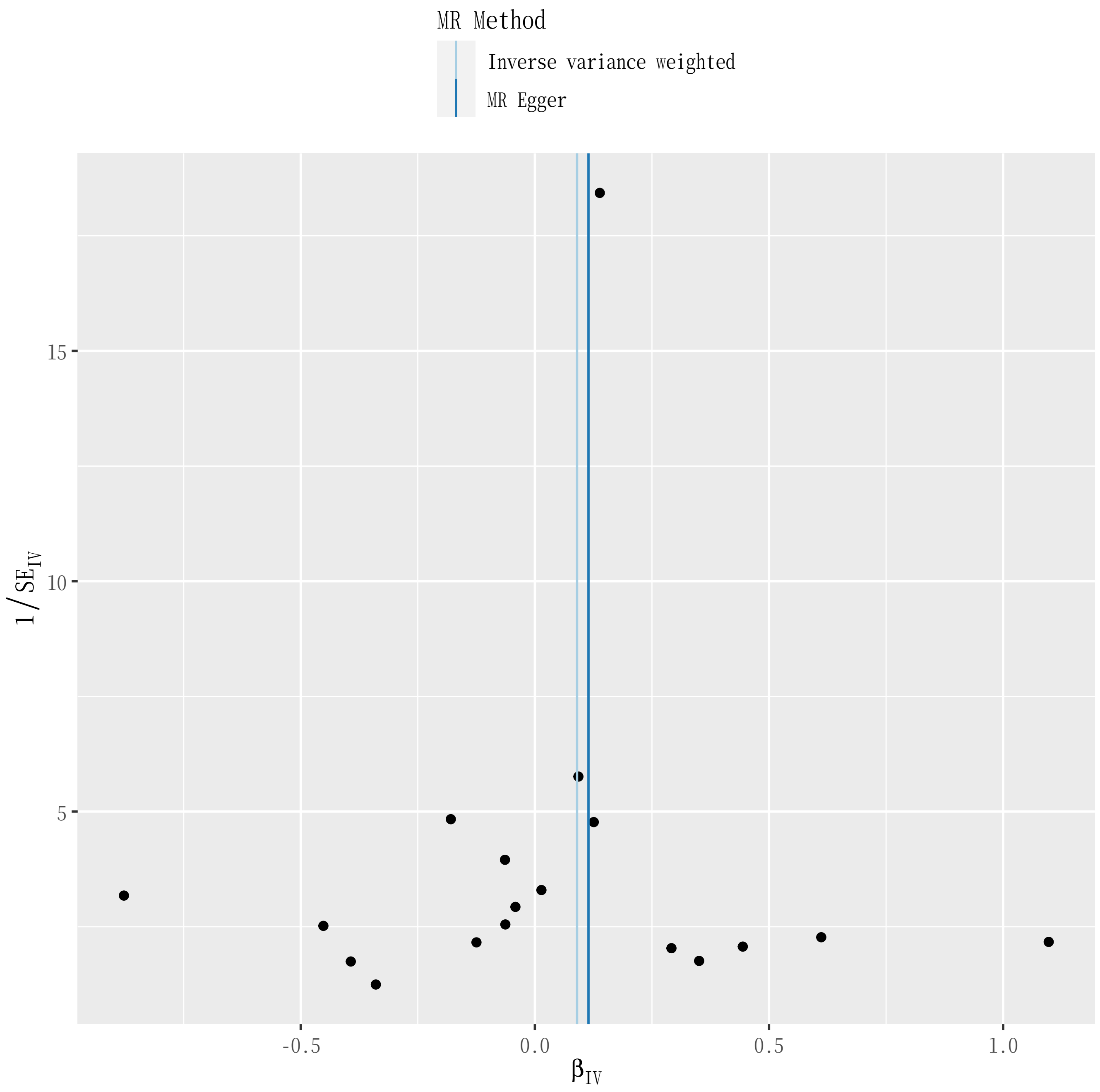

MR Method
Inverse variance weighted
MR Egger
15
10
5
$1/SE_{IV}$
-0.5
0.0
0.5
1.0
β_{IV}

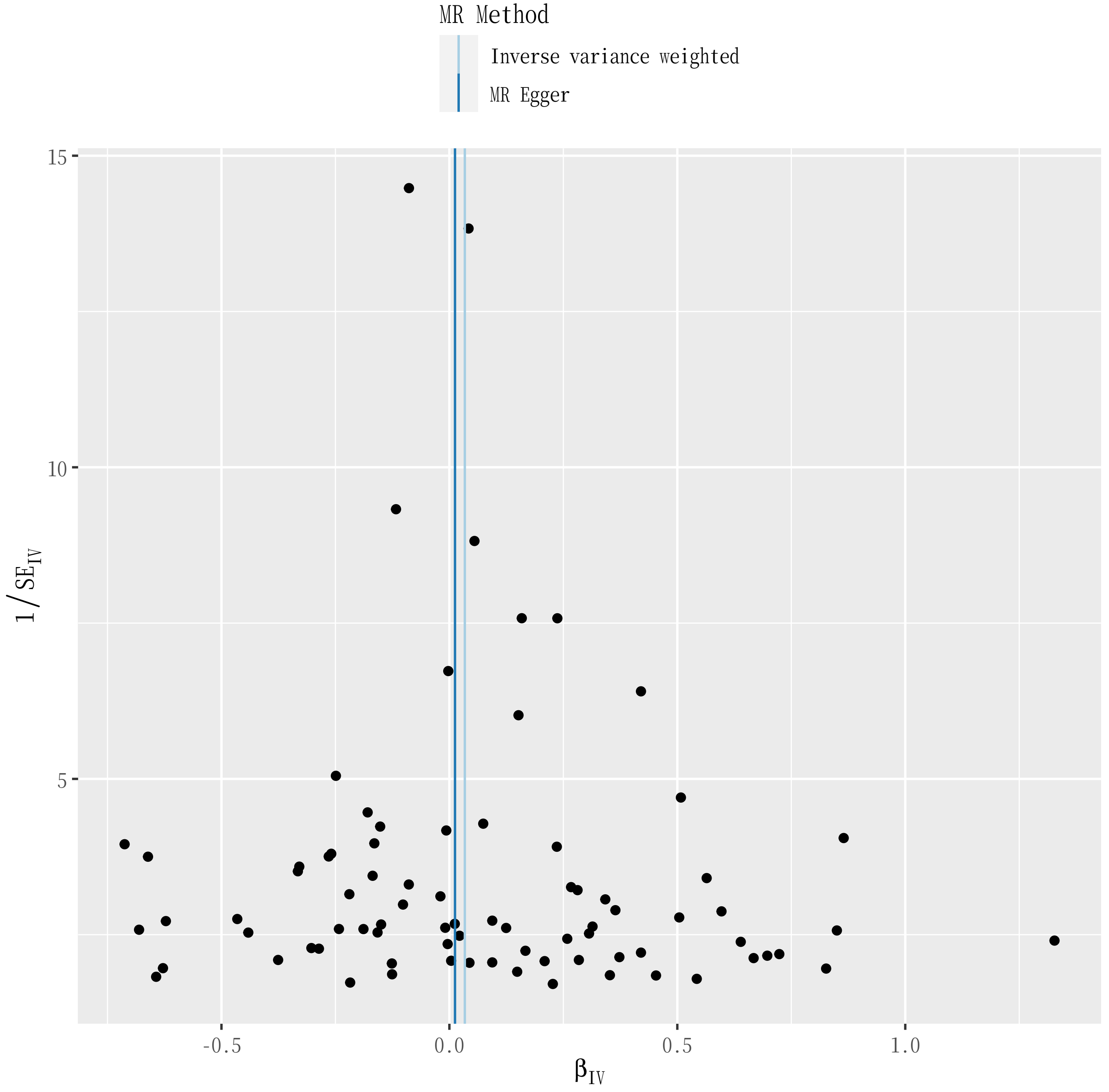

MR Method
Inverse variance weighted
MR Egger
15
10
5
-0.5
0.0
0.5
1.0
$1/SE_{IV}$
β_{IV}

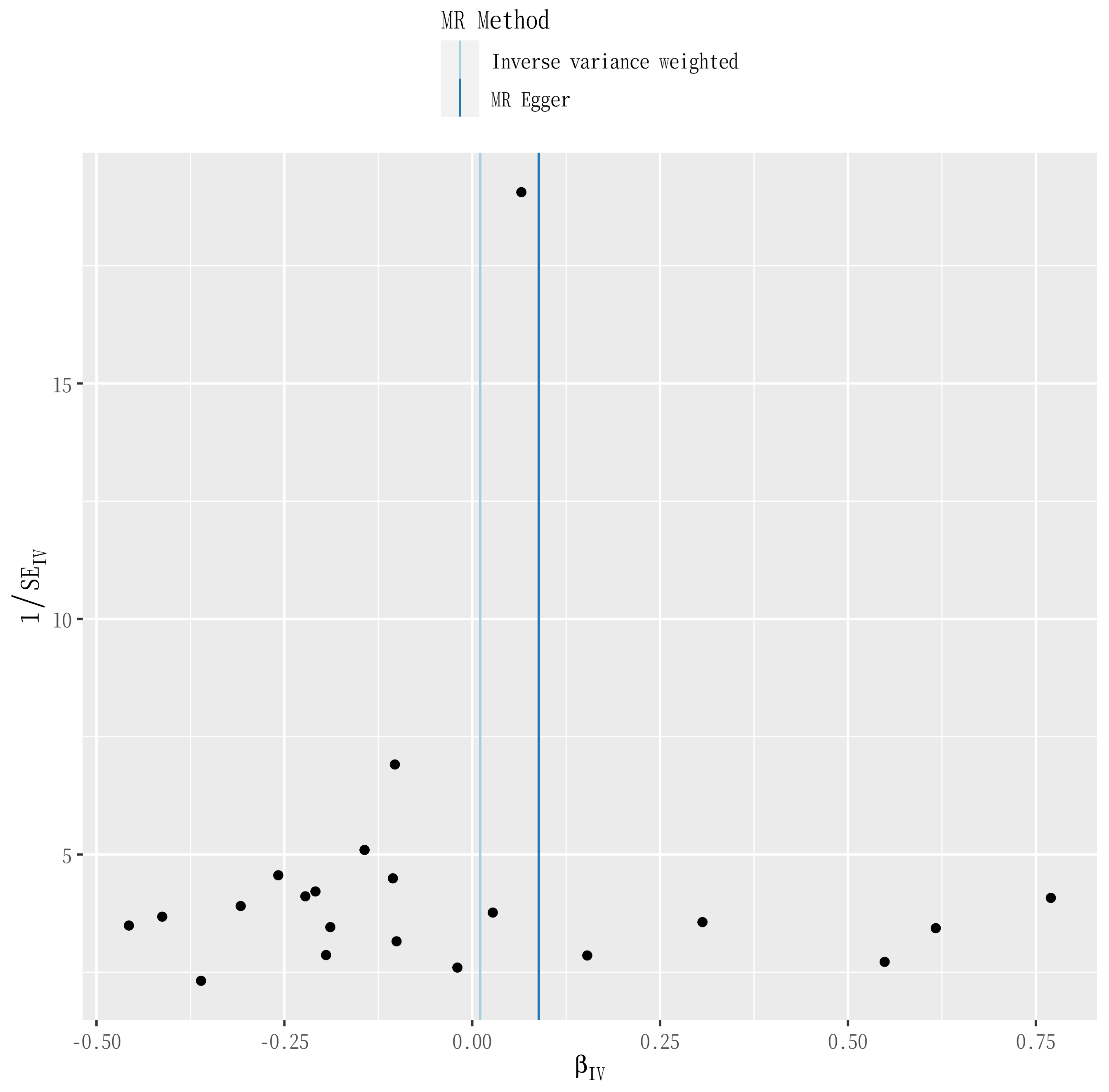

MR Method
Inverse variance weighted
MR Egger
15
10
5
$1/SE_{IV}$
-0.50
-0.25
0.00
0.25
0.50
0.75
β_{IV}

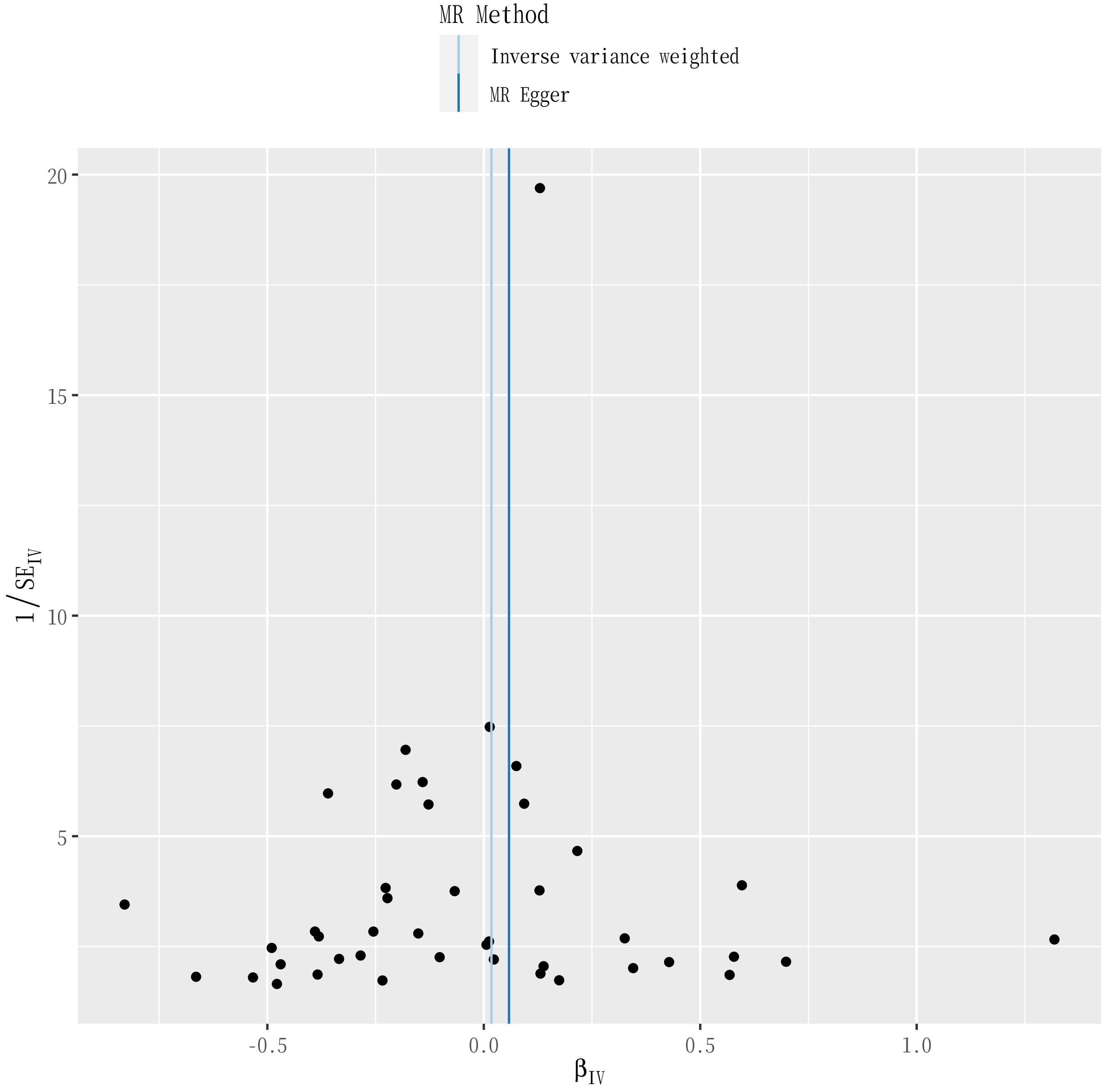

MR Method
Inverse variance weighted
MR Egger
20
15
10
5
1/SE_IV
-0.5
0.0
0.5
1.0
β_IV

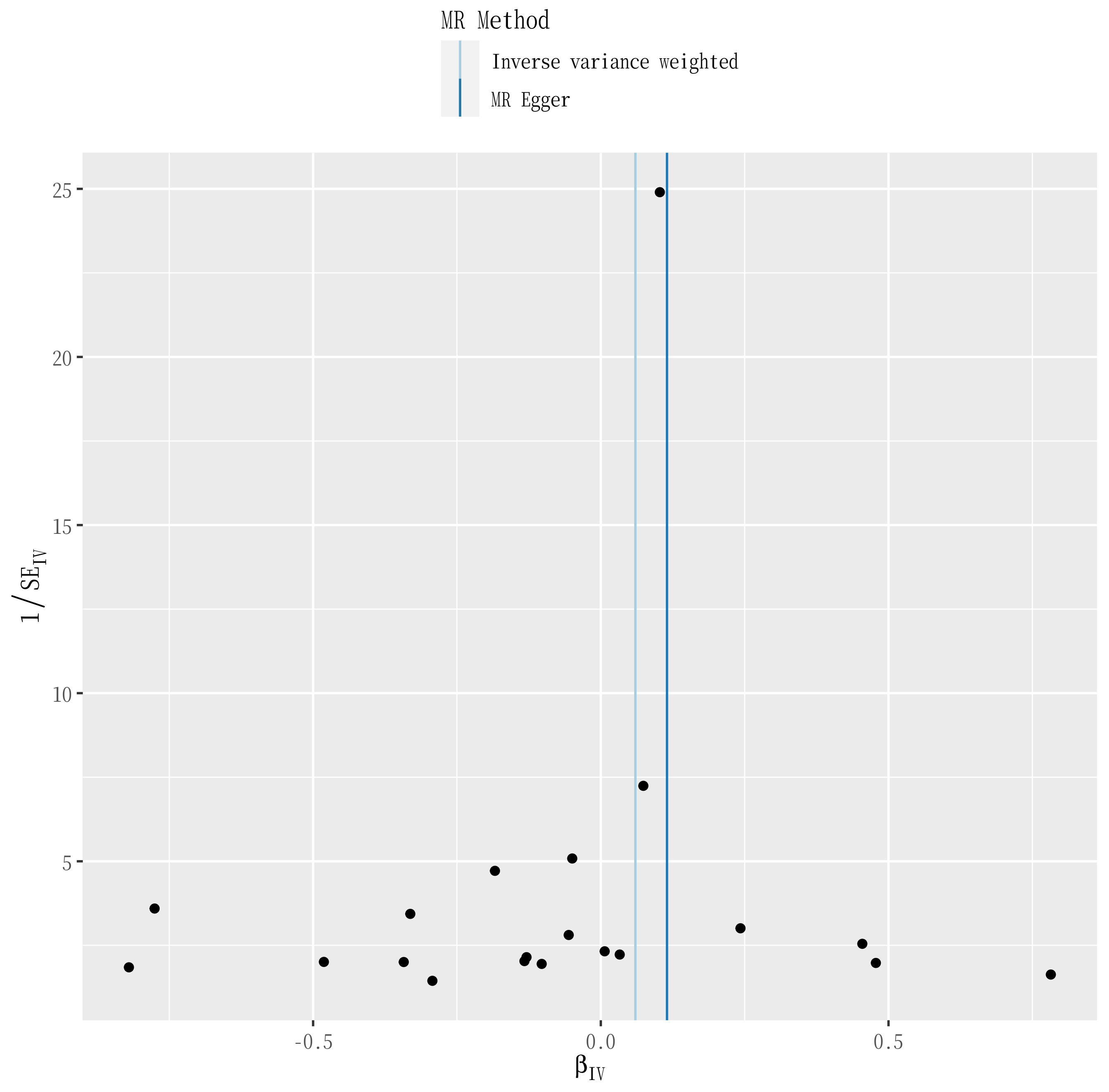

MR Method
Inverse variance weighted
MR Egger
25
20
15
10
5
1/SE$_{IV}$
-0.5
0.0
0.5
β$_{IV}$

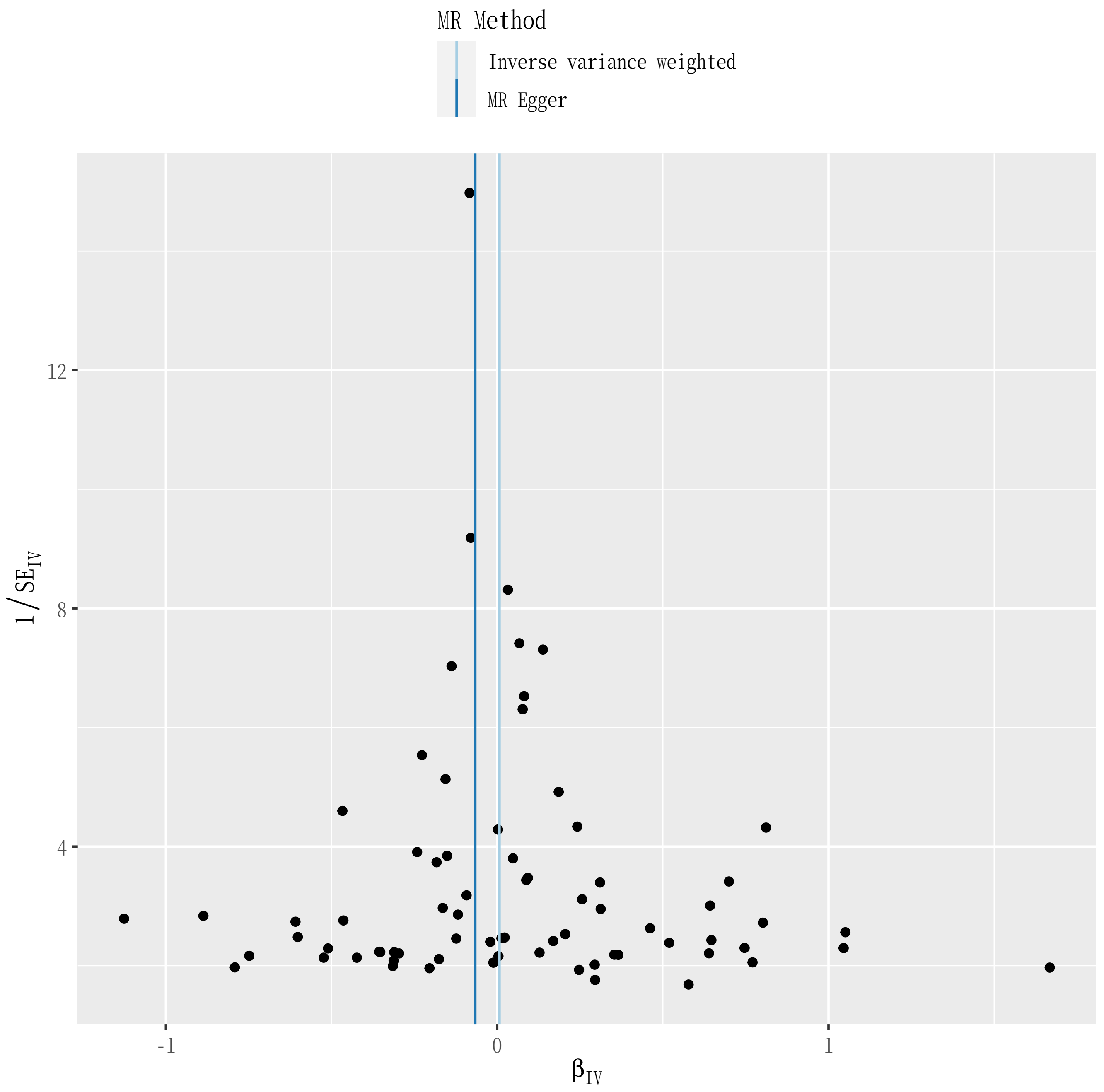

MR Method
Inverse variance weighted
MR Egger
12
8
4
-1
0
1
1/SE_IV
β_IV

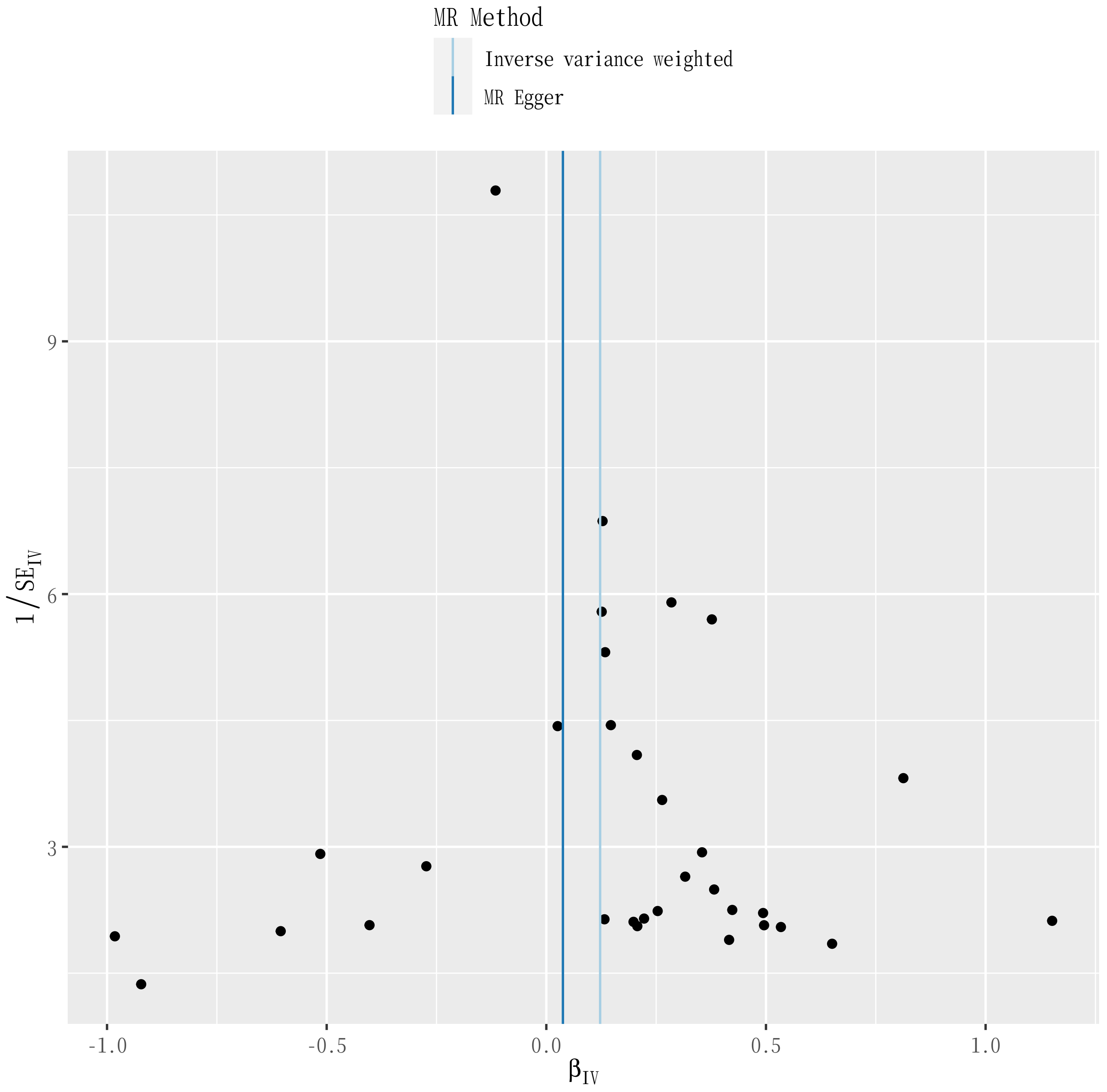

MR Method
Inverse variance weighted
MR Egger
9
6
3
1/SE_IV
-1.0
-0.5
0.0
0.5
1.0
β_IV

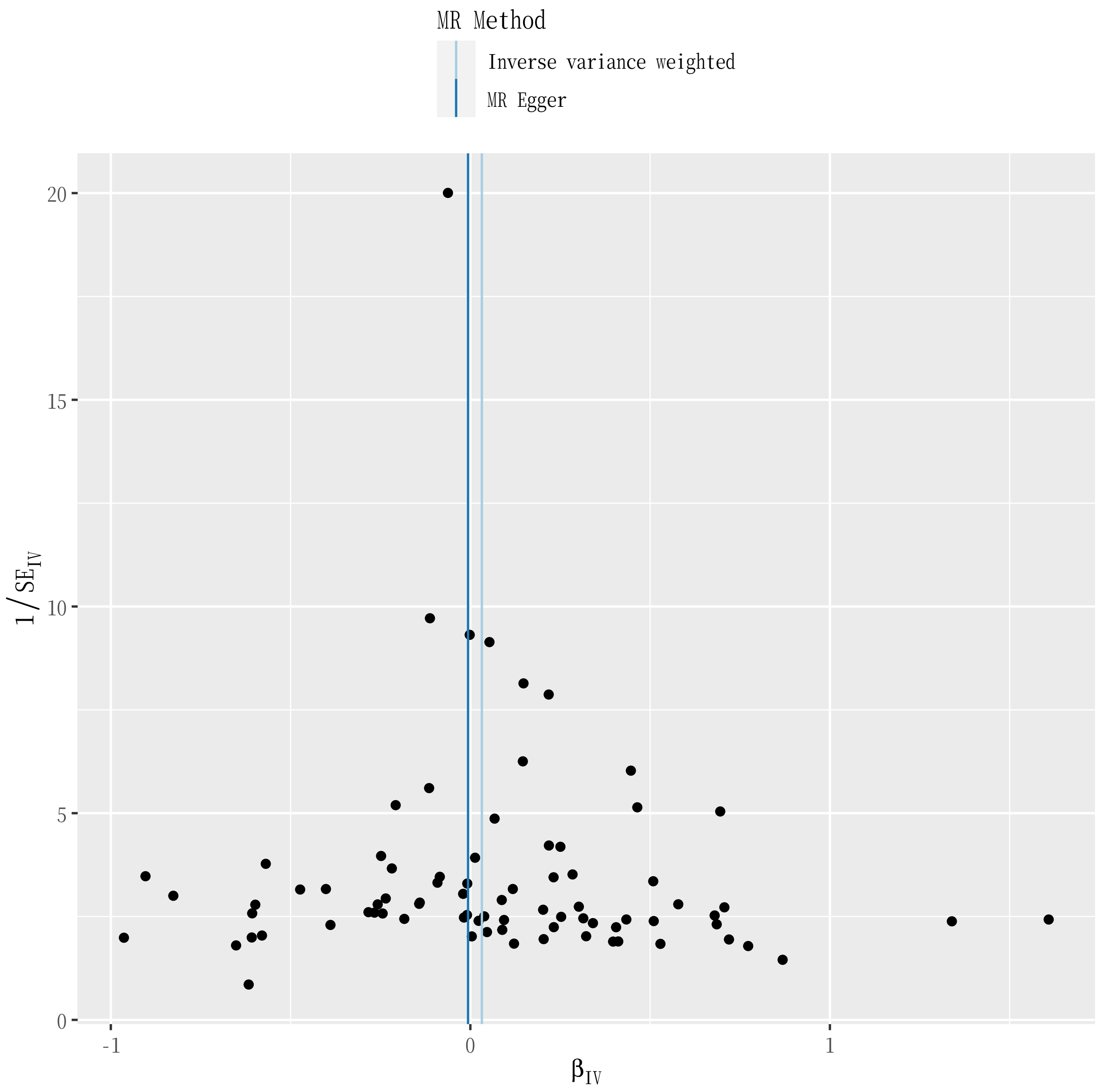
MR Method
Inverse variance weighted
MR Egger
20
15
10
5
0
1/SE_IV
-1
0
1
β_IV

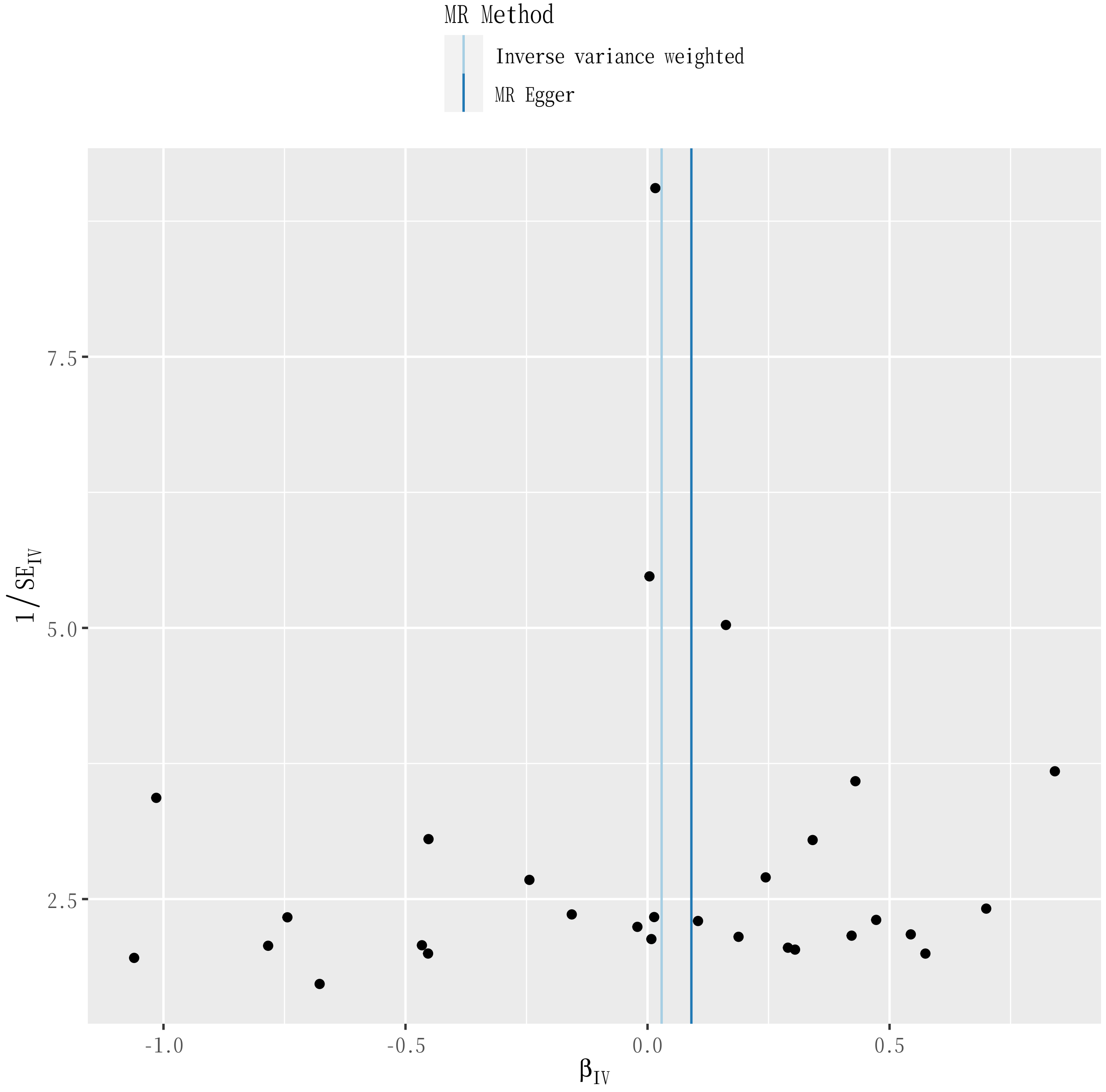

MR Method
Inverse variance weighted
MR Egger
7.5
5.0
2.5
$1/SE_{IV}$
-1.0
-0.5
0.0
0.5
β_{IV}

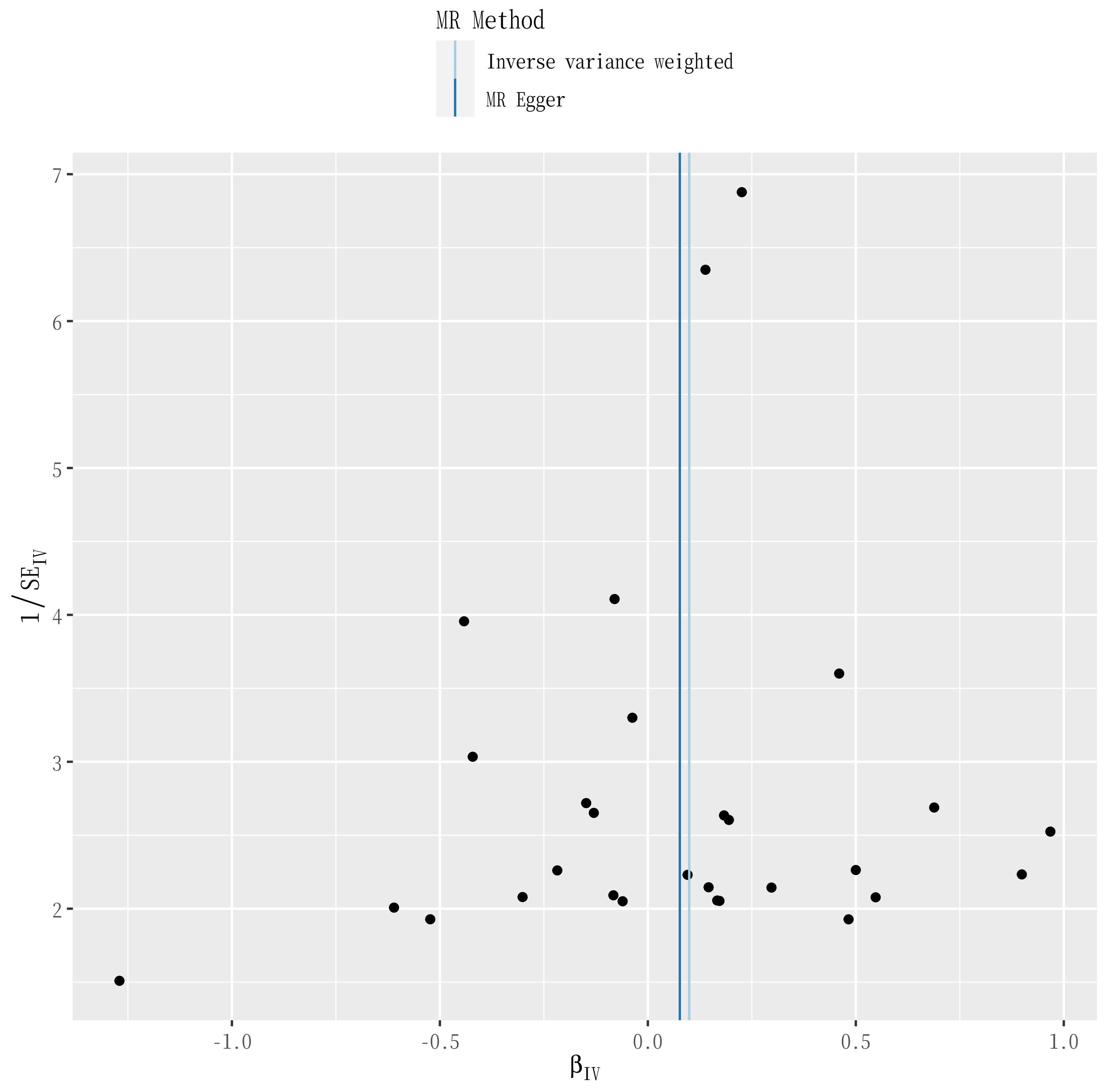

MR Method
Inverse variance weighted
MR Egger
7
6
5
4
3
2
1/SE_IV
-1.0
-0.5
0.0
0.5
1.0
β_IV

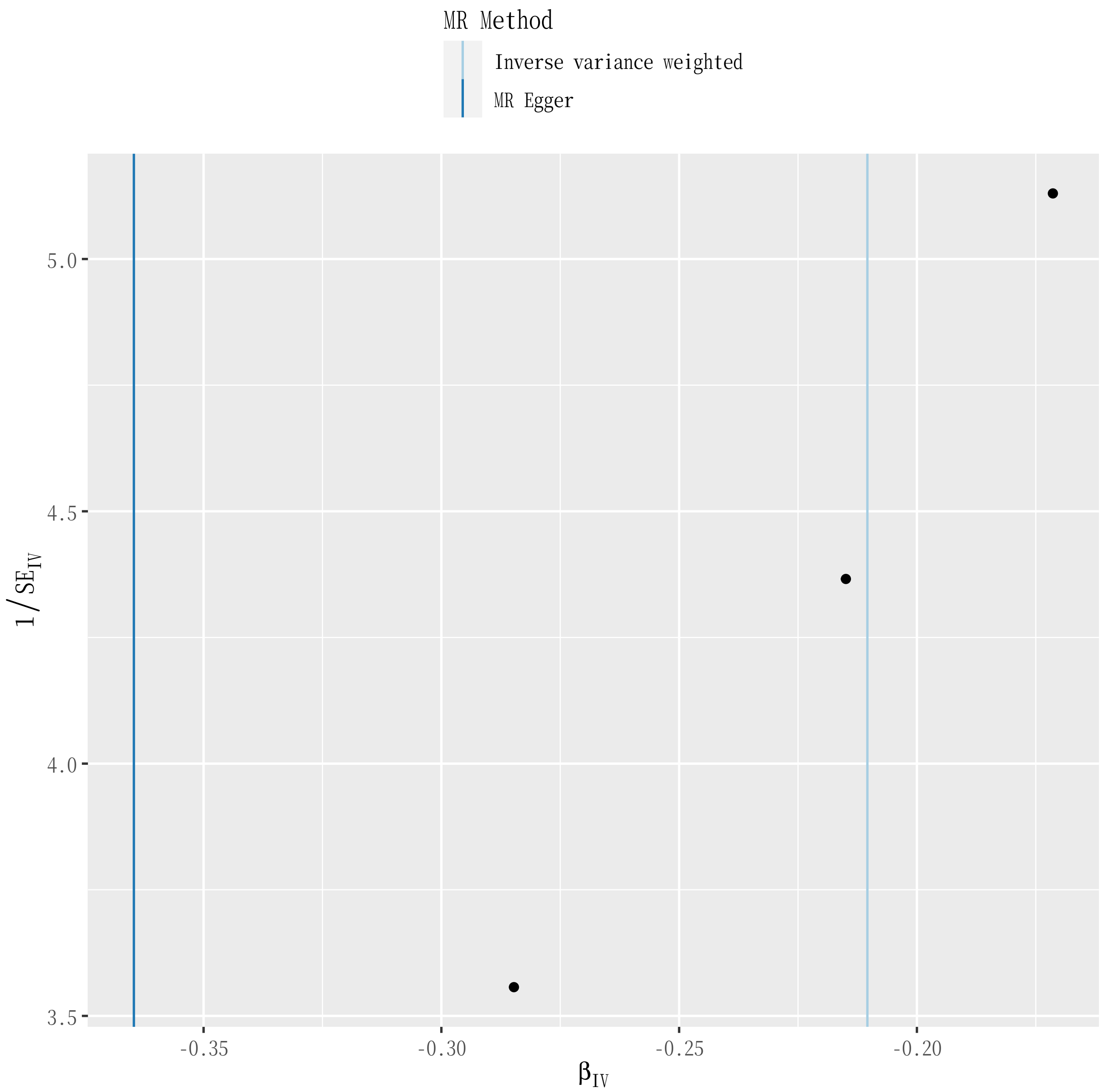
MR Method
Inverse variance weighted
MR Egger
5.0
4.5
4.0
3.5
-0.35
-0.30
-0.25
-0.20
$1/SE_{IV}$
β_{IV}

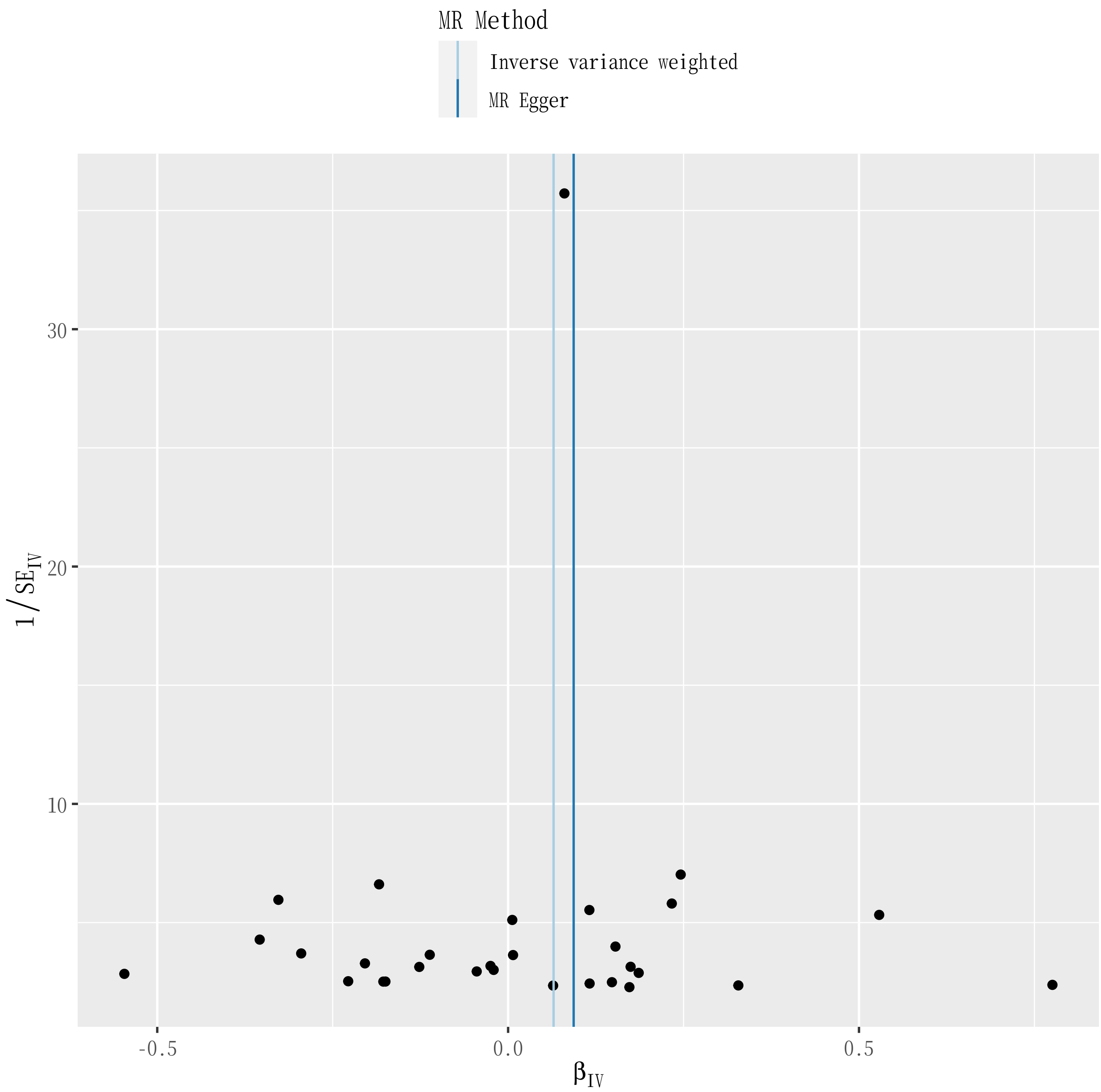
MR Method
Inverse variance weighted
MR Egger
30
20
10
1/SE_IV
-0.5
0.0
0.5
β_IV

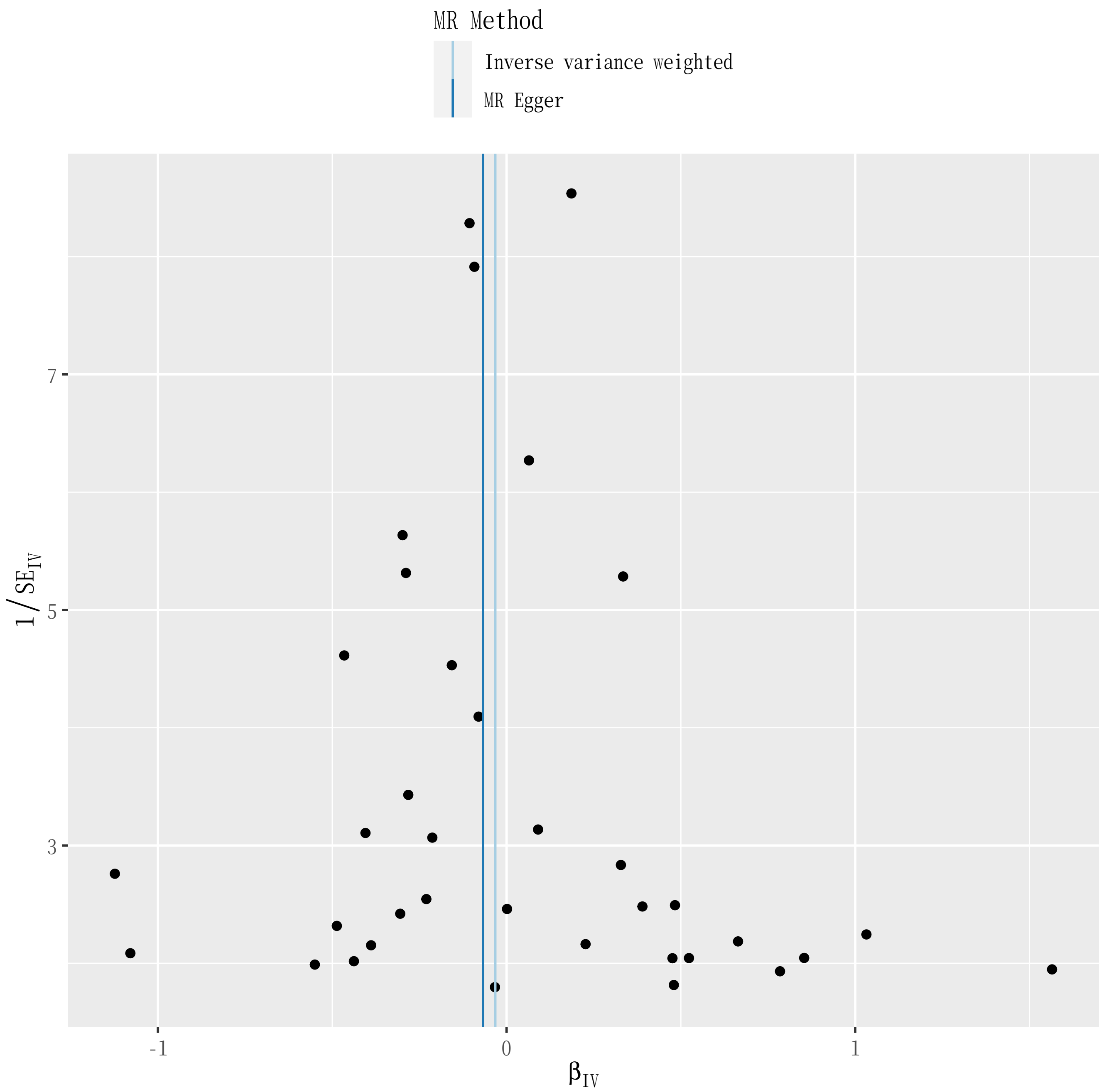
MR Method
Inverse variance weighted
MR Egger
7
5
3
$1/SE_{IV}$
-1
0
1
β_{IV}

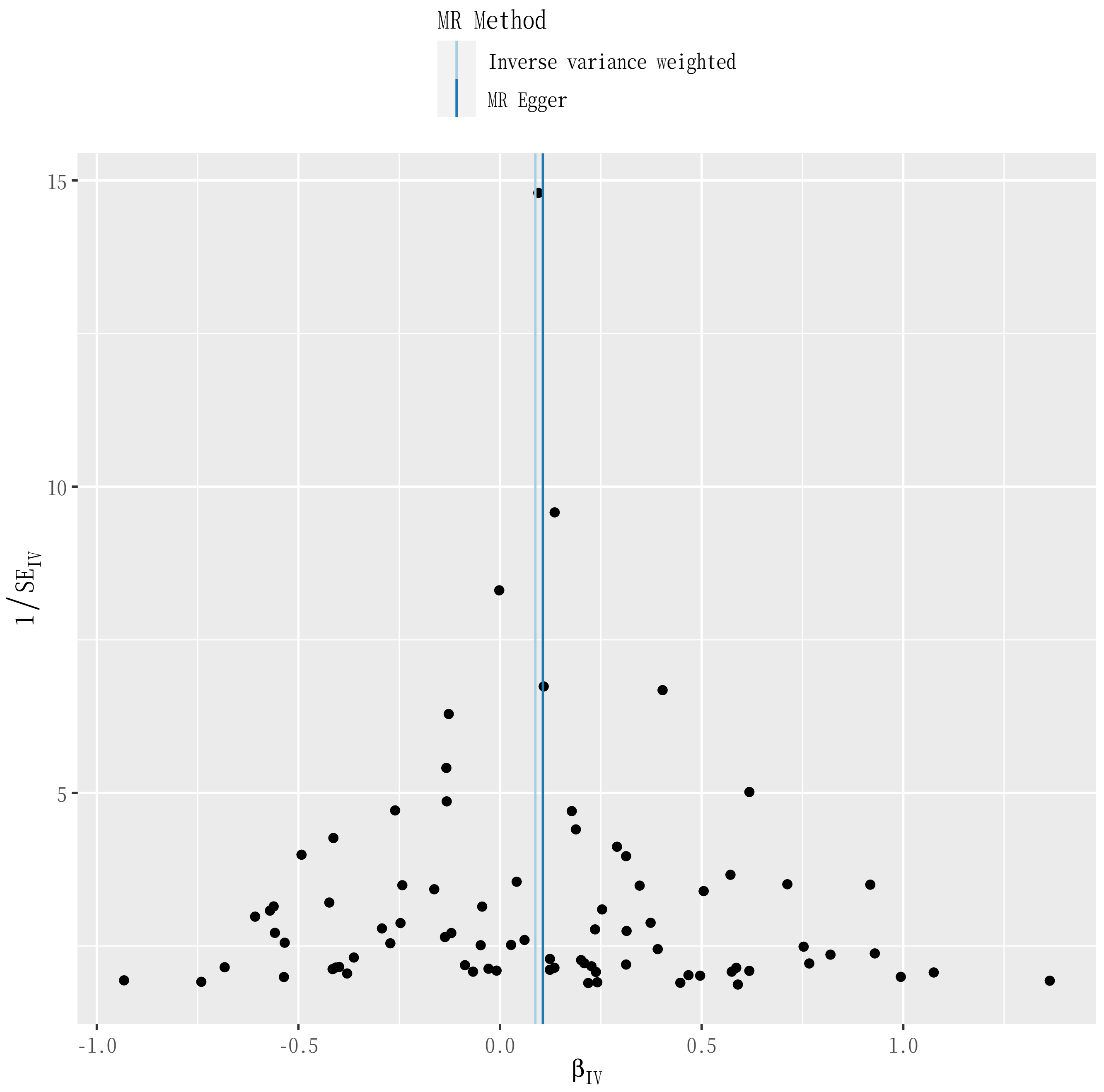
MR Method
Inverse variance weighted
MR Egger
15
10
5
-1.0
-0.5
0.0
0.5
1.0
1/SE_IV
β_IV

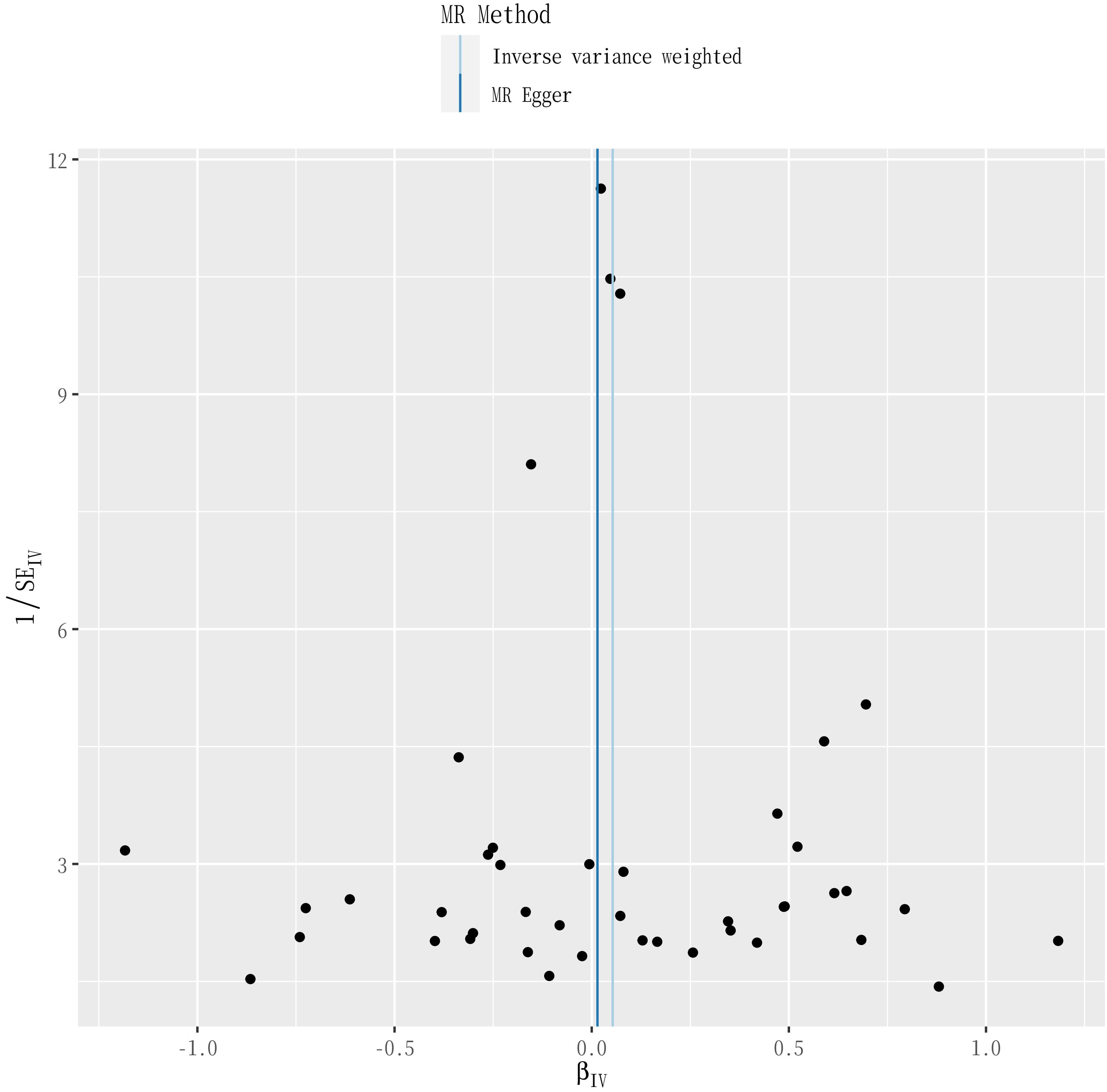
MR Method
Inverse variance weighted
MR Egger
12
9
6
3
-1.0
-0.5
0.0
0.5
1.0
$1/SE_{IV}$
β_{IV}

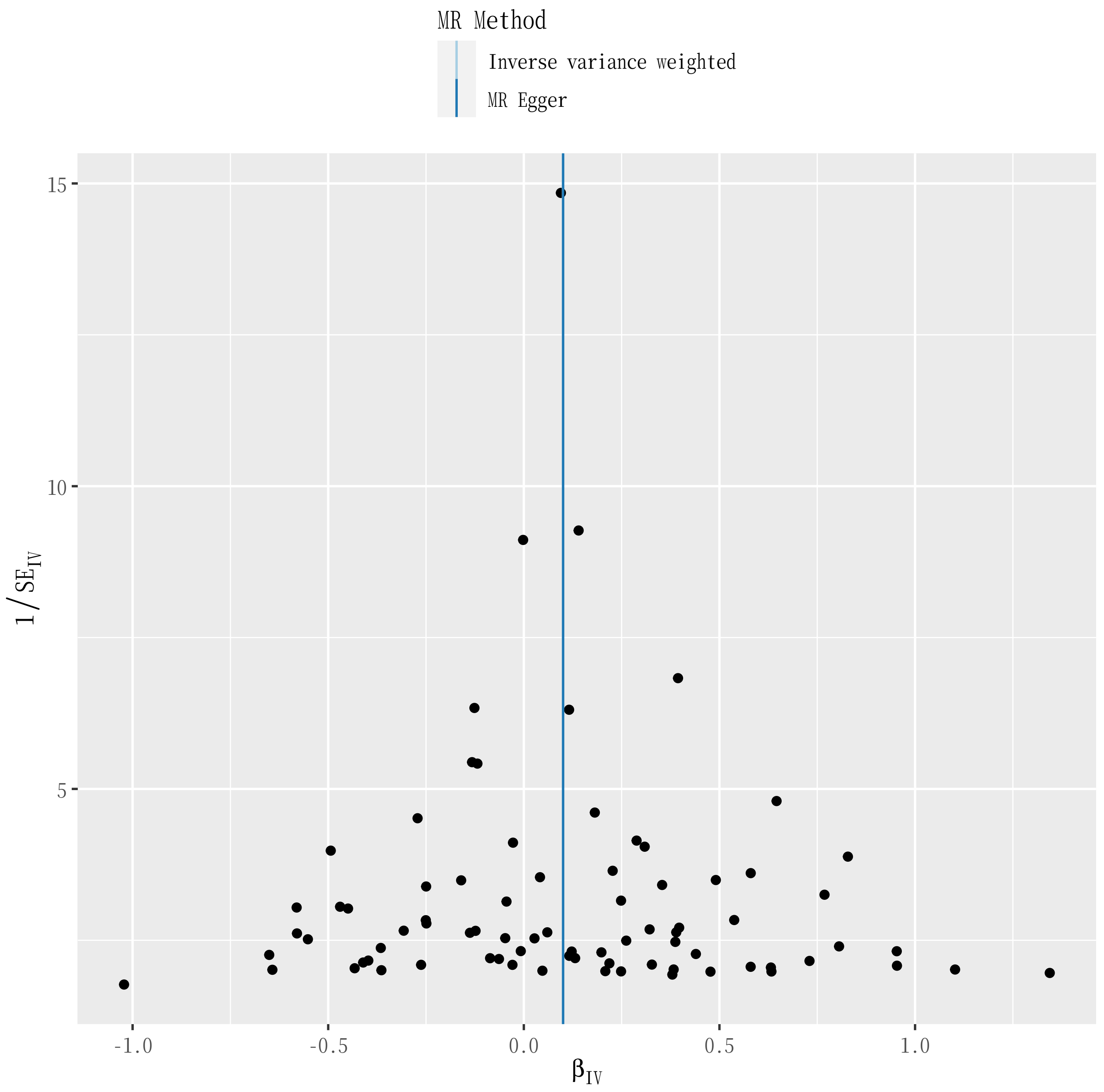

MR Method
Inverse variance weighted
MR Egger
15
10
5
1/SE_IV
-1.0
-0.5
0.0
0.5
1.0
β_IV

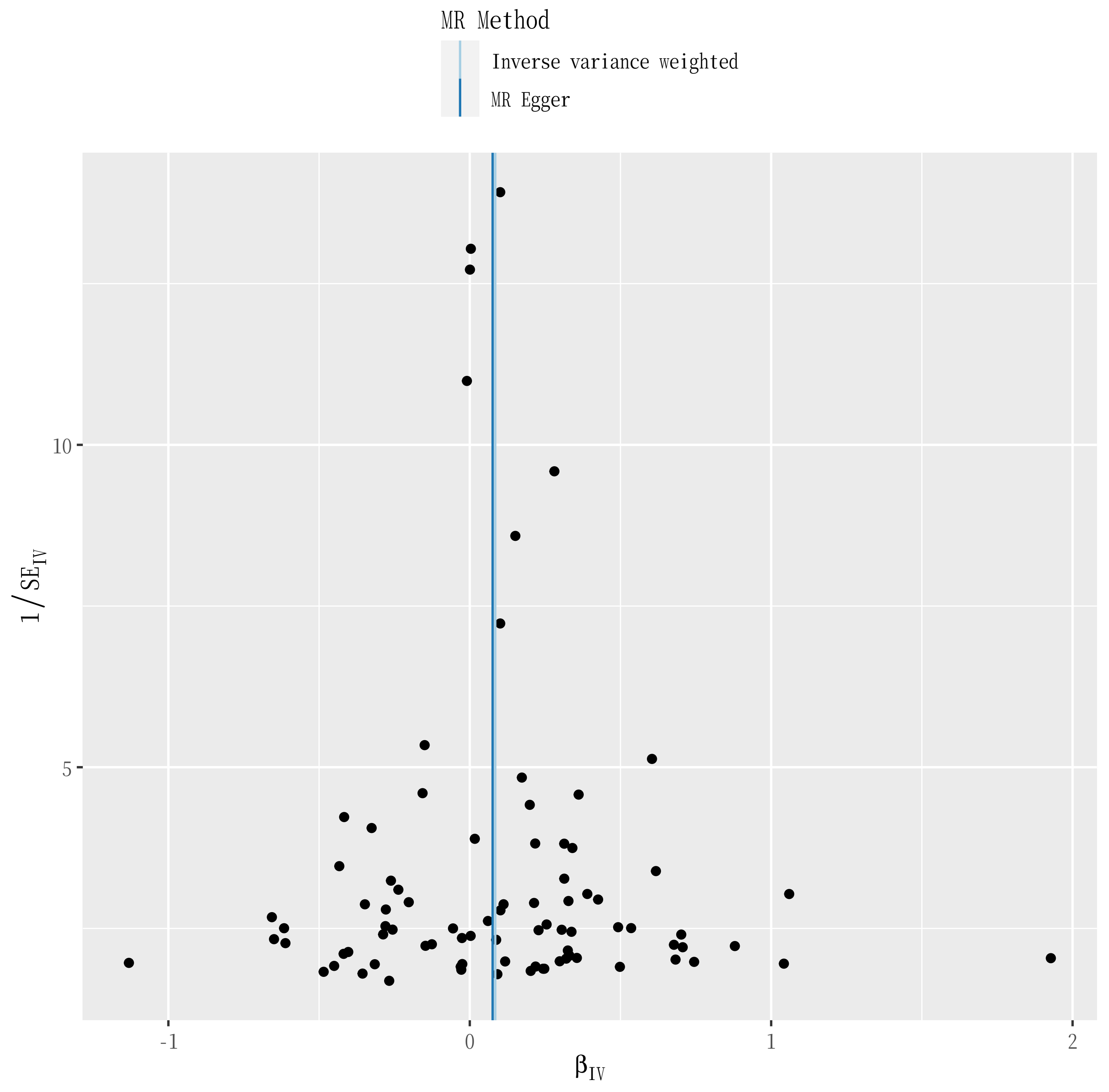

MR Method
Inverse variance weighted
MR Egger
10
5
$1/SE_{IV}$
-1
0
1
2
β_{IV}

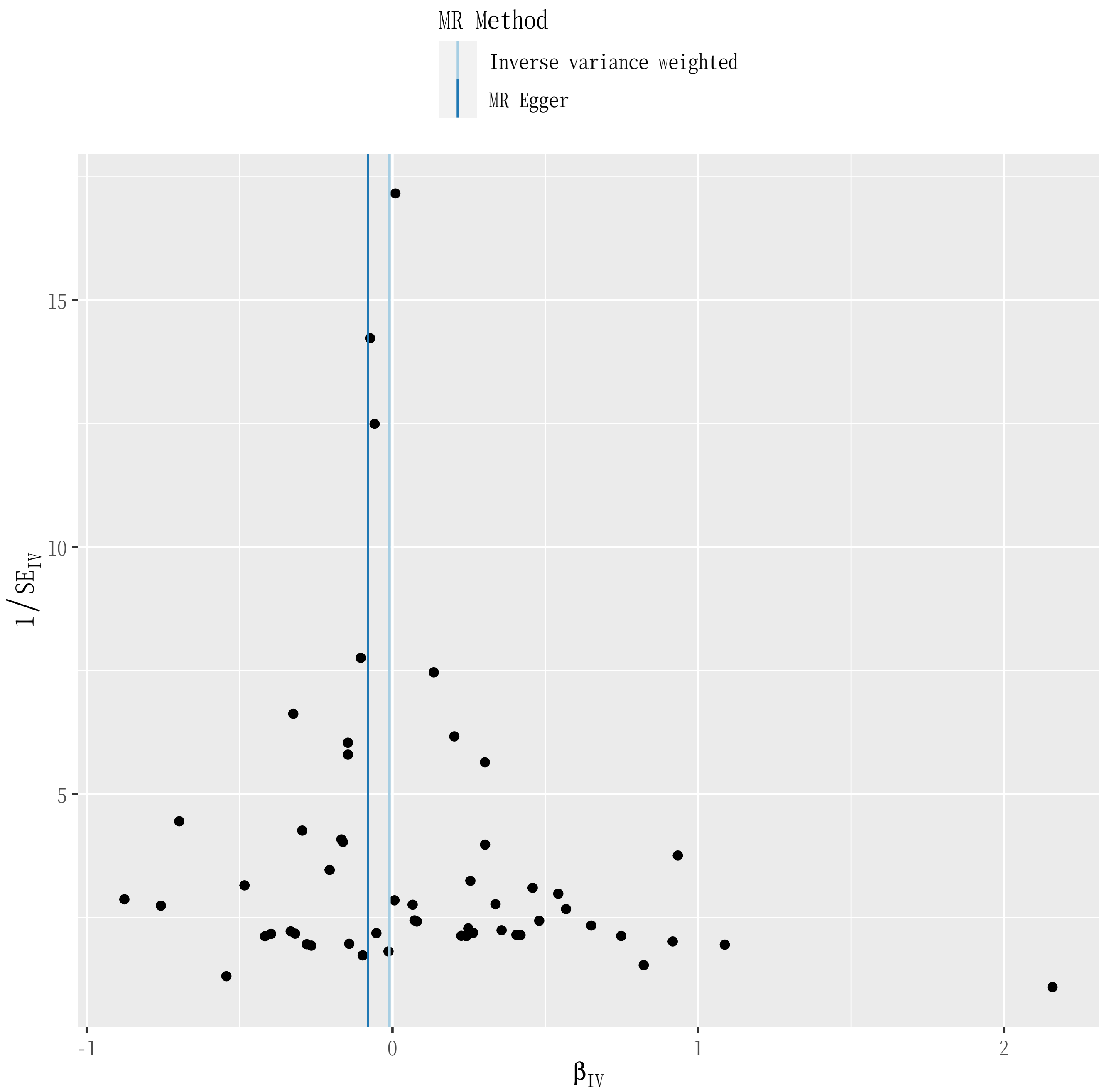

MR Method
Inverse variance weighted
MR Egger
15
10
5
1/SE_IV
-1
0
1
2
β_IV

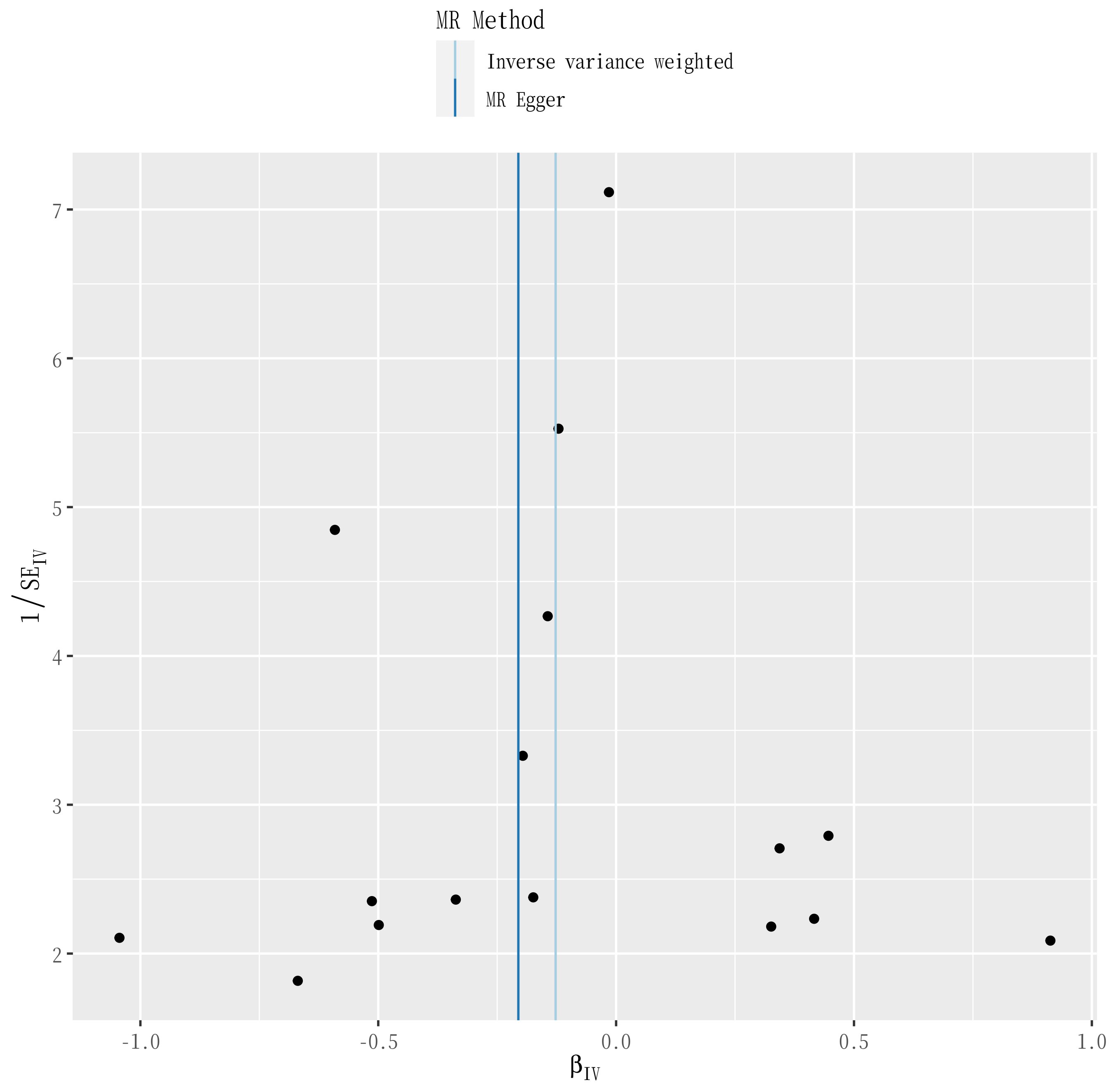
MR Method
Inverse variance weighted
MR Egger
7
6
5
4
3
2
1/SE_IV
-1.0
-0.5
0.0
0.5
1.0
β_IV

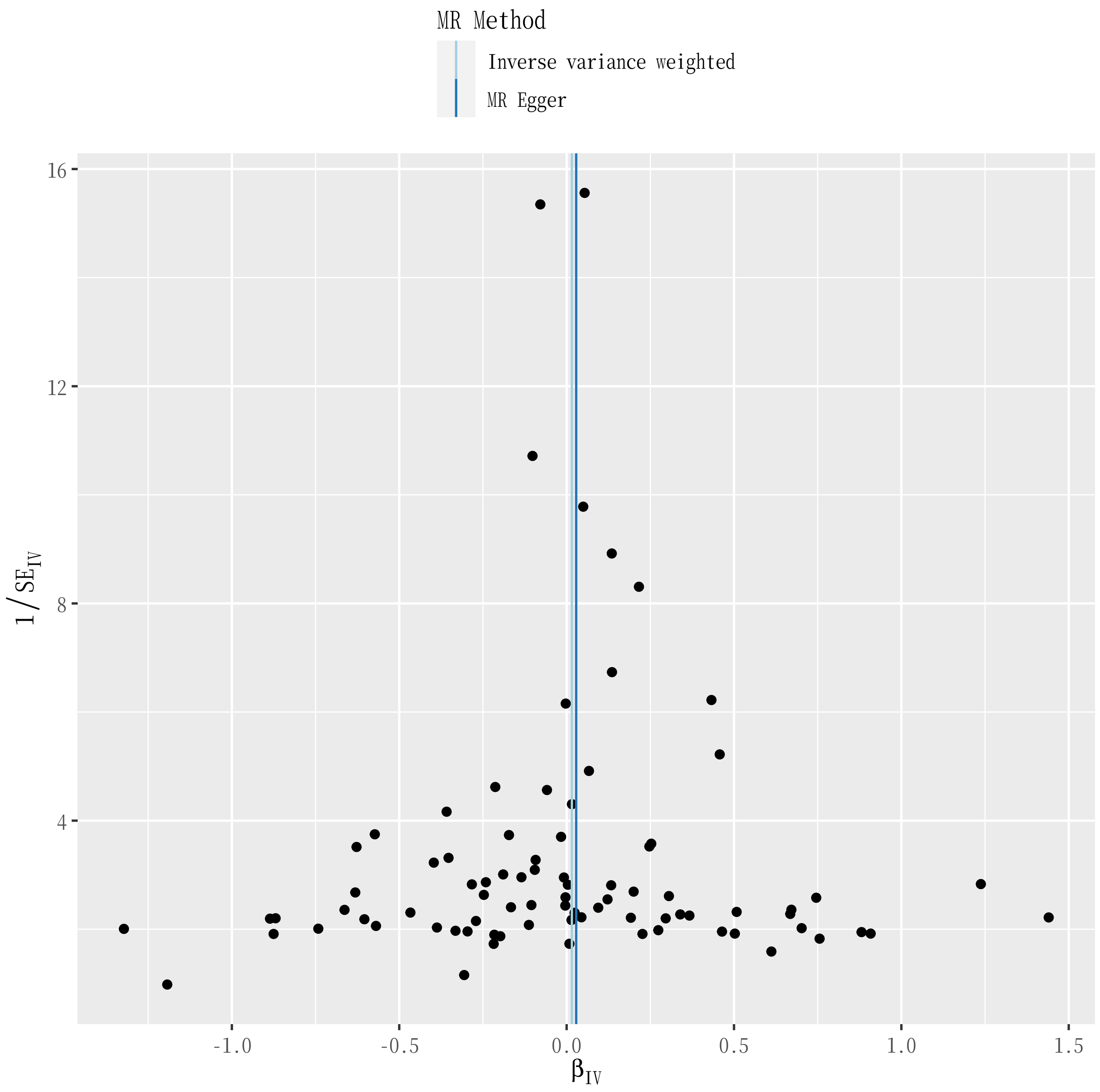

MR Method
Inverse variance weighted
MR Egger
16
12
8
4
$1/SE_{IV}$
-1.0
-0.5
0.0
0.5
1.0
1.5
β_{IV}

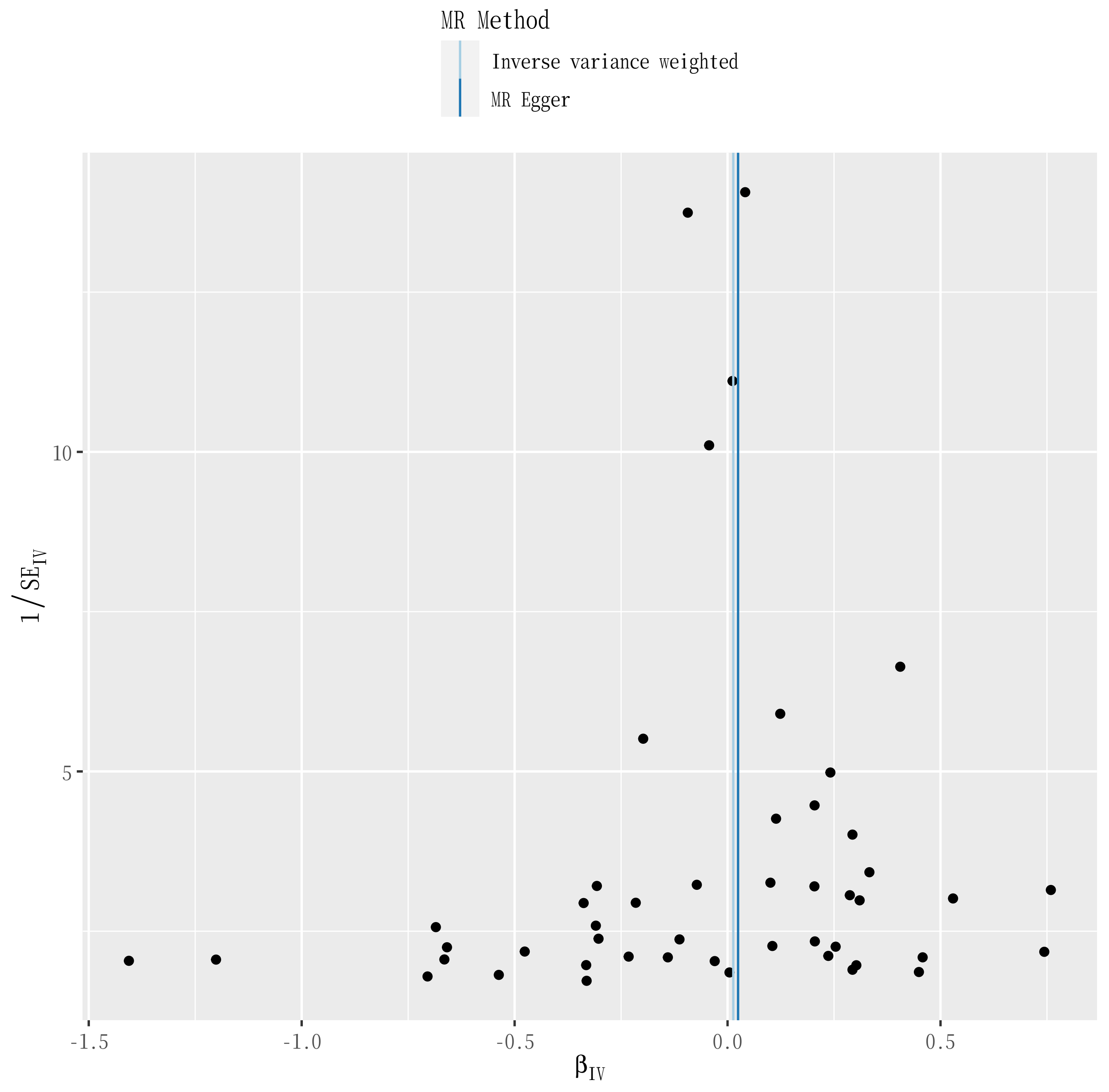
MR Method
Inverse variance weighted
MR Egger
1/SE_IV
10
5
-1.5
-1.0
-0.5
0.0
0.5
β_IV

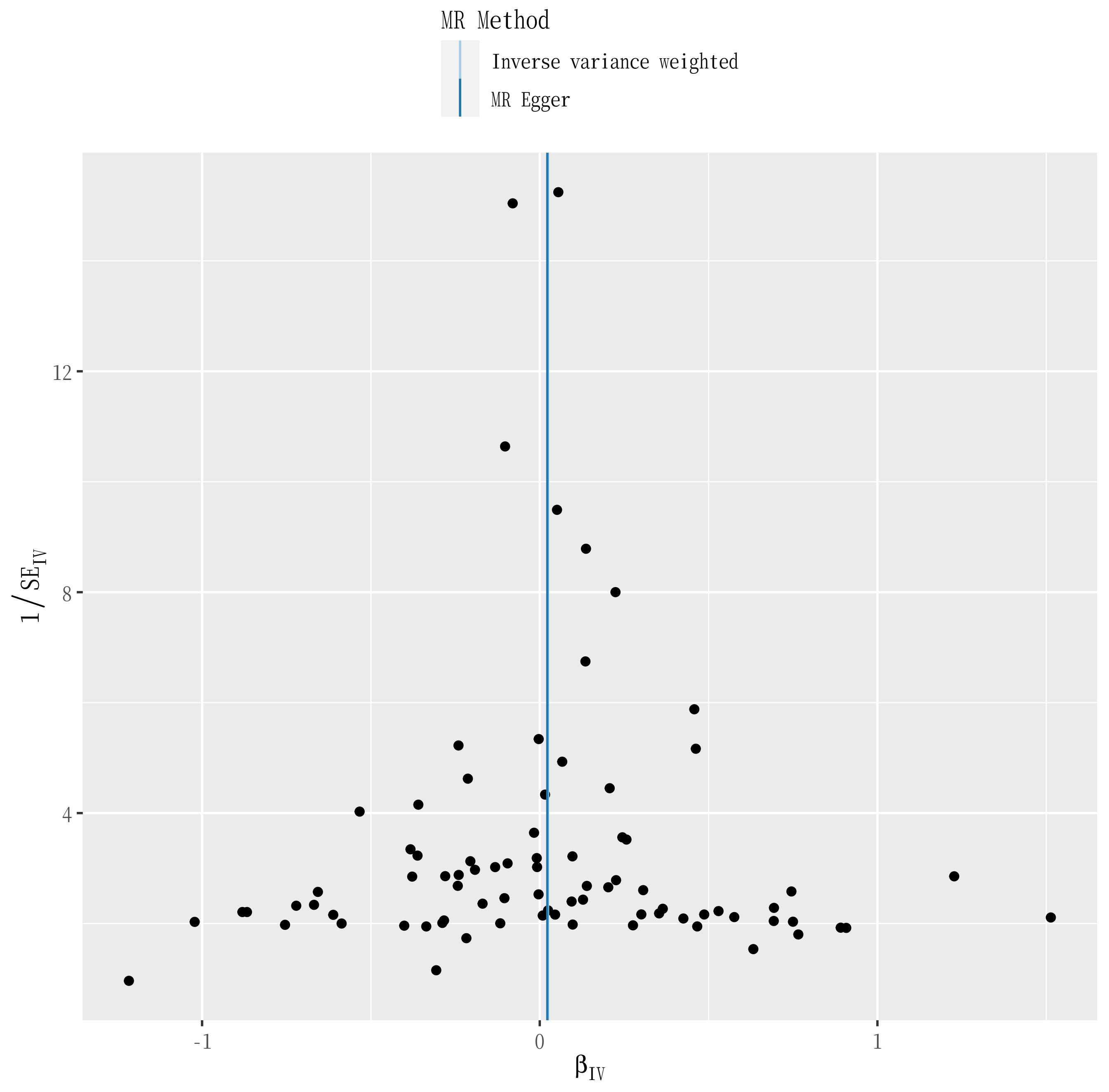

MR Method
Inverse variance weighted
MR Egger
12
8
4
1/SE_IV
-1
0
1
β_IV

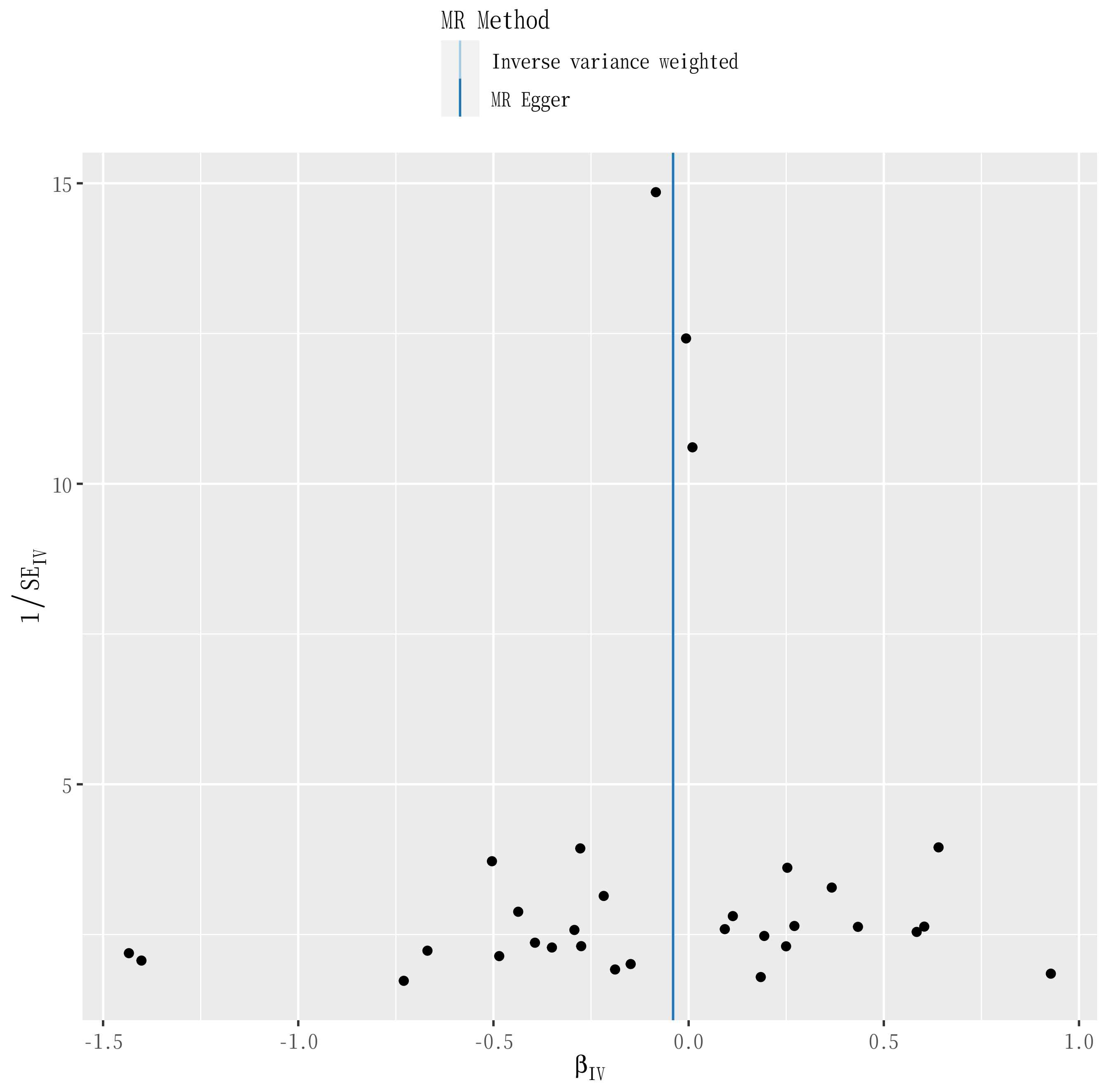
MR Method
Inverse variance weighted
MR Egger
15
10
5
1/SE_IV
-1.5
-1.0
-0.5
0.0
0.5
1.0
β_IV

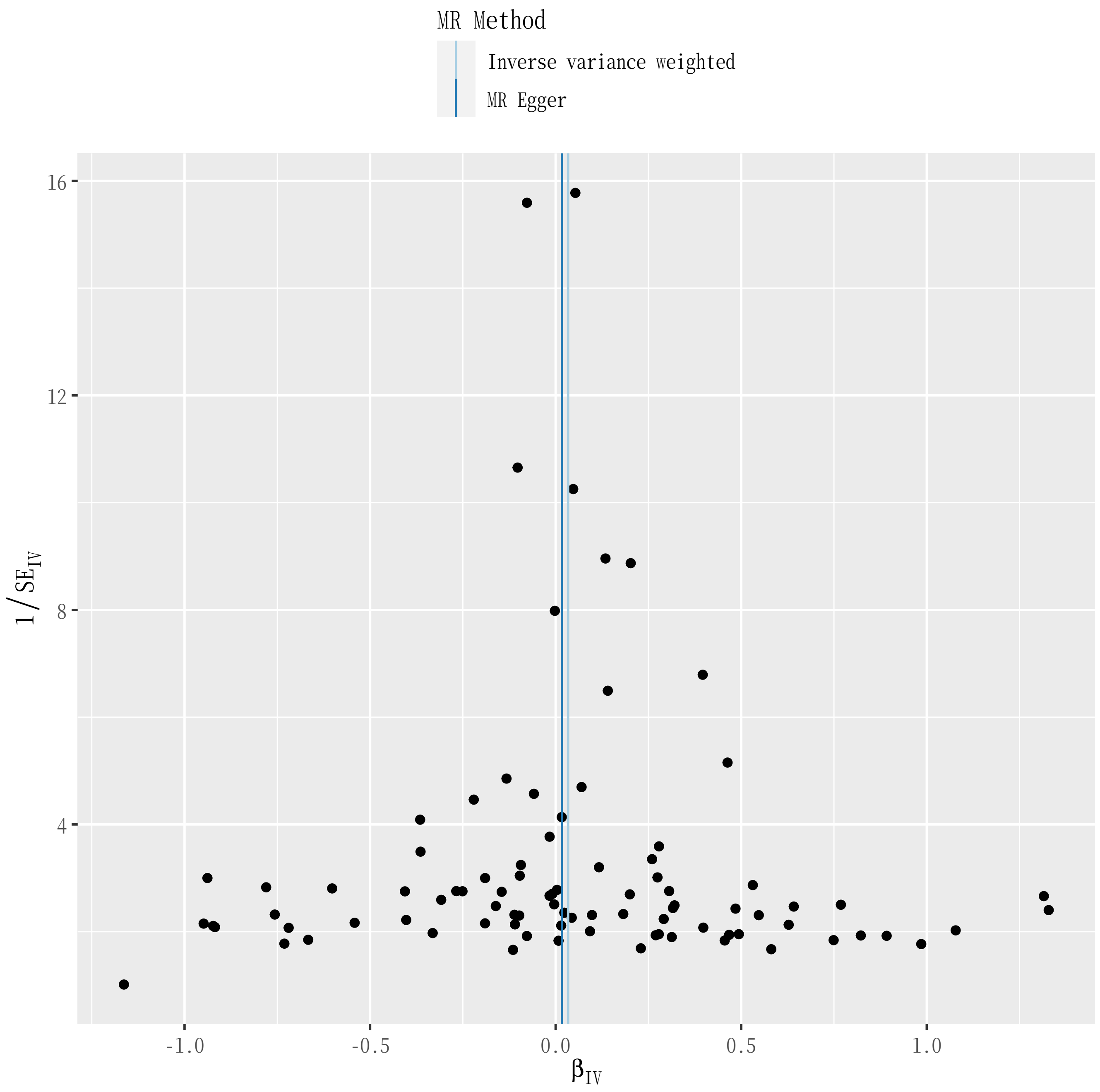
MR Method
Inverse variance weighted
MR Egger
16
12
8
4
$1/SE_{IV}$
-1.0
-0.5
0.0
0.5
1.0
β_{IV}

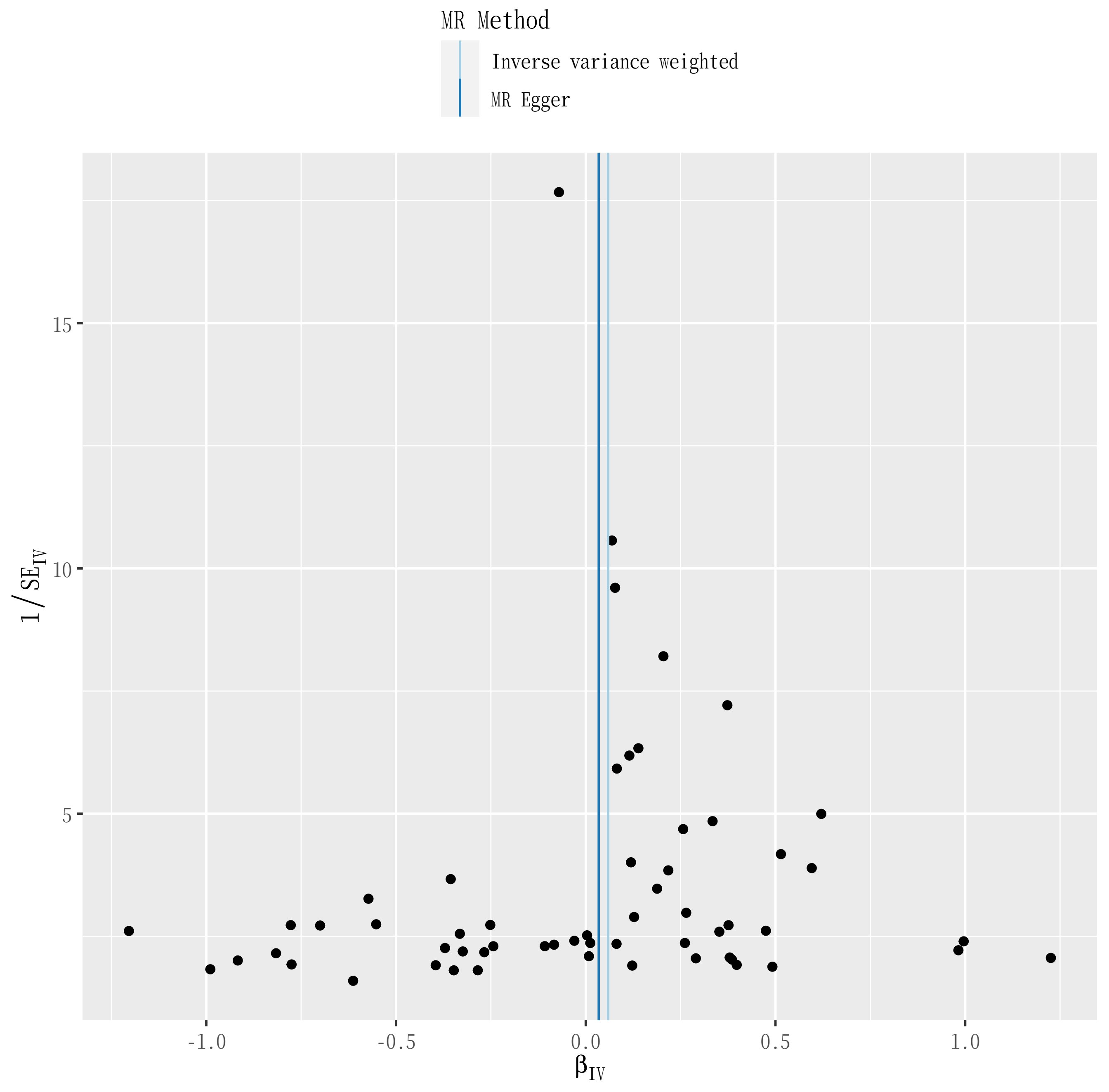
MR Method
Inverse variance weighted
MR Egger
15
10
5
1/SE_IV
-1.0
-0.5
0.0
0.5
1.0
β_IV

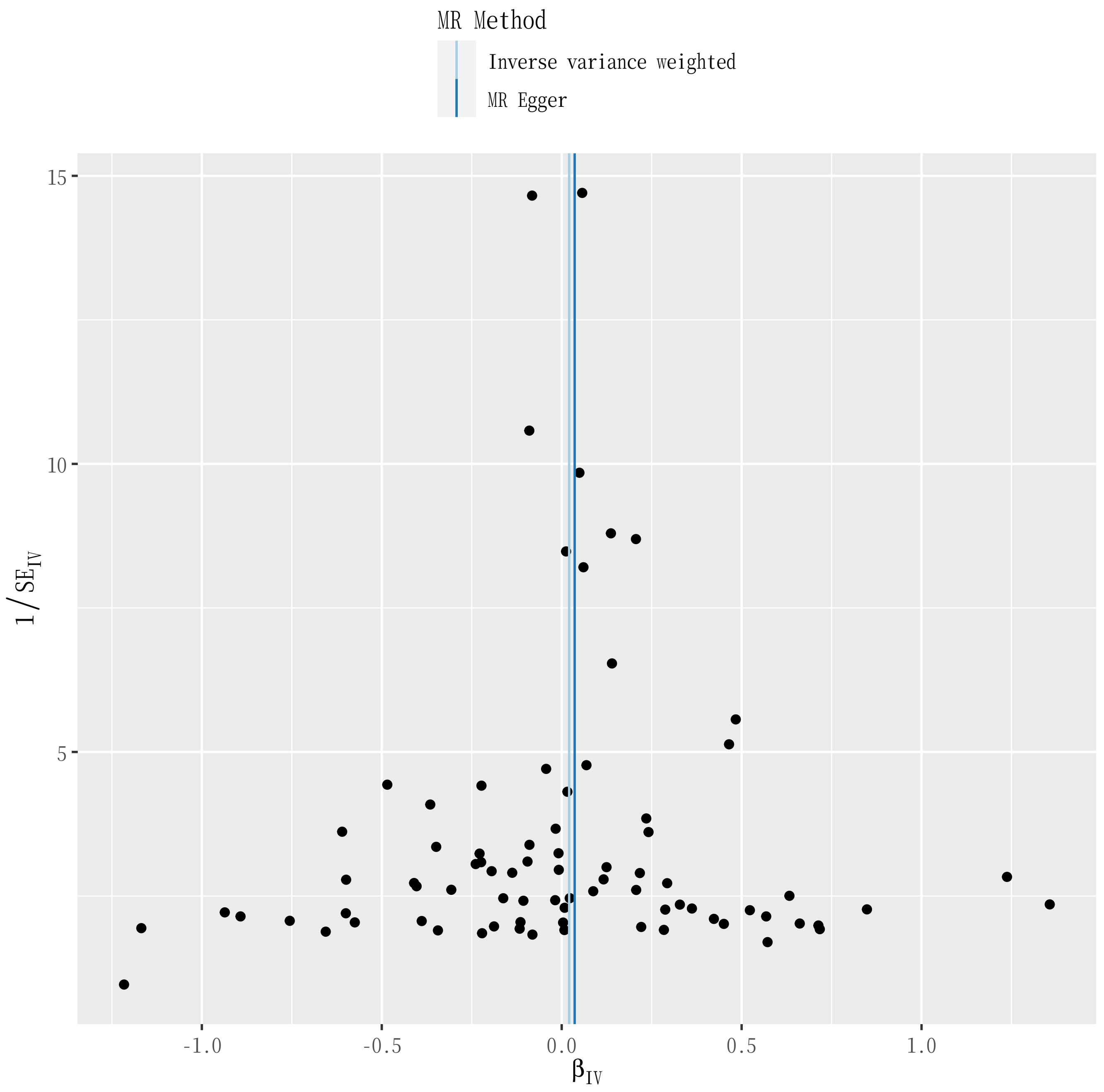
MR Method
Inverse variance weighted
MR Egger
15
10
5
$1/SE_{IV}$
-1.0
-0.5
0.0
0.5
1.0
β_{IV}

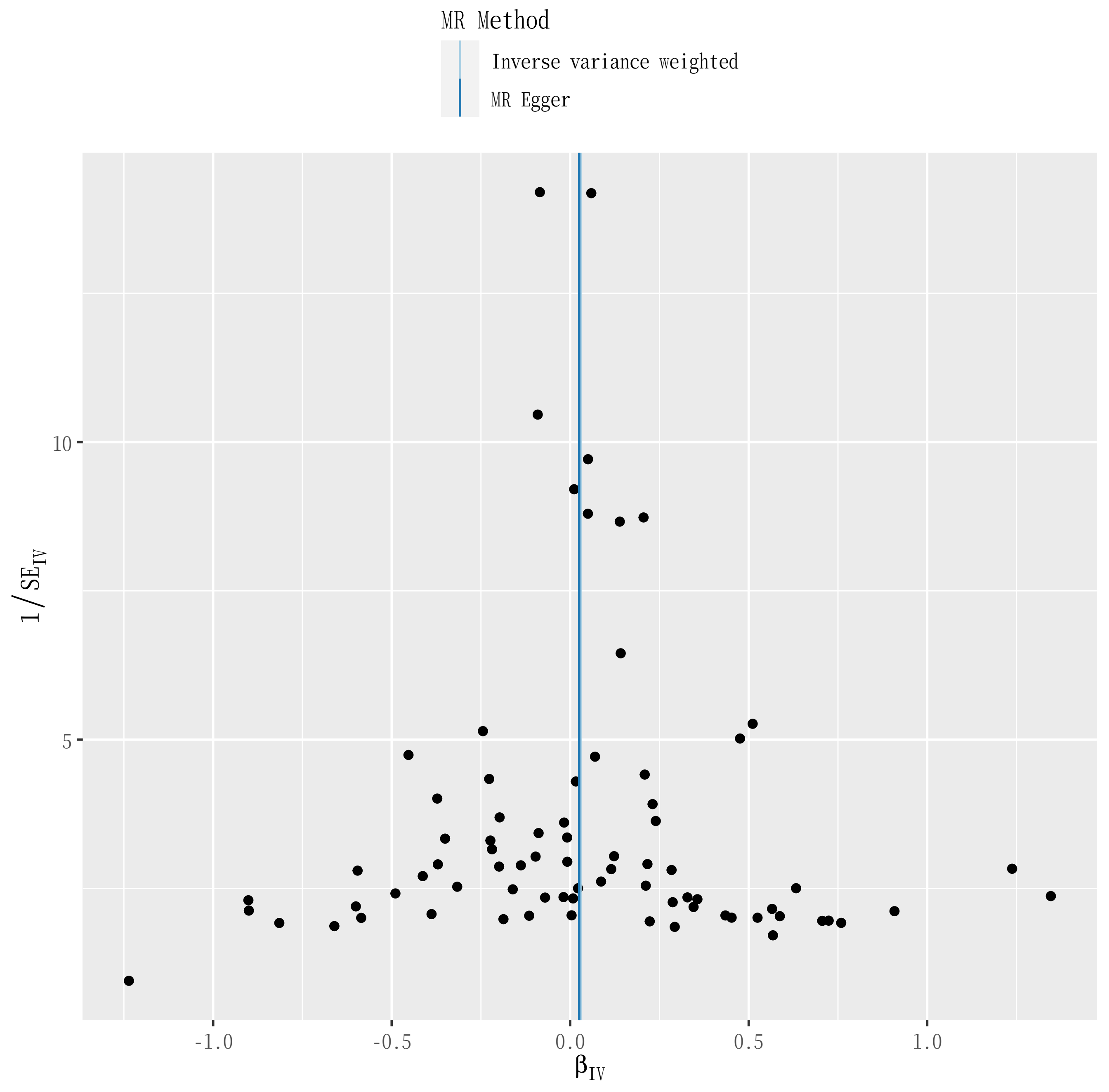
MR Method
Inverse variance weighted
MR Egger
10
5
$1/SE_{IV}$
-1.0
-0.5
0.0
0.5
1.0
β_{IV}

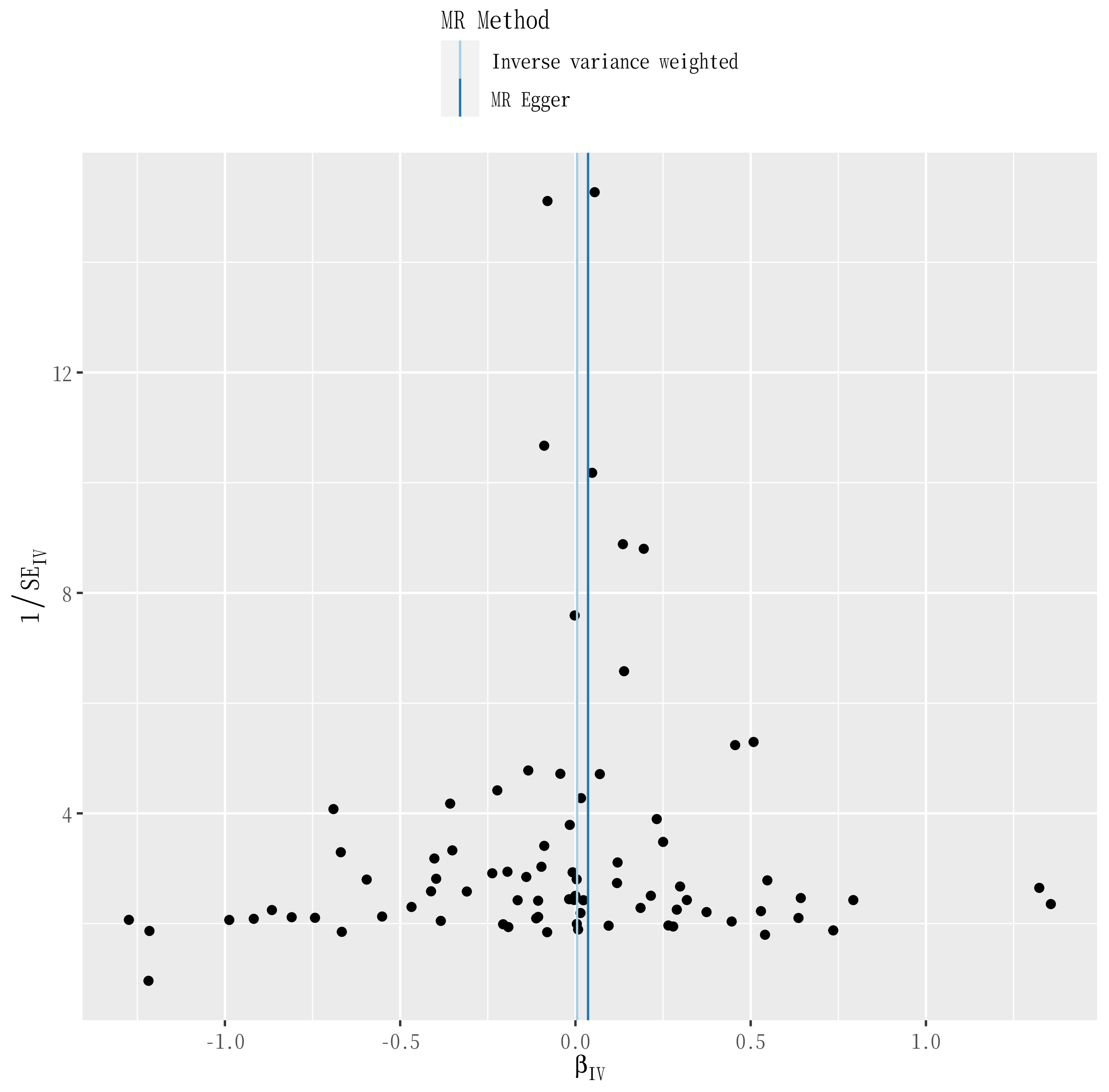
MR Method
Inverse variance weighted
MR Egger
12
8
4
$1/SE_{IV}$
-1.0
-0.5
0.0
0.5
1.0
β_{IV}

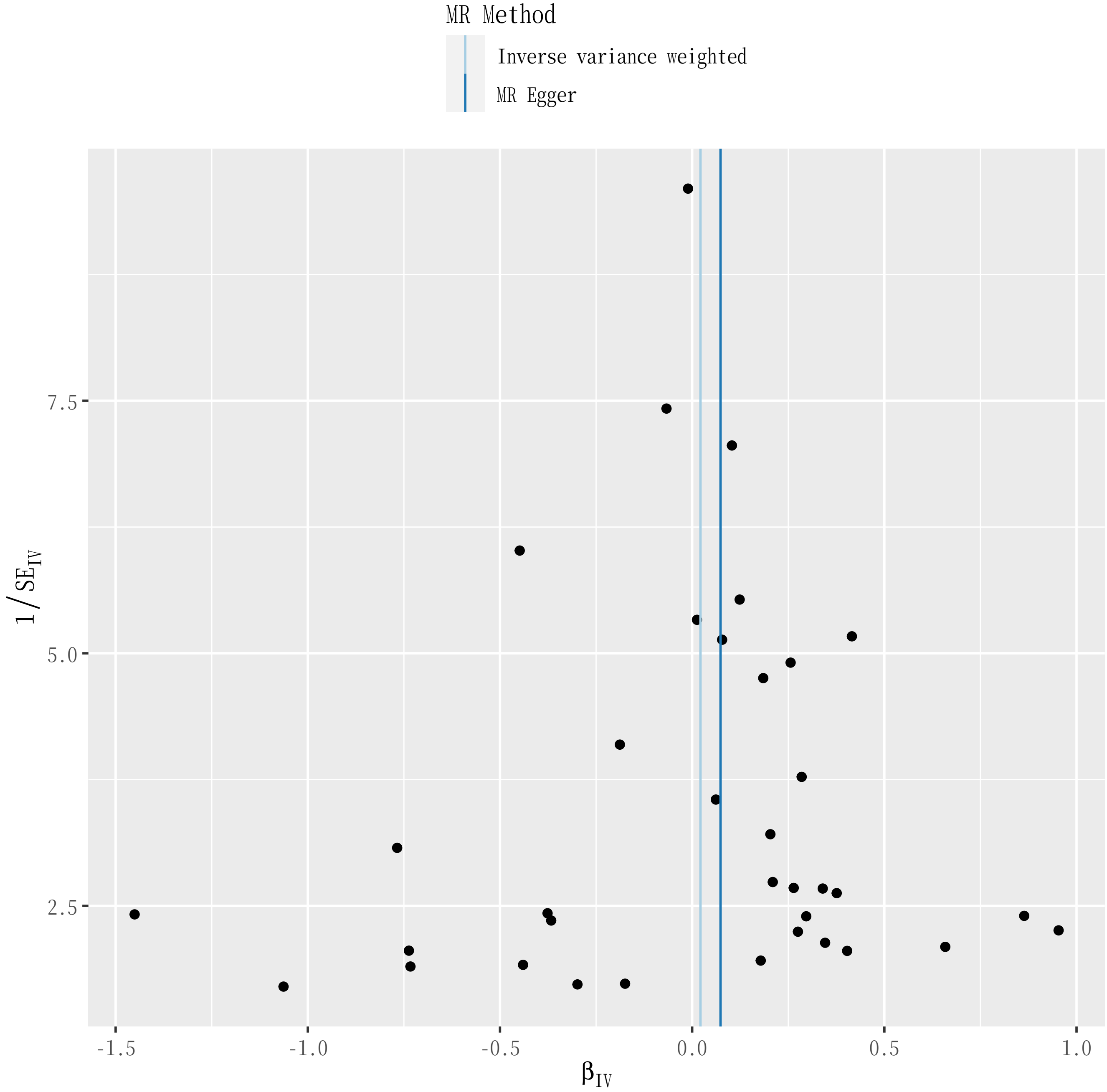
MR Method
Inverse variance weighted
MR Egger
7.5
5.0
2.5
$1/SE_{IV}$
-1.5
-1.0
-0.5
0.0
0.5
1.0
β_{IV}

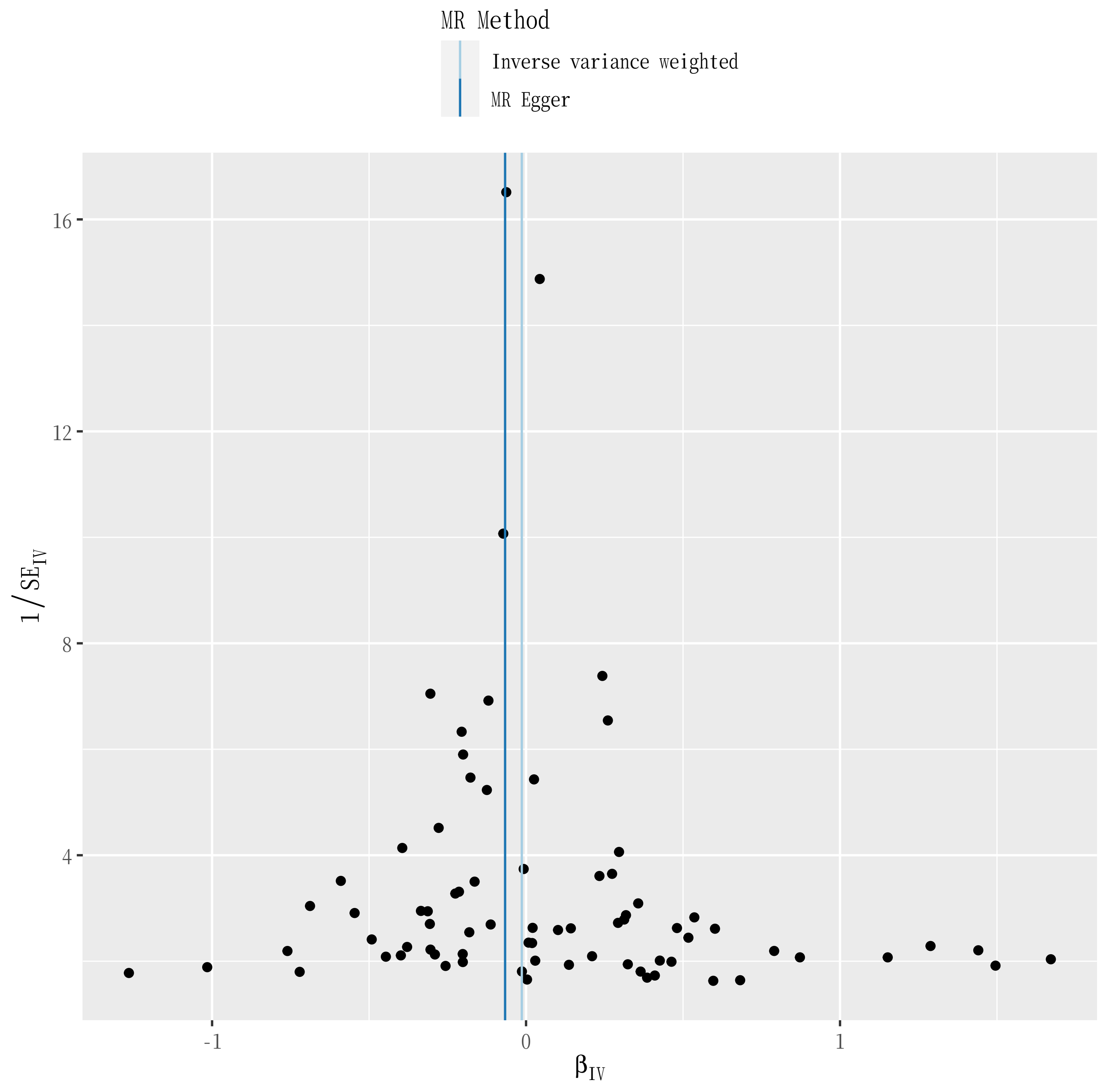
MR Method
Inverse variance weighted
MR Egger
16
12
8
4
-1
0
1
1/SE_IV
β_IV

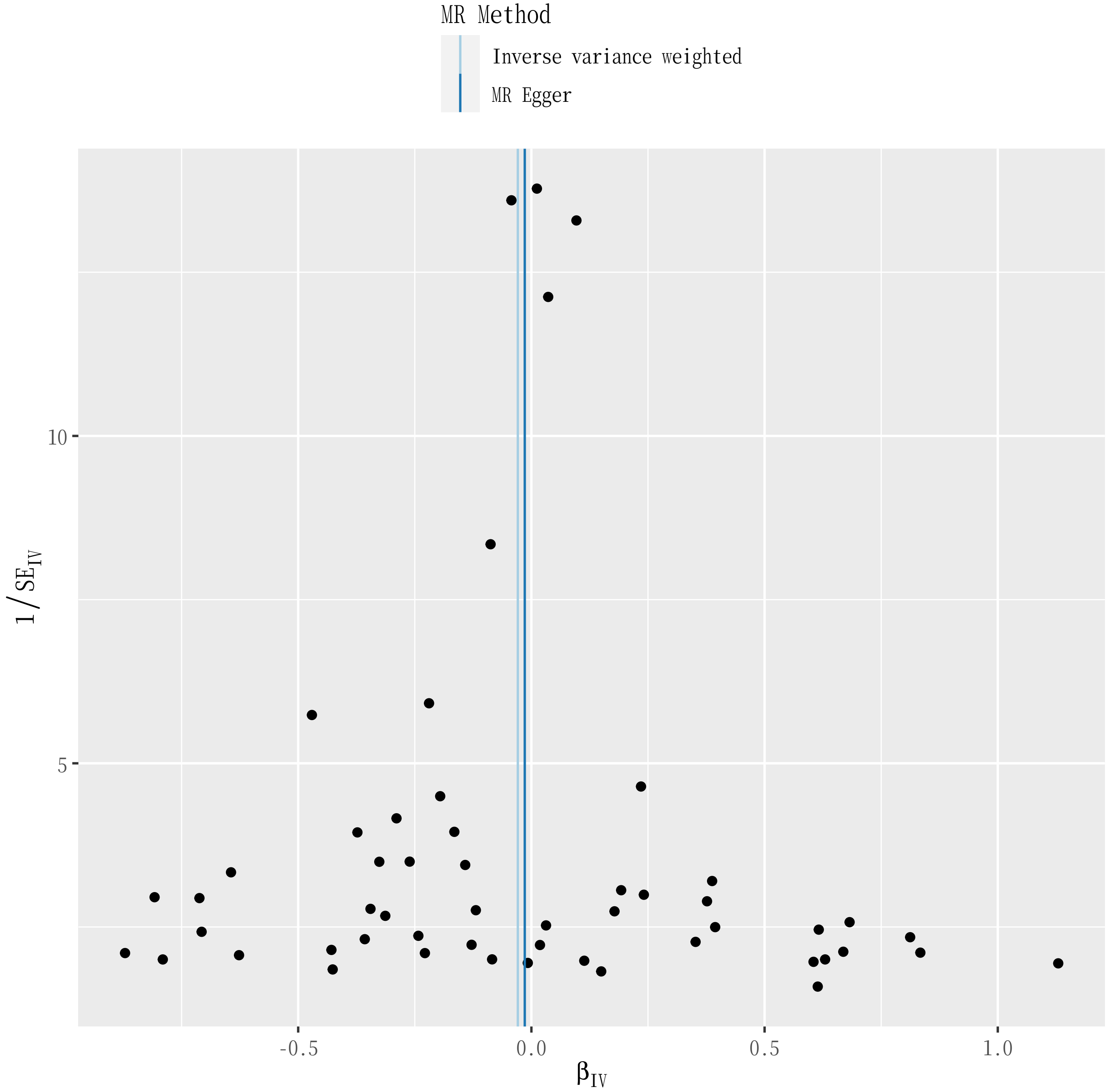

MR Method
Inverse variance weighted
MR Egger
10
5
-0.5
0.0
0.5
1.0
$1/SE_{IV}$
β_{IV}

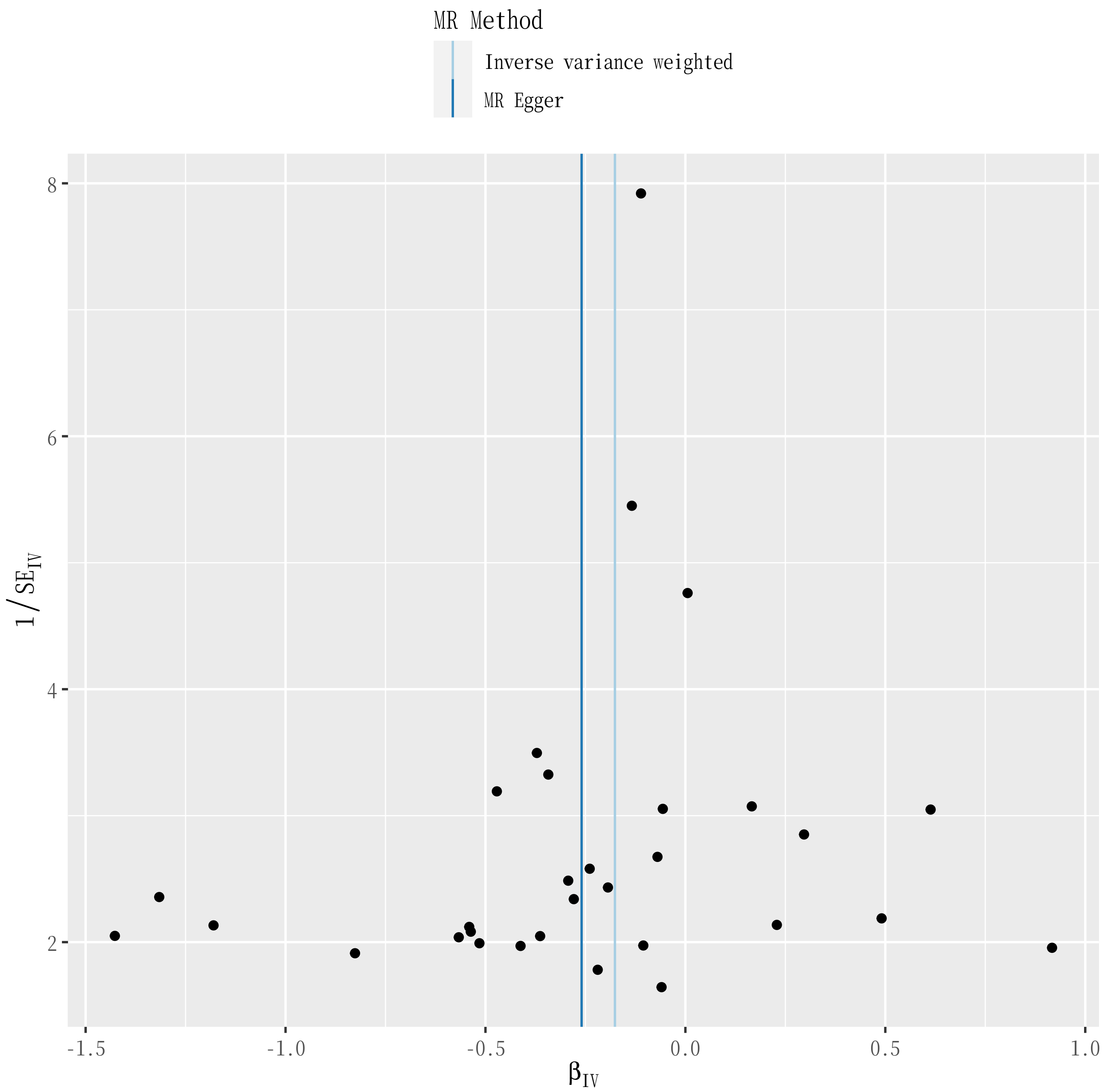
MR Method
Inverse variance weighted
MR Egger
8
6
4
2
1/SE_IV
-1.5
-1.0
-0.5
0.0
0.5
1.0
β_IV

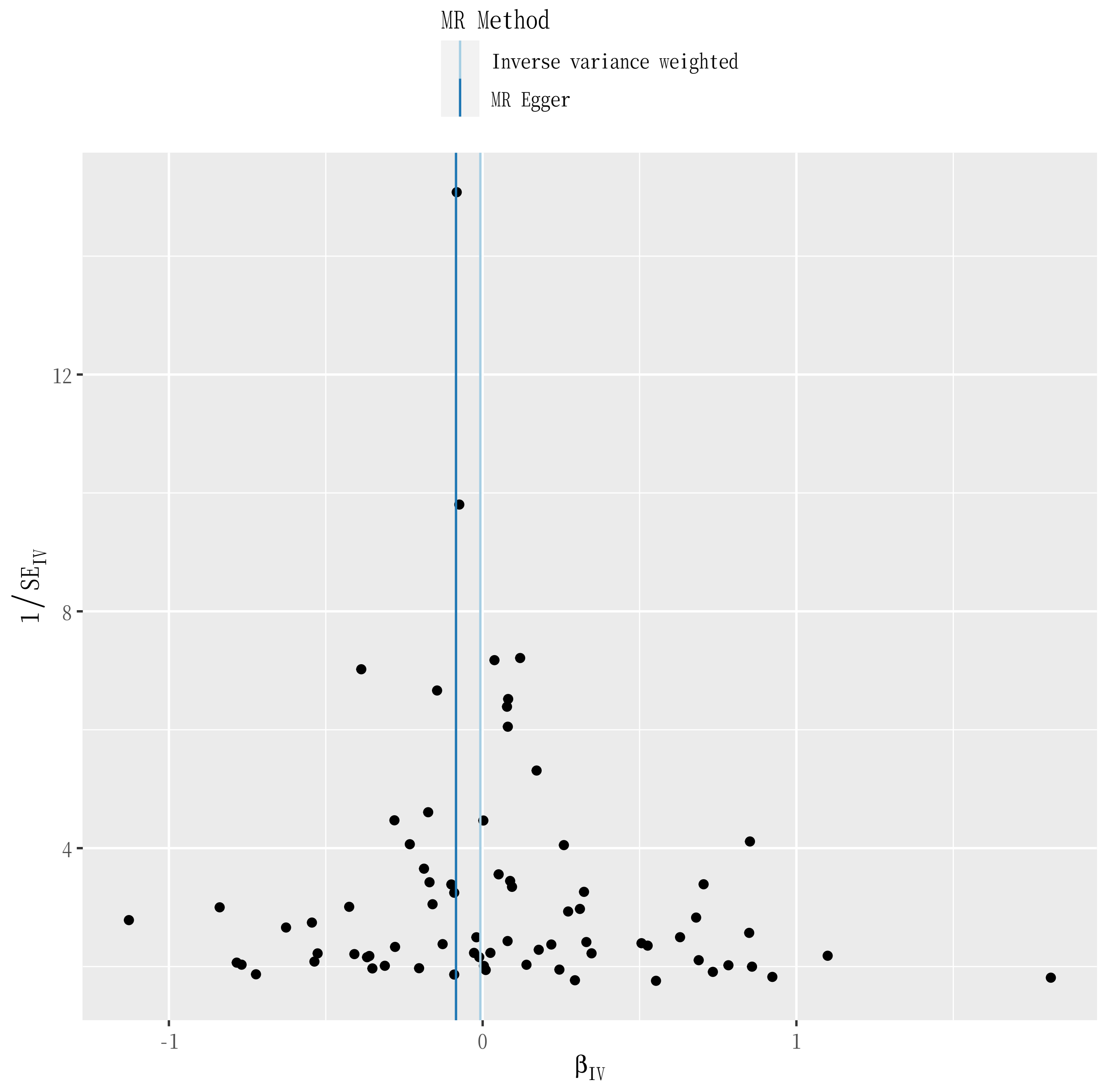
MR Method
Inverse variance weighted
MR Egger
12
8
4
1/SE_IV
-1
0
1
β_IV

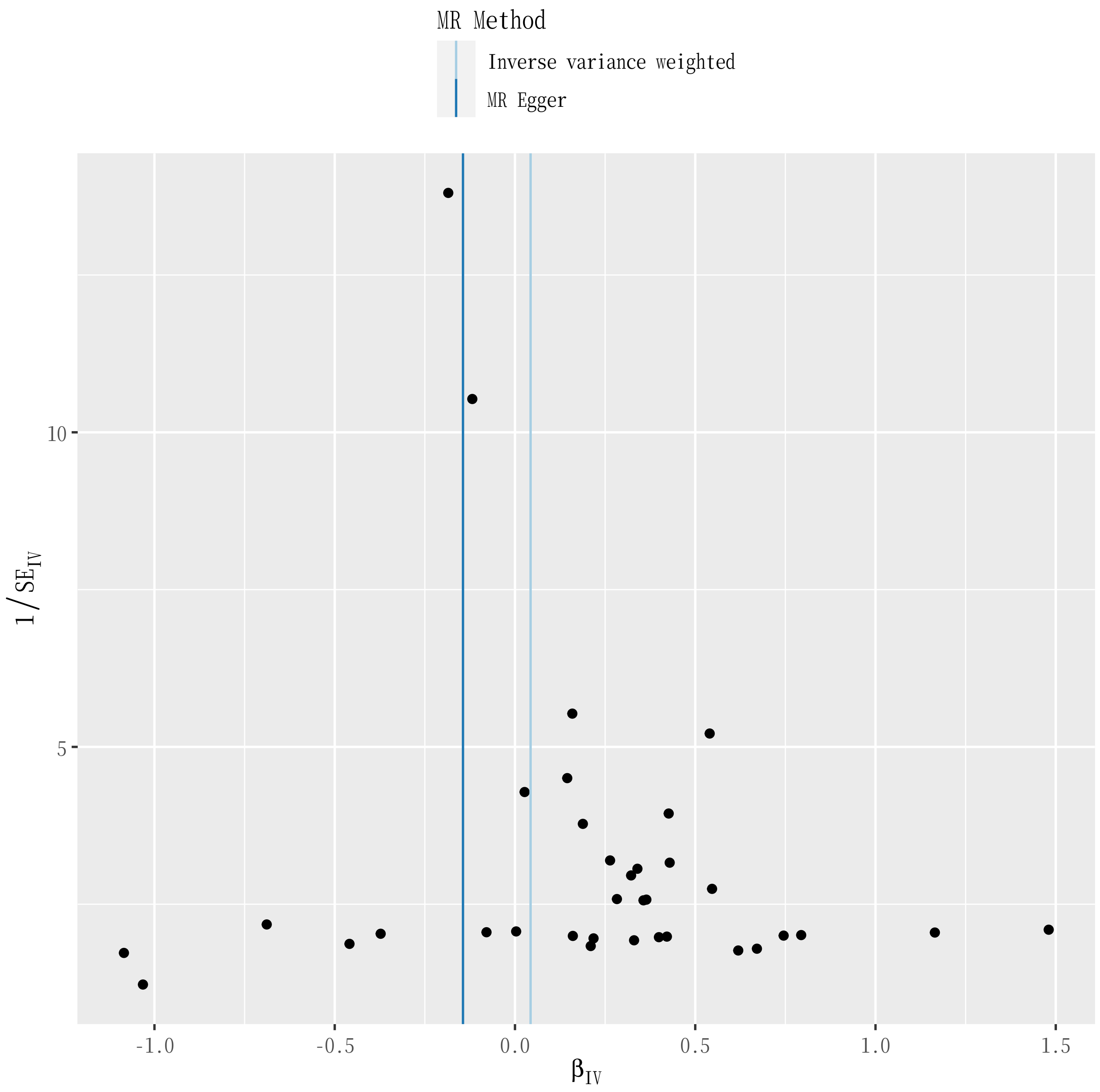
MR Method
Inverse variance weighted
MR Egger
10
5
-1.0
-0.5
0.0
0.5
1.0
1.5
$1/SE_{IV}$
β_{IV}

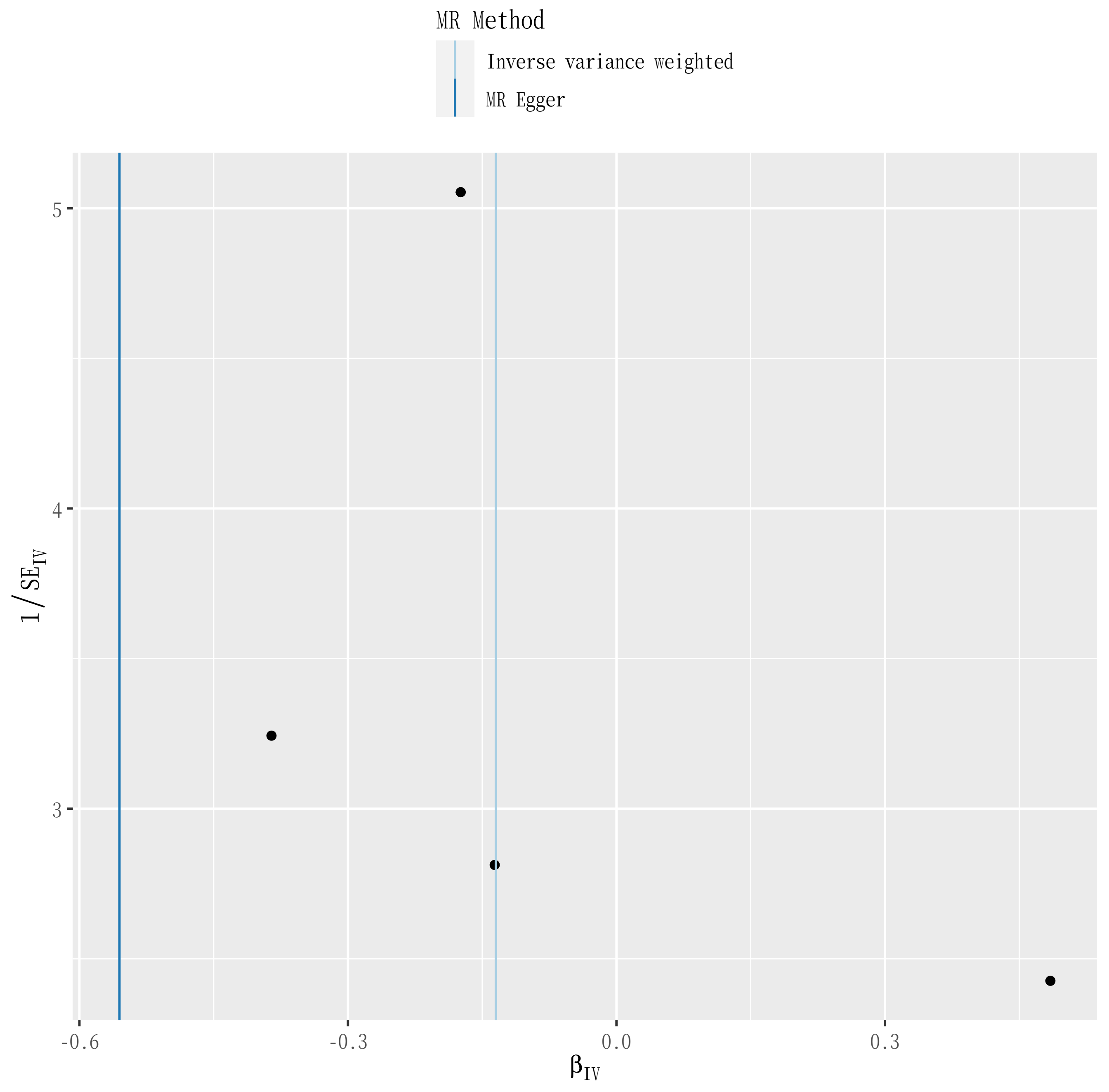
MR Method
Inverse variance weighted
MR Egger
5
4
3
1/SE_IV
-0.6
-0.3
0.0
0.3
β_IV

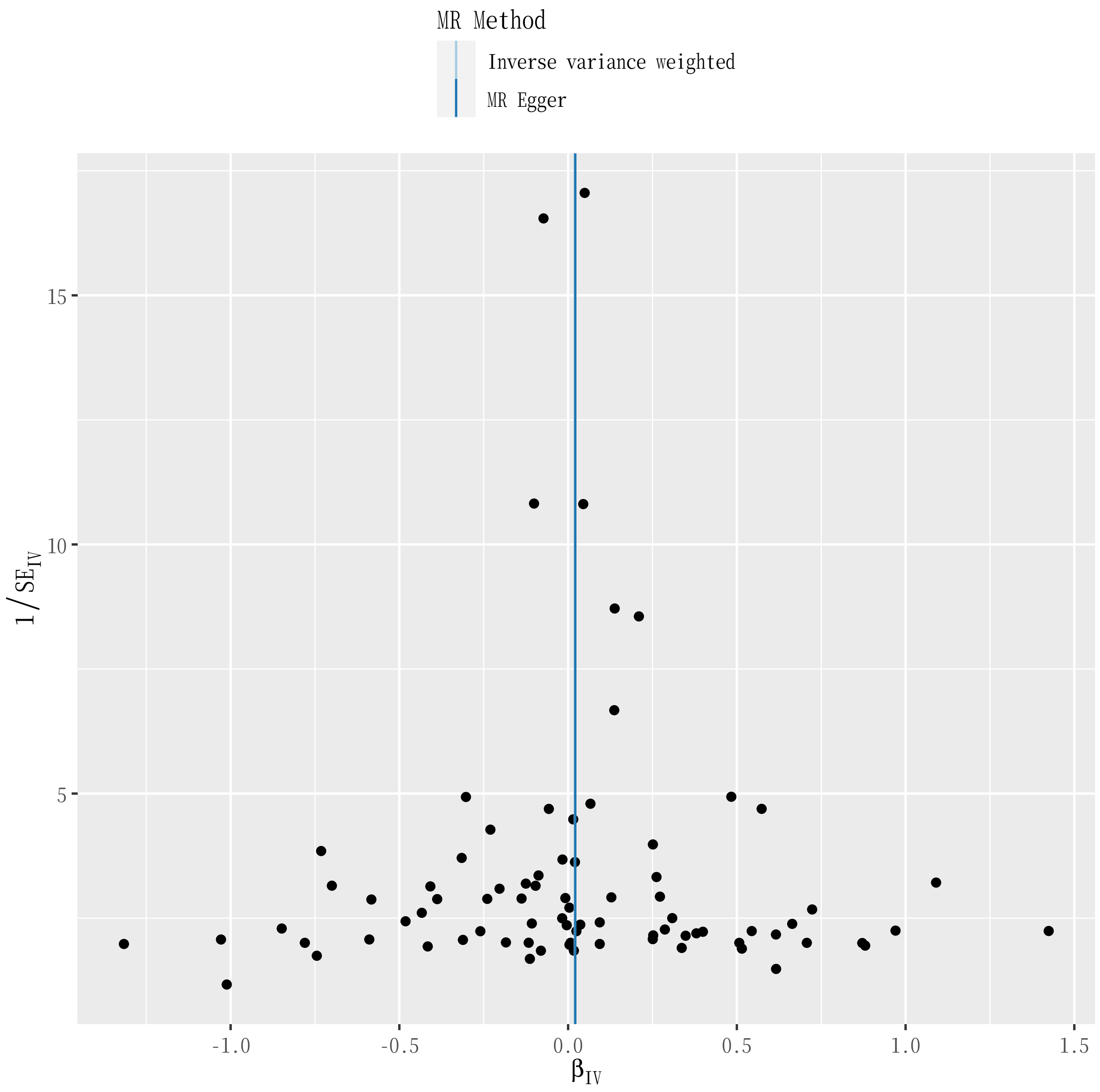
MR Method
Inverse variance weighted
MR Egger
15
10
5
1/SE_IV
-1.0
-0.5
0.0
0.5
1.0
1.5
β_IV

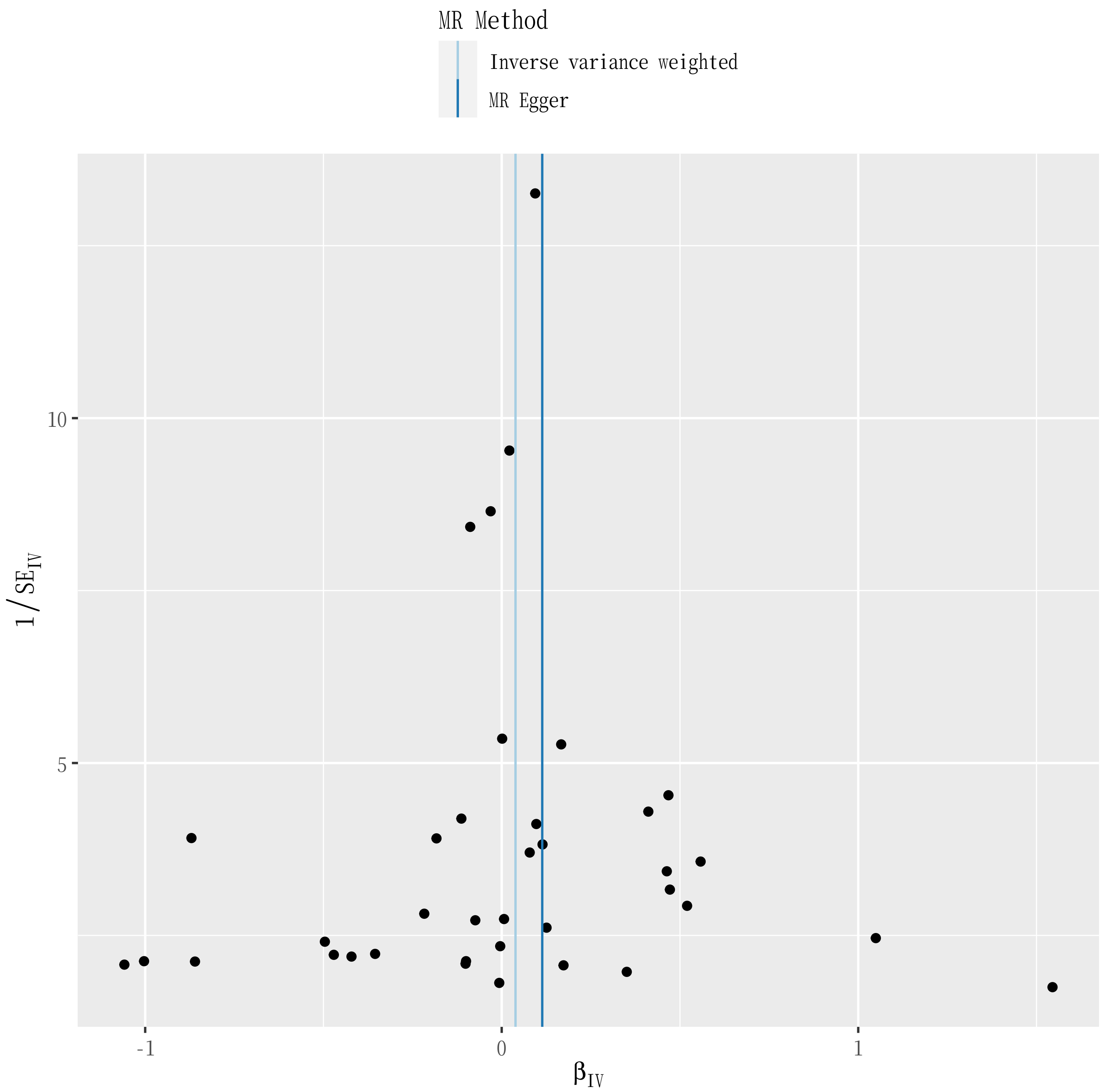
MR Method
Inverse variance weighted
MR Egger
10
5
$1/SE_{IV}$
-1
0
1
β_{IV}

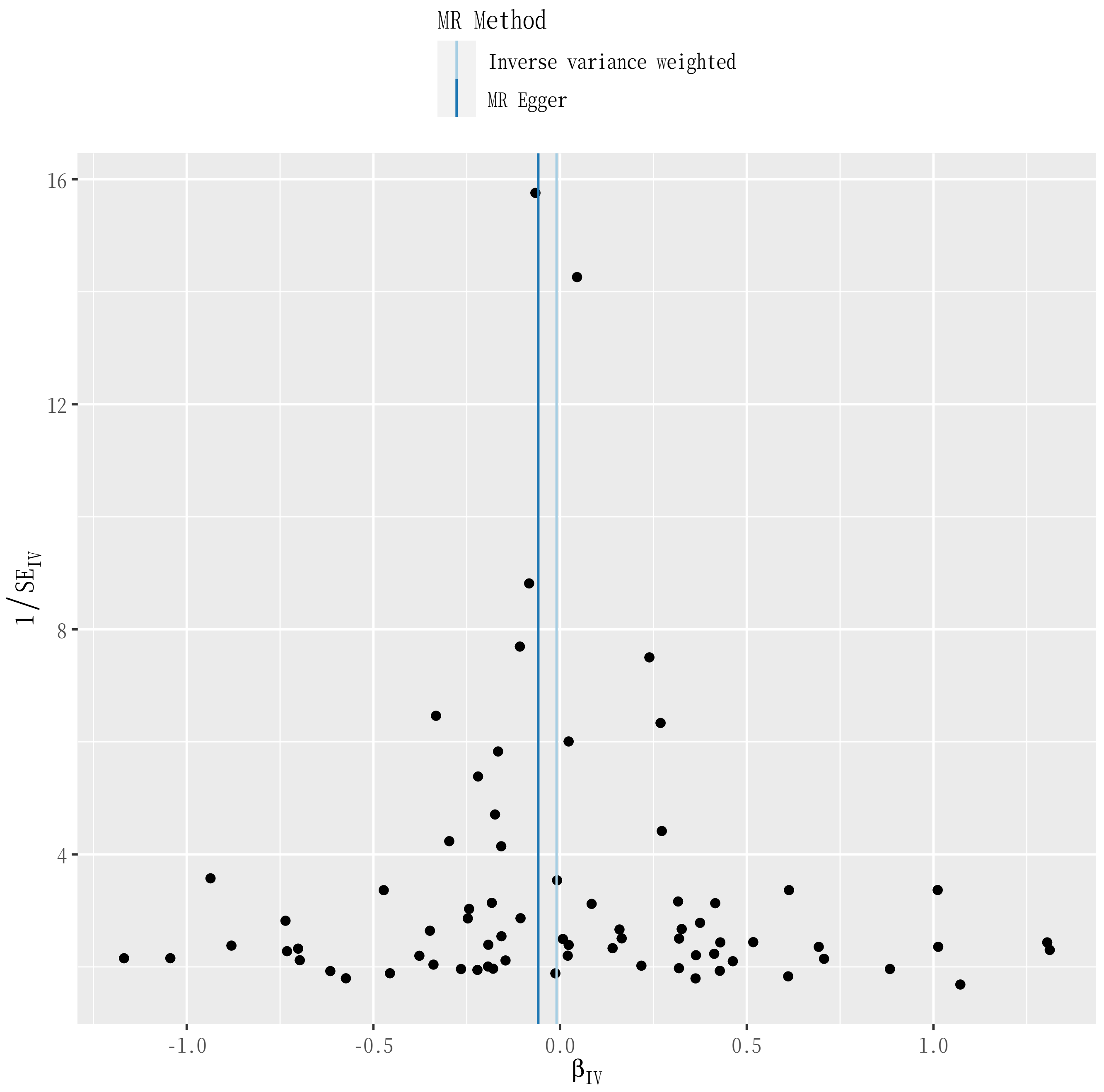

MR Method
Inverse variance weighted
MR Egger
16
12
8
4
1/SE$_{IV}$
-1.0
-0.5
0.0
0.5
1.0
β$_{IV}$

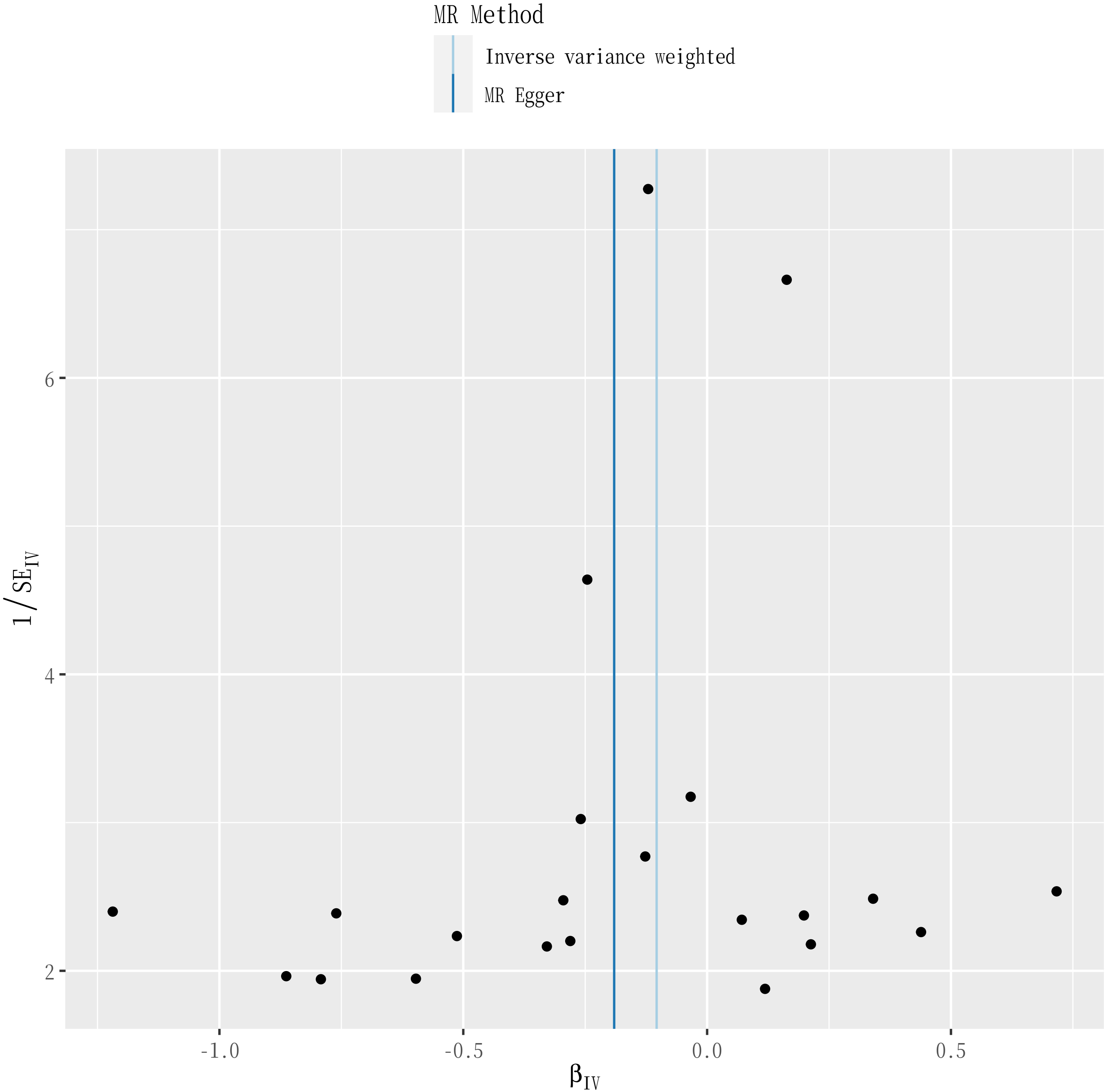

MR Method
Inverse variance weighted
MR Egger
6
4
2
1/SE_IV
-1.0
-0.5
0.0
0.5
β_IV

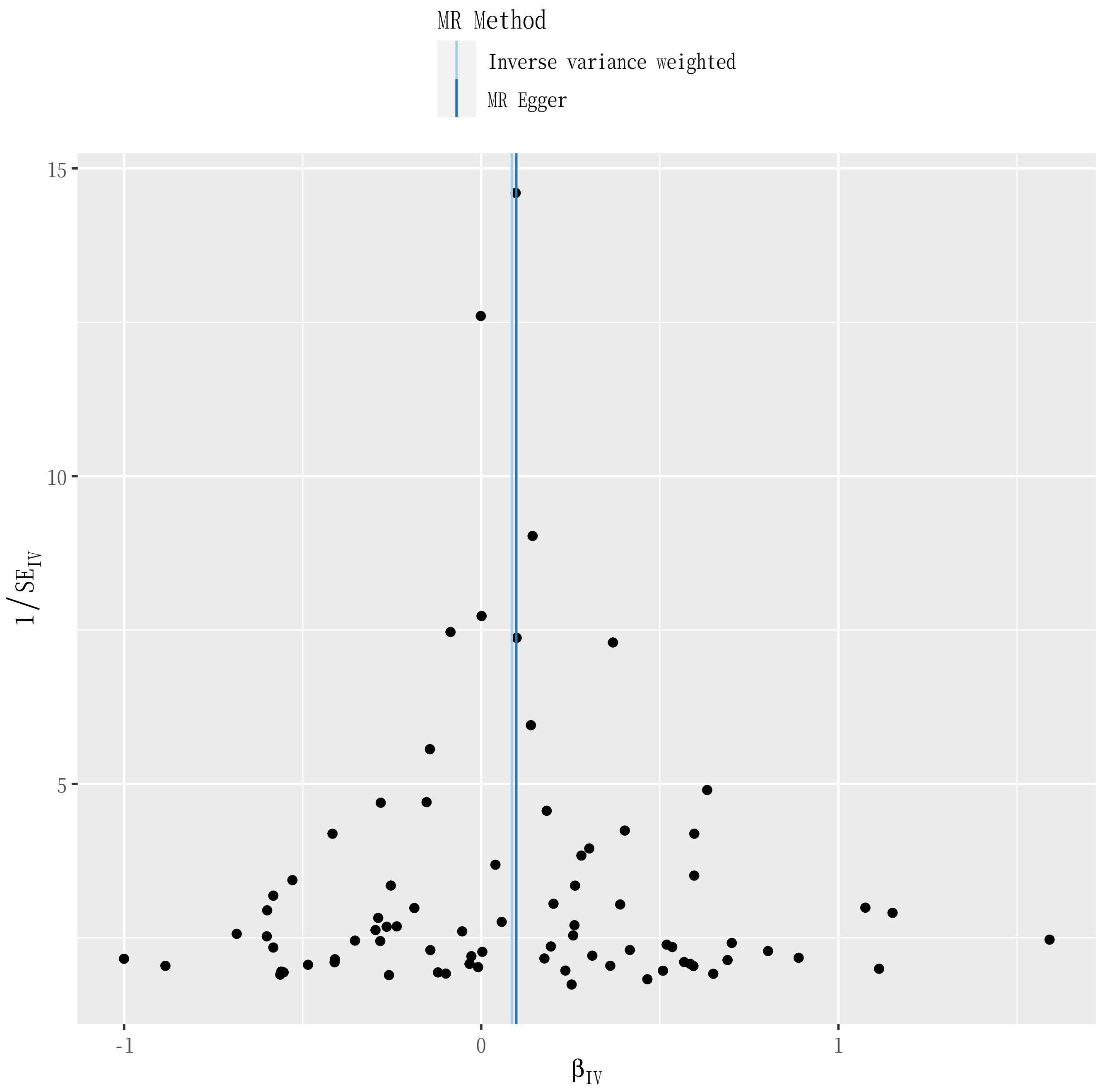

MR Method
Inverse variance weighted
MR Egger
15
10
5
-1
0
1
$1/SE_{IV}$
β_{IV}

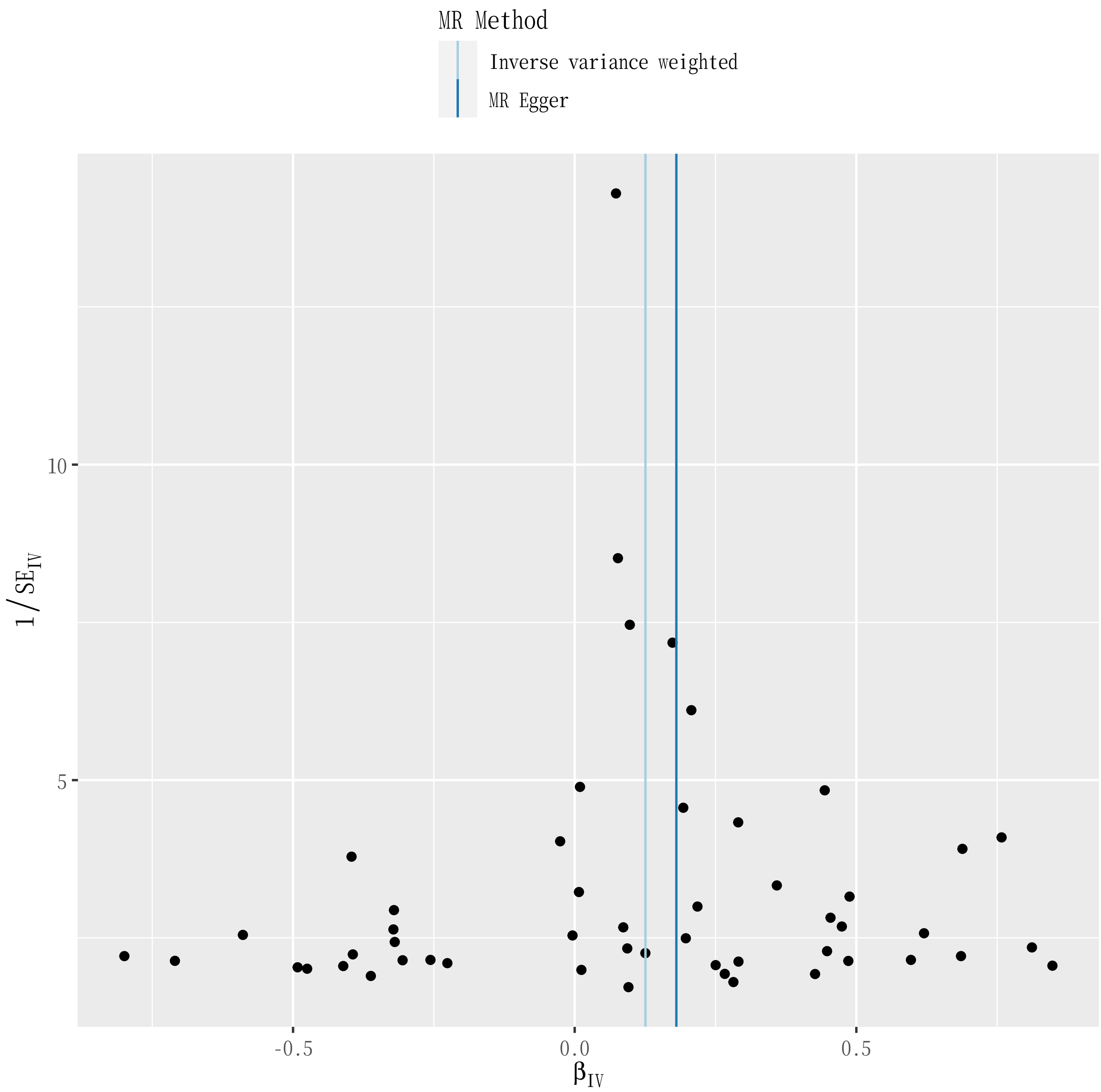

MR Method
Inverse variance weighted
MR Egger
10
5
1/SE_IV
-0.5
0.0
0.5
β_IV

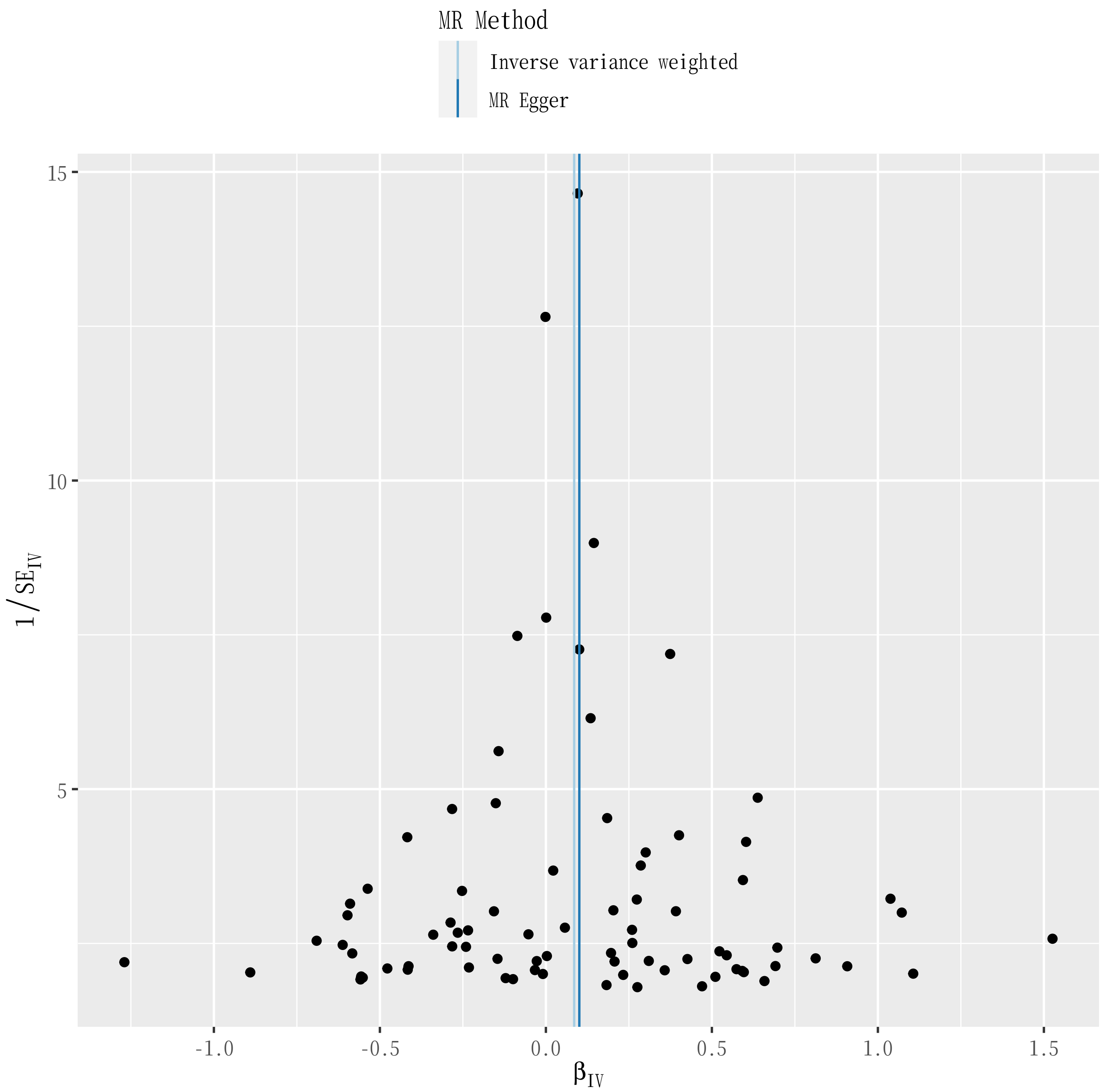
MR Method
Inverse variance weighted
MR Egger
15
10
5
1/SE$_{IV}$
-1.0
-0.5
0.0
0.5
1.0
1.5
β$_{IV}$

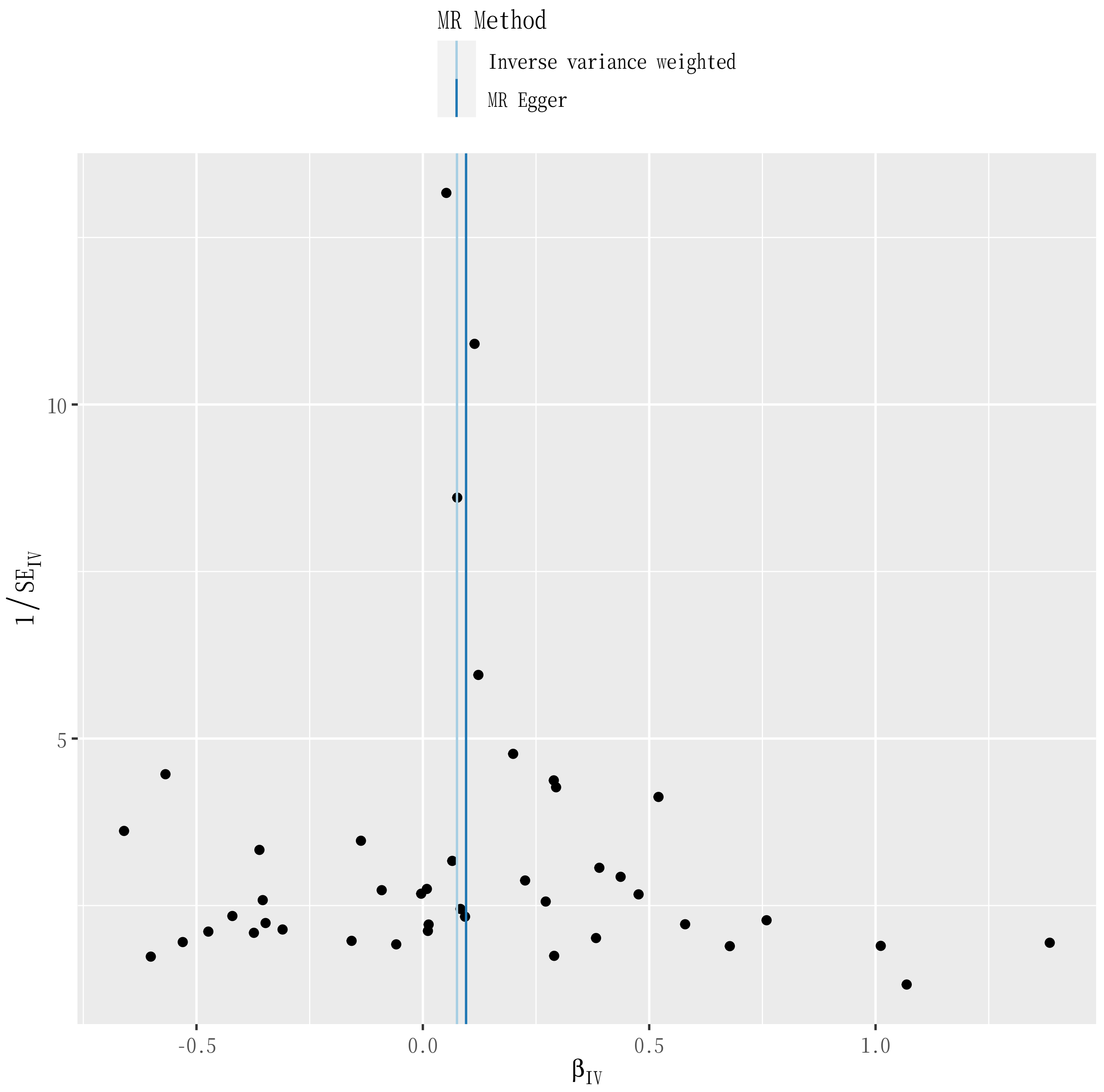

MR Method
Inverse variance weighted
MR Egger
10
5
-0.5
0.0
0.5
1.0
$1/SE_{IV}$
β_{IV}

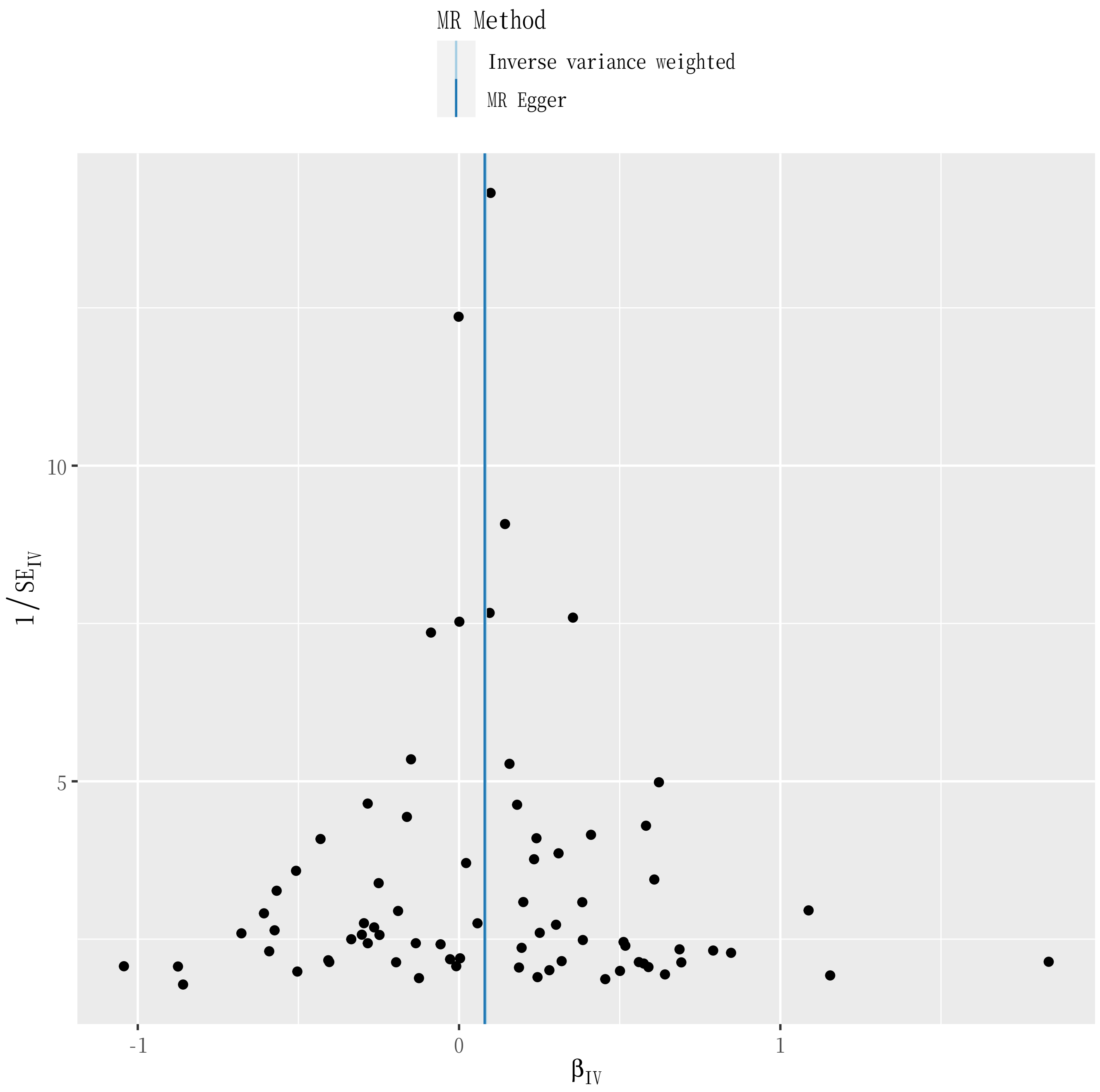

MR Method
Inverse variance weighted
MR Egger
10
5
-1
0
1
β_IV
1/SE_IV

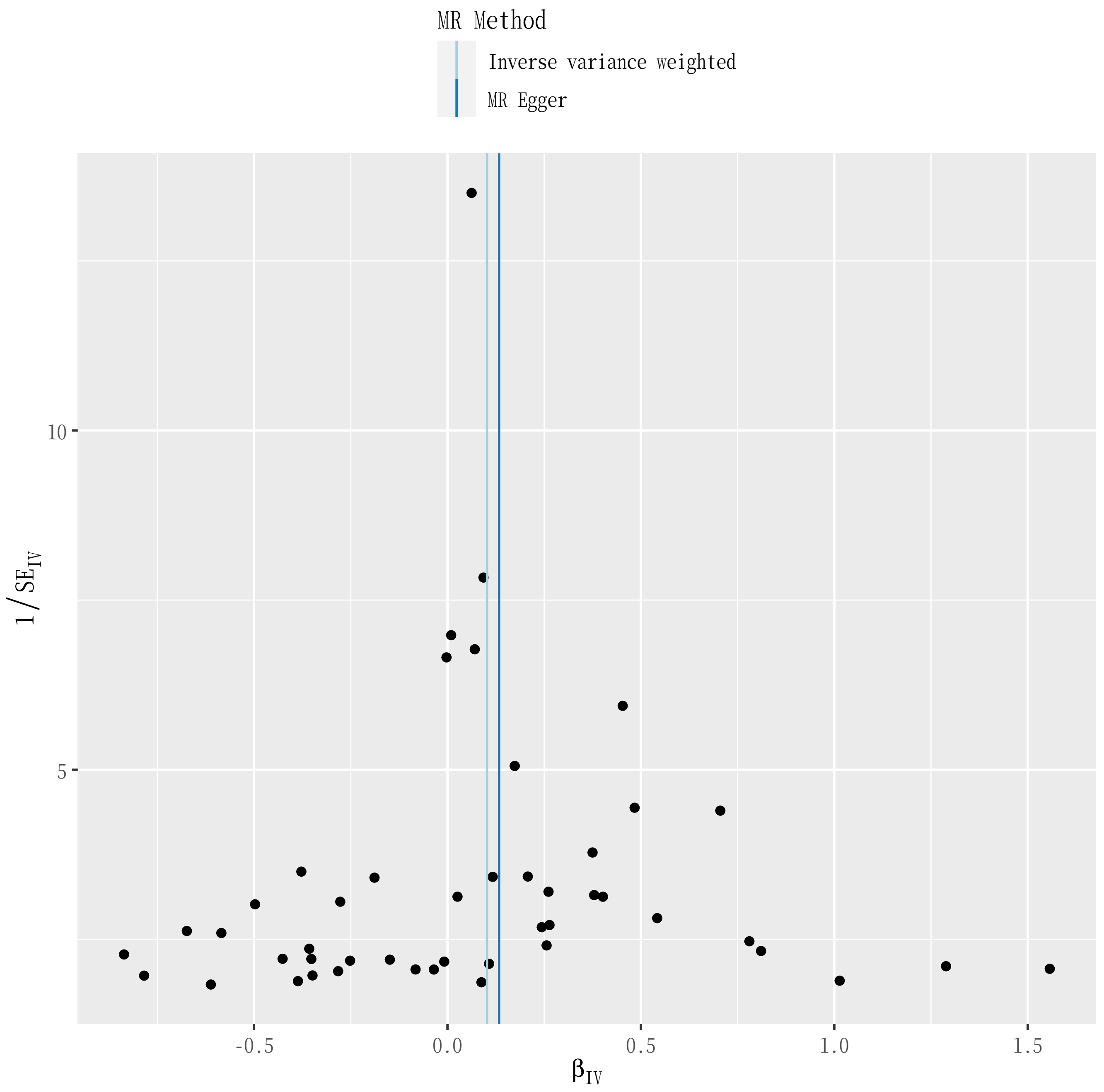

MR Method
Inverse variance weighted
MR Egger
10
5
$1/SE_{IV}$
-0.5
0.0
0.5
1.0
1.5
β_{IV}

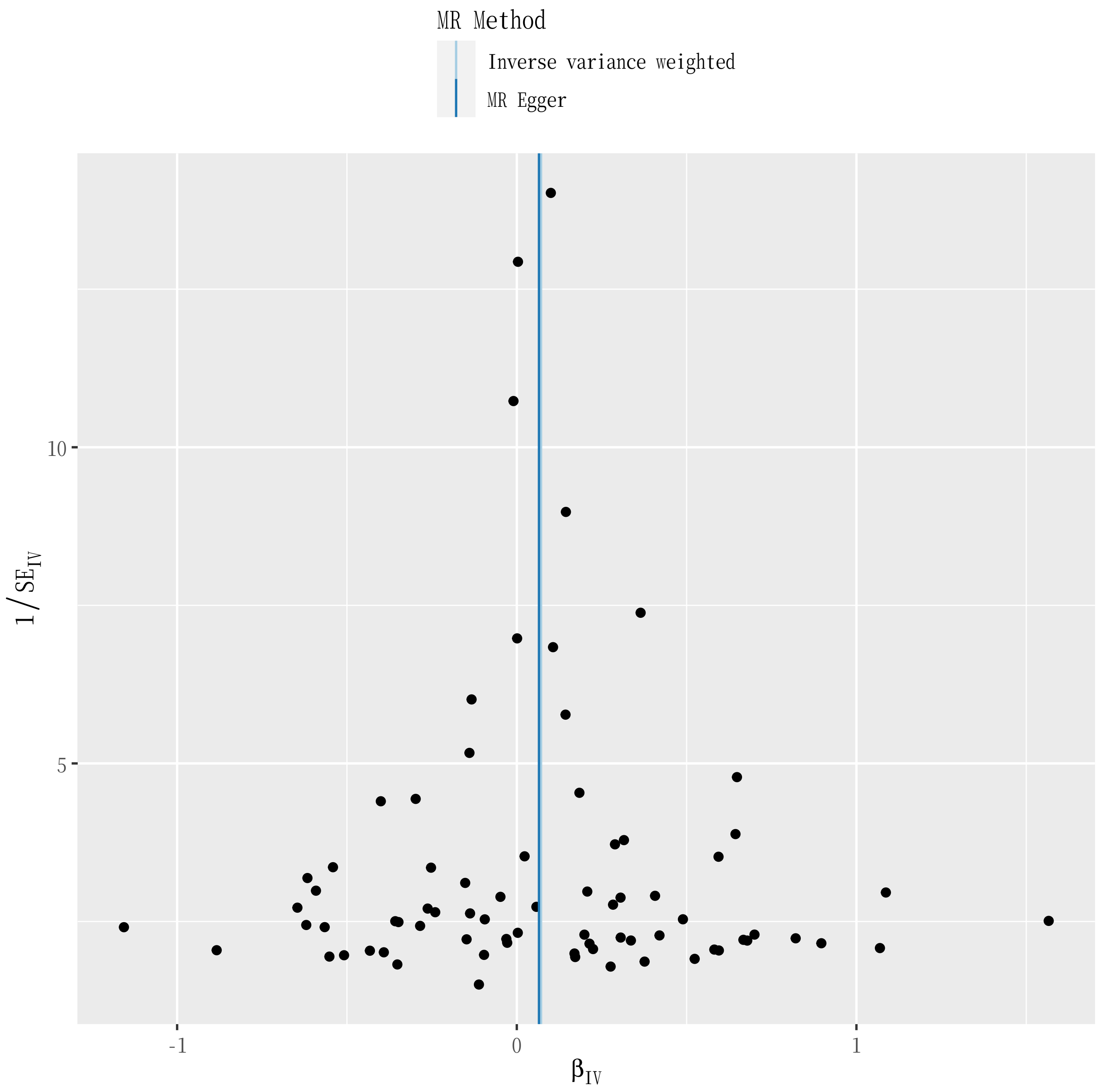
MR Method
Inverse variance weighted
MR Egger
10
5
$1/SE_{IV}$
-1
0
1
β_{IV}

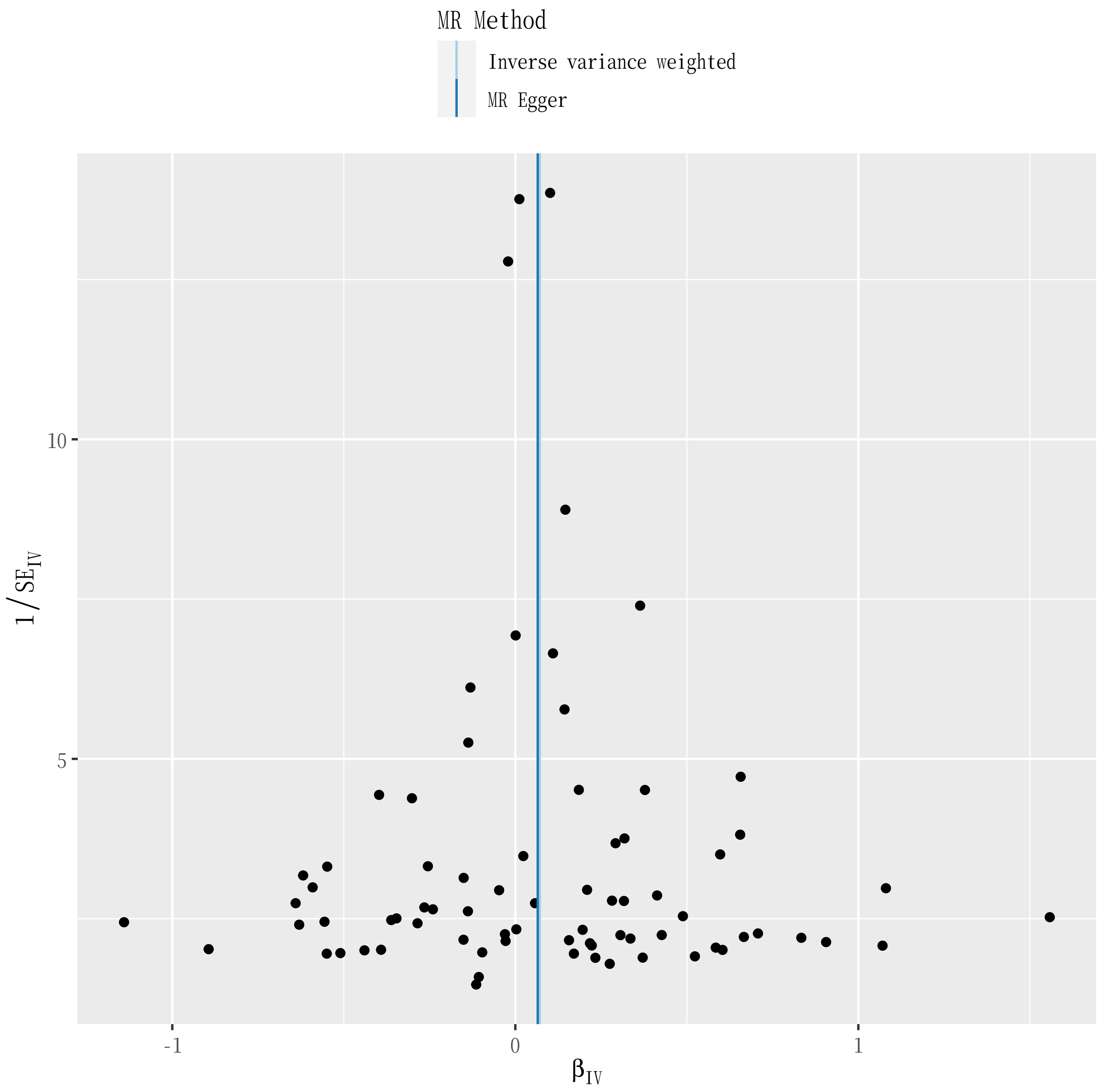

MR Method
Inverse variance weighted
MR Egger
1/SE_IV
10
5
-1
0
1
β_IV

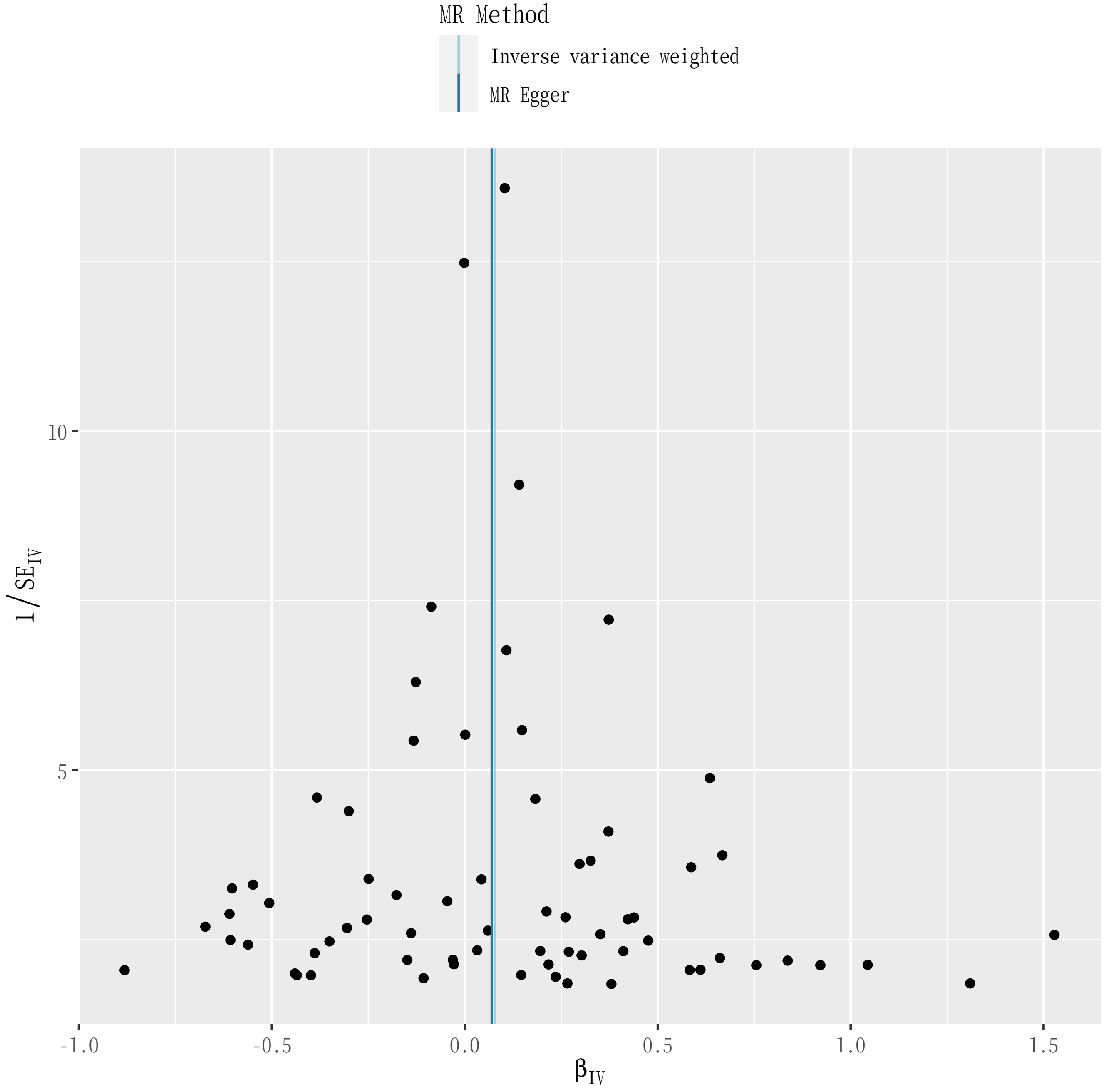

MR Method
Inverse variance weighted
MR Egger
10
5
$1/SE_{IV}$
-1.0
-0.5
0.0
0.5
1.0
1.5
β_{IV}

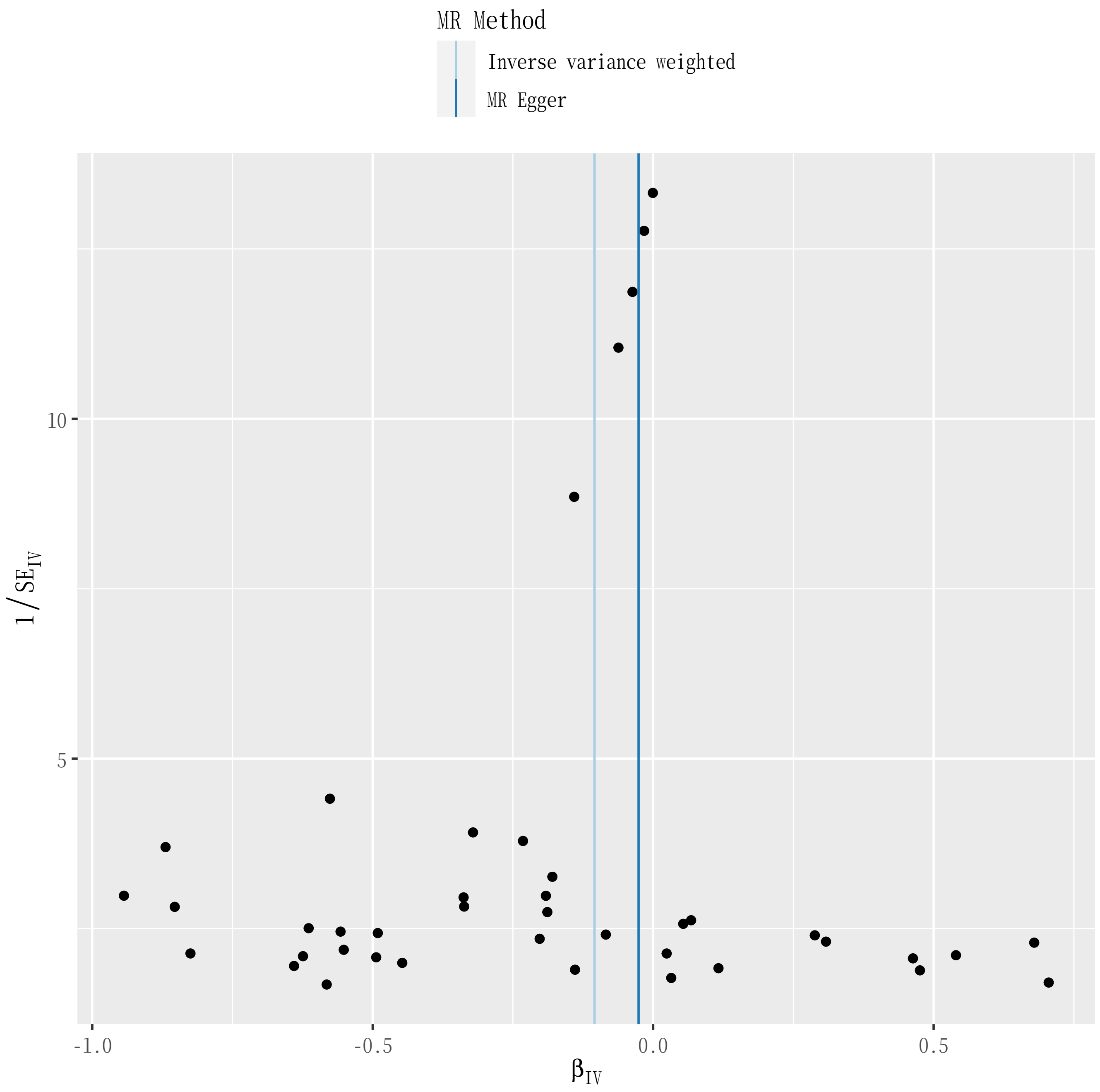

MR Method
Inverse variance weighted
MR Egger
10
5
$1/SE_{IV}$
-1.0
-0.5
0.0
0.5
β_{IV}

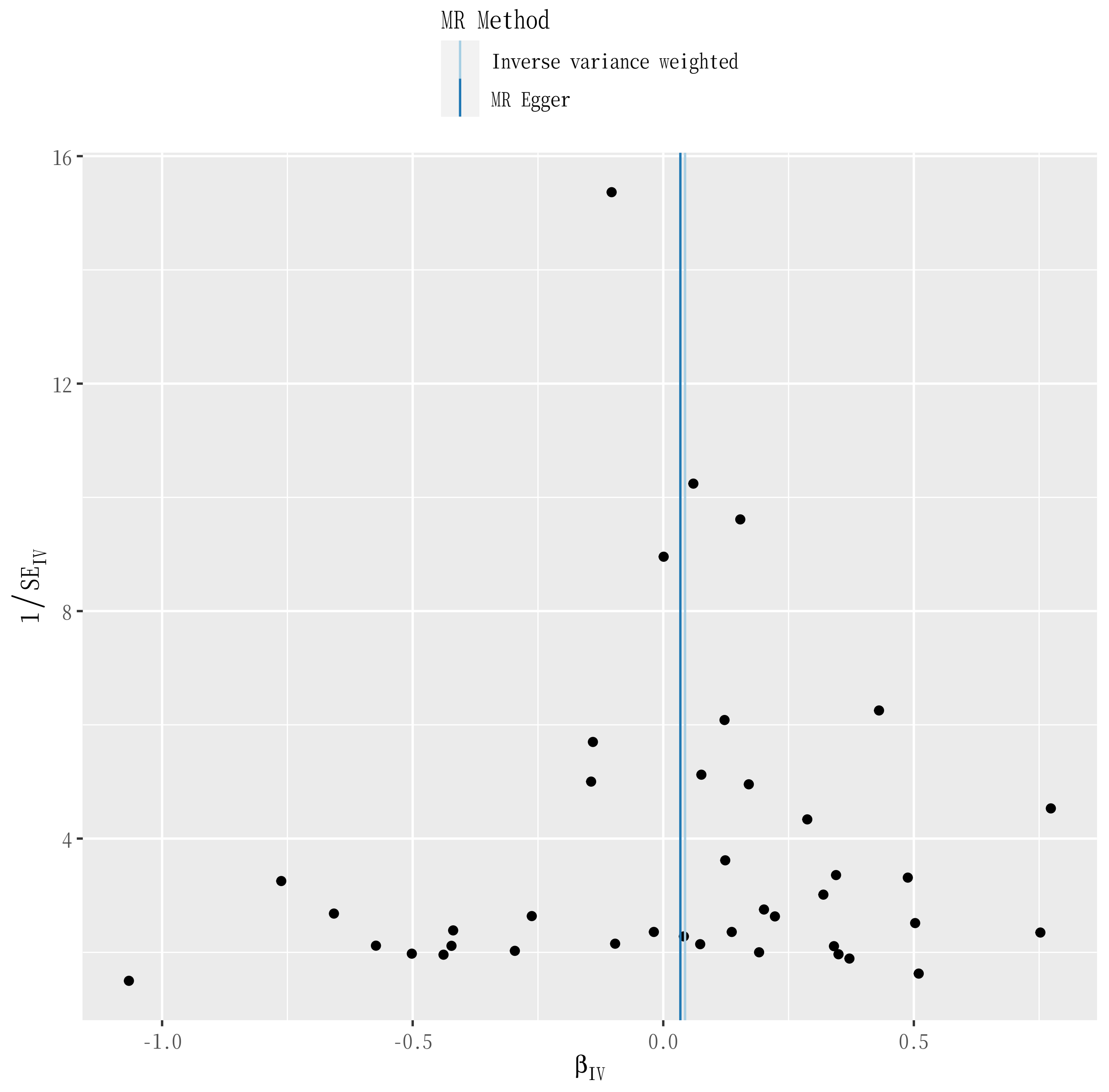

MR Method
Inverse variance weighted
MR Egger
16
12
8
4
-1.0
-0.5
0.0
0.5
$1/SE_{IV}$
β_{IV}

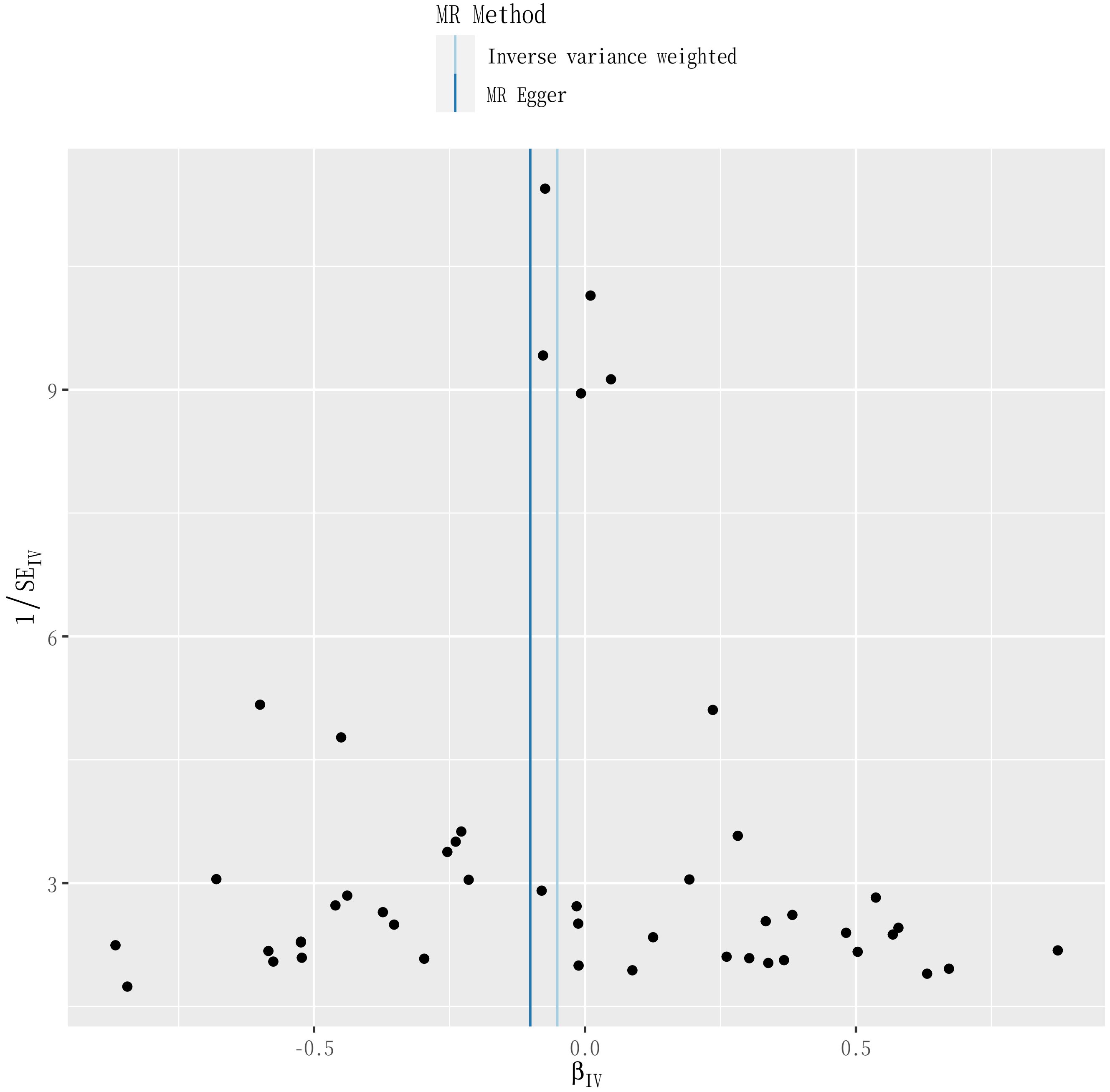

MR Method
Inverse variance weighted
MR Egger
$1/SE_{IV}$
9
6
3
-0.5
0.0
0.5
β_{IV}

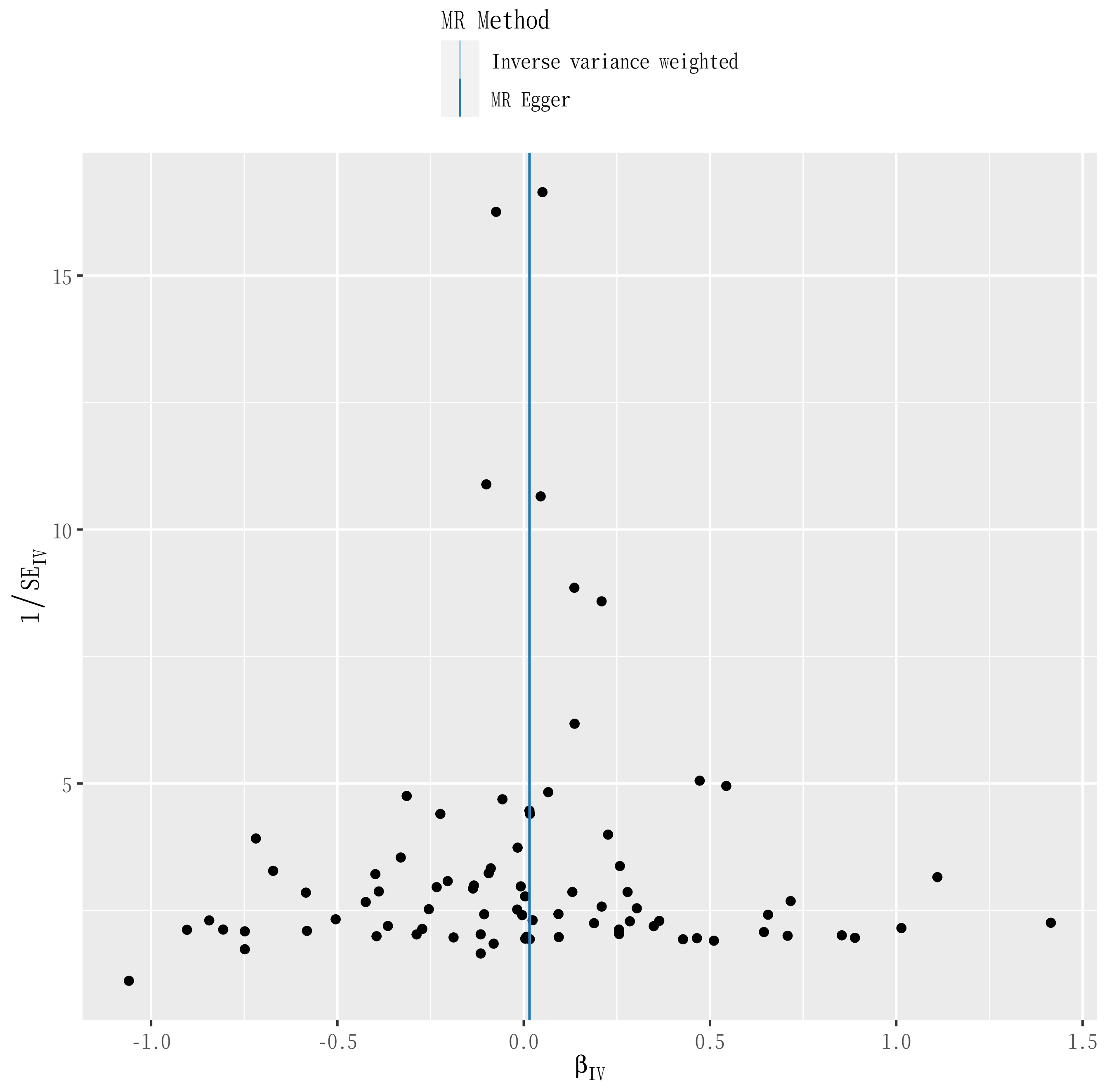

MR Method
Inverse variance weighted
MR Egger
15
10
5
1/SE_IV
-1.0
-0.5
0.0
0.5
1.0
1.5
β_IV

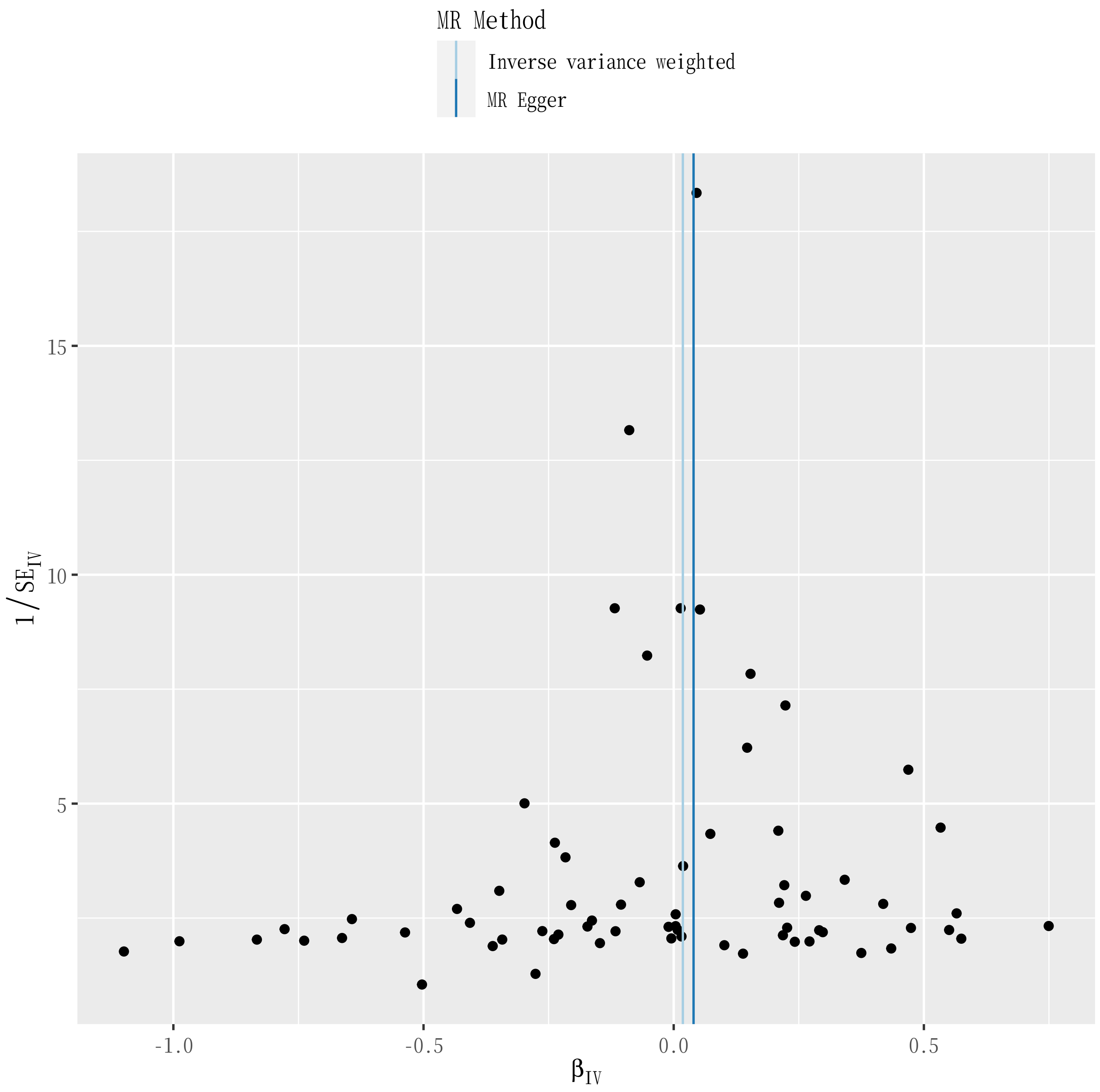
MR Method
Inverse variance weighted
MR Egger
15
10
5
1/SE_IV
-1.0
-0.5
0.0
0.5
β_IV

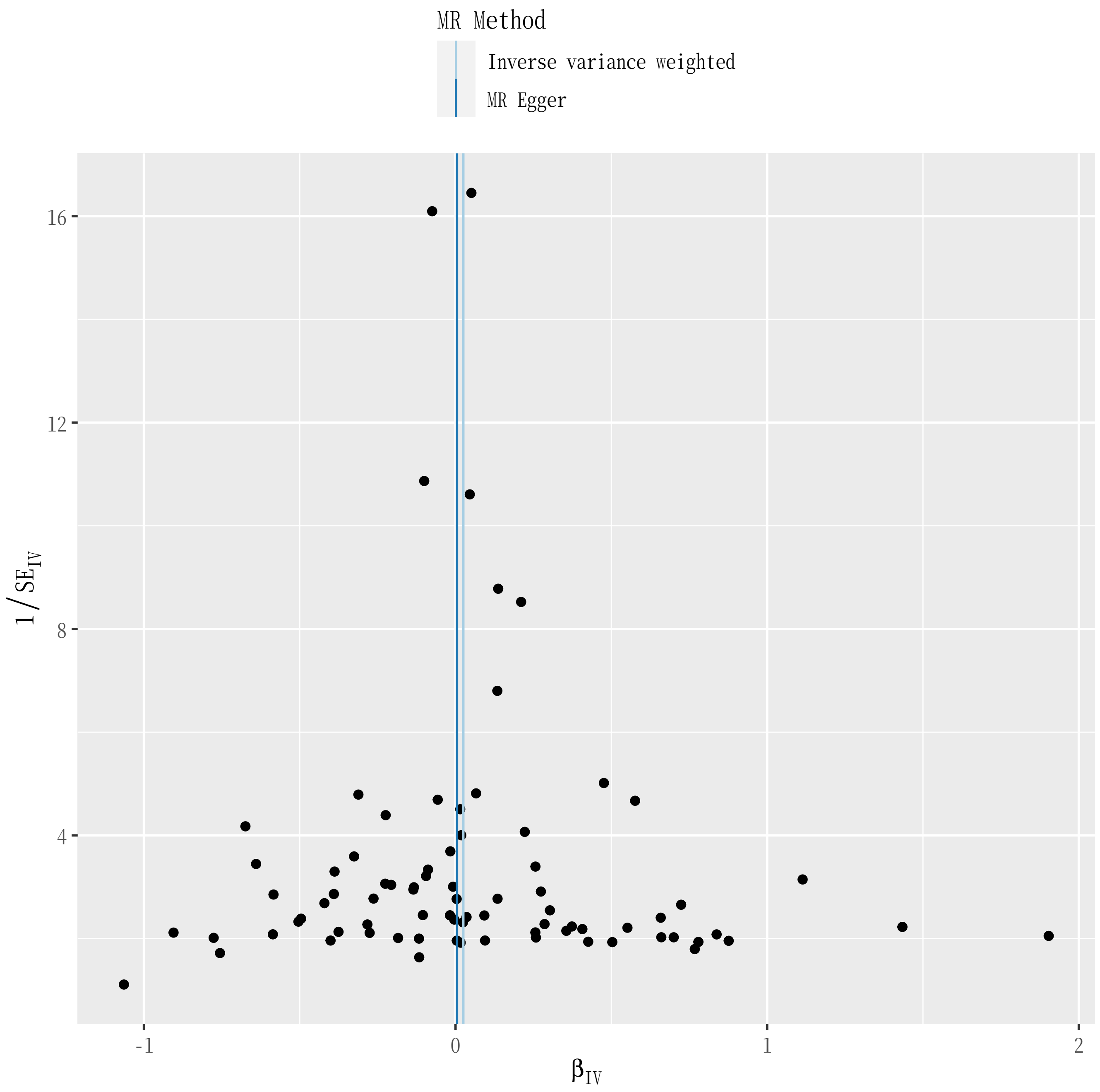

MR Method
Inverse variance weighted
MR Egger
16
12
8
4
1/SE_IV
-1
0
1
2
β_IV

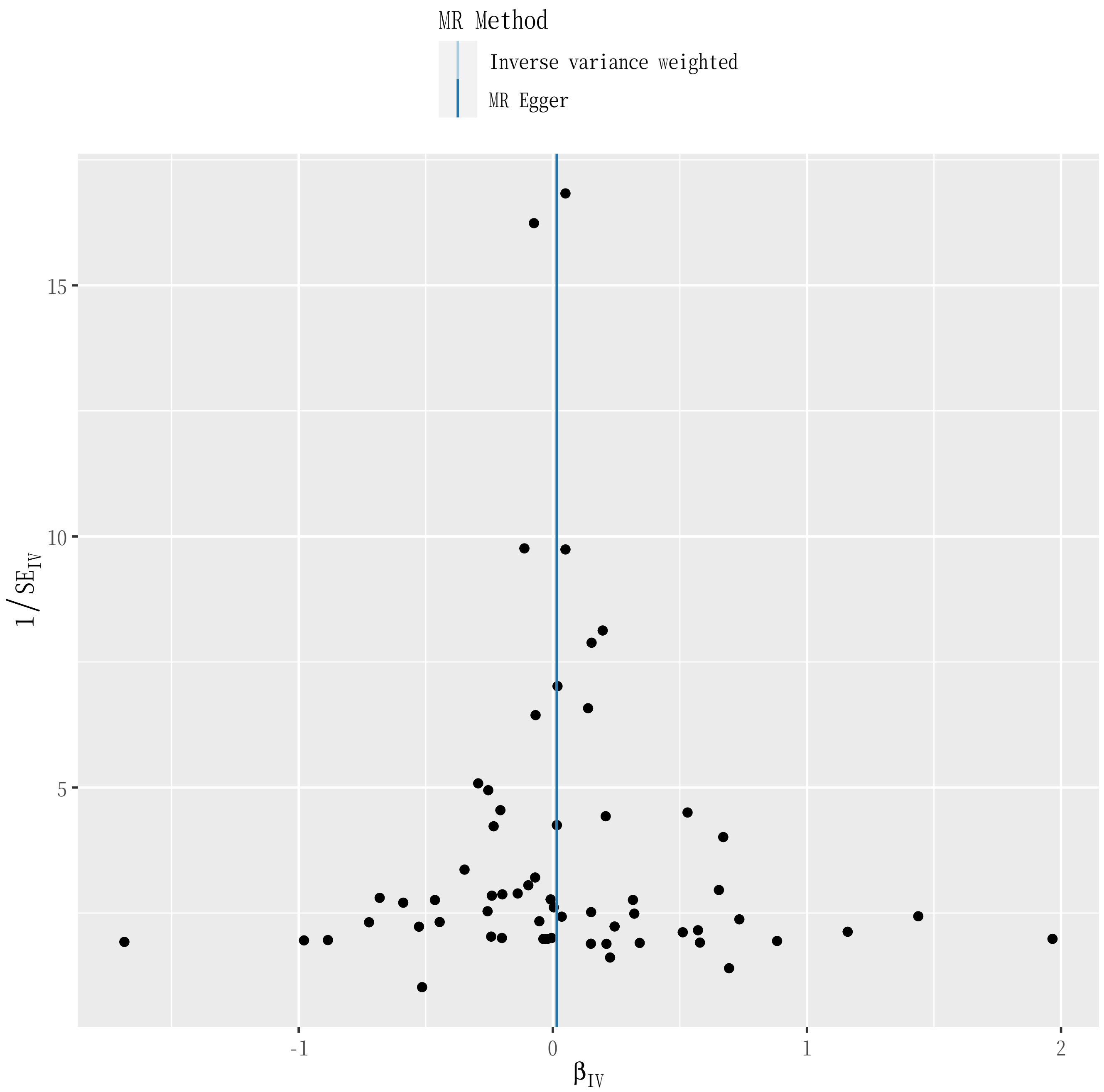
MR Method
Inverse variance weighted
MR Egger
15
10
5
-1
0
1
2
1/SE_IV
β_IV

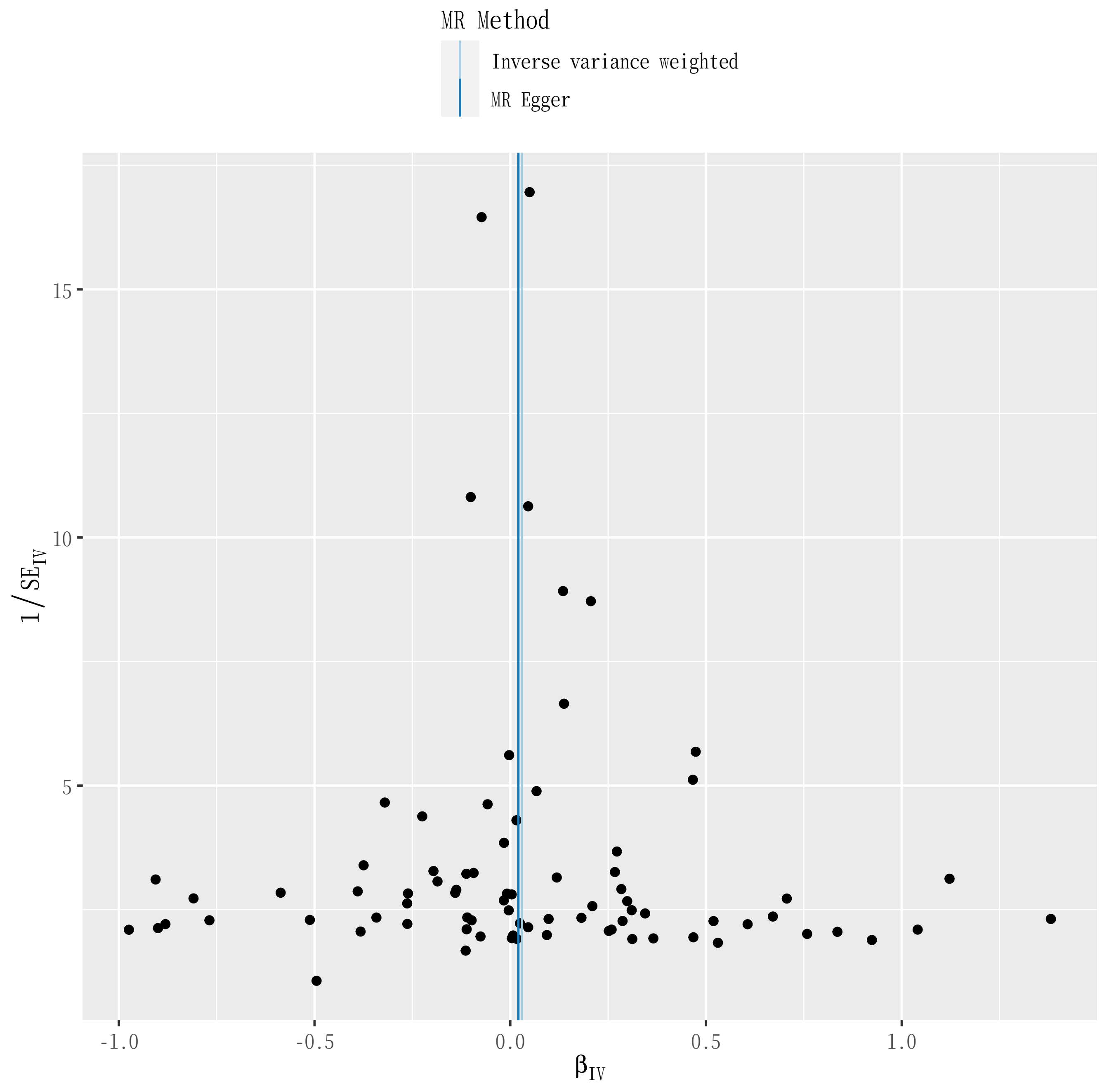
MR Method
Inverse variance weighted
MR Egger
15
10
5
$1/SE_{IV}$
-1.0
-0.5
0.0
0.5
1.0
β_{IV}

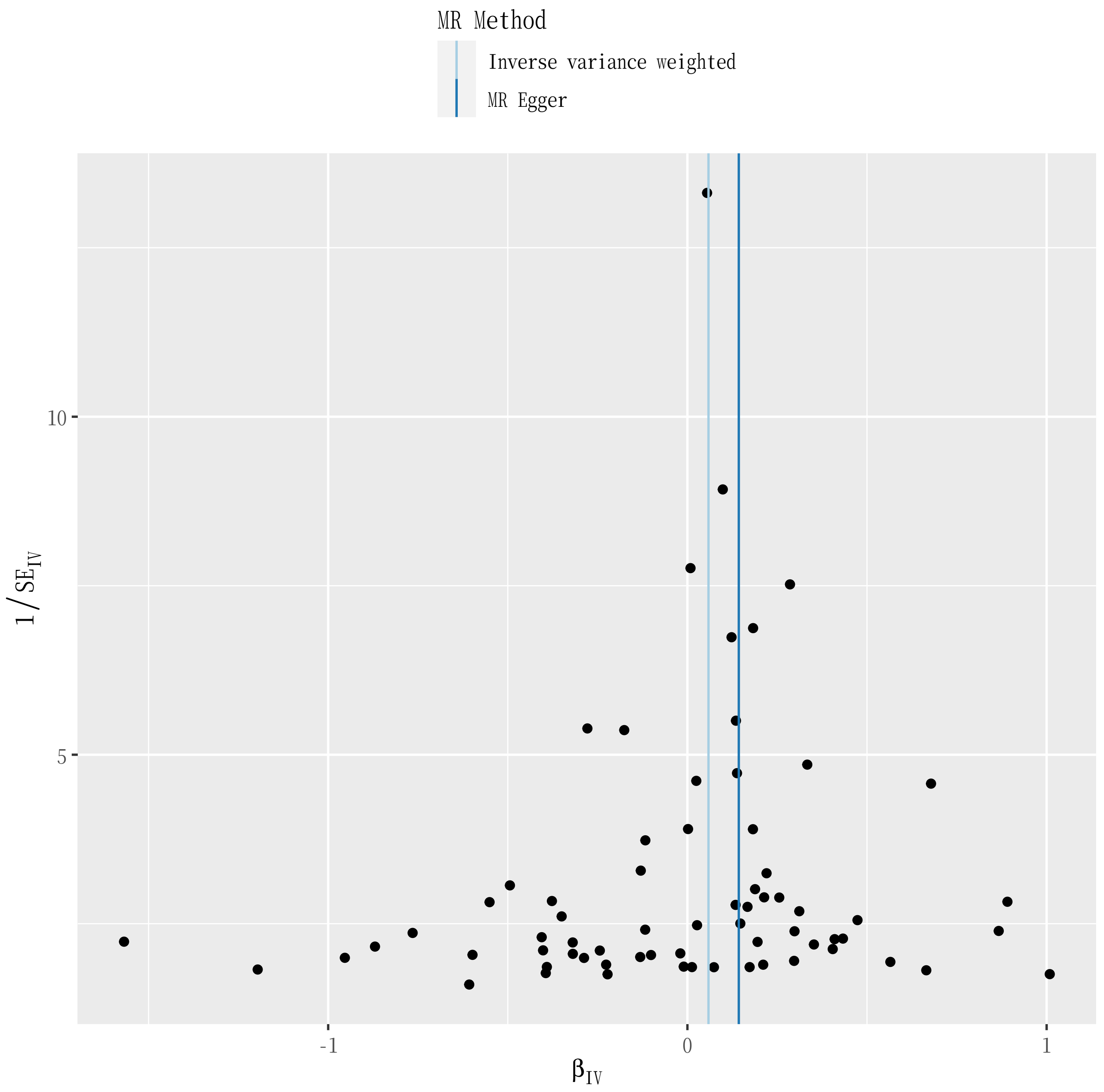

MR Method
Inverse variance weighted
MR Egger
10
5
1/SE_IV
-1
0
1
β_IV

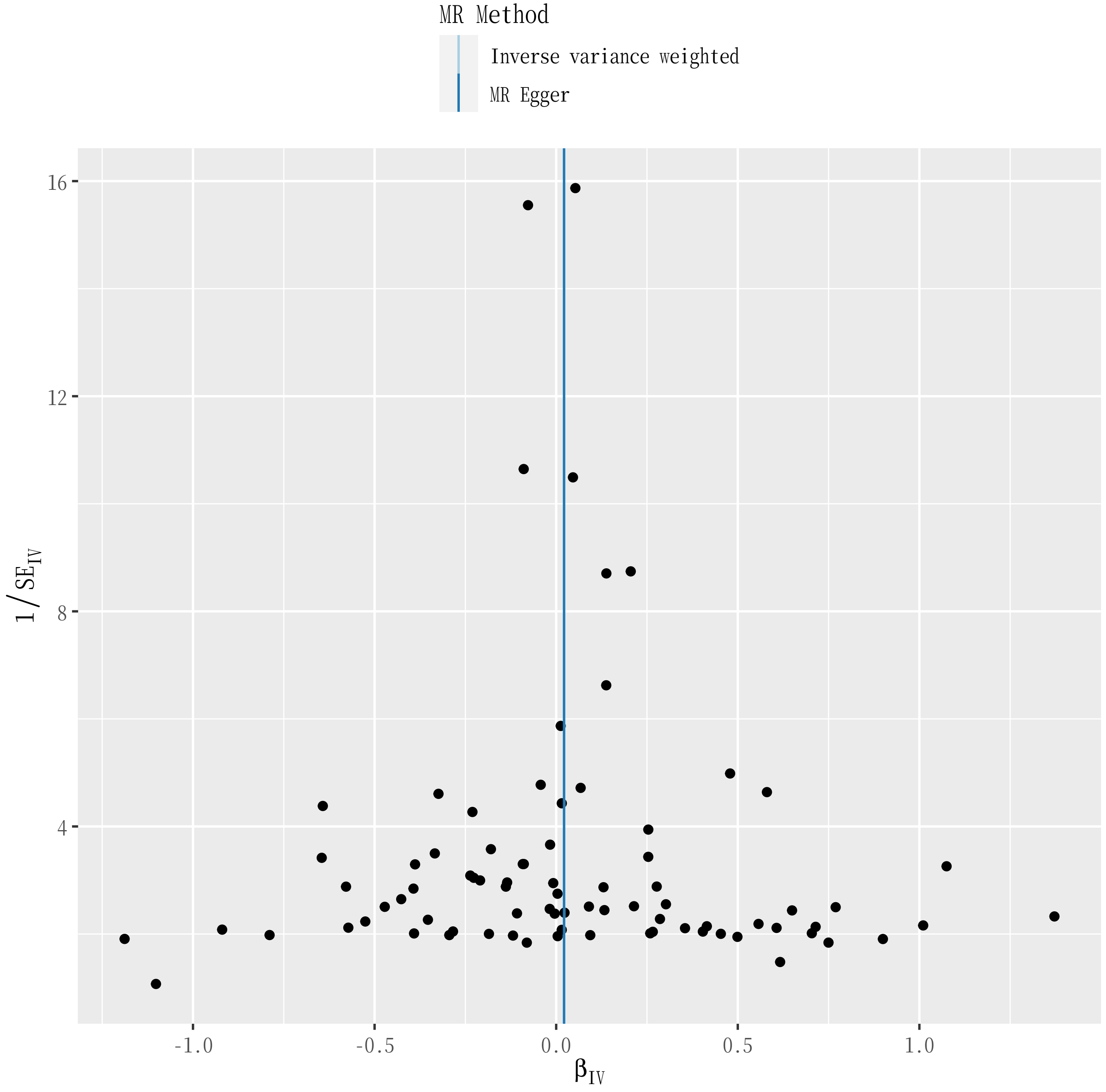

MR Method
Inverse variance weighted
MR Egger
16
12
8
4
$1/SE_{IV}$
-1.0
-0.5
0.0
0.5
1.0
β_{IV}

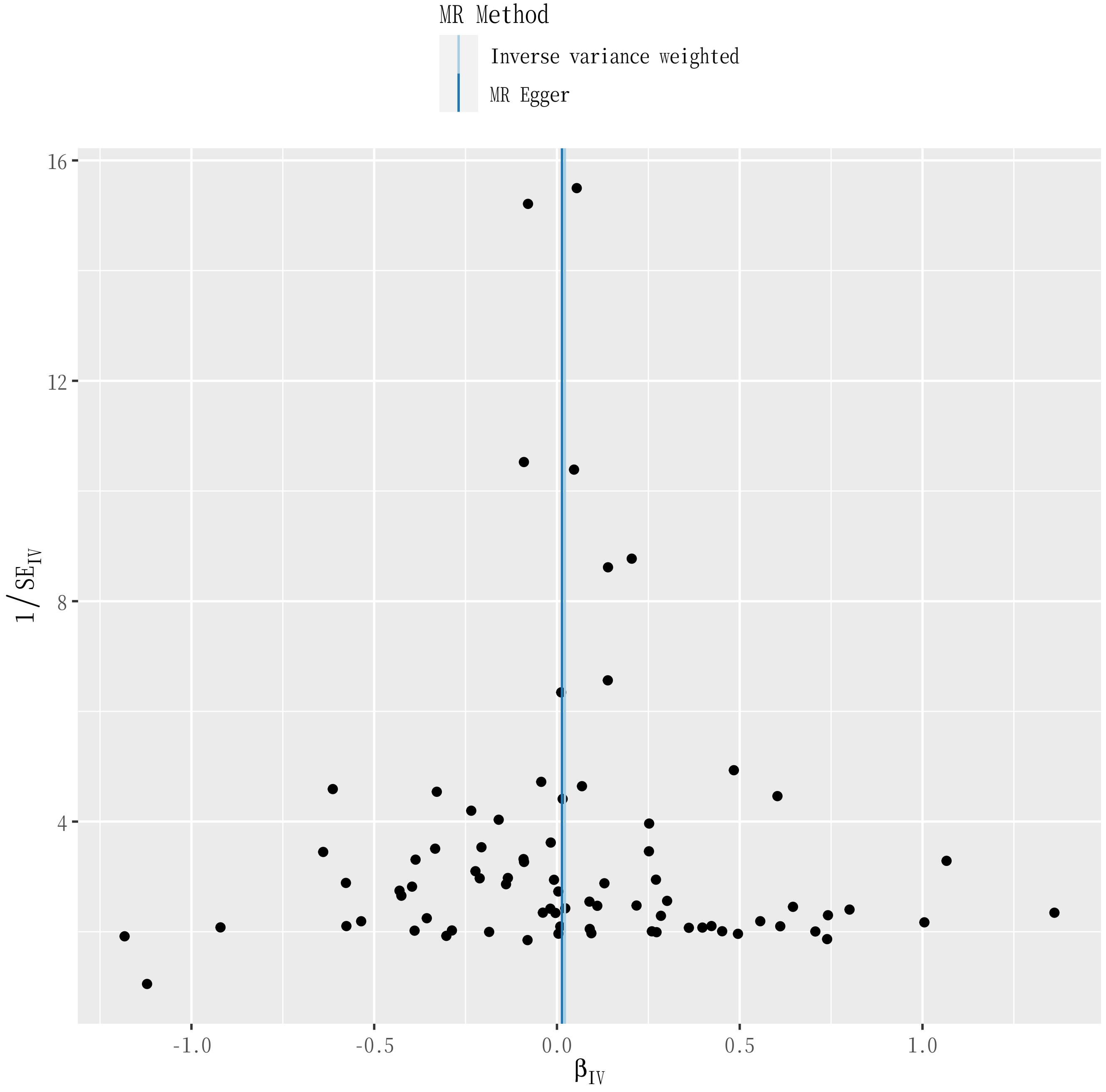
MR Method
Inverse variance weighted
MR Egger
16
12
8
4
1/SE_IV
-1.0
-0.5
0.0
0.5
1.0
β_IV

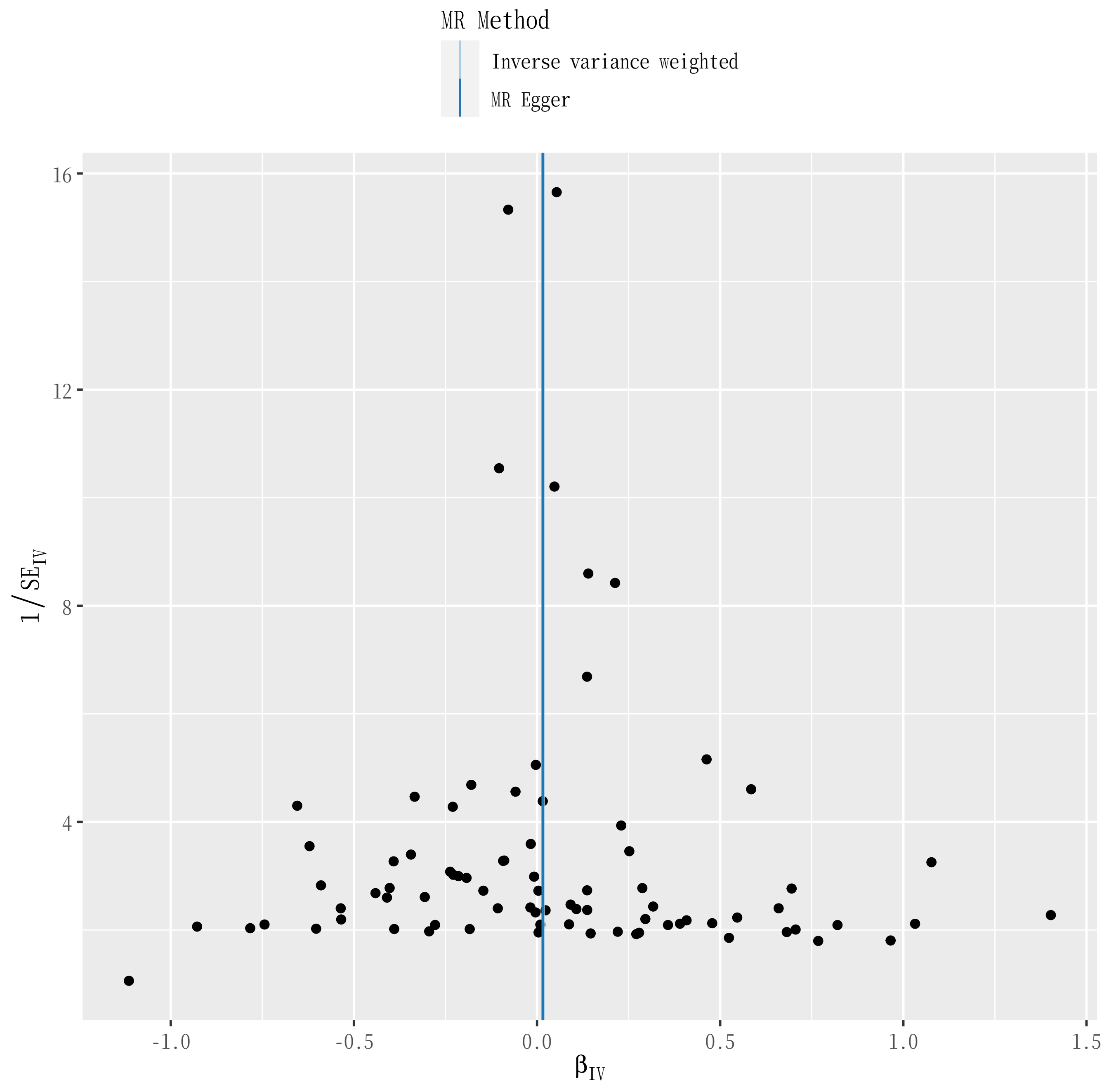

MR Method
Inverse variance weighted
MR Egger
16
12
8
4
1/SE_IV
-1.0
-0.5
0.0
0.5
1.0
1.5
β_IV

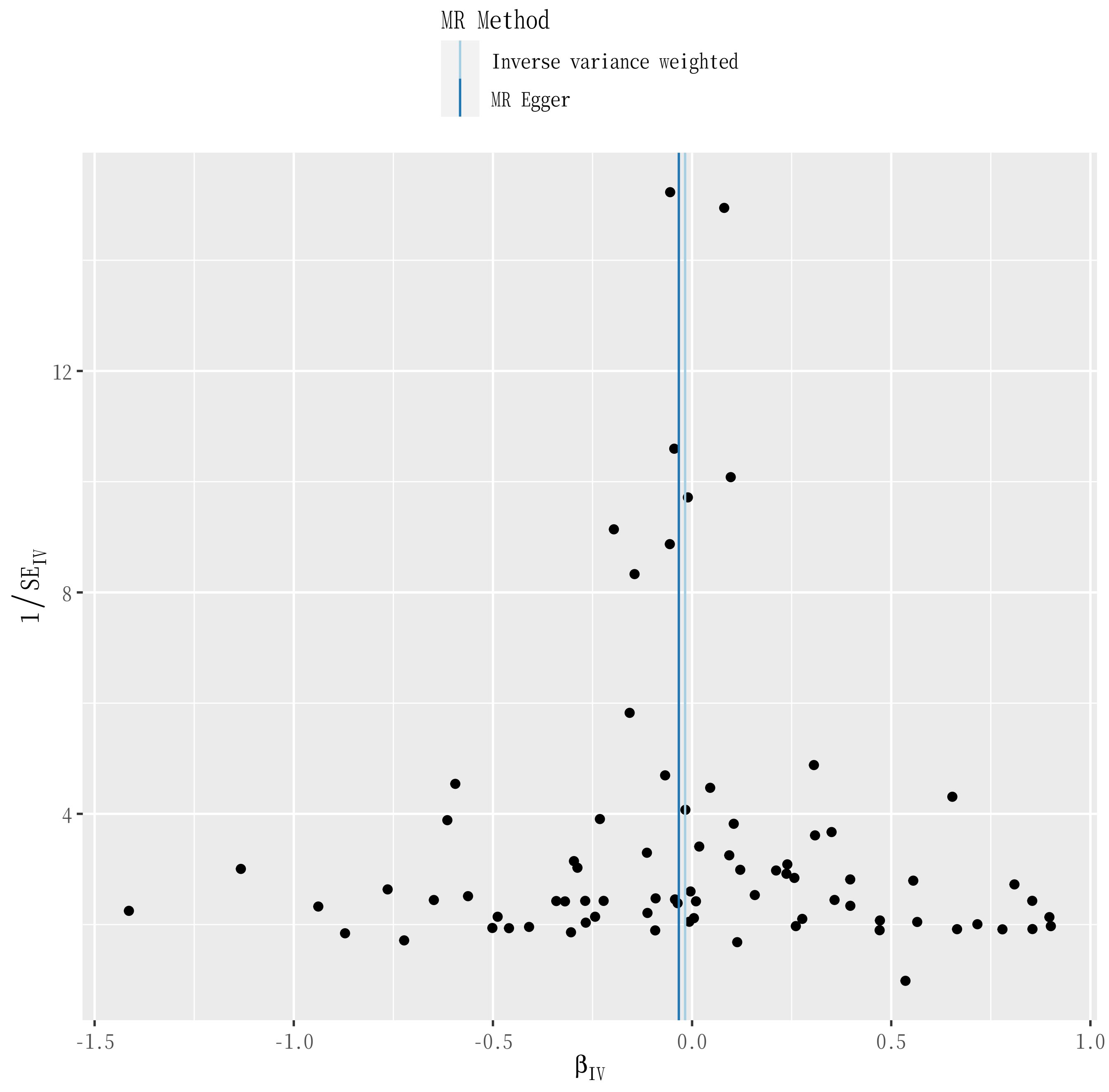
MR Method
Inverse variance weighted
MR Egger
12
8
4
$1/SE_{IV}$
-1.5
-1.0
-0.5
0.0
0.5
1.0
β_{IV}

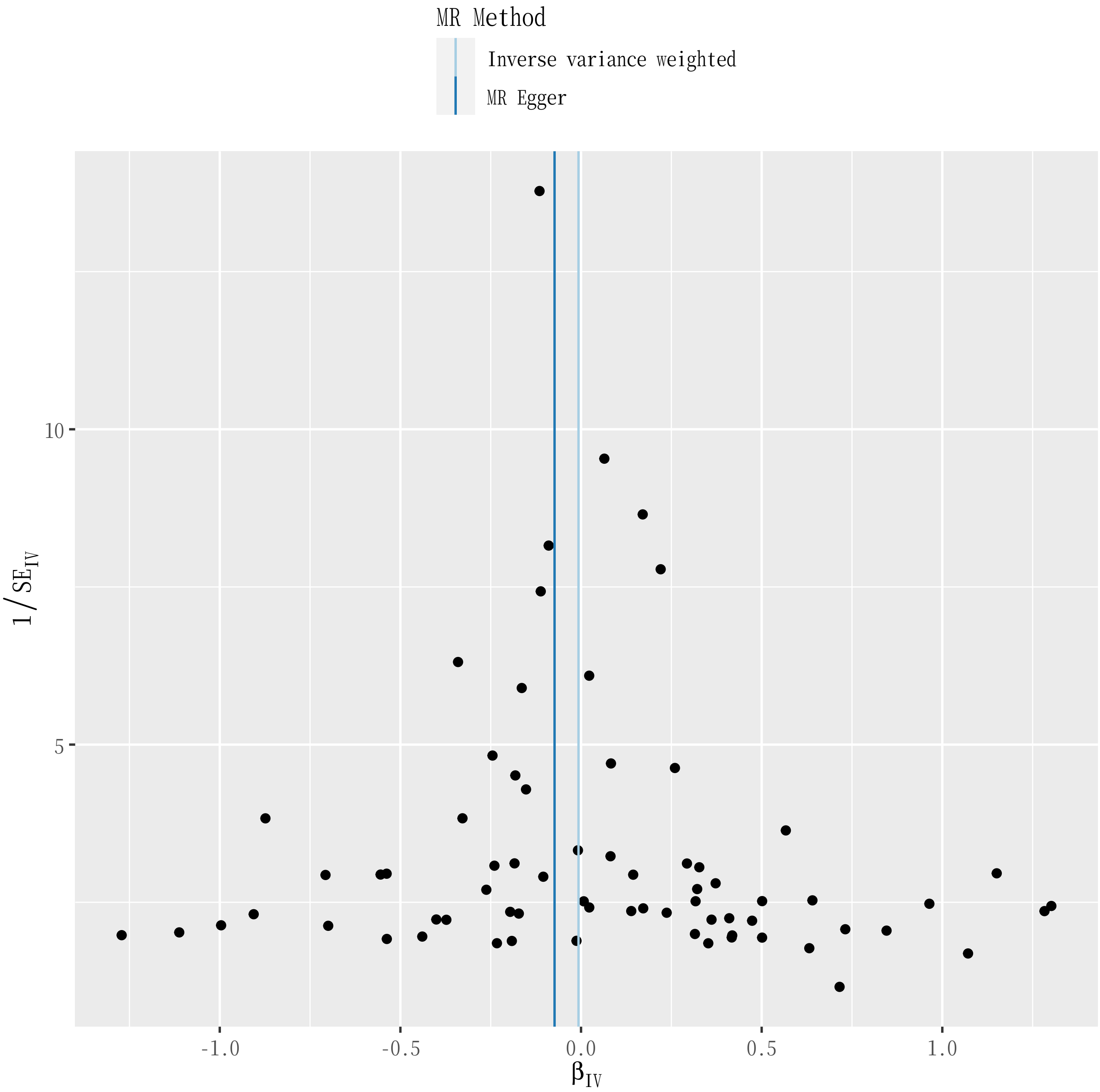

MR Method
Inverse variance weighted
MR Egger
10
5
1/SE$_{IV}$
-1.0
-0.5
0.0
0.5
1.0
β_{IV}

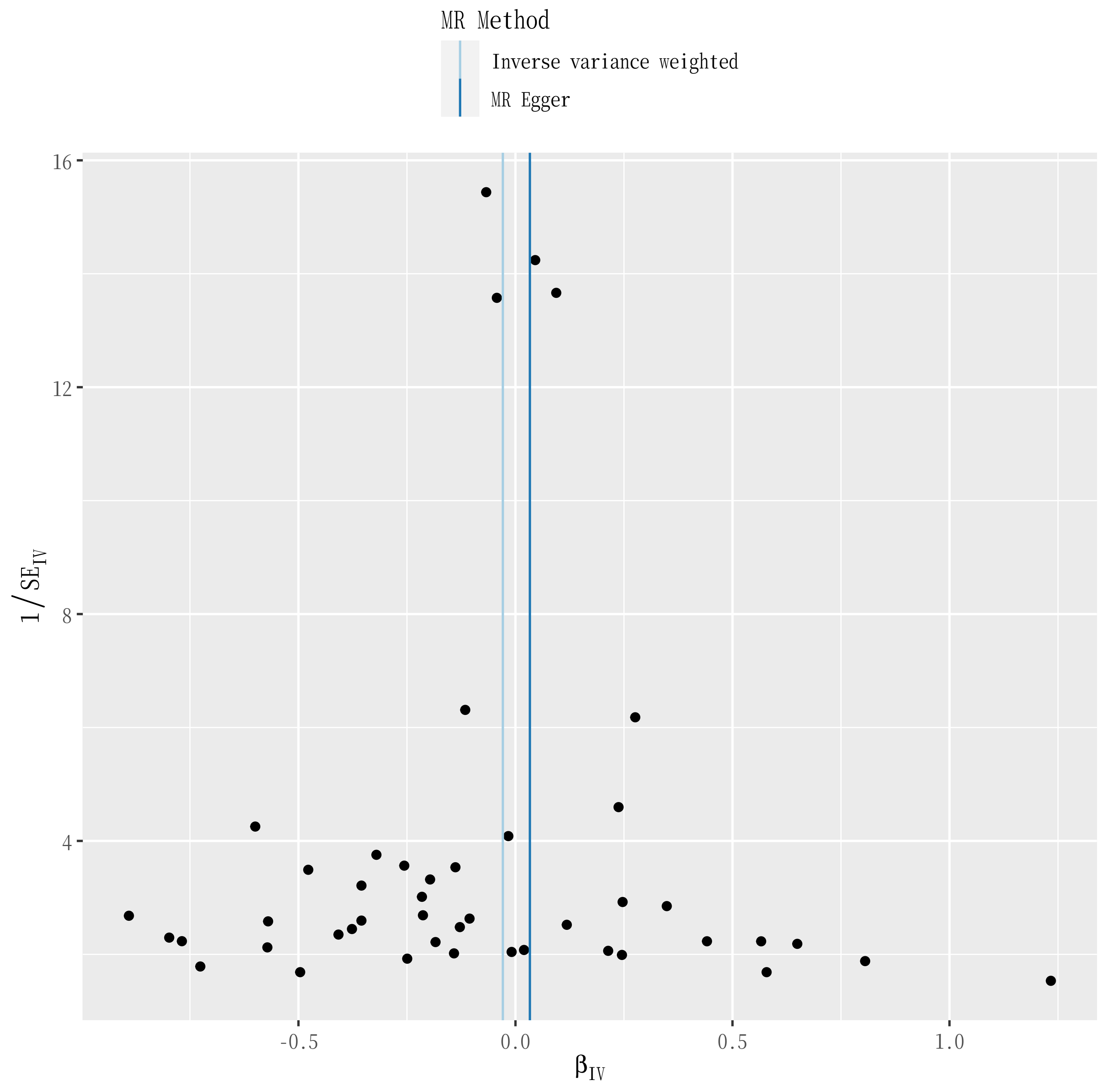
MR Method
Inverse variance weighted
MR Egger
16
12
8
4
-0.5
0.0
0.5
1.0
1/SE_IV
β_IV

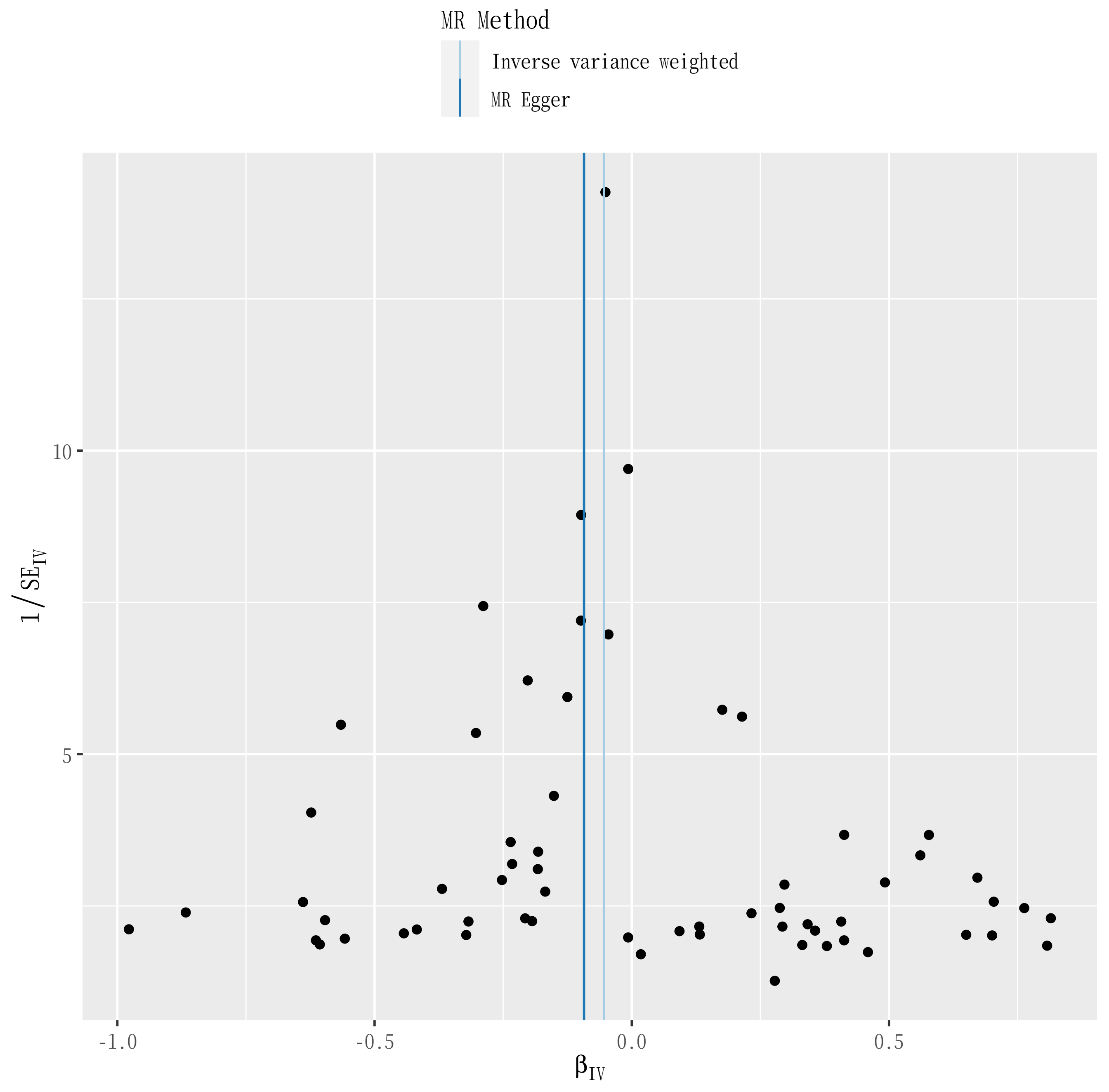
MR Method
Inverse variance weighted
MR Egger
1/SE_IV
10
5
-1.0
-0.5
0.0
0.5
β_IV

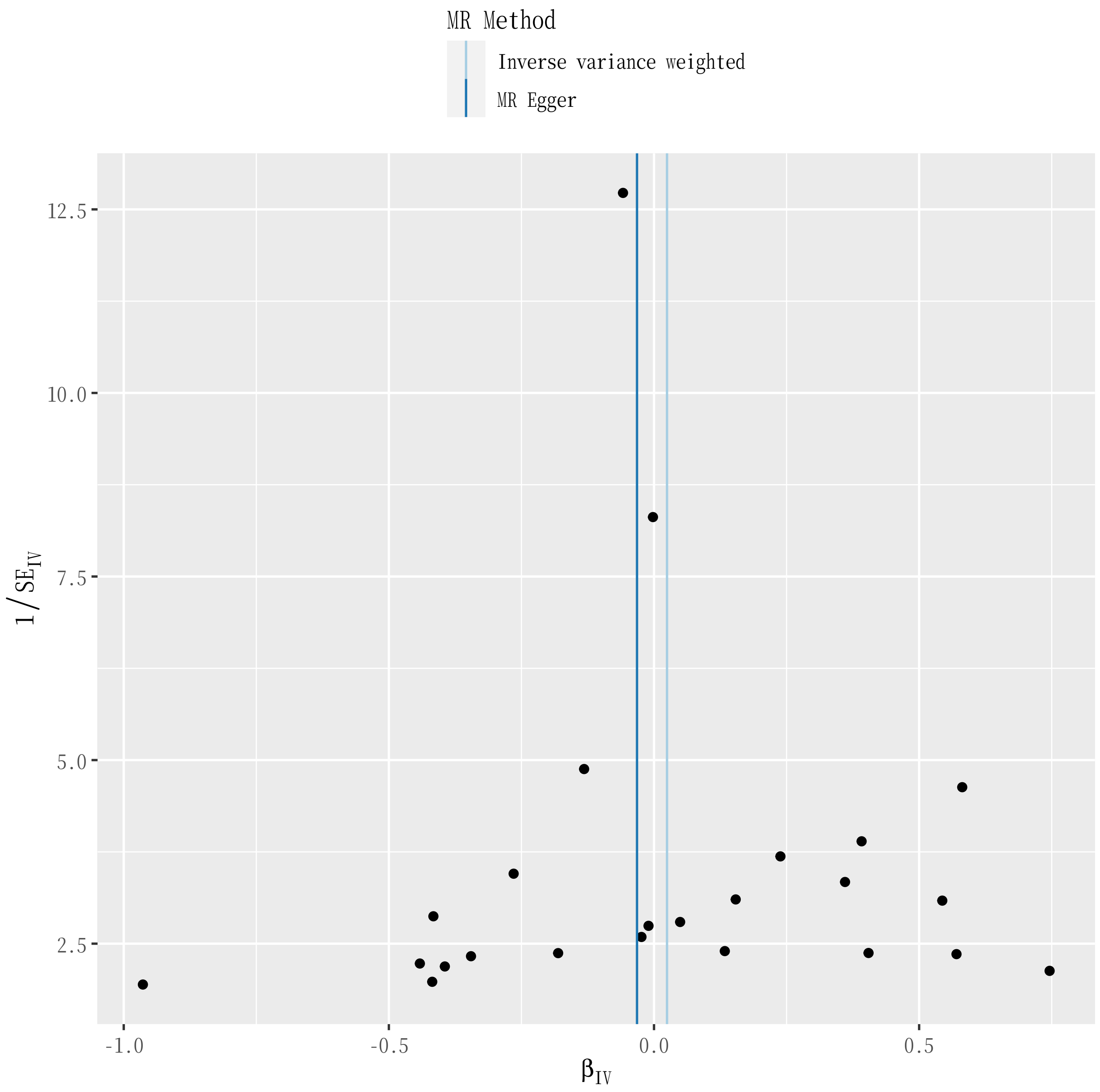
MR Method
Inverse variance weighted
MR Egger
12.5
10.0
7.5
5.0
2.5
$1/SE_{IV}$
-1.0
-0.5
0.0
0.5
β_{IV}

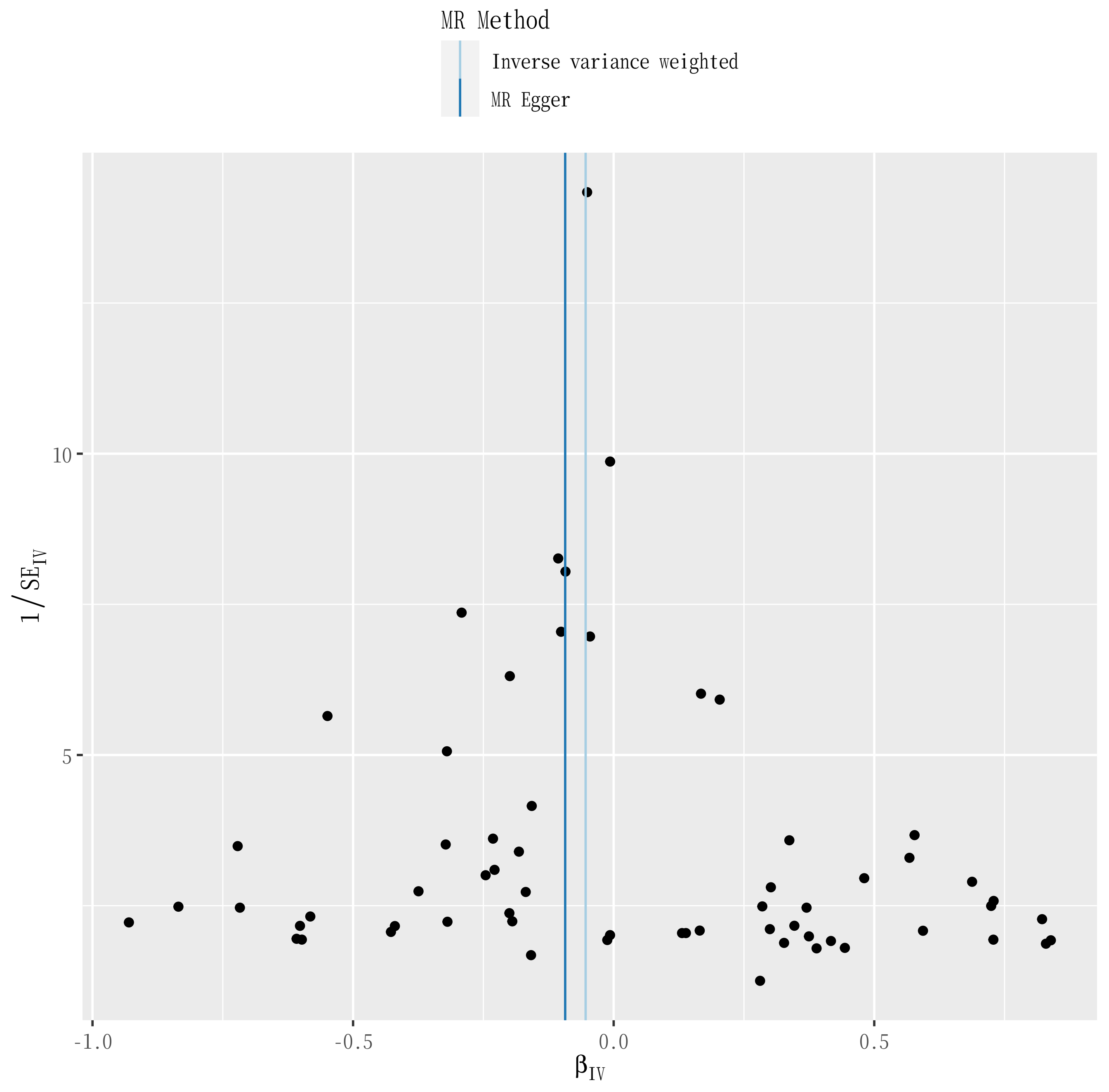
MR Method
Inverse variance weighted
MR Egger
1/SE_IV
10
5
-1.0
-0.5
0.0
0.5
β_IV

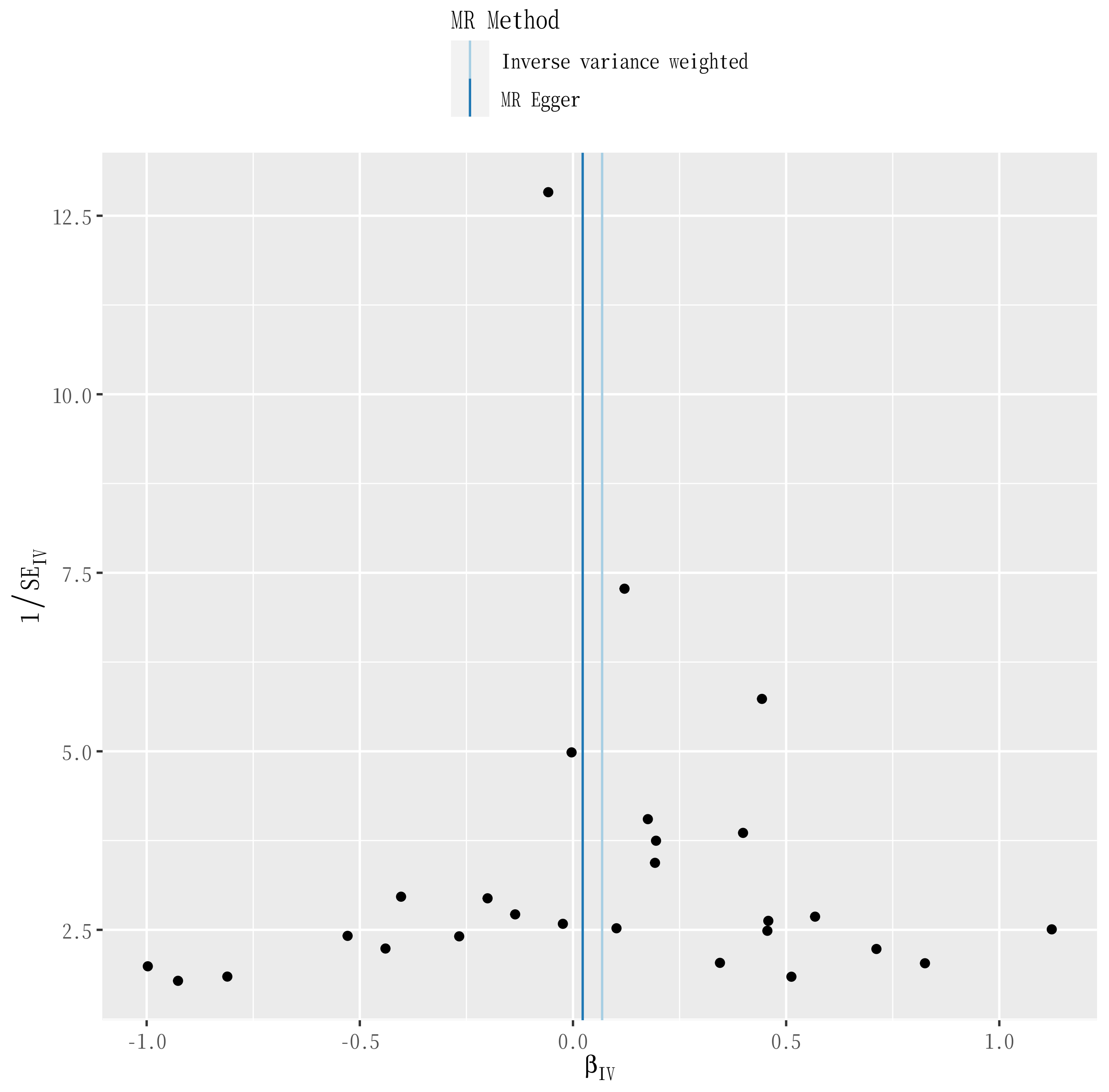

MR Method
Inverse variance weighted
MR Egger
12.5
10.0
7.5
5.0
2.5
$1/SE_{IV}$
-1.0
-0.5
0.0
0.5
1.0
β_{IV}

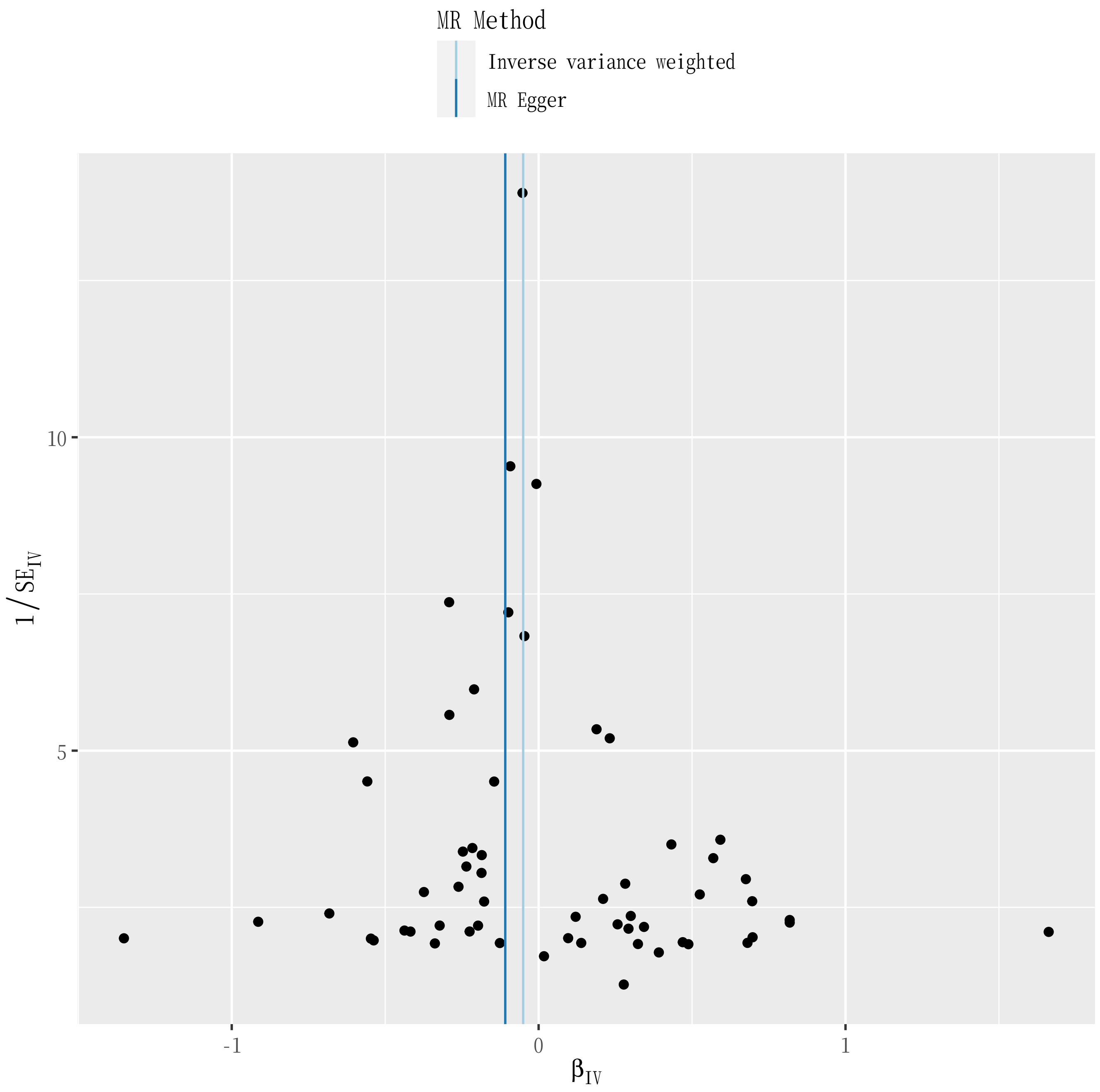
MR Method
Inverse variance weighted
MR Egger
10
5
$1/SE_{IV}$
-1
0
1
β_{IV}

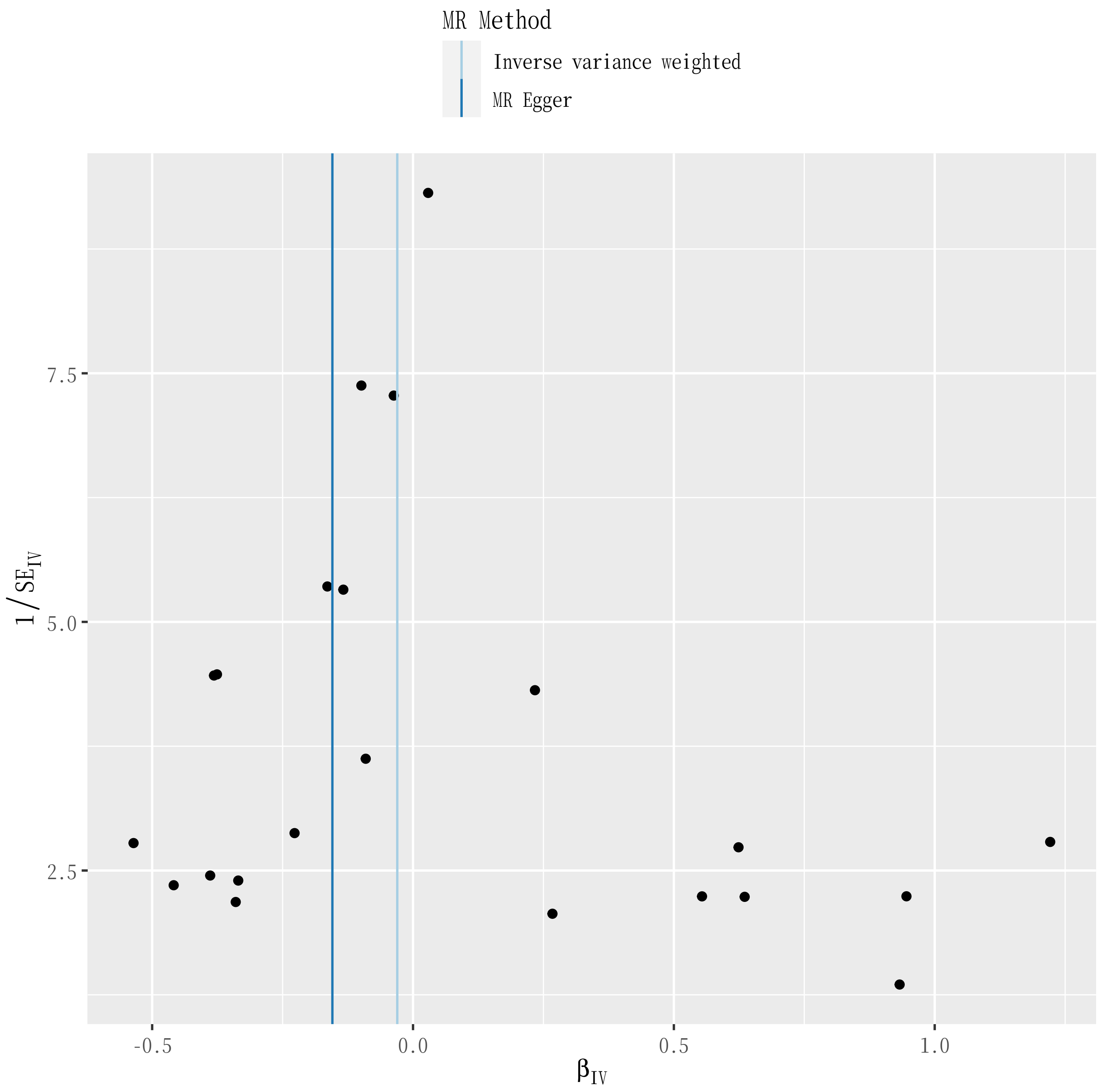

MR Method
Inverse variance weighted
MR Egger
7.5
5.0
2.5
-0.5
0.0
0.5
1.0
1/SE_IV
β_IV

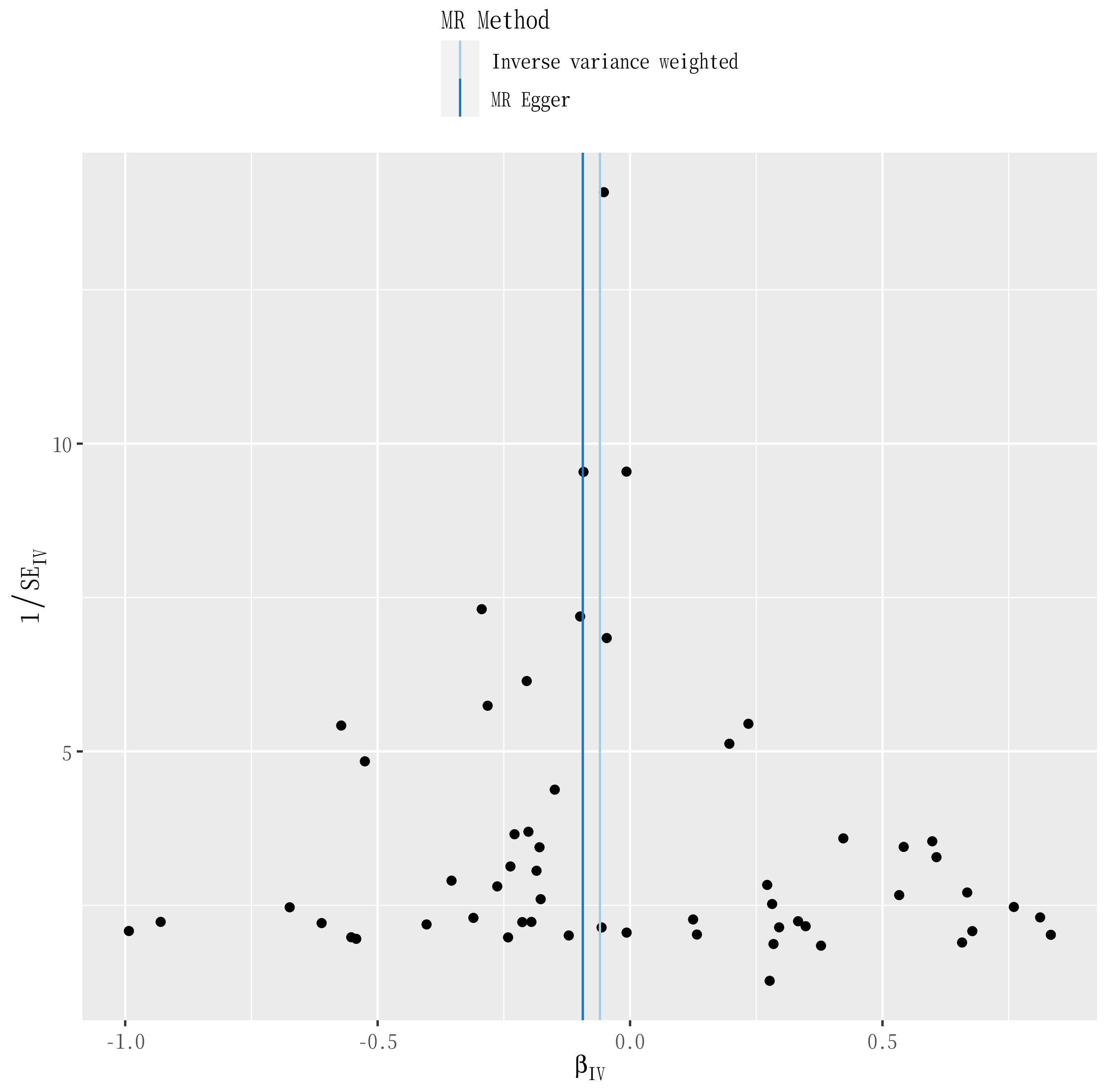
MR Method
Inverse variance weighted
MR Egger
10
5
1/SE_IV
-1.0
-0.5
0.0
0.5
β_IV

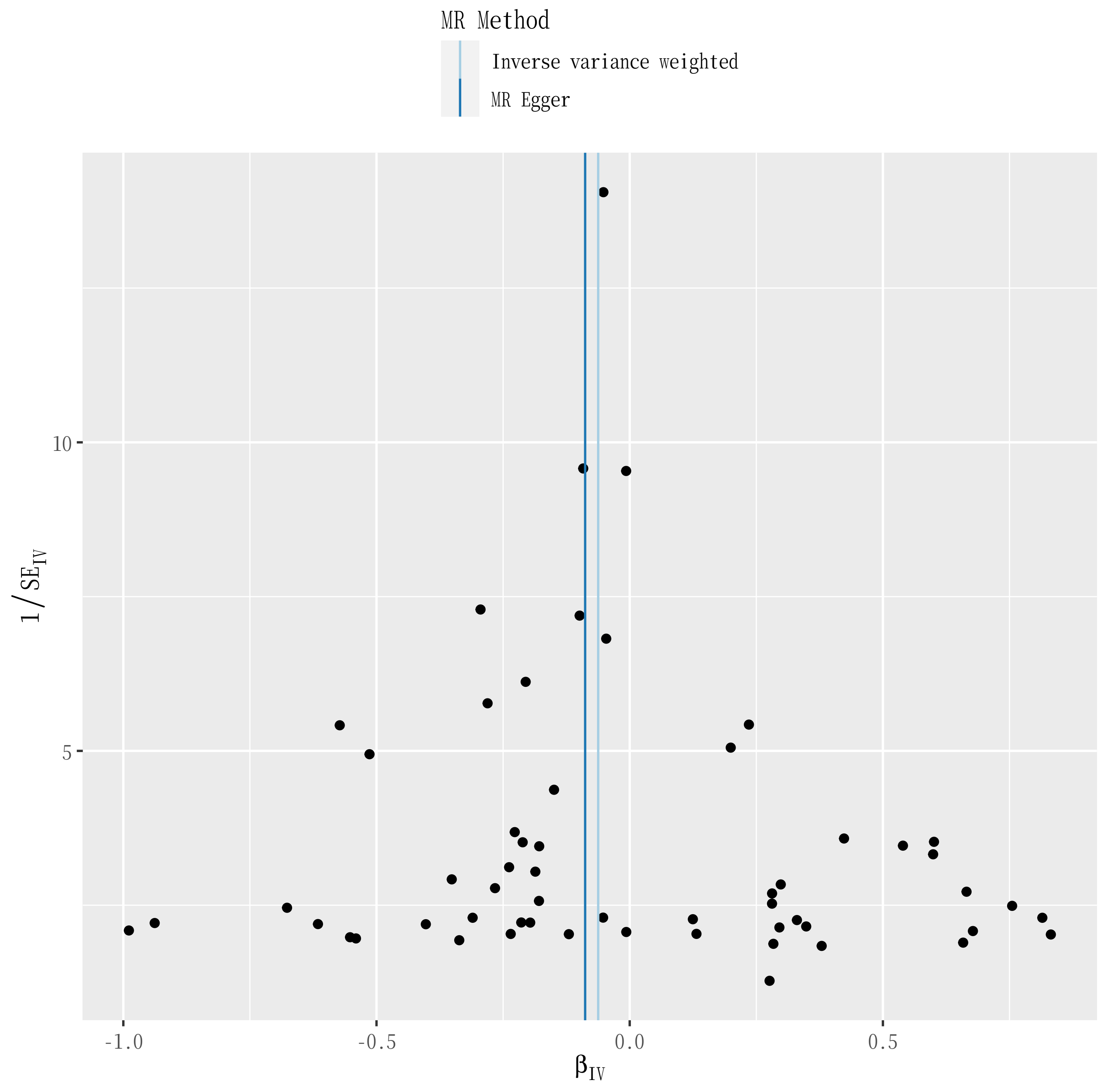

MR Method
Inverse variance weighted
MR Egger
10
5
1/SE_IV
-1.0
-0.5
0.0
0.5
β_IV

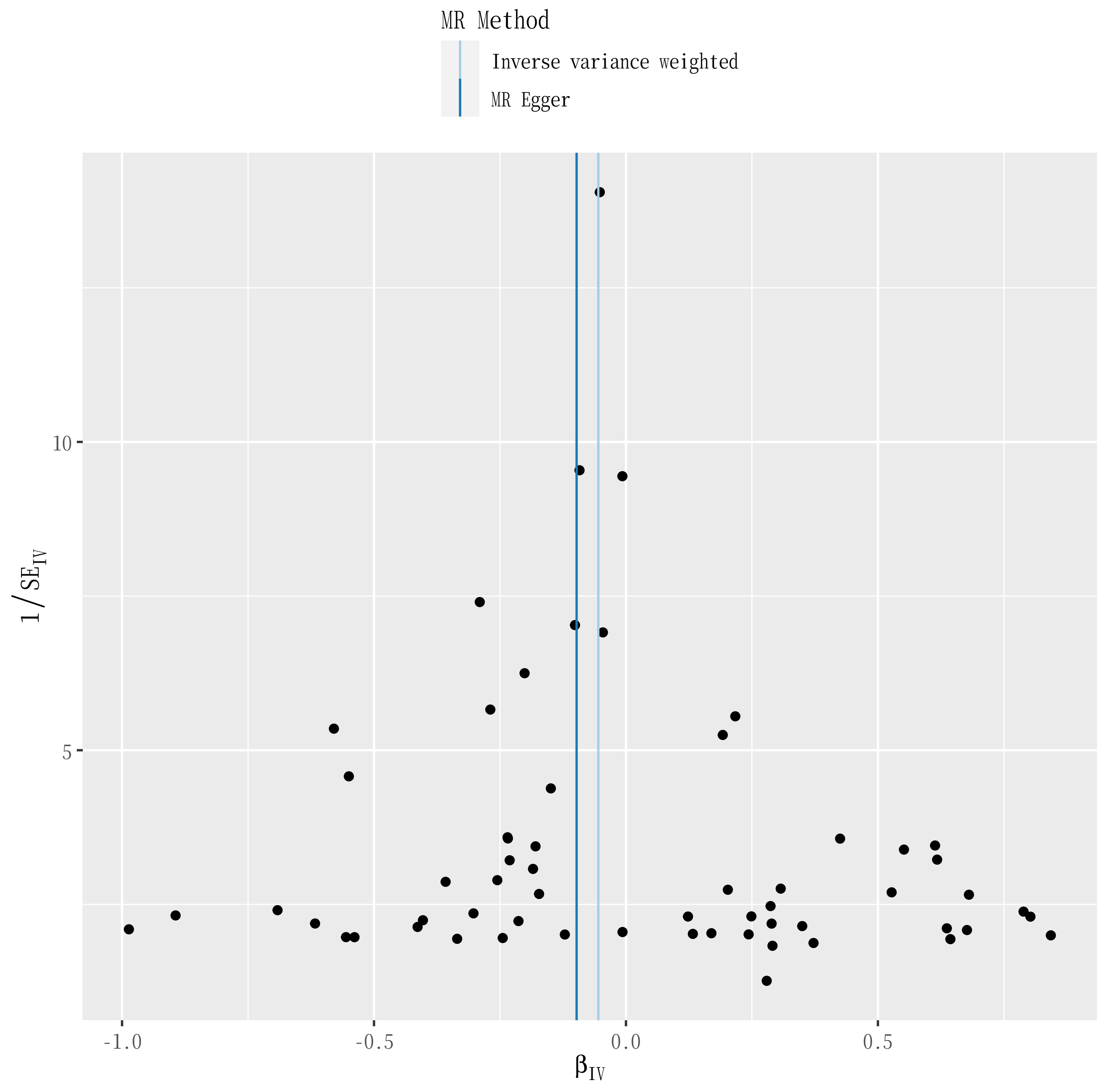
MR Method
Inverse variance weighted
MR Egger
10
5
-1.0
-0.5
0.0
0.5
1/SE_IV
β_IV

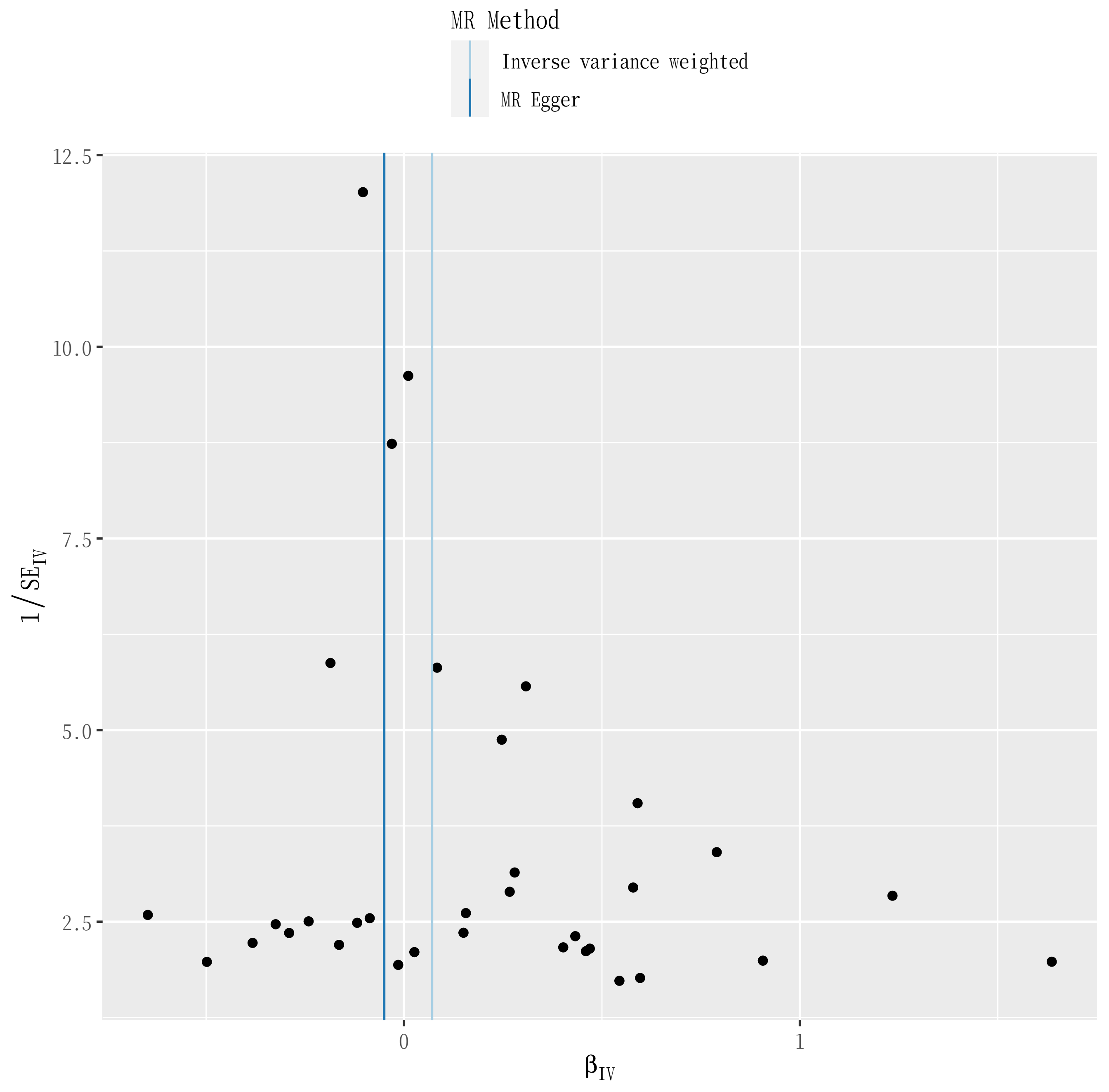

MR Method
Inverse variance weighted
MR Egger
12.5
10.0
7.5
5.0
2.5
$1/SE_{IV}$
0
1
β_{IV}

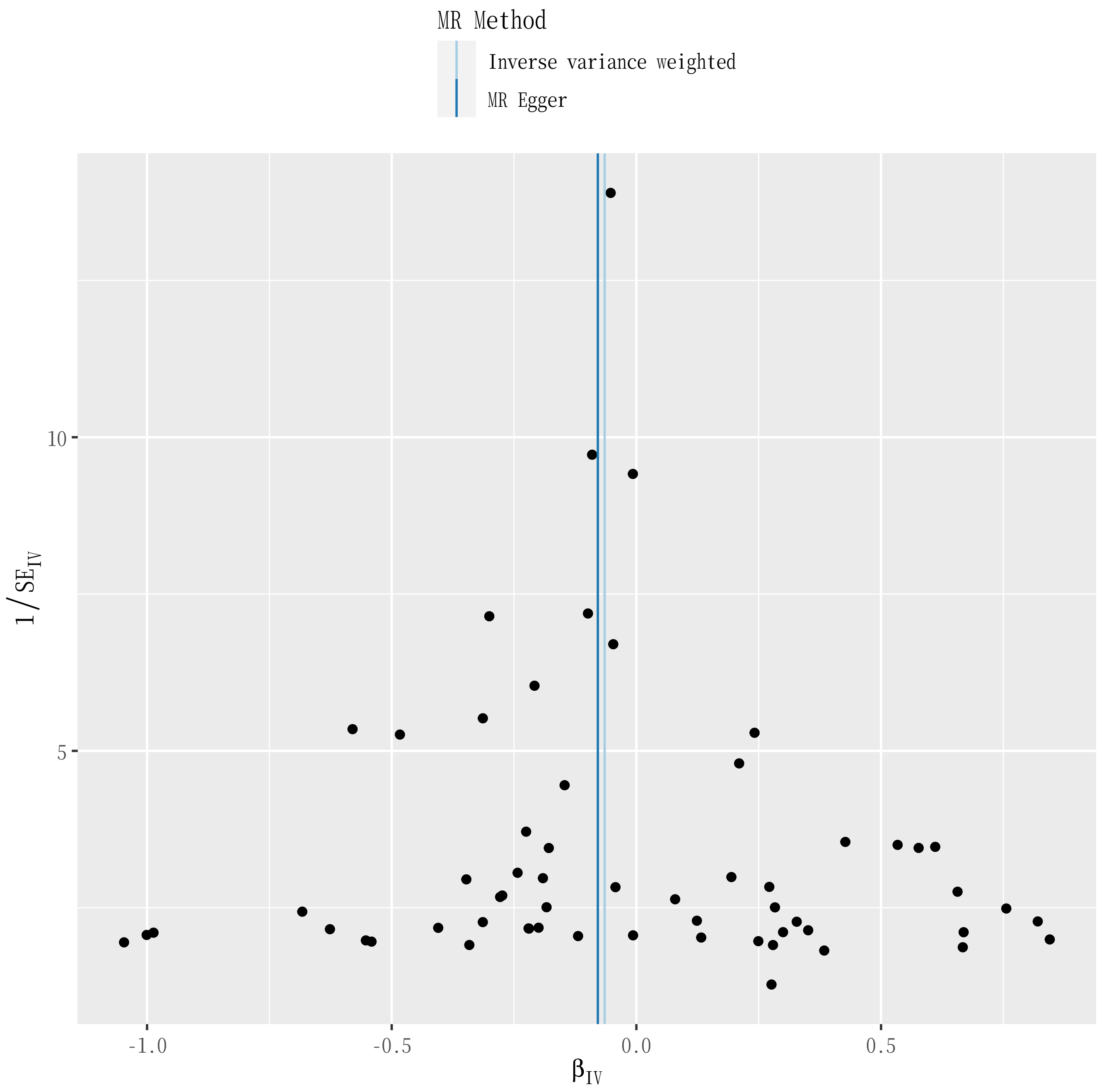

MR Method
Inverse variance weighted
MR Egger
10
5
$1/SE_{IV}$
-1.0
-0.5
0.0
0.5
β_{IV}

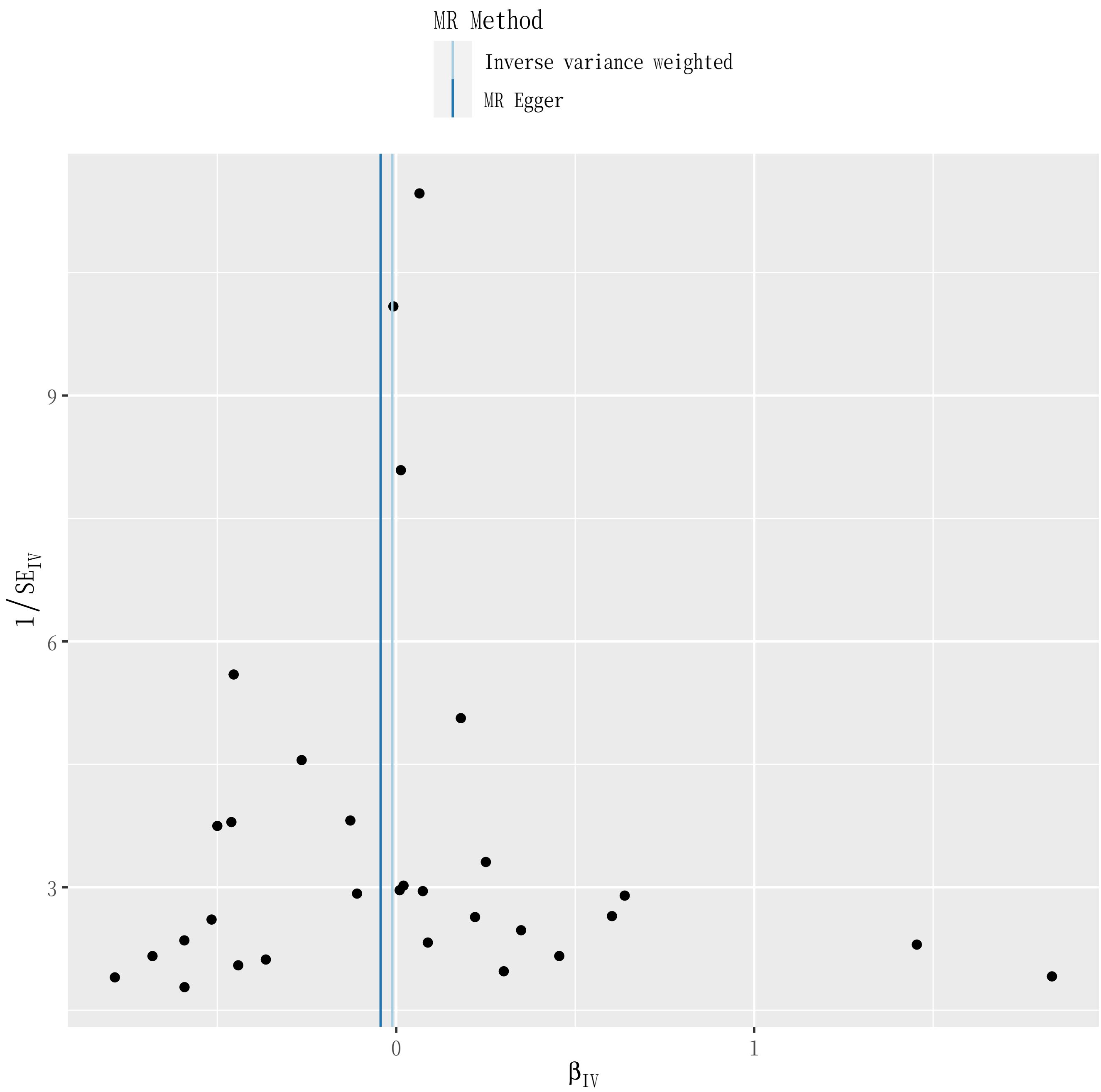

MR Method
Inverse variance weighted
MR Egger
9
6
3
1/SE_IV
0
1
β_IV

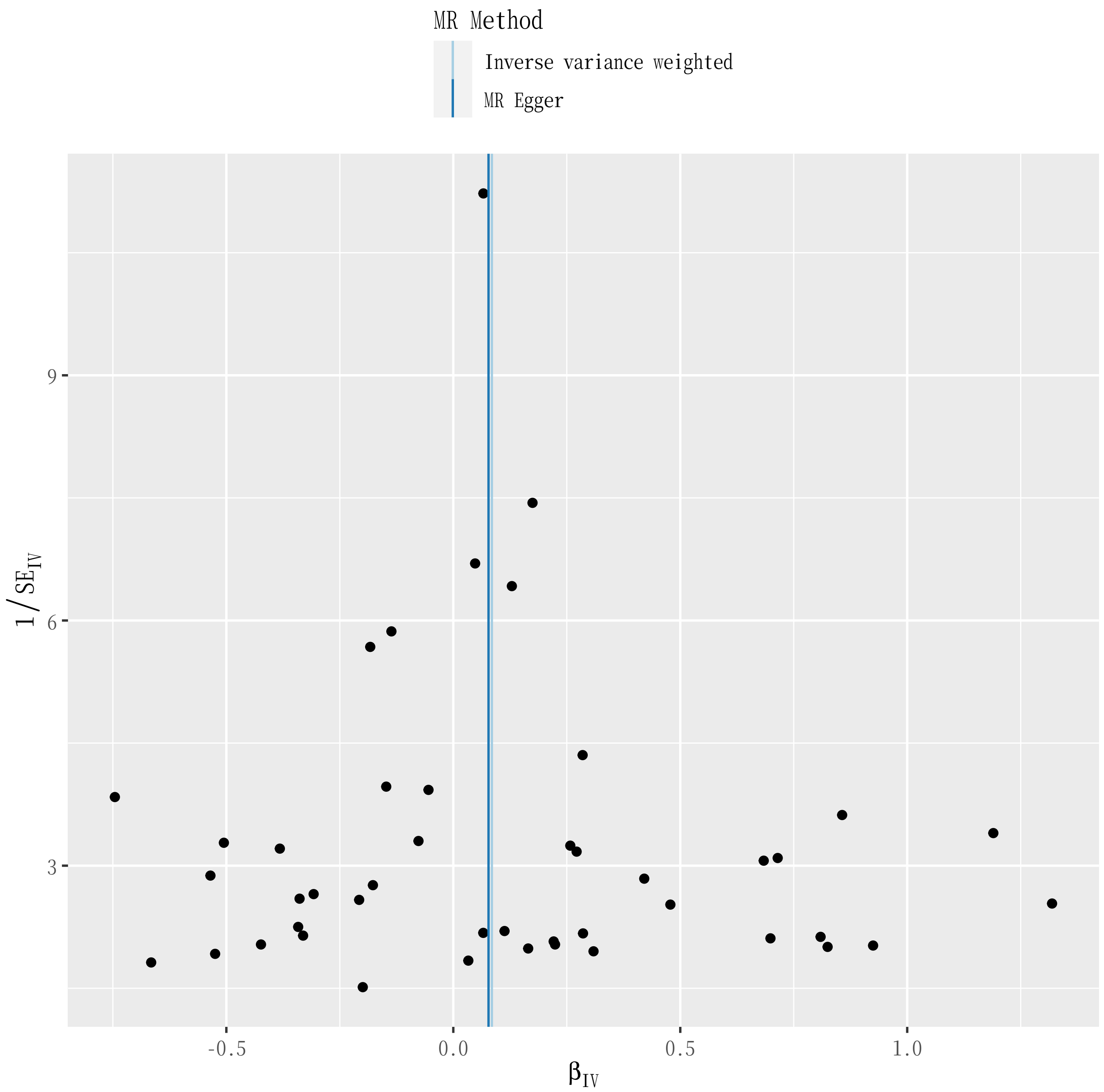

MR Method
Inverse variance weighted
MR Egger
9
6
3
-0.5
0.0
0.5
1.0
$1/SE_{IV}$
β_{IV}

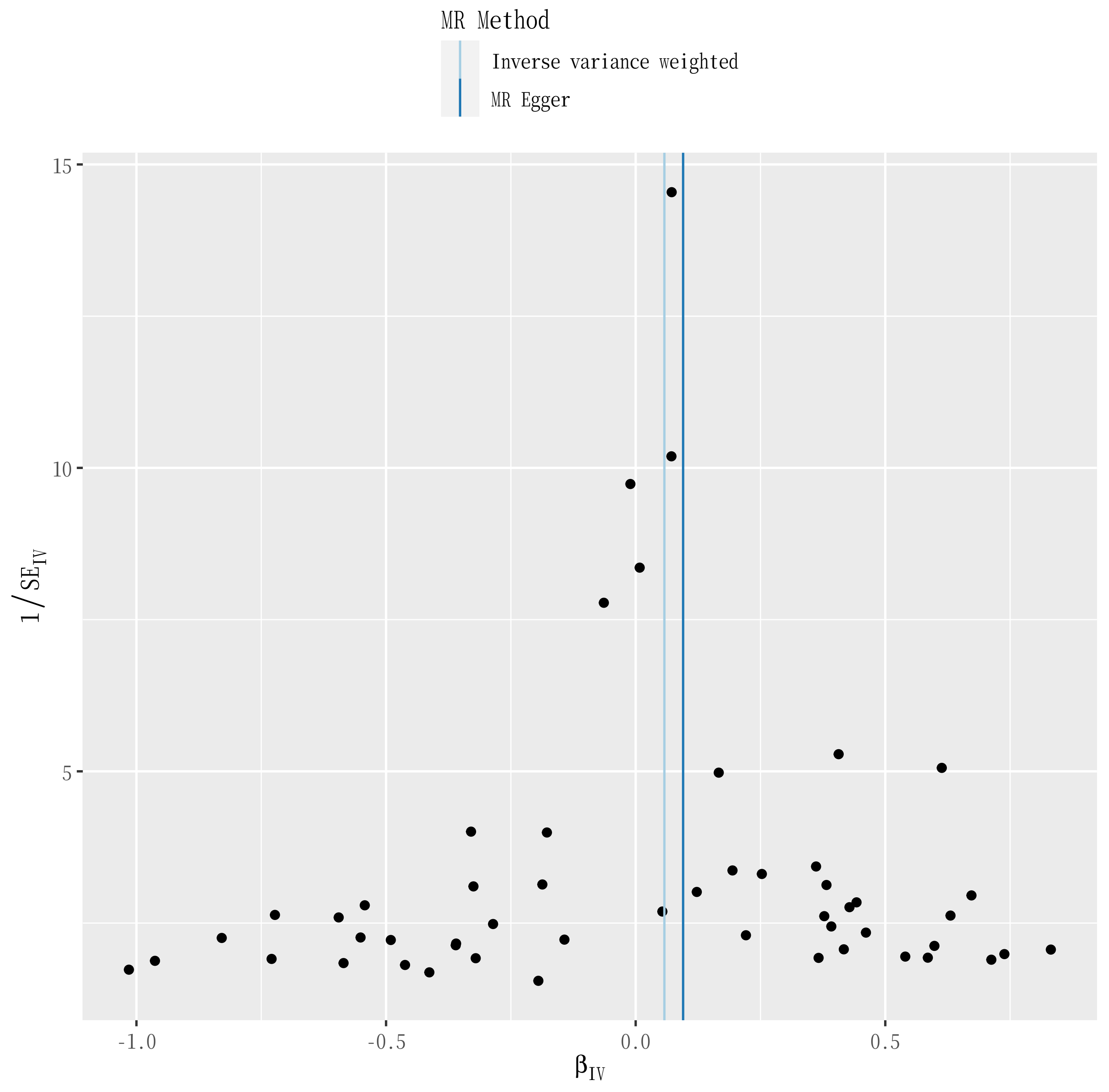

MR Method
Inverse variance weighted
MR Egger
15
10
5
-1.0
-0.5
0.0
0.5
$1/SE_{IV}$
β_{IV}

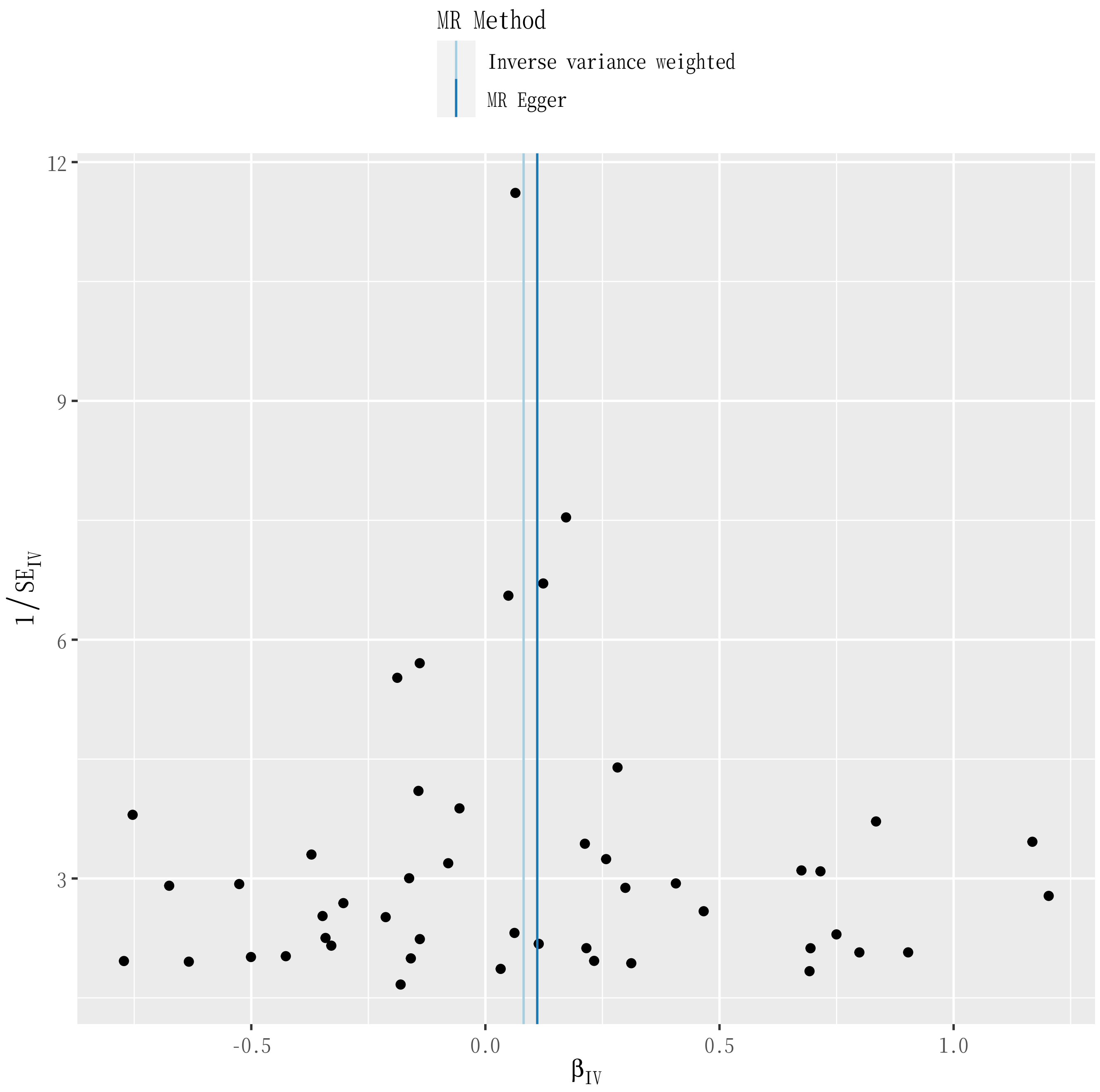
MR Method
Inverse variance weighted
MR Egger
12
9
6
3
$1/SE_{IV}$
-0.5
0.0
0.5
1.0
β_{IV}

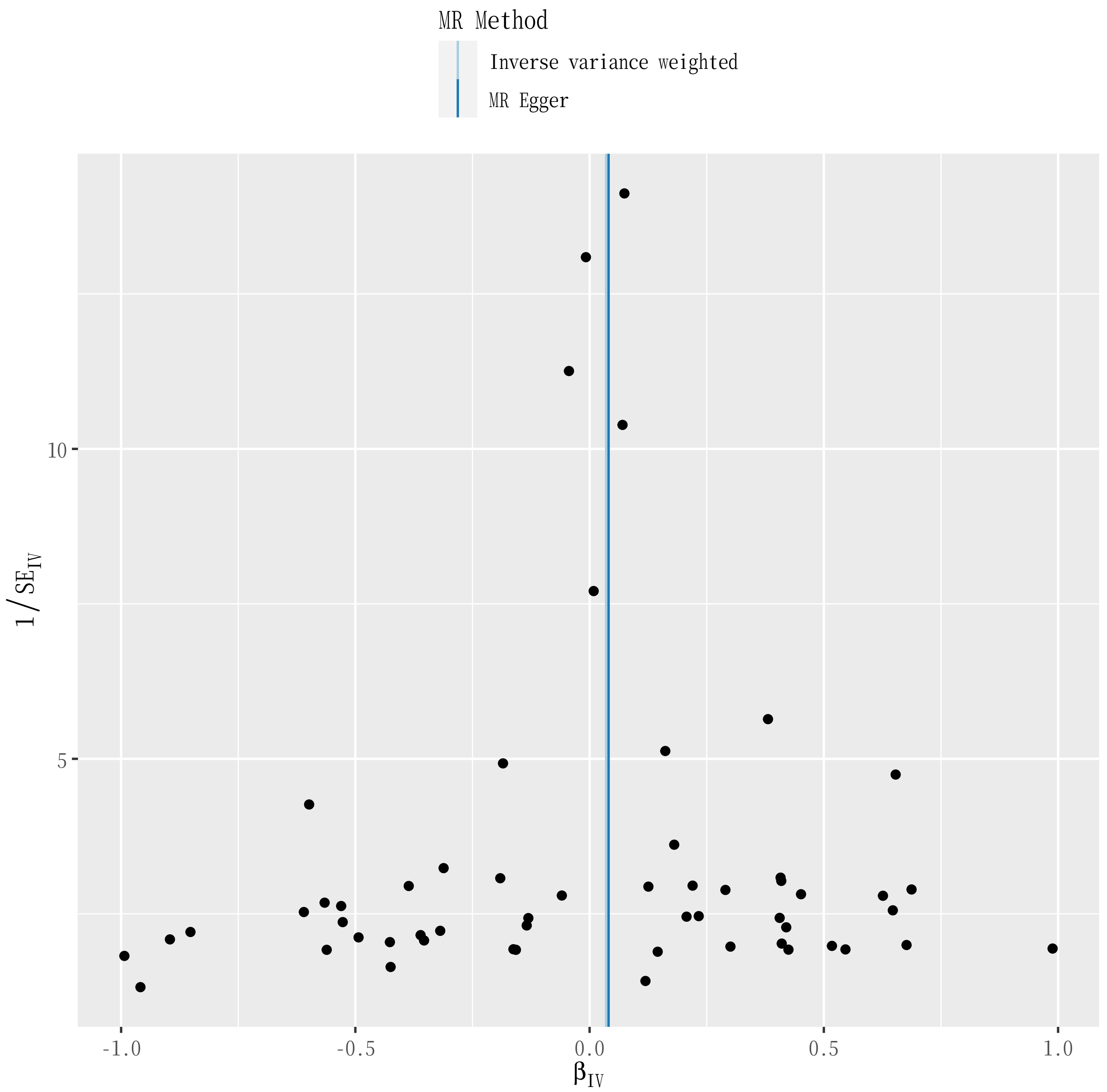

MR Method
Inverse variance weighted
MR Egger
10
5
$1/SE_{IV}$
-1.0
-0.5
0.0
0.5
1.0
β_{IV}

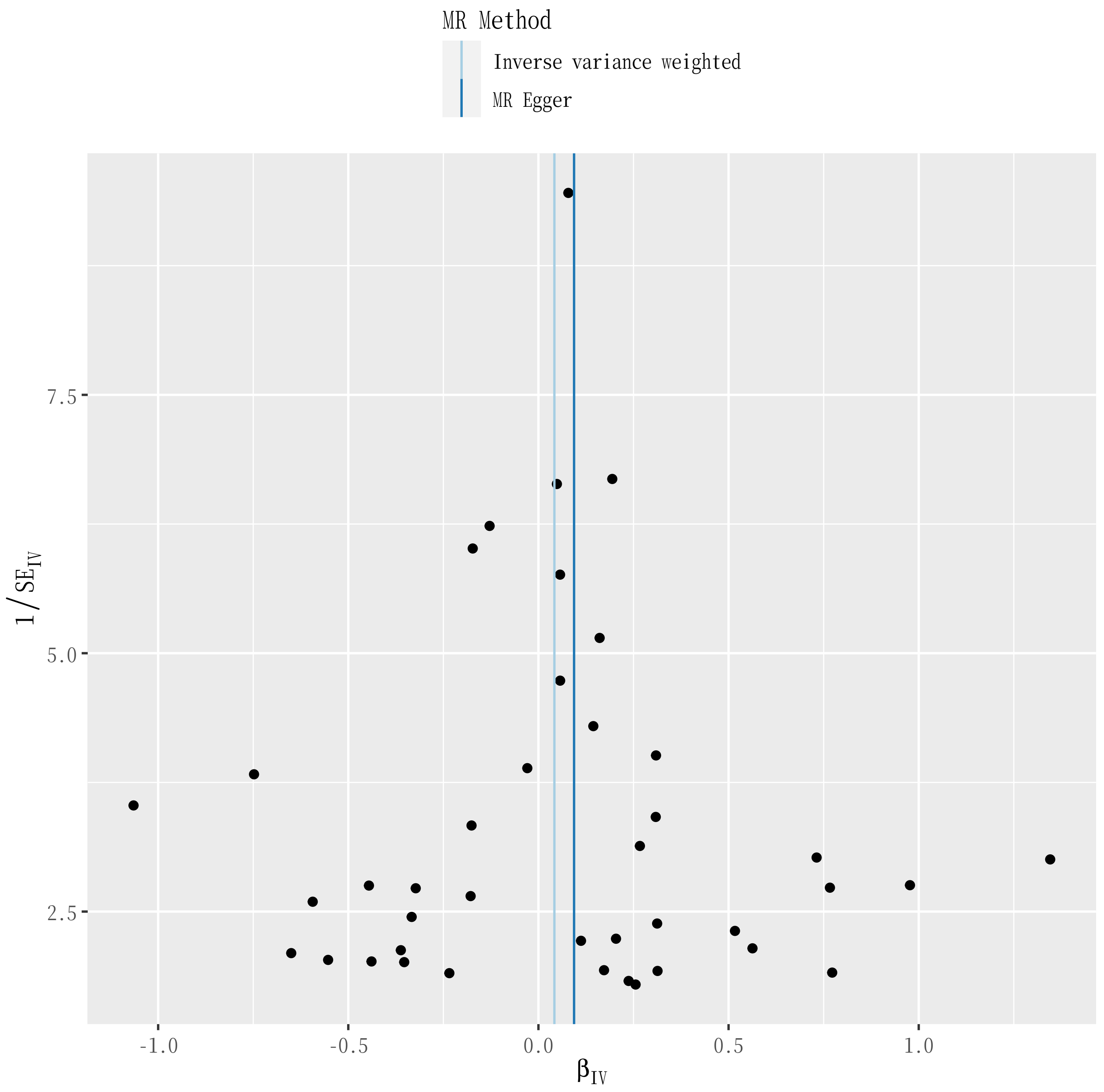

MR Method
Inverse variance weighted
MR Egger
7.5
5.0
2.5
1/SE_IV
-1.0
-0.5
0.0
0.5
1.0
β_IV

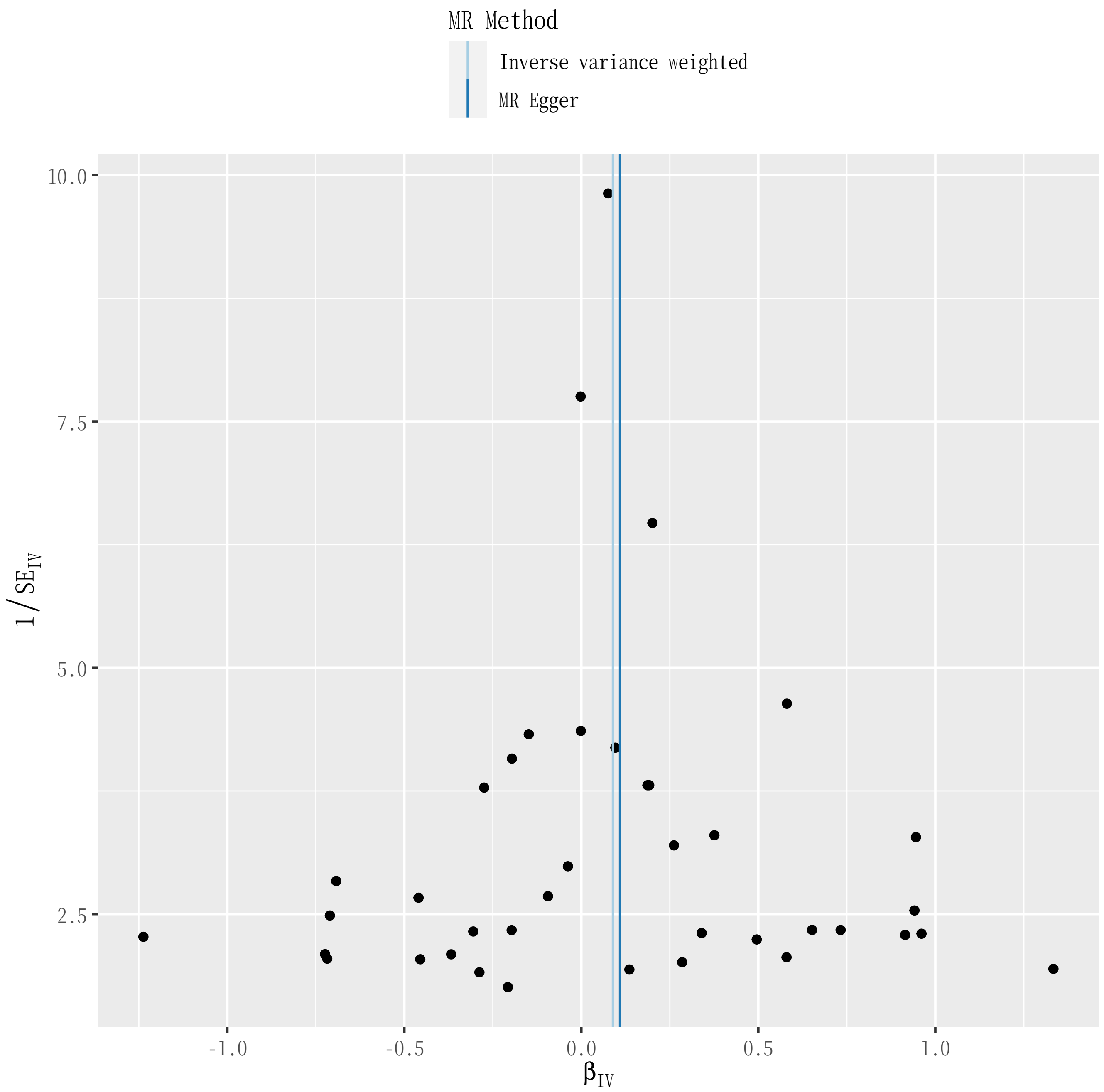

MR Method
Inverse variance weighted
MR Egger
10.0
7.5
5.0
2.5
1/SE_IV
-1.0
-0.5
0.0
0.5
1.0
β_IV

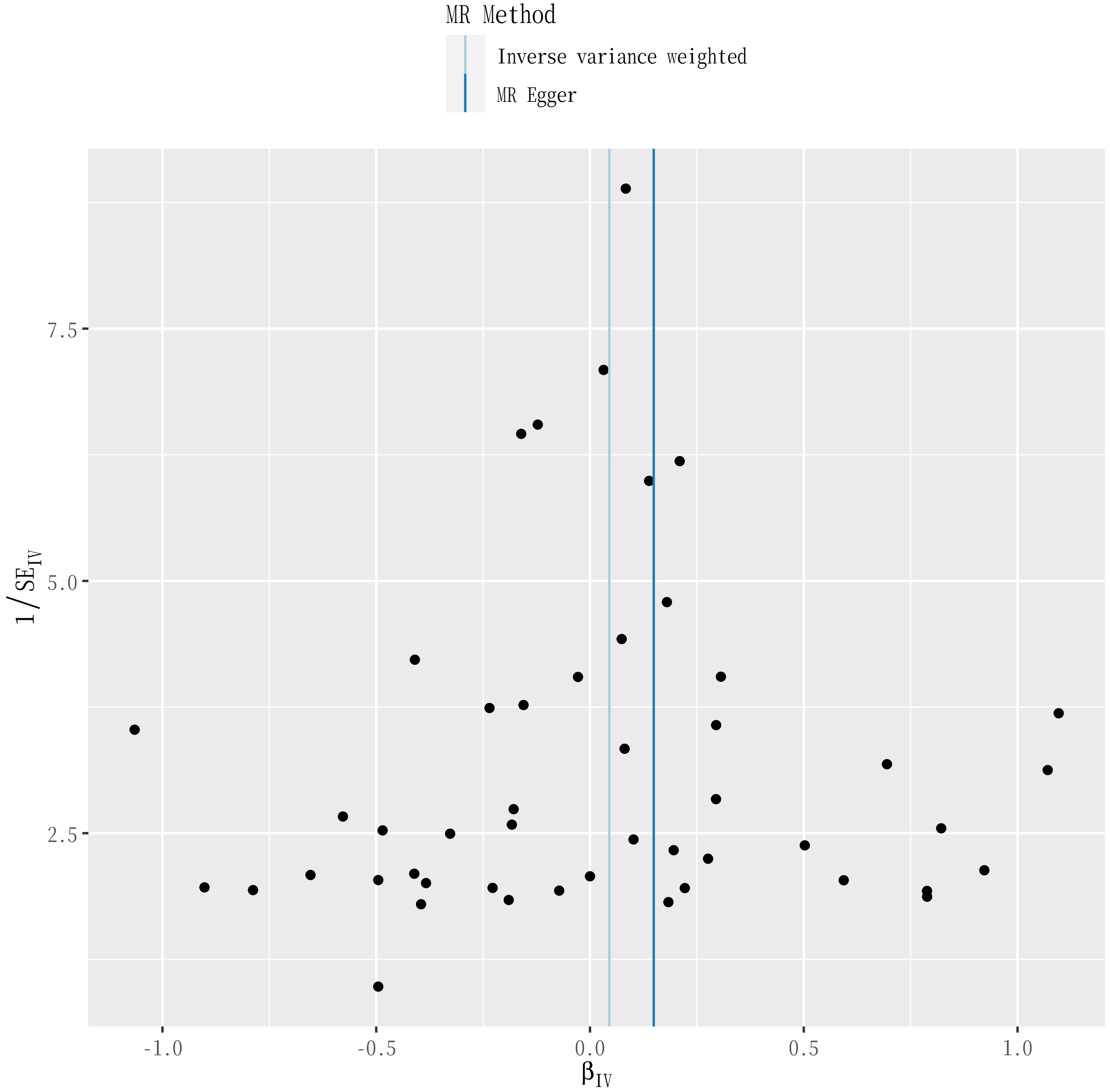

MR Method
Inverse variance weighted
MR Egger
7.5
5.0
2.5
$1/SE_{IV}$
-1.0
-0.5
0.0
0.5
1.0
β_{IV}

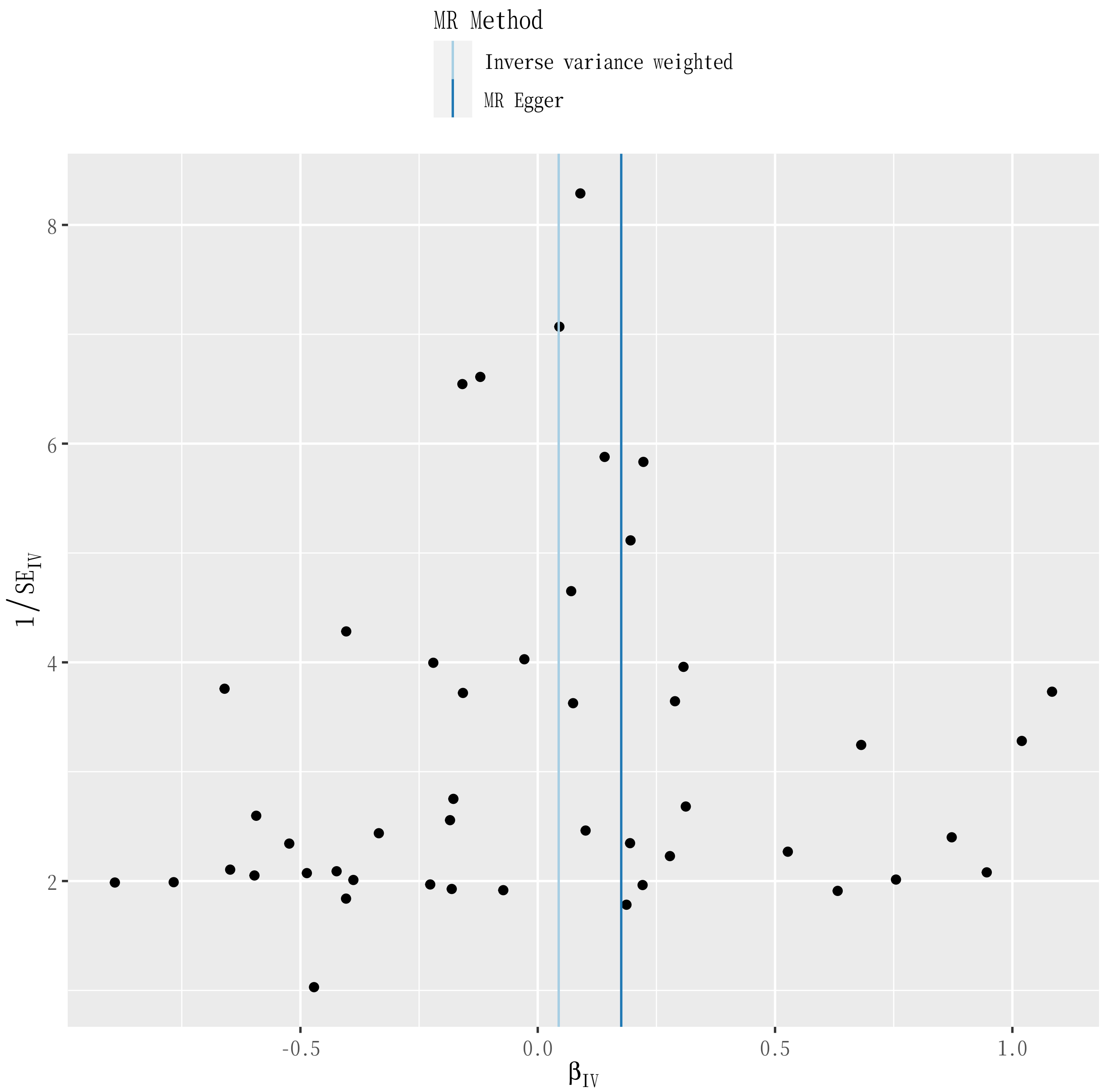
MR Method
Inverse variance weighted
MR Egger
8
6
4
2
1/SE_IV
-0.5
0.0
0.5
1.0
β_IV

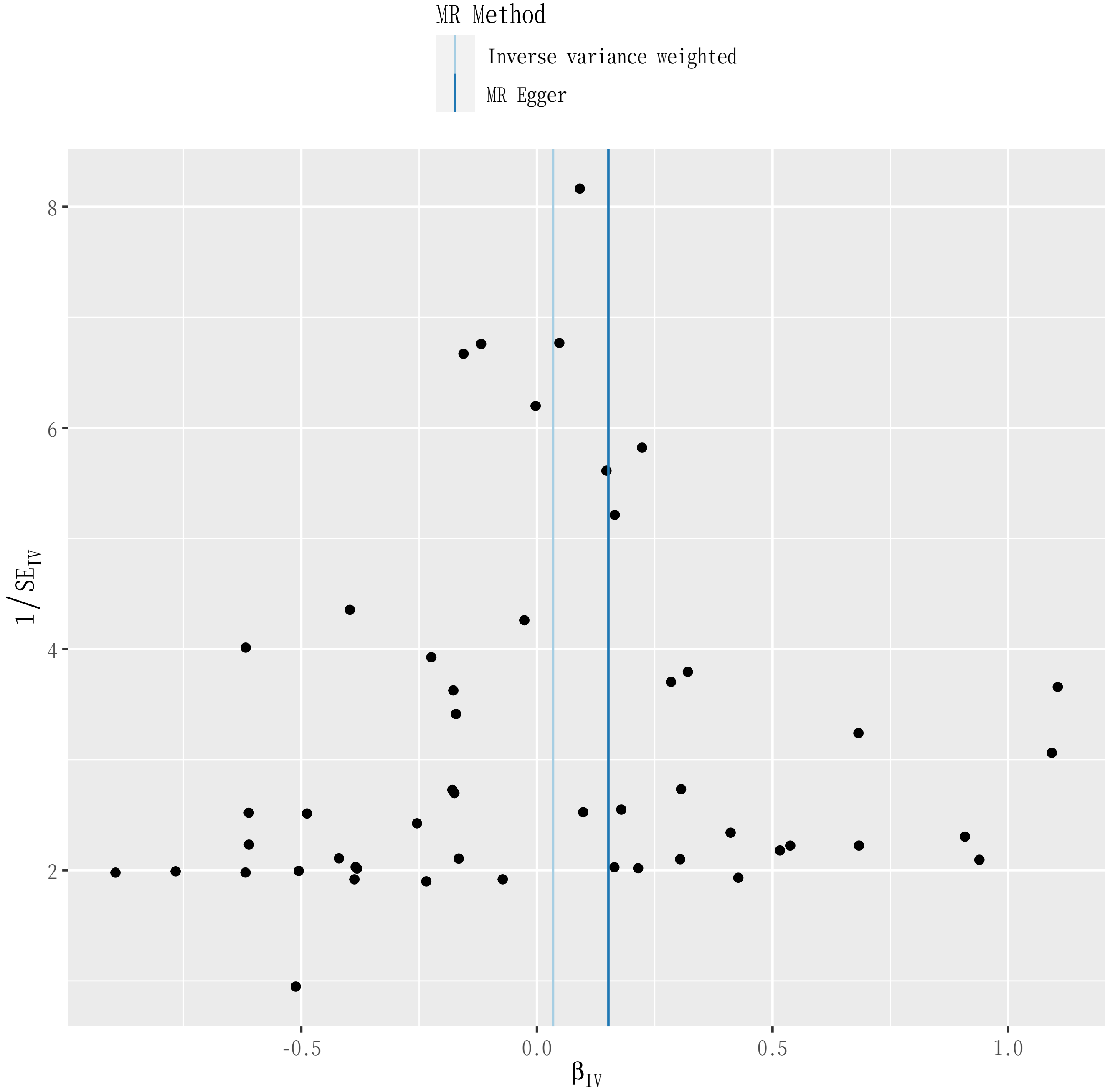
MR Method
Inverse variance weighted
MR Egger
8
6
4
2
-0.5
0.0
0.5
1.0
$1/SE_{IV}$
β_{IV}

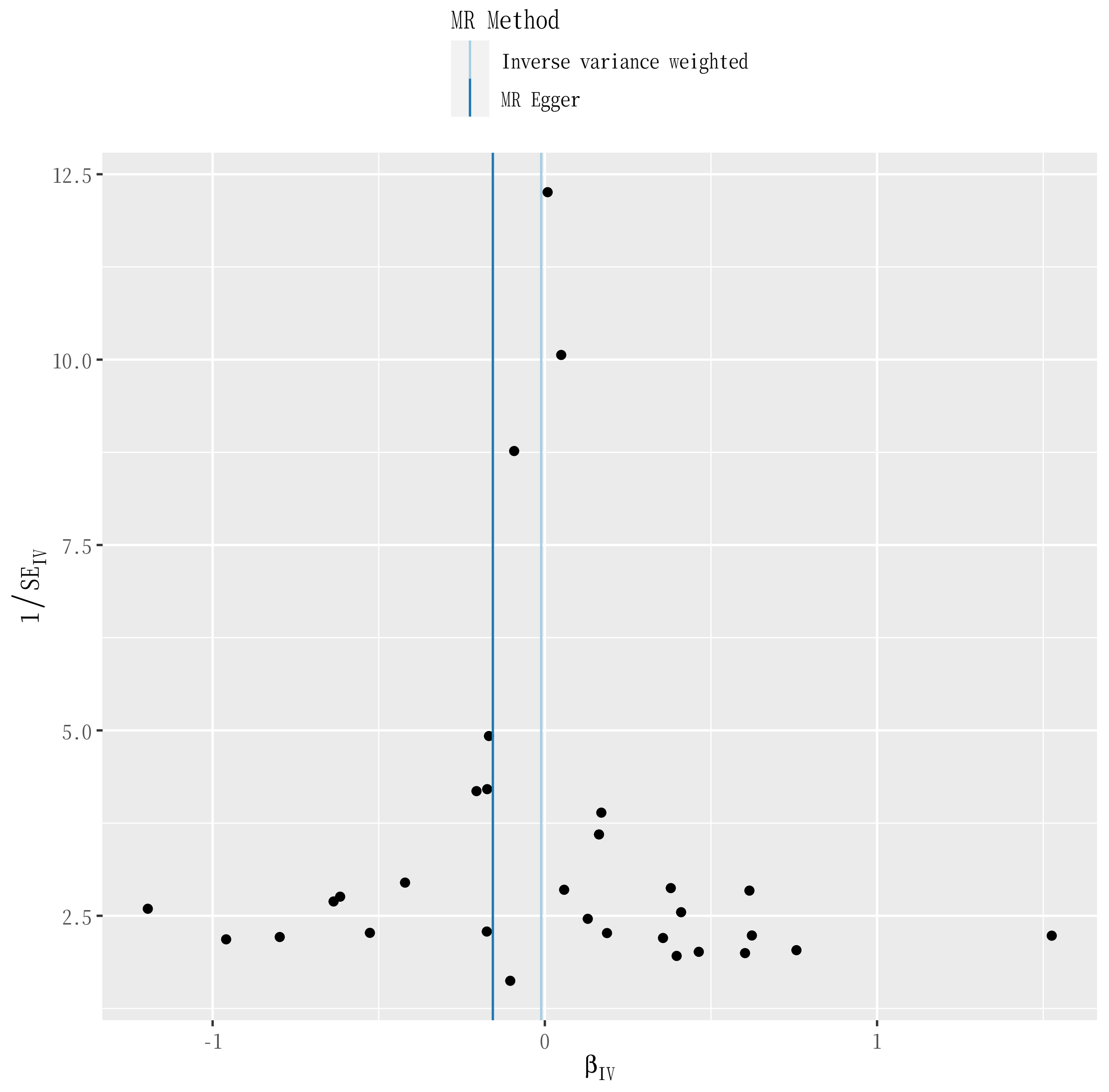

MR Method
Inverse variance weighted
MR Egger
12.5
10.0
7.5
5.0
2.5
1/SE$_{IV}$
-1
0
1
β$_{IV}$

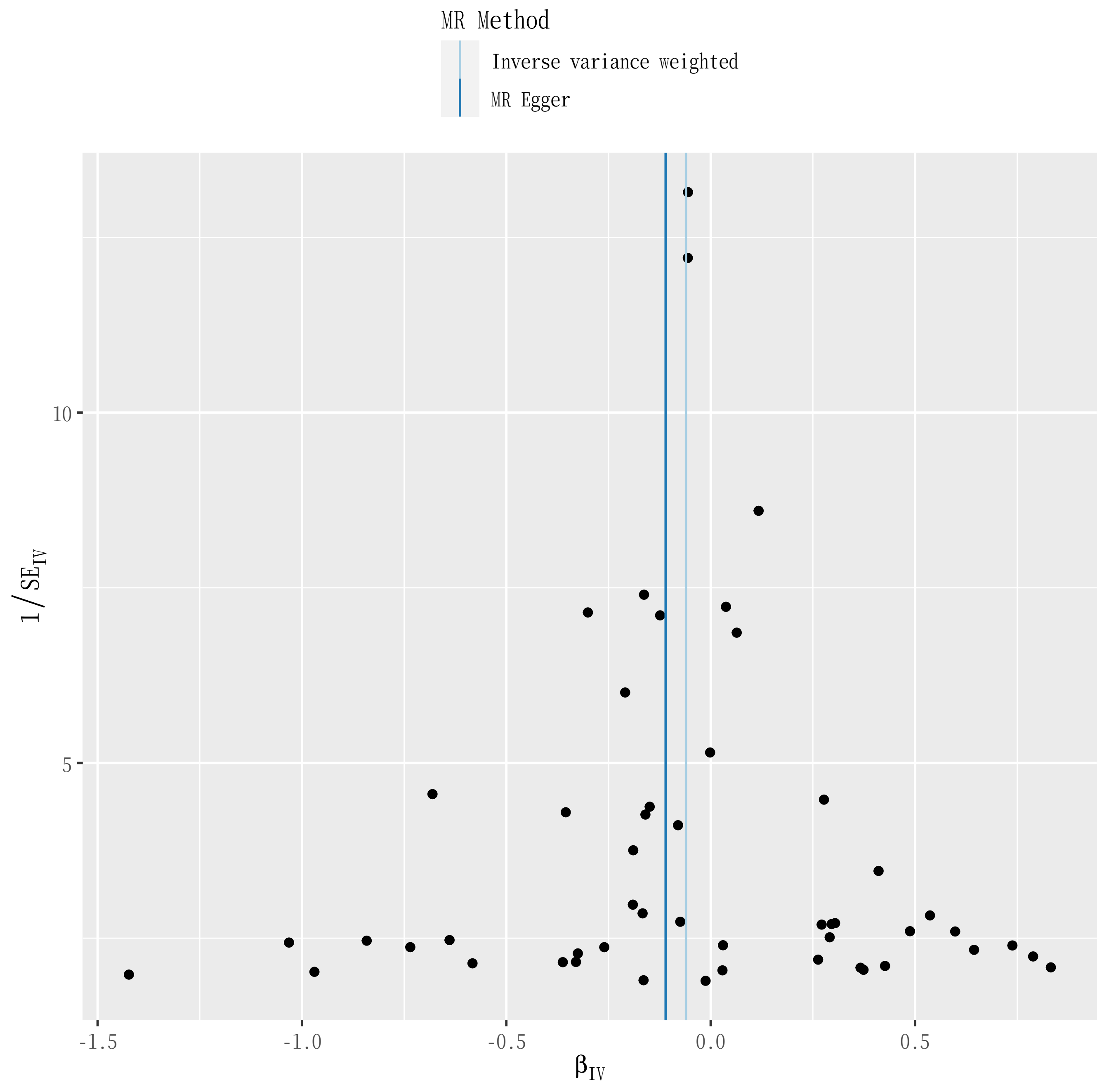

MR Method
Inverse variance weighted
MR Egger
10
5
1/SE_IV
-1.5
-1.0
-0.5
0.0
0.5
β_IV

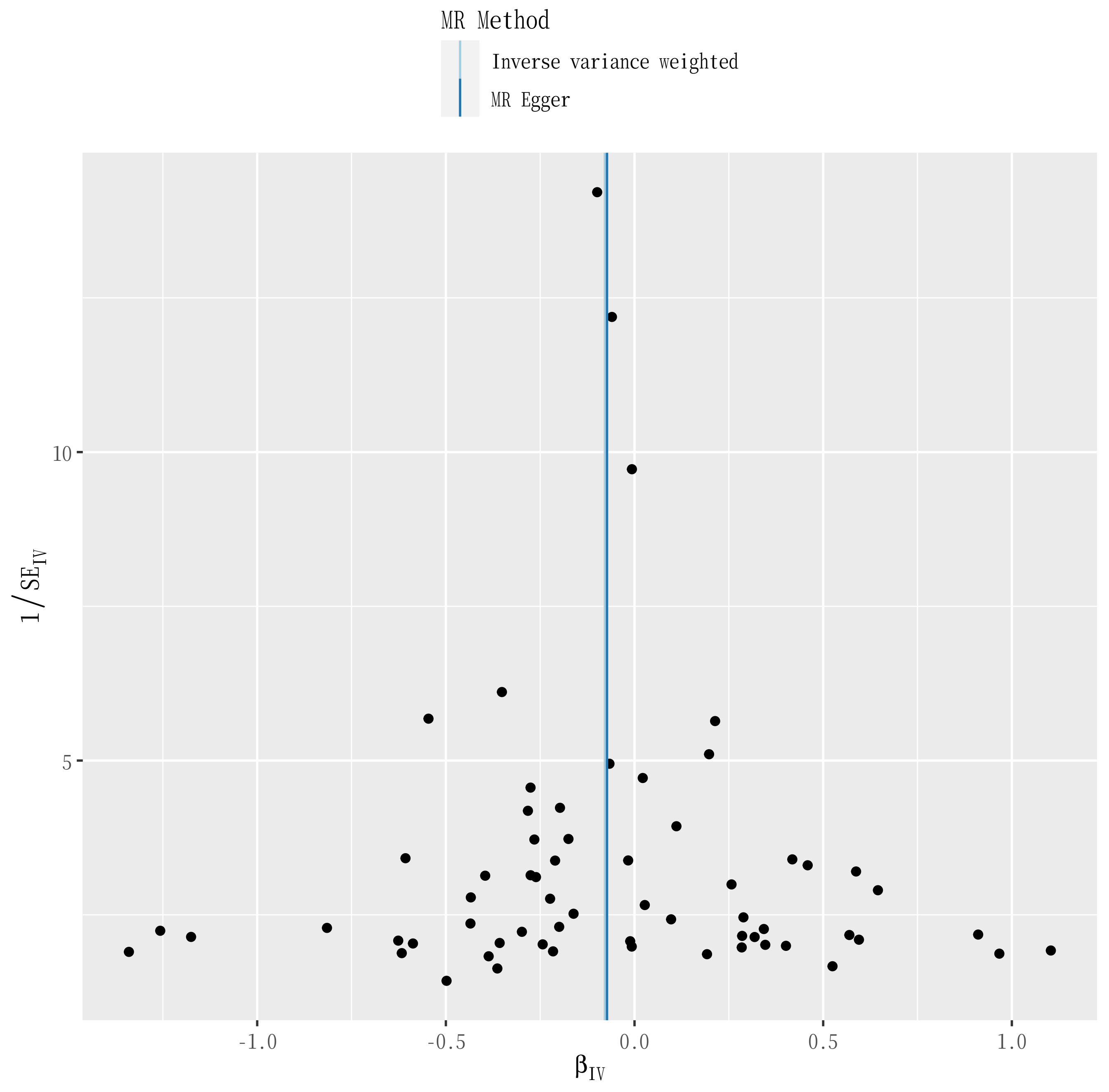

MR Method
Inverse variance weighted
MR Egger
10
5
1/SE_IV
-1.0
-0.5
0.0
0.5
1.0
β_IV

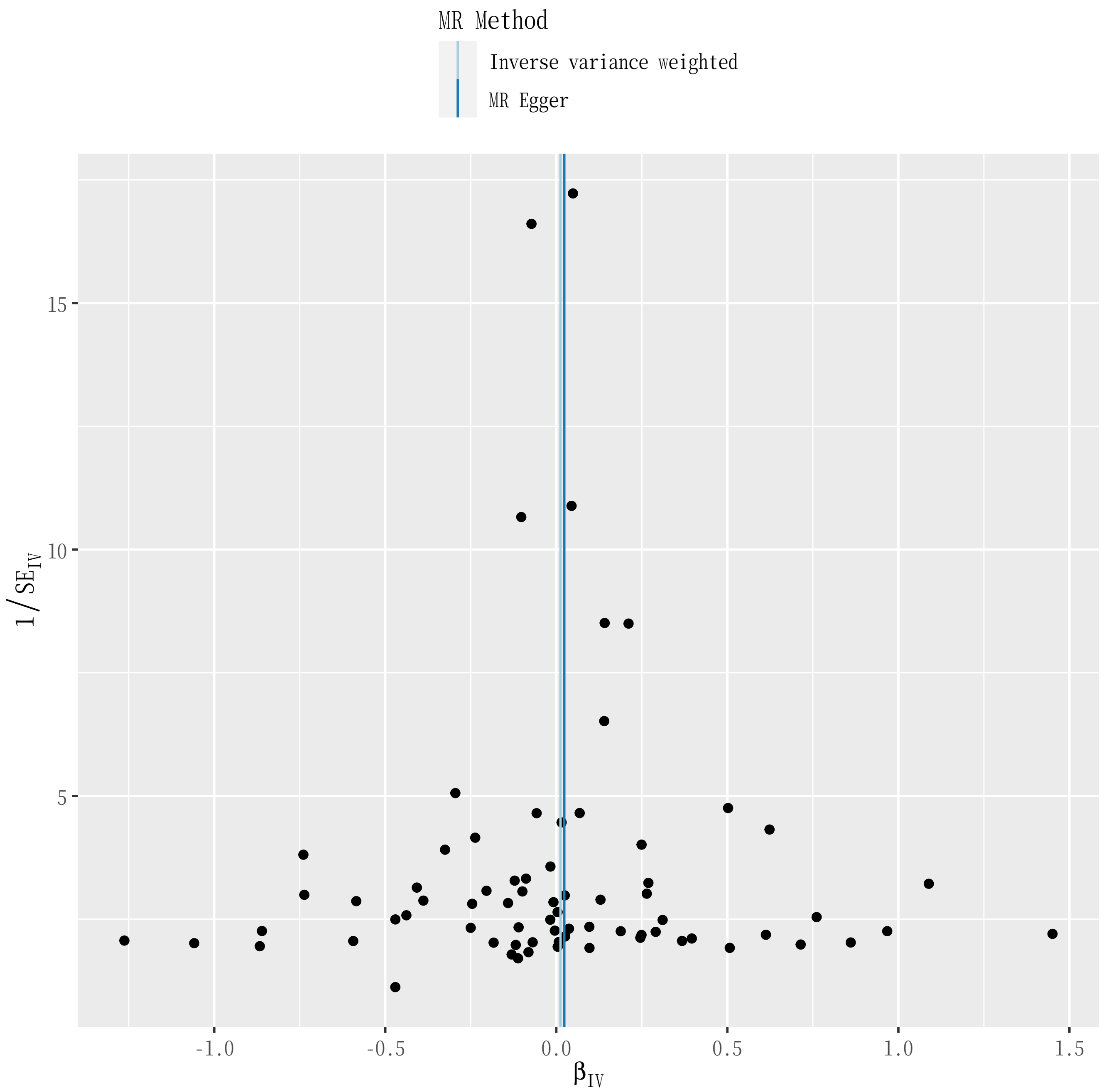

MR Method
Inverse variance weighted
MR Egger
15
10
5
$1/SE_{IV}$
-1.0
-0.5
0.0
0.5
1.0
1.5
β_{IV}

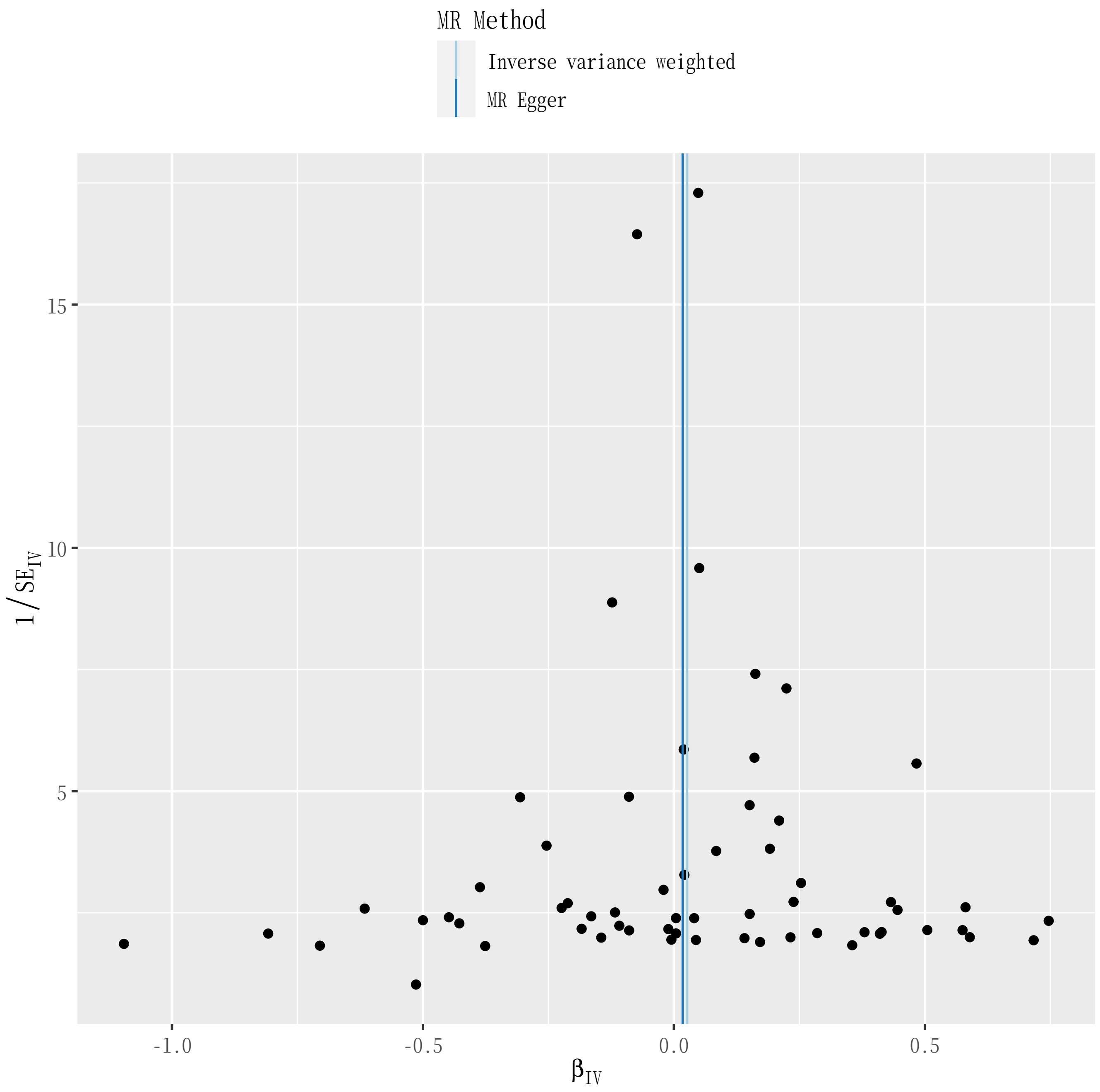
MR Method
Inverse variance weighted
MR Egger
15
10
5
1/SE_IV
-1.0
-0.5
0.0
0.5
β_IV

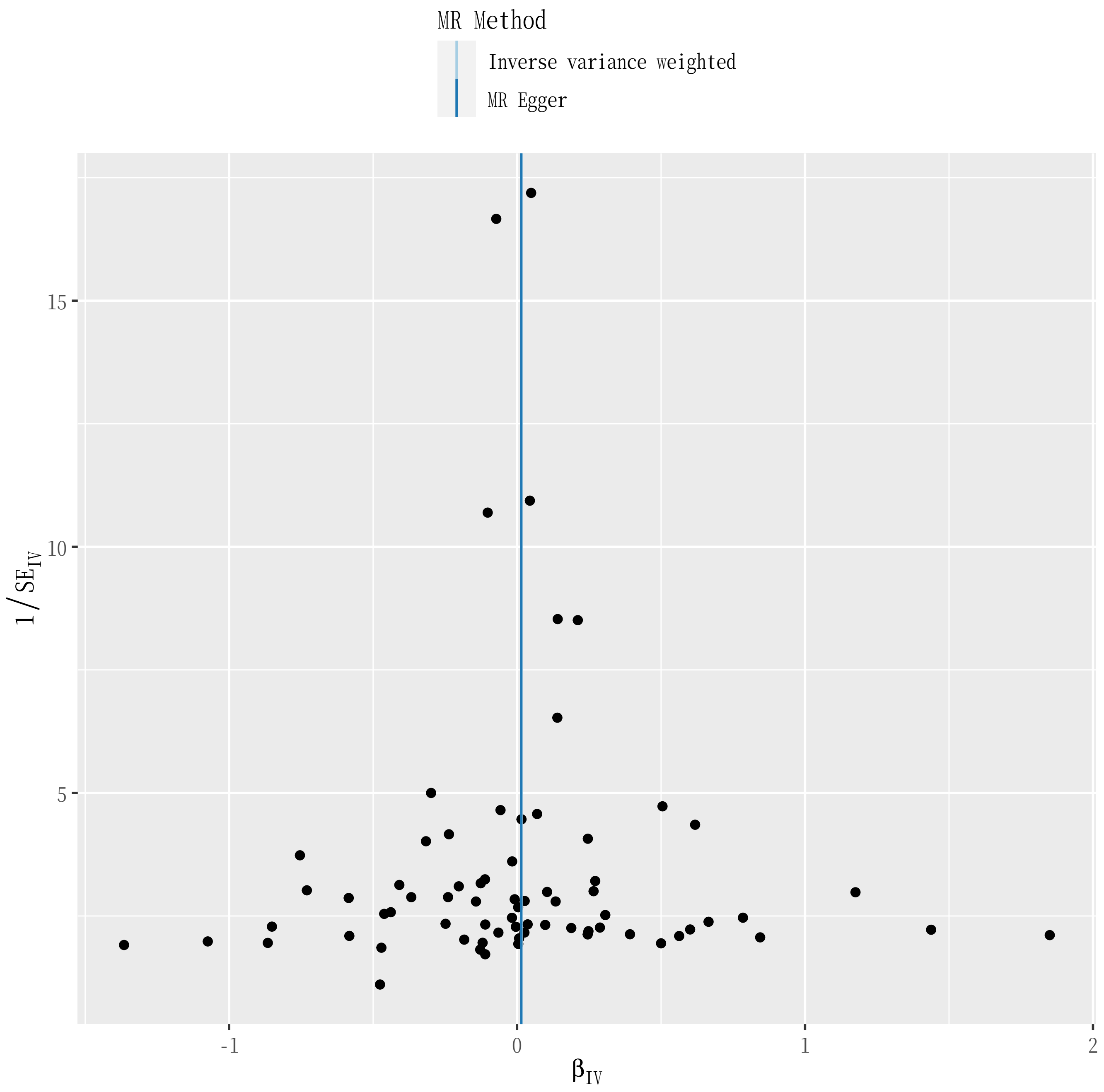

MR Method
Inverse variance weighted
MR Egger
15
10
5
1/SE$_{IV}$
-1
0
1
2
β$_{IV}$

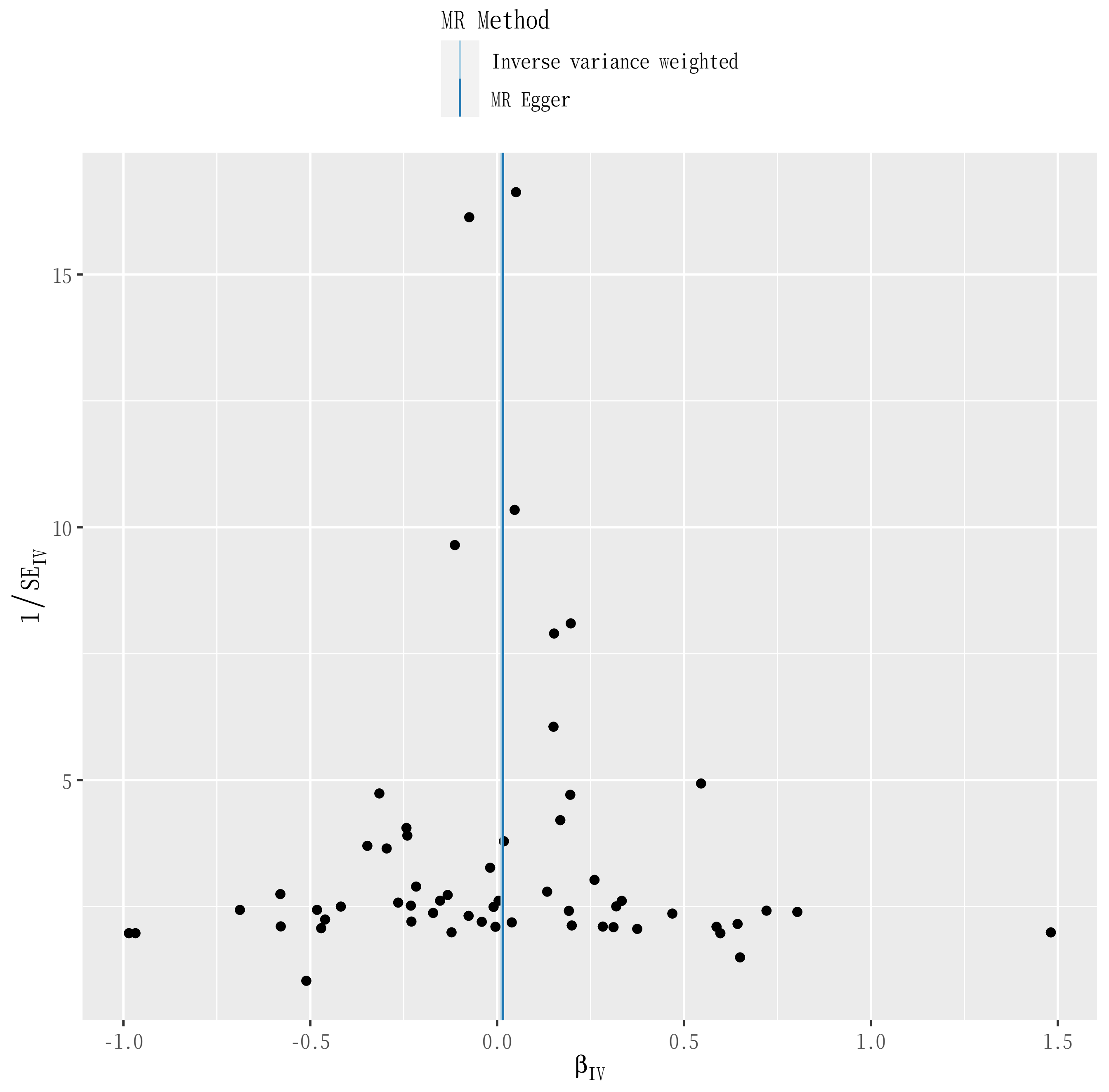
MR Method
Inverse variance weighted
MR Egger
15
10
5
1/SE_IV
-1.0
-0.5
0.0
0.5
1.0
1.5
β_IV

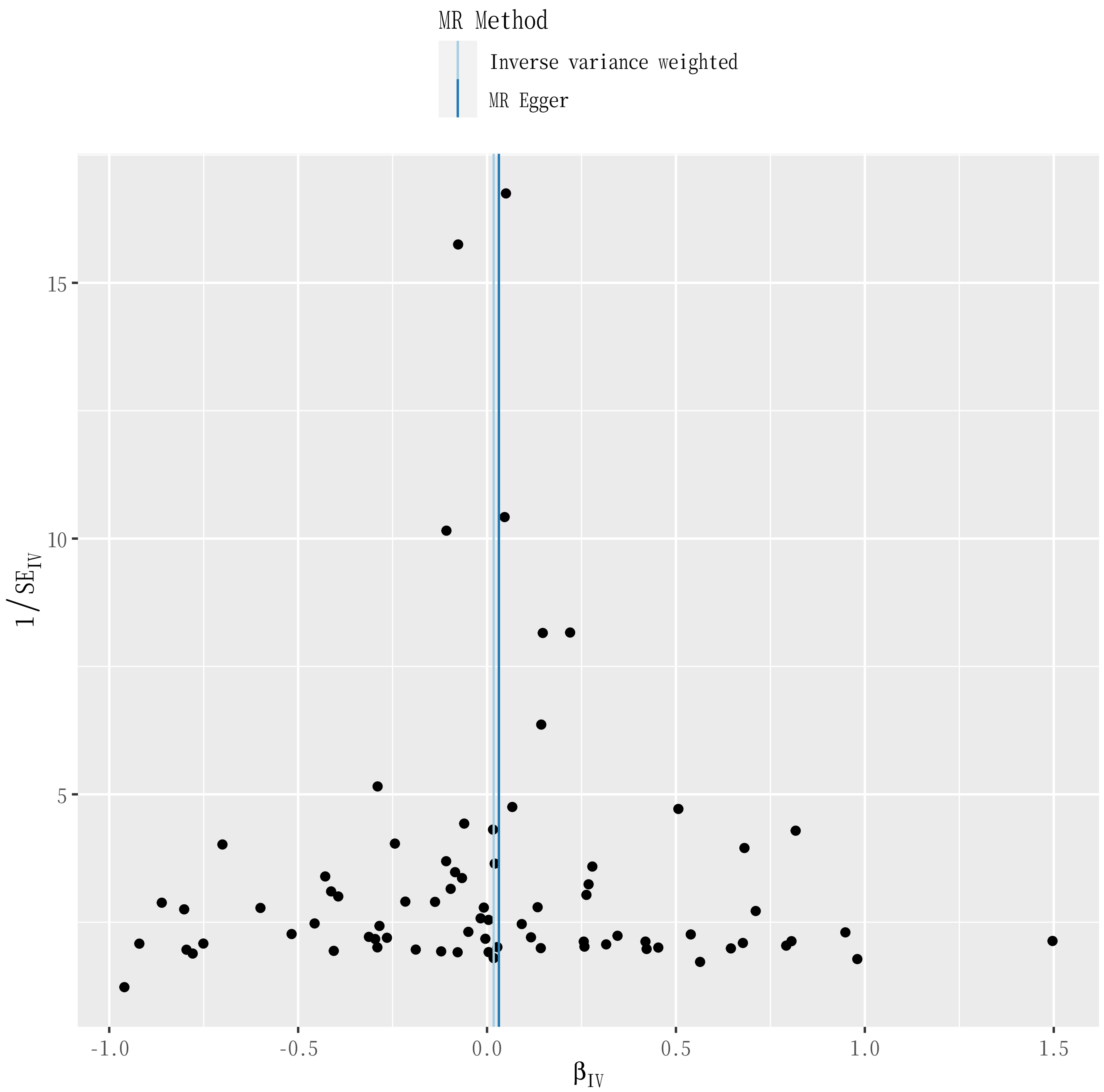

MR Method
Inverse variance weighted
MR Egger
15
10
5
1/SE_IV
-1.0
-0.5
0.0
0.5
1.0
1.5
β_IV

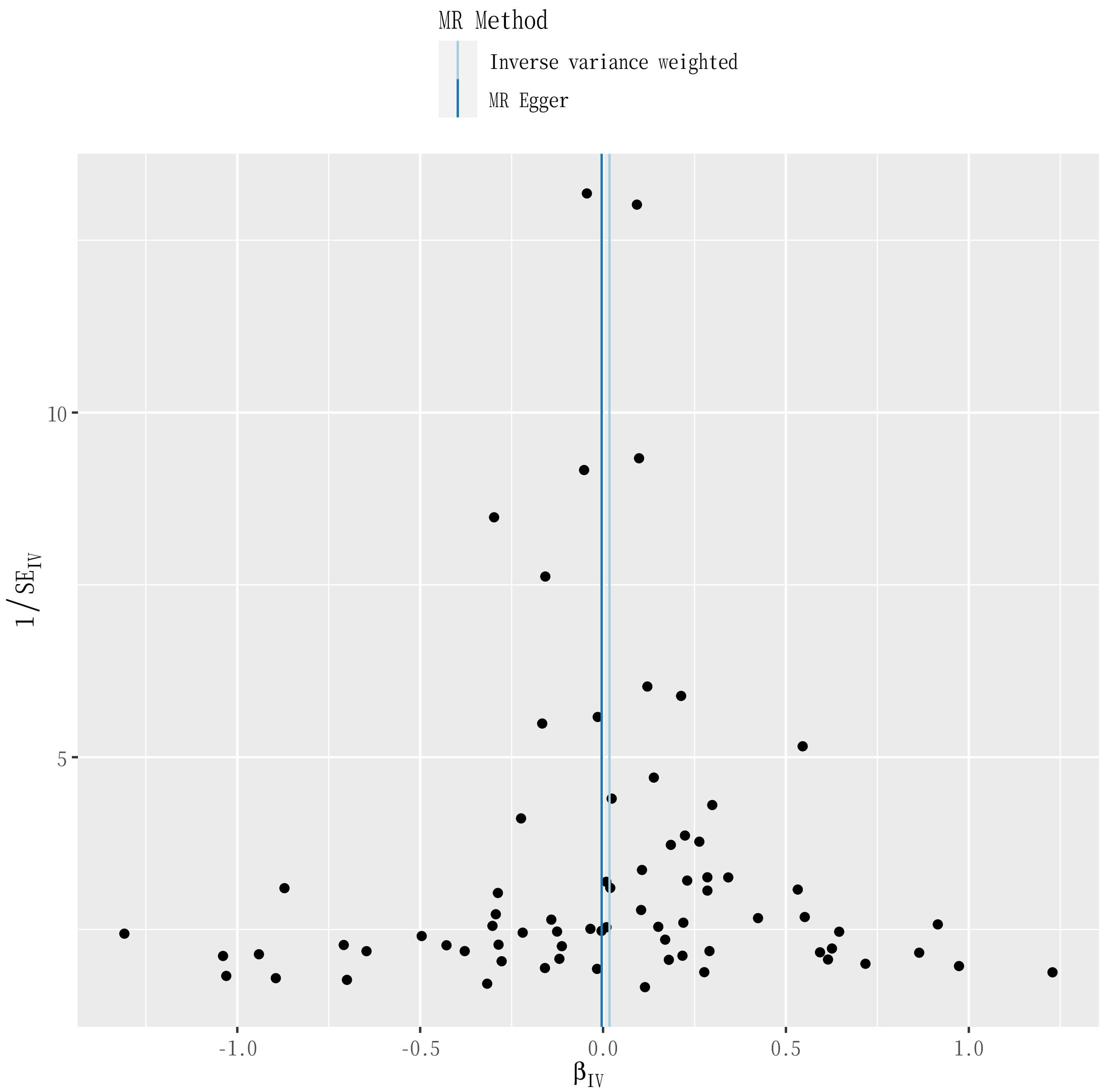
MR Method
Inverse variance weighted
MR Egger
1/SE$_{IV}$
10
5
-1.0
-0.5
0.0
0.5
1.0
β$_{IV}$

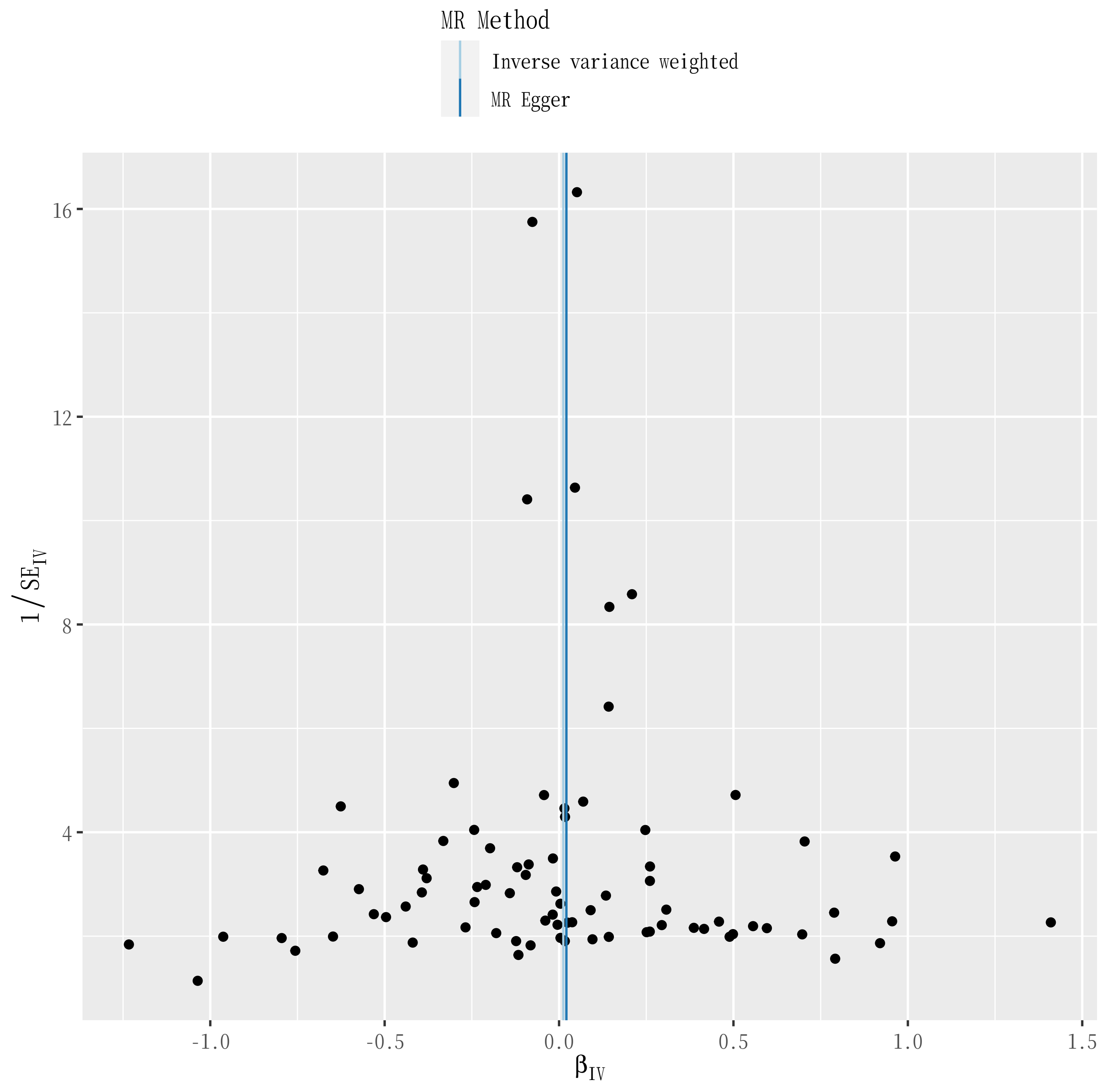

MR Method
Inverse variance weighted
MR Egger
16
12
8
4
-1.0
-0.5
0.0
0.5
1.0
1.5
$1/SE_{IV}$
β_{IV}

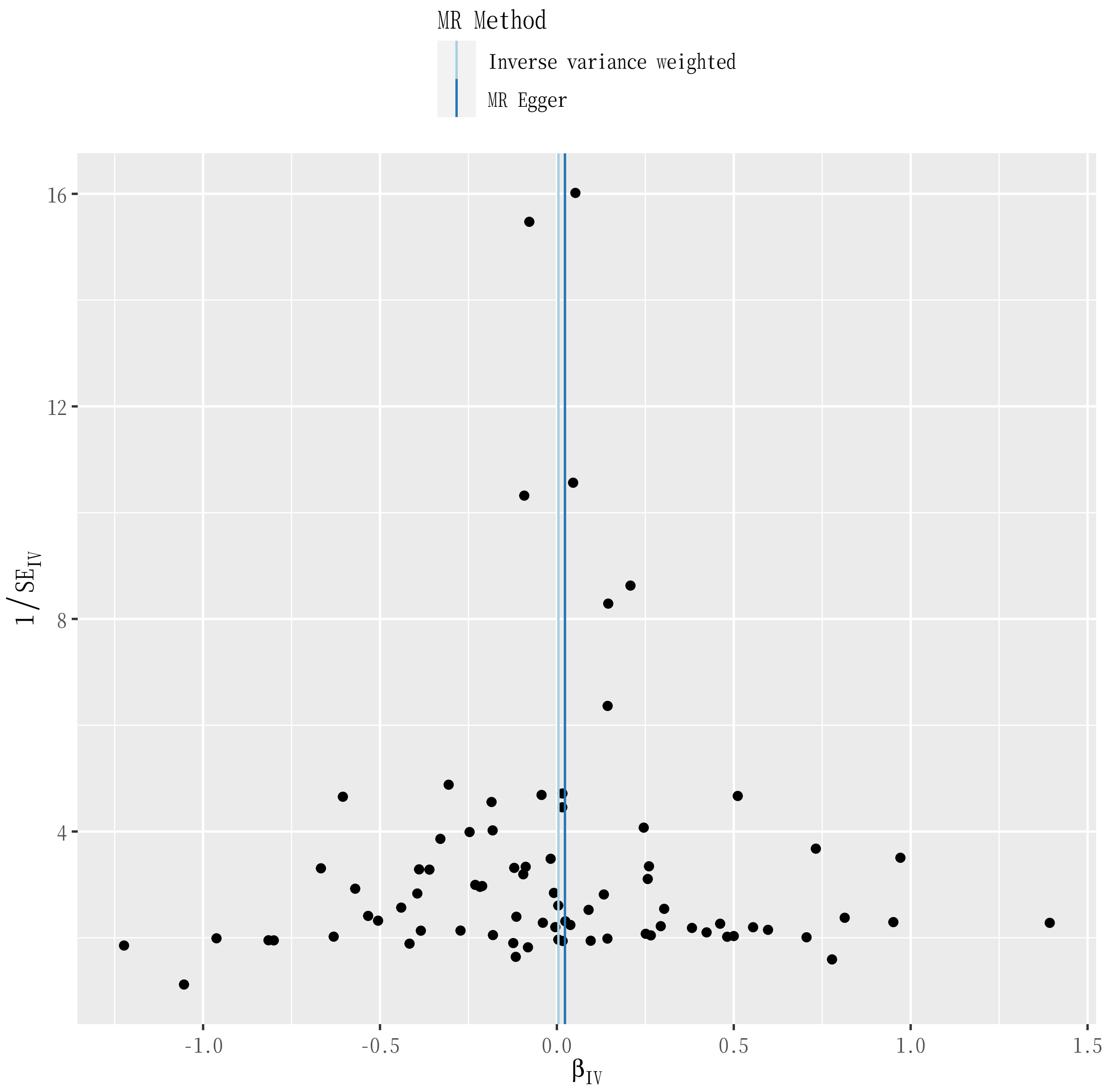

MR Method
Inverse variance weighted
MR Egger
16
12
8
4
1/SE_IV
-1.0
-0.5
0.0
0.5
1.0
1.5
β_IV

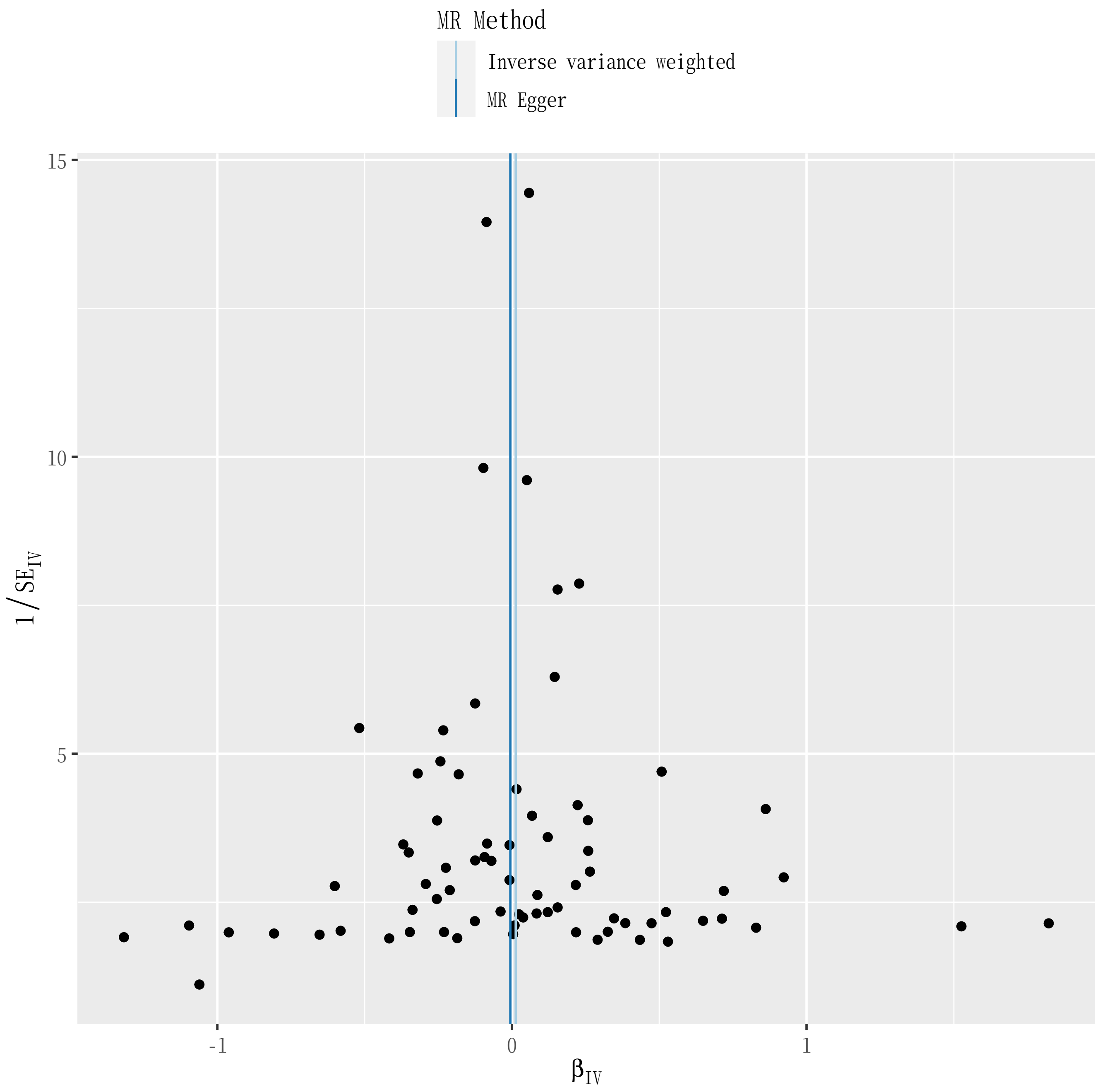
MR Method
Inverse variance weighted
MR Egger
15
10
5
-1
0
1
$1/SE_{IV}$
β_{IV}

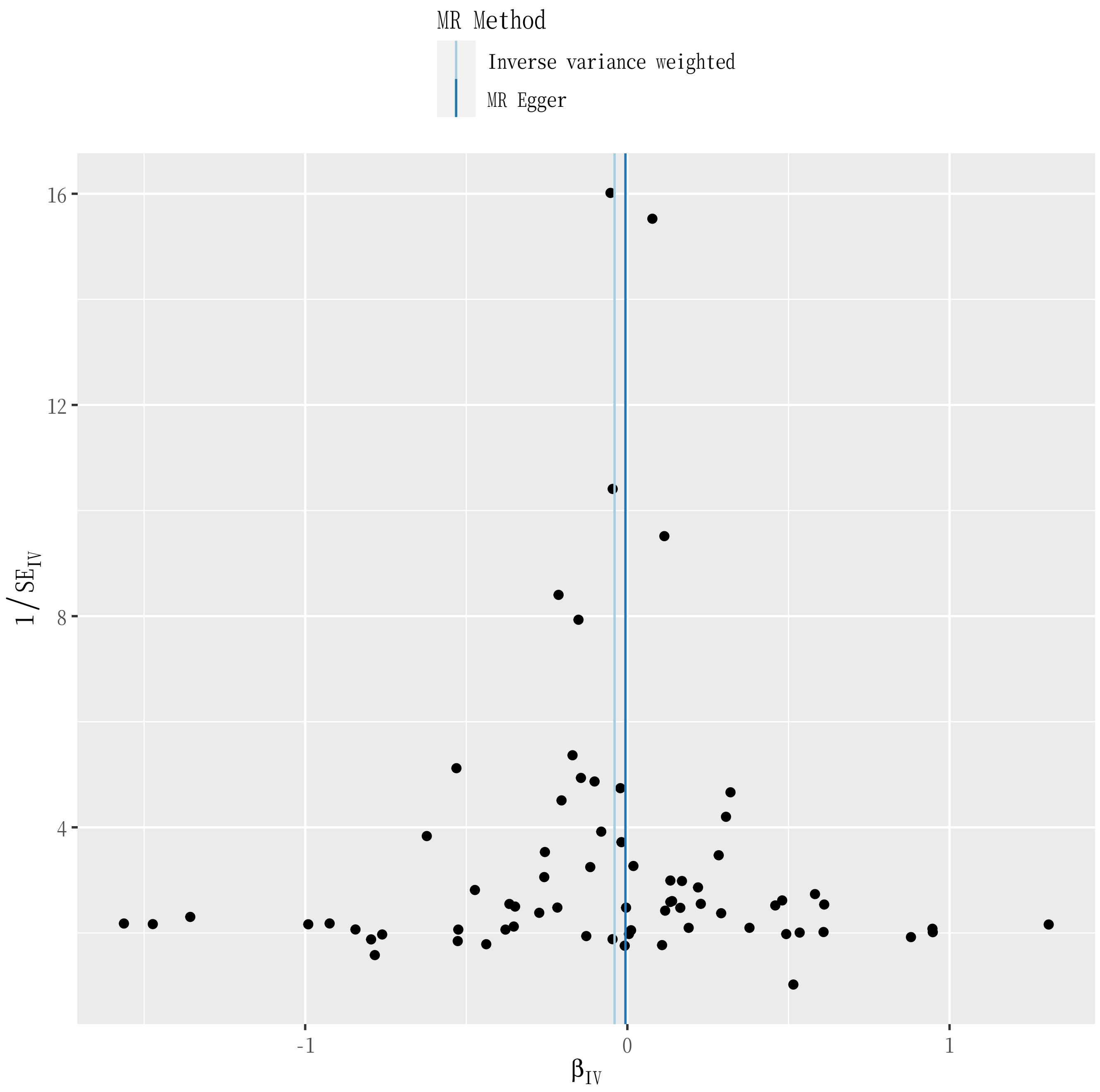

MR Method
Inverse variance weighted
MR Egger
16
12
8
4
$1/SE_{IV}$
-1
0
1
β_{IV}

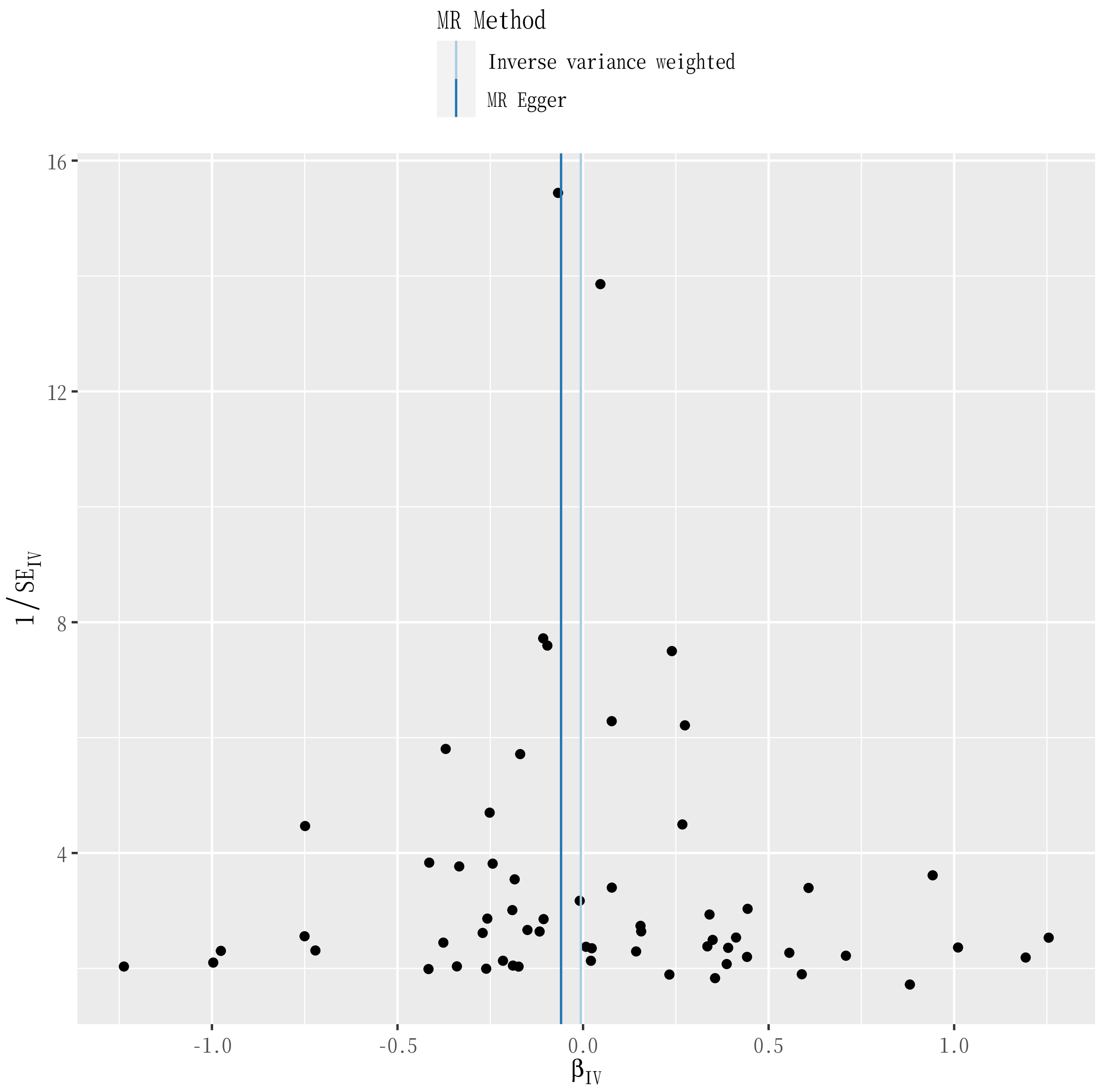

MR Method
Inverse variance weighted
MR Egger
16
12
8
4
1/SE_IV
-1.0
-0.5
0.0
0.5
1.0
β_IV

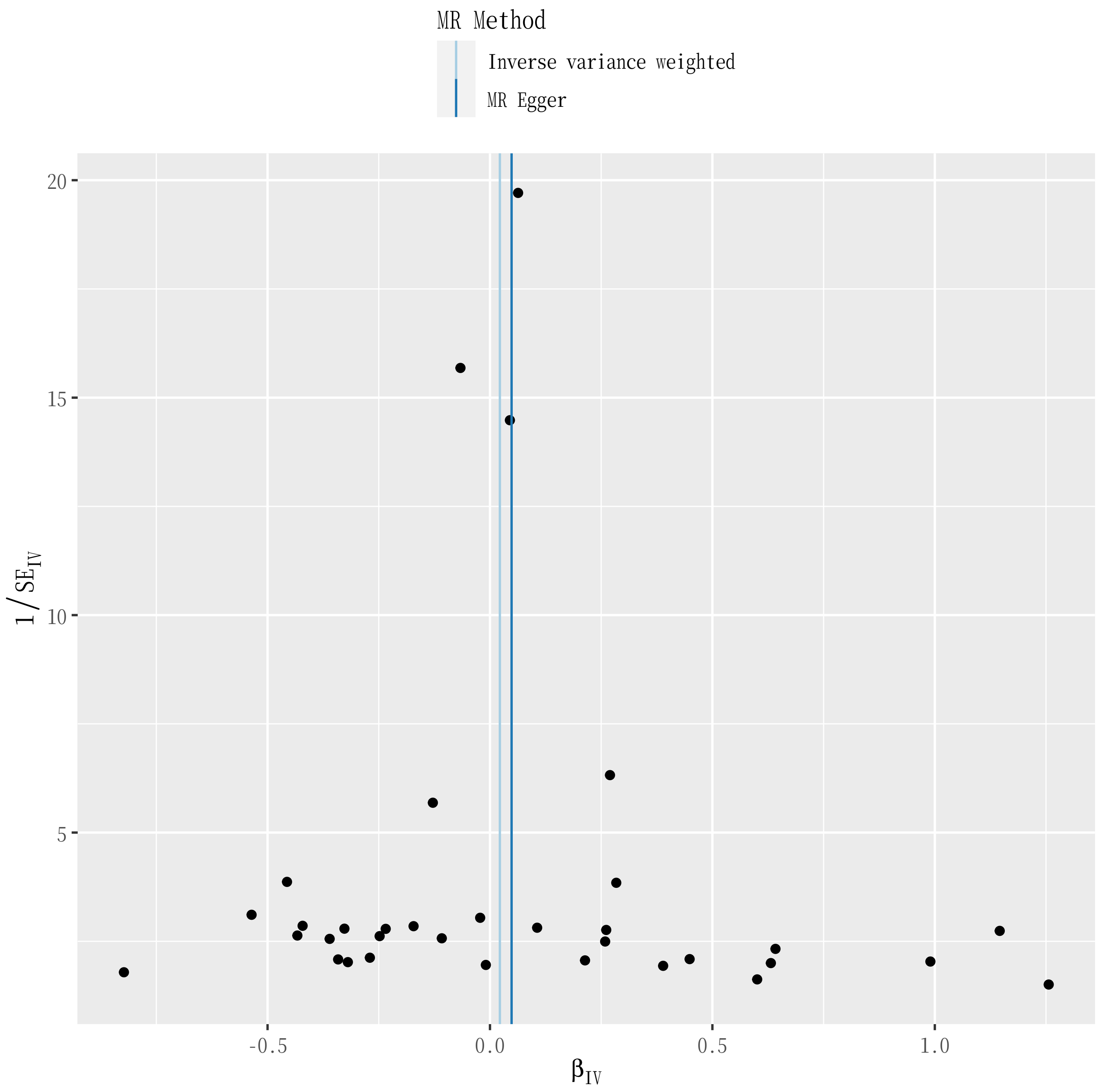
MR Method
Inverse variance weighted
MR Egger
20
15
10
5
-0.5
0.0
0.5
1.0
$1/SE_{IV}$
β_{IV}

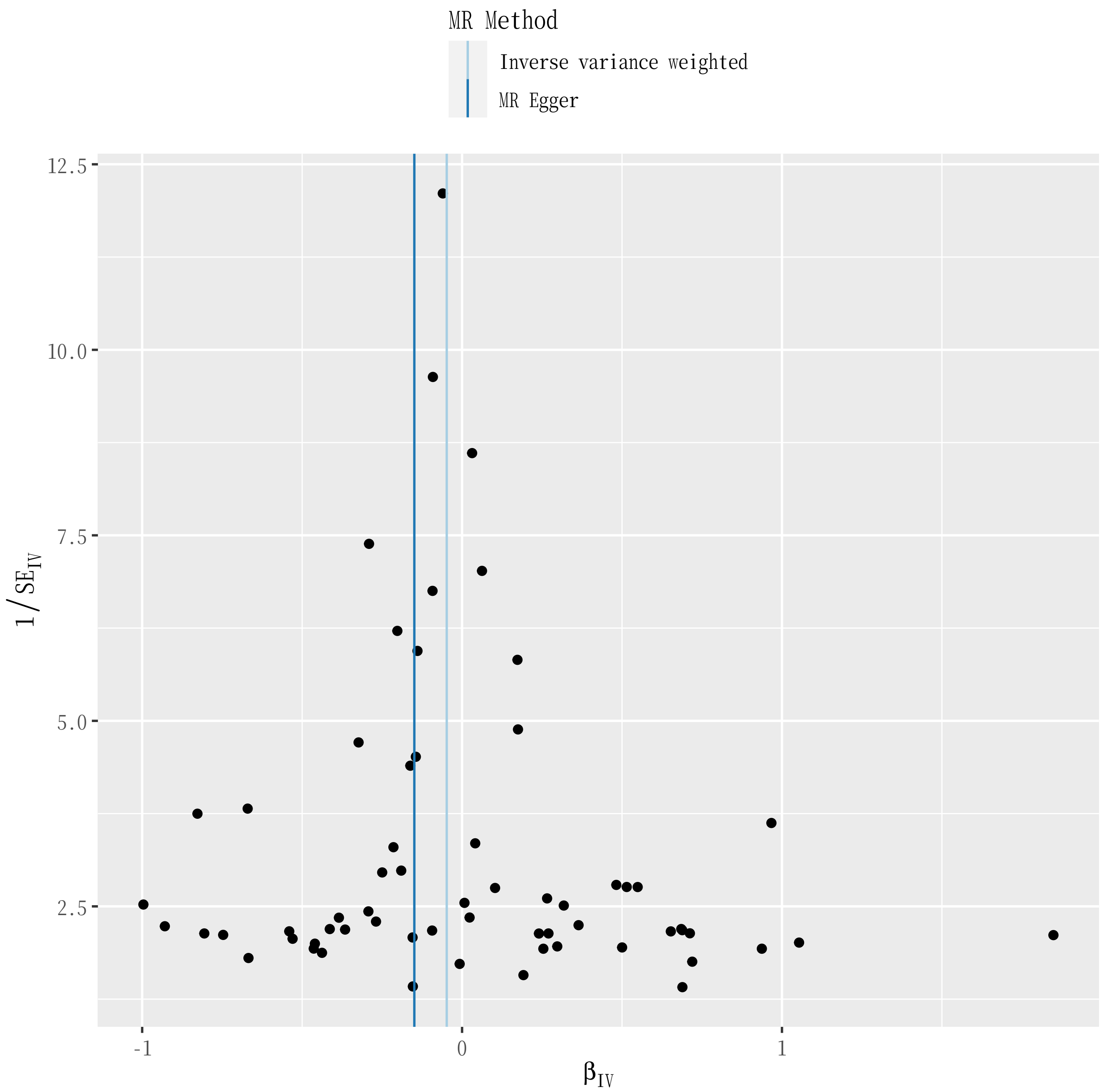

MR Method
Inverse variance weighted
MR Egger
12.5
10.0
7.5
5.0
2.5
$1/SE_{IV}$
-1
0
1
β_{IV}

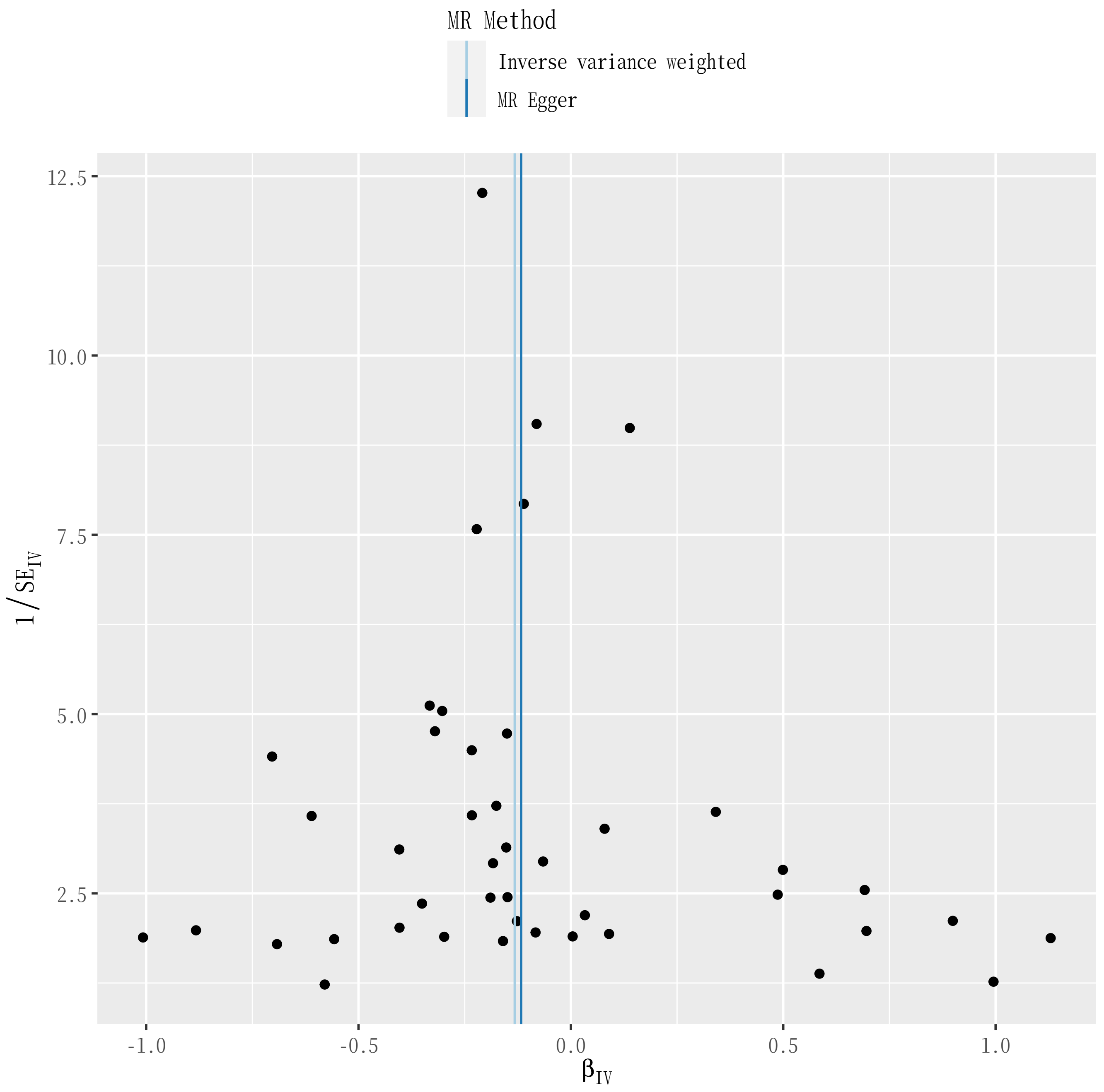

MR Method
Inverse variance weighted
MR Egger
12.5
10.0
7.5
5.0
2.5
$1/SE_{IV}$
-1.0
-0.5
0.0
0.5
1.0
β_{IV}

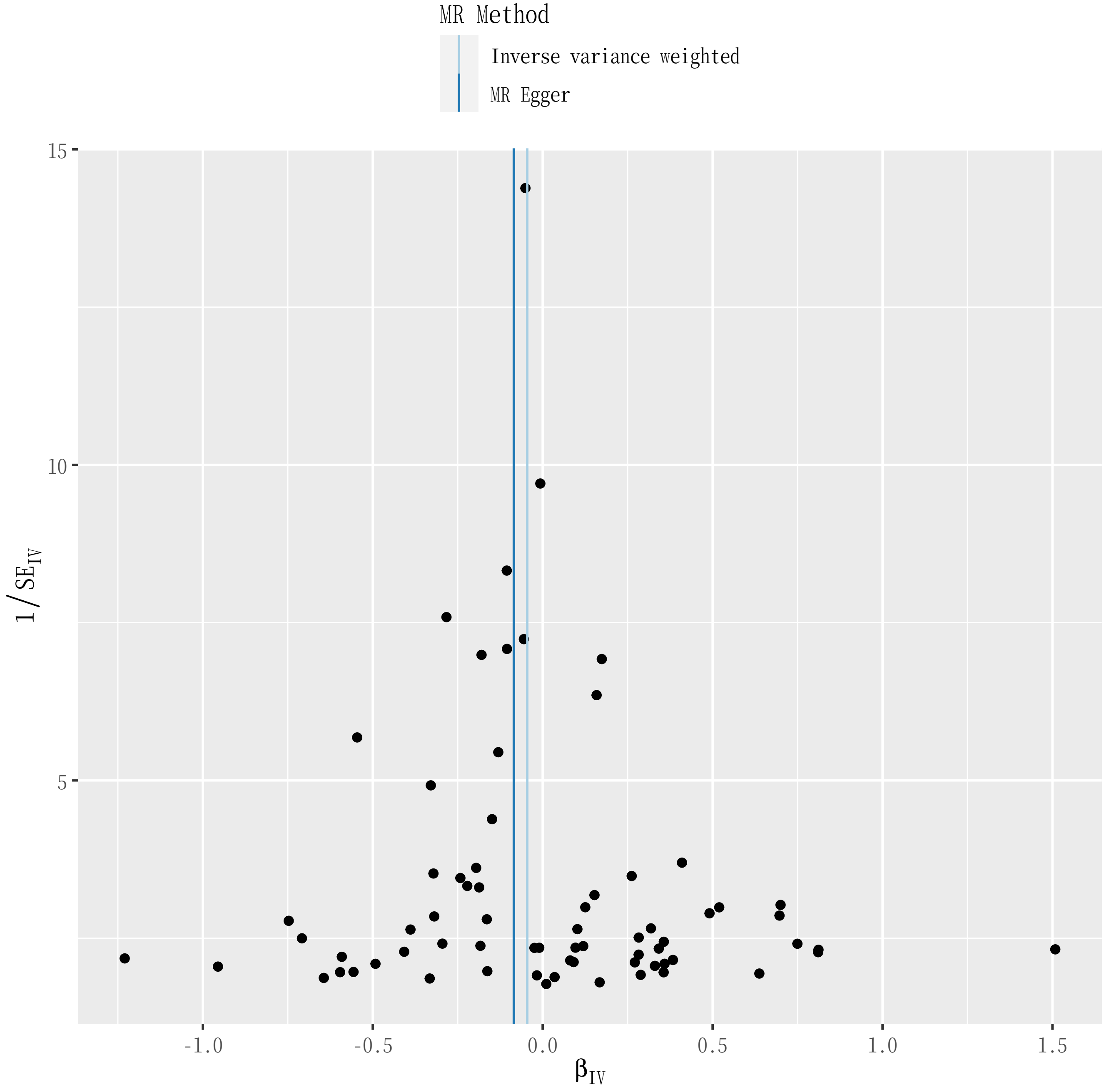

MR Method
Inverse variance weighted
MR Egger
15
10
5
$1/SE_{IV}$
-1.0
-0.5
0.0
0.5
1.0
1.5
β_{IV}

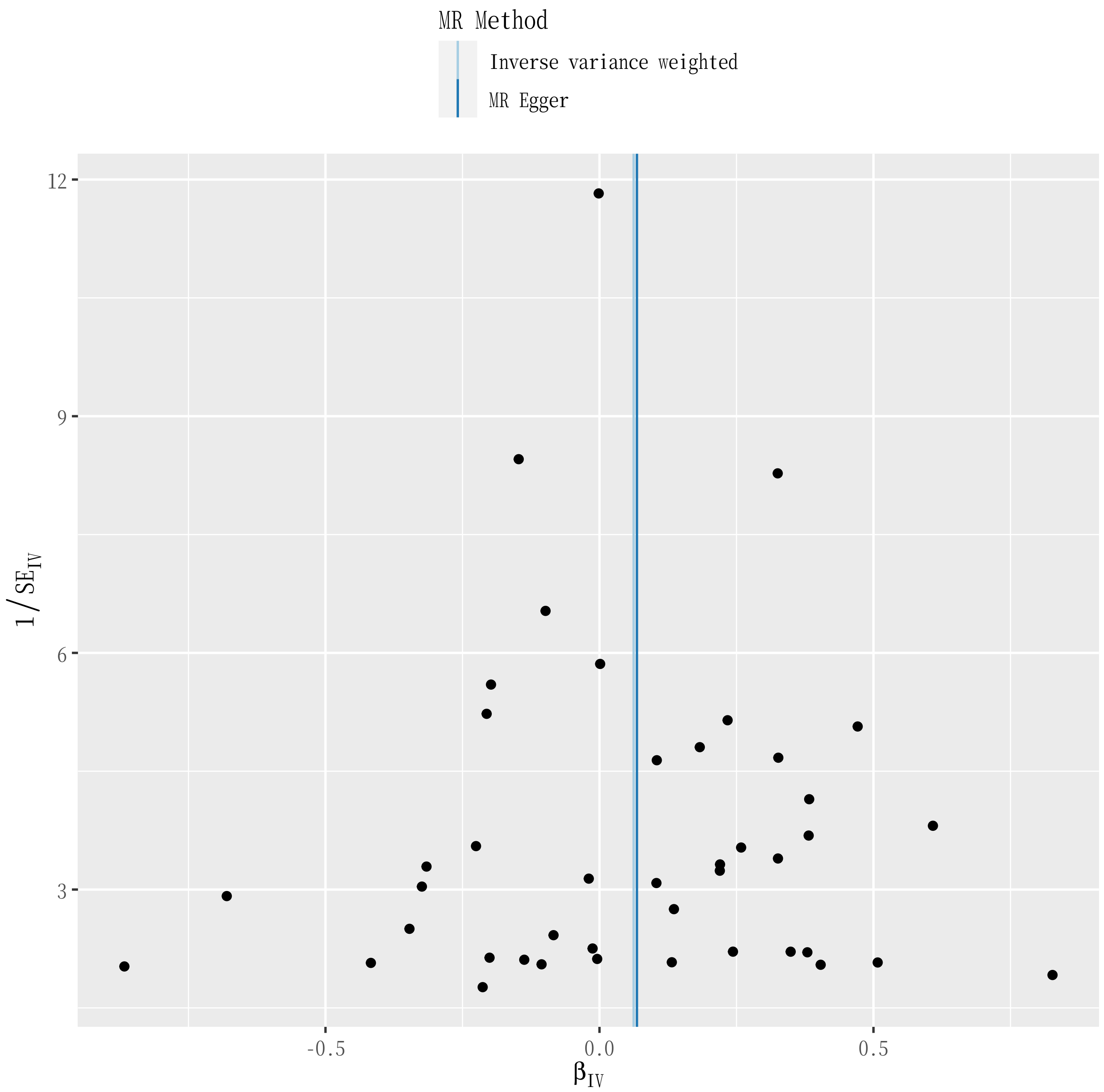

MR Method
Inverse variance weighted
MR Egger
12
9
6
3
1/SE_IV
-0.5
0.0
0.5
β_IV

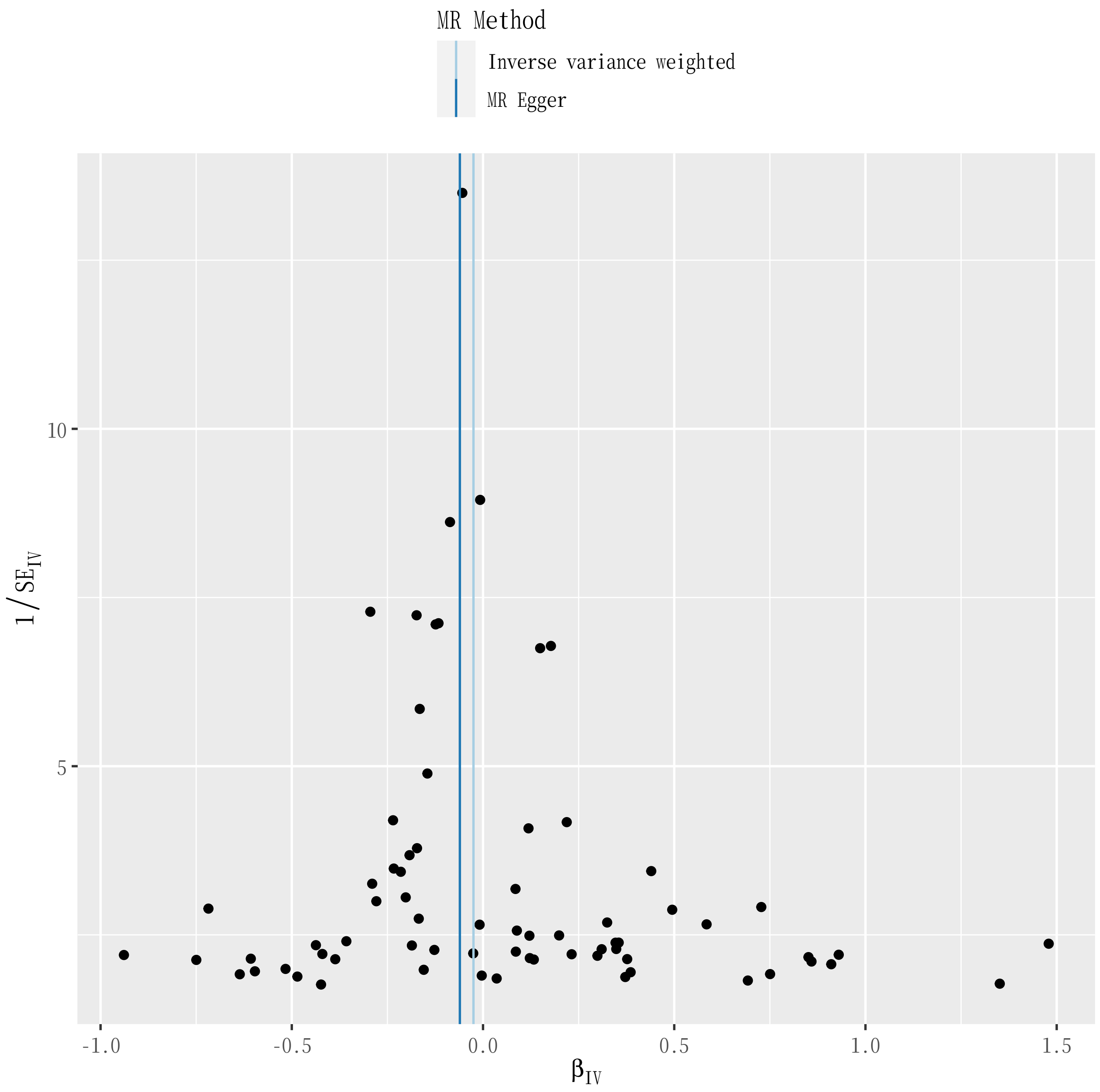

MR Method
Inverse variance weighted
MR Egger
10
5
$1/SE_{IV}$
-1.0
-0.5
0.0
0.5
1.0
1.5
β_{IV}

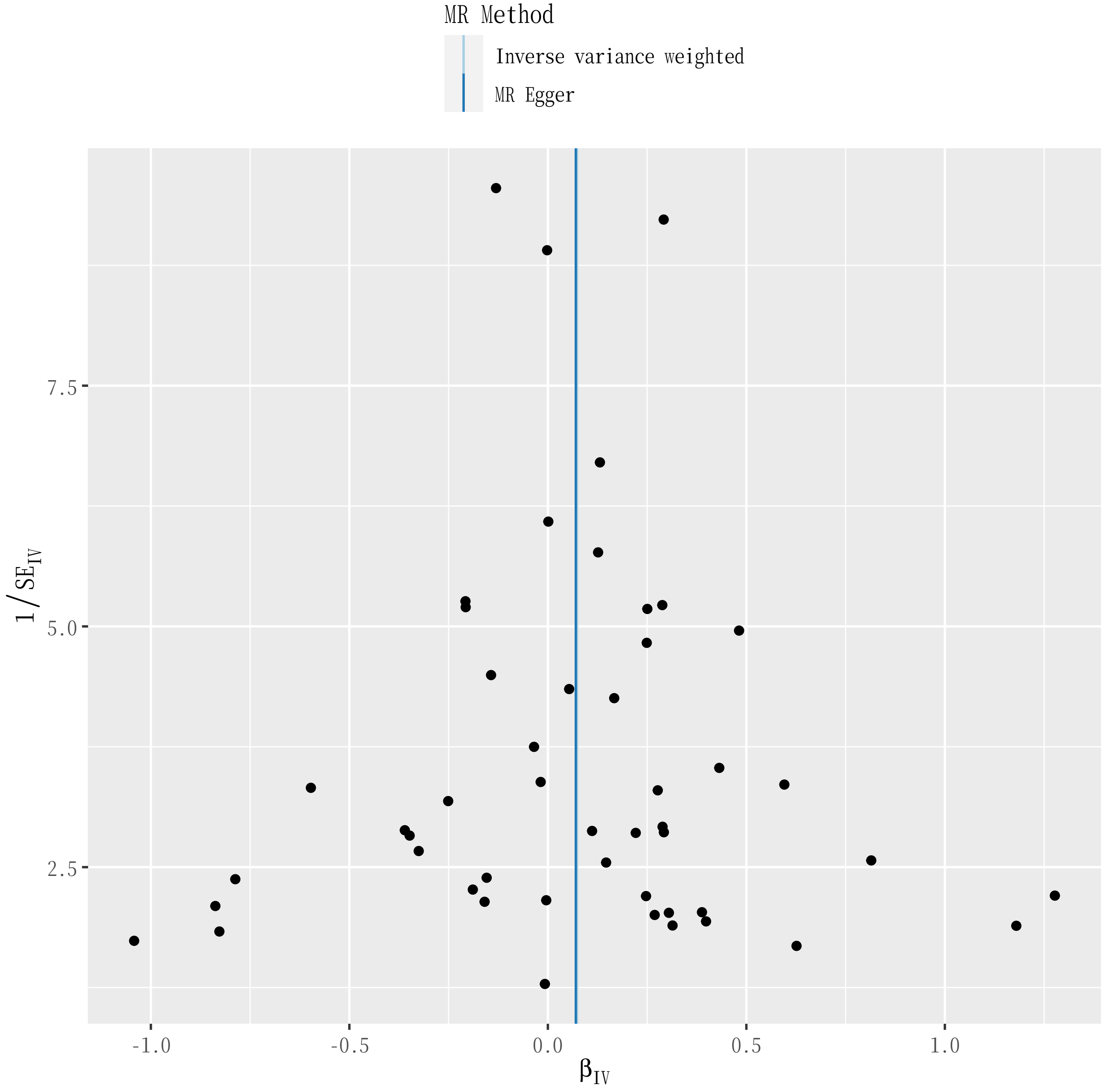

MR Method
Inverse variance weighted
MR Egger
7.5
5.0
2.5
$1/SE_{IV}$
-1.0
-0.5
0.0
0.5
1.0
β_{IV}

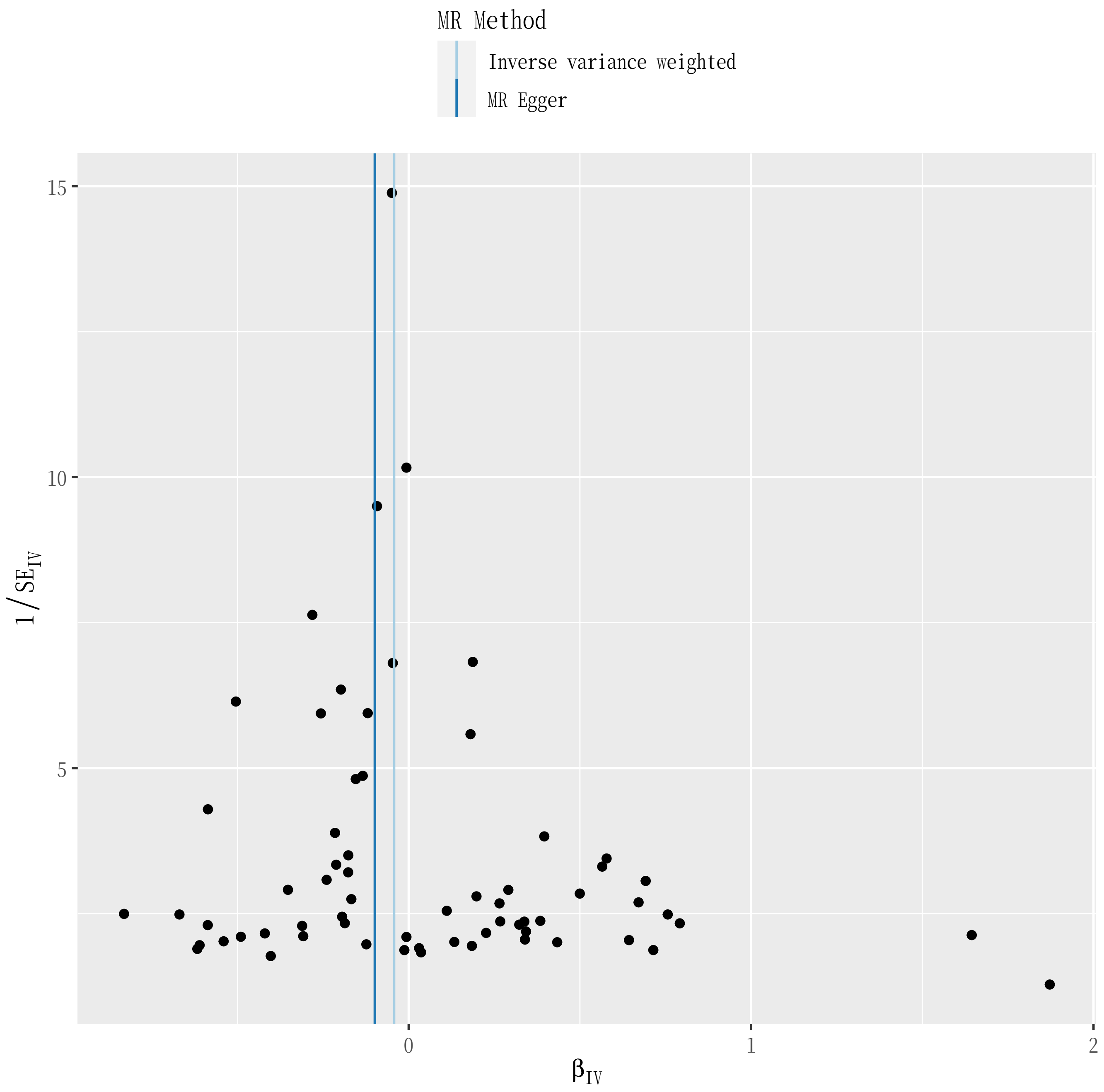
MR Method
Inverse variance weighted
MR Egger
15
10
5
0
1
2
1/SE_IV
β_IV

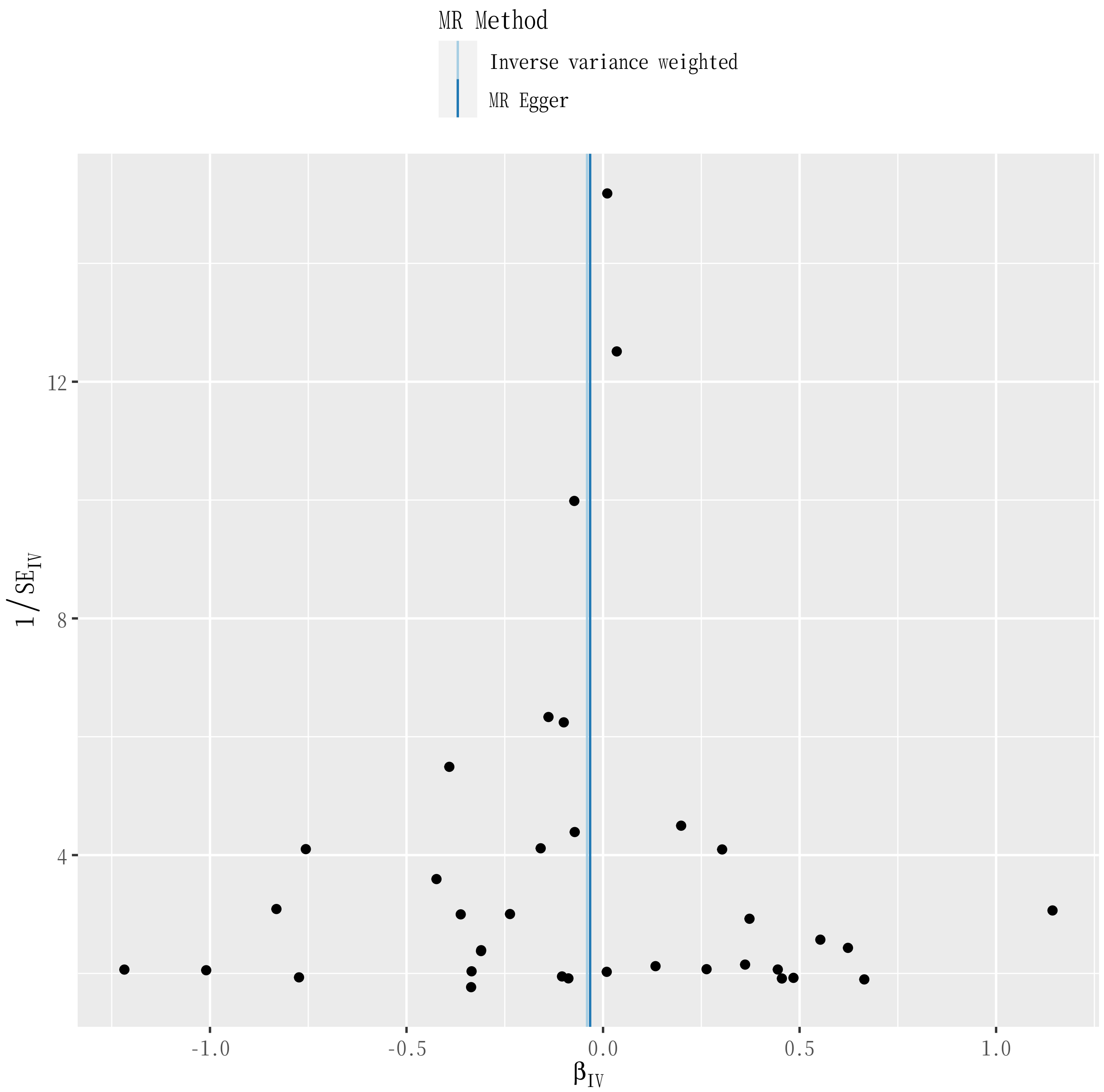
MR Method
Inverse variance weighted
MR Egger
12
8
4
-1.0
-0.5
0.0
0.5
1.0
$1/SE_{IV}$
β_{IV}

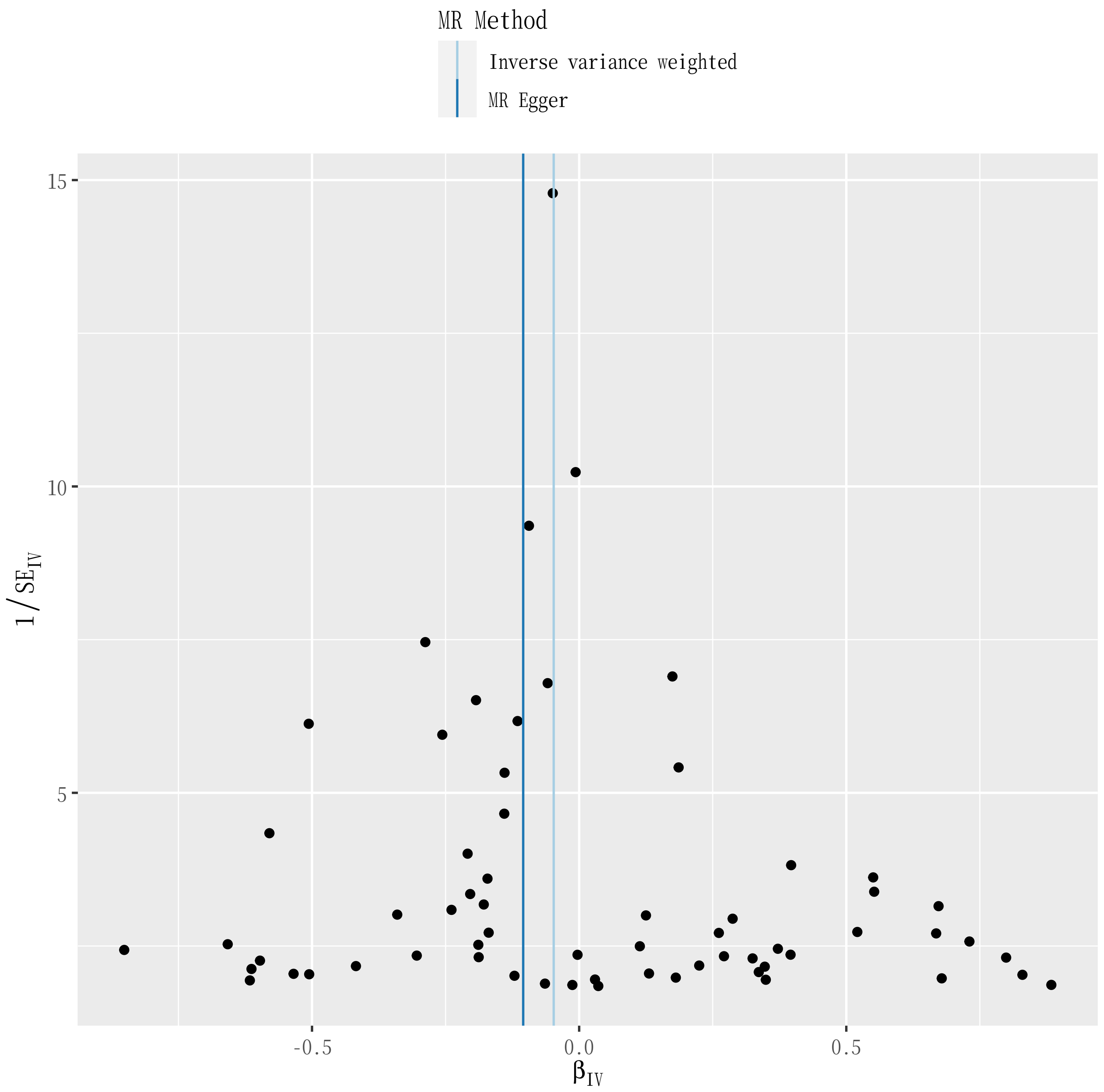
MR Method
Inverse variance weighted
MR Egger
15
10
5
-0.5
0.0
0.5
1/SE_IV
β_IV

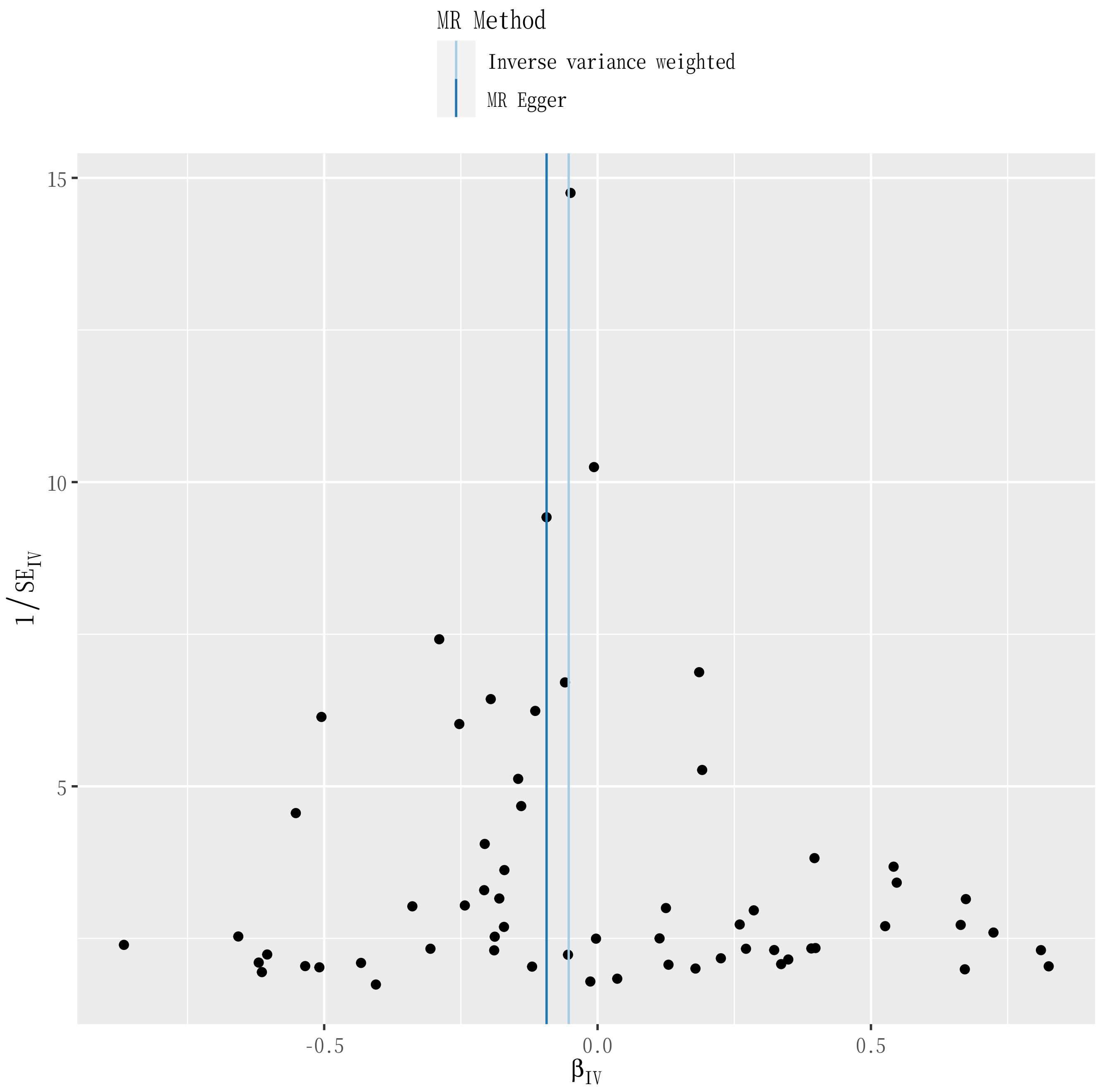
MR Method
Inverse variance weighted
MR Egger
15
10
5
-0.5
0.0
0.5
$1/SE_{IV}$
β_{IV}

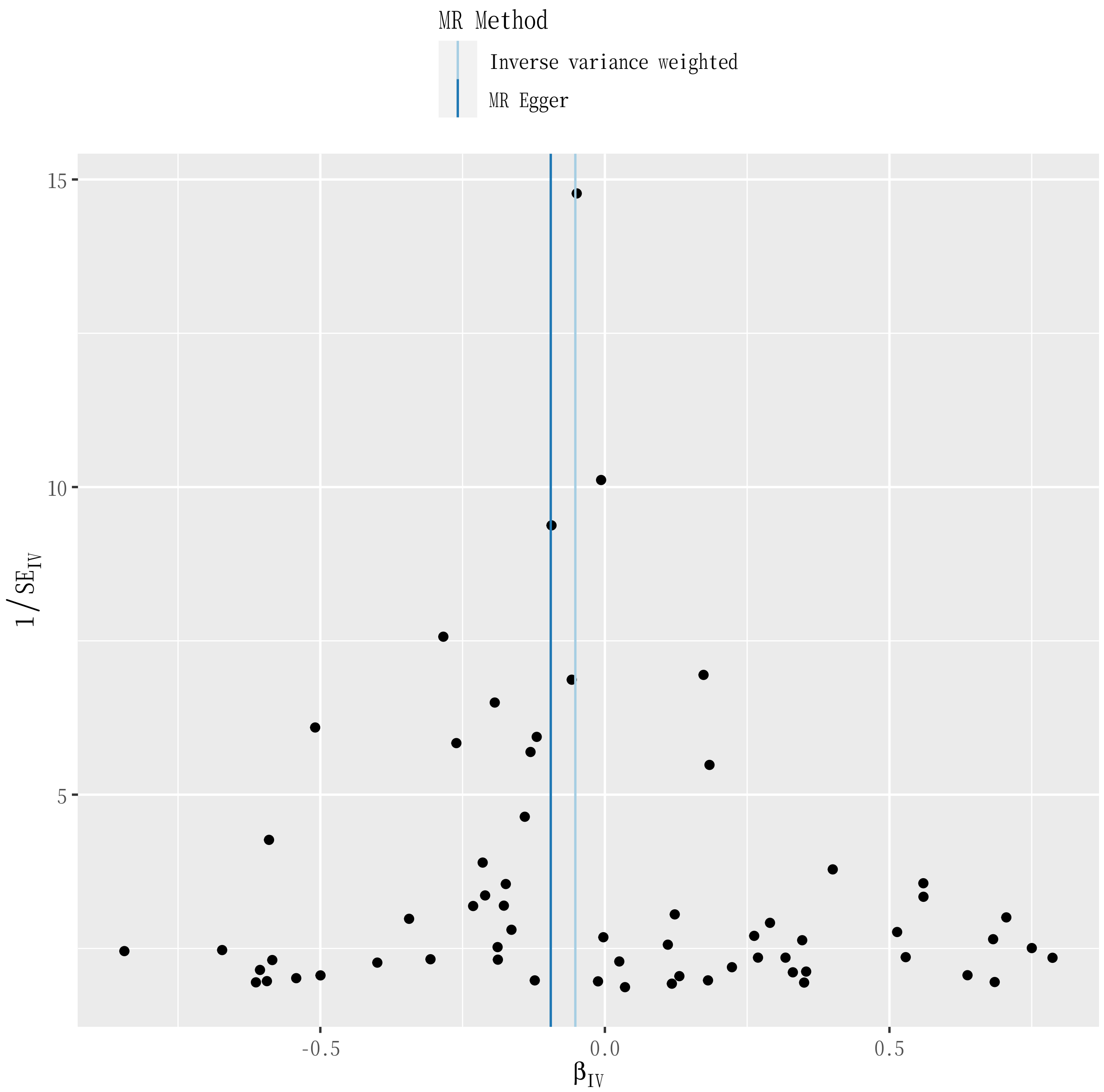

MR Method
Inverse variance weighted
MR Egger
15
10
5
-0.5
0.0
0.5
$1/SE_{IV}$
β_{IV}

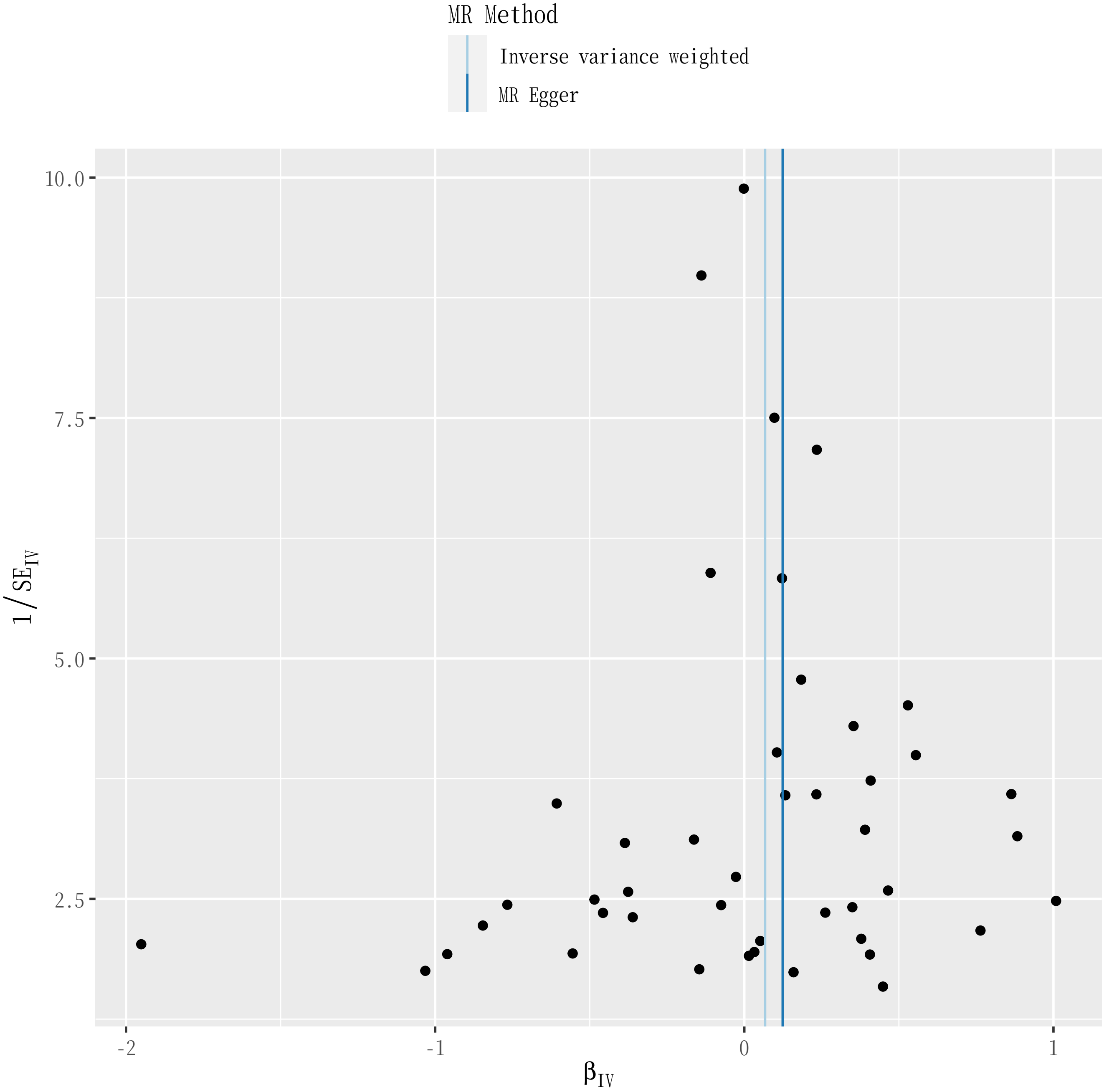
MR Method
Inverse variance weighted
MR Egger
10.0
7.5
5.0
2.5
1/SE_IV
-2
-1
0
1
β_IV

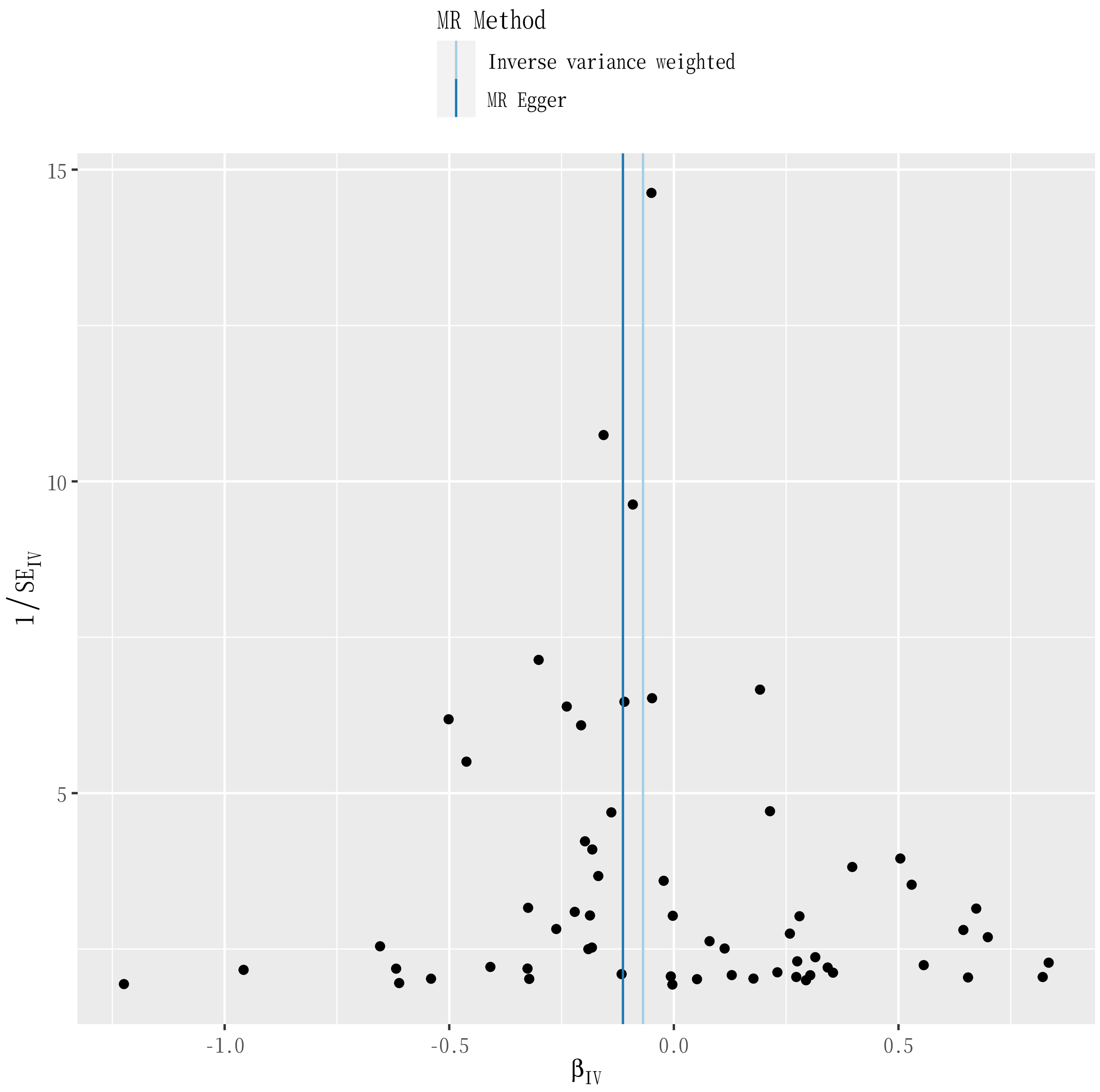

MR Method
Inverse variance weighted
MR Egger
15
10
5
1/SE_IV
-1.0
-0.5
0.0
0.5
β_IV

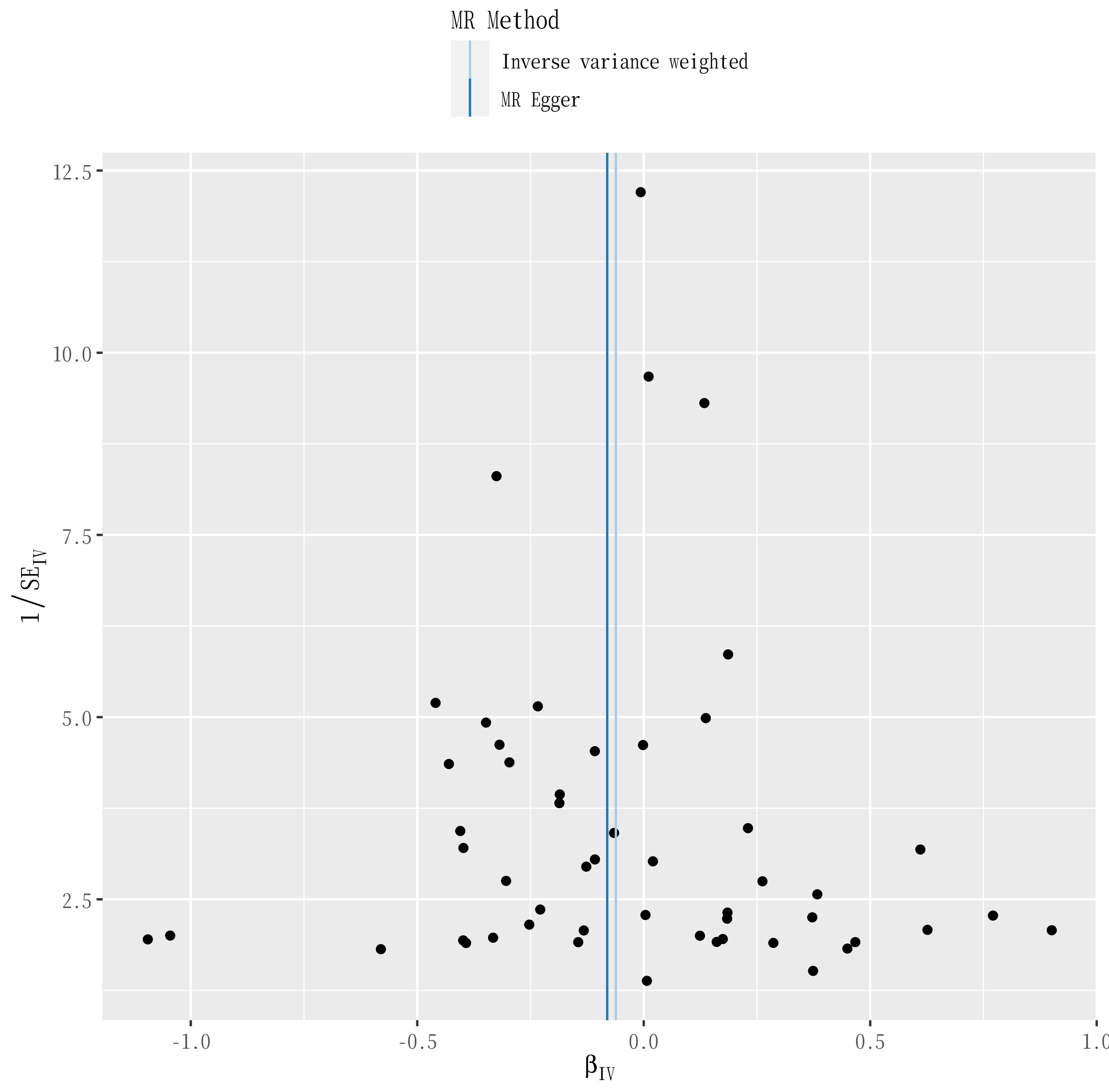

MR Method
Inverse variance weighted
MR Egger
12.5
10.0
7.5
5.0
2.5
$1/SE_{IV}$
-1.0
-0.5
0.0
0.5
1.0
β_{IV}

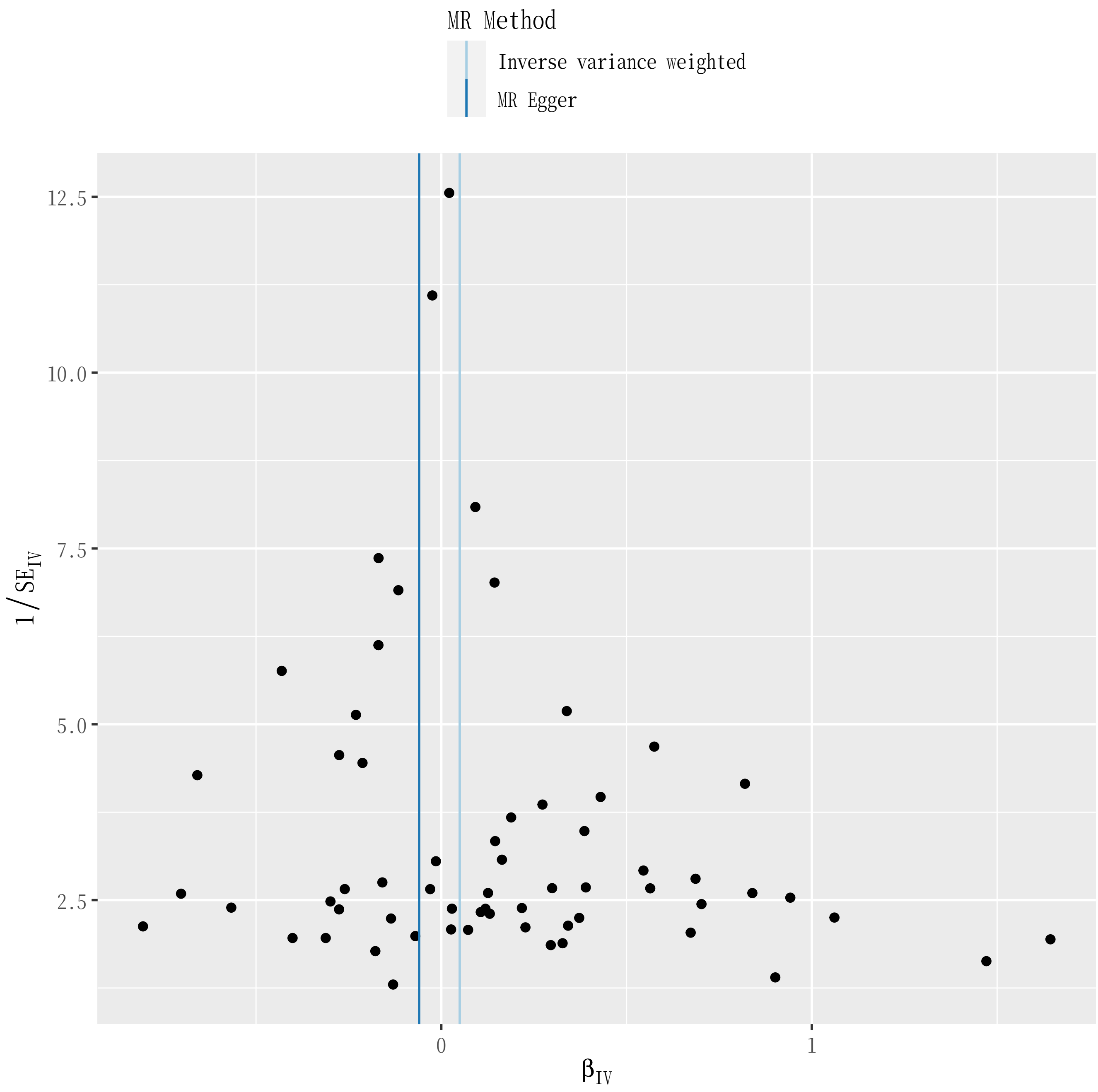

MR Method
Inverse variance weighted
MR Egger
12.5
10.0
7.5
5.0
2.5
1/SE_IV
0
1
β_IV

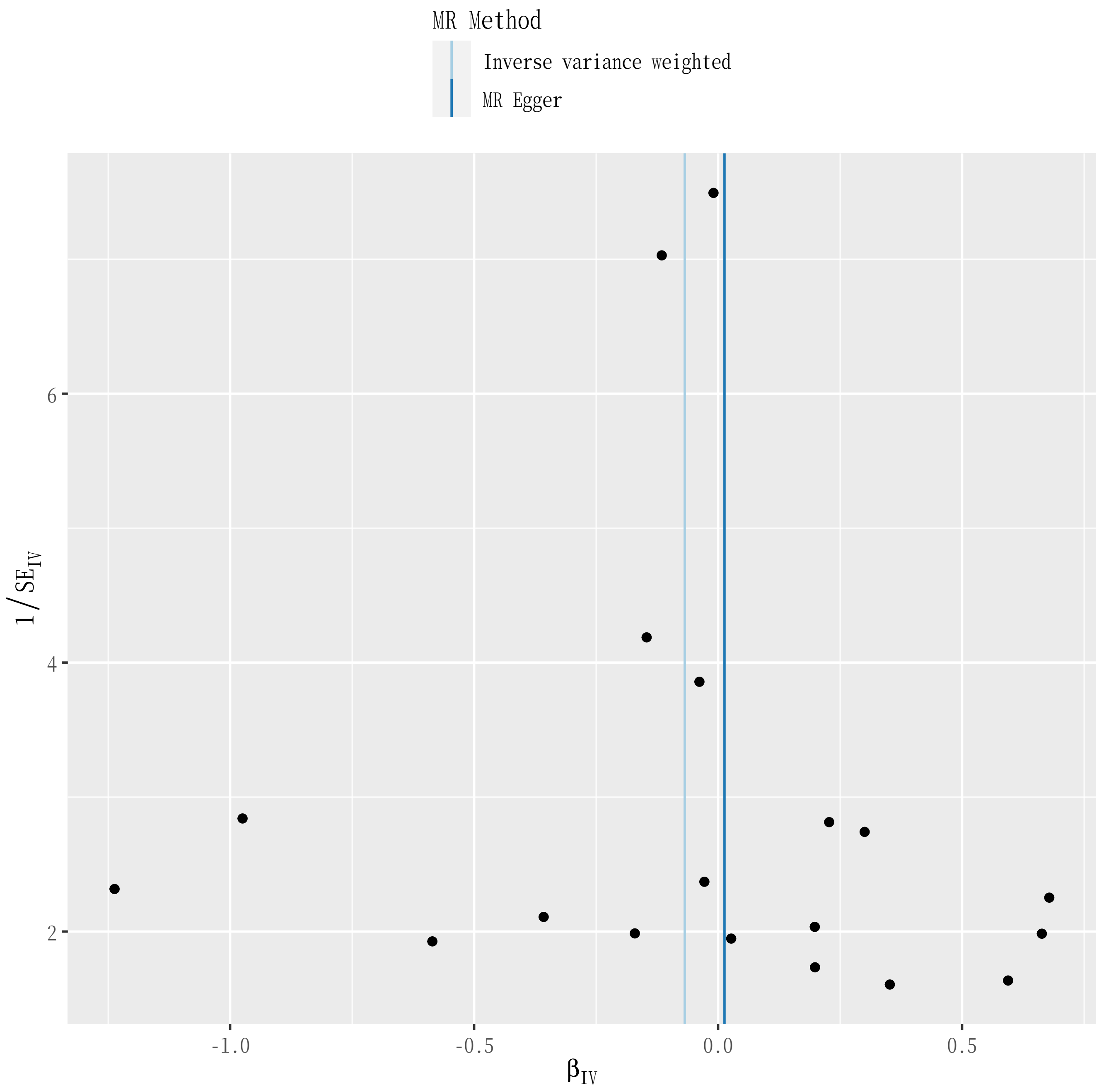

MR Method
Inverse variance weighted
MR Egger
6
4
2
1/SE$_{IV}$
-1.0
-0.5
0.0
0.5
β$_{IV}$

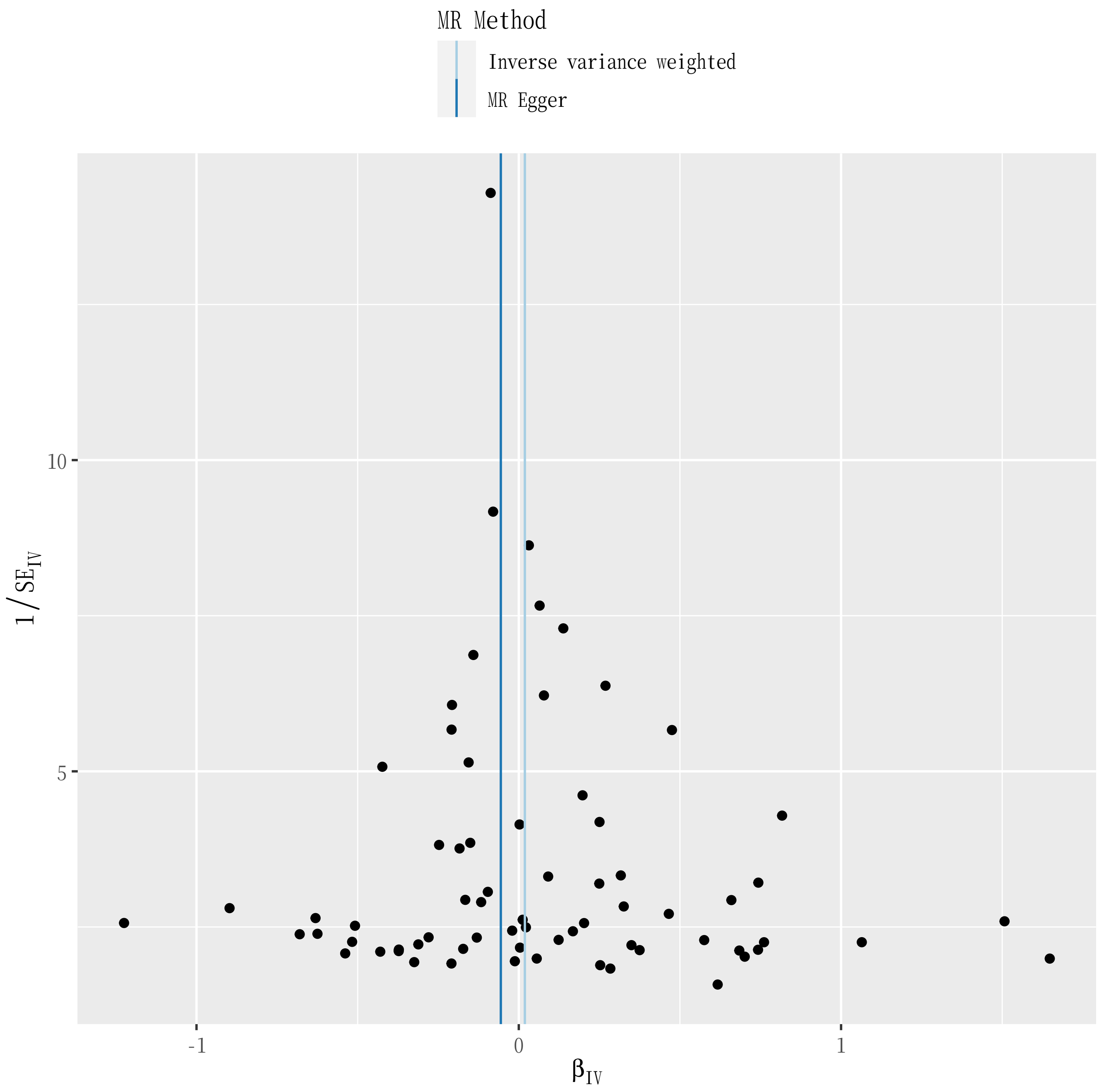

MR Method
Inverse variance weighted
MR Egger
10
5
-1
0
1
1/SE_IV
β_IV

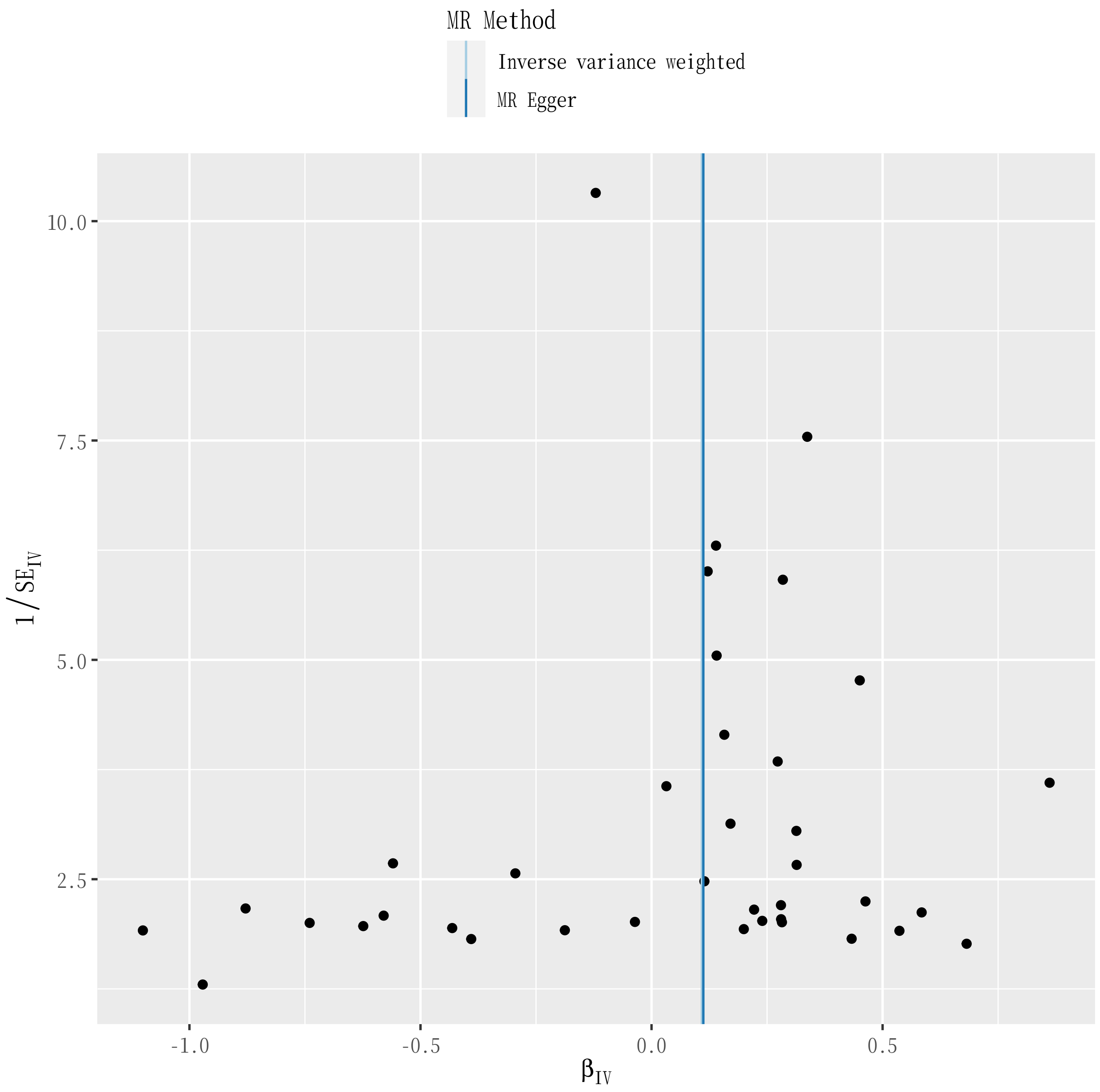

MR Method
Inverse variance weighted
MR Egger
10.0
7.5
5.0
2.5
$1/SE_{IV}$
-1.0
-0.5
0.0
0.5
β_{IV}

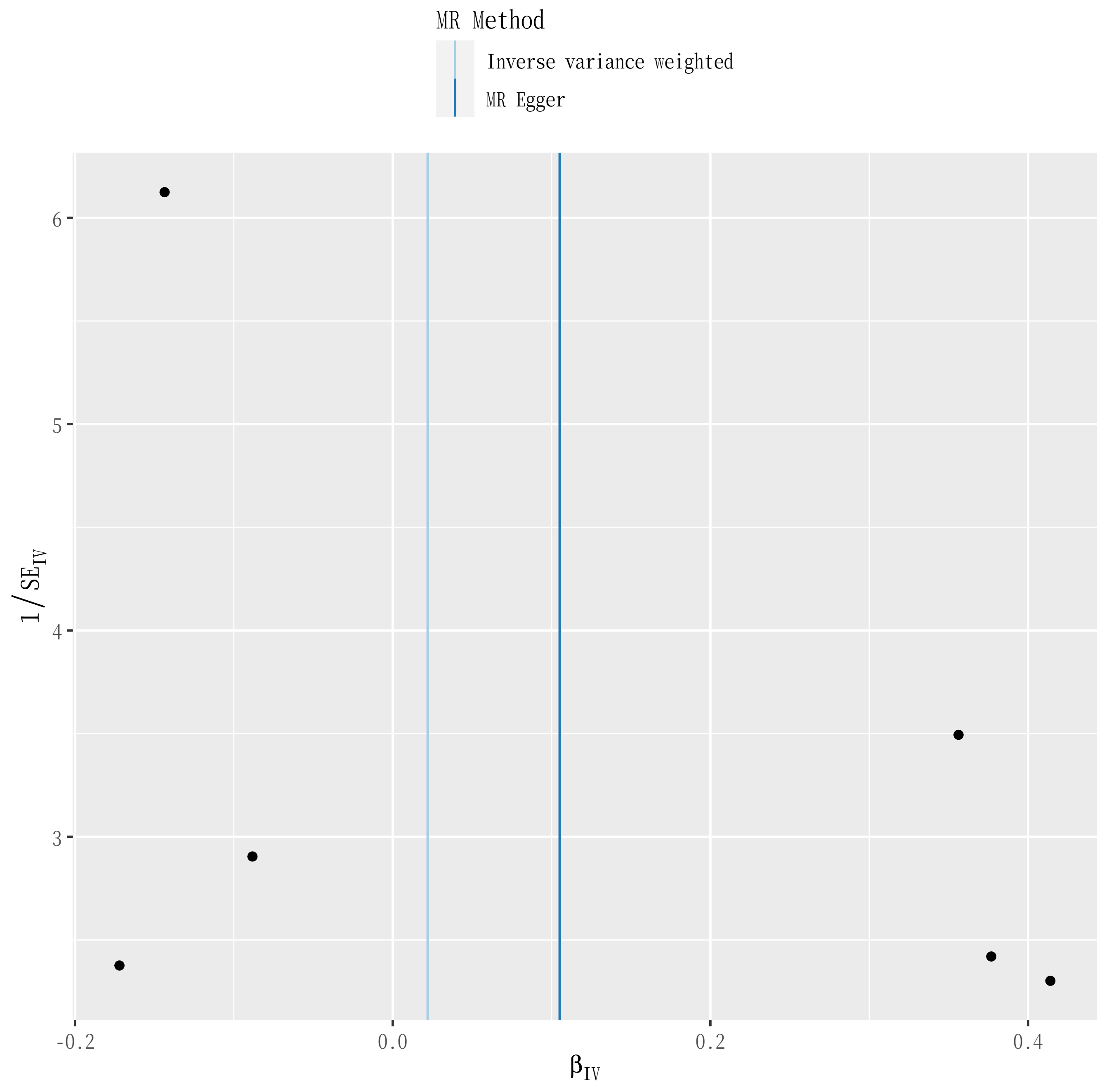
MR Method
Inverse variance weighted
MR Egger
6
5
4
3
1/SE_IV
-0.2
0.0
0.2
0.4
β_IV

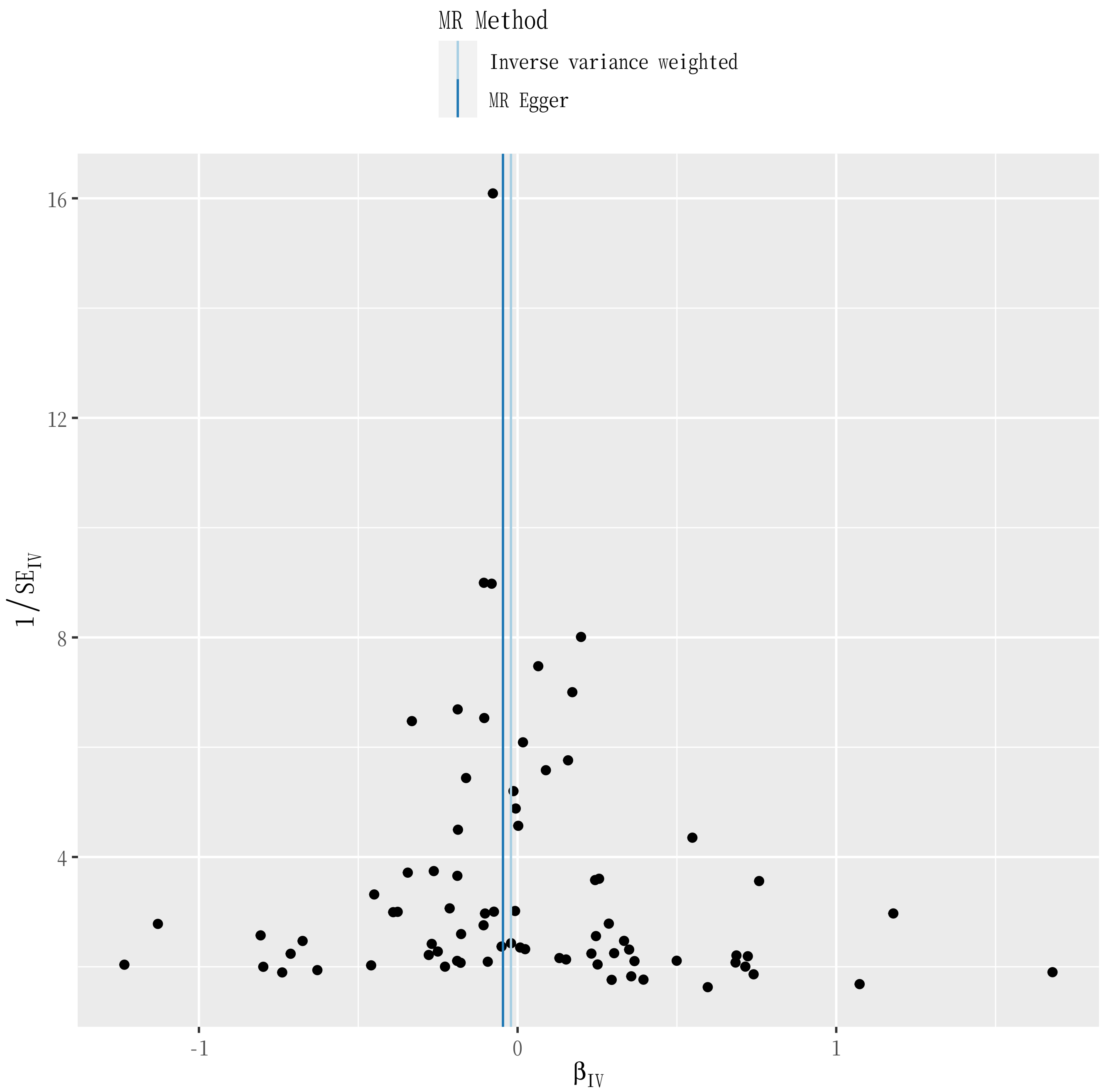

MR Method
Inverse variance weighted
MR Egger
16
12
8
4
1/SE_IV
-1
0
1
β_IV

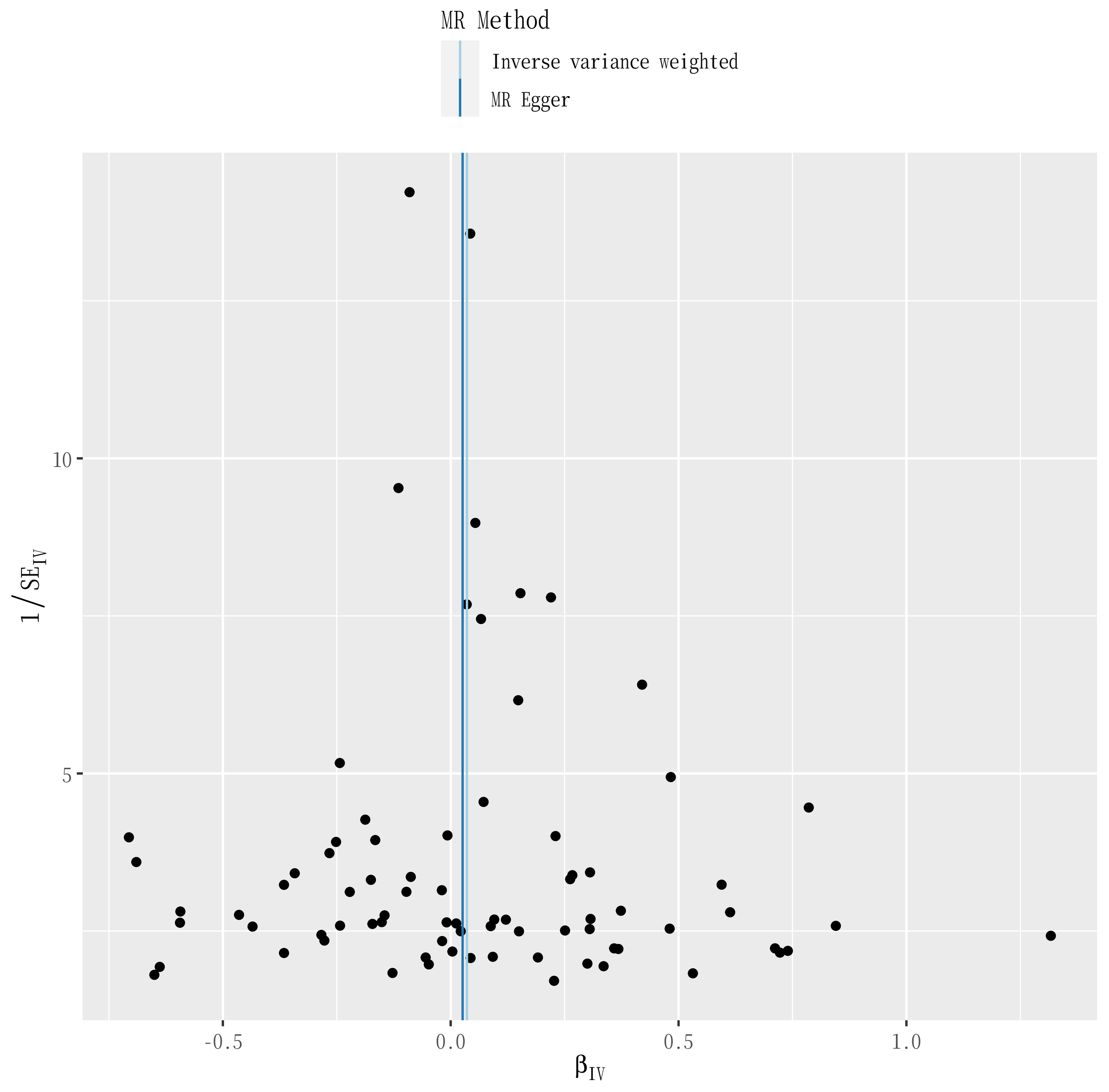
MR Method
Inverse variance weighted
MR Egger
10
5
1/SE$_{IV}$
-0.5
0.0
0.5
1.0
β$_{IV}$

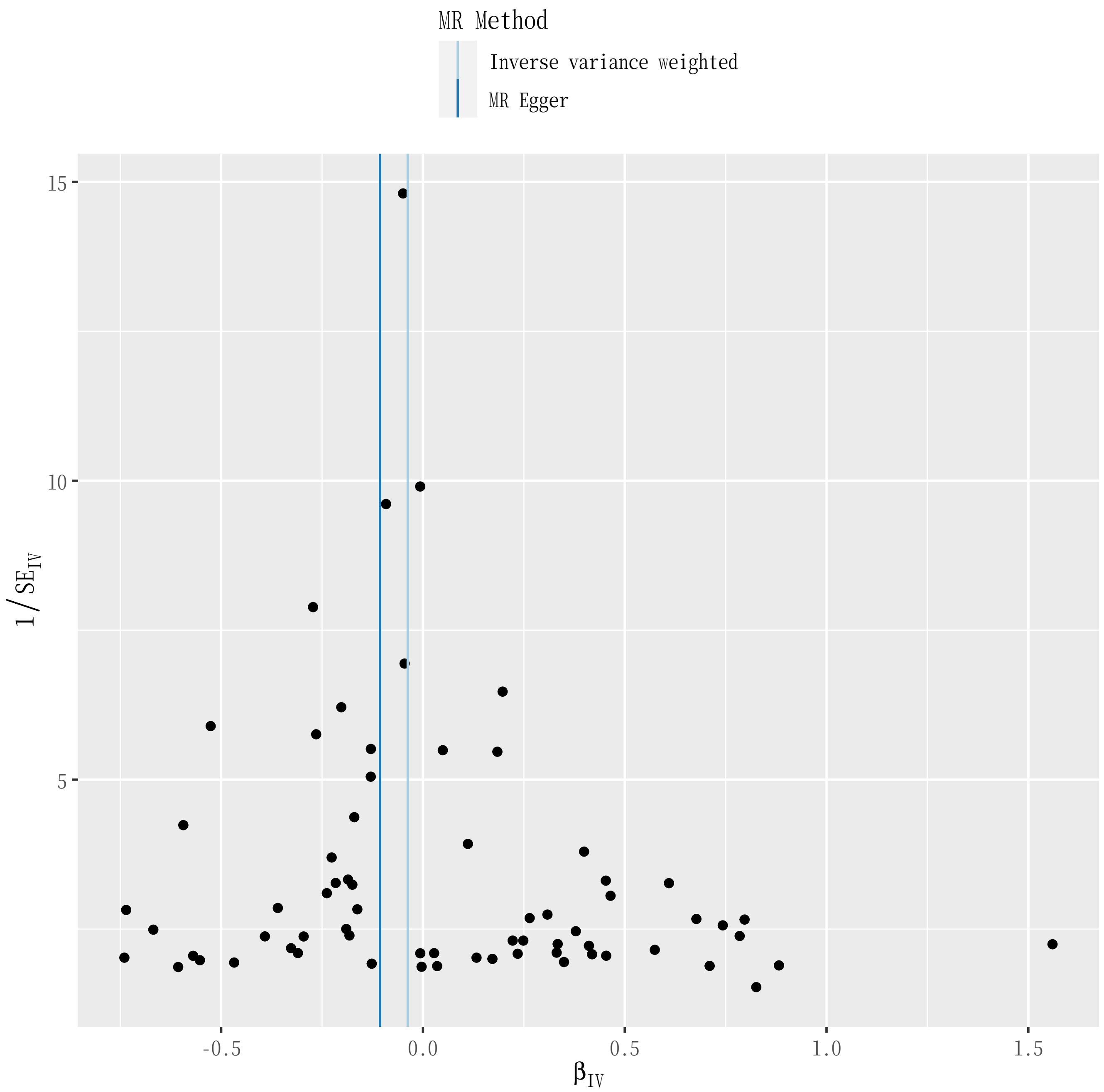

MR Method
Inverse variance weighted
MR Egger
15
10
5
1/SE_IV
-0.5
0.0
0.5
1.0
1.5
β_IV

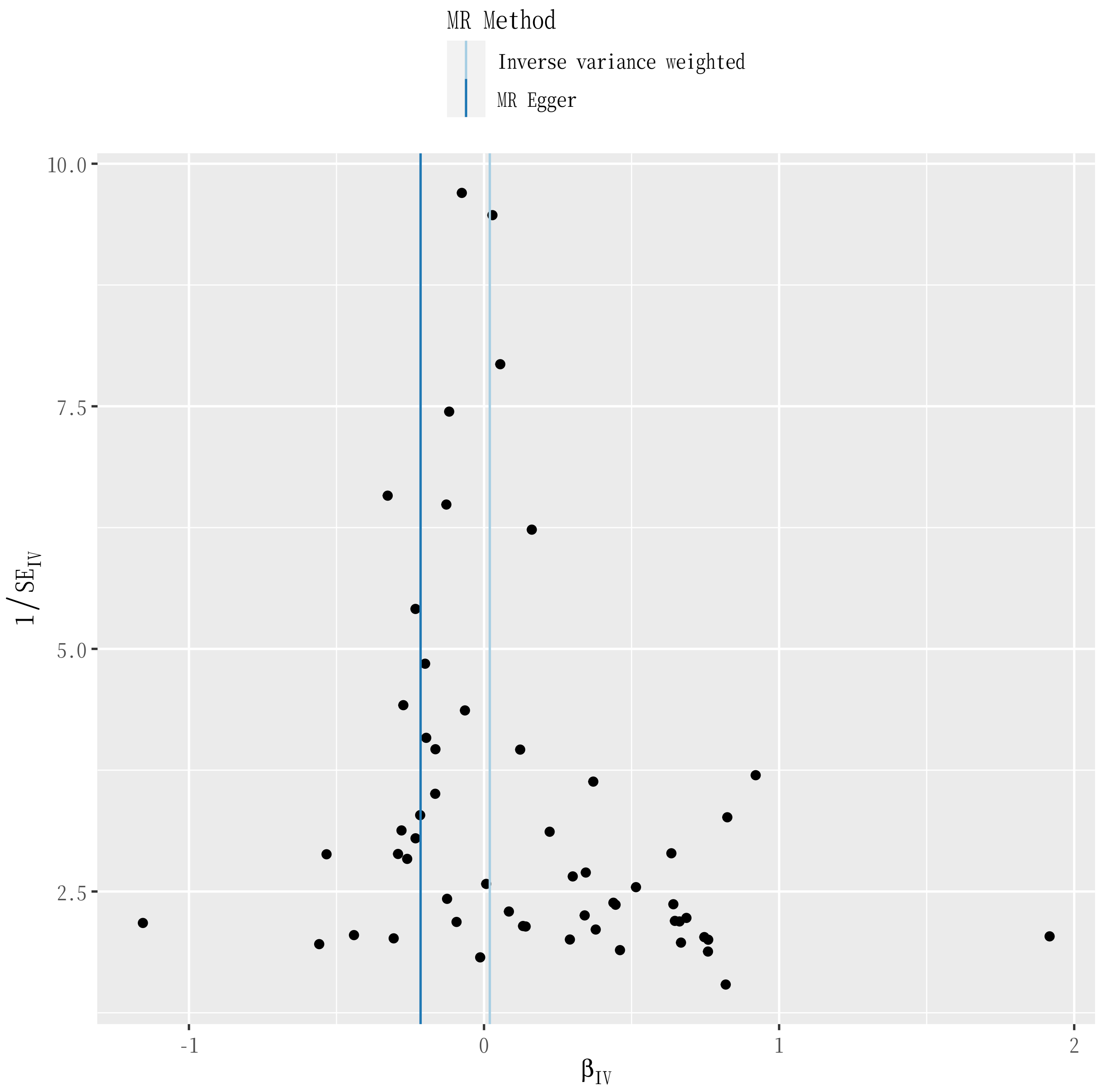
MR Method
Inverse variance weighted
MR Egger
10.0
7.5
5.0
2.5
1/SE_IV
-1
0
1
2
β_IV

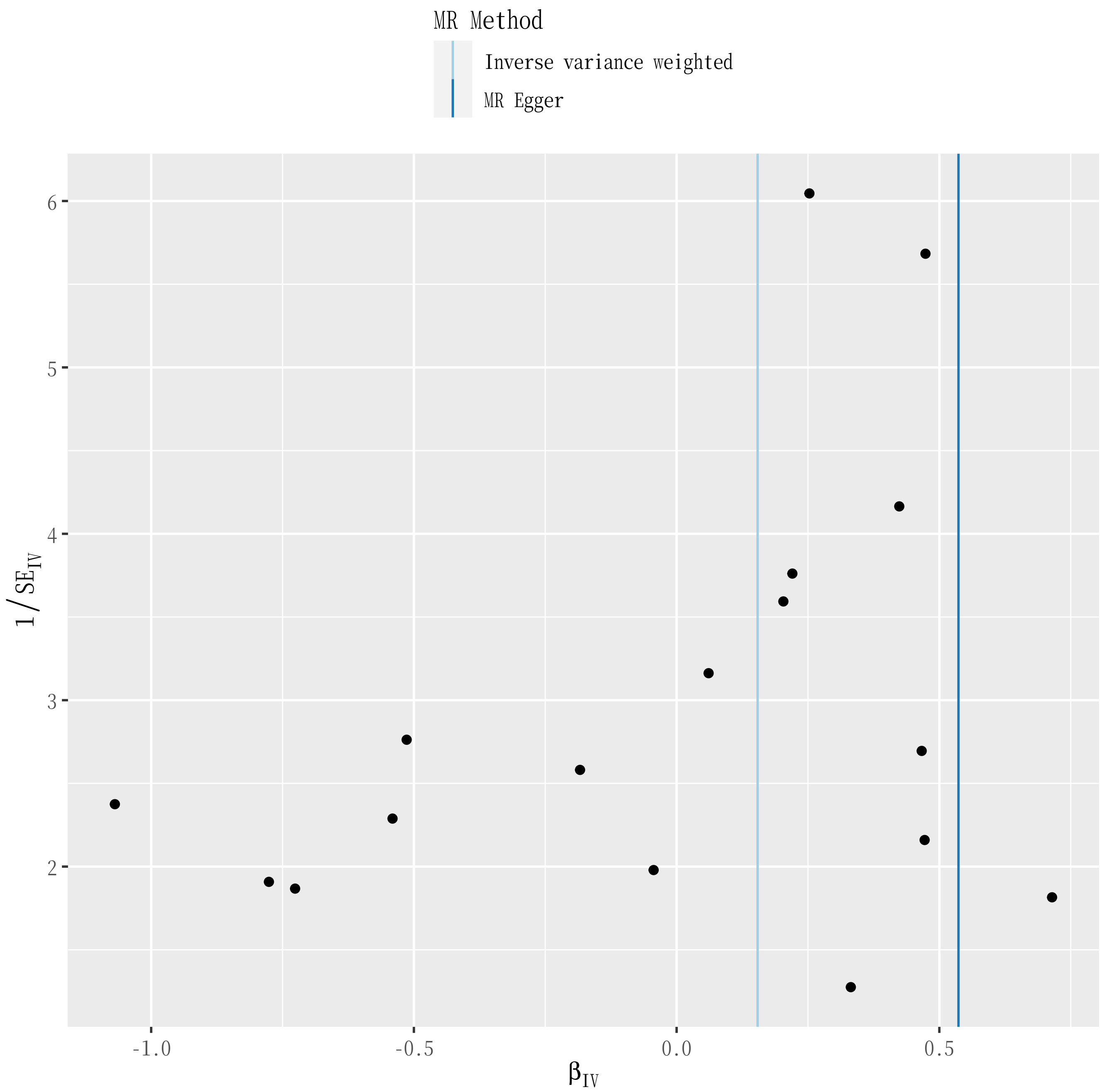
MR Method
Inverse variance weighted
MR Egger
6
5
4
3
2
1/SE_IV
-1.0
-0.5
0.0
0.5
β_IV

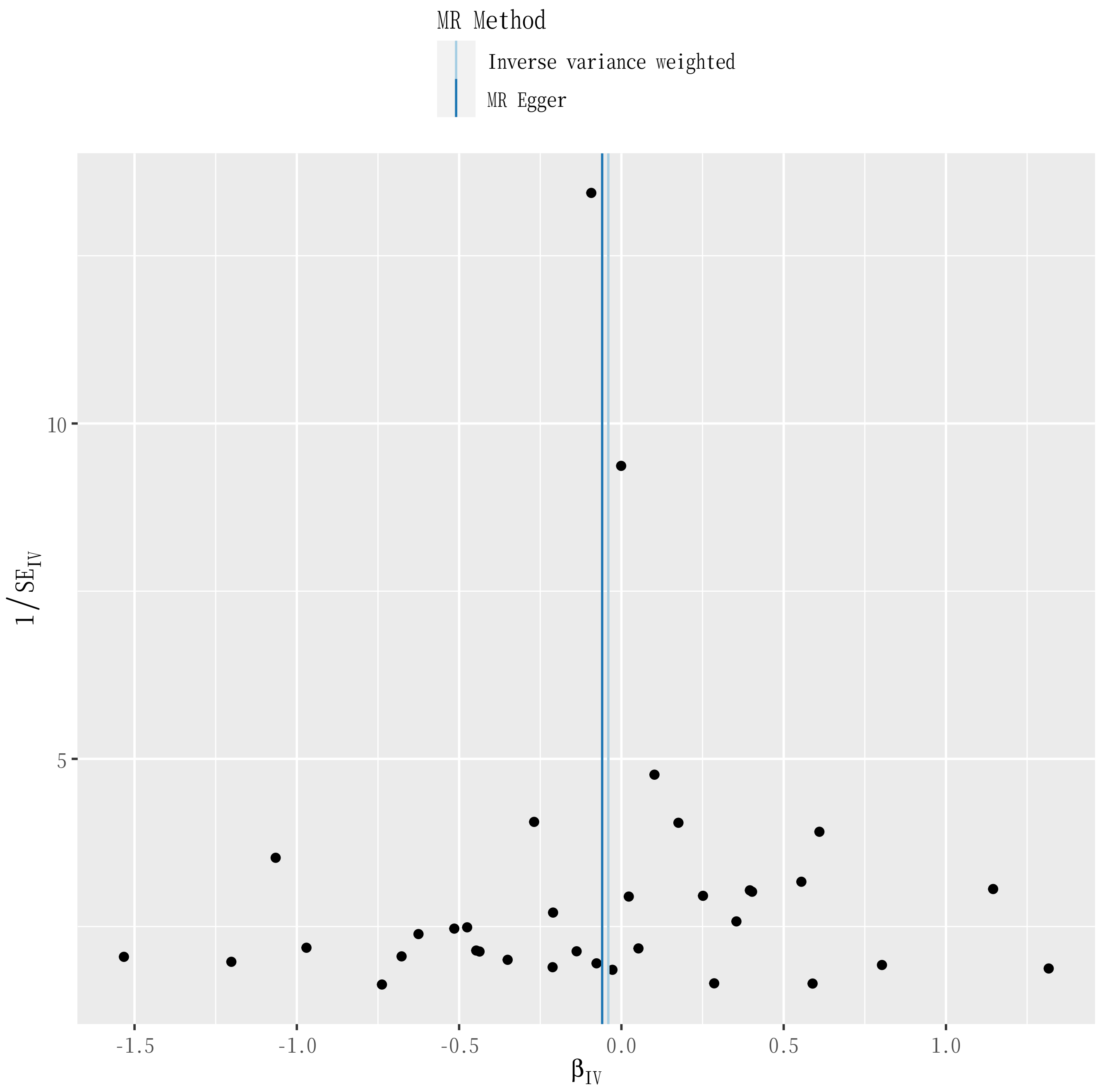

MR Method
Inverse variance weighted
MR Egger
10
5
1/SE_IV
-1.5
-1.0
-0.5
0.0
0.5
1.0
β_IV

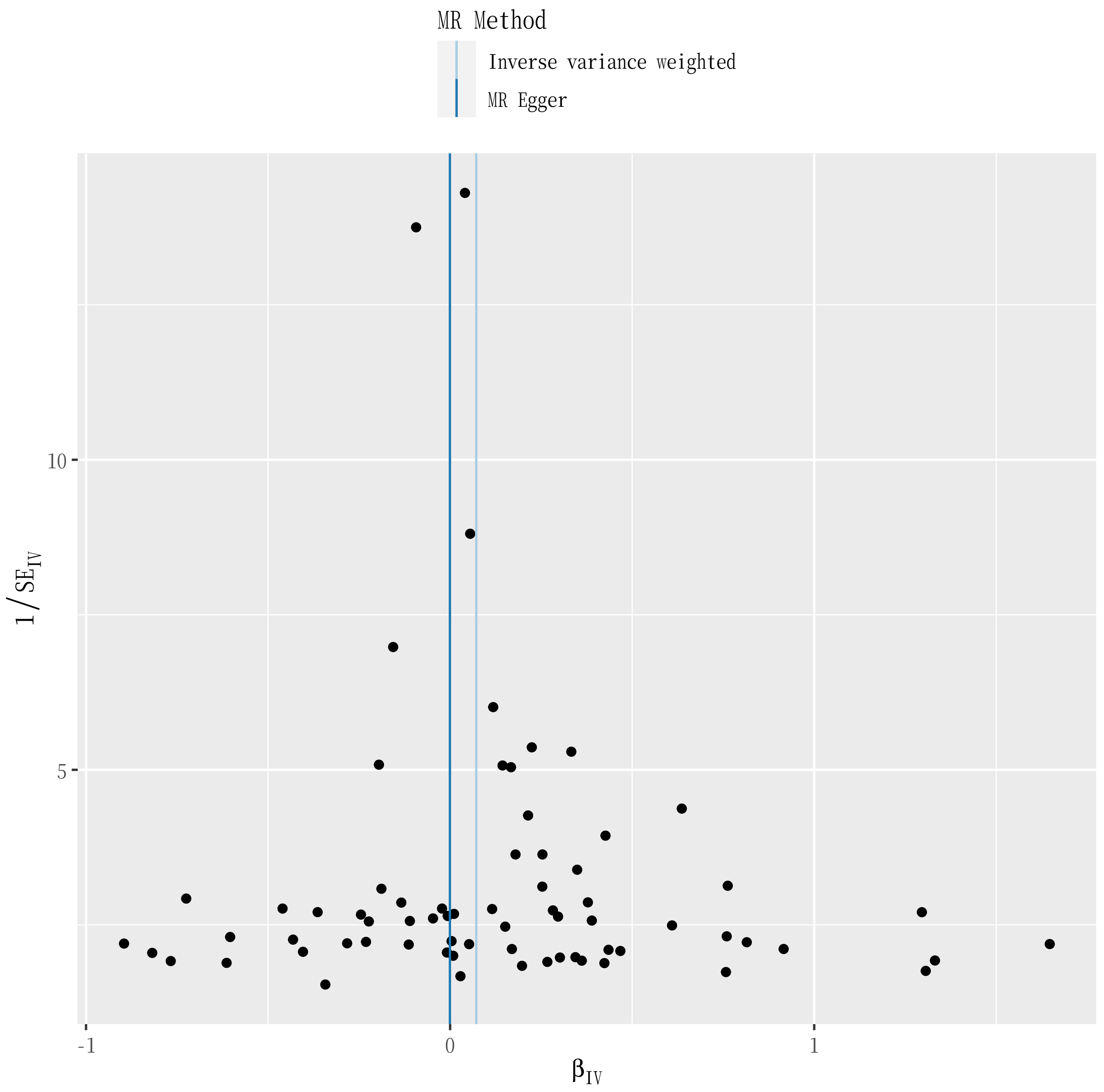
MR Method
Inverse variance weighted
MR Egger
10
5
1/SE_IV
-1
0
1
β_IV

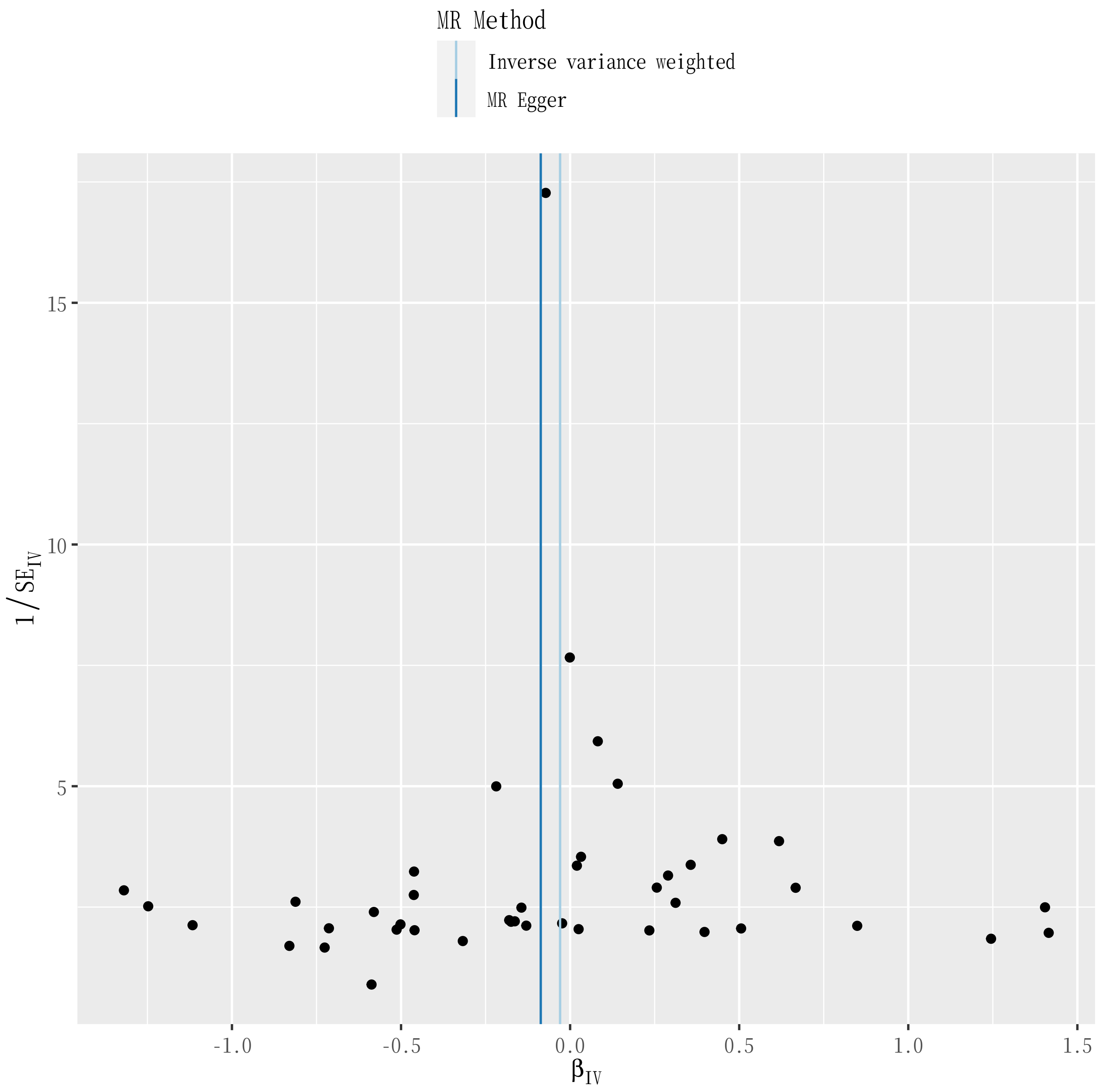

MR Method
Inverse variance weighted
MR Egger
15
10
5
1/SE_IV
-1.0
-0.5
0.0
0.5
1.0
1.5
β_IV

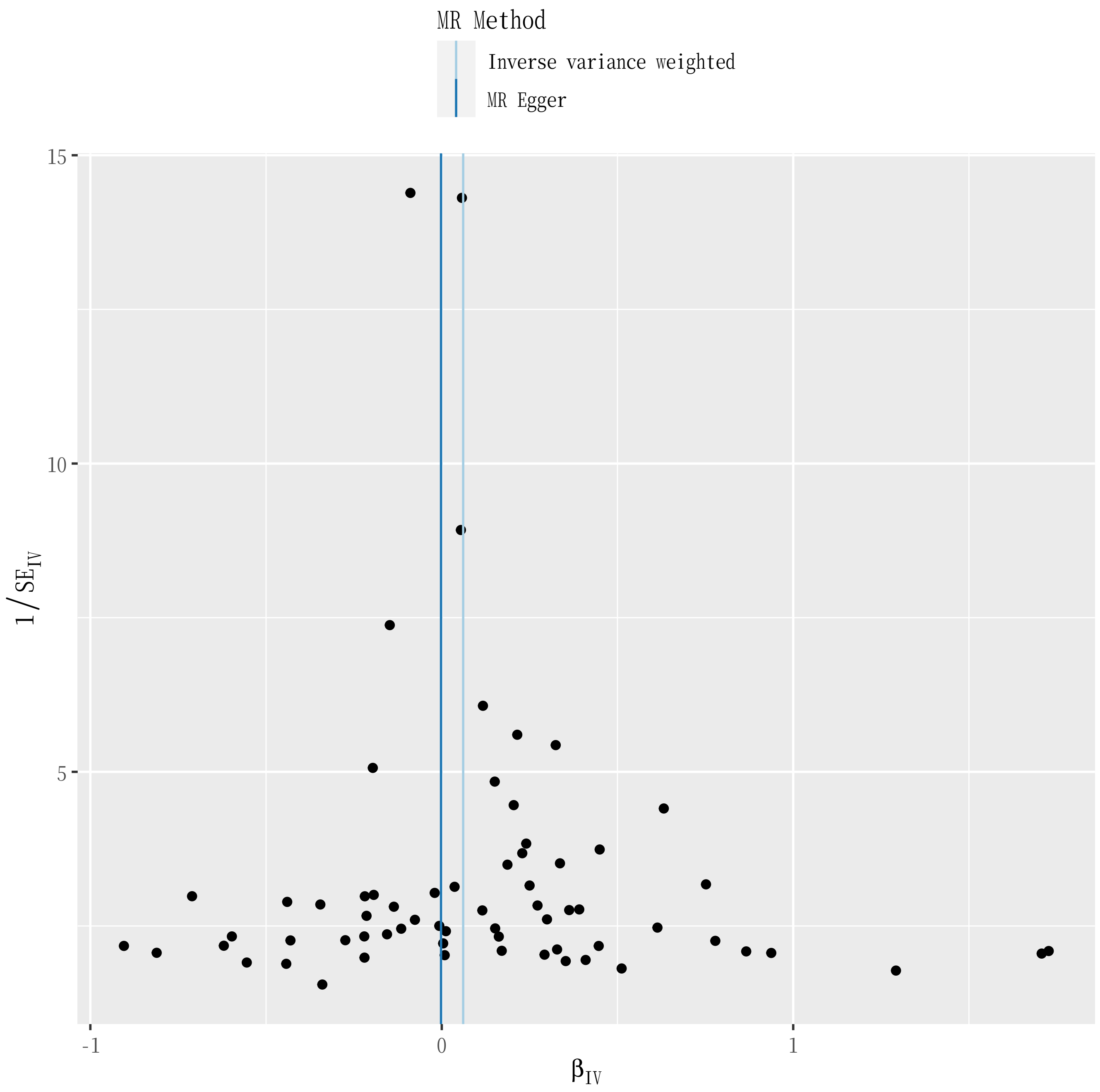
MR Method
Inverse variance weighted
MR Egger
15
10
5
1/SE_IV
-1
0
1
β_IV

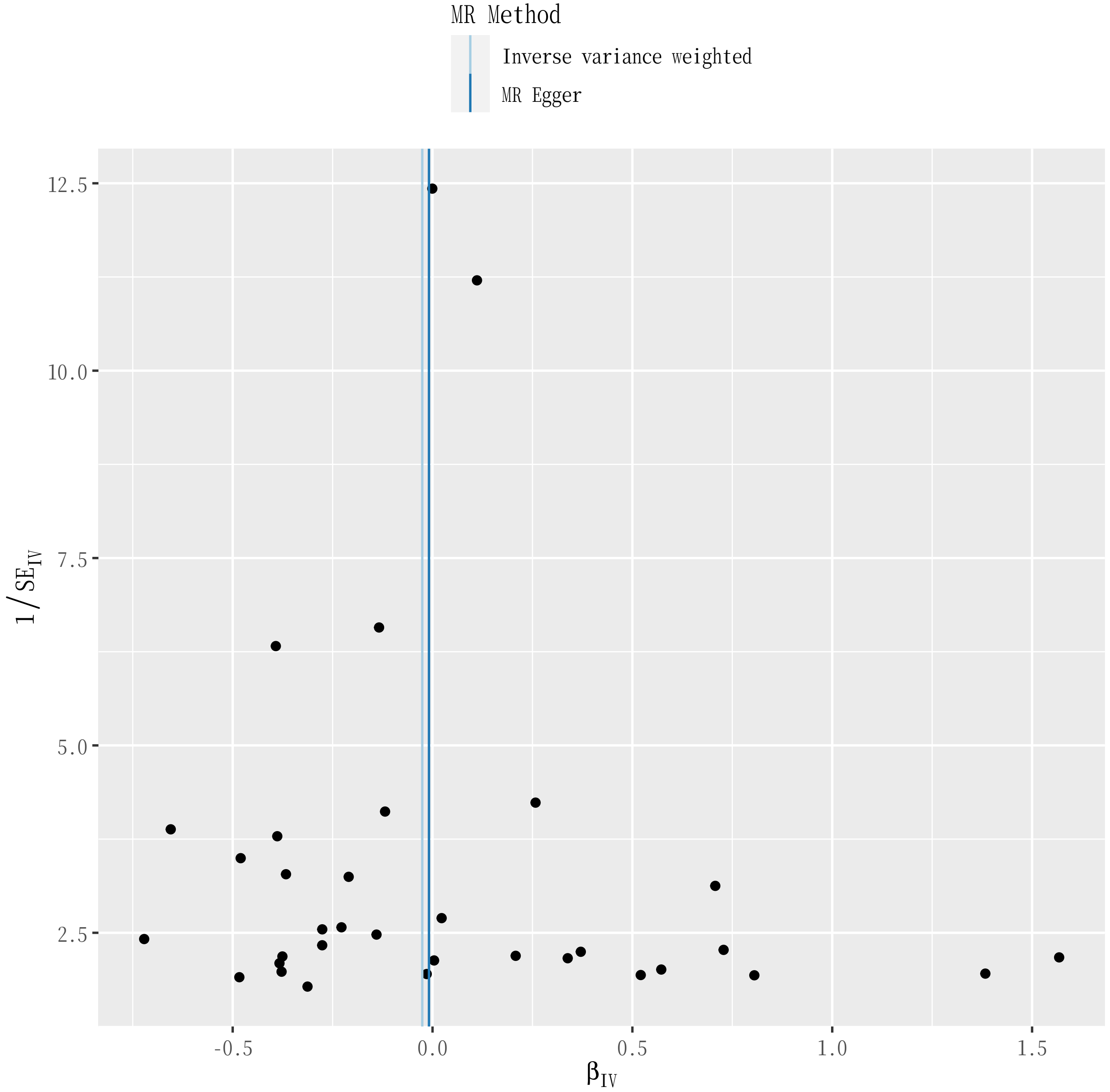

MR Method
Inverse variance weighted
MR Egger
12.5
10.0
7.5
5.0
2.5
1/SE$_{IV}$
-0.5
0.0
0.5
1.0
1.5
β$_{IV}$

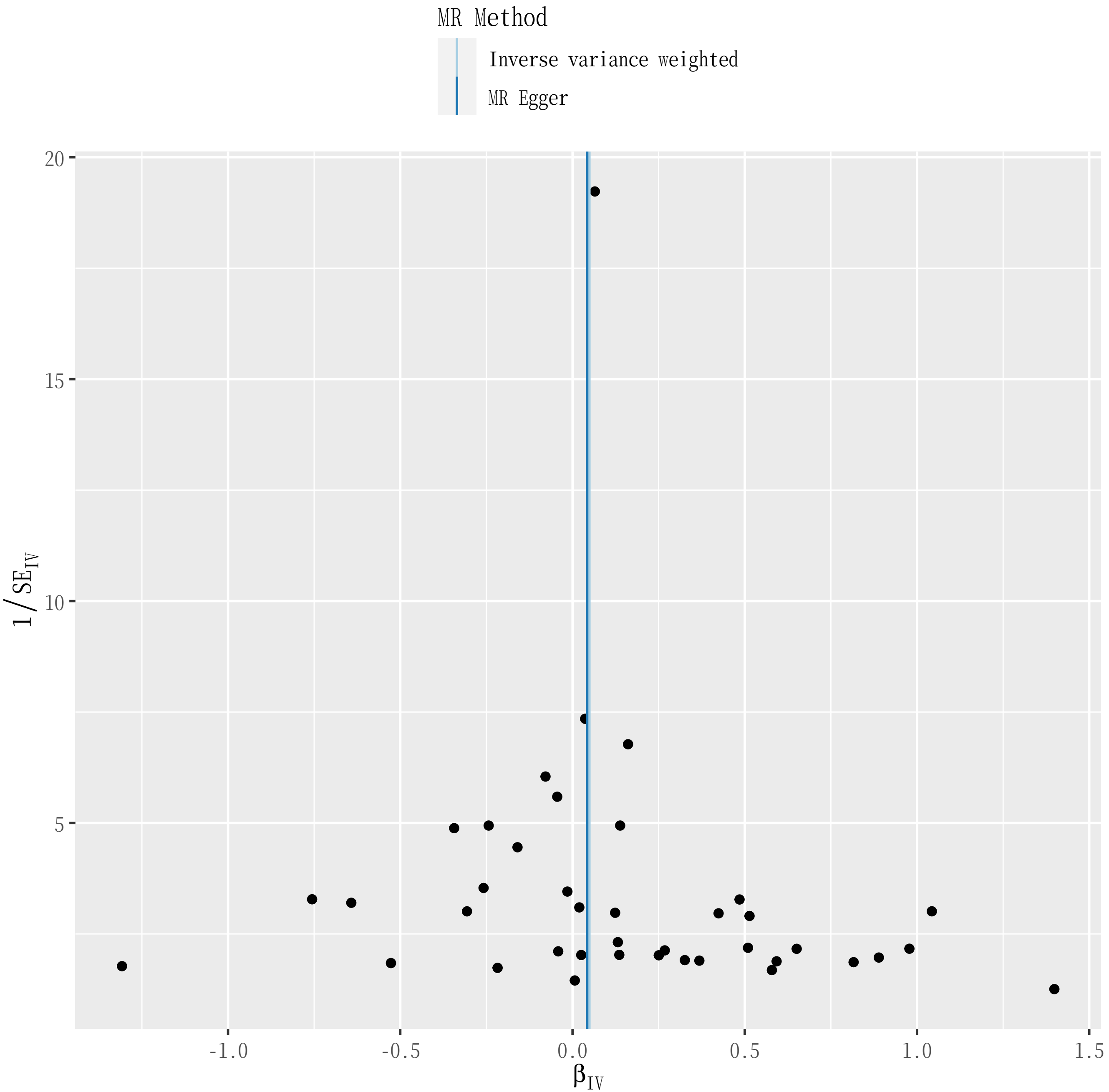

MR Method
Inverse variance weighted
MR Egger
20
15
10
5
$1/SE_{IV}$
-1.0
-0.5
0.0
0.5
1.0
1.5
β_{IV}

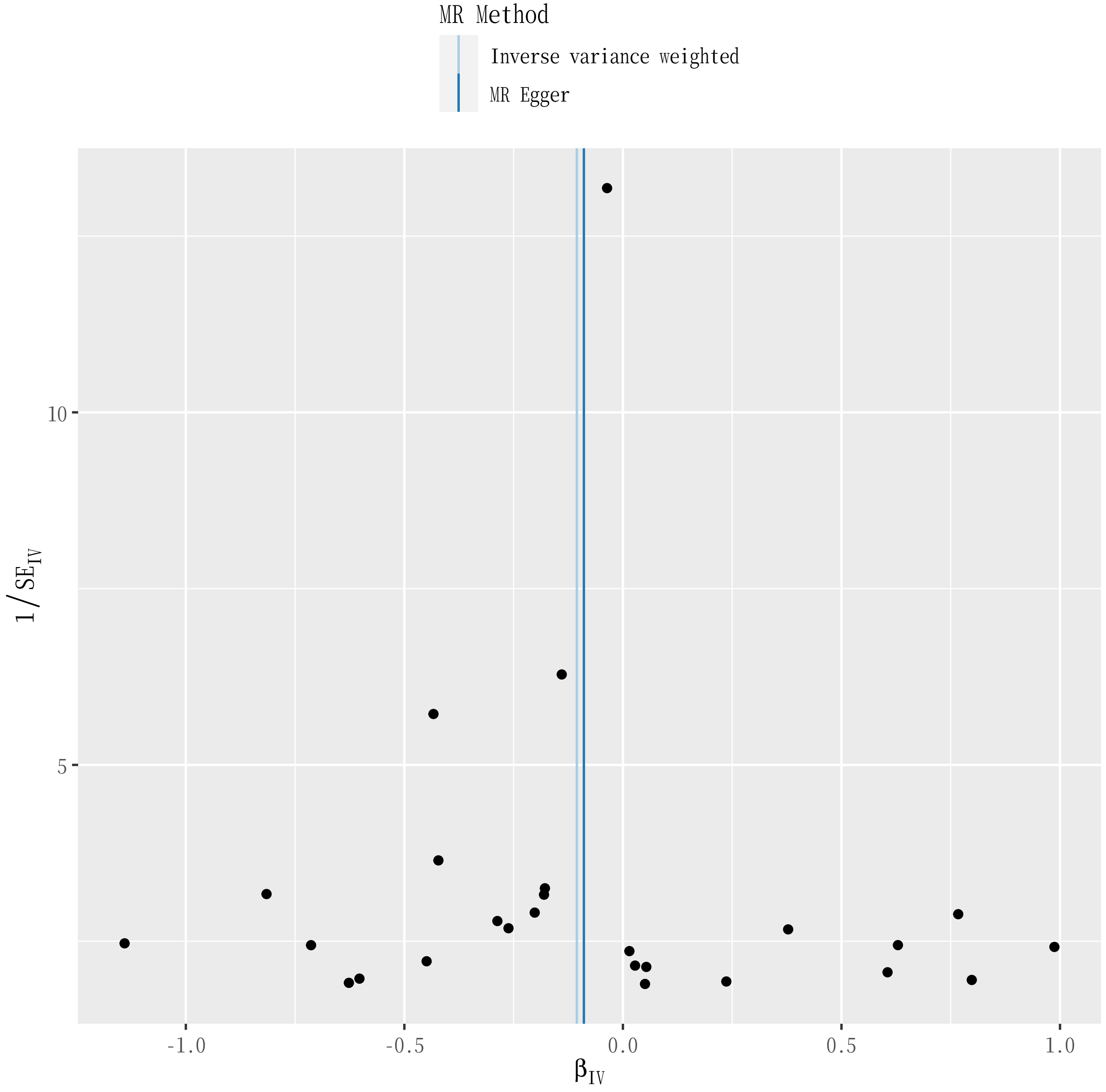
MR Method
Inverse variance weighted
MR Egger
10
5
1/SE_IV
-1.0
-0.5
0.0
0.5
1.0
β_IV

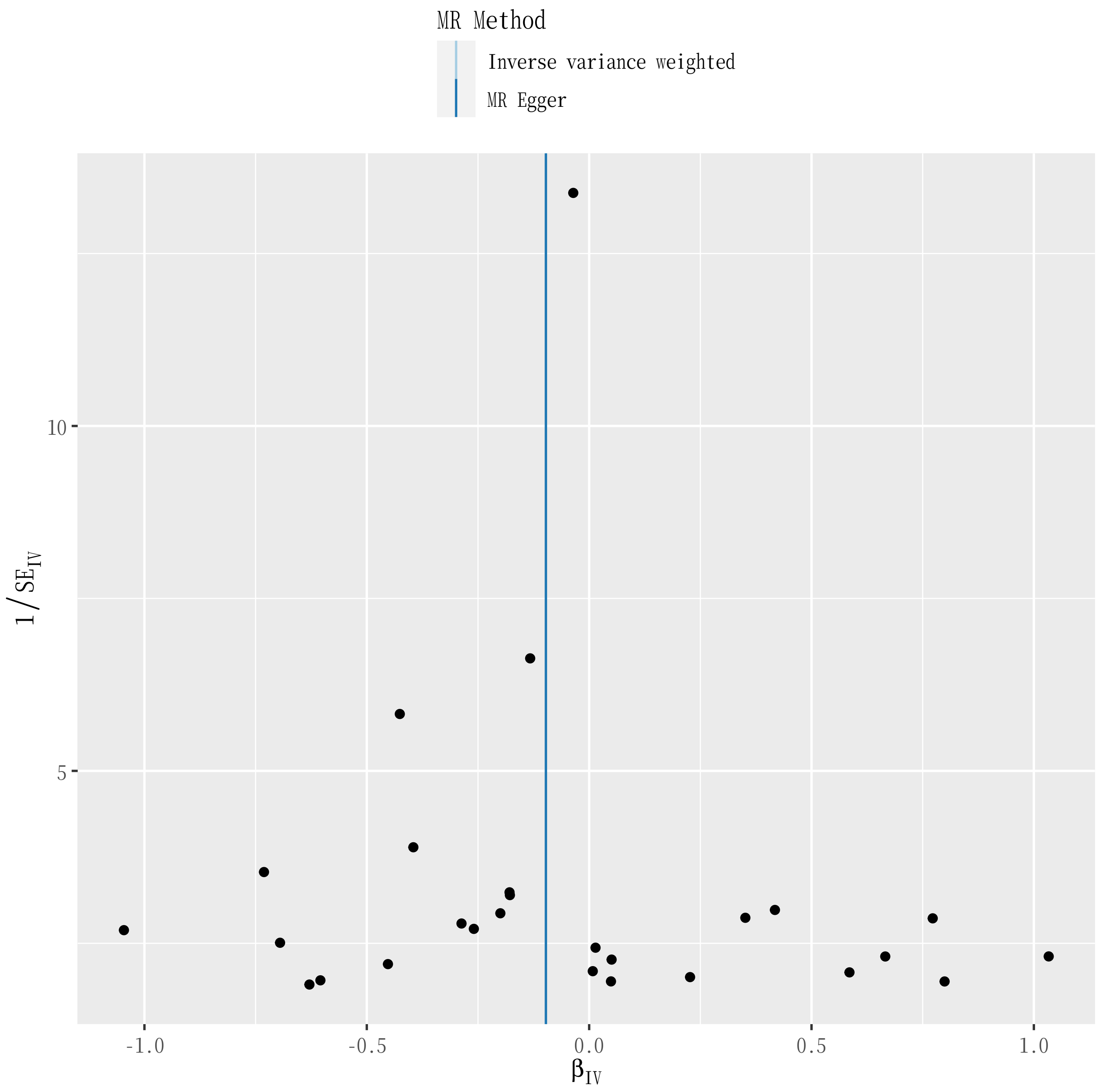
MR Method
Inverse variance weighted
MR Egger
10
5
1/SE_IV
-1.0
-0.5
0.0
0.5
1.0
β_IV

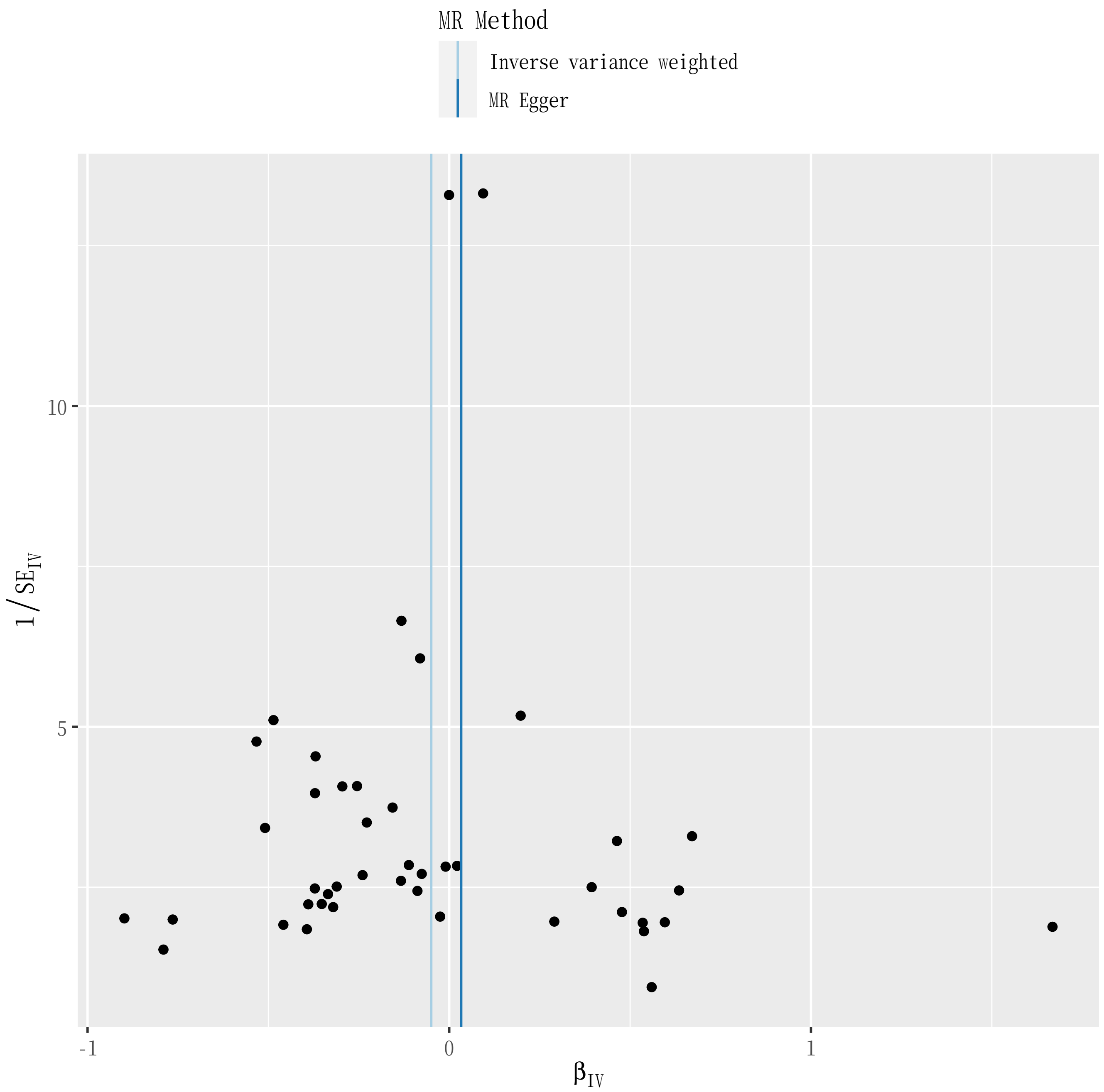
MR Method
Inverse variance weighted
MR Egger
1/SE_{IV}
10
5
β_{IV}
-1
0
1

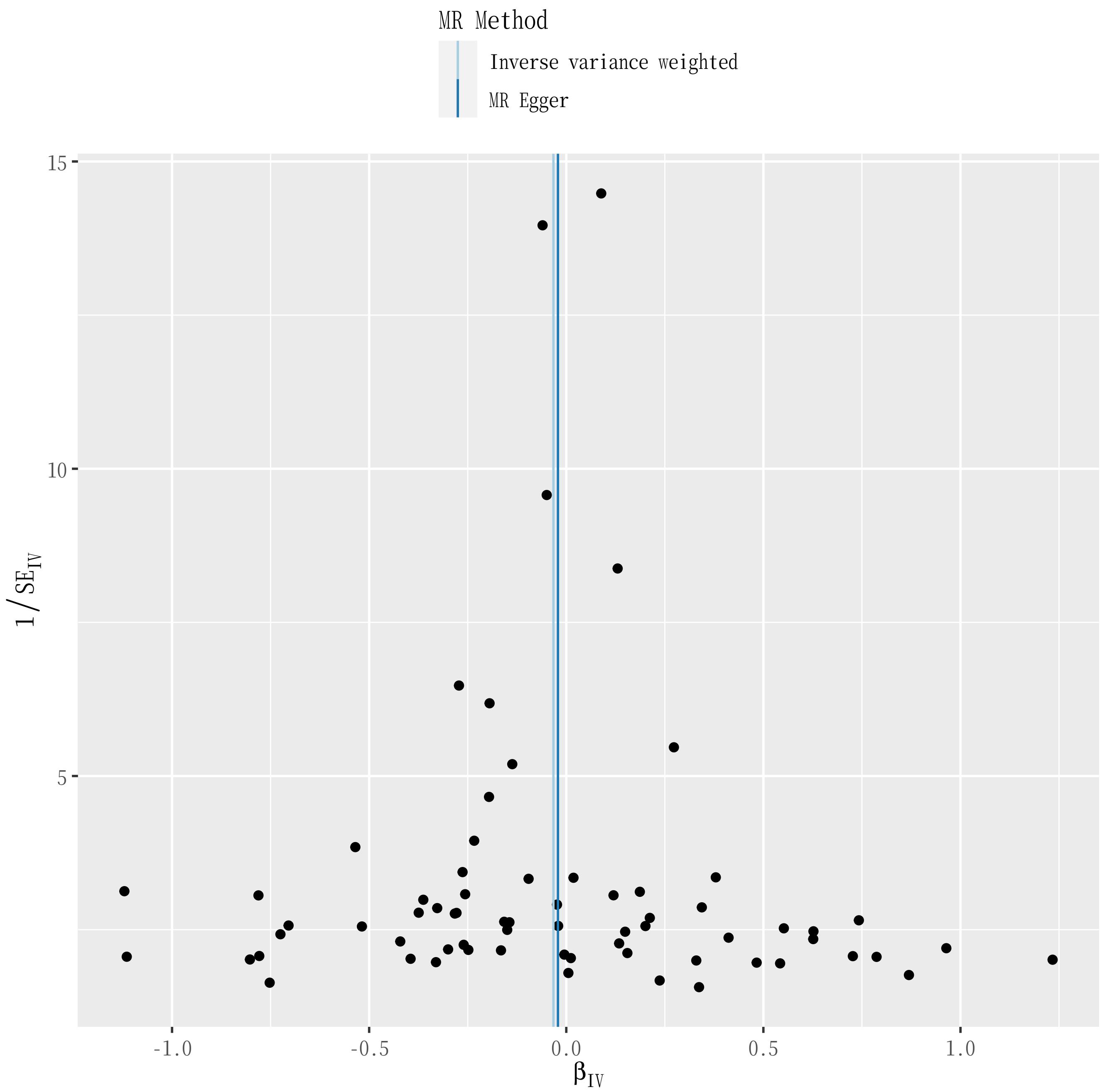

MR Method
Inverse variance weighted
MR Egger
15
10
5
$1/SE_{IV}$
-1.0
-0.5
0.0
0.5
1.0
β_{IV}

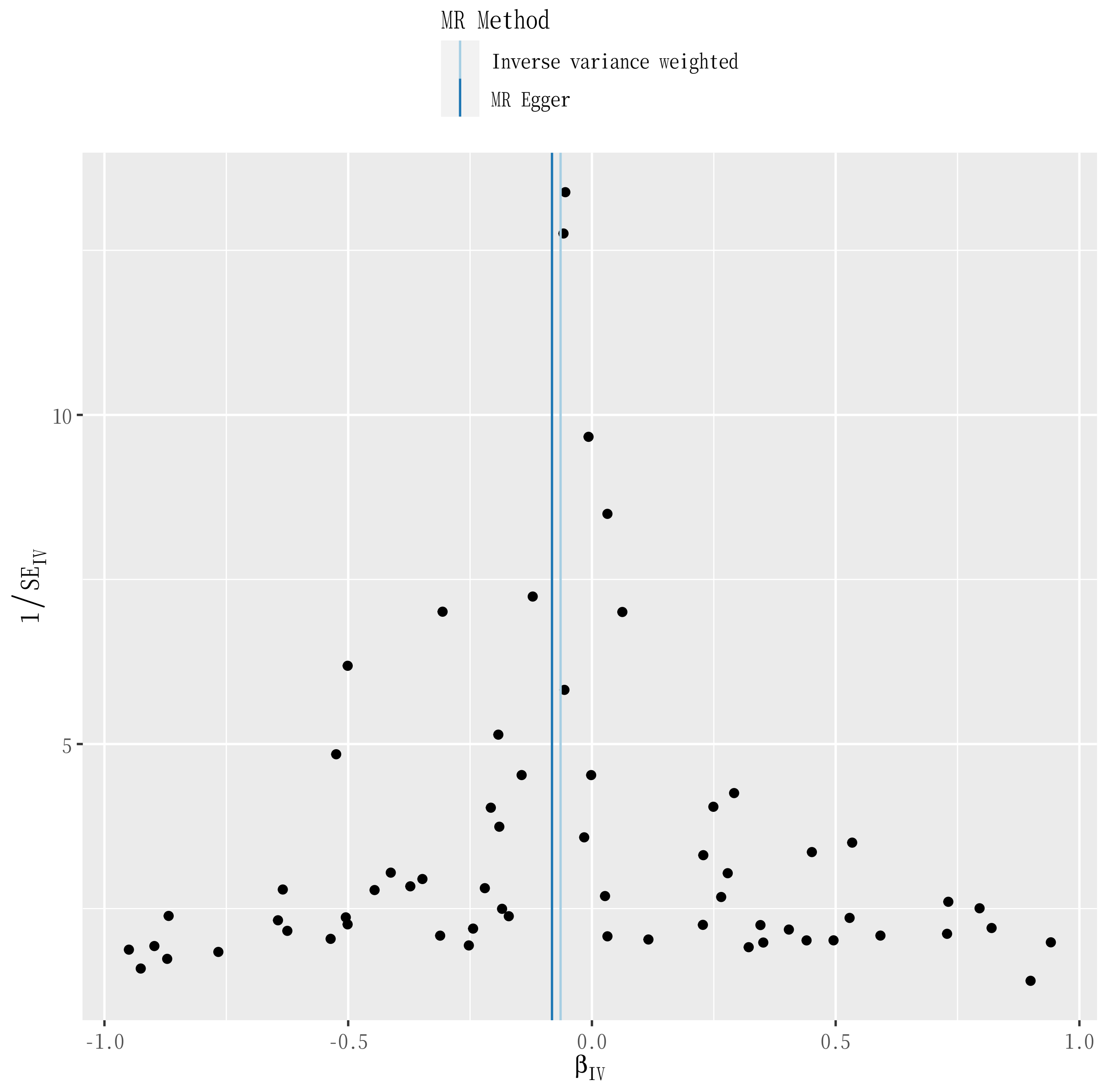

MR Method
Inverse variance weighted
MR Egger
10
5
$1/SE_{IV}$
-1.0
-0.5
0.0
0.5
1.0
β_{IV}

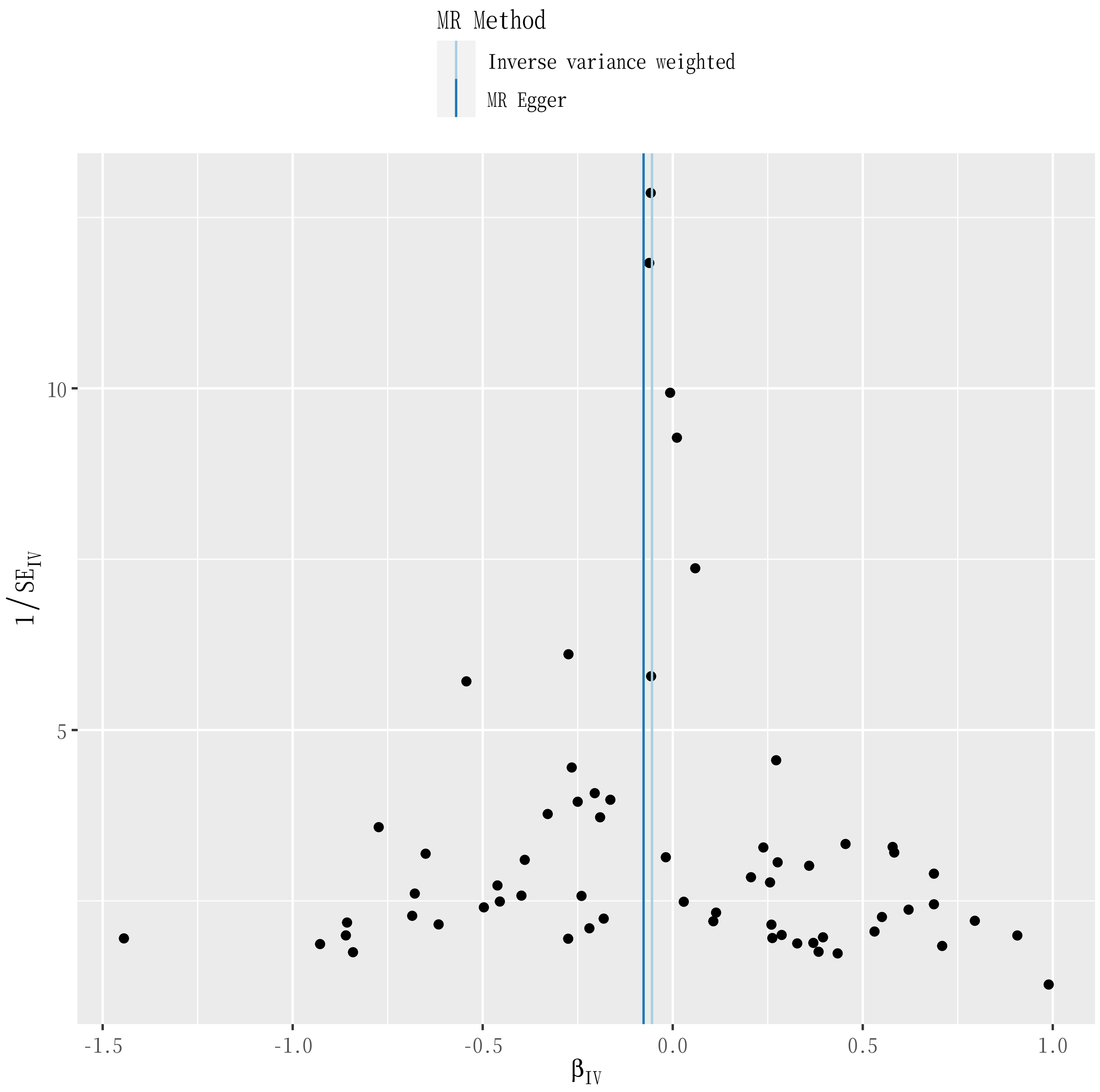
MR Method
Inverse variance weighted
MR Egger
10
5
1/SE_IV
-1.5
-1.0
-0.5
0.0
0.5
1.0
β_IV

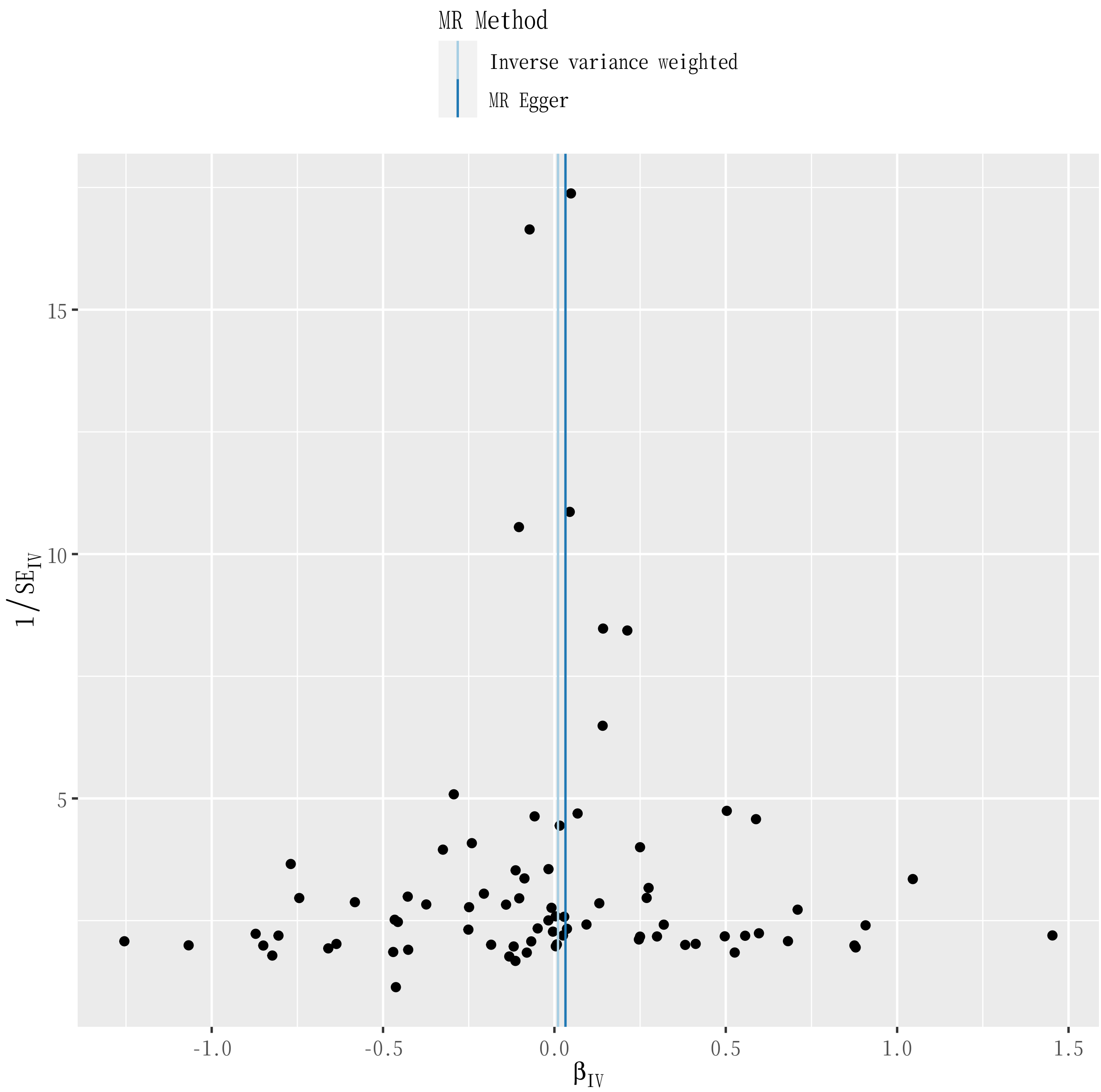
MR Method
Inverse variance weighted
MR Egger
15
10
5
$1/SE_{IV}$
-1.0
-0.5
0.0
0.5
1.0
1.5
β_{IV}

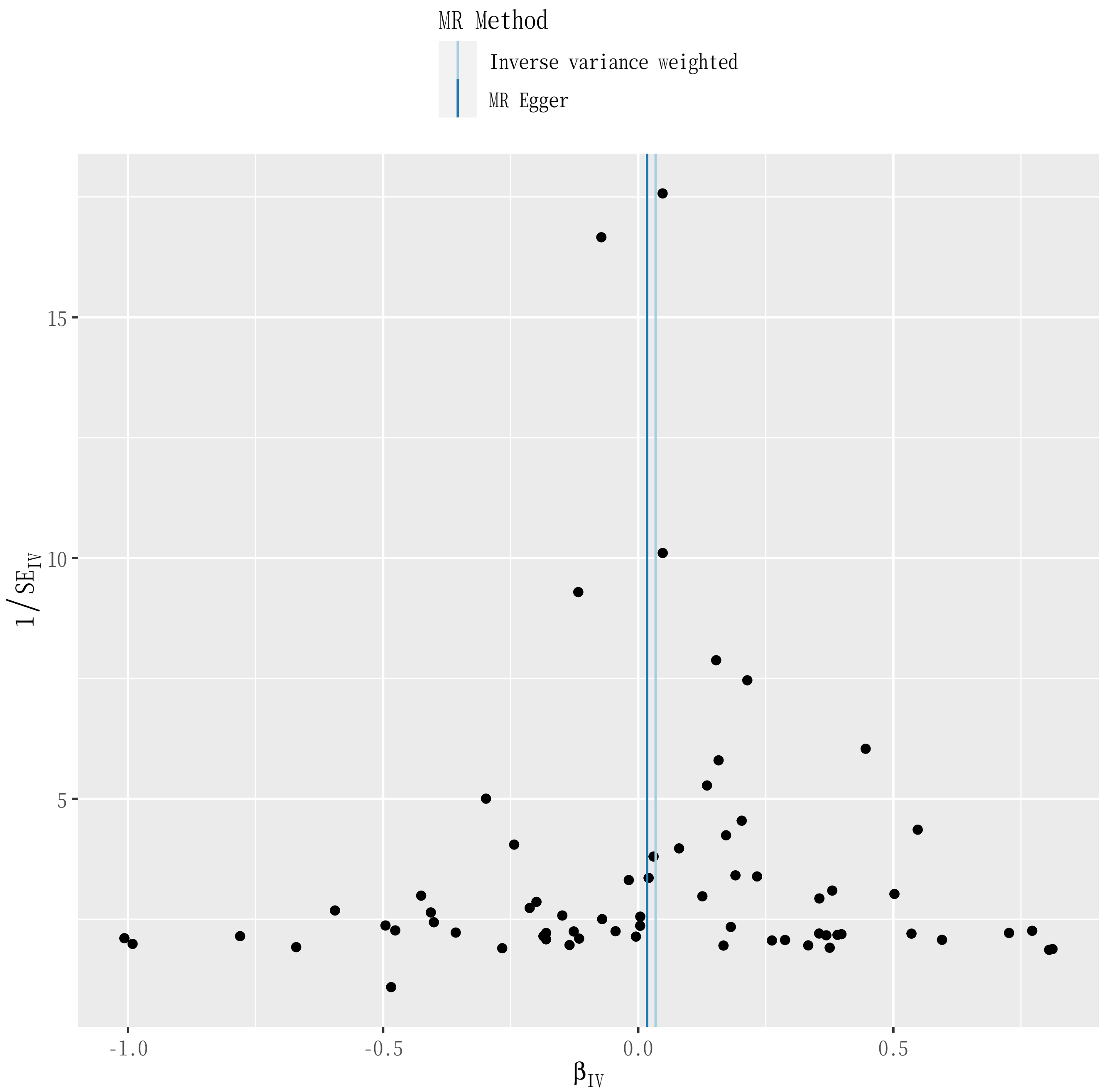

MR Method
Inverse variance weighted
MR Egger
15
10
5
$1/SE_{IV}$
-1.0
-0.5
0.0
0.5
β_{IV}

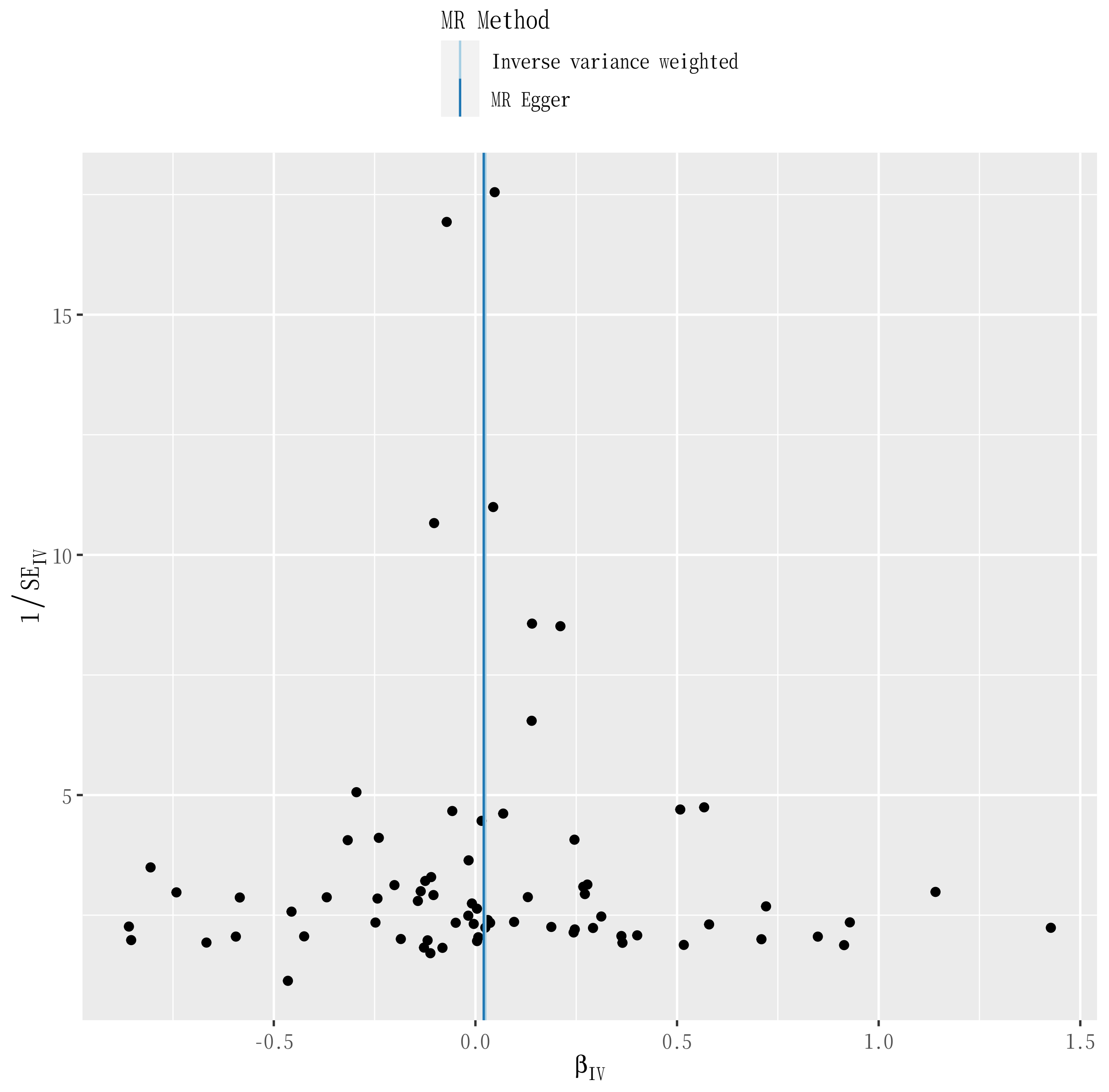
MR Method
Inverse variance weighted
MR Egger
15
10
5
1/SE_{IV}
-0.5
0.0
0.5
1.0
1.5
β_{IV}

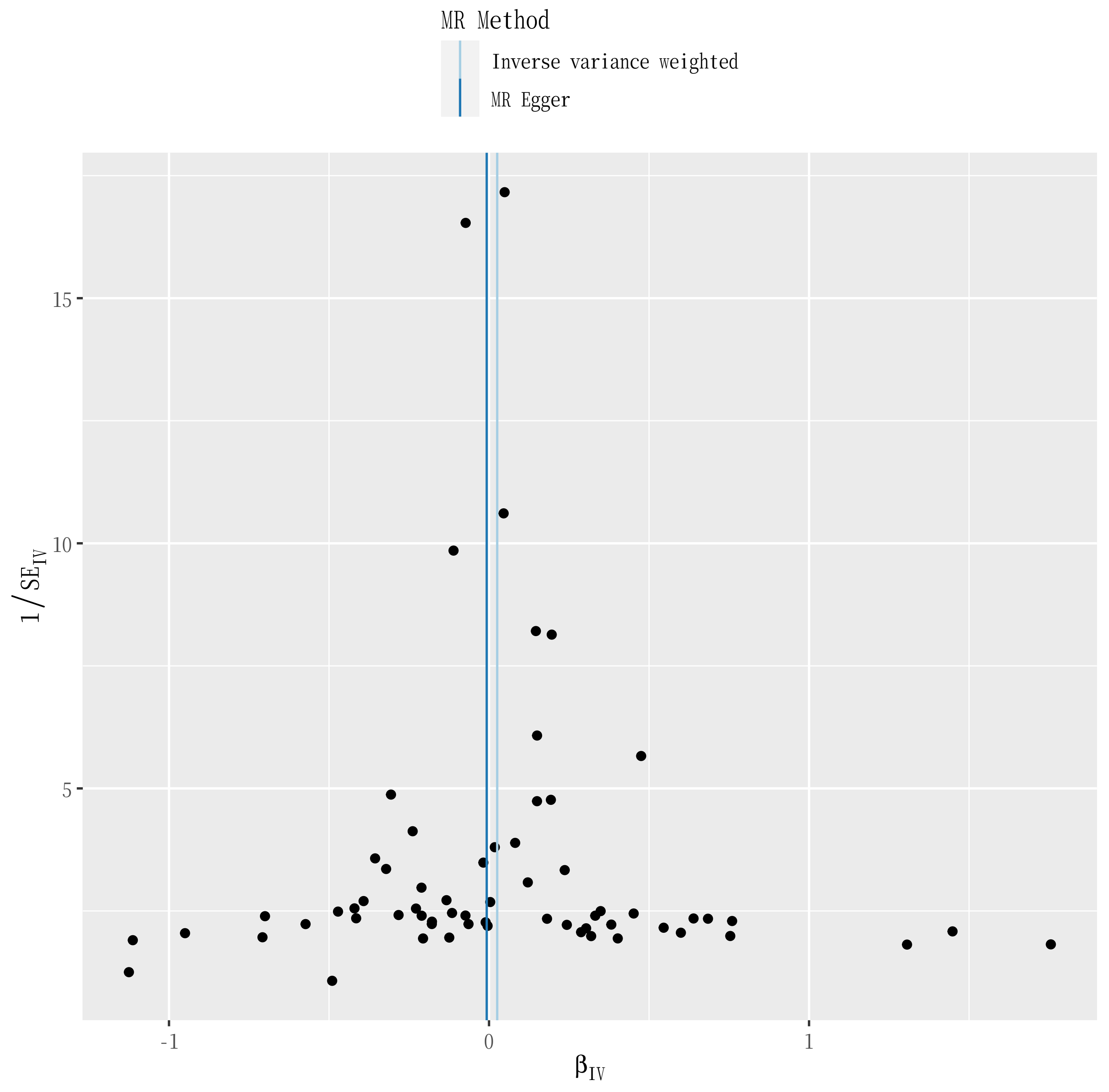
MR Method
Inverse variance weighted
MR Egger
15
10
5
-1
0
1
1/SE_IV
β_IV

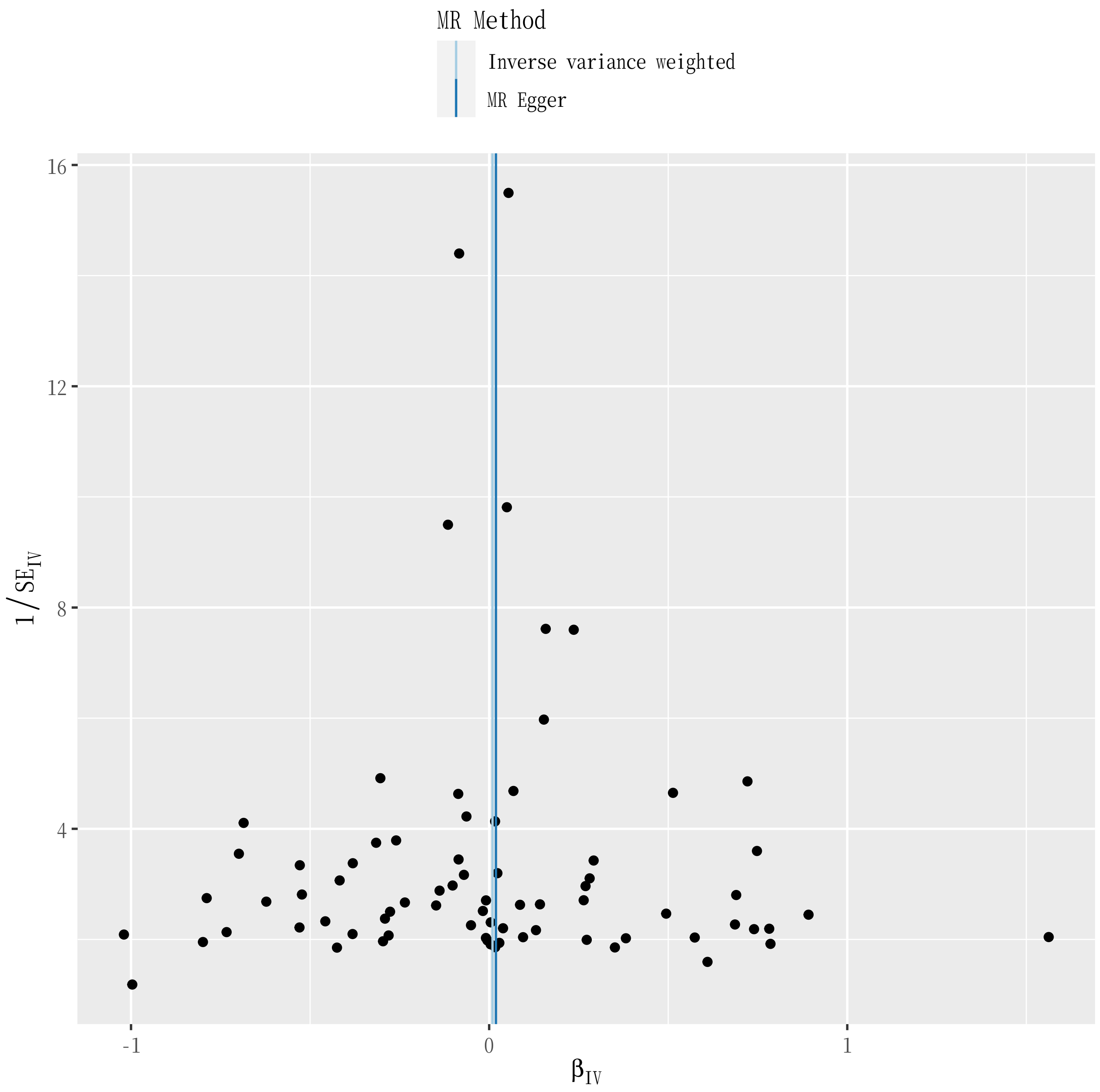
MR Method
Inverse variance weighted
MR Egger
16
12
8
4
1/SE_IV
-1
0
1
β_IV

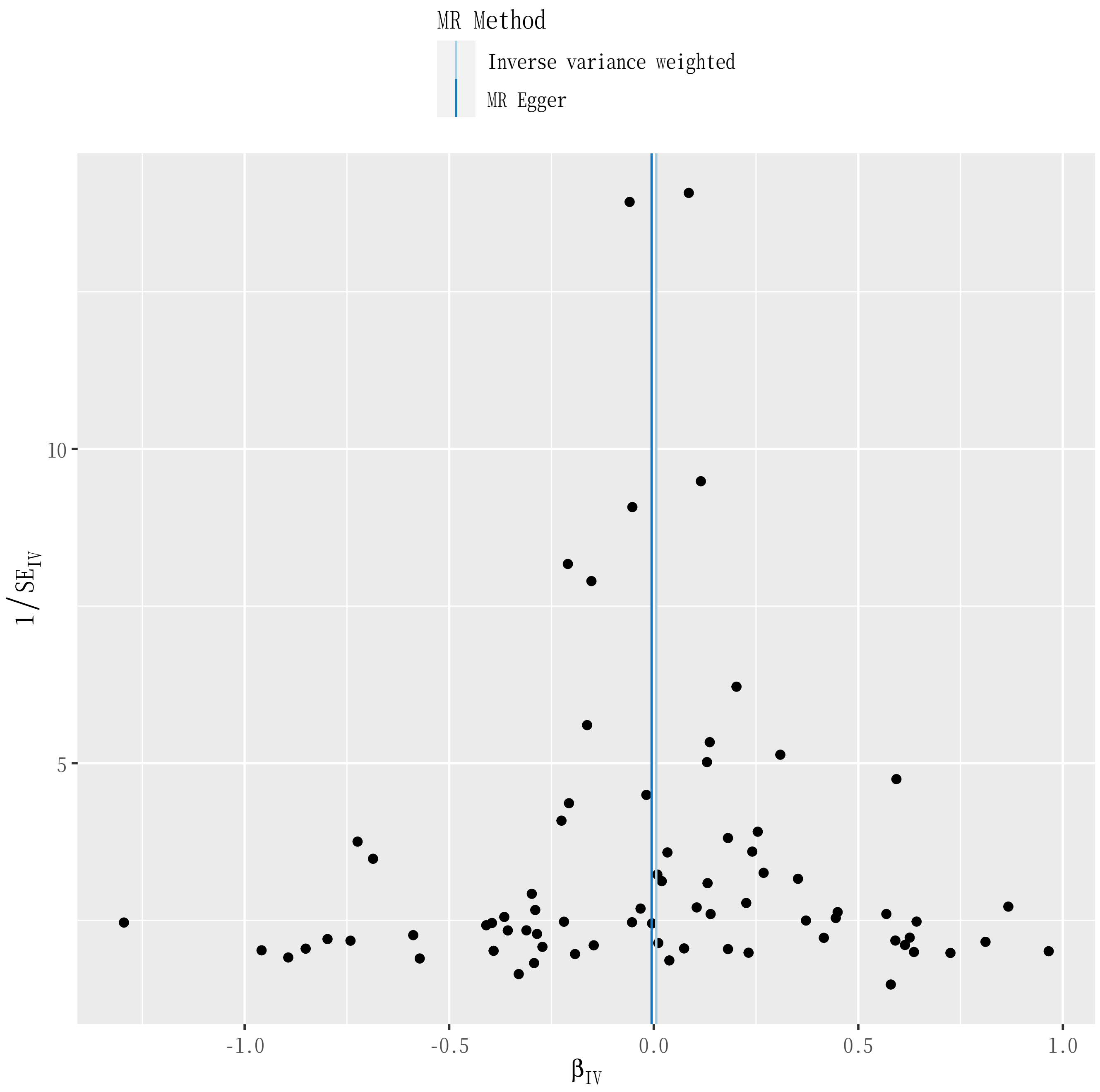

MR Method
Inverse variance weighted
MR Egger
10
5
1/SE_IV
-1.0
-0.5
0.0
0.5
1.0
β_IV

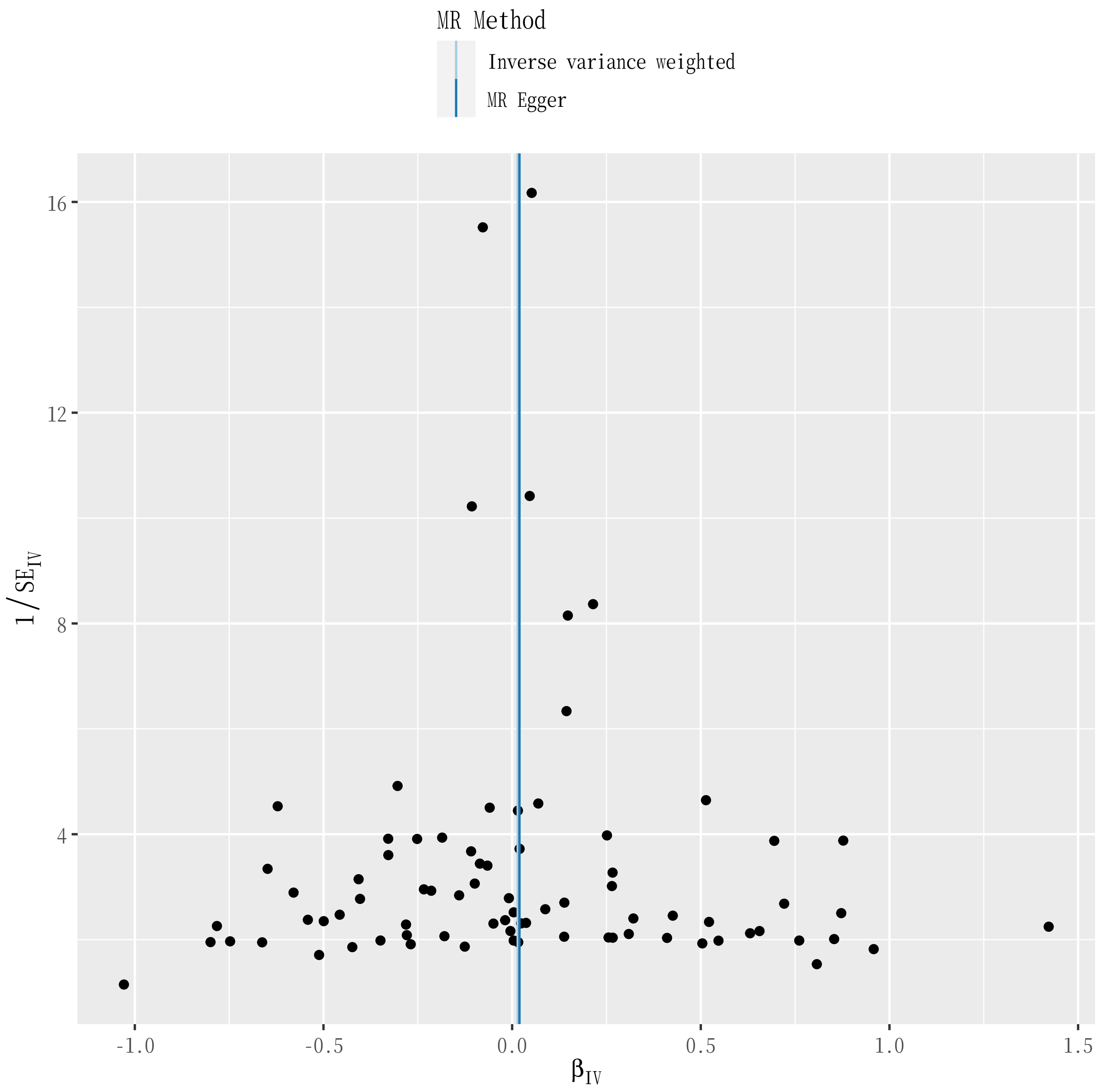
MR Method
Inverse variance weighted
MR Egger
16
12
8
4
$1/SE_{IV}$
-1.0
-0.5
0.0
0.5
1.0
1.5
β_{IV}

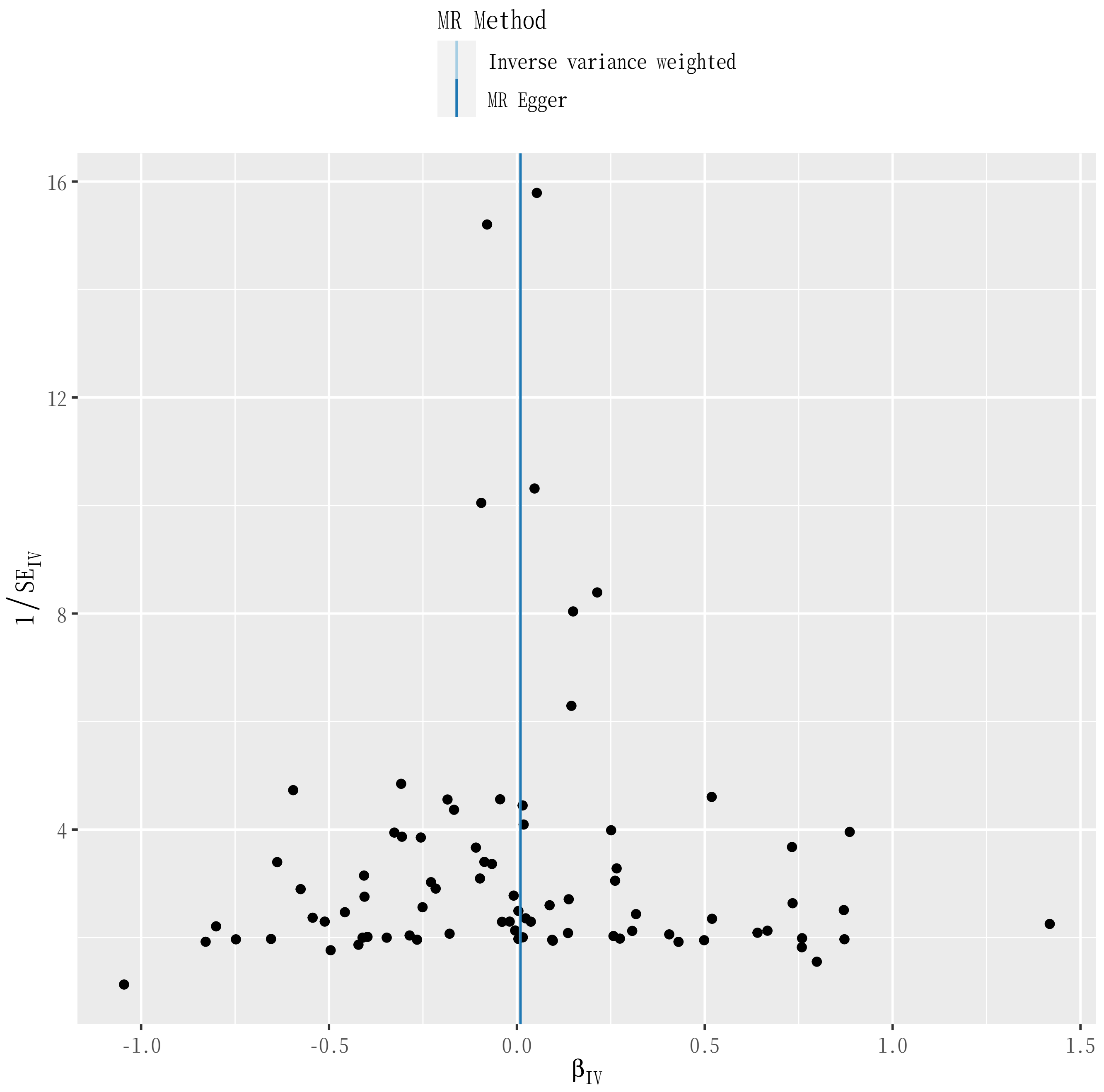
MR Method
Inverse variance weighted
MR Egger
16
12
8
4
$1/SE_{IV}$
-1.0
-0.5
0.0
0.5
1.0
1.5
β_{IV}

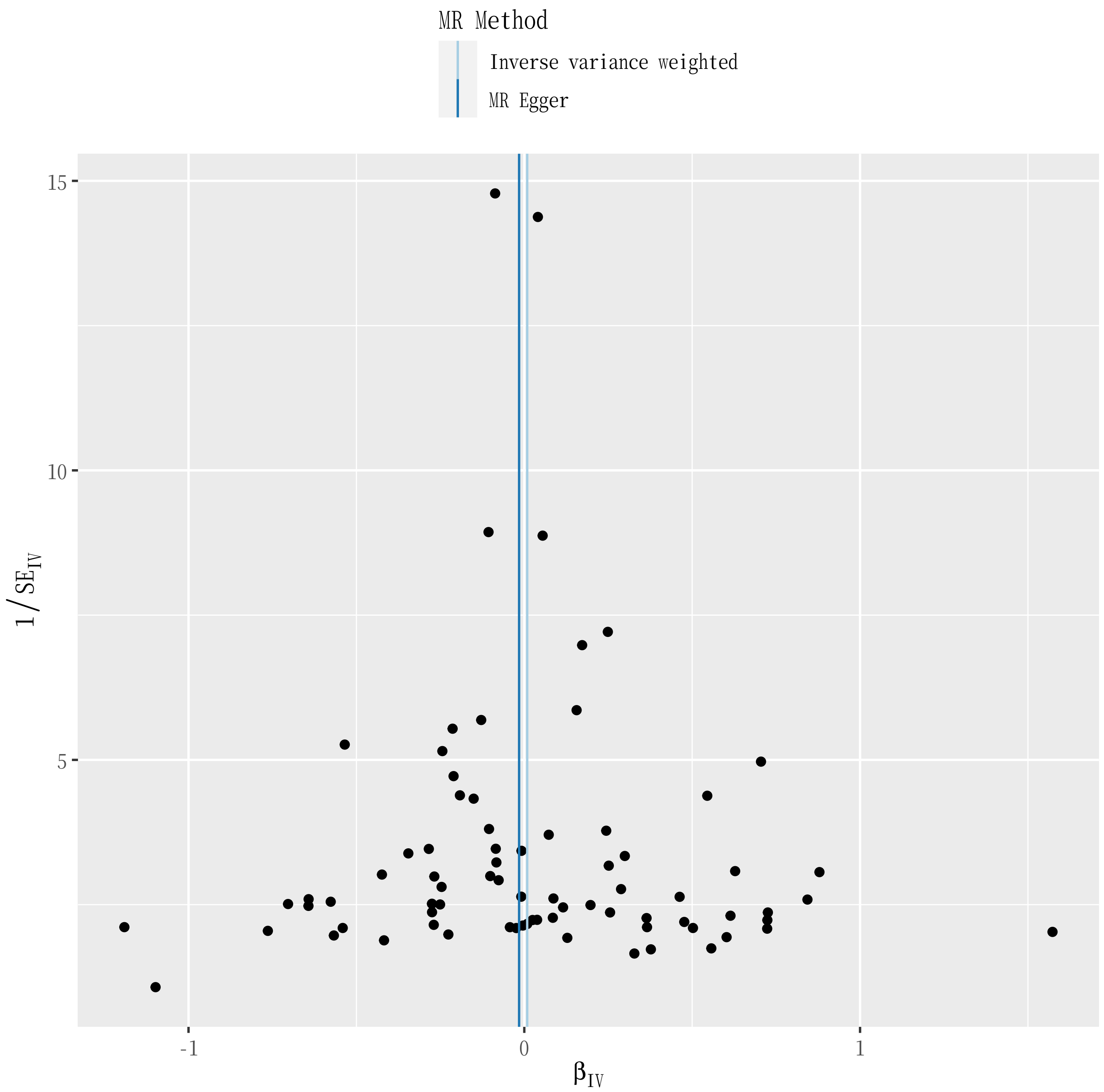
MR Method
Inverse variance weighted
MR Egger
15
10
5
-1
0
1
1/SE_IV
β_IV

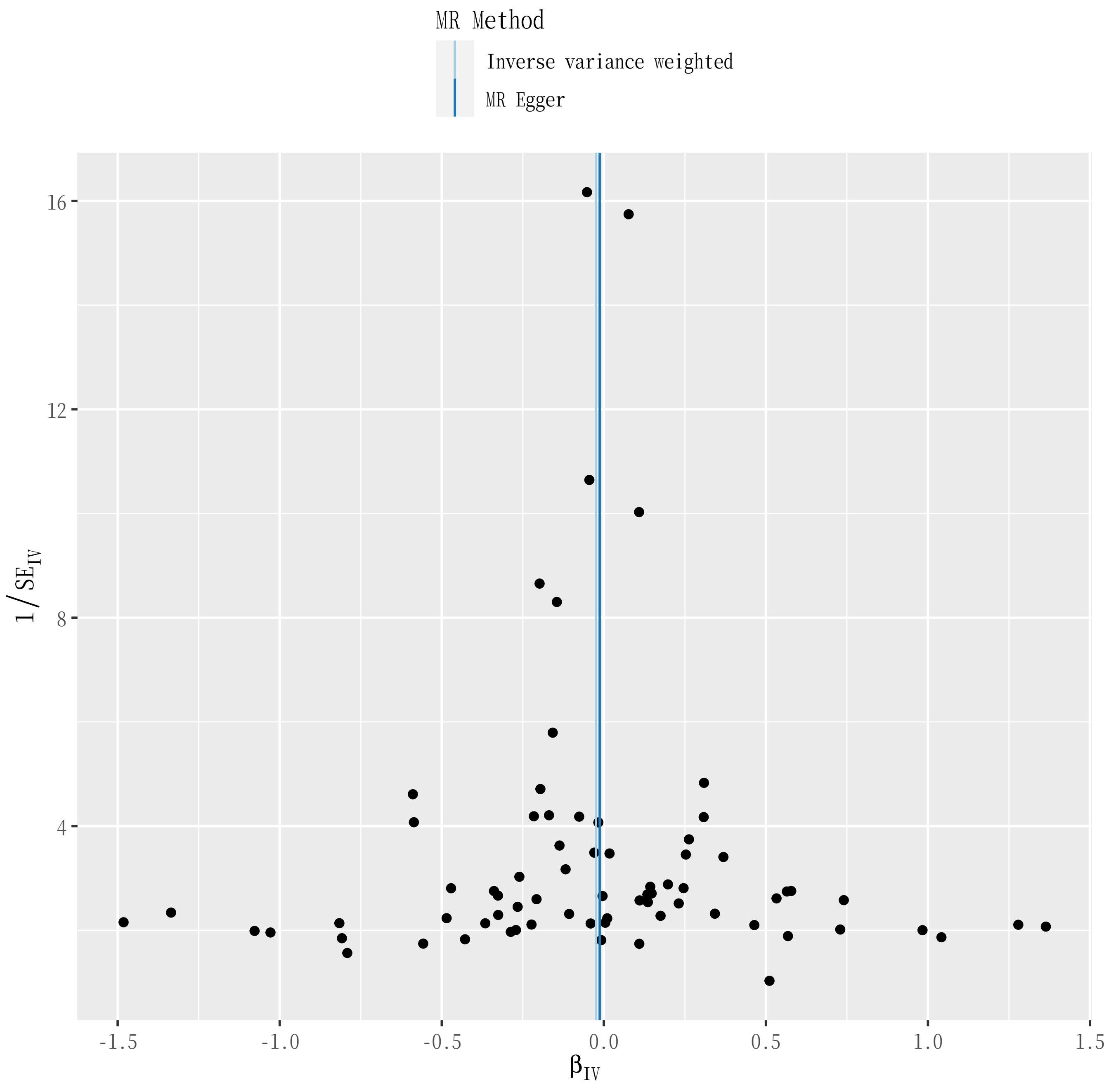

MR Method
Inverse variance weighted
MR Egger
16
12
8
4
$1/SE_{IV}$
-1.5
-1.0
-0.5
0.0
0.5
1.0
1.5
β_{IV}

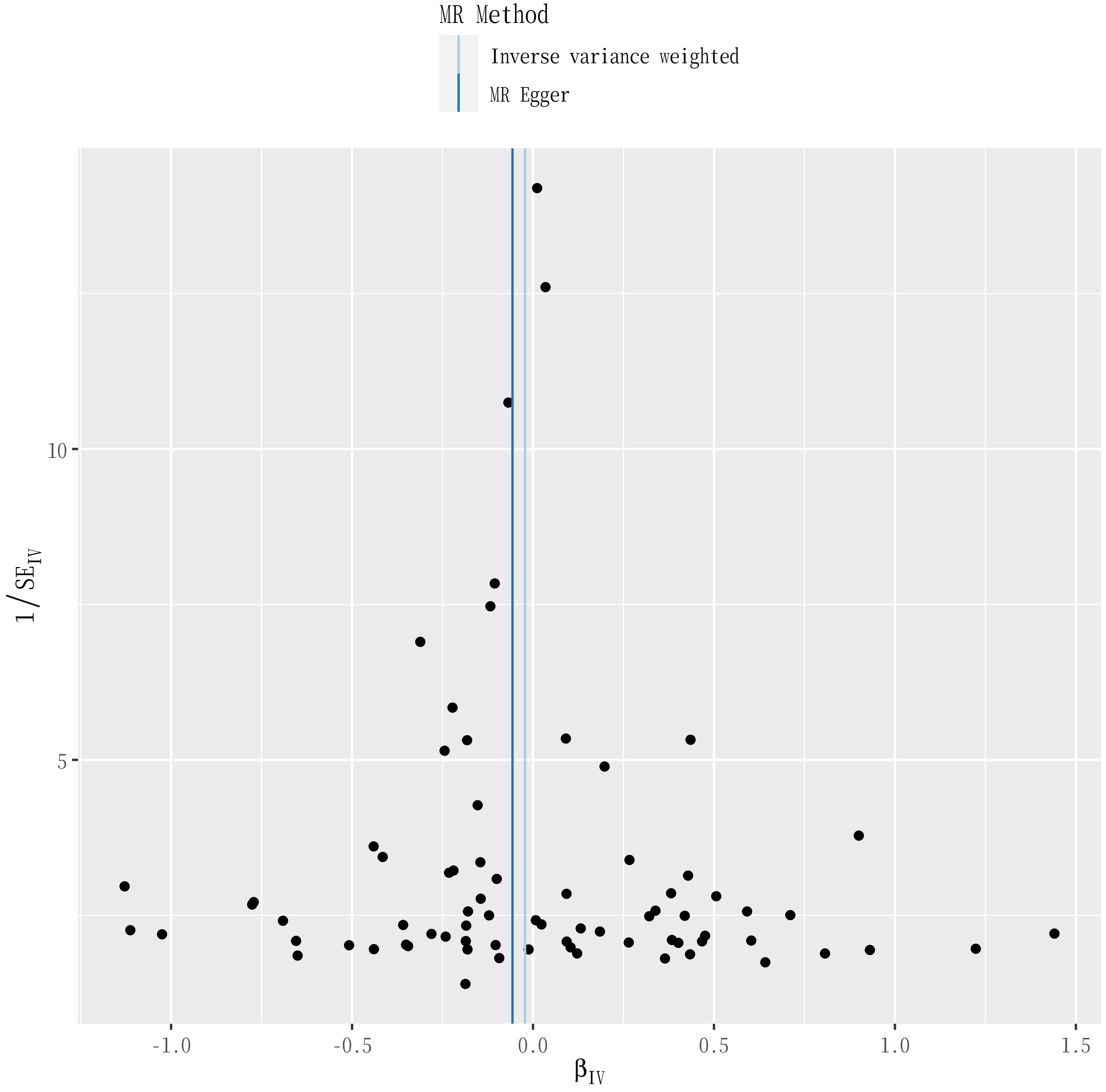

MR Method
Inverse variance weighted
MR Egger
10
5
1/SE_IV
-1.0
-0.5
0.0
0.5
1.0
1.5
β_IV

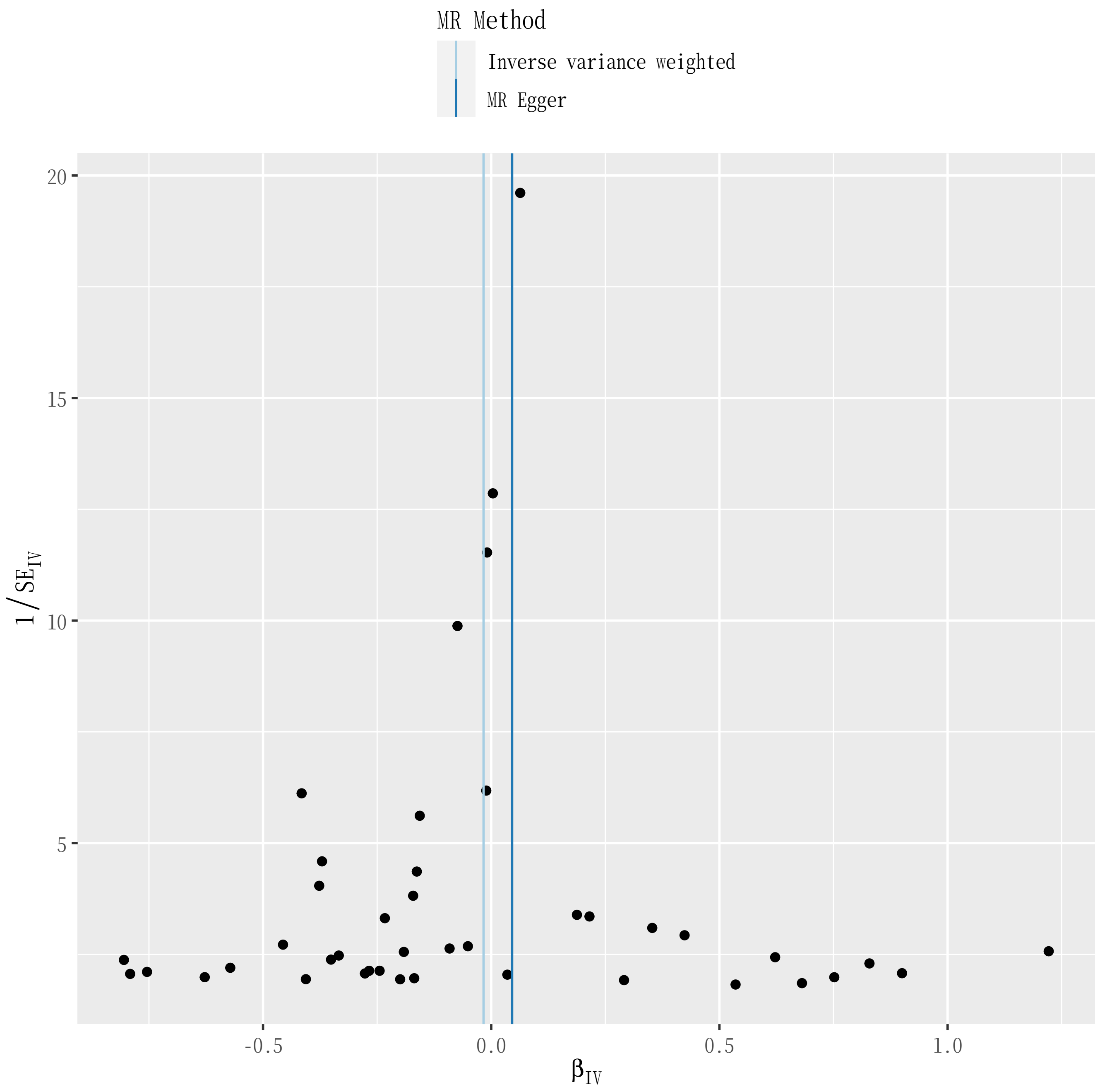

MR Method
Inverse variance weighted
MR Egger
20
15
10
5
1/SE_IV
-0.5
0.0
0.5
1.0
β_IV

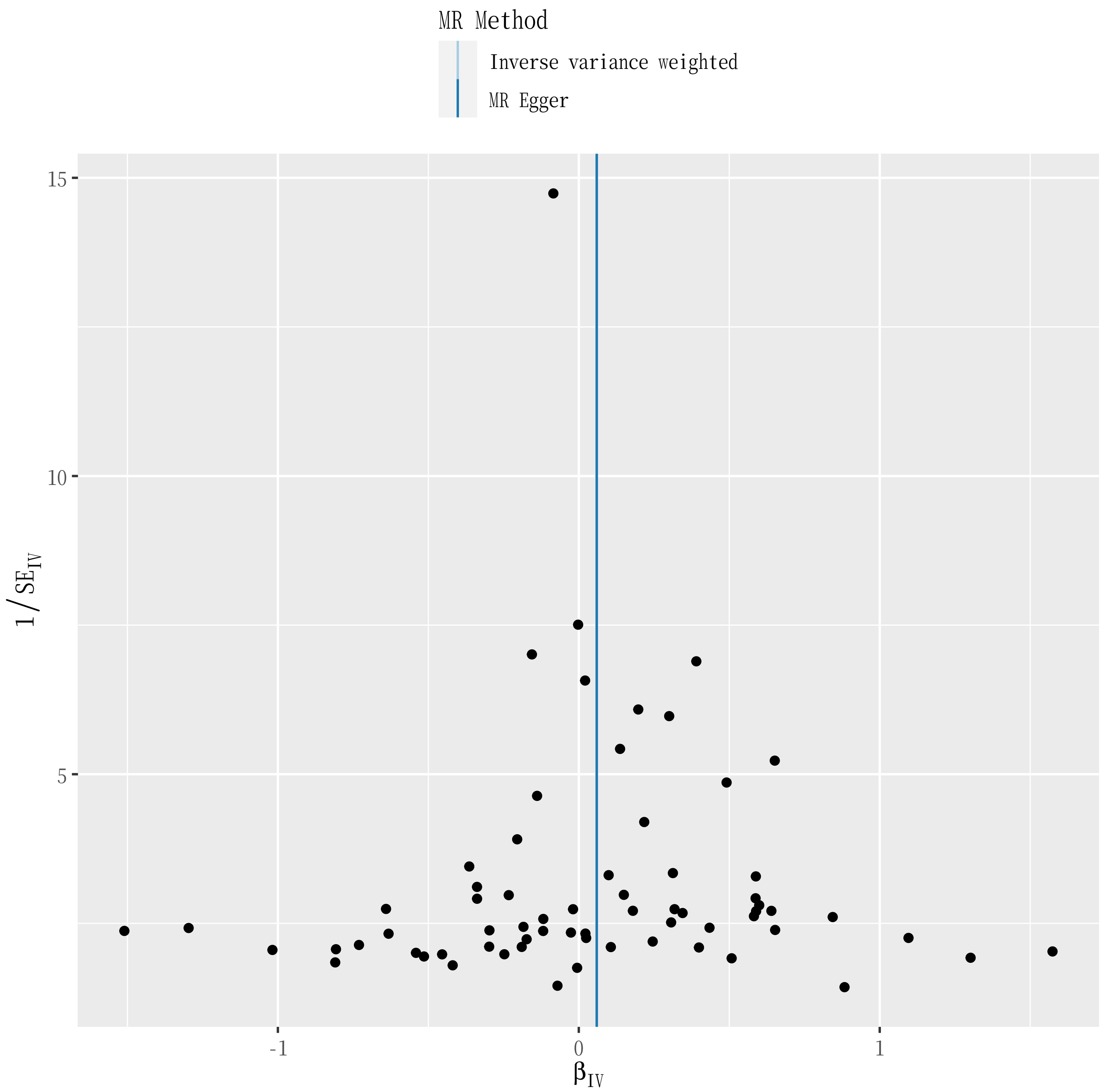

MR Method
Inverse variance weighted
MR Egger
15
10
5
1/SE_IV
-1
0
1
β_IV

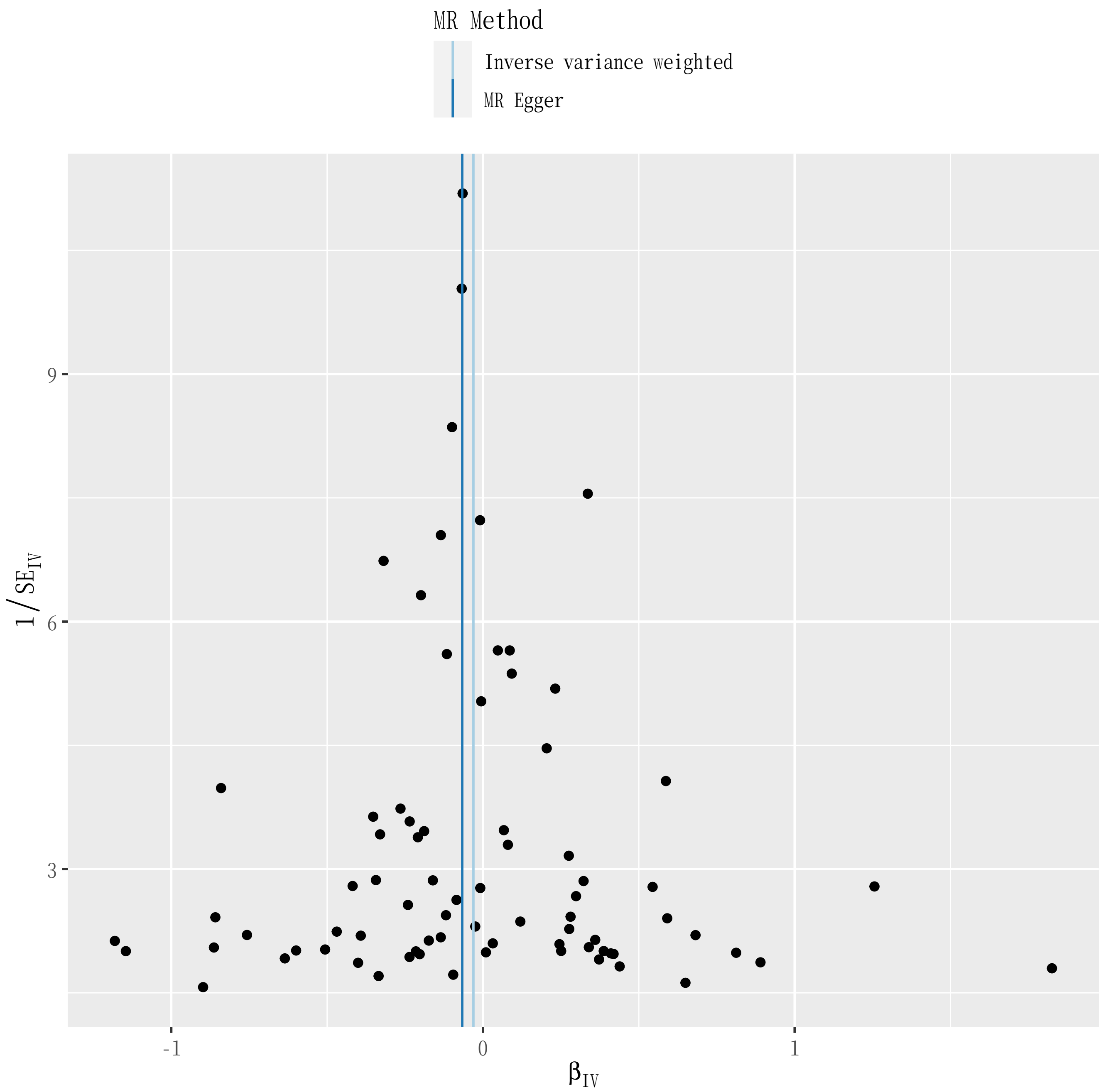
MR Method
Inverse variance weighted
MR Egger
9
6
3
-1
0
1
1/SE_IV
β_IV

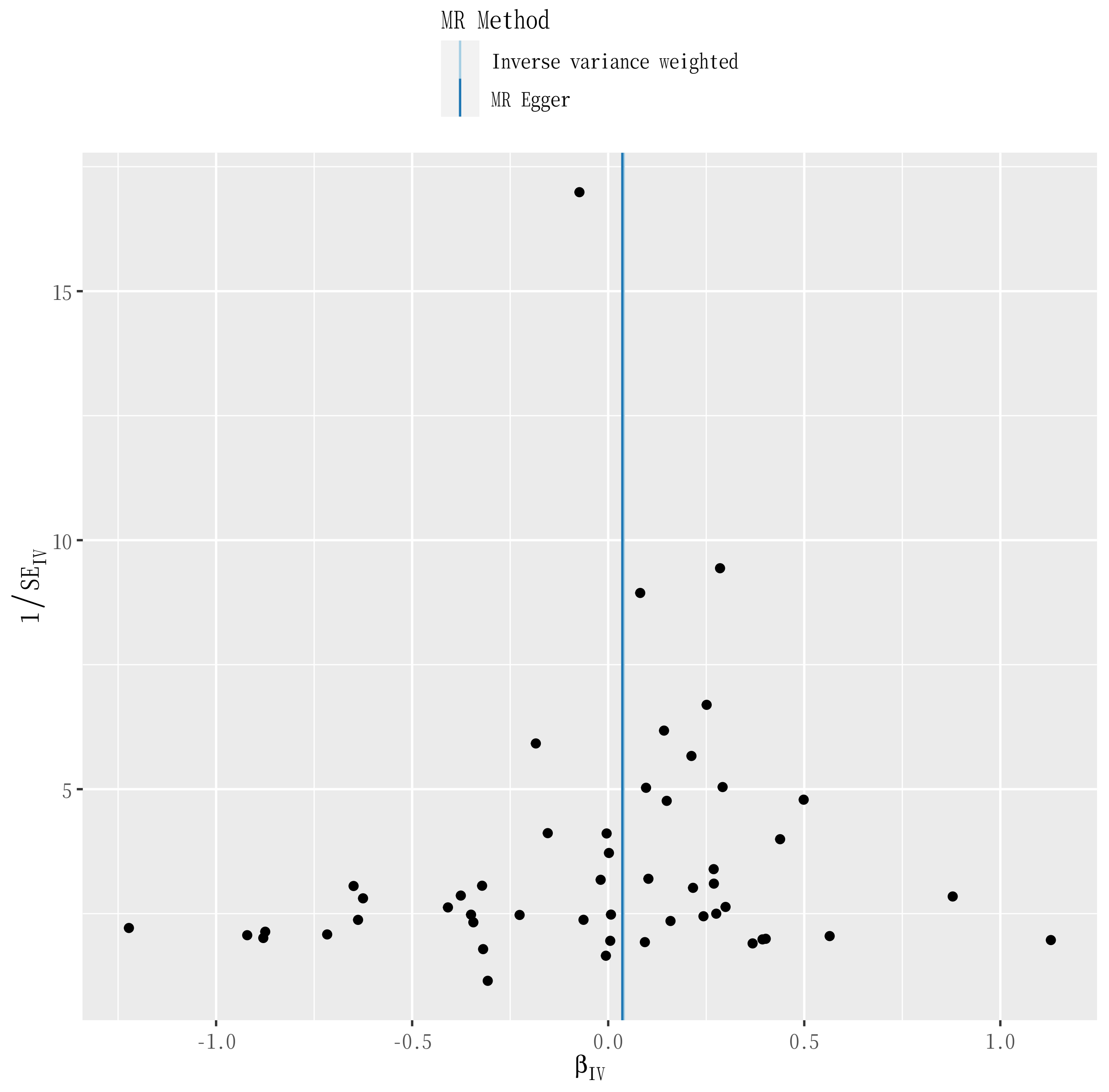

MR Method
Inverse variance weighted
MR Egger
15
10
5
$1/SE_{IV}$
-1.0
-0.5
0.0
0.5
1.0
β_{IV}

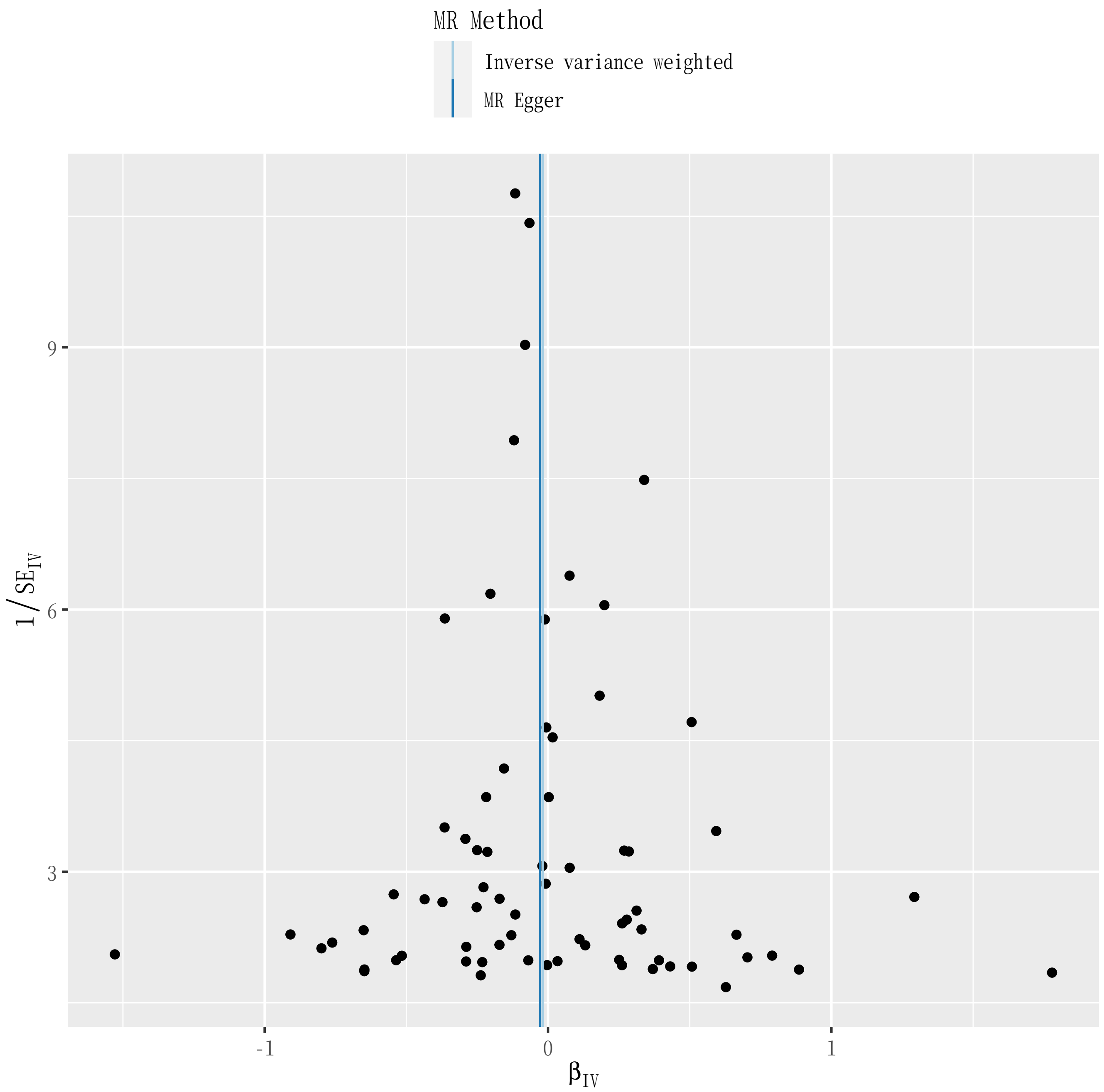
MR Method
Inverse variance weighted
MR Egger
9
6
3
-1
0
1
1/SE_IV
β_IV

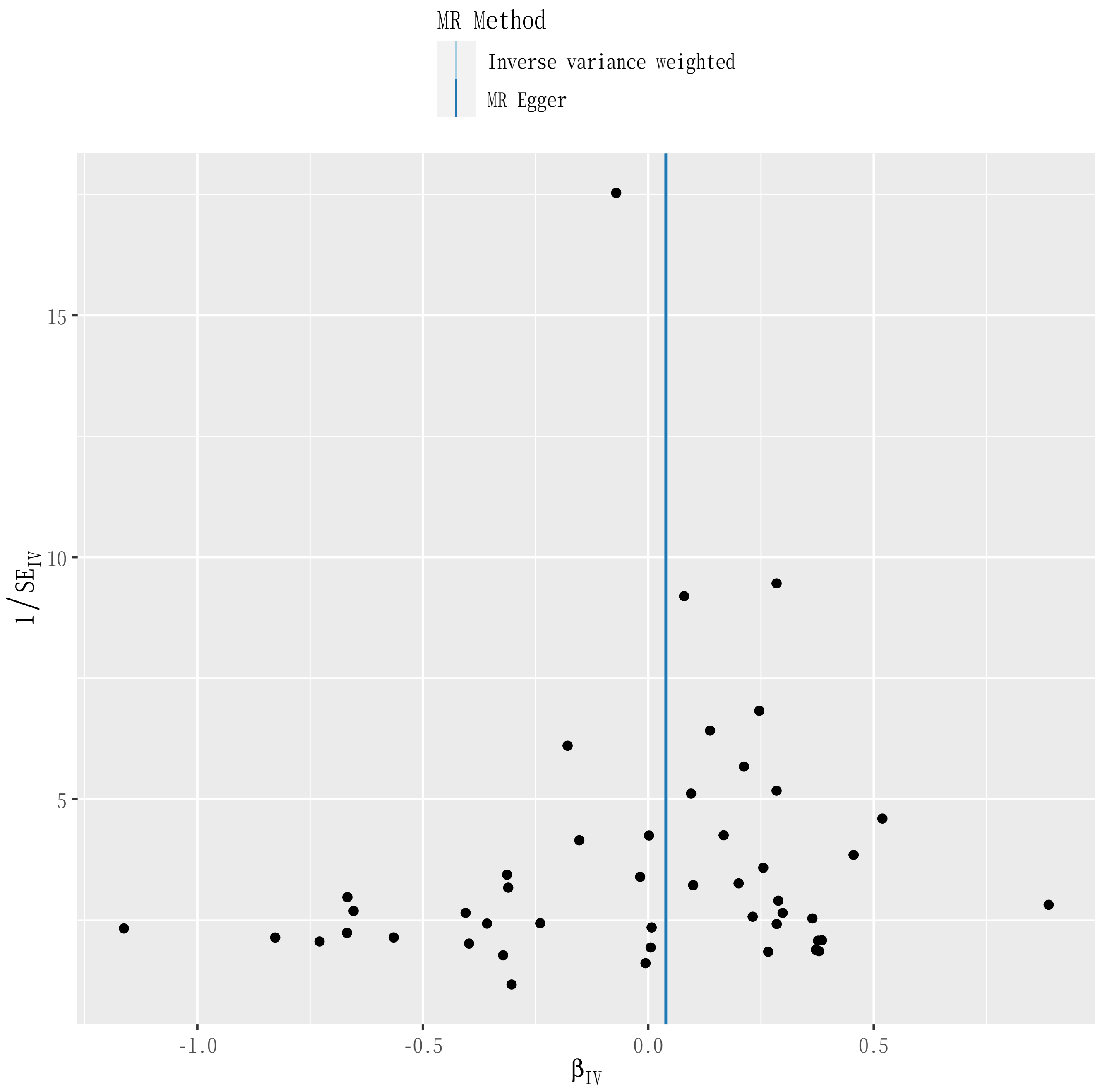

MR Method
Inverse variance weighted
MR Egger
15
10
5
1/SE$_{IV}$
-1.0
-0.5
0.0
0.5
β$_{IV}$

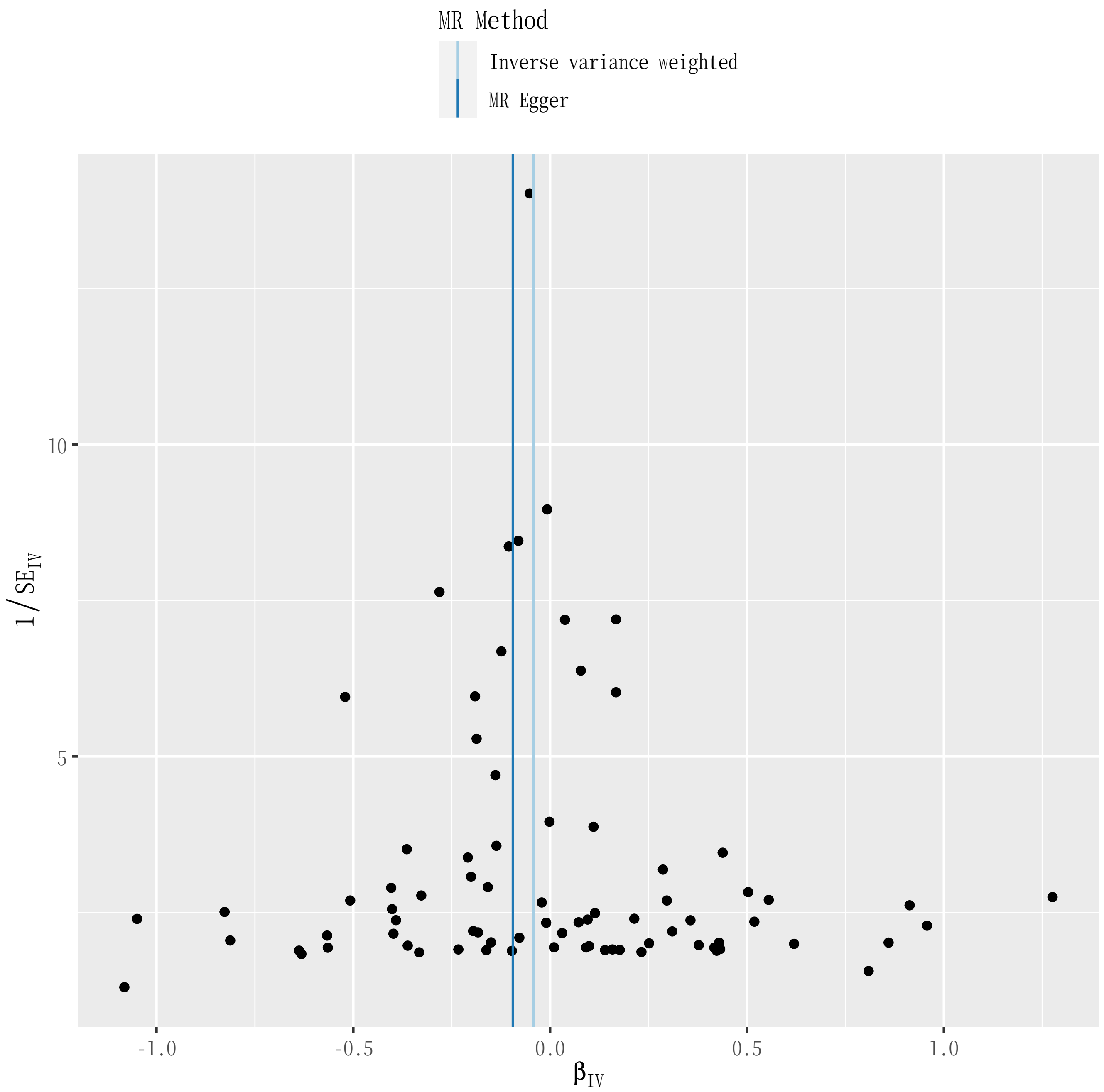

MR Method
Inverse variance weighted
MR Egger
1/SE_IV
10
5
-1.0
-0.5
0.0
0.5
1.0
β_IV

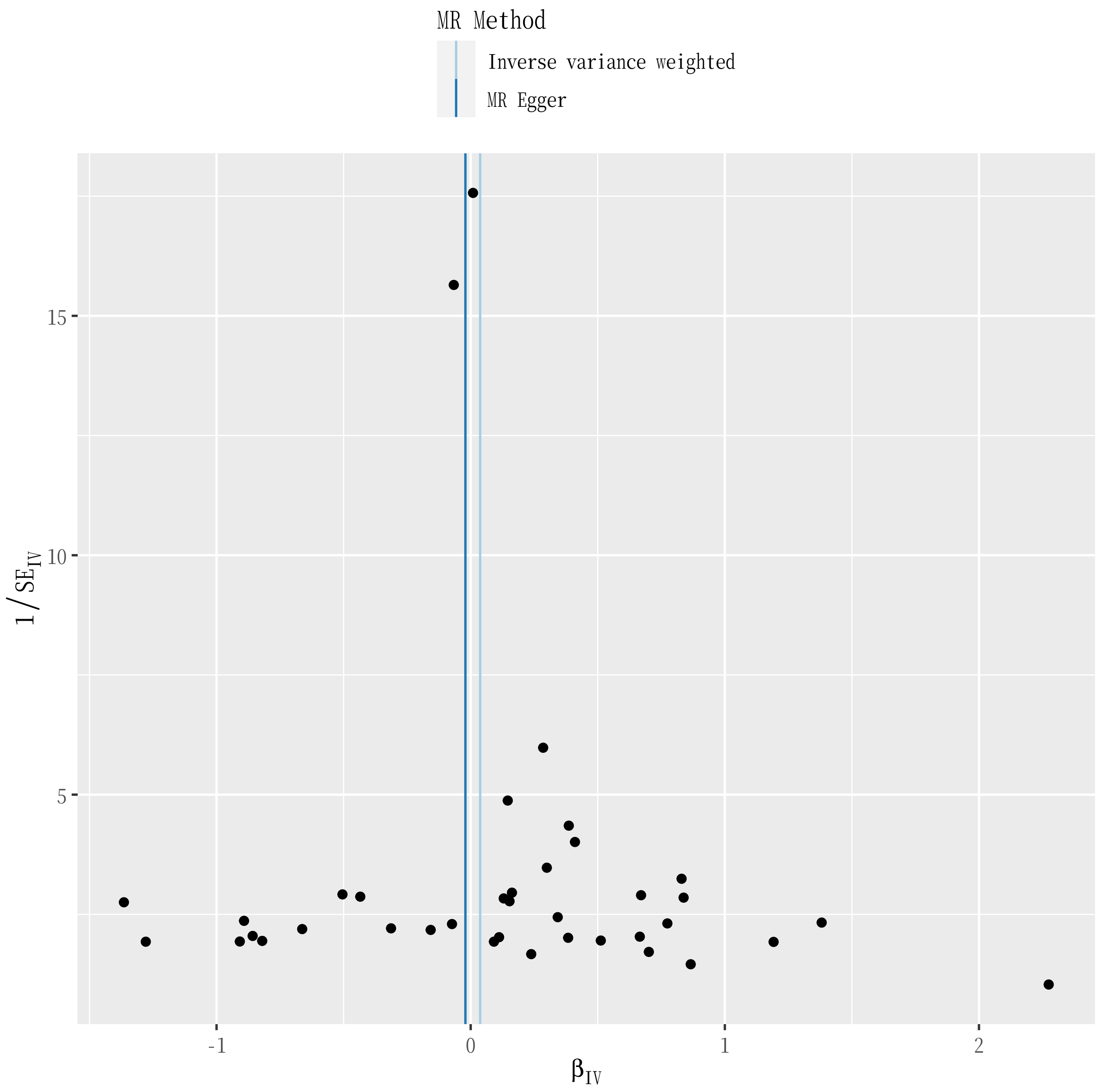

MR Method
Inverse variance weighted
MR Egger
15
10
5
$1/SE_{IV}$
-1
0
1
2
β_{IV}

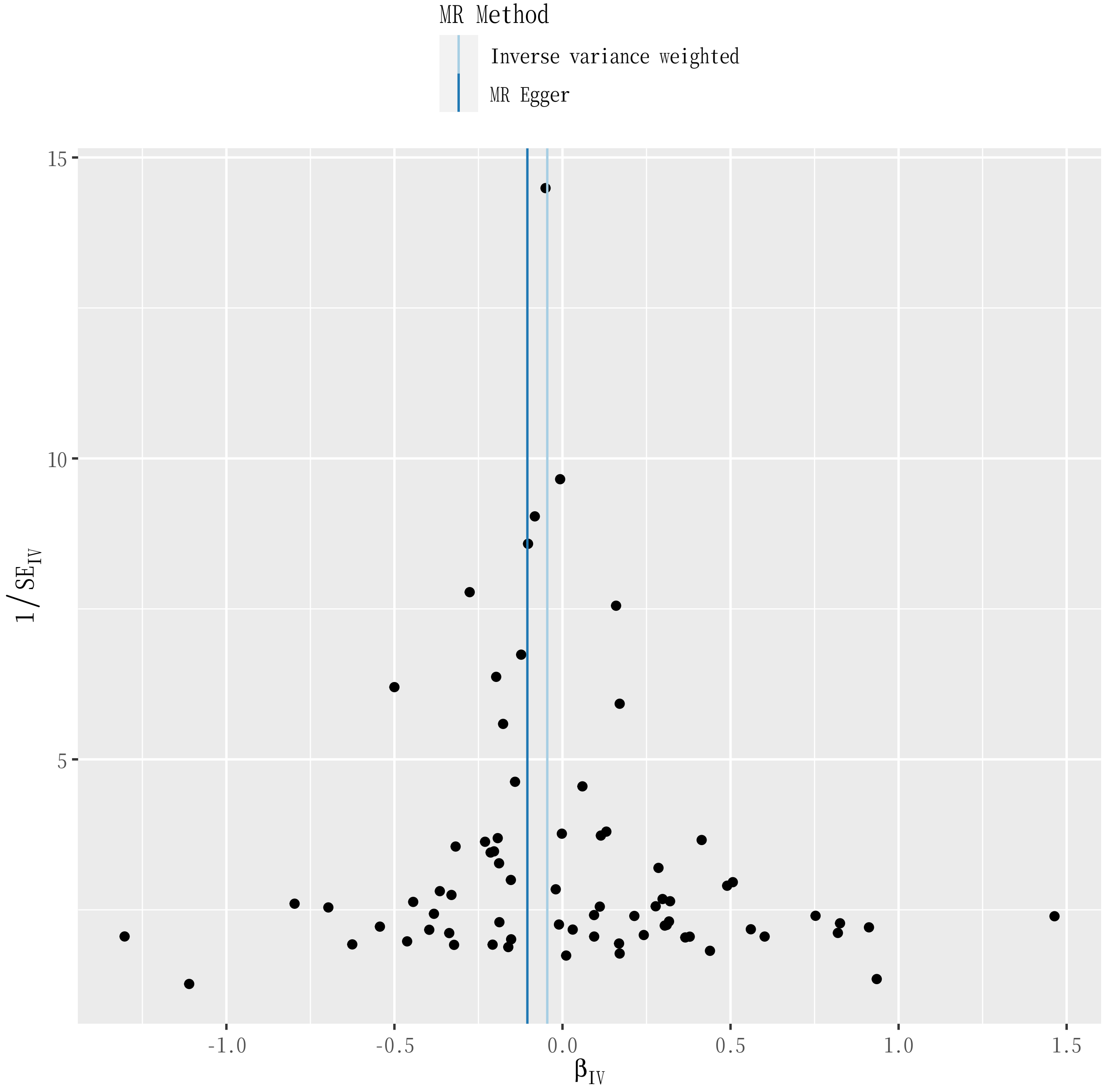
MR Method
Inverse variance weighted
MR Egger
15
10
5
-1.0
-0.5
0.0
0.5
1.0
1.5
β_{IV}
$1/SE_{IV}$

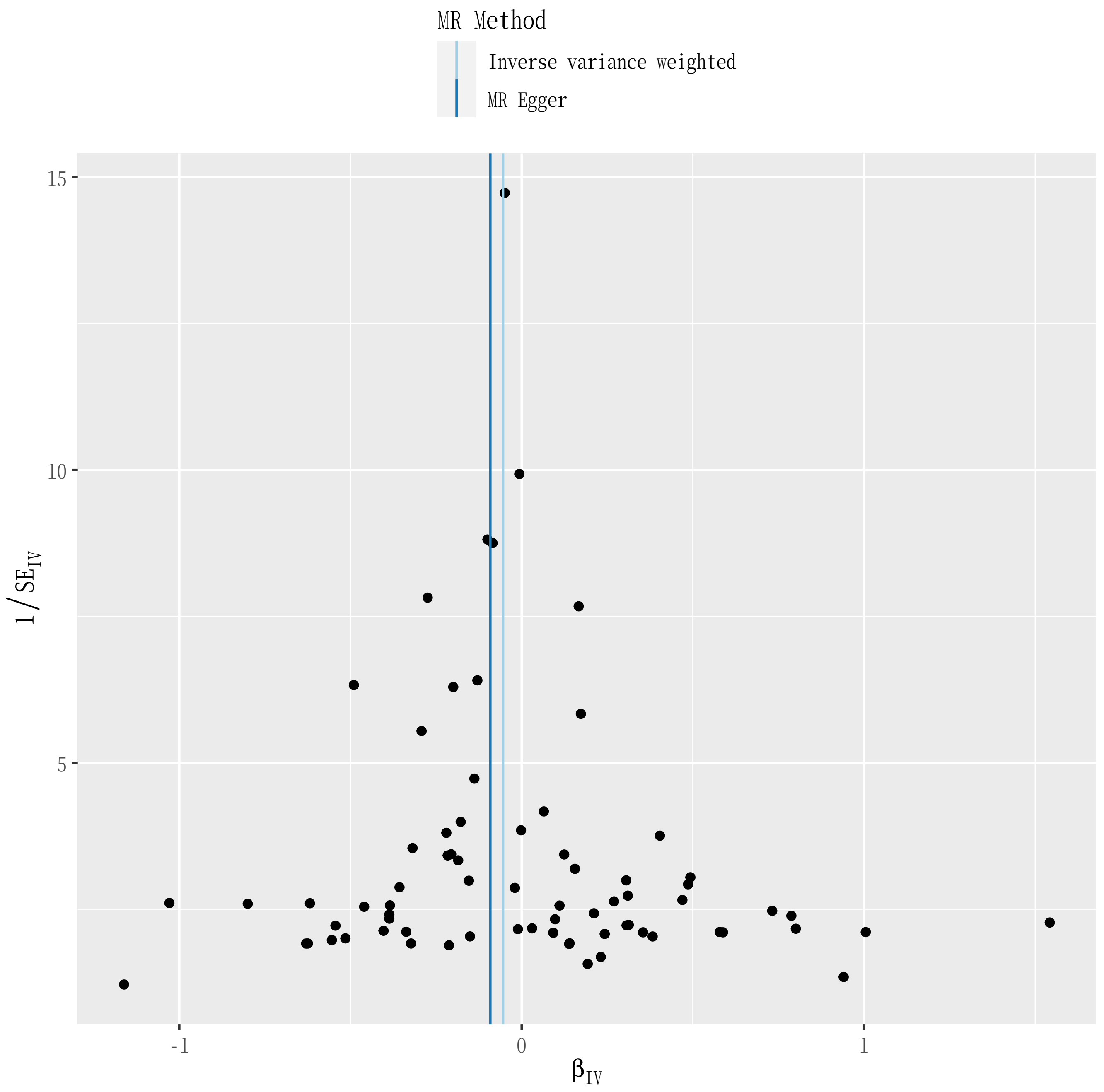
MR Method
Inverse variance weighted
MR Egger
15
10
5
1/SE$_{IV}$
-1
0
1
β$_{IV}$

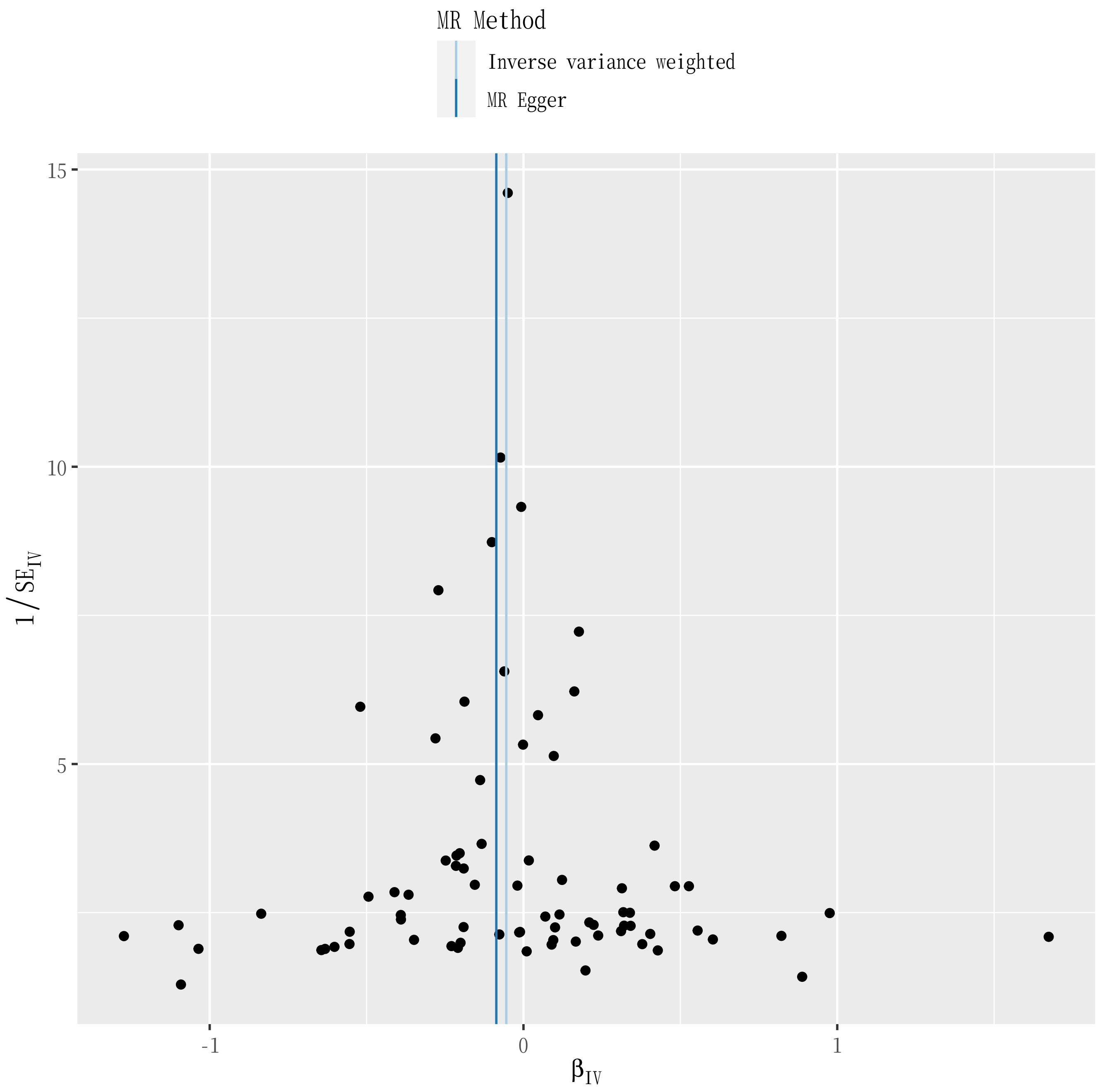

MR Method
Inverse variance weighted
MR Egger
15
10
5
$1/SE_{IV}$
-1
0
1
β_{IV}

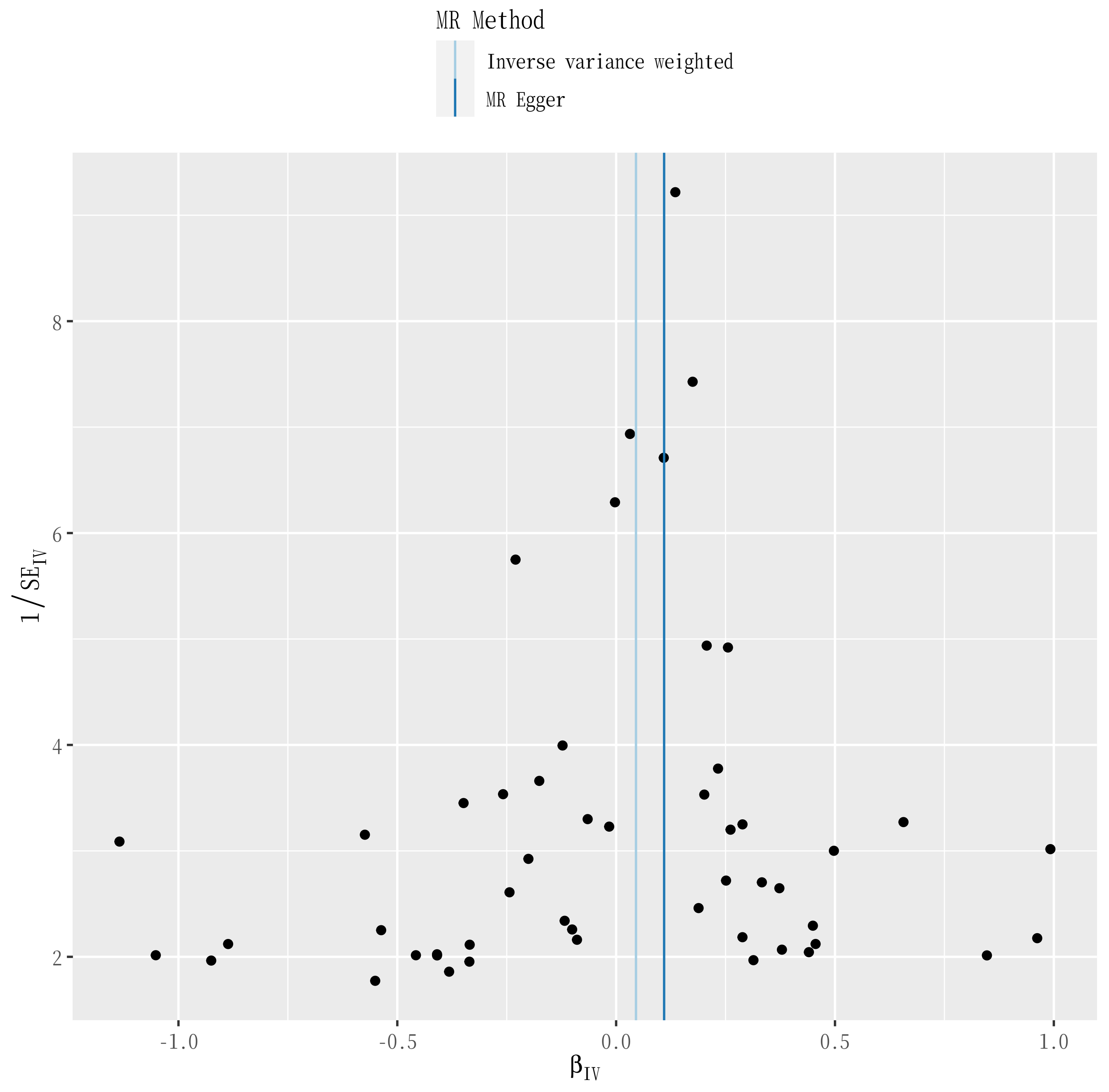
MR Method
Inverse variance weighted
MR Egger
8
6
4
2
-1.0
-0.5
0.0
0.5
1.0
$1/SE_{IV}$
β_{IV}

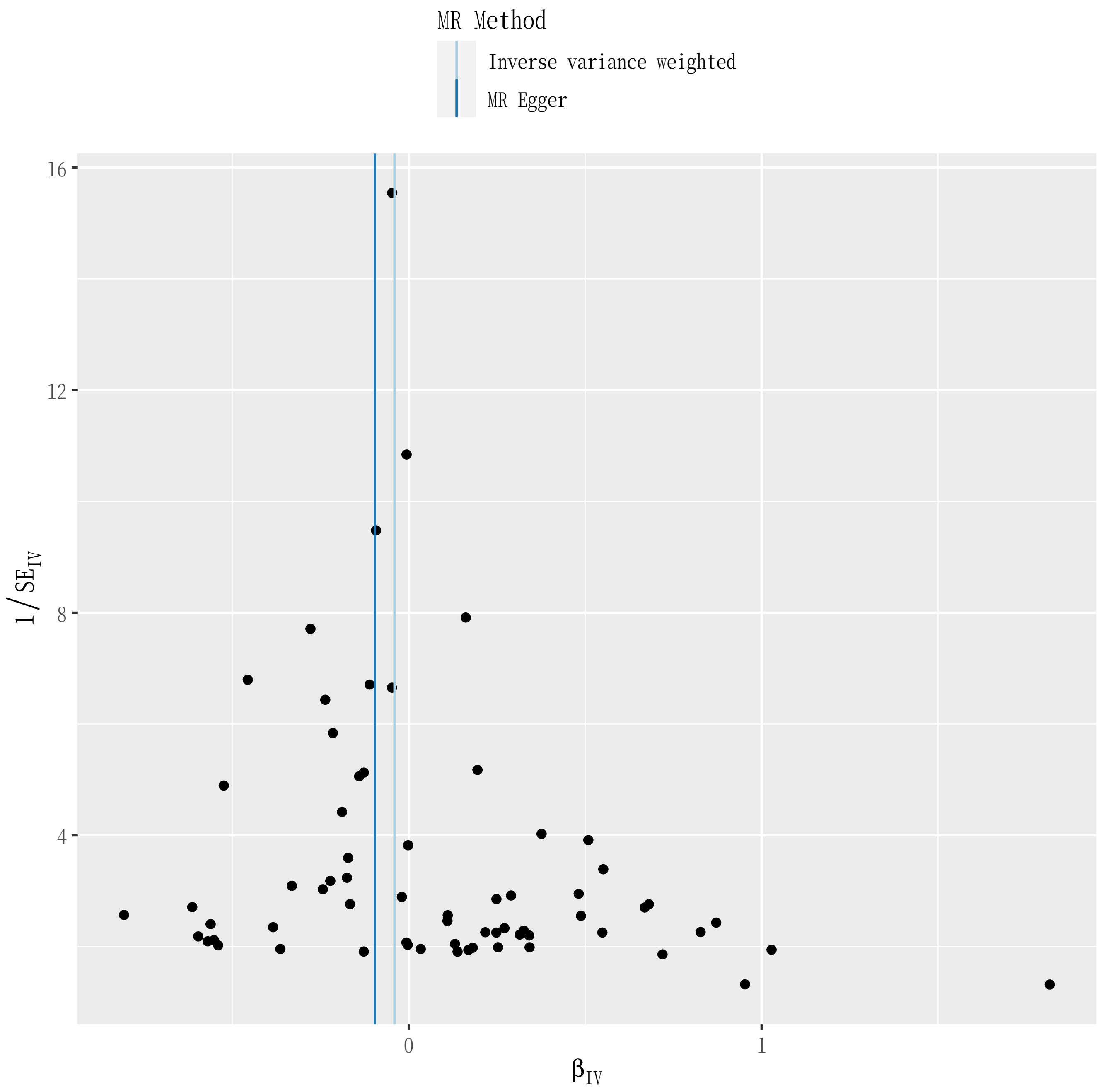

MR Method
Inverse variance weighted
MR Egger
16
12
8
4
$1/SE_{IV}$
0
1
β_{IV}

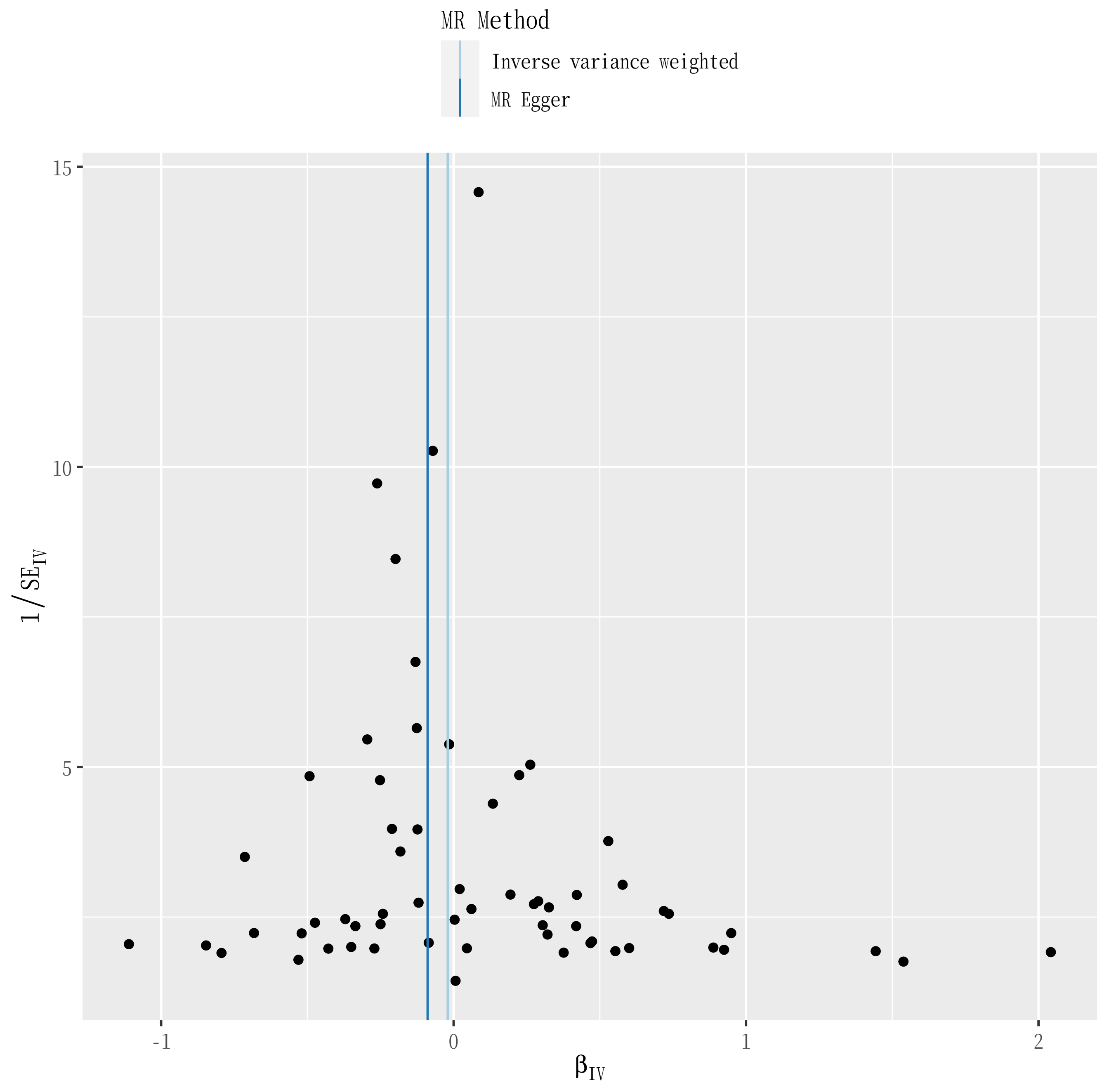

MR Method
Inverse variance weighted
MR Egger
15
10
5
$1/SE_{IV}$
-1
0
1
2
β_{IV}

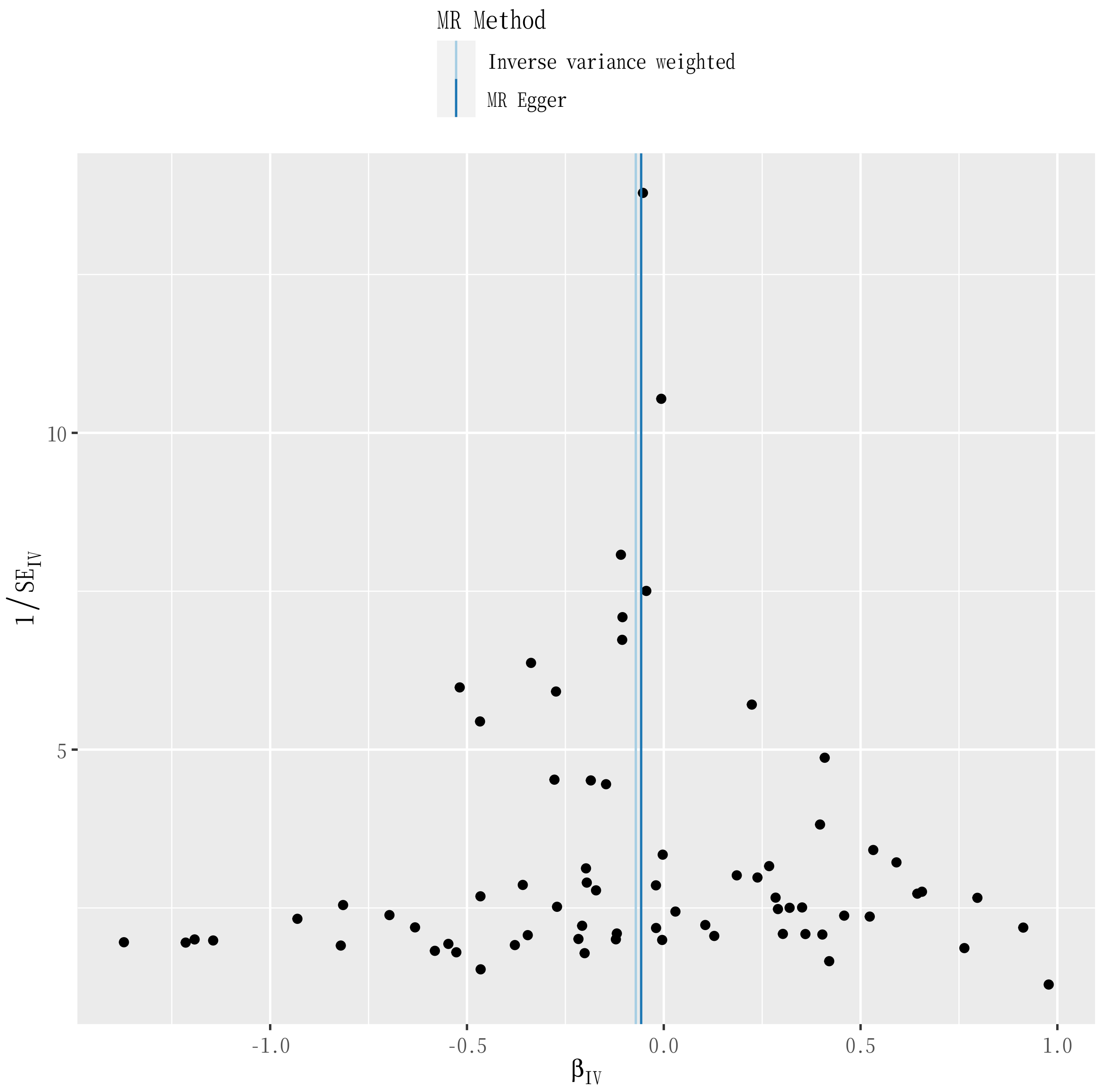

MR Method
Inverse variance weighted
MR Egger
10
5
-1.0
-0.5
0.0
0.5
1.0
β_{IV}
$1/SE_{IV}$

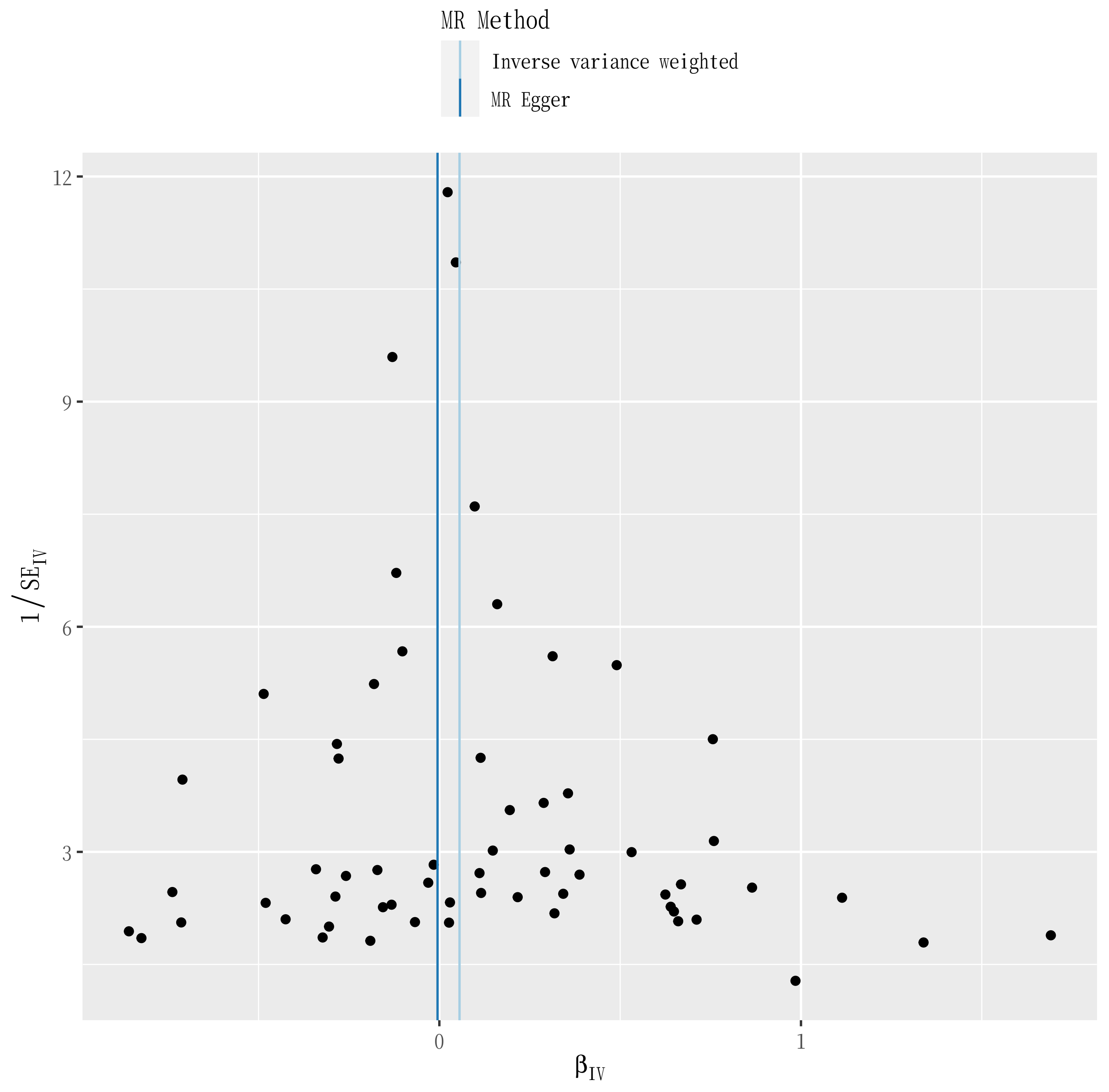
MR Method
Inverse variance weighted
MR Egger
12
9
6
3
0
1
$1/SE_{IV}$
β_{IV}

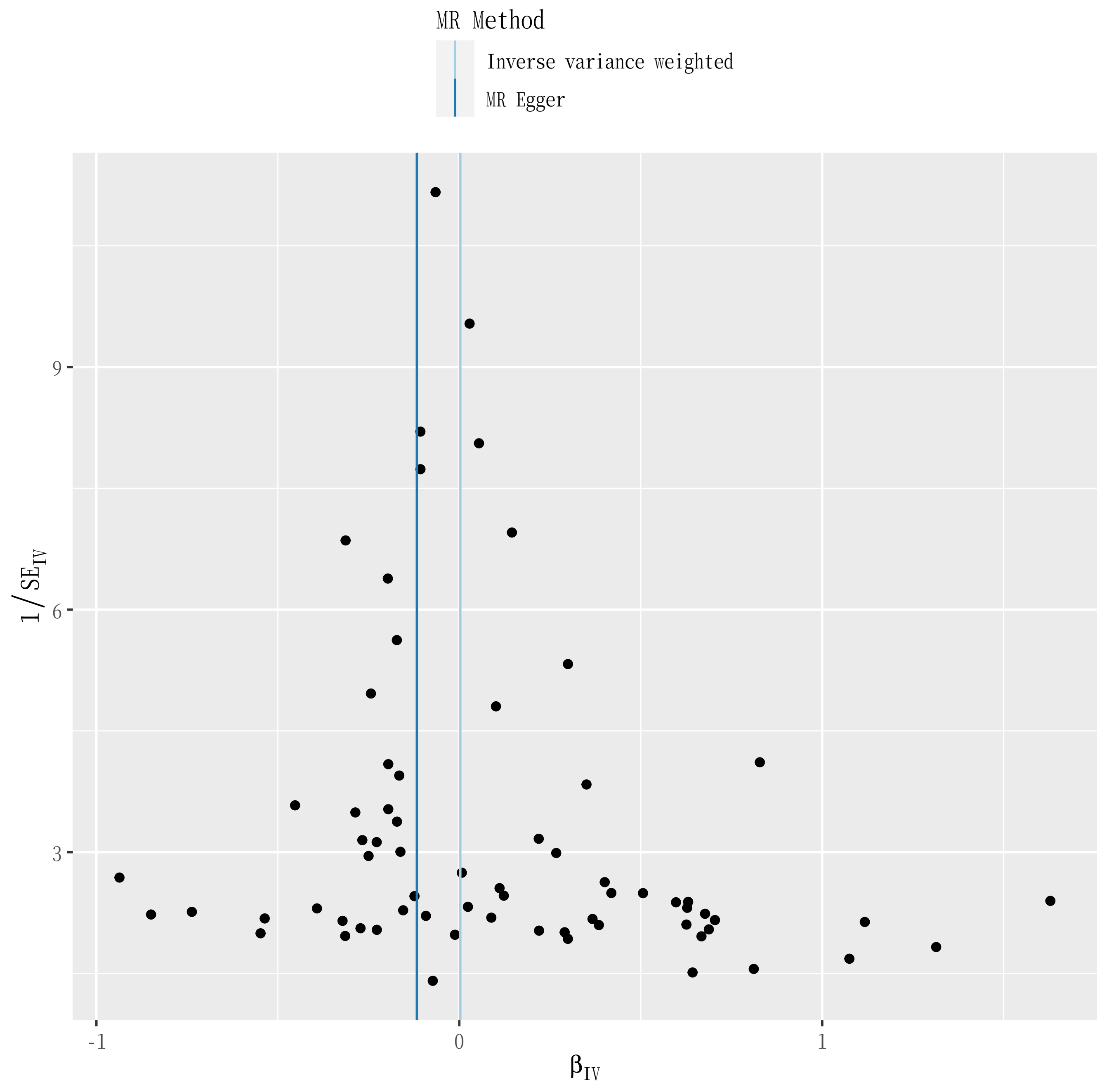

MR Method
Inverse variance weighted
MR Egger
9
6
3
$1/SE_{IV}$
-1
0
1
β_{IV}

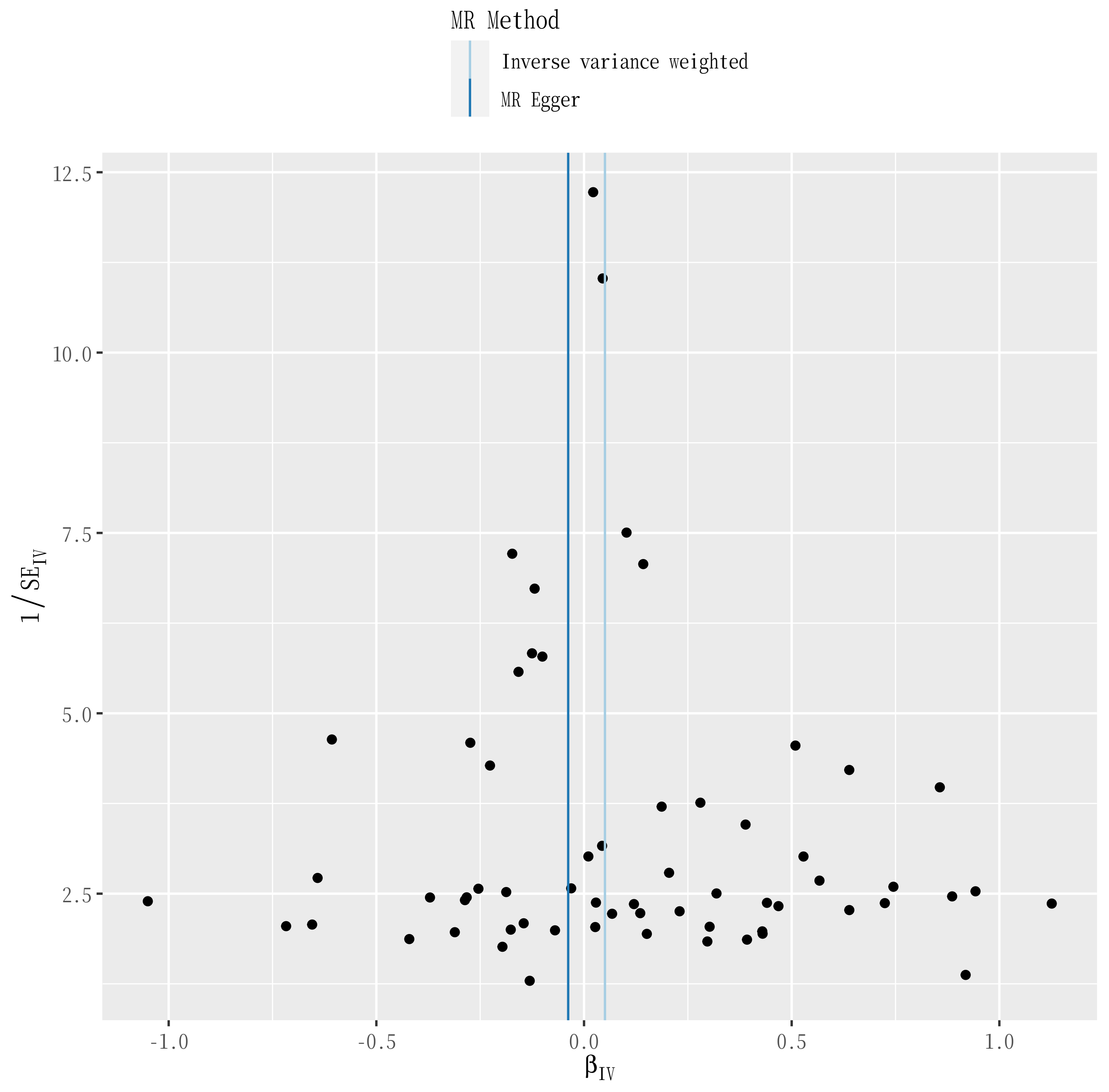

MR Method
Inverse variance weighted
MR Egger
12.5
10.0
7.5
5.0
2.5
1/SE_IV
-1.0
-0.5
0.0
0.5
1.0
β_IV

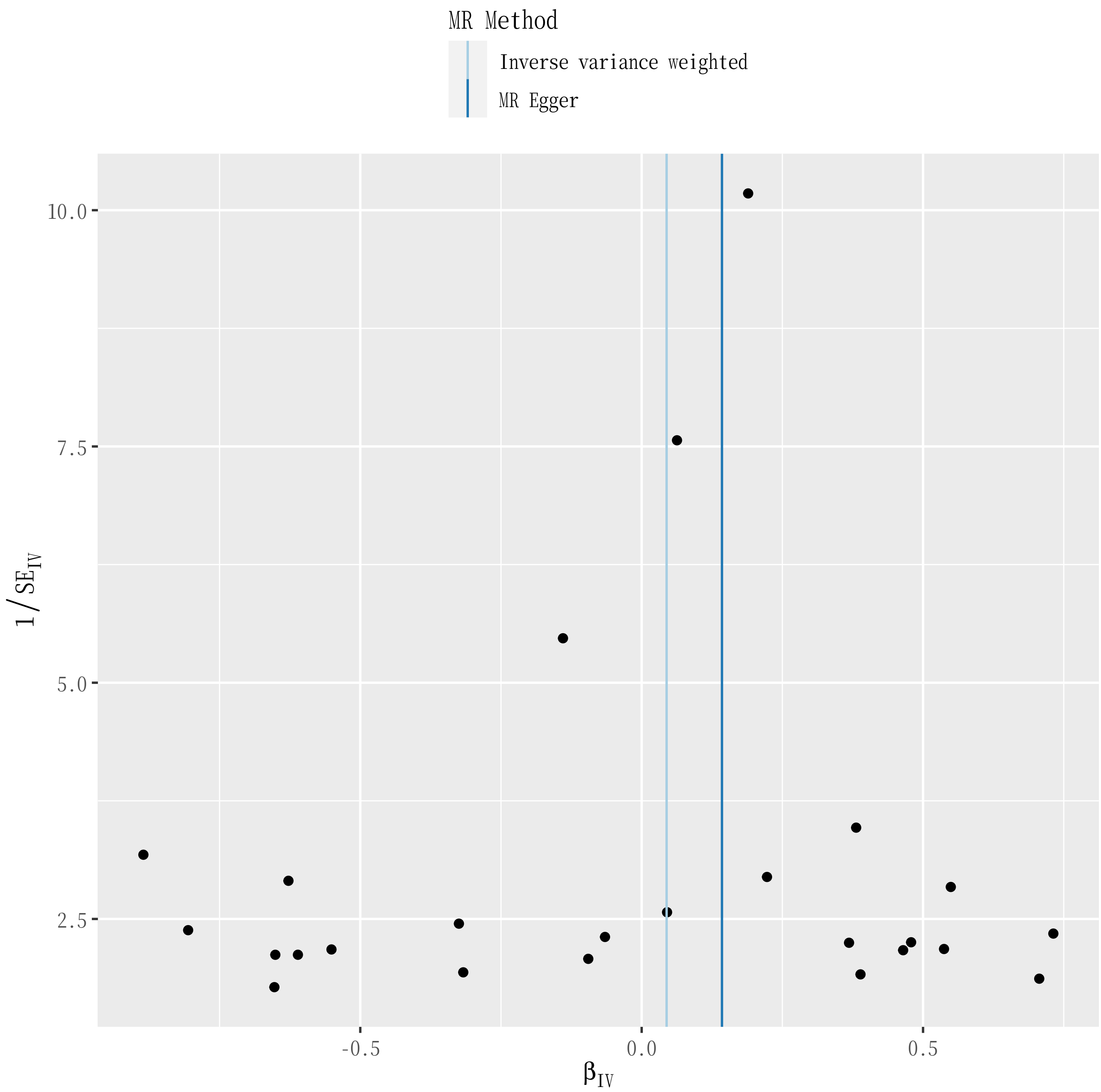

MR Method
Inverse variance weighted
MR Egger
10.0
7.5
5.0
2.5
1/SE_IV
-0.5
0.0
0.5
β_IV

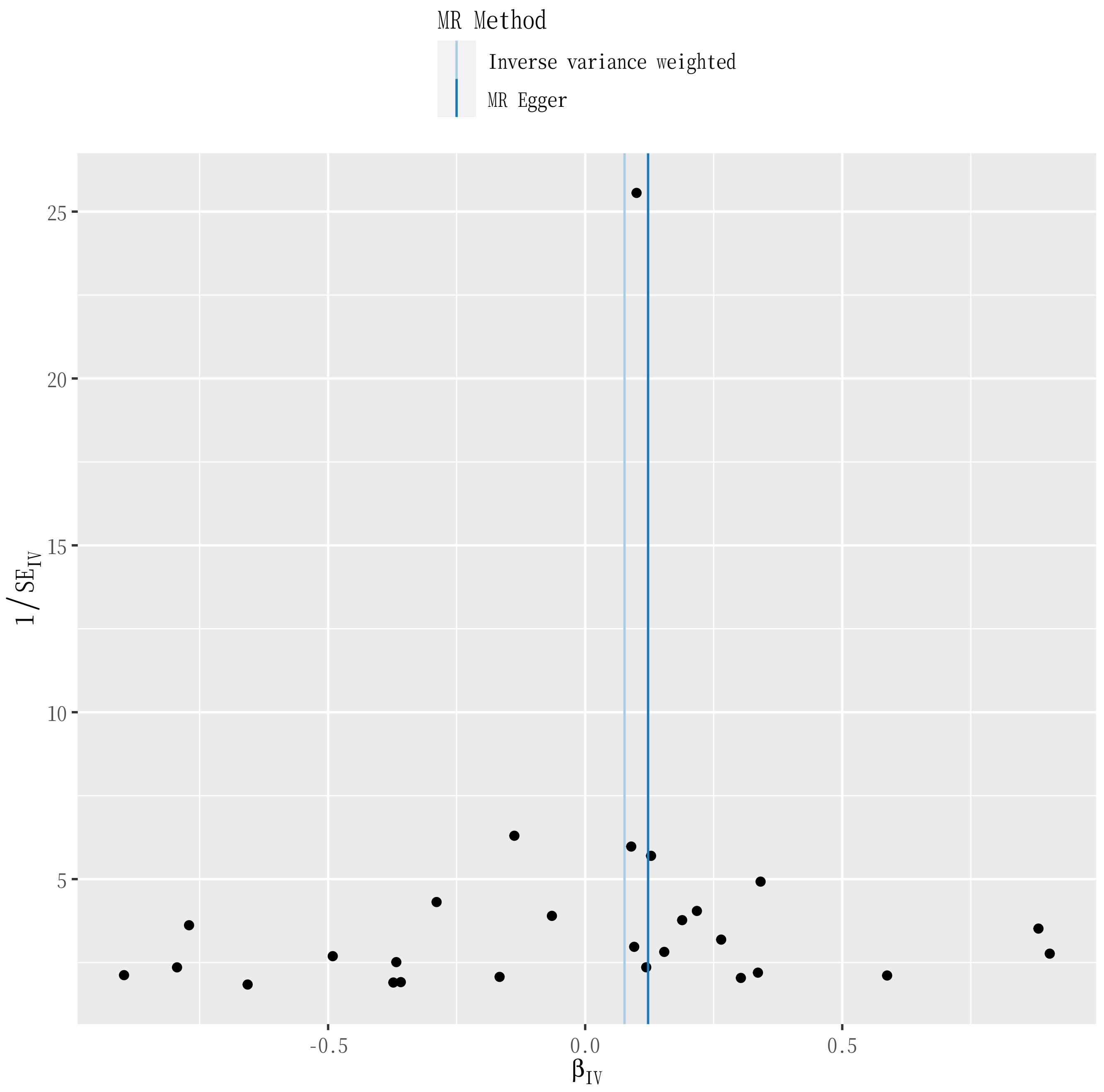

MR Method
Inverse variance weighted
MR Egger
25
20
15
10
5
1/SE_IV
-0.5
0.0
0.5
β_IV

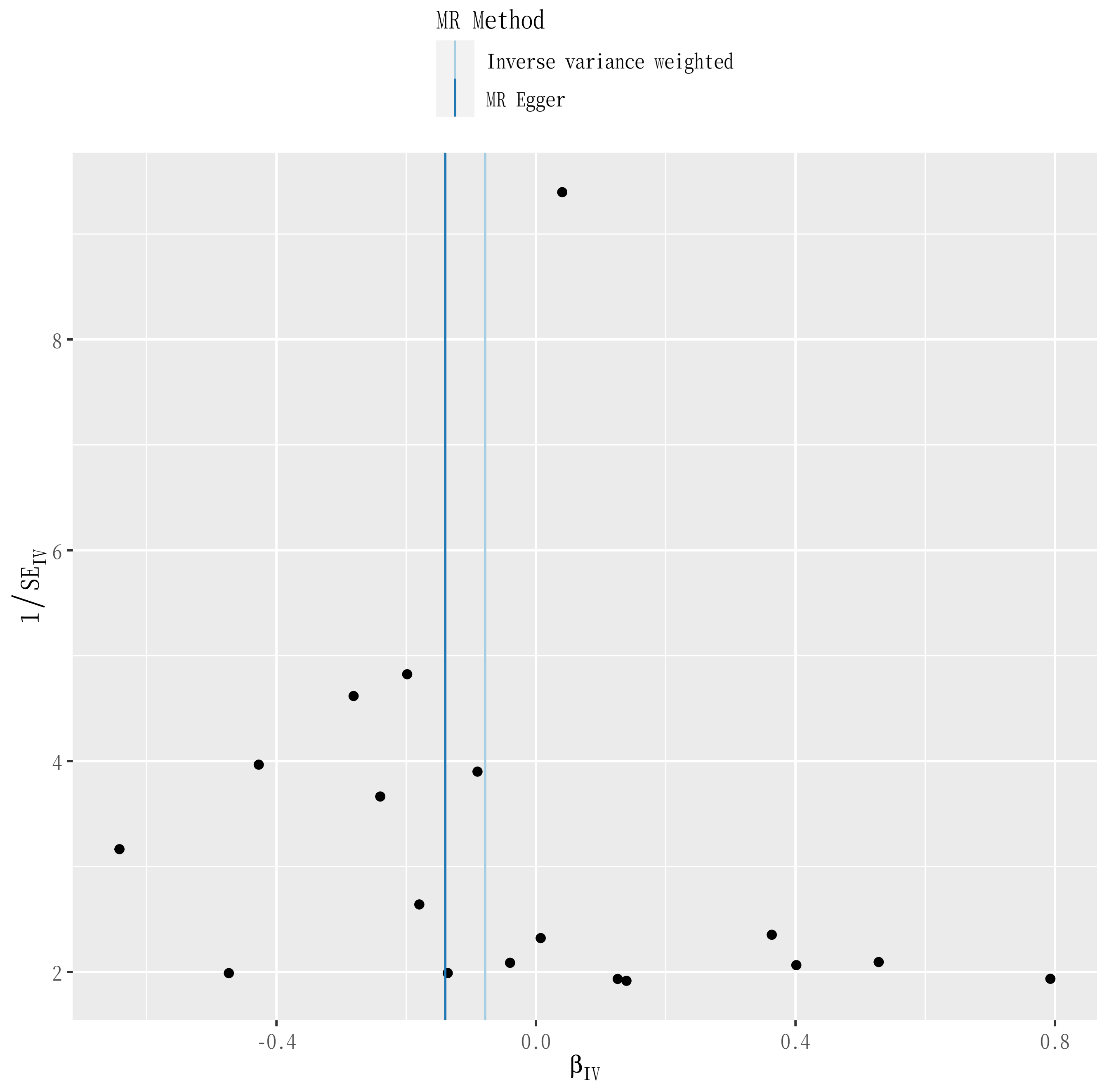
MR Method
Inverse variance weighted
MR Egger
1/SE_IV
8
6
4
2
-0.4
0.0
0.4
0.8
β_IV

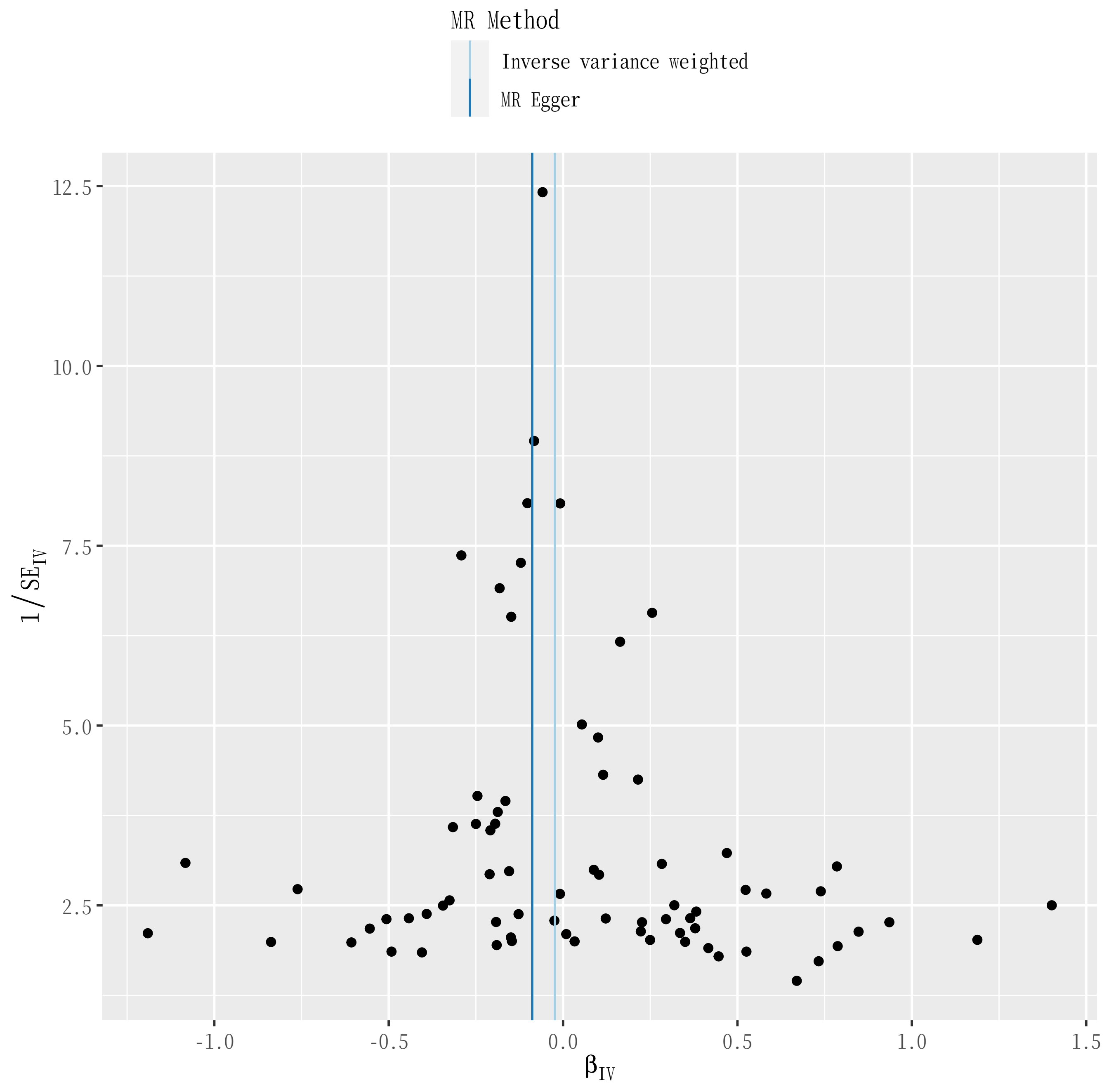
MR Method
Inverse variance weighted
MR Egger
12.5
10.0
7.5
5.0
2.5
1/SE_IV
-1.0
-0.5
0.0
0.5
1.0
1.5
β_IV

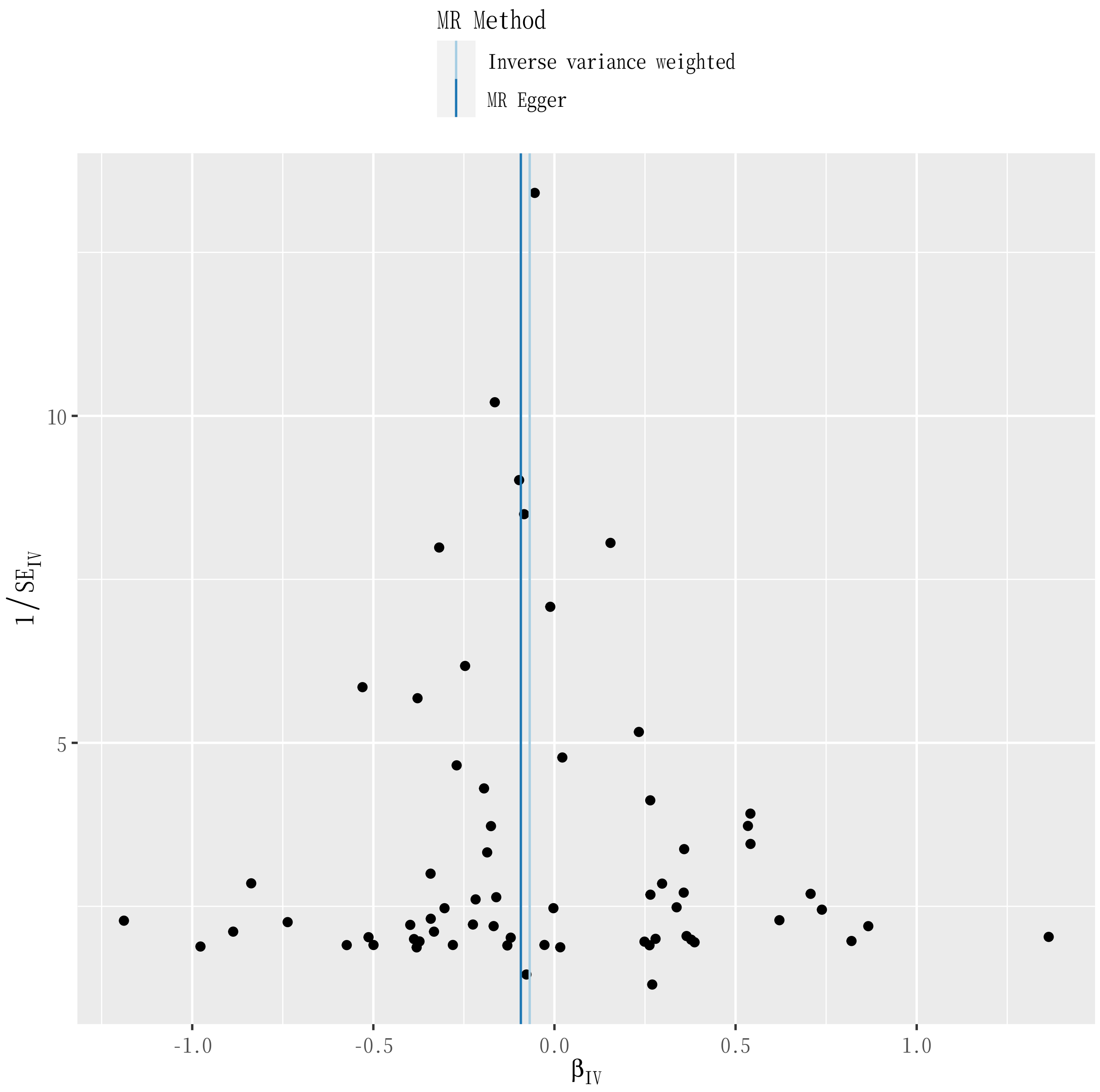
MR Method
Inverse variance weighted
MR Egger
10
5
$1/SE_{IV}$
-1.0
-0.5
0.0
0.5
1.0
β_{IV}

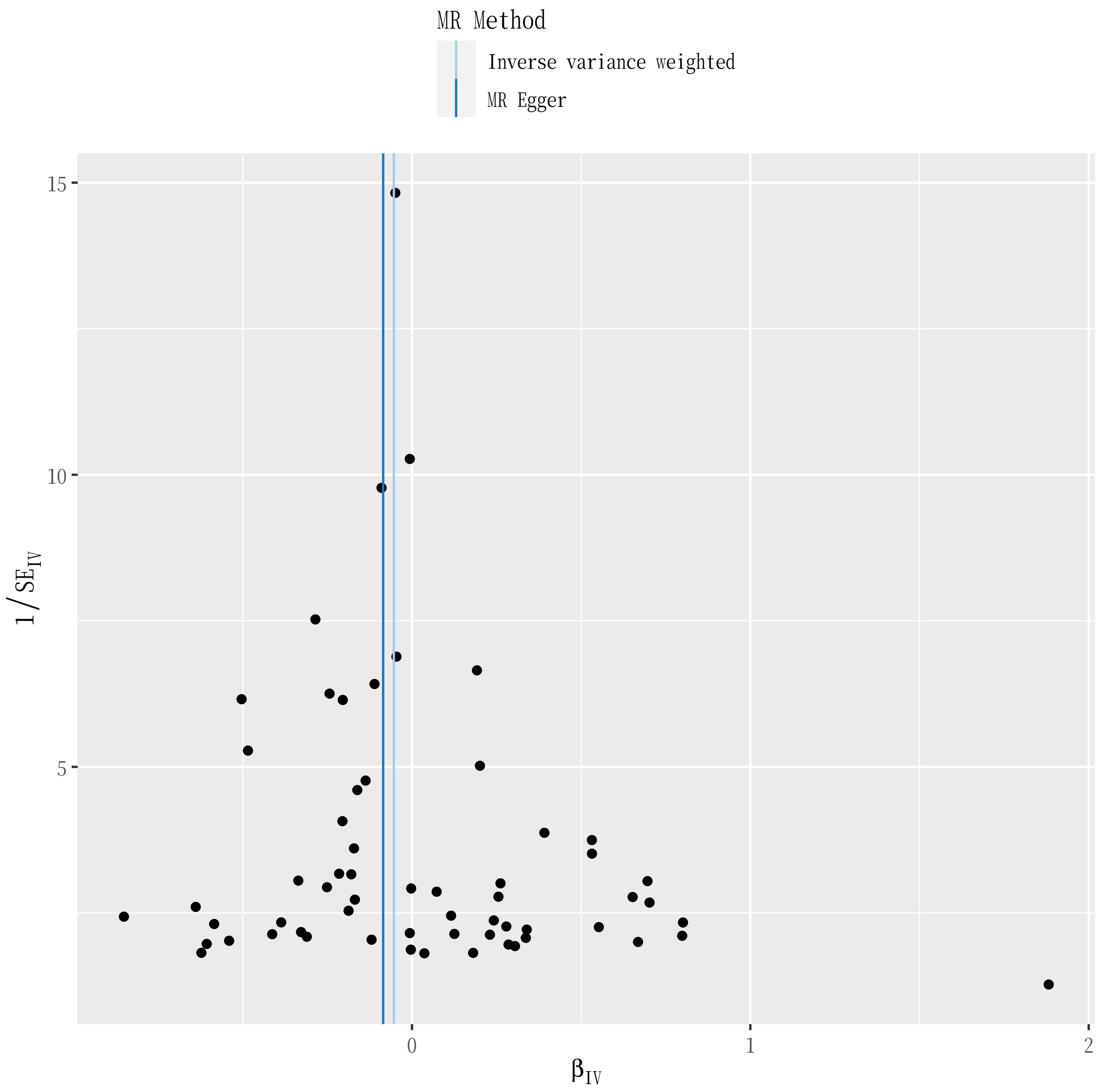
MR Method
Inverse variance weighted
MR Egger
15
10
5
0
1
2
$1/SE_{IV}$
β_{IV}

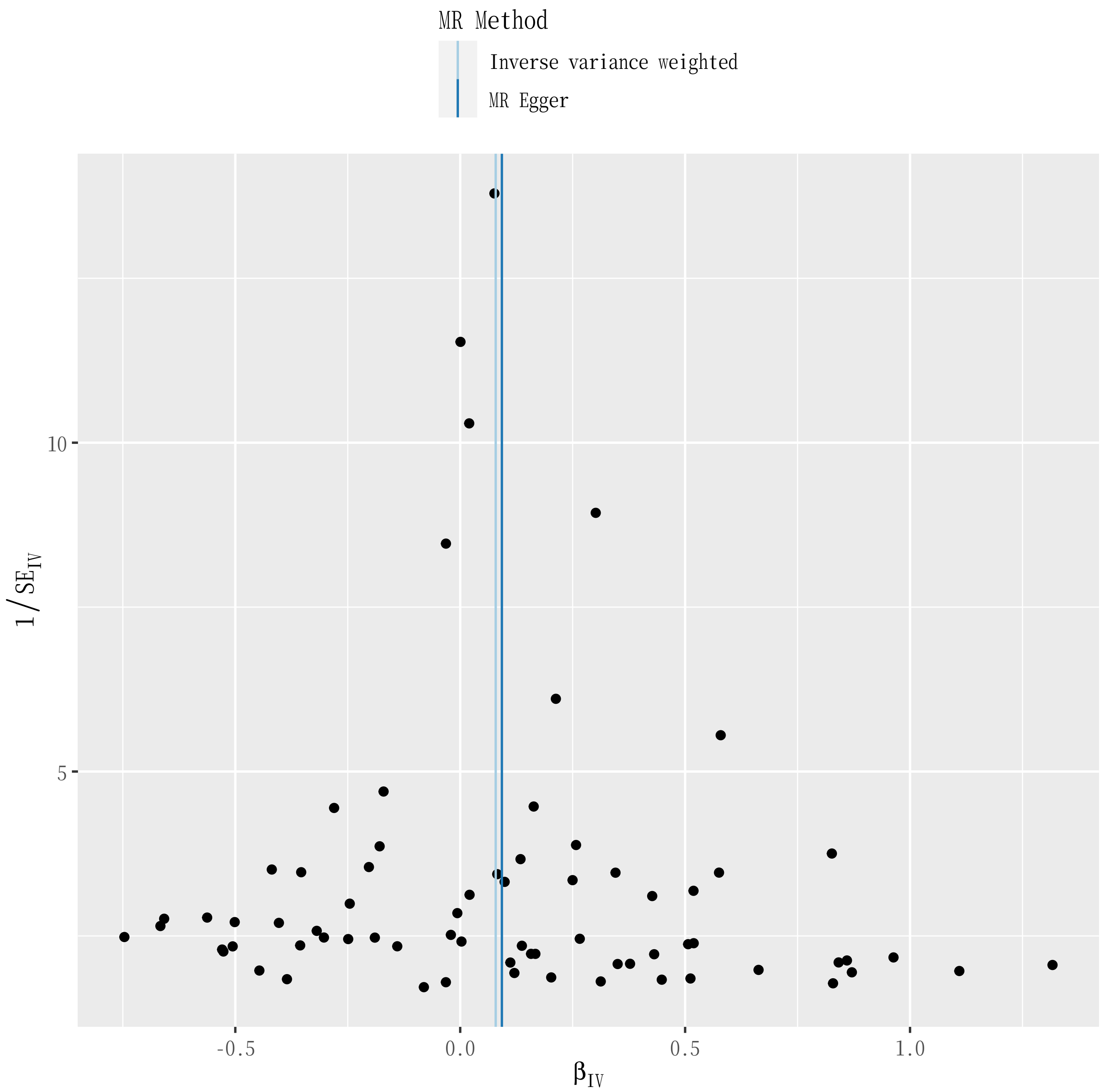
MR Method
Inverse variance weighted
MR Egger
10
5
-0.5
0.0
0.5
1.0
$1/SE_{IV}$
β_{IV}

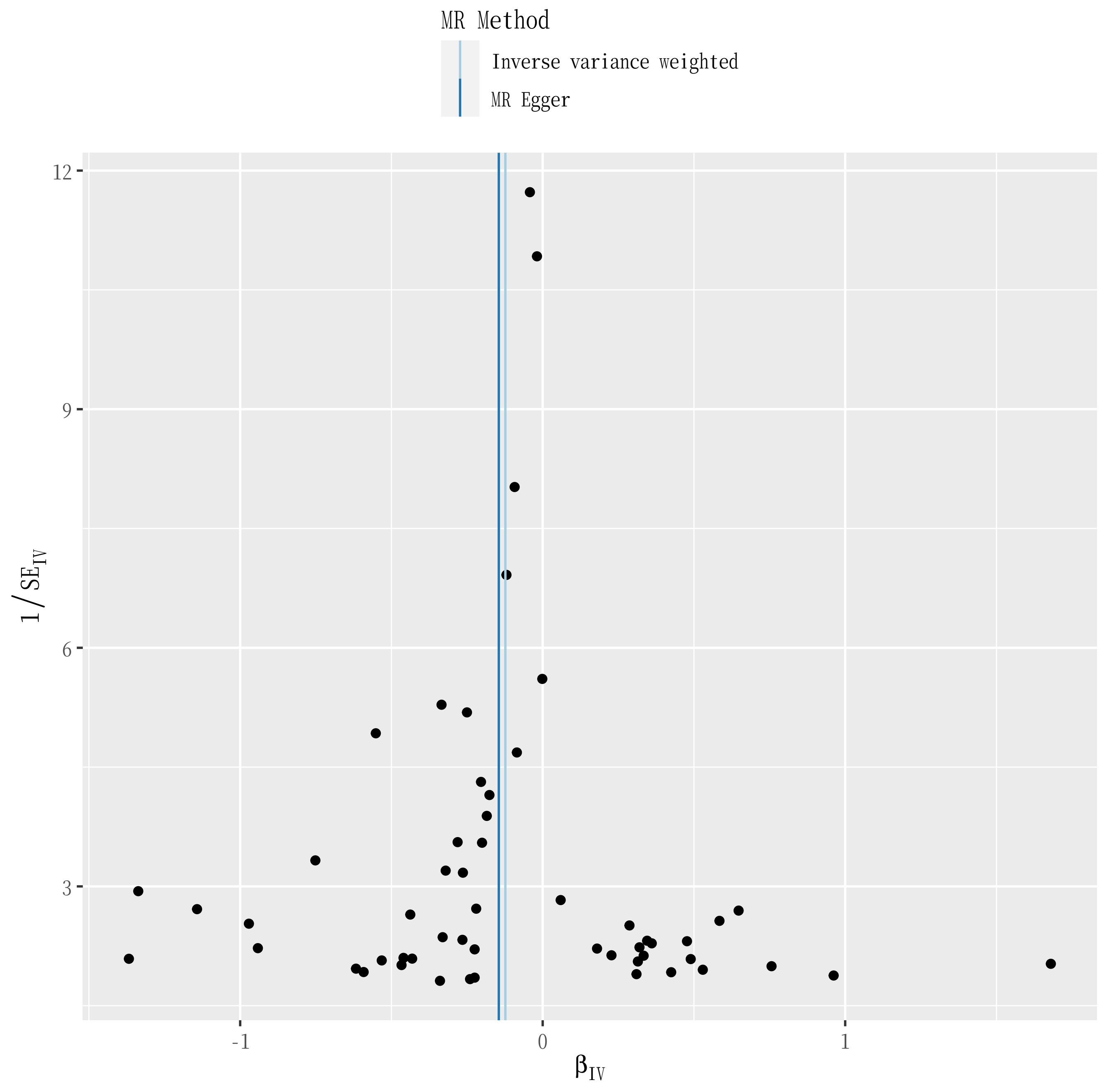
MR Method
Inverse variance weighted
MR Egger
12
9
6
3
1/SE$_{IV}$
-1
0
1
β$_{IV}$

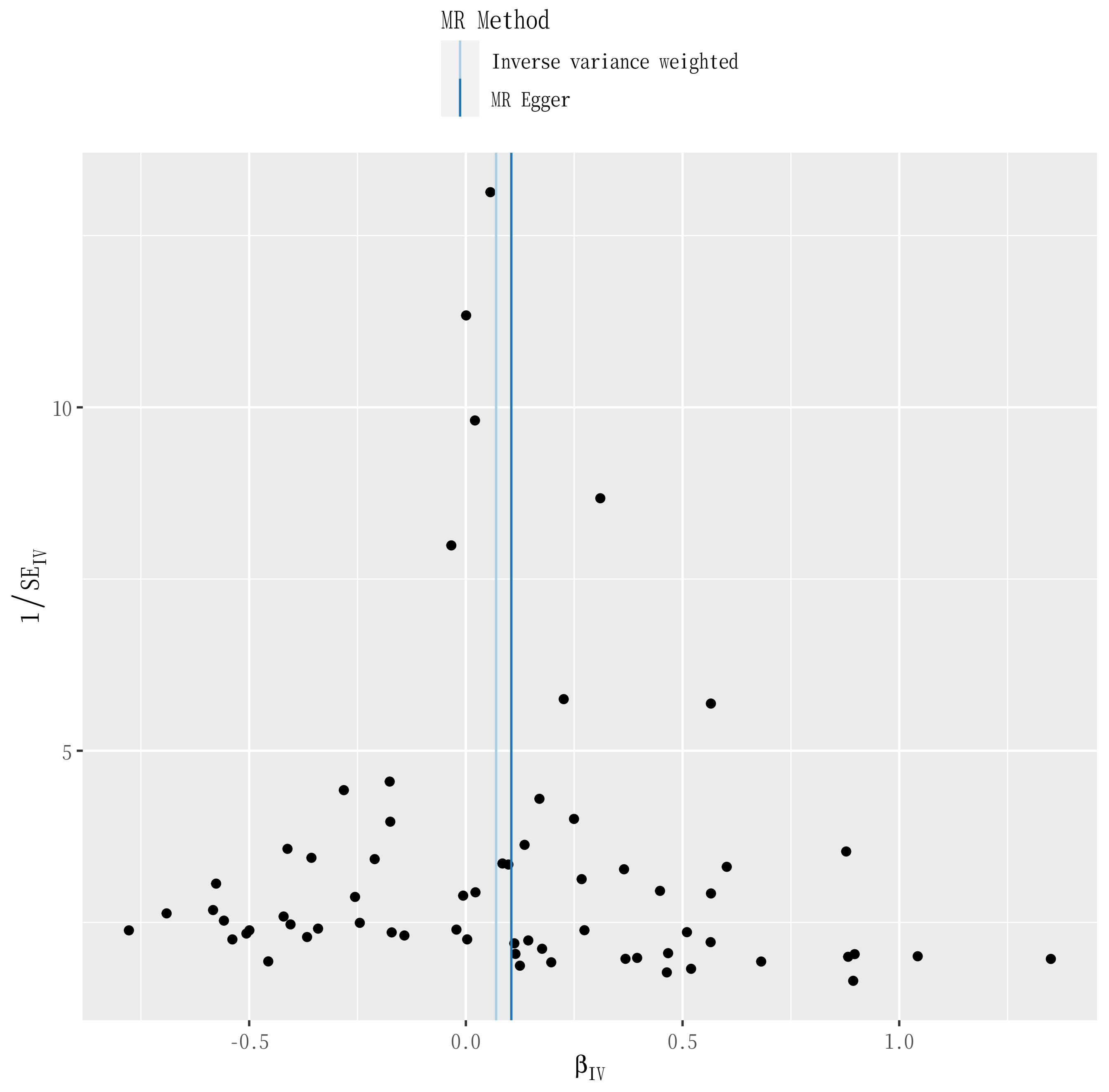

MR Method
Inverse variance weighted
MR Egger
10
5
-0.5
0.0
0.5
1.0
β_{IV}
$1/SE_{IV}$

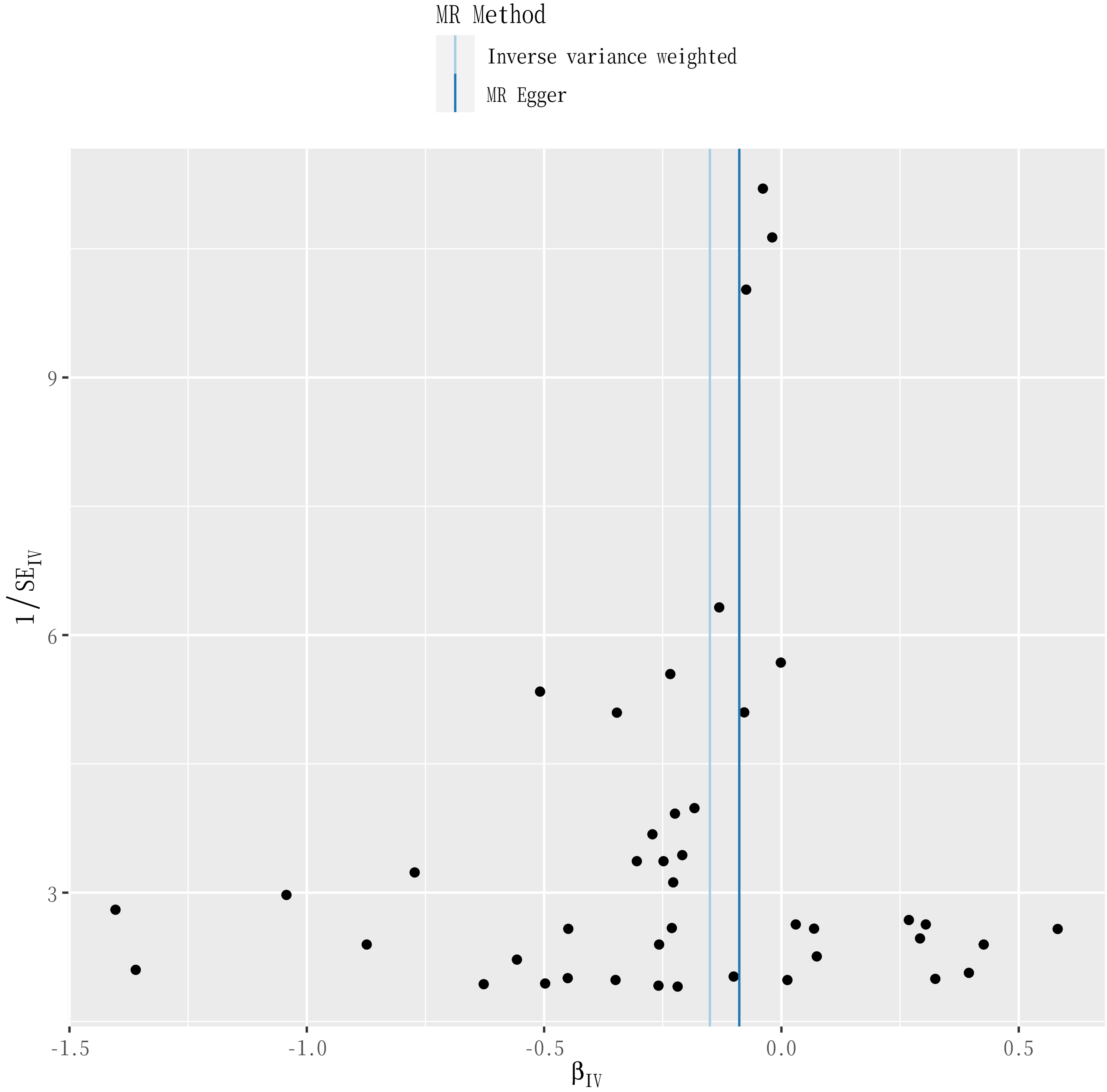
MR Method
Inverse variance weighted
MR Egger
9
6
3
$1/SE_{IV}$
-1.5
-1.0
-0.5
0.0
0.5
β_{IV}

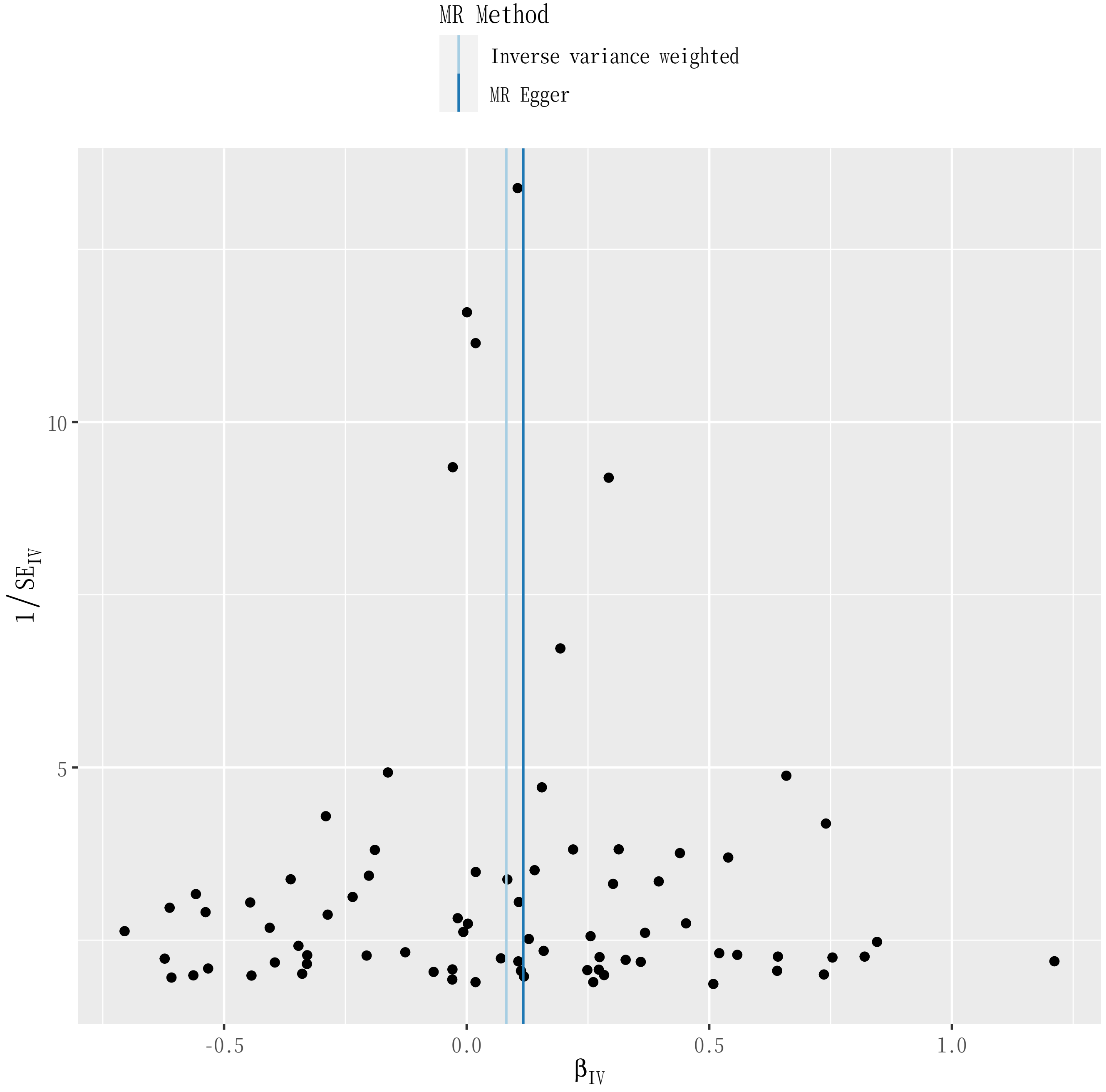
MR Method
Inverse variance weighted
MR Egger
10
5
$1/SE_{IV}$
-0.5
0.0
0.5
1.0
β_{IV}

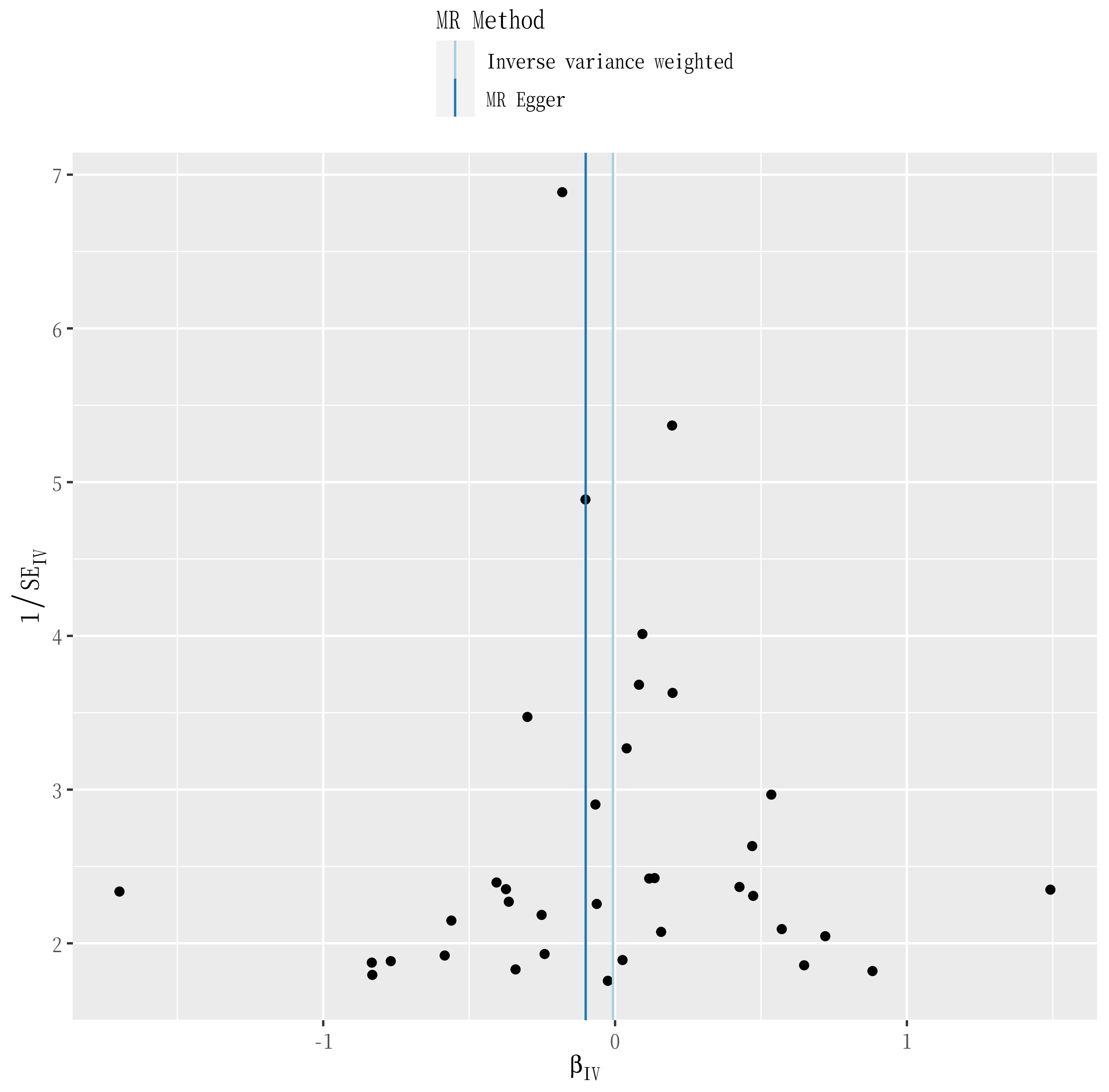

MR Method
Inverse variance weighted
MR Egger
7
6
5
4
3
2
$1/SE_{IV}$
-1
0
1
β_{IV}

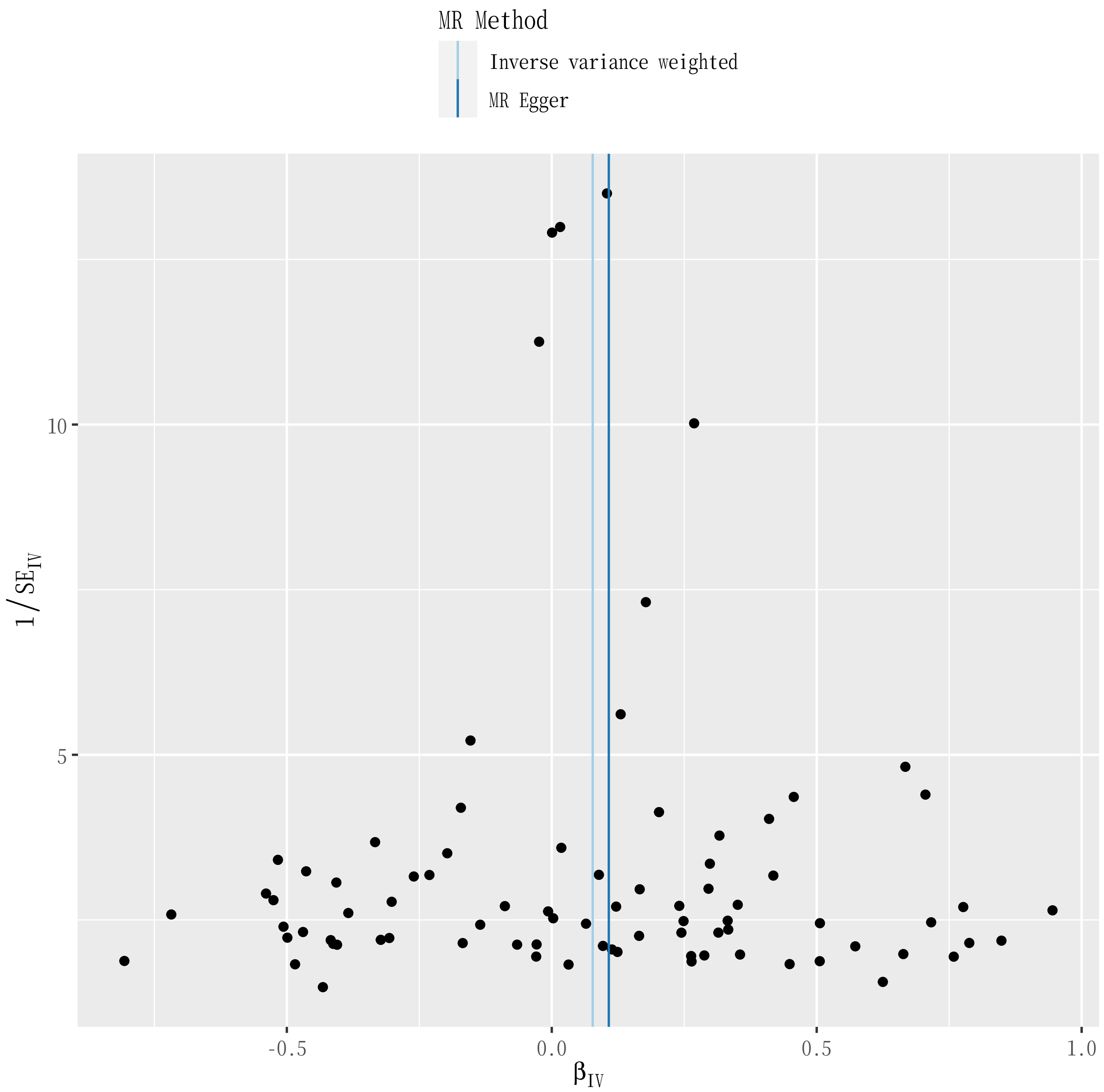

MR Method
Inverse variance weighted
MR Egger
10
5
-0.5
0.0
0.5
1.0
$1/SE_{IV}$
β_{IV}

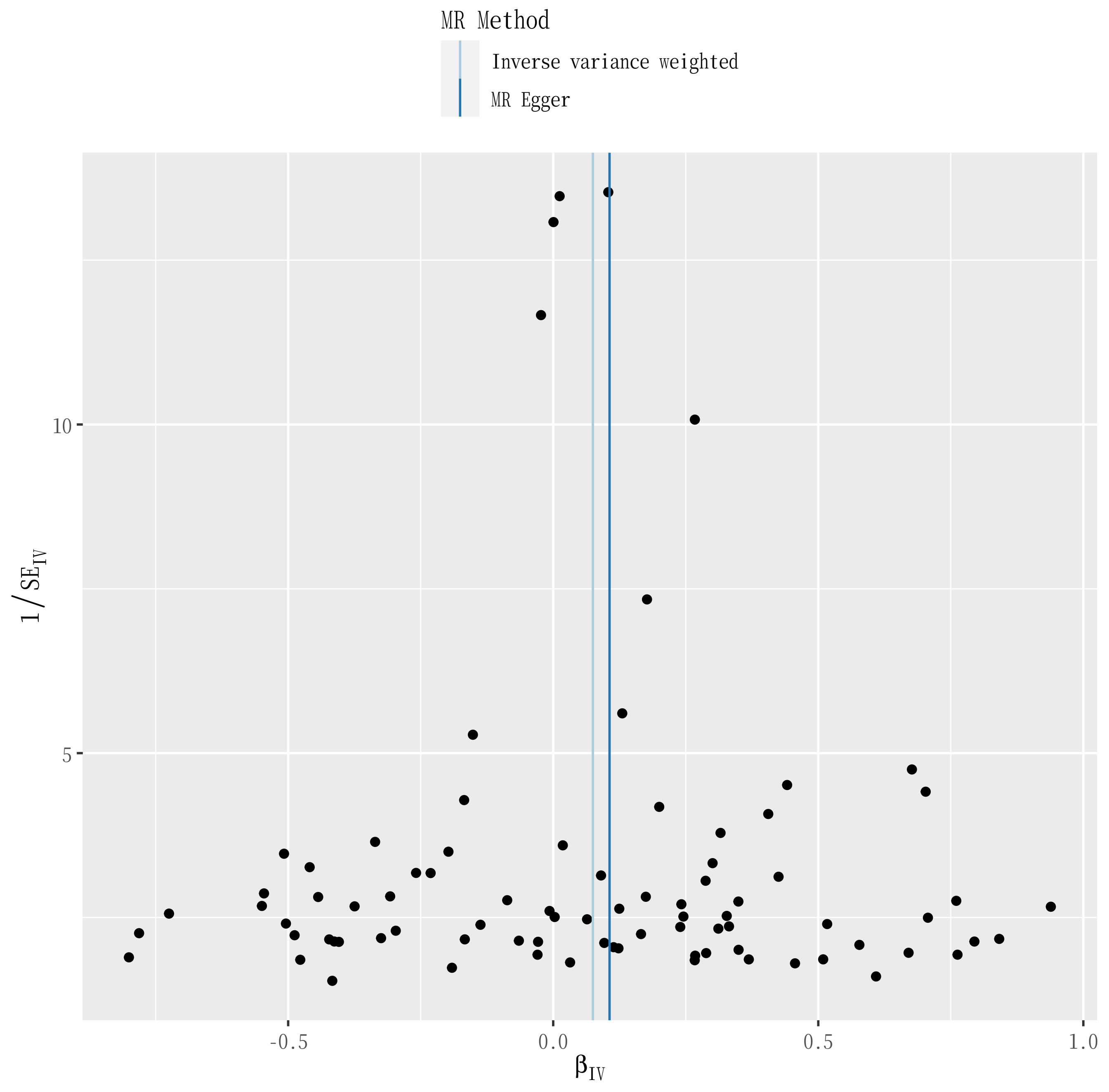

MR Method
Inverse variance weighted
MR Egger
10
5
$1/SE_{IV}$
-0.5
0.0
0.5
1.0
β_{IV}

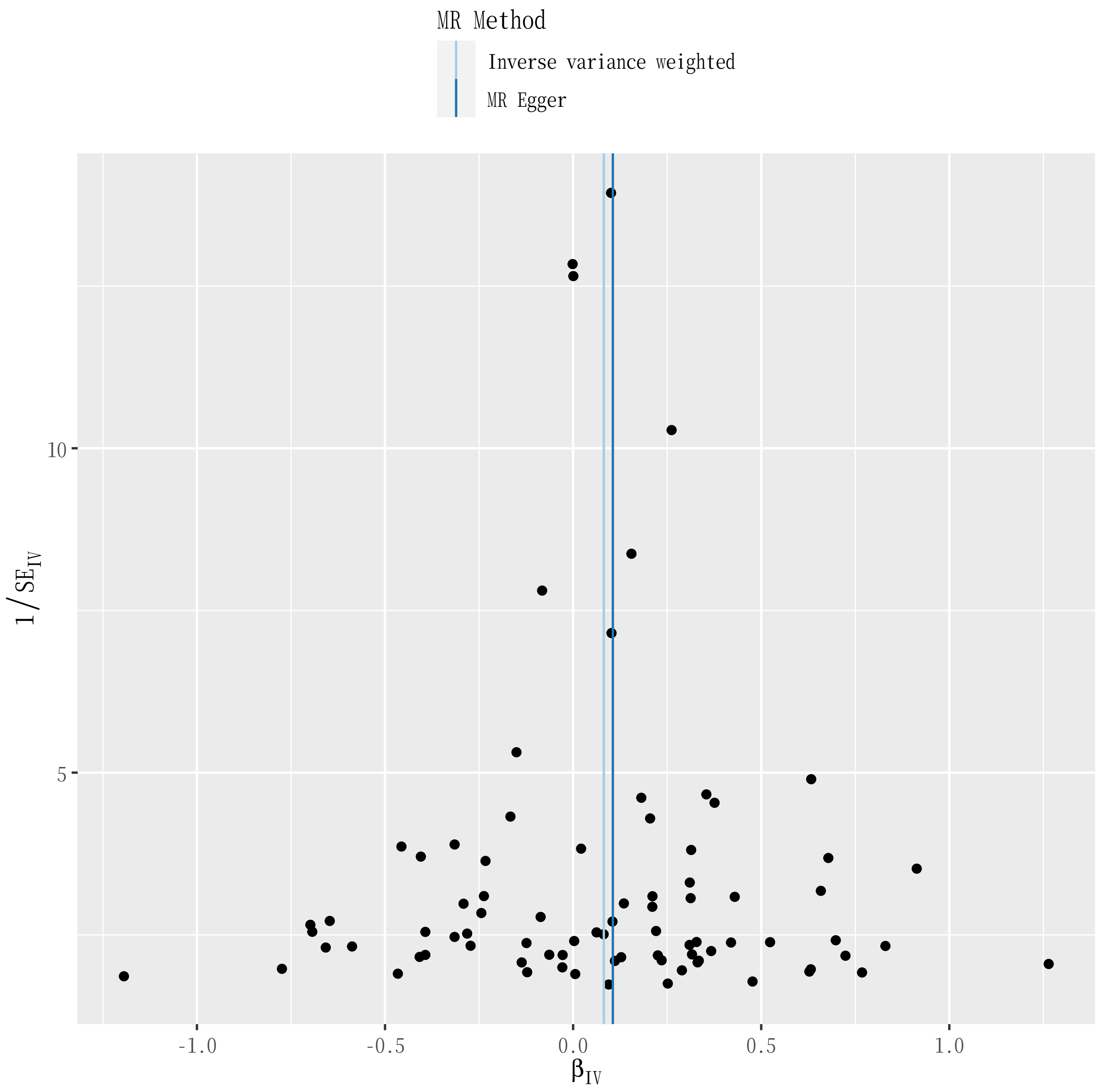
MR Method
Inverse variance weighted
MR Egger
10
5
-1.0
-0.5
0.0
0.5
1.0
$1/SE_{IV}$
β_{IV}

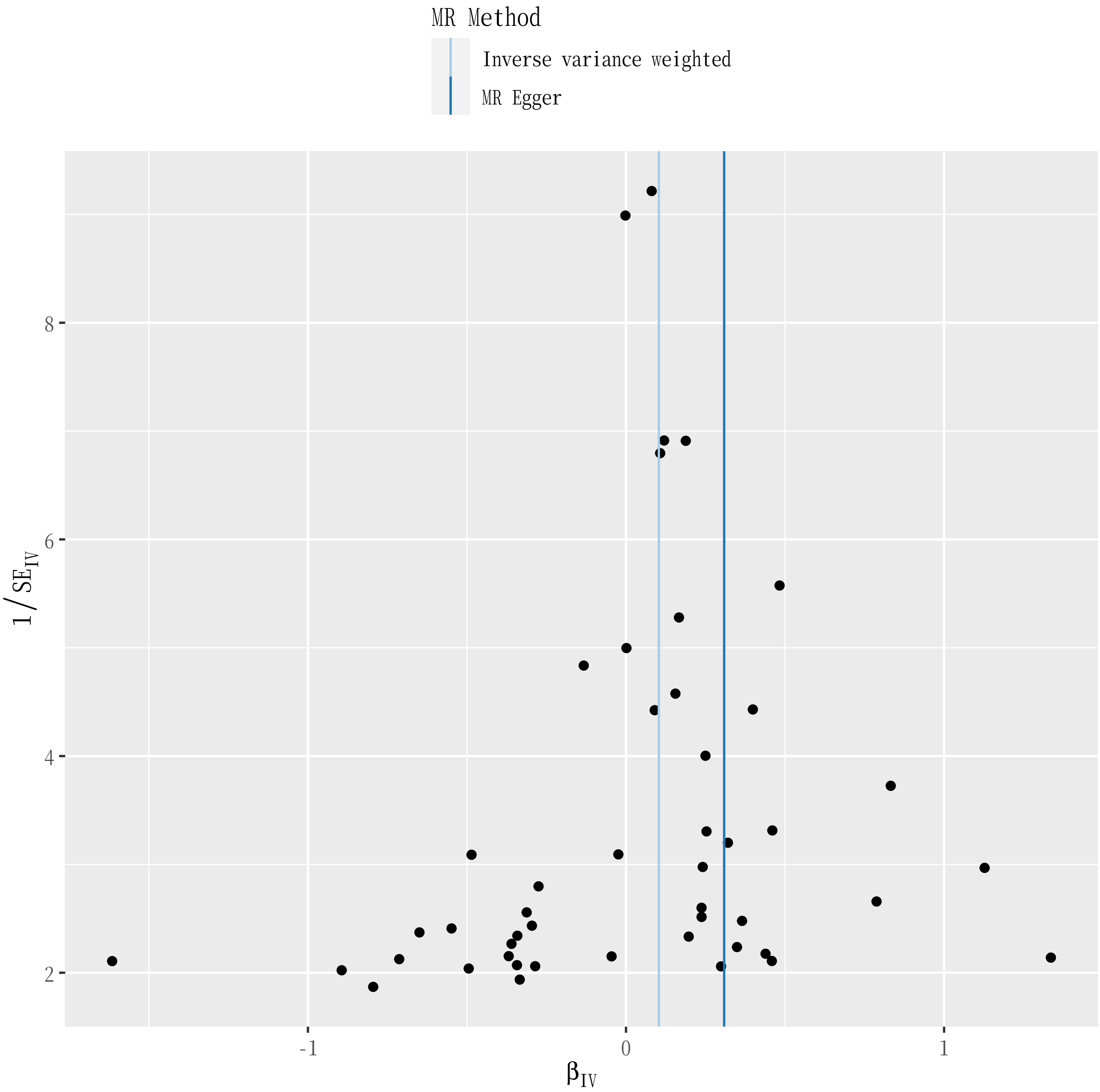

MR Method
Inverse variance weighted
MR Egger
$1/SE_{IV}$
β_{IV}
8
6
4
2
-1
0
1

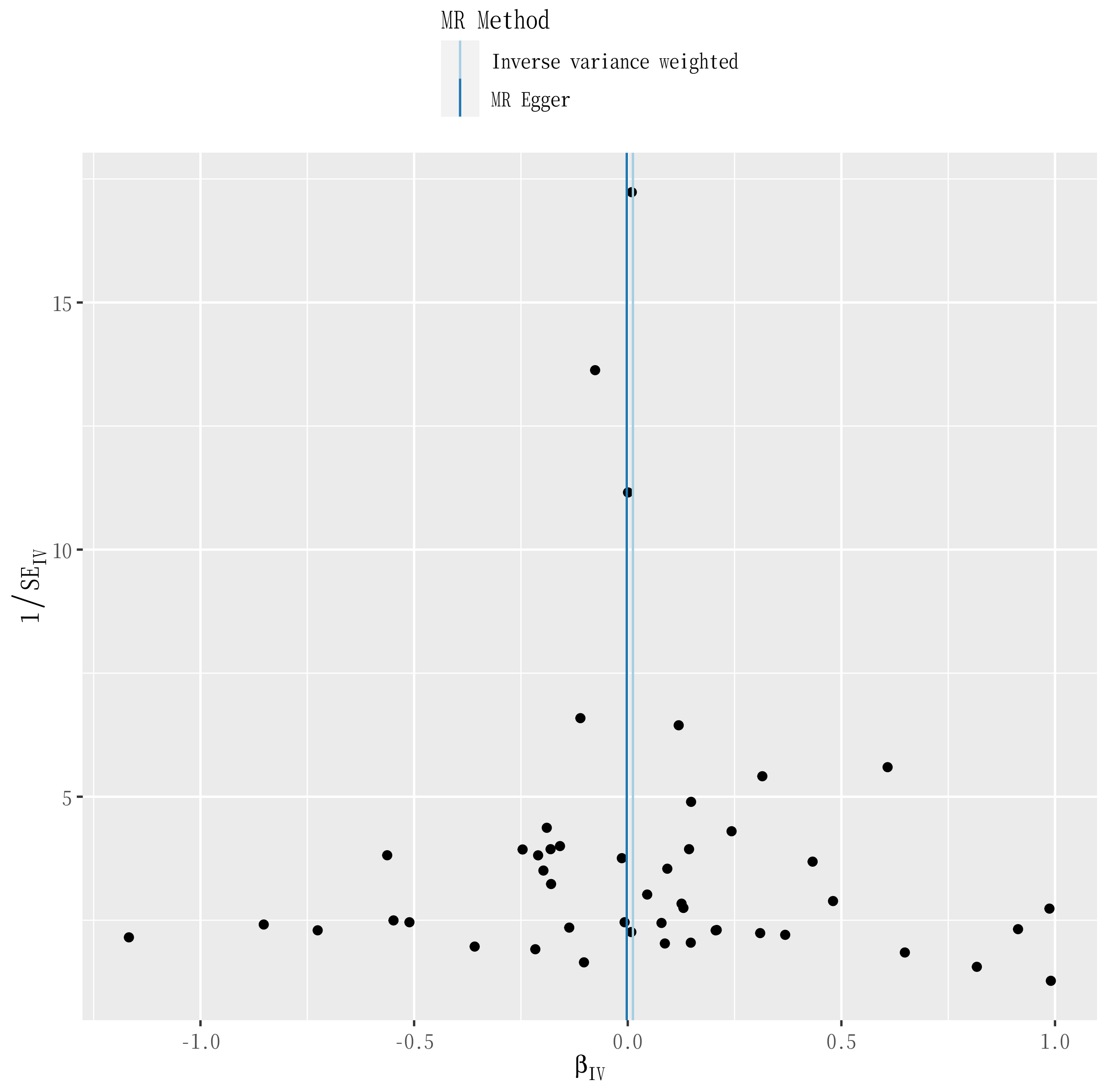
MR Method
Inverse variance weighted
MR Egger
15
10
5
1/SE_IV
-1.0
-0.5
0.0
0.5
1.0
β_IV

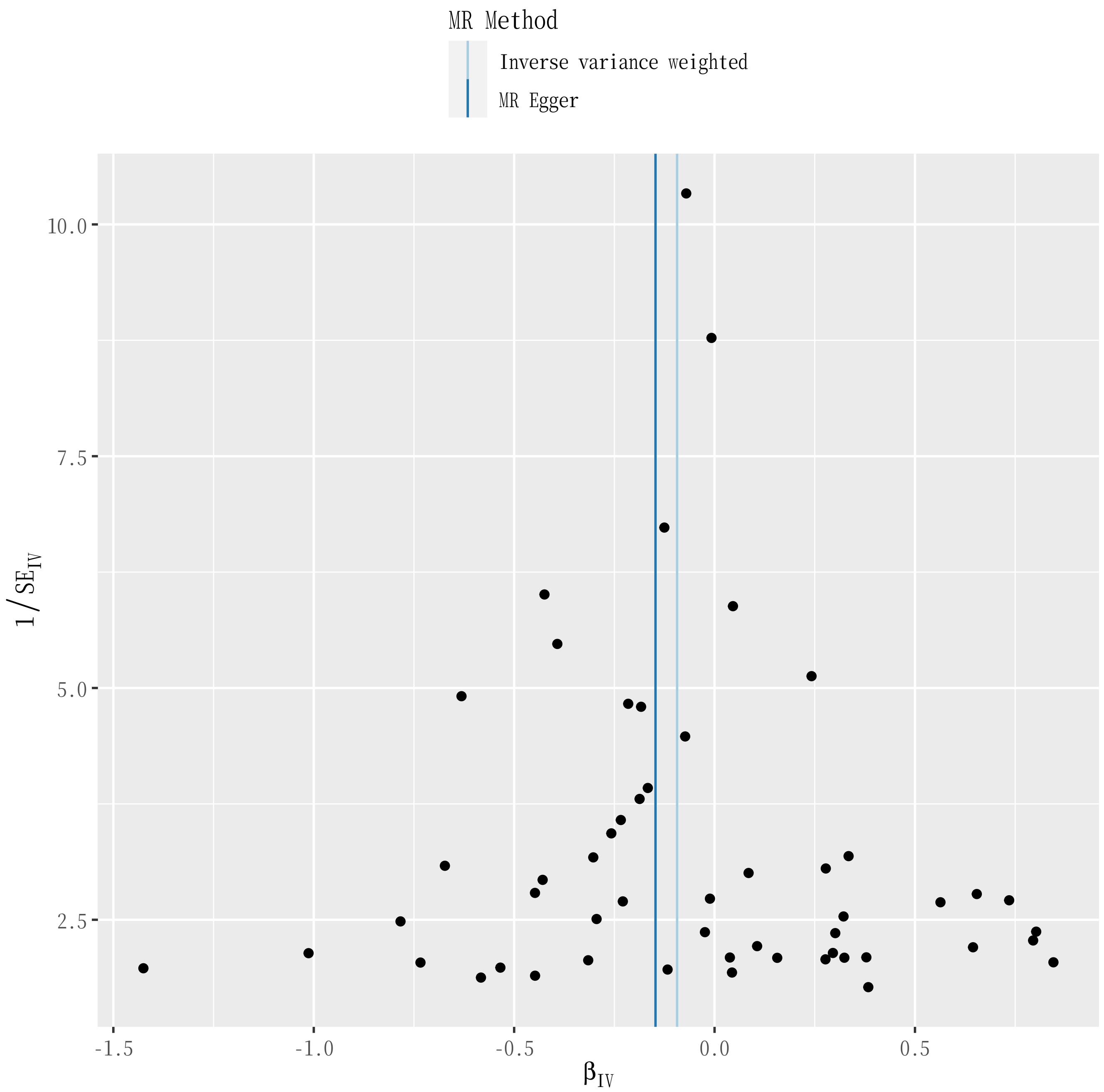

MR Method
Inverse variance weighted
MR Egger
10.0
7.5
5.0
2.5
$1/SE_{IV}$
-1.5
-1.0
-0.5
0.0
0.5
β_{IV}

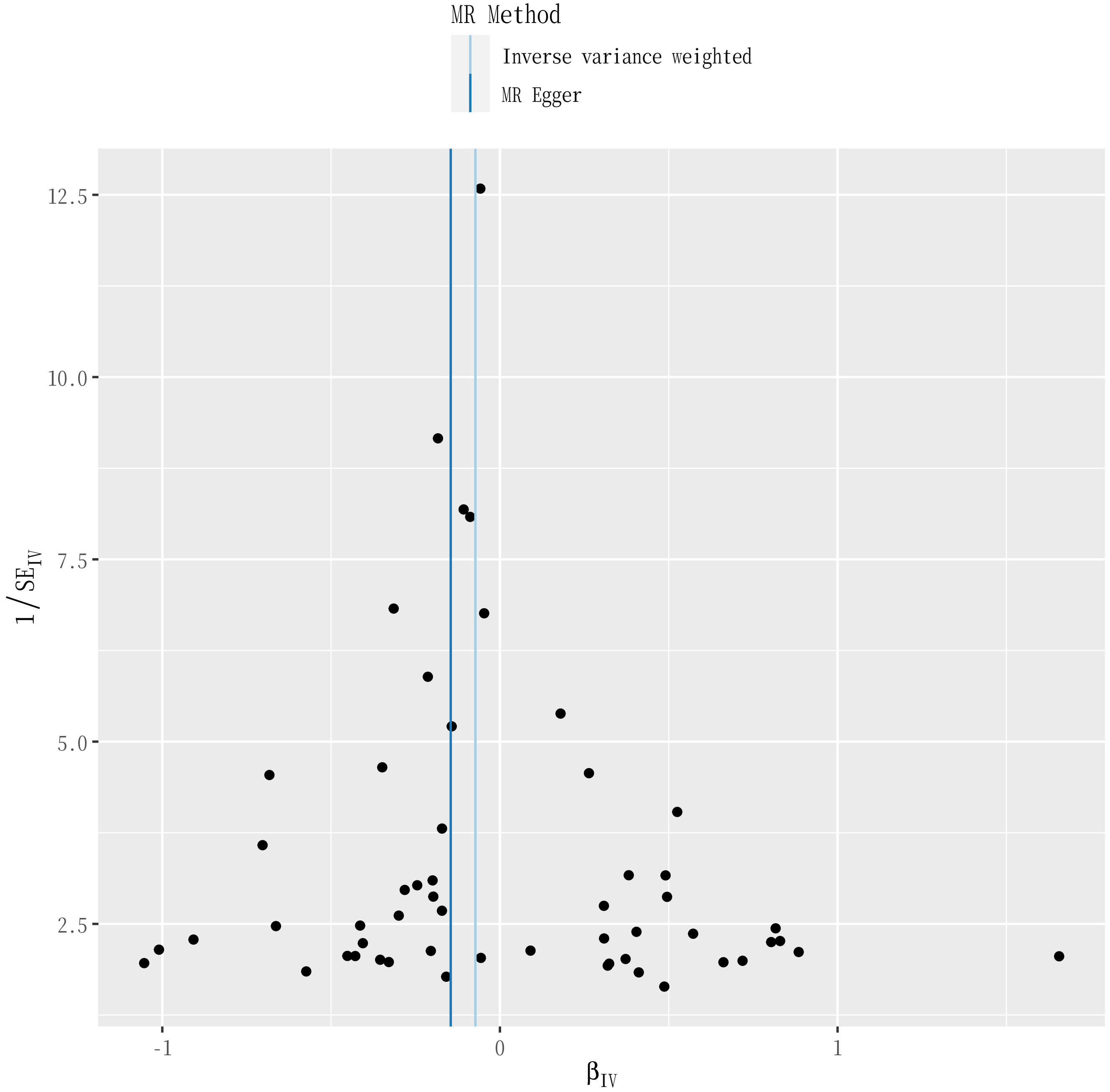

MR Method
Inverse variance weighted
MR Egger
12.5
10.0
7.5
5.0
2.5
$1/SE_{IV}$
-1
0
1
β_{IV}

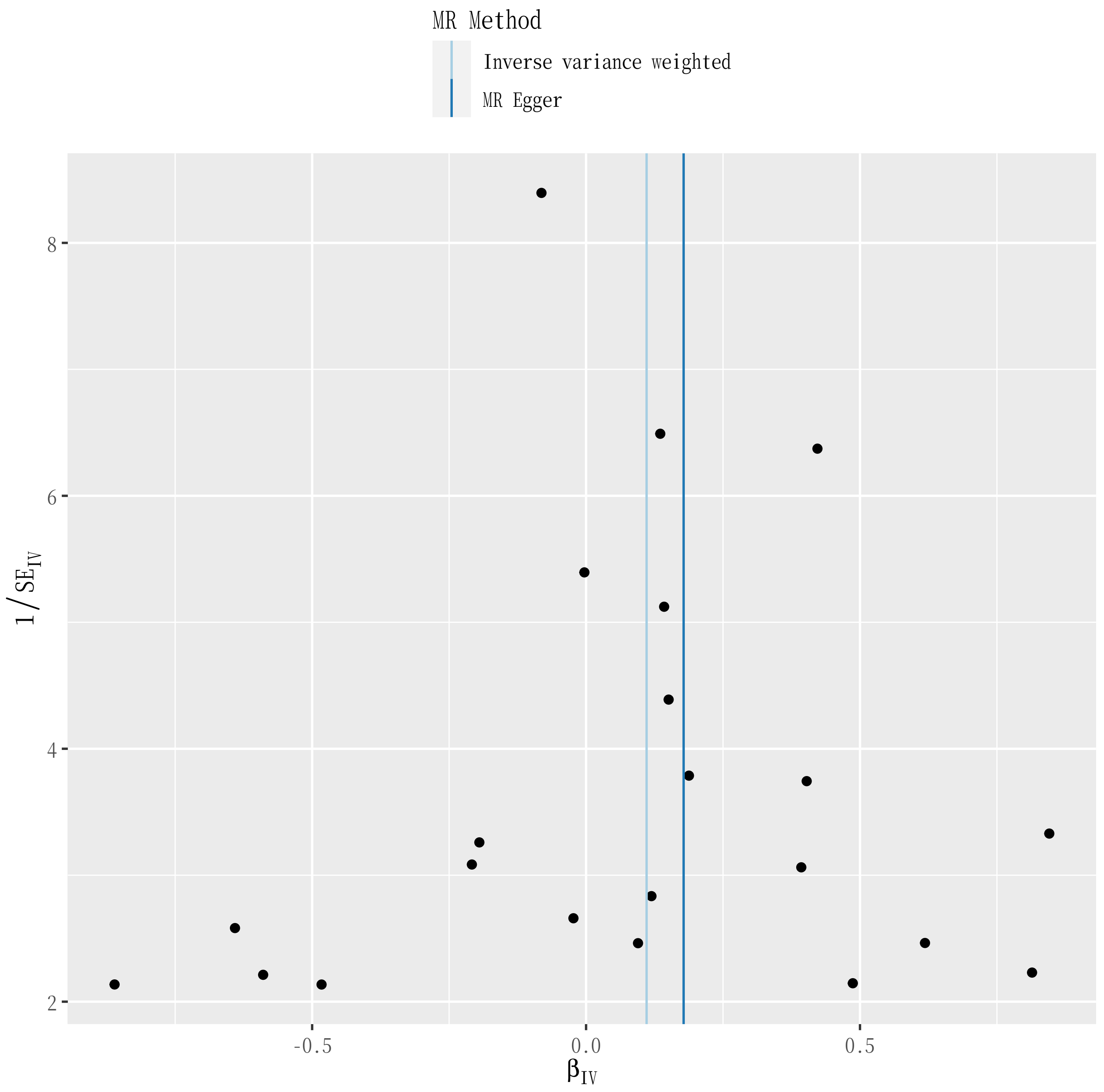

MR Method
Inverse variance weighted
MR Egger
8
6
4
2
-0.5
0.0
0.5
$1/SE_{IV}$
β_{IV}

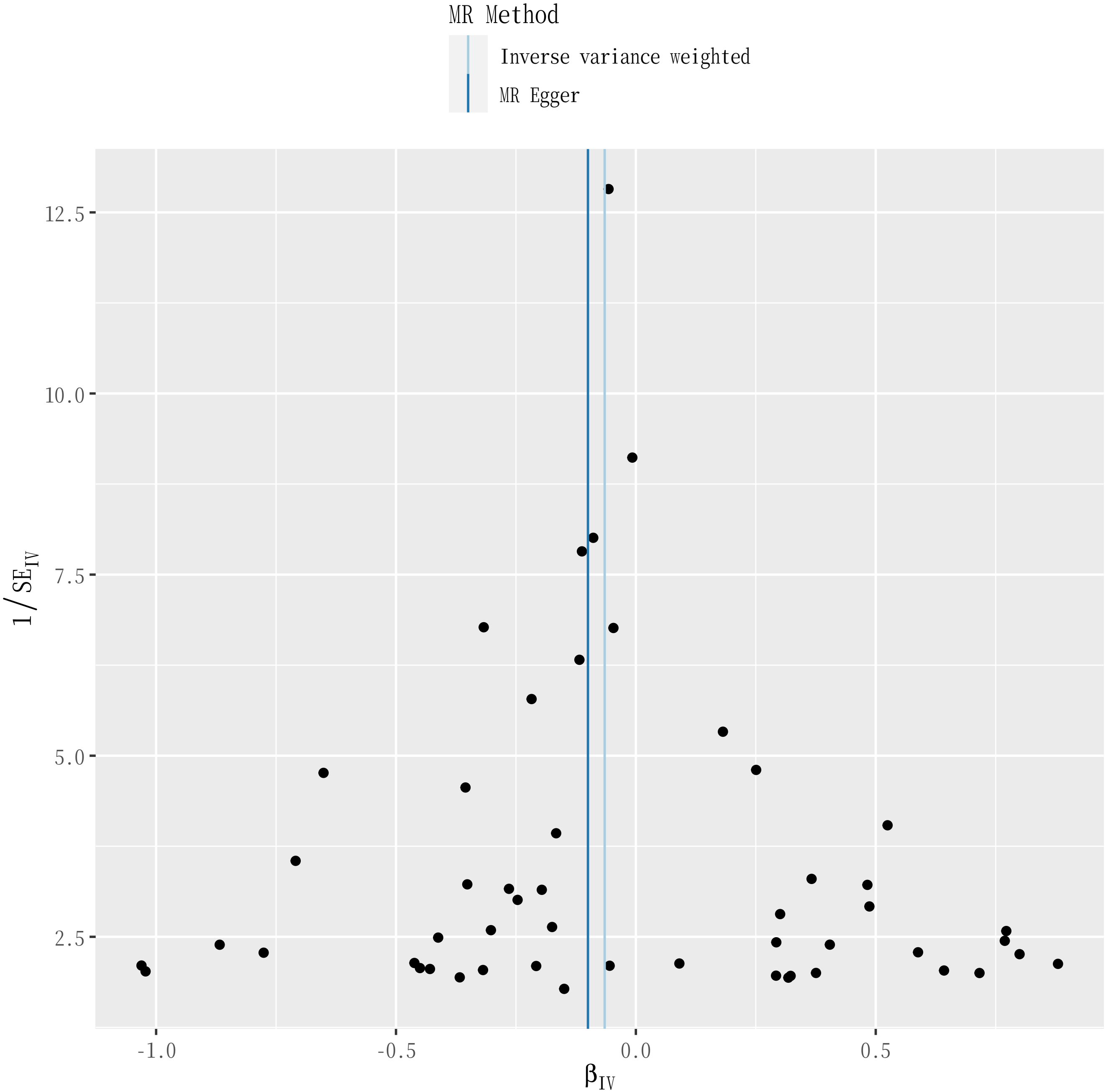

MR Method
Inverse variance weighted
MR Egger
12.5
10.0
7.5
5.0
2.5
1/SE_IV
-1.0
-0.5
0.0
0.5
β_IV

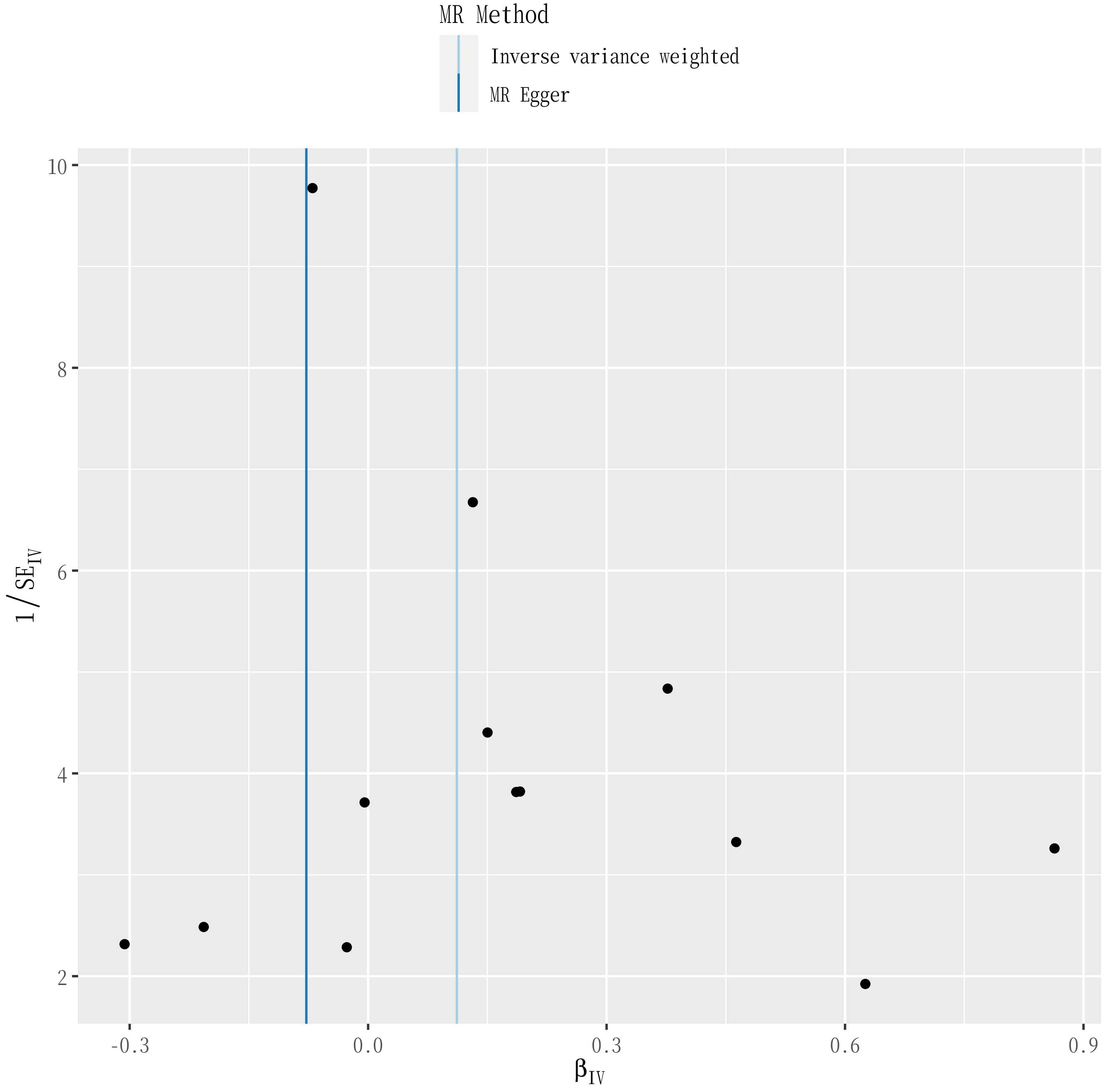
MR Method
Inverse variance weighted
MR Egger
10
8
6
4
2
$1/SE_{IV}$
-0.3
0.0
0.3
0.6
0.9
β_{IV}

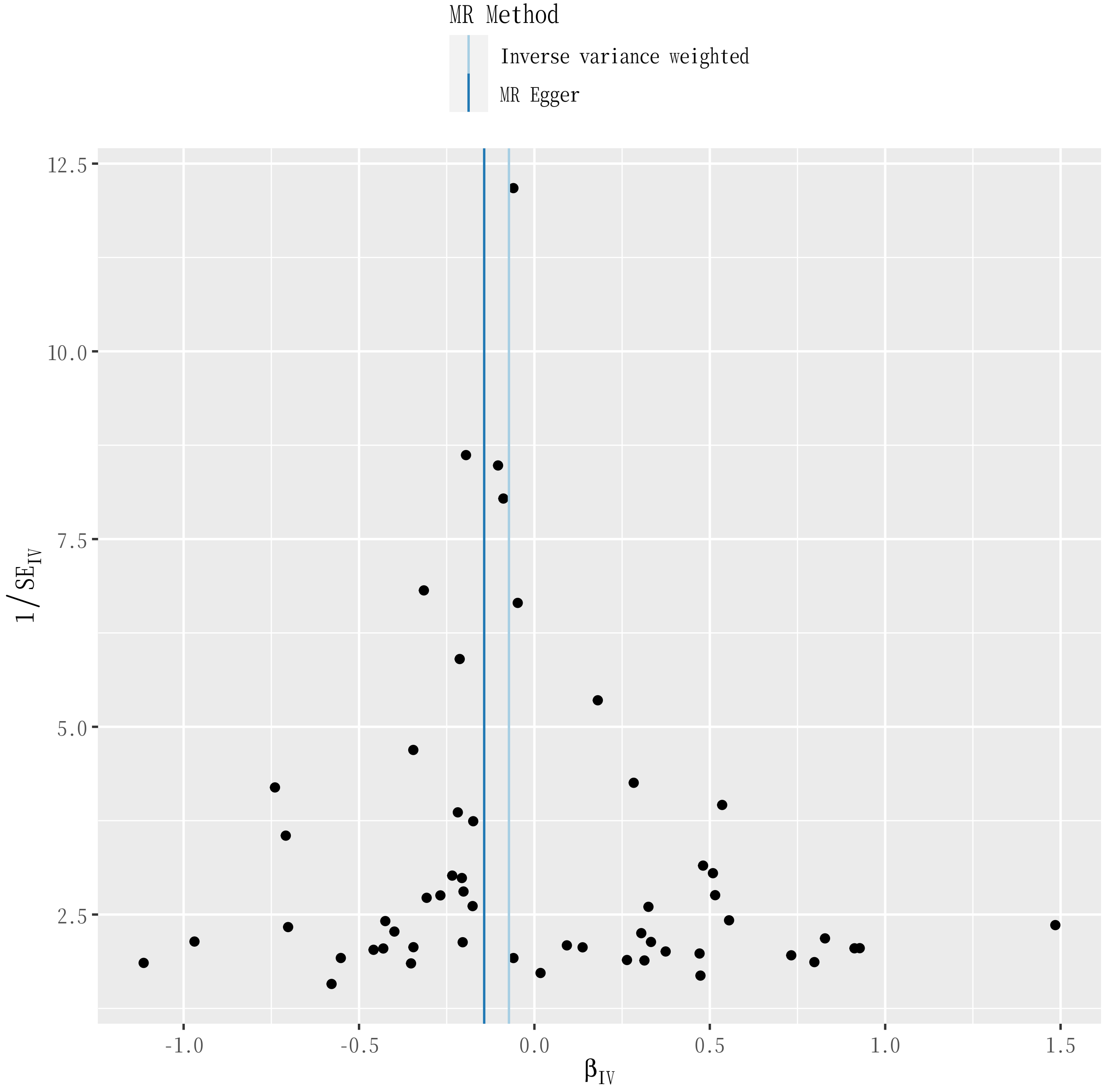

MR Method
Inverse variance weighted
MR Egger
12.5
10.0
7.5
5.0
2.5
-1.0
-0.5
0.0
0.5
1.0
1.5
$1/SE_{IV}$
β_{IV}

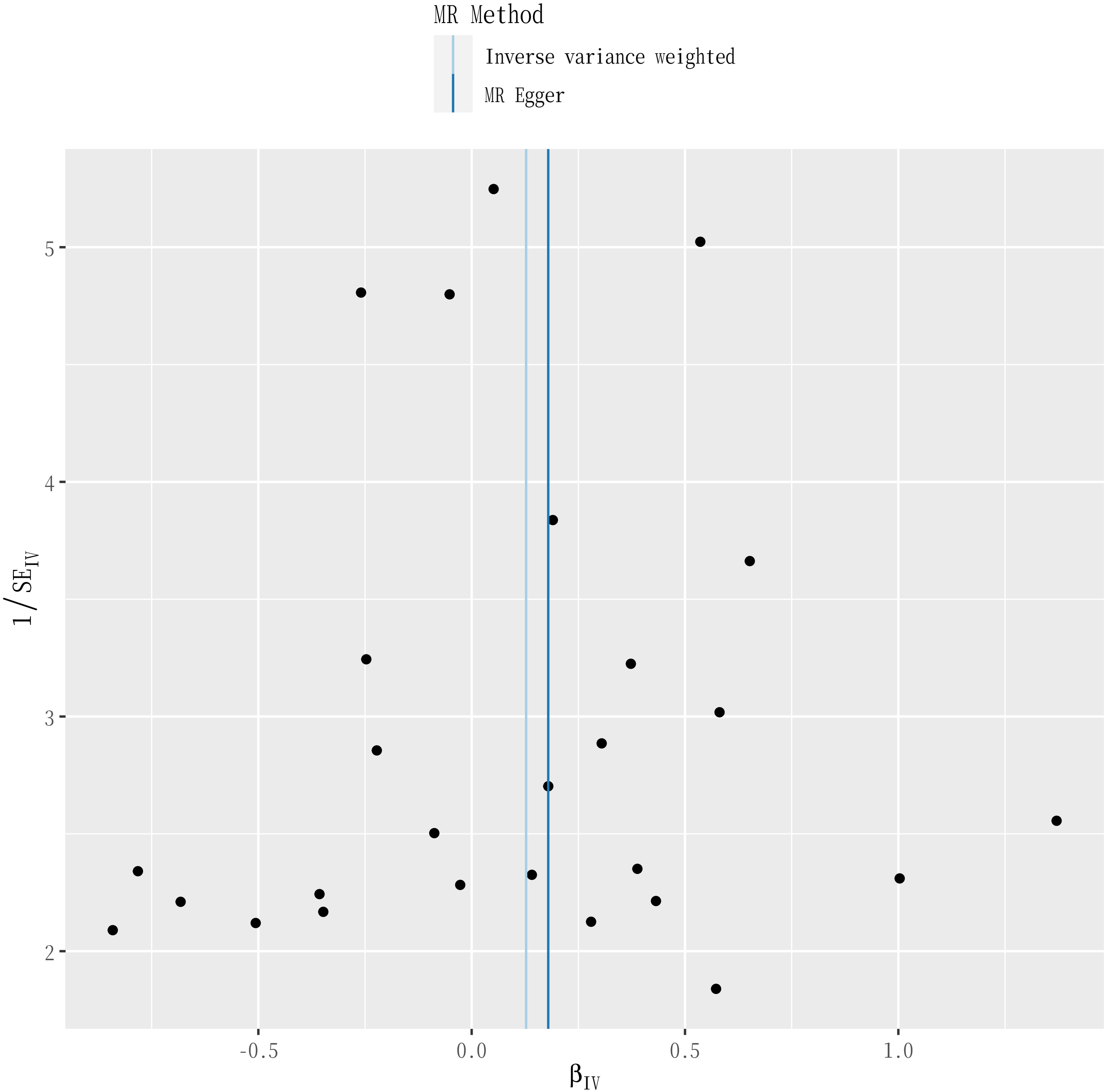

MR Method
Inverse variance weighted
MR Egger
5
4
3
2
$1/SE_{IV}$
-0.5
0.0
0.5
1.0
β_{IV}

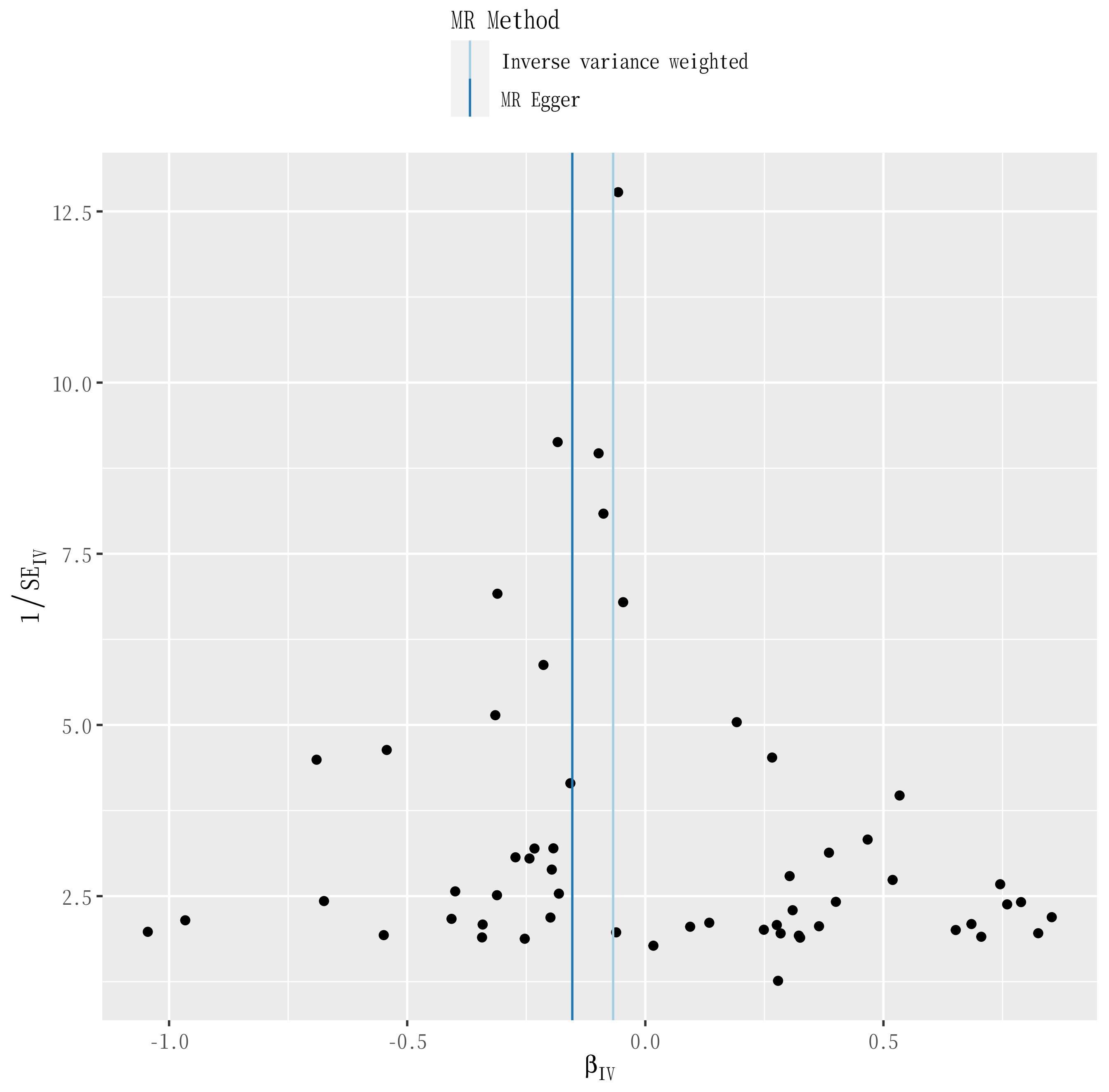
MR Method
Inverse variance weighted
MR Egger
12.5
10.0
7.5
5.0
2.5
1/SE_IV
-1.0
-0.5
0.0
0.5
β_IV

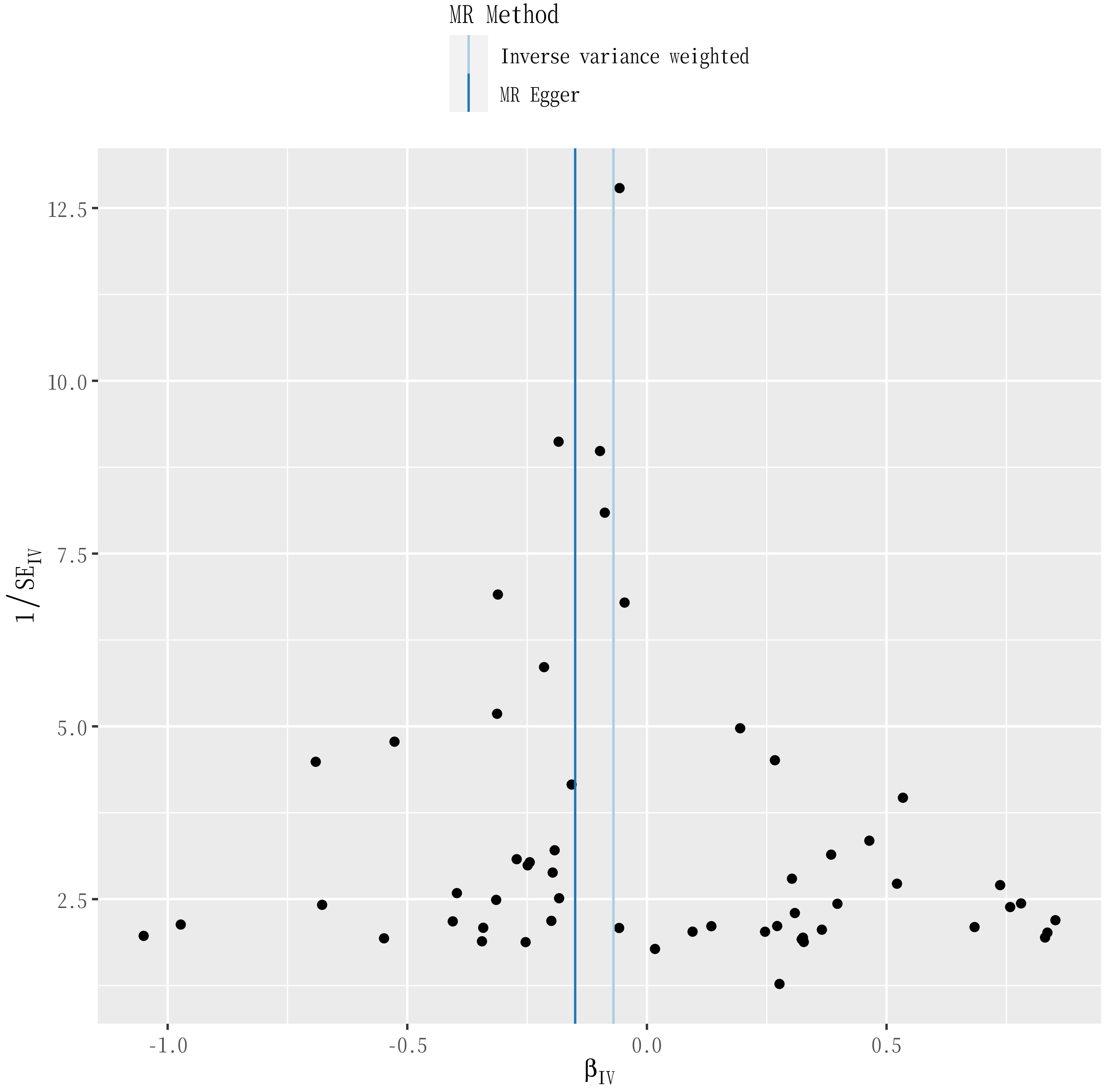

MR Method
Inverse variance weighted
MR Egger
12.5
10.0
7.5
5.0
2.5
$1/SE_{IV}$
-1.0
-0.5
0.0
0.5
β_{IV}

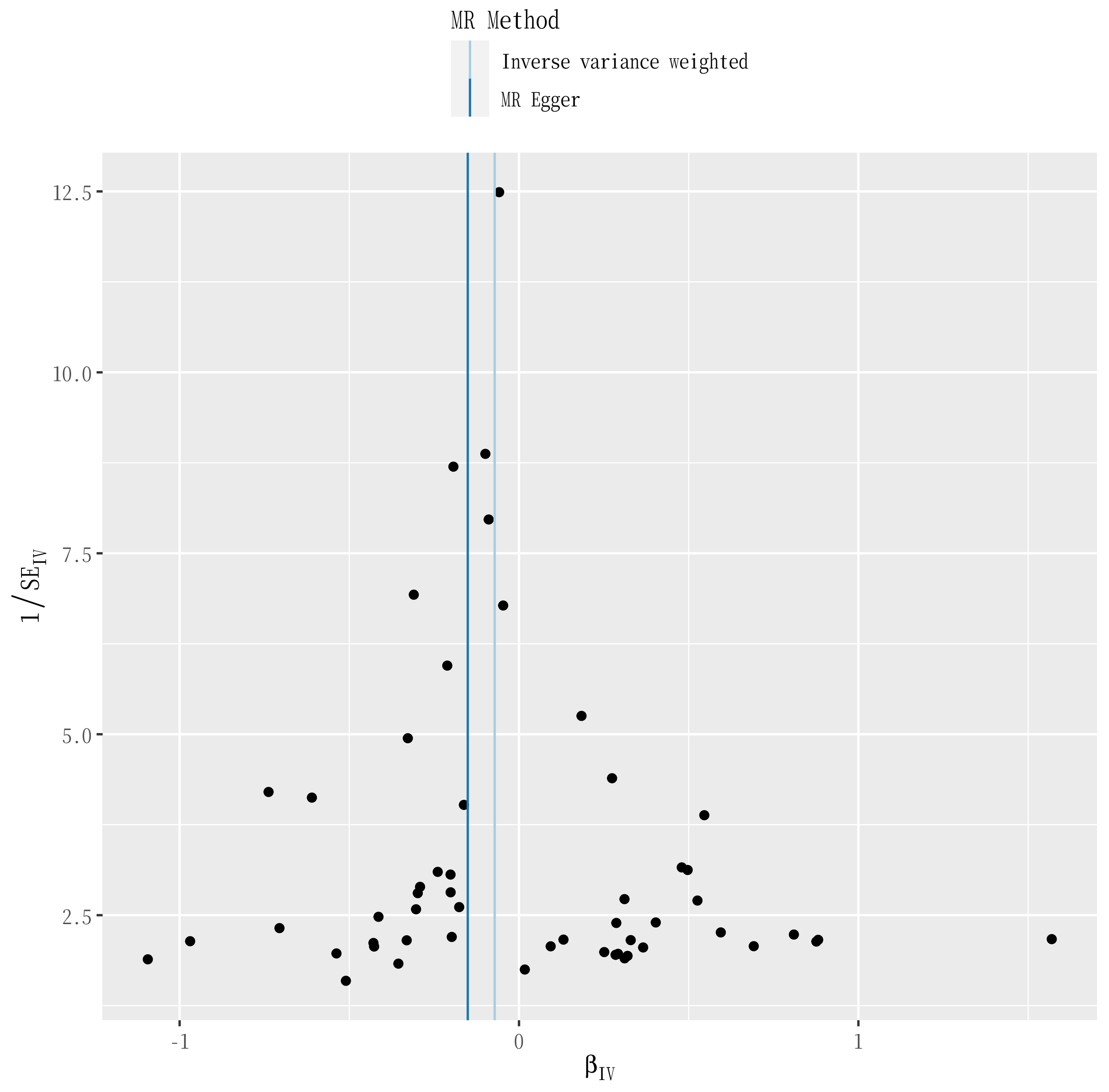
MR Method
Inverse variance weighted
MR Egger
12.5
10.0
7.5
5.0
2.5
$1/SE_{IV}$
-1
0
1
β_{IV}

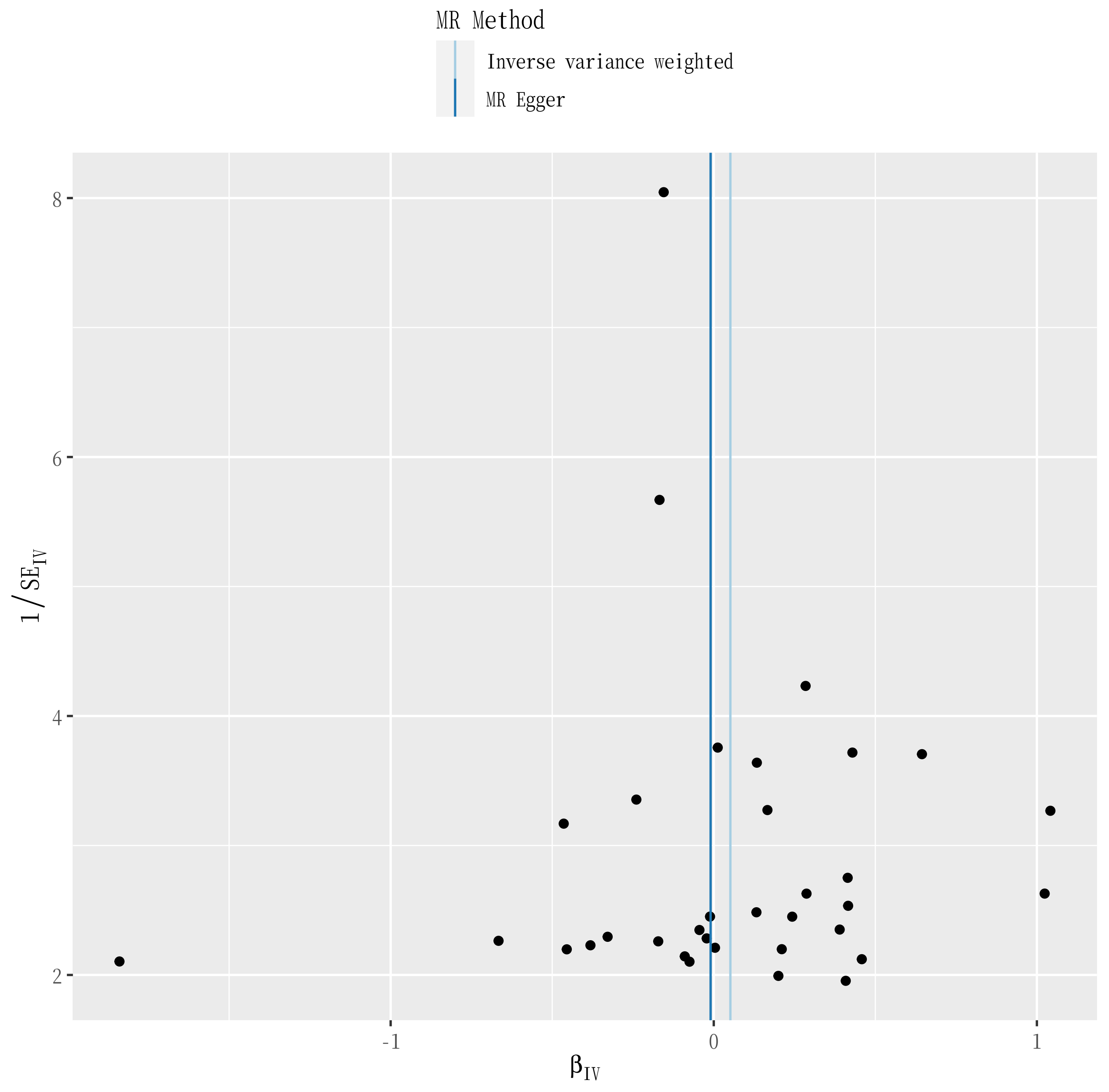
MR Method
Inverse variance weighted
MR Egger
8
6
4
2
-1
0
1
1/SE_IV
β_IV

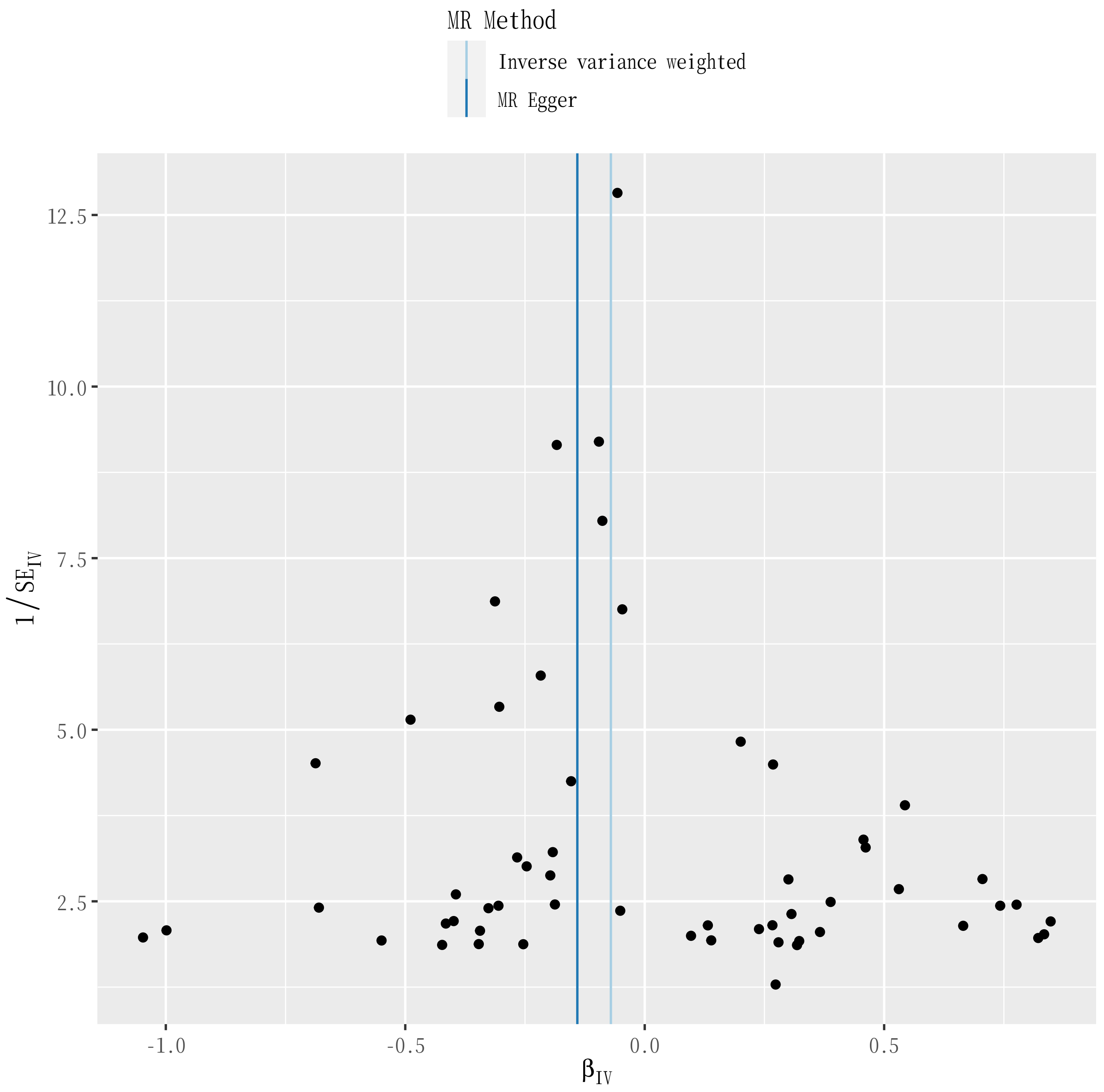

MR Method
Inverse variance weighted
MR Egger
12.5
10.0
7.5
5.0
2.5
1/SE_IV
-1.0
-0.5
0.0
0.5
β_IV

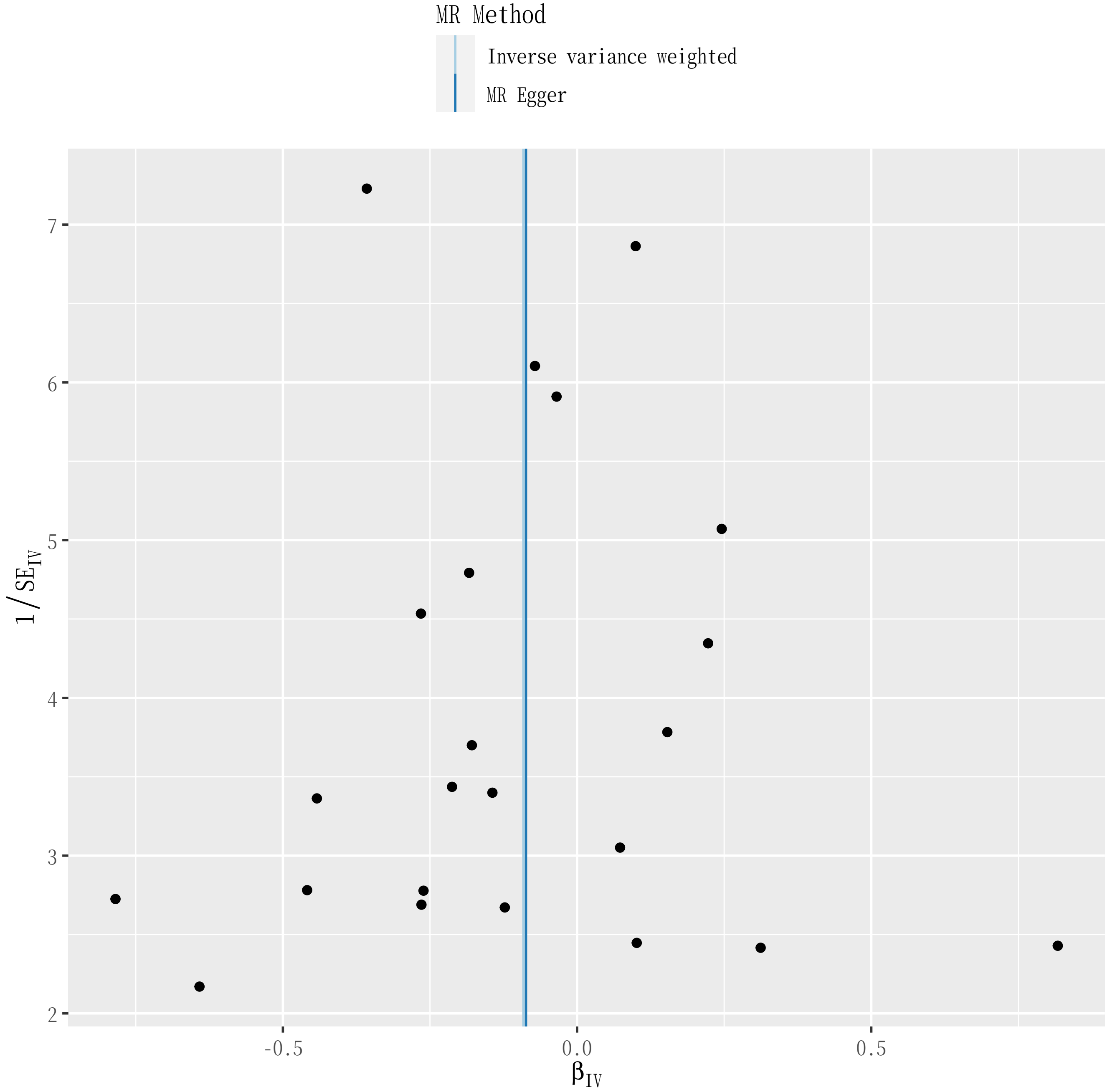

MR Method
Inverse variance weighted
MR Egger
7
6
5
4
3
2
$1/SE_{IV}$
-0.5
0.0
0.5
β_{IV}

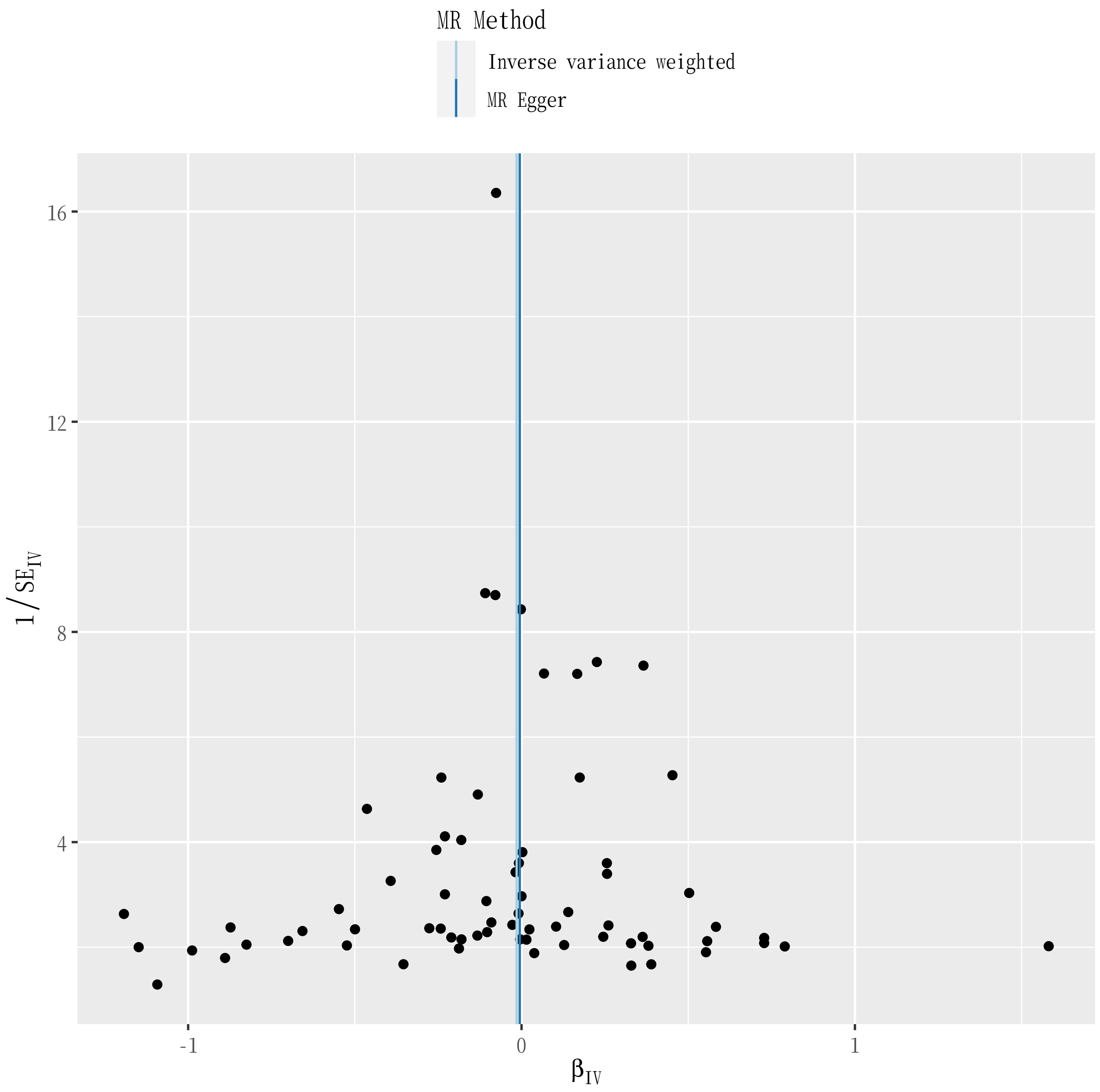
MR Method
Inverse variance weighted
MR Egger
16
12
8
4
-1
0
1
1/SE_IV
β_IV

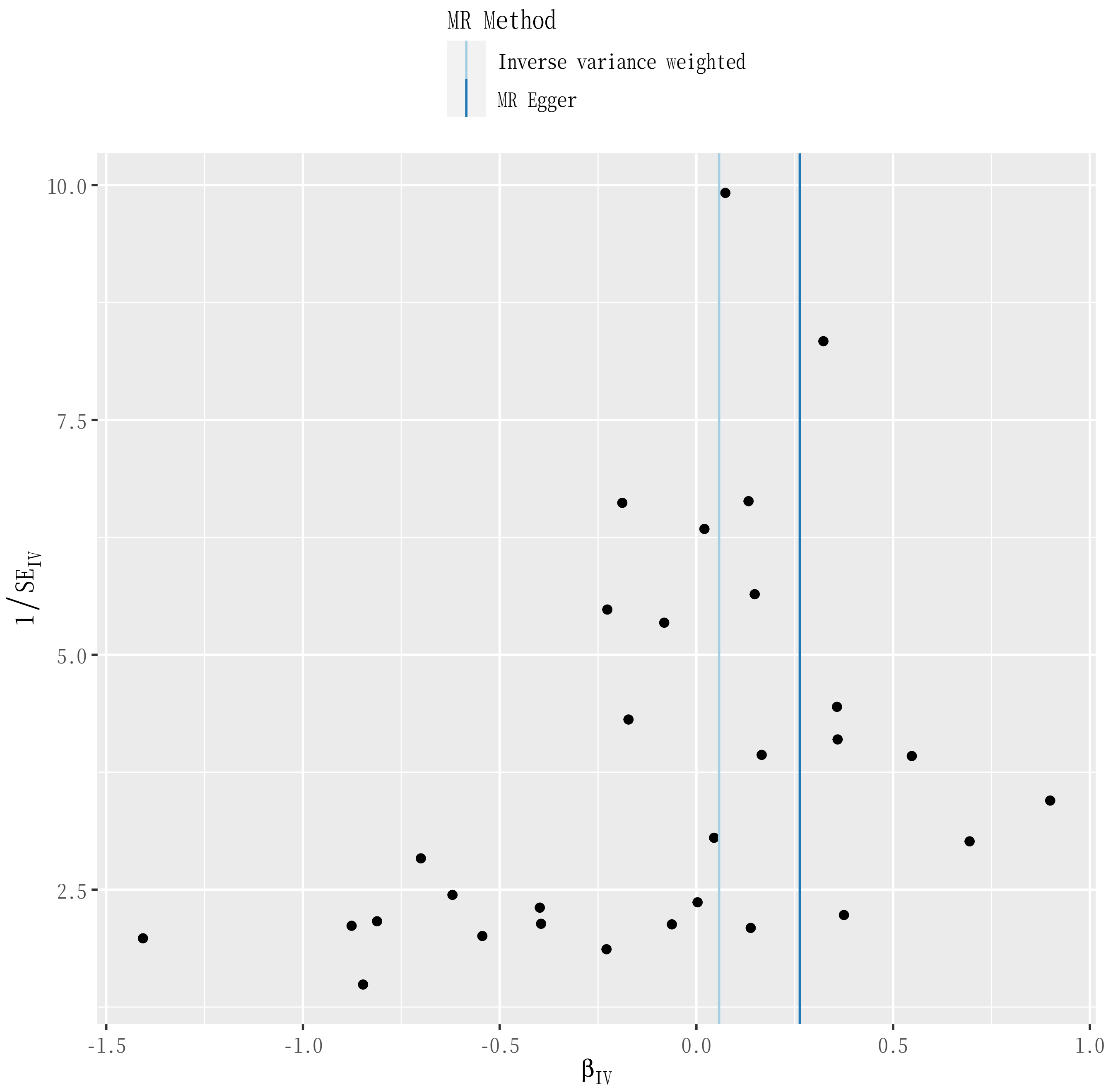
MR Method
Inverse variance weighted
MR Egger
10.0
7.5
5.0
2.5
1/SE_IV
-1.5
-1.0
-0.5
0.0
0.5
1.0
β_IV

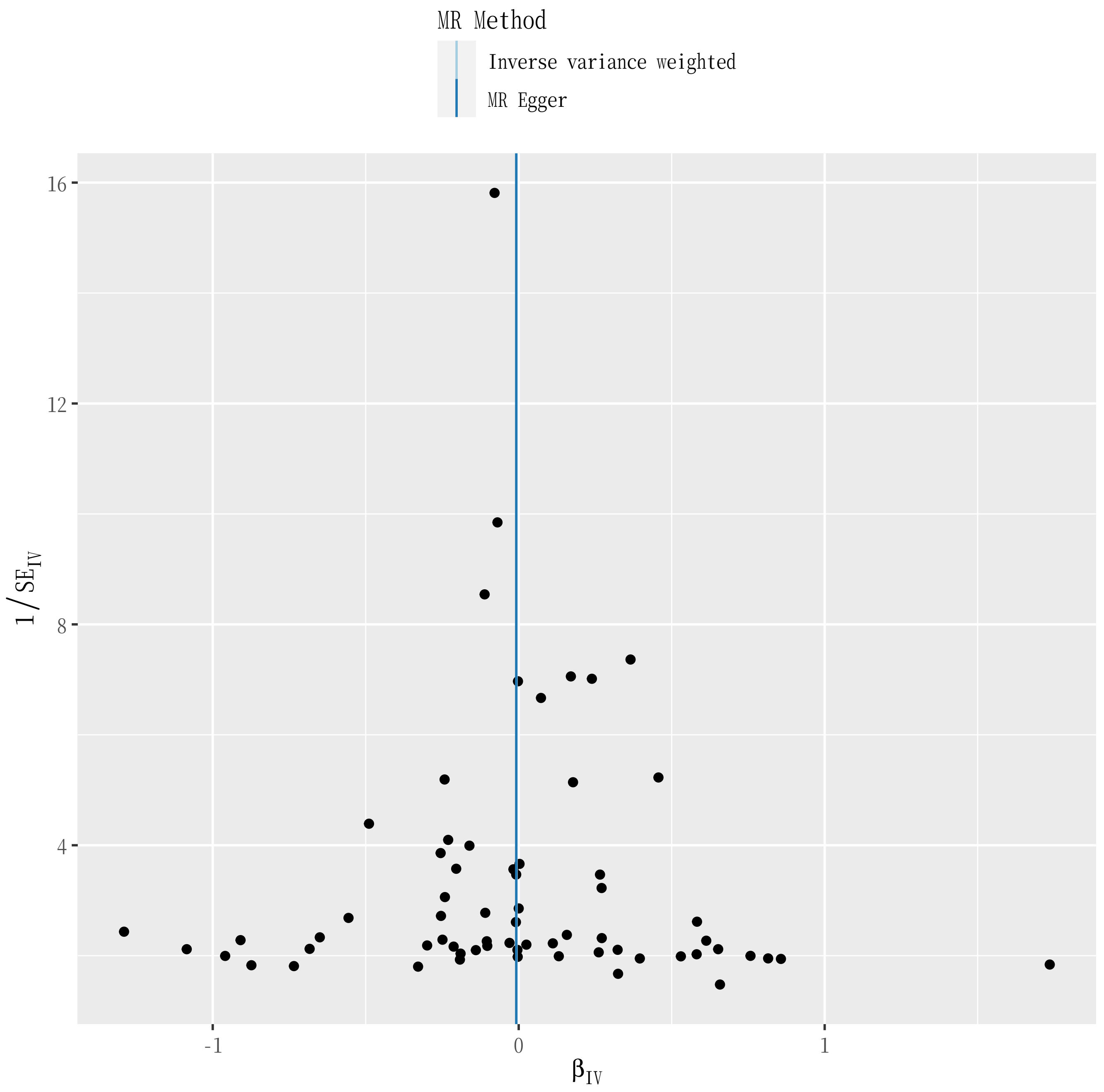
MR Method
Inverse variance weighted
MR Egger
16
12
8
4
-1
0
1
$1/SE_{IV}$
β_{IV}

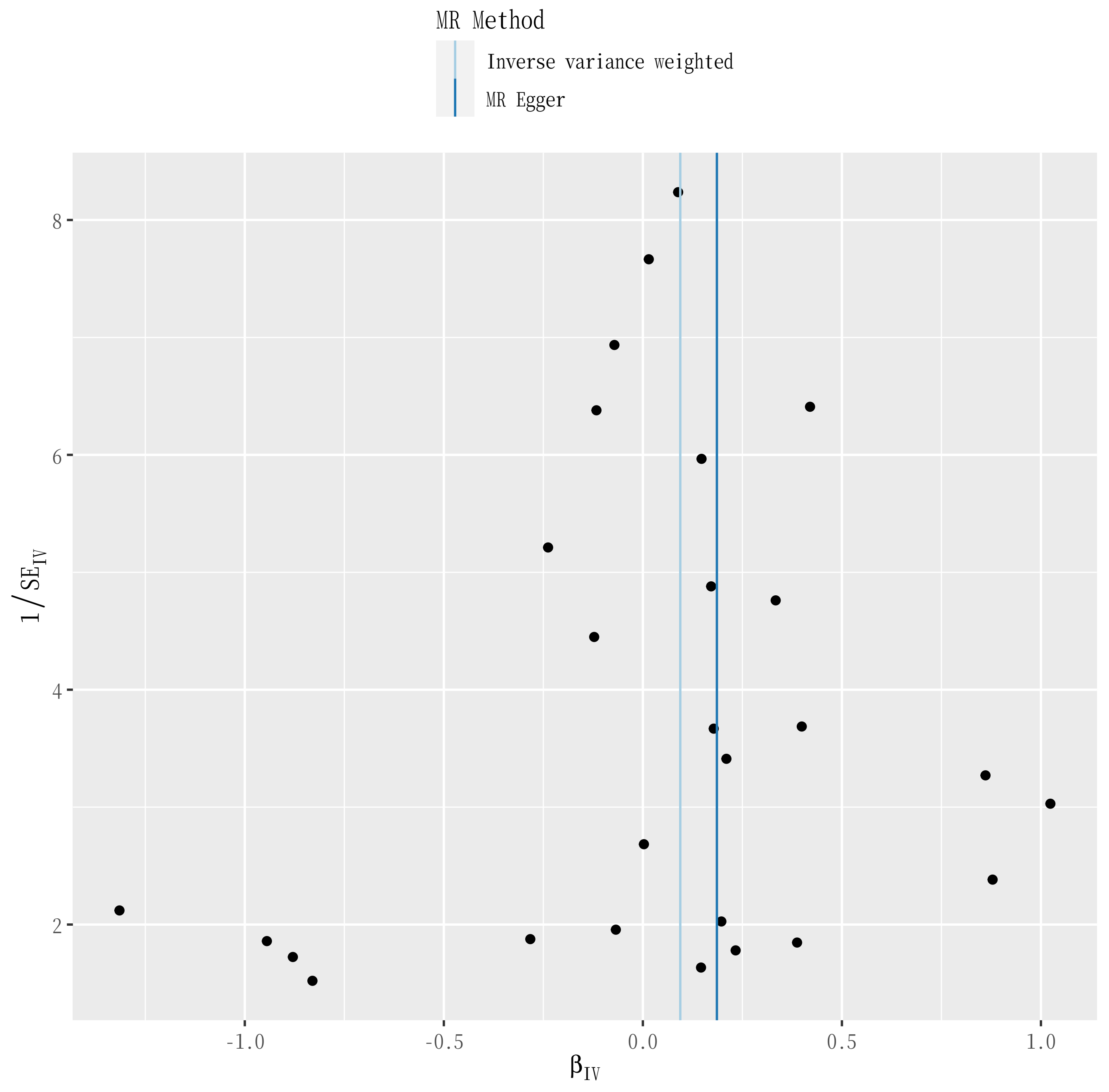
MR Method
Inverse variance weighted
MR Egger
8
6
4
2
$1/SE_{IV}$
-1.0
-0.5
0.0
0.5
1.0
β_{IV}

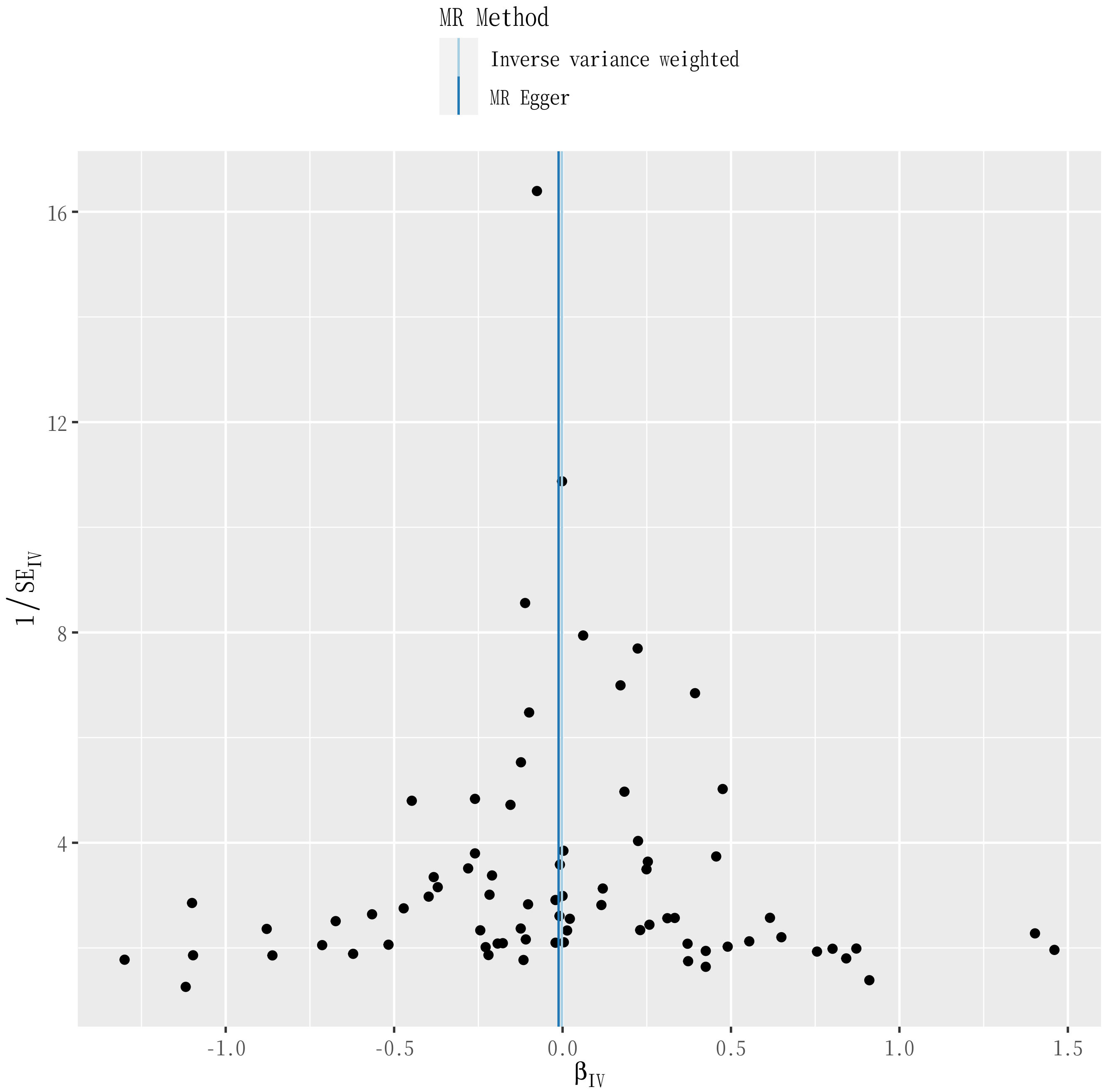

MR Method
Inverse variance weighted
MR Egger
16
12
8
4
-1.0
-0.5
0.0
0.5
1.0
1.5
β_{IV}
$1/SE_{IV}$

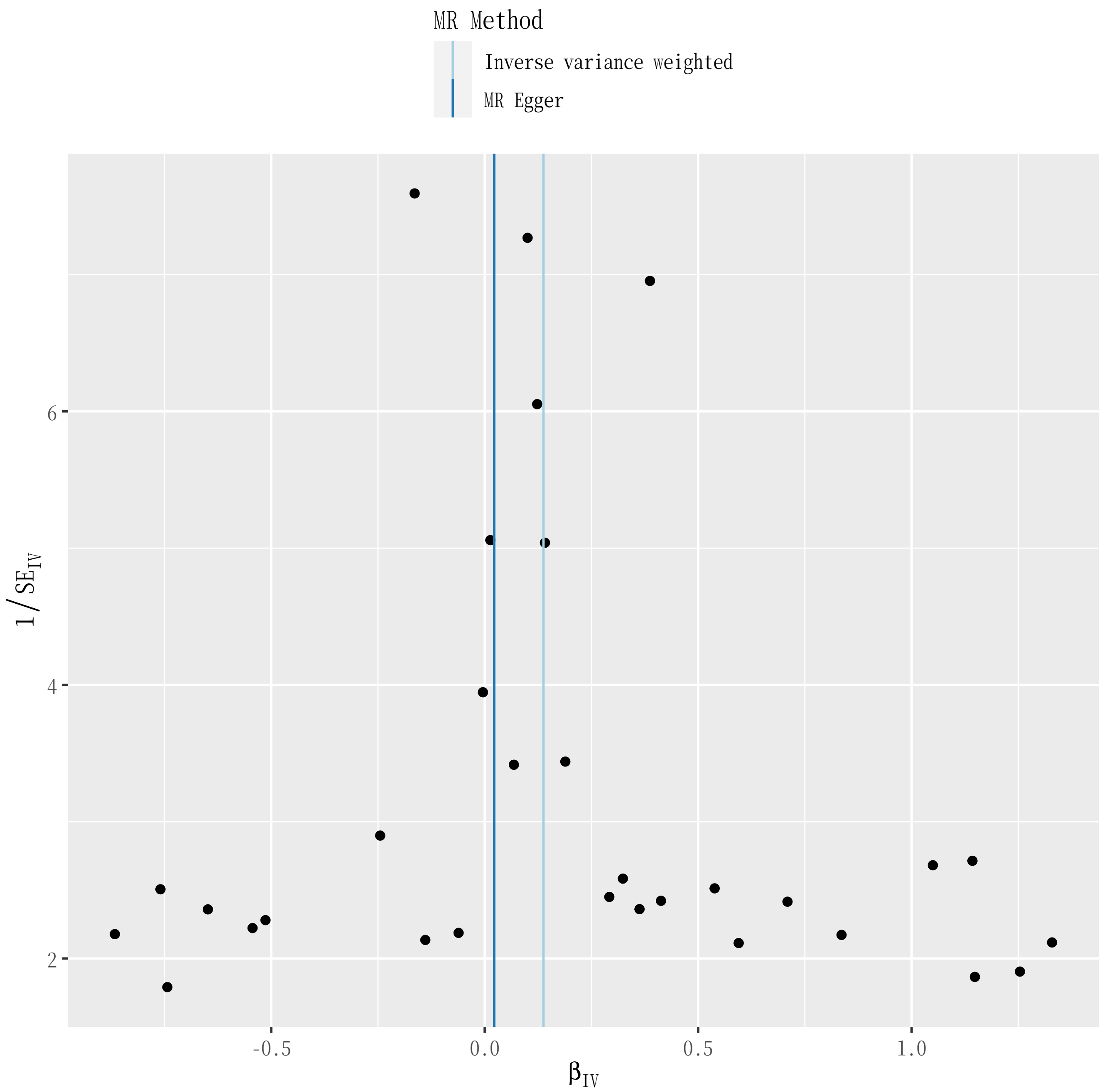
MR Method
Inverse variance weighted
MR Egger
6
4
2
1/SE_IV
-0.5
0.0
0.5
1.0
β_IV

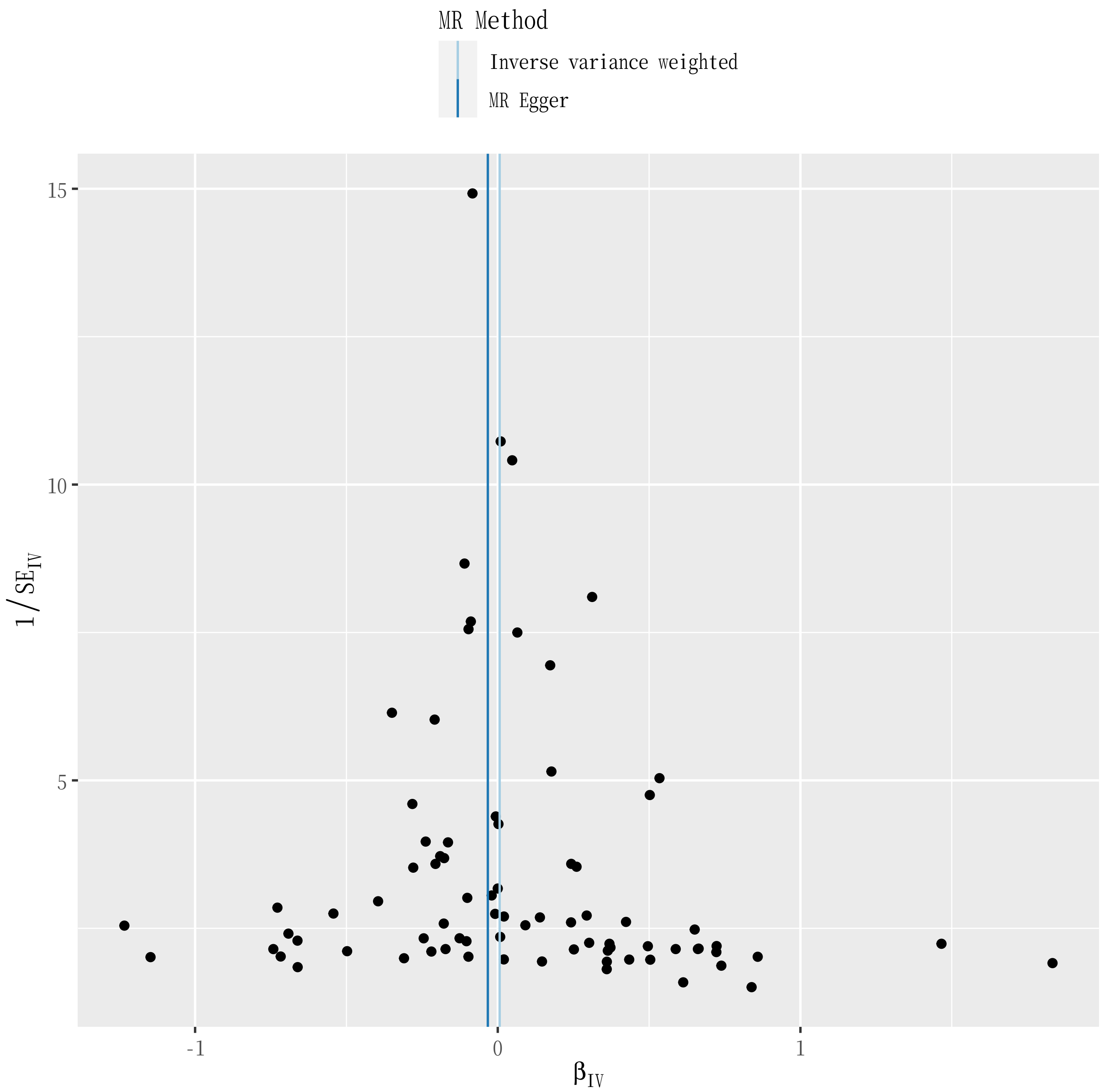
MR Method
Inverse variance weighted
MR Egger
15
10
5
-1
0
1
1/SE$_{IV}$
β$_{IV}$

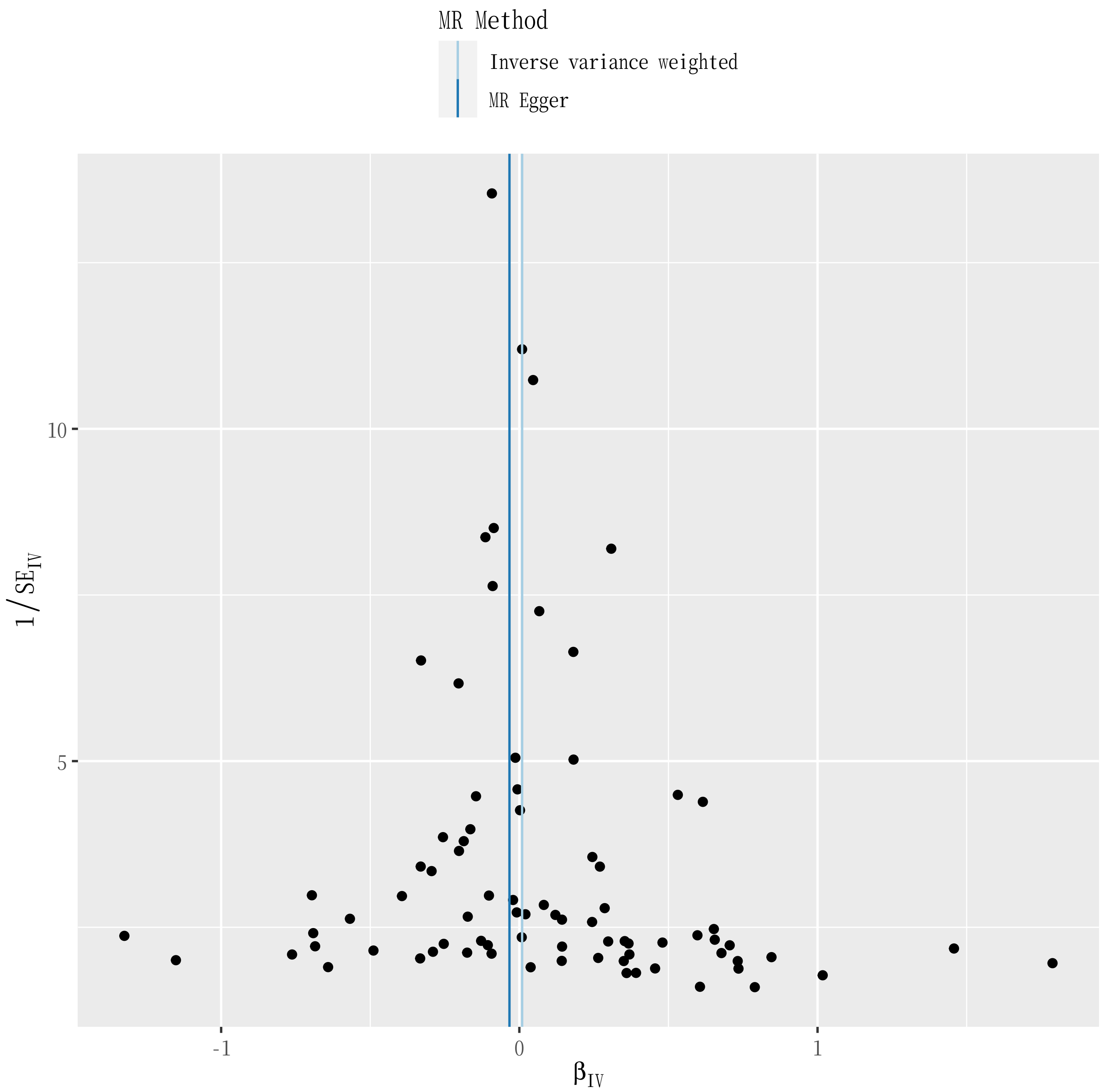
MR Method
Inverse variance weighted
MR Egger
10
5
-1
0
1
$1/SE_{IV}$
β_{IV}

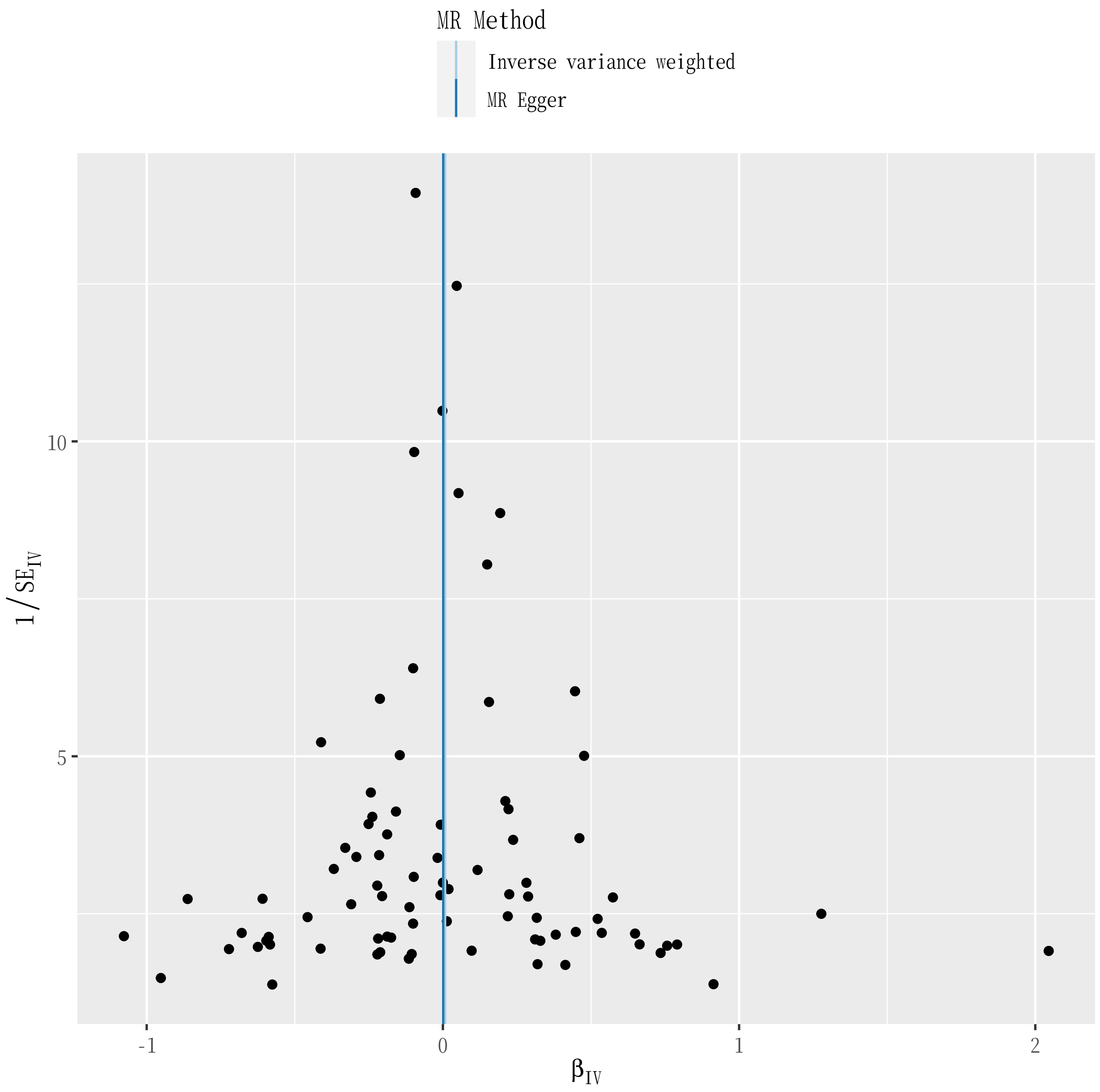
MR Method
Inverse variance weighted
MR Egger
10
5
1/SE_IV
-1
0
1
2
β_IV

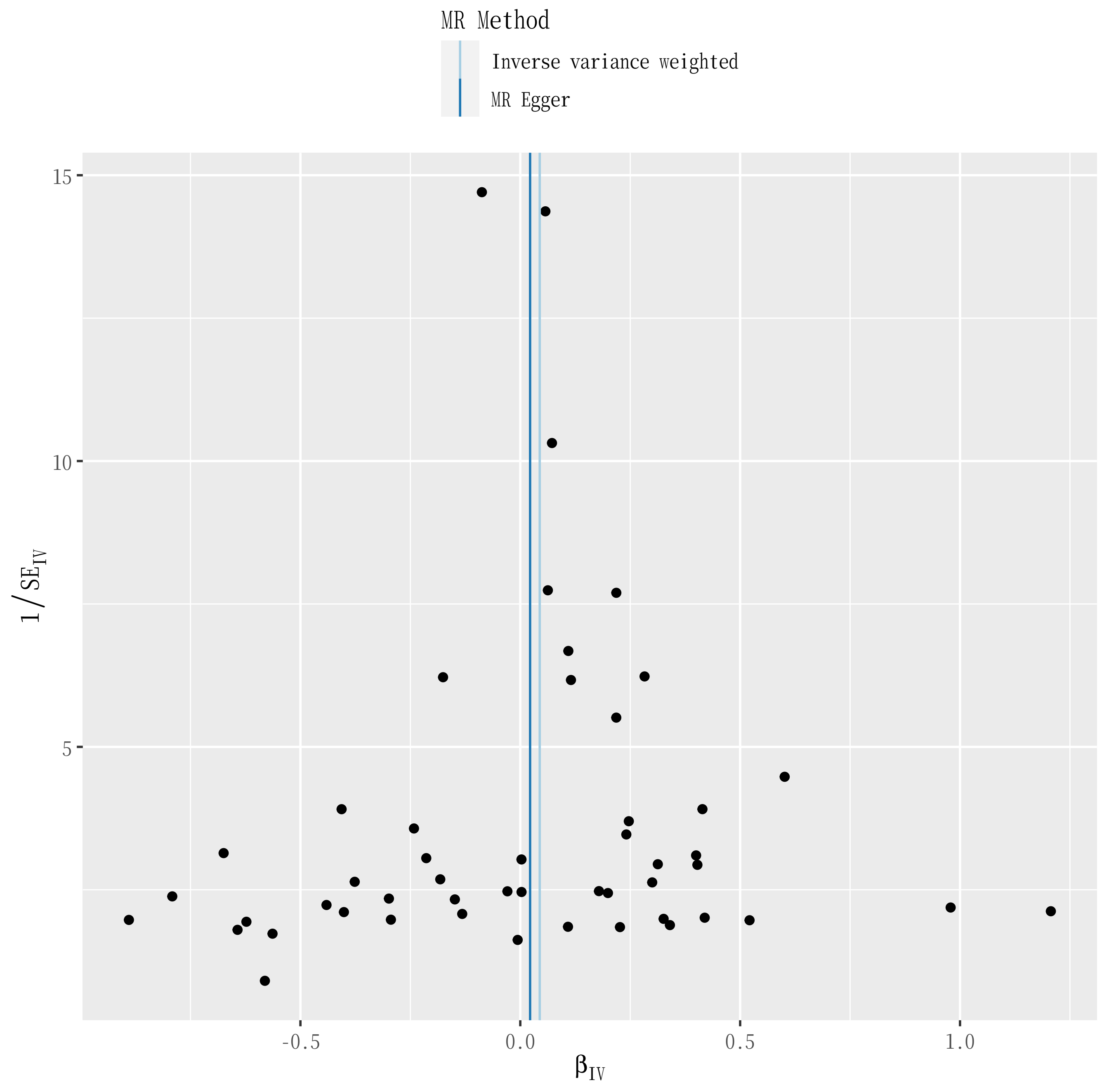

MR Method
Inverse variance weighted
MR Egger
15
10
5
-0.5
0.0
0.5
1.0
1/SE_IV
β_IV

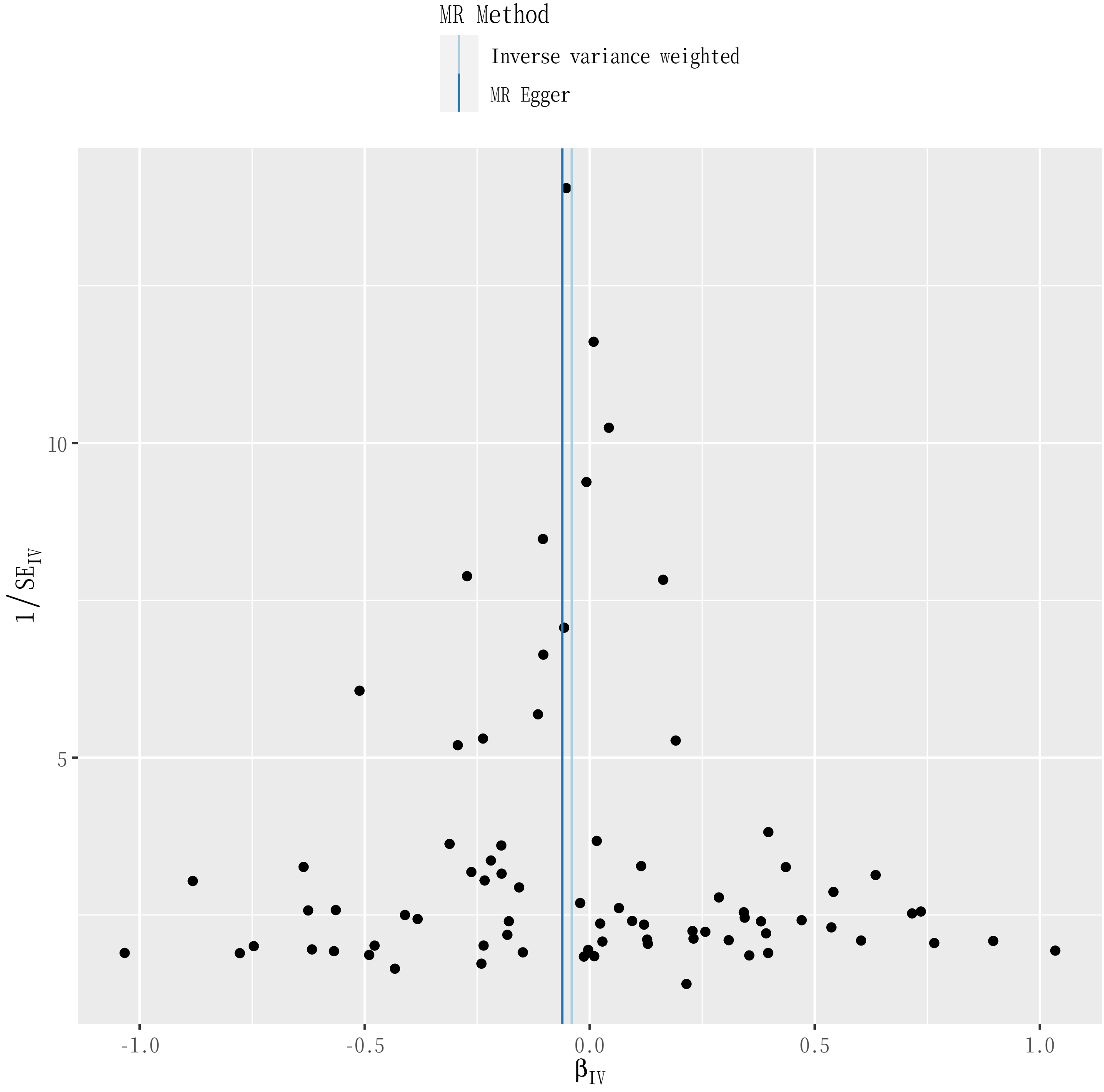

MR Method
Inverse variance weighted
MR Egger
10
5
1/SE_IV
-1.0
-0.5
0.0
0.5
1.0
β_IV

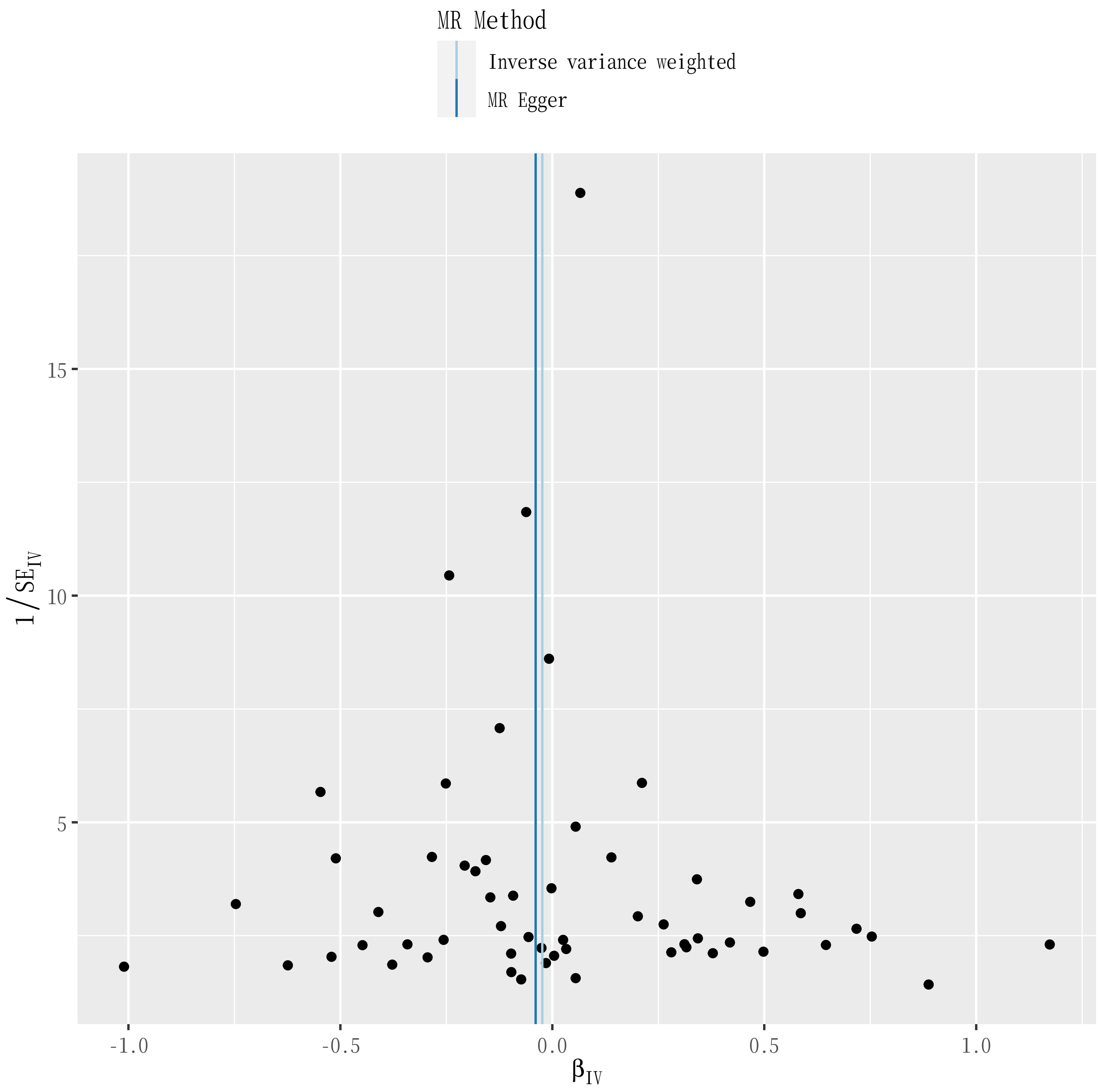
MR Method
Inverse variance weighted
MR Egger
15
10
5
-1.0
-0.5
0.0
0.5
1.0
$1/SE_{IV}$
β_{IV}

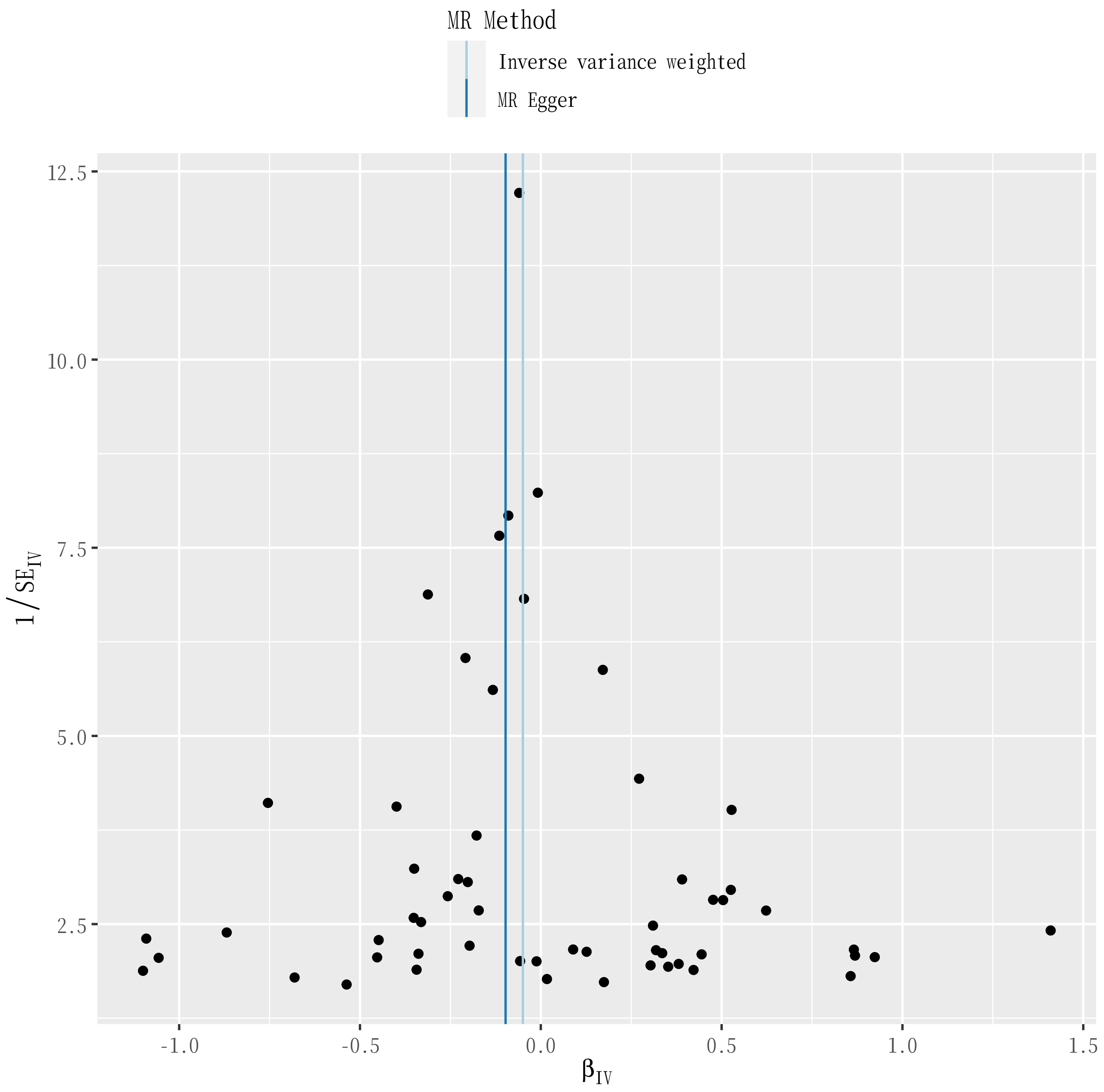

MR Method
Inverse variance weighted
MR Egger
12.5
10.0
7.5
5.0
2.5
1/SE_IV
-1.0
-0.5
0.0
0.5
1.0
1.5
β_IV

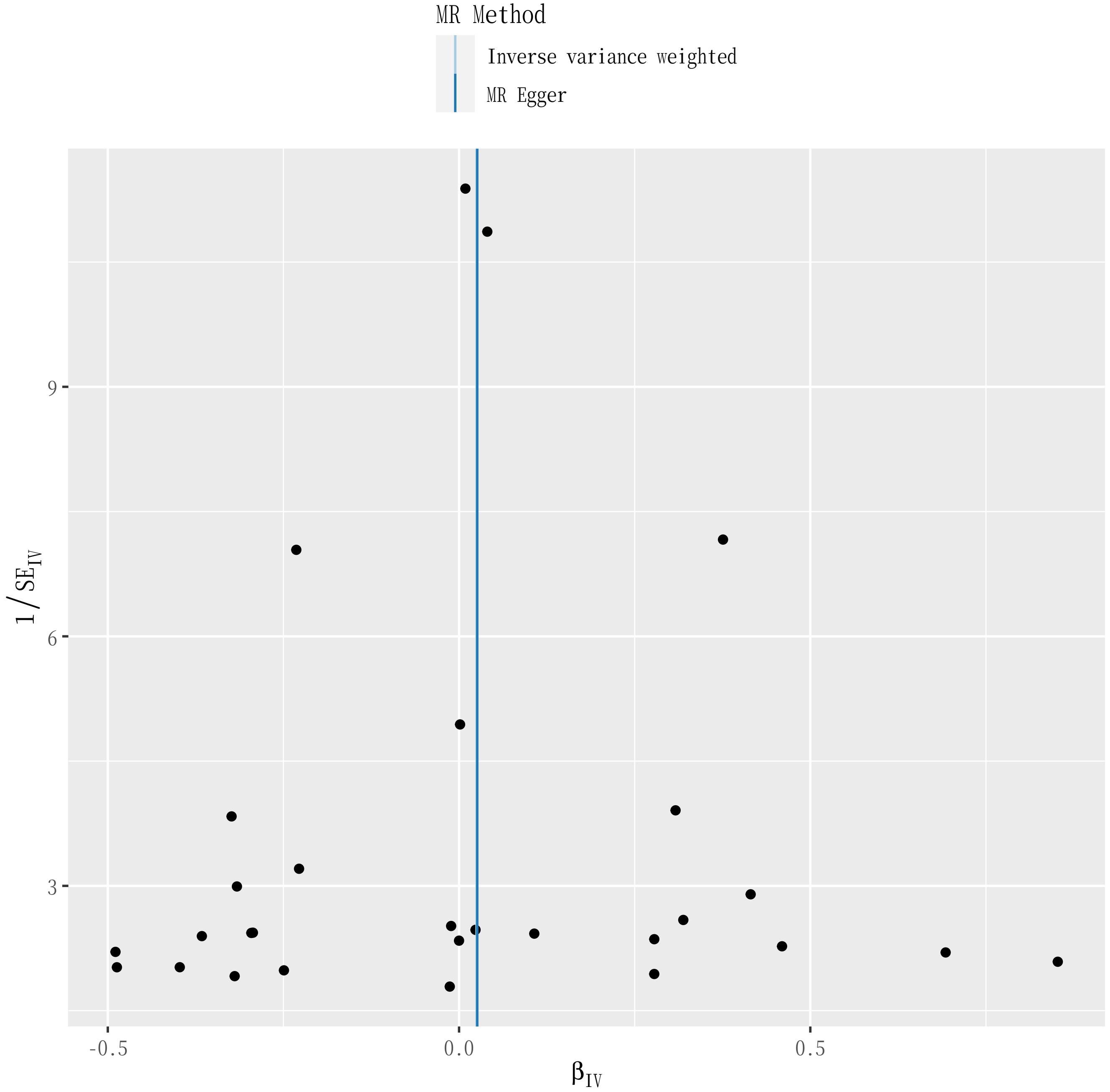

MR Method
Inverse variance weighted
MR Egger
9
6
3
1/SE$_{IV}$
-0.5
0.0
0.5
β$_{IV}$

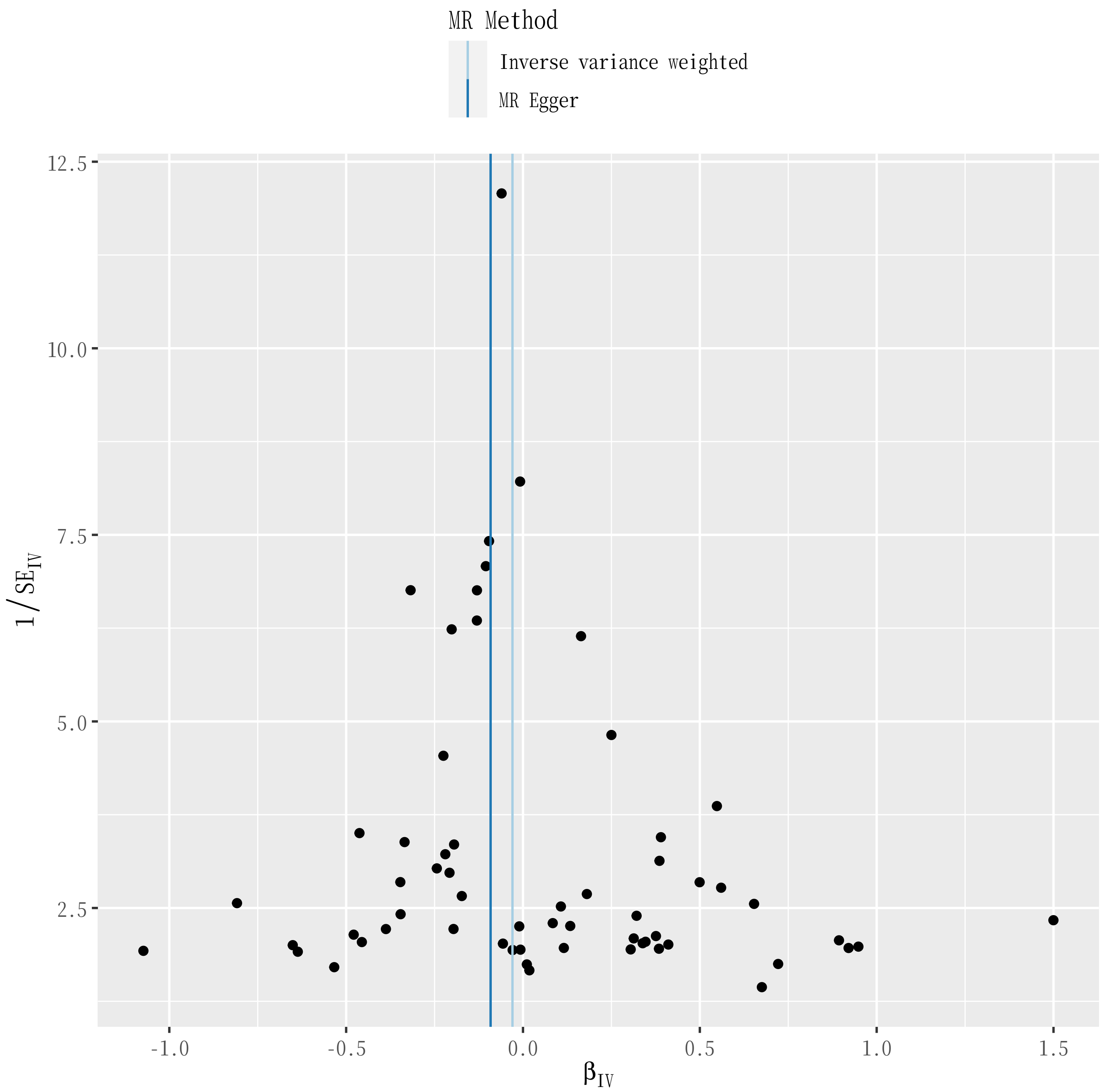
MR Method
Inverse variance weighted
MR Egger
12.5
10.0
7.5
5.0
2.5
1/SE$_{IV}$
-1.0
-0.5
0.0
0.5
1.0
1.5
β$_{IV}$

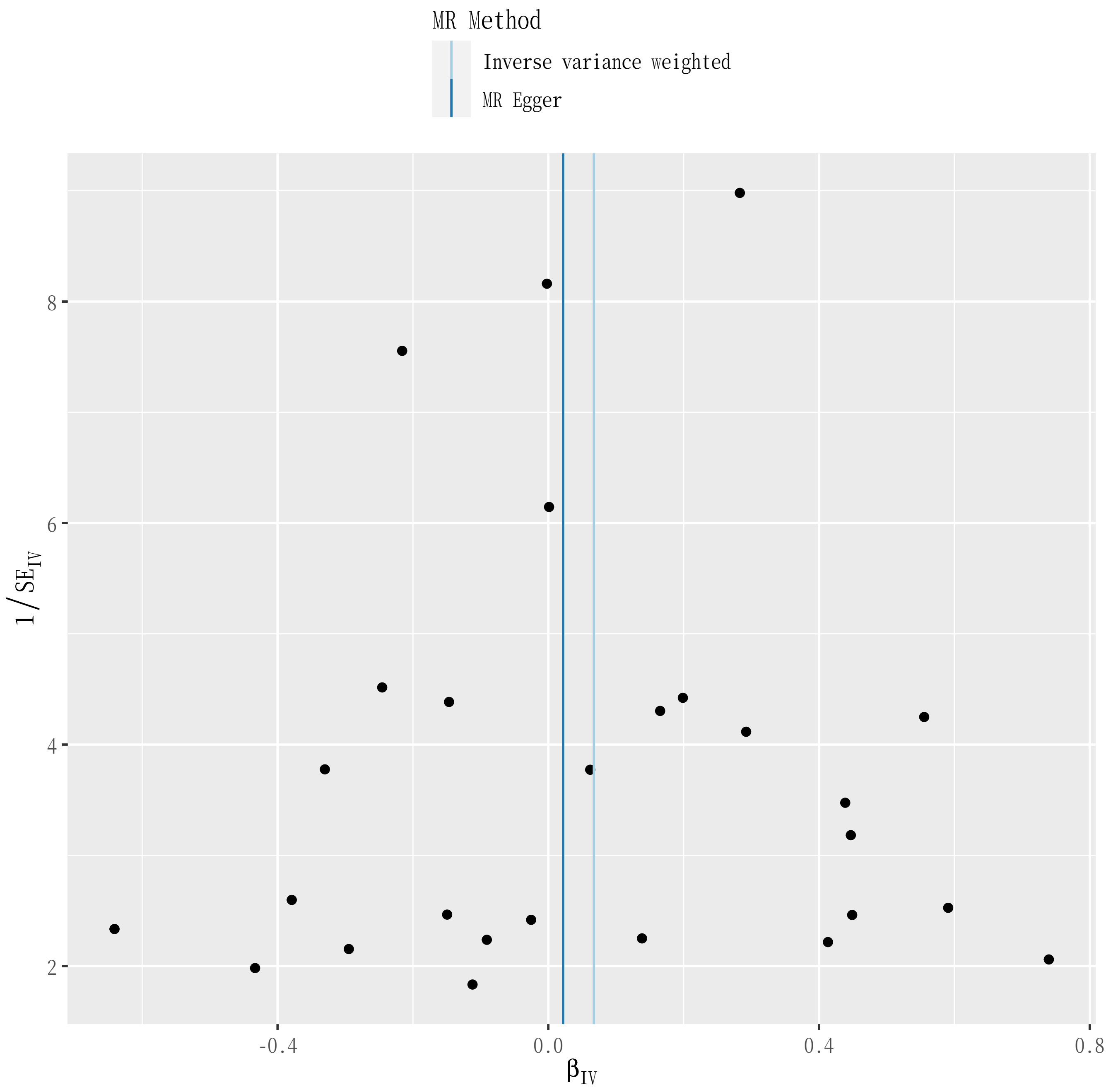
MR Method
Inverse variance weighted
MR Egger
8
6
4
2
$1/SE_{IV}$
-0.4
0.0
0.4
0.8
β_{IV}

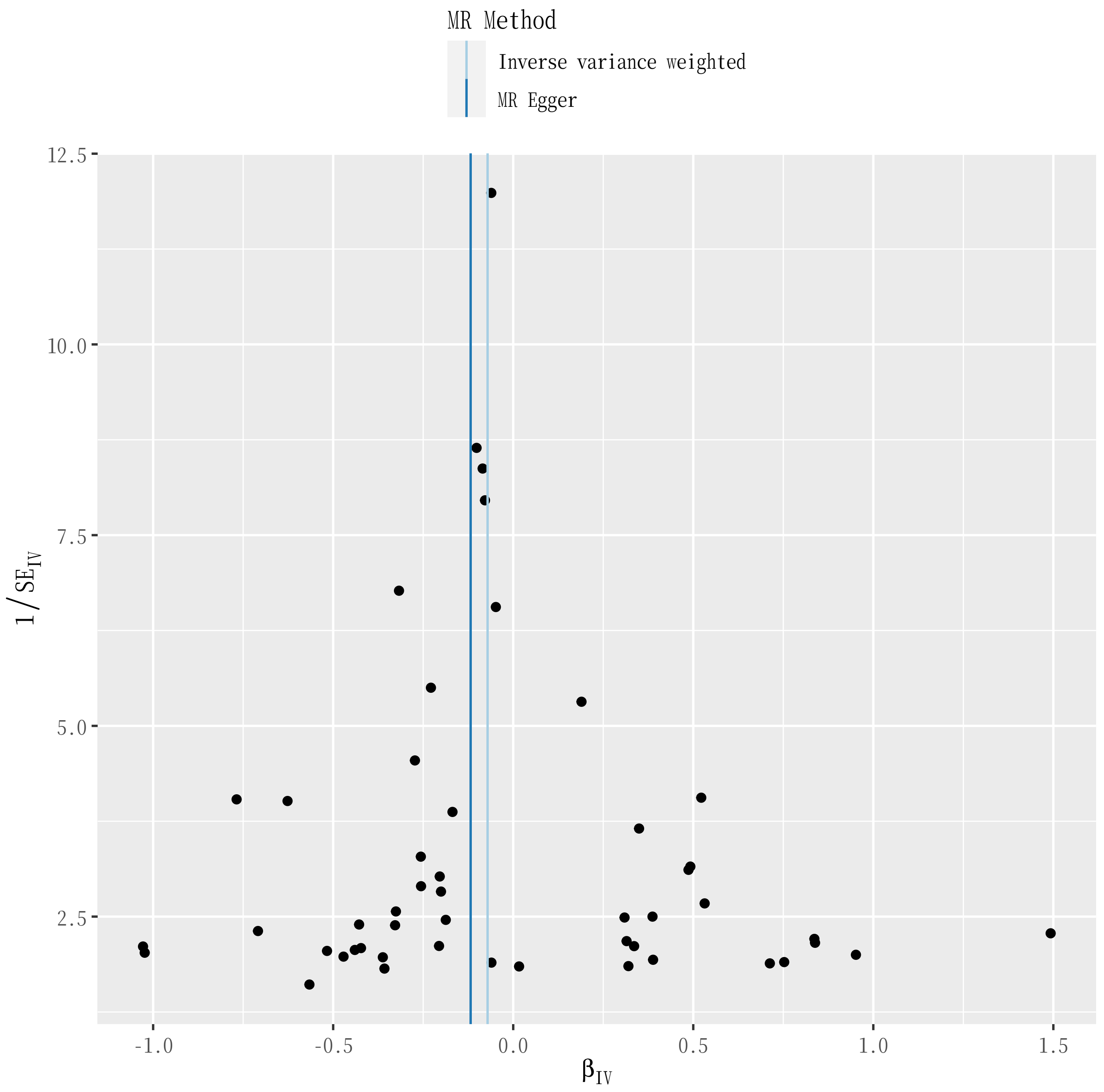

MR Method
Inverse variance weighted
MR Egger
12.5
10.0
7.5
5.0
2.5
$1/SE_{IV}$
-1.0
-0.5
0.0
0.5
1.0
1.5
β_{IV}

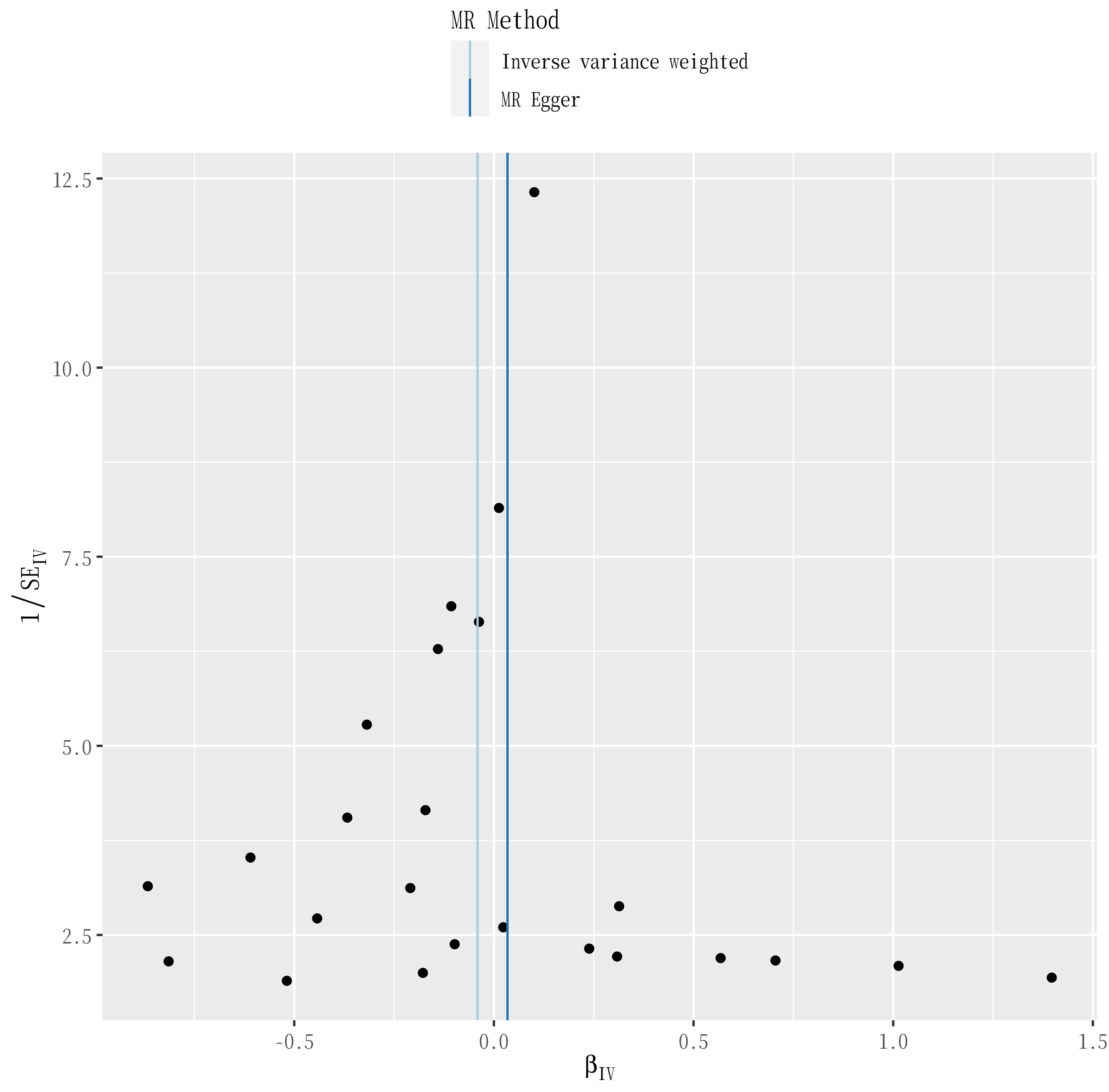
MR Method
Inverse variance weighted
MR Egger
12.5
10.0
7.5
5.0
2.5
1/SE_IV
-0.5
0.0
0.5
1.0
1.5
β_IV

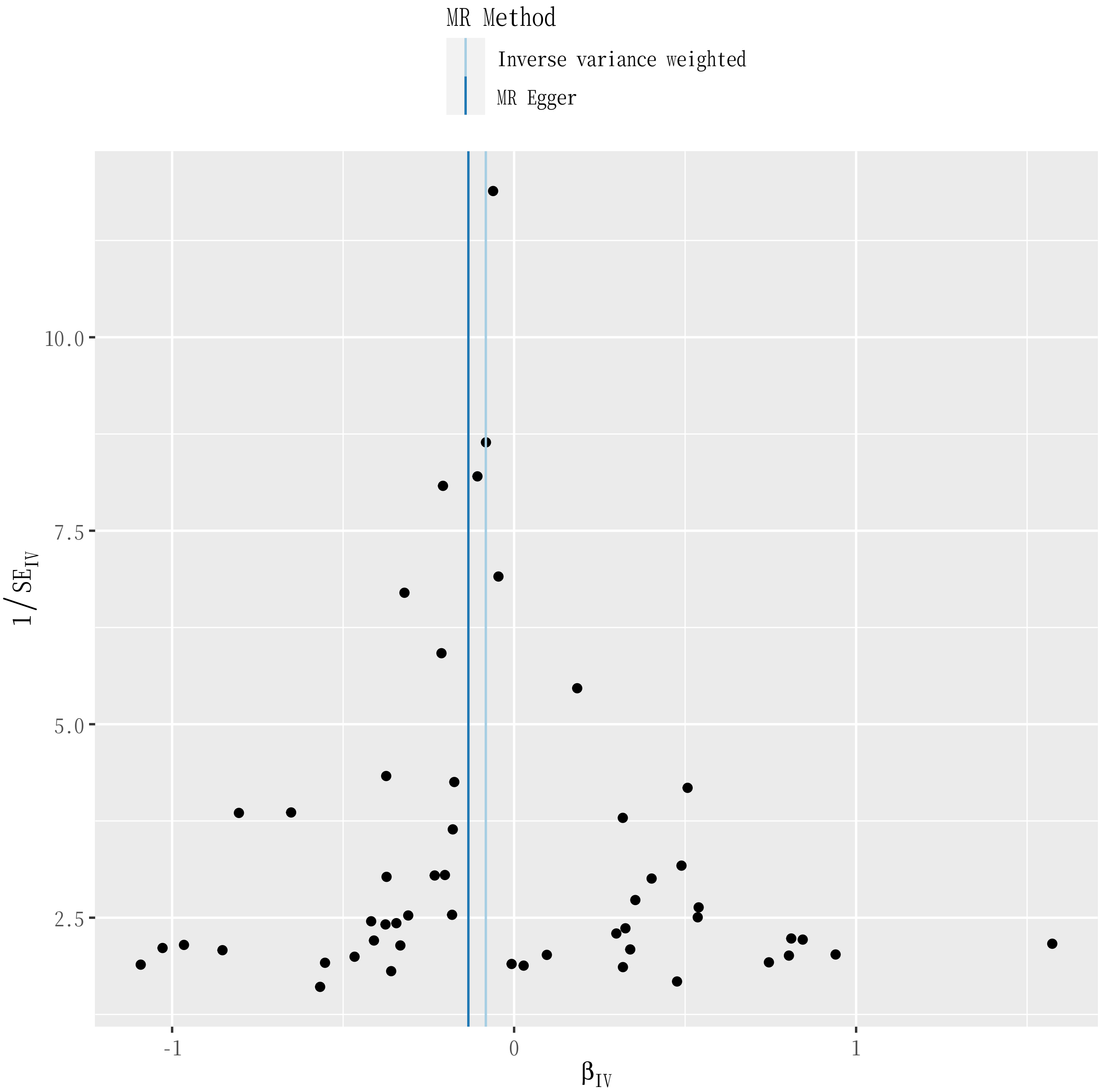

MR Method
Inverse variance weighted
MR Egger
10.0
7.5
5.0
2.5
1/SE_IV
-1
0
1
β_IV

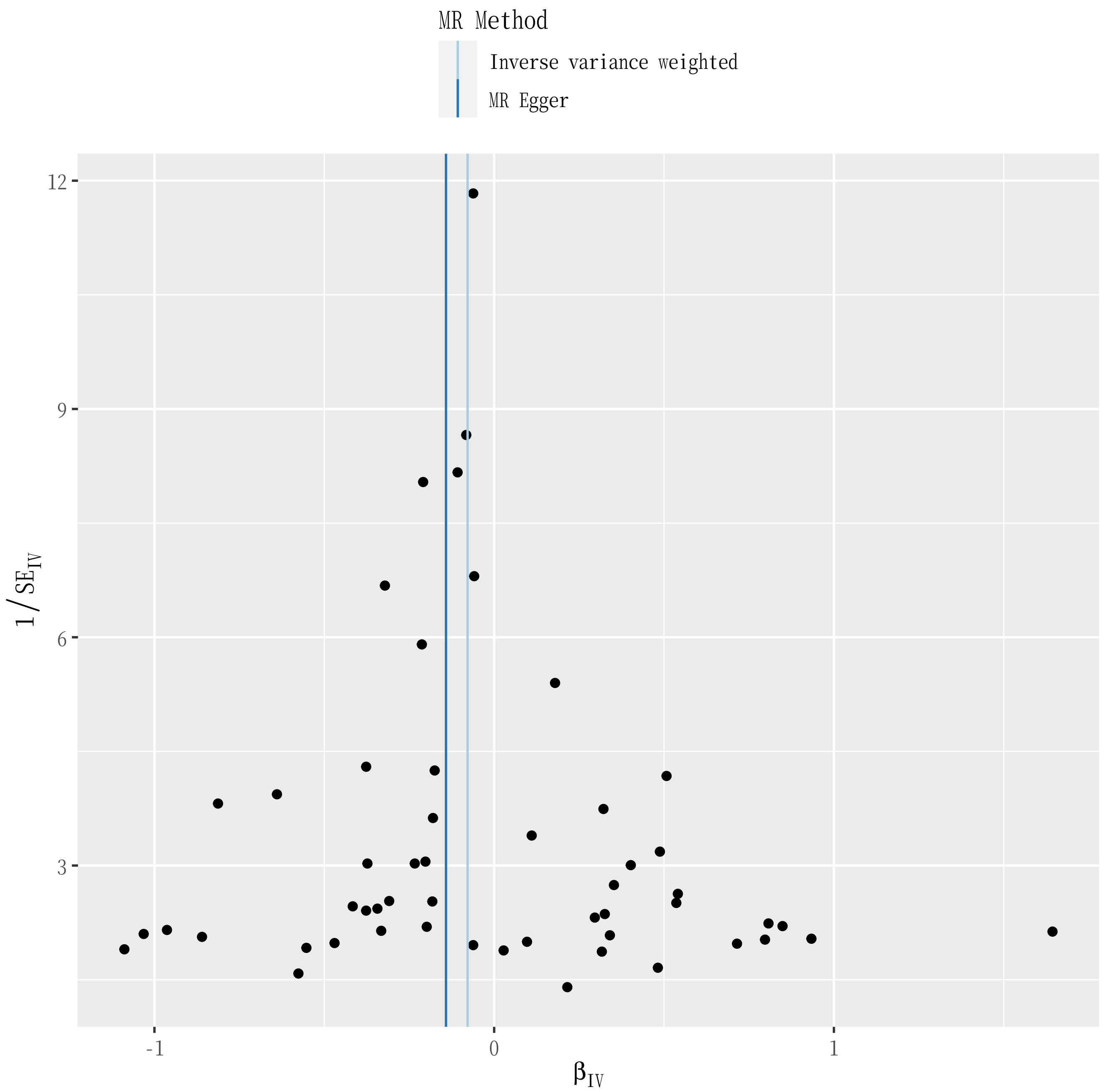

MR Method
Inverse variance weighted
MR Egger
12
9
6
3
1/SE_IV
-1
0
1
β_IV

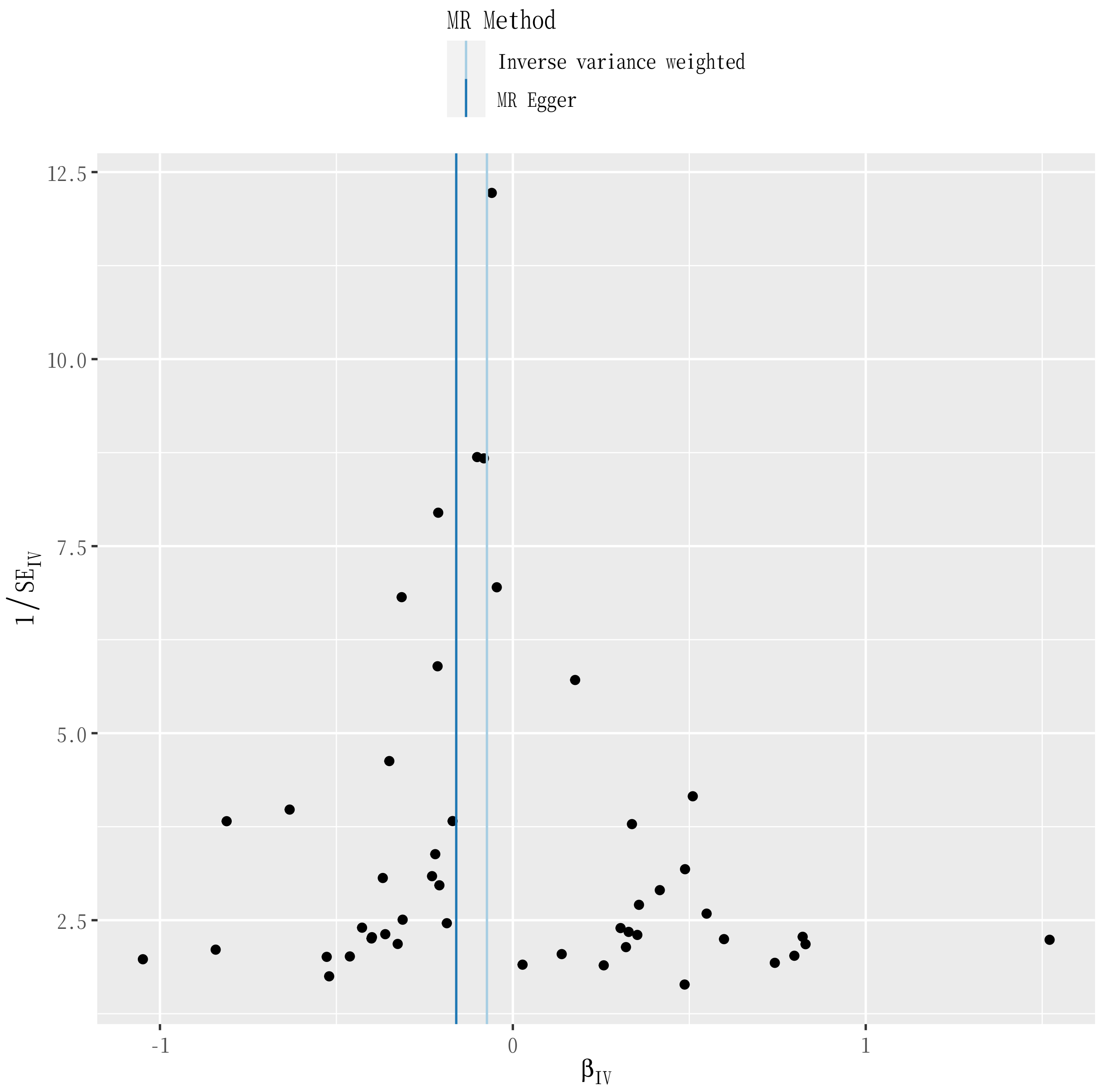

MR Method
Inverse variance weighted
MR Egger
12.5
10.0
7.5
5.0
2.5
1/SE_IV
-1
0
1
β_IV

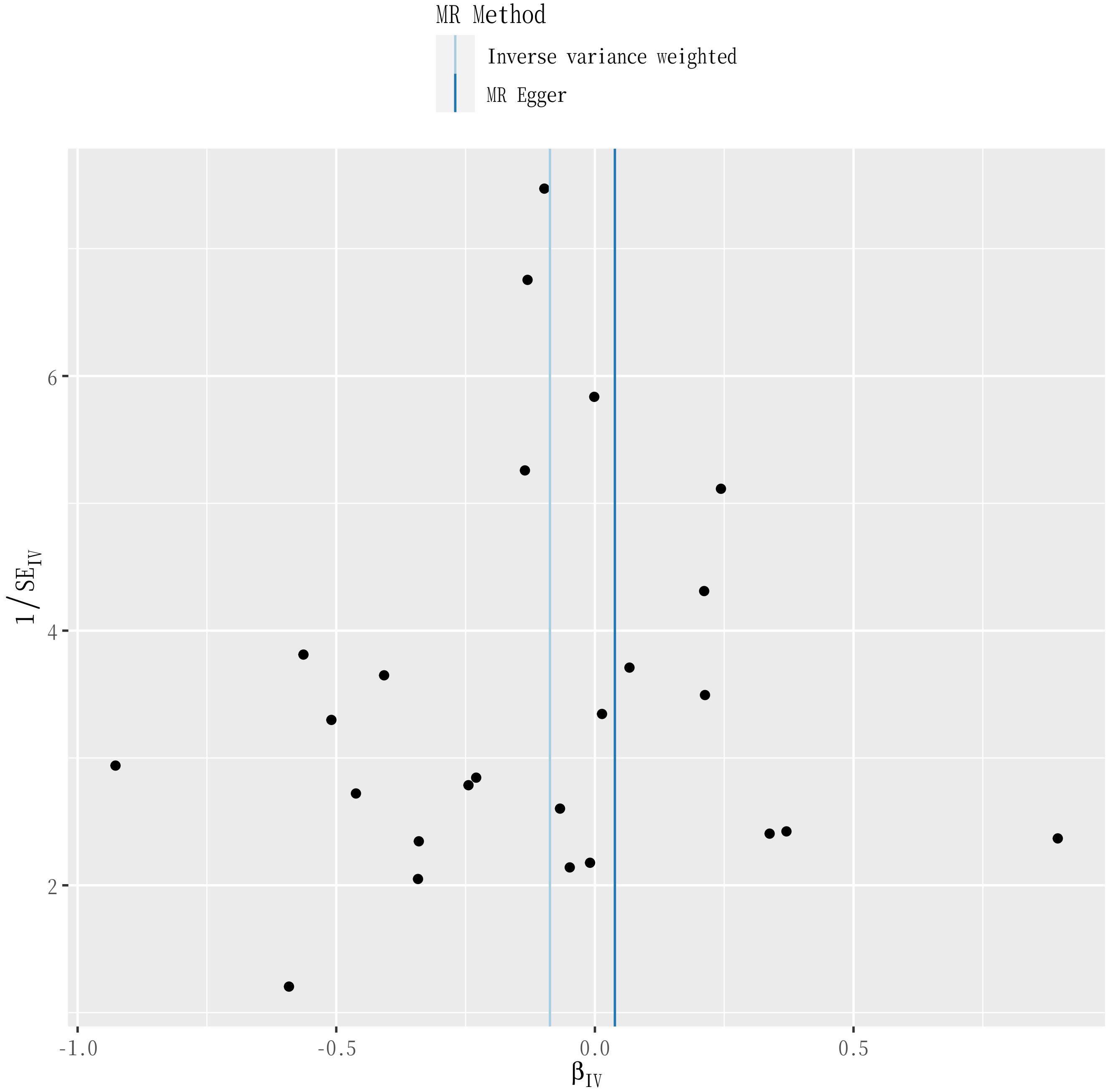
MR Method
Inverse variance weighted
MR Egger
6
4
2
$1/SE_{IV}$
-1.0
-0.5
0.0
0.5
β_{IV}

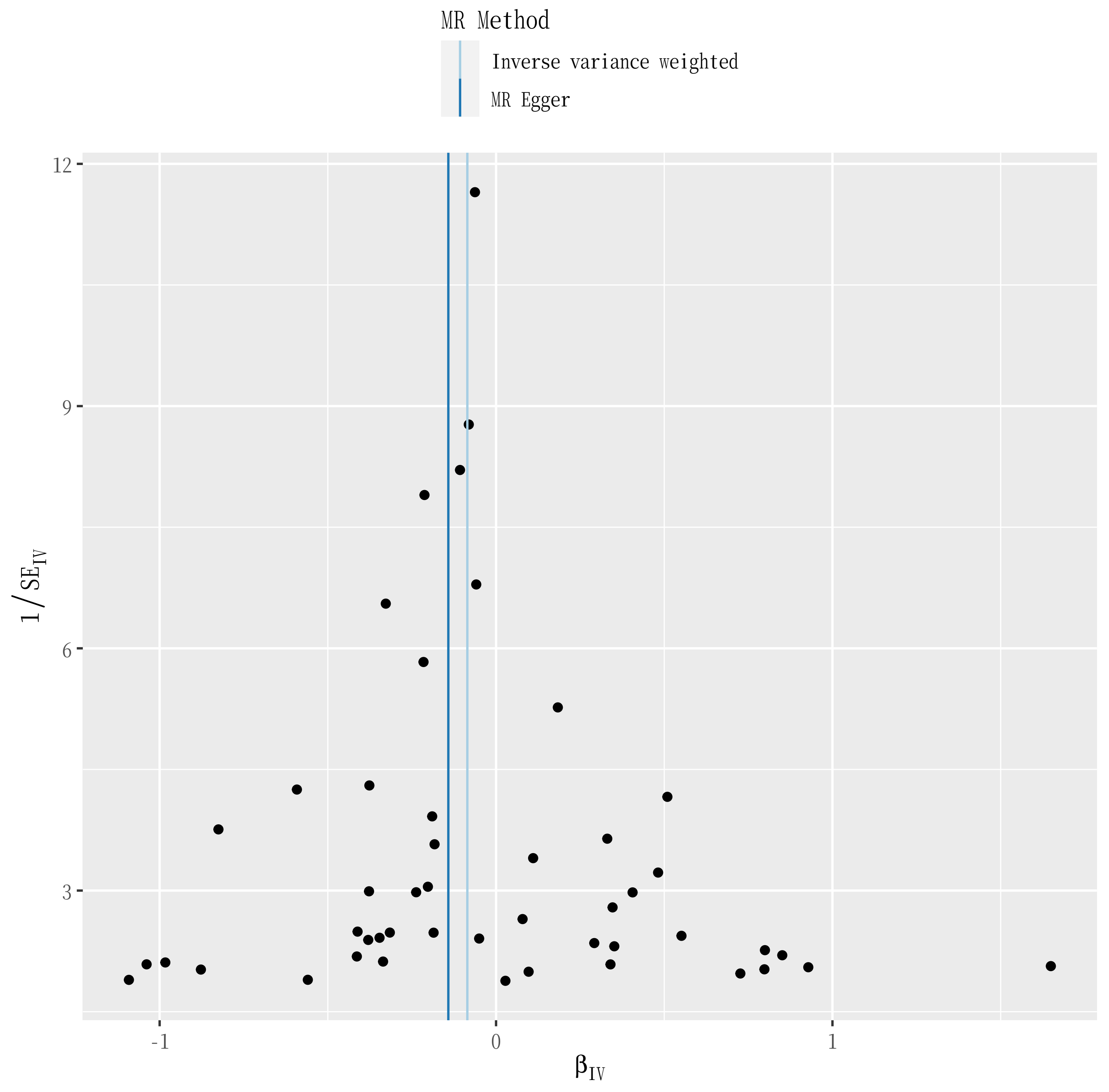
MR Method
Inverse variance weighted
MR Egger
12
9
6
3
1/SE_IV
-1
0
1
β_IV

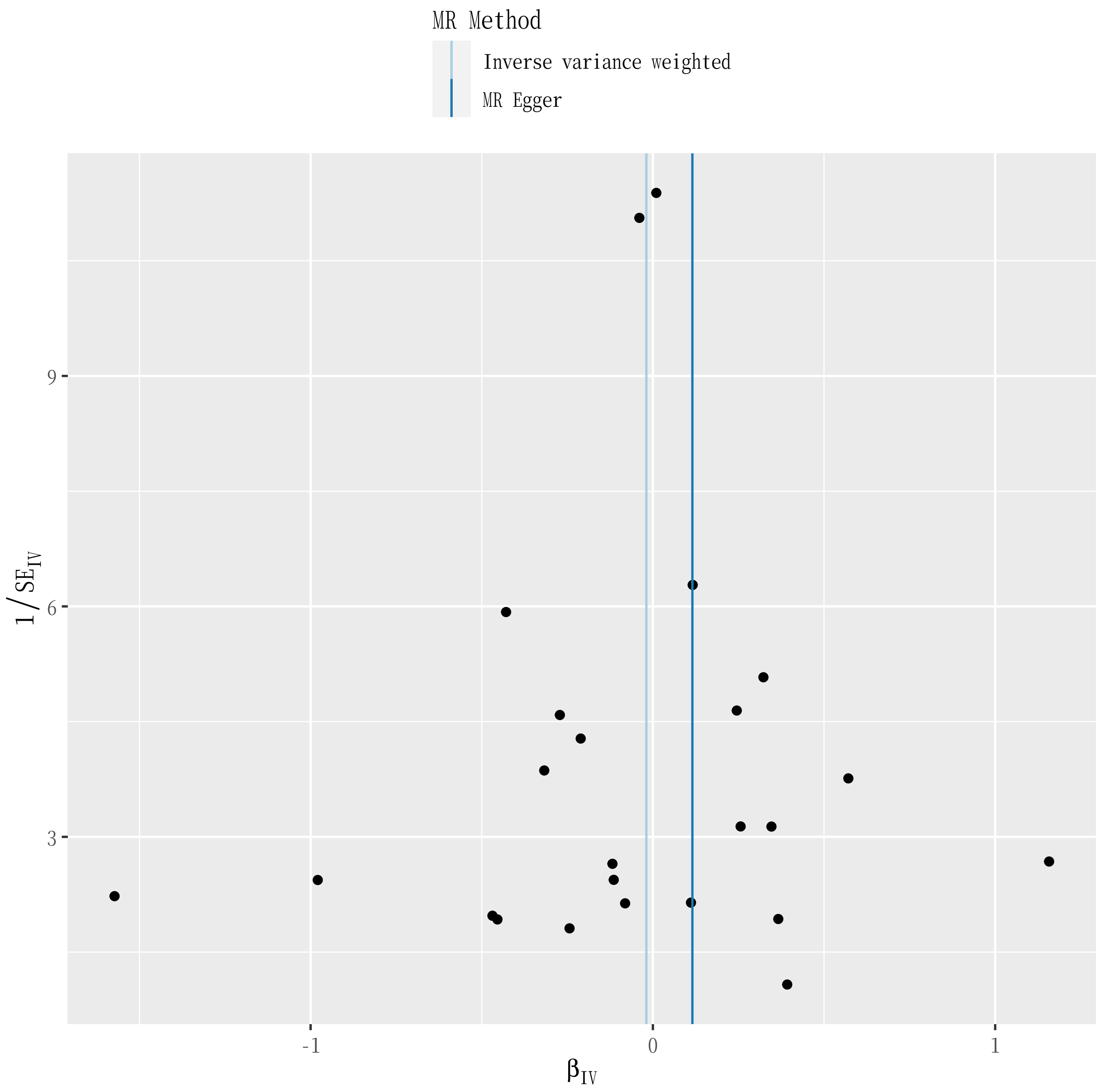

MR Method
Inverse variance weighted
MR Egger
9
6
3
$1/SE_{IV}$
-1
0
1
β_{IV}
