# Supplementary material for: Assessing the causal relationships between circulating metabolic biomarkers and breast cancer by using mendelian randomization
Source: Front Genet. 2024 Dec 18;15:1448748. doi: 10.3389/fgene.2024.1448748 (PMC11688392; doi:10.3389/fgene.2024.1448748)
Supplement: Supplementary file 7 [file Table1.docx]

Supplementary Material

Assessing the causal relationships between circulating metabolic biomarkers and breast cancer by using Mendelian Randomization

Bowen Wang^1^, Yue Ling^2^, Hui Zhang^1^, Ming Yang^1*^

*** Correspondence:** Ming Yang: yangming@jlu.edu.cn

# Supplementary materials

## Additional file 1.xlsx

Supplementary Table 1. Circulating metabolic biomarkers Index Number and Name Correspondence Table.

Supplementary Table 2. Associations between circulating metabolic biomarkers and BC from Mendelian randomization analyses.

Supplementary Table 3. Associations between circulating metabolic biomarkers and ER+ BC from Mendelian randomization analyses.

Supplementary Table 4. Associations between circulating metabolic biomarkers and ER- BC from Mendelian randomization analyses.

Supplementary Table 5. Heterogeneity in BC.

Supplementary Table 6. Heterogeneity in ER+ BC.

Supplementary Table 7. Heterogeneity in ER- BC.

Supplementary Table 8. Pleiotropy in BC.

Supplementary Table 9. Pleiotropy in ER+ BC.

Supplementary Table 10. Pleiotropy in ER- BC.

Supplementary Table 11. Details of the instrumental variables of circulating metabolic biomarkers used in MR analysis on BC.

Supplementary Table 12. Details of the instrumental variables of circulating metabolic biomarkers used in MR analysis on ER+ BC.

Supplementary Table 13. Details of the instrumental variables of circulating metabolic biomarkers used in MR analysis on ER- BC.

Supplementary Table 14. Reverse causality in BC.

Supplementary Table 15. Reverse causality in ER+ BC.

Supplementary Table 16. Reverse causality in ER- BC.

Supplementary Table 17: List of Circulating metabolic biomarkers and their Units.

## STROBE-MR-checklisk.pdf

STROBE-MR checklist of recommended items to address in reports of Mendelian randomization studies1

## Leave-one-out plot of circulating metabolic biomarkers and BC

## Leave-one-out plot of circulating metabolic biomarkers and ER+BC

## Leave-one-out plot of circulating metabolic biomarkers and ER-BC

## Funnel plot of circulating metabolic biomarkers and BC

## Funnel plot of circulating metabolic biomarkers and ER+BC

## Funnel plot of circulating metabolic biomarkers and ER-BC

## Forest plot of circulating metabolic biomarkers and BC

## Forest plot of circulating metabolic biomarkers and ER+BC

## Forest plot of circulating metabolic biomarkers and ER-BC

## Scatter plot of circulating metabolic biomarkers and BC

## Scatter plot of circulating metabolic biomarkers and ER+BC

## Scatter plot of circulating metabolic biomarkers and ER-BC
