# Supplementary material for: Assessing the causal relationships between circulating metabolic biomarkers and breast cancer by using mendelian randomization
Source: Front Genet. 2024 Dec 18;15:1448748. doi: 10.3389/fgene.2024.1448748 (PMC11688392; doi:10.3389/fgene.2024.1448748)
Supplement: Supplementary file 8 [file DataSheet11.pdf]

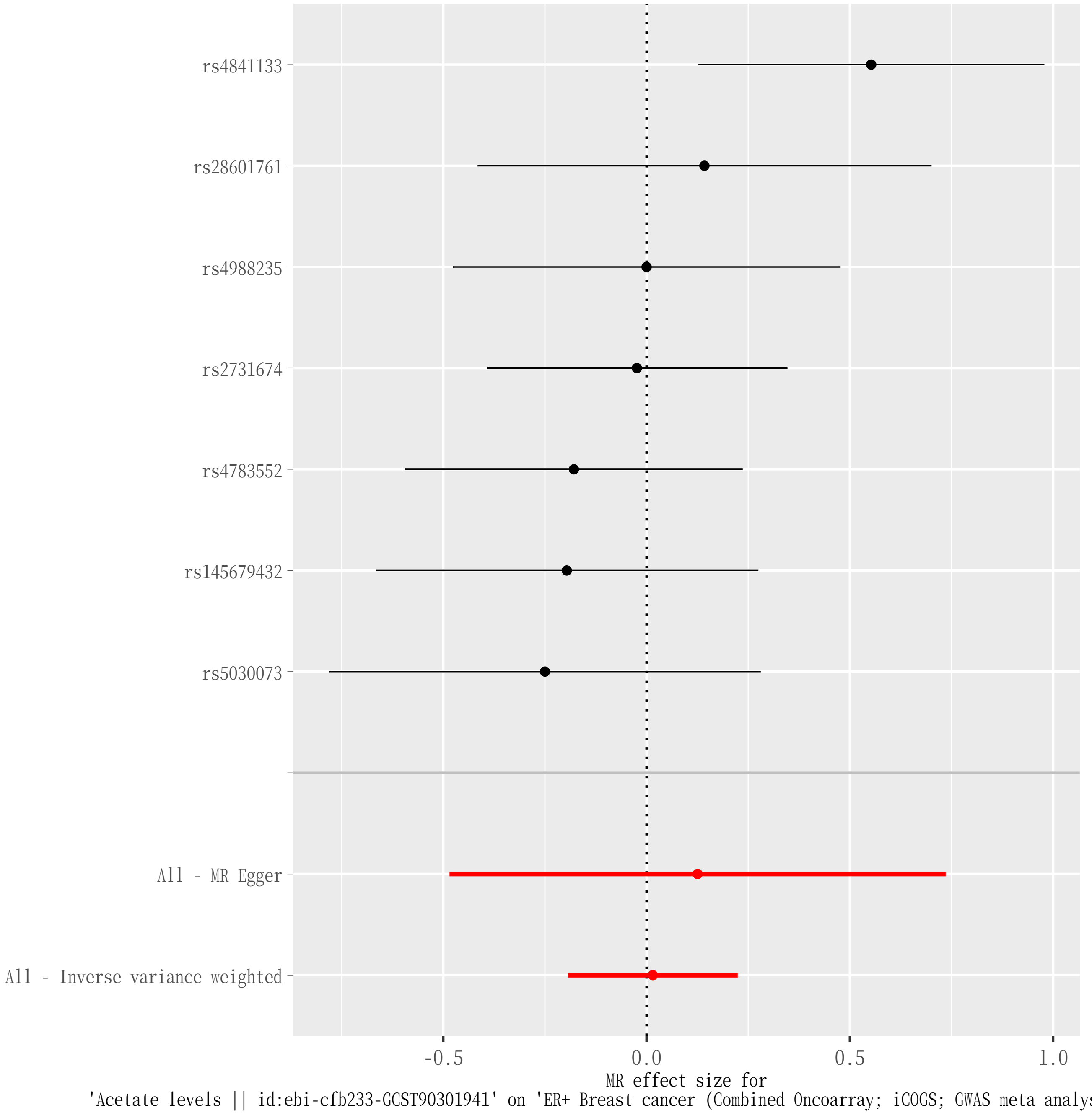
rs4841133
rs28601761
rs4988235
rs2731674
rs4783552
rs145679432
rs5030073
All - MR Egger
All - Inverse variance weighted
-0.5
0.0
0.5
1.0
MR effect size for
'Acetate levels || id:ebi-cfb233-GCST90301941' on 'ER+ Breast cancer (Combined Oncoarray; iCOGS; GWAS meta analys

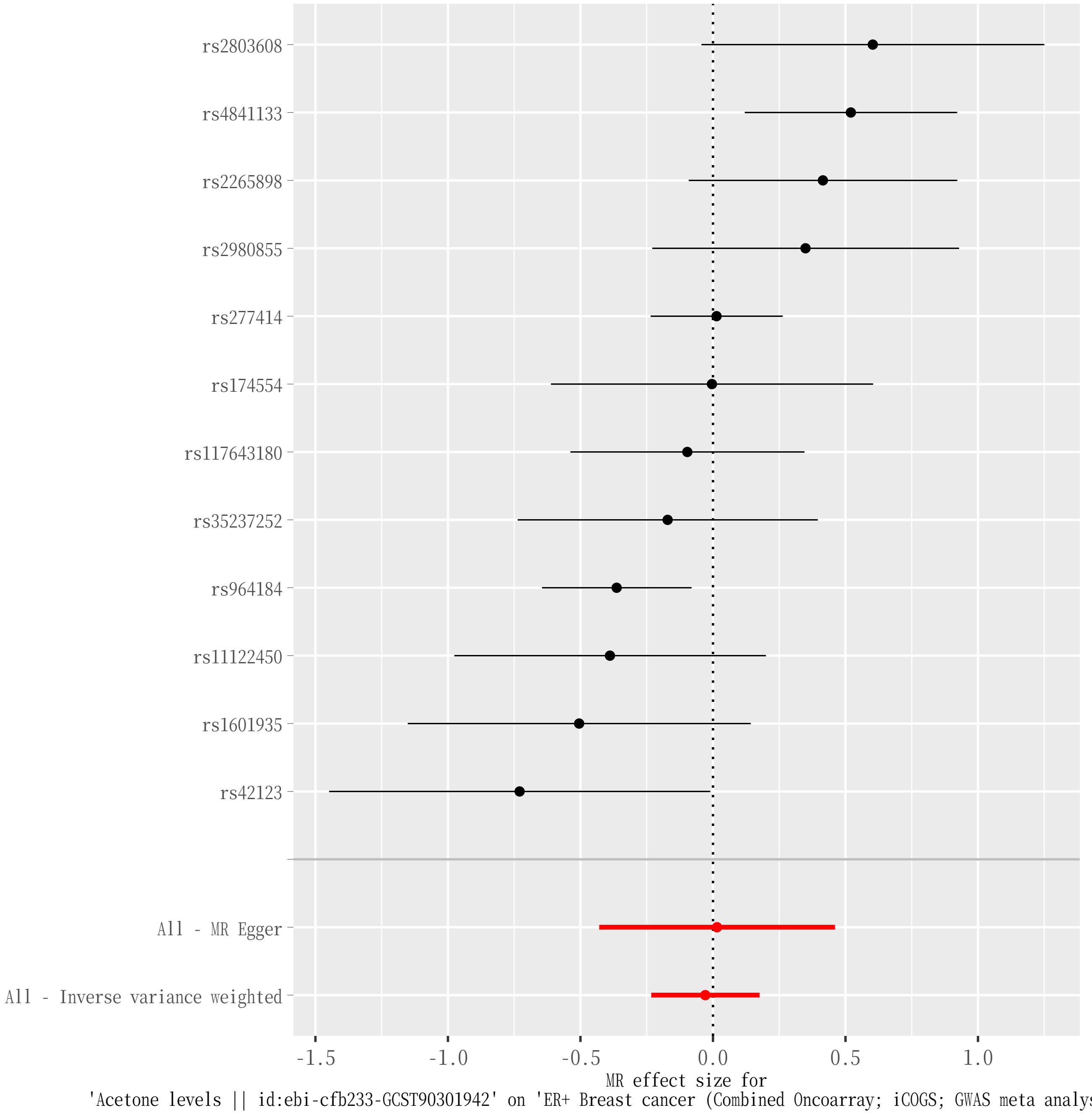

rs2803608
rs4841133
rs2265898
rs2980855
rs277414
rs174554
rs117643180
rs35237252
rs964184
rs11122450
rs1601935
rs42123
All - MR Egger
All - Inverse variance weighted
-1.5
-1.0
-0.5
0.0
0.5
1.0
MR effect size for
'Acetone levels || id:ebi-cfb233-GCST90301942' on 'ER+ Breast cancer (Combined Oncoarray; iCOGS; GWAS meta analys

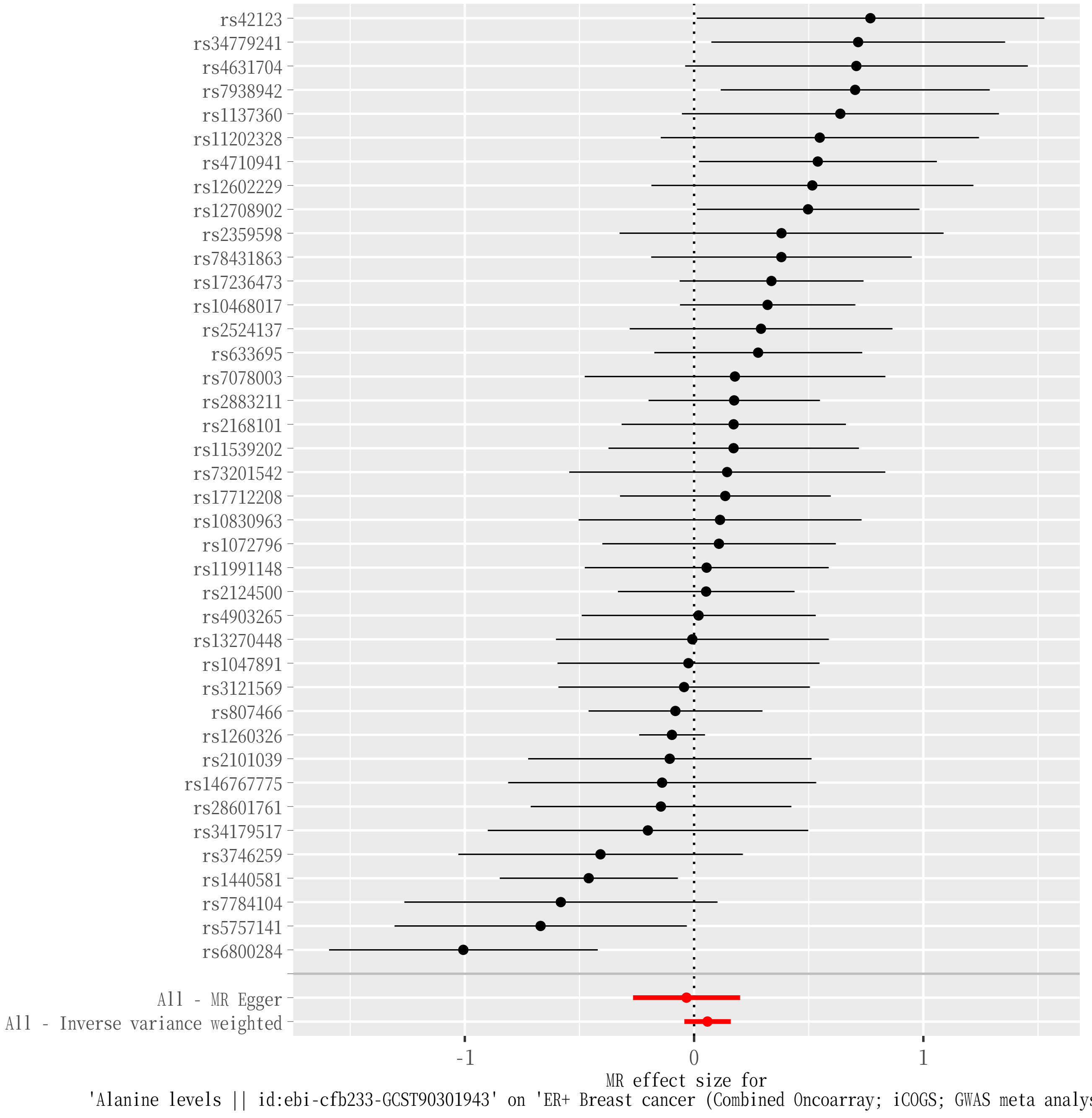

rs42123
rs34779241
rs4631704
rs7938942
rs1137360
rs11202328
rs4710941
rs12602229
rs12708902
rs2359598
rs78431863
rs17236473
rs10468017
rs2524137
rs633695
rs7078003
rs2883211
rs2168101
rs11539202
rs73201542
rs17712208
rs10830963
rs1072796
rs11991148
rs2124500
rs4903265
rs13270448
rs1047891
rs3121569
rs807466
rs1260326
rs2101039
rs146767775
rs28601761
rs34179517
rs3746259
rs1440581
rs7784104
rs5757141
rs6800284
All - MR Egger
All - Inverse variance weighted
-1
0
1
MR effect size for
'Alanine levels || id:ebi-cfb233-GCST90301943' on 'ER+ Breast cancer (Combined Oncoarray; iCOGS; GWAS meta analys

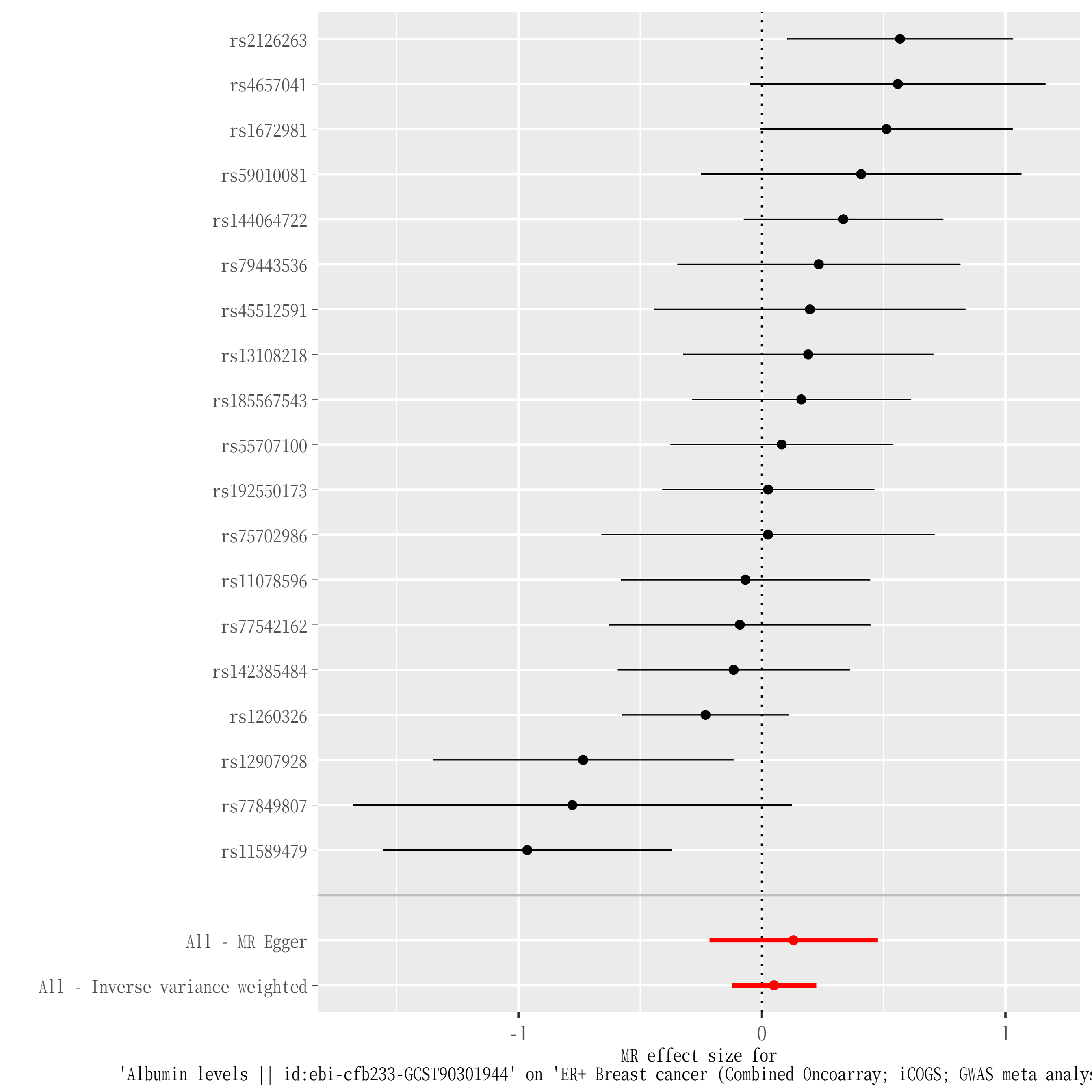
rs2126263
rs4657041
rs1672981
rs59010081
rs144064722
rs79443536
rs45512591
rs13108218
rs185567543
rs55707100
rs192550173
rs75702986
rs11078596
rs77542162
rs142385484
rs1260326
rs12907928
rs77849807
rs11589479
All - MR Egger
All - Inverse variance weighted
-1
0
1
MR effect size for
'Albumin levels || id:ebi-cfb233-GCST90301944' on 'ER+ Breast cancer (Combined Oncoarray; iCOGS; GWAS meta analys

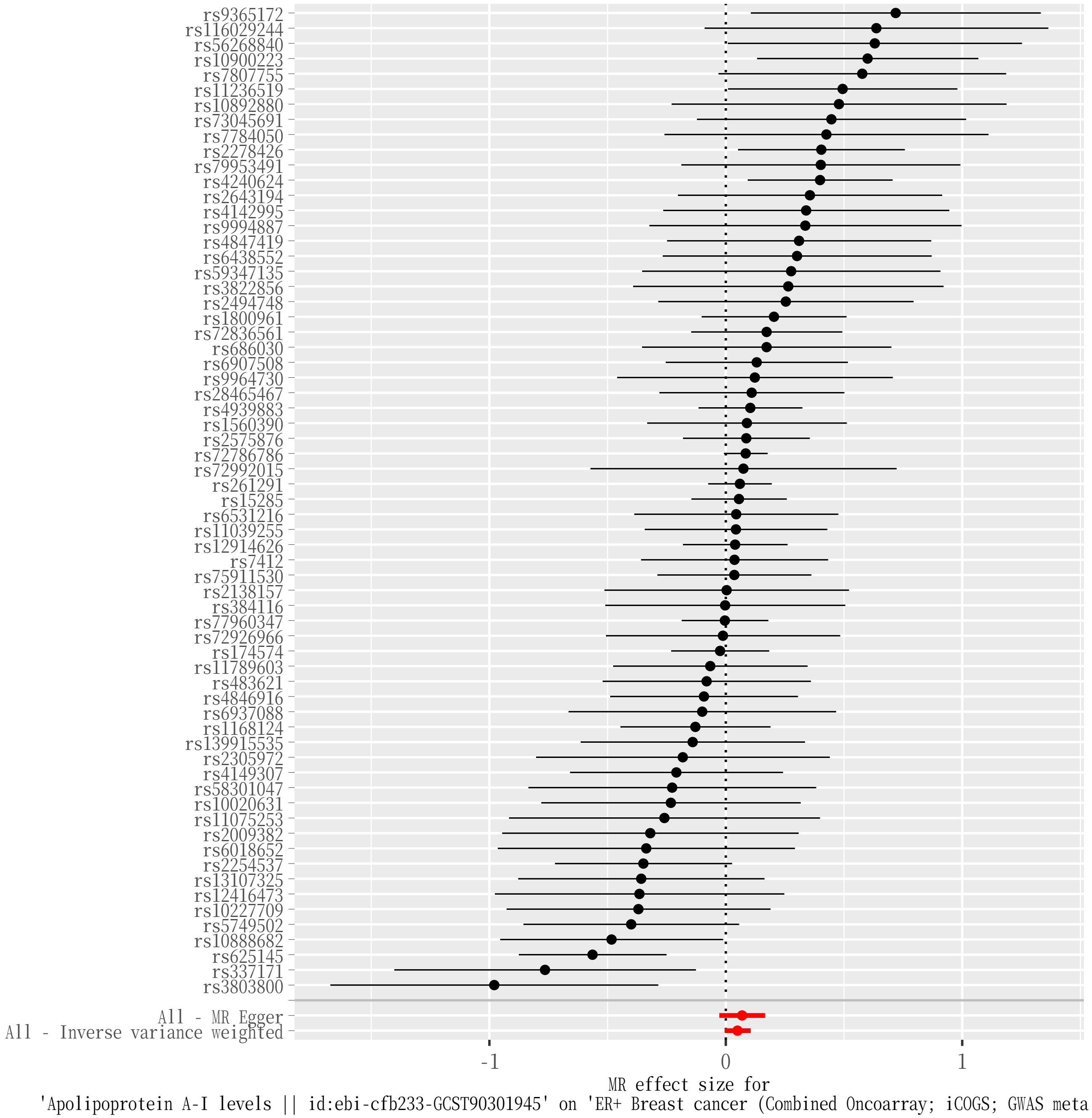
rs9365172
rs116029244
rs56268840
rs10900223
rs7807755
rs11236519
rs10892880
rs73045691
rs7784050
rs2278426
rs79953491
rs4240624
rs2643194
rs4142995
rs9994887
rs4847419
rs6438552
rs59347135
rs3822856
rs2494748
rs1800961
rs72836561
rs686030
rs6907508
rs9964730
rs28465467
rs4939883
rs1560390
rs2575876
rs72786786
rs72992015
rs261291
rs15285
rs6531216
rs11039255
rs12914626
rs7412
rs75911530
rs2138157
rs384116
rs77960347
rs72926966
rs174574
rs11789603
rs483621
rs4846916
rs6937088
rs1168124
rs139915535
rs2305972
rs4149307
rs58301047
rs10020631
rs11075253
rs2009382
rs6018652
rs2254537
rs13107325
rs12416473
rs10227709
rs5749502
rs10888682
rs625145
rs337171
rs3803800
All - MR Egger
All - Inverse variance weighted
-1
0
1
MR effect size for
'Apolipoprotein A-I levels || id:ebi-cfb233-GCST90301945' on 'ER+ Breast cancer (Combined Oncoarray; iCOGS; GWAS meta

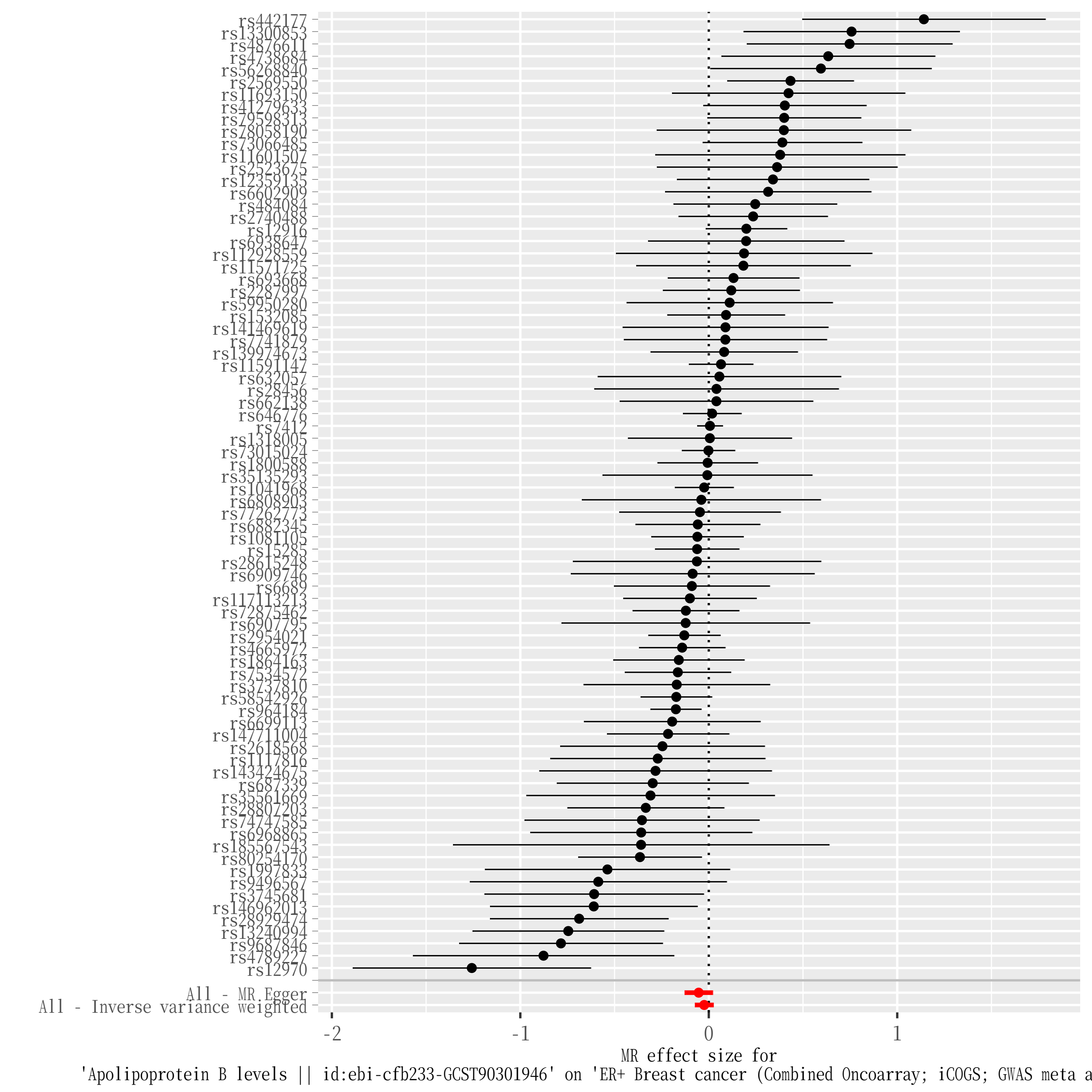

rs442177
rs13300853
rs4876611
rs4738684
rs56268840
rs2569550
rs11693150
rs41279633
rs79528313
rs78058190
rs73066485
rs11601507
rs2523675
rs12359135
rs6602909
rs484084
rs2740488
rs12916
rs6938647
rs112928559
rs11571725
rs693668
rs2287997
rs59950280
rs1532085
rs141469619
rs7741879
rs139974673
rs11591147
rs632057
rs28456
rs662138
rs646776
rs7412
rs1318005
rs73015024
rs1800588
rs35135293
rs1041968
rs6808903
rs77262773
rs6882345
rs1081105
rs15285
rs28615248
rs6909746
rs6689
rs117113213
rs72875462
rs6907795
rs2954021
rs4665972
rs1864163
rs7534572
rs3737810
rs58542926
rs964184
rs6699113
rs147711004
rs2618568
rs1117816
rs143424675
rs687339
rs35561669
rs28807203
rs74747585
rs6968865
rs185567543
rs80254170
rs1997833
rs9496567
rs3745681
rs146962013
rs28929474
rs13240994
rs9687846
rs4789227
rs12970
All - MR Egger
All - Inverse variance weighted
-2
-1
0
1
MR effect size for
'Apolipoprotein B levels || id:ebi-cfb233-GCST90301946' on 'ER+ Breast cancer (Combined Oncoarray; iCOGS; GWAS meta a

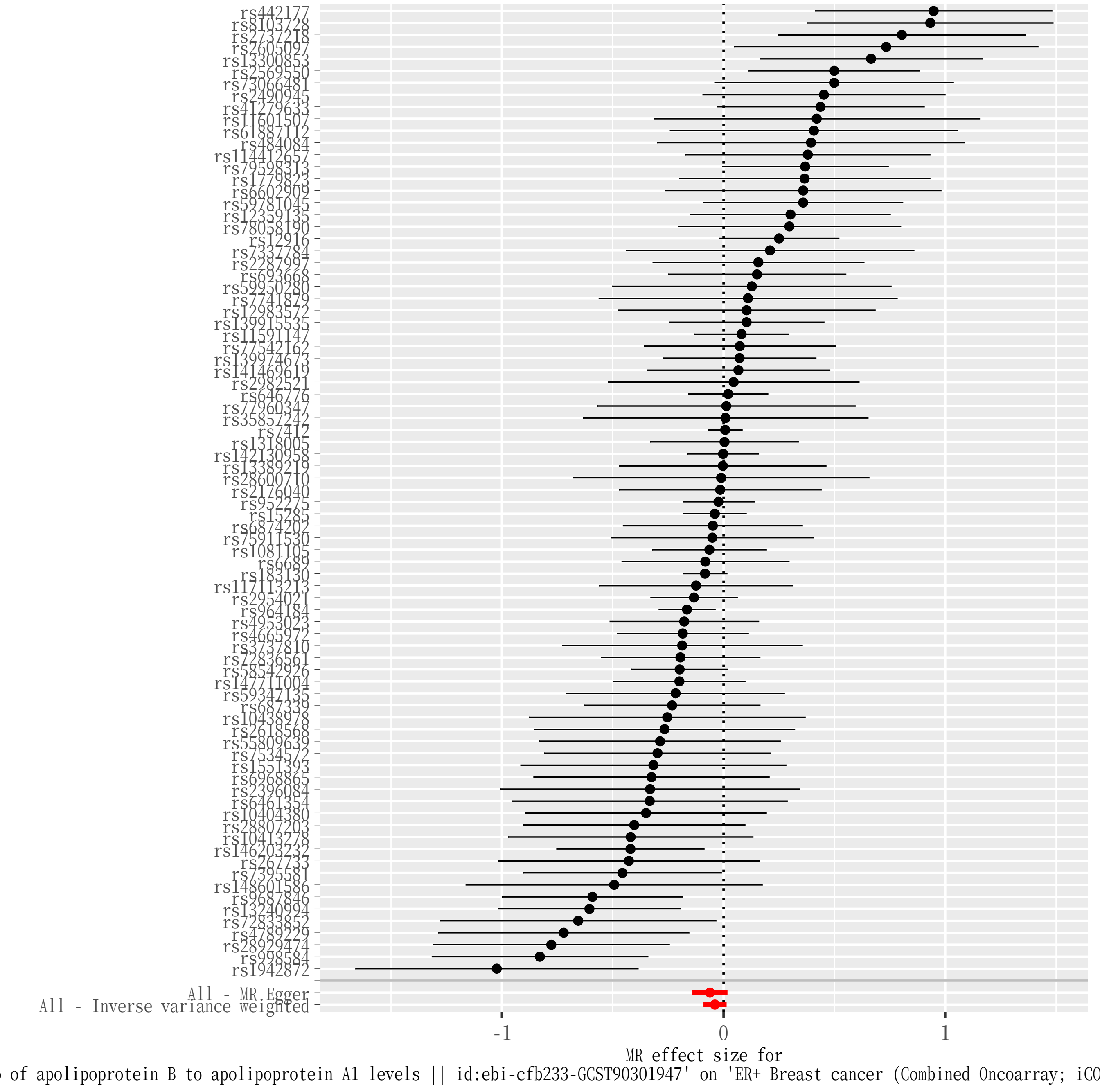
rs442177
rs8103728
rs2737218
rs2605097
rs13300853
rs2569550
rs73066481
rs2490945
rs41279633
rs11601507
rs61887112
rs484084
rs114412657
rs79598313
rs1779823
rs6602909
rs59781045
rs12359135
rs78058190
rs12916
rs7337784
rs2287927
rs693668
rs59950280
rs7741879
rs12983572
rs139915535
rs11591147
rs77542162
rs139974673
rs141469619
rs2982521
rs646776
rs77960347
rs35857242
rs7412
rs1318005
rs142130958
rs13389219
rs28600710
rs2176040
rs952275
rs15285
rs6874202
rs75911530
rs1081105
rs6689
rs183130
rs117113213
rs2954021
rs964184
rs4953023
rs4665972
rs3737810
rs72836561
rs58542926
rs147711004
rs59342135
rs687339
rs10438978
rs2618568
rs55809639
rs7534572
rs1551393
rs6968865
rs2396084
rs6461354
rs10404380
rs28807203
rs10413278
rs146203232
rs267733
rs7395581
rs148601586
rs9687846
rs13240994
rs72833852
rs4789229
rs28929474
rs998584
rs1942872
All - MR Egger
All - Inverse variance weighted
-1
0
1
MR effect size for
o of apolipoprotein B to apolipoprotein A1 levels || id:ebi-cfb233-GCST90301947' on 'ER+ Breast cancer (Combined Oncoarray; iCO

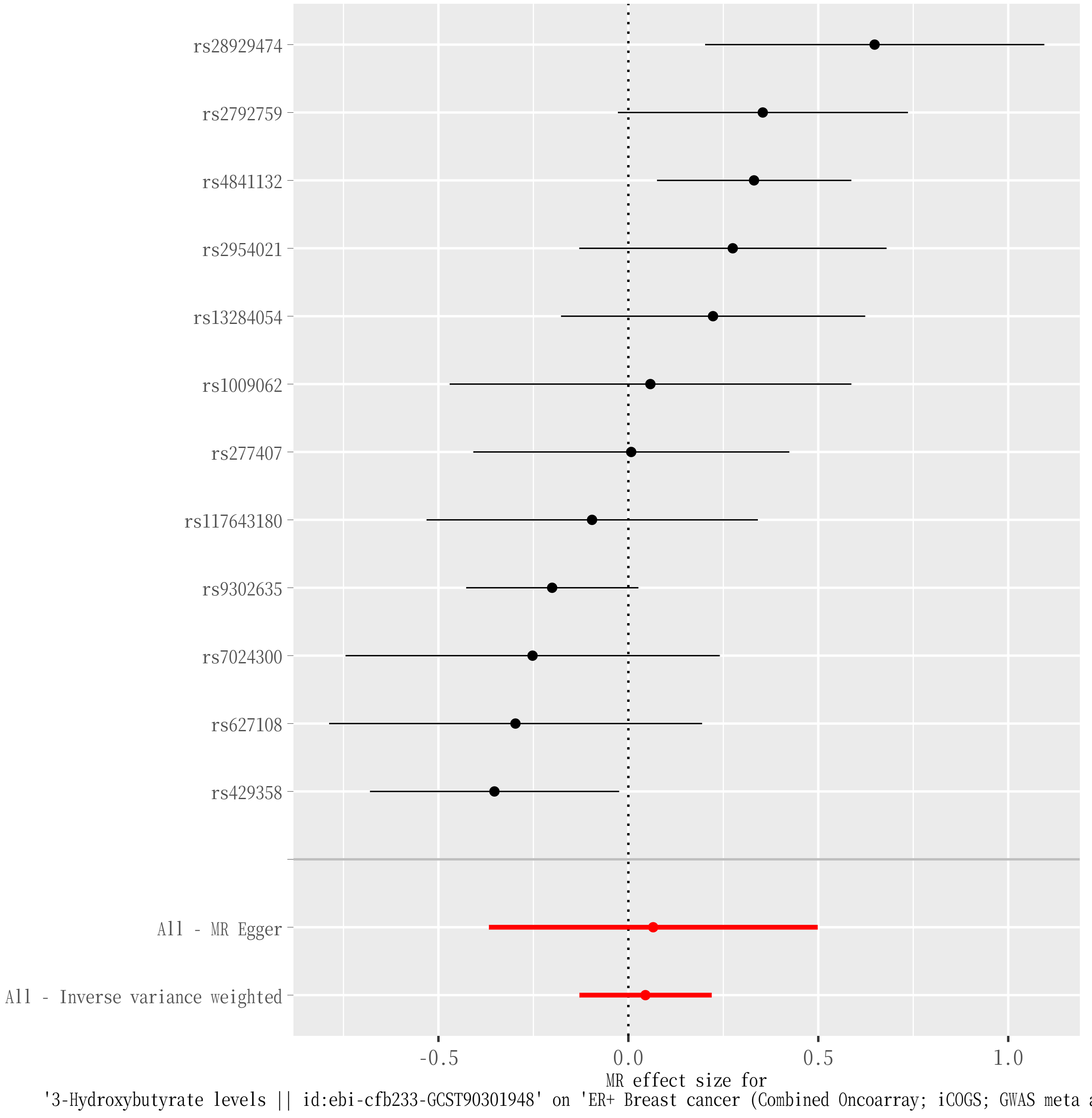

rs28929474
rs2792759
rs4841132
rs2954021
rs13284054
rs1009062
rs277407
rs117643180
rs9302635
rs7024300
rs627108
rs429358
All - MR Egger
All - Inverse variance weighted
-0.5
0.0
0.5
1.0
MR effect size for
'3-Hydroxybutyrate levels || id:ebi-cfb233-GCST90301948' on 'ER+ Breast cancer (Combined Oncoarray; iCOGS; GWAS meta a

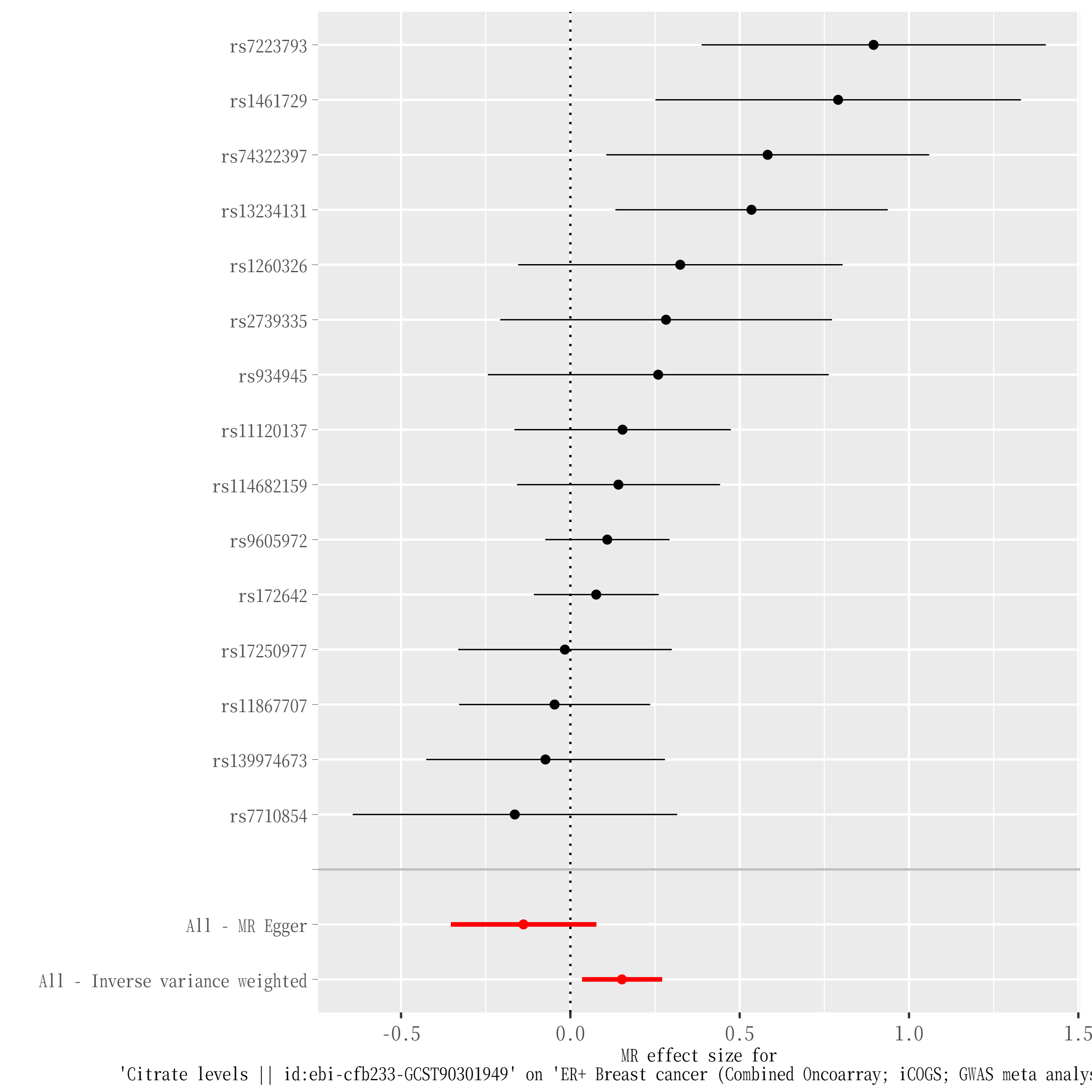
rs7223793
rs1461729
rs74322397
rs13234131
rs1260326
rs2739335
rs934945
rs11120137
rs114682159
rs9605972
rs172642
rs17250977
rs11867707
rs139974673
rs7710854
All - MR Egger
All - Inverse variance weighted
-0.5
0.0
0.5
1.0
1.5
MR effect size for
'Citrate levels || id:ebi-cfb233-GCST90301949' on 'ER+ Breast cancer (Combined Oncoarray; iCOGS; GWAS meta analys

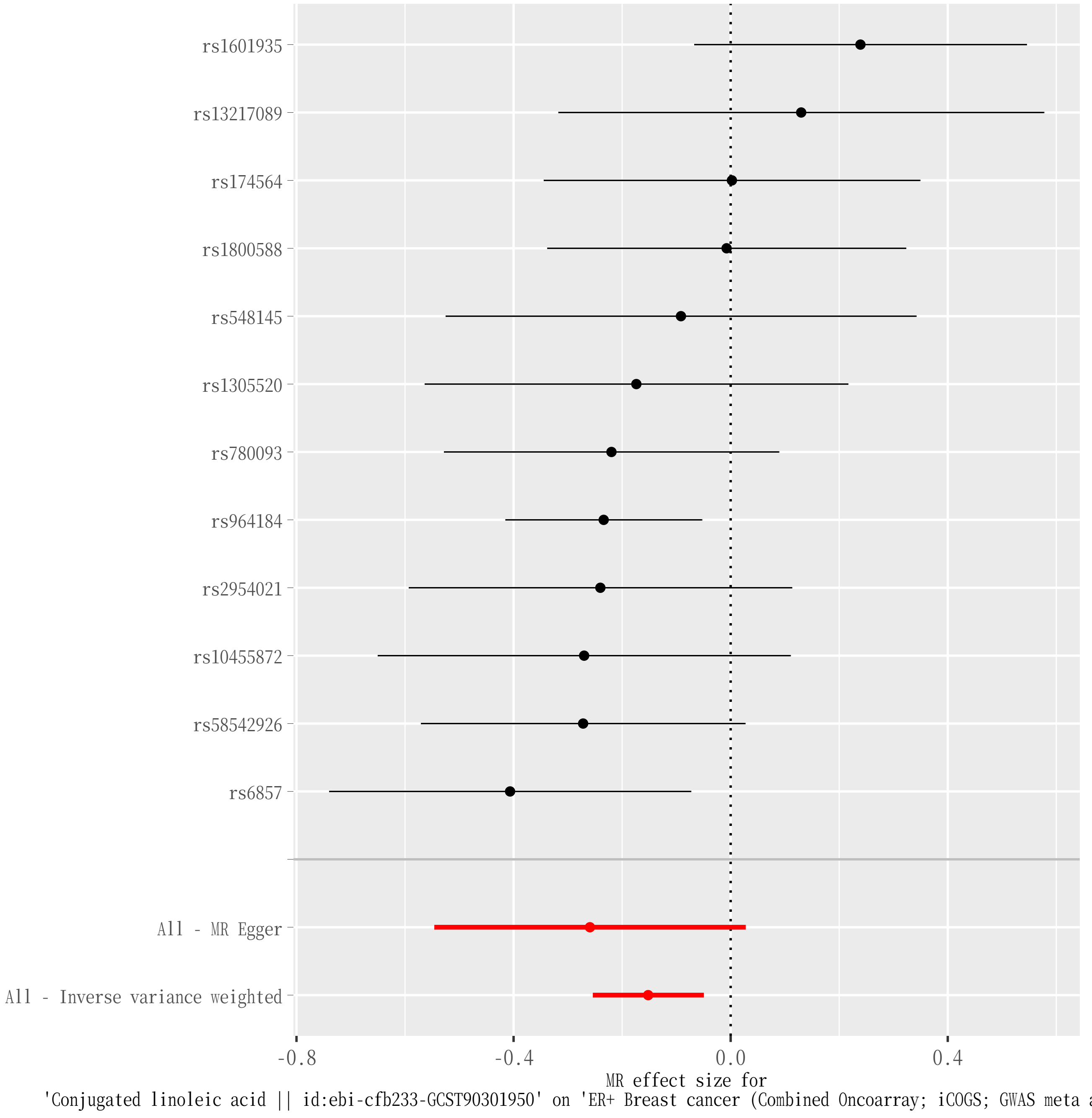
rs1601935
rs13217089
rs174564
rs1800588
rs548145
rs1305520
rs780093
rs964184
rs2954021
rs10455872
rs58542926
rs6857
All - MR Egger
All - Inverse variance weighted
-0.8
-0.4
0.0
0.4
MR effect size for
'Conjugated linoleic acid || id:ebi-cfb233-GCST90301950' on 'ER+ Breast cancer (Combined Oncoarray; iCOGS; GWAS meta a

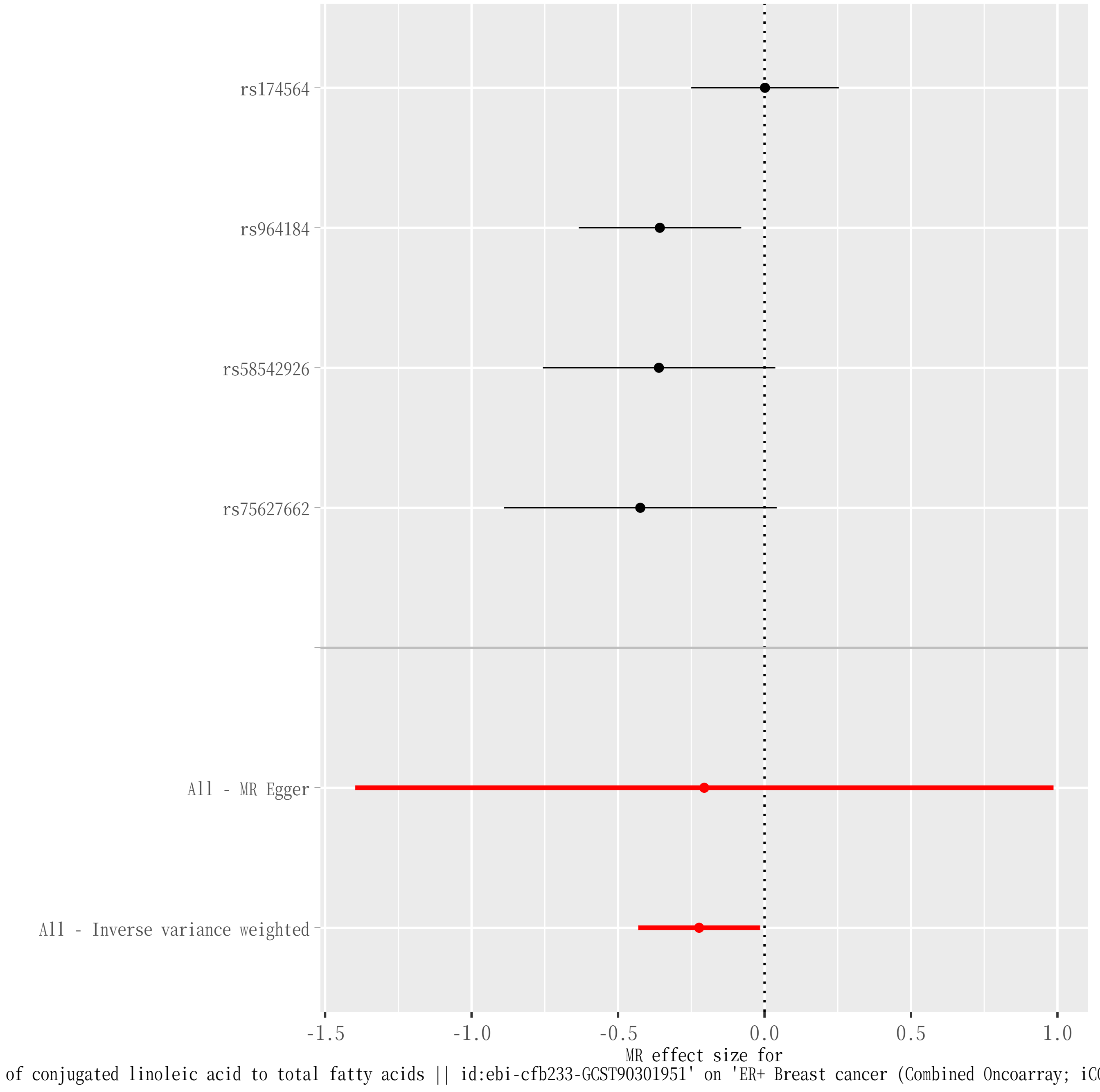

rs174564
rs964184
rs58542926
rs75627662
All - MR Egger
All - Inverse variance weighted
-1.5
-1.0
-0.5
0.0
0.5
1.0
MR effect size for
of conjugated linoleic acid to total fatty acids || id:ebi-cfb233-GCST90301951' on 'ER+ Breast cancer (Combined Oncoarray; iC

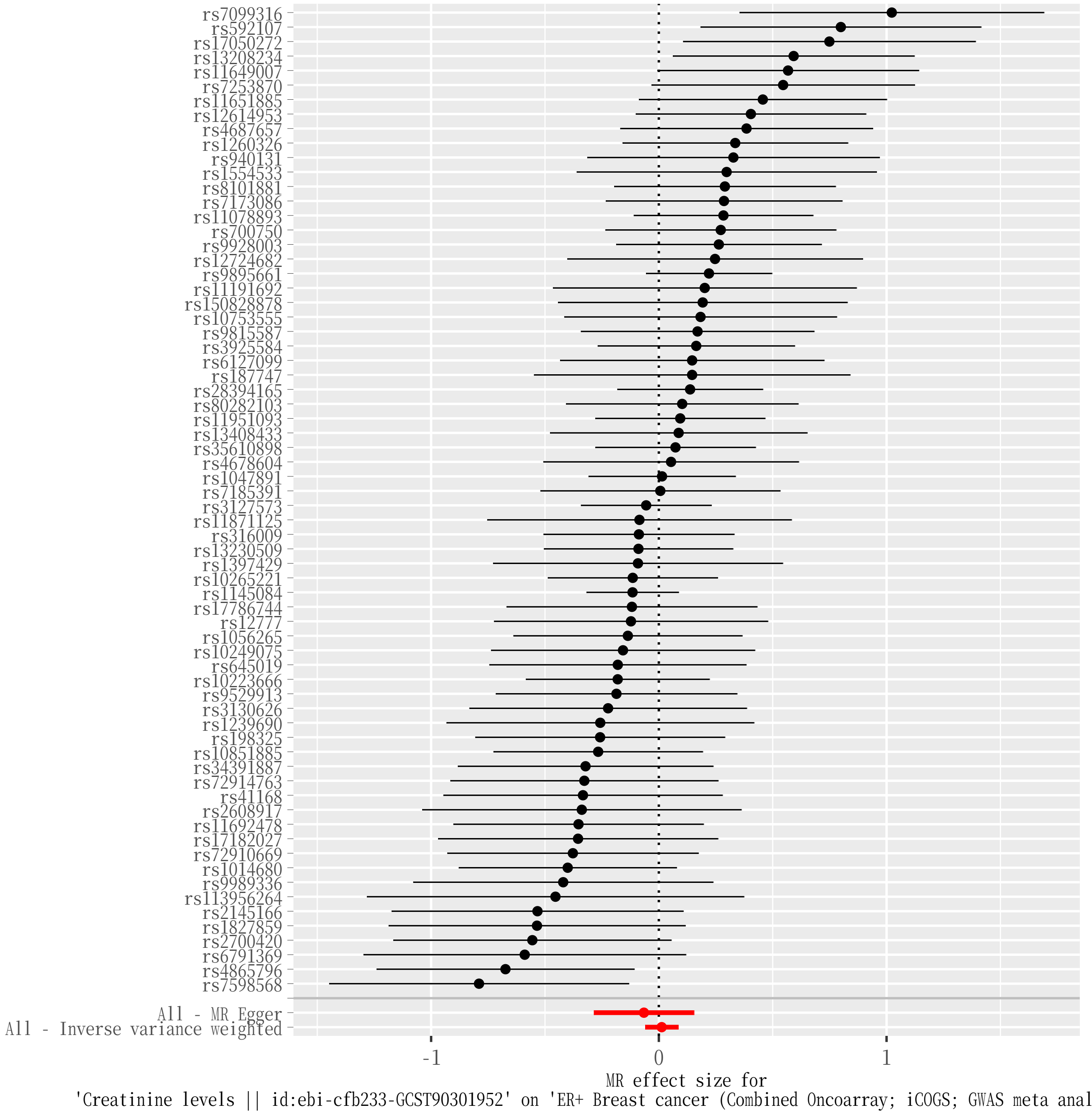
rs7099316
rs592107
rs17050272
rs13208234
rs11649007
rs7253870
rs11651885
rs12614953
rs4687657
rs1260326
rs940131
rs1554533
rs8101881
rs7173086
rs11078893
rs700750
rs9928003
rs12724682
rs9895661
rs11191692
rs150828878
rs10753555
rs9815587
rs3925584
rs6127099
rs187747
rs28394165
rs80282103
rs11951093
rs13408433
rs35610898
rs4678604
rs1047891
rs7185391
rs3127573
rs11871125
rs316009
rs13230509
rs1397429
rs10265221
rs1145084
rs17786744
rs12777
rs1056265
rs10249075
rs645019
rs10223666
rs9529913
rs3130626
rs1239690
rs198325
rs10851885
rs34391887
rs72914763
rs41168
rs2608917
rs11692478
rs17182027
rs72910669
rs1014680
rs9989336
rs113956264
rs2145166
rs1827859
rs2700420
rs6791369
rs4865796
rs7598568
All - MR Egger
All - Inverse variance weighted
-1
0
1
MR effect size for
'Creatinine levels || id:ebi-cfb233-GCST90301952' on 'ER+ Breast cancer (Combined Oncoarray; iCOGS; GWAS meta anal

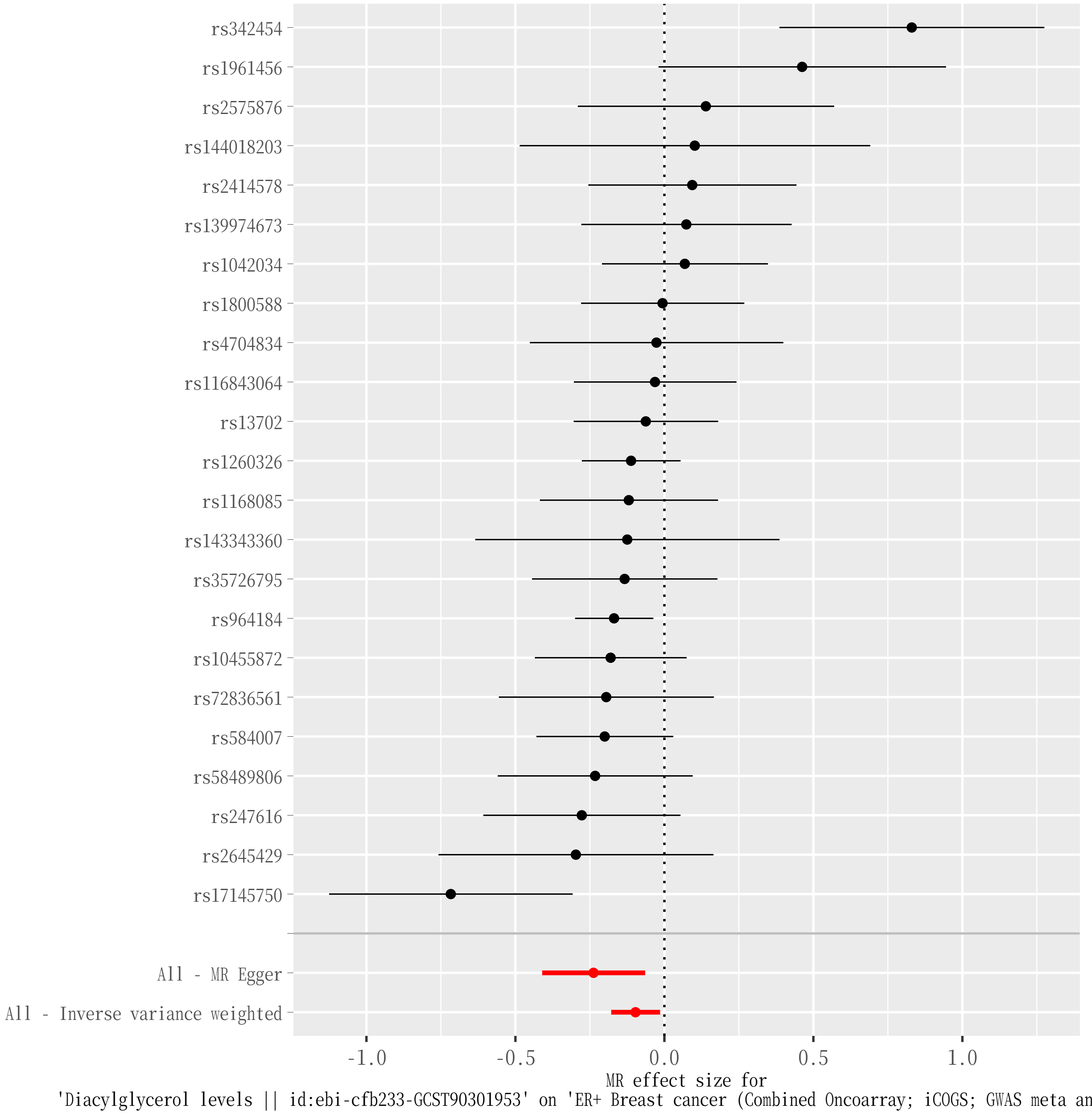

rs342454
rs1961456
rs2575876
rs144018203
rs2414578
rs139974673
rs1042034
rs1800588
rs4704834
rs116843064
rs13702
rs1260326
rs1168085
rs143343360
rs35726795
rs964184
rs10455872
rs72836561
rs584007
rs58489806
rs247616
rs2645429
rs17145750
All - MR Egger
All - Inverse variance weighted
-1.0
-0.5
0.0
0.5
1.0
MR effect size for
'Diacylglycerol levels || id:ebi-cfb233-GCST90301953' on 'ER+ Breast cancer (Combined Oncoarray; iCOGS; GWAS meta an

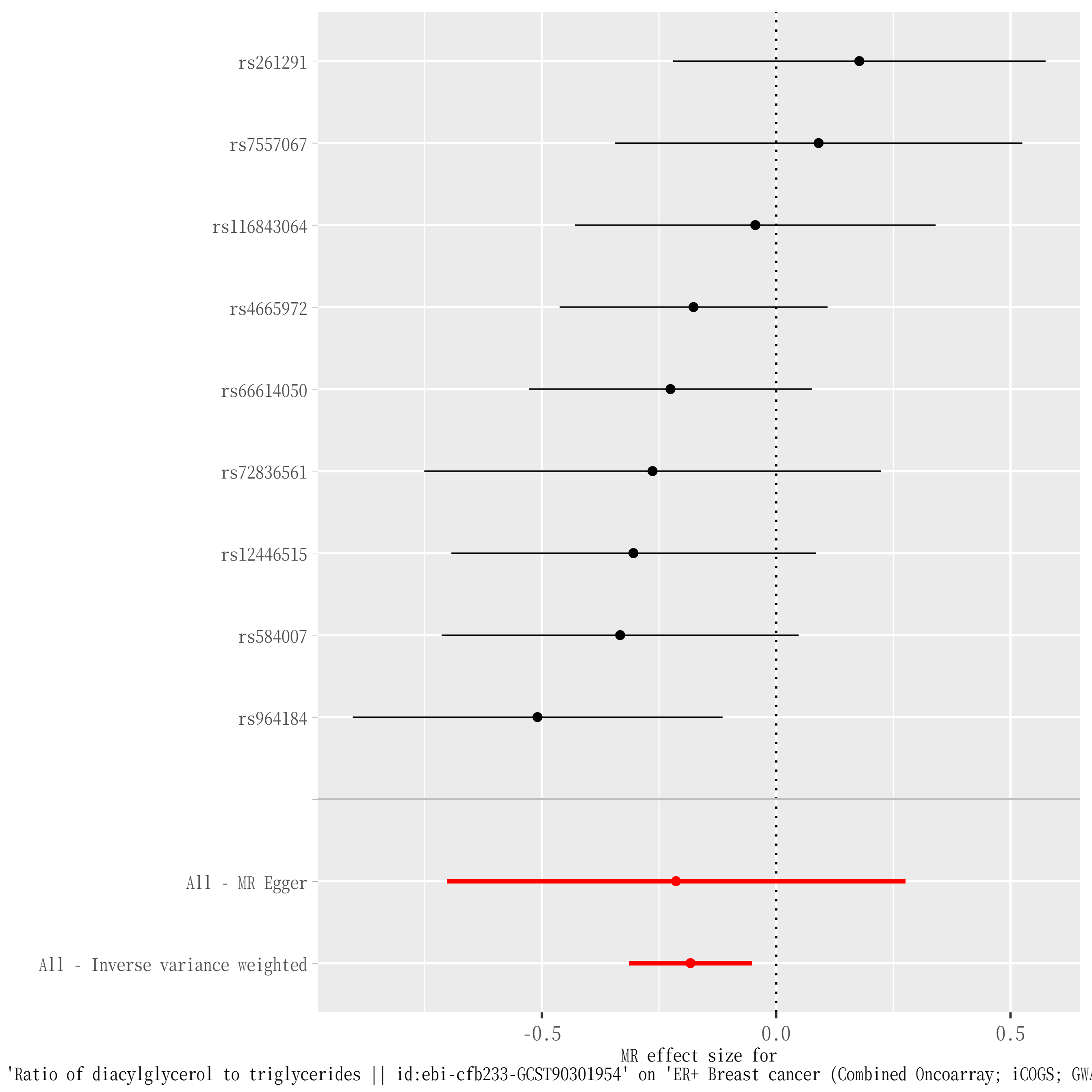
rs261291
rs7557067
rs116843064
rs4665972
rs66614050
rs72836561
rs12446515
rs584007
rs964184
All - MR Egger
All - Inverse variance weighted
-0.5
0.0
0.5
MR effect size for
'Ratio of diacylglycerol to triglycerides || id:ebi-cfb233-GCST90301954' on 'ER+ Breast cancer (Combined Oncoarray; iCOGS; GW

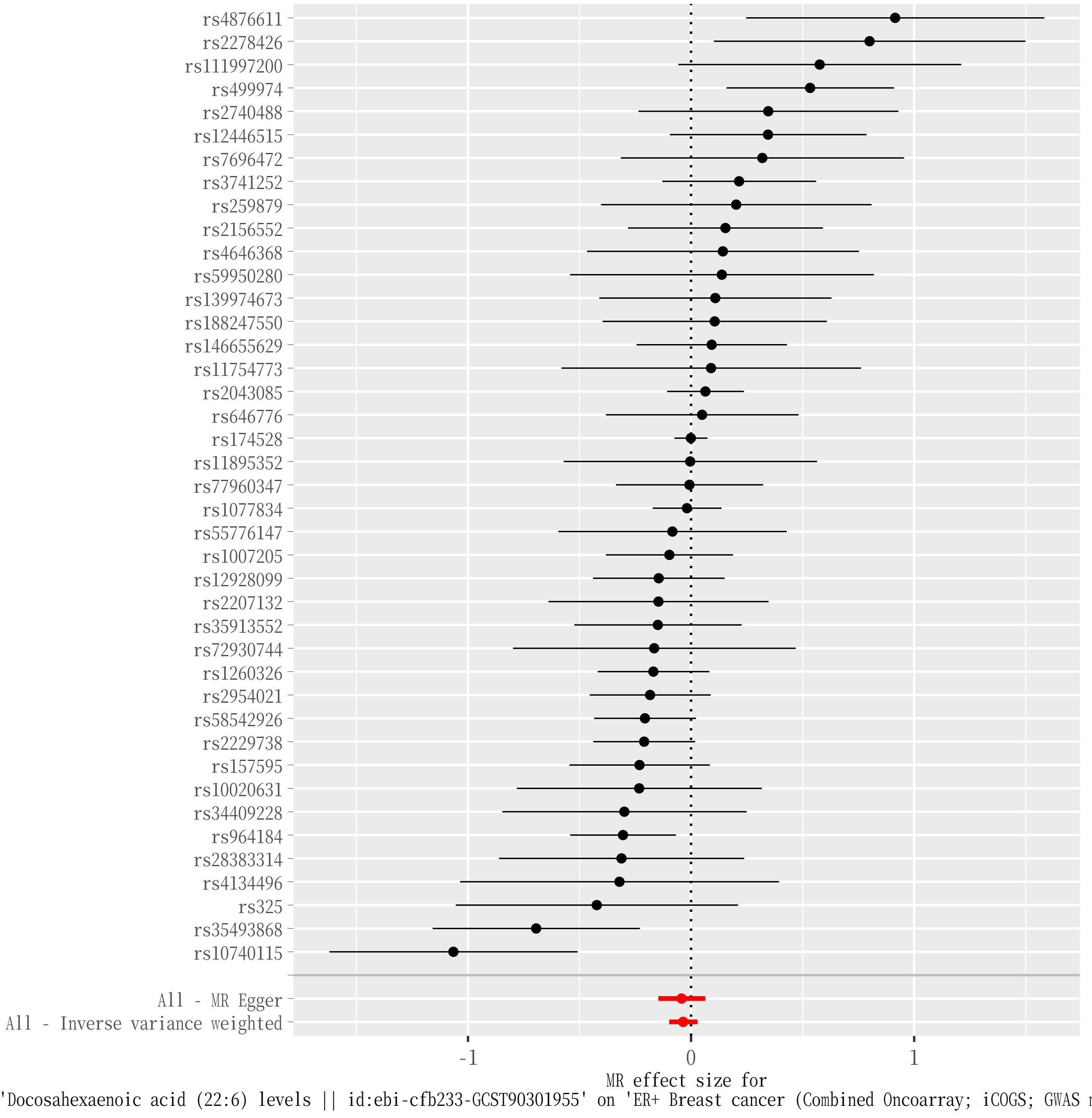

rs4876611
rs2278426
rs111997200
rs499974
rs2740488
rs12446515
rs7696472
rs3741252
rs259879
rs2156552
rs4646368
rs59950280
rs139974673
rs188247550
rs146655629
rs11754773
rs2043085
rs646776
rs174528
rs11895352
rs77960347
rs1077834
rs55776147
rs1007205
rs12928099
rs2207132
rs35913552
rs72930744
rs1260326
rs2954021
rs58542926
rs2229738
rs157595
rs10020631
rs34409228
rs964184
rs28383314
rs4134496
rs325
rs35493868
rs10740115
All - MR Egger
All - Inverse variance weighted
-1
0
1
MR effect size for
'Docosahexaenoic acid (22:6) levels || id:ebi-cfb233-GCST90301955' on 'ER+ Breast cancer (Combined Oncoarray; iCOGS; GWAS m

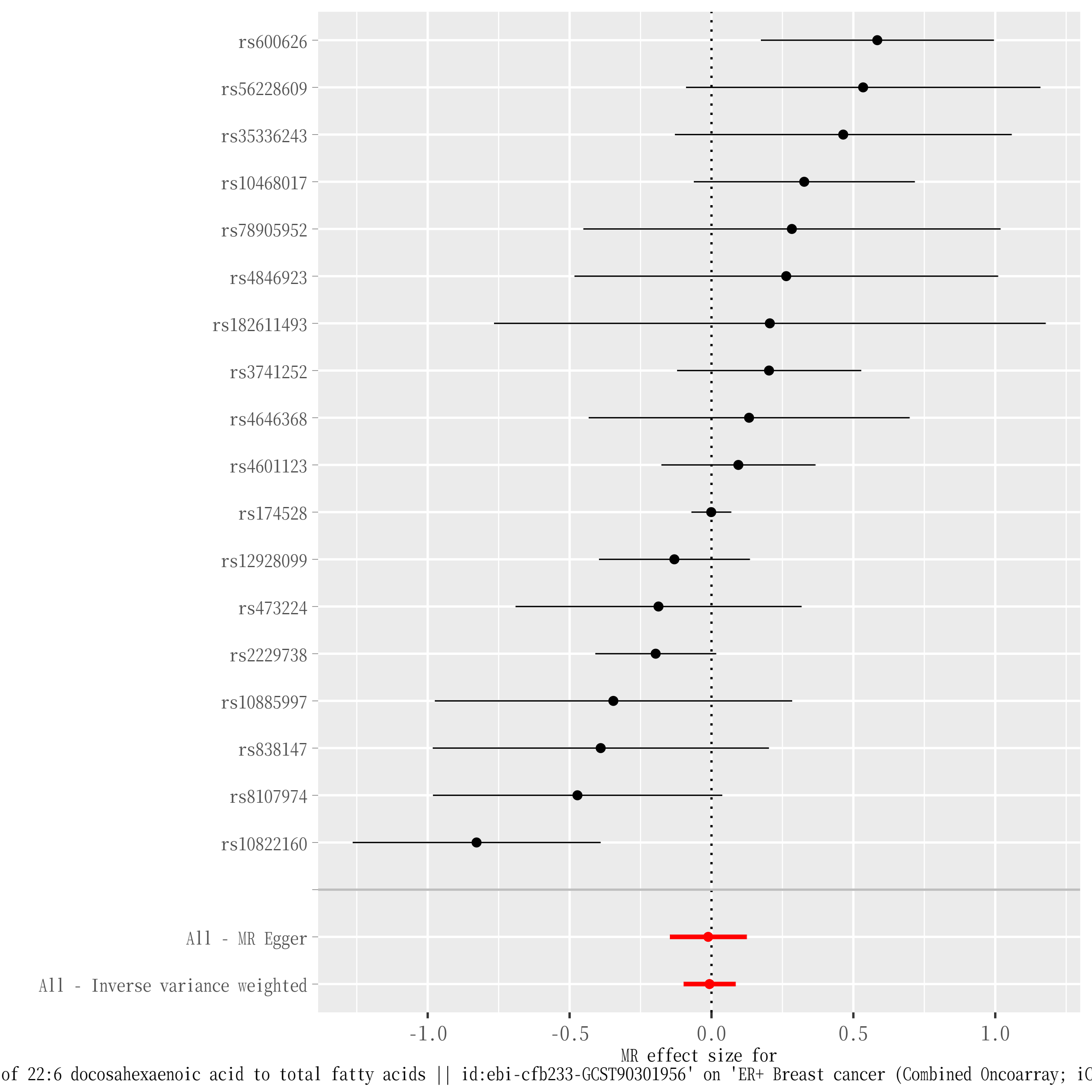

rs600626
rs56228609
rs35336243
rs10468017
rs78905952
rs4846923
rs182611493
rs3741252
rs4646368
rs4601123
rs174528
rs12928099
rs473224
rs2229738
rs10885997
rs838147
rs8107974
rs10822160
All - MR Egger
All - Inverse variance weighted
-1.0
-0.5
0.0
0.5
1.0
MR effect size for
of 22:6 docosahexaenoic acid to total fatty acids || id:ebi-cfb233-GCST90301956' on 'ER+ Breast cancer (Combined Oncoarray; iC

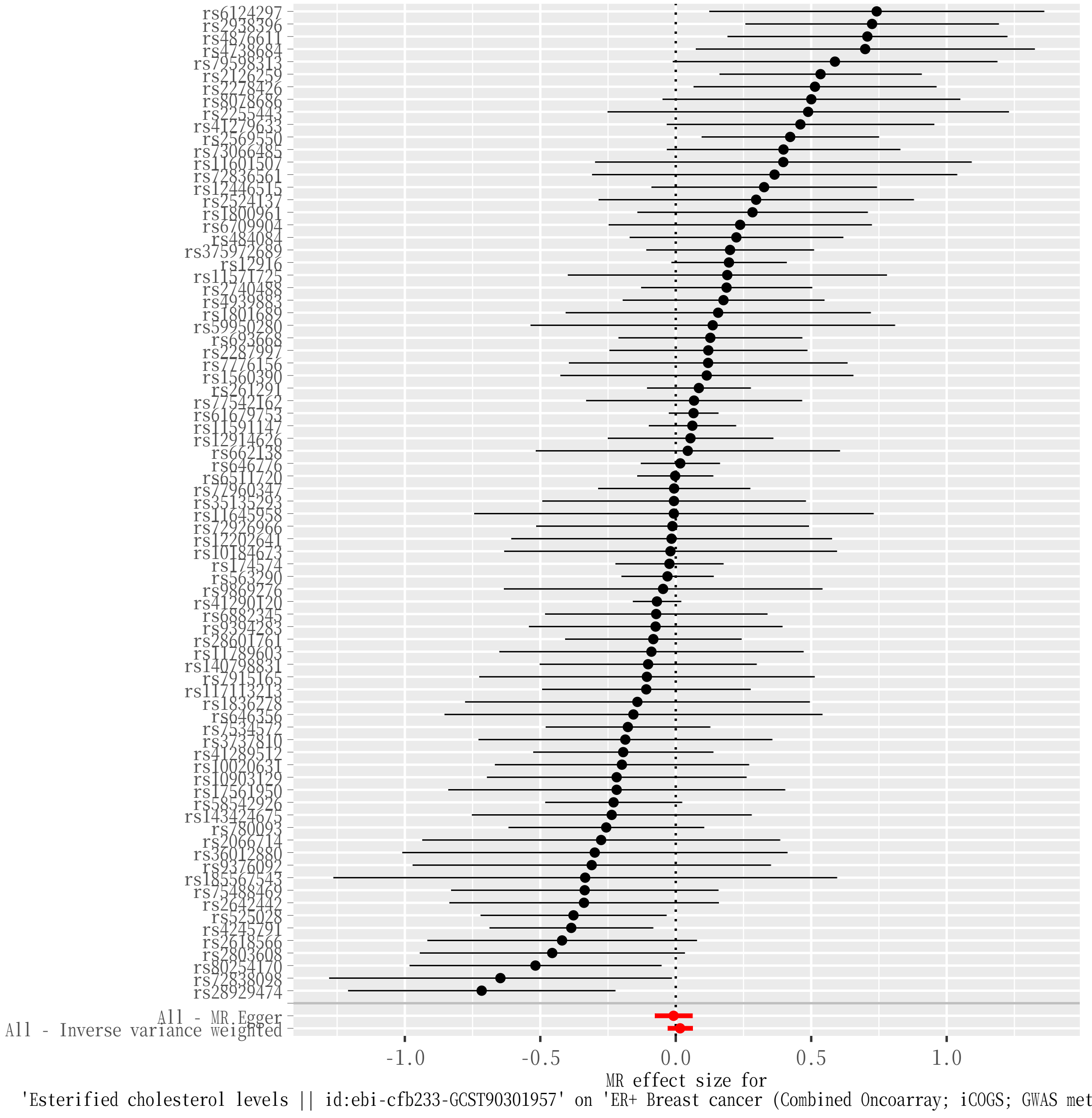
rs6124297
rs2938396
rs4876611
rs4738684
rs79598313
rs2126259
rs2278426
rs8078686
rs2255443
rs41279633
rs2569550
rs73066485
rs11601507
rs72836561
rs12446515
rs2524137
rs1800961
rs6709904
rs484084
rs375972689
rs12916
rs11571725
rs2740488
rs4939883
rs1801689
rs59950280
rs693668
rs2287927
rs7776156
rs1560390
rs261291
rs77542162
rs61679753
rs11591147
rs12914626
rs662138
rs646776
rs6511720
rs72960347
rs35135293
rs11645958
rs72926966
rs12202641
rs10184673
rs174574
rs563290
rs9869276
rs41290120
rs6882345
rs9394283
rs28601761
rs11789603
rs140798831
rs2915165
rs117113213
rs1836278
rs646356
rs7534572
rs3737810
rs41289512
rs10020631
rs10903129
rs17561950
rs58542926
rs143424675
rs780093
rs2066714
rs36012880
rs9376092
rs185567543
rs75488469
rs2642442
rs525028
rs4245791
rs2618566
rs2803608
rs80254170
rs72838098
rs28929474
All - MR Egger
All - Inverse variance weighted
-1.0
-0.5
0.0
0.5
1.0
MR effect size for
'Esterified cholesterol levels || id:ebi-cfb233-GCST90301957' on 'ER+ Breast cancer (Combined Oncoarray; iCOGS; GWAS met

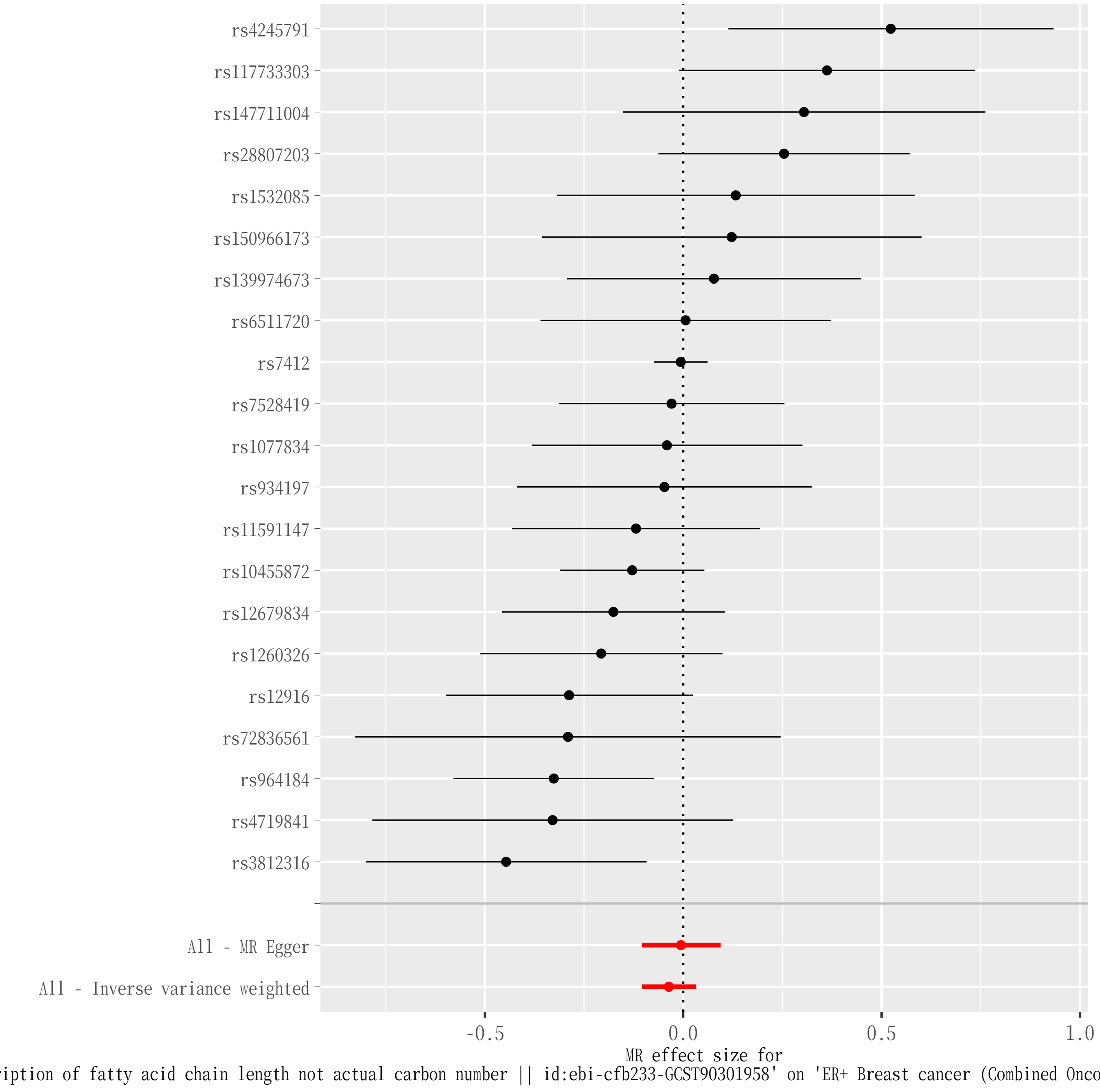

rs4245791
rs117733303
rs147711004
rs28807203
rs1532085
rs150966173
rs139974673
rs6511720
rs7412
rs7528419
rs1077834
rs934197
rs11591147
rs10455872
rs12679834
rs1260326
rs12916
rs72836561
rs964184
rs4719841
rs3812316
All - MR Egger
All - Inverse variance weighted
-0.5
0.0
0.5
1.0
MR effect size for
ription of fatty acid chain length not actual carbon number || id:ebi-cfb233-GCST90301958' on 'ER+ Breast cancer (Combined Onco

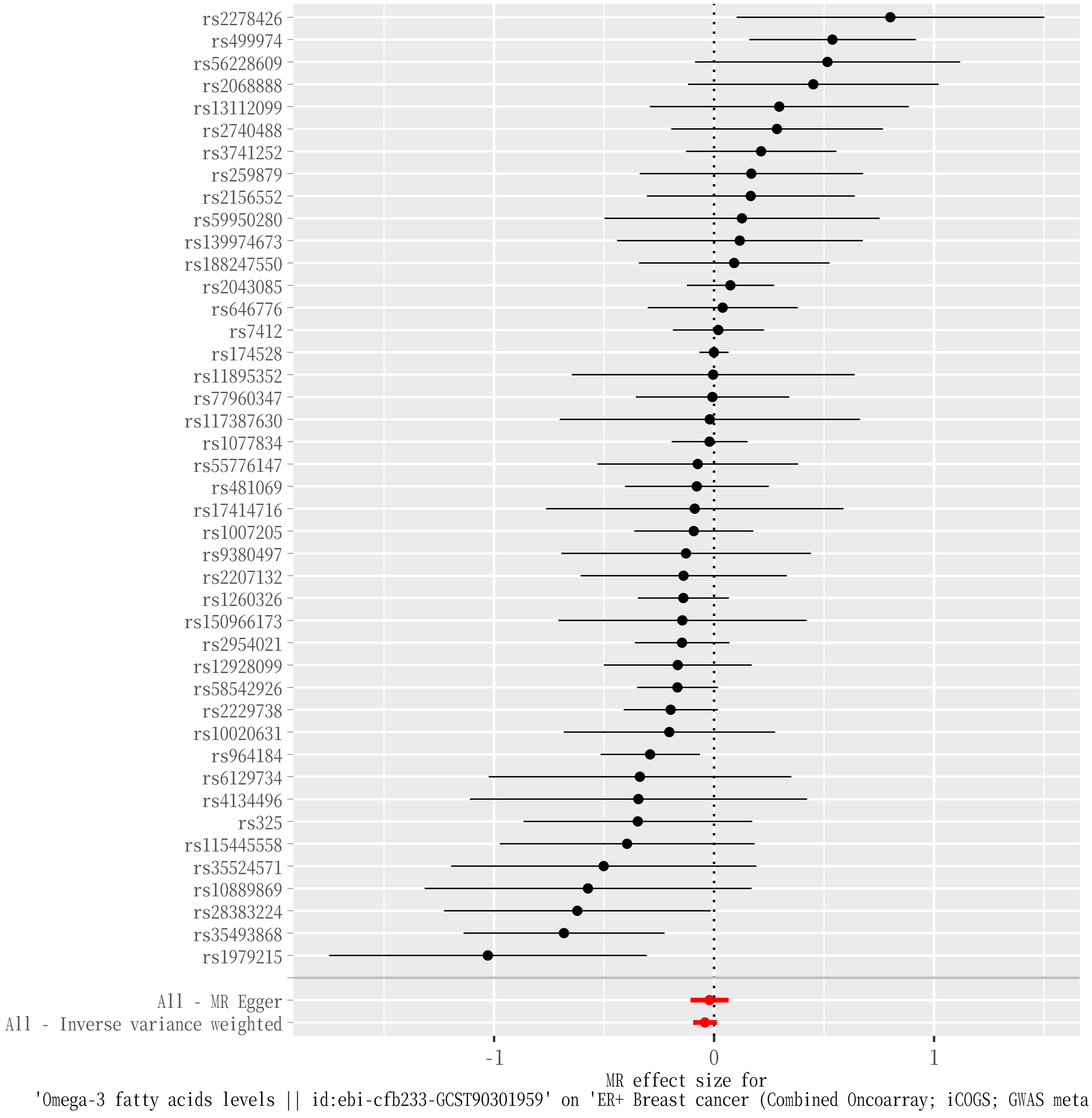
rs2278426
rs499974
rs56228609
rs2068888
rs13112099
rs2740488
rs3741252
rs259879
rs2156552
rs59950280
rs139974673
rs188247550
rs2043085
rs646776
rs7412
rs174528
rs11895352
rs77960347
rs117387630
rs1077834
rs55776147
rs481069
rs17414716
rs1007205
rs9380497
rs2207132
rs1260326
rs150966173
rs2954021
rs12928099
rs58542926
rs2229738
rs10020631
rs964184
rs6129734
rs4134496
rs325
rs115445558
rs35524571
rs10889869
rs28383224
rs35493868
rs1979215
All - MR Egger
All - Inverse variance weighted
-1
0
1
MR effect size for
'Omega-3 fatty acids levels || id:ebi-cfb233-GCST90301959' on 'ER+ Breast cancer (Combined Oncoarray; iCOGS; GWAS meta

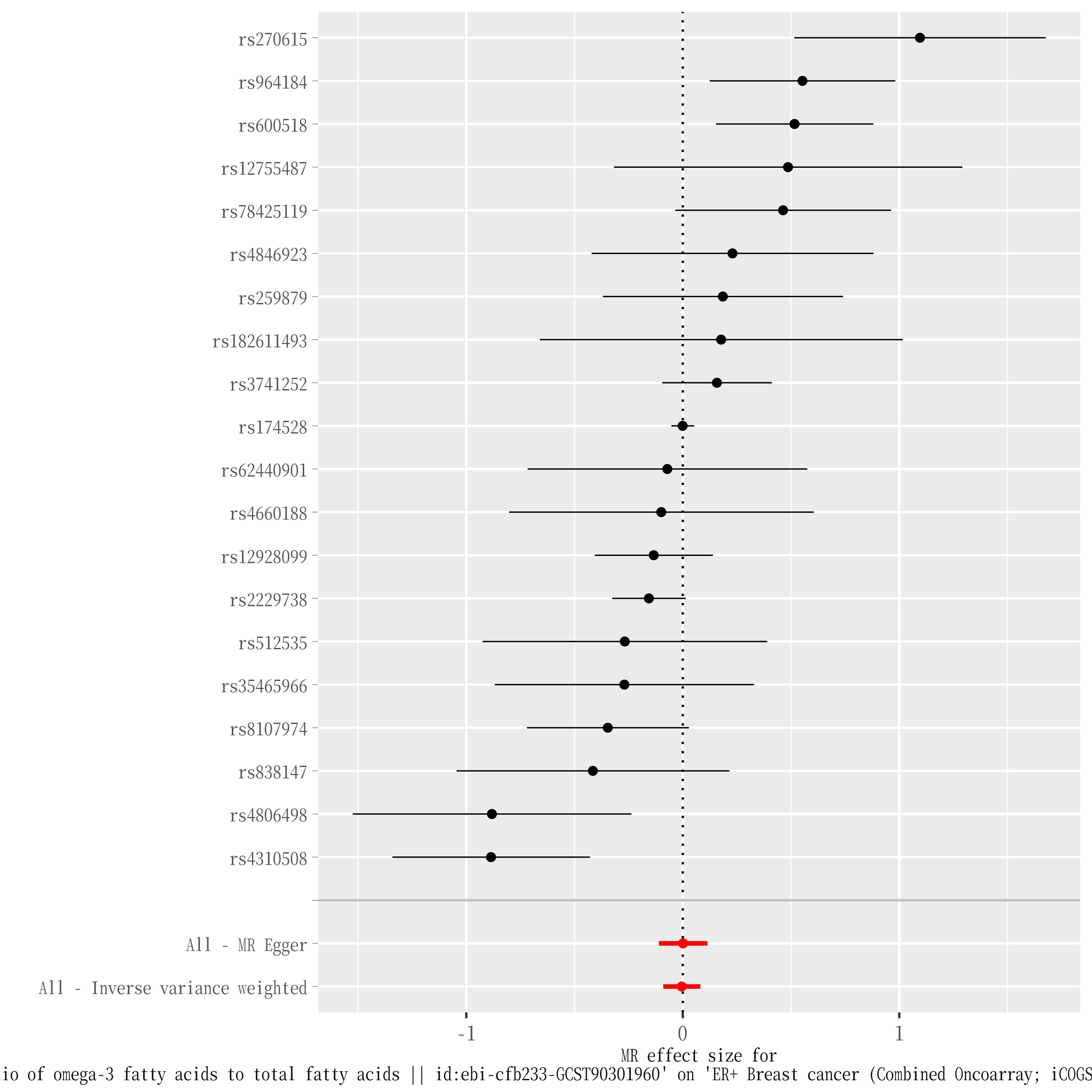
rs270615
rs964184
rs600518
rs12755487
rs78425119
rs4846923
rs259879
rs182611493
rs3741252
rs174528
rs62440901
rs4660188
rs12928099
rs2229738
rs512535
rs35465966
rs8107974
rs838147
rs4806498
rs4310508
All - MR Egger
All - Inverse variance weighted
-1
0
1
MR effect size for
io of omega-3 fatty acids to total fatty acids || id:ebi-cfb233-GCST90301960' on 'ER+ Breast cancer (Combined Oncoarray; iCOGS

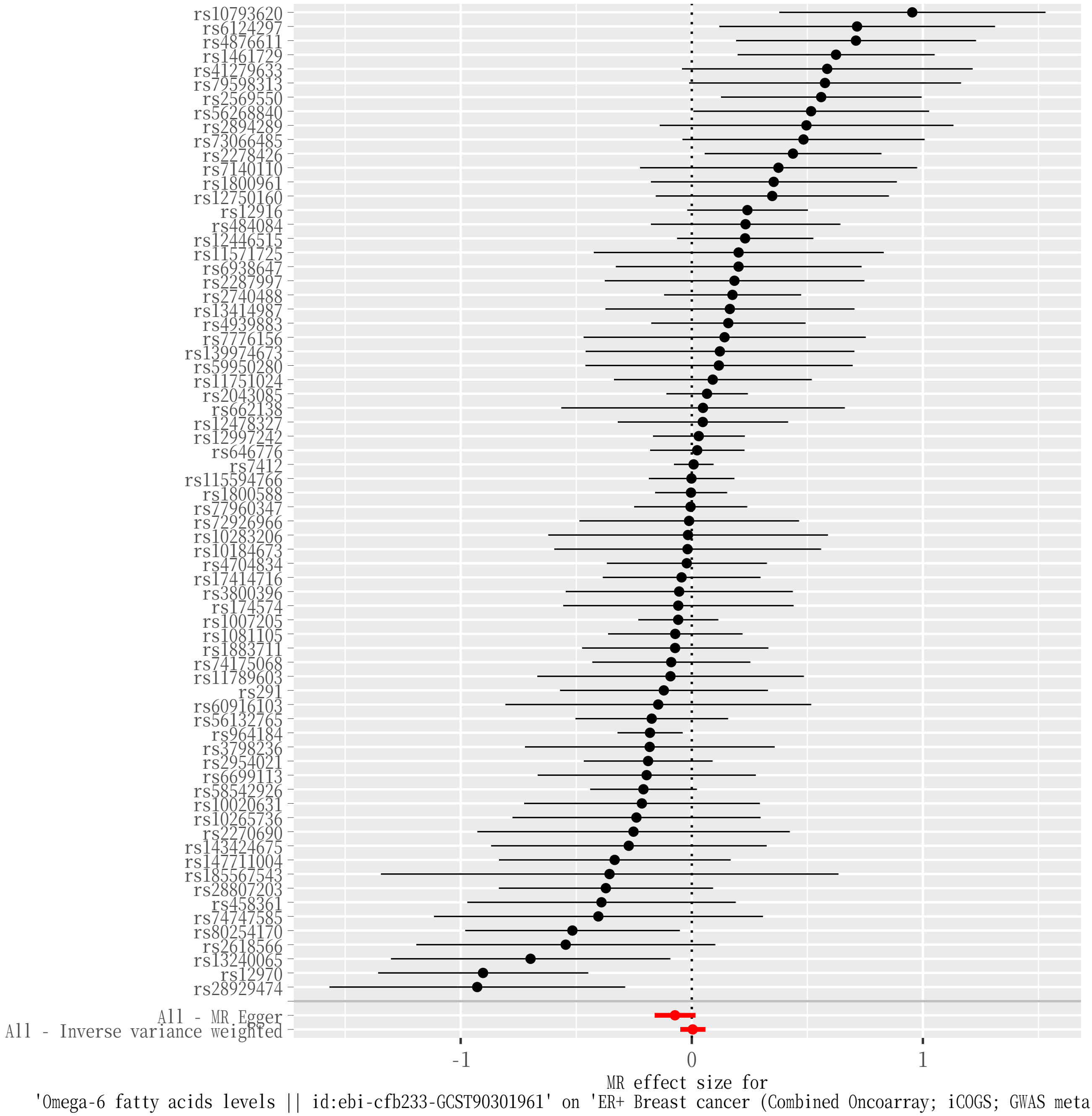
rs10793620
rs6124297
rs4876611
rs1461729
rs41279633
rs79598313
rs2569550
rs56268840
rs2894289
rs73066485
rs2278426
rs7140110
rs1800961
rs12750160
rs12916
rs484084
rs12446515
rs11571725
rs6938647
rs2287997
rs2740488
rs13414987
rs4939883
rs7776156
rs139974673
rs59950280
rs11751024
rs2043085
rs662138
rs12478327
rs12997242
rs646776
rs7412
rs115594766
rs1800588
rs77960347
rs72926966
rs10283206
rs10184673
rs4704834
rs17414716
rs3800396
rs174574
rs1007205
rs1081105
rs1883711
rs74175068
rs11789603
rs291
rs60916103
rs56132765
rs964184
rs3798236
rs2954021
rs6699113
rs58542926
rs10020631
rs10265736
rs2270690
rs143424675
rs147711004
rs185567543
rs28807203
rs458361
rs74747585
rs80254170
rs2618566
rs13240065
rs12970
rs28929474
All - MR Egger
All - Inverse variance weighted
-1
0
1
MR effect size for
'Omega-6 fatty acids levels || id:ebi-cfb233-GCST90301961' on 'ER+ Breast cancer (Combined Oncoarray; iCOGS; GWAS meta

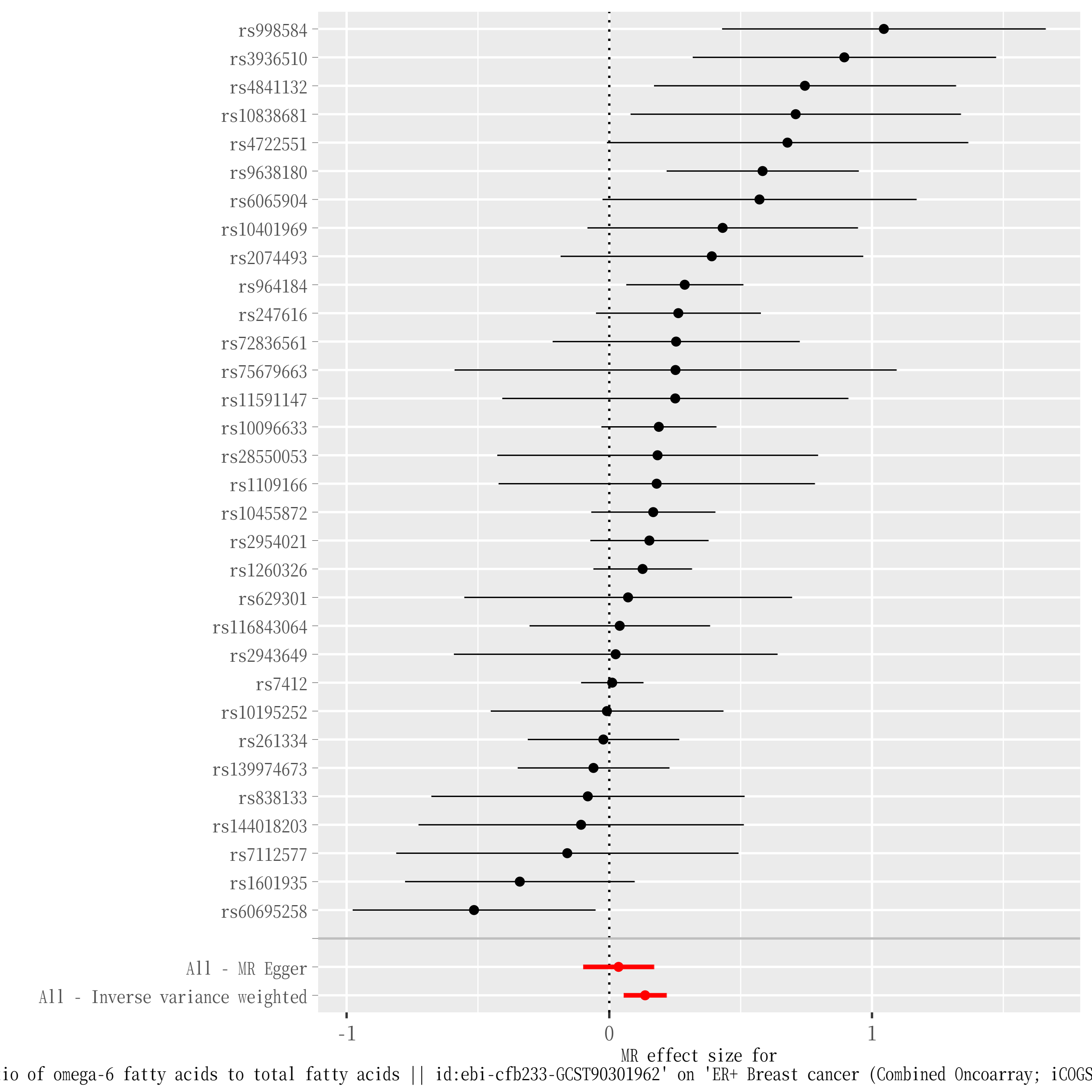

rs998584
rs3936510
rs4841132
rs10838681
rs4722551
rs9638180
rs6065904
rs10401969
rs2074493
rs964184
rs247616
rs72836561
rs75679663
rs11591147
rs10096633
rs28550053
rs1109166
rs10455872
rs2954021
rs1260326
rs629301
rs116843064
rs2943649
rs7412
rs10195252
rs261334
rs139974673
rs838133
rs144018203
rs7112577
rs1601935
rs60695258
All - MR Egger
All - Inverse variance weighted
-1
0
1
MR effect size for
io of omega-6 fatty acids to total fatty acids || id:ebi-cfb233-GCST90301962' on 'ER+ Breast cancer (Combined Oncoarray; iCOGS

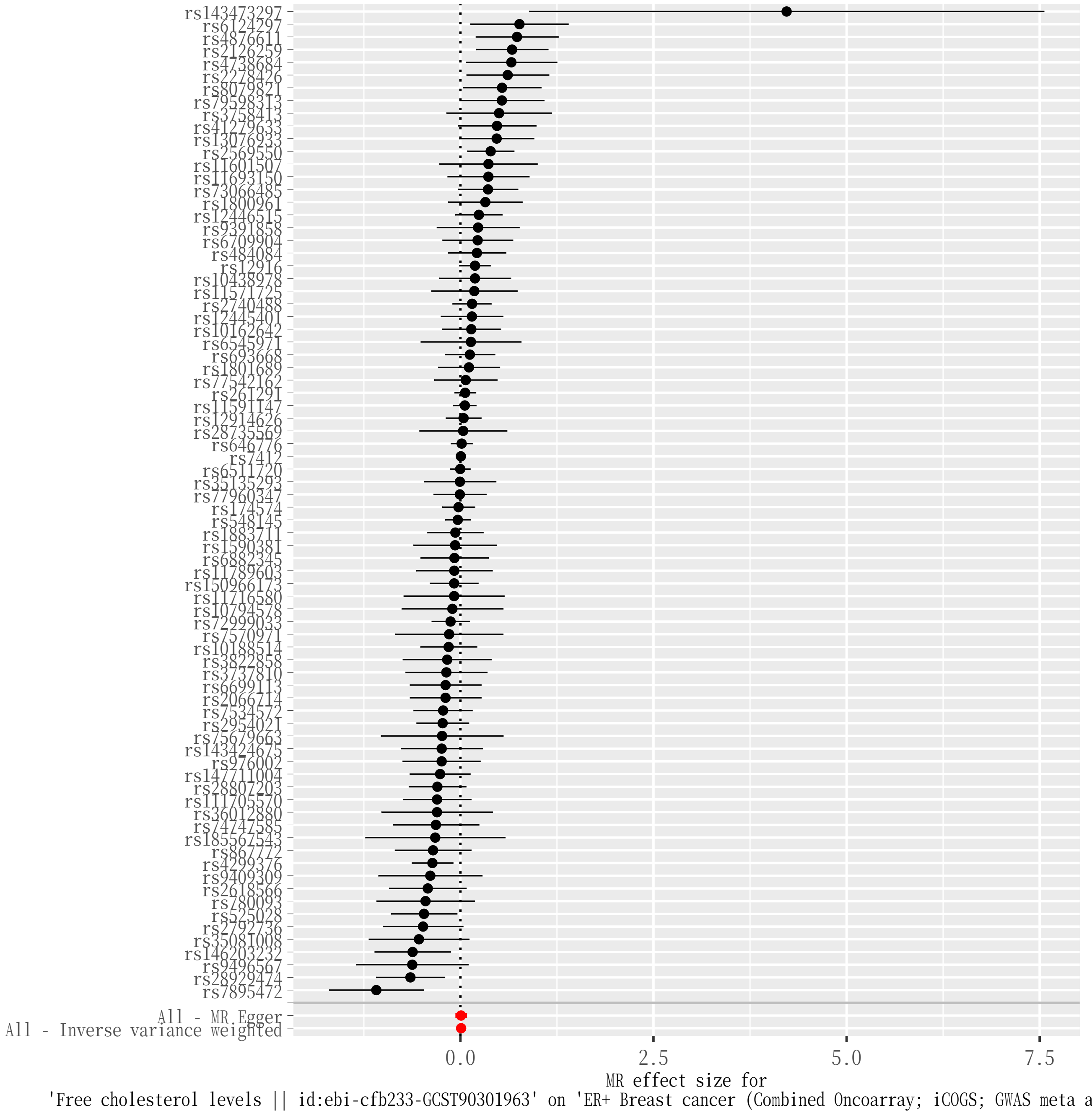
rs143473297
rs6124297
rs4876611
rs2126259
rs4738684
rs2278426
rs8079821
rs79598313
rs3758413
rs41279633
rs13076933
rs2569550
rs11601507
rs11693150
rs73066485
rs1800961
rs12446515
rs9391858
rs6709904
rs484084
rs12916
rs10438978
rs11571725
rs2740488
rs12445401
rs10162642
rs6545971
rs693668
rs1801689
rs77542162
rs261291
rs11591147
rs12914626
rs28735569
rs646776
rs7412
rs6511720
rs35135293
rs77960347
rs174574
rs548145
rs1883711
rs1590381
rs6882345
rs11789603
rs150966173
rs11716580
rs10794578
rs72999033
rs7570971
rs10188514
rs3822858
rs3737810
rs6699113
rs2066714
rs7534572
rs2954021
rs75679663
rs143424675
rs976002
rs147711004
rs28807203
rs111705570
rs36012880
rs74747585
rs185567543
rs867772
rs4299376
rs9409309
rs2618566
rs780093
rs525028
rs2792736
rs35081008
rs146203232
rs9496567
rs28929474
rs7895472
All - MR Egger
All - Inverse variance weighted
0.0
2.5
5.0
7.5
MR effect size for
'Free cholesterol levels || id:ebi-cfb233-GCST90301963' on 'ER+ Breast cancer (Combined Oncoarray; iCOGS; GWAS meta a

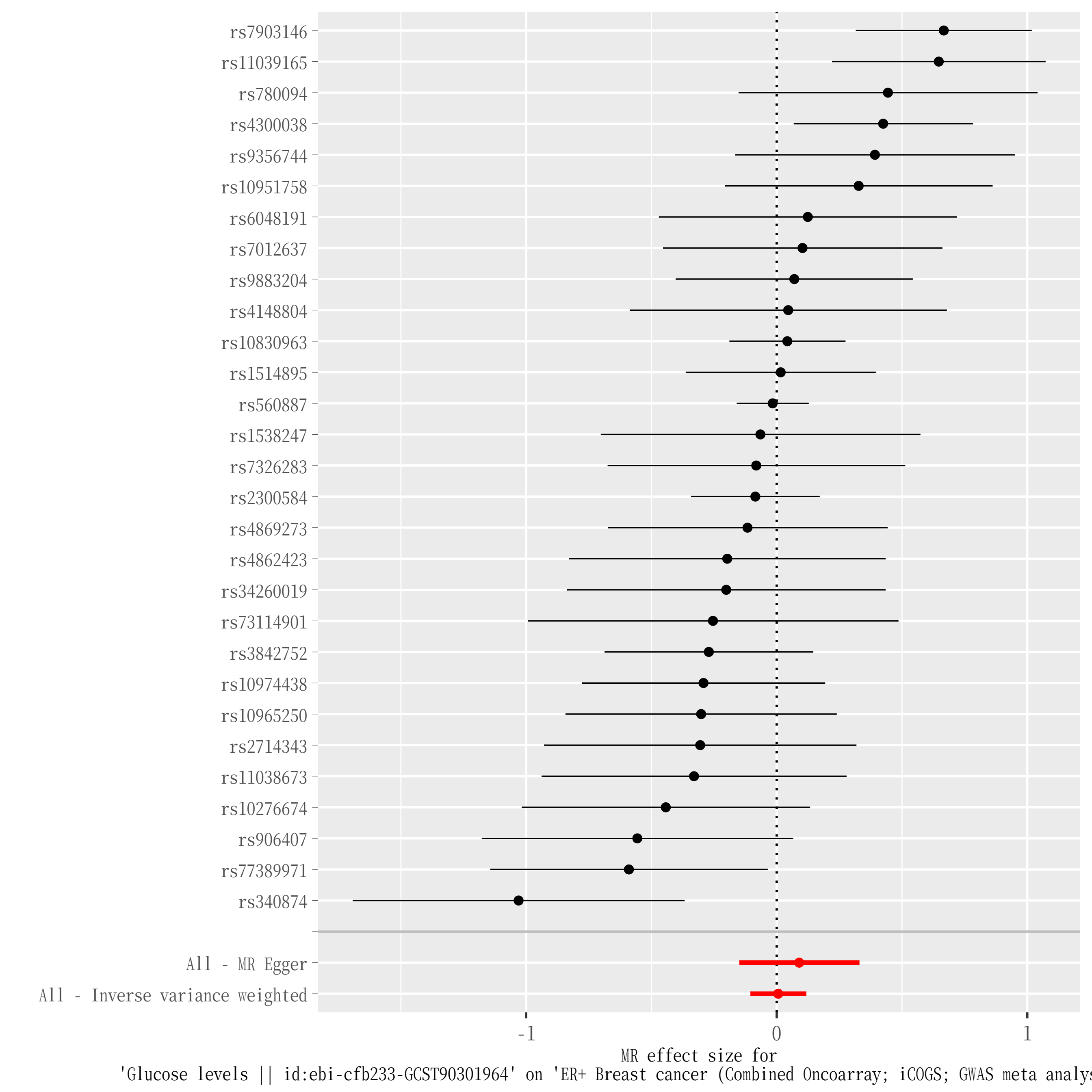

rs7903146
rs11039165
rs780094
rs4300038
rs9356744
rs10951758
rs6048191
rs7012637
rs9883204
rs4148804
rs10830963
rs1514895
rs560887
rs1538247
rs7326283
rs2300584
rs4869273
rs4862423
rs34260019
rs73114901
rs3842752
rs10974438
rs10965250
rs2714343
rs11038673
rs10276674
rs906407
rs77389971
rs340874
All - MR Egger
All - Inverse variance weighted
-1
0
1
MR effect size for
'Glucose levels || id:ebi-cfb233-GCST90301964' on 'ER+ Breast cancer (Combined Oncoarray; iCOGS; GWAS meta analys

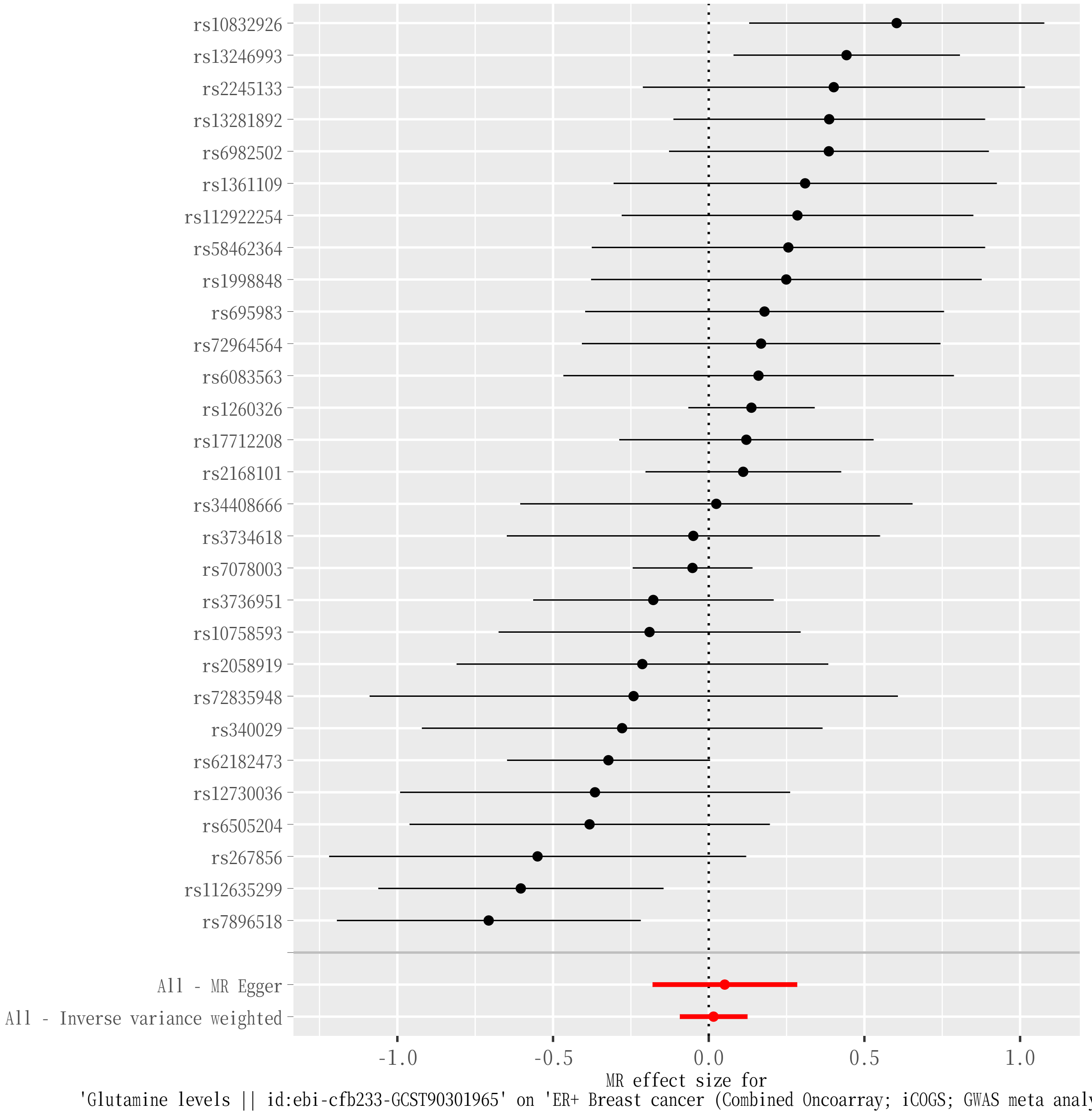
rs10832926
rs13246993
rs2245133
rs13281892
rs6982502
rs1361109
rs112922254
rs58462364
rs1998848
rs695983
rs72964564
rs6083563
rs1260326
rs17712208
rs2168101
rs34408666
rs3734618
rs7078003
rs3736951
rs10758593
rs2058919
rs72835948
rs340029
rs62182473
rs12730036
rs6505204
rs267856
rs112635299
rs7896518
All - MR Egger
All - Inverse variance weighted
-1.0
-0.5
0.0
0.5
1.0
MR effect size for
'Glutamine levels || id:ebi-cfb233-GCST90301965' on 'ER+ Breast cancer (Combined Oncoarray; iCOGS; GWAS meta analy

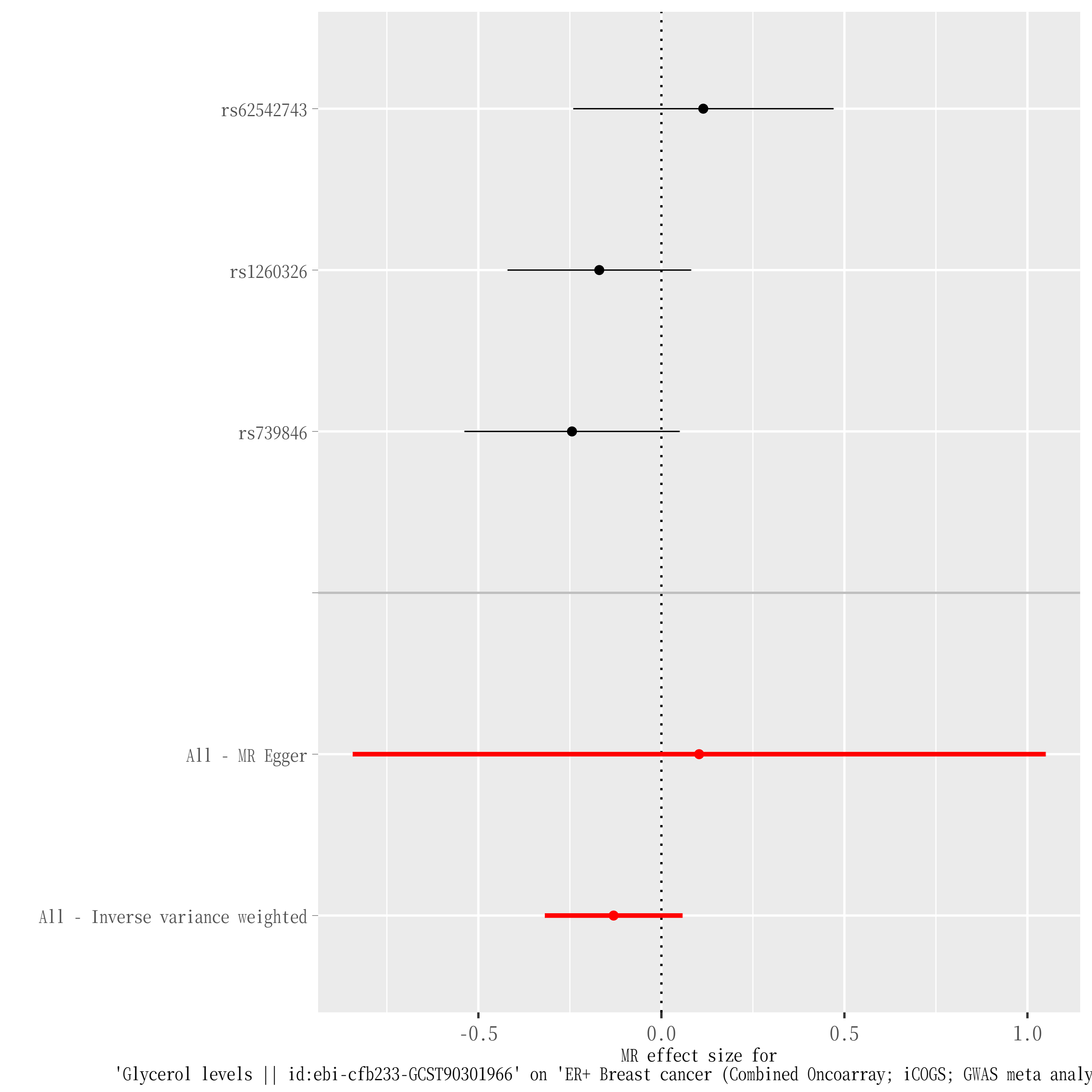
rs62542743
rs1260326
rs739846
All - MR Egger
All - Inverse variance weighted
-0.5
0.0
0.5
1.0
MR effect size for
'Glycerol levels || id:ebi-cfb233-GCST90301966' on 'ER+ Breast cancer (Combined Oncoarray; iCOGS; GWAS meta analy

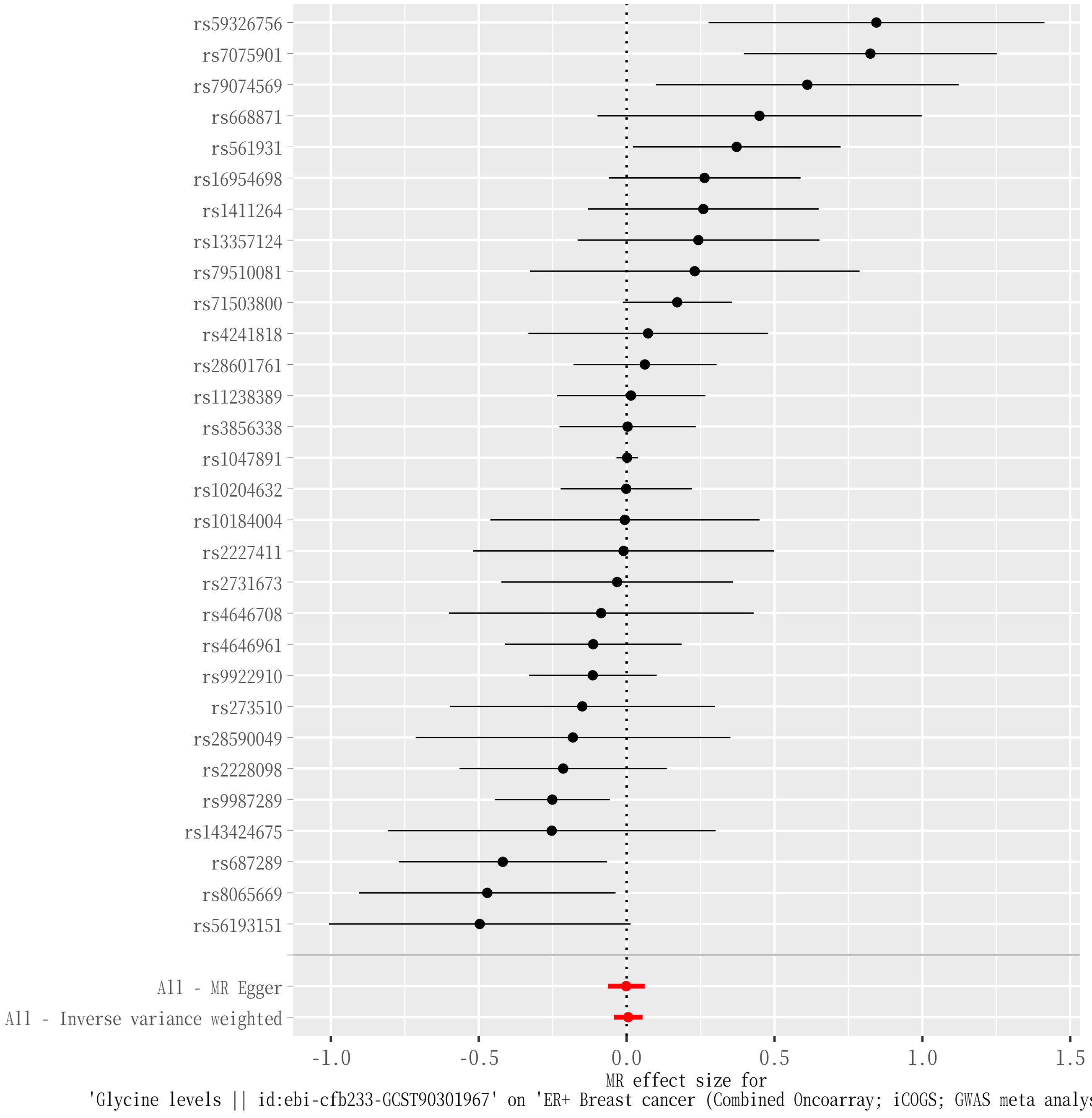
rs59326756
rs7075901
rs79074569
rs668871
rs561931
rs16954698
rs1411264
rs13357124
rs79510081
rs71503800
rs4241818
rs28601761
rs11238389
rs3856338
rs1047891
rs10204632
rs10184004
rs2227411
rs2731673
rs4646708
rs4646961
rs9922910
rs273510
rs28590049
rs2228098
rs9987289
rs143424675
rs687289
rs8065669
rs56193151
All - MR Egger
All - Inverse variance weighted
-1.0
-0.5
0.0
0.5
1.0
1.5
MR effect size for
'Glycine levels || id:ebi-cfb233-GCST90301967' on 'ER+ Breast cancer (Combined Oncoarray; iCOGS; GWAS meta analys

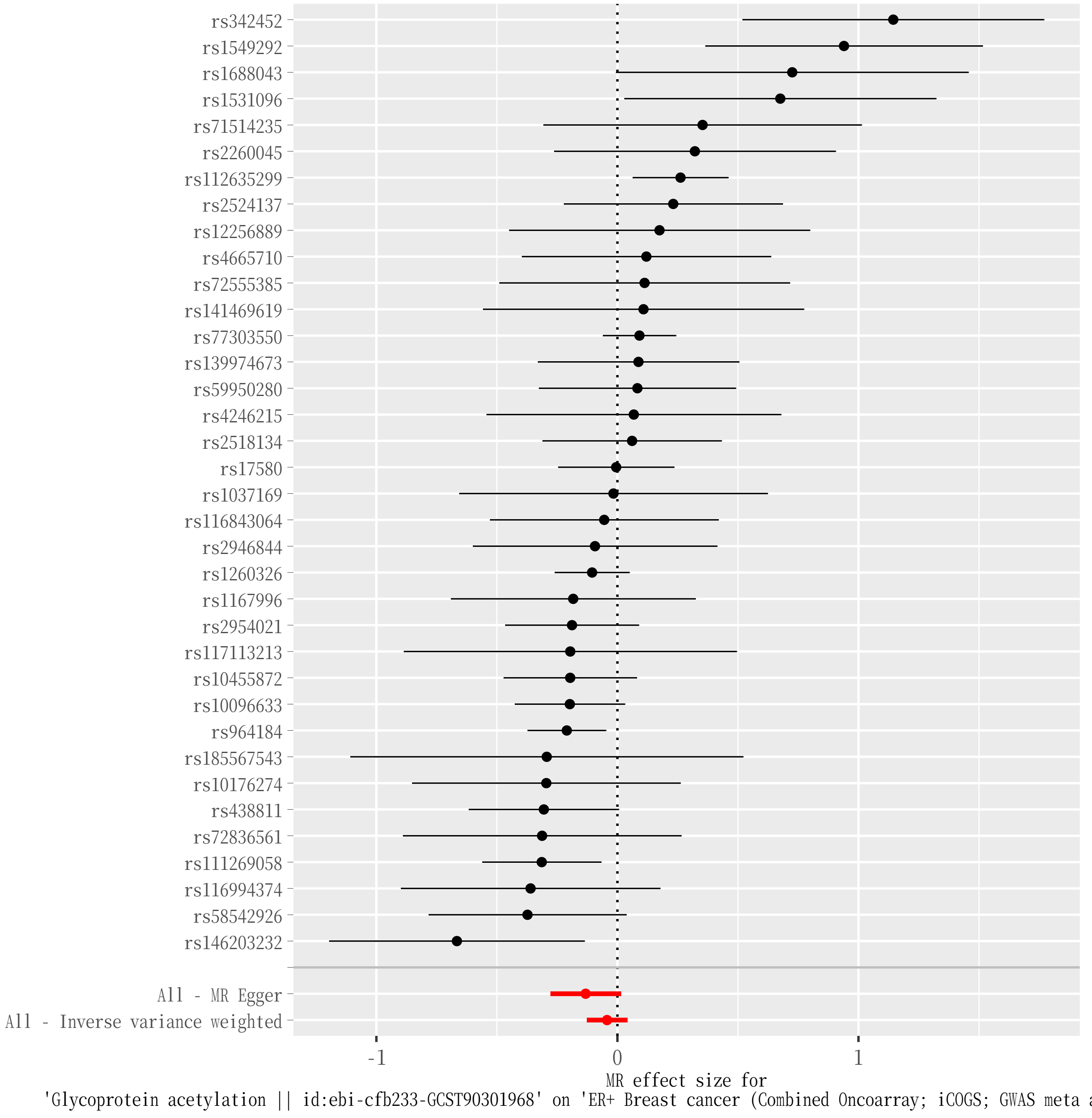
rs342452
rs1549292
rs1688043
rs1531096
rs71514235
rs2260045
rs112635299
rs2524137
rs12256889
rs4665710
rs72555385
rs141469619
rs77303550
rs139974673
rs59950280
rs4246215
rs2518134
rs17580
rs1037169
rs116843064
rs2946844
rs1260326
rs1167996
rs2954021
rs117113213
rs10455872
rs10096633
rs964184
rs185567543
rs10176274
rs438811
rs72836561
rs111269058
rs116994374
rs58542926
rs146203232
All - MR Egger
All - Inverse variance weighted
-1
0
1
MR effect size for
'Glycoprotein acetylation || id:ebi-cfb233-GCST90301968' on 'ER+ Breast cancer (Combined Oncoarray; iCOGS; GWAS meta a

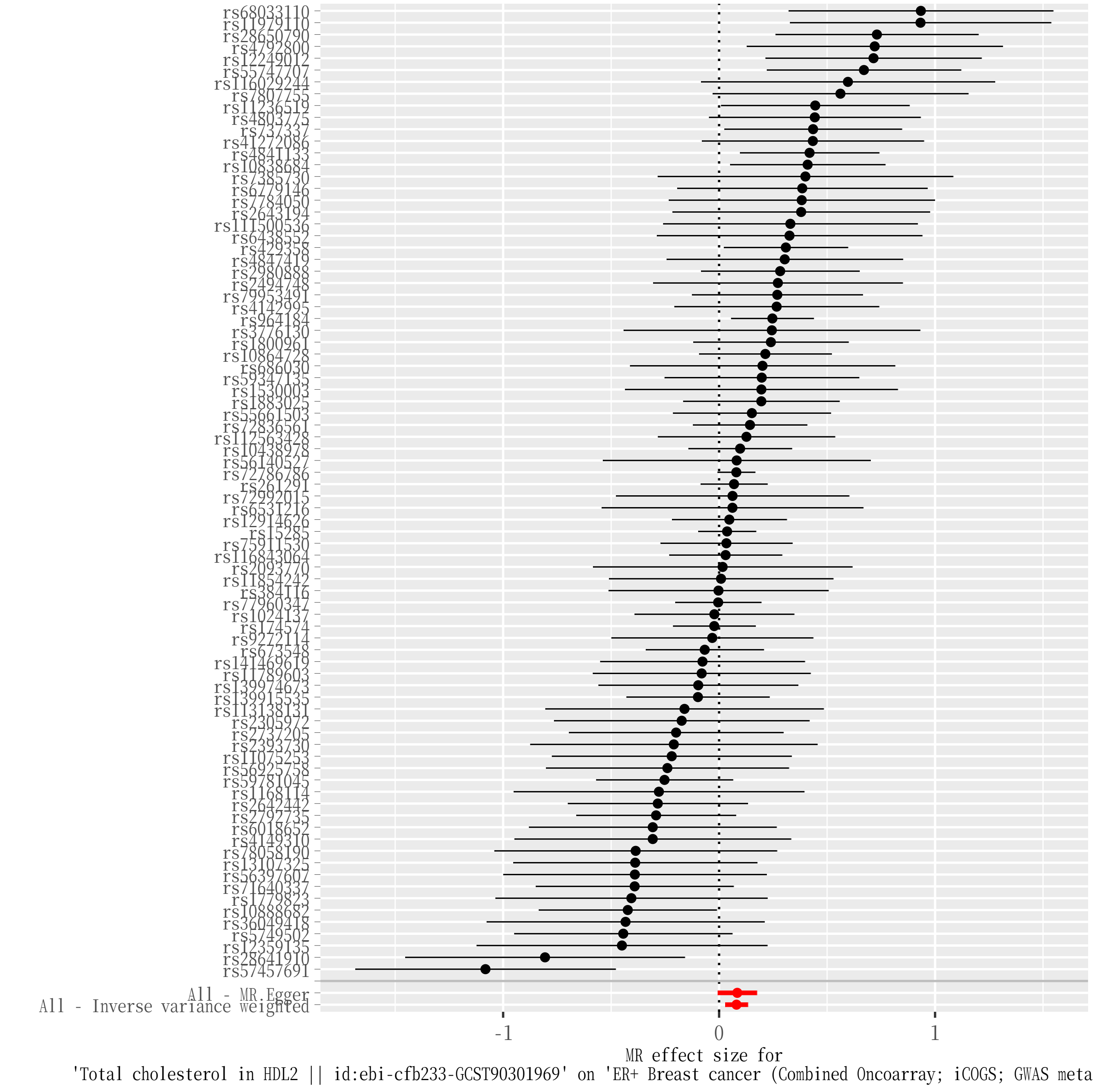

rs68033110
rs11979110
rs28650790
rs4792800
rs12249012
rs55747707
rs116029244
rs7807755
rs11236519
rs4803775
rs737337
rs41272086
rs4841133
rs10838684
rs7385730
rs6779146
rs7784050
rs2643194
rs111500536
rs6438552
rs429358
rs4847419
rs2980888
rs7494748
rs79953491
rs4142995
rs964184
rs3776130
rs1800961
rs10864728
rs686030
rs59347135
rs1530003
rs1883025
rs55661503
rs72836561
rs112563428
rs10438978
rs56140527
rs72786786
rs261291
rs72992015
rs6531216
rs12914626
rs15285
rs75911530
rs116843064
rs2093770
rs11854242
rs384116
rs77960347
rs1024137
rs174574
rs9272114
rs673548
rs141469619
rs11789603
rs139974673
rs139915535
rs113138131
rs2305972
rs2737205
rs2393730
rs11075253
rs56925758
rs59781045
rs1168114
rs2642442
rs2792735
rs6018652
rs4149310
rs78058190
rs13107325
rs56397607
rs71640337
rs1779823
rs10888682
rs36049418
rs5749502
rs12359135
rs28641910
rs57457691
All - MR Egger
All - Inverse variance weighted
-1
0
1
MR effect size for
'Total cholesterol in HDL2 || id:ebi-cfb233-GCST90301969' on 'ER+ Breast cancer (Combined Oncoarray; iCOGS; GWAS meta

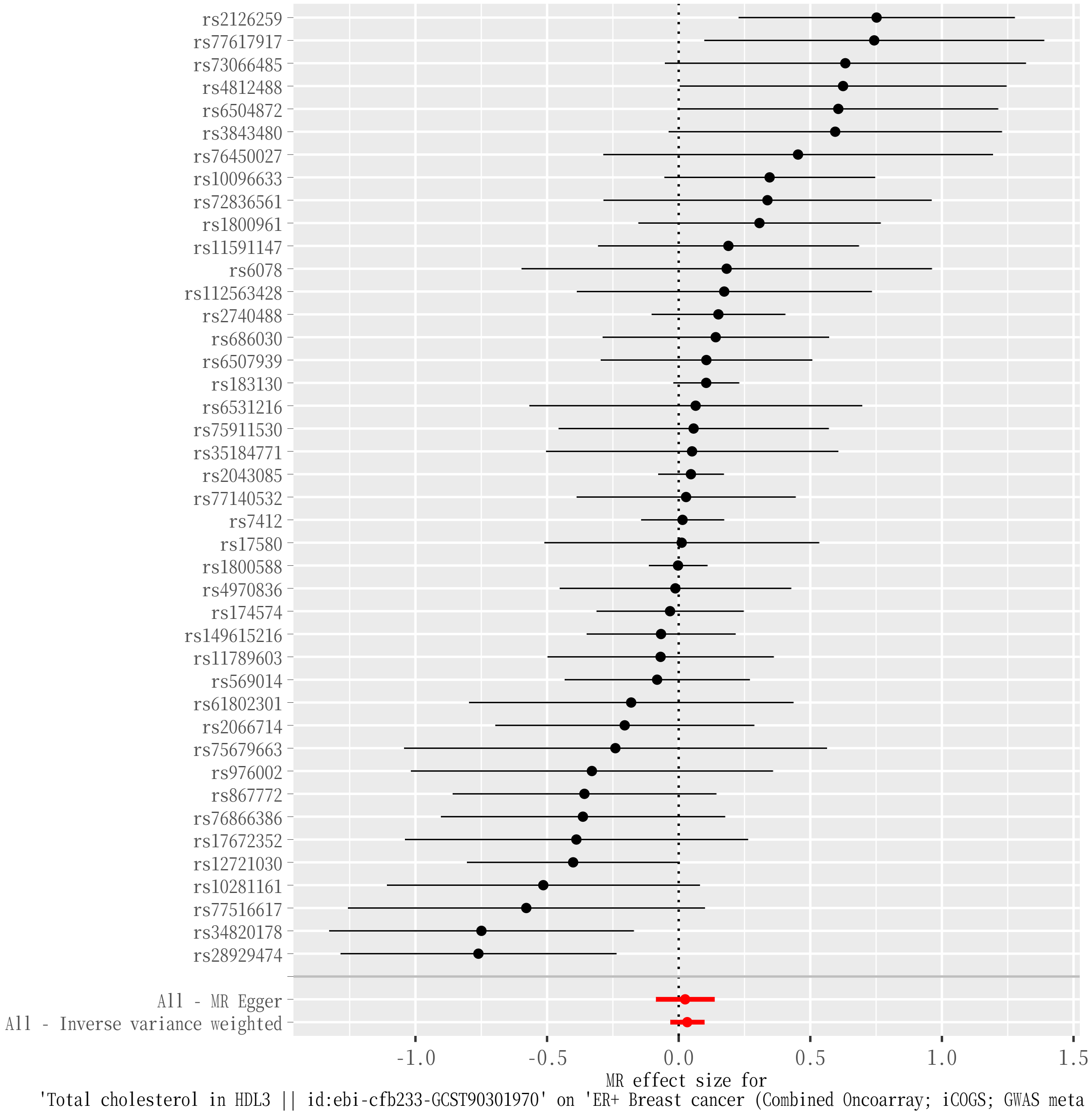

rs2126259
rs77617917
rs73066485
rs4812488
rs6504872
rs3843480
rs76450027
rs10096633
rs72836561
rs1800961
rs11591147
rs6078
rs112563428
rs2740488
rs686030
rs6507939
rs183130
rs6531216
rs75911530
rs35184771
rs2043085
rs77140532
rs7412
rs17580
rs1800588
rs4970836
rs174574
rs149615216
rs11789603
rs569014
rs61802301
rs2066714
rs75679663
rs976002
rs867772
rs76866386
rs17672352
rs12721030
rs10281161
rs77516617
rs34820178
rs28929474
All - MR Egger
All - Inverse variance weighted
-1.0
-0.5
0.0
0.5
1.0
1.5
MR effect size for
'Total cholesterol in HDL3 || id:ebi-cfb233-GCST90301970' on 'ER+ Breast cancer (Combined Oncoarray; iCOGS; GWAS meta

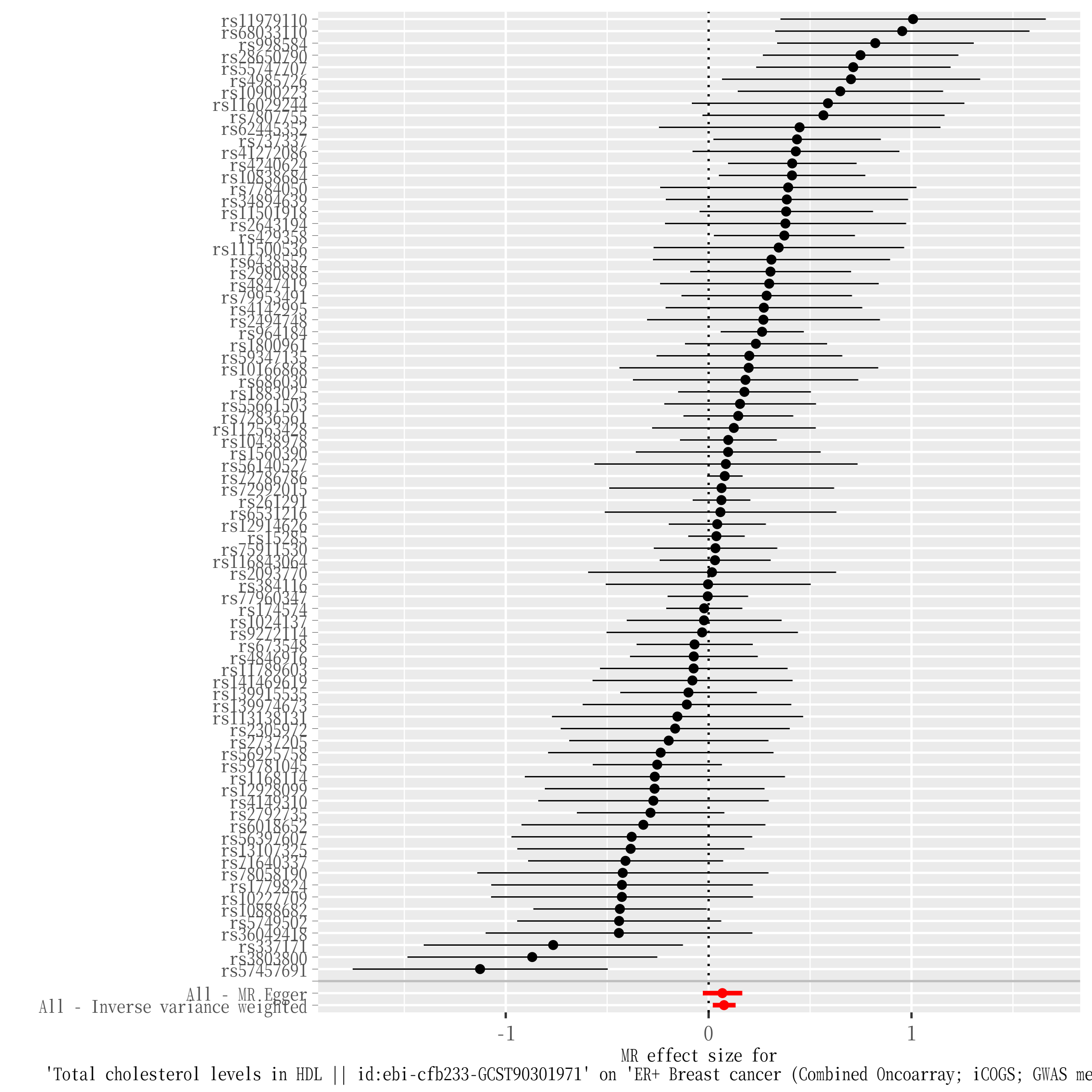
rs11979110
rs68033110
rs998584
rs28650790
rs55747707
rs4985726
rs10900223
rs116029244
rs7807755
rs62445352
rs737337
rs41272086
rs4240624
rs10838684
rs7784050
rs34894639
rs11501918
rs2643194
rs429358
rs111500536
rs6438552
rs2980888
rs4847419
rs79953491
rs4142995
rs2494748
rs964184
rs1800961
rs59347135
rs10166868
rs686030
rs1883025
rs55661503
rs72836561
rs112563428
rs10438978
rs1560390
rs56140527
rs72786786
rs72992015
rs261291
rs6531216
rs12914626
rs15285
rs75911530
rs116843064
rs2093770
rs384116
rs77960347
rs174574
rs1024137
rs9272114
rs673548
rs4846916
rs11789603
rs141469619
rs139915535
rs139974673
rs113138131
rs2305972
rs2737205
rs56925758
rs59781045
rs1168114
rs12928099
rs4149310
rs2792735
rs6018652
rs56397607
rs13107325
rs71640337
rs78058190
rs1779824
rs10227709
rs10888682
rs5749502
rs36049418
rs337171
rs3803800
rs57457691
All - MR Egger
All - Inverse variance weighted
-1
0
1
MR effect size for
'Total cholesterol levels in HDL || id:ebi-cfb233-GCST90301971' on 'ER+ Breast cancer (Combined Oncoarray; iCOGS; GWAS me

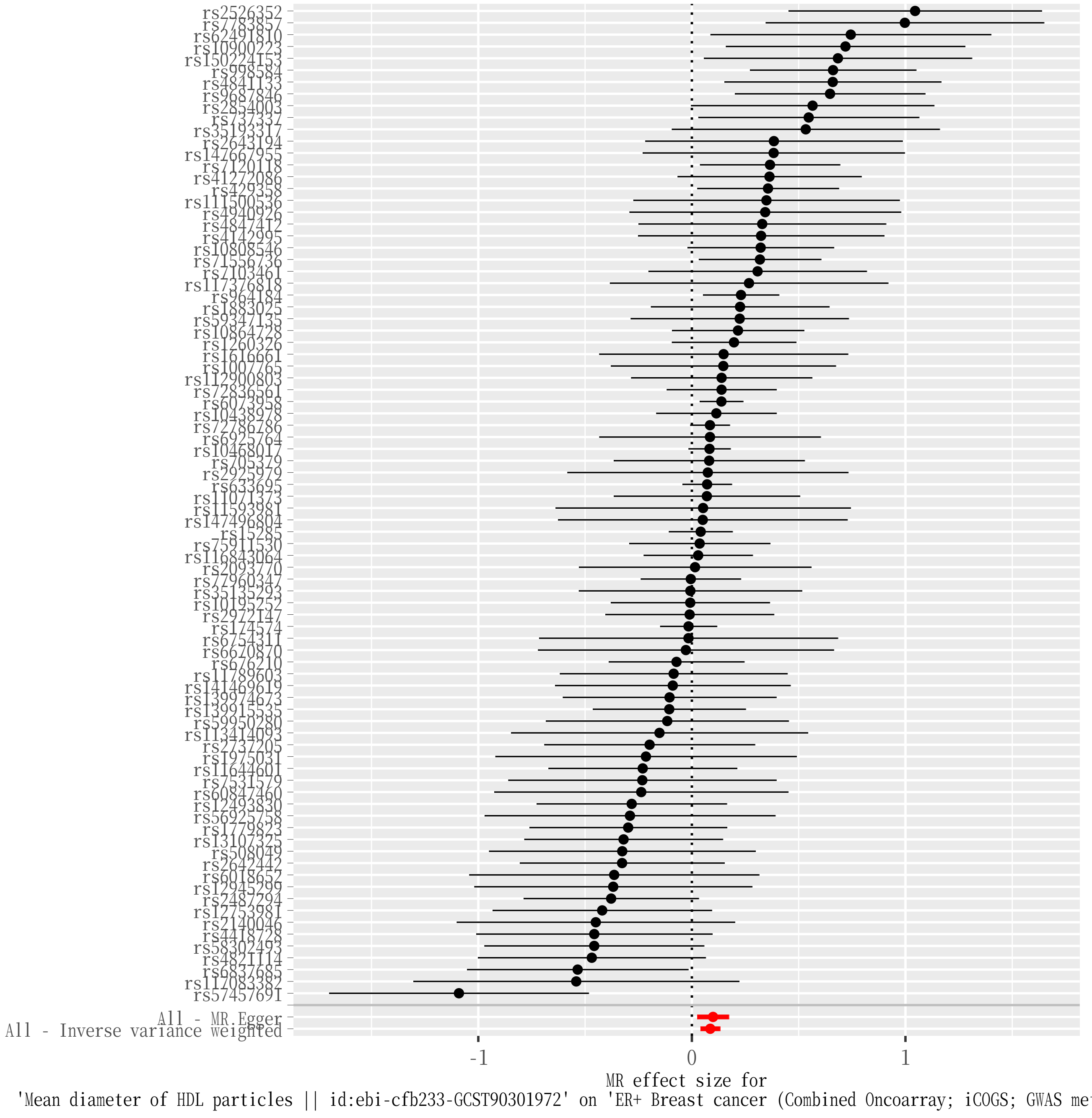

rs2526352
rs62491810
rs10900223
rs150224153
rs4841133
rs9687846
rs2854003
rs737337
rs35193317
rs2643194
rs147667955
rs7120118
rs41272086
rs429358
rs111500536
rs4940926
rs4847412
rs4142995
rs10808546
rs71556736
rs7103461
rs117376818
rs964184
rs1883025
rs59347135
rs10864728
rs1260326
rs1616661
rs1007765
rs112900803
rs72836561
rs6073958
rs10438978
rs72786786
rs6925764
rs10468017
rs705379
rs2925979
rs633695
rs11071373
rs11593981
rs147496804
rs15285
rs75911530
rs116843064
rs2093770
rs77960347
rs35135293
rs10195252
rs2972147
rs174574
rs6754311
rs6670870
rs676210
rs11789603
rs141469619
rs139974673
rs139915535
rs59950280
rs113414093
rs2737205
rs1975031
rs11644601
rs7531579
rs60847460
rs12493830
rs56925758
rs1779823
rs13107325
rs508049
rs2642442
rs6018652
rs12945299
rs2487294
rs12753981
rs2140046
rs4418728
rs58302473
rs4821114
rs6837685
rs117083382
rs57457691
All - MR Egger
All - Inverse variance weighted
-1
0
1
MR effect size for
'Mean diameter of HDL particles || id:ebi-cfb233-GCST90301972' on 'ER+ Breast cancer (Combined Oncoarray; iCOGS; GWAS me

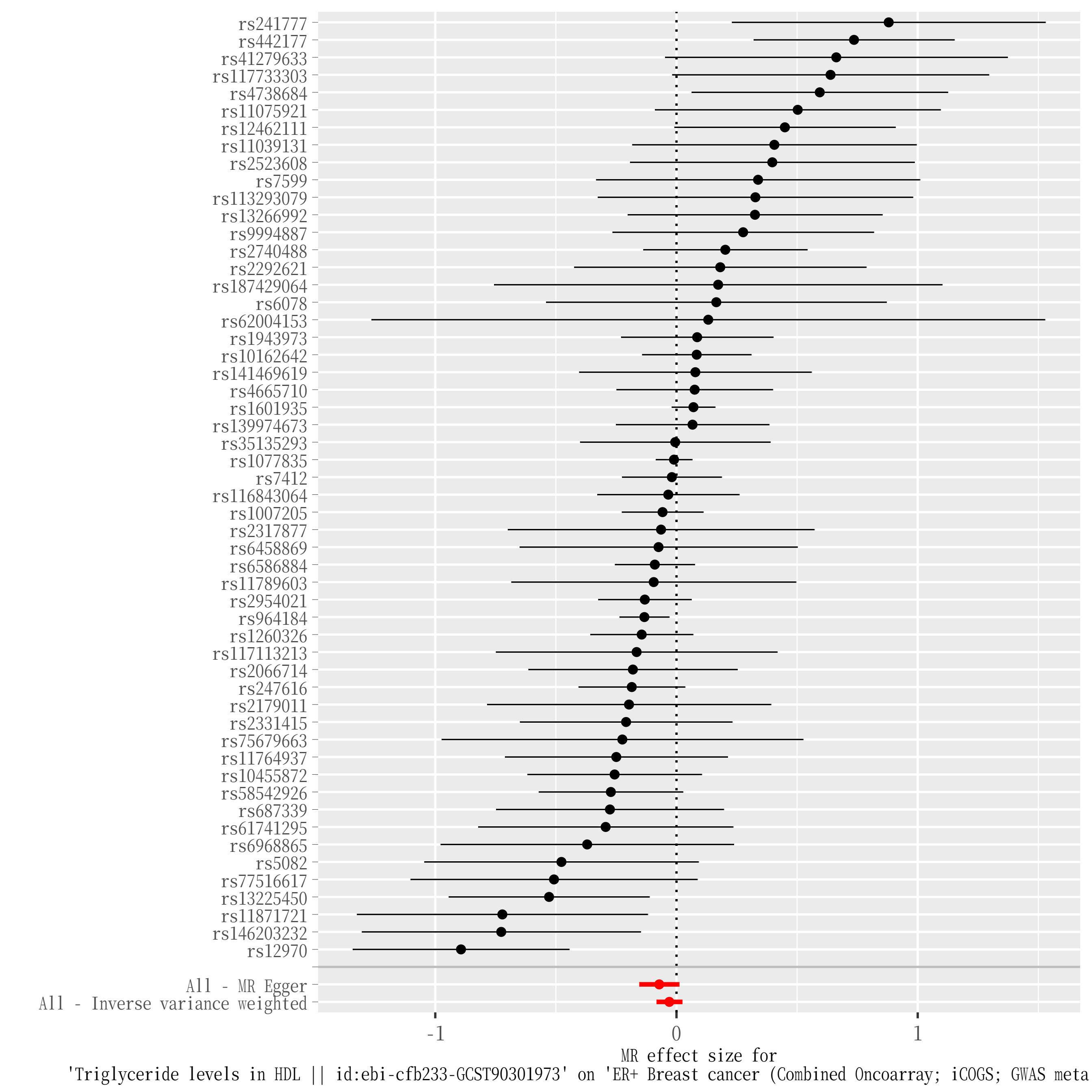
rs241777
rs442177
rs41279633
rs117733303
rs4738684
rs11075921
rs12462111
rs11039131
rs2523608
rs7599
rs113293079
rs13266992
rs9994887
rs2740488
rs2292621
rs187429064
rs6078
rs62004153
rs1943973
rs10162642
rs141469619
rs4665710
rs1601935
rs139974673
rs35135293
rs1077835
rs7412
rs116843064
rs1007205
rs2317877
rs6458869
rs6586884
rs11789603
rs2954021
rs964184
rs1260326
rs117113213
rs2066714
rs247616
rs2179011
rs2331415
rs75679663
rs11764937
rs10455872
rs58542926
rs687339
rs61741295
rs6968865
rs5082
rs77516617
rs13225450
rs11871721
rs146203232
rs12970
All - MR Egger
All - Inverse variance weighted
-1
0
1
MR effect size for
'Triglyceride levels in HDL || id:ebi-cfb233-GCST90301973' on 'ER+ Breast cancer (Combined Oncoarray; iCOGS; GWAS meta

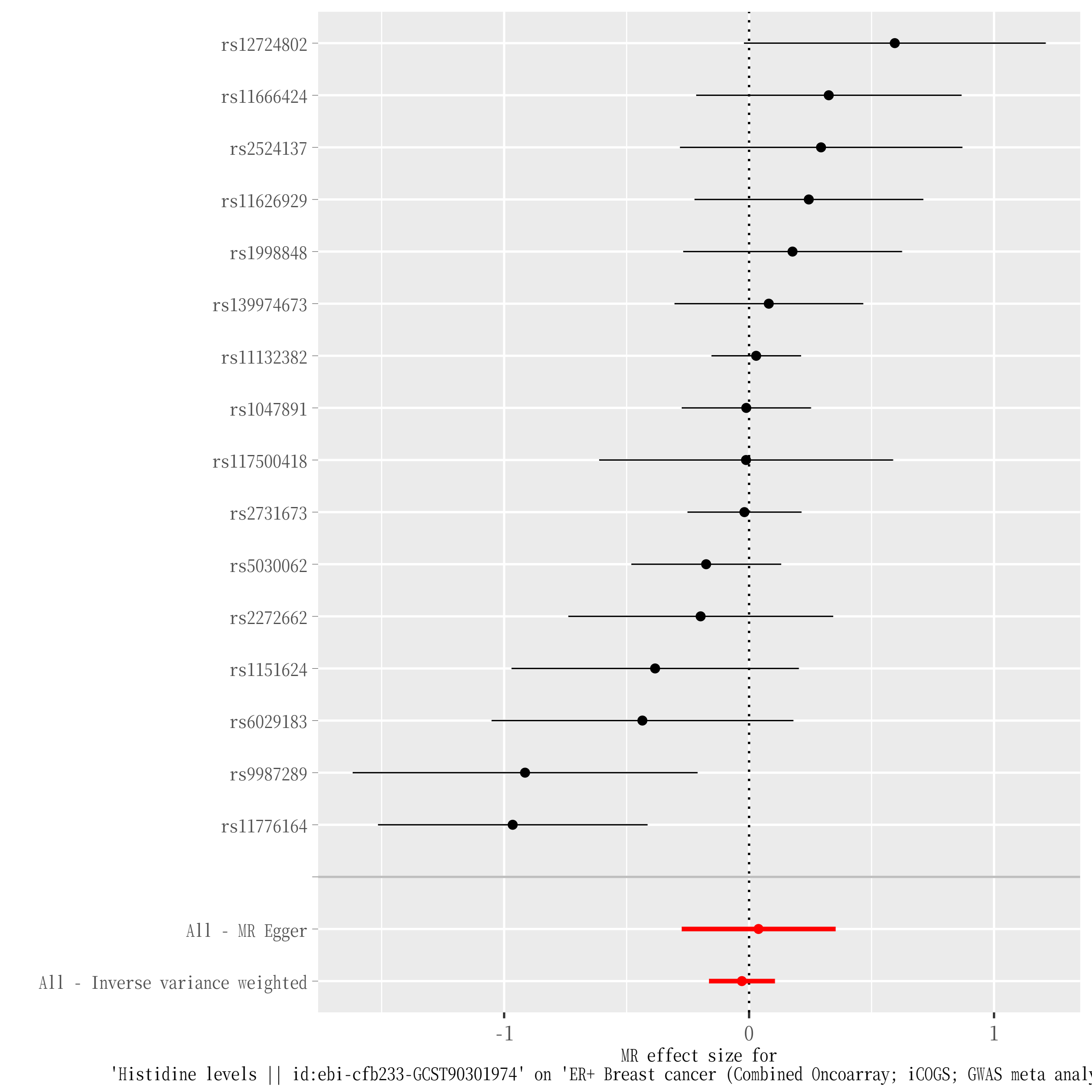

rs12724802
rs11666424
rs2524137
rs11626929
rs1998848
rs139974673
rs11132382
rs1047891
rs117500418
rs2731673
rs5030062
rs2272662
rs1151624
rs6029183
rs9987289
rs11776164
All - MR Egger
All - Inverse variance weighted
-1
0
1
MR effect size for
'Histidine levels || id:ebi-cfb233-GCST90301974' on 'ER+ Breast cancer (Combined Oncoarray; iCOGS; GWAS meta analy

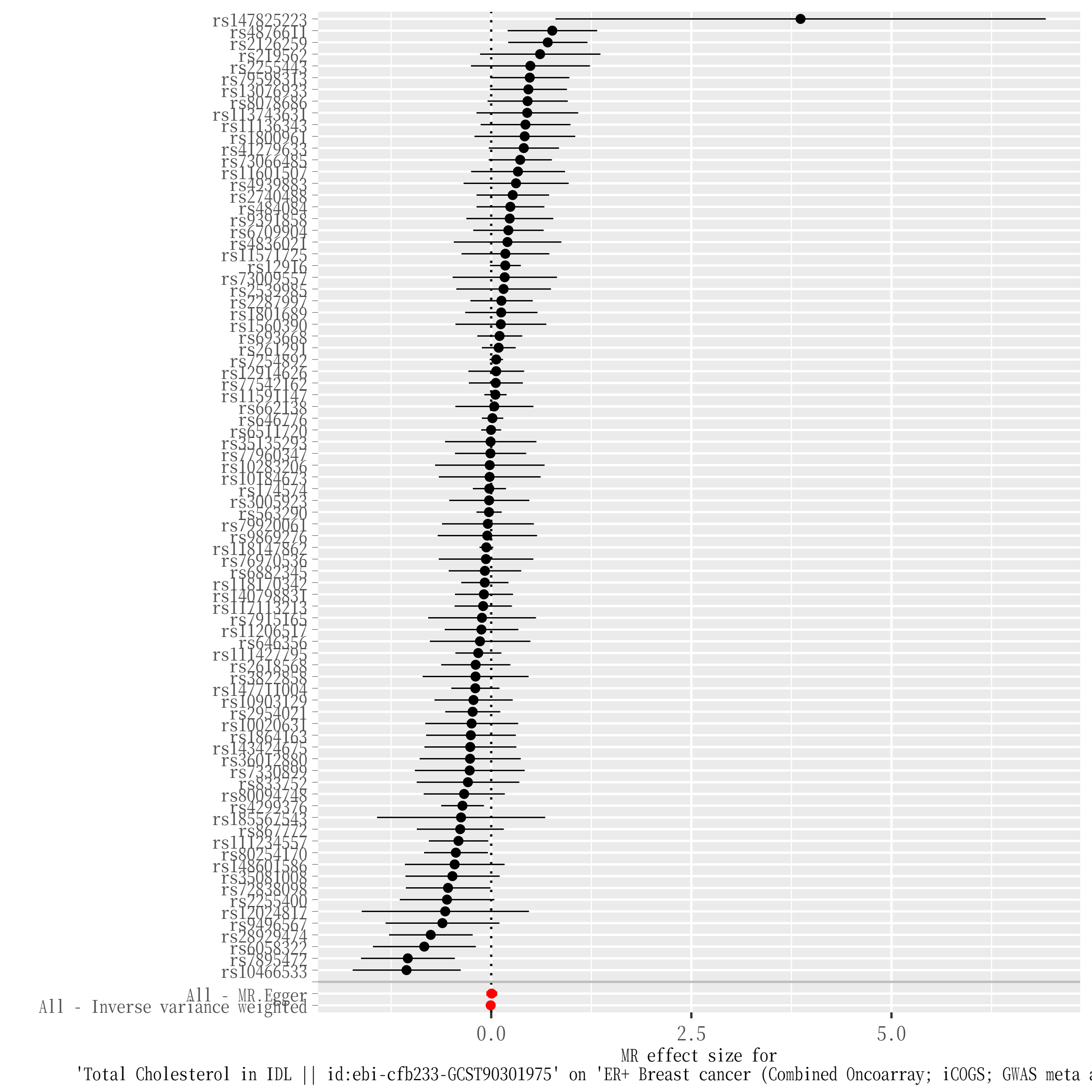
rs147825223
rs4876611
rs2126259
rs219562
rs2255443
rs79598313
rs13076933
rs8078686
rs113743631
rs11136343
rs1800961
rs41279633
rs73066485
rs11601507
rs4939883
rs2740488
rs484084
rs9391858
rs6709904
rs4836021
rs11571725
rs12916
rs73009557
rs2539985
rs2287997
rs1801689
rs1560390
rs693668
rs261291
rs7254892
rs12914626
rs77542162
rs11591147
rs662138
rs646776
rs6511720
rs35135293
rs77960347
rs10283206
rs10184673
rs174574
rs3005923
rs563290
rs79920061
rs9869276
rs118147862
rs76970536
rs6882345
rs118170342
rs140798831
rs112113213
rs7915165
rs11206517
rs646356
rs111427795
rs2618568
rs3822858
rs147711004
rs10903129
rs2954021
rs10020631
rs1864163
rs143424675
rs36012880
rs7330899
rs833752
rs80094748
rs4299376
rs185567543
rs867772
rs111234557
rs80254170
rs148601586
rs35081008
rs72838098
rs2255400
rs12024817
rs9496567
rs28929474
rs6058322
rs7895472
rs10466533
All - MR Egger
All - Inverse variance weighted
0.0
2.5
5.0
MR effect size for
'Total Cholesterol in IDL || id:ebi-cfb233-GCST90301975' on 'ER+ Breast cancer (Combined Oncoarray; iCOGS; GWAS meta a

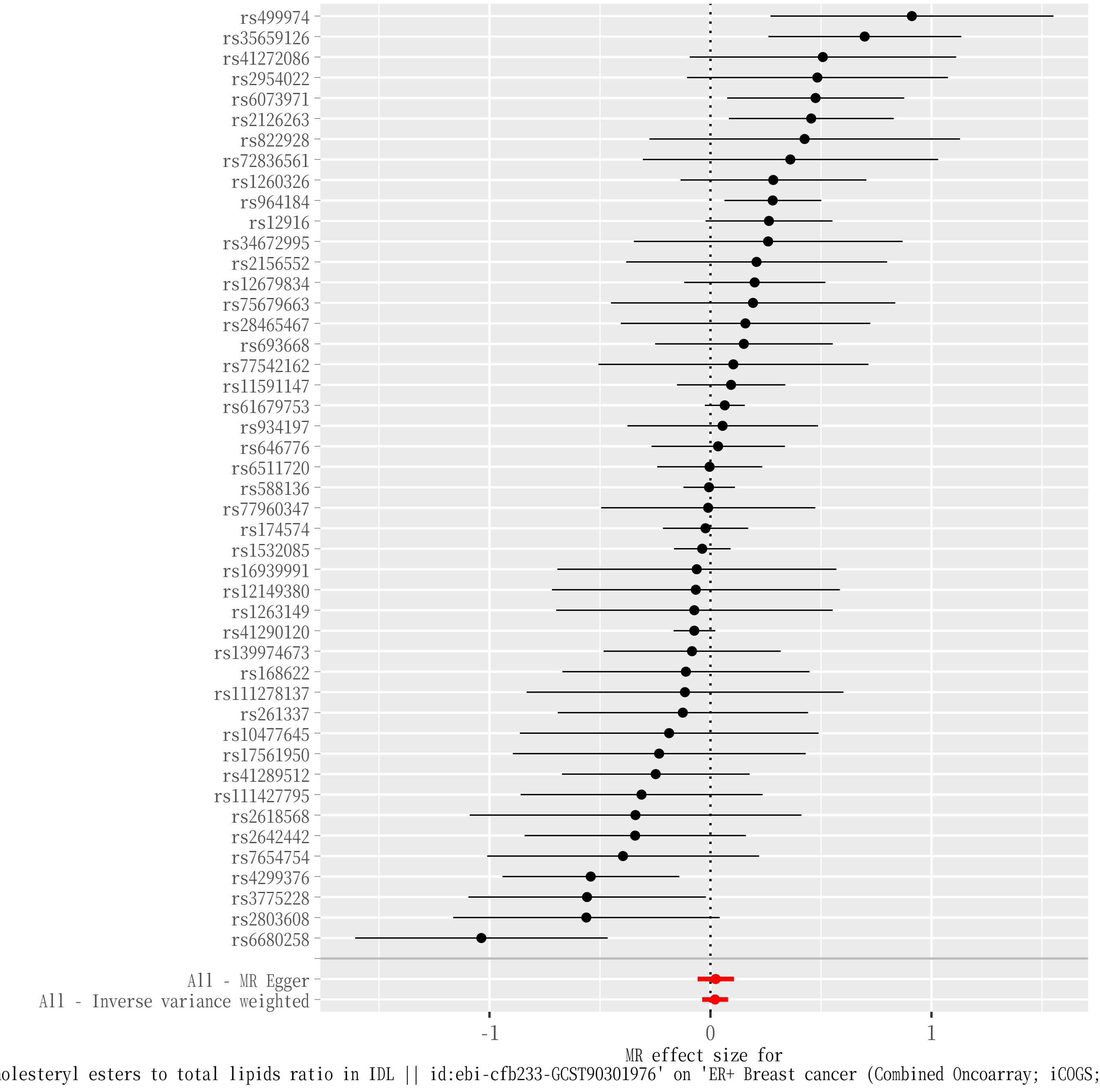

rs499974
rs35659126
rs41272086
rs2954022
rs6073971
rs2126263
rs822928
rs72836561
rs1260326
rs964184
rs12916
rs34672995
rs2156552
rs12679834
rs75679663
rs28465467
rs693668
rs77542162
rs11591147
rs61679753
rs934197
rs646776
rs6511720
rs588136
rs77960347
rs174574
rs1532085
rs16939991
rs12149380
rs1263149
rs41290120
rs139974673
rs168622
rs111278137
rs261337
rs10477645
rs17561950
rs41289512
rs111427795
rs2618568
rs2642442
rs7654754
rs4299376
rs3775228
rs2803608
rs6680258
All - MR Egger
All - Inverse variance weighted
-1
0
1
MR effect size for
holesteryl esters to total lipids ratio in IDL || id:ebi-cfb233-GCST90301976' on 'ER+ Breast cancer (Combined Oncoarray; iCOGS;

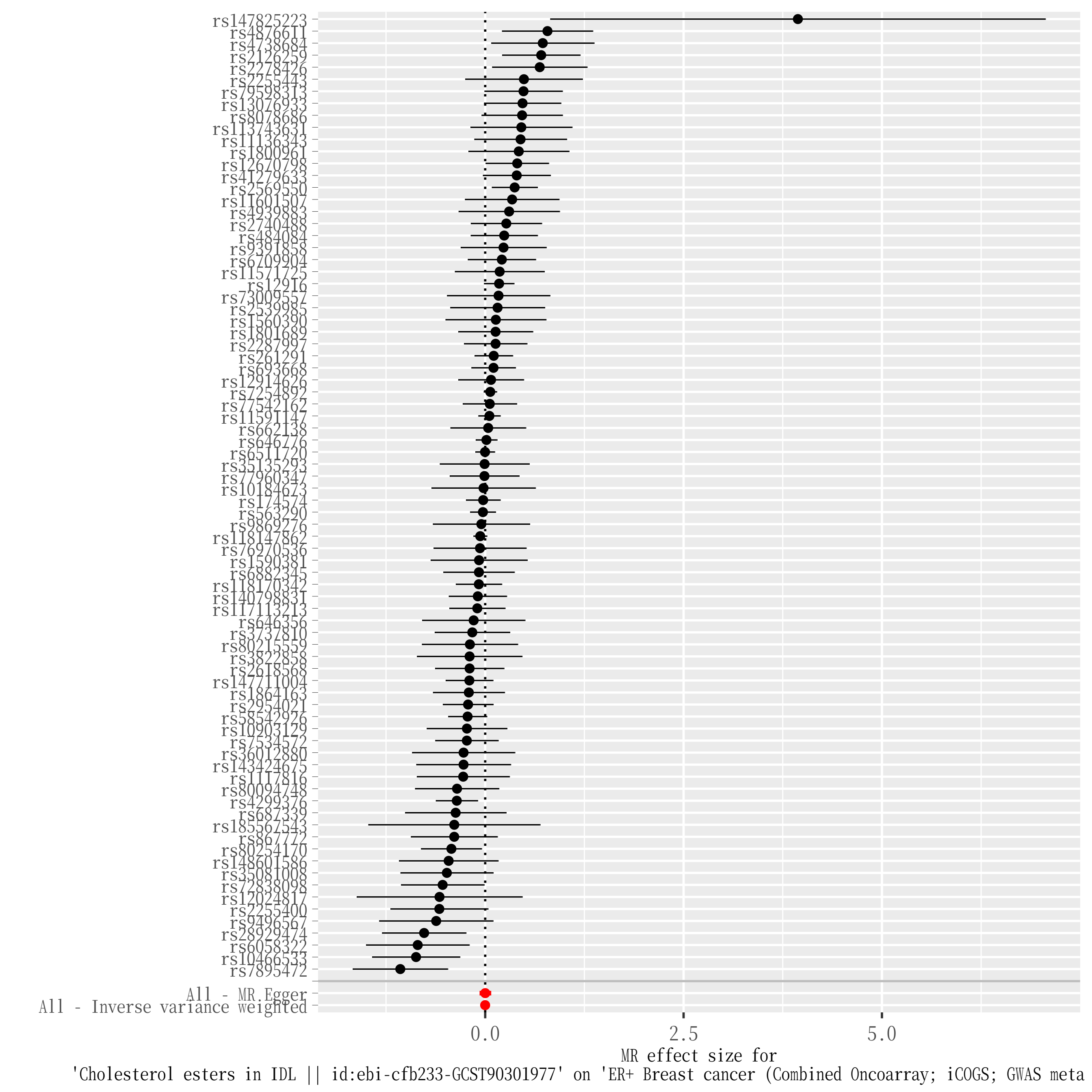

rs147825223
rs4876611
rs4738684
rs2126259
rs2278426
rs2255443
rs79598313
rs13076933
rs8078686
rs113743631
rs11136343
rs1800961
rs12670798
rs41279633
rs2569550
rs11601507
rs4939883
rs2740488
rs484084
rs9391858
rs6709904
rs11571725
rs12916
rs73009557
rs2539985
rs1560390
rs1801689
rs2287997
rs261291
rs693668
rs12914626
rs7254892
rs77542162
rs11591147
rs662138
rs646776
rs6511720
rs35135293
rs77960347
rs10184673
rs174574
rs563290
rs9869276
rs118147862
rs76970536
rs1590381
rs6882345
rs118170342
rs140798831
rs117113213
rs646356
rs3737810
rs80215559
rs3822858
rs2618568
rs147711004
rs1864163
rs2954021
rs58542926
rs10903129
rs7534572
rs36012880
rs143424675
rs1117816
rs80094748
rs4299376
rs687339
rs185567543
rs867772
rs80254170
rs148601586
rs35081008
rs72838098
rs12024817
rs2255400
rs9496567
rs28929474
rs6058322
rs10466533
rs7895472
All - MR Egger
All - Inverse variance weighted
0.0
2.5
5.0
MR effect size for
'Cholesterol esters in IDL || id:ebi-cfb233-GCST90301977' on 'ER+ Breast cancer (Combined Oncoarray; iCOGS; GWAS meta

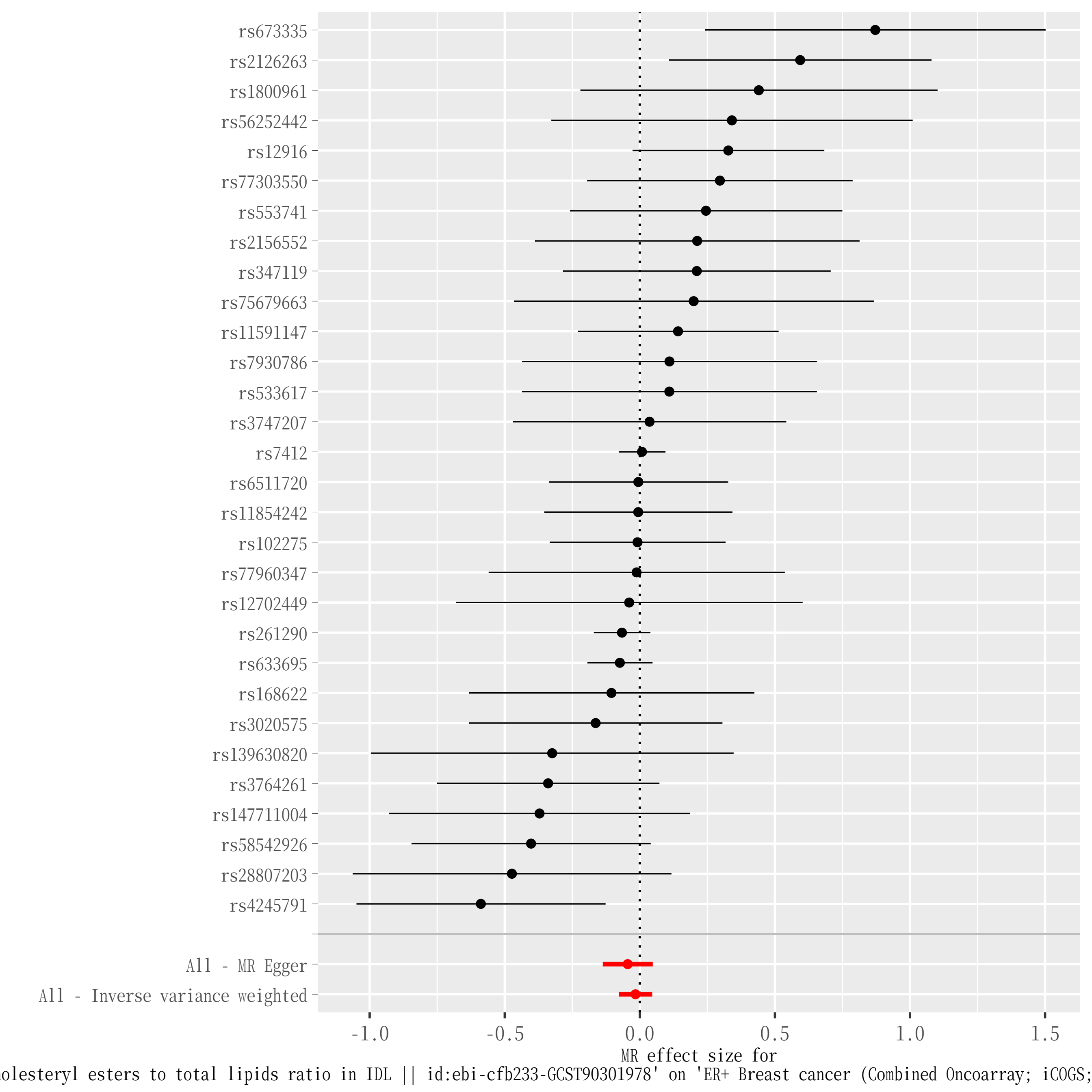

rs673335
rs2126263
rs1800961
rs56252442
rs12916
rs77303550
rs553741
rs2156552
rs347119
rs75679663
rs11591147
rs7930786
rs533617
rs3747207
rs7412
rs6511720
rs11854242
rs102275
rs77960347
rs12702449
rs261290
rs633695
rs168622
rs3020575
rs139630820
rs3764261
rs147711004
rs58542926
rs28807203
rs4245791
All - MR Egger
All - Inverse variance weighted
-1.0
-0.5
0.0
0.5
1.0
1.5
MR effect size for
olesteryl esters to total lipids ratio in IDL || id:ebi-cfb233-GCST90301978' on 'ER+ Breast cancer (Combined Oncoarray; iCOGS;

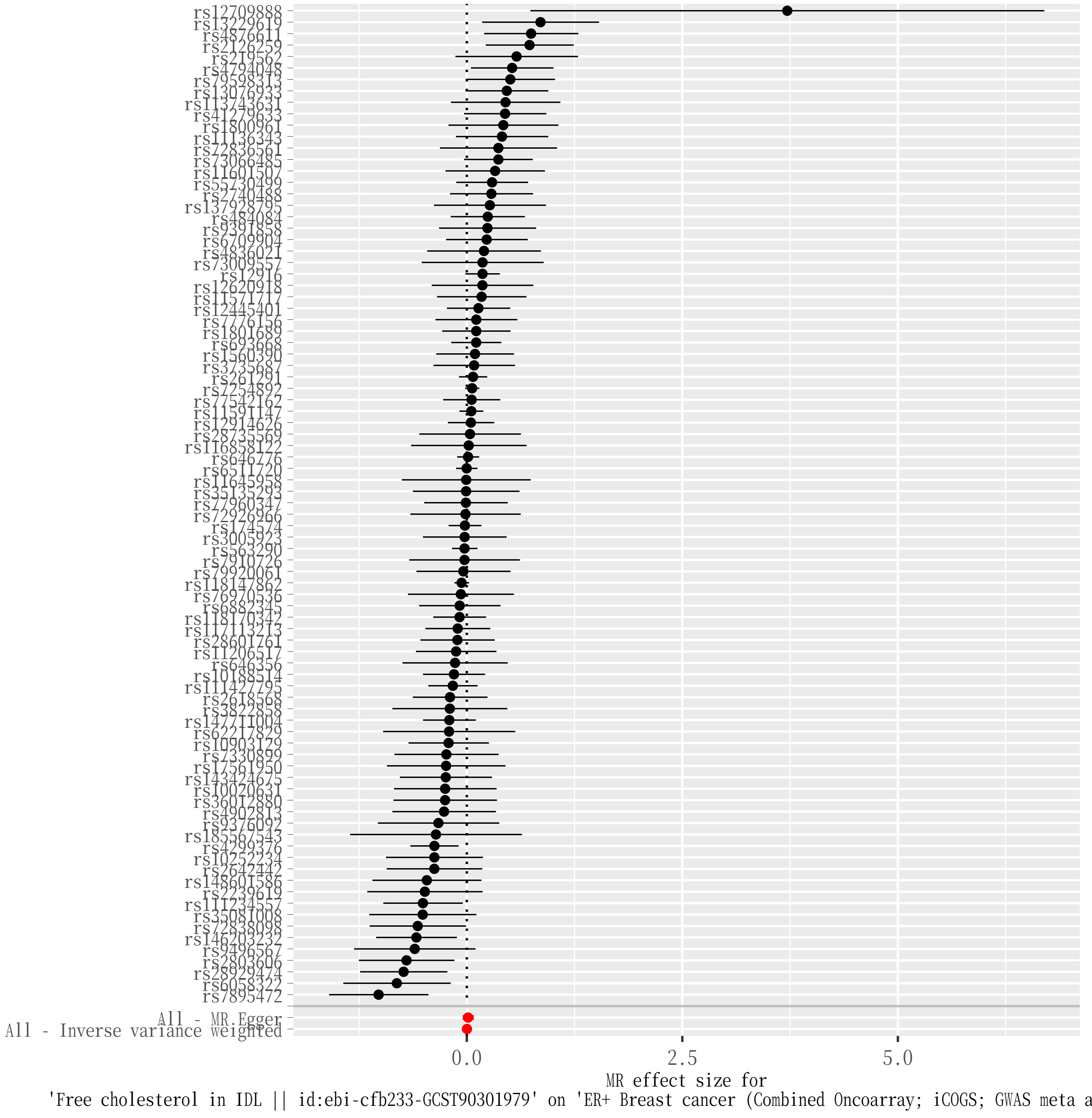

rs12709888
rs13229619
rs4876611
rs2126259
rs219562
rs4794048
rs79598313
rs13076933
rs113743631
rs41279633
rs1800961
rs11136343
rs72836561
rs73066485
rs11601507
rs55730499
rs2740488
rs137928795
rs484084
rs9391858
rs6709904
rs4836021
rs73009557
rs12916
rs12620918
rs11571717
rs12445401
rs7776156
rs1801689
rs623908
rs1560390
rs3735687
rs261291
rs7254892
rs77542162
rs11591147
rs12914626
rs28735569
rs116858122
rs646776
rs6511720
rs11645958
rs35135293
rs72960347
rs72926266
rs174574
rs3005923
rs563290
rs7910726
rs79920061
rs118147862
rs76970536
rs6882345
rs118170342
rs117113213
rs28601761
rs11206517
rs646356
rs10188514
rs111427795
rs2618568
rs3822858
rs147711004
rs62217829
rs10903129
rs7330899
rs17561950
rs143424675
rs10020631
rs36012880
rs4902813
rs9376092
rs185567543
rs4299376
rs10252234
rs2642442
rs148601586
rs2239619
rs111234557
rs35081008
rs72838098
rs146203232
rs9496567
rs2803606
rs28929474
rs6058322
rs7895472
All - MR Egger
All - Inverse variance weighted
0.0
2.5
5.0
MR effect size for
'Free cholesterol in IDL || id:ebi-cfb233-GCST90301979' on 'ER+ Breast cancer (Combined Oncoarray; iCOGS; GWAS meta a

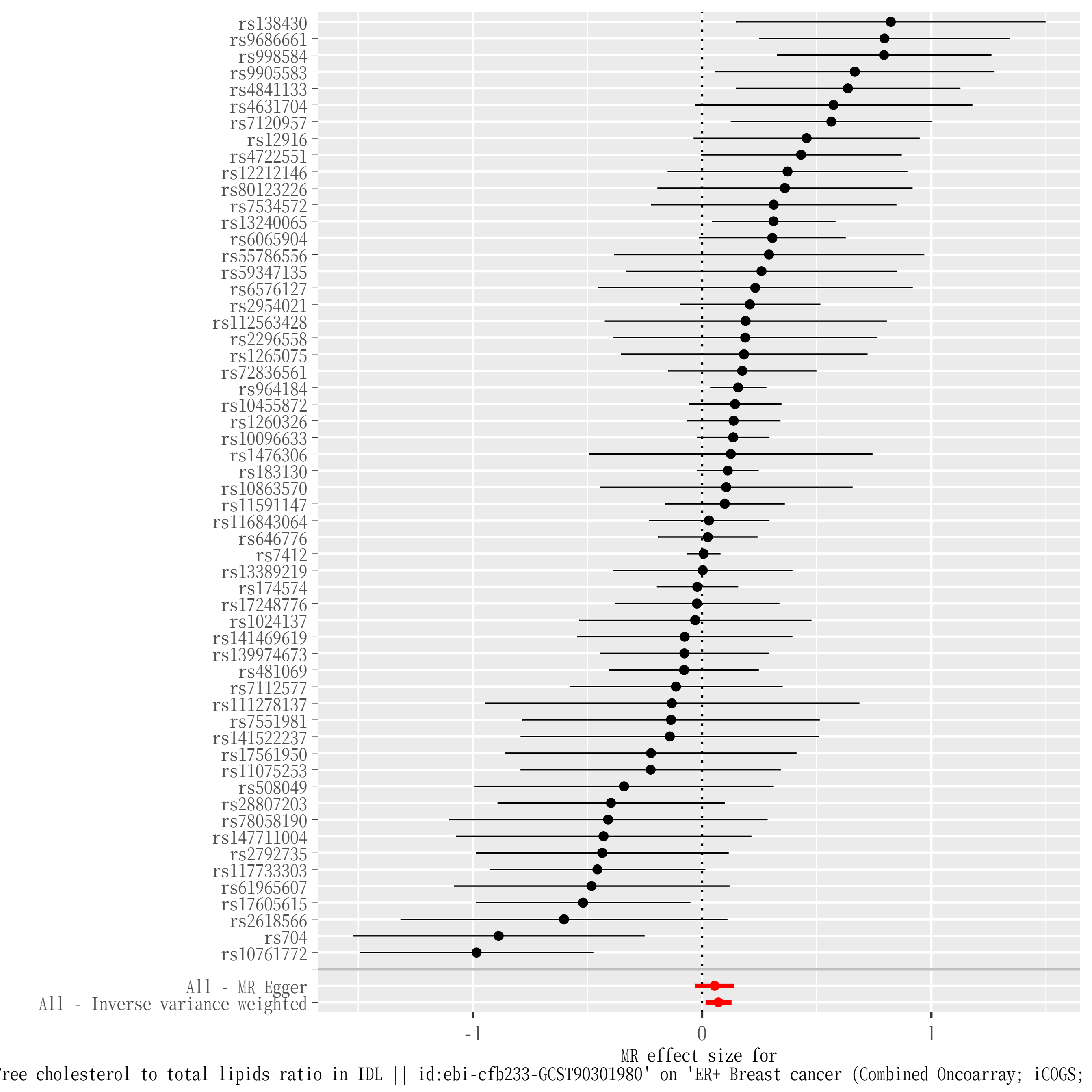

rs138430
rs9686661
rs998584
rs9905583
rs4841133
rs4631704
rs7120957
rs12916
rs4722551
rs12212146
rs80123226
rs7534572
rs13240065
rs6065904
rs55786556
rs59347135
rs6576127
rs2954021
rs112563428
rs2296558
rs1265075
rs72836561
rs964184
rs10455872
rs1260326
rs10096633
rs1476306
rs183130
rs10863570
rs11591147
rs116843064
rs646776
rs7412
rs13389219
rs174574
rs17248776
rs1024137
rs141469619
rs139974673
rs481069
rs7112577
rs111278137
rs7551981
rs141522237
rs17561950
rs11075253
rs508049
rs28807203
rs78058190
rs147711004
rs2792735
rs117733303
rs61965607
rs17605615
rs2618566
rs704
rs10761772
All - MR Egger
All - Inverse variance weighted
-1
0
1
MR effect size for
'Free cholesterol to total lipids ratio in IDL || id:ebi-cfb233-GCST90301980' on 'ER+ Breast cancer (Combined Oncoarray; iCOGS;

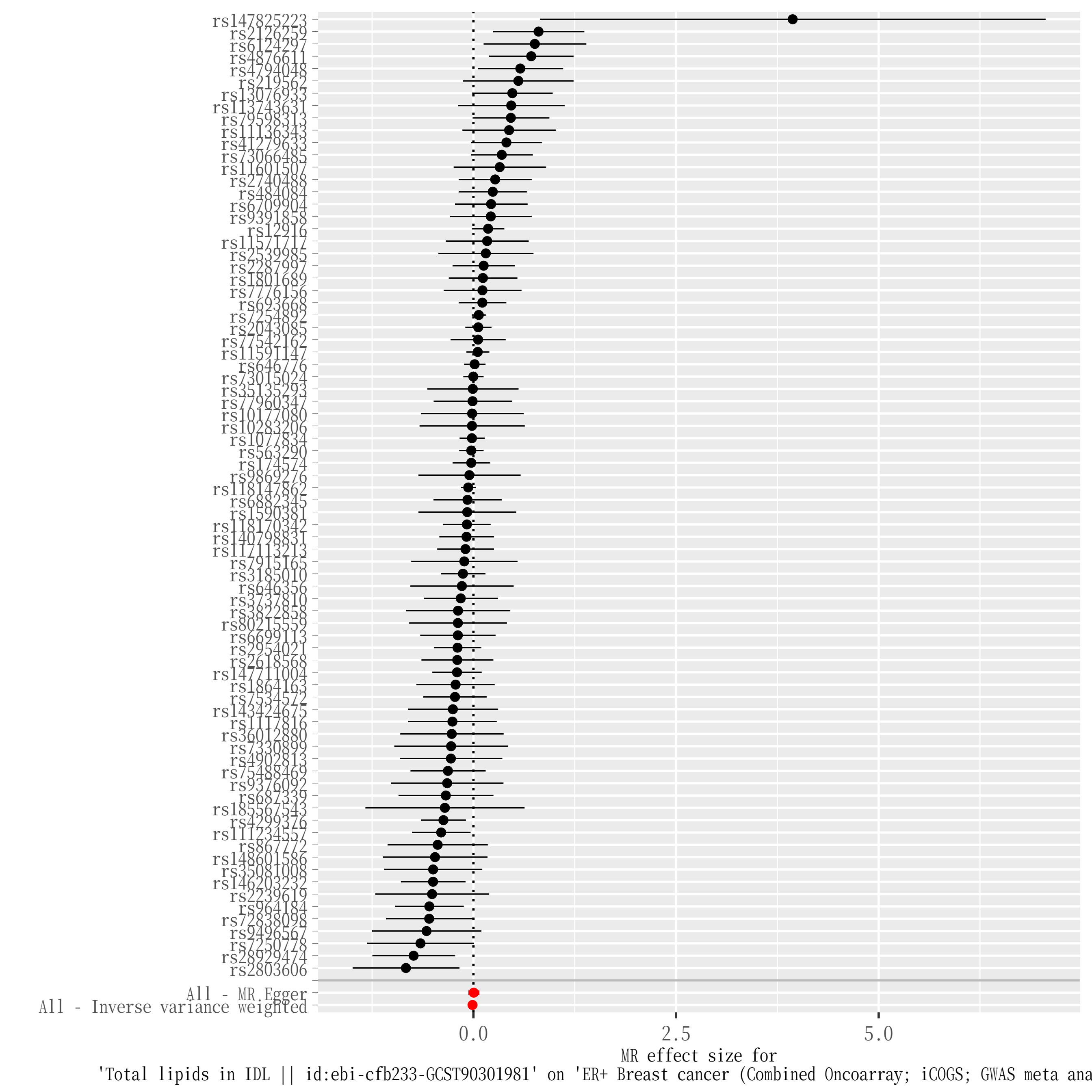

rs147825223
rs2126259
rs6124297
rs4876611
rs4794048
rs219562
rs13076933
rs113743631
rs79598313
rs11136343
rs41279633
rs73066485
rs11601507
rs2740488
rs484084
rs6709904
rs9391858
rs12916
rs11571717
rs2532985
rs2287997
rs1801689
rs7776156
rs693668
rs7254892
rs2043085
rs77542162
rs11591147
rs646776
rs73015024
rs35135293
rs77960347
rs10177080
rs10283206
rs1077834
rs563290
rs174574
rs9869276
rs118147862
rs6882345
rs1590381
rs118170342
rs140798831
rs117113213
rs7915165
rs3185010
rs646356
rs3737810
rs3822858
rs80215559
rs6699113
rs2954021
rs2618568
rs147711004
rs1864163
rs7534572
rs143424675
rs1117816
rs36012880
rs7330899
rs4902813
rs75488469
rs9376092
rs687339
rs185567543
rs4299376
rs111234557
rs867772
rs148601586
rs35081008
rs146203232
rs2239619
rs964184
rs72838098
rs9496567
rs7250778
rs28929474
rs2803606
All - MR Egger
All - Inverse variance weighted
0.0
2.5
5.0
MR effect size for
'Total lipids in IDL || id:ebi-cfb233-GCST90301981' on 'ER+ Breast cancer (Combined Oncoarray; iCOGS; GWAS meta ana

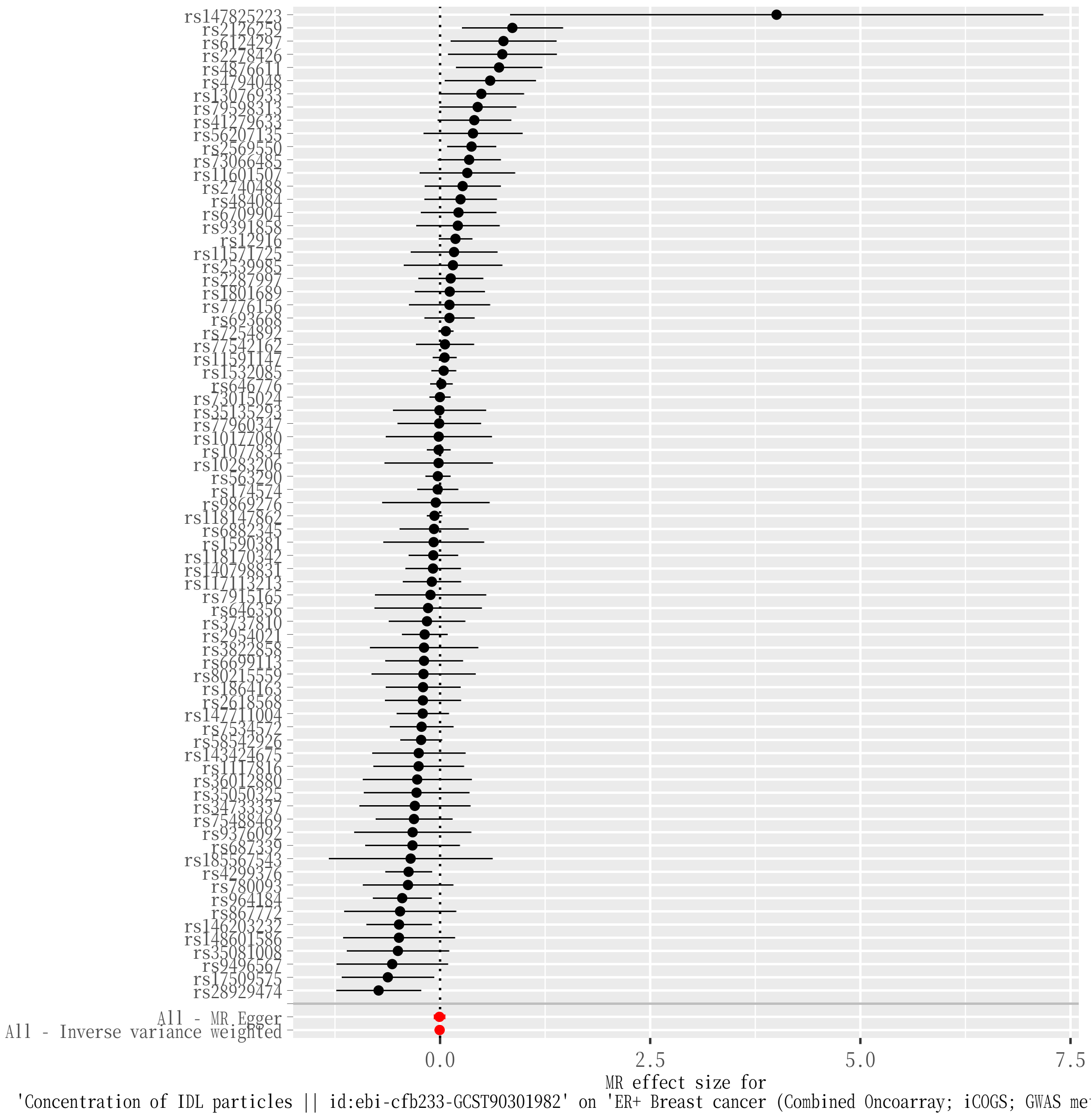
rs147825223
rs2126259
rs6124297
rs2278426
rs4876611
rs4794048
rs13076933
rs79598313
rs41279633
rs56207135
rs2569550
rs73066485
rs11601507
rs2740488
rs484084
rs6709904
rs9391858
rs12916
rs11571725
rs2539985
rs2287997
rs1801689
rs7776156
rs693668
rs7254892
rs77542162
rs11591147
rs1532085
rs646776
rs73015024
rs35135293
rs77960347
rs10177080
rs1077834
rs10283206
rs563290
rs174574
rs9869276
rs118147862
rs6882345
rs1590381
rs118170342
rs140798831
rs117113213
rs7915165
rs646356
rs3737810
rs2954021
rs3822858
rs6699113
rs80215559
rs1864163
rs2618568
rs147711004
rs7534572
rs58542926
rs143424675
rs1117816
rs36012880
rs35050325
rs34733337
rs75488469
rs9376092
rs687339
rs185567543
rs4299376
rs780093
rs964184
rs867772
rs146203232
rs148601586
rs35081008
rs2496567
rs17509575
rs28929474
All - MR Egger
All - Inverse variance weighted
0.0
2.5
5.0
7.5
MR effect size for
'Concentration of IDL particles || id:ebi-cfb233-GCST90301982' on 'ER+ Breast cancer (Combined Oncoarray; iCOGS; GWAS me

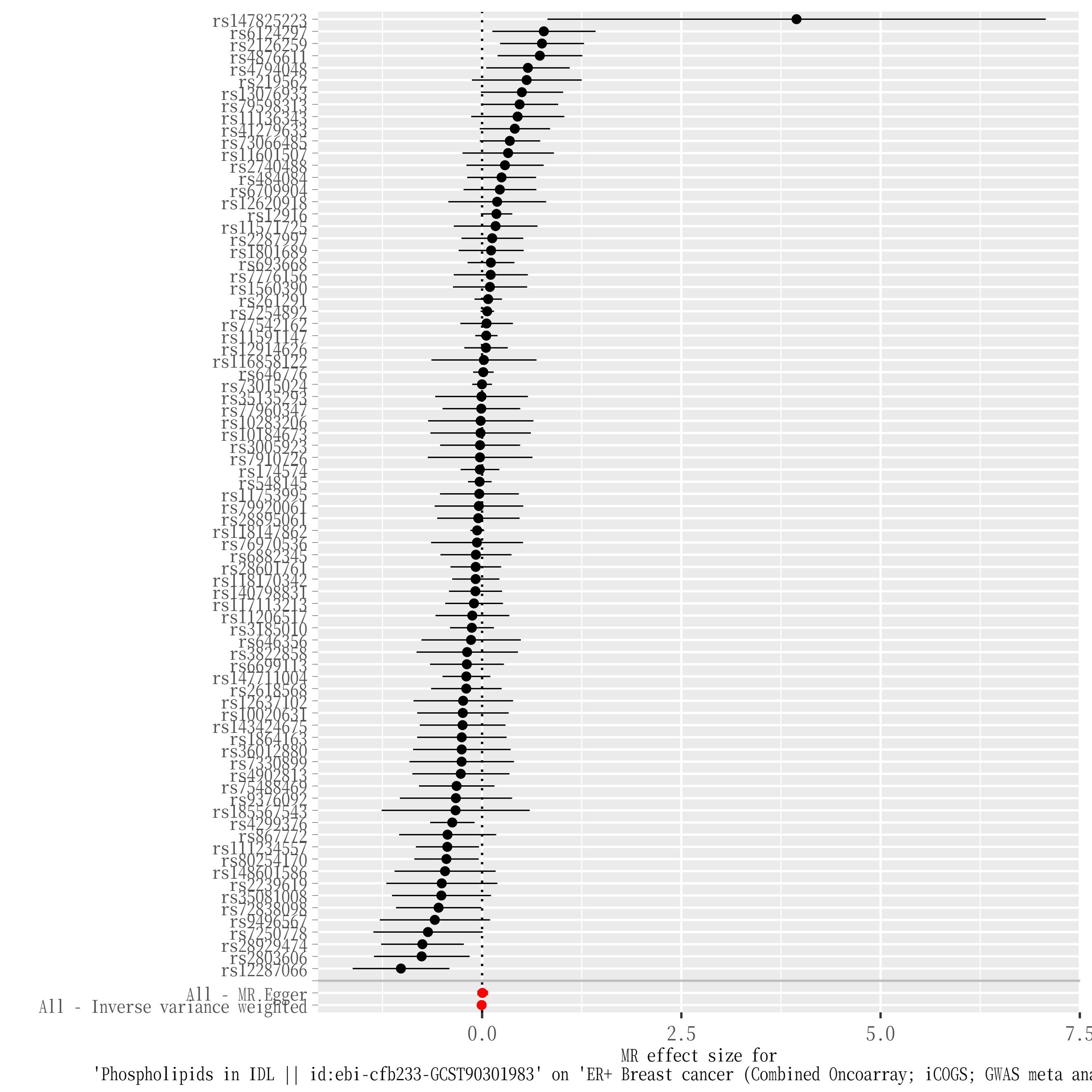
rs147825223
rs6124297
rs2126259
rs4876611
rs4794048
rs219562
rs13076933
rs79598313
rs11136343
rs41279633
rs73066485
rs11601507
rs2740488
rs484084
rs6709904
rs12620918
rs12916
rs11571725
rs2287997
rs1801689
rs623668
rs7776156
rs1560390
rs261291
rs7254892
rs77542162
rs11591147
rs12914626
rs116858122
rs646776
rs73015024
rs35135293
rs7796347
rs10283206
rs10184673
rs3005923
rs7910726
rs174574
rs548145
rs11753995
rs79920061
rs28895061
rs118147862
rs76970536
rs6882345
rs28601761
rs118170342
rs140798831
rs117113213
rs11206517
rs3185010
rs646356
rs3822858
rs6699113
rs147711004
rs2618568
rs12637102
rs10020631
rs143424675
rs1864163
rs36012880
rs7330899
rs4902813
rs75488469
rs9376092
rs185567543
rs4299376
rs867772
rs111234557
rs80254170
rs148601586
rs2239619
rs35081008
rs72838098
rs9496567
rs7250778
rs28929474
rs2803606
rs12287066
All - MR Egger
All - Inverse variance weighted
0.0
2.5
5.0
7.5
MR effect size for
'Phospholipids in IDL || id:ebi-cfb233-GCST90301983' on 'ER+ Breast cancer (Combined Oncoarray; iCOGS; GWAS meta ana

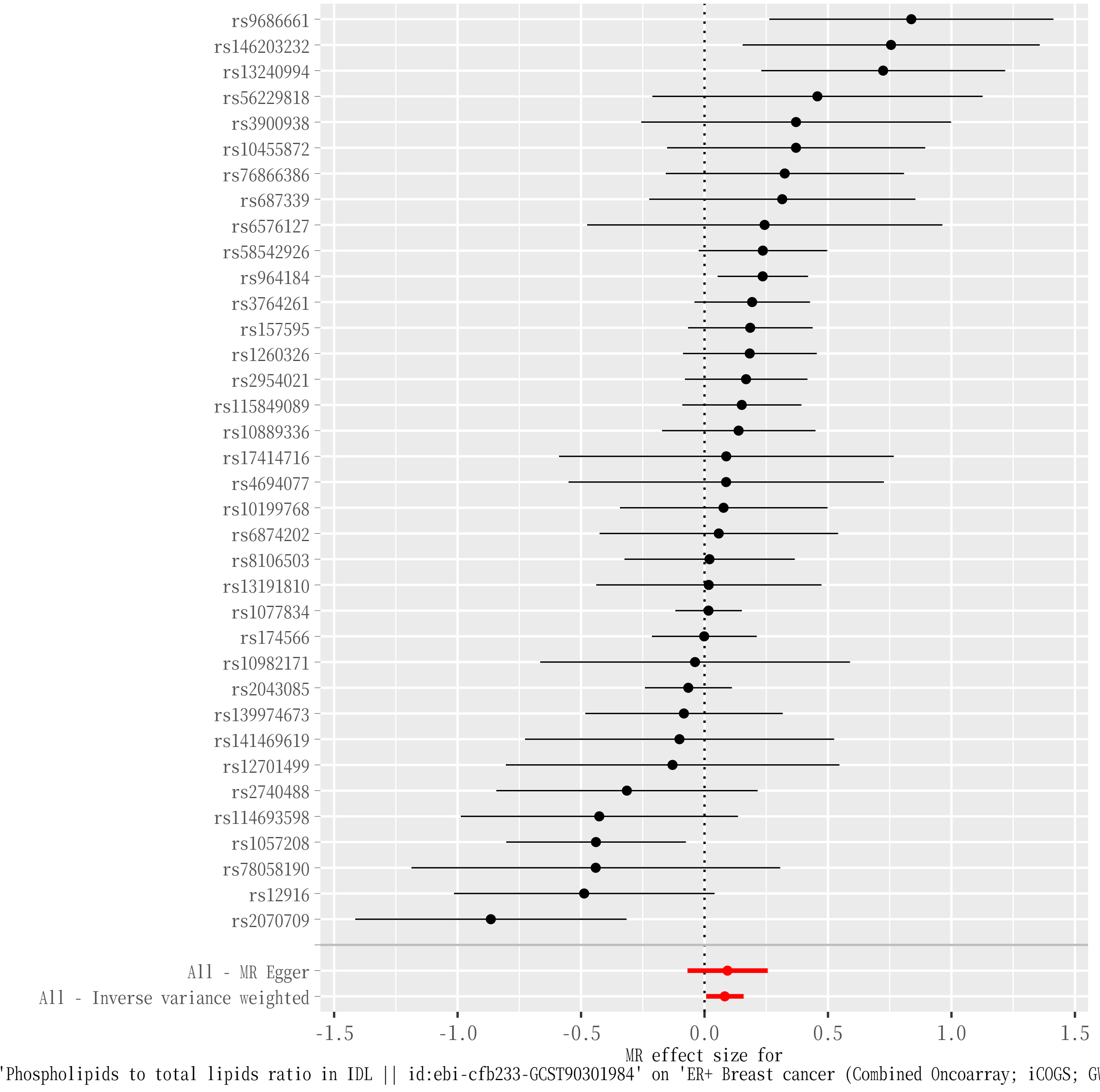

rs9686661
rs146203232
rs13240994
rs56229818
rs3900938
rs10455872
rs76866386
rs687339
rs6576127
rs58542926
rs964184
rs3764261
rs157595
rs1260326
rs2954021
rs115849089
rs10889336
rs17414716
rs4694077
rs10199768
rs6874202
rs8106503
rs13191810
rs1077834
rs174566
rs10982171
rs2043085
rs139974673
rs141469619
rs12701499
rs2740488
rs114693598
rs1057208
rs78058190
rs12916
rs2070709
All - MR Egger
All - Inverse variance weighted
-1.5
-1.0
-0.5
0.0
0.5
1.0
1.5
MR effect size for
'Phospholipids to total lipids ratio in IDL || id:ebi-cfb233-GCST90301984' on 'ER+ Breast cancer (Combined Oncoarray; iCOGS; GW

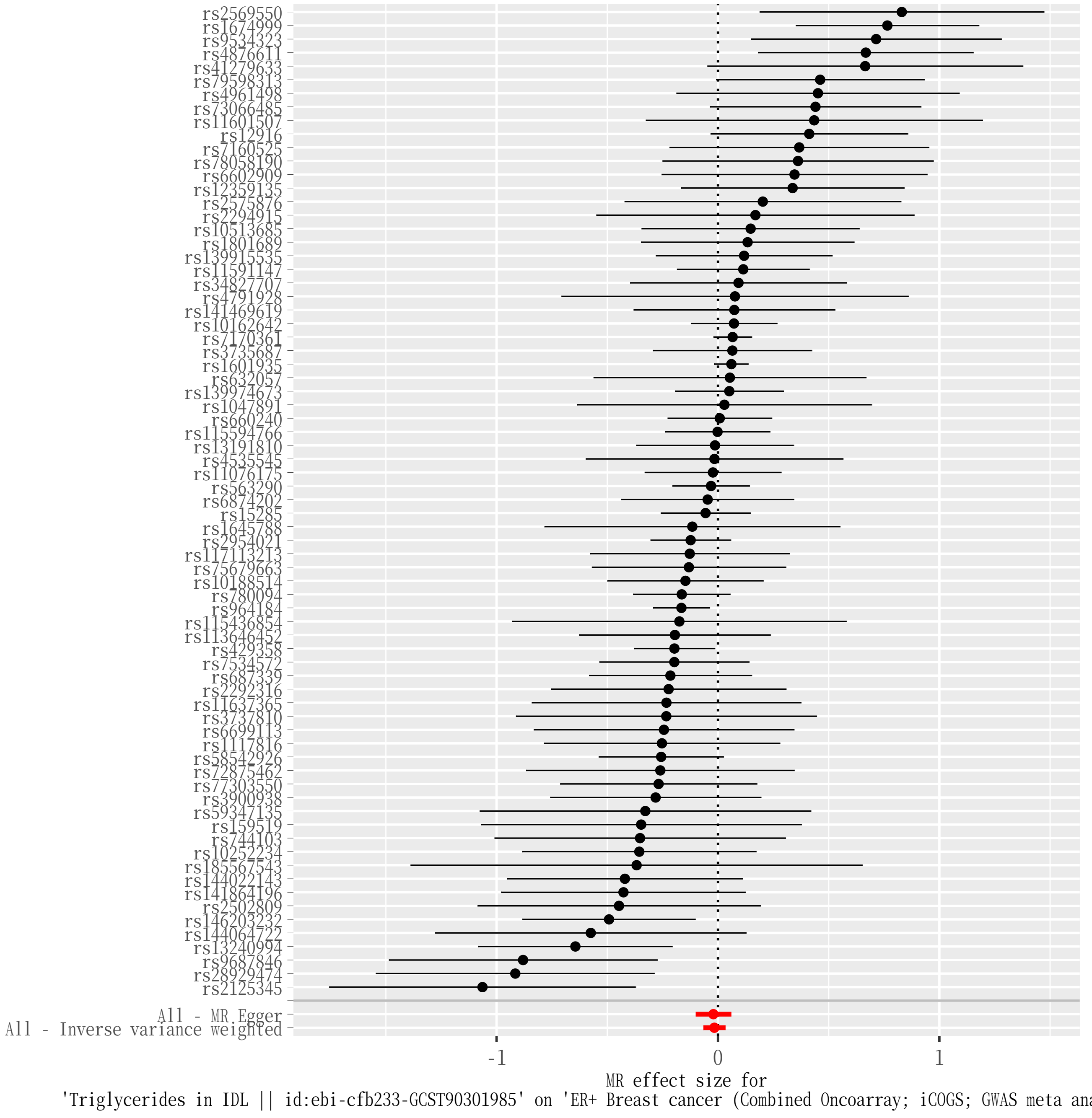

rs2569550
rs1674999
rs9534323
rs4876611
rs41279633
rs79598313
rs4961498
rs73066485
rs11601507
rs12916
rs7160525
rs78058190
rs6602909
rs12359135
rs2575876
rs2294915
rs10513685
rs1801689
rs139915535
rs11591147
rs34827707
rs4791928
rs141469619
rs10162642
rs7170361
rs3735687
rs1601935
rs632057
rs139974673
rs1047891
rs660240
rs115594766
rs13191810
rs4535545
rs11076175
rs563290
rs6874202
rs15285
rs1645788
rs2954021
rs117113213
rs75679663
rs10188514
rs780094
rs964184
rs115436854
rs113646452
rs429358
rs7534572
rs687339
rs2292316
rs11637365
rs3737810
rs6699113
rs1117816
rs58542926
rs72875462
rs77303550
rs3900938
rs59347135
rs159519
rs744103
rs10252234
rs185567543
rs144022143
rs141864196
rs2502809
rs146203232
rs144064722
rs13240994
rs9687846
rs28929474
rs2125345
All - MR Egger
All - Inverse variance weighted
-1
0
1
MR effect size for
'Triglycerides in IDL || id:ebi-cfb233-GCST90301985' on 'ER+ Breast cancer (Combined Oncoarray; iCOGS; GWAS meta ana

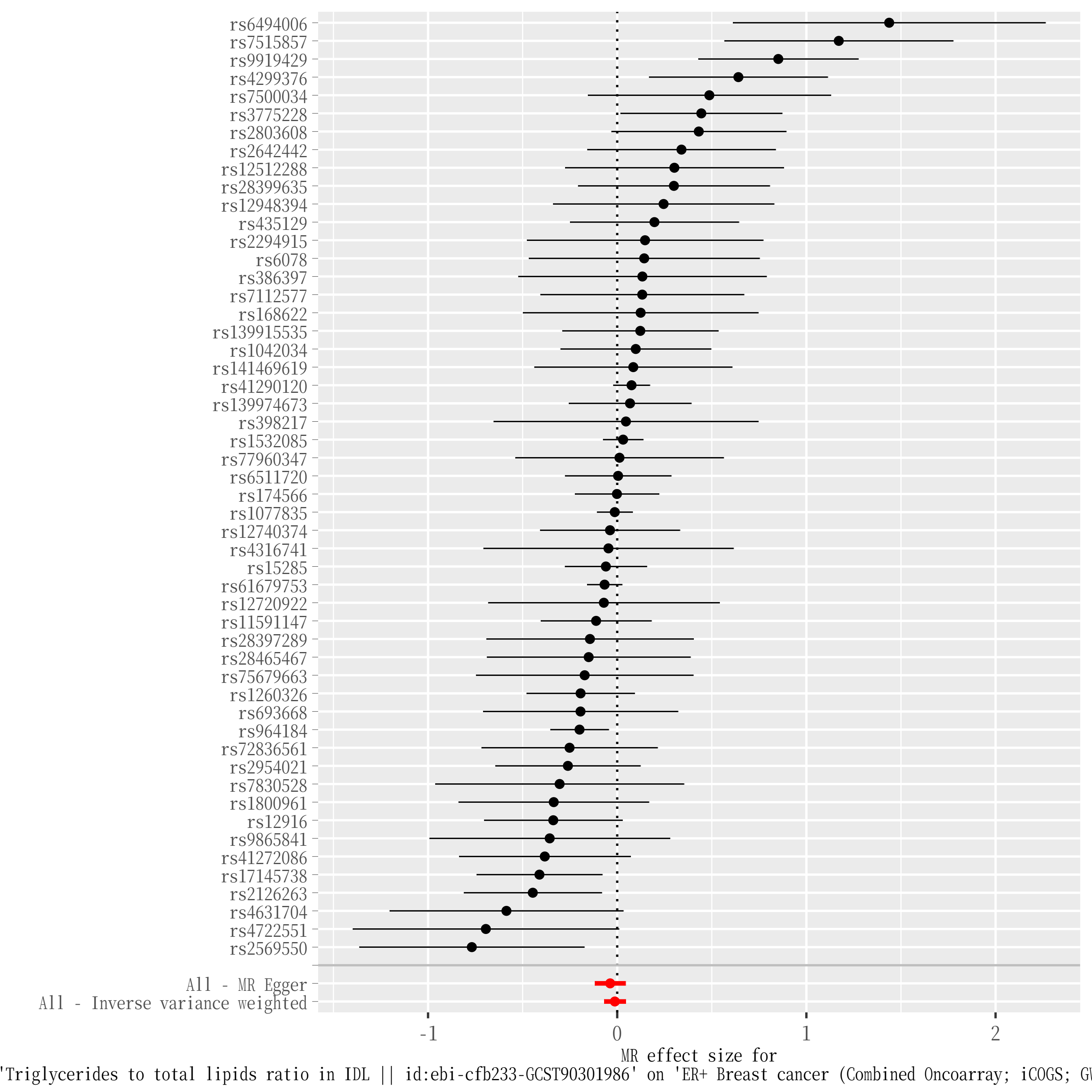
rs6494006
rs7515857
rs9919429
rs4299376
rs7500034
rs3775228
rs2803608
rs2642442
rs12512288
rs28399635
rs12948394
rs435129
rs2294915
rs6078
rs386397
rs7112577
rs168622
rs139915535
rs1042034
rs141469619
rs41290120
rs139974673
rs398217
rs1532085
rs77960347
rs6511720
rs174566
rs1077835
rs12740374
rs4316741
rs15285
rs61679753
rs12720922
rs11591147
rs28397289
rs28465467
rs75679663
rs1260326
rs693668
rs964184
rs72836561
rs2954021
rs7830528
rs1800961
rs12916
rs9865841
rs41272086
rs17145738
rs2126263
rs4631704
rs4722551
rs2569550
All - MR Egger
All - Inverse variance weighted
-1
0
1
2
MR effect size for
'Triglycerides to total lipids ratio in IDL || id:ebi-cfb233-GCST90301986' on 'ER+ Breast cancer (Combined Oncoarray; iCOGS; G

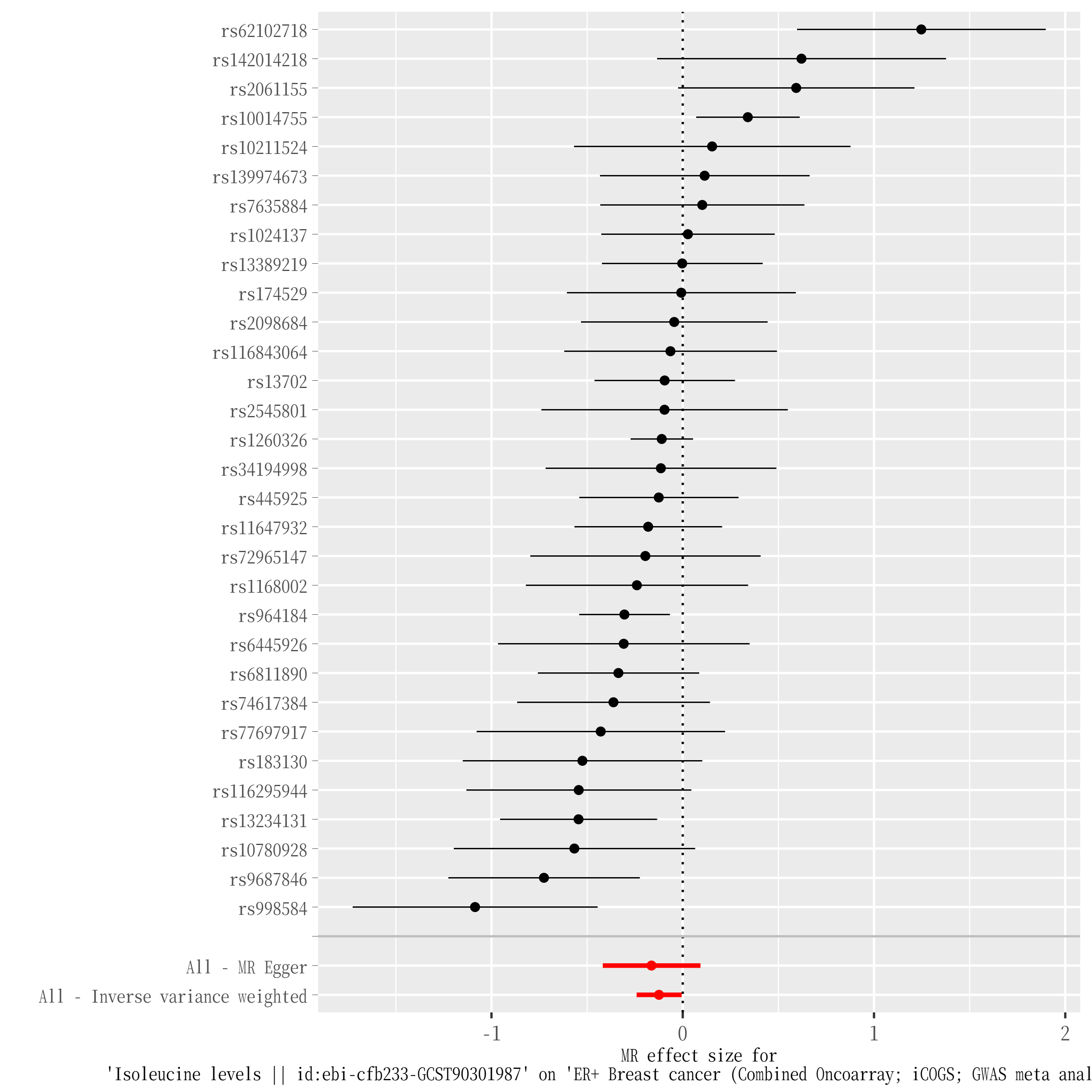

rs62102718
rs142014218
rs2061155
rs10014755
rs10211524
rs139974673
rs7635884
rs1024137
rs13389219
rs174529
rs2098684
rs116843064
rs13702
rs2545801
rs1260326
rs34194998
rs445925
rs11647932
rs72965147
rs1168002
rs964184
rs6445926
rs6811890
rs74617384
rs77697917
rs183130
rs116295944
rs13234131
rs10780928
rs9687846
rs998584
All - MR Egger
All - Inverse variance weighted
-1
0
1
2
MR effect size for
'Isoleucine levels || id:ebi-cfb233-GCST90301987' on 'ER+ Breast cancer (Combined Oncoarray; iCOGS; GWAS meta anal

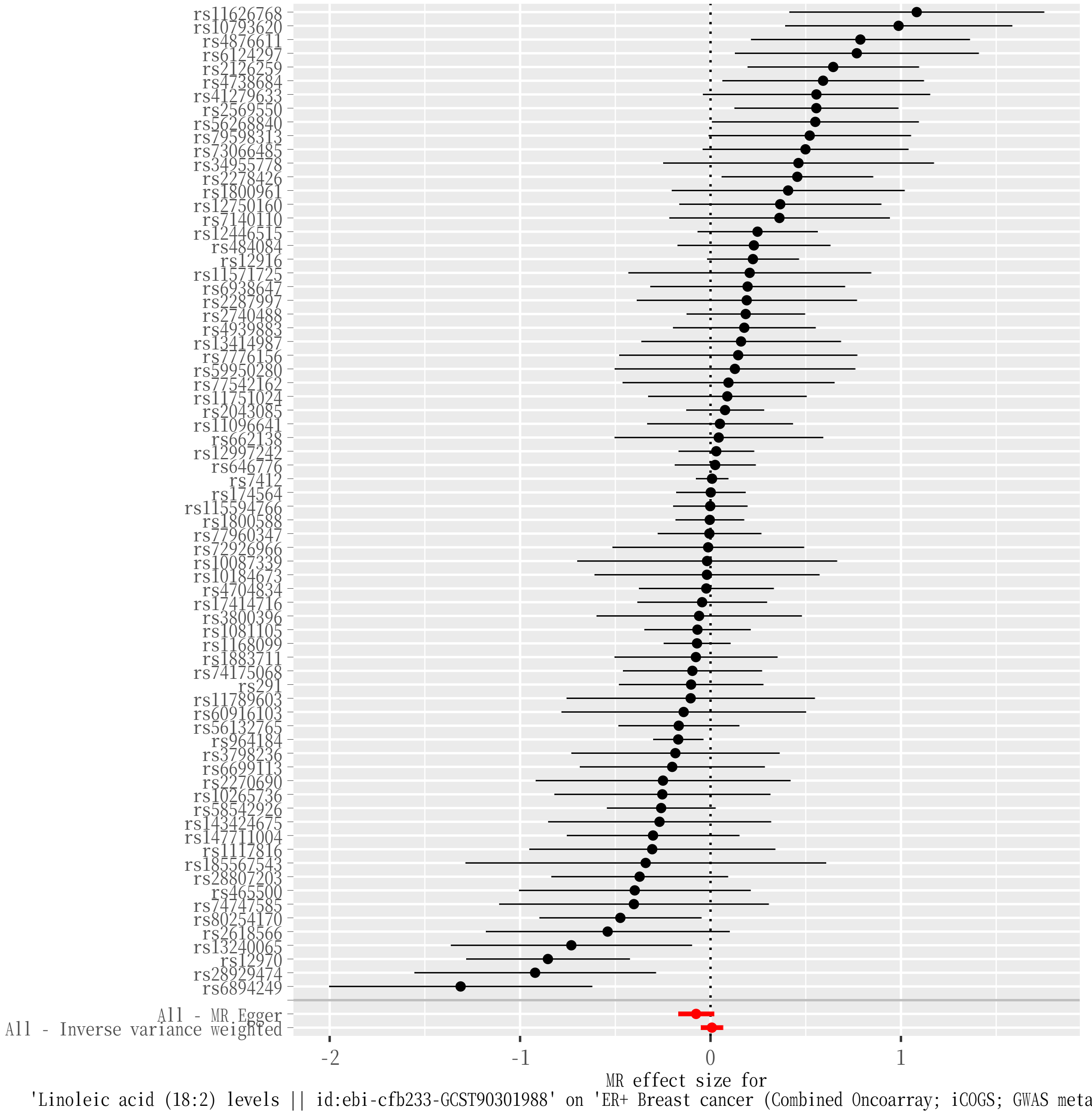
rs11626768
rs10793620
rs4876611
rs6124297
rs2126259
rs4738684
rs41279633
rs2569550
rs56268840
rs79598313
rs73066485
rs34955778
rs2278426
rs1800961
rs12750160
rs7140110
rs12446515
rs484084
rs12916
rs11571725
rs6938647
rs2287997
rs2740488
rs4939883
rs13414987
rs7776156
rs59950280
rs77542162
rs11751024
rs2043085
rs11096641
rs662138
rs12997242
rs646776
rs7412
rs174564
rs115594766
rs1800588
rs77960347
rs72926966
rs10087339
rs10184673
rs4704834
rs17414716
rs3800396
rs1081105
rs1168099
rs1883711
rs74175068
rs291
rs11789603
rs60916103
rs56132765
rs964184
rs3798236
rs6699113
rs2270690
rs10265736
rs58542926
rs143424675
rs147711004
rs1117816
rs185567543
rs28807203
rs465500
rs74747585
rs80254170
rs2618566
rs13240065
rs12970
rs28929474
rs6894249
All - MR Egger
All - Inverse variance weighted
-2
-1
0
1
MR effect size for
'Linoleic acid (18:2) levels || id:ebi-cfb233-GCST90301988' on 'ER+ Breast cancer (Combined Oncoarray; iCOGS; GWAS meta

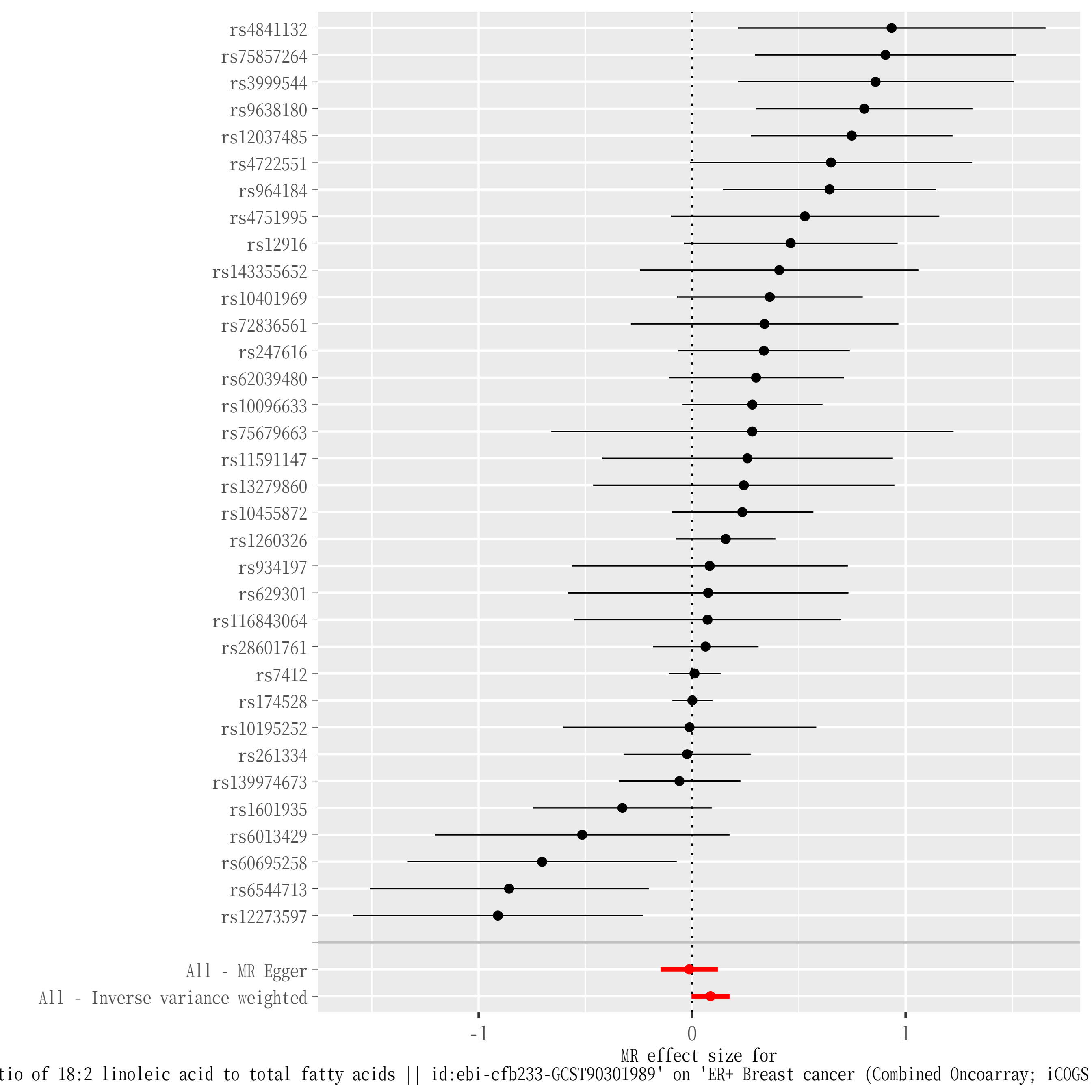
rs4841132
rs75857264
rs3999544
rs9638180
rs12037485
rs4722551
rs964184
rs4751995
rs12916
rs143355652
rs10401969
rs72836561
rs247616
rs62039480
rs10096633
rs75679663
rs11591147
rs13279860
rs10455872
rs1260326
rs934197
rs629301
rs116843064
rs28601761
rs7412
rs174528
rs10195252
rs261334
rs139974673
rs1601935
rs6013429
rs60695258
rs6544713
rs12273597
All - MR Egger
All - Inverse variance weighted
-1
0
1
MR effect size for
tio of 18:2 linoleic acid to total fatty acids || id:ebi-cfb233-GCST90301989' on 'ER+ Breast cancer (Combined Oncoarray; iCOGS

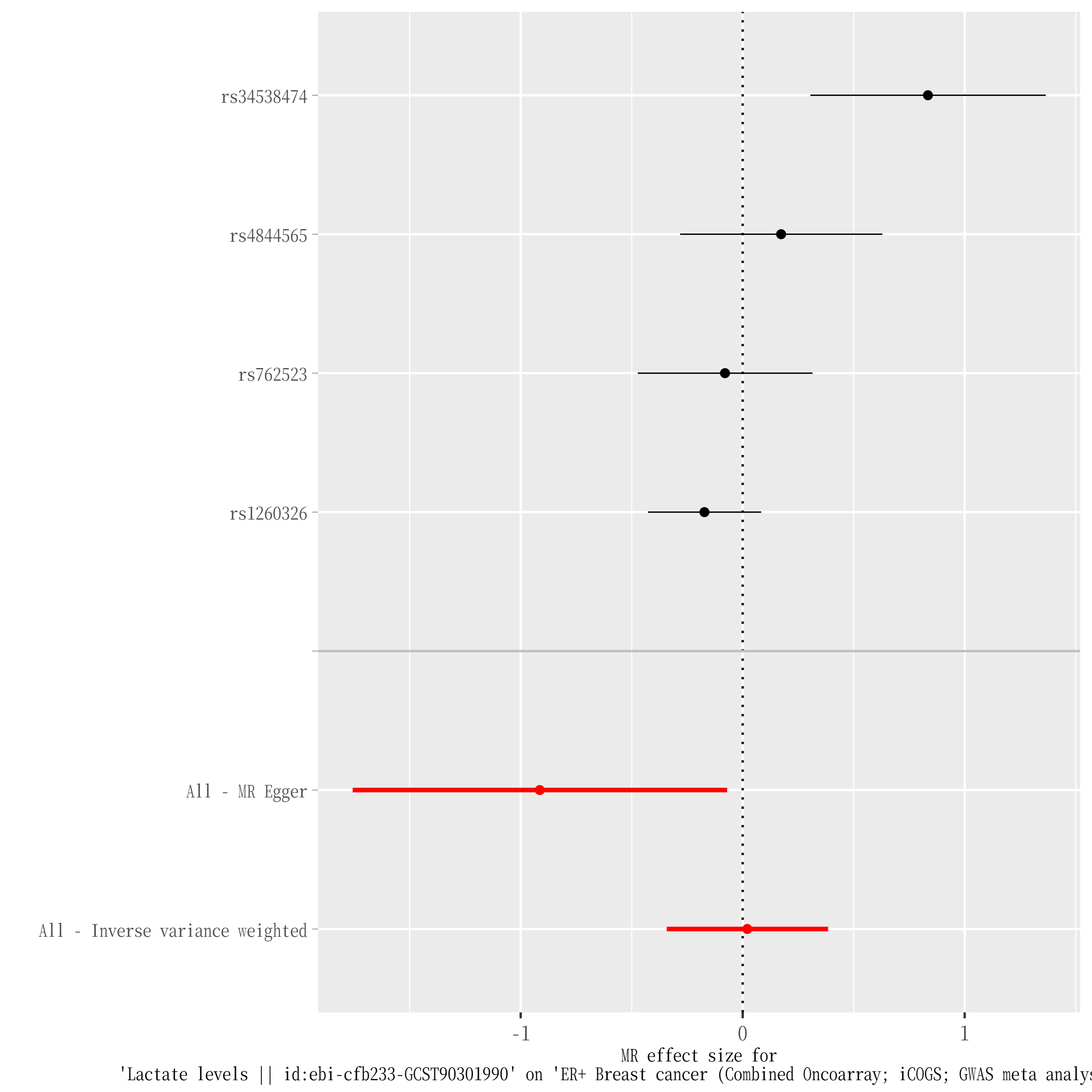

rs34538474
rs4844565
rs762523
rs1260326
All - MR Egger
All - Inverse variance weighted
-1
0
1
MR effect size for
'Lactate levels || id:ebi-cfb233-GCST90301990' on 'ER+ Breast cancer (Combined Oncoarray; iCOGS; GWAS meta analys

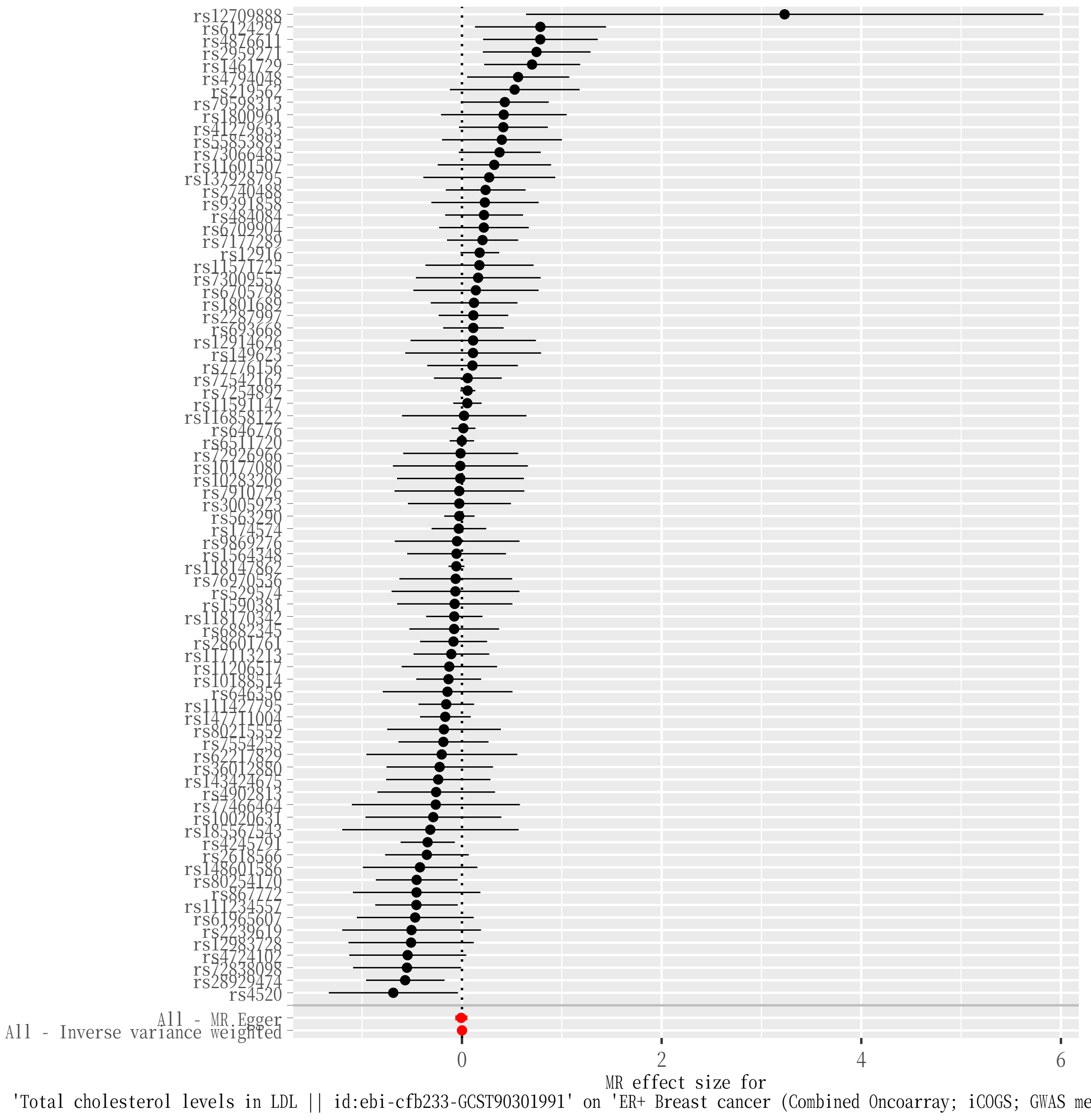

rs12709888
rs6124297
rs4876611
rs2959271
rs1461729
rs4794048
rs219562
rs79598313
rs1800961
rs41279633
rs55853893
rs73066485
rs11601507
rs137928795
rs2740488
rs9391858
rs484084
rs6709904
rs7177289
rs12916
rs11571725
rs73009557
rs6705798
rs1801689
rs2287997
rs693668
rs12914626
rs149623
rs2776156
rs7542162
rs7254892
rs11591147
rs116858122
rs646776
rs6511720
rs72926966
rs10177080
rs10283206
rs7210726
rs3005923
rs563290
rs174574
rs9869276
rs1564348
rs118147862
rs76970536
rs529574
rs1590381
rs118170342
rs6882345
rs28601761
rs117113213
rs11206517
rs10188514
rs646356
rs111427795
rs147711004
rs80215559
rs7554255
rs62217829
rs36012880
rs143424675
rs4902813
rs77466464
rs10020631
rs185567543
rs4245791
rs2618566
rs148601586
rs80254170
rs867772
rs111234557
rs61965607
rs2239619
rs12983728
rs4724102
rs72838098
rs28929474
rs4520
All - MR Egger
All - Inverse variance weighted
0
2
4
6
MR effect size for
'Total cholesterol levels in LDL || id:ebi-cfb233-GCST90301991' on 'ER+ Breast cancer (Combined Oncoarray; iCOGS; GWAS me

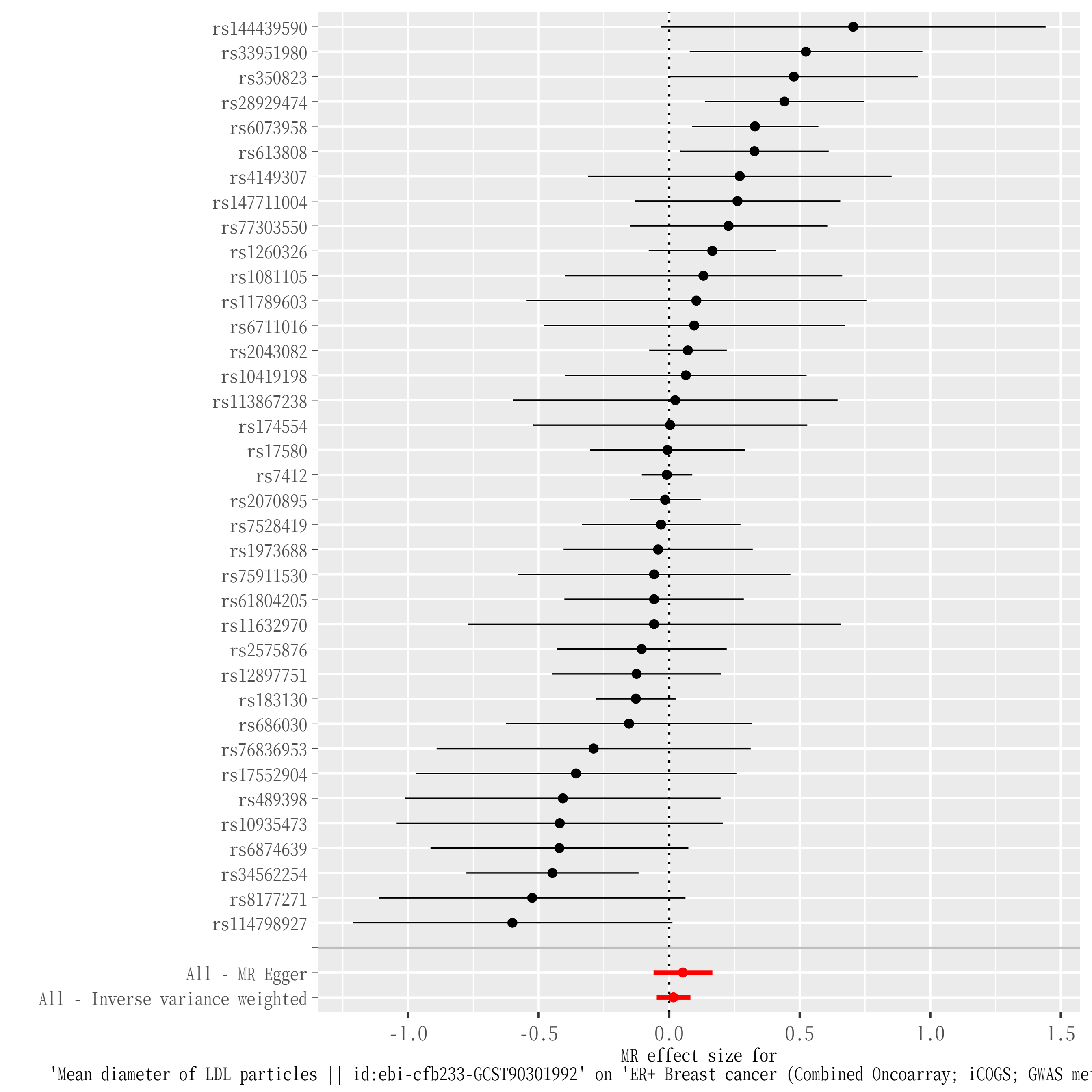

rs144439590
rs33951980
rs350823
rs28929474
rs6073958
rs613808
rs4149307
rs147711004
rs77303550
rs1260326
rs1081105
rs11789603
rs6711016
rs2043082
rs10419198
rs113867238
rs174554
rs17580
rs7412
rs2070895
rs7528419
rs1973688
rs75911530
rs61804205
rs11632970
rs2575876
rs12897751
rs183130
rs686030
rs76836953
rs17552904
rs489398
rs10935473
rs6874639
rs34562254
rs8177271
rs114798927
All - MR Egger
All - Inverse variance weighted
-1.0
-0.5
0.0
0.5
1.0
1.5
MR effect size for
'Mean diameter of LDL particles || id:ebi-cfb233-GCST90301992' on 'ER+ Breast cancer (Combined Oncoarray; iCOGS; GWAS me

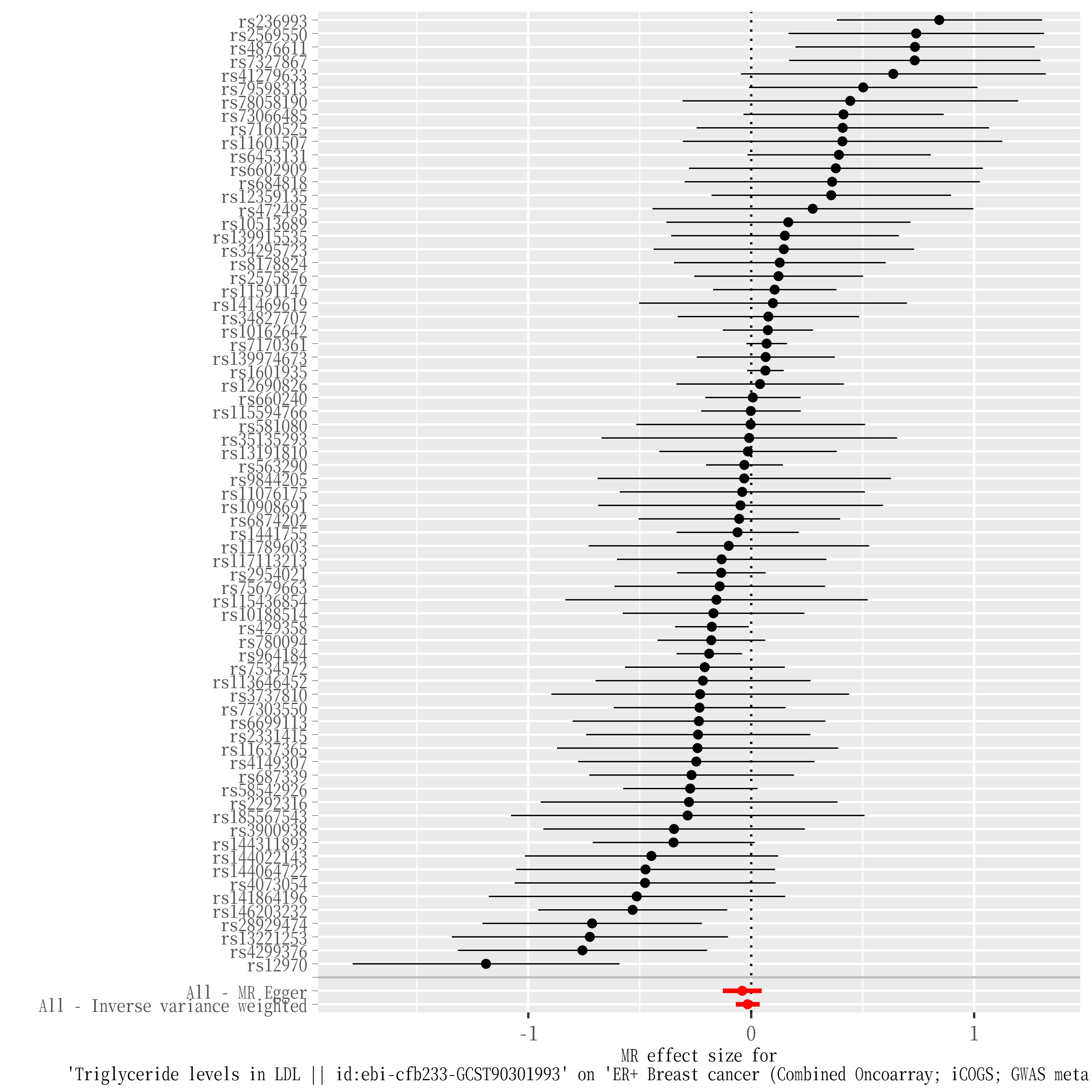
rs236993
rs2569550
rs4876611
rs7327867
rs41279633
rs79598313
rs78058190
rs73066485
rs7160525
rs11601507
rs6453131
rs6602909
rs684818
rs12359135
rs472495
rs10513689
rs139915535
rs34295723
rs8178824
rs2575876
rs11591147
rs141469619
rs34827707
rs10162642
rs7170361
rs139974673
rs1601935
rs12690826
rs660240
rs115594766
rs581080
rs35135293
rs13191810
rs563290
rs9844205
rs11076175
rs10908691
rs6874202
rs1441755
rs11789603
rs117113213
rs2954021
rs75679663
rs115436854
rs10188514
rs429358
rs780094
rs964184
rs7534572
rs113646452
rs3737810
rs77303550
rs6699113
rs2331415
rs11637365
rs4149307
rs687339
rs58542926
rs2292316
rs185567543
rs3900938
rs144311893
rs144022143
rs144064722
rs4073054
rs141864196
rs146203232
rs28929474
rs13221253
rs4299376
rs12970
All - MR Egger
All - Inverse variance weighted
-1
0
1
MR effect size for
'Triglyceride levels in LDL || id:ebi-cfb233-GCST90301993' on 'ER+ Breast cancer (Combined Oncoarray; iCOGS; GWAS meta

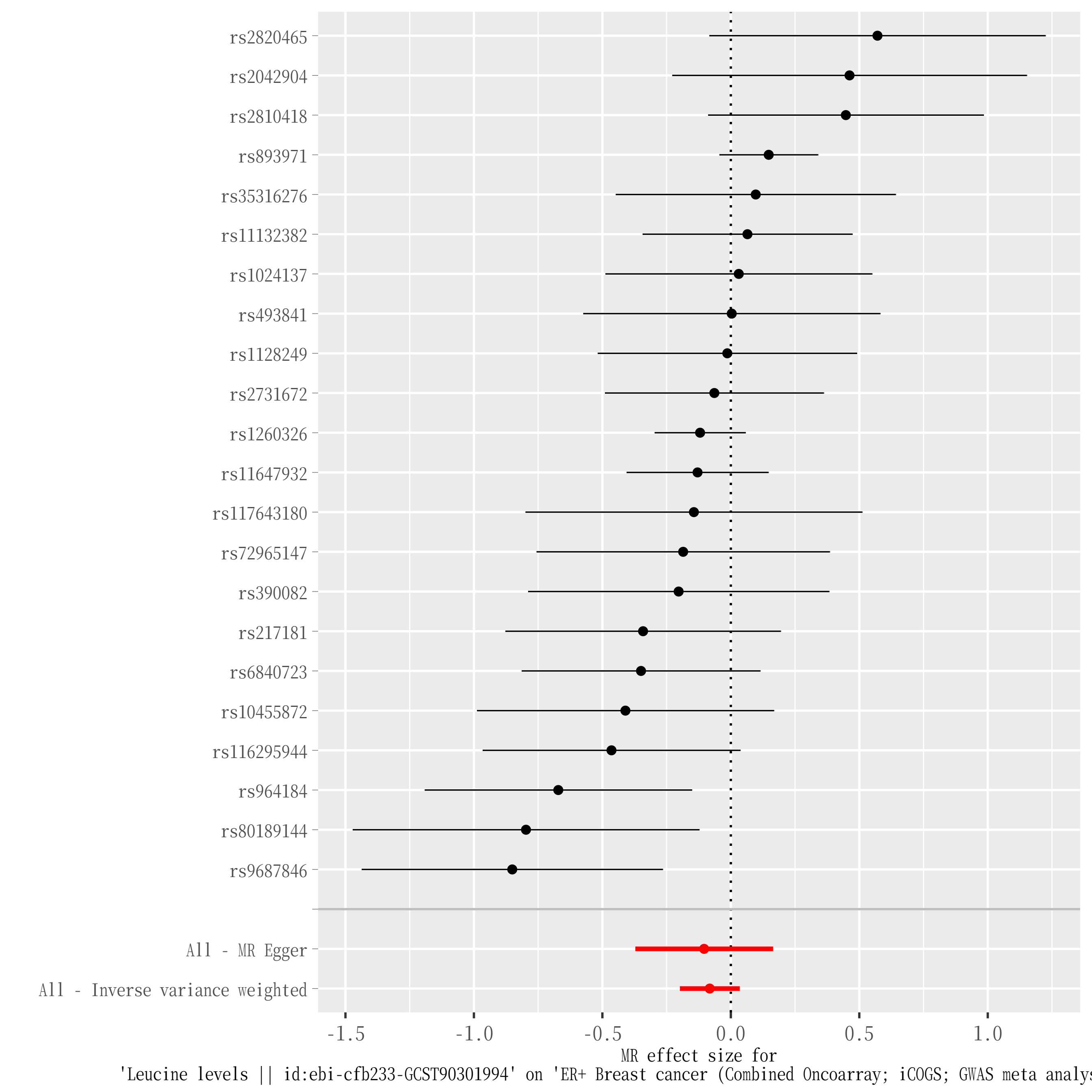

rs2820465
rs2042904
rs2810418
rs893971
rs35316276
rs11132382
rs1024137
rs493841
rs1128249
rs2731672
rs1260326
rs11647932
rs117643180
rs72965147
rs390082
rs217181
rs6840723
rs10455872
rs116295944
rs964184
rs80189144
rs9687846
All - MR Egger
All - Inverse variance weighted
-1.5
-1.0
-0.5
0.0
0.5
1.0
MR effect size for
'Leucine levels || id:ebi-cfb233-GCST90301994' on 'ER+ Breast cancer (Combined Oncoarray; iCOGS; GWAS meta analys

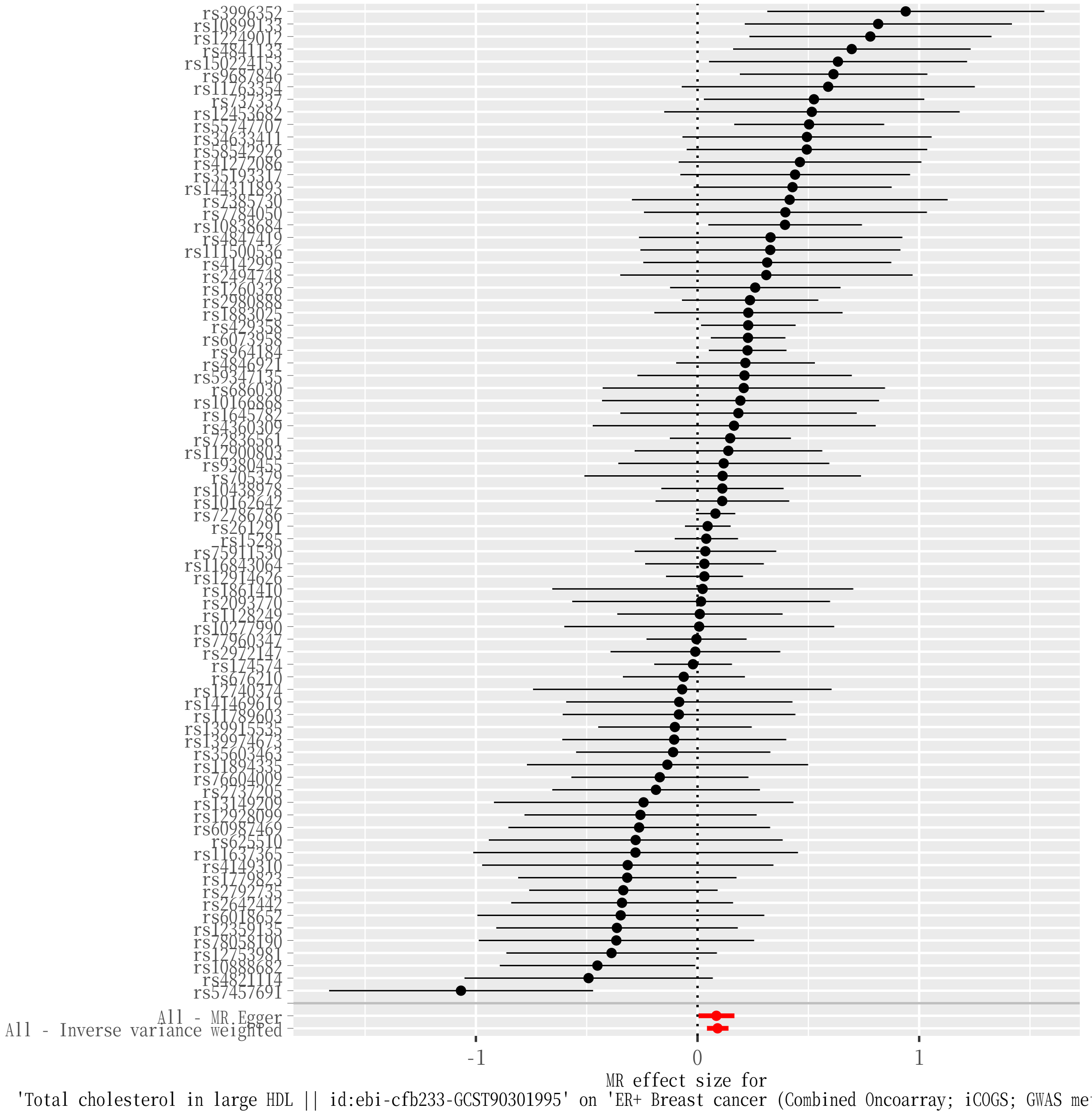

rs3996352
rs10899133
rs12249012
rs4841133
rs150224153
rs9687846
rs11763354
rs737337
rs12453682
rs55747707
rs34633411
rs58542926
rs41272086
rs35193317
rs144311893
rs7385730
rs7784050
rs10838684
rs4847419
rs111500536
rs4142995
rs2494748
rs1260326
rs2980888
rs1883025
rs429358
rs6073958
rs964184
rs4846921
rs59347135
rs686030
rs10166868
rs1645782
rs4360309
rs72836561
rs112900803
rs9380455
rs705379
rs10438978
rs10162642
rs72786786
rs261291
rs15285
rs75911530
rs116843064
rs12914626
rs1861410
rs2093770
rs1128249
rs10277990
rs77960347
rs2972147
rs174574
rs676210
rs12740374
rs141469619
rs11789603
rs139915535
rs139974673
rs35603463
rs11894335
rs76604009
rs2737205
rs13149209
rs12928099
rs60987469
rs625510
rs11637365
rs4149310
rs1779823
rs2792735
rs2642442
rs6018652
rs12359135
rs78058190
rs1275981
rs10888682
rs4821114
rs57457691
All - MR Egger
All - Inverse variance weighted
-1
0
1
MR effect size for
'Total cholesterol in large HDL || id:ebi-cfb233-GCST90301995' on 'ER+ Breast cancer (Combined Oncoarray; iCOGS; GWAS me

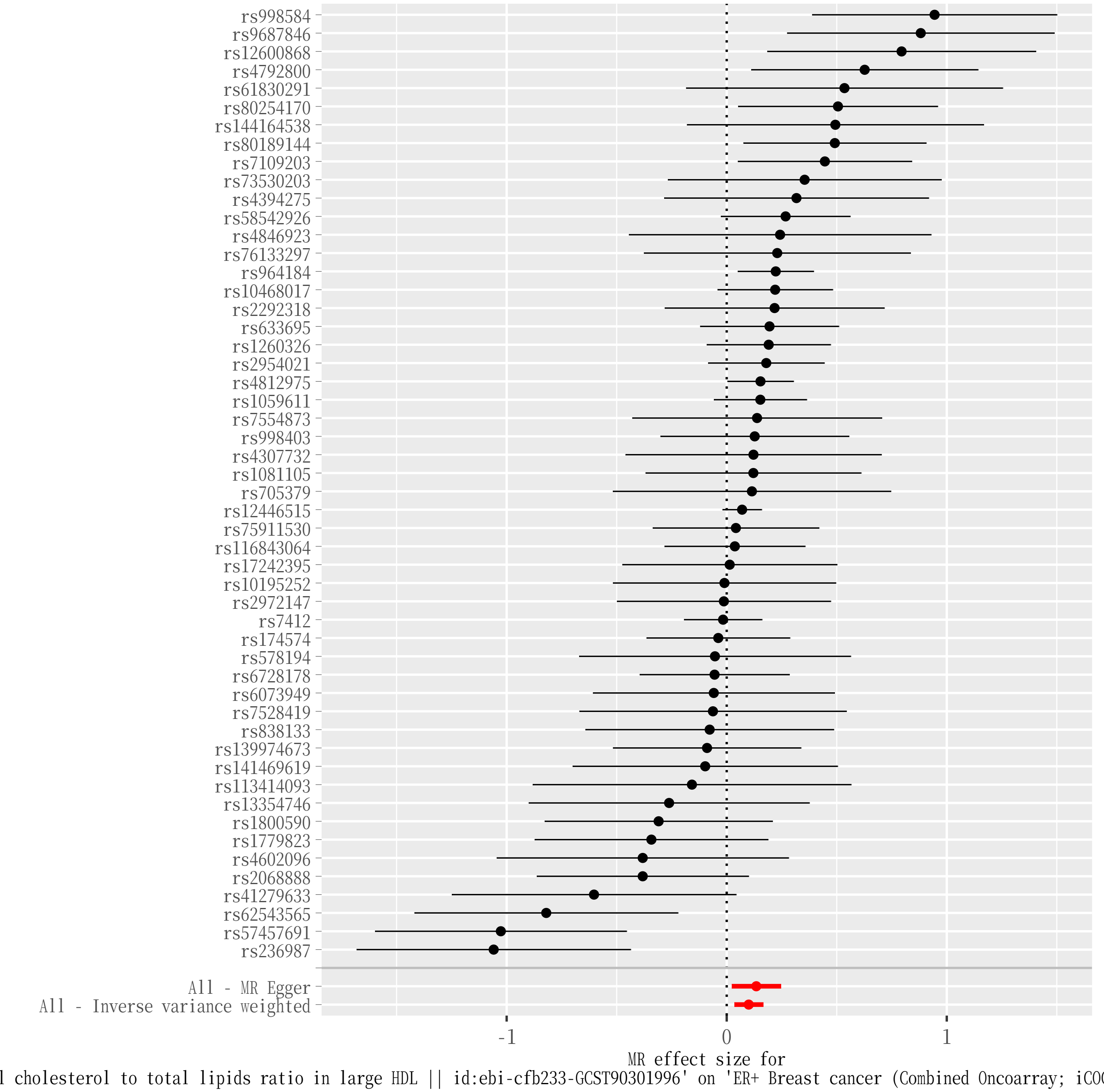

rs998584
rs9687846
rs12600868
rs4792800
rs61830291
rs80254170
rs144164538
rs80189144
rs7109203
rs73530203
rs4394275
rs58542926
rs4846923
rs76133297
rs964184
rs10468017
rs2292318
rs633695
rs1260326
rs2954021
rs4812975
rs1059611
rs7554873
rs998403
rs4307732
rs1081105
rs705379
rs12446515
rs75911530
rs116843064
rs17242395
rs10195252
rs2972147
rs7412
rs174574
rs578194
rs6728178
rs6073949
rs7528419
rs838133
rs139974673
rs141469619
rs113414093
rs13354746
rs1800590
rs1779823
rs4602096
rs2068888
rs41279633
rs62543565
rs57457691
rs236987
All - MR Egger
All - Inverse variance weighted
-1
0
1
MR effect size for
l cholesterol to total lipids ratio in large HDL || id:ebi-cfb233-GCST90301996' on 'ER+ Breast cancer (Combined Oncoarray; iCO

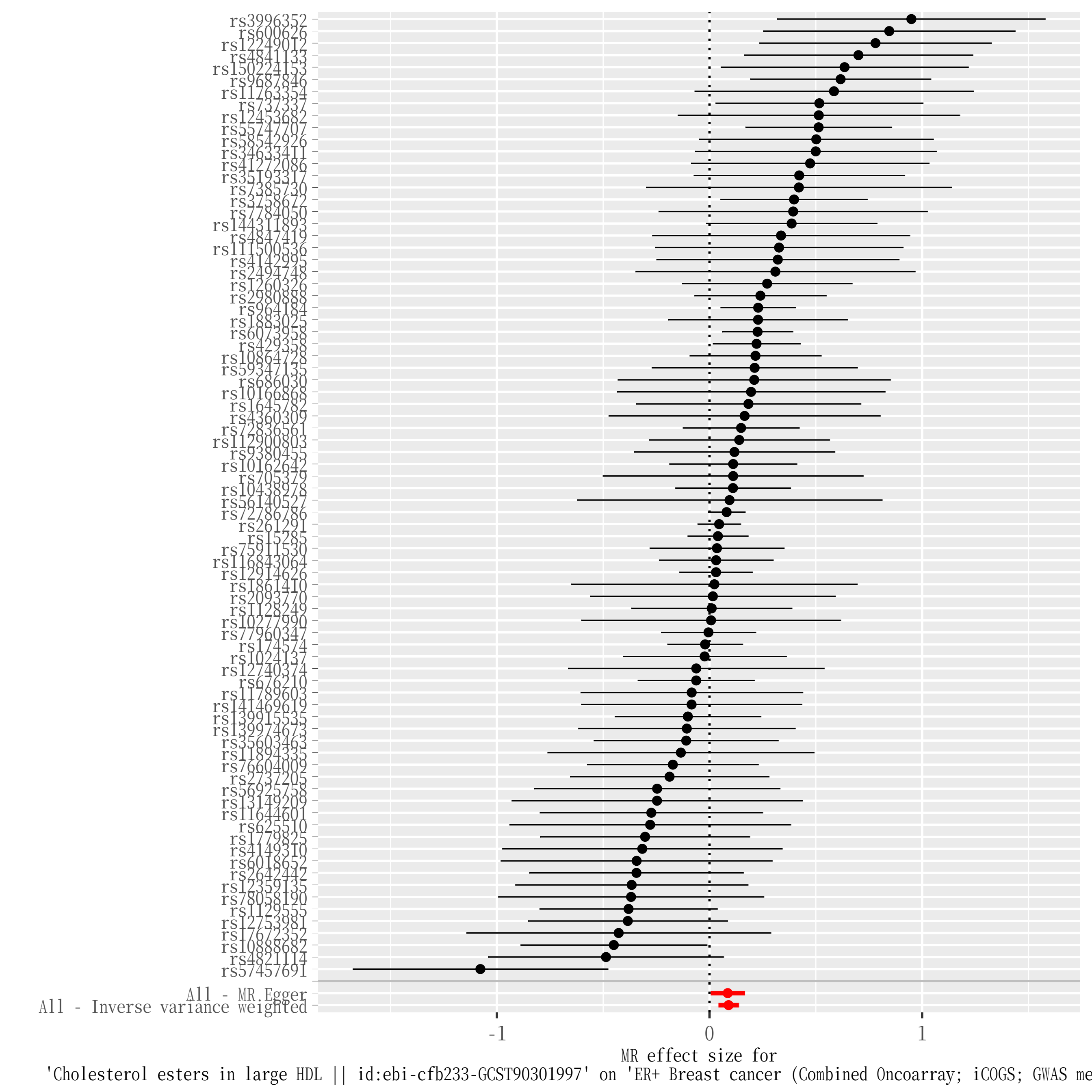

rs3996352
rs600626
rs12249012
rs4841133
rs150224153
rs9687846
rs11763354
rs737337
rs12453682
rs55747707
rs58542926
rs34633411
rs41272086
rs35193317
rs7385730
rs3758672
rs7784050
rs144311893
rs4847419
rs111500536
rs4142995
rs2494748
rs1260326
rs2980888
rs964184
rs1883025
rs6073958
rs429358
rs10864728
rs59347135
rs686030
rs10166868
rs1645782
rs4360309
rs72836561
rs112900803
rs9380455
rs10162642
rs705379
rs10438978
rs56140527
rs72786786
rs261291
rs15285
rs75911530
rs116843064
rs12914626
rs1861410
rs2093770
rs1128249
rs10277990
rs77960347
rs174574
rs1024137
rs12740374
rs676210
rs11789603
rs141462612
rs139915535
rs139974673
rs35603463
rs11894335
rs76604009
rs2737205
rs56925758
rs13149209
rs11644601
rs625510
rs1179825
rs4149310
rs6018652
rs2642442
rs12359135
rs78058190
rs1129555
rs12753981
rs17672352
rs10888682
rs4821114
rs57457691
All - MR Egger
All - Inverse variance weighted
-1
0
1
MR effect size for
'Cholesterol esters in large HDL || id:ebi-cfb233-GCST90301997' on 'ER+ Breast cancer (Combined Oncoarray; iCOGS; GWAS me

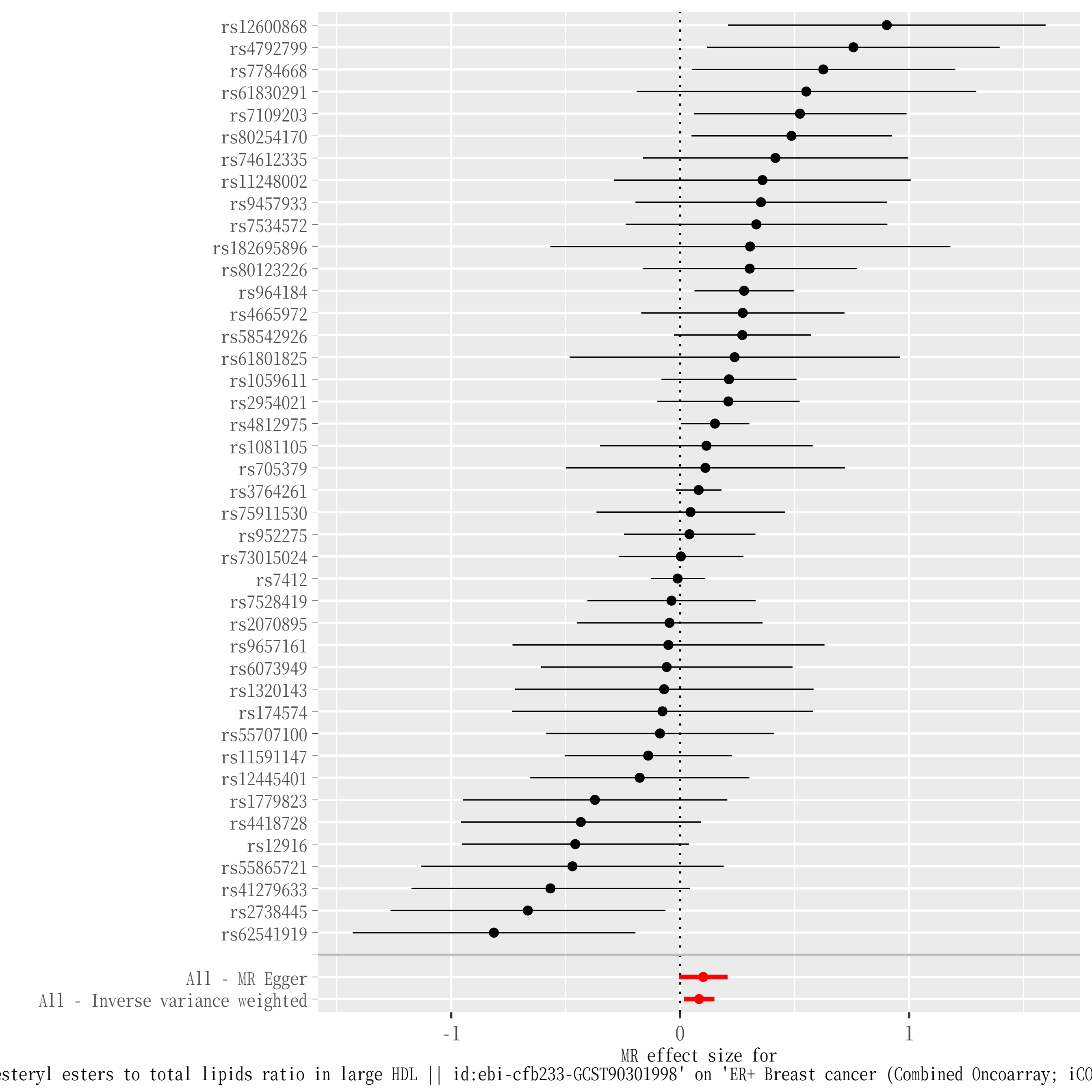

rs12600868
rs4792799
rs7784668
rs61830291
rs7109203
rs80254170
rs74612335
rs11248002
rs9457933
rs7534572
rs182695896
rs80123226
rs964184
rs4665972
rs58542926
rs61801825
rs1059611
rs2954021
rs4812975
rs1081105
rs705379
rs3764261
rs75911530
rs952275
rs73015024
rs7412
rs7528419
rs2070895
rs9657161
rs6073949
rs1320143
rs174574
rs55707100
rs11591147
rs12445401
rs1779823
rs4418728
rs12916
rs55865721
rs41279633
rs2738445
rs62541919
All - MR Egger
All - Inverse variance weighted
-1
0
1
MR effect size for
esteryl esters to total lipids ratio in large HDL || id:ebi-cfb233-GCST90301998' on 'ER+ Breast cancer (Combined Oncoarray; iCO

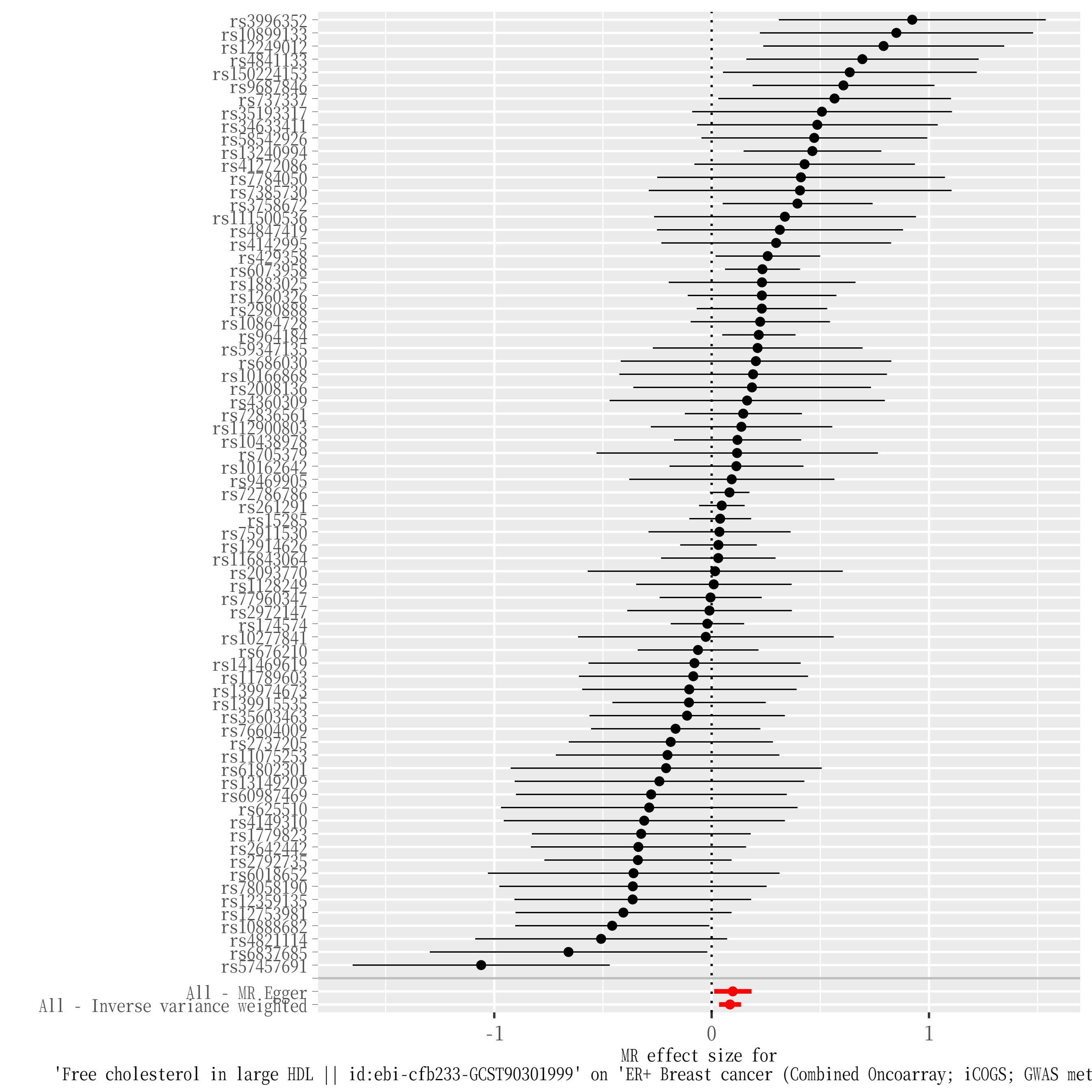
rs3996352
rs10899133
rs12249012
rs4841133
rs150224153
rs9687846
rs737337
rs35193317
rs34633411
rs58542926
rs13240994
rs41272086
rs7784050
rs7385730
rs3758672
rs111500536
rs4847419
rs4142995
rs429358
rs6073958
rs1883025
rs1260326
rs2980888
rs10864728
rs964184
rs59347135
rs686030
rs10166868
rs2008136
rs4360309
rs72836561
rs112900803
rs10438978
rs705379
rs10162642
rs9469905
rs72786786
rs261291
rs15285
rs75911530
rs12914626
rs116843064
rs2093770
rs1128249
rs77960347
rs2972147
rs174574
rs10277841
rs676210
rs141469619
rs11789603
rs139974673
rs139915535
rs35603463
rs76604009
rs2737205
rs11075253
rs61802301
rs13149209
rs60987469
rs625510
rs4149310
rs1779823
rs2642442
rs2792735
rs6018652
rs78058190
rs12359135
rs12753981
rs10888682
rs4821114
rs6837685
rs57457691
All - MR Egger
All - Inverse variance weighted
-1
0
1
MR effect size for
'Free cholesterol in large HDL || id:ebi-cfb233-GCST90301999' on 'ER+ Breast cancer (Combined Oncoarray; iCOGS; GWAS met

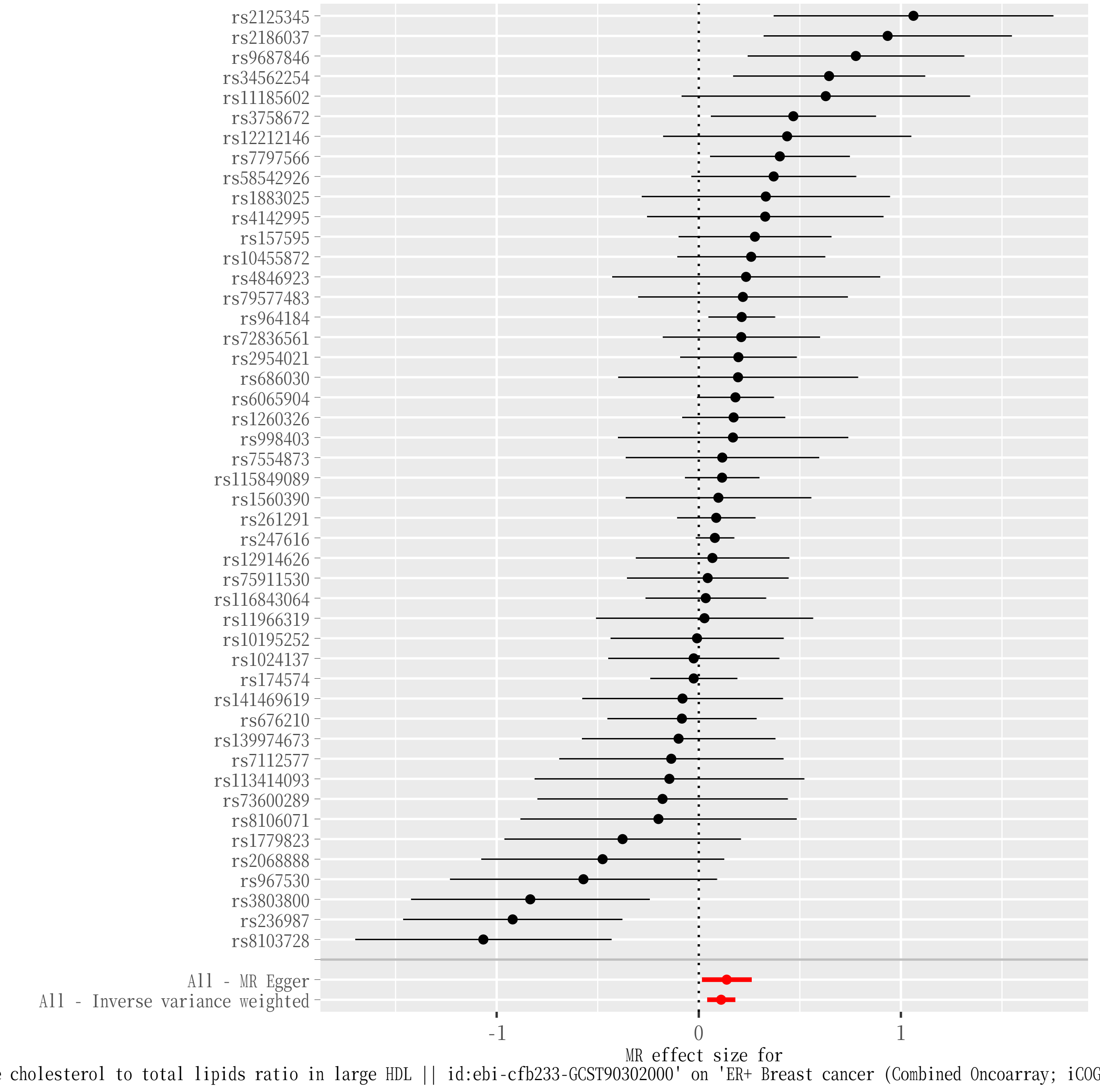

rs2125345
rs2186037
rs9687846
rs34562254
rs11185602
rs3758672
rs12212146
rs7797566
rs58542926
rs1883025
rs4142995
rs157595
rs10455872
rs4846923
rs79577483
rs964184
rs72836561
rs2954021
rs686030
rs6065904
rs1260326
rs998403
rs7554873
rs115849089
rs1560390
rs261291
rs247616
rs12914626
rs75911530
rs116843064
rs11966319
rs10195252
rs1024137
rs174574
rs141469619
rs676210
rs139974673
rs7112577
rs113414093
rs73600289
rs8106071
rs1779823
rs2068888
rs967530
rs3803800
rs236987
rs8103728
All - MR Egger
All - Inverse variance weighted
-1
0
1
MR effect size for
e cholesterol to total lipids ratio in large HDL || id:ebi-cfb233-GCST90302000' on 'ER+ Breast cancer (Combined Oncoarray; iCOG

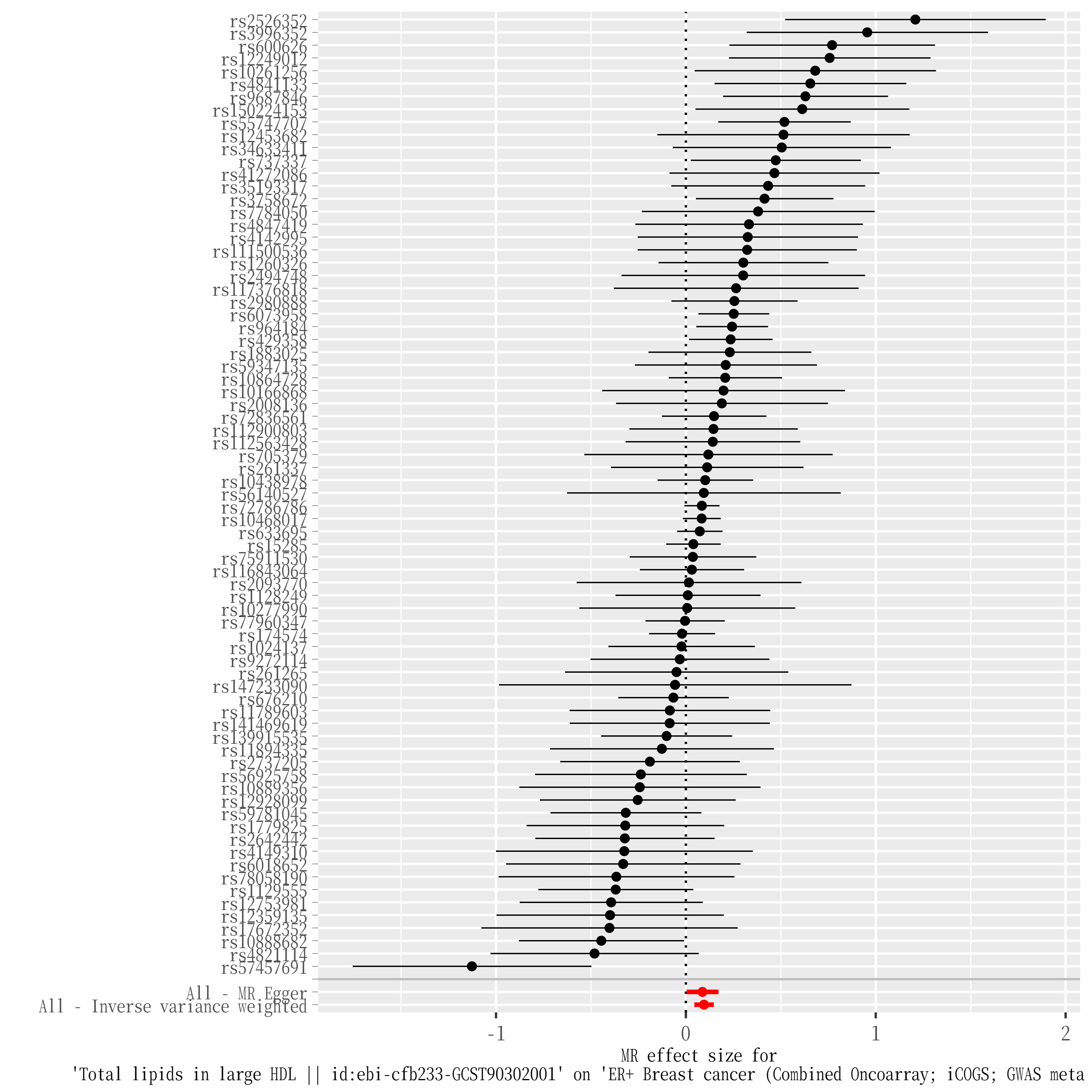
rs2526352
rs3996352
rs600626
rs12249012
rs10261256
rs4841133
rs9687846
rs150224153
rs55747707
rs12453682
rs34633411
rs737337
rs41272086
rs35193317
rs3758672
rs7784050
rs4847419
rs4142995
rs111500536
rs1260326
rs2494748
rs117376818
rs2980888
rs6073958
rs964184
rs429358
rs1883025
rs59347135
rs10864728
rs10166868
rs2008136
rs72836561
rs112900803
rs112563428
rs705379
rs261337
rs10438978
rs56140527
rs72786786
rs10468017
rs633695
rs15285
rs75911530
rs116843064
rs2093770
rs1128249
rs10277990
rs77960347
rs174574
rs1024137
rs9272114
rs261265
rs147233090
rs676210
rs11789603
rs141469619
rs139915535
rs11894335
rs2737205
rs56925758
rs10889356
rs12928099
rs59781045
rs1779825
rs2642442
rs4149310
rs6018652
rs78058190
rs1129555
rs12753981
rs12359135
rs17672352
rs10888682
rs4821114
rs57457691
All - MR Egger
All - Inverse variance weighted
-1
0
1
2
MR effect size for
'Total lipids in large HDL || id:ebi-cfb233-GCST90302001' on 'ER+ Breast cancer (Combined Oncoarray; iCOGS; GWAS meta

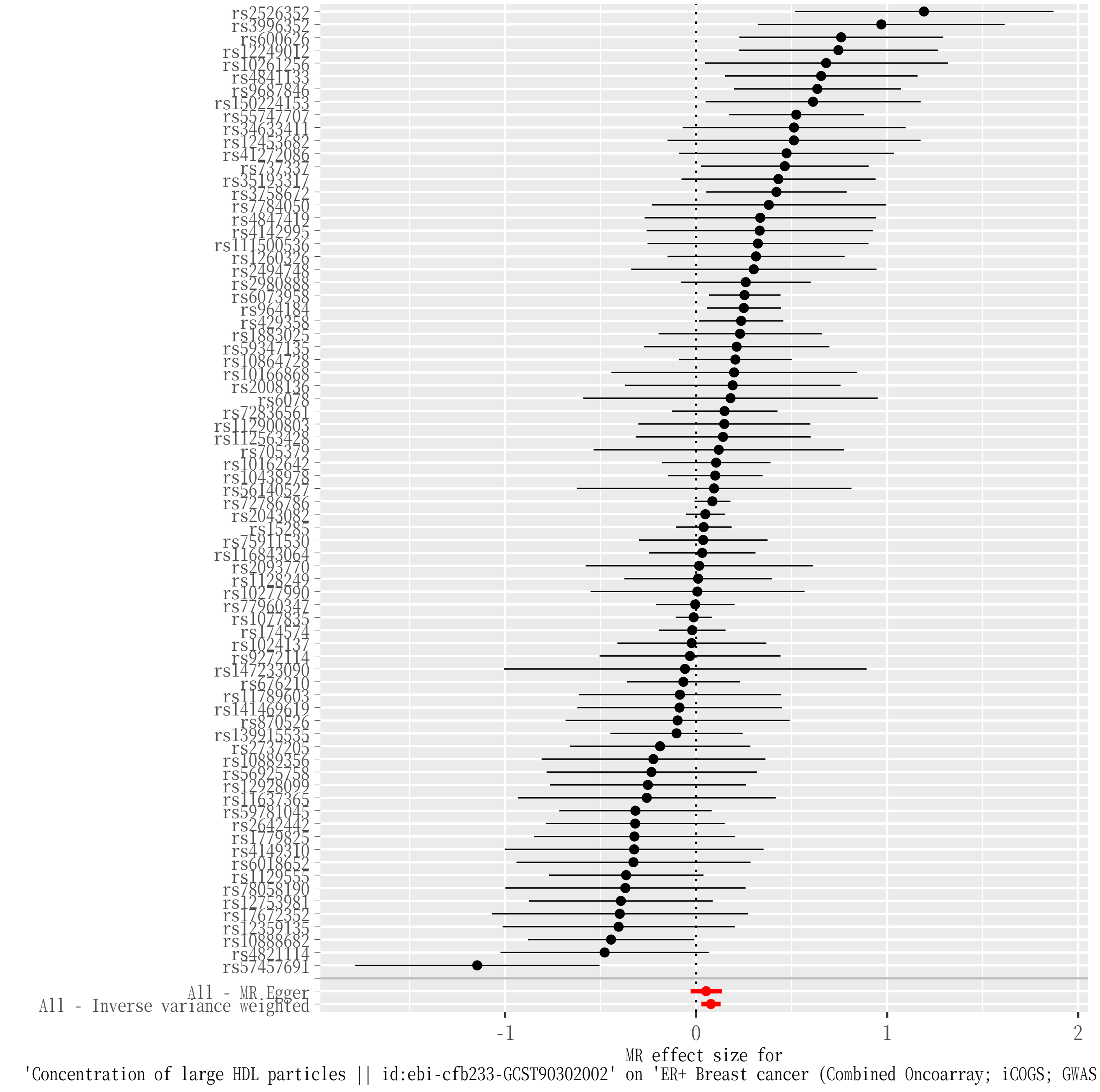
rs2526352
rs3996352
rs600626
rs12249012
rs10261256
rs4841133
rs9687846
rs150224153
rs55747707
rs34633411
rs12453682
rs41272086
rs737337
rs35193317
rs3758672
rs7784050
rs4847419
rs4142995
rs111500536
rs1260326
rs2494748
rs2980888
rs6073958
rs964184
rs429358
rs1883025
rs59347135
rs10864728
rs10166868
rs2008136
rs6078
rs72836561
rs112900803
rs112563428
rs705379
rs10162642
rs10438978
rs56140527
rs72786786
rs2043082
rs15285
rs75911530
rs116843064
rs2093770
rs1128249
rs10277990
rs77960347
rs1077835
rs174574
rs1024137
rs9272114
rs147233090
rs676210
rs11789603
rs141469619
rs870526
rs139915535
rs2737205
rs10889356
rs56925758
rs12928099
rs11637365
rs59781045
rs2642442
rs1779825
rs4149310
rs6018652
rs1129555
rs78058190
rs12753981
rs17672352
rs12359135
rs10888682
rs4821114
rs57457691
All - MR Egger
All - Inverse variance weighted
-1
0
1
2
MR effect size for
'Concentration of large HDL particles || id:ebi-cfb233-GCST90302002' on 'ER+ Breast cancer (Combined Oncoarray; iCOGS; GWAS

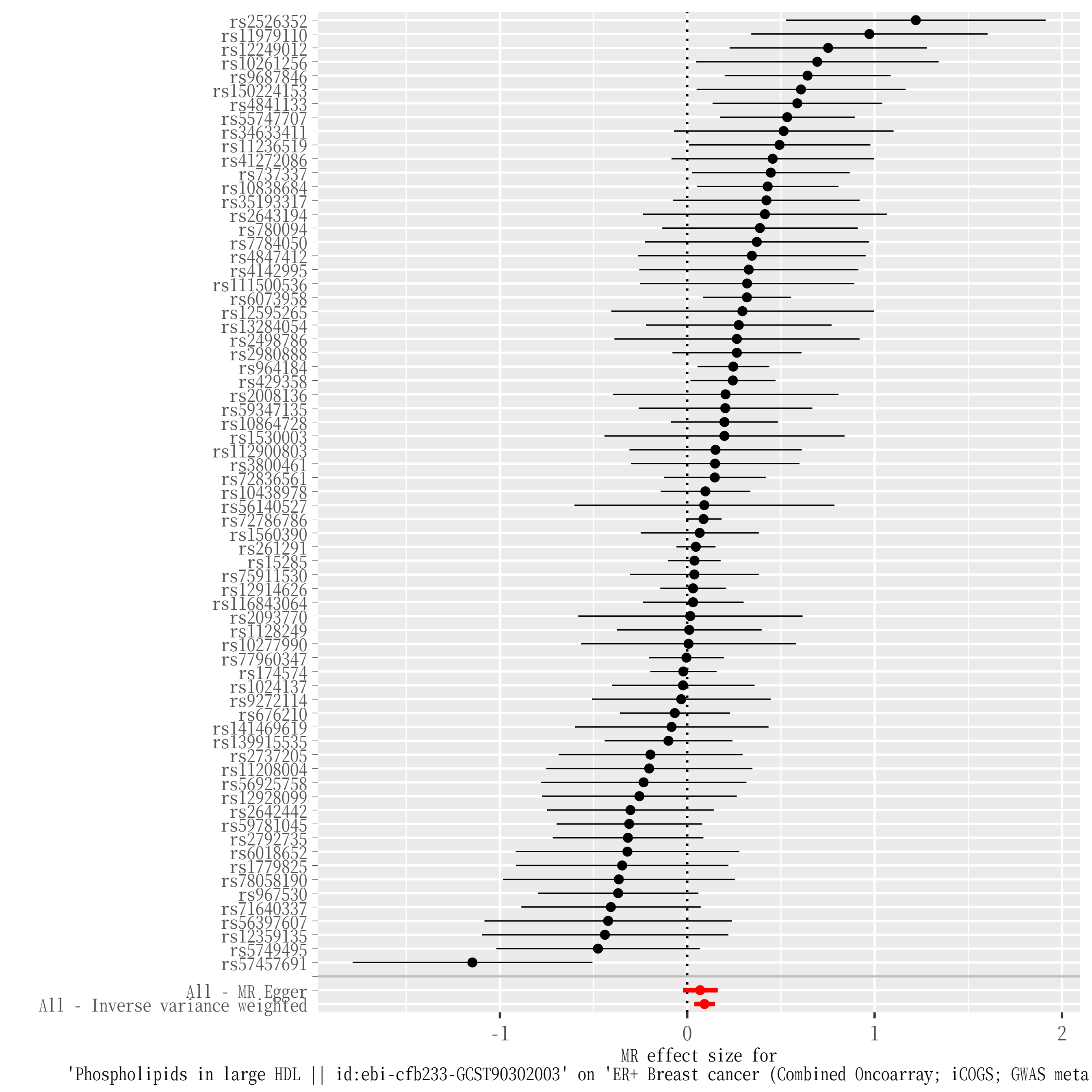

rs2526352
rs11979110
rs12249012
rs10261256
rs9687846
rs150224153
rs4841133
rs55747707
rs34633411
rs11236519
rs41272086
rs737337
rs10838684
rs35193317
rs2643194
rs780094
rs7784050
rs4847412
rs4142995
rs111500536
rs6073958
rs12595265
rs13284054
rs2498786
rs2980888
rs964184
rs429358
rs2008136
rs59347135
rs10864728
rs1530003
rs112900803
rs3800461
rs72836561
rs10438978
rs56140527
rs72786786
rs1560390
rs261291
rs15285
rs75911530
rs12914626
rs116843064
rs2093770
rs1128249
rs10277990
rs77960347
rs174574
rs1024137
rs9272114
rs676210
rs141469619
rs139915535
rs2737205
rs11208004
rs56925758
rs12928099
rs2642442
rs59781045
rs2792735
rs6018652
rs1779825
rs78058190
rs967530
rs71640337
rs56397607
rs12359135
rs5749495
rs57457691
All - MR Egger
All - Inverse variance weighted
-1
0
1
2
MR effect size for
'Phospholipids in large HDL || id:ebi-cfb233-GCST90302003' on 'ER+ Breast cancer (Combined Oncoarray; iCOGS; GWAS meta

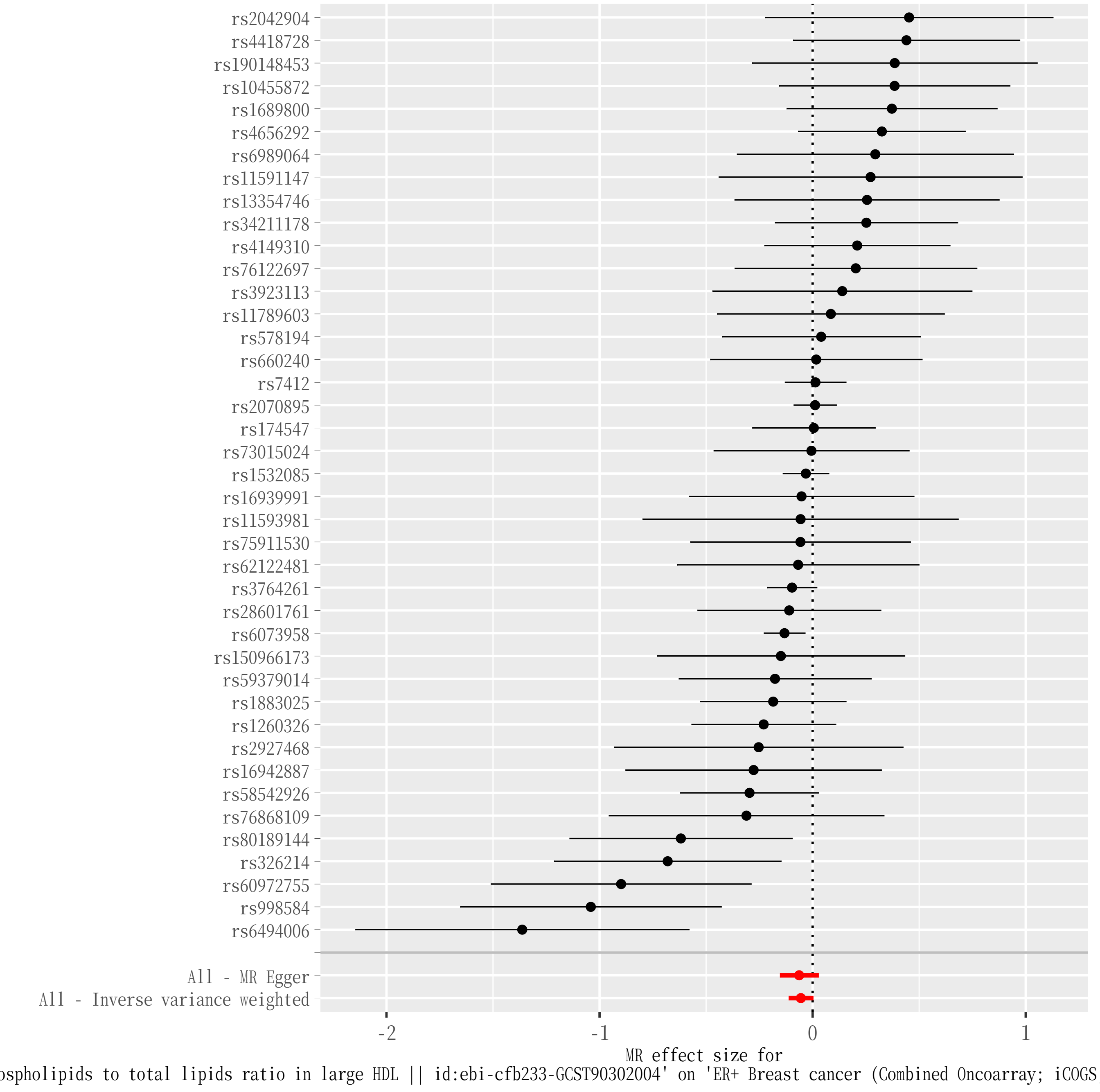
rs2042904
rs4418728
rs190148453
rs10455872
rs1689800
rs4656292
rs6989064
rs11591147
rs13354746
rs34211178
rs4149310
rs76122697
rs3923113
rs11789603
rs578194
rs660240
rs7412
rs2070895
rs174547
rs73015024
rs1532085
rs16939991
rs11593981
rs75911530
rs62122481
rs3764261
rs28601761
rs6073958
rs150966173
rs59379014
rs1883025
rs1260326
rs2927468
rs16942887
rs58542926
rs76868109
rs80189144
rs326214
rs60972755
rs998584
rs6494006
All - MR Egger
All - Inverse variance weighted
-2
-1
0
1
MR effect size for
ospholipids to total lipids ratio in large HDL || id:ebi-cfb233-GCST90302004' on 'ER+ Breast cancer (Combined Oncoarray; iCOGS

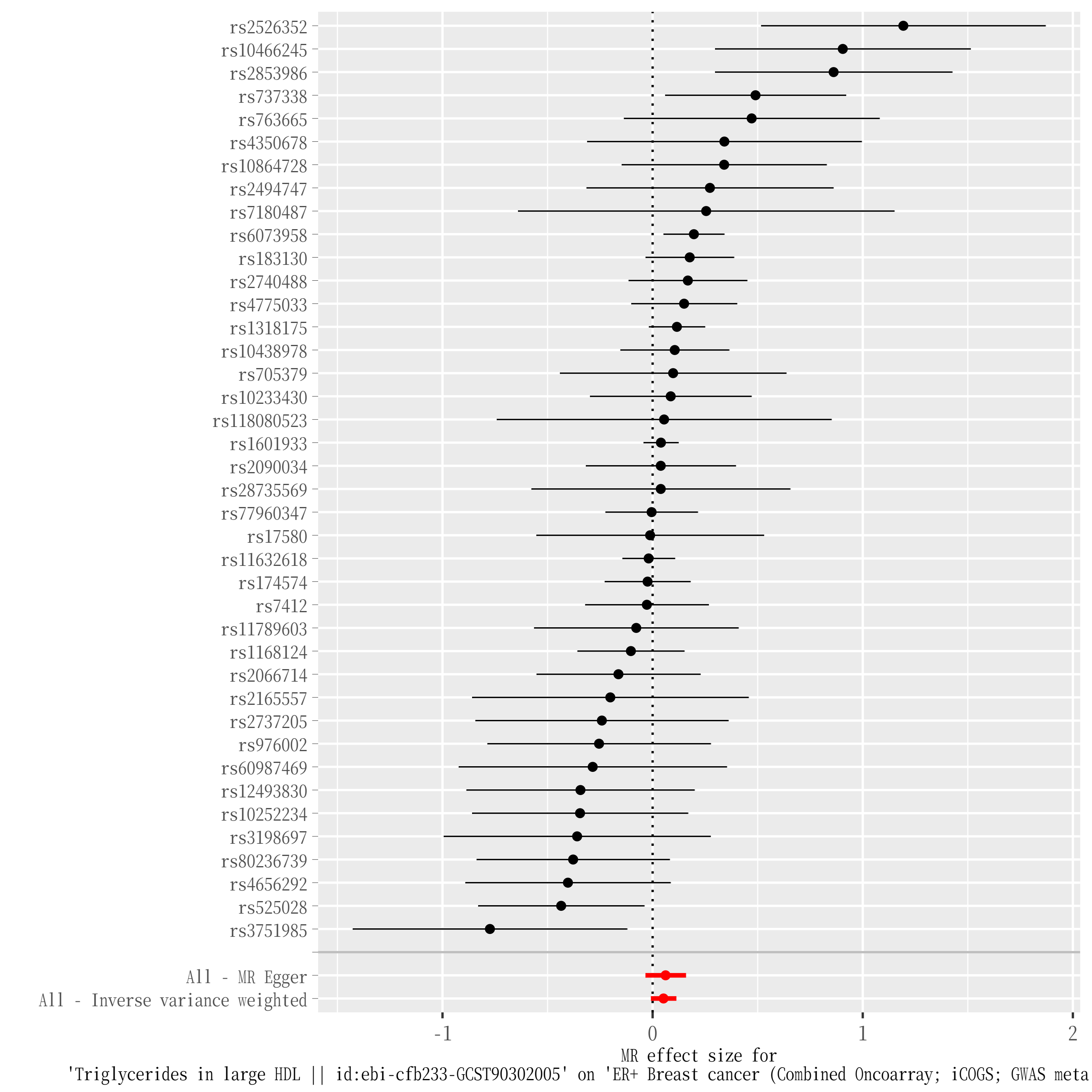

rs2526352
rs10466245
rs2853986
rs737338
rs763665
rs4350678
rs10864728
rs2494747
rs7180487
rs6073958
rs183130
rs2740488
rs4775033
rs1318175
rs10438978
rs705379
rs10233430
rs118080523
rs1601933
rs2090034
rs28735569
rs77960347
rs17580
rs11632618
rs174574
rs7412
rs11789603
rs1168124
rs2066714
rs2165557
rs2737205
rs976002
rs60987469
rs12493830
rs10252234
rs3198697
rs80236739
rs4656292
rs525028
rs3751985
All - MR Egger
All - Inverse variance weighted
-1
0
1
2
MR effect size for
'Triglycerides in large HDL || id:ebi-cfb233-GCST90302005' on 'ER+ Breast cancer (Combined Oncoarray; iCOGS; GWAS meta

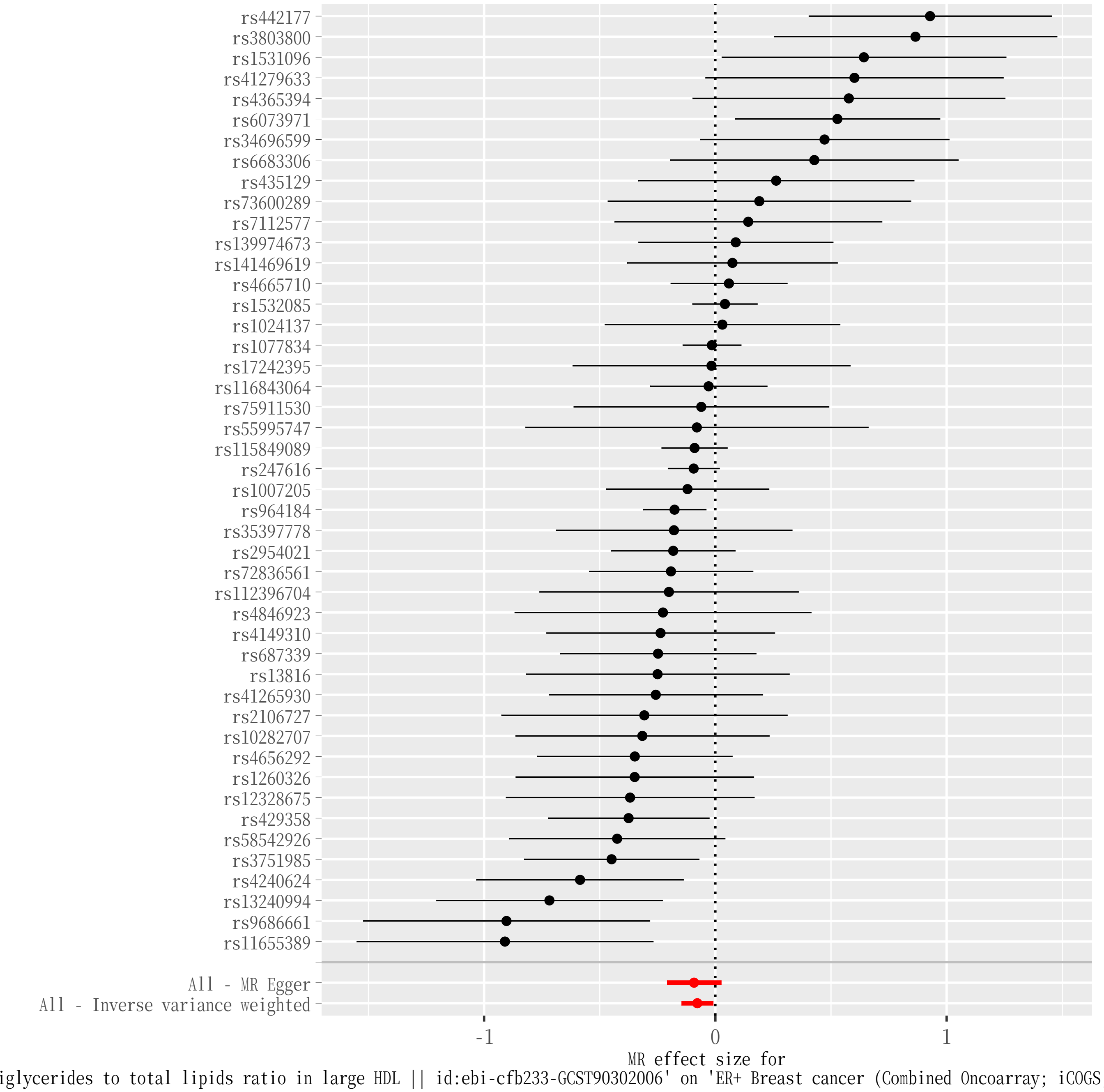
rs442177
rs3803800
rs1531096
rs41279633
rs4365394
rs6073971
rs34696599
rs6683306
rs435129
rs73600289
rs7112577
rs139974673
rs141469619
rs4665710
rs1532085
rs1024137
rs1077834
rs17242395
rs116843064
rs75911530
rs55995747
rs115849089
rs247616
rs1007205
rs964184
rs35397778
rs2954021
rs72836561
rs112396704
rs4846923
rs4149310
rs687339
rs13816
rs41265930
rs2106727
rs10282707
rs4656292
rs1260326
rs12328675
rs429358
rs58542926
rs3751985
rs4240624
rs13240994
rs9686661
rs11655389
All - MR Egger
All - Inverse variance weighted
-1
0
1
MR effect size for
iglycerides to total lipids ratio in large HDL || id:ebi-cfb233-GCST90302006' on 'ER+ Breast cancer (Combined Oncoarray; iCOGS

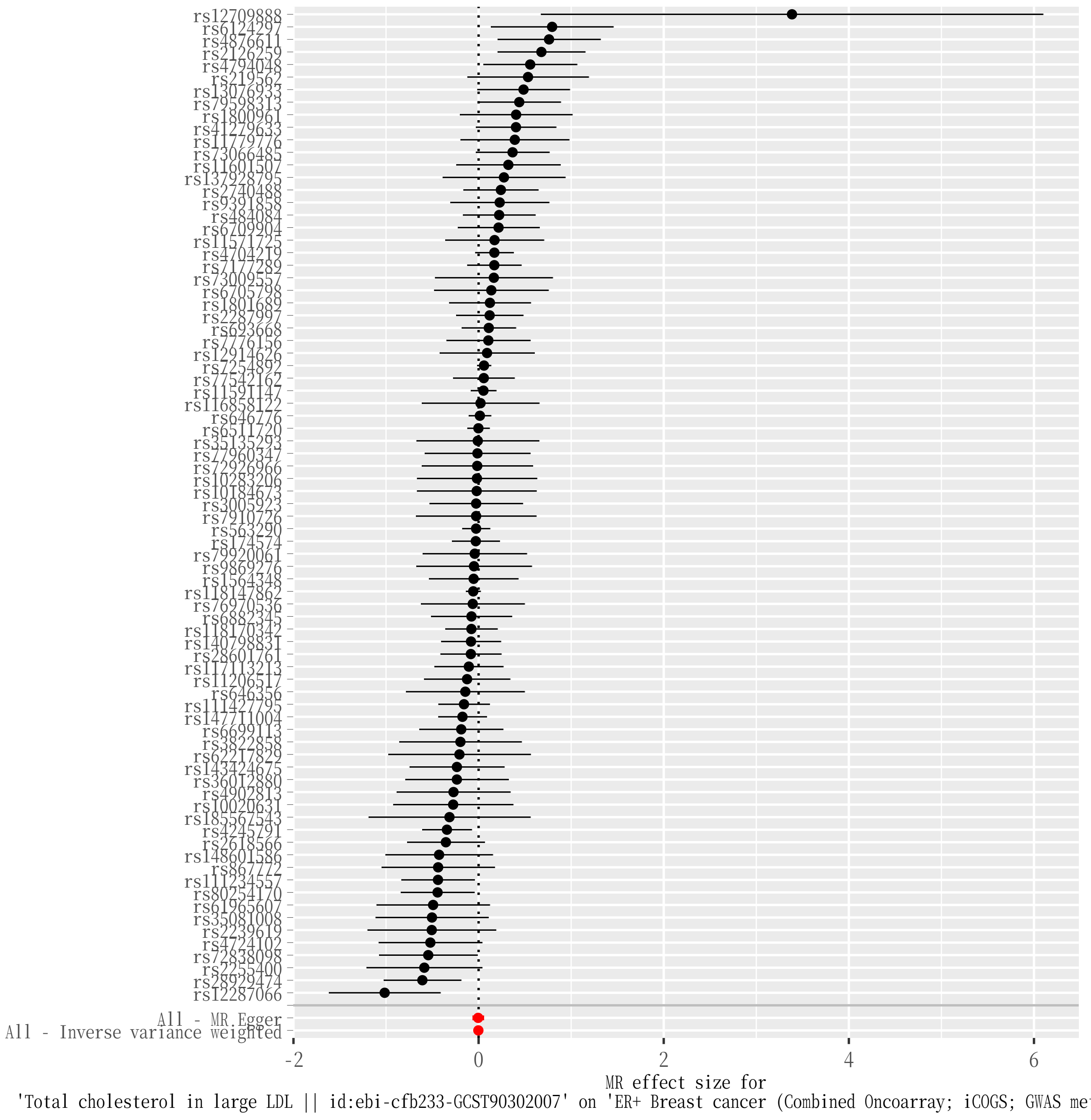

rs12709888
rs6124297
rs4876611
rs2126259
rs4794048
rs219562
rs13076933
rs79598313
rs1800961
rs41279633
rs11779776
rs73066485
rs11601507
rs137928795
rs2740488
rs9391858
rs484084
rs6709904
rs11571725
rs4704219
rs7177289
rs73009557
rs6705798
rs1801689
rs2287997
rs623668
rs7776156
rs12914626
rs7254892
rs77542162
rs11591147
rs116858122
rs646776
rs6511720
rs35135293
rs77960347
rs72926966
rs10283206
rs10184673
rs3005923
rs7210726
rs563290
rs174574
rs79920061
rs9869276
rs1564348
rs118147862
rs76970536
rs6882345
rs118170342
rs140798831
rs28601761
rs117113213
rs11206517
rs646356
rs111427795
rs147711004
rs6699113
rs3822858
rs62217829
rs143424675
rs36012880
rs4902813
rs10020631
rs185567543
rs4245791
rs2618566
rs148601586
rs867772
rs111234557
rs80254170
rs61965607
rs35081008
rs2239619
rs4724102
rs72838098
rs2255400
rs28929474
rs12287066
All - MR Egger
All - Inverse variance weighted
-2
0
2
4
6
MR effect size for
'Total cholesterol in large LDL || id:ebi-cfb233-GCST90302007' on 'ER+ Breast cancer (Combined Oncoarray; iCOGS; GWAS me

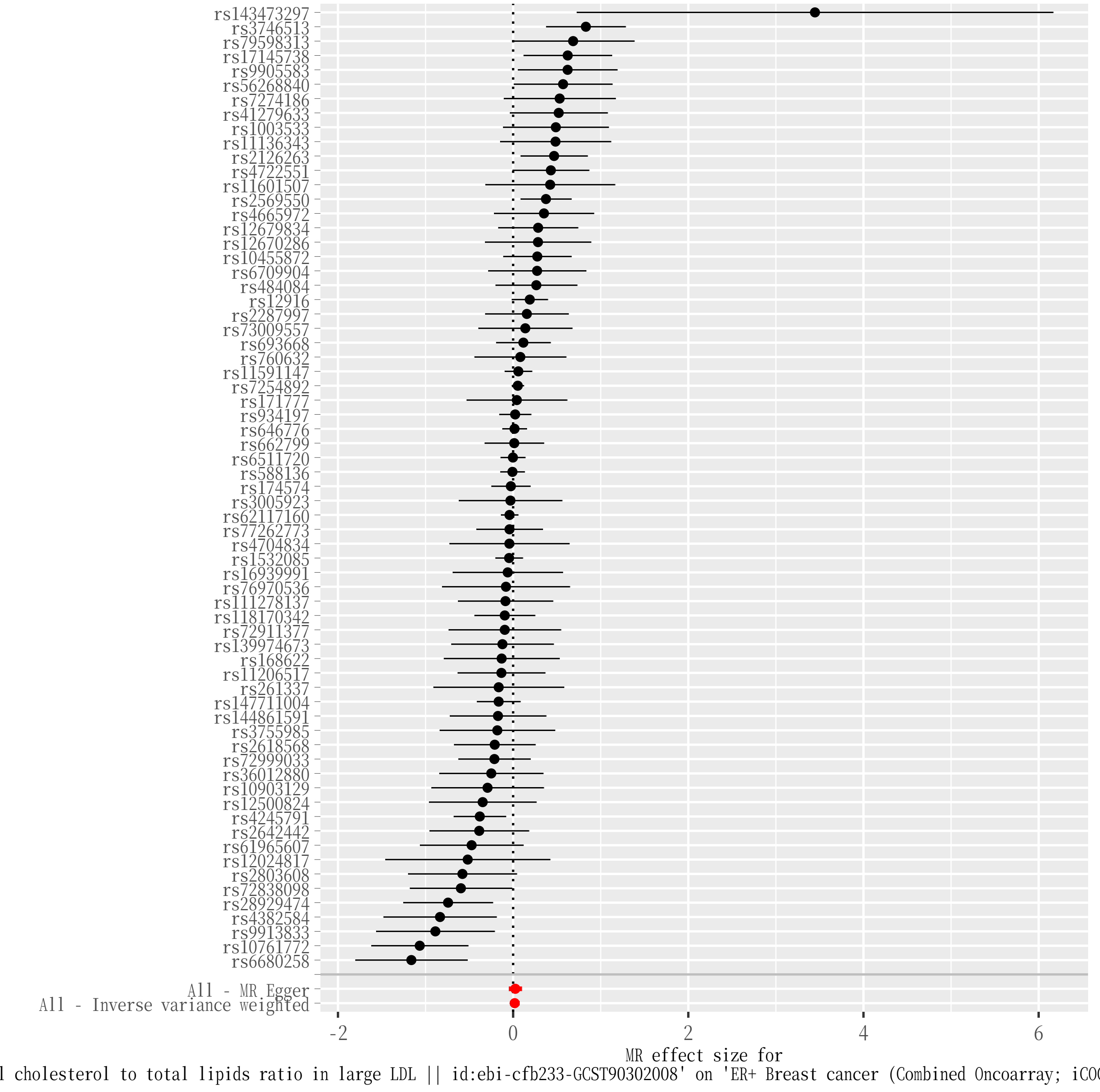

rs143473297
rs3746513
rs79598313
rs17145738
rs9905583
rs56268840
rs7274186
rs41279633
rs1003533
rs11136343
rs2126263
rs4722551
rs11601507
rs2569550
rs4665972
rs12679834
rs12670286
rs10455872
rs6709904
rs484084
rs12916
rs2287997
rs73009557
rs693668
rs760632
rs11591147
rs7254892
rs171777
rs934197
rs646776
rs662799
rs6511720
rs588136
rs174574
rs3005923
rs62117160
rs77262773
rs4704834
rs1532085
rs16939991
rs76970536
rs111278137
rs118170342
rs72911377
rs139974673
rs168622
rs11206517
rs261337
rs147711004
rs144861591
rs3755985
rs2618568
rs72999033
rs36012880
rs10903129
rs12500824
rs4245791
rs2642442
rs61965607
rs12024817
rs2803608
rs72838098
rs28929474
rs4382584
rs9913833
rs10761772
rs6680258
All - MR Egger
All - Inverse variance weighted
-2
0
2
4
6
MR effect size for
l cholesterol to total lipids ratio in large LDL || id:ebi-cfb233-GCST90302008' on 'ER+ Breast cancer (Combined Oncoarray; iCO

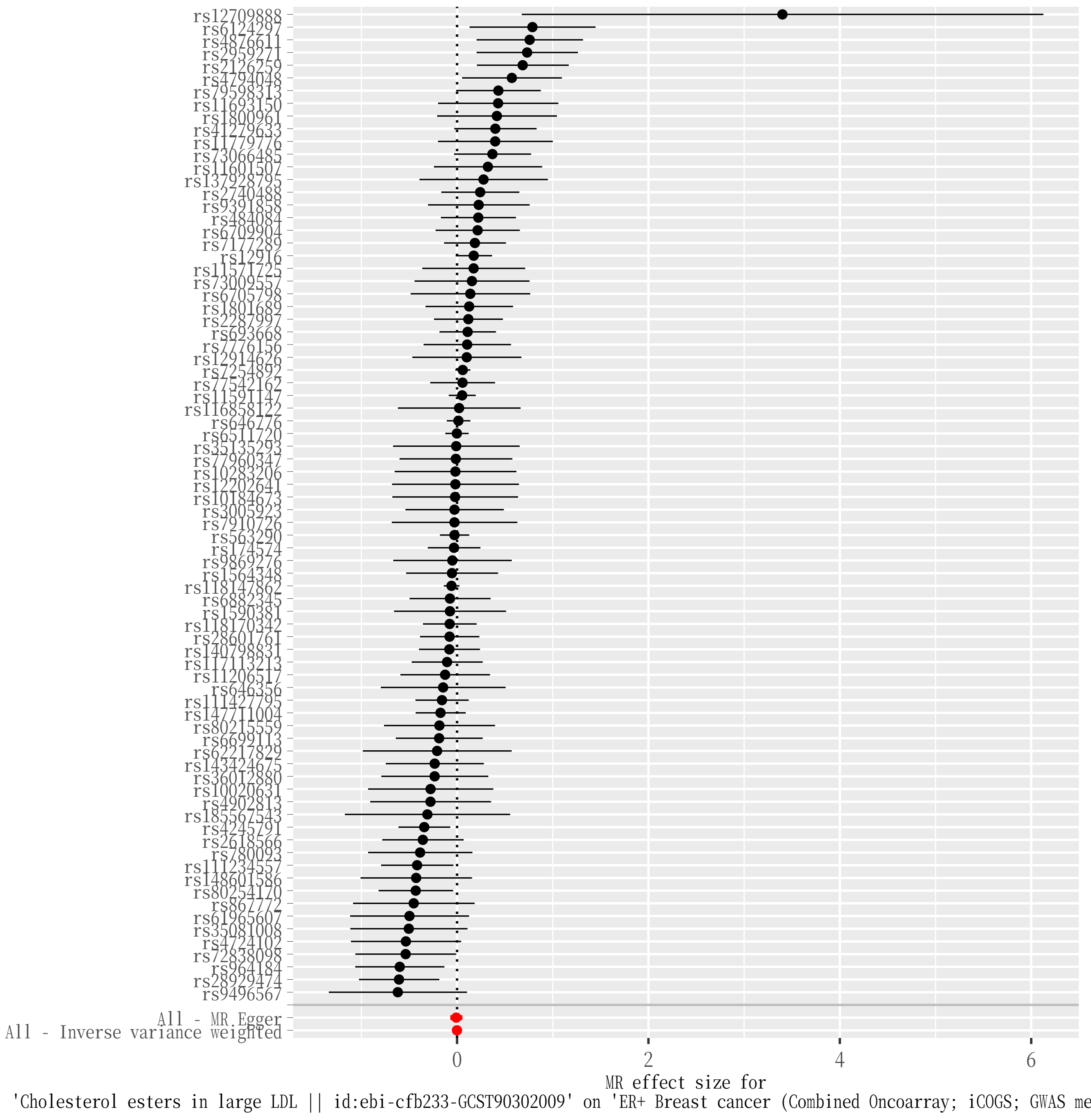

rs12709888
rs6124297
rs4876611
rs2959271
rs2126259
rs4794048
rs79598313
rs11693150
rs1800961
rs41279633
rs11779776
rs73066485
rs11601507
rs137928795
rs2740488
rs9391858
rs484084
rs6709904
rs7177289
rs12916
rs11571725
rs73009557
rs6705798
rs1801689
rs2287997
rs693668
rs7776156
rs12914626
rs7254892
rs77542162
rs11591147
rs116858122
rs646776
rs6511720
rs35135293
rs77960347
rs10283206
rs12202641
rs10184673
rs3005923
rs7910726
rs563290
rs174574
rs9869276
rs1564348
rs118147862
rs6882345
rs1590381
rs118170342
rs28601761
rs140798831
rs117113213
rs11206517
rs646356
rs111427795
rs147711004
rs80215559
rs6699113
rs62217829
rs143424675
rs36012880
rs10020631
rs4902813
rs185567543
rs4245791
rs2618566
rs780093
rs111234557
rs148601586
rs80254170
rs867772
rs61965607
rs35081008
rs4724102
rs72838098
rs964184
rs28929474
rs9496567
All - MR Egger
All - Inverse variance weighted
0
2
4
6
MR effect size for
'Cholesterol esters in large LDL || id:ebi-cfb233-GCST90302009' on 'ER+ Breast cancer (Combined Oncoarray; iCOGS; GWAS me

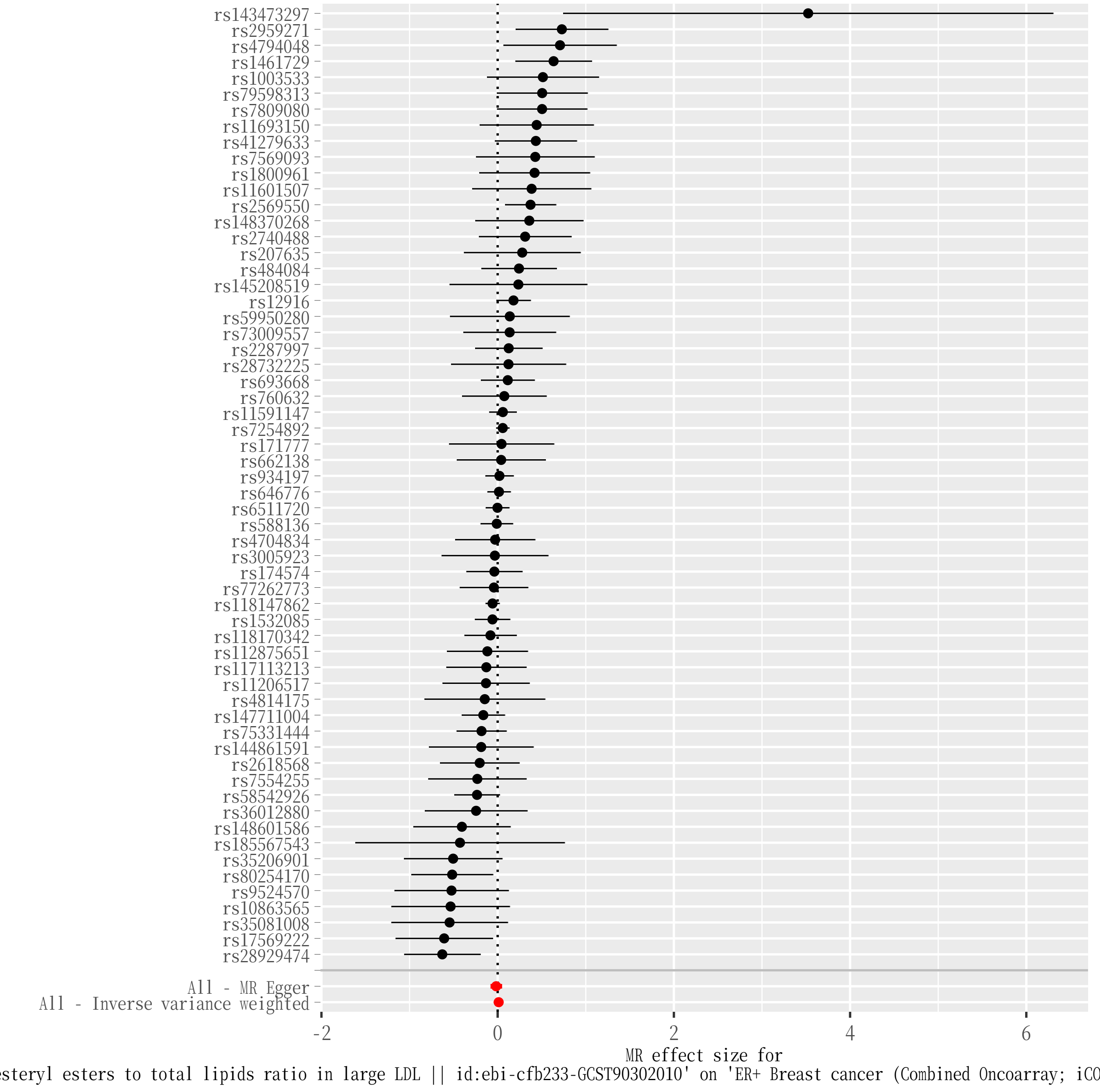

rs143473297
rs2959271
rs4794048
rs1461729
rs1003533
rs79598313
rs7809080
rs11693150
rs41279633
rs7569093
rs1800961
rs11601507
rs2569550
rs148370268
rs2740488
rs207635
rs484084
rs145208519
rs12916
rs59950280
rs73009557
rs2287997
rs28732225
rs693668
rs760632
rs11591147
rs7254892
rs171777
rs662138
rs934197
rs646776
rs6511720
rs588136
rs4704834
rs3005923
rs174574
rs77262773
rs118147862
rs1532085
rs118170342
rs112875651
rs117113213
rs11206517
rs4814175
rs147711004
rs75331444
rs144861591
rs2618568
rs7554255
rs58542926
rs36012880
rs148601586
rs185567543
rs35206901
rs80254170
rs9524570
rs10863565
rs35081008
rs17569222
rs28929474
All - MR Egger
All - Inverse variance weighted
-2
0
2
4
6
MR effect size for
esteryl esters to total lipids ratio in large LDL || id:ebi-cfb233-GCST90302010' on 'ER+ Breast cancer (Combined Oncoarray; iCO

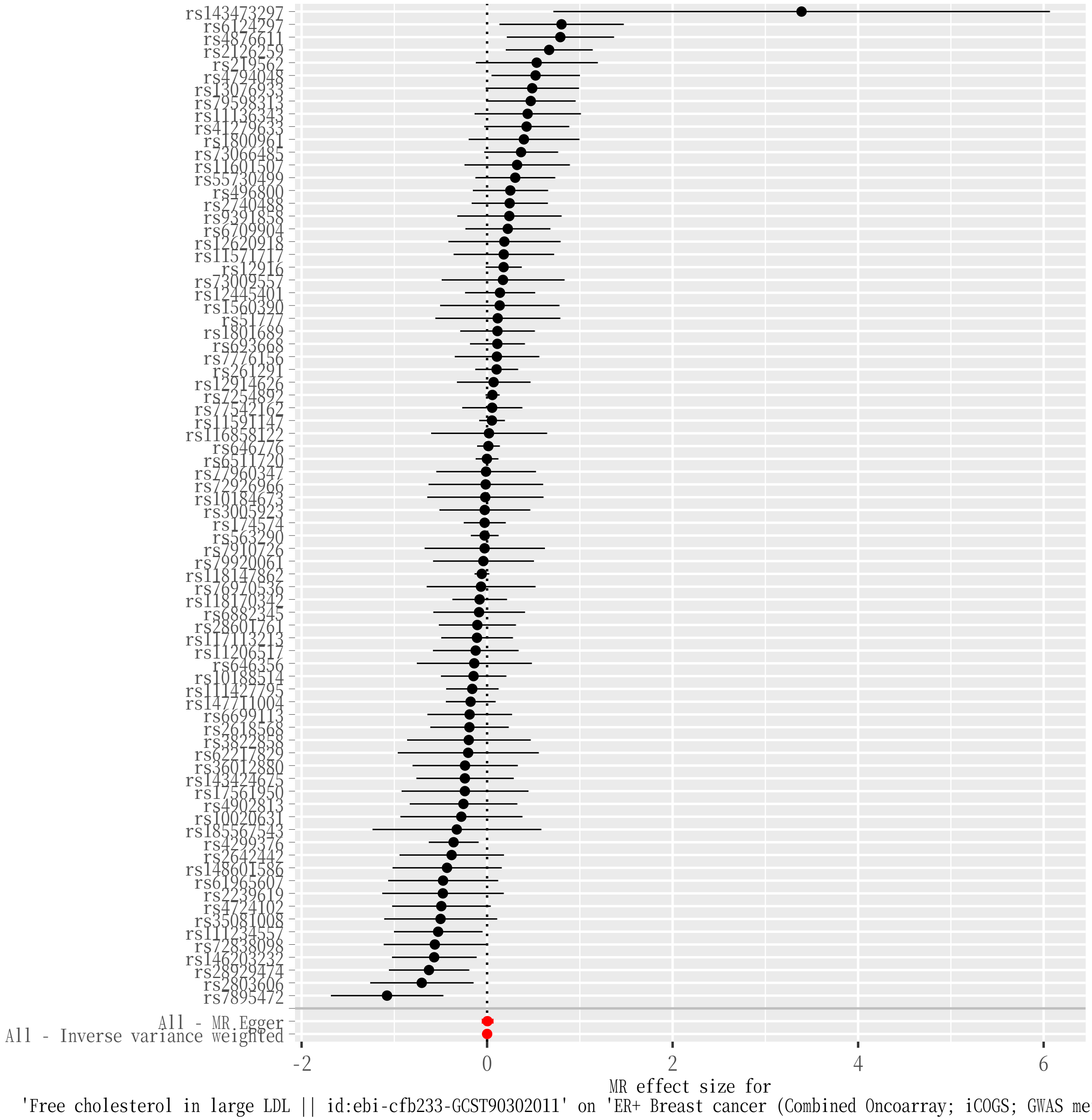

rs143473297
rs6124297
rs4876611
rs2126259
rs219562
rs4794048
rs13076933
rs79598313
rs11136343
rs41279633
rs1800961
rs73066485
rs11601507
rs55730499
rs496800
rs2740488
rs9391858
rs6709904
rs12620918
rs11571717
rs12916
rs73009557
rs12445401
rs1560390
rs51777
rs1801689
rs623668
rs7776156
rs261291
rs12914626
rs7254892
rs77542162
rs11591147
rs116858122
rs646776
rs6511720
rs77960347
rs72926966
rs10184673
rs3005923
rs174574
rs563290
rs7910726
rs79920061
rs118147862
rs76970536
rs118170342
rs6882345
rs28601761
rs117113213
rs11206517
rs646356
rs10188514
rs111427795
rs147711004
rs6699113
rs2618568
rs3822858
rs62217829
rs36012880
rs143424675
rs17561950
rs4902813
rs10020631
rs185567543
rs4299376
rs2642442
rs148601586
rs61965607
rs2239619
rs4724102
rs35081008
rs111234557
rs72838098
rs146203232
rs28929474
rs2803606
rs7895472
All - MR Egger
All - Inverse variance weighted
-2
0
2
4
6
MR effect size for
'Free cholesterol in large LDL || id:ebi-cfb233-GCST90302011' on 'ER+ Breast cancer (Combined Oncoarray; iCOGS; GWAS met

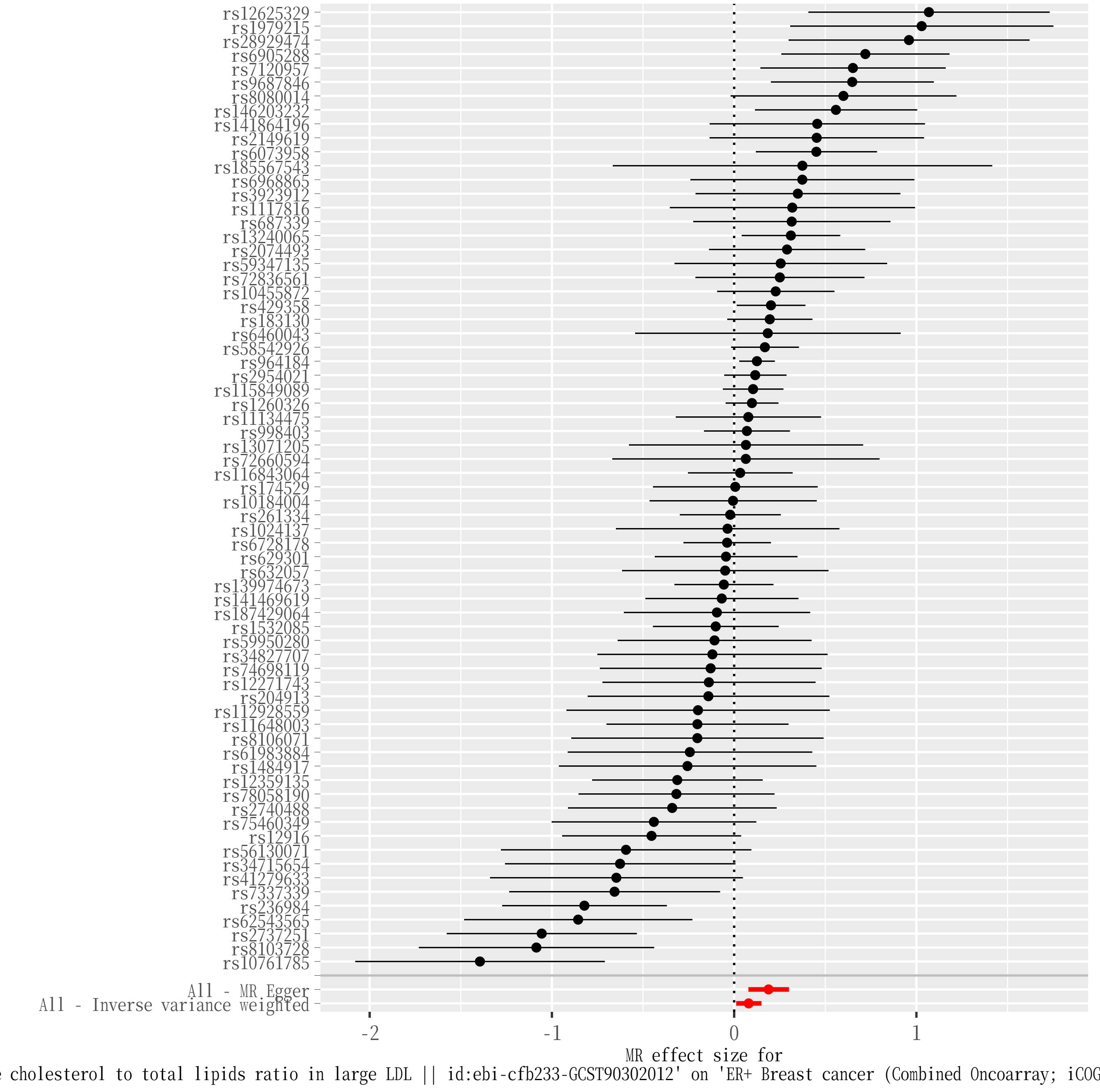
rs12625329
rs1979215
rs28929474
rs6905288
rs7120957
rs9687846
rs8080014
rs146203232
rs141864196
rs2149619
rs6073958
rs185567543
rs6968865
rs3923912
rs1117816
rs687339
rs13240065
rs2074493
rs59347135
rs72836561
rs10455872
rs429358
rs183130
rs6460043
rs58542926
rs964184
rs2954021
rs115849089
rs1260326
rs11134475
rs998403
rs13071205
rs72660594
rs116843064
rs174529
rs10184004
rs261334
rs1024137
rs6728178
rs629301
rs632057
rs139974673
rs141469619
rs187429064
rs1532085
rs59950280
rs34827707
rs74698119
rs12271743
rs204913
rs112928559
rs11648003
rs8106071
rs61983884
rs1484917
rs12359135
rs78058190
rs2740488
rs75460349
rs12916
rs56130071
rs34715654
rs41279633
rs7337339
rs236984
rs62543565
rs2737251
rs8103728
rs10761785
All - MR Egger
All - Inverse variance weighted
-2
-1
0
1
MR effect size for
e cholesterol to total lipids ratio in large LDL || id:ebi-cfb233-GCST90302012' on 'ER+ Breast cancer (Combined Oncoarray; iCOG

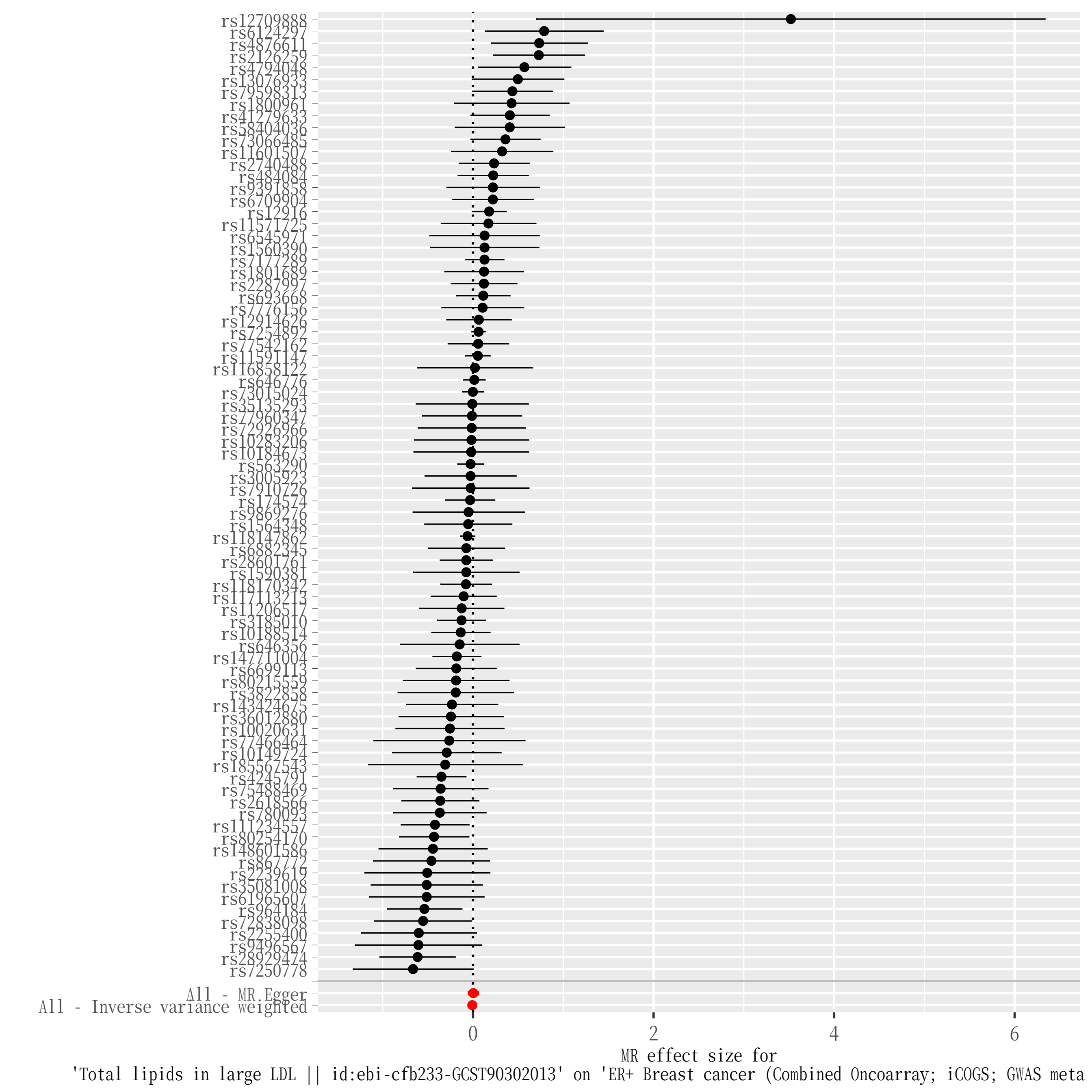
rs12709888
rs6124297
rs4876611
rs2126259
rs4794048
rs13076933
rs79598313
rs1800961
rs41279633
rs58404036
rs73066485
rs11601507
rs2740488
rs484084
rs9391858
rs6709904
rs12916
rs11571725
rs6545971
rs1560390
rs7177289
rs1801689
rs2287997
rs693668
rs7776156
rs12914626
rs7254892
rs77542162
rs11591147
rs116858122
rs646776
rs73015024
rs35135293
rs77960347
rs72926966
rs10283206
rs10184673
rs563290
rs3005923
rs7910726
rs174574
rs9869276
rs1564348
rs118147862
rs6882345
rs28601761
rs1590381
rs118170342
rs117113213
rs11206517
rs3185010
rs10188514
rs646356
rs147711004
rs6699113
rs80215559
rs3822858
rs143424675
rs36012880
rs10020631
rs77466464
rs10149724
rs185567543
rs4245791
rs75488469
rs2618566
rs780093
rs111234557
rs80254170
rs148601586
rs867772
rs2239619
rs35081008
rs61965607
rs964184
rs72838098
rs2255400
rs9496567
rs28929474
rs7250778
All - MR Egger
All - Inverse variance weighted
0
2
4
6
MR effect size for
'Total lipids in large LDL || id:ebi-cfb233-GCST90302013' on 'ER+ Breast cancer (Combined Oncoarray; iCOGS; GWAS meta

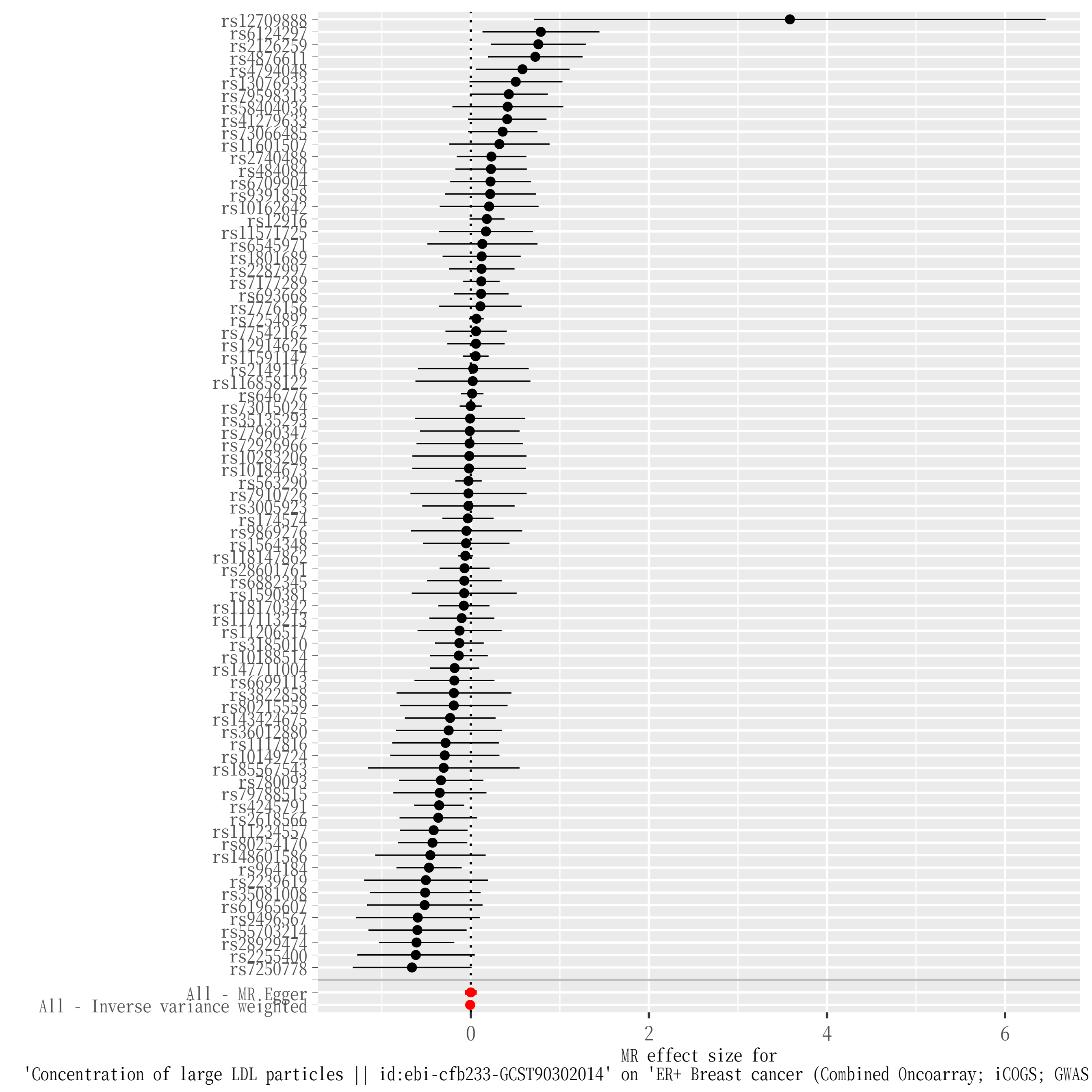

rs12709888
rs6124297
rs2126259
rs4876611
rs4794048
rs13076933
rs79598313
rs58404036
rs41279633
rs73066485
rs11601507
rs2740488
rs484084
rs6709904
rs9391858
rs10162642
rs12916
rs11571725
rs6545971
rs1801689
rs2287997
rs7177289
rs623668
rs2776156
rs7254892
rs77542162
rs12914626
rs11591147
rs2149116
rs116858122
rs646776
rs73015024
rs35135293
rs77960347
rs72926966
rs10283206
rs10184673
rs563290
rs7910726
rs3005923
rs174574
rs9869276
rs1564348
rs118147862
rs28601761
rs6882345
rs1520381
rs118170342
rs117113213
rs11206517
rs3185010
rs10188514
rs147711004
rs6699113
rs3822858
rs80215559
rs143424675
rs36012880
rs1117816
rs10149724
rs185567543
rs780093
rs79788515
rs4245791
rs2618566
rs111234557
rs80254170
rs148601586
rs964184
rs2239619
rs35081008
rs61965607
rs9496567
rs55703214
rs28929474
rs2255400
rs7250778
All - MR Egger
All - Inverse variance weighted
0
2
4
6
MR effect size for
'Concentration of large LDL particles || id:ebi-cfb233-GCST90302014' on 'ER+ Breast cancer (Combined Oncoarray; iCOGS; GWAS

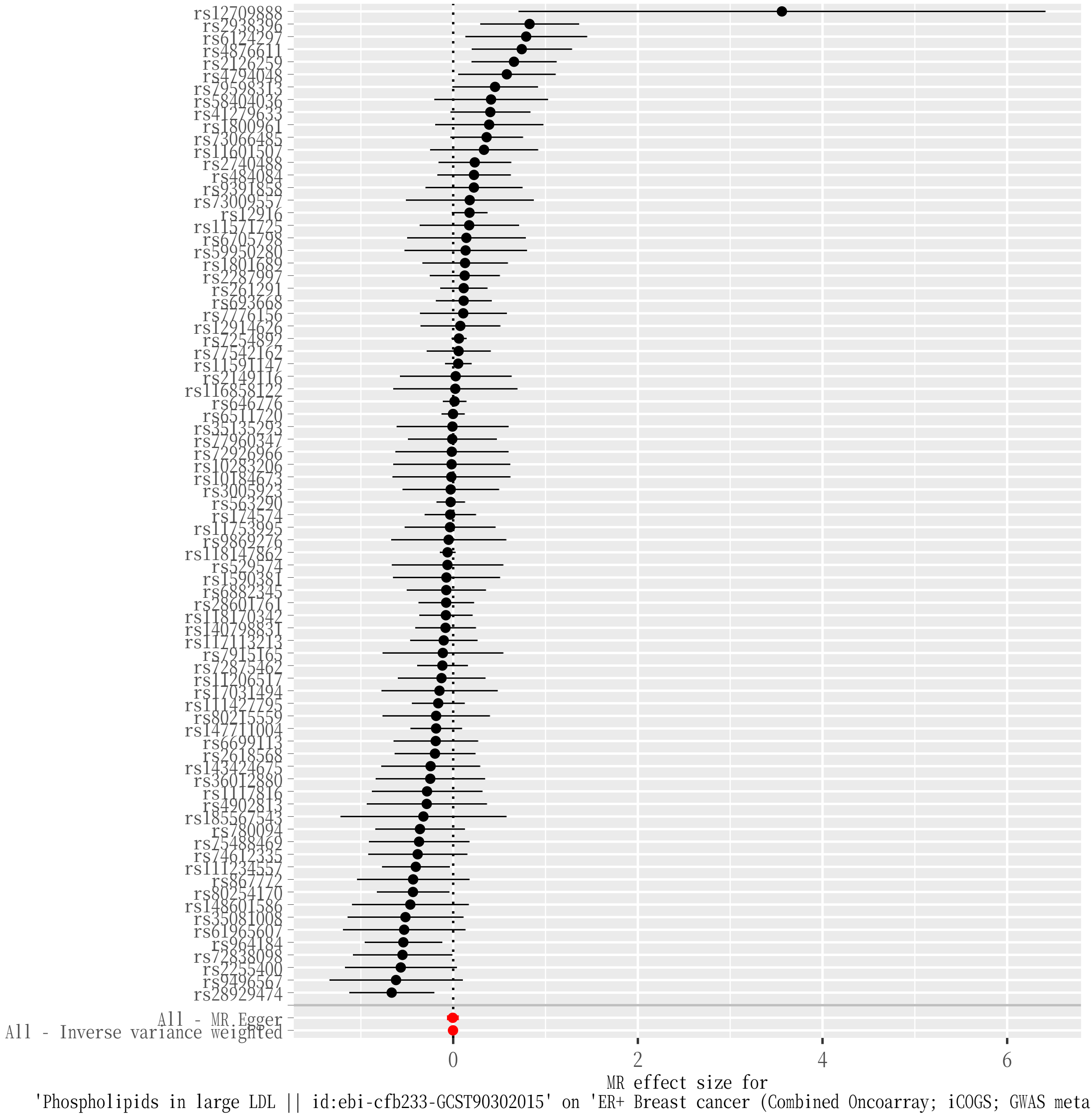
rs12709888
rs2938396
rs6124297
rs4876611
rs2126259
rs4794048
rs79598313
rs58404036
rs41279633
rs1800961
rs73066485
rs11601507
rs2740488
rs484084
rs9391858
rs73009557
rs12916
rs11571725
rs6705798
rs59950280
rs1801689
rs2287997
rs261291
rs693668
rs7776156
rs12914626
rs7254892
rs77542162
rs11591147
rs2149116
rs116858122
rs646776
rs6511720
rs35135293
rs77960347
rs72926966
rs10283206
rs10184673
rs3005923
rs563290
rs174574
rs11753995
rs9869276
rs118147862
rs529574
rs1590381
rs6882345
rs28601761
rs118170342
rs140798831
rs112113213
rs7915165
rs72875462
rs11206517
rs17031494
rs111427795
rs80215559
rs147711004
rs6699113
rs2618568
rs143424675
rs36012880
rs1117816
rs4902813
rs185567543
rs780094
rs75488469
rs74612335
rs111234557
rs867772
rs80254170
rs148601586
rs35081008
rs61965607
rs964184
rs72838098
rs2255400
rs9496567
rs28929474
All - MR Egger
All - Inverse variance weighted
0
2
4
6
MR effect size for
'Phospholipids in large LDL || id:ebi-cfb233-GCST90302015' on 'ER+ Breast cancer (Combined Oncoarray; iCOGS; GWAS meta

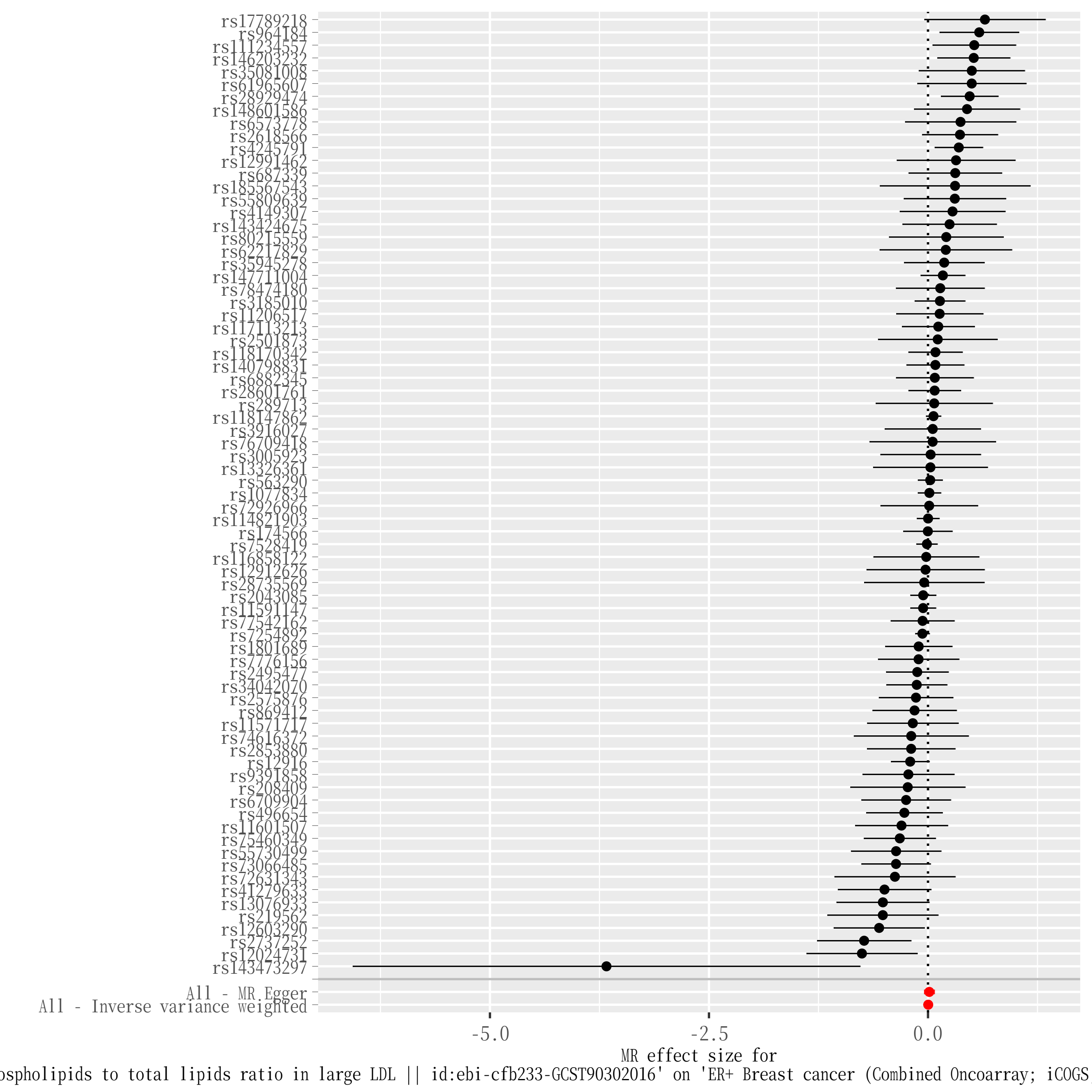
rs17789218
rs964184
rs111234557
rs146203232
rs35081008
rs61965607
rs28929474
rs148601586
rs6573778
rs2618566
rs4245791
rs12991462
rs687339
rs185567543
rs55809639
rs4149307
rs143424675
rs80215559
rs62217829
rs35945278
rs147711004
rs78474180
rs3185010
rs11206517
rs117113213
rs2501873
rs118170342
rs140798831
rs6882345
rs28601761
rs289713
rs118147862
rs3916027
rs76709418
rs3005923
rs13326361
rs563290
rs1077834
rs72926966
rs114821903
rs174566
rs7528419
rs116858122
rs12912626
rs28735569
rs2043085
rs11591147
rs77542162
rs7254892
rs1801689
rs7776156
rs2495477
rs34042070
rs2575876
rs869412
rs11571717
rs74616372
rs2853880
rs12916
rs9391858
rs208409
rs6709904
rs496654
rs11601507
rs75460349
rs55730499
rs73066485
rs72631343
rs41279633
rs13076933
rs219562
rs12603290
rs2737252
rs12024731
rs143473297
All - MR Egger
All - Inverse variance weighted
-5.0
-2.5
0.0
MR effect size for
ospholipids to total lipids ratio in large LDL || id:ebi-cfb233-GCST90302016' on 'ER+ Breast cancer (Combined Oncoarray; iCOGS

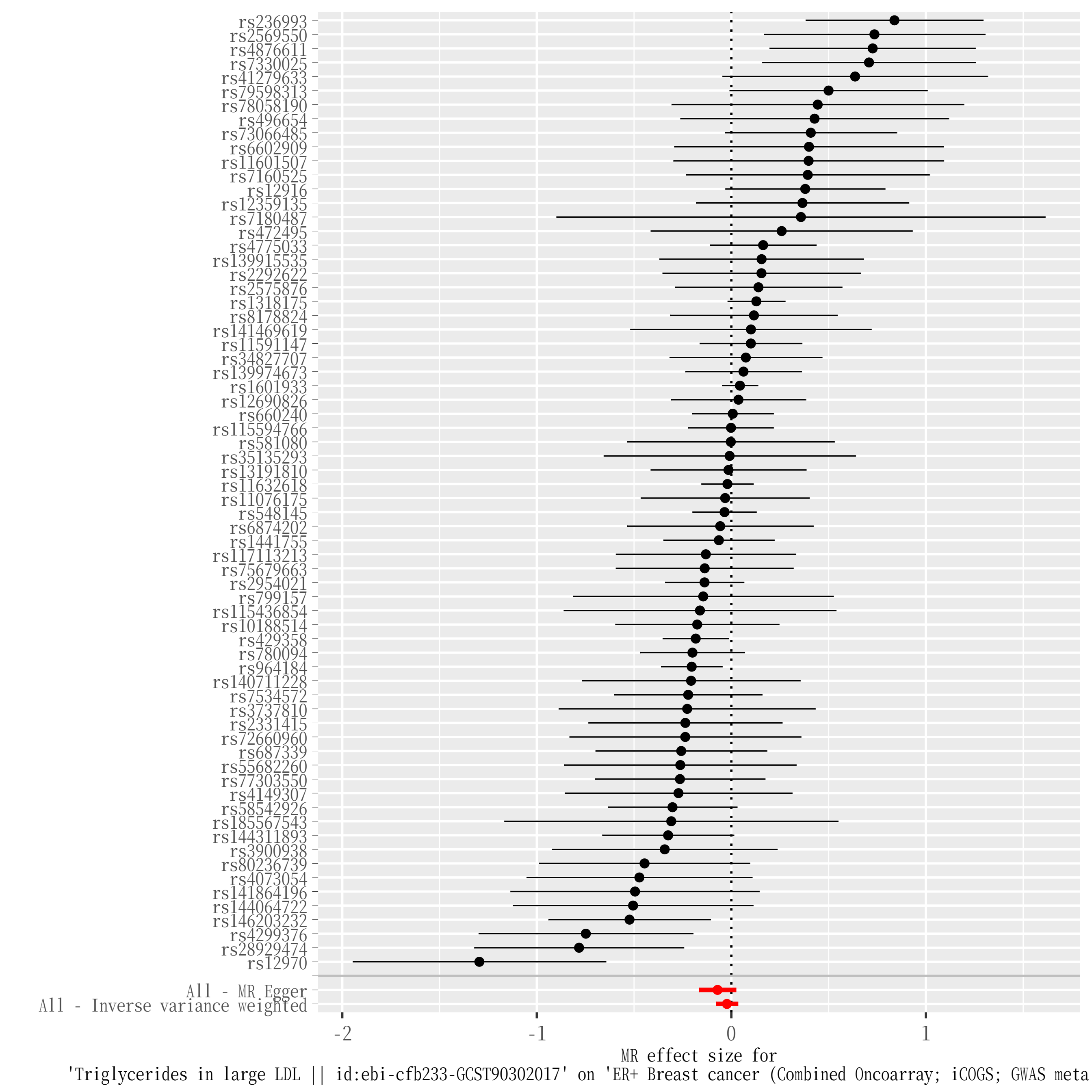
rs236993
rs2569550
rs4876611
rs7330025
rs41279633
rs79598313
rs78058190
rs496654
rs73066485
rs6602909
rs11601507
rs7160525
rs12916
rs12359135
rs7180487
rs472495
rs4775033
rs139915535
rs2292622
rs2575876
rs1318175
rs8178824
rs141469619
rs11591147
rs34827707
rs139974673
rs1601933
rs12690826
rs660240
rs115594766
rs581080
rs35135293
rs13191810
rs11632618
rs11076175
rs548145
rs6874202
rs1441755
rs117113213
rs75679663
rs2954021
rs799157
rs115436854
rs10188514
rs429358
rs780094
rs964184
rs140711228
rs7534572
rs3737810
rs2331415
rs72660960
rs687339
rs55682260
rs77303550
rs4149307
rs58542926
rs185567543
rs144311893
rs3900938
rs80236739
rs4073054
rs141864196
rs144064722
rs146203232
rs4299376
rs28929474
rs12970
All - MR Egger
All - Inverse variance weighted
-2
-1
0
1
MR effect size for
'Triglycerides in large LDL || id:ebi-cfb233-GCST90302017' on 'ER+ Breast cancer (Combined Oncoarray; iCOGS; GWAS meta

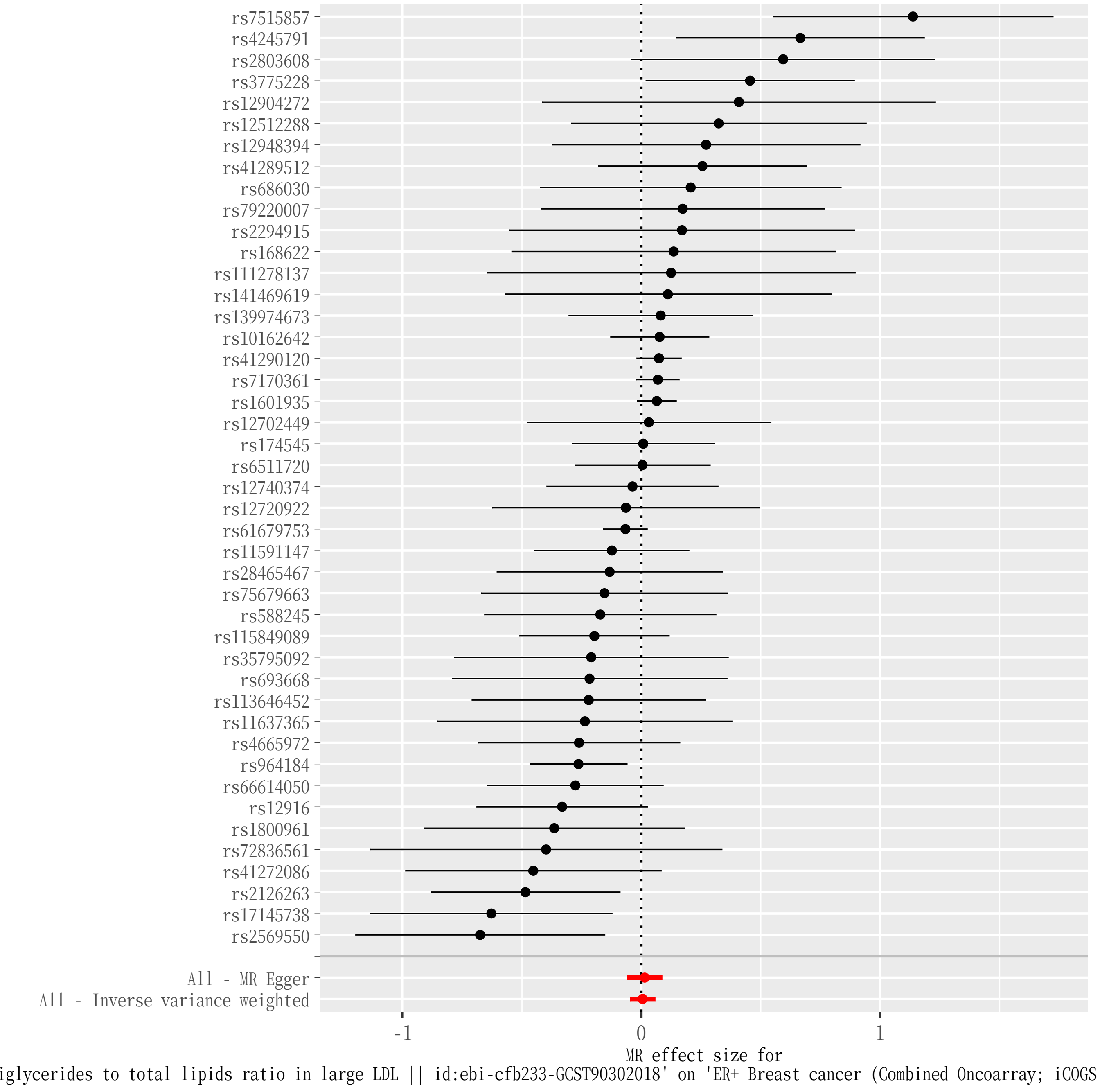
rs7515857
rs4245791
rs2803608
rs3775228
rs12904272
rs12512288
rs12948394
rs41289512
rs686030
rs79220007
rs2294915
rs168622
rs111278137
rs141469619
rs139974673
rs10162642
rs41290120
rs7170361
rs1601935
rs12702449
rs174545
rs6511720
rs12740374
rs12720922
rs61679753
rs11591147
rs28465467
rs75679663
rs588245
rs115849089
rs35795092
rs693668
rs113646452
rs11637365
rs4665972
rs964184
rs66614050
rs12916
rs1800961
rs72836561
rs41272086
rs2126263
rs17145738
rs2569550
All - MR Egger
All - Inverse variance weighted
-1
0
1
MR effect size for
iglycerides to total lipids ratio in large LDL || id:ebi-cfb233-GCST90302018' on 'ER+ Breast cancer (Combined Oncoarray; iCOGS

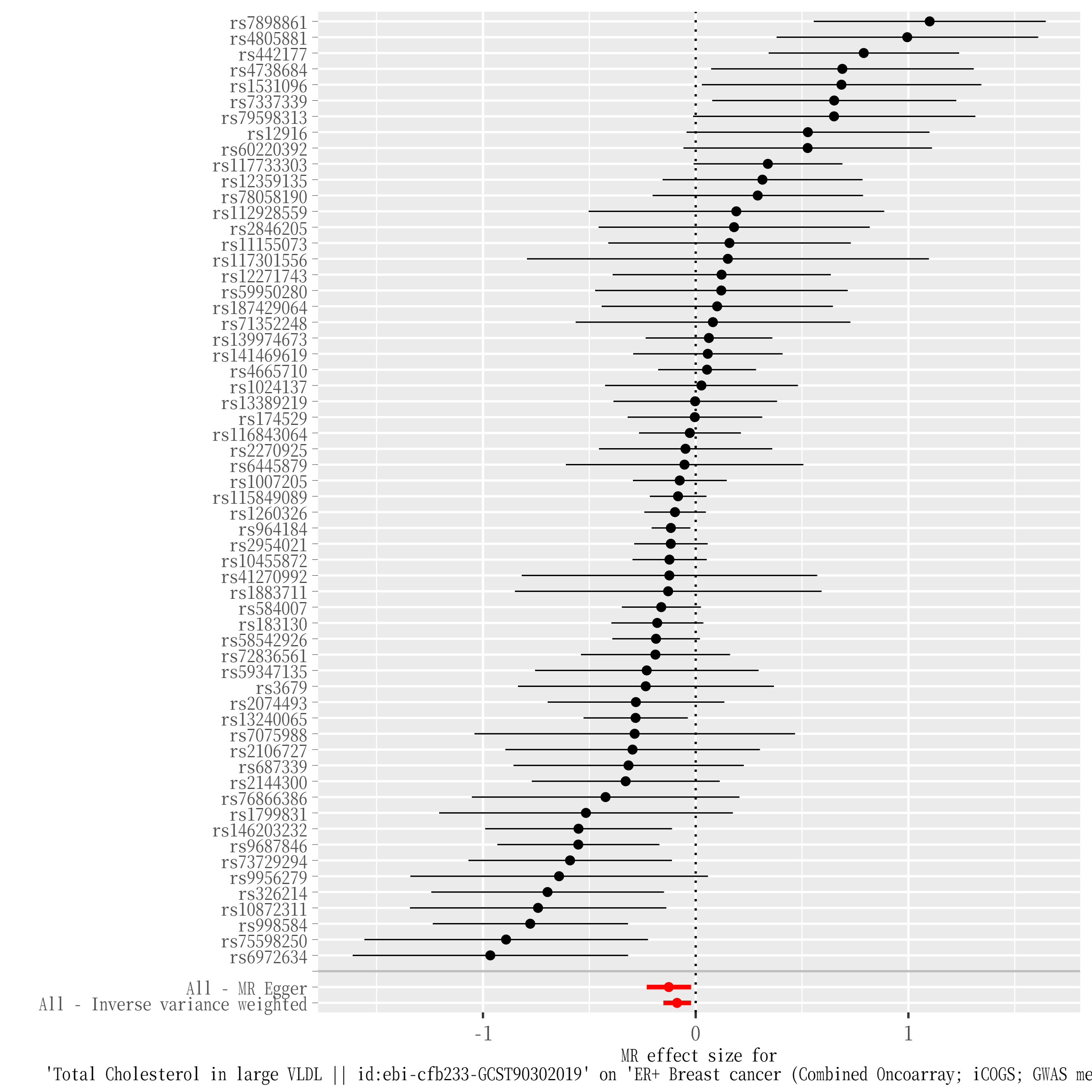

rs7898861
rs4805881
rs442177
rs4738684
rs1531096
rs7337339
rs79598313
rs12916
rs60220392
rs117733303
rs12359135
rs78058190
rs112928559
rs2846205
rs11155073
rs117301556
rs12271743
rs59950280
rs187429064
rs71352248
rs139974673
rs141469619
rs4665710
rs1024137
rs13389219
rs174529
rs116843064
rs2270925
rs6445879
rs1007205
rs115849089
rs1260326
rs964184
rs2954021
rs10455872
rs41270992
rs1883711
rs584007
rs183130
rs58542926
rs72836561
rs59347135
rs3679
rs2074493
rs13240065
rs7075988
rs2106727
rs687339
rs2144300
rs76866386
rs1799831
rs146203232
rs9687846
rs73729294
rs9956279
rs326214
rs10872311
rs998584
rs75598250
rs6972634
All - MR Egger
All - Inverse variance weighted
-1
0
1
MR effect size for
'Total Cholesterol in large VLDL || id:ebi-cfb233-GCST90302019' on 'ER+ Breast cancer (Combined Oncoarray; iCOGS; GWAS me

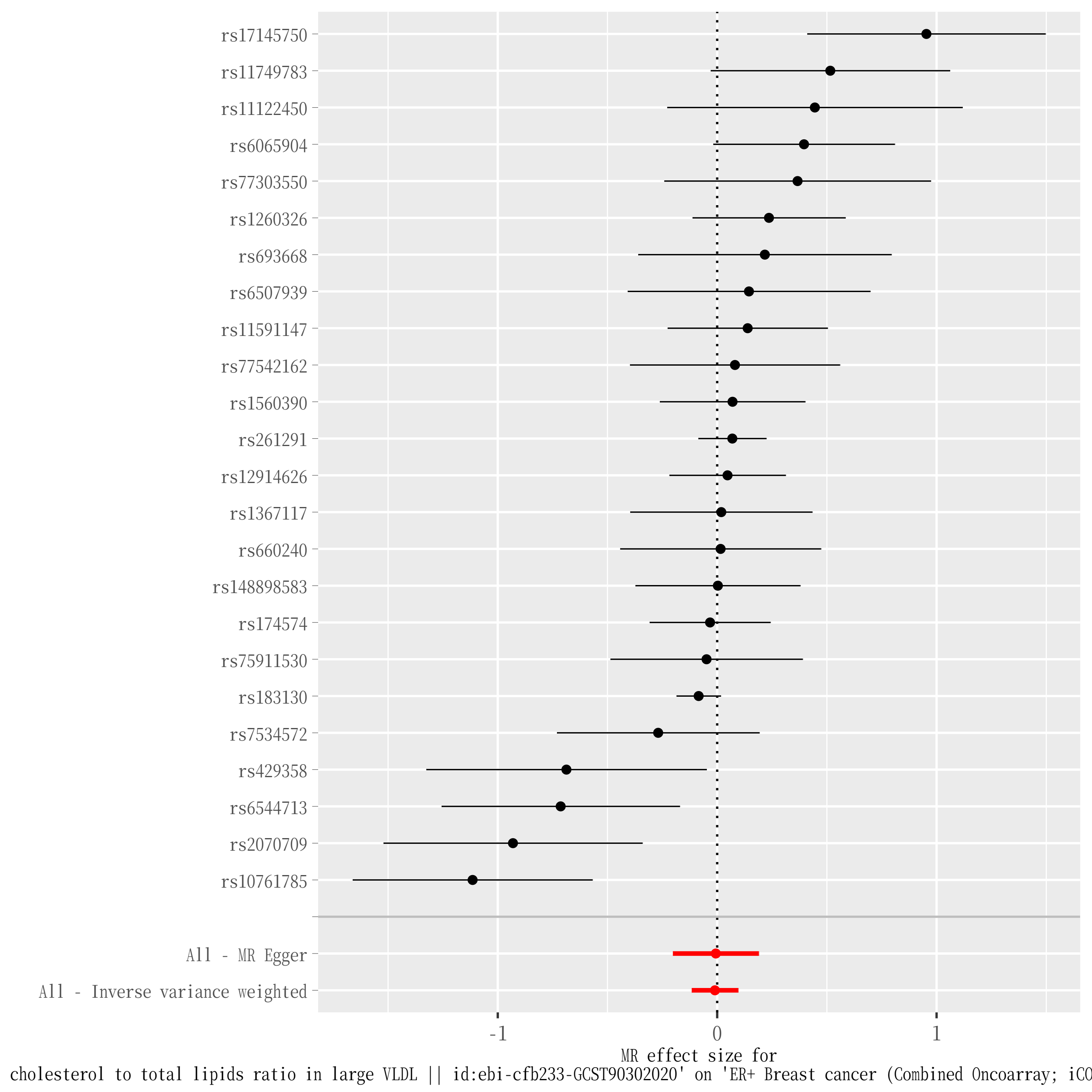
rs17145750
rs11749783
rs11122450
rs6065904
rs77303550
rs1260326
rs693668
rs6507939
rs11591147
rs77542162
rs1560390
rs261291
rs12914626
rs1367117
rs660240
rs148898583
rs174574
rs75911530
rs183130
rs7534572
rs429358
rs6544713
rs2070709
rs10761785
All - MR Egger
All - Inverse variance weighted
-1
0
1
MR effect size for
cholesterol to total lipids ratio in large VLDL || id:ebi-cfb233-GCST90302020' on 'ER+ Breast cancer (Combined Oncoarray; iCO

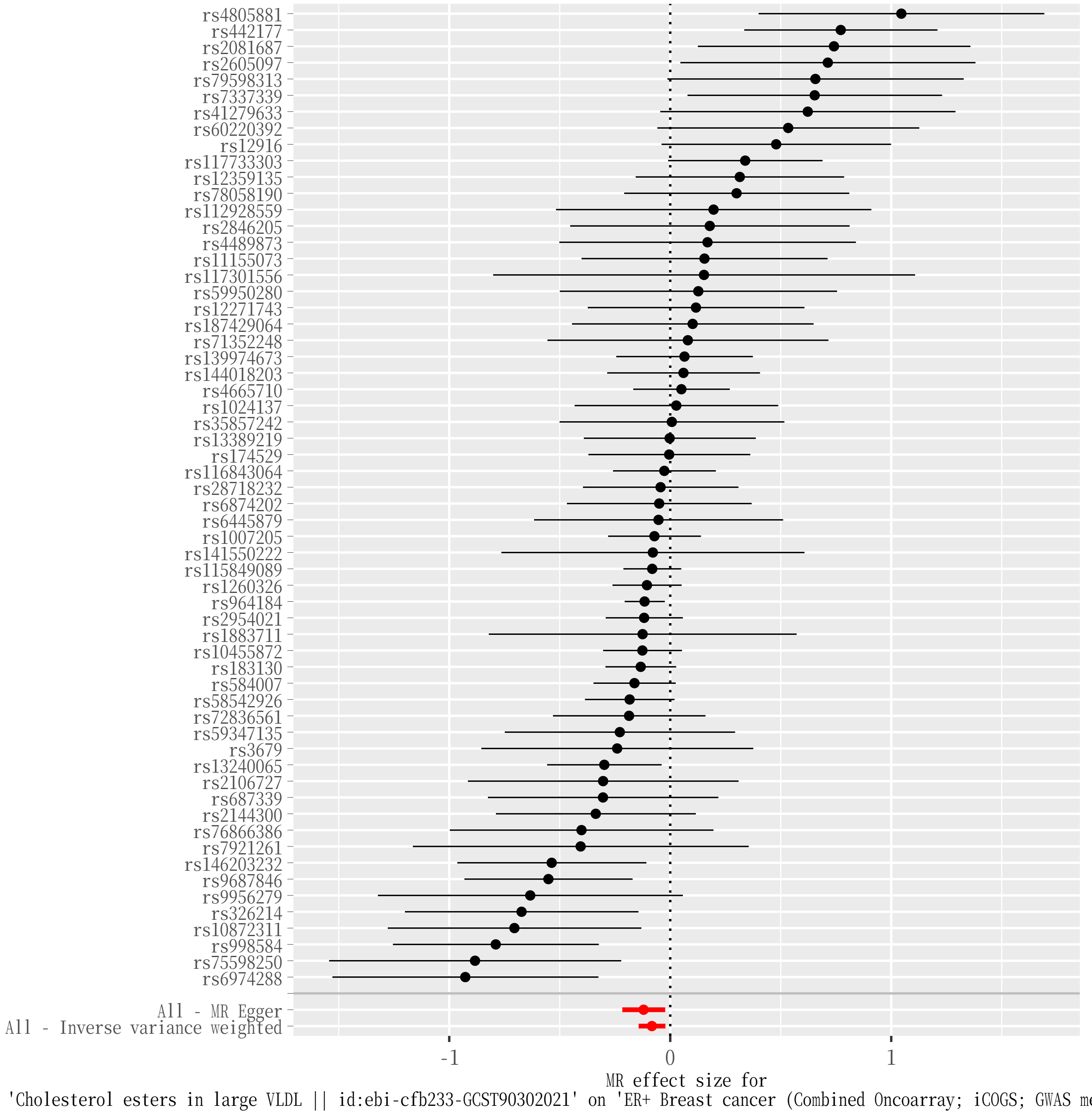

rs4805881
rs442177
rs2081687
rs2605097
rs79598313
rs7337339
rs41279633
rs60220392
rs12916
rs117733303
rs12359135
rs78058190
rs112928559
rs2846205
rs4489873
rs11155073
rs117301556
rs59950280
rs12271743
rs187429064
rs71352248
rs139974673
rs144018203
rs4665710
rs1024137
rs35857242
rs13389219
rs174529
rs116843064
rs28718232
rs6874202
rs6445879
rs1007205
rs141550222
rs115849089
rs1260326
rs964184
rs2954021
rs1883711
rs10455872
rs183130
rs584007
rs58542926
rs72836561
rs59347135
rs3679
rs13240065
rs2106727
rs687339
rs2144300
rs76866386
rs7921261
rs146203232
rs9687846
rs9956279
rs326214
rs10872311
rs998584
rs75598250
rs6974288
All - MR Egger
All - Inverse variance weighted
-1
0
1
MR effect size for
'Cholesterol esters in large VLDL || id:ebi-cfb233-GCST90302021' on 'ER+ Breast cancer (Combined Oncoarray; iCOGS; GWAS me

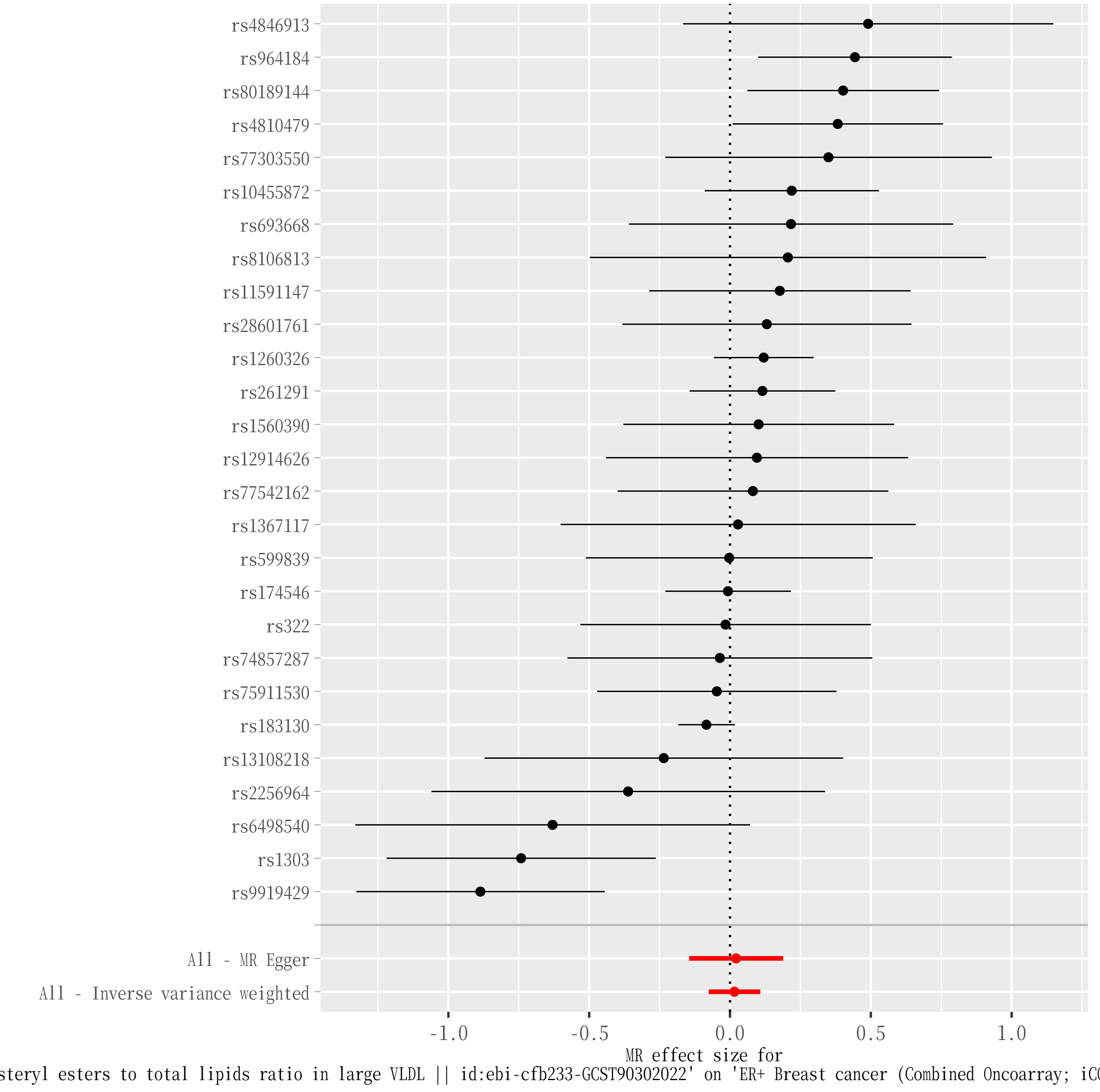

rs4846913
rs964184
rs80189144
rs4810479
rs77303550
rs10455872
rs693668
rs8106813
rs11591147
rs28601761
rs1260326
rs261291
rs1560390
rs12914626
rs77542162
rs1367117
rs599839
rs174546
rs322
rs74857287
rs75911530
rs183130
rs13108218
rs2256964
rs6498540
rs1303
rs9919429
All - MR Egger
All - Inverse variance weighted
-1.0
-0.5
0.0
0.5
1.0
MR effect size for
steryl esters to total lipids ratio in large VLDL || id:ebi-cfb233-GCST90302022' on 'ER+ Breast cancer (Combined Oncoarray; iC

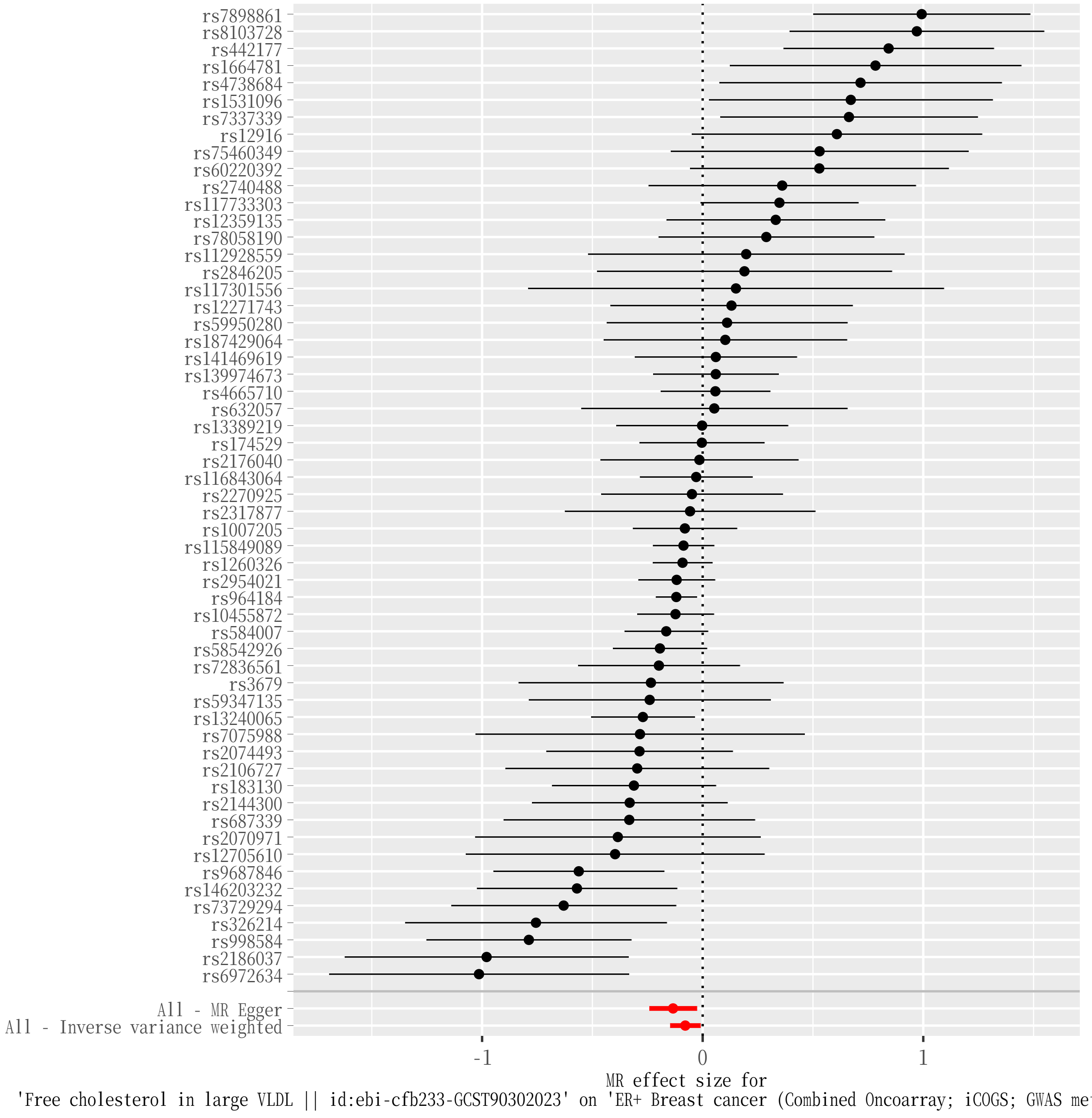

rs7898861
rs8103728
rs442177
rs1664781
rs4738684
rs1531096
rs7337339
rs12916
rs75460349
rs60220392
rs2740488
rs117733303
rs12359135
rs78058190
rs112928559
rs2846205
rs117301556
rs12271743
rs59950280
rs187429064
rs141469619
rs139974673
rs4665710
rs632057
rs13389219
rs174529
rs2176040
rs116843064
rs2270925
rs2317877
rs1007205
rs115849089
rs1260326
rs2954021
rs964184
rs10455872
rs584007
rs58542926
rs72836561
rs3679
rs59347135
rs13240065
rs7075988
rs2074493
rs2106727
rs183130
rs2144300
rs687339
rs2070971
rs12705610
rs9687846
rs146203232
rs73729294
rs326214
rs998584
rs2186037
rs6972634
All - MR Egger
All - Inverse variance weighted
-1
0
1
MR effect size for
'Free cholesterol in large VLDL || id:ebi-cfb233-GCST90302023' on 'ER+ Breast cancer (Combined Oncoarray; iCOGS; GWAS me

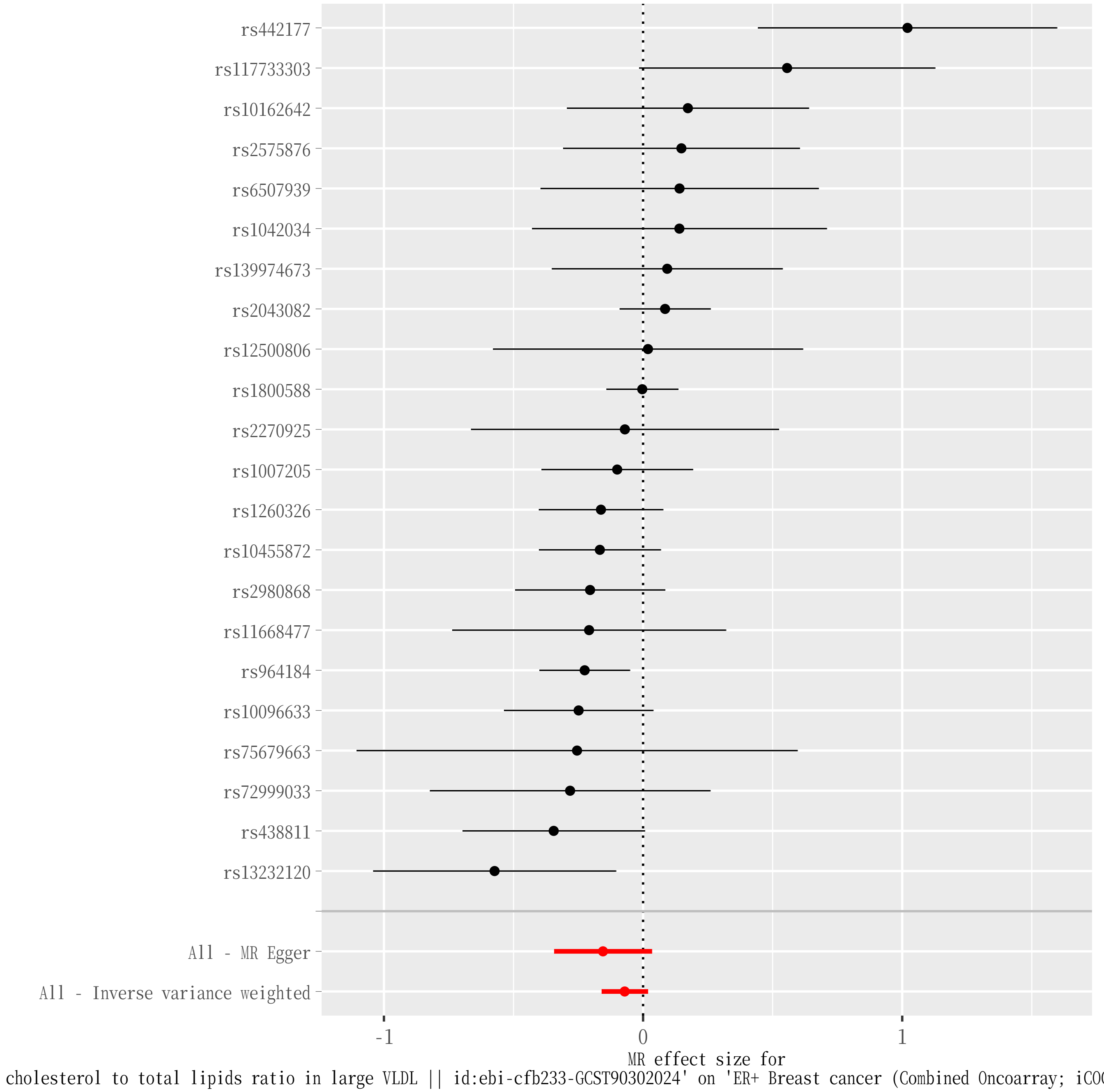
rs442177
rs117733303
rs10162642
rs2575876
rs6507939
rs1042034
rs139974673
rs2043082
rs12500806
rs1800588
rs2270925
rs1007205
rs1260326
rs10455872
rs2980868
rs11668477
rs964184
rs10096633
rs75679663
rs72999033
rs438811
rs13232120
All - MR Egger
All - Inverse variance weighted
-1
0
1
MR effect size for
cholesterol to total lipids ratio in large VLDL || id:ebi-cfb233-GCST90302024' on 'ER+ Breast cancer (Combined Oncoarray; iCO

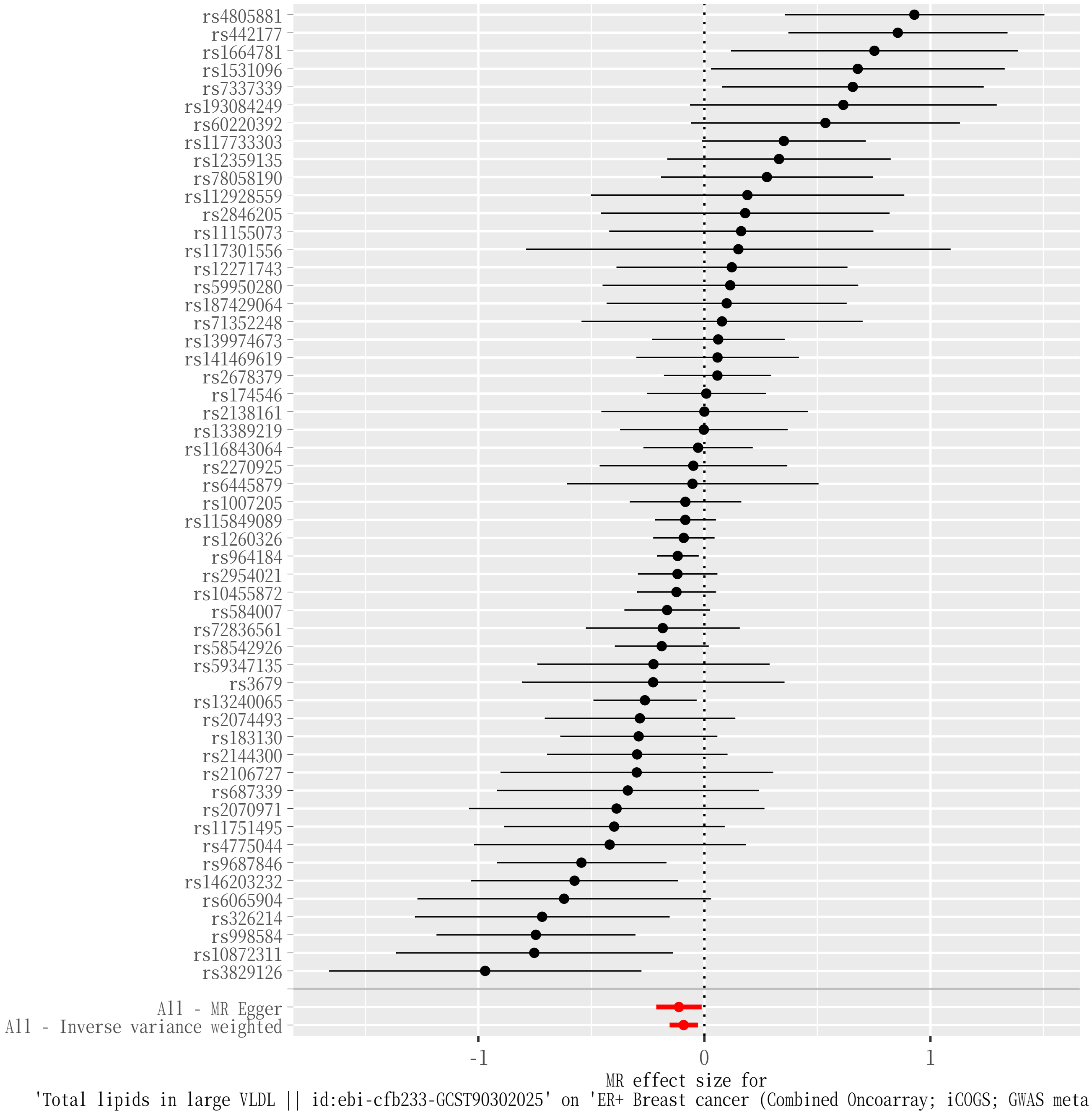

rs4805881
rs442177
rs1664781
rs1531096
rs7337339
rs193084249
rs60220392
rs117733303
rs12359135
rs78058190
rs112928559
rs2846205
rs11155073
rs117301556
rs12271743
rs59950280
rs187429064
rs71352248
rs139974673
rs141469619
rs2678379
rs174546
rs2138161
rs13389219
rs116843064
rs2270925
rs6445879
rs1007205
rs115849089
rs1260326
rs964184
rs2954021
rs10455872
rs584007
rs72836561
rs58542926
rs59347135
rs3679
rs13240065
rs2074493
rs183130
rs2144300
rs2106727
rs687339
rs2070971
rs11751495
rs4775044
rs9687846
rs146203232
rs6065904
rs326214
rs998584
rs10872311
rs3829126
All - MR Egger
All - Inverse variance weighted
-1
0
1
MR effect size for
'Total lipids in large VLDL || id:ebi-cfb233-GCST90302025' on 'ER+ Breast cancer (Combined Oncoarray; iCOGS; GWAS meta

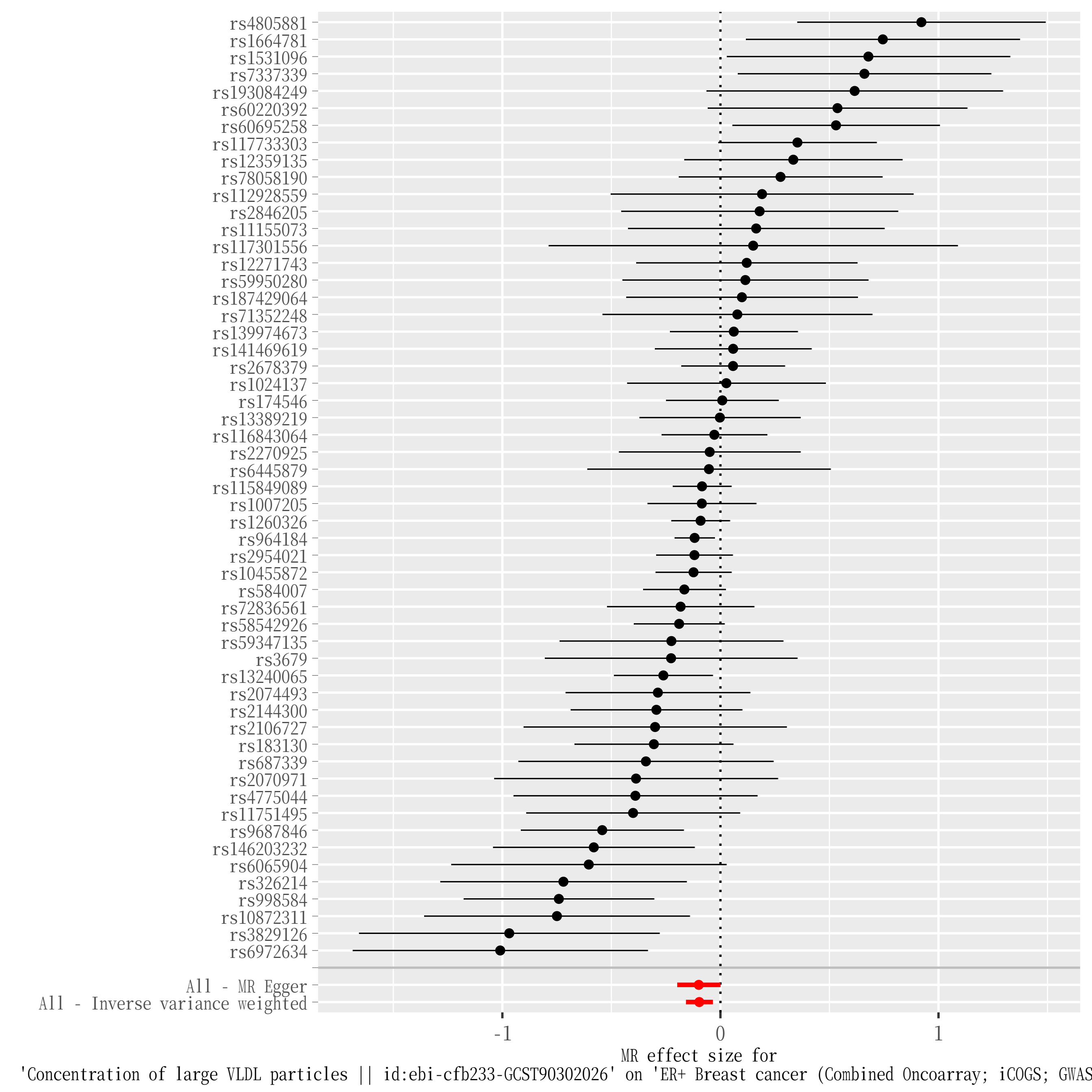
rs4805881
rs1664781
rs1531096
rs7337339
rs193084249
rs60220392
rs60695258
rs117733303
rs12359135
rs78058190
rs112928559
rs2846205
rs11155073
rs117301556
rs12271743
rs59950280
rs187429064
rs71352248
rs139974673
rs141469619
rs2678379
rs1024137
rs174546
rs13389219
rs116843064
rs2270925
rs6445879
rs115849089
rs1007205
rs1260326
rs964184
rs2954021
rs10455872
rs584007
rs72836561
rs58542926
rs59347135
rs3679
rs13240065
rs2074493
rs2144300
rs2106727
rs183130
rs687339
rs2070971
rs4775044
rs11751495
rs9687846
rs146203232
rs6065904
rs326214
rs998584
rs10872311
rs3829126
rs6972634
All - MR Egger
All - Inverse variance weighted
-1
0
1
MR effect size for
'Concentration of large VLDL particles || id:ebi-cfb233-GCST90302026' on 'ER+ Breast cancer (Combined Oncoarray; iCOGS; GWAS

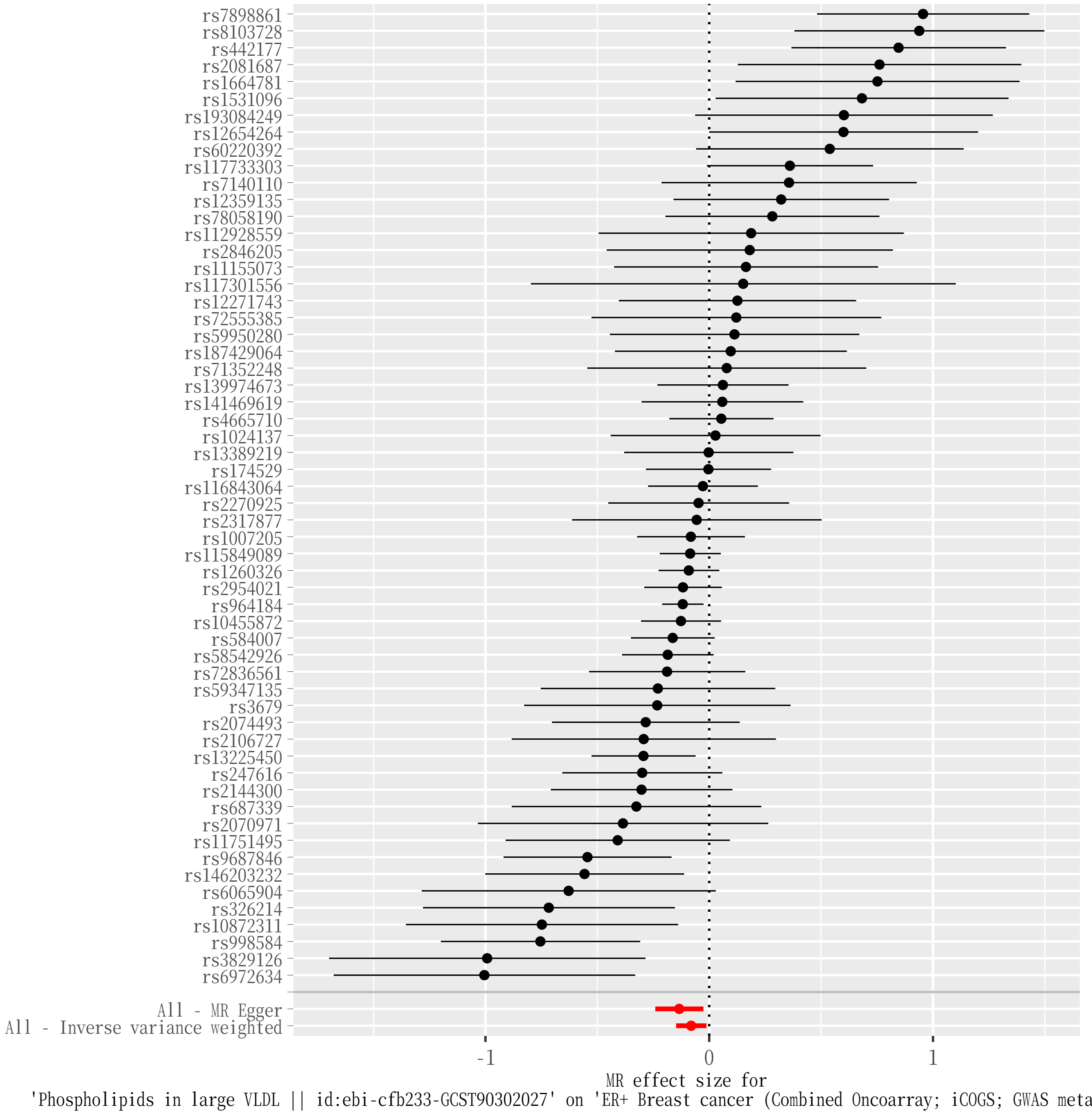

rs7898861
rs8103728
rs442177
rs2081687
rs1664781
rs1531096
rs193084249
rs12654264
rs60220392
rs117733303
rs7140110
rs12359135
rs78058190
rs112928559
rs2846205
rs11155073
rs117301556
rs12271743
rs72555385
rs59950280
rs187429064
rs71352248
rs139974673
rs141469619
rs4665710
rs1024137
rs13389219
rs174529
rs116843064
rs2270925
rs2317877
rs1007205
rs115849089
rs1260326
rs2954021
rs964184
rs10455872
rs584007
rs58542926
rs72836561
rs59347135
rs3679
rs2074493
rs2106727
rs13225450
rs247616
rs2144300
rs687339
rs2070971
rs11751495
rs9687846
rs146203232
rs6065904
rs326214
rs10872311
rs998584
rs3829126
rs6972634
All - MR Egger
All - Inverse variance weighted
-1
0
1
MR effect size for
'Phospholipids in large VLDL || id:ebi-cfb233-GCST90302027' on 'ER+ Breast cancer (Combined Oncoarray; iCOGS; GWAS meta

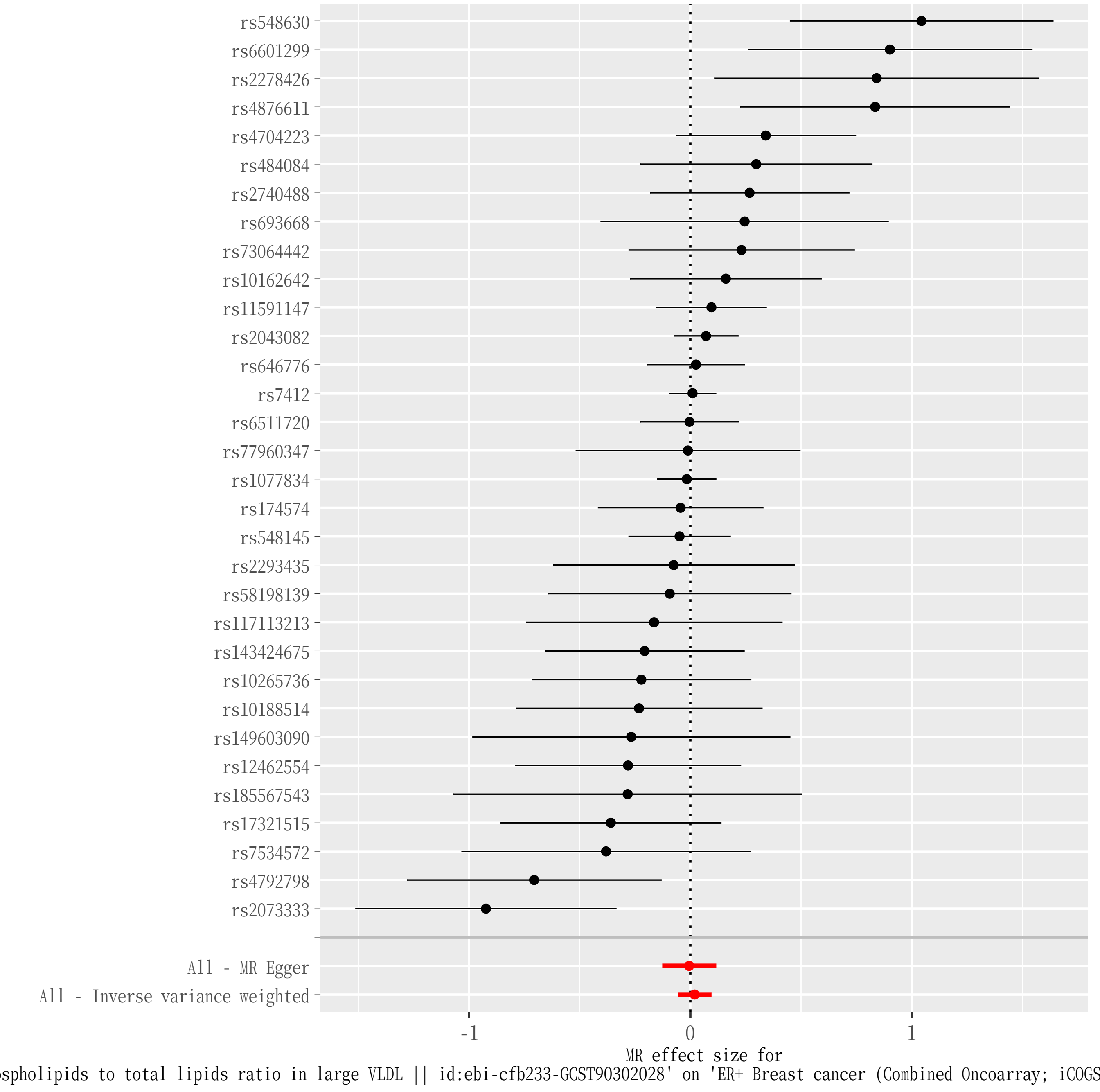

rs548630
rs6601299
rs2278426
rs4876611
rs4704223
rs484084
rs2740488
rs693668
rs73064442
rs10162642
rs11591147
rs2043082
rs646776
rs7412
rs6511720
rs77960347
rs1077834
rs174574
rs548145
rs2293435
rs58198139
rs117113213
rs143424675
rs10265736
rs10188514
rs149603090
rs12462554
rs185567543
rs17321515
rs7534572
rs4792798
rs2073333
All - MR Egger
All - Inverse variance weighted
-1
0
1
MR effect size for
ospholipids to total lipids ratio in large VLDL || id:ebi-cfb233-GCST90302028' on 'ER+ Breast cancer (Combined Oncoarray; iCOGS

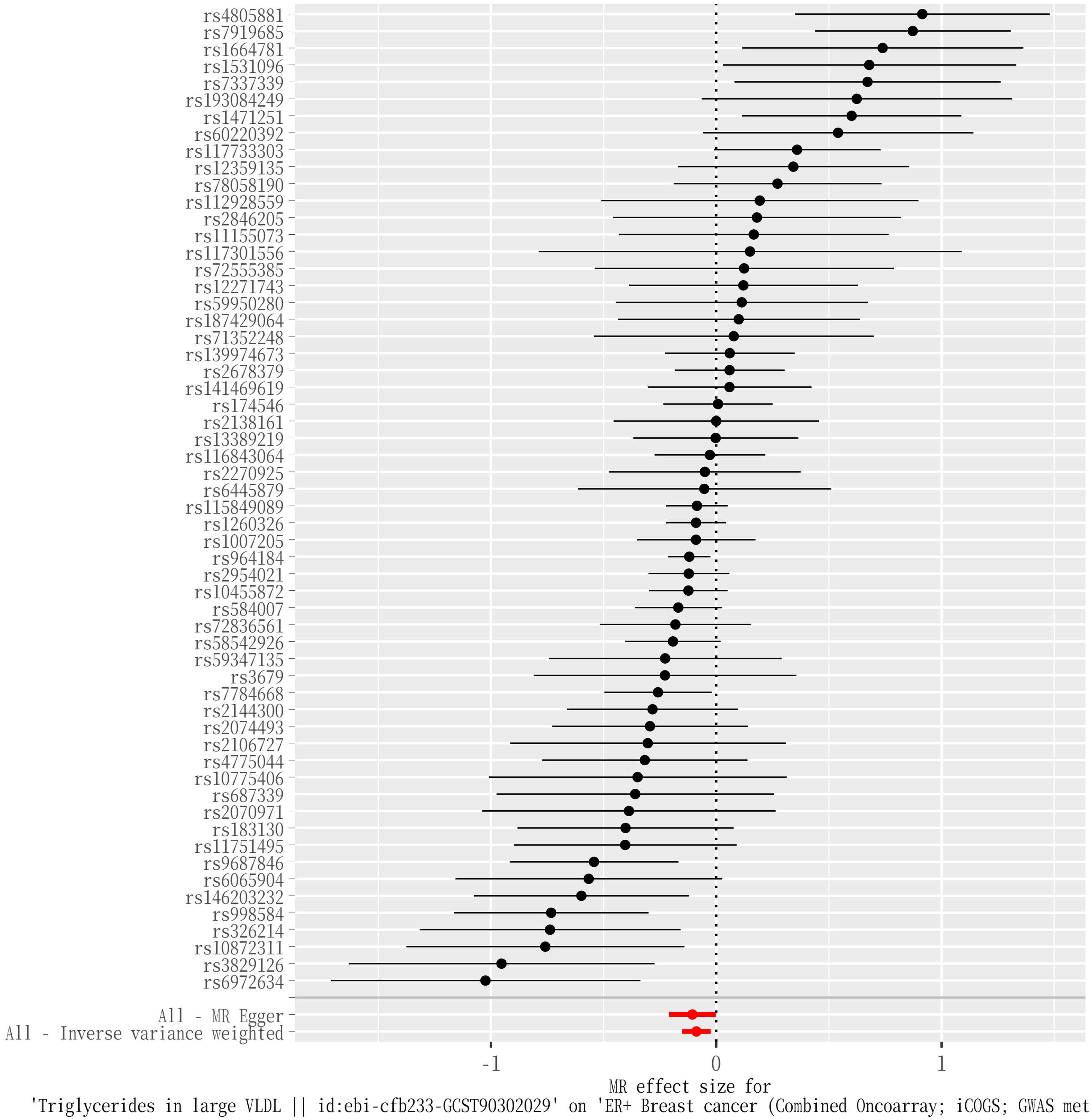
rs4805881
rs7919685
rs1664781
rs1531096
rs7337339
rs193084249
rs1471251
rs60220392
rs117733303
rs12359135
rs78058190
rs112928559
rs2846205
rs11155073
rs117301556
rs72555385
rs12271743
rs59950280
rs187429064
rs71352248
rs139974673
rs2678379
rs141469619
rs174546
rs2138161
rs13389219
rs116843064
rs2270925
rs6445879
rs115849089
rs1260326
rs1007205
rs964184
rs2954021
rs10455872
rs584007
rs72836561
rs58542926
rs59347135
rs3679
rs7784668
rs2144300
rs2074493
rs2106727
rs4775044
rs10775406
rs687339
rs2070971
rs183130
rs11751495
rs9687846
rs6065904
rs146203232
rs998584
rs326214
rs10872311
rs3829126
rs6972634
All - MR Egger
All - Inverse variance weighted
-1
0
1
MR effect size for
'Triglycerides in large VLDL || id:ebi-cfb233-GCST90302029' on 'ER+ Breast cancer (Combined Oncoarray; iCOGS; GWAS meta

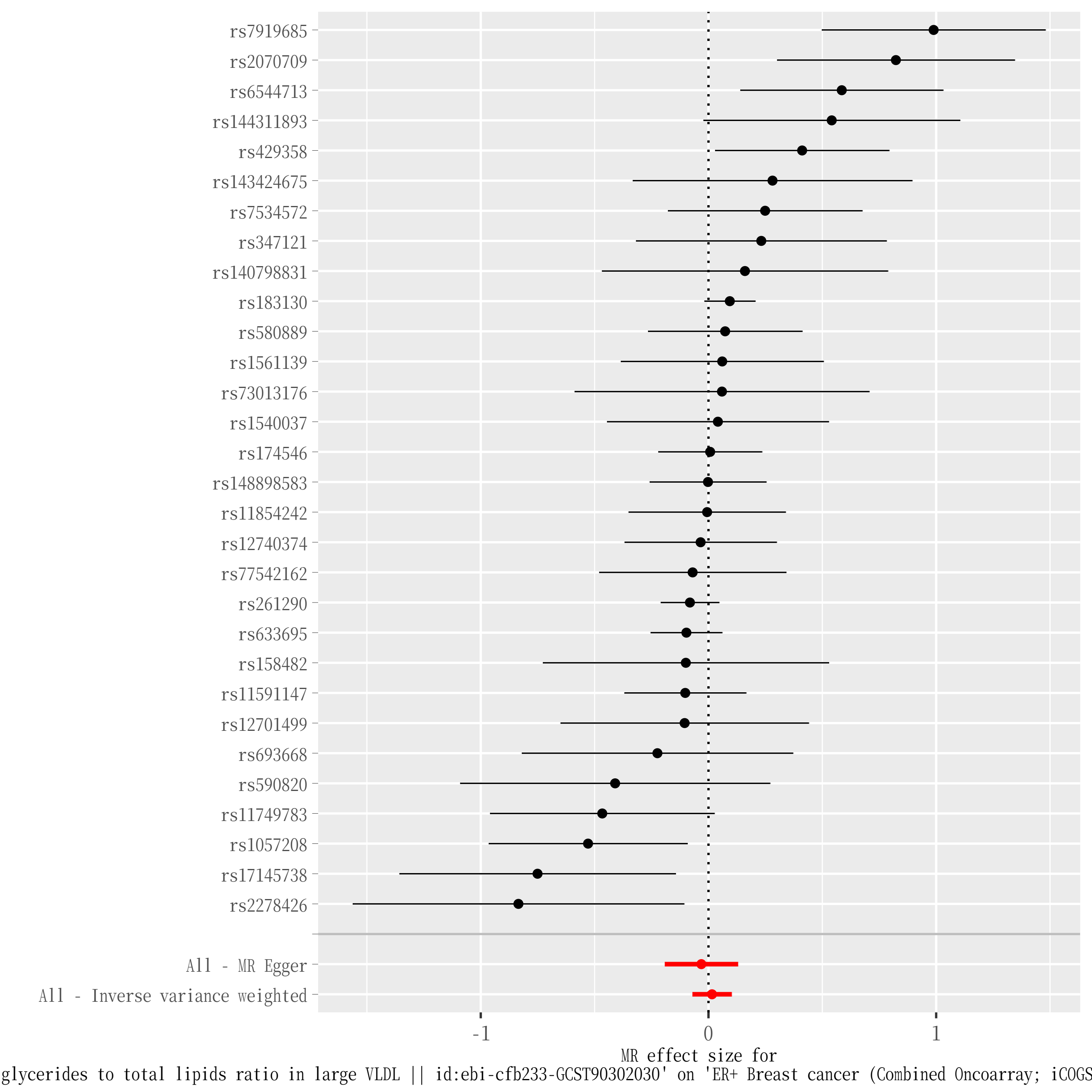
rs7919685
rs2070709
rs6544713
rs144311893
rs429358
rs143424675
rs7534572
rs347121
rs140798831
rs183130
rs580889
rs1561139
rs73013176
rs1540037
rs174546
rs148898583
rs11854242
rs12740374
rs77542162
rs261290
rs633695
rs158482
rs11591147
rs12701499
rs693668
rs590820
rs11749783
rs1057208
rs17145738
rs2278426
All - MR Egger
All - Inverse variance weighted
-1
0
1
MR effect size for
glycerides to total lipids ratio in large VLDL || id:ebi-cfb233-GCST90302030' on 'ER+ Breast cancer (Combined Oncoarray; iCOGS

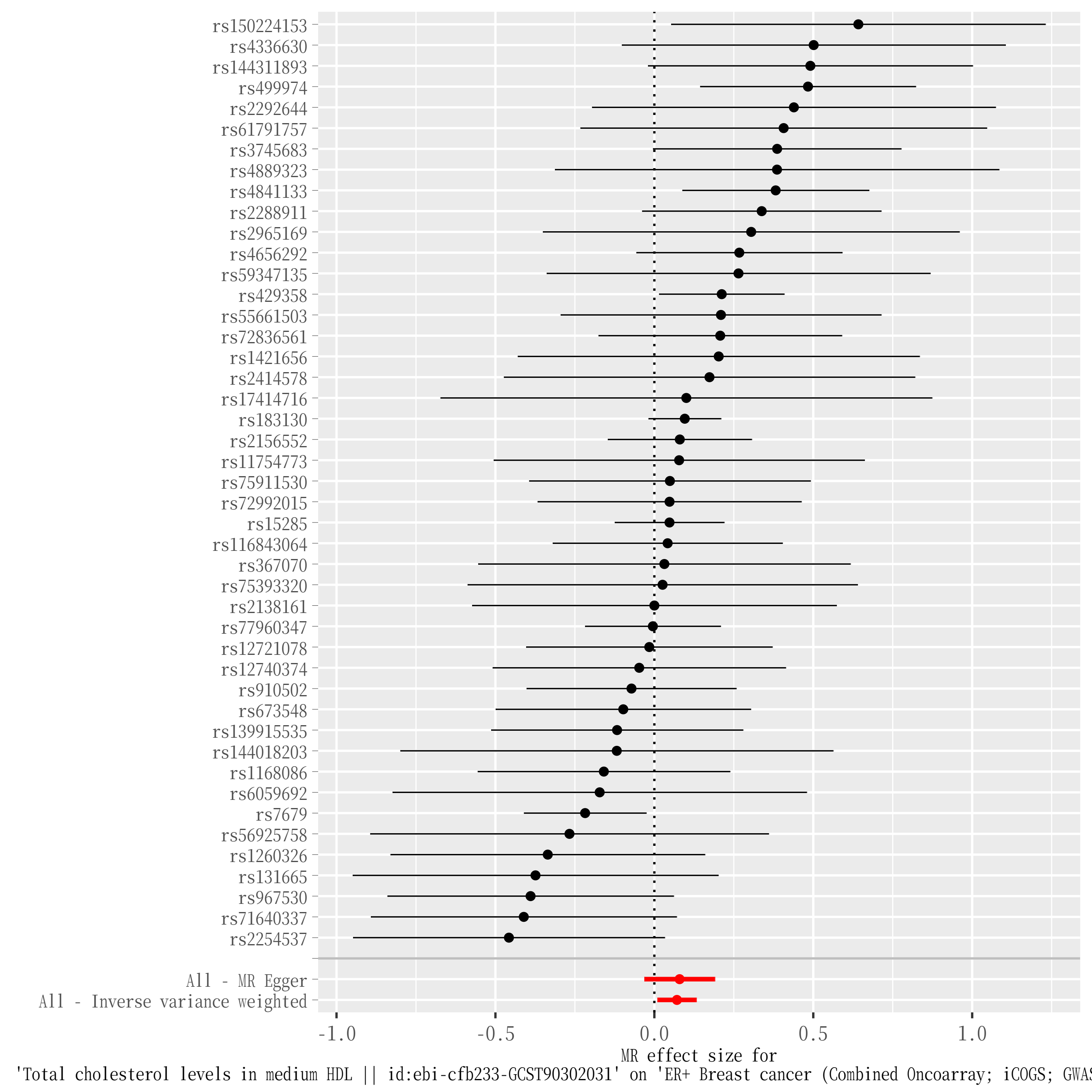
rs150224153
rs4336630
rs144311893
rs499974
rs2292644
rs61791757
rs3745683
rs4889323
rs4841133
rs2288911
rs2965169
rs4656292
rs59347135
rs429358
rs55661503
rs72836561
rs1421656
rs2414578
rs17414716
rs183130
rs2156552
rs11754773
rs75911530
rs72992015
rs15285
rs116843064
rs367070
rs75393320
rs2138161
rs77960347
rs12721078
rs12740374
rs910502
rs673548
rs139915535
rs144018203
rs1168086
rs6059692
rs7679
rs56925758
rs1260326
rs131665
rs967530
rs71640337
rs2254537
All - MR Egger
All - Inverse variance weighted
-1.0
-0.5
0.0
0.5
1.0
MR effect size for
'Total cholesterol levels in medium HDL || id:ebi-cfb233-GCST90302031' on 'ER+ Breast cancer (Combined Oncoarray; iCOGS; GWAS

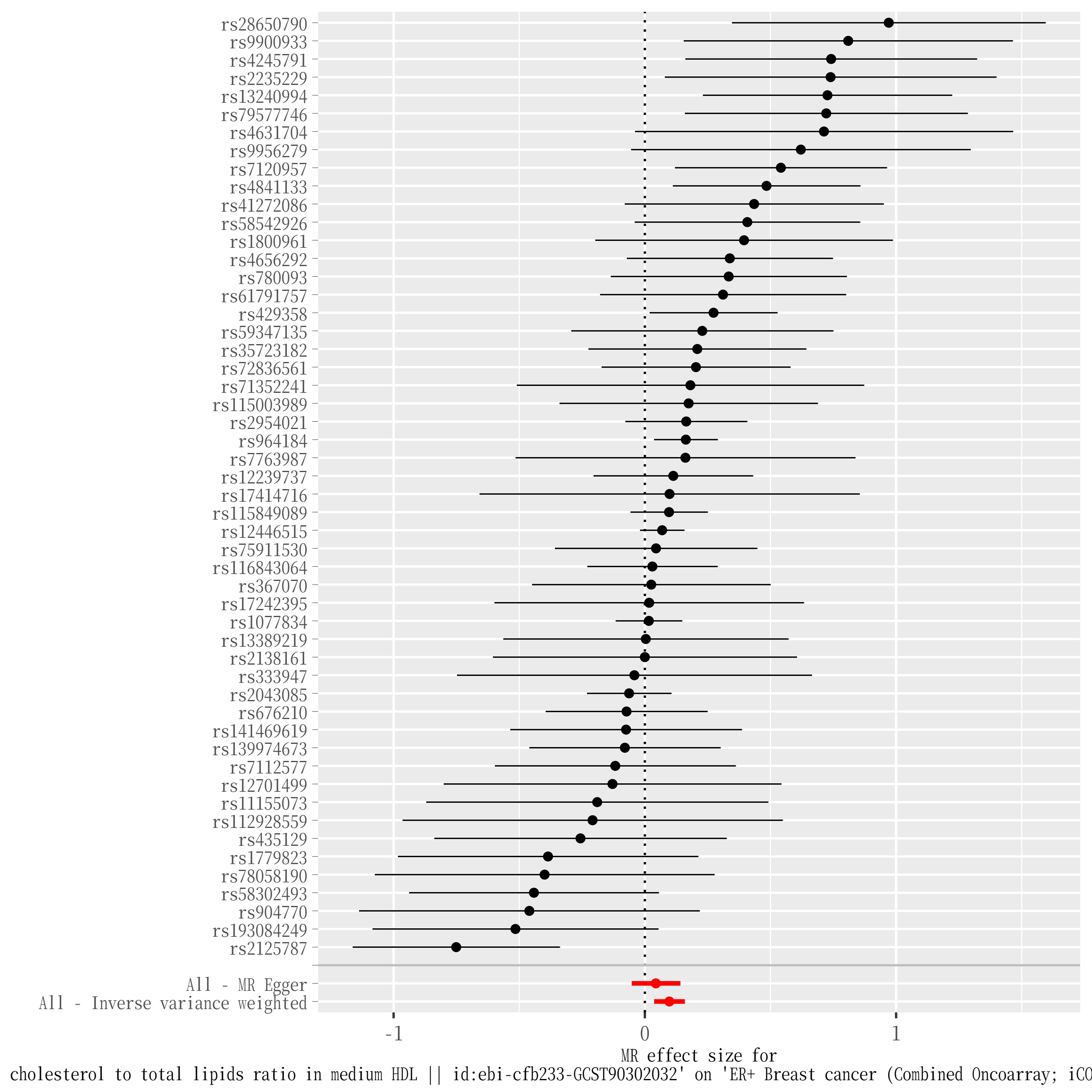

rs28650790
rs9900933
rs4245791
rs2235229
rs13240994
rs79577746
rs4631704
rs9956279
rs7120957
rs4841133
rs41272086
rs58542926
rs1800961
rs4656292
rs780093
rs61791757
rs429358
rs59347135
rs35723182
rs72836561
rs71352241
rs115003989
rs2954021
rs964184
rs7763987
rs12239737
rs17414716
rs115849089
rs12446515
rs75911530
rs116843064
rs367070
rs17242395
rs1077834
rs13389219
rs2138161
rs333947
rs2043085
rs676210
rs141469619
rs139974673
rs7112577
rs12701499
rs11155073
rs112928559
rs435129
rs1779823
rs78058190
rs58302493
rs904770
rs193084249
rs2125787
All - MR Egger
All - Inverse variance weighted
-1
0
1
MR effect size for
cholesterol to total lipids ratio in medium HDL || id:ebi-cfb233-GCST90302032' on 'ER+ Breast cancer (Combined Oncoarray; iCO

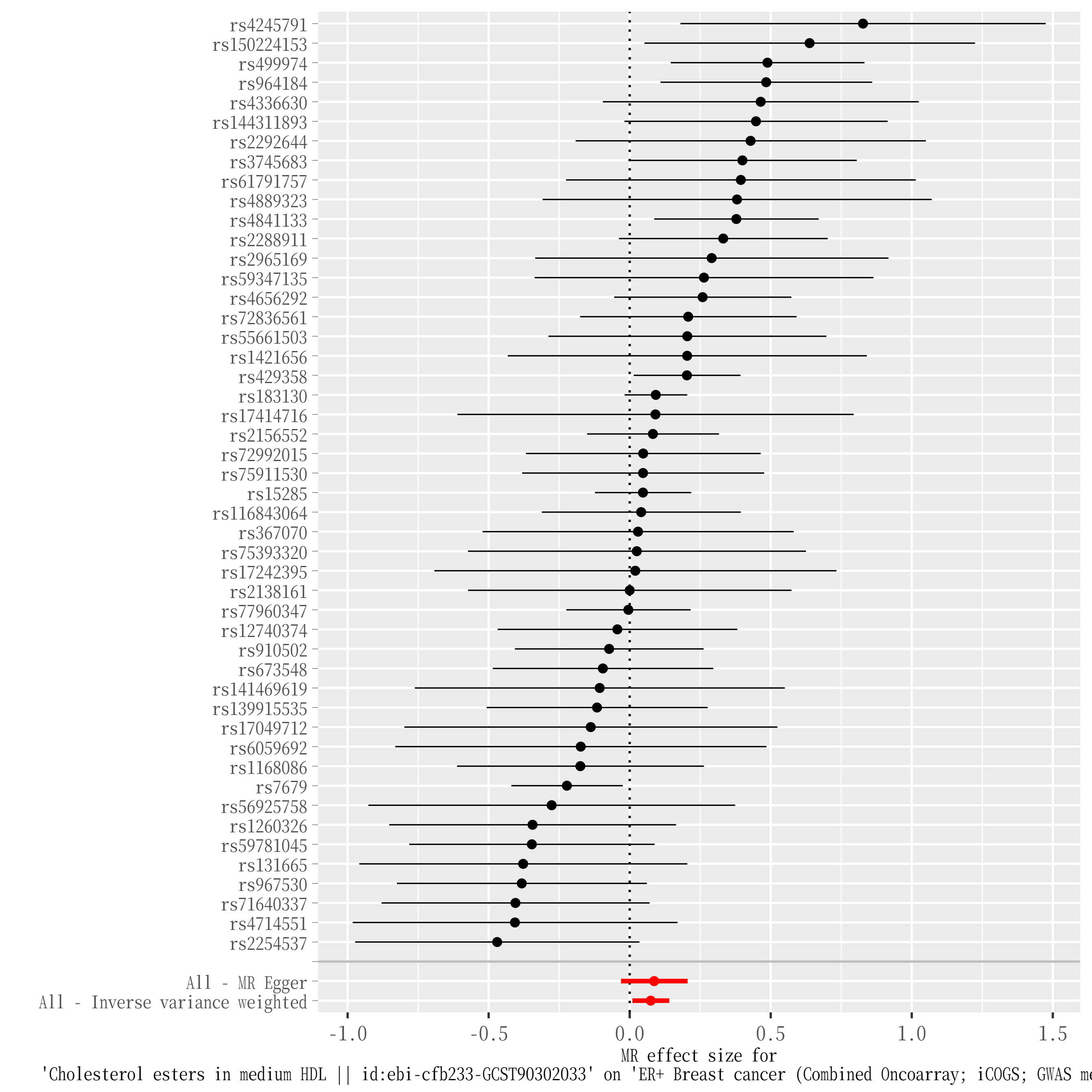

rs4245791
rs150224153
rs499974
rs964184
rs4336630
rs144311893
rs2292644
rs3745683
rs61791757
rs4889323
rs4841133
rs2288911
rs2965169
rs59347135
rs4656292
rs72836561
rs55661503
rs1421656
rs429358
rs183130
rs17414716
rs2156552
rs72992015
rs75911530
rs15285
rs116843064
rs367070
rs75393320
rs17242395
rs2138161
rs77960347
rs12740374
rs910502
rs673548
rs141469619
rs139915535
rs17049712
rs6059692
rs1168086
rs7679
rs56925758
rs1260326
rs59781045
rs131665
rs967530
rs71640337
rs4714551
rs2254537
All - MR Egger
All - Inverse variance weighted
-1.0
-0.5
0.0
0.5
1.0
1.5
MR effect size for
'Cholesterol esters in medium HDL || id:ebi-cfb233-GCST90302033' on 'ER+ Breast cancer (Combined Oncoarray; iCOGS; GWAS me

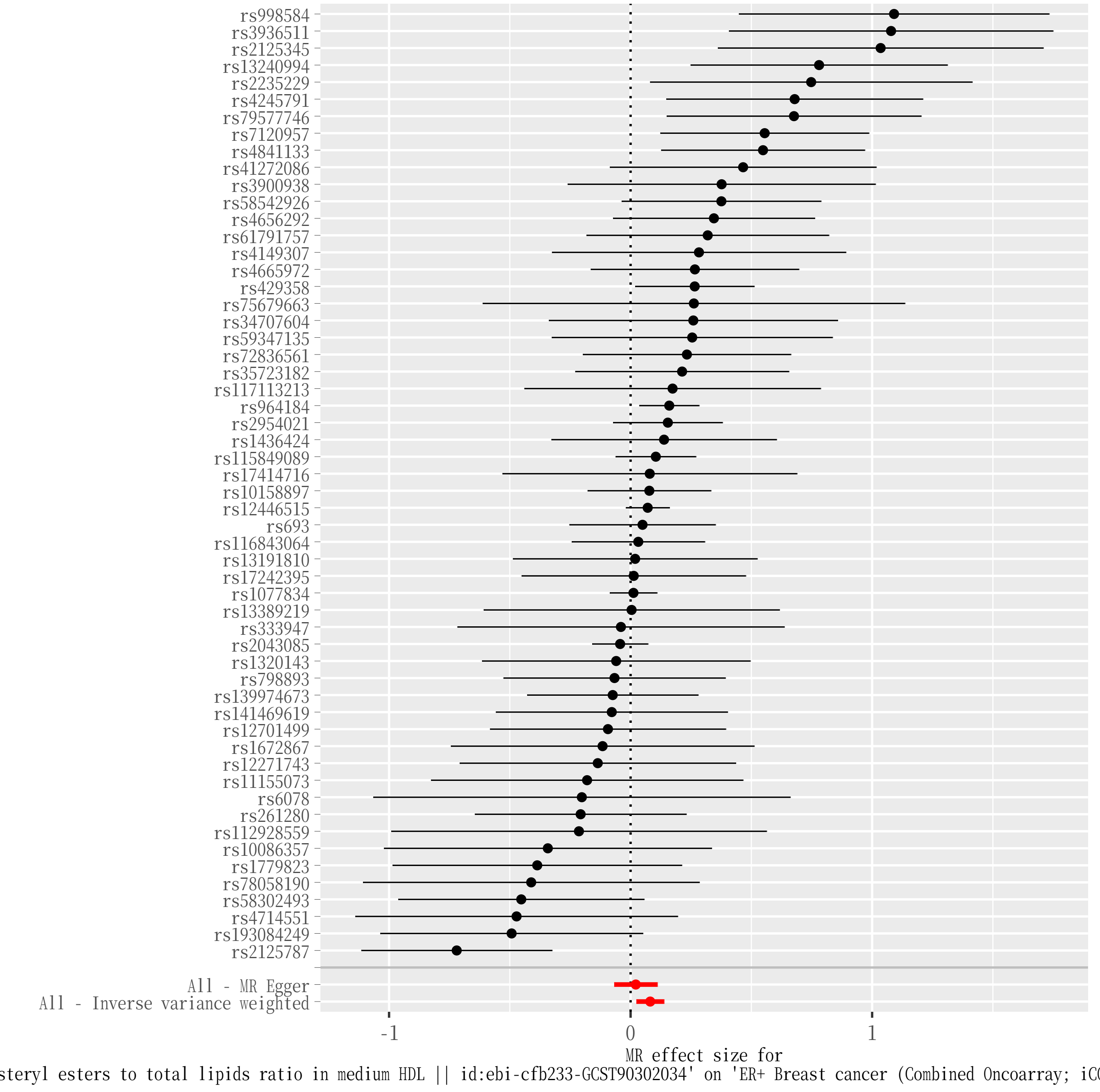

rs998584
rs3936511
rs2125345
rs13240994
rs2235229
rs4245791
rs79577746
rs7120957
rs4841133
rs41272086
rs3900938
rs58542926
rs4656292
rs61791757
rs4149307
rs4665972
rs429358
rs75679663
rs34707604
rs59347135
rs72836561
rs35723182
rs117113213
rs964184
rs2954021
rs1436424
rs115849089
rs17414716
rs10158897
rs12446515
rs693
rs116843064
rs13191810
rs17242395
rs1077834
rs13389219
rs333947
rs2043085
rs1320143
rs798893
rs139974673
rs141469619
rs12701499
rs1672867
rs12271743
rs11155073
rs6078
rs261280
rs112928559
rs10086357
rs1779823
rs78058190
rs58302493
rs4714551
rs193084249
rs2125787
All - MR Egger
All - Inverse variance weighted
-1
0
1
MR effect size for
steryl esters to total lipids ratio in medium HDL || id:ebi-cfb233-GCST90302034' on 'ER+ Breast cancer (Combined Oncoarray; iC

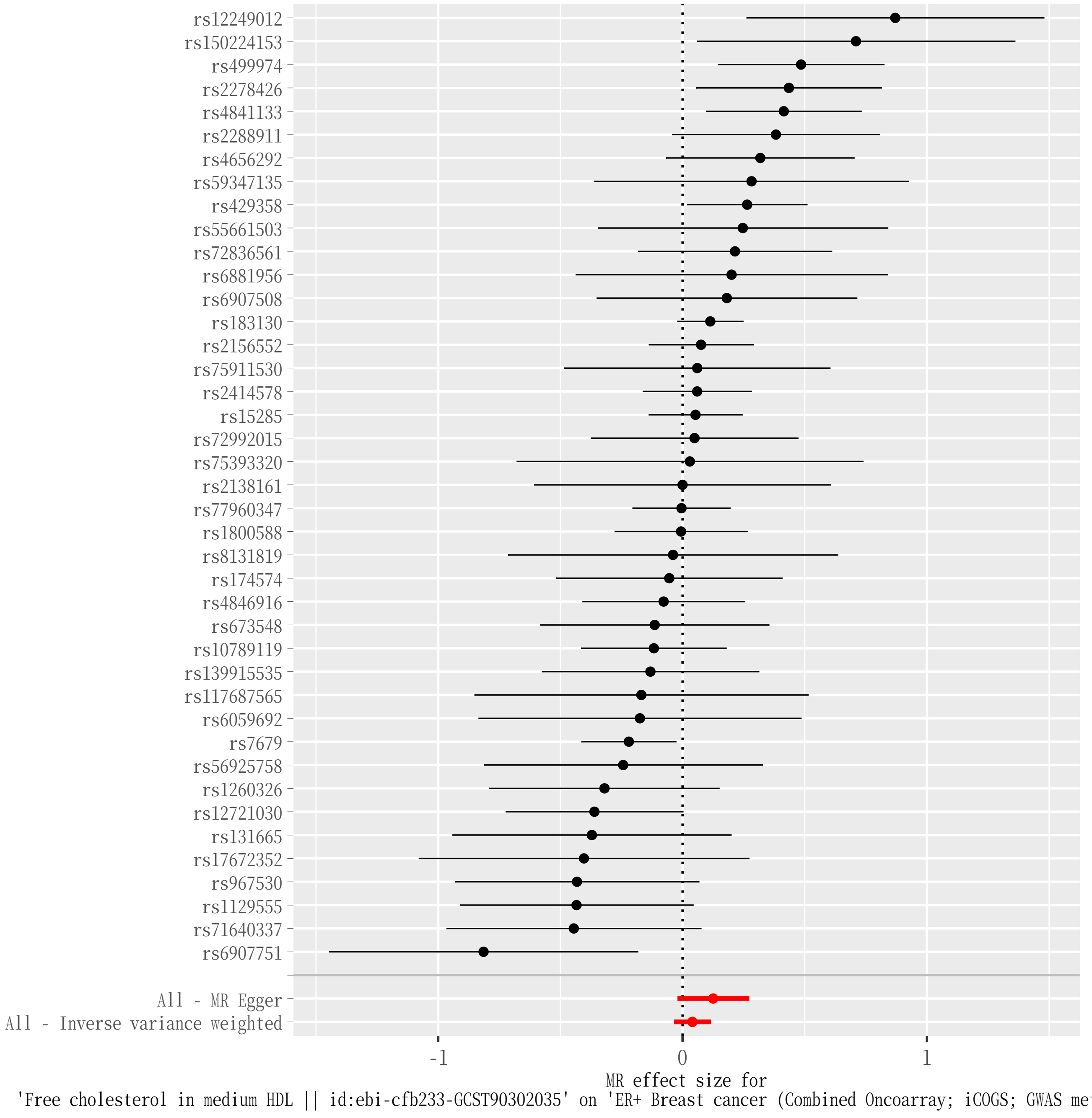

rs12249012
rs150224153
rs499974
rs2278426
rs4841133
rs2288911
rs4656292
rs59347135
rs429358
rs55661503
rs72836561
rs6881956
rs6907508
rs183130
rs2156552
rs75911530
rs2414578
rs15285
rs72992015
rs75393320
rs2138161
rs77960347
rs1800588
rs8131819
rs174574
rs4846916
rs673548
rs10789119
rs139915535
rs117687565
rs6059692
rs7679
rs56925758
rs1260326
rs12721030
rs131665
rs17672352
rs967530
rs1129555
rs71640337
rs6907751
All - MR Egger
All - Inverse variance weighted
-1
0
1
MR effect size for
'Free cholesterol in medium HDL || id:ebi-cfb233-GCST90302035' on 'ER+ Breast cancer (Combined Oncoarray; iCOGS; GWAS me

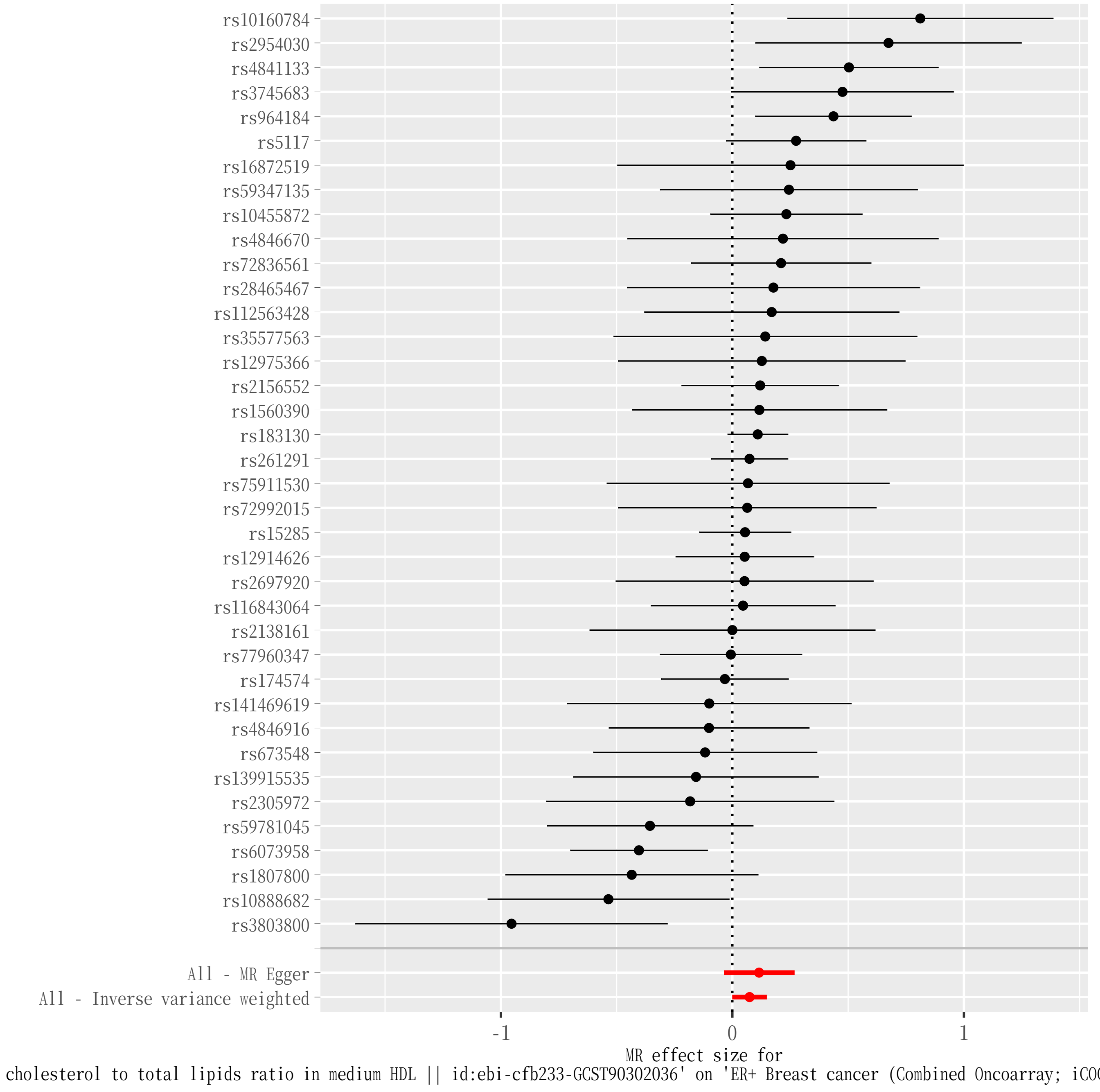
rs10160784
rs2954030
rs4841133
rs3745683
rs964184
rs5117
rs16872519
rs59347135
rs10455872
rs4846670
rs72836561
rs28465467
rs112563428
rs35577563
rs12975366
rs2156552
rs1560390
rs183130
rs261291
rs75911530
rs72992015
rs15285
rs12914626
rs2697920
rs116843064
rs2138161
rs77960347
rs174574
rs141469619
rs4846916
rs673548
rs139915535
rs2305972
rs59781045
rs6073958
rs1807800
rs10888682
rs3803800
All - MR Egger
All - Inverse variance weighted
-1
0
1
MR effect size for
cholesterol to total lipids ratio in medium HDL || id:ebi-cfb233-GCST90302036' on 'ER+ Breast cancer (Combined Oncoarray; iCO

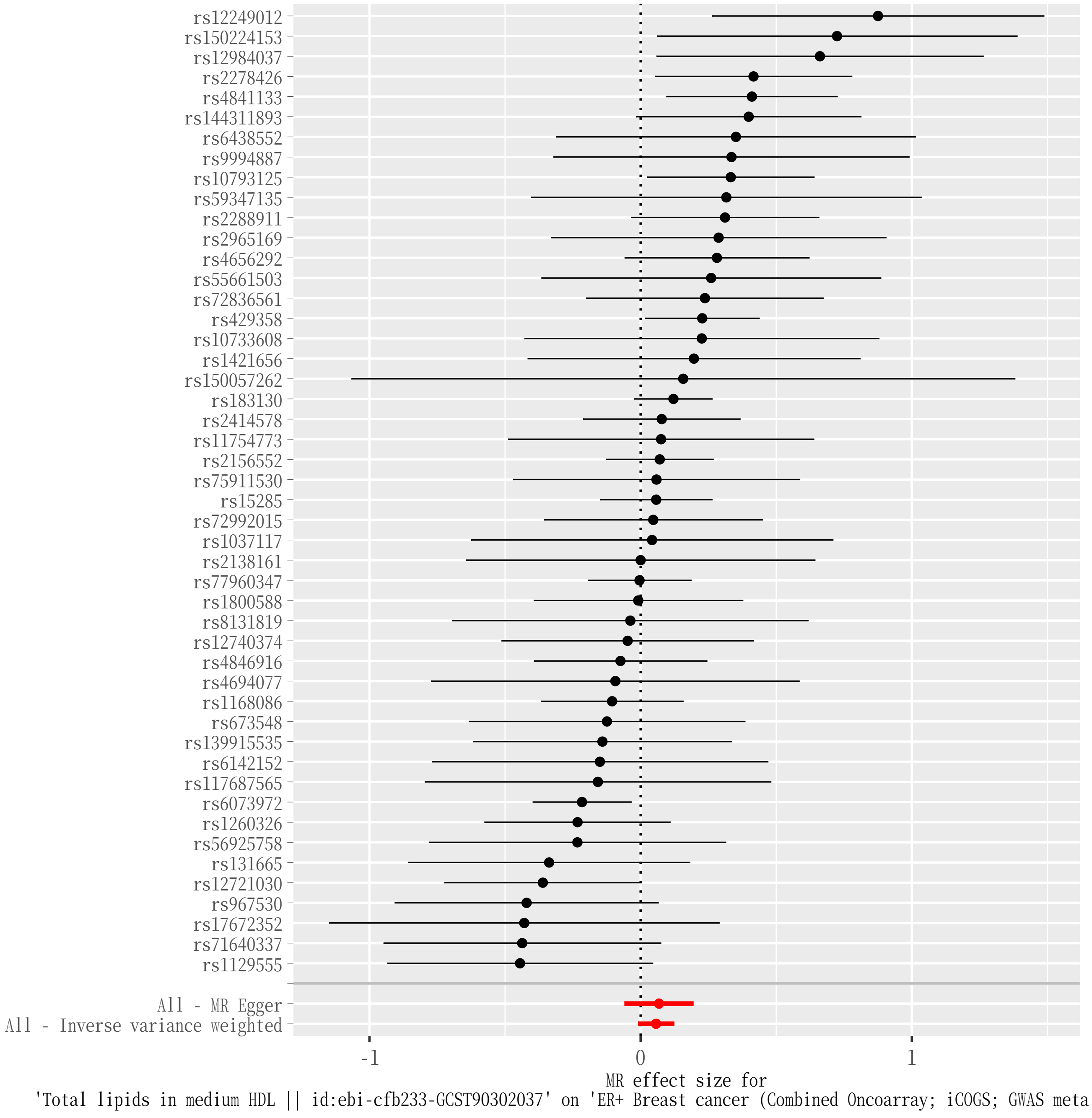

rs12249012
rs150224153
rs12984037
rs2278426
rs4841133
rs144311893
rs6438552
rs9994887
rs10793125
rs59347135
rs2288911
rs2965169
rs4656292
rs55661503
rs72836561
rs429358
rs10733608
rs1421656
rs150057262
rs183130
rs2414578
rs11754773
rs2156552
rs75911530
rs15285
rs72992015
rs1037117
rs2138161
rs77960347
rs1800588
rs8131819
rs12740374
rs4846916
rs4694077
rs1168086
rs673548
rs139915535
rs6142152
rs117687565
rs6073972
rs1260326
rs56925758
rs131665
rs12721030
rs967530
rs17672352
rs71640337
rs1129555
All - MR Egger
All - Inverse variance weighted
-1
0
1
MR effect size for
'Total lipids in medium HDL || id:ebi-cfb233-GCST90302037' on 'ER+ Breast cancer (Combined Oncoarray; iCOGS; GWAS meta

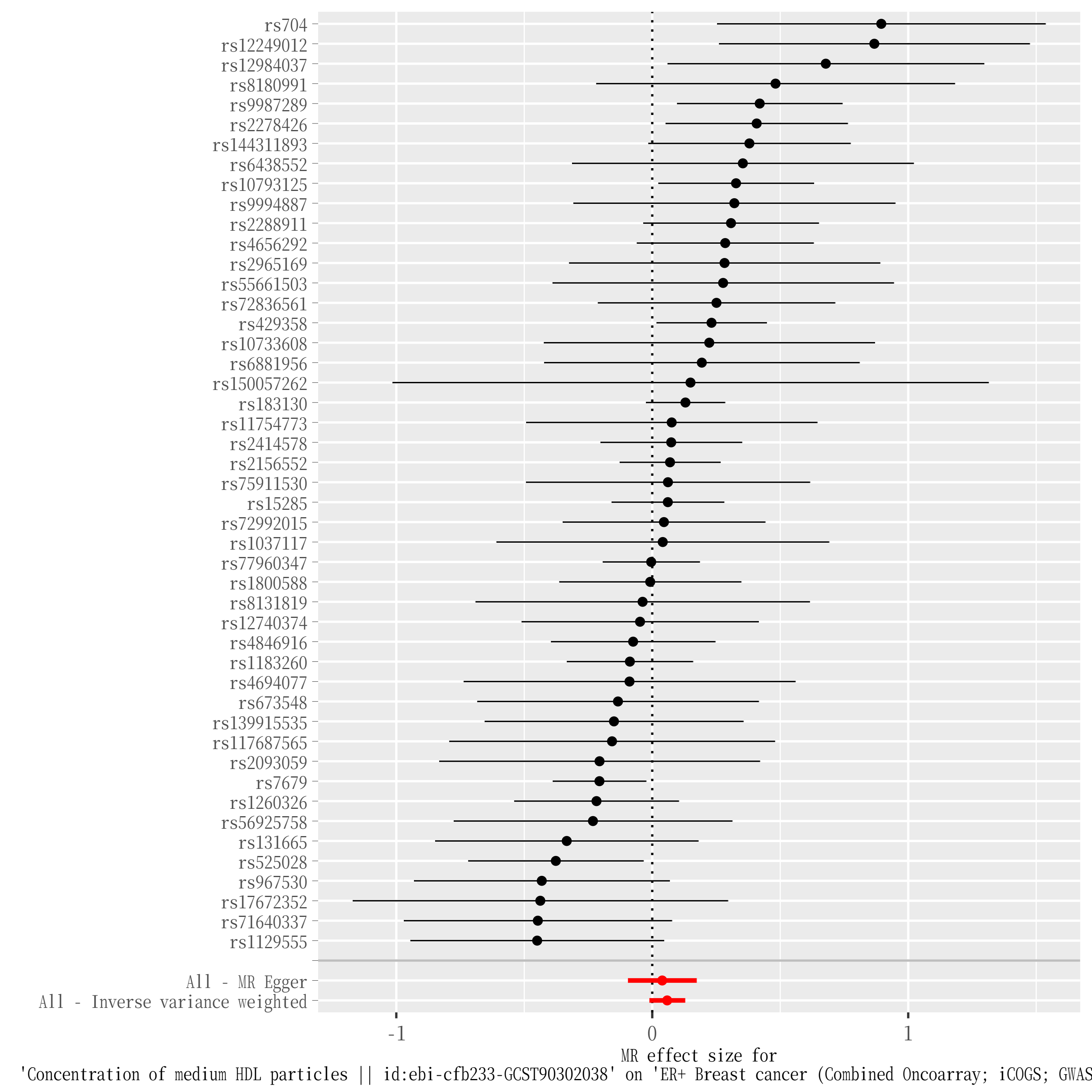
rs704
rs12249012
rs12984037
rs8180991
rs9987289
rs2278426
rs144311893
rs6438552
rs10793125
rs9994887
rs2288911
rs4656292
rs2965169
rs55661503
rs72836561
rs429358
rs10733608
rs6881956
rs150057262
rs183130
rs11754773
rs2414578
rs2156552
rs75911530
rs15285
rs72992015
rs1037117
rs77960347
rs1800588
rs8131819
rs12740374
rs4846916
rs1183260
rs4694077
rs673548
rs139915535
rs117687565
rs2093059
rs7679
rs1260326
rs56925758
rs131665
rs525028
rs967530
rs17672352
rs71640337
rs1129555
All - MR Egger
All - Inverse variance weighted
-1
0
1
MR effect size for
'Concentration of medium HDL particles || id:ebi-cfb233-GCST90302038' on 'ER+ Breast cancer (Combined Oncoarray; iCOGS; GWAS

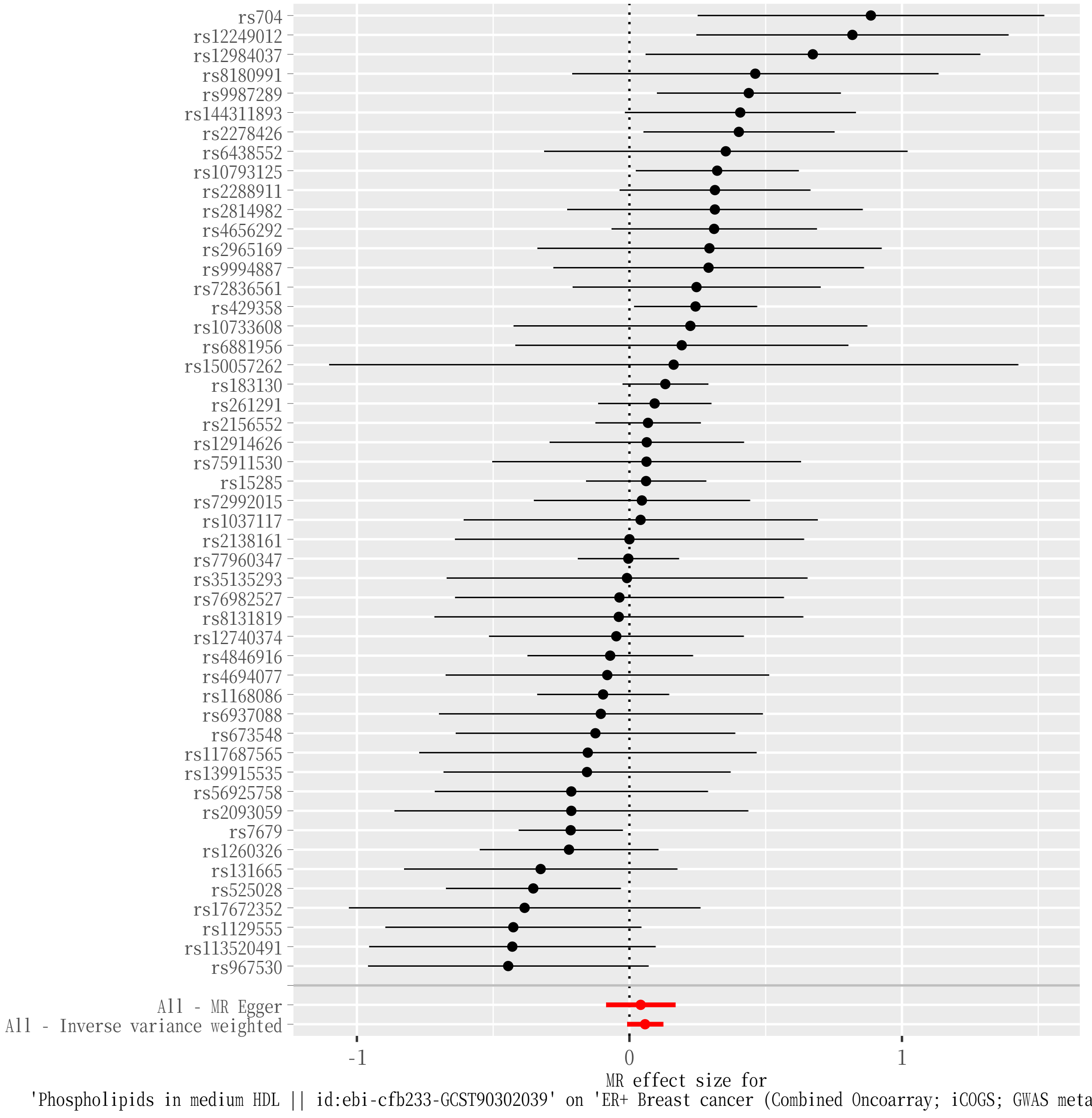

rs704
rs12249012
rs12984037
rs8180991
rs9987289
rs144311893
rs2278426
rs6438552
rs10793125
rs2288911
rs2814982
rs4656292
rs2965169
rs9994887
rs72836561
rs429358
rs10733608
rs6881956
rs150057262
rs183130
rs261291
rs2156552
rs12914626
rs75911530
rs15285
rs72992015
rs1037117
rs2138161
rs77960347
rs35135293
rs76982527
rs8131819
rs12740374
rs4846916
rs4694077
rs1168086
rs6937088
rs673548
rs117687565
rs139915535
rs56925758
rs2093059
rs7679
rs1260326
rs131665
rs525028
rs17672352
rs1129555
rs113520491
rs967530
All - MR Egger
All - Inverse variance weighted
-1
0
1
MR effect size for
'Phospholipids in medium HDL || id:ebi-cfb233-GCST90302039' on 'ER+ Breast cancer (Combined Oncoarray; iCOGS; GWAS meta

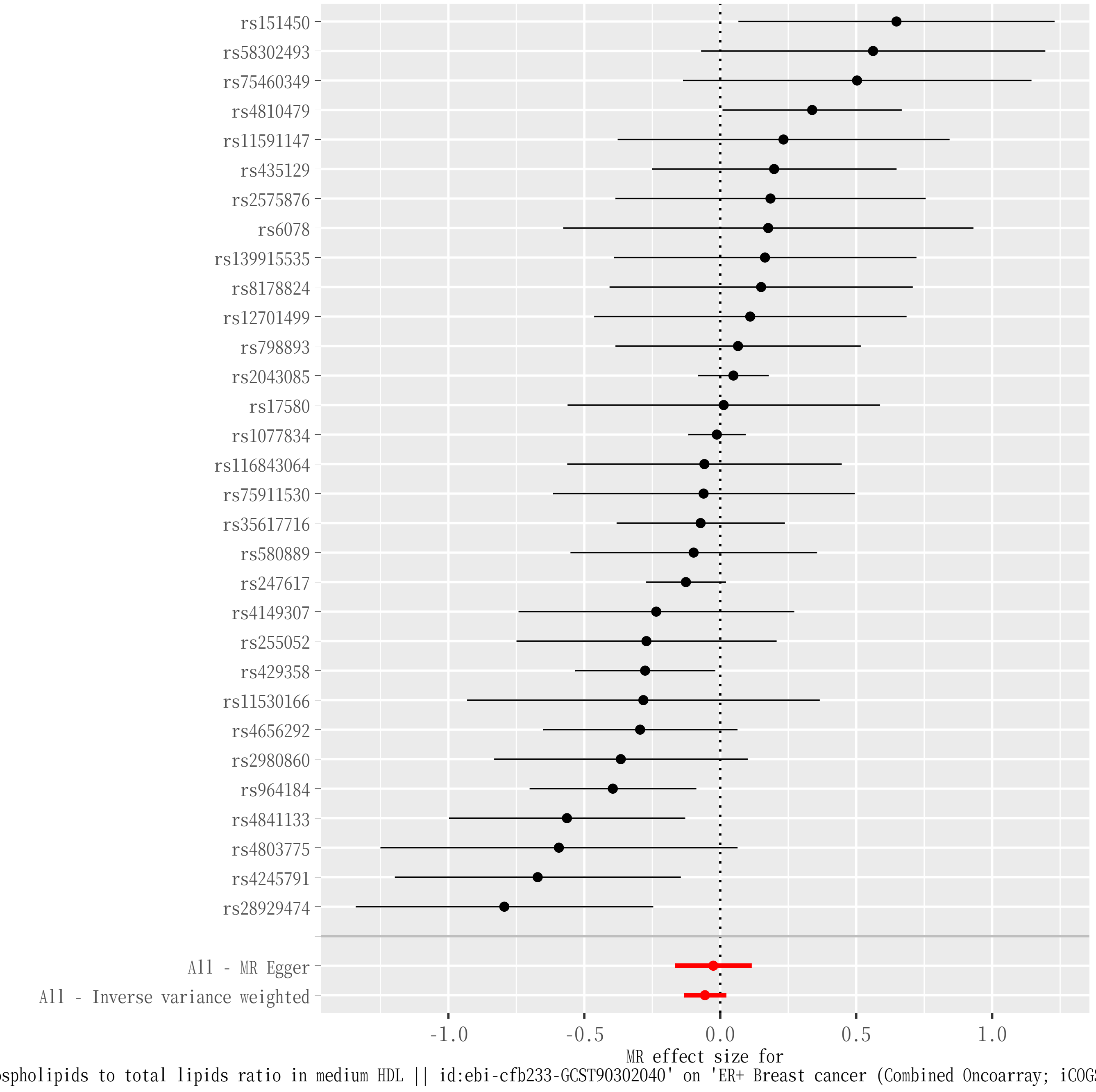

rs151450
rs58302493
rs75460349
rs4810479
rs11591147
rs435129
rs2575876
rs6078
rs139915535
rs8178824
rs12701499
rs798893
rs2043085
rs17580
rs1077834
rs116843064
rs75911530
rs35617716
rs580889
rs247617
rs4149307
rs255052
rs429358
rs11530166
rs4656292
rs2980860
rs964184
rs4841133
rs4803775
rs4245791
rs28929474
All - MR Egger
All - Inverse variance weighted
-1.0
-0.5
0.0
0.5
1.0
MR effect size for
ospholipids to total lipids ratio in medium HDL || id:ebi-cfb233-GCST90302040' on 'ER+ Breast cancer (Combined Oncoarray; iCOGS

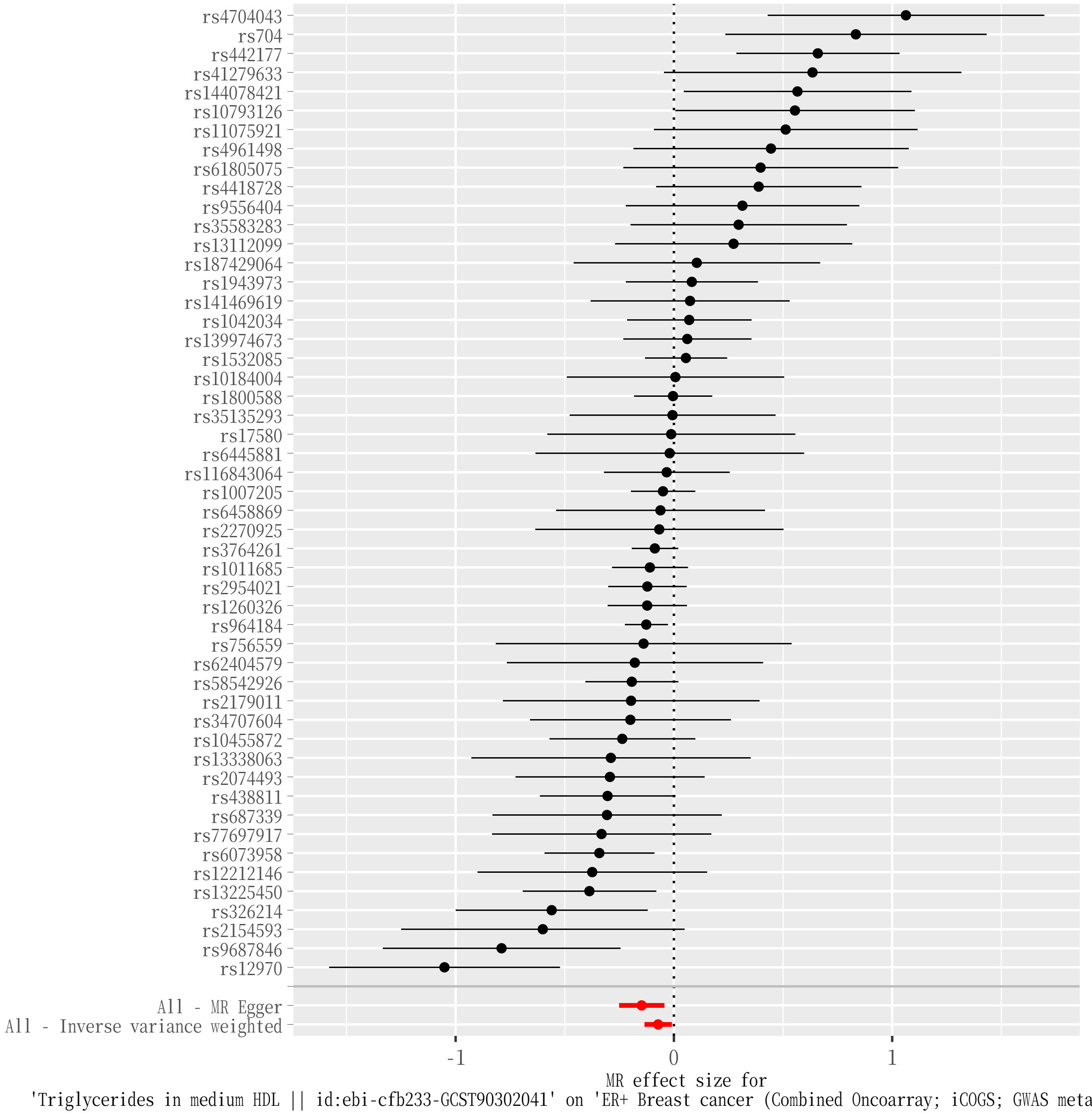
rs4704043
rs704
rs442177
rs41279633
rs144078421
rs10793126
rs11075921
rs4961498
rs61805075
rs4418728
rs9556404
rs35583283
rs13112099
rs187429064
rs1943973
rs141469619
rs1042034
rs139974673
rs1532085
rs10184004
rs1800588
rs35135293
rs17580
rs6445881
rs116843064
rs1007205
rs6458869
rs2270925
rs3764261
rs1011685
rs2954021
rs1260326
rs964184
rs756559
rs62404579
rs58542926
rs2179011
rs34707604
rs10455872
rs13338063
rs2074493
rs438811
rs687339
rs77697917
rs6073958
rs12212146
rs13225450
rs326214
rs2154593
rs9687846
rs12970
All - MR Egger
All - Inverse variance weighted
-1
0
1
MR effect size for
'Triglycerides in medium HDL || id:ebi-cfb233-GCST90302041' on 'ER+ Breast cancer (Combined Oncoarray; iCOGS; GWAS meta

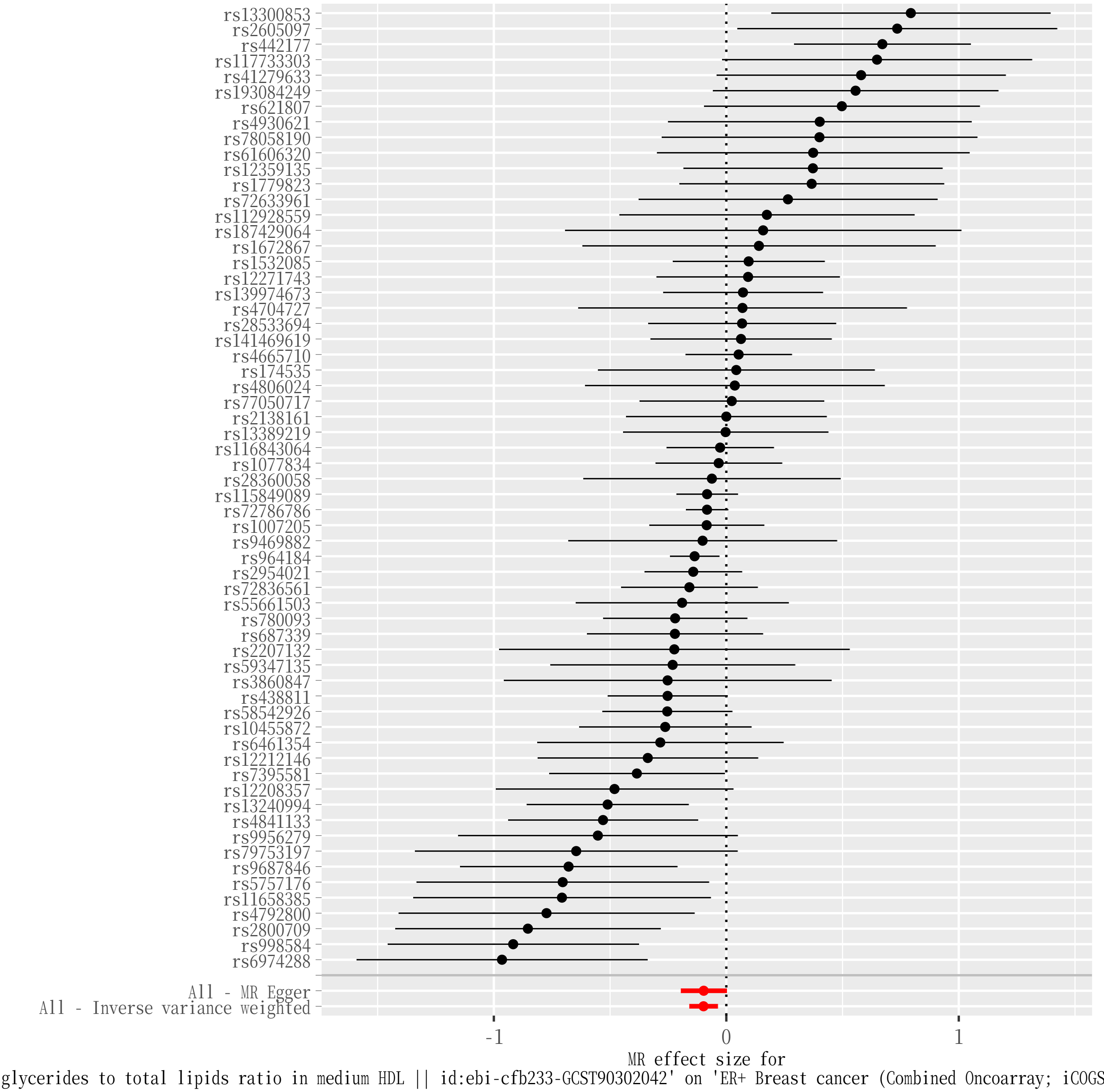

rs13300853
rs2605097
rs442177
rs117733303
rs41279633
rs193084249
rs621807
rs4930621
rs78058190
rs61606320
rs12359135
rs1779823
rs72633961
rs112928559
rs187429064
rs1672867
rs1532085
rs12271743
rs139974673
rs4704727
rs28533694
rs141469619
rs4665710
rs174535
rs4806024
rs77050717
rs2138161
rs13389219
rs116843064
rs1077834
rs28360058
rs115849089
rs72786786
rs1007205
rs9469882
rs964184
rs2954021
rs72836561
rs55661503
rs780093
rs687339
rs2207132
rs59347135
rs3860847
rs438811
rs58542926
rs10455872
rs6461354
rs12212146
rs7395581
rs12208357
rs13240994
rs4841133
rs9956279
rs79753197
rs9687846
rs5757176
rs11658385
rs4792800
rs2800709
rs998584
rs6974288
All - MR Egger
All - Inverse variance weighted
-1
0
1
MR effect size for
glycerides to total lipids ratio in medium HDL || id:ebi-cfb233-GCST90302042' on 'ER+ Breast cancer (Combined Oncoarray; iCOGS

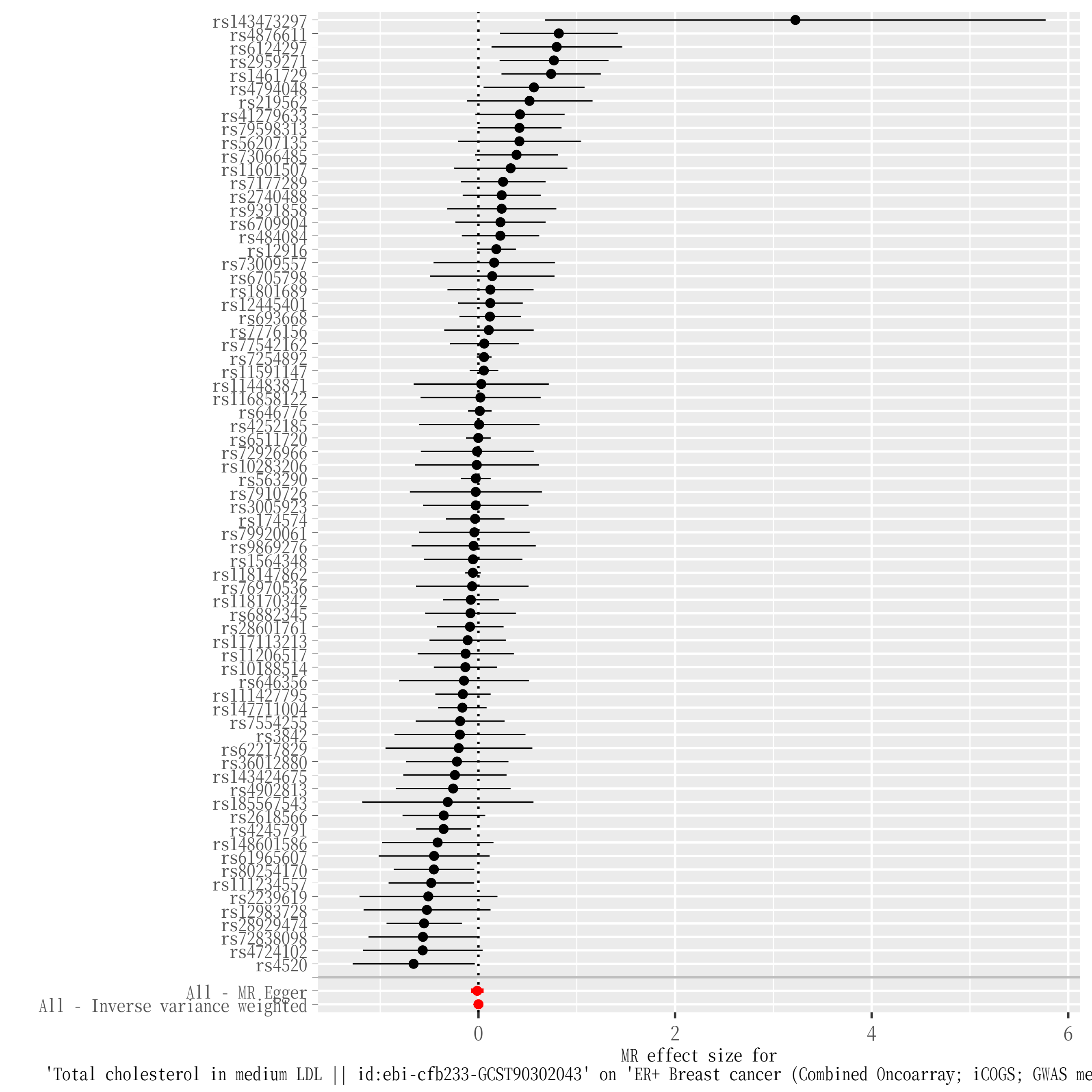
rs143473297
rs4876611
rs6124297
rs2959271
rs1461729
rs4794048
rs219562
rs41279633
rs79598313
rs56207135
rs73066485
rs11601507
rs7177289
rs2740488
rs9391858
rs6709904
rs484084
rs12916
rs73009557
rs6705798
rs1801689
rs12445401
rs693668
rs7776156
rs77542162
rs7254892
rs11591147
rs114483871
rs116858122
rs646776
rs4252185
rs6511720
rs72926966
rs10283206
rs563290
rs7910726
rs3005923
rs174574
rs79920061
rs9869276
rs1564348
rs118147862
rs76970536
rs118170342
rs6882345
rs28601761
rs117113213
rs11206517
rs10188514
rs646356
rs111427795
rs147711004
rs7554255
rs3842
rs62217829
rs36012880
rs143424675
rs4902813
rs185567543
rs2618566
rs4245791
rs148601586
rs61965607
rs80254170
rs111234557
rs2239619
rs12983728
rs28929474
rs72838098
rs4724102
rs4520
All - MR Egger
All - Inverse variance weighted
0
2
4
6
MR effect size for
'Total cholesterol in medium LDL || id:ebi-cfb233-GCST90302043' on 'ER+ Breast cancer (Combined Oncoarray; iCOGS; GWAS me

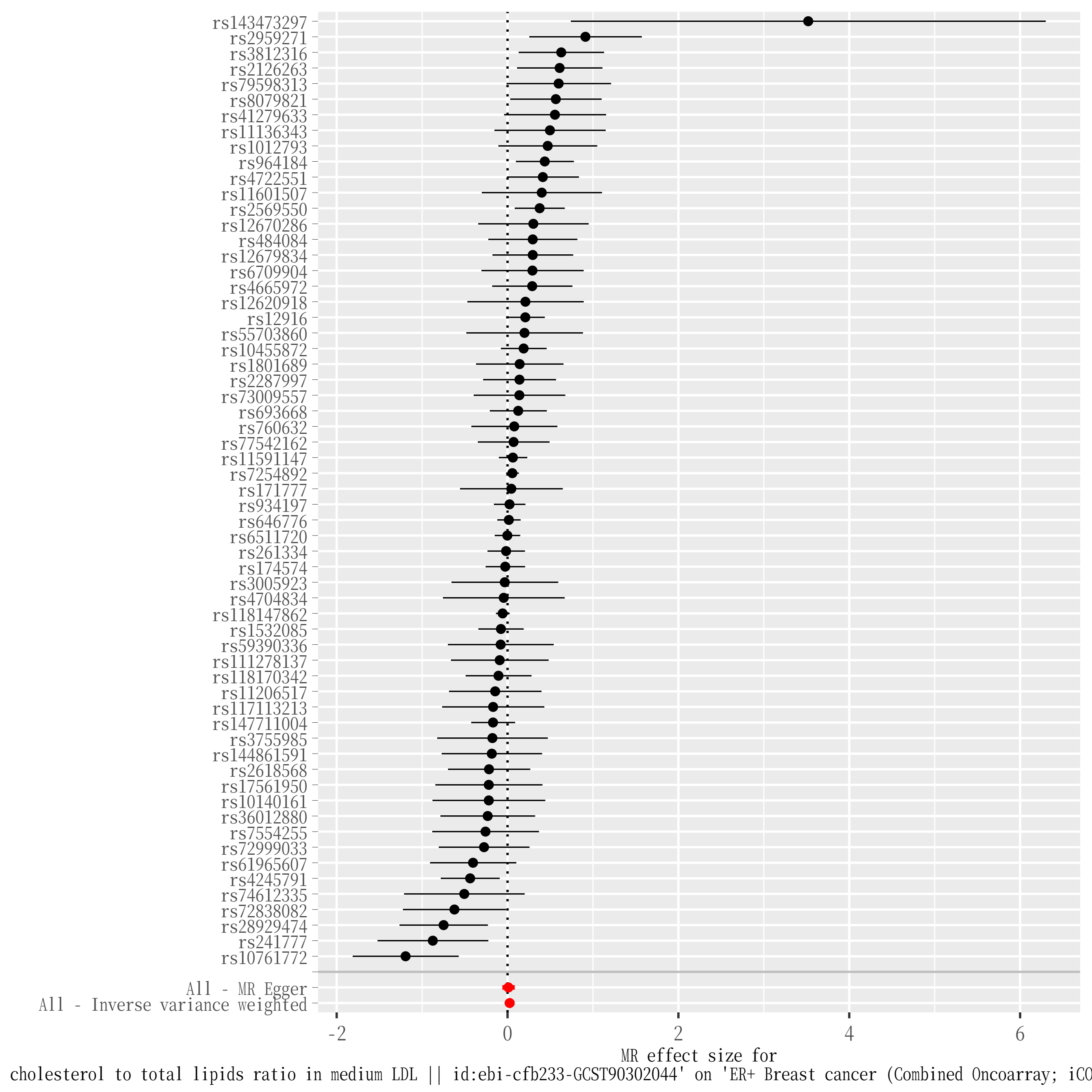

rs143473297
rs2959271
rs3812316
rs2126263
rs79598313
rs8079821
rs41279633
rs11136343
rs1012793
rs964184
rs4722551
rs11601507
rs2569550
rs12670286
rs484084
rs12679834
rs6709904
rs4665972
rs12620918
rs12916
rs55703860
rs10455872
rs1801689
rs2287997
rs73009557
rs693668
rs760632
rs77542162
rs11591147
rs7254892
rs171777
rs934197
rs646776
rs6511720
rs261334
rs174574
rs3005923
rs4704834
rs118147862
rs1532085
rs59390336
rs111278137
rs118170342
rs11206517
rs117113213
rs147711004
rs3755985
rs144861591
rs2618568
rs17561950
rs10140161
rs36012880
rs7554255
rs72999033
rs61965607
rs4245791
rs74612335
rs72838082
rs28929474
rs241777
rs10761772
All - MR Egger
All - Inverse variance weighted
-2
0
2
4
6
MR effect size for
cholesterol to total lipids ratio in medium LDL || id:ebi-cfb233-GCST90302044' on 'ER+ Breast cancer (Combined Oncoarray; iCO

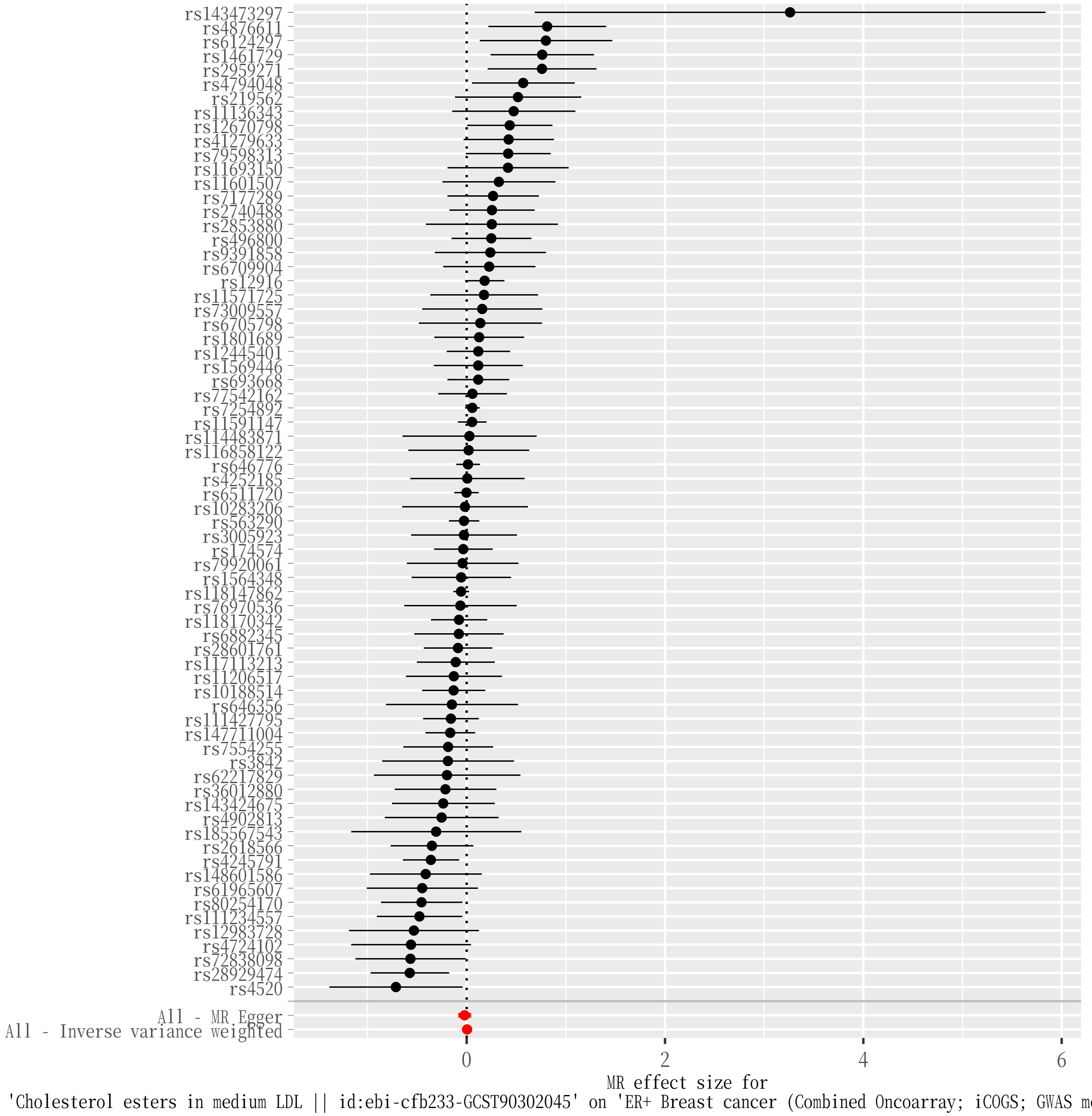

rs143473297
rs4876611
rs6124297
rs1461729
rs2959271
rs4794048
rs219562
rs11136343
rs12670798
rs41279633
rs79598313
rs11693150
rs11601507
rs7177289
rs2740488
rs2853880
rs496800
rs9391858
rs6709904
rs12916
rs11571725
rs73009557
rs6705798
rs1801689
rs12445401
rs1569446
rs693668
rs77542162
rs7254892
rs11591147
rs114483871
rs116858122
rs646776
rs4252185
rs6511720
rs10283206
rs563290
rs3005923
rs174574
rs79920061
rs1564348
rs118147862
rs76970536
rs118170342
rs6882345
rs28601761
rs117113213
rs11206517
rs10188514
rs646356
rs111427795
rs147711004
rs7554255
rs3842
rs62217829
rs36012880
rs143424675
rs4902813
rs185567543
rs2618566
rs4245791
rs148601586
rs61965607
rs80254170
rs111234557
rs12983728
rs4724102
rs72838098
rs28929474
rs4520
All - MR Egger
All - Inverse variance weighted
0
2
4
6
MR effect size for
'Cholesterol esters in medium LDL || id:ebi-cfb233-GCST90302045' on 'ER+ Breast cancer (Combined Oncoarray; iCOGS; GWAS me

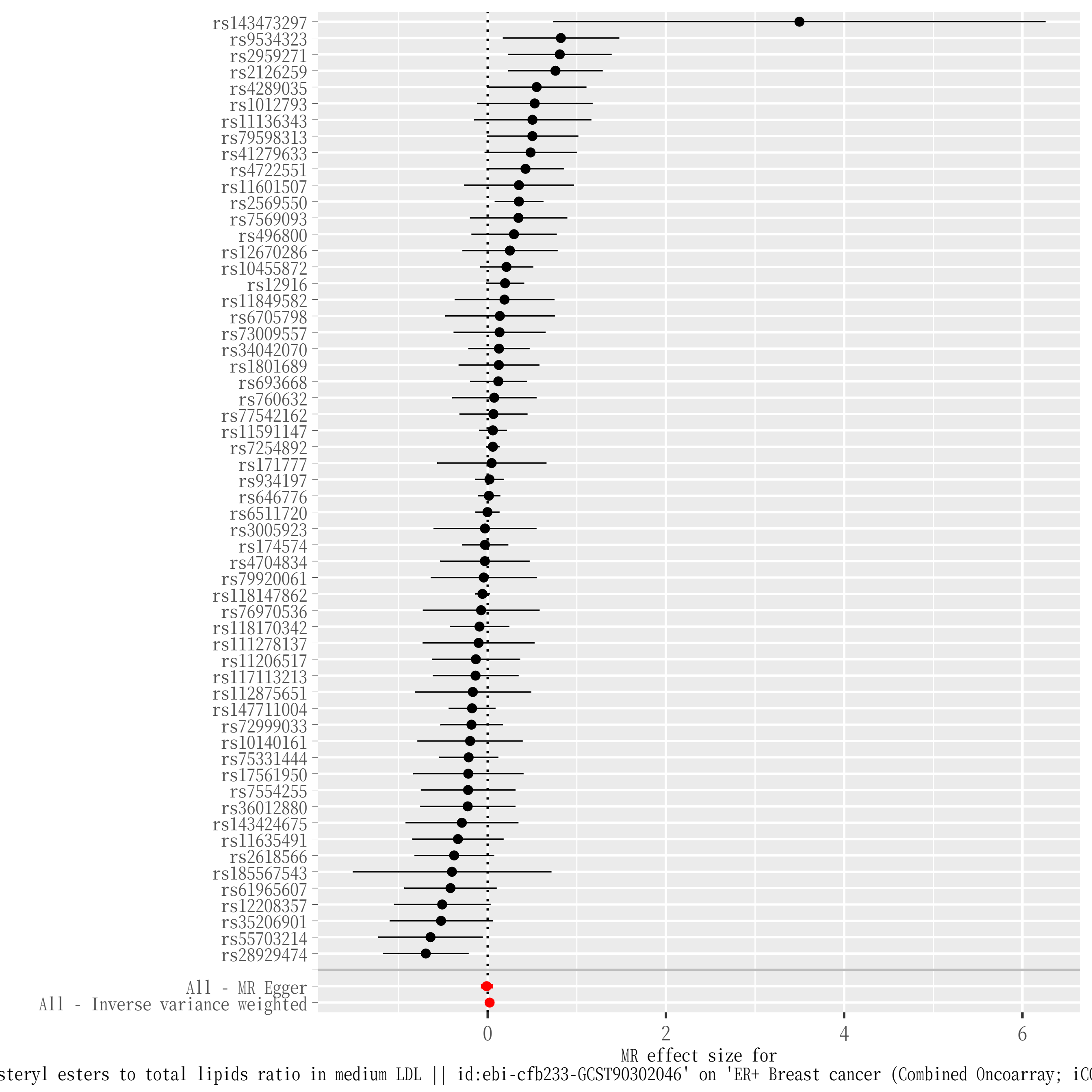

rs143473297
rs9534323
rs2959271
rs2126259
rs4289035
rs1012793
rs11136343
rs79598313
rs41279633
rs4722551
rs11601507
rs2569550
rs7569093
rs496800
rs12670286
rs10455872
rs12916
rs11849582
rs6705798
rs73009557
rs34042070
rs1801689
rs693668
rs760632
rs77542162
rs11591147
rs7254892
rs171777
rs934197
rs646776
rs6511720
rs3005923
rs174574
rs4704834
rs79920061
rs118147862
rs76970536
rs118170342
rs111278137
rs11206517
rs117113213
rs112875651
rs147711004
rs72999033
rs10140161
rs75331444
rs17561950
rs7554255
rs36012880
rs143424675
rs11635491
rs2618566
rs185567543
rs61965607
rs12208357
rs35206901
rs55703214
rs28929474
All - MR Egger
All - Inverse variance weighted
0
2
4
6
MR effect size for
steryl esters to total lipids ratio in medium LDL || id:ebi-cfb233-GCST90302046' on 'ER+ Breast cancer (Combined Oncoarray; iC

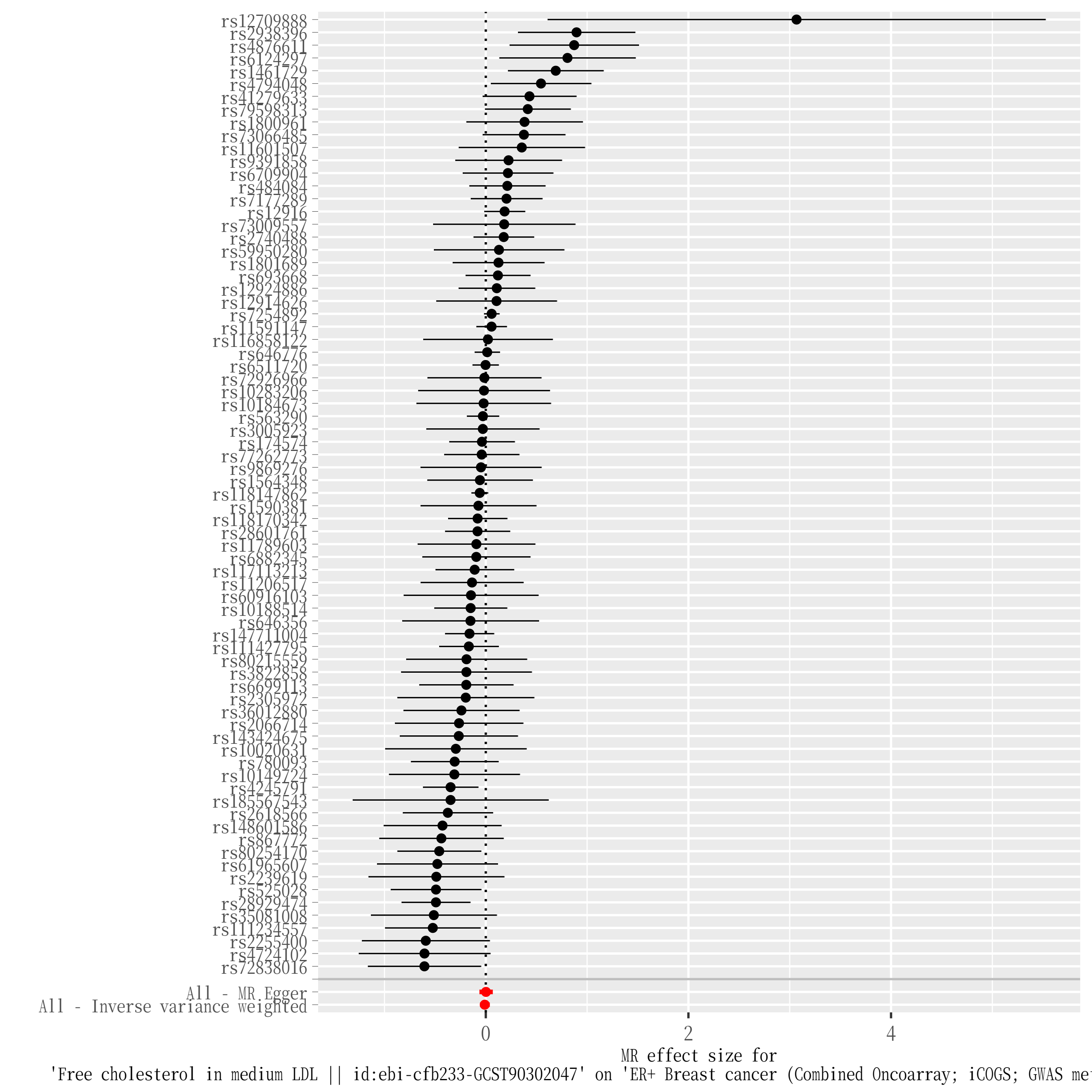

rs12709888
rs2938396
rs4876611
rs6124297
rs1461729
rs4794048
rs41279633
rs79598313
rs1800961
rs73066485
rs11601507
rs9391858
rs6709904
rs484084
rs7177289
rs12916
rs73009557
rs2740488
rs59950280
rs1801689
rs693668
rs12924886
rs12914626
rs7254892
rs11591147
rs116858122
rs646776
rs6511720
rs72926966
rs10283206
rs10184673
rs563290
rs3005923
rs174574
rs77262773
rs9869276
rs1564348
rs118147862
rs1590381
rs118170342
rs28601761
rs11789603
rs6882345
rs117113213
rs11206517
rs60916103
rs10188514
rs646356
rs147711004
rs111427795
rs80215559
rs3822858
rs6699113
rs2305972
rs36012880
rs2066714
rs143424675
rs10020631
rs780093
rs10149724
rs4245791
rs185567543
rs2618566
rs148601586
rs867772
rs80254170
rs61965607
rs2239619
rs525028
rs28929474
rs35081008
rs111234557
rs2255400
rs4724102
rs72838016
All - MR Egger
All - Inverse variance weighted
0
2
4
MR effect size for
'Free cholesterol in medium LDL || id:ebi-cfb233-GCST90302047' on 'ER+ Breast cancer (Combined Oncoarray; iCOGS; GWAS me

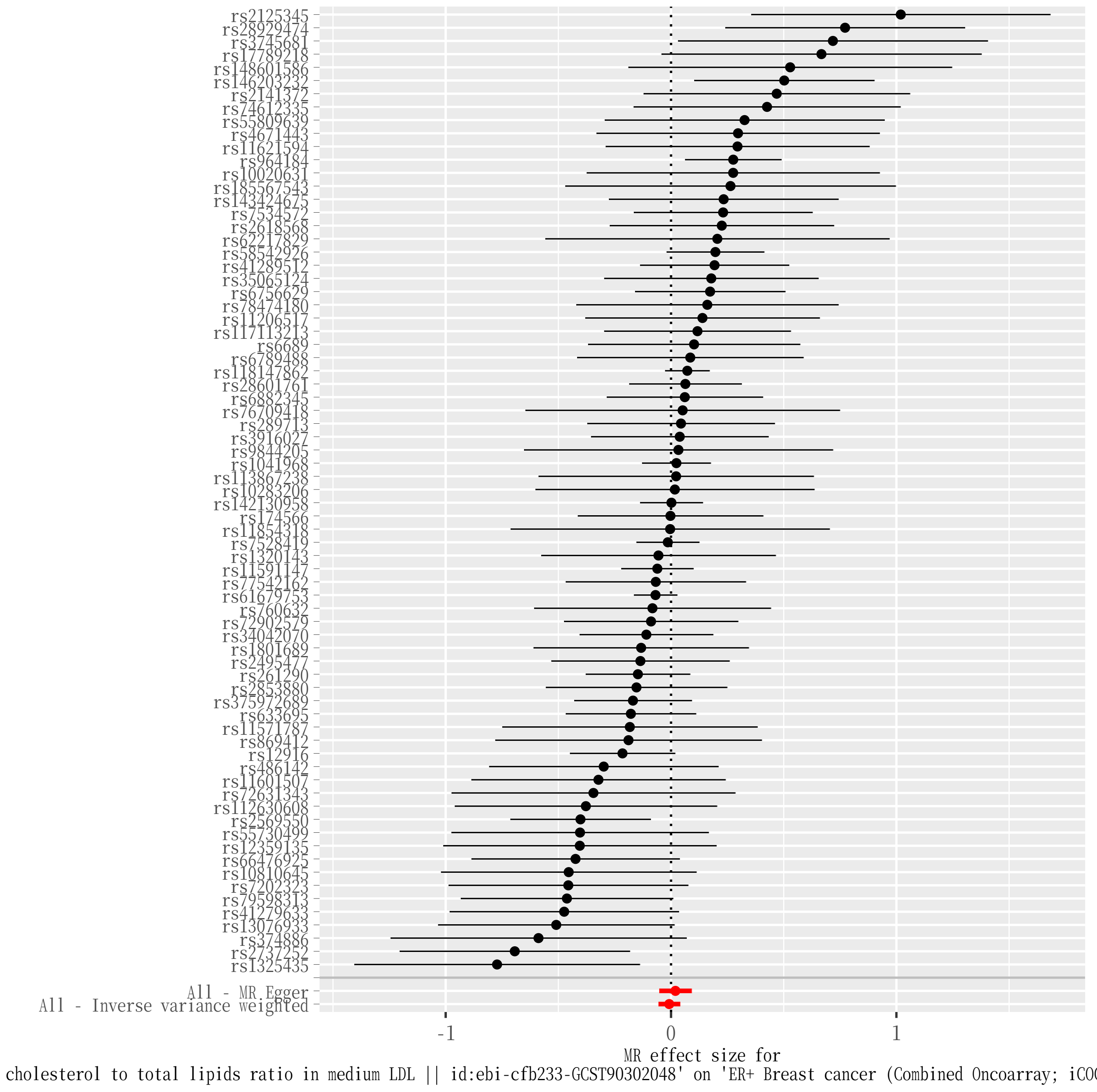

rs2125345
rs28929474
rs3745681
rs17789218
rs148601586
rs146203232
rs2141372
rs74612335
rs55809639
rs4671443
rs11621594
rs964184
rs10020631
rs185567543
rs143424675
rs7534572
rs2618568
rs62217829
rs58542926
rs41289512
rs35065124
rs6756629
rs78474180
rs11206517
rs117113213
rs6689
rs6789488
rs118147862
rs28601761
rs6882345
rs76709418
rs289713
rs3916027
rs9844205
rs1041968
rs113867238
rs10283206
rs142130958
rs174566
rs11854318
rs7528419
rs1320143
rs11591147
rs77542162
rs61679753
rs760632
rs72902579
rs34042070
rs1801689
rs2495477
rs261290
rs2853880
rs375972689
rs633695
rs11571787
rs869412
rs12916
rs486142
rs11601507
rs72631343
rs112630608
rs2569550
rs55730499
rs12359135
rs66476925
rs10810645
rs7202323
rs79598313
rs41279633
rs13076933
rs374886
rs2737252
rs1325435
All - MR Egger
All - Inverse variance weighted
-1
0
1
MR effect size for
cholesterol to total lipids ratio in medium LDL || id:ebi-cfb233-GCST90302048' on 'ER+ Breast cancer (Combined Oncoarray; iCO

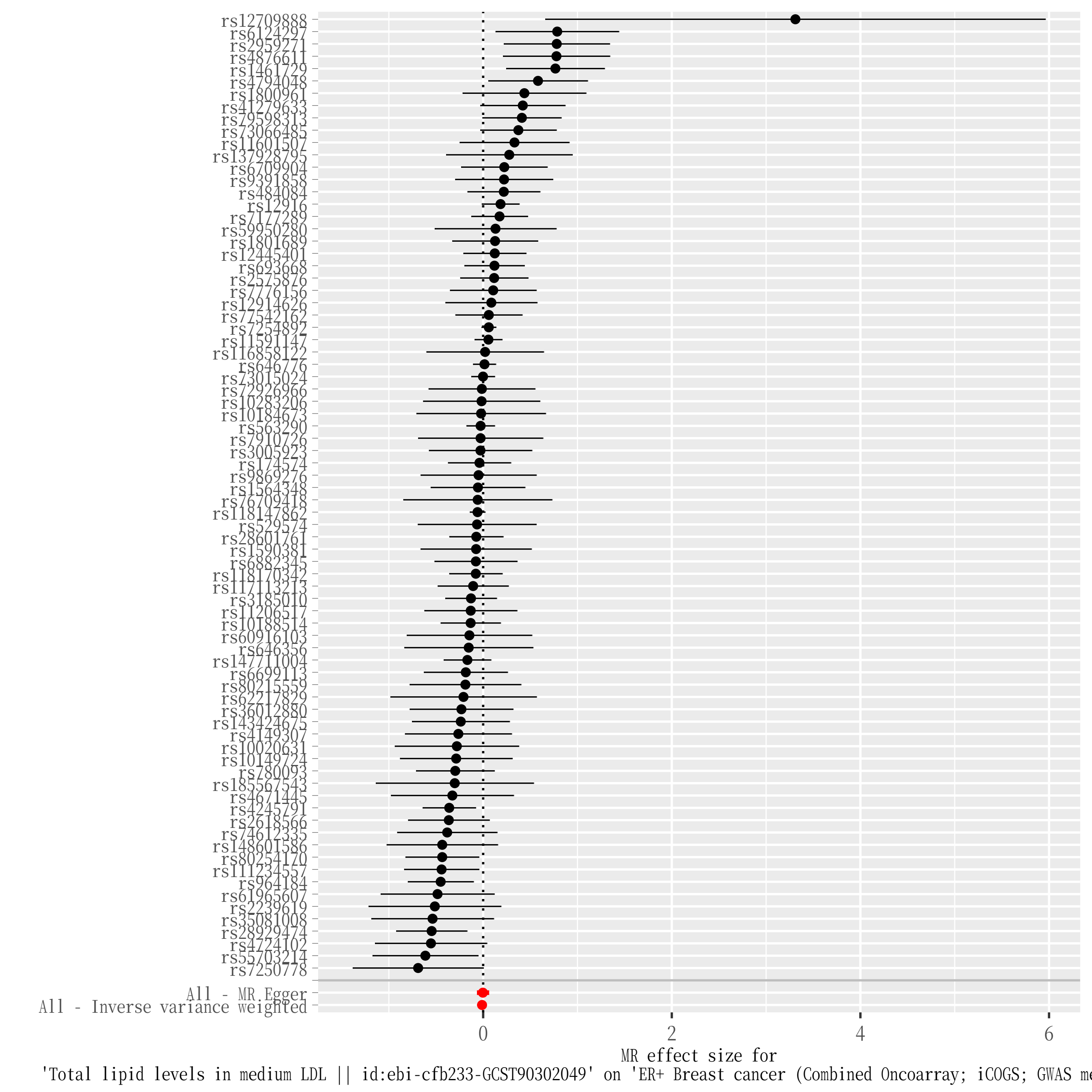
rs12709888
rs6124297
rs2959271
rs4876611
rs1461729
rs4794048
rs1800961
rs41279633
rs79598313
rs73066485
rs11601507
rs137928795
rs6709904
rs9391858
rs484084
rs12916
rs7177289
rs59950280
rs1801689
rs12445401
rs693668
rs2575876
rs7776156
rs12914626
rs77542162
rs7254892
rs11591147
rs116858122
rs646776
rs73015024
rs72926966
rs10283206
rs10184673
rs563290
rs7910726
rs3005923
rs174574
rs9869276
rs1564348
rs76709418
rs118147862
rs529574
rs28601761
rs1590381
rs6882345
rs118170342
rs117113213
rs3185010
rs11206517
rs10188514
rs60916103
rs646356
rs147711004
rs6699113
rs80215559
rs62217829
rs36012880
rs143424675
rs4149307
rs10020631
rs10149724
rs780093
rs185567543
rs4671445
rs4245791
rs2618566
rs74612335
rs148601586
rs80254170
rs111234557
rs964184
rs61965607
rs2239619
rs35081008
rs28929474
rs4724102
rs55703214
rs7250778
All - MR Egger
All - Inverse variance weighted
0
2
4
6
MR effect size for
'Total lipid levels in medium LDL || id:ebi-cfb233-GCST90302049' on 'ER+ Breast cancer (Combined Oncoarray; iCOGS; GWAS me

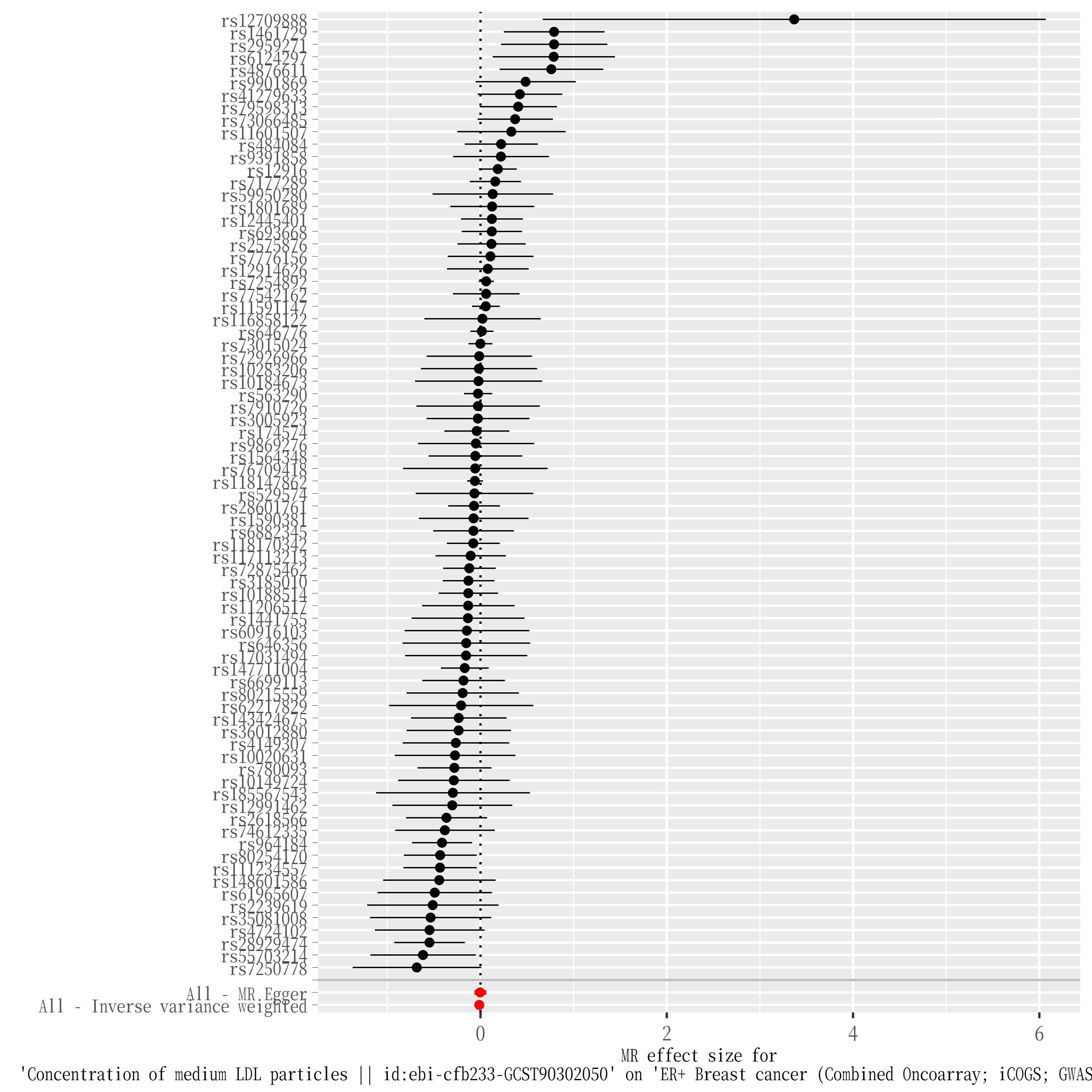

rs12709888
rs1461729
rs2959271
rs6124297
rs4876611
rs9901869
rs41279633
rs79598313
rs73066485
rs11601507
rs484084
rs9391858
rs12916
rs7177289
rs59950280
rs1801689
rs12445401
rs693668
rs2575876
rs7776156
rs12914626
rs7254892
rs77542162
rs11591147
rs116858122
rs646776
rs73015024
rs72926966
rs10283206
rs10184673
rs563290
rs7910726
rs3005923
rs174574
rs9869276
rs1564348
rs76709418
rs118147862
rs529574
rs28601761
rs1590381
rs6882345
rs118170342
rs117113213
rs7287546
rs3185010
rs10188514
rs11206517
rs1441755
rs60916103
rs646356
rs17031494
rs147711004
rs6699113
rs80215559
rs62217829
rs143424675
rs36012880
rs4149307
rs10020631
rs780093
rs10149724
rs185567543
rs12991462
rs2618566
rs74612335
rs964184
rs80254170
rs11234557
rs148601586
rs61965607
rs2239619
rs35081008
rs4724102
rs28929474
rs55703214
rs7250778
All - MR Egger
All - Inverse variance weighted
0
2
4
6
MR effect size for
'Concentration of medium LDL particles || id:ebi-cfb233-GCST90302050' on 'ER+ Breast cancer (Combined Oncoarray; iCOGS; GWAS

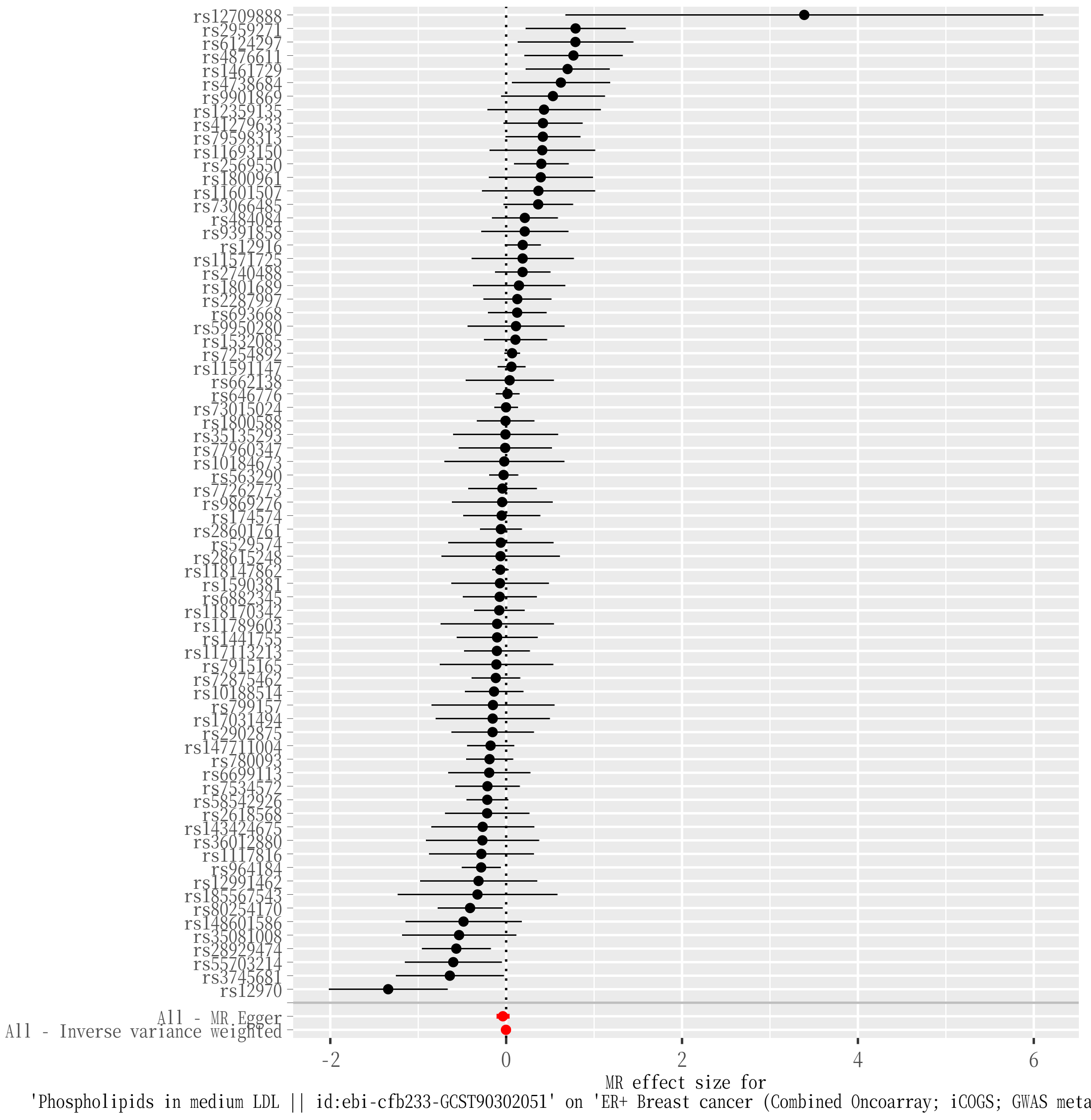

rs12709888
rs2959271
rs6124297
rs4876611
rs1461729
rs4738684
rs9901869
rs12359135
rs41279633
rs79598313
rs11693150
rs2569550
rs1800961
rs11601507
rs73066485
rs484084
rs9391858
rs12916
rs11571725
rs2740488
rs1801689
rs2287997
rs693668
rs59950280
rs1532085
rs7254892
rs11591147
rs662138
rs646776
rs73015024
rs1800588
rs35135293
rs77960347
rs10184673
rs563290
rs77262773
rs9869276
rs174574
rs28601761
rs529574
rs28615248
rs118147862
rs1590381
rs6882345
rs118170342
rs11789603
rs1441755
rs117113213
rs7915165
rs72875462
rs10188514
rs799157
rs17031494
rs2902875
rs147711004
rs780093
rs6699113
rs7534572
rs58542926
rs2618568
rs143424675
rs36012880
rs1117816
rs964184
rs12991462
rs185567543
rs80254170
rs148601586
rs35081008
rs28929474
rs55703214
rs3745681
rs12970
All - MR Egger
All - Inverse variance weighted
-2
0
2
4
6
MR effect size for
'Phospholipids in medium LDL || id:ebi-cfb233-GCST90302051' on 'ER+ Breast cancer (Combined Oncoarray; iCOGS; GWAS meta

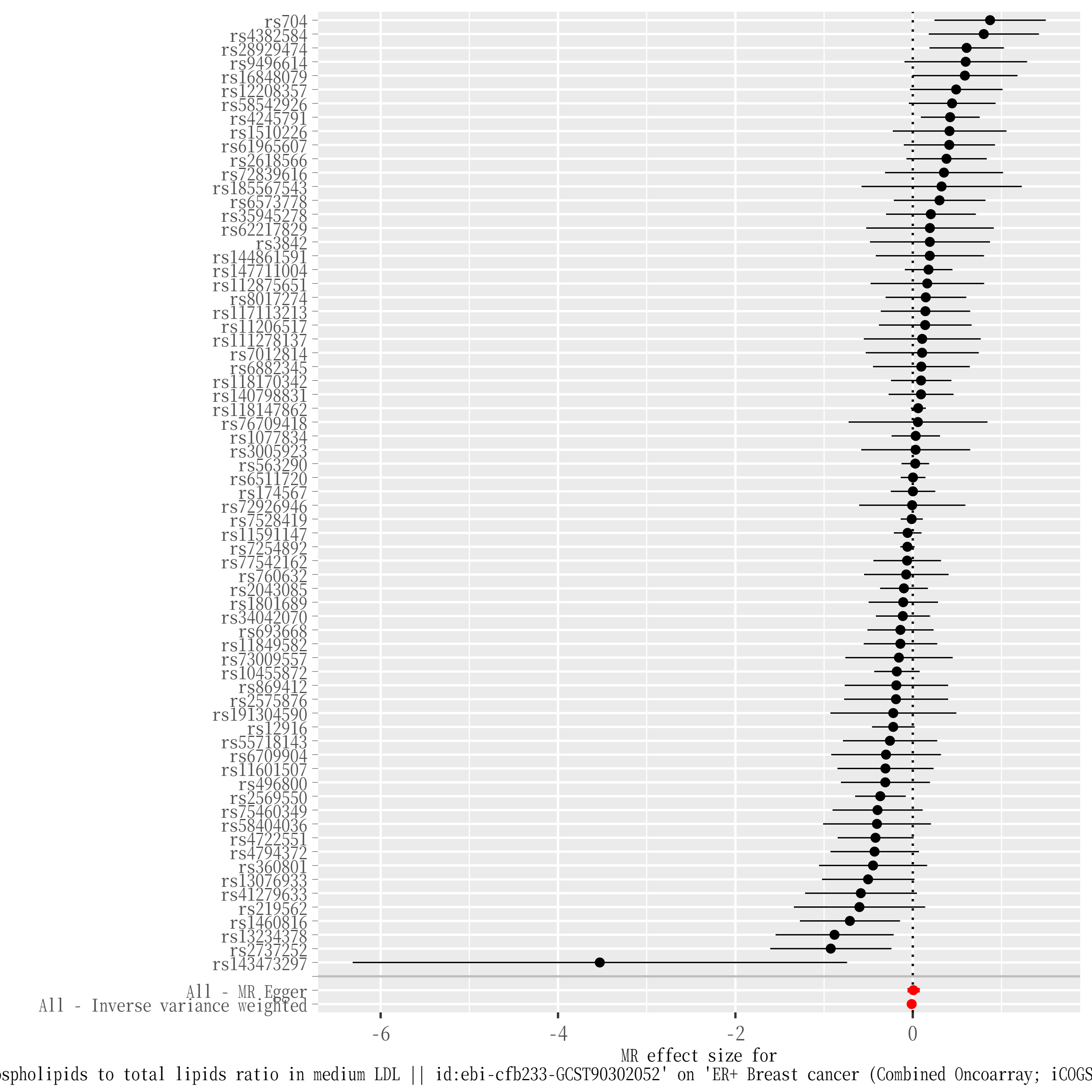
rs704
rs4382584
rs28929474
rs9496614
rs16848079
rs12208357
rs58542926
rs4245791
rs1510226
rs61965607
rs2618566
rs72839616
rs185567543
rs6573778
rs35945278
rs62217829
rs3842
rs144861591
rs147711004
rs112875651
rs8017274
rs117113213
rs11206517
rs111278137
rs7012814
rs6882345
rs118170342
rs140798831
rs118147862
rs76709418
rs1077834
rs3005923
rs563290
rs6511720
rs174567
rs72926946
rs7528419
rs11591147
rs7254892
rs77542162
rs760632
rs2043085
rs1801689
rs34042070
rs693668
rs11849582
rs73009557
rs10455872
rs869412
rs2575876
rs191304590
rs12916
rs55718143
rs6709904
rs11601507
rs496800
rs2569550
rs75460349
rs58404036
rs4722551
rs4794372
rs360801
rs13076933
rs41279633
rs219562
rs1460816
rs13234378
rs2737252
rs143473297
All - MR Egger
All - Inverse variance weighted
-6
-4
-2
0
MR effect size for
ospholipids to total lipids ratio in medium LDL || id:ebi-cfb233-GCST90302052' on 'ER+ Breast cancer (Combined Oncoarray; iCOGS

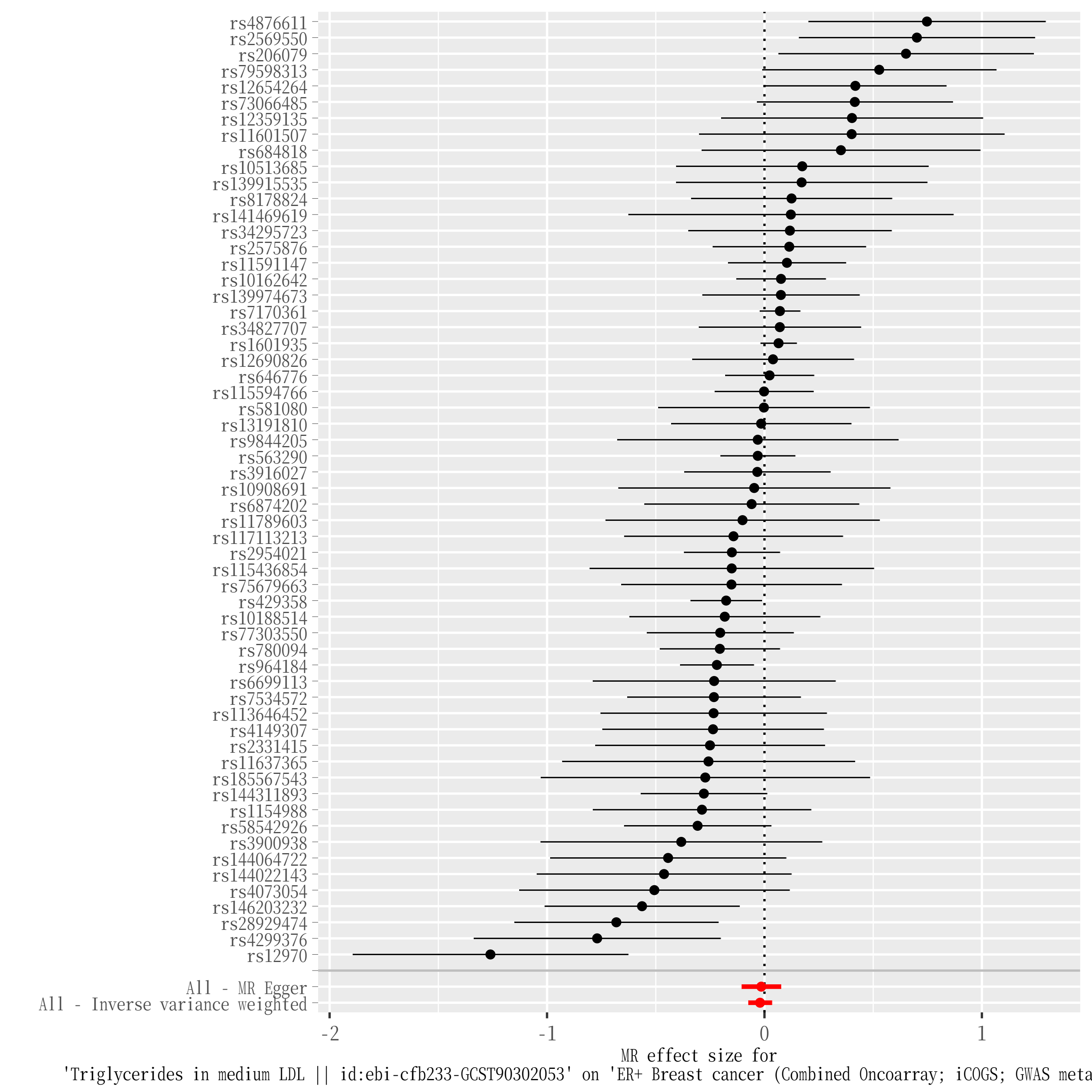
rs4876611
rs2569550
rs206079
rs79598313
rs12654264
rs73066485
rs12359135
rs11601507
rs684818
rs10513685
rs139915535
rs8178824
rs141469619
rs34295723
rs2575876
rs11591147
rs10162642
rs139974673
rs7170361
rs34827707
rs1601935
rs12690826
rs646776
rs115594766
rs581080
rs13191810
rs9844205
rs563290
rs3916027
rs10908691
rs6874202
rs11789603
rs117113213
rs2954021
rs115436854
rs75679663
rs429358
rs10188514
rs77303550
rs780094
rs964184
rs6699113
rs7534572
rs113646452
rs4149307
rs2331415
rs11637365
rs185567543
rs144311893
rs1154988
rs58542926
rs3900938
rs144064722
rs144022143
rs4073054
rs146203232
rs28929474
rs4299376
rs12970
All - MR Egger
All - Inverse variance weighted
-2
-1
0
1
MR effect size for
'Triglycerides in medium LDL || id:ebi-cfb233-GCST90302053' on 'ER+ Breast cancer (Combined Oncoarray; iCOGS; GWAS meta

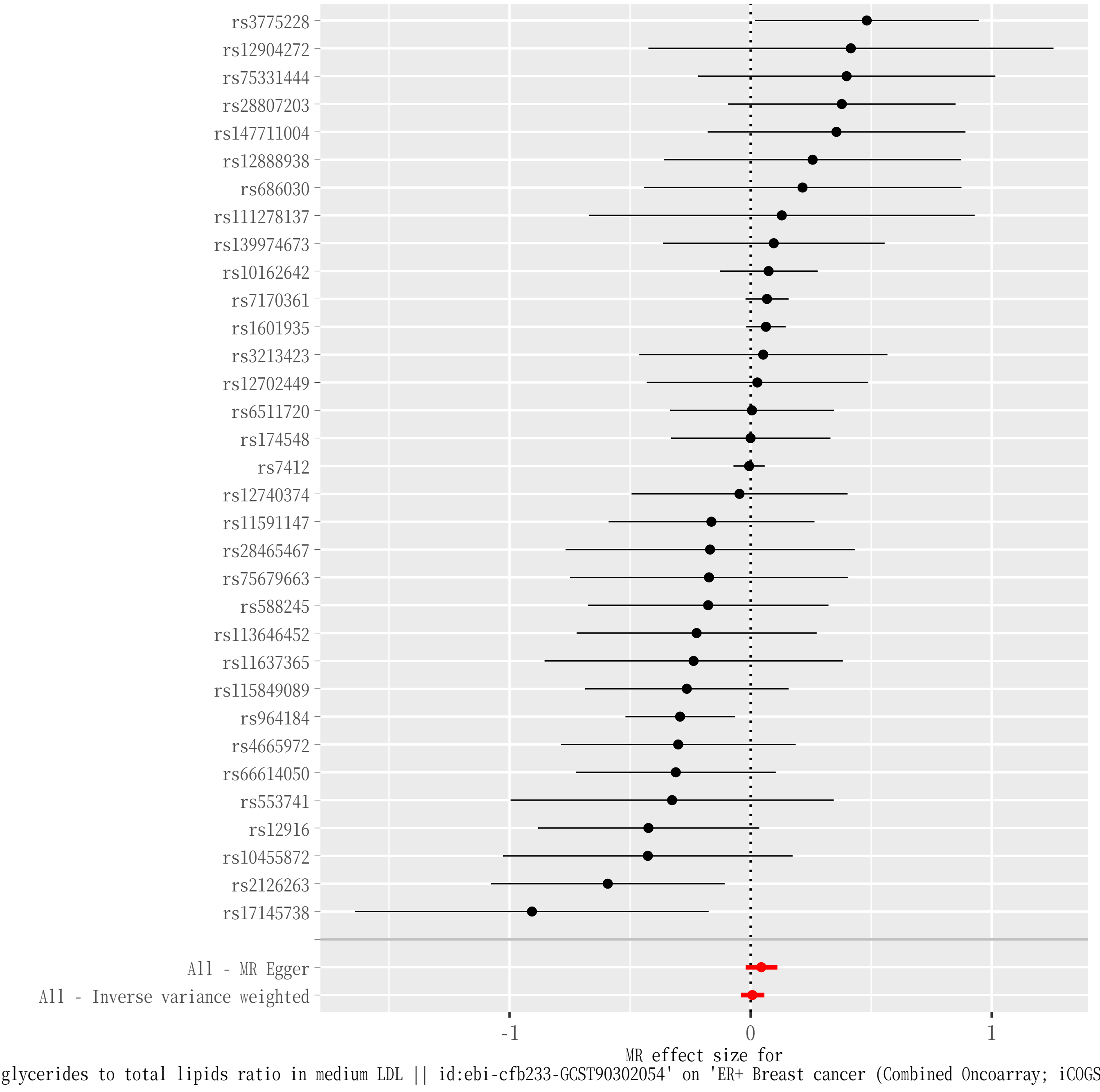

rs3775228
rs12904272
rs75331444
rs28807203
rs147711004
rs12888938
rs686030
rs111278137
rs139974673
rs10162642
rs7170361
rs1601935
rs3213423
rs12702449
rs6511720
rs174548
rs7412
rs12740374
rs11591147
rs28465467
rs75679663
rs588245
rs113646452
rs11637365
rs115849089
rs964184
rs4665972
rs66614050
rs553741
rs12916
rs10455872
rs2126263
rs17145738
All - MR Egger
All - Inverse variance weighted
-1
0
1
MR effect size for
glycerides to total lipids ratio in medium LDL || id:ebi-cfb233-GCST90302054' on 'ER+ Breast cancer (Combined Oncoarray; iCOGS

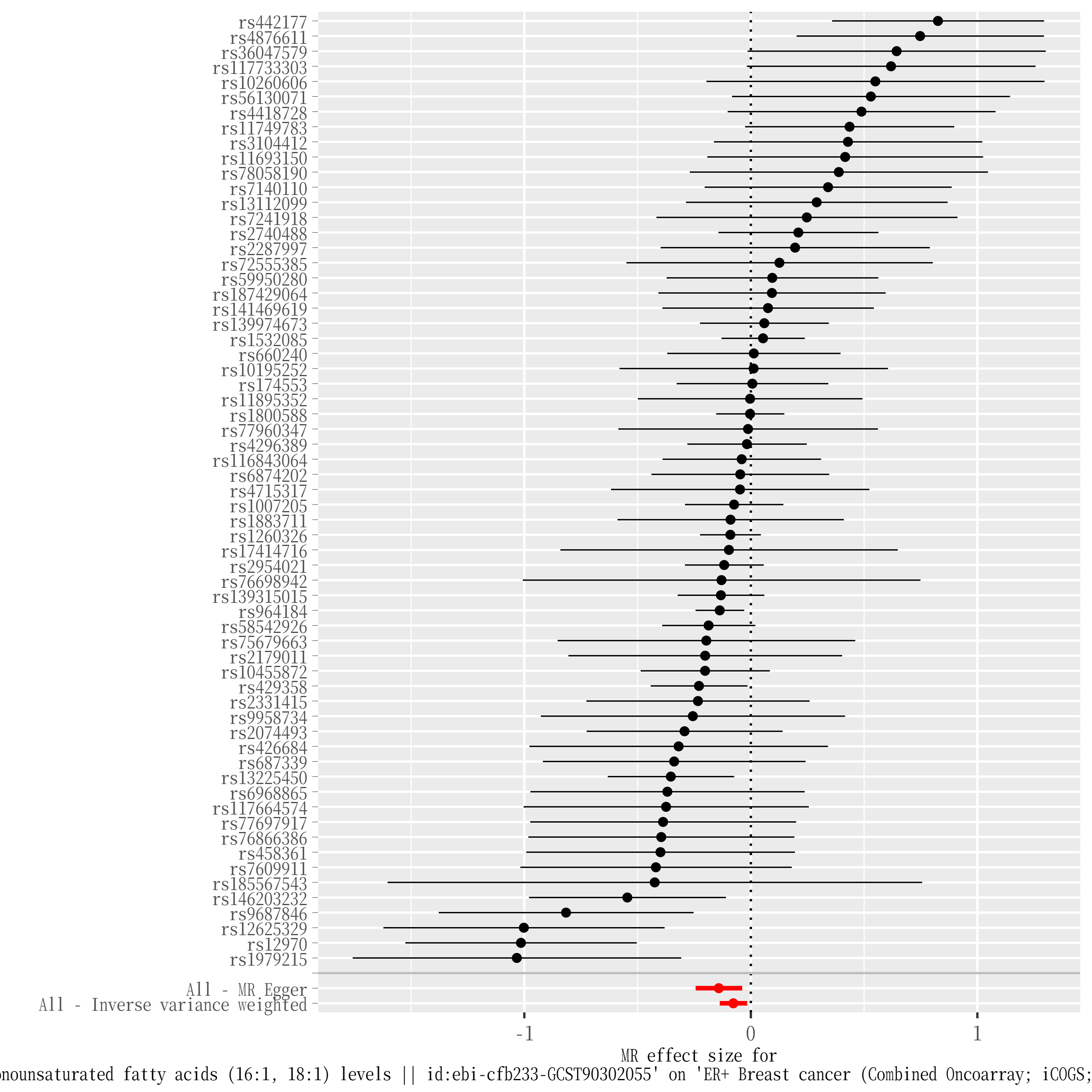
rs442177
rs4876611
rs36047579
rs117733303
rs10260606
rs56130071
rs4418728
rs11749783
rs3104412
rs11693150
rs78058190
rs7140110
rs13112099
rs7241918
rs2740488
rs2287997
rs72555385
rs59950280
rs187429064
rs141469619
rs139974673
rs1532085
rs660240
rs10195252
rs174553
rs11895352
rs1800588
rs77960347
rs4296389
rs116843064
rs6874202
rs4715317
rs1007205
rs1883711
rs1260326
rs17414716
rs2954021
rs76698942
rs139315015
rs964184
rs58542926
rs75679663
rs2179011
rs10455872
rs429358
rs2331415
rs9958734
rs2074493
rs426684
rs687339
rs13225450
rs6968865
rs117664574
rs77697917
rs76866386
rs458361
rs7609911
rs185567543
rs146203232
rs9687846
rs12625329
rs12970
rs1979215
All - MR Egger
All - Inverse variance weighted
-1
0
1
MR effect size for
onounsaturated fatty acids (16:1, 18:1) levels || id:ebi-cfb233-GCST90302055' on 'ER+ Breast cancer (Combined Oncoarray; iCOGS;

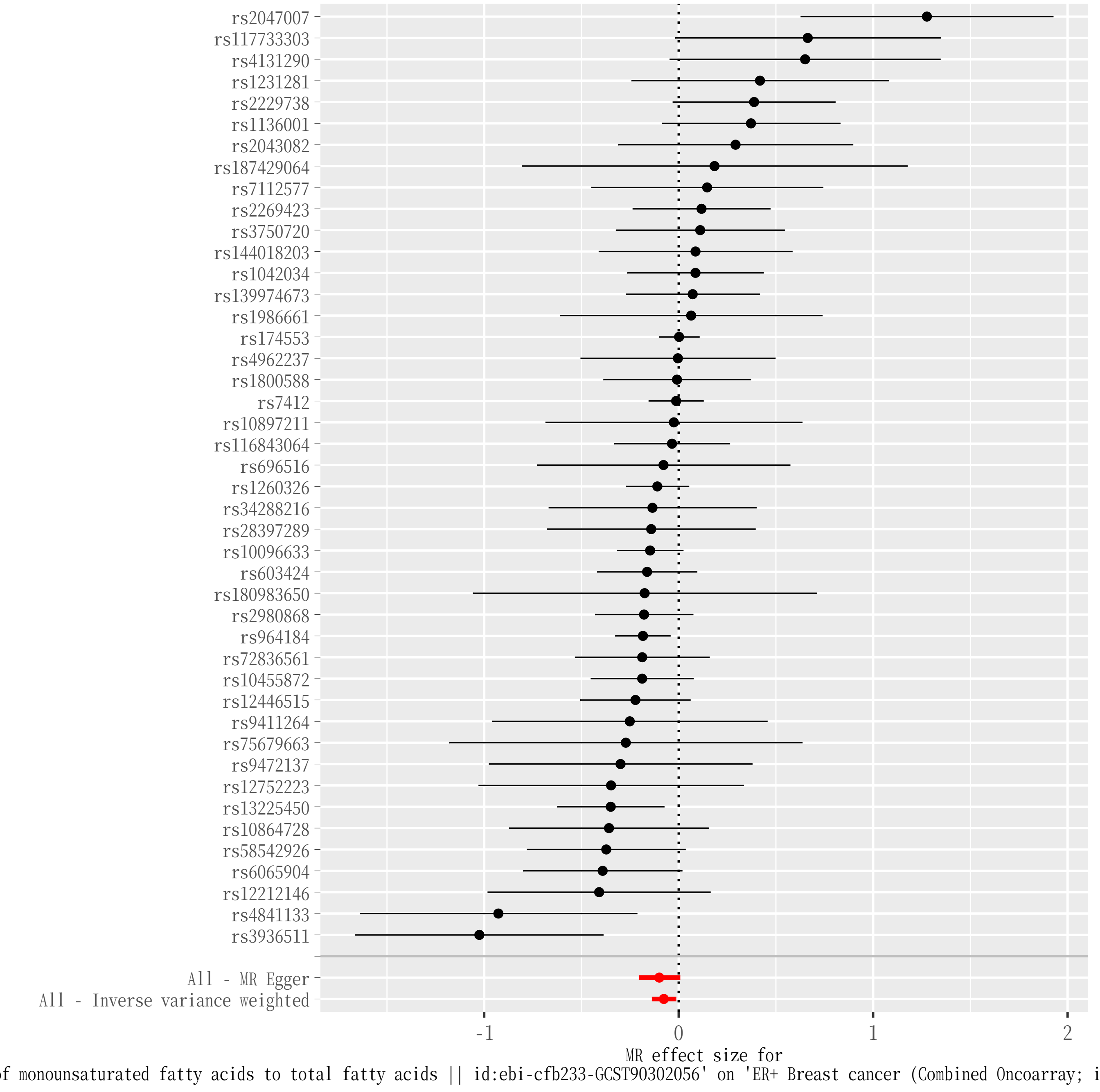
rs2047007
rs117733303
rs4131290
rs1231281
rs2229738
rs1136001
rs2043082
rs187429064
rs7112577
rs2269423
rs3750720
rs144018203
rs1042034
rs139974673
rs1986661
rs174553
rs4962237
rs1800588
rs7412
rs10897211
rs116843064
rs696516
rs1260326
rs34288216
rs28397289
rs10096633
rs603424
rs180983650
rs2980868
rs964184
rs72836561
rs10455872
rs12446515
rs9411264
rs75679663
rs9472137
rs12752223
rs13225450
rs10864728
rs58542926
rs6065904
rs12212146
rs4841133
rs3936511
All - MR Egger
All - Inverse variance weighted
-1
0
1
2
MR effect size for
of monounsaturated fatty acids to total fatty acids || id:ebi-cfb233-GCST90302056' on 'ER+ Breast cancer (Combined Oncoarray; i

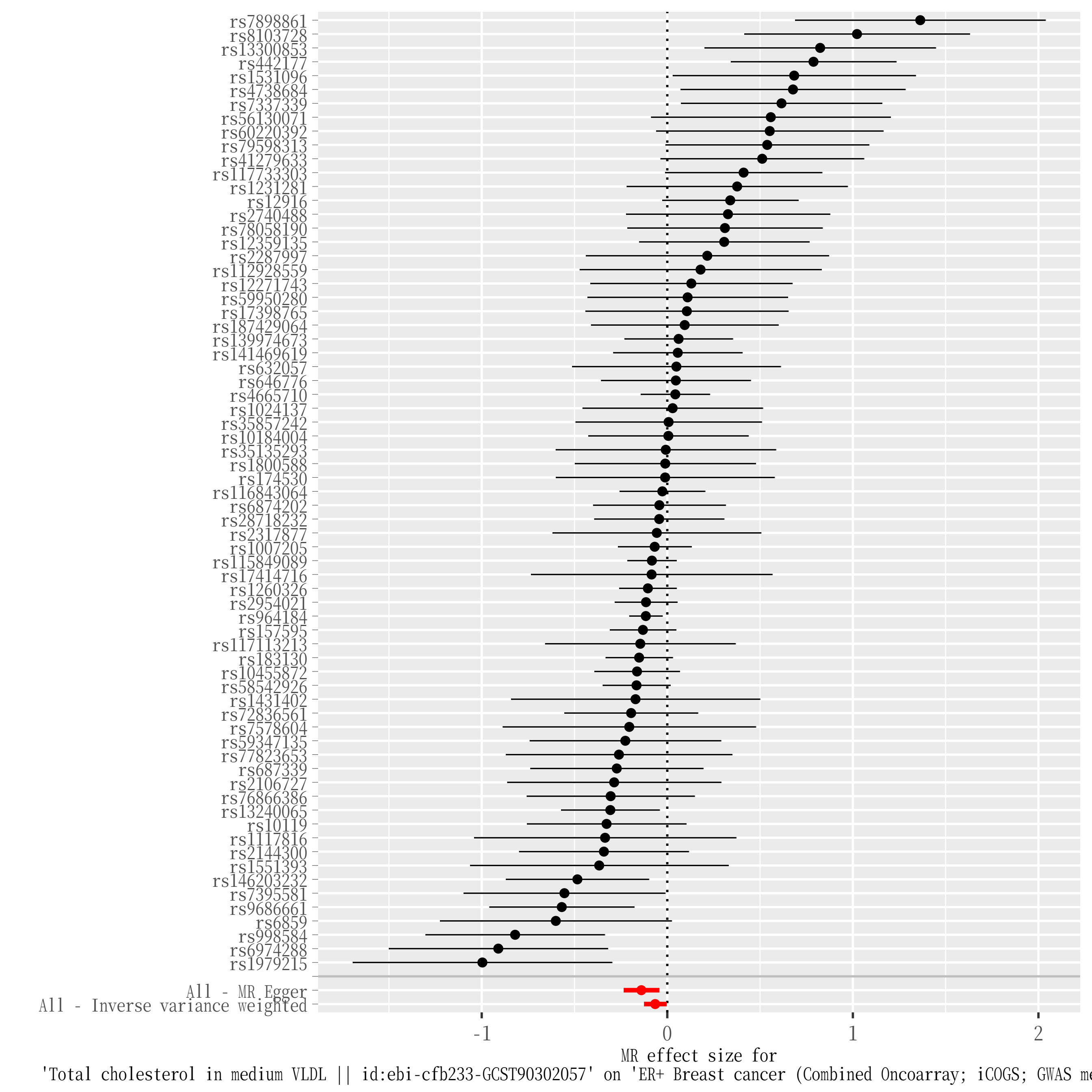
rs7898861
rs8103728
rs13300853
rs442177
rs1531096
rs4738684
rs7337339
rs56130071
rs60220392
rs79598313
rs41279633
rs117733303
rs1231281
rs12916
rs2740488
rs78058190
rs12359135
rs2287997
rs112928559
rs12271743
rs59950280
rs17398765
rs187429064
rs139974673
rs141469619
rs632057
rs646776
rs4665710
rs1024137
rs35857242
rs10184004
rs35135293
rs1800588
rs174530
rs116843064
rs6874202
rs28718232
rs2317877
rs1007205
rs115849089
rs17414716
rs1260326
rs2954021
rs964184
rs157595
rs117113213
rs183130
rs10455872
rs58542926
rs1431402
rs72836561
rs7578604
rs59347135
rs77823653
rs687339
rs2106727
rs76866386
rs13240065
rs10119
rs1117816
rs2144300
rs1551393
rs146203232
rs7395581
rs9686661
rs6859
rs998584
rs6974288
rs1979215
All - MR Egger
All - Inverse variance weighted
-1
0
1
2
MR effect size for
'Total cholesterol in medium VLDL || id:ebi-cfb233-GCST90302057' on 'ER+ Breast cancer (Combined Oncoarray; iCOGS; GWAS me

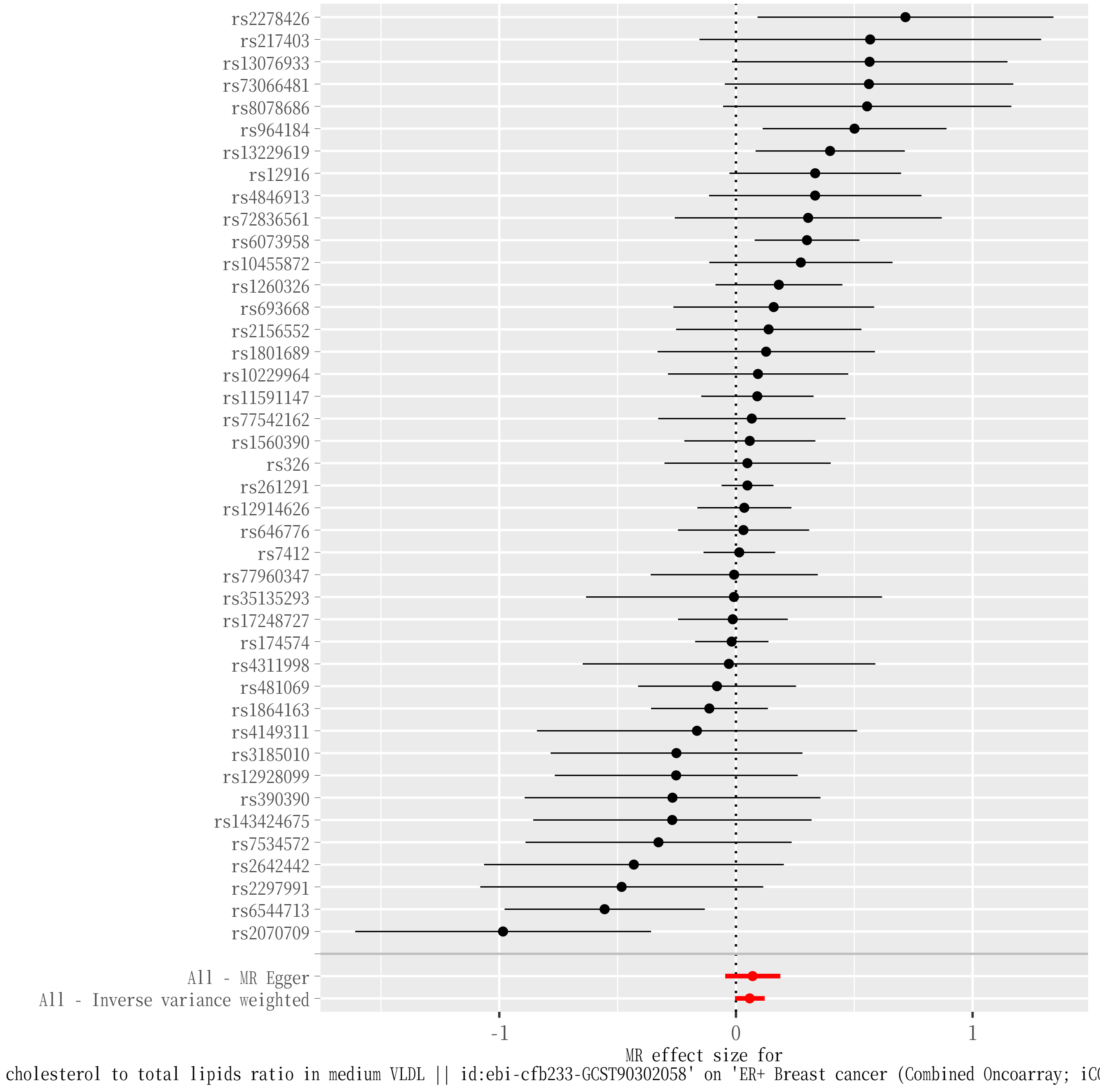

rs2278426
rs217403
rs13076933
rs73066481
rs8078686
rs964184
rs13229619
rs12916
rs4846913
rs72836561
rs6073958
rs10455872
rs1260326
rs693668
rs2156552
rs1801689
rs10229964
rs11591147
rs77542162
rs1560390
rs326
rs261291
rs12914626
rs646776
rs7412
rs77960347
rs35135293
rs17248727
rs174574
rs4311998
rs481069
rs1864163
rs4149311
rs3185010
rs12928099
rs390390
rs143424675
rs7534572
rs2642442
rs2297991
rs6544713
rs2070709
All - MR Egger
All - Inverse variance weighted
-1
0
1
MR effect size for
cholesterol to total lipids ratio in medium VLDL || id:ebi-cfb233-GCST90302058' on 'ER+ Breast cancer (Combined Oncoarray; iC

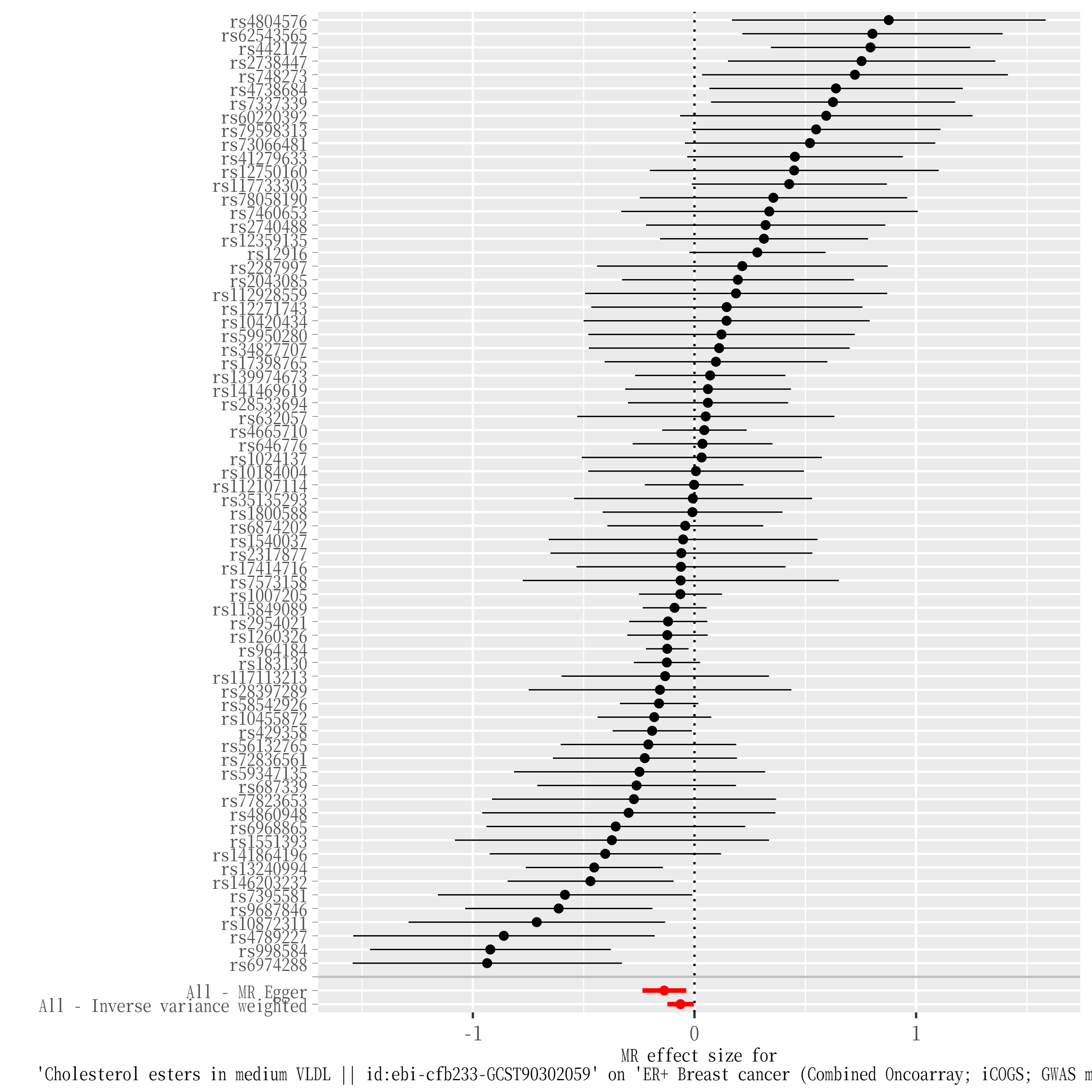

rs4804576
rs62543565
rs442177
rs2738447
rs748273
rs4738684
rs7337339
rs60220392
rs79598313
rs73066481
rs41279633
rs12750160
rs117733303
rs78058190
rs7460653
rs2740488
rs12359135
rs12916
rs2287997
rs2043085
rs112928559
rs12271743
rs10420434
rs59950280
rs34827707
rs17398765
rs139974673
rs141469619
rs28533694
rs632057
rs4665710
rs646776
rs1024137
rs10184004
rs112107114
rs35135293
rs1800588
rs6874202
rs1540037
rs2317877
rs17414716
rs7573158
rs1007205
rs115849089
rs2954021
rs1260326
rs964184
rs183130
rs117113213
rs28397289
rs58542926
rs10455872
rs429358
rs56132765
rs72836561
rs59347135
rs687339
rs77823653
rs4860948
rs6968865
rs1551393
rs141864196
rs13240994
rs146203232
rs7395581
rs9687846
rs10872311
rs4789227
rs998584
rs6974288
All - MR Egger
All - Inverse variance weighted
-1
0
1
MR effect size for
'Cholesterol esters in medium VLDL || id:ebi-cfb233-GCST90302059' on 'ER+ Breast cancer (Combined Oncoarray; iCOGS; GWAS m

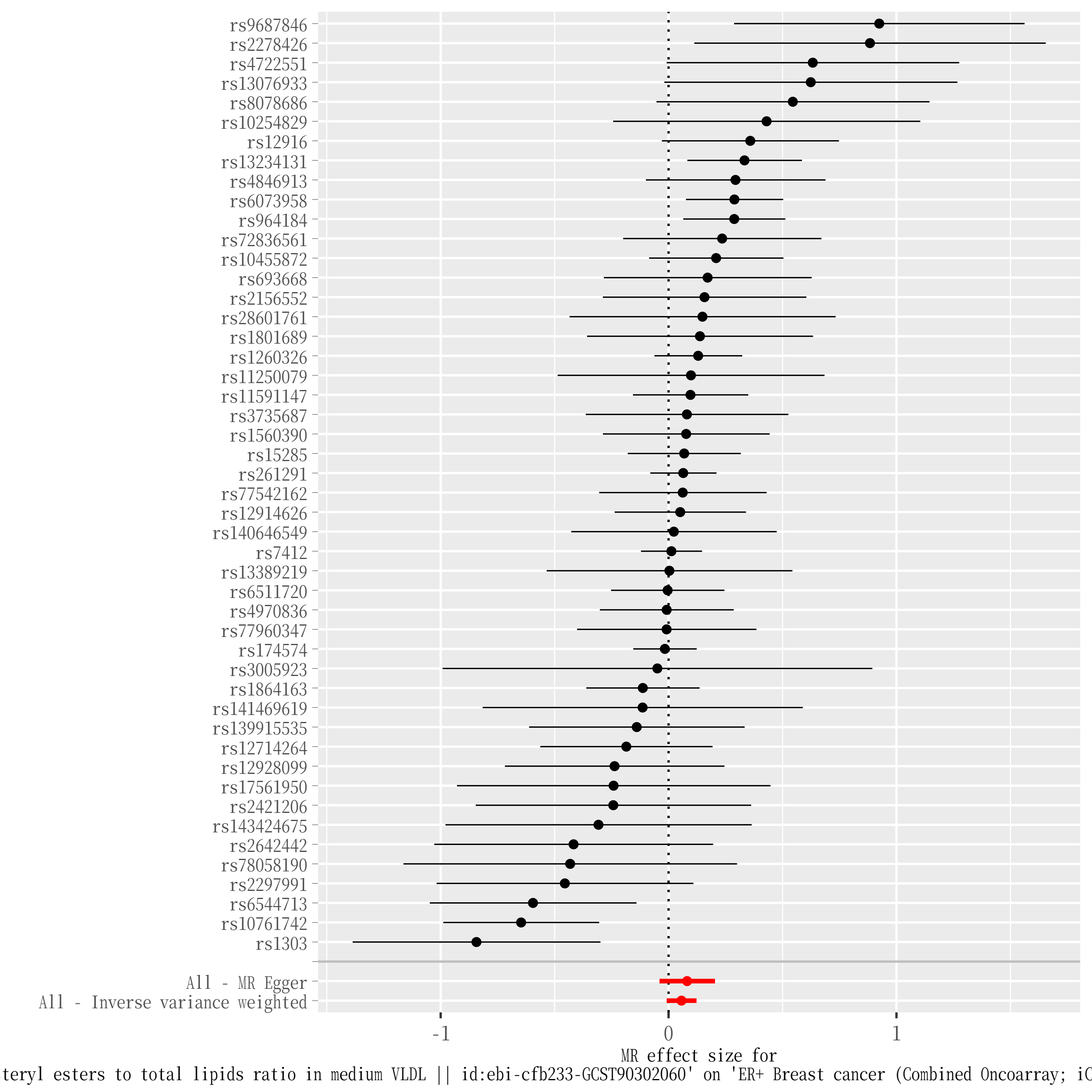
rs9687846
rs2278426
rs4722551
rs13076933
rs8078686
rs10254829
rs12916
rs13234131
rs4846913
rs6073958
rs964184
rs72836561
rs10455872
rs693668
rs2156552
rs28601761
rs1801689
rs1260326
rs11250079
rs11591147
rs3735687
rs1560390
rs15285
rs261291
rs77542162
rs12914626
rs140646549
rs7412
rs13389219
rs6511720
rs4970836
rs77960347
rs174574
rs3005923
rs1864163
rs141469619
rs139915535
rs12714264
rs12928099
rs17561950
rs2421206
rs143424675
rs2642442
rs78058190
rs2297991
rs6544713
rs10761742
rs1303
All - MR Egger
All - Inverse variance weighted
-1
0
1
MR effect size for
teryl esters to total lipids ratio in medium VLDL || id:ebi-cfb233-GCST90302060' on 'ER+ Breast cancer (Combined Oncoarray; iC

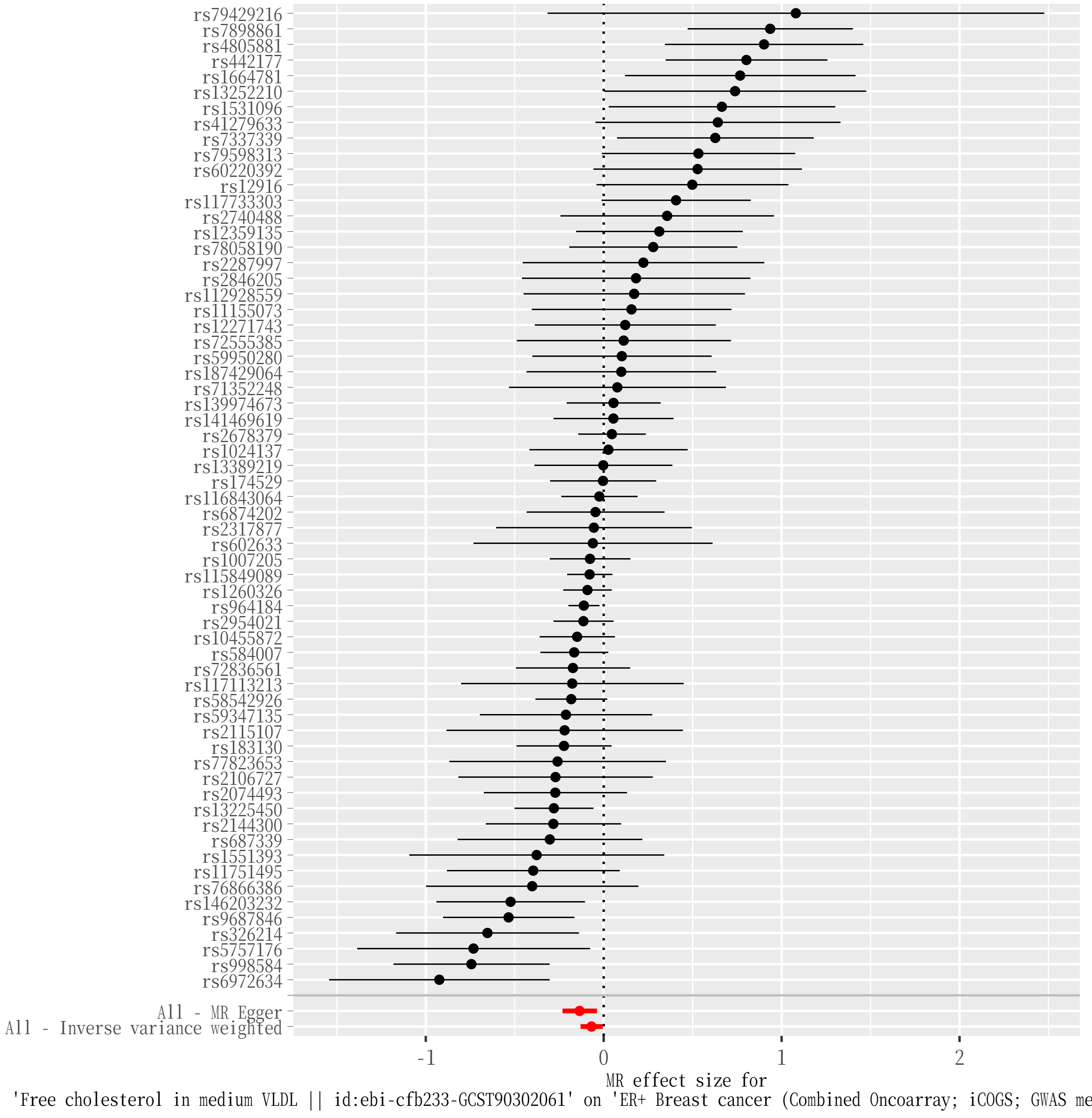

rs79429216
rs7898861
rs4805881
rs442177
rs1664781
rs13252210
rs1531096
rs41279633
rs7337339
rs79598313
rs60220392
rs12916
rs117733303
rs2740488
rs12359135
rs78058190
rs2287997
rs2846205
rs112928559
rs11155073
rs12271743
rs72555385
rs59950280
rs187429064
rs71352248
rs139974673
rs141469619
rs2678379
rs1024137
rs13389219
rs174529
rs116843064
rs6874202
rs2317877
rs602633
rs1007205
rs115849089
rs1260326
rs964184
rs2954021
rs10455872
rs584007
rs72836561
rs117113213
rs58542926
rs59347135
rs2115107
rs183130
rs77823653
rs2106727
rs2074493
rs13225450
rs2144300
rs687339
rs1551393
rs11751495
rs76866386
rs146203232
rs9687846
rs326214
rs5757176
rs998584
rs6972634
All - MR Egger
All - Inverse variance weighted
-1
0
1
2
MR effect size for
'Free cholesterol in medium VLDL || id:ebi-cfb233-GCST90302061' on 'ER+ Breast cancer (Combined Oncoarray; iCOGS; GWAS me

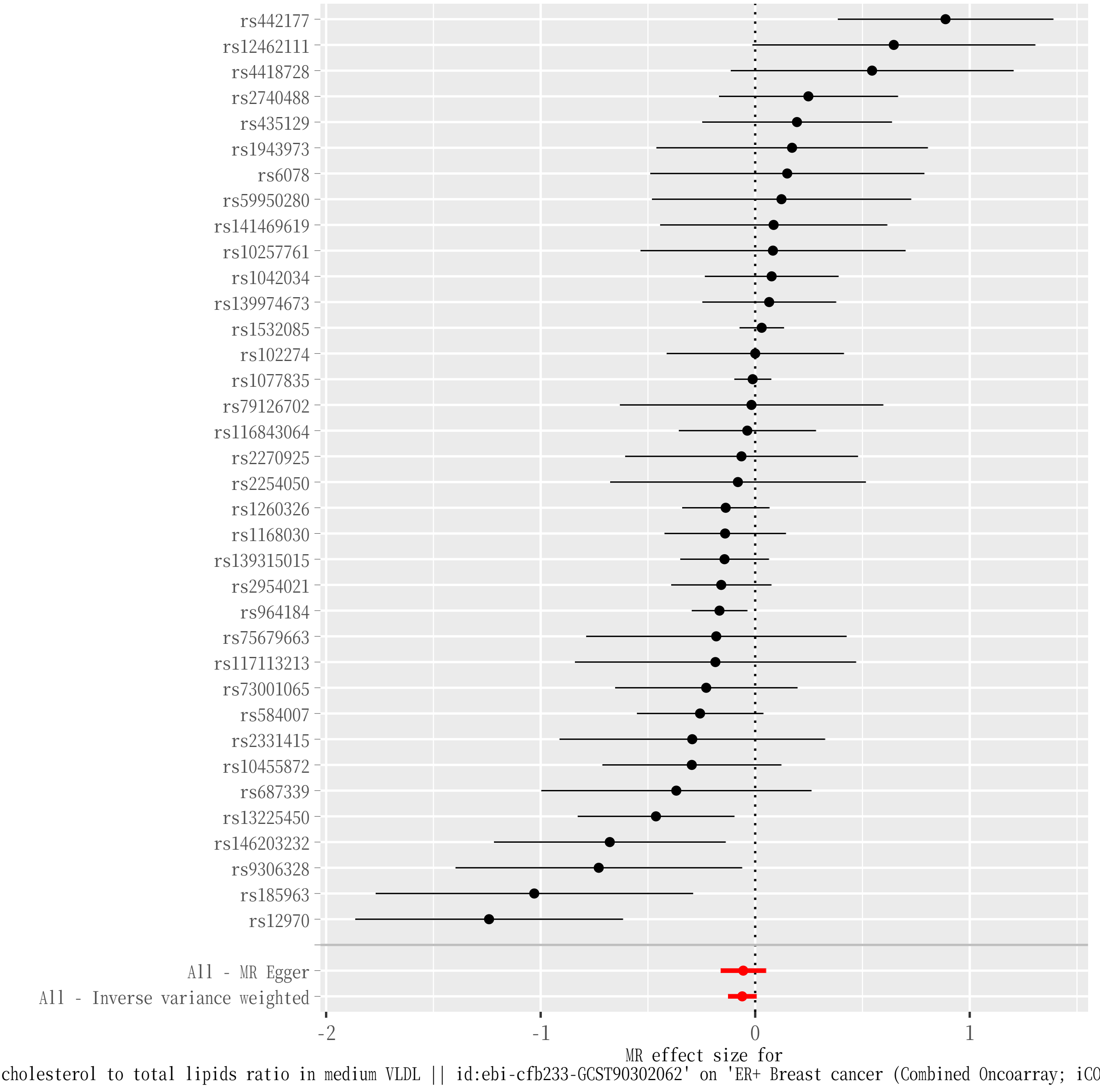
rs442177
rs12462111
rs4418728
rs2740488
rs435129
rs1943973
rs6078
rs59950280
rs141469619
rs10257761
rs1042034
rs139974673
rs1532085
rs102274
rs1077835
rs79126702
rs116843064
rs2270925
rs2254050
rs1260326
rs1168030
rs139315015
rs2954021
rs964184
rs75679663
rs117113213
rs73001065
rs584007
rs2331415
rs10455872
rs687339
rs13225450
rs146203232
rs9306328
rs185963
rs12970
All - MR Egger
All - Inverse variance weighted
-2
-1
0
1
MR effect size for
cholesterol to total lipids ratio in medium VLDL || id:ebi-cfb233-GCST90302062' on 'ER+ Breast cancer (Combined Oncoarray; iCO

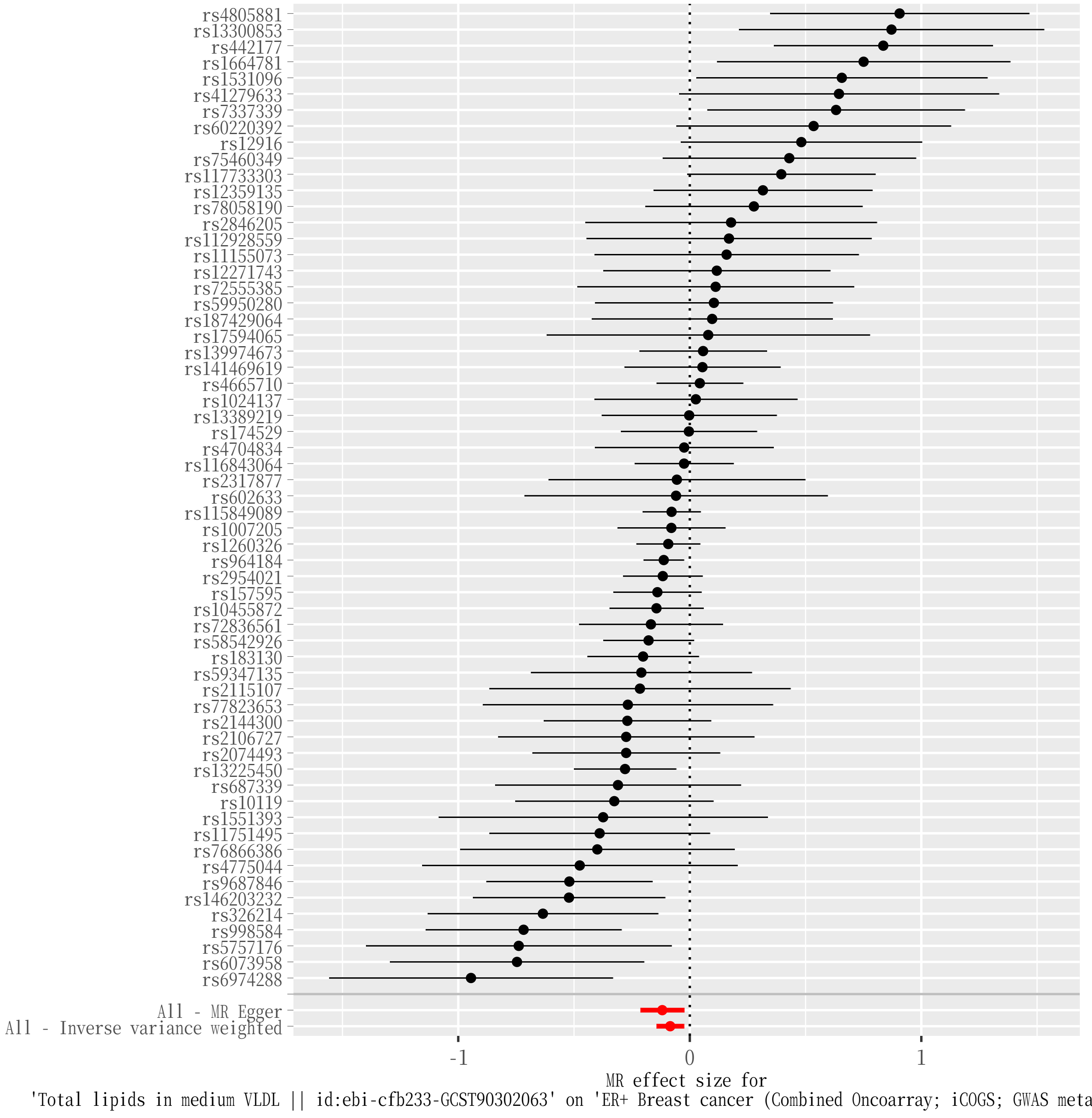

rs4805881
rs13300853
rs442177
rs1664781
rs1531096
rs41279633
rs7337339
rs60220392
rs12916
rs75460349
rs117733303
rs12359135
rs78058190
rs2846205
rs112928559
rs11155073
rs12271743
rs72555385
rs59950280
rs187429064
rs17594065
rs139974673
rs141469619
rs4665710
rs1024137
rs13389219
rs174529
rs4704834
rs116843064
rs2317877
rs602633
rs115849089
rs1007205
rs1260326
rs964184
rs2954021
rs157595
rs10455872
rs72836561
rs58542926
rs183130
rs59347135
rs2115107
rs77823653
rs2144300
rs2106727
rs2074493
rs13225450
rs687339
rs10119
rs1551393
rs11751495
rs76866386
rs4775044
rs9687846
rs146203232
rs326214
rs998584
rs5757176
rs6073958
rs6974288
All - MR Egger
All - Inverse variance weighted
-1
0
1
MR effect size for
'Total lipids in medium VLDL || id:ebi-cfb233-GCST90302063' on 'ER+ Breast cancer (Combined Oncoarray; iCOGS; GWAS meta

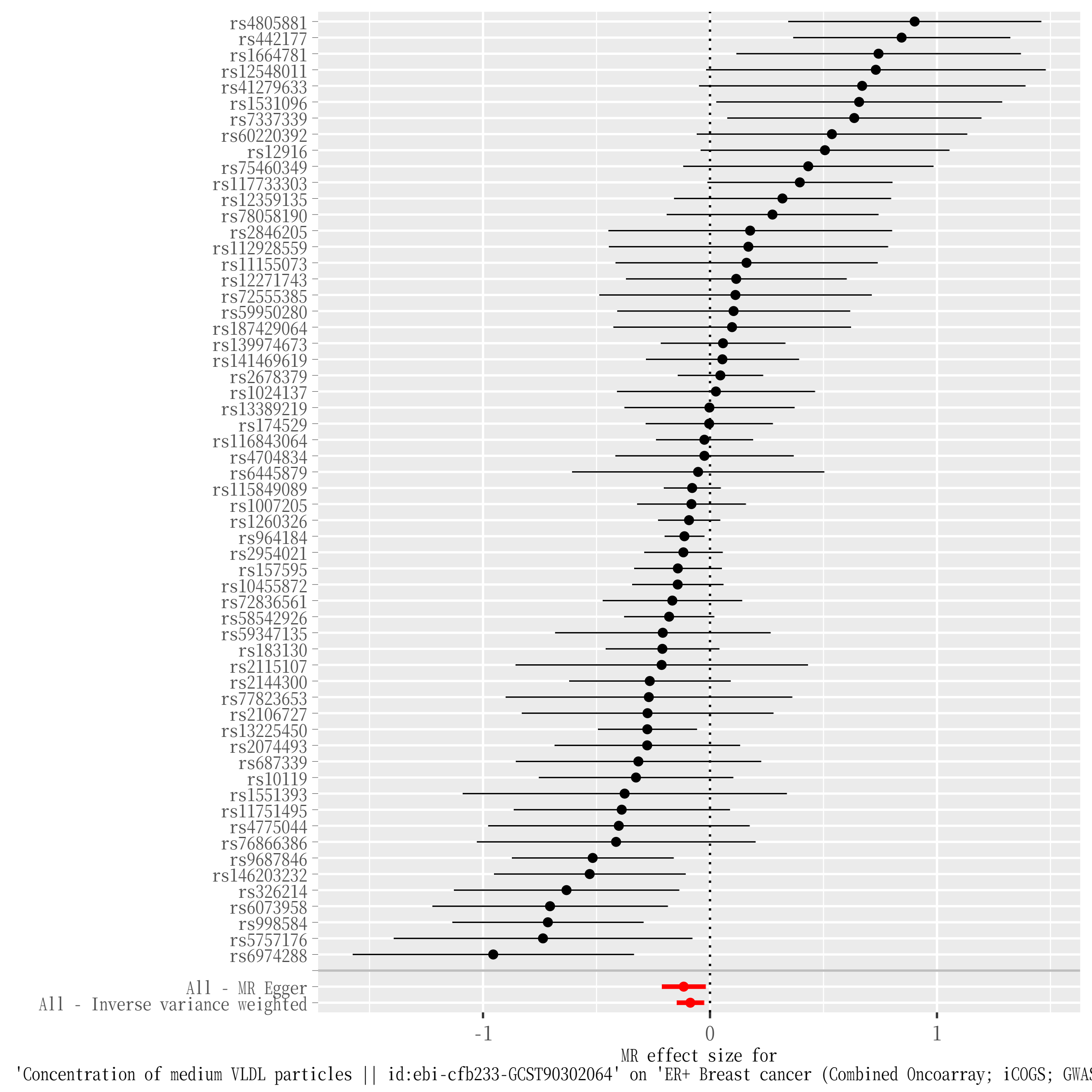

rs4805881
rs442177
rs1664781
rs12548011
rs41279633
rs1531096
rs7337339
rs60220392
rs12916
rs75460349
rs117733303
rs12359135
rs78058190
rs2846205
rs112928559
rs11155073
rs12271743
rs72555385
rs59950280
rs187429064
rs139974673
rs141469619
rs2678379
rs1024137
rs13389219
rs174529
rs116843064
rs4704834
rs6445879
rs115849089
rs1007205
rs1260326
rs964184
rs2954021
rs157595
rs10455872
rs72836561
rs58542926
rs59347135
rs183130
rs2115107
rs2144300
rs77823653
rs2106727
rs13225450
rs2074493
rs687339
rs10119
rs1551393
rs11751495
rs4775044
rs76866386
rs9687846
rs146203232
rs326214
rs6073958
rs998584
rs5757176
rs6974288
All - MR Egger
All - Inverse variance weighted
-1
0
1
MR effect size for
'Concentration of medium VLDL particles || id:ebi-cfb233-GCST90302064' on 'ER+ Breast cancer (Combined Oncoarray; iCOGS; GWAS

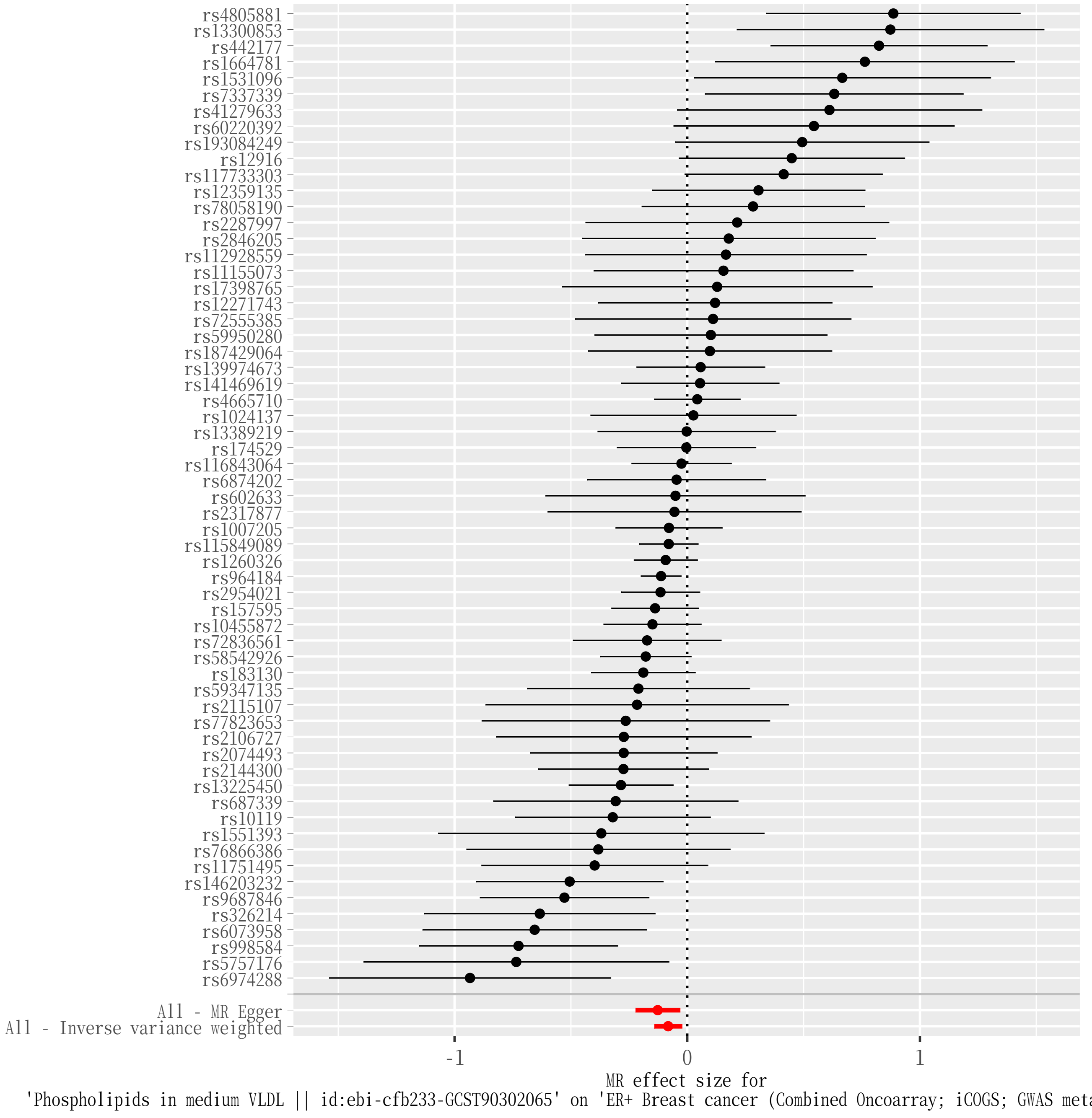

rs4805881
rs13300853
rs442177
rs1664781
rs1531096
rs7337339
rs41279633
rs60220392
rs193084249
rs12916
rs117733303
rs12359135
rs78058190
rs2287997
rs2846205
rs112928559
rs11155073
rs17398765
rs12271743
rs72555385
rs59950280
rs187429064
rs139974673
rs141469619
rs4665710
rs1024137
rs13389219
rs174529
rs116843064
rs6874202
rs602633
rs2317877
rs1007205
rs115849089
rs1260326
rs964184
rs2954021
rs157595
rs10455872
rs72836561
rs58542926
rs183130
rs59347135
rs2115107
rs77823653
rs2106727
rs2074493
rs2144300
rs13225450
rs687339
rs10119
rs1551393
rs76866386
rs11751495
rs146203232
rs9687846
rs326214
rs6073958
rs998584
rs5757176
rs6974288
All - MR Egger
All - Inverse variance weighted
-1
0
1
MR effect size for
'Phospholipids in medium VLDL || id:ebi-cfb233-GCST90302065' on 'ER+ Breast cancer (Combined Oncoarray; iCOGS; GWAS meta

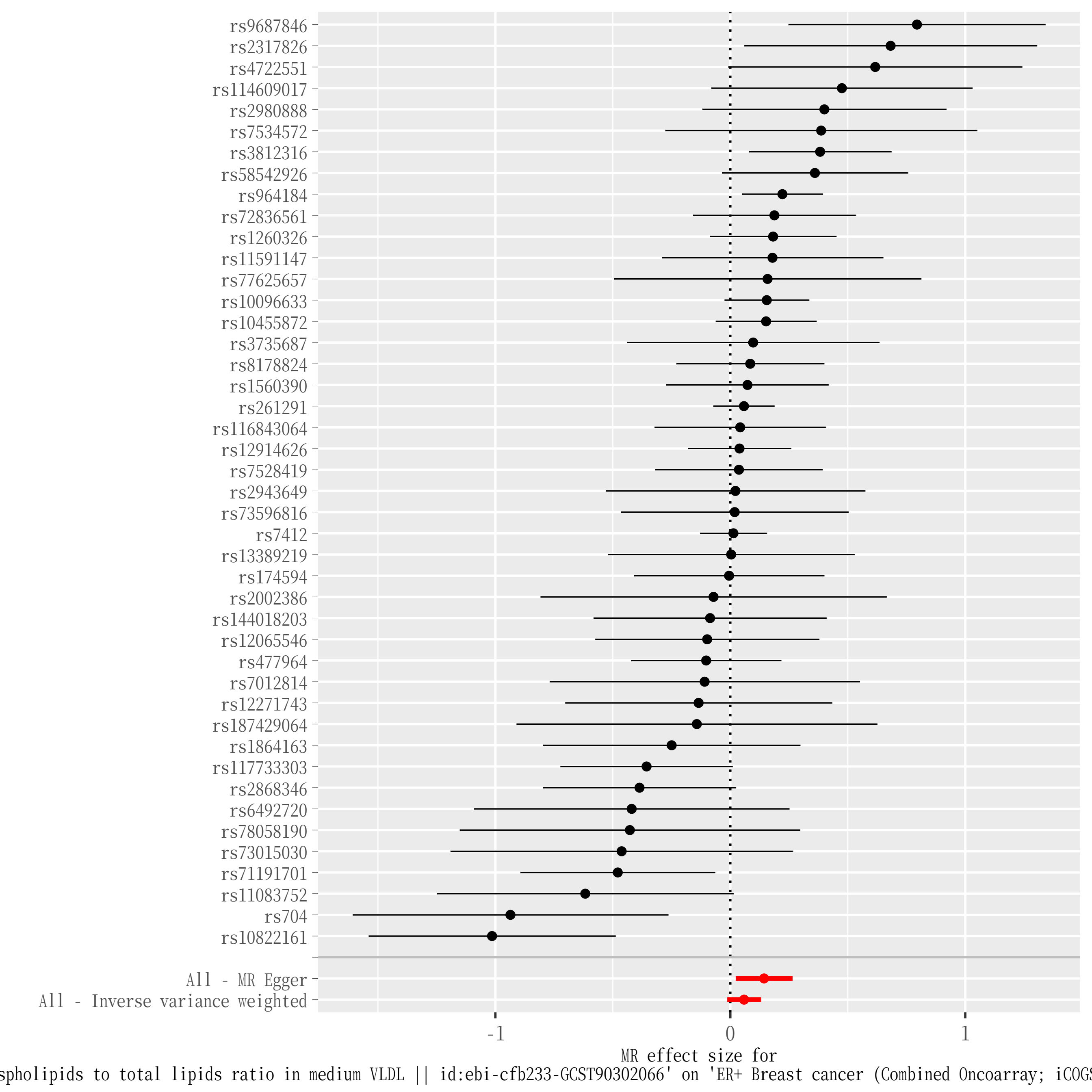

rs9687846
rs2317826
rs4722551
rs114609017
rs2980888
rs7534572
rs3812316
rs58542926
rs964184
rs72836561
rs1260326
rs11591147
rs77625657
rs10096633
rs10455872
rs3735687
rs8178824
rs1560390
rs261291
rs116843064
rs12914626
rs7528419
rs2943649
rs73596816
rs7412
rs13389219
rs174594
rs2002386
rs144018203
rs12065546
rs477964
rs7012814
rs12271743
rs187429064
rs1864163
rs117733303
rs2868346
rs6492720
rs78058190
rs73015030
rs71191701
rs11083752
rs704
rs10822161
All - MR Egger
All - Inverse variance weighted
-1
0
1
MR effect size for
spholipids to total lipids ratio in medium VLDL || id:ebi-cfb233-GCST90302066' on 'ER+ Breast cancer (Combined Oncoarray; iCOGS

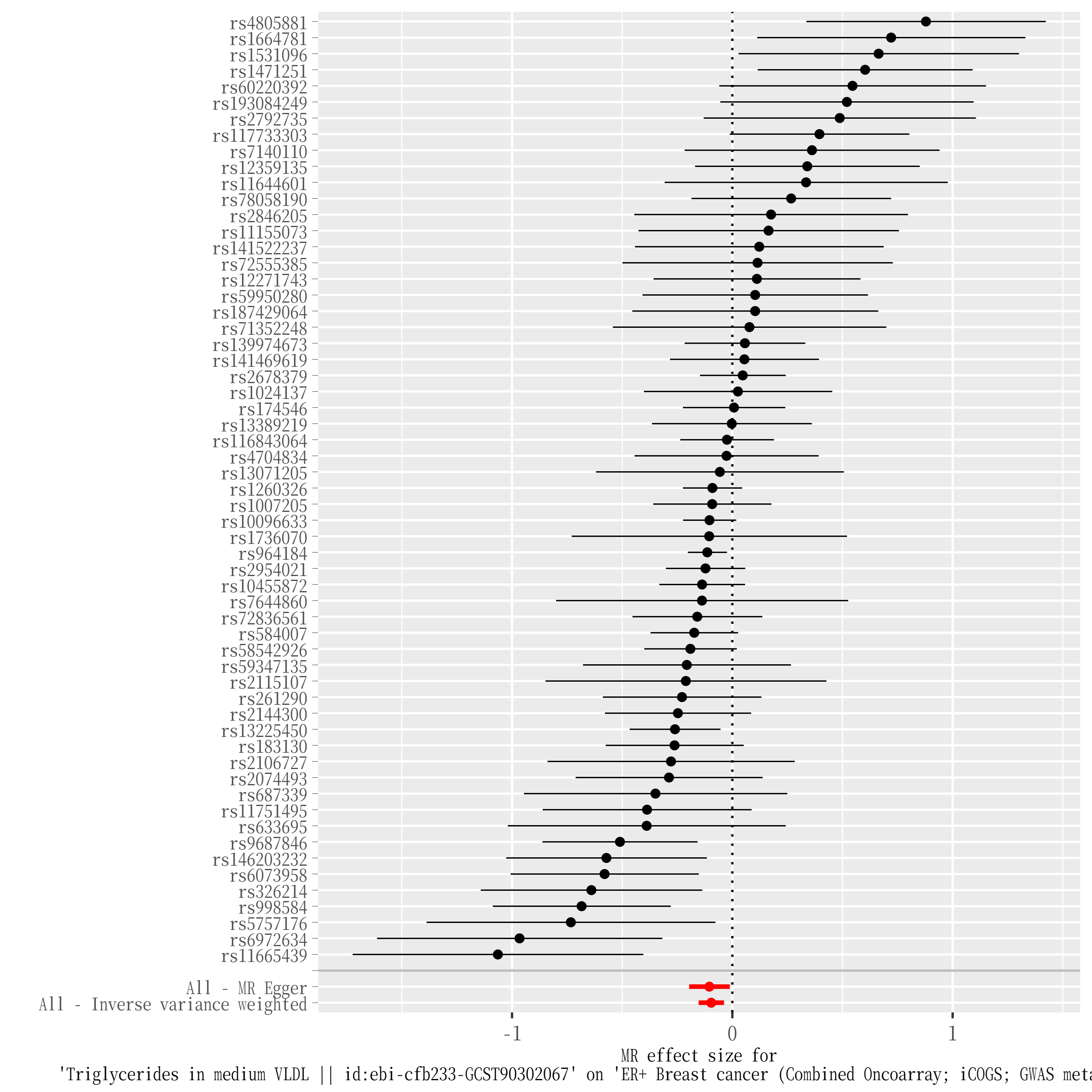
rs4805881
rs1664781
rs1531096
rs1471251
rs60220392
rs193084249
rs2792735
rs117733303
rs7140110
rs12359135
rs11644601
rs78058190
rs2846205
rs11155073
rs141522237
rs72555385
rs12271743
rs59950280
rs187429064
rs71352248
rs139974673
rs141469619
rs2678379
rs1024137
rs174546
rs13389219
rs116843064
rs4704834
rs13071205
rs1260326
rs1007205
rs10096633
rs1736070
rs964184
rs2954021
rs10455872
rs7644860
rs72836561
rs584007
rs58542926
rs59347135
rs2115107
rs261290
rs2144300
rs13225450
rs183130
rs2106727
rs2074493
rs687339
rs11751495
rs633695
rs9687846
rs146203232
rs6073958
rs326214
rs998584
rs5757176
rs6972634
rs11665439
All - MR Egger
All - Inverse variance weighted
-1
0
1
MR effect size for
'Triglycerides in medium VLDL || id:ebi-cfb233-GCST90302067' on 'ER+ Breast cancer (Combined Oncoarray; iCOGS; GWAS meta

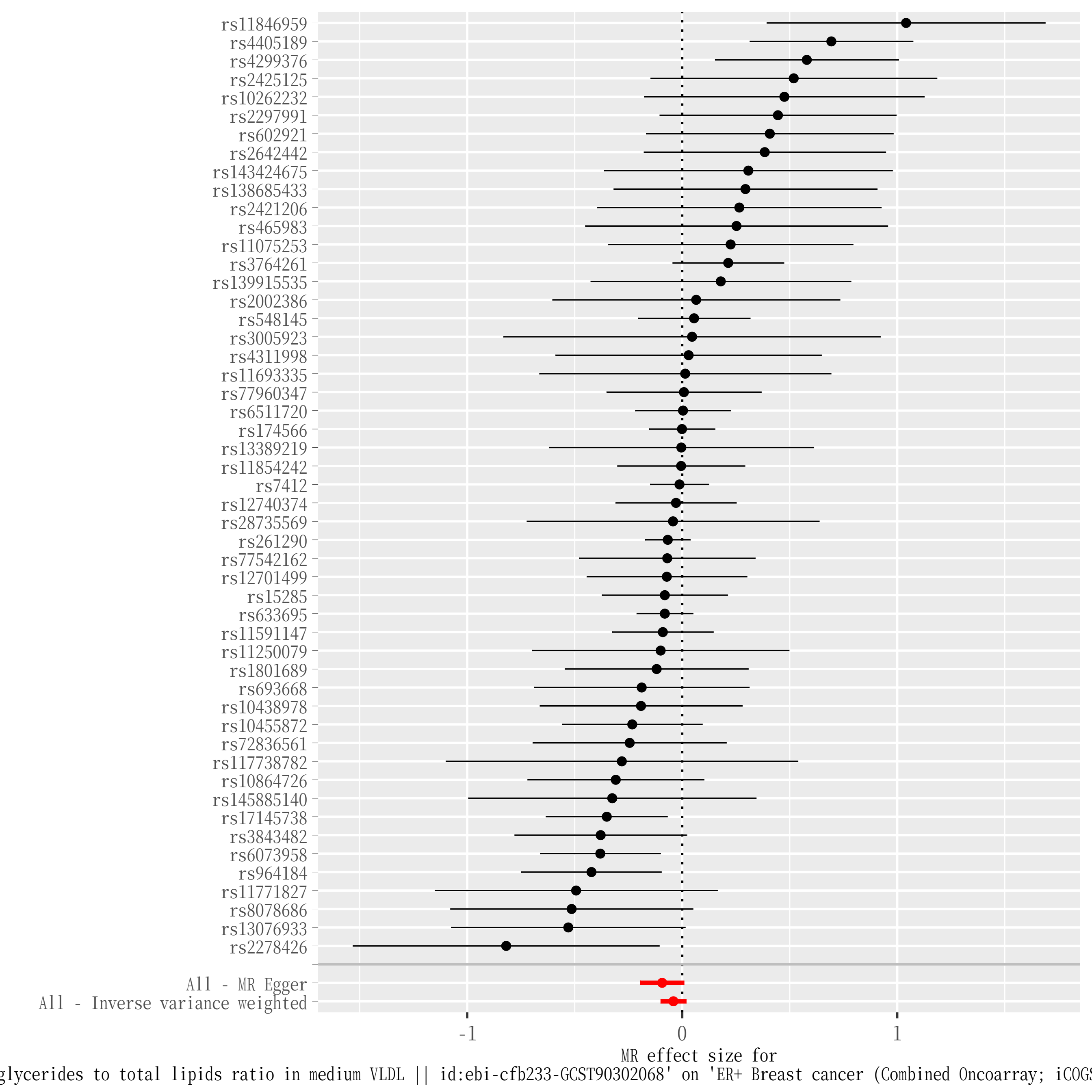
rs11846959
rs4405189
rs4299376
rs2425125
rs10262232
rs2297991
rs602921
rs2642442
rs143424675
rs138685433
rs2421206
rs465983
rs11075253
rs3764261
rs139915535
rs2002386
rs548145
rs3005923
rs4311998
rs11693335
rs77960347
rs6511720
rs174566
rs13389219
rs11854242
rs7412
rs12740374
rs28735569
rs261290
rs77542162
rs12701499
rs15285
rs633695
rs11591147
rs11250079
rs1801689
rs693668
rs10438978
rs10455872
rs72836561
rs117738782
rs10864726
rs145885140
rs17145738
rs3843482
rs6073958
rs964184
rs11771827
rs8078686
rs13076933
rs2278426
All - MR Egger
All - Inverse variance weighted
-1
0
1
MR effect size for
glycerides to total lipids ratio in medium VLDL || id:ebi-cfb233-GCST90302068' on 'ER+ Breast cancer (Combined Oncoarray; iCOGS

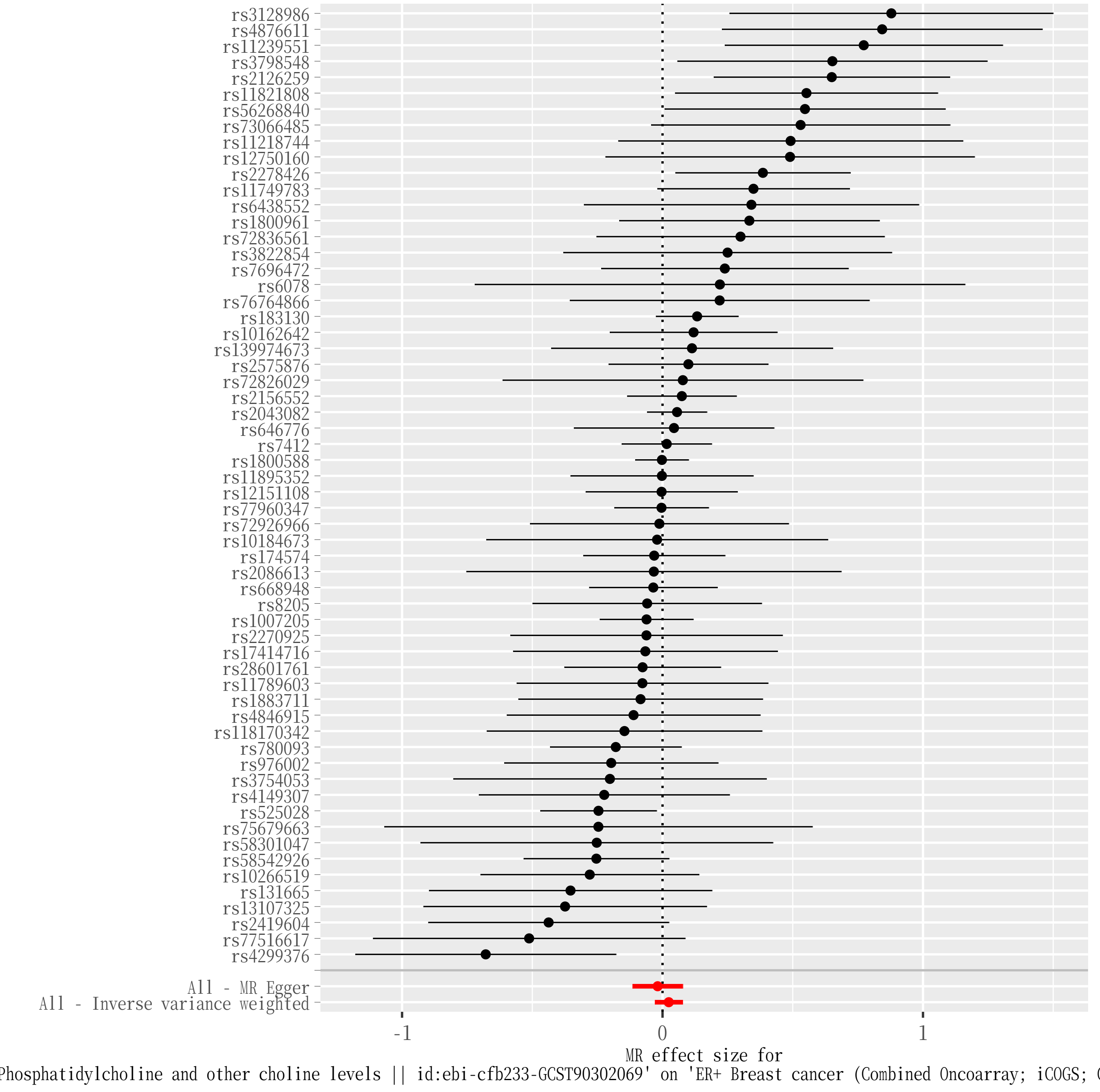
rs3128986
rs4876611
rs11239551
rs3798548
rs2126259
rs11821808
rs56268840
rs73066485
rs11218744
rs12750160
rs2278426
rs11749783
rs6438552
rs1800961
rs72836561
rs3822854
rs7696472
rs6078
rs76764866
rs183130
rs10162642
rs139974673
rs2575876
rs72826029
rs2156552
rs2043082
rs646776
rs7412
rs1800588
rs11895352
rs12151108
rs77960347
rs72926966
rs10184673
rs174574
rs2086613
rs668948
rs8205
rs1007205
rs2270925
rs17414716
rs28601761
rs11789603
rs1883711
rs4846915
rs118170342
rs780093
rs976002
rs3754053
rs4149307
rs525028
rs75679663
rs58301047
rs58542926
rs10266519
rs131665
rs13107325
rs2419604
rs77516617
rs4299376
All - MR Egger
All - Inverse variance weighted
-1
0
1
MR effect size for
'Phosphatidylcholine and other choline levels || id:ebi-cfb233-GCST90302069' on 'ER+ Breast cancer (Combined Oncoarray; iCOGS; G

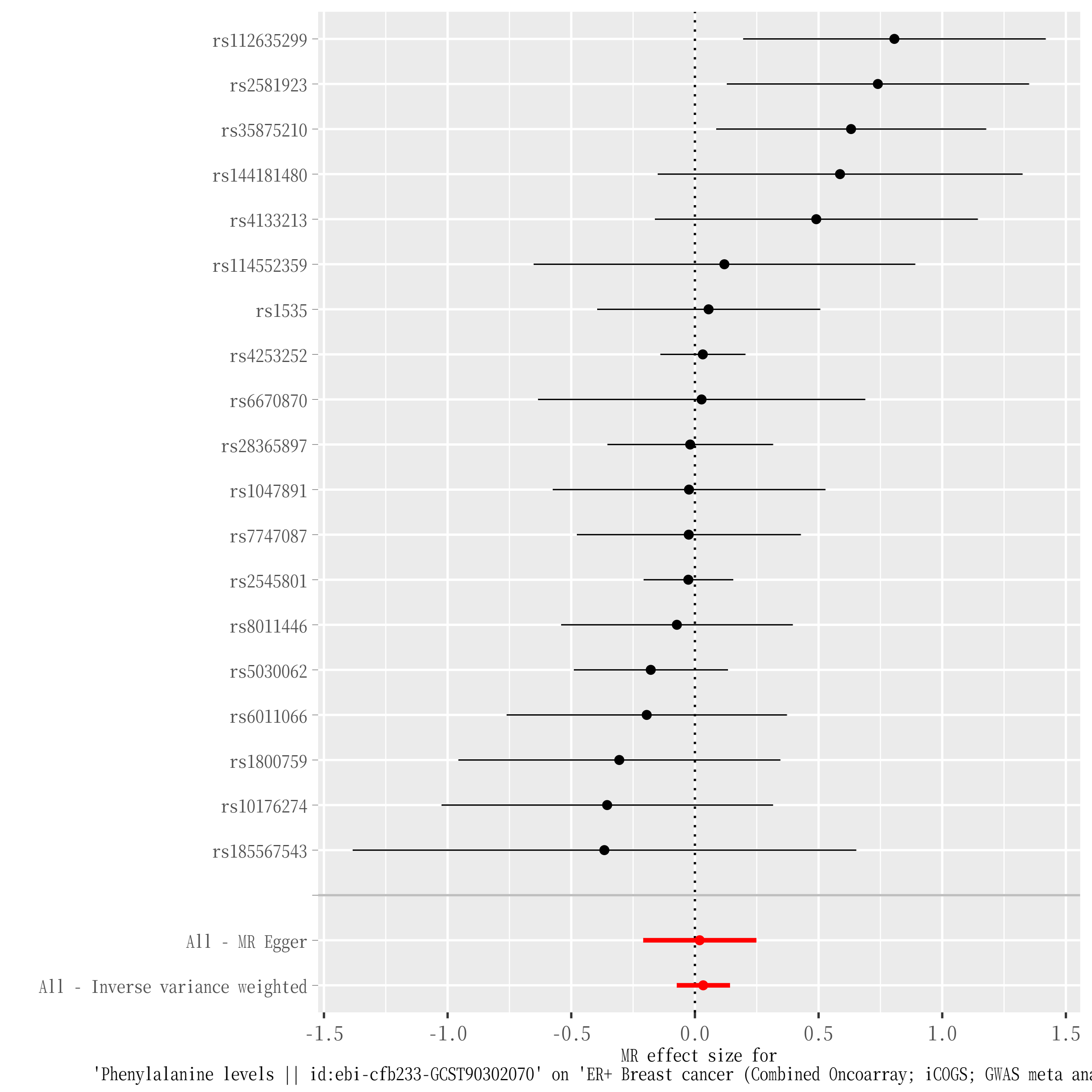

rs112635299
rs2581923
rs35875210
rs144181480
rs4133213
rs114552359
rs1535
rs4253252
rs6670870
rs28365897
rs1047891
rs7747087
rs2545801
rs8011446
rs5030062
rs6011066
rs1800759
rs10176274
rs185567543
All - MR Egger
All - Inverse variance weighted
-1.5
-1.0
-0.5
0.0
0.5
1.0
1.5
MR effect size for
'Phenylalanine levels || id:ebi-cfb233-GCST90302070' on 'ER+ Breast cancer (Combined Oncoarray; iCOGS; GWAS meta ana

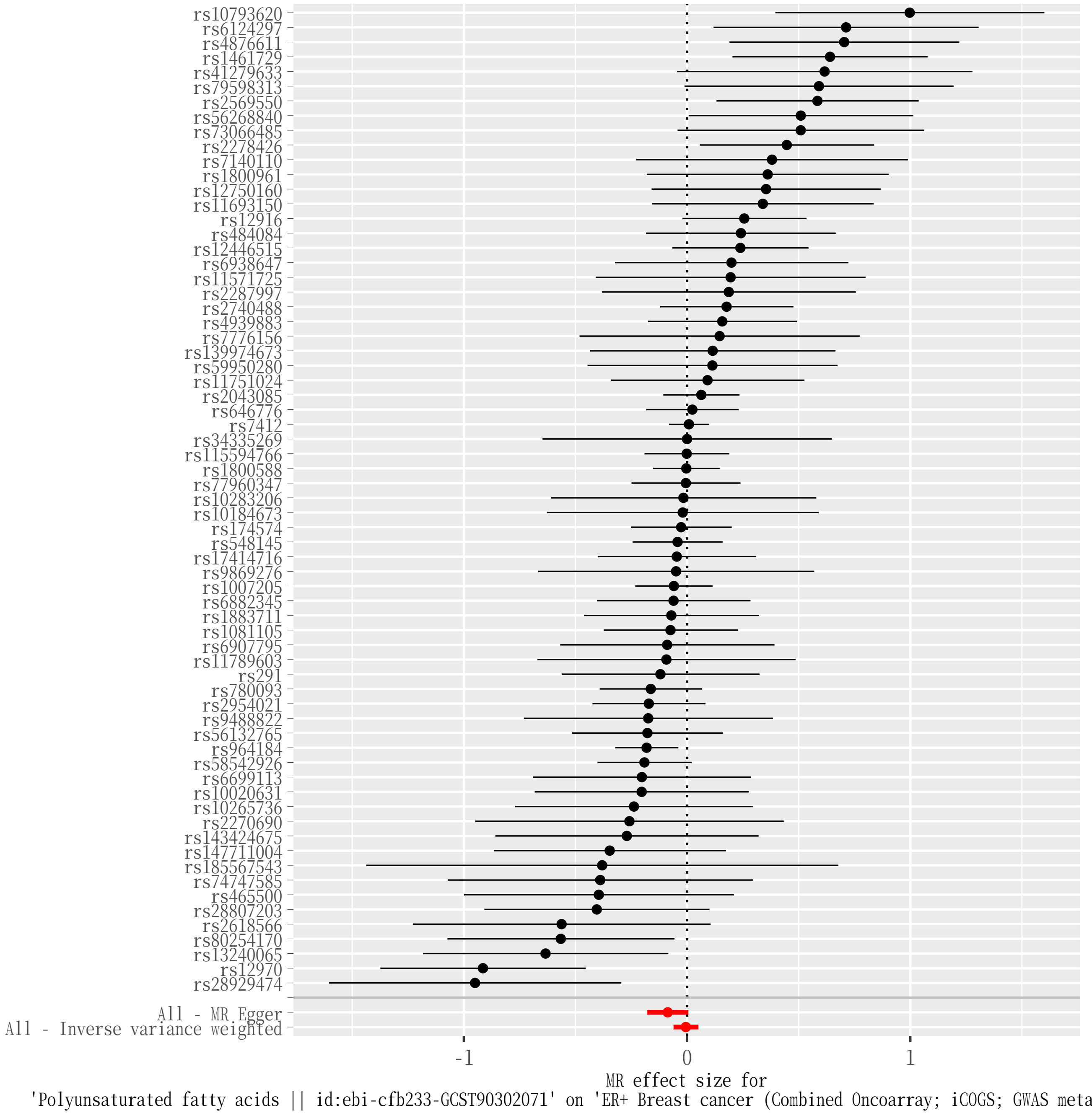
rs10793620
rs6124297
rs4876611
rs1461729
rs41279633
rs79598313
rs2569550
rs56268840
rs73066485
rs2278426
rs7140110
rs1800961
rs12750160
rs11693150
rs12916
rs484084
rs12446515
rs6938647
rs11571725
rs2287997
rs2740488
rs4939883
rs7776156
rs139974673
rs59950280
rs11751024
rs2043085
rs646776
rs7412
rs34335269
rs115594766
rs1800588
rs77960347
rs10283206
rs10184673
rs174574
rs548145
rs17414716
rs9869276
rs1007205
rs6882345
rs1883711
rs1081105
rs6907795
rs11789603
rs291
rs780093
rs2954021
rs9488822
rs56132765
rs964184
rs58542926
rs6699113
rs10020631
rs10265736
rs2270690
rs143424675
rs147711004
rs185567543
rs74747585
rs465500
rs28807203
rs2618566
rs80254170
rs13240065
rs12970
rs28929474
All - MR Egger
All - Inverse variance weighted
-1
0
1
MR effect size for
'Polyunsaturated fatty acids || id:ebi-cfb233-GCST90302071' on 'ER+ Breast cancer (Combined Oncoarray; iCOGS; GWAS meta

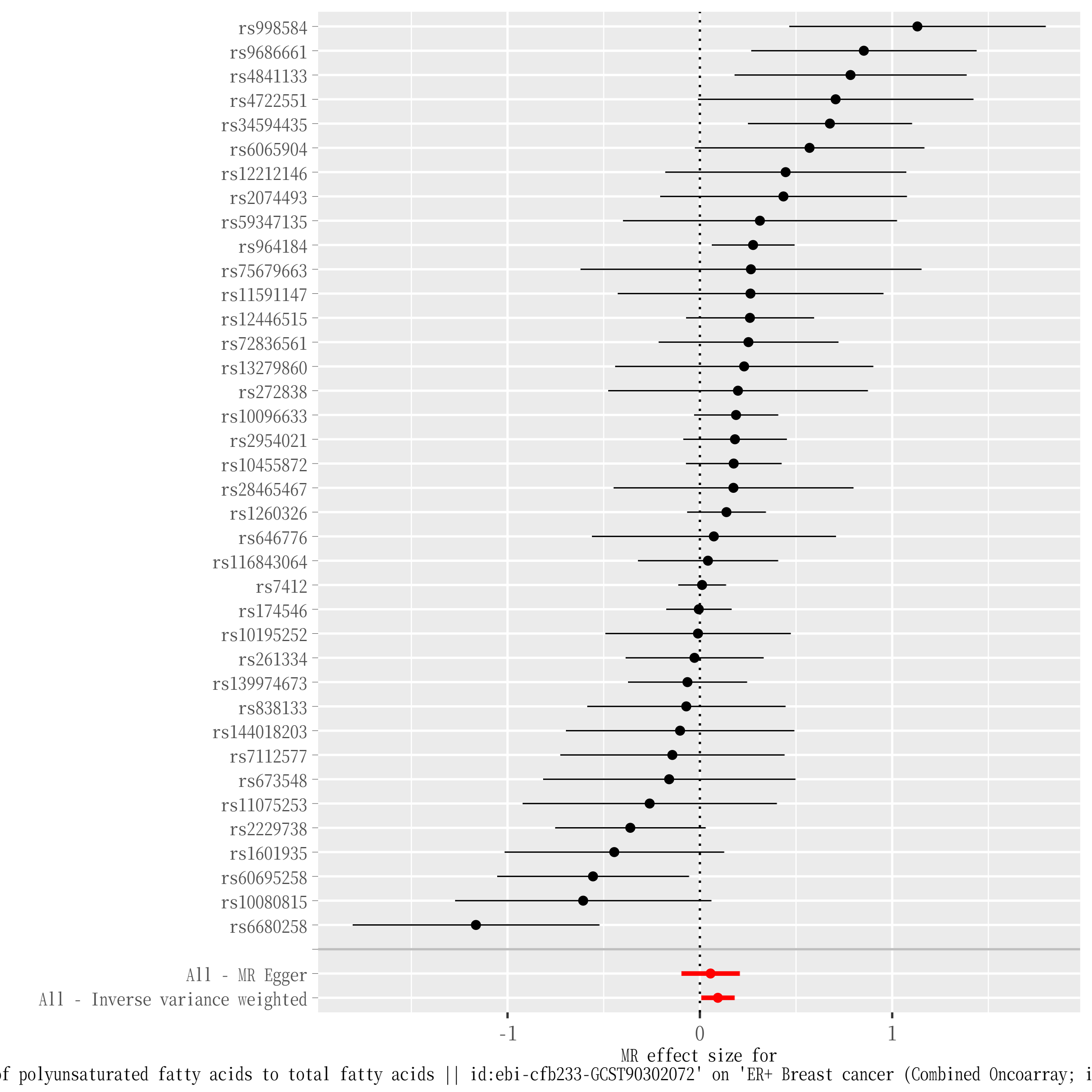
rs998584
rs9686661
rs4841133
rs4722551
rs34594435
rs6065904
rs12212146
rs2074493
rs59347135
rs964184
rs75679663
rs11591147
rs12446515
rs72836561
rs13279860
rs272838
rs10096633
rs2954021
rs10455872
rs28465467
rs1260326
rs646776
rs116843064
rs7412
rs174546
rs10195252
rs261334
rs139974673
rs838133
rs144018203
rs7112577
rs673548
rs11075253
rs2229738
rs1601935
rs60695258
rs10080815
rs6680258
All - MR Egger
All - Inverse variance weighted
-1
0
1
MR effect size for
of polyunsaturated fatty acids to total fatty acids || id:ebi-cfb233-GCST90302072' on 'ER+ Breast cancer (Combined Oncoarray; i

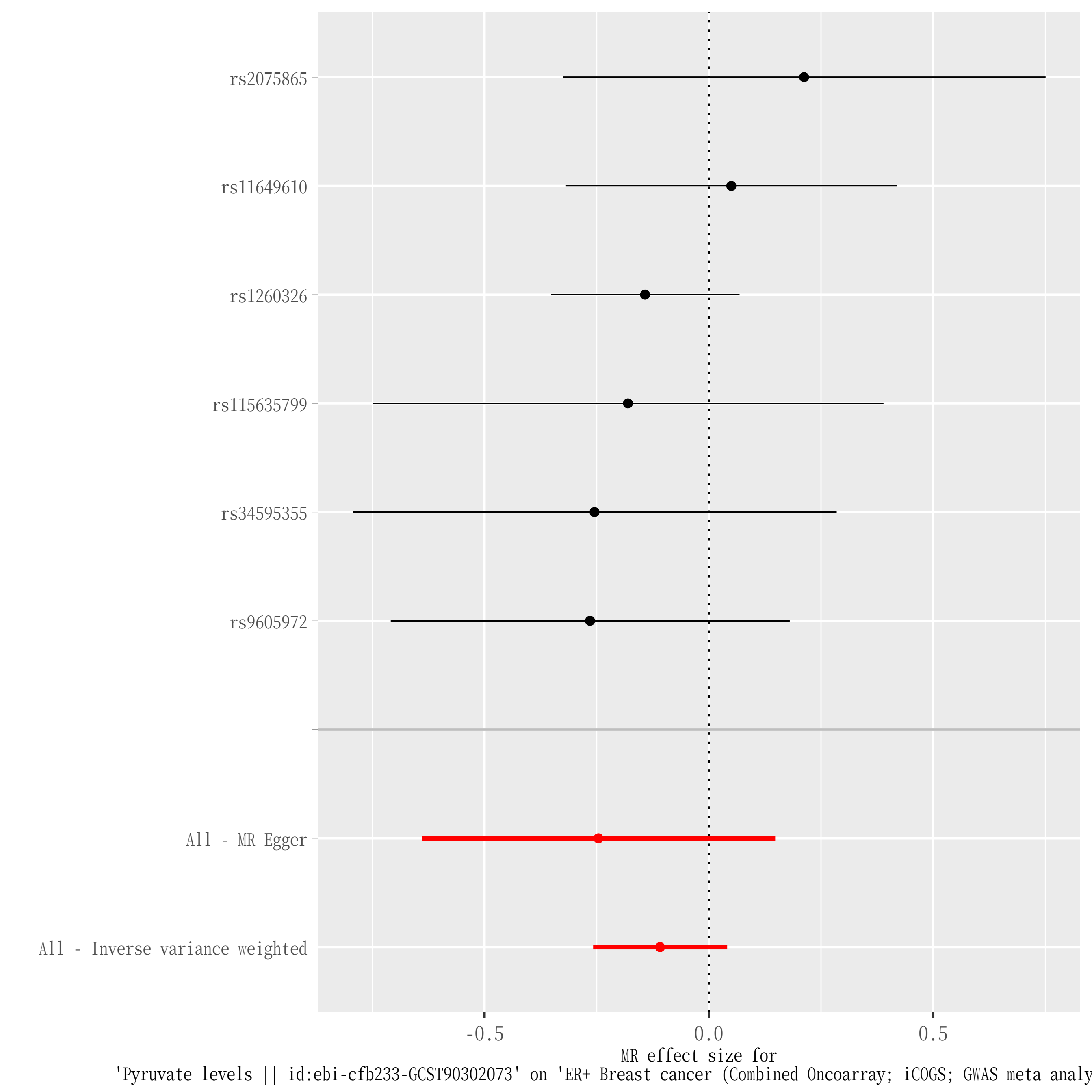
rs2075865
rs11649610
rs1260326
rs115635799
rs34595355
rs9605972
All - MR Egger
All - Inverse variance weighted
-0.5
0.0
0.5
MR effect size for
'Pyruvate levels || id:ebi-cfb233-GCST90302073' on 'ER+ Breast cancer (Combined Oncoarray; iCOGS; GWAS meta analy

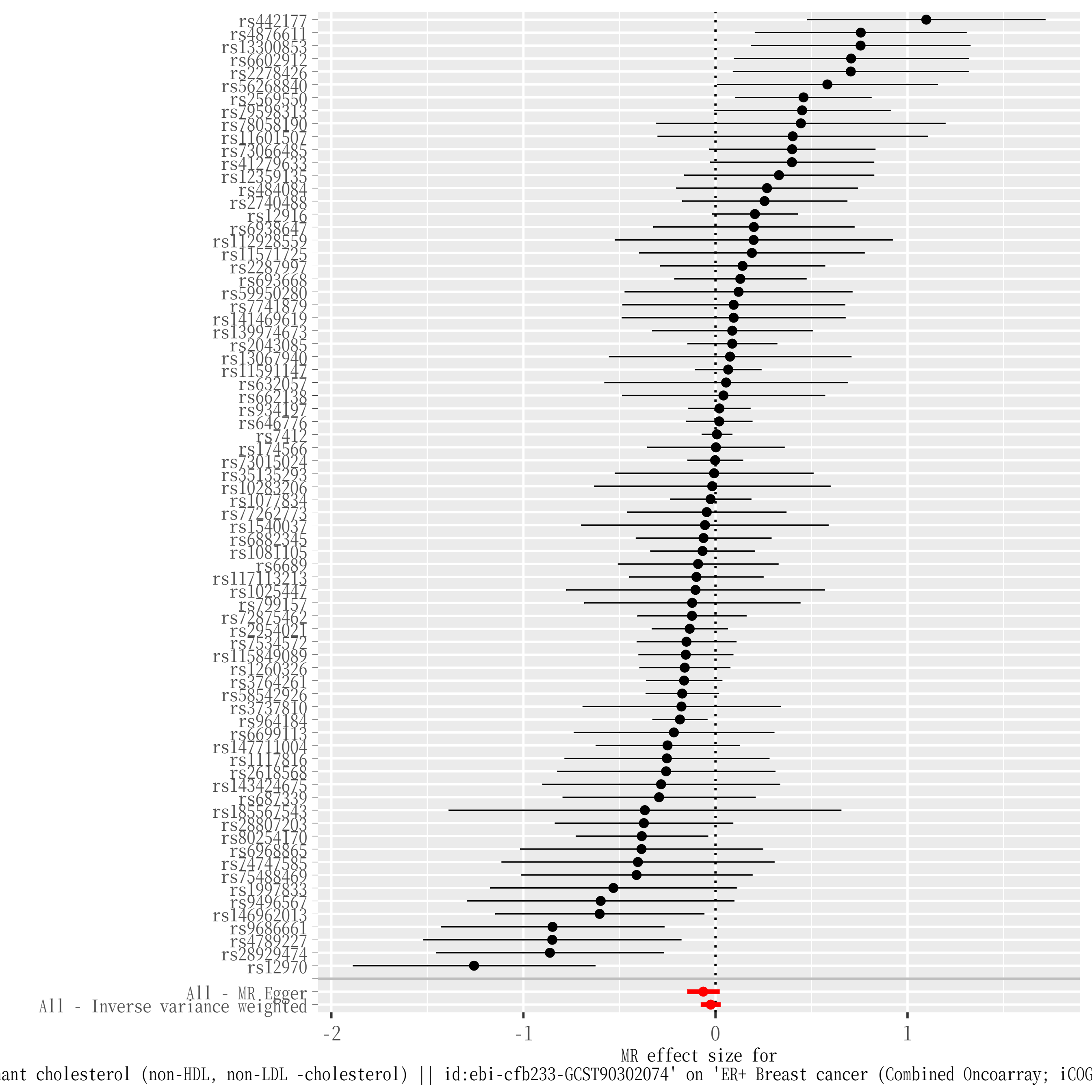
rs442177
rs4876611
rs13300853
rs6602912
rs2278426
rs56268840
rs2569550
rs79598313
rs78058190
rs11601507
rs73066485
rs41279633
rs12359135
rs484084
rs2740488
rs12916
rs6938647
rs112928559
rs11571725
rs2287997
rs693668
rs59950280
rs7741879
rs141469619
rs139974673
rs2043085
rs13067940
rs11591147
rs632057
rs662138
rs934197
rs646776
rs7412
rs174566
rs73015024
rs35135293
rs10283206
rs1077834
rs77262773
rs1540037
rs6882345
rs1081105
rs6689
rs117113213
rs1025447
rs799157
rs72875462
rs2954021
rs7534572
rs115849089
rs1260326
rs3764261
rs58542926
rs3737810
rs964184
rs6699113
rs147711004
rs1117816
rs2618568
rs143424675
rs687339
rs185567543
rs28807203
rs80254170
rs6968865
rs74747585
rs75488469
rs1997833
rs9496567
rs146962013
rs9686661
rs4789227
rs28929474
rs12970
All - MR Egger
All - Inverse variance weighted
-2
-1
0
1
MR effect size for
ant cholesterol (non-HDL, non-LDL -cholesterol) || id:ebi-cfb233-GCST90302074' on 'ER+ Breast cancer (Combined Oncoarray; iCOG

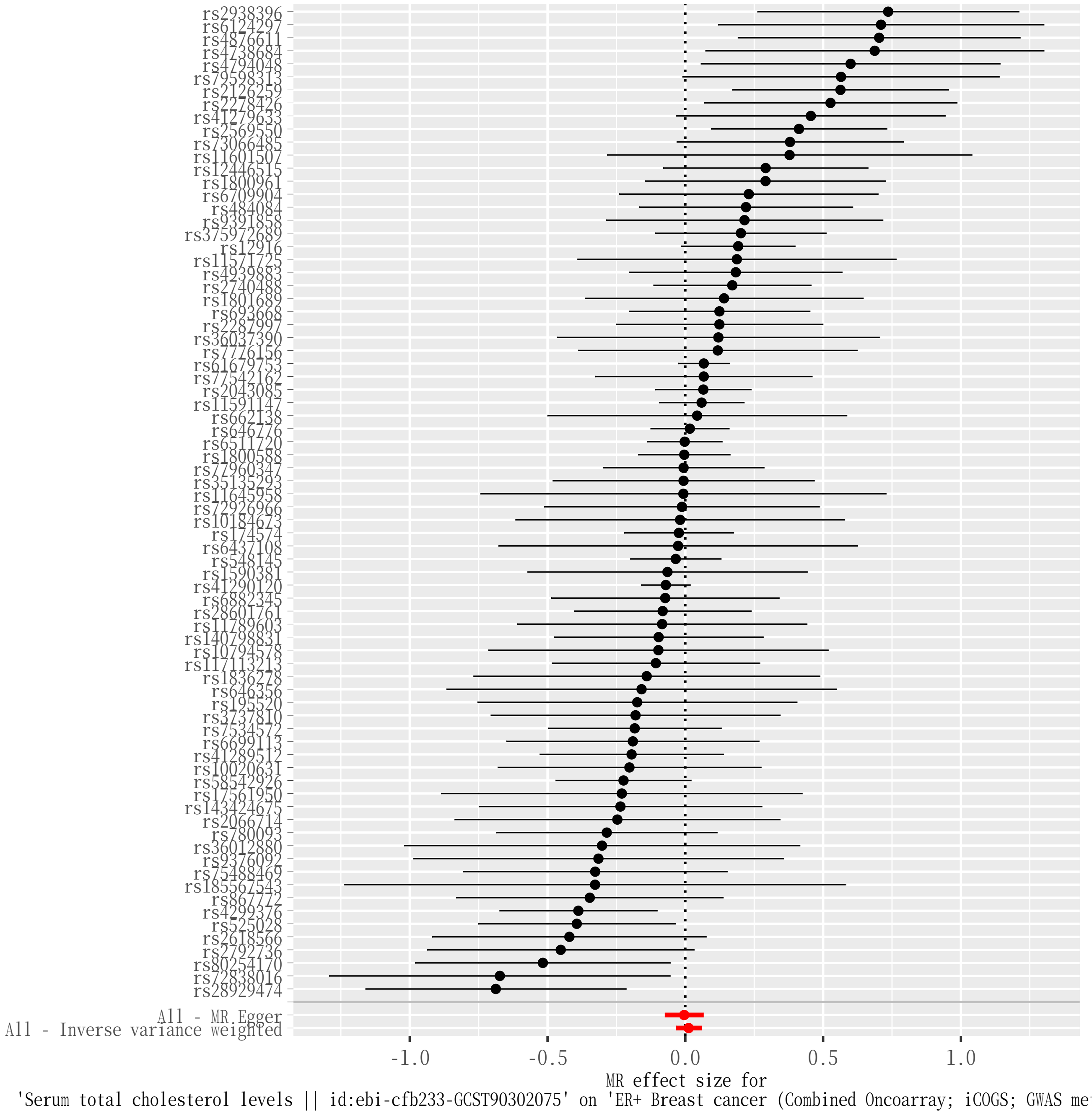
rs2938396
rs6124297
rs4876611
rs4738684
rs4794048
rs79598313
rs2126259
rs2278426
rs41279633
rs2569550
rs73066485
rs11601507
rs12446515
rs1800961
rs6709904
rs484084
rs9321858
rs375972689
rs12916
rs11571725
rs4939883
rs2740488
rs1801689
rs693668
rs2287997
rs36037390
rs7776156
rs61679753
rs77542162
rs2043085
rs11591147
rs662138
rs646776
rs6511720
rs1800588
rs77960347
rs35135293
rs11645958
rs72926966
rs10184673
rs174574
rs6437108
rs548145
rs1590381
rs41290120
rs6882345
rs28601761
rs11789603
rs140798831
rs10794578
rs117113213
rs1836278
rs646356
rs195520
rs3737810
rs7534572
rs6699113
rs41289512
rs10020631
rs58542926
rs17561950
rs143424675
rs2066714
rs780093
rs36012880
rs9376092
rs75488469
rs185567543
rs867772
rs4299376
rs525028
rs2618566
rs2792736
rs80254170
rs72838016
rs28929474
All - MR Egger
All - Inverse variance weighted
-1.0
-0.5
0.0
0.5
1.0
MR effect size for
'Serum total cholesterol levels || id:ebi-cfb233-GCST90302075' on 'ER+ Breast cancer (Combined Oncoarray; iCOGS; GWAS me

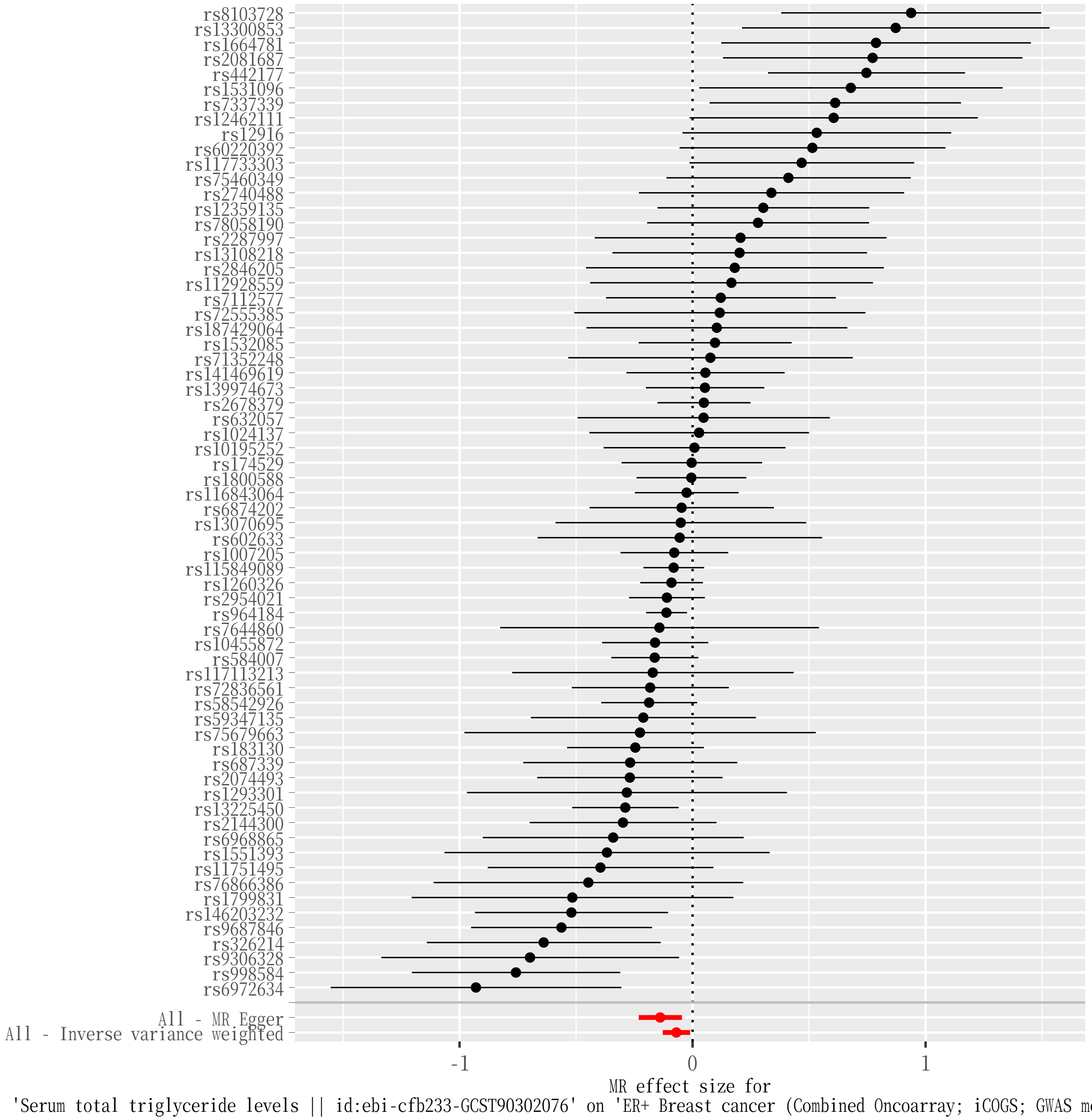

rs8103728
rs13300853
rs1664781
rs2081687
rs442177
rs1531096
rs7337339
rs12462111
rs12916
rs60220392
rs117733303
rs75460349
rs2740488
rs12359135
rs78058190
rs2287997
rs13108218
rs2846205
rs112928559
rs7112577
rs72555385
rs187429064
rs1532085
rs71352248
rs141469619
rs139974673
rs2678379
rs632057
rs1024137
rs10195252
rs174529
rs1800588
rs116843064
rs6874202
rs13070695
rs602633
rs1007205
rs115849089
rs1260326
rs2954021
rs964184
rs7644860
rs10455872
rs584007
rs117113213
rs72836561
rs58542926
rs59347135
rs75679663
rs183130
rs687339
rs2074493
rs1293301
rs13225450
rs2144300
rs6968865
rs1551393
rs11751495
rs76866386
rs1799831
rs146203232
rs9687846
rs326214
rs9306328
rs998584
rs6972634
All - MR Egger
All - Inverse variance weighted
-1
0
1
MR effect size for
'Serum total triglyceride levels || id:ebi-cfb233-GCST90302076' on 'ER+ Breast cancer (Combined Oncoarray; iCOGS; GWAS me

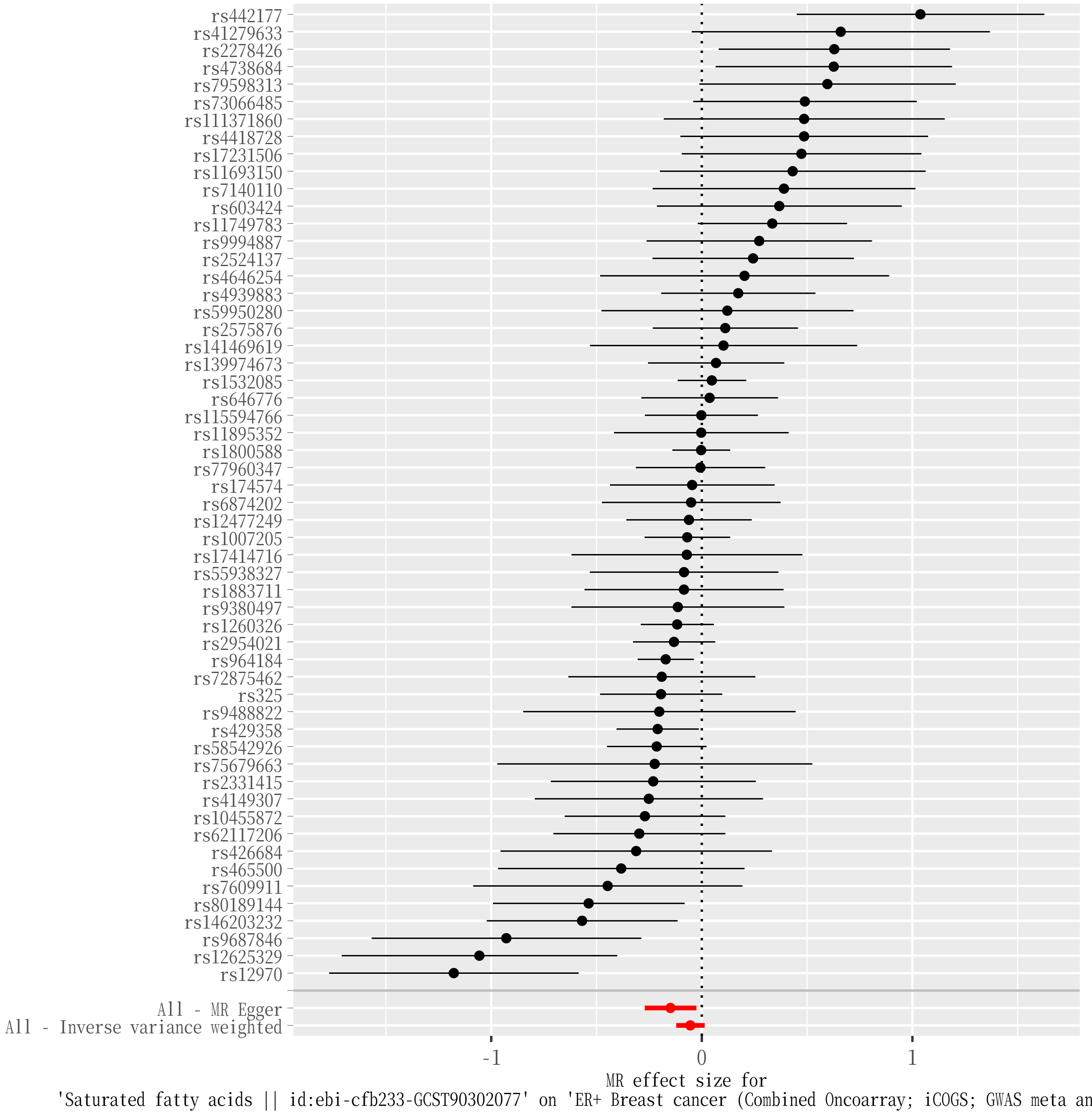
rs442177
rs41279633
rs2278426
rs4738684
rs79598313
rs73066485
rs111371860
rs4418728
rs17231506
rs11693150
rs7140110
rs603424
rs11749783
rs9994887
rs2524137
rs4646254
rs4939883
rs59950280
rs2575876
rs141469619
rs139974673
rs1532085
rs646776
rs115594766
rs11895352
rs1800588
rs77960347
rs174574
rs6874202
rs12477249
rs1007205
rs17414716
rs55938327
rs1883711
rs9380497
rs1260326
rs2954021
rs964184
rs72875462
rs325
rs9488822
rs429358
rs58542926
rs75679663
rs2331415
rs4149307
rs10455872
rs62117206
rs426684
rs465500
rs7609911
rs80189144
rs146203232
rs9687846
rs12625329
rs12970
All - MR Egger
All - Inverse variance weighted
-1
0
1
MR effect size for
'Saturated fatty acids || id:ebi-cfb233-GCST90302077' on 'ER+ Breast cancer (Combined Oncoarray; iCOGS; GWAS meta an

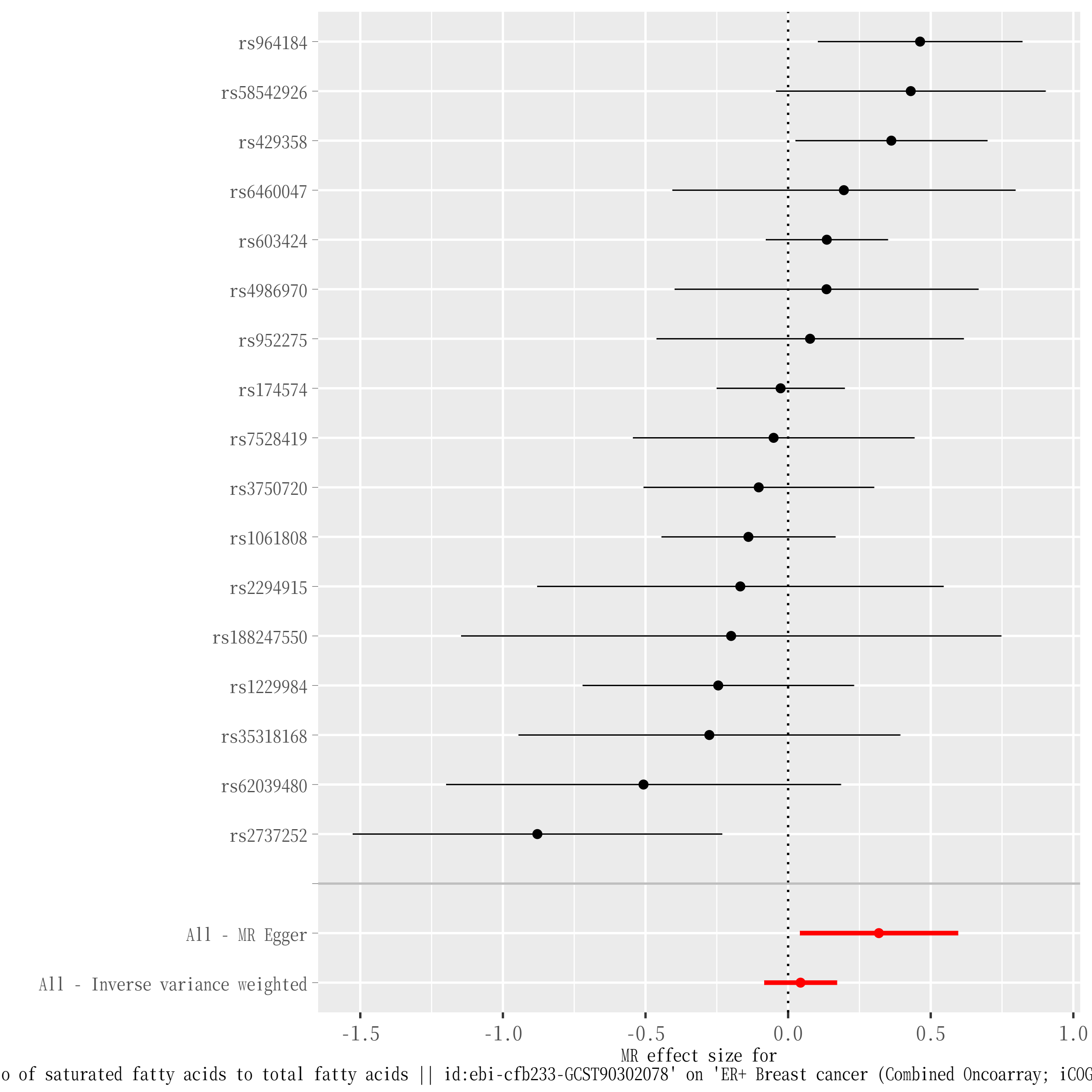

rs964184
rs58542926
rs429358
rs6460047
rs603424
rs4986970
rs952275
rs174574
rs7528419
rs3750720
rs1061808
rs2294915
rs188247550
rs1229984
rs35318168
rs62039480
rs2737252
All - MR Egger
All - Inverse variance weighted
-1.5
-1.0
-0.5
0.0
0.5
1.0
MR effect size for
o of saturated fatty acids to total fatty acids || id:ebi-cfb233-GCST90302078' on 'ER+ Breast cancer (Combined Oncoarray; iCOG

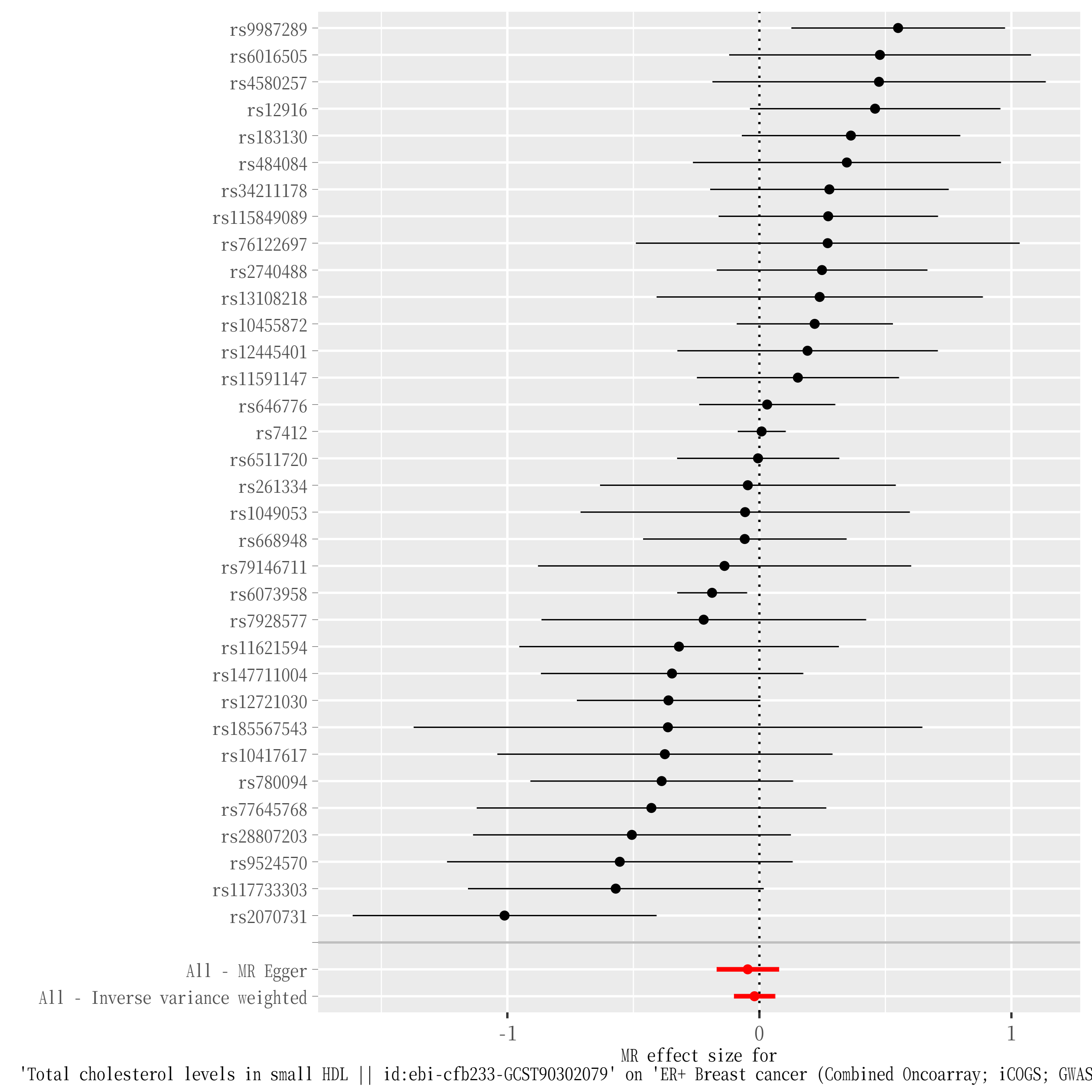

rs9987289
rs6016505
rs4580257
rs12916
rs183130
rs484084
rs34211178
rs115849089
rs76122697
rs2740488
rs13108218
rs10455872
rs12445401
rs11591147
rs646776
rs7412
rs6511720
rs261334
rs1049053
rs668948
rs79146711
rs6073958
rs7928577
rs11621594
rs147711004
rs12721030
rs185567543
rs10417617
rs780094
rs77645768
rs28807203
rs9524570
rs117733303
rs2070731
All - MR Egger
All - Inverse variance weighted
-1
0
1
MR effect size for
'Total cholesterol levels in small HDL || id:ebi-cfb233-GCST90302079' on 'ER+ Breast cancer (Combined Oncoarray; iCOGS; GWAS

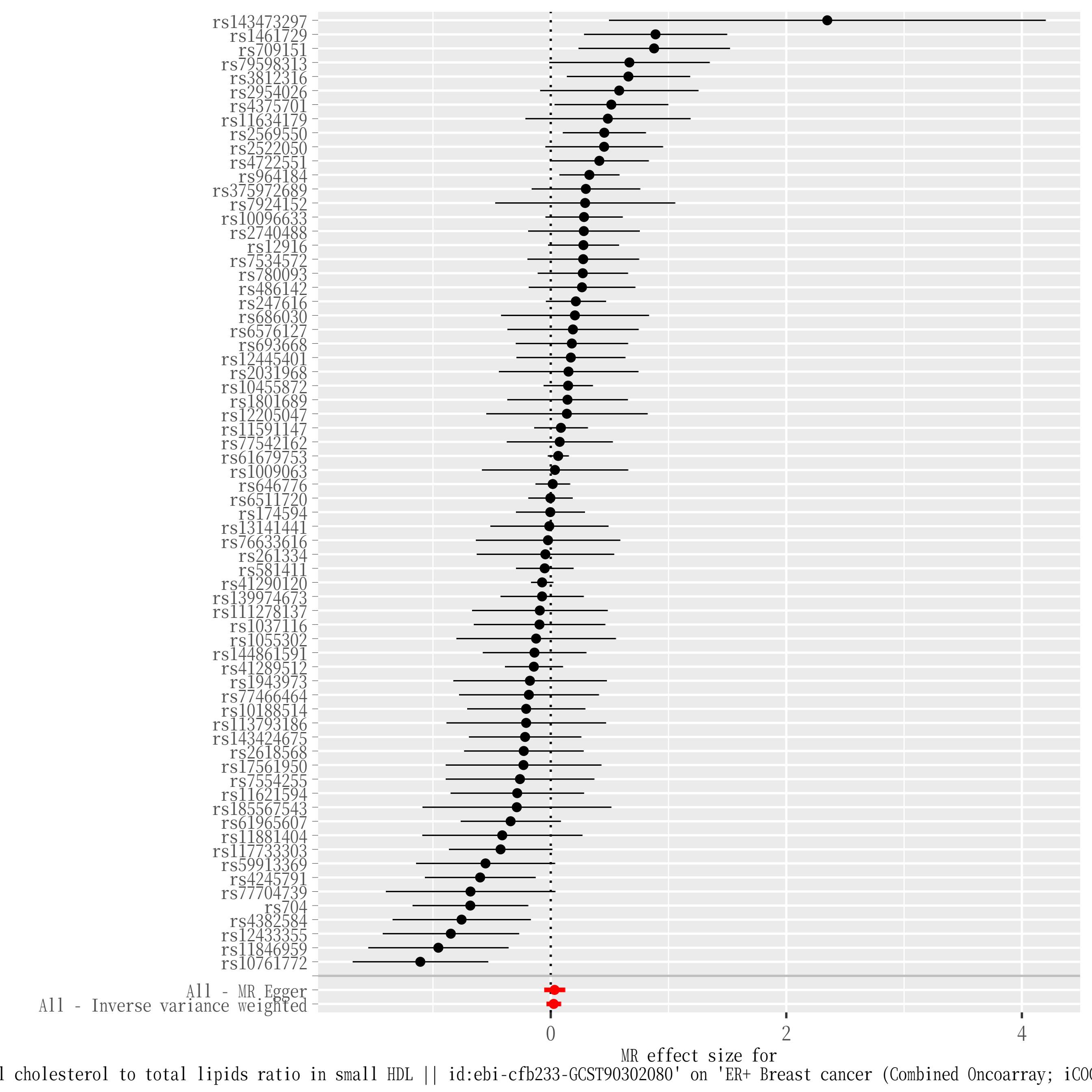

rs143473297
rs1461729
rs709151
rs79598313
rs3812316
rs2954026
rs4375701
rs11634179
rs2569550
rs2522050
rs4722551
rs964184
rs375972689
rs7924152
rs10096633
rs2740488
rs12916
rs7534572
rs780093
rs486142
rs247616
rs686030
rs6576127
rs693668
rs12445401
rs2031968
rs10455872
rs1801689
rs12205047
rs11591147
rs77542162
rs61679753
rs1009063
rs646776
rs6511720
rs174594
rs13141441
rs76633616
rs261334
rs581411
rs41290120
rs139974673
rs111278137
rs1037116
rs1055302
rs144861591
rs41289512
rs1943973
rs77466464
rs10188514
rs113793186
rs143424675
rs2618568
rs17561950
rs7554255
rs11621594
rs185567543
rs61965607
rs11881404
rs117733303
rs59913369
rs4245791
rs77704739
rs704
rs4382584
rs12433355
rs11846959
rs10761772
All - MR Egger
All - Inverse variance weighted
0
2
4
MR effect size for
l cholesterol to total lipids ratio in small HDL || id:ebi-cfb233-GCST90302080' on 'ER+ Breast cancer (Combined Oncoarray; iCOG

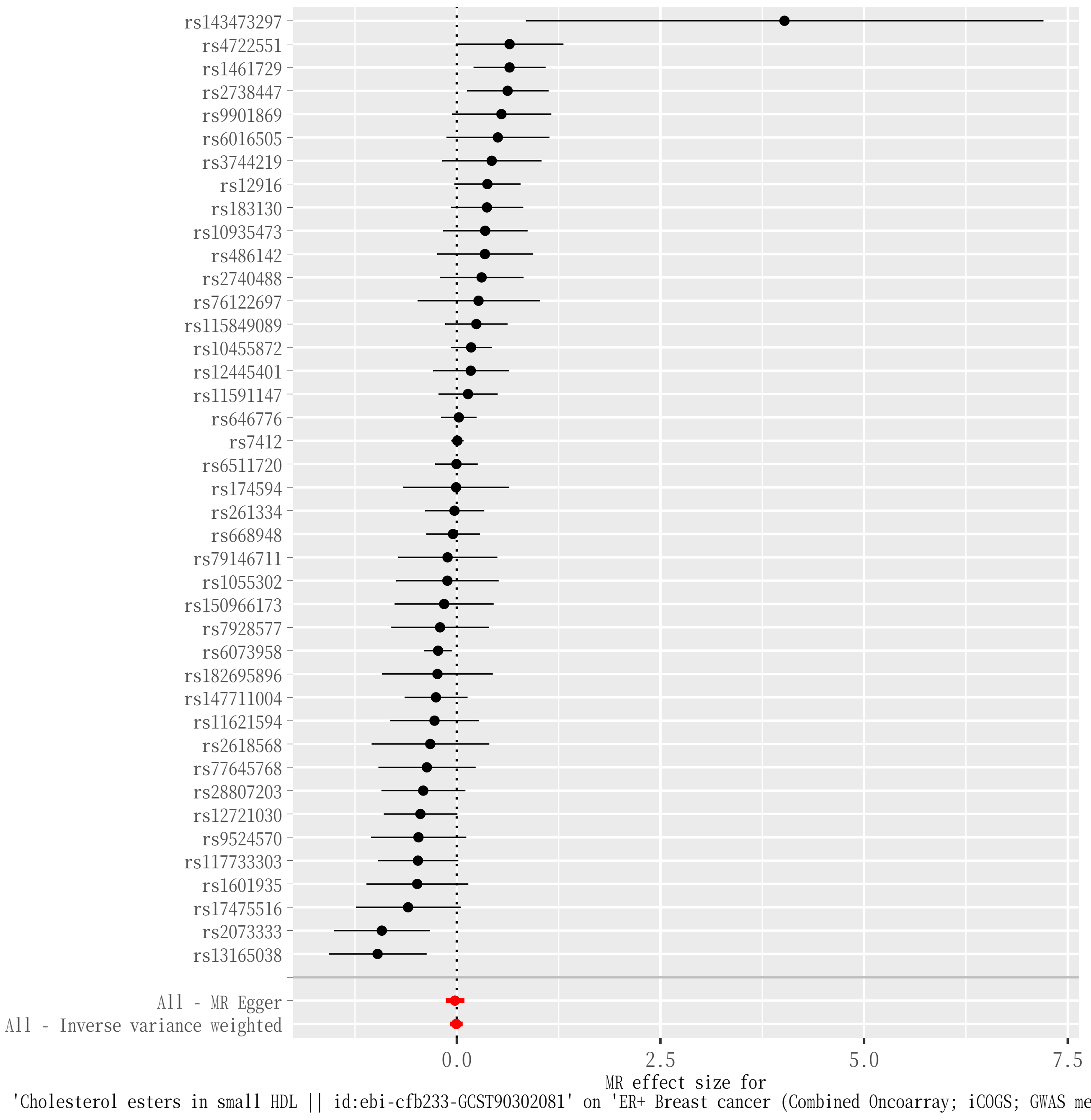

rs143473297
rs4722551
rs1461729
rs2738447
rs9901869
rs6016505
rs3744219
rs12916
rs183130
rs10935473
rs486142
rs2740488
rs76122697
rs115849089
rs10455872
rs12445401
rs11591147
rs646776
rs7412
rs6511720
rs174594
rs261334
rs668948
rs79146711
rs1055302
rs150966173
rs7928577
rs6073958
rs182695896
rs147711004
rs11621594
rs2618568
rs77645768
rs28807203
rs12721030
rs9524570
rs117733303
rs1601935
rs17475516
rs2073333
rs13165038
All - MR Egger
All - Inverse variance weighted
0.0
2.5
5.0
7.5
MR effect size for
'Cholesterol esters in small HDL || id:ebi-cfb233-GCST90302081' on 'ER+ Breast cancer (Combined Oncoarray; iCOGS; GWAS me

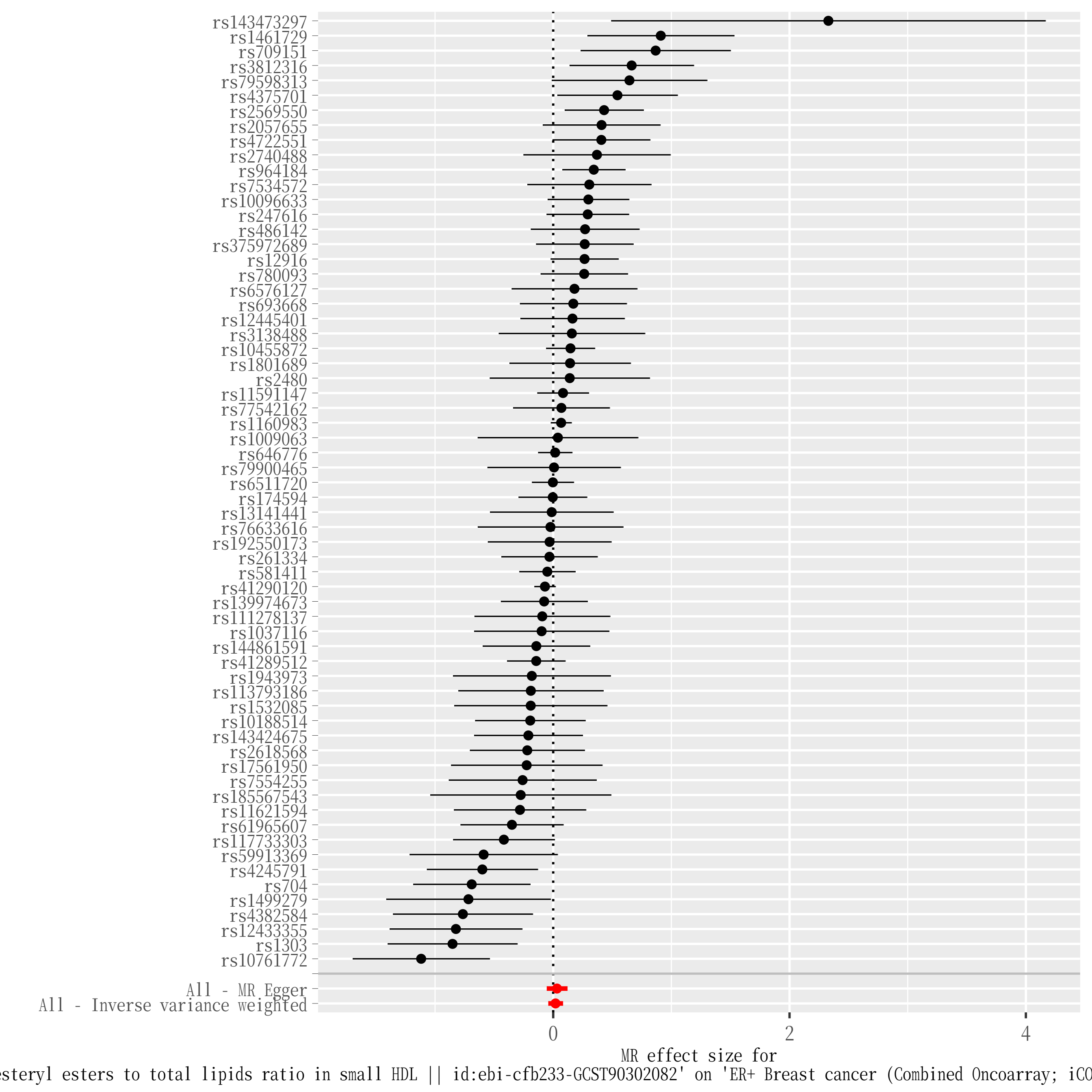
rs143473297
rs1461729
rs709151
rs3812316
rs79598313
rs4375701
rs2569550
rs2057655
rs4722551
rs2740488
rs964184
rs7534572
rs10096633
rs247616
rs486142
rs375972689
rs12916
rs780093
rs6576127
rs693668
rs12445401
rs3138488
rs10455872
rs1801689
rs2480
rs11591147
rs77542162
rs1160983
rs1009063
rs646776
rs79900465
rs6511720
rs174594
rs13141441
rs76633616
rs192550173
rs261334
rs581411
rs41290120
rs139974673
rs111278137
rs1037116
rs144861591
rs41289512
rs1943973
rs113793186
rs1532085
rs10188514
rs143424675
rs2618568
rs17561950
rs7554255
rs185567543
rs11621594
rs61965607
rs117733303
rs59913369
rs4245791
rs704
rs1499279
rs4382584
rs12433355
rs1303
rs10761772
All - MR Egger
All - Inverse variance weighted
0
2
4
MR effect size for
esteryl esters to total lipids ratio in small HDL || id:ebi-cfb233-GCST90302082' on 'ER+ Breast cancer (Combined Oncoarray; iCO

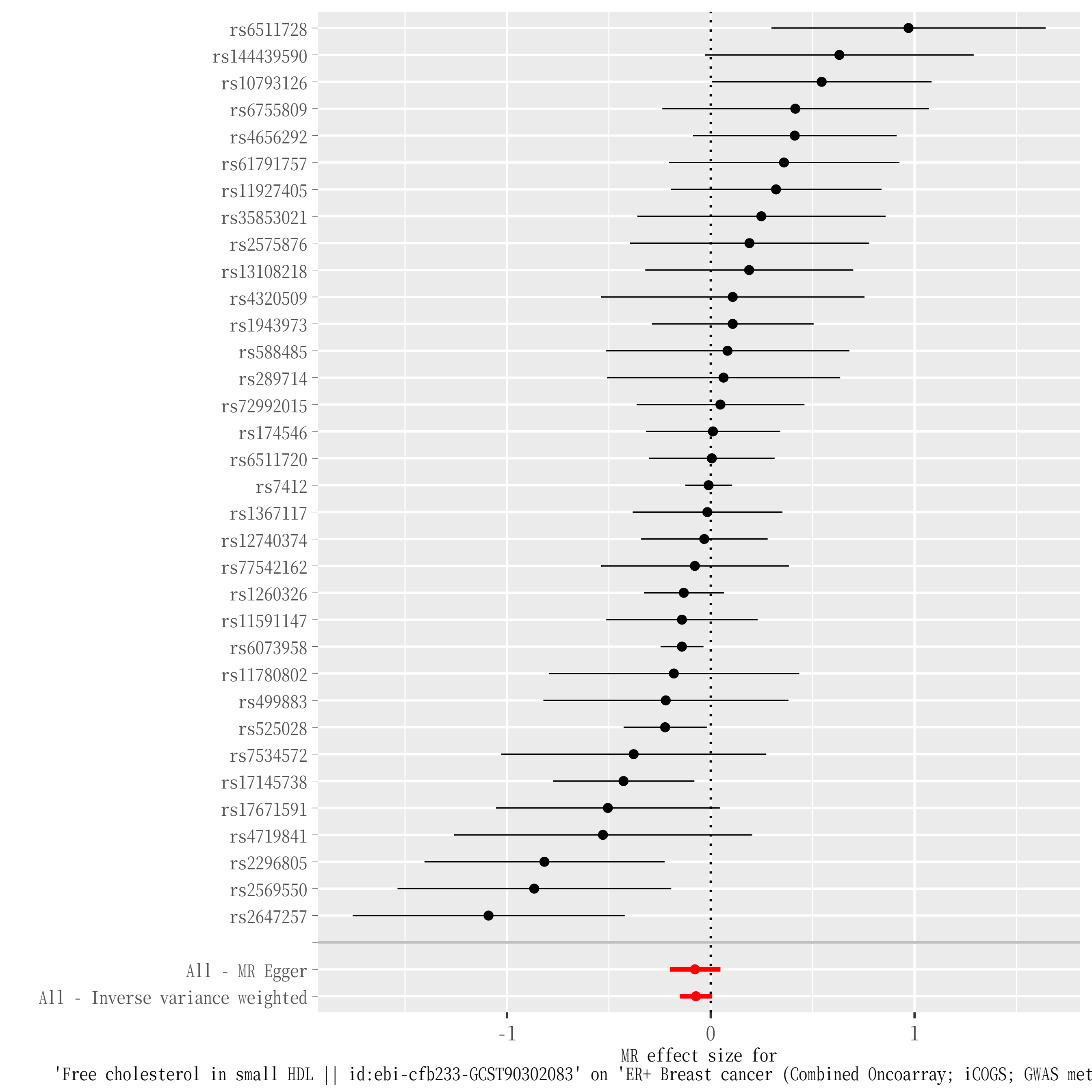

rs6511728
rs144439590
rs10793126
rs6755809
rs4656292
rs61791757
rs11927405
rs35853021
rs2575876
rs13108218
rs4320509
rs1943973
rs588485
rs289714
rs72992015
rs174546
rs6511720
rs7412
rs1367117
rs12740374
rs77542162
rs1260326
rs11591147
rs6073958
rs11780802
rs499883
rs525028
rs7534572
rs17145738
rs17671591
rs4719841
rs2296805
rs2569550
rs2647257
All - MR Egger
All - Inverse variance weighted
-1
0
1
MR effect size for
'Free cholesterol in small HDL || id:ebi-cfb233-GCST90302083' on 'ER+ Breast cancer (Combined Oncoarray; iCOGS; GWAS met

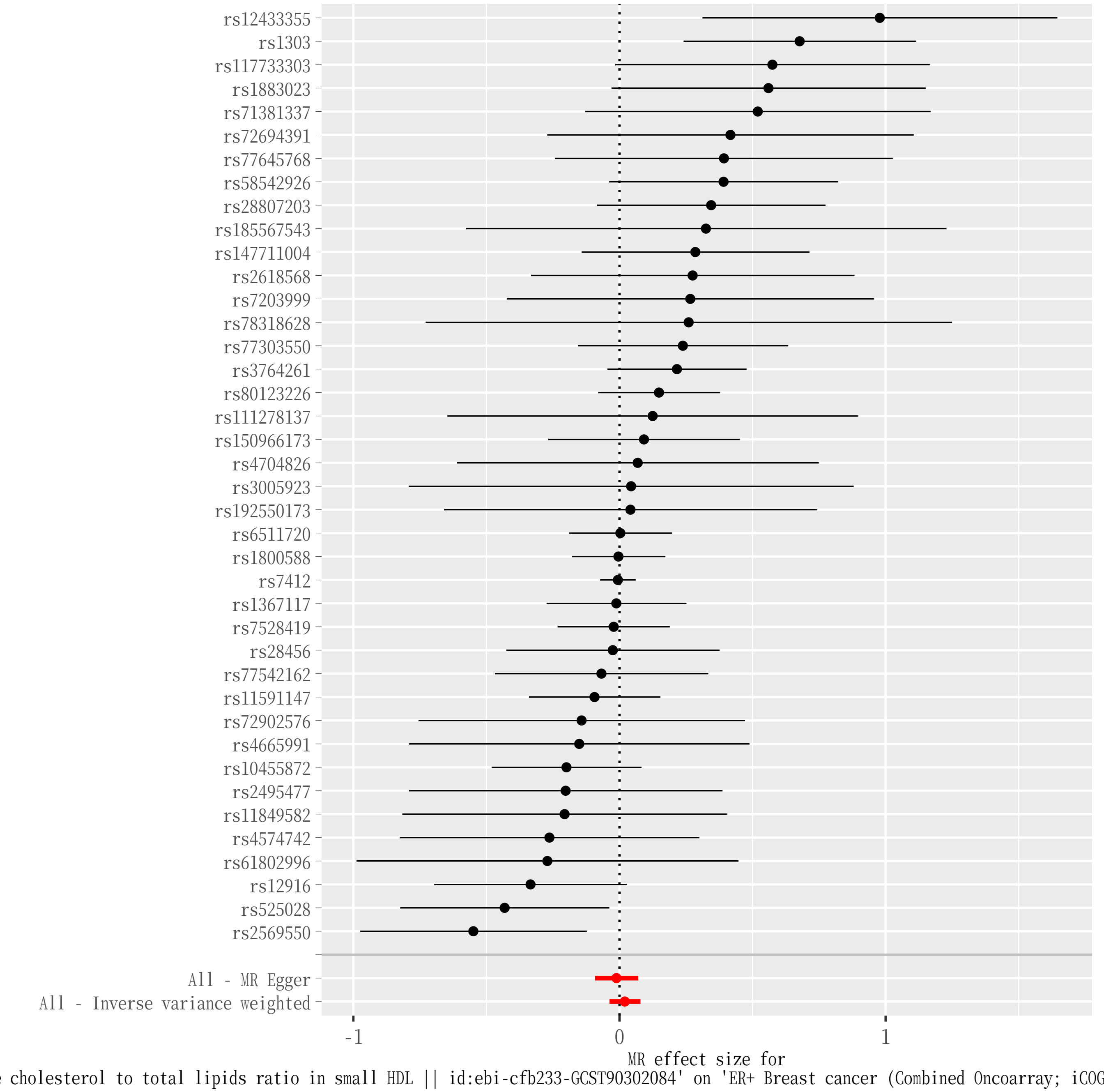
rs12433355
rs1303
rs117733303
rs1883023
rs71381337
rs72694391
rs77645768
rs58542926
rs28807203
rs185567543
rs147711004
rs2618568
rs7203999
rs78318628
rs77303550
rs3764261
rs80123226
rs111278137
rs150966173
rs4704826
rs3005923
rs192550173
rs6511720
rs1800588
rs7412
rs1367117
rs7528419
rs28456
rs77542162
rs11591147
rs72902576
rs4665991
rs10455872
rs2495477
rs11849582
rs4574742
rs61802996
rs12916
rs525028
rs2569550
All - MR Egger
All - Inverse variance weighted
-1
0
1
MR effect size for
e cholesterol to total lipids ratio in small HDL || id:ebi-cfb233-GCST90302084' on 'ER+ Breast cancer (Combined Oncoarray; iCOG

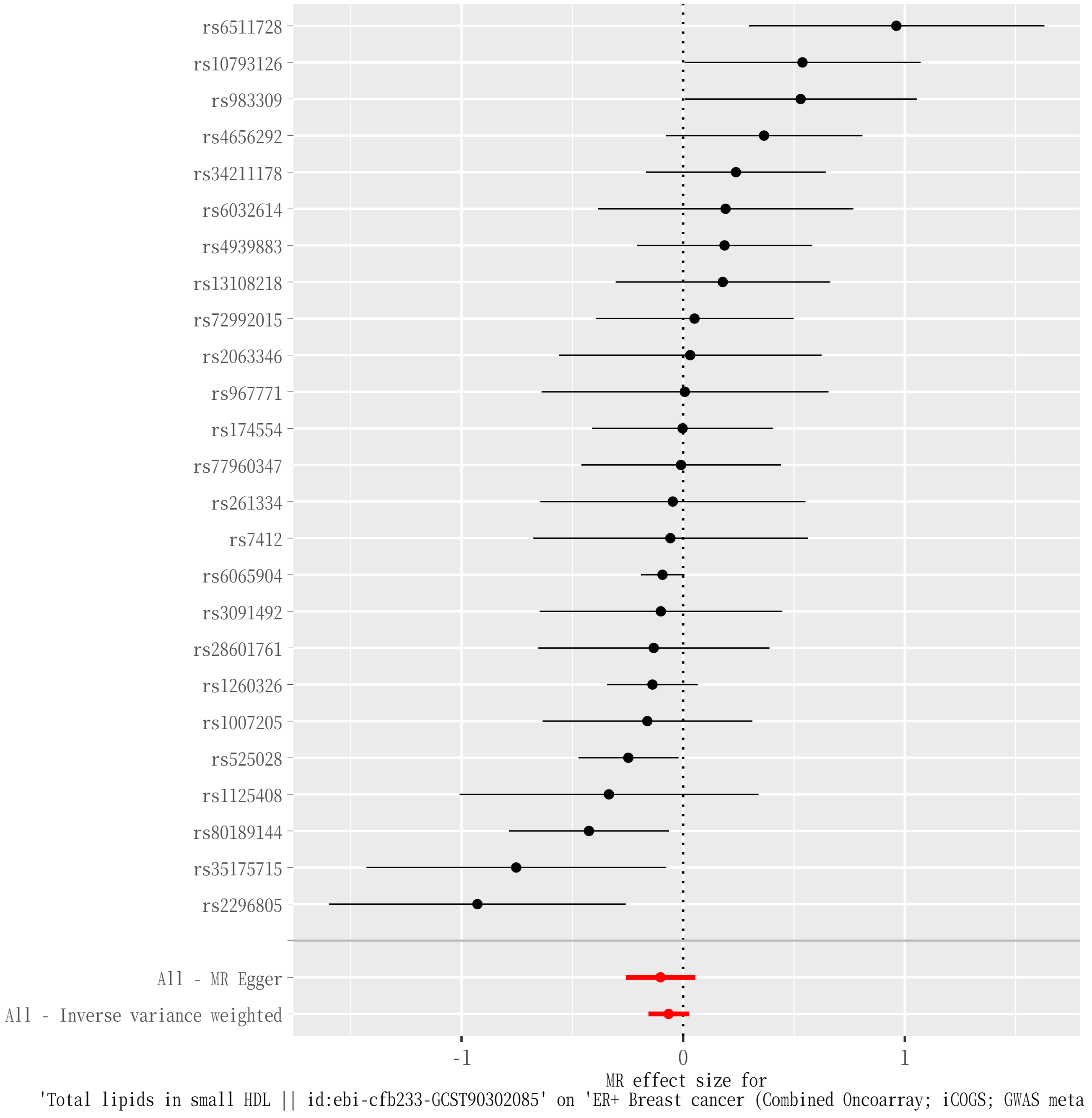
rs6511728
rs10793126
rs983309
rs4656292
rs34211178
rs6032614
rs4939883
rs13108218
rs72992015
rs2063346
rs967771
rs174554
rs77960347
rs261334
rs7412
rs6065904
rs3091492
rs28601761
rs1260326
rs1007205
rs525028
rs1125408
rs80189144
rs35175715
rs2296805
All - MR Egger
All - Inverse variance weighted
-1
0
1
MR effect size for
'Total lipids in small HDL || id:ebi-cfb233-GCST90302085' on 'ER+ Breast cancer (Combined Oncoarray; iCOGS; GWAS meta

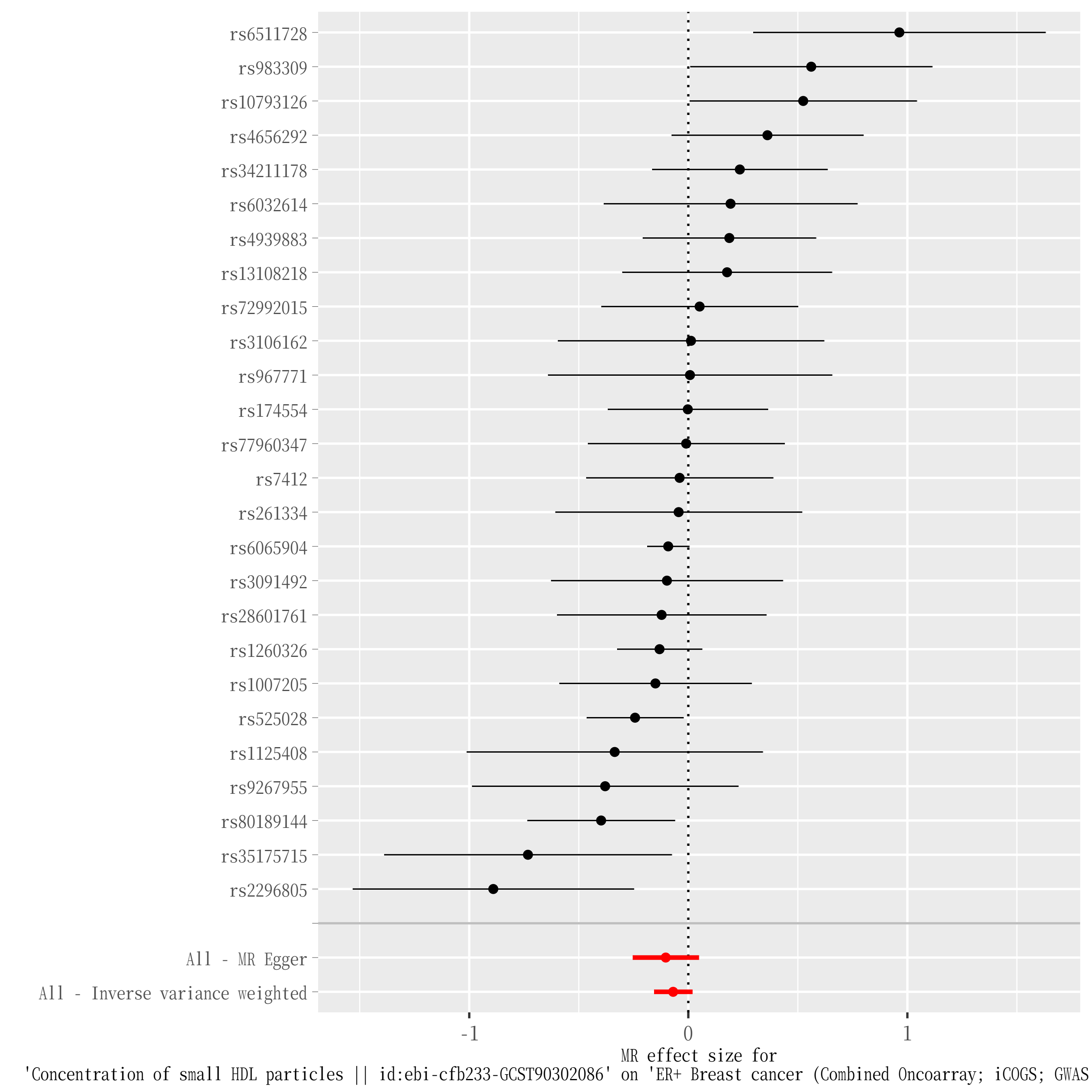

rs6511728
rs983309
rs10793126
rs4656292
rs34211178
rs6032614
rs4939883
rs13108218
rs72992015
rs3106162
rs967771
rs174554
rs77960347
rs7412
rs261334
rs6065904
rs3091492
rs28601761
rs1260326
rs1007205
rs525028
rs1125408
rs9267955
rs80189144
rs35175715
rs2296805
All - MR Egger
All - Inverse variance weighted
-1
0
1
MR effect size for
'Concentration of small HDL particles || id:ebi-cfb233-GCST90302086' on 'ER+ Breast cancer (Combined Oncoarray; iCOGS; GWAS

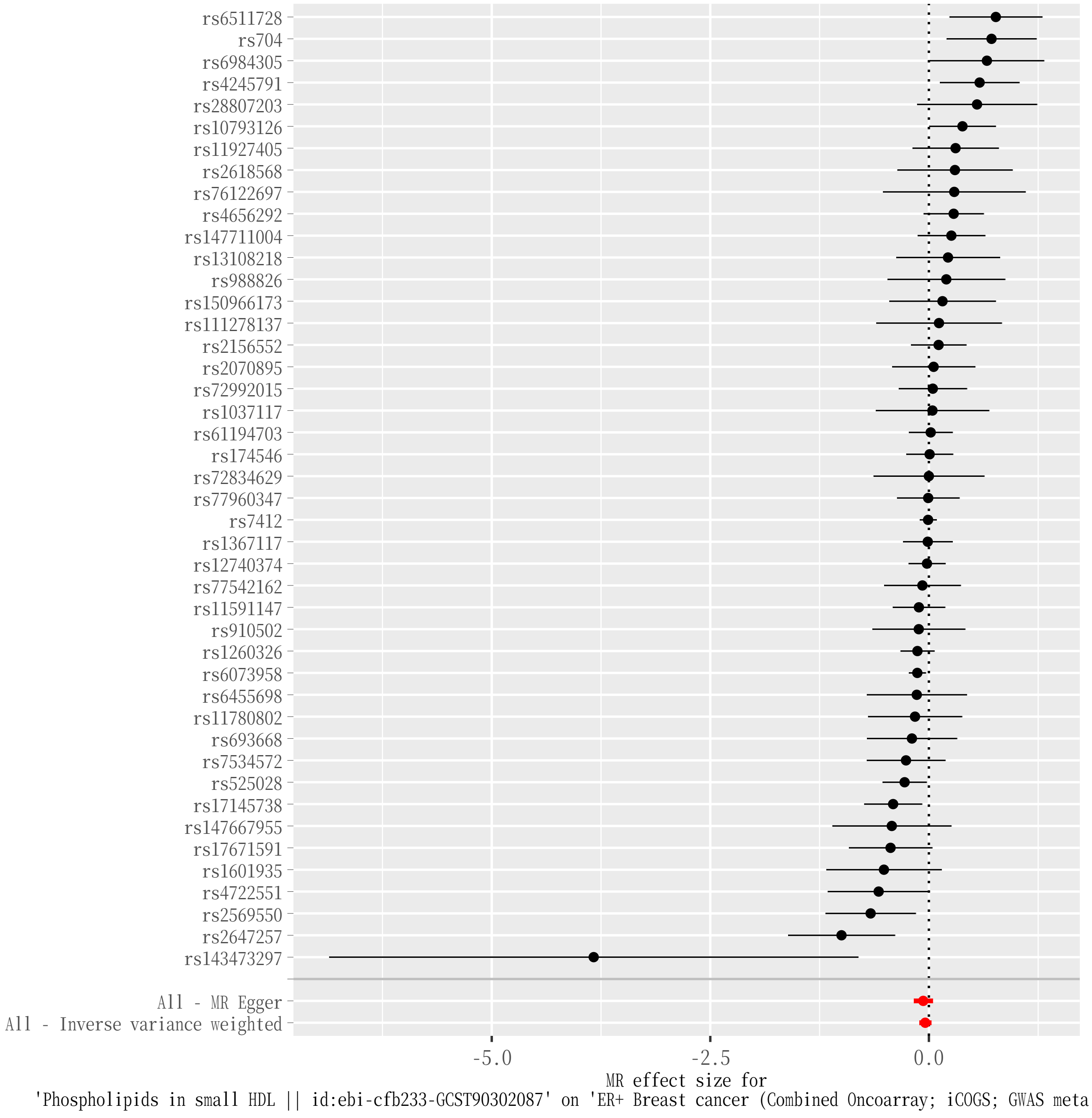
rs6511728
rs704
rs6984305
rs4245791
rs28807203
rs10793126
rs11927405
rs2618568
rs76122697
rs4656292
rs147711004
rs13108218
rs988826
rs150966173
rs111278137
rs2156552
rs2070895
rs72992015
rs1037117
rs61194703
rs174546
rs72834629
rs77960347
rs7412
rs1367117
rs12740374
rs77542162
rs11591147
rs910502
rs1260326
rs6073958
rs6455698
rs11780802
rs693668
rs7534572
rs525028
rs17145738
rs147667955
rs17671591
rs1601935
rs4722551
rs2569550
rs2647257
rs143473297
All - MR Egger
All - Inverse variance weighted
-5.0
-2.5
0.0
MR effect size for
'Phospholipids in small HDL || id:ebi-cfb233-GCST90302087' on 'ER+ Breast cancer (Combined Oncoarray; iCOGS; GWAS meta

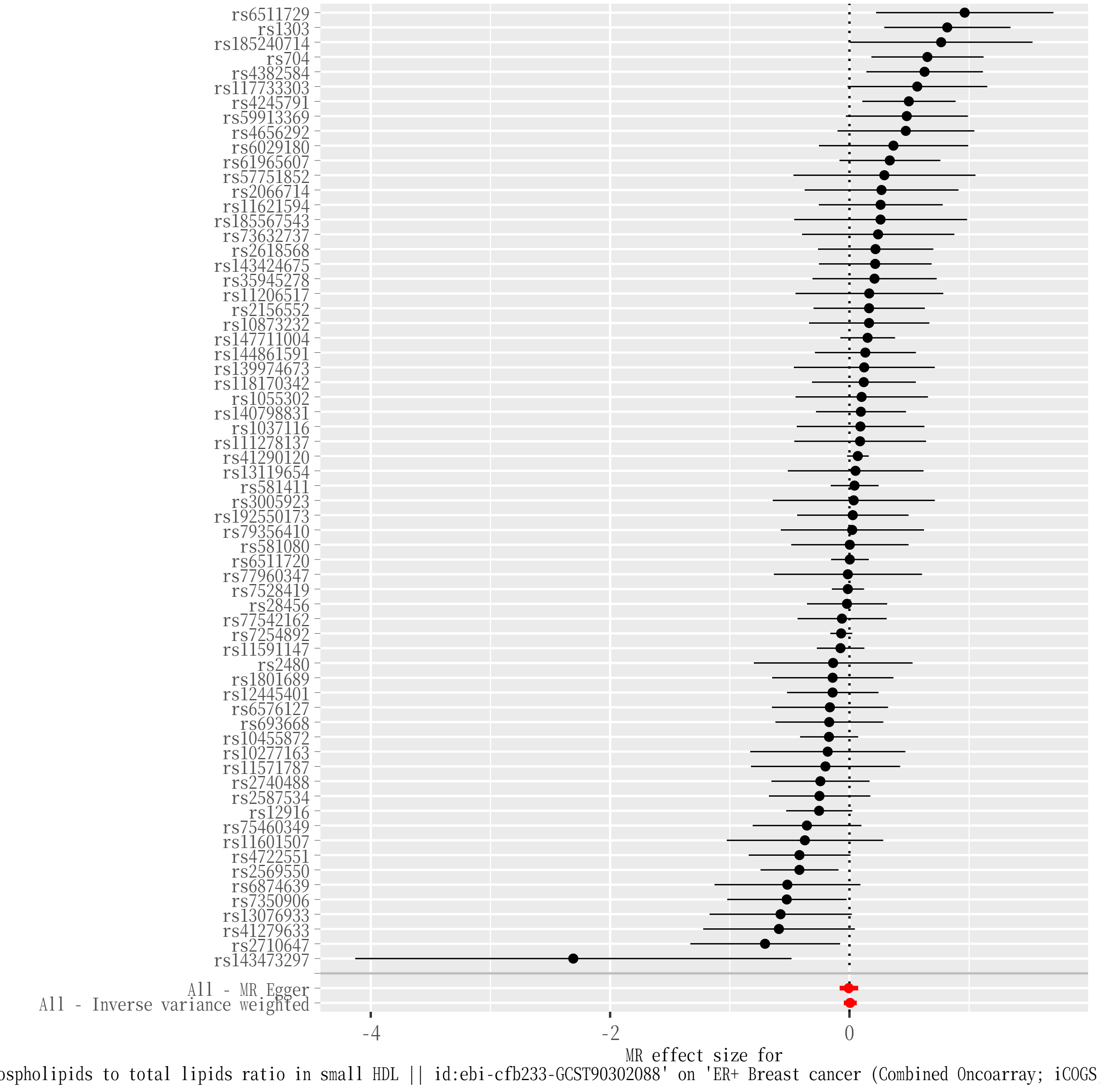

rs6511729
rs1303
rs185240714
rs704
rs4382584
rs117733303
rs4245791
rs59913369
rs4656292
rs6029180
rs61965607
rs57751852
rs2066714
rs11621594
rs185567543
rs73632737
rs2618568
rs143424675
rs35945278
rs11206517
rs2156552
rs10873232
rs147711004
rs144861591
rs139974673
rs118170342
rs1055302
rs140798831
rs1037116
rs111278137
rs41290120
rs13119654
rs581411
rs3005923
rs192550173
rs79356410
rs581080
rs6511720
rs77960347
rs7528419
rs28456
rs77542162
rs7254892
rs11591147
rs2480
rs1801689
rs12445401
rs6576127
rs693668
rs10455872
rs10277163
rs11571787
rs2740488
rs2587534
rs12916
rs75460349
rs11601507
rs4722551
rs2569550
rs6874639
rs7350906
rs13076933
rs41279633
rs2710647
rs143473297
All - MR Egger
All - Inverse variance weighted
-4
-2
0
MR effect size for
ospholipids to total lipids ratio in small HDL || id:ebi-cfb233-GCST90302088' on 'ER+ Breast cancer (Combined Oncoarray; iCOGS

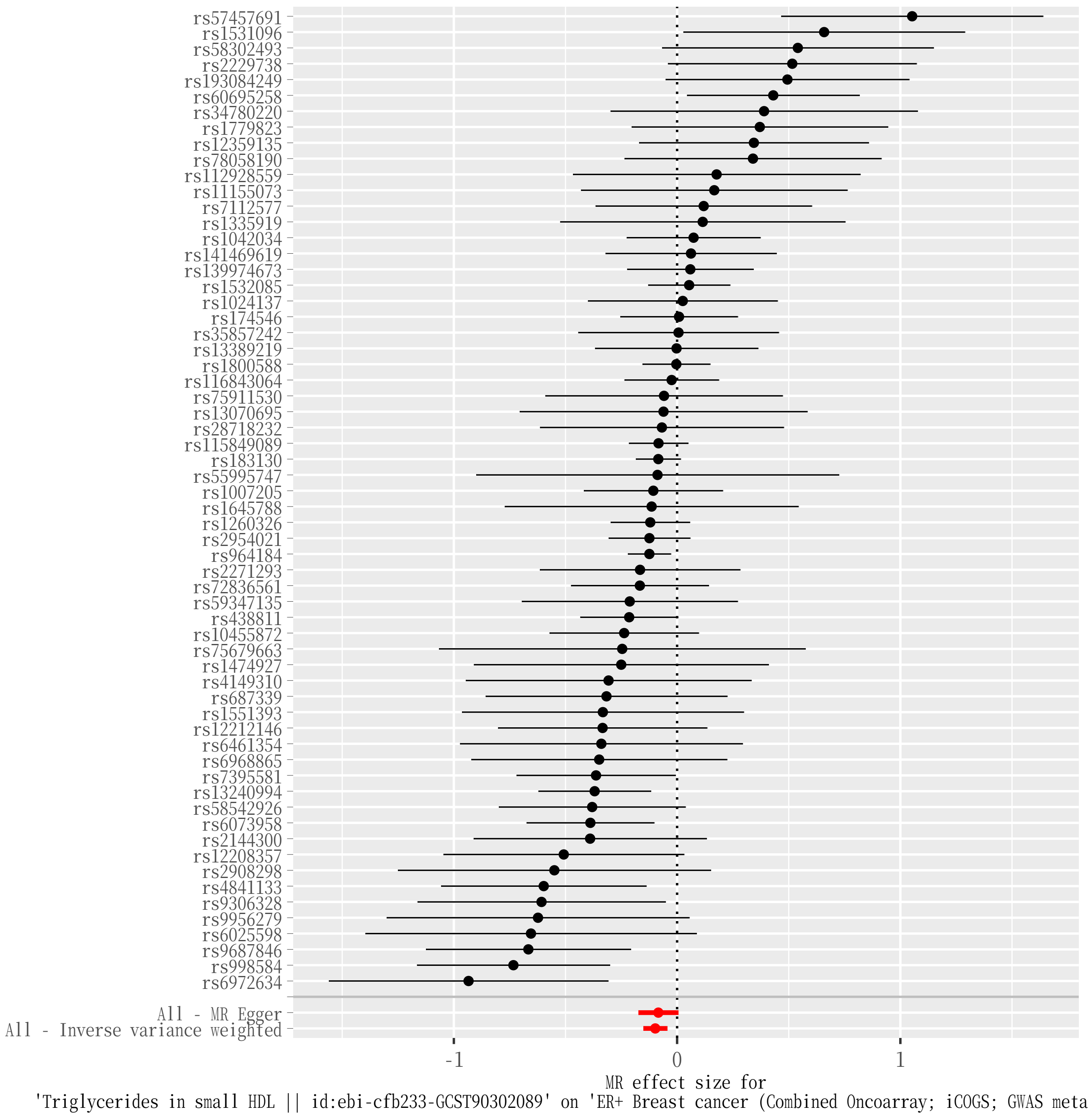
rs57457691
rs1531096
rs58302493
rs2229738
rs193084249
rs60695258
rs34780220
rs1779823
rs12359135
rs78058190
rs112928559
rs11155073
rs7112577
rs1335919
rs1042034
rs141469619
rs139974673
rs1532085
rs1024137
rs174546
rs35857242
rs13389219
rs1800588
rs116843064
rs75911530
rs13070695
rs28718232
rs115849089
rs183130
rs55995747
rs1007205
rs1645788
rs1260326
rs2954021
rs964184
rs2271293
rs72836561
rs59347135
rs438811
rs10455872
rs75679663
rs1474927
rs4149310
rs687339
rs1551393
rs12212146
rs6461354
rs6968865
rs7395581
rs13240994
rs58542926
rs6073958
rs2144300
rs12208357
rs2908298
rs4841133
rs9306328
rs9956279
rs6025598
rs9687846
rs998584
rs6972634
All - MR Egger
All - Inverse variance weighted
-1
0
1
MR effect size for
'Triglycerides in small HDL || id:ebi-cfb233-GCST90302089' on 'ER+ Breast cancer (Combined Oncoarray; iCOGS; GWAS meta

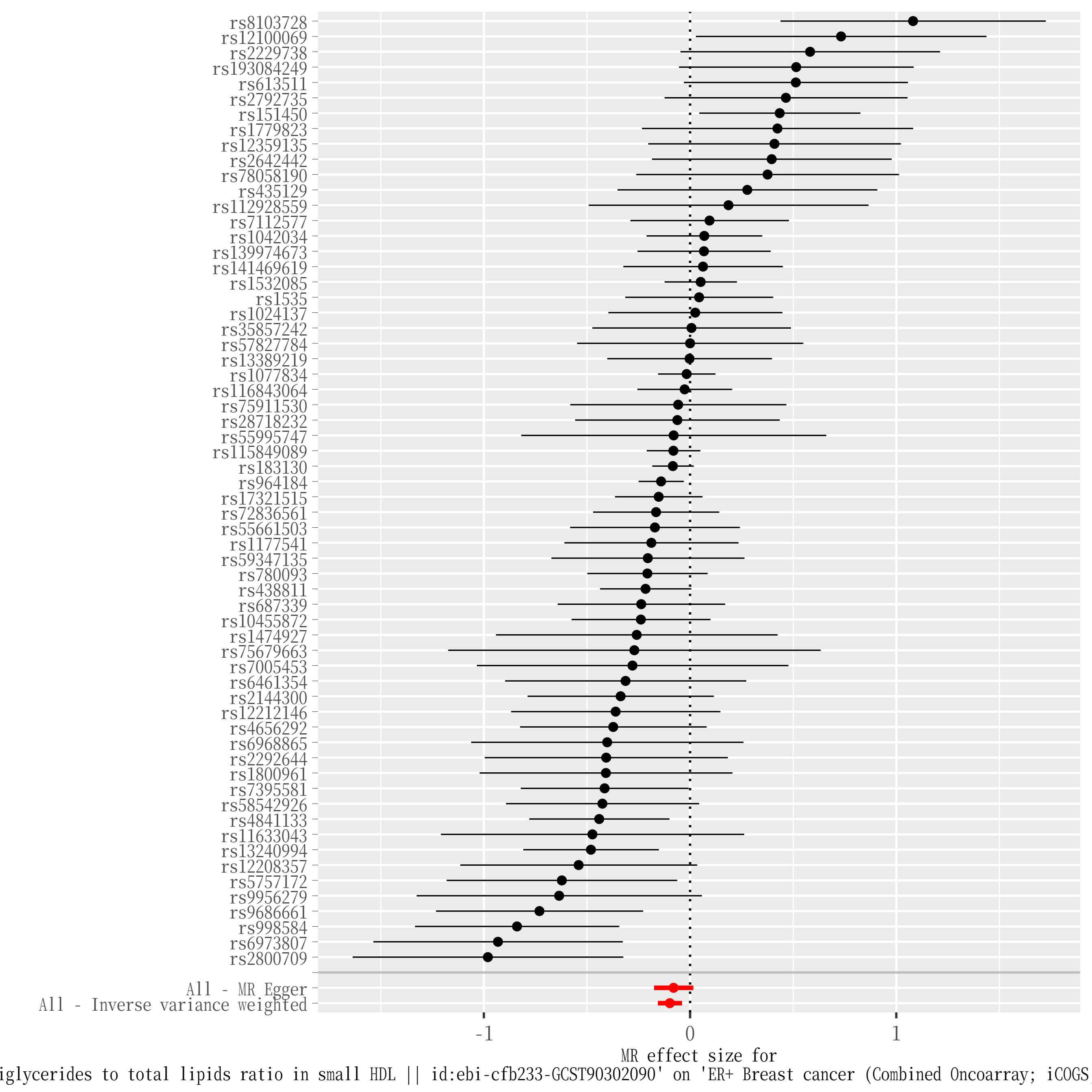

rs8103728
rs12100069
rs2229738
rs193084249
rs613511
rs2792735
rs151450
rs1779823
rs12359135
rs2642442
rs78058190
rs435129
rs112928559
rs7112577
rs1042034
rs139974673
rs141469619
rs1532085
rs1535
rs1024137
rs35857242
rs57827784
rs13389219
rs1077834
rs116843064
rs75911530
rs28718232
rs55995747
rs115849089
rs183130
rs964184
rs17321515
rs72836561
rs55661503
rs1177541
rs59347135
rs780093
rs438811
rs687339
rs10455872
rs1474927
rs75679663
rs7005453
rs6461354
rs2144300
rs12212146
rs4656292
rs6968865
rs2292644
rs1800961
rs7395581
rs58542926
rs4841133
rs11633043
rs13240994
rs12208357
rs5757172
rs9956279
rs9686661
rs998584
rs6973807
rs2800709
All - MR Egger
All - Inverse variance weighted
-1
0
1
MR effect size for
iglycerides to total lipids ratio in small HDL || id:ebi-cfb233-GCST90302090' on 'ER+ Breast cancer (Combined Oncoarray; iCOGS

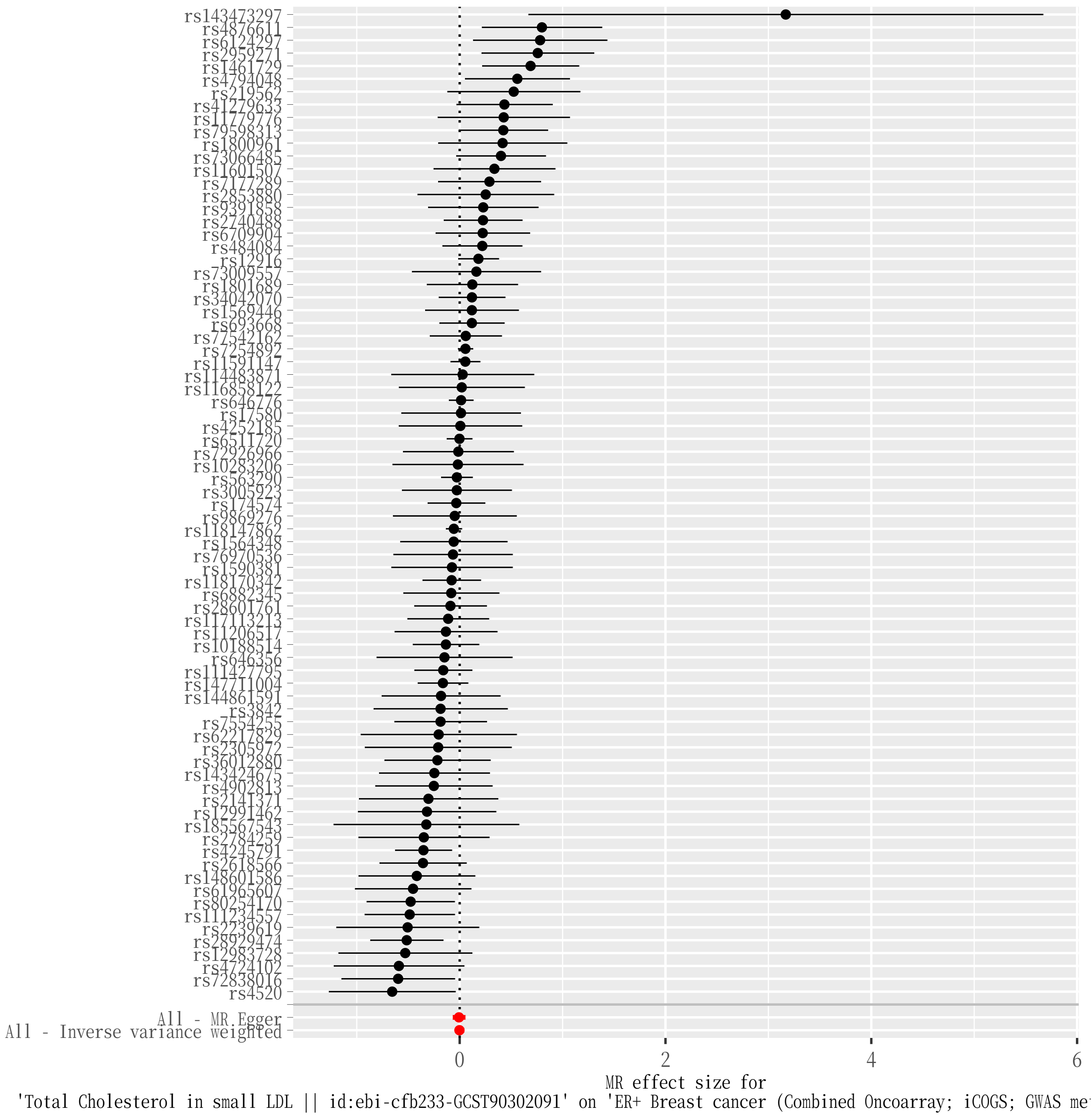
rs143473297
rs4876611
rs6124297
rs2959271
rs1461729
rs4794048
rs219562
rs41279633
rs11779776
rs79598313
rs1800961
rs73066485
rs11601507
rs7177289
rs2853880
rs9391858
rs2740488
rs6709904
rs484084
rs12916
rs73009557
rs1801689
rs34042070
rs1569446
rs693668
rs77542162
rs7254892
rs11591147
rs114483871
rs116858122
rs646776
rs17580
rs4252185
rs6511720
rs72926966
rs10283206
rs563290
rs3005923
rs174574
rs9869276
rs118147862
rs1564348
rs76970536
rs1590381
rs118170342
rs6882345
rs28601761
rs117113213
rs11206517
rs10188514
rs646356
rs111427795
rs147711004
rs144861591
rs3842
rs7554255
rs62217829
rs2305972
rs36012880
rs143424675
rs4902813
rs2141371
rs12991462
rs185567543
rs2784259
rs4245791
rs2618566
rs148601586
rs61965607
rs80254170
rs111234557
rs2239619
rs28929474
rs12983728
rs4724102
rs72838016
rs4520
All - MR Egger
All - Inverse variance weighted
0
2
4
6
MR effect size for
'Total Cholesterol in small LDL || id:ebi-cfb233-GCST90302091' on 'ER+ Breast cancer (Combined Oncoarray; iCOGS; GWAS me

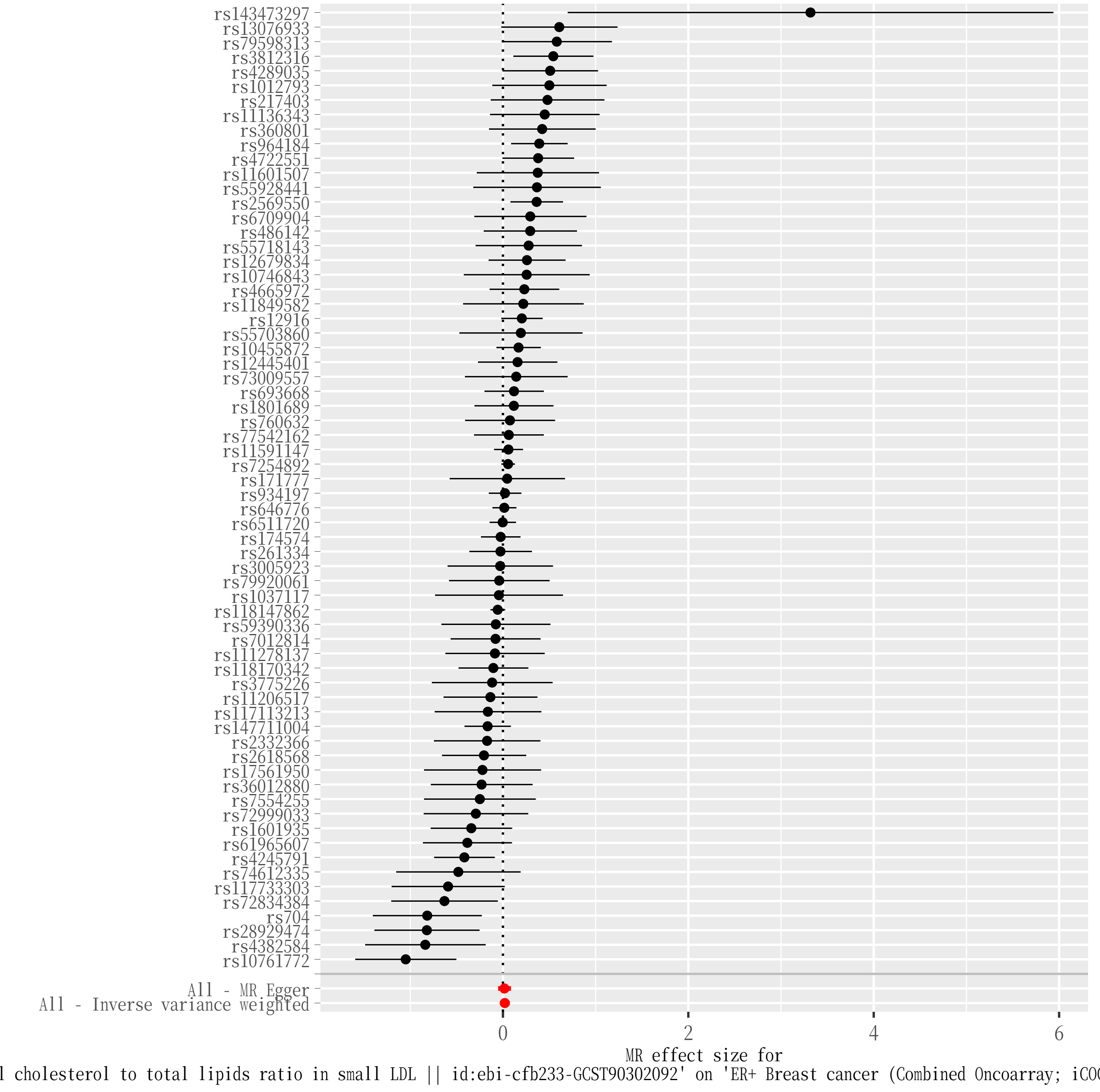

rs143473297
rs13076933
rs79598313
rs3812316
rs4289035
rs1012793
rs217403
rs11136343
rs360801
rs964184
rs4722551
rs11601507
rs55928441
rs2569550
rs6709904
rs486142
rs55718143
rs12679834
rs10746843
rs4665972
rs11849582
rs12916
rs55703860
rs10455872
rs12445401
rs73009557
rs693668
rs1801689
rs760632
rs77542162
rs11591147
rs7254892
rs171777
rs934197
rs646776
rs6511720
rs174574
rs261334
rs3005923
rs79920061
rs1037117
rs118147862
rs59390336
rs7012814
rs111278137
rs118170342
rs3775226
rs11206517
rs117113213
rs147711004
rs2332366
rs2618568
rs17561950
rs36012880
rs7554255
rs72999033
rs1601935
rs61965607
rs4245791
rs74612335
rs117733303
rs72834384
rs704
rs28929474
rs4382584
rs10761772
All - MR Egger
All - Inverse variance weighted
0
2
4
6
MR effect size for
l cholesterol to total lipids ratio in small LDL || id:ebi-cfb233-GCST90302092' on 'ER+ Breast cancer (Combined Oncoarray; iCO

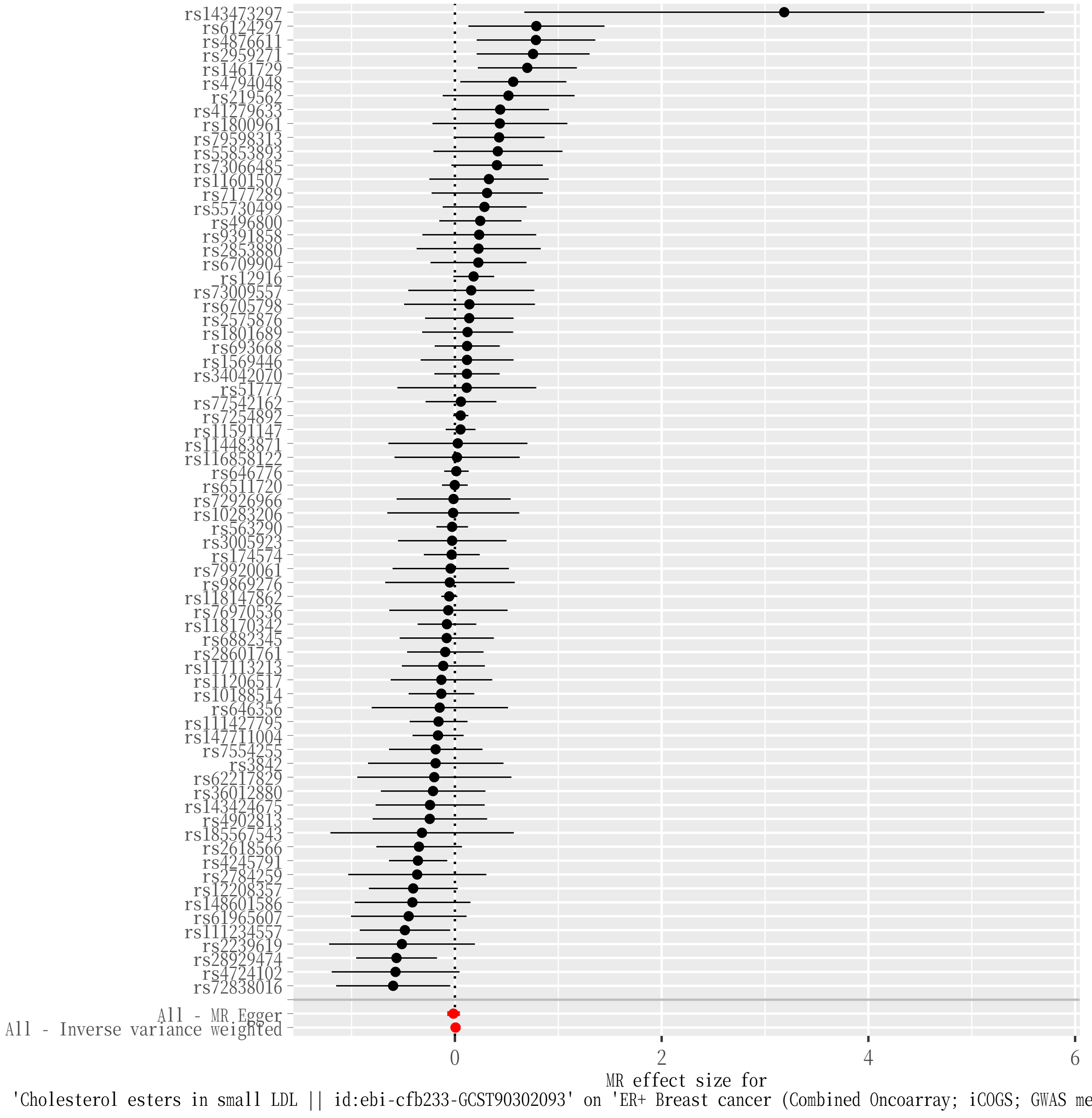
rs143473297
rs6124297
rs4876611
rs2959271
rs1461729
rs4794048
rs219562
rs41279633
rs1800961
rs79598313
rs55853893
rs73066485
rs11601507
rs7177289
rs55730499
rs496800
rs9391858
rs2853880
rs6709904
rs12916
rs73009557
rs6705798
rs2575876
rs1801689
rs693668
rs1569446
rs34042070
rs51777
rs77542162
rs7254892
rs11591147
rs114483871
rs116858122
rs646776
rs6511720
rs72926966
rs10283206
rs563290
rs3005923
rs174574
rs79920061
rs9869276
rs118147862
rs76970536
rs118170342
rs6882345
rs28601761
rs117113213
rs11206517
rs10188514
rs646356
rs111427795
rs147711004
rs7554255
rs3842
rs62217829
rs36012880
rs143424675
rs4902813
rs185567543
rs2618566
rs4245791
rs2784259
rs12208357
rs148601586
rs61965607
rs111234557
rs2239619
rs28929474
rs4724102
rs72838016
All - MR Egger
All - Inverse variance weighted
0
2
4
6
MR effect size for
'Cholesterol esters in small LDL || id:ebi-cfb233-GCST90302093' on 'ER+ Breast cancer (Combined Oncoarray; iCOGS; GWAS me

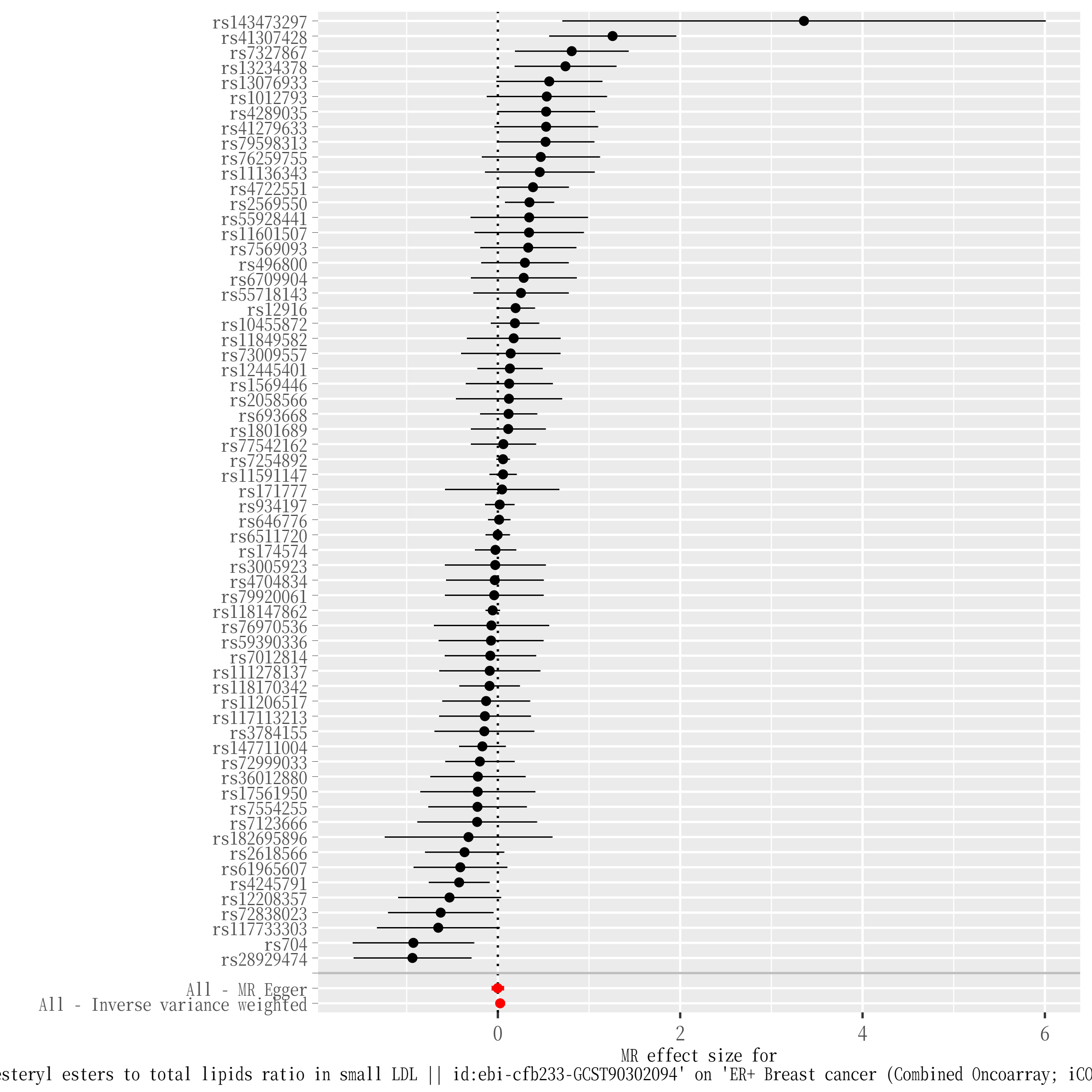

rs143473297
rs41307428
rs7327867
rs13234378
rs13076933
rs1012793
rs4289035
rs41279633
rs79598313
rs76259755
rs11136343
rs4722551
rs2569550
rs55928441
rs11601507
rs7569093
rs496800
rs6709904
rs55718143
rs12916
rs10455872
rs11849582
rs73009557
rs12445401
rs1569446
rs2058566
rs693668
rs1801689
rs77542162
rs7254892
rs11591147
rs171777
rs934197
rs646776
rs6511720
rs174574
rs3005923
rs4704834
rs79920061
rs118147862
rs76970536
rs59390336
rs7012814
rs111278137
rs118170342
rs11206517
rs117113213
rs3784155
rs147711004
rs72999033
rs36012880
rs17561950
rs7554255
rs7123666
rs182695896
rs2618566
rs61965607
rs4245791
rs12208357
rs72838023
rs117733303
rs704
rs28929474
All - MR Egger
All - Inverse variance weighted
0
2
4
6
MR effect size for
esteryl esters to total lipids ratio in small LDL || id:ebi-cfb233-GCST90302094' on 'ER+ Breast cancer (Combined Oncoarray; iCO

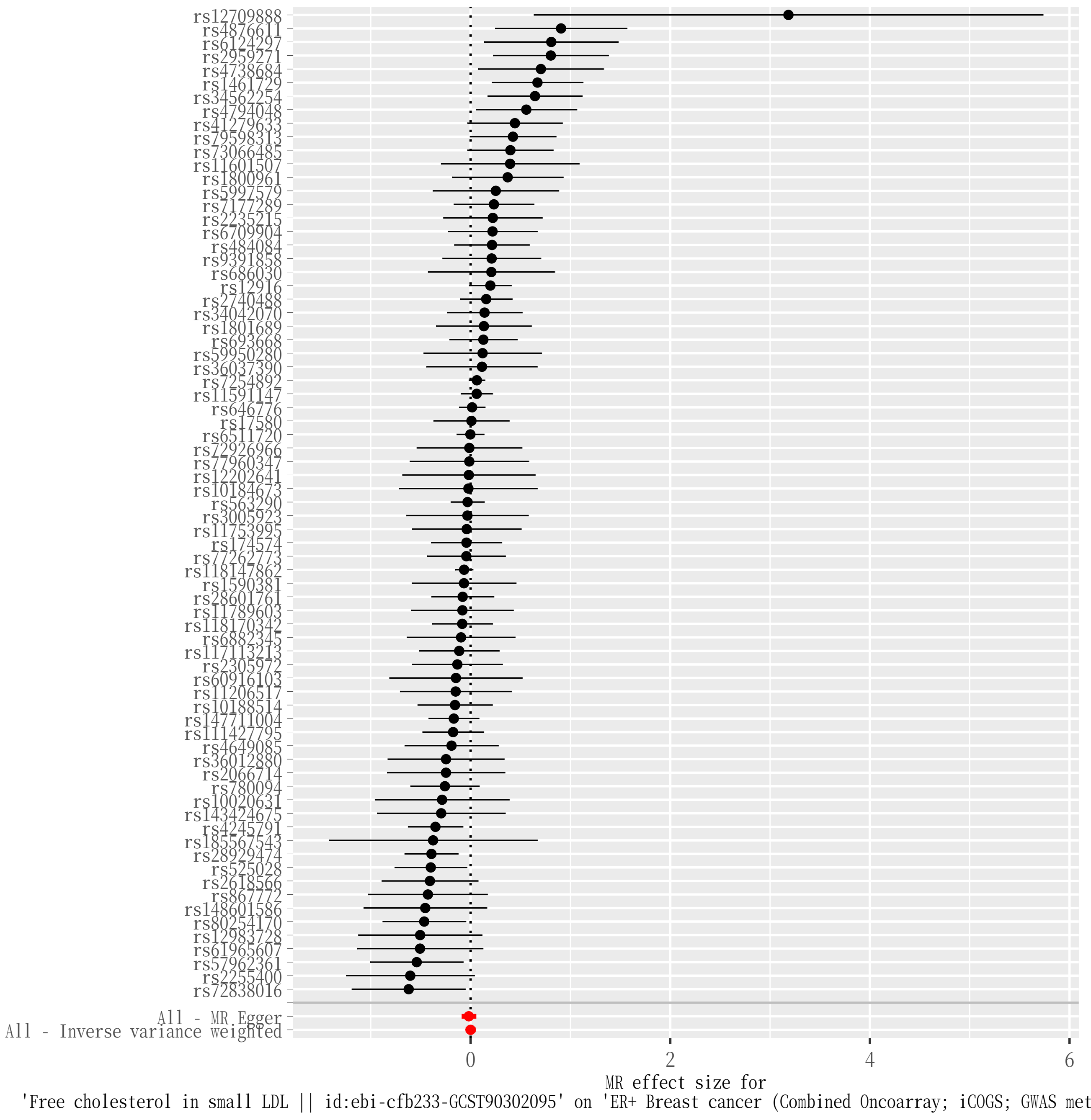
rs12709888
rs4876611
rs6124297
rs2959271
rs4738684
rs1461729
rs34562254
rs4794048
rs41279633
rs79598313
rs73066485
rs11601507
rs1800961
rs5997579
rs7177289
rs2235215
rs6709904
rs484084
rs9391858
rs686030
rs12916
rs2740488
rs34042070
rs1801689
rs693668
rs59950280
rs36037390
rs7254892
rs11591147
rs646776
rs17580
rs6511720
rs72926966
rs77960347
rs12202641
rs10184673
rs563290
rs3005923
rs11753995
rs174574
rs77262773
rs118147862
rs1590381
rs28601761
rs11789603
rs118170342
rs6882345
rs117113213
rs2305972
rs60916103
rs11206517
rs10188514
rs147711004
rs111427795
rs4649085
rs36012880
rs2066714
rs780094
rs10020631
rs143424675
rs4245791
rs185567543
rs28929474
rs525028
rs2618566
rs867772
rs148601586
rs80254170
rs12983728
rs61965607
rs57962361
rs2255400
rs72838016
All - MR Egger
All - Inverse variance weighted
0
2
4
6
MR effect size for
'Free cholesterol in small LDL || id:ebi-cfb233-GCST90302095' on 'ER+ Breast cancer (Combined Oncoarray; iCOGS; GWAS met

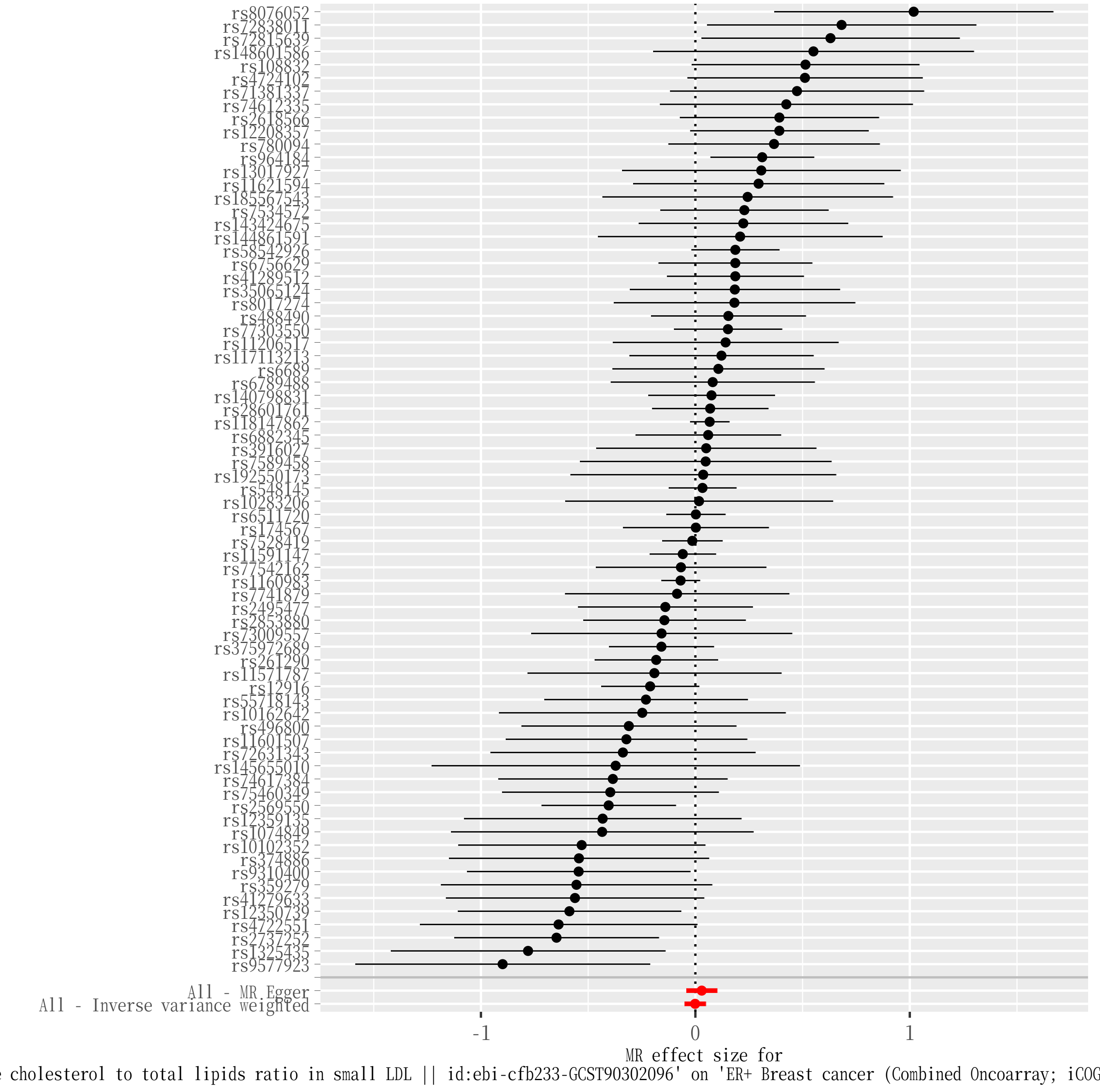
rs8076052
rs72838011
rs72815639
rs148601586
rs108832
rs4724102
rs71381337
rs74612335
rs2618566
rs12208357
rs780094
rs964184
rs13017927
rs11621594
rs185567543
rs7534572
rs143424675
rs144861591
rs58542926
rs6756629
rs41289512
rs35065124
rs8017274
rs488490
rs77303550
rs11206517
rs117113213
rs6689
rs6789488
rs140798831
rs28601761
rs118147862
rs6882345
rs3916027
rs7589458
rs192550173
rs548145
rs10283206
rs6511720
rs174567
rs7528419
rs11591147
rs77542162
rs1160983
rs7741879
rs2495477
rs2853880
rs73009557
rs375972689
rs261290
rs11571787
rs12916
rs55718143
rs10162642
rs496800
rs11601507
rs72631343
rs145655010
rs74617384
rs75460349
rs2569550
rs12359135
rs1074849
rs10102352
rs374886
rs9310400
rs359279
rs41279633
rs12350739
rs4722551
rs2737252
rs1325435
rs9577923
All - MR Egger
All - Inverse variance weighted
-1
0
1
MR effect size for
e cholesterol to total lipids ratio in small LDL || id:ebi-cfb233-GCST90302096' on 'ER+ Breast cancer (Combined Oncoarray; iCOG

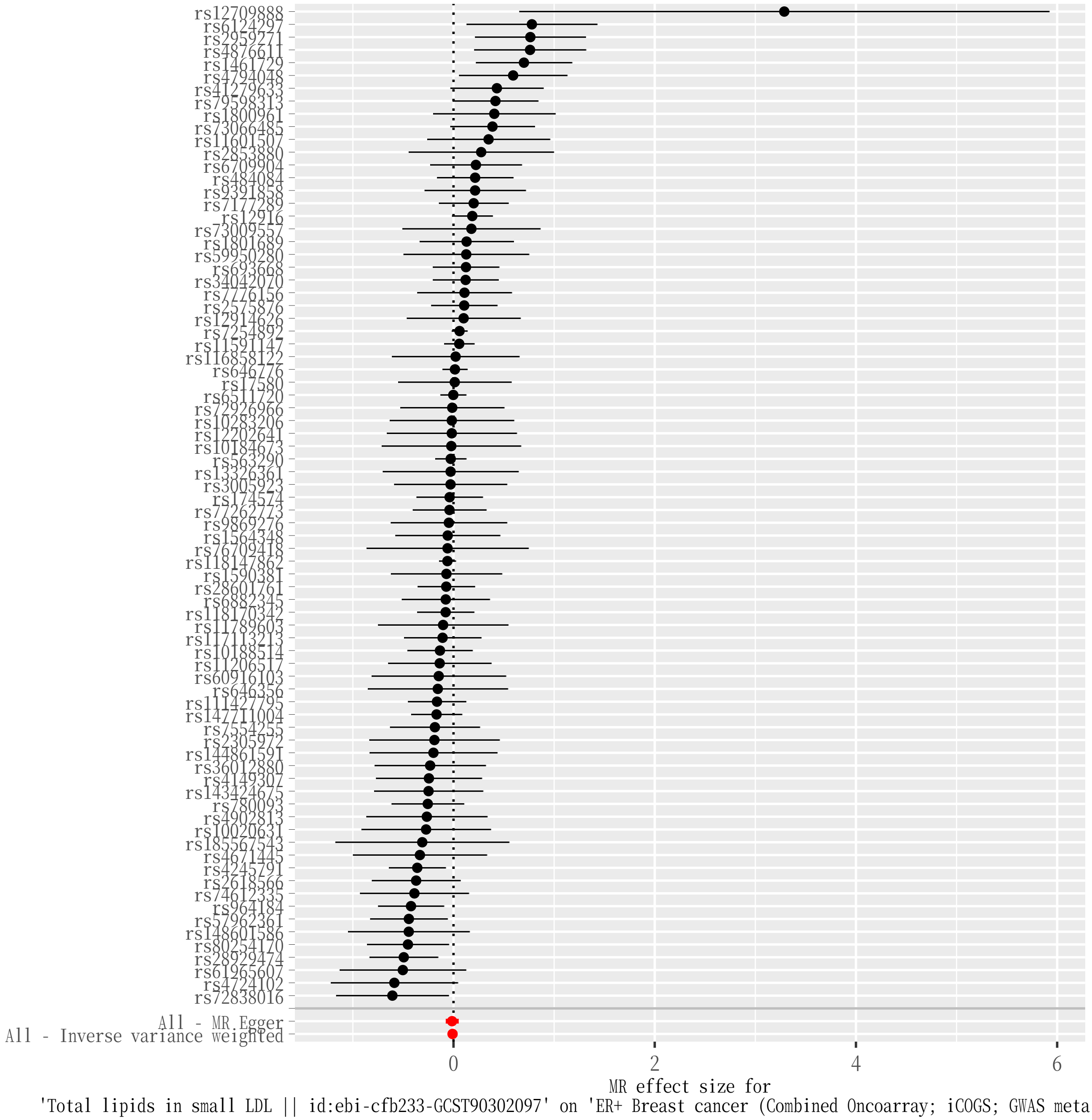
rs12709888
rs6124297
rs2959271
rs4876611
rs1461729
rs4794048
rs41279633
rs79598313
rs1800961
rs73066485
rs11601507
rs2853880
rs6709904
rs484084
rs9391858
rs7177289
rs12916
rs73009557
rs1801689
rs59950280
rs693668
rs34042070
rs7776156
rs2575876
rs12914626
rs7254892
rs11591147
rs116858122
rs646776
rs17580
rs6511720
rs72926966
rs10283206
rs1220264l
rs10184673
rs563290
rs13326361
rs3005923
rs174574
rs77262773
rs9869276
rs1564348
rs76709418
rs118147862
rs1590381
rs28601761
rs6882345
rs118170342
rs11789603
rs117113213
rs10188514
rs11206517
rs60916103
rs646356
rs111427795
rs147711004
rs7554255
rs2305972
rs144861591
rs36012880
rs4149307
rs143424675
rs780093
rs4902813
rs10020631
rs185567543
rs4671445
rs4245791
rs2618566
rs74612335
rs964184
rs57962361
rs148601586
rs80254170
rs28929474
rs61965607
rs4724102
rs72838016
All - MR Egger
All - Inverse variance weighted
0
2
4
6
MR effect size for
'Total lipids in small LDL || id:ebi-cfb233-GCST90302097' on 'ER+ Breast cancer (Combined Oncoarray; iCOGS; GWAS meta

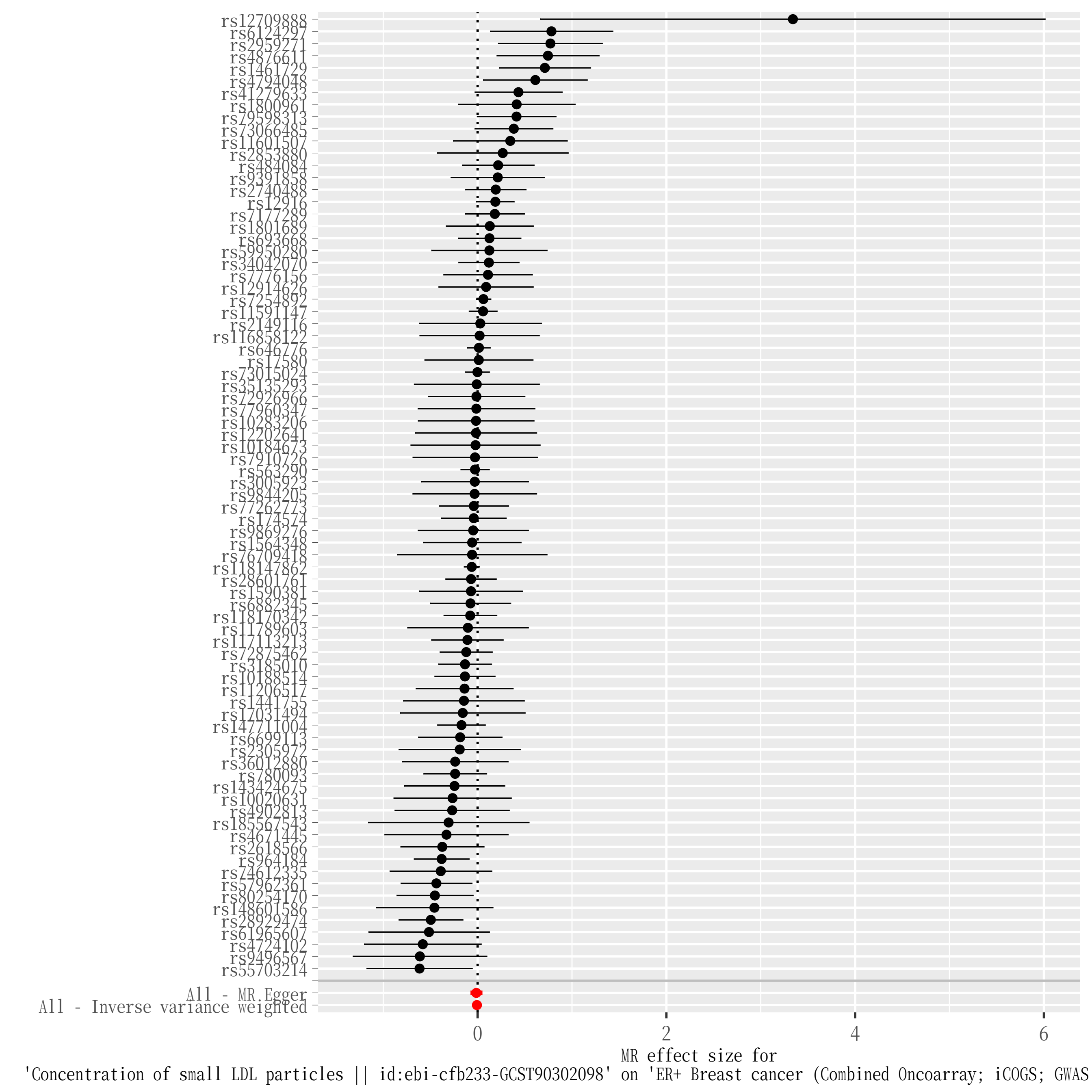
rs12709888
rs6124297
rs2959271
rs4876611
rs1461729
rs4794048
rs41279633
rs1800961
rs79598313
rs73066485
rs11601507
rs2853880
rs484084
rs9391858
rs2740488
rs12916
rs7177289
rs1801689
rs693668
rs59950280
rs34042070
rs7776156
rs12914626
rs7254892
rs11591147
rs2149116
rs116858122
rs646776
rs17580
rs73015024
rs35135293
rs72926966
rs77960347
rs10283206
rs12202641
rs10184673
rs7910726
rs563290
rs3005923
rs9844205
rs77262773
rs174574
rs9869276
rs1564348
rs76709418
rs118147862
rs28601761
rs1590381
rs6882345
rs118170342
rs11789603
rs117113213
rs72875462
rs3185010
rs10188514
rs11206517
rs1441755
rs17031494
rs147711004
rs6699113
rs2305972
rs36012880
rs780093
rs143424675
rs10020631
rs4902813
rs185567543
rs4671445
rs2618566
rs964184
rs74612335
rs57962361
rs80254170
rs148601586
rs28929474
rs61965607
rs4724102
rs9496567
rs55703214
All - MR Egger
All - Inverse variance weighted
0
2
4
6
MR effect size for
'Concentration of small LDL particles || id:ebi-cfb233-GCST90302098' on 'ER+ Breast cancer (Combined Oncoarray; iCOGS; GWAS

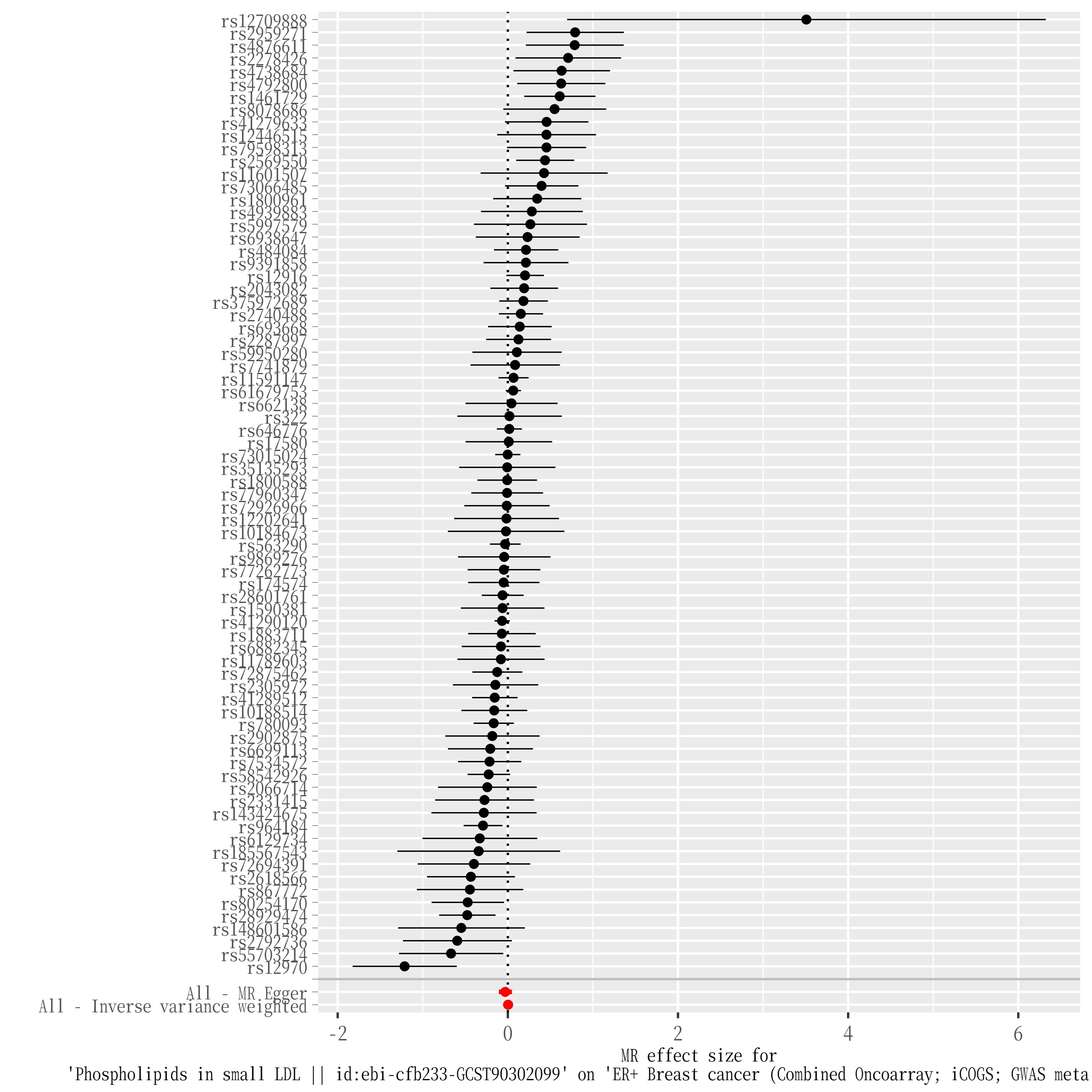
rs12709888
rs2959271
rs4876611
rs2278426
rs4738684
rs4792800
rs1461729
rs8078686
rs41279633
rs12446515
rs79598313
rs2569550
rs11601507
rs73066485
rs1800961
rs4939883
rs5997579
rs6938647
rs484084
rs9391858
rs12916
rs2043082
rs375972689
rs2740488
rs693668
rs2287997
rs59950280
rs7741879
rs11591147
rs61679753
rs662138
rs322
rs646776
rs17580
rs73015024
rs35135293
rs1800588
rs77960347
rs72926966
rs12202641
rs10184673
rs563290
rs9869276
rs77262773
rs174574
rs28601761
rs1590381
rs41290120
rs1883711
rs6882345
rs11789603
rs72875462
rs2305972
rs41289512
rs10188514
rs780093
rs2902875
rs6699113
rs7534572
rs58542926
rs2066714
rs2331415
rs143424675
rs964184
rs6129734
rs185567543
rs72694391
rs2618566
rs867772
rs80254170
rs28929474
rs148601586
rs2792736
rs55703214
rs12970
All - MR Egger
All - Inverse variance weighted
-2
0
2
4
6
MR effect size for
'Phospholipids in small LDL || id:ebi-cfb233-GCST90302099' on 'ER+ Breast cancer (Combined Oncoarray; iCOGS; GWAS meta

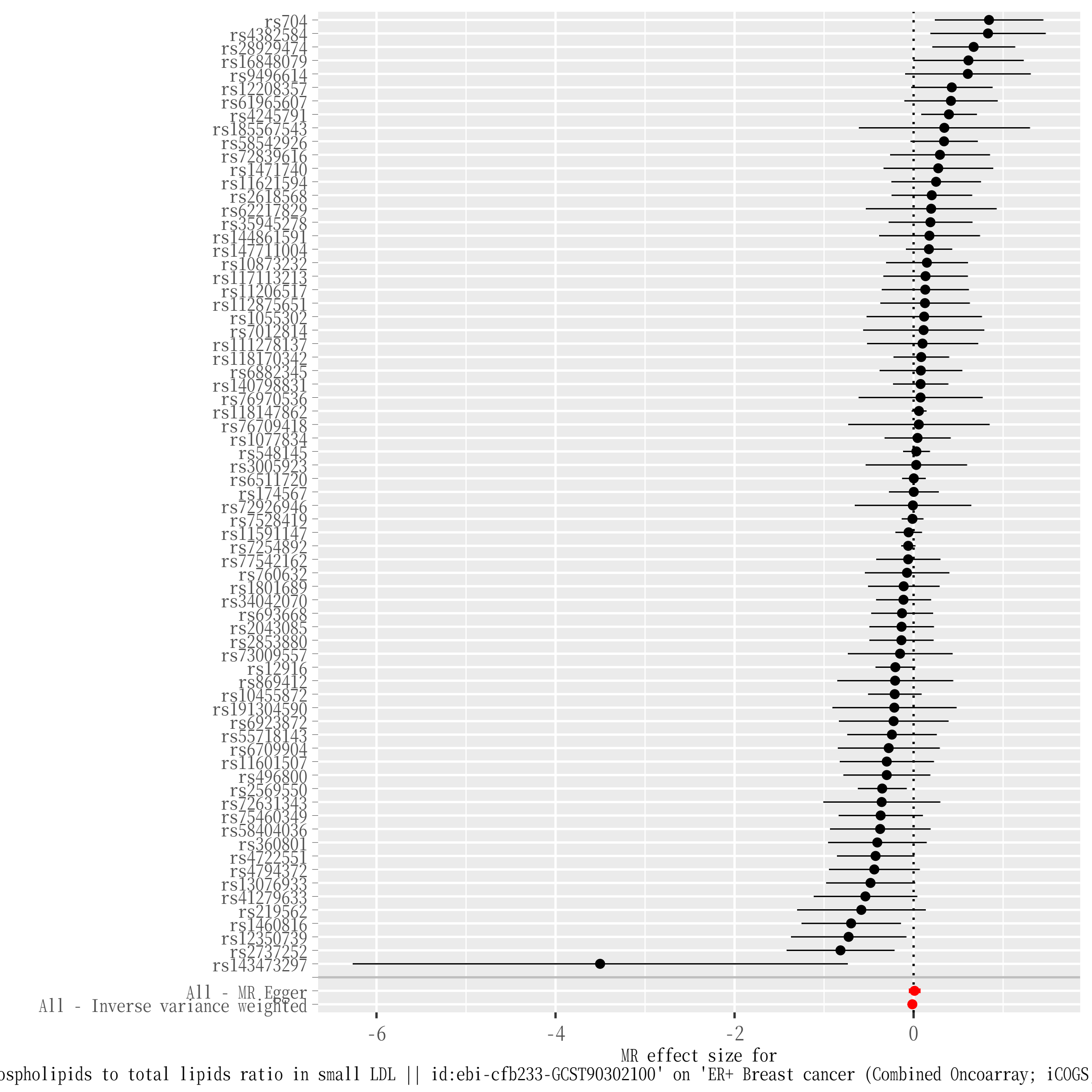
rs704
rs4382584
rs28929474
rs16848079
rs9496614
rs12208357
rs61965607
rs4245791
rs185567543
rs58542926
rs72839616
rs1471740
rs11621594
rs2618568
rs62217829
rs35945278
rs144861591
rs147711004
rs10873232
rs117113213
rs11206517
rs112875651
rs1055302
rs7012814
rs111278137
rs118170342
rs6882345
rs140798831
rs76970536
rs118147862
rs76709418
rs1077834
rs548145
rs3005923
rs6511720
rs174567
rs72926946
rs7528419
rs11591147
rs7254892
rs77542162
rs760632
rs1801689
rs34042070
rs693668
rs2043085
rs2853880
rs73009557
rs12916
rs869412
rs10455872
rs191304590
rs6923872
rs55718143
rs6709904
rs11601507
rs496800
rs2569550
rs72631343
rs75460349
rs58404036
rs360801
rs4722551
rs4794372
rs13076933
rs41279633
rs219562
rs1460816
rs12350739
rs2737252
rs143473297
All - MR Egger
All - Inverse variance weighted
-6
-4
-2
0
MR effect size for
ospholipids to total lipids ratio in small LDL || id:ebi-cfb233-GCST90302100' on 'ER+ Breast cancer (Combined Oncoarray; iCOGS

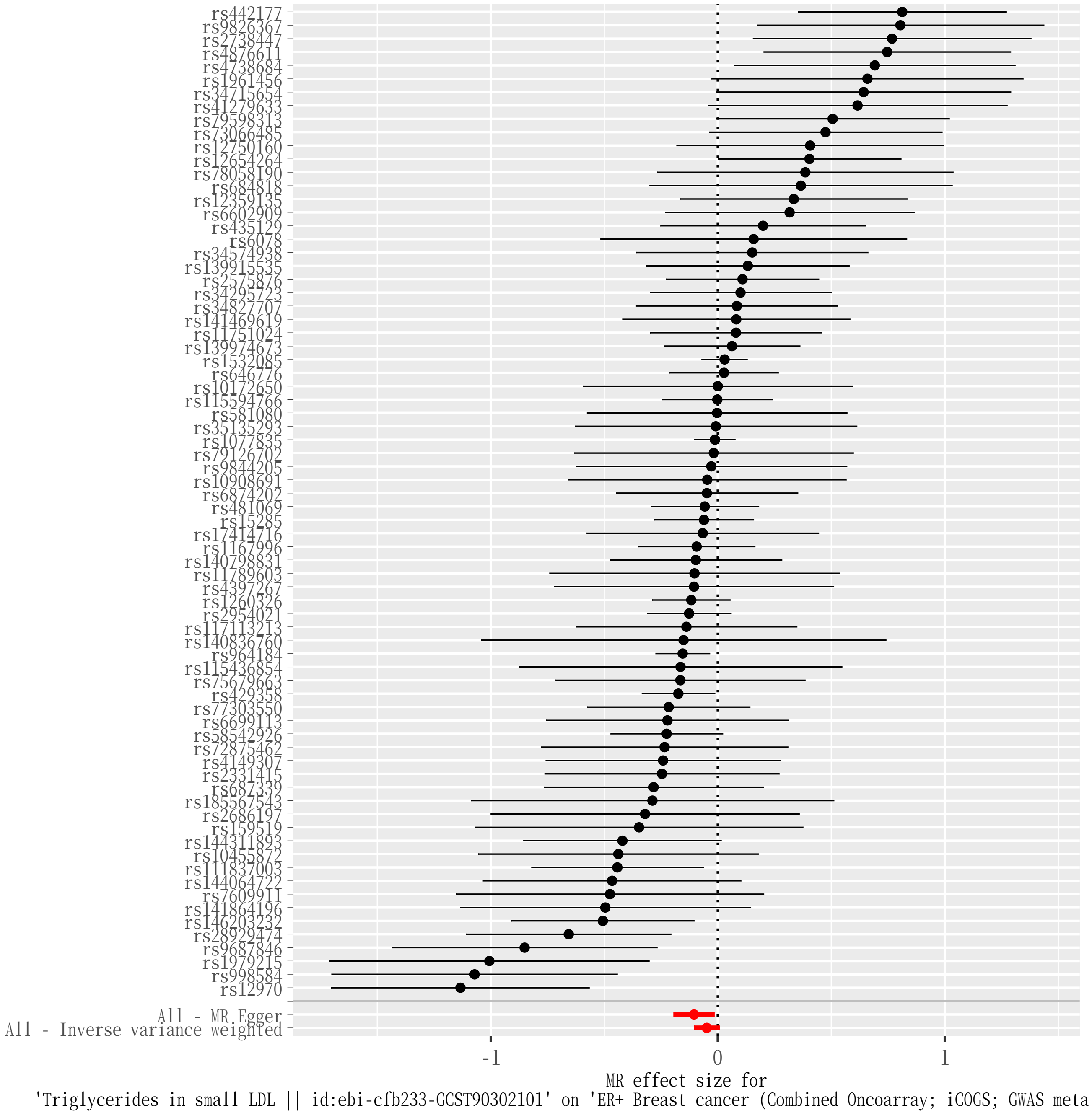
rs442177
rs9826367
rs2738447
rs4876611
rs4738684
rs1961456
rs34715654
rs41279633
rs79598313
rs73066485
rs12750160
rs12654264
rs78058190
rs684818
rs12359135
rs6602909
rs435129
rs6078
rs34574938
rs139915535
rs2575876
rs34295723
rs34827707
rs141469619
rs11751024
rs139974673
rs1532085
rs646776
rs10172650
rs115594766
rs581080
rs35135293
rs1077835
rs79126702
rs9844205
rs10908691
rs6874202
rs481069
rs15285
rs17414716
rs1167996
rs140798831
rs11789603
rs4397267
rs1260326
rs2954021
rs117113213
rs140836760
rs964184
rs115436854
rs75679663
rs429358
rs77303550
rs6699113
rs58542926
rs72875462
rs4149307
rs2331415
rs687339
rs185567543
rs2686197
rs159519
rs144311893
rs10455872
rs111837003
rs144064722
rs7609911
rs141864196
rs146203232
rs28929474
rs9687846
rs1979215
rs998584
rs12970
All - MR Egger
All - Inverse variance weighted
-1
0
1
MR effect size for
'Triglycerides in small LDL || id:ebi-cfb233-GCST90302101' on 'ER+ Breast cancer (Combined Oncoarray; iCOGS; GWAS meta

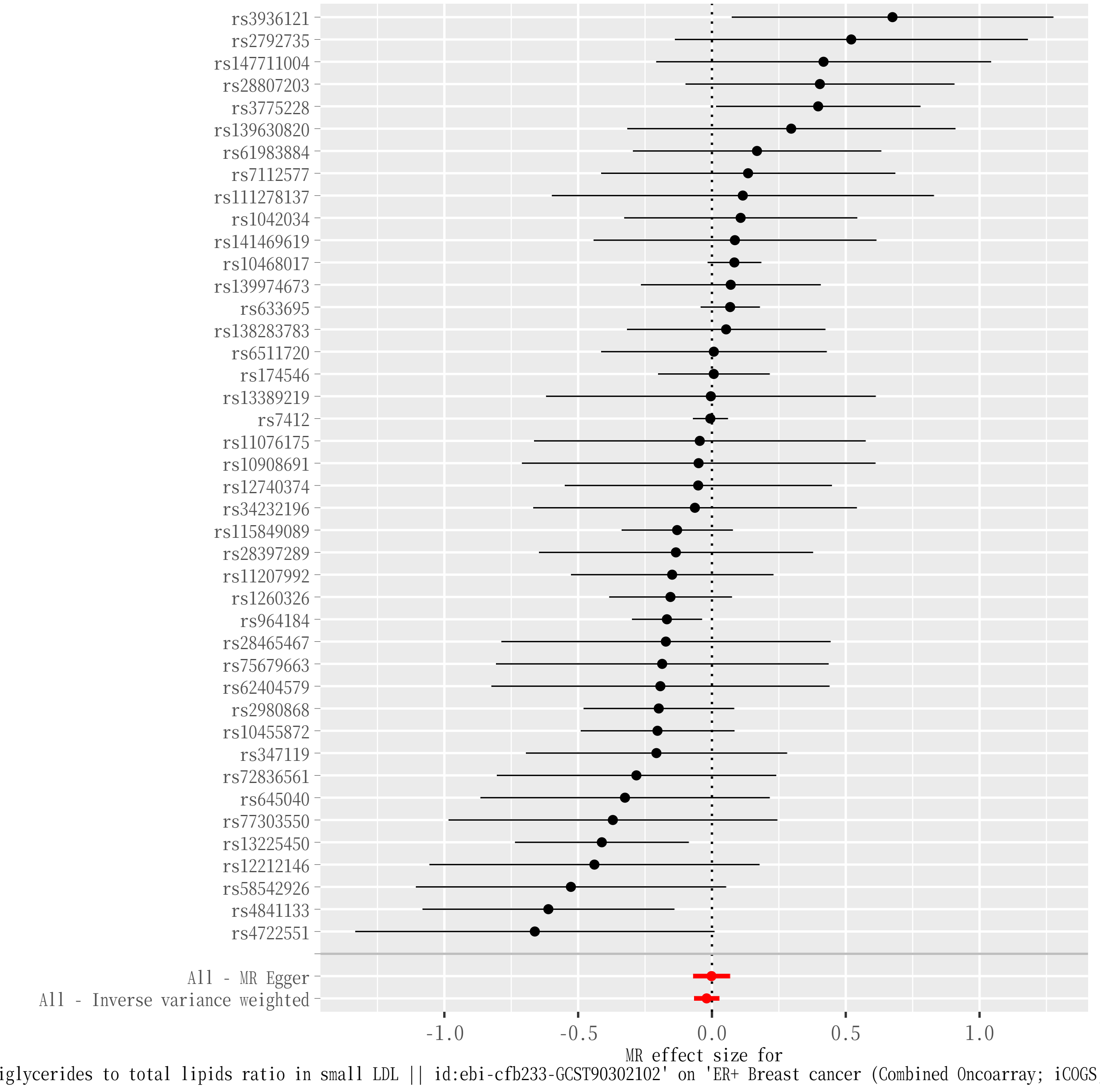
rs3936121
rs2792735
rs147711004
rs28807203
rs3775228
rs139630820
rs61983884
rs7112577
rs111278137
rs1042034
rs141469619
rs10468017
rs139974673
rs633695
rs138283783
rs6511720
rs174546
rs13389219
rs7412
rs11076175
rs10908691
rs12740374
rs34232196
rs115849089
rs28397289
rs11207992
rs1260326
rs964184
rs28465467
rs75679663
rs62404579
rs2980868
rs10455872
rs347119
rs72836561
rs645040
rs77303550
rs13225450
rs12212146
rs58542926
rs4841133
rs4722551
All - MR Egger
All - Inverse variance weighted
-1.0
-0.5
0.0
0.5
1.0
MR effect size for
iglycerides to total lipids ratio in small LDL || id:ebi-cfb233-GCST90302102' on 'ER+ Breast cancer (Combined Oncoarray; iCOGS

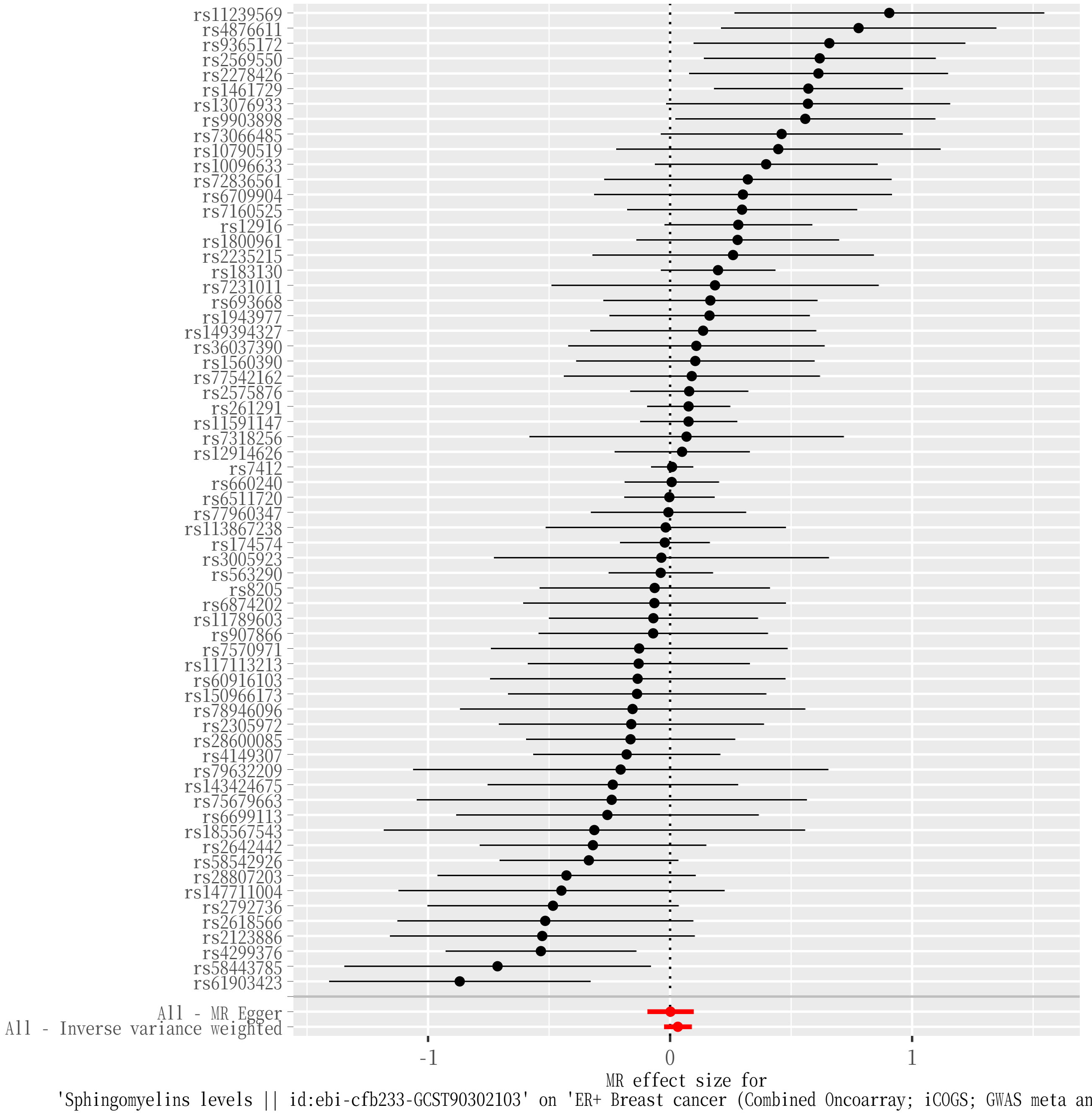
rs11239569
rs4876611
rs9365172
rs2569550
rs2278426
rs1461729
rs13076933
rs9903898
rs73066485
rs10790519
rs10096633
rs72836561
rs6709904
rs7160525
rs12916
rs1800961
rs2235215
rs183130
rs7231011
rs693668
rs1943977
rs149394327
rs36037390
rs1560390
rs77542162
rs2575876
rs261291
rs11591147
rs7318256
rs12914626
rs7412
rs660240
rs6511720
rs77960347
rs113867238
rs174574
rs3005923
rs563290
rs8205
rs6874202
rs11789603
rs907866
rs7570971
rs117113213
rs60916103
rs150966173
rs78946096
rs2305972
rs28600085
rs4149307
rs79632209
rs143424675
rs75679663
rs6699113
rs185567543
rs2642442
rs58542926
rs28807203
rs147711004
rs2792736
rs2618566
rs2123886
rs4299376
rs58443785
rs61903423
All - MR Egger
All - Inverse variance weighted
-1
0
1
MR effect size for
'Sphingomyelins levels || id:ebi-cfb233-GCST90302103' on 'ER+ Breast cancer (Combined Oncoarray; iCOGS; GWAS meta an

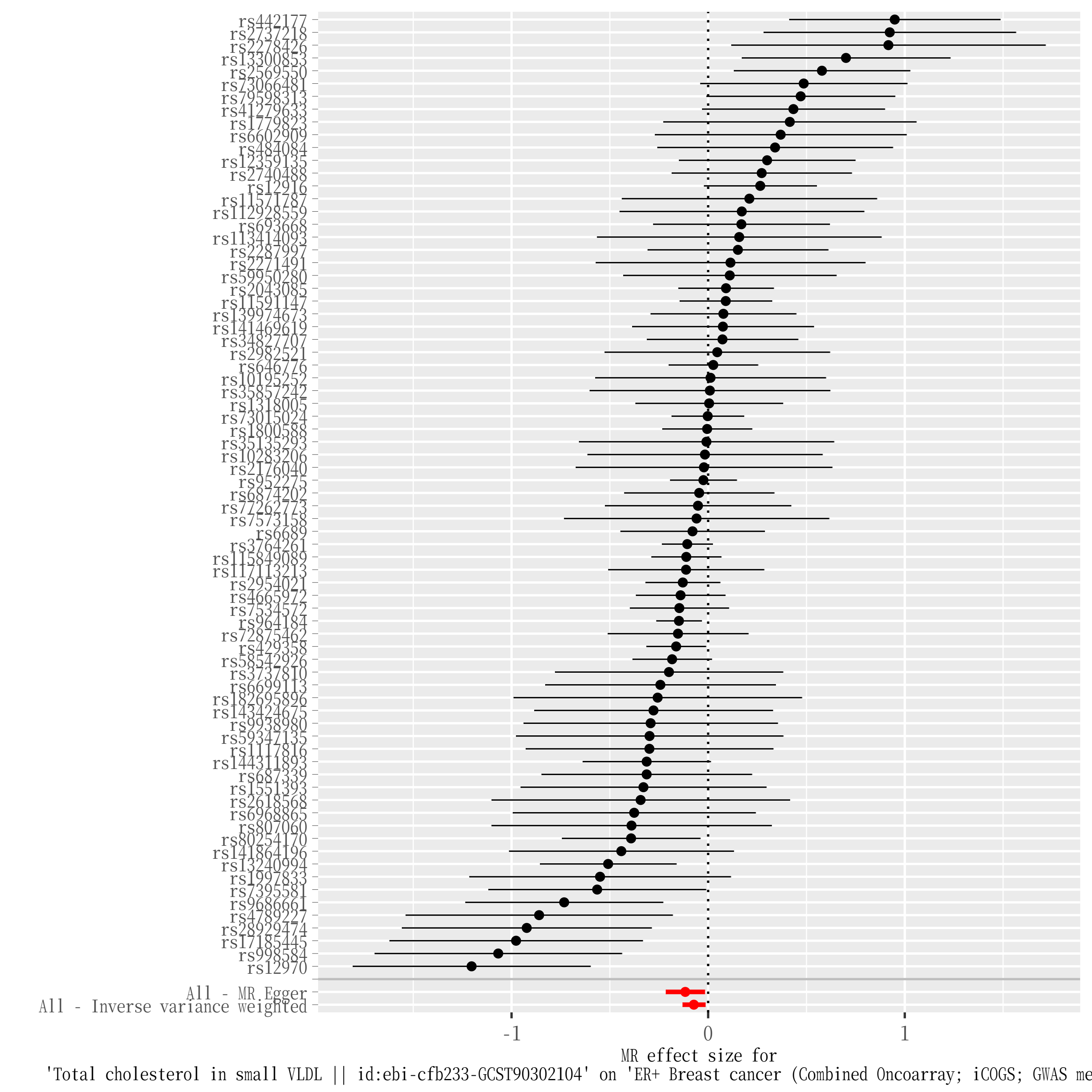
rs442177
rs2737218
rs2278426
rs13300853
rs2569550
rs73066481
rs79598313
rs41279633
rs1779823
rs6602909
rs484084
rs12359135
rs2740488
rs12916
rs11571787
rs112928559
rs693668
rs113414093
rs2287997
rs2271491
rs59950280
rs2043085
rs11591147
rs139974673
rs141469619
rs34827707
rs2982521
rs646776
rs10195252
rs35857242
rs1318005
rs73015024
rs1800588
rs35135293
rs10283206
rs2176040
rs952275
rs6874202
rs77262773
rs7573158
rs6689
rs3764261
rs115849089
rs117113213
rs2954021
rs4665972
rs7534572
rs964184
rs72875462
rs429358
rs58542926
rs3737810
rs6699113
rs182695896
rs143424675
rs9938980
rs59347135
rs1117816
rs144311893
rs687339
rs1551393
rs2618568
rs6968865
rs807060
rs80254170
rs141864196
rs13240994
rs1997833
rs7395581
rs9686661
rs4789227
rs28929474
rs17185445
rs998584
rs12970
All - MR Egger
All - Inverse variance weighted
-1
0
1
MR effect size for
'Total cholesterol in small VLDL || id:ebi-cfb233-GCST90302104' on 'ER+ Breast cancer (Combined Oncoarray; iCOGS; GWAS me

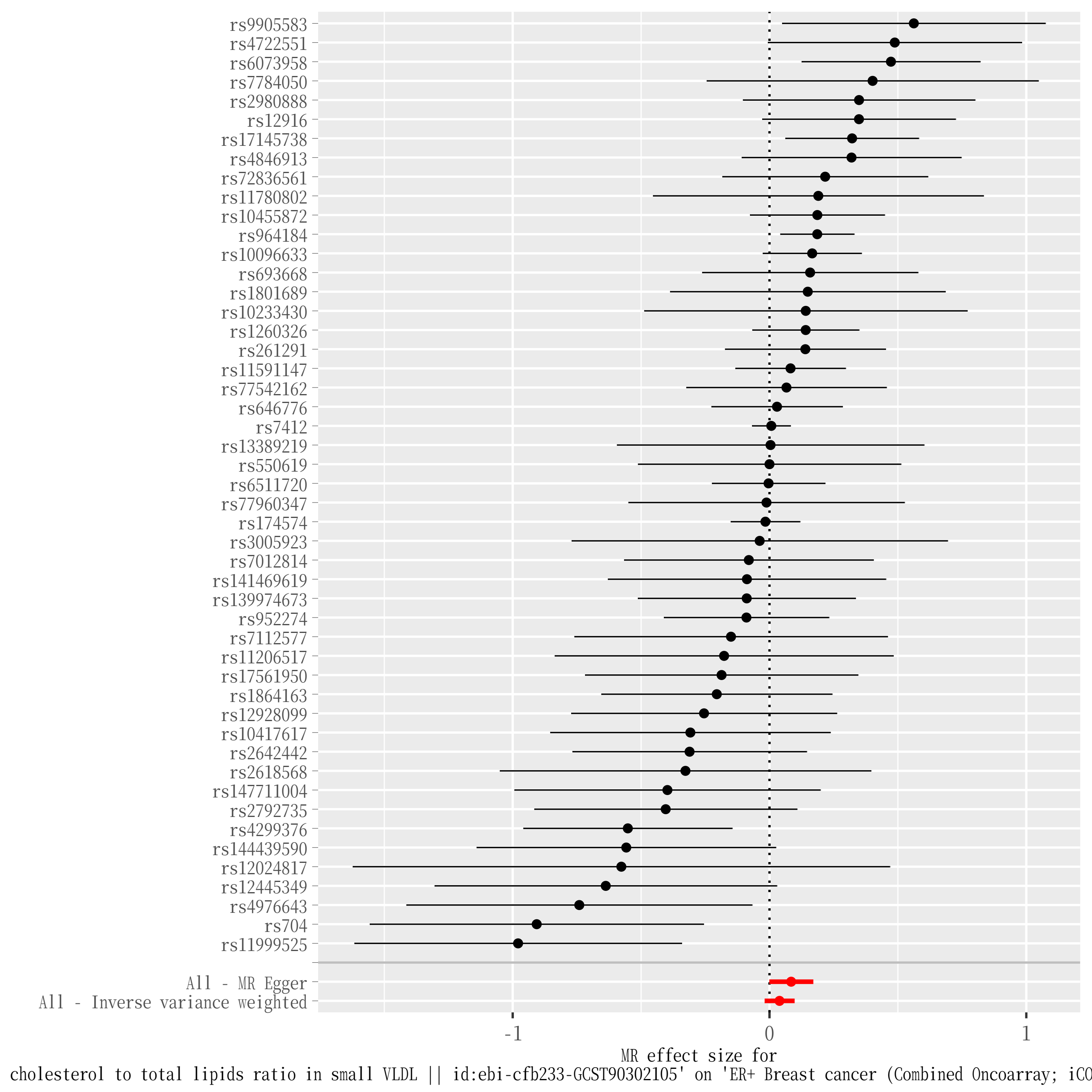

rs9905583
rs4722551
rs6073958
rs7784050
rs2980888
rs12916
rs17145738
rs4846913
rs72836561
rs11780802
rs10455872
rs964184
rs10096633
rs693668
rs1801689
rs10233430
rs1260326
rs261291
rs11591147
rs77542162
rs646776
rs7412
rs13389219
rs550619
rs6511720
rs77960347
rs174574
rs3005923
rs7012814
rs141469619
rs139974673
rs952274
rs7112577
rs11206517
rs17561950
rs1864163
rs12928099
rs10417617
rs2642442
rs2618568
rs147711004
rs2792735
rs4299376
rs144439590
rs12024817
rs12445349
rs4976643
rs704
rs11999525
All - MR Egger
All - Inverse variance weighted
-1
0
1
MR effect size for
cholesterol to total lipids ratio in small VLDL || id:ebi-cfb233-GCST90302105' on 'ER+ Breast cancer (Combined Oncoarray; iCO

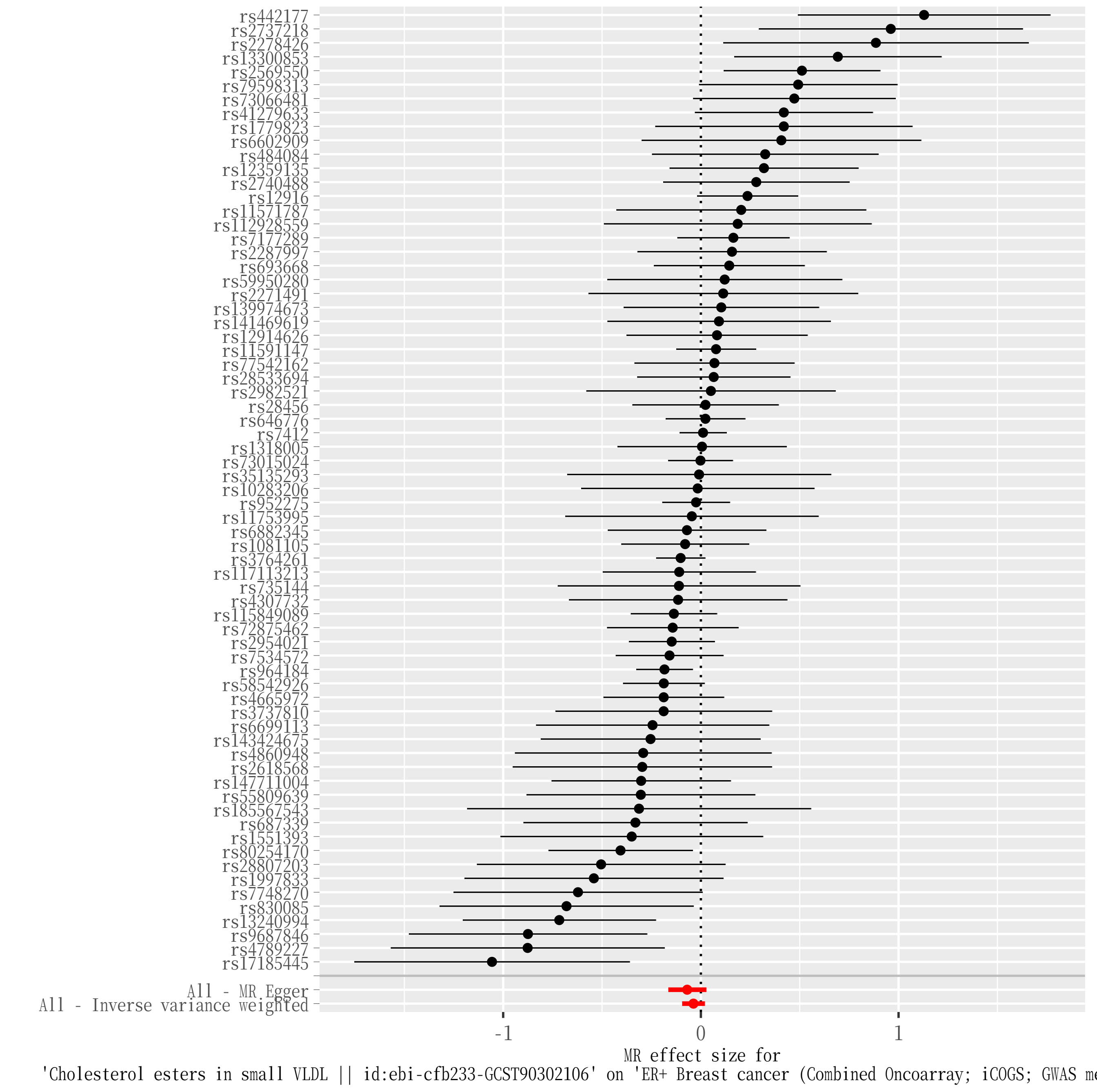

rs442177
rs2737218
rs2278426
rs13300853
rs2569550
rs79598313
rs73066481
rs41279633
rs1779823
rs6602909
rs484084
rs12359135
rs2740488
rs12916
rs11571787
rs112928559
rs7177289
rs2287997
rs693668
rs59950280
rs2271491
rs139974673
rs141469619
rs12914626
rs11591147
rs77542162
rs28533694
rs2982521
rs28456
rs646776
rs7412
rs1318005
rs73015024
rs35135293
rs10283206
rs952275
rs11753995
rs6882345
rs1081105
rs3764261
rs117113213
rs735144
rs4307732
rs115849089
rs72875462
rs2954021
rs7534572
rs964184
rs58542926
rs4665972
rs3737810
rs6699113
rs143424675
rs4860948
rs2618568
rs147711004
rs55809639
rs185567543
rs687339
rs1551393
rs80254170
rs28807203
rs1997833
rs7748270
rs830085
rs13240994
rs9687846
rs4789227
rs17185445
All - MR Egger
All - Inverse variance weighted
-1
0
1
MR effect size for
'Cholesterol esters in small VLDL || id:ebi-cfb233-GCST90302106' on 'ER+ Breast cancer (Combined Oncoarray; iCOGS; GWAS me

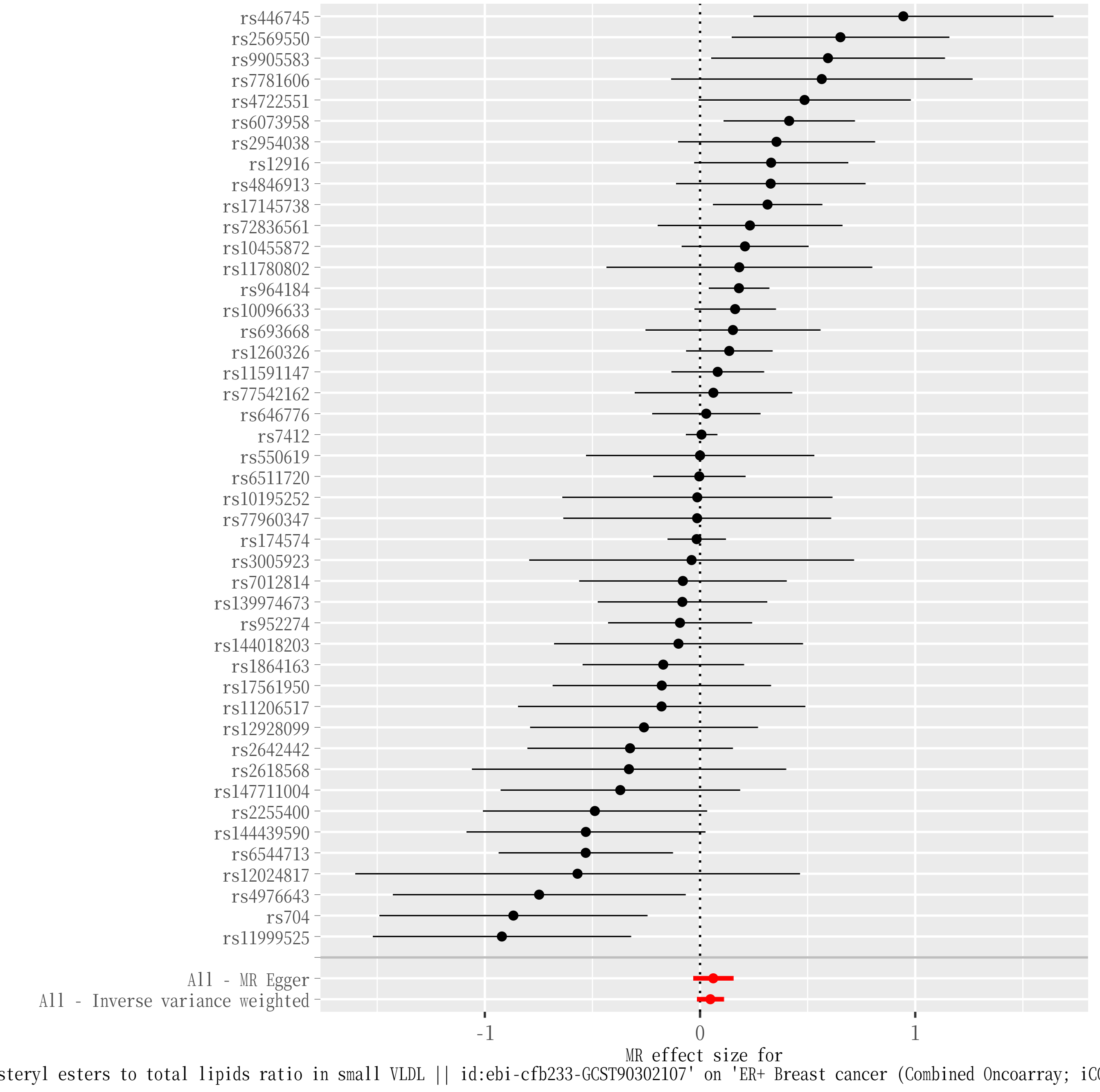

rs446745
rs2569550
rs9905583
rs7781606
rs4722551
rs6073958
rs2954038
rs12916
rs4846913
rs17145738
rs72836561
rs10455872
rs11780802
rs964184
rs10096633
rs693668
rs1260326
rs11591147
rs77542162
rs646776
rs7412
rs550619
rs6511720
rs10195252
rs77960347
rs174574
rs3005923
rs7012814
rs139974673
rs952274
rs144018203
rs1864163
rs17561950
rs11206517
rs12928099
rs2642442
rs2618568
rs147711004
rs2255400
rs144439590
rs6544713
rs12024817
rs4976643
rs704
rs11999525
All - MR Egger
All - Inverse variance weighted
-1
0
1
MR effect size for
steryl esters to total lipids ratio in small VLDL || id:ebi-cfb233-GCST90302107' on 'ER+ Breast cancer (Combined Oncoarray; iC

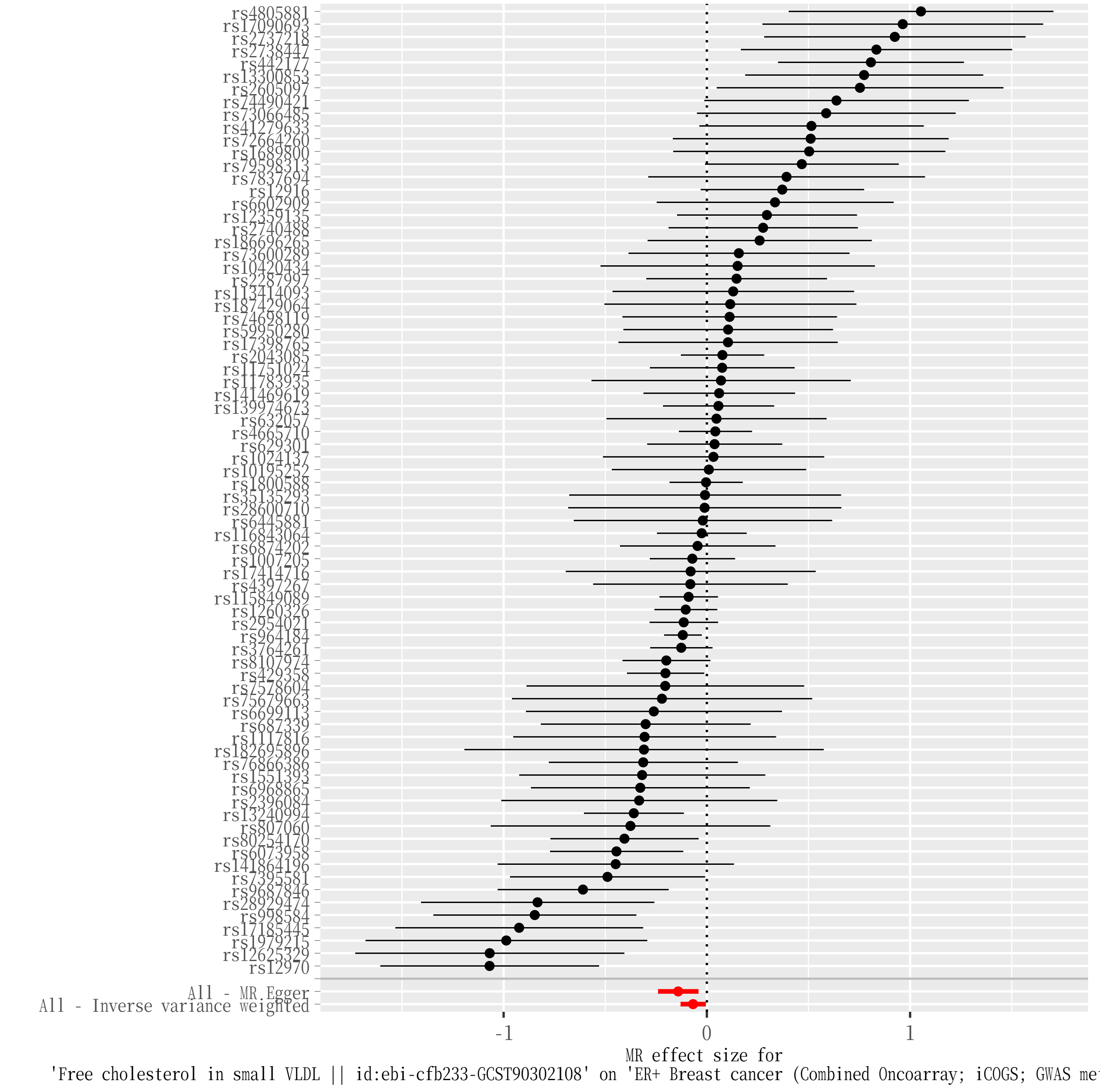
rs4805881
rs17090693
rs2737218
rs2738447
rs442177
rs13300853
rs2605097
rs74490421
rs73066485
rs41279633
rs72664260
rs1689800
rs79598313
rs7837694
rs12916
rs6602909
rs12359135
rs2740488
rs186696265
rs73600289
rs10420434
rs2287997
rs113414093
rs187429064
rs74628119
rs59950280
rs17398765
rs2043085
rs11751024
rs11783935
rs141469619
rs139974673
rs632057
rs4665710
rs629301
rs1024137
rs10195252
rs1800588
rs35135293
rs28600710
rs6445881
rs116843064
rs6874202
rs1007205
rs17414716
rs4397267
rs115849089
rs1260326
rs2954021
rs964184
rs3764261
rs8107974
rs429358
rs7528604
rs75679663
rs6699113
rs687339
rs1117816
rs182695896
rs76866386
rs1551393
rs6968865
rs2396084
rs13240994
rs807060
rs80254170
rs6073958
rs141864196
rs7395581
rs9687846
rs28929474
rs998584
rs17185445
rs1979215
rs12625329
rs12970
All - MR Egger
All - Inverse variance weighted
-1
0
1
MR effect size for
'Free cholesterol in small VLDL || id:ebi-cfb233-GCST90302108' on 'ER+ Breast cancer (Combined Oncoarray; iCOGS; GWAS me

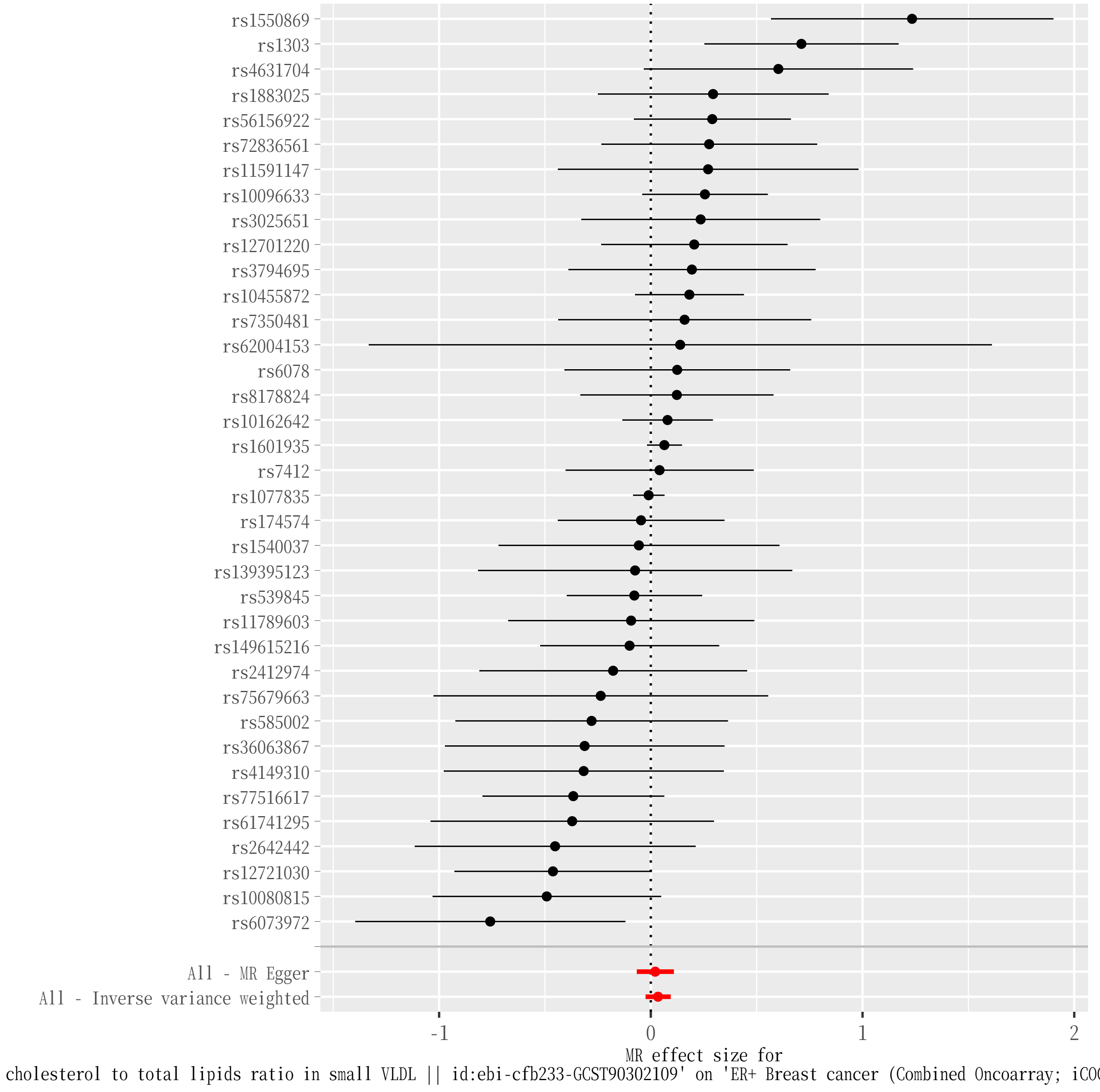

rs1550869
rs1303
rs4631704
rs1883025
rs56156922
rs72836561
rs11591147
rs10096633
rs3025651
rs12701220
rs3794695
rs10455872
rs7350481
rs62004153
rs6078
rs8178824
rs10162642
rs1601935
rs7412
rs1077835
rs174574
rs1540037
rs139395123
rs539845
rs11789603
rs149615216
rs2412974
rs75679663
rs585002
rs36063867
rs4149310
rs77516617
rs61741295
rs2642442
rs12721030
rs10080815
rs6073972
All - MR Egger
All - Inverse variance weighted
-1
0
1
2
MR effect size for
cholesterol to total lipids ratio in small VLDL || id:ebi-cfb233-GCST90302109' on 'ER+ Breast cancer (Combined Oncoarray; iCO

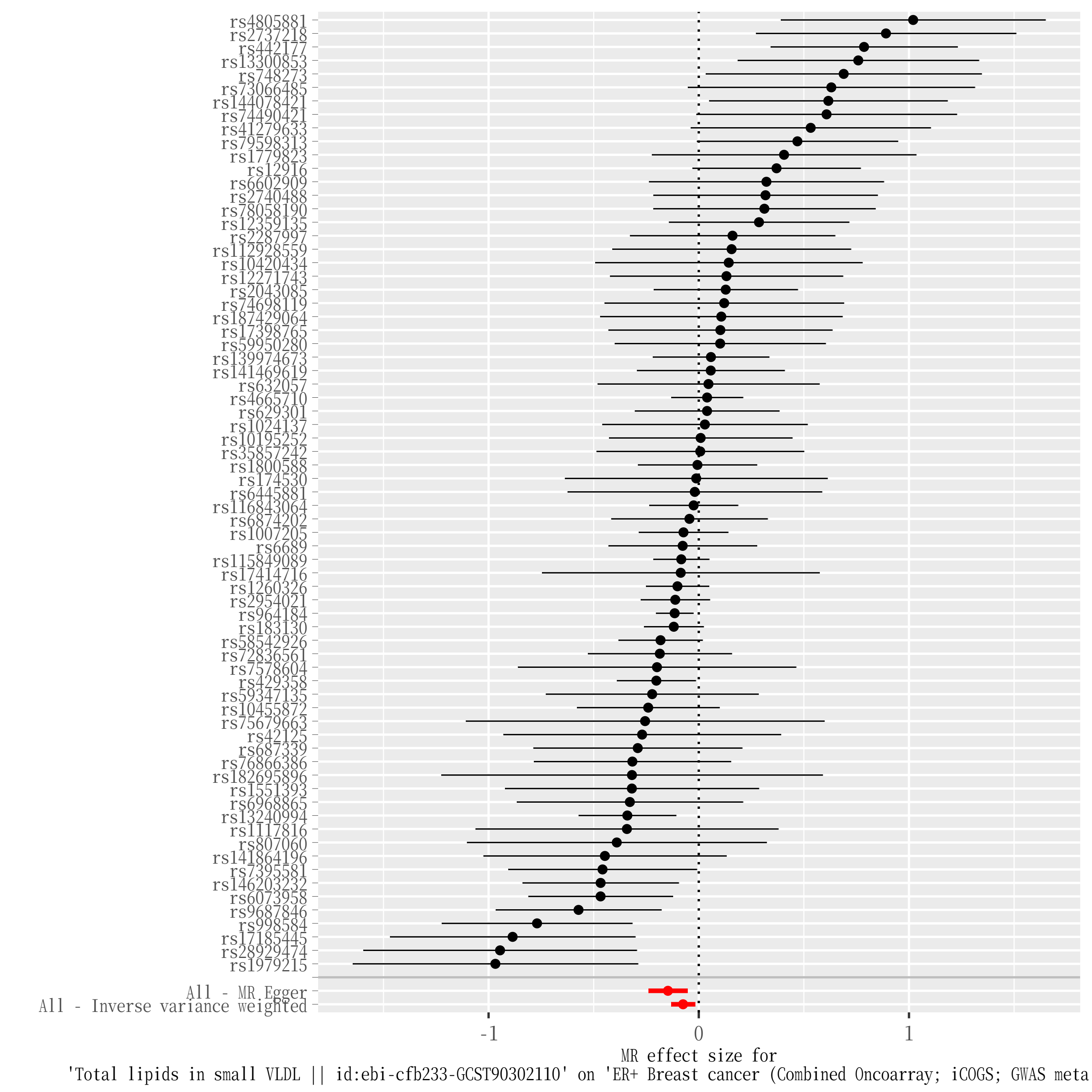
rs4805881
rs2737218
rs442177
rs13300853
rs748273
rs73066485
rs144078421
rs74490421
rs41279633
rs79598313
rs1779823
rs12916
rs6602909
rs2740488
rs78058190
rs12359135
rs2287997
rs112928559
rs10420434
rs12271743
rs2043085
rs74698119
rs187429064
rs17398765
rs59950280
rs139974673
rs141469619
rs632057
rs4665710
rs629301
rs1024137
rs10195252
rs35857242
rs1800588
rs174530
rs6445881
rs116843064
rs6874202
rs1007205
rs6689
rs115849089
rs17414716
rs1260326
rs2954021
rs964184
rs183130
rs58542926
rs72836561
rs7578604
rs429358
rs59347135
rs10455872
rs75679663
rs42125
rs687339
rs76866386
rs182695896
rs1551393
rs6968865
rs13240994
rs1117816
rs807060
rs141864196
rs7395581
rs146203232
rs6073958
rs9687846
rs998584
rs17185445
rs28929474
rs1979215
All - MR Egger
All - Inverse variance weighted
-1
0
1
MR effect size for
'Total lipids in small VLDL || id:ebi-cfb233-GCST90302110' on 'ER+ Breast cancer (Combined Oncoarray; iCOGS; GWAS meta

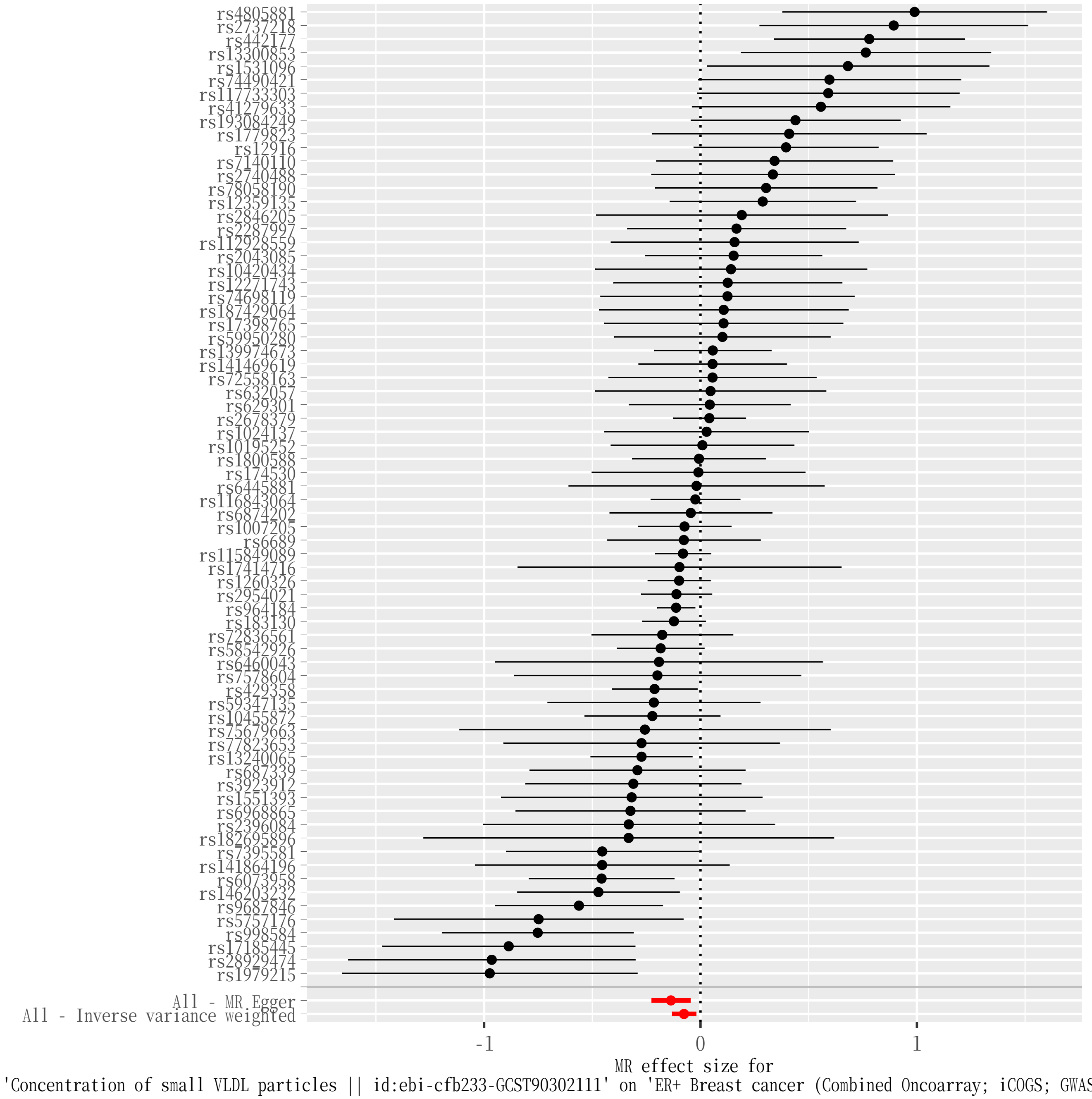
rs4805881
rs2737218
rs442177
rs13300853
rs1531096
rs74490421
rs117733303
rs41279633
rs193084249
rs1779823
rs12916
rs7140110
rs2740488
rs78058190
rs12359135
rs2846205
rs2287997
rs112928559
rs2043085
rs10420434
rs12271743
rs74698119
rs187429064
rs17398765
rs59950280
rs139974673
rs141469619
rs72558163
rs632057
rs629301
rs2678379
rs1024137
rs10195252
rs1800588
rs174530
rs6445881
rs116843064
rs6874202
rs1007205
rs6689
rs115849089
rs17414716
rs1260326
rs2954021
rs964184
rs183130
rs72836561
rs58542926
rs6460043
rs7578604
rs429358
rs59347135
rs10455872
rs75679663
rs77823653
rs13240065
rs687339
rs3923912
rs1551393
rs6968865
rs2396084
rs182695896
rs7395581
rs141864196
rs6073958
rs146203232
rs9687846
rs5757176
rs998584
rs17185445
rs28929474
rs1979215
All - MR Egger
All - Inverse variance weighted
-1
0
1
MR effect size for
'Concentration of small VLDL particles || id:ebi-cfb233-GCST90302111' on 'ER+ Breast cancer (Combined Oncoarray; iCOGS; GWAS

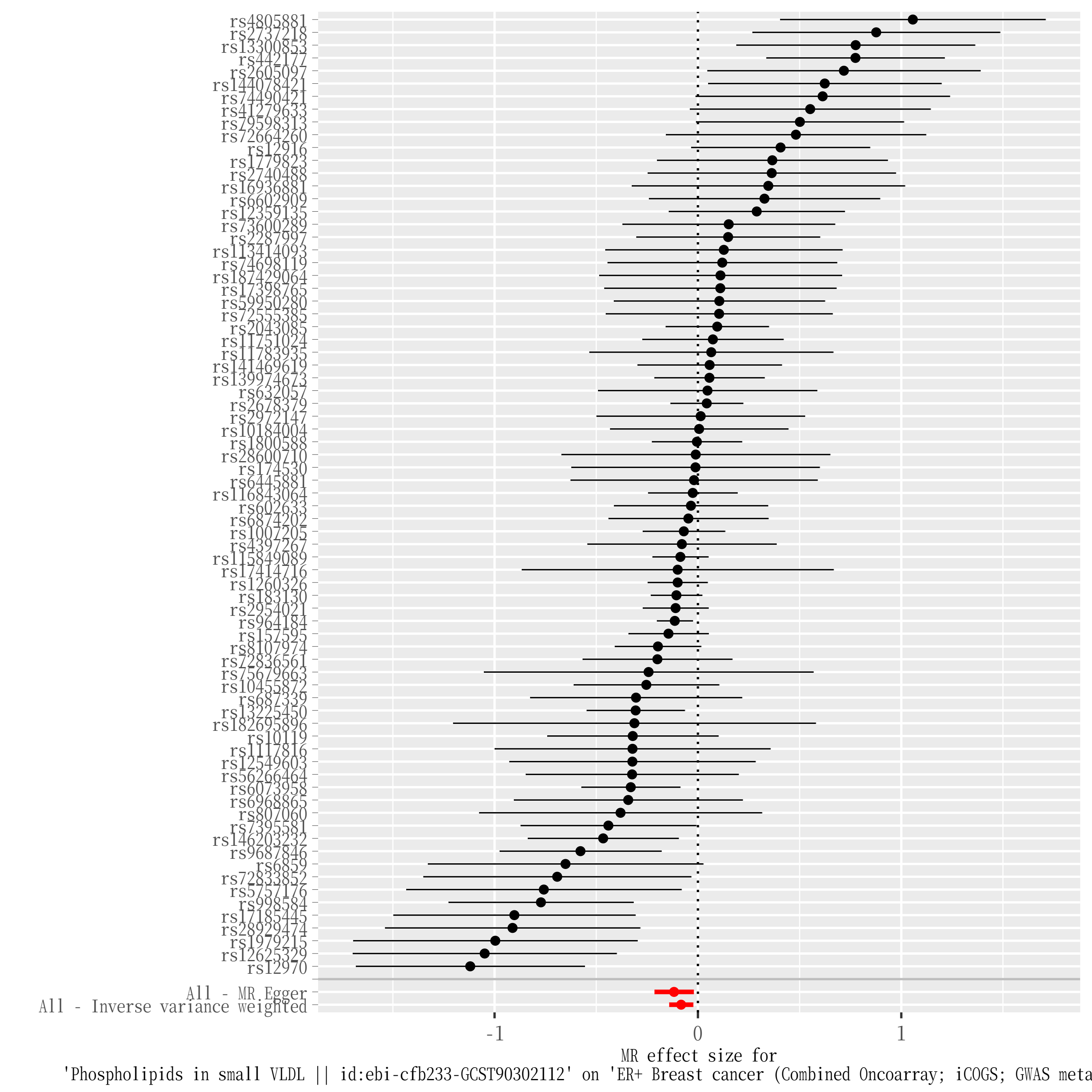
rs4805881
rs2737218
rs13300853
rs442177
rs2605097
rs144078421
rs74490421
rs41279633
rs79598313
rs72664260
rs12916
rs1779823
rs2740488
rs16936881
rs6602909
rs12359135
rs73600289
rs2287997
rs113414093
rs74698119
rs187429064
rs17398765
rs59950280
rs72555385
rs2043085
rs11751024
rs11783935
rs141469619
rs139974673
rs632057
rs2678379
rs2972147
rs10184004
rs1800588
rs28600710
rs174530
rs6445881
rs116843064
rs602633
rs6874202
rs1007205
rs4397267
rs115849089
rs17414716
rs1260326
rs183130
rs2954021
rs964184
rs157595
rs8107974
rs72836561
rs75679663
rs10455872
rs687339
rs13225450
rs182695896
rs10119
rs1117816
rs12549603
rs56266464
rs6073958
rs6968865
rs807060
rs7395581
rs146203232
rs9687846
rs6859
rs72833852
rs5757176
rs998584
rs17185445
rs28929474
rs1979215
rs12625329
rs12970
All - MR Egger
All - Inverse variance weighted
-1
0
1
MR effect size for
'Phospholipids in small VLDL || id:ebi-cfb233-GCST90302112' on 'ER+ Breast cancer (Combined Oncoarray; iCOGS; GWAS meta

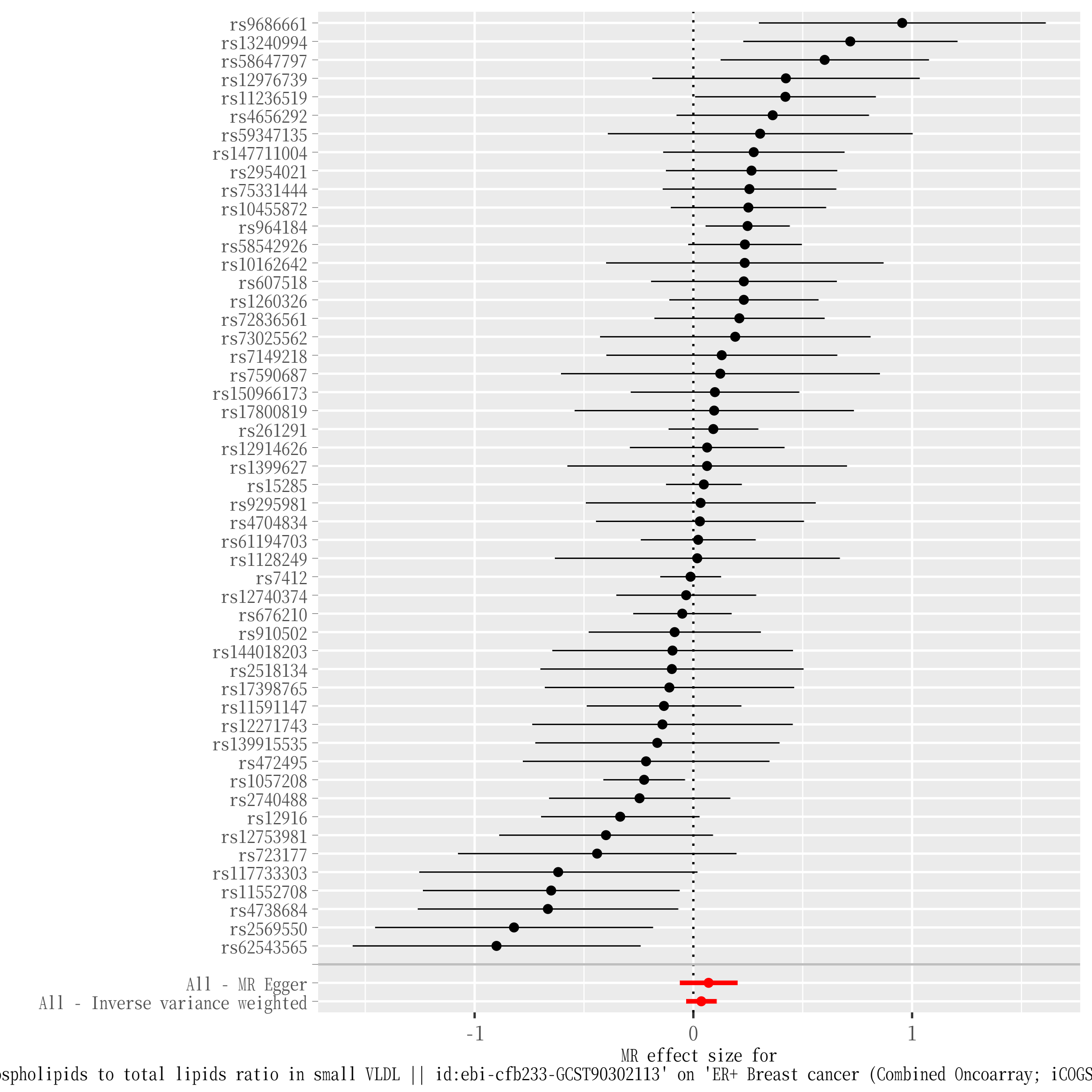

rs9686661
rs13240994
rs58647797
rs12976739
rs11236519
rs4656292
rs59347135
rs147711004
rs2954021
rs75331444
rs10455872
rs964184
rs58542926
rs10162642
rs607518
rs1260326
rs72836561
rs73025562
rs7149218
rs7590687
rs150966173
rs17800819
rs261291
rs12914626
rs1399627
rs15285
rs9295981
rs4704834
rs61194703
rs1128249
rs7412
rs12740374
rs676210
rs910502
rs144018203
rs2518134
rs17398765
rs11591147
rs12271743
rs139915535
rs472495
rs1057208
rs2740488
rs12916
rs12753981
rs723177
rs117733303
rs11552708
rs4738684
rs2569550
rs62543565
All - MR Egger
All - Inverse variance weighted
-1
0
1
MR effect size for
ospholipids to total lipids ratio in small VLDL || id:ebi-cfb233-GCST90302113' on 'ER+ Breast cancer (Combined Oncoarray; iCOGS

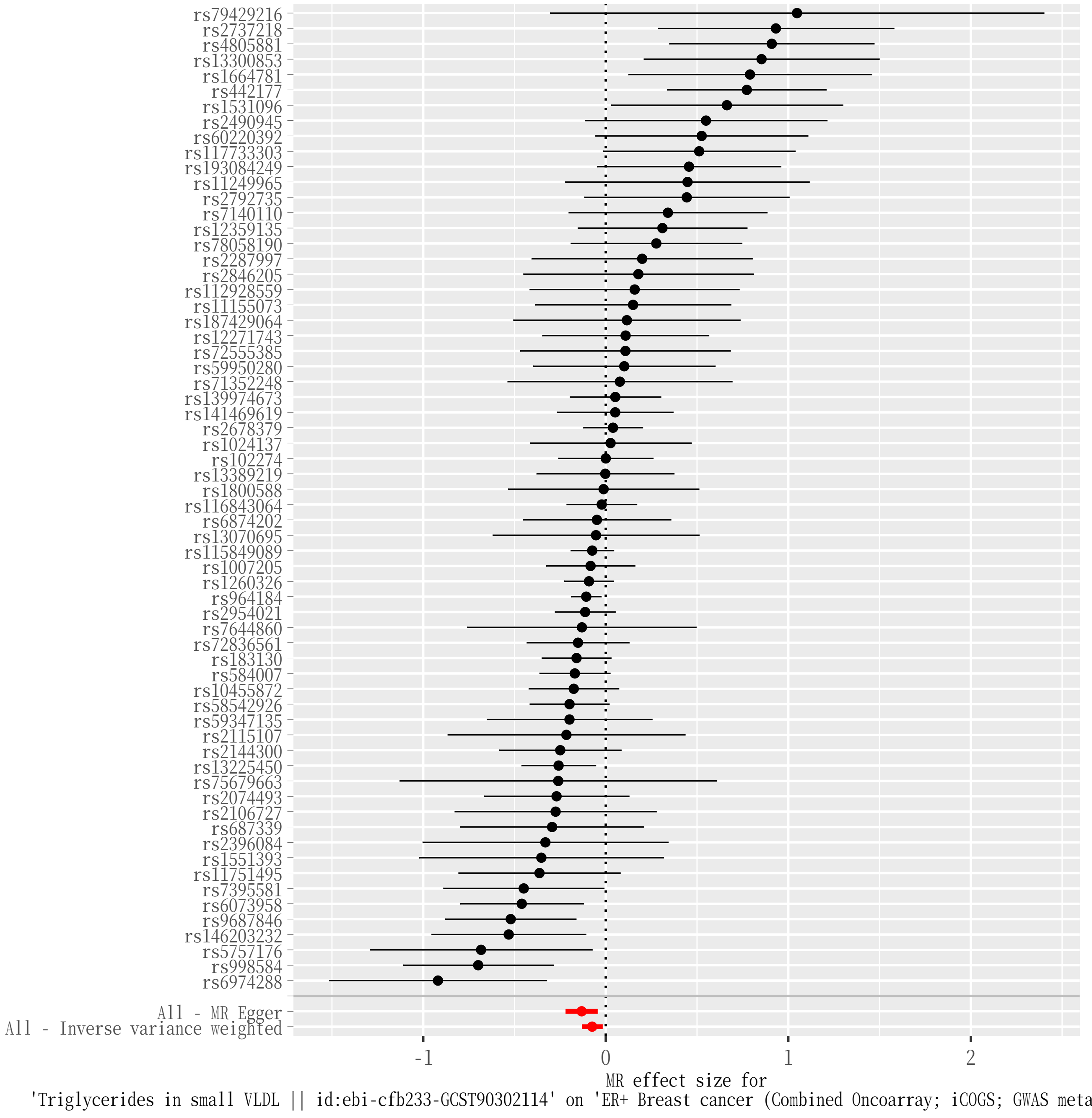
rs79429216
rs2737218
rs4805881
rs13300853
rs1664781
rs442177
rs1531096
rs2490945
rs60220392
rs117733303
rs193084249
rs11249965
rs2792735
rs7140110
rs12359135
rs78058190
rs2287997
rs2846205
rs112928559
rs11155073
rs187429064
rs12271743
rs72555385
rs59950280
rs71352248
rs139974673
rs141469619
rs2678379
rs1024137
rs102274
rs13389219
rs1800588
rs116843064
rs6874202
rs13070695
rs115849089
rs1007205
rs1260326
rs964184
rs2954021
rs7644860
rs72836561
rs183130
rs584007
rs10455872
rs58542926
rs59347135
rs2115107
rs2144300
rs13225450
rs75679663
rs2074493
rs2106727
rs687339
rs2396084
rs1551393
rs11751495
rs7395581
rs6073958
rs9687846
rs146203232
rs5757176
rs998584
rs6974288
All - MR Egger
All - Inverse variance weighted
-1
0
1
2
MR effect size for
'Triglycerides in small VLDL || id:ebi-cfb233-GCST90302114' on 'ER+ Breast cancer (Combined Oncoarray; iCOGS; GWAS meta

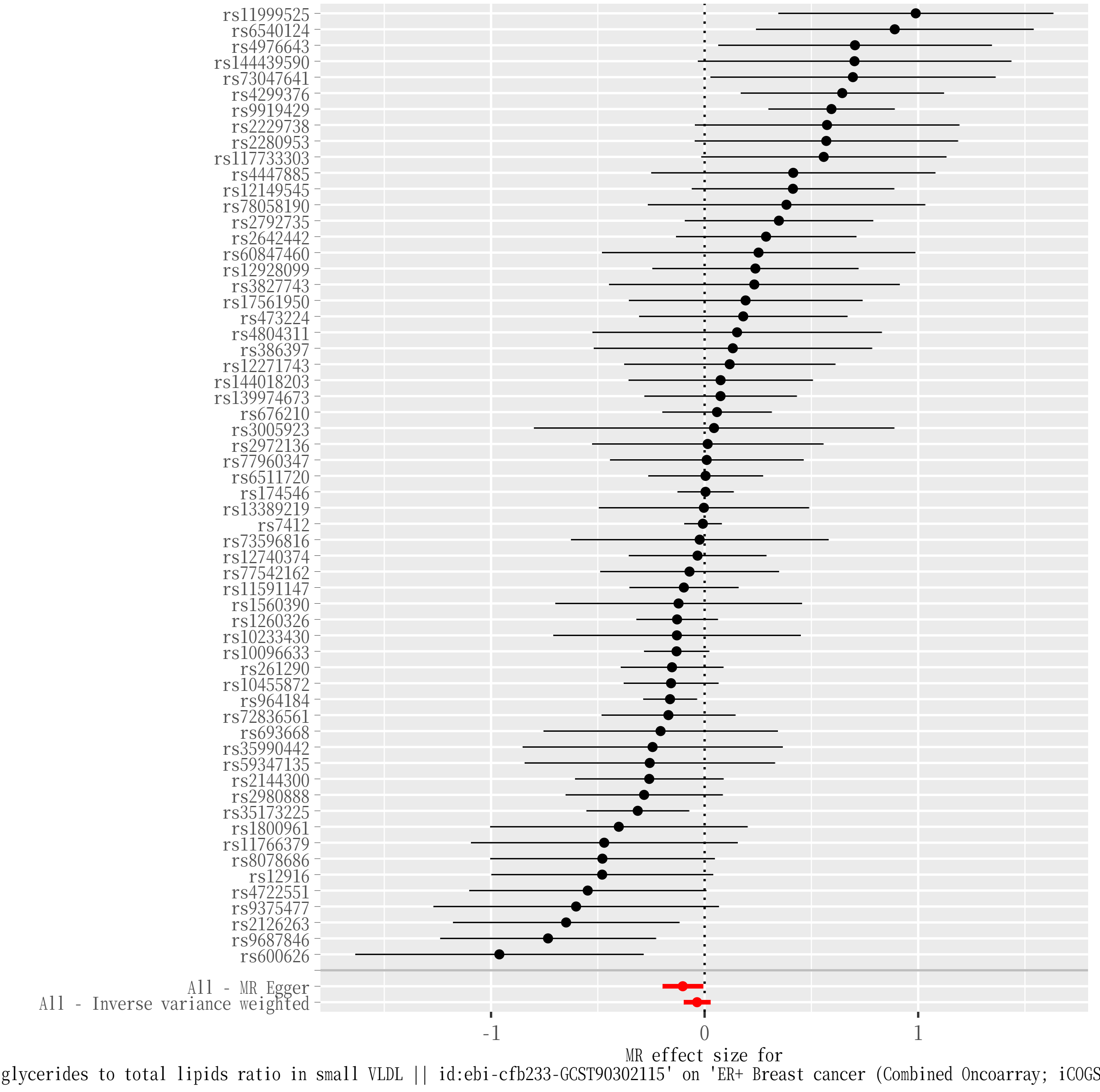

rs11999525
rs6540124
rs4976643
rs144439590
rs73047641
rs4299376
rs9919429
rs2229738
rs2280953
rs117733303
rs4447885
rs12149545
rs78058190
rs2792735
rs2642442
rs60847460
rs12928099
rs3827743
rs17561950
rs473224
rs4804311
rs386397
rs12271743
rs144018203
rs139974673
rs676210
rs3005923
rs2972136
rs77960347
rs6511720
rs174546
rs13389219
rs7412
rs73596816
rs12740374
rs77542162
rs11591147
rs1560390
rs1260326
rs10233430
rs10096633
rs261290
rs10455872
rs964184
rs72836561
rs693668
rs35990442
rs59347135
rs2144300
rs2980888
rs35173225
rs1800961
rs11766379
rs8078686
rs12916
rs4722551
rs9375477
rs2126263
rs9687846
rs600626
All - MR Egger
All - Inverse variance weighted
-1
0
1
MR effect size for
glycerides to total lipids ratio in small VLDL || id:ebi-cfb233-GCST90302115' on 'ER+ Breast cancer (Combined Oncoarray; iCOGS

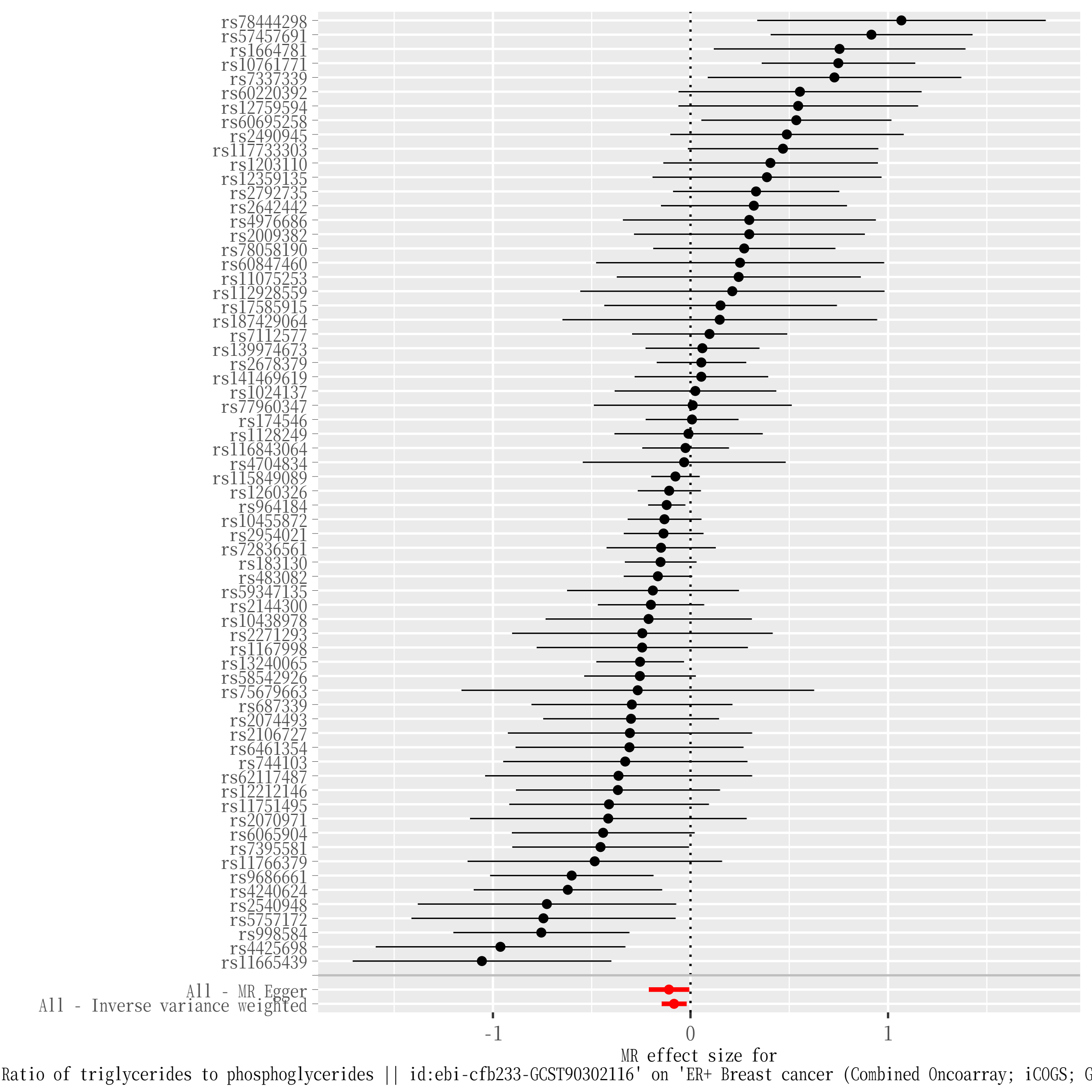
rs78444298
rs57457691
rs1664781
rs10761771
rs7337339
rs60220392
rs12759594
rs60695258
rs2490945
rs117733303
rs1203110
rs12359135
rs2792735
rs2642442
rs4976686
rs2009382
rs78058190
rs60847460
rs11075253
rs112928559
rs17585915
rs187429064
rs7112577
rs139974673
rs2678379
rs141469619
rs1024137
rs77960347
rs174546
rs1128249
rs116843064
rs4704834
rs115849089
rs1260326
rs964184
rs10455872
rs2954021
rs72836561
rs183130
rs483082
rs59347135
rs2144300
rs10438978
rs2271293
rs1167998
rs13240065
rs58542926
rs75679663
rs687339
rs2074493
rs2106727
rs6461354
rs744103
rs62117487
rs12212146
rs11751495
rs2070971
rs6065904
rs7395581
rs11766379
rs9686661
rs4240624
rs2540948
rs5757172
rs998584
rs4425698
rs11665439
All - MR Egger
All - Inverse variance weighted
-1
0
1
MR effect size for
'Ratio of triglycerides to phosphoglycerides || id:ebi-cfb233-GCST90302116' on 'ER+ Breast cancer (Combined Oncoarray; iCOGS; G

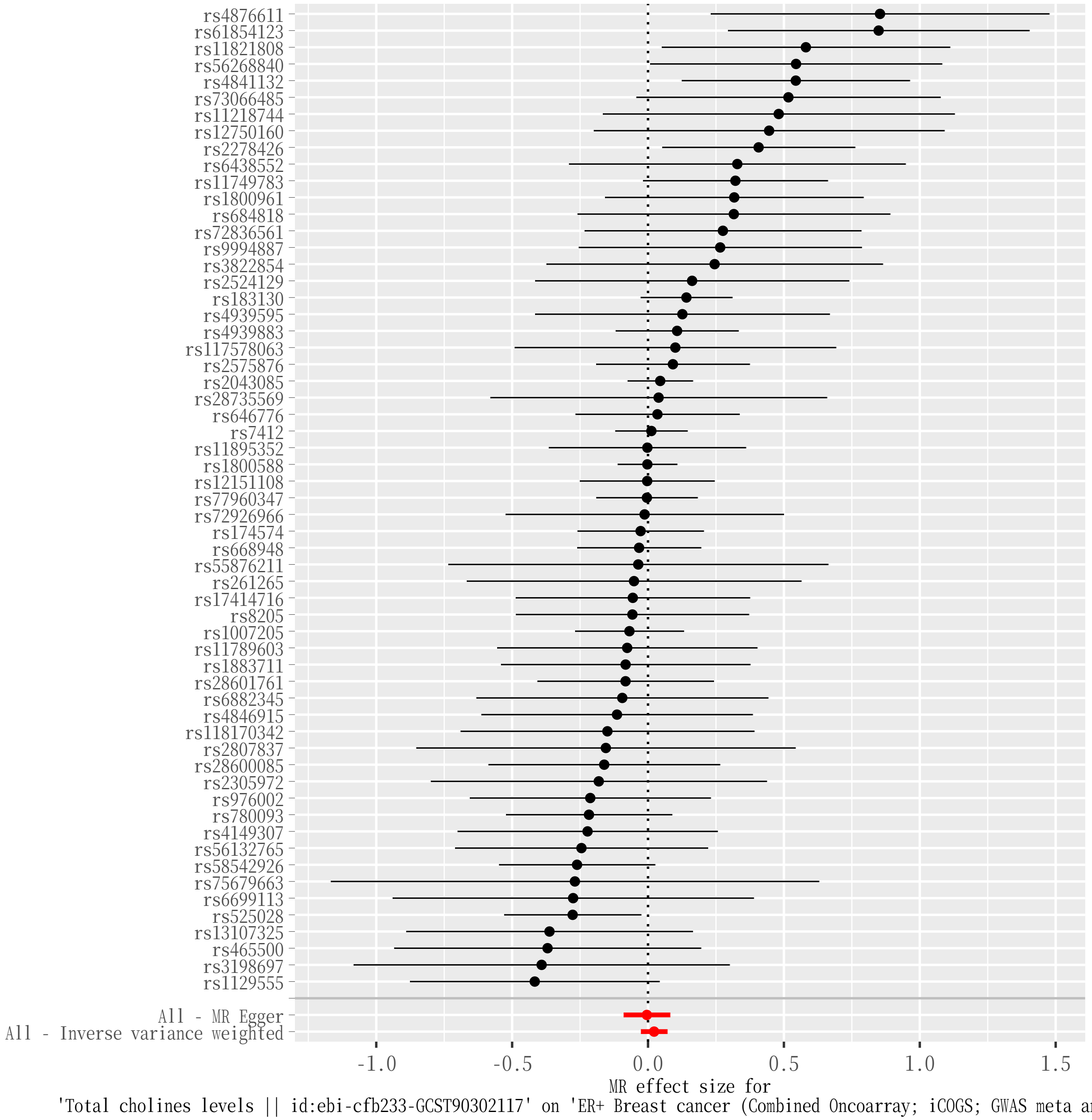
rs4876611
rs61854123
rs11821808
rs56268840
rs4841132
rs73066485
rs11218744
rs12750160
rs2278426
rs6438552
rs11749783
rs1800961
rs684818
rs72836561
rs9994887
rs3822854
rs2524129
rs183130
rs4939595
rs4939883
rs117578063
rs2575876
rs2043085
rs28735569
rs646776
rs7412
rs11895352
rs1800588
rs12151108
rs77960347
rs72926966
rs174574
rs668948
rs55876211
rs261265
rs17414716
rs8205
rs1007205
rs11789603
rs1883711
rs28601761
rs6882345
rs4846915
rs118170342
rs2807837
rs28600085
rs2305972
rs976002
rs780093
rs4149307
rs56132765
rs58542926
rs75679663
rs6699113
rs525028
rs13107325
rs465500
rs3198697
rs1129555
All - MR Egger
All - Inverse variance weighted
-1.0
-0.5
0.0
0.5
1.0
1.5
MR effect size for
'Total cholines levels || id:ebi-cfb233-GCST90302117' on 'ER+ Breast cancer (Combined Oncoarray; iCOGS; GWAS meta an

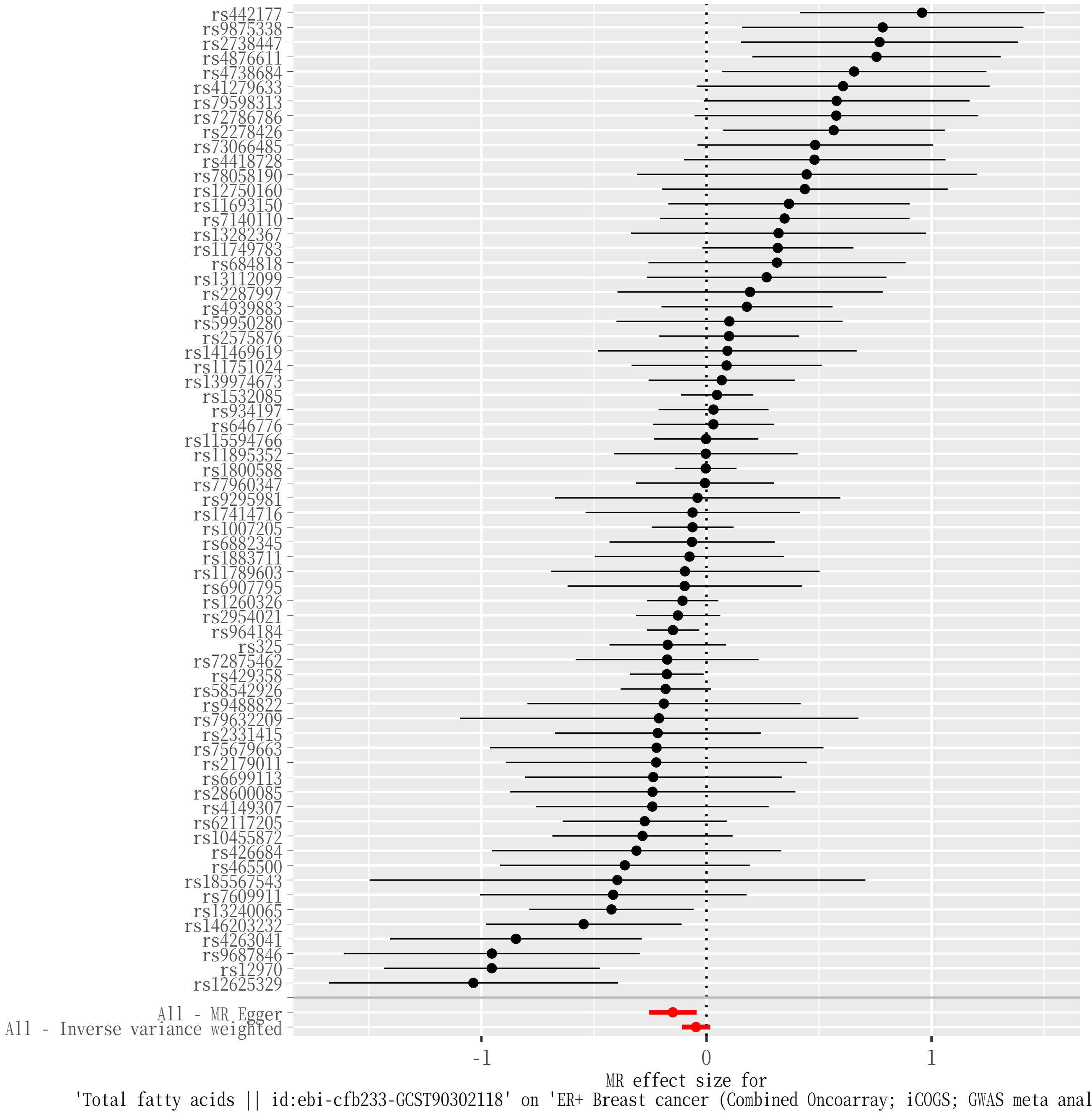
rs442177
rs9875338
rs2738447
rs4876611
rs4738684
rs41279633
rs79598313
rs72786786
rs2278426
rs73066485
rs4418728
rs78058190
rs12750160
rs11693150
rs7140110
rs13282367
rs11749783
rs684818
rs13112099
rs2287997
rs4939883
rs59950280
rs2575876
rs141469619
rs11751024
rs139974673
rs1532085
rs934197
rs646776
rs115594766
rs11895352
rs1800588
rs77960347
rs9295981
rs17414716
rs1007205
rs6882345
rs1883711
rs11789603
rs6907795
rs1260326
rs2954021
rs964184
rs325
rs72875462
rs429358
rs58542926
rs9488822
rs79632209
rs2331415
rs75679663
rs2179011
rs6699113
rs28600085
rs4149307
rs62117205
rs10455872
rs426684
rs465500
rs185567543
rs7609911
rs13240065
rs146203232
rs4263041
rs9687846
rs12970
rs12625329
All - MR Egger
All - Inverse variance weighted
-1
0
1
MR effect size for
'Total fatty acids || id:ebi-cfb233-GCST90302118' on 'ER+ Breast cancer (Combined Oncoarray; iCOGS; GWAS meta anal

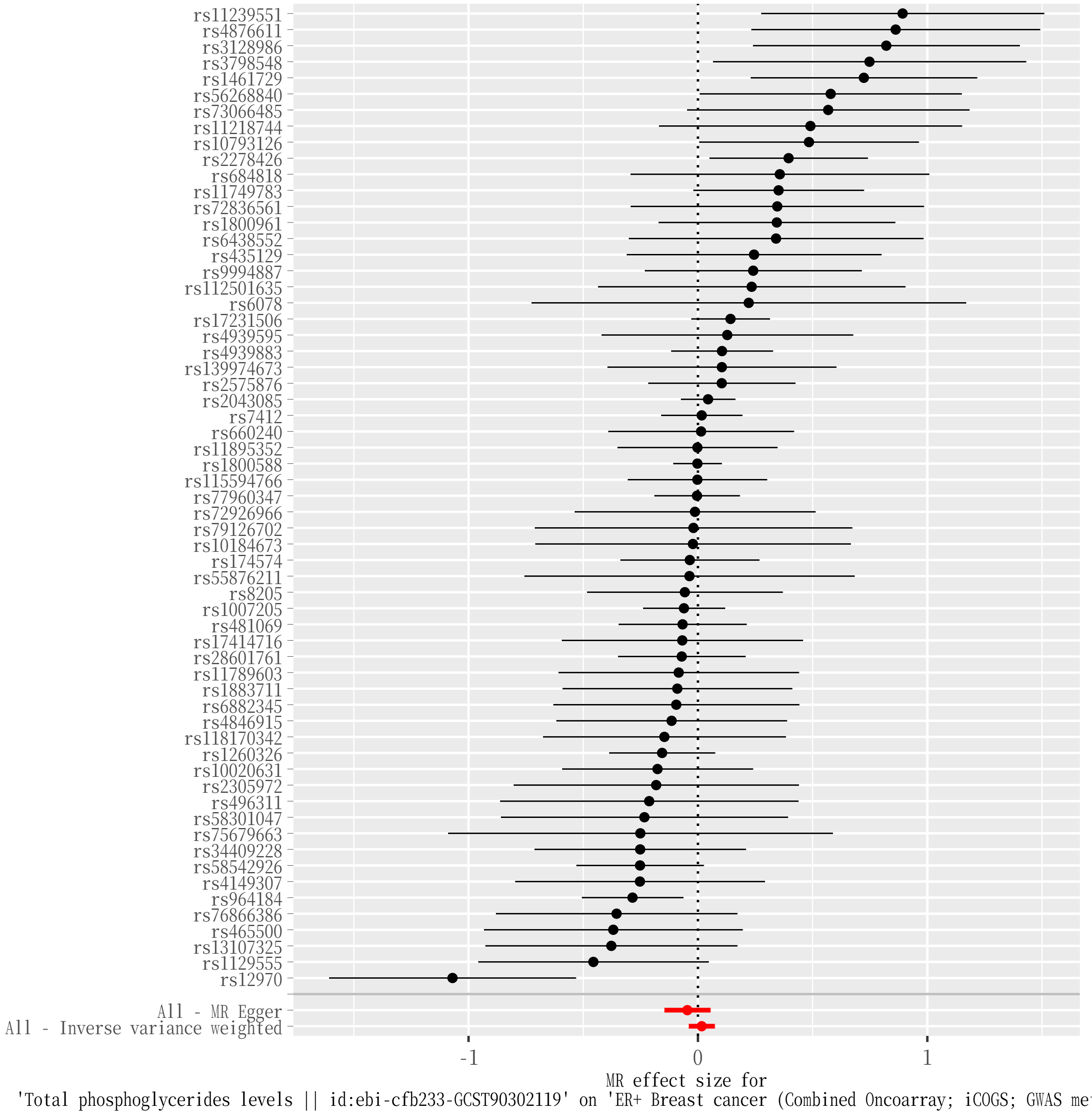

rs11239551
rs4876611
rs3128986
rs3798548
rs1461729
rs56268840
rs73066485
rs11218744
rs10793126
rs2278426
rs684818
rs11749783
rs72836561
rs1800961
rs6438552
rs435129
rs9994887
rs112501635
rs6078
rs17231506
rs4939595
rs4939883
rs139974673
rs2575876
rs2043085
rs7412
rs660240
rs11895352
rs1800588
rs115594766
rs77960347
rs72926966
rs79126702
rs10184673
rs174574
rs55876211
rs8205
rs1007205
rs481069
rs17414716
rs28601761
rs11789603
rs1883711
rs6882345
rs4846915
rs118170342
rs1260326
rs10020631
rs2305972
rs496311
rs58301047
rs75679663
rs34409228
rs58542926
rs4149307
rs964184
rs76866386
rs465500
rs13107325
rs1129555
rs12970
All - MR Egger
All - Inverse variance weighted
-1
0
1
MR effect size for
'Total phosphoglycerides levels || id:ebi-cfb233-GCST90302119' on 'ER+ Breast cancer (Combined Oncoarray; iCOGS; GWAS me

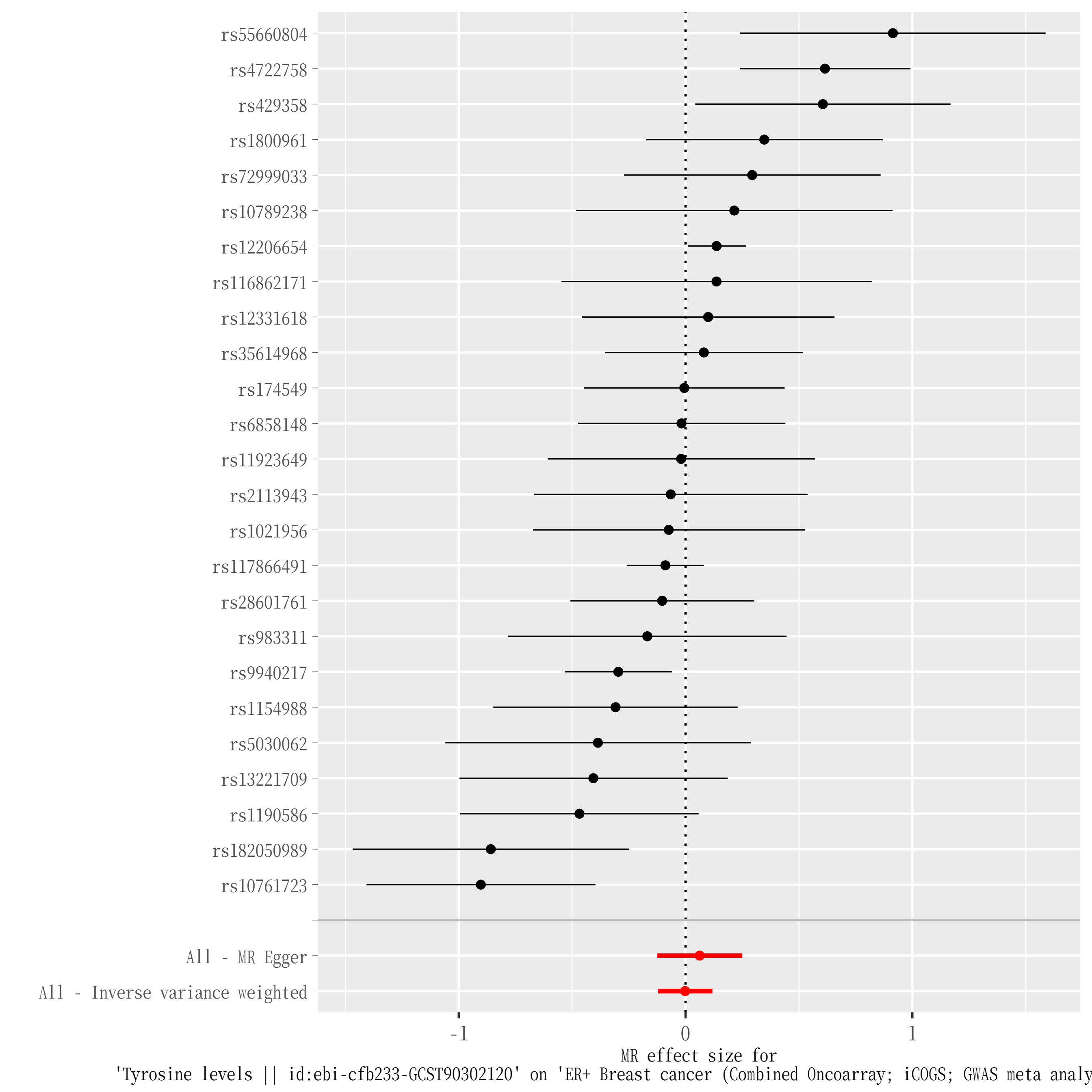
rs55660804
rs4722758
rs429358
rs1800961
rs72999033
rs10789238
rs12206654
rs116862171
rs12331618
rs35614968
rs174549
rs6858148
rs11923649
rs2113943
rs1021956
rs117866491
rs28601761
rs983311
rs9940217
rs1154988
rs5030062
rs13221709
rs1190586
rs182050989
rs10761723
All - MR Egger
All - Inverse variance weighted
-1
0
1
MR effect size for
'Tyrosine levels || id:ebi-cfb233-GCST90302120' on 'ER+ Breast cancer (Combined Oncoarray; iCOGS; GWAS meta analy

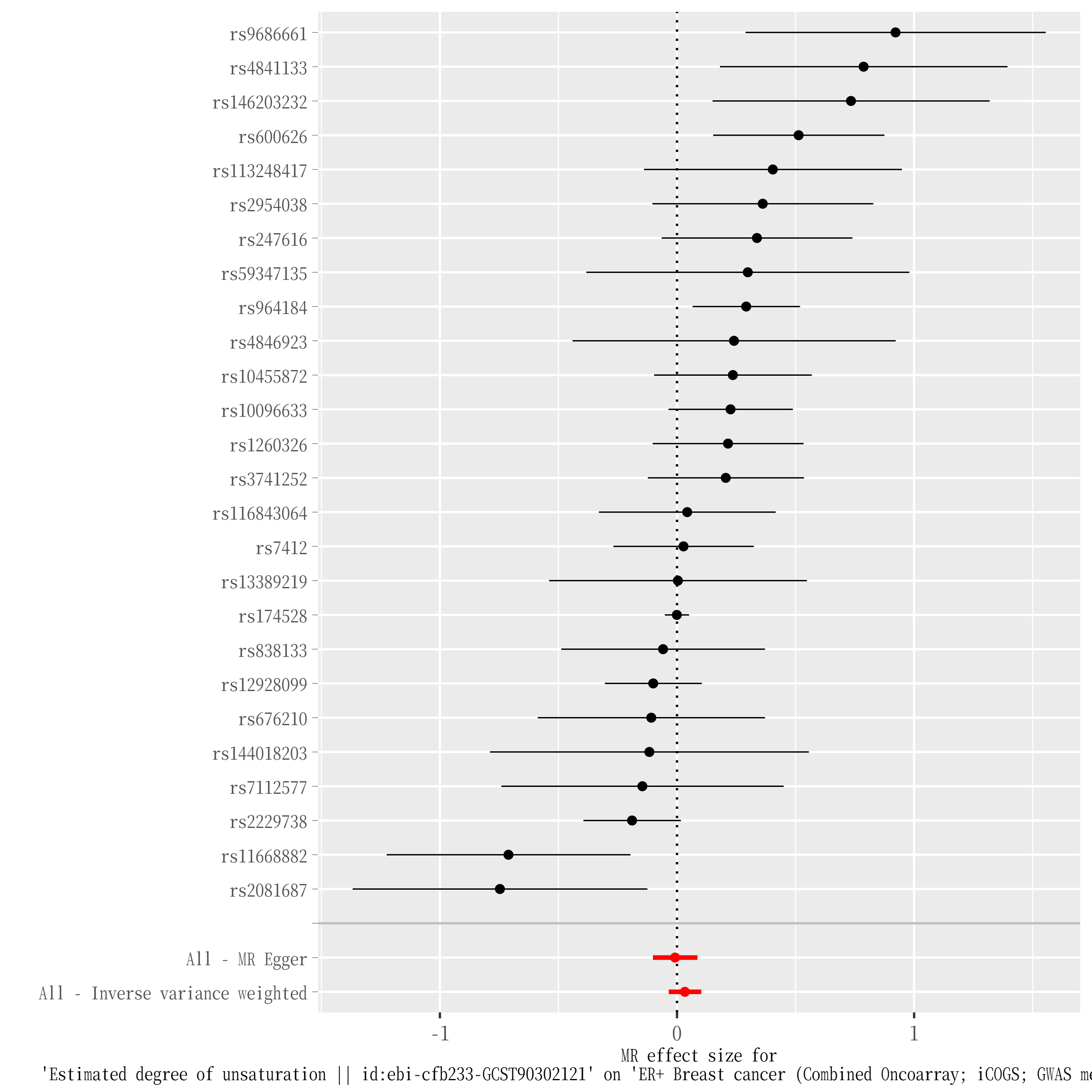

rs9686661
rs4841133
rs146203232
rs600626
rs113248417
rs2954038
rs247616
rs59347135
rs964184
rs4846923
rs10455872
rs10096633
rs1260326
rs3741252
rs116843064
rs7412
rs13389219
rs174528
rs838133
rs12928099
rs676210
rs144018203
rs7112577
rs2229738
rs11668882
rs2081687
All - MR Egger
All - Inverse variance weighted
-1
0
1
MR effect size for
'Estimated degree of unsaturation || id:ebi-cfb233-GCST90302121' on 'ER+ Breast cancer (Combined Oncoarray; iCOGS; GWAS me

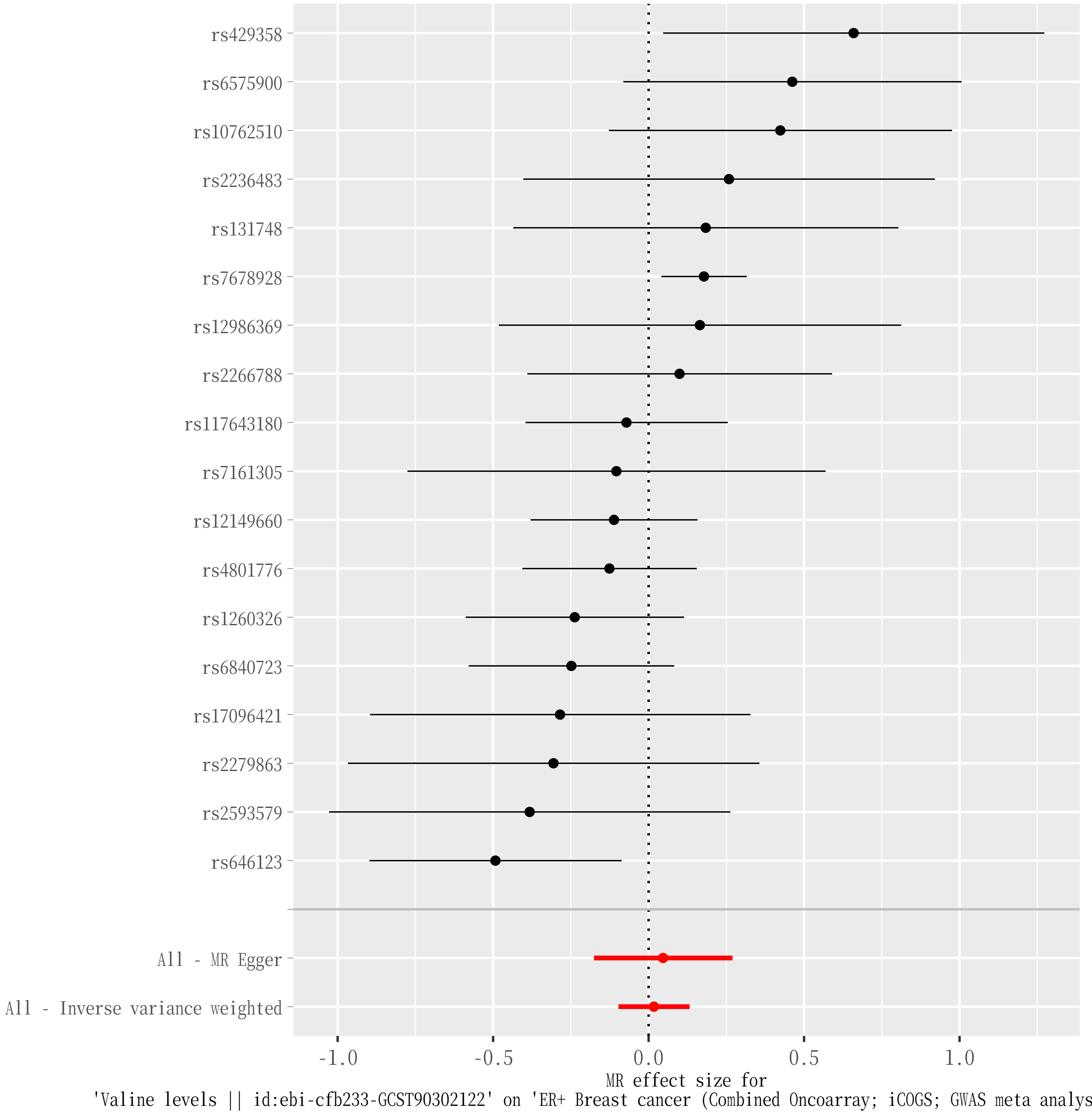

rs429358
rs6575900
rs10762510
rs2236483
rs131748
rs7678928
rs12986369
rs2266788
rs117643180
rs7161305
rs12149660
rs4801776
rs1260326
rs6840723
rs17096421
rs2279863
rs2593579
rs646123
All - MR Egger
All - Inverse variance weighted
-1.0
-0.5
0.0
0.5
1.0
MR effect size for
'Valine levels || id:ebi-cfb233-GCST90302122' on 'ER+ Breast cancer (Combined Oncoarray; iCOGS; GWAS meta analys

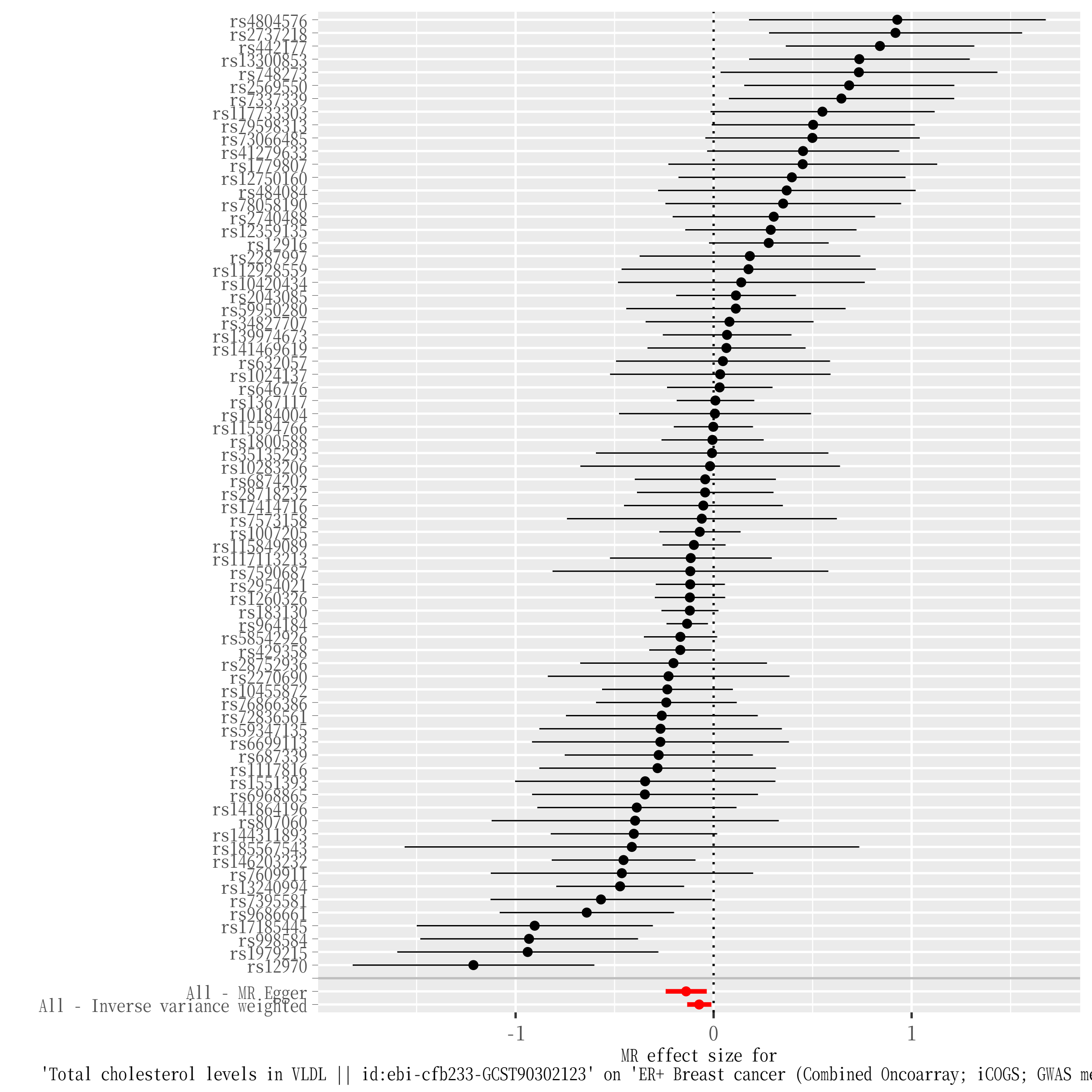

rs4804576
rs2737218
rs442177
rs13300853
rs748273
rs2569550
rs7337339
rs117733303
rs79598313
rs73066485
rs41279633
rs1779807
rs12750160
rs484084
rs78058190
rs2740488
rs12359135
rs12916
rs2287997
rs112928559
rs10420434
rs2043085
rs59950280
rs34827707
rs139974673
rs141469619
rs632057
rs1024137
rs646776
rs1367117
rs10184004
rs115594766
rs1800588
rs35135293
rs10283206
rs6874202
rs28718232
rs17414716
rs7573158
rs1007205
rs115849089
rs117113213
rs7590687
rs2954021
rs1260326
rs183130
rs964184
rs58542926
rs429358
rs28752936
rs2270690
rs10455872
rs76866386
rs72836561
rs59347135
rs6699113
rs687339
rs1117816
rs1551393
rs6968865
rs141864196
rs807060
rs144311893
rs185567543
rs146203232
rs7609911
rs13240994
rs7395581
rs9686661
rs17185445
rs998584
rs1979215
rs12970
All - MR Egger
All - Inverse variance weighted
-1
0
1
MR effect size for
'Total cholesterol levels in VLDL || id:ebi-cfb233-GCST90302123' on 'ER+ Breast cancer (Combined Oncoarray; iCOGS; GWAS me

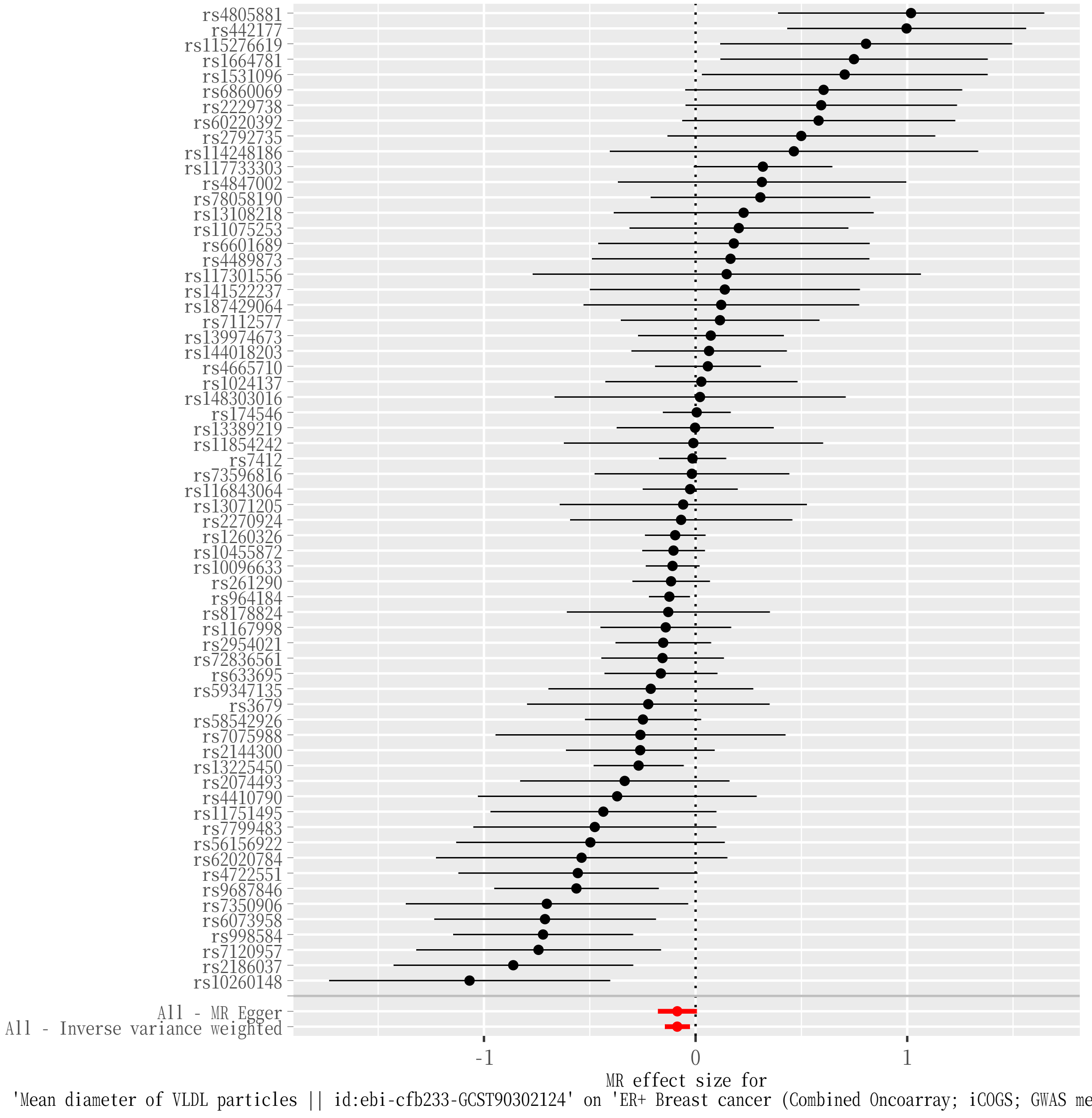
rs4805881
rs442177
rs115276619
rs1664781
rs1531096
rs6860069
rs2229738
rs60220392
rs2792735
rs114248186
rs117733303
rs4847002
rs78058190
rs13108218
rs11075253
rs6601689
rs4489873
rs117301556
rs141522237
rs187429064
rs7112577
rs139974673
rs144018203
rs4665710
rs1024137
rs148303016
rs174546
rs13389219
rs11854242
rs7412
rs73596816
rs116843064
rs13071205
rs2270924
rs1260326
rs10455872
rs10096633
rs261290
rs964184
rs8178824
rs1167998
rs2954021
rs72836561
rs633695
rs59347135
rs3679
rs58542926
rs7075988
rs2144300
rs13225450
rs2074493
rs4410790
rs11751495
rs7799483
rs56156922
rs62020784
rs4722551
rs9687846
rs7350906
rs6073958
rs998584
rs7120957
rs2186037
rs10260148
All - MR Egger
All - Inverse variance weighted
-1
0
1
MR effect size for
'Mean diameter of VLDL particles || id:ebi-cfb233-GCST90302124' on 'ER+ Breast cancer (Combined Oncoarray; iCOGS; GWAS me

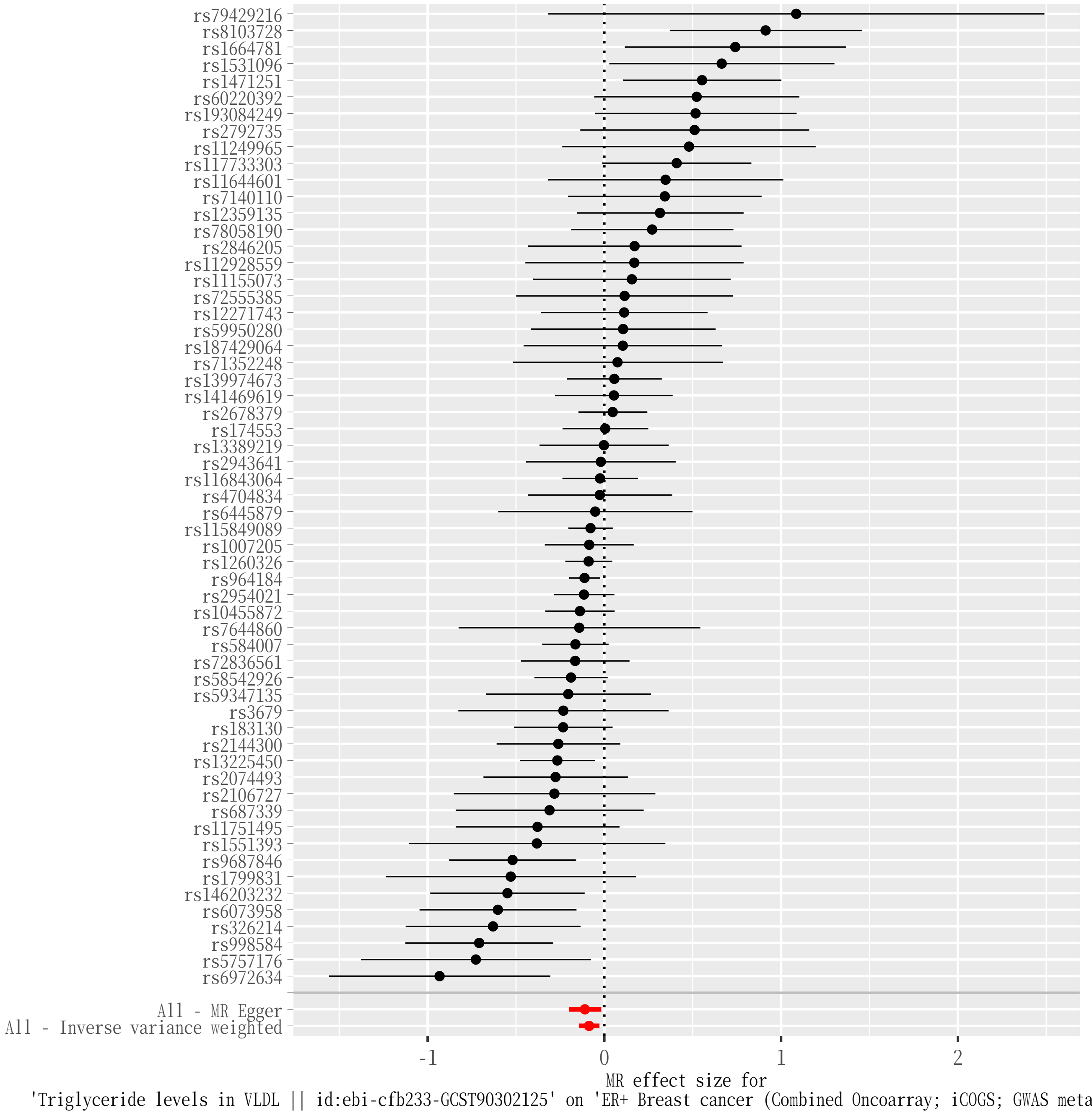
rs79429216
rs8103728
rs1664781
rs1531096
rs1471251
rs60220392
rs193084249
rs2792735
rs11249965
rs117733303
rs11644601
rs7140110
rs12359135
rs78058190
rs2846205
rs112928559
rs11155073
rs72555385
rs12271743
rs59950280
rs187429064
rs71352248
rs139974673
rs141469619
rs2678379
rs174553
rs13389219
rs2943641
rs116843064
rs4704834
rs6445879
rs115849089
rs1007205
rs1260326
rs964184
rs2954021
rs10455872
rs7644860
rs584007
rs72836561
rs58542926
rs59347135
rs3679
rs183130
rs2144300
rs13225450
rs2074493
rs2106727
rs687339
rs11751495
rs1551393
rs9687846
rs1799831
rs146203232
rs6073958
rs326214
rs998584
rs5757176
rs6972634
All - MR Egger
All - Inverse variance weighted
-1
0
1
2
MR effect size for
'Triglyceride levels in VLDL || id:ebi-cfb233-GCST90302125' on 'ER+ Breast cancer (Combined Oncoarray; iCOGS; GWAS meta

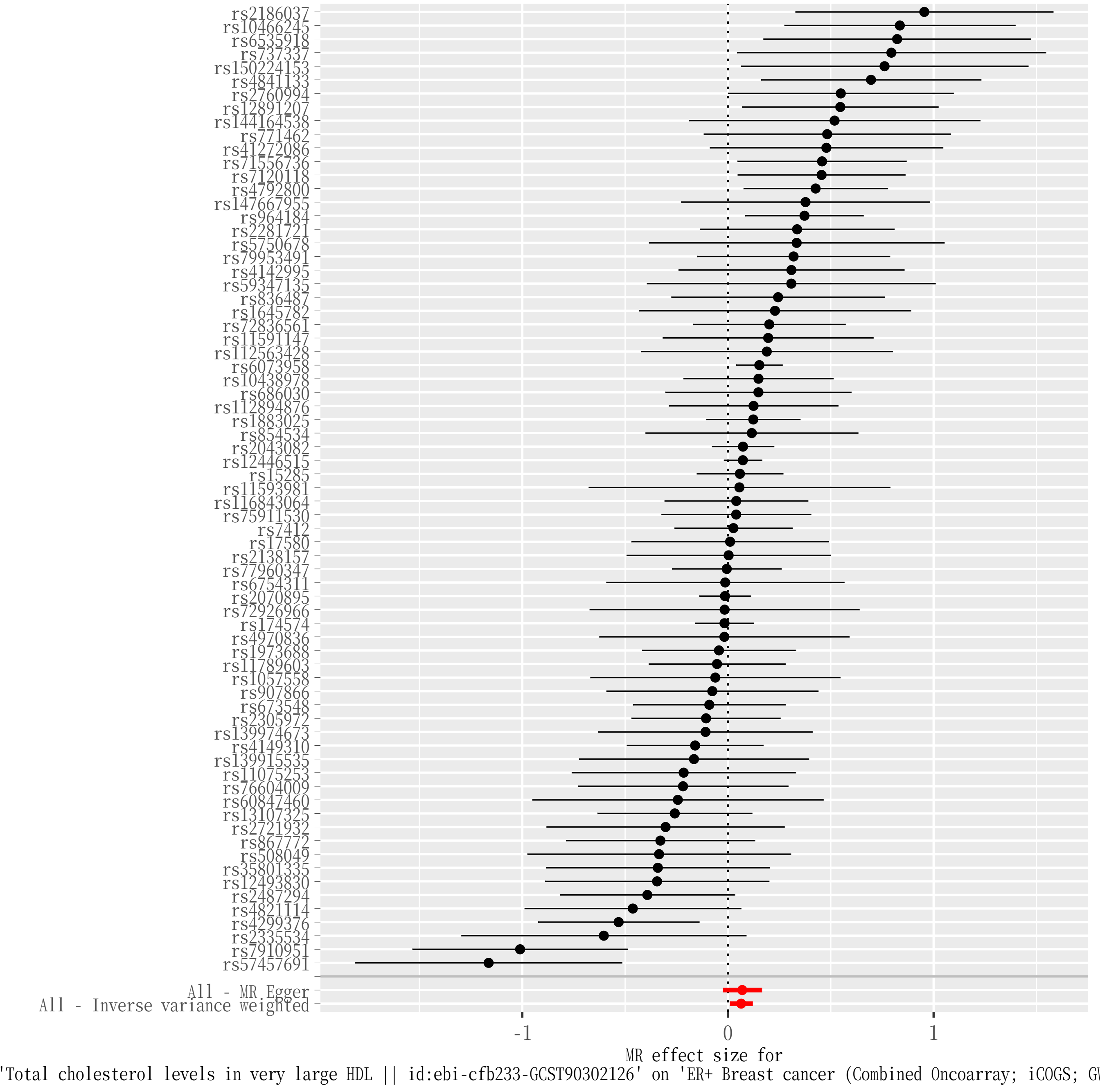

rs2186037
rs10466245
rs6535918
rs737337
rs150224153
rs4841133
rs2760994
rs12891207
rs144164538
rs771462
rs41272086
rs71556736
rs7120118
rs4792800
rs147667955
rs964184
rs2281721
rs5750678
rs79953491
rs4142995
rs59347135
rs836487
rs1645782
rs72836561
rs11591147
rs112563428
rs6073958
rs10438978
rs686030
rs112894876
rs1883025
rs854534
rs2043082
rs12446515
rs15285
rs11593981
rs116843064
rs75911530
rs7412
rs17580
rs2138157
rs77960347
rs6754311
rs2070895
rs72926966
rs174574
rs4970836
rs1973688
rs11789603
rs1057558
rs907866
rs673548
rs2305972
rs139974673
rs4149310
rs139915535
rs11075253
rs76604009
rs60847460
rs13107325
rs2721932
rs867772
rs508049
rs35801335
rs12493830
rs2487294
rs4821114
rs4299376
rs2335534
rs7910951
rs57457691
All - MR Egger
All - Inverse variance weighted
-1
0
1
MR effect size for
'Total cholesterol levels in very large HDL || id:ebi-cfb233-GCST90302126' on 'ER+ Breast cancer (Combined Oncoarray; iCOGS; GW

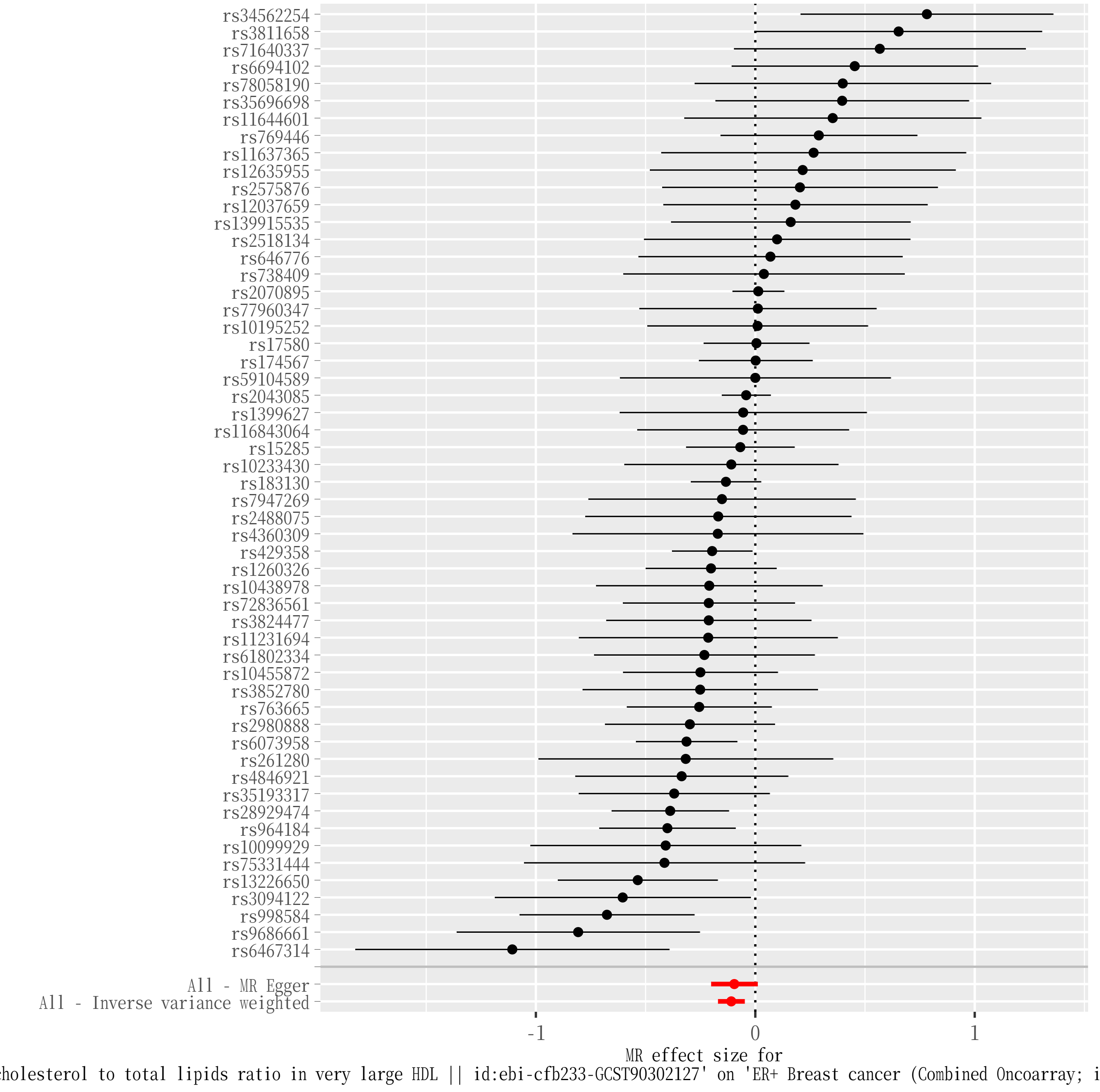
rs34562254
rs3811658
rs71640337
rs6694102
rs78058190
rs35696698
rs11644601
rs769446
rs11637365
rs12635955
rs2575876
rs12037659
rs139915535
rs2518134
rs646776
rs738409
rs2070895
rs77960347
rs10195252
rs17580
rs174567
rs59104589
rs2043085
rs1399627
rs116843064
rs15285
rs10233430
rs183130
rs7947269
rs2488075
rs4360309
rs429358
rs1260326
rs10438978
rs72836561
rs3824477
rs11231694
rs61802334
rs10455872
rs3852780
rs763665
rs2980888
rs6073958
rs261280
rs4846921
rs35193317
rs28929474
rs964184
rs10099929
rs75331444
rs13226650
rs3094122
rs998584
rs9686661
rs6467314
All - MR Egger
All - Inverse variance weighted
-1
0
1
MR effect size for
cholesterol to total lipids ratio in very large HDL || id:ebi-cfb233-GCST90302127' on 'ER+ Breast cancer (Combined Oncoarray; i

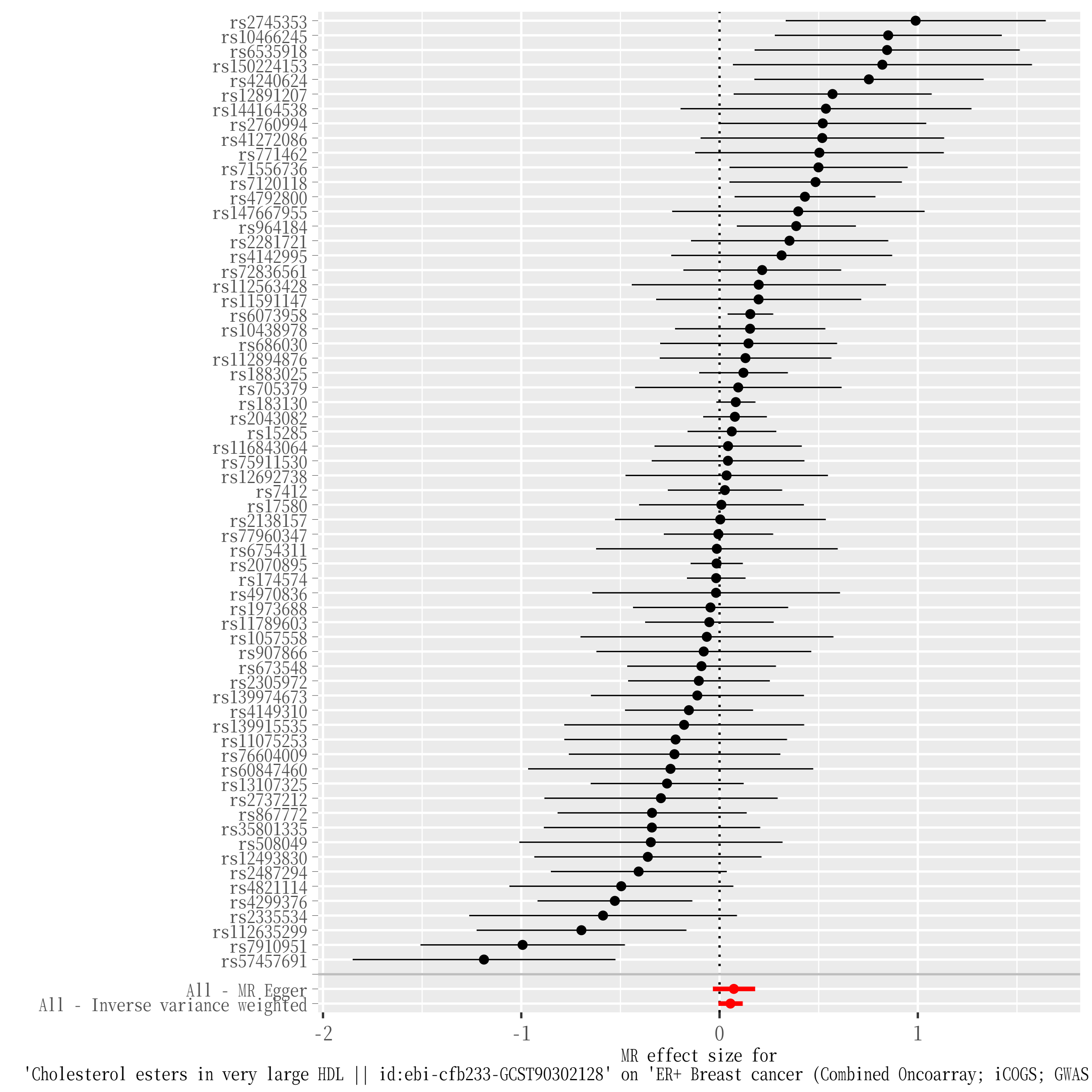
rs2745353
rs10466245
rs6535918
rs150224153
rs4240624
rs12891207
rs144164538
rs2760994
rs41272086
rs771462
rs71556736
rs7120118
rs4792800
rs147667955
rs964184
rs2281721
rs4142995
rs72836561
rs112563428
rs11591147
rs6073958
rs10438978
rs686030
rs112894876
rs1883025
rs705379
rs183130
rs2043082
rs15285
rs116843064
rs75911530
rs12692738
rs7412
rs17580
rs2138157
rs77960347
rs6754311
rs2070895
rs174574
rs4970836
rs1973688
rs11789603
rs1057558
rs907866
rs673548
rs2305972
rs139974673
rs4149310
rs139915535
rs11075253
rs76604009
rs60847460
rs13107325
rs2737212
rs867772
rs35801335
rs508049
rs12493830
rs2487294
rs4821114
rs4299376
rs2335534
rs112635299
rs7910951
rs57457691
All - MR Egger
All - Inverse variance weighted
-2
-1
0
1
MR effect size for
'Cholesterol esters in very large HDL || id:ebi-cfb233-GCST90302128' on 'ER+ Breast cancer (Combined Oncoarray; iCOGS; GWAS

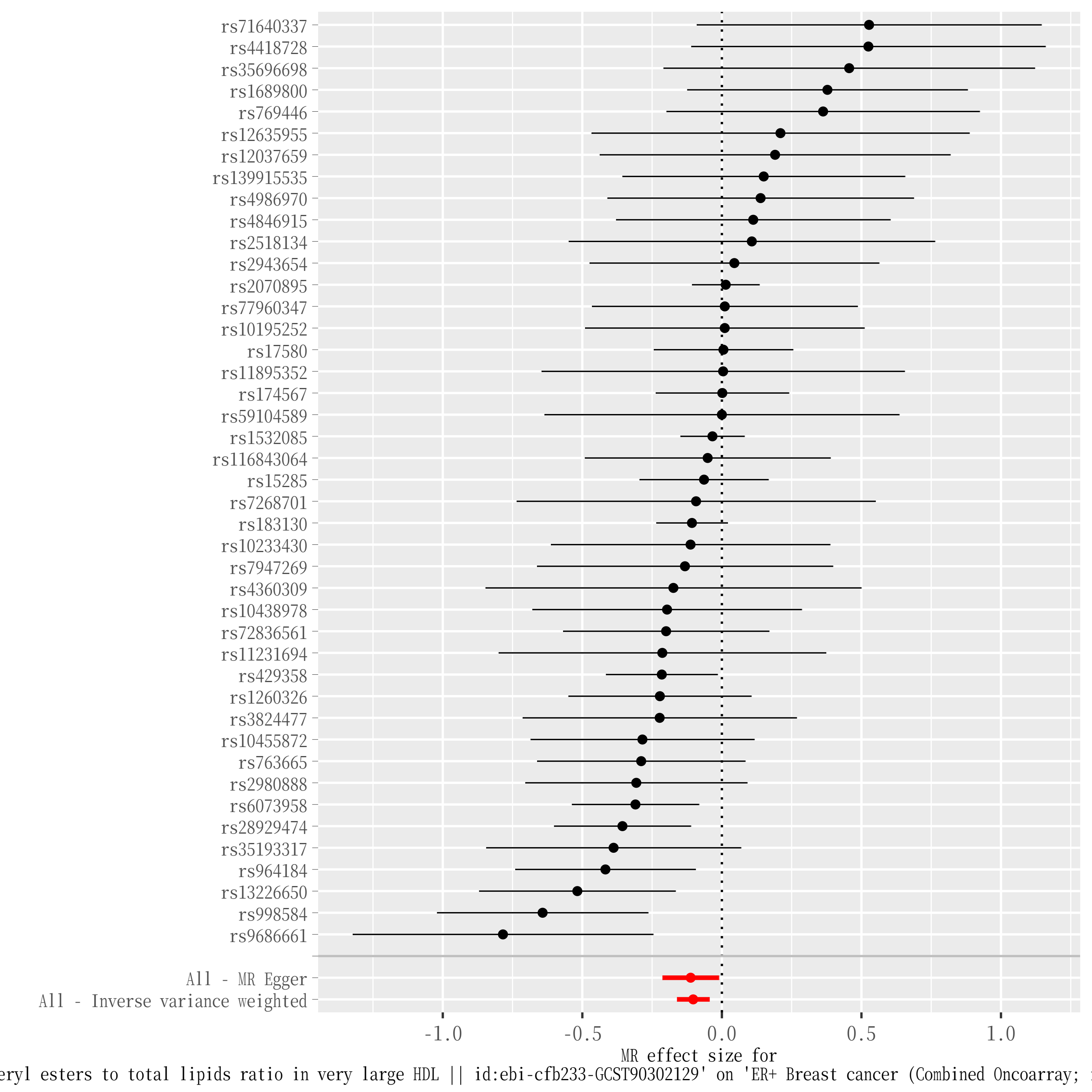

rs71640337
rs4418728
rs35696698
rs1689800
rs769446
rs12635955
rs12037659
rs139915535
rs4986970
rs4846915
rs2518134
rs2943654
rs2070895
rs77960347
rs10195252
rs17580
rs11895352
rs174567
rs59104589
rs1532085
rs116843064
rs15285
rs7268701
rs183130
rs10233430
rs7947269
rs4360309
rs10438978
rs72836561
rs11231694
rs429358
rs1260326
rs3824477
rs10455872
rs763665
rs2980888
rs6073958
rs28929474
rs35193317
rs964184
rs13226650
rs998584
rs9686661
All - MR Egger
All - Inverse variance weighted
-1.0
-0.5
0.0
0.5
1.0
MR effect size for
eryl esters to total lipids ratio in very large HDL || id:ebi-cfb233-GCST90302129' on 'ER+ Breast cancer (Combined Oncoarray;

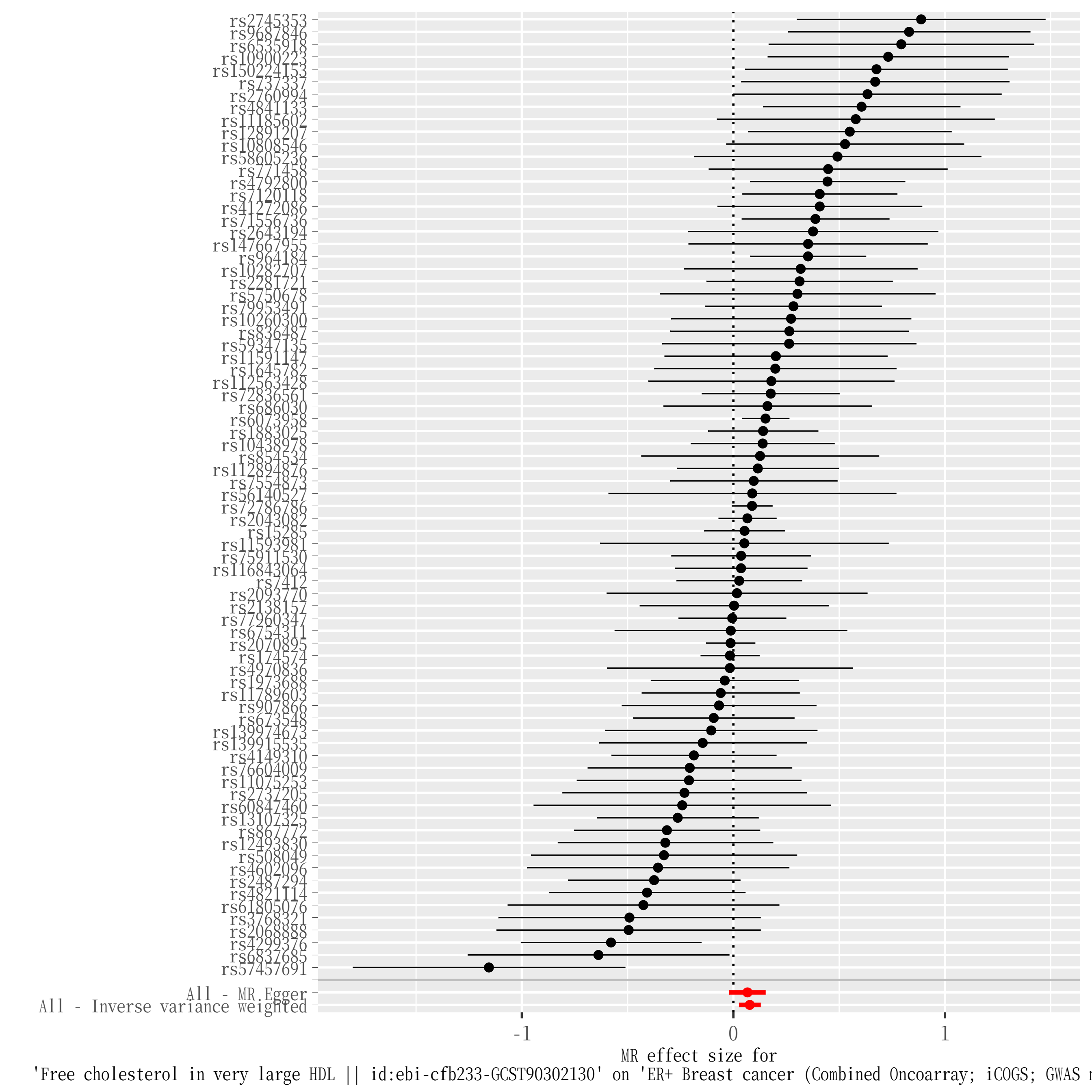
rs2745353
rs9687846
rs6535918
rs10900223
rs150224153
rs737337
rs2760994
rs4841133
rs11185602
rs12891207
rs10808546
rs58605236
rs771458
rs4792800
rs7120118
rs41272086
rs71556736
rs2643194
rs147667955
rs964184
rs10282707
rs2281721
rs5750678
rs79953491
rs10260300
rs836487
rs59347135
rs11591147
rs1645782
rs112563428
rs72836561
rs686030
rs6073958
rs1883025
rs10438978
rs854534
rs112894876
rs7554873
rs56140527
rs72786786
rs2043082
rs15285
rs11593981
rs75911530
rs116843064
rs7412
rs2093770
rs2138157
rs77960347
rs6754311
rs2070895
rs174574
rs4970836
rs1973688
rs11789603
rs907866
rs673548
rs139974673
rs139915535
rs4149310
rs76604009
rs11075253
rs2737205
rs60847460
rs13107325
rs867772
rs12493830
rs508049
rs4602096
rs2487294
rs4821114
rs61805076
rs3768321
rs2068888
rs4299376
rs6837685
rs57457691
All - MR Egger
All - Inverse variance weighted
-1
0
1
MR effect size for
'Free cholesterol in very large HDL || id:ebi-cfb233-GCST90302130' on 'ER+ Breast cancer (Combined Oncoarray; iCOGS; GWAS m

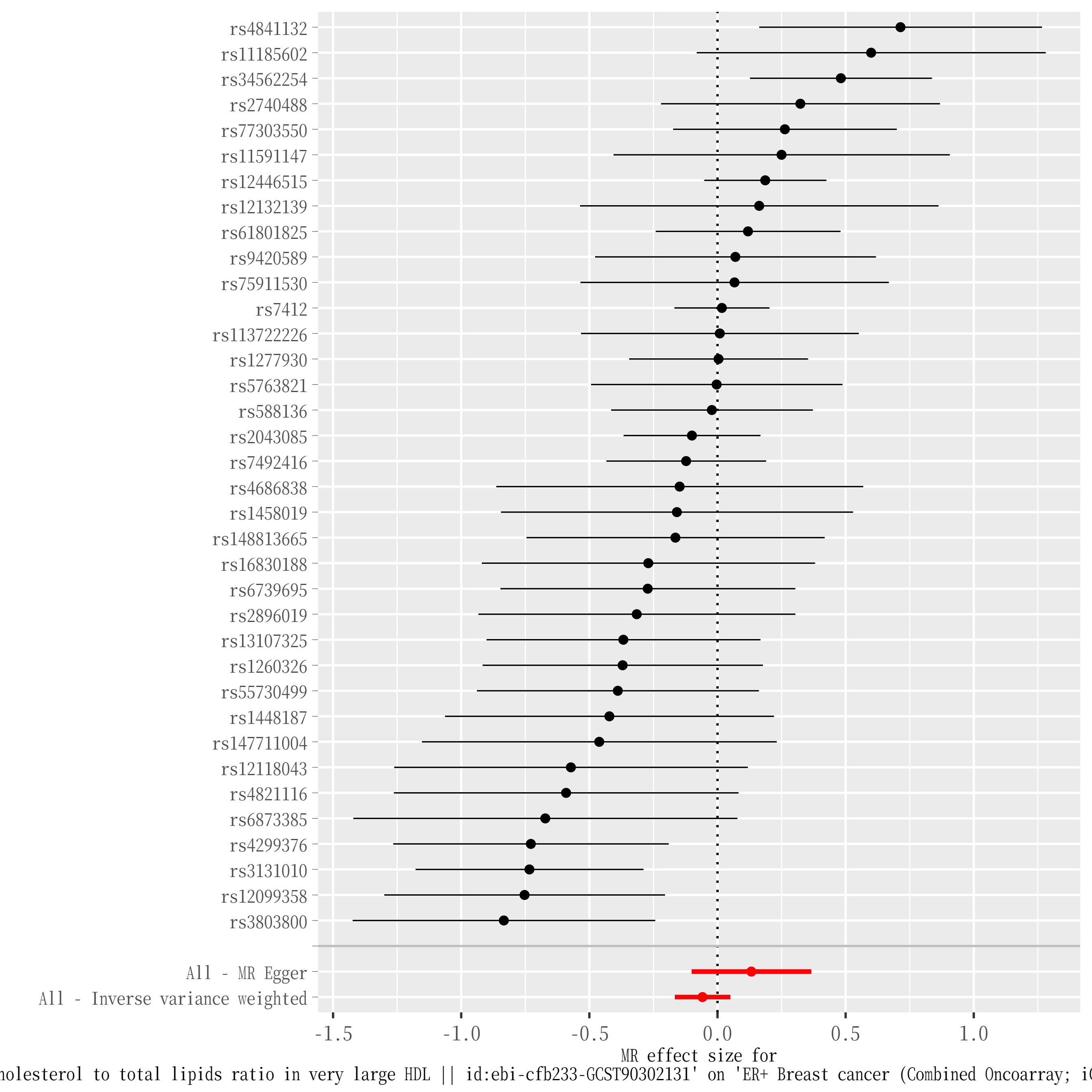
rs4841132
rs11185602
rs34562254
rs2740488
rs77303550
rs11591147
rs12446515
rs12132139
rs61801825
rs9420589
rs75911530
rs7412
rs113722226
rs1277930
rs5763821
rs588136
rs2043085
rs7492416
rs4686838
rs1458019
rs148813665
rs16830188
rs6739695
rs2896019
rs13107325
rs1260326
rs55730499
rs1448187
rs147711004
rs12118043
rs4821116
rs6873385
rs4299376
rs3131010
rs12099358
rs3803800
All - MR Egger
All - Inverse variance weighted
-1.5
-1.0
-0.5
0.0
0.5
1.0
MR effect size for
holesterol to total lipids ratio in very large HDL || id:ebi-cfb233-GCST90302131' on 'ER+ Breast cancer (Combined Oncoarray; i

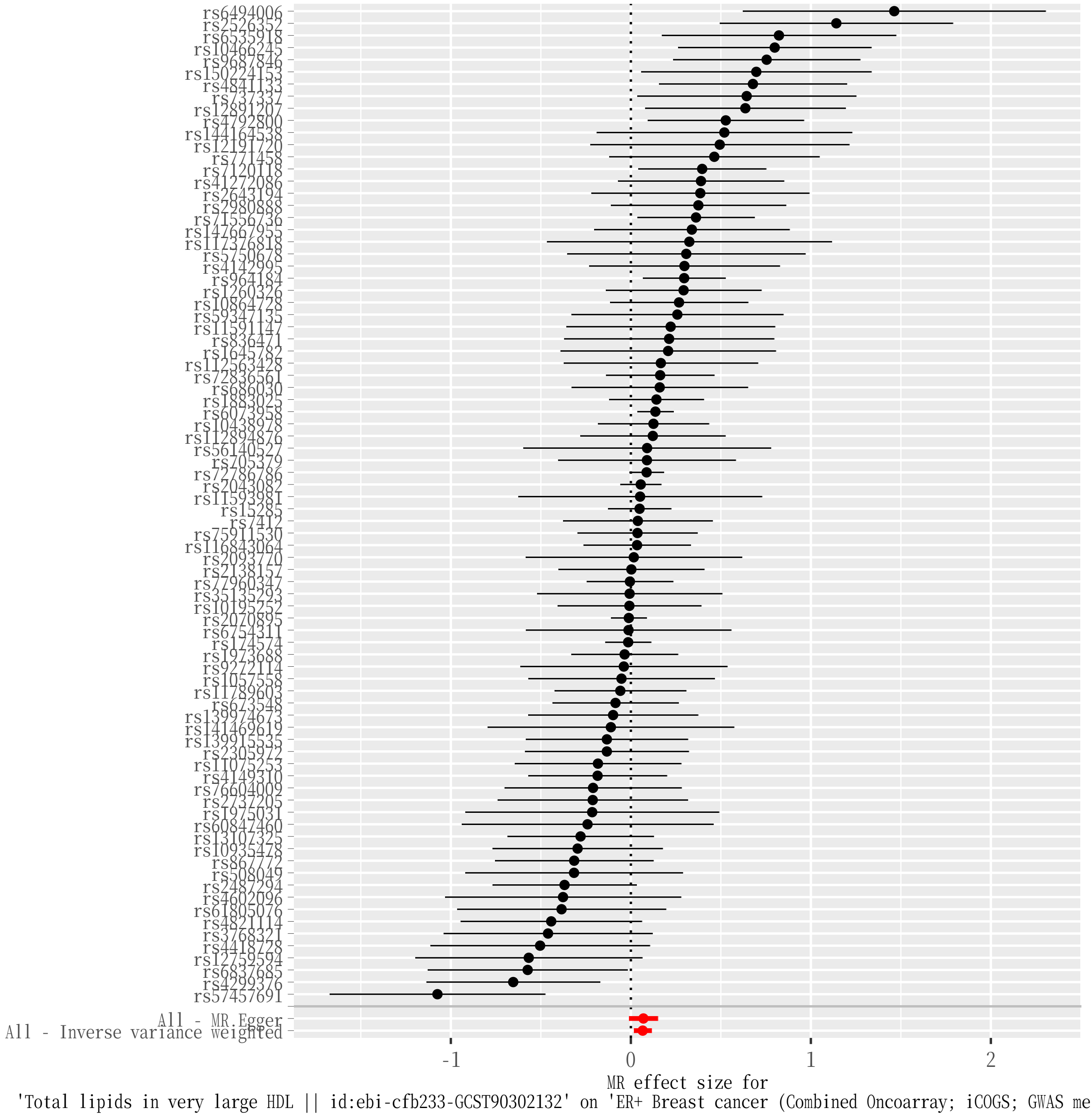

rs6494006
rs2526352
rs6535918
rs10466245
rs9687846
rs150224153
rs4841133
rs737337
rs12891207
rs4792800
rs144164538
rs12191720
rs771458
rs7120118
rs41272086
rs2643194
rs2980888
rs71556736
rs147667955
rs117376818
rs5750678
rs4142995
rs964184
rs1260326
rs10864728
rs59347135
rs11591147
rs836471
rs1645782
rs112563428
rs72836561
rs686030
rs1883025
rs6073958
rs10438978
rs112894876
rs56140527
rs705379
rs72786786
rs2043082
rs11593981
rs15285
rs7412
rs75911530
rs116843064
rs2093770
rs2138157
rs77960347
rs35135293
rs10195252
rs2070895
rs6754311
rs174574
rs1973688
rs9272114
rs1057558
rs11789603
rs673548
rs139974673
rs141469619
rs139915535
rs2305972
rs11075253
rs4149310
rs76604009
rs2737205
rs1975031
rs60847460
rs13107325
rs10935478
rs867772
rs508049
rs2487294
rs4602096
rs61805076
rs4821114
rs3768321
rs4418728
rs12759594
rs6837685
rs4299376
rs57457691
All - MR Egger
All - Inverse variance weighted
-1
0
1
2
MR effect size for
'Total lipids in very large HDL || id:ebi-cfb233-GCST90302132' on 'ER+ Breast cancer (Combined Oncoarray; iCOGS; GWAS me

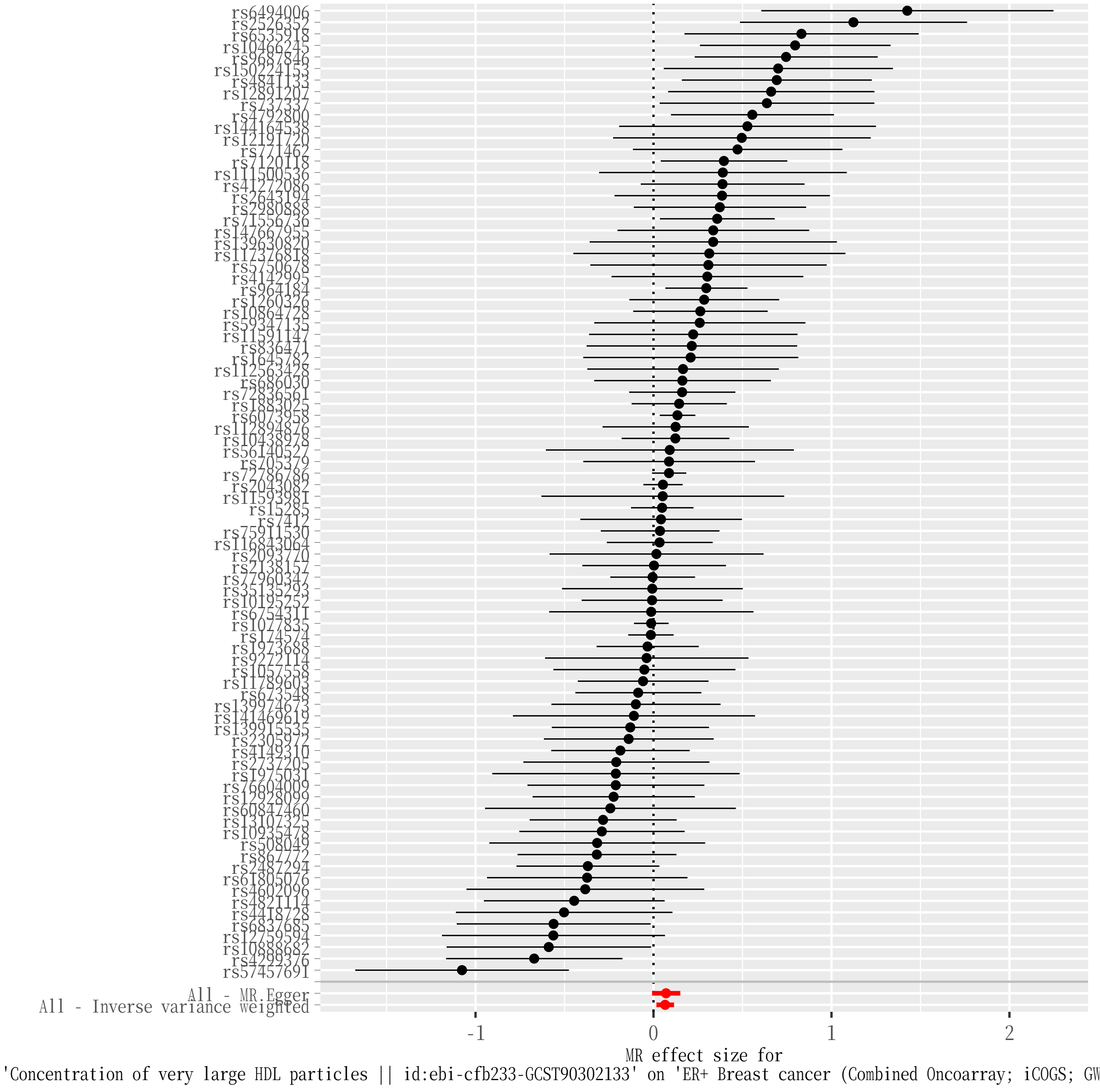
rs6494006
rs2526352
rs6535918
rs10466245
rs9687846
rs150224153
rs4841133
rs12891207
rs737337
rs4792800
rs144164538
rs12191720
rs771462
rs7120118
rs111500536
rs41272086
rs2643194
rs2980888
rs71556736
rs147667955
rs139630820
rs117376818
rs5750678
rs4142995
rs964184
rs1260326
rs10864728
rs59347135
rs11591147
rs836471
rs1645782
rs112563428
rs686030
rs72836561
rs1883025
rs6073958
rs112894876
rs10438978
rs56140527
rs705379
rs72786786
rs2043082
rs11593981
rs15285
rs7412
rs75911530
rs116843064
rs2093770
rs2138157
rs77960347
rs35135293
rs10195252
rs6754311
rs1077835
rs174574
rs1973688
rs9272114
rs1057558
rs11789603
rs673548
rs139974673
rs141469619
rs139915535
rs2305972
rs4149310
rs2737205
rs1975031
rs76604009
rs12928099
rs60847460
rs13107325
rs10935478
rs508049
rs867772
rs2487294
rs61805076
rs4602096
rs4821114
rs4418728
rs6837685
rs12759594
rs10888682
rs4299376
rs57457691
All - MR Egger
All - Inverse variance weighted
-1
0
1
2
MR effect size for
'Concentration of very large HDL particles || id:ebi-cfb233-GCST90302133' on 'ER+ Breast cancer (Combined Oncoarray; iCOGS; GW

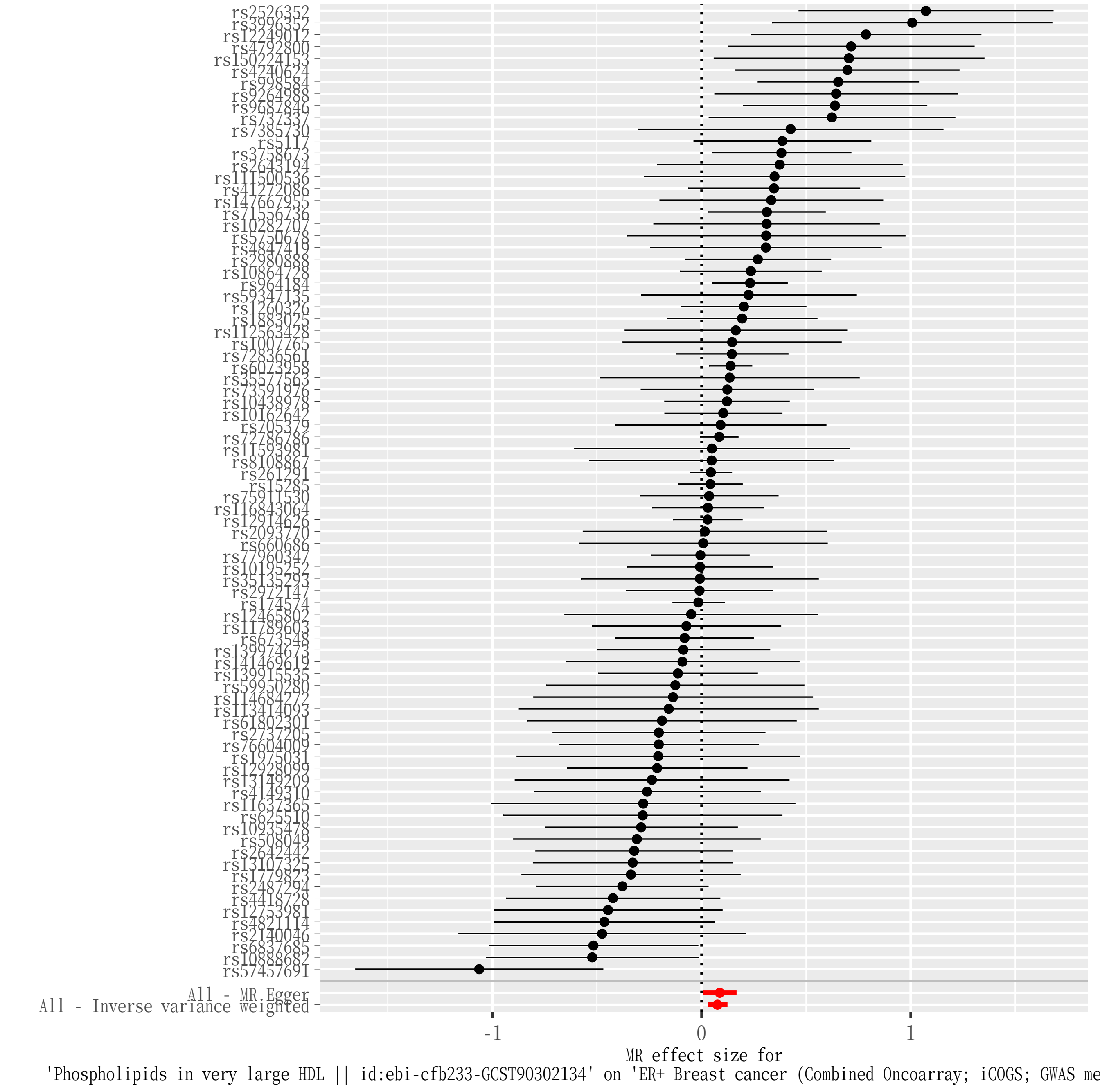

rs2526352
rs12249012
rs4792800
rs150224153
rs4240624
rs998584
rs9264988
rs9687846
rs737337
rs7385730
rs5117
rs3758673
rs2643194
rs111500536
rs41272086
rs147667955
rs71556736
rs10282707
rs5750678
rs4847419
rs2980888
rs10864728
rs964184
rs59347135
rs1260326
rs1883025
rs112563428
rs1007765
rs72836561
rs6073958
rs35577563
rs73591976
rs10438978
rs10162642
rs705379
rs72786786
rs11593981
rs8108867
rs261291
rs15285
rs75911530
rs116843064
rs12914626
rs2093770
rs660686
rs77960347
rs10195252
rs35135293
rs2972147
rs174574
rs12465802
rs11789603
rs673548
rs139974673
rs141469619
rs139915535
rs59950280
rs114684272
rs113414093
rs61802301
rs2737205
rs76604009
rs1975031
rs12928099
rs13149209
rs4149310
rs11637365
rs625510
rs10935478
rs508049
rs2642442
rs13107325
rs1779823
rs2487294
rs4418728
rs12753981
rs4821114
rs2140046
rs6837685
rs10888682
rs57457691
All - MR Egger
All - Inverse variance weighted
-1
0
1
MR effect size for
'Phospholipids in very large HDL || id:ebi-cfb233-GCST90302134' on 'ER+ Breast cancer (Combined Oncoarray; iCOGS; GWAS me

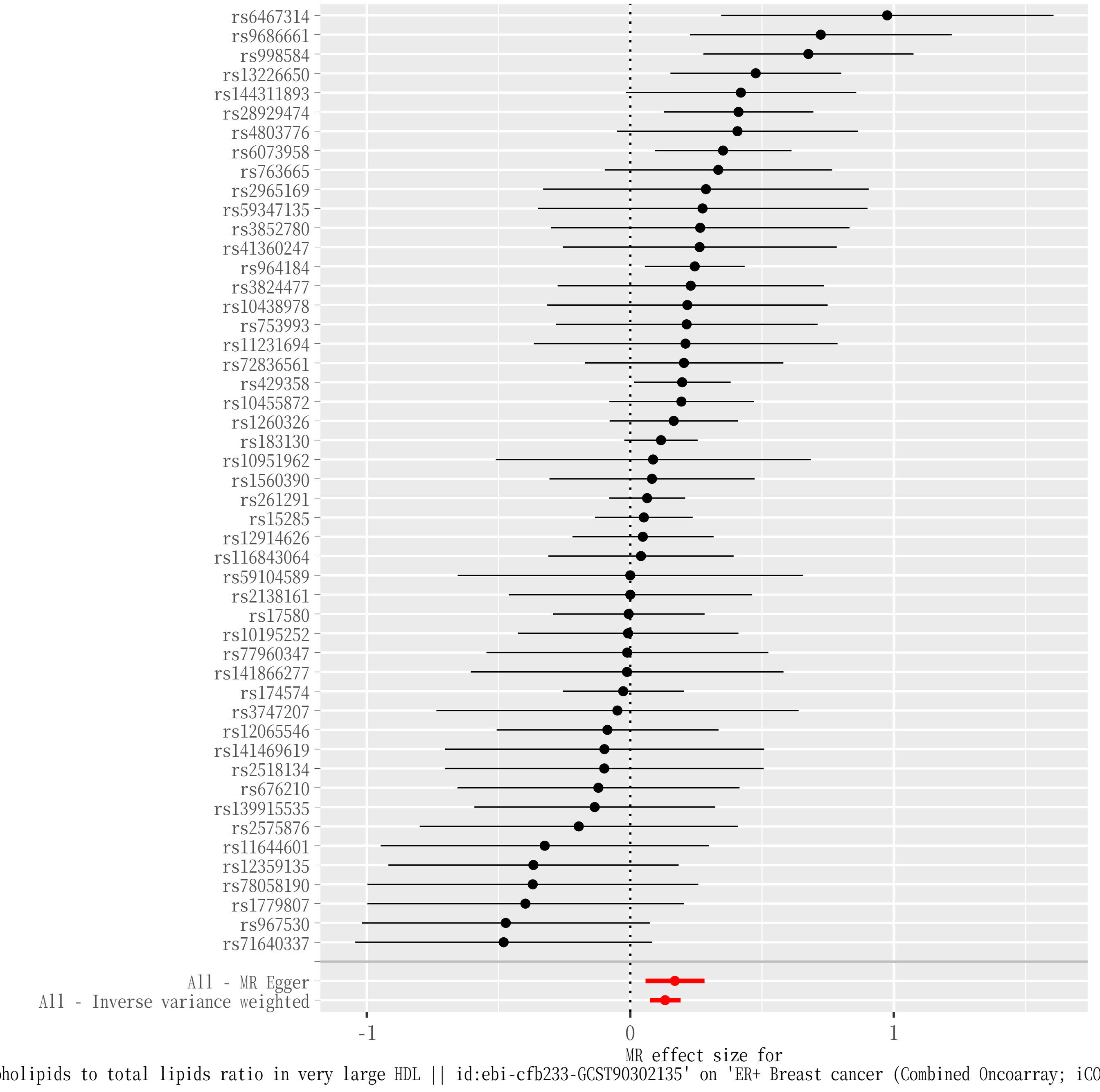
rs6467314
rs9686661
rs998584
rs13226650
rs144311893
rs28929474
rs4803776
rs6073958
rs763665
rs2965169
rs59347135
rs3852780
rs41360247
rs964184
rs3824477
rs10438978
rs753993
rs11231694
rs72836561
rs429358
rs10455872
rs1260326
rs183130
rs10951962
rs1560390
rs261291
rs15285
rs12914626
rs116843064
rs59104589
rs2138161
rs17580
rs10195252
rs77960347
rs141866277
rs174574
rs3747207
rs12065546
rs141469619
rs2518134
rs676210
rs139915535
rs2575876
rs11644601
rs12359135
rs78058190
rs1779807
rs967530
rs71640337
All - MR Egger
All - Inverse variance weighted
-1
0
1
MR effect size for
pholipids to total lipids ratio in very large HDL || id:ebi-cfb233-GCST90302135' on 'ER+ Breast cancer (Combined Oncoarray; iCO

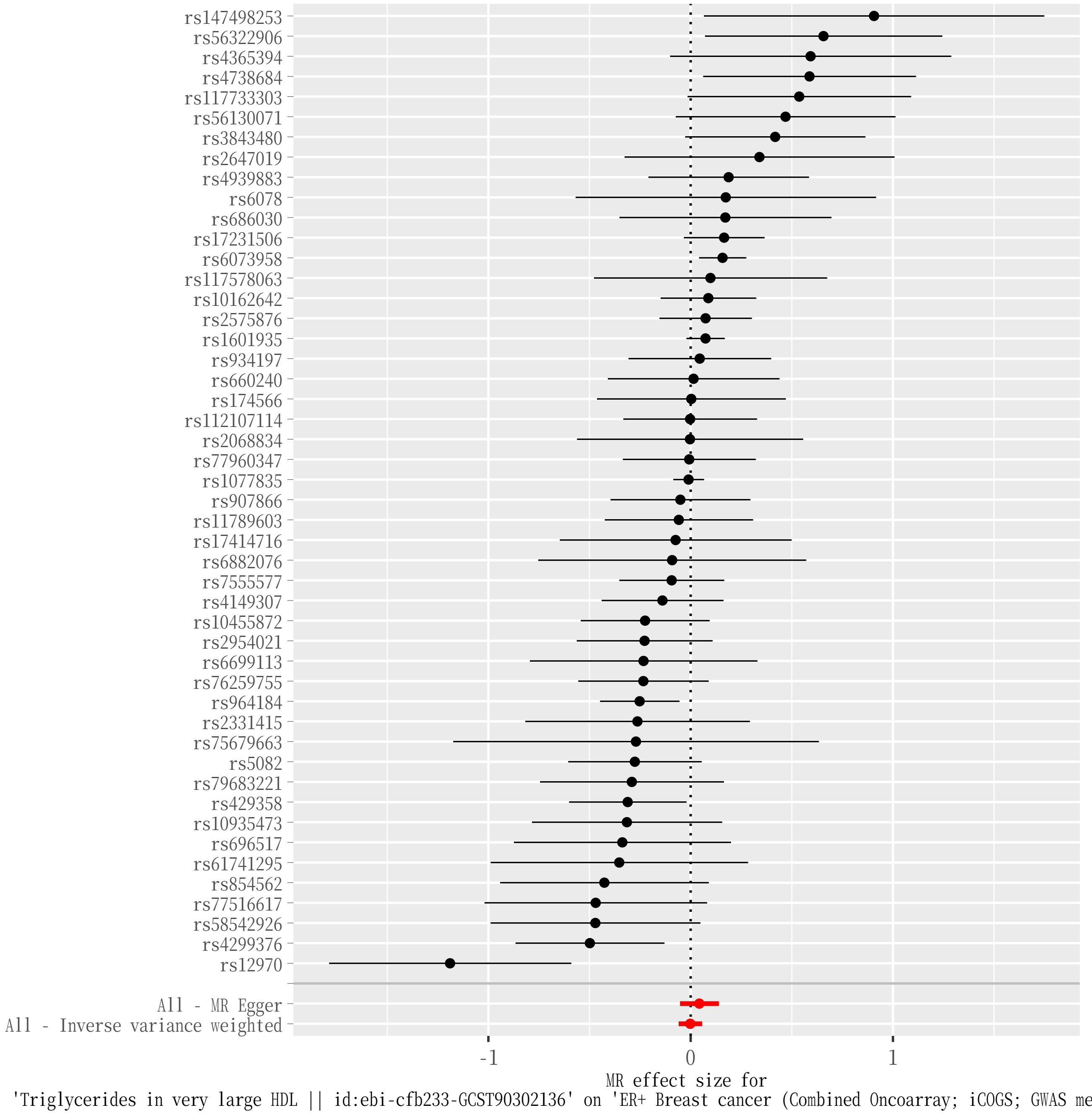
rs147498253
rs56322906
rs4365394
rs4738684
rs117733303
rs56130071
rs3843480
rs2647019
rs4939883
rs6078
rs686030
rs17231506
rs6073958
rs117578063
rs10162642
rs2575876
rs1601935
rs934197
rs660240
rs174566
rs112107114
rs2068834
rs77960347
rs1077835
rs907866
rs11789603
rs17414716
rs6882076
rs7555577
rs4149307
rs10455872
rs2954021
rs6699113
rs76259755
rs964184
rs2331415
rs75679663
rs5082
rs79683221
rs429358
rs10935473
rs696517
rs61741295
rs854562
rs77516617
rs58542926
rs4299376
rs12970
All - MR Egger
All - Inverse variance weighted
-1
0
1
MR effect size for
'Triglycerides in very large HDL || id:ebi-cfb233-GCST90302136' on 'ER+ Breast cancer (Combined Oncoarray; iCOGS; GWAS me

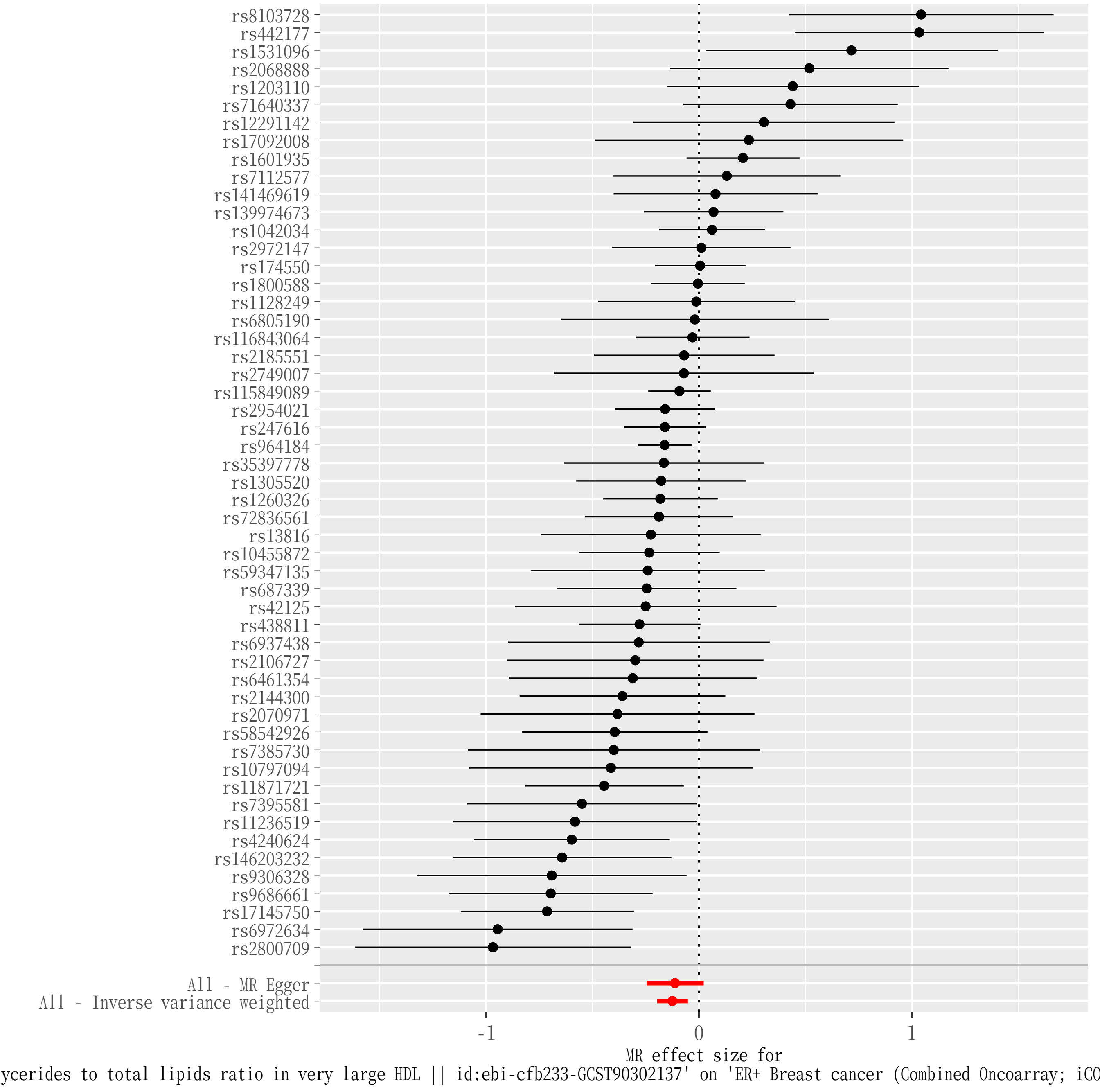
rs8103728
rs442177
rs1531096
rs2068888
rs1203110
rs71640337
rs12291142
rs17092008
rs1601935
rs7112577
rs141469619
rs139974673
rs1042034
rs2972147
rs174550
rs1800588
rs1128249
rs6805190
rs116843064
rs2185551
rs2749007
rs115849089
rs2954021
rs247616
rs964184
rs35397778
rs1305520
rs1260326
rs72836561
rs13816
rs10455872
rs59347135
rs687339
rs42125
rs438811
rs6937438
rs2106727
rs6461354
rs2144300
rs2070971
rs58542926
rs7385730
rs10797094
rs11871721
rs7395581
rs11236519
rs4240624
rs146203232
rs9306328
rs9686661
rs17145750
rs6972634
rs2800709
All - MR Egger
All - Inverse variance weighted
-1
0
1
MR effect size for
ycerides to total lipids ratio in very large HDL || id:ebi-cfb233-GCST90302137' on 'ER+ Breast cancer (Combined Oncoarray; iCO

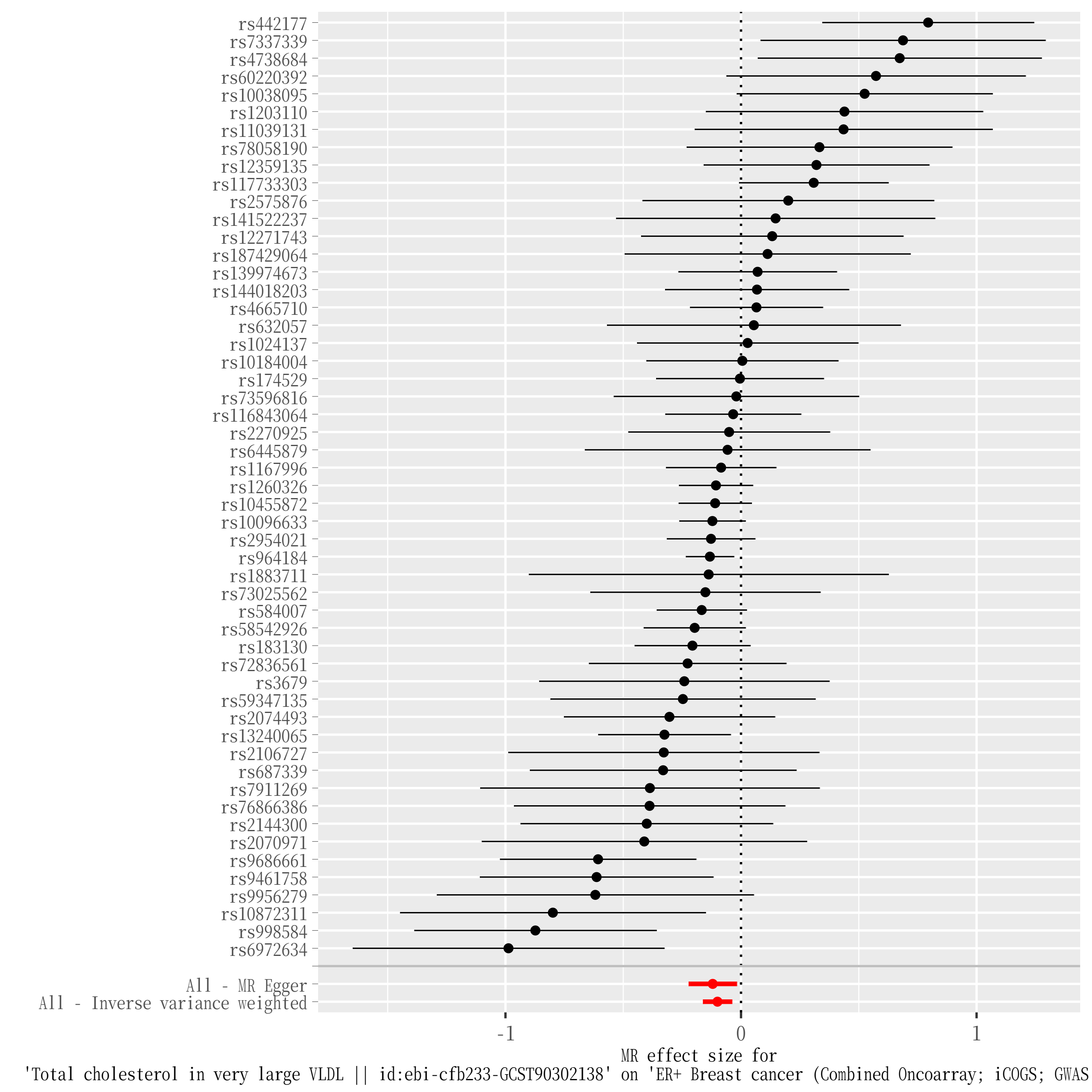
rs442177
rs7337339
rs4738684
rs60220392
rs10038095
rs1203110
rs11039131
rs78058190
rs12359135
rs117733303
rs2575876
rs141522237
rs12271743
rs187429064
rs139974673
rs144018203
rs4665710
rs632057
rs1024137
rs10184004
rs174529
rs73596816
rs116843064
rs2270925
rs6445879
rs1167996
rs1260326
rs10455872
rs10096633
rs2954021
rs964184
rs1883711
rs73025562
rs584007
rs58542926
rs183130
rs72836561
rs3679
rs59347135
rs2074493
rs13240065
rs2106727
rs687339
rs7911269
rs76866386
rs2144300
rs2070971
rs9686661
rs9461758
rs9956279
rs10872311
rs998584
rs6972634
All - MR Egger
All - Inverse variance weighted
-1
0
1
MR effect size for
'Total cholesterol in very large VLDL || id:ebi-cfb233-GCST90302138' on 'ER+ Breast cancer (Combined Oncoarray; iCOGS; GWAS

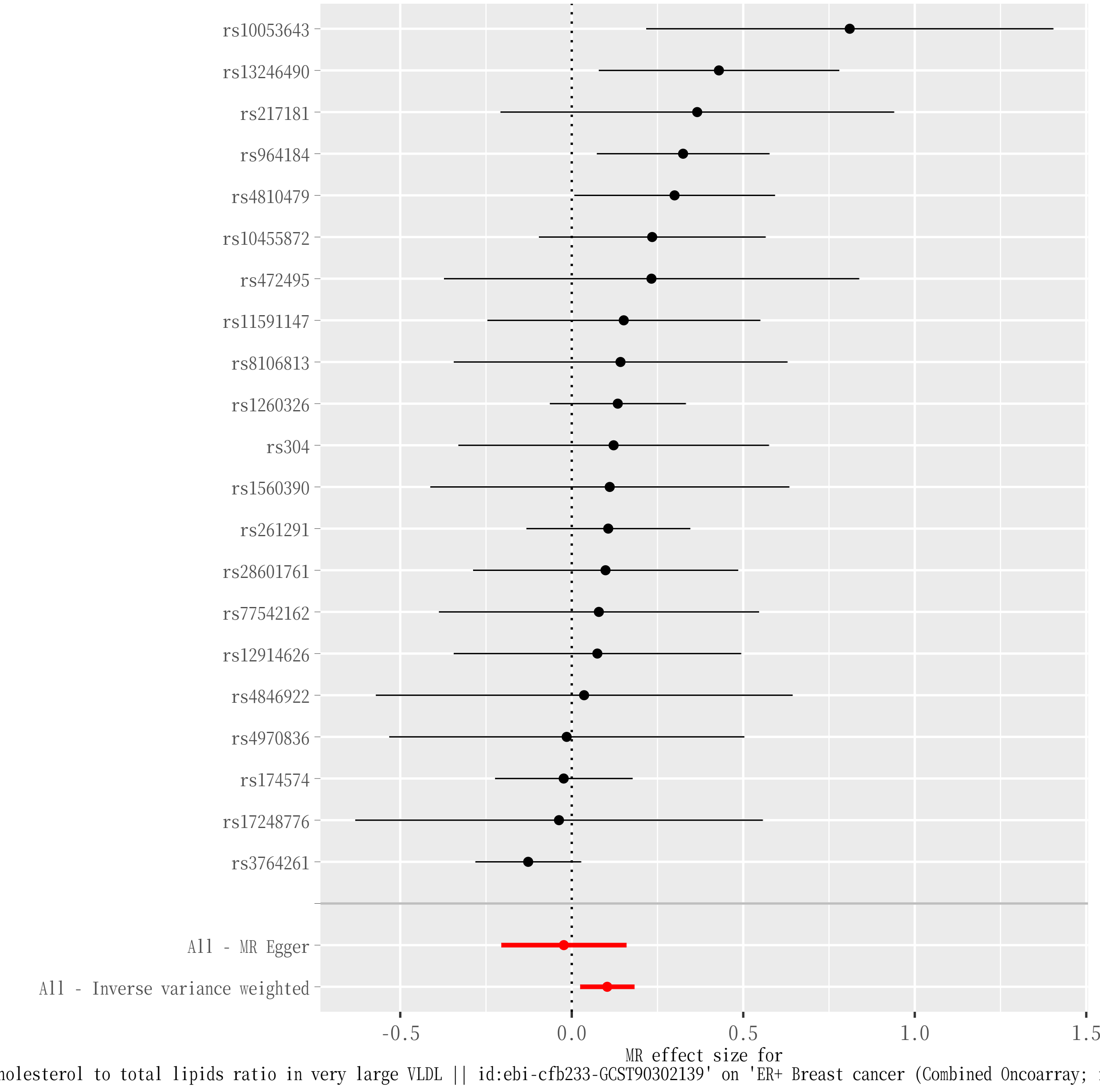
rs10053643
rs13246490
rs217181
rs964184
rs4810479
rs10455872
rs472495
rs11591147
rs8106813
rs1260326
rs304
rs1560390
rs261291
rs28601761
rs77542162
rs12914626
rs4846922
rs4970836
rs174574
rs17248776
rs3764261
All - MR Egger
All - Inverse variance weighted
-0.5
0.0
0.5
1.0
1.5
MR effect size for
holesterol to total lipids ratio in very large VLDL || id:ebi-cfb233-GCST90302139' on 'ER+ Breast cancer (Combined Oncoarray;

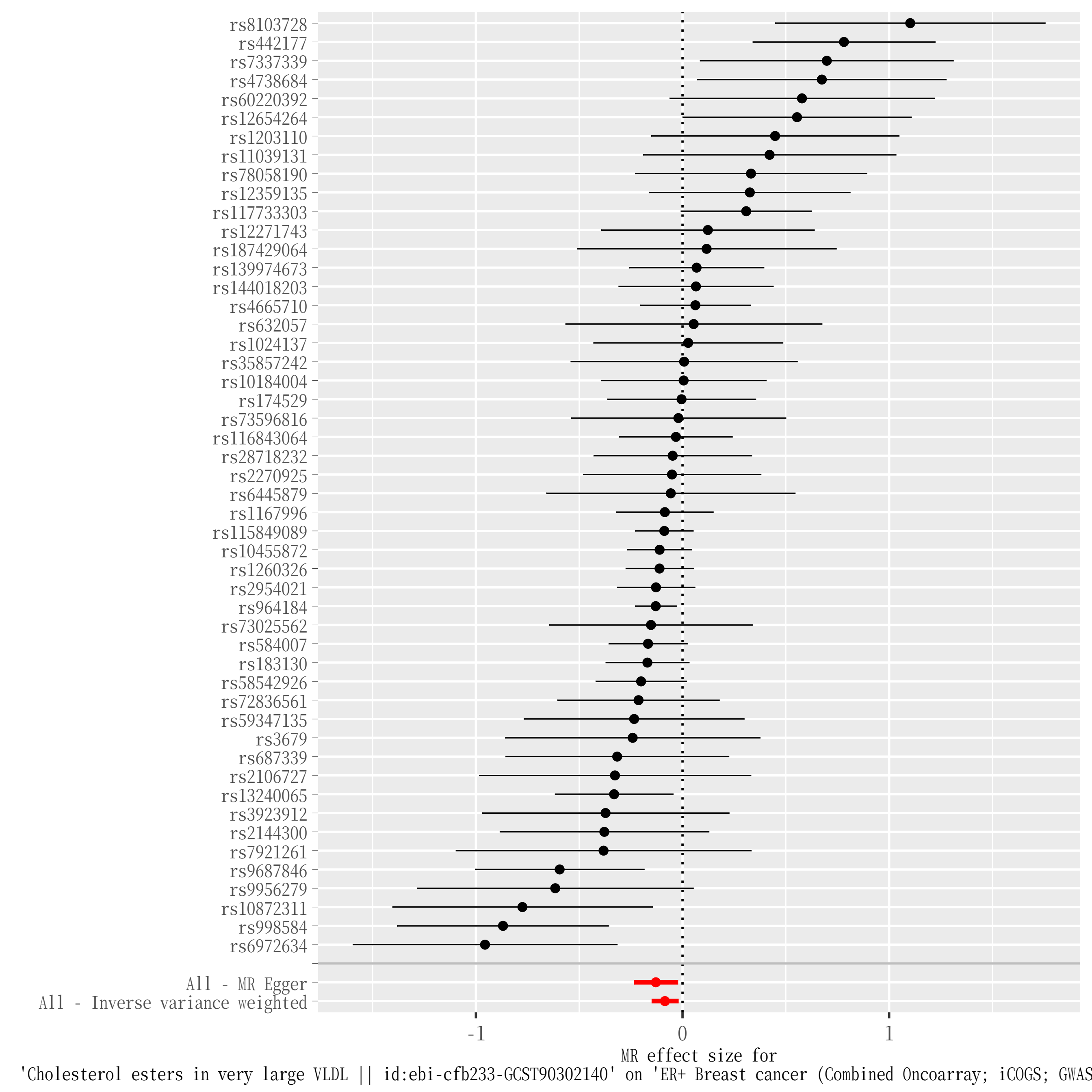
rs8103728
rs442177
rs7337339
rs4738684
rs60220392
rs12654264
rs1203110
rs11039131
rs78058190
rs12359135
rs117733303
rs12271743
rs187429064
rs139974673
rs144018203
rs4665710
rs632057
rs1024137
rs35857242
rs10184004
rs174529
rs73596816
rs116843064
rs28718232
rs2270925
rs6445879
rs1167996
rs115849089
rs10455872
rs1260326
rs2954021
rs964184
rs73025562
rs584007
rs183130
rs58542926
rs72836561
rs59347135
rs3679
rs687339
rs2106727
rs13240065
rs3923912
rs2144300
rs7921261
rs9687846
rs9956279
rs10872311
rs998584
rs6972634
All - MR Egger
All - Inverse variance weighted
-1
0
1
MR effect size for
'Cholesterol esters in very large VLDL || id:ebi-cfb233-GCST90302140' on 'ER+ Breast cancer (Combined Oncoarray; iCOGS; GWAS

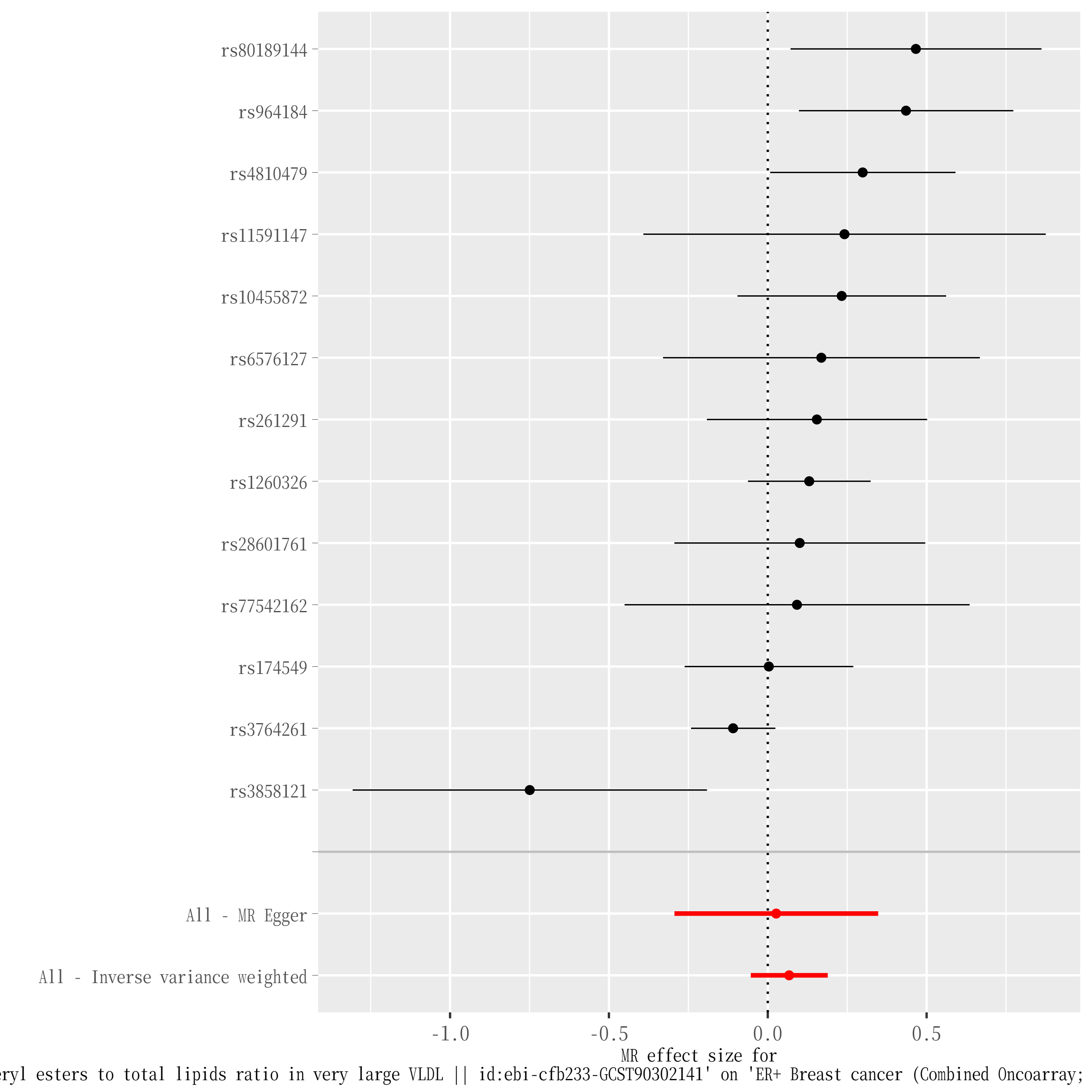

rs80189144
rs964184
rs4810479
rs11591147
rs10455872
rs6576127
rs261291
rs1260326
rs28601761
rs77542162
rs174549
rs3764261
rs3858121
All - MR Egger
All - Inverse variance weighted
-1.0
-0.5
0.0
0.5
MR effect size for
eryl esters to total lipids ratio in very large VLDL || id:ebi-cfb233-GCST90302141' on 'ER+ Breast cancer (Combined Oncoarray;

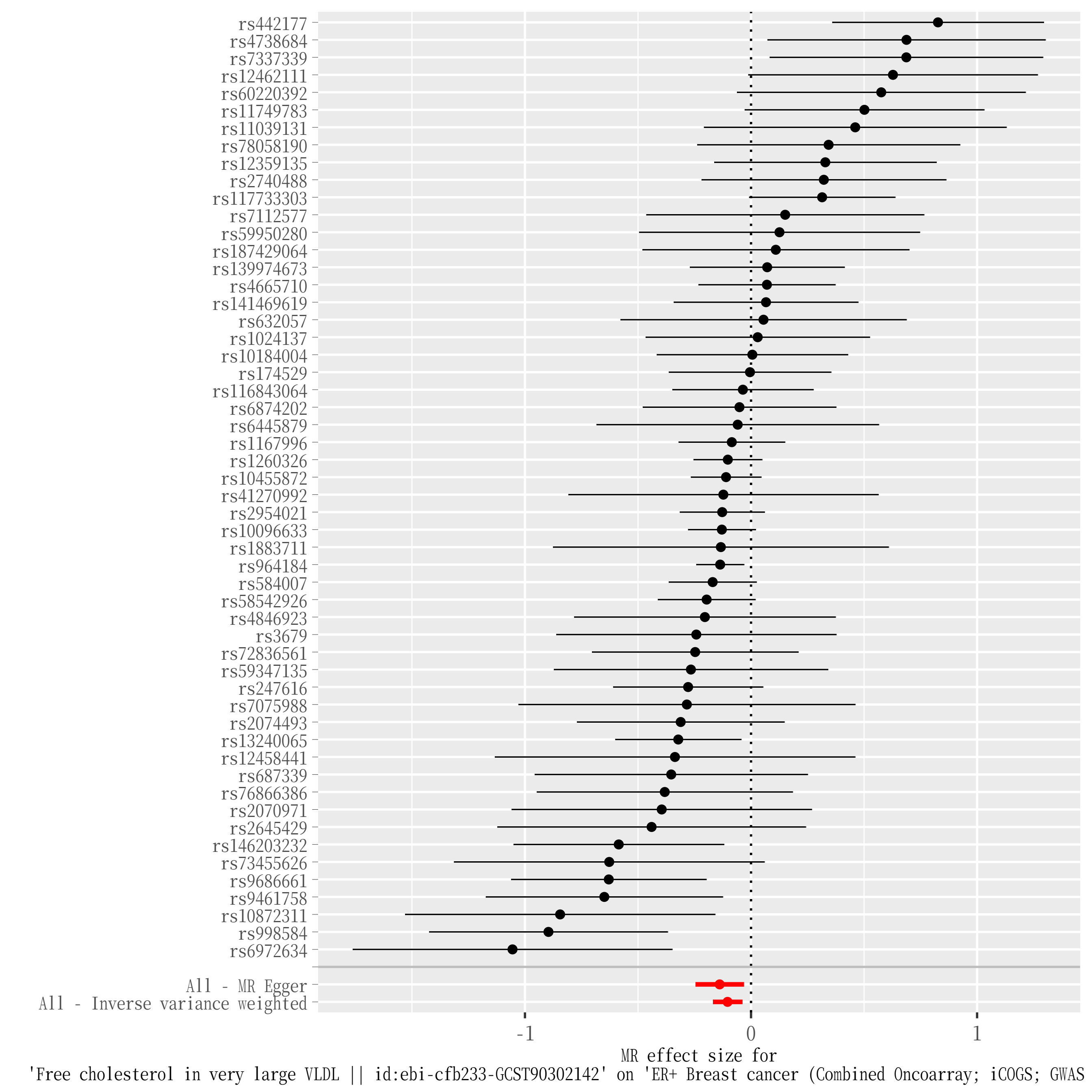

rs442177
rs4738684
rs7337339
rs12462111
rs60220392
rs11749783
rs11039131
rs78058190
rs12359135
rs2740488
rs117733303
rs7112577
rs59950280
rs187429064
rs139974673
rs4665710
rs141469619
rs632057
rs1024137
rs10184004
rs174529
rs116843064
rs6874202
rs6445879
rs1167996
rs1260326
rs10455872
rs41270992
rs2954021
rs10096633
rs1883711
rs964184
rs584007
rs58542926
rs4846923
rs3679
rs72836561
rs59347135
rs247616
rs7075988
rs2074493
rs13240065
rs12458441
rs687339
rs76866386
rs2070971
rs2645429
rs146203232
rs73455626
rs9686661
rs9461758
rs10872311
rs998584
rs6972634
All - MR Egger
All - Inverse variance weighted
-1
0
1
MR effect size for
'Free cholesterol in very large VLDL || id:ebi-cfb233-GCST90302142' on 'ER+ Breast cancer (Combined Oncoarray; iCOGS; GWAS

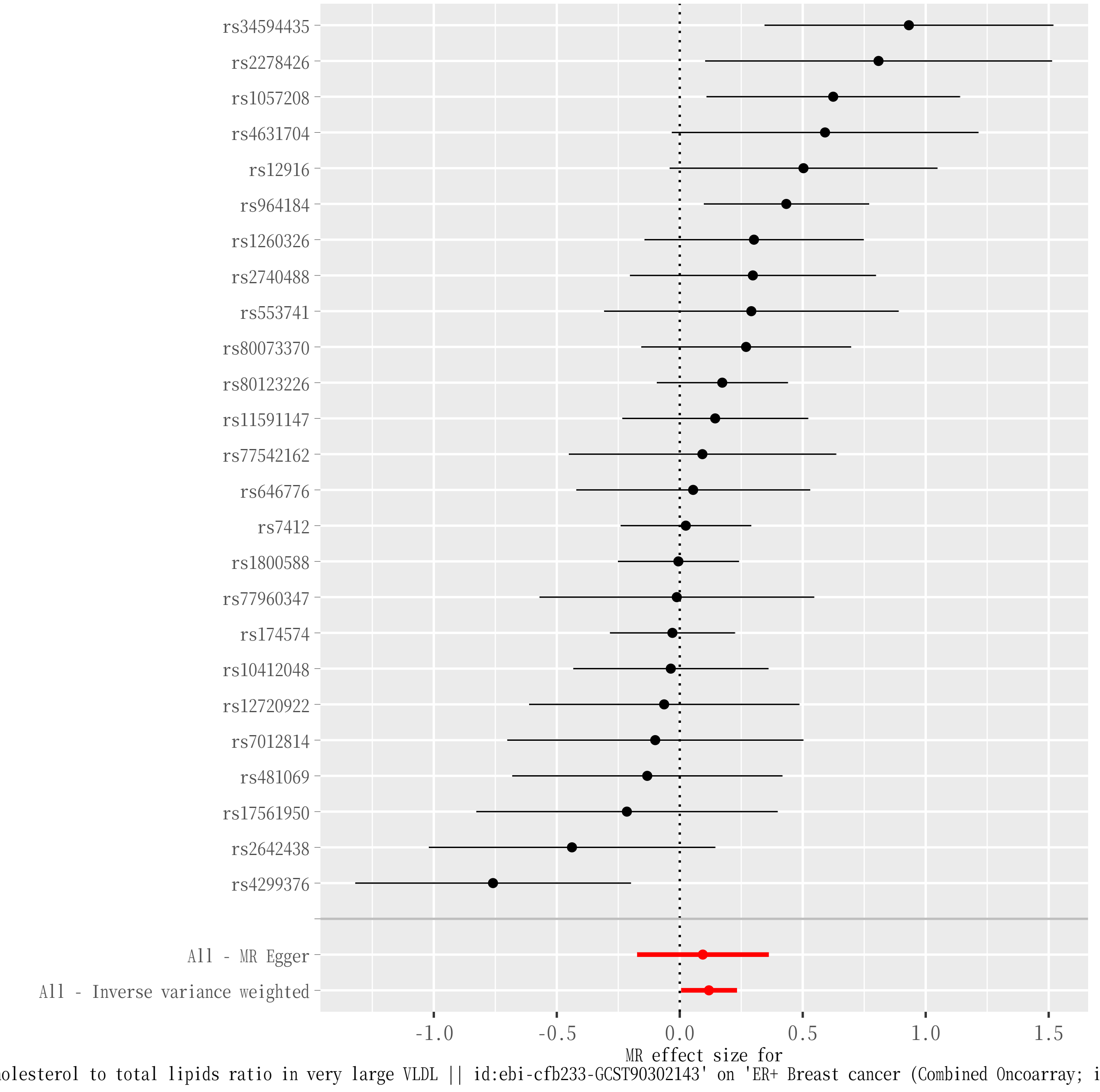

rs34594435
rs2278426
rs1057208
rs4631704
rs12916
rs964184
rs1260326
rs2740488
rs553741
rs80073370
rs80123226
rs11591147
rs77542162
rs646776
rs7412
rs1800588
rs77960347
rs174574
rs10412048
rs12720922
rs7012814
rs481069
rs17561950
rs2642438
rs4299376
All - MR Egger
All - Inverse variance weighted
-1.0
-0.5
0.0
0.5
1.0
1.5
MR effect size for
holesterol to total lipids ratio in very large VLDL || id:ebi-cfb233-GCST90302143' on 'ER+ Breast cancer (Combined Oncoarray; i

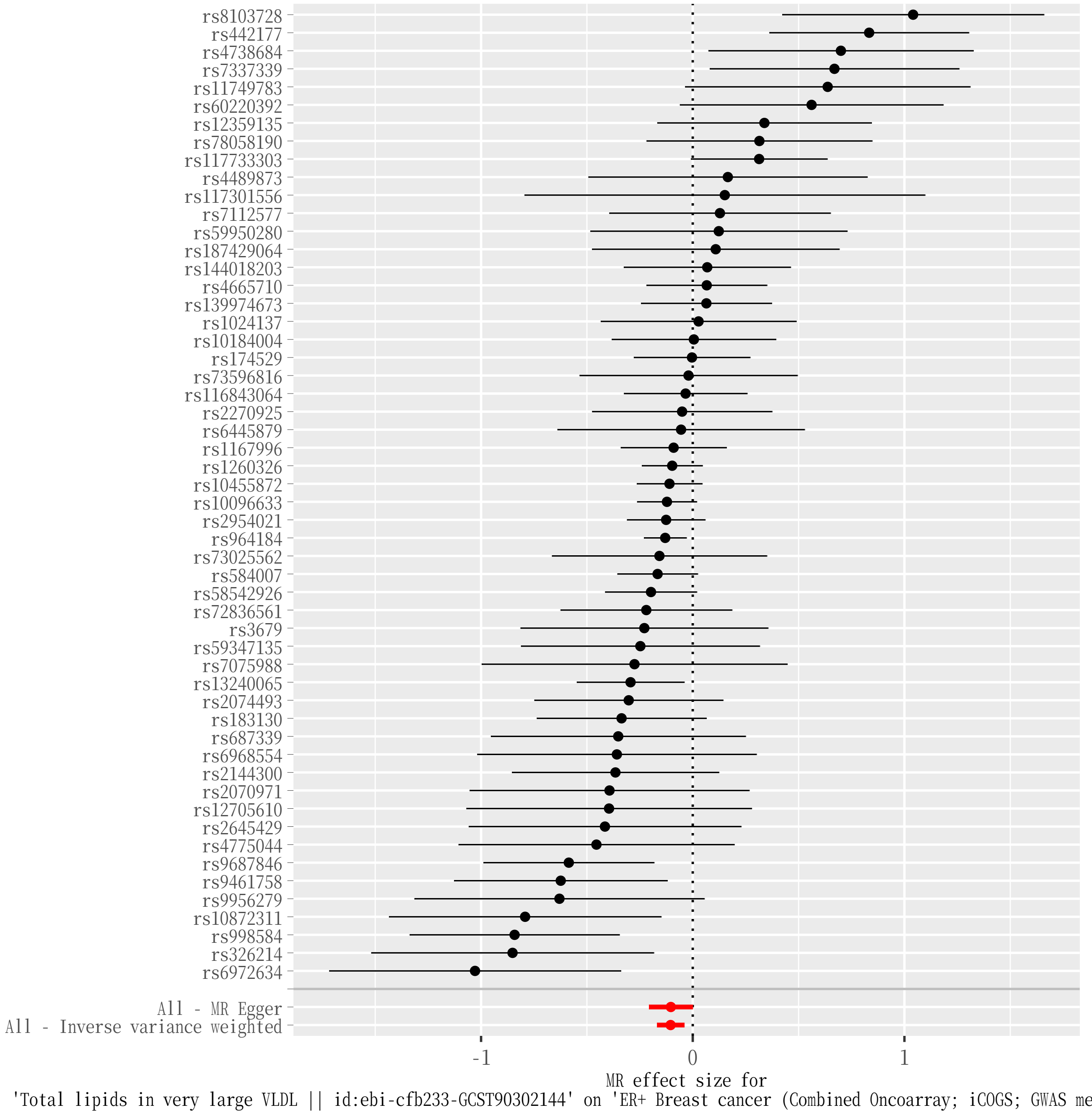

rs8103728
rs442177
rs4738684
rs7337339
rs11749783
rs60220392
rs12359135
rs78058190
rs117733303
rs4489873
rs117301556
rs7112577
rs59950280
rs187429064
rs144018203
rs4665710
rs139974673
rs1024137
rs10184004
rs174529
rs73596816
rs116843064
rs2270925
rs6445879
rs1167996
rs1260326
rs10455872
rs10096633
rs2954021
rs964184
rs73025562
rs584007
rs58542926
rs72836561
rs3679
rs59347135
rs7075988
rs13240065
rs2074493
rs183130
rs687339
rs6968554
rs2144300
rs2070971
rs12705610
rs2645429
rs4775044
rs9687846
rs9461758
rs9956279
rs10872311
rs998584
rs326214
rs6972634
All - MR Egger
All - Inverse variance weighted
-1
0
1
MR effect size for
'Total lipids in very large VLDL || id:ebi-cfb233-GCST90302144' on 'ER+ Breast cancer (Combined Oncoarray; iCOGS; GWAS me

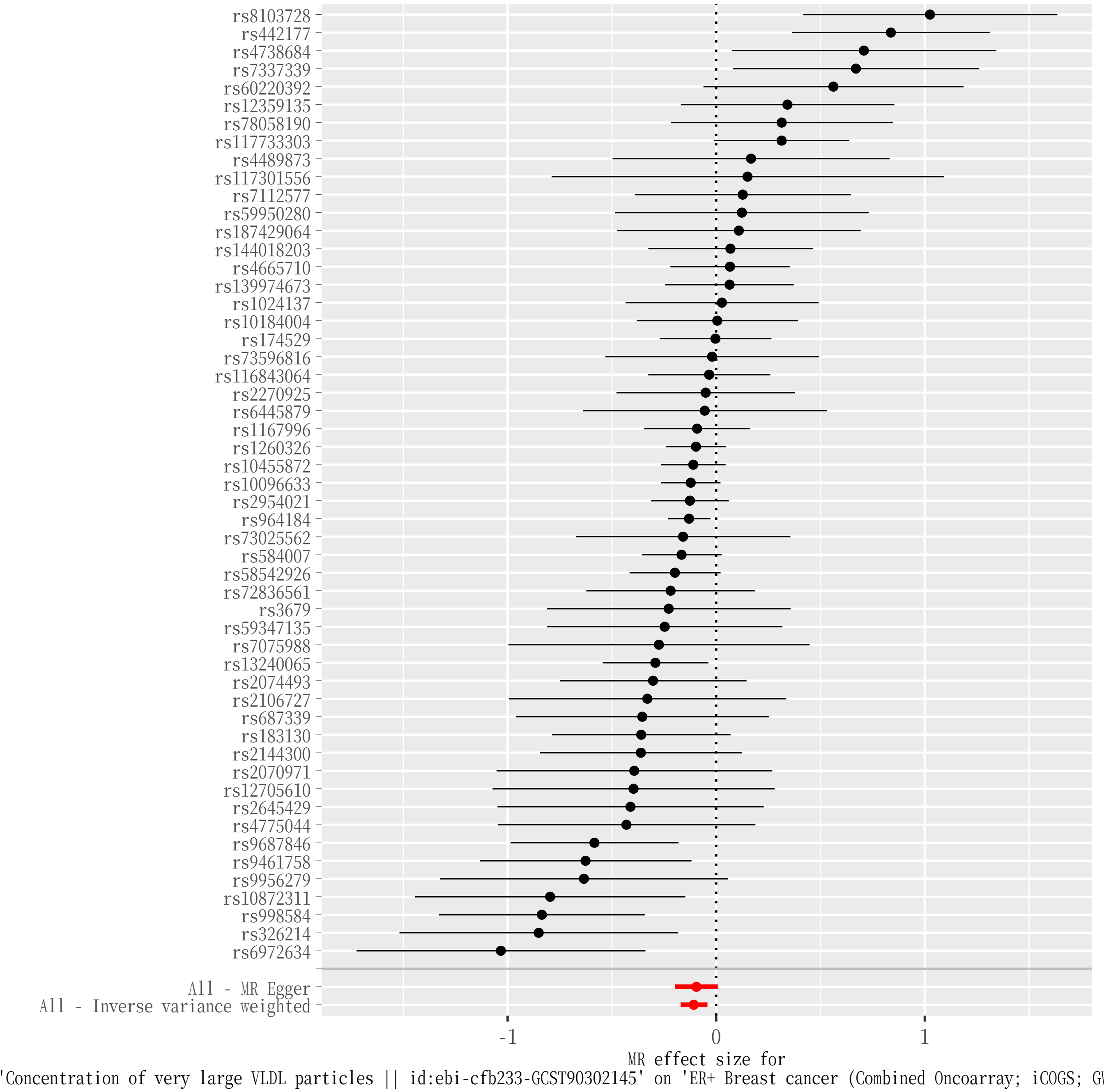

rs8103728
rs442177
rs4738684
rs7337339
rs60220392
rs12359135
rs78058190
rs117733303
rs4489873
rs117301556
rs7112577
rs59950280
rs187429064
rs144018203
rs4665710
rs139974673
rs1024137
rs10184004
rs174529
rs73596816
rs116843064
rs2270925
rs6445879
rs1167996
rs1260326
rs10455872
rs10096633
rs2954021
rs964184
rs73025562
rs584007
rs58542926
rs72836561
rs3679
rs59347135
rs7075988
rs13240065
rs2074493
rs2106727
rs687339
rs183130
rs2144300
rs2070971
rs12705610
rs2645429
rs4775044
rs9687846
rs9461758
rs9956279
rs10872311
rs998584
rs326214
rs6972634
All - MR Egger
All - Inverse variance weighted
-1
0
1
MR effect size for
'Concentration of very large VLDL particles || id:ebi-cfb233-GCST90302145' on 'ER+ Breast cancer (Combined Oncoarray; iCOGS; GW

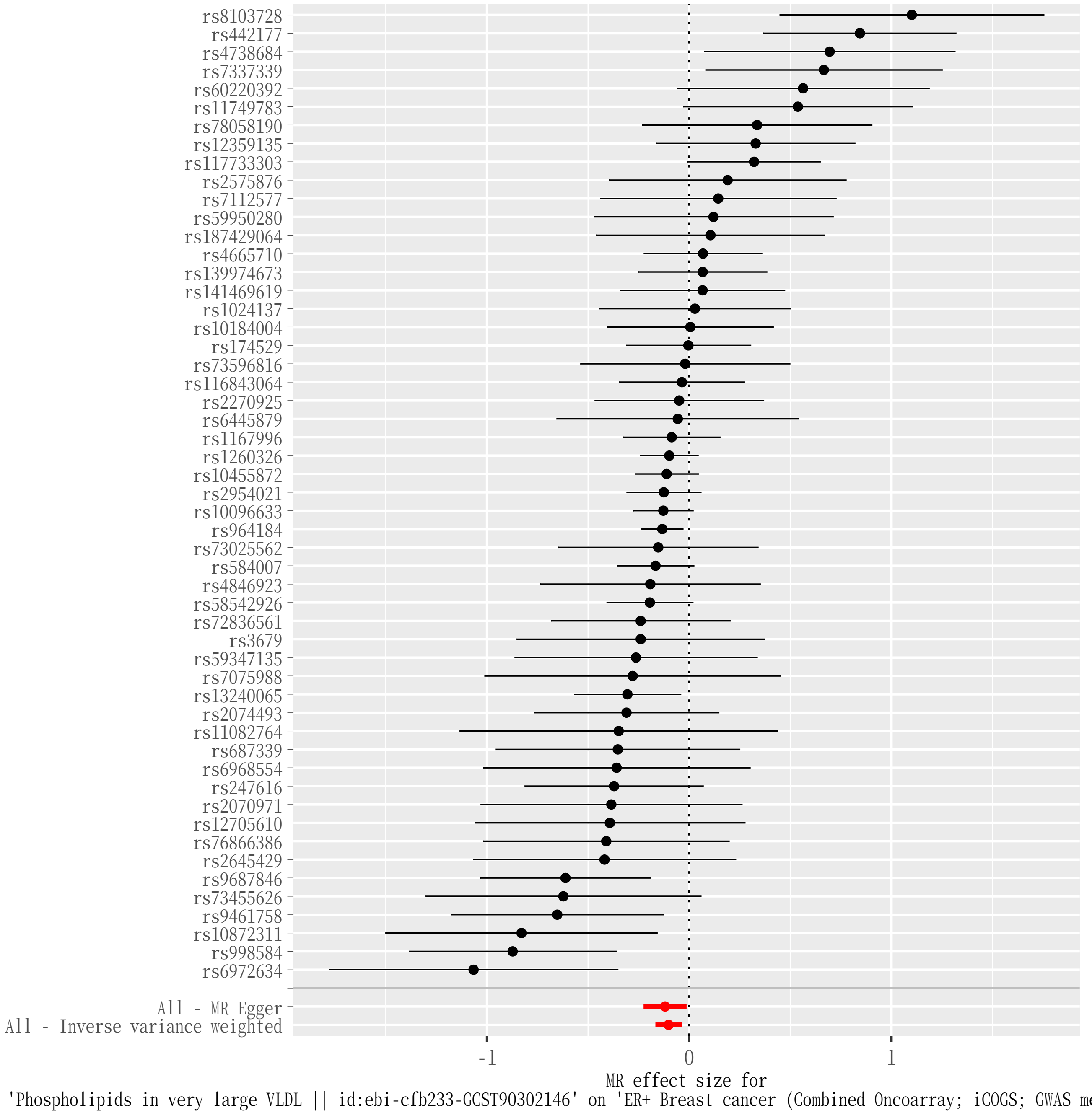
rs8103728
rs442177
rs4738684
rs7337339
rs60220392
rs11749783
rs78058190
rs12359135
rs117733303
rs2575876
rs7112577
rs59950280
rs187429064
rs4665710
rs139974673
rs141469619
rs1024137
rs10184004
rs174529
rs73596816
rs116843064
rs2270925
rs6445879
rs1167996
rs1260326
rs10455872
rs2954021
rs10096633
rs964184
rs73025562
rs584007
rs4846923
rs58542926
rs72836561
rs3679
rs59347135
rs7075988
rs13240065
rs2074493
rs11082764
rs687339
rs6968554
rs247616
rs2070971
rs12705610
rs76866386
rs2645429
rs9687846
rs73455626
rs9461758
rs10872311
rs998584
rs6972634
All - MR Egger
All - Inverse variance weighted
-1
0
1
MR effect size for
'Phospholipids in very large VLDL || id:ebi-cfb233-GCST90302146' on 'ER+ Breast cancer (Combined Oncoarray; iCOGS; GWAS me

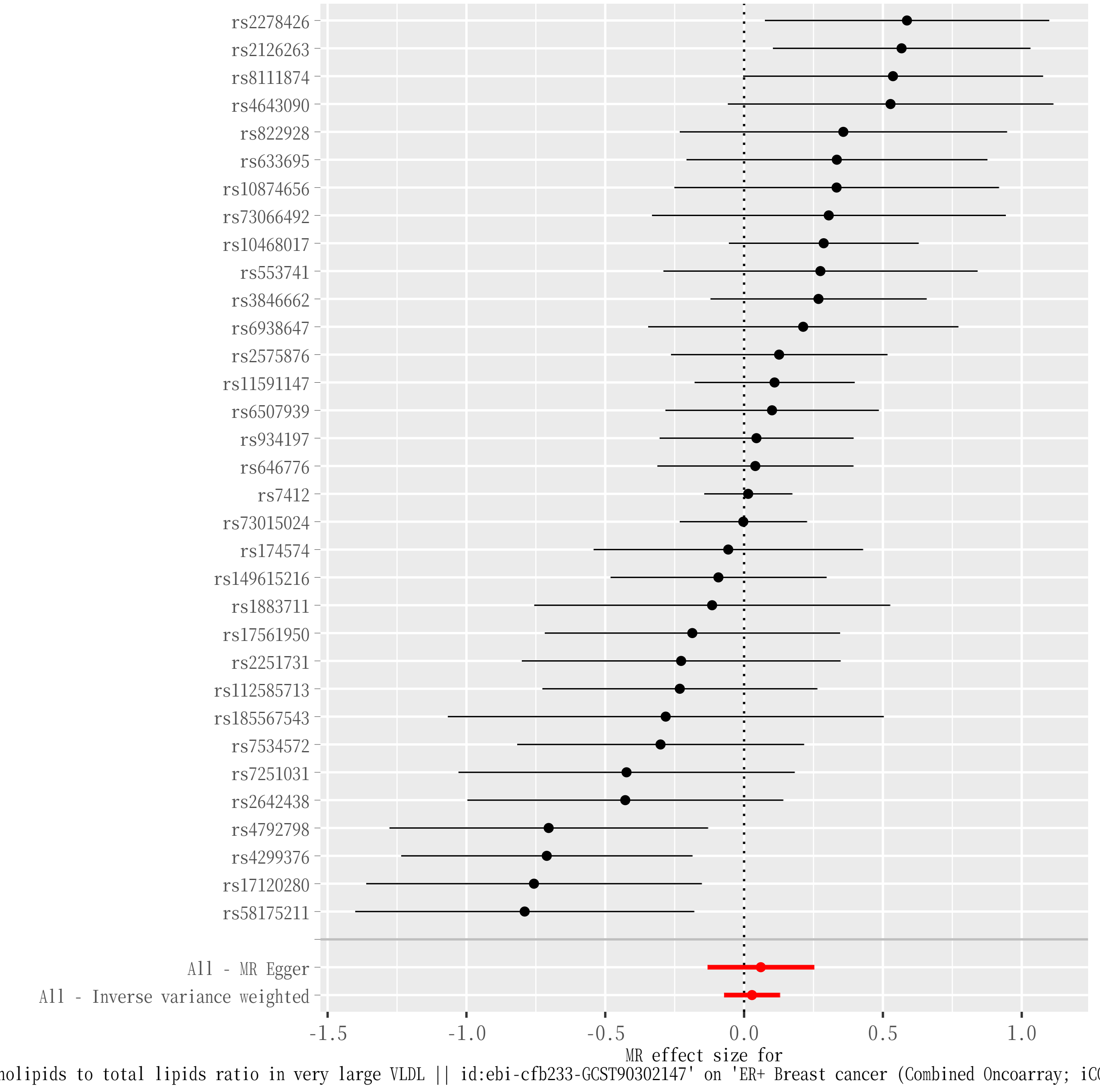
rs2278426
rs2126263
rs8111874
rs4643090
rs822928
rs633695
rs10874656
rs73066492
rs10468017
rs553741
rs3846662
rs6938647
rs2575876
rs11591147
rs6507939
rs934197
rs646776
rs7412
rs73015024
rs174574
rs149615216
rs1883711
rs17561950
rs2251731
rs112585713
rs185567543
rs7534572
rs7251031
rs2642438
rs4792798
rs4299376
rs17120280
rs58175211
All - MR Egger
All - Inverse variance weighted
-1.5
-1.0
-0.5
0.0
0.5
1.0
MR effect size for
holipids to total lipids ratio in very large VLDL || id:ebi-cfb233-GCST90302147' on 'ER+ Breast cancer (Combined Oncoarray; iC

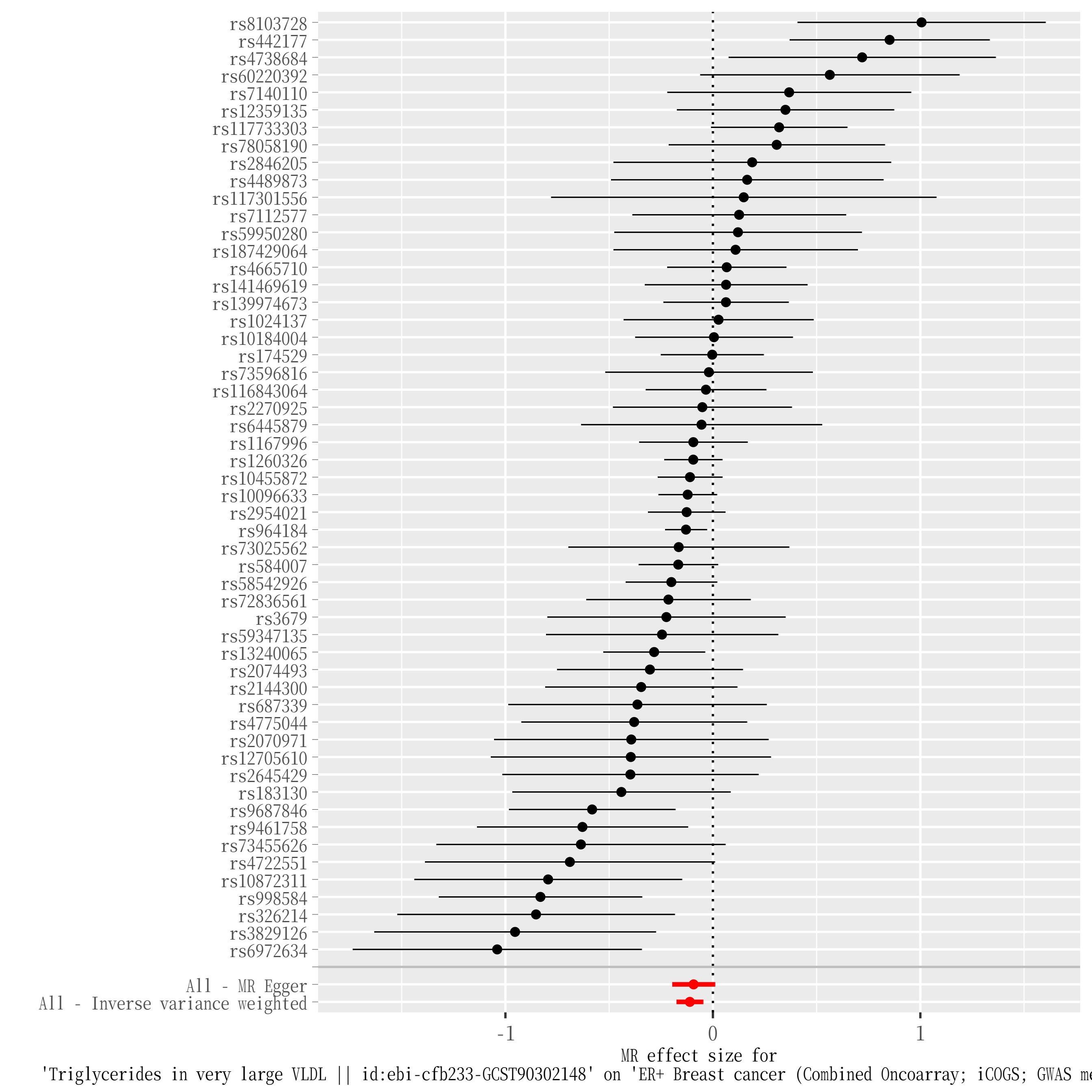

rs8103728
rs442177
rs4738684
rs60220392
rs7140110
rs12359135
rs117733303
rs78058190
rs2846205
rs4489873
rs117301556
rs7112577
rs59950280
rs187429064
rs4665710
rs141469619
rs139974673
rs1024137
rs10184004
rs174529
rs73596816
rs116843064
rs2270925
rs6445879
rs1167996
rs1260326
rs10455872
rs10096633
rs2954021
rs964184
rs73025562
rs584007
rs58542926
rs72836561
rs3679
rs59347135
rs13240065
rs2074493
rs2144300
rs687339
rs4775044
rs2070971
rs12705610
rs2645429
rs183130
rs9687846
rs9461758
rs73455626
rs4722551
rs10872311
rs998584
rs326214
rs3829126
rs6972634
All - MR Egger
All - Inverse variance weighted
-1
0
1
MR effect size for
'Triglycerides in very large VLDL || id:ebi-cfb233-GCST90302148' on 'ER+ Breast cancer (Combined Oncoarray; iCOGS; GWAS me

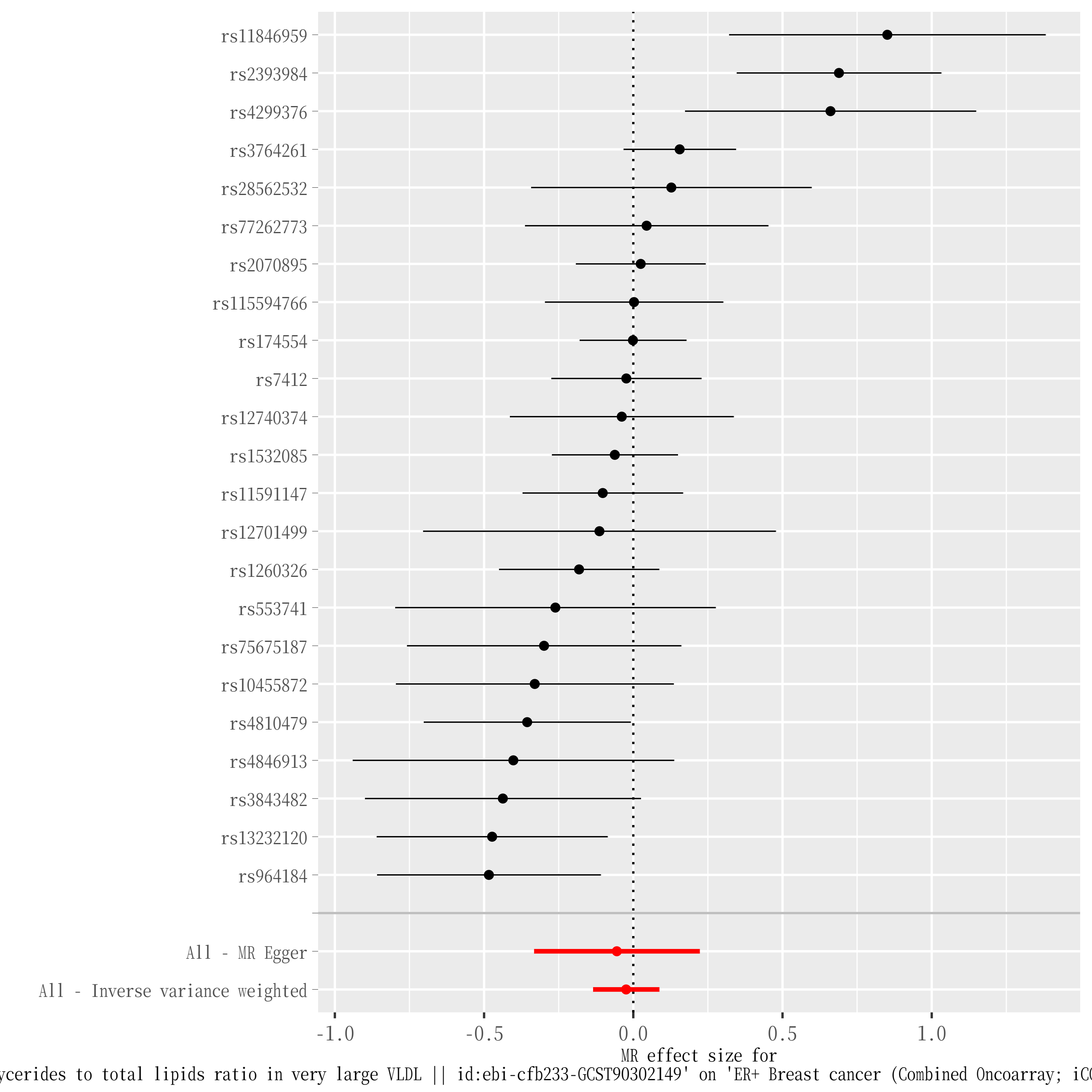

rs11846959
rs2393984
rs4299376
rs3764261
rs28562532
rs77262773
rs2070895
rs115594766
rs174554
rs7412
rs12740374
rs1532085
rs11591147
rs12701499
rs1260326
rs553741
rs75675187
rs10455872
rs4810479
rs4846913
rs3843482
rs13232120
rs964184
All - MR Egger
All - Inverse variance weighted
-1.0
-0.5
0.0
0.5
1.0
MR effect size for
ycerides to total lipids ratio in very large VLDL || id:ebi-cfb233-GCST90302149' on 'ER+ Breast cancer (Combined Oncoarray; iC

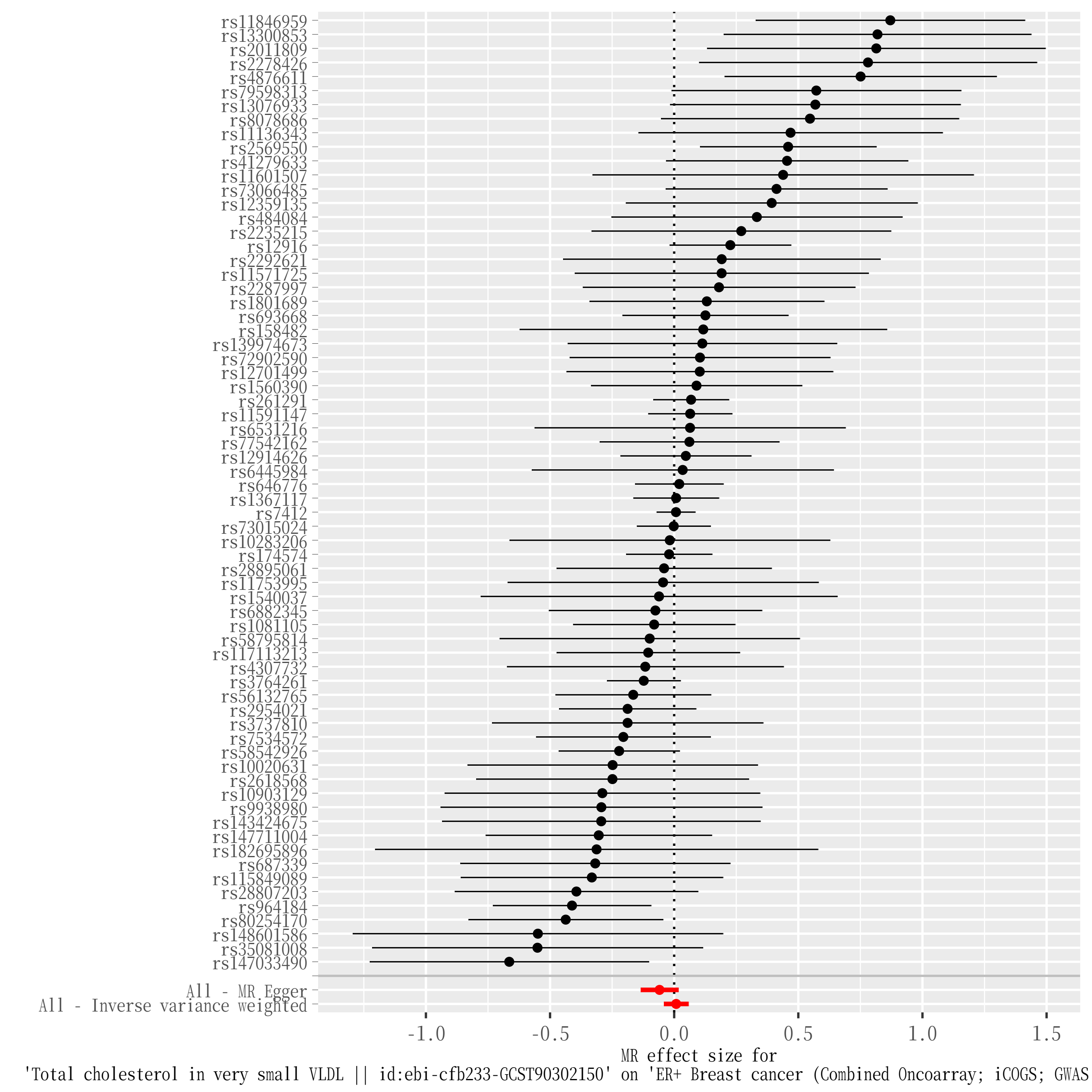

rs11846959
rs13300853
rs2011809
rs2278426
rs4876611
rs79598313
rs13076933
rs8078686
rs11136343
rs2569550
rs41279633
rs11601507
rs73066485
rs12359135
rs484084
rs2235215
rs12916
rs2292621
rs11571725
rs2287997
rs1801689
rs693668
rs158482
rs139974673
rs72902590
rs12701499
rs1560390
rs261291
rs11591147
rs6531216
rs77542162
rs12914626
rs6445984
rs646776
rs1367117
rs7412
rs73015024
rs10283206
rs174574
rs28895061
rs11753995
rs1540037
rs6882345
rs1081105
rs58795814
rs117113213
rs4307732
rs3764261
rs56132765
rs2954021
rs3737810
rs7534572
rs58542926
rs10020631
rs2618568
rs10903129
rs9938980
rs143424675
rs147711004
rs182695896
rs687339
rs115849089
rs28807203
rs964184
rs80254170
rs148601586
rs35081008
rs147033490
All - MR Egger
All - Inverse variance weighted
-1.0
-0.5
0.0
0.5
1.0
1.5
MR effect size for
'Total cholesterol in very small VLDL || id:ebi-cfb233-GCST90302150' on 'ER+ Breast cancer (Combined Oncoarray; iCOGS; GWAS

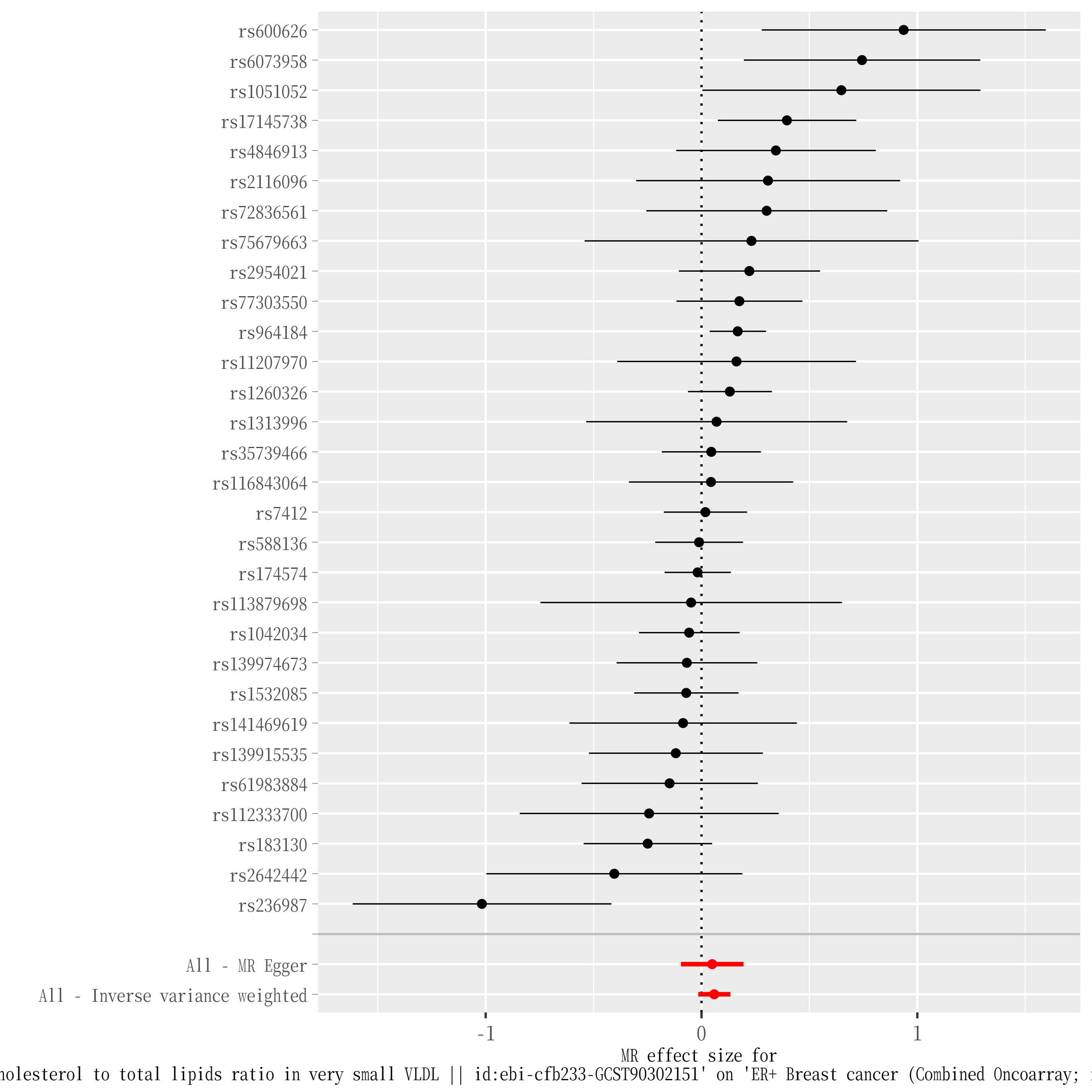

rs600626
rs6073958
rs1051052
rs17145738
rs4846913
rs2116096
rs72836561
rs75679663
rs2954021
rs77303550
rs964184
rs11207970
rs1260326
rs1313996
rs35739466
rs116843064
rs7412
rs588136
rs174574
rs113879698
rs1042034
rs139974673
rs1532085
rs141469619
rs139915535
rs61983884
rs112333700
rs183130
rs2642442
rs236987
All - MR Egger
All - Inverse variance weighted
-1
0
1
MR effect size for
holesterol to total lipids ratio in very small VLDL || id:ebi-cfb233-GCST90302151' on 'ER+ Breast cancer (Combined Oncoarray;

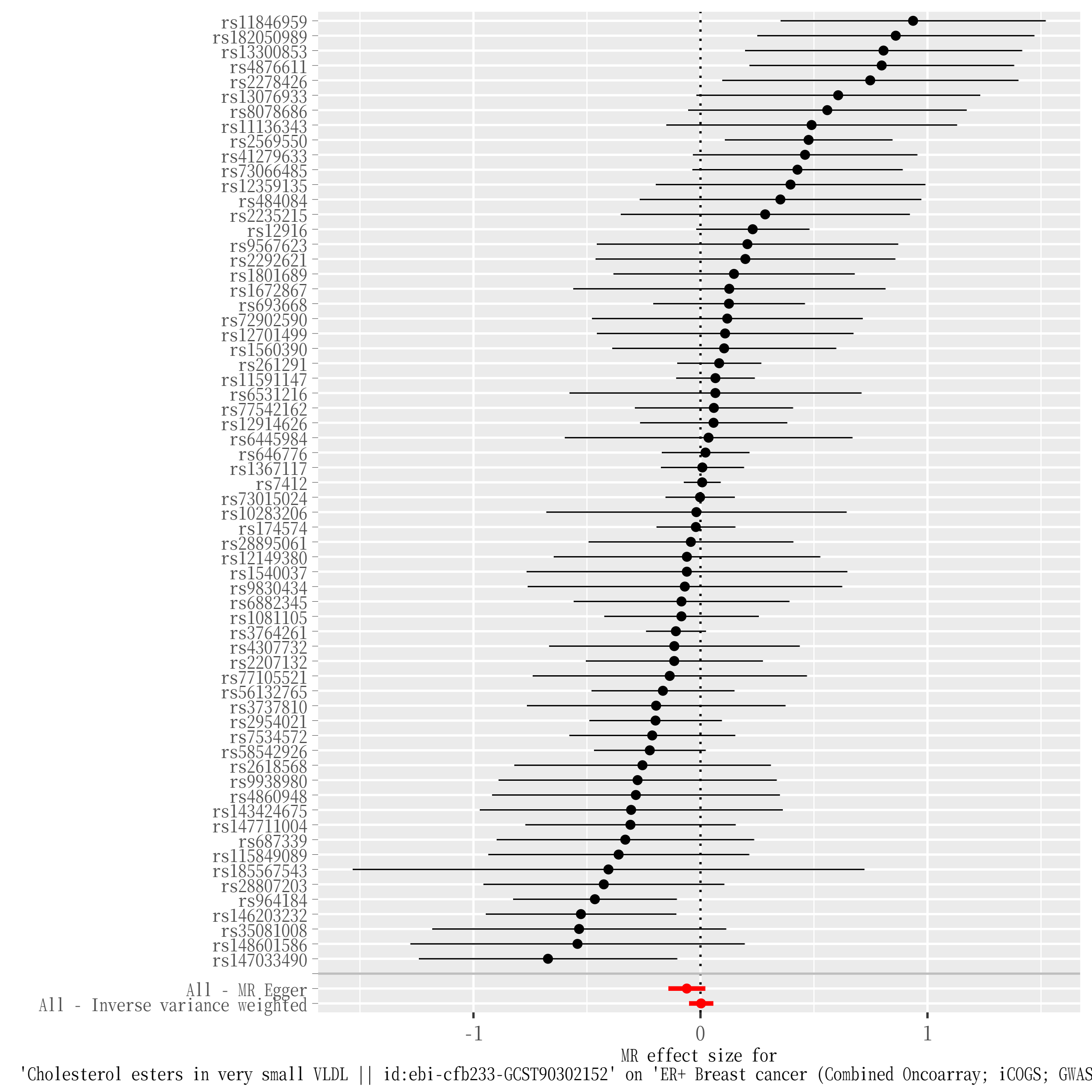
rs11846959
rs182050989
rs13300853
rs4876611
rs2278426
rs13076933
rs8078686
rs11136343
rs2569550
rs41279633
rs73066485
rs12359135
rs484084
rs2235215
rs12916
rs9567623
rs2292621
rs1801689
rs1672867
rs693668
rs72902590
rs12701499
rs1560390
rs261291
rs11591147
rs6531216
rs77542162
rs12914626
rs6445984
rs646776
rs1367117
rs7412
rs73015024
rs10283206
rs174574
rs28895061
rs12149380
rs1540037
rs9830434
rs6882345
rs1081105
rs3764261
rs4307732
rs2207132
rs77105521
rs56132765
rs3737810
rs2954021
rs7534572
rs58542926
rs2618568
rs9938980
rs4860948
rs143424675
rs147711004
rs687339
rs115849089
rs185567543
rs28807203
rs964184
rs146203232
rs35081008
rs148601586
rs147033490
All - MR Egger
All - Inverse variance weighted
-1
0
1
MR effect size for
'Cholesterol esters in very small VLDL || id:ebi-cfb233-GCST90302152' on 'ER+ Breast cancer (Combined Oncoarray; iCOGS; GWAS

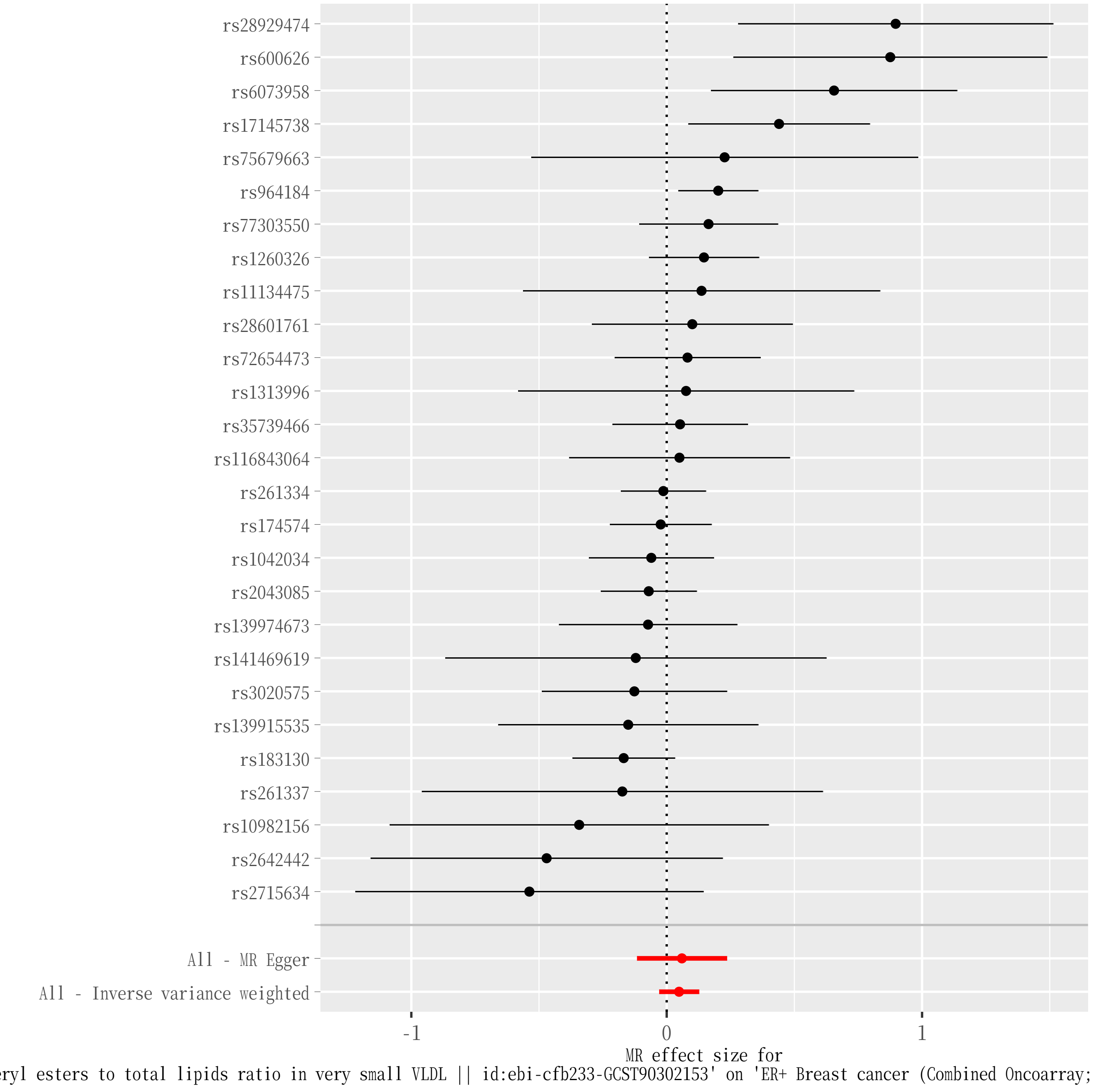
rs28929474
rs600626
rs6073958
rs17145738
rs75679663
rs964184
rs77303550
rs1260326
rs11134475
rs28601761
rs72654473
rs1313996
rs35739466
rs116843064
rs261334
rs174574
rs1042034
rs2043085
rs139974673
rs141469619
rs3020575
rs139915535
rs183130
rs261337
rs10982156
rs2642442
rs2715634
All - MR Egger
All - Inverse variance weighted
-1
0
1
MR effect size for
eryl esters to total lipids ratio in very small VLDL || id:ebi-cfb233-GCST90302153' on 'ER+ Breast cancer (Combined Oncoarray;

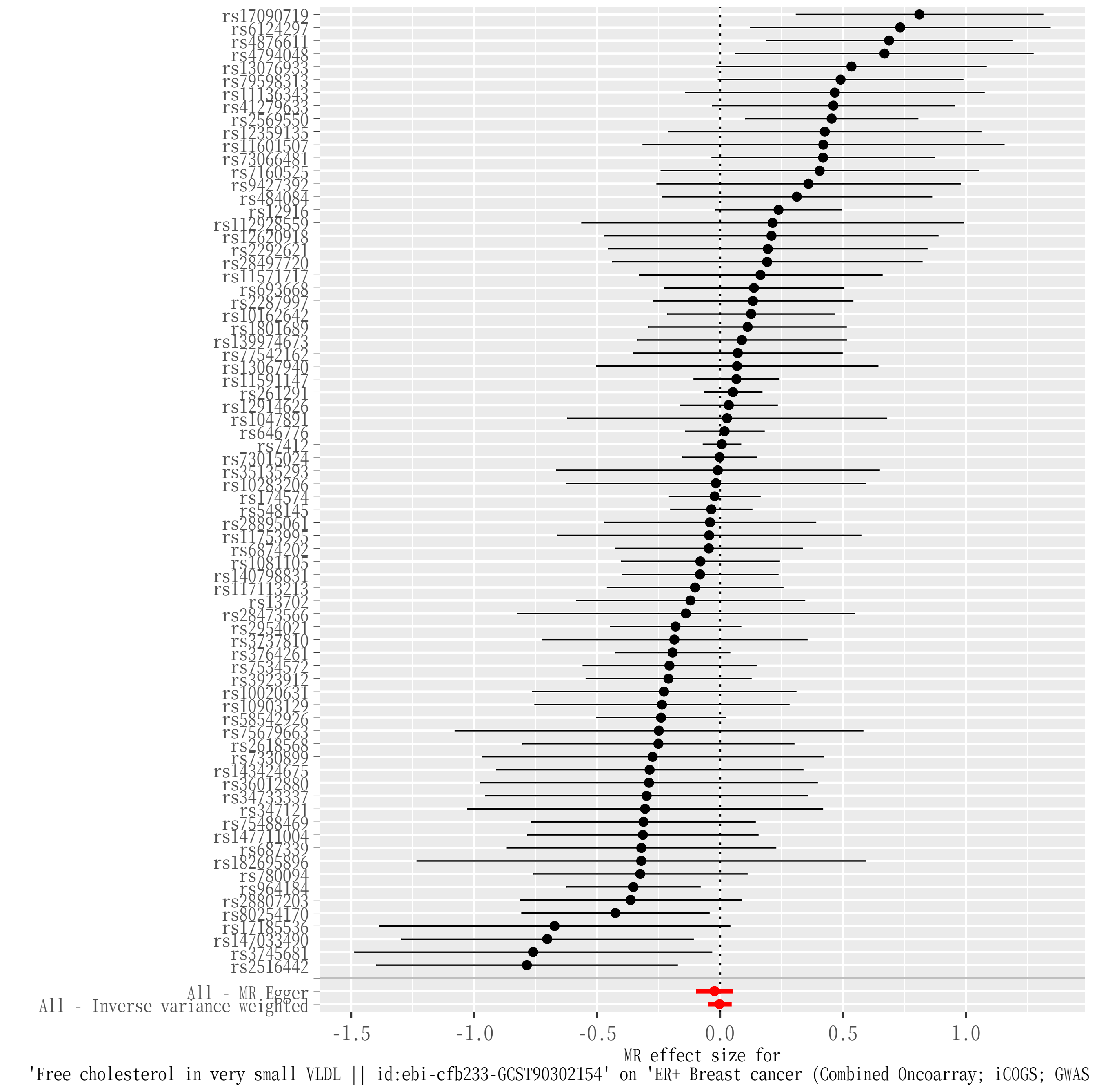

rs17090719
rs6124297
rs4876611
rs4794048
rs13076933
rs79598313
rs11136343
rs41279633
rs2569550
rs12359135
rs11601507
rs73066481
rs7160525
rs9427392
rs484084
rs12916
rs112928559
rs12620918
rs2292621
rs28497720
rs11571717
rs693668
rs2287997
rs10162642
rs1801689
rs139974673
rs77542162
rs13067940
rs11591147
rs261291
rs12914626
rs1047891
rs646776
rs7412
rs73015024
rs35135293
rs10283206
rs174574
rs548145
rs28895061
rs11753995
rs6874202
rs1081105
rs140798831
rs117113213
rs13702
rs28473566
rs2954021
rs3737810
rs3764261
rs7534572
rs3923912
rs10020631
rs10903129
rs58542926
rs75679663
rs2618568
rs7330899
rs143424675
rs36012880
rs34733337
rs347121
rs75488469
rs147711004
rs687339
rs182695896
rs780094
rs964184
rs28807203
rs80254170
rs17185536
rs147033490
rs3745681
rs2516442
All - MR Egger
All - Inverse variance weighted
-1.5
-1.0
-0.5
0.0
0.5
1.0
MR effect size for
'Free cholesterol in very small VLDL || id:ebi-cfb233-GCST90302154' on 'ER+ Breast cancer (Combined Oncoarray; iCOGS; GWAS

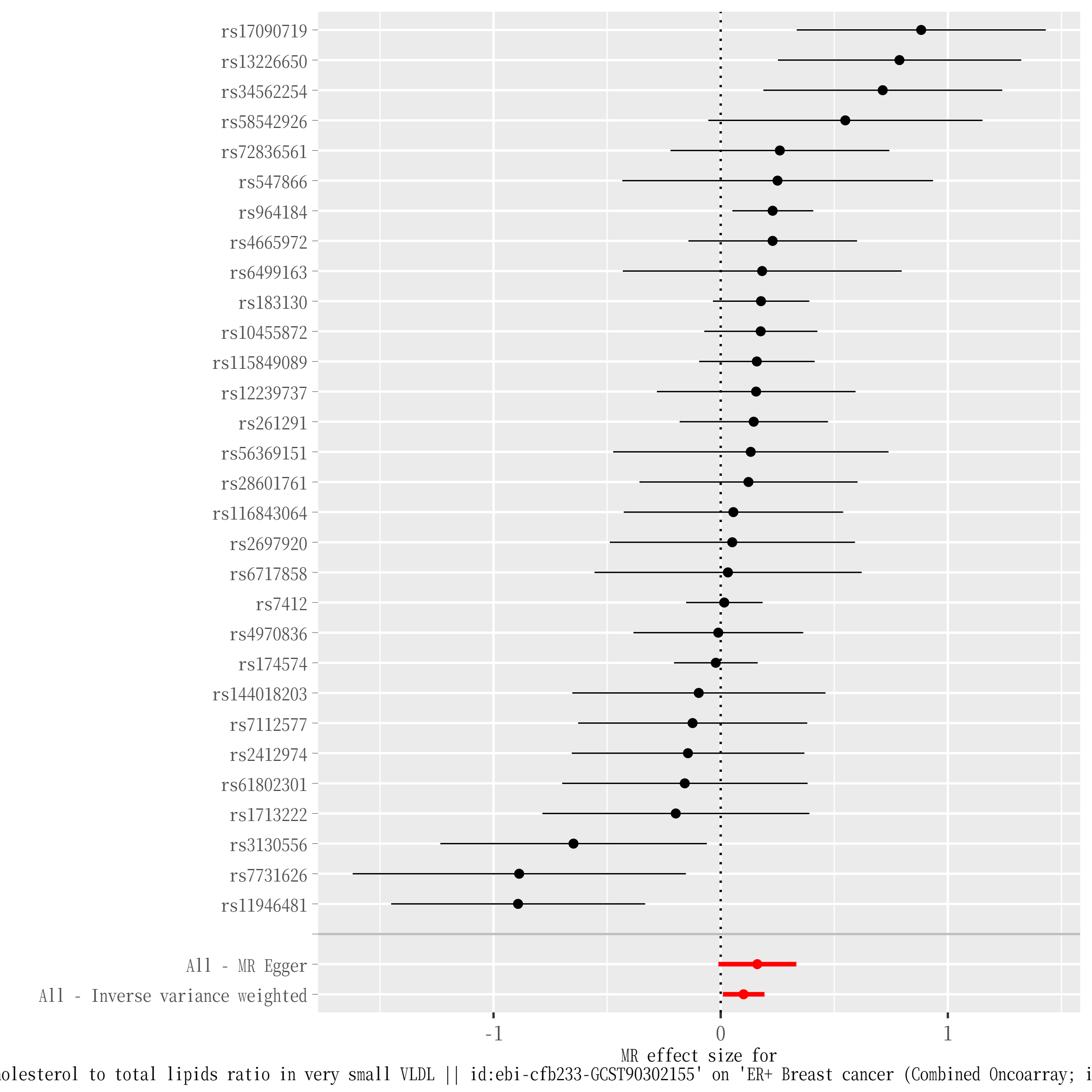

rs17090719
rs13226650
rs34562254
rs58542926
rs72836561
rs547866
rs964184
rs4665972
rs6499163
rs183130
rs10455872
rs115849089
rs12239737
rs261291
rs56369151
rs28601761
rs116843064
rs2697920
rs6717858
rs7412
rs4970836
rs174574
rs144018203
rs7112577
rs2412974
rs61802301
rs1713222
rs3130556
rs7731626
rs11946481
All - MR Egger
All - Inverse variance weighted
-1
0
1
MR effect size for
holesterol to total lipids ratio in very small VLDL || id:ebi-cfb233-GCST90302155' on 'ER+ Breast cancer (Combined Oncoarray; i

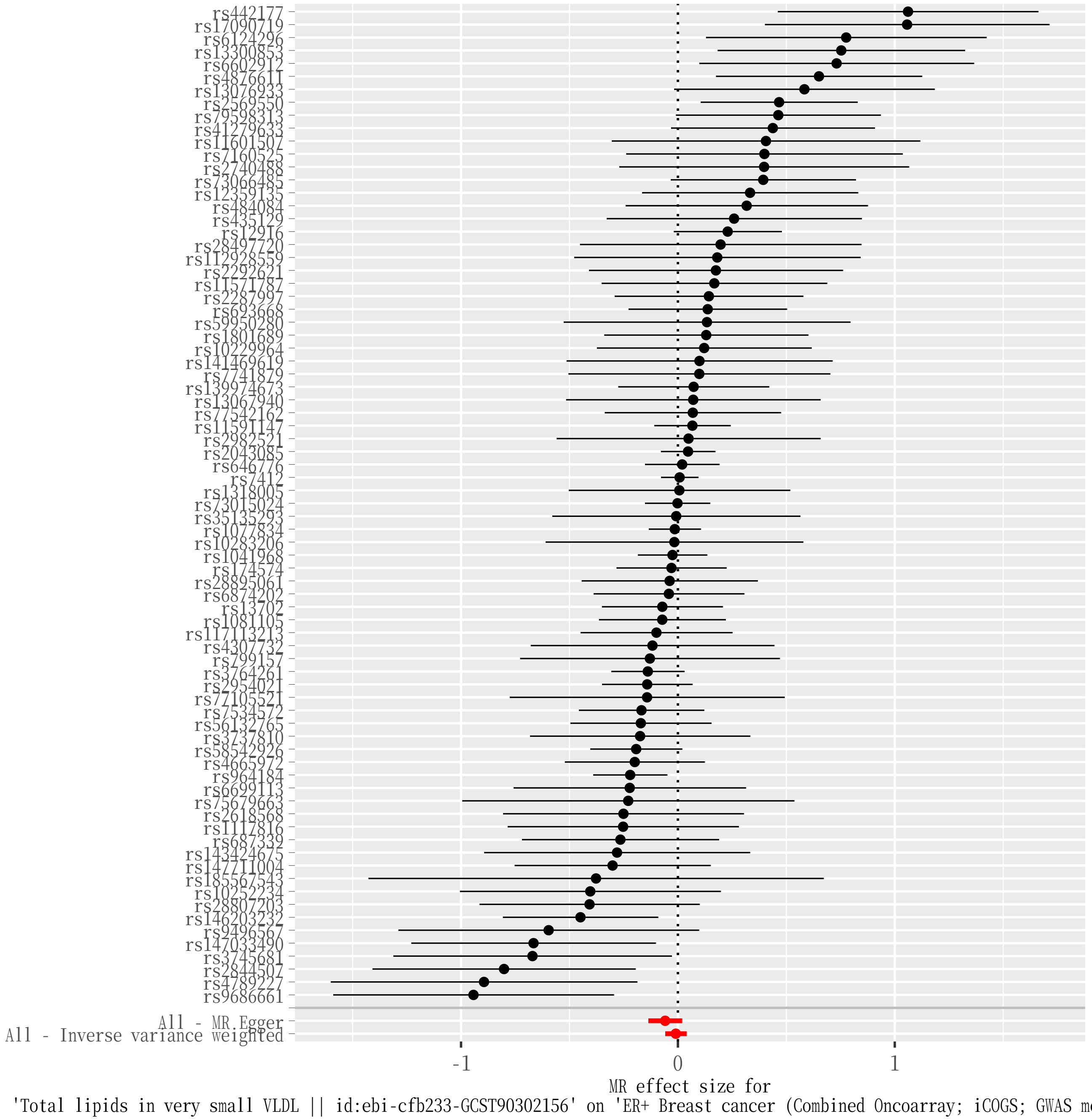

rs442177
rs17090719
rs6124296
rs13300853
rs6602912
rs4876611
rs13076933
rs2569550
rs79528313
rs41279633
rs11601507
rs7160525
rs2740488
rs73066485
rs12359135
rs484084
rs435129
rs12916
rs28497720
rs112928559
rs2292621
rs11571787
rs2287997
rs693668
rs59950280
rs1801689
rs10229964
rs141469619
rs7741879
rs139974673
rs13067940
rs77542162
rs11591147
rs2982521
rs2043085
rs646776
rs7412
rs1318005
rs73015024
rs35135293
rs1077834
rs10283206
rs1041968
rs174574
rs28895061
rs6874202
rs13702
rs1081105
rs117113213
rs4307732
rs799157
rs3764261
rs2954021
rs77105521
rs7534572
rs56132765
rs3737810
rs58542926
rs4665972
rs964184
rs6629113
rs75679663
rs2618568
rs1117816
rs687339
rs143424675
rs147711004
rs185567543
rs10252234
rs28807203
rs146203232
rs9496567
rs147033490
rs3745681
rs2844507
rs4789227
rs9686661
All - MR Egger
All - Inverse variance weighted
-1
0
1
MR effect size for
'Total lipids in very small VLDL || id:ebi-cfb233-GCST90302156' on 'ER+ Breast cancer (Combined Oncoarray; iCOGS; GWAS me

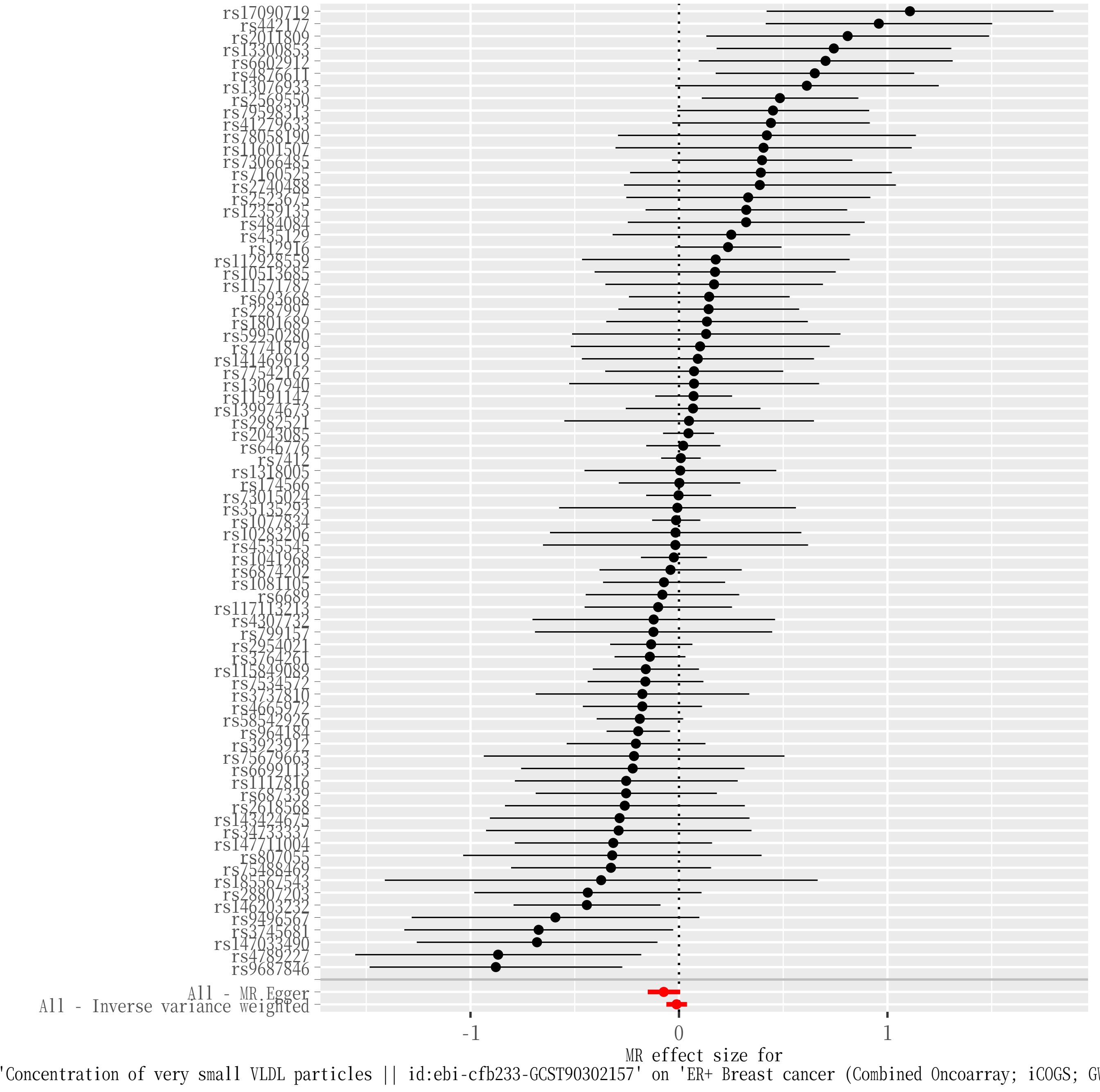
rs17090719
rs442177
rs2011809
rs13300853
rs6602912
rs4876611
rs13076933
rs2569550
rs79598313
rs41279633
rs78058190
rs11601507
rs73066485
rs7160525
rs2740488
rs2523675
rs12359135
rs484084
rs435129
rs12916
rs112928559
rs10513685
rs11571787
rs693668
rs2287997
rs1801689
rs59950280
rs7741879
rs141469619
rs77542162
rs13067940
rs11591147
rs139974673
rs2982521
rs2043085
rs646776
rs7412
rs1318005
rs174566
rs73015024
rs35135293
rs1077834
rs10283206
rs4535545
rs1041968
rs6874202
rs1081105
rs6689
rs117113213
rs4307732
rs799157
rs2954021
rs3764261
rs115849089
rs7534572
rs3737810
rs4665972
rs58542926
rs964184
rs3923912
rs75679663
rs6699113
rs1117816
rs687339
rs2618568
rs143424675
rs34233337
rs147711004
rs807055
rs75488469
rs185567543
rs28807203
rs146203232
rs9496567
rs3745681
rs147033490
rs4789227
rs9687846
All - MR Egger
All - Inverse variance weighted
-1
0
1
MR effect size for
'Concentration of very small VLDL particles || id:ebi-cfb233-GCST90302157' on 'ER+ Breast cancer (Combined Oncoarray; iCOGS; GW

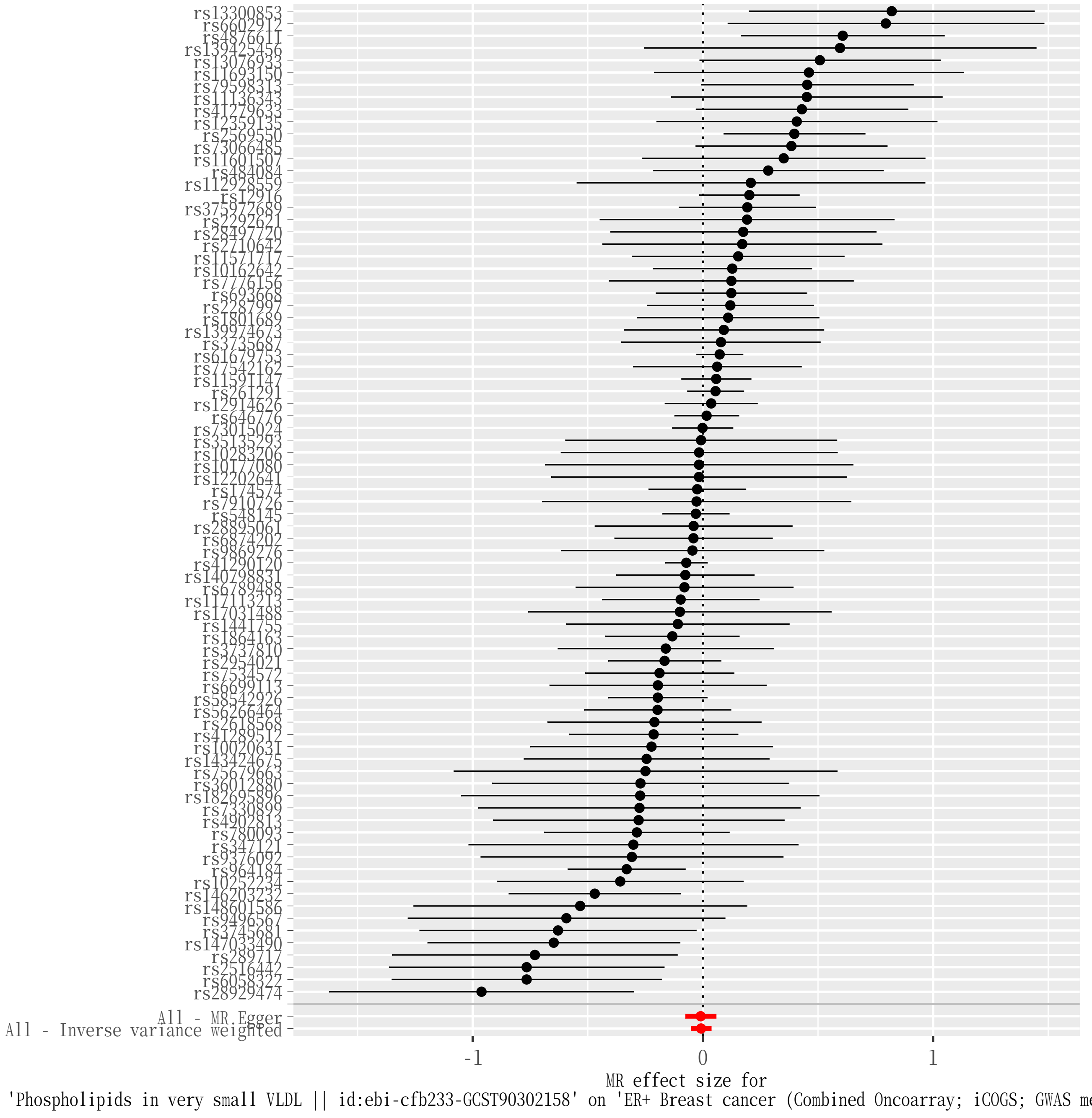

rs13300853
rs6602912
rs4876611
rs139425456
rs13076933
rs11693150
rs79598313
rs11136343
rs41279633
rs12359135
rs2569550
rs73066485
rs11601507
rs484084
rs112928559
rs12916
rs375972689
rs2292621
rs28497720
rs2710642
rs11571717
rs10162642
rs7776156
rs693668
rs2287997
rs1801689
rs139974673
rs3735687
rs61679753
rs77542162
rs11591147
rs261291
rs12914626
rs646776
rs73015024
rs35135293
rs10283206
rs10177080
rs12202641
rs174574
rs7910726
rs548145
rs28895061
rs6874202
rs9869276
rs41290120
rs140798831
rs6789488
rs117113213
rs17031488
rs1441755
rs1864163
rs3737810
rs2954021
rs7534572
rs6699113
rs58542926
rs56266464
rs2618568
rs41289512
rs10020631
rs143424675
rs75679663
rs36012880
rs182695896
rs7330899
rs4902813
rs780093
rs347121
rs9376092
rs964184
rs10252234
rs146203232
rs148601586
rs9496567
rs3745681
rs147033490
rs289717
rs2516442
rs6058322
rs28929474
All - MR.Egger
All - Inverse variance weighted
-1
0
1
MR effect size for
'Phospholipids in very small VLDL || id:ebi-cfb233-GCST90302158' on 'ER+ Breast cancer (Combined Oncoarray; iCOGS; GWAS me

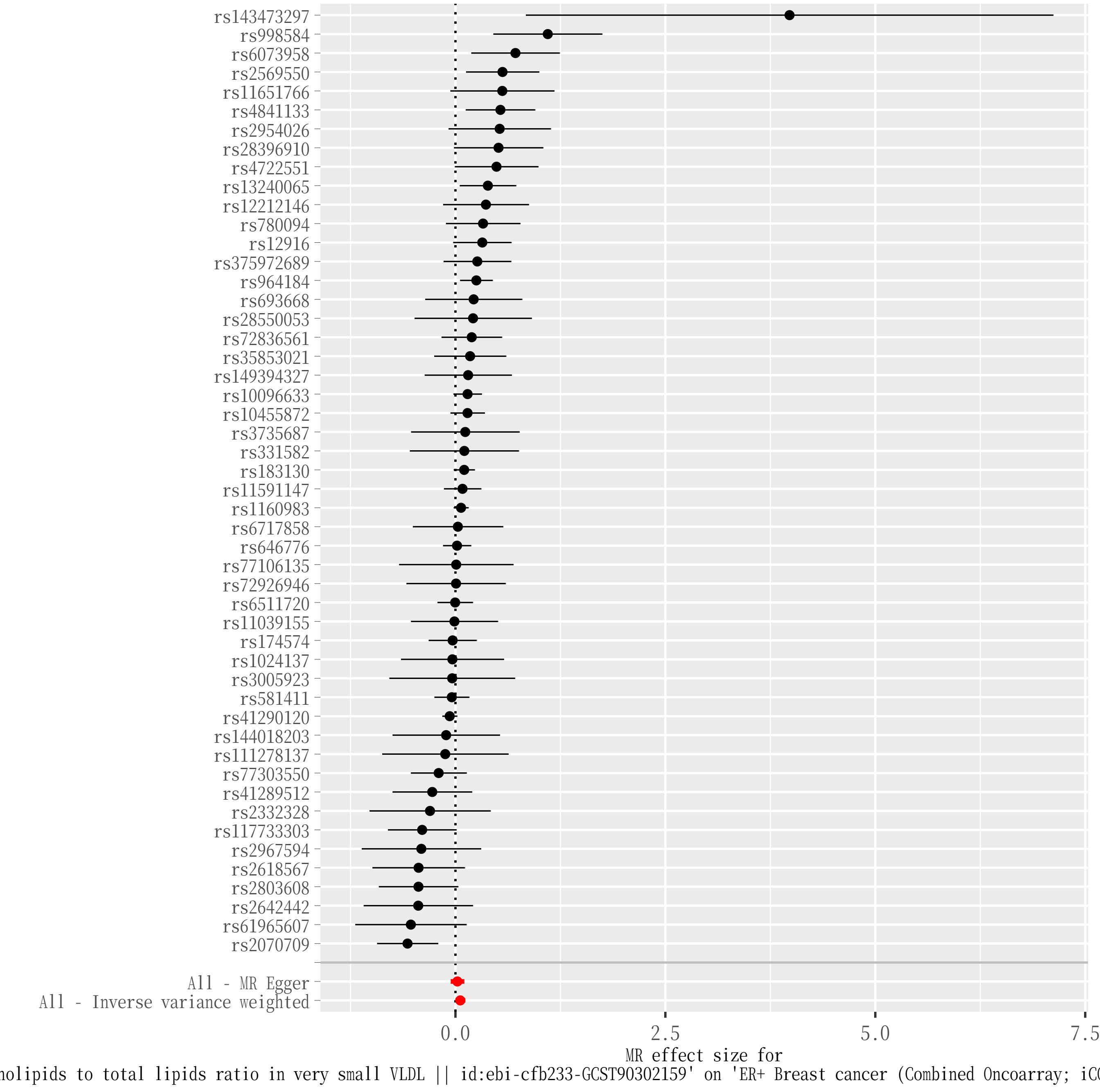
rs143473297
rs998584
rs6073958
rs2569550
rs11651766
rs4841133
rs2954026
rs28396910
rs4722551
rs13240065
rs12212146
rs780094
rs12916
rs375972689
rs964184
rs693668
rs28550053
rs72836561
rs35853021
rs149394327
rs10096633
rs10455872
rs3735687
rs331582
rs183130
rs11591147
rs1160983
rs6717858
rs646776
rs77106135
rs72926946
rs6511720
rs11039155
rs174574
rs1024137
rs3005923
rs581411
rs41290120
rs144018203
rs111278137
rs77303550
rs41289512
rs2332328
rs117733303
rs2967594
rs2618567
rs2803608
rs2642442
rs61965607
rs2070709
All - MR Egger
All - Inverse variance weighted
0.0
2.5
5.0
7.5
MR effect size for
holipids to total lipids ratio in very small VLDL || id:ebi-cfb233-GCST90302159' on 'ER+ Breast cancer (Combined Oncoarray; iC

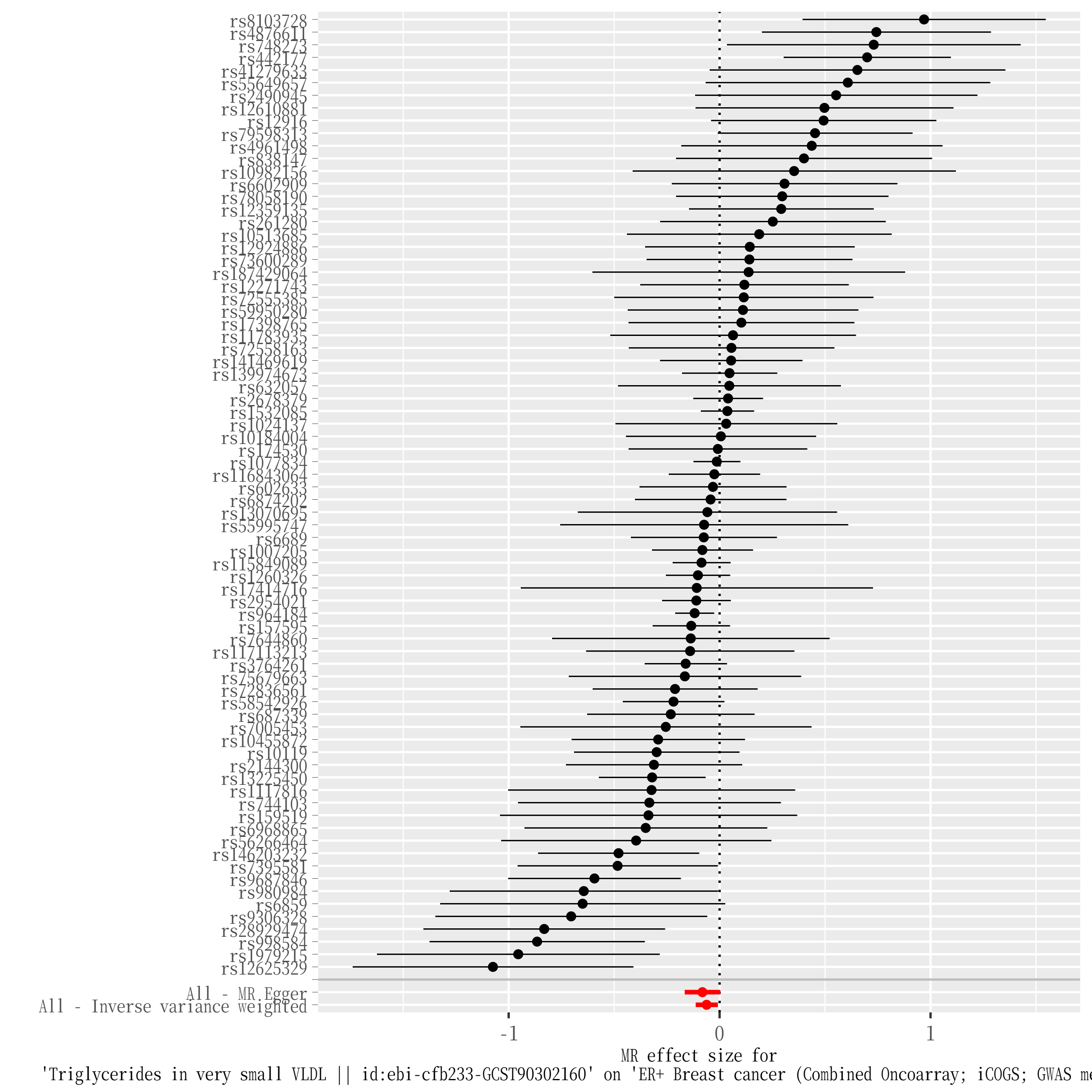
rs8103728
rs4876611
rs748273
rs442177
rs41279633
rs55649657
rs2490945
rs12610881
rs12916
rs79598313
rs4961498
rs838147
rs10982156
rs6602909
rs78058190
rs12359135
rs261280
rs10513685
rs12924886
rs73600289
rs187429064
rs12271743
rs72555385
rs59950280
rs17398765
rs11783935
rs72558163
rs141469619
rs139974673
rs632057
rs2678379
rs1532085
rs1024137
rs10184004
rs174530
rs1077834
rs116843064
rs602633
rs6874202
rs13070695
rs55995747
rs6689
rs1007205
rs115849089
rs1260326
rs17414716
rs2954021
rs964184
rs157595
rs7644860
rs117113213
rs3764261
rs75679663
rs72836561
rs58542926
rs687339
rs7005453
rs10455872
rs10119
rs2144300
rs13225450
rs1117816
rs744103
rs159519
rs6968865
rs56266464
rs146203232
rs7395581
rs9687846
rs980984
rs6859
rs9306328
rs28929474
rs928584
rs1979215
rs12625329
All - MR Egger
All - Inverse variance weighted
-1
0
1
MR effect size for
'Triglycerides in very small VLDL || id:ebi-cfb233-GCST90302160' on 'ER+ Breast cancer (Combined Oncoarray; iCOGS; GWAS me

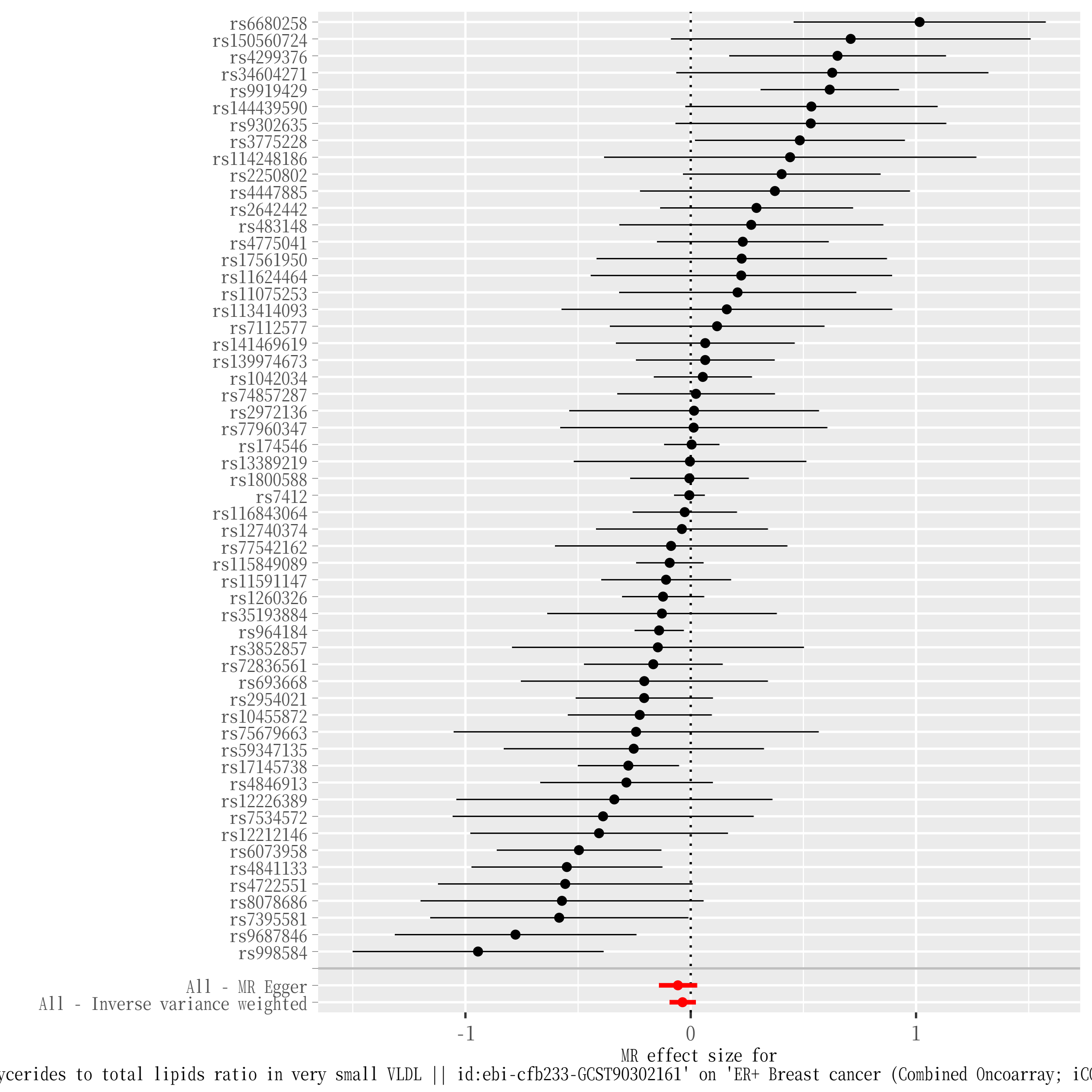
rs6680258
rs150560724
rs4299376
rs34604271
rs9919429
rs144439590
rs9302635
rs3775228
rs114248186
rs2250802
rs4447885
rs2642442
rs483148
rs4775041
rs17561950
rs11624464
rs11075253
rs113414093
rs7112577
rs141469619
rs139974673
rs1042034
rs74857287
rs2972136
rs77960347
rs174546
rs13389219
rs1800588
rs7412
rs116843064
rs12740374
rs77542162
rs115849089
rs11591147
rs1260326
rs35193884
rs964184
rs3852857
rs72836561
rs693668
rs2954021
rs10455872
rs75679663
rs59347135
rs17145738
rs4846913
rs12226389
rs7534572
rs12212146
rs6073958
rs4841133
rs4722551
rs8078686
rs7395581
rs9687846
rs998584
All - MR Egger
All - Inverse variance weighted
-1
0
1
MR effect size for
ycerides to total lipids ratio in very small VLDL || id:ebi-cfb233-GCST90302161' on 'ER+ Breast cancer (Combined Oncoarray; iC

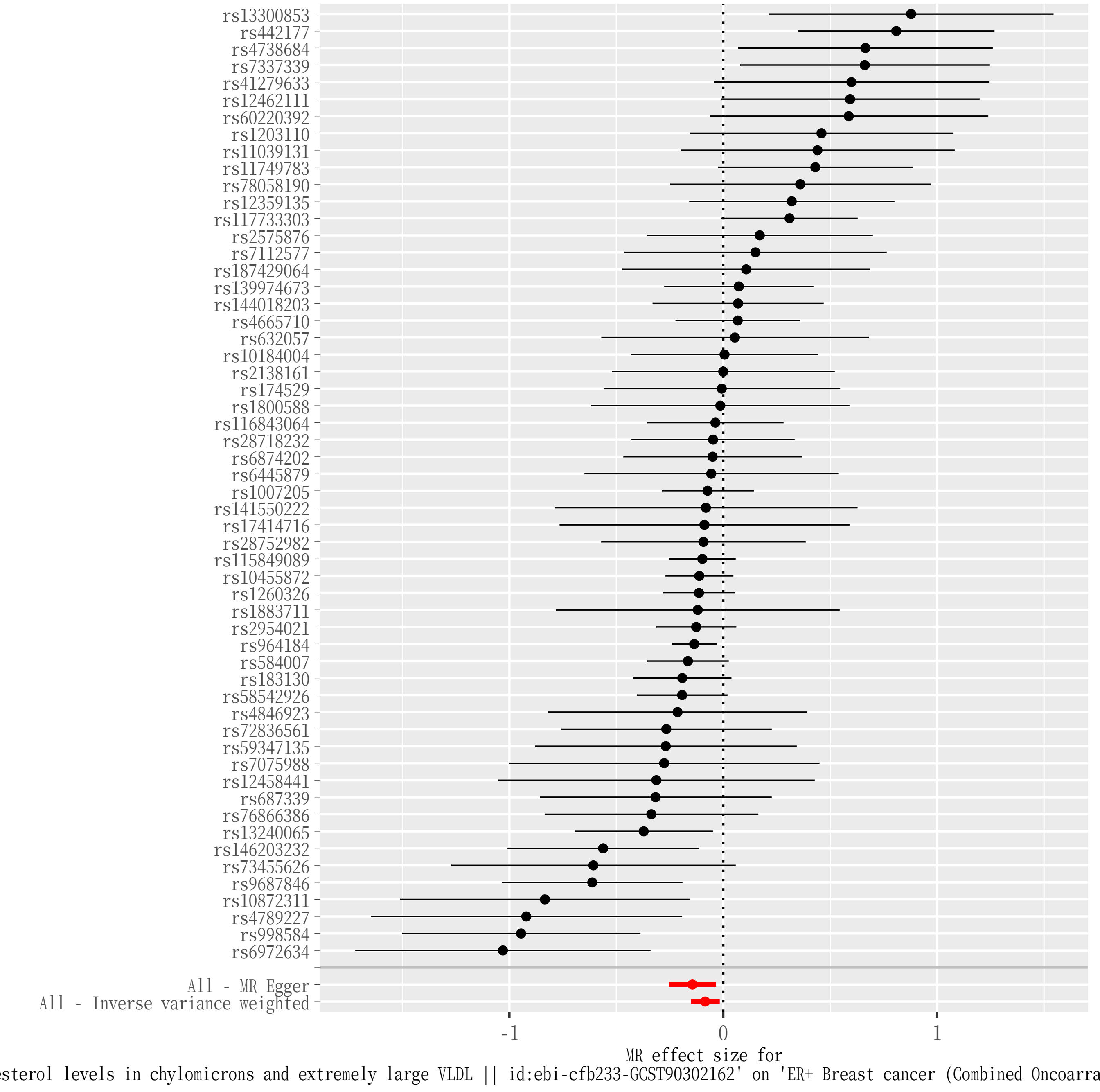

rs13300853
rs442177
rs4738684
rs7337339
rs41279633
rs12462111
rs60220392
rs1203110
rs11039131
rs11749783
rs78058190
rs12359135
rs117733303
rs2575876
rs7112577
rs187429064
rs139974673
rs144018203
rs4665710
rs632057
rs10184004
rs2138161
rs174529
rs1800588
rs116843064
rs28718232
rs6874202
rs6445879
rs1007205
rs141550222
rs17414716
rs28752982
rs115849089
rs10455872
rs1260326
rs1883711
rs2954021
rs964184
rs584007
rs183130
rs58542926
rs4846923
rs72836561
rs59347135
rs7075988
rs12458441
rs687339
rs76866386
rs13240065
rs146203232
rs73455626
rs9687846
rs10872311
rs4789227
rs998584
rs6972634
All - MR Egger
All - Inverse variance weighted
-1
0
1
MR effect size for
esterol levels in chylomicrons and extremely large VLDL || id:ebi-cfb233-GCST90302162' on 'ER+ Breast cancer (Combined Oncoarra

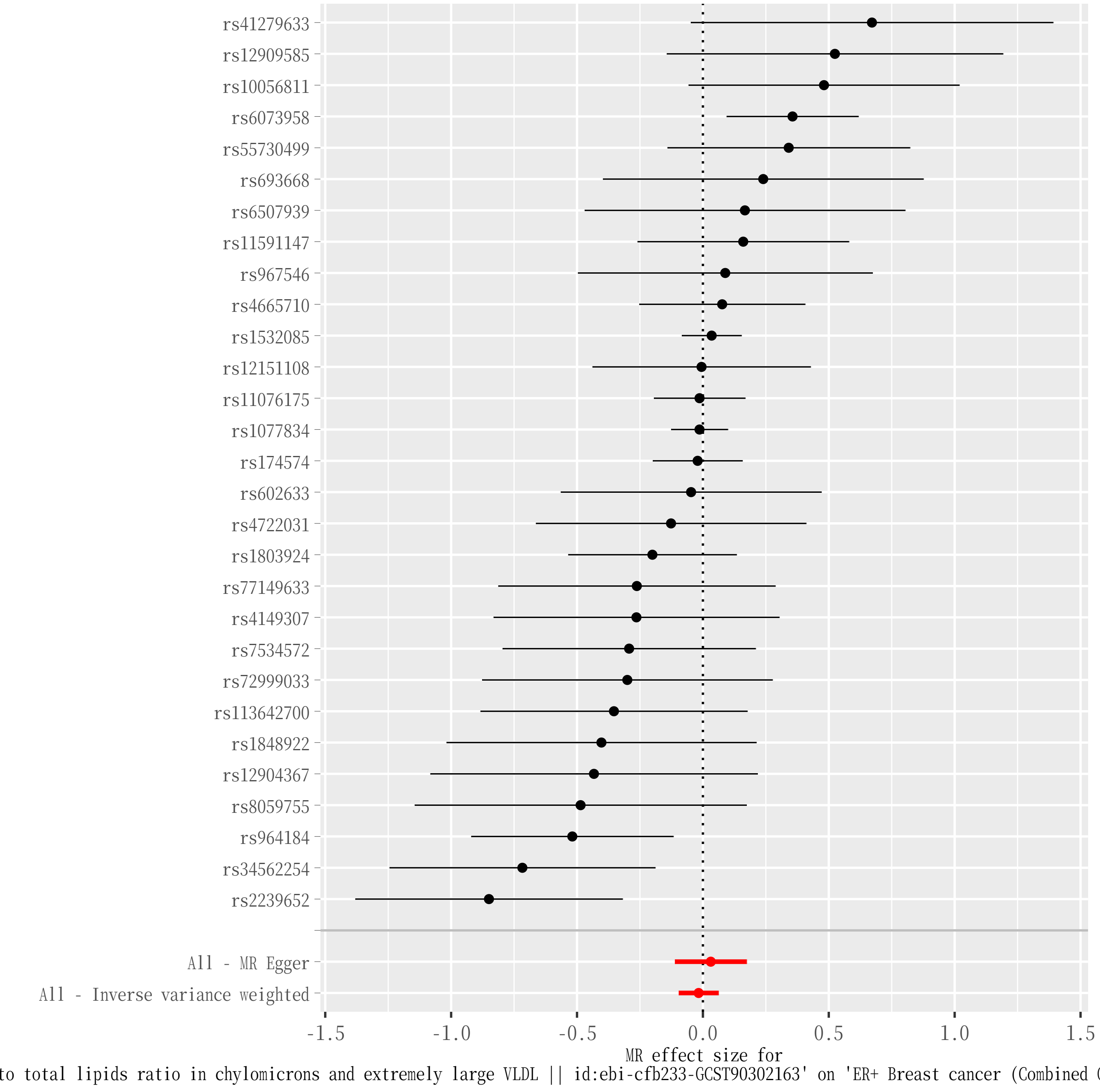
rs41279633
rs12909585
rs10056811
rs6073958
rs55730499
rs693668
rs6507939
rs11591147
rs967546
rs4665710
rs1532085
rs12151108
rs11076175
rs1077834
rs174574
rs602633
rs4722031
rs1803924
rs77149633
rs4149307
rs7534572
rs72999033
rs113642700
rs1848922
rs12904367
rs8059755
rs964184
rs34562254
rs2239652
All - MR Egger
All - Inverse variance weighted
-1.5
-1.0
-0.5
0.0
0.5
1.0
1.5
MR effect size for
to total lipids ratio in chylomicrons and extremely large VLDL || id:ebi-cfb233-GCST90302163' on 'ER+ Breast cancer (Combined (

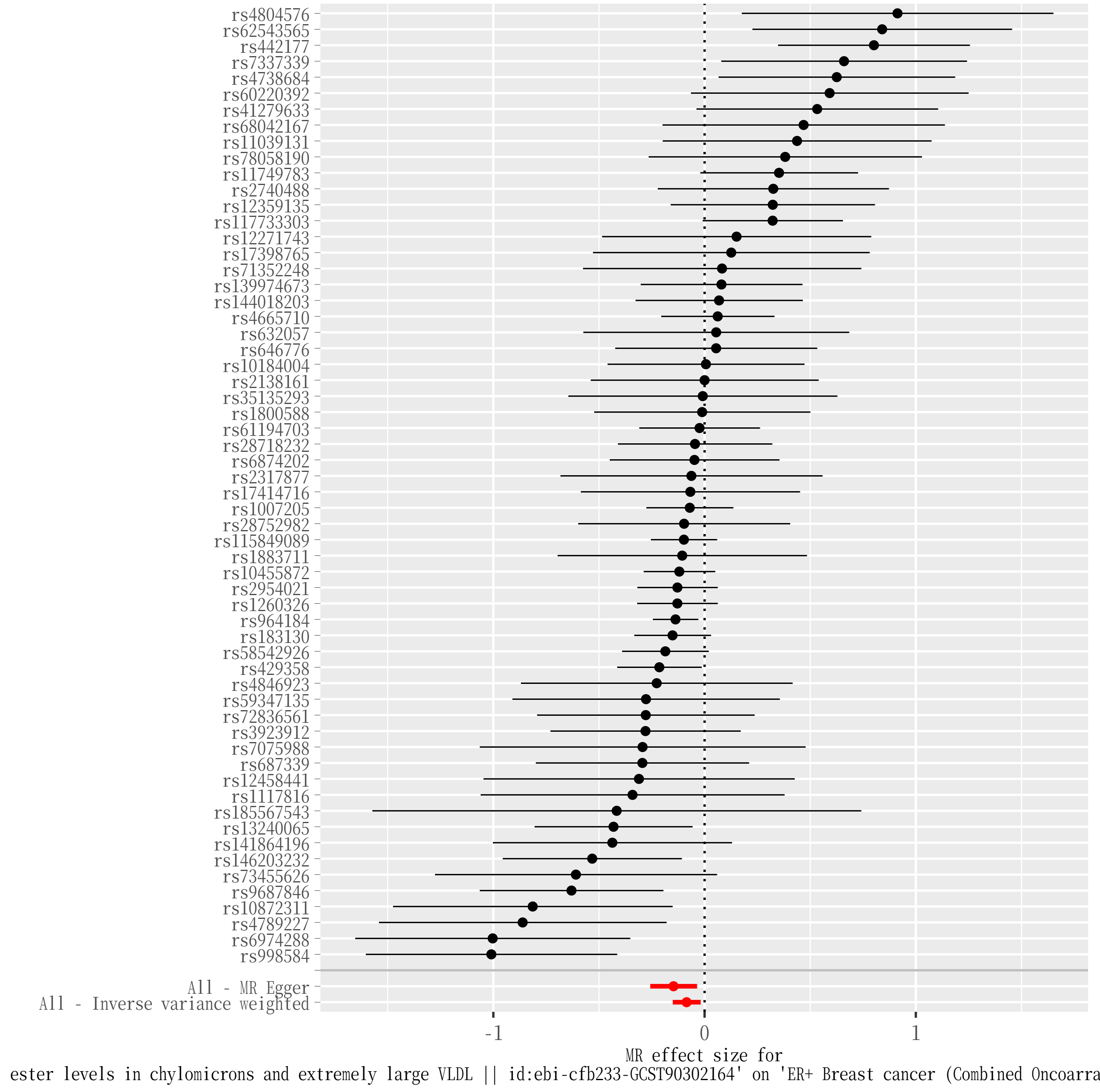
rs4804576
rs62543565
rs442177
rs7337339
rs4738684
rs60220392
rs41279633
rs68042167
rs11039131
rs78058190
rs11749783
rs2740488
rs12359135
rs117733303
rs12271743
rs17398765
rs71352248
rs139974673
rs144018203
rs4665710
rs632057
rs646776
rs10184004
rs2138161
rs35135293
rs1800588
rs61194703
rs28718232
rs6874202
rs2317877
rs17414716
rs1007205
rs28752982
rs115849089
rs1883711
rs10455872
rs2954021
rs1260326
rs964184
rs183130
rs58542926
rs429358
rs4846923
rs59347135
rs72836561
rs3923912
rs7075988
rs687339
rs12458441
rs1117816
rs185567543
rs13240065
rs141864196
rs146203232
rs73455626
rs9687846
rs10872311
rs4789227
rs6974288
rs998584
All - MR Egger
All - Inverse variance weighted
-1
0
1
MR effect size for
ester levels in chylomicrons and extremely large VLDL || id:ebi-cfb233-GCST90302164' on 'ER+ Breast cancer (Combined Oncoarra

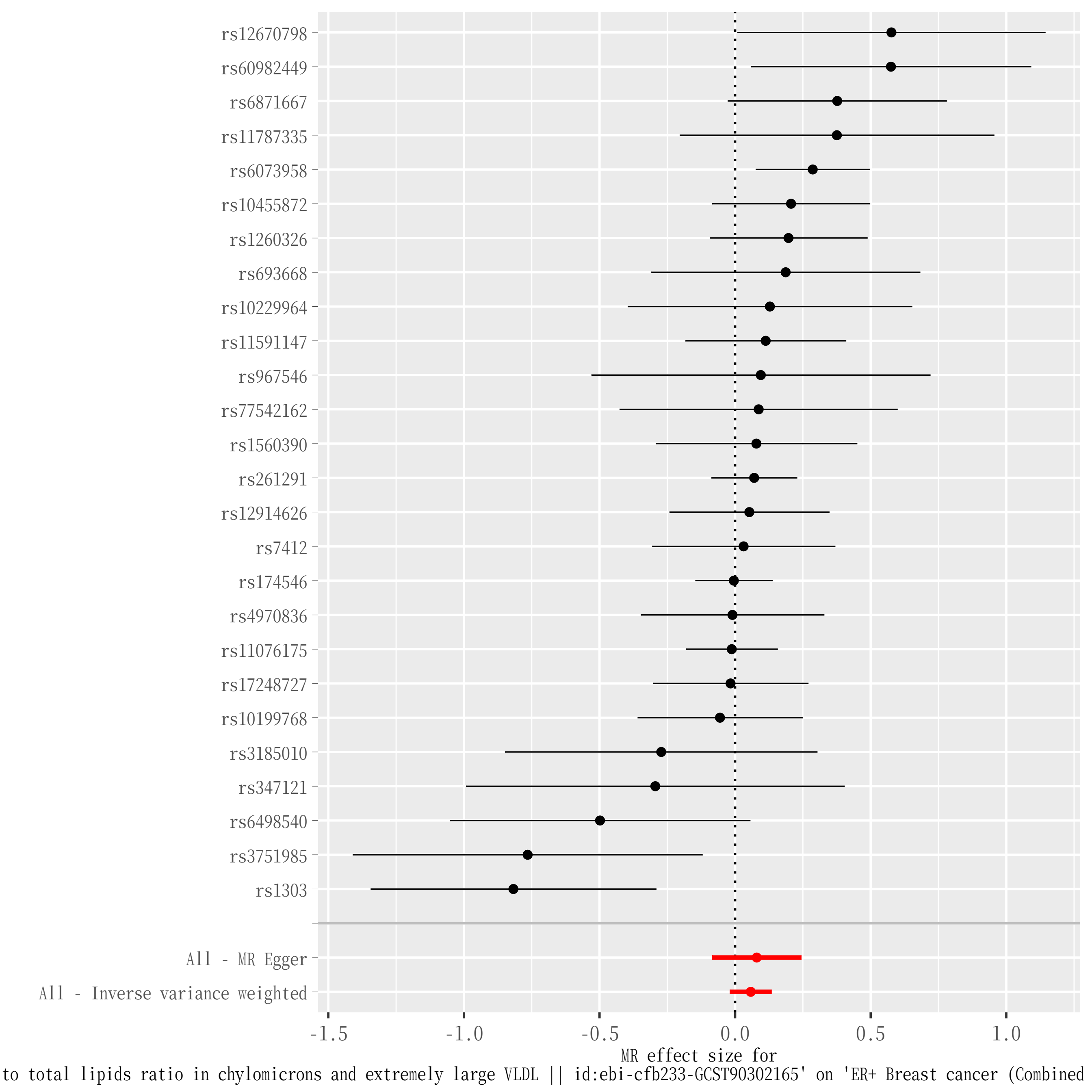
rs12670798
rs60982449
rs6871667
rs11787335
rs6073958
rs10455872
rs1260326
rs693668
rs10229964
rs11591147
rs967546
rs77542162
rs1560390
rs261291
rs12914626
rs7412
rs174546
rs4970836
rs11076175
rs17248727
rs10199768
rs3185010
rs347121
rs6498540
rs3751985
rs1303
All - MR Egger
All - Inverse variance weighted
-1.5
-1.0
-0.5
0.0
0.5
1.0
MR effect size for
to total lipids ratio in chylomicrons and extremely large VLDL || id:ebi-cfb233-GCST90302165' on 'ER+ Breast cancer (Combined

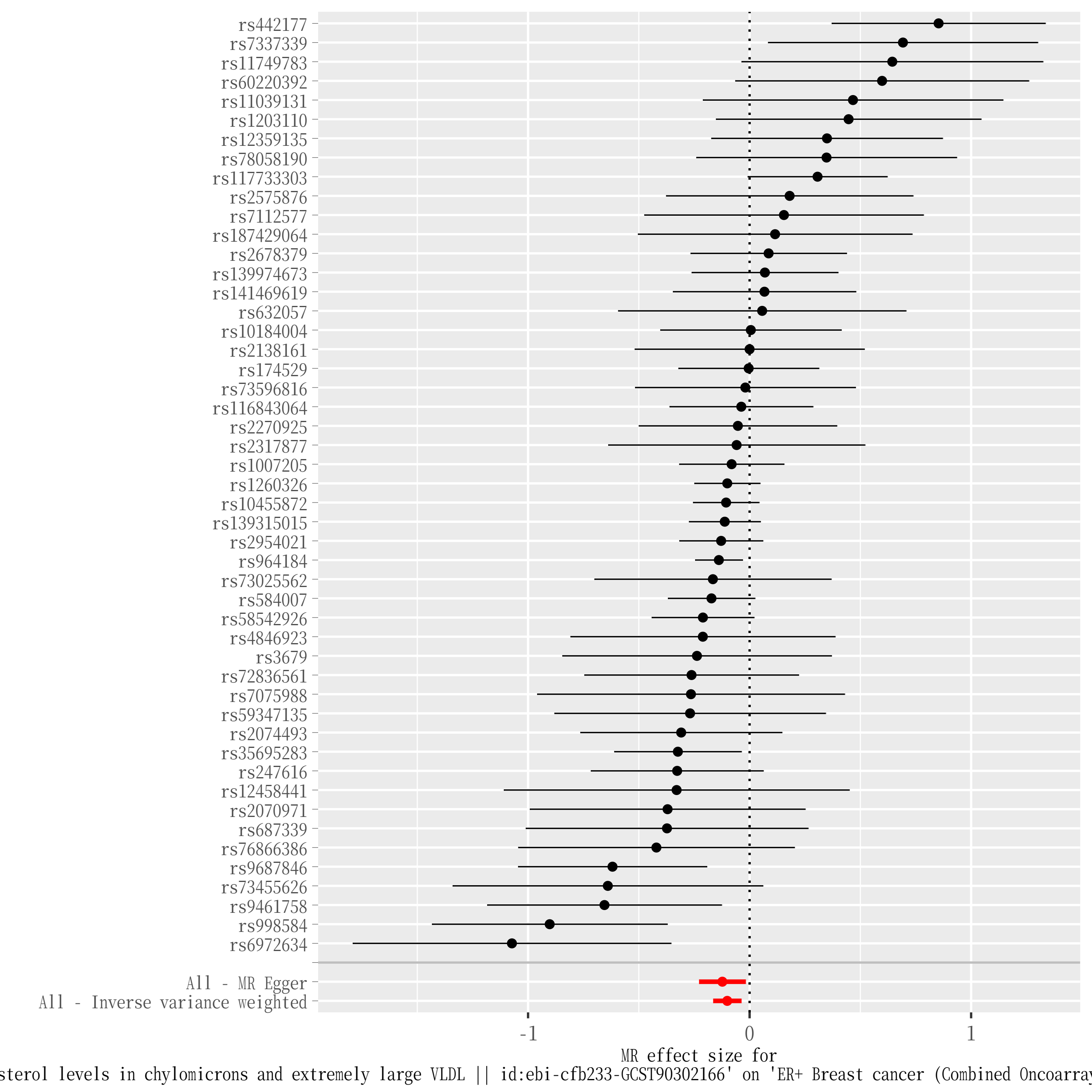

rs442177
rs7337339
rs11749783
rs60220392
rs11039131
rs1203110
rs12359135
rs78058190
rs117733303
rs2575876
rs7112577
rs187429064
rs2678379
rs139974673
rs141469619
rs632057
rs10184004
rs2138161
rs174529
rs73596816
rs116843064
rs2270925
rs2317877
rs1007205
rs1260326
rs10455872
rs139315015
rs2954021
rs964184
rs73025562
rs584007
rs58542926
rs4846923
rs3679
rs72836561
rs7075988
rs59347135
rs2074493
rs35695283
rs247616
rs12458441
rs2070971
rs687339
rs76866386
rs9687846
rs73455626
rs9461758
rs998584
rs6972634
All - MR Egger
All - Inverse variance weighted
-1
0
1
MR effect size for
sterol levels in chylomicrons and extremely large VLDL || id:ebi-cfb233-GCST90302166' on 'ER+ Breast cancer (Combined Oncoarray

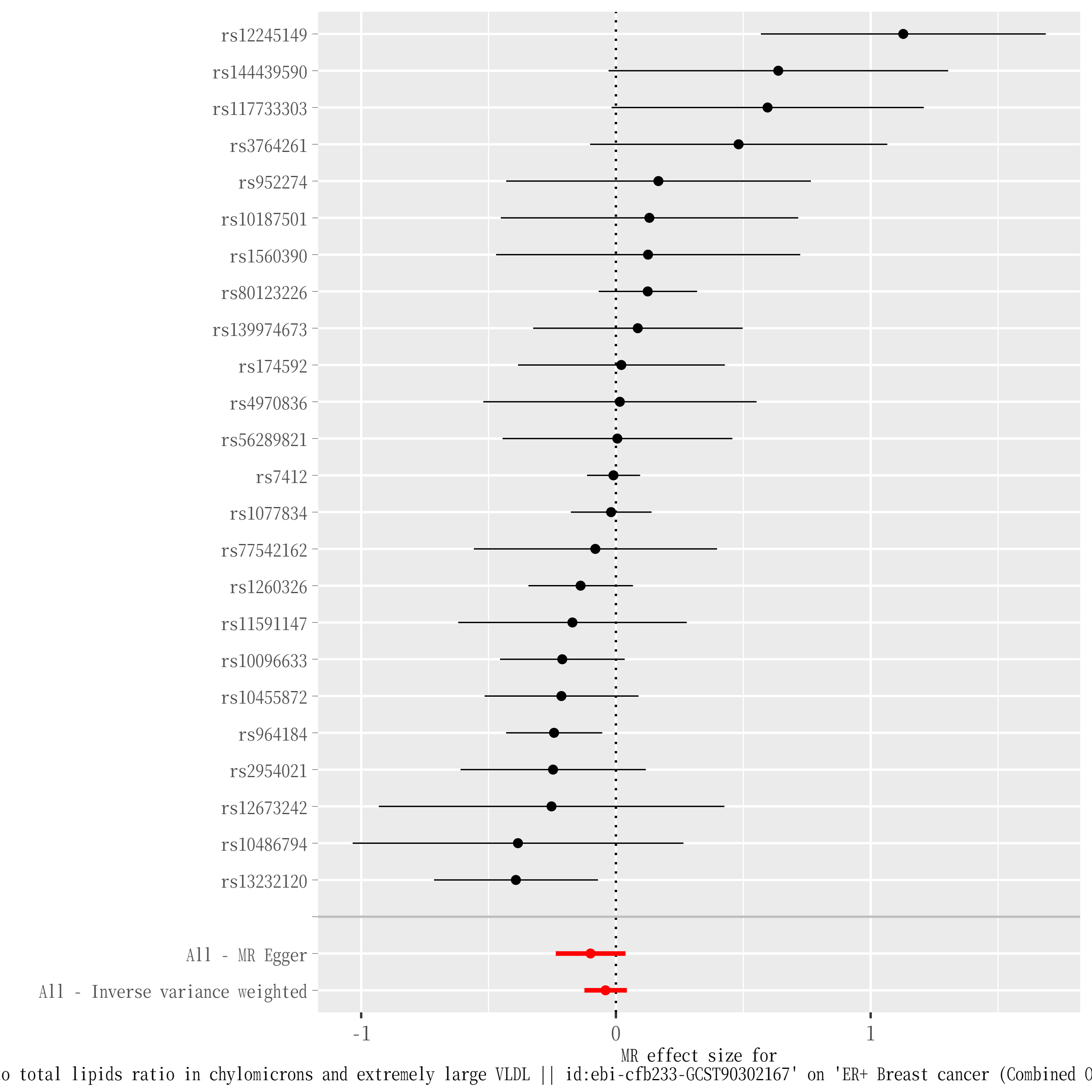
rs12245149
rs144439590
rs117733303
rs3764261
rs952274
rs10187501
rs1560390
rs80123226
rs139974673
rs174592
rs4970836
rs56289821
rs7412
rs1077834
rs77542162
rs1260326
rs11591147
rs10096633
rs10455872
rs964184
rs2954021
rs12673242
rs10486794
rs13232120
All - MR Egger
All - Inverse variance weighted
-1
0
1
MR effect size for
o total lipids ratio in chylomicrons and extremely large VLDL || id:ebi-cfb233-GCST90302167' on 'ER+ Breast cancer (Combined O

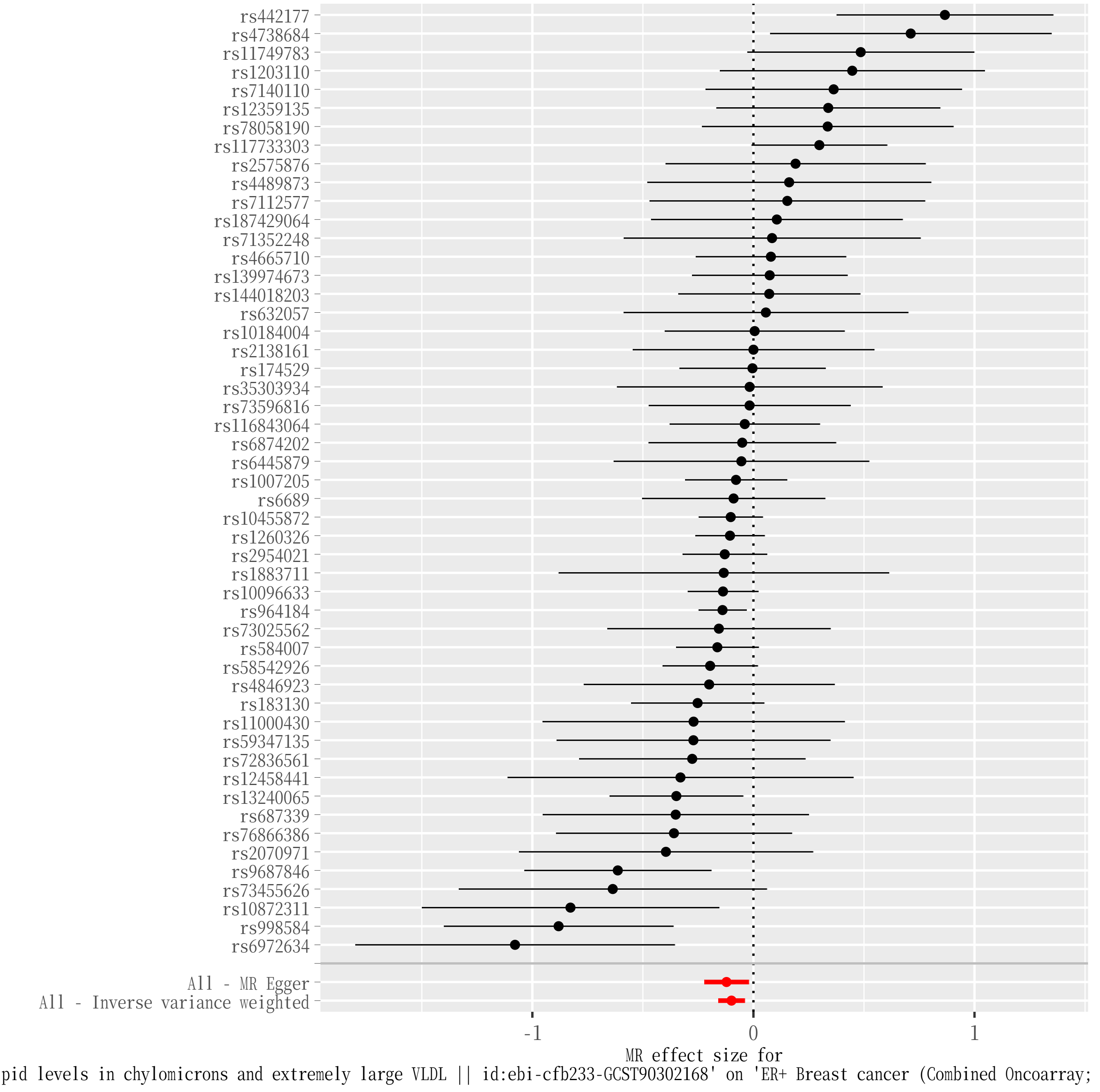
rs442177
rs4738684
rs11749783
rs1203110
rs7140110
rs12359135
rs78058190
rs117733303
rs2575876
rs4489873
rs7112577
rs187429064
rs71352248
rs4665710
rs139974673
rs144018203
rs632057
rs10184004
rs2138161
rs174529
rs35303934
rs73596816
rs116843064
rs6874202
rs6445879
rs1007205
rs6689
rs10455872
rs1260326
rs2954021
rs1883711
rs10096633
rs964184
rs73025562
rs584007
rs58542926
rs4846923
rs183130
rs11000430
rs59347135
rs72836561
rs12458441
rs13240065
rs687339
rs76866386
rs2070971
rs9687846
rs73455626
rs10872311
rs998584
rs6972634
All - MR Egger
All - Inverse variance weighted
-1
0
1
MR effect size for
pid levels in chylomicrons and extremely large VLDL || id:ebi-cfb233-GCST90302168' on 'ER+ Breast cancer (Combined Oncoarray;

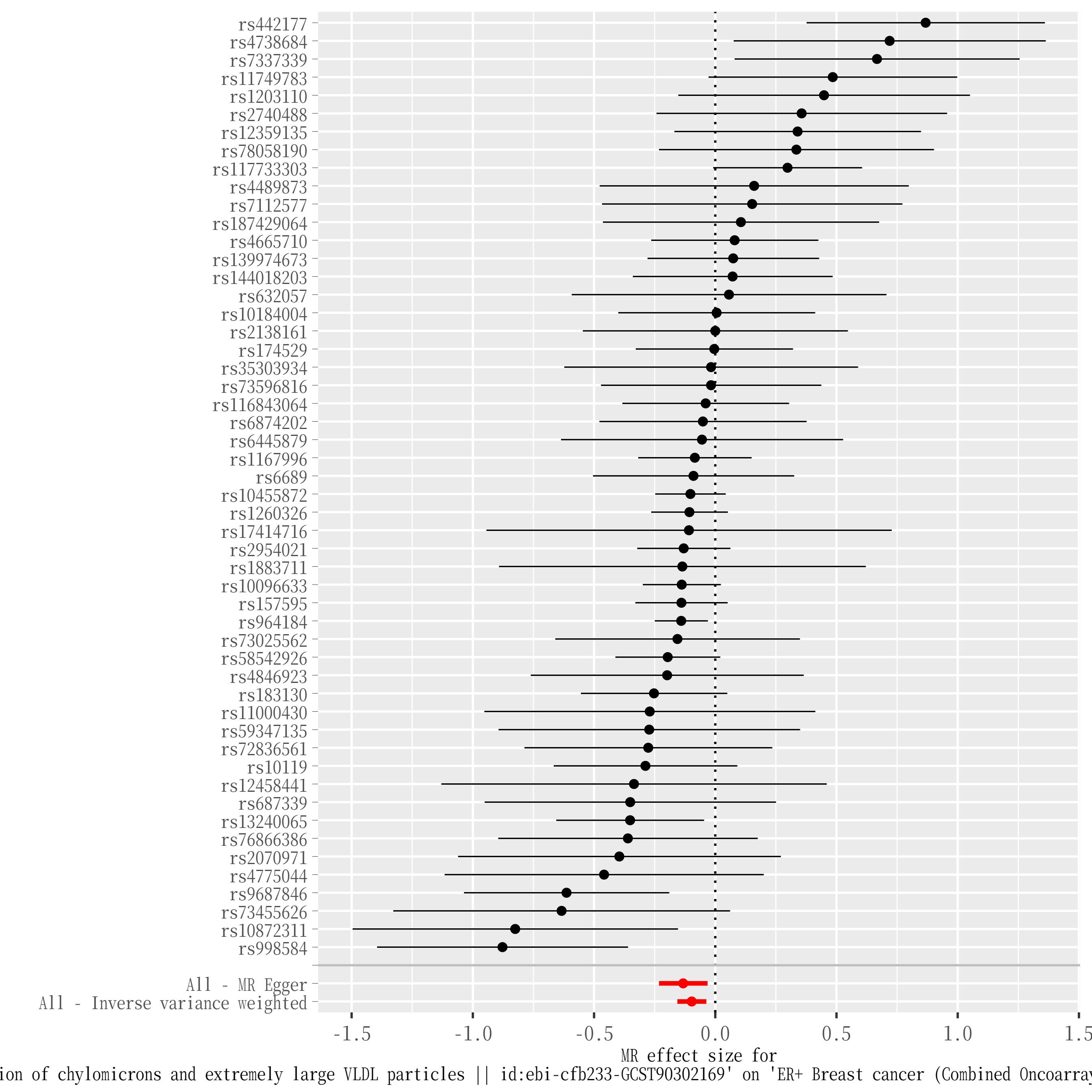
rs442177
rs4738684
rs7337339
rs11749783
rs1203110
rs2740488
rs12359135
rs78058190
rs117733303
rs4489873
rs7112577
rs187429064
rs4665710
rs139974673
rs144018203
rs632057
rs10184004
rs2138161
rs174529
rs35303934
rs73596816
rs116843064
rs6874202
rs6445879
rs1167996
rs6689
rs10455872
rs1260326
rs17414716
rs2954021
rs1883711
rs10096633
rs157595
rs964184
rs73025562
rs58542926
rs4846923
rs183130
rs11000430
rs59347135
rs72836561
rs10119
rs12458441
rs687339
rs13240065
rs76866386
rs2070971
rs4775044
rs9687846
rs73455626
rs10872311
rs998584
All - MR Egger
All - Inverse variance weighted
-1.5
-1.0
-0.5
0.0
0.5
1.0
1.5
MR effect size for
ion of chylomicrons and extremely large VLDL particles || id:ebi-cfb233-GCST90302169' on 'ER+ Breast cancer (Combined Oncoarray

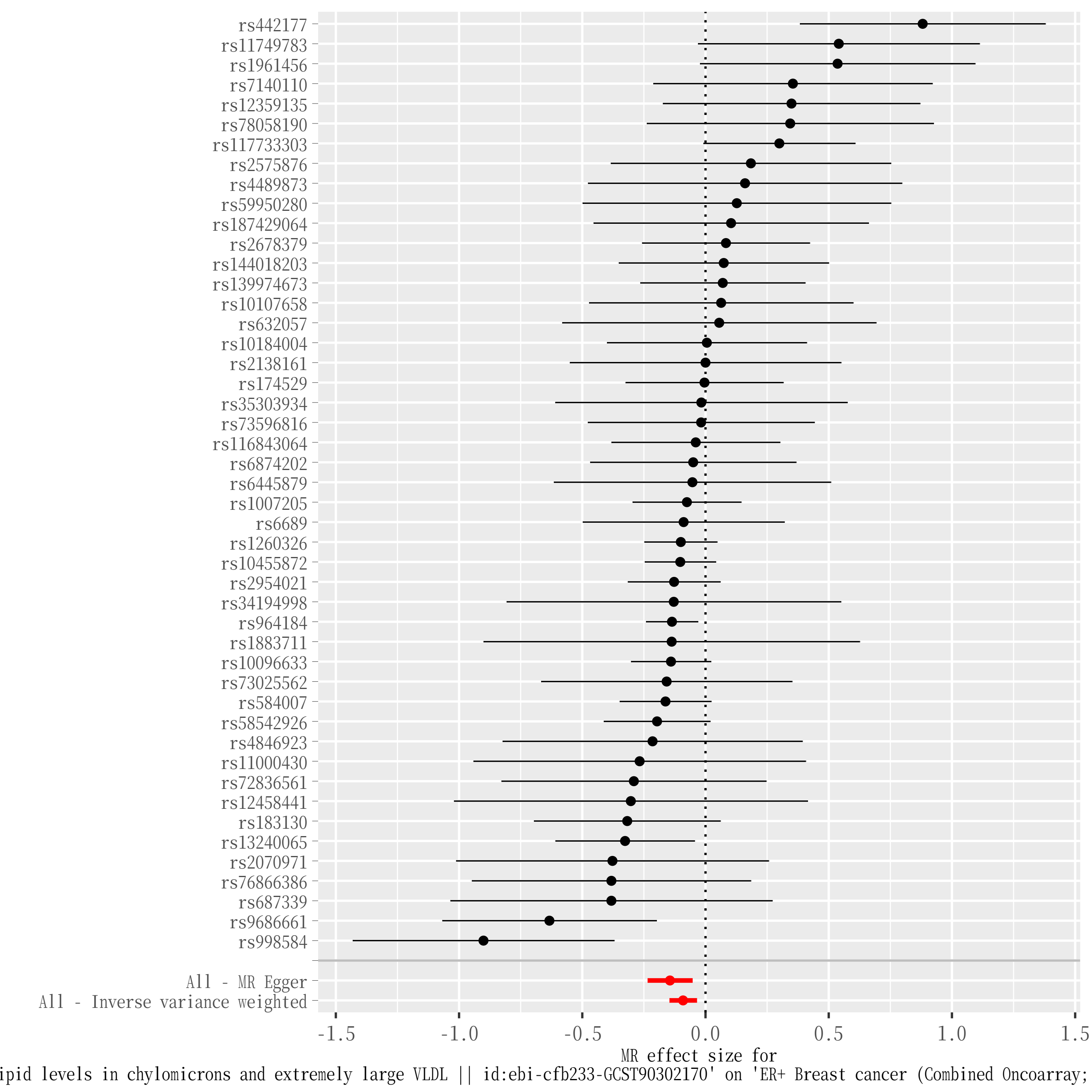

rs442177
rs11749783
rs1961456
rs7140110
rs12359135
rs78058190
rs117733303
rs2575876
rs4489873
rs59950280
rs187429064
rs2678379
rs144018203
rs139974673
rs10107658
rs632057
rs10184004
rs2138161
rs174529
rs35303934
rs73596816
rs116843064
rs6874202
rs6445879
rs1007205
rs6689
rs1260326
rs10455872
rs2954021
rs34194998
rs964184
rs1883711
rs10096633
rs73025562
rs584007
rs58542926
rs4846923
rs11000430
rs72836561
rs12458441
rs183130
rs13240065
rs2070971
rs76866386
rs687339
rs9686661
rs998584
All - MR Egger
All - Inverse variance weighted
-1.5
-1.0
-0.5
0.0
0.5
1.0
1.5
MR effect size for
ipid levels in chylomicrons and extremely large VLDL || id:ebi-cfb233-GCST90302170' on 'ER+ Breast cancer (Combined Oncoarray;

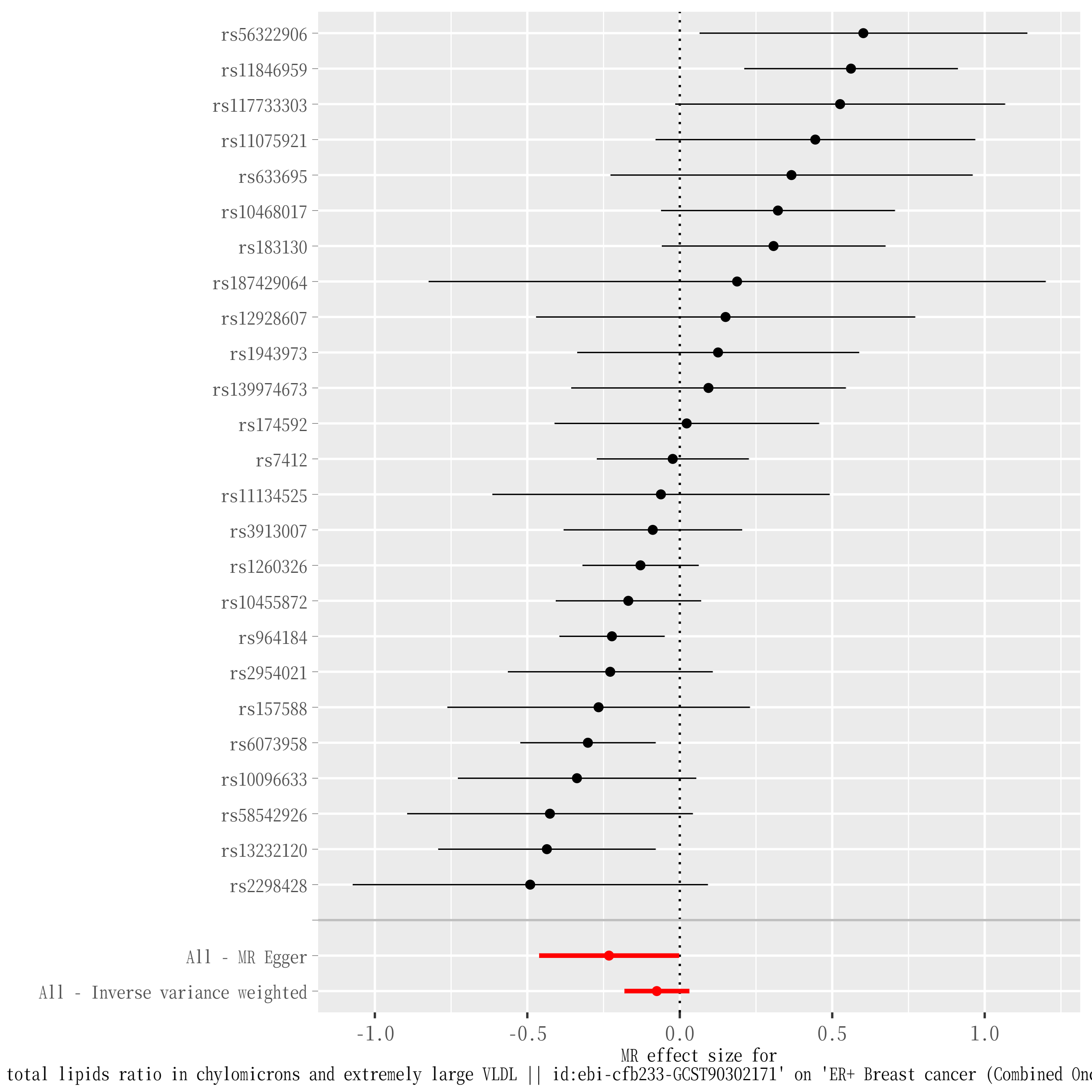
rs56322906
rs11846959
rs117733303
rs11075921
rs633695
rs10468017
rs183130
rs187429064
rs12928607
rs1943973
rs139974673
rs174592
rs7412
rs11134525
rs3913007
rs1260326
rs10455872
rs964184
rs2954021
rs157588
rs6073958
rs10096633
rs58542926
rs13232120
rs2298428
All - MR Egger
All - Inverse variance weighted
-1.0
-0.5
0.0
0.5
1.0
MR effect size for
total lipids ratio in chylomicrons and extremely large VLDL || id:ebi-cfb233-GCST90302171' on 'ER+ Breast cancer (Combined On

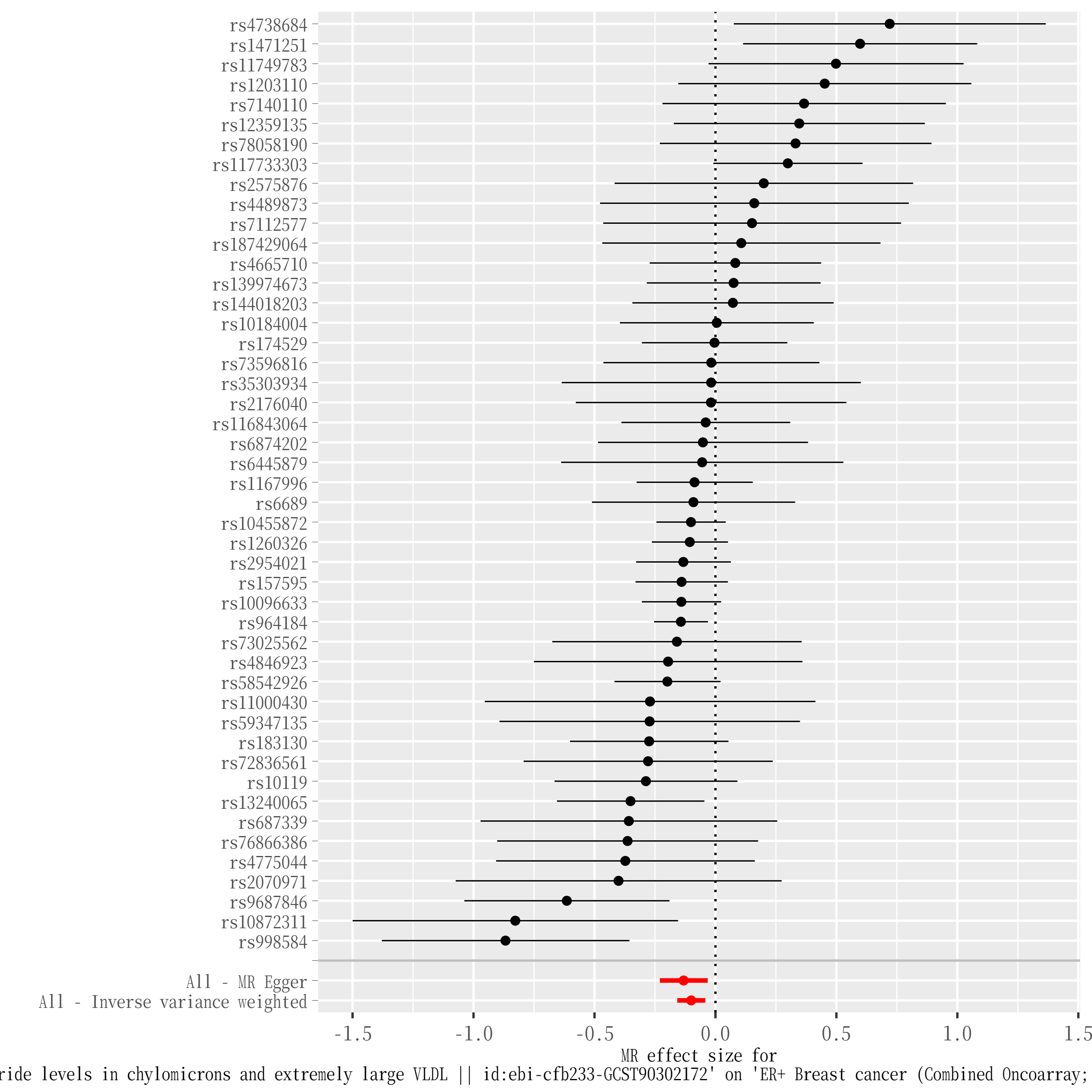
rs4738684
rs1471251
rs11749783
rs1203110
rs7140110
rs12359135
rs78058190
rs117733303
rs2575876
rs4489873
rs7112577
rs187429064
rs4665710
rs139974673
rs144018203
rs10184004
rs174529
rs73596816
rs35303934
rs2176040
rs116843064
rs6874202
rs6445879
rs1167996
rs6689
rs10455872
rs1260326
rs2954021
rs157595
rs10096633
rs964184
rs73025562
rs4846923
rs58542926
rs11000430
rs59347135
rs183130
rs72836561
rs10119
rs13240065
rs687339
rs76866386
rs4775044
rs2070971
rs9687846
rs10872311
rs998584
All - MR Egger
All - Inverse variance weighted
-1.5
-1.0
-0.5
0.0
0.5
1.0
1.5
MR effect size for
ride levels in chylomicrons and extremely large VLDL || id:ebi-cfb233-GCST90302172' on 'ER+ Breast cancer (Combined Oncoarray;

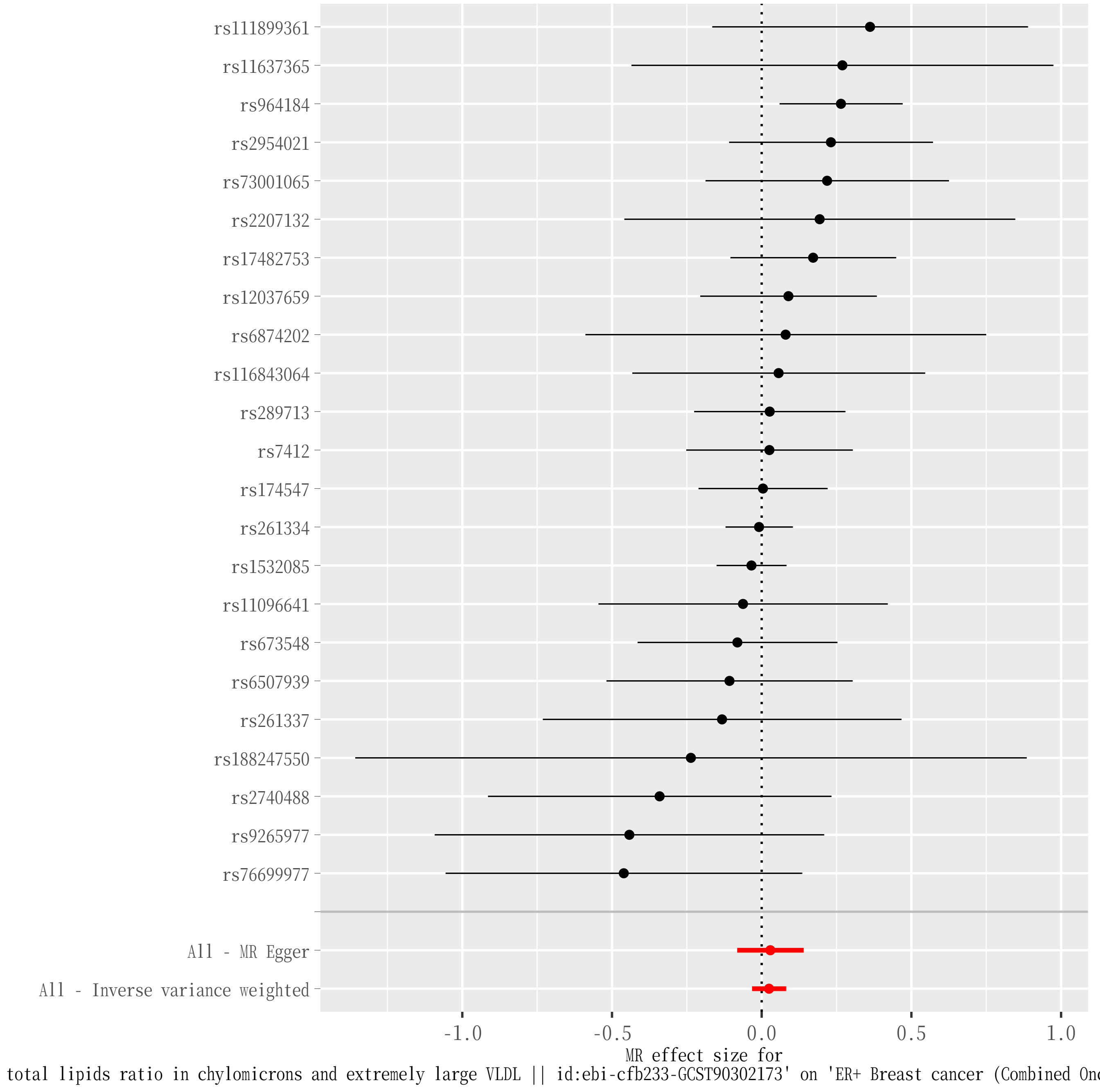
rs111899361
rs11637365
rs964184
rs2954021
rs73001065
rs2207132
rs17482753
rs12037659
rs6874202
rs116843064
rs289713
rs7412
rs174547
rs261334
rs1532085
rs11096641
rs673548
rs6507939
rs261337
rs188247550
rs2740488
rs9265977
rs76699977
All - MR Egger
All - Inverse variance weighted
-1.0
-0.5
0.0
0.5
1.0
MR effect size for
total lipids ratio in chylomicrons and extremely large VLDL || id:ebi-cfb233-GCST90302173' on 'ER+ Breast cancer (Combined On
