# Supplementary material for: Assessing the causal relationships between circulating metabolic biomarkers and breast cancer by using mendelian randomization
Source: Front Genet. 2024 Dec 18;15:1448748. doi: 10.3389/fgene.2024.1448748 (PMC11688392; doi:10.3389/fgene.2024.1448748)
Supplement: Supplementary file 10 [file DataSheet3.pdf]

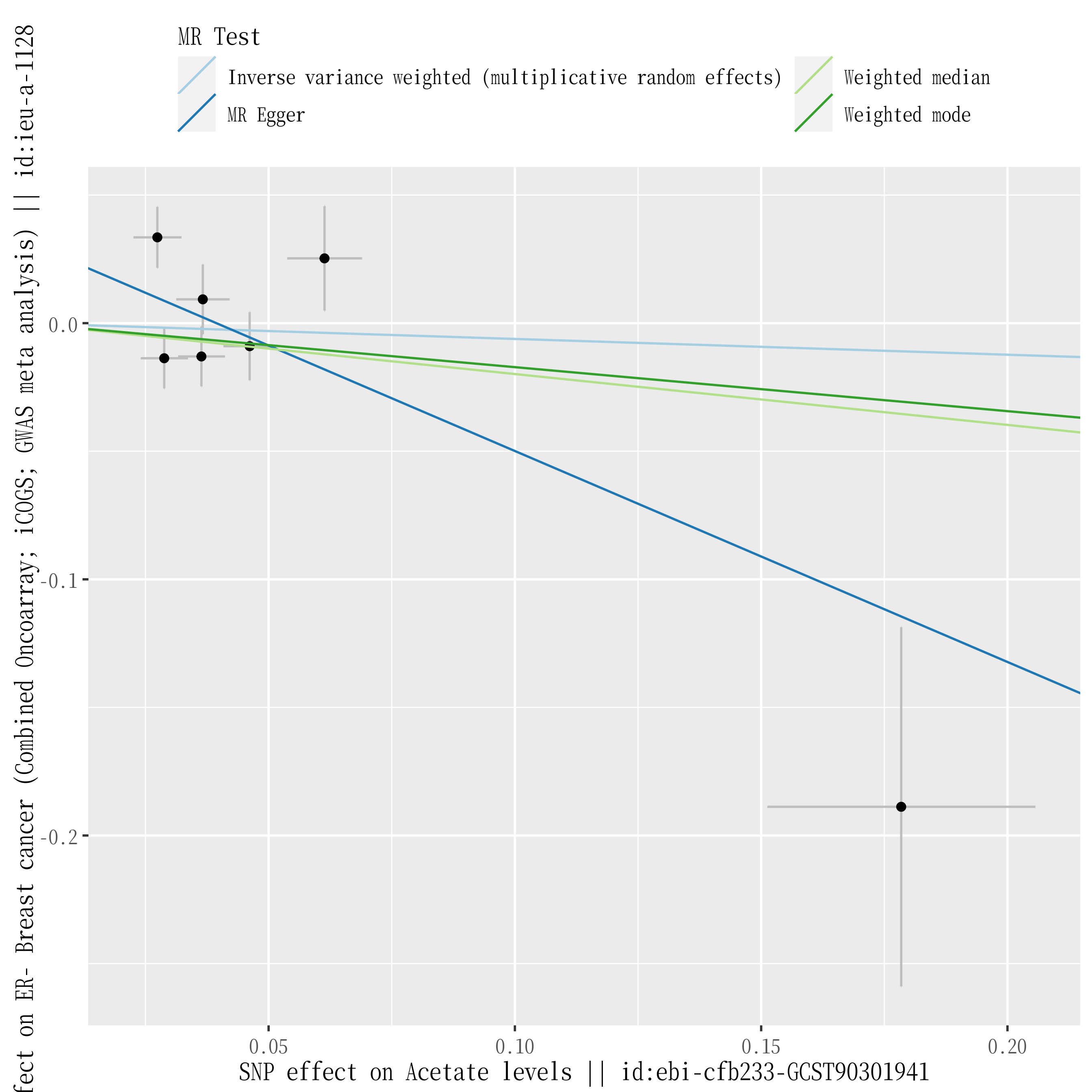
MR Test
Inverse variance weighted (multiplicative random effects)
MR Egger
Weighted median
Weighted mode
0.0
-0.1
-0.2
0.05
0.10
0.15
0.20
SNP effect on Acetate levels || id:ebi-cfb233-GCST90301941
fect on ER- Breast cancer (Combined Oncoarray; iCOGS; GWAS meta analysis) || id:ieu-a-1128

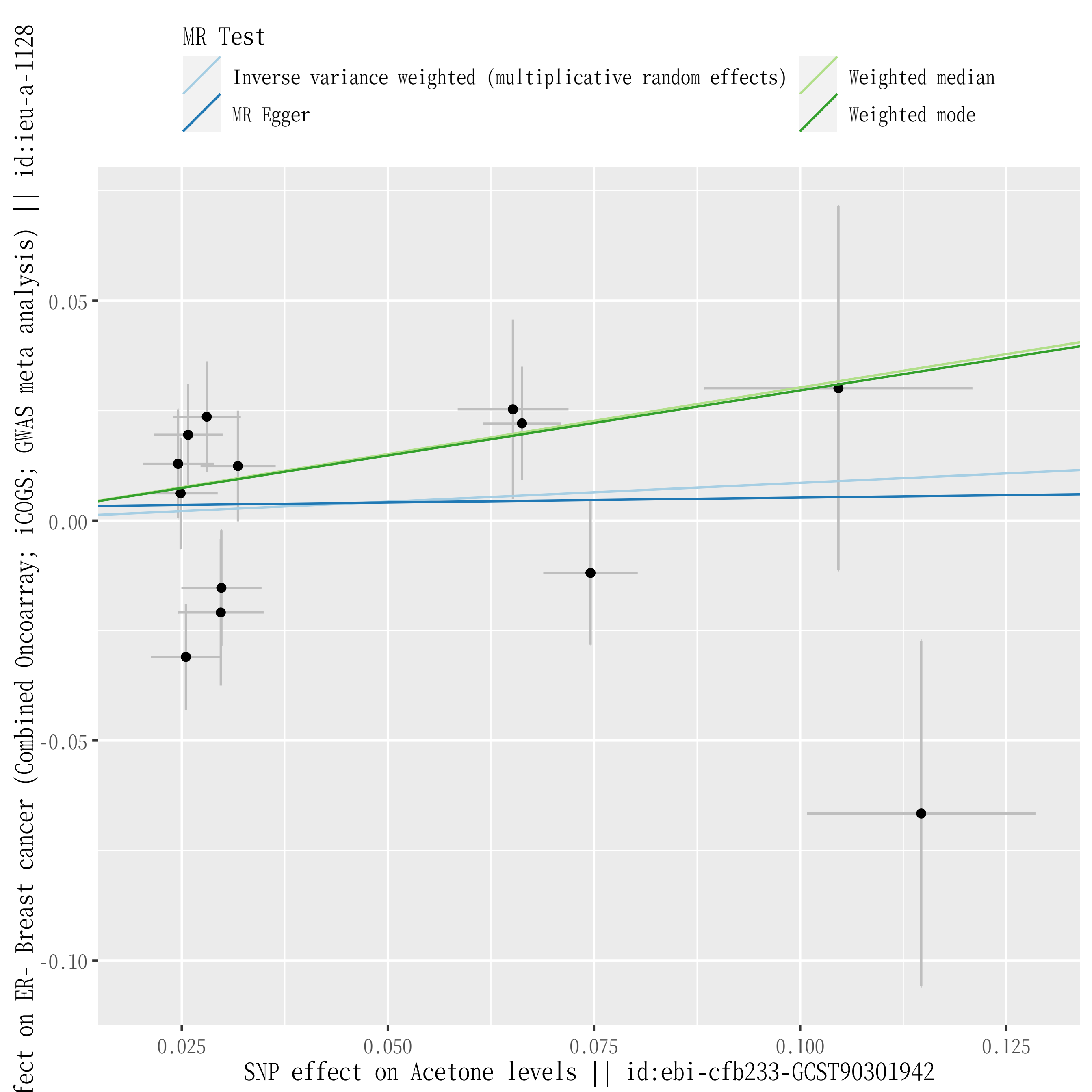
MR Test
Inverse variance weighted (multiplicative random effects)
MR Egger
Weighted median
Weighted mode
0.05
0.00
-0.05
-0.10
0.025
0.050
0.075
0.100
0.125
SNP effect on Acetone levels || id:ebi-cfb233-GCST90301942
fect on ER- Breast cancer (Combined Oncoarray; iCOGS; GWAS meta analysis) || id:ieu-a-1128

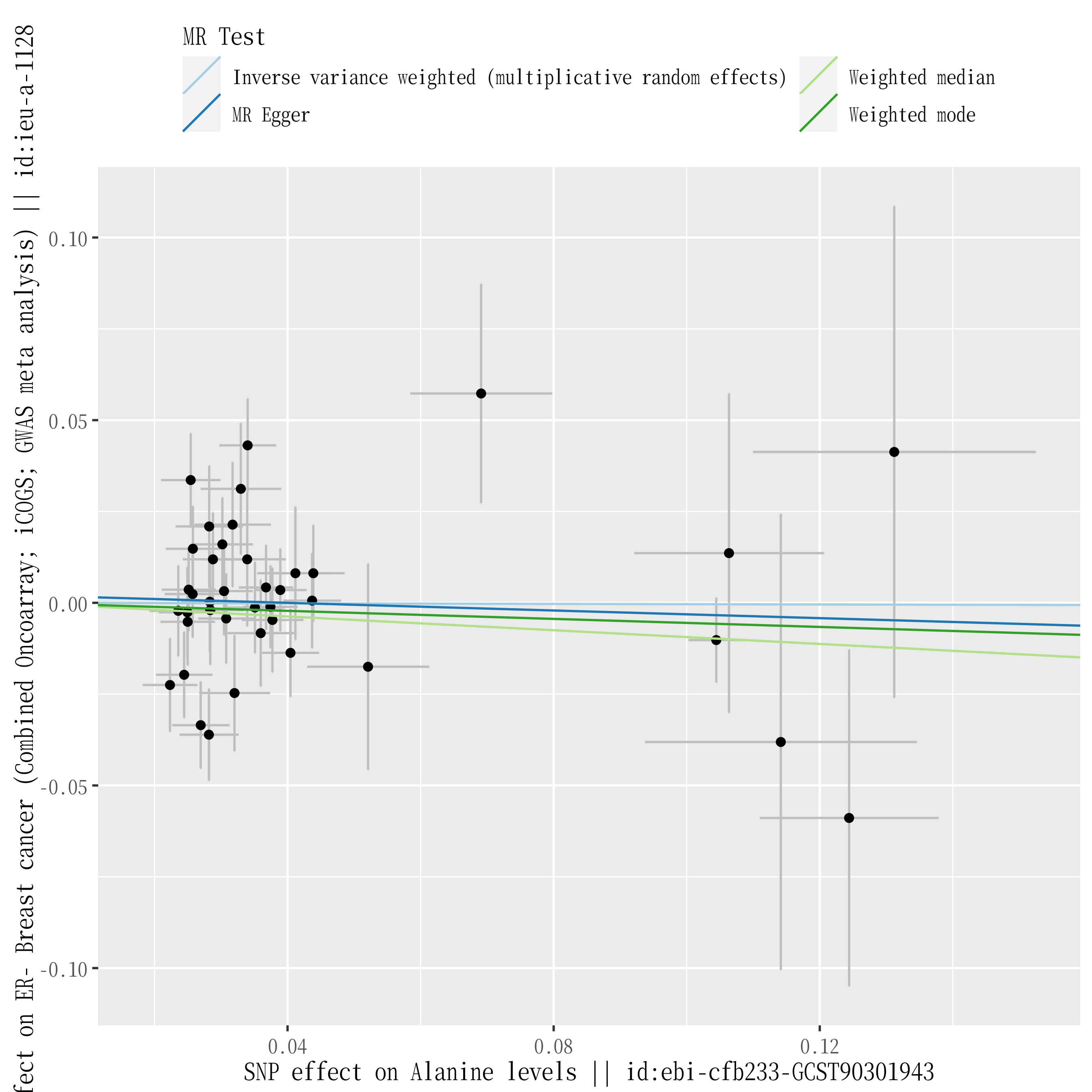

MR Test
Inverse variance weighted (multiplicative random effects)
MR Egger
Weighted median
Weighted mode
0.10
0.05
0.00
-0.05
-0.10
0.04
0.08
0.12
SNP effect on Alanine levels || id:ebi-cfb233-GCST90301943
fect on ER- Breast cancer (Combined Oncoarray; iCOGS; GWAS meta analysis) || id:ieu-a-1128

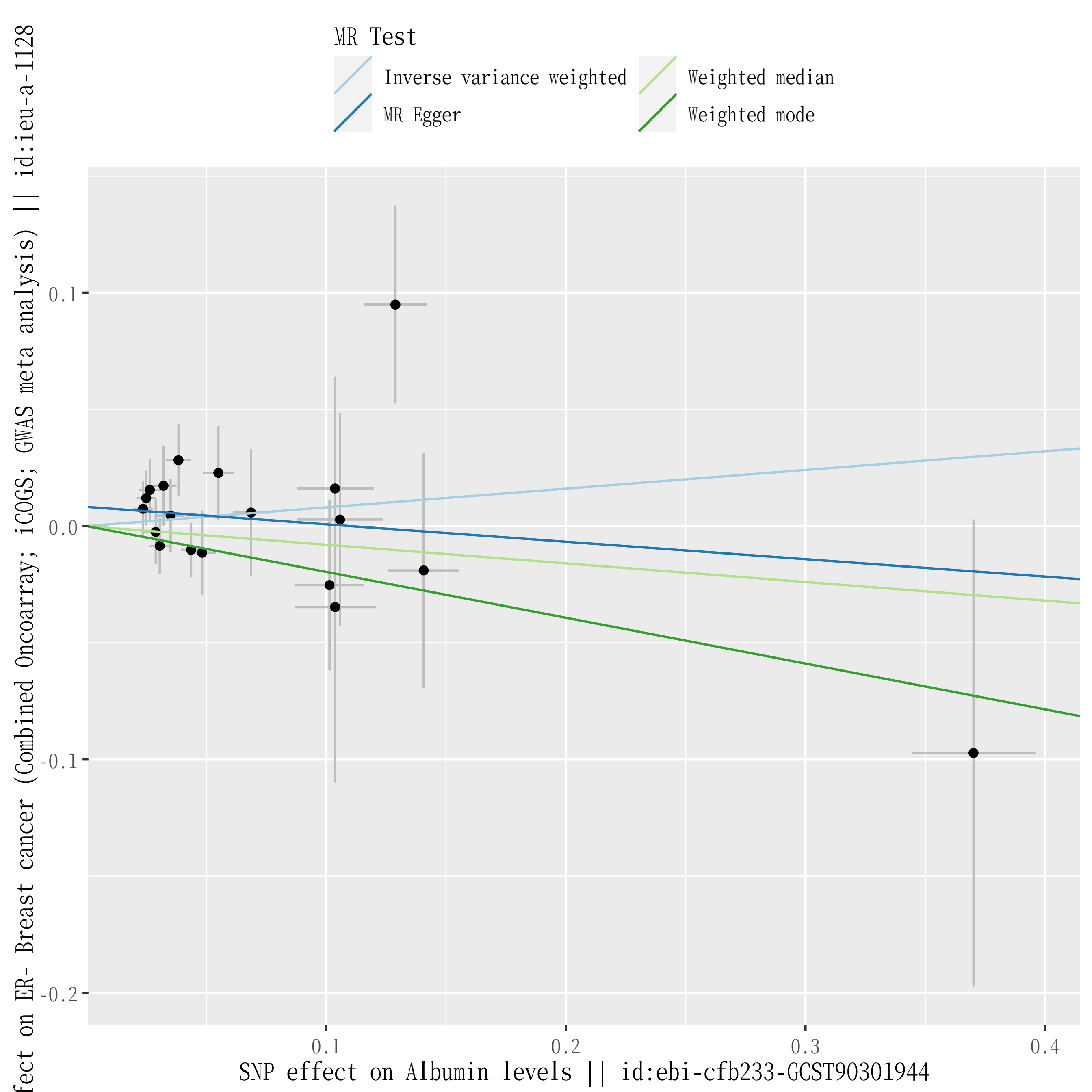

MR Test
Inverse variance weighted
MR Egger
Weighted median
Weighted mode
0.1
0.0
-0.1
-0.2
0.1
0.2
0.3
0.4
SNP effect on Albumin levels || id:ebi-cfb233-GCST90301944
fect on ER- Breast cancer (Combined Oncoarray; iCOGS; GWAS meta analysis) || id:ieu-a-1128

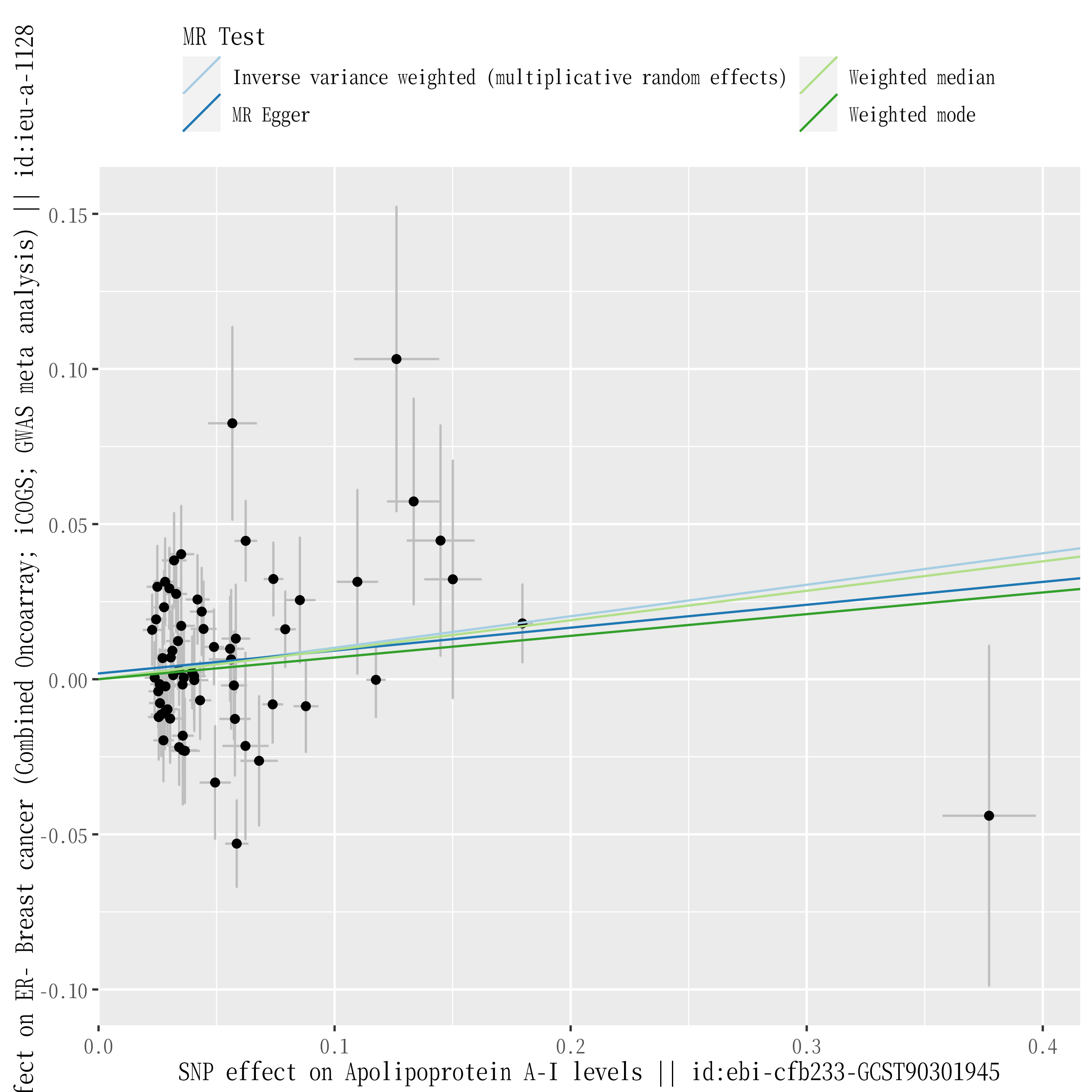

MR Test
Inverse variance weighted (multiplicative random effects)
MR Egger
Weighted median
Weighted mode
fect on ER- Breast cancer (Combined Oncoarray; iCOGS; GWAS meta analysis) || id:ieu-a-1128
0.15
0.10
0.05
0.00
-0.05
-0.10
0.0
0.1
0.2
0.3
0.4
SNP effect on Apolipoprotein A-I levels || id:ebi-cfb233-GCST90301945

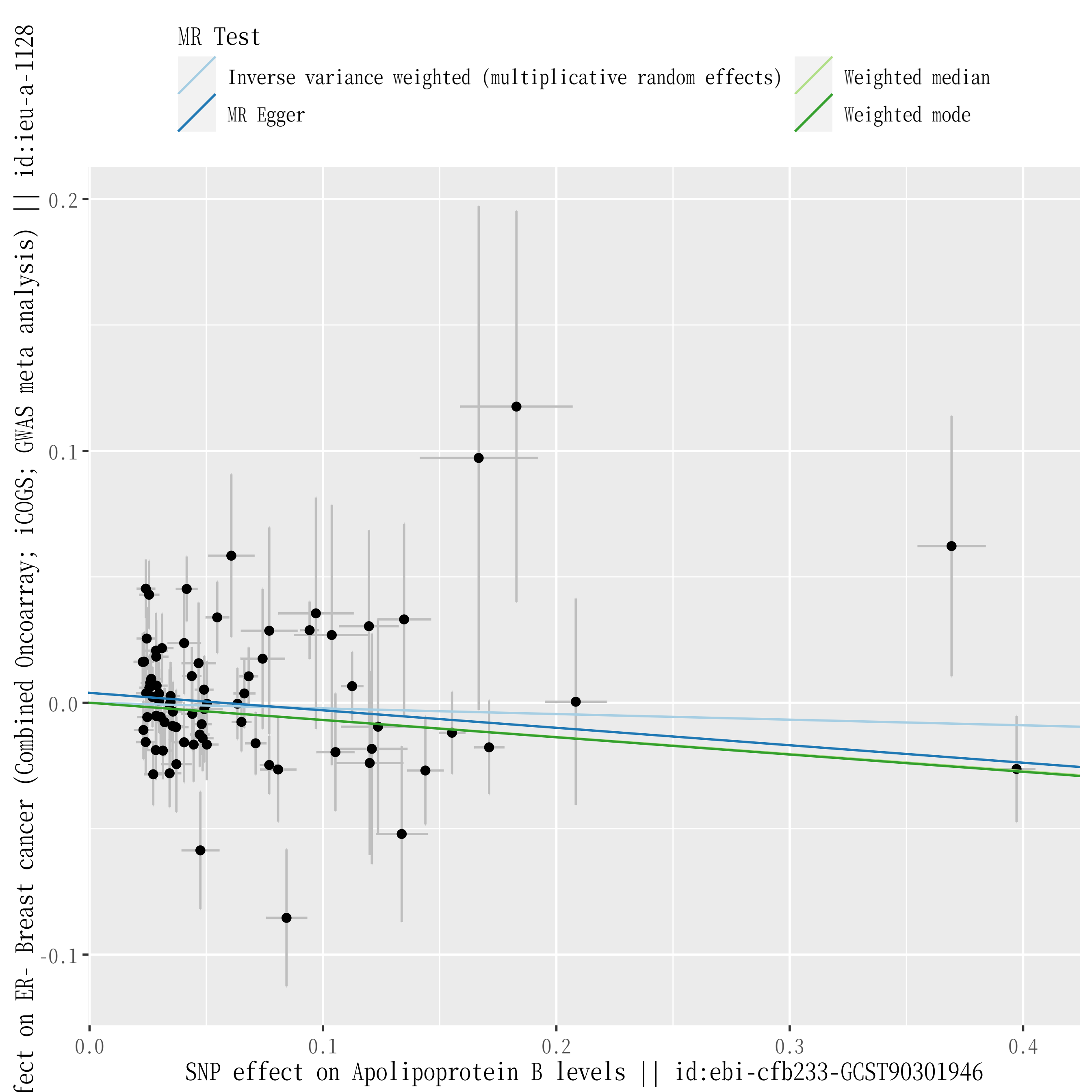
MR Test
Inverse variance weighted (multiplicative random effects)
MR Egger
Weighted median
Weighted mode
0.2
0.1
0.0
-0.1
0.0
0.1
0.2
0.3
0.4
SNP effect on Apolipoprotein B levels || id:ebi-cfb233-GCST90301946
fect on ER- Breast cancer (Combined Oncoarray; iCOGS; GWAS meta analysis) || id:ieu-a-1128

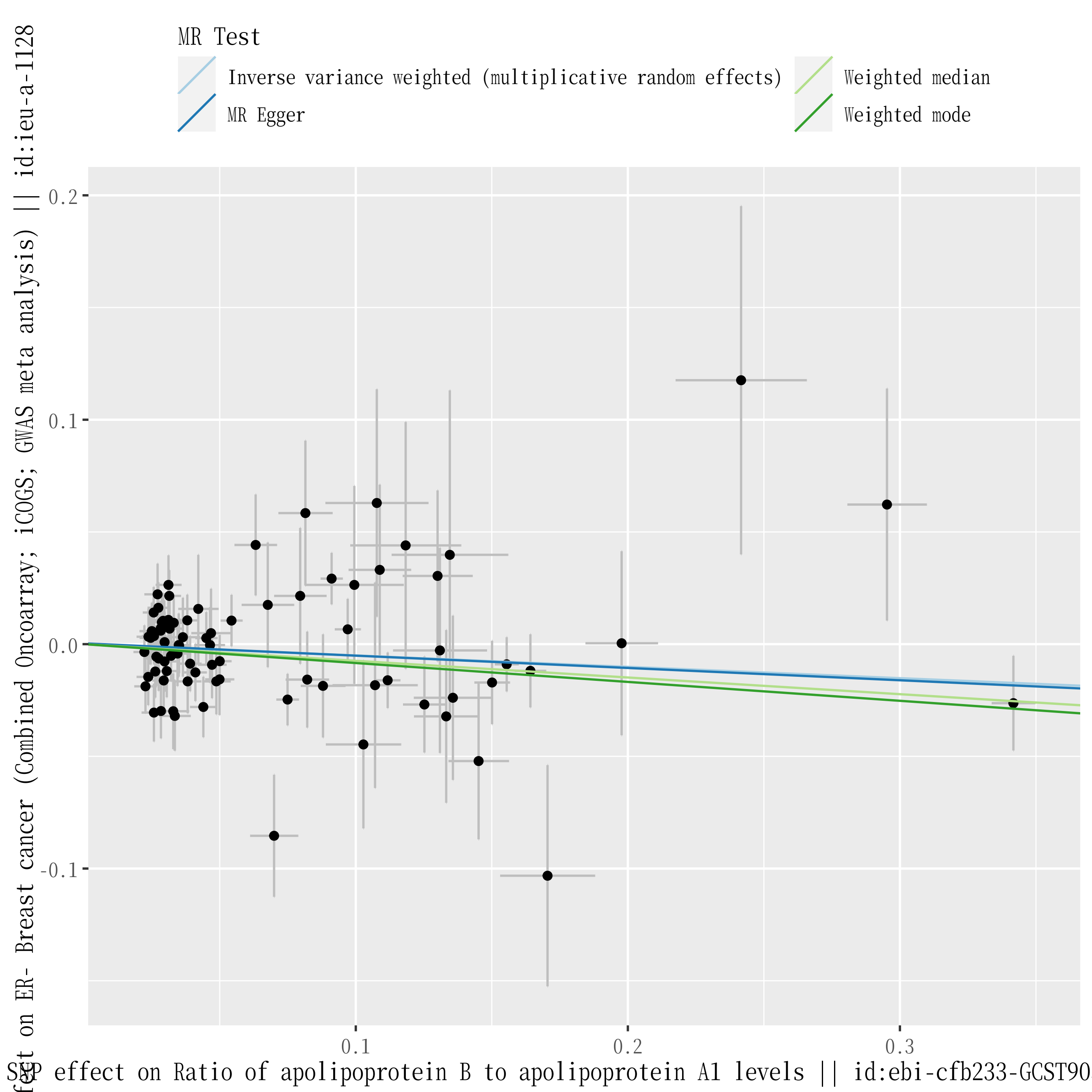

MR Test
Inverse variance weighted (multiplicative random effects)
MR Egger
Weighted median
Weighted mode
0.2
0.1
0.0
-0.1
0.1
0.2
0.3
SNP effect on Ratio of apolipoprotein B to apolipoprotein A1 levels || id:ebi-cfb233-GCST90
fect on ER- Breast cancer (Combined Oncoarray; iCOGS; GWAS meta analysis) || id:ieu-a-1128

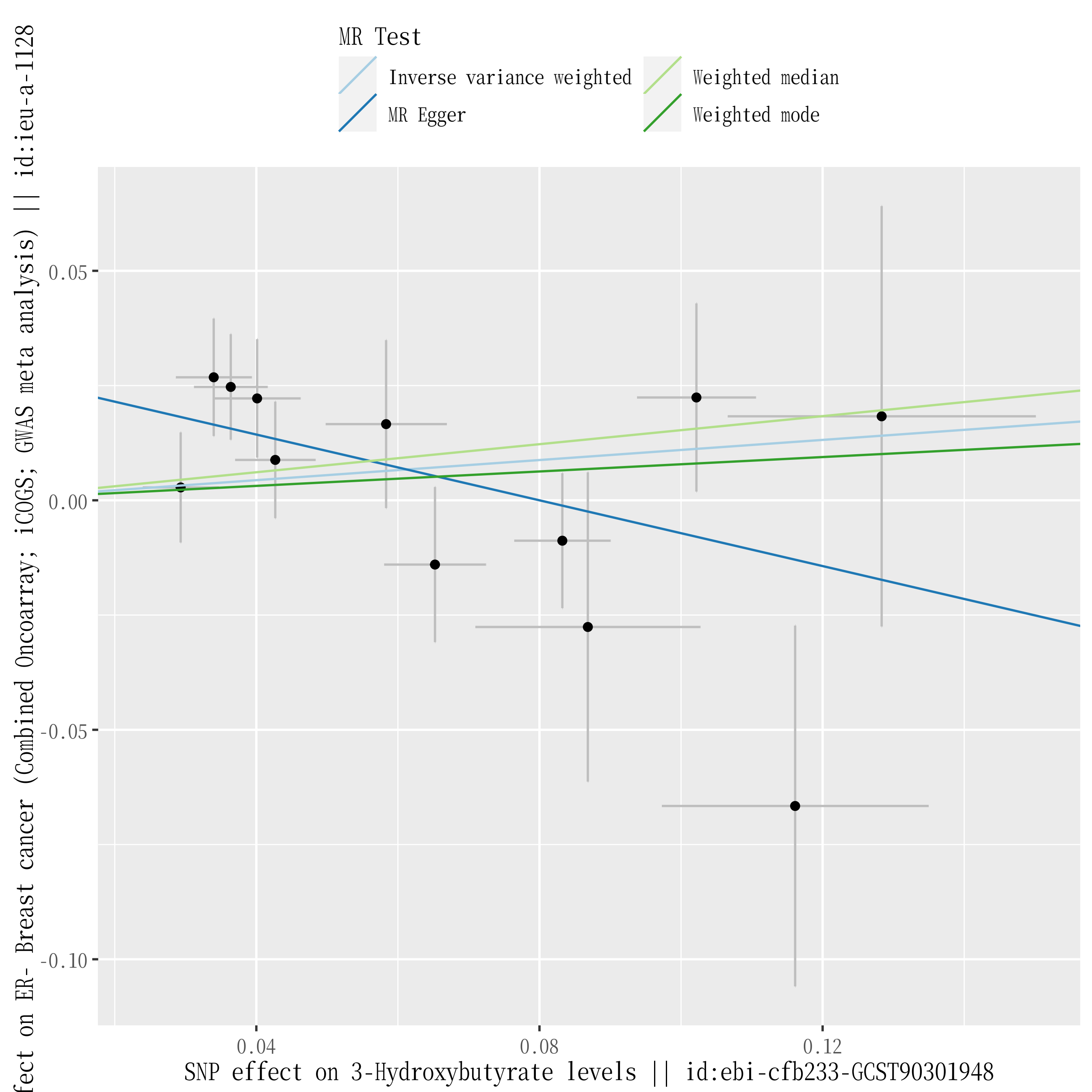

MR Test
Inverse variance weighted
MR Egger
Weighted median
Weighted mode
0.05
0.00
-0.05
-0.10
0.04
0.08
0.12
SNP effect on 3-Hydroxybutyrate levels || id:ebi-cfb233-GCST90301948
fect on ER- Breast cancer (Combined Oncoarray; iCOGS; GWAS meta analysis) || id:ieu-a-1128

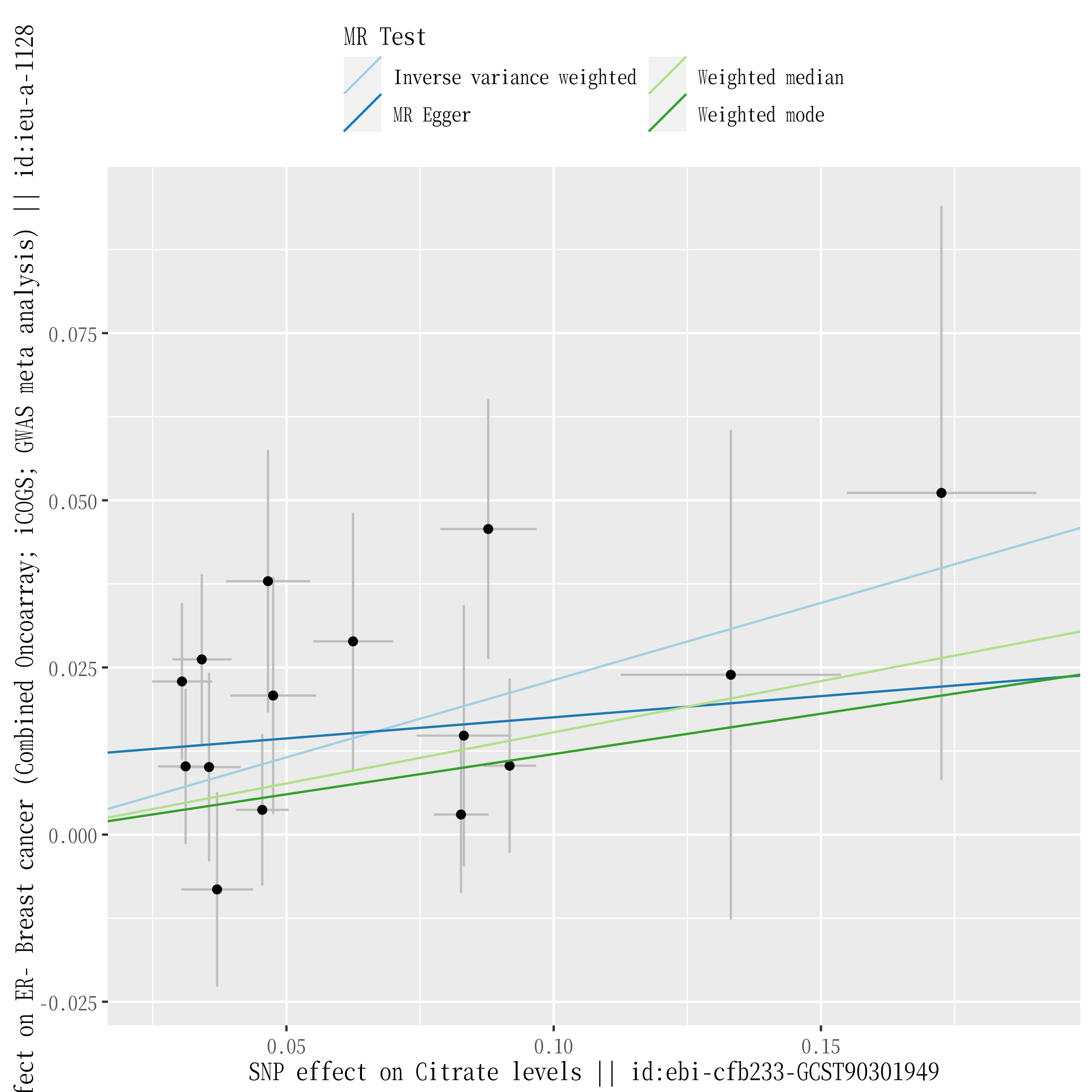

MR Test
Inverse variance weighted
MR Egger
Weighted median
Weighted mode
0.075
0.050
0.025
0.000
-0.025
0.05
0.10
0.15
SNP effect on Citrate levels || id:ebi-cfb233-GCST90301949
fect on ER- Breast cancer (Combined Oncoarray; iCOGS; GWAS meta analysis) || id:ieu-a-1128

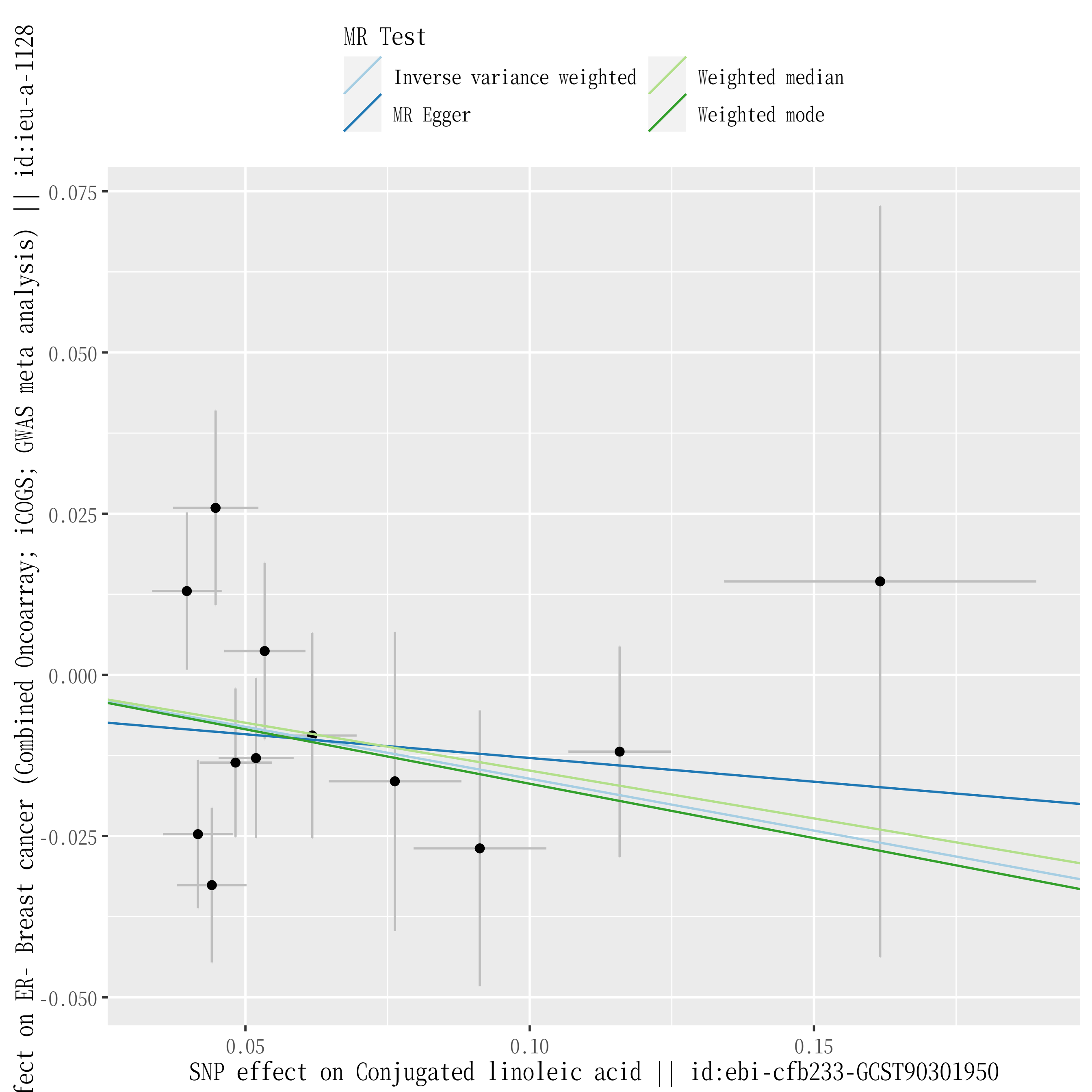

MR Test
Inverse variance weighted
MR Egger
Weighted median
Weighted mode
0.075
0.050
0.025
0.000
-0.025
-0.050
0.05
0.10
0.15
SNP effect on Conjugated linoleic acid || id:ebi-cfb233-GCST90301950
fect on ER- Breast cancer (Combined Oncoarray; iCOGS; GWAS meta analysis) || id:ieu-a-1128

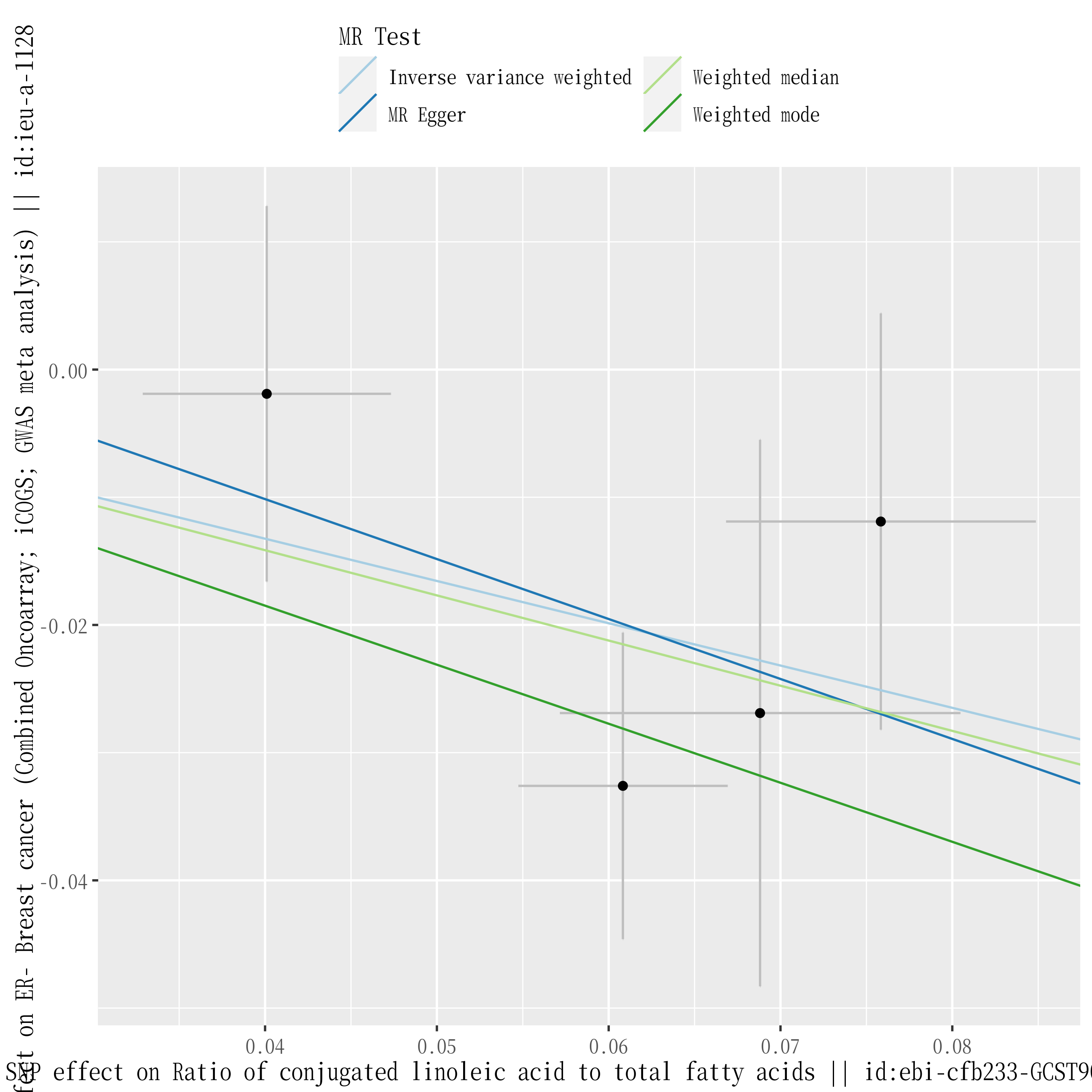

MR Test
Inverse variance weighted
MR Egger
Weighted median
Weighted mode
0.00
-0.02
-0.04
0.04
0.05
0.06
0.07
0.08
SNP effect on Ratio of conjugated linoleic acid to total fatty acids || id:ebi-cfb233-GCST90
fect on ER- Breast cancer (Combined Oncoarray; iCOGS; GWAS meta analysis) || id:ieu-a-1128

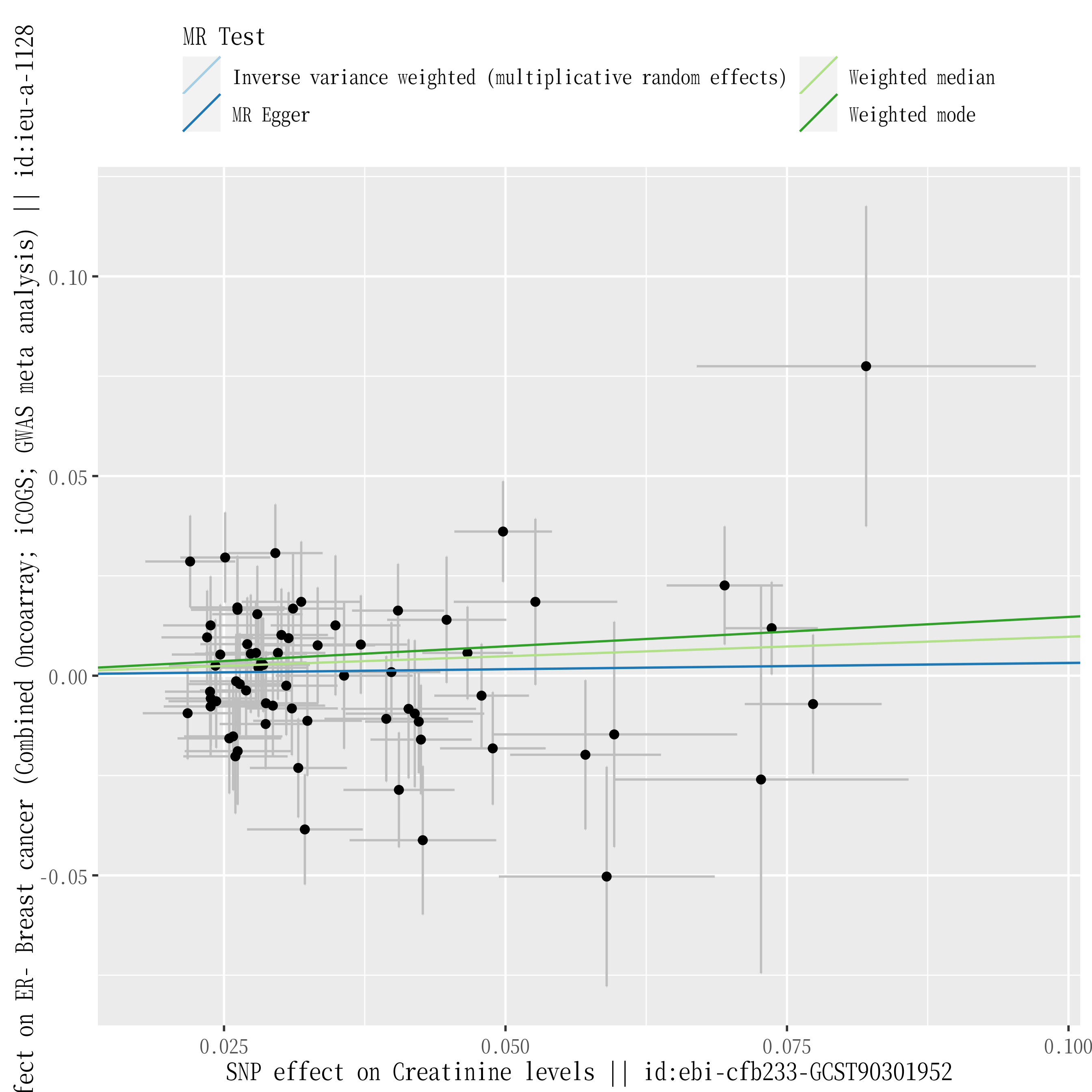
MR Test
Inverse variance weighted (multiplicative random effects)
MR Egger
Weighted median
Weighted mode
0.10
0.05
0.00
-0.05
0.025
0.050
0.075
0.100
SNP effect on Creatinine levels || id:ebi-cfb233-GCST90301952
fect on ER- Breast cancer (Combined Oncoarray; iCOGS; GWAS meta analysis) || id:ieu-a-1128

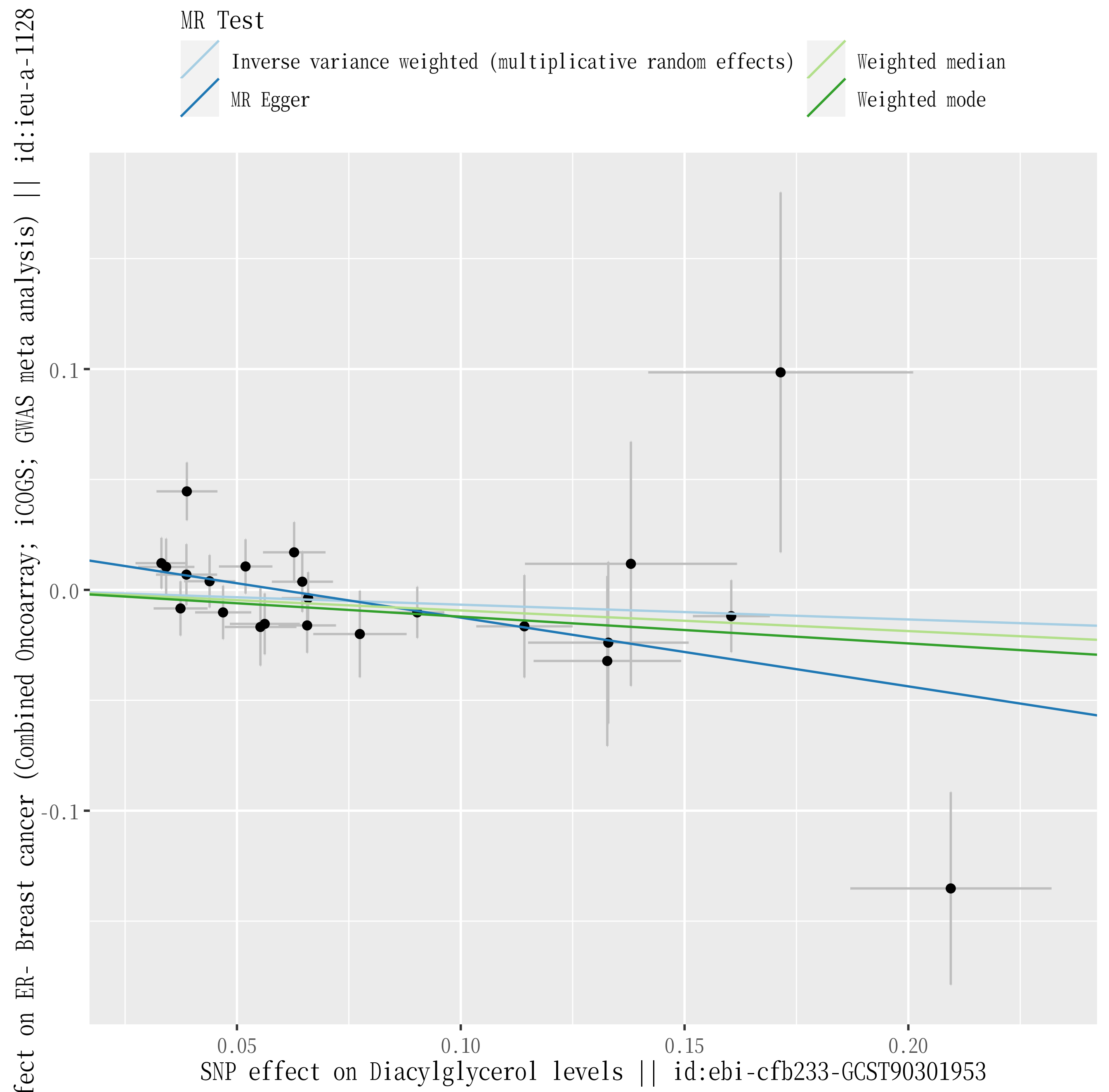

MR Test
Inverse variance weighted (multiplicative random effects)
MR Egger
Weighted median
Weighted mode
0.1
0.0
-0.1
0.05
0.10
0.15
0.20
SNP effect on Diacylglycerol levels || id:ebi-cfb233-GCST90301953
fect on ER- Breast cancer (Combined Oncoarray; iCOGS; GWAS meta analysis) || id:ieu-a-1128

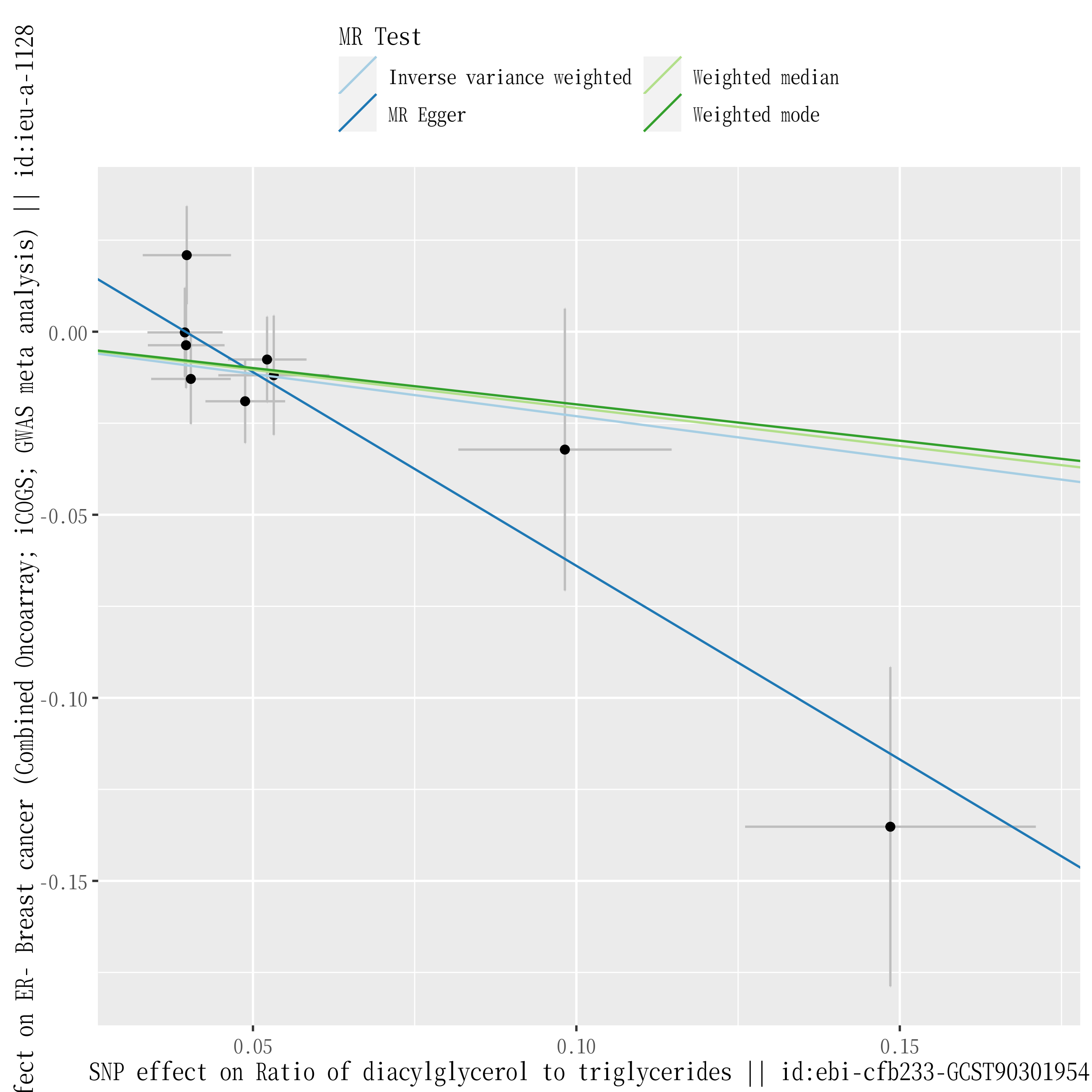
MR Test
Inverse variance weighted
MR Egger
Weighted median
Weighted mode
0.00
-0.05
-0.10
-0.15
0.05
0.10
0.15
SNP effect on Ratio of diacylglycerol to triglycerides || id:ebi-cfb233-GCST90301954
fect on ER- Breast cancer (Combined Oncoarray; iCOGS; GWAS meta analysis) || id:ieu-a-1128

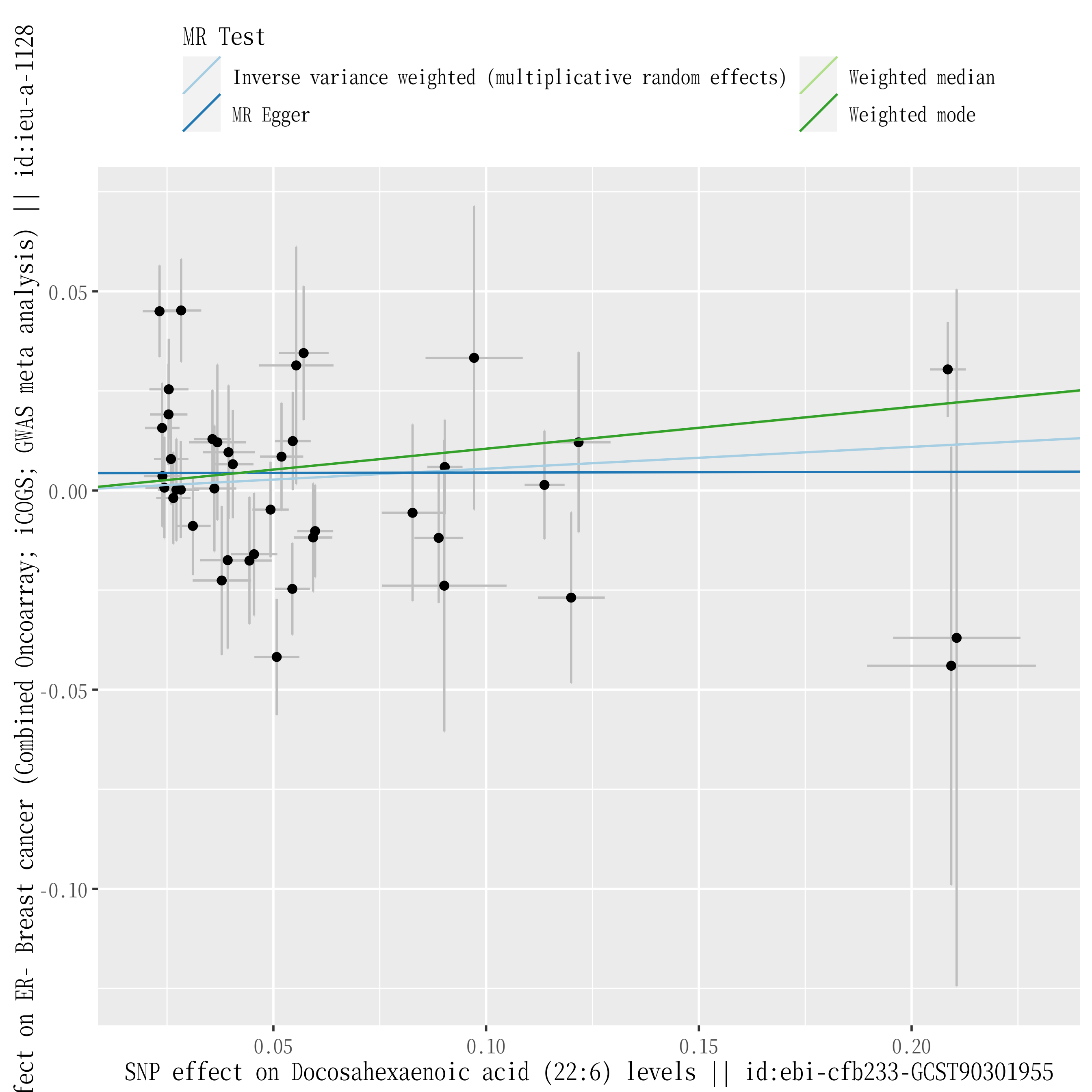
MR Test
Inverse variance weighted (multiplicative random effects)
MR Egger
Weighted median
Weighted mode
0.05
0.00
-0.05
-0.10
0.05
0.10
0.15
0.20
SNP effect on Docosahexaenoic acid (22:6) levels || id:ebi-cfb233-GCST90301955
fect on ER- Breast cancer (Combined Oncoarray; iCOGS; GWAS meta analysis) || id:ieu-a-1128

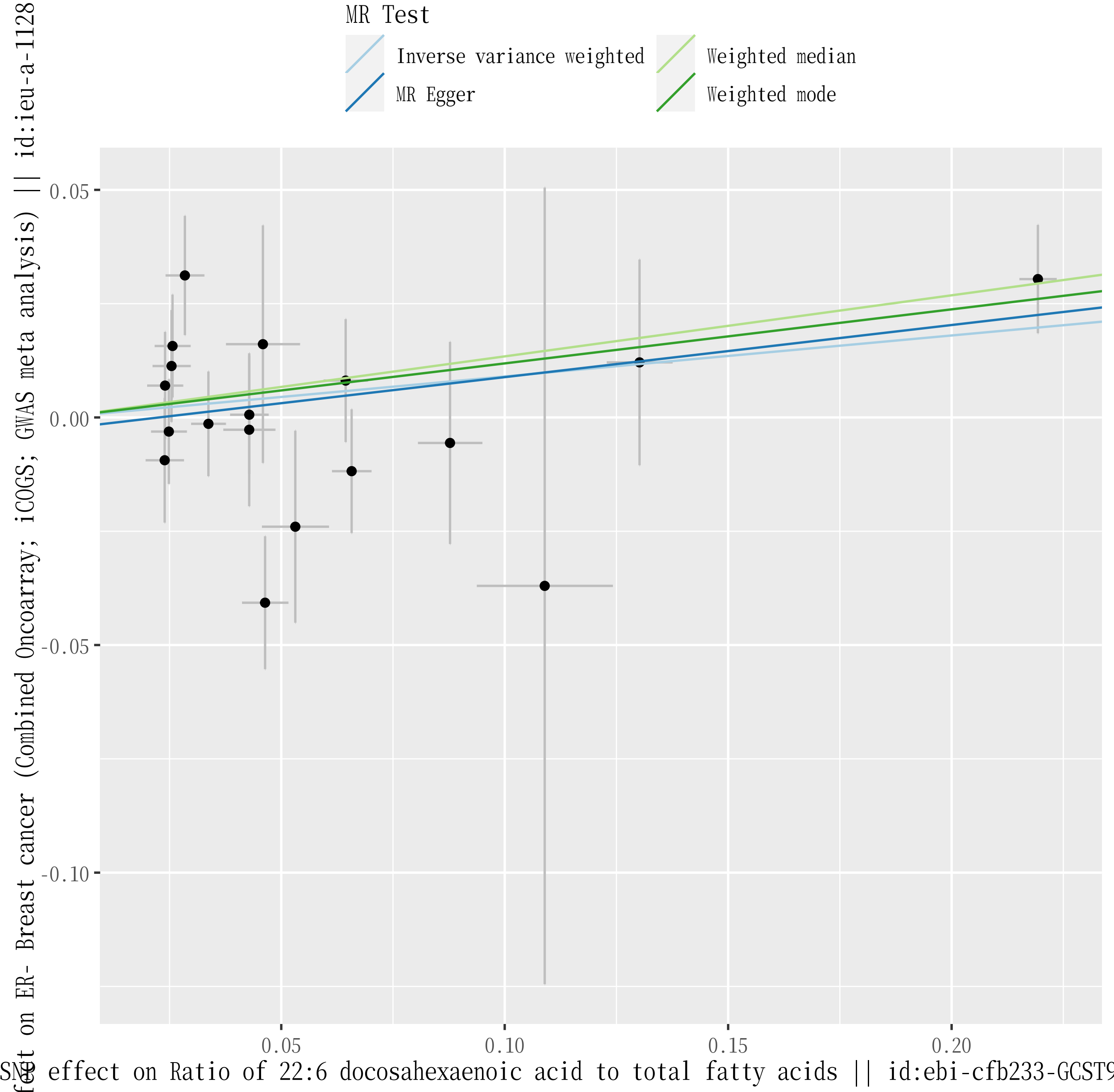
MR Test
Inverse variance weighted
MR Egger
Weighted median
Weighted mode
0.05
0.00
-0.05
-0.10
0.05
0.10
0.15
0.20
SNP effect on Ratio of 22:6 docosahexaenoic acid to total fatty acids || id:ebi-cfb233-GCST9
fect on ER- Breast cancer (Combined Oncoarray; iCOGS; GWAS meta analysis) || id:ieu-a-1128

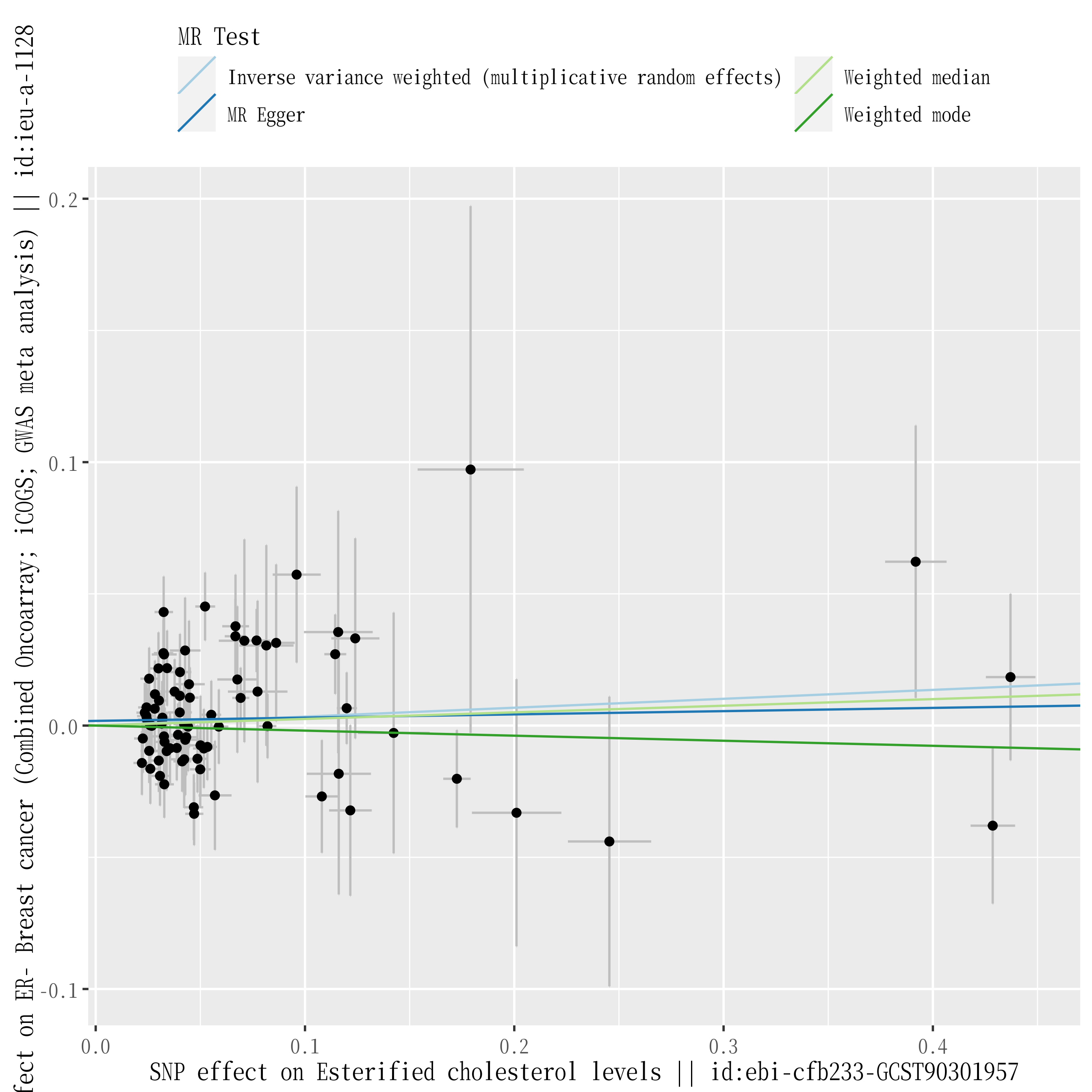
MR Test
Inverse variance weighted (multiplicative random effects)
MR Egger
Weighted median
Weighted mode
0.2
0.1
0.0
-0.1
0.0
0.1
0.2
0.3
0.4
SNP effect on Esterified cholesterol levels || id:ebi-cfb233-GCST90301957
fect on ER- Breast cancer (Combined Oncoarray; iCOGS; GWAS meta analysis) || id:ieu-a-1128

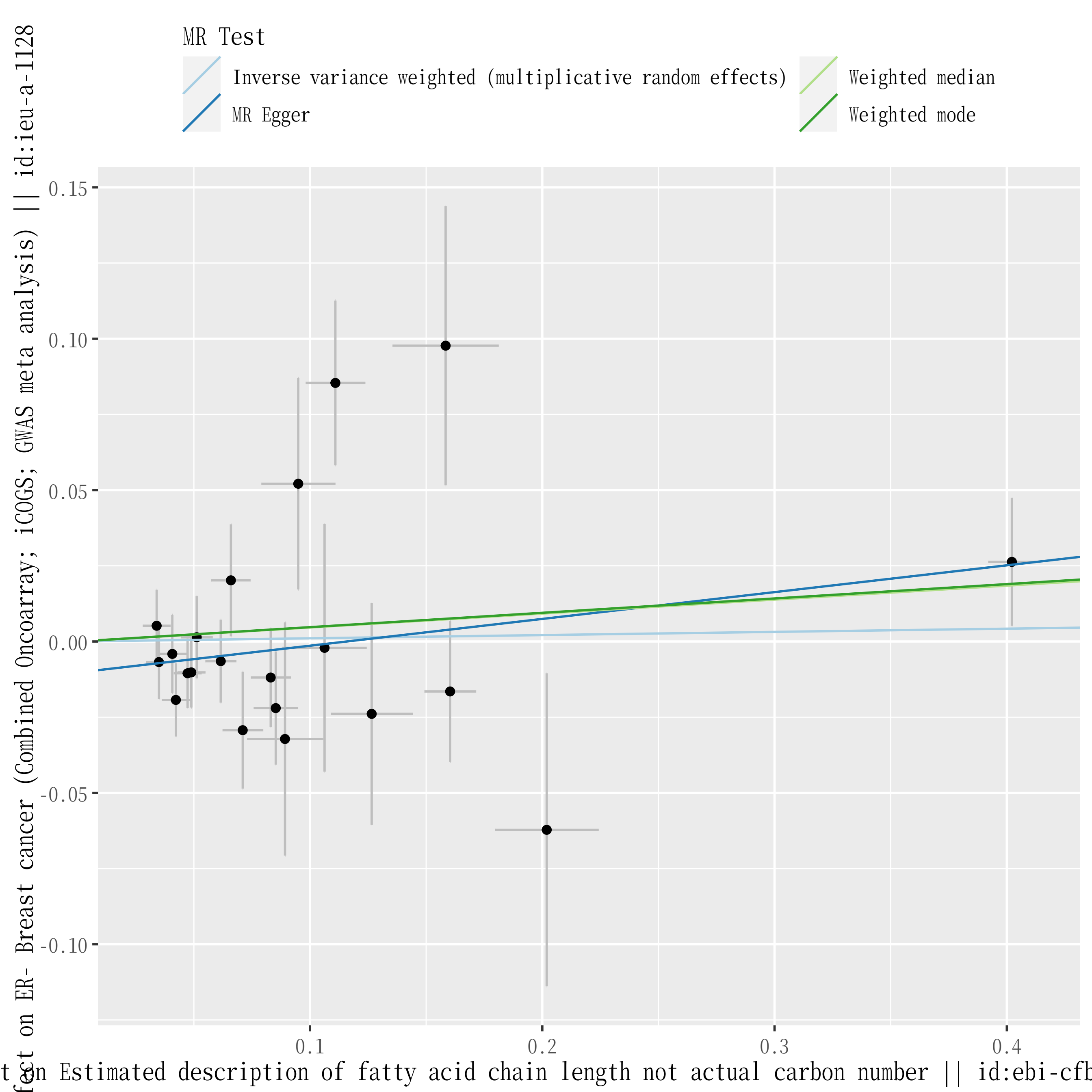

MR Test
Inverse variance weighted (multiplicative random effects)
MR Egger
Weighted median
Weighted mode
0.15
0.10
0.05
0.00
-0.05
-0.10
0.1
0.2
0.3
0.4
fect on ER- Breast cancer (Combined Oncoarray; iCOGS; GWAS meta analysis) || id:ieu-a-1128
t on Estimated description of fatty acid chain length not actual carbon number || id:ebi-cfb

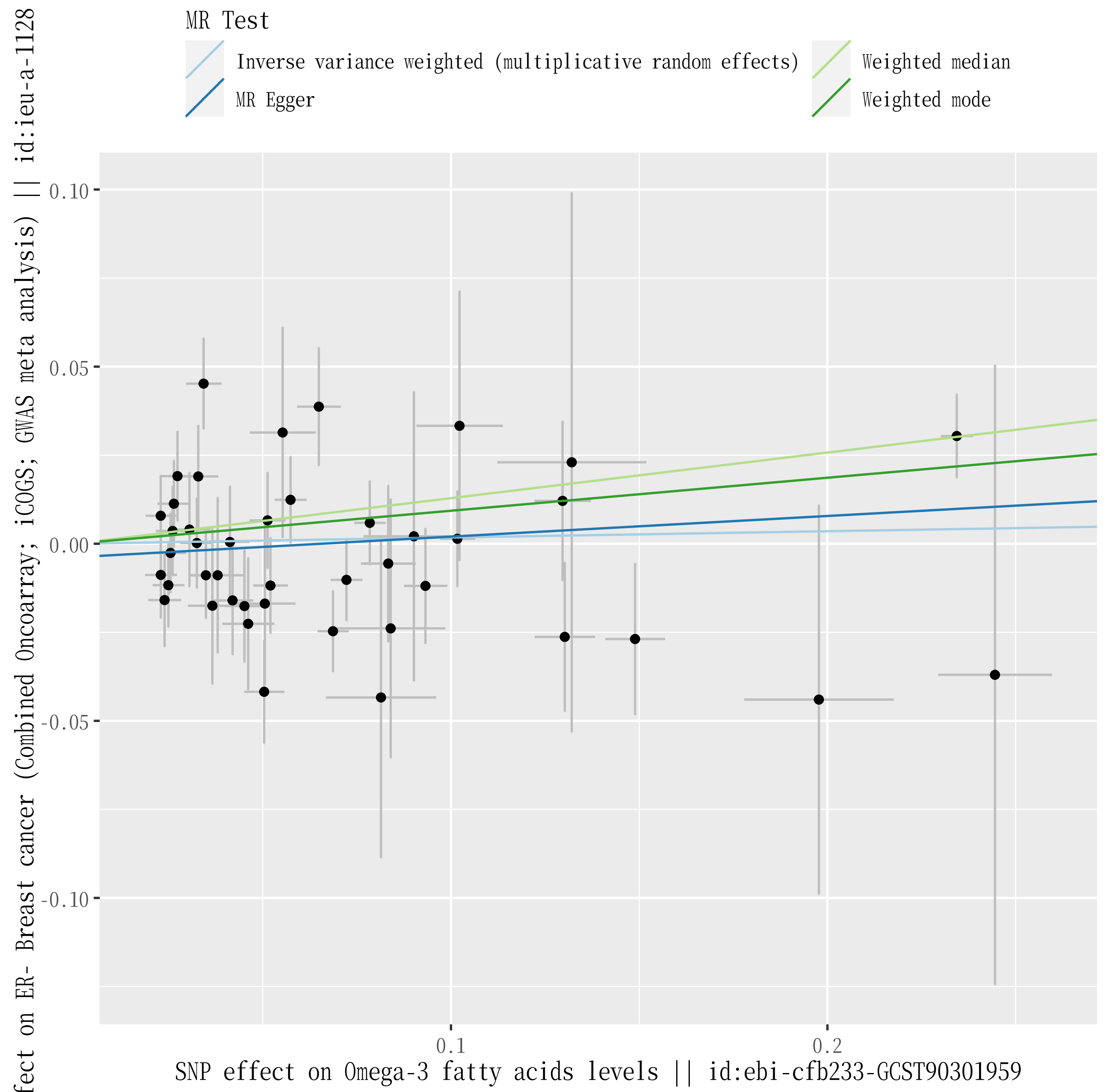
MR Test
Inverse variance weighted (multiplicative random effects)
MR Egger
Weighted median
Weighted mode
0.10
0.05
0.00
-0.05
-0.10
0.1
0.2
SNP effect on Omega-3 fatty acids levels || id:ebi-cfb233-GCST90301959
fect on ER- Breast cancer (Combined Oncoarray; iCOGS; GWAS meta analysis) || id:ieu-a-1128

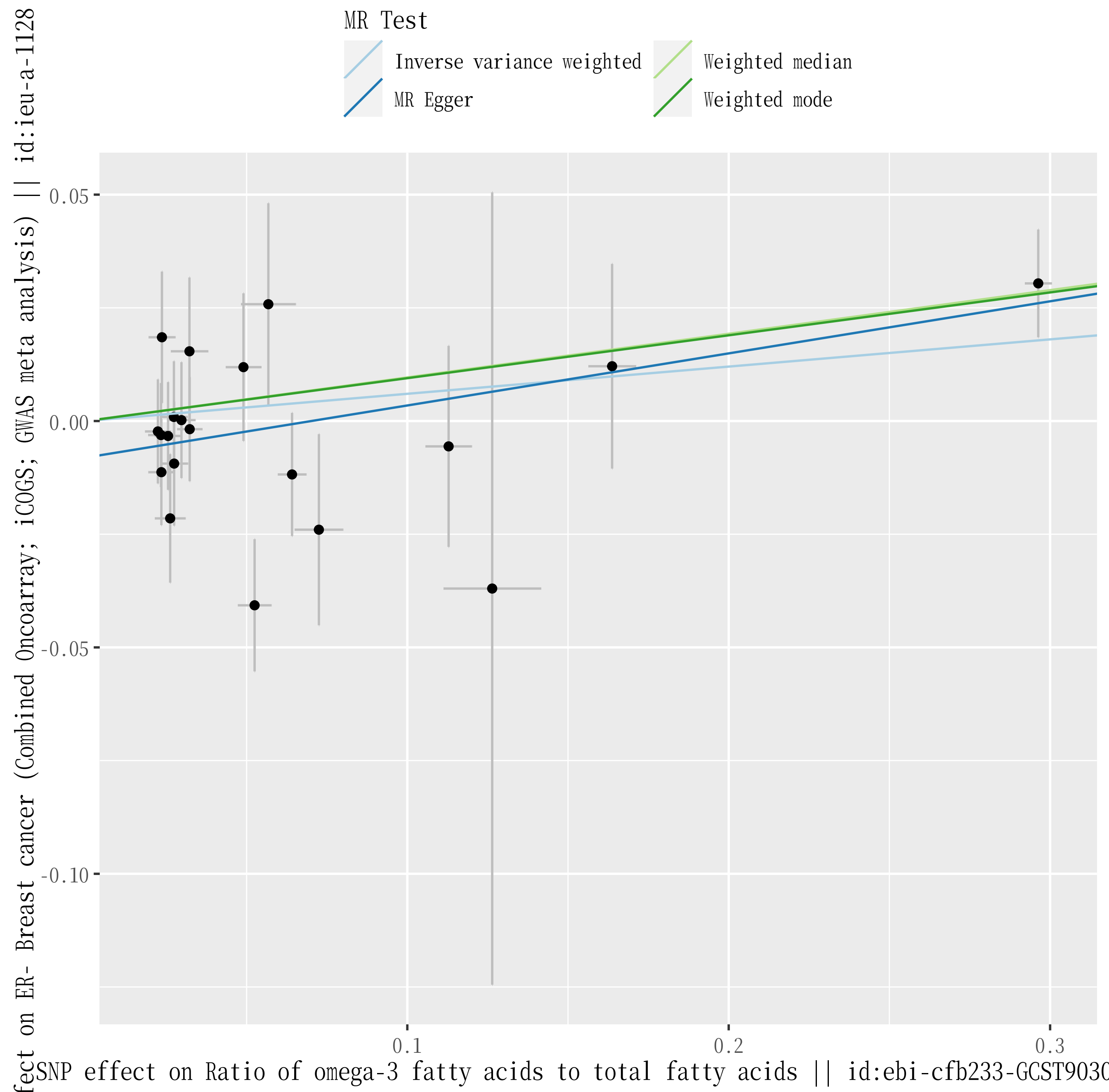

MR Test
Inverse variance weighted
MR Egger
Weighted median
Weighted mode
0.05
0.00
-0.05
-0.10
0.1
0.2
0.3
SNP effect on Ratio of omega-3 fatty acids to total fatty acids || id:ebi-cfb233-GCST9030
fect on ER- Breast cancer (Combined Oncoarray; iCOGS; GWAS meta analysis) || id:ieu-a-1128

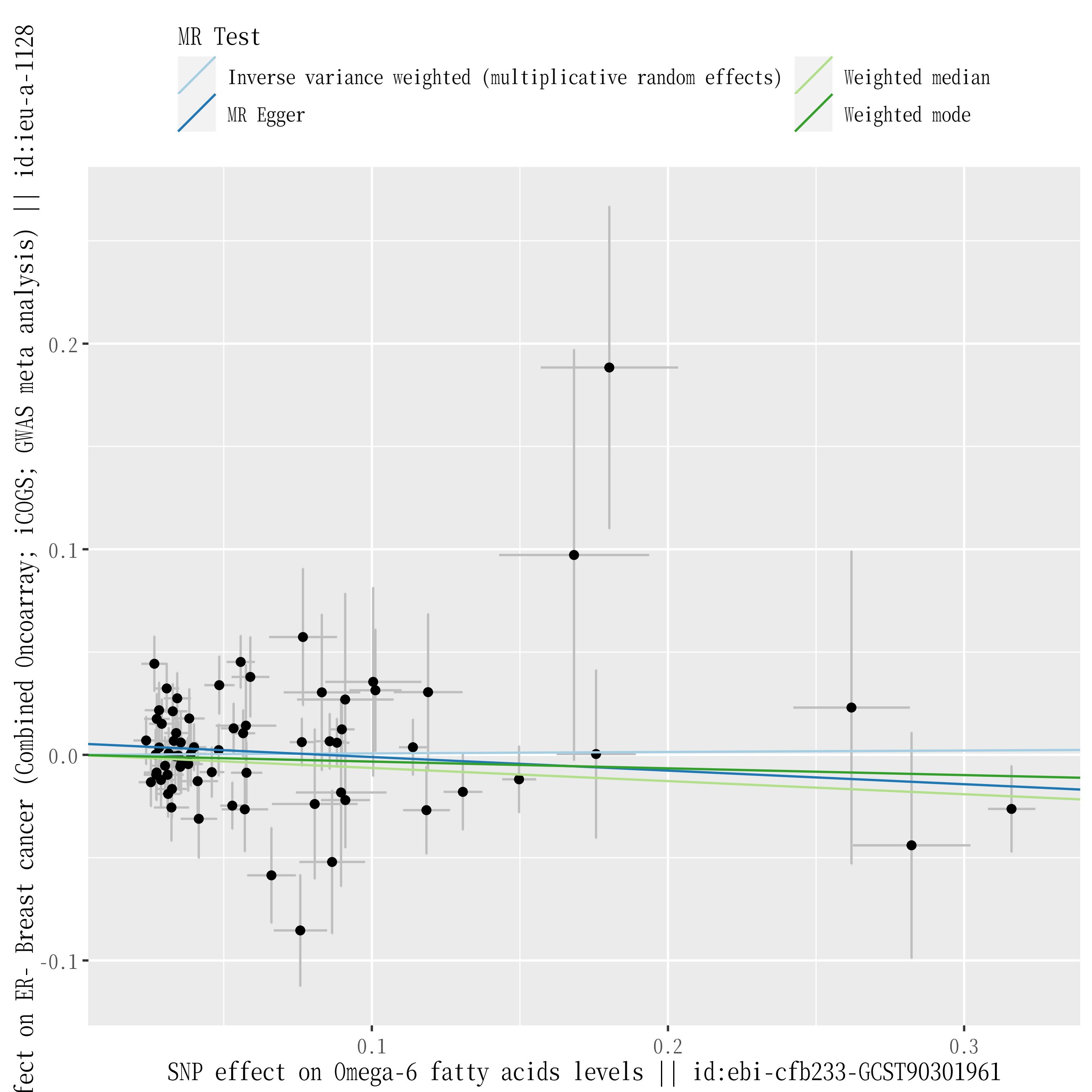
MR Test
Inverse variance weighted (multiplicative random effects)
MR Egger
Weighted median
Weighted mode
0.2
0.1
0.0
-0.1
0.1
0.2
0.3
SNP effect on Omega-6 fatty acids levels || id:ebi-cfb233-GCST90301961
fect on ER- Breast cancer (Combined Oncoarray; iCOGS; GWAS meta analysis) || id:ieu-a-1128

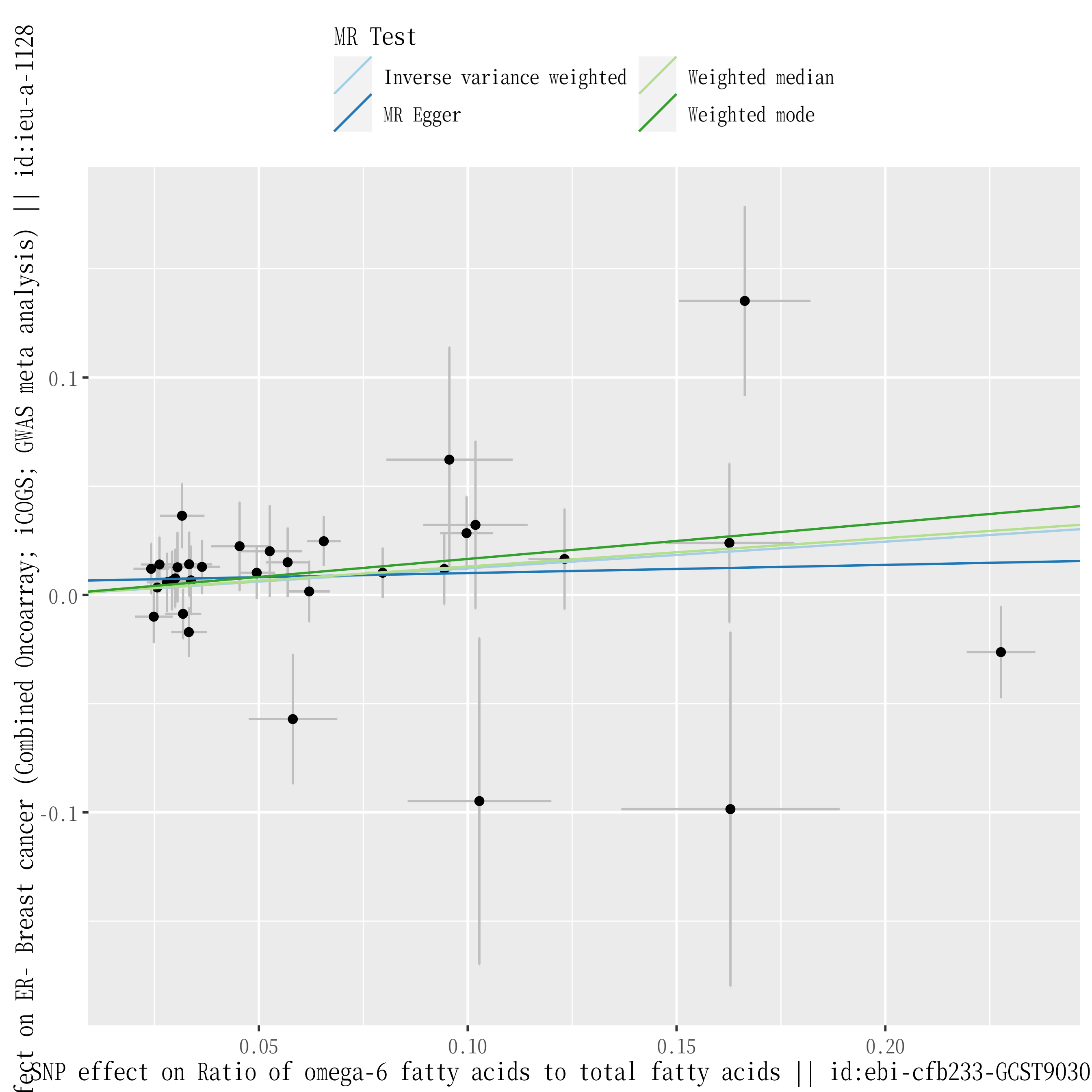

MR Test
Inverse variance weighted
MR Egger
Weighted median
Weighted mode
0.1
0.0
-0.1
0.05
0.10
0.15
0.20
SNP effect on Ratio of omega-6 fatty acids to total fatty acids || id:ebi-cfb233-GCST9030
fect on ER- Breast cancer (Combined Oncoarray; iCOGS; GWAS meta analysis) || id:ieu-a-1128

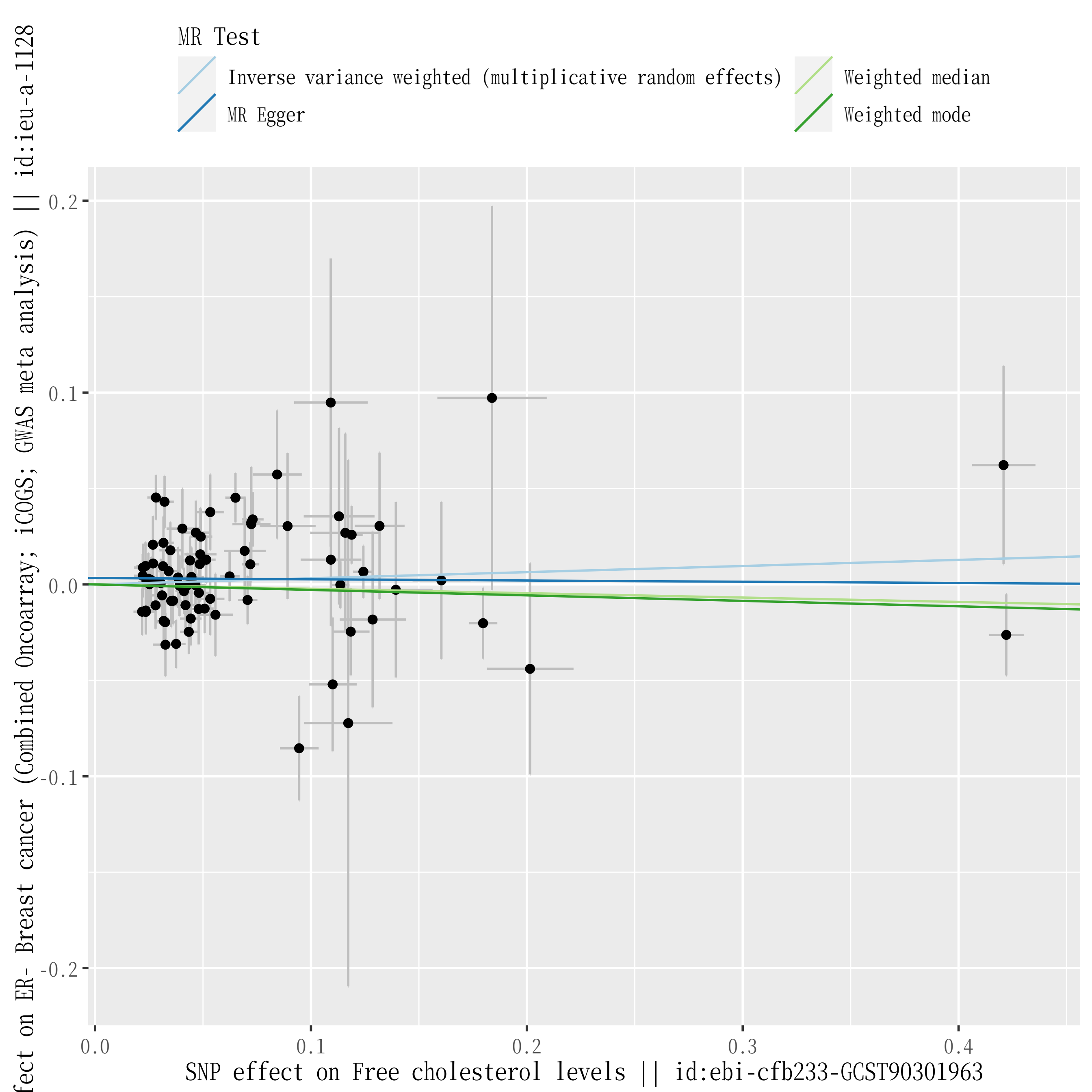
MR Test
Inverse variance weighted (multiplicative random effects)
MR Egger
Weighted median
Weighted mode
0.2
0.1
0.0
-0.1
-0.2
0.0
0.1
0.2
0.3
0.4
SNP effect on Free cholesterol levels || id:ebi-cfb233-GCST90301963
fect on ER- Breast cancer (Combined Oncoarray; iCOGS; GWAS meta analysis) || id:ieu-a-1128

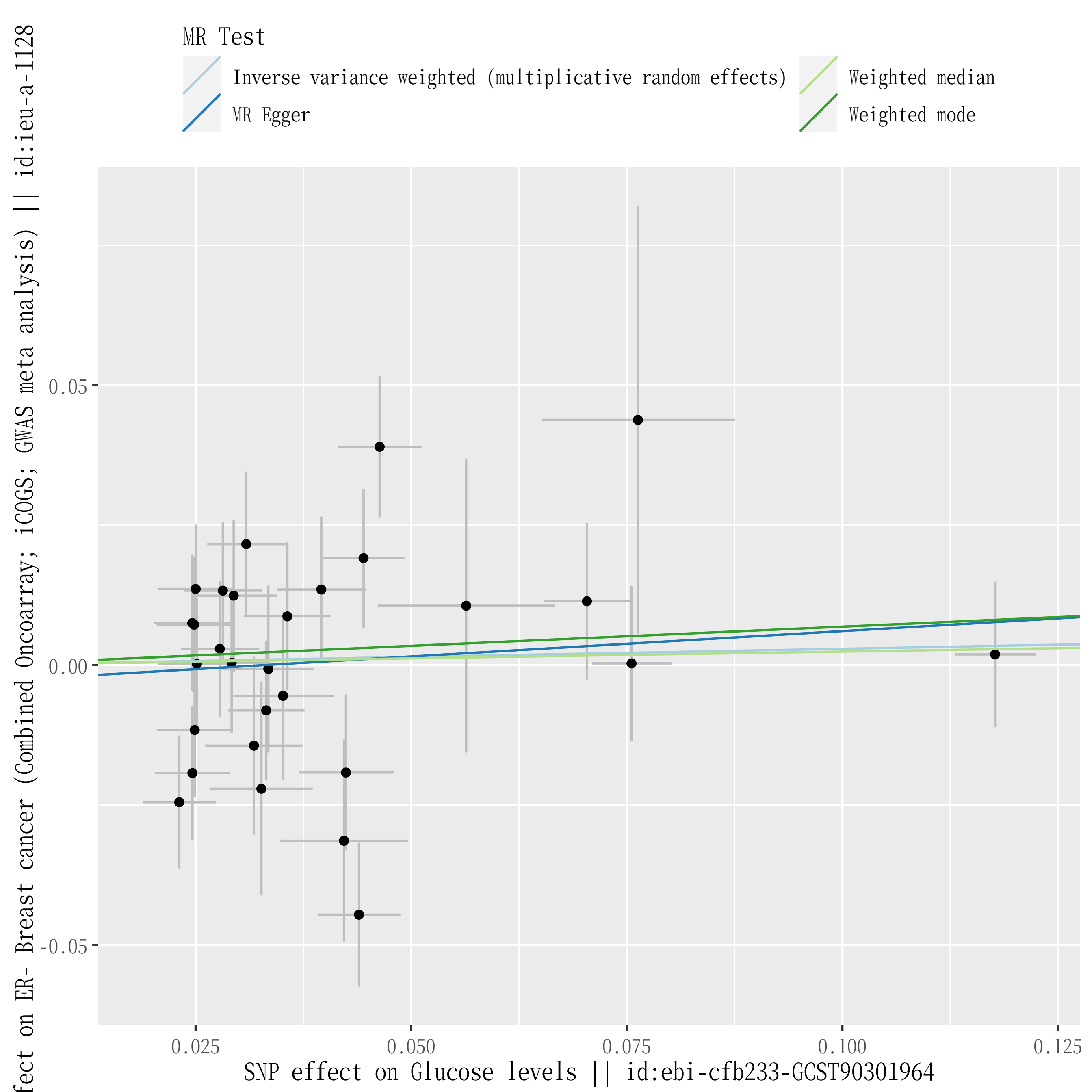

MR Test
Inverse variance weighted (multiplicative random effects)
MR Egger
Weighted median
Weighted mode
0.05
0.00
-0.05
0.025
0.050
0.075
0.100
0.125
SNP effect on Glucose levels || id:ebi-cfb233-GCST90301964
fect on ER- Breast cancer (Combined Oncoarray; iCOGS; GWAS meta analysis) || id:ieu-a-1128

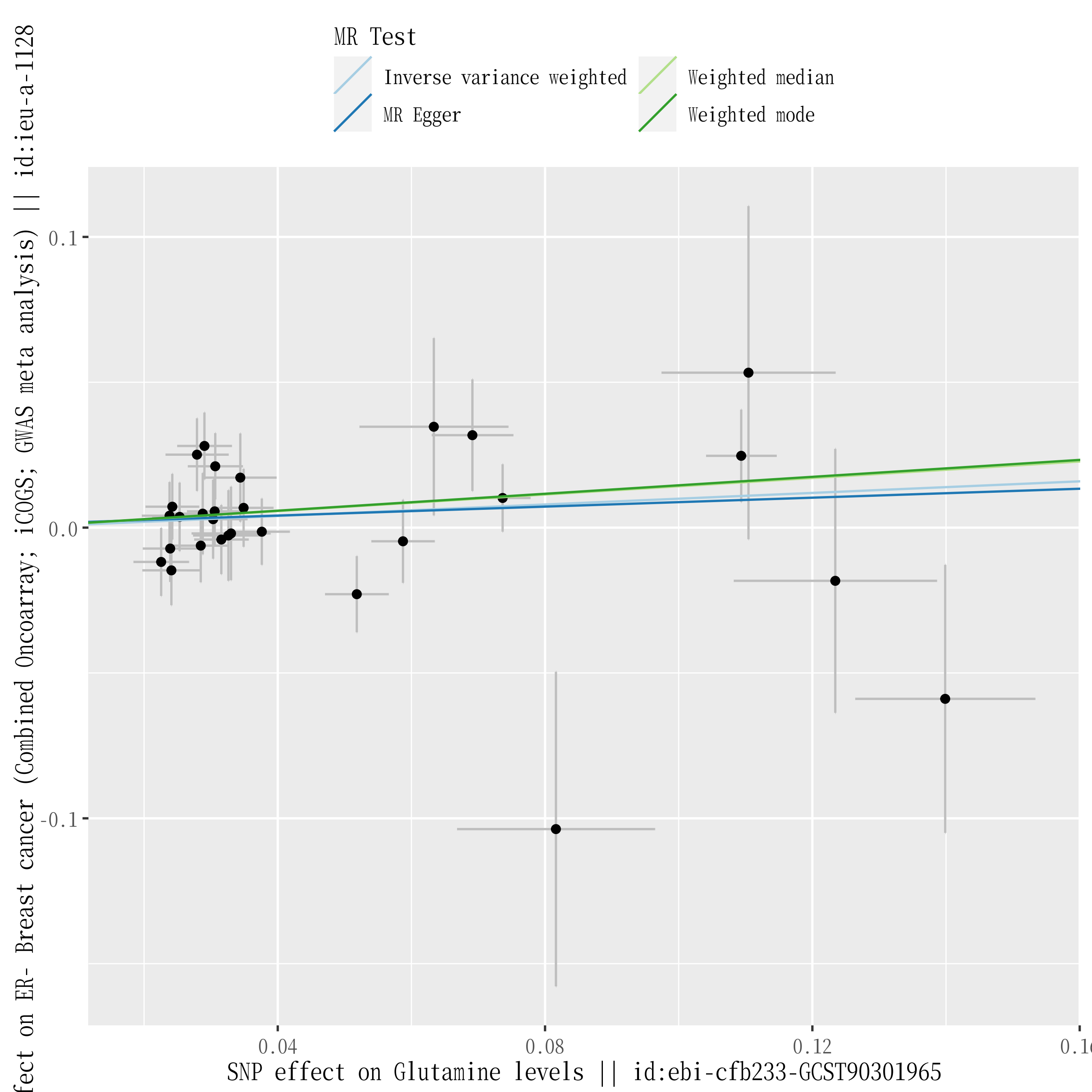

MR Test
Inverse variance weighted
MR Egger
Weighted median
Weighted mode
0.1
0.0
-0.1
0.04
0.08
0.12
SNP effect on Glutamine levels || id:ebi-cfb233-GCST90301965
fect on ER- Breast cancer (Combined Oncoarray; iCOGS; GWAS meta analysis) || id:ieu-a-1128

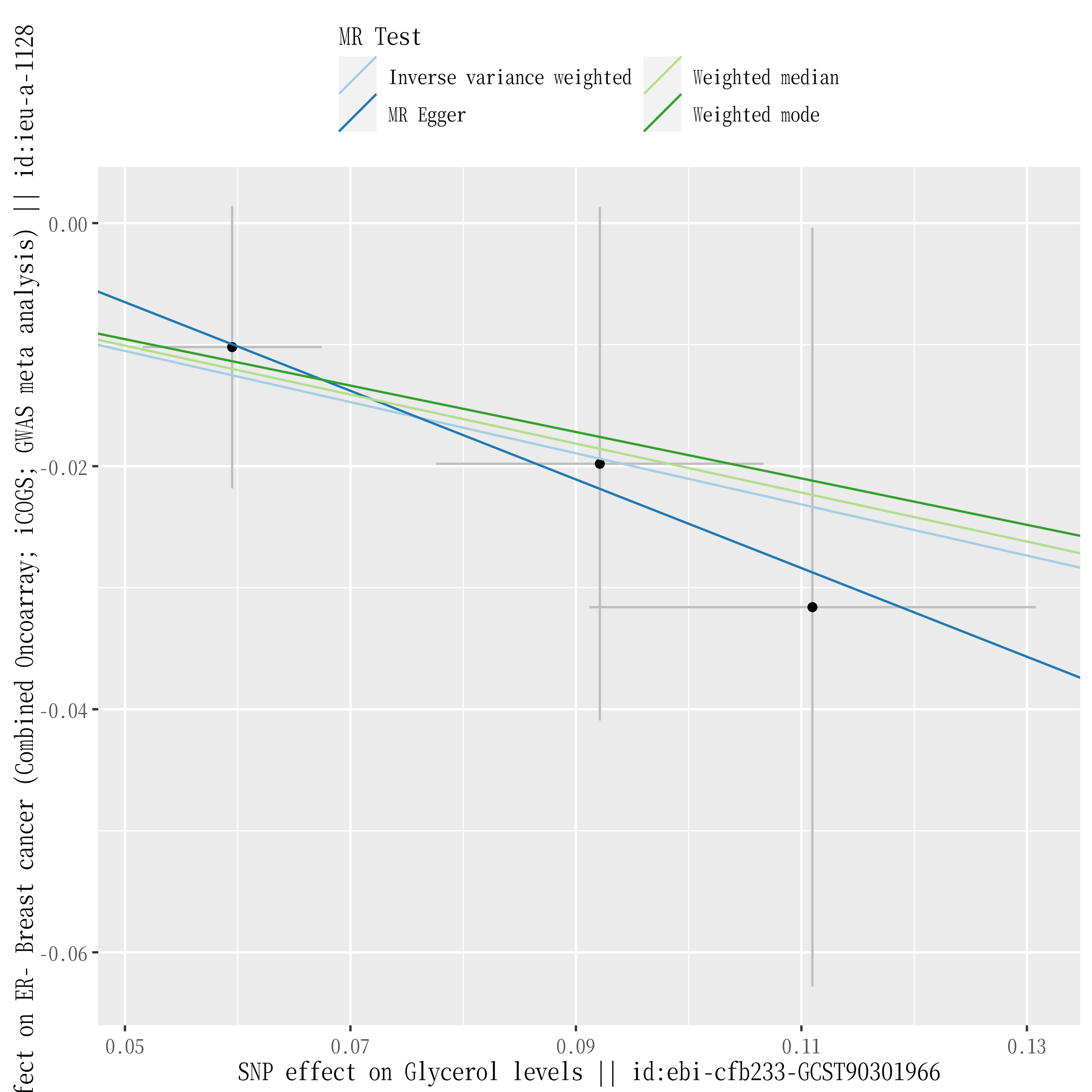

MR Test
Inverse variance weighted
MR Egger
Weighted median
Weighted mode
0.00
-0.02
-0.04
-0.06
0.05
0.07
0.09
0.11
0.13
SNP effect on Glycerol levels || id:ebi-cfb233-GCST90301966
fect on ER- Breast cancer (Combined Oncoarray; iCOGS; GWAS meta analysis) || id:ieu-a-1128

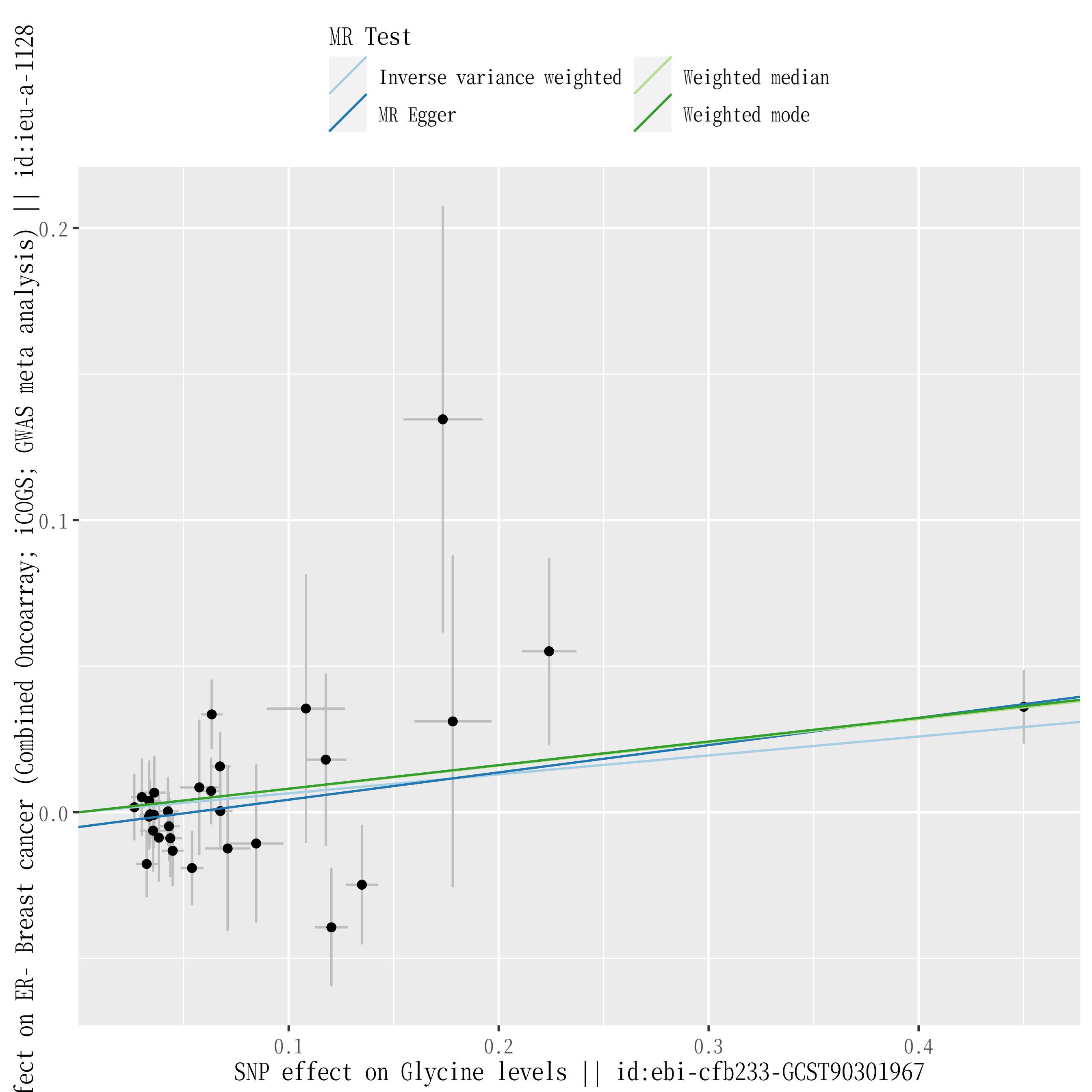
MR Test
Inverse variance weighted
MR Egger
Weighted median
Weighted mode
0.2
0.1
0.0
0.1
0.2
0.3
0.4
SNP effect on Glycine levels || id:ebi-cfb233-GCST90301967
fect on ER- Breast cancer (Combined Oncoarray; iCOGS; GWAS meta analysis) || id:ieu-a-1128

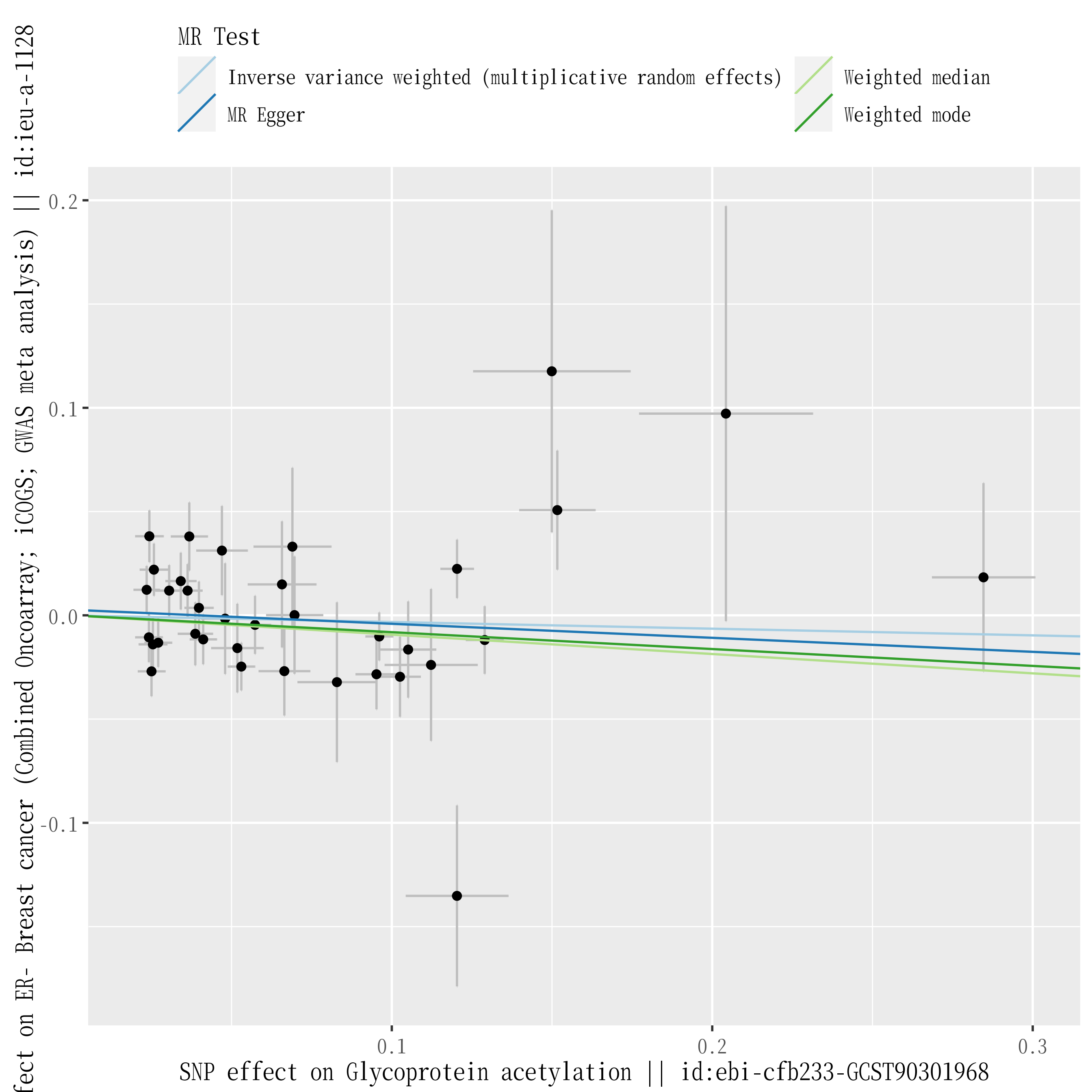

MR Test
Inverse variance weighted (multiplicative random effects)
MR Egger
Weighted median
Weighted mode
0.2
0.1
0.0
-0.1
0.1
0.2
0.3
SNP effect on Glycoprotein acetylation || id:ebi-cfb233-GCST90301968
fect on ER- Breast cancer (Combined Oncoarray; iCOGS; GWAS meta analysis) || id:ieu-a-1128

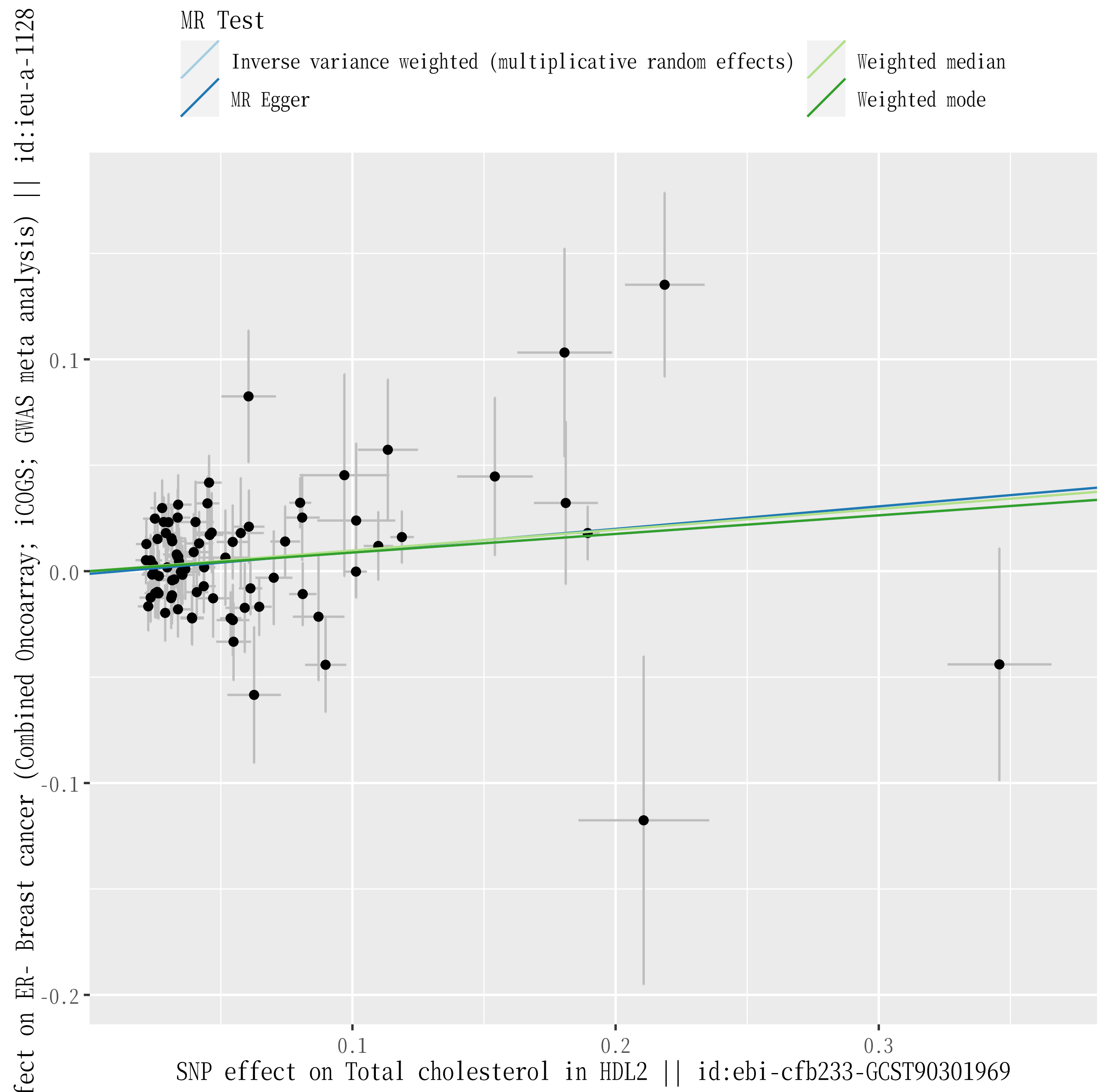

MR Test
Inverse variance weighted (multiplicative random effects)
MR Egger
Weighted median
Weighted mode
fect on ER- Breast cancer (Combined Oncoarray; iCOGS; GWAS meta analysis) || id:ieu-a-1128
0.1
0.0
-0.1
-0.2
0.1
0.2
0.3
SNP effect on Total cholesterol in HDL2 || id:ebi-cfb233-GCST90301969

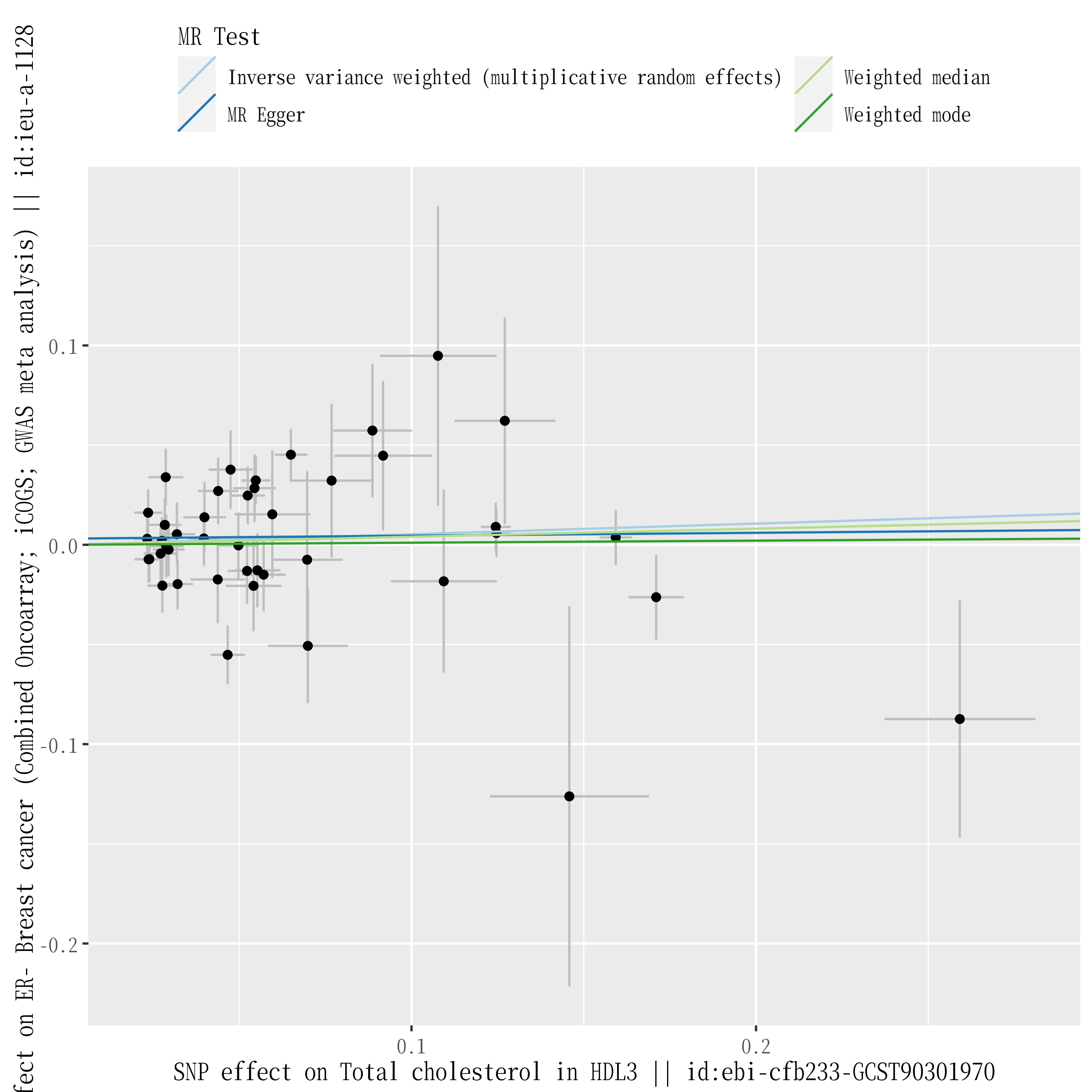
MR Test
Inverse variance weighted (multiplicative random effects)
MR Egger
Weighted median
Weighted mode
0.1
0.0
-0.1
-0.2
0.1
0.2
SNP effect on Total cholesterol in HDL3 || id:ebi-cfb233-GCST90301970
fect on ER- Breast cancer (Combined Oncoarray; iCOGS; GWAS meta analysis) || id:ieu-a-1128

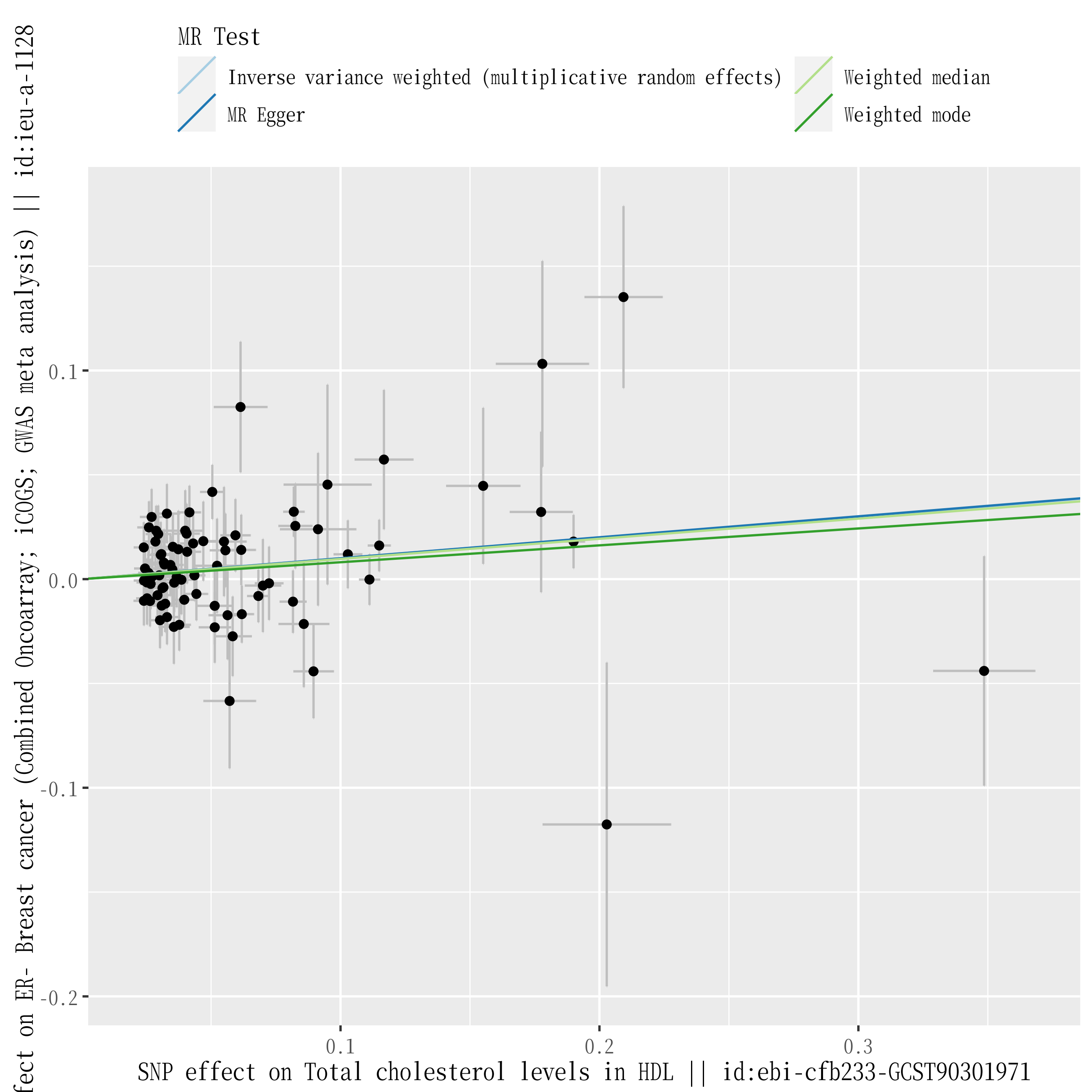
MR Test
Inverse variance weighted (multiplicative random effects)
MR Egger
Weighted median
Weighted mode
0.1
0.0
-0.1
-0.2
0.1
0.2
0.3
SNP effect on Total cholesterol levels in HDL || id:ebi-cfb233-GCST90301971
fect on ER- Breast cancer (Combined Oncoarray; iCOGS; GWAS meta analysis) || id:ieu-a-1128

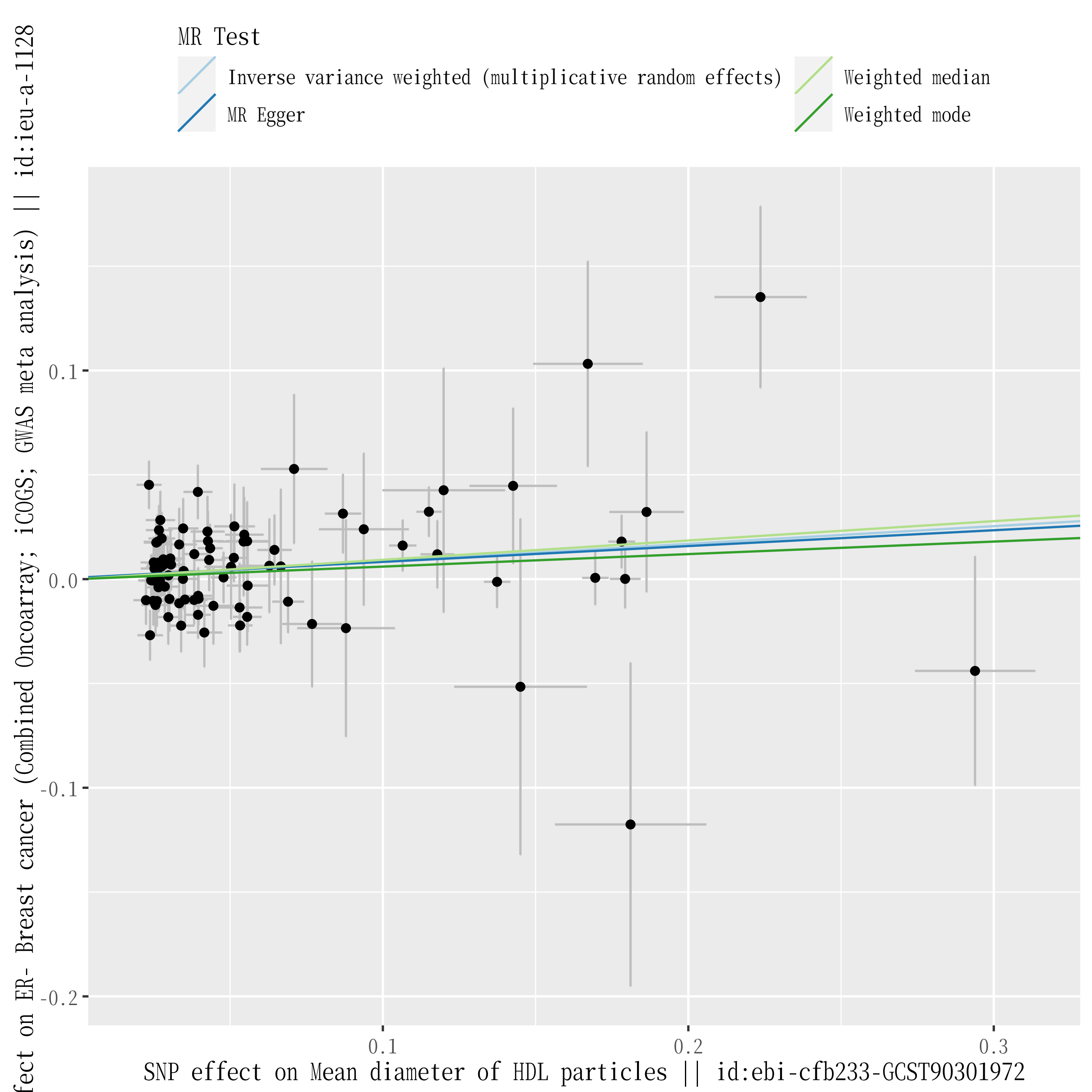

MR Test
Inverse variance weighted (multiplicative random effects)
MR Egger
Weighted median
Weighted mode
0.1
0.0
-0.1
-0.2
0.1
0.2
0.3
SNP effect on Mean diameter of HDL particles || id:ebi-cfb233-GCST90301972
fect on ER- Breast cancer (Combined Oncoarray; iCOGS; GWAS meta analysis) || id:ieu-a-1128

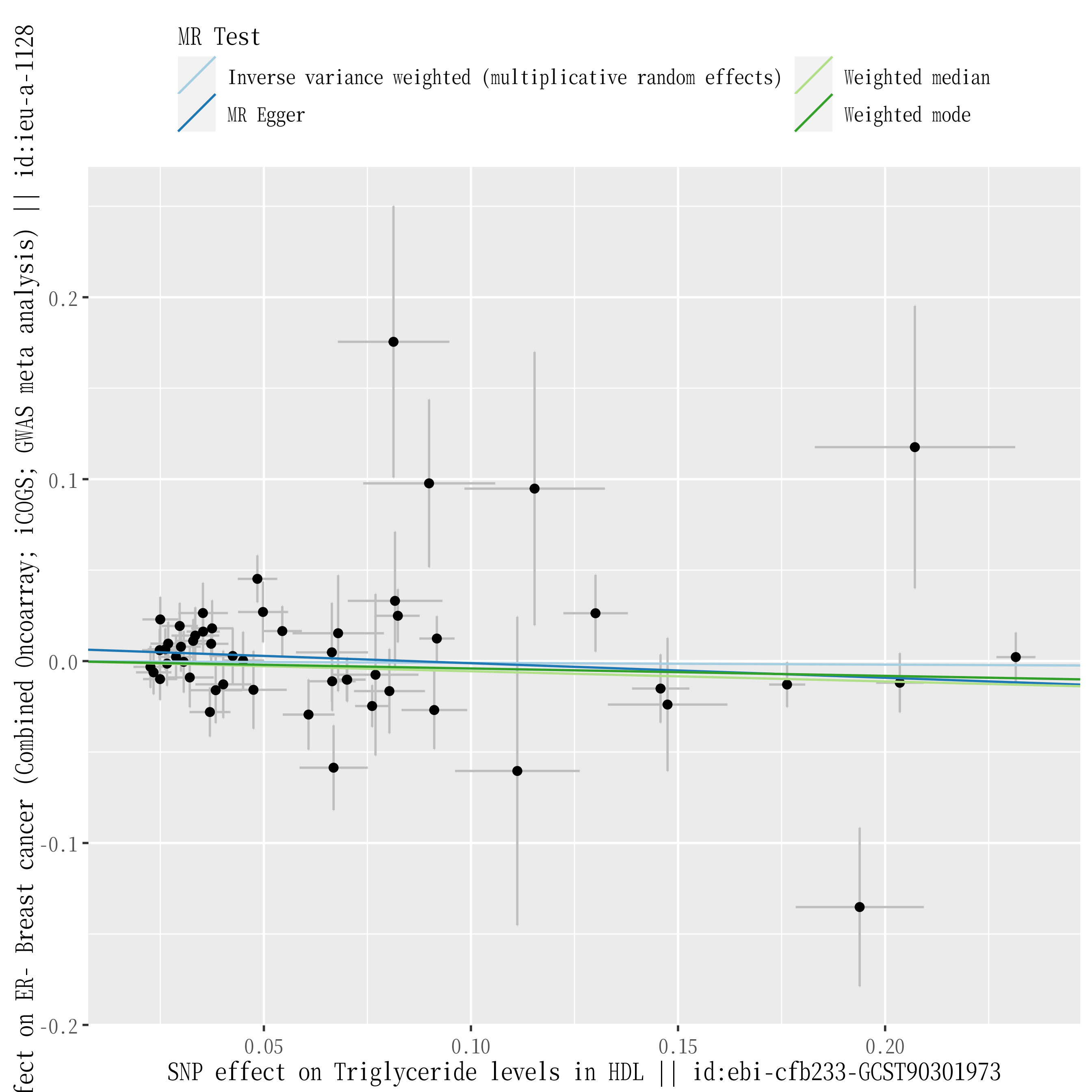
MR Test
Inverse variance weighted (multiplicative random effects)
MR Egger
Weighted median
Weighted mode
0.2
0.1
0.0
-0.1
-0.2
0.05
0.10
0.15
0.20
SNP effect on Triglyceride levels in HDL || id:ebi-cfb233-GCST90301973
fect on ER- Breast cancer (Combined Oncoarray; iCOGS; GWAS meta analysis) || id:ieu-a-1128

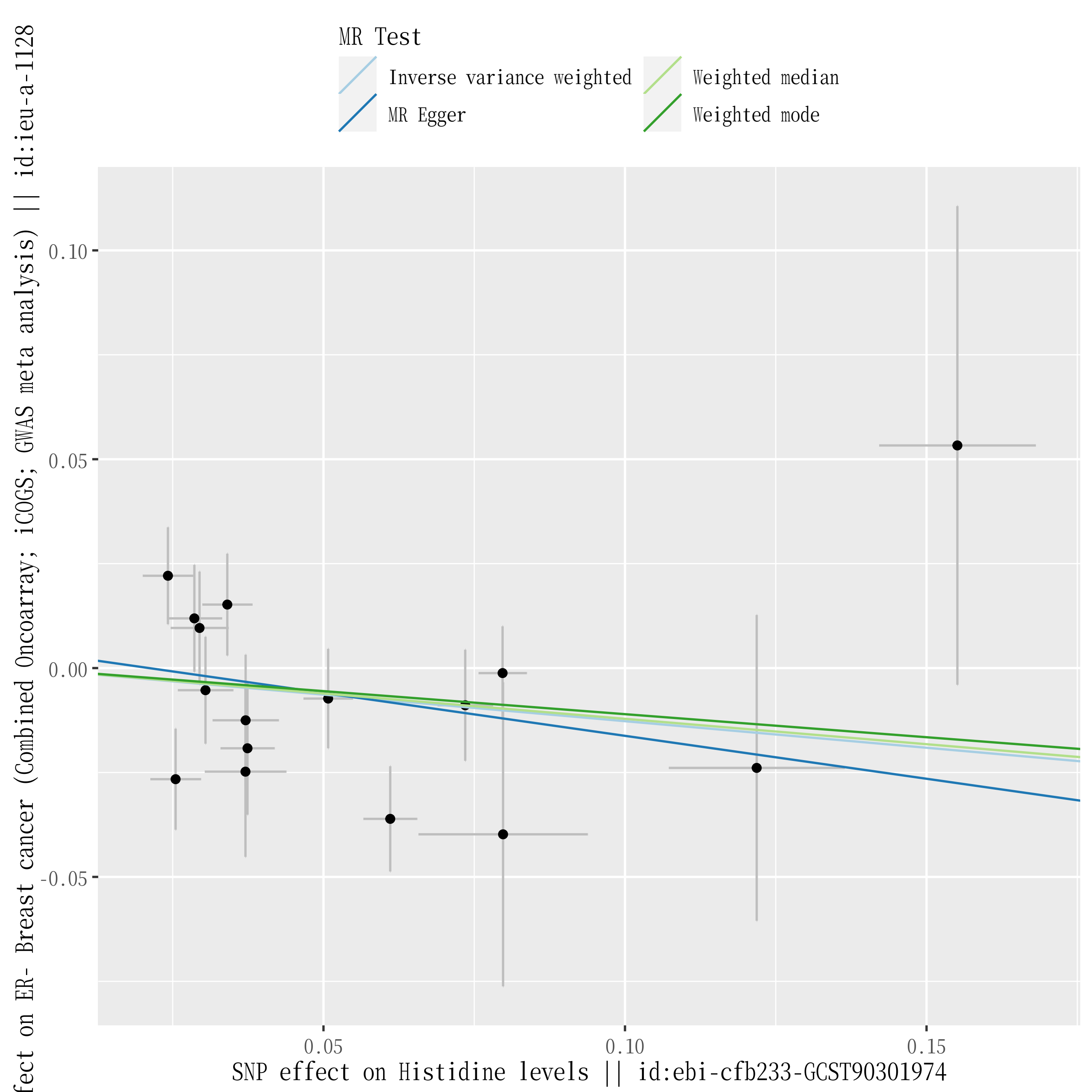

MR Test
Inverse variance weighted
MR Egger
Weighted median
Weighted mode
0.10
0.05
0.00
-0.05
0.05
0.10
0.15
SNP effect on Histidine levels || id:ebi-cfb233-GCST90301974
fect on ER- Breast cancer (Combined Oncoarray; iCOGS; GWAS meta analysis) || id:ieu-a-1128

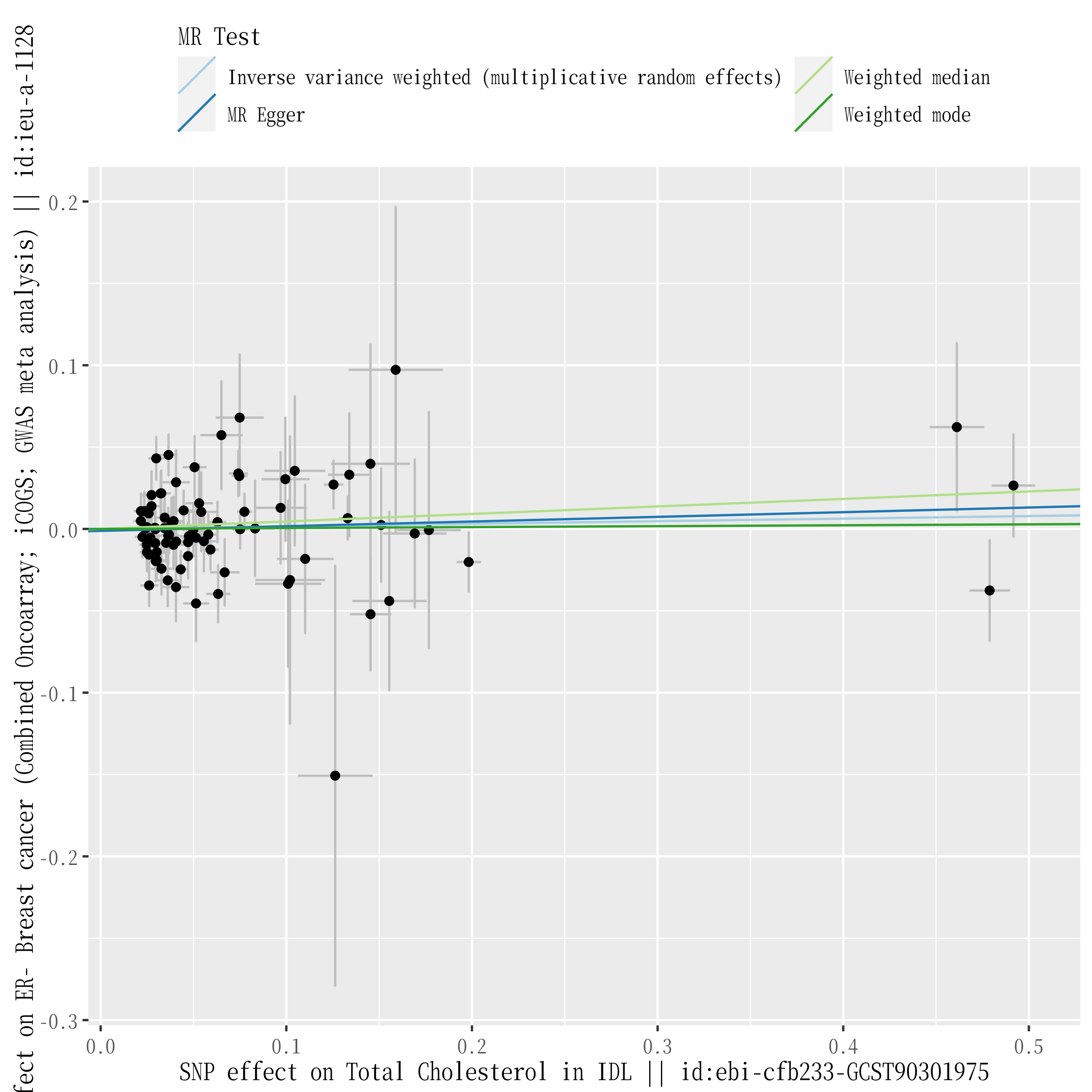

MR Test
Inverse variance weighted (multiplicative random effects)
MR Egger
Weighted median
Weighted mode
fect on ER- Breast cancer (Combined Oncoarray; iCOGS; GWAS meta analysis) || id:ieu-a-1128
0.2
0.1
0.0
-0.1
-0.2
-0.3
0.0
0.1
0.2
0.3
0.4
0.5
SNP effect on Total Cholesterol in IDL || id:ebi-cfb233-GCST90301975

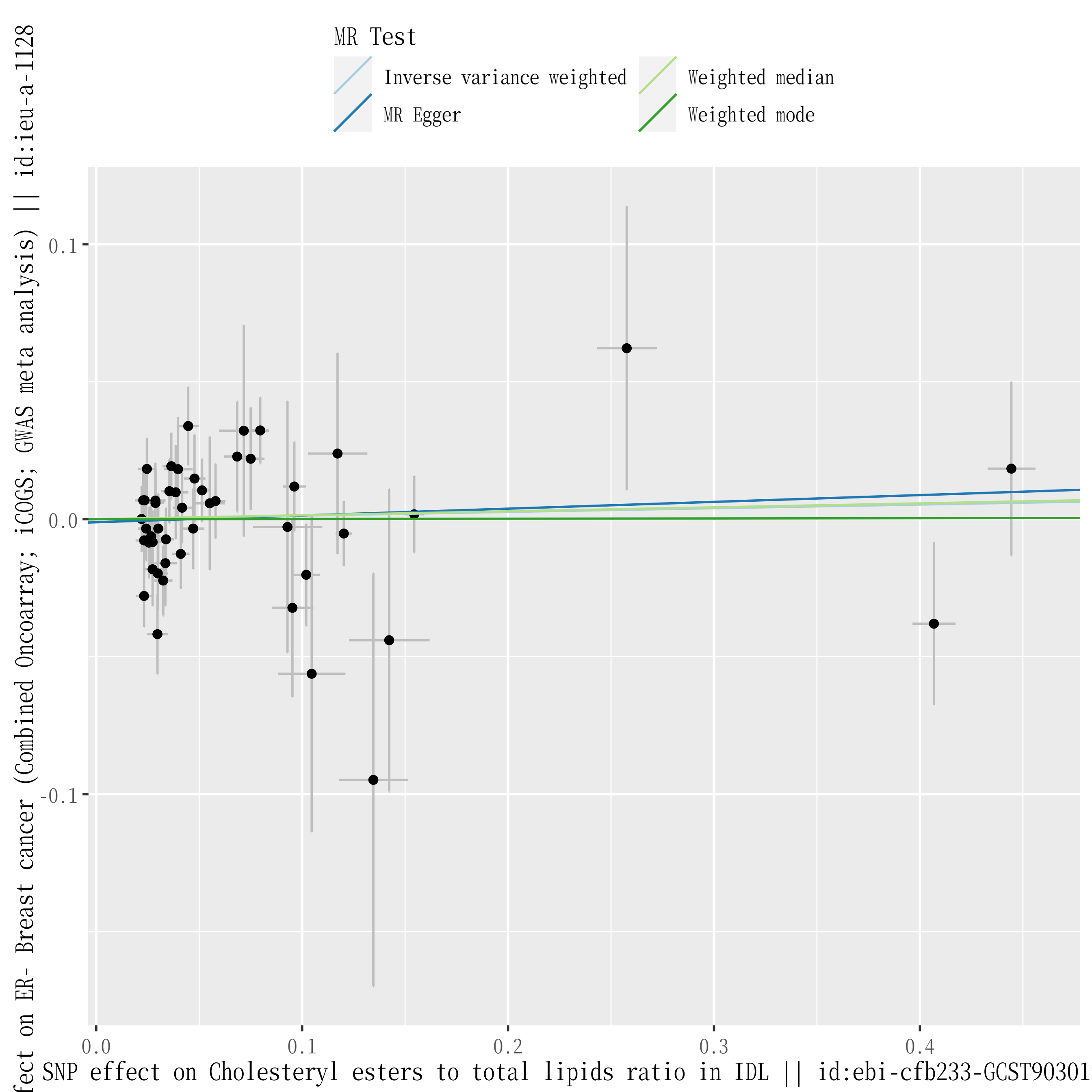
MR Test
Inverse variance weighted
MR Egger
Weighted median
Weighted mode
0.1
0.0
-0.1
0.0
0.1
0.2
0.3
0.4
fect on ER- Breast cancer (Combined Oncoarray; iCOGS; GWAS meta analysis) || id:ieu-a-1128
SNP effect on Cholesteryl esters to total lipids ratio in IDL || id:ebi-cfb233-GCST90301

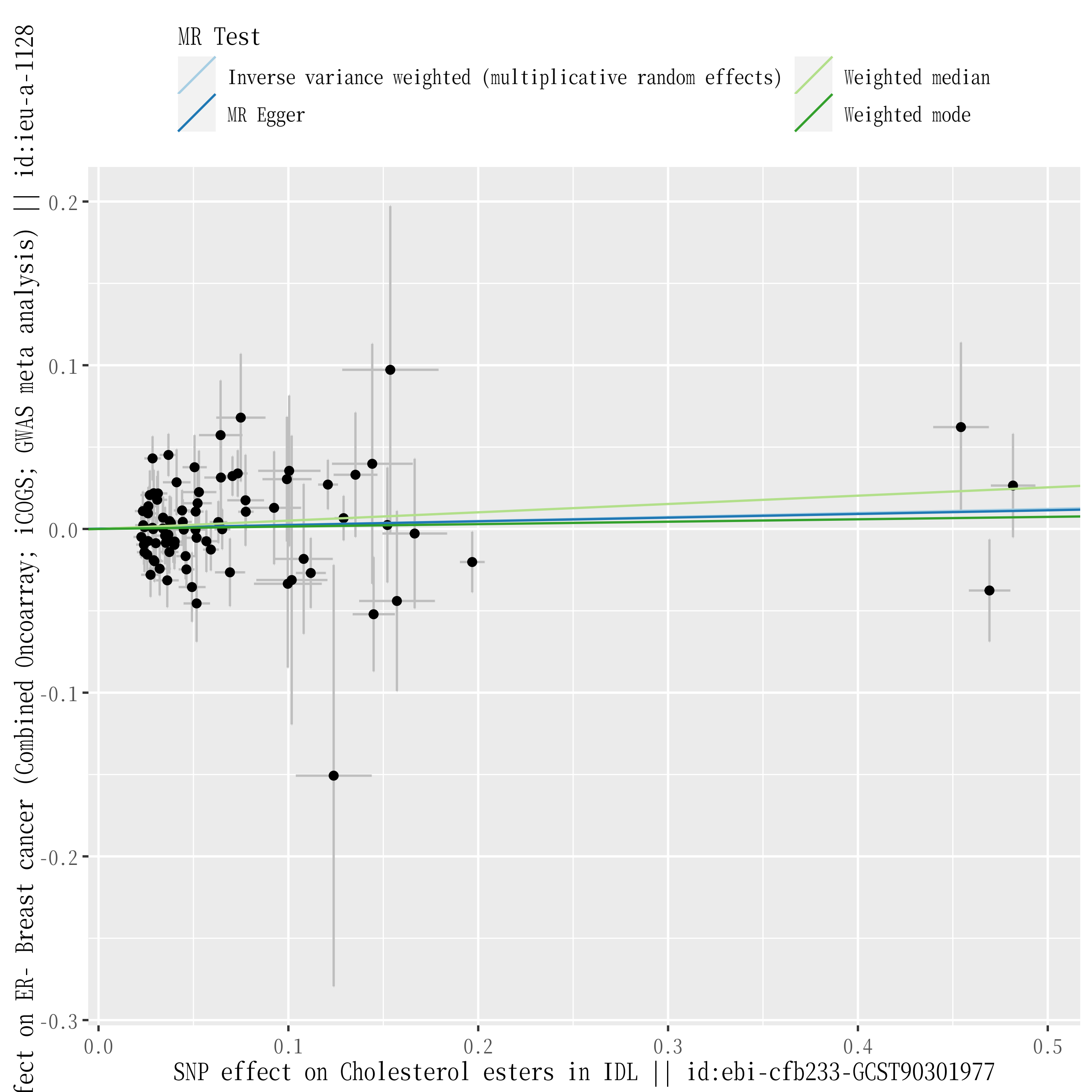
MR Test
Inverse variance weighted (multiplicative random effects)
MR Egger
Weighted median
Weighted mode
fect on ER- Breast cancer (Combined Oncoarray; iCOGS; GWAS meta analysis) || id:ieu-a-1128
0.2
0.1
0.0
-0.1
-0.2
-0.3
0.0
0.1
0.2
0.3
0.4
0.5
SNP effect on Cholesterol esters in IDL || id:ebi-cfb233-GCST90301977

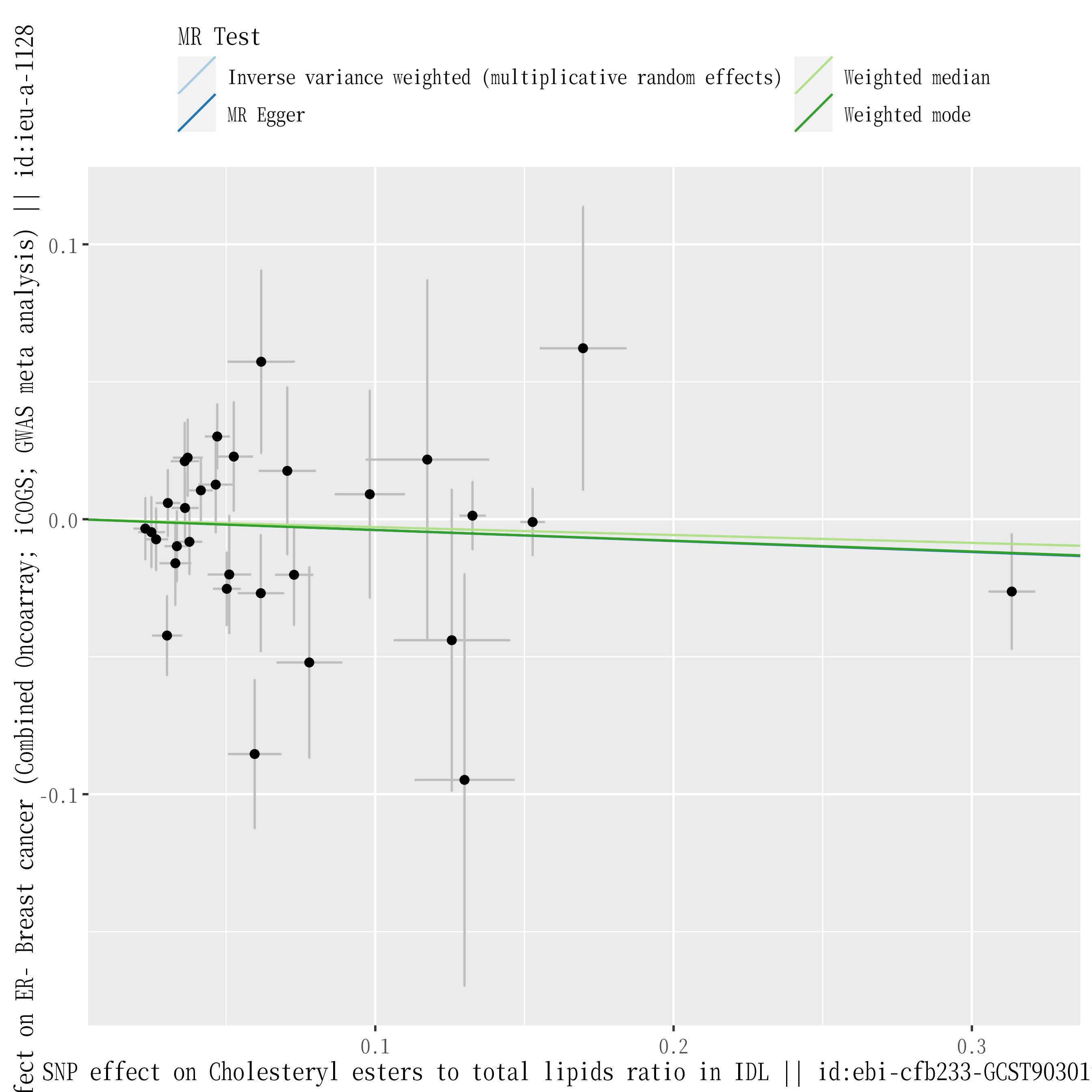
MR Test
Inverse variance weighted (multiplicative random effects)
MR Egger
Weighted median
Weighted mode
0.1
0.0
-0.1
0.1
0.2
0.3
SNP effect on Cholesteryl esters to total lipids ratio in IDL || id:ebi-cfb233-GCST90301
fect on ER- Breast cancer (Combined Oncoarray; iCOGS; GWAS meta analysis) || id:ieu-a-1128

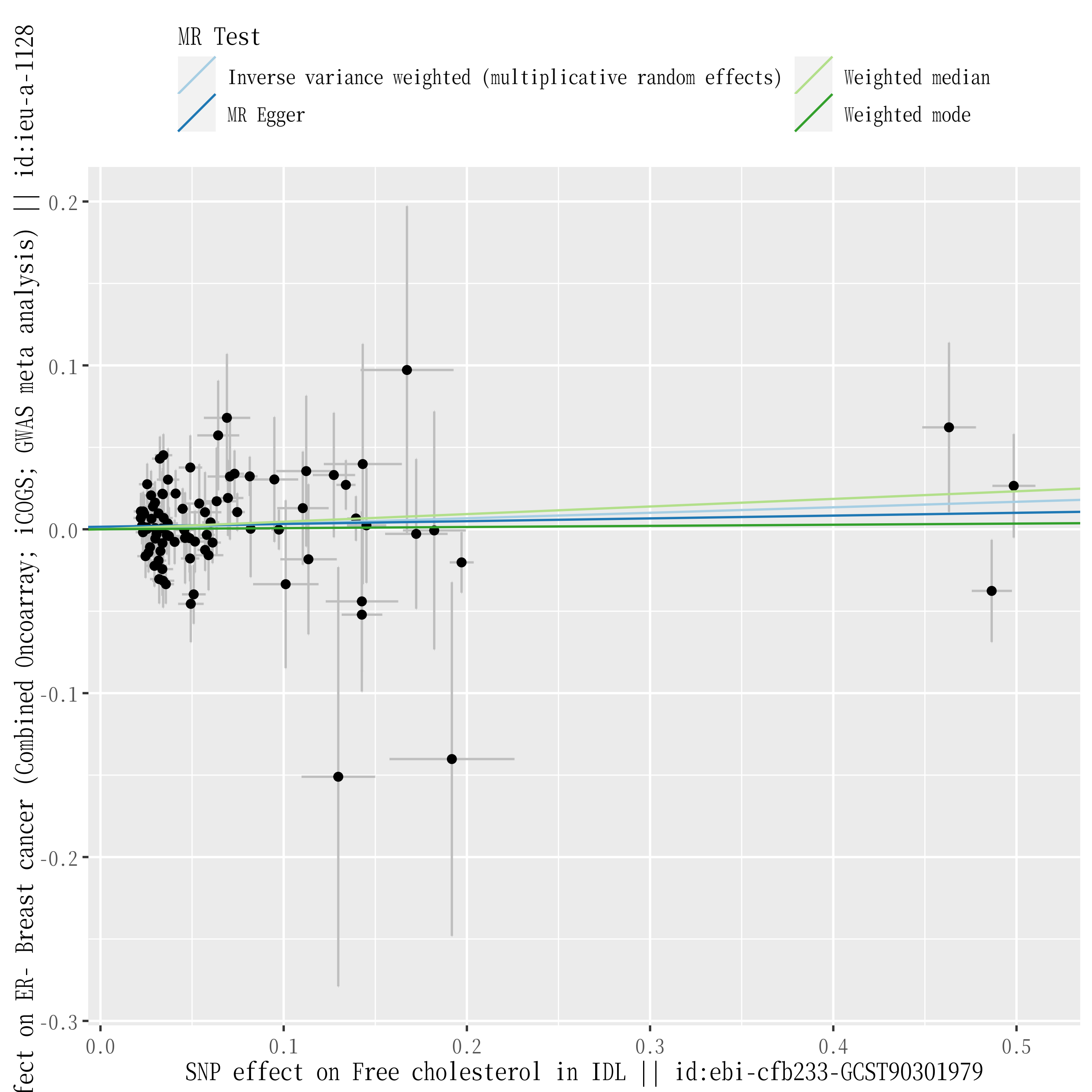

MR Test
Inverse variance weighted (multiplicative random effects)
MR Egger
Weighted median
Weighted mode
0.2
0.1
0.0
-0.1
-0.2
-0.3
0.0
0.1
0.2
0.3
0.4
0.5
SNP effect on Free cholesterol in IDL || id:ebi-cfb233-GCST90301979
fect on ER- Breast cancer (Combined Oncoarray; iCOGS; GWAS meta analysis) || id:ieu-a-1128

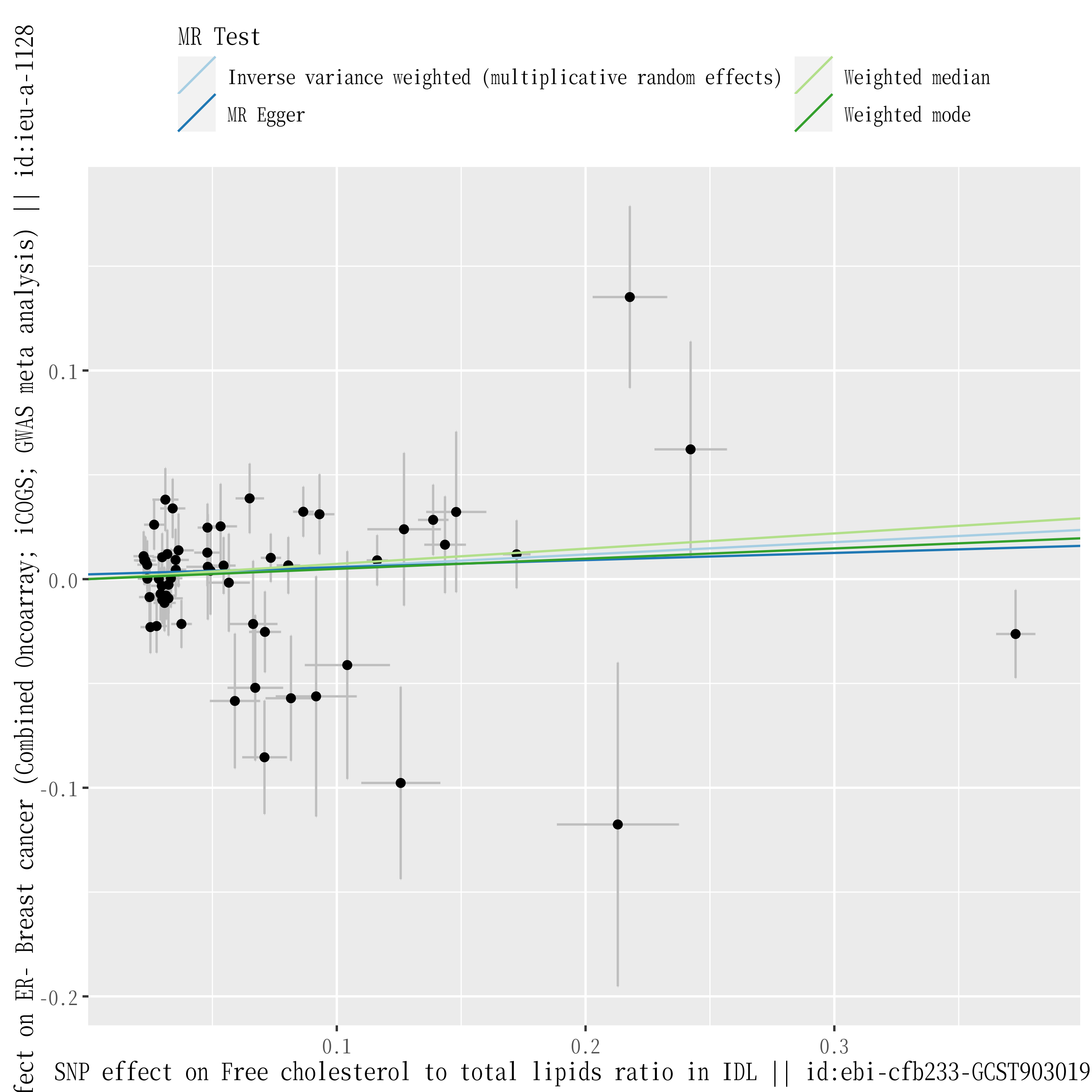
MR Test
Inverse variance weighted (multiplicative random effects)
MR Egger
Weighted median
Weighted mode
0.1
0.0
-0.1
-0.2
0.1
0.2
0.3
SNP effect on Free cholesterol to total lipids ratio in IDL || id:ebi-cfb233-GCST903019
fect on ER- Breast cancer (Combined Oncoarray; iCOGS; GWAS meta analysis) || id:ieu-a-1128

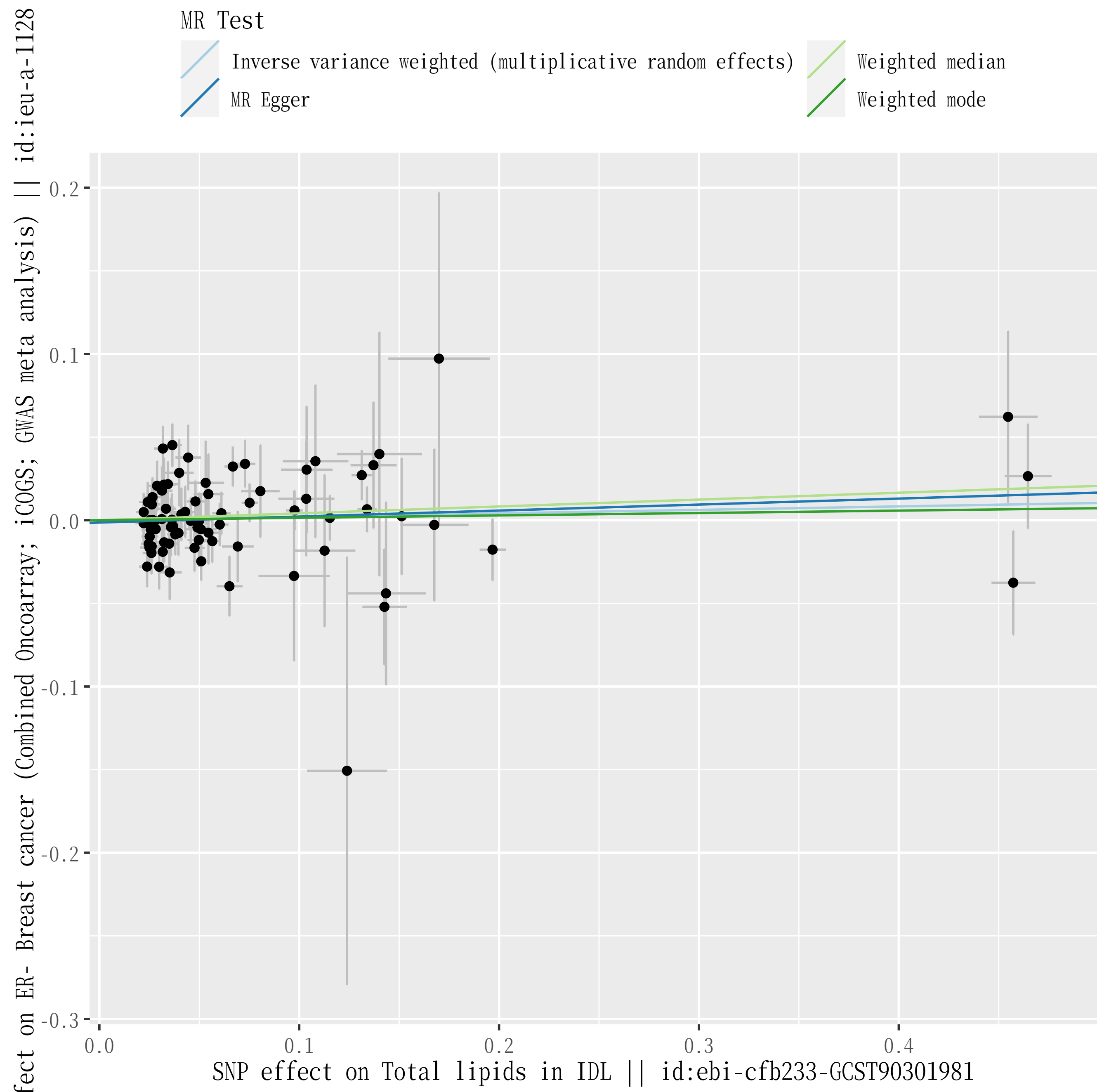

MR Test
Inverse variance weighted (multiplicative random effects)
MR Egger
Weighted median
Weighted mode
0.2
0.1
0.0
-0.1
-0.2
-0.3
0.0
0.1
0.2
0.3
0.4
fect on ER- Breast cancer (Combined Oncoarray; iCOGS; GWAS meta analysis) || id:ieu-a-1128
SNP effect on Total lipids in IDL || id:ebi-cfb233-GCST90301981

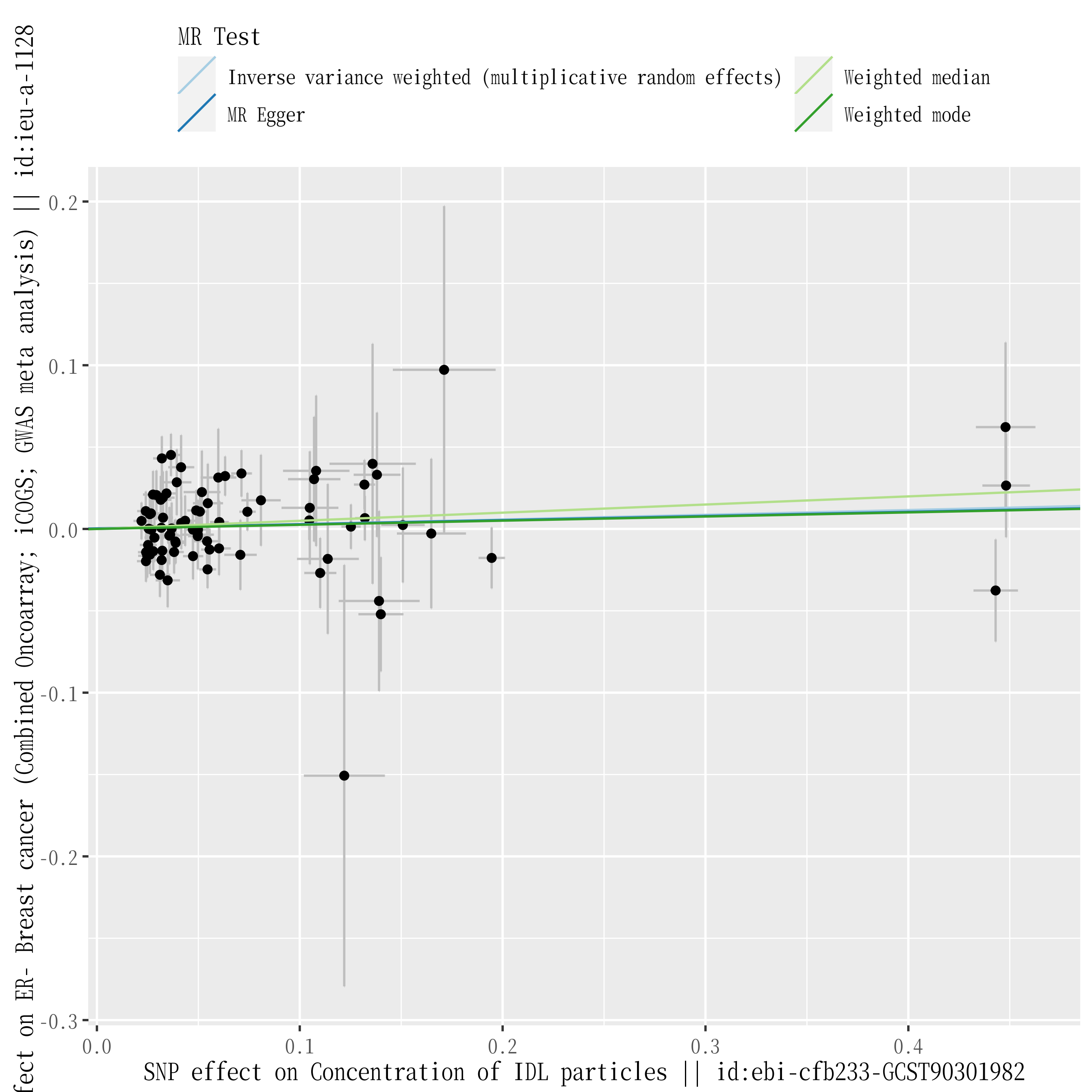

MR Test
Inverse variance weighted (multiplicative random effects)
MR Egger
Weighted median
Weighted mode
0.2
0.1
0.0
-0.1
-0.2
-0.3
0.0
0.1
0.2
0.3
0.4
SNP effect on Concentration of IDL particles || id:ebi-cfb233-GCST90301982
fect on ER- Breast cancer (Combined Oncoarray; iCOGS; GWAS meta analysis) || id:ieu-a-1128

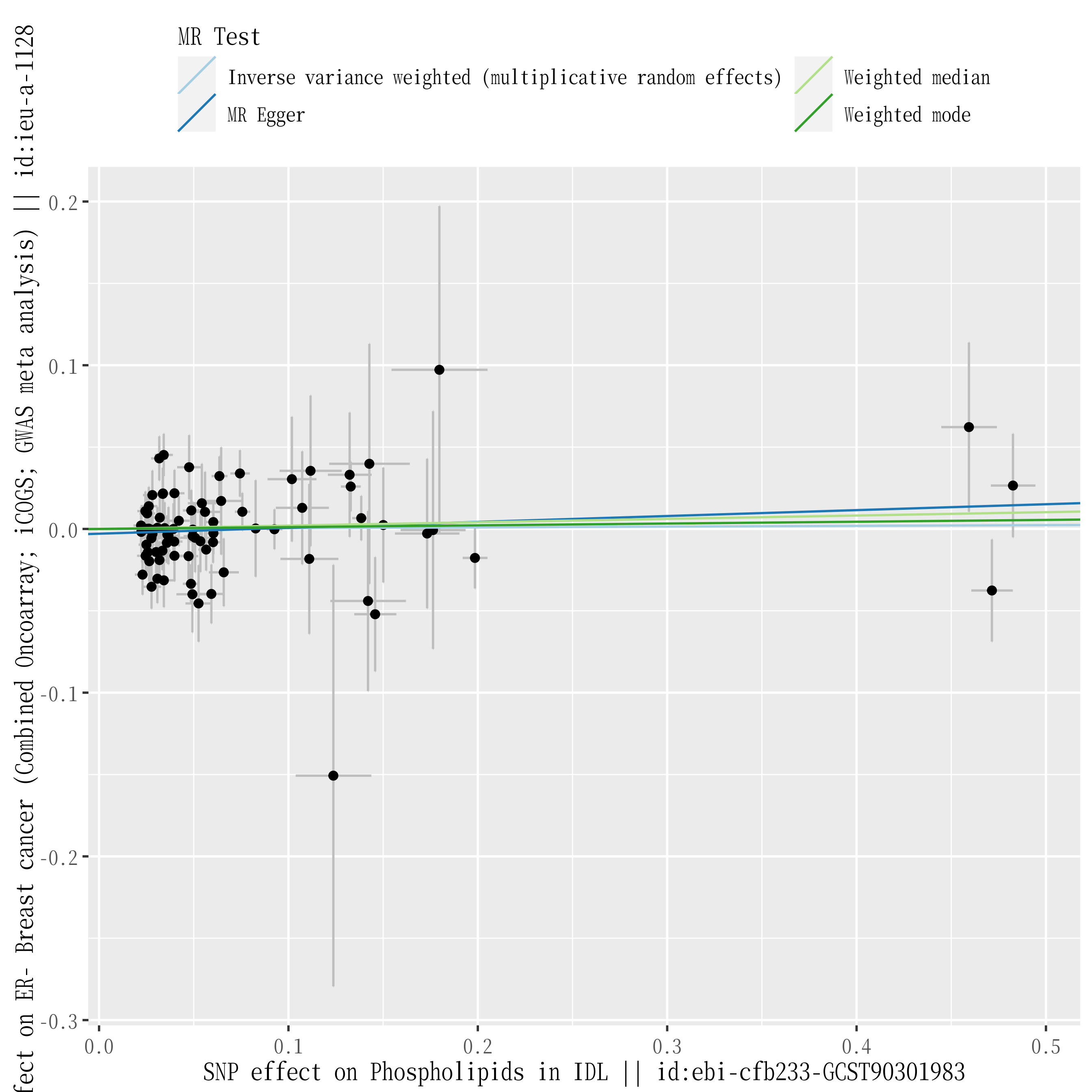

MR Test
Inverse variance weighted (multiplicative random effects)
MR Egger
Weighted median
Weighted mode
0.2
0.1
0.0
-0.1
-0.2
-0.3
0.0
0.1
0.2
0.3
0.4
0.5
SNP effect on Phospholipids in IDL || id:ebi-cfb233-GCST90301983
fect on ER- Breast cancer (Combined Oncoarray; iCOGS; GWAS meta analysis) || id:ieu-a-1128

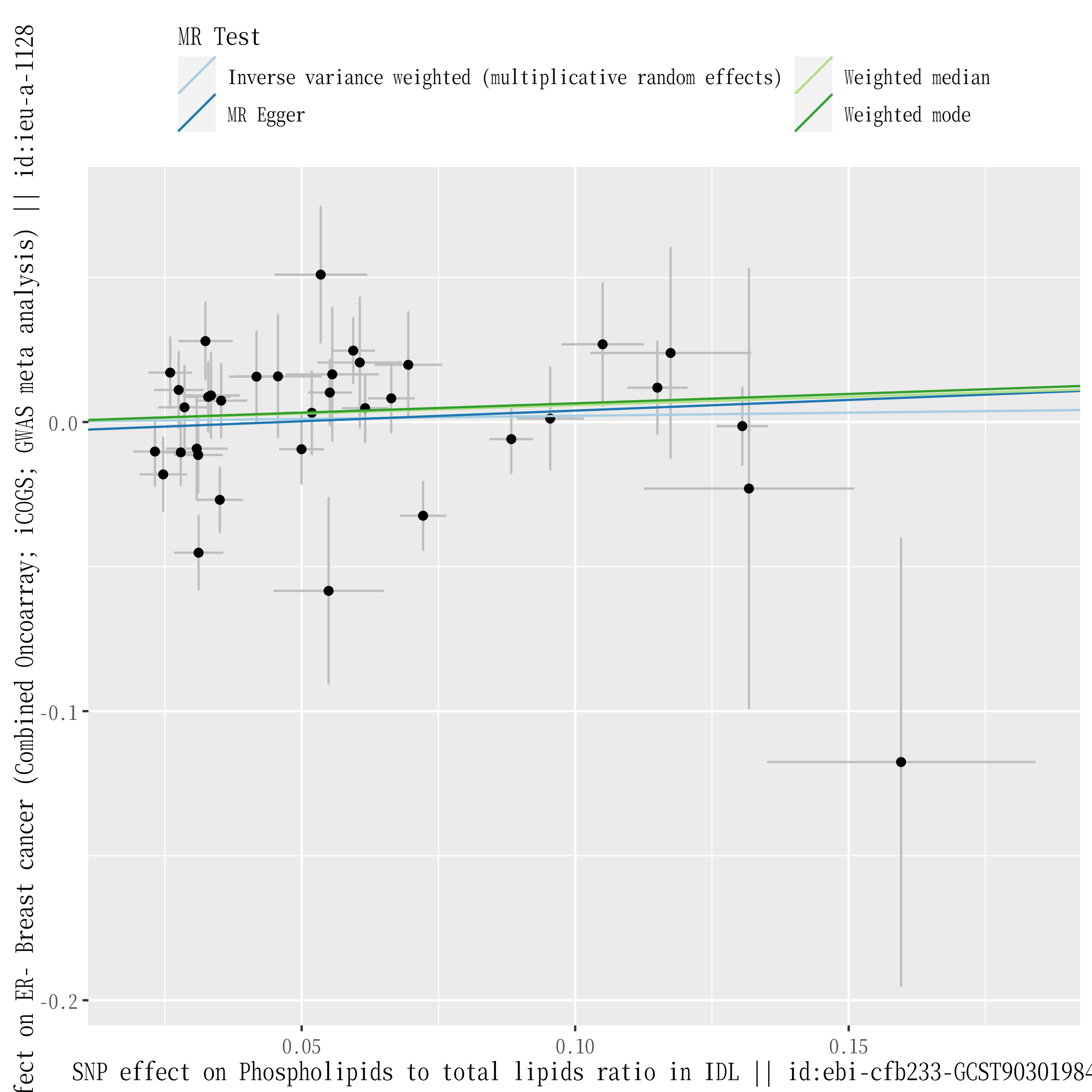
MR Test
Inverse variance weighted (multiplicative random effects)
MR Egger
Weighted median
Weighted mode
0.0
-0.1
-0.2
0.05
0.10
0.15
SNP effect on Phospholipids to total lipids ratio in IDL || id:ebi-cfb233-GCST9030198
fect on ER- Breast cancer (Combined Oncoarray; iCOGS; GWAS meta analysis) || id:ieu-a-1128

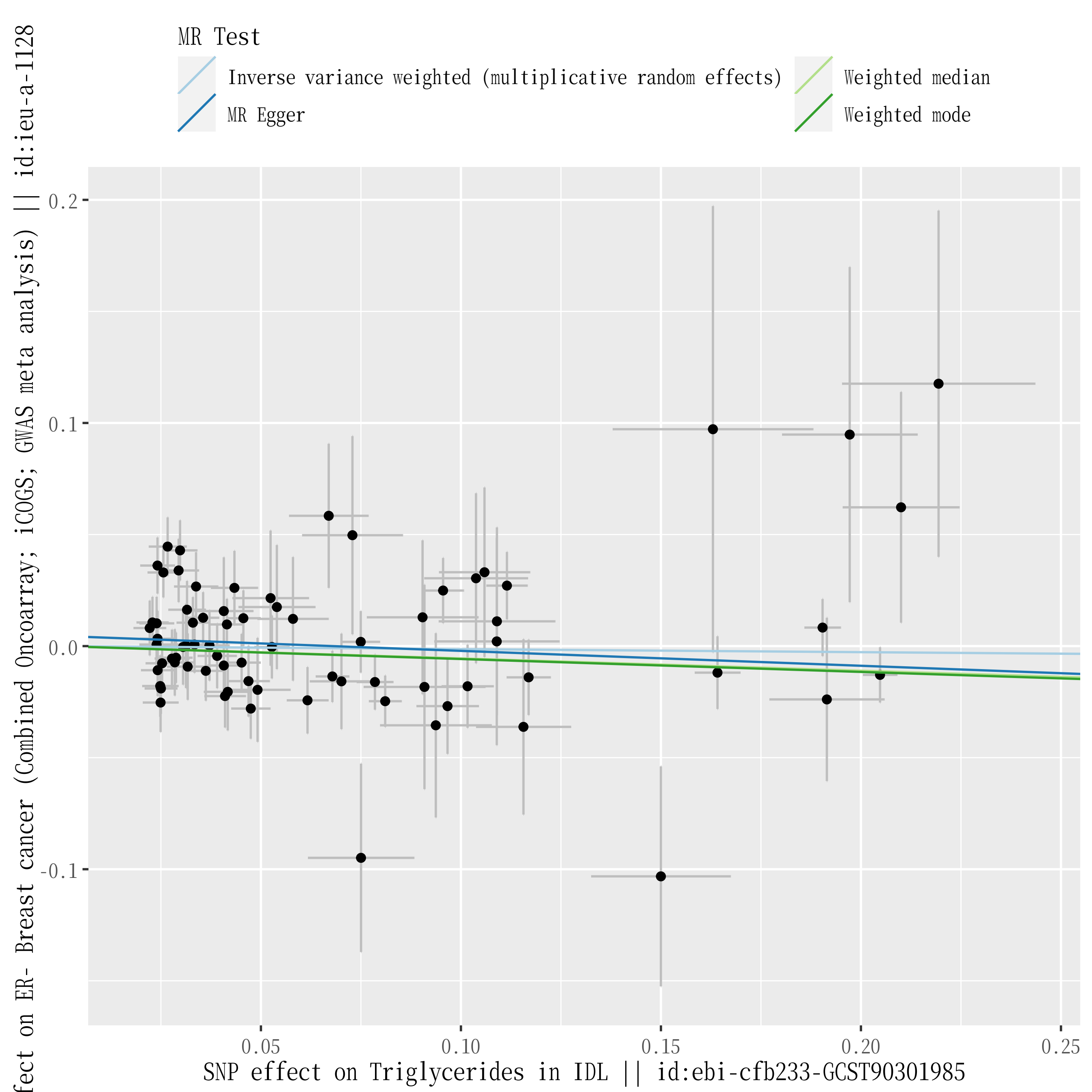
MR Test
Inverse variance weighted (multiplicative random effects)
MR Egger
Weighted median
Weighted mode
0.2
0.1
0.0
-0.1
0.05
0.10
0.15
0.20
0.25
SNP effect on Triglycerides in IDL || id:ebi-cfb233-GCST90301985
fect on ER- Breast cancer (Combined Oncoarray; iCOGS; GWAS meta analysis) || id:ieu-a-1128

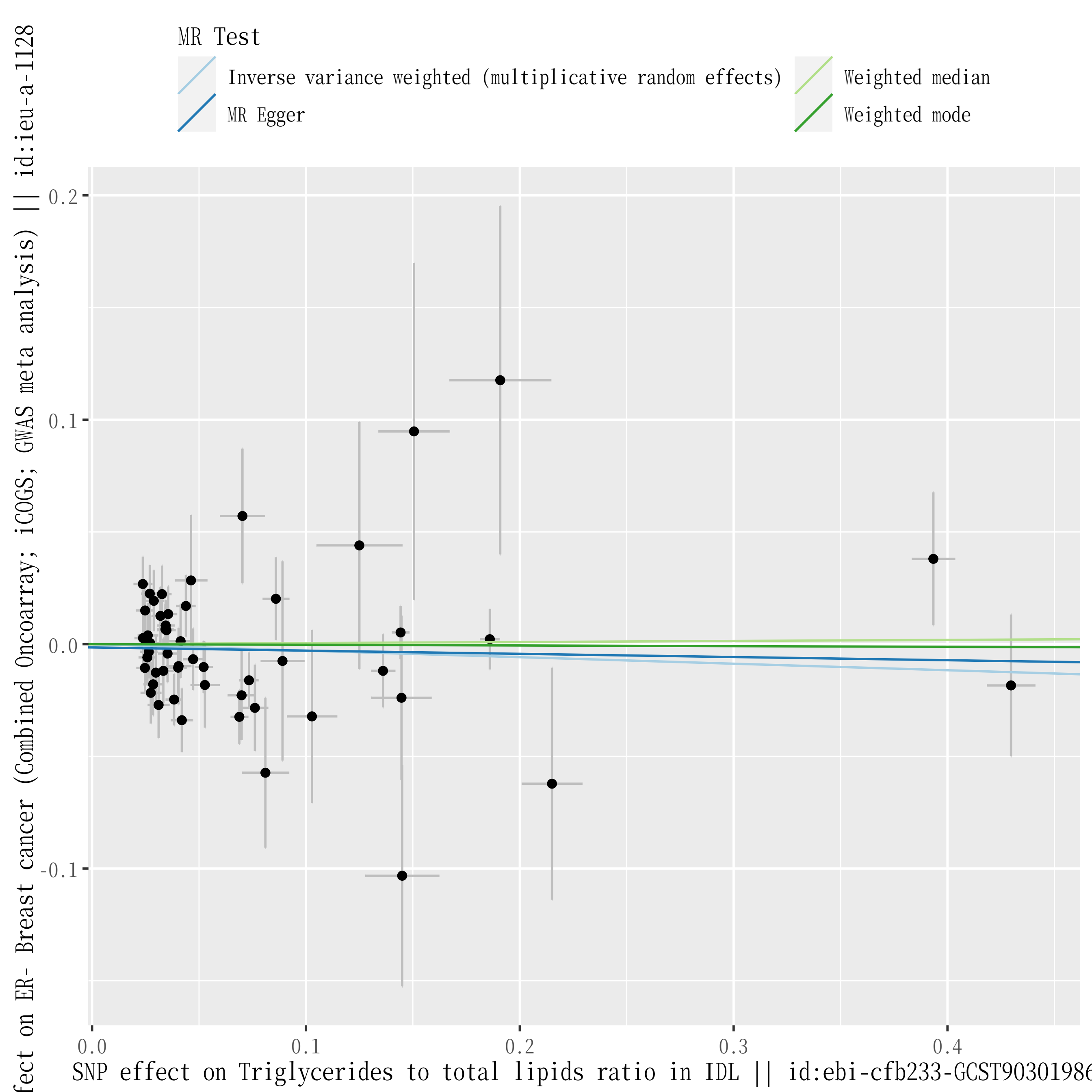
MR Test
Inverse variance weighted (multiplicative random effects)
MR Egger
Weighted median
Weighted mode
0.2
0.1
0.0
-0.1
0.0
0.1
0.2
0.3
0.4
SNP effect on Triglycerides to total lipids ratio in IDL || id:ebi-cfb233-GCST9030198
fect on ER- Breast cancer (Combined Oncoarray; iCOGS; GWAS meta analysis) || id:ieu-a-1128

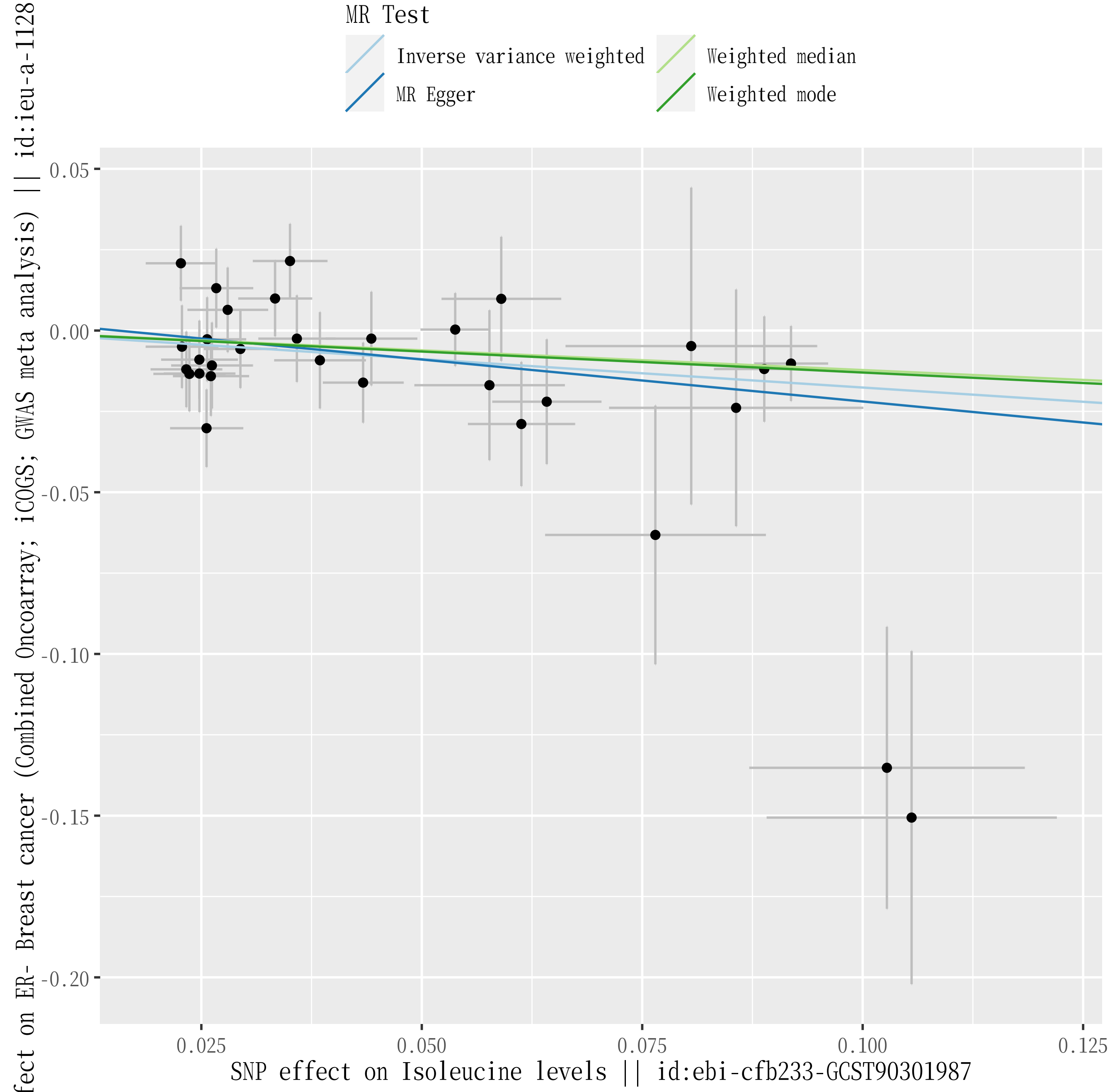

MR Test
Inverse variance weighted
MR Egger
Weighted median
Weighted mode
0.05
0.00
-0.05
-0.10
-0.15
-0.20
0.025
0.050
0.075
0.100
0.125
SNP effect on Isoleucine levels || id:ebi-cfb233-GCST90301987
fect on ER- Breast cancer (Combined Oncoarray; iCOGS; GWAS meta analysis) || id:ieu-a-1128

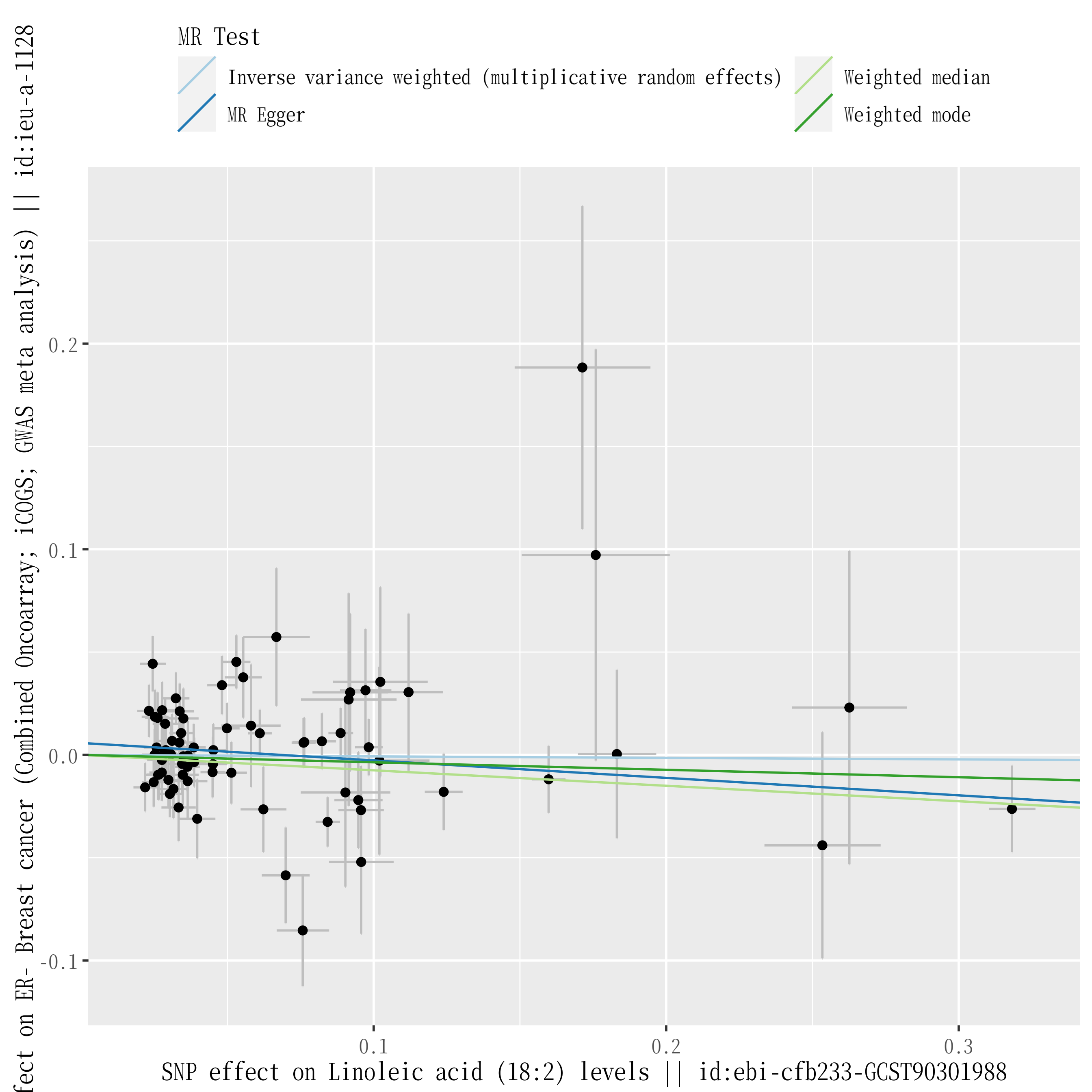
MR Test
Inverse variance weighted (multiplicative random effects)
MR Egger
Weighted median
Weighted mode
0.2
0.1
0.0
-0.1
0.1
0.2
0.3
SNP effect on Linoleic acid (18:2) levels || id:ebi-cfb233-GCST90301988
fect on ER- Breast cancer (Combined Oncoarray; iCOGS; GWAS meta analysis) || id:ieu-a-1128

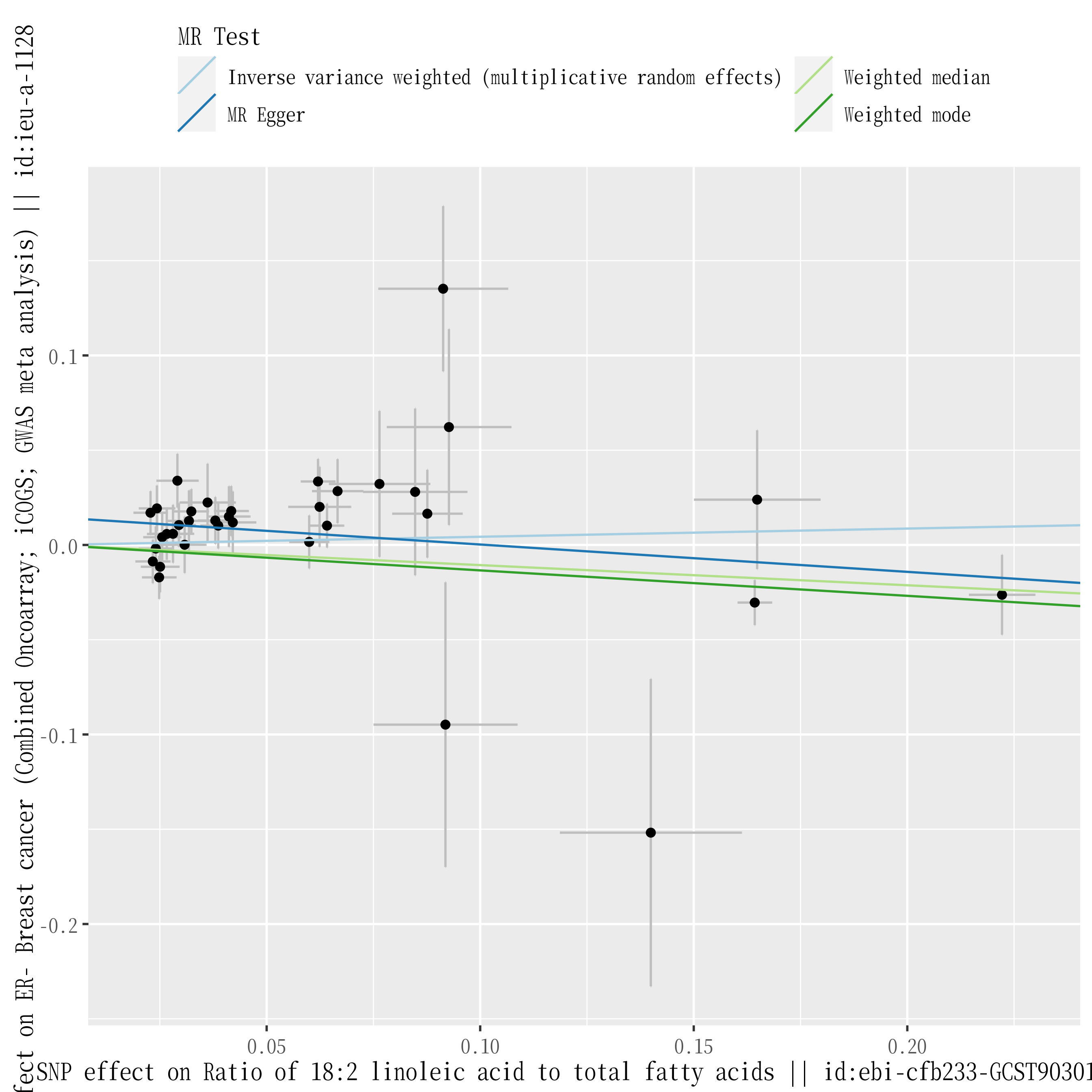

MR Test
Inverse variance weighted (multiplicative random effects)
MR Egger
Weighted median
Weighted mode
0.1
0.0
-0.1
-0.2
0.05
0.10
0.15
0.20
SNP effect on Ratio of 18:2 linoleic acid to total fatty acids || id:ebi-cfb233-GCST90301
fect on ER- Breast cancer (Combined Oncoarray; iCOGS; GWAS meta analysis) || id:ieu-a-1128

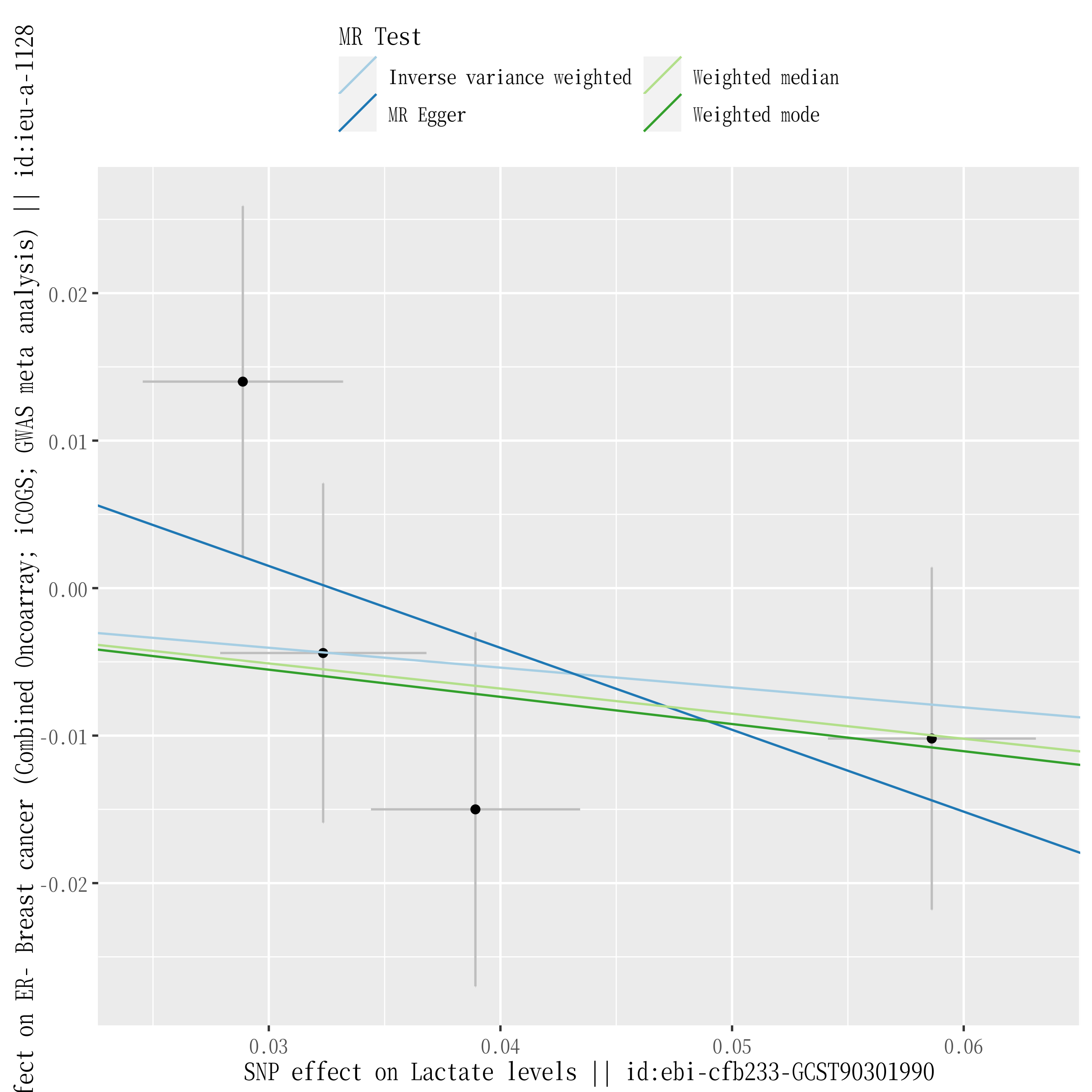
MR Test
Inverse variance weighted
MR Egger
Weighted median
Weighted mode
0.02
0.01
0.00
-0.01
-0.02
0.03
0.04
0.05
0.06
SNP effect on Lactate levels || id:ebi-cfb233-GCST90301990
fect on ER- Breast cancer (Combined Oncoarray; iCOGS; GWAS meta analysis) || id:ieu-a-1128

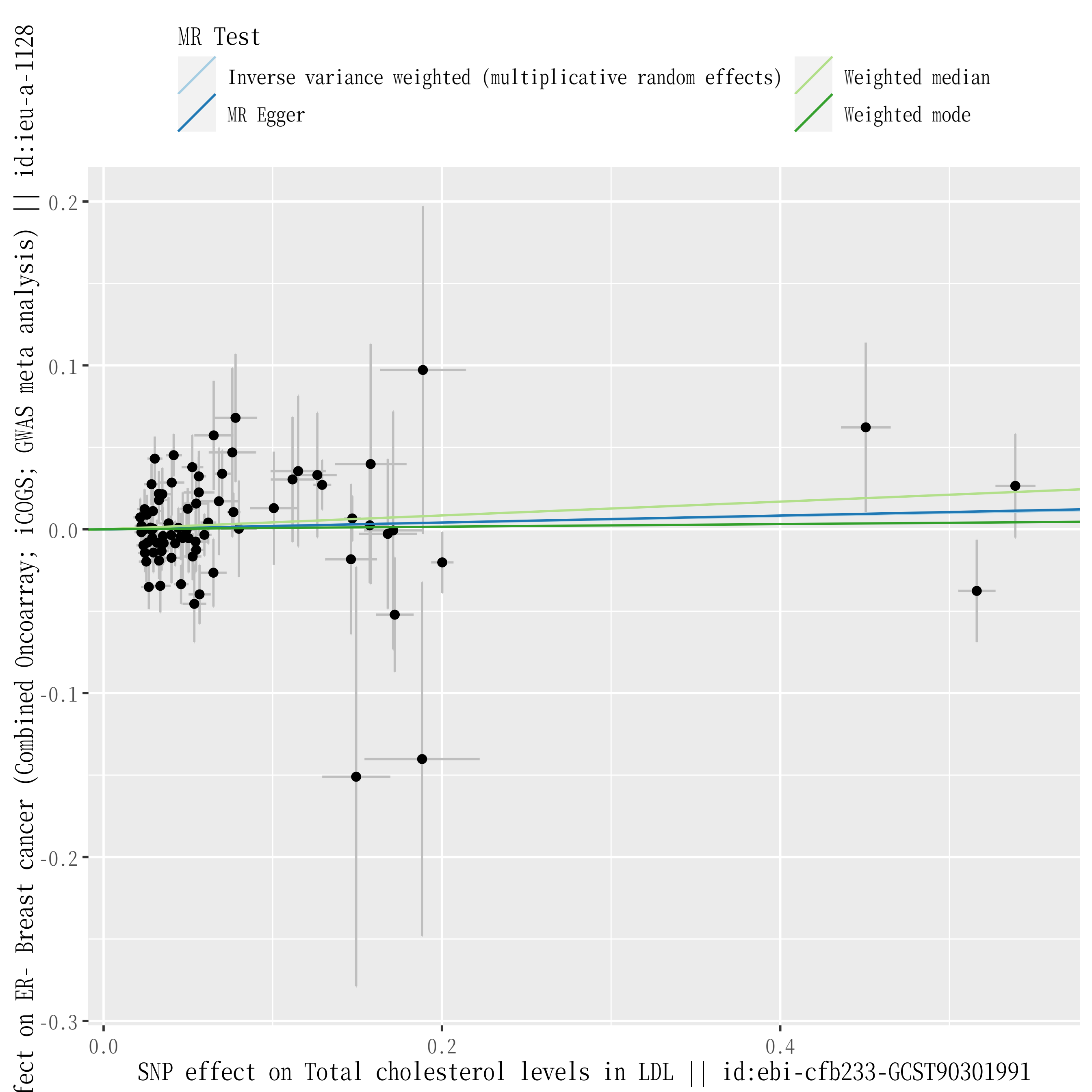
MR Test
Inverse variance weighted (multiplicative random effects)
MR Egger
Weighted median
Weighted mode
fect on ER- Breast cancer (Combined Oncoarray; iCOGS; GWAS meta analysis) || id:ieu-a-1128
0.2
0.1
0.0
-0.1
-0.2
-0.3
0.0
0.2
0.4
SNP effect on Total cholesterol levels in LDL || id:ebi-cfb233-GCST90301991

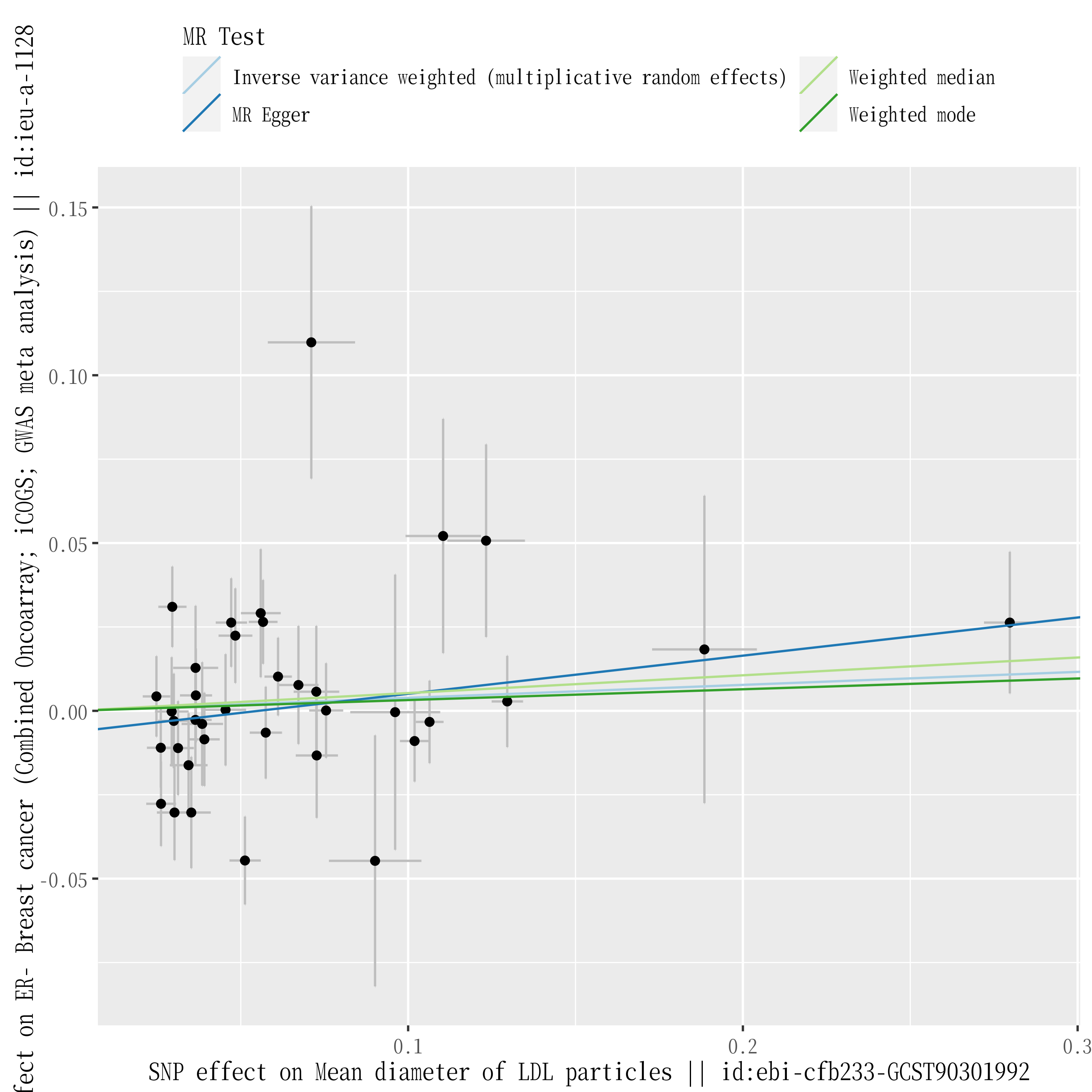
MR Test
Inverse variance weighted (multiplicative random effects)
MR Egger
Weighted median
Weighted mode
0.15
0.10
0.05
0.00
-0.05
0.1
0.2
0.3
SNP effect on Mean diameter of LDL particles || id:ebi-cfb233-GCST90301992
fect on ER- Breast cancer (Combined Oncoarray; iCOGS; GWAS meta analysis) || id:ieu-a-1128

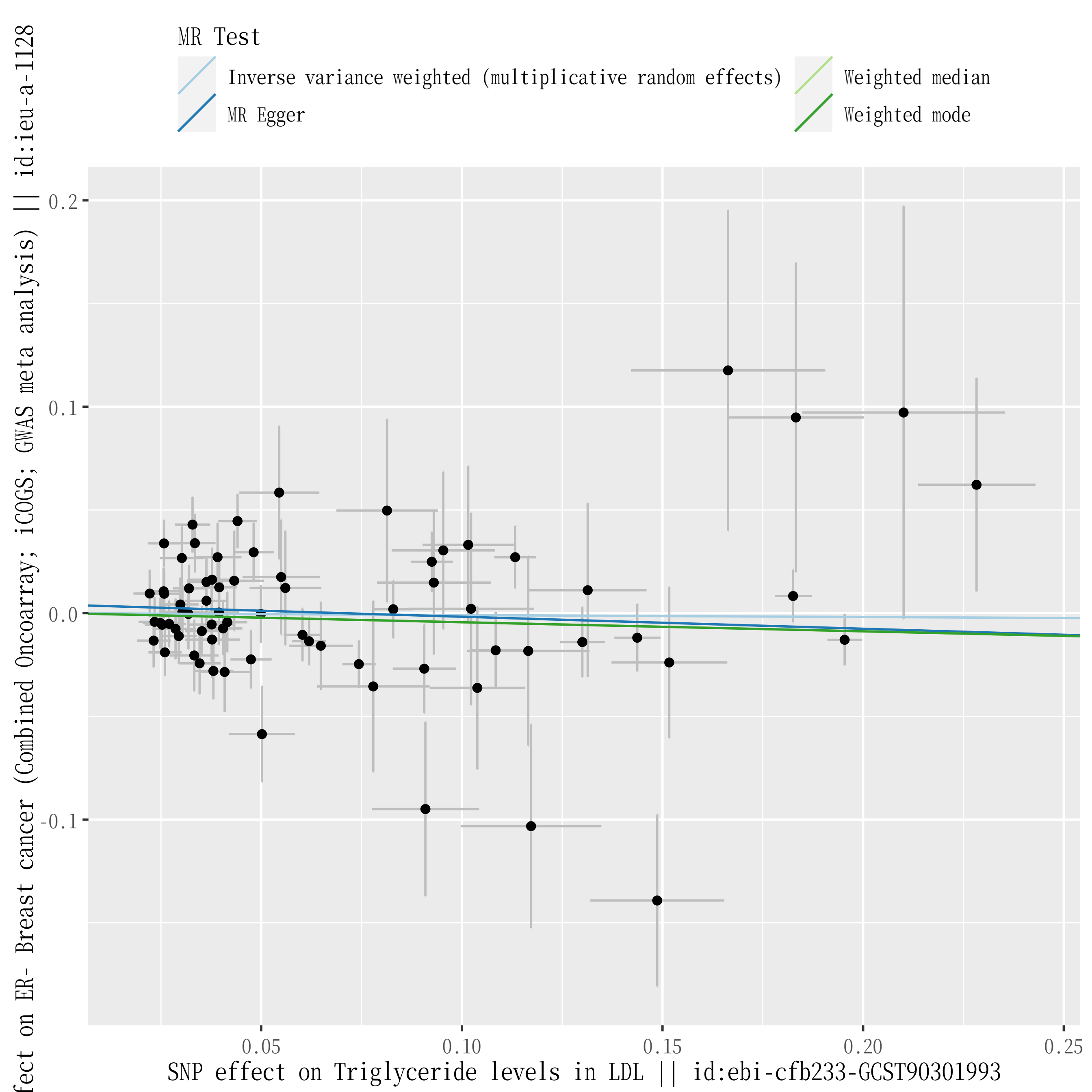
MR Test
Inverse variance weighted (multiplicative random effects)
MR Egger
Weighted median
Weighted mode
0.2
0.1
0.0
-0.1
0.05
0.10
0.15
0.20
0.25
SNP effect on Triglyceride levels in LDL || id:ebi-cfb233-GCST90301993
fect on ER- Breast cancer (Combined Oncoarray; iCOGS; GWAS meta analysis) || id:ieu-a-1128

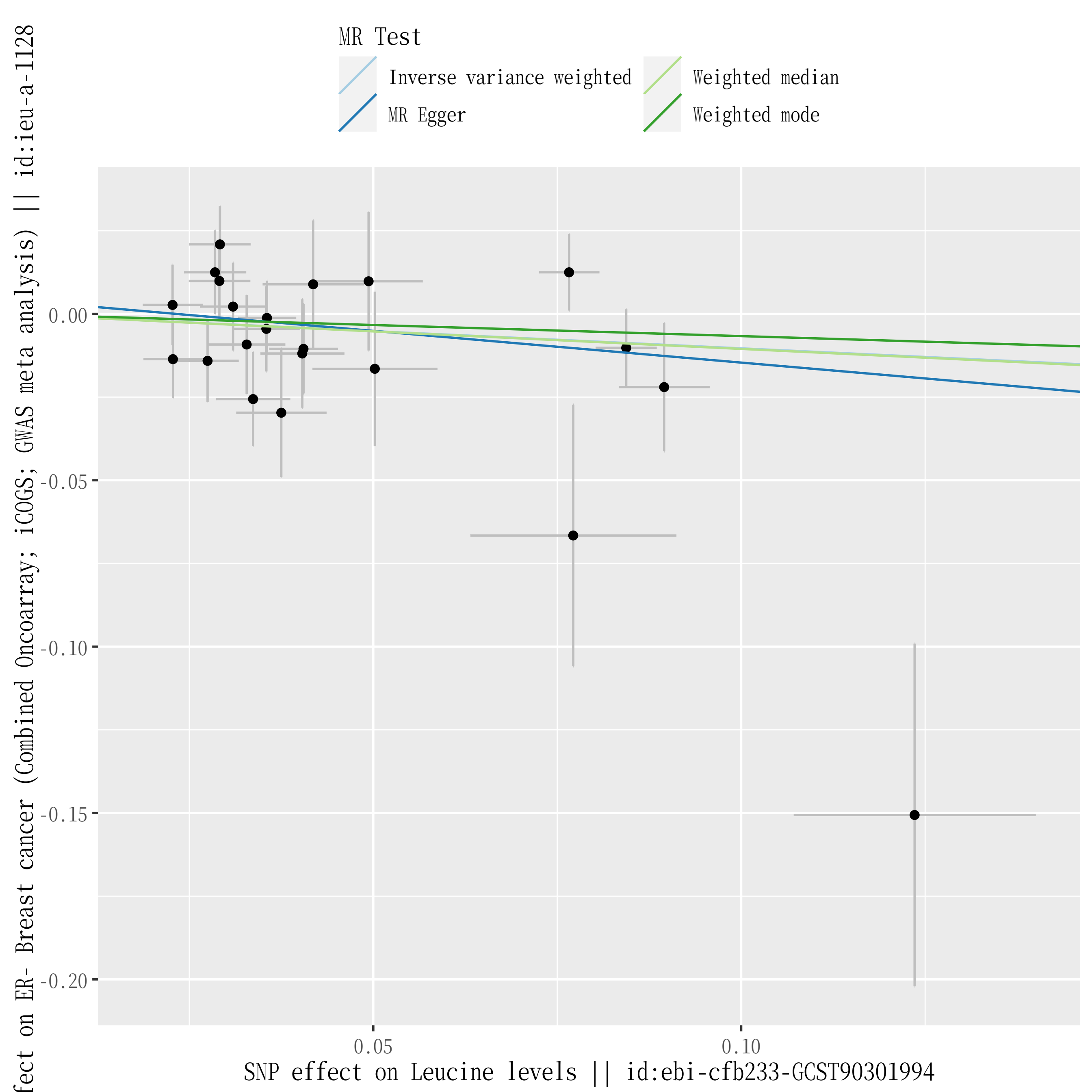

MR Test
Inverse variance weighted
MR Egger
Weighted median
Weighted mode
0.00
-0.05
-0.10
-0.15
-0.20
0.05
0.10
SNP effect on Leucine levels || id:ebi-cfb233-GCST90301994
fect on ER- Breast cancer (Combined Oncoarray; iCOGS; GWAS meta analysis) || id:ieu-a-1128

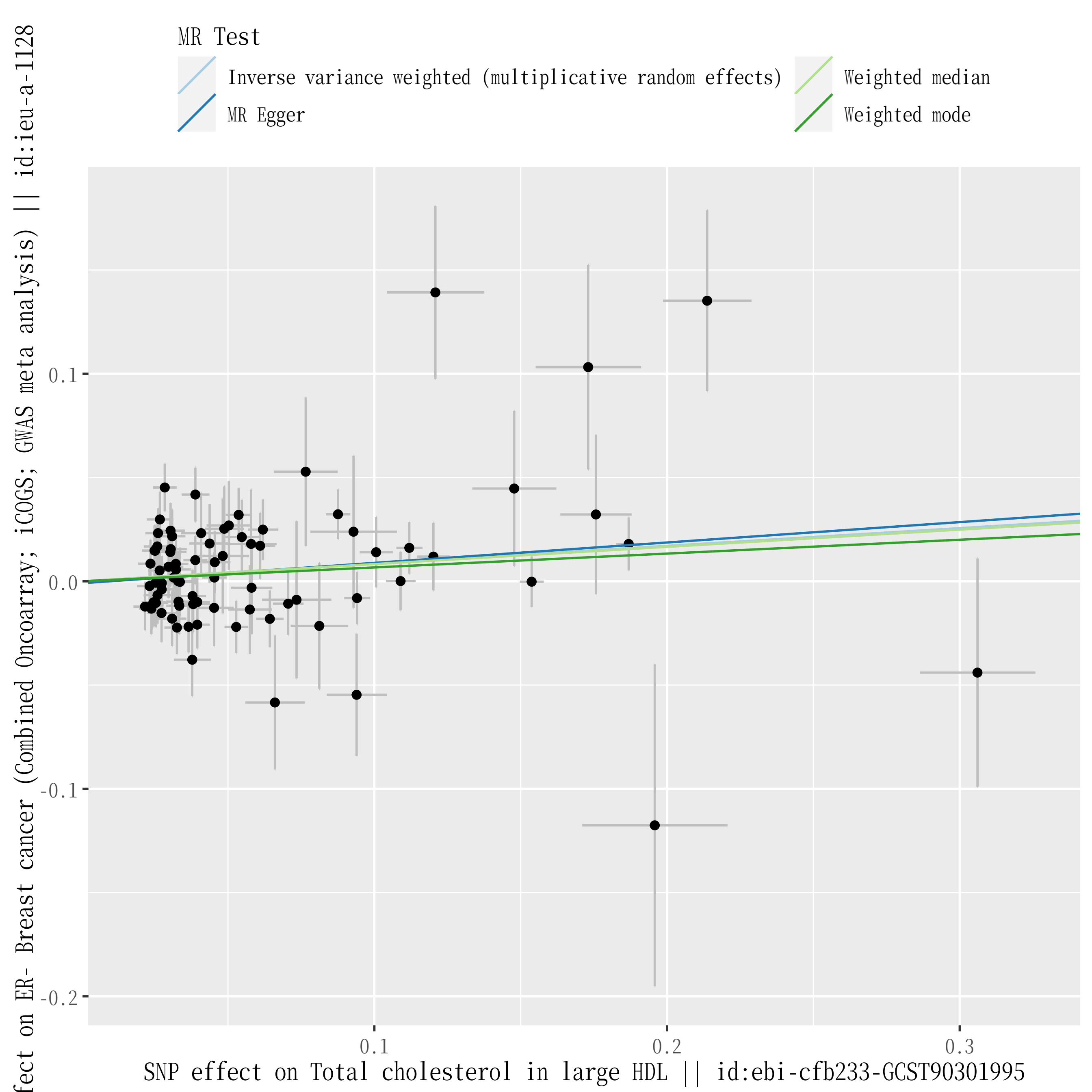
MR Test
Inverse variance weighted (multiplicative random effects)
MR Egger
Weighted median
Weighted mode
0.1
0.0
-0.1
-0.2
0.1
0.2
0.3
SNP effect on Total cholesterol in large HDL || id:ebi-cfb233-GCST90301995
fect on ER- Breast cancer (Combined Oncoarray; iCOGS; GWAS meta analysis) || id:ieu-a-1128

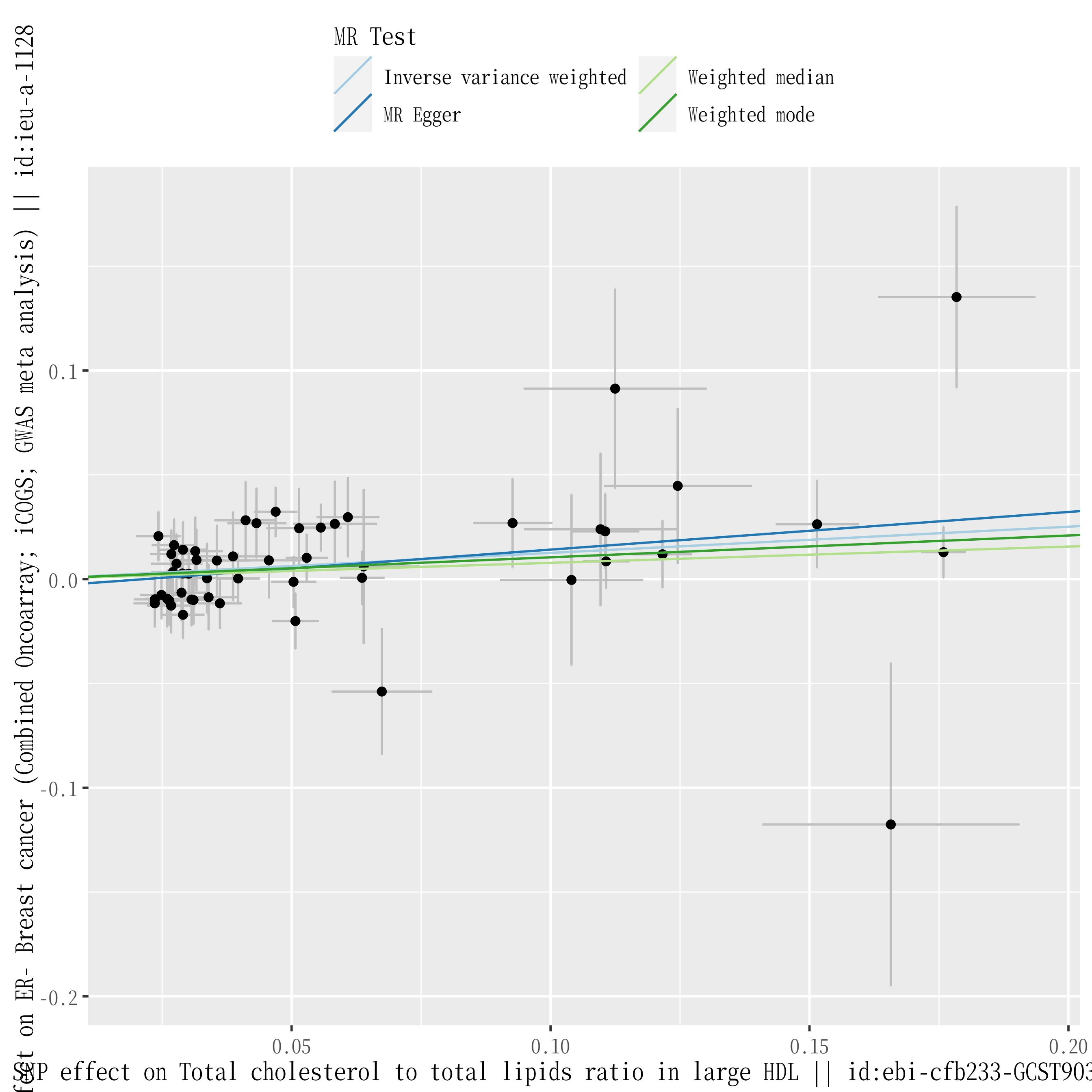

MR Test
Inverse variance weighted
MR Egger
Weighted median
Weighted mode
fect on ER- Breast cancer (Combined Oncoarray; iCOGS; GWAS meta analysis) || id:ieu-a-1128
0.1
0.0
-0.1
-0.2
0.05
0.10
0.15
0.20
SNP effect on Total cholesterol to total lipids ratio in large HDL || id:ebi-cfb233-GCST903

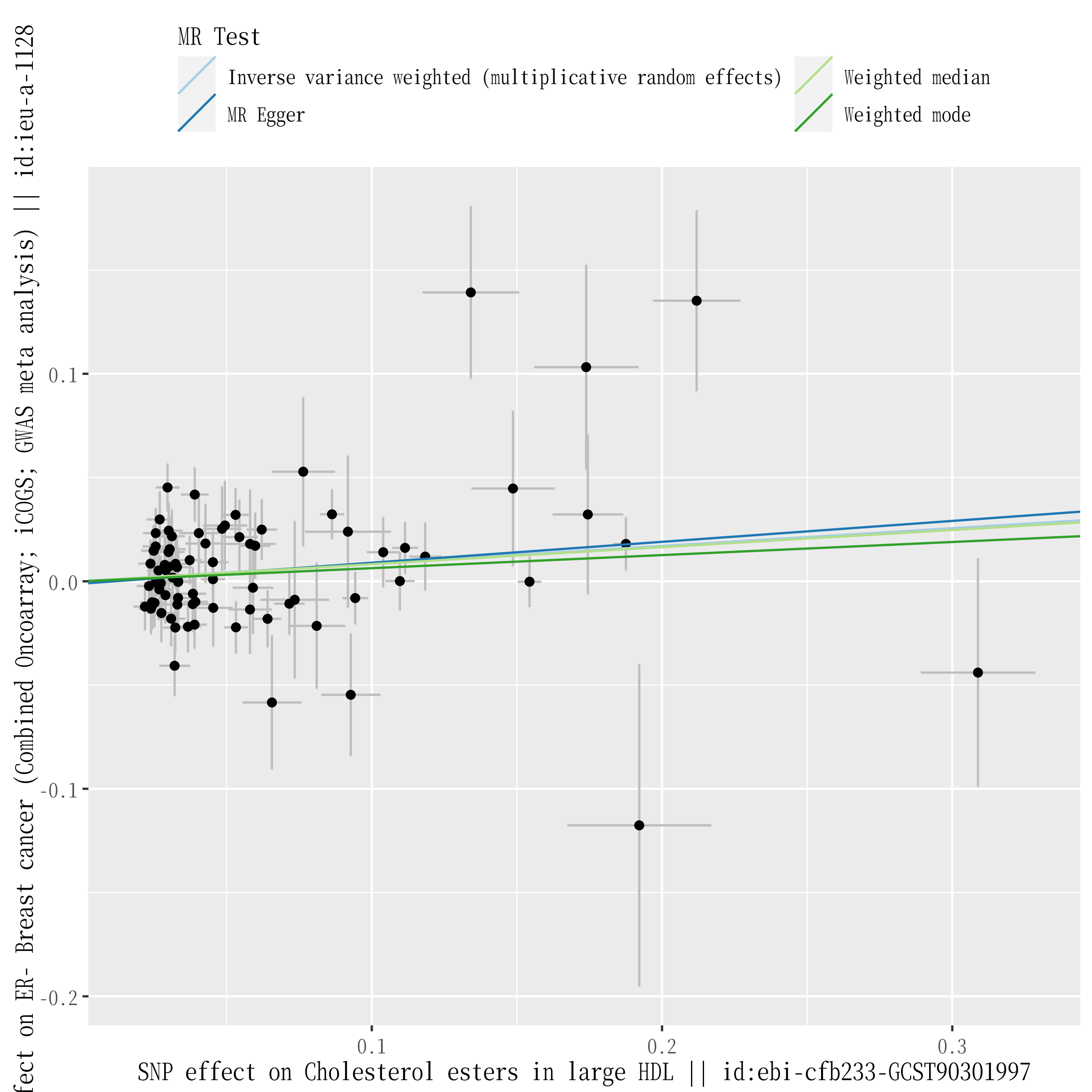

MR Test
Inverse variance weighted (multiplicative random effects)
MR Egger
Weighted median
Weighted mode
0.1
0.0
-0.1
-0.2
0.1
0.2
0.3
SNP effect on Cholesterol esters in large HDL || id:ebi-cfb233-GCST90301997
fect on ER- Breast cancer (Combined Oncoarray; iCOGS; GWAS meta analysis) || id:ieu-a-1128

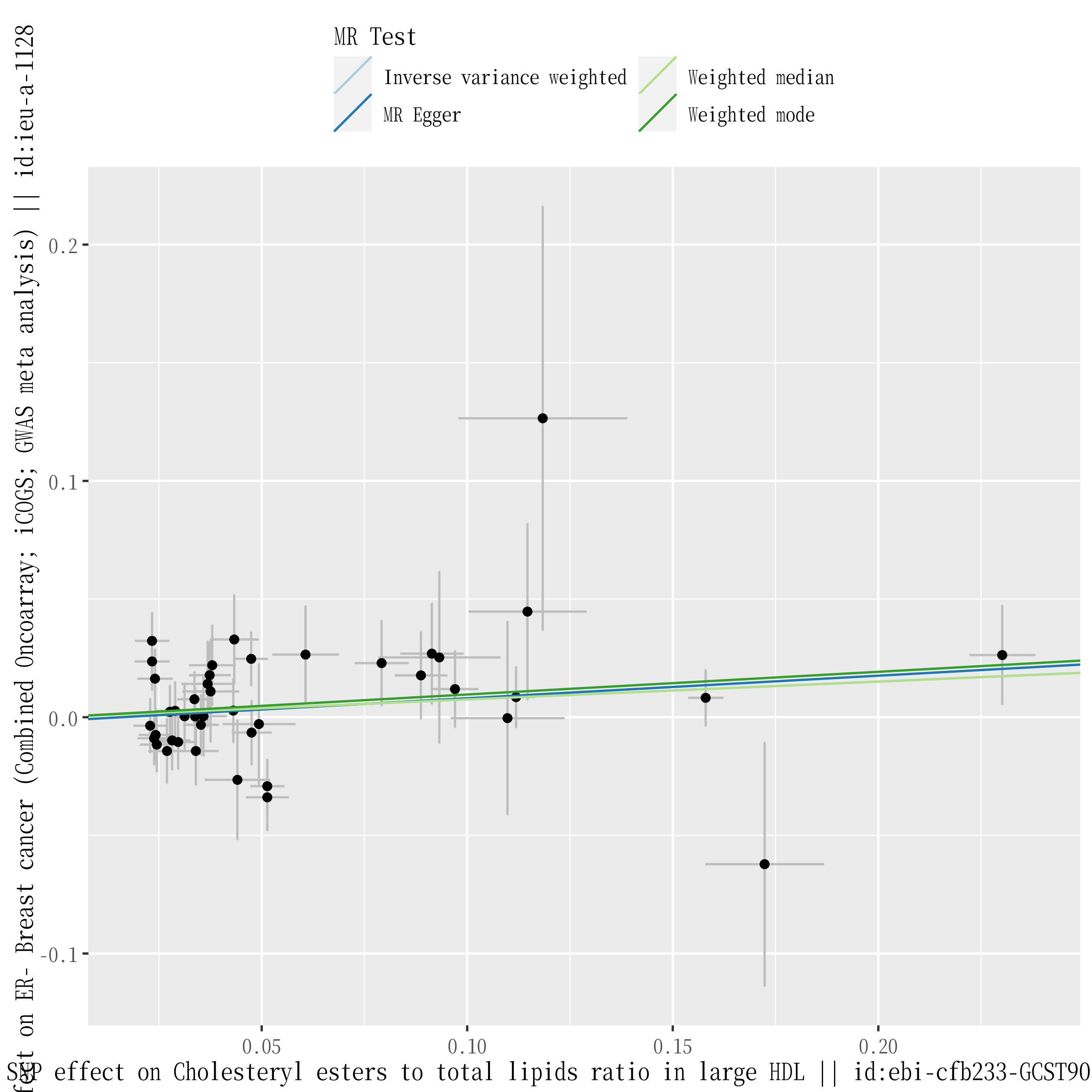
MR Test
Inverse variance weighted
MR Egger
Weighted median
Weighted mode
0.2
0.1
0.0
-0.1
0.05
0.10
0.15
0.20
SNP effect on Cholesteryl esters to total lipids ratio in large HDL || id:ebi-cfb233-GCST90
fect on ER- Breast cancer (Combined Oncoarray; iCOGS; GWAS meta analysis) || id:ieu-a-1128

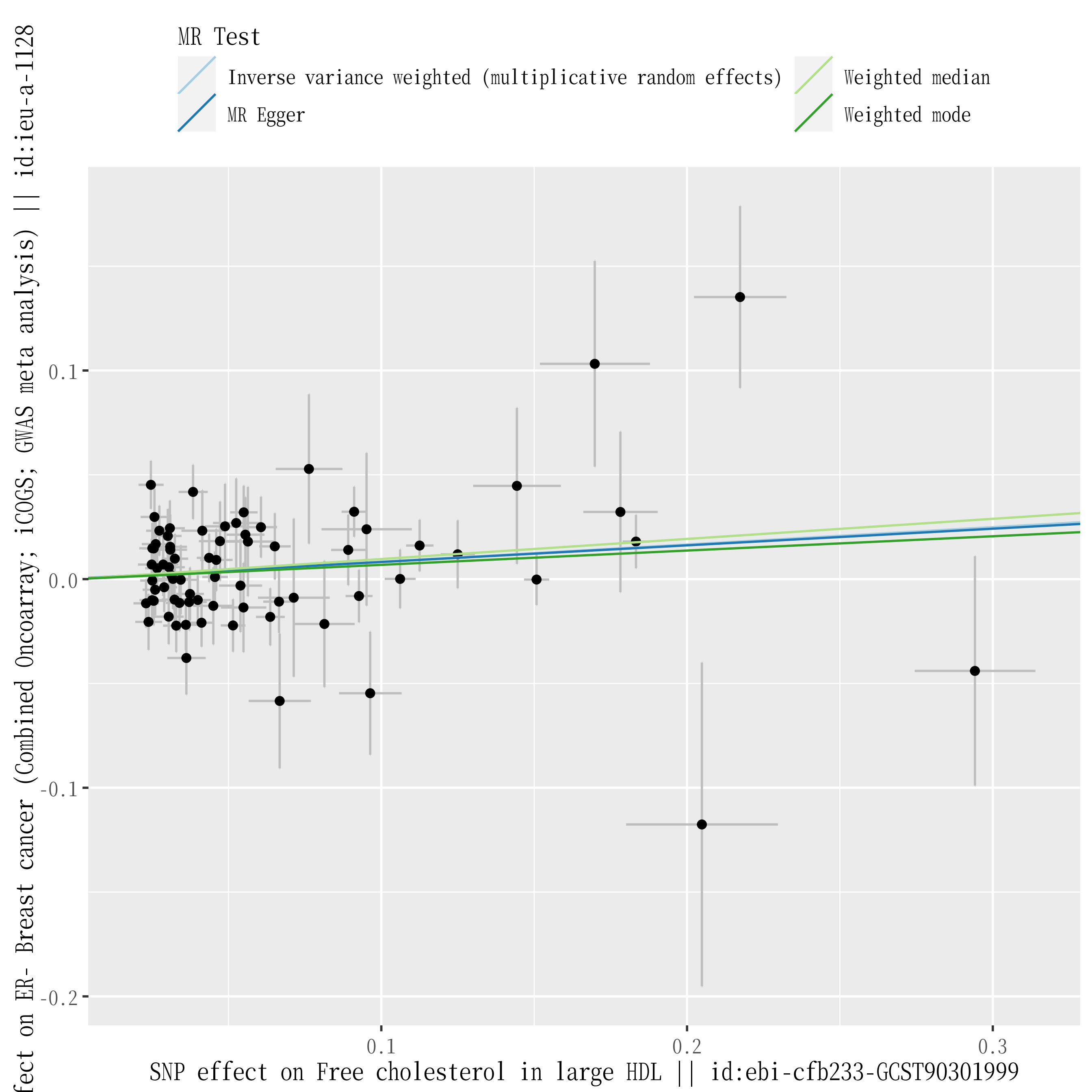
MR Test
Inverse variance weighted (multiplicative random effects)
MR Egger
Weighted median
Weighted mode
0.1
0.0
-0.1
-0.2
0.1
0.2
0.3
SNP effect on Free cholesterol in large HDL || id:ebi-cfb233-GCST90301999
fect on ER- Breast cancer (Combined Oncoarray; iCOGS; GWAS meta analysis) || id:ieu-a-1128

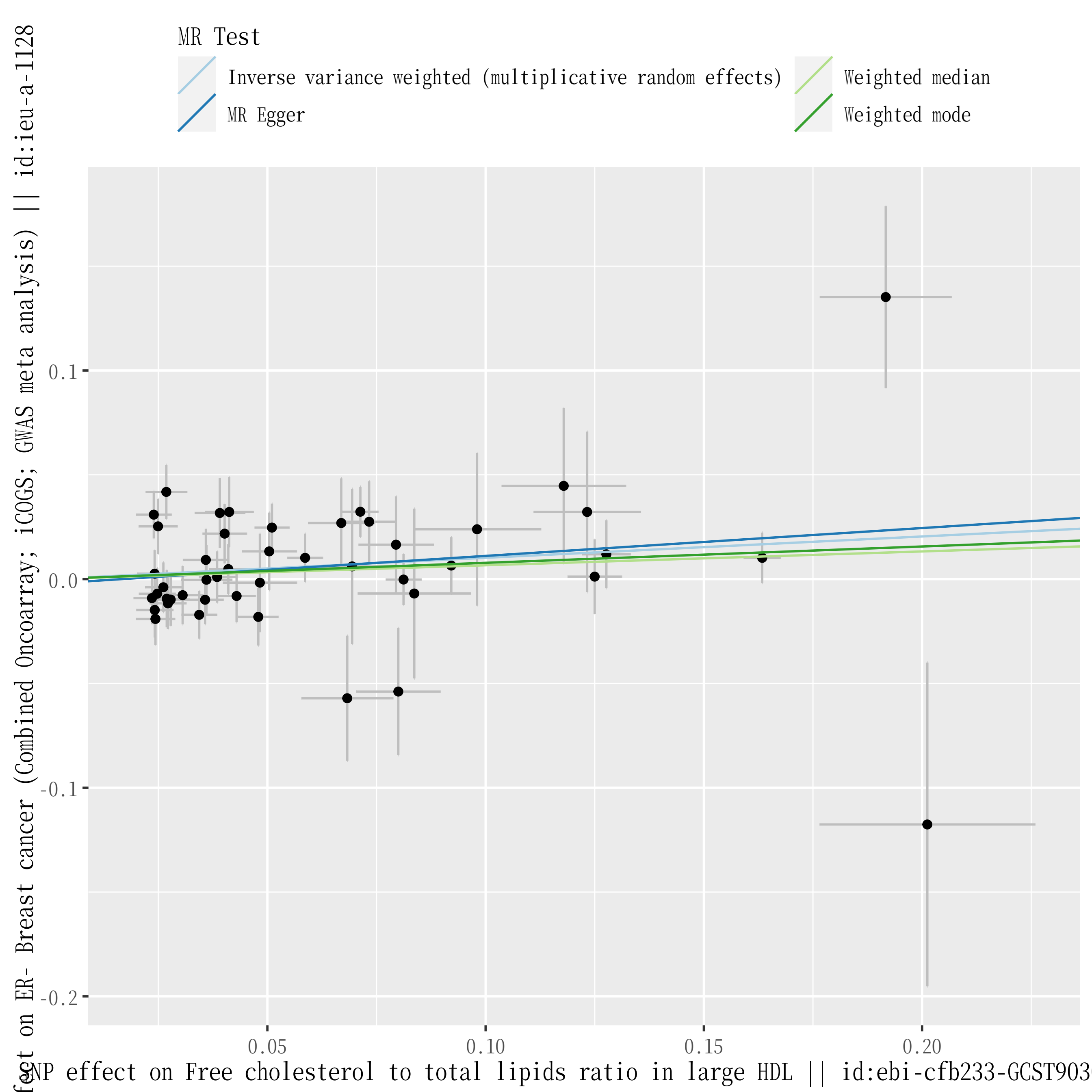

MR Test
Inverse variance weighted (multiplicative random effects)
MR Egger
Weighted median
Weighted mode
0.1
0.0
-0.1
-0.2
0.05
0.10
0.15
0.20
SNP effect on Free cholesterol to total lipids ratio in large HDL || id:ebi-cfb233-GCST903
fect on ER- Breast cancer (Combined Oncoarray; iCOGS; GWAS meta analysis) || id:ieu-a-1128

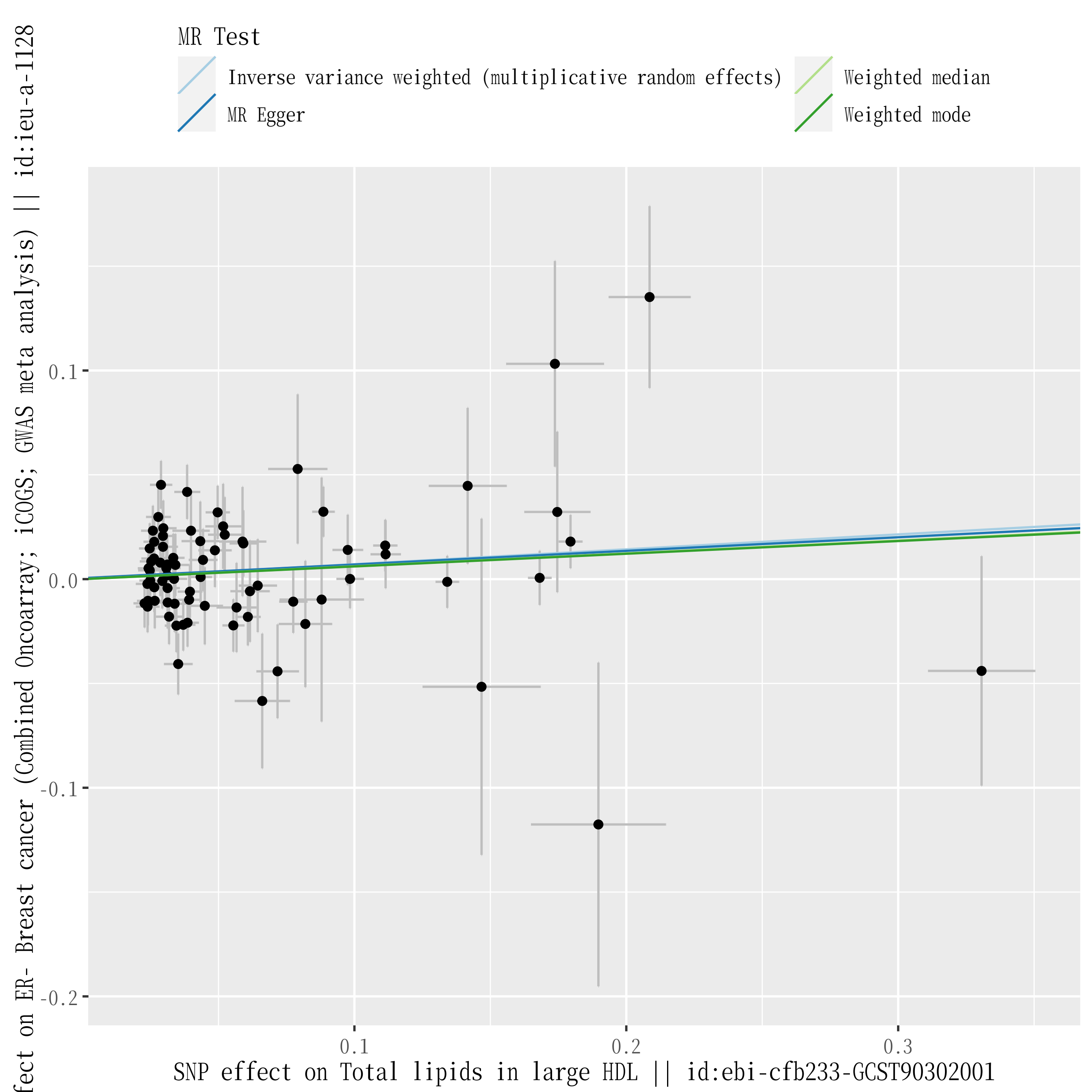
MR Test
Inverse variance weighted (multiplicative random effects)
MR Egger
Weighted median
Weighted mode
0.1
0.0
-0.1
-0.2
0.1
0.2
0.3
SNP effect on Total lipids in large HDL || id:ebi-cfb233-GCST90302001
fect on ER- Breast cancer (Combined Oncoarray; iCOGS; GWAS meta analysis) || id:ieu-a-1128

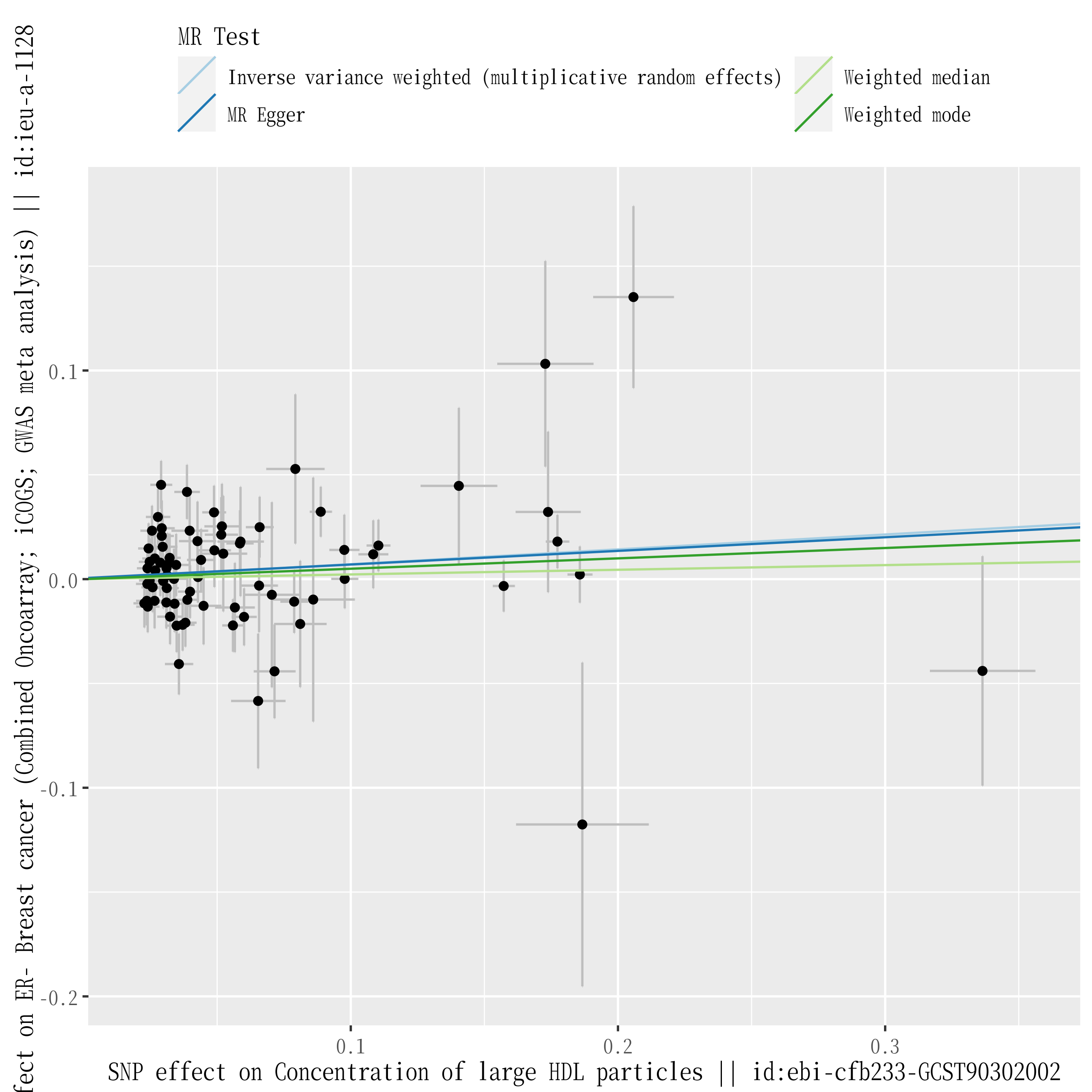
MR Test
Inverse variance weighted (multiplicative random effects)
MR Egger
Weighted median
Weighted mode
fect on ER- Breast cancer (Combined Oncoarray; iCOGS; GWAS meta analysis) || id:ieu-a-1128
0.1
0.0
-0.1
-0.2
0.1
0.2
0.3
SNP effect on Concentration of large HDL particles || id:ebi-cfb233-GCST90302002

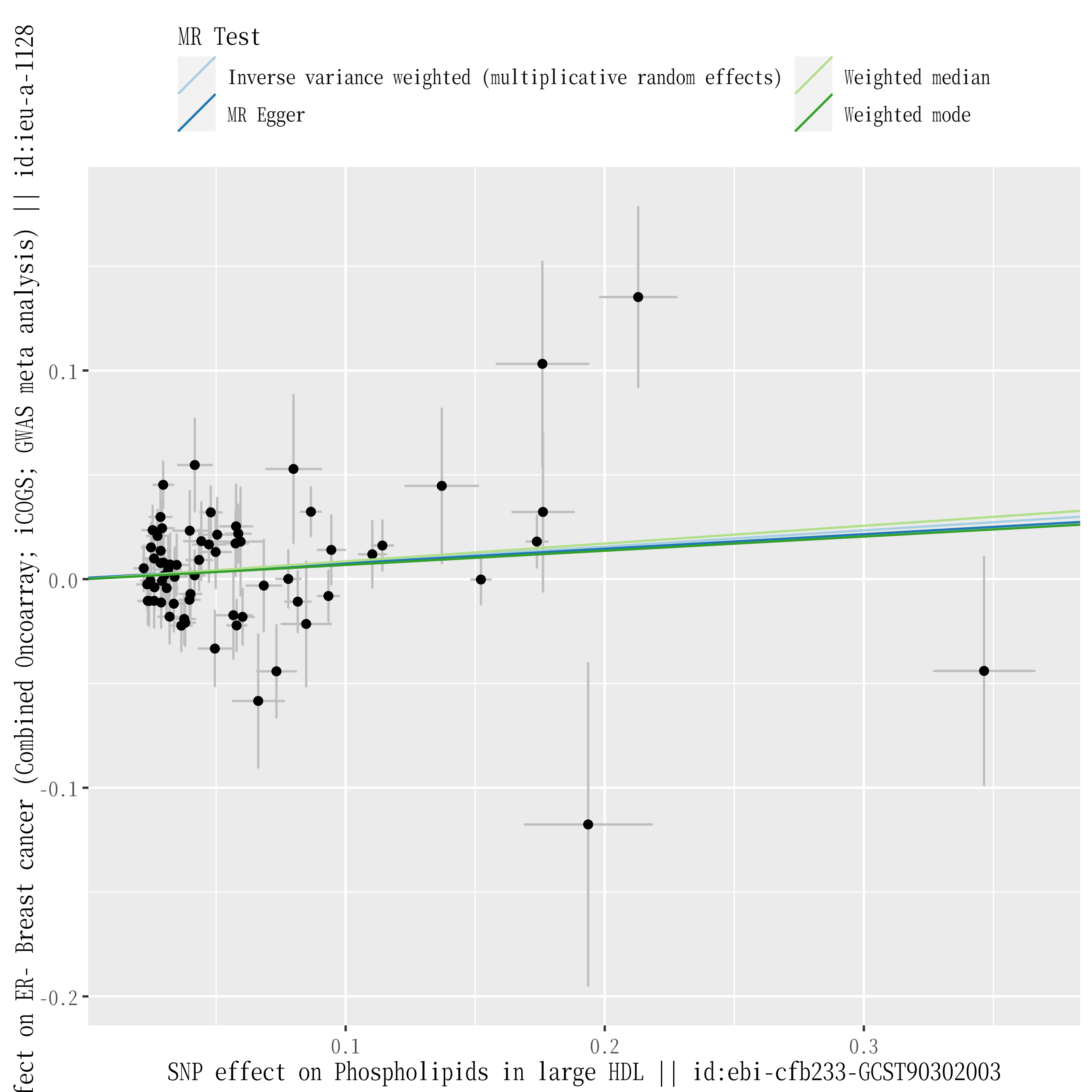

MR Test
Inverse variance weighted (multiplicative random effects)
MR Egger
Weighted median
Weighted mode
0.1
0.0
-0.1
-0.2
0.1
0.2
0.3
SNP effect on Phospholipids in large HDL || id:ebi-cfb233-GCST90302003
fect on ER- Breast cancer (Combined Oncoarray; iCOGS; GWAS meta analysis) || id:ieu-a-1128

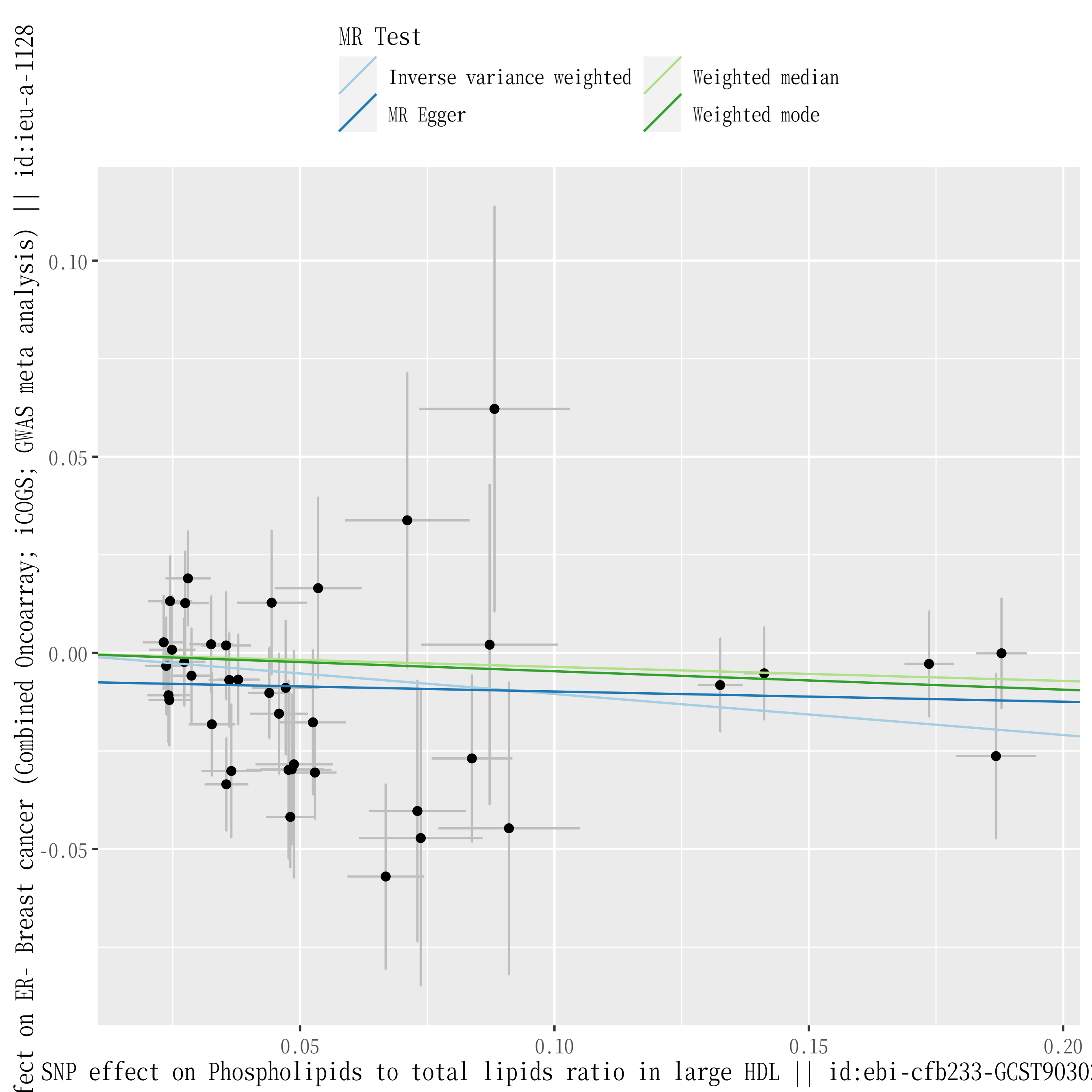
MR Test
Inverse variance weighted
MR Egger
Weighted median
Weighted mode
0.10
0.05
0.00
-0.05
0.05
0.10
0.15
0.20
SNP effect on Phospholipids to total lipids ratio in large HDL || id:ebi-cfb233-GCST9030
fect on ER- Breast cancer (Combined Oncoarray; iCOGS; GWAS meta analysis) || id:ieu-a-1128

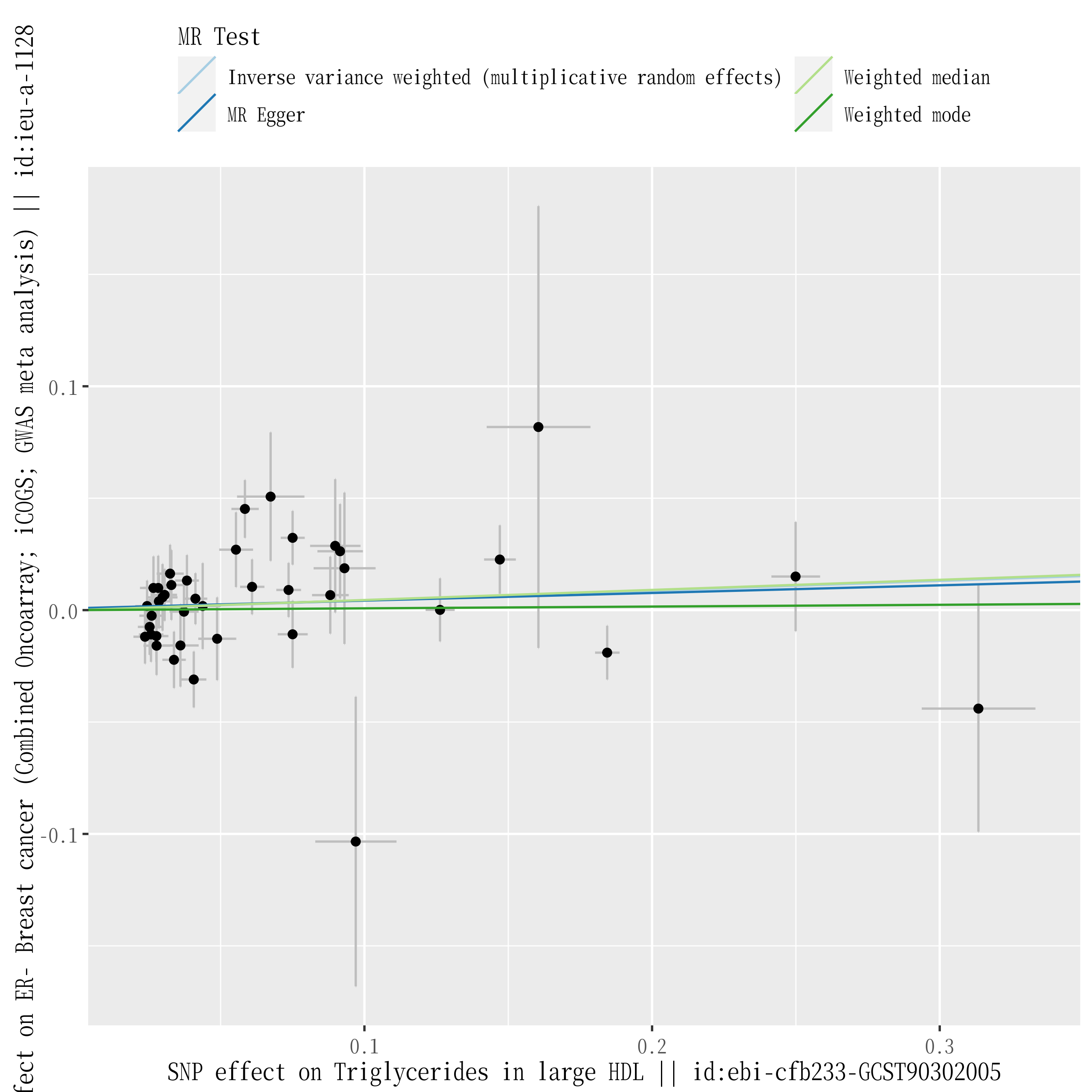

MR Test
Inverse variance weighted (multiplicative random effects)
MR Egger
Weighted median
Weighted mode
0.1
0.0
-0.1
0.1
0.2
0.3
SNP effect on Triglycerides in large HDL || id:ebi-cfb233-GCST90302005
fect on ER- Breast cancer (Combined Oncoarray; iCOGS; GWAS meta analysis) || id:ieu-a-1128

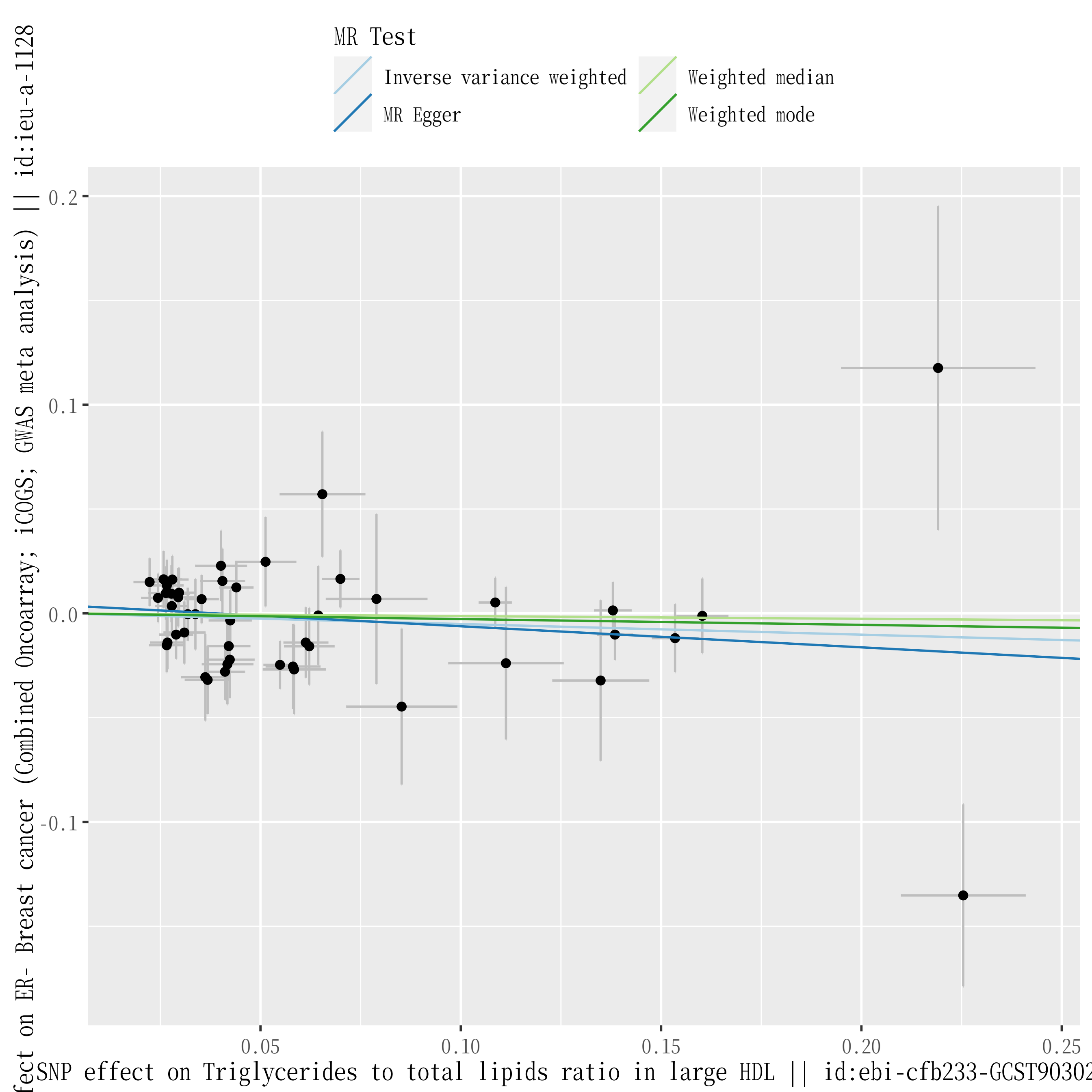

MR Test
Inverse variance weighted
MR Egger
Weighted median
Weighted mode
0.2
0.1
0.0
-0.1
0.05
0.10
0.15
0.20
0.25
SNP effect on Triglycerides to total lipids ratio in large HDL || id:ebi-cfb233-GCST90302
fect on ER- Breast cancer (Combined Oncoarray; iCOGS; GWAS meta analysis) || id:ieu-a-1128

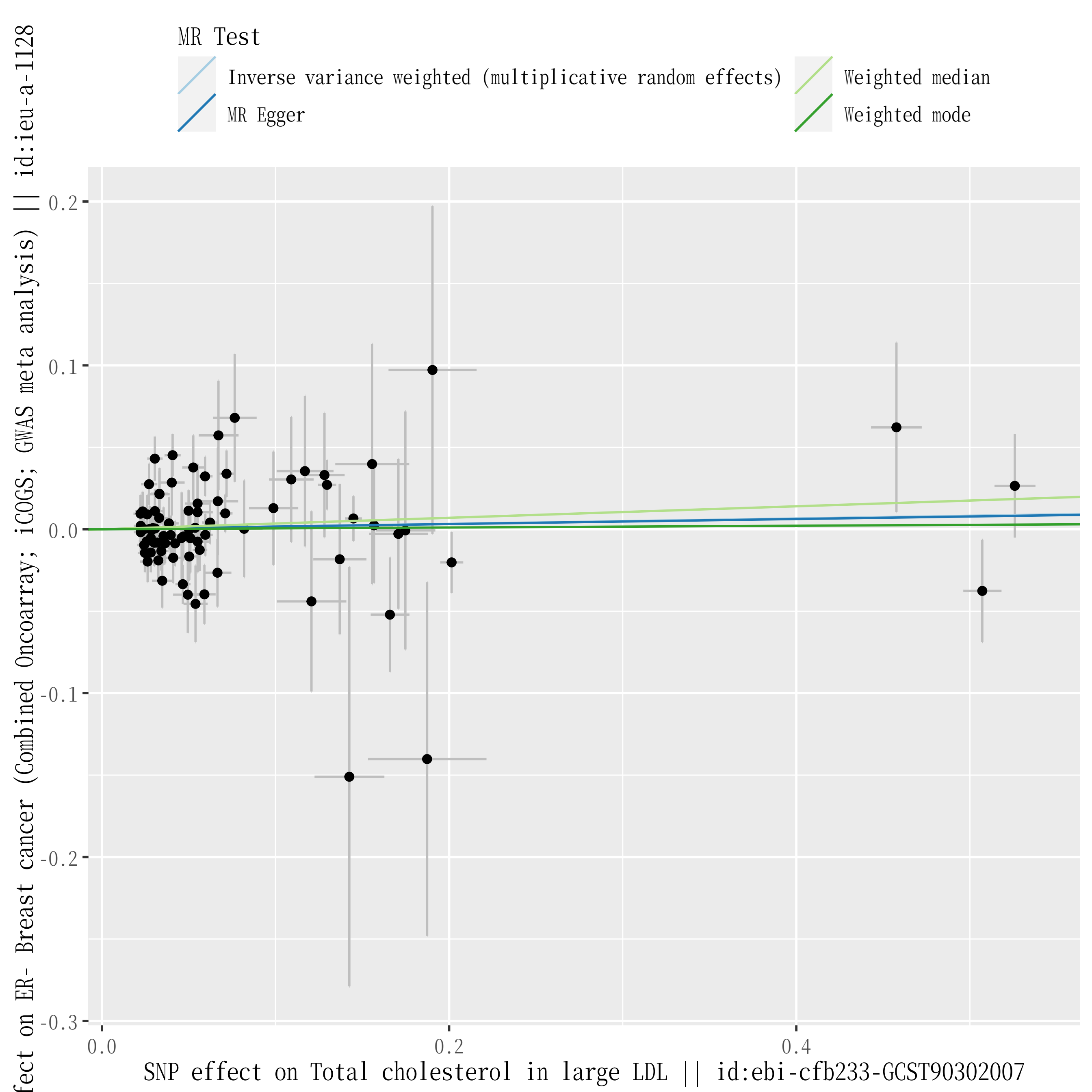
MR Test
Inverse variance weighted (multiplicative random effects)
MR Egger
Weighted median
Weighted mode
fect on ER- Breast cancer (Combined Oncoarray; iCOGS; GWAS meta analysis) || id:ieu-a-1128
0.2
0.1
0.0
-0.1
-0.2
-0.3
0.0
0.2
0.4
SNP effect on Total cholesterol in large LDL || id:ebi-cfb233-GCST90302007

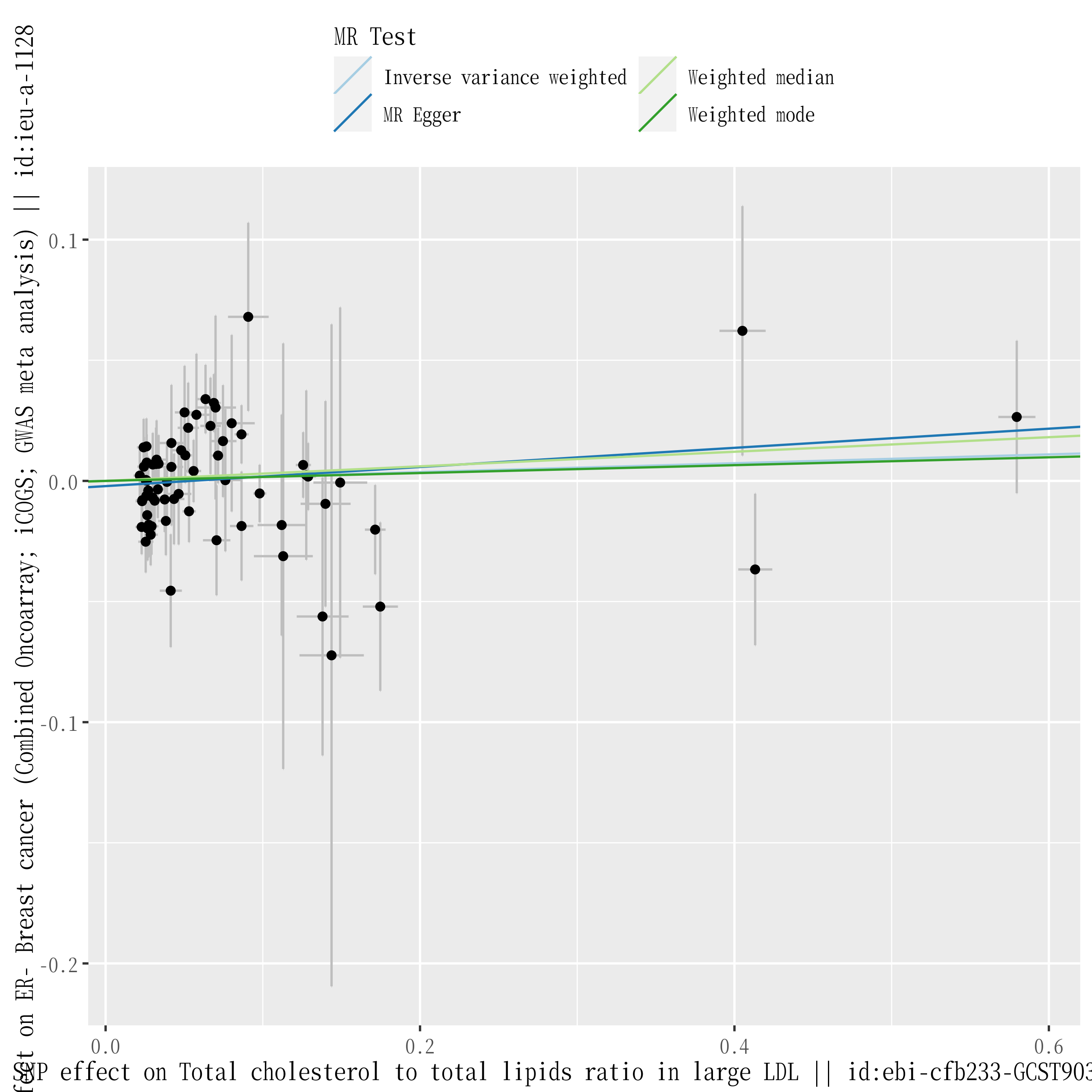
MR Test
Inverse variance weighted
MR Egger
Weighted median
Weighted mode
0.1
0.0
-0.1
-0.2
0.0
0.2
0.4
0.6
fect on ER- Breast cancer (Combined Oncoarray; iCOGS; GWAS meta analysis) || id:ieu-a-1128
SNP effect on Total cholesterol to total lipids ratio in large LDL || id:ebi-cfb233-GCST903

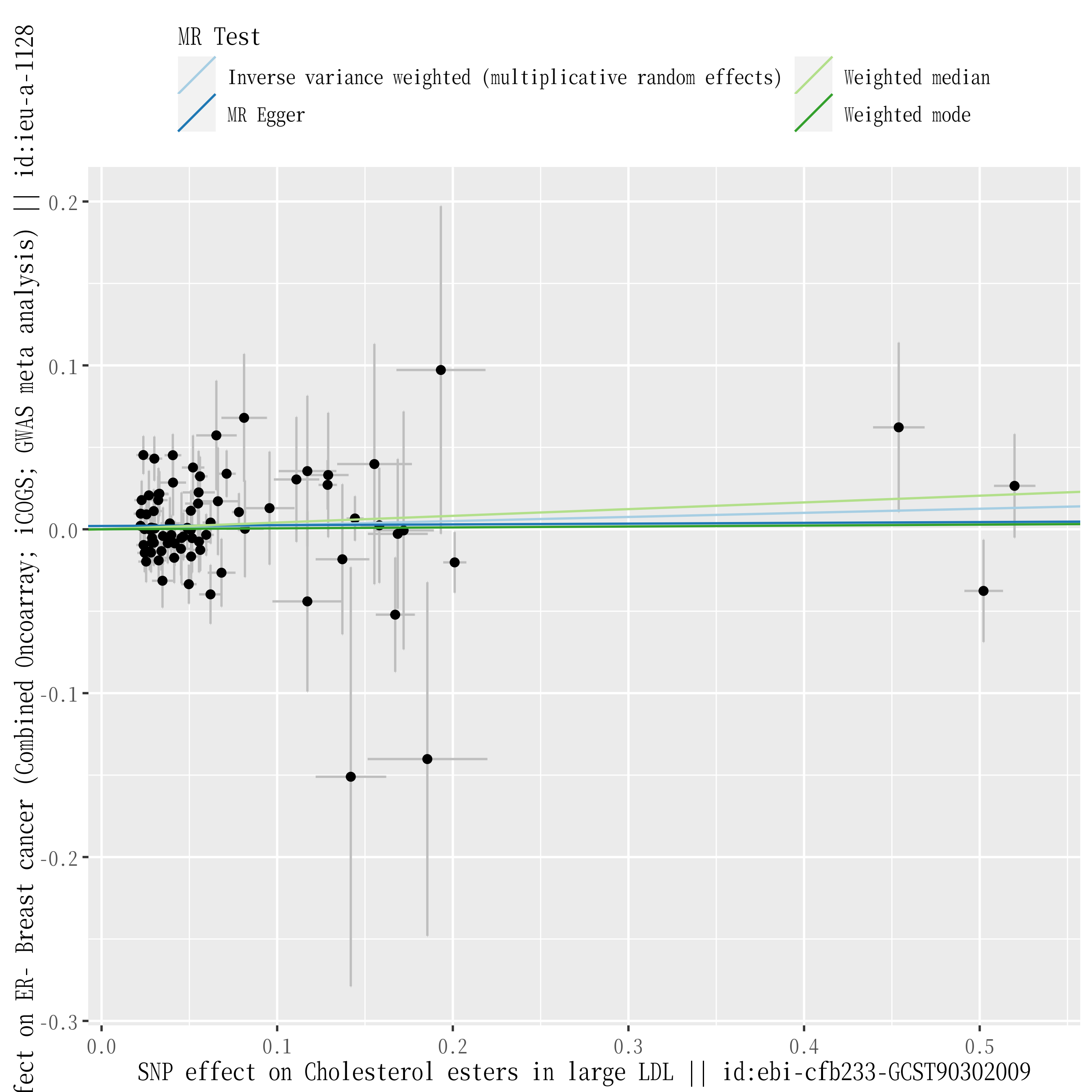
MR Test
Inverse variance weighted (multiplicative random effects)
MR Egger
Weighted median
Weighted mode
0.2
0.1
0.0
-0.1
-0.2
-0.3
0.0
0.1
0.2
0.3
0.4
0.5
SNP effect on Cholesterol esters in large LDL || id:ebi-cfb233-GCST90302009
fect on ER- Breast cancer (Combined Oncoarray; iCOGS; GWAS meta analysis) || id:ieu-a-1128

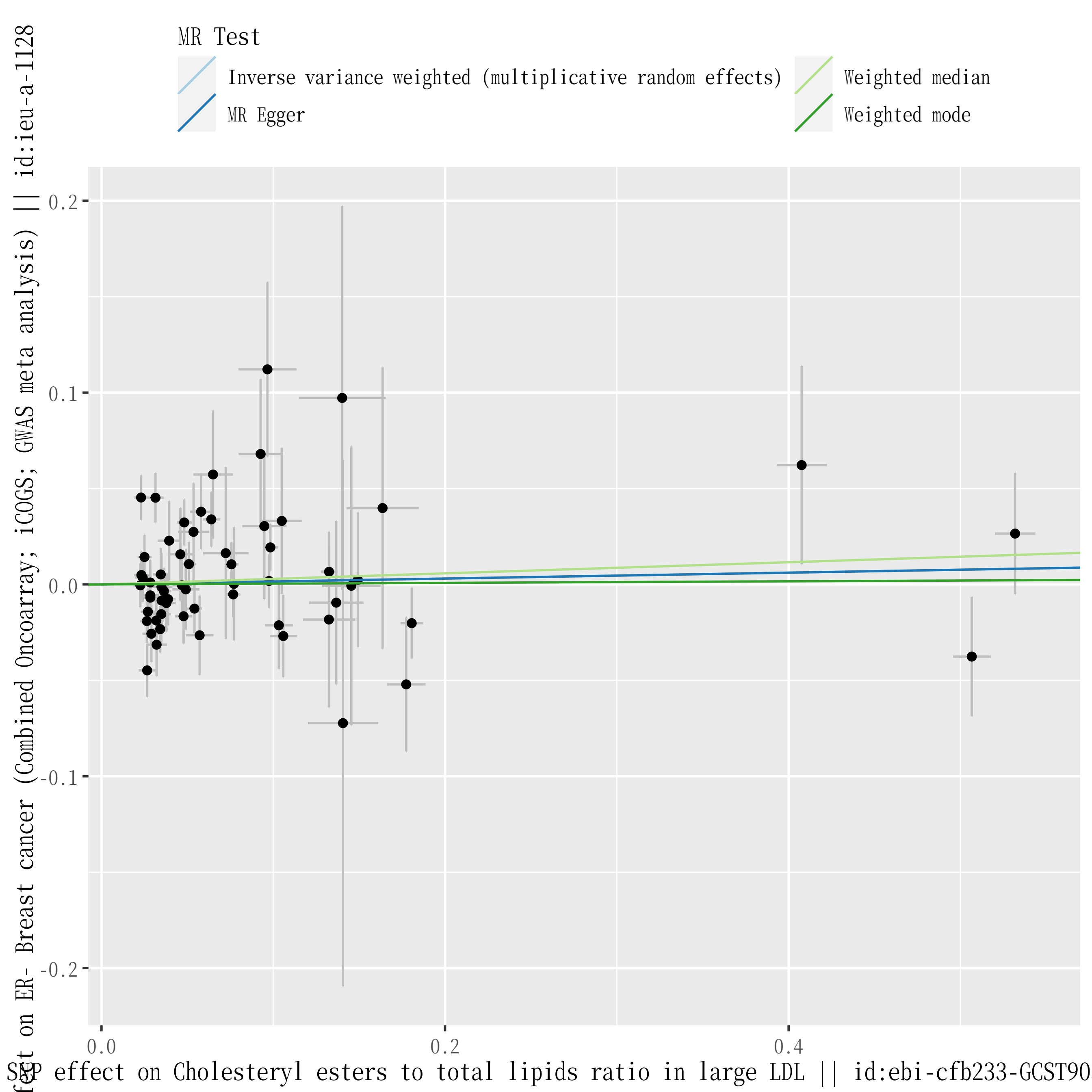

MR Test
Inverse variance weighted (multiplicative random effects)
MR Egger
Weighted median
Weighted mode
0.2
0.1
0.0
-0.1
-0.2
0.0
0.2
0.4
SNP effect on Cholesteryl esters to total lipids ratio in large LDL || id:ebi-cfb233-GCST90
fect on ER- Breast cancer (Combined Oncoarray; iCOGS; GWAS meta analysis) || id:ieu-a-1128

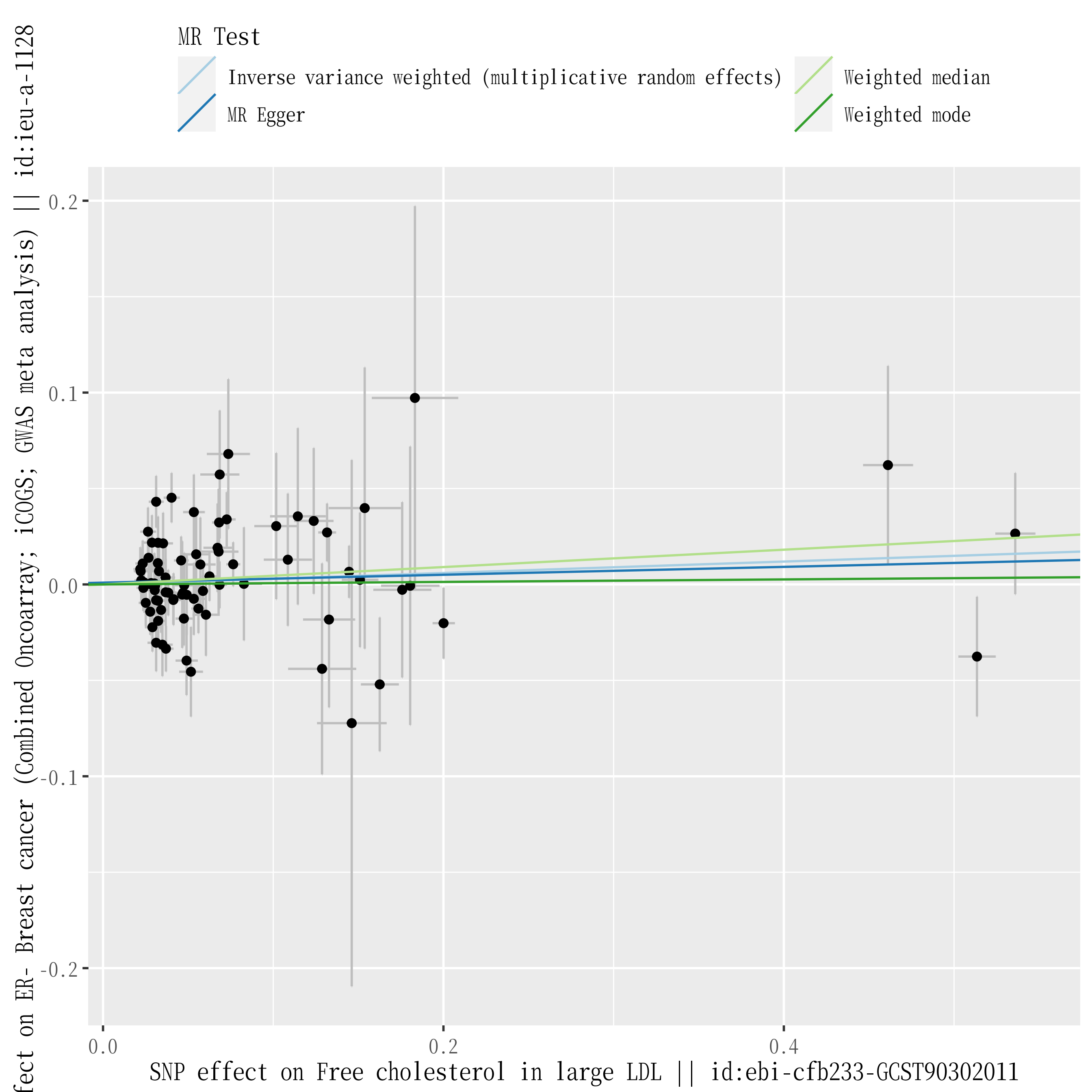
MR Test
Inverse variance weighted (multiplicative random effects)
MR Egger
Weighted median
Weighted mode
fect on ER- Breast cancer (Combined Oncoarray; iCOGS; GWAS meta analysis) || id:ieu-a-1128
0.2
0.1
0.0
-0.1
-0.2
0.0
0.2
0.4
SNP effect on Free cholesterol in large LDL || id:ebi-cfb233-GCST90302011

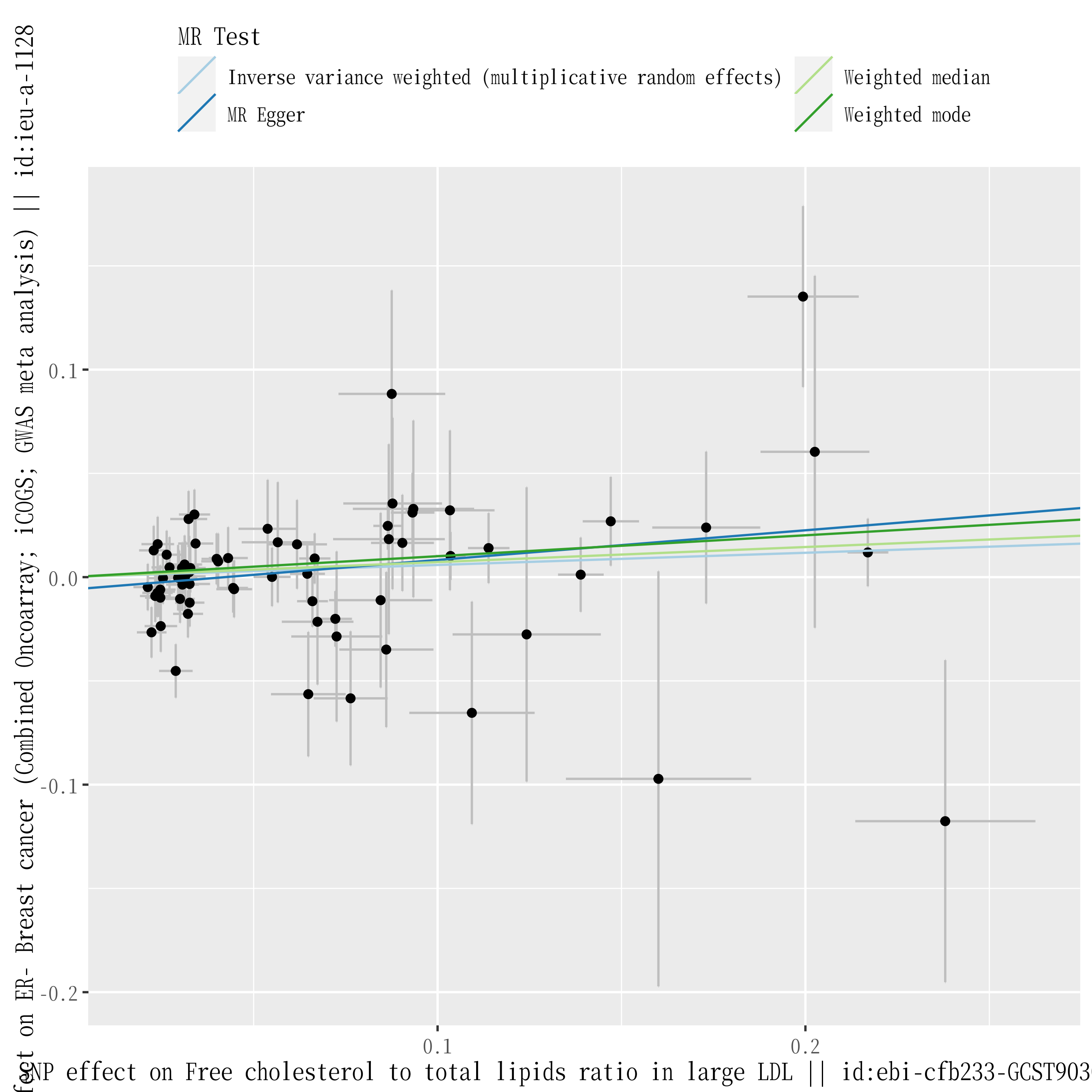
MR Test
Inverse variance weighted (multiplicative random effects)
MR Egger
Weighted median
Weighted mode
0.1
0.0
-0.1
-0.2
0.1
0.2
SNP effect on Free cholesterol to total lipids ratio in large LDL || id:ebi-cfb233-GCST903
SNP effect on ER- Breast cancer (Combined Oncoarray; iCOGS; GWAS meta analysis) || id:ieu-a-1128

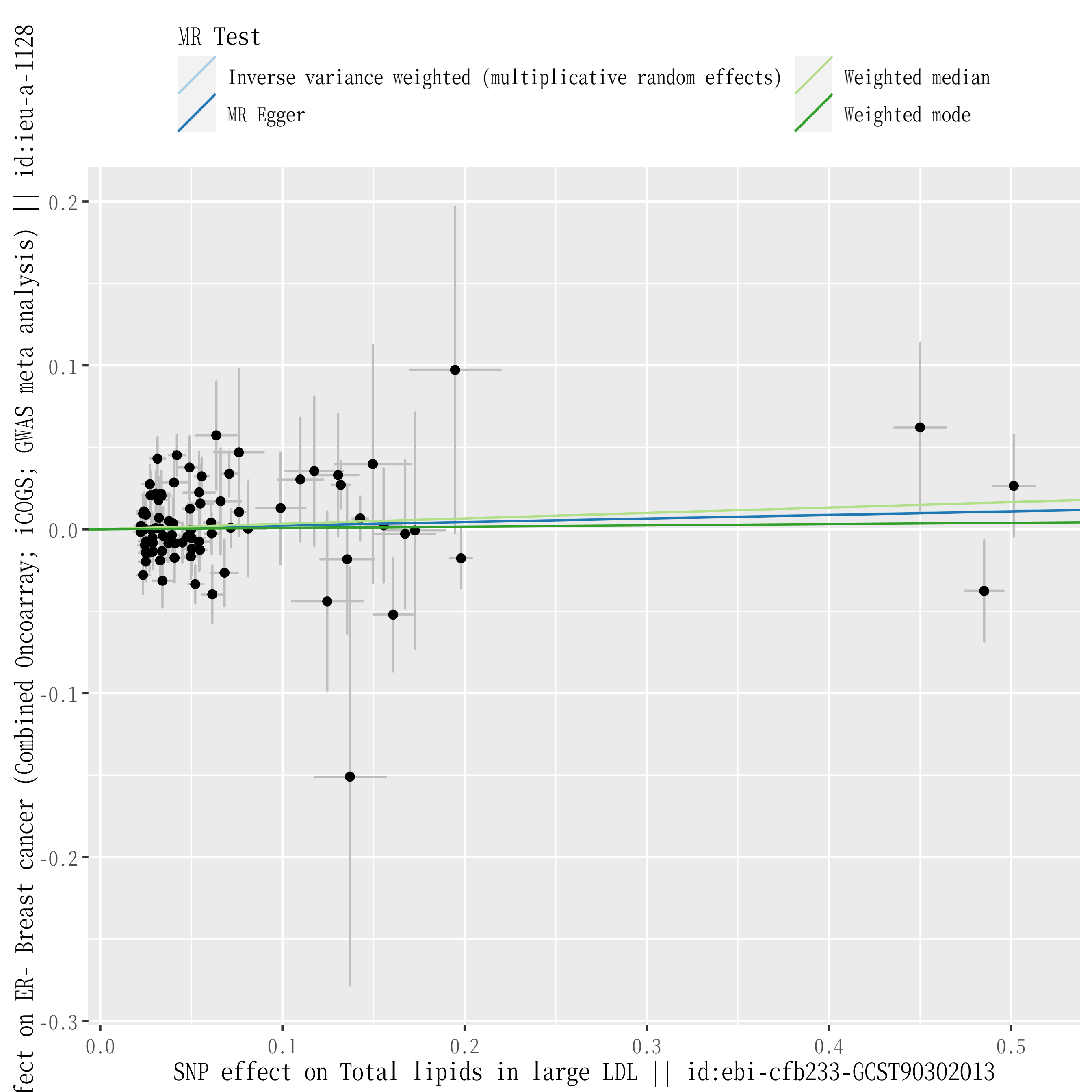
MR Test
Inverse variance weighted (multiplicative random effects)
MR Egger
Weighted median
Weighted mode
fect on ER- Breast cancer (Combined Oncoarray; iCOGS; GWAS meta analysis) || id:ieu-a-1128
0.2
0.1
0.0
-0.1
-0.2
-0.3
0.0
0.1
0.2
0.3
0.4
0.5
SNP effect on Total lipids in large LDL || id:ebi-cfb233-GCST90302013

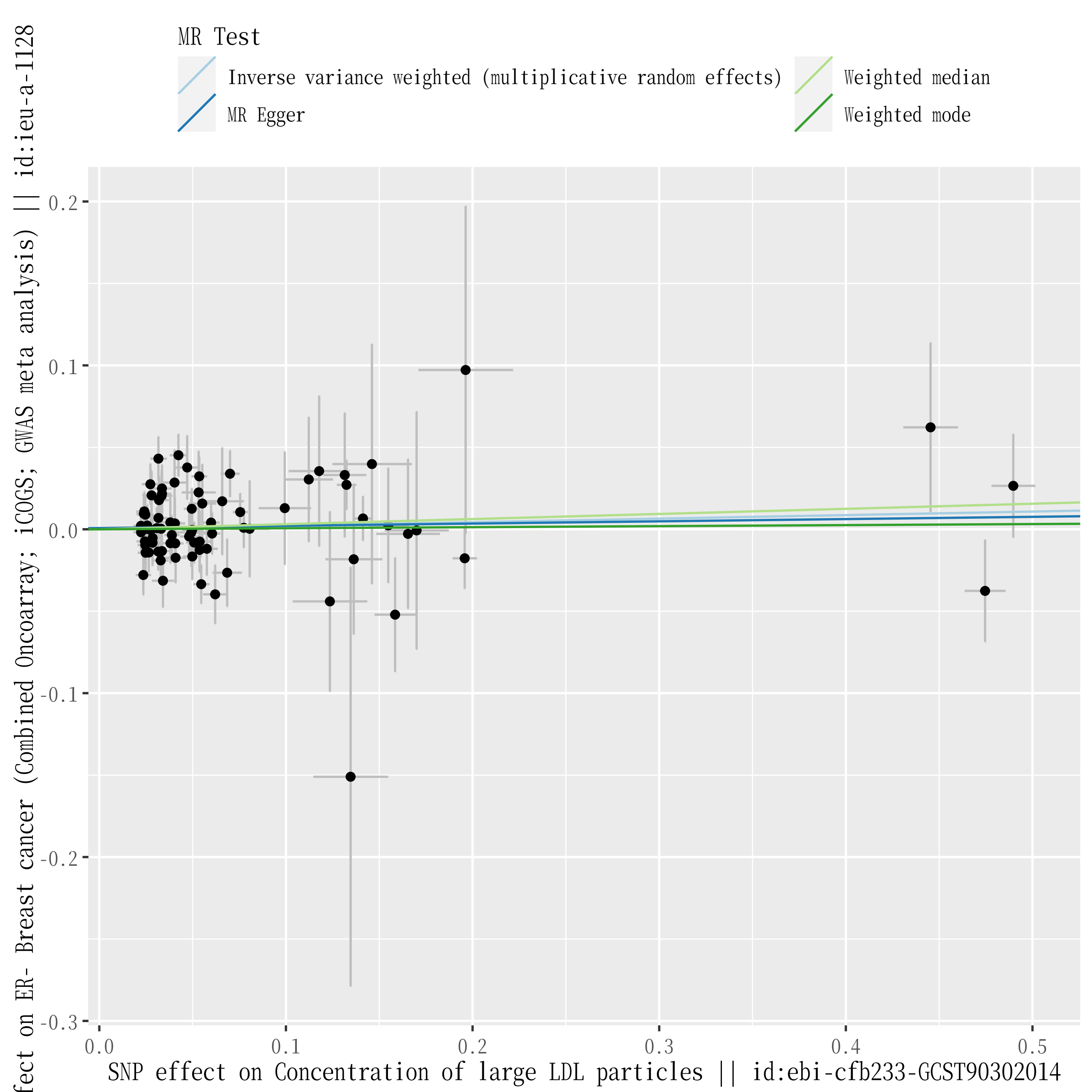

MR Test
Inverse variance weighted (multiplicative random effects)
MR Egger
Weighted median
Weighted mode
0.2
0.1
0.0
-0.1
-0.2
-0.3
0.0
0.1
0.2
0.3
0.4
0.5
SNP effect on Concentration of large LDL particles || id:ebi-cfb233-GCST90302014
fect on ER- Breast cancer (Combined Oncoarray; iCOGS; GWAS meta analysis) || id:ieu-a-1128

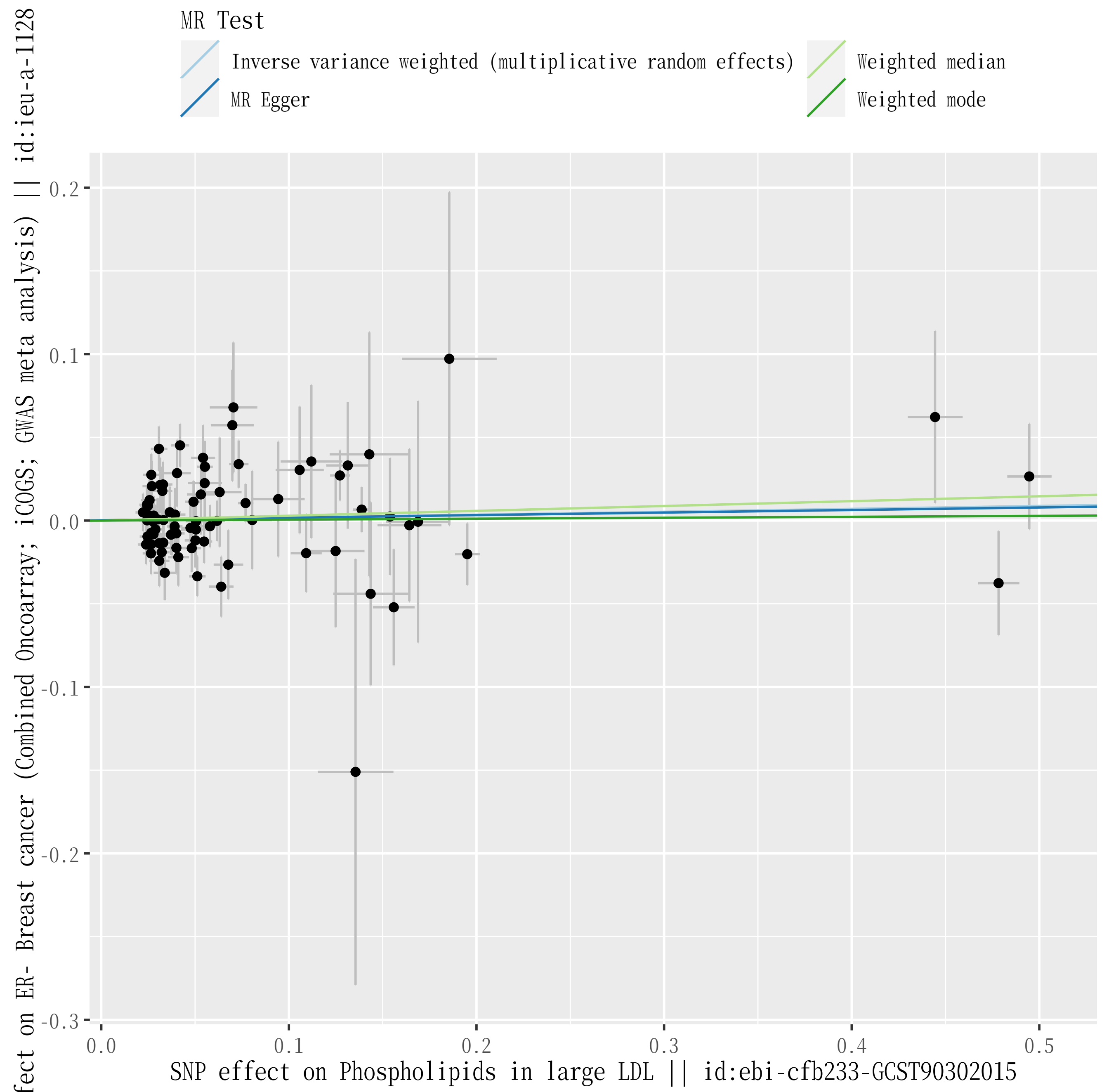

MR Test
Inverse variance weighted (multiplicative random effects)
MR Egger
Weighted median
Weighted mode
0.2
0.1
0.0
-0.1
-0.2
-0.3
0.0
0.1
0.2
0.3
0.4
0.5
SNP effect on Phospholipids in large LDL || id:ebi-cfb233-GCST90302015
fect on ER- Breast cancer (Combined Oncoarray; iCOGS; GWAS meta analysis) || id:ieu-a-1128

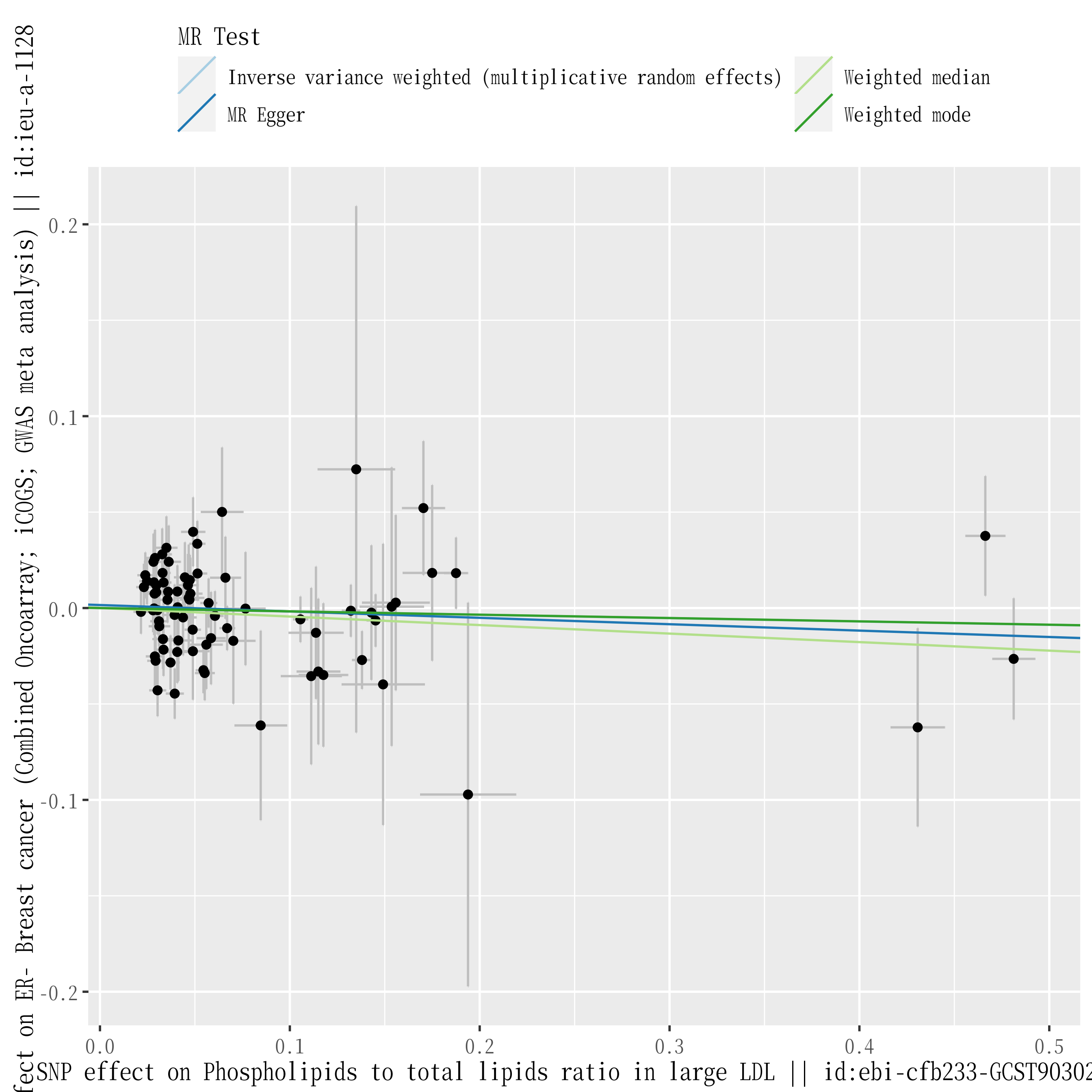

MR Test
Inverse variance weighted (multiplicative random effects)
MR Egger
Weighted median
Weighted mode
0.2
0.1
0.0
-0.1
-0.2
0.0
0.1
0.2
0.3
0.4
0.5
fect on ER- Breast cancer (Combined Oncoarray; iCOGS; GWAS meta analysis) || id:ieu-a-1128
SNP effect on Phospholipids to total lipids ratio in large LDL || id:ebi-cfb233-GCST90302

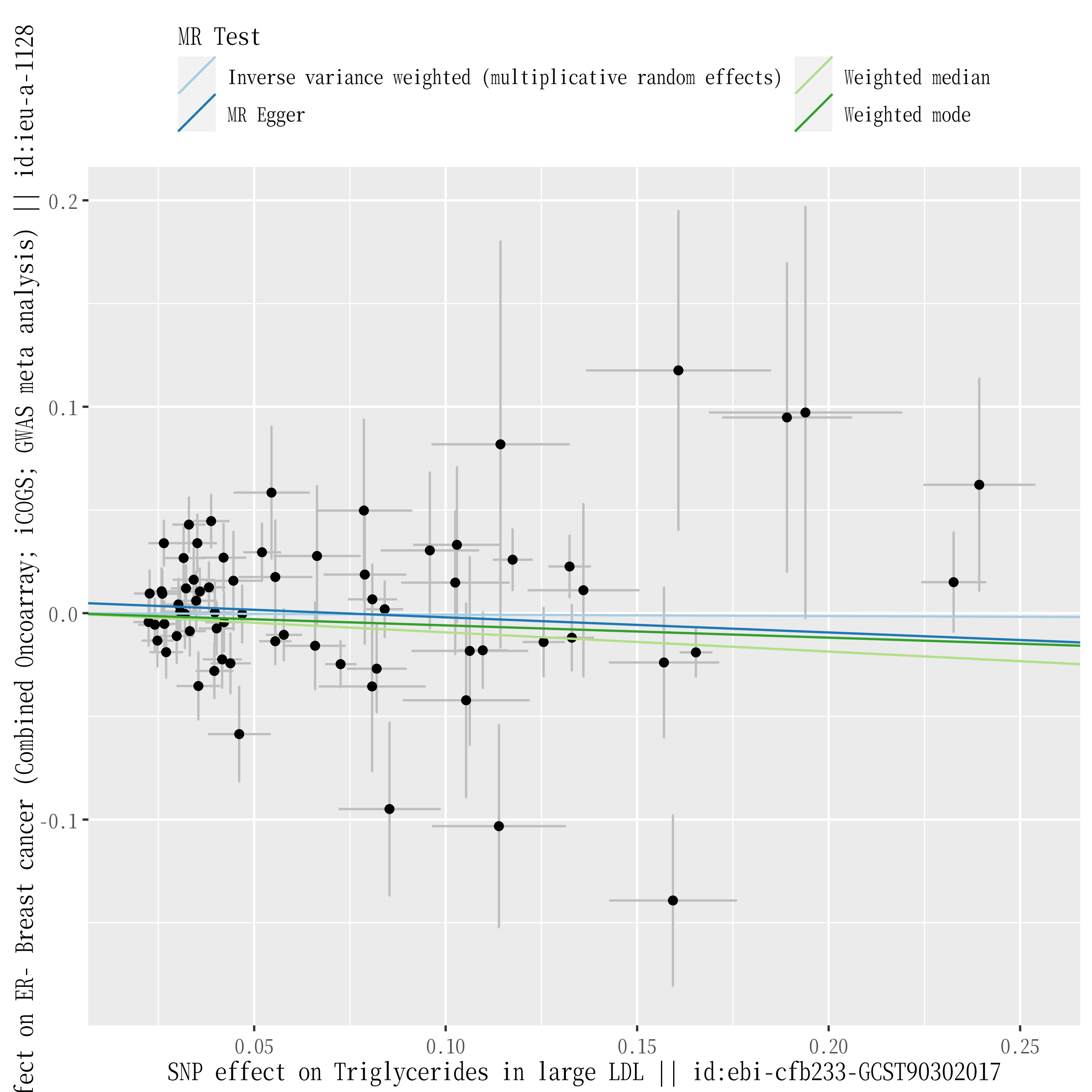
MR Test
Inverse variance weighted (multiplicative random effects)
MR Egger
Weighted median
Weighted mode
0.2
0.1
0.0
-0.1
0.05
0.10
0.15
0.20
0.25
SNP effect on Triglycerides in large LDL || id:ebi-cfb233-GCST90302017
fect on ER- Breast cancer (Combined Oncoarray; iCOGS; GWAS meta analysis) || id:ieu-a-1128

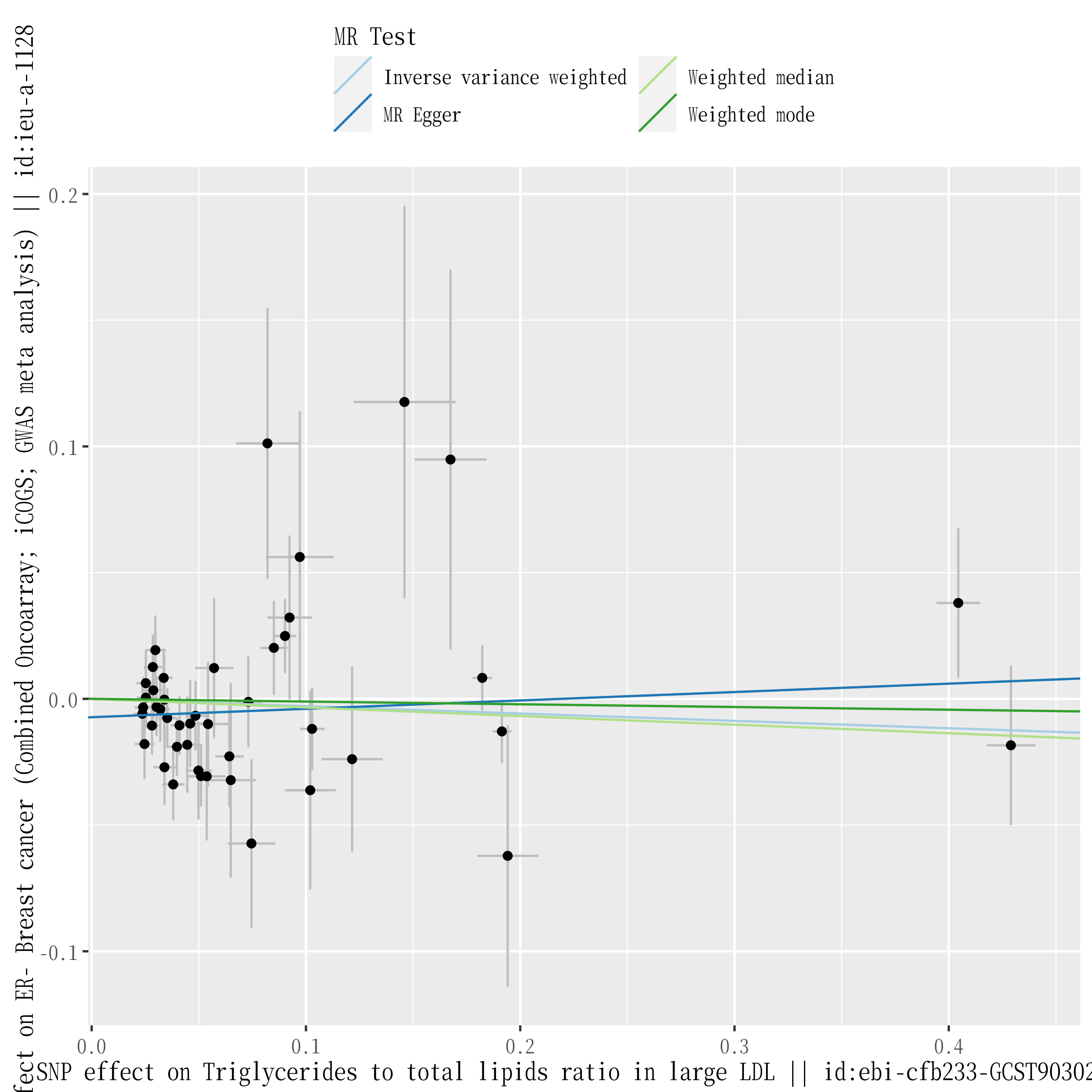

MR Test
Inverse variance weighted
MR Egger
Weighted median
Weighted mode
0.2
0.1
0.0
-0.1
0.0
0.1
0.2
0.3
0.4
fect on ER- Breast cancer (Combined Oncoarray; iCOGS; GWAS meta analysis) || id:ieu-a-1128
SNP effect on Triglycerides to total lipids ratio in large LDL || id:ebi-cfb233-GCST90302

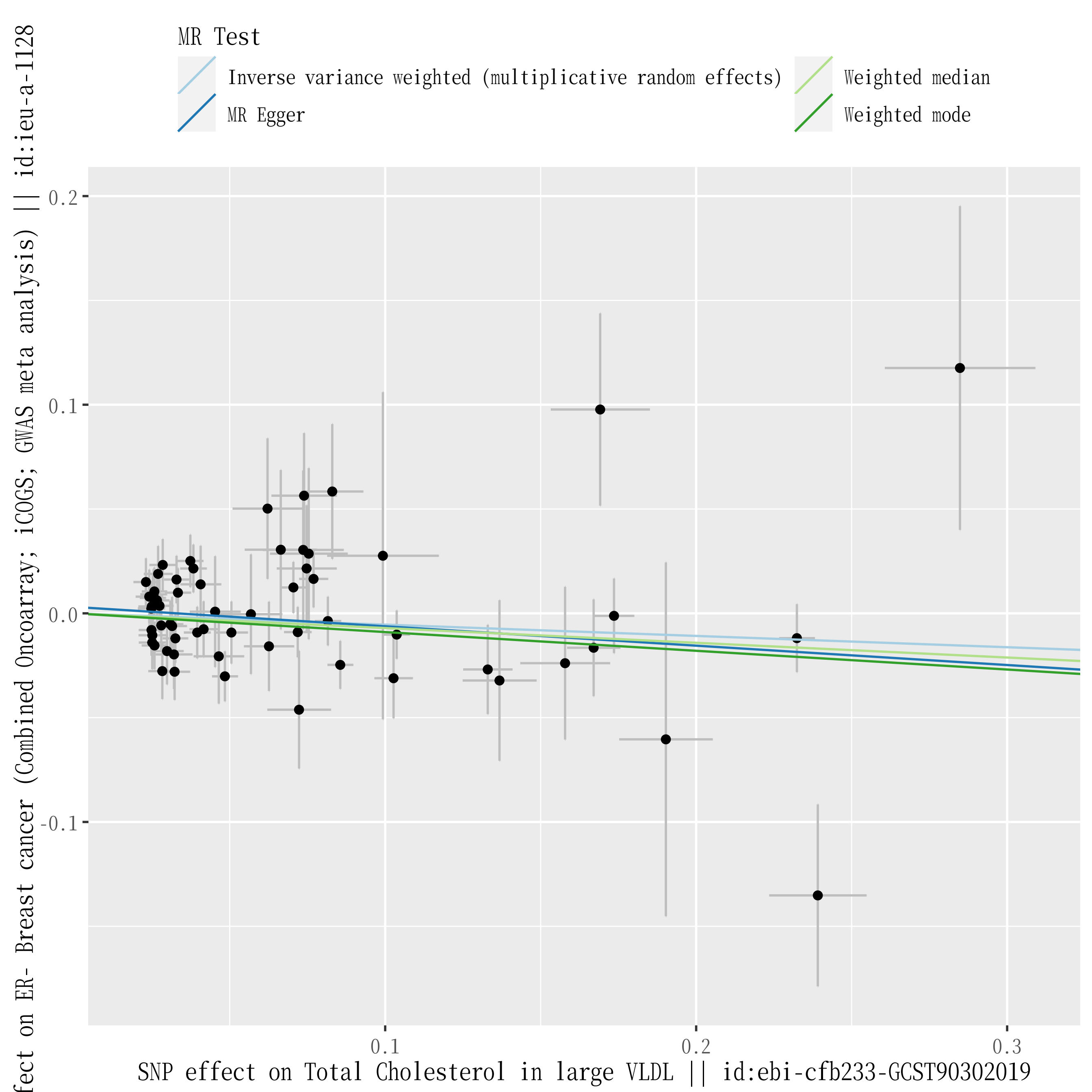
MR Test
Inverse variance weighted (multiplicative random effects)
MR Egger
Weighted median
Weighted mode
0.2
0.1
0.0
-0.1
0.1
0.2
0.3
SNP effect on Total Cholesterol in large VLDL || id:ebi-cfb233-GCST90302019
fect on ER- Breast cancer (Combined Oncoarray; iCOGS; GWAS meta analysis) || id:ieu-a-1128

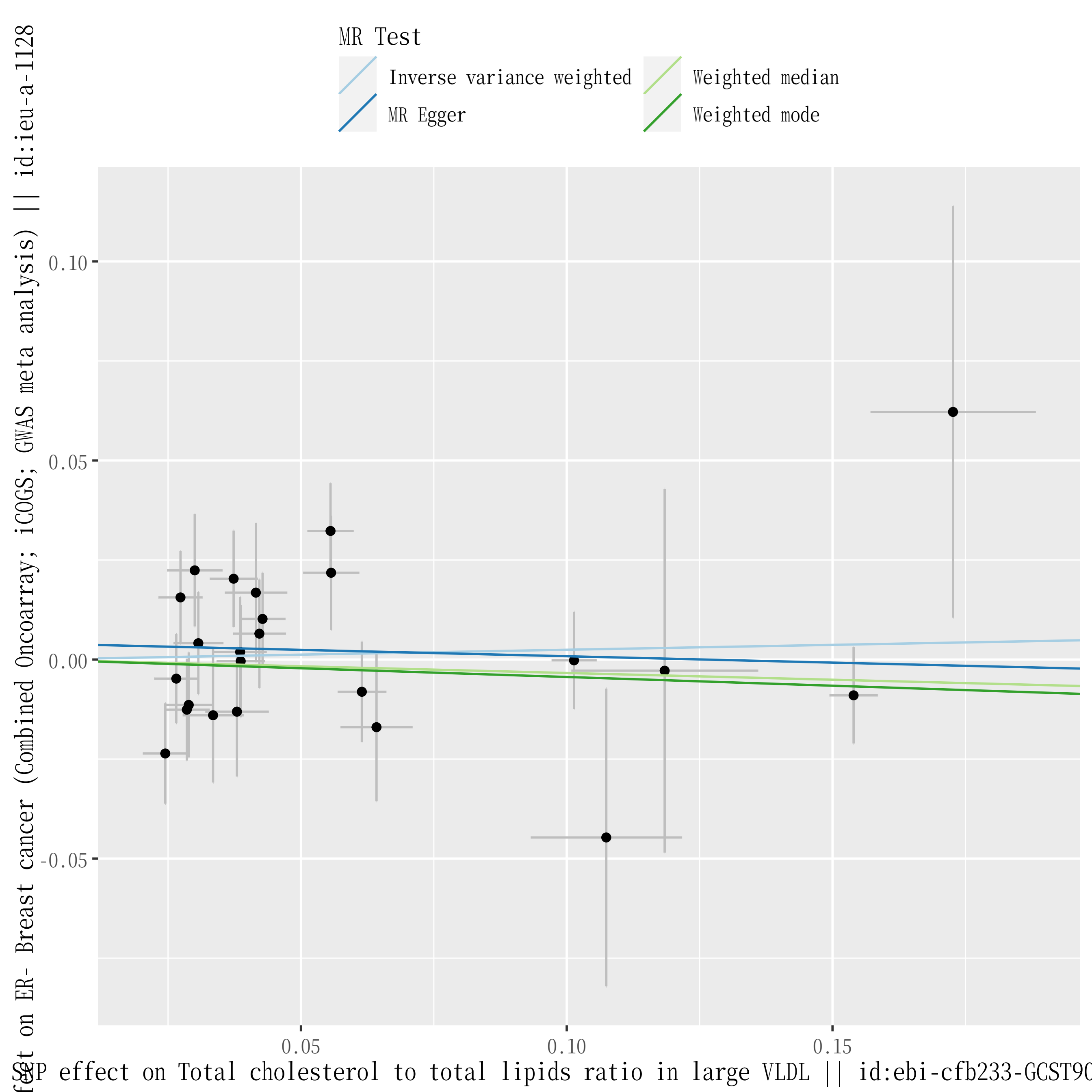

MR Test
Inverse variance weighted
MR Egger
Weighted median
Weighted mode
0.10
0.05
0.00
-0.05
0.05
0.10
0.15
SNP effect on Total cholesterol to total lipids ratio in large VLDL || id:ebi-cfb233-GCST90
fect on ER- Breast cancer (Combined Oncoarray; iCOGS; GWAS meta analysis) || id:ieu-a-1128

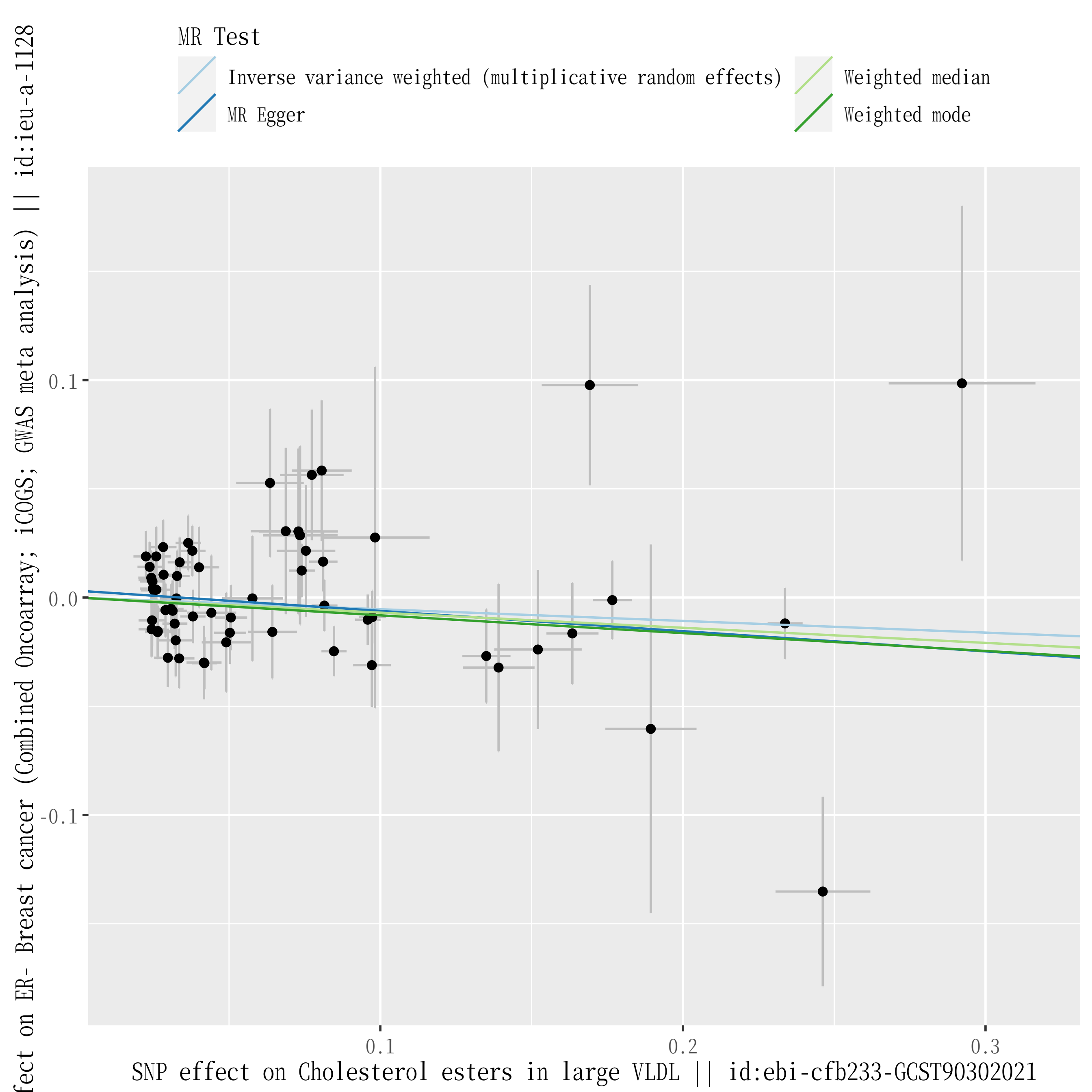

MR Test
Inverse variance weighted (multiplicative random effects)
MR Egger
Weighted median
Weighted mode
0.1
0.0
-0.1
0.1
0.2
0.3
SNP effect on Cholesterol esters in large VLDL || id:ebi-cfb233-GCST90302021
fect on ER- Breast cancer (Combined Oncoarray; iCOGS; GWAS meta analysis) || id:ieu-a-1128

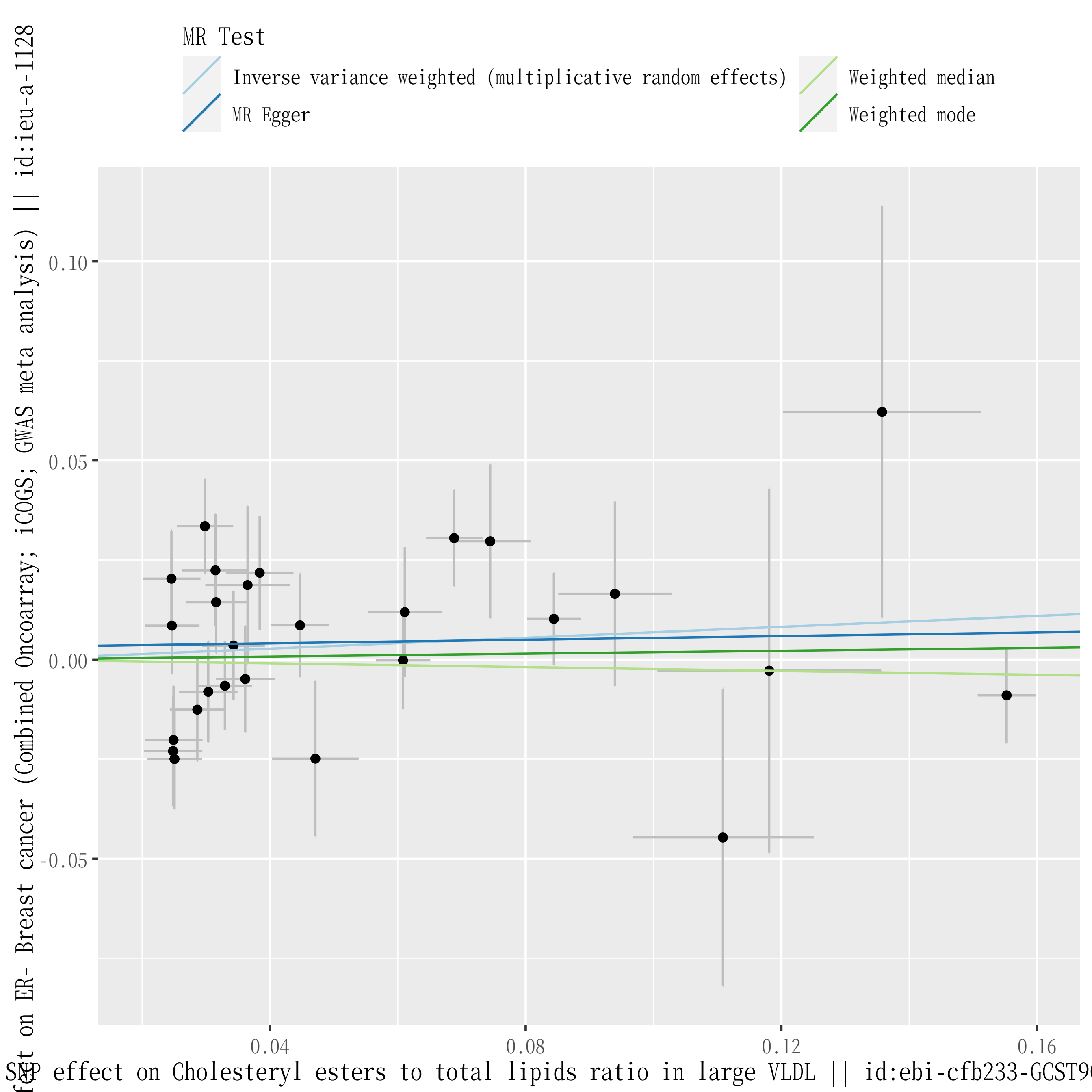
MR Test
Inverse variance weighted (multiplicative random effects)
MR Egger
Weighted median
Weighted mode
0.10
0.05
0.00
-0.05
0.04
0.08
0.12
0.16
SNP effect on Cholesteryl esters to total lipids ratio in large VLDL || id:ebi-cfb233-GCST9
fect on ER- Breast cancer (Combined Oncoarray; iCOGS; GWAS meta analysis) || id:ieu-a-1128

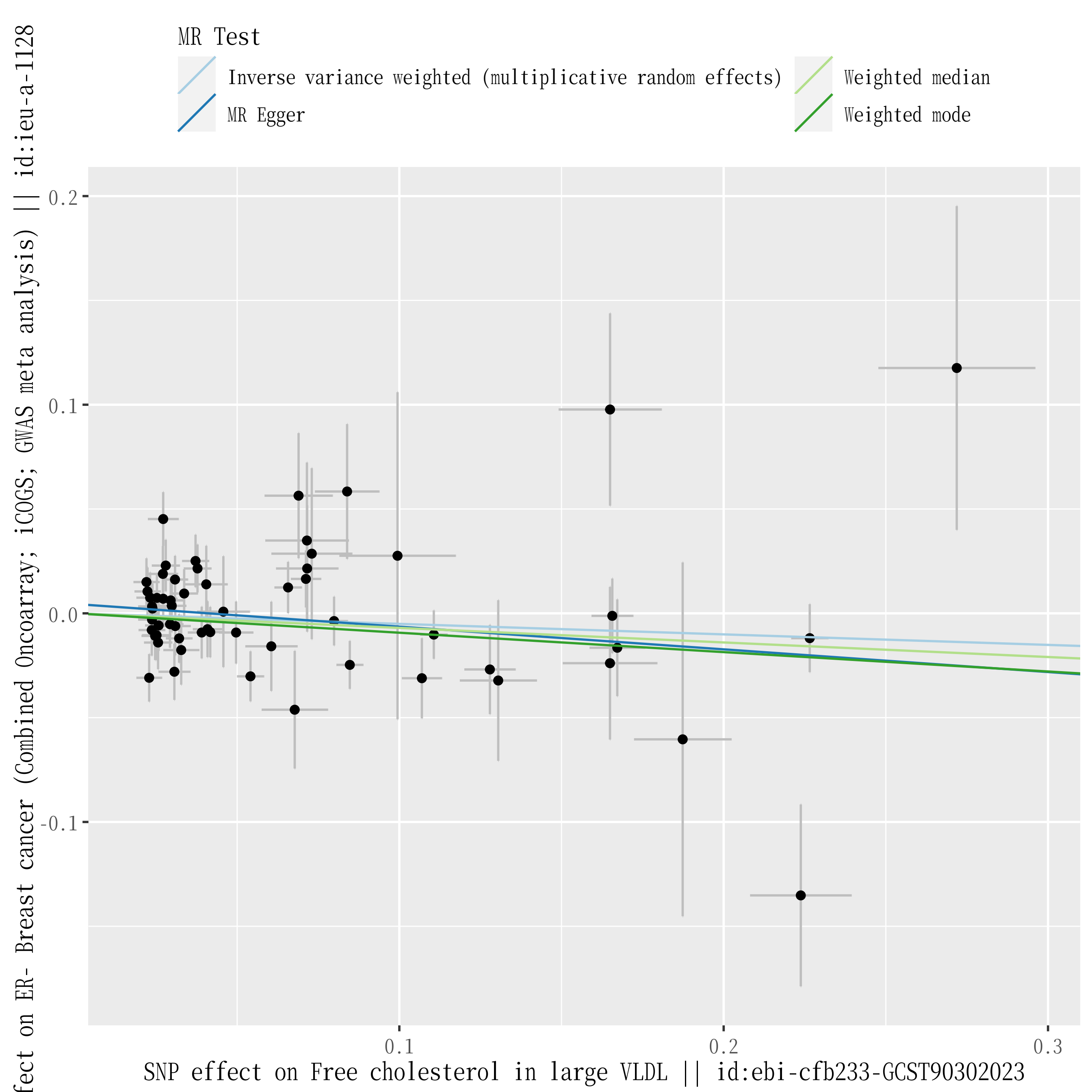
MR Test
Inverse variance weighted (multiplicative random effects)
MR Egger
Weighted median
Weighted mode
0.2
0.1
0.0
-0.1
0.1
0.2
0.3
SNP effect on Free cholesterol in large VLDL || id:ebi-cfb233-GCST90302023
fect on ER- Breast cancer (Combined Oncoarray; iCOGS; GWAS meta analysis) || id:ieu-a-1128

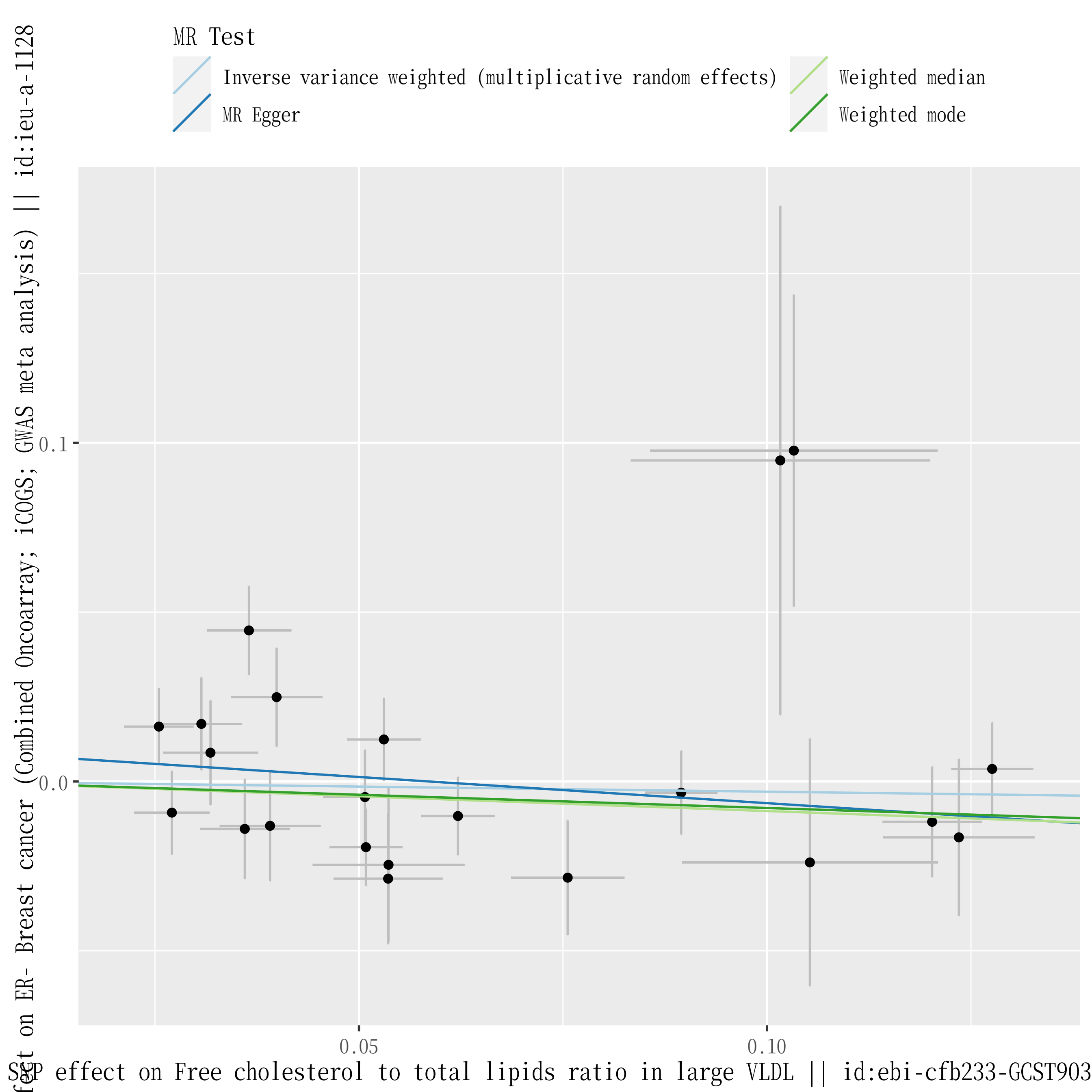

MR Test
Inverse variance weighted (multiplicative random effects)
MR Egger
Weighted median
Weighted mode
0.1
0.0
0.05
0.10
SNP effect on Free cholesterol to total lipids ratio in large VLDL || id:ebi-cfb233-GCST903
fect on ER- Breast cancer (Combined Oncoarray; iCOGS; GWAS meta analysis) || id:ieu-a-1128

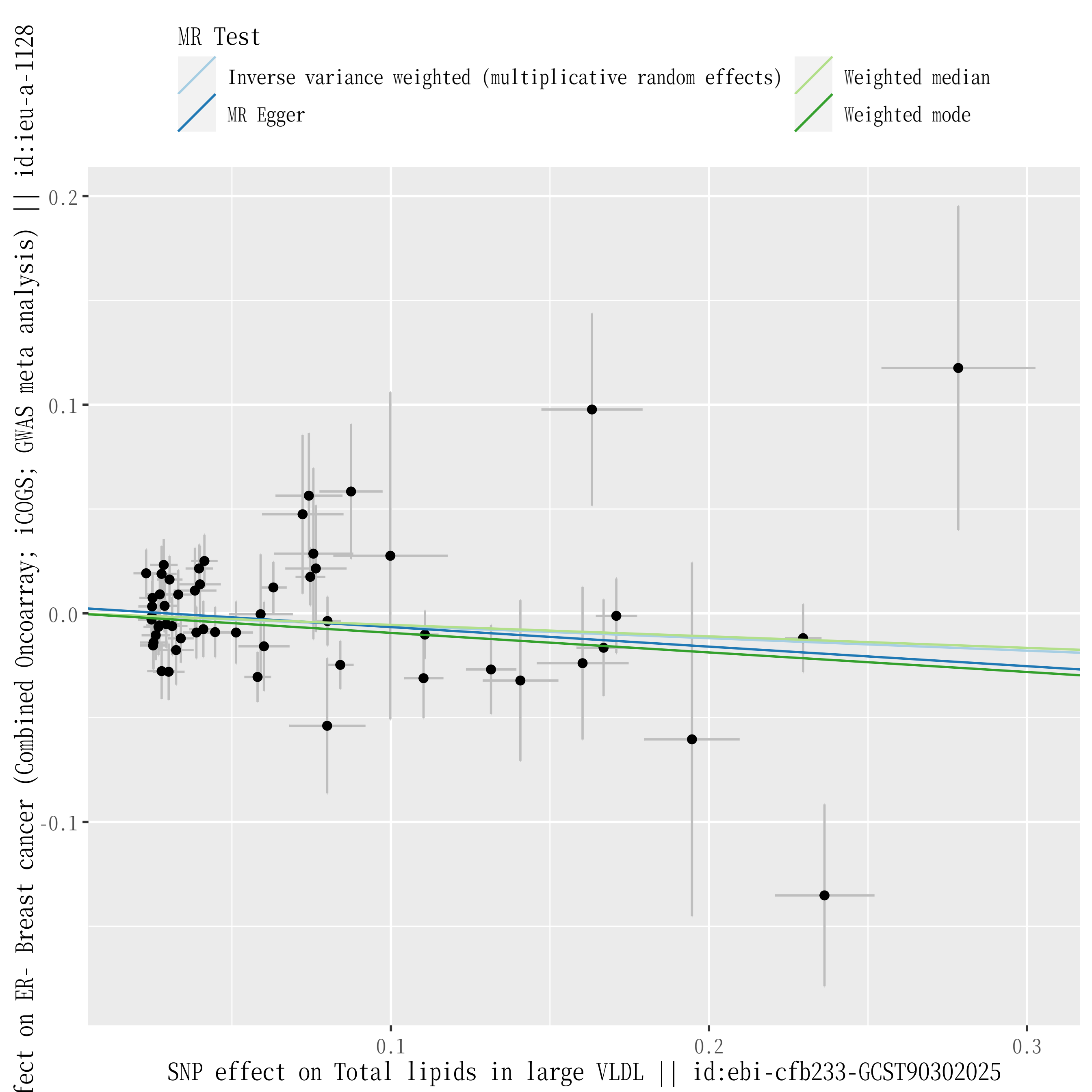

MR Test
Inverse variance weighted (multiplicative random effects)
MR Egger
Weighted median
Weighted mode
0.2
0.1
0.0
-0.1
0.1
0.2
0.3
fect on ER- Breast cancer (Combined Oncoarray; iCOGS; GWAS meta analysis) || id:ieu-a-1128
SNP effect on Total lipids in large VLDL || id:ebi-cfb233-GCST90302025

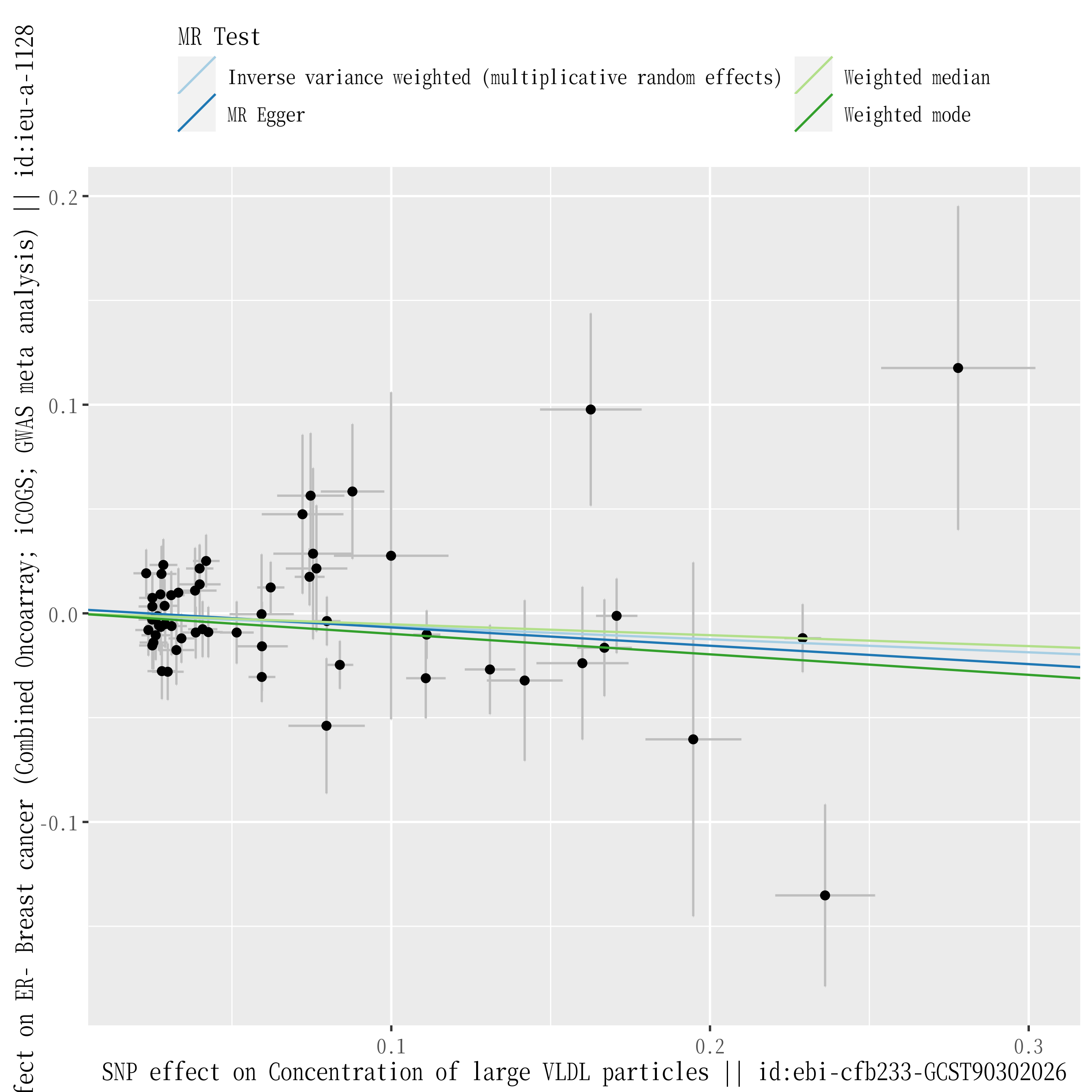

MR Test
Inverse variance weighted (multiplicative random effects)
MR Egger
Weighted median
Weighted mode
0.2
0.1
0.0
-0.1
0.1
0.2
0.3
SNP effect on Concentration of large VLDL particles || id:ebi-cfb233-GCST90302026
fect on ER- Breast cancer (Combined Oncoarray; iCOGS; GWAS meta analysis) || id:ieu-a-1128

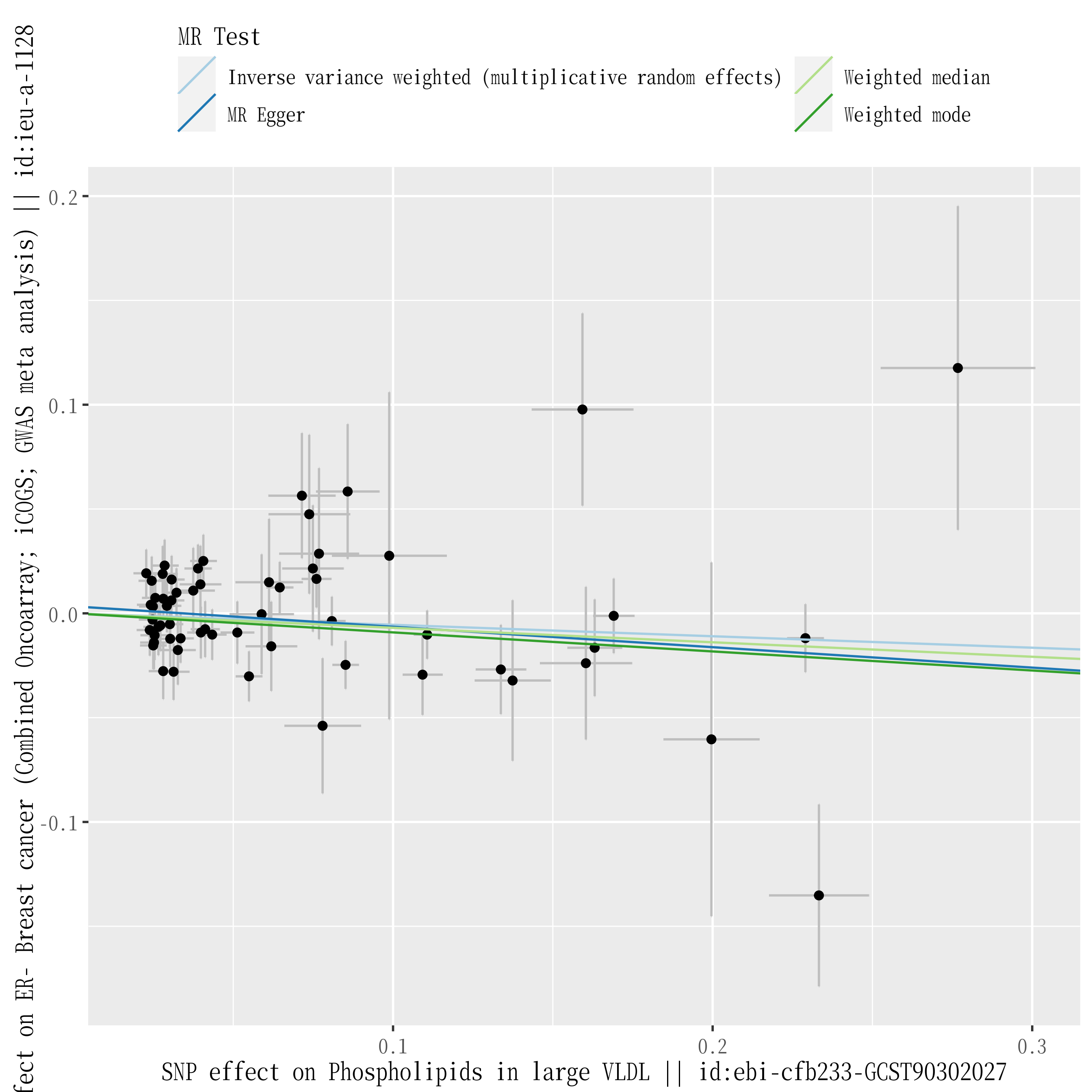

MR Test
Inverse variance weighted (multiplicative random effects)
MR Egger
Weighted median
Weighted mode
0.2
0.1
0.0
-0.1
0.1
0.2
0.3
SNP effect on Phospholipids in large VLDL || id:ebi-cfb233-GCST90302027
fect on ER- Breast cancer (Combined Oncoarray; iCOGS; GWAS meta analysis) || id:ieu-a-1128

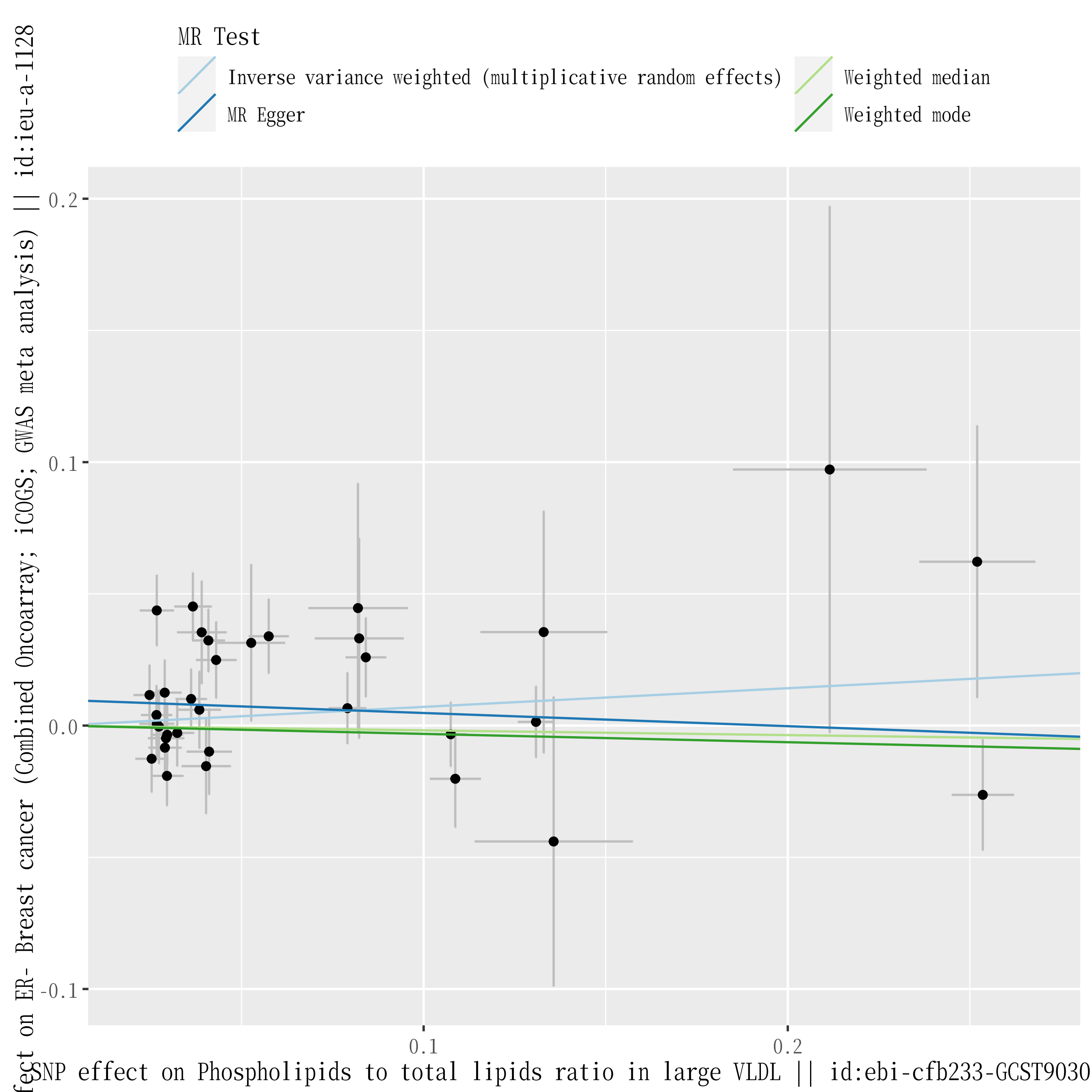

MR Test
Inverse variance weighted (multiplicative random effects)
MR Egger
Weighted median
Weighted mode
0.2
0.1
0.0
-0.1
0.1
0.2
SNP effect on Phospholipids to total lipids ratio in large VLDL || id:ebi-cfb233-GCST9030
fect on ER- Breast cancer (Combined Oncoarray; iCOGS; GWAS meta analysis) || id:ieu-a-1128

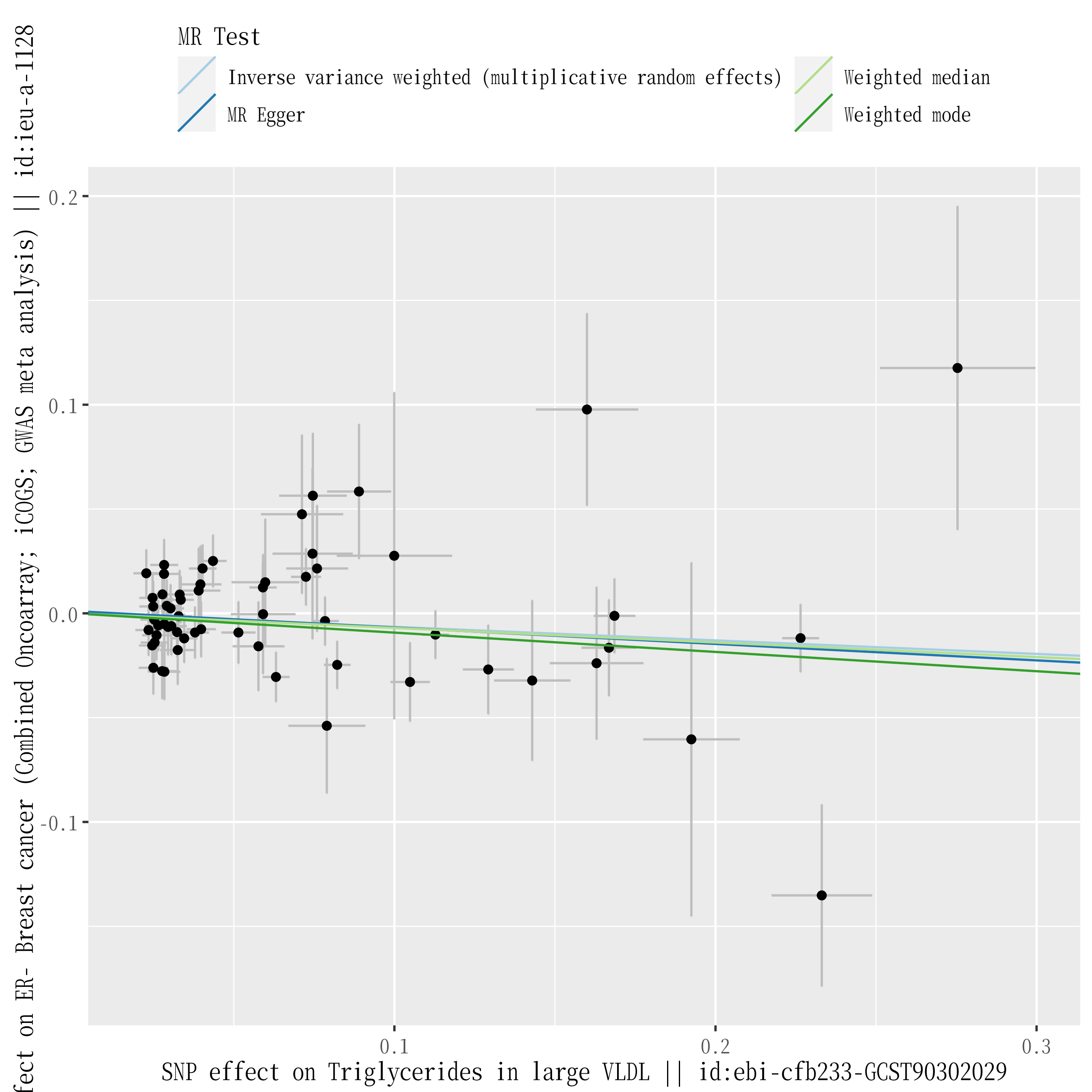
MR Test
Inverse variance weighted (multiplicative random effects)
MR Egger
Weighted median
Weighted mode
0.2
0.1
0.0
-0.1
0.1
0.2
0.3
fect on ER- Breast cancer (Combined Oncoarray; iCOGS; GWAS meta analysis) || id:ieu-a-1128
SNP effect on Triglycerides in large VLDL || id:ebi-cfb233-GCST90302029

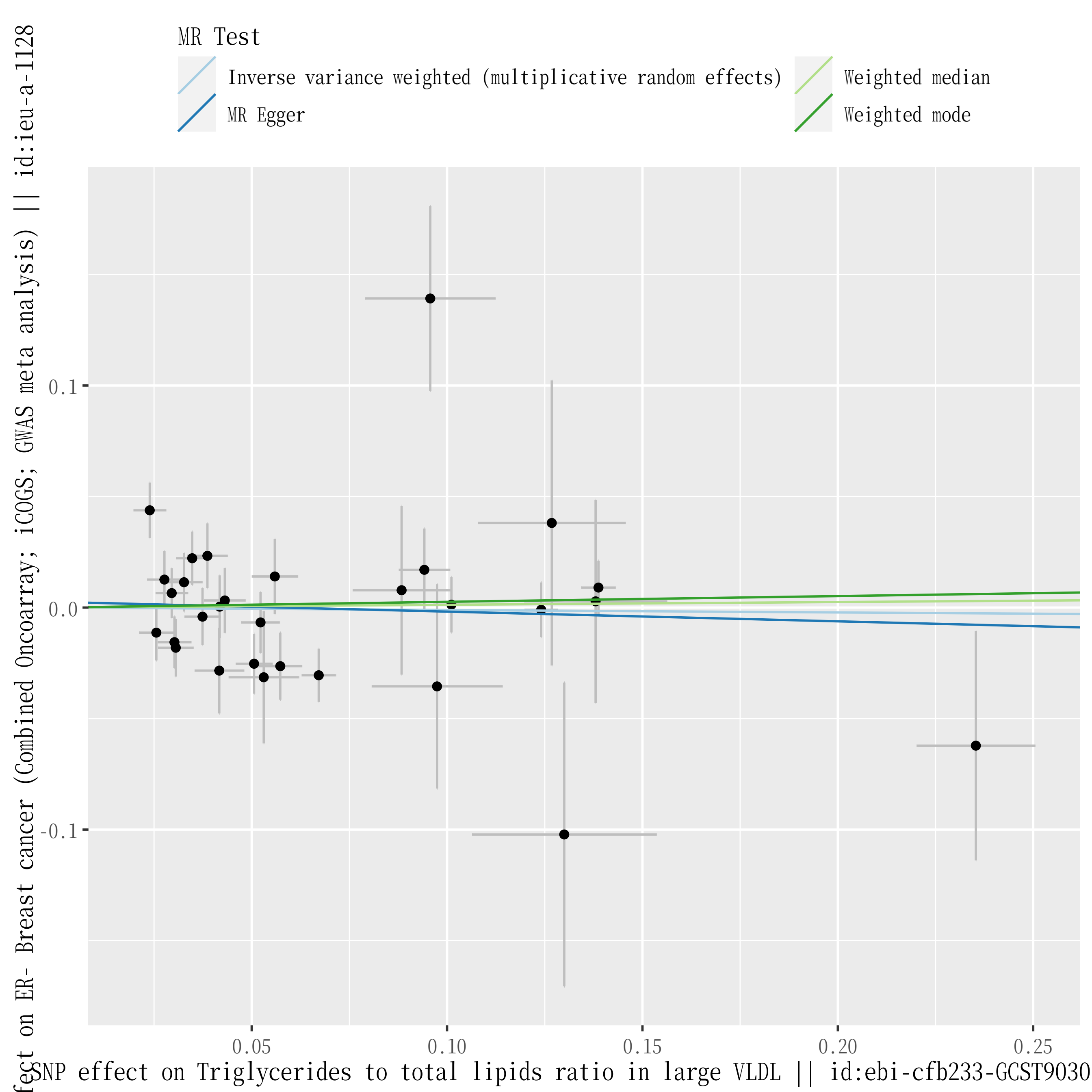

MR Test
Inverse variance weighted (multiplicative random effects)
MR Egger
Weighted median
Weighted mode
0.1
0.0
-0.1
0.05
0.10
0.15
0.20
0.25
SNP effect on Triglycerides to total lipids ratio in large VLDL || id:ebi-cfb233-GCST9030
fect on ER- Breast cancer (Combined Oncoarray; iCOGS; GWAS meta analysis) || id:ieu-a-1128

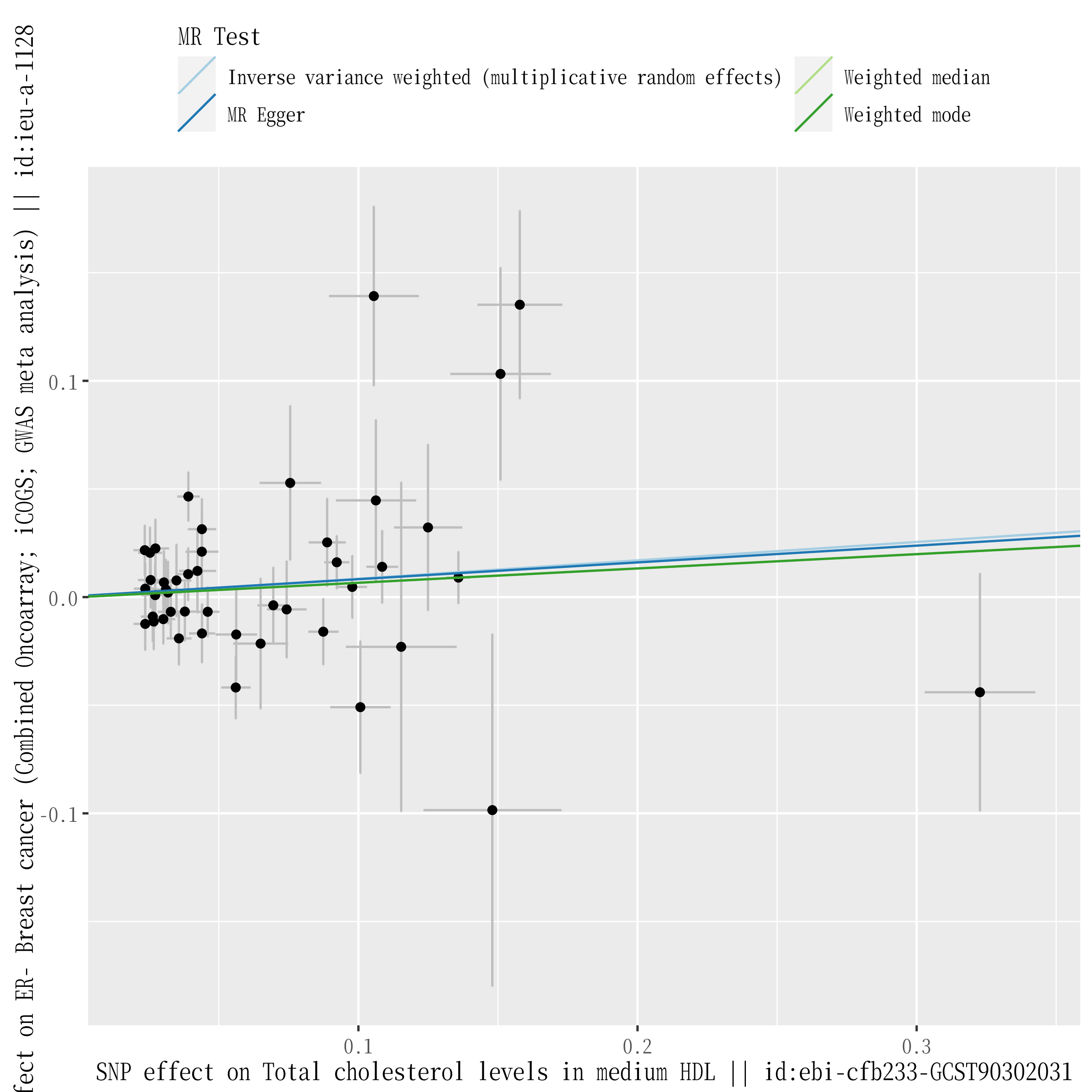
MR Test
Inverse variance weighted (multiplicative random effects)
MR Egger
Weighted median
Weighted mode
0.1
0.0
-0.1
0.1
0.2
0.3
SNP effect on Total cholesterol levels in medium HDL || id:ebi-cfb233-GCST90302031
fect on ER- Breast cancer (Combined Oncoarray; iCOGS; GWAS meta analysis) || id:ieu-a-1128

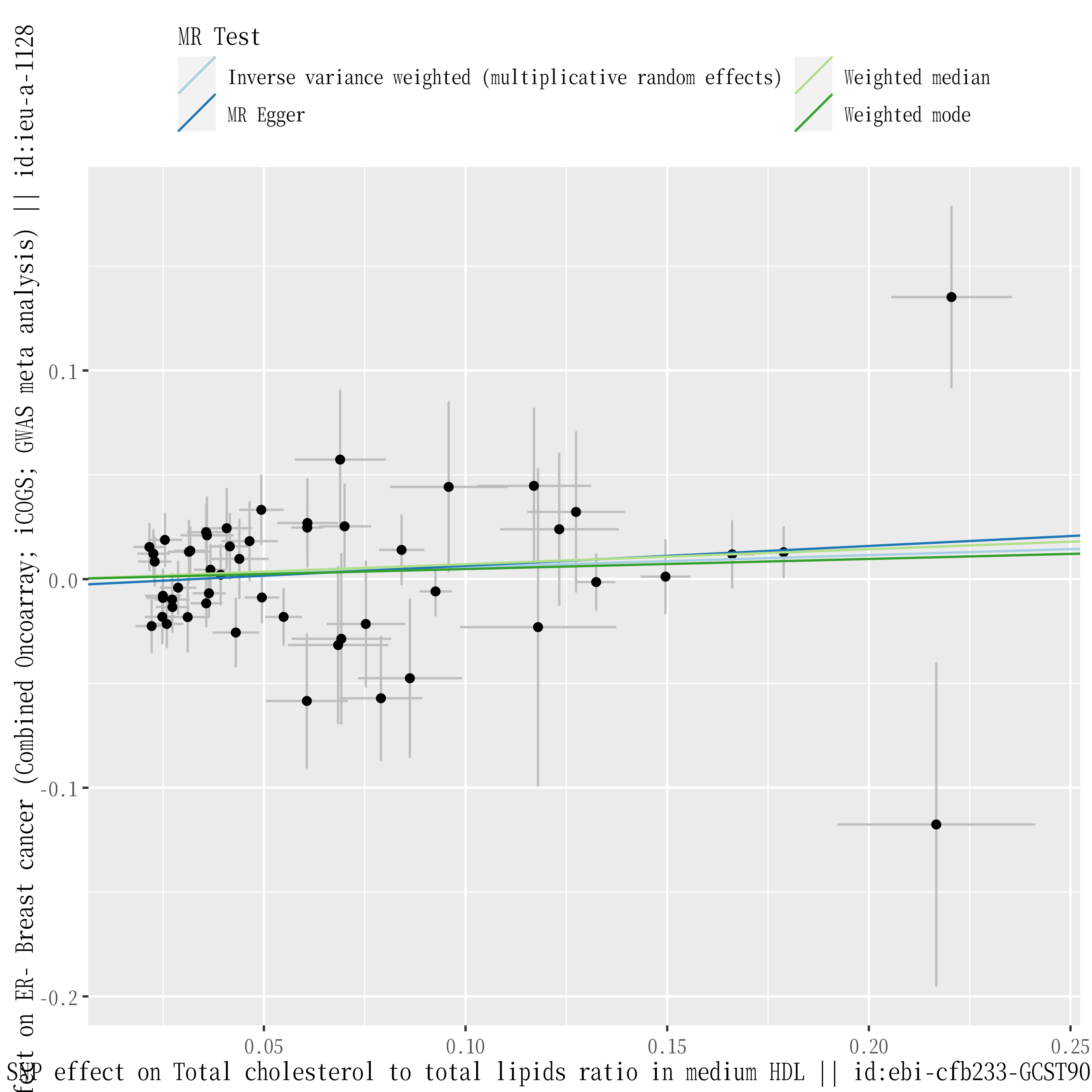

MR Test
Inverse variance weighted (multiplicative random effects)
MR Egger
Weighted median
Weighted mode
0.1
0.0
-0.1
-0.2
0.05
0.10
0.15
0.20
0.25
SNP effect on Total cholesterol to total lipids ratio in medium HDL || id:ebi-cfb233-GCST90
fect on ER- Breast cancer (Combined Oncoarray; iCOGS; GWAS meta analysis) || id:ieu-a-1128

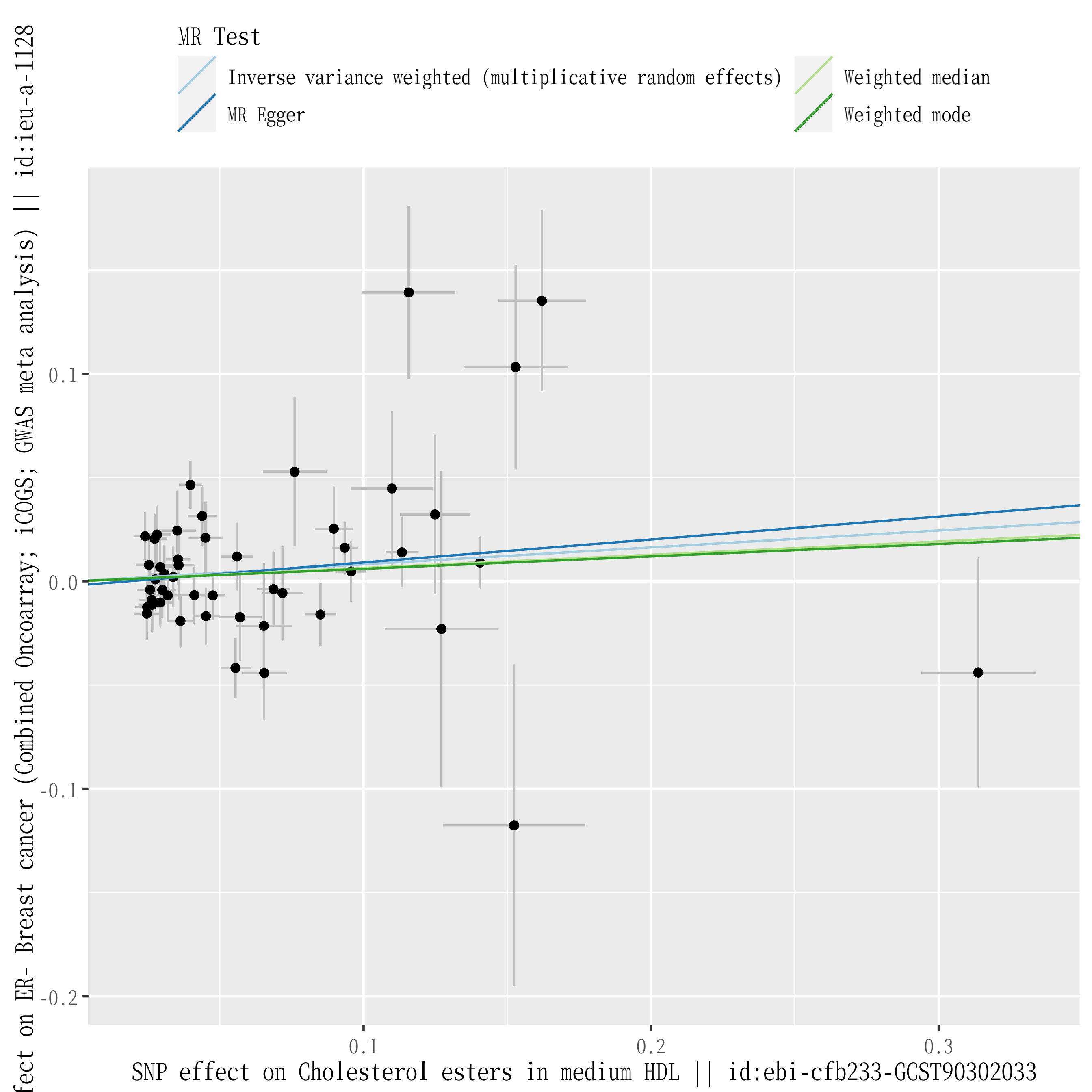

MR Test
Inverse variance weighted (multiplicative random effects)
MR Egger
Weighted median
Weighted mode
0.1
0.0
-0.1
-0.2
0.1
0.2
0.3
SNP effect on Cholesterol esters in medium HDL || id:ebi-cfb233-GCST90302033
fect on ER- Breast cancer (Combined Oncoarray; iCOGS; GWAS meta analysis) || id:ieu-a-1128

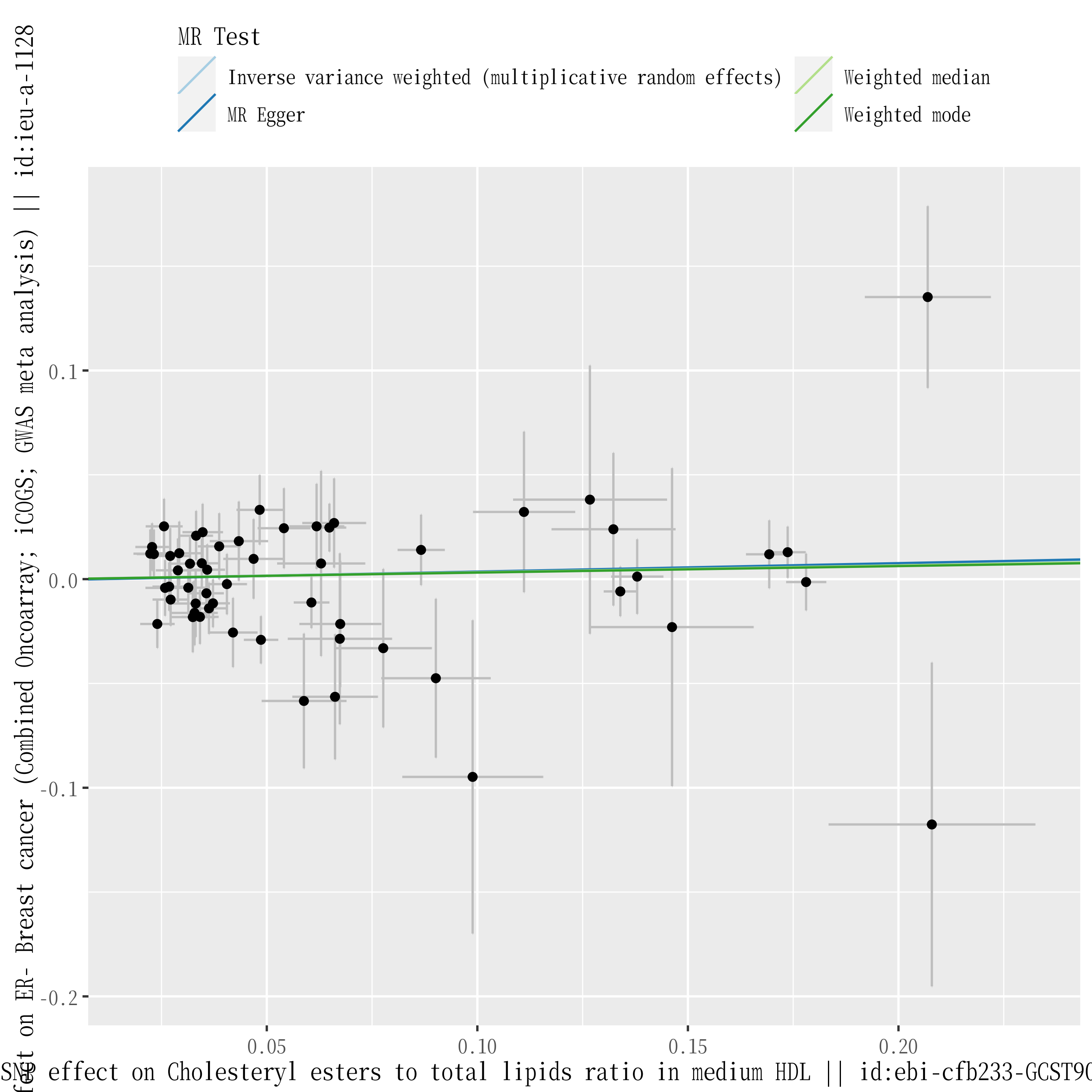
MR Test
Inverse variance weighted (multiplicative random effects)
MR Egger
Weighted median
Weighted mode
0.1
0.0
-0.1
-0.2
0.05
0.10
0.15
0.20
SNP effect on Cholesteryl esters to total lipids ratio in medium HDL || id:ebi-cfb233-GCST90
fect on ER- Breast cancer (Combined Oncoarray; iCOGS; GWAS meta analysis) || id:ieu-a-1128

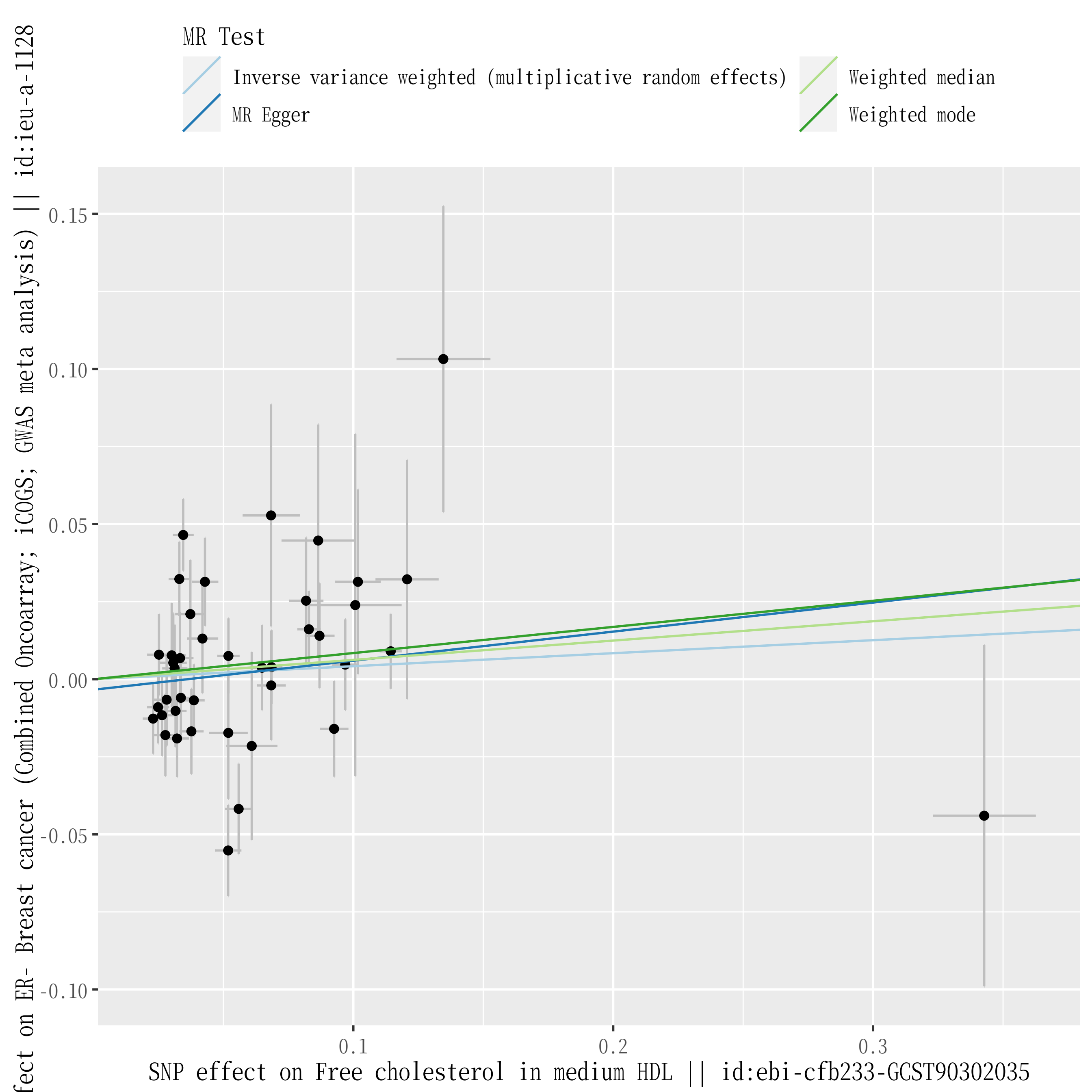

MR Test
Inverse variance weighted (multiplicative random effects)
MR Egger
Weighted median
Weighted mode
0.15
0.10
0.05
0.00
-0.05
-0.10
0.1
0.2
0.3
SNP effect on Free cholesterol in medium HDL || id:ebi-cfb233-GCST90302035
fect on ER- Breast cancer (Combined Oncoarray; iCOGS; GWAS meta analysis) || id:ieu-a-1128

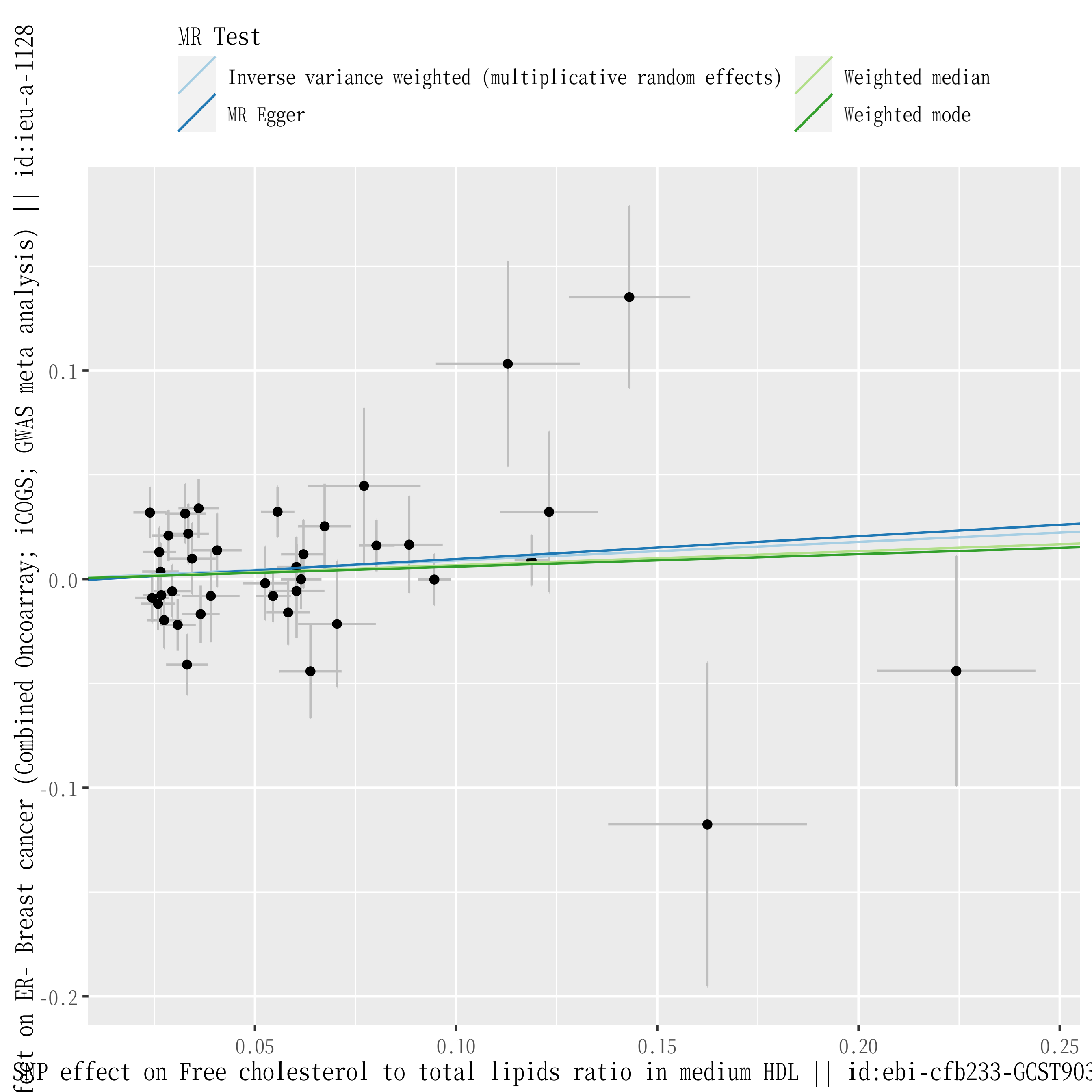

MR Test
Inverse variance weighted (multiplicative random effects)
MR Egger
Weighted median
Weighted mode
0.1
0.0
-0.1
-0.2
0.05
0.10
0.15
0.20
0.25
SNP effect on Free cholesterol to total lipids ratio in medium HDL || id:ebi-cfb233-GCST903
fect on ER- Breast cancer (Combined Oncoarray; iCOGS; GWAS meta analysis) || id:ieu-a-1128

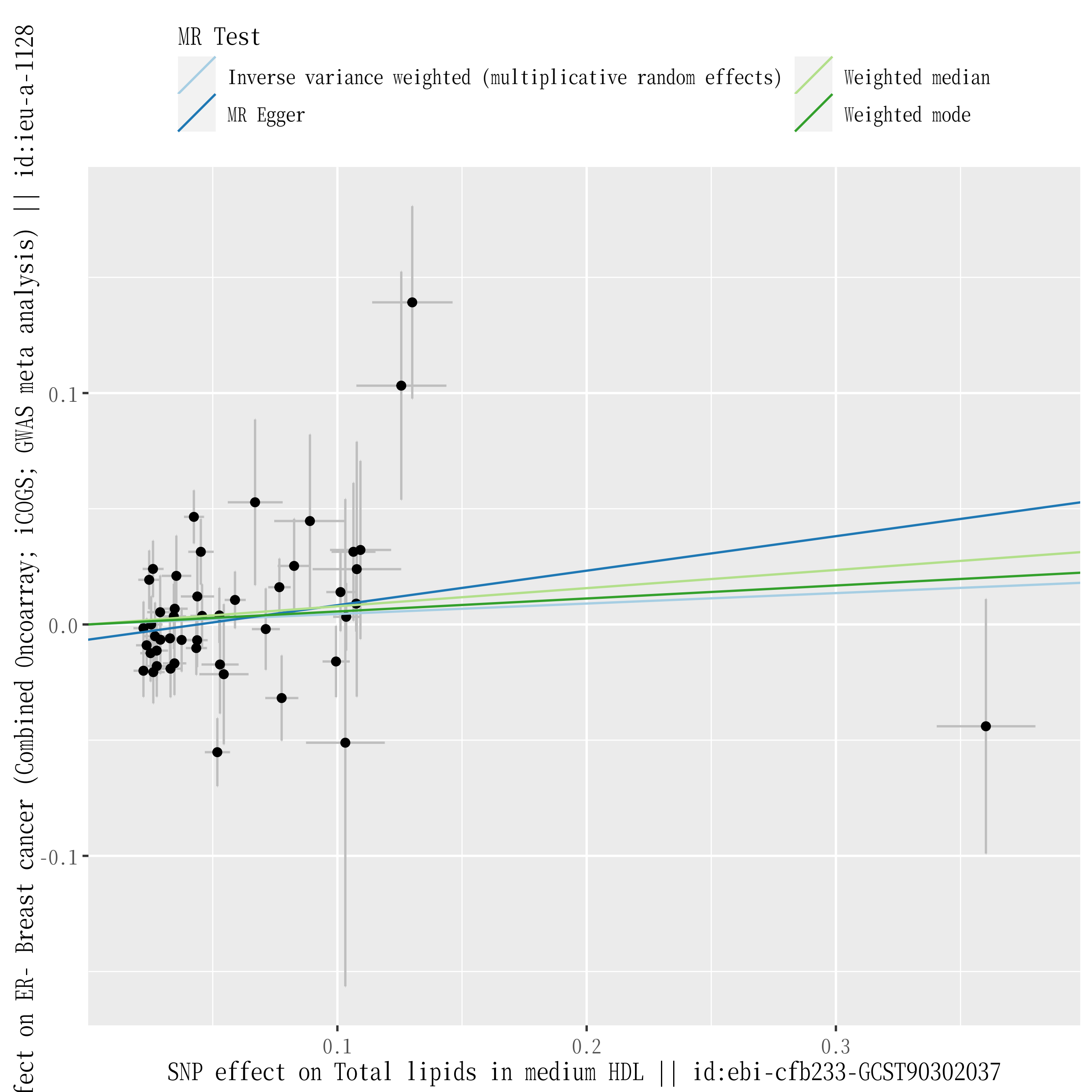

MR Test
Inverse variance weighted (multiplicative random effects)
MR Egger
Weighted median
Weighted mode
0.1
0.0
-0.1
0.1
0.2
0.3
SNP effect on Total lipids in medium HDL || id:ebi-cfb233-GCST90302037
fect on ER- Breast cancer (Combined Oncoarray; iCOGS; GWAS meta analysis) || id:ieu-a-1128

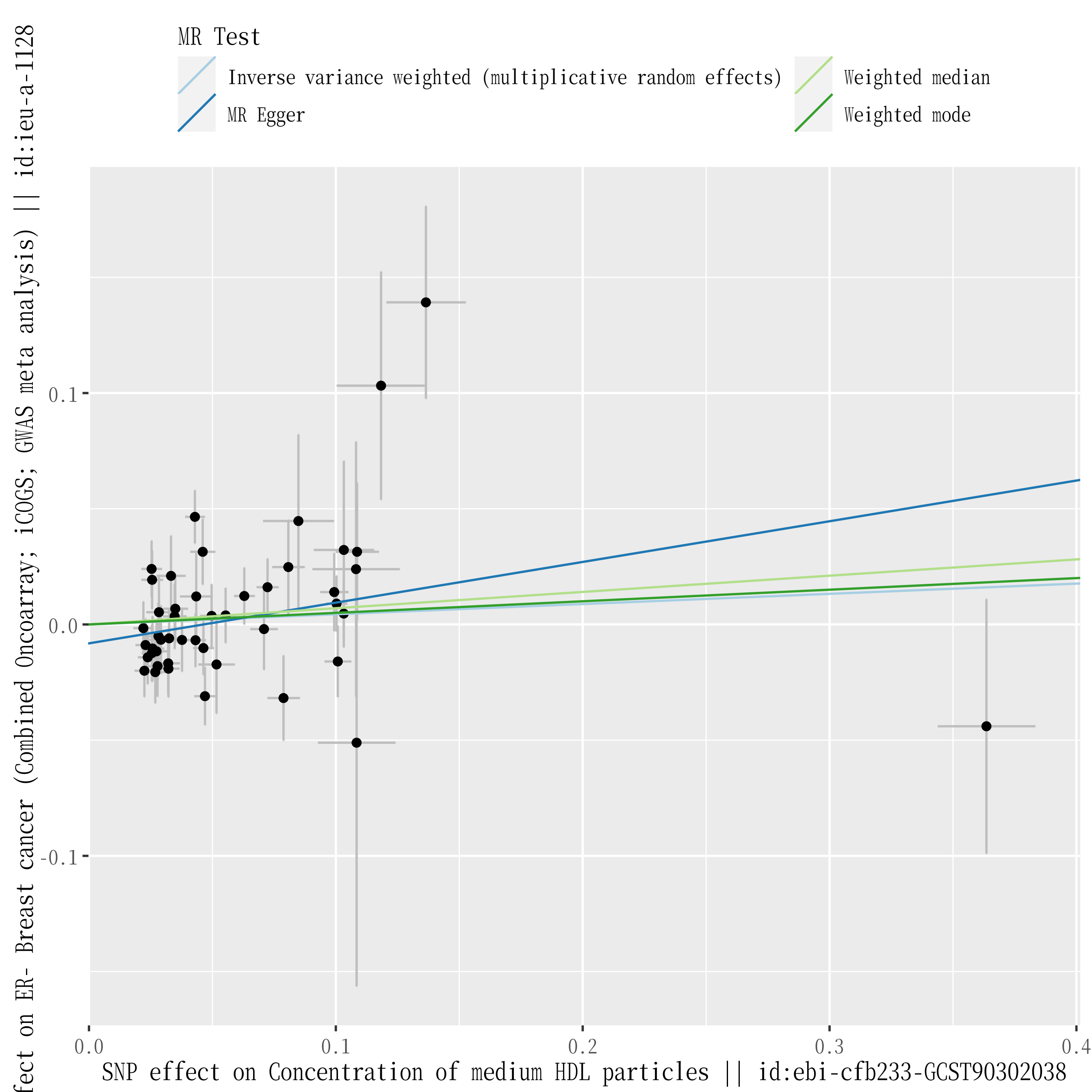
MR Test
Inverse variance weighted (multiplicative random effects)
MR Egger
Weighted median
Weighted mode
0.1
0.0
-0.1
0.0
0.1
0.2
0.3
0.4
SNP effect on Concentration of medium HDL particles || id:ebi-cfb233-GCST90302038
fect on ER- Breast cancer (Combined Oncoarray; iCOGS; GWAS meta analysis) || id:ieu-a-1128

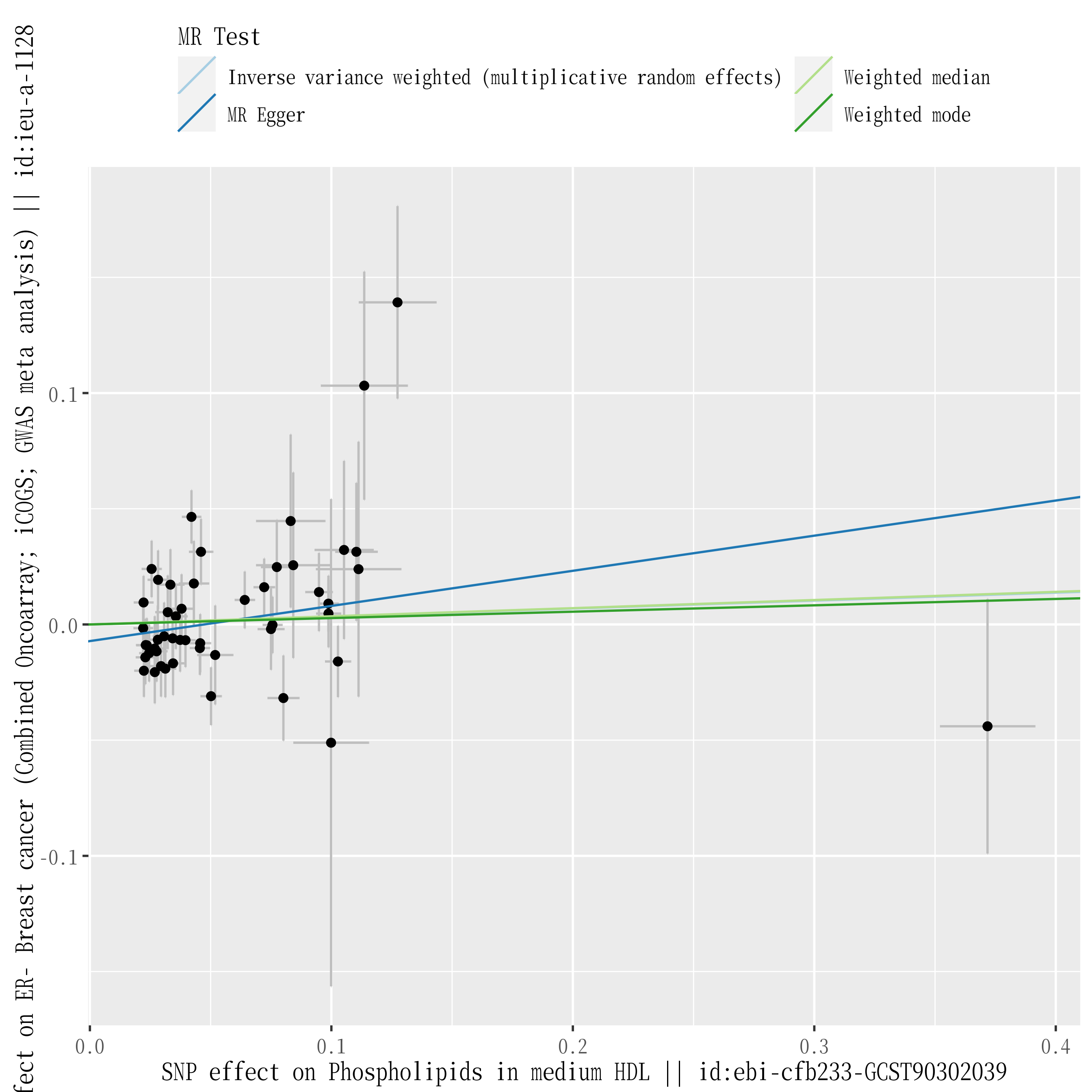
MR Test
Inverse variance weighted (multiplicative random effects)
MR Egger
Weighted median
Weighted mode
0.1
0.0
-0.1
0.0
0.1
0.2
0.3
0.4
SNP effect on Phospholipids in medium HDL || id:ebi-cfb233-GCST90302039
fect on ER- Breast cancer (Combined Oncoarray; iCOGS; GWAS meta analysis) || id:ieu-a-1128

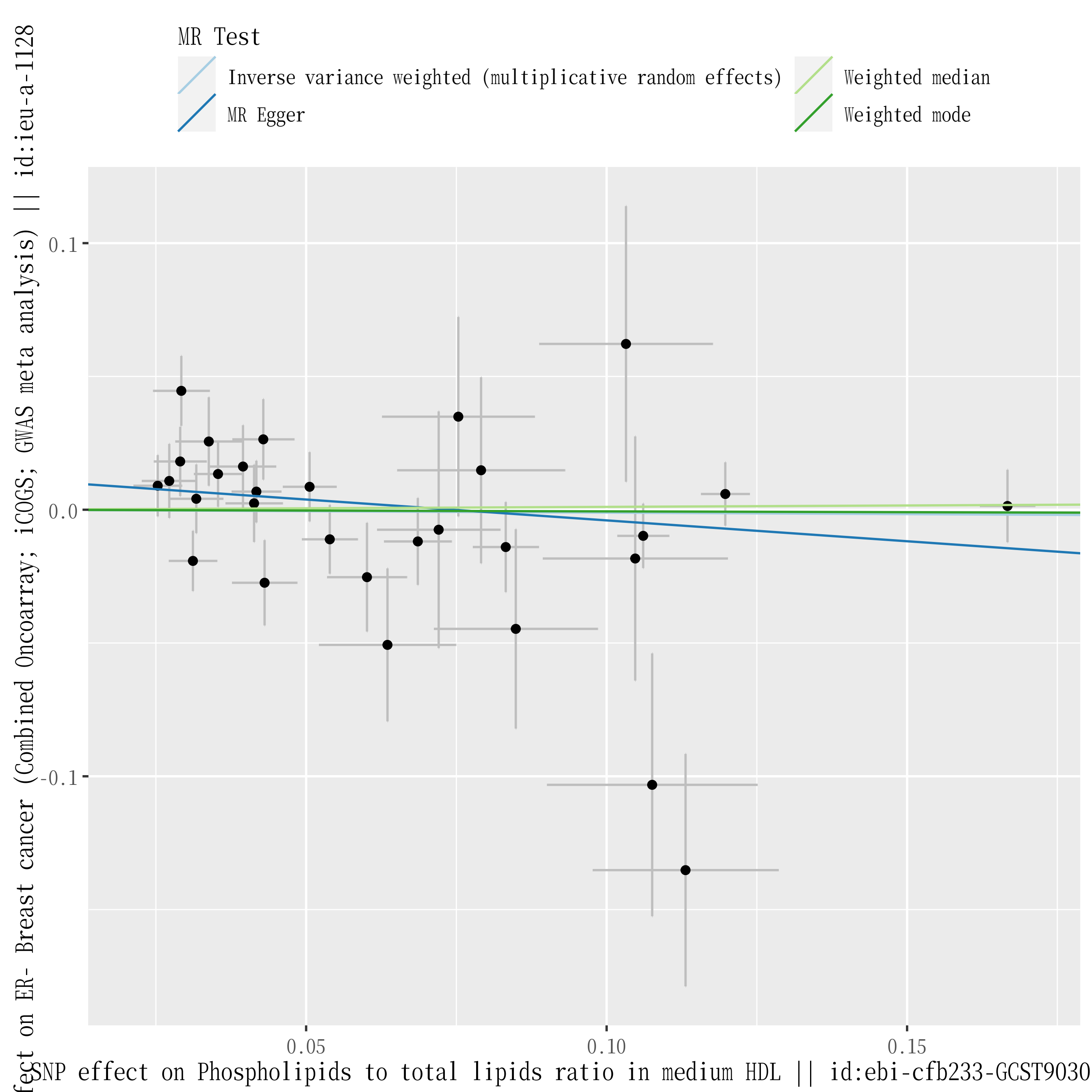

MR Test
Inverse variance weighted (multiplicative random effects)
MR Egger
Weighted median
Weighted mode
0.1
0.0
-0.1
0.05
0.10
0.15
SNP effect on Phospholipids to total lipids ratio in medium HDL || id:ebi-cfb233-GCST9030
fect on ER- Breast cancer (Combined Oncoarray; iCOGS; GWAS meta analysis) || id:ieu-a-1128

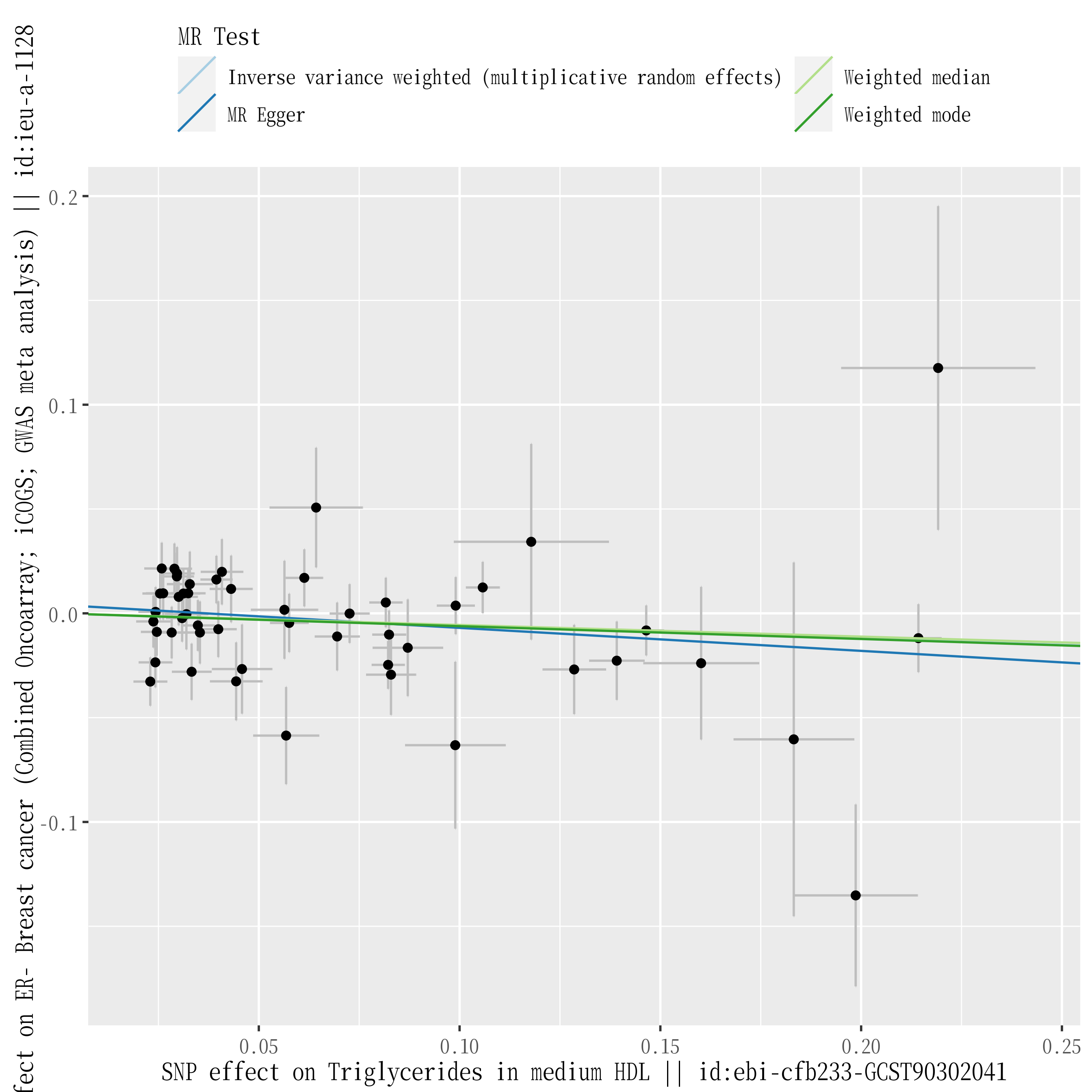
MR Test
Inverse variance weighted (multiplicative random effects)
MR Egger
Weighted median
Weighted mode
0.2
0.1
0.0
-0.1
0.05
0.10
0.15
0.20
0.25
SNP effect on Triglycerides in medium HDL || id:ebi-cfb233-GCST90302041
fect on ER- Breast cancer (Combined Oncoarray; iCOGS; GWAS meta analysis) || id:ieu-a-1128

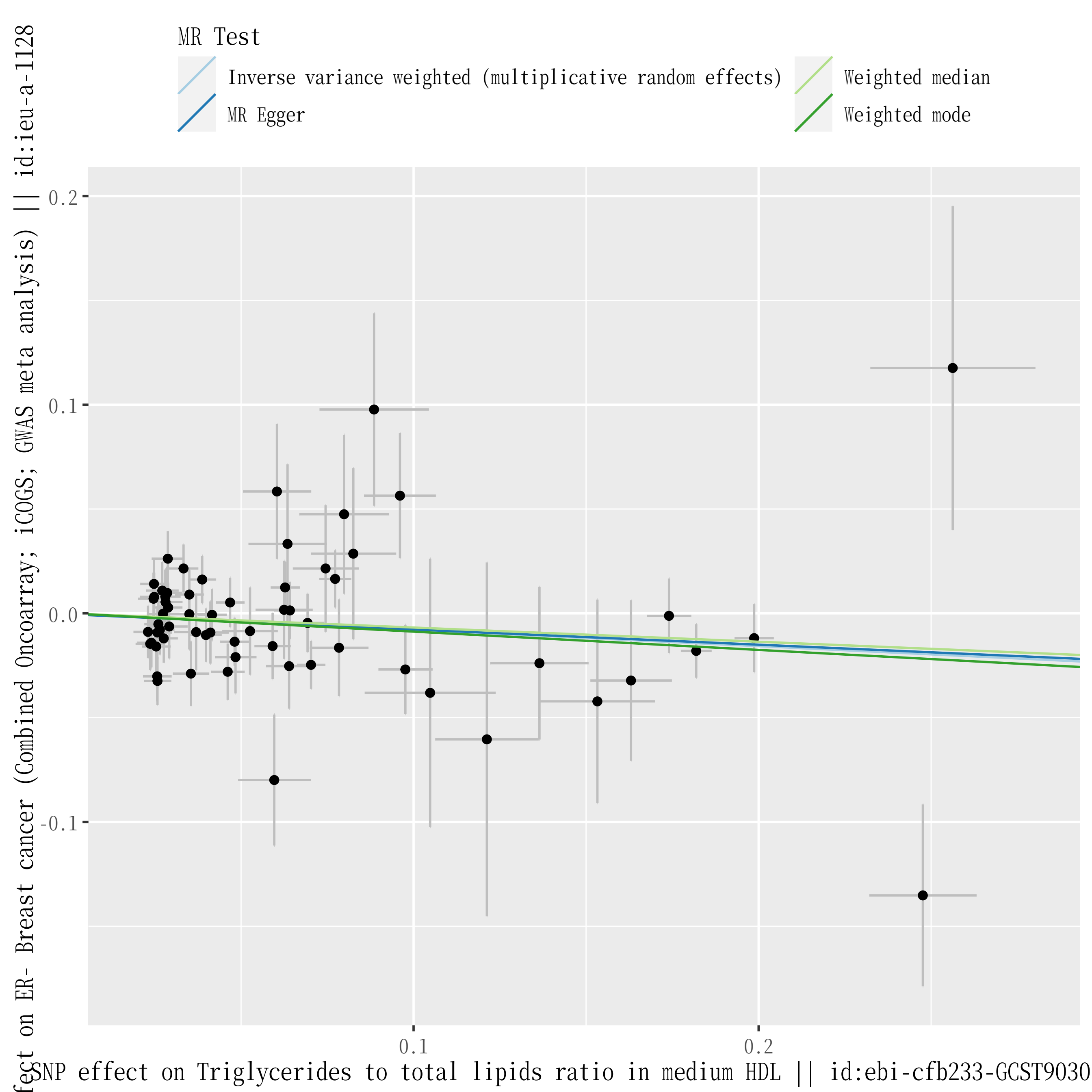

MR Test
Inverse variance weighted (multiplicative random effects)
MR Egger
Weighted median
Weighted mode
0.2
0.1
0.0
-0.1
0.1
0.2
fect on ER- Breast cancer (Combined Oncoarray; iCOGS; GWAS meta analysis) || id:ieu-a-1128
SNP effect on Triglycerides to total lipids ratio in medium HDL || id:ebi-cfb233-GCST9030

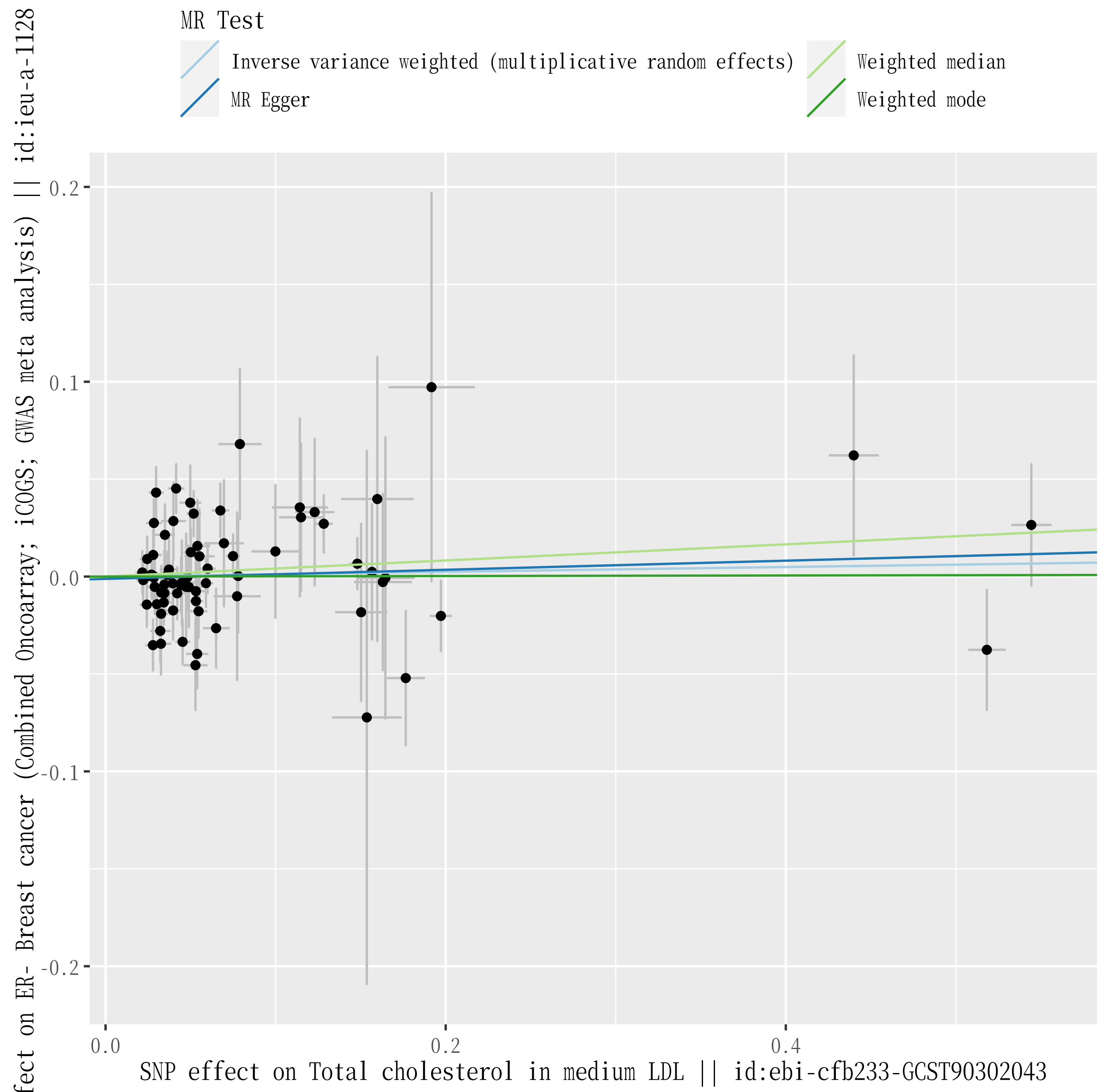
MR Test
Inverse variance weighted (multiplicative random effects)
MR Egger
Weighted median
Weighted mode
0.2
0.1
0.0
-0.1
-0.2
0.0
0.2
0.4
SNP effect on Total cholesterol in medium LDL || id:ebi-cfb233-GCST90302043
fect on ER- Breast cancer (Combined Oncoarray; iCOGS; GWAS meta analysis) || id:ieu-a-1128

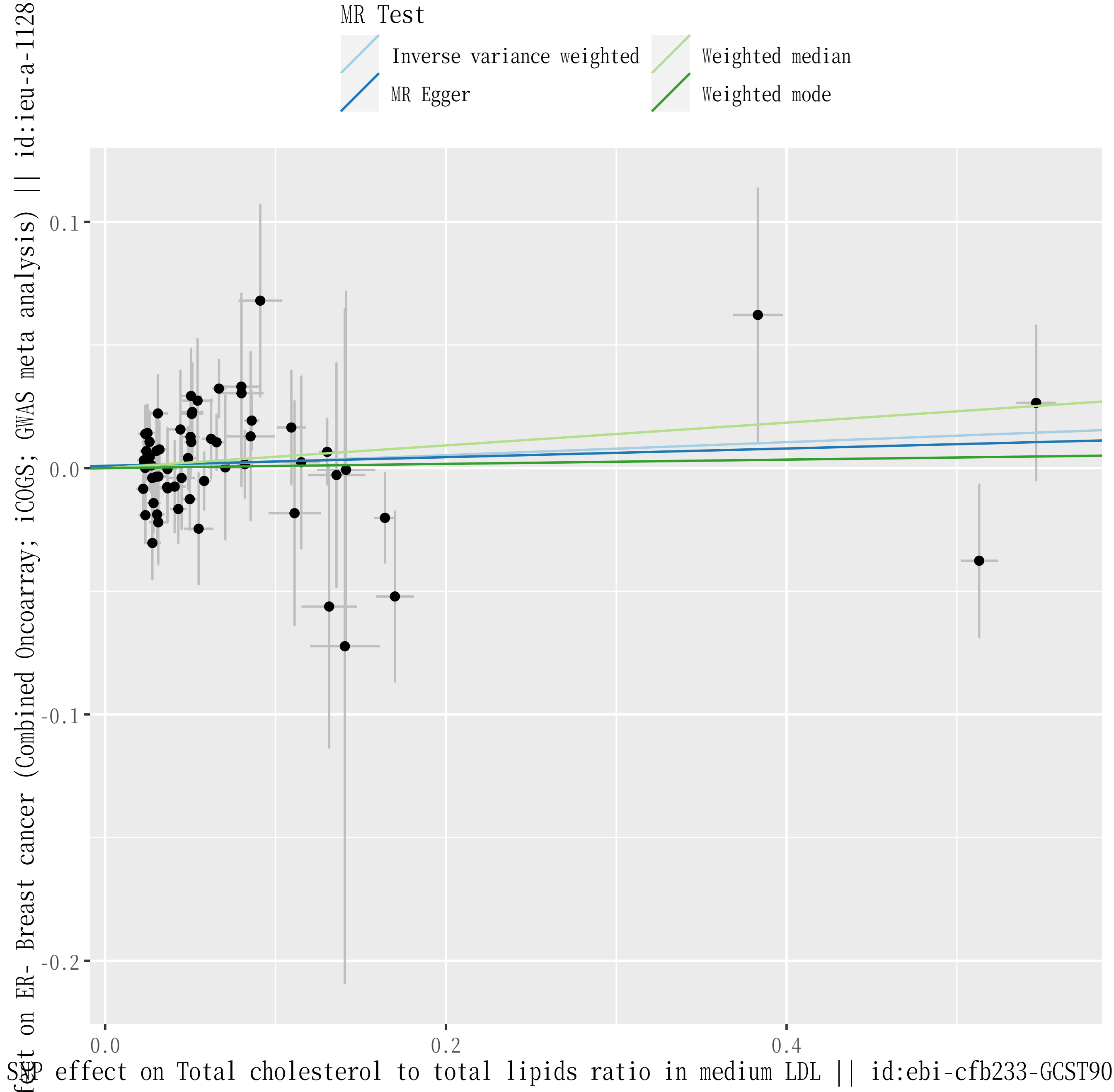
MR Test
Inverse variance weighted
MR Egger
Weighted median
Weighted mode
0.1
0.0
-0.1
-0.2
0.0
0.2
0.4
SNP effect on Total cholesterol to total lipids ratio in medium LDL || id:ebi-cfb233-GCST90
fect on ER- Breast cancer (Combined Oncoarray; iCOGS; GWAS meta analysis) || id:ieu-a-1128

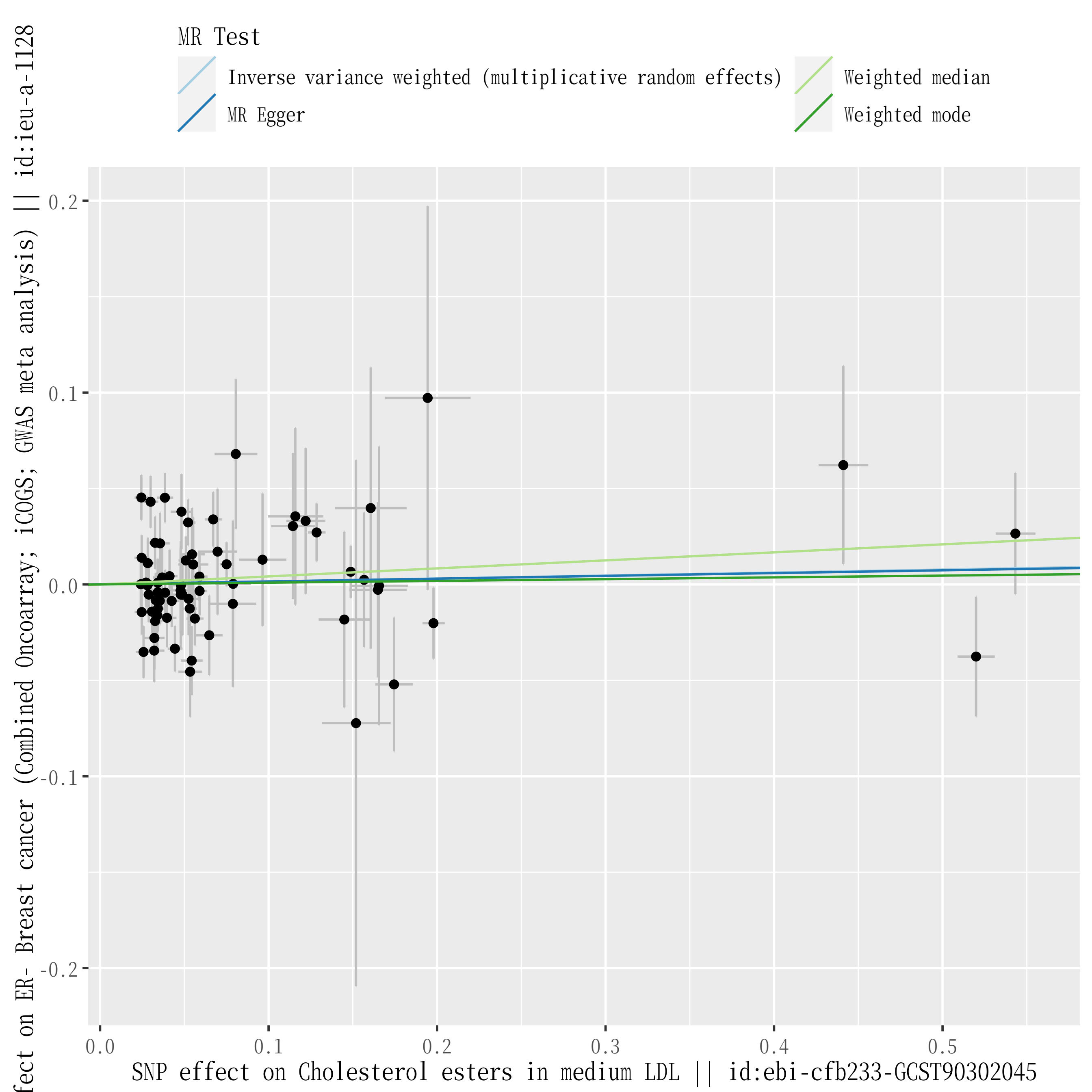
MR Test
Inverse variance weighted (multiplicative random effects)
MR Egger
Weighted median
Weighted mode
fect on ER- Breast cancer (Combined Oncoarray; iCOGS; GWAS meta analysis) || id:ieu-a-1128
0.2
0.1
0.0
-0.1
-0.2
0.0
0.1
0.2
0.3
0.4
0.5
SNP effect on Cholesterol esters in medium LDL || id:ebi-cfb233-GCST90302045

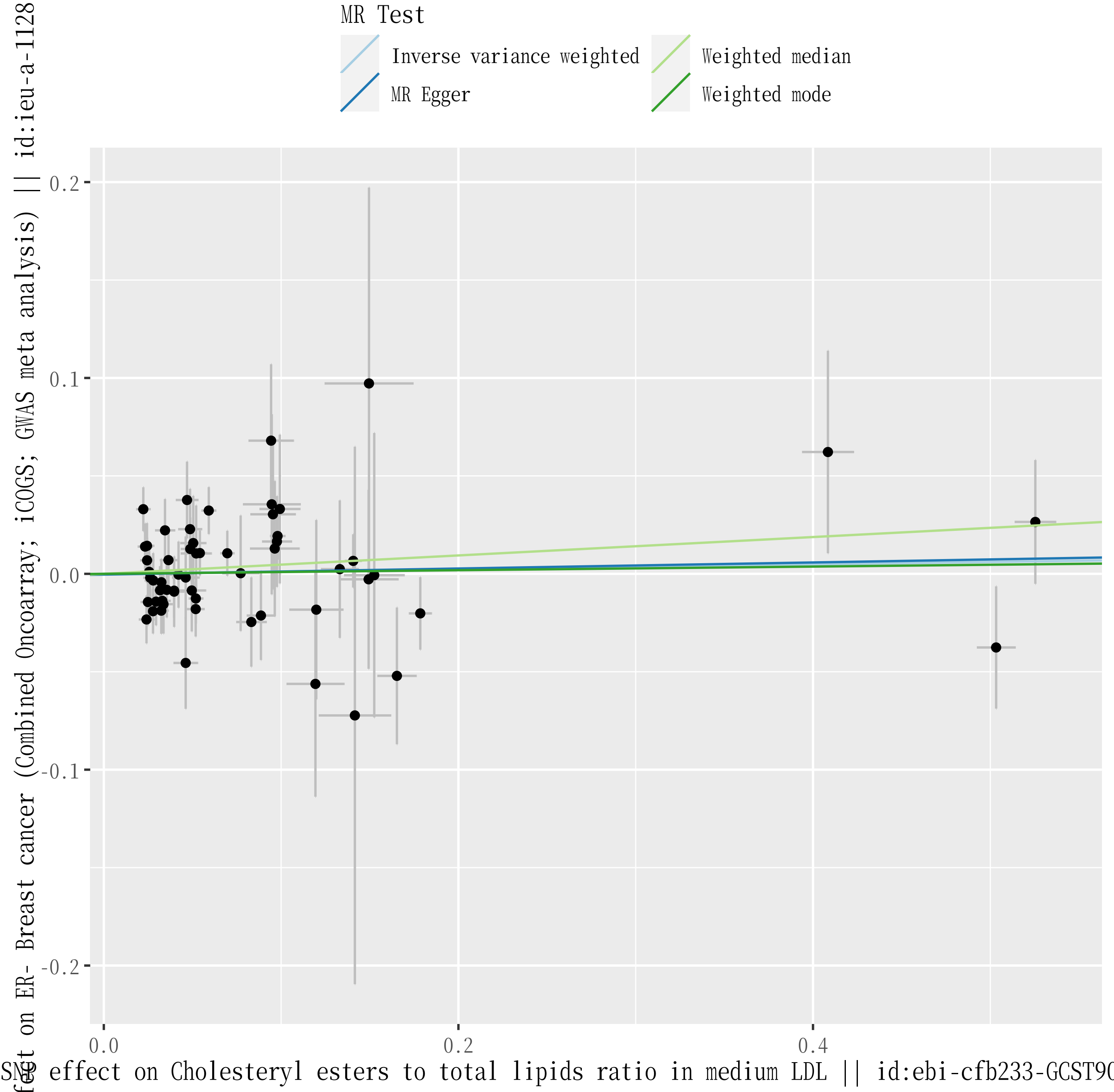
MR Test
Inverse variance weighted
Weighted median
MR Egger
Weighted mode
0.2
0.1
0.0
-0.1
-0.2
0.0
0.2
0.4
SNP effect on Cholesteryl esters to total lipids ratio in medium LDL || id:ebi-cfb233-GCST90
fect on ER- Breast cancer (Combined Oncoarray; iCOGS; GWAS meta analysis) || id:ieu-a-1128

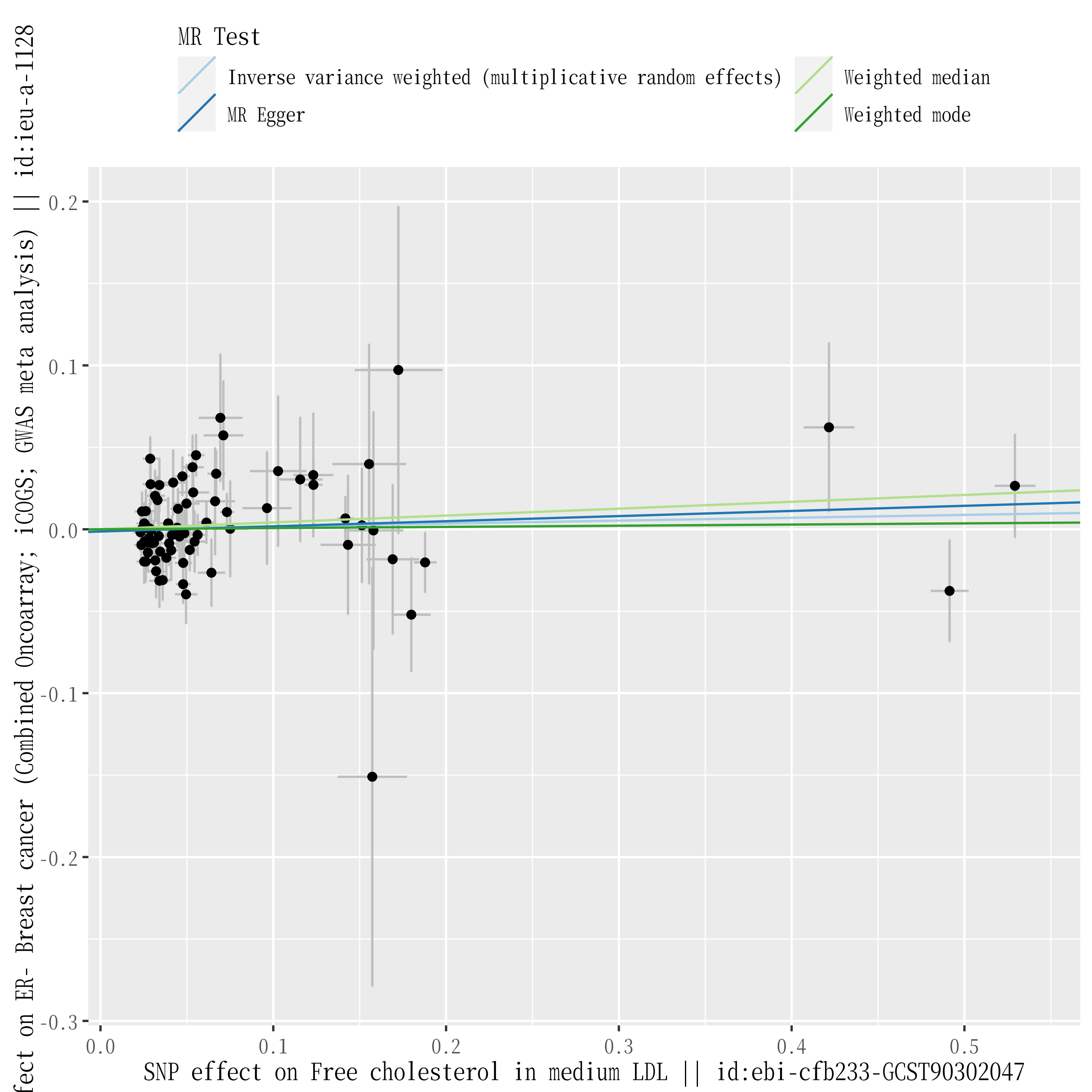
MR Test
Inverse variance weighted (multiplicative random effects)
MR Egger
Weighted median
Weighted mode
0.2
0.1
0.0
-0.1
-0.2
-0.3
0.0
0.1
0.2
0.3
0.4
0.5
SNP effect on Free cholesterol in medium LDL || id:ebi-cfb233-GCST90302047
fect on ER- Breast cancer (Combined Oncoarray; iCOGS; GWAS meta analysis) || id:ieu-a-1128

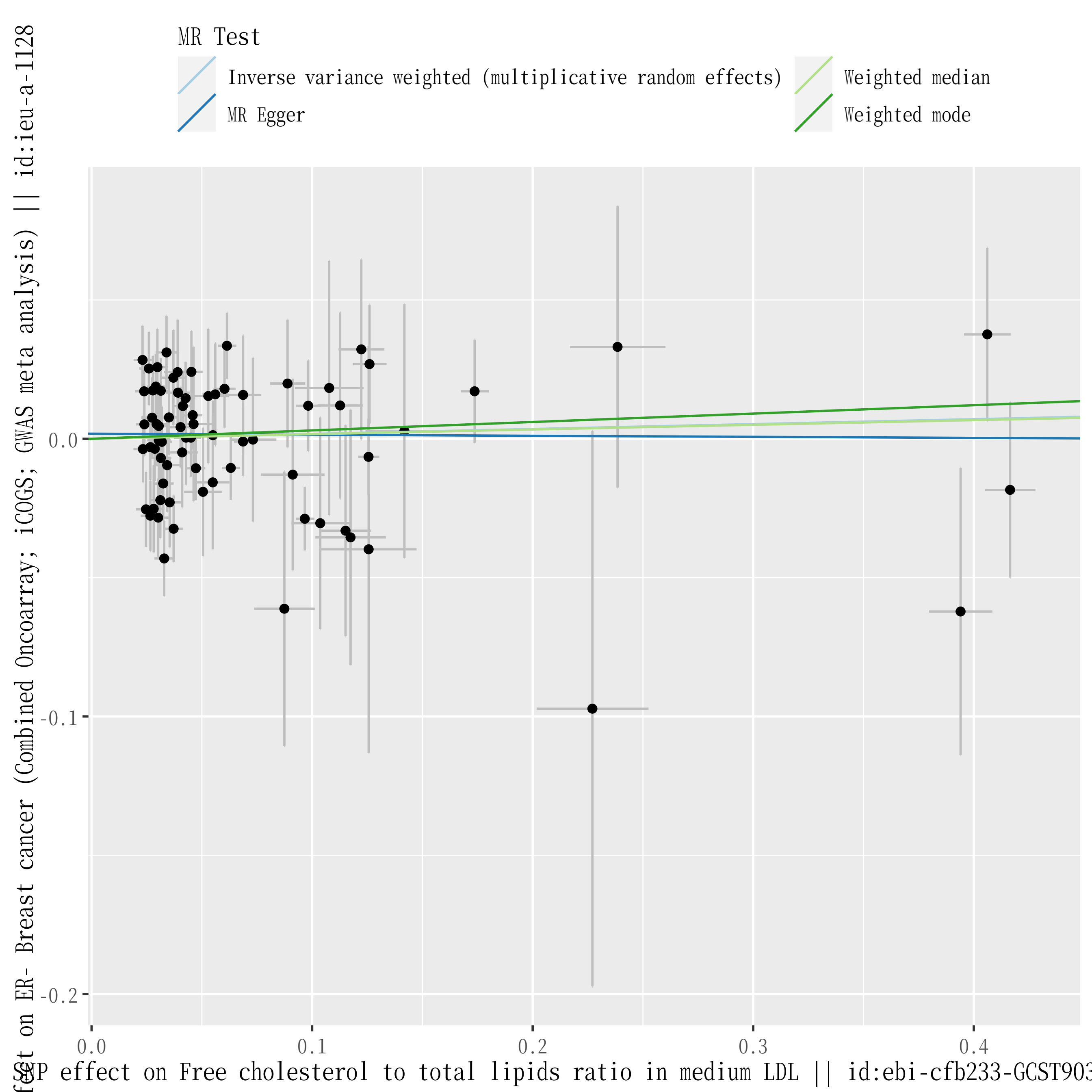
MR Test
Inverse variance weighted (multiplicative random effects)
MR Egger
Weighted median
Weighted mode
0.0
-0.1
-0.2
0.0
0.1
0.2
0.3
0.4
SNP effect on Free cholesterol to total lipids ratio in medium LDL || id:ebi-cfb233-GCST903
SNP effect on ER- Breast cancer (Combined Oncoarray; iCOGS; GWAS meta analysis) || id:ieu-a-1128

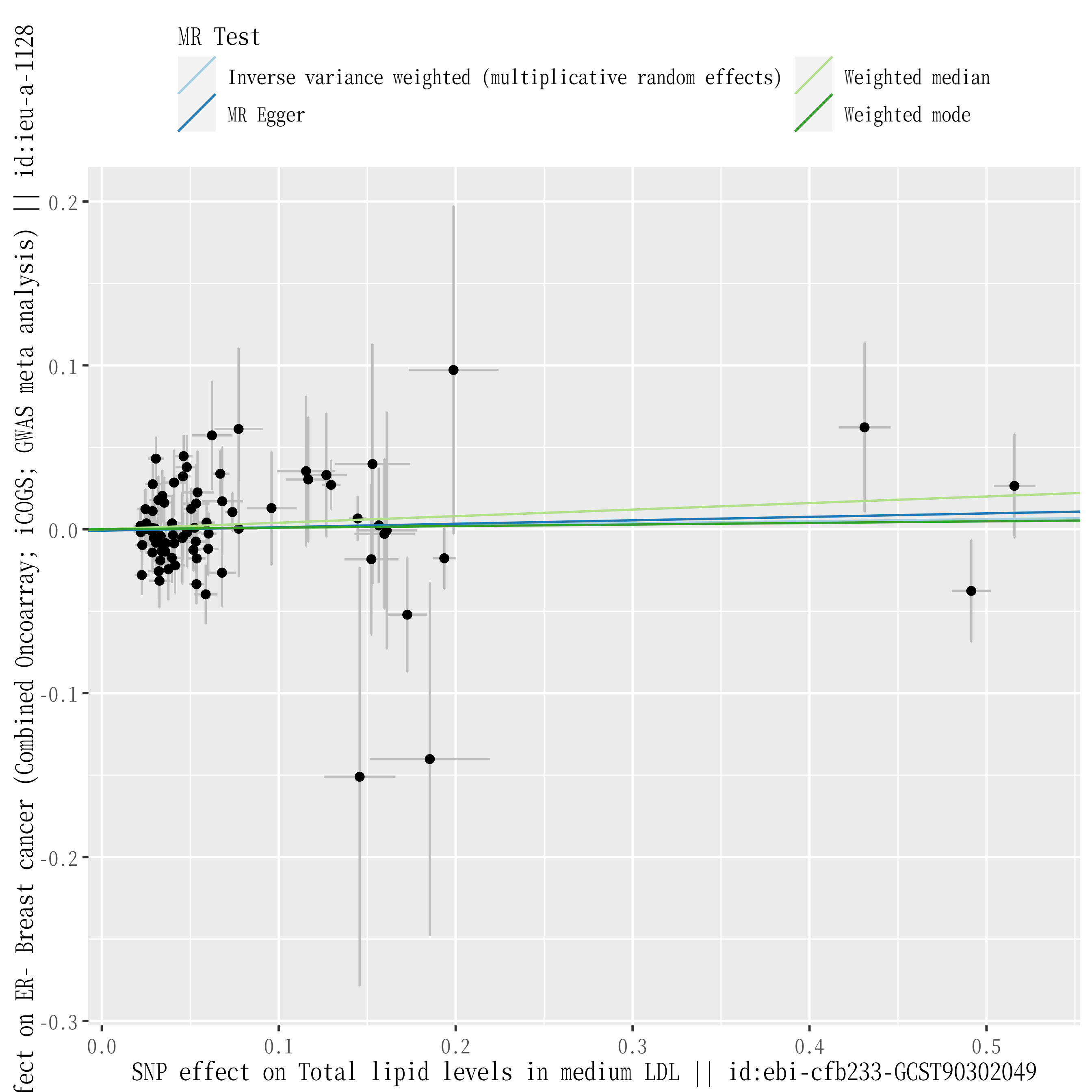

MR Test
Inverse variance weighted (multiplicative random effects)
MR Egger
Weighted median
Weighted mode
0.2
0.1
0.0
-0.1
-0.2
-0.3
0.0
0.1
0.2
0.3
0.4
0.5
SNP effect on Total lipid levels in medium LDL || id:ebi-cfb233-GCST90302049
fect on ER- Breast cancer (Combined Oncoarray; iCOGS; GWAS meta analysis) || id:ieu-a-1128

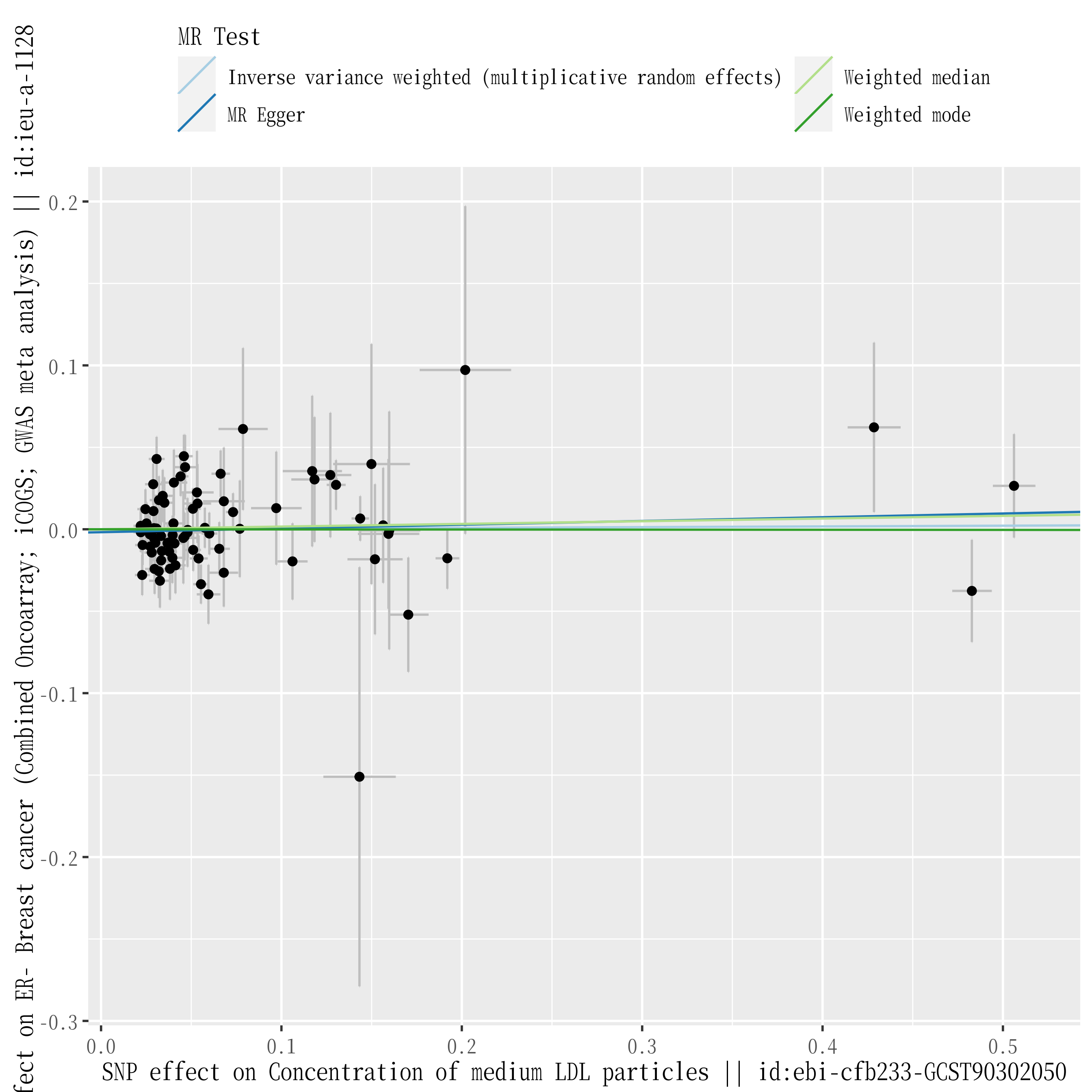

MR Test
Inverse variance weighted (multiplicative random effects)
MR Egger
Weighted median
Weighted mode
0.2
0.1
0.0
-0.1
-0.2
-0.3
0.0
0.1
0.2
0.3
0.4
0.5
SNP effect on Concentration of medium LDL particles || id:ebi-cfb233-GCST90302050
fect on ER- Breast cancer (Combined Oncoarray; iCOGS; GWAS meta analysis) || id:ieu-a-1128

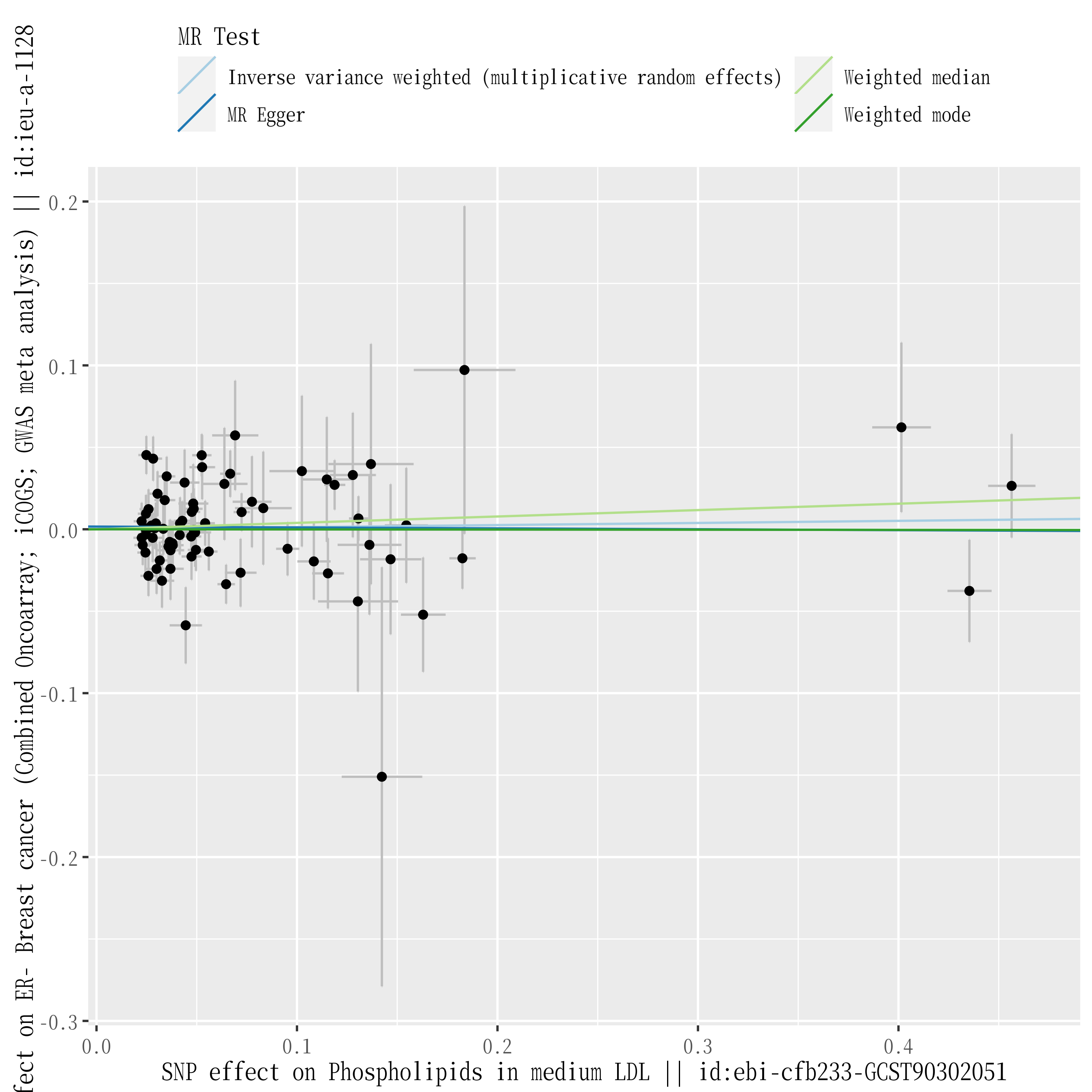
MR Test
Inverse variance weighted (multiplicative random effects)
MR Egger
Weighted median
Weighted mode
0.2
0.1
0.0
-0.1
-0.2
-0.3
0.0
0.1
0.2
0.3
0.4
SNP effect on Phospholipids in medium LDL || id:ebi-cfb233-GCST90302051
fect on ER- Breast cancer (Combined Oncoarray; iCOGS; GWAS meta analysis) || id:ieu-a-1128

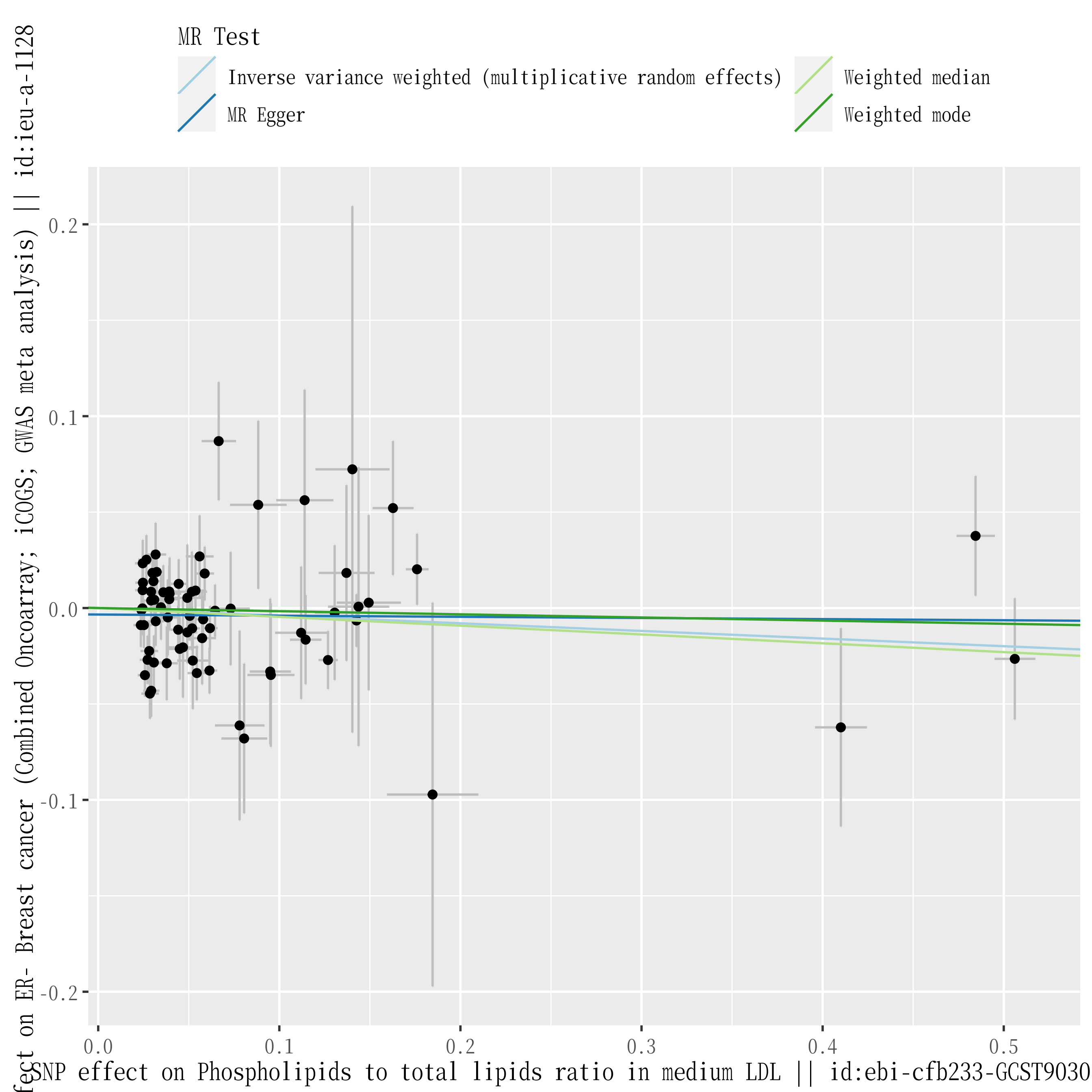

MR Test
Inverse variance weighted (multiplicative random effects)
MR Egger
Weighted median
Weighted mode
fect on ER- Breast cancer (Combined Oncoarray; iCOGS; GWAS meta analysis) || id:ieu-a-1128
0.2
0.1
0.0
-0.1
-0.2
0.0
0.1
0.2
0.3
0.4
0.5
SNP effect on Phospholipids to total lipids ratio in medium LDL || id:ebi-cfb233-GCST9030

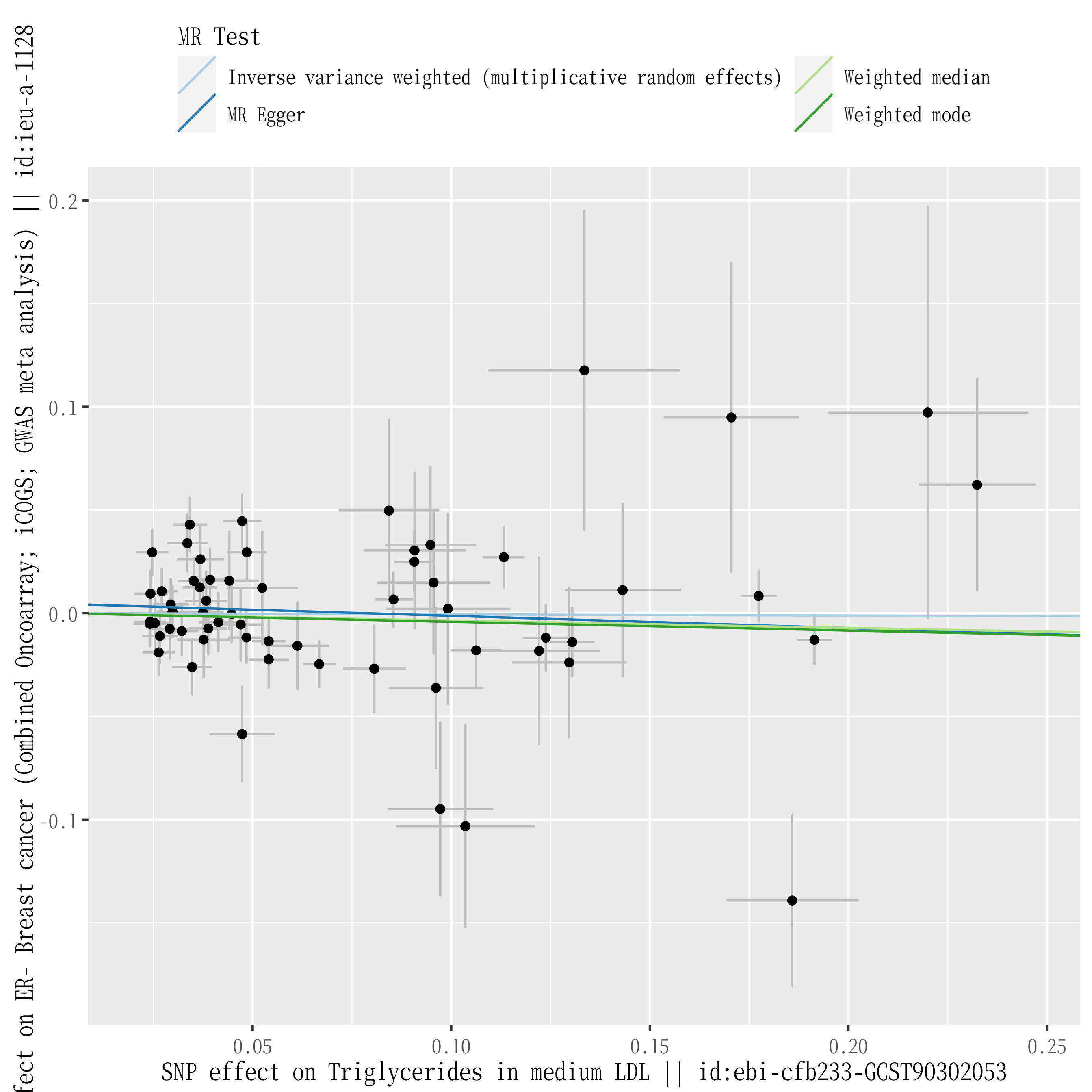

MR Test
Inverse variance weighted (multiplicative random effects)
MR Egger
Weighted median
Weighted mode
0.2
0.1
0.0
-0.1
0.05
0.10
0.15
0.20
0.25
SNP effect on Triglycerides in medium LDL || id:ebi-cfb233-GCST90302053
fect on ER- Breast cancer (Combined Oncoarray; iCOGS; GWAS meta analysis) || id:ieu-a-1128

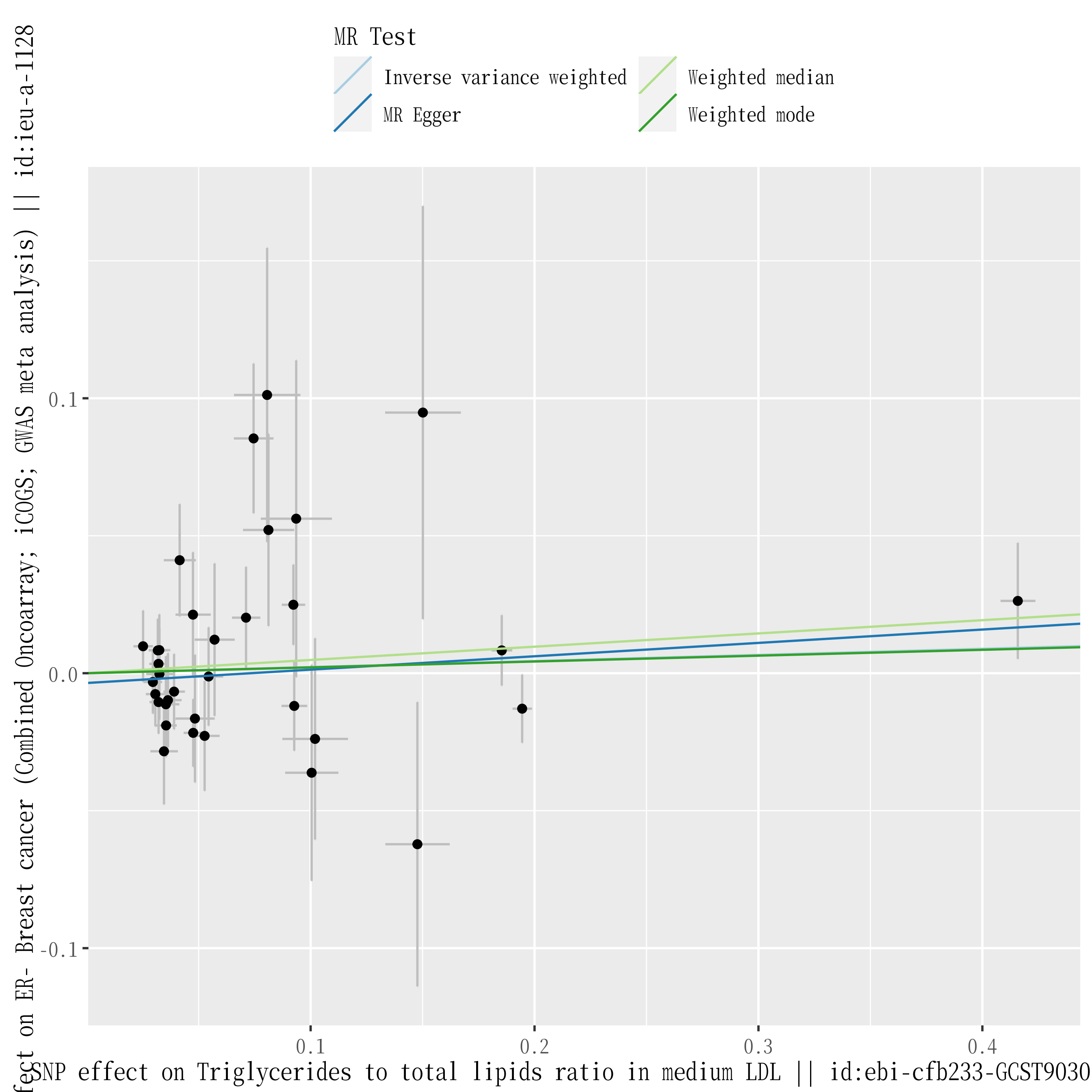
MR Test
Inverse variance weighted
MR Egger
Weighted median
Weighted mode
0.1
0.0
-0.1
0.1
0.2
0.3
0.4
SNP effect on Triglycerides to total lipids ratio in medium LDL || id:ebi-cfb233-GCST9030
fect on ER- Breast cancer (Combined Oncoarray; iCOGS; GWAS meta analysis) || id:ieu-a-1128

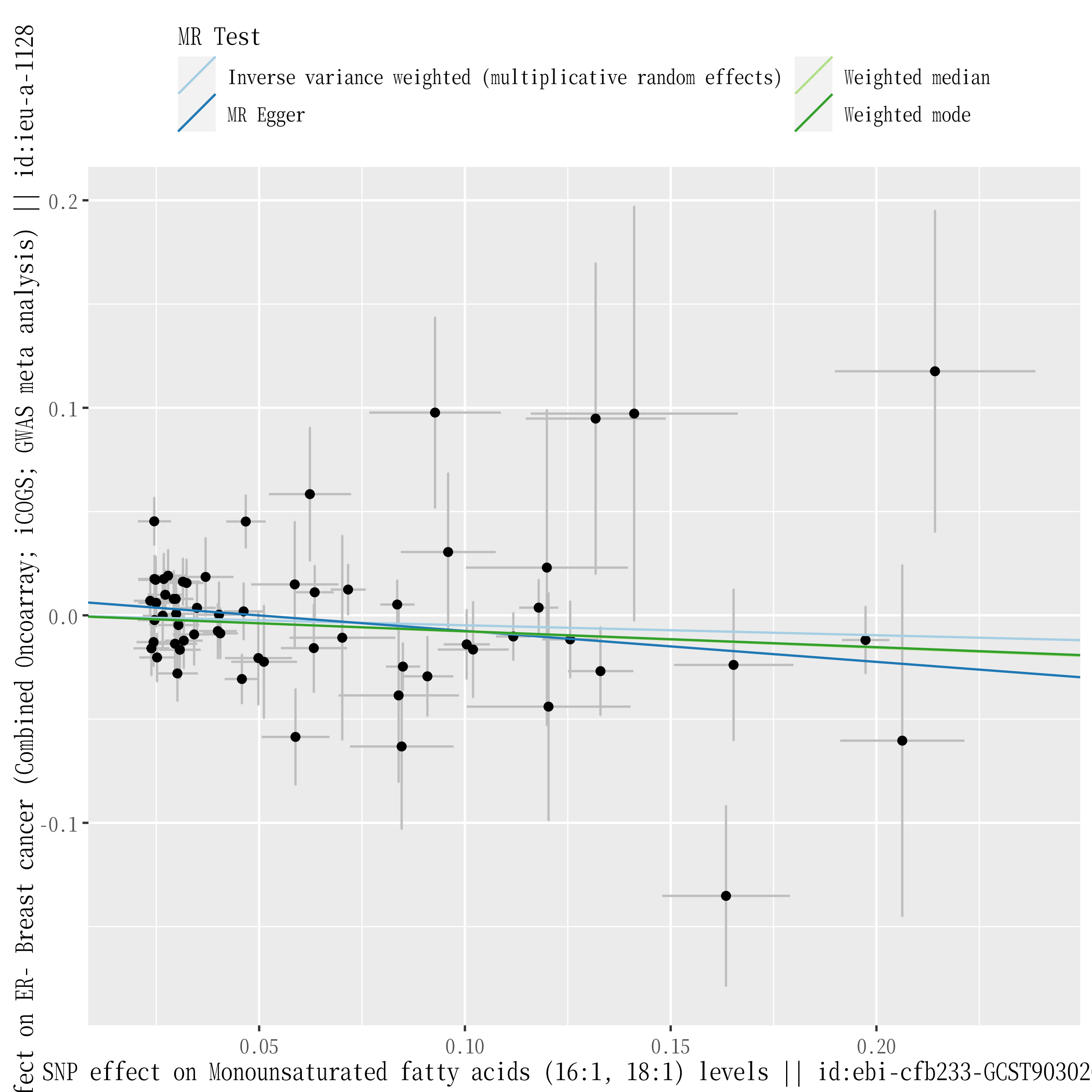
MR Test
Inverse variance weighted (multiplicative random effects)
MR Egger
Weighted median
Weighted mode
0.2
0.1
0.0
-0.1
0.05
0.10
0.15
0.20
SNP effect on Monounsaturated fatty acids (16:1, 18:1) levels || id:ebi-cfb233-GCST90302
fect on ER- Breast cancer (Combined Oncoarray; iCOGS; GWAS meta analysis) || id:ieu-a-1128

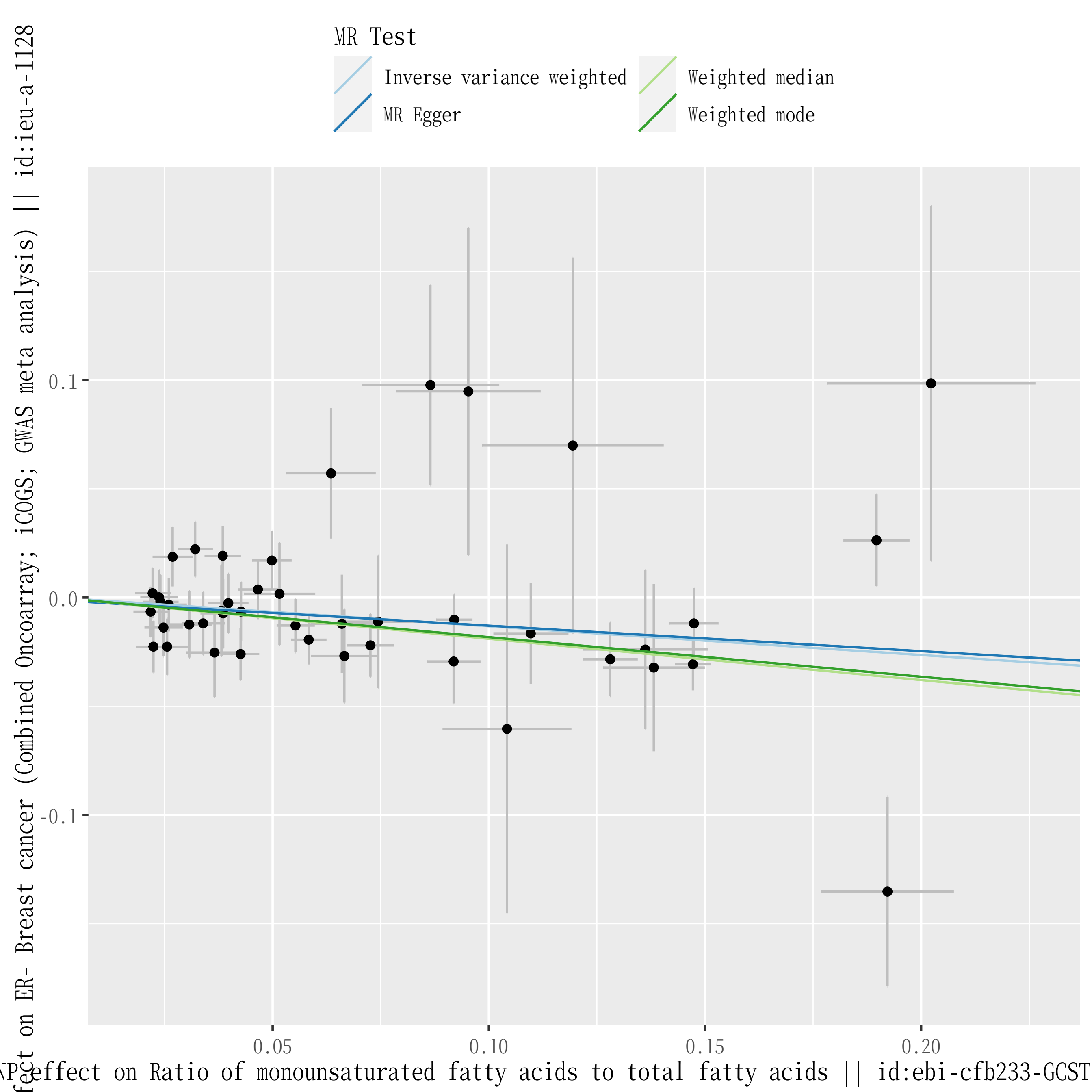

MR Test
Inverse variance weighted
MR Egger
Weighted median
Weighted mode
0.1
0.0
-0.1
0.05
0.10
0.15
0.20
SNP effect on Ratio of monounsaturated fatty acids to total fatty acids || id:ebi-cfb233-GCST
SNP effect on ER- Breast cancer (Combined Oncoarray; iCOGS; GWAS meta analysis) || id:ieu-a-1128

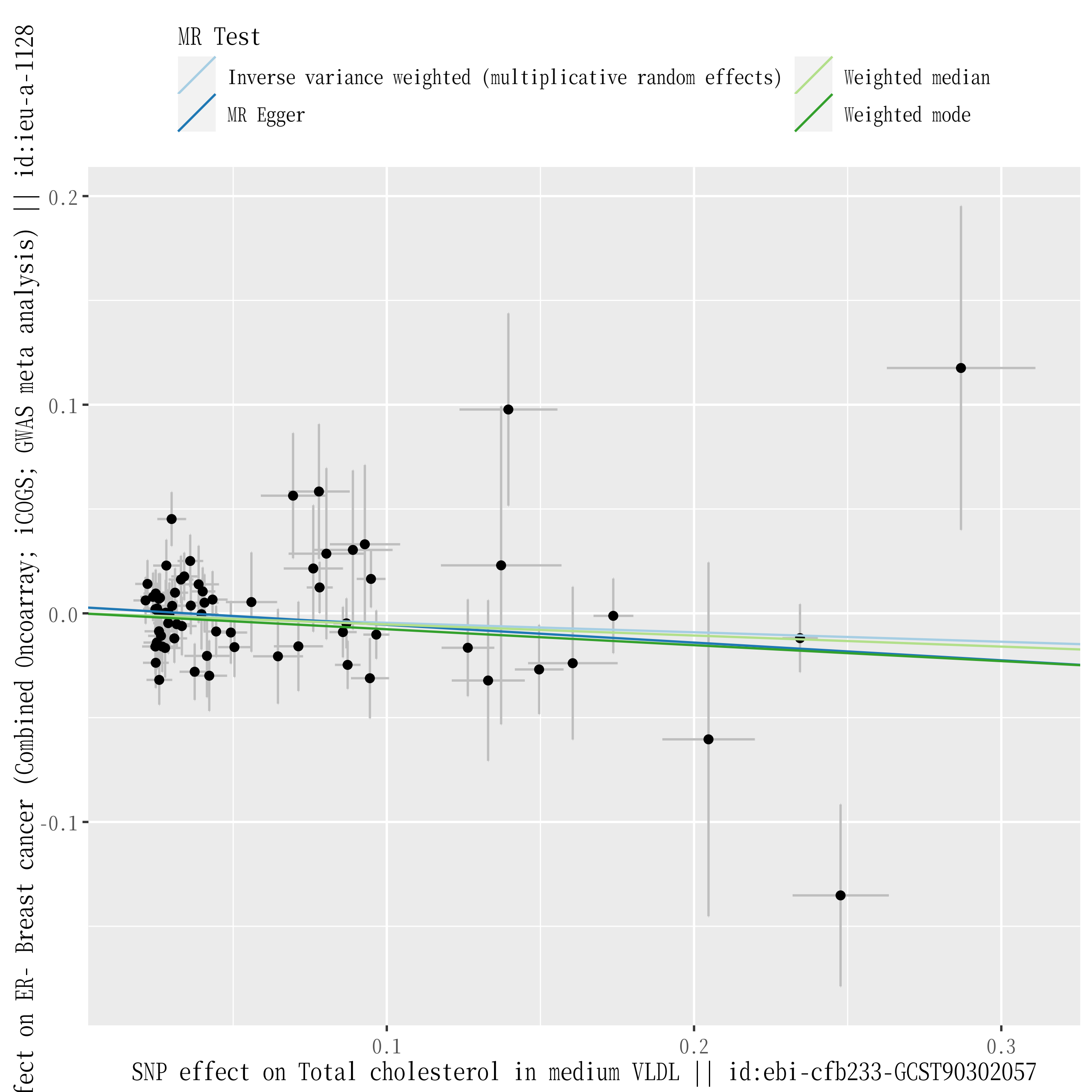
MR Test
Inverse variance weighted (multiplicative random effects)
MR Egger
Weighted median
Weighted mode
fect on ER- Breast cancer (Combined Oncoarray; iCOGS; GWAS meta analysis) || id:ieu-a-1128
0.2
0.1
0.0
-0.1
0.1
0.2
0.3
SNP effect on Total cholesterol in medium VLDL || id:ebi-cfb233-GCST90302057

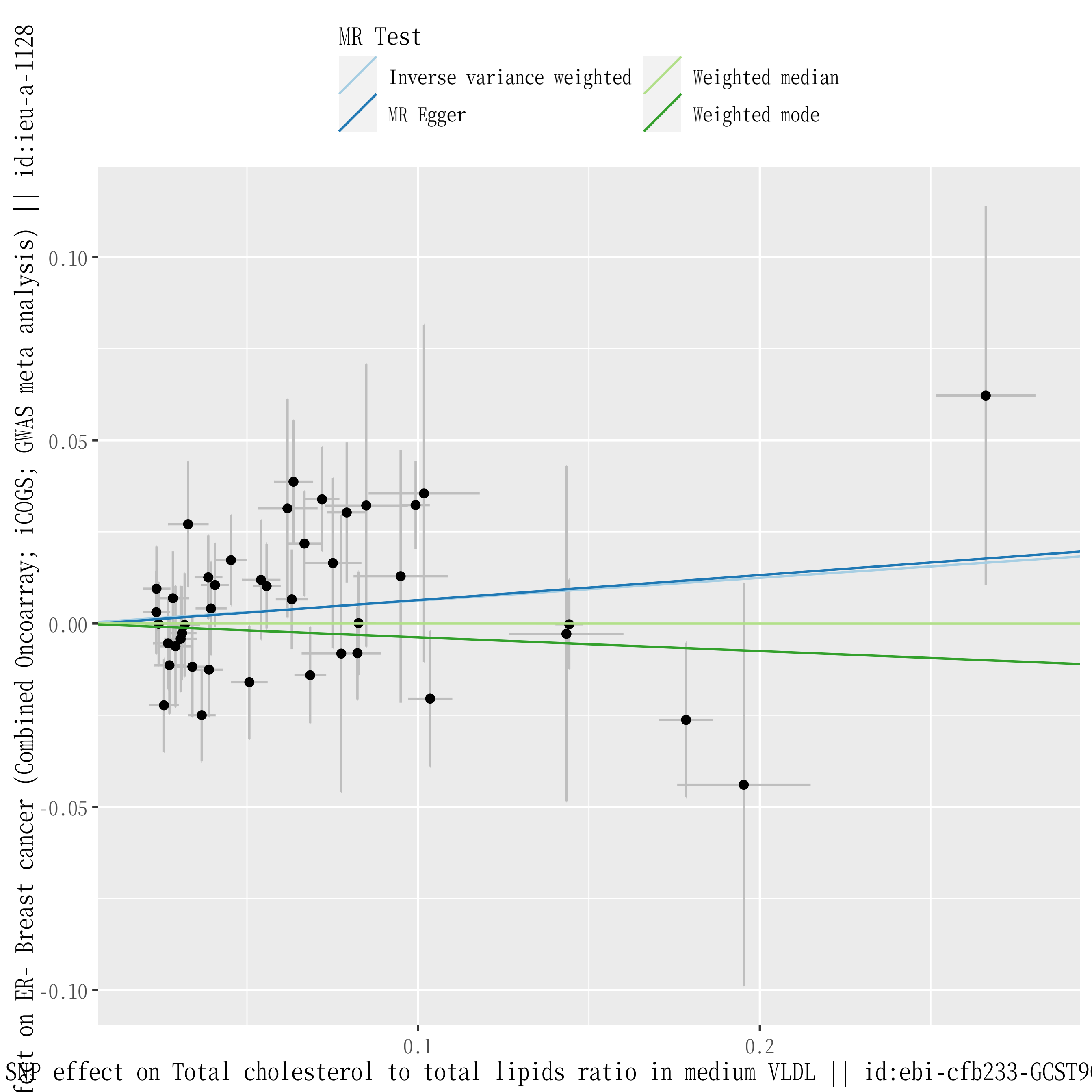

MR Test
Inverse variance weighted
MR Egger
Weighted median
Weighted mode
0.10
0.05
0.00
-0.05
-0.10
0.1
0.2
SNP effect on Total cholesterol to total lipids ratio in medium VLDL || id:ebi-cfb233-GCST9
fect on ER- Breast cancer (Combined Oncoarray; iCOGS; GWAS meta analysis) || id:ieu-a-1128

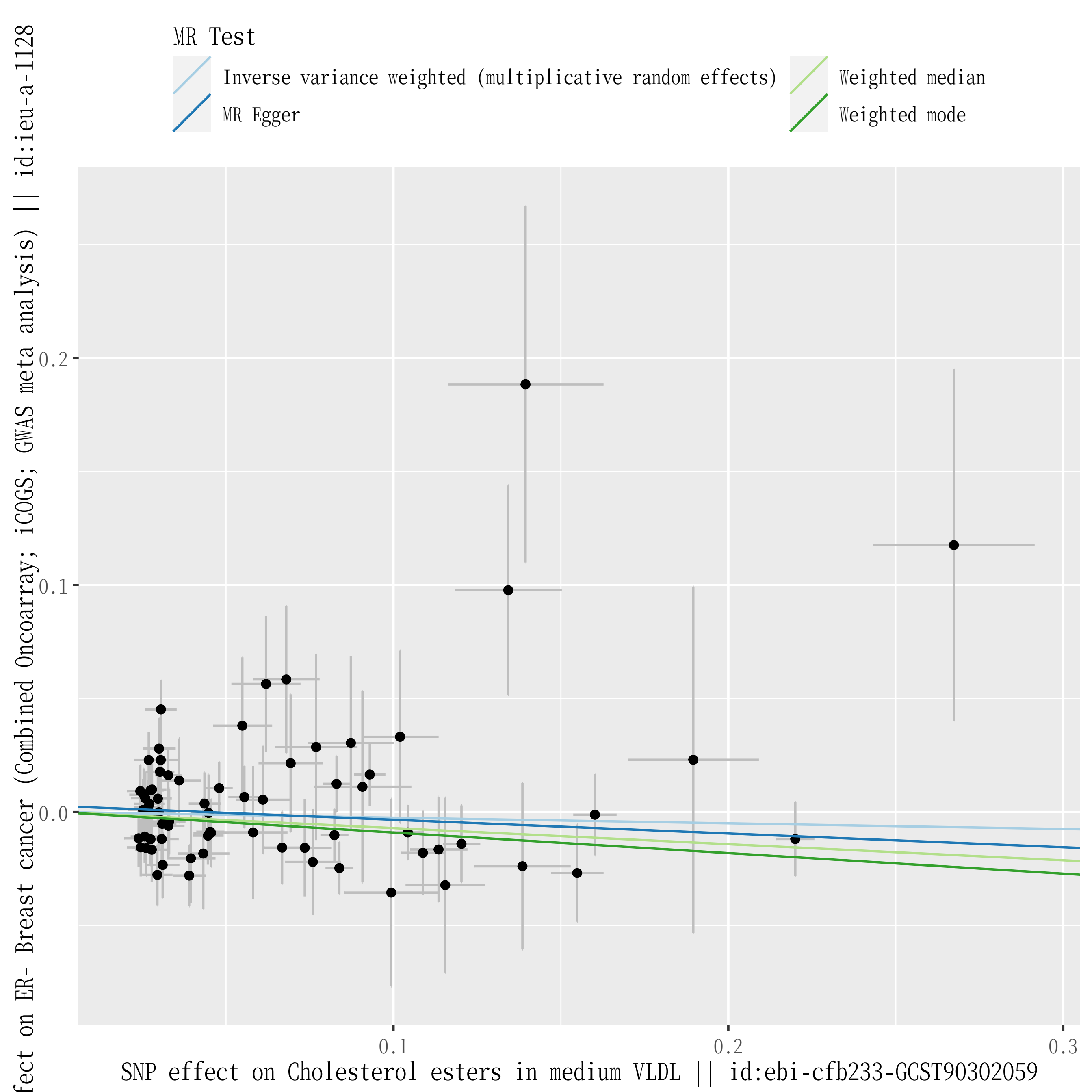
MR Test
Inverse variance weighted (multiplicative random effects)
MR Egger
Weighted median
Weighted mode
0.2
0.1
0.0
0.1
0.2
0.3
SNP effect on Cholesterol esters in medium VLDL || id:ebi-cfb233-GCST90302059
fect on ER- Breast cancer (Combined Oncoarray; iCOGS; GWAS meta analysis) || id:ieu-a-1128

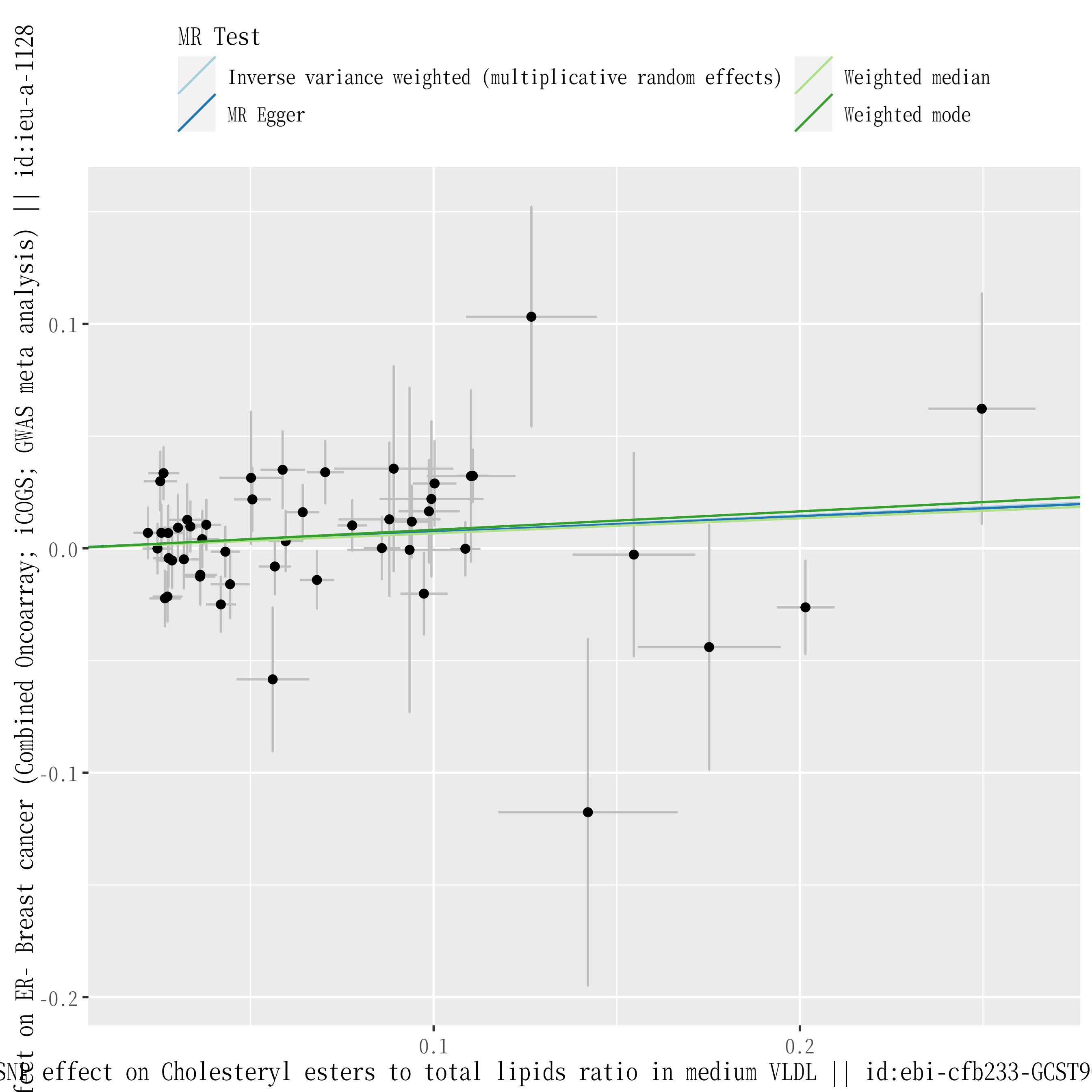

MR Test
Inverse variance weighted (multiplicative random effects)
MR Egger
Weighted median
Weighted mode
0.1
0.0
-0.1
-0.2
0.1
0.2
SNP effect on Cholesteryl esters to total lipids ratio in medium VLDL || id:ebi-cfb233-GCST9
fect on ER- Breast cancer (Combined Oncoarray; iCOGS; GWAS meta analysis) || id:ieu-a-1128

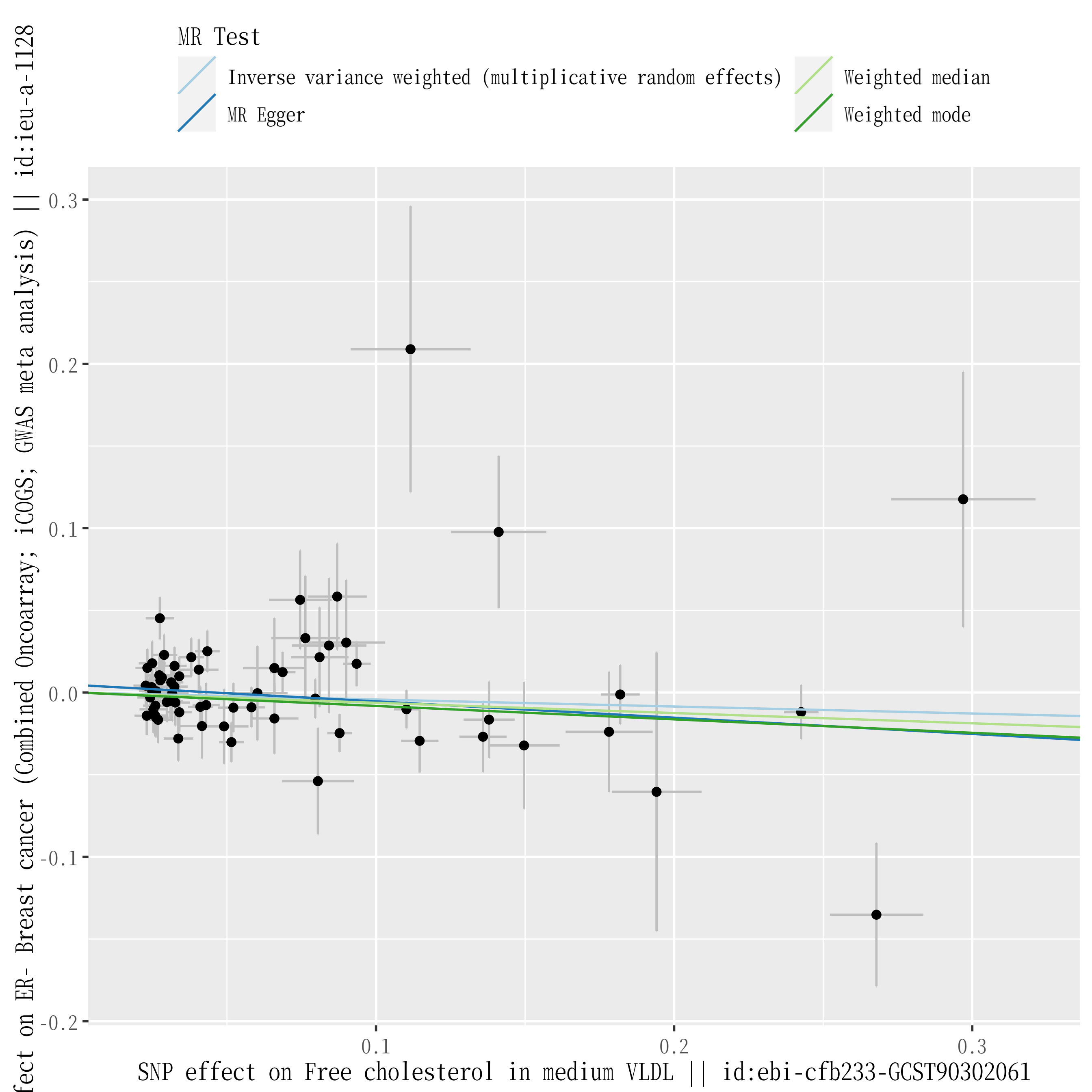
MR Test
Inverse variance weighted (multiplicative random effects)
MR Egger
Weighted median
Weighted mode
SNP effect on ER- Breast cancer (Combined Oncoarray; iCOGS; GWAS meta analysis) || id:ieu-a-1128
0.3
0.2
0.1
0.0
-0.1
-0.2
0.1
0.2
0.3
SNP effect on Free cholesterol in medium VLDL || id:ebi-cfb233-GCST90302061

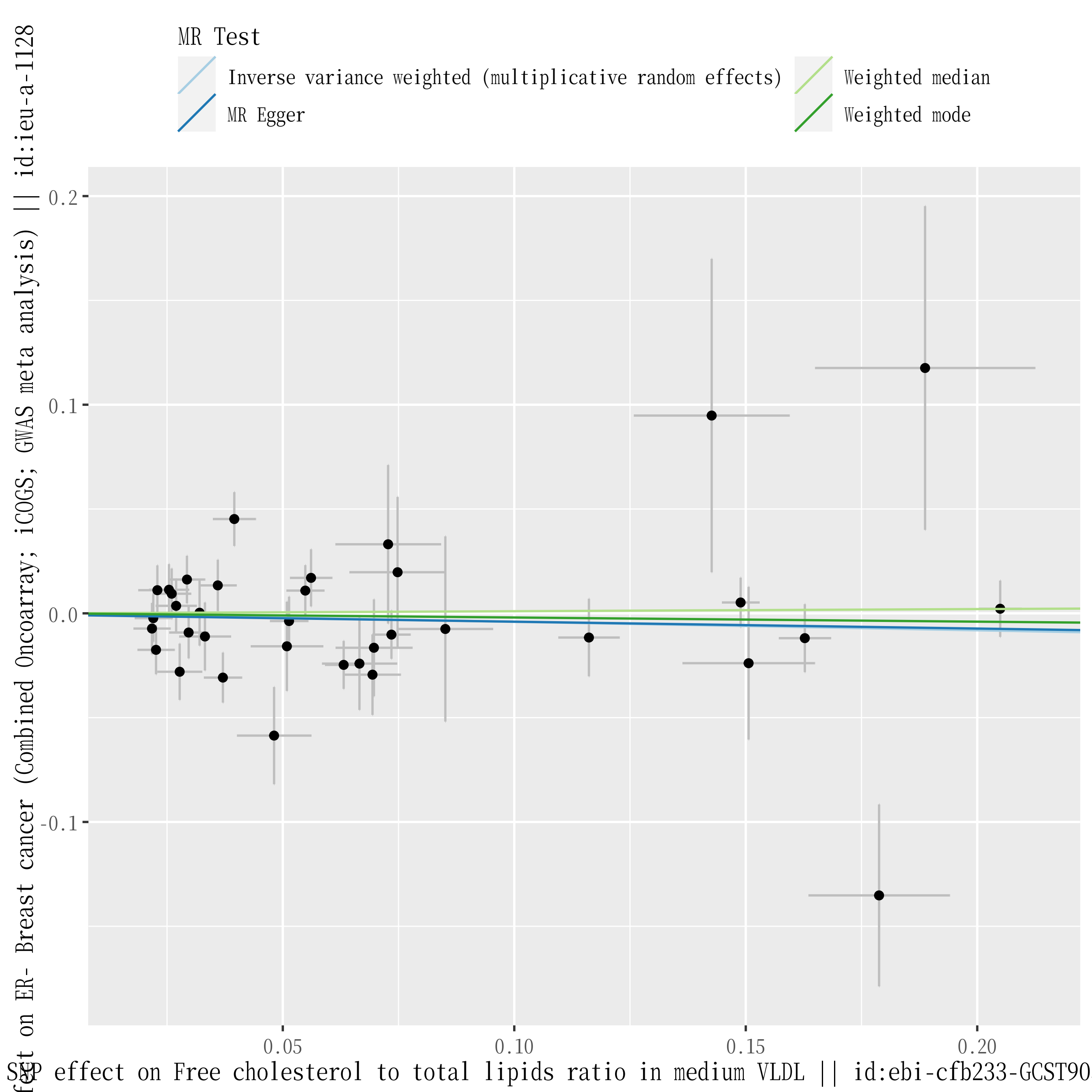

MR Test
Inverse variance weighted (multiplicative random effects)
MR Egger
Weighted median
Weighted mode
0.2
0.1
0.0
-0.1
0.05
0.10
0.15
0.20
SNP effect on Free cholesterol to total lipids ratio in medium VLDL || id:ebi-cfb233-GCST90
SNP effect on ER- Breast cancer (Combined Oncoarray; iCOGS; GWAS meta analysis) || id:ieu-a-1128

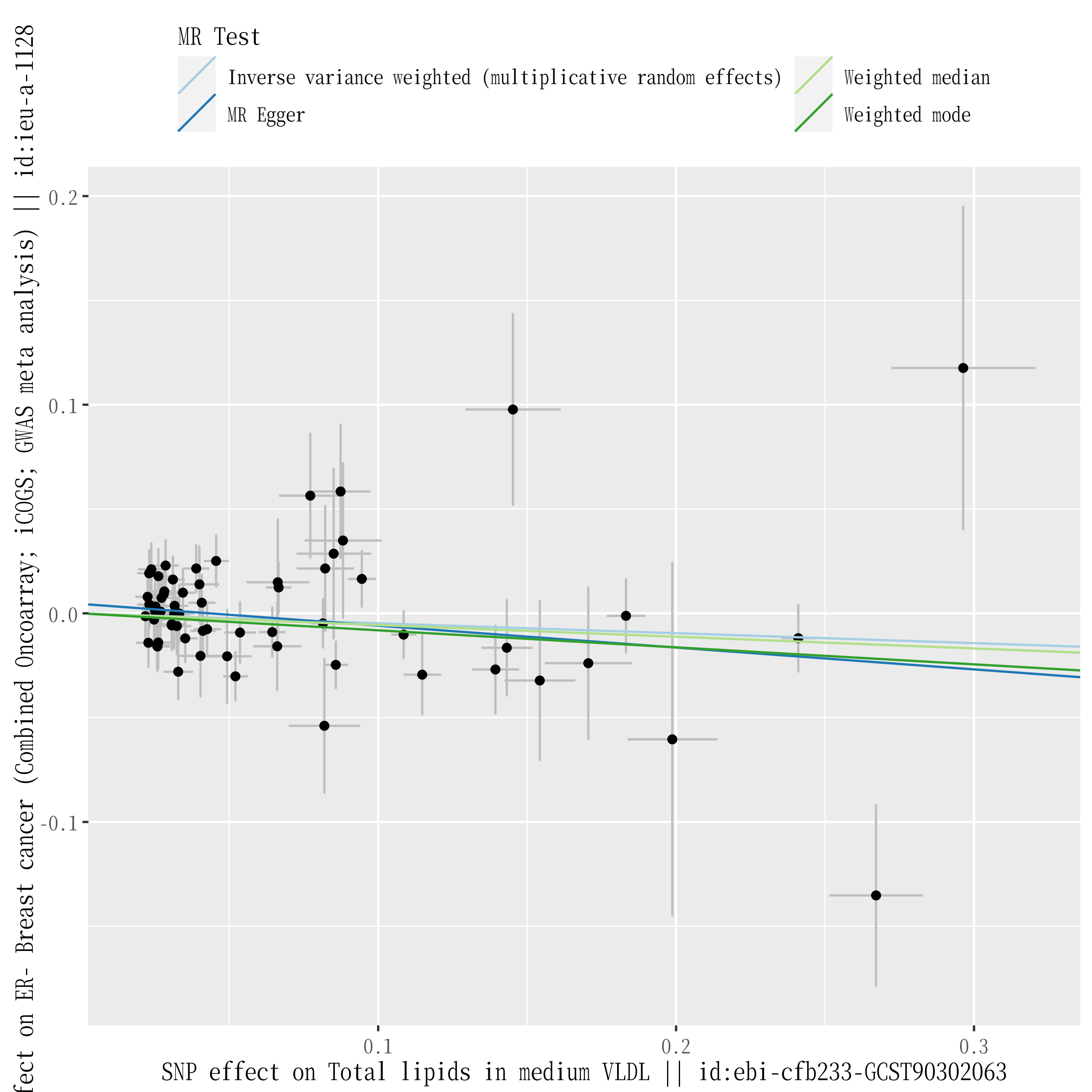

MR Test
Inverse variance weighted (multiplicative random effects)
MR Egger
Weighted median
Weighted mode
0.2
0.1
0.0
-0.1
0.1
0.2
0.3
SNP effect on Total lipids in medium VLDL || id:ebi-cfb233-GCST90302063
fect on ER- Breast cancer (Combined Oncoarray; iCOGS; GWAS meta analysis) || id:ieu-a-1128

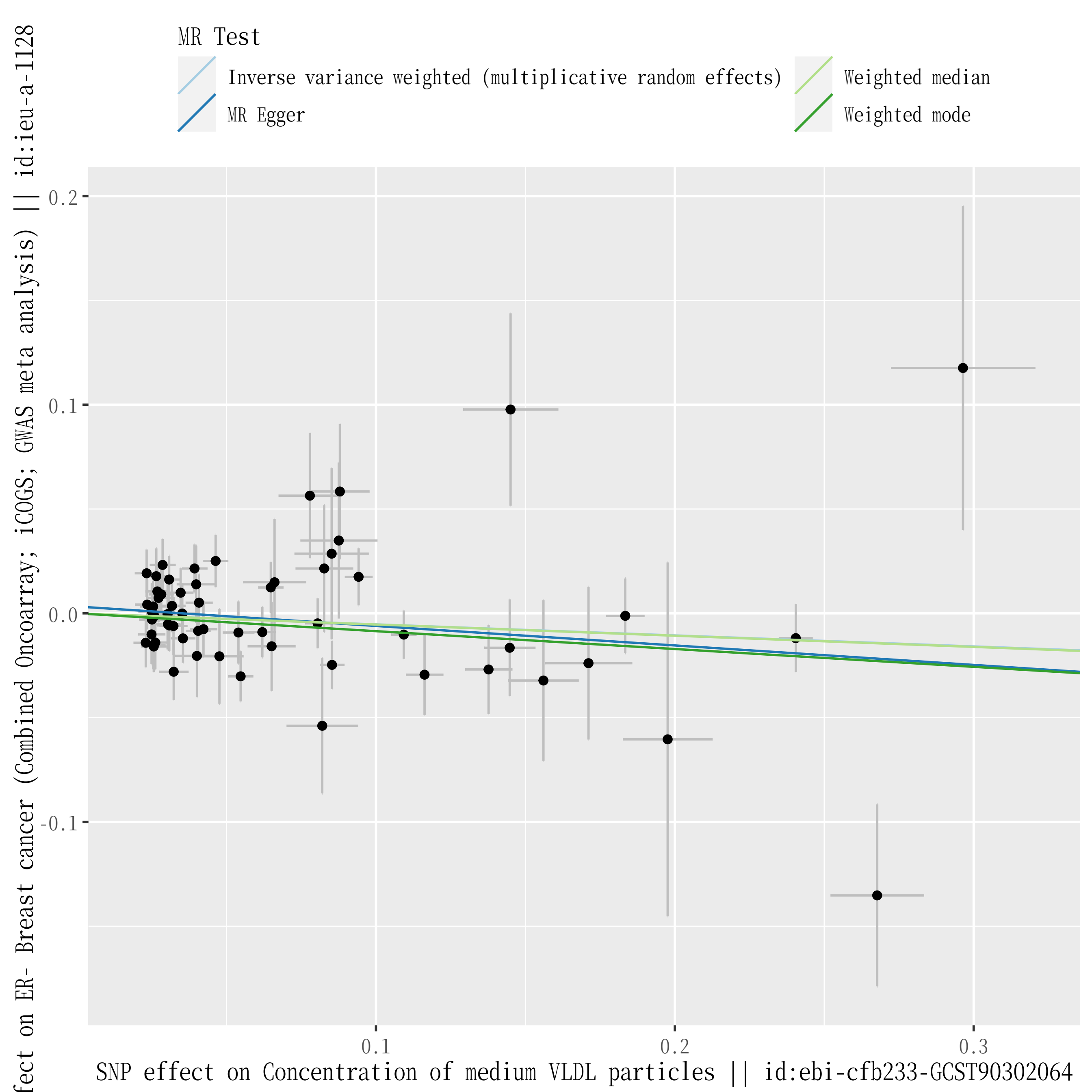
MR Test
Inverse variance weighted (multiplicative random effects)
MR Egger
Weighted median
Weighted mode
0.2
0.1
0.0
-0.1
0.1
0.2
0.3
SNP effect on Concentration of medium VLDL particles || id:ebi-cfb233-GCST90302064
fect on ER- Breast cancer (Combined Oncoarray; iCOGS; GWAS meta analysis) || id:ieu-a-1128

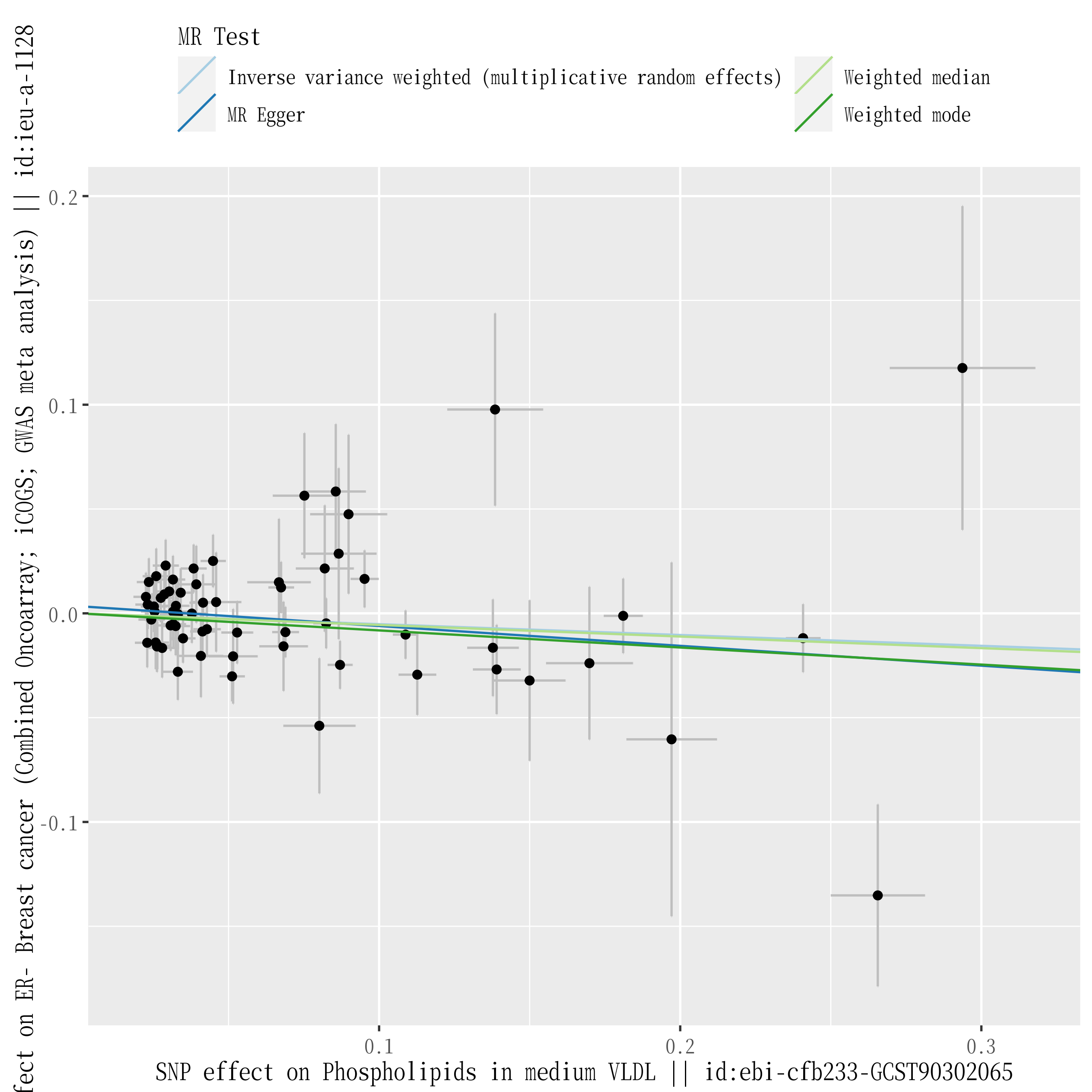
MR Test
Inverse variance weighted (multiplicative random effects)
MR Egger
Weighted median
Weighted mode
0.2
0.1
0.0
-0.1
0.1
0.2
0.3
SNP effect on Phospholipids in medium VLDL || id:ebi-cfb233-GCST90302065
fect on ER- Breast cancer (Combined Oncoarray; iCOGS; GWAS meta analysis) || id:ieu-a-1128

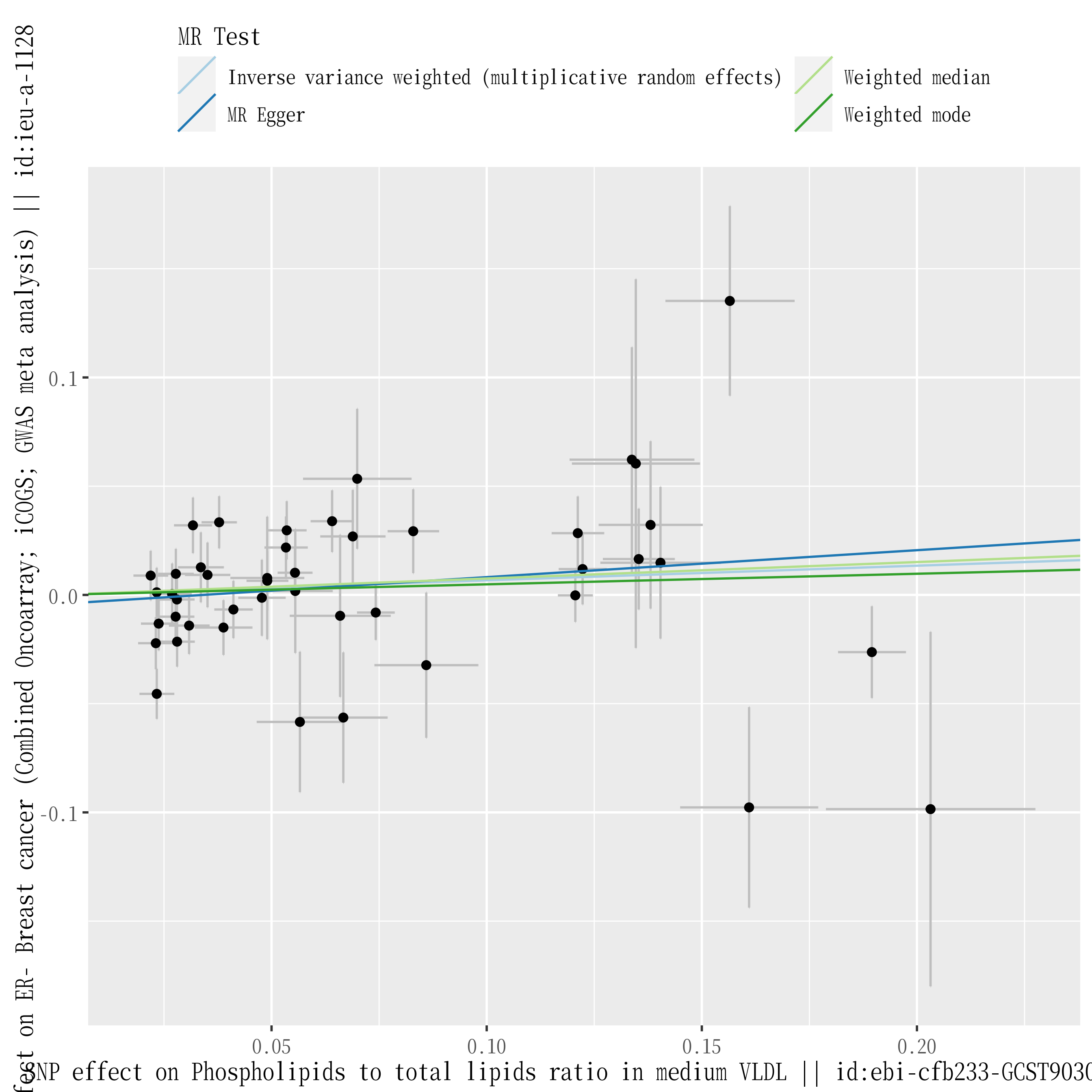
MR Test
Inverse variance weighted (multiplicative random effects)
MR Egger
Weighted median
Weighted mode
0.1
0.0
-0.1
0.05
0.10
0.15
0.20
SNP effect on Phospholipids to total lipids ratio in medium VLDL || id:ebi-cfb233-GCST9030
fect on ER- Breast cancer (Combined Oncoarray; iCOGS; GWAS meta analysis) || id:ieu-a-1128

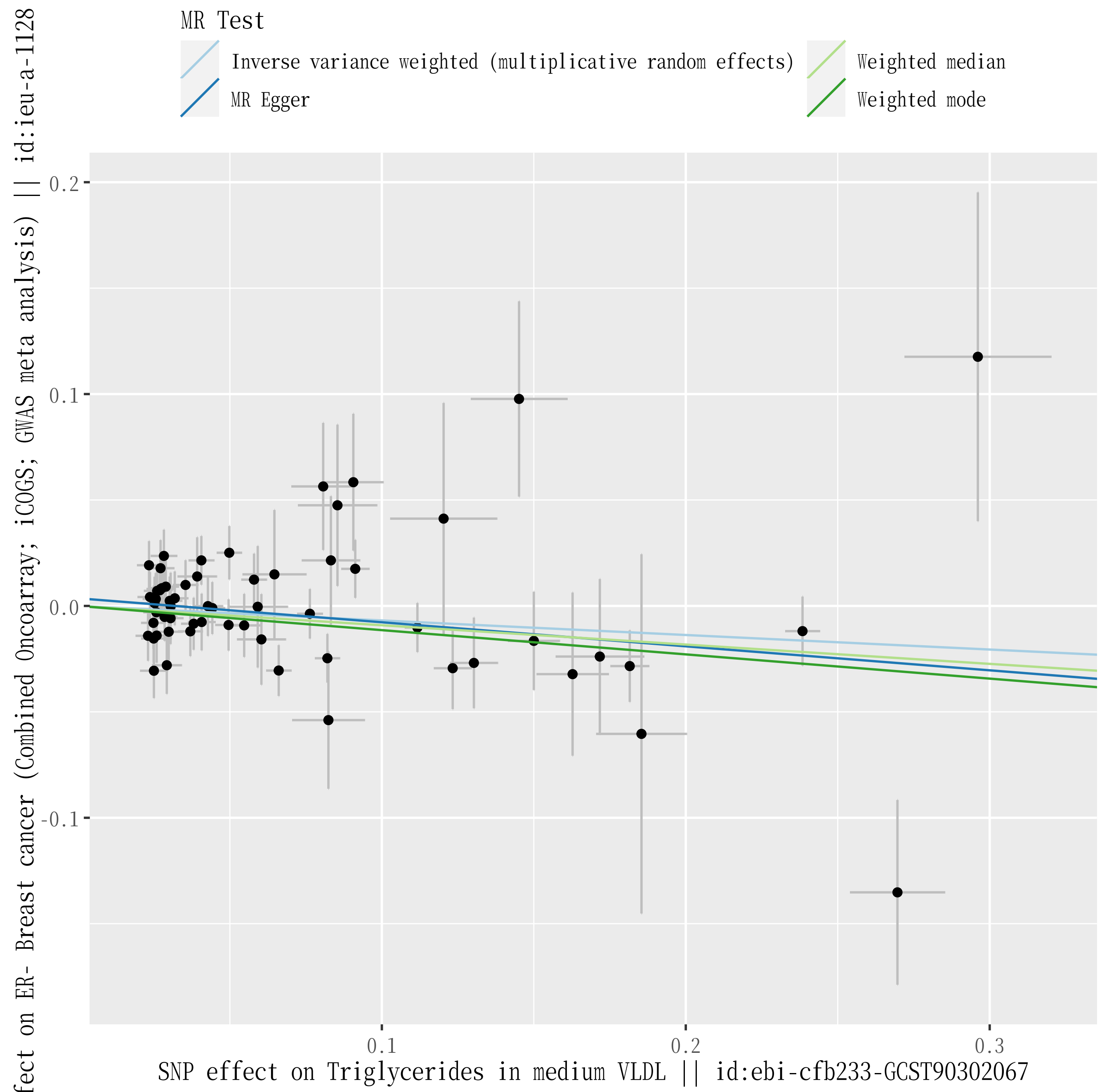
MR Test
Inverse variance weighted (multiplicative random effects)
MR Egger
Weighted median
Weighted mode
0.2
0.1
0.0
-0.1
0.1
0.2
0.3
SNP effect on Triglycerides in medium VLDL || id:ebi-cfb233-GCST90302067
fect on ER- Breast cancer (Combined Oncoarray; iCOGS; GWAS meta analysis) || id:ieu-a-1128

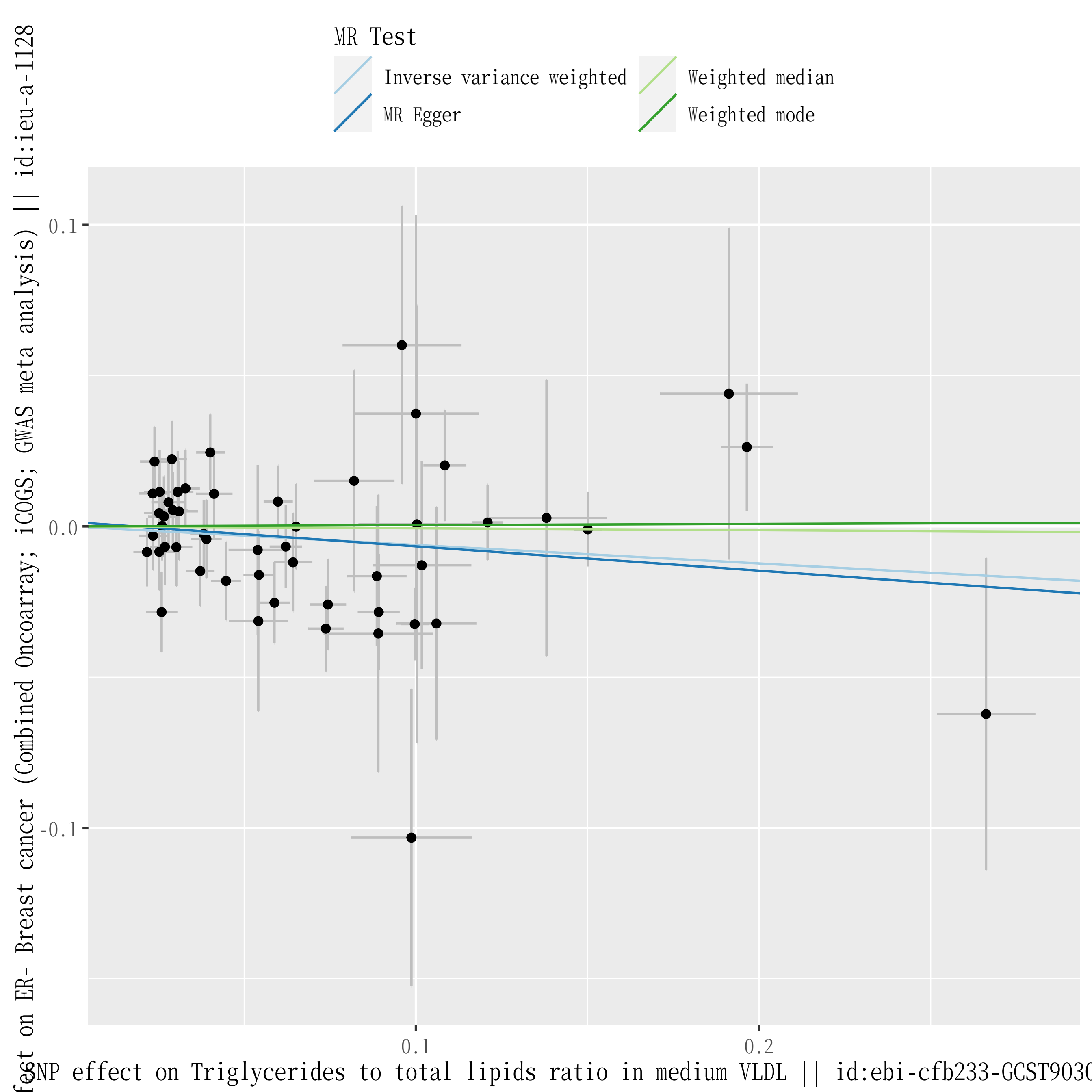

MR Test
Inverse variance weighted
MR Egger
Weighted median
Weighted mode
0.1
0.0
-0.1
0.1
0.2
SNP effect on Triglycerides to total lipids ratio in medium VLDL || id:ebi-cfb233-GCST9030
fect on ER- Breast cancer (Combined Oncoarray; iCOGS; GWAS meta analysis) || id:ieu-a-1128

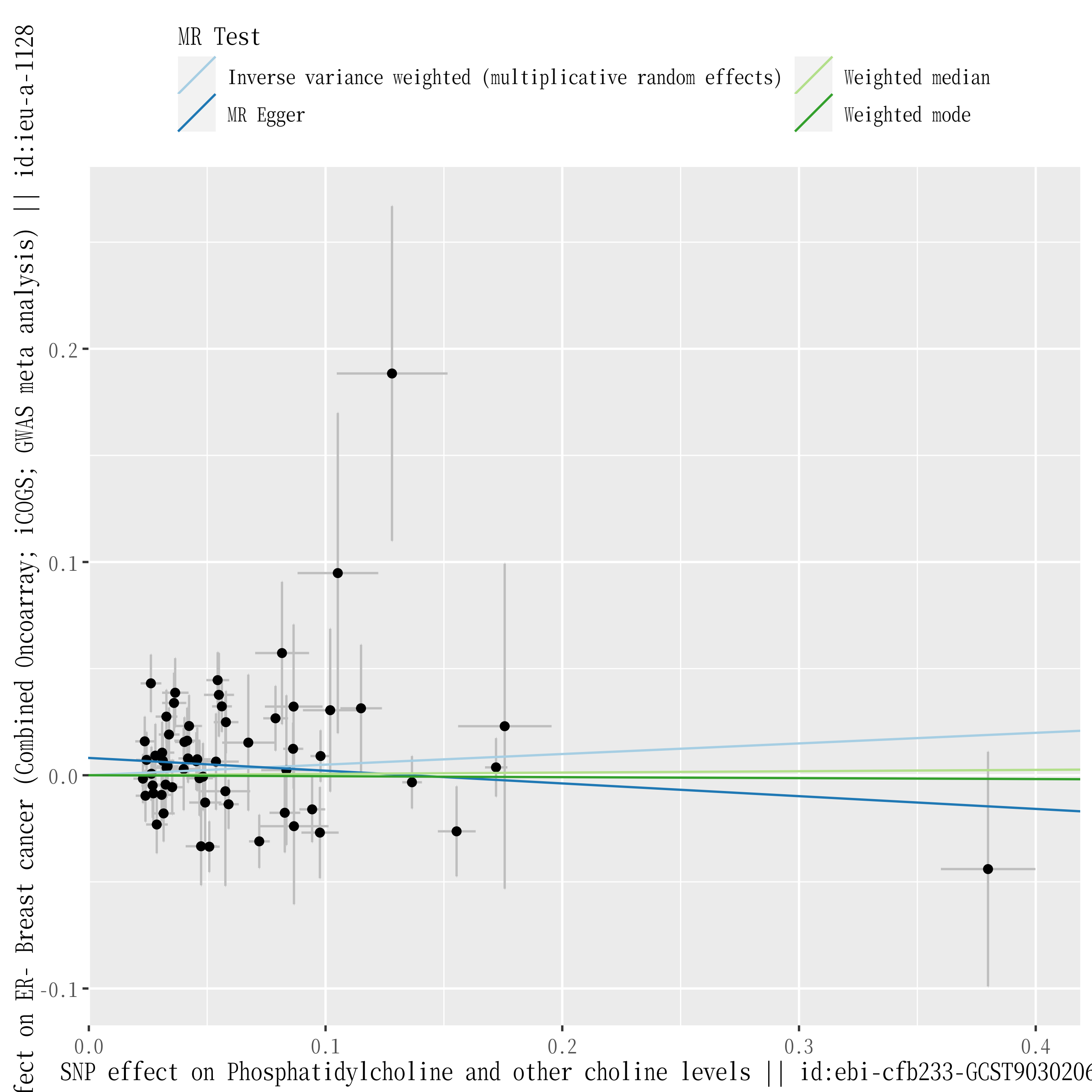

MR Test
Inverse variance weighted (multiplicative random effects)
MR Egger
Weighted median
Weighted mode
0.2
0.1
0.0
-0.1
0.0
0.1
0.2
0.3
0.4
SNP effect on Phosphatidylcholine and other choline levels || id:ebi-cfb233-GCST903020
fect on ER- Breast cancer (Combined Oncoarray; iCOGS; GWAS meta analysis) || id:ieu-a-1128

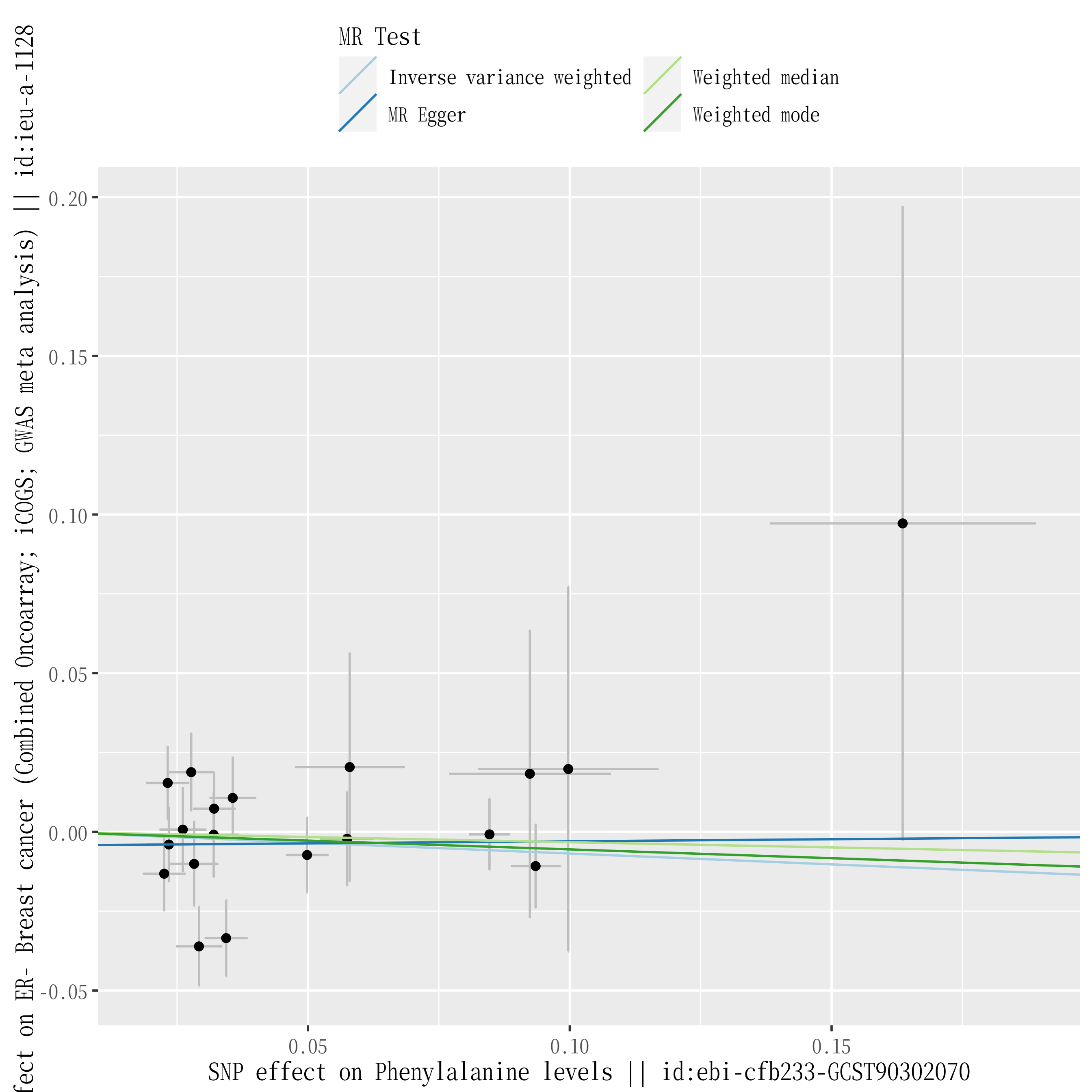

MR Test
Inverse variance weighted
MR Egger
Weighted median
Weighted mode
0.20
0.15
0.10
0.05
0.00
-0.05
0.05
0.10
0.15
SNP effect on Phenylalanine levels || id:ebi-cfb233-GCST90302070
fect on ER- Breast cancer (Combined Oncoarray; iCOGS; GWAS meta analysis) || id:ieu-a-1128

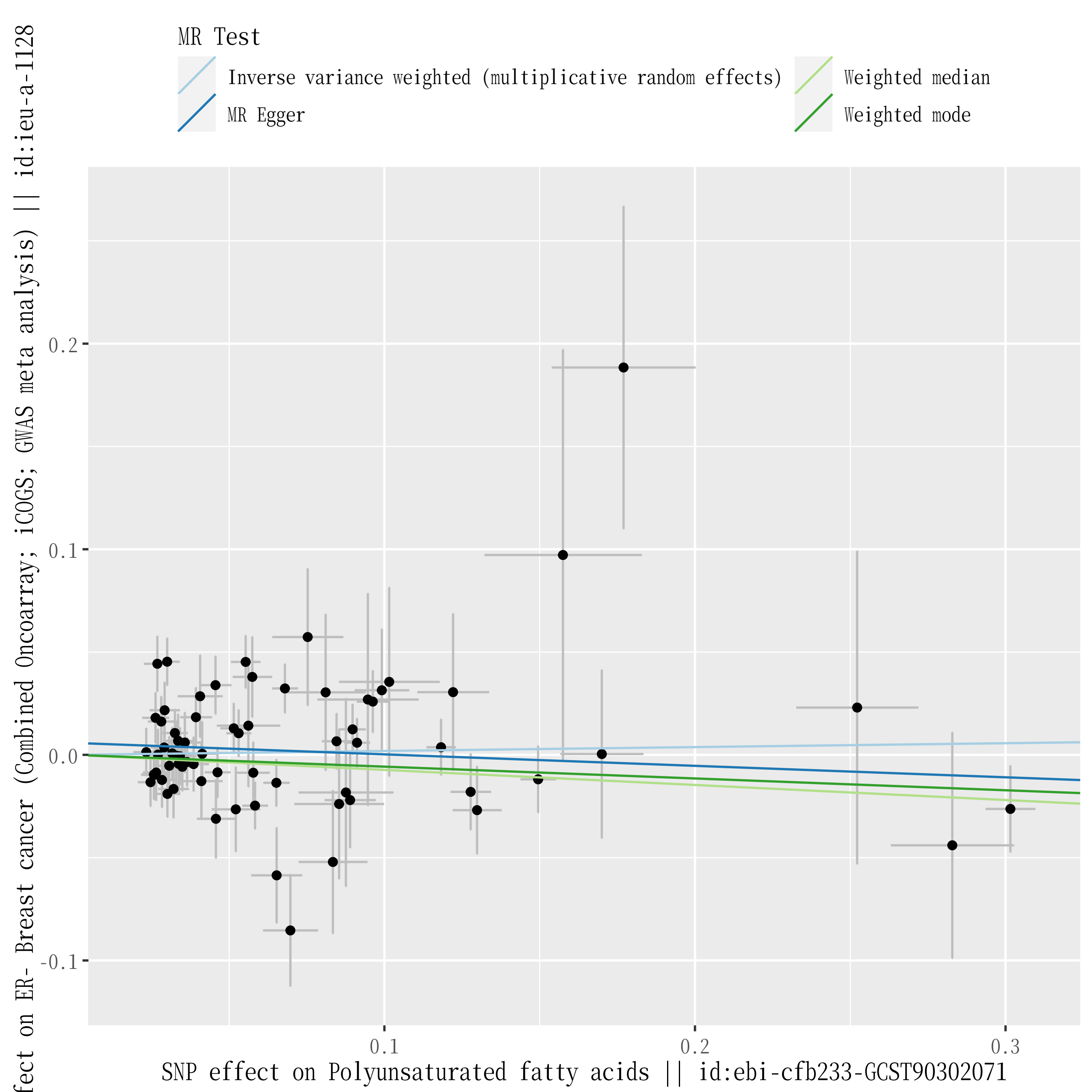

MR Test
Inverse variance weighted (multiplicative random effects)
MR Egger
Weighted median
Weighted mode
fect on ER- Breast cancer (Combined Oncoarray; iCOGS; GWAS meta analysis) || id:ieu-a-1128
0.2
0.1
0.0
-0.1
0.1
0.2
0.3
SNP effect on Polyunsaturated fatty acids || id:ebi-cfb233-GCST90302071

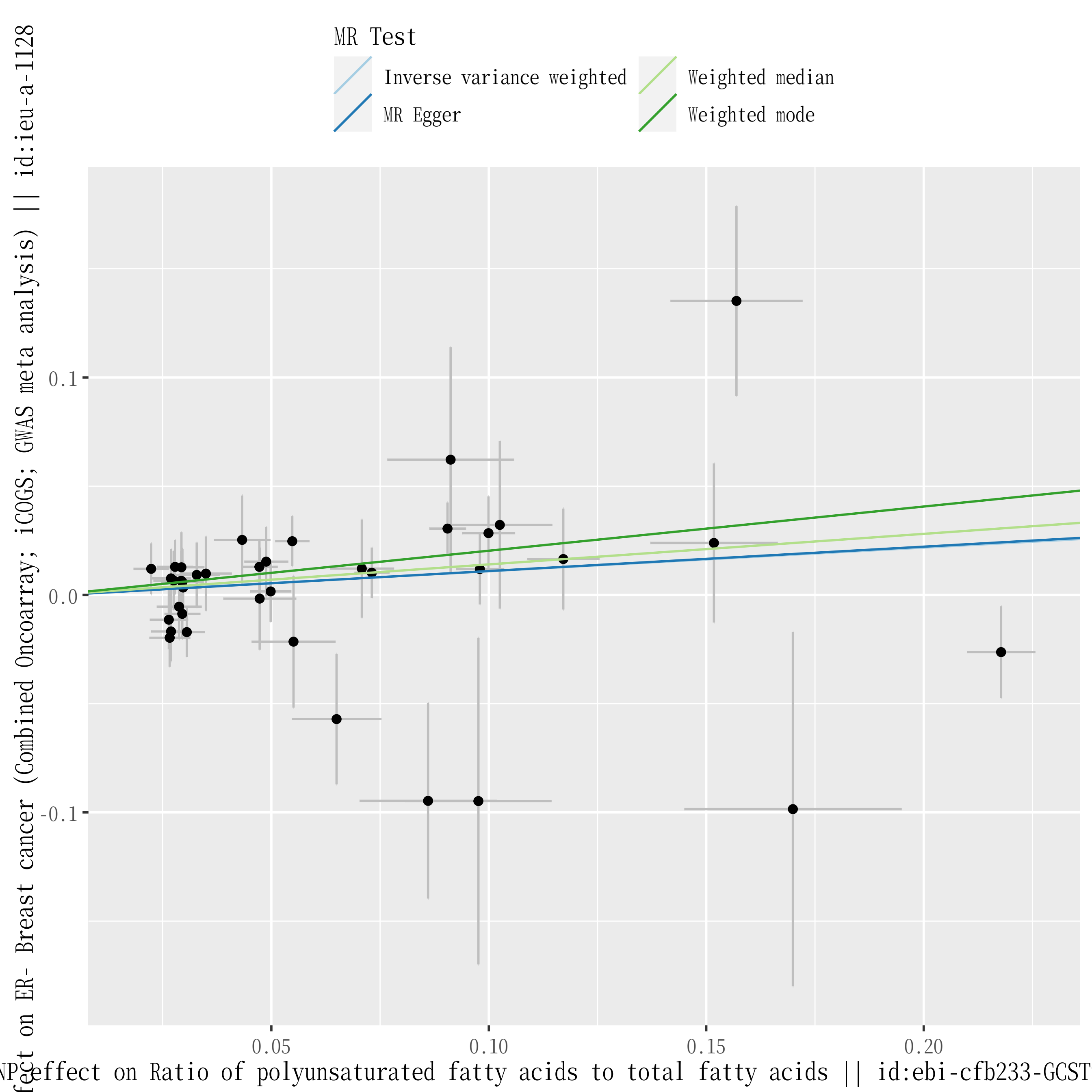

MR Test
Inverse variance weighted
MR Egger
Weighted median
Weighted mode
0.1
0.0
-0.1
0.05
0.10
0.15
0.20
fect on ER- Breast cancer (Combined Oncoarray; iCOGS; GWAS meta analysis) || id:ieu-a-1128
NP effect on Ratio of polyunsaturated fatty acids to total fatty acids || id:ebi-cfb233-GCST

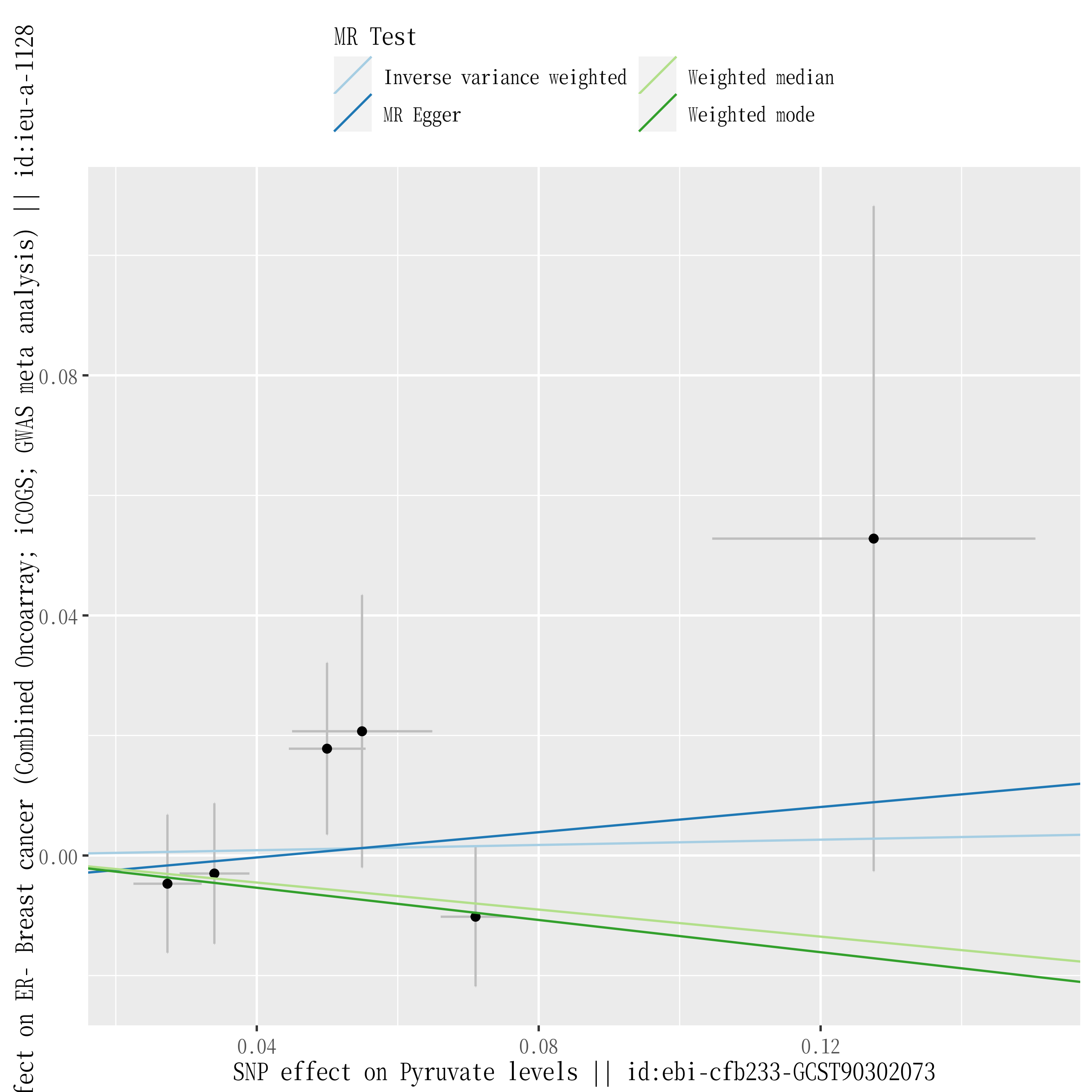

MR Test
Inverse variance weighted
MR Egger
Weighted median
Weighted mode
0.08
0.04
0.00
0.04
0.08
0.12
SNP effect on Pyruvate levels || id:ebi-cfb233-GCST90302073
fect on ER- Breast cancer (Combined Oncoarray; iCOGS; GWAS meta analysis) || id:ieu-a-1128

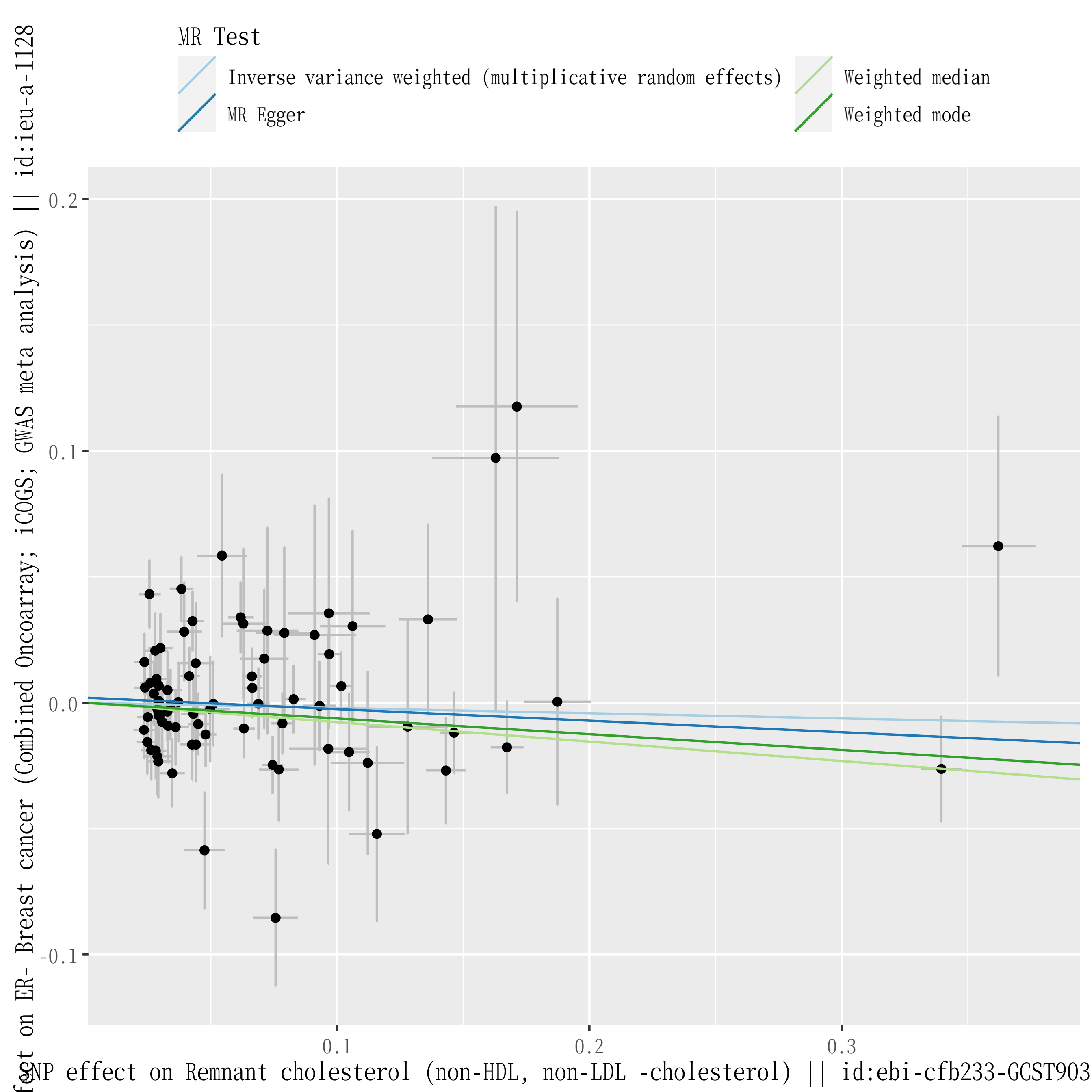

MR Test
Inverse variance weighted (multiplicative random effects)
MR Egger
Weighted median
Weighted mode
0.2
0.1
0.0
-0.1
0.1
0.2
0.3
SNP effect on Remnant cholesterol (non-HDL, non-LDL -cholesterol) || id:ebi-cfb233-GCST903
SNP effect on ER- Breast cancer (Combined Oncoarray; iCOGS; GWAS meta analysis) || id:ieu-a-1128

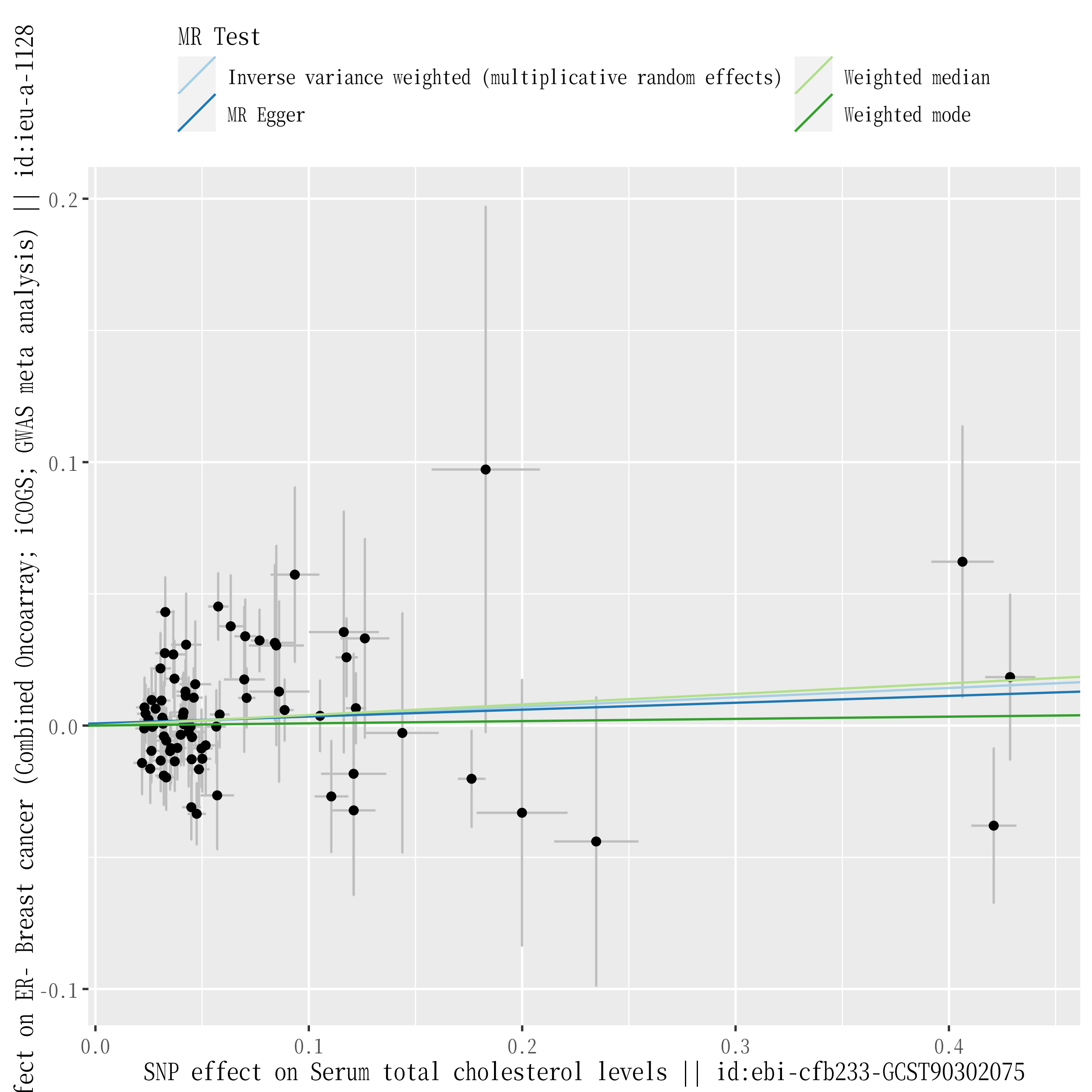
MR Test
Inverse variance weighted (multiplicative random effects)
MR Egger
Weighted median
Weighted mode
0.2
0.1
0.0
-0.1
0.0
0.1
0.2
0.3
0.4
SNP effect on Serum total cholesterol levels || id:ebi-cfb233-GCST90302075
fect on ER- Breast cancer (Combined Oncoarray; iCOGS; GWAS meta analysis) || id:ieu-a-1128

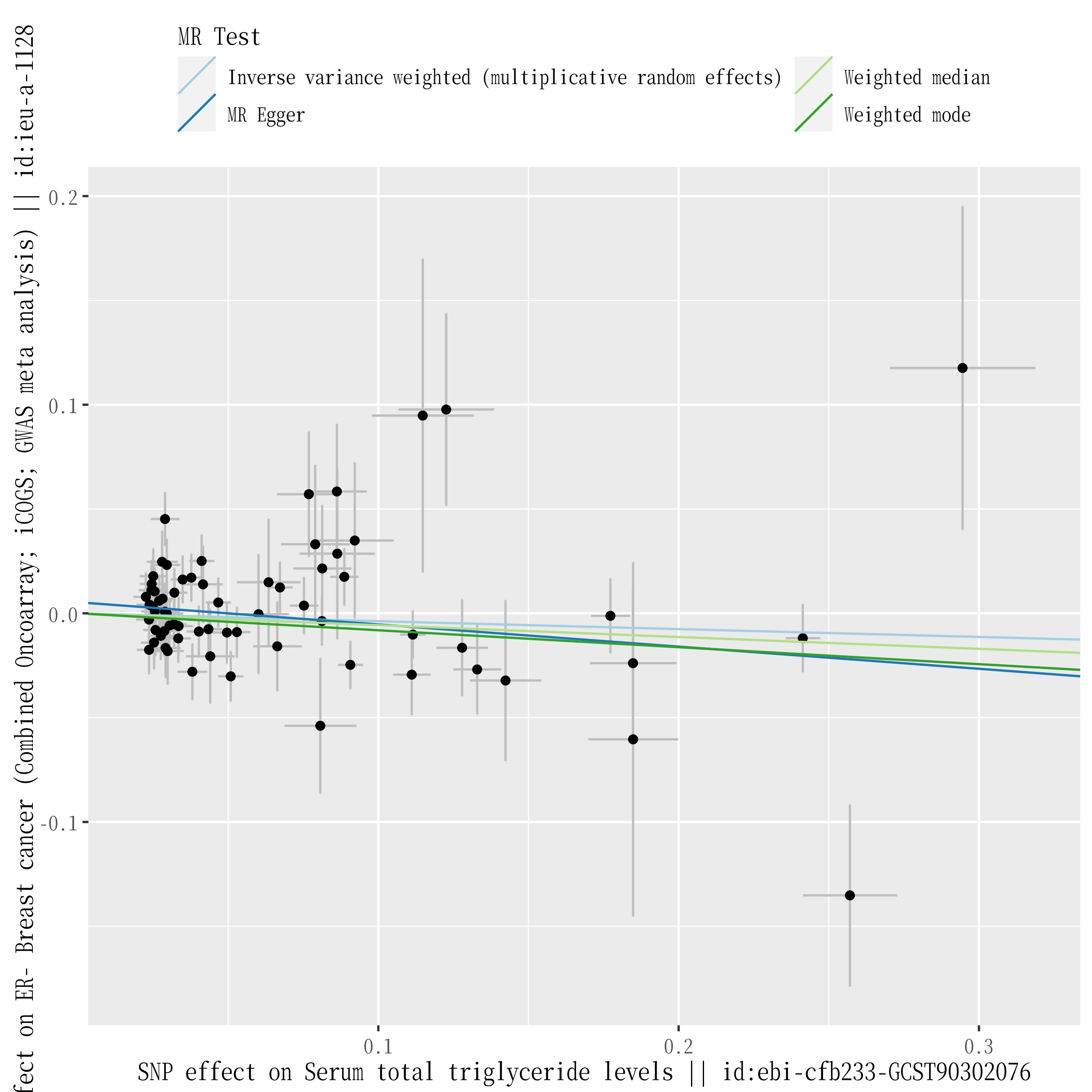

MR Test
Inverse variance weighted (multiplicative random effects)
MR Egger
Weighted median
Weighted mode
0.2
0.1
0.0
-0.1
0.1
0.2
0.3
fect on ER- Breast cancer (Combined Oncoarray; iCOGS; GWAS meta analysis) || id:ieu-a-1128
SNP effect on Serum total triglyceride levels || id:ebi-cfb233-GCST90302076

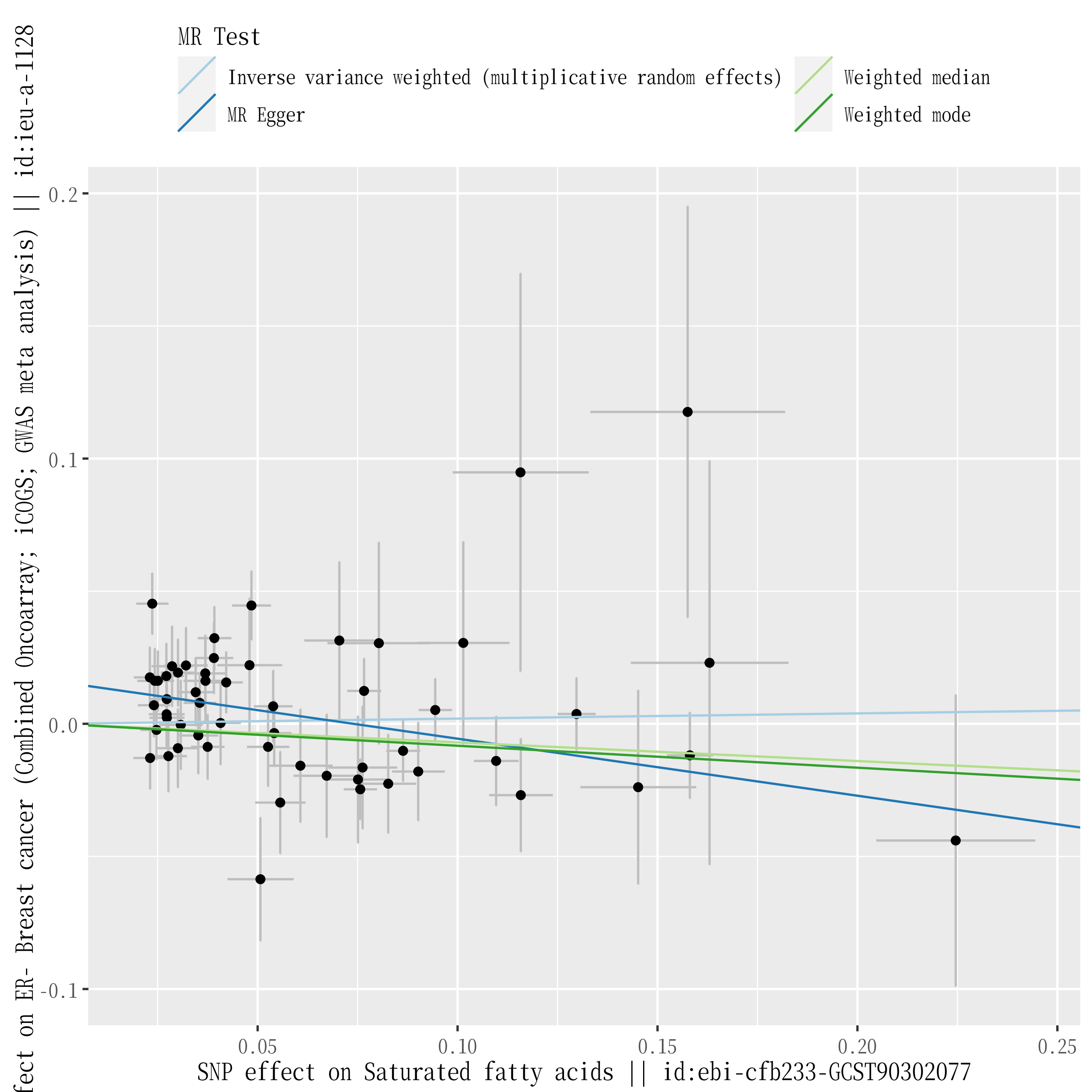

MR Test
Inverse variance weighted (multiplicative random effects)
MR Egger
Weighted median
Weighted mode
0.2
0.1
0.0
-0.1
0.05
0.10
0.15
0.20
0.25
SNP effect on Saturated fatty acids || id:ebi-cfb233-GCST90302077
fect on ER- Breast cancer (Combined Oncoarray; iCOGS; GWAS meta analysis) || id:ieu-a-1128

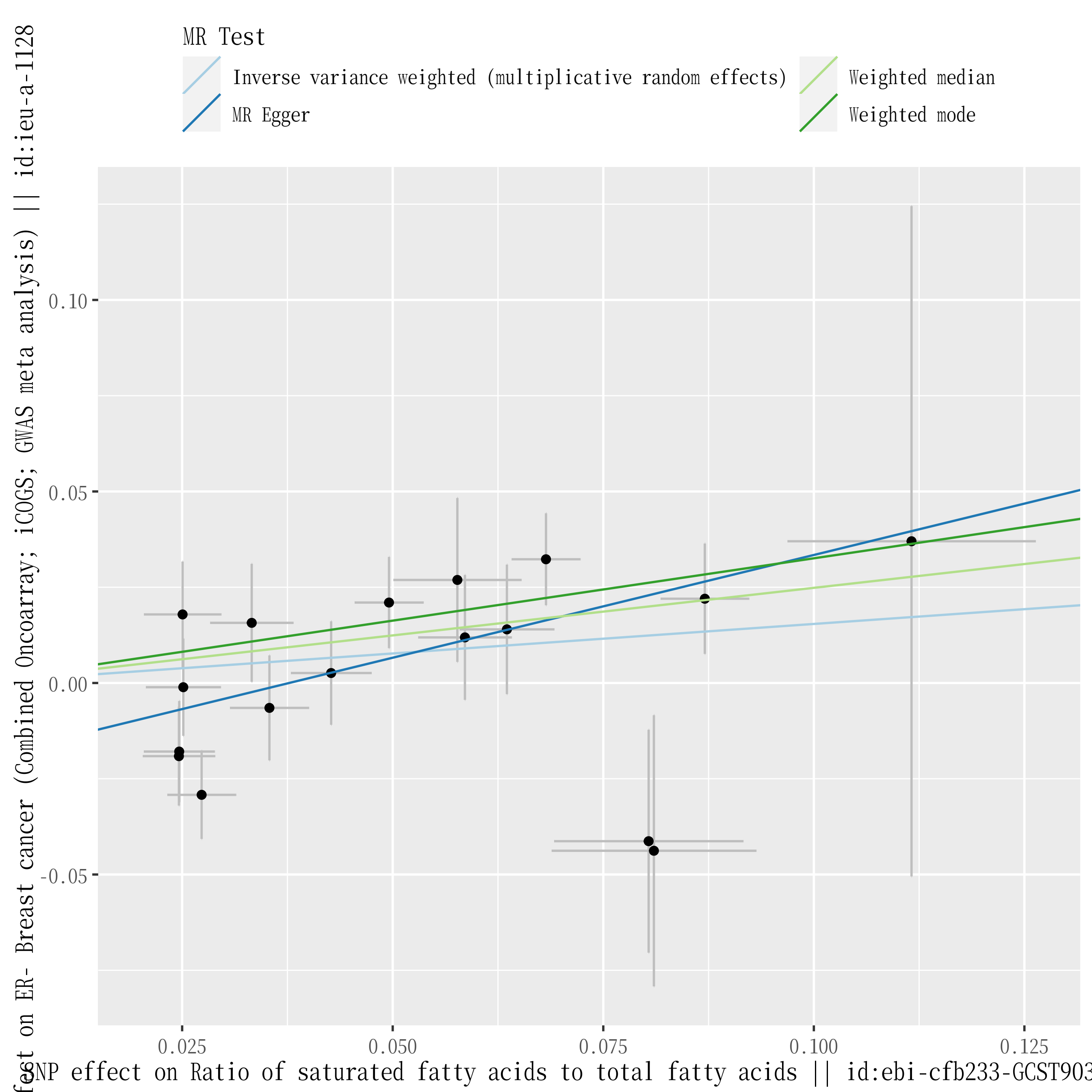
MR Test
Inverse variance weighted (multiplicative random effects)
MR Egger
Weighted median
Weighted mode
0.10
0.05
0.00
-0.05
0.025
0.050
0.075
0.100
0.125
fect on ER- Breast cancer (Combined Oncoarray; iCOGS; GWAS meta analysis) || id:ieu-a-1128
SNP effect on Ratio of saturated fatty acids to total fatty acids || id:ebi-cfb233-GCST903

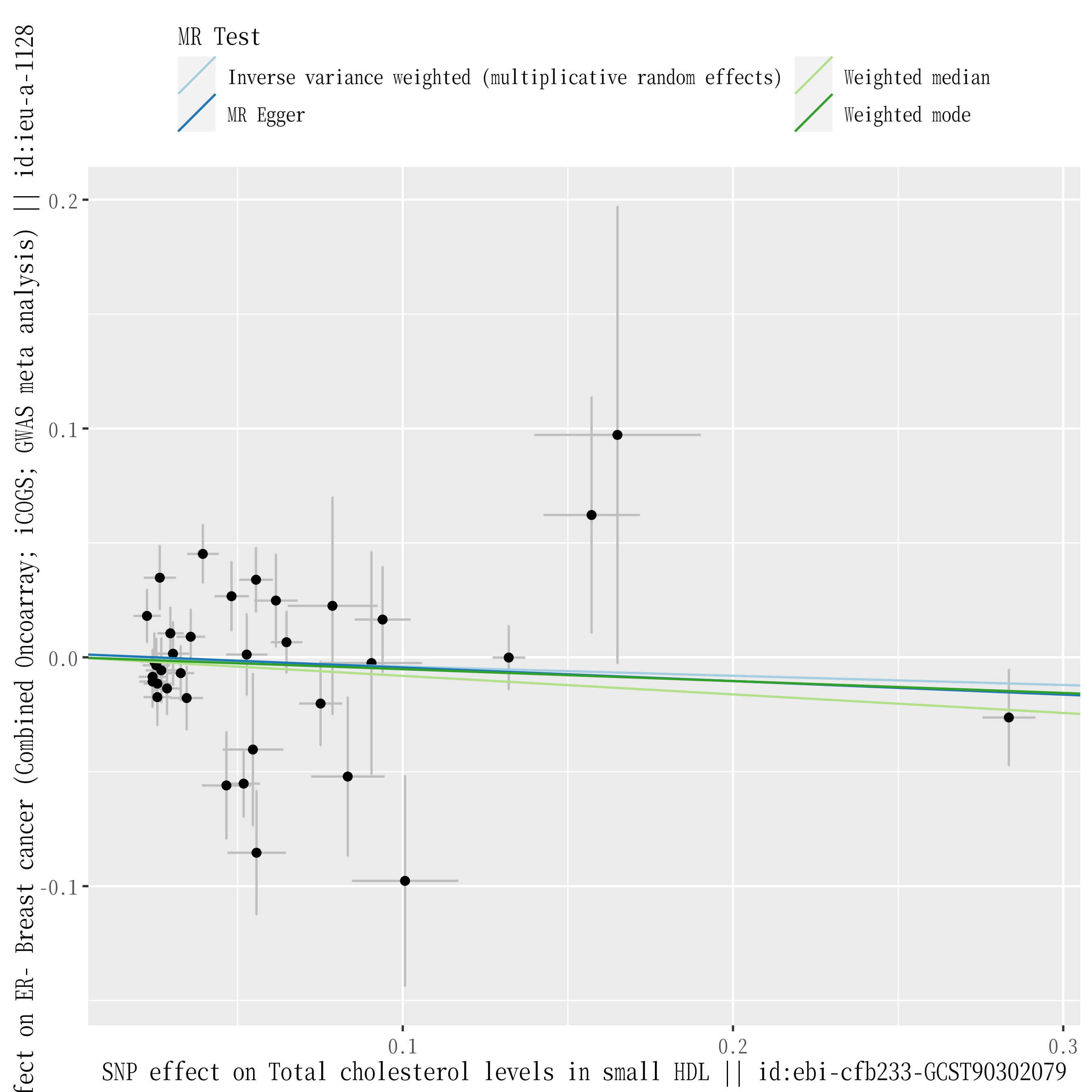
MR Test
Inverse variance weighted (multiplicative random effects)
MR Egger
Weighted median
Weighted mode
0.2
0.1
0.0
-0.1
0.1
0.2
0.3
SNP effect on Total cholesterol levels in small HDL || id:ebi-cfb233-GCST90302079
fect on ER- Breast cancer (Combined Oncoarray; iCOGS; GWAS meta analysis) || id:ieu-a-1128

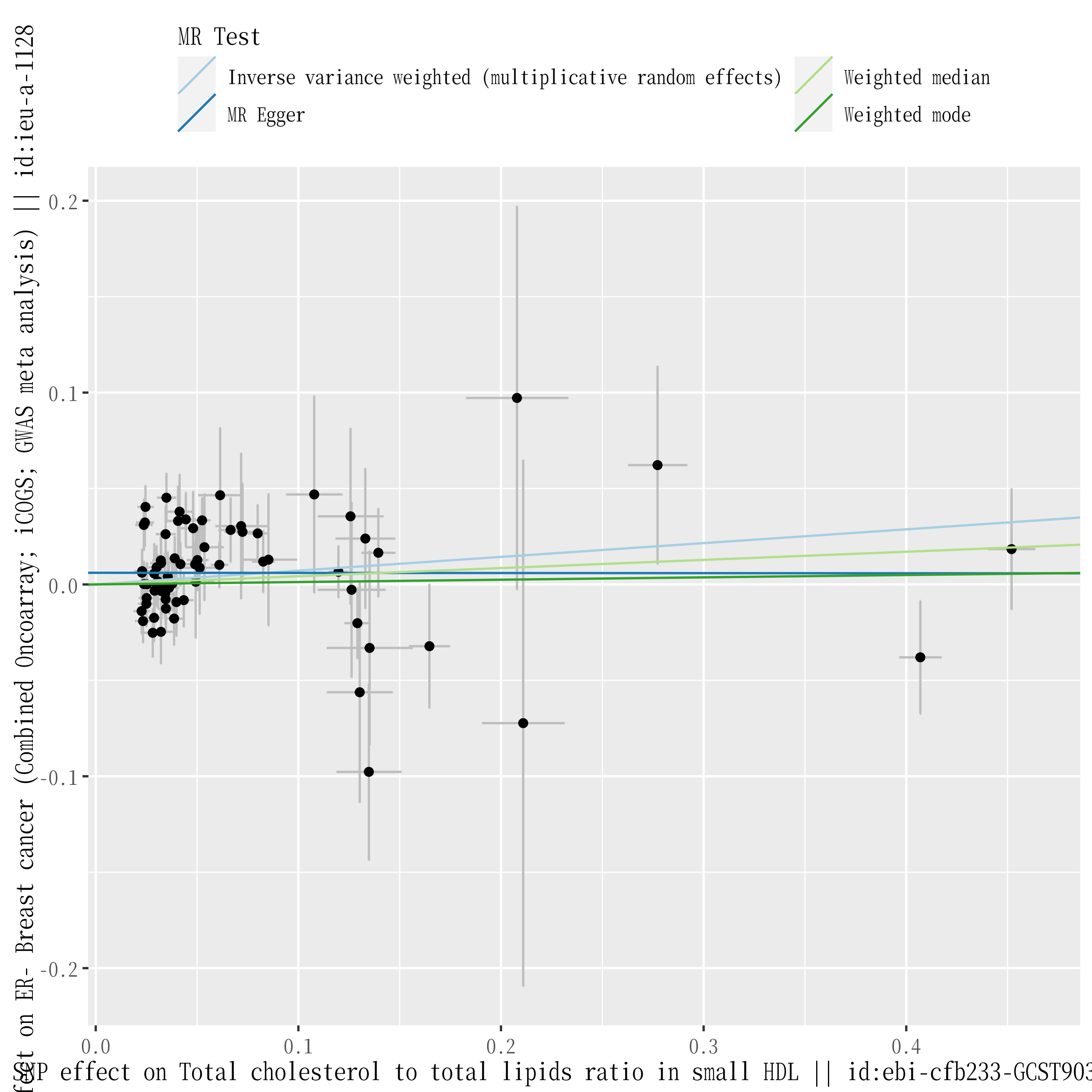
MR Test
Inverse variance weighted (multiplicative random effects)
MR Egger
Weighted median
Weighted mode
0.2
0.1
0.0
-0.1
-0.2
0.0
0.1
0.2
0.3
0.4
SNP effect on Total cholesterol to total lipids ratio in small HDL || id:ebi-cfb233-GCST903
fect on ER- Breast cancer (Combined Oncoarray; iCOGS; GWAS meta analysis) || id:ieu-a-1128

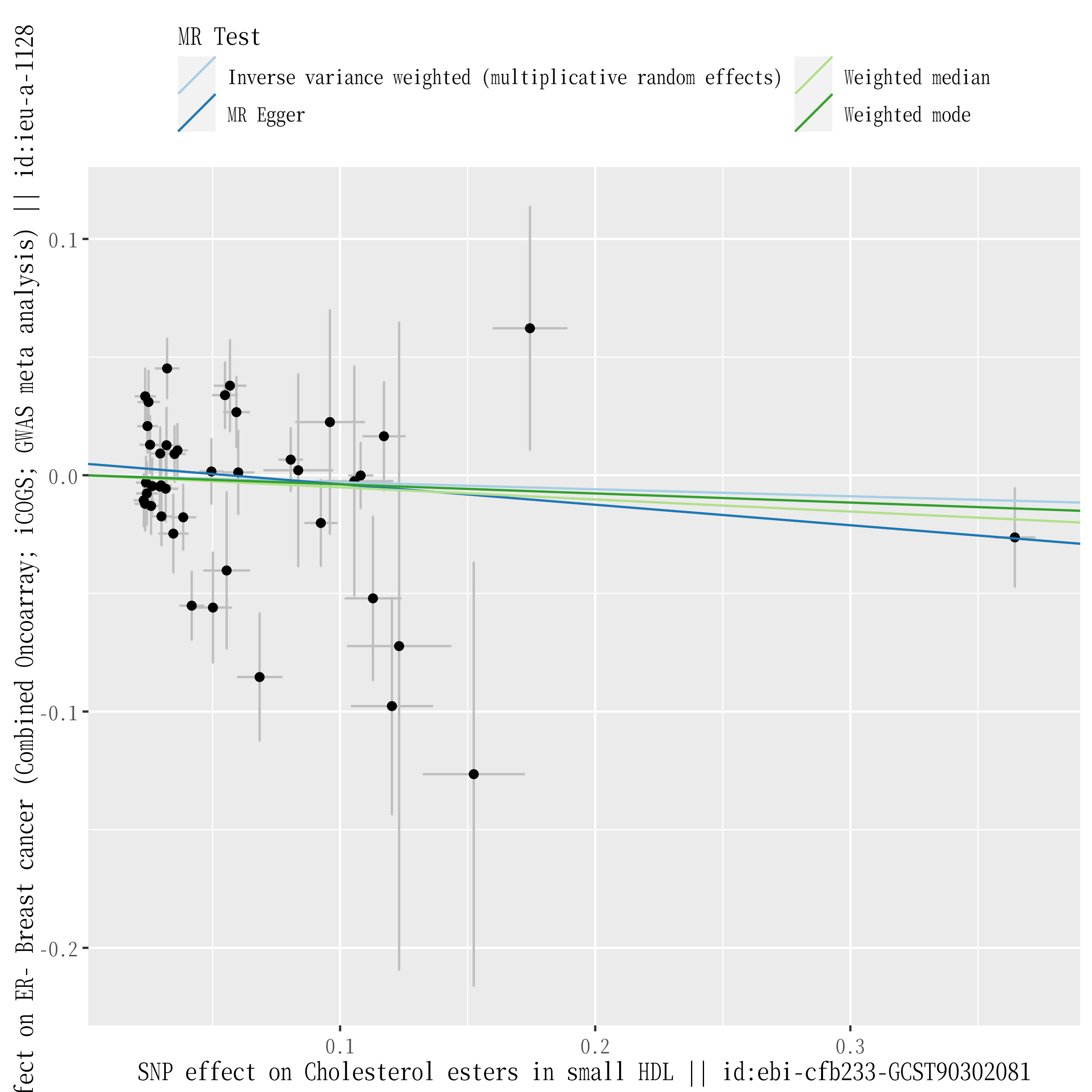

MR Test
Inverse variance weighted (multiplicative random effects)
MR Egger
Weighted median
Weighted mode
0.1
0.0
-0.1
-0.2
0.1
0.2
0.3
SNP effect on Cholesterol esters in small HDL || id:ebi-cfb233-GCST90302081
fect on ER- Breast cancer (Combined Oncoarray; iCOGS; GWAS meta analysis) || id:ieu-a-1128

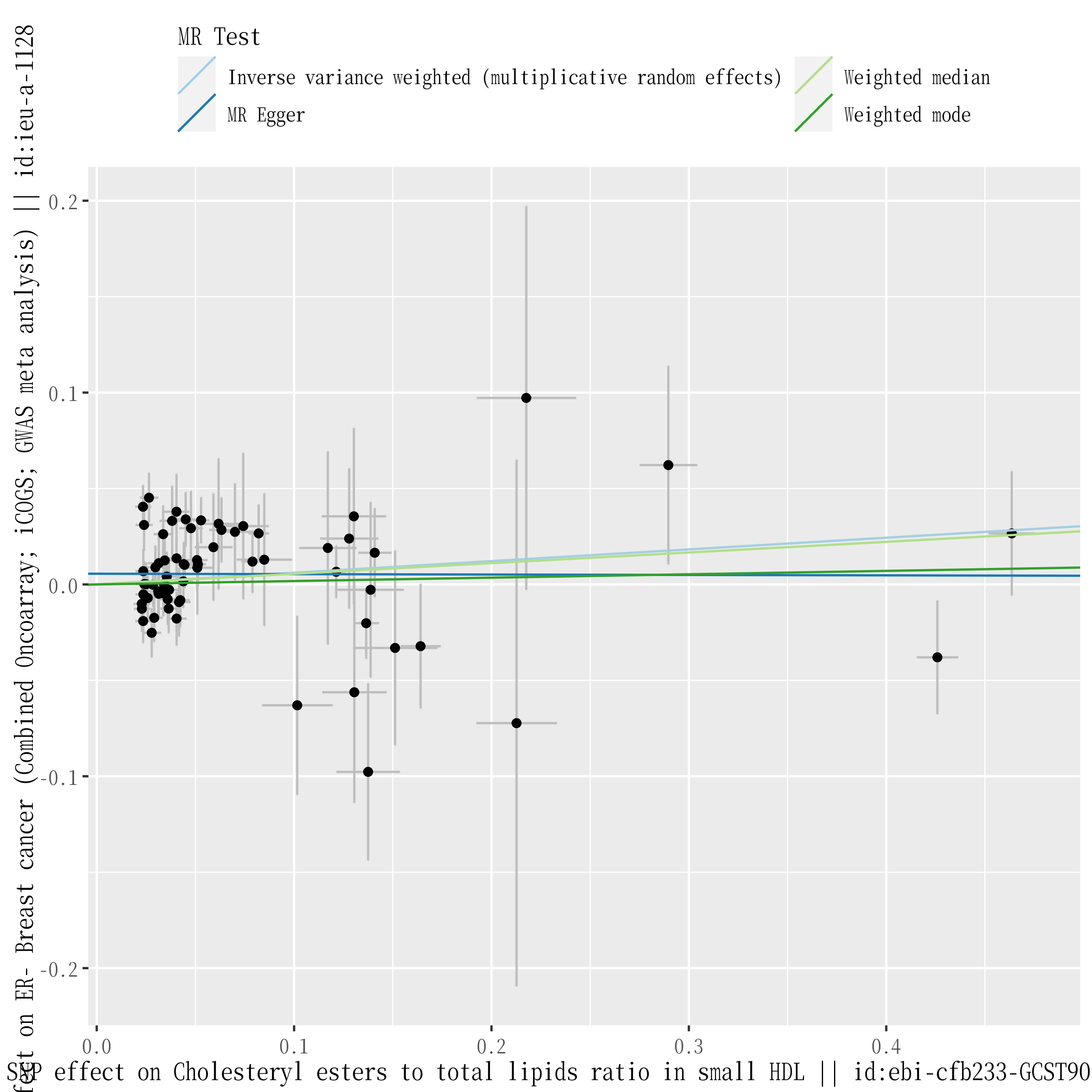

MR Test
Inverse variance weighted (multiplicative random effects)
MR Egger
Weighted median
Weighted mode
0.2
0.1
0.0
-0.1
-0.2
0.0
0.1
0.2
0.3
0.4
SNP effect on Cholesteryl esters to total lipids ratio in small HDL || id:ebi-cfb233-GCST90
fect on ER- Breast cancer (Combined Oncoarray; iCOGS; GWAS meta analysis) || id:ieu-a-1128

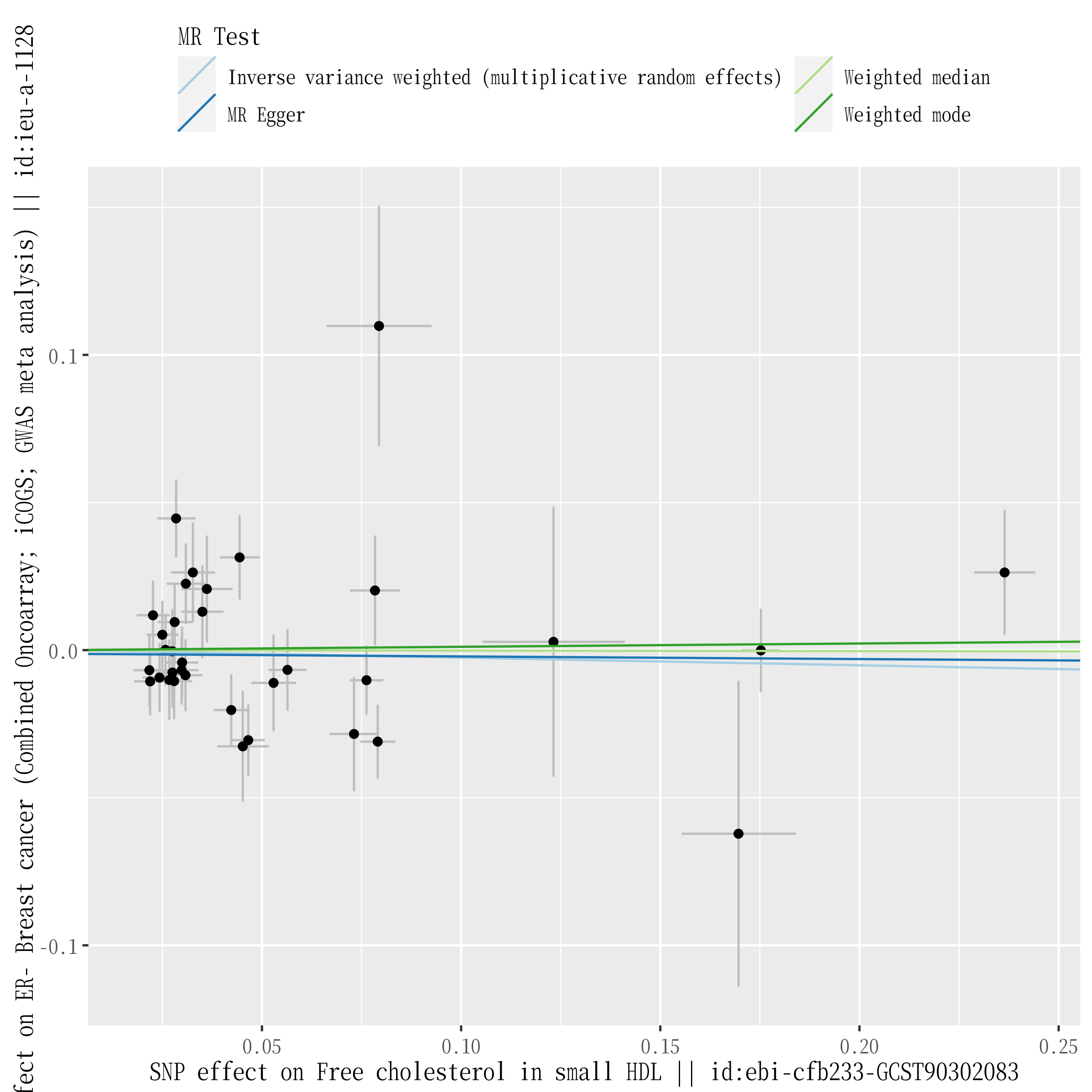
MR Test
Inverse variance weighted (multiplicative random effects)
MR Egger
Weighted median
Weighted mode
0.1
0.0
-0.1
0.05
0.10
0.15
0.20
0.25
SNP effect on Free cholesterol in small HDL || id:ebi-cfb233-GCST90302083
fect on ER- Breast cancer (Combined Oncoarray; iCOGS; GWAS meta analysis) || id:ieu-a-1128

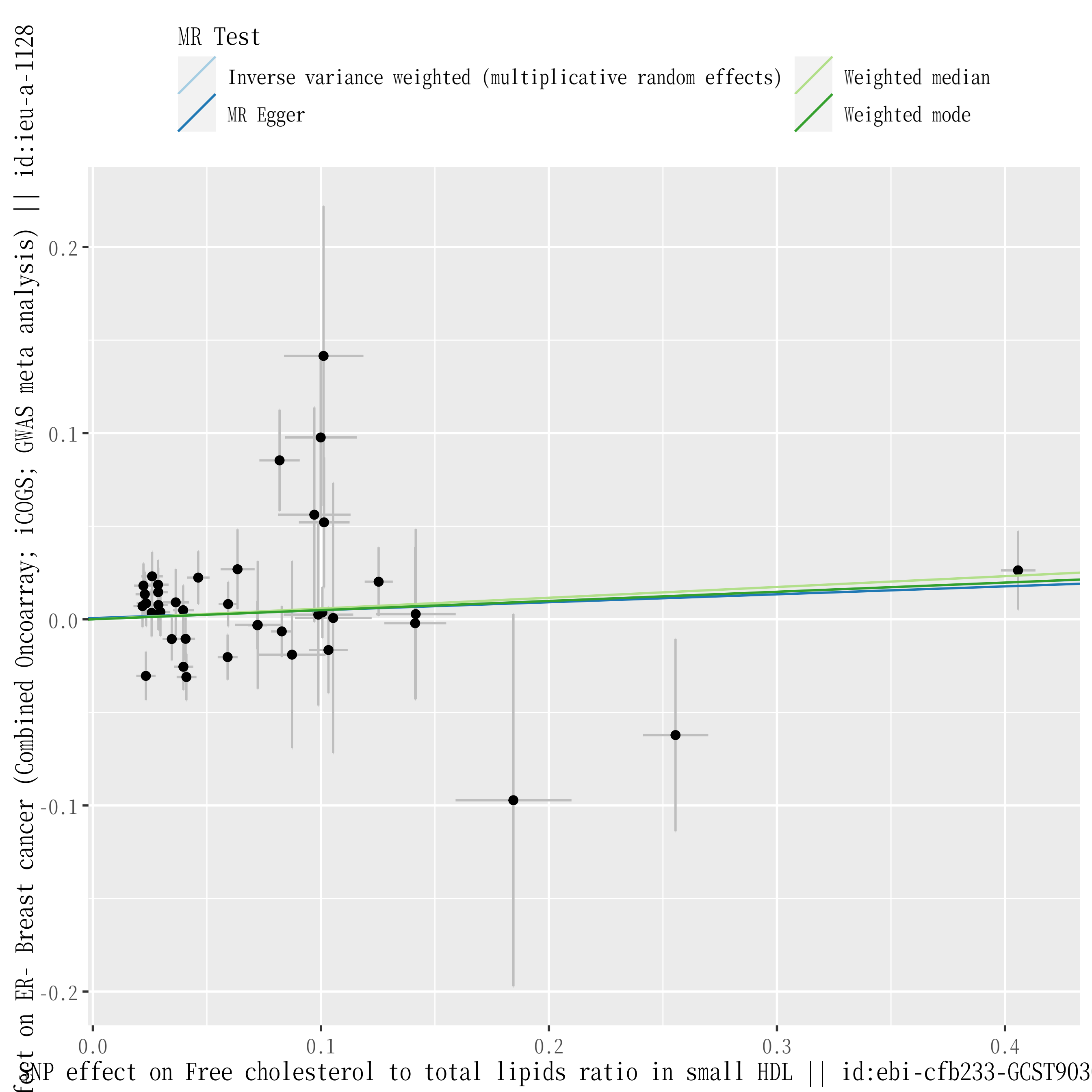
MR Test
Inverse variance weighted (multiplicative random effects)
MR Egger
Weighted median
Weighted mode
0.2
0.1
0.0
-0.1
-0.2
0.0
0.1
0.2
0.3
0.4
SNP effect on Free cholesterol to total lipids ratio in small HDL || id:ebi-cfb233-GCST903
fect on ER- Breast cancer (Combined Oncoarray; iCOGS; GWAS meta analysis) || id:ieu-a-1128

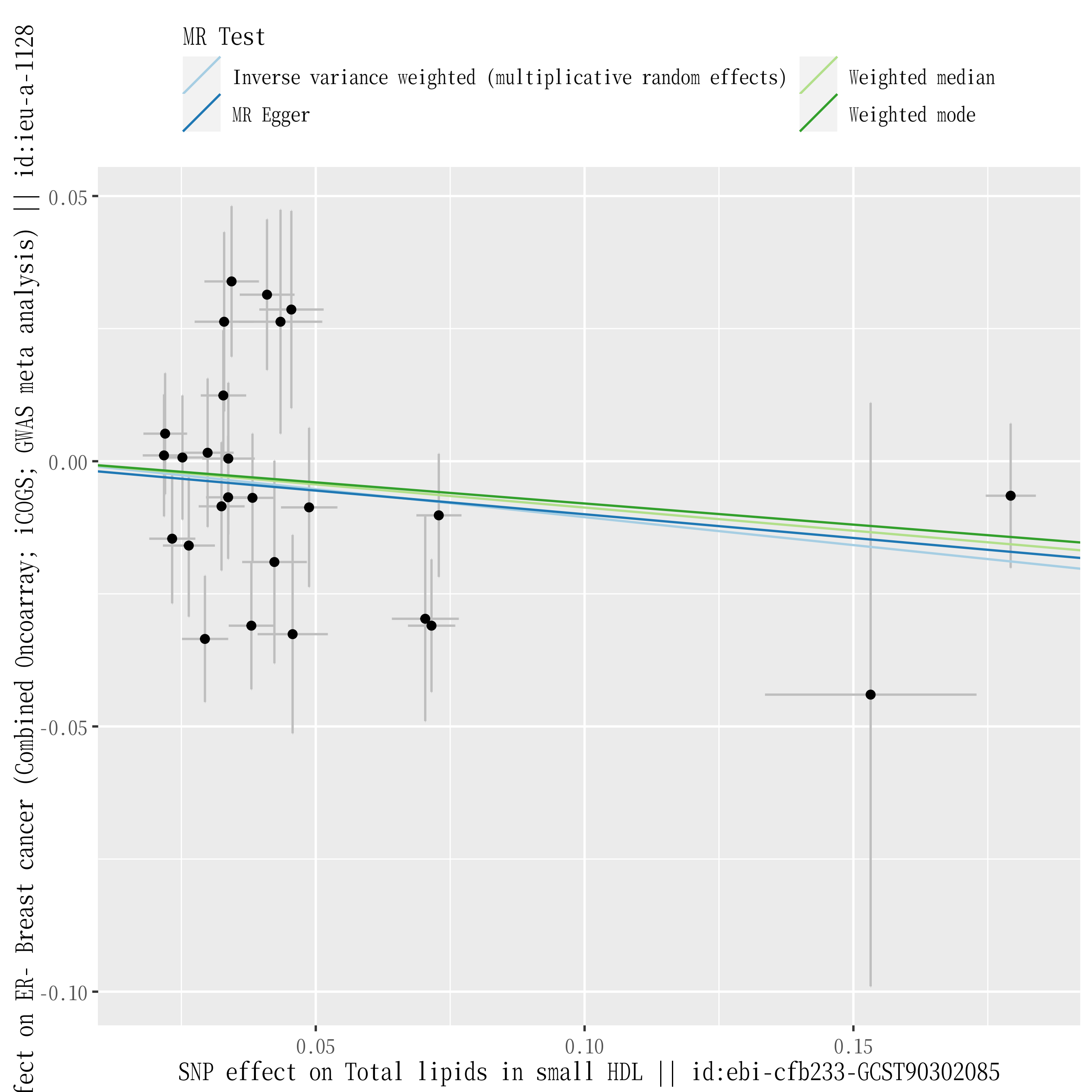
MR Test
Inverse variance weighted (multiplicative random effects)
MR Egger
Weighted median
Weighted mode
0.05
0.00
-0.05
-0.10
0.05
0.10
0.15
SNP effect on Total lipids in small HDL || id:ebi-cfb233-GCST90302085
fect on ER- Breast cancer (Combined Oncoarray; iCOGS; GWAS meta analysis) || id:ieu-a-1128

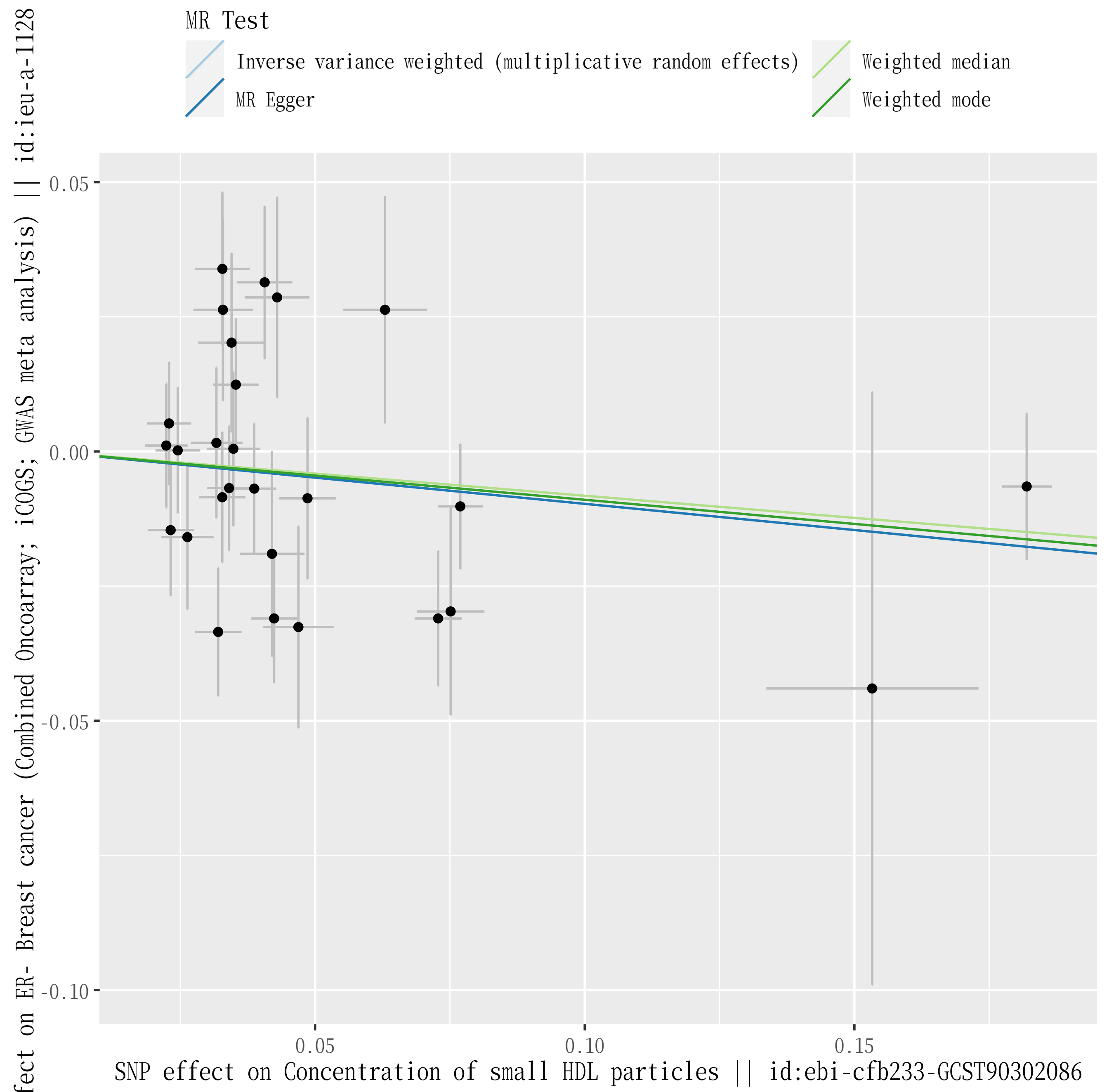

MR Test
Inverse variance weighted (multiplicative random effects)
MR Egger
Weighted median
Weighted mode
0.05
0.00
-0.05
-0.10
0.05
0.10
0.15
SNP effect on Concentration of small HDL particles || id:ebi-cfb233-GCST90302086
fect on ER- Breast cancer (Combined Oncoarray; iCOGS; GWAS meta analysis) || id:ieu-a-1128

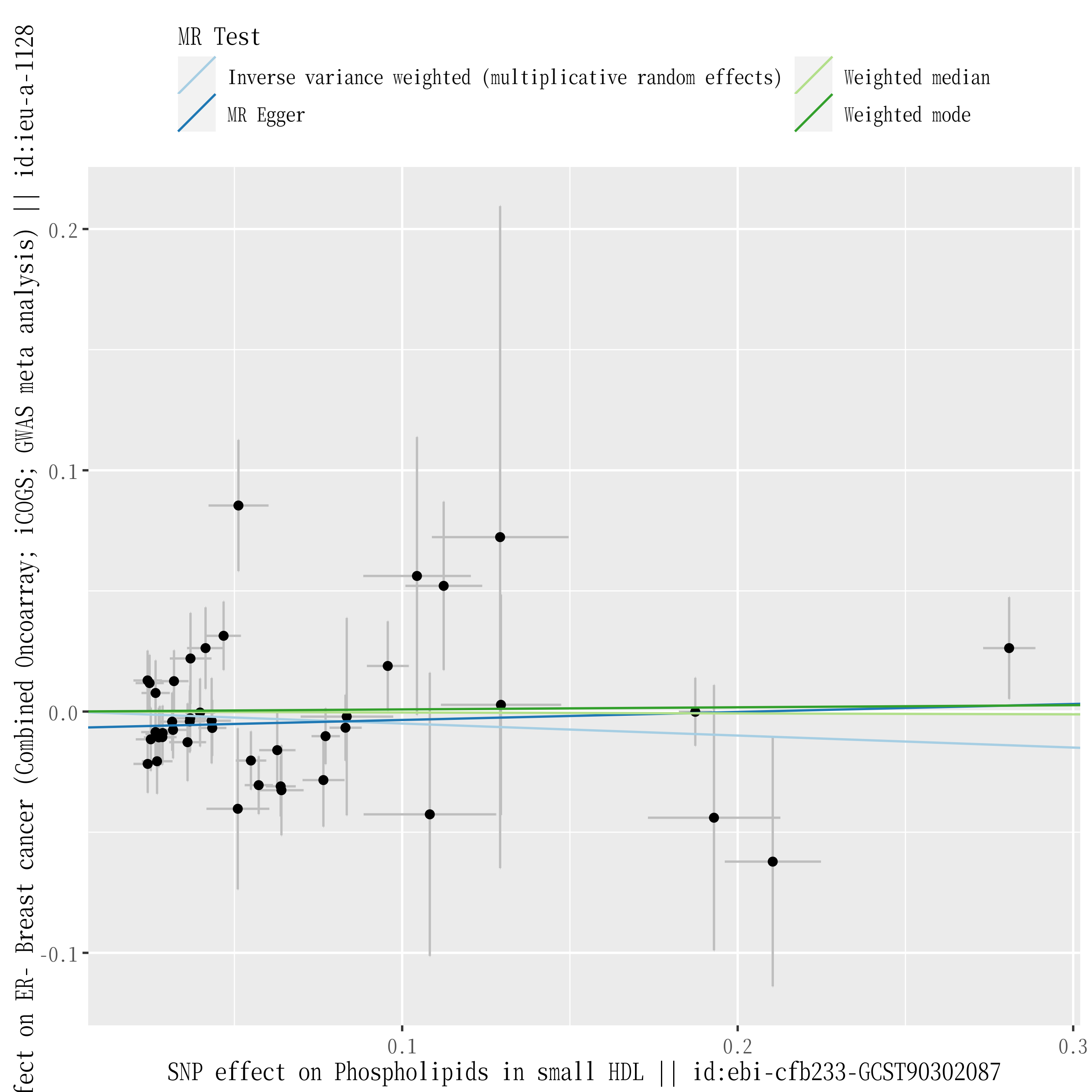

MR Test
Inverse variance weighted (multiplicative random effects)
MR Egger
Weighted median
Weighted mode
0.2
0.1
0.0
-0.1
0.1
0.2
0.3
SNP effect on Phospholipids in small HDL || id:ebi-cfb233-GCST90302087
fect on ER- Breast cancer (Combined Oncoarray; iCOGS; GWAS meta analysis) || id:ieu-a-1128

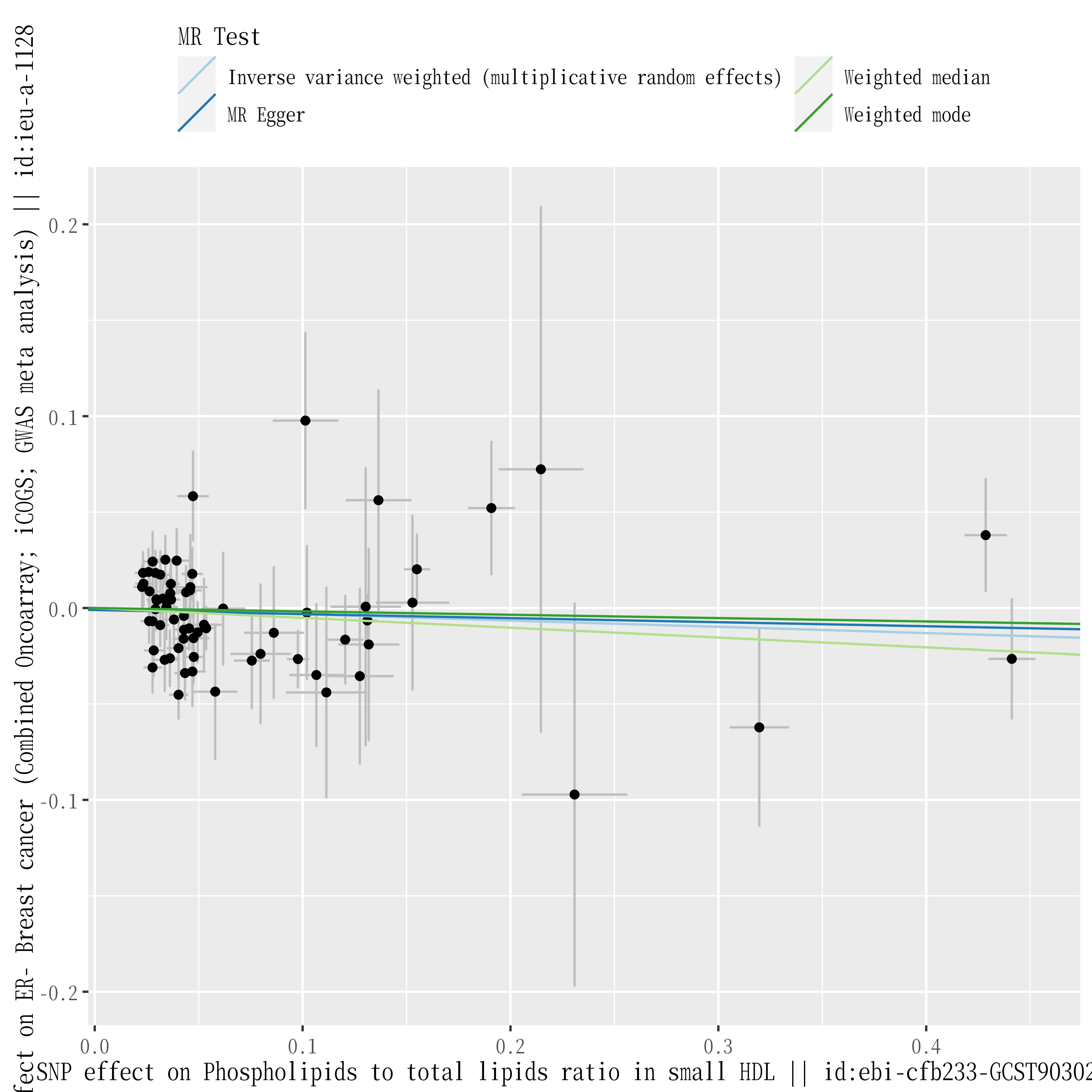
MR Test
Inverse variance weighted (multiplicative random effects)
MR Egger
Weighted median
Weighted mode
fect on ER- Breast cancer (Combined Oncoarray; iCOGS; GWAS meta analysis) || id:ieu-a-1128
0.2
0.1
0.0
-0.1
-0.2
0.0
0.1
0.2
0.3
0.4
SNP effect on Phospholipids to total lipids ratio in small HDL || id:ebi-cfb233-GCST90302

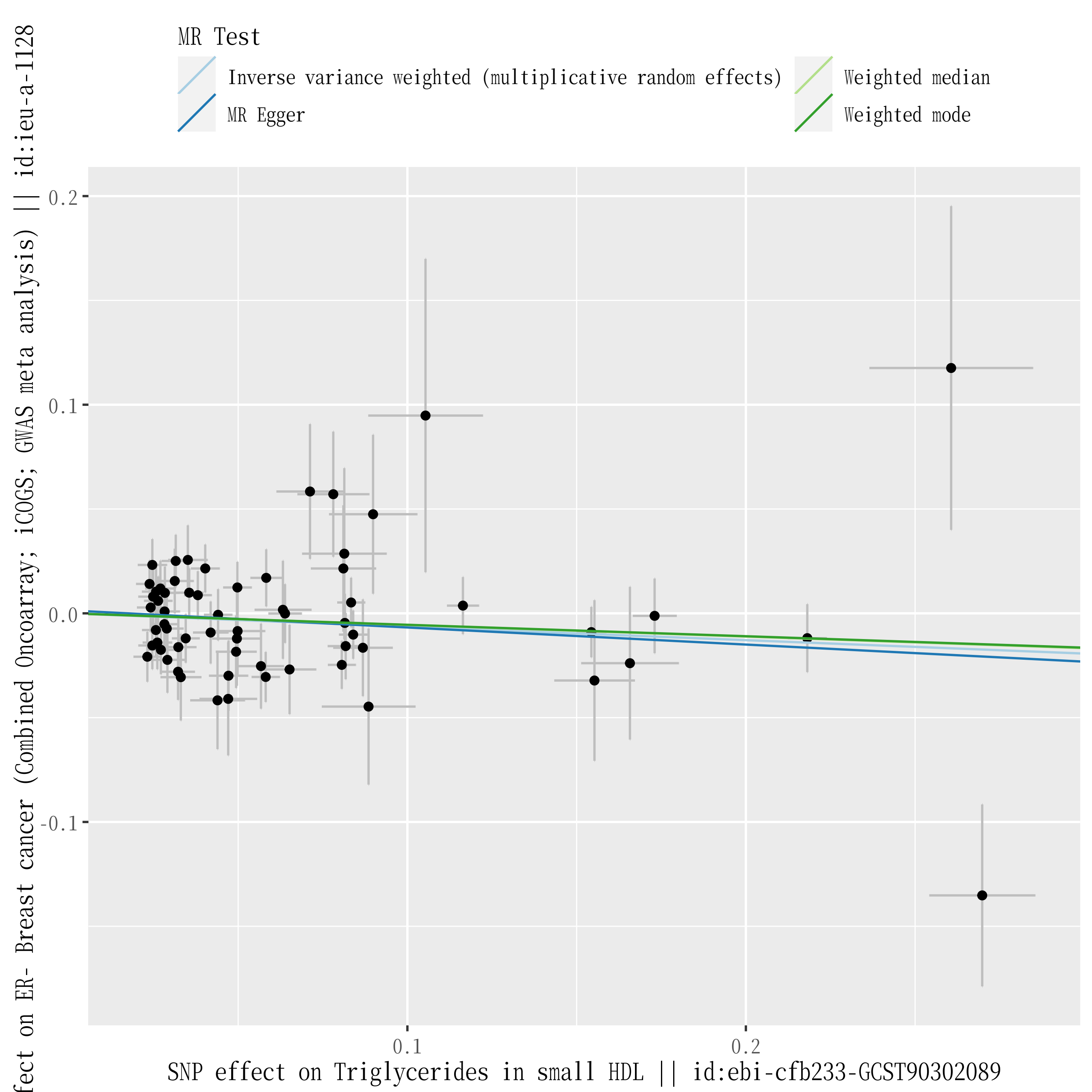
MR Test
Inverse variance weighted (multiplicative random effects)
MR Egger
Weighted median
Weighted mode
0.2
0.1
0.0
-0.1
0.1
0.2
SNP effect on Triglycerides in small HDL || id:ebi-cfb233-GCST90302089
fect on ER- Breast cancer (Combined Oncoarray; iCOGS; GWAS meta analysis) || id:ieu-a-1128

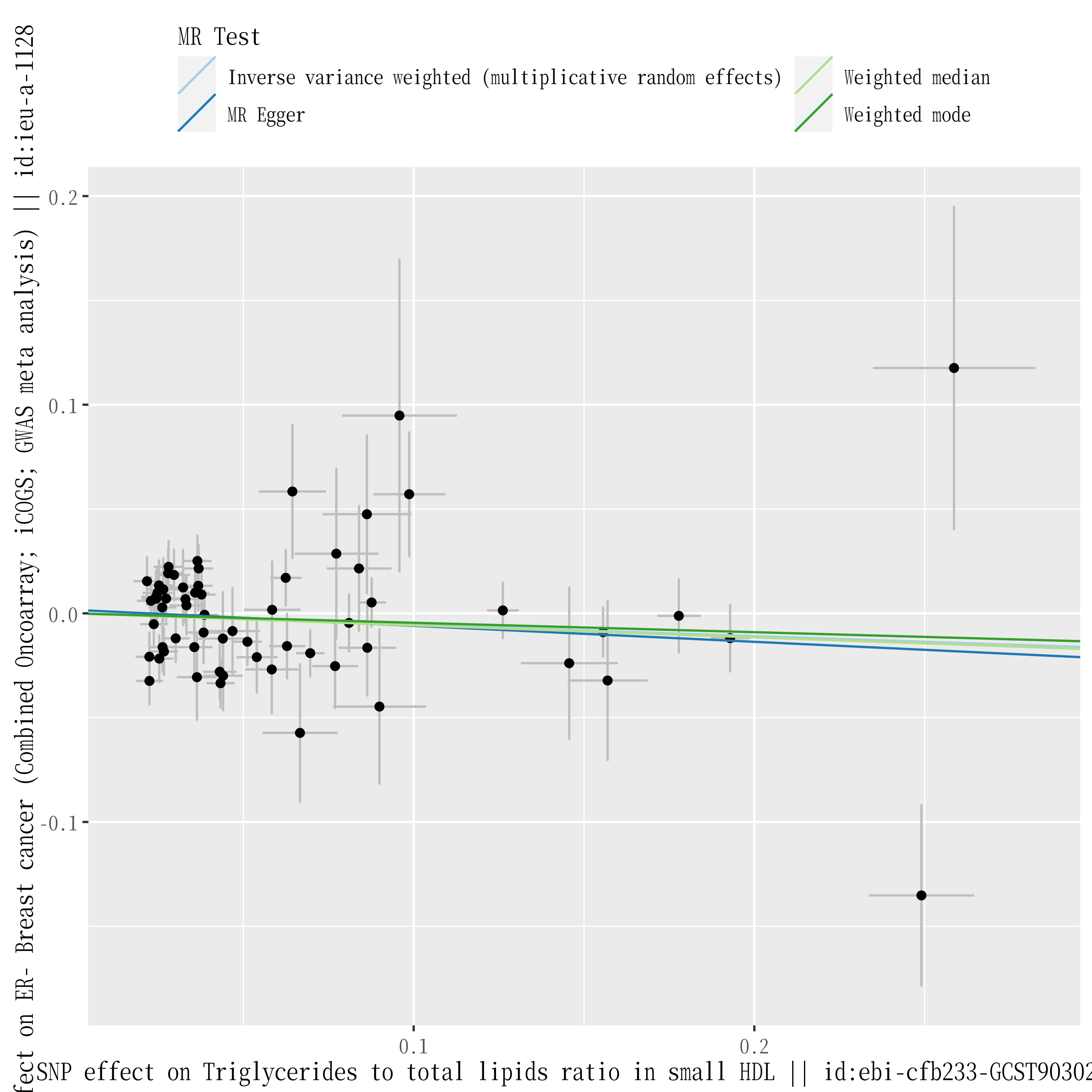
MR Test
Inverse variance weighted (multiplicative random effects)
MR Egger
Weighted median
Weighted mode
0.2
0.1
0.0
-0.1
0.1
0.2
SNP effect on Triglycerides to total lipids ratio in small HDL || id:ebi-cfb233-GCST90302
fect on ER- Breast cancer (Combined Oncoarray; iCOGS; GWAS meta analysis) || id:ieu-a-1128

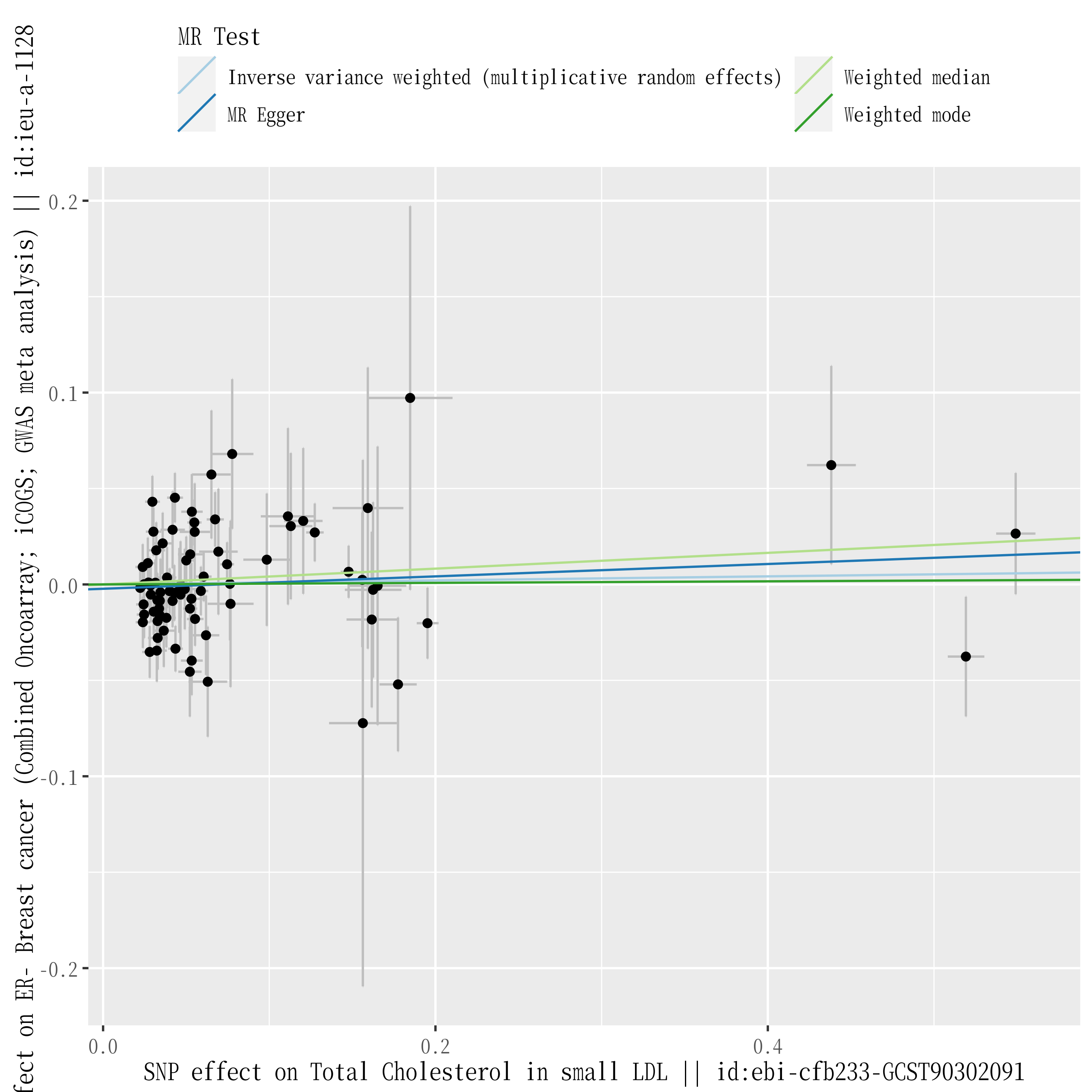
MR Test
Inverse variance weighted (multiplicative random effects)
MR Egger
Weighted median
Weighted mode
SNP effect on ER- Breast cancer (Combined Oncoarray; iCOGS; GWAS meta analysis) || id:ieu-a-1128
0.2
0.1
0.0
-0.1
-0.2
0.0
0.2
0.4
SNP effect on Total Cholesterol in small LDL || id:ebi-cfb233-GCST90302091

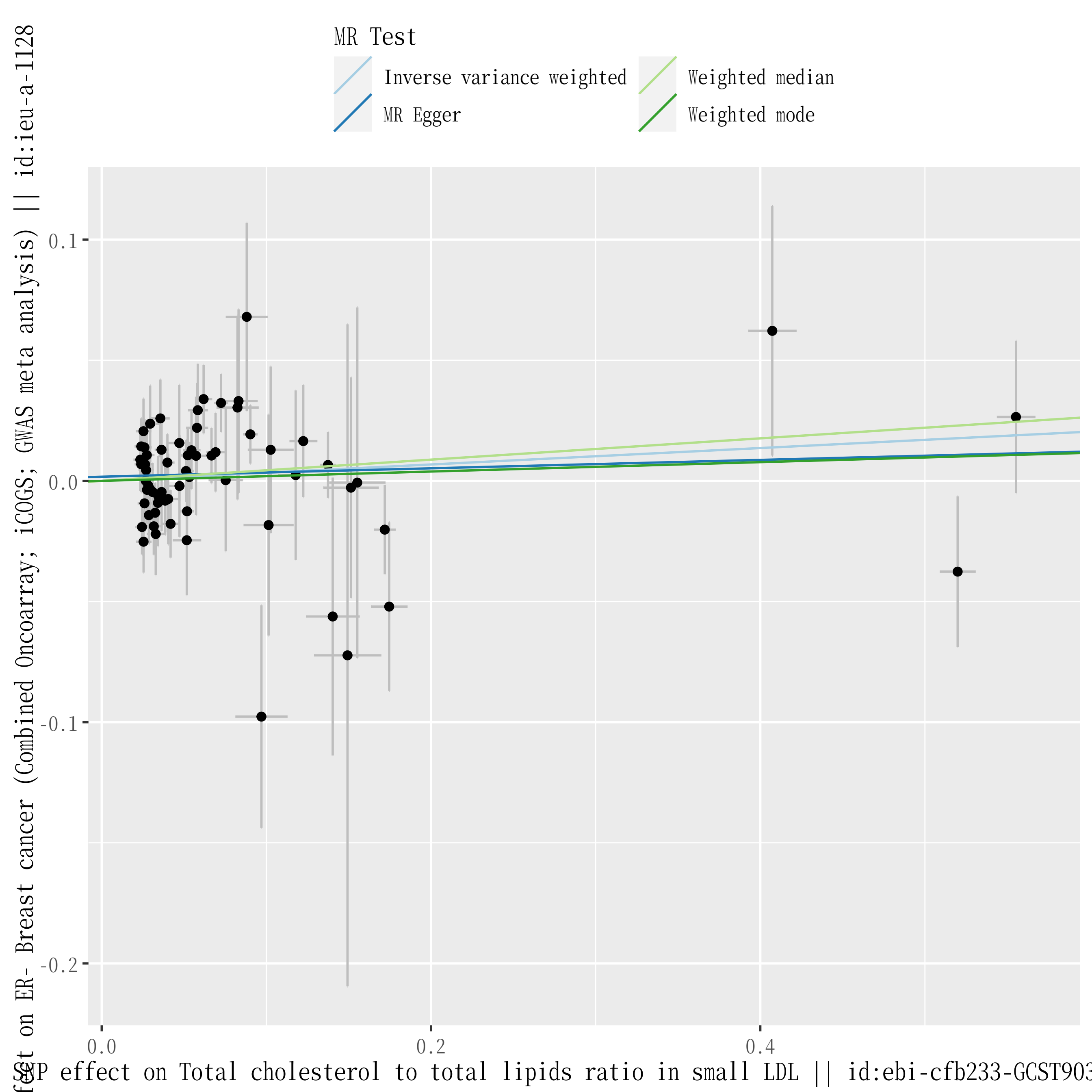

MR Test
Inverse variance weighted
MR Egger
Weighted median
Weighted mode
0.1
0.0
-0.1
-0.2
0.0
0.2
0.4
SNP effect on Total cholesterol to total lipids ratio in small LDL || id:ebi-cfb233-GCST903
SNP effect on ER- Breast cancer (Combined Oncoarray; iCOGS; GWAS meta analysis) || id:ieu-a-1128

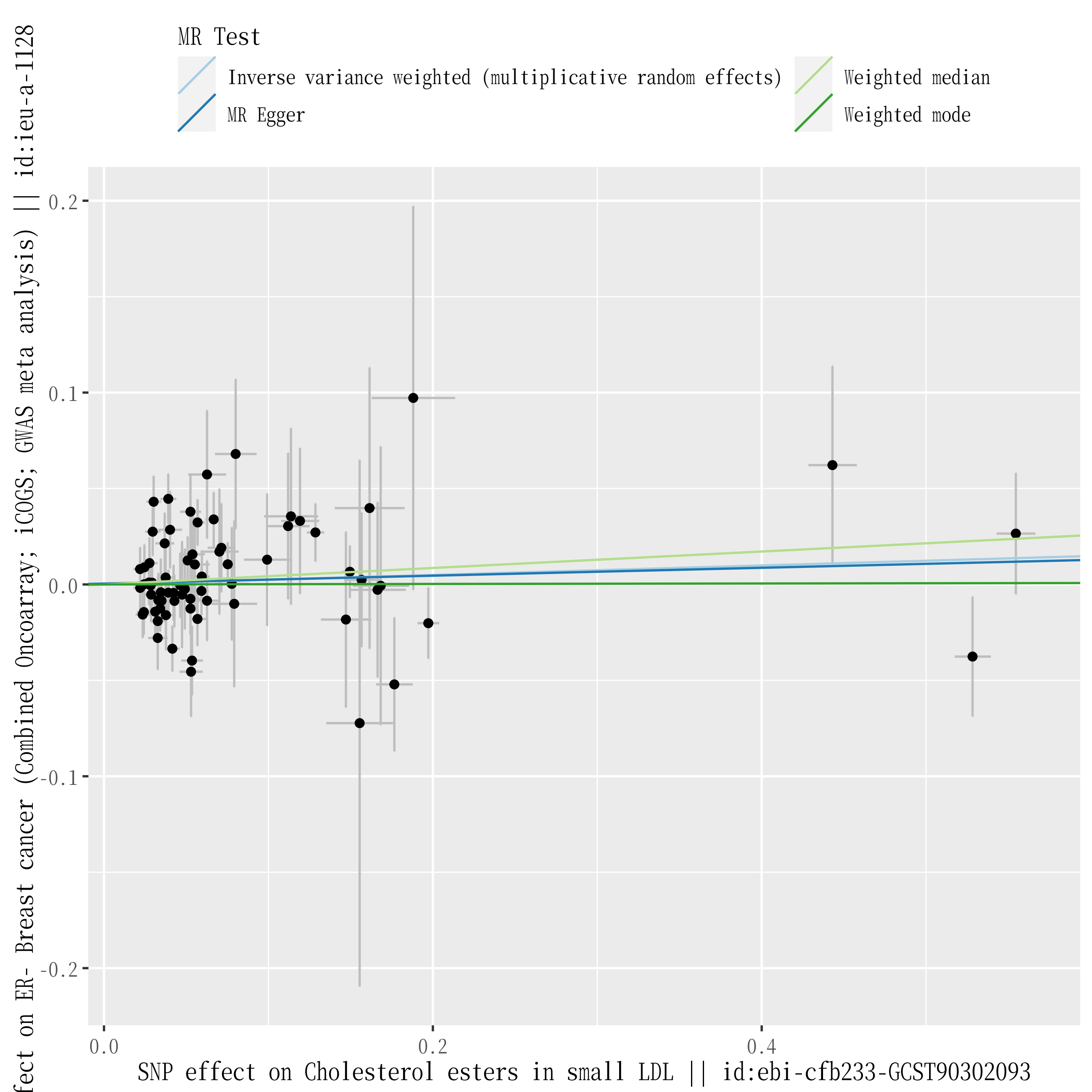

MR Test
Inverse variance weighted (multiplicative random effects)
MR Egger
Weighted median
Weighted mode
0.2
0.1
0.0
-0.1
-0.2
0.0
0.2
0.4
SNP effect on Cholesterol esters in small LDL || id:ebi-cfb233-GCST90302093
fect on ER- Breast cancer (Combined Oncoarray; iCOGS; GWAS meta analysis) || id:ieu-a-1128

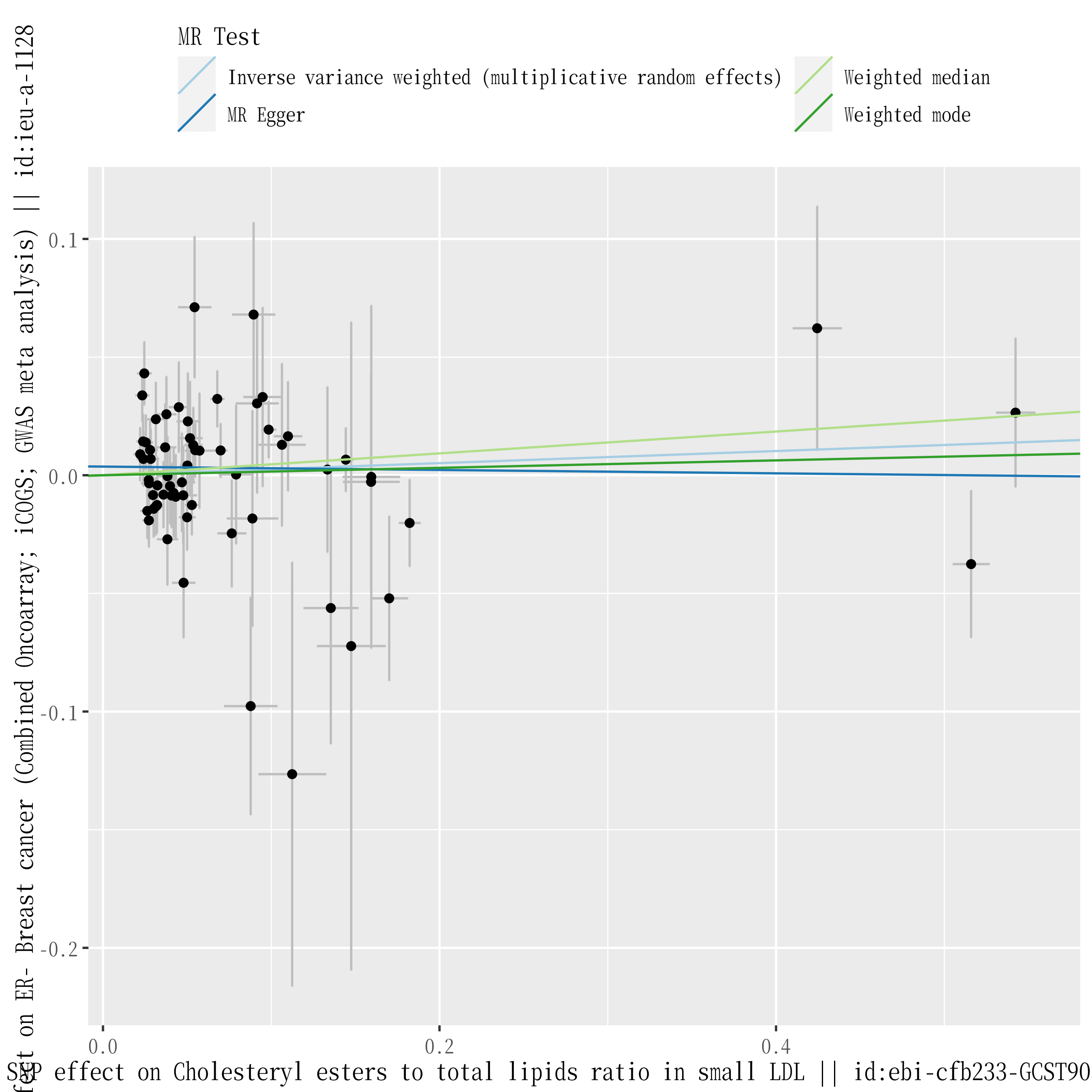
MR Test
Inverse variance weighted (multiplicative random effects)
MR Egger
Weighted median
Weighted mode
0.1
0.0
-0.1
-0.2
0.0
0.2
0.4
SNP effect on Cholesteryl esters to total lipids ratio in small LDL || id:ebi-cfb233-GCST90
Breast cancer (Combined Oncoarray; iCOGS; GWAS meta analysis) || id:ieu-a-1128

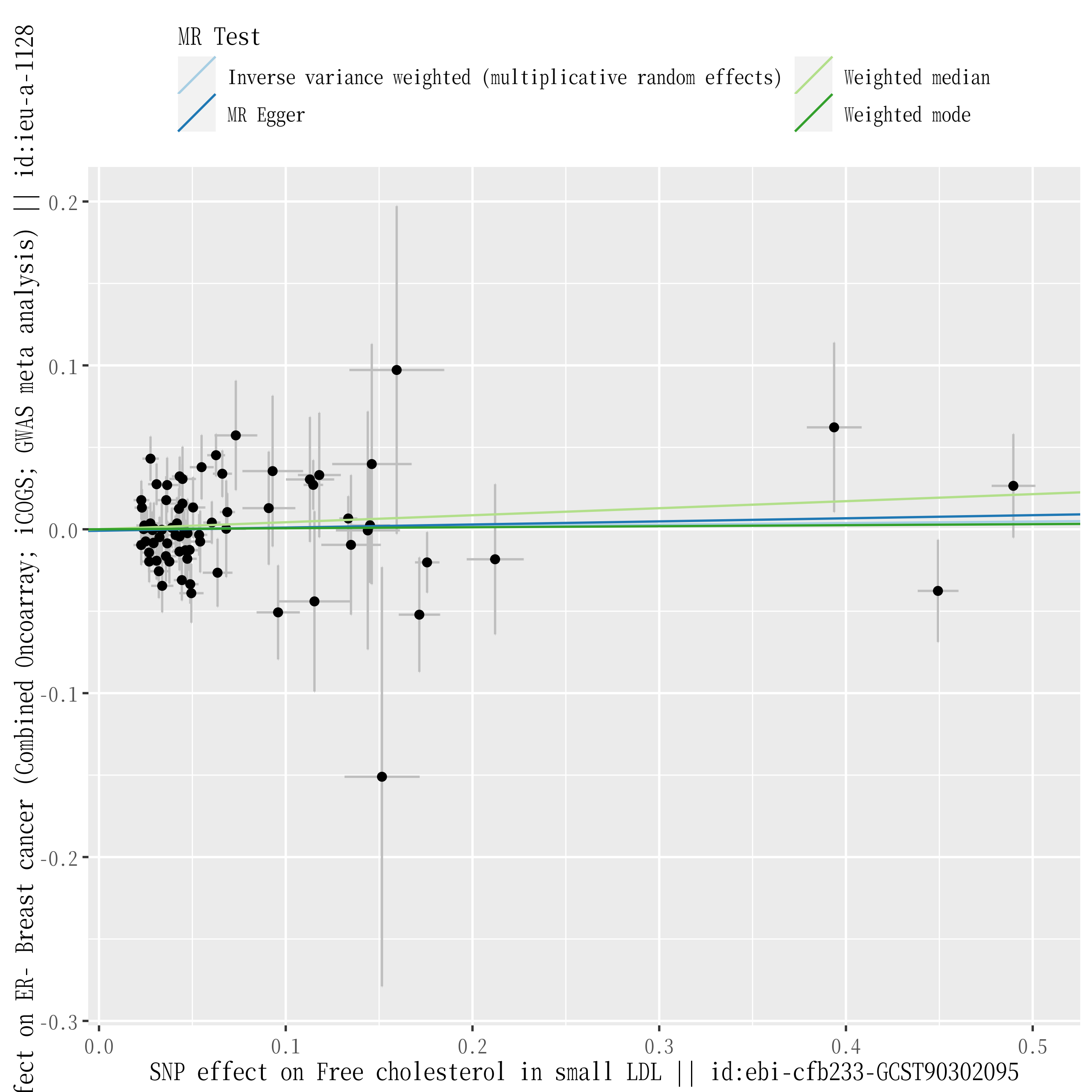

MR Test
Inverse variance weighted (multiplicative random effects)
MR Egger
Weighted median
Weighted mode
0.2
0.1
0.0
-0.1
-0.2
-0.3
0.0
0.1
0.2
0.3
0.4
0.5
SNP effect on Free cholesterol in small LDL || id:ebi-cfb233-GCST90302095
fect on ER- Breast cancer (Combined Oncoarray; iCOGS; GWAS meta analysis) || id:ieu-a-1128

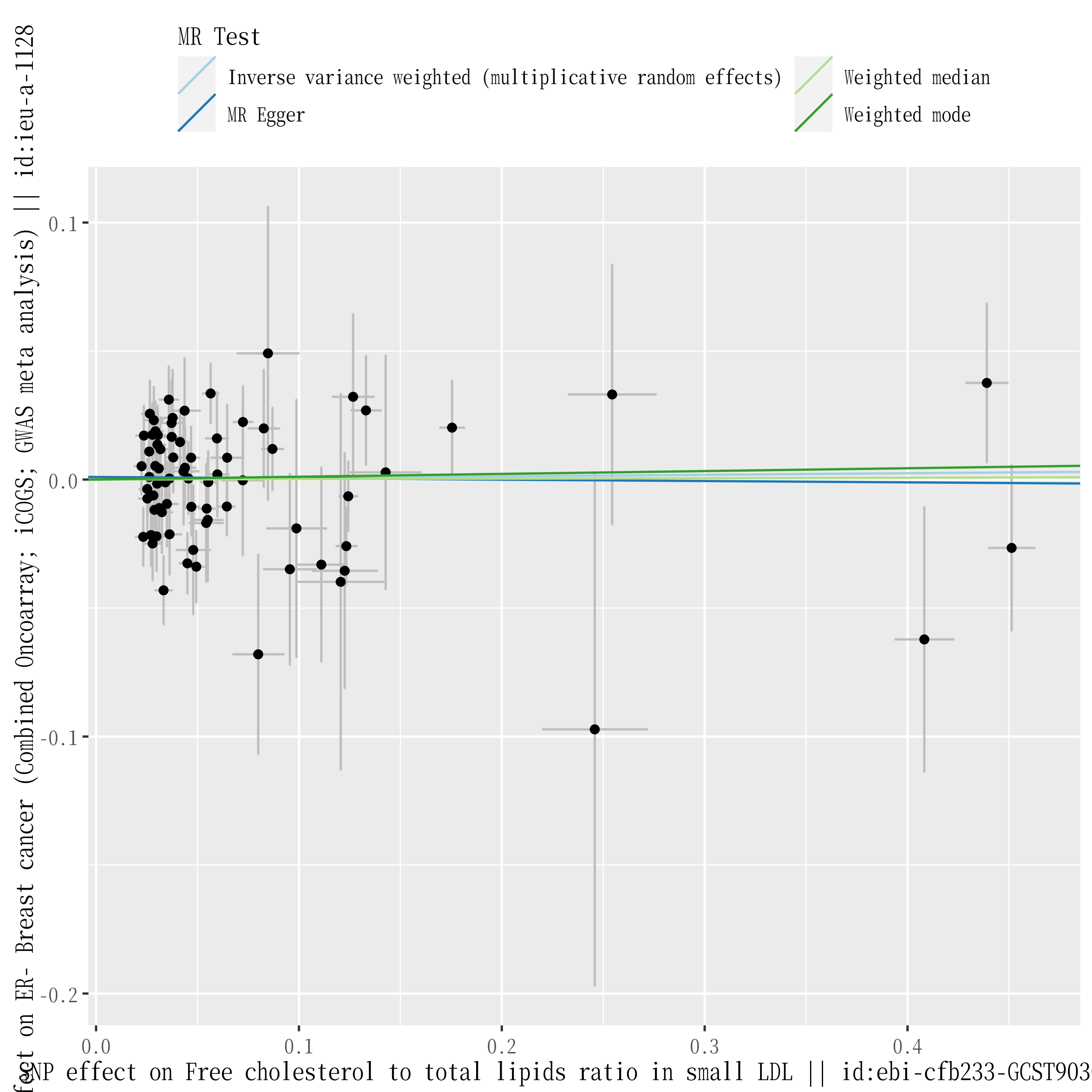
MR Test
Inverse variance weighted (multiplicative random effects)
MR Egger
Weighted median
Weighted mode
0.1
0.0
-0.1
-0.2
0.0
0.1
0.2
0.3
0.4
SNP effect on Free cholesterol to total lipids ratio in small LDL || id:ebi-cfb233-GCST903
SNP effect on ER- Breast cancer (Combined Oncoarray; iCOGS; GWAS meta analysis) || id:ieu-a-1128

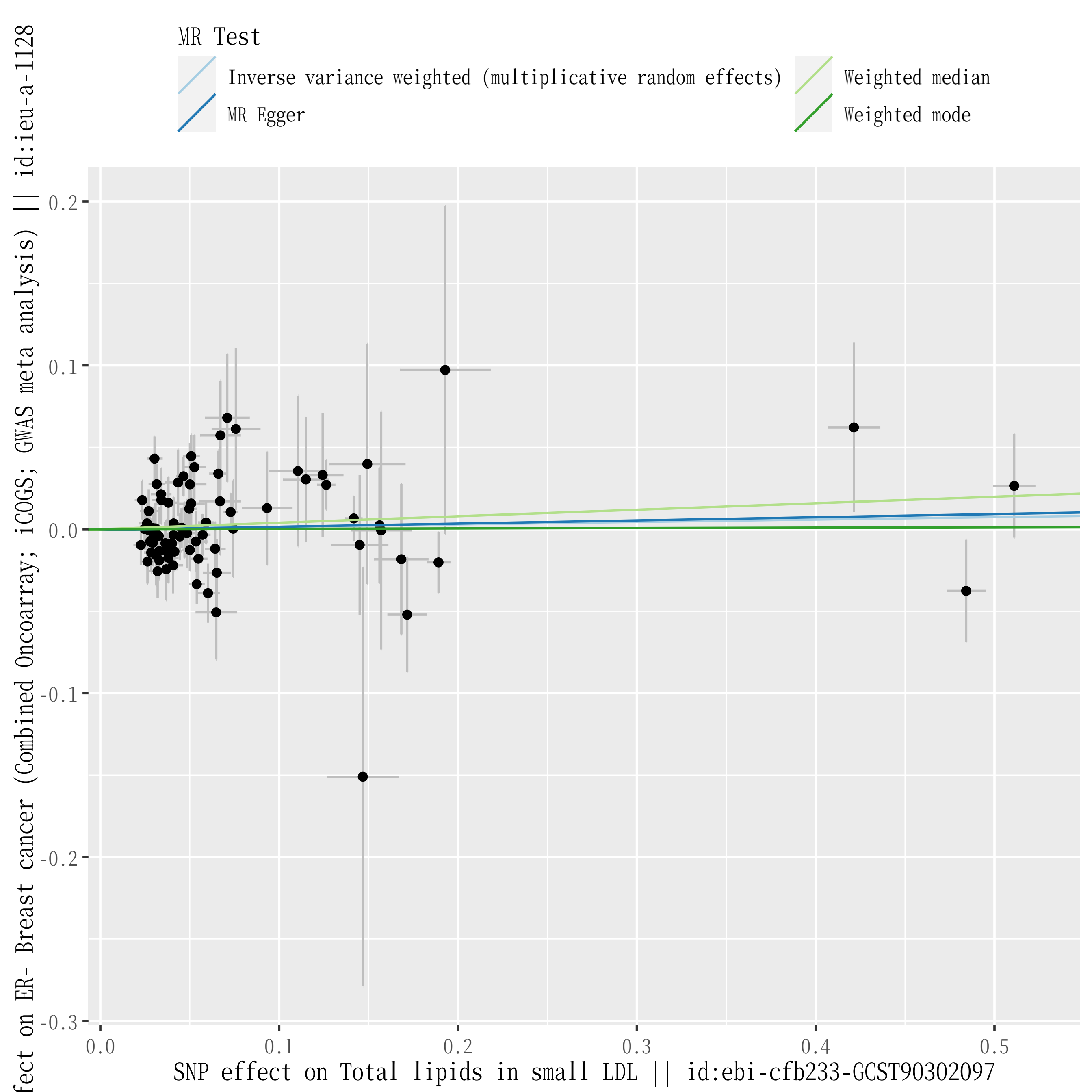
MR Test
Inverse variance weighted (multiplicative random effects)
MR Egger
Weighted median
Weighted mode
0.2
0.1
0.0
-0.1
-0.2
-0.3
0.0
0.1
0.2
0.3
0.4
0.5
SNP effect on Total lipids in small LDL || id:ebi-cfb233-GCST90302097
fect on ER- Breast cancer (Combined Oncoarray; iCOGS; GWAS meta analysis) || id:ieu-a-1128

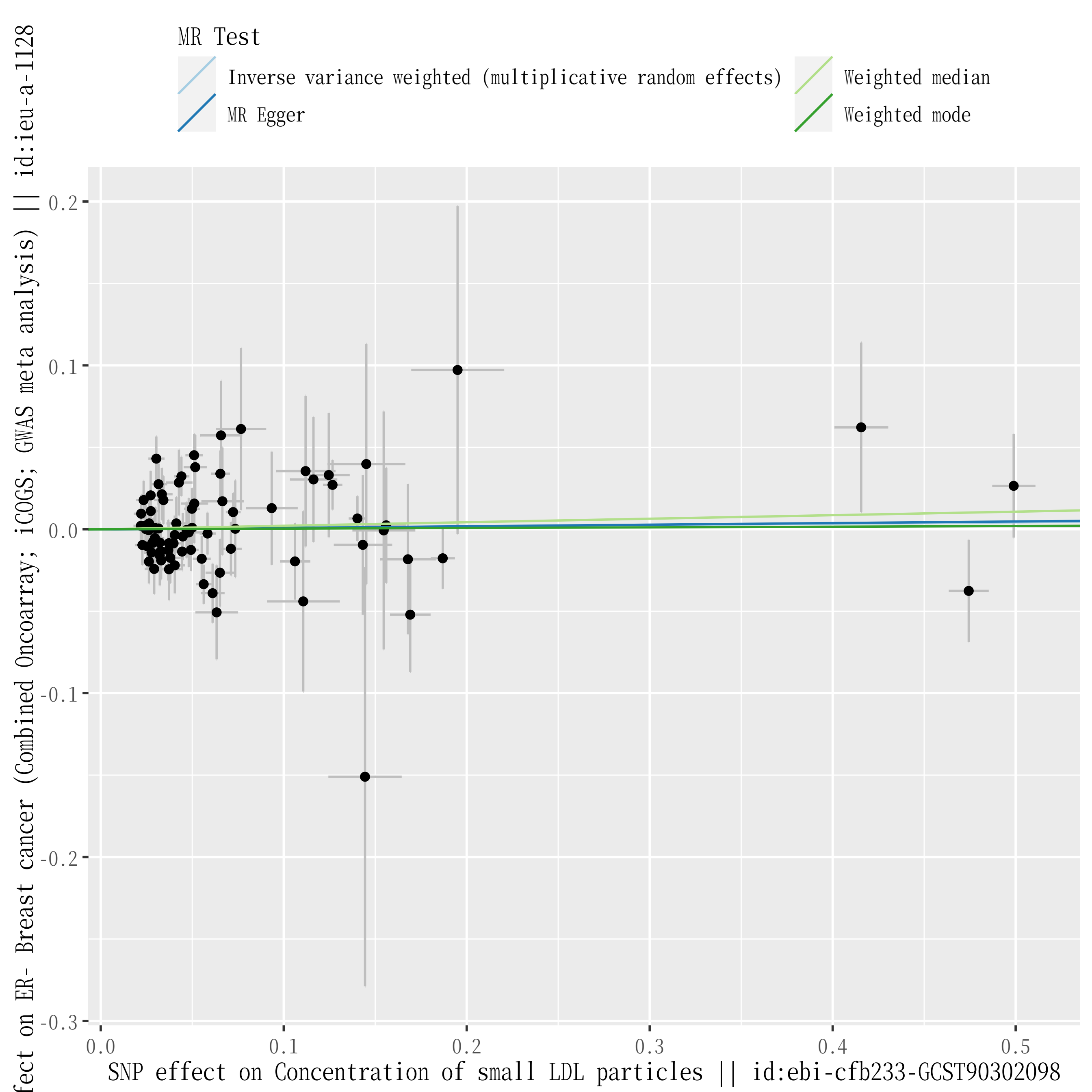
MR Test
Inverse variance weighted (multiplicative random effects)
MR Egger
Weighted median
Weighted mode
fect on ER- Breast cancer (Combined Oncoarray; iCOGS; GWAS meta analysis) || id:ieu-a-1128
SNP effect on Concentration of small LDL particles || id:ebi-cfb233-GCST90302098
0.2
0.1
0.0
-0.1
-0.2
-0.3
0.0
0.1
0.2
0.3
0.4
0.5

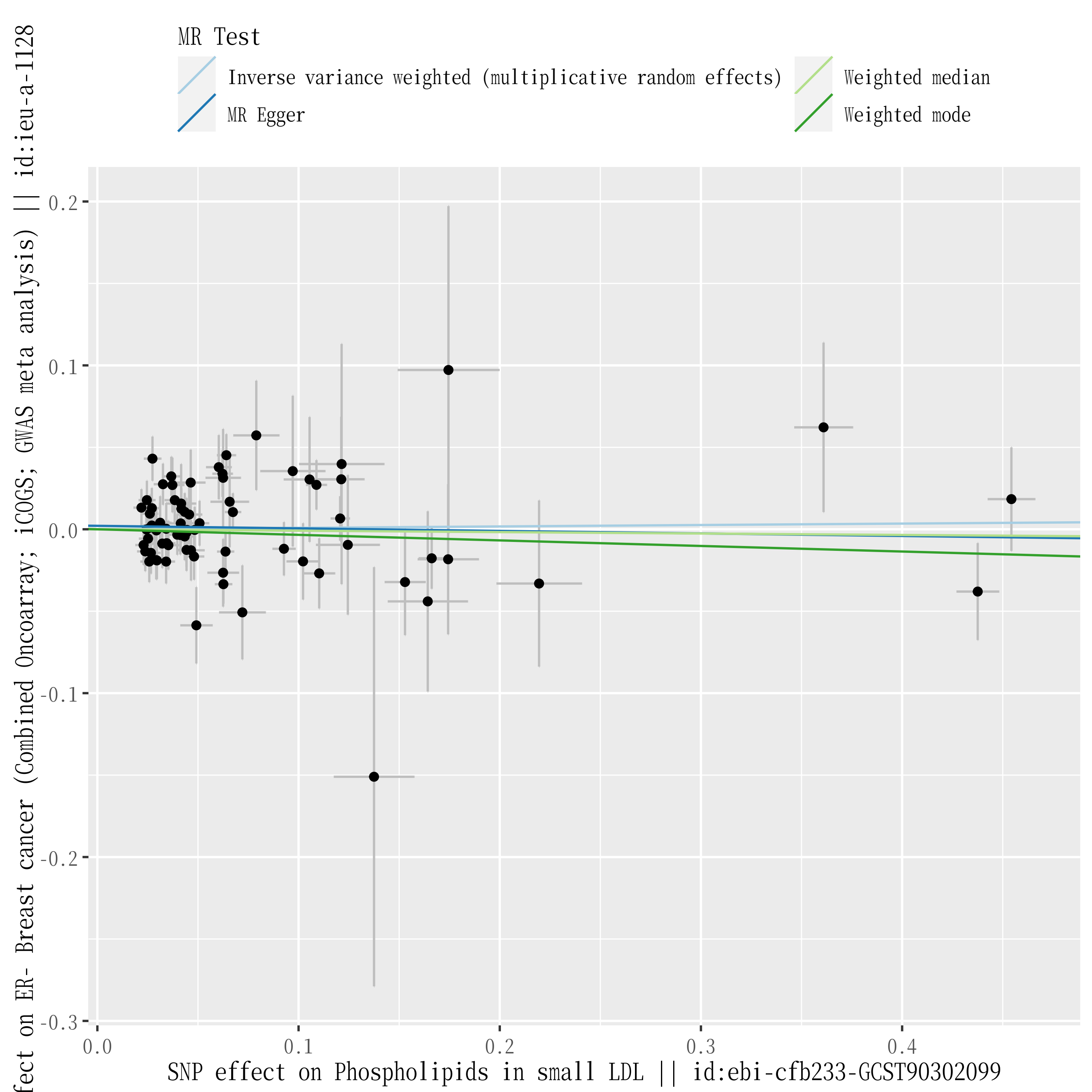
MR Test
Inverse variance weighted (multiplicative random effects)
MR Egger
Weighted median
Weighted mode
SNP effect on ER- Breast cancer (Combined Oncoarray; iCOGS; GWAS meta analysis) || id:ieu-a-1128
SNP effect on Phospholipids in small LDL || id:ebi-cfb233-GCST90302099
0.2
0.1
0.0
-0.1
-0.2
-0.3
0.0
0.1
0.2
0.3
0.4

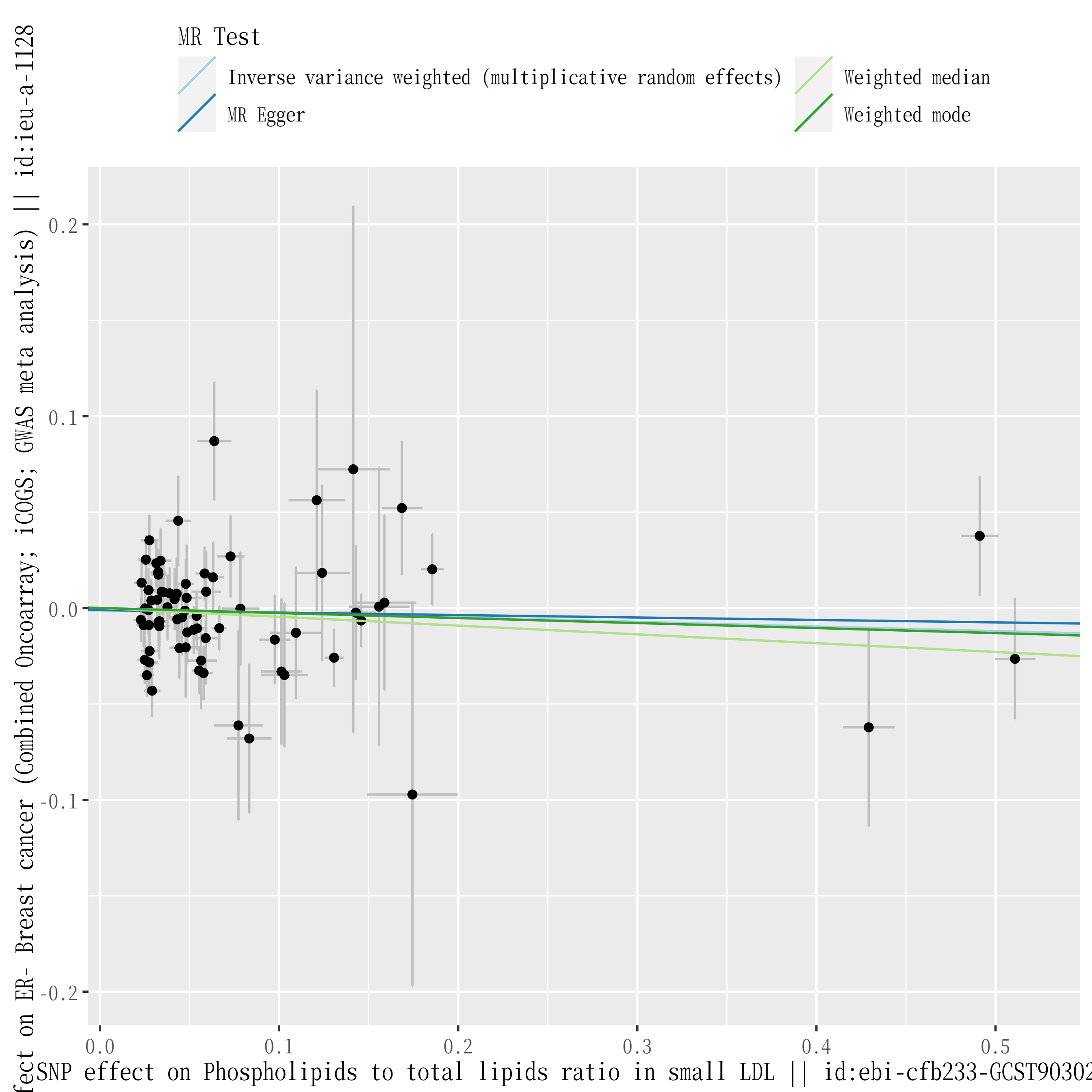
MR Test
Inverse variance weighted (multiplicative random effects)
MR Egger
Weighted median
Weighted mode
0.2
0.1
0.0
-0.1
-0.2
0.0
0.1
0.2
0.3
0.4
0.5
SNP effect on Phospholipids to total lipids ratio in small LDL || id:ebi-cfb233-GCST90302
fect on ER- Breast cancer (Combined Oncoarray; iCOGS; GWAS meta analysis) || id:ieu-a-1128

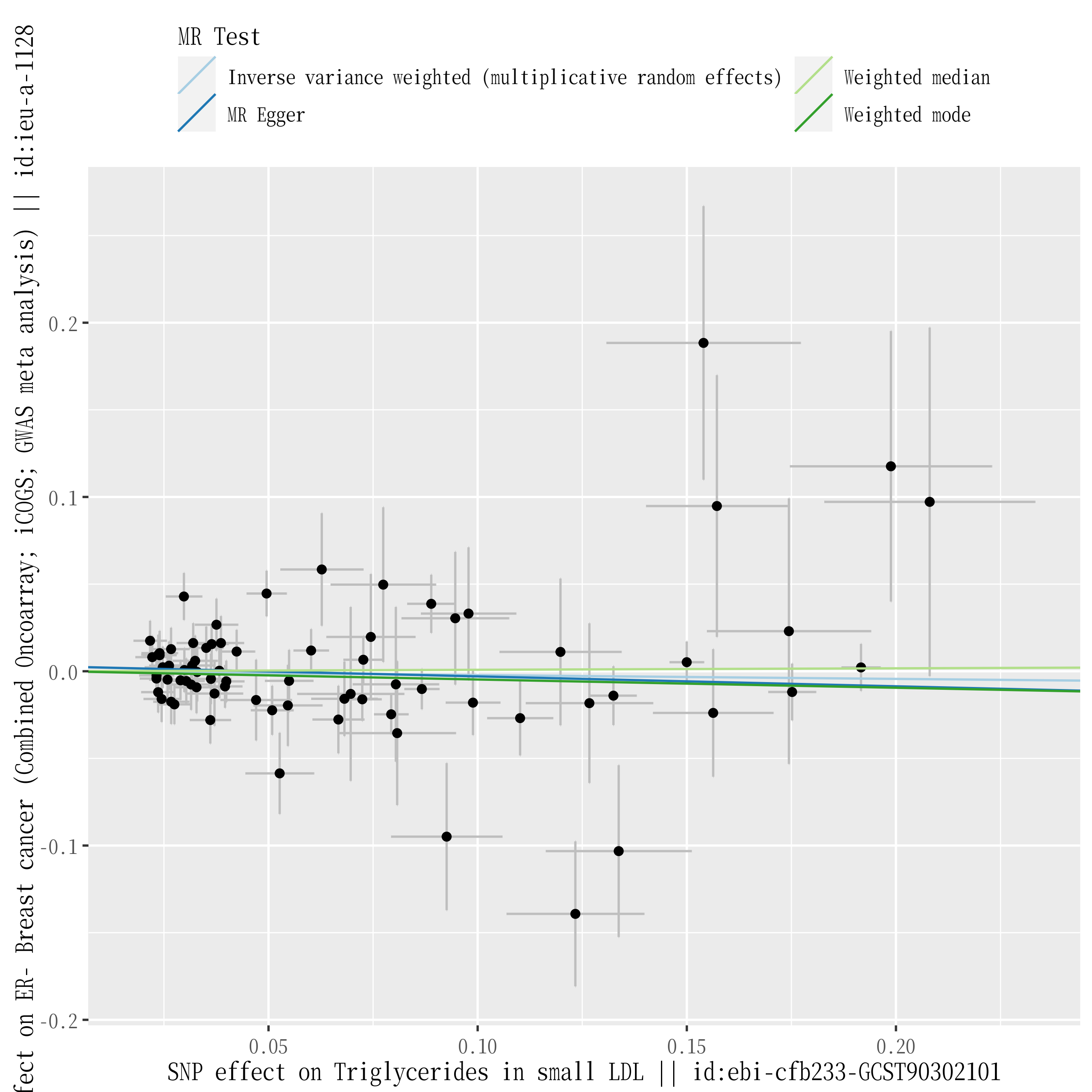
MR Test
Inverse variance weighted (multiplicative random effects)
MR Egger
Weighted median
Weighted mode
SNP effect on ER- Breast cancer (Combined Oncoarray; iCOGS; GWAS meta analysis) || id:ieu-a-1128
0.2
0.1
0.0
-0.1
-0.2
0.05
0.10
0.15
0.20
SNP effect on Triglycerides in small LDL || id:ebi-cfb233-GCST90302101

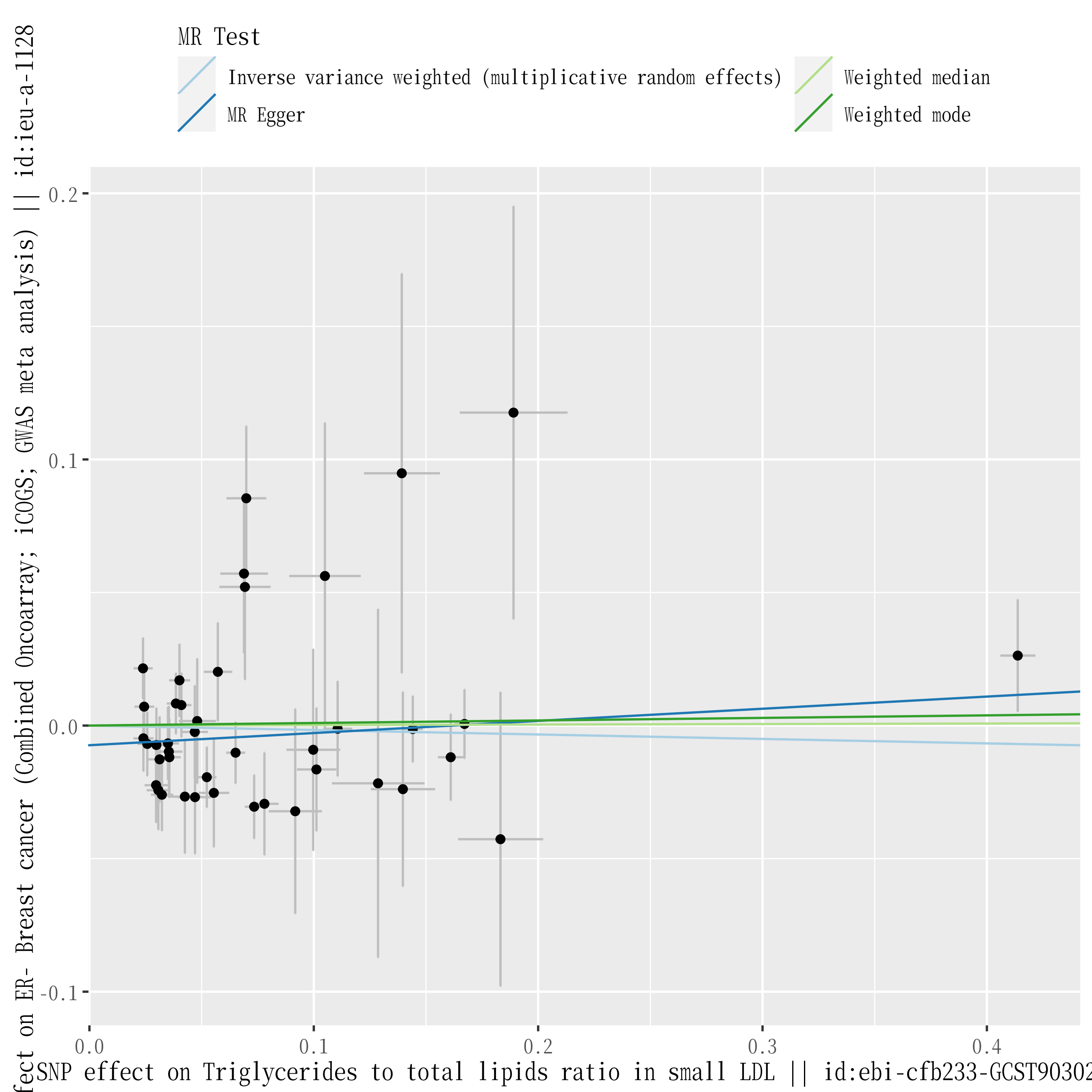

MR Test
Inverse variance weighted (multiplicative random effects)
MR Egger
Weighted median
Weighted mode
0.2
0.1
0.0
-0.1
0.0
0.1
0.2
0.3
0.4
SNP effect on Triglycerides to total lipids ratio in small LDL || id:ebi-cfb233-GCST90302
fect on ER- Breast cancer (Combined Oncoarray; iCOGS; GWAS meta analysis) || id:ieu-a-1128

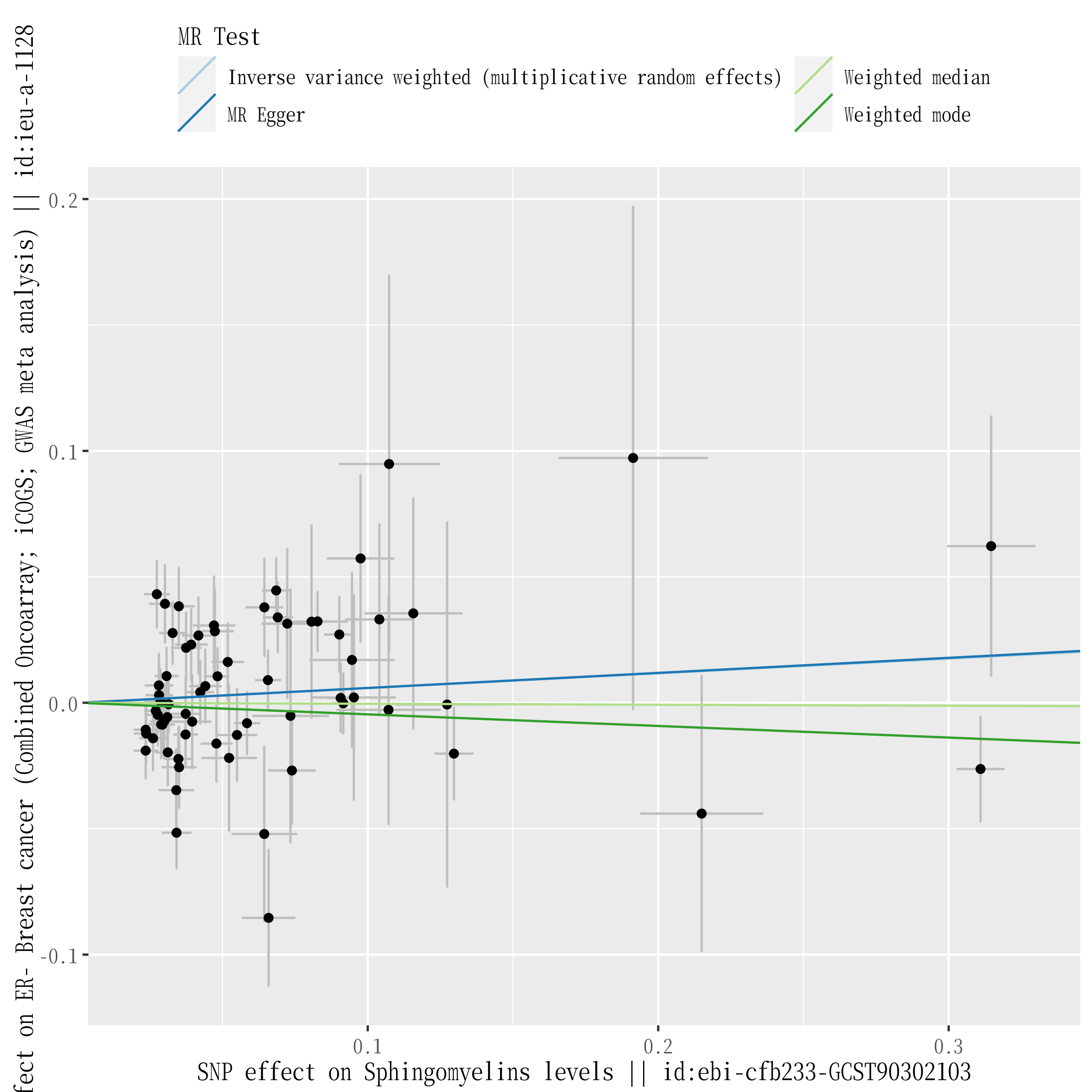
MR Test
Inverse variance weighted (multiplicative random effects)
MR Egger
Weighted median
Weighted mode
0.2
0.1
0.0
-0.1
0.1
0.2
0.3
SNP effect on Sphingomyelins levels || id:ebi-cfb233-GCST90302103
fect on ER- Breast cancer (Combined Oncoarray; iCOGS; GWAS meta analysis) || id:ieu-a-1128

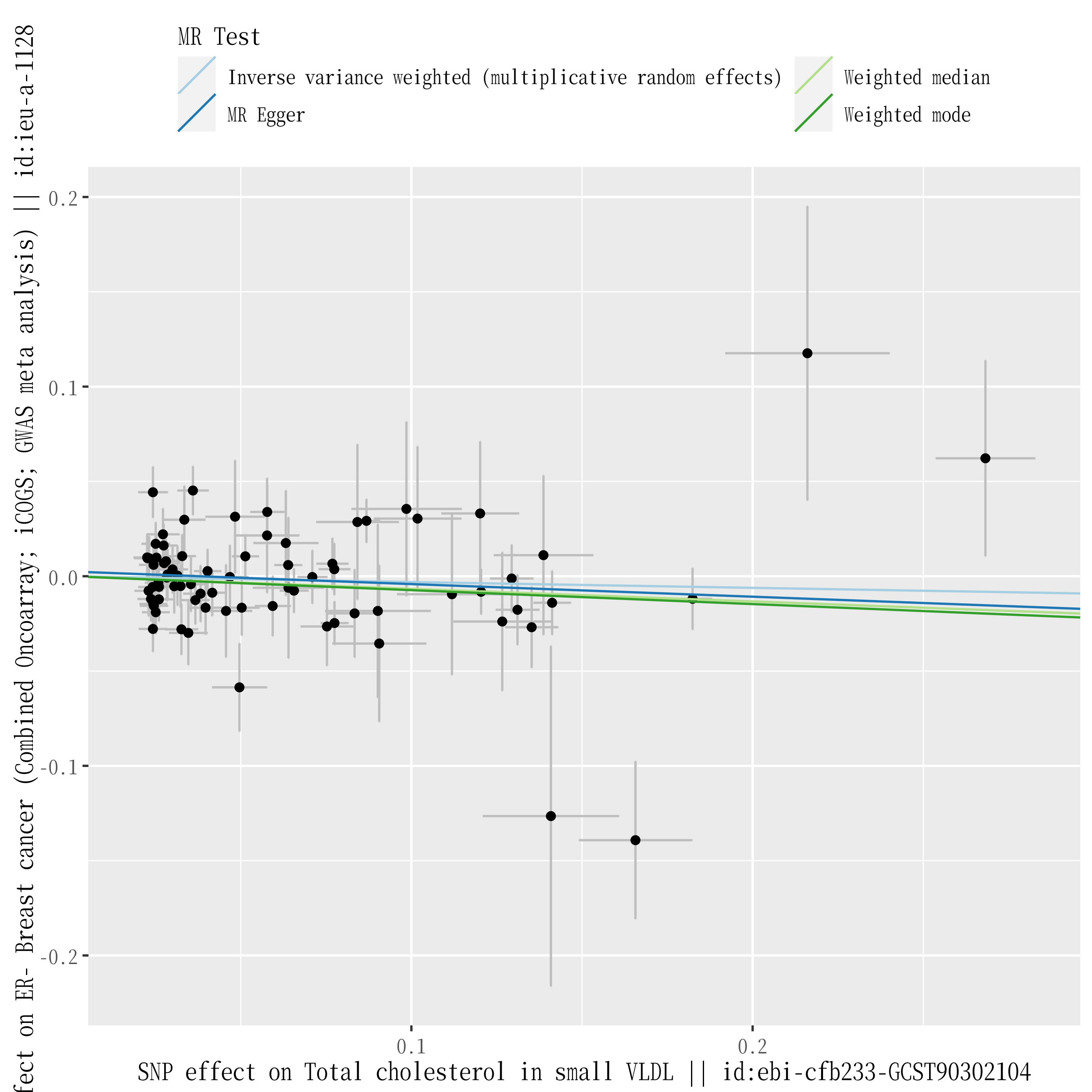

MR Test
Inverse variance weighted (multiplicative random effects)
MR Egger
Weighted median
Weighted mode
0.2
0.1
0.0
-0.1
-0.2
0.1
0.2
SNP effect on Total cholesterol in small VLDL || id:ebi-cfb233-GCST90302104
fect on ER- Breast cancer (Combined Oncoarray; iCOGS; GWAS meta analysis) || id:ieu-a-1128

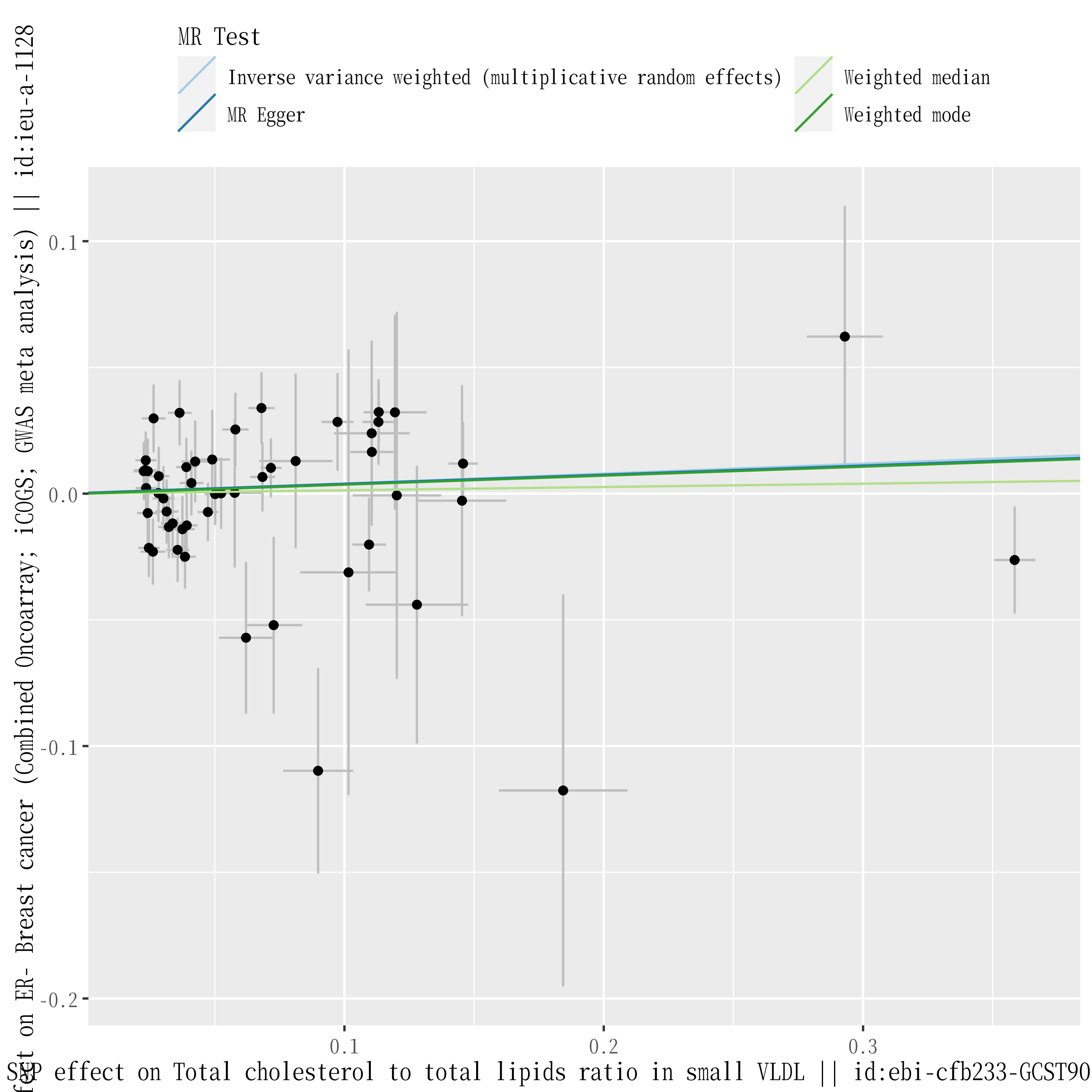

MR Test
Inverse variance weighted (multiplicative random effects)
MR Egger
Weighted median
Weighted mode
0.1
0.0
-0.1
-0.2
0.1
0.2
0.3
SNP effect on Total cholesterol to total lipids ratio in small VLDL || id:ebi-cfb233-GCST90
fect on ER- Breast cancer (Combined Oncoarray; iCOGS; GWAS meta analysis) || id:ieu-a-1128

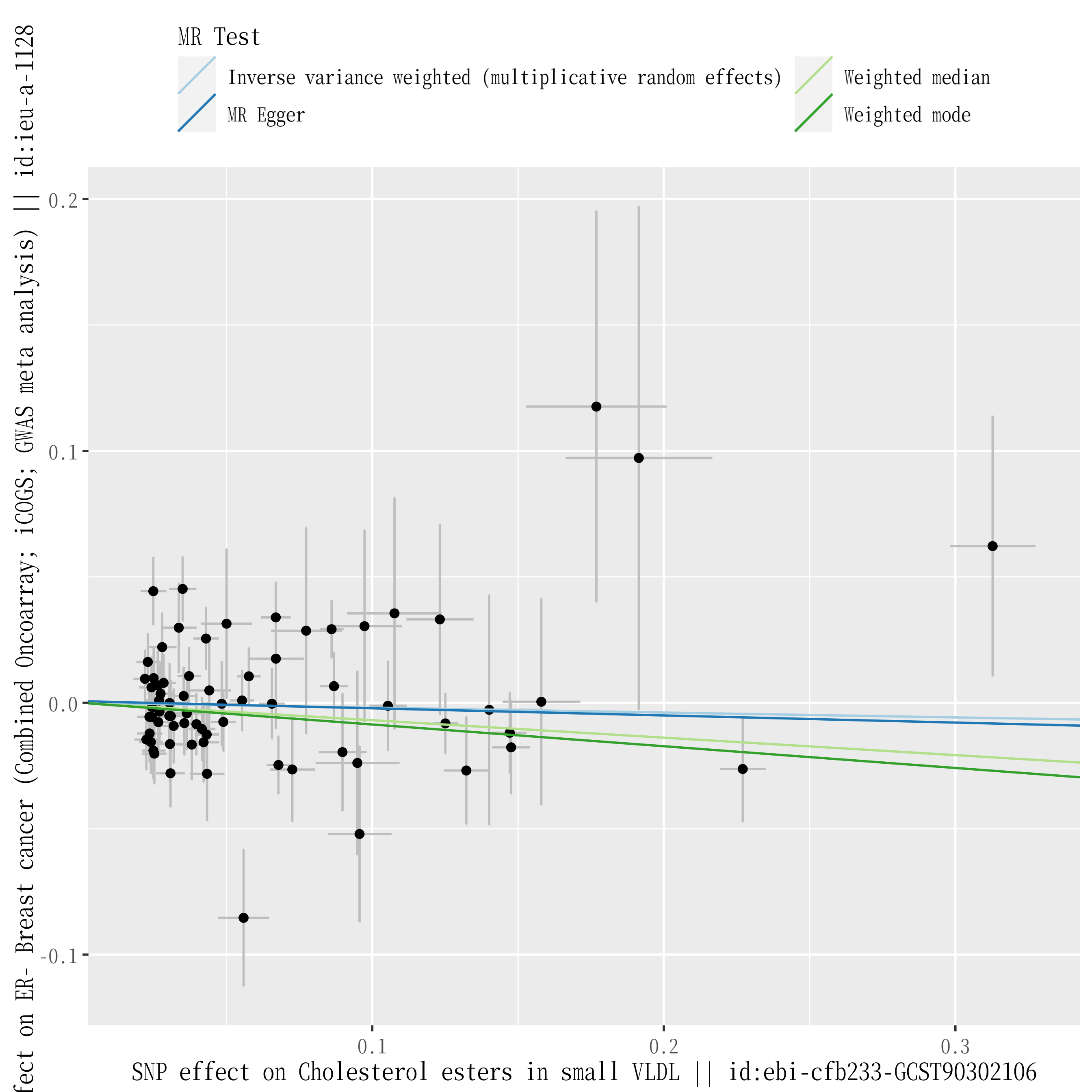

MR Test
Inverse variance weighted (multiplicative random effects)
MR Egger
Weighted median
Weighted mode
0.2
0.1
0.0
-0.1
0.1
0.2
0.3
SNP effect on Cholesterol esters in small VLDL || id:ebi-cfb233-GCST90302106
fect on ER- Breast cancer (Combined Oncoarray; iCOGS; GWAS meta analysis) || id:ieu-a-1128

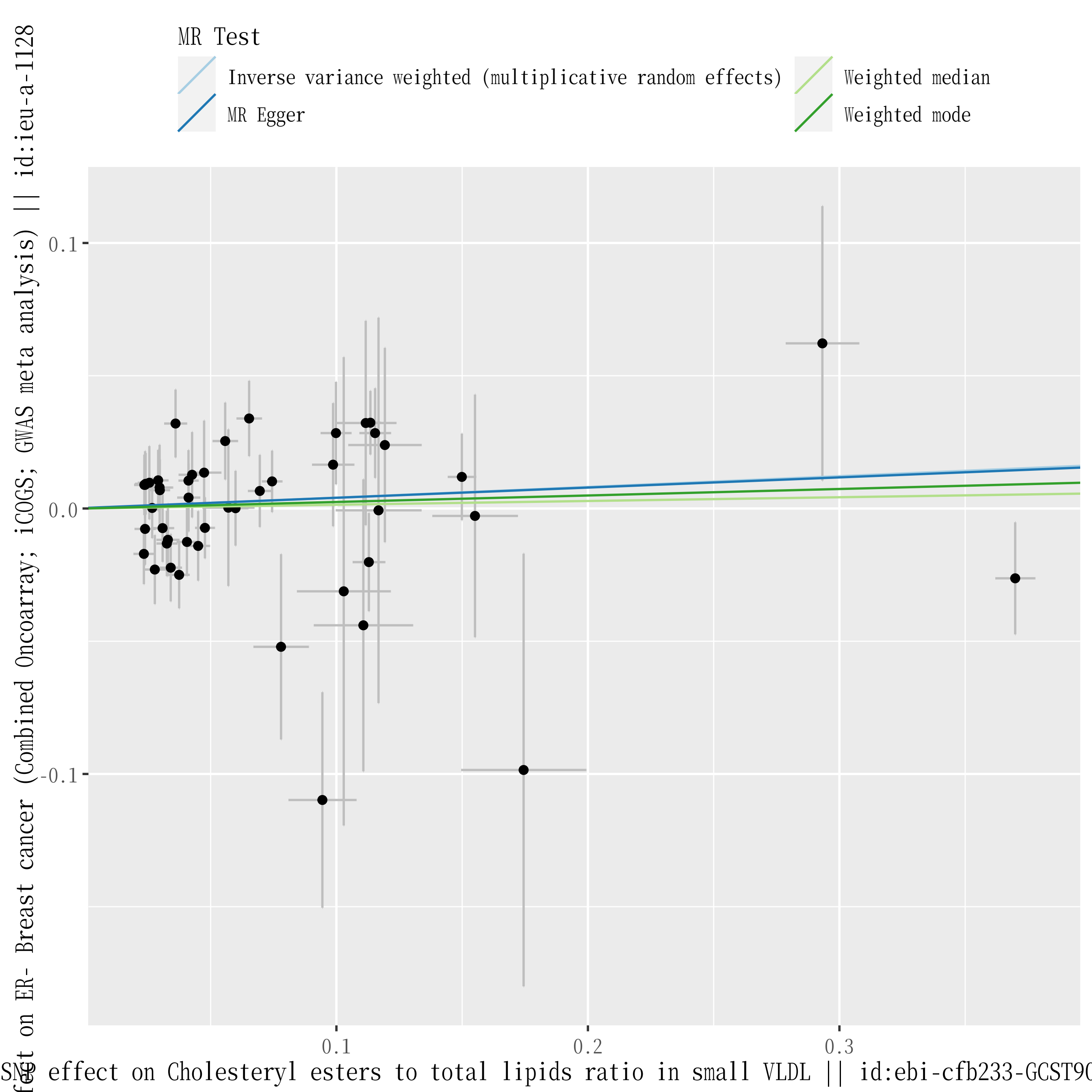
MR Test
Inverse variance weighted (multiplicative random effects)
MR Egger
Weighted median
Weighted mode
0.1
0.0
-0.1
0.1
0.2
0.3
SNP effect on Cholesteryl esters to total lipids ratio in small VLDL || id:ebi-cfb233-GCST90
SNP effect on ER- Breast cancer (Combined Oncoarray; iCOGS; GWAS meta analysis) || id:ieu-a-1128

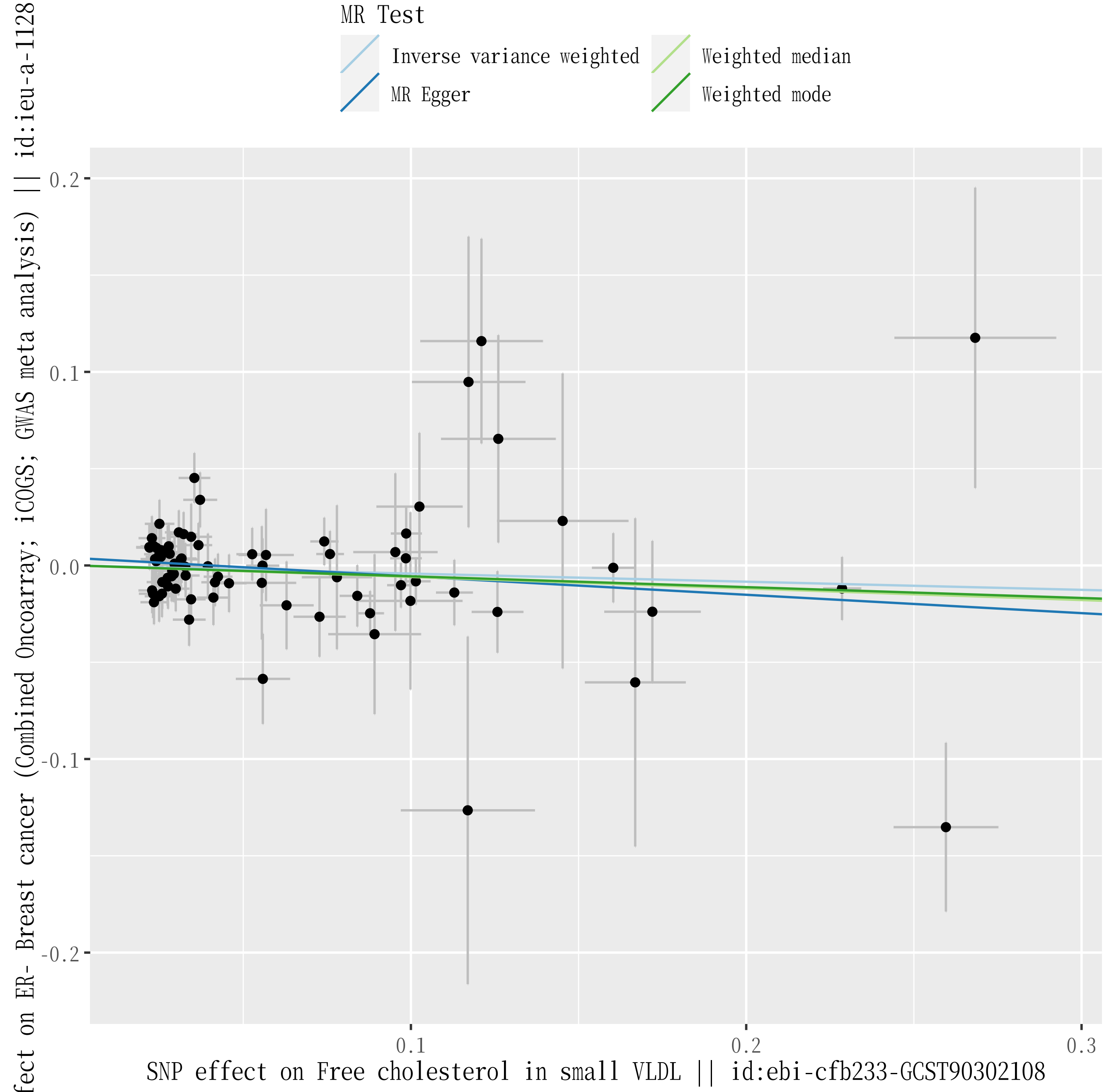

MR Test
Inverse variance weighted
MR Egger
Weighted median
Weighted mode
0.2
0.1
0.0
-0.1
-0.2
0.1
0.2
0.3
fect on ER- Breast cancer (Combined Oncoarray; iCOGS; GWAS meta analysis) || id:ieu-a-1128
SNP effect on Free cholesterol in small VLDL || id:ebi-cfb233-GCST90302108

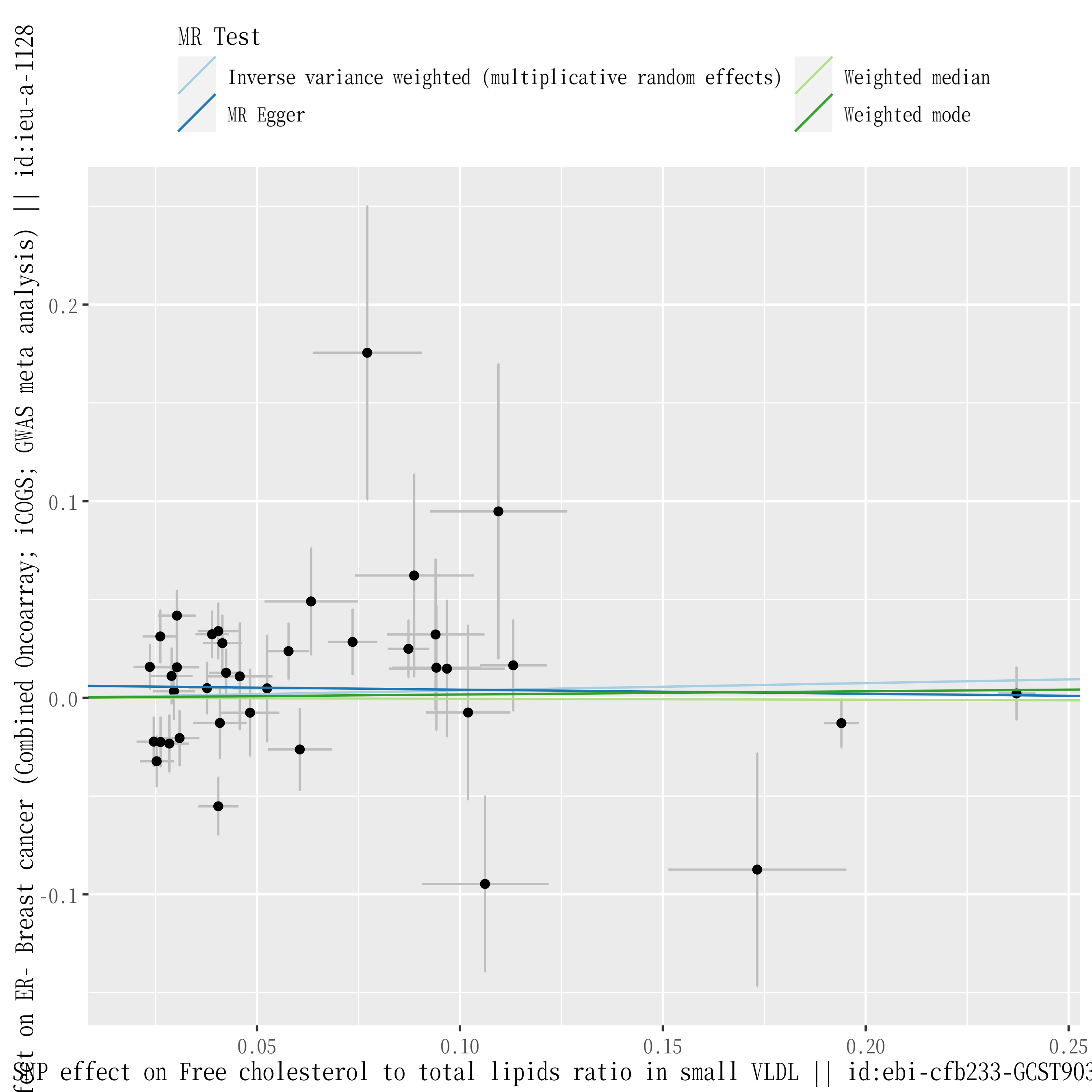

MR Test
Inverse variance weighted (multiplicative random effects)
MR Egger
Weighted median
Weighted mode
0.2
0.1
0.0
-0.1
0.05
0.10
0.15
0.20
0.25
SNP effect on Free cholesterol to total lipids ratio in small VLDL || id:ebi-cfb233-GCST903
fect on ER- Breast cancer (Combined Oncoarray; iCOGS; GWAS meta analysis) || id:ieu-a-1128

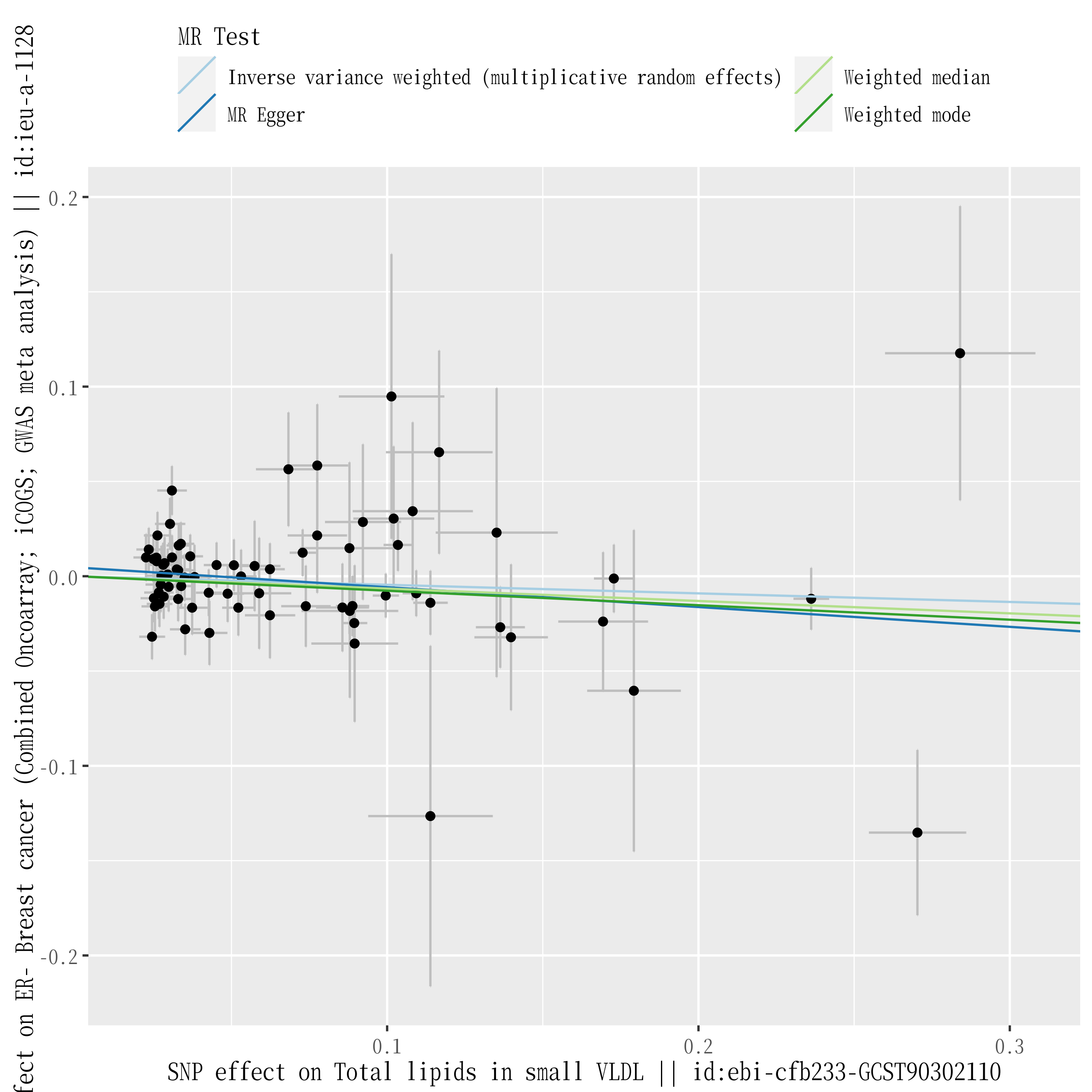
MR Test
Inverse variance weighted (multiplicative random effects)
MR Egger
Weighted median
Weighted mode
0.2
0.1
0.0
-0.1
-0.2
0.1
0.2
0.3
SNP effect on Total lipids in small VLDL || id:ebi-cfb233-GCST90302110
fect on ER- Breast cancer (Combined Oncoarray; iCOGS; GWAS meta analysis) || id:ieu-a-1128

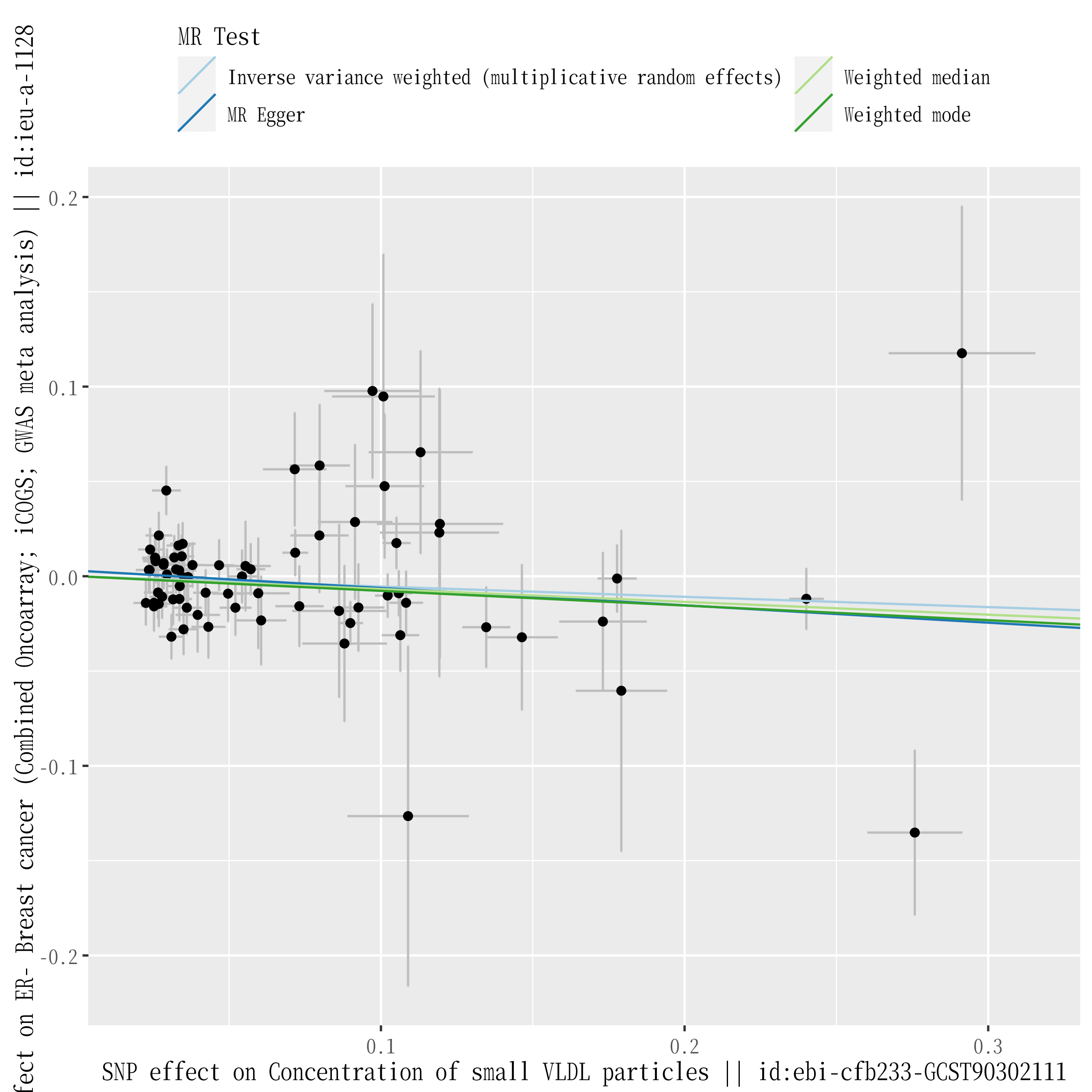

MR Test
Inverse variance weighted (multiplicative random effects)
MR Egger
Weighted median
Weighted mode
0.2
0.1
0.0
-0.1
-0.2
0.1
0.2
0.3
SNP effect on Concentration of small VLDL particles || id:ebi-cfb233-GCST90302111
fect on ER- Breast cancer (Combined Oncoarray; iCOGS; GWAS meta analysis) || id:ieu-a-1128

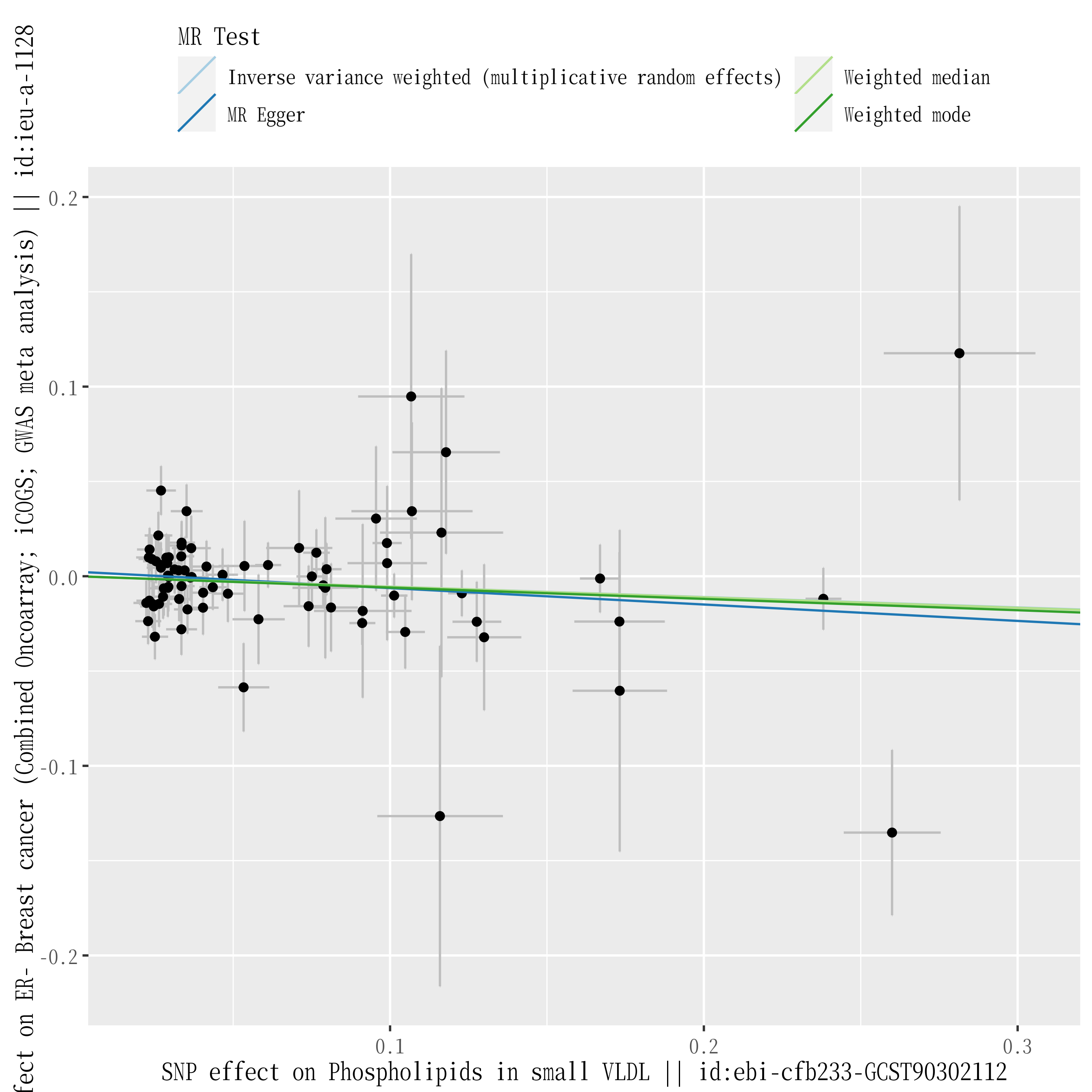

MR Test
Inverse variance weighted (multiplicative random effects)
MR Egger
Weighted median
Weighted mode
0.2
0.1
0.0
-0.1
-0.2
0.1
0.2
0.3
SNP effect on Phospholipids in small VLDL || id:ebi-cfb233-GCST90302112
fect on ER- Breast cancer (Combined Oncoarray; iCOGS; GWAS meta analysis) || id:ieu-a-1128

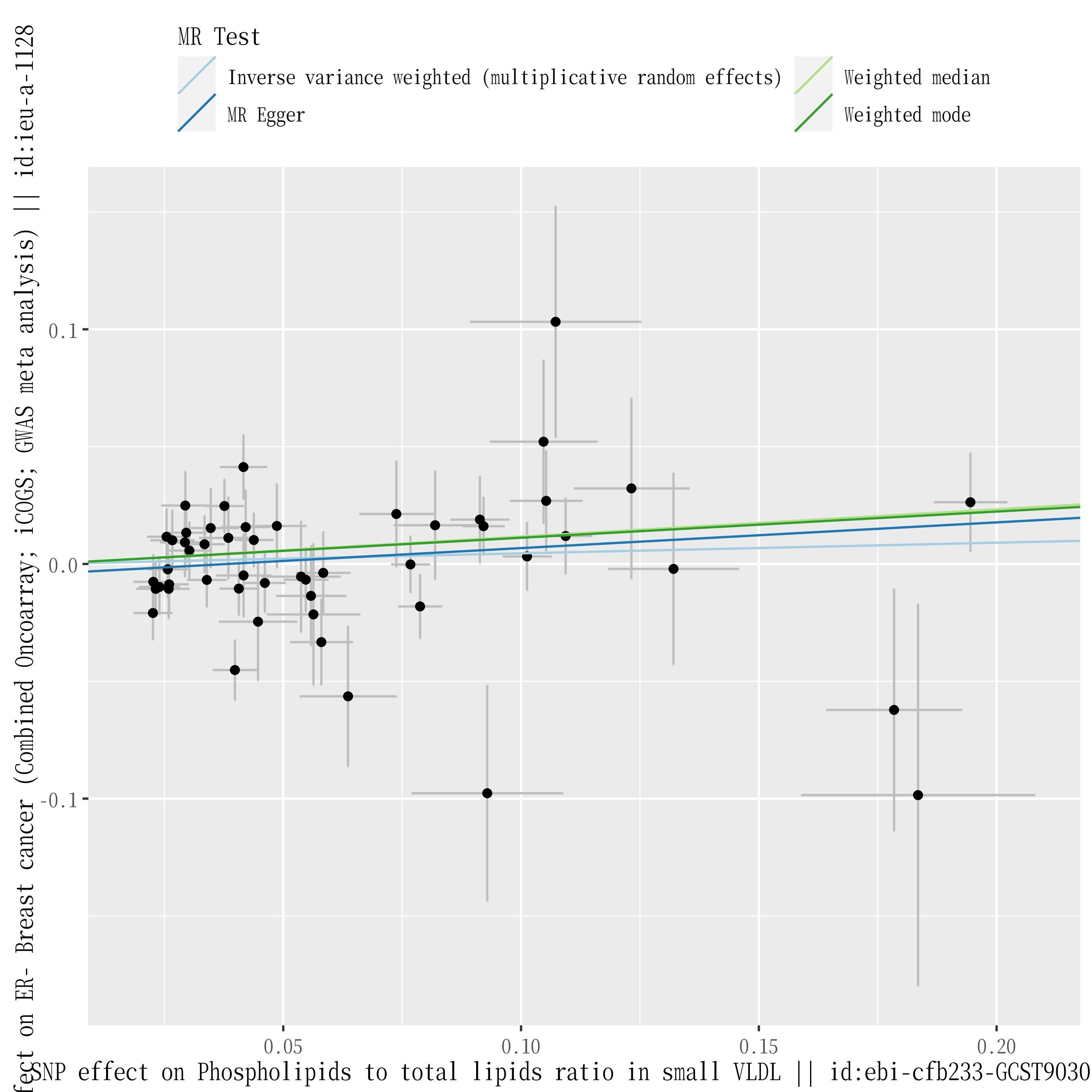

MR Test
Inverse variance weighted (multiplicative random effects)
MR Egger
Weighted median
Weighted mode
0.1
0.0
-0.1
0.05
0.10
0.15
0.20
SNP effect on Phospholipids to total lipids ratio in small VLDL || id:ebi-cfb233-GCST9030
fect on ER- Breast cancer (Combined Oncoarray; iCOGS; GWAS meta analysis) || id:ieu-a-1128

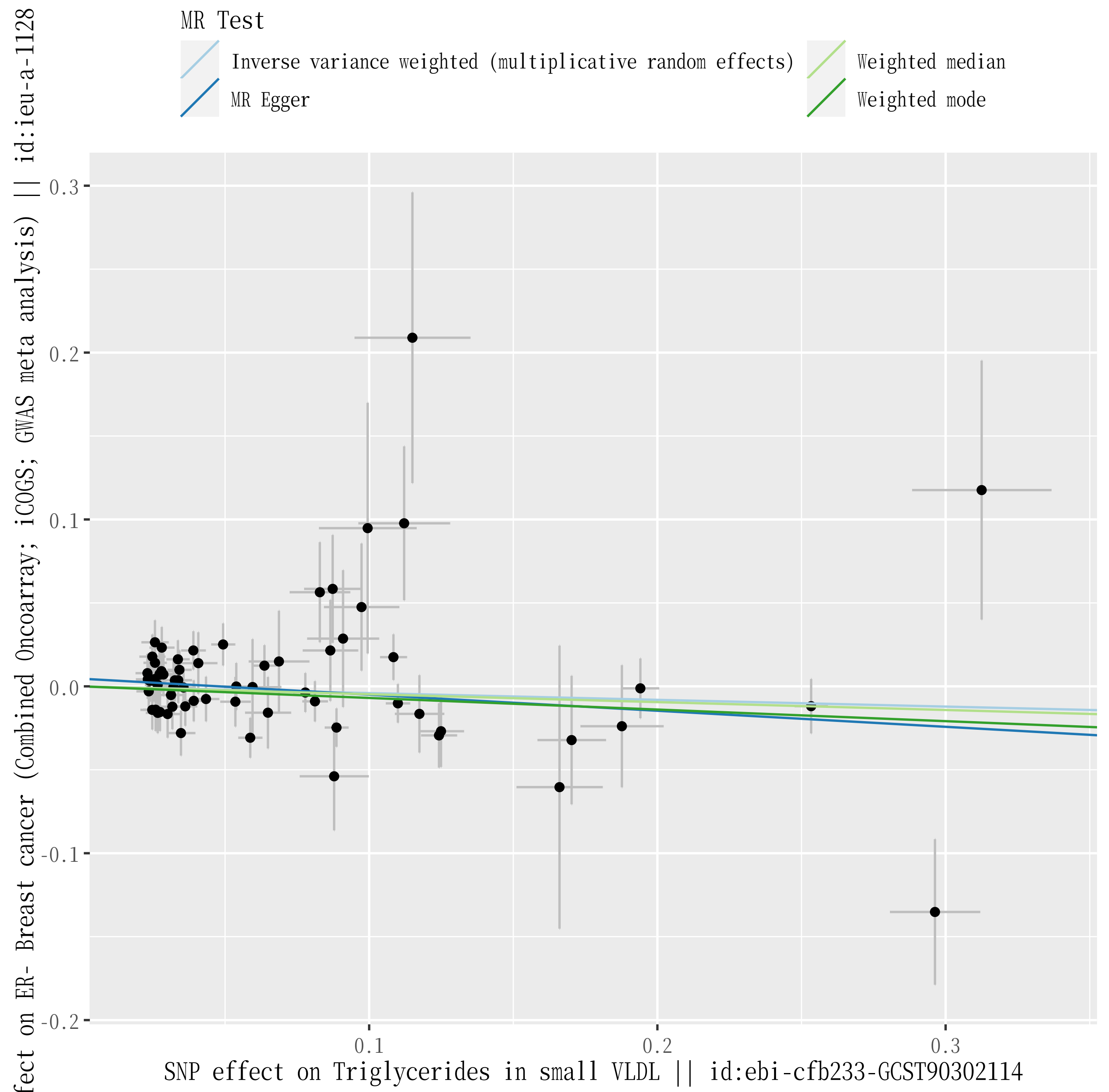

MR Test
Inverse variance weighted (multiplicative random effects)
MR Egger
Weighted median
Weighted mode
0.3
0.2
0.1
0.0
-0.1
-0.2
0.1
0.2
0.3
SNP effect on Triglycerides in small VLDL || id:ebi-cfb233-GCST90302114
fect on ER- Breast cancer (Combined Oncoarray; iCOGS; GWAS meta analysis) || id:ieu-a-1128

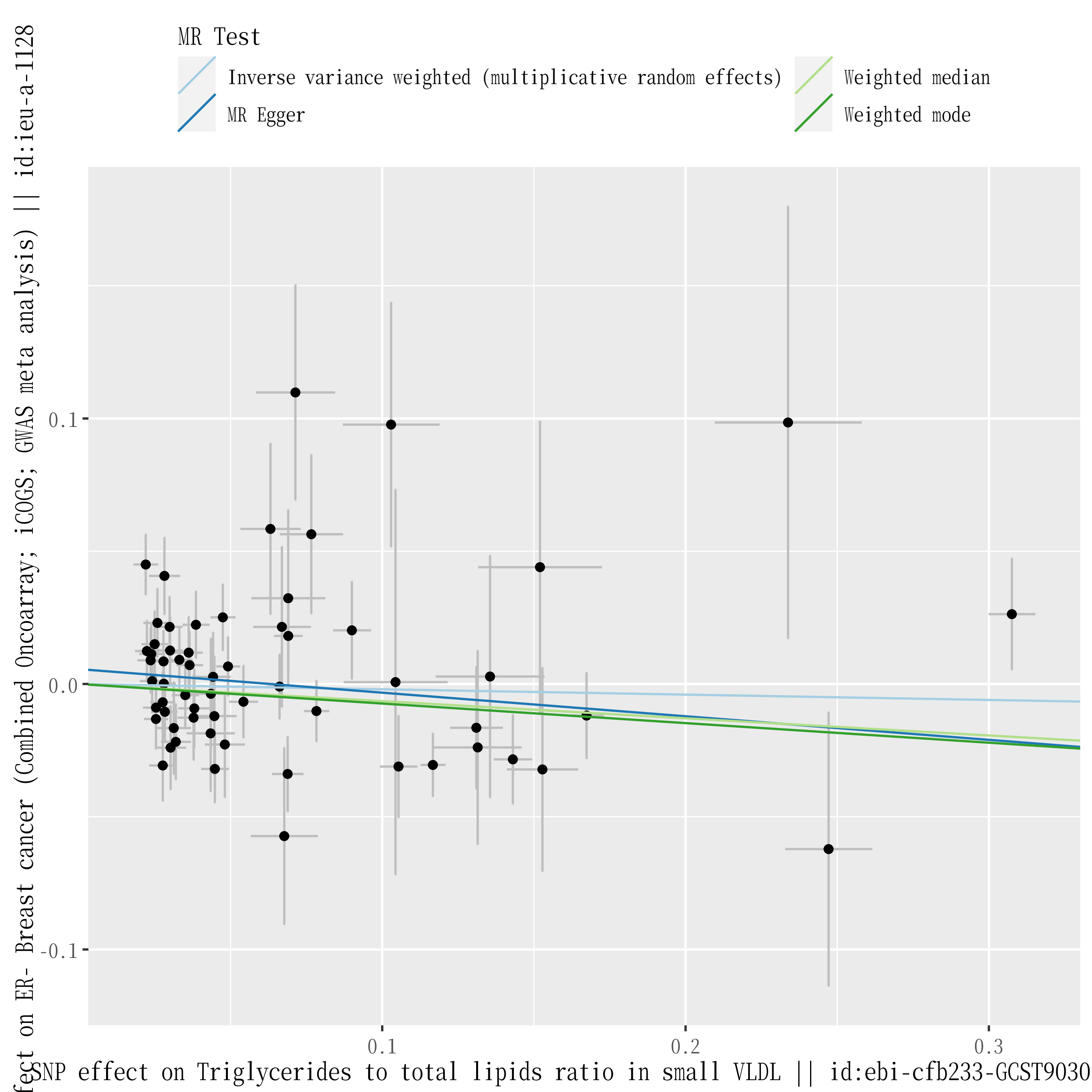
MR Test
Inverse variance weighted (multiplicative random effects)
MR Egger
Weighted median
Weighted mode
0.1
0.0
-0.1
0.1
0.2
0.3
SNP effect on Triglycerides to total lipids ratio in small VLDL || id:ebi-cfb233-GCST9030
fect on ER- Breast cancer (Combined Oncoarray; iCOGS; GWAS meta analysis) || id:ieu-a-1128

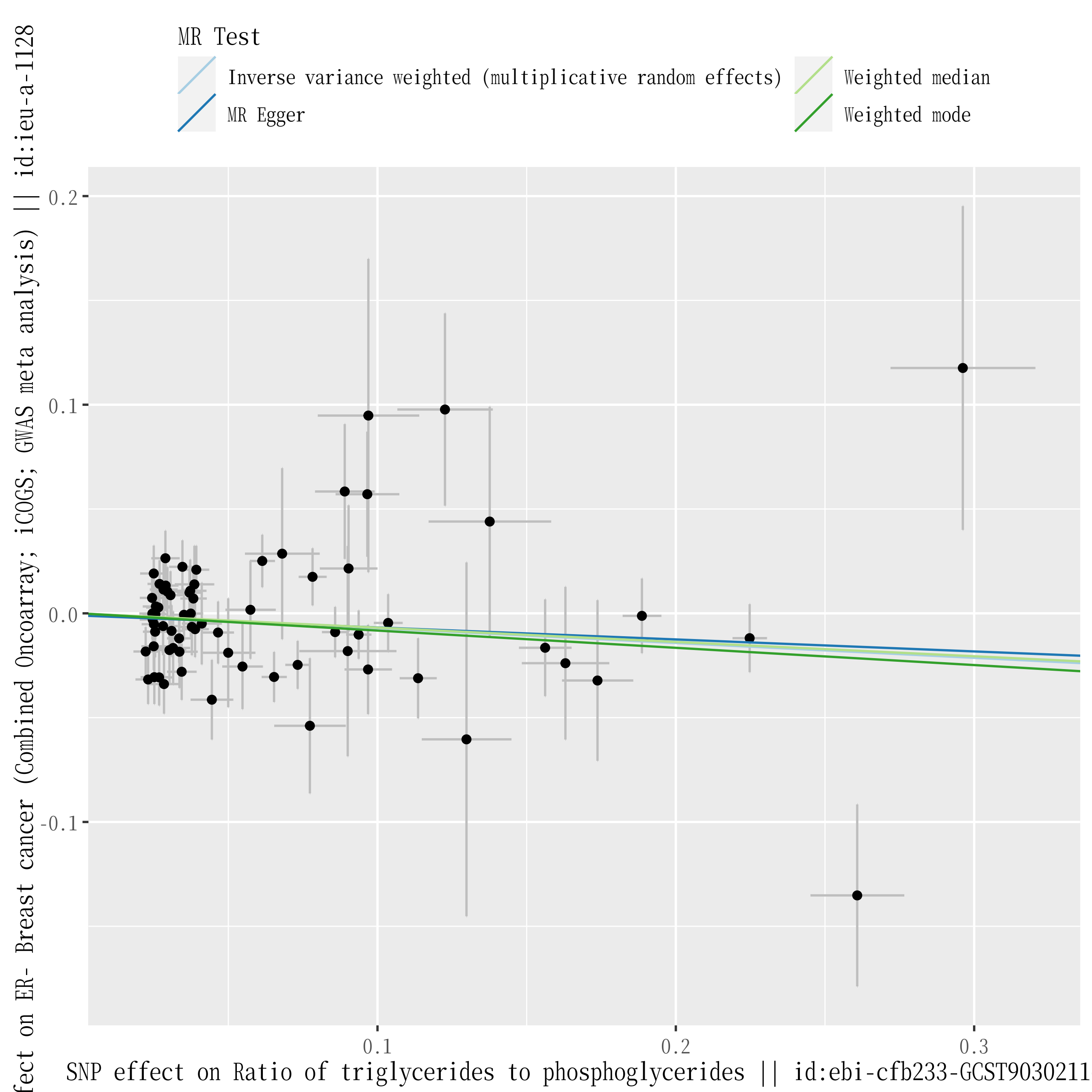
MR Test
Inverse variance weighted (multiplicative random effects)
MR Egger
Weighted median
Weighted mode
0.2
0.1
0.0
-0.1
0.1
0.2
0.3
SNP effect on Ratio of triglycerides to phosphoglycerides || id:ebi-cfb233-GCST9030211
fect on ER- Breast cancer (Combined Oncoarray; iCOGS; GWAS meta analysis) || id:ieu-a-1128

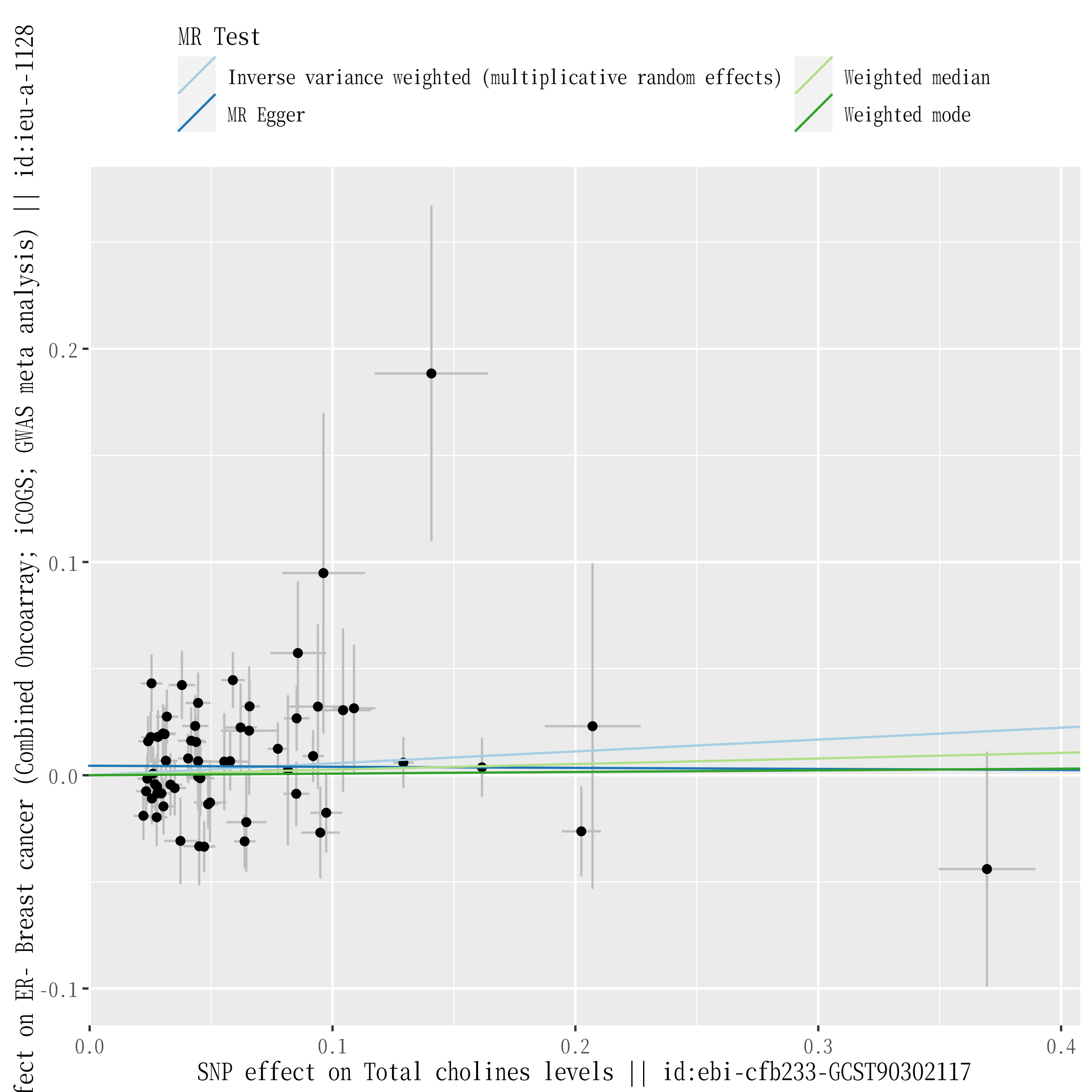
MR Test
Inverse variance weighted (multiplicative random effects)
MR Egger
Weighted median
Weighted mode
fect on ER- Breast cancer (Combined Oncoarray; iCOGS; GWAS meta analysis) || id:ieu-a-1128
0.2
0.1
0.0
-0.1
0.0
0.1
0.2
0.3
0.4
SNP effect on Total cholines levels || id:ebi-cfb233-GCST90302117

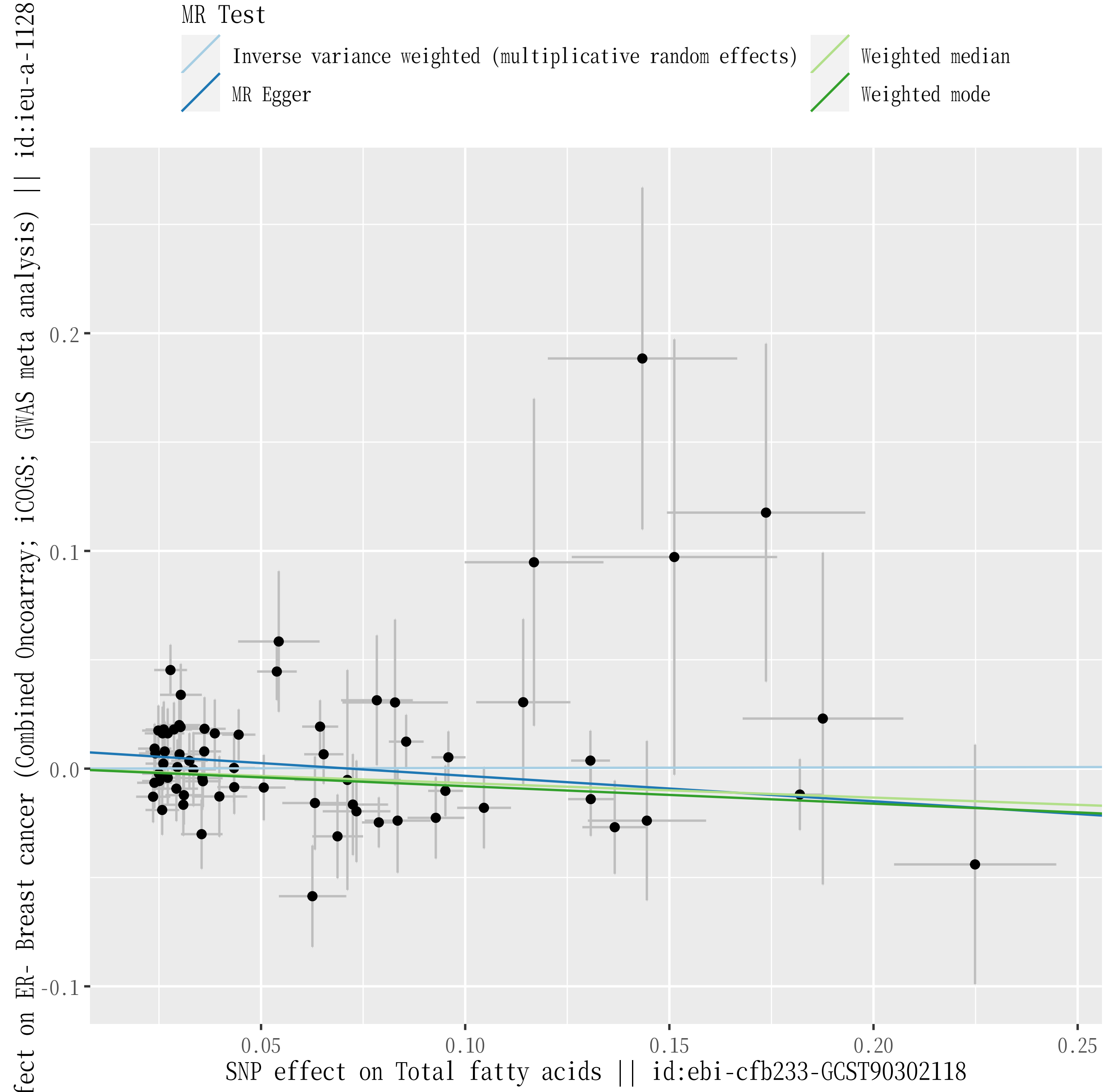
MR Test
Inverse variance weighted (multiplicative random effects)
MR Egger
Weighted median
Weighted mode
fect on ER- Breast cancer (Combined Oncoarray; iCOGS; GWAS meta analysis) || id:ieu-a-1128
0.2
0.1
0.0
-0.1
0.05
0.10
0.15
0.20
0.25
SNP effect on Total fatty acids || id:ebi-cfb233-GCST90302118

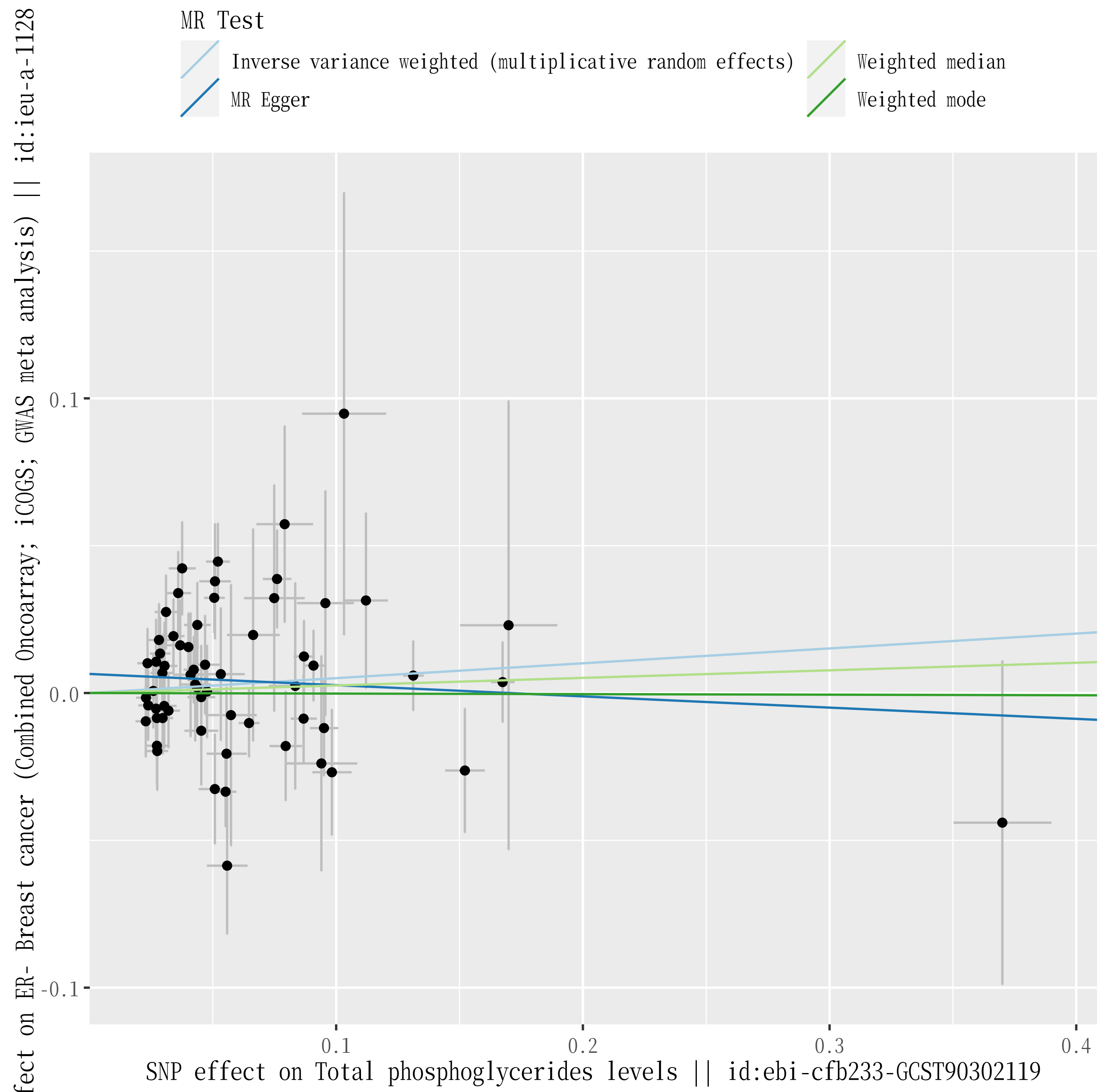

MR Test
Inverse variance weighted (multiplicative random effects)
MR Egger
Weighted median
Weighted mode
0.1
0.0
-0.1
0.1
0.2
0.3
0.4
SNP effect on Total phosphoglycerides levels || id:ebi-cfb233-GCST90302119
fect on ER- Breast cancer (Combined Oncoarray; iCOGS; GWAS meta analysis) || id:ieu-a-1128

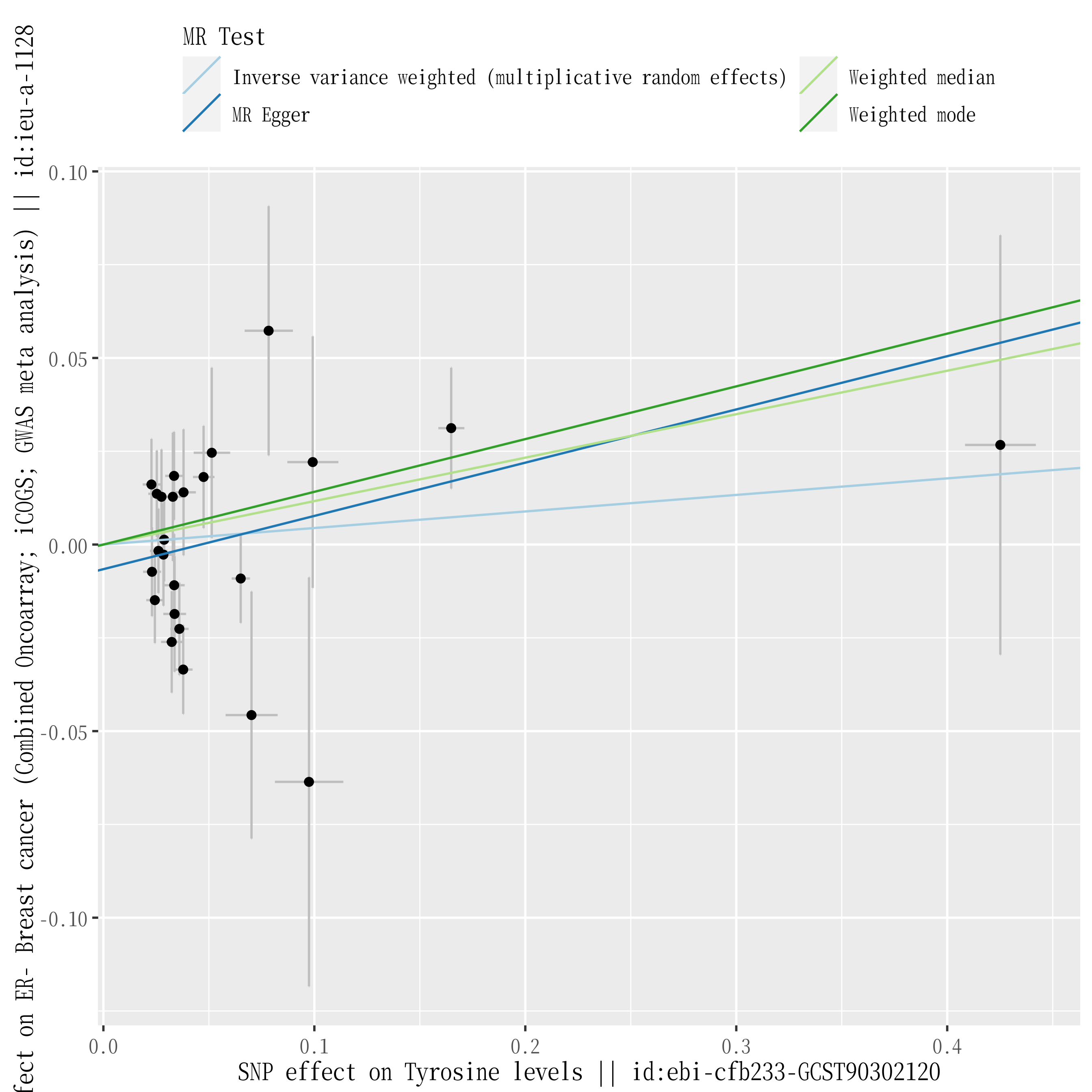
MR Test
Inverse variance weighted (multiplicative random effects)
MR Egger
Weighted median
Weighted mode
0.10
0.05
0.00
-0.05
-0.10
0.0
0.1
0.2
0.3
0.4
SNP effect on Tyrosine levels || id:ebi-cfb233-GCST90302120
fect on ER- Breast cancer (Combined Oncoarray; iCOGS; GWAS meta analysis) || id:ieu-a-1128

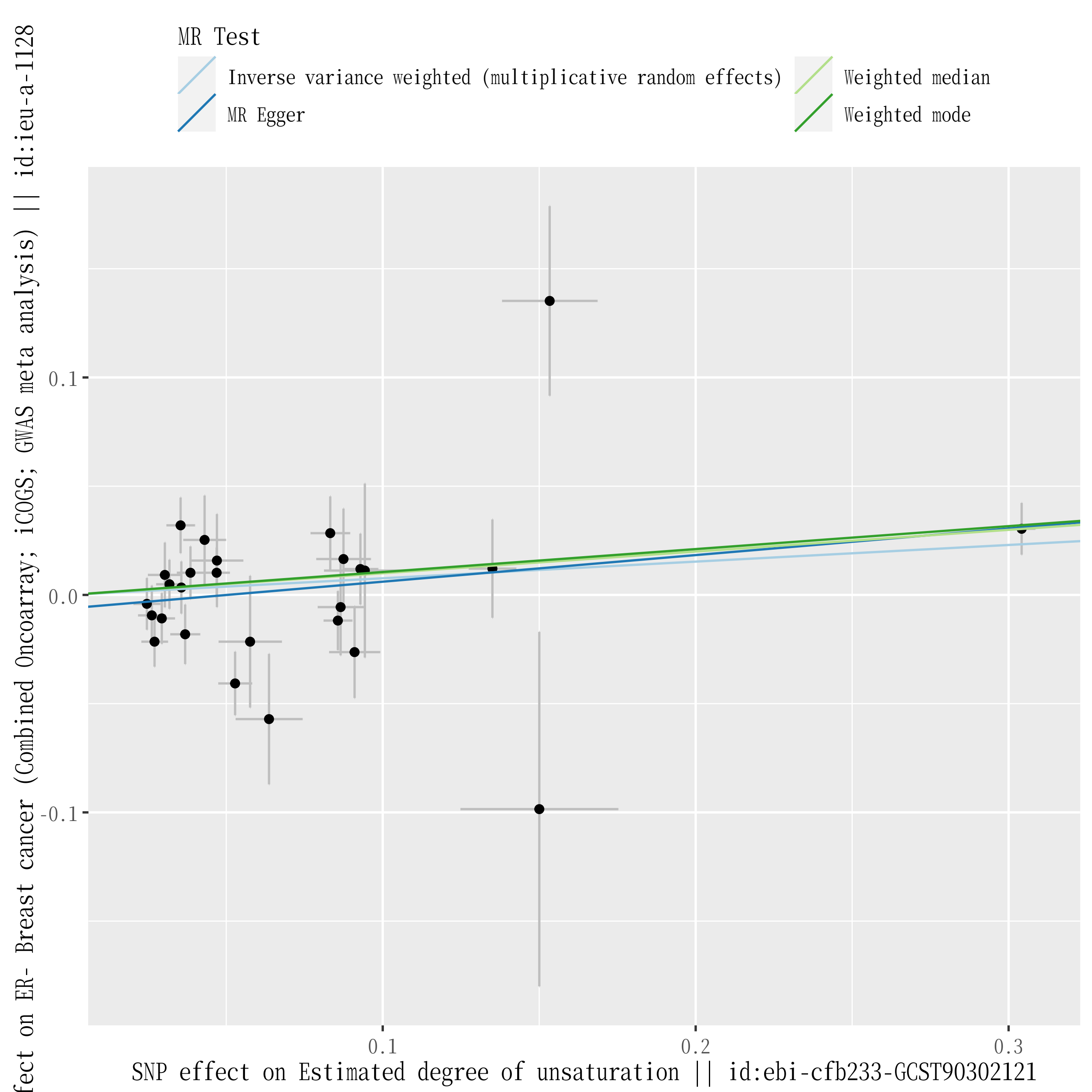
MR Test
Inverse variance weighted (multiplicative random effects)
MR Egger
Weighted median
Weighted mode
0.1
0.0
-0.1
0.1
0.2
0.3
SNP effect on Estimated degree of unsaturation || id:ebi-cfb233-GCST90302121
fect on ER- Breast cancer (Combined Oncoarray; iCOGS; GWAS meta analysis) || id:ieu-a-1128

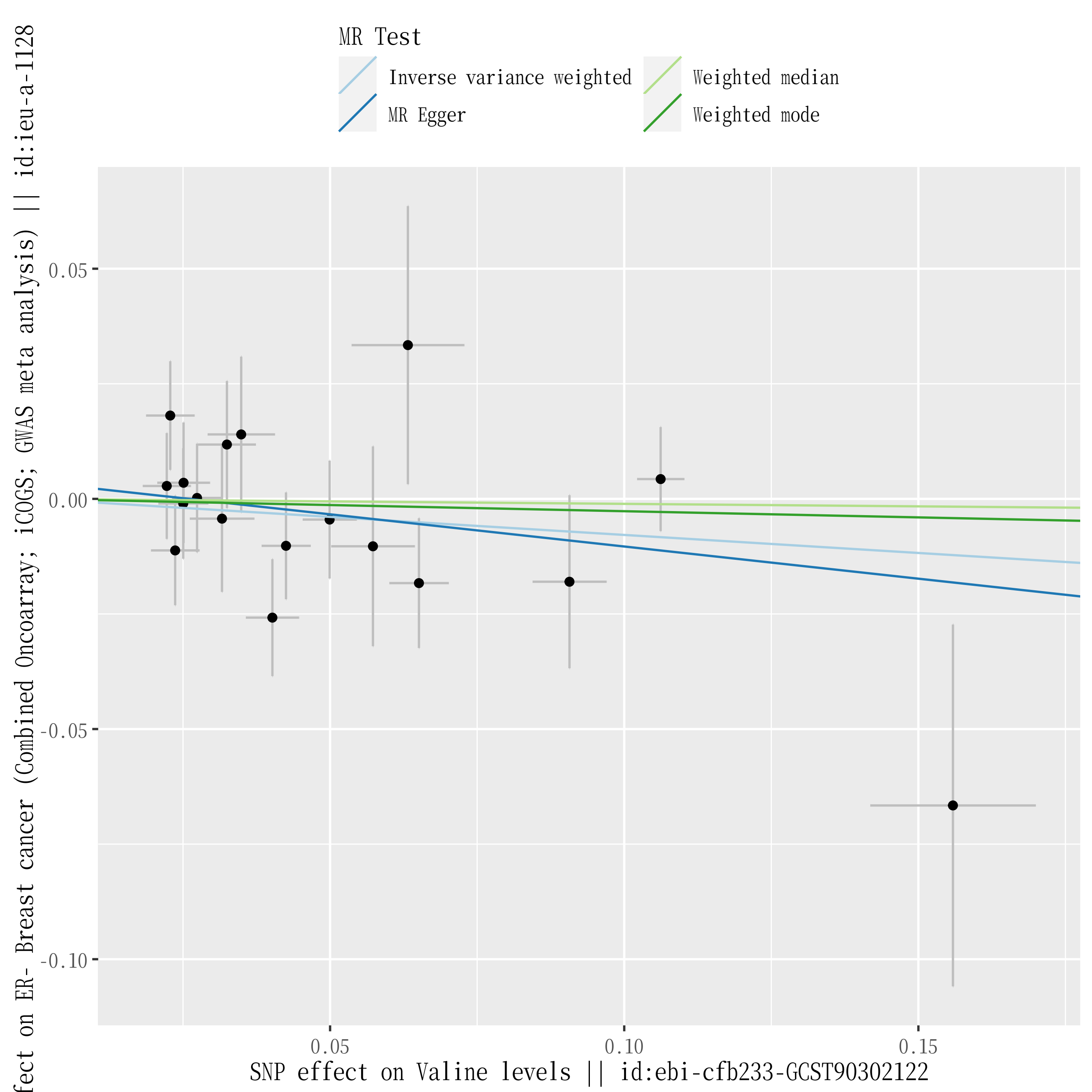
MR Test
Inverse variance weighted
MR Egger
Weighted median
Weighted mode
0.05
0.00
-0.05
-0.10
0.05
0.10
0.15
SNP effect on Valine levels || id:ebi-cfb233-GCST90302122
fect on ER- Breast cancer (Combined Oncoarray; iCOGS; GWAS meta analysis) || id:ieu-a-1128

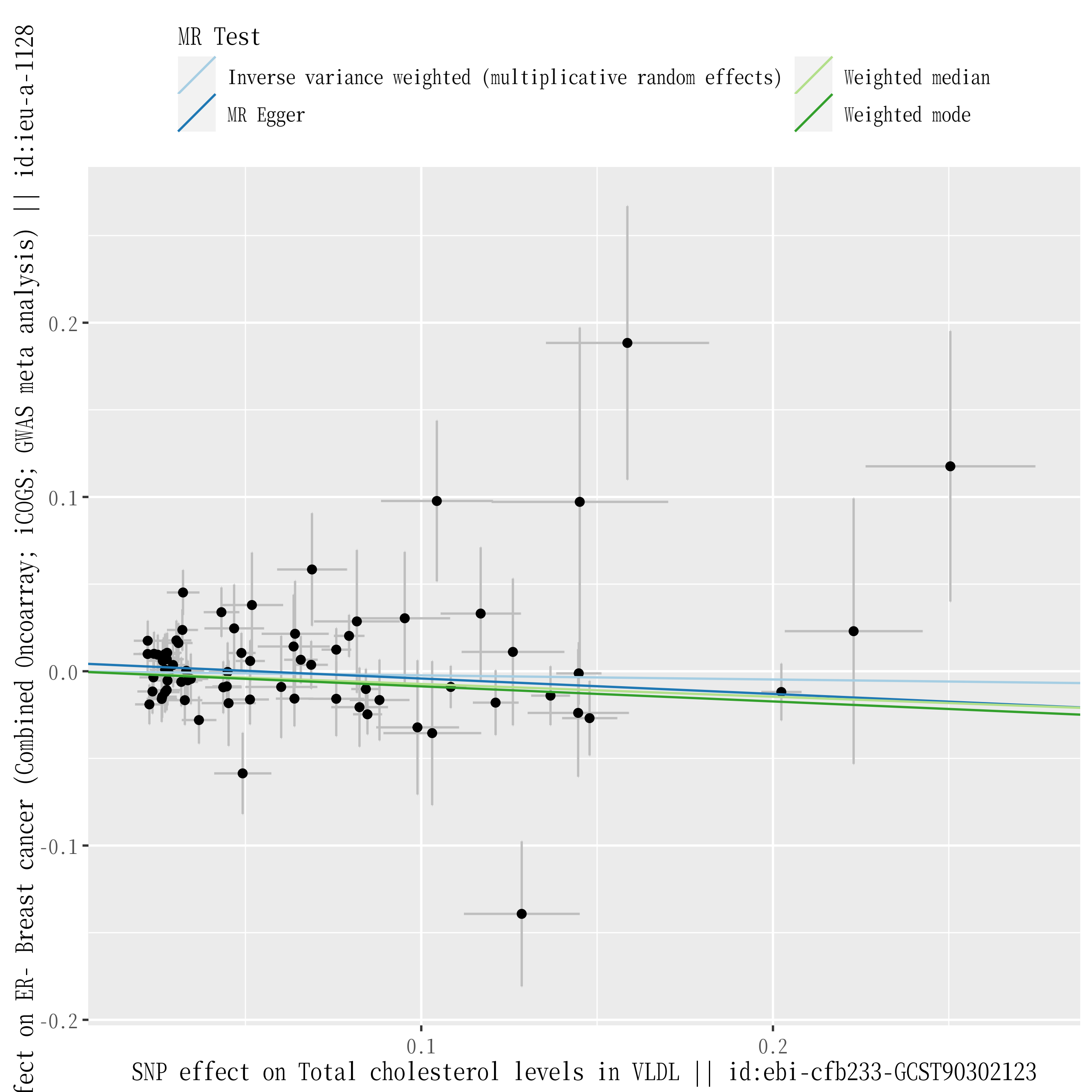
MR Test
Inverse variance weighted (multiplicative random effects)
MR Egger
Weighted median
Weighted mode
0.2
0.1
0.0
-0.1
-0.2
0.1
0.2
SNP effect on Total cholesterol levels in VLDL || id:ebi-cfb233-GCST90302123
fect on ER- Breast cancer (Combined Oncoarray; iCOGS; GWAS meta analysis) || id:ieu-a-1128

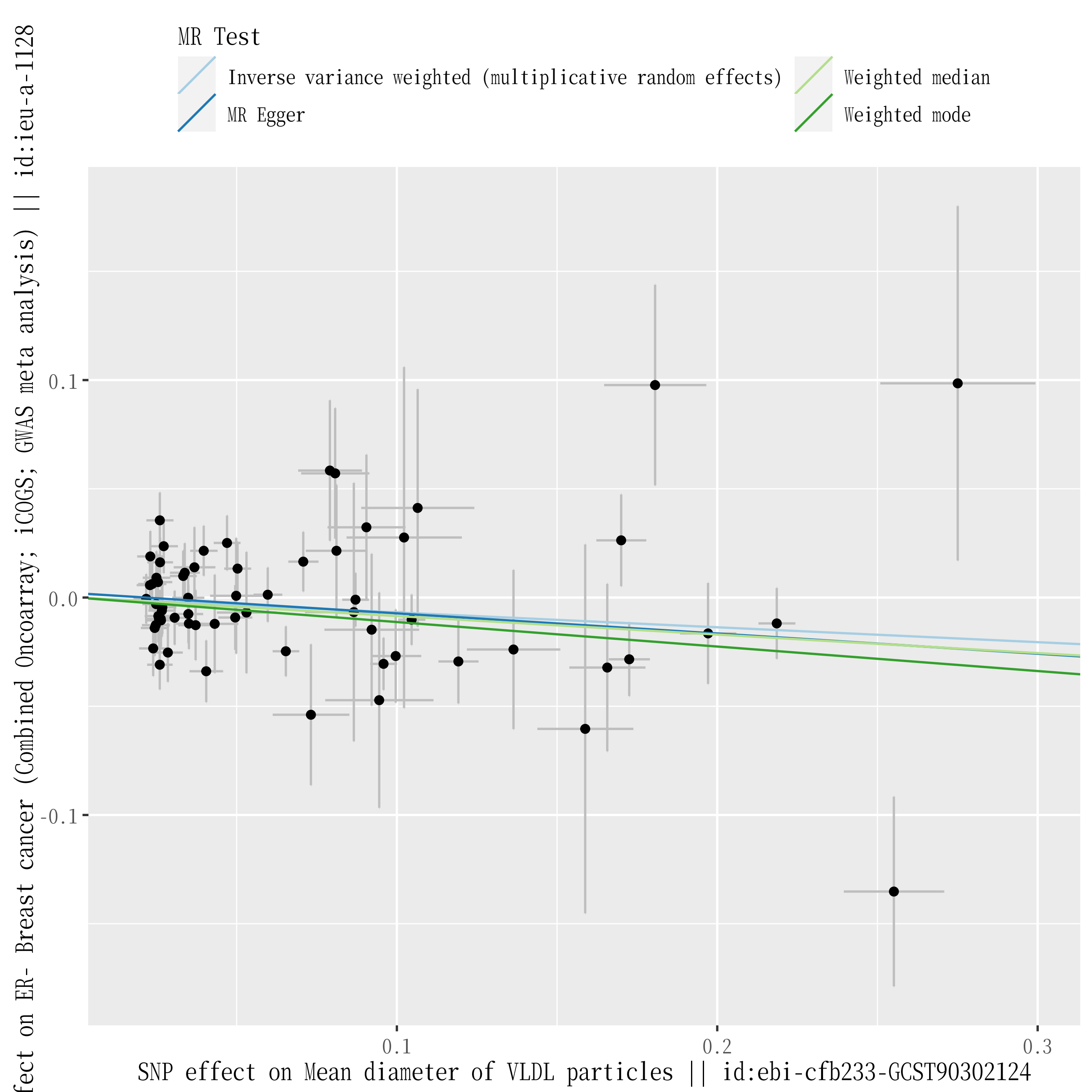

MR Test
Inverse variance weighted (multiplicative random effects)
MR Egger
Weighted median
Weighted mode
0.1
0.0
-0.1
0.1
0.2
0.3
SNP effect on Mean diameter of VLDL particles || id:ebi-cfb233-GCST90302124
fect on ER- Breast cancer (Combined Oncoarray; iCOGS; GWAS meta analysis) || id:ieu-a-1128

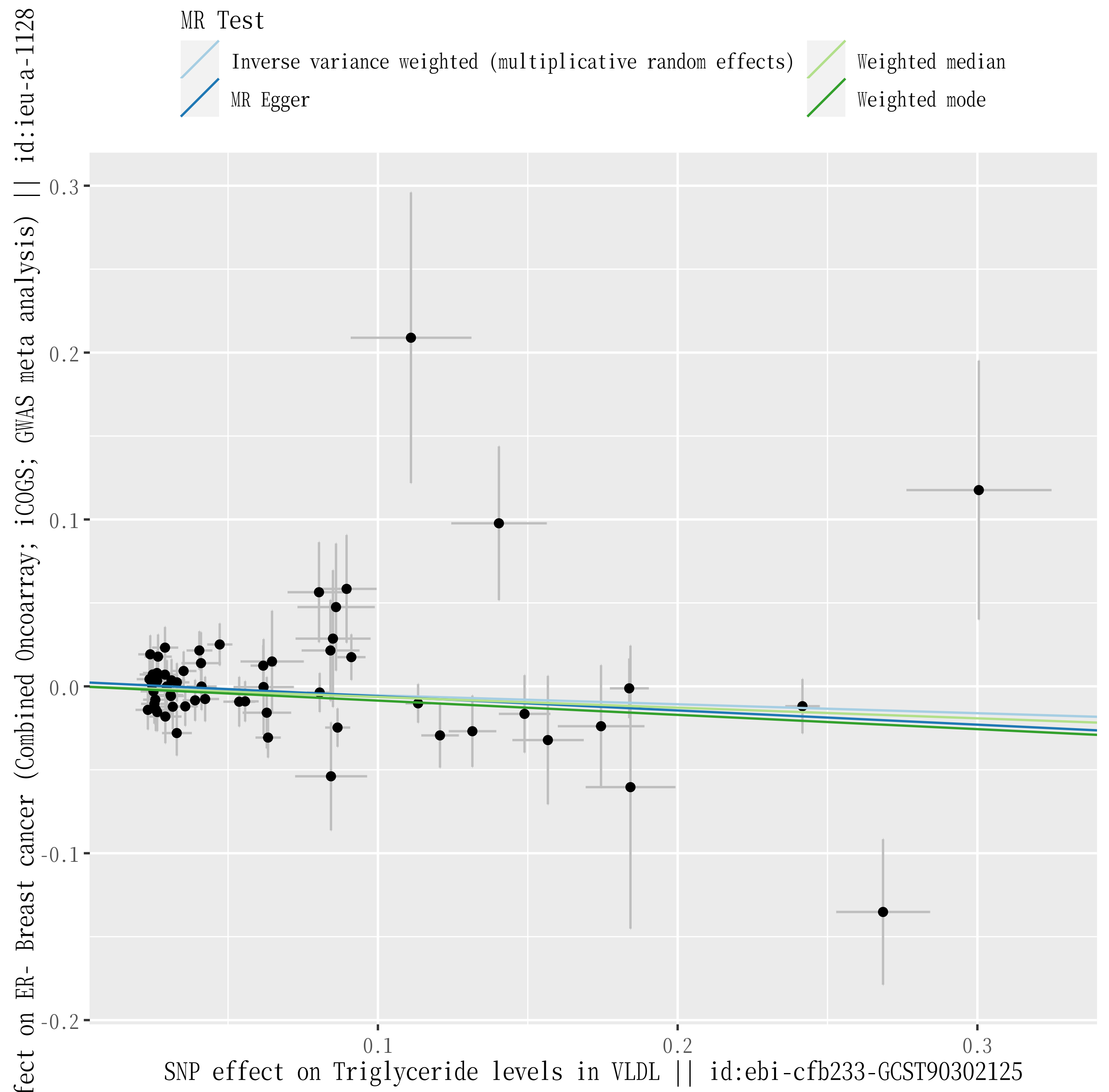

MR Test
Inverse variance weighted (multiplicative random effects)
MR Egger
Weighted median
Weighted mode
0.3
0.2
0.1
0.0
-0.1
-0.2
0.1
0.2
0.3
SNP effect on Triglyceride levels in VLDL || id:ebi-cfb233-GCST90302125
fect on ER- Breast cancer (Combined Oncoarray; iCOGS; GWAS meta analysis) || id:ieu-a-1128

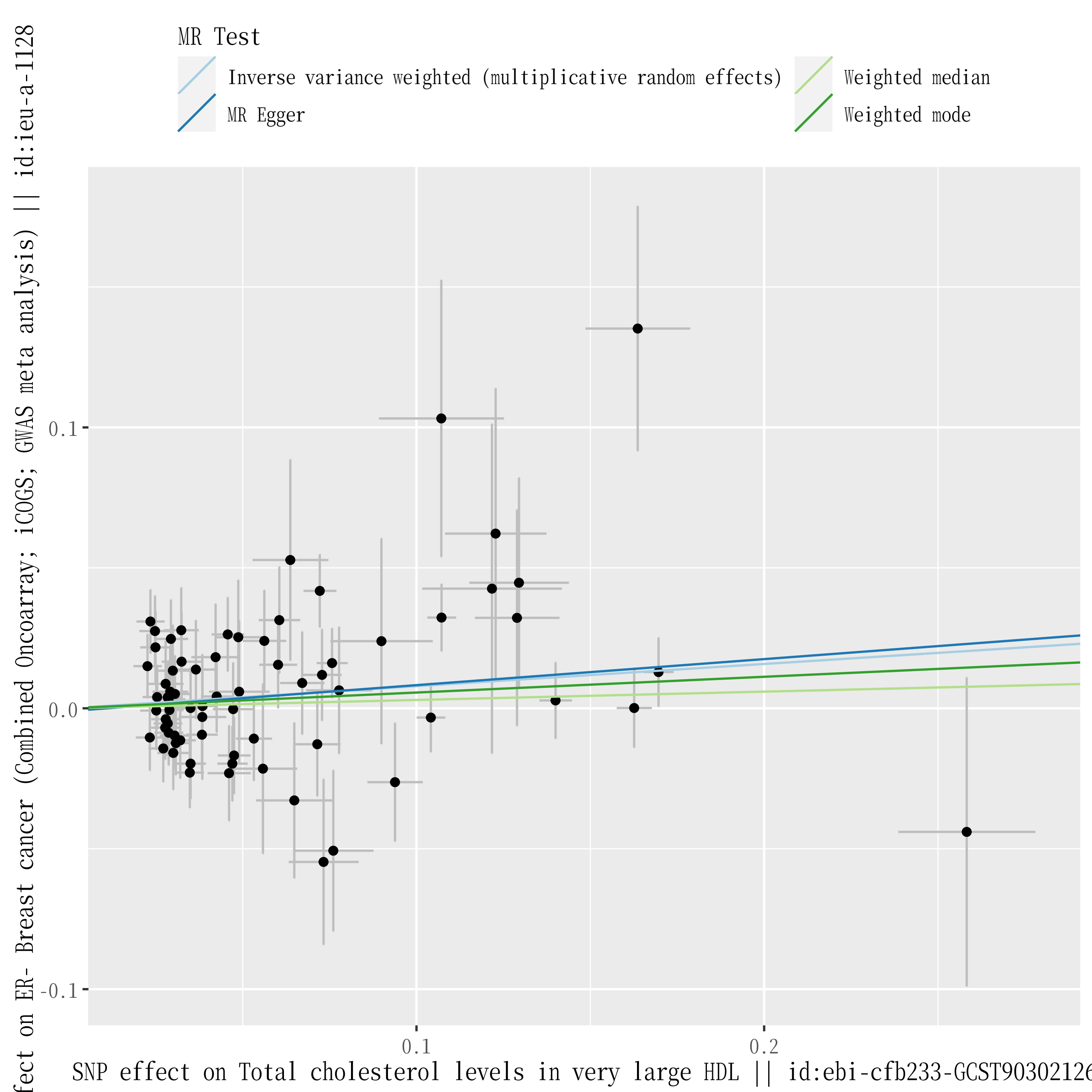
MR Test
Inverse variance weighted (multiplicative random effects)
MR Egger
Weighted median
Weighted mode
0.1
0.0
-0.1
0.1
0.2
fect on ER- Breast cancer (Combined Oncoarray; iCOGS; GWAS meta analysis) || id:ieu-a-1128
SNP effect on Total cholesterol levels in very large HDL || id:ebi-cfb233-GCST9030212

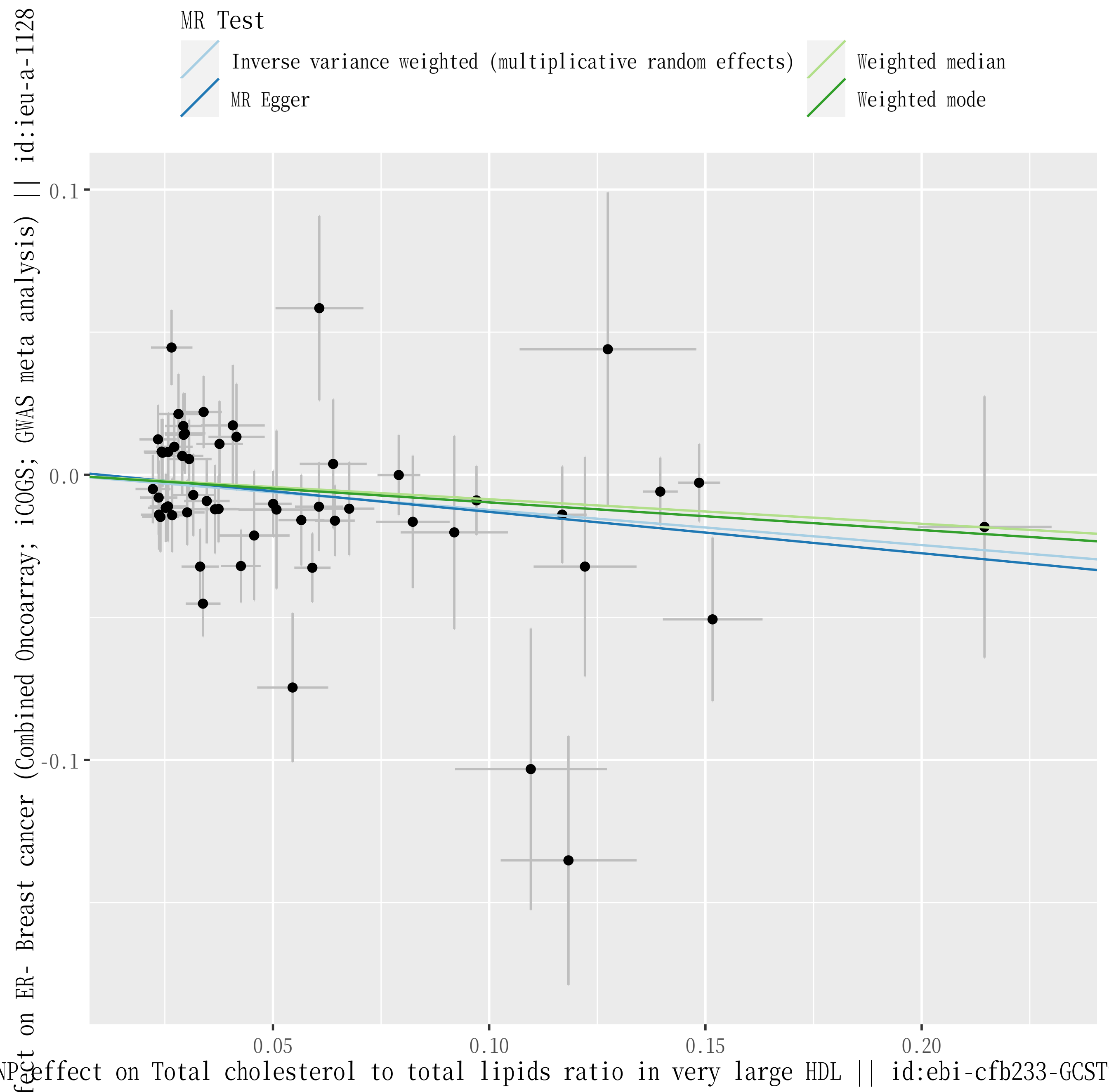
MR Test
Inverse variance weighted (multiplicative random effects)
MR Egger
Weighted median
Weighted mode
0.1
0.0
-0.1
0.05
0.10
0.15
0.20
NP effect on Total cholesterol to total lipids ratio in very large HDL || id:ebi-cfb233-GCST
fect on ER- Breast cancer (Combined Oncoarray; iCOGS; GWAS meta analysis) || id:ieu-a-1128

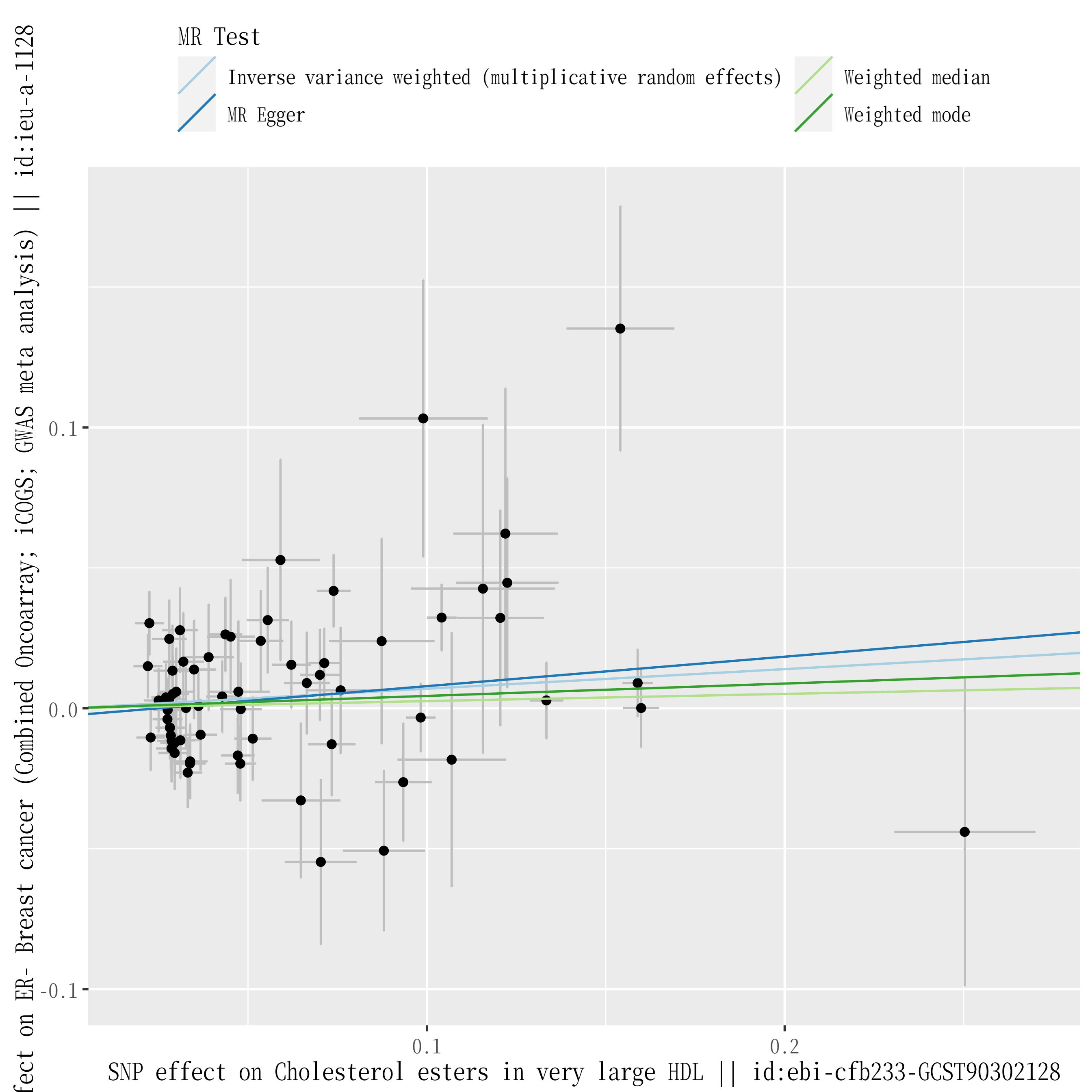

MR Test
Inverse variance weighted (multiplicative random effects)
MR Egger
Weighted median
Weighted mode
0.1
0.0
-0.1
0.1
0.2
SNP effect on Cholesterol esters in very large HDL || id:ebi-cfb233-GCST90302128
fect on ER- Breast cancer (Combined Oncoarray; iCOGS; GWAS meta analysis) || id:ieu-a-1128

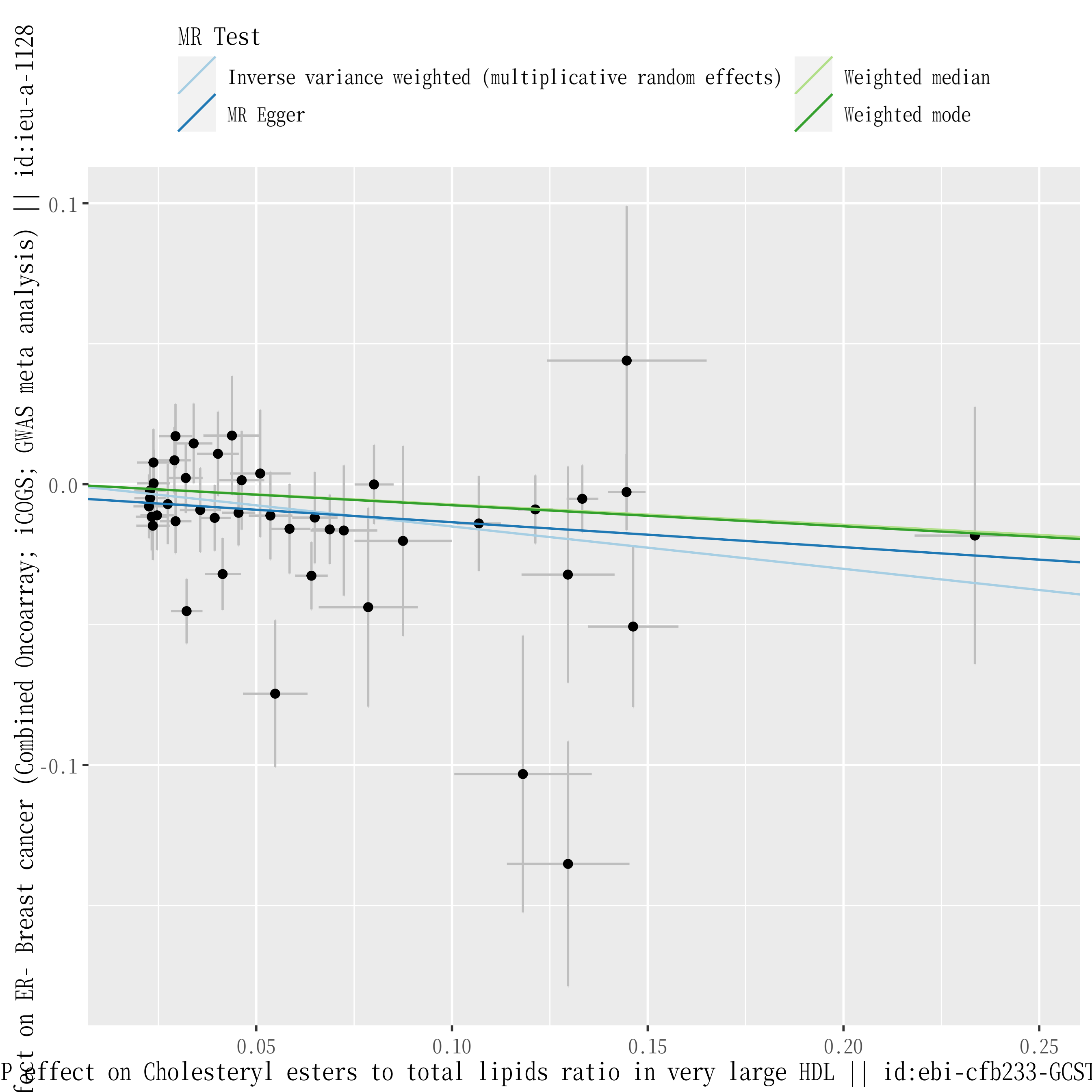
MR Test
Inverse variance weighted (multiplicative random effects)
MR Egger
Weighted median
Weighted mode
0.1
0.0
-0.1
0.05
0.10
0.15
0.20
0.25
P effect on Cholesteryl esters to total lipids ratio in very large HDL || id:ebi-cfb233-GCST
fect on ER- Breast cancer (Combined Oncoarray; iCOGS; GWAS meta analysis) || id:ieu-a-1128

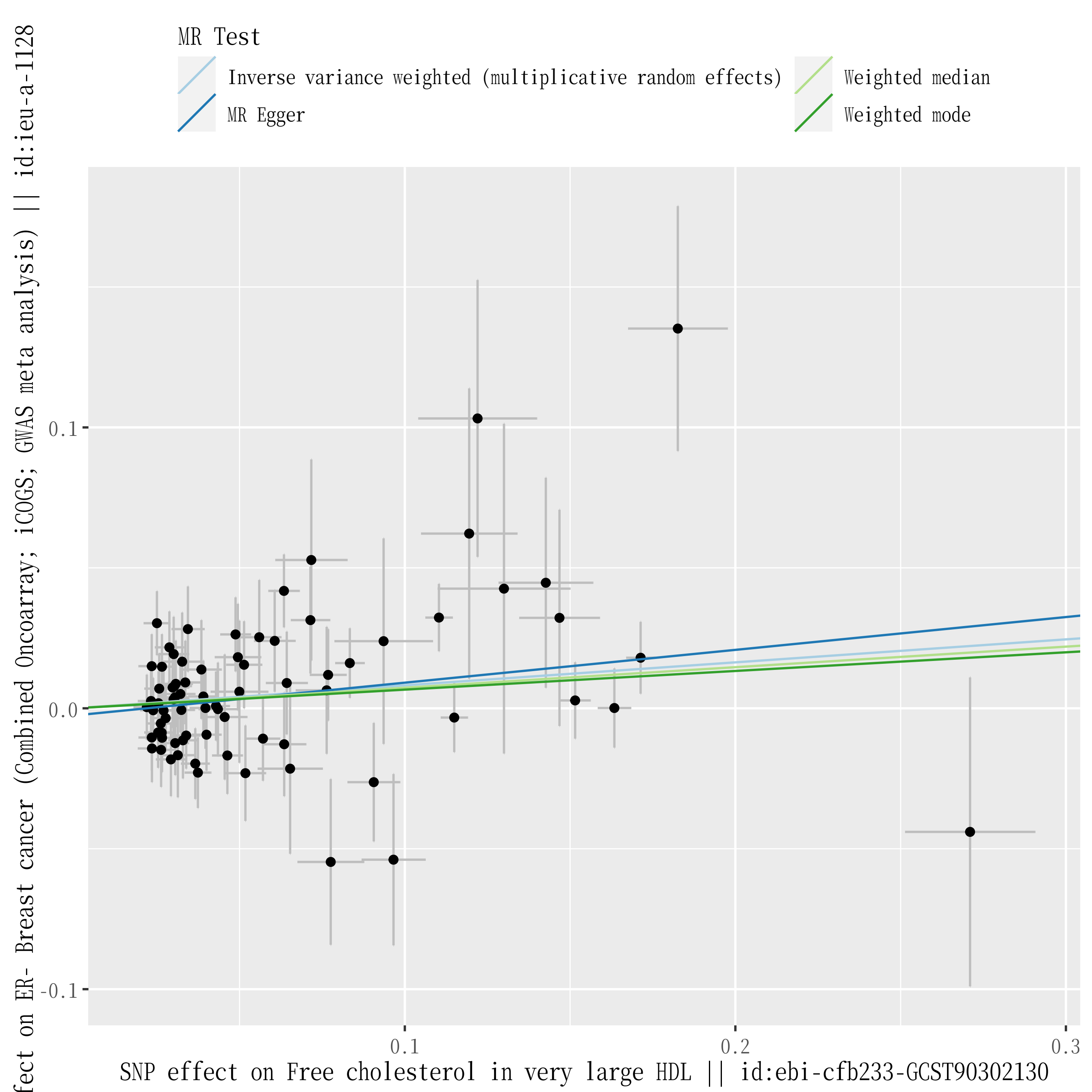

MR Test
Inverse variance weighted (multiplicative random effects)
MR Egger
Weighted median
Weighted mode
0.1
0.0
-0.1
0.1
0.2
0.3
SNP effect on Free cholesterol in very large HDL || id:ebi-cfb233-GCST90302130
fect on ER- Breast cancer (Combined Oncoarray; iCOGS; GWAS meta analysis) || id:ieu-a-1128

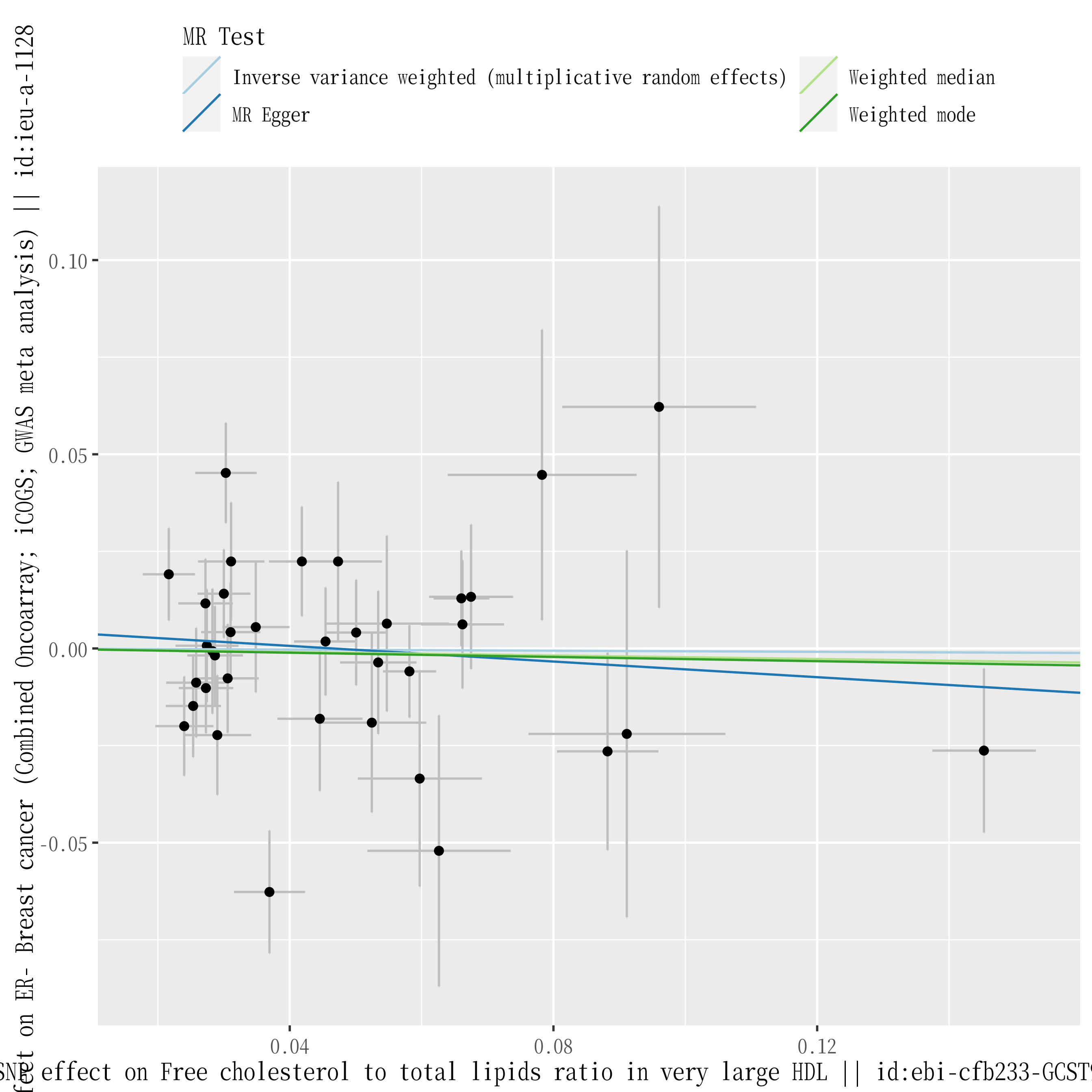
MR Test
Inverse variance weighted (multiplicative random effects)
MR Egger
Weighted median
Weighted mode
0.10
0.05
0.00
-0.05
0.04
0.08
0.12
SNP effect on Free cholesterol to total lipids ratio in very large HDL || id:ebi-cfb233-GCST
fect on ER- Breast cancer (Combined Oncoarray; iCOGS; GWAS meta analysis) || id:ieu-a-1128

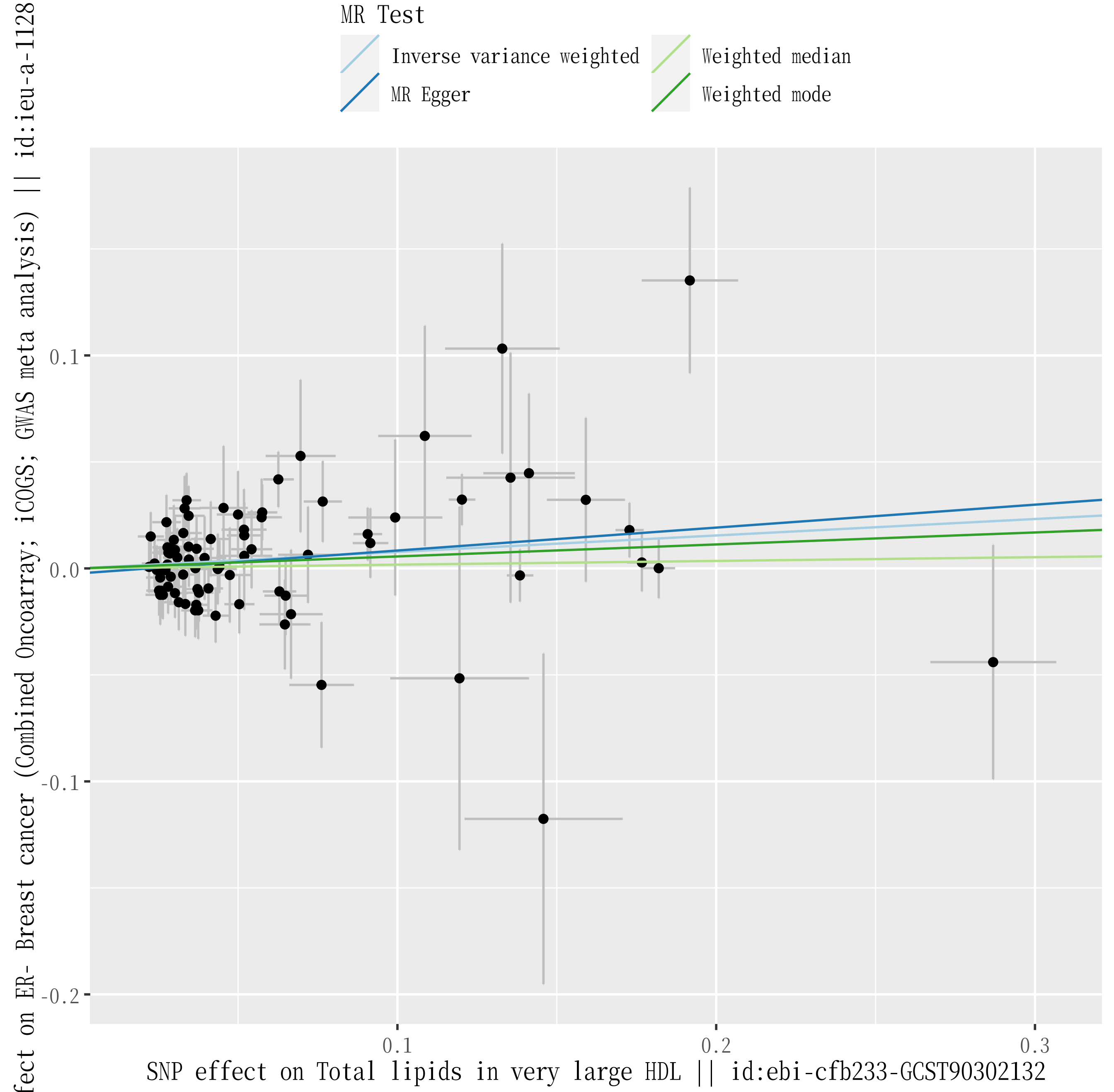
MR Test
Inverse variance weighted
MR Egger
Weighted median
Weighted mode
0.1
0.0
-0.1
-0.2
0.1
0.2
0.3
SNP effect on Total lipids in very large HDL || id:ebi-cfb233-GCST90302132
fect on ER- Breast cancer (Combined Oncoarray; iCOGS; GWAS meta analysis) || id:ieu-a-1128

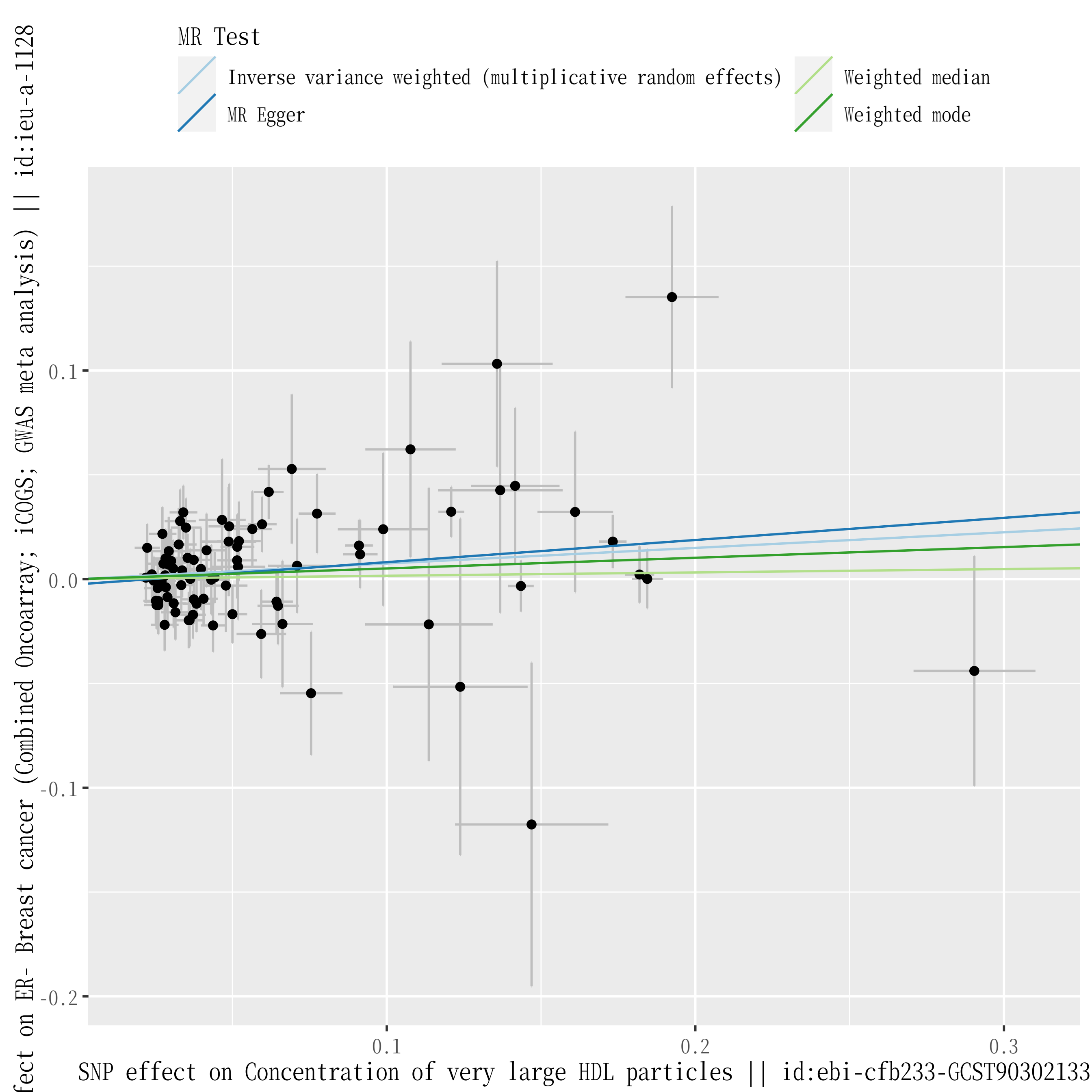
MR Test
Inverse variance weighted (multiplicative random effects)
MR Egger
Weighted median
Weighted mode
0.1
0.0
-0.1
-0.2
0.1
0.2
0.3
SNP effect on Concentration of very large HDL particles || id:ebi-cfb233-GCST90302133
fect on ER- Breast cancer (Combined Oncoarray; iCOGS; GWAS meta analysis) || id:ieu-a-1128

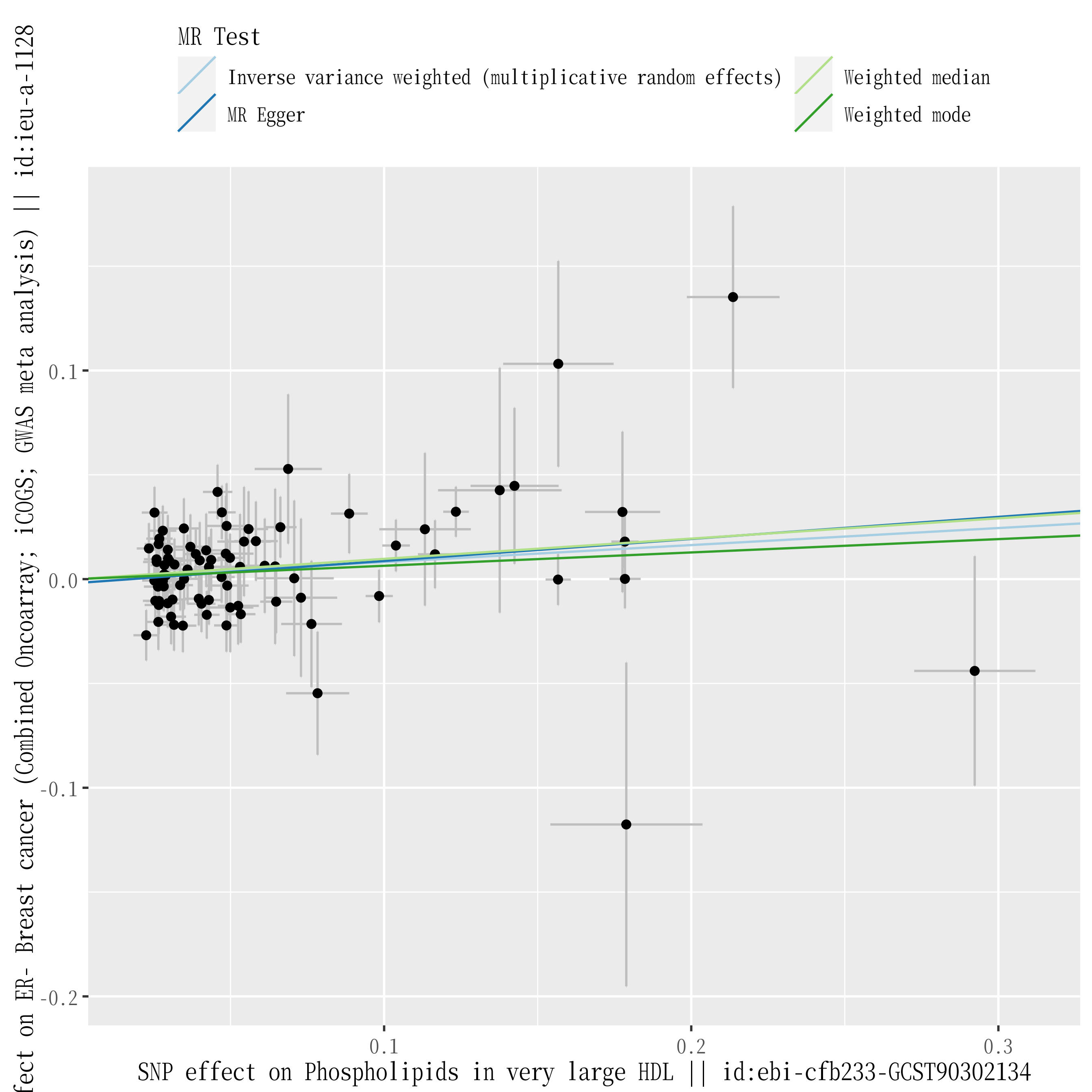

MR Test
Inverse variance weighted (multiplicative random effects)
MR Egger
Weighted median
Weighted mode
0.1
0.0
-0.1
-0.2
0.1
0.2
0.3
SNP effect on Phospholipids in very large HDL || id:ebi-cfb233-GCST90302134
fect on ER- Breast cancer (Combined Oncoarray; iCOGS; GWAS meta analysis) || id:ieu-a-1128

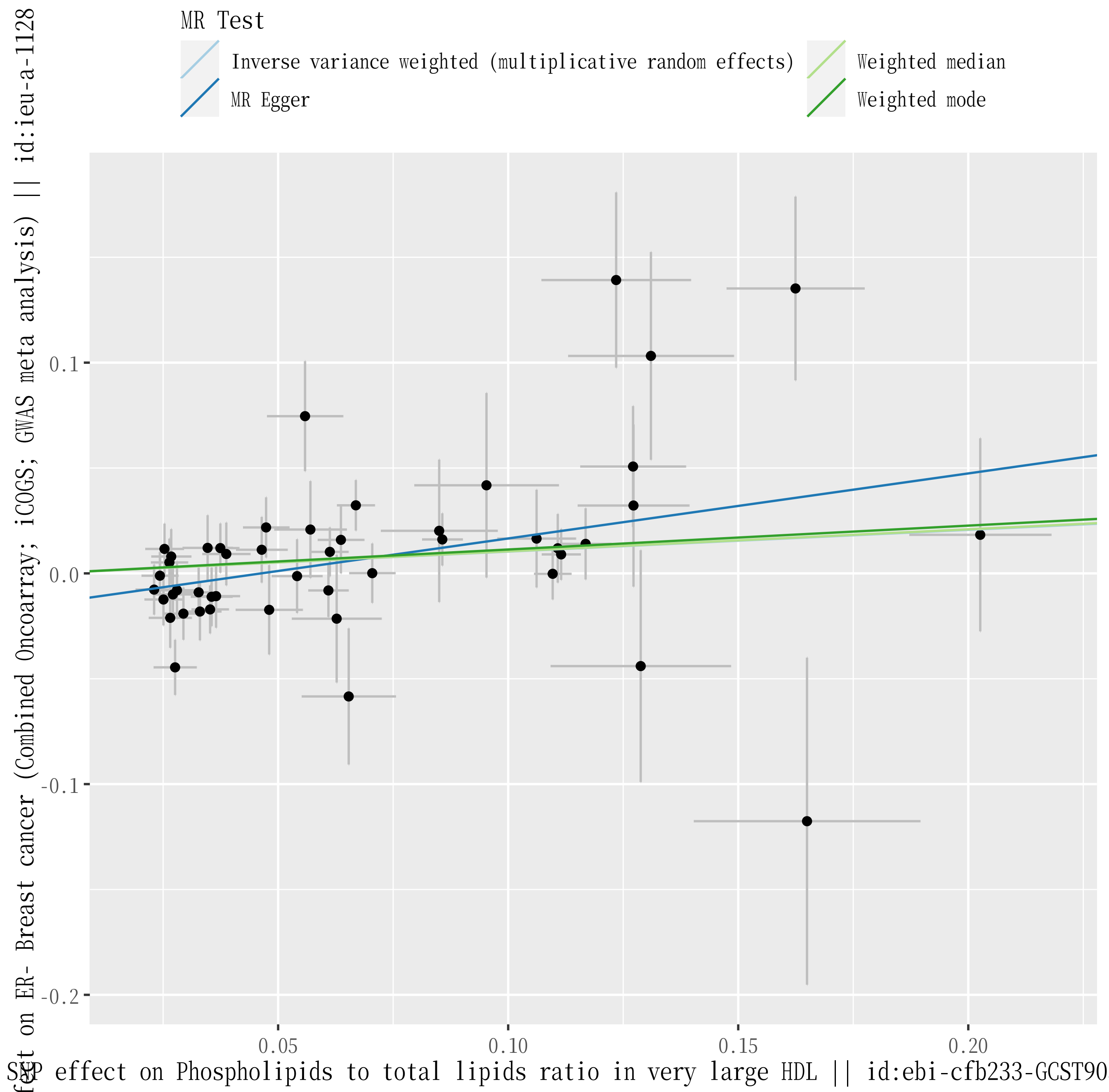

MR Test
Inverse variance weighted (multiplicative random effects)
MR Egger
Weighted median
Weighted mode
0.1
0.0
-0.1
-0.2
0.05
0.10
0.15
0.20
SNP effect on Phospholipids to total lipids ratio in very large HDL || id:ebi-cfb233-GCST90
fect on ER- Breast cancer (Combined Oncoarray; iCOGS; GWAS meta analysis) || id:ieu-a-1128

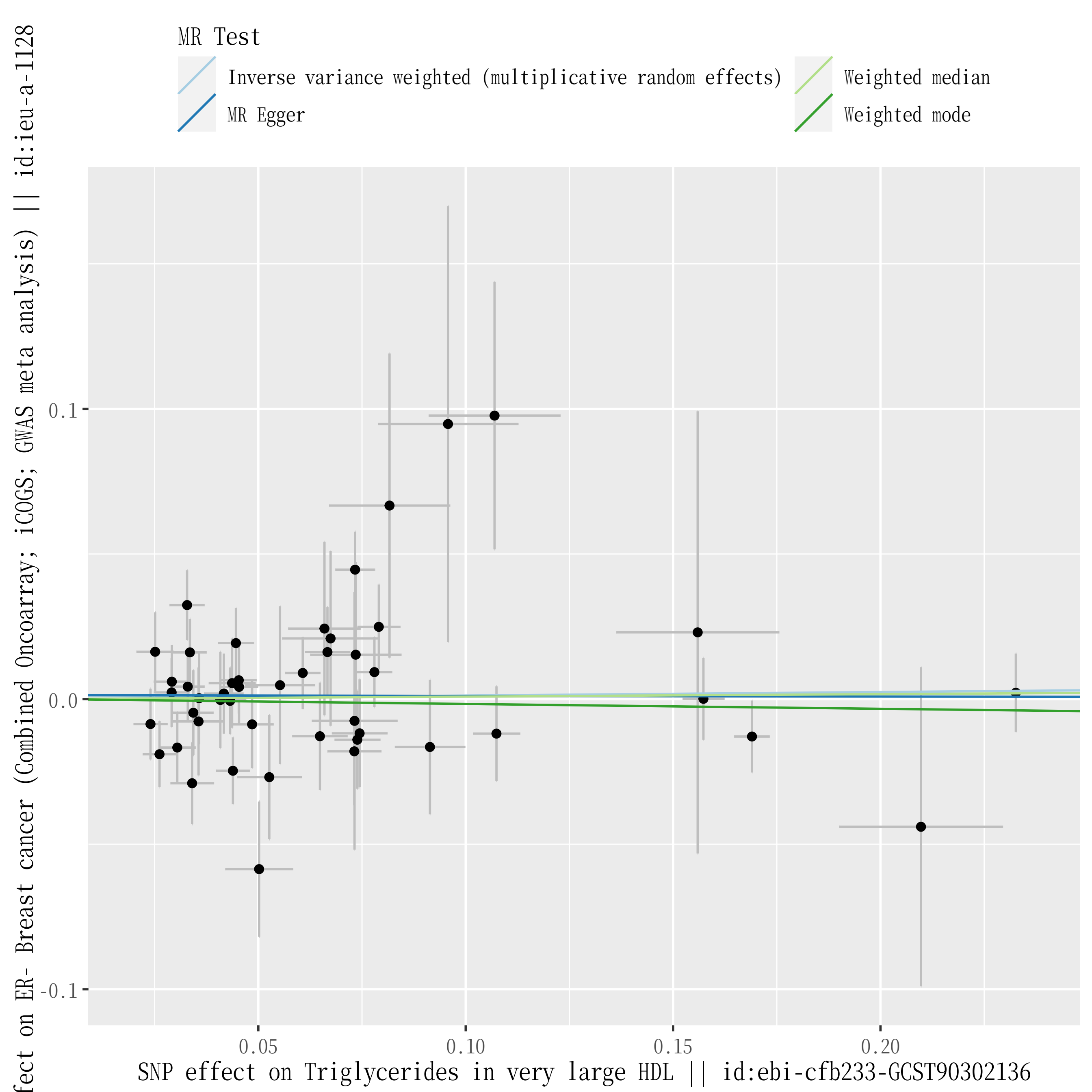
MR Test
Inverse variance weighted (multiplicative random effects)
MR Egger
Weighted median
Weighted mode
0.1
0.0
-0.1
0.05
0.10
0.15
0.20
SNP effect on Triglycerides in very large HDL || id:ebi-cfb233-GCST90302136
fect on ER- Breast cancer (Combined Oncoarray; iCOGS; GWAS meta analysis) || id:ieu-a-1128

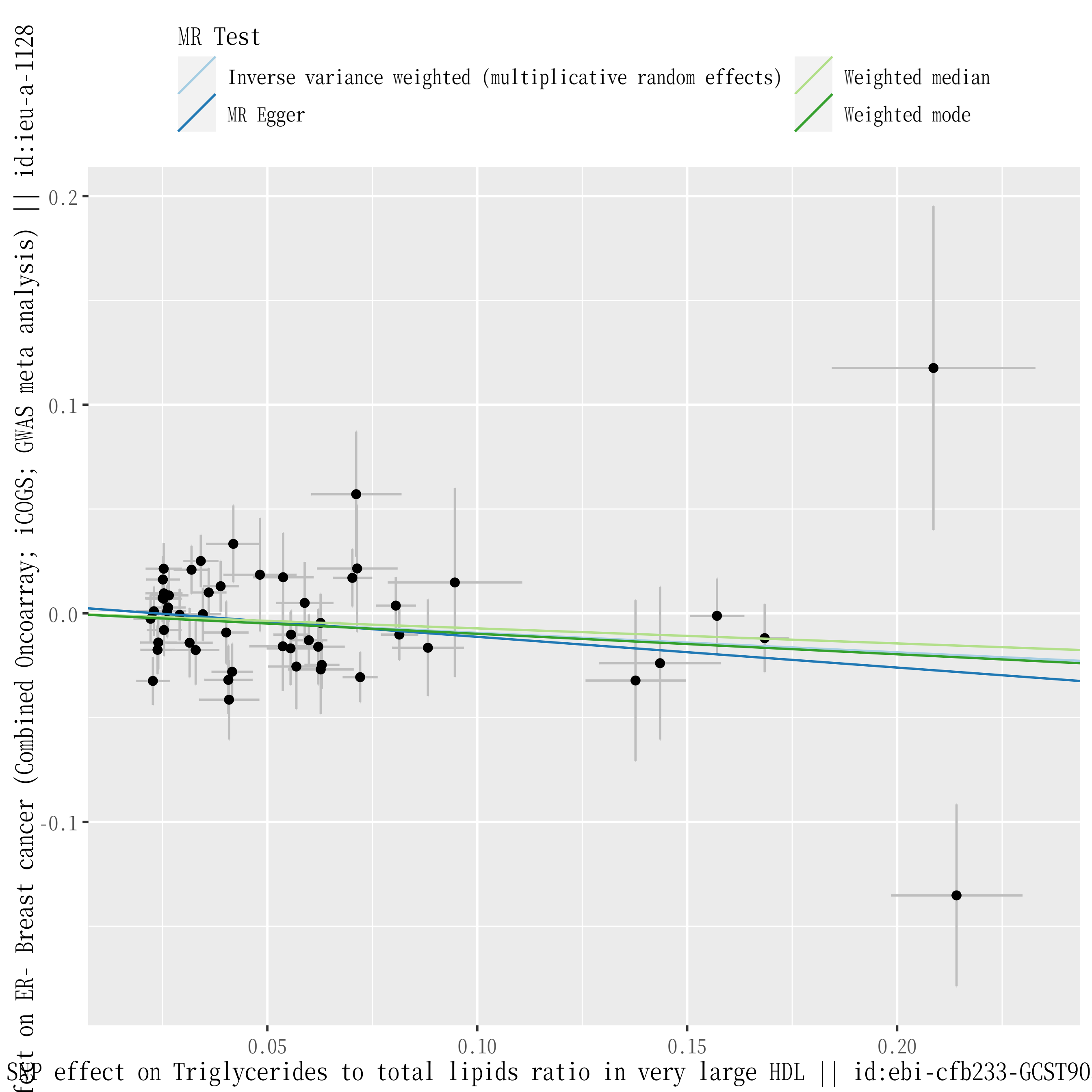
MR Test
Inverse variance weighted (multiplicative random effects)
MR Egger
Weighted median
Weighted mode
0.2
0.1
0.0
-0.1
0.05
0.10
0.15
0.20
SNP effect on Triglycerides to total lipids ratio in very large HDL || id:ebi-cfb233-GCST90
fect on ER- Breast cancer (Combined Oncoarray; iCOGS; GWAS meta analysis) || id:ieu-a-1128

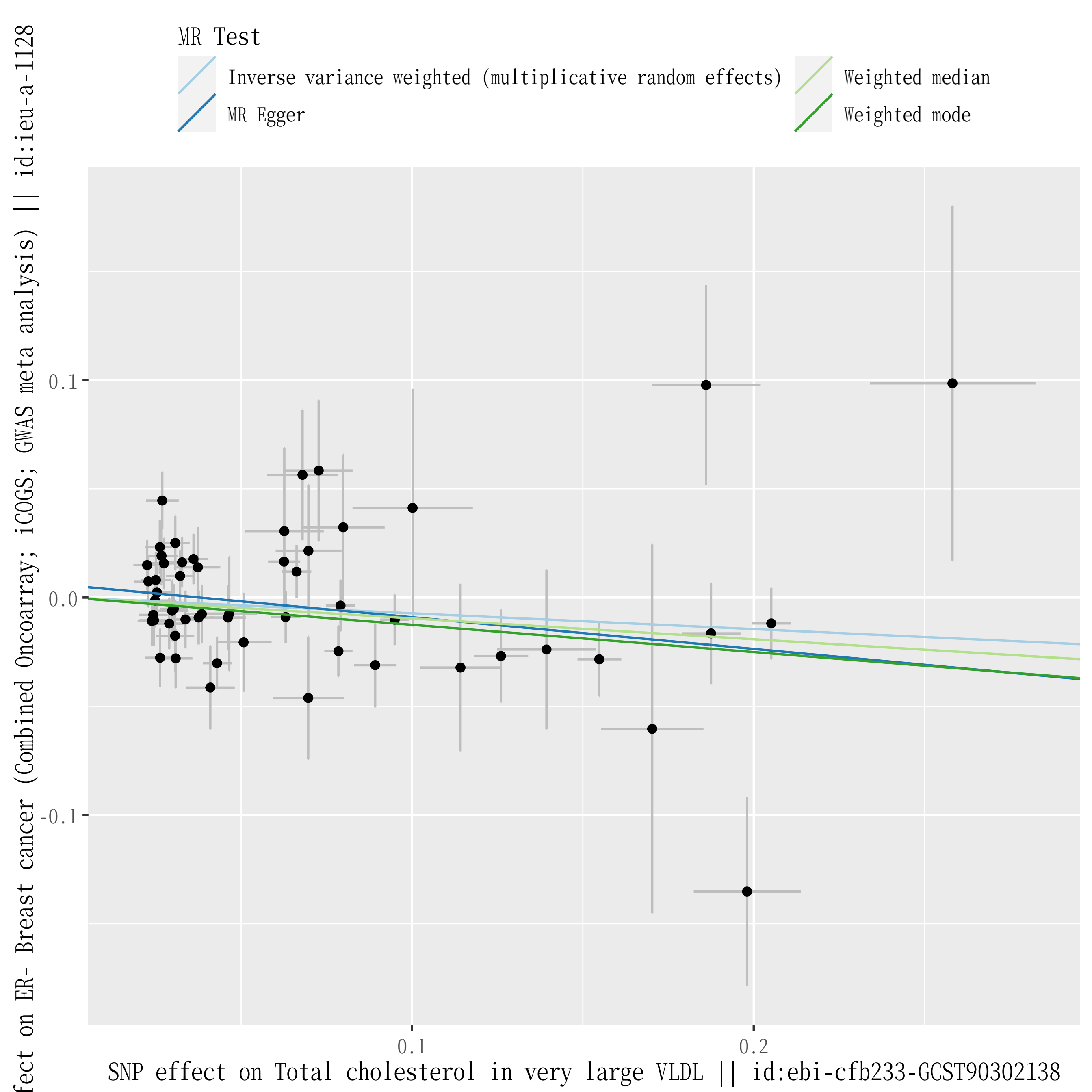
MR Test
Inverse variance weighted (multiplicative random effects)
MR Egger
Weighted median
Weighted mode
0.1
0.0
-0.1
0.1
0.2
SNP effect on Total cholesterol in very large VLDL || id:ebi-cfb233-GCST90302138
fect on ER- Breast cancer (Combined Oncoarray; iCOGS; GWAS meta analysis) || id:ieu-a-1128

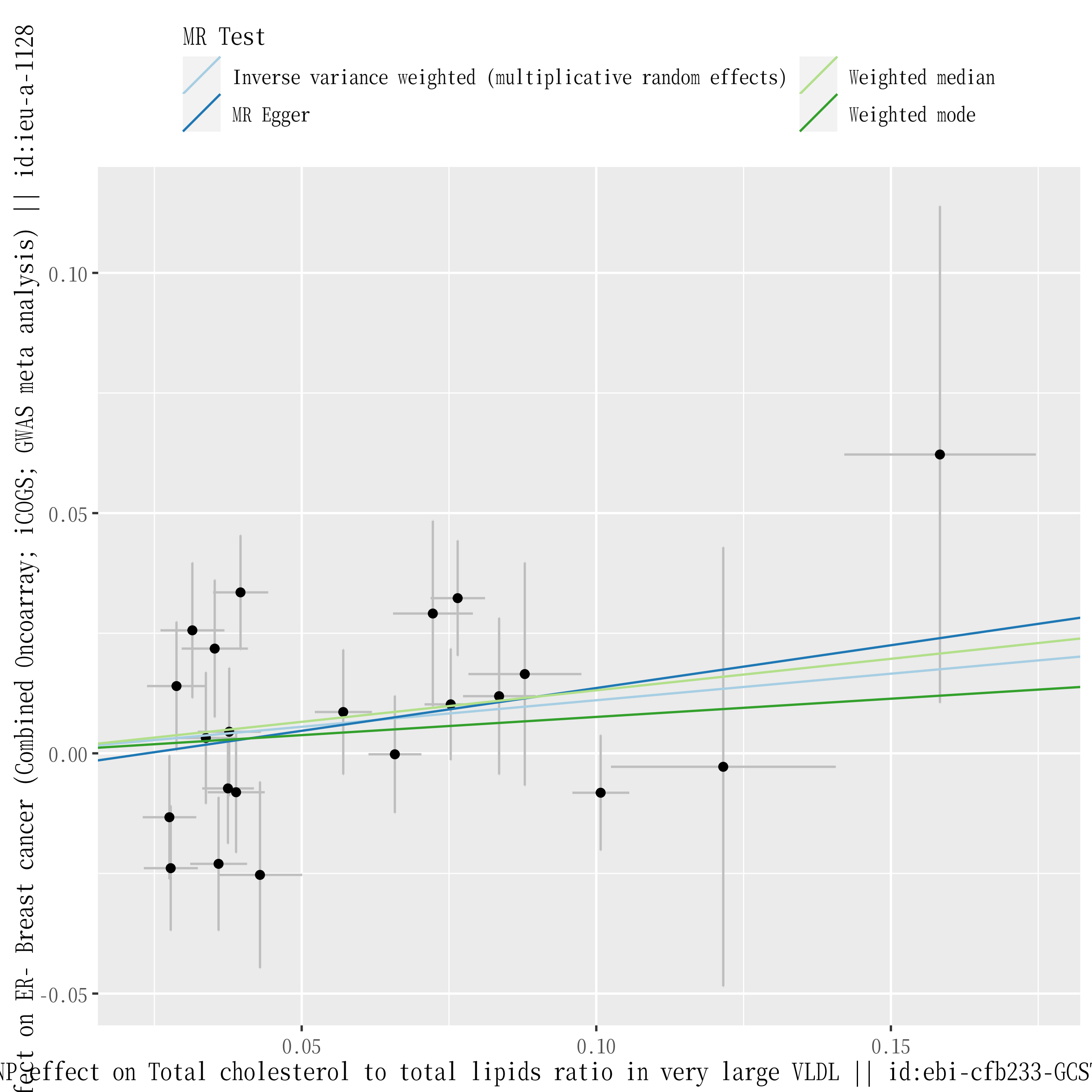

MR Test
Inverse variance weighted (multiplicative random effects)
MR Egger
Weighted median
Weighted mode
0.10
0.05
0.00
-0.05
0.05
0.10
0.15
fect on ER- Breast cancer (Combined Oncoarray; iCOGS; GWAS meta analysis) || id:ieu-a-1128
NP effect on Total cholesterol to total lipids ratio in very large VLDL || id:ebi-cfb233-GCS

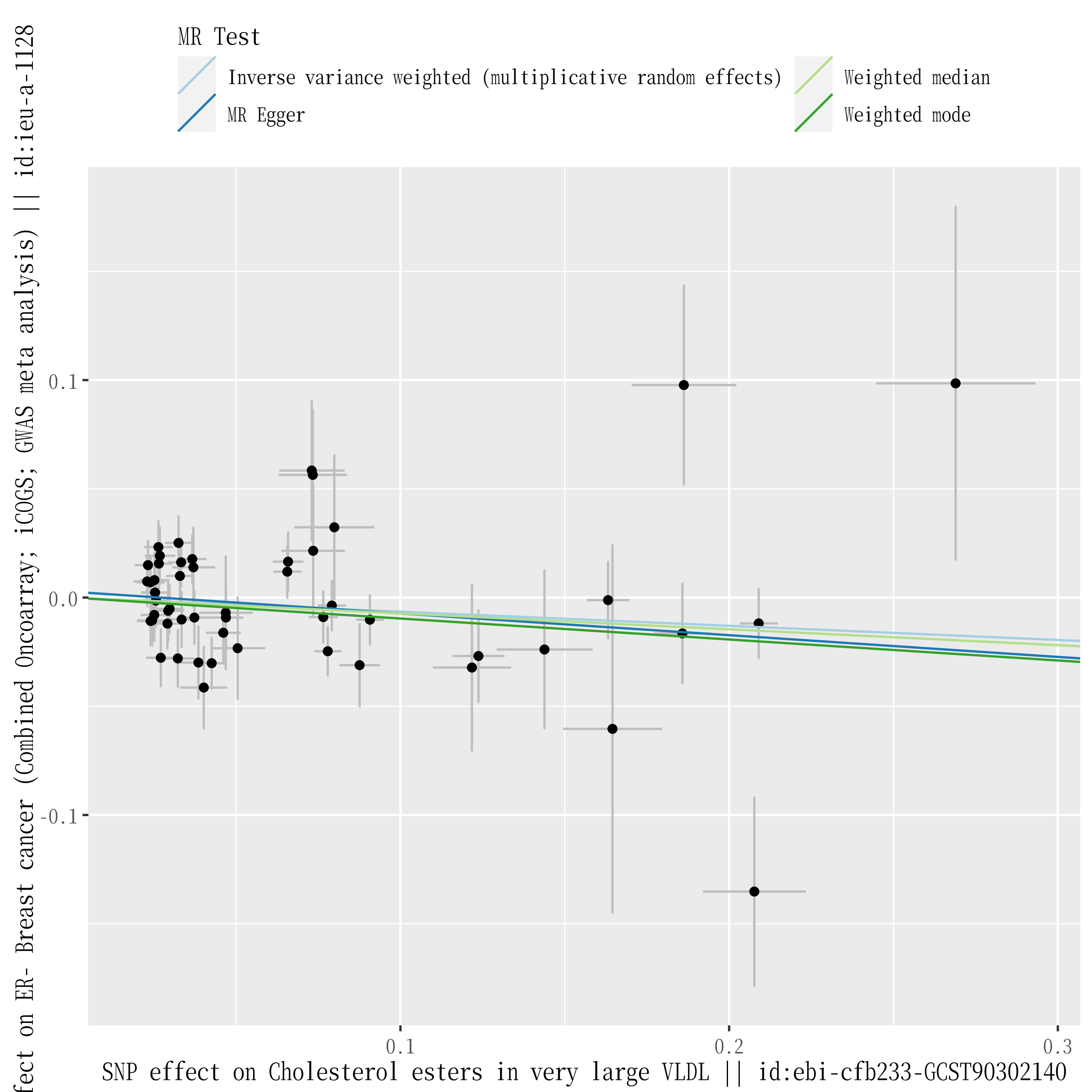
MR Test
Inverse variance weighted (multiplicative random effects)
MR Egger
Weighted median
Weighted mode
0.1
0.0
-0.1
0.1
0.2
0.3
SNP effect on Cholesterol esters in very large VLDL || id:ebi-cfb233-GCST90302140
fect on ER- Breast cancer (Combined Oncoarray; iCOGS; GWAS meta analysis) || id:ieu-a-1128

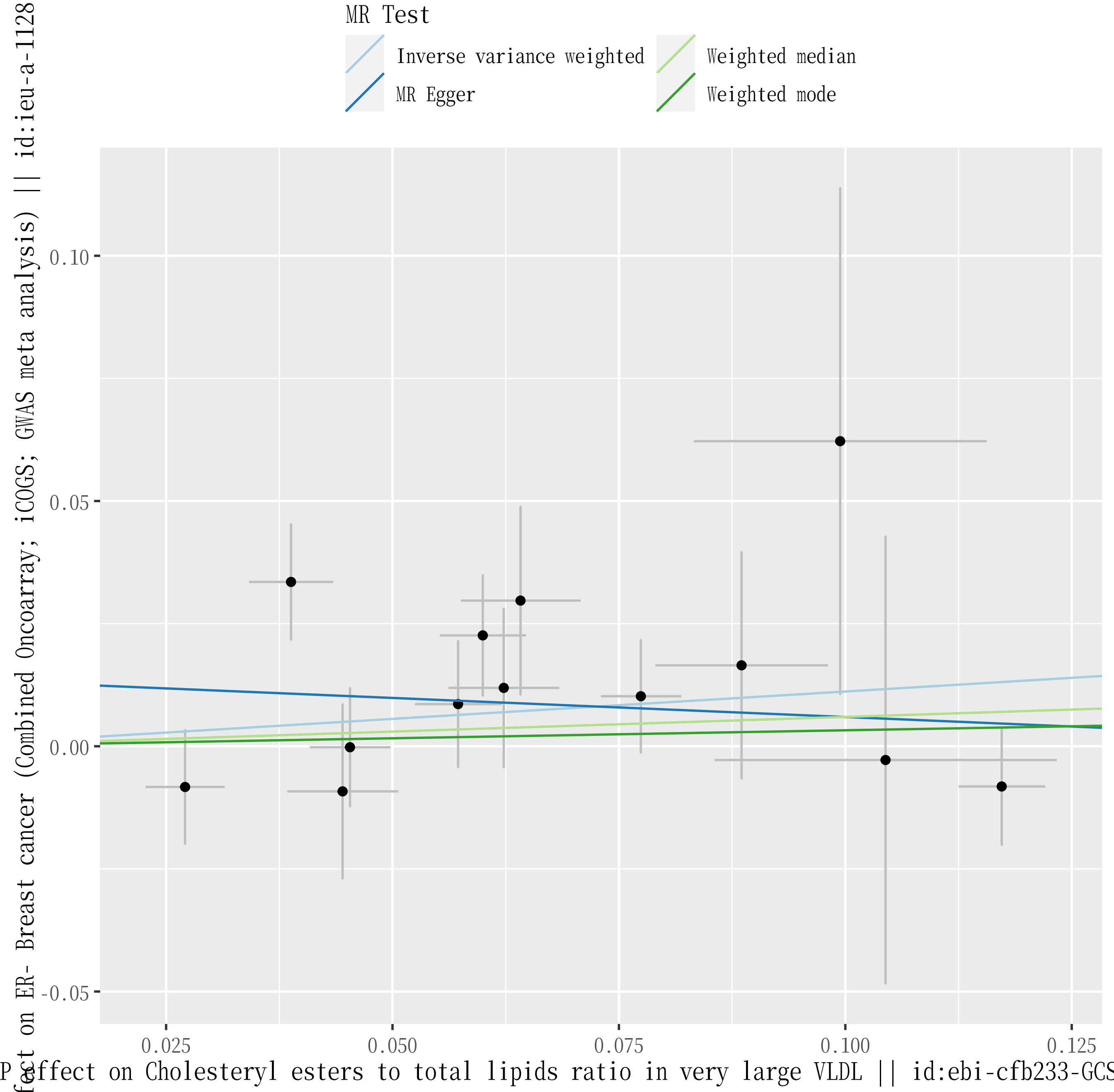

MR Test
Inverse variance weighted
MR Egger
Weighted median
Weighted mode
0.10
0.05
0.00
-0.05
0.025
0.050
0.075
0.100
0.125
P effect on Cholesteryl esters to total lipids ratio in very large VLDL || id:ebi-cfb233-GCS
fect on ER- Breast cancer (Combined Oncoarray; iCOGS; GWAS meta analysis) || id:ieu-a-1128

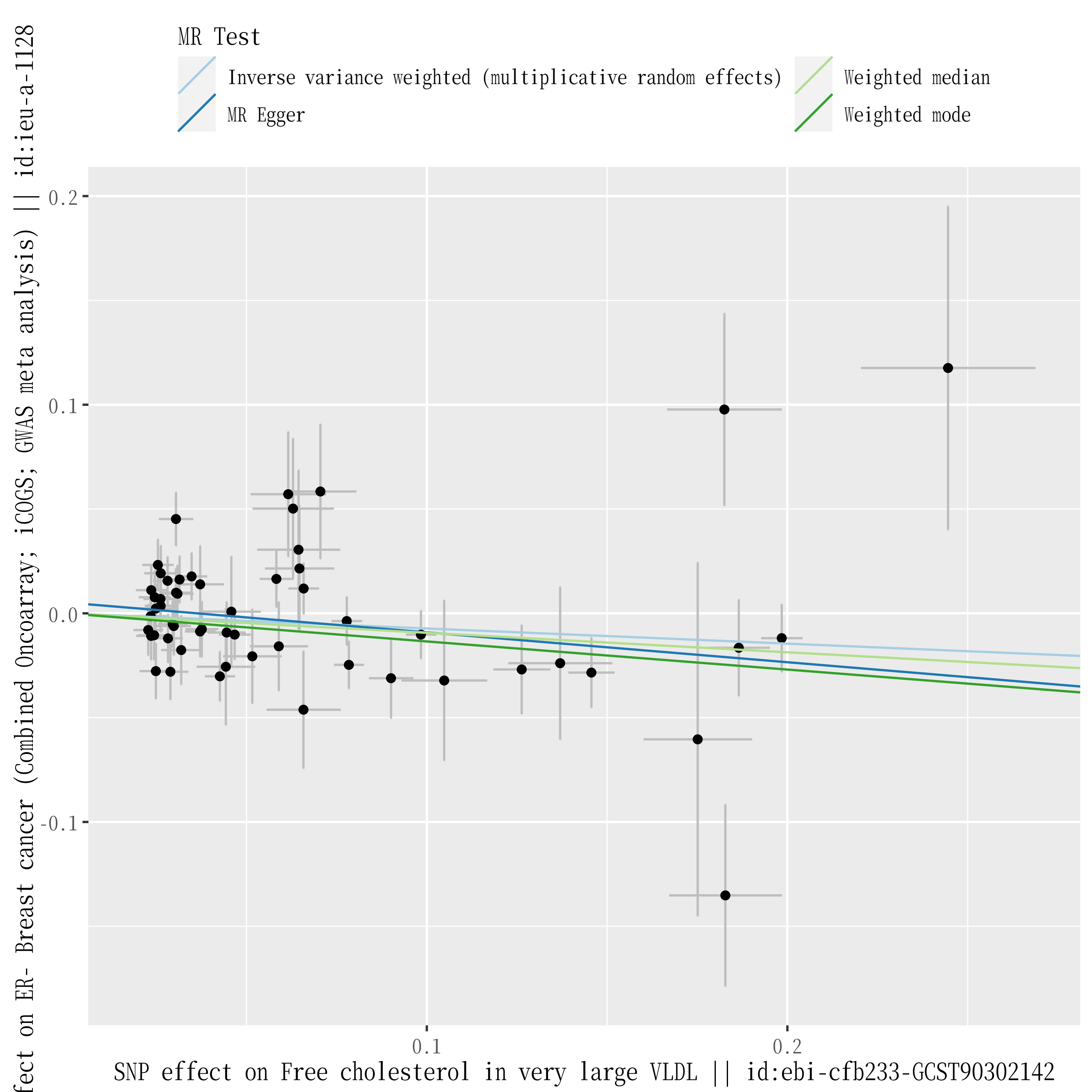
MR Test
Inverse variance weighted (multiplicative random effects)
MR Egger
Weighted median
Weighted mode
0.2
0.1
0.0
-0.1
0.1
0.2
SNP effect on Free cholesterol in very large VLDL || id:ebi-cfb233-GCST90302142
fect on ER- Breast cancer (Combined Oncoarray; iCOGS; GWAS meta analysis) || id:ieu-a-1128

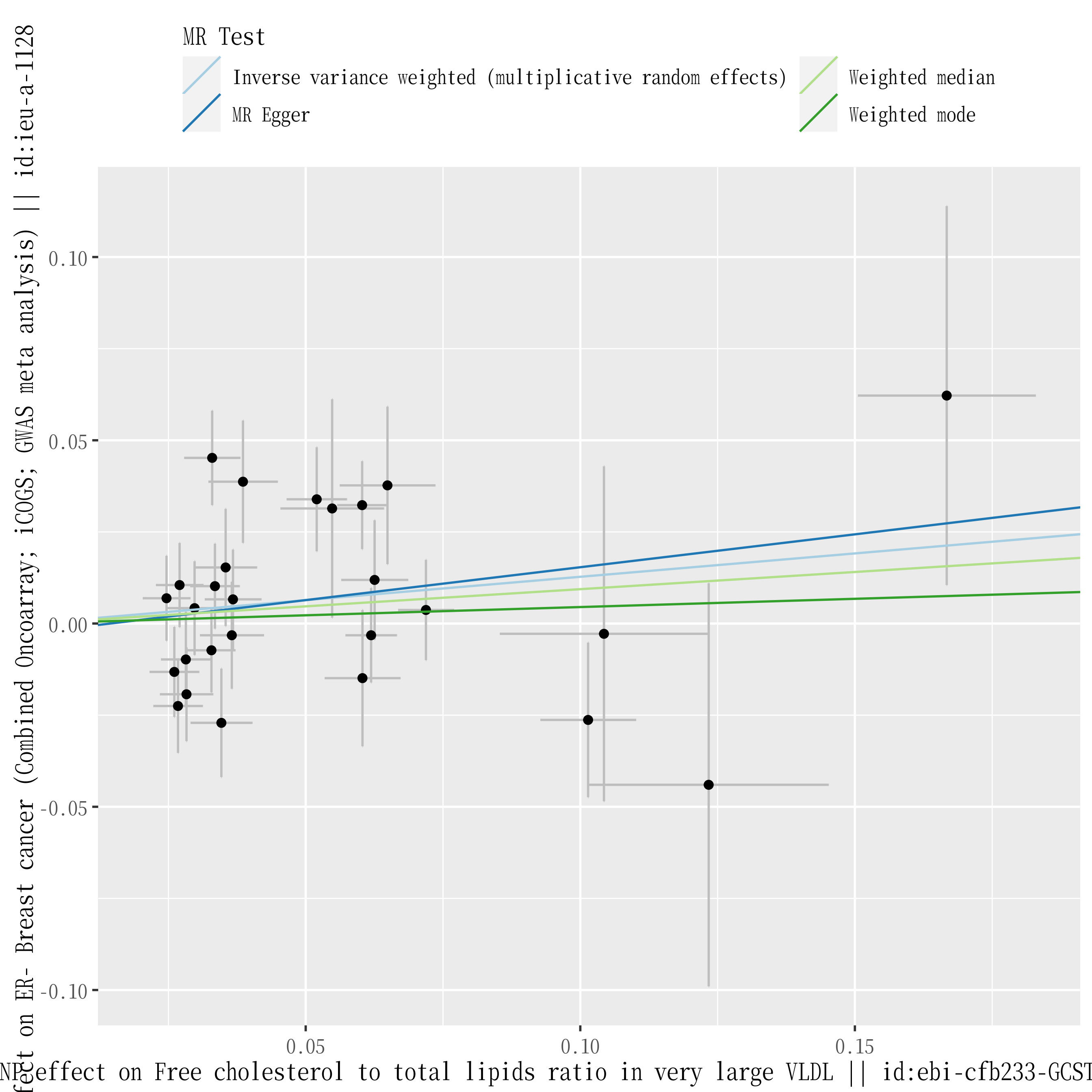
MR Test
Inverse variance weighted (multiplicative random effects)
MR Egger
Weighted median
Weighted mode
0.10
0.05
0.00
-0.05
-0.10
0.05
0.10
0.15
SNP effect on Free cholesterol to total lipids ratio in very large VLDL || id:ebi-a-GCST
SNP effect on ER- Breast cancer (Combined Oncoarray; iCOGS; GWAS meta analysis) || id:ieu-a-1128

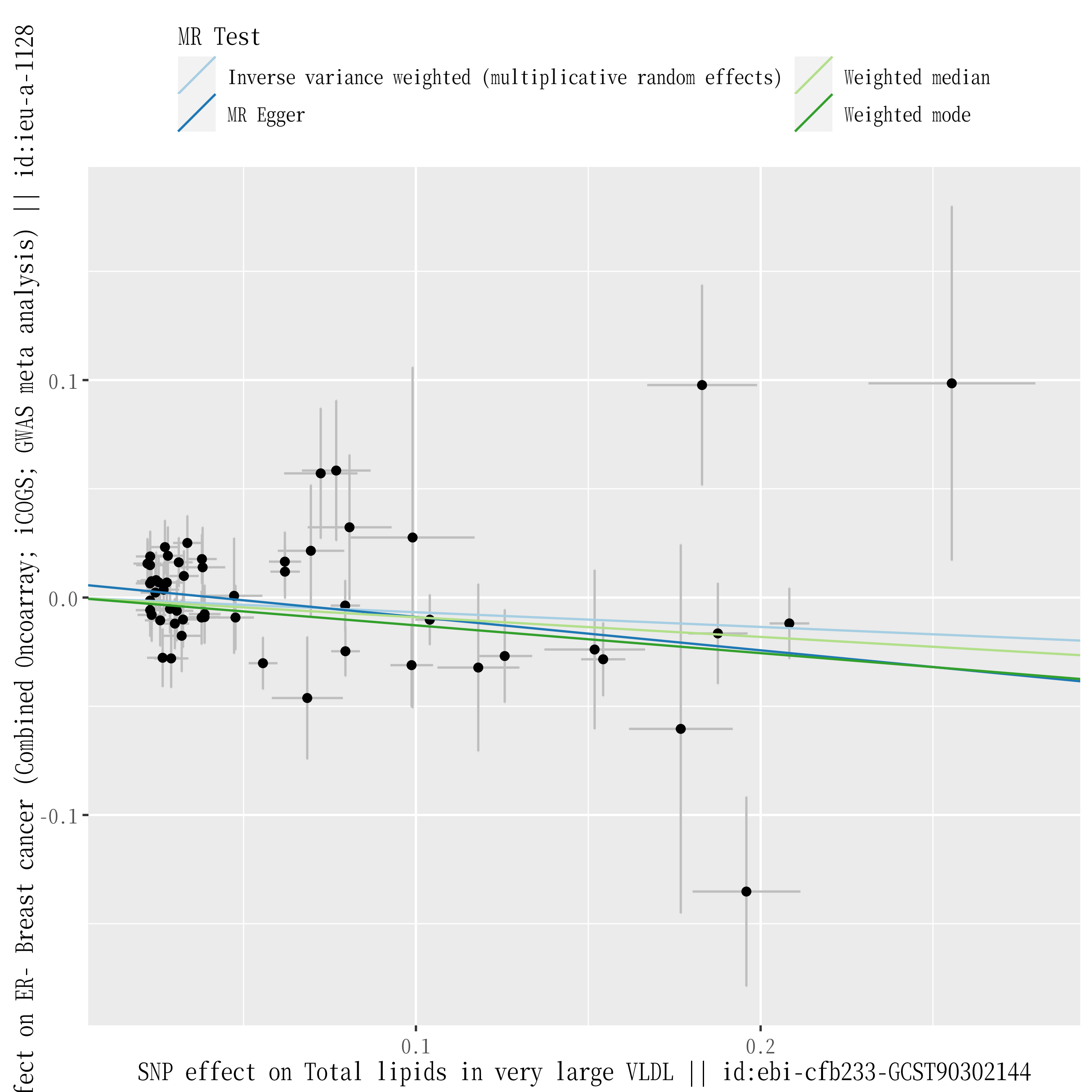
MR Test
Inverse variance weighted (multiplicative random effects)
MR Egger
Weighted median
Weighted mode
0.1
0.0
-0.1
0.1
0.2
SNP effect on Total lipids in very large VLDL || id:ebi-cfb233-GCST90302144
fect on ER- Breast cancer (Combined Oncoarray; iCOGS; GWAS meta analysis) || id:ieu-a-1128

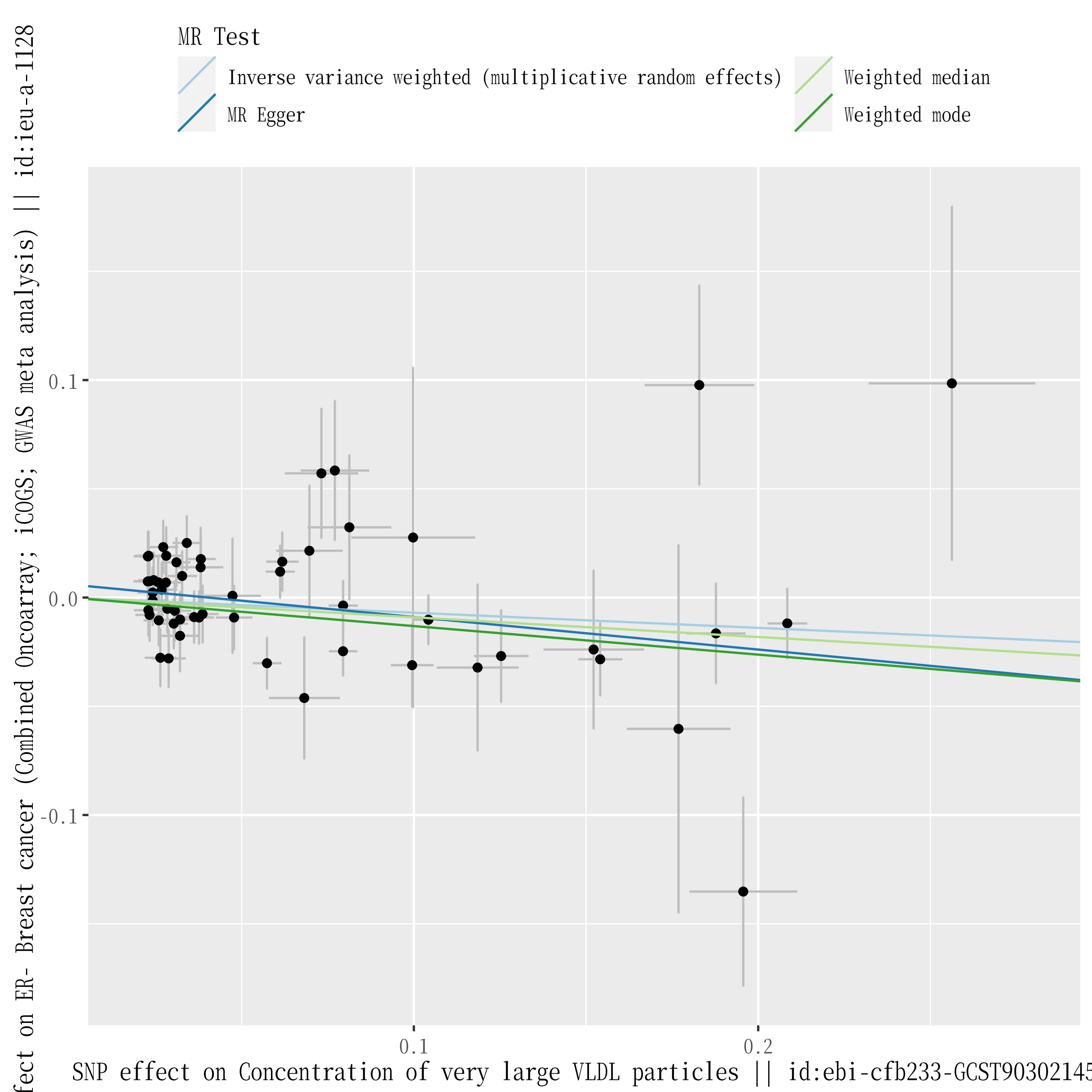

MR Test
Inverse variance weighted (multiplicative random effects)
MR Egger
Weighted median
Weighted mode
0.1
0.0
-0.1
0.1
0.2
SNP effect on Concentration of very large VLDL particles || id:ebi-cfb233-GCST90302145
fect on ER- Breast cancer (Combined Oncoarray; iCOGS; GWAS meta analysis) || id:ieu-a-1128

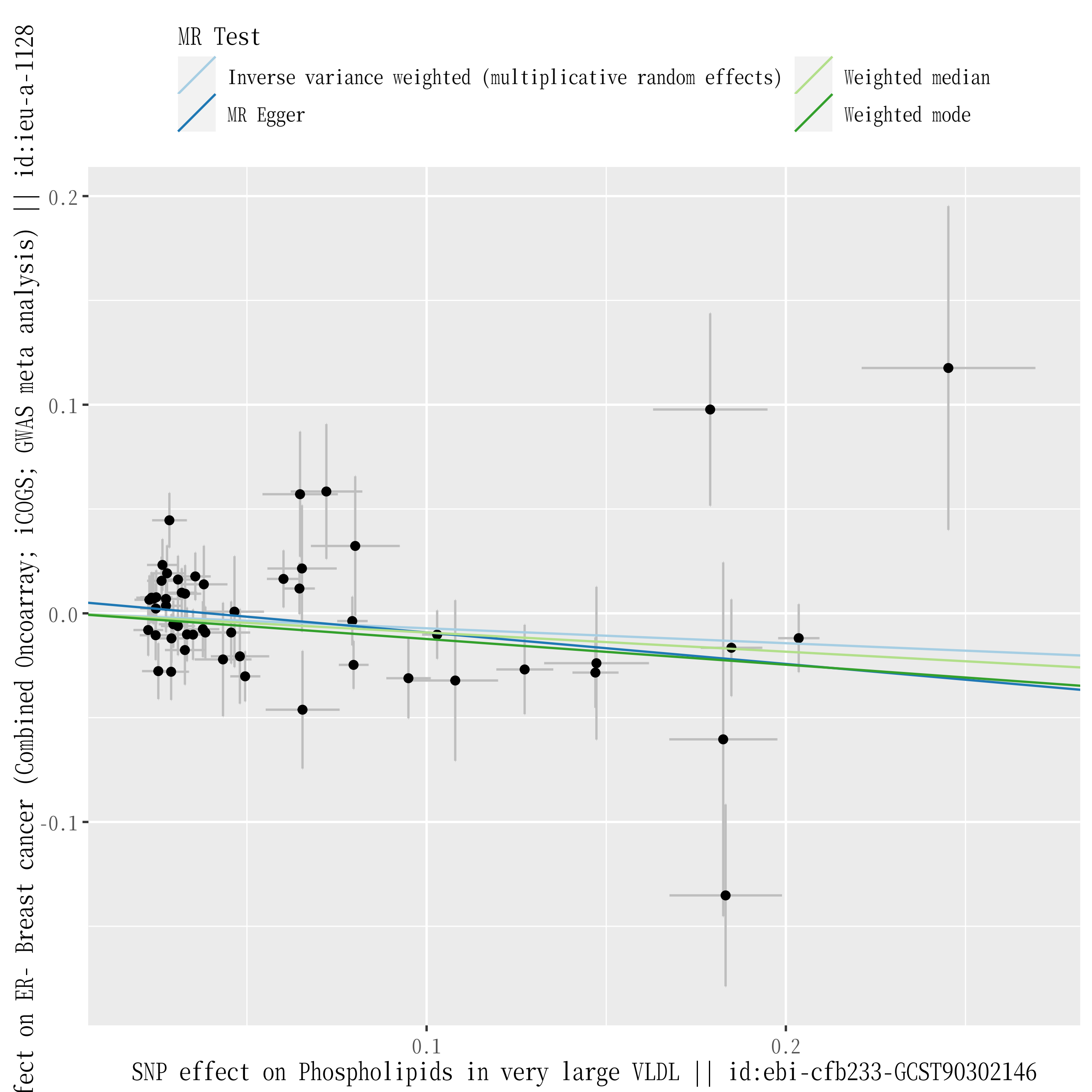
MR Test
Inverse variance weighted (multiplicative random effects)
MR Egger
Weighted median
Weighted mode
0.2
0.1
0.0
-0.1
0.1
0.2
SNP effect on Phospholipids in very large VLDL || id:ebi-cfb233-GCST90302146
fect on ER- Breast cancer (Combined Oncoarray; iCOGS; GWAS meta analysis) || id:ieu-a-1128

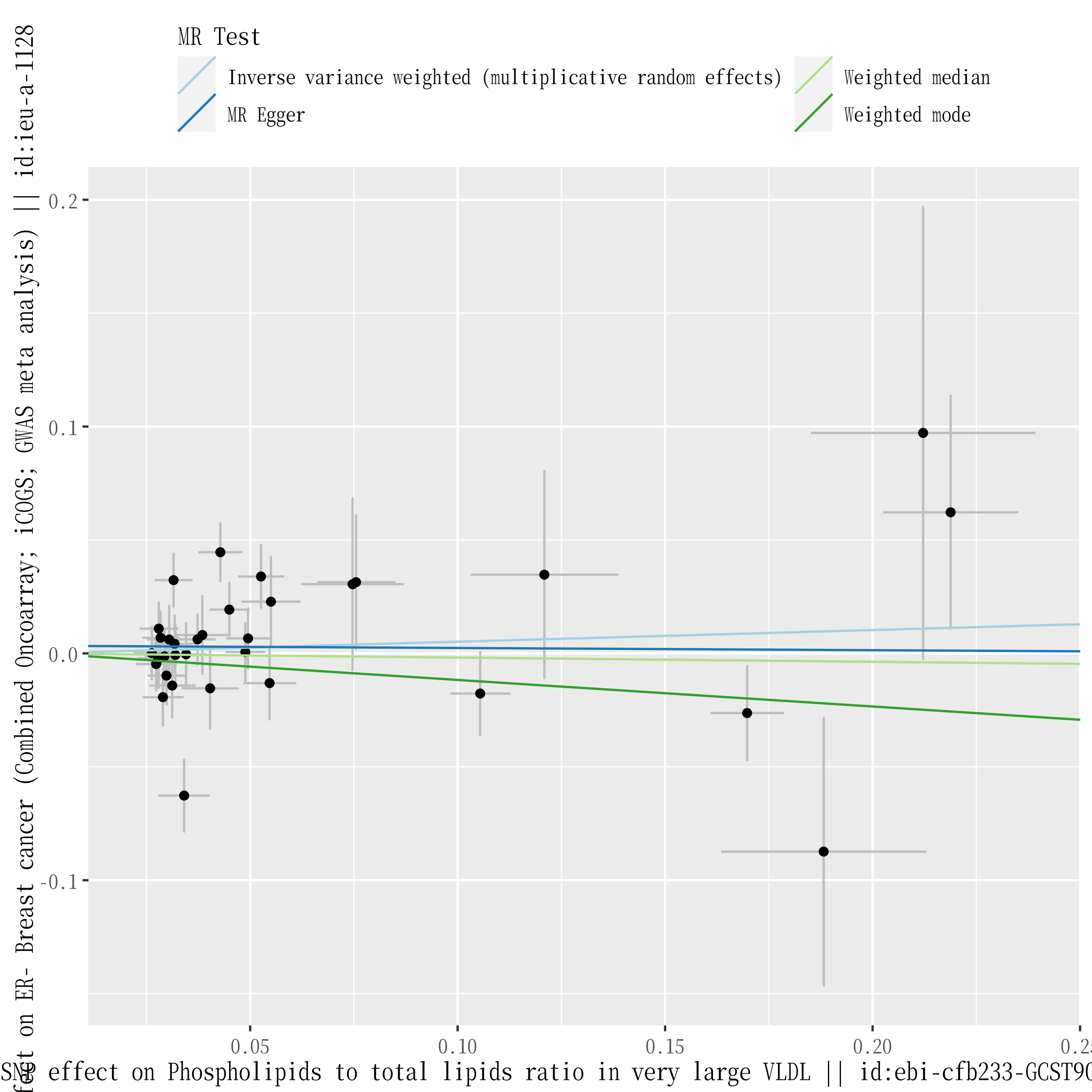

MR Test
Inverse variance weighted (multiplicative random effects)
MR Egger
Weighted median
Weighted mode
0.2
0.1
0.0
-0.1
0.05
0.10
0.15
0.20
SNP effect on Phospholipids to total lipids ratio in very large VLDL || id:ebi-cfb233-GCST90
SNP effect on ER- Breast cancer (Combined Oncoarray; iCOGS; GWAS meta analysis) || id:ieu-a-1128

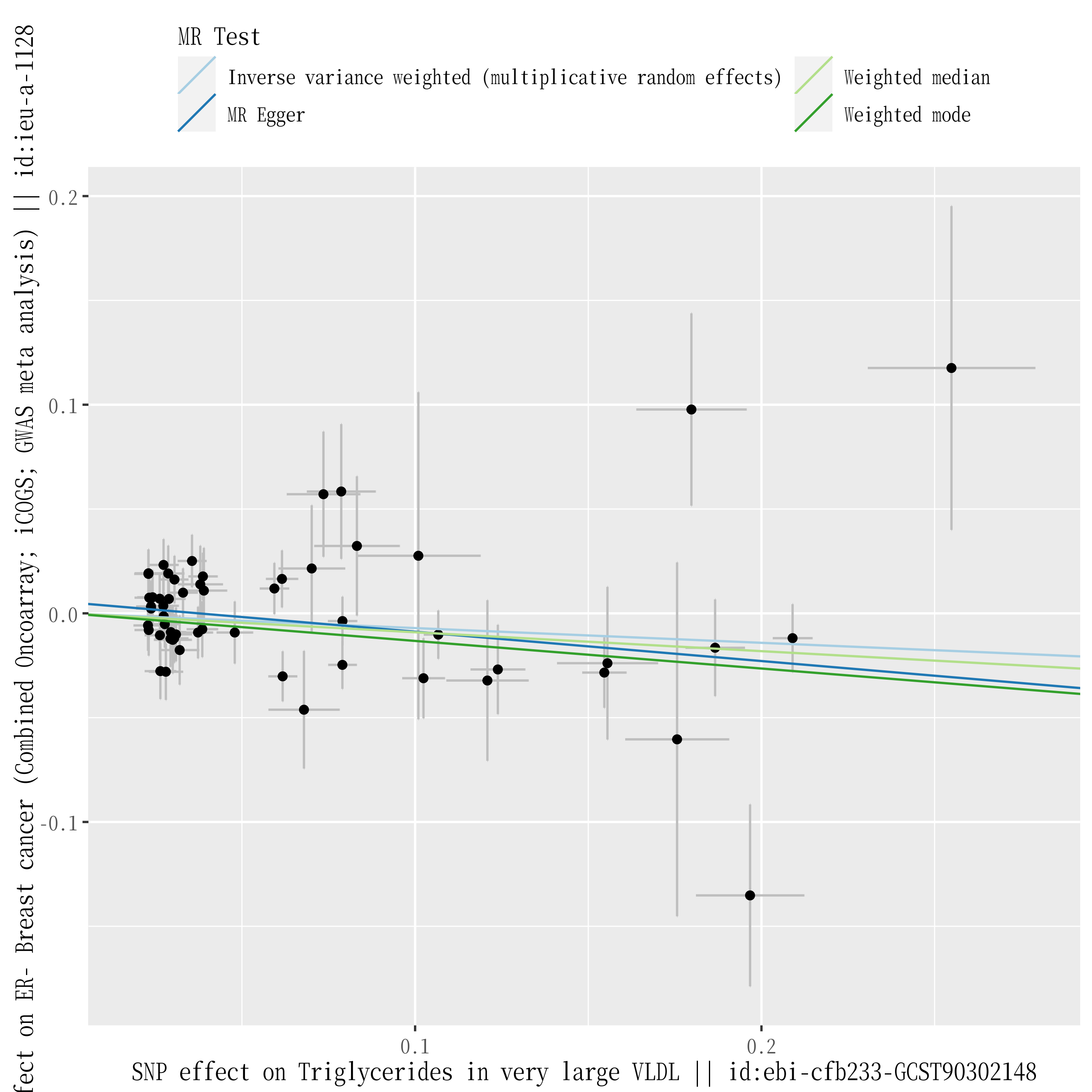

MR Test
Inverse variance weighted (multiplicative random effects)
MR Egger
Weighted median
Weighted mode
0.2
0.1
0.0
-0.1
0.1
0.2
SNP effect on Triglycerides in very large VLDL || id:ebi-cfb233-GCST90302148
fect on ER- Breast cancer (Combined Oncoarray; iCOGS; GWAS meta analysis) || id:ieu-a-1128

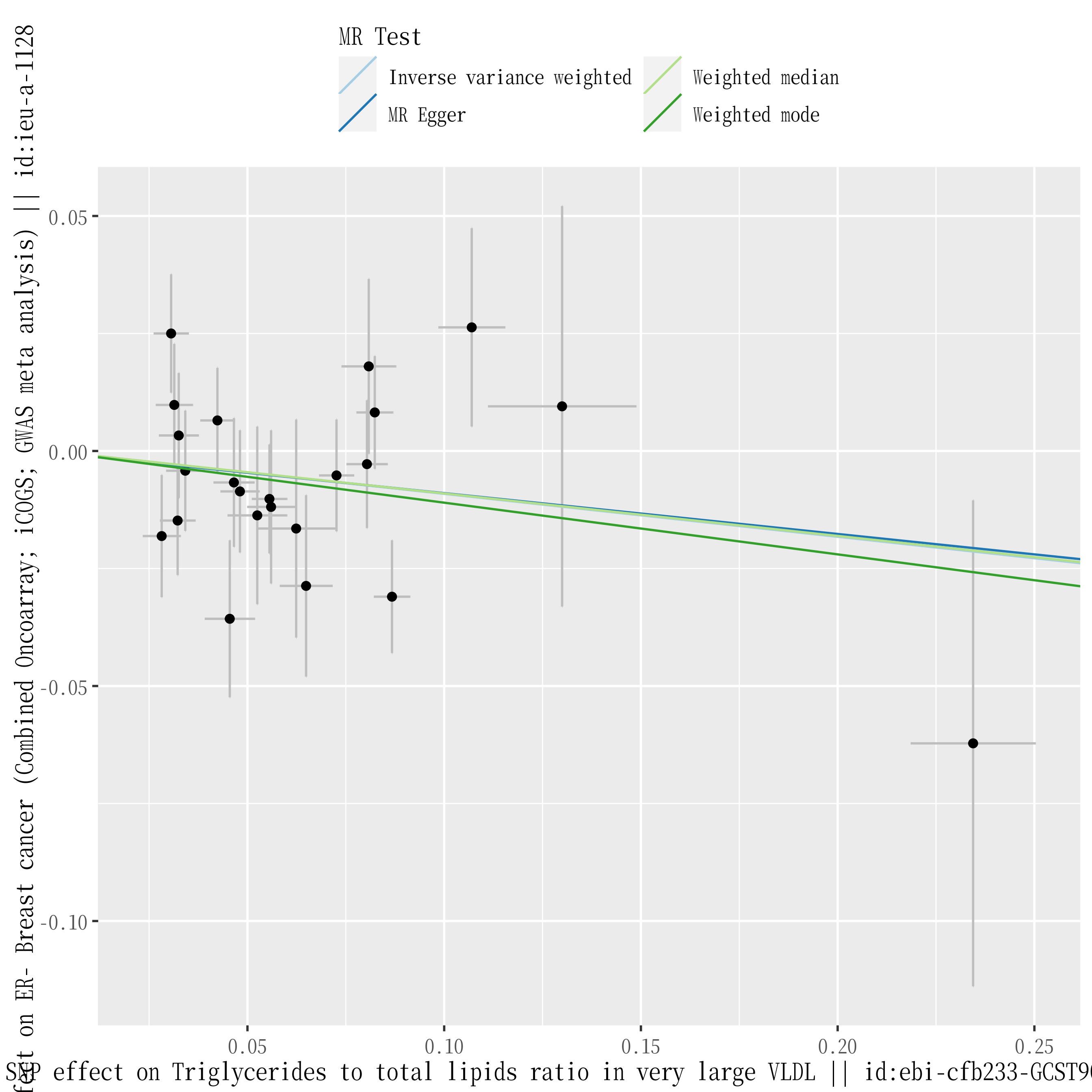
MR Test
Inverse variance weighted
MR Egger
Weighted median
Weighted mode
0.05
0.00
-0.05
-0.10
0.05
0.10
0.15
0.20
0.25
SNP effect on Triglycerides to total lipids ratio in very large VLDL || id:ebi-cfb233-GCST90
SNP effect on ER- Breast cancer (Combined Oncoarray; iCOGS; GWAS meta analysis) || id:ieu-a-1128

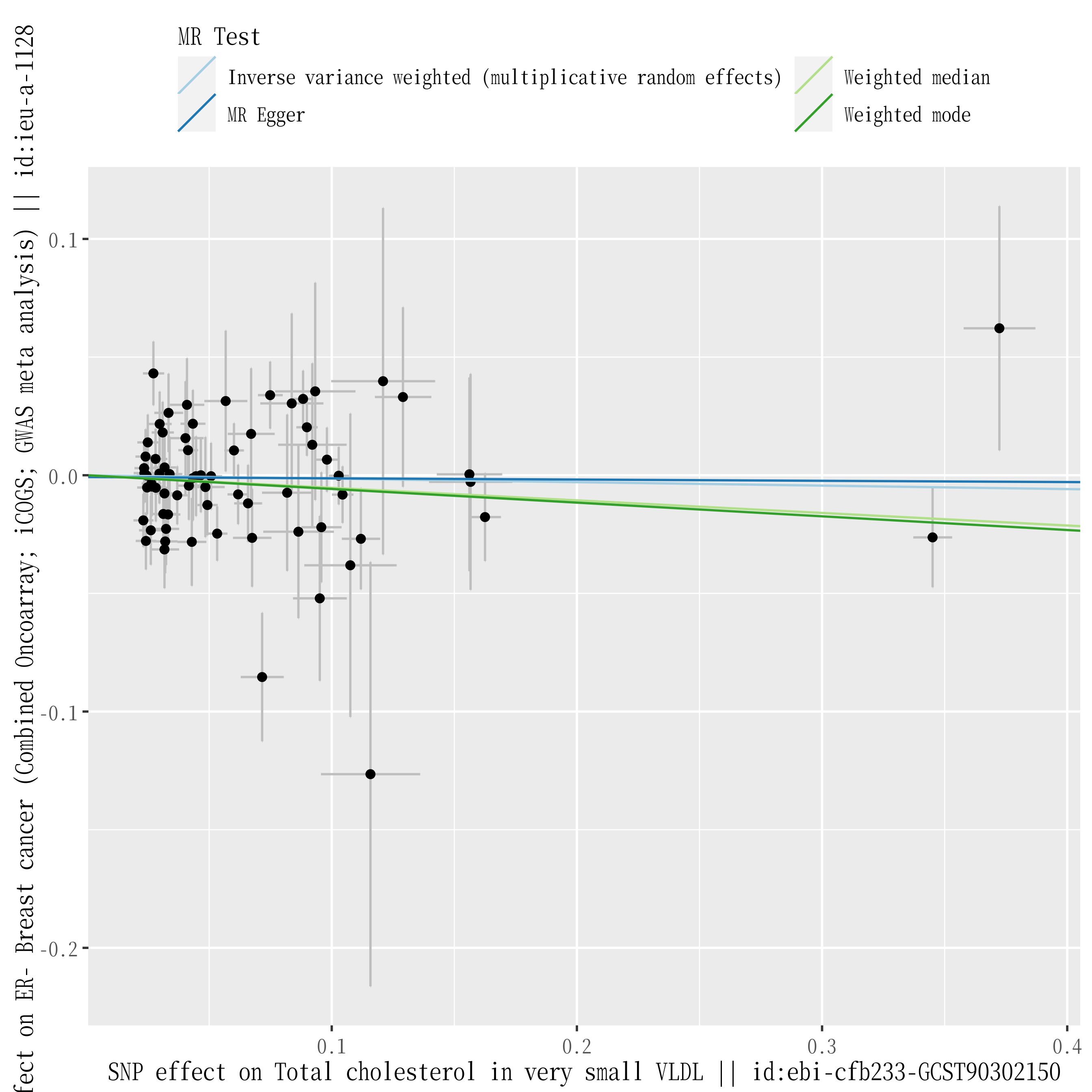
MR Test
Inverse variance weighted (multiplicative random effects)
MR Egger
Weighted median
Weighted mode
0.1
0.0
-0.1
-0.2
0.1
0.2
0.3
0.4
SNP effect on Total cholesterol in very small VLDL || id:ebi-cfb233-GCST90302150
fect on ER- Breast cancer (Combined Oncoarray; iCOGS; GWAS meta analysis) || id:ieu-a-1128

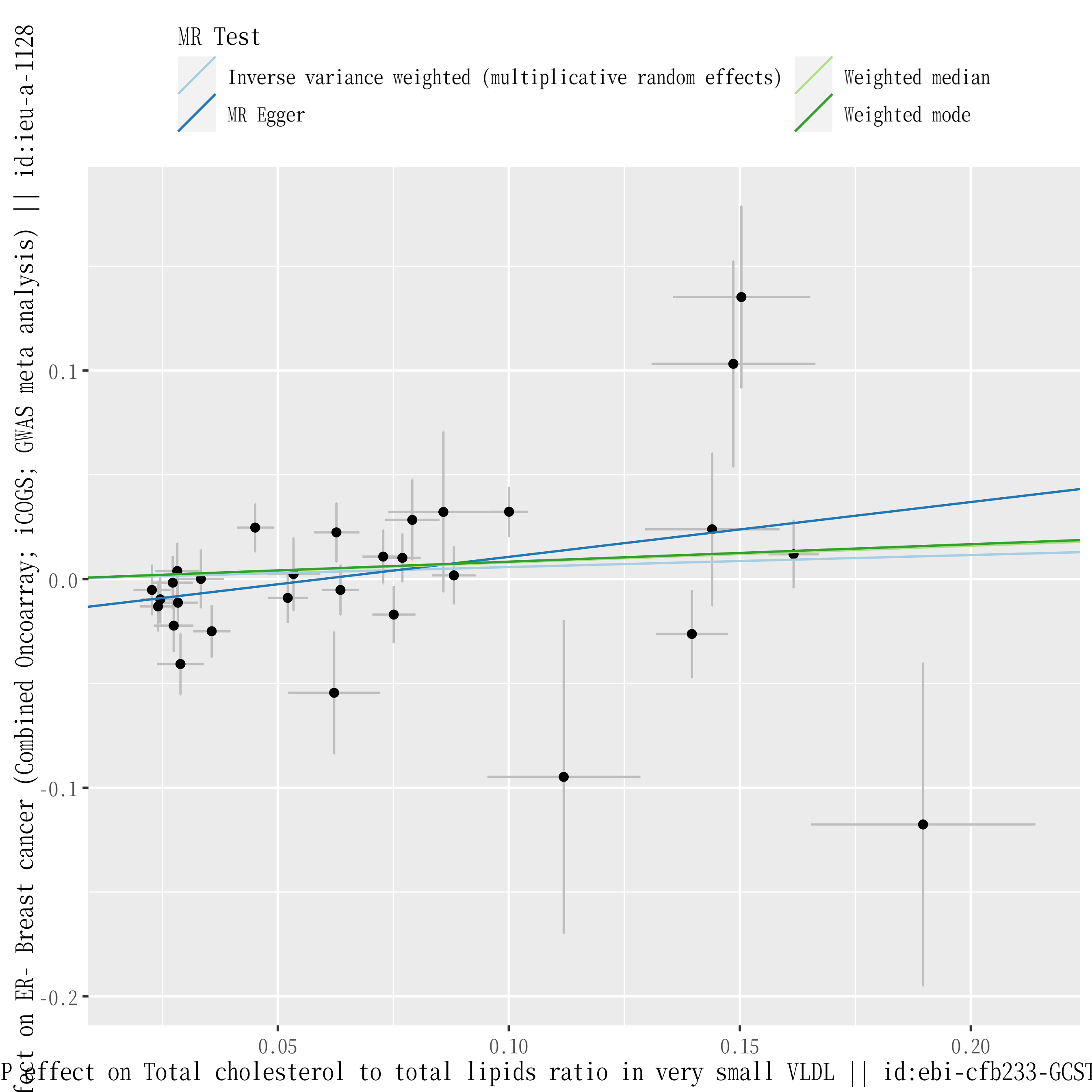

MR Test
Inverse variance weighted (multiplicative random effects)
MR Egger
Weighted median
Weighted mode
0.1
0.0
-0.1
-0.2
0.05
0.10
0.15
0.20
P effect on Total cholesterol to total lipids ratio in very small VLDL || id:ebi-cfb233-GCST
fect on ER- Breast cancer (Combined Oncoarray; iCOGS; GWAS meta analysis) || id:ieu-a-1128

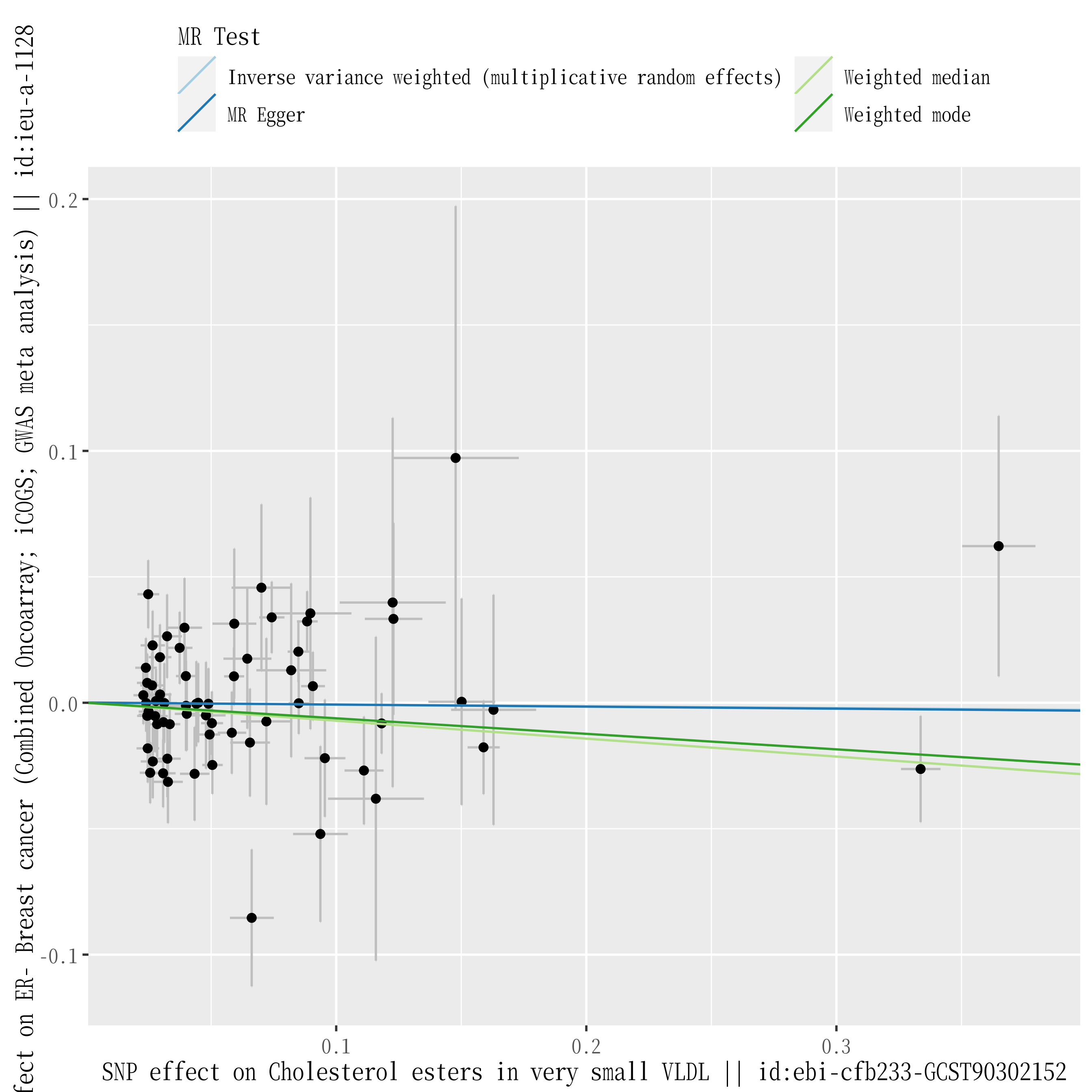

MR Test
Inverse variance weighted (multiplicative random effects)
MR Egger
Weighted median
Weighted mode
0.2
0.1
0.0
-0.1
0.1
0.2
0.3
SNP effect on Cholesterol esters in very small VLDL || id:ebi-cfb233-GCST90302152
fect on ER- Breast cancer (Combined Oncoarray; iCOGS; GWAS meta analysis) || id:ieu-a-1128

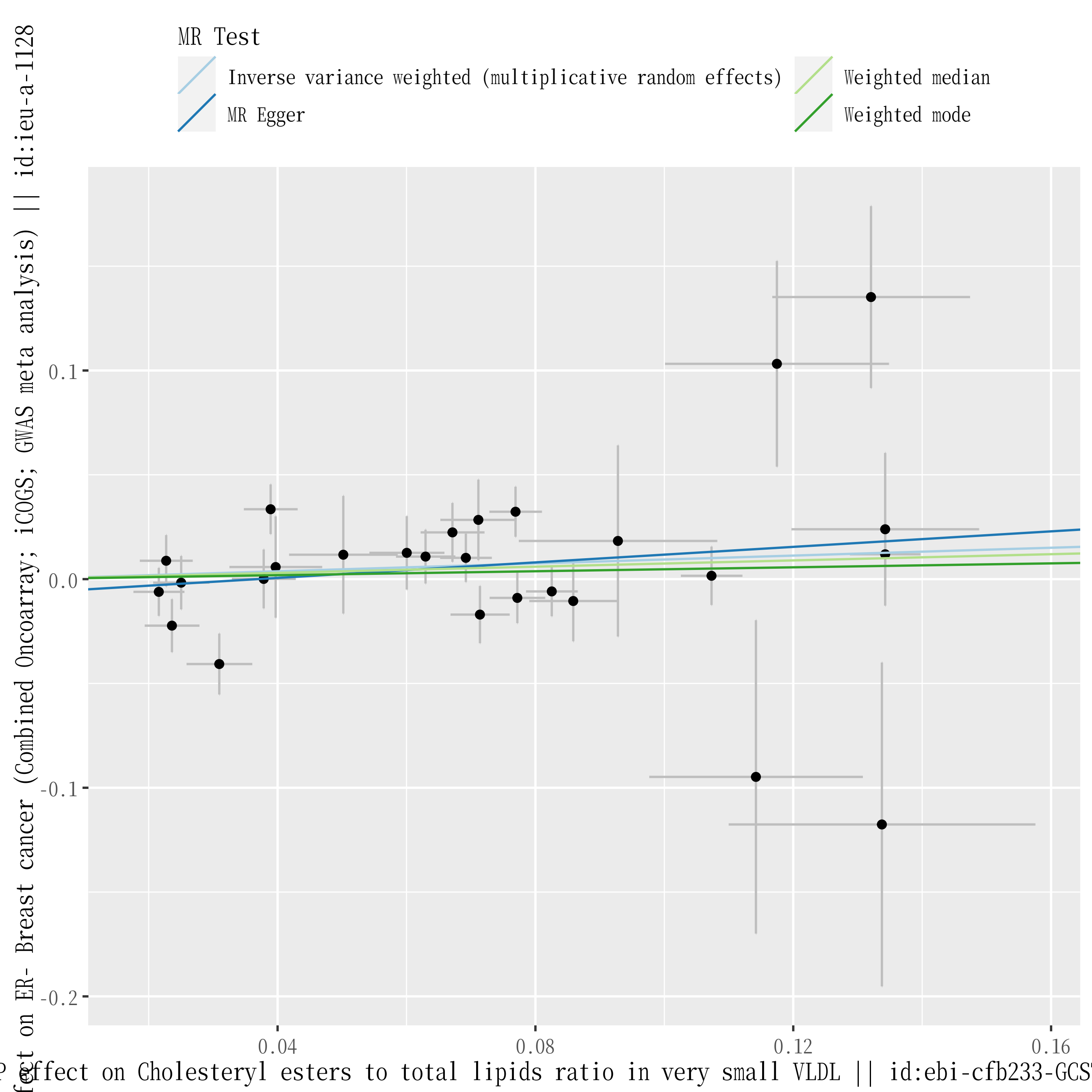

MR Test
Inverse variance weighted (multiplicative random effects)
MR Egger
Weighted median
Weighted mode
0.1
0.0
-0.1
-0.2
0.04
0.08
0.12
0.16
SNP effect on Cholesteryl esters to total lipids ratio in very small VLDL || id:ebi-cfb233-GCST
SNP effect on ER- Breast cancer (Combined Oncoarray; iCOGS; GWAS meta analysis) || id:ieu-a-1128

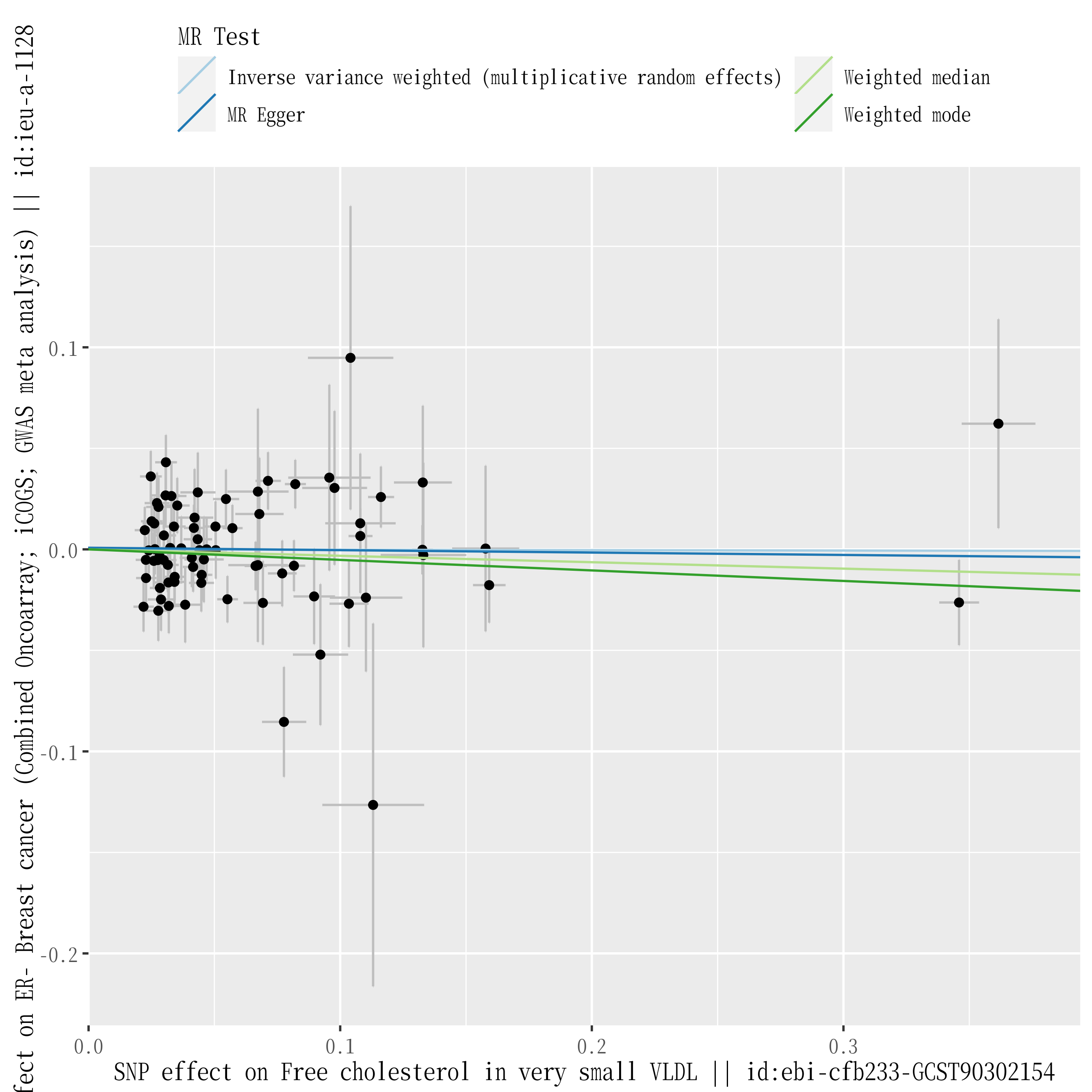
MR Test
Inverse variance weighted (multiplicative random effects)
MR Egger
Weighted median
Weighted mode
0.1
0.0
-0.1
-0.2
0.0
0.1
0.2
0.3
SNP effect on Free cholesterol in very small VLDL || id:ebi-cfb233-GCST90302154
fect on ER- Breast cancer (Combined Oncoarray; iCOGS; GWAS meta analysis) || id:ieu-a-1128

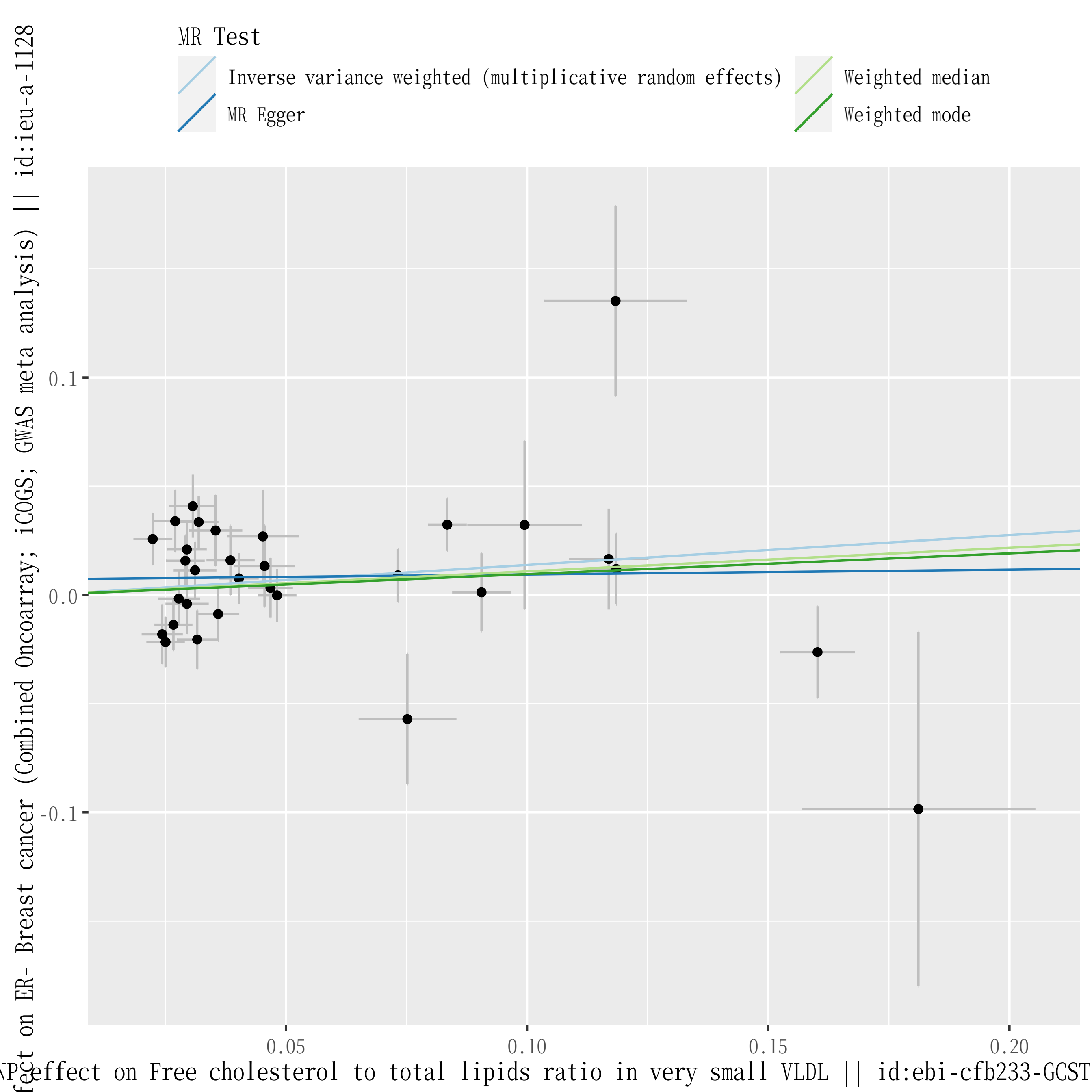
MR Test
Inverse variance weighted (multiplicative random effects)
MR Egger
Weighted median
Weighted mode
0.1
0.0
-0.1
0.05
0.10
0.15
0.20
NP effect on Free cholesterol to total lipids ratio in very small VLDL || id:ebi-cfb233-GCST
fect on ER- Breast cancer (Combined Oncoarray; iCOGS; GWAS meta analysis) || id:ieu-a-1128

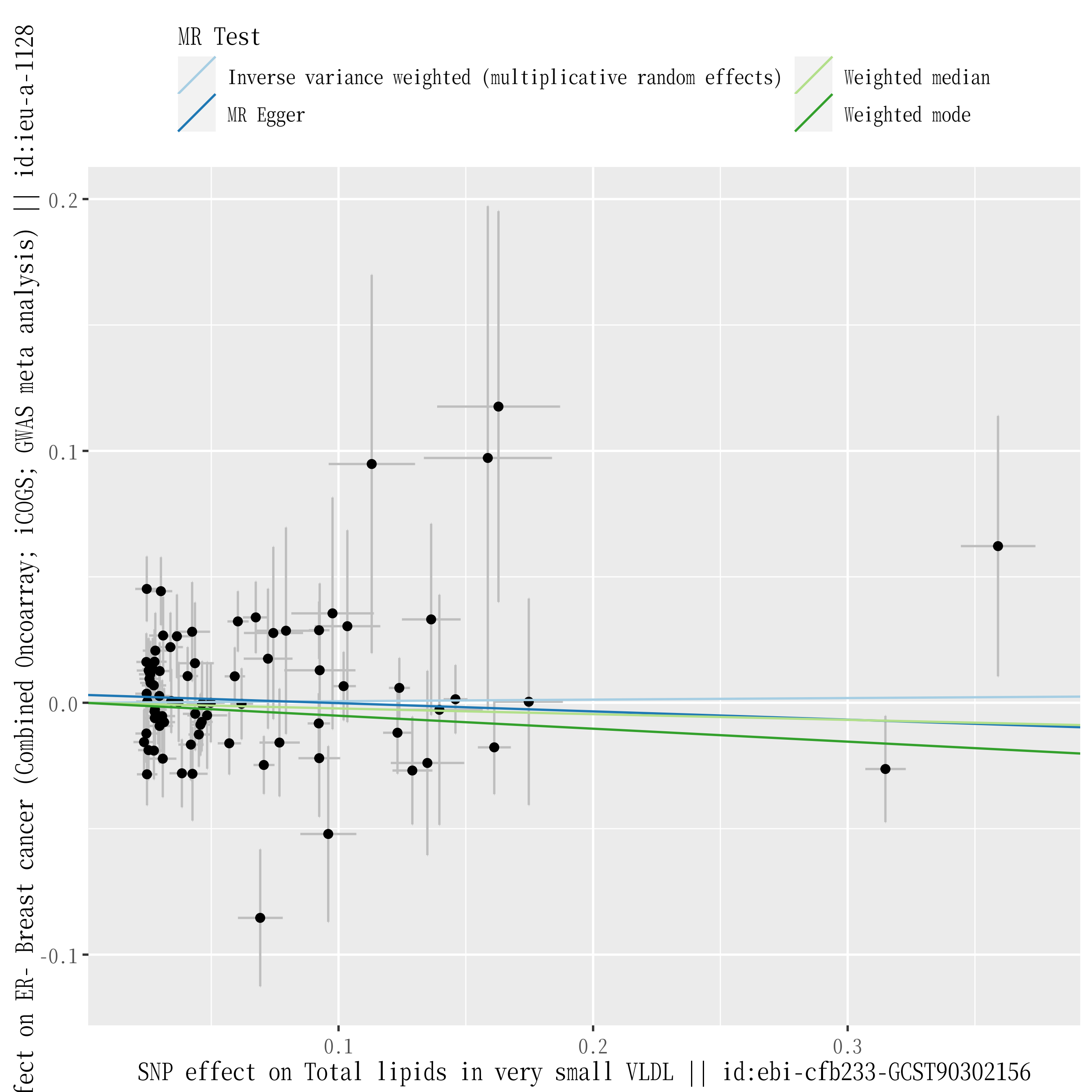

MR Test
Inverse variance weighted (multiplicative random effects)
MR Egger
Weighted median
Weighted mode
0.2
0.1
0.0
-0.1
0.1
0.2
0.3
SNP effect on Total lipids in very small VLDL || id:ebi-cfb233-GCST90302156
fect on ER- Breast cancer (Combined Oncoarray; iCOGS; GWAS meta analysis) || id:ieu-a-1128

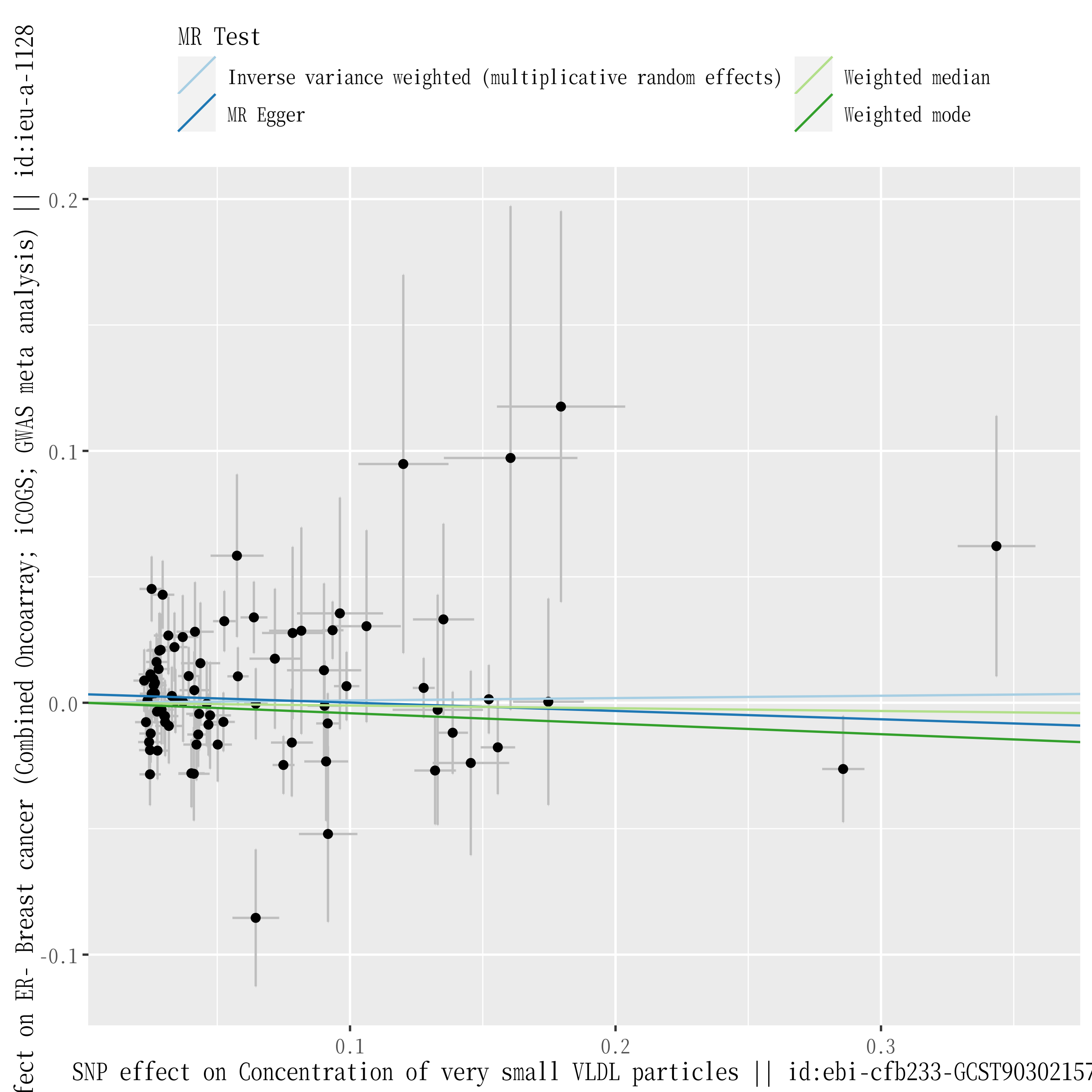
MR Test
Inverse variance weighted (multiplicative random effects)
MR Egger
Weighted median
Weighted mode
0.2
0.1
0.0
-0.1
0.1
0.2
0.3
SNP effect on Concentration of very small VLDL particles || id:ebi-cfb233-GCST90302157
fect on ER- Breast cancer (Combined Oncoarray; iCOGS; GWAS meta analysis) || id:ieu-a-1128

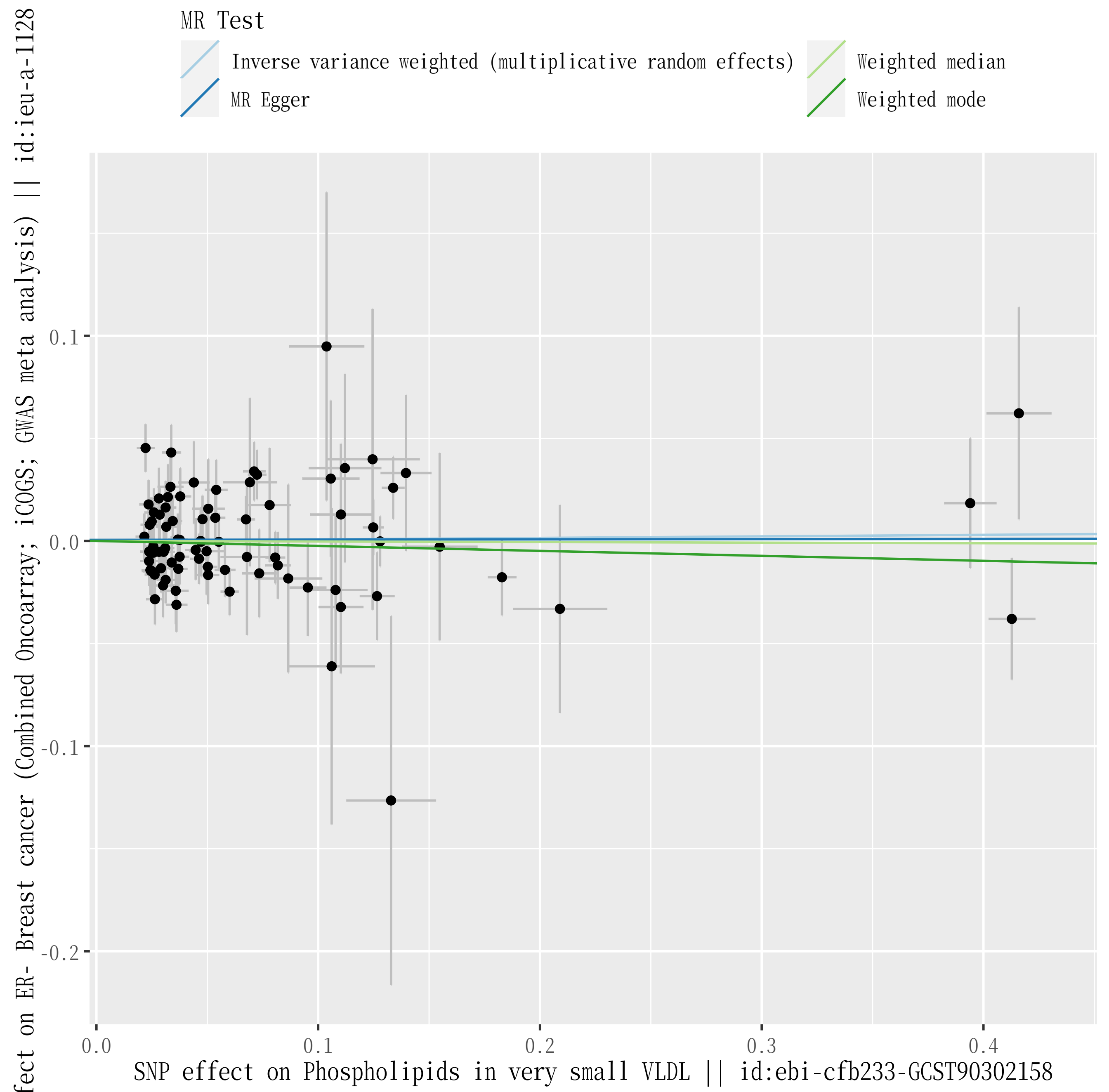
MR Test
Inverse variance weighted (multiplicative random effects)
MR Egger
Weighted median
Weighted mode
0.1
0.0
-0.1
-0.2
0.0
0.1
0.2
0.3
0.4
SNP effect on Phospholipids in very small VLDL || id:ebi-cfb233-GCST90302158
fect on ER- Breast cancer (Combined Oncoarray; iCOGS; GWAS meta analysis) || id:ieu-a-1128

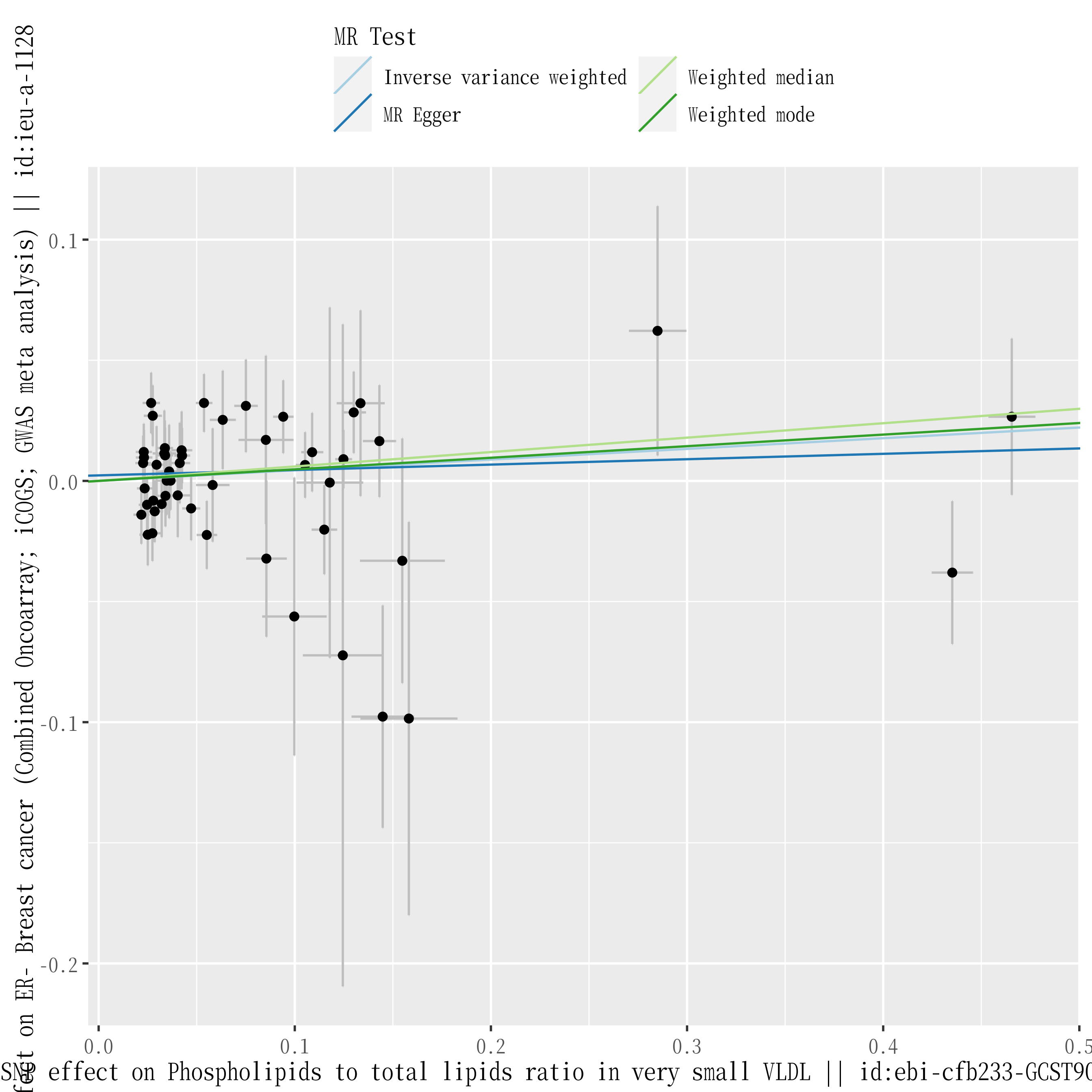

MR Test
Inverse variance weighted
MR Egger
Weighted median
Weighted mode
0.1
0.0
-0.1
-0.2
0.0
0.1
0.2
0.3
0.4
0.5
SNP effect on Phospholipids to total lipids ratio in very small VLDL || id:ebi-cfb233-GCST90
SNP effect on ER- Breast cancer (Combined Oncoarray; iCOGS; GWAS meta analysis) || id:ieu-a-1128

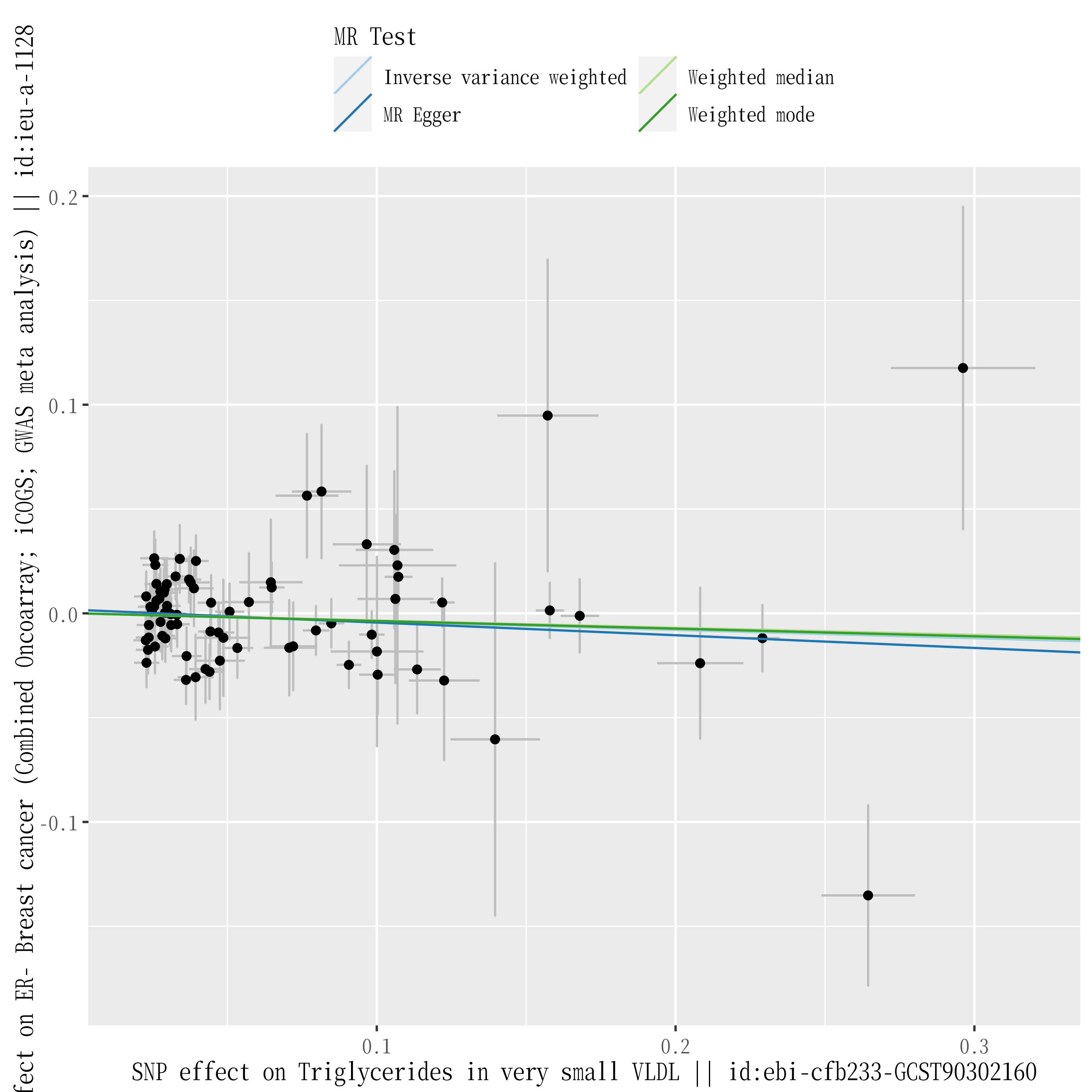

MR Test
Inverse variance weighted
MR Egger
Weighted median
Weighted mode
0.2
0.1
0.0
-0.1
0.1
0.2
0.3
fect on ER- Breast cancer (Combined Oncoarray; iCOGS; GWAS meta analysis) || id:ieu-a-1128
SNP effect on Triglycerides in very small VLDL || id:ebi-cfb233-GCST90302160

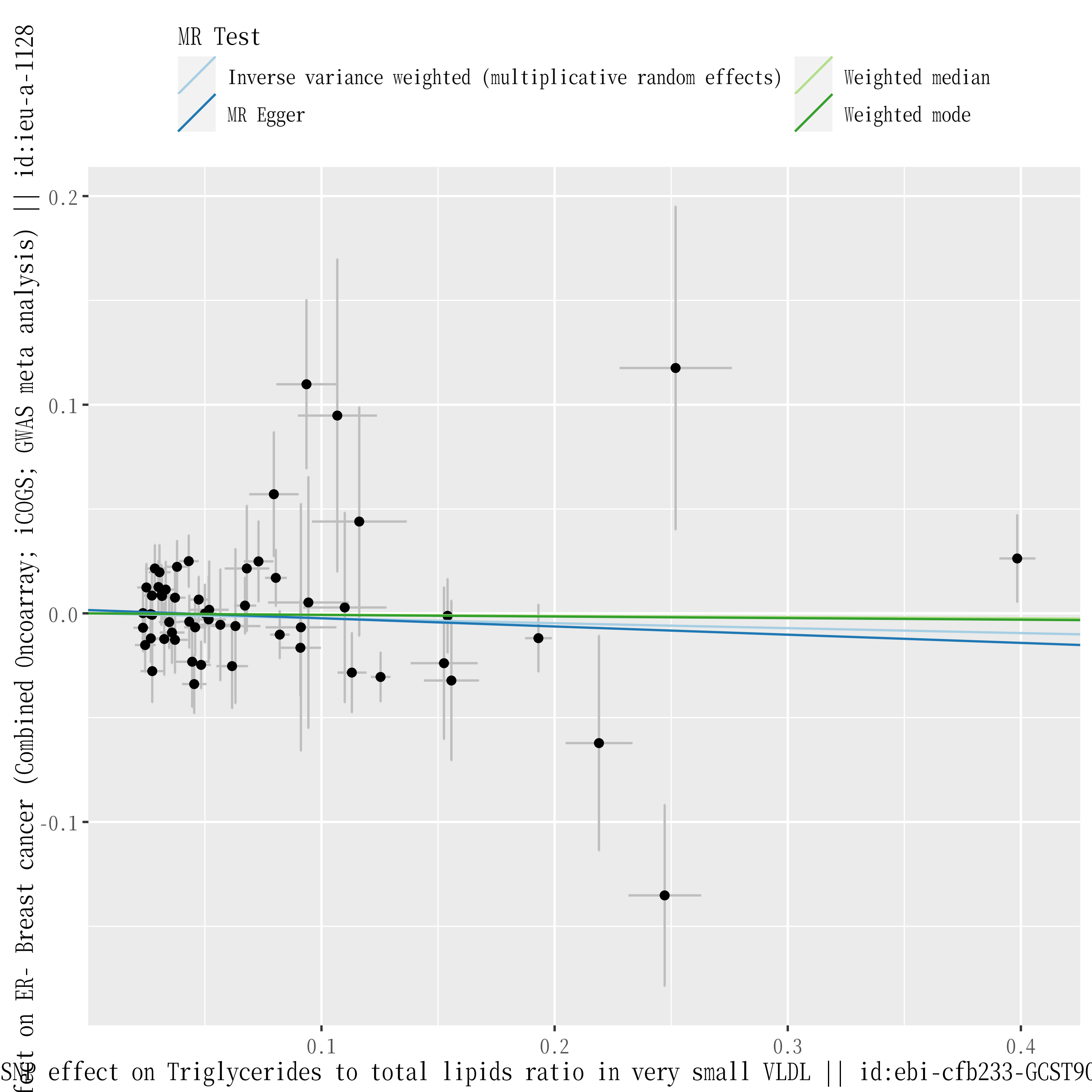

MR Test
Inverse variance weighted (multiplicative random effects)
MR Egger
Weighted median
Weighted mode
0.2
0.1
0.0
-0.1
0.1
0.2
0.3
0.4
SNP effect on Triglycerides to total lipids ratio in very small VLDL || id:ebi-cfb233-GCST90
fect on ER- Breast cancer (Combined Oncoarray; iCOGS; GWAS meta analysis) || id:ieu-a-1128

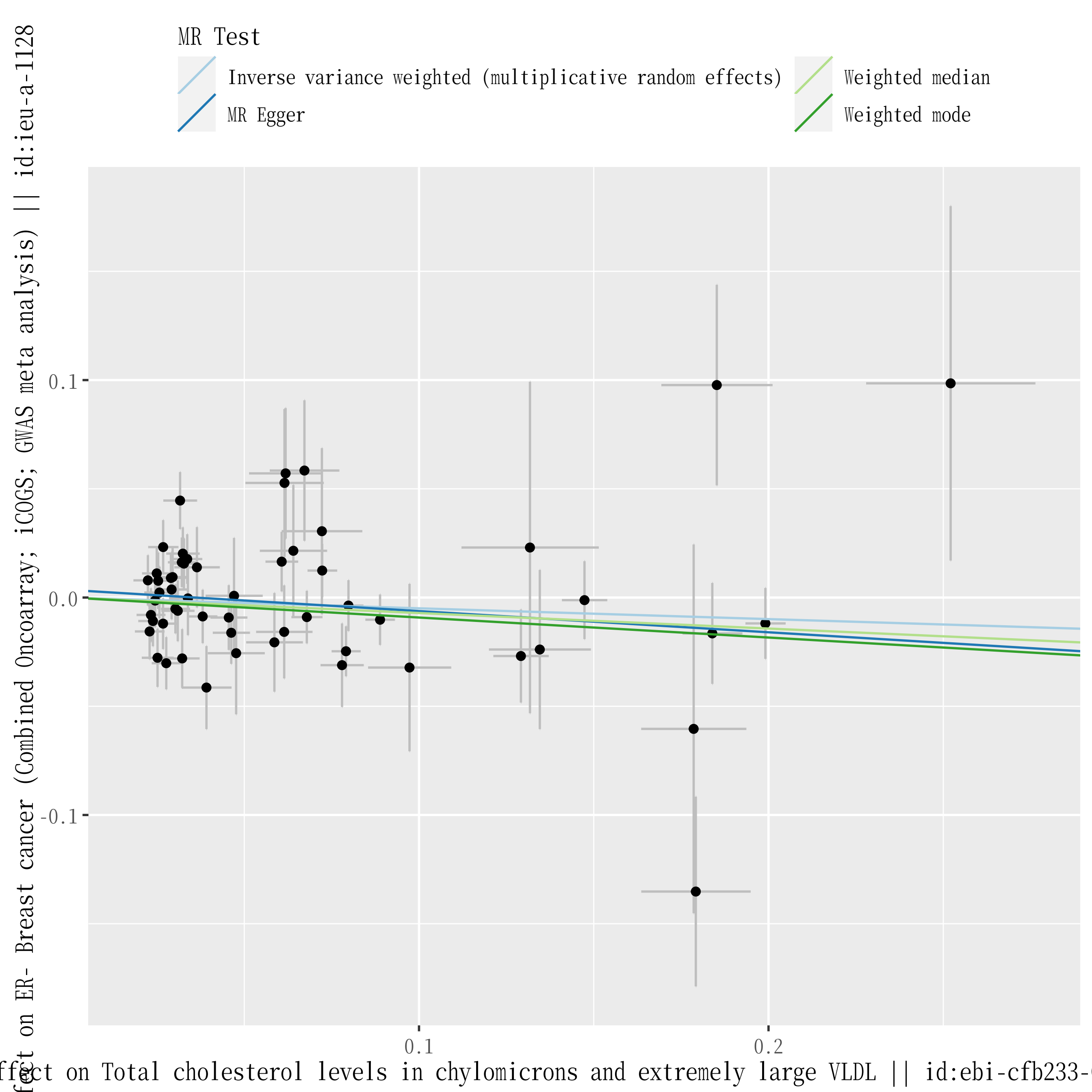

MR Test
Inverse variance weighted (multiplicative random effects)
MR Egger
Weighted median
Weighted mode
0.1
0.0
-0.1
0.1
0.2
ffect on Total cholesterol levels in chylomicrons and extremely large VLDL || id:ebi-cfb233-
fect on ER- Breast cancer (Combined Oncoarray; iCOGS; GWAS meta analysis) || id:ieu-a-1128

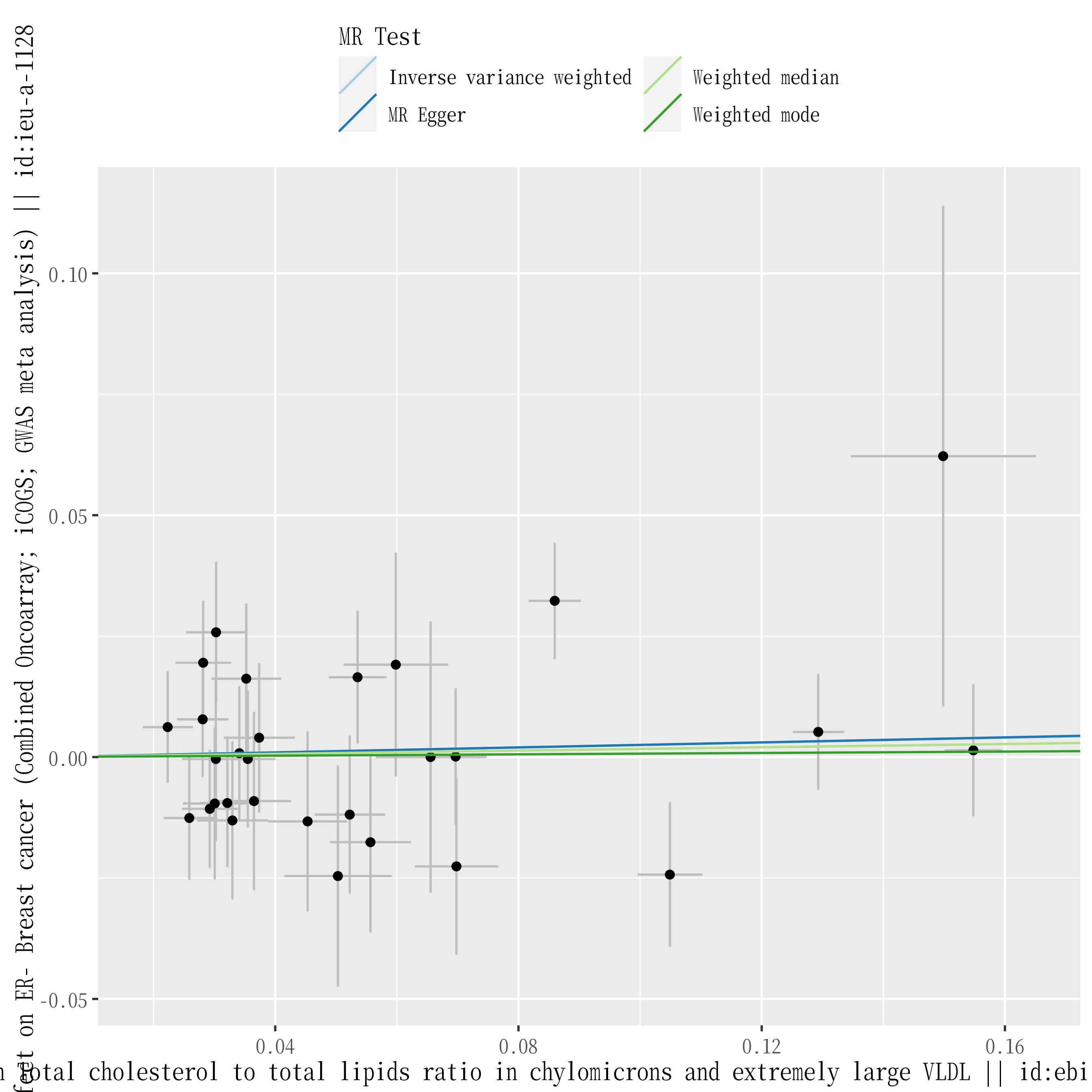

MR Test
Inverse variance weighted
MR Egger
Weighted median
Weighted mode
0.10
0.05
0.00
-0.05
0.04
0.08
0.12
0.16
n total cholesterol to total lipids ratio in chylomicrons and extremely large VLDL || id:ebi
fect on ER- Breast cancer (Combined Oncoarray; iCOGS; GWAS meta analysis) || id:ieu-a-1128

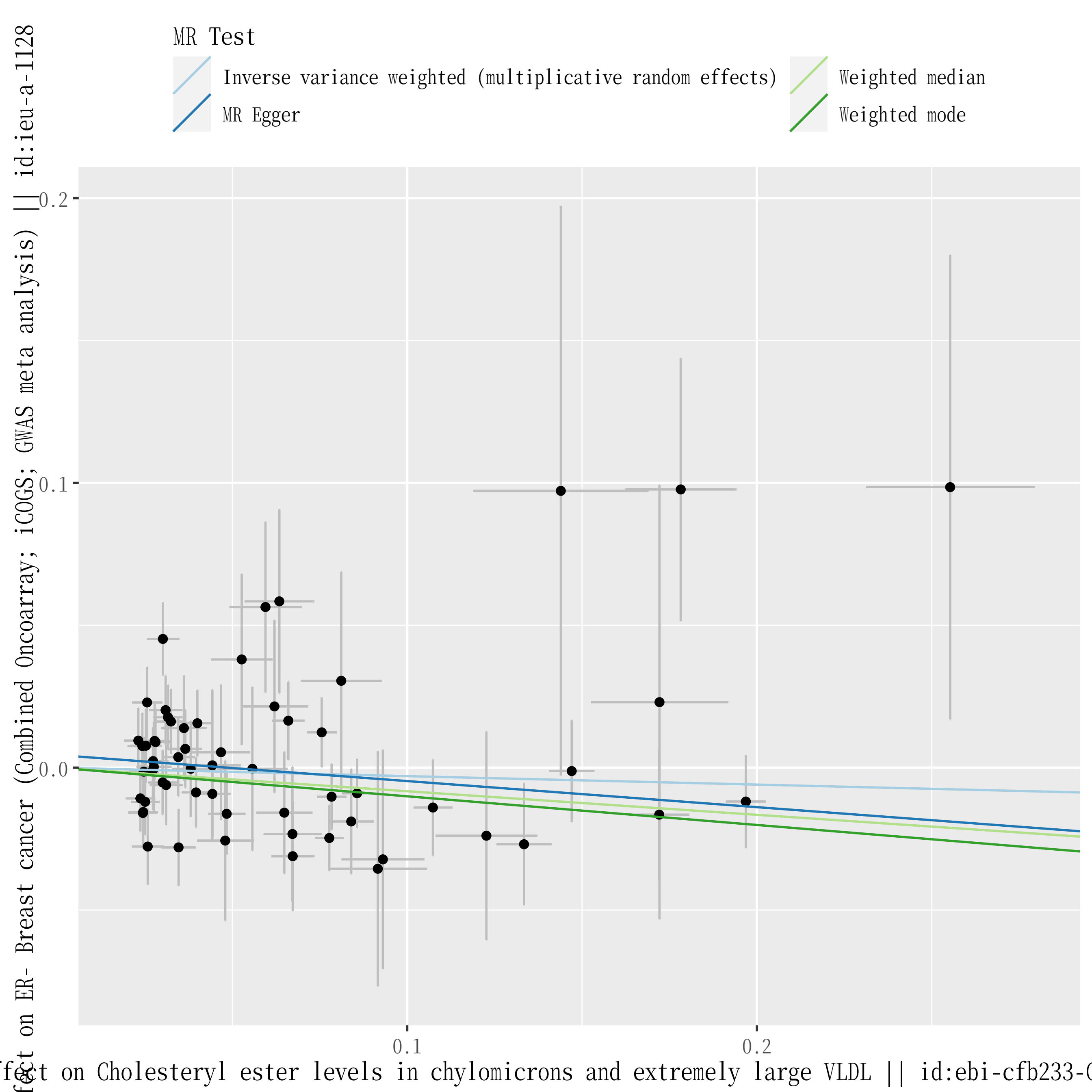
MR Test
Inverse variance weighted (multiplicative random effects)
MR Egger
Weighted median
Weighted mode
0.2
0.1
0.0
0.1
0.2
fect on Cholesteryl ester levels in chylomicrons and extremely large VLDL || id:ebi-cfb233-0
fect on ER- Breast cancer (Combined Oncoarray; iCOGS; GWAS meta analysis) || id:ieu-a-1128

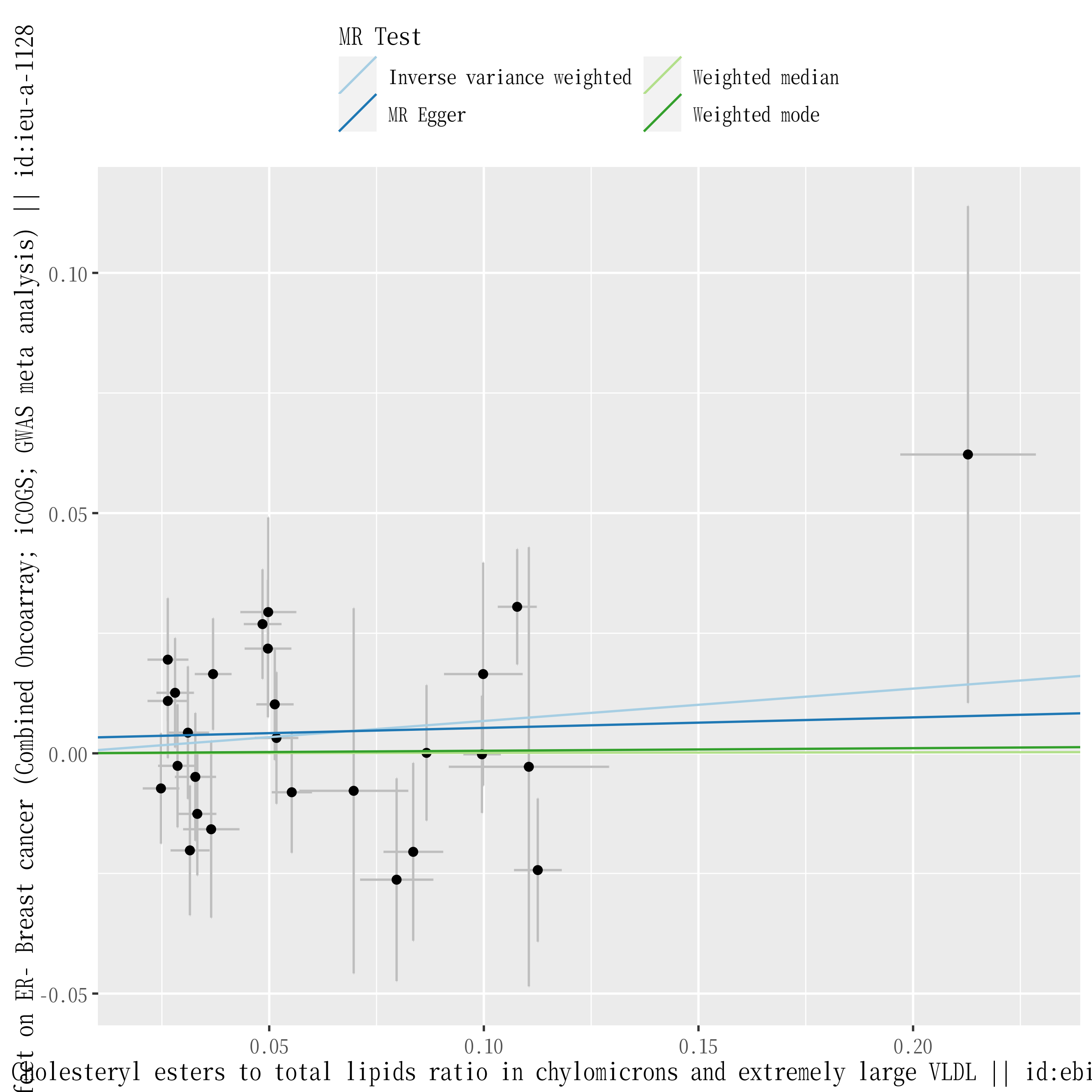
MR Test
Inverse variance weighted
MR Egger
Weighted median
Weighted mode
0.10
0.05
0.00
-0.05
0.05
0.10
0.15
0.20
fect on ER- Breast cancer (Combined Oncoarray; iCOGS; GWAS meta analysis) || id:ieu-a-1128
Cholesteryl esters to total lipids ratio in chylomicrons and extremely large VLDL || id:ebi

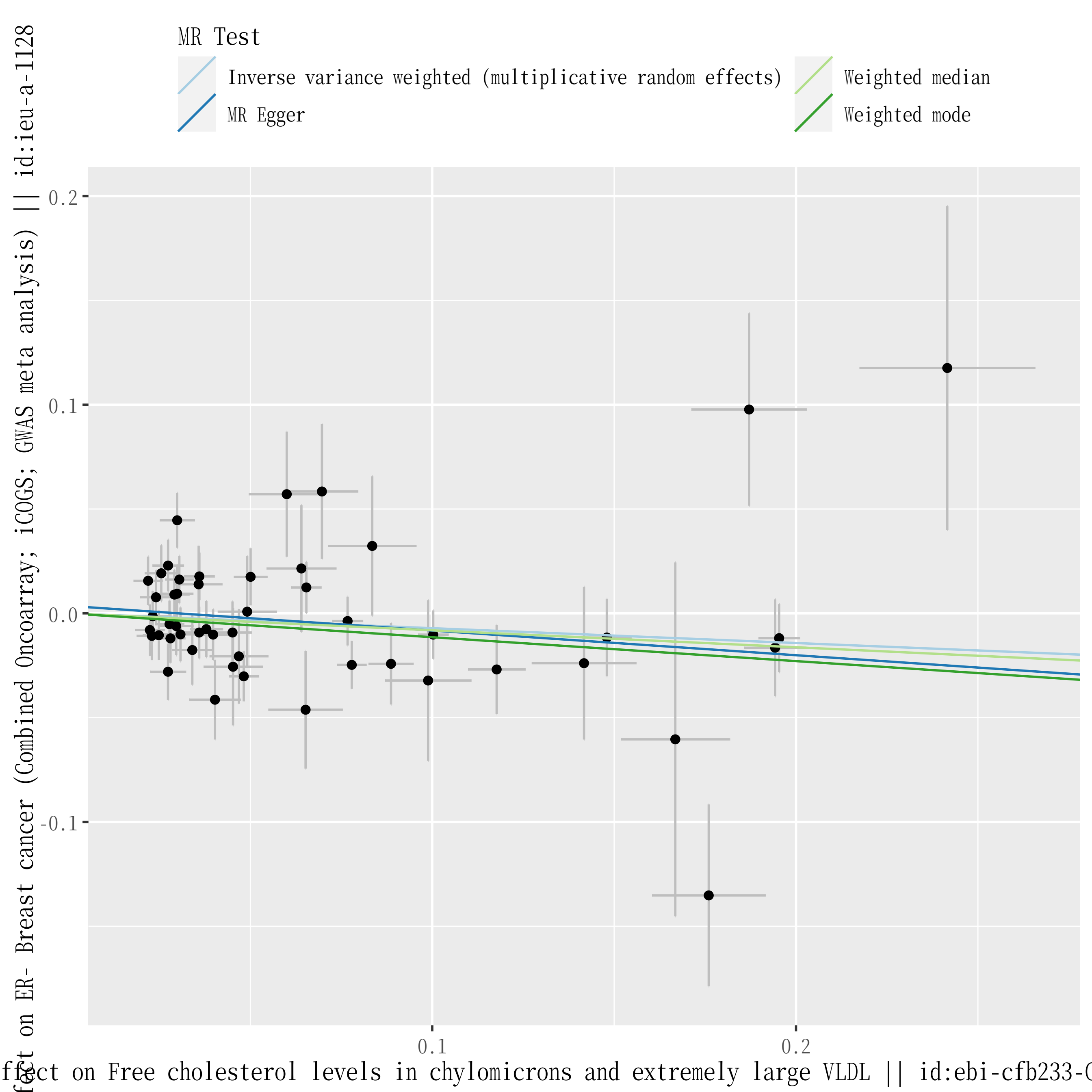
MR Test
Inverse variance weighted (multiplicative random effects)
MR Egger
Weighted median
Weighted mode
0.2
0.1
0.0
-0.1
0.1
0.2
fect on Free cholesterol levels in chylomicrons and extremely large VLDL || id:ebi-cfb233-0
fect on ER- Breast cancer (Combined Oncoarray; iCOGS; GWAS meta analysis) || id:ieu-a-1128

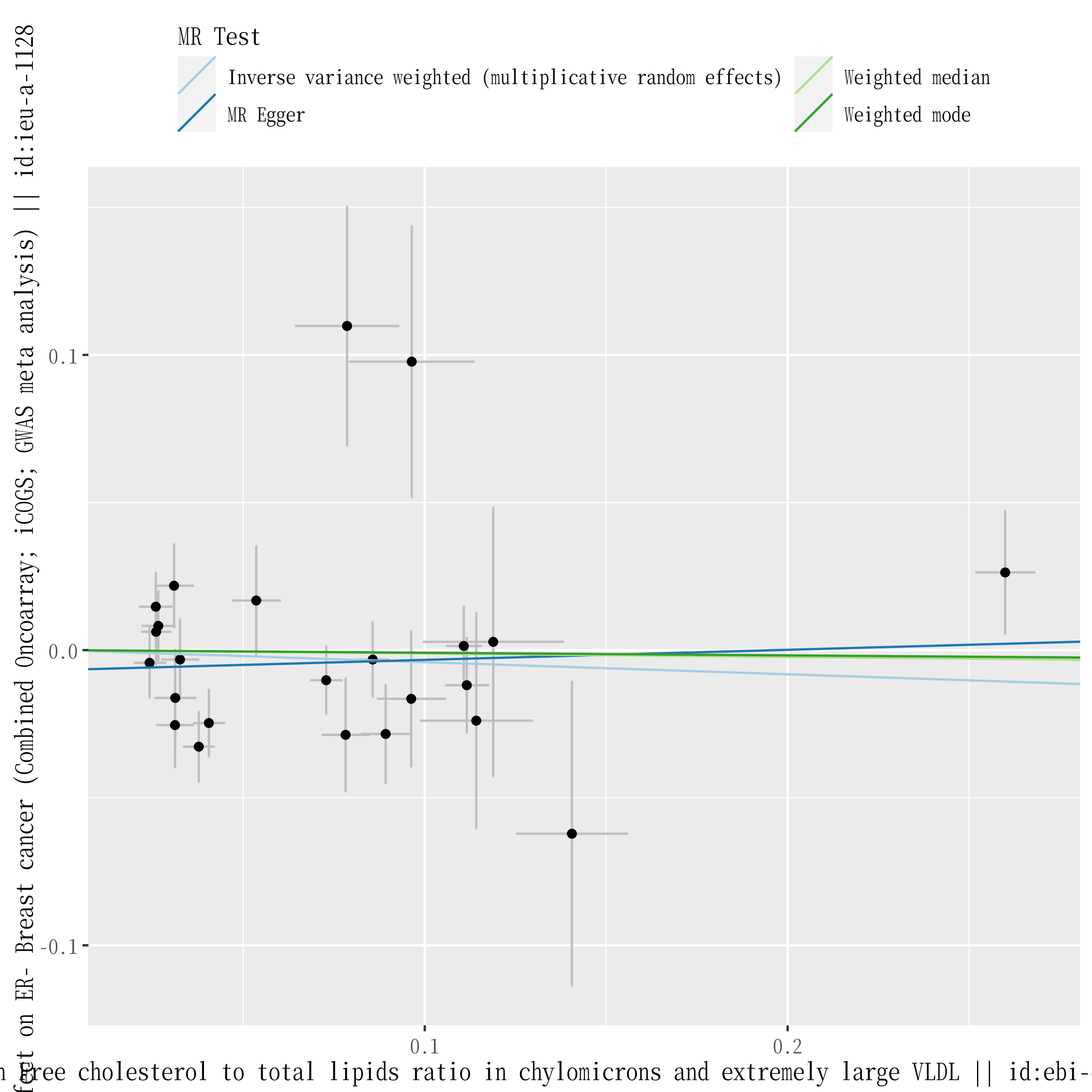

MR Test
Inverse variance weighted (multiplicative random effects)
MR Egger
Weighted median
Weighted mode
0.1
0.0
-0.1
0.1
0.2
fect on ER- Breast cancer (Combined Oncoarray; iCOGS; GWAS meta analysis) || id:ieu-a-1128
n Free cholesterol to total lipids ratio in chylomicrons and extremely large VLDL || id:ebi-

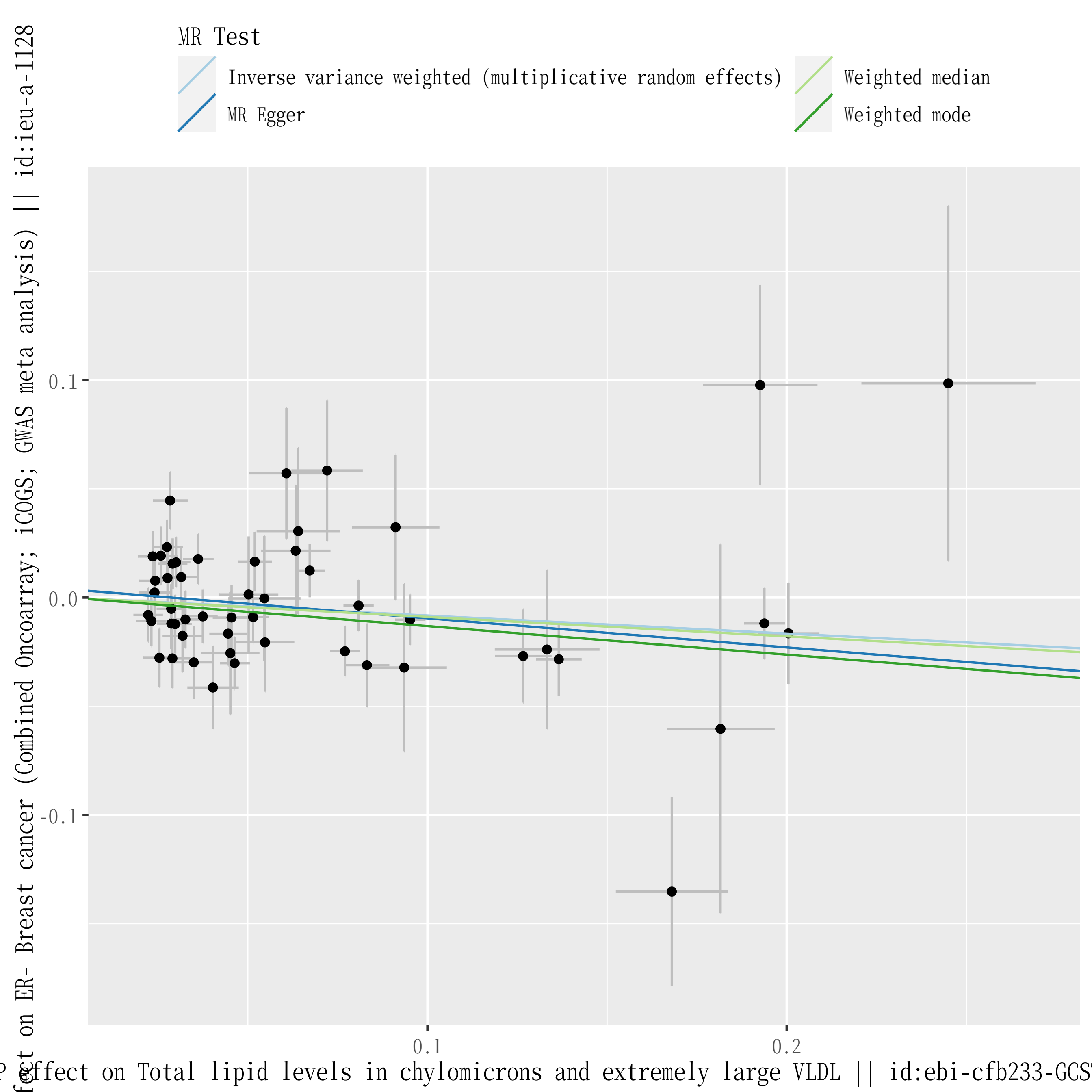

MR Test
Inverse variance weighted (multiplicative random effects)
MR Egger
Weighted median
Weighted mode
0.1
0.0
-0.1
0.1
0.2
SNP effect on Total lipid levels in chylomicrons and extremely large VLDL || id:ebi-cfb233-GCST
SNP effect on ER- Breast cancer (Combined Oncoarray; iCOGS; GWAS meta analysis) || id:ieu-a-1128

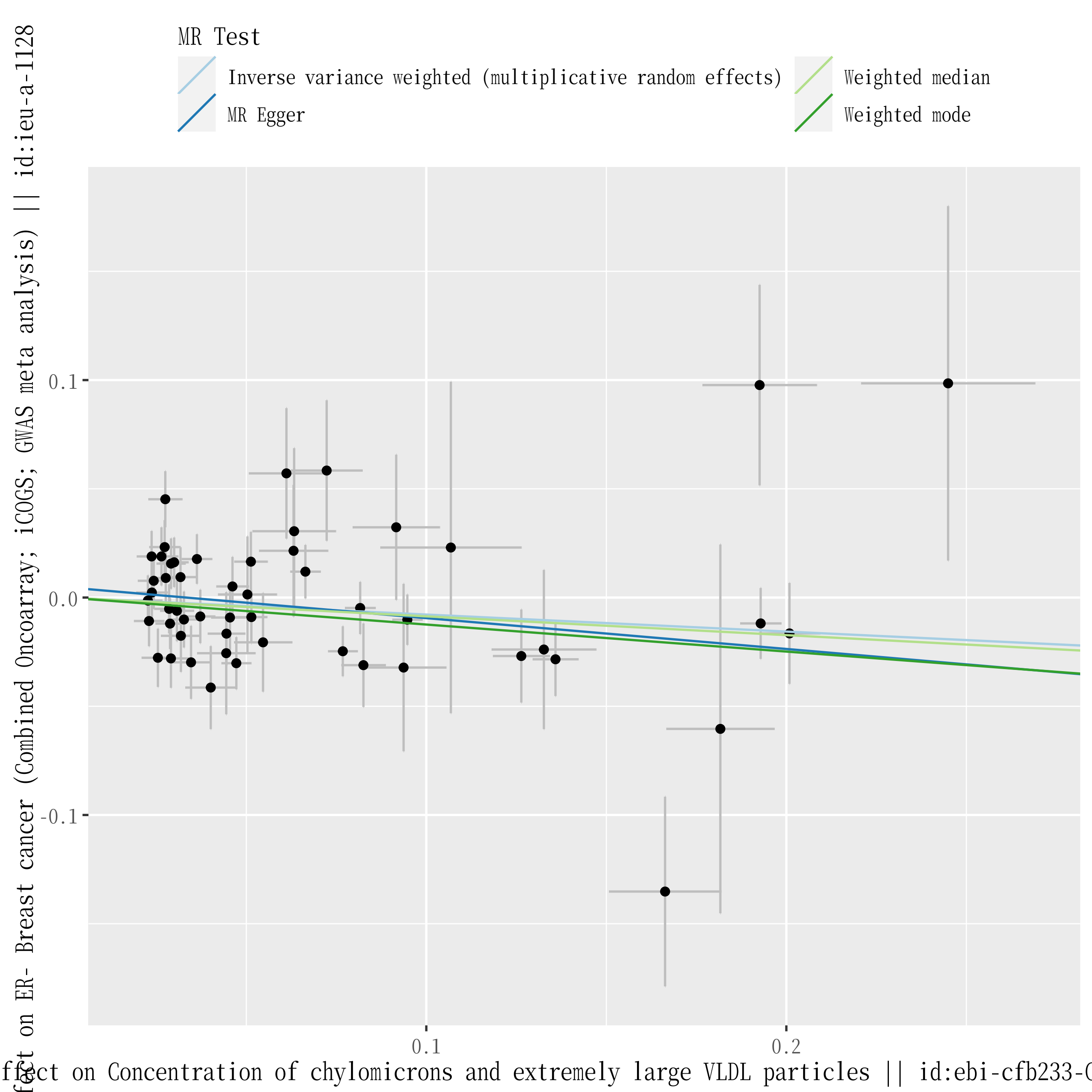

MR Test
Inverse variance weighted (multiplicative random effects)
MR Egger
Weighted median
Weighted mode
0.1
0.0
-0.1
0.1
0.2
fect on ER- Breast cancer (Combined Oncoarray; iCOGS; GWAS meta analysis) || id:ieu-a-1128
ffect on Concentration of chylomicrons and extremely large VLDL particles || id:ebi-cfb233-C

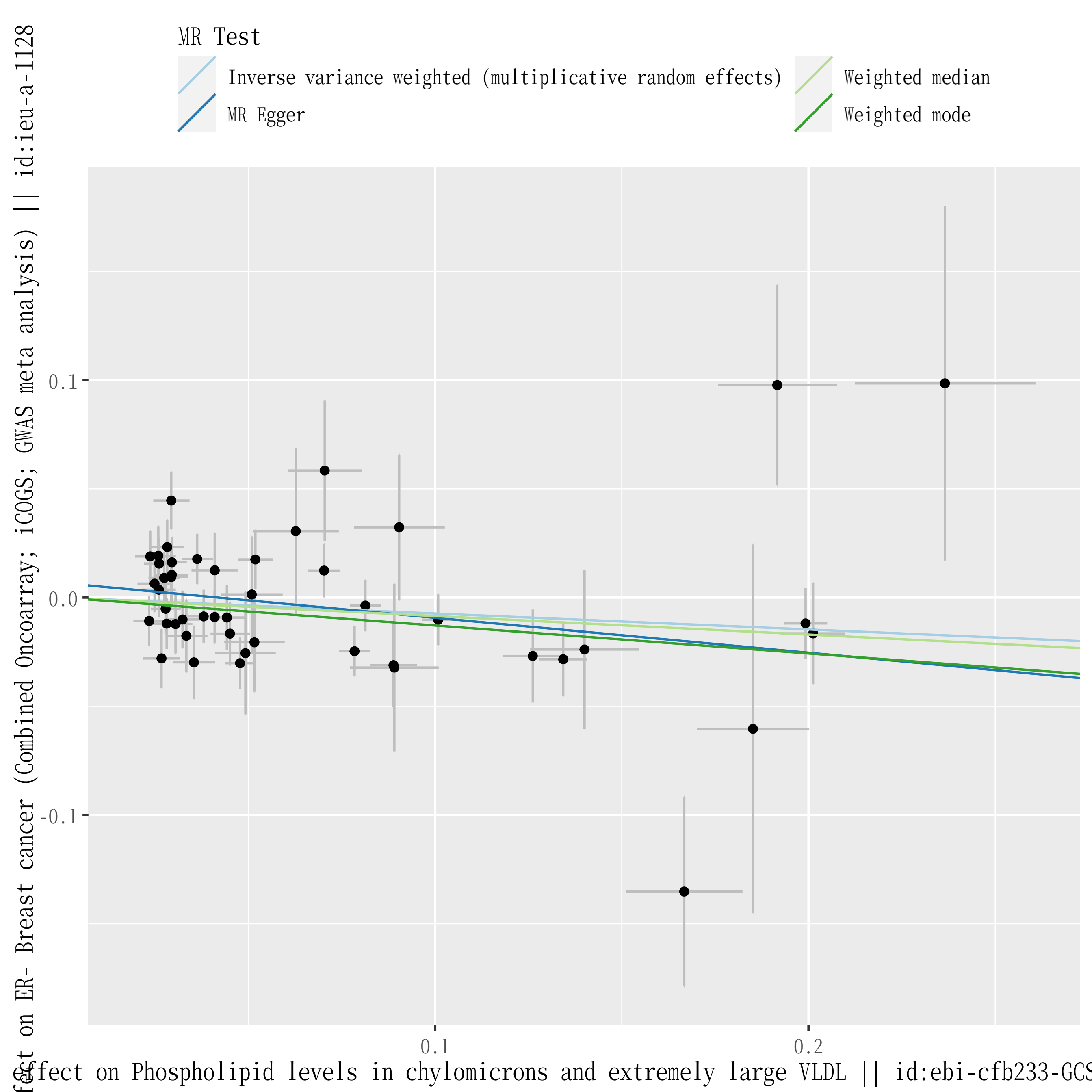
MR Test
Inverse variance weighted (multiplicative random effects)
MR Egger
Weighted median
Weighted mode
0.1
0.0
-0.1
0.1
0.2
effect on Phospholipid levels in chylomicrons and extremely large VLDL || id:ebi-cfb233-GCS
fect on ER- Breast cancer (Combined Oncoarray; iCOGS; GWAS meta analysis) || id:ieu-a-1128

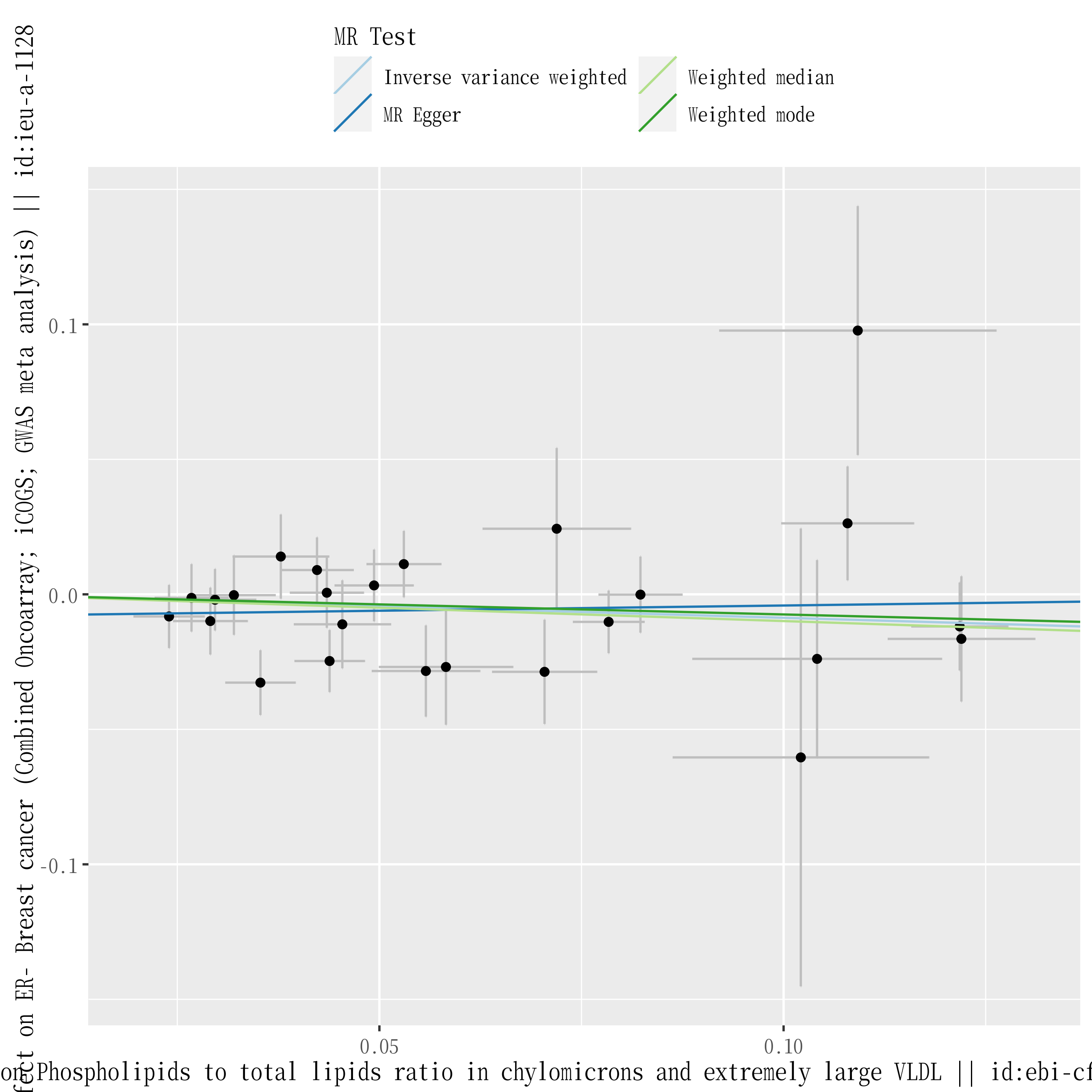

MR Test
Inverse variance weighted
Weighted median
MR Egger
Weighted mode
0.1
0.0
-0.1
0.05
0.10
fect on ER- Breast cancer (Combined Oncoarray; iCOGS; GWAS meta analysis) || id:ieu-a-1128
on Phospholipids to total lipids ratio in chylomicrons and extremely large VLDL || id:ebi-c

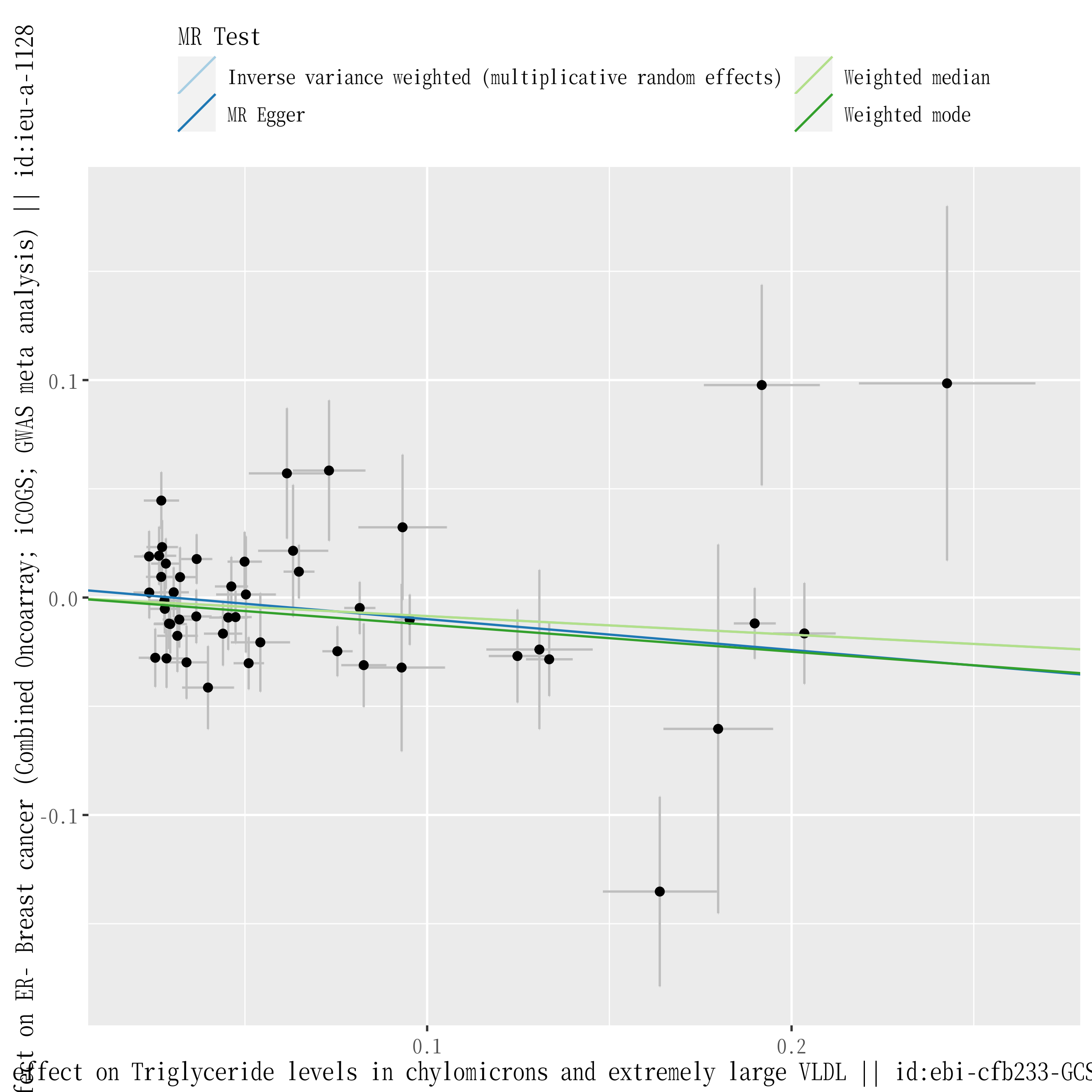
MR Test
Inverse variance weighted (multiplicative random effects)
MR Egger
Weighted median
Weighted mode
0.1
0.0
-0.1
0.1
0.2
fect on ER- Breast cancer (Combined Oncoarray; iCOGS; GWAS meta analysis) || id:ieu-a-1128
effect on Triglyceride levels in chylomicrons and extremely large VLDL || id:ebi-cfb233-GCS

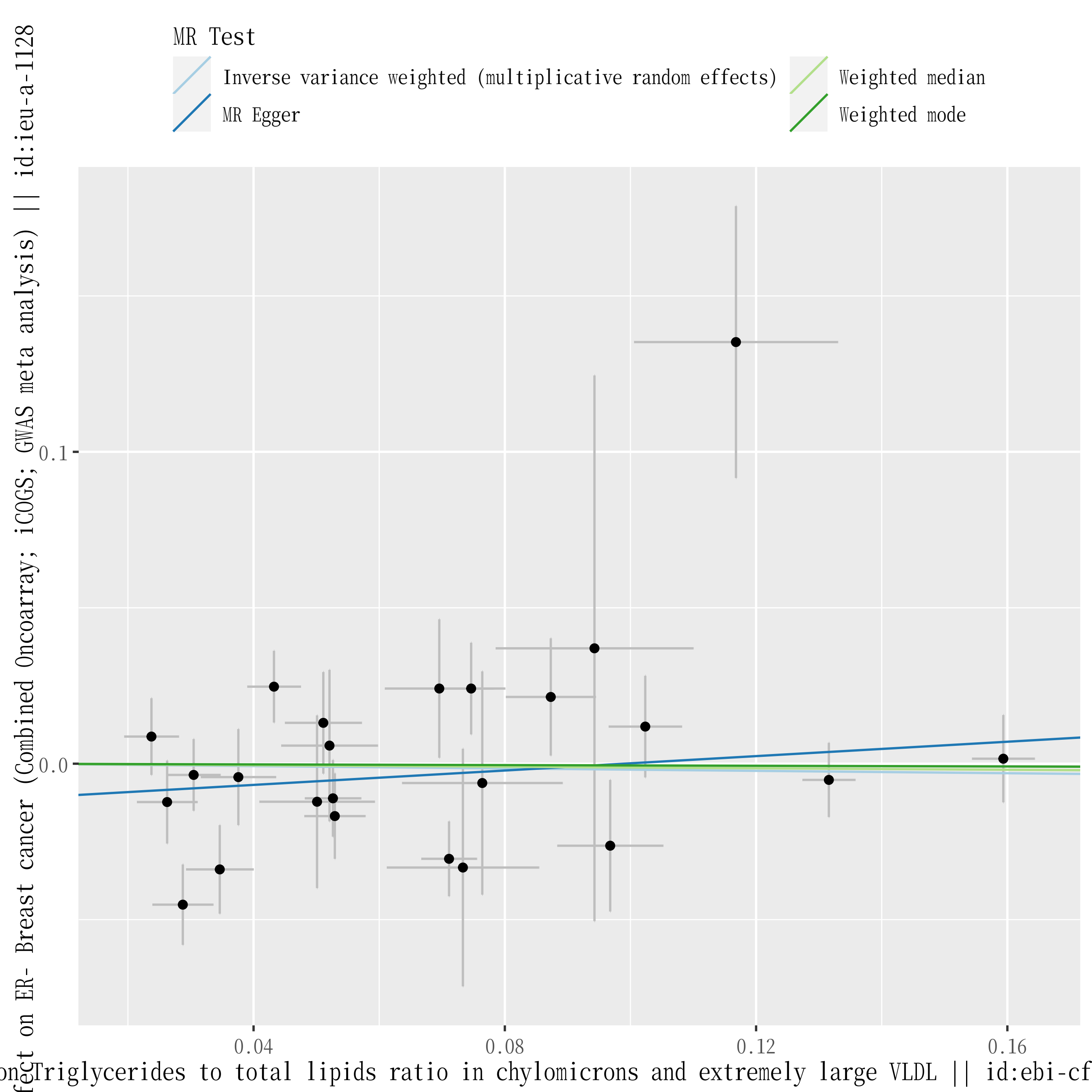
MR Test
Inverse variance weighted (multiplicative random effects)
MR Egger
Weighted median
Weighted mode
0.1
0.0
0.04
0.08
0.12
0.16
fect on ER- Breast cancer (Combined Oncoarray; iCOGS; GWAS meta analysis) || id:ieu-a-1128
on Triglycerides to total lipids ratio in chylomicrons and extremely large VLDL || id:ebi-cf
