# Supplementary material for: Assessing the causal relationships between circulating metabolic biomarkers and breast cancer by using mendelian randomization
Source: Front Genet. 2024 Dec 18;15:1448748. doi: 10.3389/fgene.2024.1448748 (PMC11688392; doi:10.3389/fgene.2024.1448748)
Supplement: Supplementary file 12 [file DataSheet5.pdf]

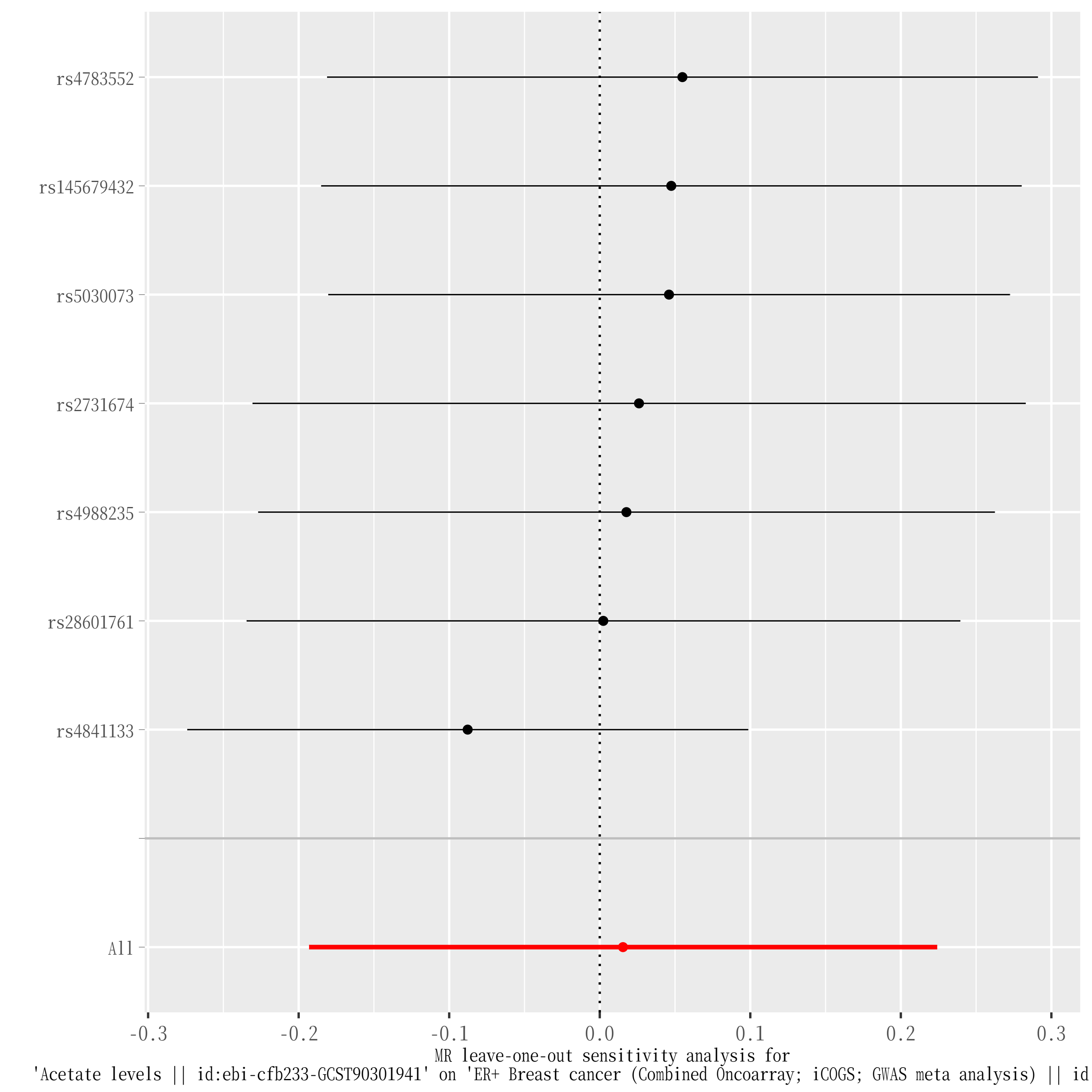
rs4783552
rs145679432
rs5030073
rs2731674
rs4988235
rs28601761
rs4841133
All
-0.3
-0.2
-0.1
0.0
0.1
0.2
0.3
MR leave-one-out sensitivity analysis for
'Acetate levels || id:ebi-cfb233-GCST90301941' on 'ER+ Breast cancer (Combined Oncoarray; iCOGS; GWAS meta analysis) || id

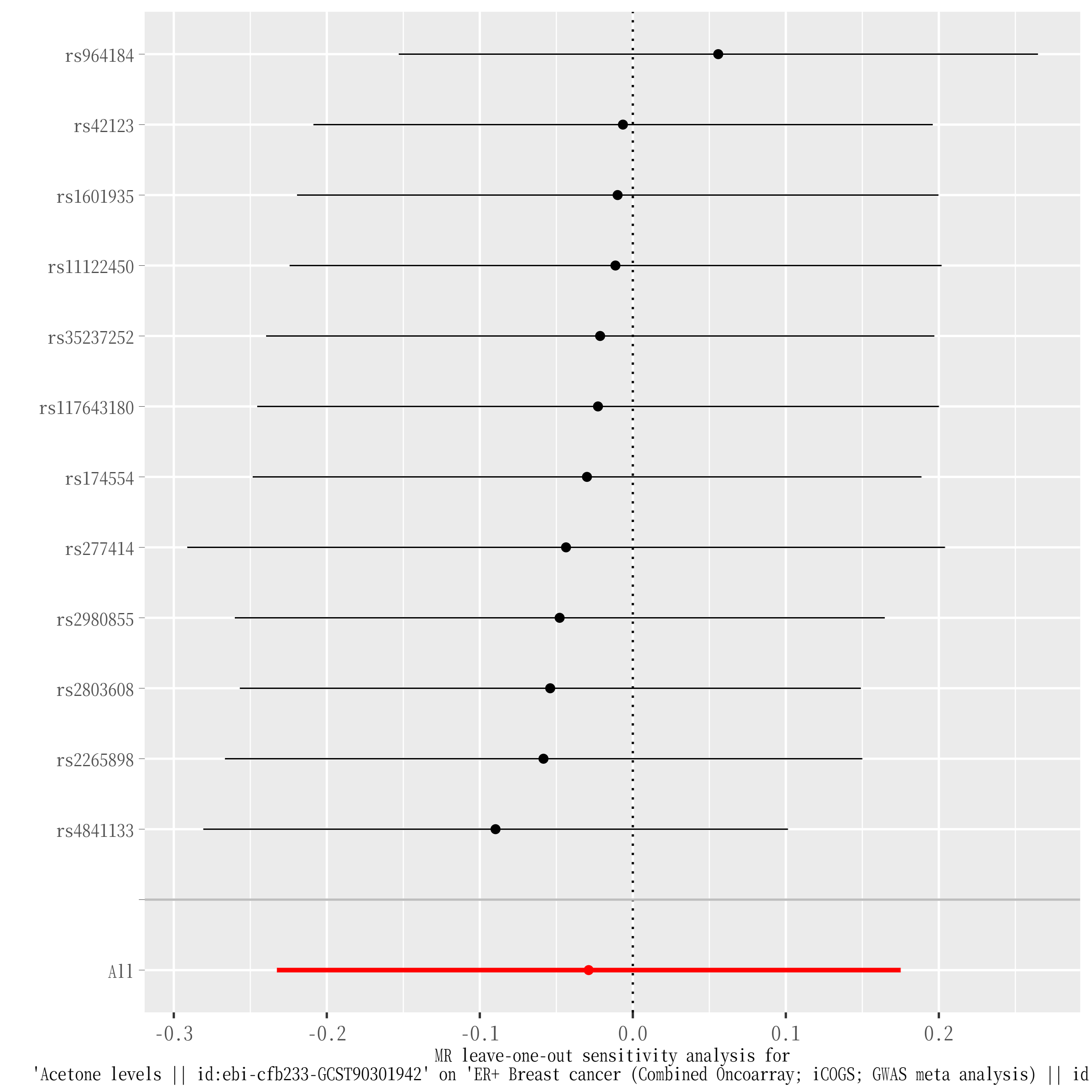
rs964184
rs42123
rs1601935
rs11122450
rs35237252
rs117643180
rs174554
rs277414
rs2980855
rs2803608
rs2265898
rs4841133
All
-0.3
-0.2
-0.1
0.0
0.1
0.2
MR leave-one-out sensitivity analysis for
'Acetone levels || id:ebi-cfb233-GCST90301942' on 'ER+ Breast cancer (Combined Oncoarray; iCOGS; GWAS meta analysis) || id

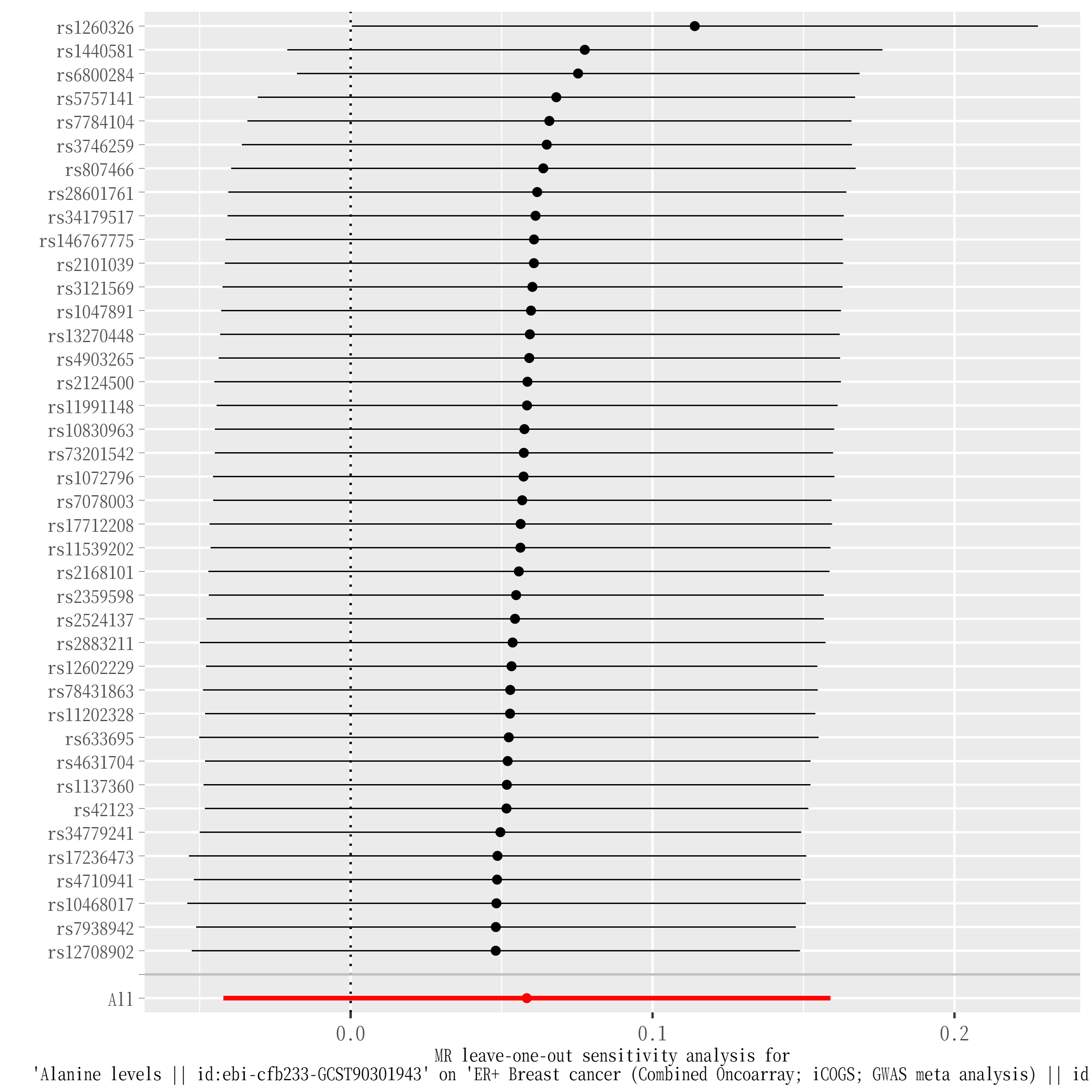

rs1260326
rs1440581
rs6800284
rs5757141
rs7784104
rs3746259
rs807466
rs28601761
rs34179517
rs146767775
rs2101039
rs3121569
rs1047891
rs13270448
rs4903265
rs2124500
rs11991148
rs10830963
rs73201542
rs1072796
rs7078003
rs17712208
rs11539202
rs2168101
rs2359598
rs2524137
rs2883211
rs12602229
rs78431863
rs11202328
rs633695
rs4631704
rs1137360
rs42123
rs34779241
rs17236473
rs4710941
rs10468017
rs7938942
rs12708902
All
0.0
0.1
0.2
MR leave-one-out sensitivity analysis for
'Alanine levels || id:ebi-cfb233-GCST90301943' on 'ER+ Breast cancer (Combined Oncoarray; iCOGS; GWAS meta analysis) || id

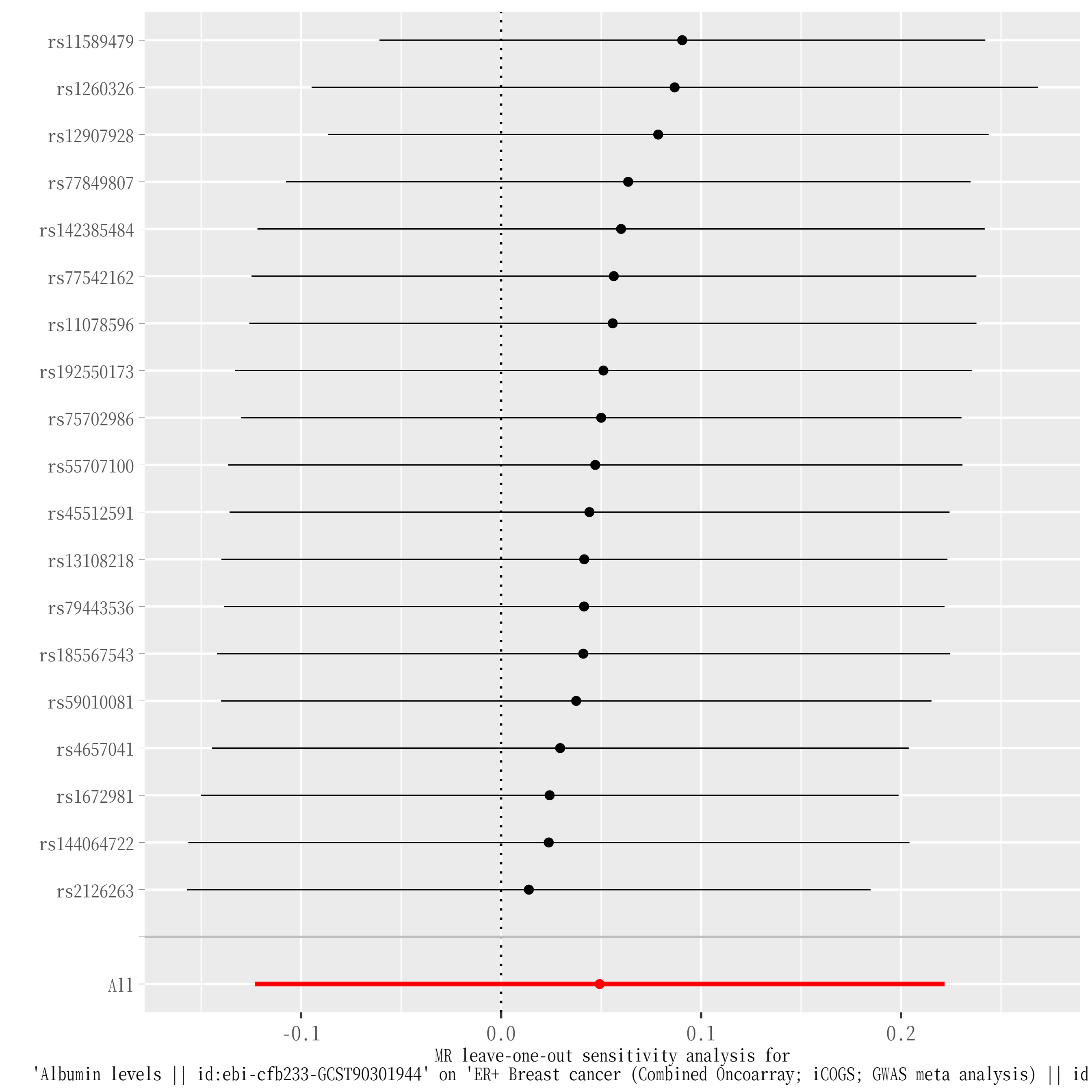
rs11589479
rs1260326
rs12907928
rs77849807
rs142385484
rs77542162
rs11078596
rs192550173
rs75702986
rs55707100
rs45512591
rs13108218
rs79443536
rs185567543
rs59010081
rs4657041
rs1672981
rs144064722
rs2126263
All
-0.1
0.0
0.1
0.2
MR leave-one-out sensitivity analysis for
'Albumin levels || id:ebi-cfb233-GCST90301944' on 'ER+ Breast cancer (Combined Oncoarray; iCOGS; GWAS meta analysis) || id

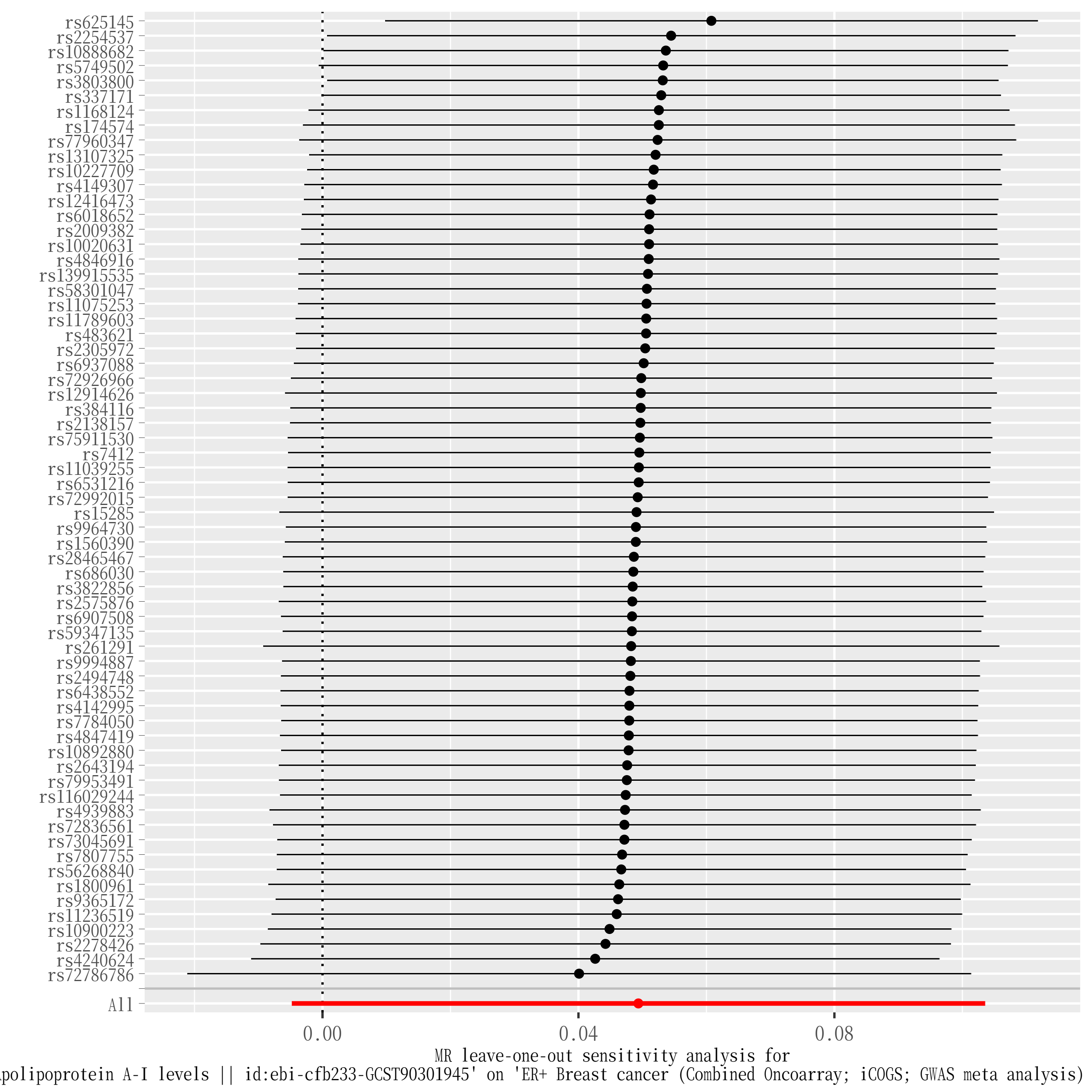
rs625145
rs2254537
rs10888682
rs5749502
rs3803800
rs337171
rs1168124
rs174574
rs77960347
rs13107325
rs10227709
rs4149307
rs12416473
rs6018652
rs2009382
rs10020631
rs4846916
rs139915535
rs58301047
rs11075253
rs11789603
rs483621
rs2305972
rs6937088
rs72926966
rs12914626
rs384116
rs2138157
rs75911530
rs7412
rs11039255
rs6531216
rs72992015
rs15285
rs9964730
rs1560390
rs28465467
rs686030
rs3822856
rs2575876
rs6907508
rs59347135
rs261291
rs9994887
rs2494748
rs6438552
rs4142995
rs7784050
rs4847419
rs10892880
rs2643194
rs79953491
rs116029244
rs4939883
rs72836561
rs73045691
rs7807755
rs56268840
rs1800961
rs9365172
rs11236519
rs10900223
rs2278426
rs4240624
rs72786786
All
0.00
0.04
0.08
MR leave-one-out sensitivity analysis for
'Apolipoprotein A-I levels || id:ebi-cfb233-GCST90301945' on 'ER+ Breast cancer (Combined Oncoarray; iCOGS; GWAS meta analysis)'

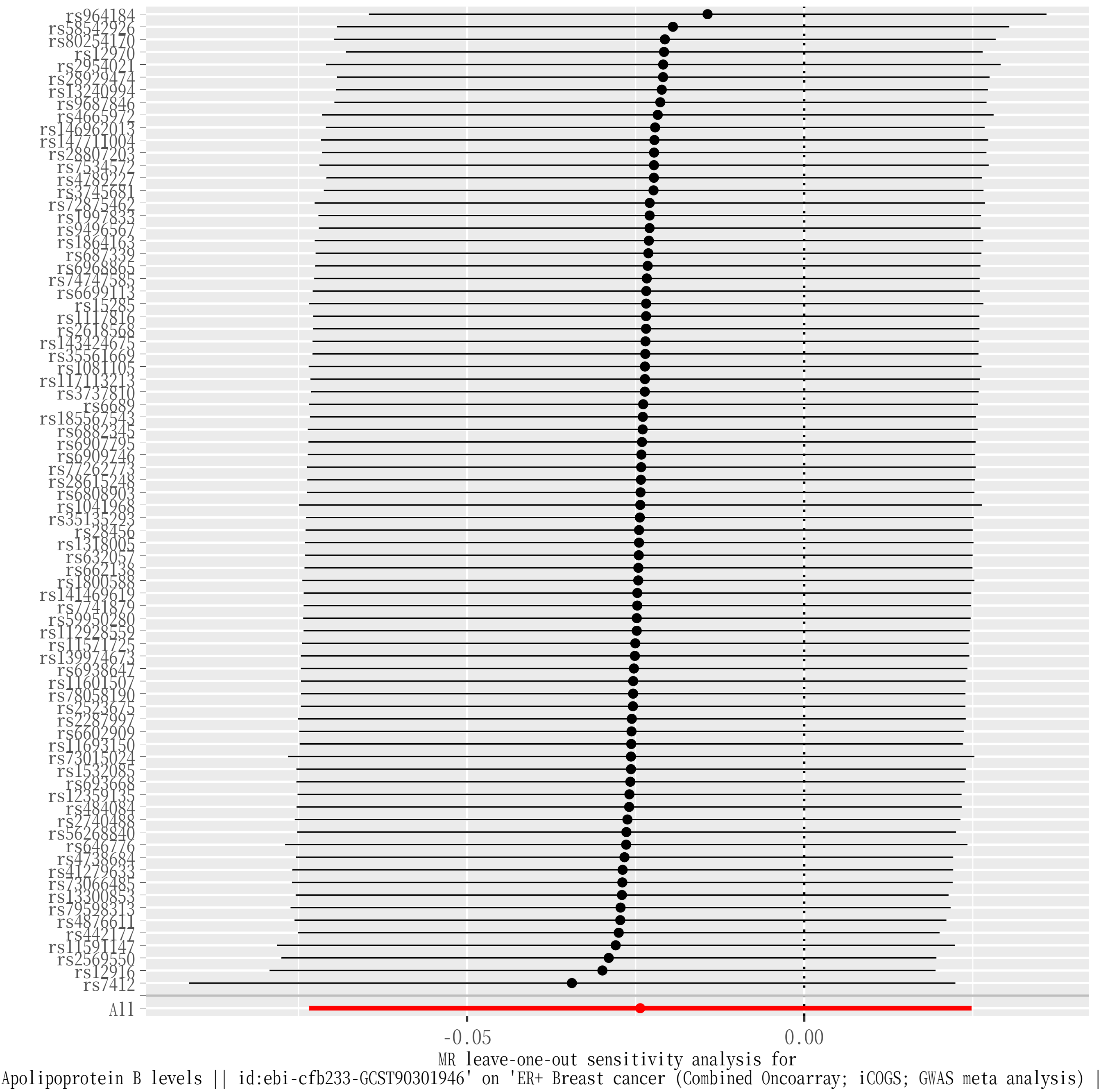
rs964184
rs58542926
rs80254170
rs12970
rs2954021
rs28929474
rs13240994
rs9687846
rs4665972
rs146962013
rs147711004
rs28807203
rs7534572
rs4782227
rs3745681
rs72875462
rs1997833
rs9496567
rs1864163
rs687339
rs6268865
rs74747585
rs6699113
rs15285
rs1117816
rs2618568
rs143424675
rs35561669
rs1081105
rs117113213
rs3737810
rs6689
rs185567543
rs6882345
rs6907795
rs6909746
rs77262773
rs28615248
rs6808903
rs1041968
rs35135293
rs28456
rs1318005
rs632057
rs662138
rs1800588
rs141469619
rs7741879
rs59950280
rs112928559
rs11571725
rs139974673
rs6938647
rs11601507
rs78058190
rs2523675
rs2287997
rs6602909
rs11693150
rs73015024
rs1532085
rs693668
rs12359135
rs484084
rs2740488
rs56268840
rs646776
rs4738684
rs41279633
rs73066485
rs13300853
rs79598313
rs4876611
rs442177
rs11591147
rs2569550
rs12916
rs7412
All
-0.05
0.00
MR leave-one-out sensitivity analysis for
Apolipoprotein B levels || id:ebi-cfb233-GCST90301946' on 'ER+ Breast cancer (Combined Oncoarray; iCOGS; GWAS meta analysis) |

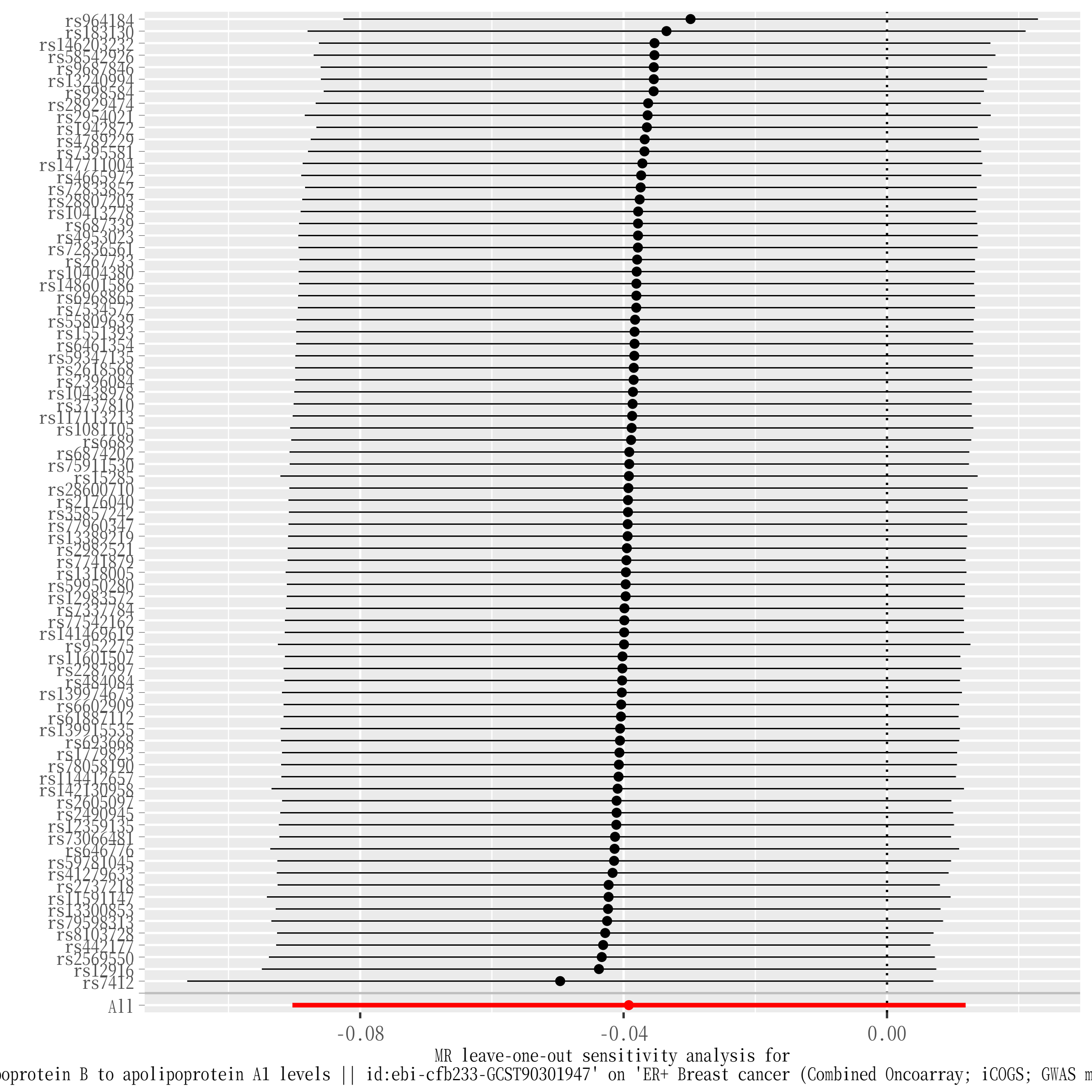
rs964184
rs183130
rs146203232
rs58542926
rs9687846
rs13240994
rs998584
rs28929474
rs2954021
rs1942872
rs4789229
rs7395581
rs147711004
rs4665972
rs72833852
rs28807203
rs10413278
rs687339
rs4953023
rs72836561
rs267733
rs10404380
rs148601586
rs6968865
rs7534572
rs55809639
rs1551323
rs6461354
rs59347135
rs2618568
rs2396084
rs10438978
rs3737810
rs117113213
rs1081105
rs6689
rs6874202
rs75911530
rs15285
rs28600710
rs2176040
rs35857242
rs77960347
rs13389219
rs2982521
rs7741879
rs1318005
rs59950280
rs12983572
rs7337784
rs77542162
rs141469619
rs952275
rs11601507
rs2287997
rs484084
rs139974673
rs6602909
rs61887112
rs139915535
rs693668
rs1779823
rs78058190
rs114412657
rs142130958
rs2605097
rs7490945
rs12359135
rs73066481
rs646776
rs59781045
rs41279633
rs2737218
rs11591147
rs13300853
rs79598313
rs8103728
rs442177
rs2569550
rs12916
rs7412
All
-0.08
-0.04
0.00
MR leave-one-out sensitivity analysis for
oprotein B to apolipoprotein A1 levels || id:ebi-cfb233-GCST90301947' on 'ER+ Breast cancer (Combined Oncoarray; iCOGS; GWAS m

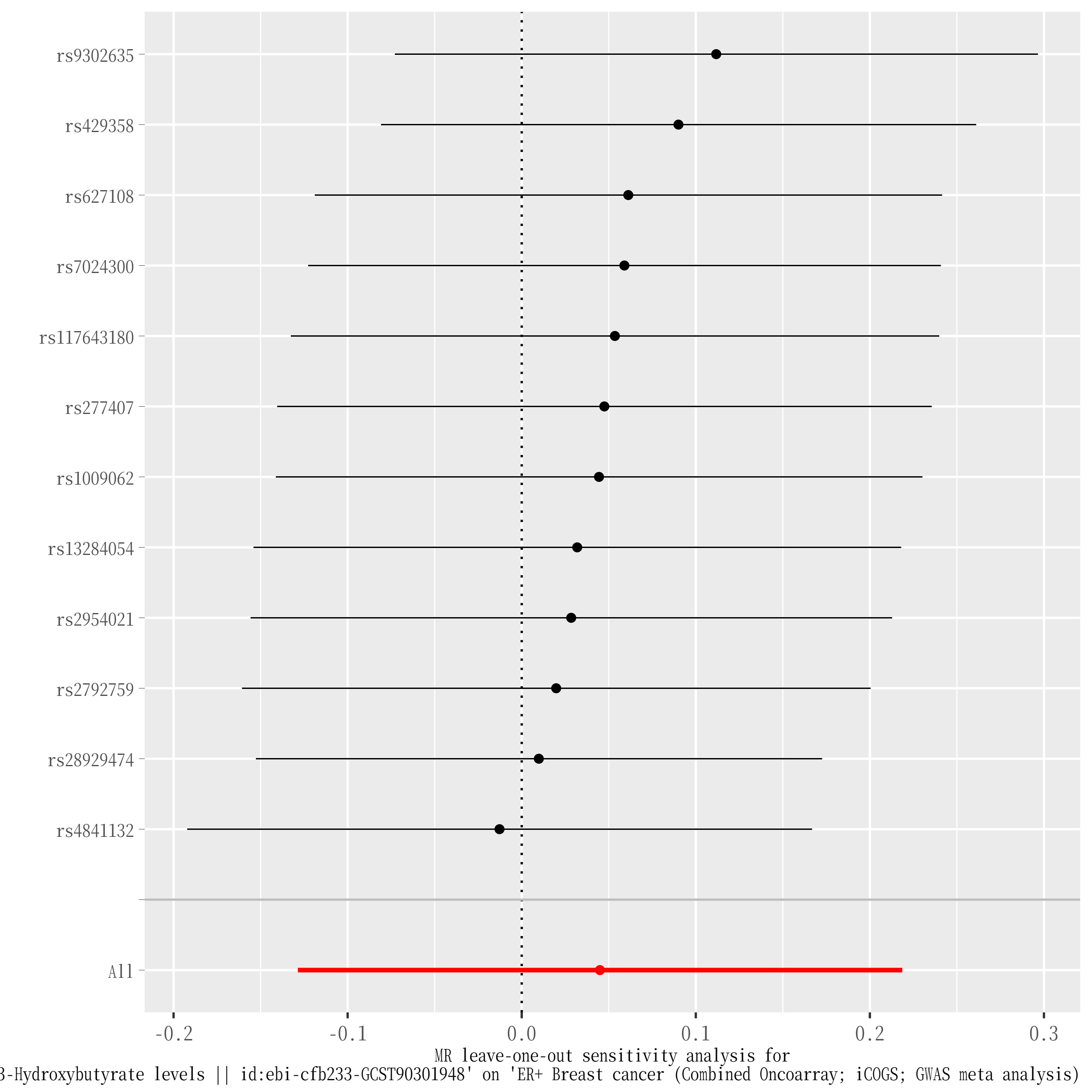

rs9302635
rs429358
rs627108
rs7024300
rs117643180
rs277407
rs1009062
rs13284054
rs2954021
rs2792759
rs28929474
rs4841132
All
-0.2
-0.1
0.0
0.1
0.2
0.3
MR leave-one-out sensitivity analysis for
3-Hydroxybutyrate levels || id:ebi-cfb233-GCST90301948' on 'ER+ Breast cancer (Combined Oncoarray; iCOGS; GWAS meta analysis)

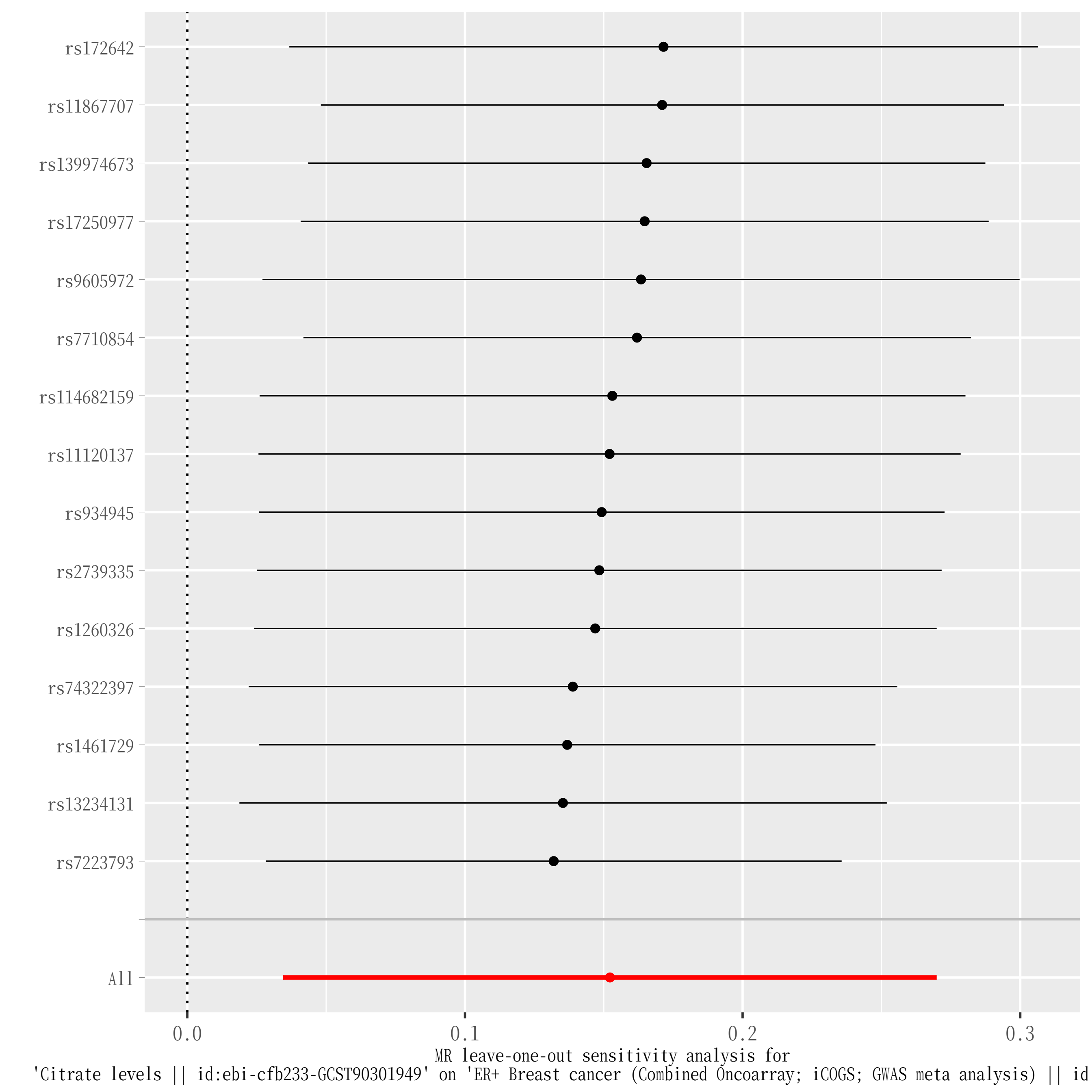
rs172642
rs11867707
rs139974673
rs17250977
rs9605972
rs7710854
rs114682159
rs11120137
rs934945
rs2739335
rs1260326
rs74322397
rs1461729
rs13234131
rs7223793
All
0.0
0.1
0.2
0.3
MR leave-one-out sensitivity analysis for
'Citrate levels || id:ebi-cfb233-GCST90301949' on 'ER+ Breast cancer (Combined Oncoarray; iCOGS; GWAS meta analysis) || id

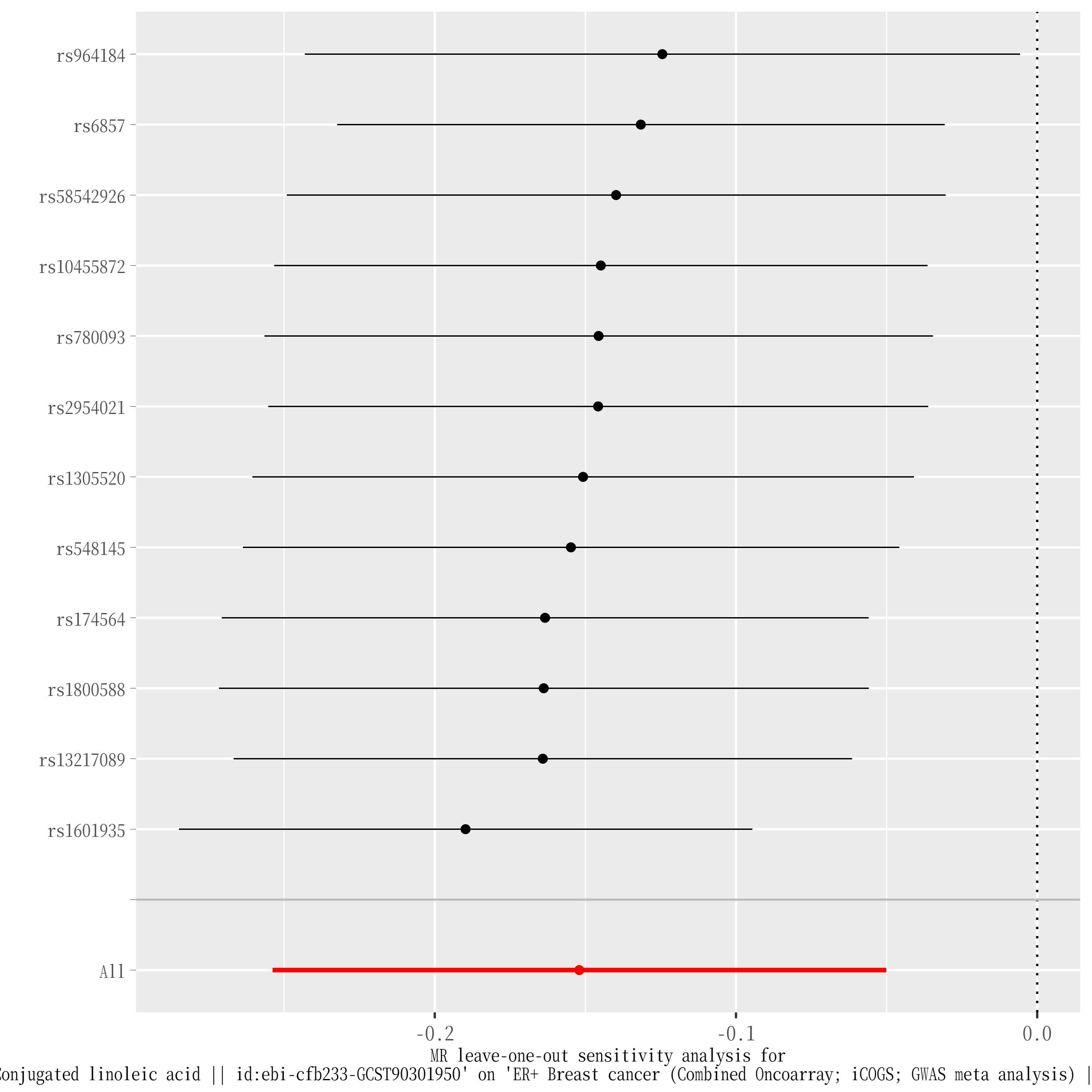
rs964184
rs6857
rs58542926
rs10455872
rs780093
rs2954021
rs1305520
rs548145
rs174564
rs1800588
rs13217089
rs1601935
All
-0.2
-0.1
0.0
MR leave-one-out sensitivity analysis for
Conjugated linoleic acid || id:ebi-cfb233-GCST90301950' on 'ER+ Breast cancer (Combined Oncoarray; iCOGS; GWAS meta analysis) |

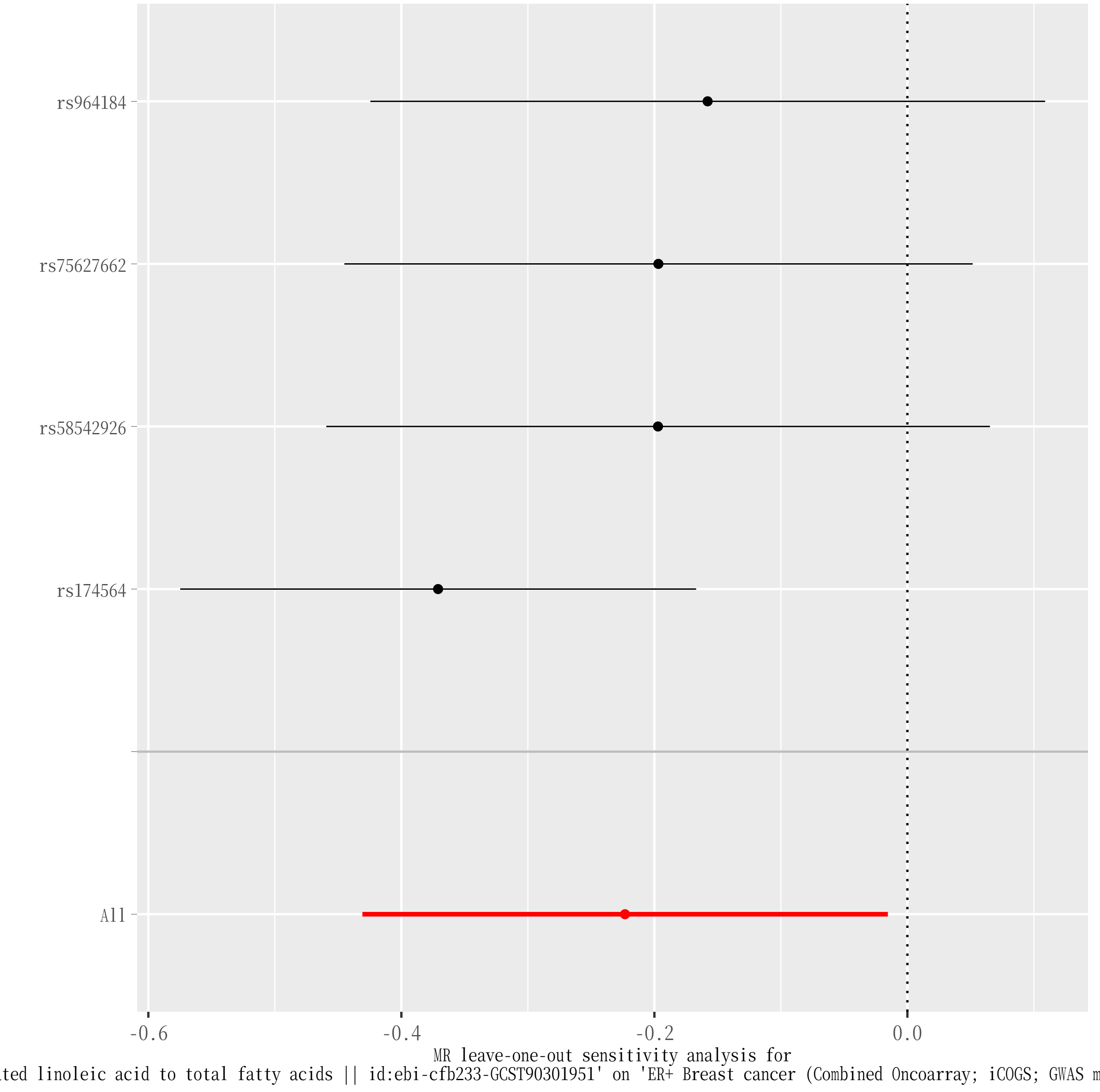
rs964184
rs75627662
rs58542926
rs174564
All
-0.6
-0.4
-0.2
0.0
MR leave-one-out sensitivity analysis for
ted linoleic acid to total fatty acids || id:ebi-cfb233-GCST90301951' on 'ER+ Breast cancer (Combined Oncoarray; iCOGS; GWAS m

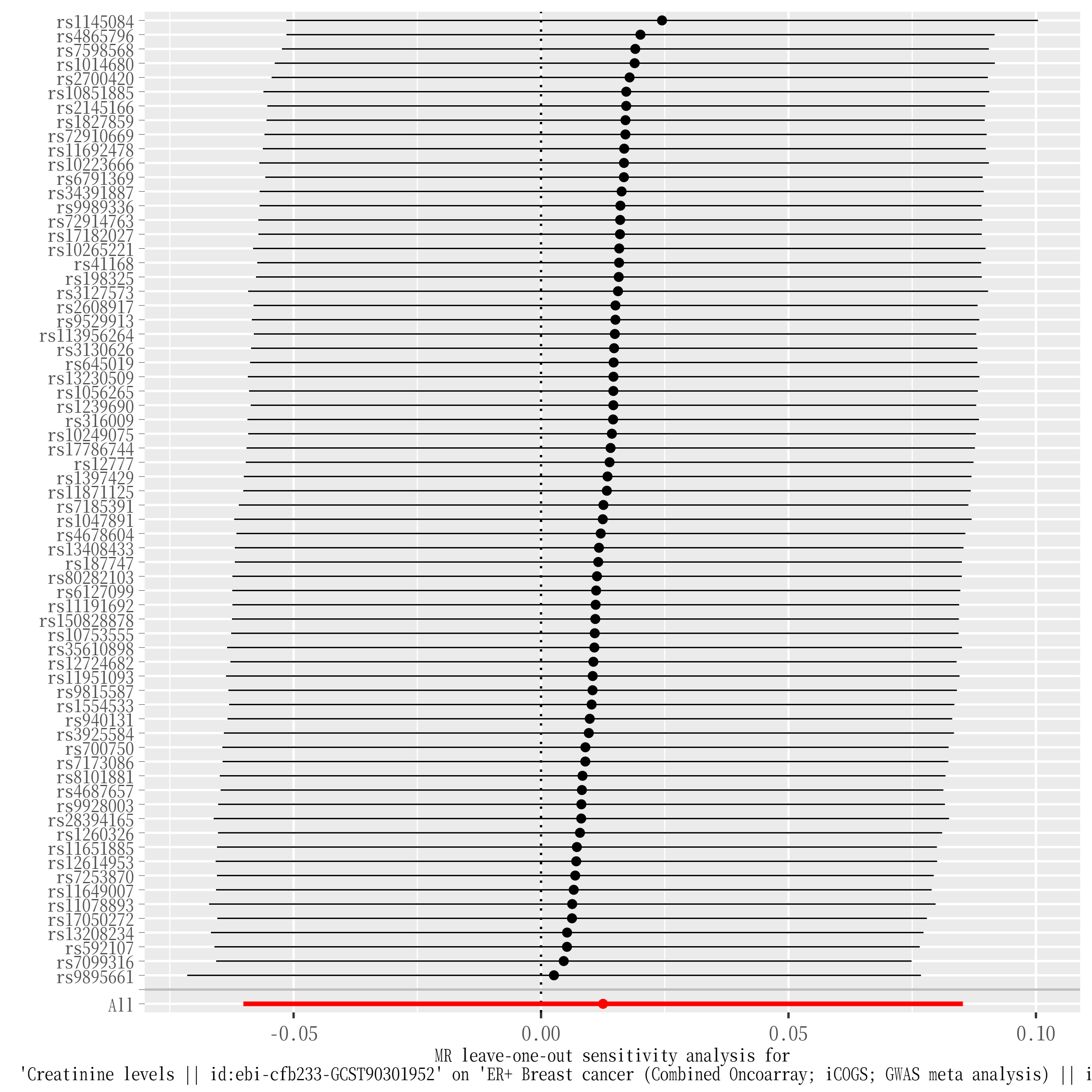

rs1145084
rs4865796
rs7598568
rs1014680
rs2700420
rs10851885
rs2145166
rs1827859
rs72910669
rs11692478
rs10223666
rs6791369
rs34391887
rs9989336
rs72914763
rs17182027
rs10265221
rs41168
rs198325
rs3127573
rs2608917
rs9529913
rs113956264
rs3130626
rs645019
rs13230509
rs1056265
rs1239690
rs316009
rs10249075
rs17786744
rs12777
rs1397429
rs11871125
rs7185391
rs1047891
rs4678604
rs13408433
rs187747
rs80282103
rs6127099
rs11191692
rs150828878
rs10753555
rs35610898
rs12724682
rs11951093
rs9815587
rs1554533
rs940131
rs3925584
rs700750
rs7173086
rs8101881
rs4687657
rs9928003
rs28394165
rs1260326
rs11651885
rs12614953
rs7253870
rs11649007
rs11078893
rs17050272
rs13208234
rs592107
rs7099316
rs9895661
All
-0.05
0.00
0.05
0.10
MR leave-one-out sensitivity analysis for
'Creatinine levels || id:ebi-cfb233-GCST90301952' on 'ER+ Breast cancer (Combined Oncoarray; iCOGS; GWAS meta analysis) || i

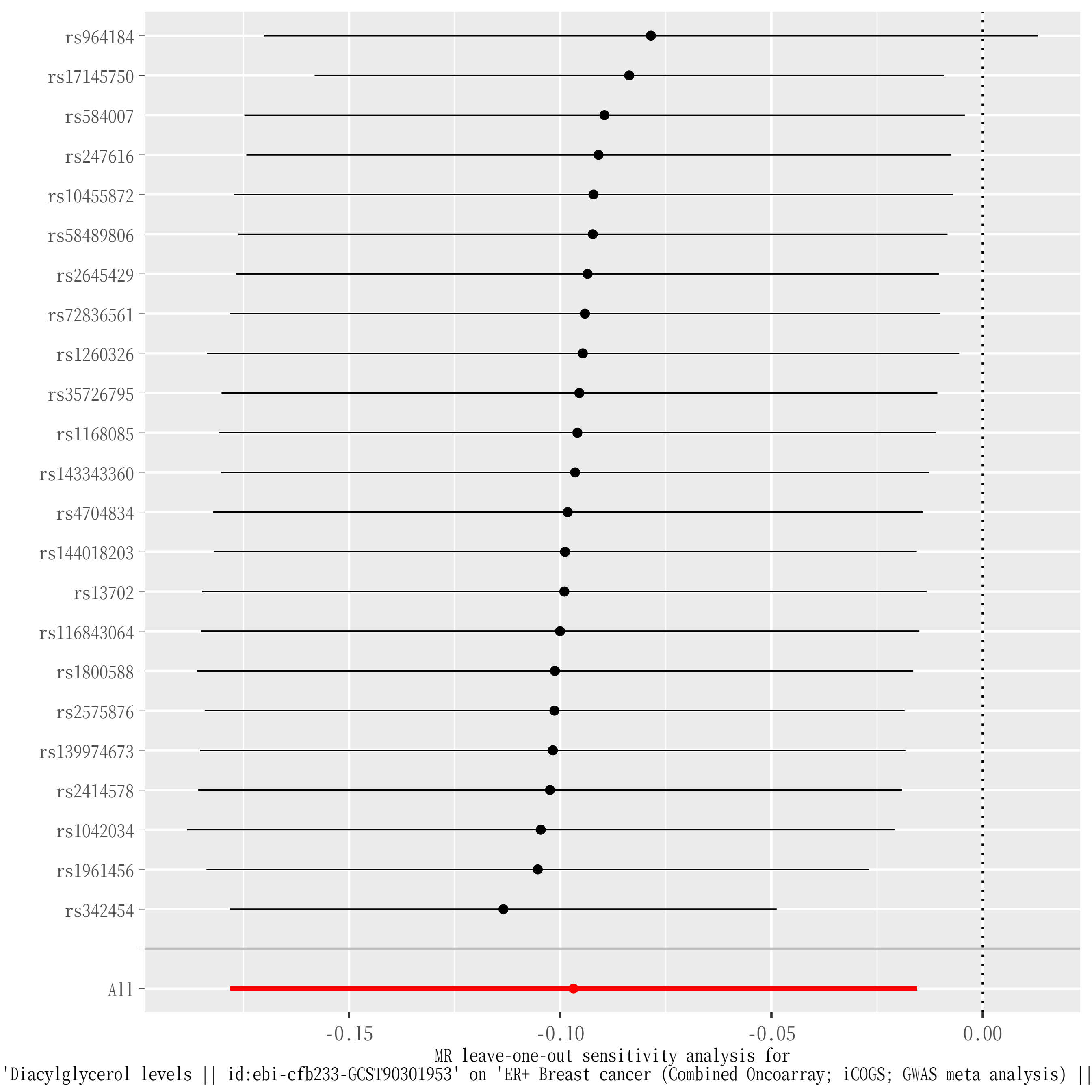
rs964184
rs17145750
rs584007
rs247616
rs10455872
rs58489806
rs2645429
rs72836561
rs1260326
rs35726795
rs1168085
rs143343360
rs4704834
rs144018203
rs13702
rs116843064
rs1800588
rs2575876
rs139974673
rs2414578
rs1042034
rs1961456
rs342454
All
-0.15
-0.10
-0.05
0.00
MR leave-one-out sensitivity analysis for
'Diacylglycerol levels || id:ebi-cfb233-GCST90301953' on 'ER+ Breast cancer (Combined Oncoarray; iCOGS; GWAS meta analysis) ||

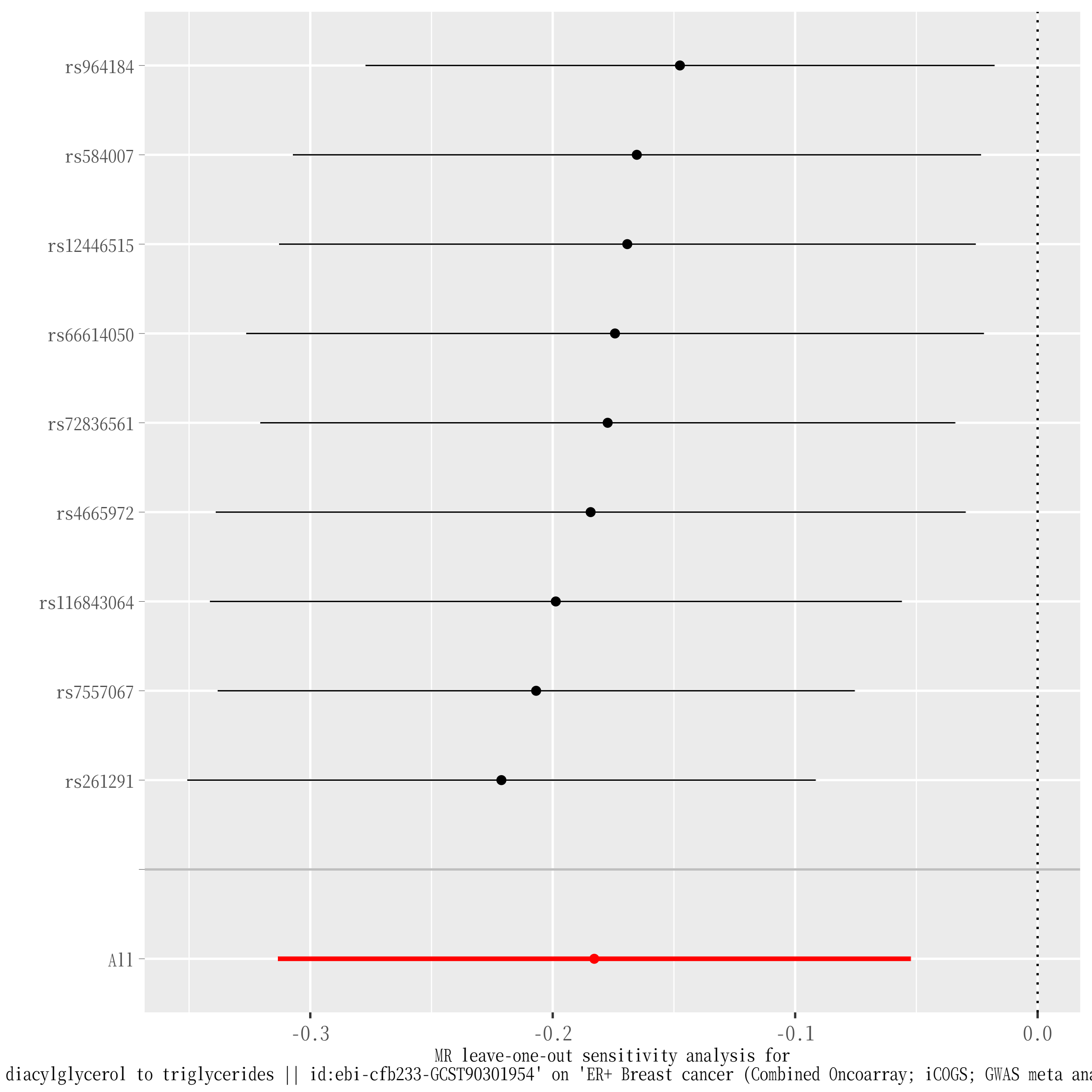
rs964184
rs584007
rs12446515
rs66614050
rs72836561
rs4665972
rs116843064
rs7557067
rs261291
All
-0.3
-0.2
-0.1
0.0
MR leave-one-out sensitivity analysis for
diacylglycerol to triglycerides || id:ebi-cfb233-GCST90301954' on 'ER+ Breast cancer (Combined Oncoarray; iCOGS; GWAS meta ana

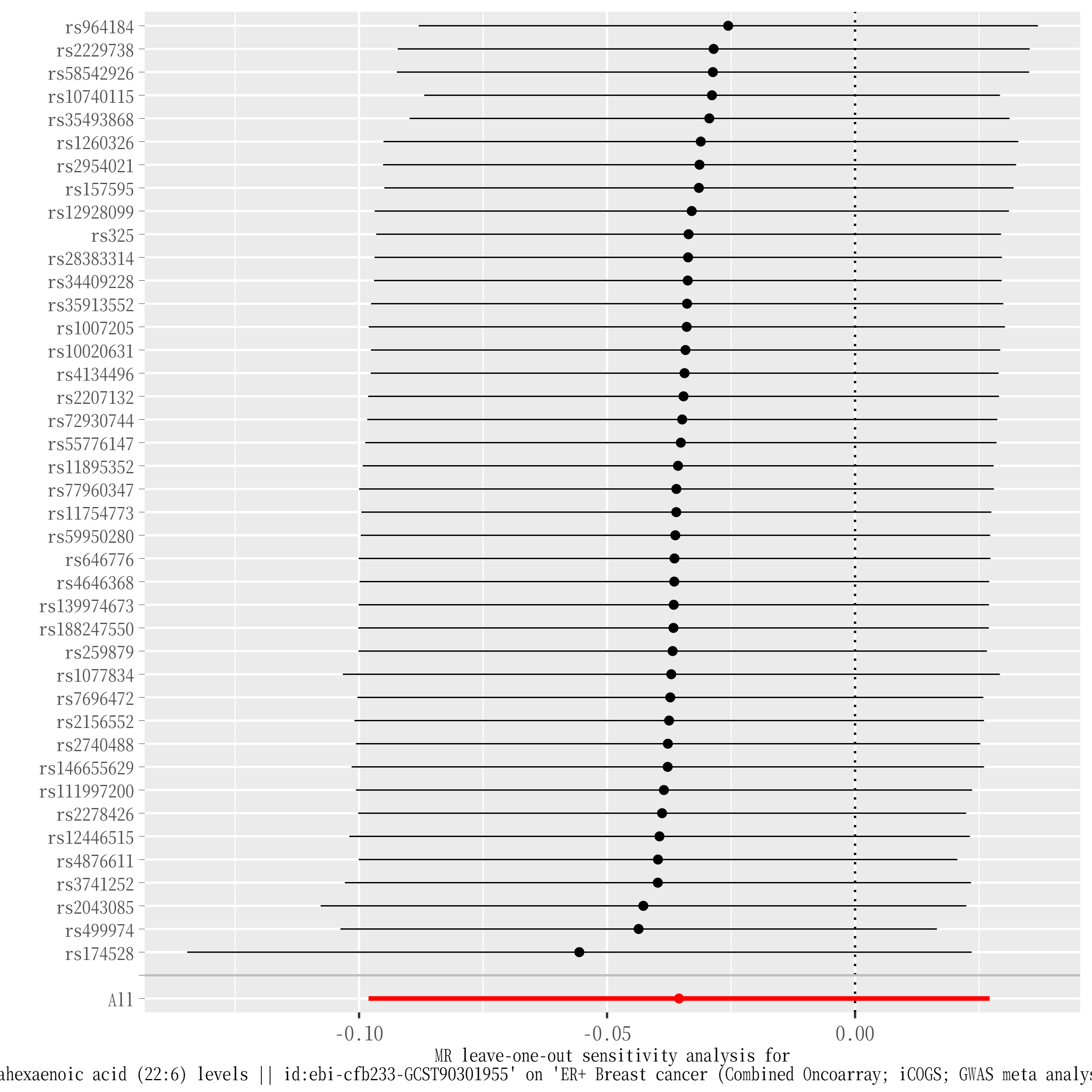

rs964184
rs2229738
rs58542926
rs10740115
rs35493868
rs1260326
rs2954021
rs157595
rs12928099
rs325
rs28383314
rs34409228
rs35913552
rs1007205
rs10020631
rs4134496
rs2207132
rs72930744
rs55776147
rs11895352
rs77960347
rs11754773
rs59950280
rs646776
rs4646368
rs139974673
rs188247550
rs259879
rs1077834
rs7696472
rs2156552
rs2740488
rs146655629
rs111997200
rs2278426
rs12446515
rs4876611
rs3741252
rs2043085
rs499974
rs174528
All
-0.10
-0.05
0.00
MR leave-one-out sensitivity analysis for
ahexaenoic acid (22:6) levels || id:ebi-cfb233-GCST90301955' on 'ER+ Breast cancer (Combined Oncoarray; iCOGS; GWAS meta analys

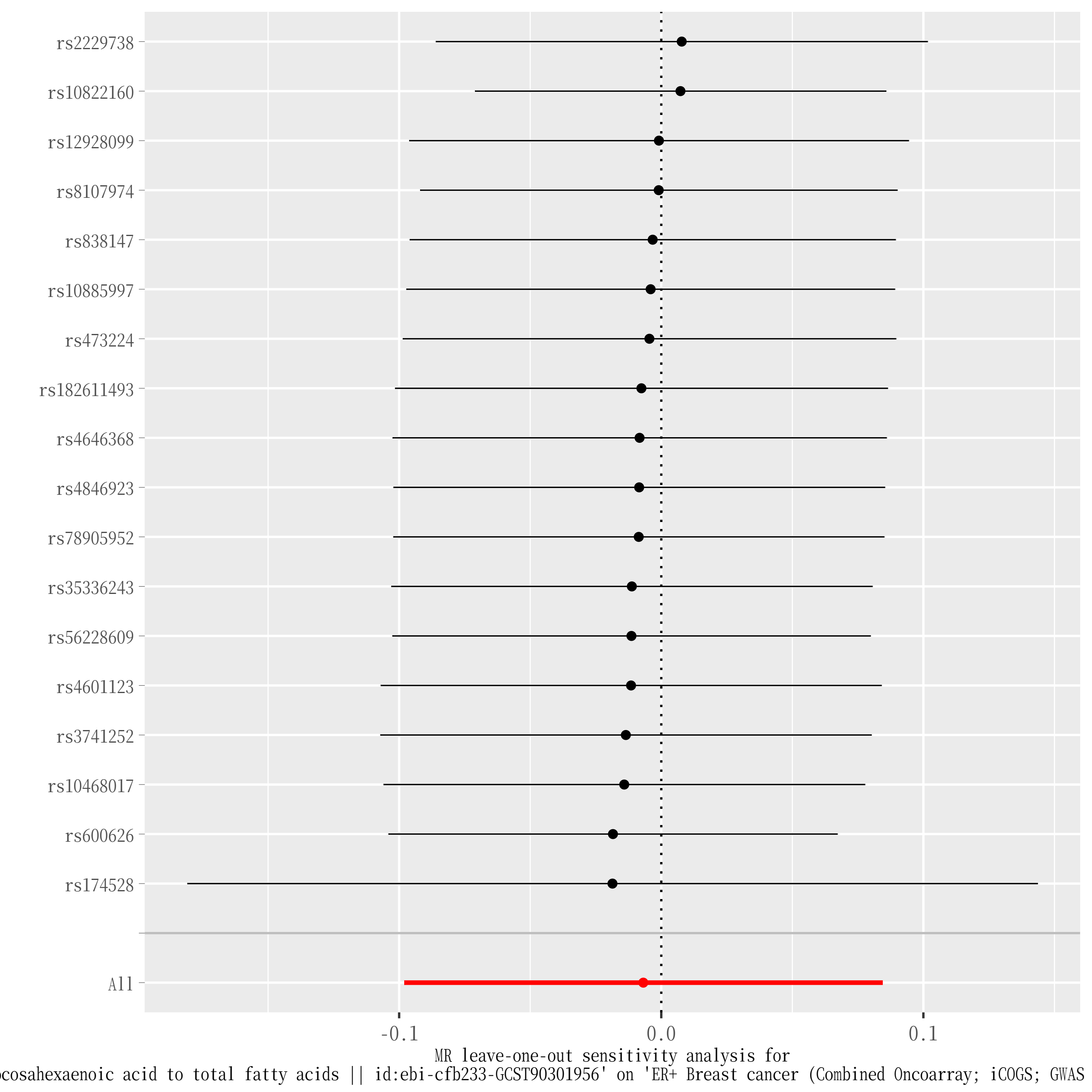
rs2229738
rs10822160
rs12928099
rs8107974
rs838147
rs10885997
rs473224
rs182611493
rs4646368
rs4846923
rs78905952
rs35336243
rs56228609
rs4601123
rs3741252
rs10468017
rs600626
rs174528
All
-0.1
0.0
0.1
MR leave-one-out sensitivity analysis for
ocosahexaenoic acid to total fatty acids || id:ebi-cfb233-GCST90301956' on 'ER+ Breast cancer (Combined Oncoarray; iCOGS; GWAS

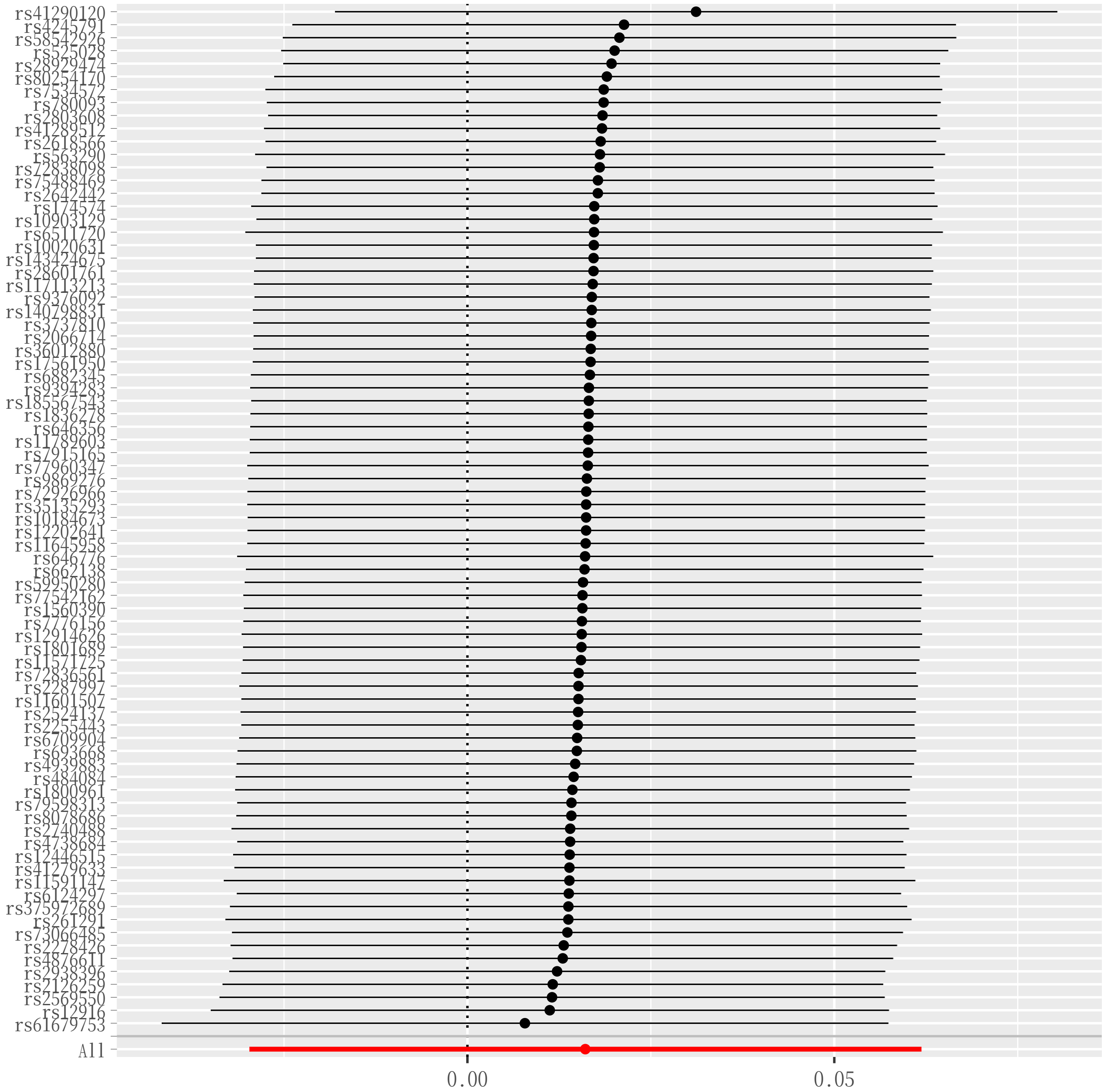

MR leave-one-out sensitivity analysis for
erified cholesterol levels || id:ebi-cfb233-GCST90301957' on 'ER+ Breast cancer (Combined Oncoarray; iCOGS; GWAS meta analysis

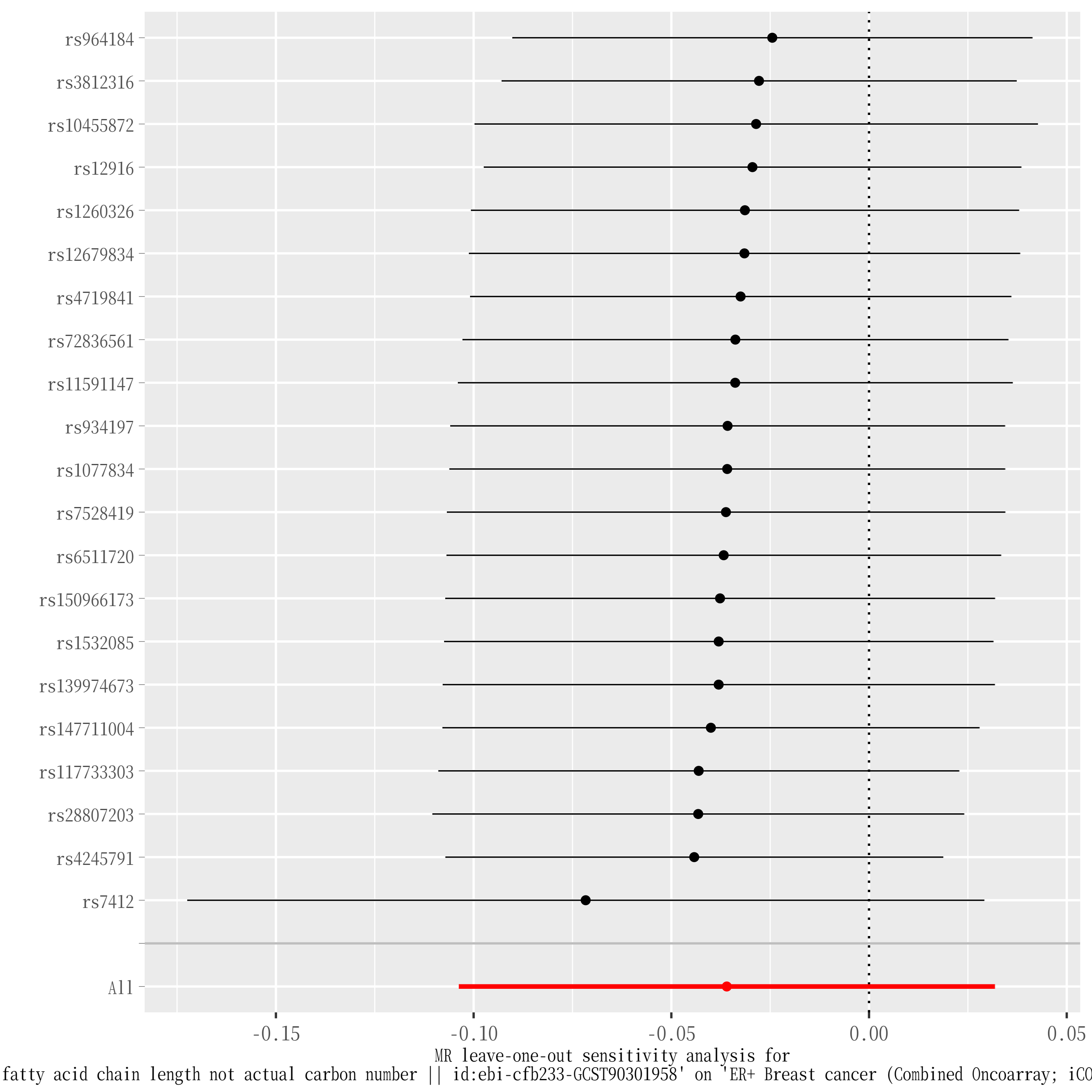
rs964184
rs3812316
rs10455872
rs12916
rs1260326
rs12679834
rs4719841
rs72836561
rs11591147
rs934197
rs1077834
rs7528419
rs6511720
rs150966173
rs1532085
rs139974673
rs147711004
rs117733303
rs28807203
rs4245791
rs7412
All
-0.15
-0.10
-0.05
0.00
0.05
MR leave-one-out sensitivity analysis for
fatty acid chain length not actual carbon number || id:ebi-cfb233-GCST90301958' on 'ER+ Breast cancer (Combined Oncoarray; iC

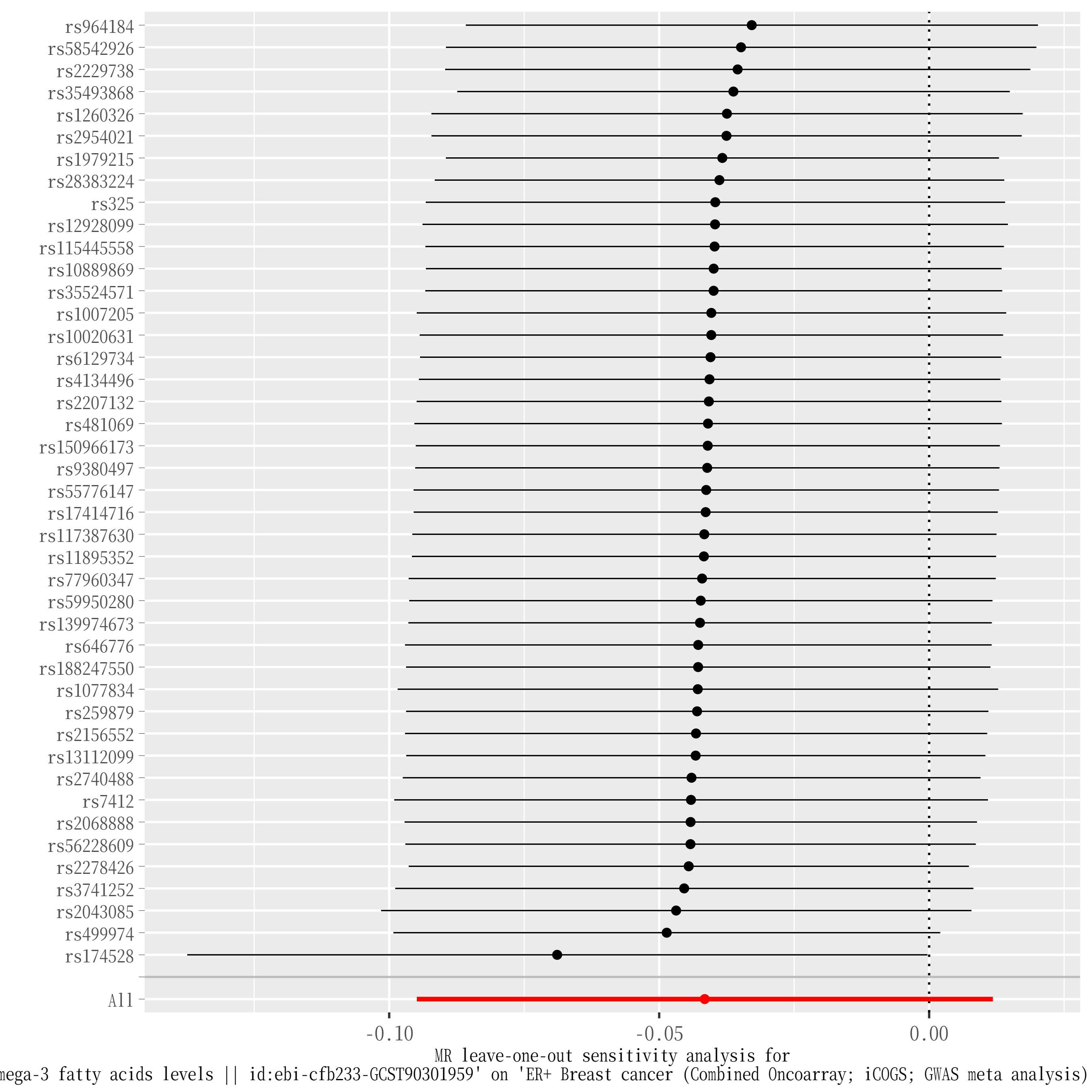
rs964184
rs58542926
rs2229738
rs35493868
rs1260326
rs2954021
rs1979215
rs28383224
rs325
rs12928099
rs115445558
rs10889869
rs35524571
rs1007205
rs10020631
rs6129734
rs4134496
rs2207132
rs481069
rs150966173
rs9380497
rs55776147
rs17414716
rs117387630
rs11895352
rs77960347
rs59950280
rs139974673
rs646776
rs188247550
rs1077834
rs259879
rs2156552
rs13112099
rs2740488
rs7412
rs2068888
rs56228609
rs2278426
rs3741252
rs2043085
rs499974
rs174528
All
-0.10
-0.05
0.00
MR leave-one-out sensitivity analysis for
mega-3 fatty acids levels || id:ebi-cfb233-GCST90301959' on 'ER+ Breast cancer (Combined Oncoarray; iCOGS; GWAS meta analysis)

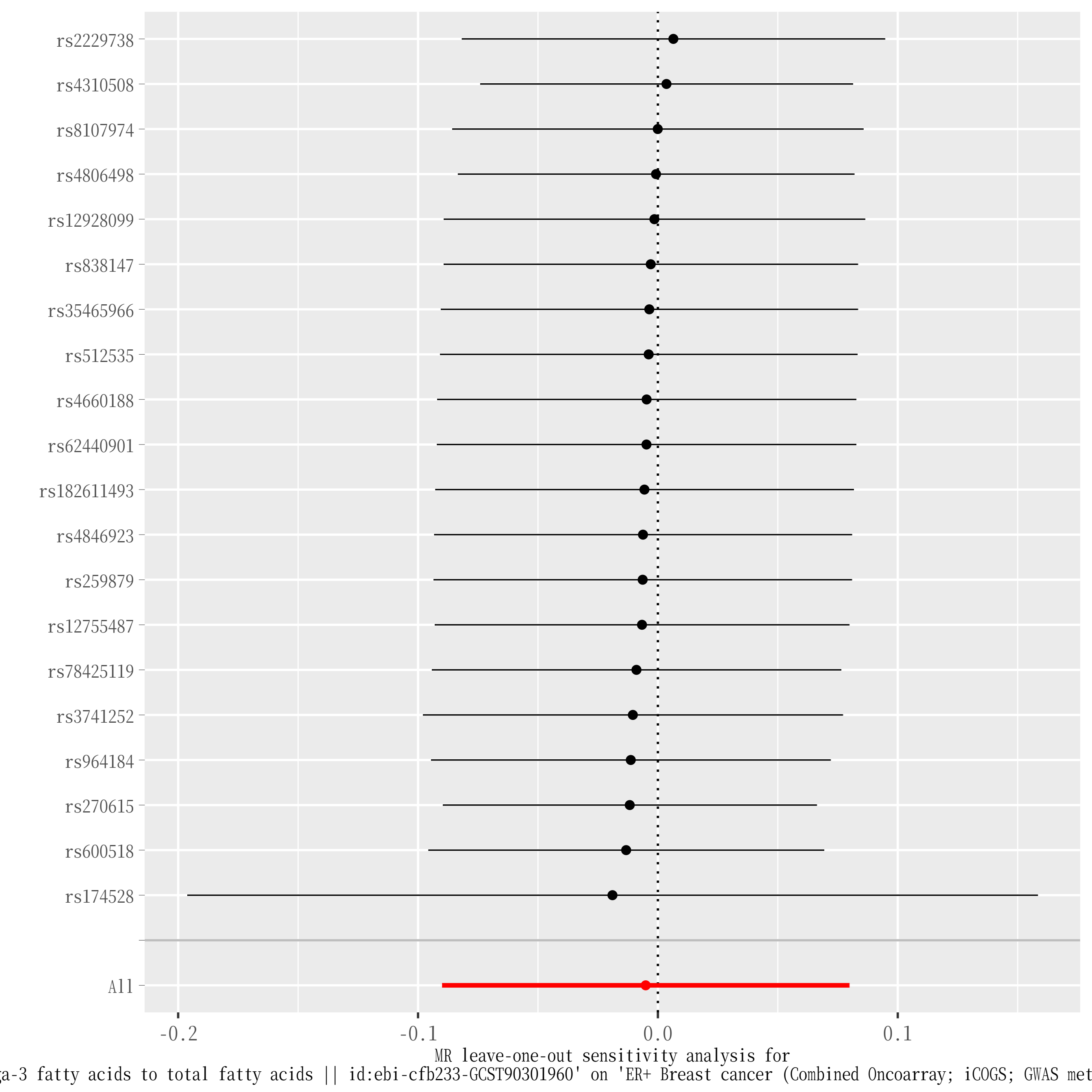
rs2229738
rs4310508
rs8107974
rs4806498
rs12928099
rs838147
rs35465966
rs512535
rs4660188
rs62440901
rs182611493
rs4846923
rs259879
rs12755487
rs78425119
rs3741252
rs964184
rs270615
rs600518
rs174528
All
-0.2
-0.1
0.0
0.1
MR leave-one-out sensitivity analysis for
a-3 fatty acids to total fatty acids || id:ebi-cfb233-GCST90301960' on 'ER+ Breast cancer (Combined Oncoarray; iCOGS; GWAS met

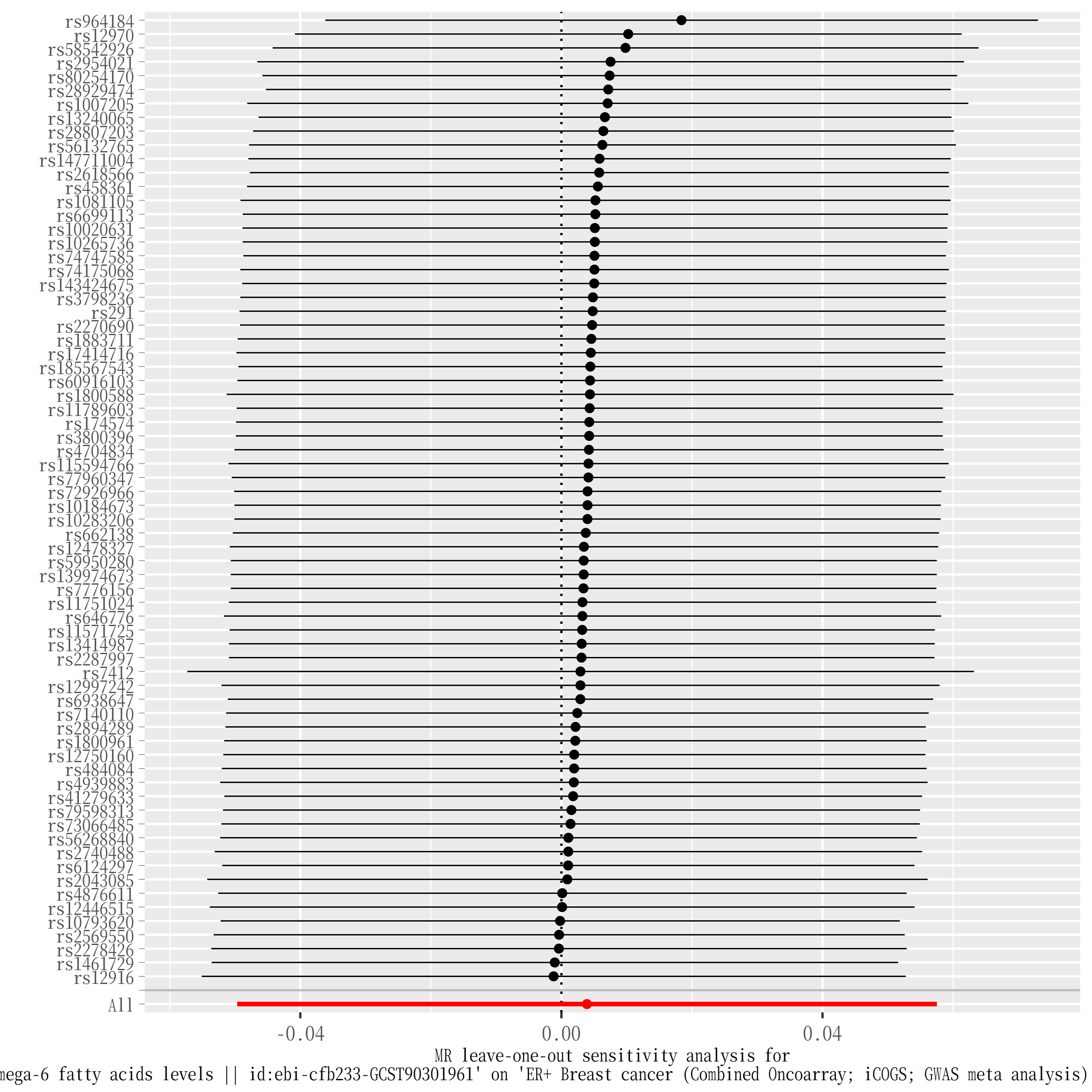
rs964184
rs12970
rs58542926
rs2954021
rs80254170
rs28929474
rs1007205
rs13240065
rs28807203
rs56132765
rs147711004
rs2618566
rs458361
rs1081105
rs6699113
rs10020631
rs10265736
rs74747585
rs74175068
rs143424675
rs3798236
rs291
rs2270690
rs1883711
rs17414716
rs185567543
rs60916103
rs1800588
rs11789603
rs174574
rs3800396
rs4704834
rs115594766
rs77960347
rs72926966
rs10184673
rs10283206
rs662138
rs12478327
rs59950280
rs139974673
rs7776156
rs11751024
rs646776
rs11571725
rs13414987
rs2287997
rs7412
rs12997242
rs6938647
rs7140110
rs2894289
rs1800961
rs12750160
rs484084
rs4939883
rs41279633
rs79598313
rs73066485
rs56268840
rs2740488
rs6124297
rs2043085
rs4876611
rs12446515
rs10793620
rs2569550
rs2278426
rs1461729
rs12916
All
-0.04
0.00
0.04
MR leave-one-out sensitivity analysis for
mega-6 fatty acids levels || id:ebi-cfb233-GCST90301961' on 'ER+ Breast cancer (Combined Oncoarray; iCOGS; GWAS meta analysis)

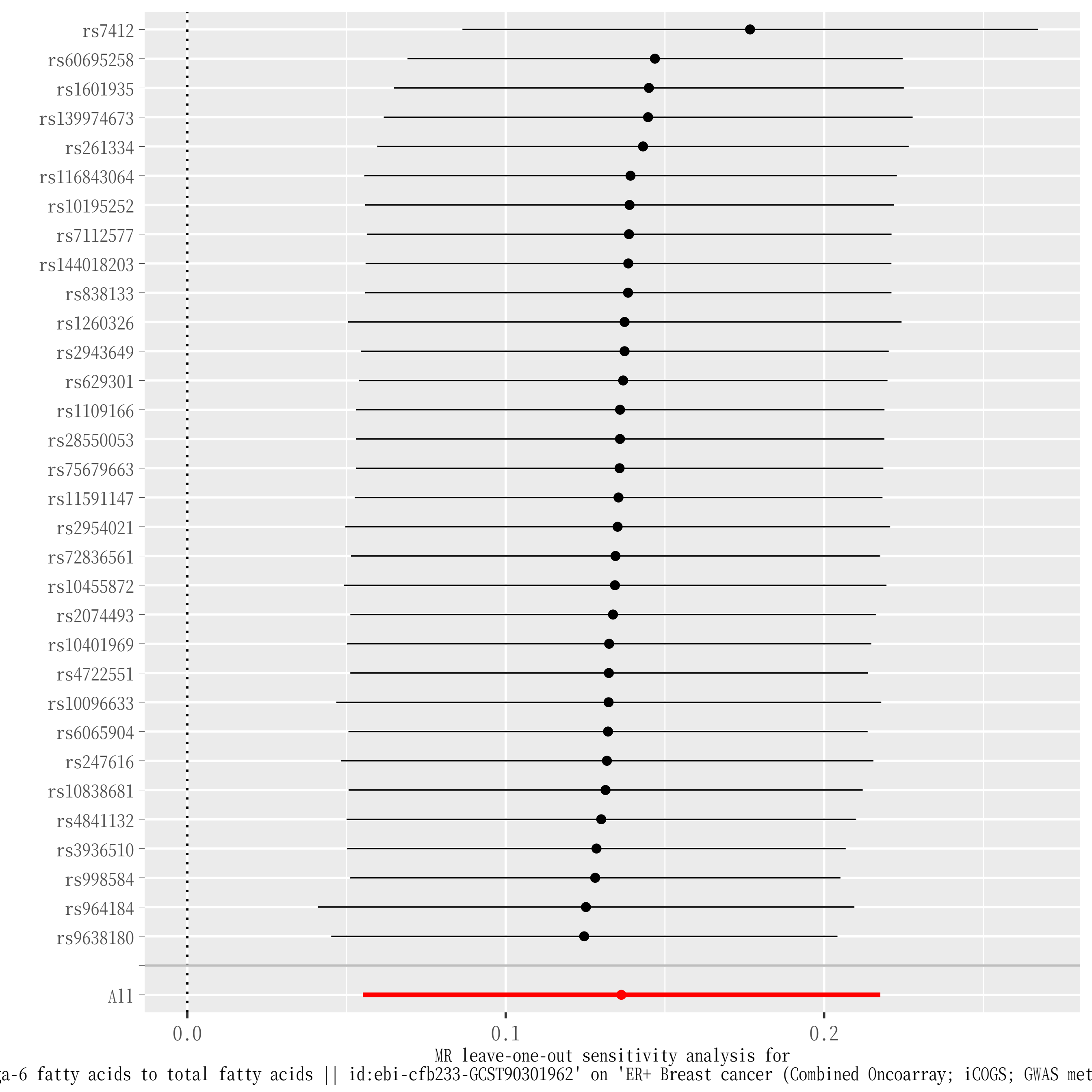

rs7412
rs60695258
rs1601935
rs139974673
rs261334
rs116843064
rs10195252
rs7112577
rs144018203
rs838133
rs1260326
rs2943649
rs629301
rs1109166
rs28550053
rs75679663
rs11591147
rs2954021
rs72836561
rs10455872
rs2074493
rs10401969
rs4722551
rs10096633
rs6065904
rs247616
rs10838681
rs4841132
rs3936510
rs998584
rs964184
rs9638180
All
0.0
0.1
0.2
MR leave-one-out sensitivity analysis for
a-6 fatty acids to total fatty acids || id:ebi-cfb233-GCST90301962' on 'ER+ Breast cancer (Combined Oncoarray; iCOGS; GWAS met

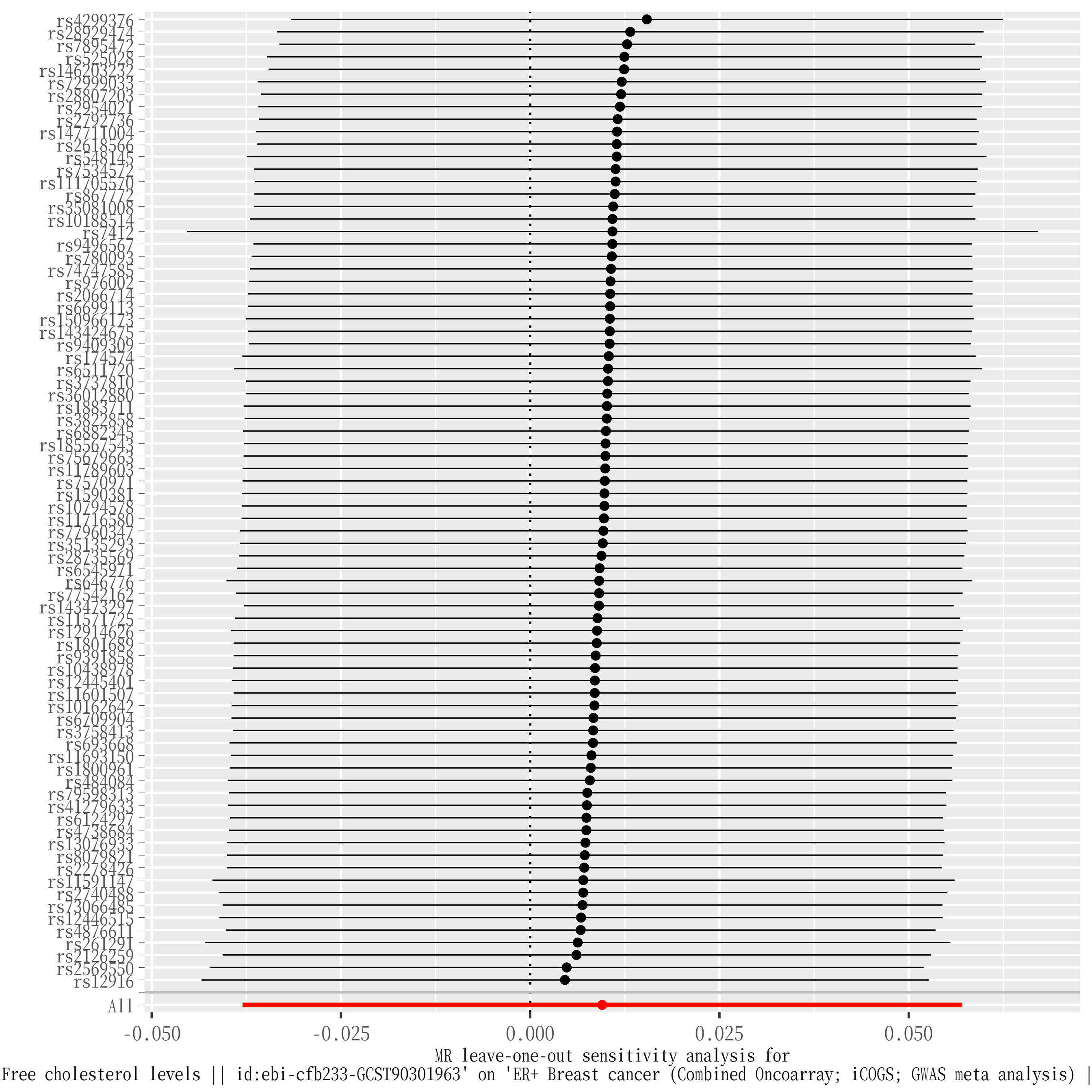

rs4299376
rs28929474
rs7895472
rs525028
rs146203232
rs72999033
rs28807203
rs2954021
rs2792736
rs147711004
rs2618566
rs548145
rs7534572
rs111705570
rs867772
rs35081008
rs10188514
rs7412
rs9496567
rs780093
rs74747585
rs976002
rs2066714
rs6699113
rs150966173
rs143424675
rs9409309
rs174574
rs6511720
rs3737810
rs36012880
rs1883711
rs3822858
rs6882345
rs185567543
rs75679663
rs11789603
rs7570971
rs1590381
rs10794578
rs11716580
rs77960347
rs35135293
rs28735569
rs6545971
rs646776
rs77542162
rs143473297
rs11571725
rs12914626
rs1801689
rs9391858
rs10438978
rs12445401
rs11601507
rs10162642
rs6709904
rs3758413
rs693668
rs11693150
rs1800961
rs484084
rs79598313
rs41279633
rs6124297
rs4738684
rs13076933
rs8079821
rs2278426
rs11591147
rs2740488
rs73066485
rs12446515
rs4876611
rs261291
rs2126259
rs2569550
rs12916
All
-0.050
-0.025
0.000
0.025
0.050
MR leave-one-out sensitivity analysis for
'Free cholesterol levels || id:ebi-cfb233-GCST90301963' on 'ER+ Breast cancer (Combined Oncoarray; iCOGS; GWAS meta analysis) |

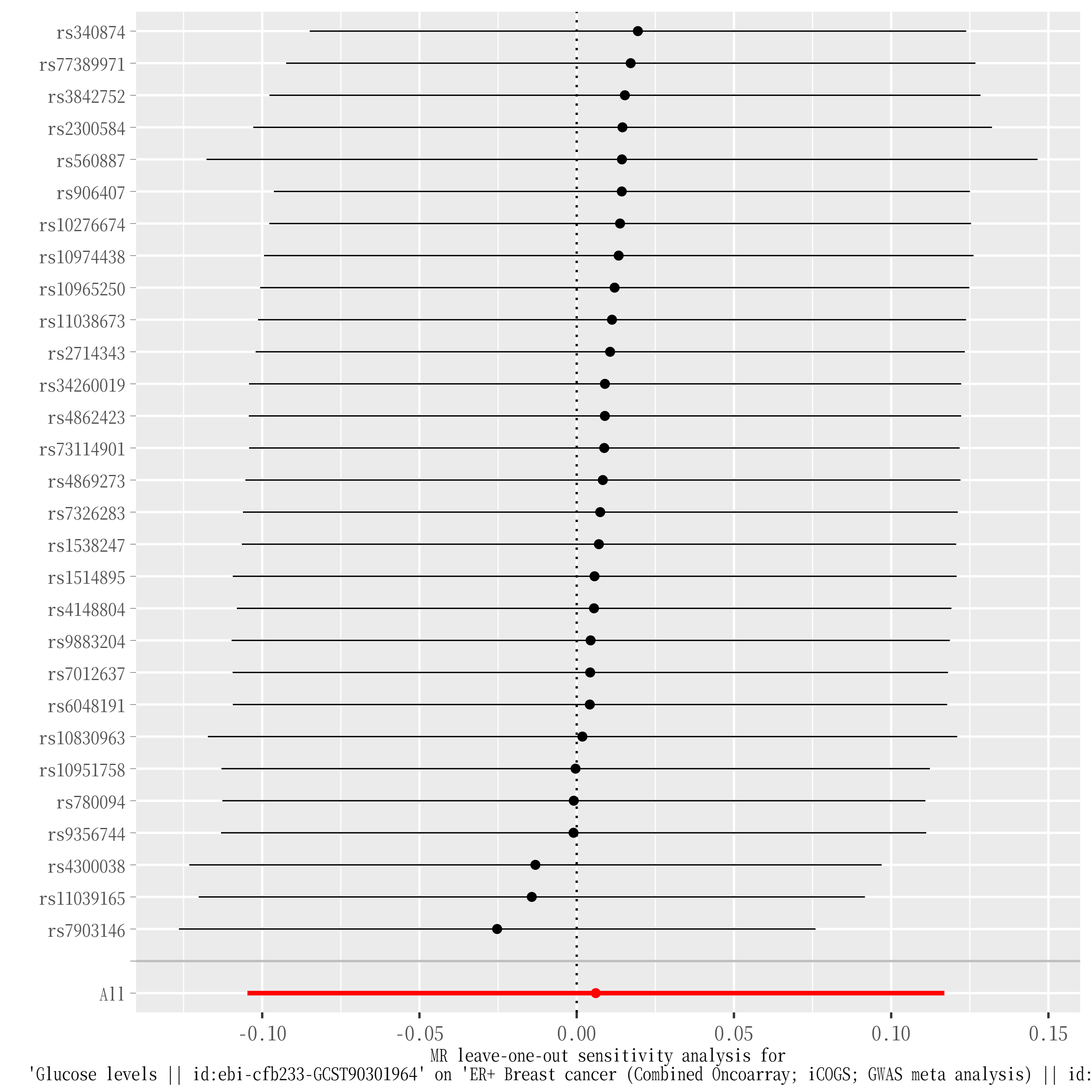

rs340874
rs77389971
rs3842752
rs2300584
rs560887
rs906407
rs10276674
rs10974438
rs10965250
rs11038673
rs2714343
rs34260019
rs4862423
rs73114901
rs4869273
rs7326283
rs1538247
rs1514895
rs4148804
rs9883204
rs7012637
rs6048191
rs10830963
rs10951758
rs780094
rs9356744
rs4300038
rs11039165
rs7903146
All
-0.10
-0.05
0.00
0.05
0.10
0.15
MR leave-one-out sensitivity analysis for
'Glucose levels || id:ebi-cfb233-GCST90301964' on 'ER+ Breast cancer (Combined Oncoarray; iCOGS; GWAS meta analysis) || id:

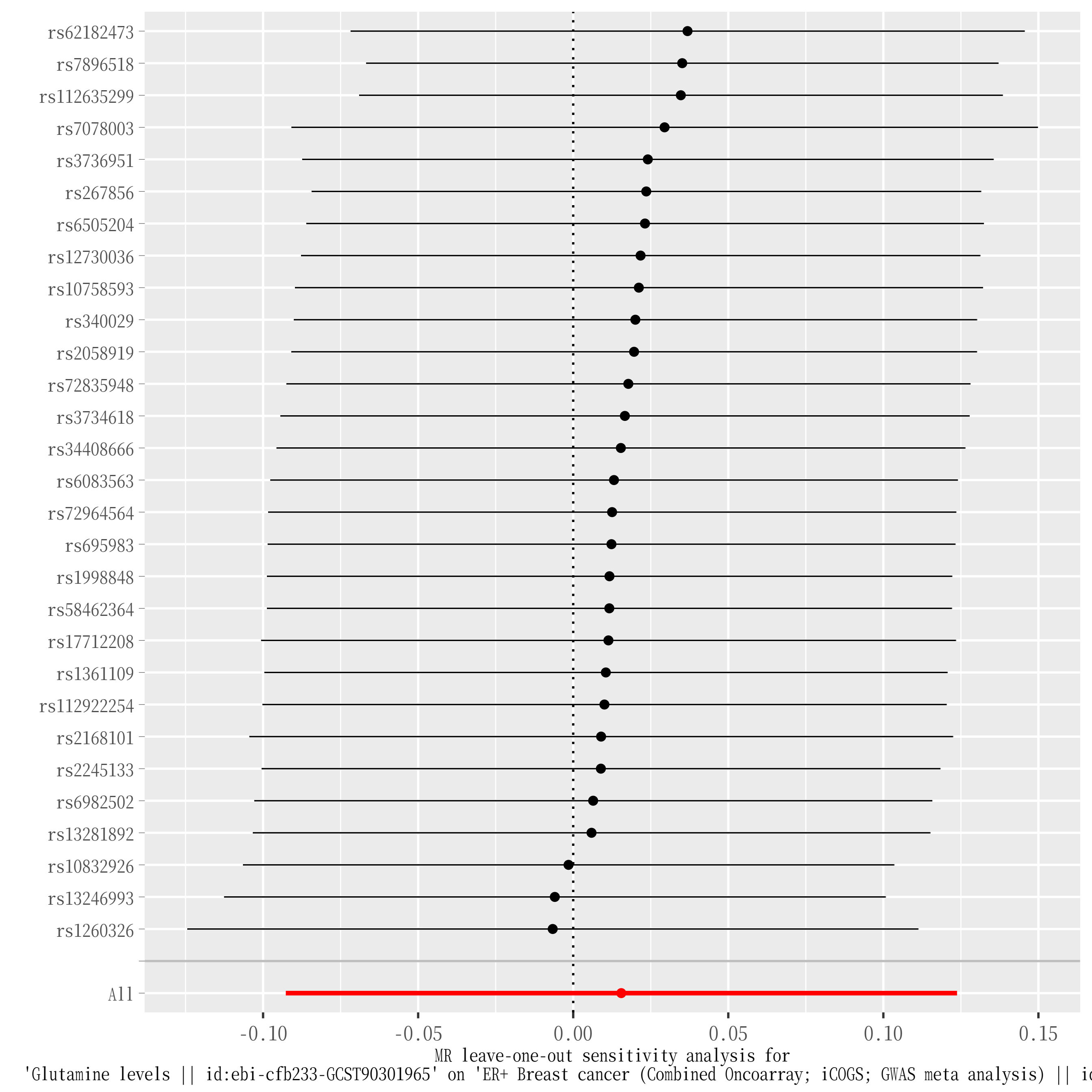
rs62182473
rs7896518
rs112635299
rs7078003
rs3736951
rs267856
rs6505204
rs12730036
rs10758593
rs340029
rs2058919
rs72835948
rs3734618
rs34408666
rs6083563
rs72964564
rs695983
rs1998848
rs58462364
rs17712208
rs1361109
rs112922254
rs2168101
rs2245133
rs6982502
rs13281892
rs10832926
rs13246993
rs1260326
All
-0.10
-0.05
0.00
0.05
0.10
0.15
MR leave-one-out sensitivity analysis for
'Glutamine levels || id:ebi-cfb233-GCST90301965' on 'ER+ Breast cancer (Combined Oncoarray; iCOGS; GWAS meta analysis) || id

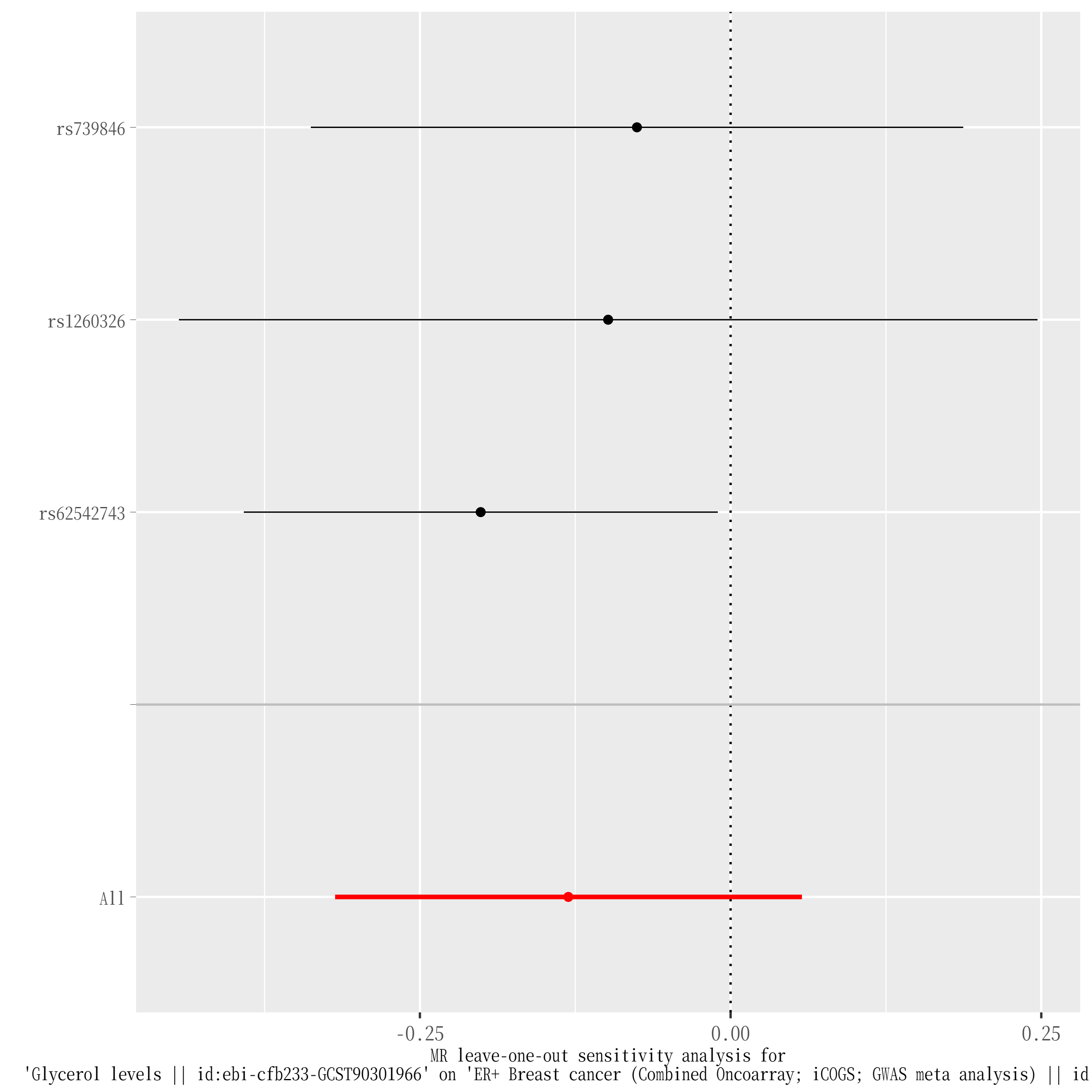

rs739846
rs1260326
rs62542743
All
-0.25
0.00
0.25
MR leave-one-out sensitivity analysis for
'Glycerol levels || id:ebi-cfb233-GCST90301966' on 'ER+ Breast cancer (Combined Oncoarray; iCOGS; GWAS meta analysis) || id

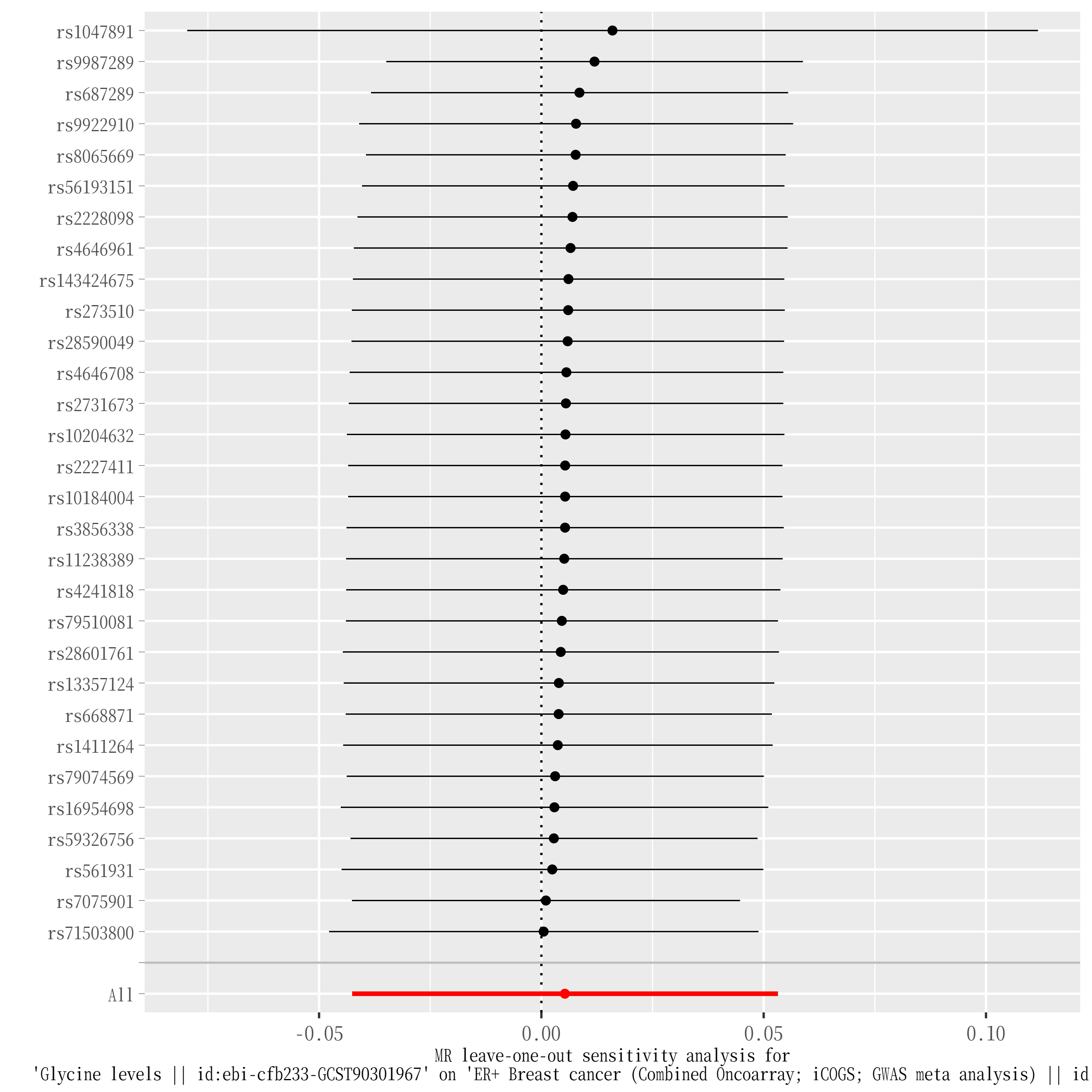
rs1047891
rs9987289
rs687289
rs9922910
rs8065669
rs56193151
rs2228098
rs4646961
rs143424675
rs273510
rs28590049
rs4646708
rs2731673
rs10204632
rs2227411
rs10184004
rs3856338
rs11238389
rs4241818
rs79510081
rs28601761
rs13357124
rs668871
rs1411264
rs79074569
rs16954698
rs59326756
rs561931
rs7075901
rs71503800
All
-0.05
0.00
0.05
0.10
MR leave-one-out sensitivity analysis for
'Glycine levels || id:ebi-cfb233-GCST90301967' on 'ER+ Breast cancer (Combined Oncoarray; iCOGS; GWAS meta analysis) || id

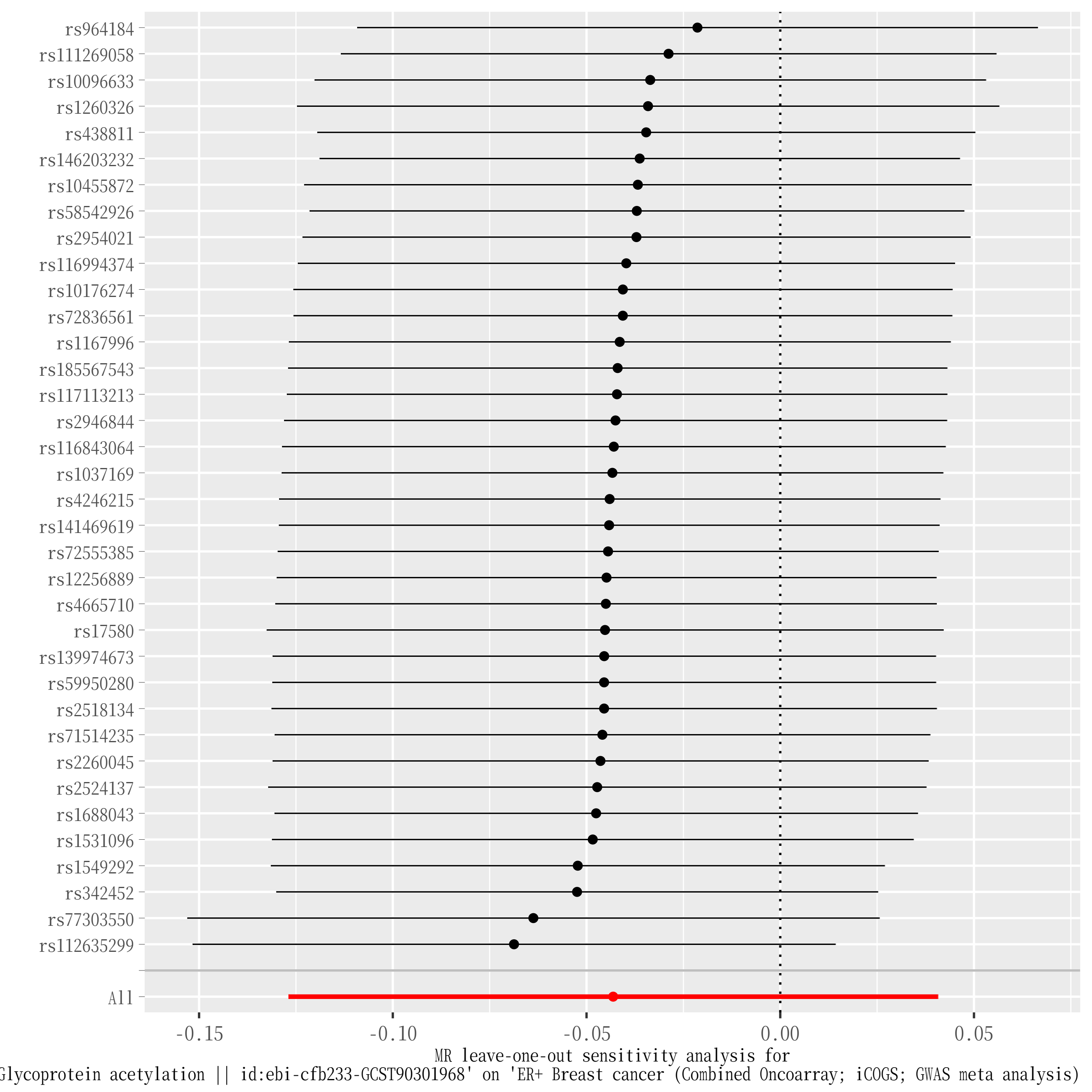
rs964184
rs111269058
rs10096633
rs1260326
rs438811
rs146203232
rs10455872
rs58542926
rs2954021
rs116994374
rs10176274
rs72836561
rs1167996
rs185567543
rs117113213
rs2946844
rs116843064
rs1037169
rs4246215
rs141469619
rs72555385
rs12256889
rs4665710
rs17580
rs139974673
rs59950280
rs2518134
rs71514235
rs2260045
rs2524137
rs1688043
rs1531096
rs1549292
rs342452
rs77303550
rs112635299
All
-0.15
-0.10
-0.05
0.00
0.05
MR leave-one-out sensitivity analysis for
'Glycoprotein acetylation || id:ebi-cfb233-GCST90301968' on 'ER+ Breast cancer (Combined Oncoarray; iCOGS; GWAS meta analysis)'

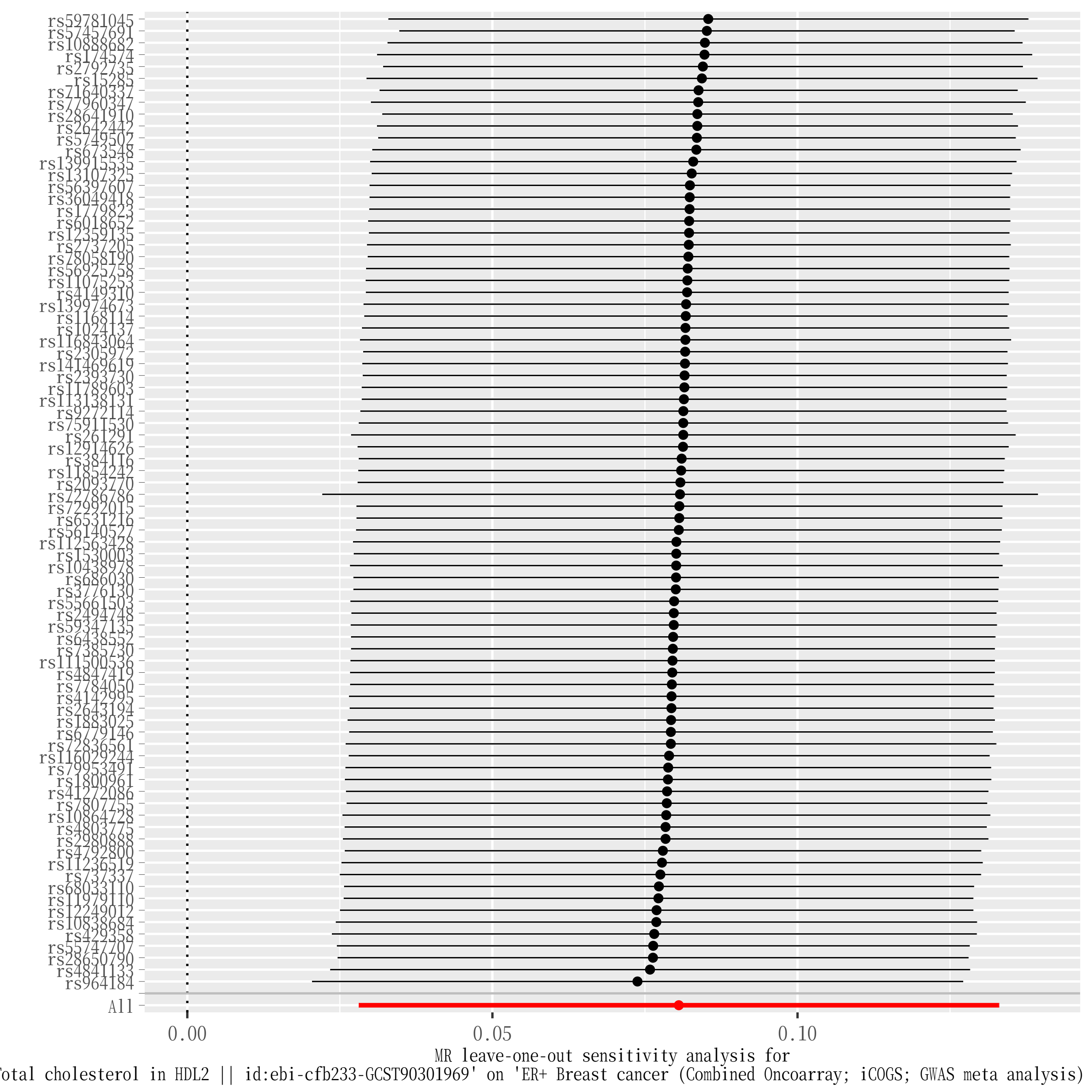
rs59781045
rs57457691
rs10888682
rs174574
rs2792735
rs15285
rs71640337
rs77960347
rs28641910
rs2642442
rs5749502
rs673548
rs139915535
rs13107325
rs56397607
rs36049418
rs1779823
rs6018652
rs12359135
rs2737205
rs78058190
rs56925758
rs11075253
rs4149310
rs139974673
rs1168114
rs1024137
rs116843064
rs2305972
rs141469619
rs2393730
rs11789603
rs113138131
rs9272114
rs75911530
rs261291
rs12914626
rs384116
rs11854242
rs2093770
rs72786786
rs72992015
rs6531216
rs56140527
rs112563428
rs1530003
rs10438978
rs686030
rs3776130
rs55661503
rs2494748
rs59347135
rs6438552
rs7385730
rs111500536
rs4847419
rs7784050
rs4142995
rs2643194
rs1883025
rs6779146
rs72836561
rs116029244
rs79953491
rs1800961
rs41272086
rs7807755
rs10864728
rs4803775
rs2980888
rs4792800
rs11236519
rs737337
rs68033110
rs11979110
rs12249012
rs10838684
rs429358
rs55747707
rs28650790
rs4841133
rs964184
All
0.00
0.05
0.10
MR leave-one-out sensitivity analysis for
'Total cholesterol in HDL2 || id:ebi-cfb233-GCST90301969' on 'ER+ Breast cancer (Combined Oncoarray; iCOGS; GWAS meta analysis)

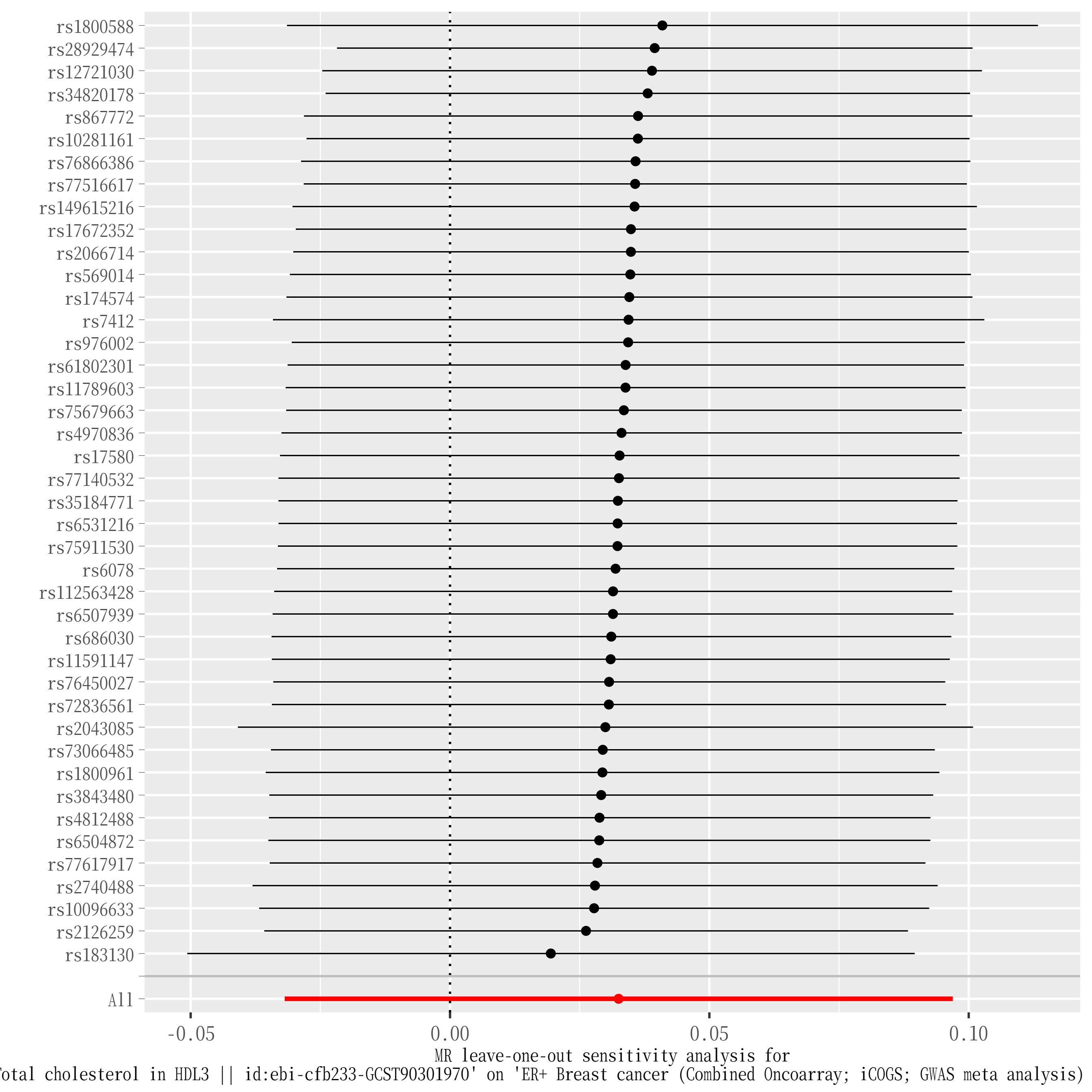
rs1800588
rs28929474
rs12721030
rs34820178
rs867772
rs10281161
rs76866386
rs77516617
rs149615216
rs17672352
rs2066714
rs569014
rs174574
rs7412
rs976002
rs61802301
rs11789603
rs75679663
rs4970836
rs17580
rs77140532
rs35184771
rs6531216
rs75911530
rs6078
rs112563428
rs6507939
rs686030
rs11591147
rs76450027
rs72836561
rs2043085
rs73066485
rs1800961
rs3843480
rs4812488
rs6504872
rs77617917
rs2740488
rs10096633
rs2126259
rs183130
All
-0.05
0.00
0.05
0.10
MR leave-one-out sensitivity analysis for
otal cholesterol in HDL3 || id:ebi-cfb233-GCST90301970' on 'ER+ Breast cancer (Combined Oncoarray; iCOGS; GWAS meta analysis)

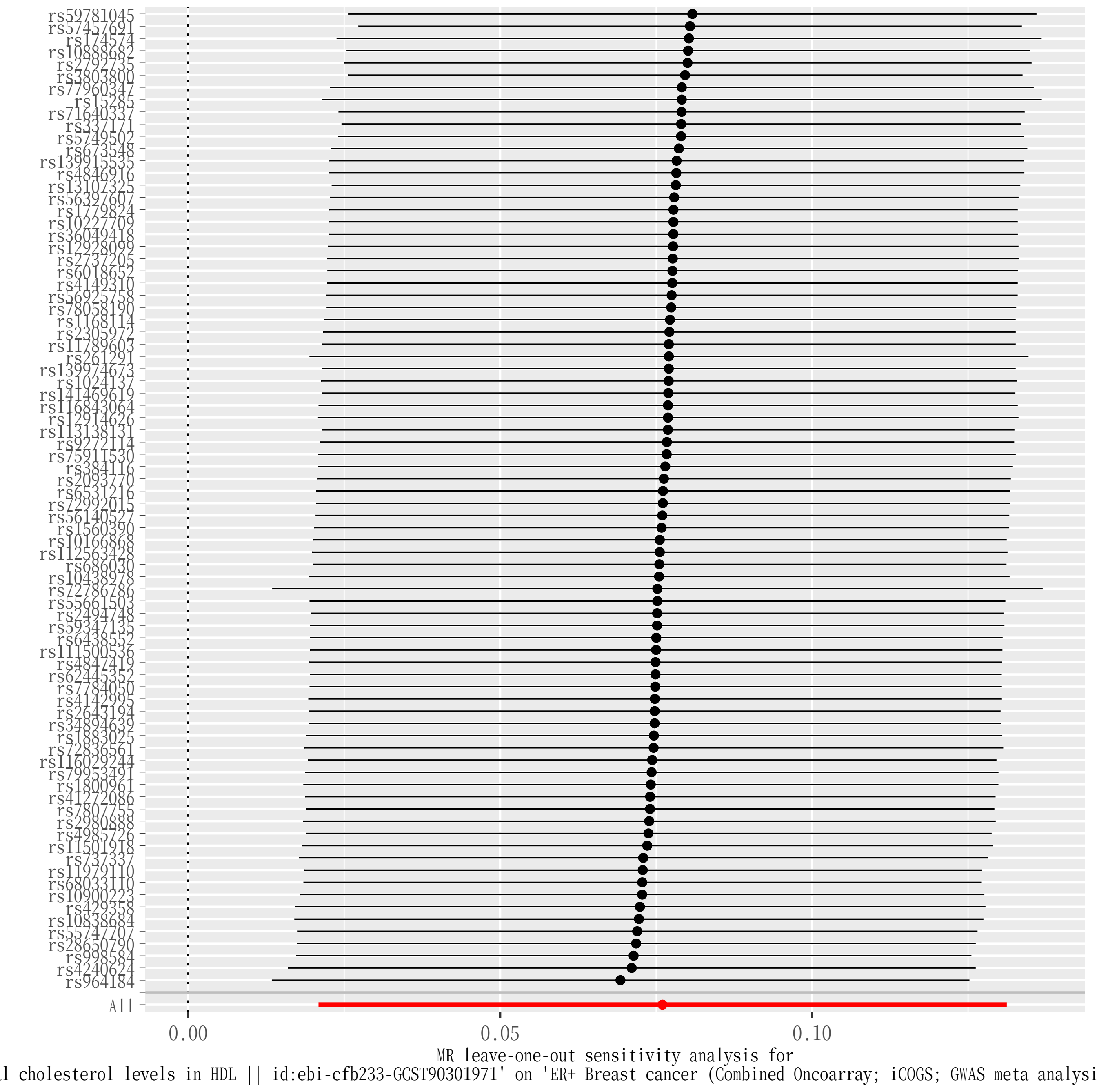
rs59781045
rs57457621
rs174574
rs10888682
rs2792735
rs3803800
rs77960347
rs15285
rs71640337
rs337171
rs5749502
rs673548
rs139915535
rs4846916
rs13107325
rs56397607
rs1779824
rs10227709
rs36049418
rs12928099
rs2737205
rs6018652
rs4149310
rs56925758
rs78058190
rs1168114
rs2305972
rs11789603
rs261291
rs139974673
rs1024137
rs141469619
rs116843064
rs12914626
rs113138131
rs9272114
rs75911530
rs384116
rs2093770
rs6531216
rs72992015
rs56140527
rs1560390
rs10166868
rs112563428
rs686030
rs10438978
rs72786786
rs55661503
rs2494748
rs59347135
rs6438552
rs111500536
rs4847419
rs62445352
rs7784050
rs4142995
rs2643194
rs34894639
rs1883025
rs72836561
rs116029244
rs79953491
rs1800961
rs41272086
rs7807755
rs2980888
rs4985726
rs11501918
rs737337
rs11979110
rs68033110
rs10900223
rs429358
rs10838684
rs55747707
rs28650790
rs998584
rs4240624
rs964184
All
0.00
0.05
0.10
MR leave-one-out sensitivity analysis for
l cholesterol levels in HDL || id:ebi-cfb233-GCST90301971' on 'ER+ Breast cancer (Combined Oncoarray; iCOGS; GWAS meta analysi

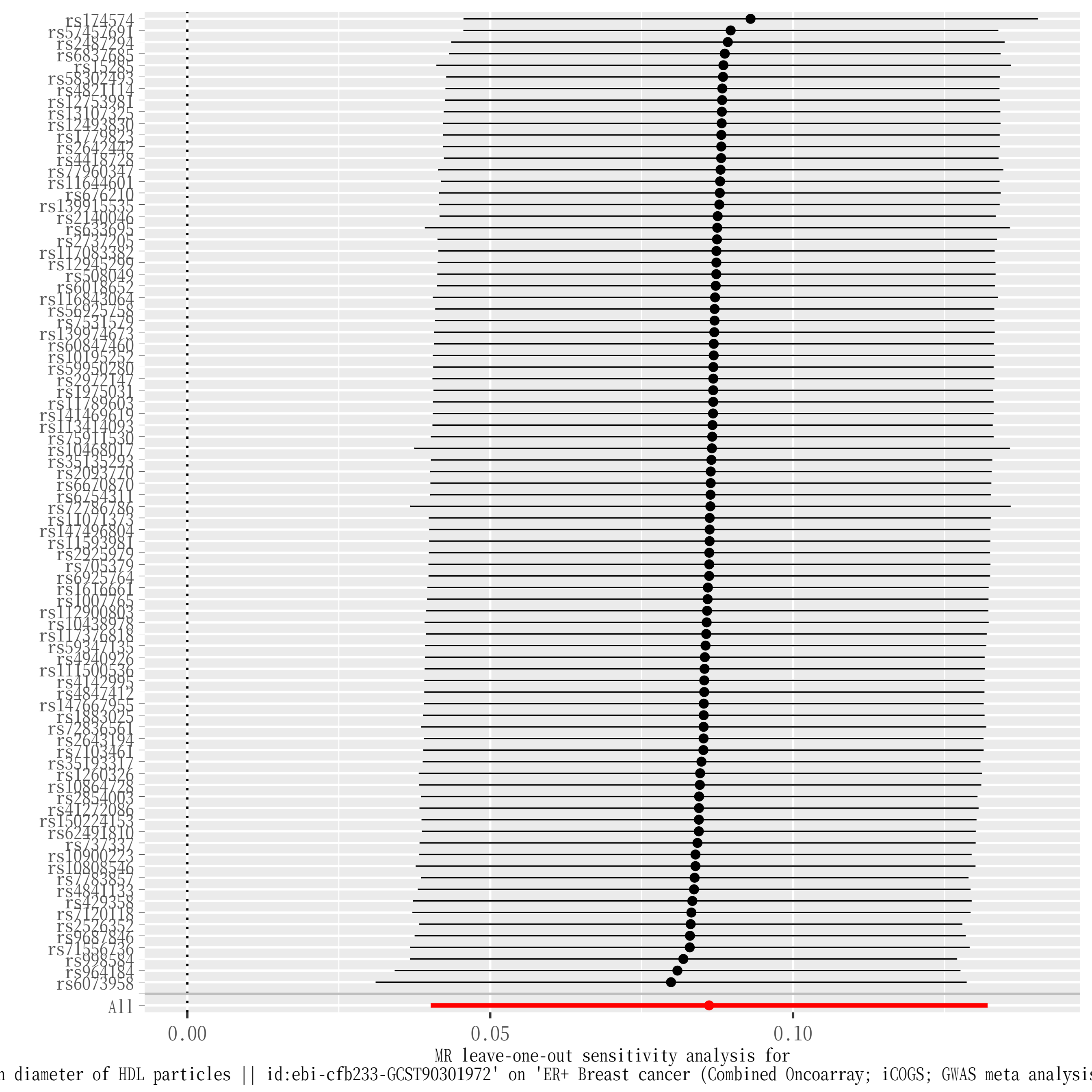

rs174574
rs57457691
rs2487294
rs6837685
rs15285
rs58302493
rs4821114
rs12753981
rs13107325
rs12493830
rs1772823
rs2642442
rs4418728
rs77960347
rs11644601
rs676210
rs139915535
rs2140046
rs633695
rs2737205
rs117083382
rs12945299
rs508049
rs6018652
rs116843064
rs56925758
rs7531579
rs139974673
rs60847460
rs10195252
rs59950280
rs2972147
rs1975031
rs11789603
rs141469619
rs113414093
rs75911530
rs10468017
rs35135293
rs2093770
rs6670870
rs6754311
rs72786786
rs11071373
rs147496804
rs11593981
rs2925979
rs705379
rs6925764
rs1616661
rs1007765
rs112900803
rs10438978
rs117376818
rs59347135
rs4940926
rs111500536
rs4142995
rs4847412
rs147667955
rs1883025
rs72836561
rs2643194
rs7103461
rs35193317
rs1260326
rs10864728
rs2854003
rs41272086
rs150224153
rs62491810
rs737337
rs10900223
rs10808546
rs7783857
rs4841133
rs429358
rs7120118
rs2576352
rs9687846
rs71556736
rs998584
rs964184
rs6073958
All
0.00
0.05
0.10
MR leave-one-out sensitivity analysis for
n diameter of HDL particles || id:ebi-cfb233-GCST90301972' on 'ER+ Breast cancer (Combined Oncoarray; iCOGS; GWAS meta analysis

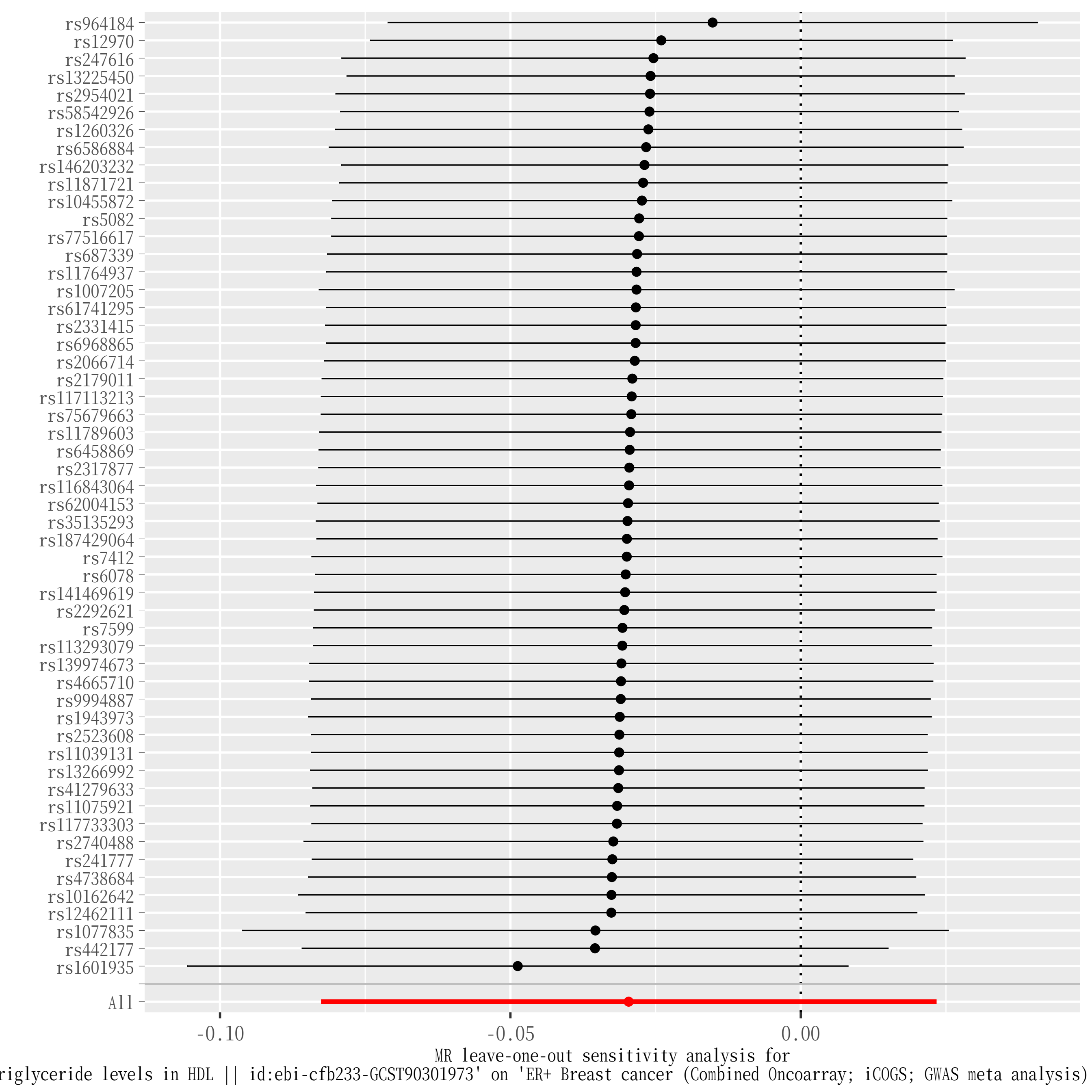
rs964184
rs12970
rs247616
rs13225450
rs2954021
rs58542926
rs1260326
rs6586884
rs146203232
rs11871721
rs10455872
rs5082
rs77516617
rs687339
rs11764937
rs1007205
rs61741295
rs2331415
rs6968865
rs2066714
rs2179011
rs117113213
rs75679663
rs11789603
rs6458869
rs2317877
rs116843064
rs62004153
rs35135293
rs187429064
rs7412
rs6078
rs141469619
rs2292621
rs7599
rs113293079
rs139974673
rs4665710
rs9994887
rs1943973
rs2523608
rs11039131
rs13266992
rs41279633
rs11075921
rs117733303
rs2740488
rs241777
rs4738684
rs10162642
rs12462111
rs1077835
rs442177
rs1601935
All
-0.10
-0.05
0.00
MR leave-one-out sensitivity analysis for
riglyceride levels in HDL || id:ebi-cfb233-GCST90301973' on 'ER+ Breast cancer (Combined Oncoarray; iCOGS; GWAS meta analysis)

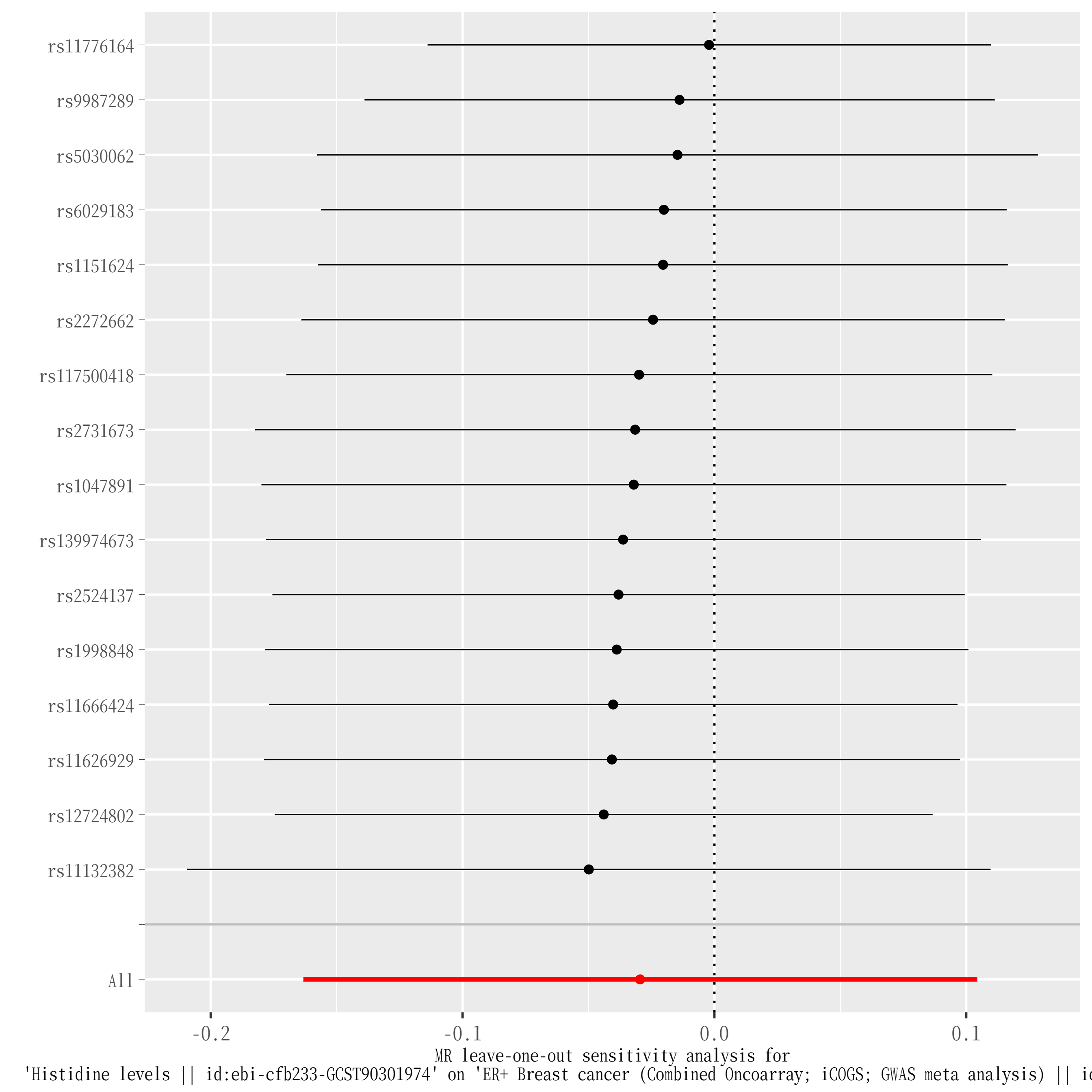
rs11776164
rs9987289
rs5030062
rs6029183
rs1151624
rs2272662
rs117500418
rs2731673
rs1047891
rs139974673
rs2524137
rs1998848
rs11666424
rs11626929
rs12724802
rs11132382
All
-0.2
-0.1
0.0
0.1
MR leave-one-out sensitivity analysis for
'Histidine levels || id:ebi-cfb233-GCST90301974' on 'ER+ Breast cancer (Combined Oncoarray; iCOGS; GWAS meta analysis) || id

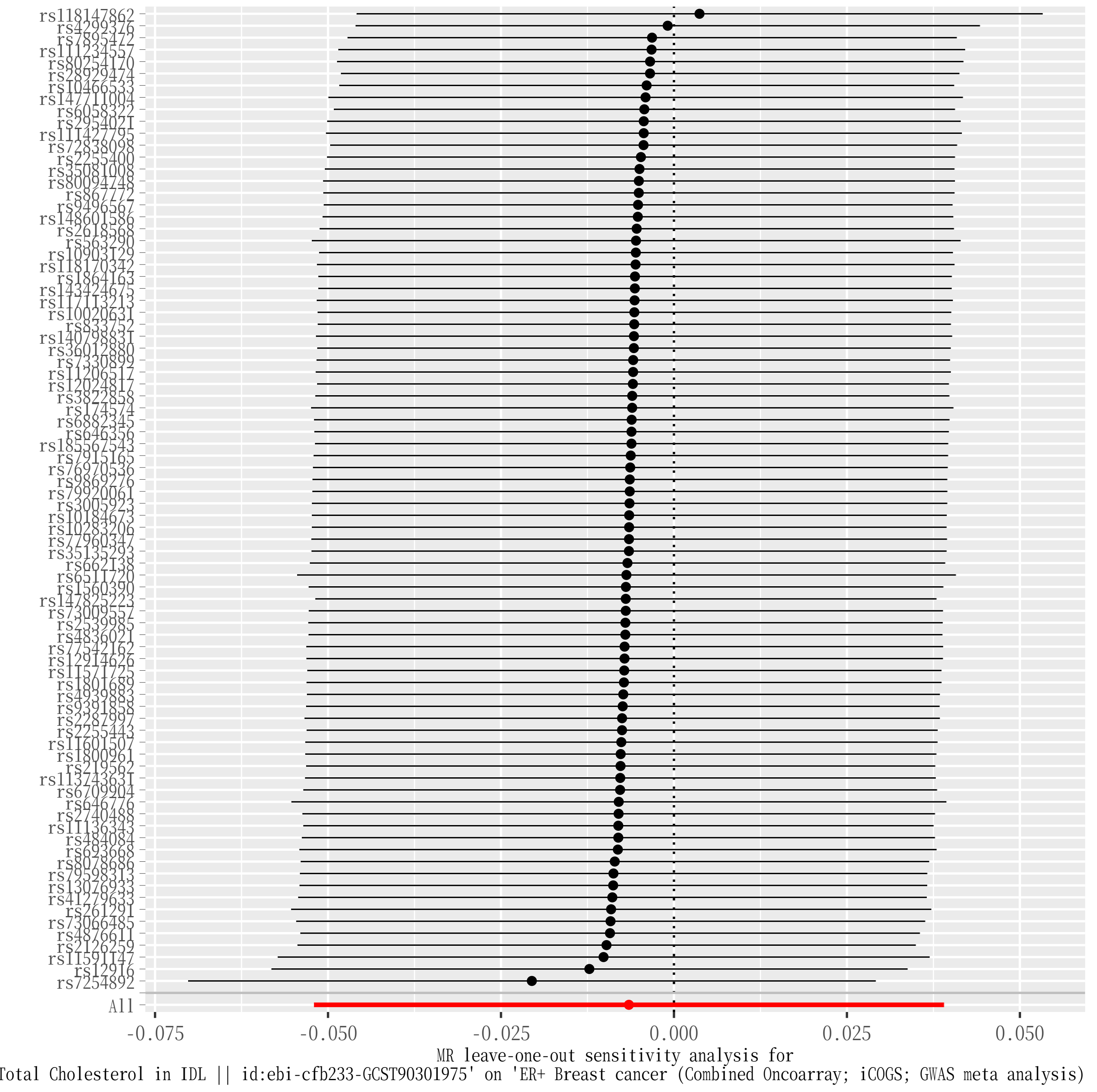
rs118147862
rs4299376
rs7895472
rs111234557
rs80254170
rs28929474
rs10466533
rs147711004
rs6058322
rs2954021
rs111427795
rs72838098
rs2255400
rs35081008
rs80094748
rs867772
rs9496567
rs148601586
rs2618568
rs563290
rs10903129
rs118170342
rs1864163
rs143424675
rs117113213
rs10020631
rs833752
rs140798831
rs36012880
rs7330892
rs11206517
rs12024817
rs3822858
rs174574
rs6882345
rs646356
rs185567543
rs7915165
rs76970536
rs9869276
rs79920061
rs3005923
rs10184673
rs10283206
rs77960347
rs35135293
rs662138
rs6511720
rs1560390
rs147825223
rs73009557
rs2539985
rs4836021
rs77542162
rs12914626
rs11571725
rs1801689
rs4939883
rs9391858
rs2287997
rs2255443
rs11601507
rs1800961
rs219562
rs113743631
rs6709904
rs646776
rs2740488
rs11136343
rs484084
rs693668
rs8078686
rs79598313
rs13026933
rs41279633
rs261291
rs73066485
rs4876611
rs2126259
rs11591147
rs12916
rs7254892
All
-0.075
-0.050
-0.025
0.000
0.025
0.050
MR leave-one-out sensitivity analysis for
Total Cholesterol in IDL || id:ebi-cfb233-GCST90301975' on 'ER+ Breast cancer (Combined Oncoarray; iCOGS; GWAS meta analysis)

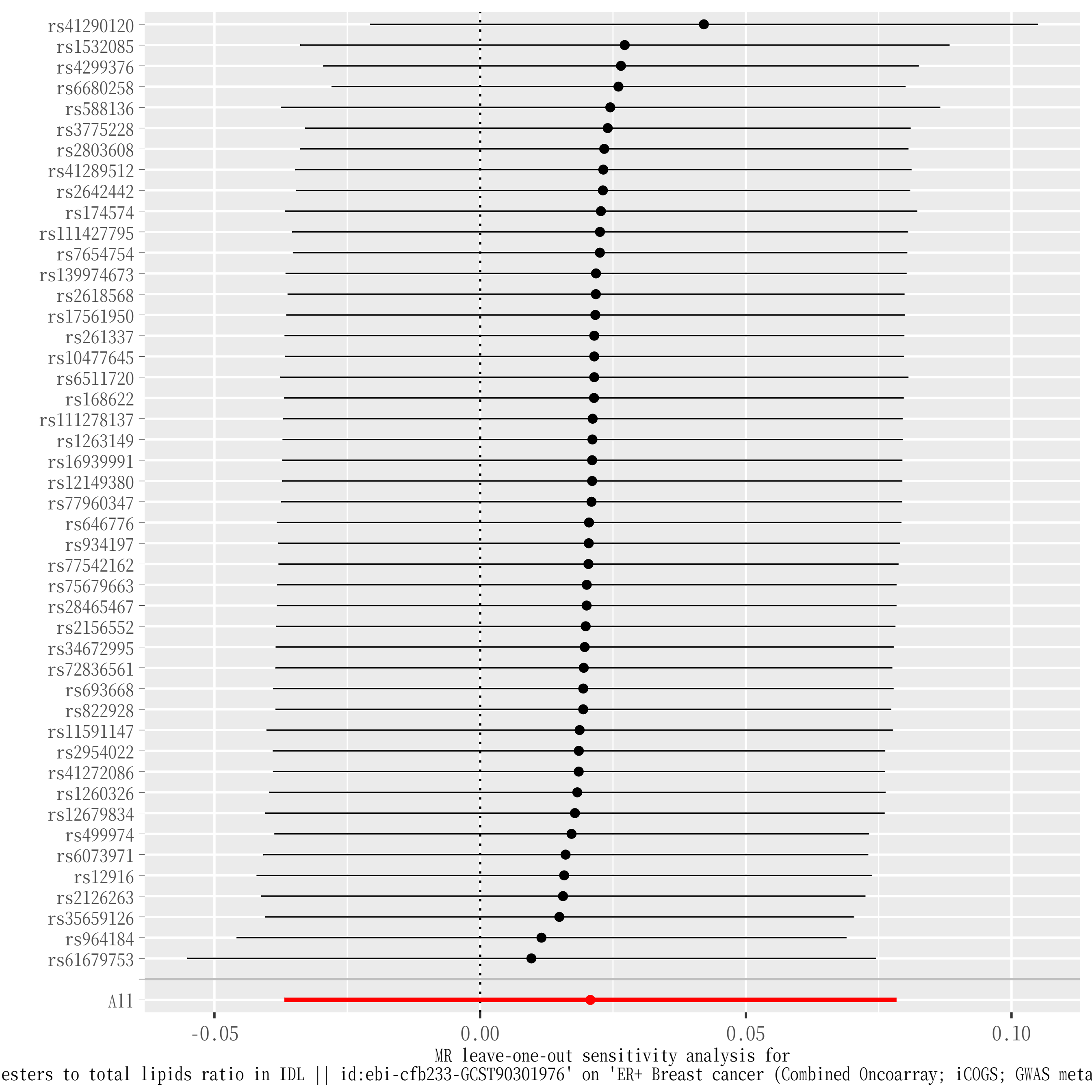
rs41290120
rs1532085
rs4299376
rs6680258
rs588136
rs3775228
rs2803608
rs41289512
rs2642442
rs174574
rs111427795
rs7654754
rs139974673
rs2618568
rs17561950
rs261337
rs10477645
rs6511720
rs168622
rs111278137
rs1263149
rs16939991
rs12149380
rs77960347
rs646776
rs934197
rs77542162
rs75679663
rs28465467
rs2156552
rs34672995
rs72836561
rs693668
rs822928
rs11591147
rs2954022
rs41272086
rs1260326
rs12679834
rs499974
rs6073971
rs12916
rs2126263
rs35659126
rs964184
rs61679753
All
-0.05
0.00
0.05
0.10
MR leave-one-out sensitivity analysis for
esters to total lipids ratio in IDL || id:ebi-cfb233-GCST90301976' on 'ER+ Breast cancer (Combined Oncoarray; iCOGS; GWAS meta

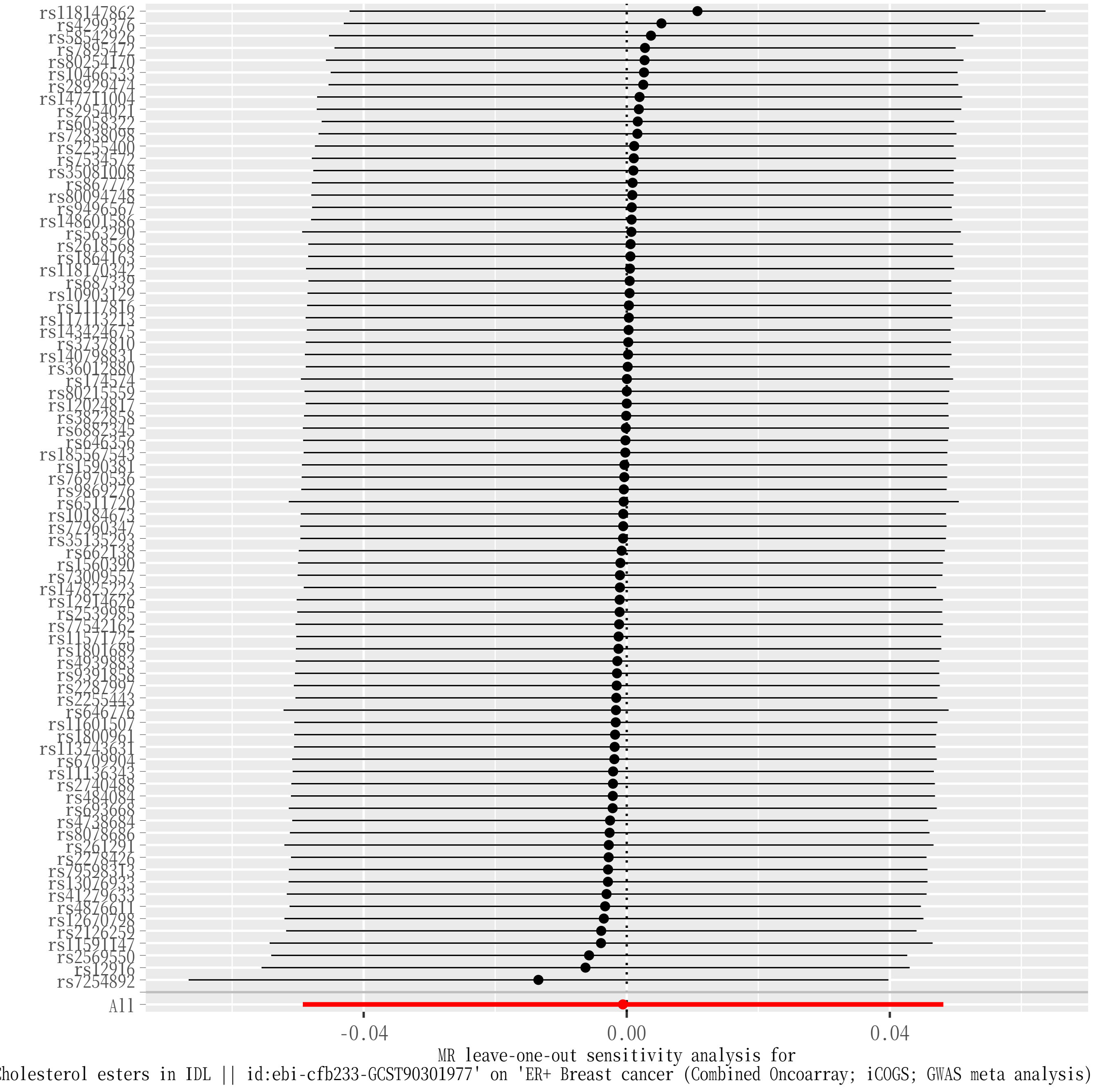
rs118147862
rs4299376
rs58542926
rs7825472
rs80254170
rs10466533
rs28929474
rs147711004
rs2954021
rs6058322
rs72838098
rs2255400
rs7534572
rs35081008
rs867772
rs80094748
rs9496567
rs148601586
rs563290
rs2618568
rs1864163
rs118170342
rs687339
rs10903129
rs1117816
rs117113213
rs143424675
rs3737810
rs140798831
rs36012880
rs174574
rs80215559
rs12024817
rs3822858
rs6882345
rs646356
rs185567543
rs1590381
rs76970536
rs9869276
rs6511720
rs10184673
rs77960347
rs35135293
rs662138
rs1560390
rs73009557
rs147825223
rs12914626
rs2539985
rs77542162
rs11571725
rs1801689
rs4939883
rs9391858
rs2287997
rs2255443
rs646776
rs11601507
rs1800961
rs113743631
rs6709904
rs11136343
rs2740488
rs484084
rs693668
rs4738684
rs8078686
rs261291
rs2278426
rs79598313
rs13076933
rs41279633
rs4876611
rs12670798
rs2126259
rs11591147
rs2569550
rs12916
rs7254892
All
-0.04
0.00
0.04
MR leave-one-out sensitivity analysis for
Cholesterol esters in IDL || id:ebi-cfb233-GCST90301977' on 'ER+ Breast cancer (Combined Oncoarray; iCOGS; GWAS meta analysis)

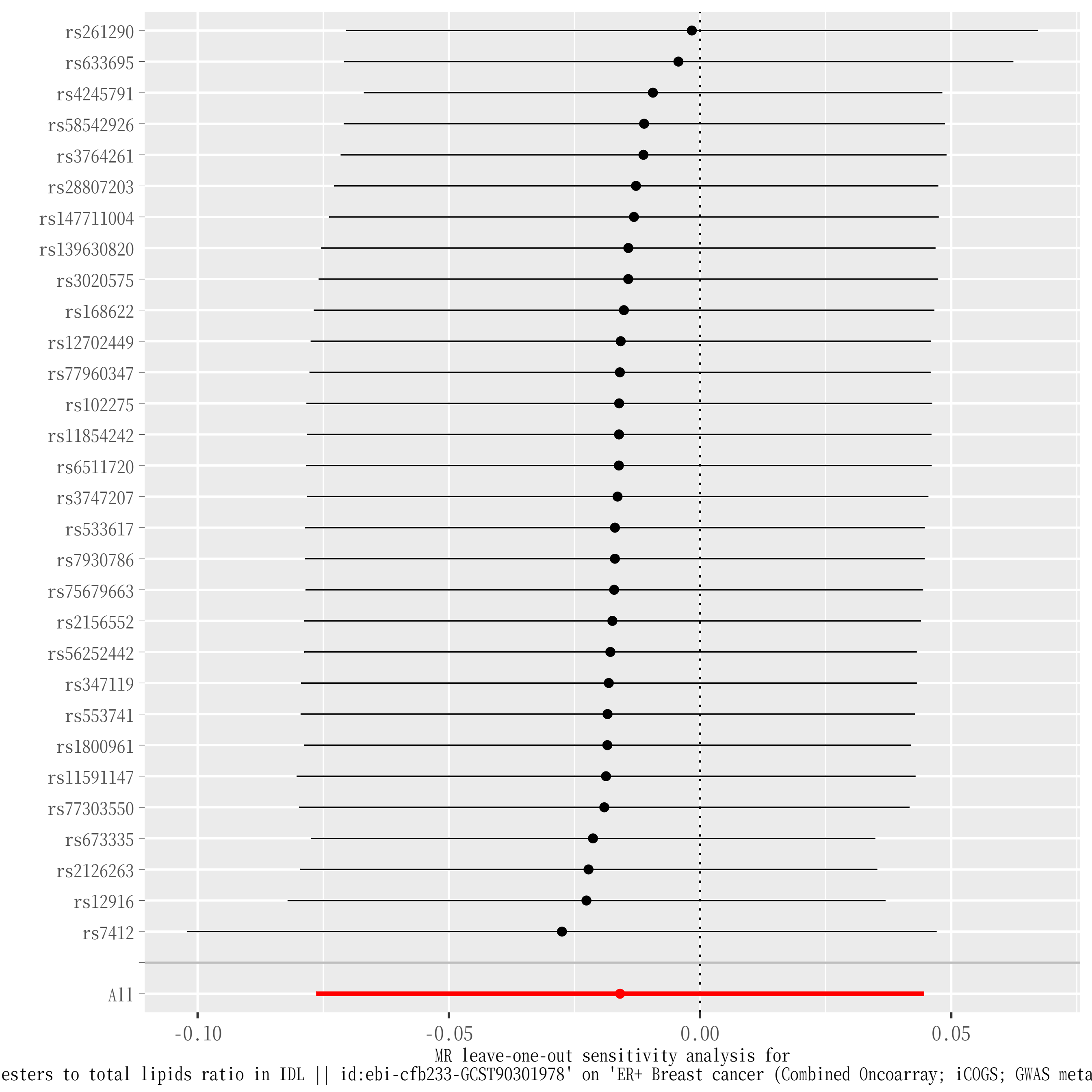
rs261290
rs633695
rs4245791
rs58542926
rs3764261
rs28807203
rs147711004
rs139630820
rs3020575
rs168622
rs12702449
rs77960347
rs102275
rs11854242
rs6511720
rs3747207
rs533617
rs7930786
rs75679663
rs2156552
rs56252442
rs347119
rs553741
rs1800961
rs11591147
rs77303550
rs673335
rs2126263
rs12916
rs7412
All
-0.10
-0.05
0.00
0.05
MR leave-one-out sensitivity analysis for
esters to total lipids ratio in IDL || id:ebi-cfb233-GCST90301978' on 'ER+ Breast cancer (Combined Oncoarray; iCOGS; GWAS meta

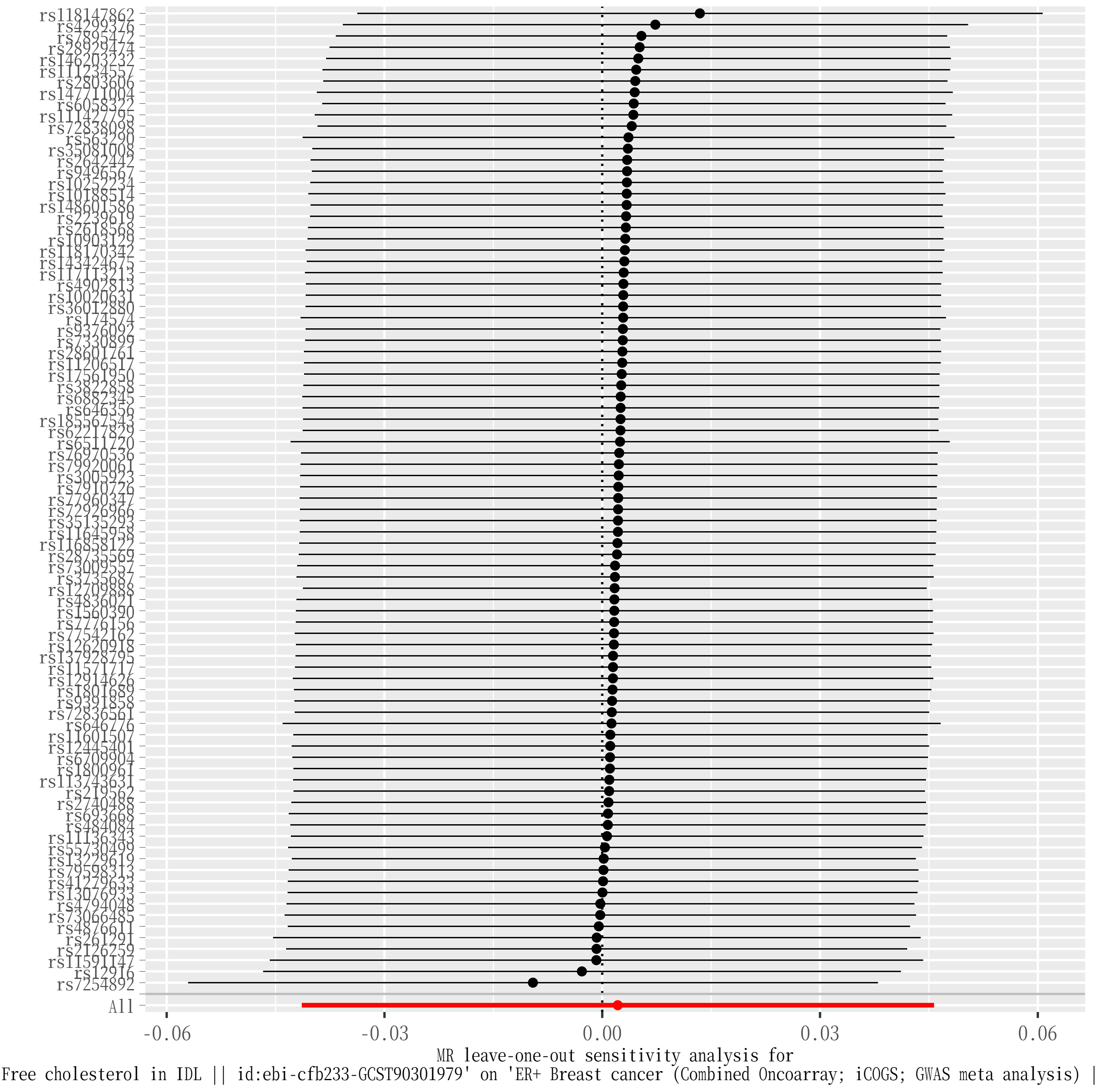
rs118147862
rs4299376
rs7895472
rs28929474
rs146203232
rs111234557
rs2803606
rs147711004
rs6058322
rs111427795
rs72838098
rs563290
rs35081008
rs2642442
rs9496567
rs10252234
rs10188514
rs148601586
rs2239619
rs2618568
rs10903129
rs118170342
rs143424675
rs117113213
rs4902813
rs10020631
rs36012880
rs174574
rs9376092
rs7330899
rs28601761
rs11206517
rs17561950
rs3822858
rs6882345
rs646356
rs185567543
rs62217829
rs6511720
rs76970536
rs79920061
rs3005923
rs7910726
rs77960347
rs72926966
rs35135293
rs11645958
rs116858122
rs28735569
rs73009557
rs3735687
rs12709888
rs4836021
rs1560390
rs7776156
rs77542162
rs12620918
rs137928795
rs11571717
rs12914626
rs1801689
rs9391858
rs72836561
rs646776
rs11601507
rs12445401
rs6709904
rs1800961
rs113743631
rs219562
rs2740488
rs693668
rs484084
rs11136343
rs55730499
rs13229619
rs79598313
rs41279633
rs13076933
rs4794048
rs73066485
rs4876611
rs261291
rs2126259
rs11591147
rs12916
rs7254892
All
-0.06
-0.03
0.00
0.03
0.06
MR leave-one-out sensitivity analysis for
Free cholesterol in IDL || id:ebi-cfb233-GCST90301979' on 'ER+ Breast cancer (Combined Oncoarray; iCOGS; GWAS meta analysis) |

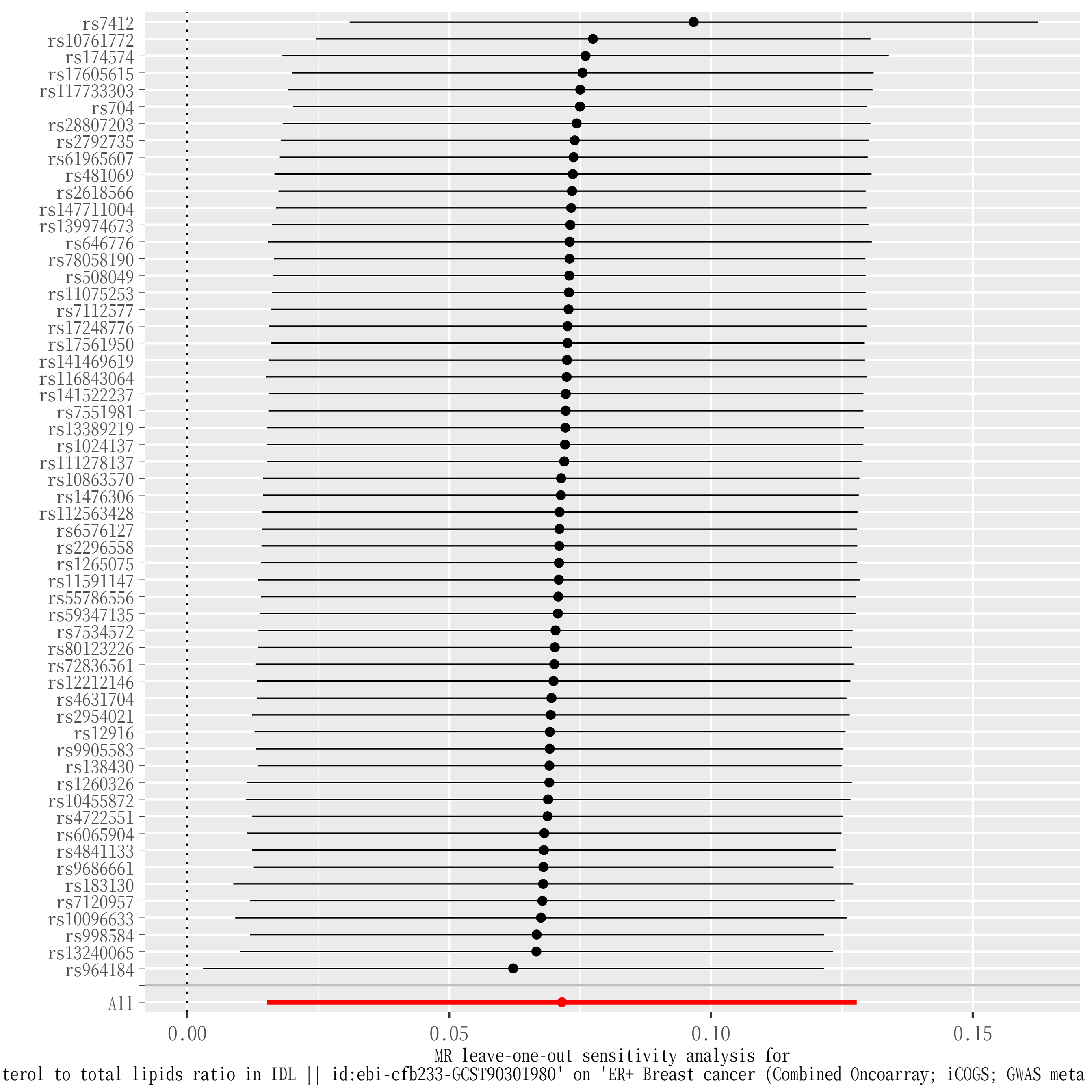
rs7412
rs10761772
rs174574
rs17605615
rs117733303
rs704
rs28807203
rs2792735
rs61965607
rs481069
rs2618566
rs147711004
rs139974673
rs646776
rs78058190
rs508049
rs11075253
rs7112577
rs17248776
rs17561950
rs141469619
rs116843064
rs141522237
rs7551981
rs13389219
rs1024137
rs111278137
rs10863570
rs1476306
rs112563428
rs6576127
rs2296558
rs1265075
rs11591147
rs55786556
rs59347135
rs7534572
rs80123226
rs72836561
rs12212146
rs4631704
rs2954021
rs12916
rs9905583
rs138430
rs1260326
rs10455872
rs4722551
rs6065904
rs4841133
rs9686661
rs183130
rs7120957
rs10096633
rs998584
rs13240065
rs964184
All
0.00
0.05
0.10
0.15
MR leave-one-out sensitivity analysis for
terol to total lipids ratio in IDL || id:ebi-cfb233-GCST90301980' on 'ER+ Breast cancer (Combined Oncoarray; iCOGS; GWAS meta

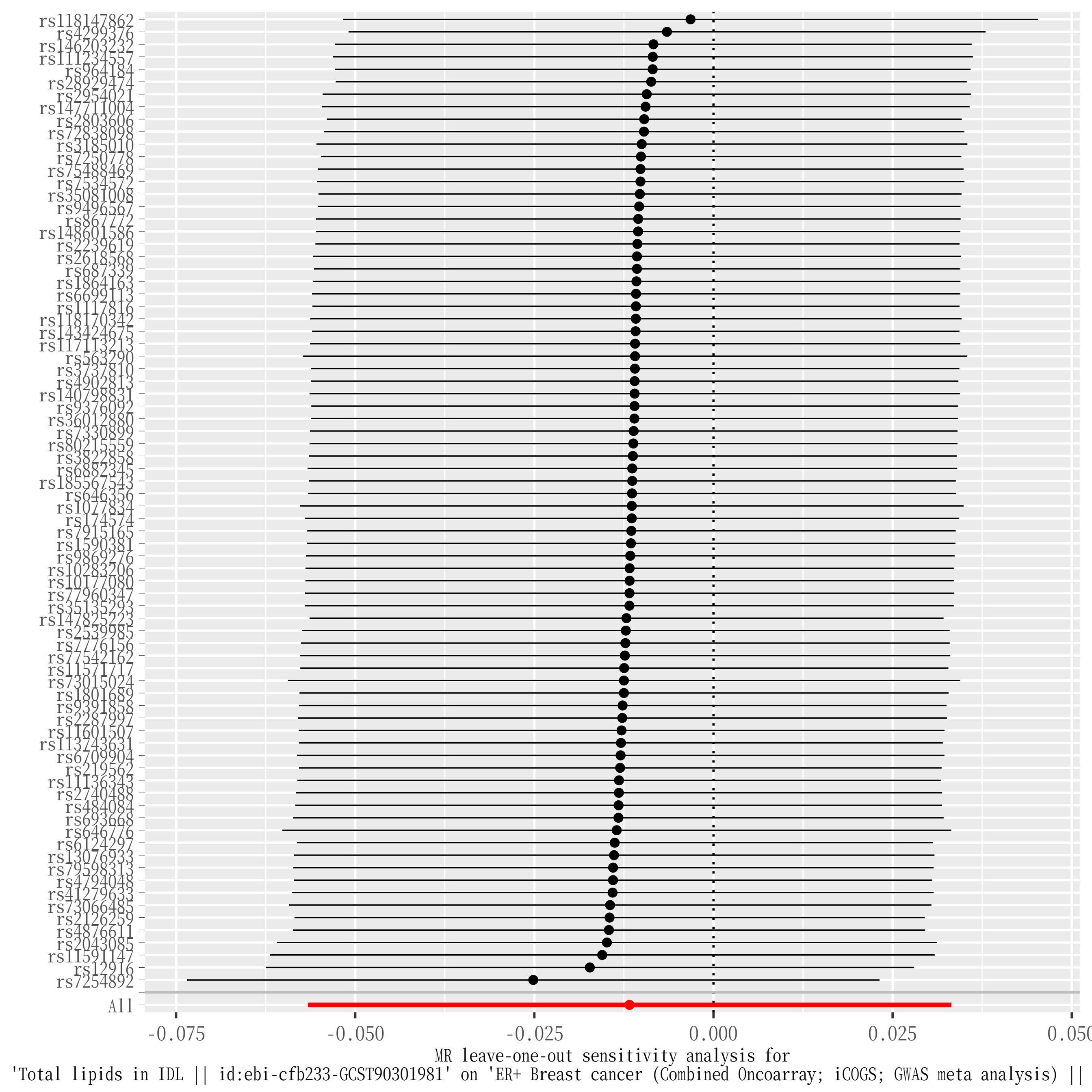

rs118147862
rs4299376
rs146203232
rs111234557
rs964184
rs28929474
rs2954021
rs147711004
rs2803606
rs72838098
rs3185010
rs7250778
rs75488469
rs7534572
rs35081008
rs9496567
rs867772
rs148601586
rs2239619
rs2618568
rs687339
rs1864163
rs6699113
rs1117816
rs118170342
rs143424675
rs117113213
rs563290
rs3737810
rs4902813
rs140728831
rs9376092
rs36012880
rs7330899
rs80215559
rs3822858
rs6882345
rs185567543
rs646356
rs1077834
rs174574
rs7915165
rs1590381
rs9869276
rs10283206
rs10177080
rs77960347
rs35135293
rs147825223
rs2539985
rs7776156
rs77542162
rs11571717
rs73015024
rs1801689
rs9391858
rs2287997
rs11601507
rs113743631
rs6709904
rs219562
rs11136343
rs2740488
rs484084
rs693668
rs646776
rs6124297
rs13076933
rs79598313
rs4794048
rs41279633
rs73066485
rs2126259
rs4876611
rs2043085
rs11591147
rs12916
rs7254892
All
-0.075
-0.050
-0.025
0.000
0.025
0.050
MR leave-one-out sensitivity analysis for
'Total lipids in IDL || id:ebi-cfb233-GCST90301981' on 'ER+ Breast cancer (Combined Oncoarray; iCOGS; GWAS meta analysis) ||

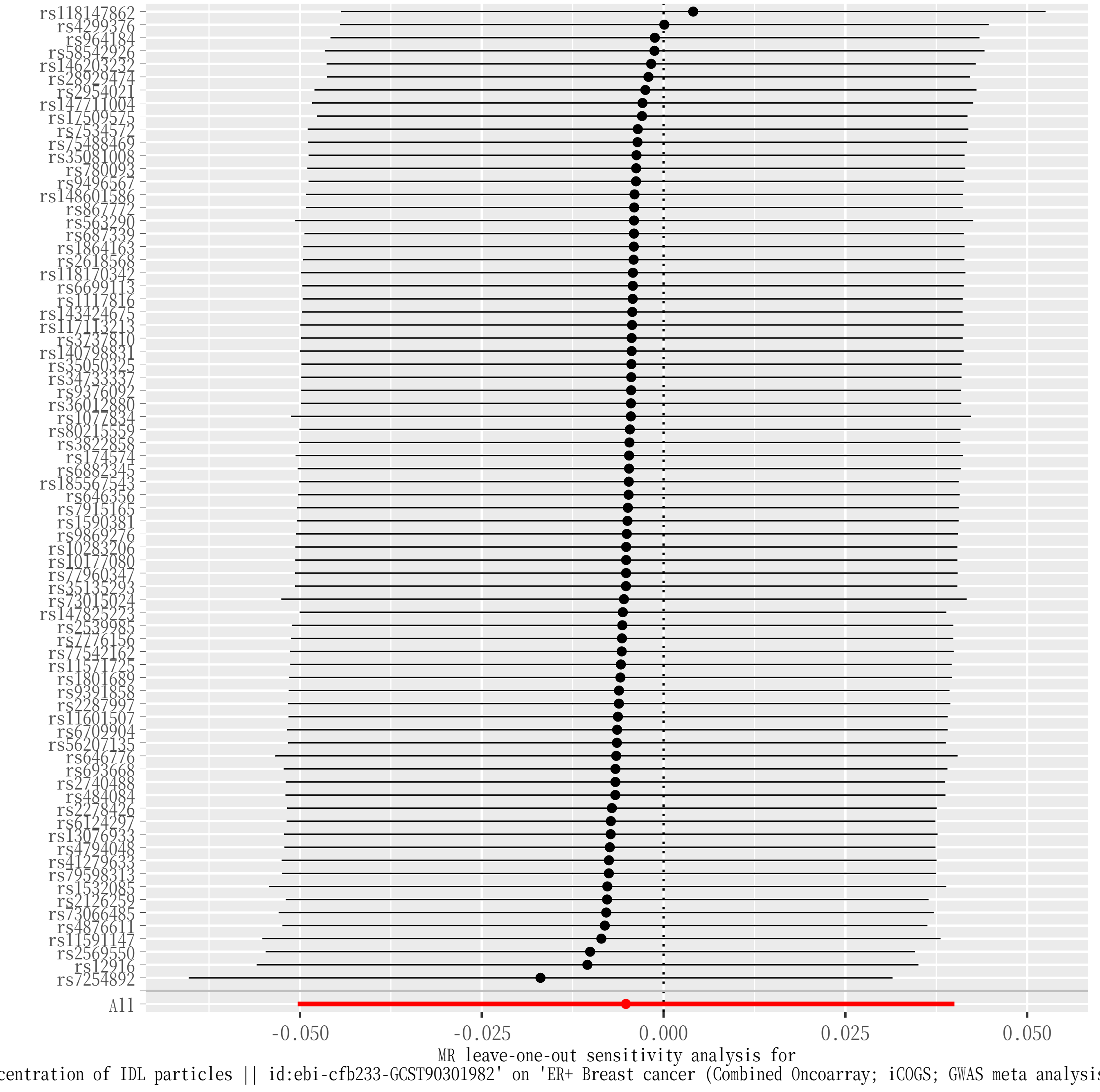

rs118147862
rs4299376
rs964184
rs58542926
rs146203232
rs28929474
rs2954021
rs147711004
rs17509575
rs7534572
rs75488469
rs35081008
rs780093
rs9496567
rs148601586
rs867772
rs563290
rs687339
rs1864163
rs2618568
rs118170342
rs6699113
rs1117816
rs143424675
rs117113213
rs3737810
rs140798831
rs35050325
rs34733337
rs9376092
rs36012880
rs1077834
rs80215559
rs3822858
rs174574
rs6882345
rs185567543
rs646356
rs7915165
rs1590381
rs9869276
rs10283206
rs10177080
rs77960347
rs35135293
rs73015024
rs147825223
rs2539985
rs7776156
rs77542162
rs11571725
rs1801689
rs9391858
rs2287997
rs11601507
rs6709904
rs56207135
rs646776
rs693668
rs2740488
rs484084
rs2278426
rs6124297
rs13076933
rs4794048
rs41279633
rs79598313
rs1532085
rs2126259
rs73066485
rs4876611
rs11591147
rs2569550
rs12916
rs7254892
All
-0.050
-0.025
0.000
0.025
0.050
MR leave-one-out sensitivity analysis for
centration of IDL particles || id:ebi-cfb233-GCST90301982' on 'ER+ Breast cancer (Combined Oncoarray; iCOGS; GWAS meta analysis

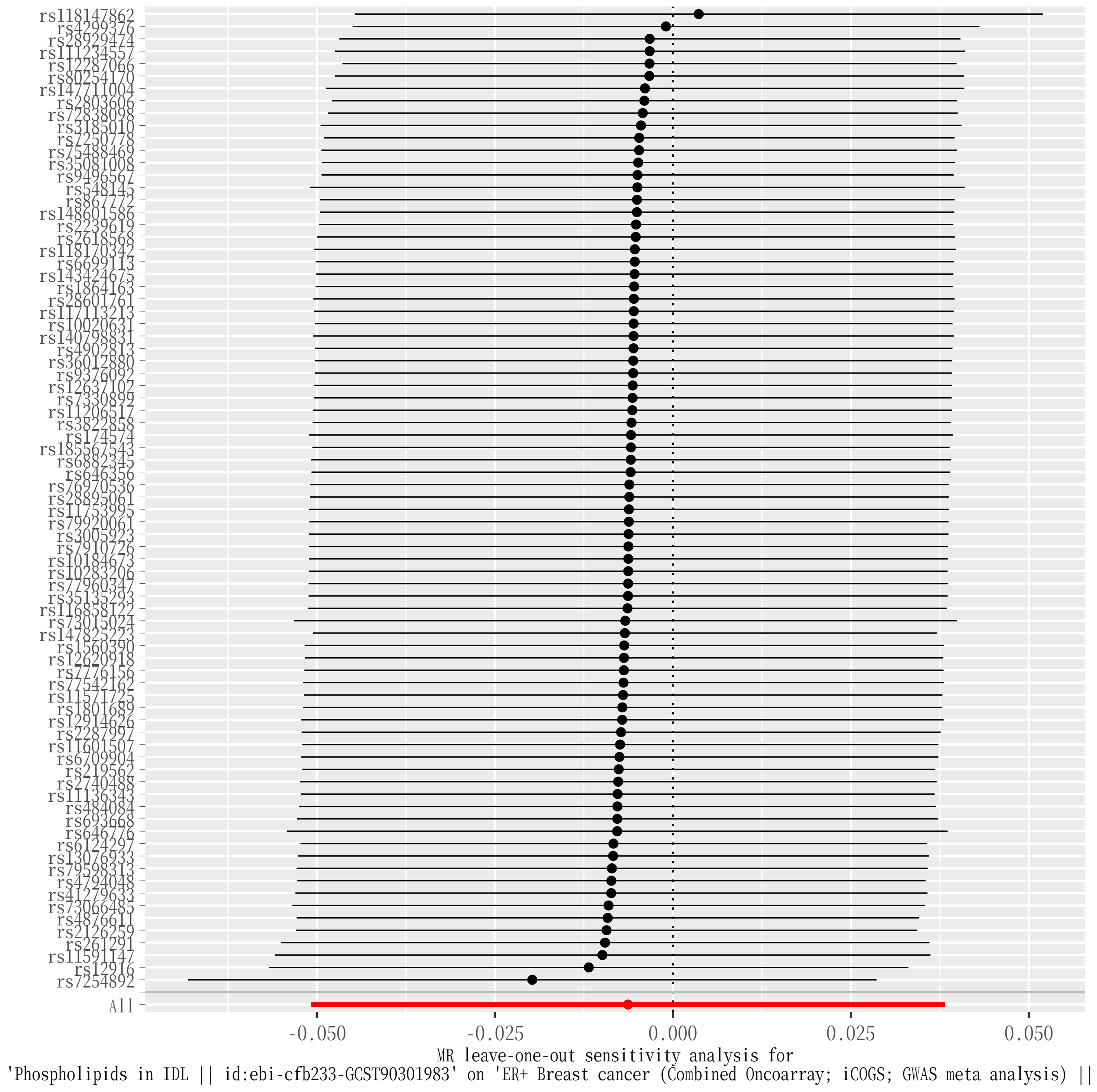
rs118147862
rs4299376
rs28929474
rs111234557
rs12287066
rs80254170
rs147711004
rs2803606
rs72838098
rs3185010
rs7250778
rs75488469
rs35081008
rs9496567
rs548145
rs867772
rs148601586
rs2239619
rs2618568
rs118170342
rs6699113
rs143424675
rs1864163
rs28601761
rs117113213
rs10020631
rs140798831
rs4902813
rs36012880
rs9376092
rs12637102
rs7330899
rs11206517
rs3822858
rs174574
rs185567543
rs6882345
rs646356
rs76970536
rs28895061
rs11753995
rs79920061
rs3005923
rs7910726
rs10184673
rs10283206
rs77960347
rs35135293
rs116858122
rs73015024
rs147825223
rs1560390
rs12620918
rs7776156
rs77542162
rs11571725
rs1801689
rs12914626
rs2287997
rs11601507
rs6709904
rs219562
rs2740488
rs11136343
rs484084
rs693668
rs646776
rs6124297
rs13076933
rs79598313
rs4794048
rs41279633
rs73066485
rs4876611
rs2126259
rs261291
rs11591147
rs12916
rs7254892
All
-0.050
-0.025
0.000
0.025
0.050
MR leave-one-out sensitivity analysis for
'Phospholipids in IDL || id:ebi-cfb233-GCST90301983' on 'ER+ Breast cancer (Combined Oncoarray; iCOGS; GWAS meta analysis) ||

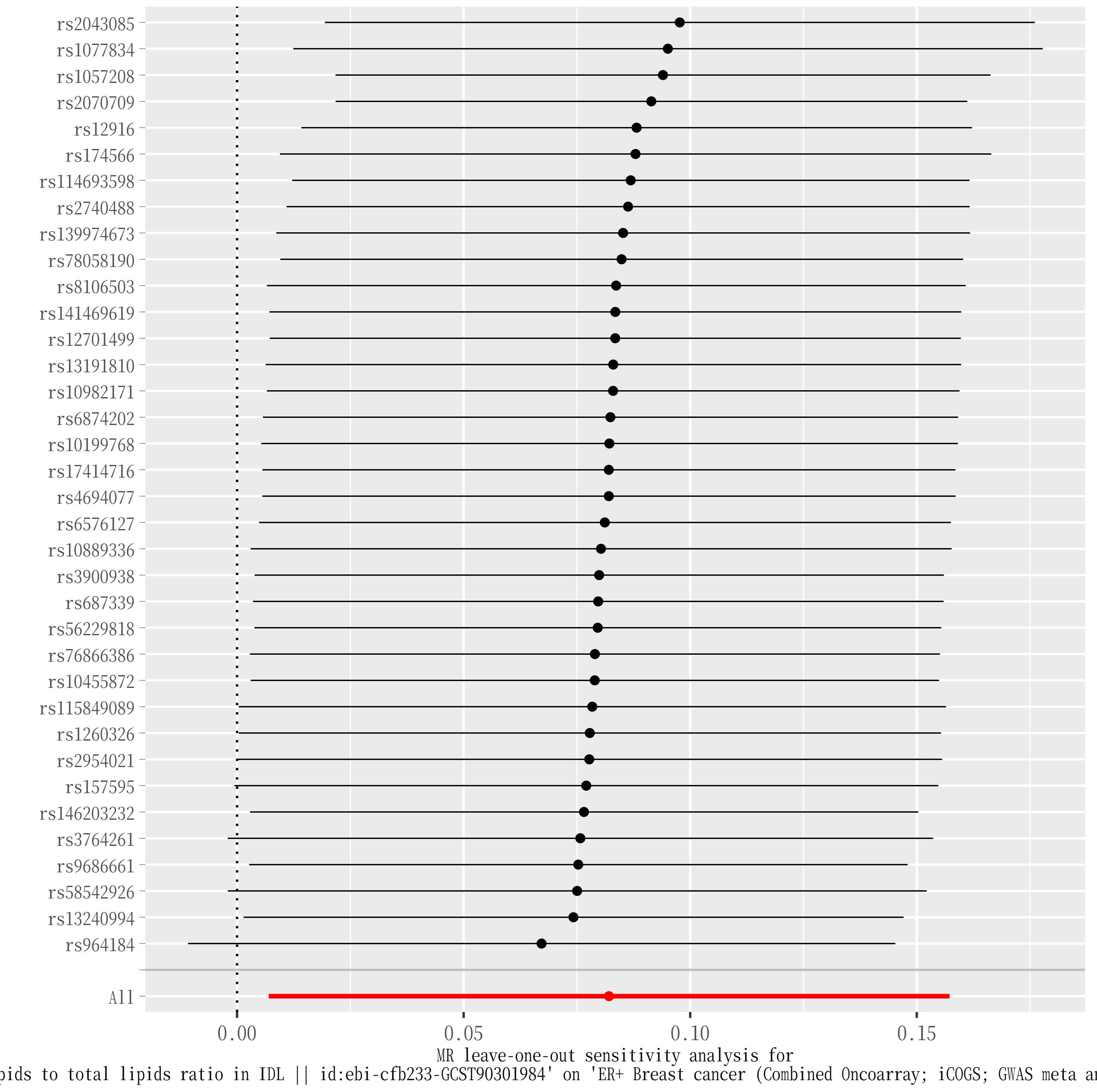
rs2043085
rs1077834
rs1057208
rs2070709
rs12916
rs174566
rs114693598
rs2740488
rs139974673
rs78058190
rs8106503
rs141469619
rs12701499
rs13191810
rs10982171
rs6874202
rs10199768
rs17414716
rs4694077
rs6576127
rs10889336
rs3900938
rs687339
rs56229818
rs76866386
rs10455872
rs115849089
rs1260326
rs2954021
rs157595
rs146203232
rs3764261
rs9686661
rs58542926
rs13240994
rs964184
All
0.00
0.05
0.10
0.15
MR leave-one-out sensitivity analysis for
pids to total lipids ratio in IDL || id:ebi-cfb233-GCST90301984' on 'ER+ Breast cancer (Combined Oncoarray; iCOGS; GWAS meta an

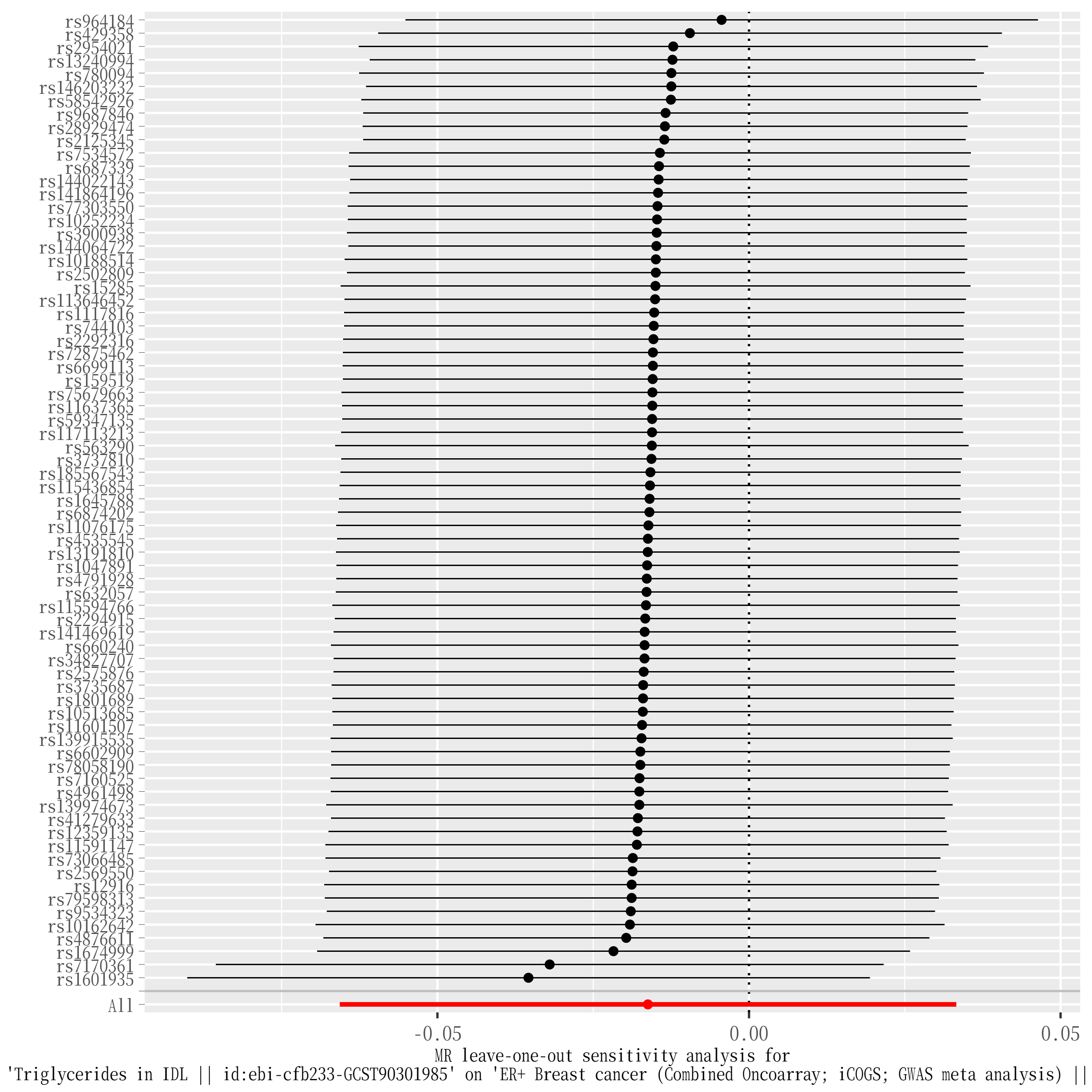
rs964184
rs429358
rs2954021
rs13240994
rs780094
rs146203232
rs58542926
rs9687846
rs28929474
rs2125345
rs7534572
rs687339
rs144022143
rs141864196
rs77303550
rs10252234
rs3900938
rs144064722
rs10188514
rs2502809
rs15285
rs113646452
rs1117816
rs744103
rs2292316
rs72875462
rs6699113
rs159519
rs75679663
rs11637365
rs59347135
rs117113213
rs563290
rs3737810
rs185567543
rs115436854
rs1645788
rs6874202
rs11076175
rs4535545
rs13191810
rs1047891
rs4791928
rs632057
rs115594766
rs2294915
rs141469619
rs660240
rs34827707
rs2575876
rs3735687
rs1801689
rs10513685
rs11601507
rs139915535
rs6602909
rs78058190
rs7160525
rs4961498
rs139974673
rs41279633
rs12359135
rs11591147
rs73066485
rs2569550
rs12916
rs79598313
rs9534323
rs10162642
rs4876611
rs1674999
rs7170361
rs1601935
All
-0.05
0.00
0.05
MR leave-one-out sensitivity analysis for
'Triglycerides in IDL || id:ebi-cfb233-GCST90301985' on 'ER+ Breast cancer (Combined Oncoarray; iCOGS; GWAS meta analysis) ||

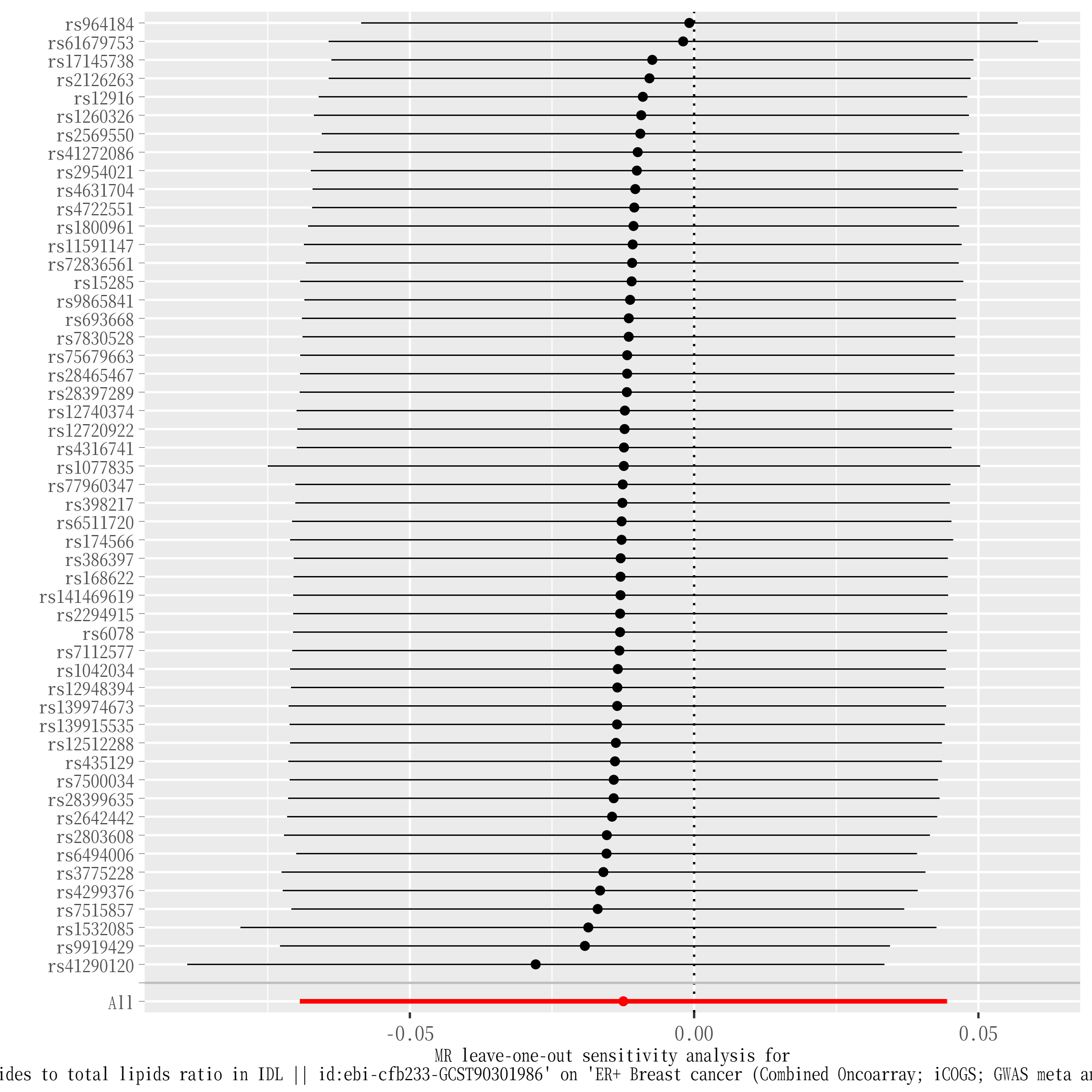
rs964184
rs61679753
rs17145738
rs2126263
rs12916
rs1260326
rs2569550
rs41272086
rs2954021
rs4631704
rs4722551
rs1800961
rs11591147
rs72836561
rs15285
rs9865841
rs693668
rs7830528
rs75679663
rs28465467
rs28397289
rs12740374
rs12720922
rs4316741
rs1077835
rs77960347
rs398217
rs6511720
rs174566
rs386397
rs168622
rs141469619
rs2294915
rs6078
rs7112577
rs1042034
rs12948394
rs139974673
rs139915535
rs12512288
rs435129
rs7500034
rs28399635
rs2642442
rs2803608
rs6494006
rs3775228
rs4299376
rs7515857
rs1532085
rs9919429
rs41290120
All
-0.05
0.00
0.05
MR leave-one-out sensitivity analysis for
ides to total lipids ratio in IDL || id:ebi-cfb233-GCST90301986' on 'ER+ Breast cancer (Combined Oncoarray; iCOGS; GWAS meta an

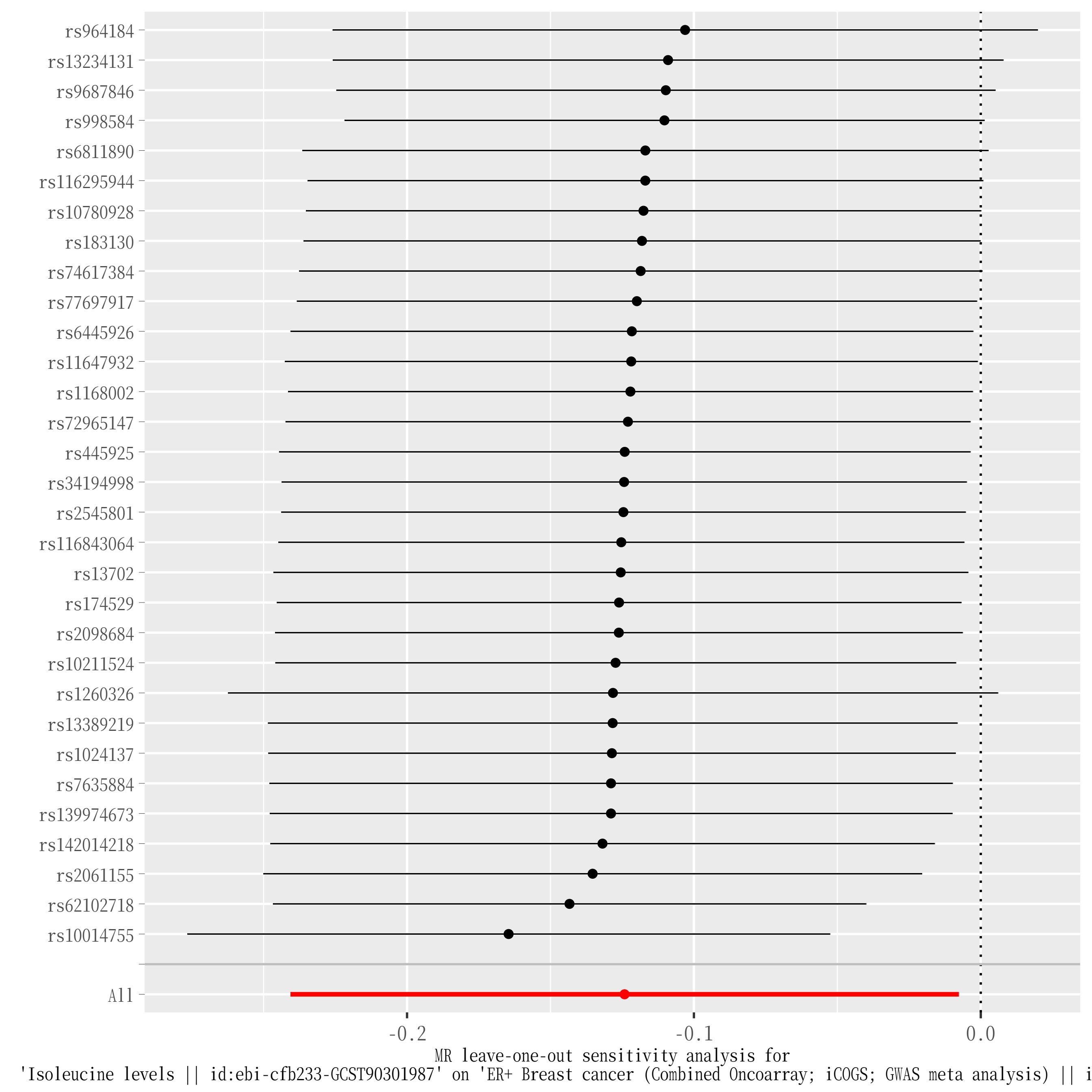
rs964184
rs13234131
rs9687846
rs998584
rs6811890
rs116295944
rs10780928
rs183130
rs74617384
rs77697917
rs6445926
rs11647932
rs1168002
rs72965147
rs445925
rs34194998
rs2545801
rs116843064
rs13702
rs174529
rs2098684
rs10211524
rs1260326
rs13389219
rs1024137
rs7635884
rs139974673
rs142014218
rs2061155
rs62102718
rs10014755
All
-0.2
-0.1
0.0
MR leave-one-out sensitivity analysis for
'Isoleucine levels || id:ebi-cfb233-GCST90301987' on 'ER+ Breast cancer (Combined Oncoarray; iCOGS; GWAS meta analysis) || i

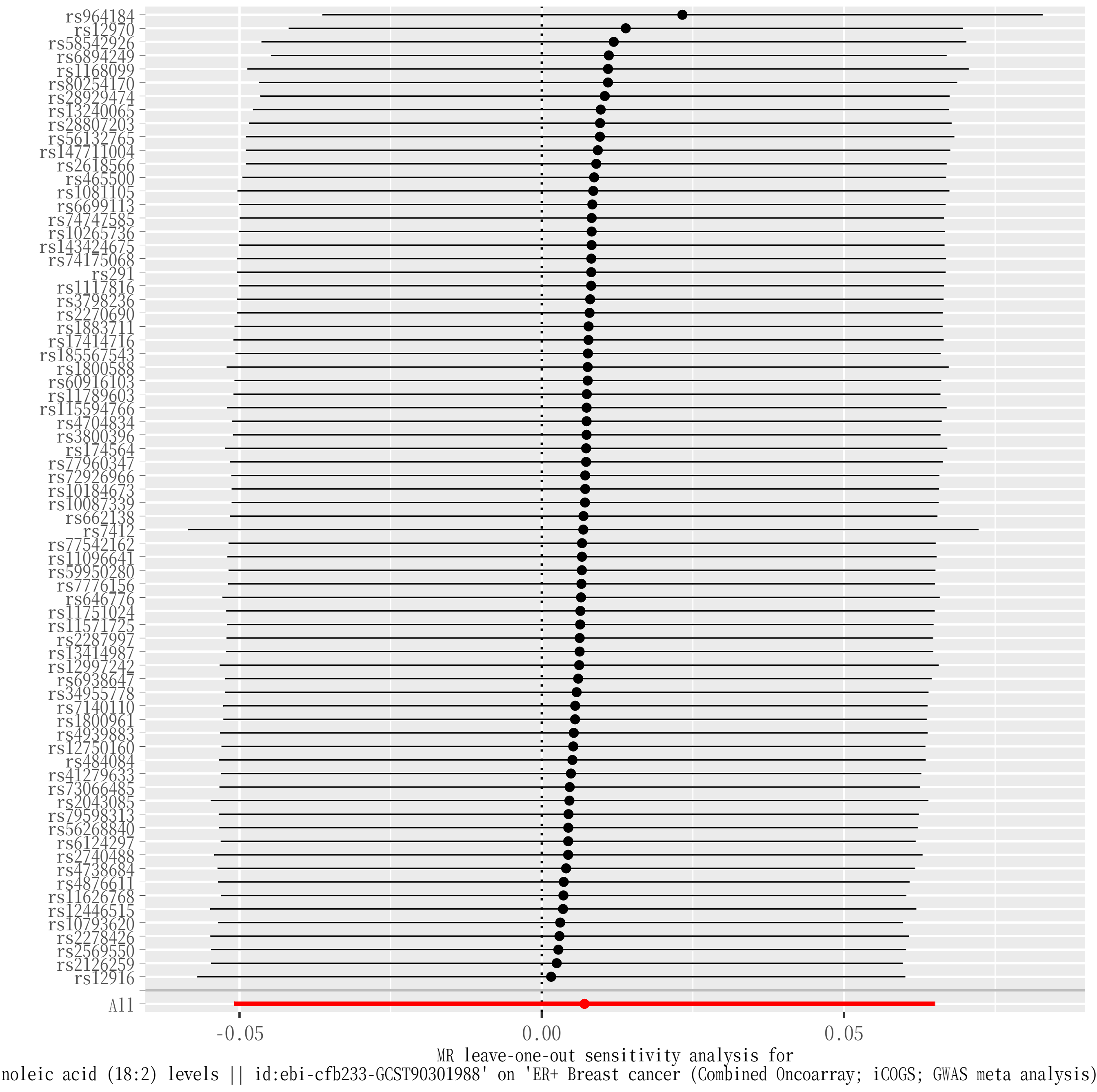
rs964184
rs12970
rs58542926
rs6894249
rs1168099
rs80254170
rs28929474
rs13240065
rs28807203
rs56132765
rs147711004
rs2618566
rs465500
rs1081105
rs6699113
rs74747585
rs10265736
rs143424675
rs74175068
rs291
rs1117816
rs3798236
rs2270690
rs1883711
rs17414716
rs185567543
rs1800588
rs60916103
rs11789603
rs115594766
rs4704834
rs3800396
rs174564
rs77960347
rs72926966
rs10184673
rs10087339
rs662138
rs7412
rs77542162
rs11096641
rs59950280
rs7776156
rs646776
rs11751024
rs11571725
rs2287997
rs13414987
rs12997242
rs6938647
rs34955778
rs7140110
rs1800961
rs4939883
rs12750160
rs484084
rs41279633
rs73066485
rs2043085
rs79598313
rs56268840
rs6124297
rs2740488
rs4738684
rs4876611
rs11626768
rs12446515
rs10793620
rs2278426
rs2569550
rs2126259
rs12916
All
-0.05
0.00
0.05
MR leave-one-out sensitivity analysis for
noleic acid (18:2) levels || id:ebi-cfb233-GCST90301988' on 'ER+ Breast cancer (Combined Oncoarray; iCOGS; GWAS meta analysis)

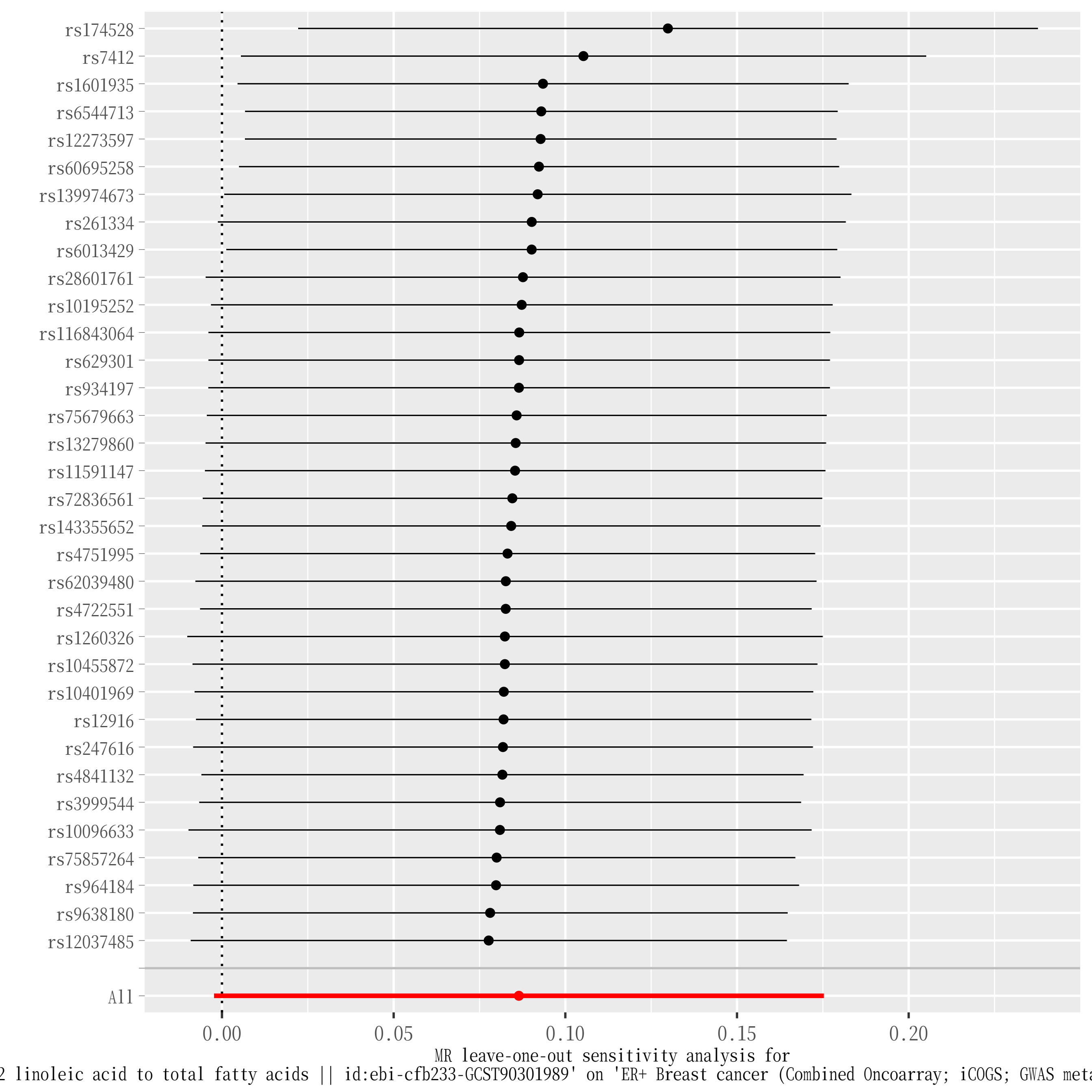
rs174528
rs7412
rs1601935
rs6544713
rs12273597
rs60695258
rs139974673
rs261334
rs6013429
rs28601761
rs10195252
rs116843064
rs629301
rs934197
rs75679663
rs13279860
rs11591147
rs72836561
rs143355652
rs4751995
rs62039480
rs4722551
rs1260326
rs10455872
rs10401969
rs12916
rs247616
rs4841132
rs3999544
rs10096633
rs75857264
rs964184
rs9638180
rs12037485
All
0.00
0.05
0.10
0.15
0.20
MR leave-one-out sensitivity analysis for
2 linoleic acid to total fatty acids || id:ebi-cfb233-GCST90301989' on 'ER+ Breast cancer (Combined Oncoarray; iCOGS; GWAS meta

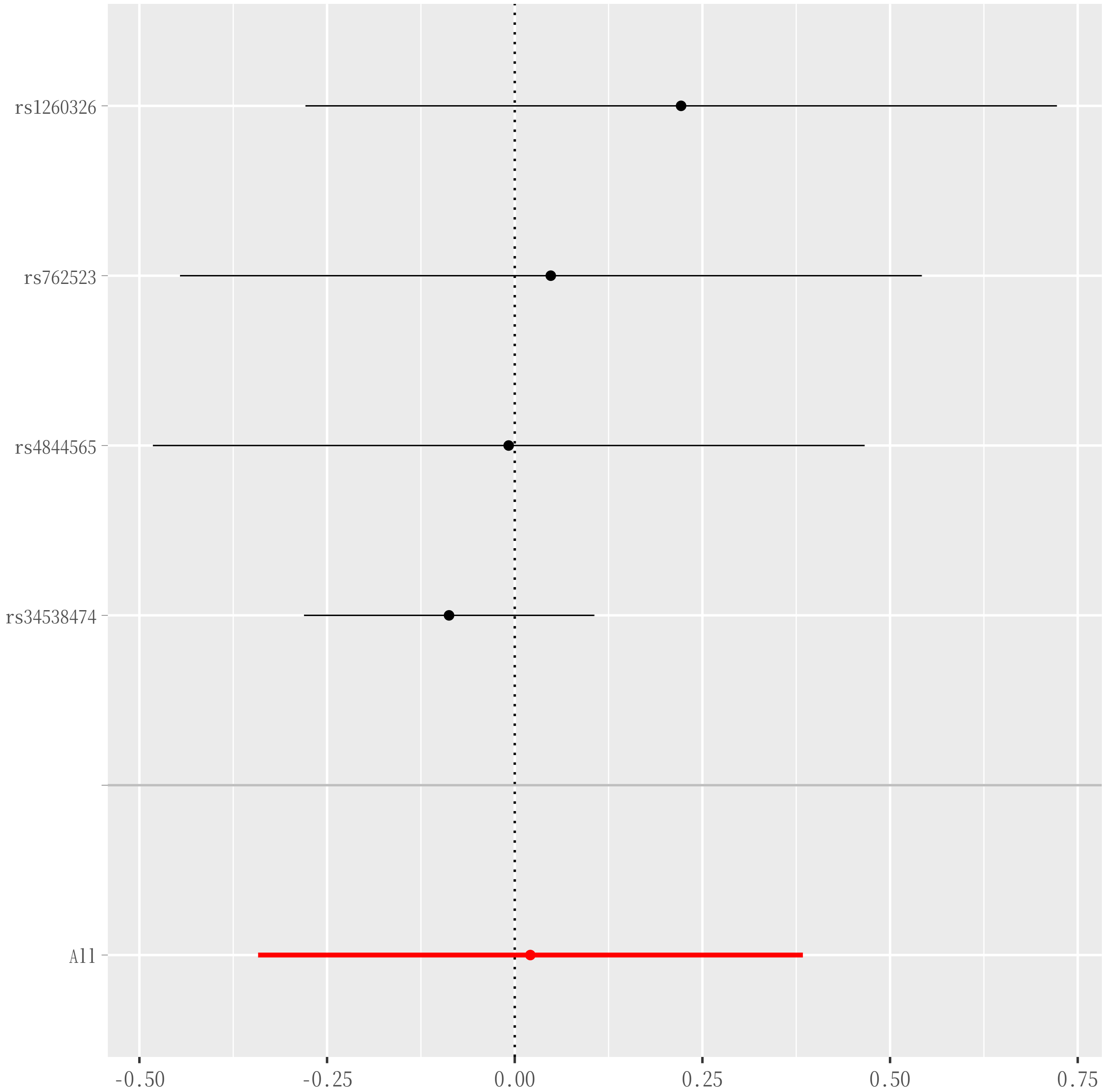

MR leave-one-out sensitivity analysis for
'Lactate levels || id:ebi-cfb233-GCST90301990' on 'ER+ Breast cancer (Combined Oncoarray; iCOGS; GWAS meta analysis) || id:

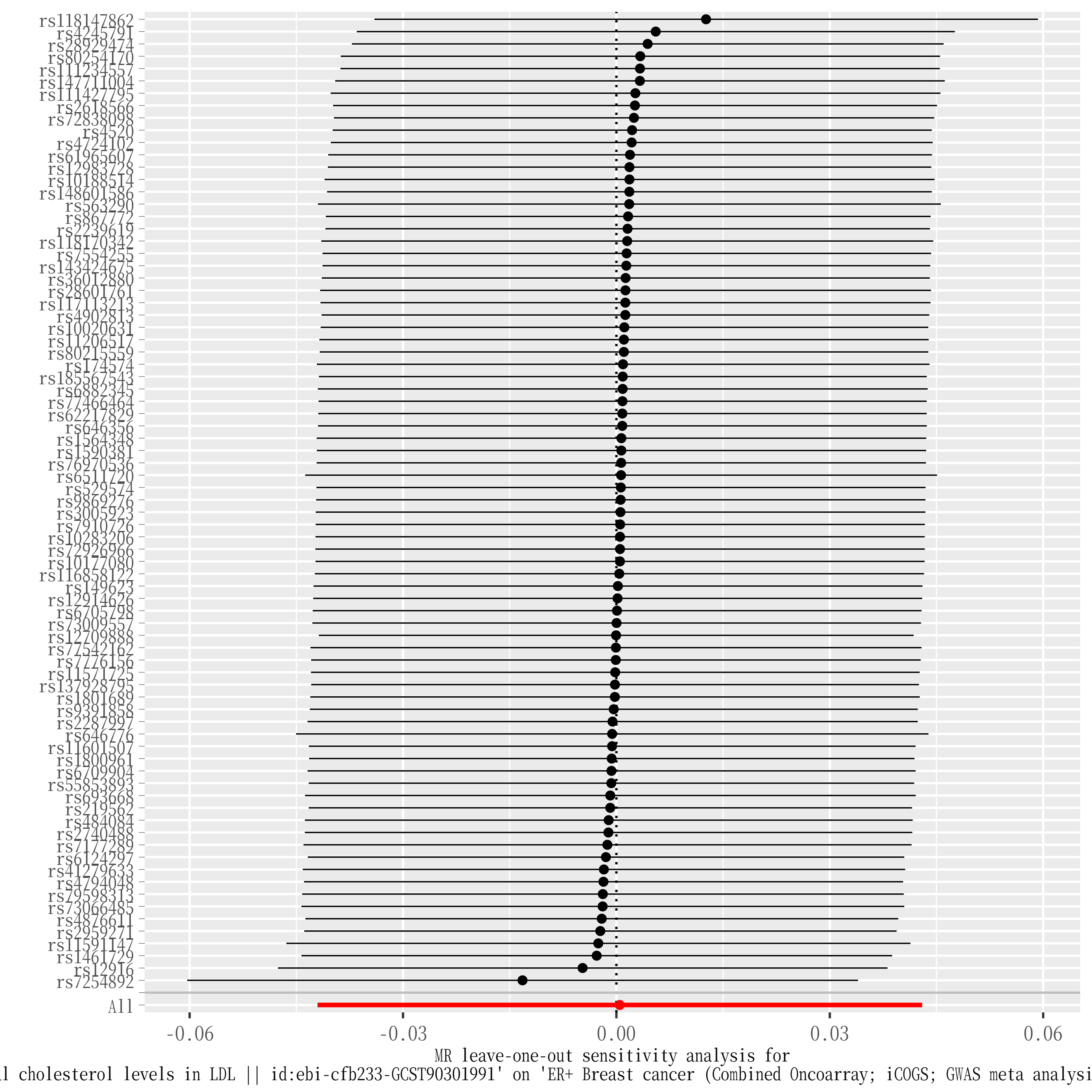
rs118147862
rs4245791
rs28929474
rs80254170
rs111234557
rs147711004
rs111427795
rs2618566
rs72838098
rs4520
rs4724102
rs61965607
rs12983728
rs10188514
rs148601586
rs563290
rs867772
rs2239619
rs118170342
rs7554255
rs143424675
rs36012880
rs28601761
rs117113213
rs4902813
rs10020631
rs11206517
rs80215559
rs174574
rs185567543
rs6882345
rs77466464
rs62217829
rs646356
rs1564348
rs1520381
rs76970536
rs6511720
rs529574
rs9869276
rs3005923
rs7910726
rs10283206
rs72926966
rs10177080
rs116858122
rs149623
rs12914626
rs6705798
rs73009557
rs12709888
rs77542162
rs7776156
rs11571725
rs137928795
rs1801689
rs9391858
rs2287997
rs646776
rs11601507
rs1800961
rs6709904
rs55853893
rs693668
rs219562
rs484084
rs2740488
rs7177289
rs6124297
rs41279633
rs4794048
rs79598313
rs73066485
rs4876611
rs2959271
rs11591147
rs1461729
rs12916
rs7254892
All
-0.06
-0.03
0.00
0.03
0.06
MR leave-one-out sensitivity analysis for
l cholesterol levels in LDL || id:ebi-cfb233-GCST90301991' on 'ER+ Breast cancer (Combined Oncoarray; iCOGS; GWAS meta analysis

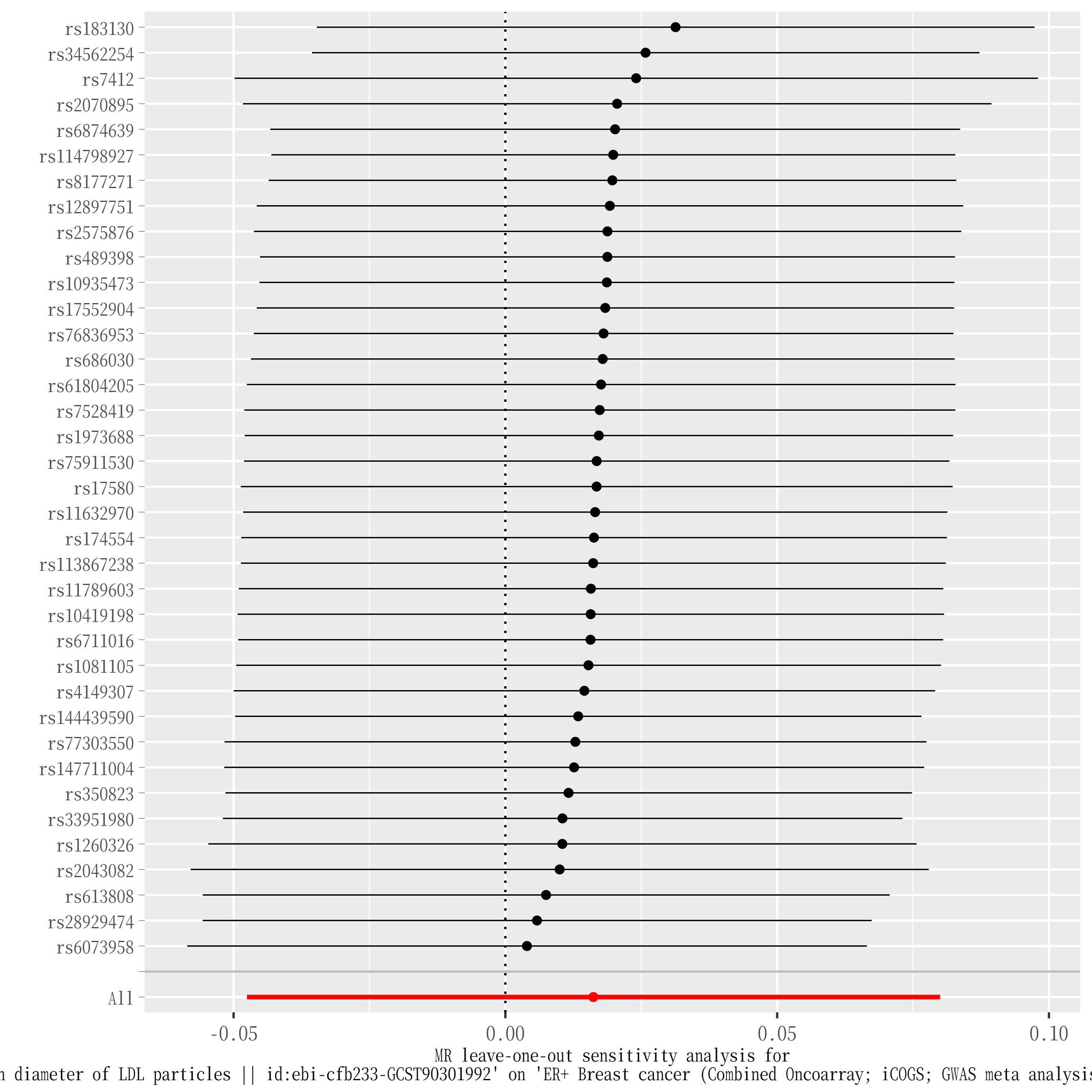
rs183130
rs34562254
rs7412
rs2070895
rs6874639
rs114798927
rs8177271
rs12897751
rs2575876
rs489398
rs10935473
rs17552904
rs76836953
rs686030
rs61804205
rs7528419
rs1973688
rs75911530
rs17580
rs11632970
rs174554
rs113867238
rs11789603
rs10419198
rs6711016
rs1081105
rs4149307
rs144439590
rs77303550
rs147711004
rs350823
rs33951980
rs1260326
rs2043082
rs613808
rs28929474
rs6073958
All
-0.05
0.00
0.05
0.10
MR leave-one-out sensitivity analysis for
n diameter of LDL particles || id:ebi-cfb233-GCST90301992' on 'ER+ Breast cancer (Combined Oncoarray; iCOGS; GWAS meta analysis

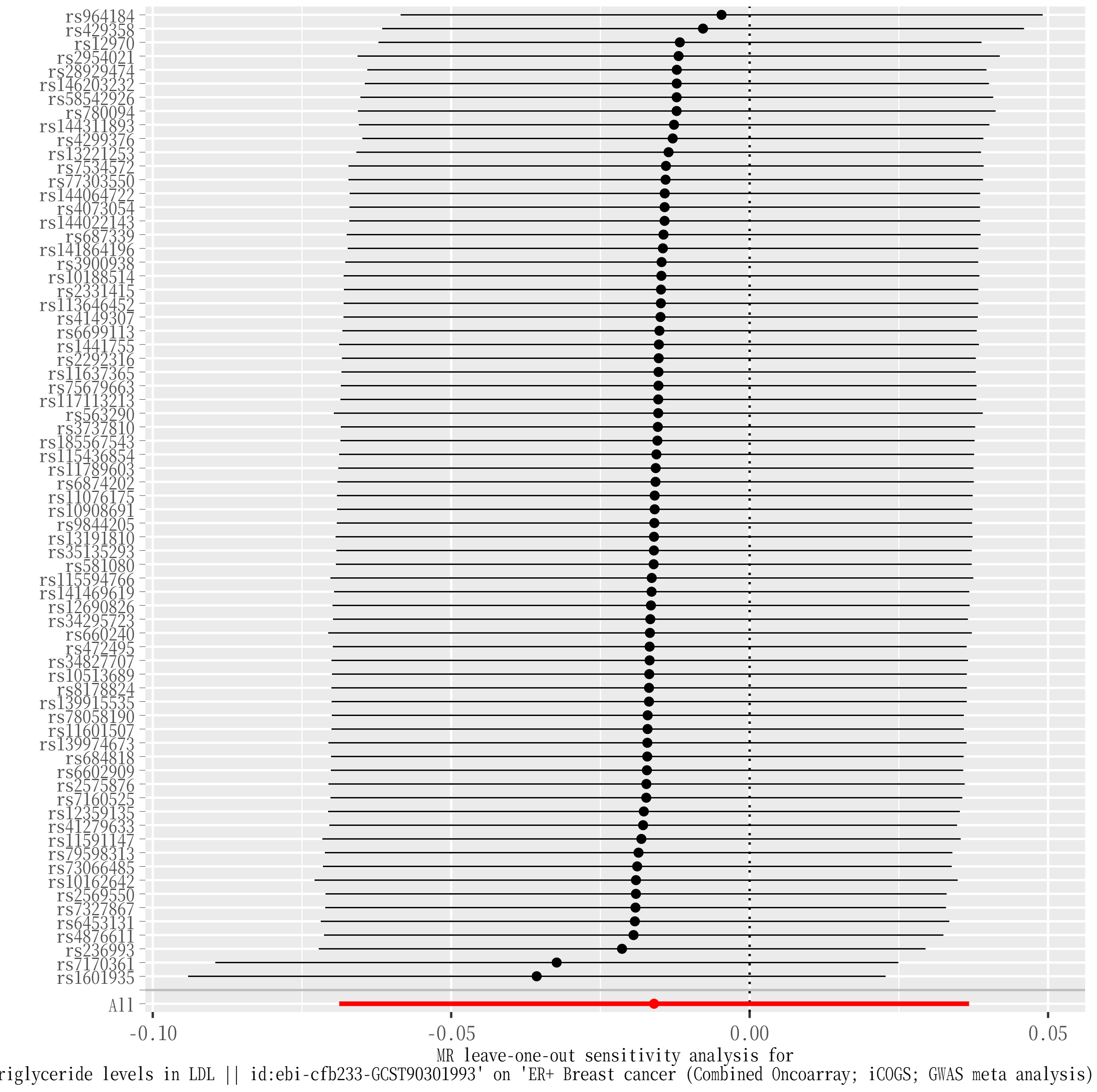
rs964184
rs429358
rs12970
rs2954021
rs28929474
rs146203232
rs58542926
rs780094
rs144311893
rs4299376
rs13221253
rs7534572
rs77303550
rs144064722
rs4073054
rs144022143
rs687339
rs141864196
rs3900938
rs10188514
rs2331415
rs113646452
rs4149307
rs6699113
rs1441755
rs2292316
rs11637365
rs75679663
rs117113213
rs563290
rs3737810
rs185567543
rs115436854
rs11789603
rs6874202
rs11076175
rs10908691
rs9844205
rs13191810
rs35135293
rs581080
rs115594766
rs141469619
rs12690826
rs34295723
rs660240
rs472495
rs34827707
rs10513689
rs8178824
rs139915535
rs78058190
rs11601507
rs139974673
rs684818
rs6602909
rs2575876
rs7160525
rs12359135
rs41279633
rs11591147
rs79598313
rs73066485
rs10162642
rs2569550
rs7327867
rs6453131
rs4876611
rs236993
rs7170361
rs1601935
All
-0.10
-0.05
0.00
0.05
MR leave-one-out sensitivity analysis for
riglyceride levels in LDL || id:ebi-cfb233-GCST90301993' on 'ER+ Breast cancer (Combined Oncoarray; iCOGS; GWAS meta analysis)

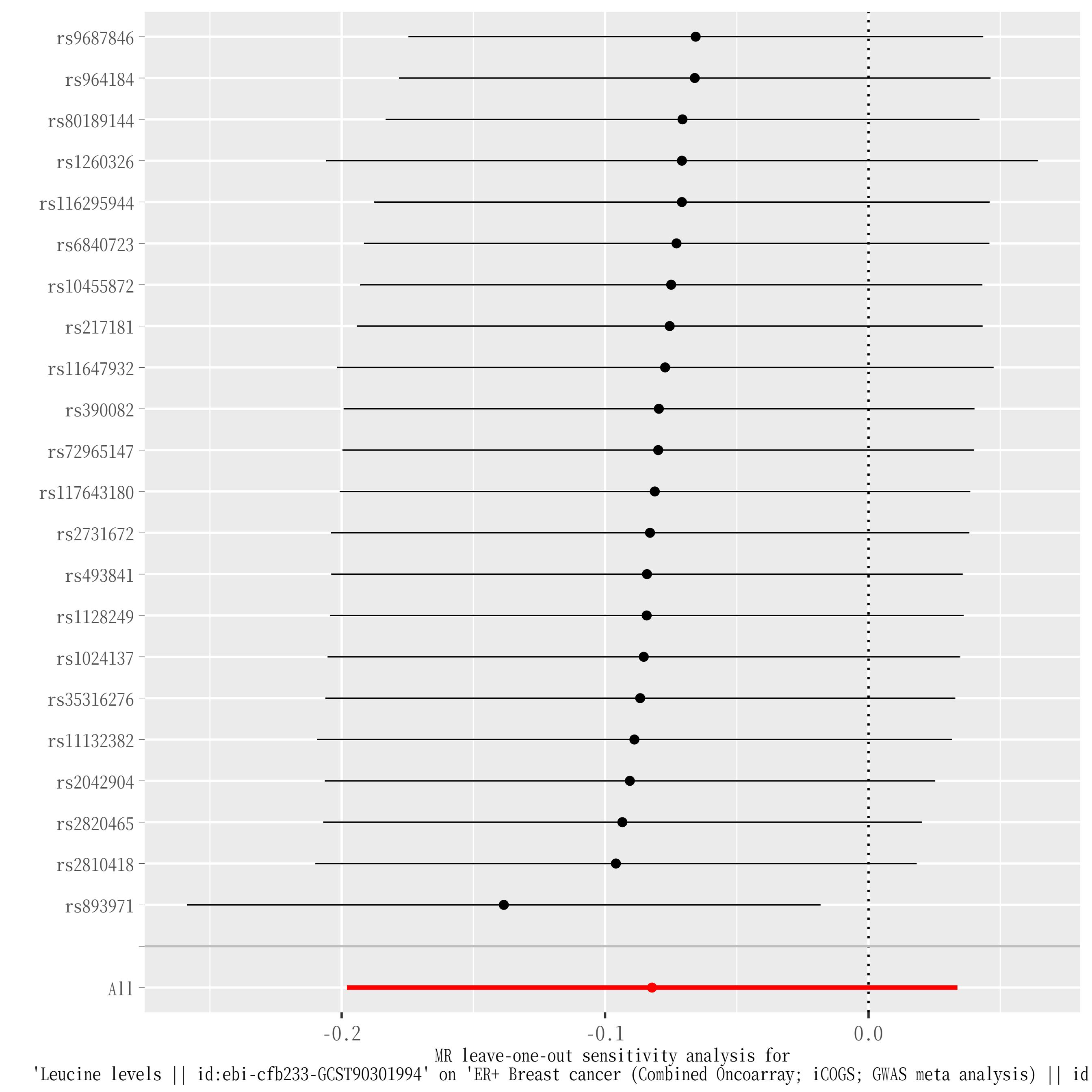

rs9687846
rs964184
rs80189144
rs1260326
rs116295944
rs6840723
rs10455872
rs217181
rs11647932
rs390082
rs72965147
rs117643180
rs2731672
rs493841
rs1128249
rs1024137
rs35316276
rs11132382
rs2042904
rs2820465
rs2810418
rs893971
All
-0.2
-0.1
0.0
MR leave-one-out sensitivity analysis for
'Leucine levels || id:ebi-cfb233-GCST90301994' on 'ER+ Breast cancer (Combined Oncoarray; iCOGS; GWAS meta analysis) || id

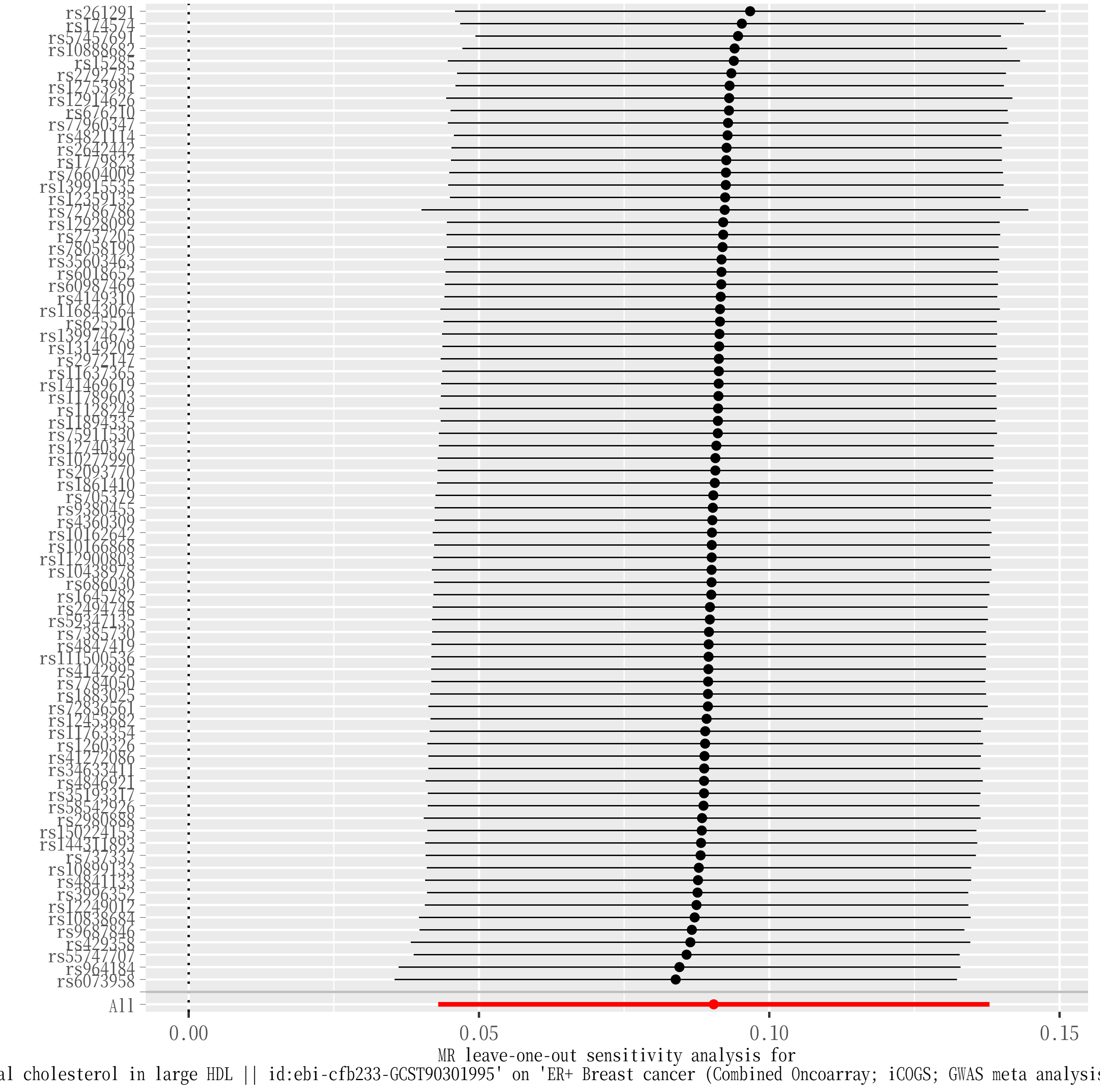

rs261291
rs174574
rs57457691
rs10888682
rs15285
rs2792735
rs12753981
rs12914626
rs676210
rs77960347
rs4821114
rs2642442
rs1779823
rs76604009
rs139915535
rs12359135
rs72786786
rs12928099
rs2737205
rs78058190
rs35603463
rs6018652
rs60987469
rs4149310
rs116843064
rs625510
rs139974673
rs13149209
rs2972147
rs11637365
rs141469619
rs11789603
rs1128249
rs11894335
rs75911530
rs12740374
rs10277990
rs2093770
rs1861410
rs705379
rs9380455
rs4360309
rs10162642
rs10166868
rs112900803
rs10438978
rs686030
rs1645782
rs2494748
rs59347135
rs7385730
rs4847419
rs111500536
rs4142995
rs7784050
rs1883025
rs72836561
rs12453682
rs11763354
rs1260326
rs41272086
rs34633411
rs4846921
rs35193317
rs58542926
rs2980888
rs150224153
rs144311893
rs737337
rs10899133
rs4841133
rs3996352
rs12249012
rs10838684
rs9687846
rs429358
rs55747707
rs964184
rs6073958
All
0.00
0.05
0.10
0.15
MR leave-one-out sensitivity analysis for
al cholesterol in large HDL || id:ebi-cfb233-GCST90301995' on 'ER+ Breast cancer (Combined Oncoarray; iCOGS; GWAS meta analysis

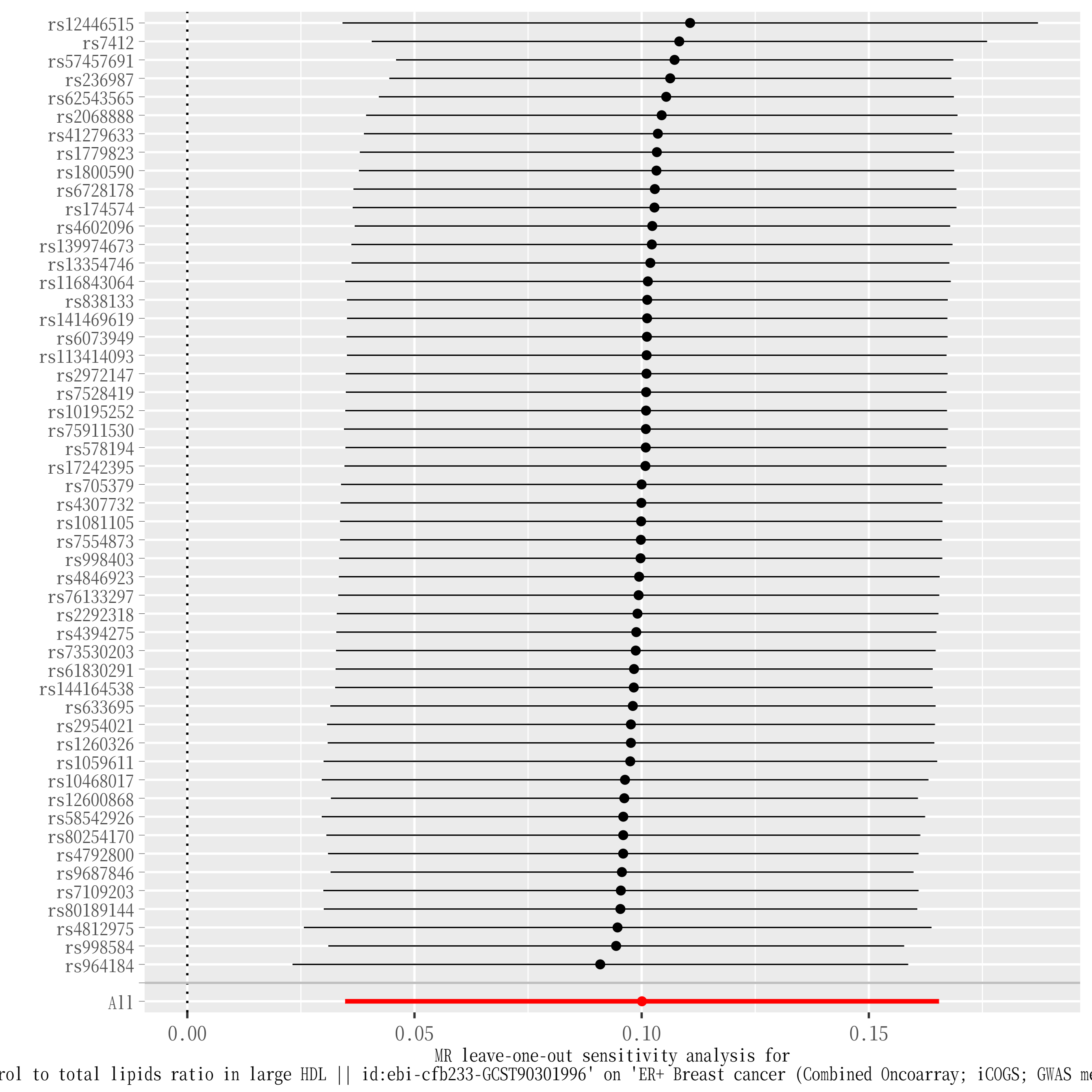
rs12446515
rs7412
rs57457691
rs236987
rs62543565
rs2068888
rs41279633
rs1779823
rs1800590
rs6728178
rs174574
rs4602096
rs139974673
rs13354746
rs116843064
rs838133
rs141469619
rs6073949
rs113414093
rs2972147
rs7528419
rs10195252
rs75911530
rs578194
rs17242395
rs705379
rs4307732
rs1081105
rs7554873
rs998403
rs4846923
rs76133297
rs2292318
rs4394275
rs73530203
rs61830291
rs144164538
rs633695
rs2954021
rs1260326
rs1059611
rs10468017
rs12600868
rs58542926
rs80254170
rs4792800
rs9687846
rs7109203
rs80189144
rs4812975
rs998584
rs964184
All
0.00
0.05
0.10
0.15
MR leave-one-out sensitivity analysis for
rol to total lipids ratio in large HDL || id:ebi-cfb233-GCST90301996' on 'ER+ Breast cancer (Combined Oncoarray; iCOGS; GWAS me

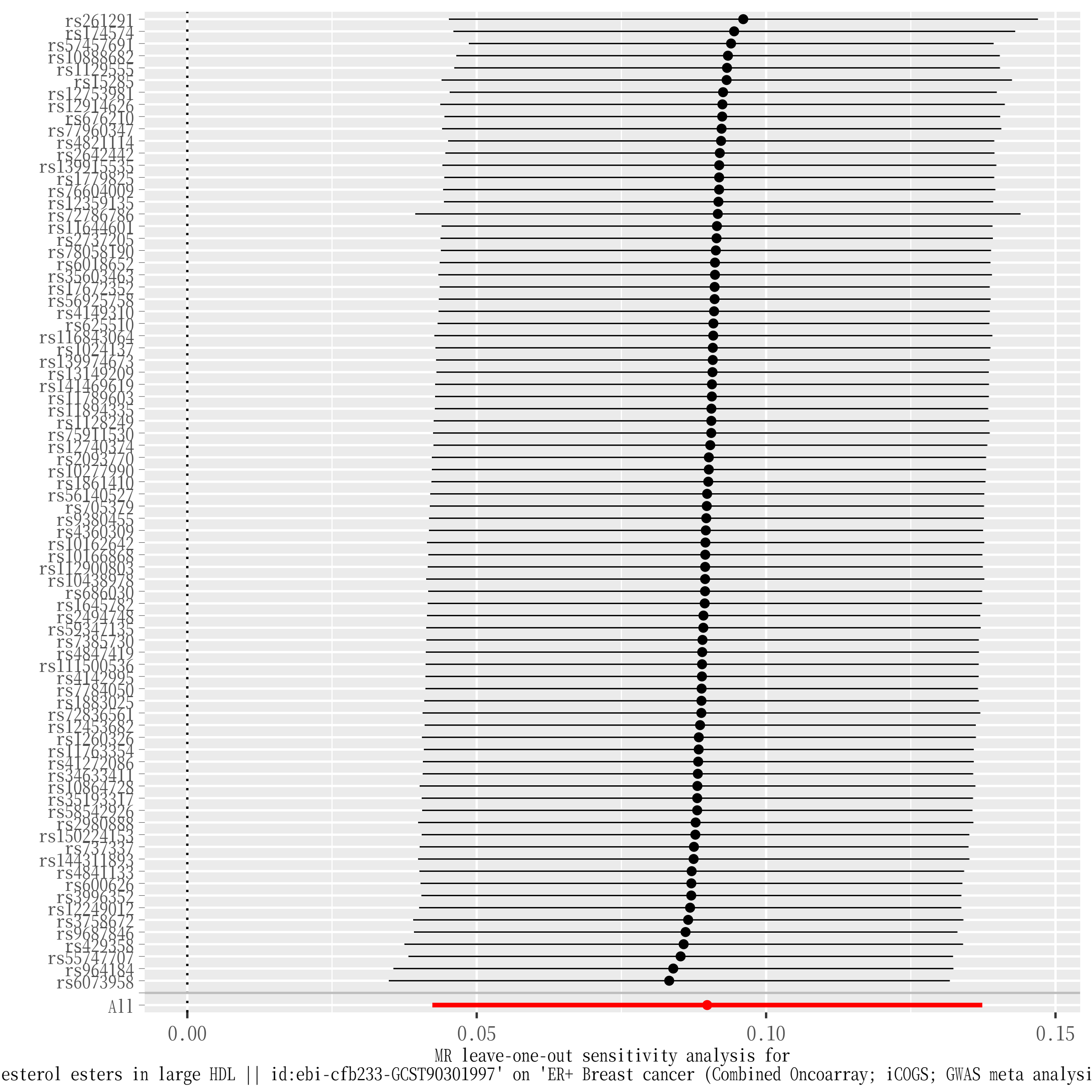
rs261291
rs174574
rs57457691
rs10888682
rs1129555
rs15285
rs12753981
rs12914626
rs676210
rs77960347
rs4821114
rs2642442
rs139215535
rs1779825
rs76604009
rs12359135
rs72786786
rs11644601
rs2737205
rs78058190
rs6018652
rs35603463
rs17672352
rs56925758
rs4149310
rs625510
rs116843064
rs1024137
rs139974673
rs13149209
rs141469619
rs11789603
rs11894335
rs1128249
rs75211530
rs12740374
rs2093770
rs10277990
rs1861410
rs56140527
rs705379
rs9380455
rs4360309
rs10162642
rs10166868
rs112900803
rs10438978
rs686030
rs1645782
rs2494748
rs59347135
rs7385730
rs4847419
rs111500536
rs4142995
rs7784050
rs1883025
rs72836561
rs12453682
rs1260326
rs11763354
rs41272086
rs34633411
rs10864728
rs35193317
rs58542926
rs2980888
rs150224153
rs737337
rs144311893
rs4841133
rs600626
rs3996352
rs12249012
rs3758672
rs9687846
rs429358
rs55747707
rs964184
rs6073958
All
0.00
0.05
0.10
0.15
MR leave-one-out sensitivity analysis for
esterol esters in large HDL || id:ebi-cfb233-GCST90301997' on 'ER+ Breast cancer (Combined Oncoarray; iCOGS; GWAS meta analysi

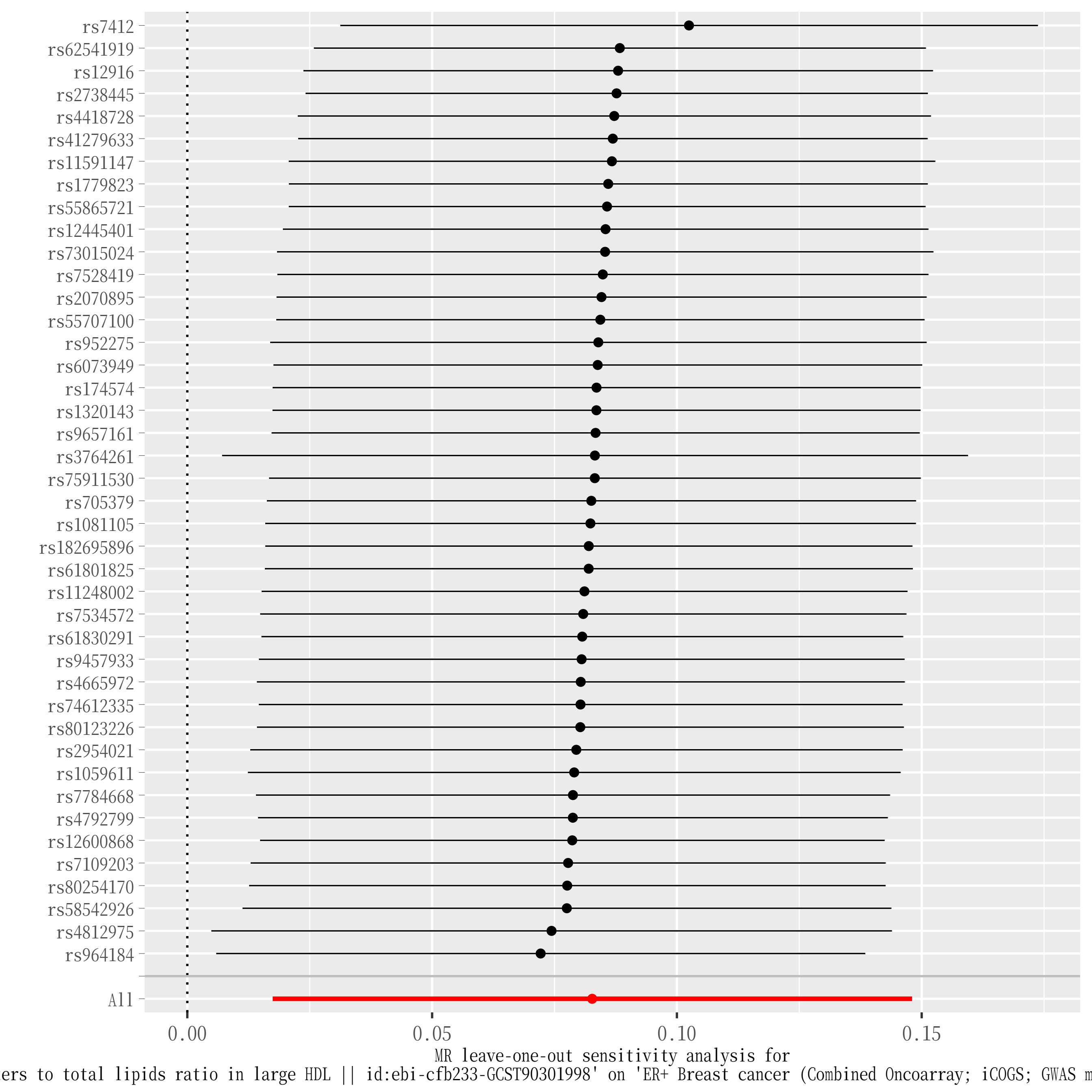
rs7412
rs62541919
rs12916
rs2738445
rs4418728
rs41279633
rs11591147
rs1779823
rs55865721
rs12445401
rs73015024
rs7528419
rs2070895
rs55707100
rs952275
rs6073949
rs174574
rs1320143
rs9657161
rs3764261
rs75911530
rs705379
rs1081105
rs182695896
rs61801825
rs11248002
rs7534572
rs61830291
rs9457933
rs4665972
rs74612335
rs80123226
rs2954021
rs1059611
rs7784668
rs4792799
rs12600868
rs7109203
rs80254170
rs58542926
rs4812975
rs964184
All
0.00
0.05
0.10
0.15
MR leave-one-out sensitivity analysis for
ers to total lipids ratio in large HDL || id:ebi-cfb233-GCST90301998' on 'ER+ Breast cancer (Combined Oncoarray; iCOGS; GWAS m

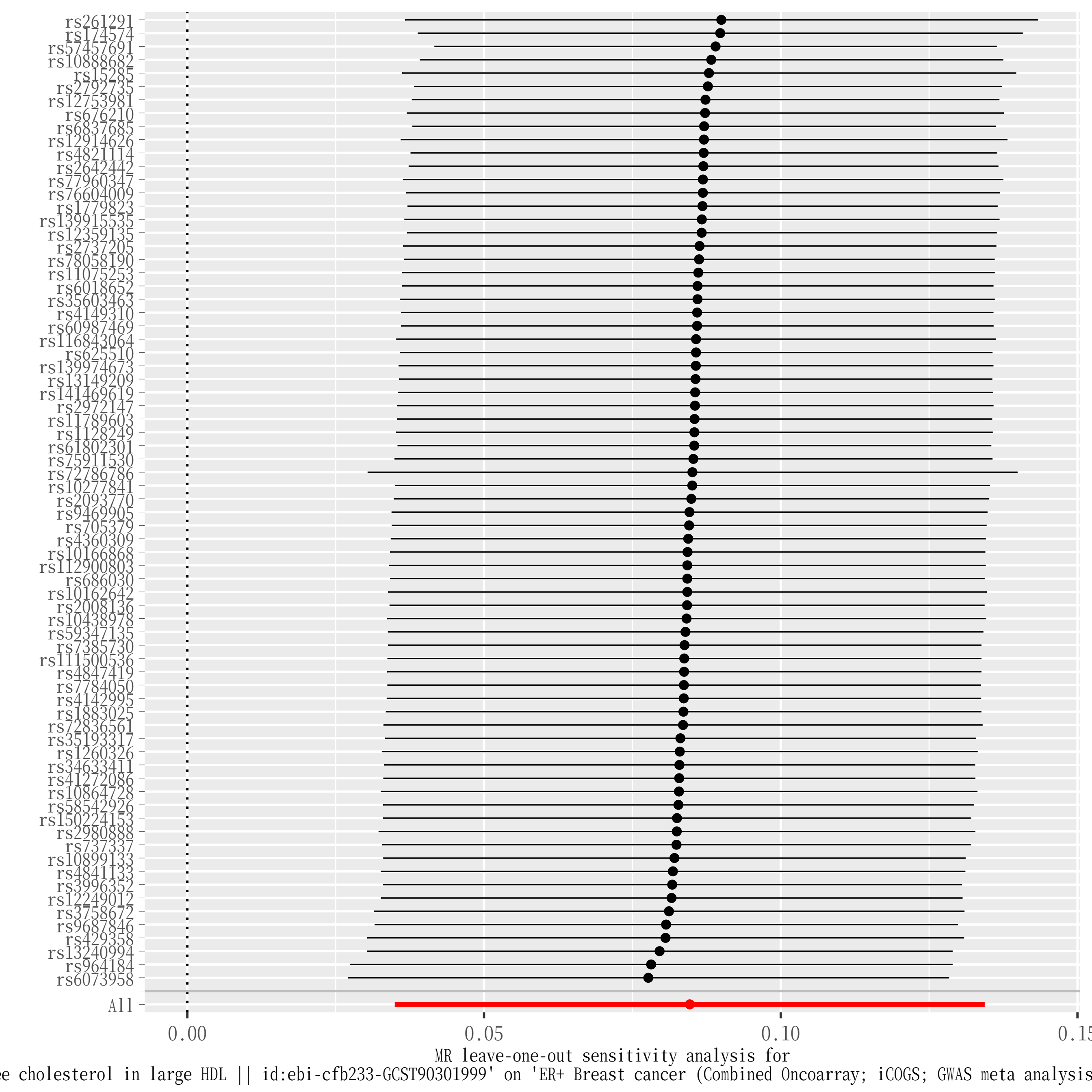
rs261291
rs174574
rs57457691
rs10888682
rs15285
rs2792735
rs12753981
rs676210
rs6837685
rs12914626
rs4821114
rs2642442
rs77960347
rs76604009
rs1779823
rs139915535
rs12359135
rs2737205
rs78058190
rs11075253
rs6018652
rs35603463
rs4149310
rs60987469
rs116843064
rs625510
rs139974673
rs13149209
rs141469619
rs2972147
rs11789603
rs1128249
rs61802301
rs75911530
rs72786786
rs10277841
rs2093770
rs9469905
rs705379
rs4360309
rs10166868
rs112900803
rs686030
rs10162642
rs2008136
rs10438978
rs59347135
rs7385730
rs111500536
rs4847419
rs7784050
rs4142995
rs1883025
rs72836561
rs35193317
rs1260326
rs34633411
rs41272086
rs10864728
rs58542926
rs150224153
rs2980888
rs737337
rs10899133
rs4841133
rs3996352
rs12249012
rs3758672
rs9687846
rs429358
rs13240994
rs964184
rs6073958
All
0.00
0.05
0.10
MR leave-one-out sensitivity analysis for
cholesterol in large HDL || id:ebi-cfb233-GCST90301999' on 'ER+ Breast cancer (Combined Oncoarray; iCOGS; GWAS meta analysis

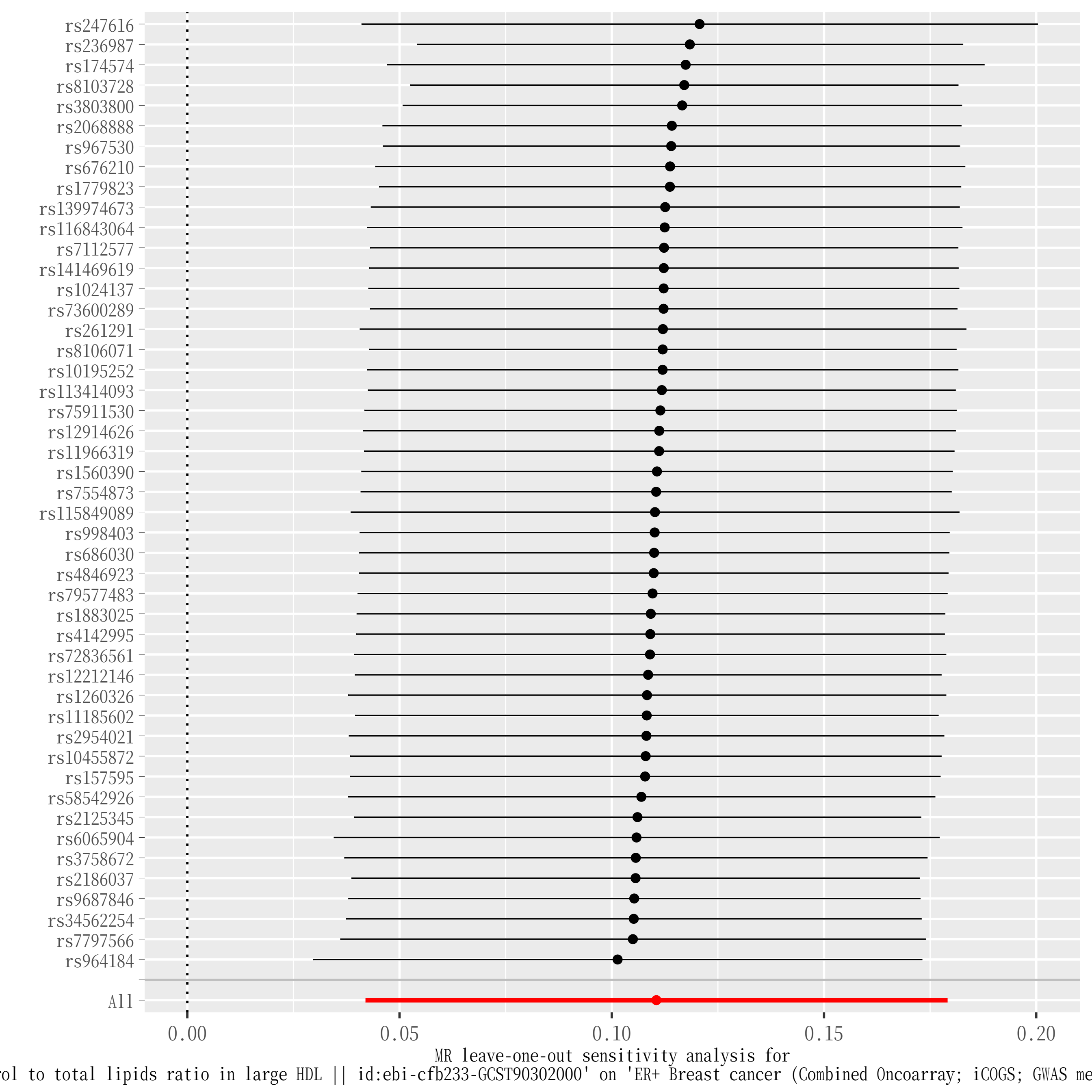
rs247616
rs236987
rs174574
rs8103728
rs3803800
rs2068888
rs967530
rs676210
rs1779823
rs139974673
rs116843064
rs7112577
rs141469619
rs1024137
rs73600289
rs261291
rs8106071
rs10195252
rs113414093
rs75911530
rs12914626
rs11966319
rs1560390
rs7554873
rs115849089
rs998403
rs686030
rs4846923
rs79577483
rs1883025
rs4142995
rs72836561
rs12212146
rs1260326
rs11185602
rs2954021
rs10455872
rs157595
rs58542926
rs2125345
rs6065904
rs3758672
rs2186037
rs9687846
rs34562254
rs7797566
rs964184
All
0.00
0.05
0.10
0.15
0.20
MR leave-one-out sensitivity analysis for
rol to total lipids ratio in large HDL || id:ebi-cfb233-GCST90302000' on 'ER+ Breast cancer (Combined Oncoarray; iCOGS; GWAS me

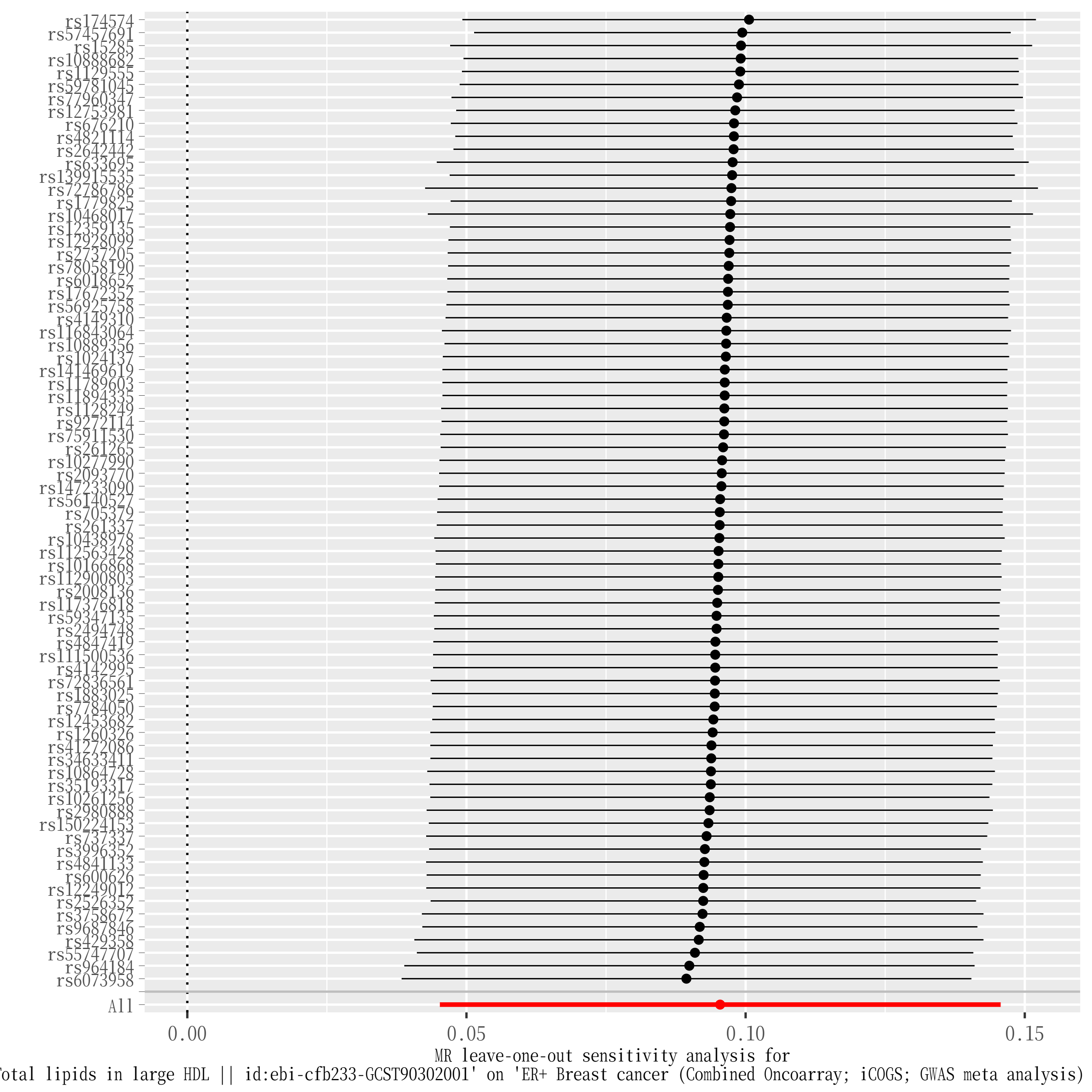
rs174574
rs57457691
rs15285
rs10888682
rs1129555
rs59781045
rs77960347
rs12753981
rs676210
rs4821114
rs2642442
rs633695
rs139915535
rs72786786
rs1779825
rs10468017
rs12359135
rs12928099
rs2737205
rs78058190
rs6018652
rs17672352
rs56925758
rs4149310
rs116843064
rs10889356
rs1024137
rs141469619
rs11789603
rs11894335
rs1128249
rs9272114
rs75911530
rs261265
rs10277990
rs2093770
rs147233090
rs56140527
rs705379
rs261337
rs10438978
rs112563428
rs10166868
rs112900803
rs2008136
rs117376818
rs59347135
rs2494748
rs4847419
rs111500536
rs4142995
rs72836561
rs1883025
rs7784050
rs12453682
rs1260326
rs41272086
rs34633411
rs10864728
rs35193317
rs10261256
rs2980888
rs150224153
rs737337
rs3996352
rs4841133
rs600626
rs12249012
rs2526352
rs3758672
rs9687846
rs429358
rs55747707
rs964184
rs6073958
All
0.00
0.05
0.10
0.15
MR leave-one-out sensitivity analysis for
'Total lipids in large HDL || id:ebi-cfb233-GCST90302001' on 'ER+ Breast cancer (Combined Oncoarray; iCOGS; GWAS meta analysis)'

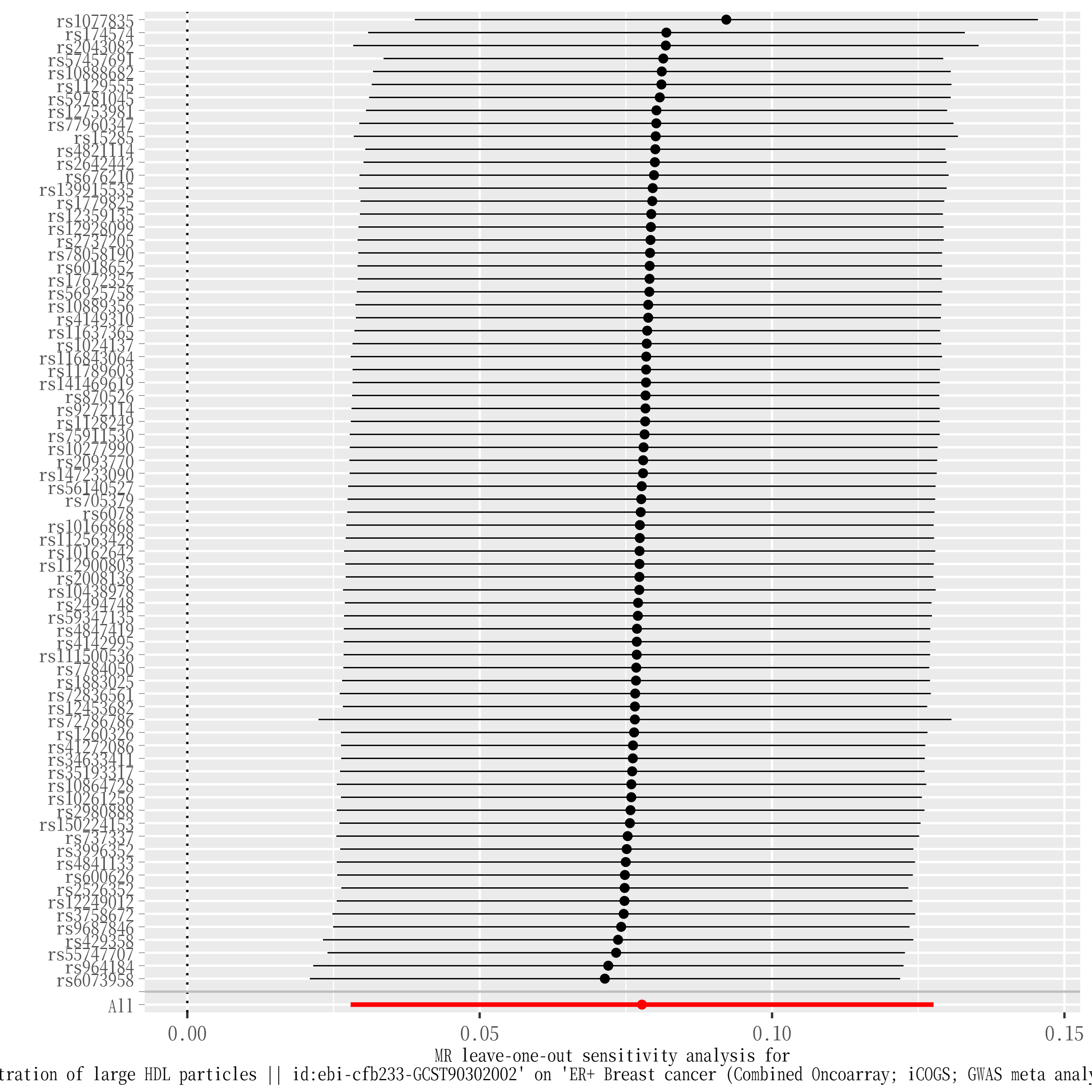

rs1077835
rs174574
rs2043082
rs57457691
rs10888682
rs1129555
rs59781045
rs12753981
rs77960347
rs15285
rs4821114
rs2642442
rs676210
rs139915535
rs1779825
rs12359135
rs12928099
rs2737205
rs78058190
rs6018652
rs17672352
rs56925758
rs10889356
rs4149310
rs11637365
rs1024137
rs116843064
rs11789603
rs141469619
rs870526
rs9272114
rs1128249
rs75911530
rs10277990
rs2093770
rs147233090
rs56140527
rs705379
rs6078
rs10166868
rs112563428
rs10162642
rs112900803
rs2008136
rs10438978
rs2494748
rs59347135
rs4847419
rs4142995
rs111500536
rs7784050
rs1883025
rs72836561
rs12453682
rs72786786
rs1260326
rs41272086
rs34633411
rs35193317
rs10864728
rs10261256
rs2980888
rs150224153
rs737337
rs3996352
rs4841133
rs600626
rs2526352
rs12249012
rs3758672
rs9687846
rs429358
rs55747707
rs964184
rs6073958
All
0.00
0.05
0.10
0.15
MR leave-one-out sensitivity analysis for
tration of large HDL particles || id:ebi-cfb233-GCST90302002' on 'ER+ Breast cancer (Combined Oncoarray; iCOGS; GWAS meta analy

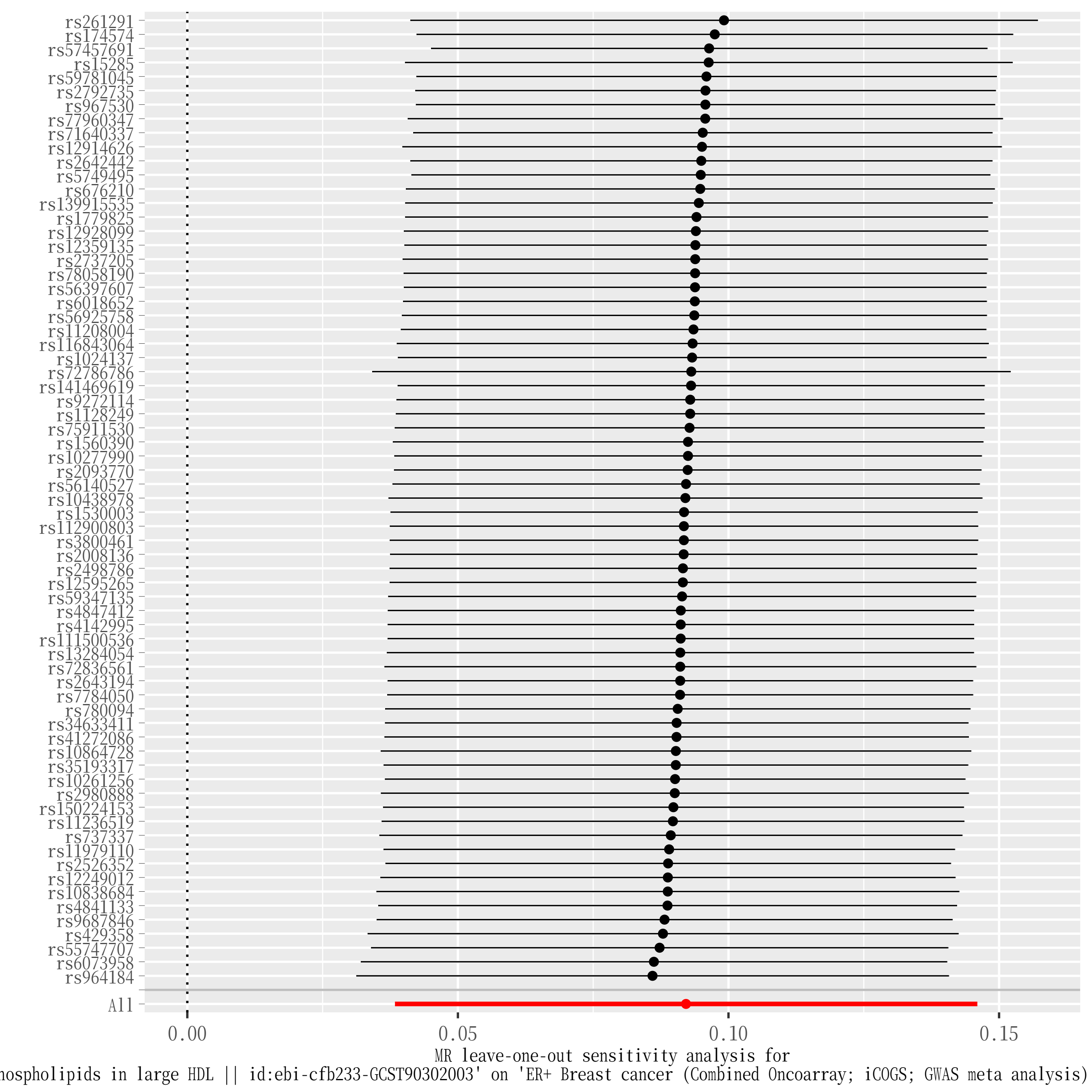
rs261291
rs174574
rs57457691
rs15285
rs59781045
rs2792735
rs967530
rs77960347
rs71640337
rs12914626
rs2642442
rs5749495
rs676210
rs139915535
rs1779825
rs12928099
rs12359135
rs2737205
rs78058190
rs56397607
rs6018652
rs56925758
rs11208004
rs116843064
rs1024137
rs72786786
rs141469619
rs9272114
rs1128249
rs75911530
rs1560390
rs10277990
rs2093770
rs56140527
rs10438978
rs1530003
rs112900803
rs3800461
rs2008136
rs2498786
rs12595265
rs59347135
rs4847412
rs4142995
rs111500536
rs13284054
rs72836561
rs2643194
rs7784050
rs780094
rs34633411
rs41272086
rs10864728
rs35193317
rs10261256
rs2980888
rs150224153
rs11236519
rs737337
rs11979110
rs2526352
rs12249012
rs10838684
rs4841133
rs9687846
rs429358
rs55747707
rs6073958
rs964184
All
0.00
0.05
0.10
0.15
MR leave-one-out sensitivity analysis for
hospholipids in large HDL || id:ebi-cfb233-GCST90302003' on 'ER+ Breast cancer (Combined Oncoarray; iCOGS; GWAS meta analysis)

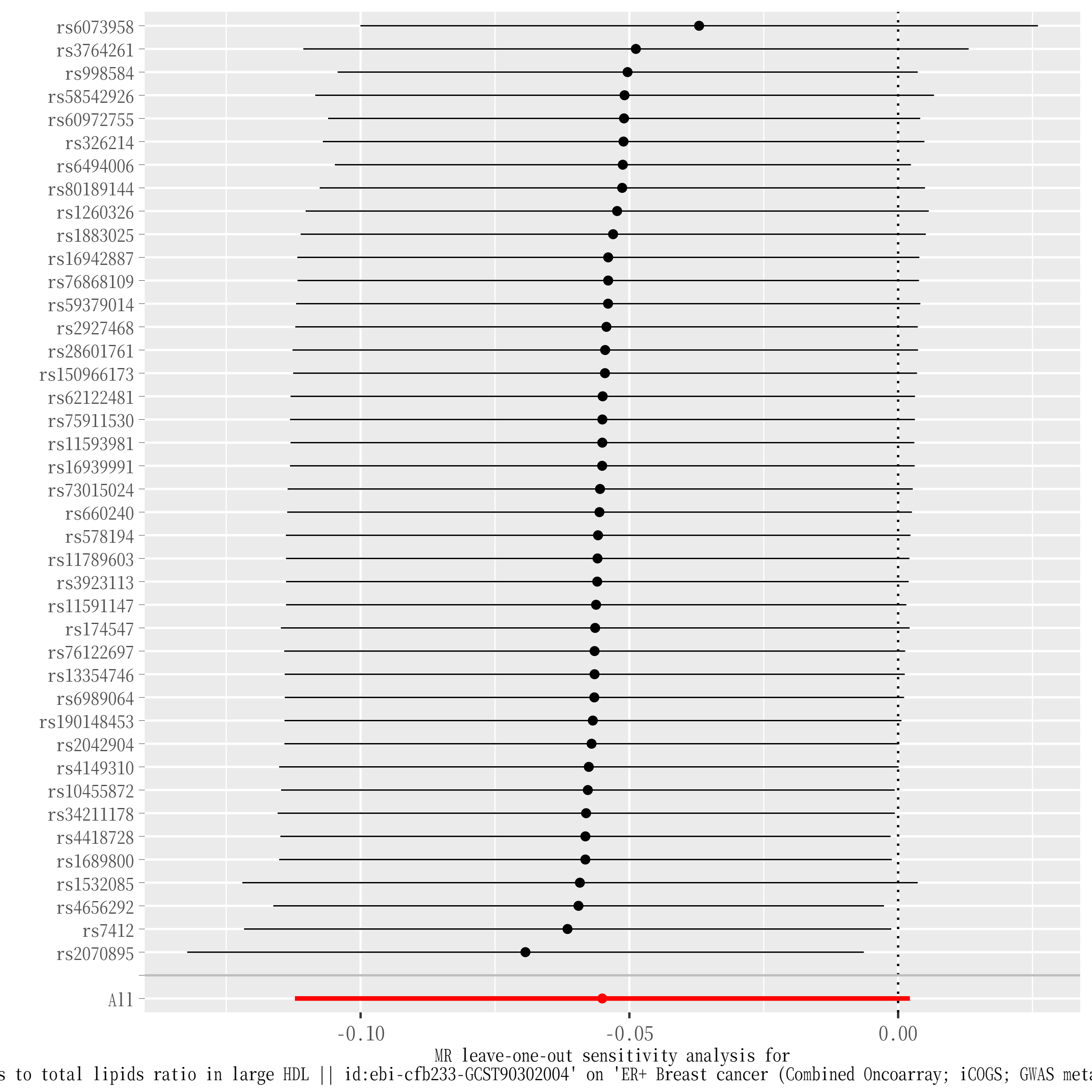

rs6073958
rs3764261
rs998584
rs58542926
rs60972755
rs326214
rs6494006
rs80189144
rs1260326
rs1883025
rs16942887
rs76868109
rs59379014
rs2927468
rs28601761
rs150966173
rs62122481
rs75911530
rs11593981
rs16939991
rs73015024
rs660240
rs578194
rs11789603
rs3923113
rs11591147
rs174547
rs76122697
rs13354746
rs6989064
rs190148453
rs2042904
rs4149310
rs10455872
rs34211178
rs4418728
rs1689800
rs1532085
rs4656292
rs7412
rs2070895
All
-0.10
-0.05
0.00
MR leave-one-out sensitivity analysis for
s to total lipids ratio in large HDL || id:ebi-cfb233-GCST90302004' on 'ER+ Breast cancer (Combined Oncoarray; iCOGS; GWAS meta

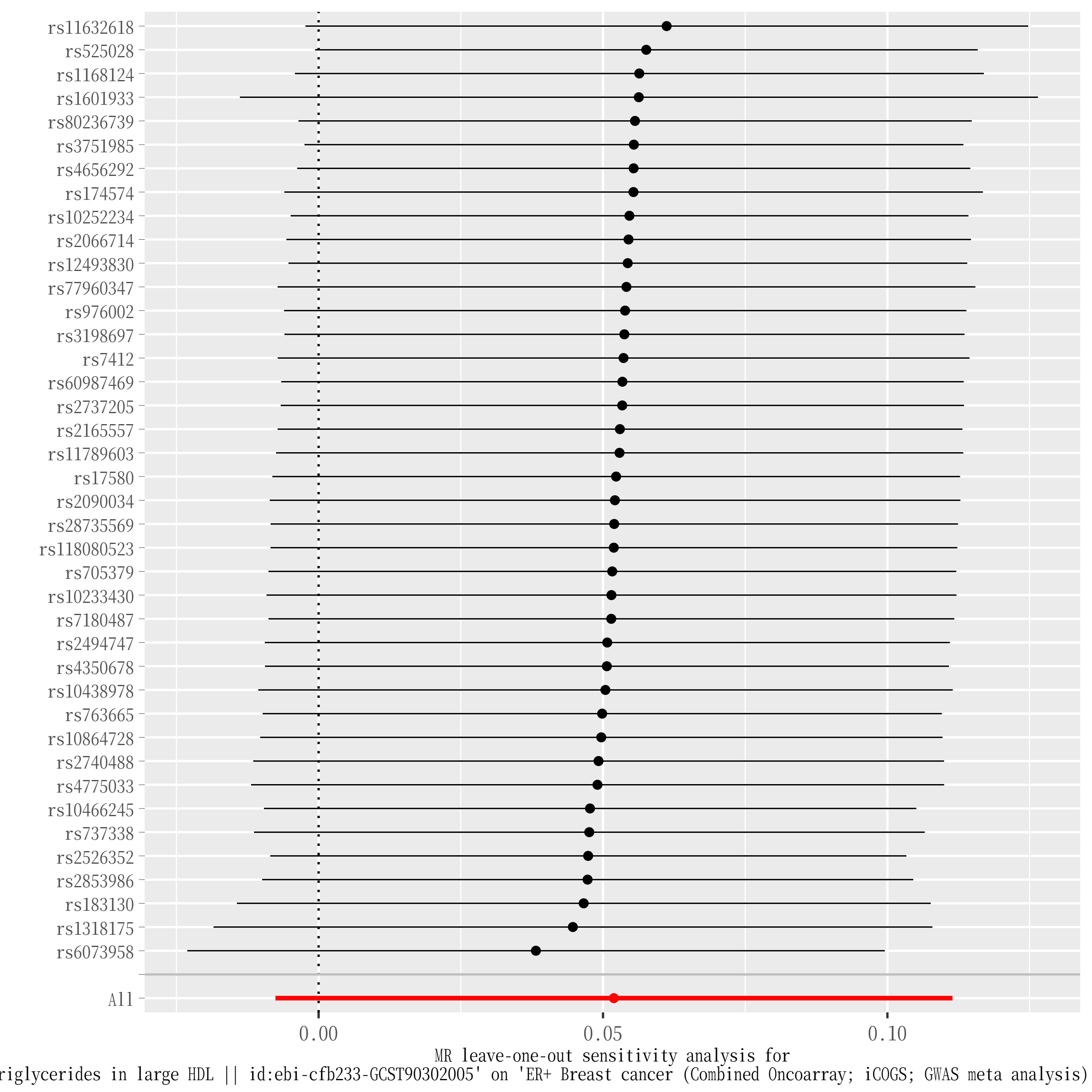
rs11632618
rs525028
rs1168124
rs1601933
rs80236739
rs3751985
rs4656292
rs174574
rs10252234
rs2066714
rs12493830
rs77960347
rs976002
rs3198697
rs7412
rs60987469
rs2737205
rs2165557
rs11789603
rs17580
rs2090034
rs28735569
rs118080523
rs705379
rs10233430
rs7180487
rs2494747
rs4350678
rs10438978
rs763665
rs10864728
rs2740488
rs4775033
rs10466245
rs737338
rs2526352
rs2853986
rs183130
rs1318175
rs6073958
All
0.00
0.05
0.10
MR leave-one-out sensitivity analysis for
riglycerides in large HDL || id:ebi-cfb233-GCST90302005' on 'ER+ Breast cancer (Combined Oncoarray; iCOGS; GWAS meta analysis)

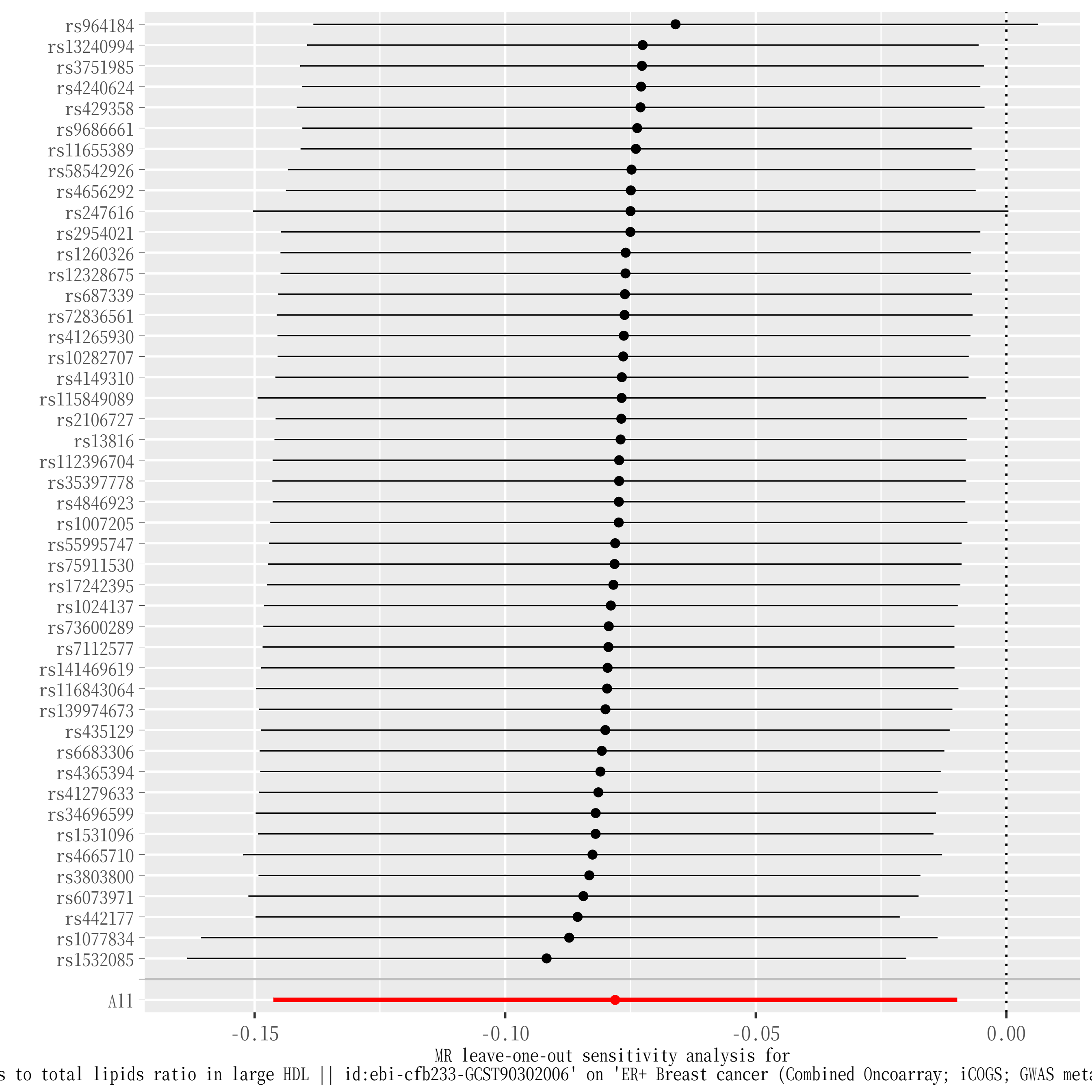
rs964184
rs13240994
rs3751985
rs4240624
rs429358
rs9686661
rs11655389
rs58542926
rs4656292
rs247616
rs2954021
rs1260326
rs12328675
rs687339
rs72836561
rs41265930
rs10282707
rs4149310
rs115849089
rs2106727
rs13816
rs112396704
rs35397778
rs4846923
rs1007205
rs55995747
rs75911530
rs17242395
rs1024137
rs73600289
rs7112577
rs141469619
rs116843064
rs139974673
rs435129
rs6683306
rs4365394
rs41279633
rs34696599
rs1531096
rs4665710
rs3803800
rs6073971
rs442177
rs1077834
rs1532085
All
-0.15
-0.10
-0.05
0.00
MR leave-one-out sensitivity analysis for
s to total lipids ratio in large HDL || id:ebi-cfb233-GCST90302006' on 'ER+ Breast cancer (Combined Oncoarray; iCOGS; GWAS meta

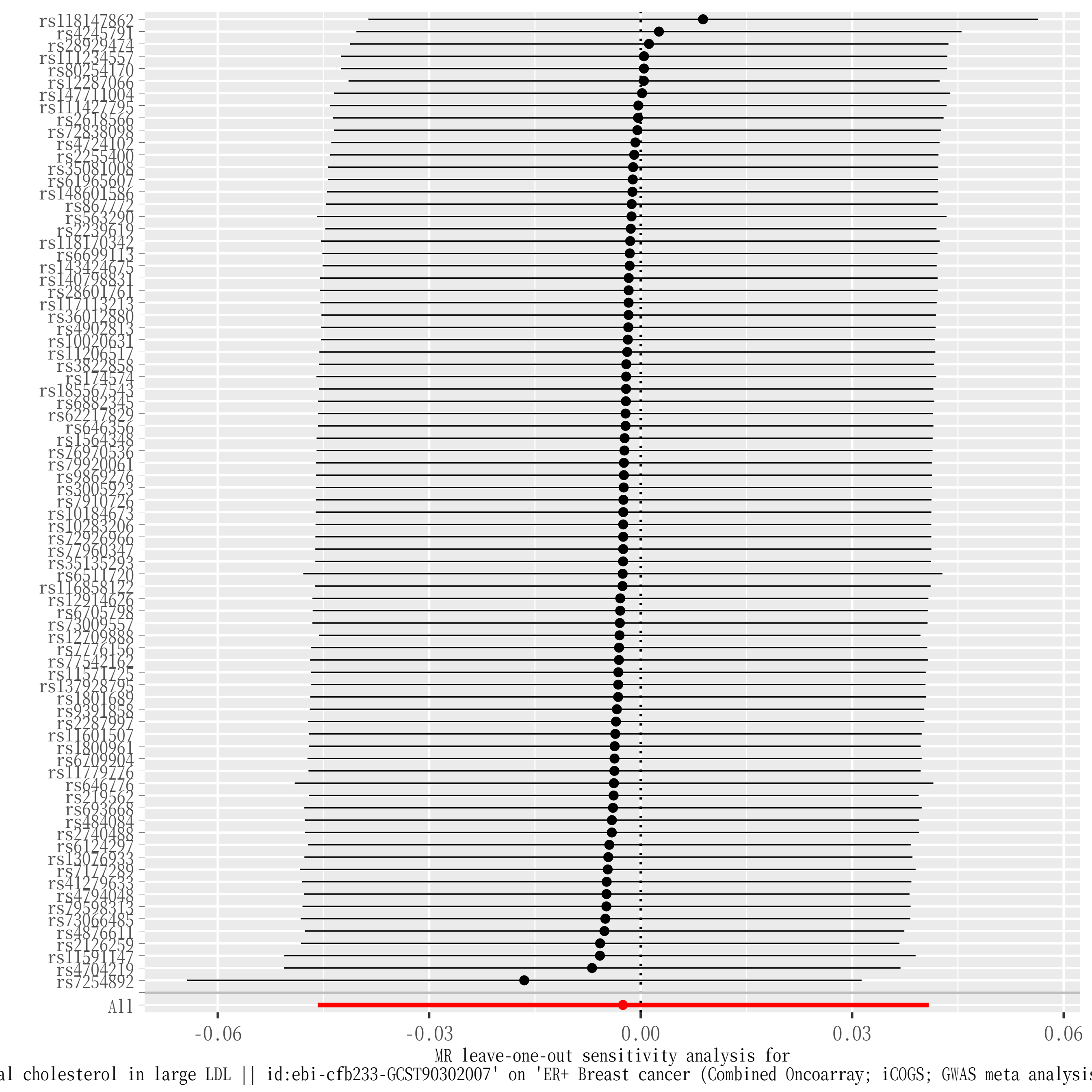
rs118147862
rs4245791
rs28929474
rs111234557
rs80254170
rs12287066
rs147711004
rs111427795
rs2618566
rs72838098
rs4724102
rs2255400
rs35081008
rs61965607
rs148601586
rs867772
rs563290
rs2239619
rs118170342
rs6699113
rs143424675
rs140798831
rs28601761
rs117113213
rs36012880
rs4902813
rs10020631
rs11206517
rs3822858
rs174574
rs185567543
rs6882345
rs62217829
rs646356
rs1564348
rs76970536
rs79920061
rs9869276
rs3005923
rs7910726
rs10184673
rs10283206
rs72926966
rs77960347
rs35135293
rs6511720
rs116858122
rs12914626
rs6705798
rs73009557
rs12709888
rs7776156
rs77542162
rs11571725
rs137928795
rs1801689
rs9391858
rs2287997
rs11601507
rs1800961
rs6709904
rs11779776
rs646776
rs219562
rs693668
rs484084
rs2740488
rs6124297
rs13076933
rs7177289
rs41279633
rs4794048
rs79598313
rs73066485
rs4876611
rs2126259
rs11591147
rs4704219
rs7254892
All
-0.06
-0.03
0.00
0.03
0.06
MR leave-one-out sensitivity analysis for
al cholesterol in large LDL || id:ebi-cfb233-GCST90302007' on 'ER+ Breast cancer (Combined Oncoarray; iCOGS; GWAS meta analysis

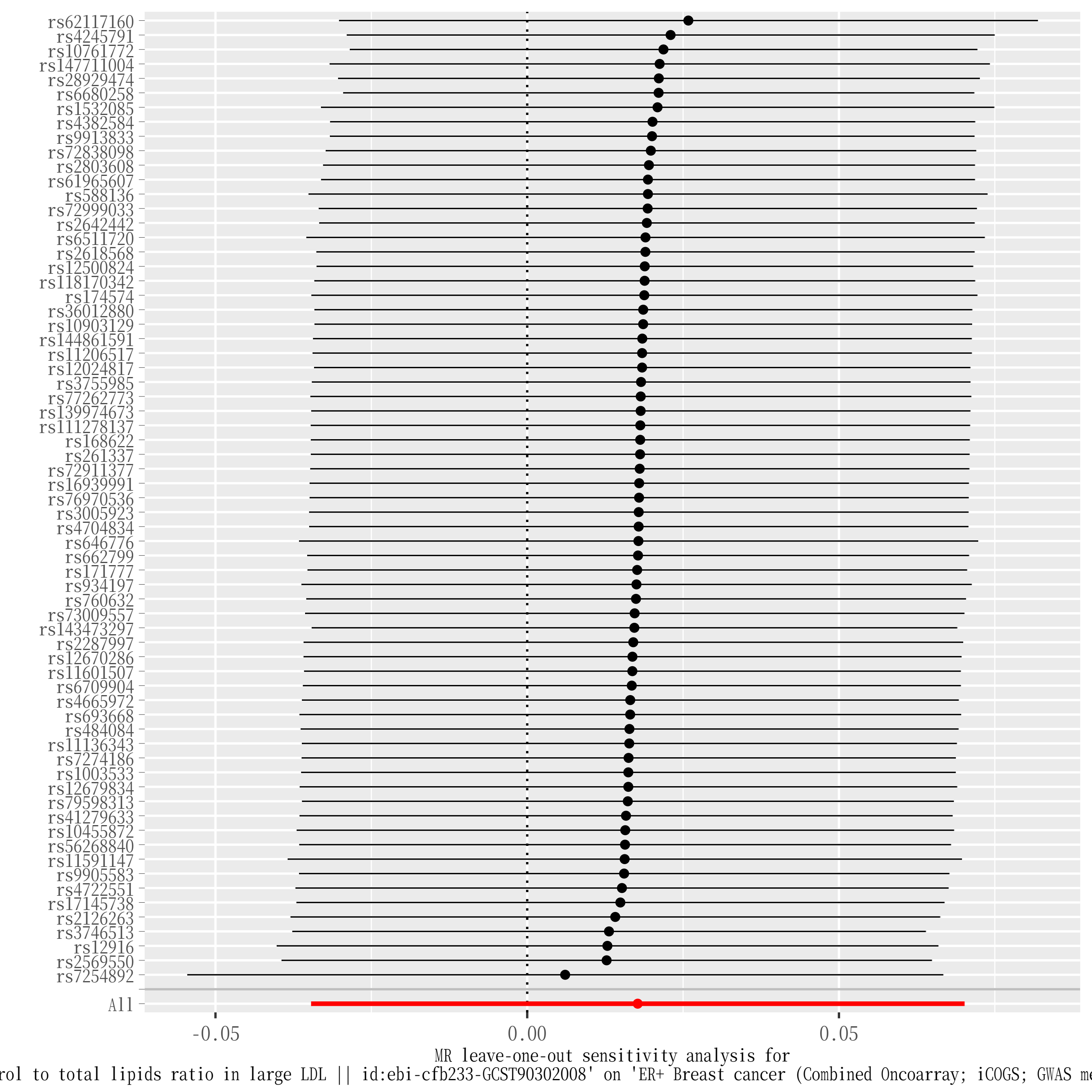
rs62117160
rs4245791
rs10761772
rs147711004
rs28929474
rs6680258
rs1532085
rs4382584
rs9913833
rs72838098
rs2803608
rs61965607
rs588136
rs72999033
rs2642442
rs6511720
rs2618568
rs12500824
rs118170342
rs174574
rs36012880
rs10903129
rs144861591
rs11206517
rs12024817
rs3755985
rs77262773
rs139974673
rs111278137
rs168622
rs261337
rs72911377
rs16939991
rs76970536
rs3005923
rs4704834
rs646776
rs662799
rs171777
rs934197
rs760632
rs73009557
rs143473297
rs2287997
rs12670286
rs11601507
rs6709904
rs4665972
rs693668
rs484084
rs11136343
rs7274186
rs1003533
rs12679834
rs79598313
rs41279633
rs10455872
rs56268840
rs11591147
rs9905583
rs4722551
rs17145738
rs2126263
rs3746513
rs12916
rs2569550
rs7254892
All
-0.05
0.00
0.05
MR leave-one-out sensitivity analysis for
rol to total lipids ratio in large LDL || id:ebi-cfb233-GCST90302008' on 'ER+ Breast cancer (Combined Oncoarray; iCOGS; GWAS me

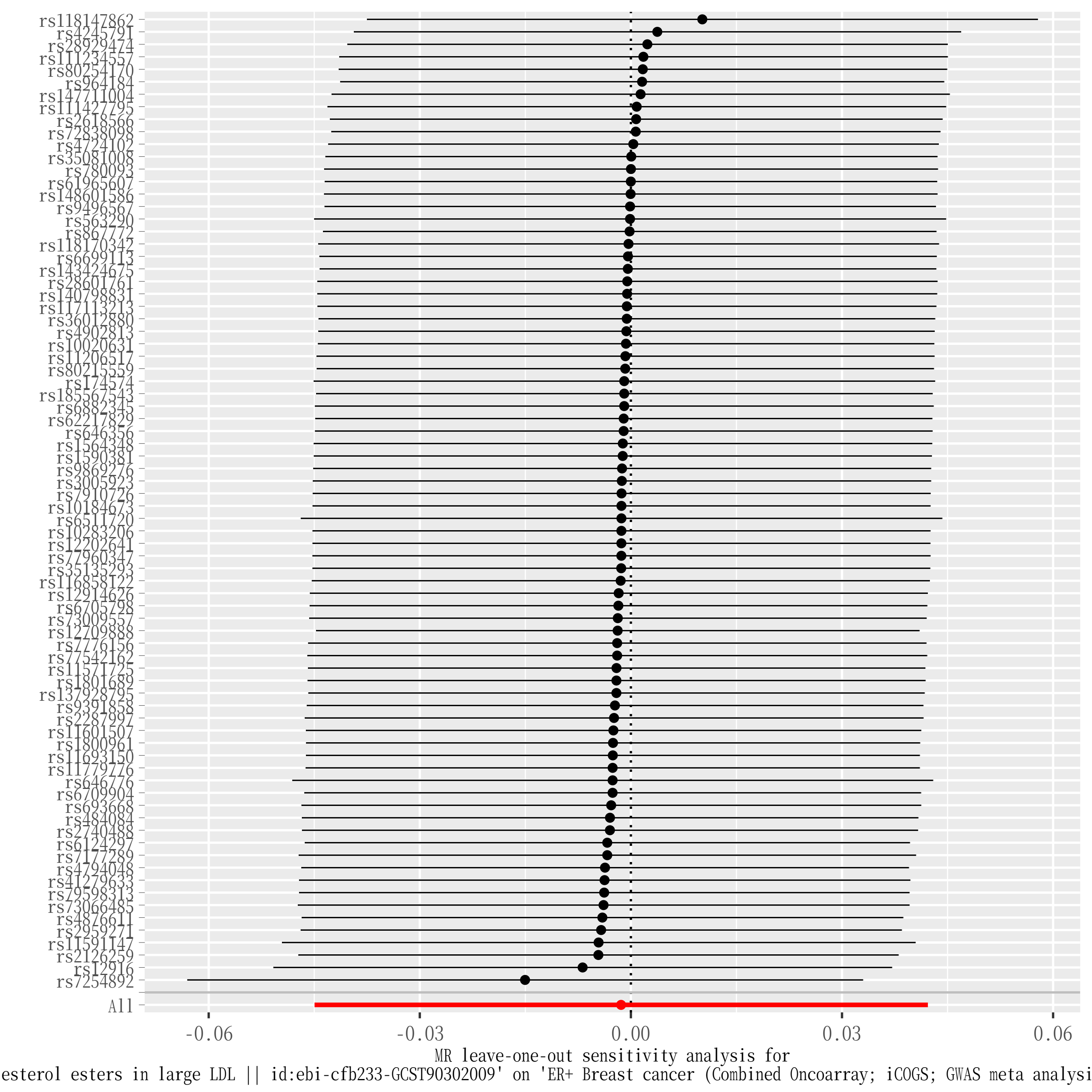
rs118147862
rs4245791
rs28929474
rs111234557
rs80254170
rs964184
rs147711004
rs111427795
rs2618566
rs72838098
rs4724102
rs35081008
rs780093
rs61965607
rs148601586
rs9496567
rs563290
rs867772
rs118170342
rs6699113
rs143424675
rs28601761
rs140798831
rs117113213
rs36012880
rs4902813
rs10020631
rs11206517
rs80215559
rs174574
rs185567543
rs6882345
rs62217829
rs646356
rs1564348
rs1590381
rs9869276
rs3005923
rs7910726
rs10184673
rs6511720
rs10283206
rs12202641
rs77960347
rs35135293
rs116858122
rs12914626
rs6705798
rs73009557
rs12709888
rs7776156
rs77542162
rs11571725
rs1801689
rs137928795
rs9391858
rs2287997
rs11601507
rs1800961
rs11693150
rs11779776
rs646776
rs6709904
rs693668
rs484084
rs2740488
rs6124297
rs7177289
rs4794048
rs41279633
rs79598313
rs73066485
rs4876611
rs2959271
rs11591147
rs2126259
rs12916
rs7254892
All
-0.06
-0.03
0.00
0.03
0.06
MR leave-one-out sensitivity analysis for
esterol esters in large LDL || id:ebi-cfb233-GCST90302009' on 'ER+ Breast cancer (Combined Oncoarray; iCOGS; GWAS meta analysi

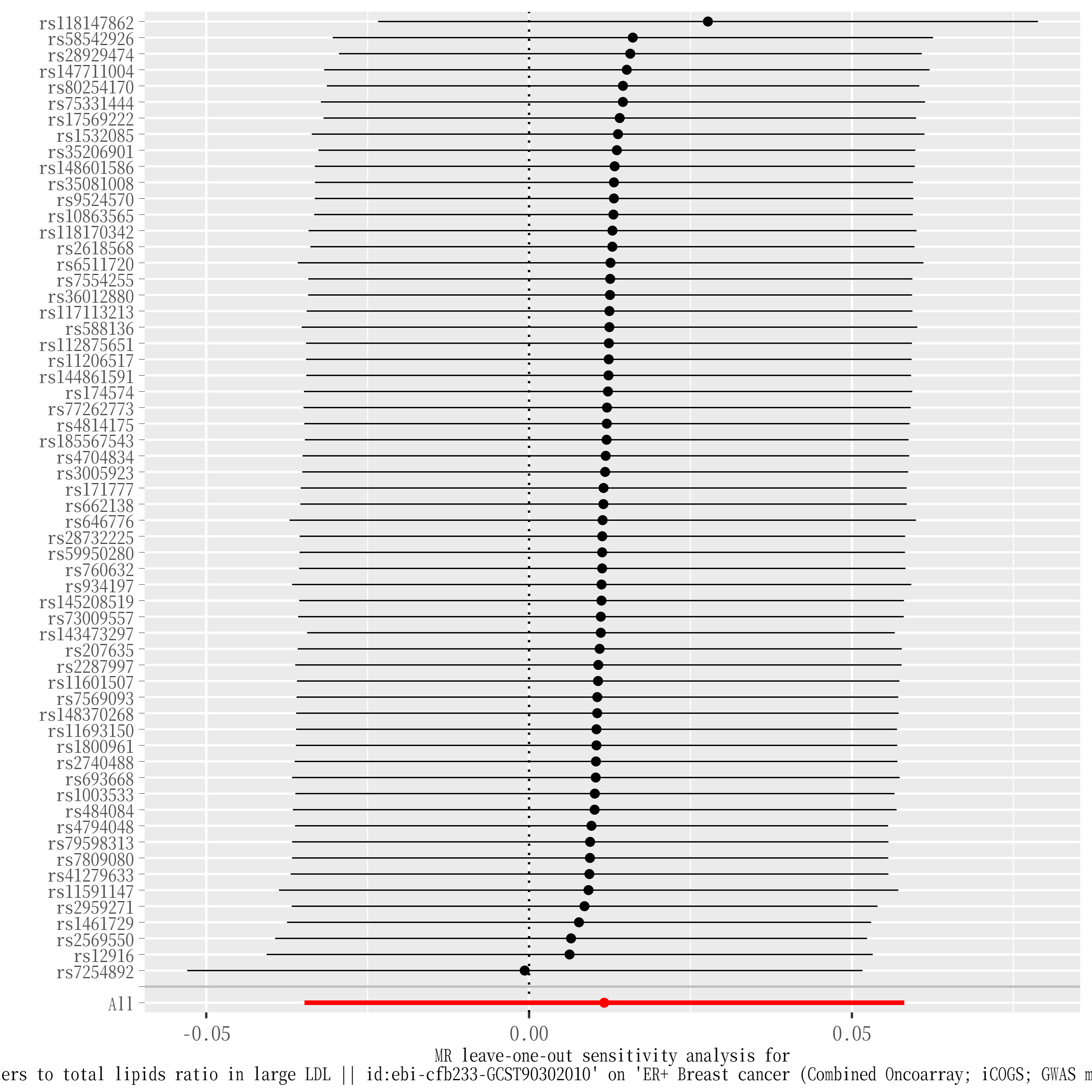

rs118147862
rs58542926
rs28929474
rs147711004
rs80254170
rs75331444
rs17569222
rs1532085
rs35206901
rs148601586
rs35081008
rs9524570
rs10863565
rs118170342
rs2618568
rs6511720
rs7554255
rs36012880
rs117113213
rs588136
rs112875651
rs11206517
rs144861591
rs174574
rs77262773
rs4814175
rs185567543
rs4704834
rs3005923
rs171777
rs662138
rs646776
rs28732225
rs59950280
rs760632
rs934197
rs145208519
rs73009557
rs143473297
rs207635
rs2287997
rs11601507
rs7569093
rs148370268
rs11693150
rs1800961
rs2740488
rs693668
rs1003533
rs484084
rs4794048
rs79598313
rs7809080
rs41279633
rs11591147
rs2959271
rs1461729
rs2569550
rs12916
rs7254892
All
-0.05
0.00
0.05
MR leave-one-out sensitivity analysis for
ers to total lipids ratio in large LDL || id:ebi-cfb233-GCST90302010' on 'ER+ Breast cancer (Combined Oncoarray; iCOGS; GWAS m

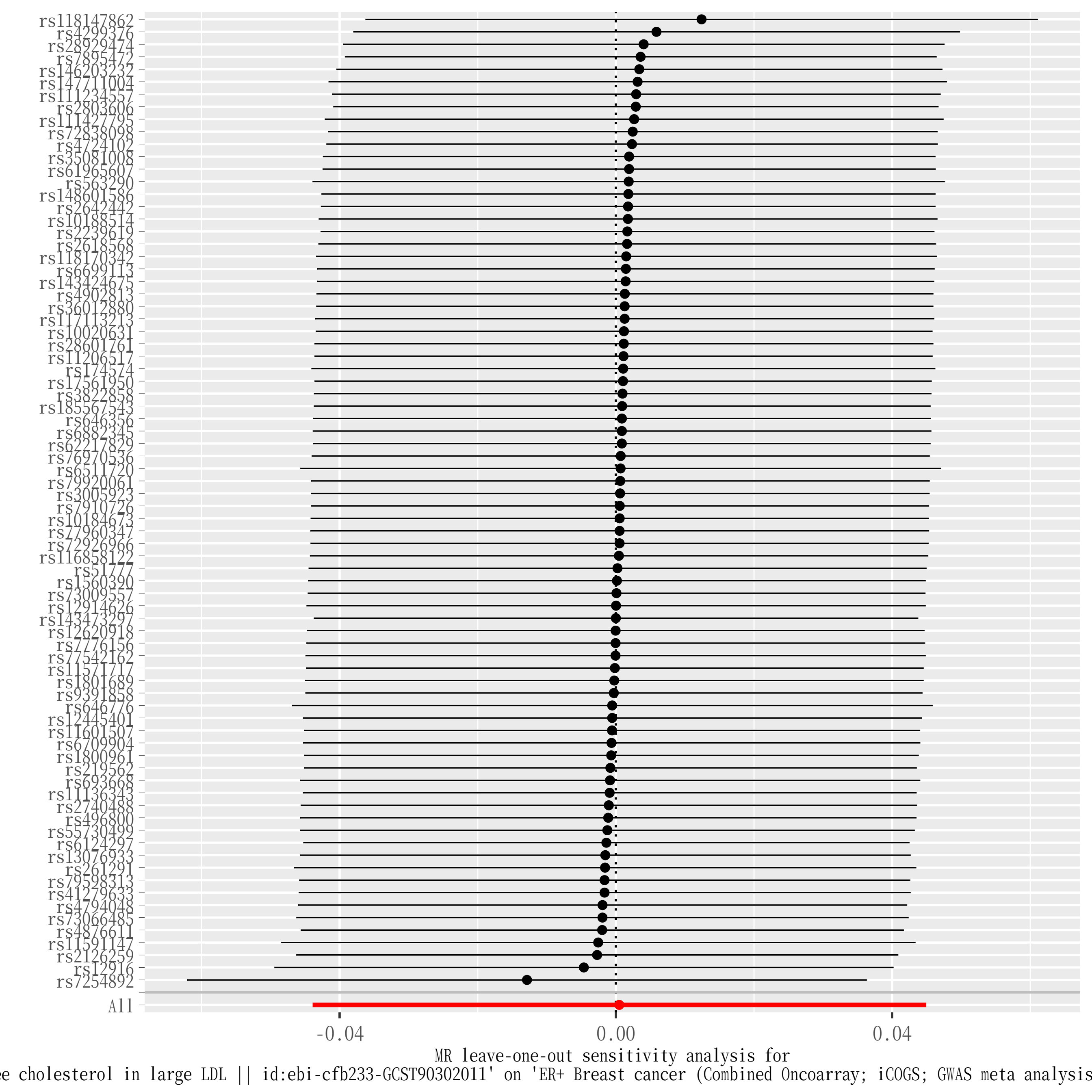
rs118147862
rs4299376
rs28929474
rs7895472
rs146203232
rs147711004
rs111234557
rs2803606
rs111427795
rs72838098
rs4724102
rs35081008
rs61965607
rs563290
rs148601586
rs2642442
rs10188514
rs2239619
rs2618568
rs118170342
rs6699113
rs143424675
rs4902813
rs36012880
rs117113213
rs10020631
rs28601761
rs11206517
rs174574
rs17561950
rs3822858
rs185567543
rs646356
rs6882345
rs62217829
rs76970536
rs6511720
rs79920061
rs3005923
rs7910726
rs10184673
rs77960347
rs72926966
rs116858122
rs51777
rs1560390
rs73009557
rs12914626
rs143473297
rs12620918
rs7776156
rs77542162
rs11571717
rs1801689
rs9391858
rs646776
rs12445401
rs11601507
rs6709904
rs1800961
rs219562
rs693668
rs11136343
rs2740488
rs496800
rs55730499
rs6124297
rs13076933
rs261291
rs79598313
rs41279633
rs4794048
rs73066485
rs4876611
rs11591147
rs2126259
rs12916
rs7254892
All
-0.04
0.00
0.04
MR leave-one-out sensitivity analysis for
ee cholesterol in large LDL || id:ebi-cfb233-GCST90302011' on 'ER+ Breast cancer (Combined Oncoarray; iCOGS; GWAS meta analysis

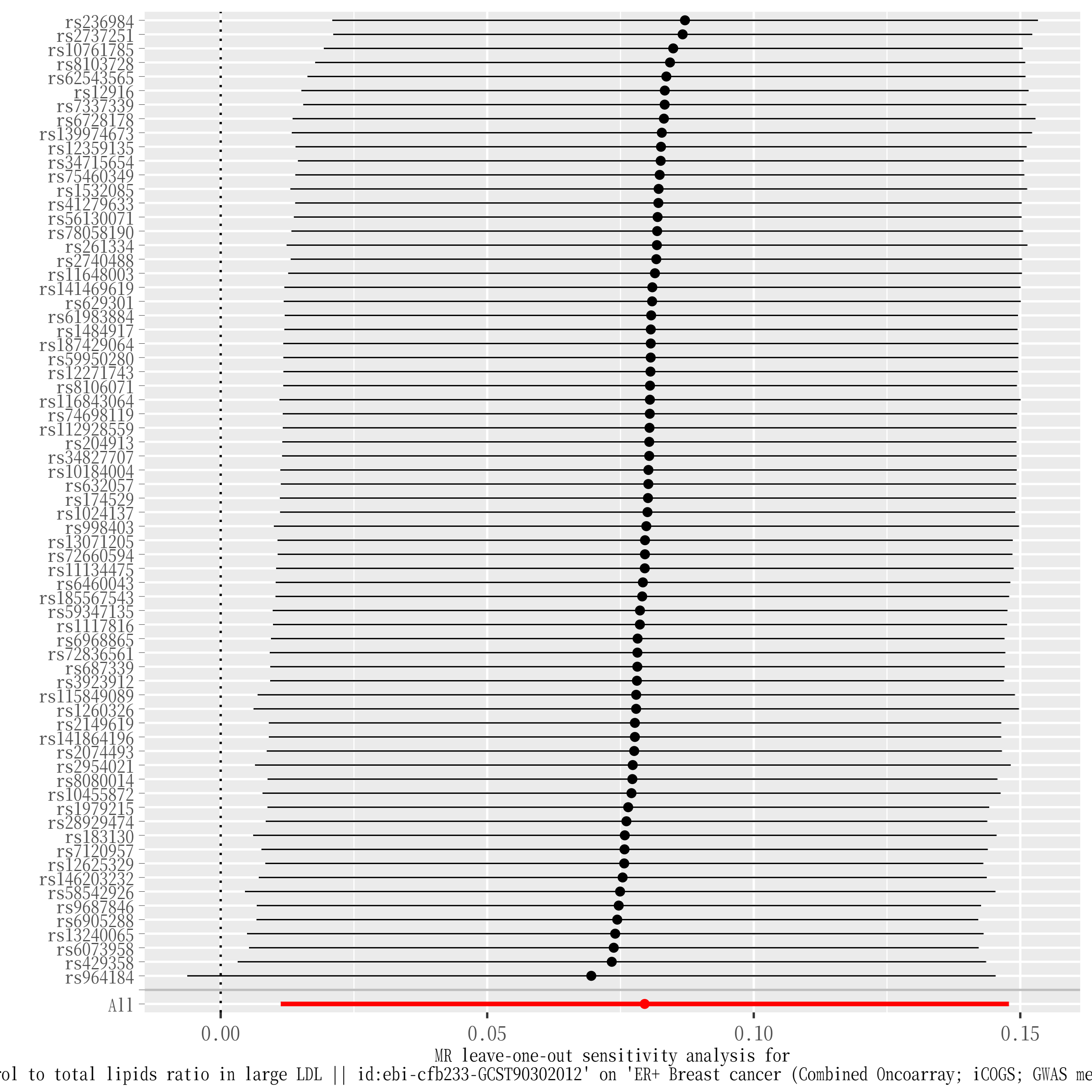
rs236984
rs2737251
rs10761785
rs8103728
rs62543565
rs12916
rs7337339
rs6728178
rs139974673
rs12359135
rs34715654
rs75460349
rs1532085
rs41279633
rs56130071
rs78058190
rs261334
rs2740488
rs11648003
rs141469619
rs629301
rs61983884
rs1484917
rs187429064
rs59950280
rs12271743
rs8106071
rs116843064
rs74698119
rs112928559
rs204913
rs34827707
rs10184004
rs632057
rs174529
rs1024137
rs998403
rs13071205
rs72660594
rs11134475
rs6460043
rs185567543
rs59347135
rs1117816
rs6968865
rs72836561
rs687339
rs3923912
rs115849089
rs1260326
rs2149619
rs141864196
rs2074493
rs2954021
rs8080014
rs10455872
rs1979215
rs28929474
rs183130
rs7120957
rs12625329
rs146203232
rs58542926
rs9687846
rs6905288
rs13240065
rs6073958
rs429358
rs964184
All
0.00
0.05
0.10
0.15
MR leave-one-out sensitivity analysis for
rol to total lipids ratio in large LDL || id:ebi-cfb233-GCST90302012' on 'ER+ Breast cancer (Combined Oncoarray; iCOGS; GWAS me

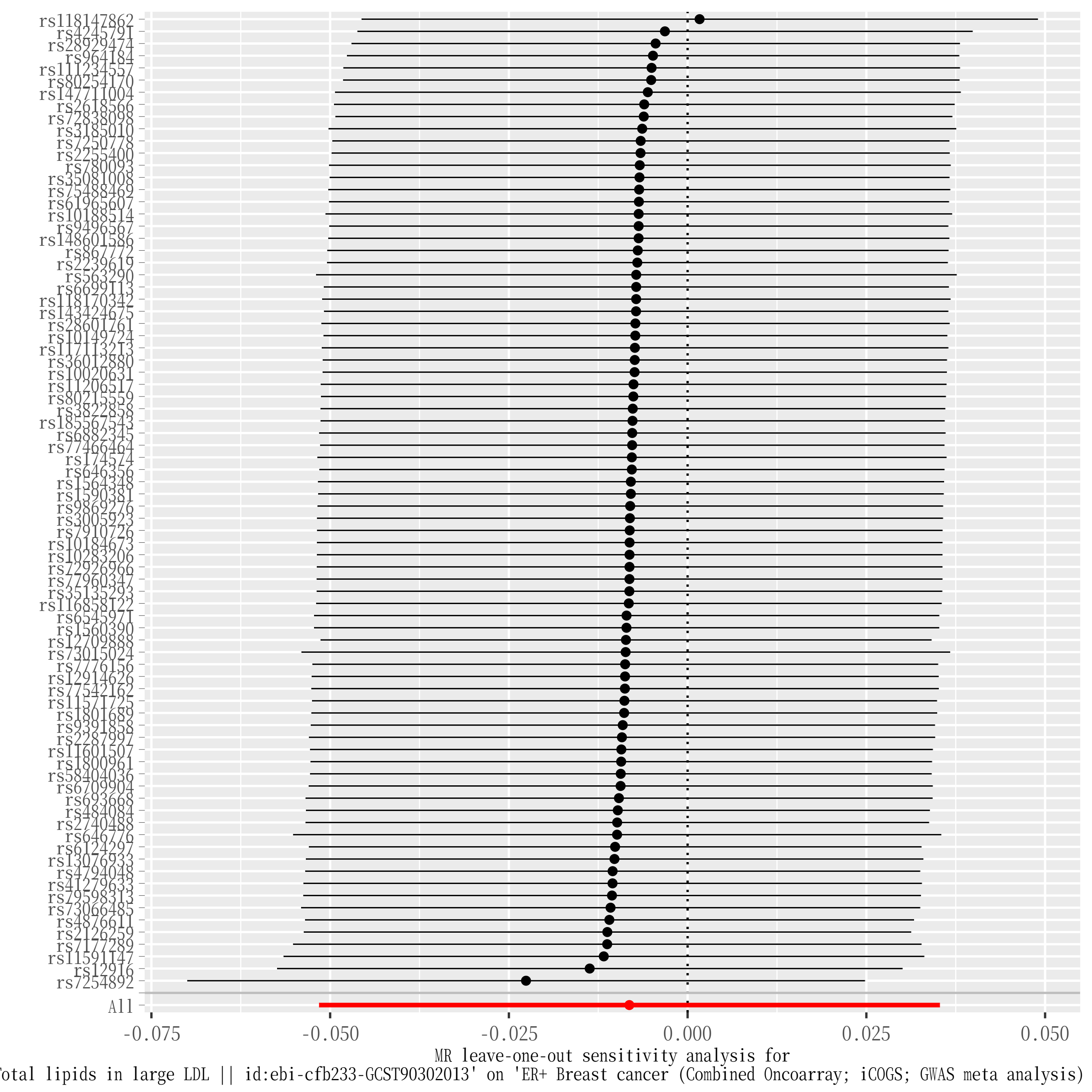
rs118147862
rs4245791
rs28929474
rs964184
rs111234557
rs80254170
rs147711004
rs2618566
rs72838098
rs3185010
rs7250778
rs2255400
rs780093
rs35081008
rs75488462
rs61965607
rs10188514
rs9496567
rs148601586
rs867772
rs2239619
rs563290
rs6699113
rs118170342
rs143424675
rs28601761
rs10149724
rs117113213
rs36012880
rs10020631
rs11206517
rs80215559
rs3822858
rs185567543
rs6882345
rs77466464
rs174574
rs646356
rs1564348
rs1590381
rs9869276
rs3005923
rs7910726
rs10184673
rs10283206
rs72926966
rs77960347
rs35135293
rs116858122
rs6545971
rs1560390
rs12709888
rs73015024
rs7776156
rs12914626
rs77542162
rs11571725
rs1801689
rs9391858
rs2287997
rs11601507
rs1800961
rs58404036
rs6709904
rs693668
rs484084
rs2740488
rs646776
rs6124297
rs13076933
rs4794048
rs41279633
rs79598313
rs73066485
rs4876611
rs2126259
rs7177289
rs11591147
rs12916
rs7254892
All
-0.075
-0.050
-0.025
0.000
0.025
0.050
MR leave-one-out sensitivity analysis for
'Total lipids in large LDL || id:ebi-cfb233-GCST90302013' on 'ER+ Breast cancer (Combined Oncoarray; iCOGS; GWAS meta analysis)'

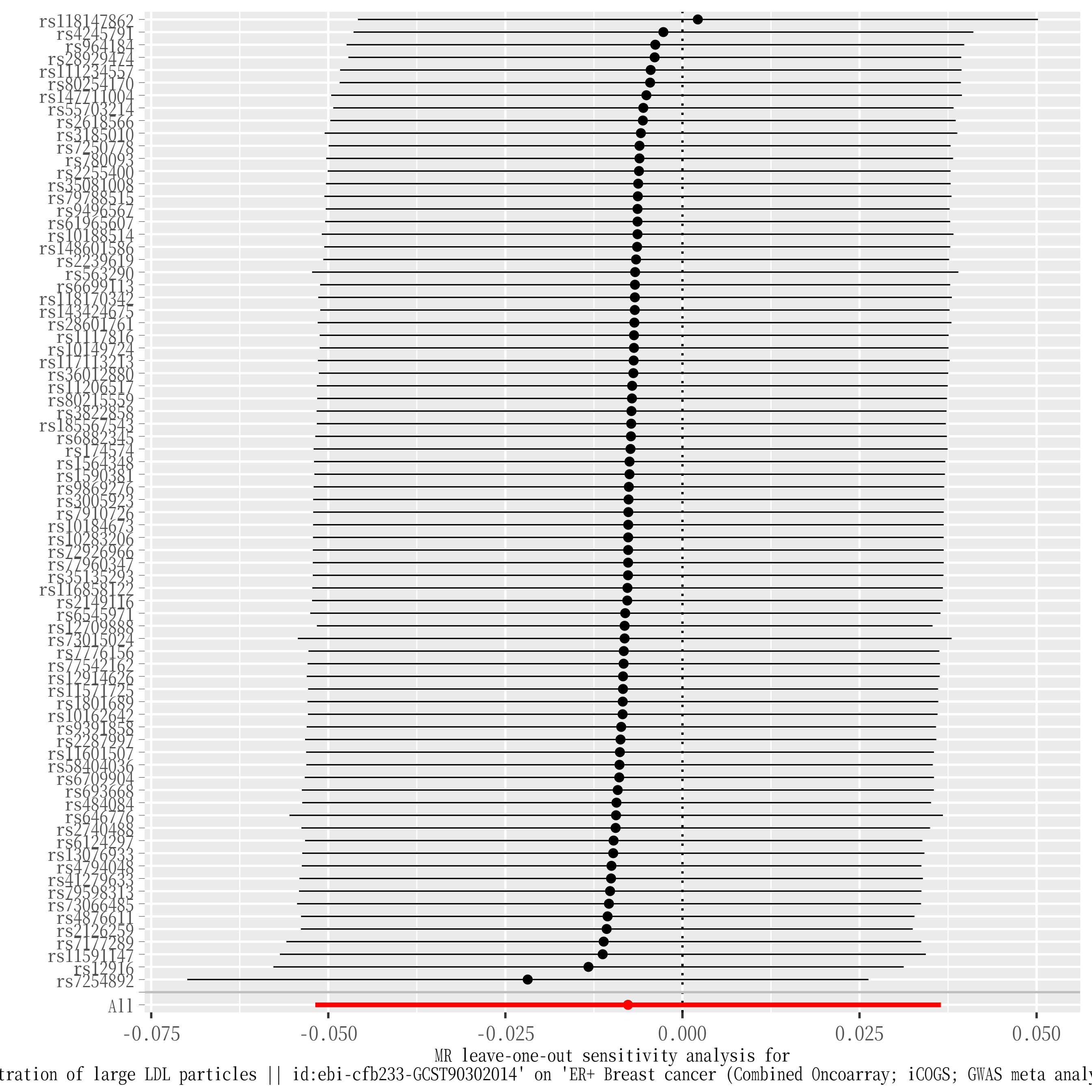

rs118147862
rs4245791
rs964184
rs28929474
rs111234557
rs80254170
rs147711004
rs55703214
rs2618566
rs3185010
rs7250778
rs780093
rs2255400
rs35081008
rs79788515
rs9496567
rs61965607
rs10188514
rs148601586
rs2239619
rs563290
rs6699113
rs118170342
rs143424675
rs28601761
rs1117816
rs10149724
rs117113213
rs36012880
rs11206517
rs80215559
rs3822858
rs185567543
rs6882345
rs174574
rs1564348
rs1590381
rs9869276
rs3005923
rs7910726
rs10184673
rs10283206
rs72926966
rs77960347
rs35135293
rs116858122
rs2149116
rs6545971
rs12709888
rs73015024
rs7776156
rs77542162
rs12914626
rs11571725
rs1801689
rs10162642
rs9391858
rs2287997
rs11601507
rs58404036
rs6709904
rs693668
rs484084
rs646776
rs2740488
rs6124297
rs13076933
rs4794048
rs41279633
rs79598313
rs73066485
rs4876611
rs2126259
rs7177289
rs11591147
rs12916
rs7254892
All
-0.075
-0.050
-0.025
0.000
0.025
0.050
MR leave-one-out sensitivity analysis for
tration of large LDL particles || id:ebi-cfb233-GCST90302014' on 'ER+ Breast cancer (Combined Oncoarray; iCOGS; GWAS meta analy

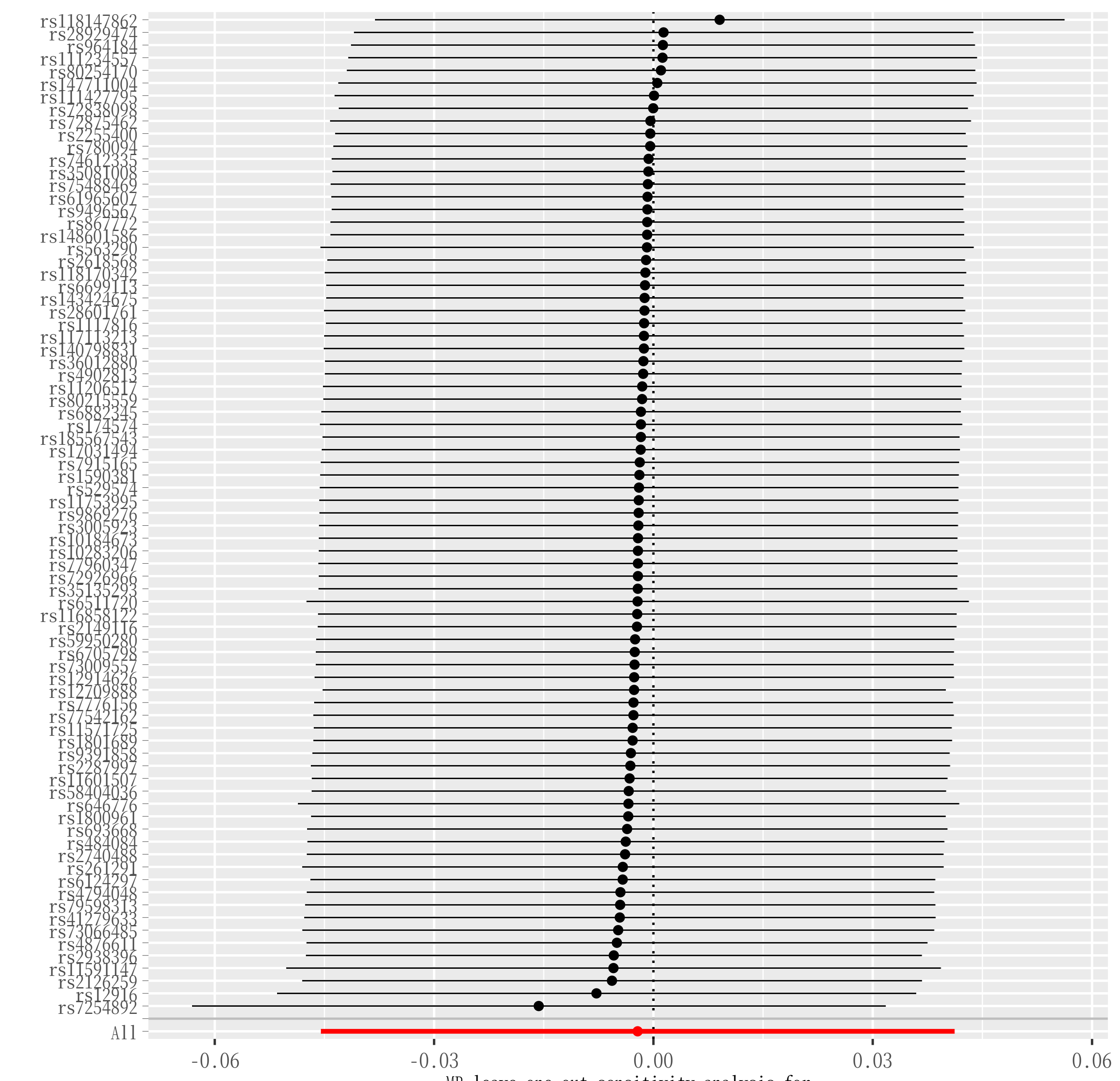

MR leave-one-out sensitivity analysis for
'Phospholipids in large LDL || id:ebi-cfb233-GCST90302015' on 'ER+ Breast cancer (Combined Oncoarray; iCOGS; GWAS meta analysis)'

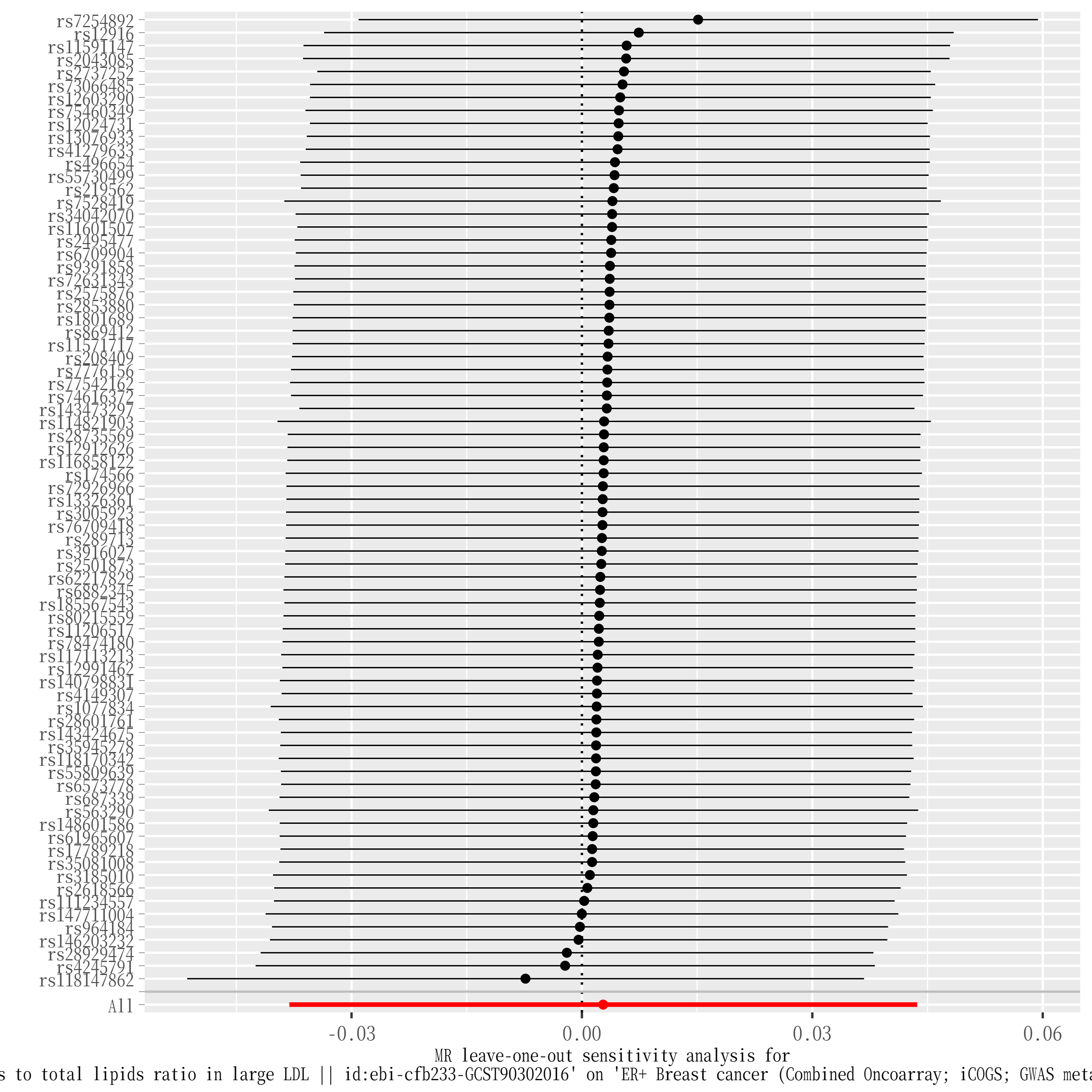

rs7254892
rs12916
rs11591147
rs2043085
rs2737252
rs73066485
rs12603290
rs75460349
rs12024731
rs13076933
rs41279633
rs496654
rs55730499
rs219562
rs7528419
rs34042070
rs11601507
rs2495477
rs6709904
rs9391858
rs72631343
rs2575876
rs2853880
rs1801689
rs869412
rs11571717
rs208409
rs7776156
rs77542162
rs74616372
rs143473297
rs114821903
rs28735569
rs12912626
rs116858122
rs174566
rs72926966
rs13326361
rs3005923
rs76709418
rs289713
rs3916027
rs2501873
rs62217829
rs6882345
rs185567543
rs80215559
rs11206517
rs78474180
rs117113213
rs12991462
rs140798831
rs4149307
rs1077834
rs28601761
rs143424675
rs35945278
rs118170342
rs55809639
rs6573778
rs687339
rs563290
rs148601586
rs61965607
rs17789218
rs35081008
rs3185010
rs2618566
rs111234557
rs147711004
rs964184
rs146203232
rs28929474
rs4245791
rs118147862
All
-0.03
0.00
0.03
0.06
MR leave-one-out sensitivity analysis for
s to total lipids ratio in large LDL || id:ebi-cfb233-GCST90302016' on 'ER+ Breast cancer (Combined Oncoarray; iCOGS; GWAS meta

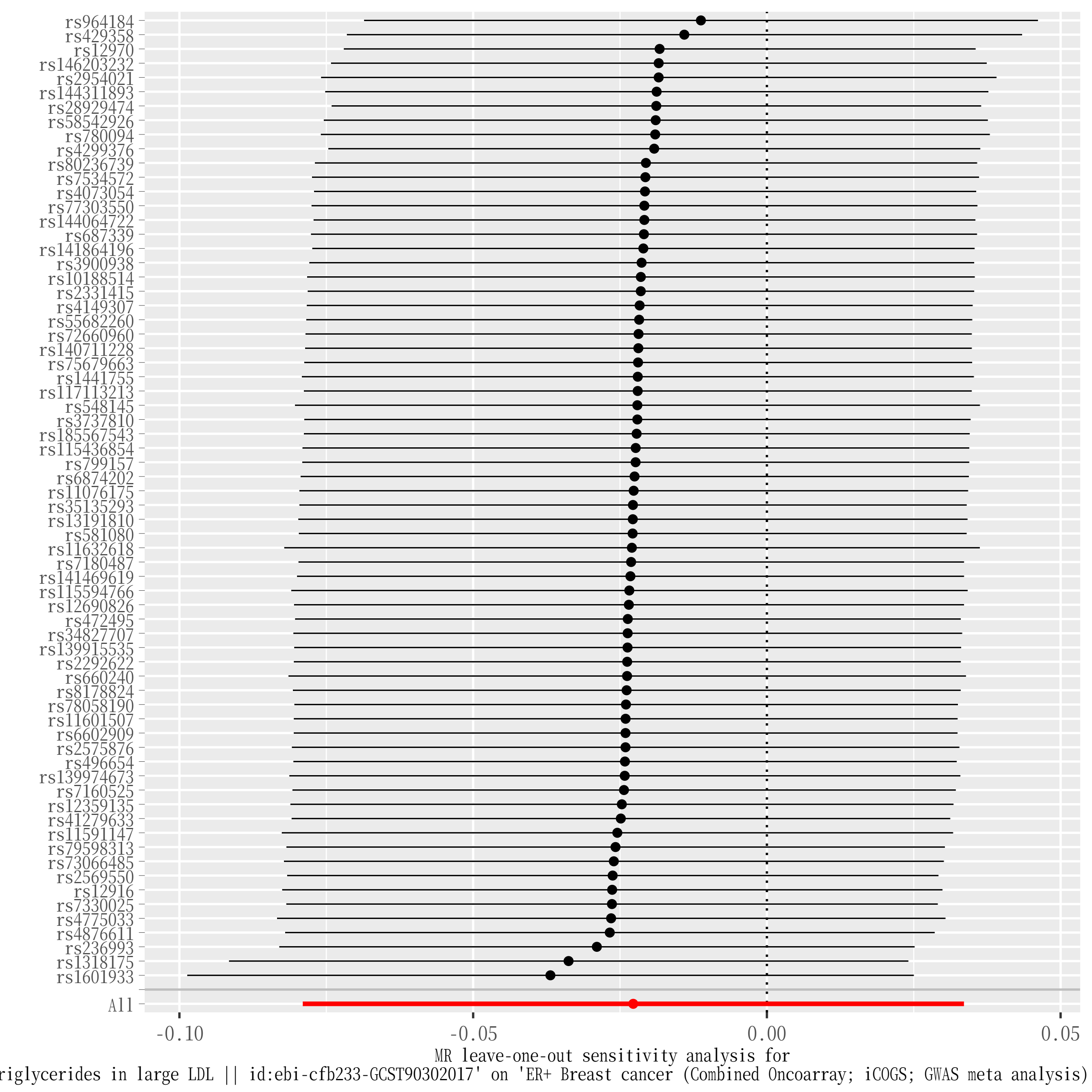
rs964184
rs429358
rs12970
rs146203232
rs2954021
rs144311893
rs28929474
rs58542926
rs780094
rs4299376
rs80236739
rs7534572
rs4073054
rs77303550
rs144064722
rs687339
rs141864196
rs3900938
rs10188514
rs2331415
rs4149307
rs55682260
rs72660960
rs140711228
rs75679663
rs1441755
rs117113213
rs548145
rs3737810
rs185567543
rs115436854
rs799157
rs6874202
rs11076175
rs35135293
rs13191810
rs581080
rs11632618
rs7180487
rs141469619
rs115594766
rs12690826
rs472495
rs34827707
rs139915535
rs2292622
rs660240
rs8178824
rs78058190
rs11601507
rs6602909
rs2575876
rs496654
rs139974673
rs7160525
rs12359135
rs41279633
rs11591147
rs79598313
rs73066485
rs2569550
rs12916
rs7330025
rs4775033
rs4876611
rs236993
rs1318175
rs1601933
All
-0.10
-0.05
0.00
0.05
MR leave-one-out sensitivity analysis for
riglycerides in large LDL || id:ebi-cfb233-GCST90302017' on 'ER+ Breast cancer (Combined Oncoarray; iCOGS; GWAS meta analysis)

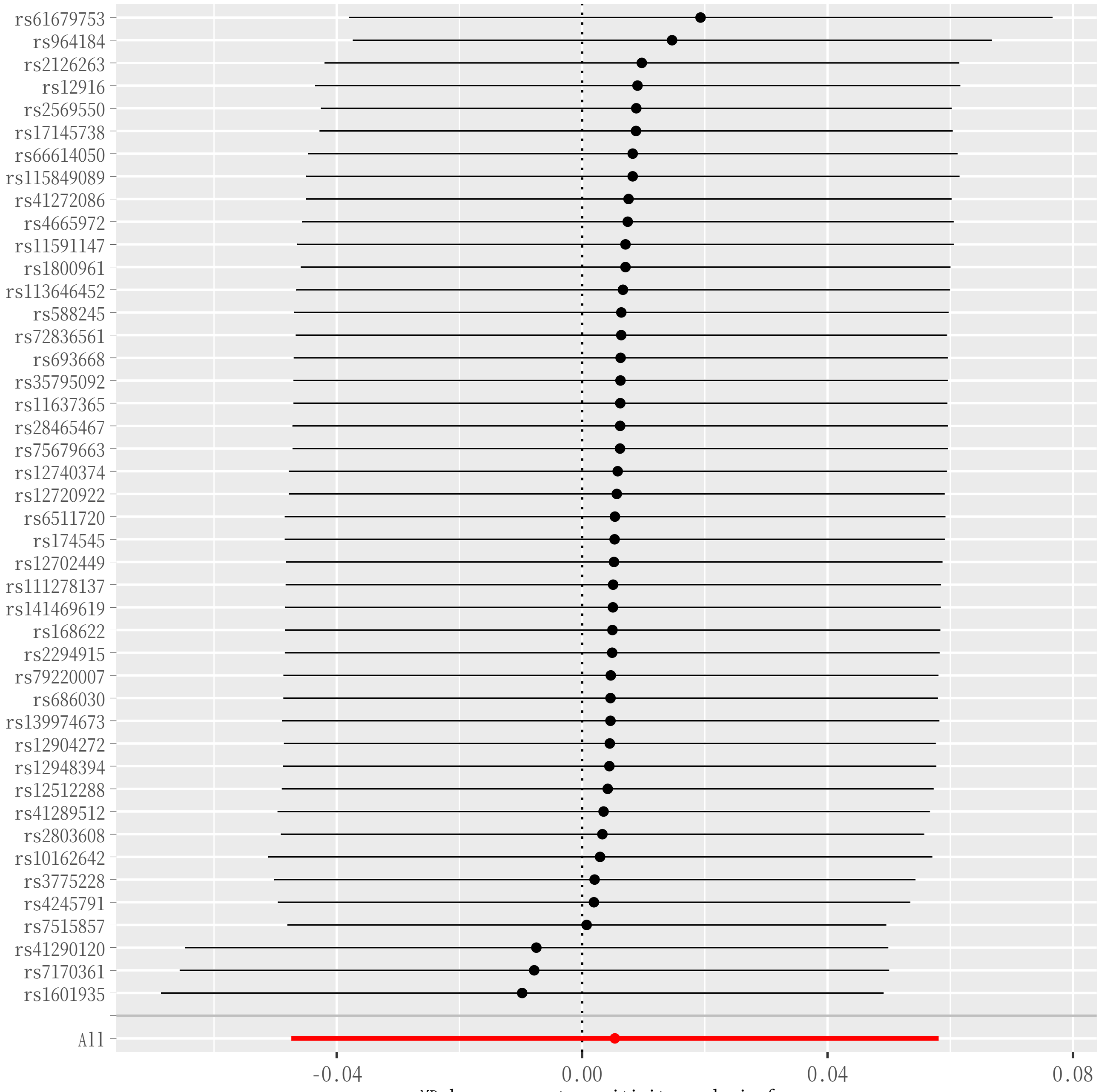

MR leave-one-out sensitivity analysis for
s to total lipids ratio in large LDL || id:ebi-cfb233-GCST90302018' on 'ER+ Breast cancer (Combined Oncoarray; iCOGS; GWAS meta

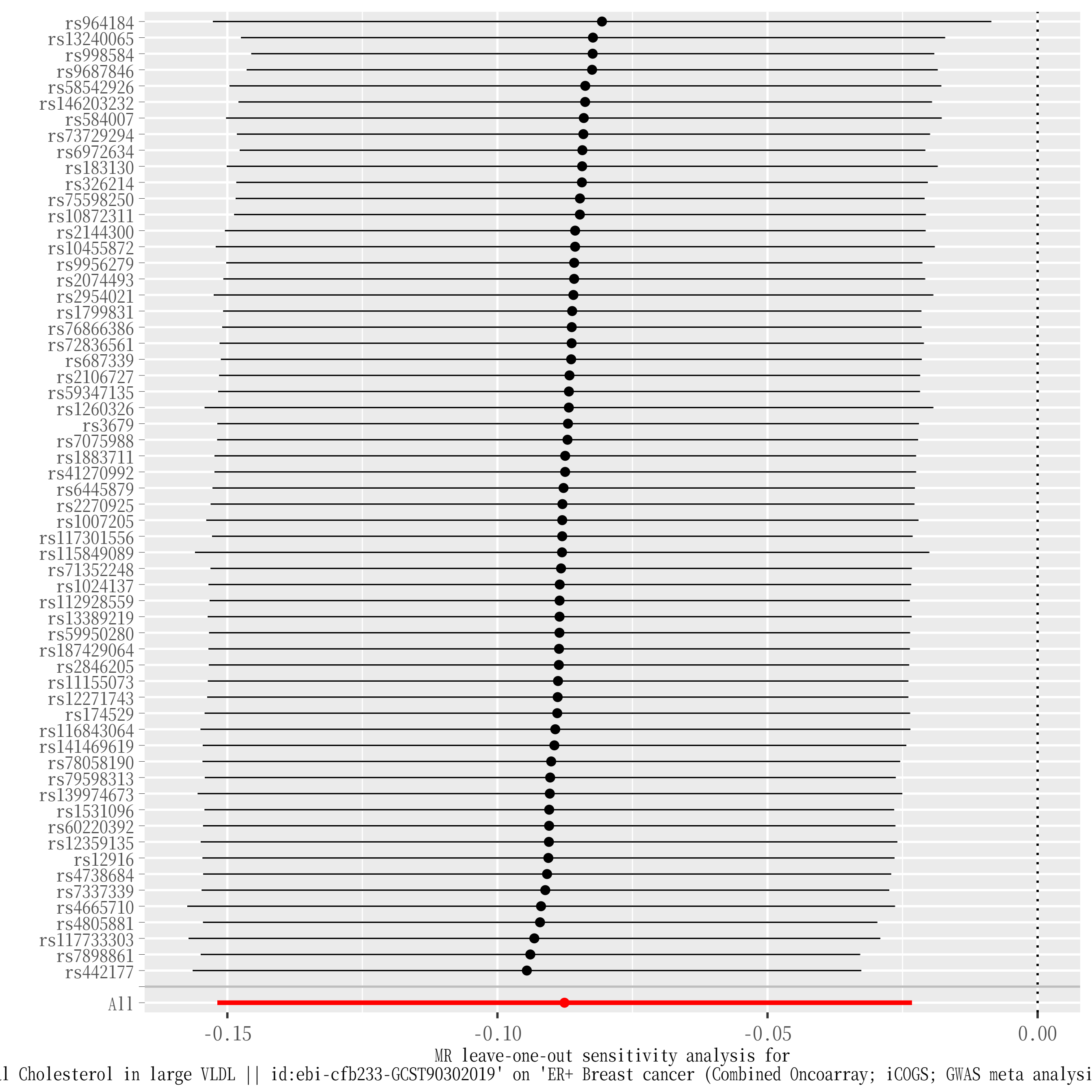
rs964184
rs13240065
rs998584
rs9687846
rs58542926
rs146203232
rs584007
rs73729294
rs6972634
rs183130
rs326214
rs75598250
rs10872311
rs2144300
rs10455872
rs9956279
rs2074493
rs2954021
rs1799831
rs76866386
rs72836561
rs687339
rs2106727
rs59347135
rs1260326
rs3679
rs7075988
rs1883711
rs41270992
rs6445879
rs2270925
rs1007205
rs117301556
rs115849089
rs71352248
rs1024137
rs112928559
rs13389219
rs59950280
rs187429064
rs2846205
rs11155073
rs12271743
rs174529
rs116843064
rs141469619
rs78058190
rs79598313
rs139974673
rs1531096
rs60220392
rs12359135
rs12916
rs4738684
rs7337339
rs4665710
rs4805881
rs117733303
rs7898861
rs442177
All
-0.15
-0.10
-0.05
0.00
MR leave-one-out sensitivity analysis for
l Cholesterol in large VLDL || id:ebi-cfb233-GCST90302019' on 'ER+ Breast cancer (Combined Oncoarray; iCOGS; GWAS meta analysi

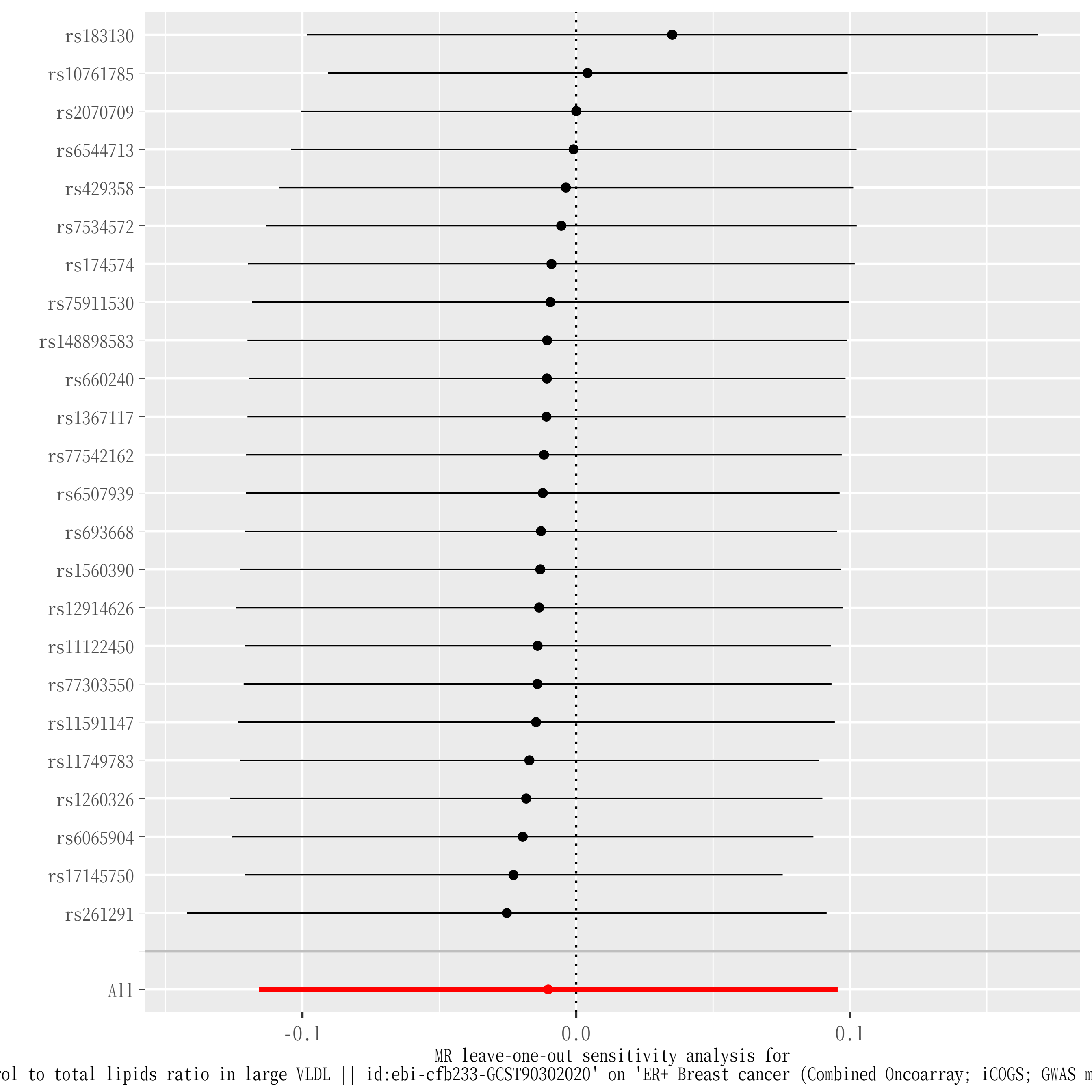
rs183130
rs10761785
rs2070709
rs6544713
rs429358
rs7534572
rs174574
rs75911530
rs148898583
rs660240
rs1367117
rs77542162
rs6507939
rs693668
rs1560390
rs12914626
rs11122450
rs77303550
rs11591147
rs11749783
rs1260326
rs6065904
rs17145750
rs261291
All
-0.1
0.0
0.1
MR leave-one-out sensitivity analysis for
rol to total lipids ratio in large VLDL || id:ebi-cfb233-GCST90302020' on 'ER+ Breast cancer (Combined Oncoarray; iCOGS; GWAS m

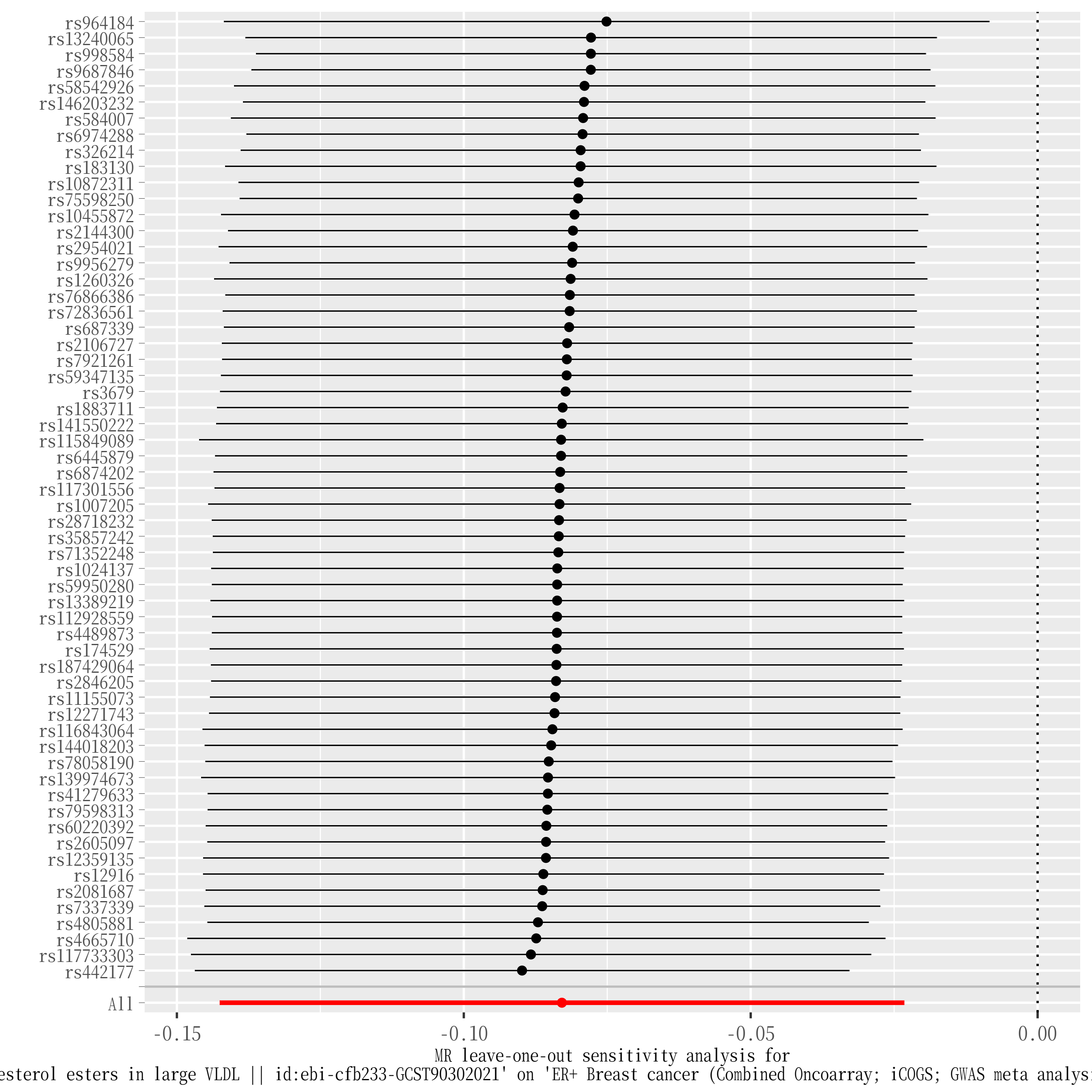
rs964184
rs13240065
rs998584
rs9687846
rs58542926
rs146203232
rs584007
rs6974288
rs326214
rs183130
rs10872311
rs75598250
rs10455872
rs2144300
rs2954021
rs9956279
rs1260326
rs76866386
rs72836561
rs687339
rs2106727
rs7921261
rs59347135
rs3679
rs1883711
rs141550222
rs115849089
rs6445879
rs6874202
rs117301556
rs1007205
rs28718232
rs35857242
rs71352248
rs1024137
rs59950280
rs13389219
rs112928559
rs4489873
rs174529
rs187429064
rs2846205
rs11155073
rs12271743
rs116843064
rs144018203
rs78058190
rs139974673
rs41279633
rs79598313
rs60220392
rs2605097
rs12359135
rs12916
rs2081687
rs7337339
rs4805881
rs4665710
rs117733303
rs442177
All
-0.15
-0.10
-0.05
0.00
MR leave-one-out sensitivity analysis for
esterol esters in large VLDL || id:ebi-cfb233-GCST90302021' on 'ER+ Breast cancer (Combined Oncoarray; iCOGS; GWAS meta analys

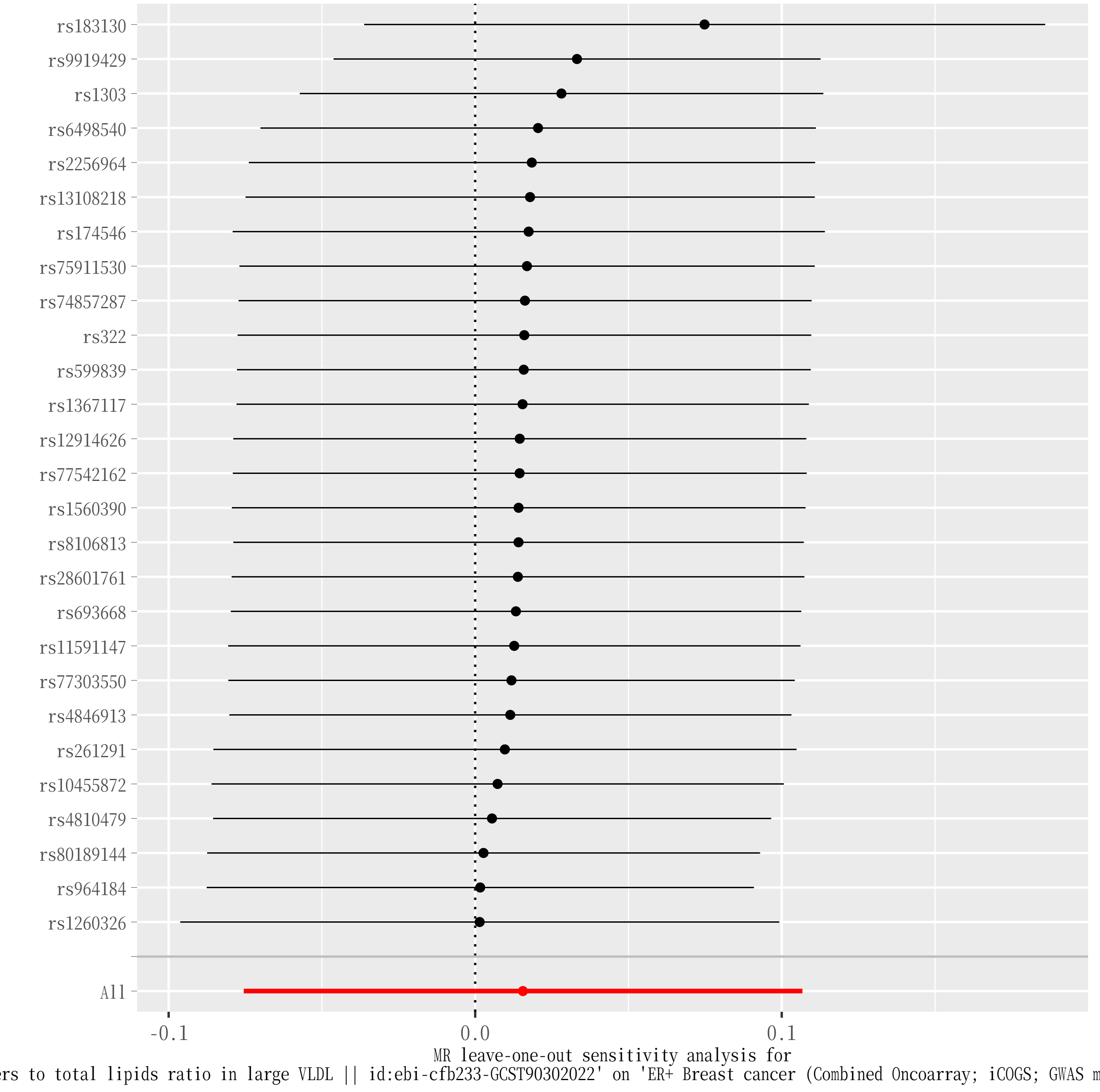

rs183130
rs9919429
rs1303
rs6498540
rs2256964
rs13108218
rs174546
rs75911530
rs74857287
rs322
rs599839
rs1367117
rs12914626
rs77542162
rs1560390
rs8106813
rs28601761
rs693668
rs11591147
rs77303550
rs4846913
rs261291
rs10455872
rs4810479
rs80189144
rs964184
rs1260326
All
-0.1
0.0
0.1
MR leave-one-out sensitivity analysis for
ers to total lipids ratio in large VLDL || id:ebi-cfb233-GCST90302022' on 'ER+ Breast cancer (Combined Oncoarray; iCOGS; GWAS m

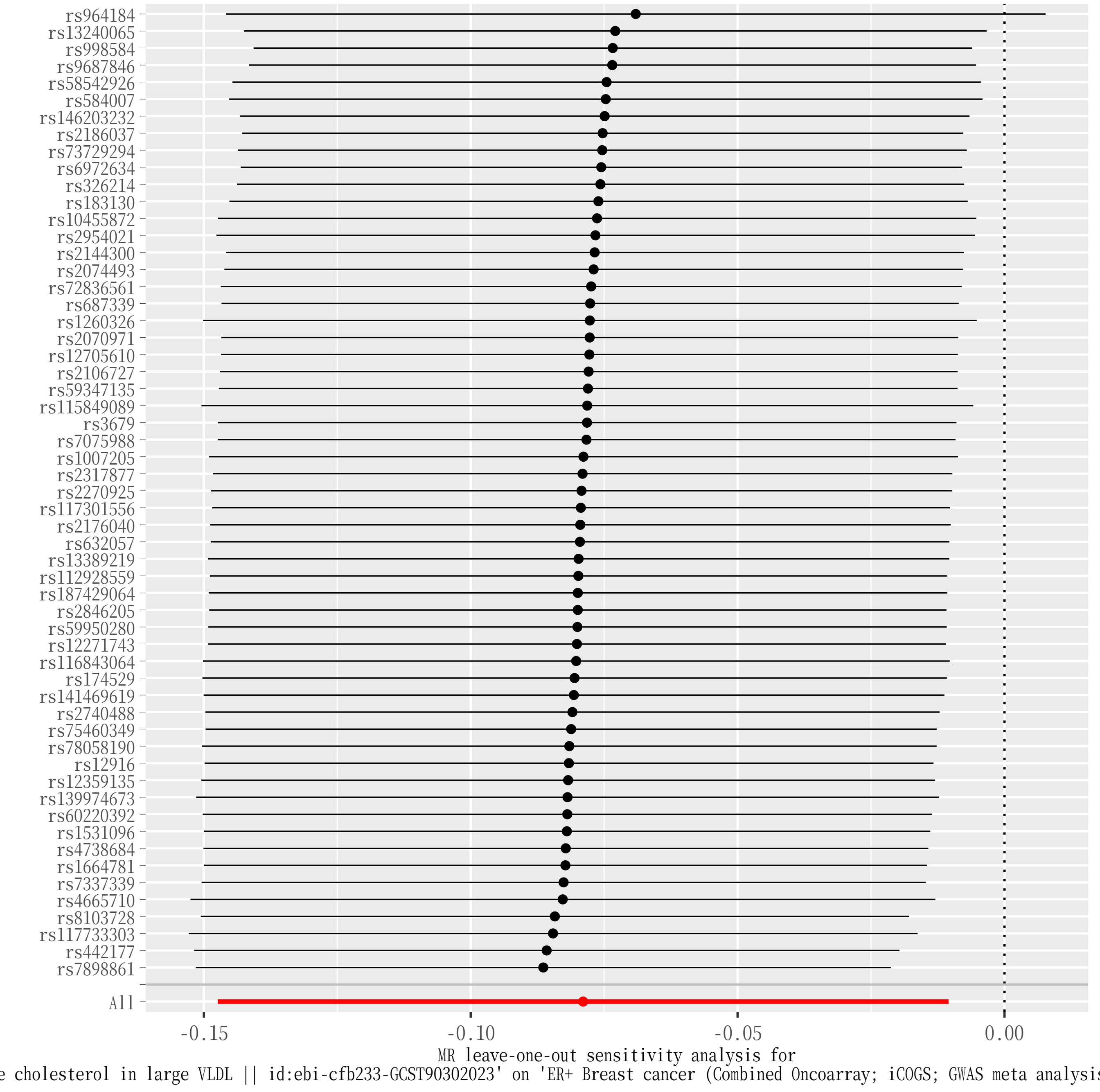
rs964184
rs13240065
rs998584
rs9687846
rs58542926
rs584007
rs146203232
rs2186037
rs73729294
rs6972634
rs326214
rs183130
rs10455872
rs2954021
rs2144300
rs2074493
rs72836561
rs687339
rs1260326
rs2070971
rs12705610
rs2106727
rs59347135
rs115849089
rs3679
rs7075988
rs1007205
rs2317877
rs2270925
rs117301556
rs2176040
rs632057
rs13389219
rs112928559
rs187429064
rs2846205
rs59950280
rs12271743
rs116843064
rs174529
rs141469619
rs2740488
rs75460349
rs78058190
rs12916
rs12359135
rs139974673
rs60220392
rs1531096
rs4738684
rs1664781
rs7337339
rs4665710
rs8103728
rs117733303
rs442177
rs7898861
All
-0.15
-0.10
-0.05
0.00
MR leave-one-out sensitivity analysis for
e cholesterol in large VLDL || id:ebi-cfb233-GCST90302023' on 'ER+ Breast cancer (Combined Oncoarray; iCOGS; GWAS meta analysis

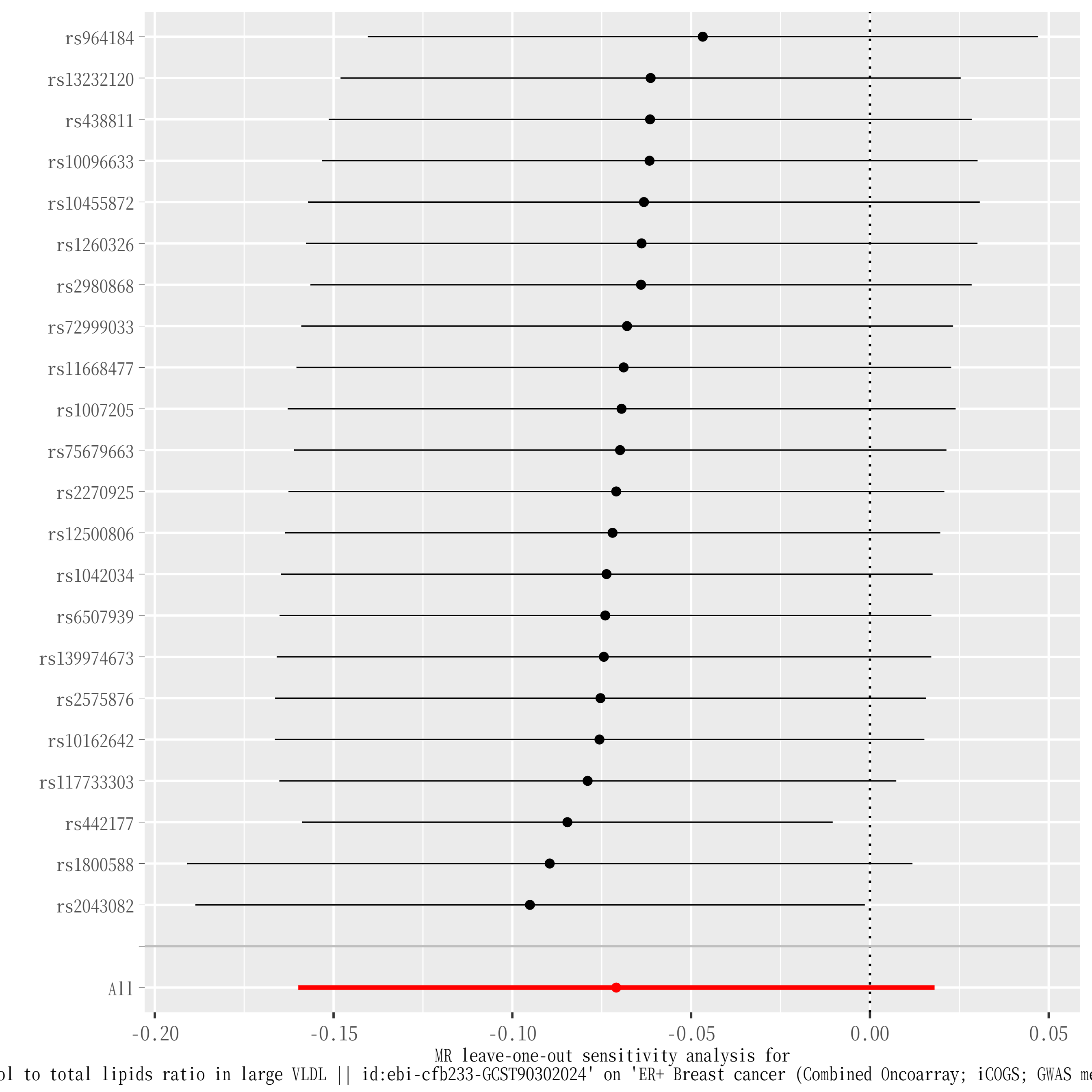
rs964184
rs13232120
rs438811
rs10096633
rs10455872
rs1260326
rs2980868
rs72999033
rs11668477
rs1007205
rs75679663
rs2270925
rs12500806
rs1042034
rs6507939
rs139974673
rs2575876
rs10162642
rs117733303
rs442177
rs1800588
rs2043082
All
-0.20
-0.15
-0.10
-0.05
0.00
0.05
MR leave-one-out sensitivity analysis for
ol to total lipids ratio in large VLDL || id:ebi-cfb233-GCST90302024' on 'ER+ Breast cancer (Combined Oncoarray; iCOGS; GWAS me

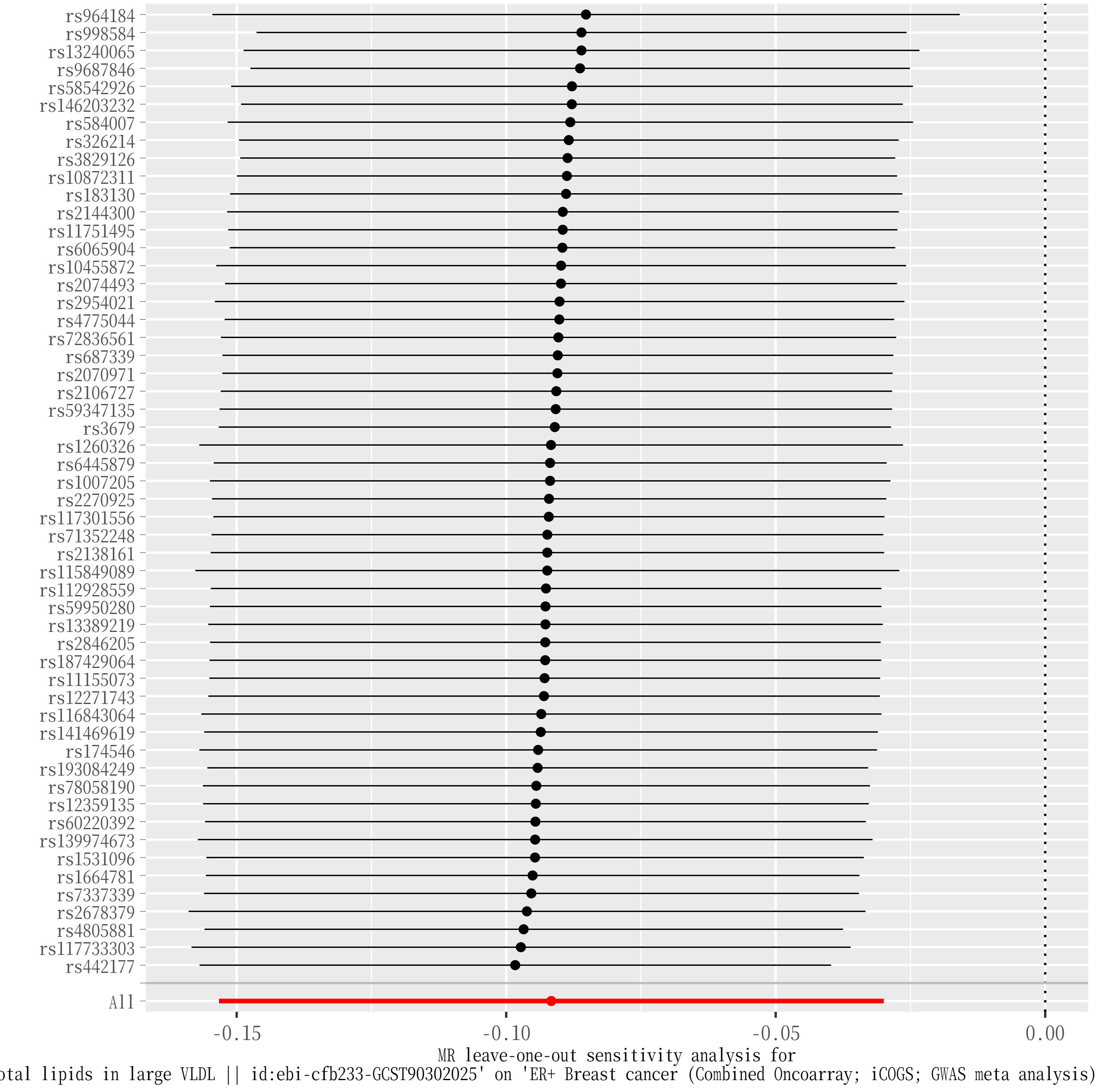
rs964184
rs998584
rs13240065
rs9687846
rs58542926
rs146203232
rs584007
rs326214
rs3829126
rs10872311
rs183130
rs2144300
rs11751495
rs6065904
rs10455872
rs2074493
rs2954021
rs4775044
rs72836561
rs687339
rs2070971
rs2106727
rs59347135
rs3679
rs1260326
rs6445879
rs1007205
rs2270925
rs117301556
rs71352248
rs2138161
rs115849089
rs112928559
rs59950280
rs13389219
rs2846205
rs187429064
rs11155073
rs12271743
rs116843064
rs141469619
rs174546
rs193084249
rs78058190
rs12359135
rs60220392
rs139974673
rs1531096
rs1664781
rs7337339
rs2678379
rs4805881
rs117733303
rs442177
All
-0.15
-0.10
-0.05
0.00
MR leave-one-out sensitivity analysis for
otal lipids in large VLDL || id:ebi-cfb233-GCST90302025' on 'ER+ Breast cancer (Combined Oncoarray; iCOGS; GWAS meta analysis)

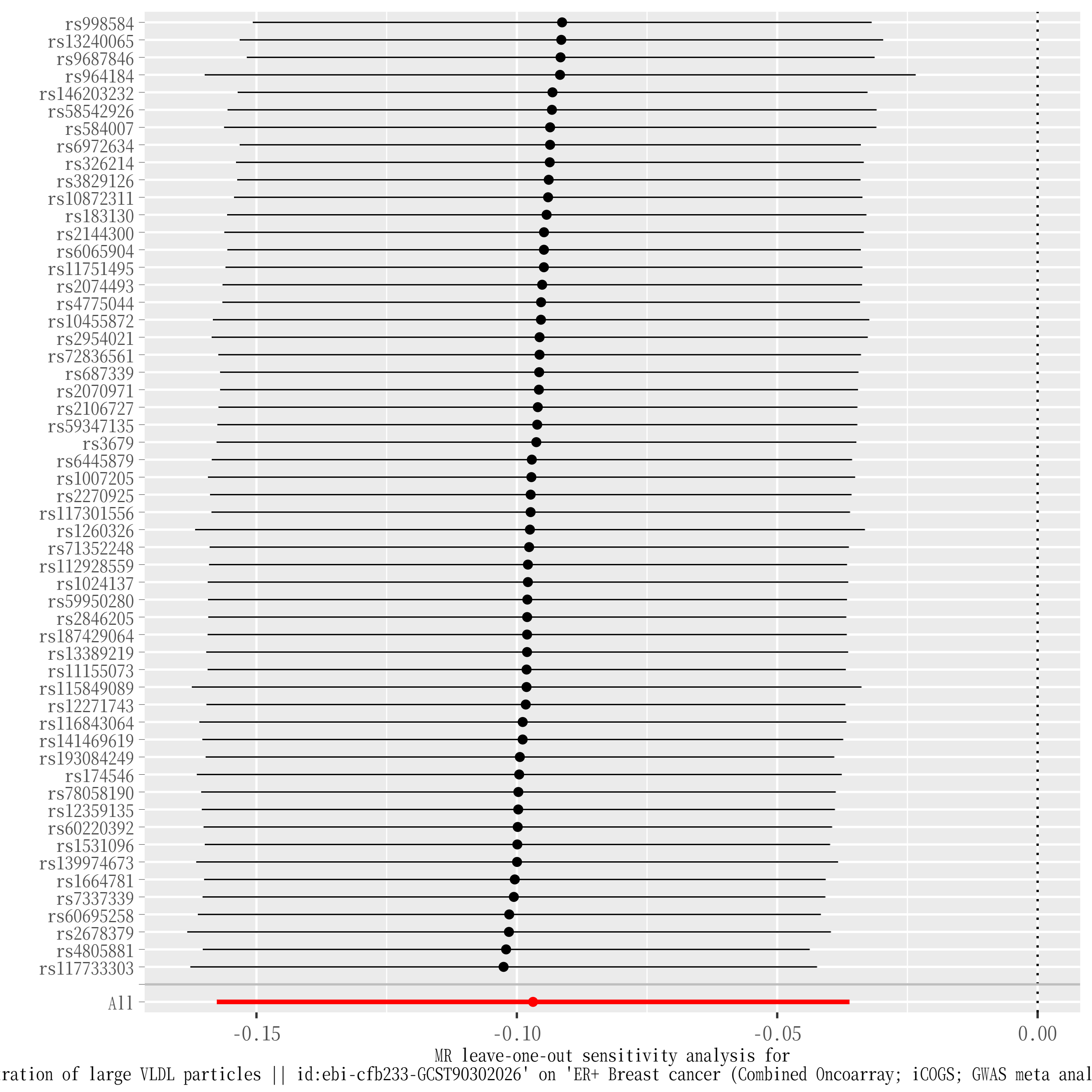
rs998584
rs13240065
rs9687846
rs964184
rs146203232
rs58542926
rs584007
rs6972634
rs326214
rs3829126
rs10872311
rs183130
rs2144300
rs6065904
rs11751495
rs2074493
rs4775044
rs10455872
rs2954021
rs72836561
rs687339
rs2070971
rs2106727
rs59347135
rs3679
rs6445879
rs1007205
rs2270925
rs117301556
rs1260326
rs71352248
rs112928559
rs1024137
rs59950280
rs2846205
rs187429064
rs13389219
rs11155073
rs115849089
rs12271743
rs116843064
rs141469619
rs193084249
rs174546
rs78058190
rs12359135
rs60220392
rs1531096
rs139974673
rs1664781
rs7337339
rs60695258
rs2678379
rs4805881
rs117733303
All
-0.15
-0.10
-0.05
0.00
MR leave-one-out sensitivity analysis for
ration of large VLDL particles || id:ebi-cfb233-GCST90302026' on 'ER+ Breast cancer (Combined Oncoarray; iCOGS; GWAS meta anal

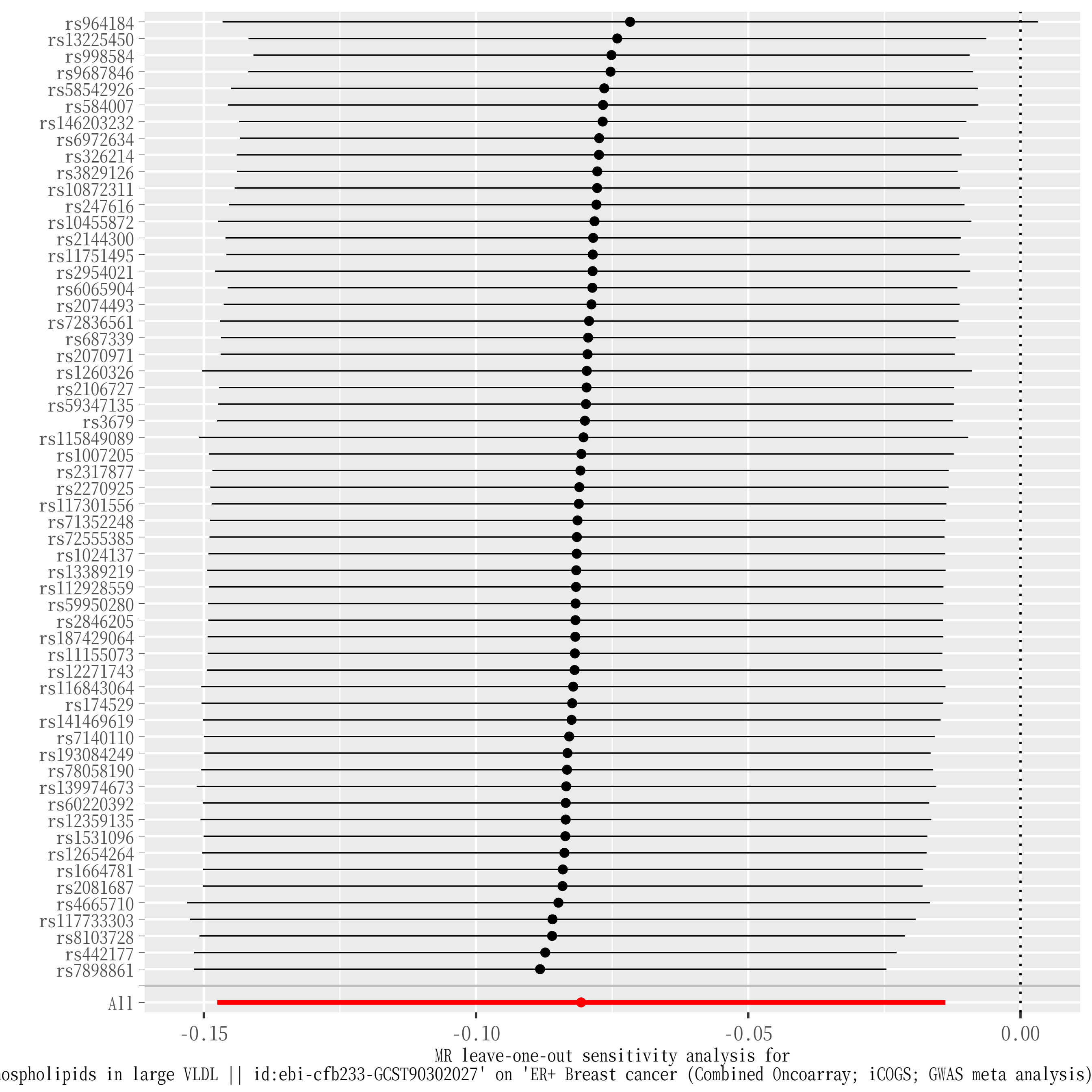
rs964184
rs13225450
rs998584
rs9687846
rs58542926
rs584007
rs146203232
rs6972634
rs326214
rs3829126
rs10872311
rs247616
rs10455872
rs2144300
rs11751495
rs2954021
rs6065904
rs2074493
rs72836561
rs687339
rs2070971
rs1260326
rs2106727
rs59347135
rs3679
rs115849089
rs1007205
rs2317877
rs2270925
rs117301556
rs71352248
rs72555385
rs1024137
rs13389219
rs112928559
rs59950280
rs2846205
rs187429064
rs11155073
rs12271743
rs116843064
rs174529
rs141469619
rs7140110
rs193084249
rs78058190
rs139974673
rs60220392
rs12359135
rs1531096
rs12654264
rs1664781
rs2081687
rs4665710
rs117733303
rs8103728
rs442177
rs7898861
All
-0.15
-0.10
-0.05
0.00
MR leave-one-out sensitivity analysis for
hospholipids in large VLDL || id:ebi-cfb233-GCST90302027' on 'ER+ Breast cancer (Combined Oncoarray; iCOGS; GWAS meta analysis)

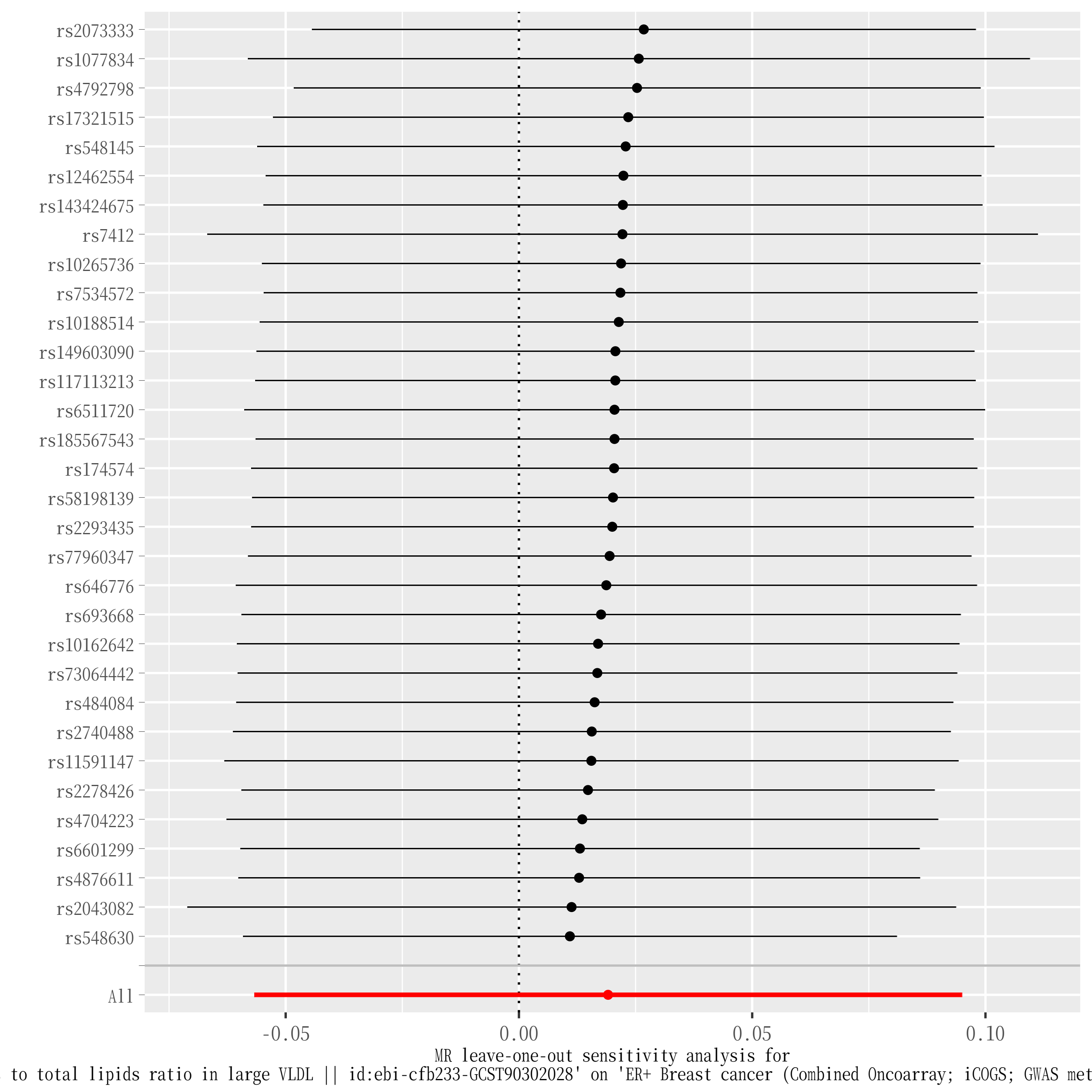
rs2073333
rs1077834
rs4792798
rs17321515
rs548145
rs12462554
rs143424675
rs7412
rs10265736
rs7534572
rs10188514
rs149603090
rs117113213
rs6511720
rs185567543
rs174574
rs58198139
rs2293435
rs77960347
rs646776
rs693668
rs10162642
rs73064442
rs484084
rs2740488
rs11591147
rs2278426
rs4704223
rs6601299
rs4876611
rs2043082
rs548630
All
-0.05
0.00
0.05
0.10
MR leave-one-out sensitivity analysis for
to total lipids ratio in large VLDL || id:ebi-cfb233-GCST90302028' on 'ER+ Breast cancer (Combined Oncoarray; iCOGS; GWAS met

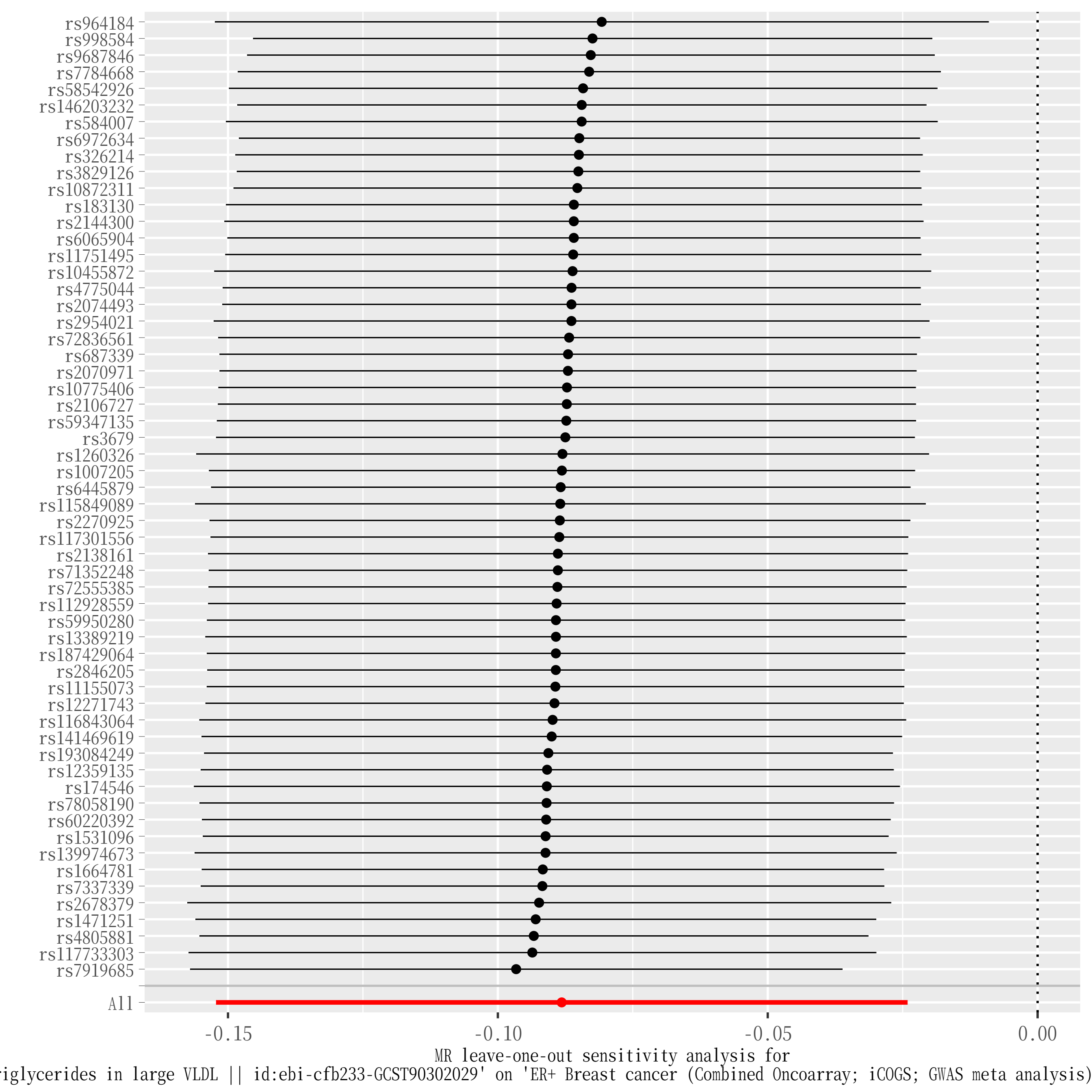
rs964184
rs998584
rs9687846
rs7784668
rs58542926
rs146203232
rs584007
rs6972634
rs326214
rs3829126
rs10872311
rs183130
rs2144300
rs6065904
rs11751495
rs10455872
rs4775044
rs2074493
rs2954021
rs72836561
rs687339
rs2070971
rs10775406
rs2106727
rs59347135
rs3679
rs1260326
rs1007205
rs6445879
rs115849089
rs2270925
rs117301556
rs2138161
rs71352248
rs72555385
rs112928559
rs59950280
rs13389219
rs187429064
rs2846205
rs11155073
rs12271743
rs116843064
rs141469619
rs193084249
rs12359135
rs174546
rs78058190
rs60220392
rs1531096
rs139974673
rs1664781
rs7337339
rs2678379
rs1471251
rs4805881
rs117733303
rs7919685
All
-0.15
-0.10
-0.05
0.00
MR leave-one-out sensitivity analysis for
riglycerides in large VLDL || id:ebi-cfb233-GCST90302029' on 'ER+ Breast cancer (Combined Oncoarray; iCOGS; GWAS meta analysis)

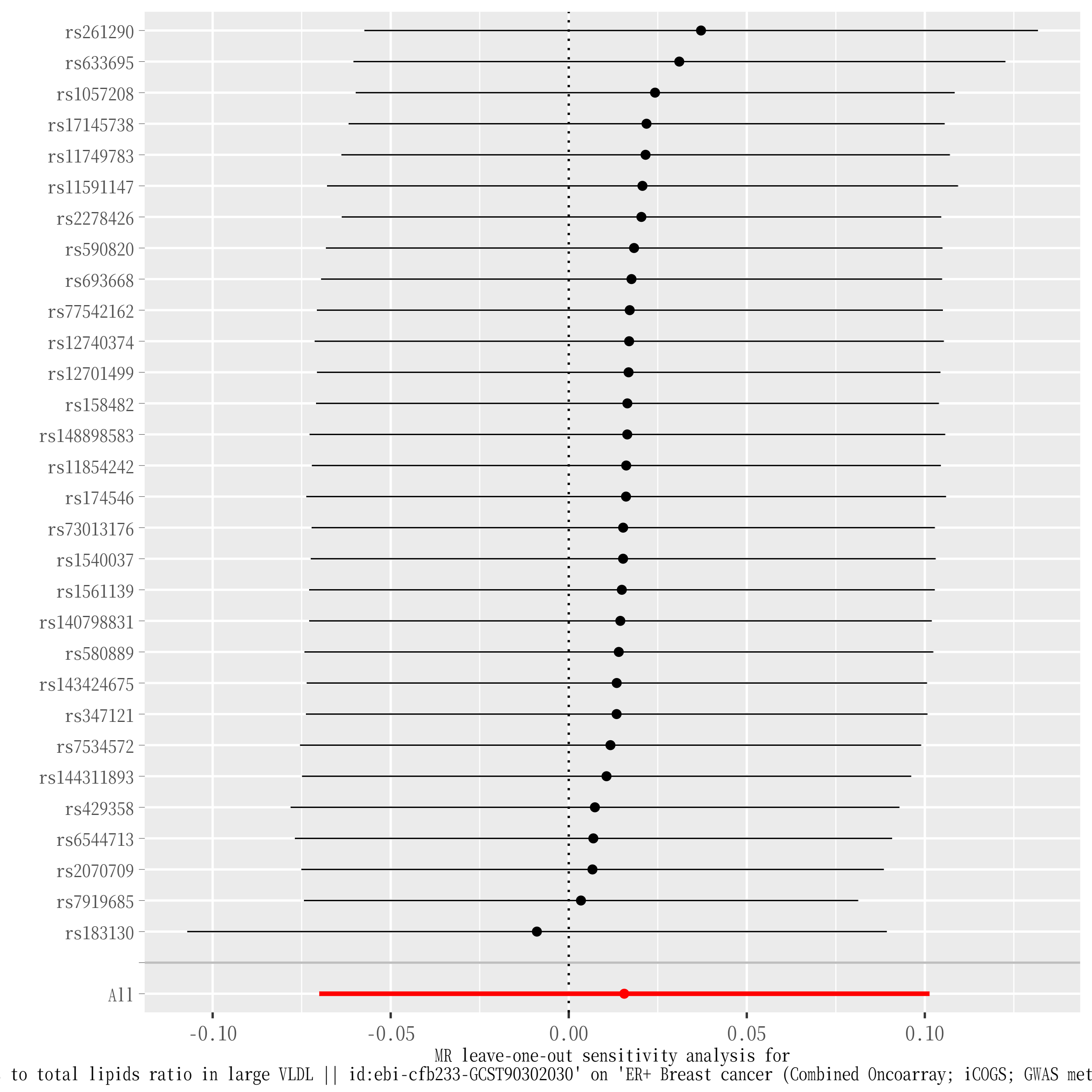
rs261290
rs633695
rs1057208
rs17145738
rs11749783
rs11591147
rs2278426
rs590820
rs693668
rs77542162
rs12740374
rs12701499
rs158482
rs148898583
rs11854242
rs174546
rs73013176
rs1540037
rs1561139
rs140798831
rs580889
rs143424675
rs347121
rs7534572
rs144311893
rs429358
rs6544713
rs2070709
rs7919685
rs183130
All
-0.10
-0.05
0.00
0.05
0.10
MR leave-one-out sensitivity analysis for
to total lipids ratio in large VLDL || id:ebi-cfb233-GCST90302030' on 'ER+ Breast cancer (Combined Oncoarray; iCOGS; GWAS met

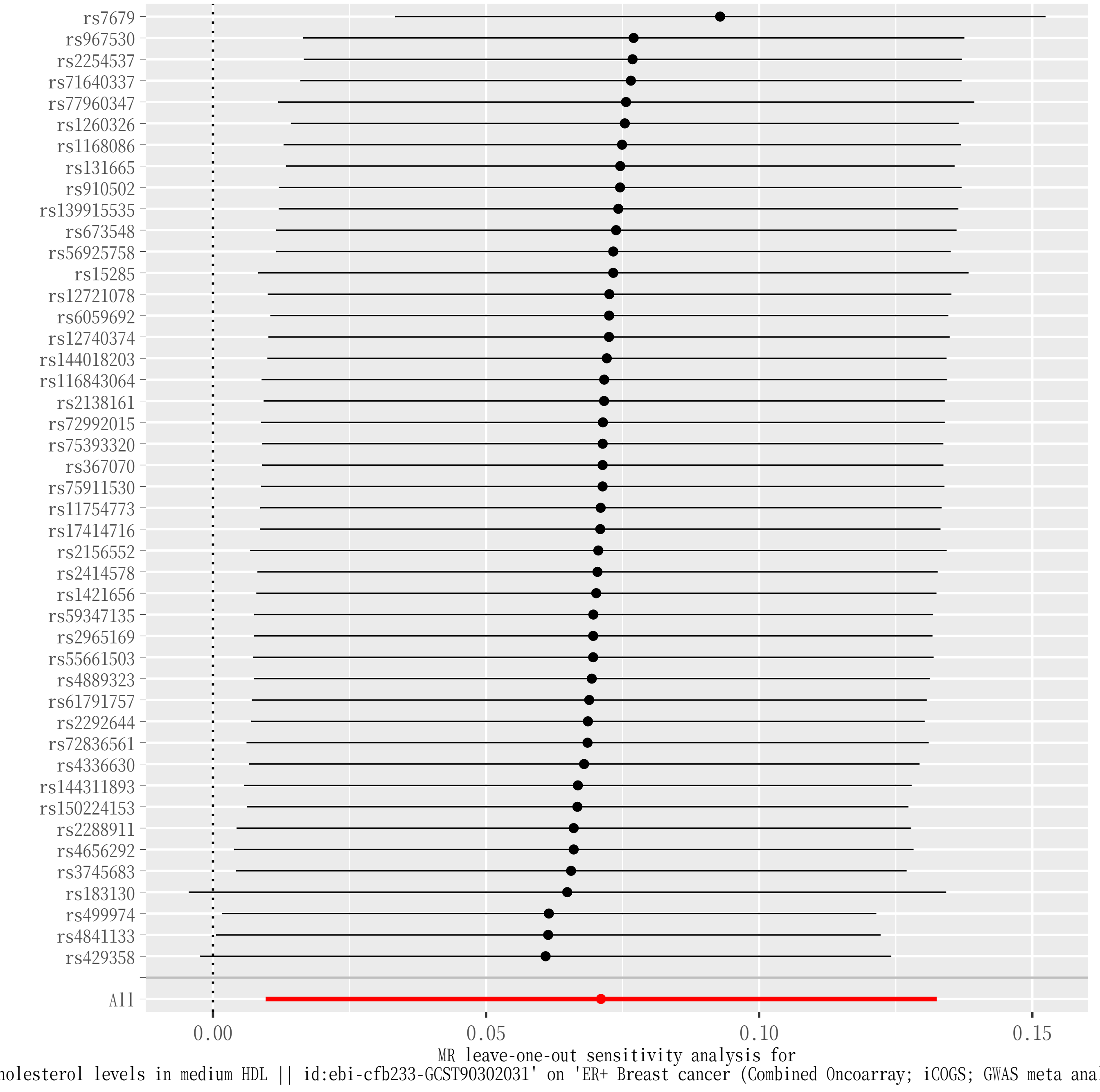
rs7679
rs967530
rs2254537
rs71640337
rs77960347
rs1260326
rs1168086
rs131665
rs910502
rs139915535
rs673548
rs56925758
rs15285
rs12721078
rs6059692
rs12740374
rs144018203
rs116843064
rs2138161
rs72992015
rs75393320
rs367070
rs75911530
rs11754773
rs17414716
rs2156552
rs2414578
rs1421656
rs59347135
rs2965169
rs55661503
rs4889323
rs61791757
rs2292644
rs72836561
rs4336630
rs144311893
rs150224153
rs2288911
rs4656292
rs3745683
rs183130
rs499974
rs4841133
rs429358
All
0.00
0.05
0.10
0.15
MR leave-one-out sensitivity analysis for
holesterol levels in medium HDL || id:ebi-cfb233-GCST90302031' on 'ER+ Breast cancer (Combined Oncoarray; iCOGS; GWAS meta anal

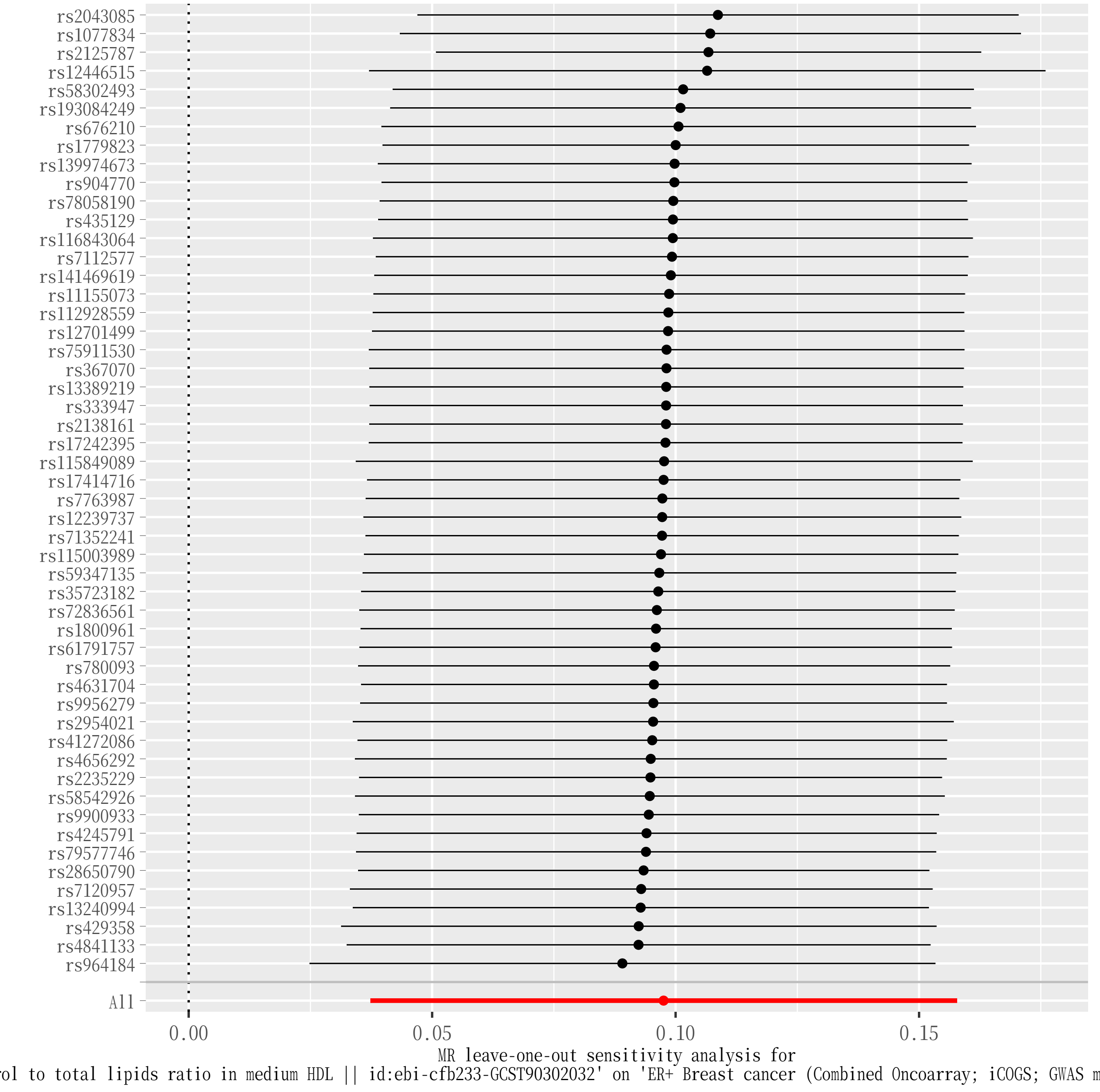
rs2043085
rs1077834
rs2125787
rs12446515
rs58302493
rs193084249
rs676210
rs1779823
rs139974673
rs904770
rs78058190
rs435129
rs116843064
rs7112577
rs141469619
rs11155073
rs112928559
rs12701499
rs75911530
rs367070
rs13389219
rs333947
rs2138161
rs17242395
rs115849089
rs17414716
rs7763987
rs12239737
rs71352241
rs115003989
rs59347135
rs35723182
rs72836561
rs1800961
rs61791757
rs780093
rs4631704
rs9956279
rs2954021
rs41272086
rs4656292
rs2235229
rs58542926
rs9900933
rs4245791
rs79577746
rs28650790
rs7120957
rs13240994
rs429358
rs4841133
rs964184
All
0.00
0.05
0.10
0.15
MR leave-one-out sensitivity analysis for
rol to total lipids ratio in medium HDL || id:ebi-cfb233-GCST90302032' on 'ER+ Breast cancer (Combined Oncoarray; iCOGS; GWAS m

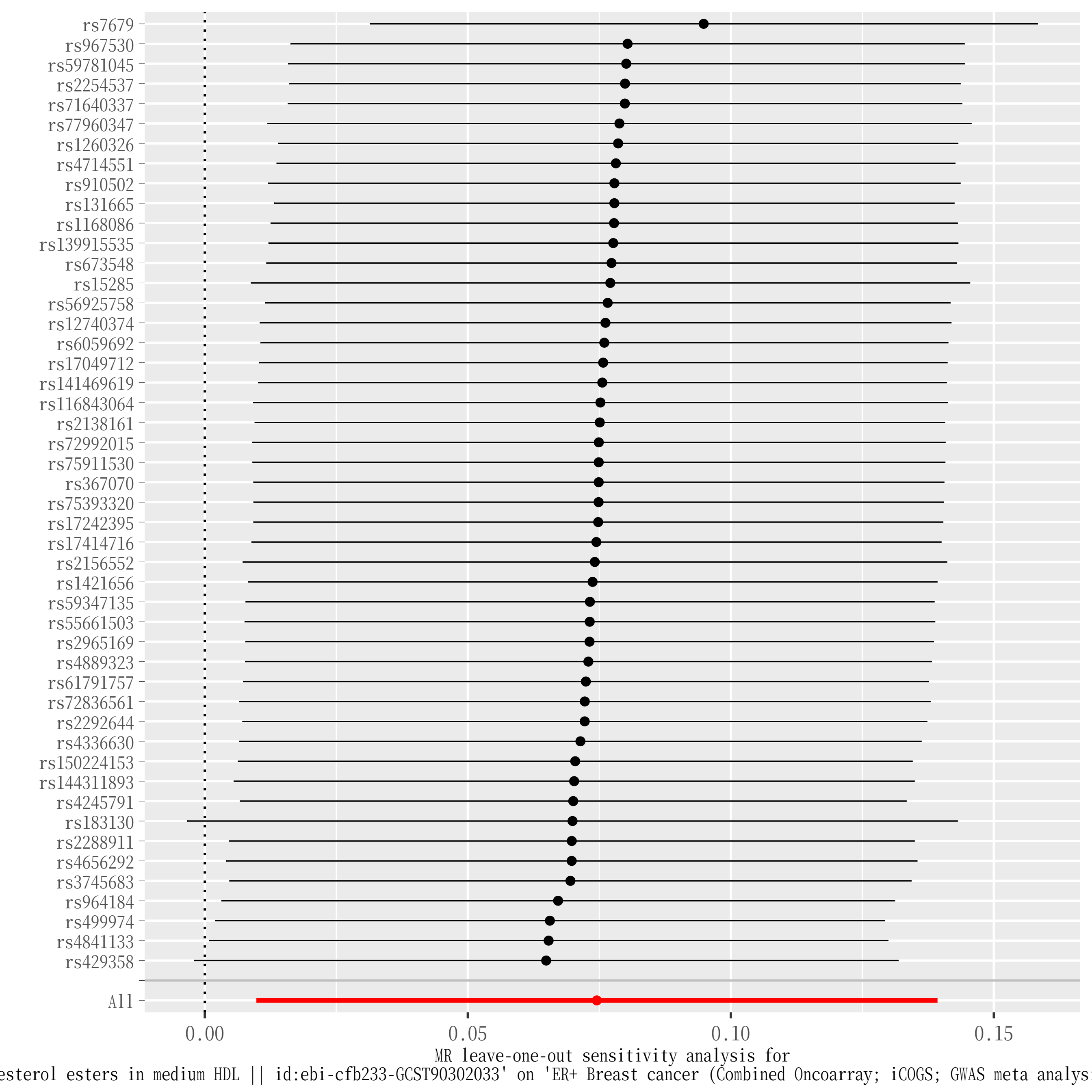
rs7679
rs967530
rs59781045
rs2254537
rs71640337
rs77960347
rs1260326
rs4714551
rs910502
rs131665
rs1168086
rs139915535
rs673548
rs15285
rs56925758
rs12740374
rs6059692
rs17049712
rs141469619
rs116843064
rs2138161
rs72992015
rs75911530
rs367070
rs75393320
rs17242395
rs17414716
rs2156552
rs1421656
rs59347135
rs55661503
rs2965169
rs4889323
rs61791757
rs72836561
rs2292644
rs4336630
rs150224153
rs144311893
rs4245791
rs183130
rs2288911
rs4656292
rs3745683
rs964184
rs499974
rs4841133
rs429358
All
0.00
0.05
0.10
0.15
MR leave-one-out sensitivity analysis for
esterol esters in medium HDL || id:ebi-cfb233-GCST90302033' on 'ER+ Breast cancer (Combined Oncoarray; iCOGS; GWAS meta analys

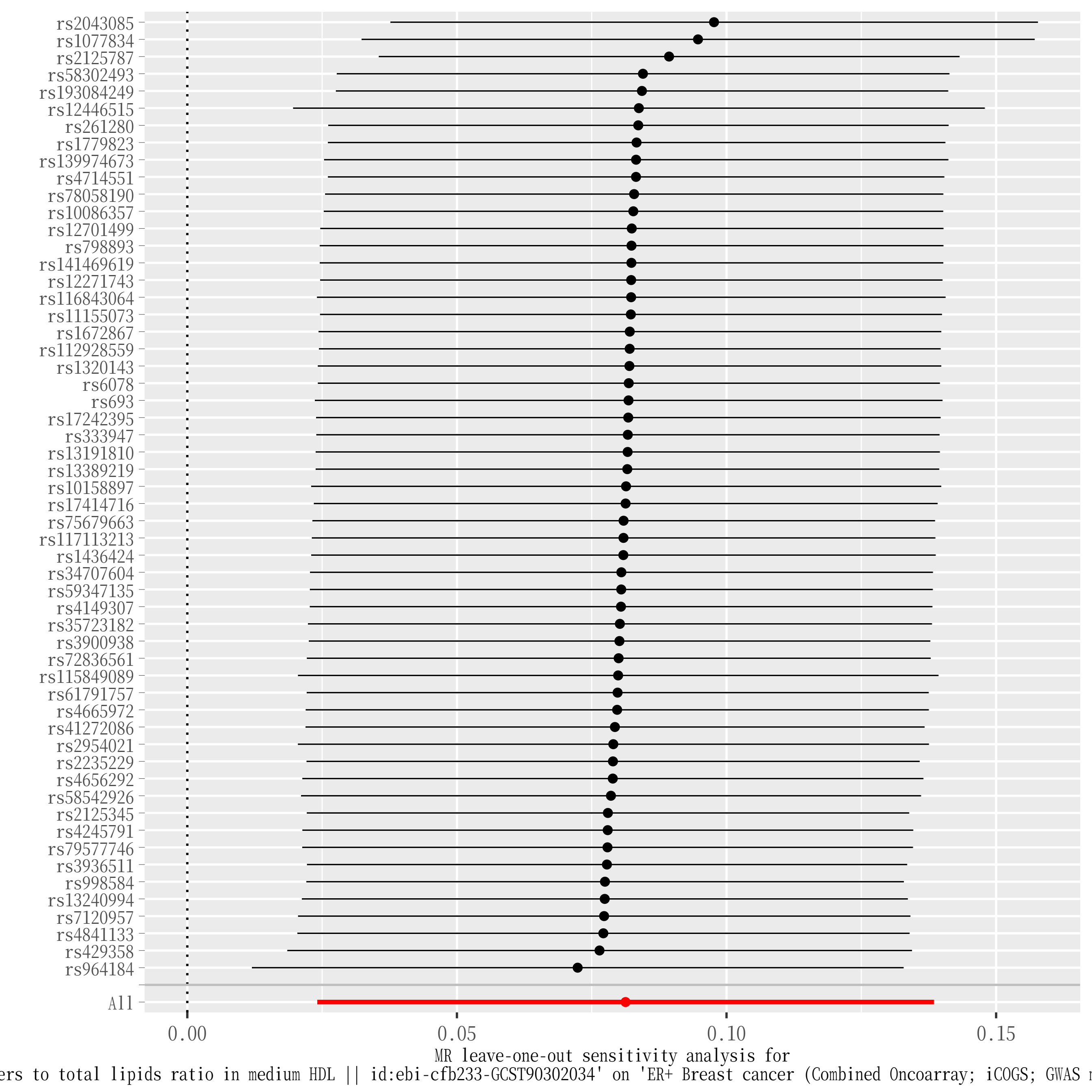
rs2043085
rs1077834
rs2125787
rs58302493
rs193084249
rs12446515
rs261280
rs1779823
rs139974673
rs4714551
rs78058190
rs10086357
rs12701499
rs798893
rs141469619
rs12271743
rs116843064
rs11155073
rs1672867
rs112928559
rs1320143
rs6078
rs693
rs17242395
rs333947
rs13191810
rs13389219
rs10158897
rs17414716
rs75679663
rs117113213
rs1436424
rs34707604
rs59347135
rs4149307
rs35723182
rs3900938
rs72836561
rs115849089
rs61791757
rs4665972
rs41272086
rs2954021
rs2235229
rs4656292
rs58542926
rs2125345
rs4245791
rs79577746
rs3936511
rs998584
rs13240994
rs7120957
rs4841133
rs429358
rs964184
All
0.00
0.05
0.10
0.15
MR leave-one-out sensitivity analysis for
ers to total lipids ratio in medium HDL || id:ebi-cfb233-GCST90302034' on 'ER+ Breast cancer (Combined Oncoarray; iCOGS; GWAS m

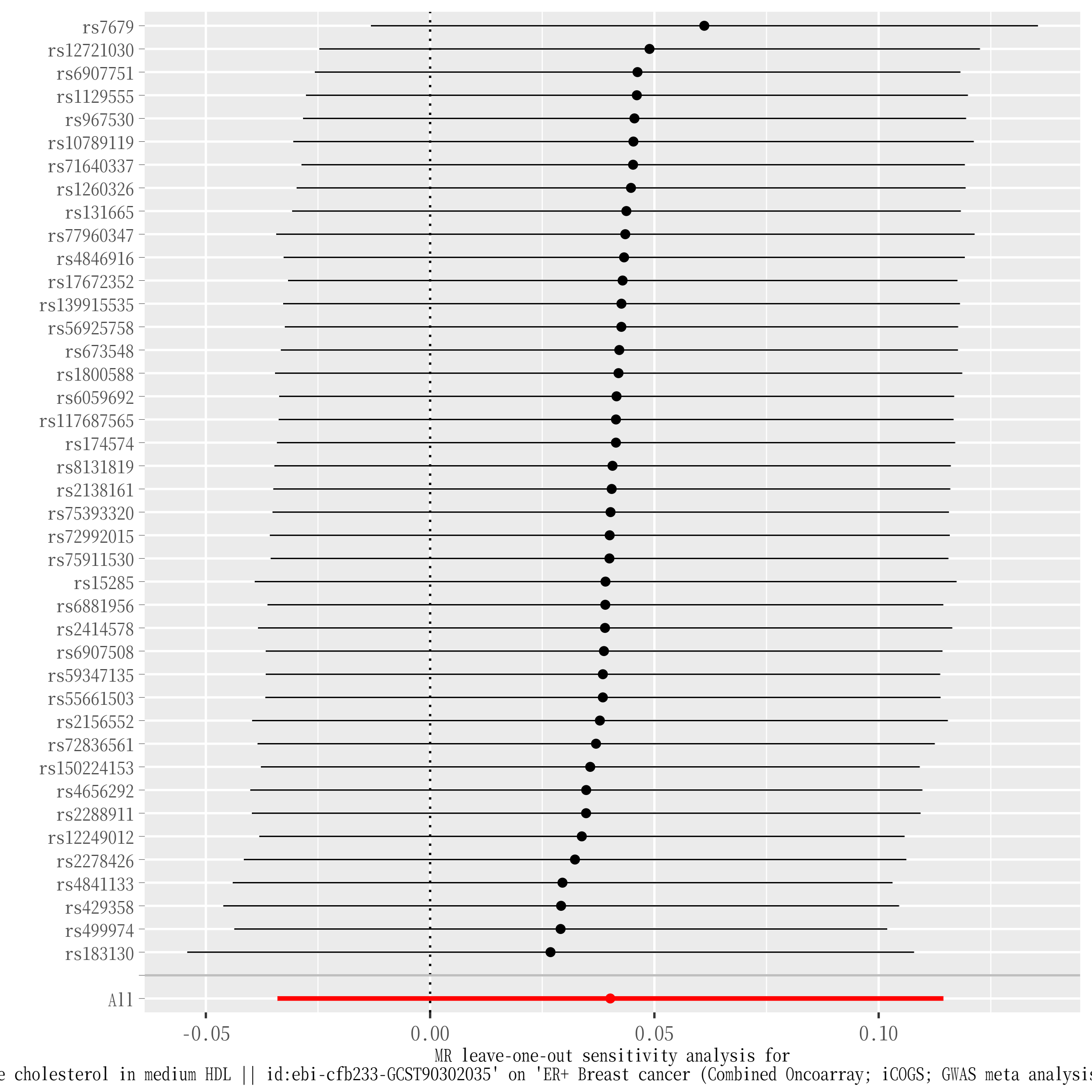
rs7679
rs12721030
rs6907751
rs1129555
rs967530
rs10789119
rs71640337
rs1260326
rs131665
rs77960347
rs4846916
rs17672352
rs139915535
rs56925758
rs673548
rs1800588
rs6059692
rs117687565
rs174574
rs8131819
rs2138161
rs75393320
rs72992015
rs75911530
rs15285
rs6881956
rs2414578
rs6907508
rs59347135
rs55661503
rs2156552
rs72836561
rs150224153
rs4656292
rs2288911
rs12249012
rs2278426
rs4841133
rs429358
rs499974
rs183130
All
-0.05
0.00
0.05
0.10
MR leave-one-out sensitivity analysis for
e cholesterol in medium HDL || id:ebi-cfb233-GCST90302035' on 'ER+ Breast cancer (Combined Oncoarray; iCOGS; GWAS meta analysis

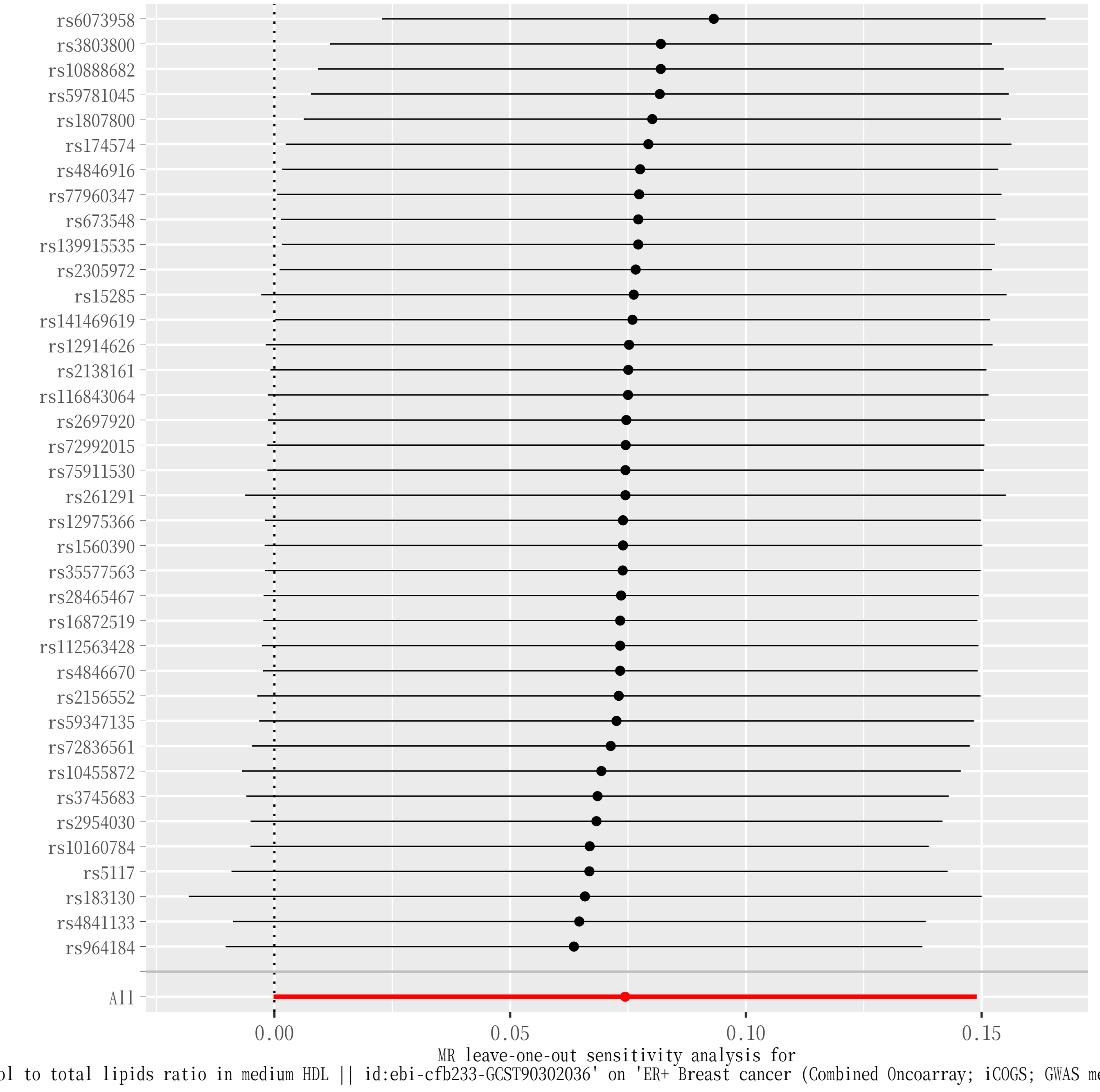
rs6073958
rs3803800
rs10888682
rs59781045
rs1807800
rs174574
rs4846916
rs77960347
rs673548
rs139915535
rs2305972
rs15285
rs141469619
rs12914626
rs2138161
rs116843064
rs2697920
rs72992015
rs75911530
rs261291
rs12975366
rs1560390
rs35577563
rs28465467
rs16872519
rs112563428
rs4846670
rs2156552
rs59347135
rs72836561
rs10455872
rs3745683
rs2954030
rs10160784
rs5117
rs183130
rs4841133
rs964184
All
0.00
0.05
0.10
0.15
MR leave-one-out sensitivity analysis for
ol to total lipids ratio in medium HDL || id:ebi-cfb233-GCST90302036' on 'ER+ Breast cancer (Combined Oncoarray; iCOGS; GWAS me

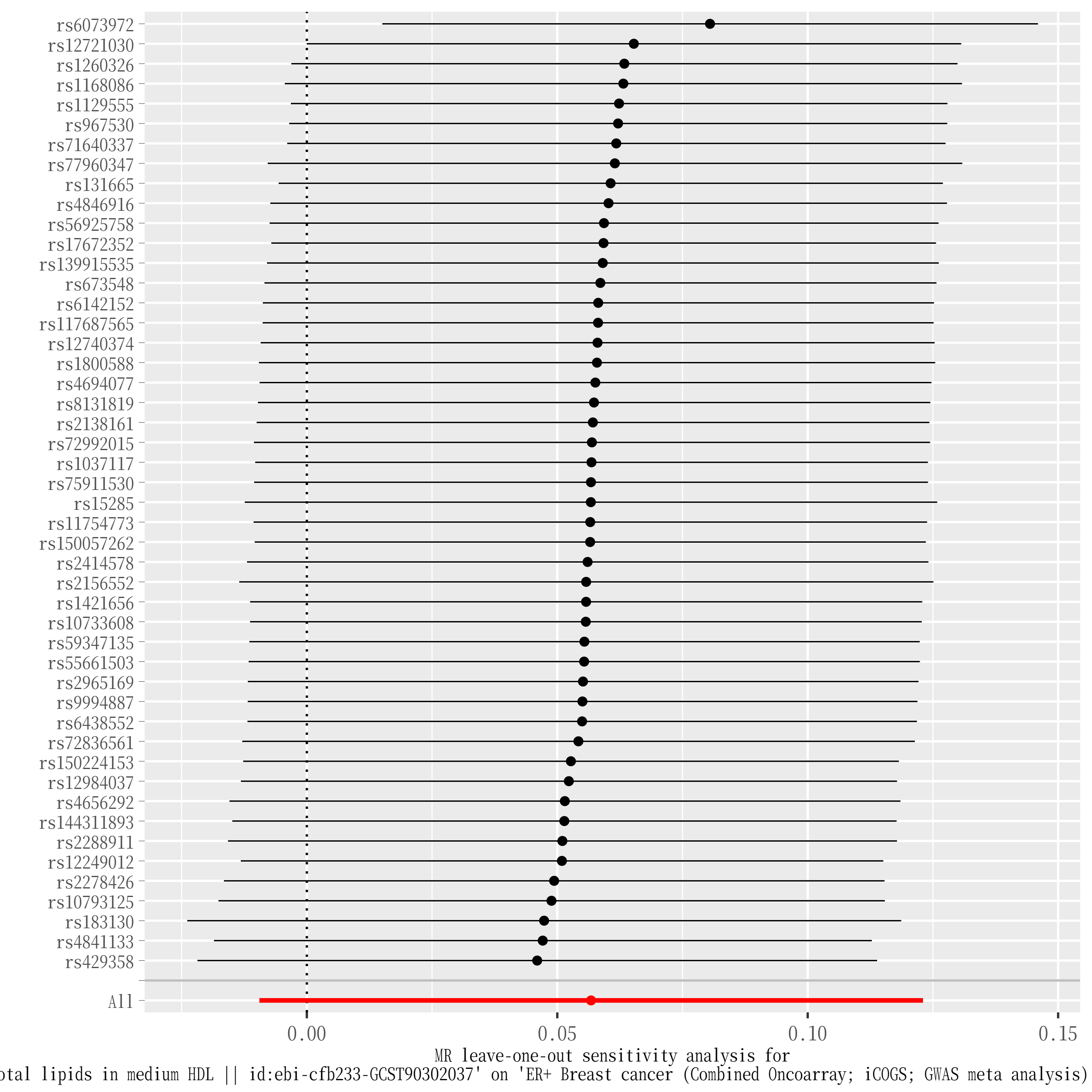
rs6073972
rs12721030
rs1260326
rs1168086
rs1129555
rs967530
rs71640337
rs77960347
rs131665
rs4846916
rs56925758
rs17672352
rs139915535
rs673548
rs6142152
rs117687565
rs12740374
rs1800588
rs4694077
rs8131819
rs2138161
rs72992015
rs1037117
rs75911530
rs15285
rs11754773
rs150057262
rs2414578
rs2156552
rs1421656
rs10733608
rs59347135
rs55661503
rs2965169
rs9994887
rs6438552
rs72836561
rs150224153
rs12984037
rs4656292
rs144311893
rs2288911
rs12249012
rs2278426
rs10793125
rs183130
rs4841133
rs429358
All
0.00
0.05
0.10
0.15
MR leave-one-out sensitivity analysis for
otal lipids in medium HDL || id:ebi-cfb233-GCST90302037' on 'ER+ Breast cancer (Combined Oncoarray; iCOGS; GWAS meta analysis)

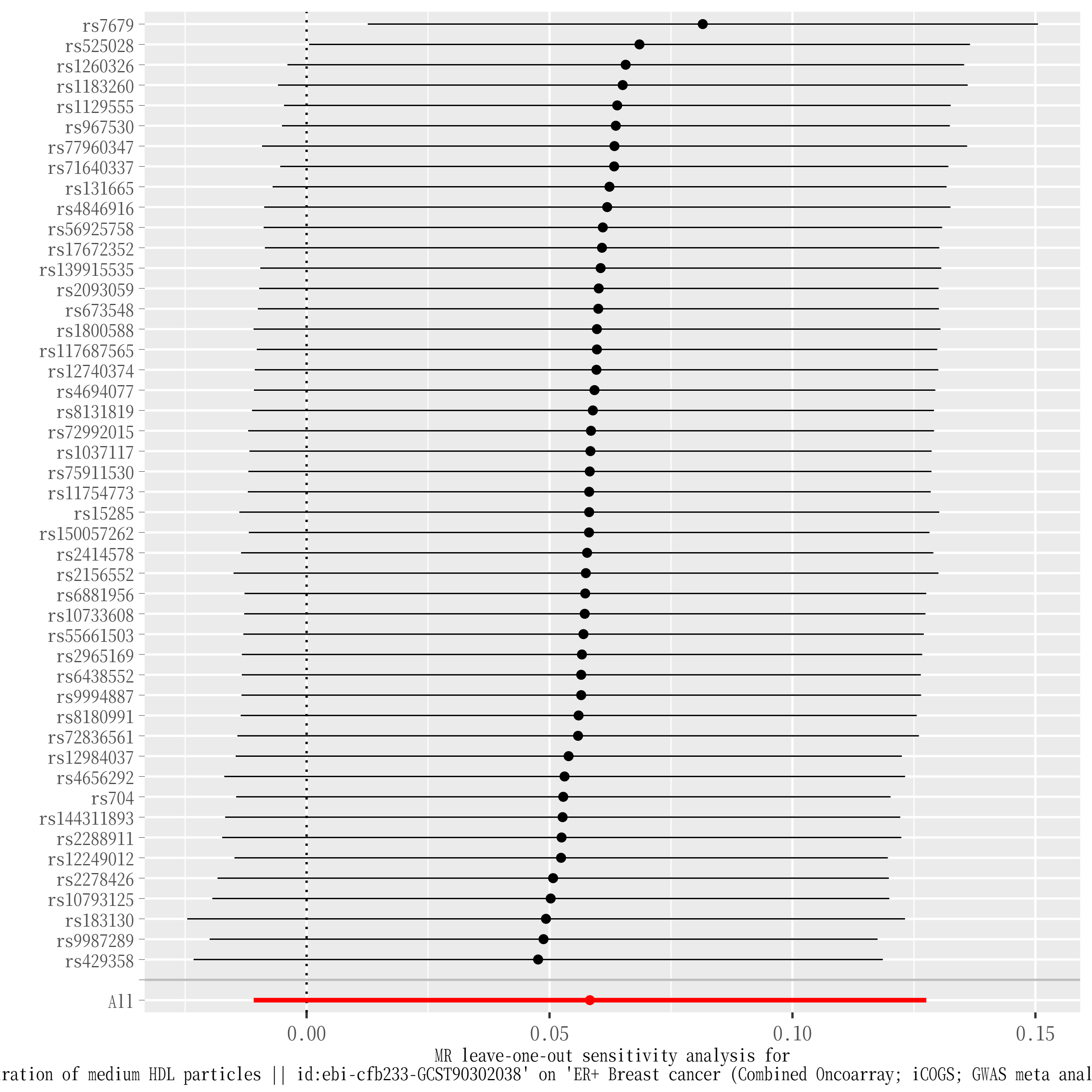
rs7679
rs525028
rs1260326
rs1183260
rs1129555
rs967530
rs77960347
rs71640337
rs131665
rs4846916
rs56925758
rs17672352
rs139915535
rs2093059
rs673548
rs1800588
rs117687565
rs12740374
rs4694077
rs8131819
rs72992015
rs1037117
rs75911530
rs11754773
rs15285
rs150057262
rs2414578
rs2156552
rs6881956
rs10733608
rs55661503
rs2965169
rs6438552
rs9994887
rs8180991
rs72836561
rs12984037
rs4656292
rs704
rs144311893
rs2288911
rs12249012
rs2278426
rs10793125
rs183130
rs9987289
rs429358
All
0.00
0.05
0.10
0.15
MR leave-one-out sensitivity analysis for
ration of medium HDL particles || id:ebi-cfb233-GCST90302038' on 'ER+ Breast cancer (Combined Oncoarray; iCOGS; GWAS meta anal

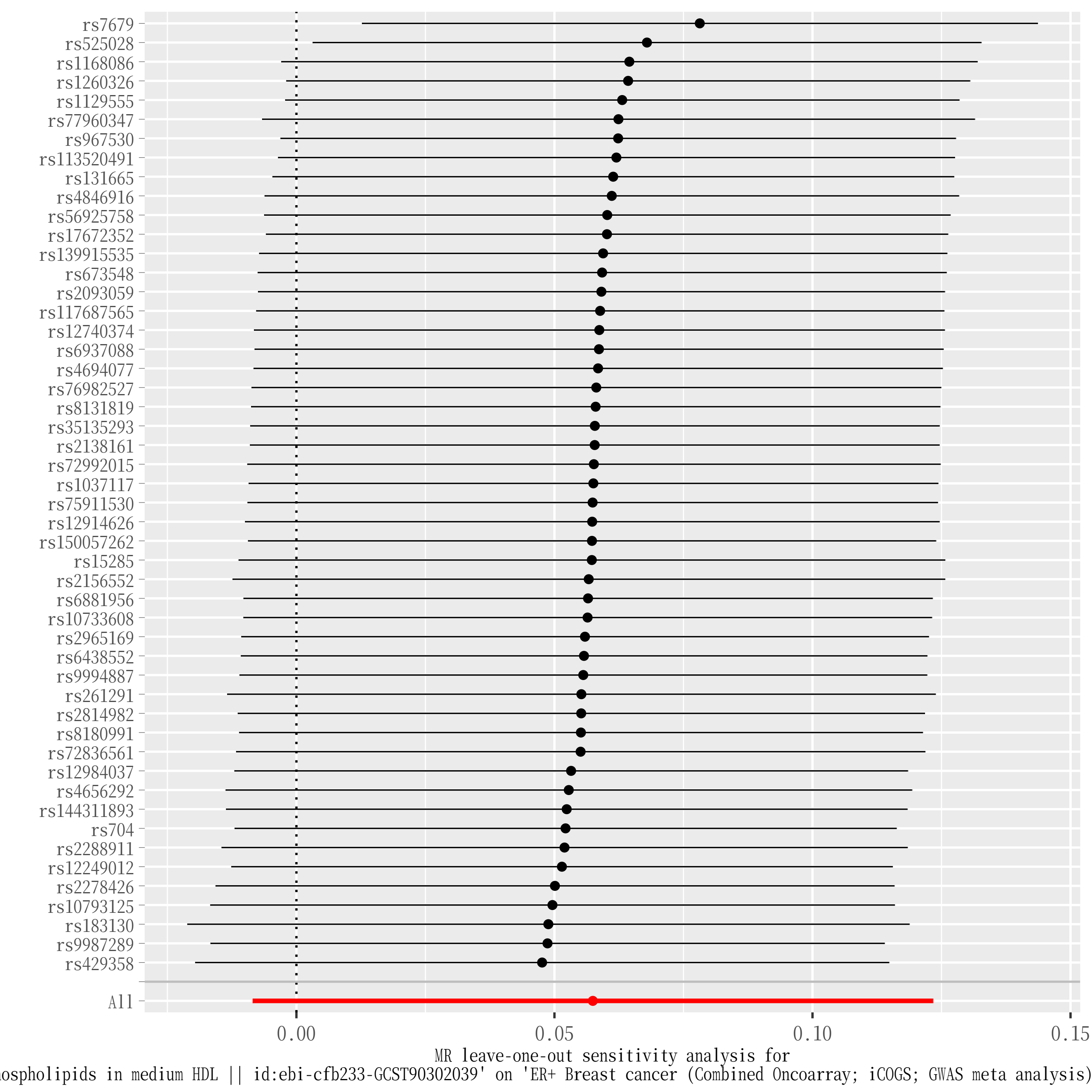
rs7679
rs525028
rs1168086
rs1260326
rs1129555
rs77960347
rs967530
rs113520491
rs131665
rs4846916
rs56925758
rs17672352
rs139915535
rs673548
rs2093059
rs117687565
rs12740374
rs6937088
rs4694077
rs76982527
rs8131819
rs35135293
rs2138161
rs72992015
rs1037117
rs75911530
rs12914626
rs150057262
rs15285
rs2156552
rs6881956
rs10733608
rs2965169
rs6438552
rs9994887
rs261291
rs2814982
rs8180991
rs72836561
rs12984037
rs4656292
rs144311893
rs704
rs2288911
rs12249012
rs2278426
rs10793125
rs183130
rs9987289
rs429358
All
0.00
0.05
0.10
0.15
MR leave-one-out sensitivity analysis for
ospholipids in medium HDL || id:ebi-cfb233-GCST90302039' on 'ER+ Breast cancer (Combined Oncoarray; iCOGS; GWAS meta analysis)

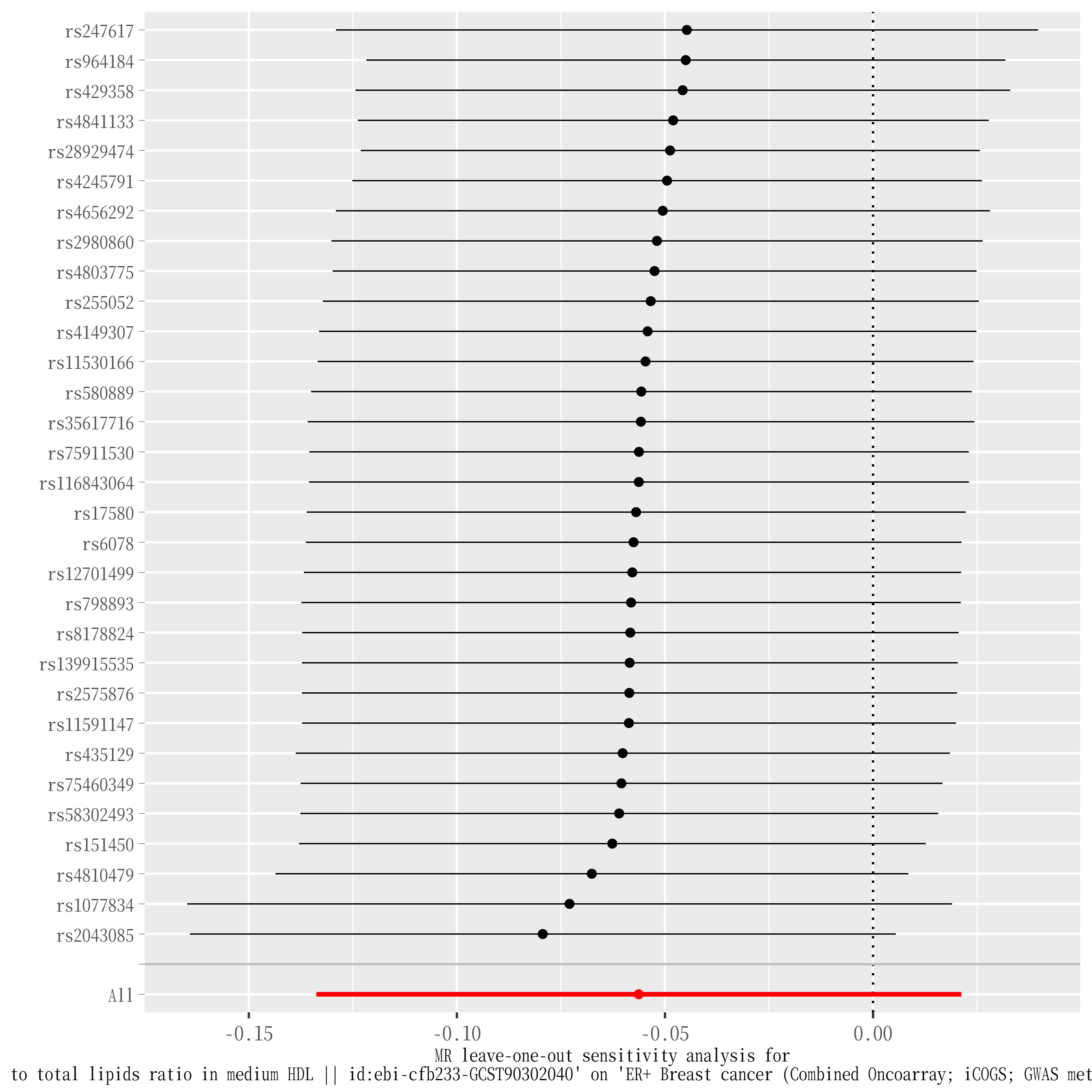
rs247617
rs964184
rs429358
rs4841133
rs28929474
rs4245791
rs4656292
rs2980860
rs4803775
rs255052
rs4149307
rs11530166
rs580889
rs35617716
rs75911530
rs116843064
rs17580
rs6078
rs12701499
rs798893
rs8178824
rs139915535
rs2575876
rs11591147
rs435129
rs75460349
rs58302493
rs151450
rs4810479
rs1077834
rs2043085
All
-0.15
-0.10
-0.05
0.00
MR leave-one-out sensitivity analysis for
to total lipids ratio in medium HDL || id:ebi-cfb233-GCST90302040' on 'ER+ Breast cancer (Combined Oncoarray; iCOGS; GWAS met

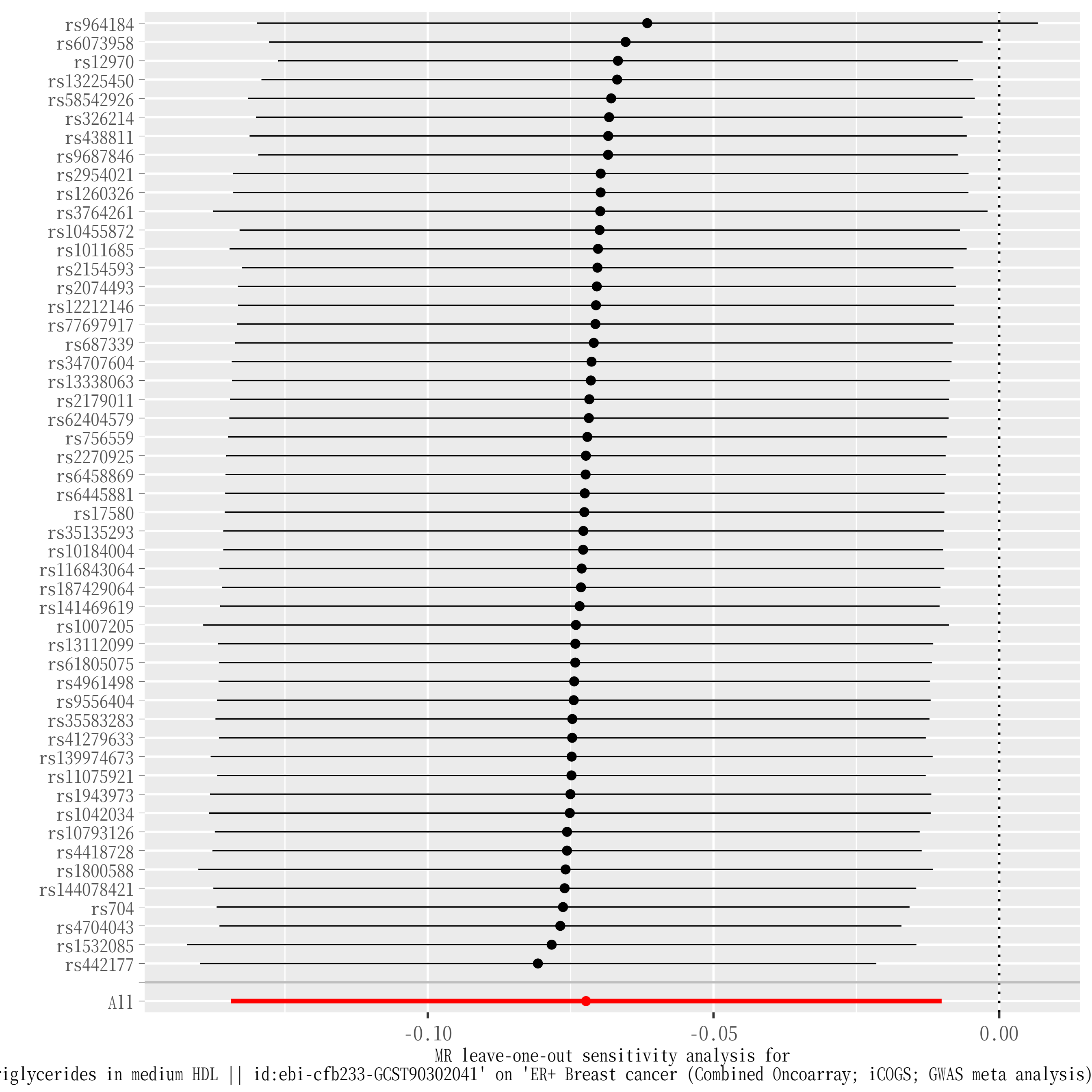
rs964184
rs6073958
rs12970
rs13225450
rs58542926
rs326214
rs438811
rs9687846
rs2954021
rs1260326
rs3764261
rs10455872
rs1011685
rs2154593
rs2074493
rs12212146
rs77697917
rs687339
rs34707604
rs13338063
rs2179011
rs62404579
rs756559
rs2270925
rs6458869
rs6445881
rs17580
rs35135293
rs10184004
rs116843064
rs187429064
rs141469619
rs1007205
rs13112099
rs61805075
rs4961498
rs9556404
rs35583283
rs41279633
rs139974673
rs11075921
rs1943973
rs1042034
rs10793126
rs4418728
rs1800588
rs144078421
rs704
rs4704043
rs1532085
rs442177
All
-0.10
-0.05
0.00
MR leave-one-out sensitivity analysis for
riglycerides in medium HDL || id:ebi-cfb233-GCST90302041' on 'ER+ Breast cancer (Combined Oncoarray; iCOGS; GWAS meta analysis)

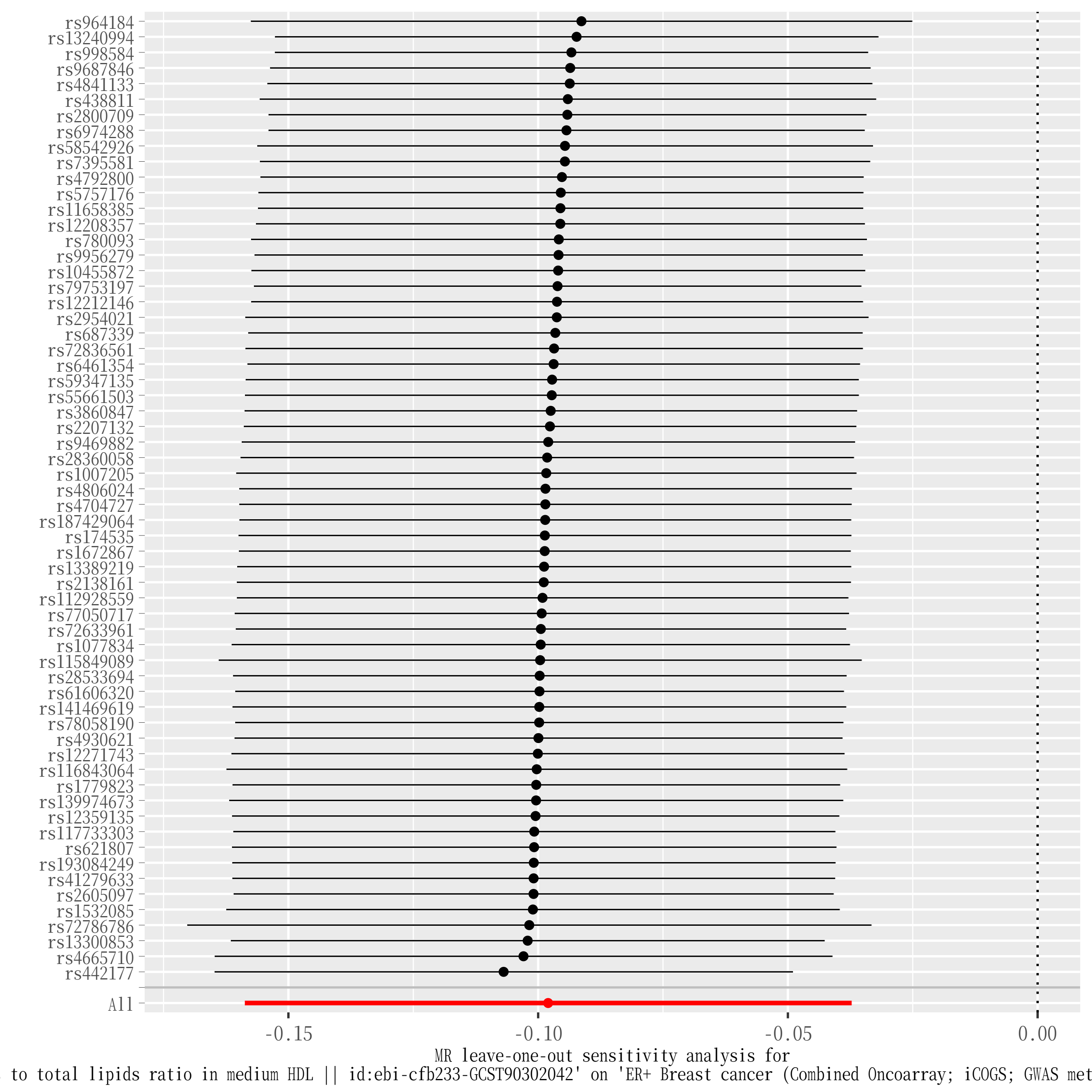
rs964184
rs13240994
rs998584
rs9687846
rs4841133
rs438811
rs2800709
rs6974288
rs58542926
rs7395581
rs4792800
rs5757176
rs11658385
rs12208357
rs780093
rs9956279
rs10455872
rs79753197
rs12212146
rs2954021
rs687339
rs72836561
rs6461354
rs59347135
rs55661503
rs3860847
rs2207132
rs9469882
rs28360058
rs1007205
rs4806024
rs4704727
rs187429064
rs174535
rs1672867
rs13389219
rs2138161
rs112928559
rs77050717
rs72633961
rs1077834
rs115849089
rs28533694
rs61606320
rs141469619
rs78058190
rs4930621
rs12271743
rs116843064
rs1779823
rs139974673
rs12359135
rs117733303
rs621807
rs193084249
rs41279633
rs2605097
rs1532085
rs72786786
rs13300853
rs4665710
rs442177
All
-0.15
-0.10
-0.05
0.00
MR leave-one-out sensitivity analysis for
to total lipids ratio in medium HDL || id:ebi-cfb233-GCST90302042' on 'ER+ Breast cancer (Combined Oncoarray; iCOGS; GWAS met

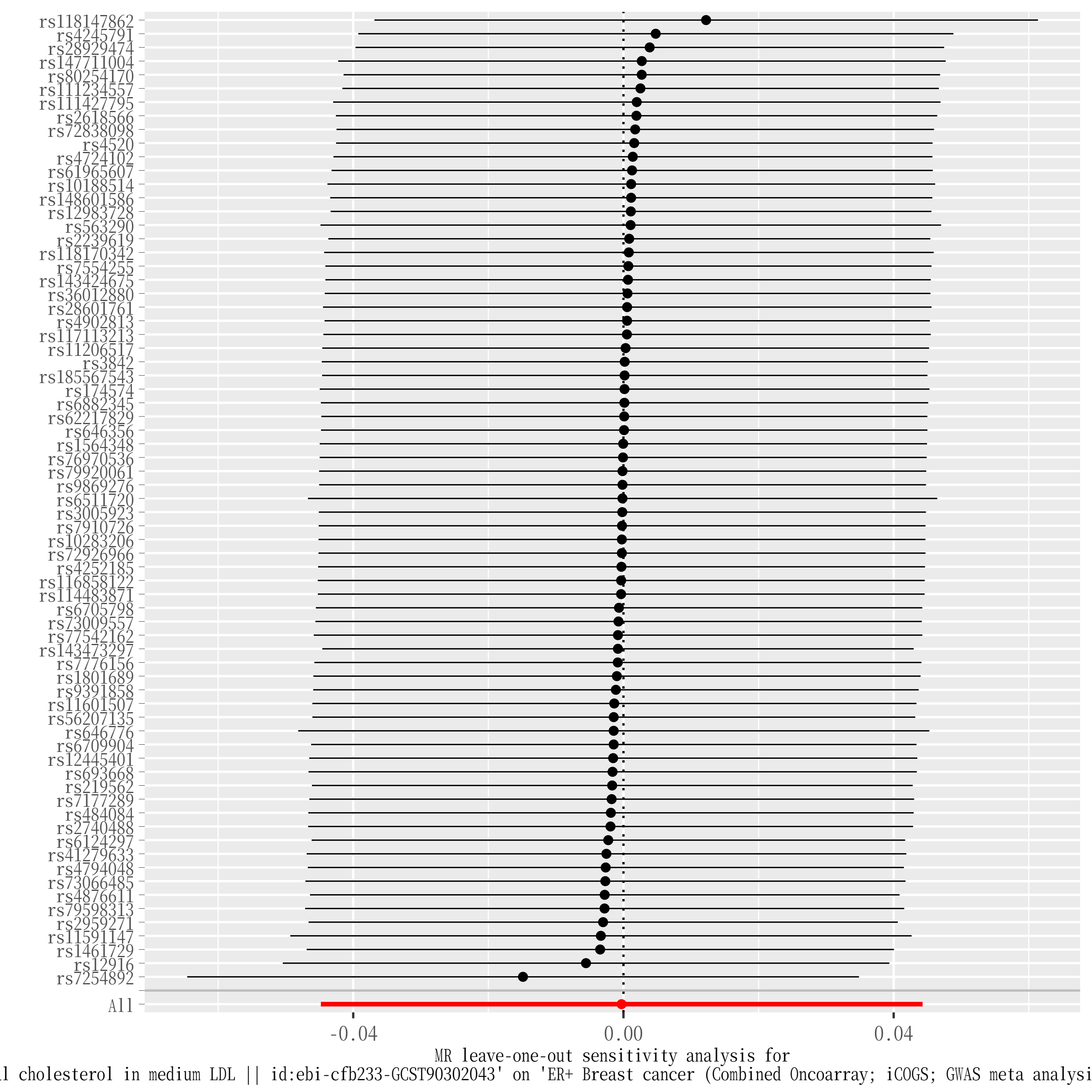
rs118147862
rs4245791
rs28929474
rs147711004
rs80254170
rs111234557
rs111427795
rs2618566
rs72838098
rs4520
rs4724102
rs61965607
rs10188514
rs148601586
rs12983728
rs563290
rs2239619
rs118170342
rs7554255
rs143424675
rs36012880
rs28601761
rs4902813
rs117113213
rs11206517
rs3842
rs185567543
rs174574
rs6882345
rs62217829
rs646356
rs1564348
rs76970536
rs79920061
rs9869276
rs6511720
rs3005923
rs7910726
rs10283206
rs72926966
rs4252185
rs116858122
rs114483871
rs6705798
rs73009557
rs77542162
rs143473297
rs7776156
rs1801689
rs9391858
rs11601507
rs56207135
rs646776
rs6709904
rs12445401
rs693668
rs219562
rs7177289
rs484084
rs2740488
rs6124297
rs41279633
rs4794048
rs73066485
rs4876611
rs79598313
rs2959271
rs11591147
rs1461729
rs12916
rs7254892
All
-0.04
0.00
0.04
MR leave-one-out sensitivity analysis for
l cholesterol in medium LDL || id:ebi-cfb233-GCST90302043' on 'ER+ Breast cancer (Combined Oncoarray; iCOGS; GWAS meta analysi

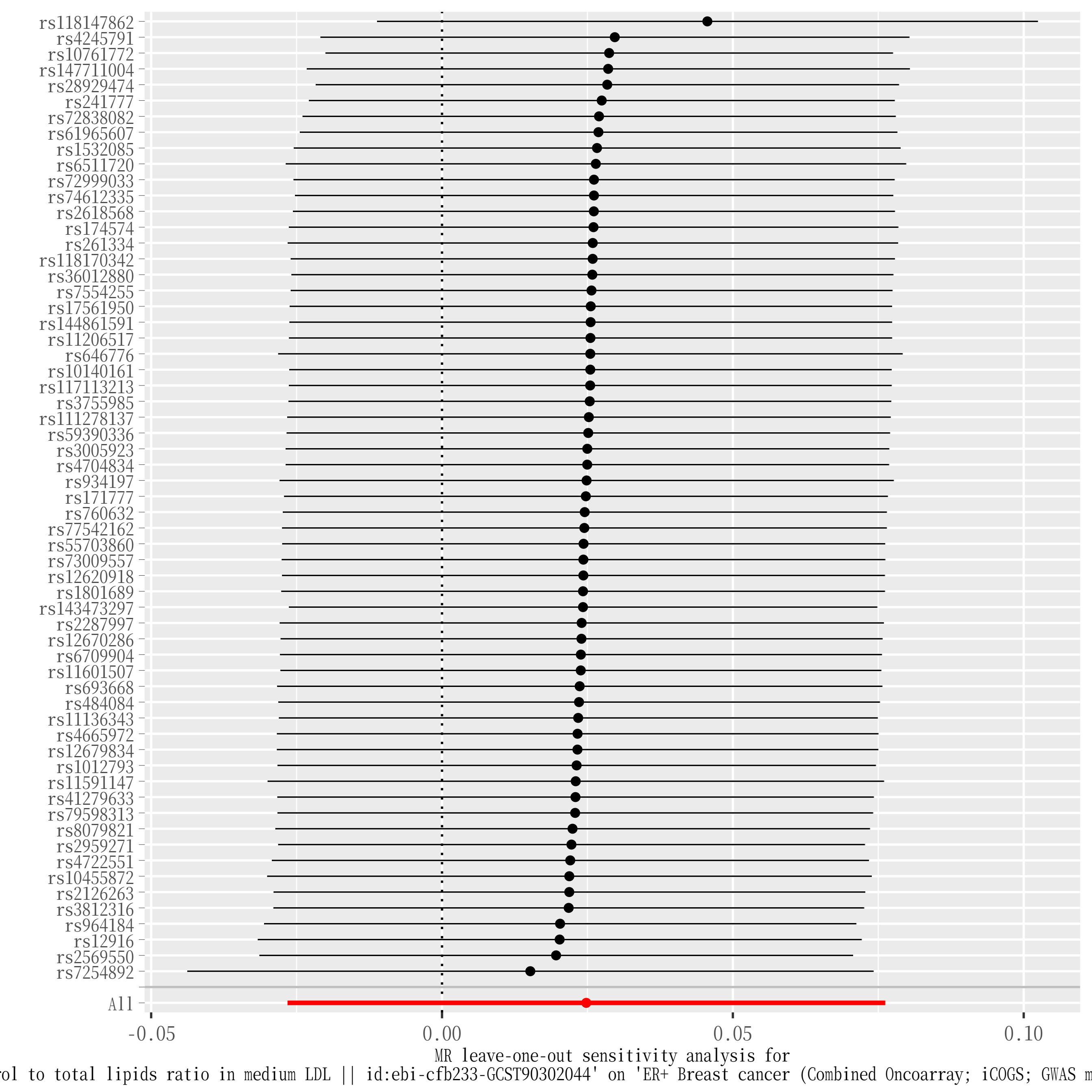
rs118147862
rs4245791
rs10761772
rs147711004
rs28929474
rs241777
rs72838082
rs61965607
rs1532085
rs6511720
rs72999033
rs74612335
rs2618568
rs174574
rs261334
rs118170342
rs36012880
rs7554255
rs17561950
rs144861591
rs11206517
rs646776
rs10140161
rs117113213
rs3755985
rs111278137
rs59390336
rs3005923
rs4704834
rs934197
rs171777
rs760632
rs77542162
rs55703860
rs73009557
rs12620918
rs1801689
rs143473297
rs2287997
rs12670286
rs6709904
rs11601507
rs693668
rs484084
rs11136343
rs4665972
rs12679834
rs1012793
rs11591147
rs41279633
rs79598313
rs8079821
rs2959271
rs4722551
rs10455872
rs2126263
rs3812316
rs964184
rs12916
rs2569550
rs7254892
All
-0.05
0.00
0.05
0.10
MR leave-one-out sensitivity analysis for
rol to total lipids ratio in medium LDL || id:ebi-cfb233-GCST90302044' on 'ER+ Breast cancer (Combined Oncoarray; iCOGS; GWAS m

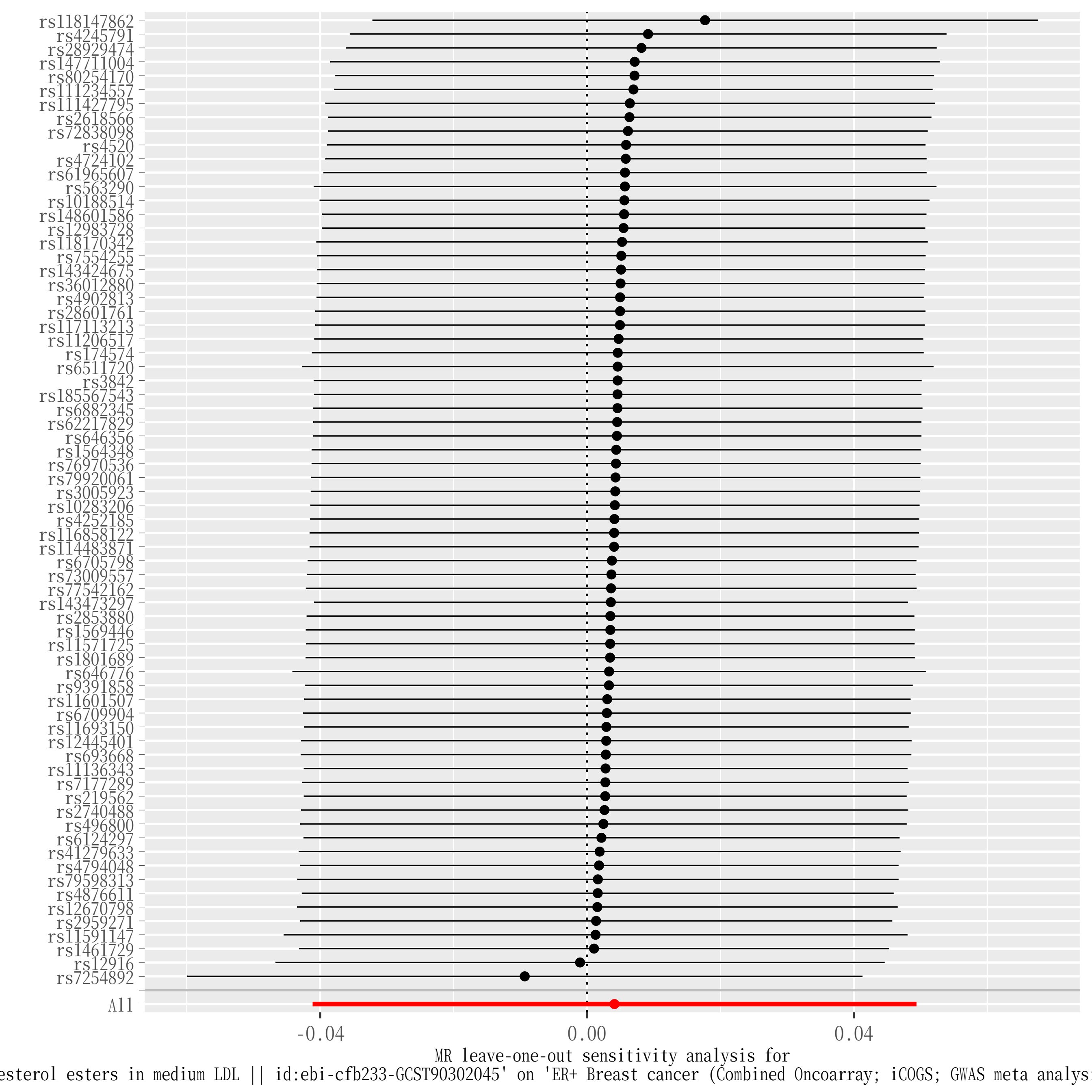

rs118147862
rs4245791
rs28929474
rs147711004
rs80254170
rs111234557
rs111427795
rs2618566
rs72838098
rs4520
rs4724102
rs61965607
rs563290
rs10188514
rs148601586
rs12983728
rs118170342
rs7554255
rs143424675
rs36012880
rs4902813
rs28601761
rs117113213
rs11206517
rs174574
rs6511720
rs3842
rs185567543
rs6882345
rs62217829
rs646356
rs1564348
rs76970536
rs79920061
rs3005923
rs10283206
rs4252185
rs116858122
rs114483871
rs6705798
rs73009557
rs77542162
rs143473297
rs2853880
rs1569446
rs11571725
rs1801689
rs646776
rs9391858
rs11601507
rs6709904
rs11693150
rs12445401
rs693668
rs11136343
rs7177289
rs219562
rs2740488
rs496800
rs6124297
rs41279633
rs4794048
rs79598313
rs4876611
rs12670798
rs2959271
rs11591147
rs1461729
rs12916
rs7254892
All
-0.04
0.00
0.04
MR leave-one-out sensitivity analysis for
esterol esters in medium LDL || id:ebi-cfb233-GCST90302045' on 'ER+ Breast cancer (Combined Oncoarray; iCOGS; GWAS meta analys

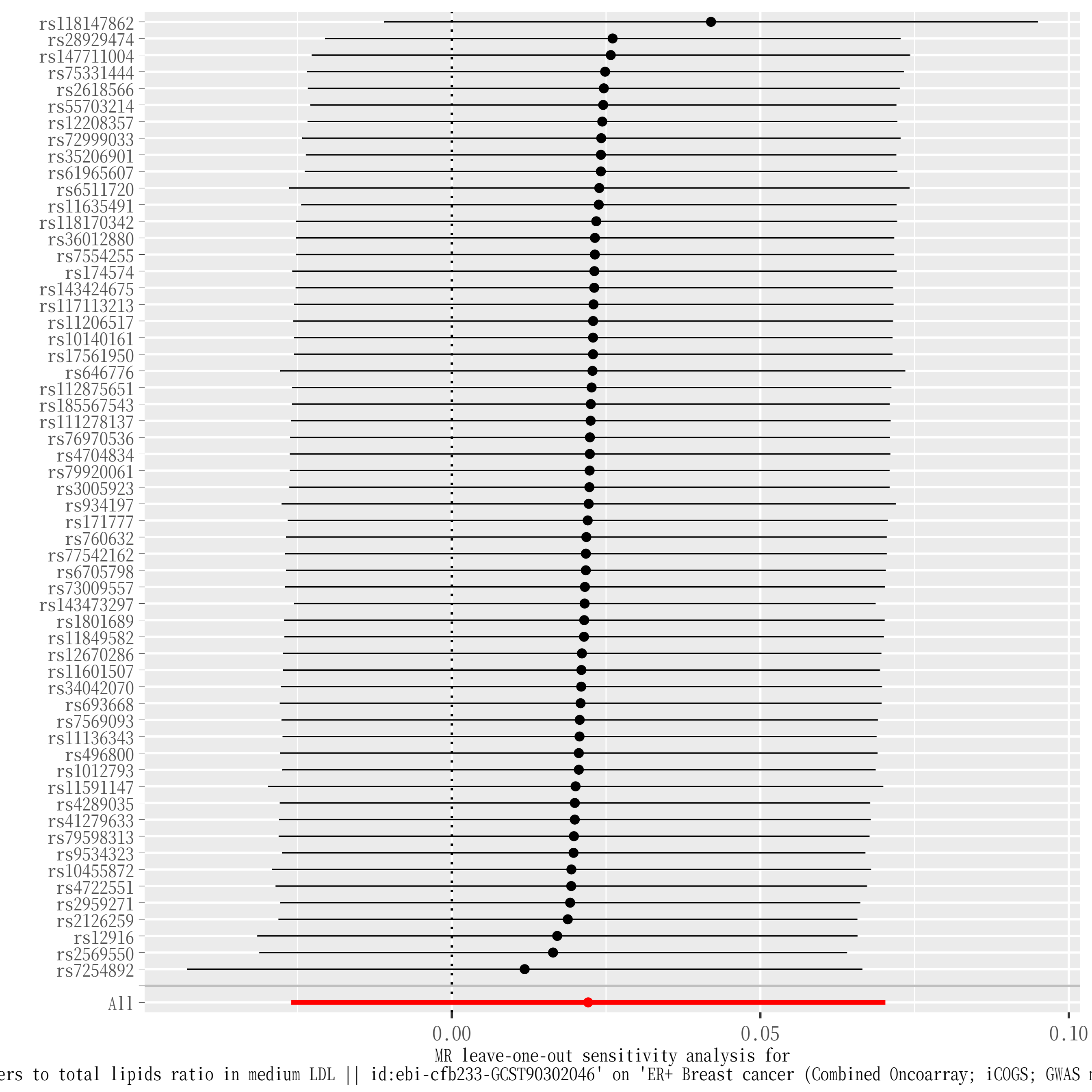
rs118147862
rs28929474
rs147711004
rs75331444
rs2618566
rs55703214
rs12208357
rs72999033
rs35206901
rs61965607
rs6511720
rs11635491
rs118170342
rs36012880
rs7554255
rs174574
rs143424675
rs117113213
rs11206517
rs10140161
rs17561950
rs646776
rs112875651
rs185567543
rs111278137
rs76970536
rs4704834
rs79920061
rs3005923
rs934197
rs171777
rs760632
rs77542162
rs6705798
rs73009557
rs143473297
rs1801689
rs11849582
rs12670286
rs11601507
rs34042070
rs693668
rs7569093
rs11136343
rs496800
rs1012793
rs11591147
rs4289035
rs41279633
rs79598313
rs9534323
rs10455872
rs4722551
rs2959271
rs2126259
rs12916
rs2569550
rs7254892
All
0.00
0.05
0.10
MR leave-one-out sensitivity analysis for
ers to total lipids ratio in medium LDL || id:ebi-cfb233-GCST90302046' on 'ER+ Breast cancer (Combined Oncoarray; iCOGS; GWAS m

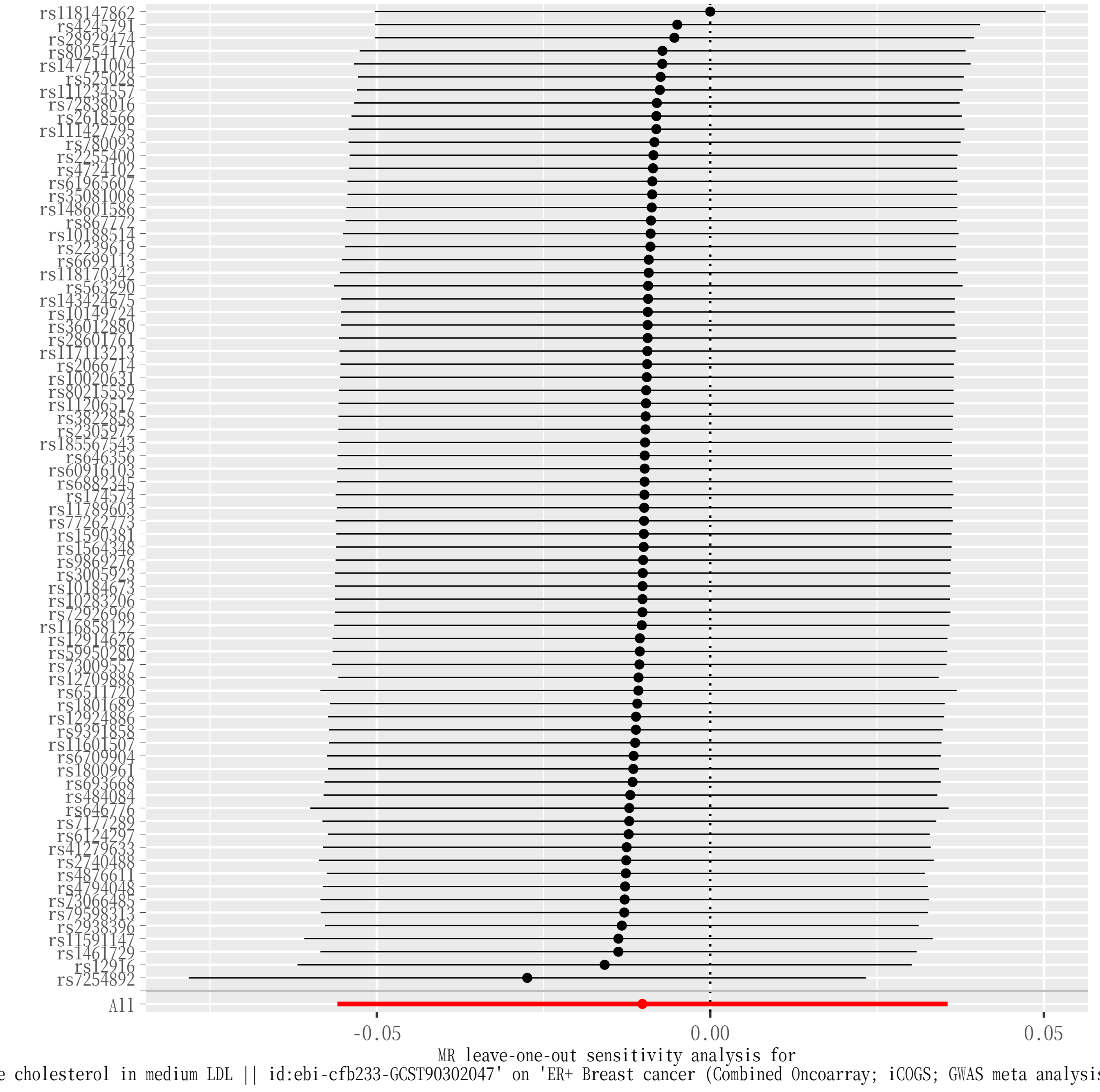
rs118147862
rs4245791
rs28929474
rs80254170
rs147711004
rs525028
rs111234557
rs72838016
rs2618566
rs111427795
rs780093
rs2255400
rs4724102
rs61965607
rs35081008
rs148601586
rs867772
rs10188514
rs2239619
rs6699113
rs118170342
rs563290
rs143424675
rs10149724
rs36012880
rs28601761
rs117113213
rs2066714
rs10020631
rs80215559
rs11206517
rs3822858
rs2305972
rs185567543
rs646356
rs60916103
rs6882345
rs174574
rs11789603
rs77262773
rs1590381
rs1564348
rs9869276
rs3005923
rs10184673
rs10283206
rs72926966
rs116858122
rs12914626
rs59950280
rs73009557
rs12709888
rs6511720
rs1801689
rs12924886
rs9391858
rs11601507
rs6709904
rs1800961
rs693668
rs484084
rs646776
rs7177289
rs6124297
rs41279633
rs2740488
rs4876611
rs4794048
rs73066485
rs79598313
rs2938396
rs11591147
rs1461729
rs12916
rs7254892
All
-0.05
0.00
0.05
MR leave-one-out sensitivity analysis for
e cholesterol in medium LDL || id:ebi-cfb233-GCST90302047' on 'ER+ Breast cancer (Combined Oncoarray; iCOGS; GWAS meta analysis

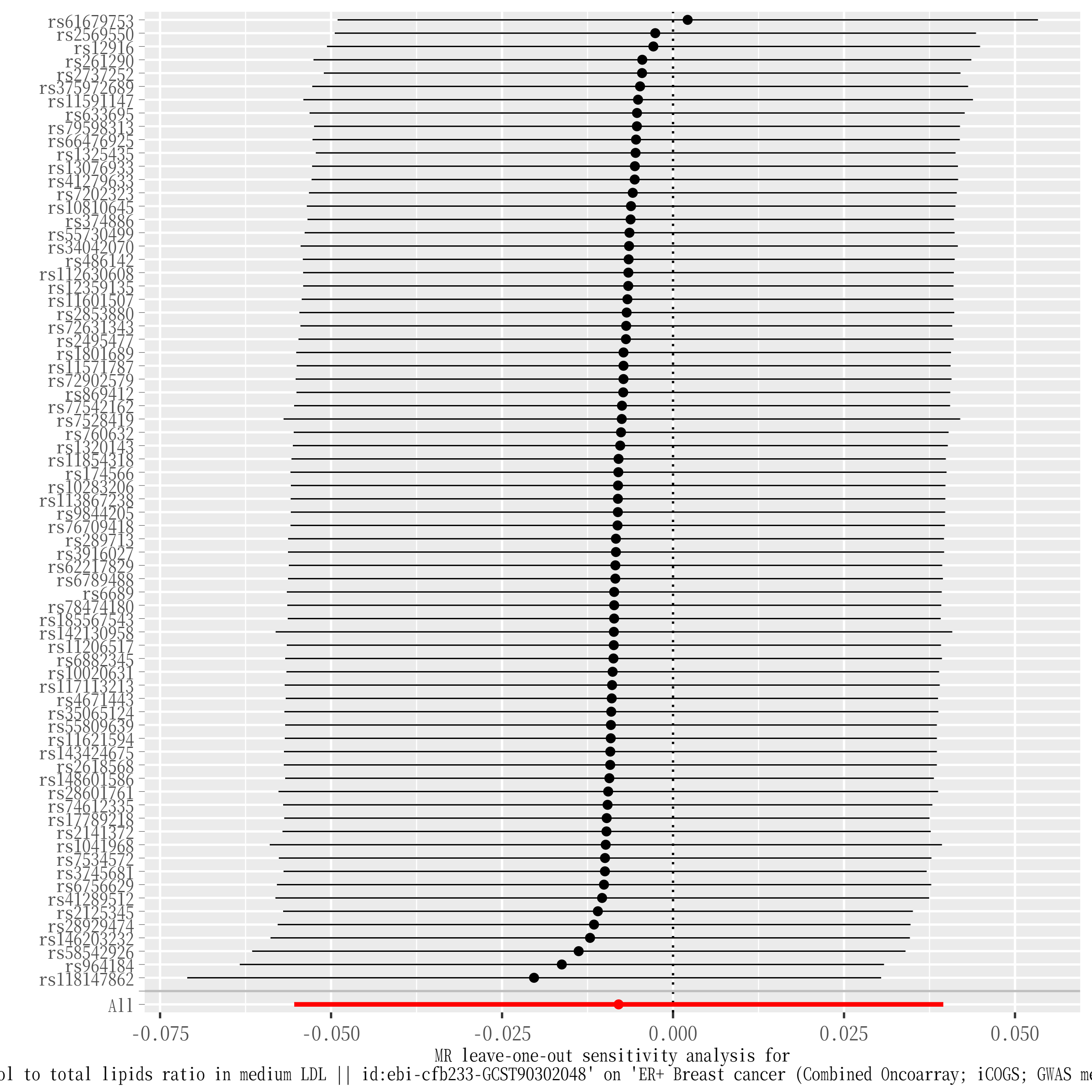
rs61679753
rs2569550
rs12916
rs261290
rs2737252
rs375972689
rs11591147
rs633695
rs79598313
rs66476925
rs1325435
rs13076933
rs41279633
rs7202323
rs10810645
rs374886
rs55730499
rs34042070
rs486142
rs112630608
rs12359135
rs11601507
rs2853880
rs72631343
rs2495477
rs1801689
rs11571787
rs72902579
rs869412
rs77542162
rs7528419
rs760632
rs1320143
rs11854318
rs174566
rs10283206
rs113867238
rs9844205
rs76709418
rs289713
rs3916027
rs62217829
rs6789488
rs6689
rs78474180
rs185567543
rs142130958
rs11206517
rs6882345
rs10020631
rs117113213
rs4671443
rs35065124
rs55809639
rs11621594
rs143424675
rs2618568
rs148601586
rs28601761
rs74612335
rs17789218
rs2141372
rs1041968
rs7534572
rs3745681
rs6756629
rs41289512
rs2125345
rs28929474
rs146203232
rs58542926
rs964184
rs118147862
All
-0.075
-0.050
-0.025
0.000
0.025
0.050
MR leave-one-out sensitivity analysis for
ol to total lipids ratio in medium LDL || id:ebi-cfb233-GCST90302048' on 'ER+ Breast cancer (Combined Oncoarray; iCOGS; GWAS me

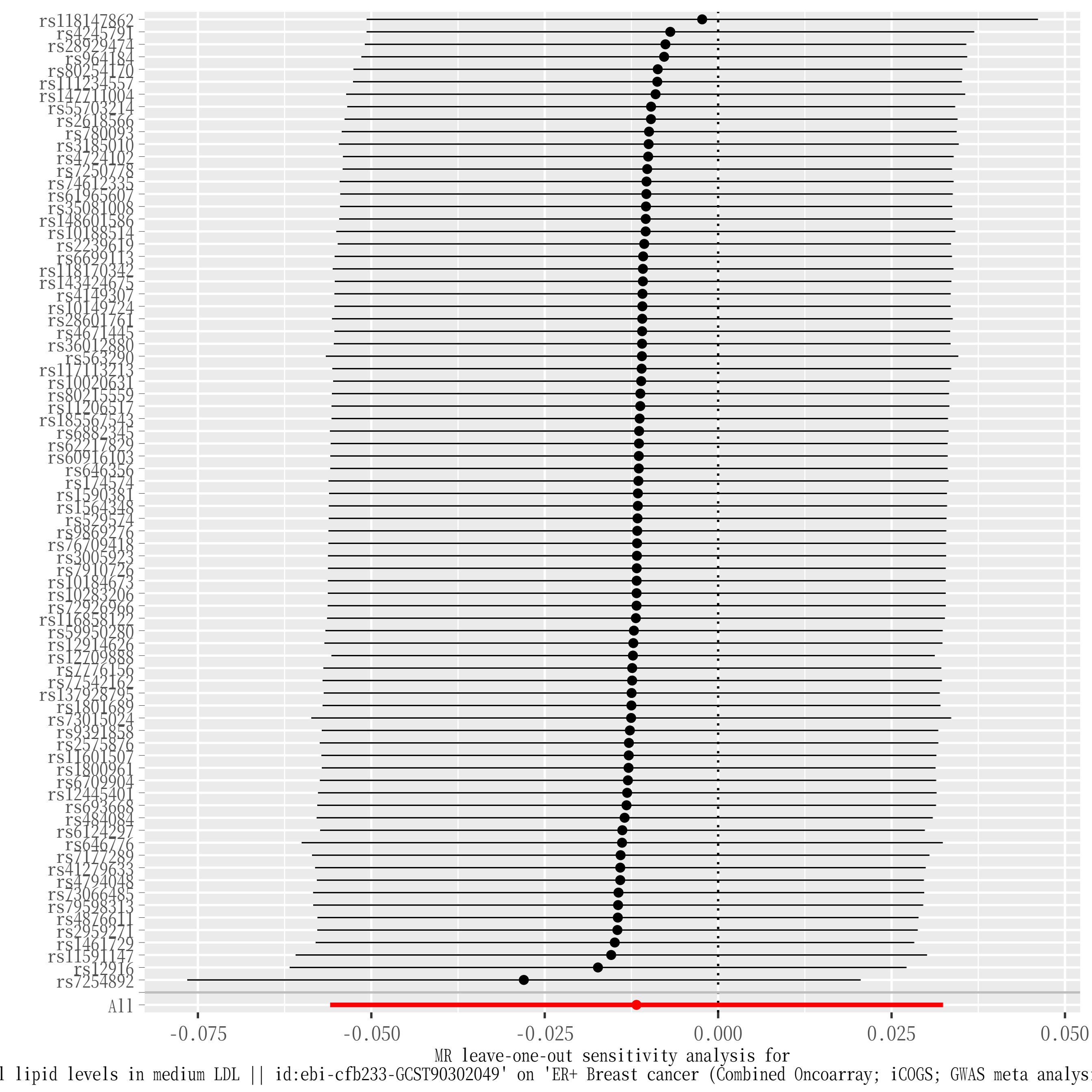

rs118147862
rs4245791
rs28929474
rs964184
rs80254170
rs111234557
rs147711004
rs55703214
rs2618566
rs780093
rs3185010
rs4724102
rs7250778
rs74612335
rs61965607
rs35081008
rs148601586
rs10188514
rs2239619
rs6699113
rs118170342
rs143424675
rs4149307
rs10149724
rs28601761
rs4671445
rs36012880
rs563290
rs117113213
rs10020631
rs80215559
rs11206517
rs185567543
rs6882345
rs62217829
rs60916103
rs646356
rs174574
rs1590381
rs1564348
rs529574
rs9869276
rs76709418
rs3005923
rs7910726
rs10184673
rs10283206
rs72926966
rs116858122
rs59950280
rs12914626
rs12709888
rs7776156
rs77542162
rs137928795
rs1801689
rs73015024
rs9391858
rs2575876
rs11601507
rs1800961
rs6709904
rs12445401
rs693668
rs484084
rs6124297
rs646776
rs7177289
rs41279633
rs4794048
rs73066485
rs79598313
rs4876611
rs2959271
rs1461729
rs11591147
rs12916
rs7254892
All
-0.075
-0.050
-0.025
0.000
0.025
0.050
MR leave-one-out sensitivity analysis for

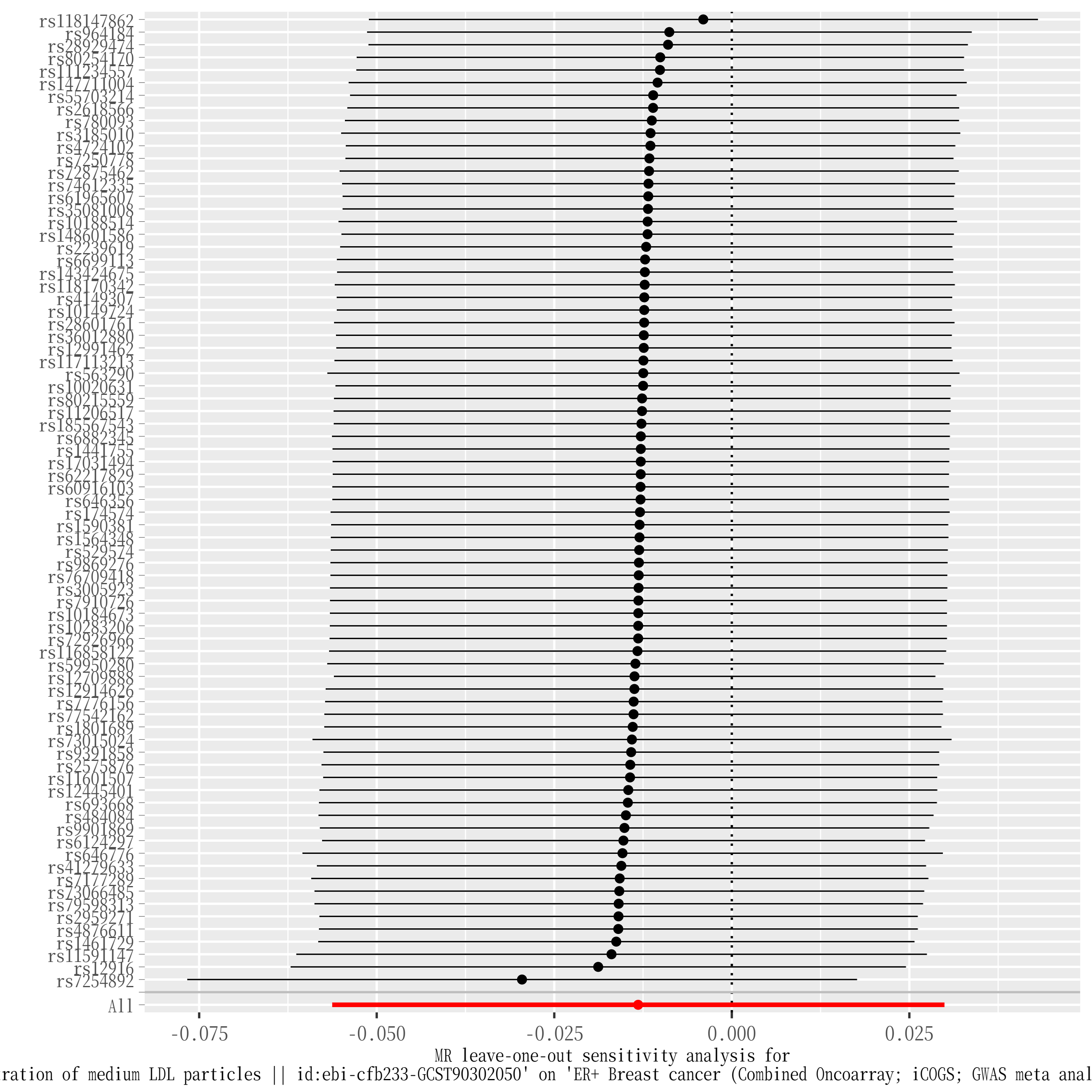
rs118147862
rs964184
rs28929474
rs80254170
rs111234557
rs147711004
rs55703214
rs2618566
rs780093
rs3185010
rs4724102
rs7250778
rs72875462
rs74612335
rs61965607
rs35081008
rs10188514
rs148601586
rs2239619
rs6699113
rs143424675
rs118170342
rs4149307
rs10149724
rs28601761
rs36012880
rs12991462
rs117113213
rs563290
rs10020631
rs80215559
rs11206517
rs185567543
rs6882345
rs1441755
rs17031494
rs62217829
rs60916103
rs646356
rs174574
rs1590381
rs1564348
rs529574
rs9869276
rs76709418
rs3005923
rs7910726
rs10184673
rs10283206
rs72926966
rs116858122
rs59950280
rs12709888
rs12914626
rs7776156
rs77542162
rs1801689
rs73015024
rs9321858
rs2575876
rs11601507
rs12445401
rs693668
rs484084
rs9901869
rs6124297
rs646776
rs41279633
rs7177282
rs73066485
rs79598313
rs2959271
rs4876611
rs1461729
rs11591147
rs12916
rs7254892
All
-0.075
-0.050
-0.025
0.000
0.025
MR leave-one-out sensitivity analysis for
ration of medium LDL particles || id:ebi-cfb233-GCST90302050' on 'ER+ Breast cancer (Combined Oncoarray; iCOGS; GWAS meta anal

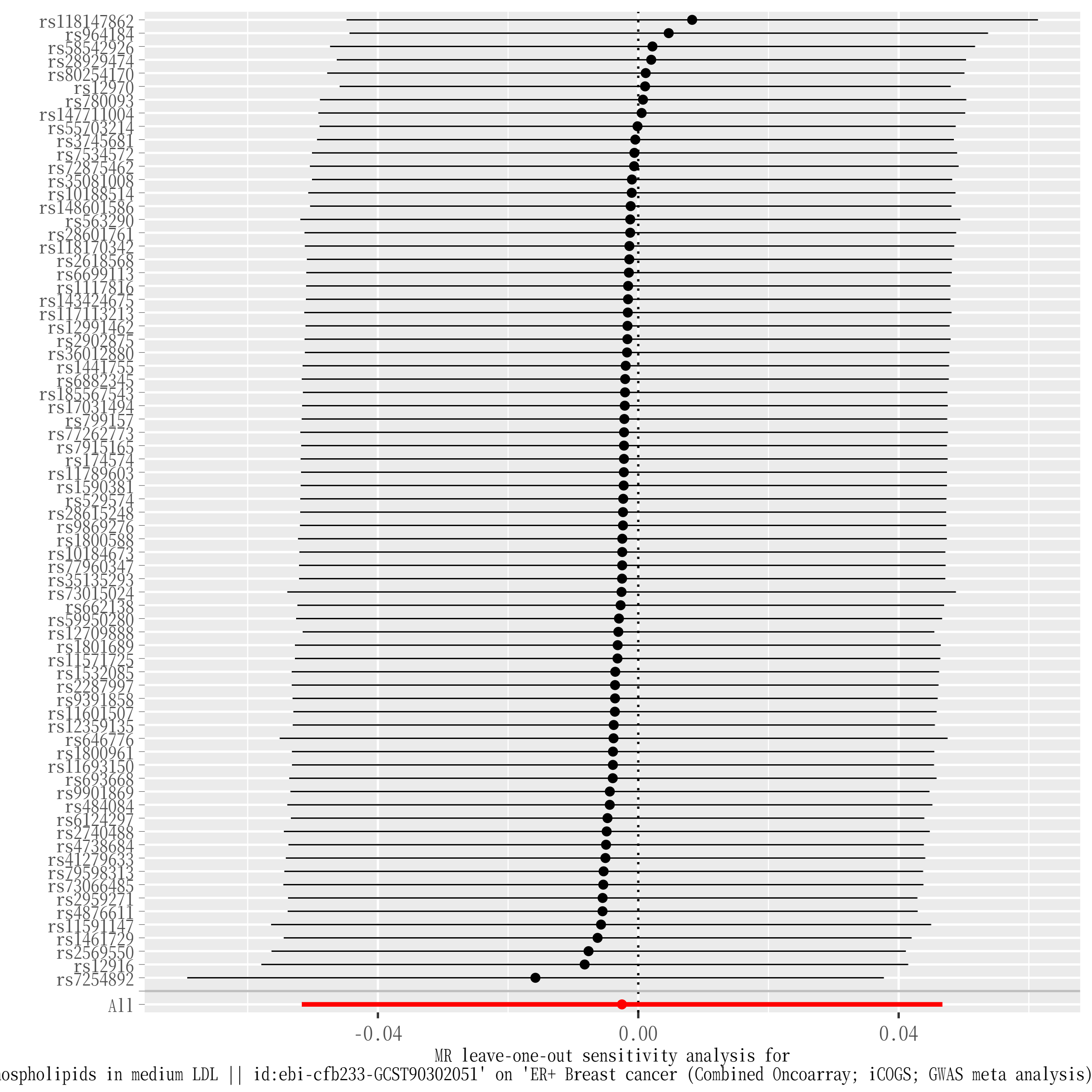
rs118147862
rs964184
rs58542926
rs28929474
rs80254170
rs12970
rs780093
rs147711004
rs55703214
rs3745681
rs7534572
rs72875462
rs35081008
rs10188514
rs148601586
rs563290
rs28601761
rs118170342
rs2618568
rs6699113
rs1117816
rs143424675
rs117113213
rs12991462
rs2902875
rs36012880
rs1441755
rs6882345
rs185567543
rs17031494
rs799157
rs77262773
rs7915165
rs174574
rs11789603
rs1590381
rs529574
rs28615248
rs9869276
rs1800588
rs10184673
rs77960347
rs35135293
rs73015024
rs662138
rs59950280
rs12709888
rs1801689
rs11571725
rs1532085
rs2287997
rs9391858
rs11601507
rs12359135
rs646776
rs1800961
rs11693150
rs693668
rs9901869
rs484084
rs6124297
rs2740488
rs4738684
rs41279633
rs79598313
rs73066485
rs2959271
rs4876611
rs11591147
rs1461729
rs2569550
rs12916
rs7254892
All
-0.04
0.00
0.04
MR leave-one-out sensitivity analysis for
ospholipids in medium LDL || id:ebi-cfb233-GCST90302051' on 'ER+ Breast cancer (Combined Oncoarray; iCOGS; GWAS meta analysis)

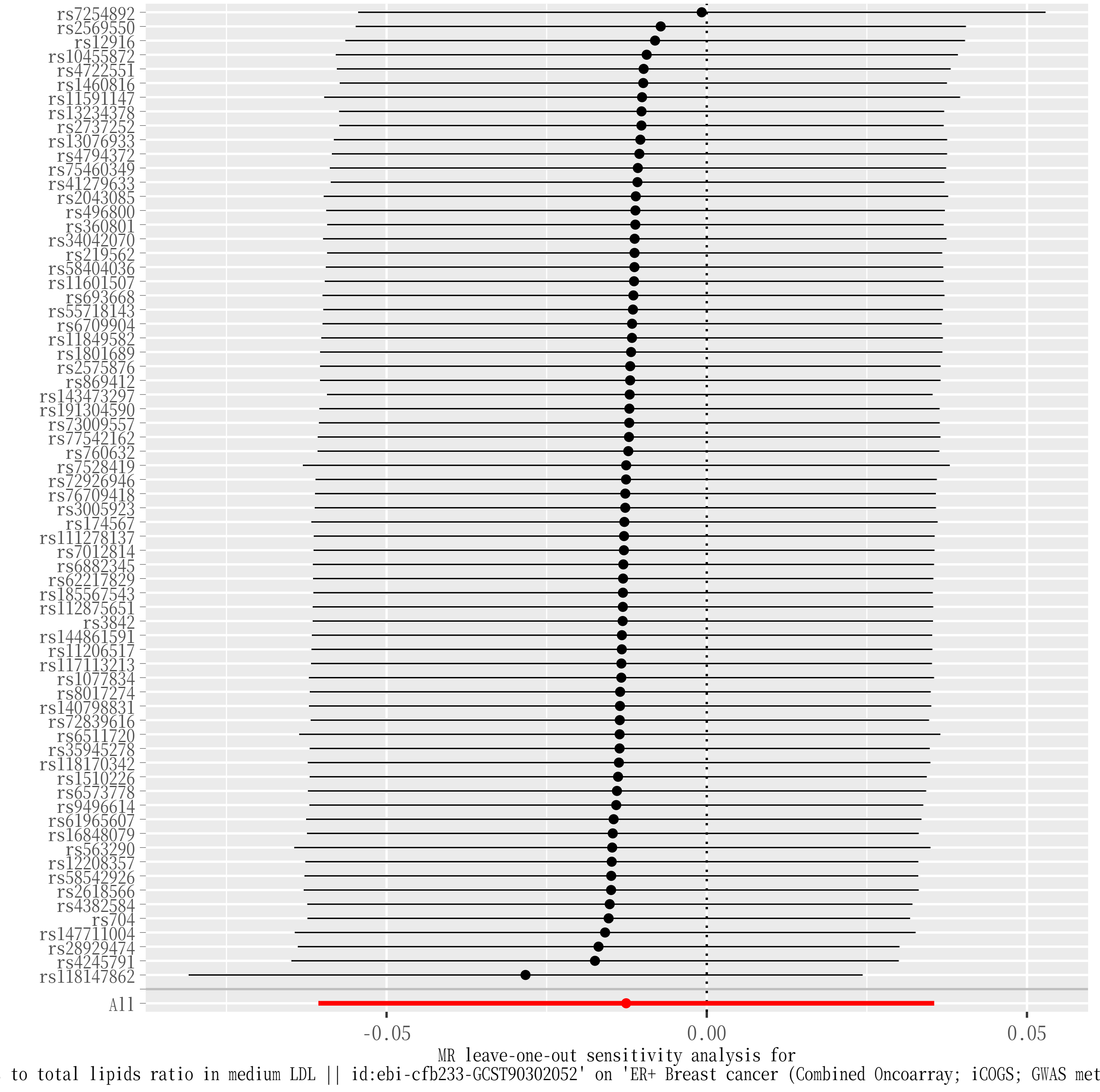
rs7254892
rs2569550
rs12916
rs10455872
rs4722551
rs1460816
rs11591147
rs13234378
rs2737252
rs13076933
rs4794372
rs75460349
rs41279633
rs2043085
rs496800
rs360801
rs34042070
rs219562
rs58404036
rs11601507
rs693668
rs55718143
rs6709904
rs11849582
rs1801689
rs2575876
rs869412
rs143473297
rs191304590
rs73009557
rs77542162
rs760632
rs7528419
rs72926946
rs76709418
rs3005923
rs174567
rs111278137
rs7012814
rs6882345
rs62217829
rs185567543
rs112875651
rs3842
rs144861591
rs11206517
rs117113213
rs1077834
rs8017274
rs140798831
rs72839616
rs6511720
rs35945278
rs118170342
rs1510226
rs6573778
rs9496614
rs61965607
rs16848079
rs563290
rs12208357
rs58542926
rs2618566
rs4382584
rs704
rs147711004
rs28929474
rs4245791
rs118147862
All
-0.05
0.00
0.05
MR leave-one-out sensitivity analysis for
to total lipids ratio in medium LDL || id:ebi-cfb233-GCST90302052' on 'ER+ Breast cancer (Combined Oncoarray; iCOGS; GWAS met

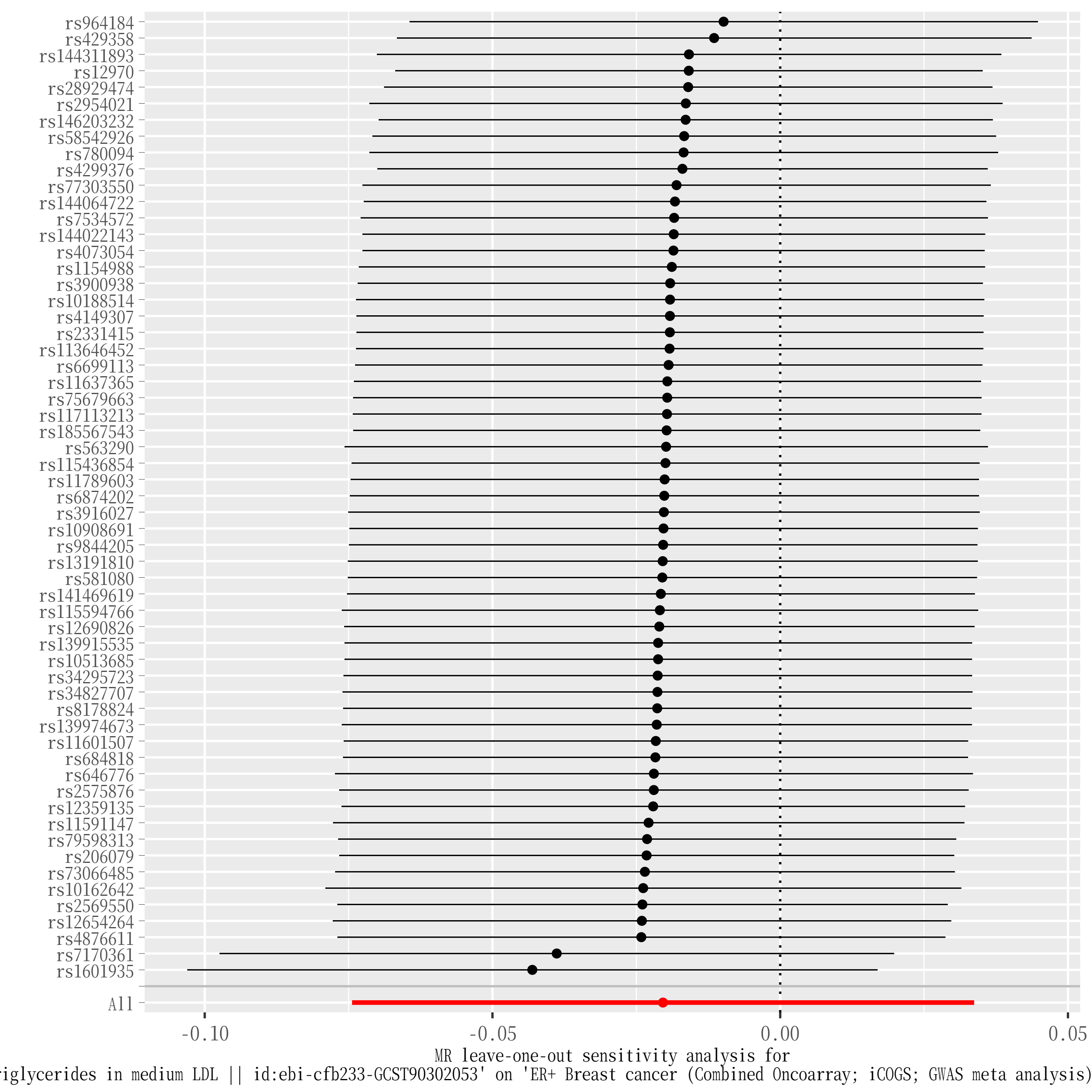
rs964184
rs429358
rs144311893
rs12970
rs28929474
rs2954021
rs146203232
rs58542926
rs780094
rs4299376
rs77303550
rs144064722
rs7534572
rs144022143
rs4073054
rs1154988
rs3900938
rs10188514
rs4149307
rs2331415
rs113646452
rs6699113
rs11637365
rs75679663
rs117113213
rs185567543
rs563290
rs115436854
rs11789603
rs6874202
rs3916027
rs10908691
rs9844205
rs13191810
rs581080
rs141469619
rs115594766
rs12690826
rs139915535
rs10513685
rs34295723
rs34827707
rs8178824
rs139974673
rs11601507
rs684818
rs646776
rs2575876
rs12359135
rs11591147
rs79598313
rs206079
rs73066485
rs10162642
rs2569550
rs12654264
rs4876611
rs7170361
rs1601935
All
-0.10
-0.05
0.00
0.05
MR leave-one-out sensitivity analysis for
riglycerides in medium LDL || id:ebi-cfb233-GCST90302053' on 'ER+ Breast cancer (Combined Oncoarray; iCOGS; GWAS meta analysis)

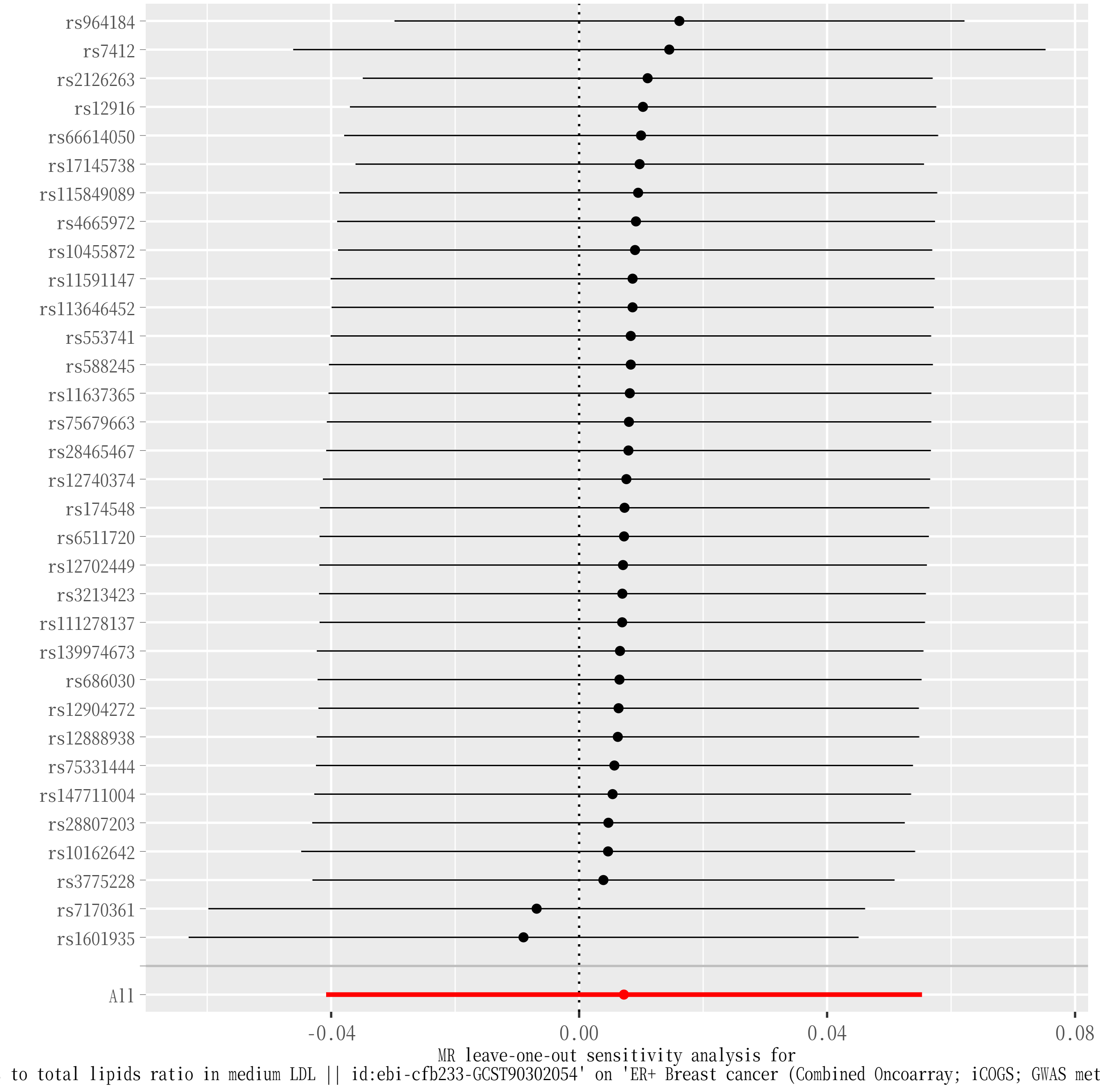
rs964184
rs7412
rs2126263
rs12916
rs66614050
rs17145738
rs115849089
rs4665972
rs10455872
rs11591147
rs113646452
rs553741
rs588245
rs11637365
rs75679663
rs28465467
rs12740374
rs174548
rs6511720
rs12702449
rs3213423
rs111278137
rs139974673
rs686030
rs12904272
rs12888938
rs75331444
rs147711004
rs28807203
rs10162642
rs3775228
rs7170361
rs1601935
All
-0.04
0.00
0.04
0.08
MR leave-one-out sensitivity analysis for
to total lipids ratio in medium LDL || id:ebi-cfb233-GCST90302054' on 'ER+ Breast cancer (Combined Oncoarray; iCOGS; GWAS met

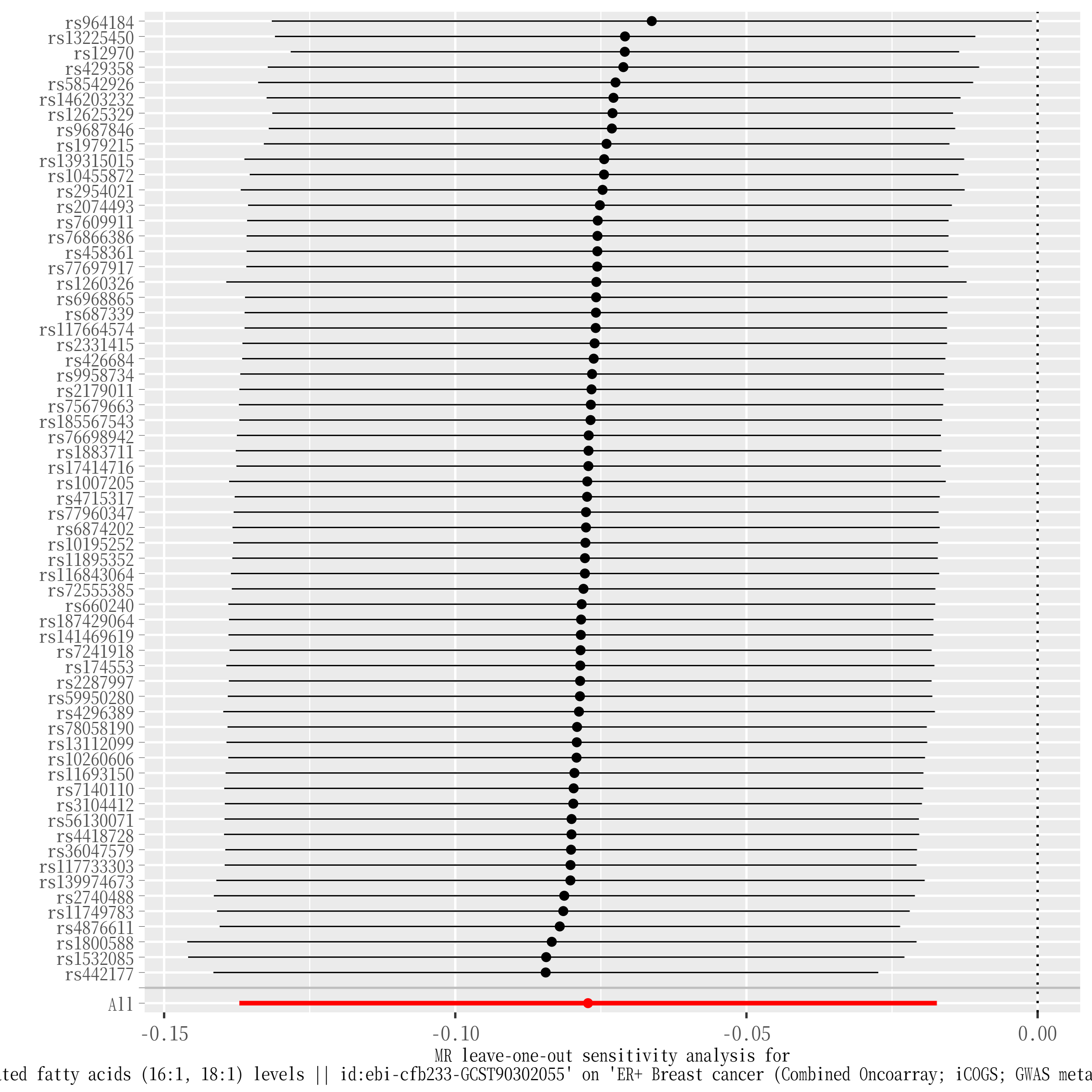

rs964184
rs13225450
rs12970
rs429358
rs58542926
rs146203232
rs12625329
rs9687846
rs1979215
rs139315015
rs10455872
rs2954021
rs2074493
rs7609911
rs76866386
rs458361
rs77697917
rs1260326
rs6968865
rs687339
rs117664574
rs2331415
rs426684
rs9958734
rs2179011
rs75679663
rs185567543
rs76698942
rs1883711
rs17414716
rs1007205
rs4715317
rs77960347
rs6874202
rs10195252
rs11895352
rs116843064
rs72555385
rs660240
rs187429064
rs141469619
rs7241918
rs174553
rs2287997
rs59950280
rs4296389
rs78058190
rs13112099
rs10260606
rs11693150
rs7140110
rs3104412
rs56130071
rs4418728
rs36047579
rs117733303
rs139974673
rs2740488
rs11749783
rs4876611
rs1800588
rs1532085
rs442177
All
-0.15
-0.10
-0.05
0.00
MR leave-one-out sensitivity analysis for
ted fatty acids (16:1, 18:1) levels || id:ebi-cfb233-GCST90302055' on 'ER+ Breast cancer (Combined Oncoarray; iCOGS; GWAS meta

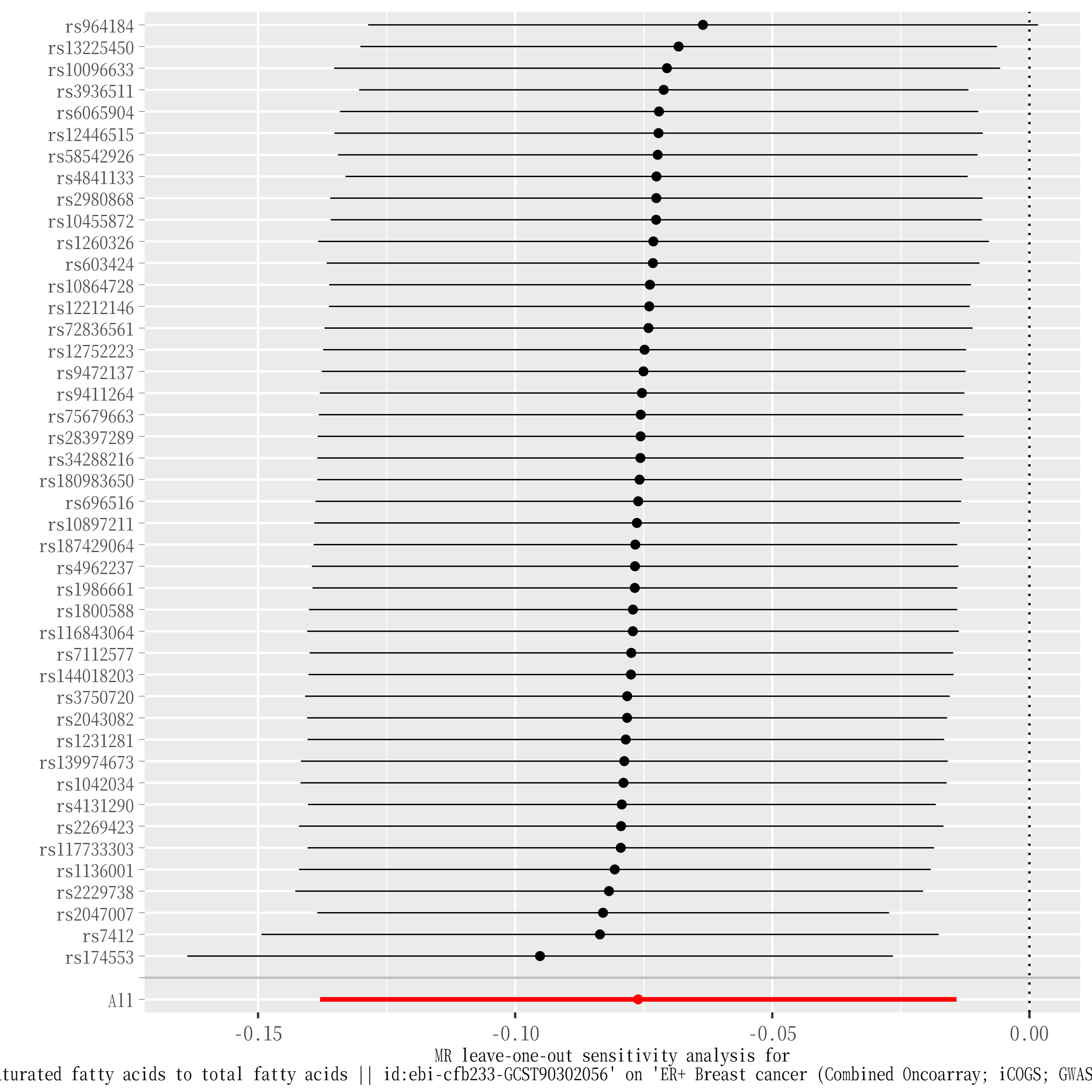
rs964184
rs13225450
rs10096633
rs3936511
rs6065904
rs12446515
rs58542926
rs4841133
rs2980868
rs10455872
rs1260326
rs603424
rs10864728
rs12212146
rs72836561
rs12752223
rs9472137
rs9411264
rs75679663
rs28397289
rs34288216
rs180983650
rs696516
rs10897211
rs187429064
rs4962237
rs1986661
rs1800588
rs116843064
rs7112577
rs144018203
rs3750720
rs2043082
rs1231281
rs139974673
rs1042034
rs4131290
rs2269423
rs117733303
rs1136001
rs2229738
rs2047007
rs7412
rs174553
All
-0.15
-0.10
-0.05
0.00
MR leave-one-out sensitivity analysis for
turated fatty acids to total fatty acids || id:ebi-cfb233-GCST90302056' on 'ER+ Breast cancer (Combined Oncoarray; iCOGS; GWAS

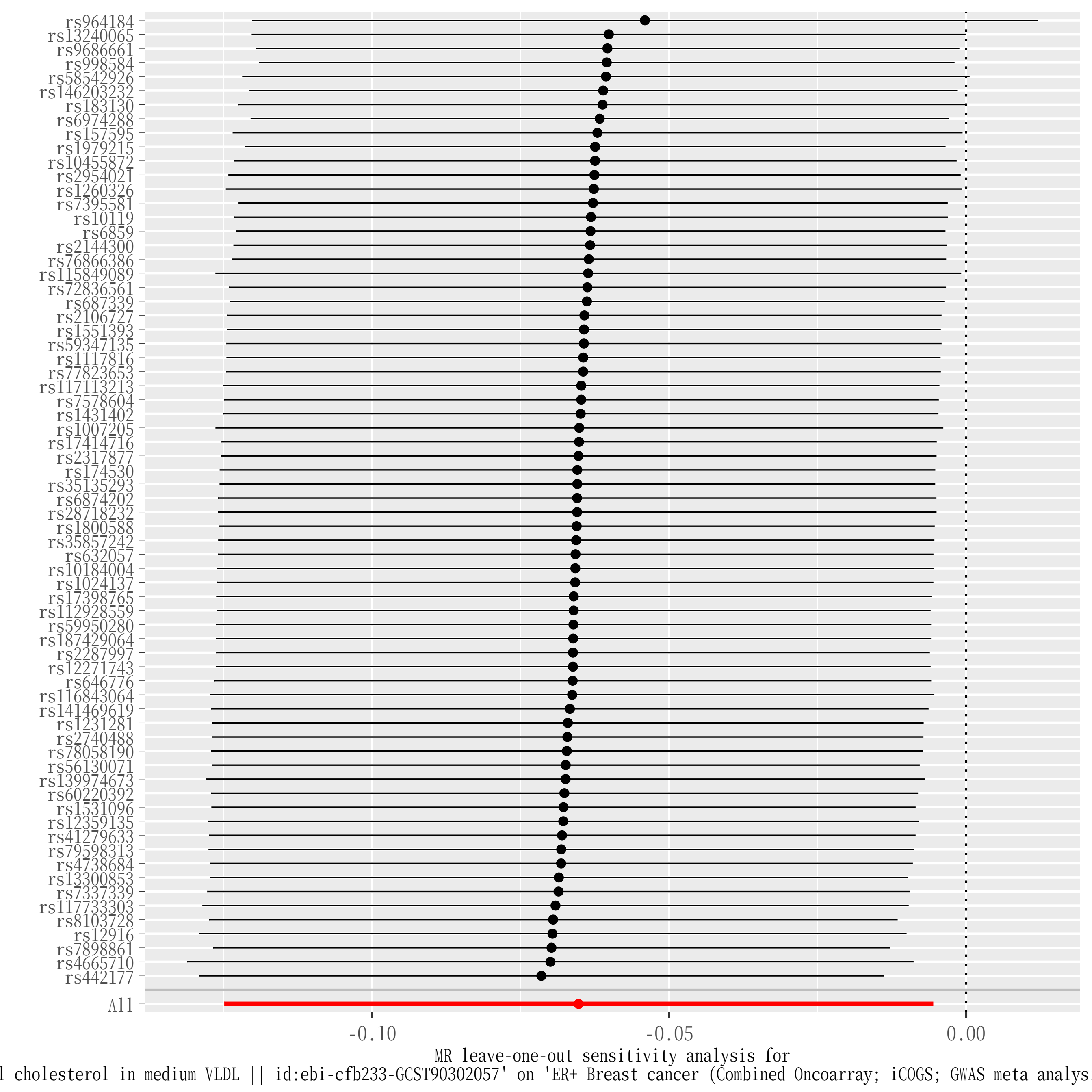
rs964184
rs13240065
rs9686661
rs998584
rs58542926
rs146203232
rs183130
rs6974288
rs157595
rs1979215
rs10455872
rs2954021
rs1260326
rs7395581
rs10119
rs6859
rs2144300
rs76866386
rs115849089
rs72836561
rs687339
rs2106727
rs1551393
rs59347135
rs1117816
rs77823653
rs117113213
rs7578604
rs1431402
rs1007205
rs17414716
rs2317877
rs174530
rs35135293
rs6874202
rs28718232
rs1800588
rs35857242
rs632057
rs10184004
rs1024137
rs17398765
rs112928559
rs59950280
rs187429064
rs2287997
rs12271743
rs646776
rs116843064
rs141469619
rs1231281
rs2740488
rs78058190
rs56130071
rs139974673
rs60220392
rs1531096
rs12359135
rs41279633
rs79598313
rs4738684
rs13300853
rs7337339
rs117733303
rs8103728
rs12916
rs7898861
rs4665710
rs442177
All
-0.10
-0.05
0.00
MR leave-one-out sensitivity analysis for
l cholesterol in medium VLDL || id:ebi-cfb233-GCST90302057' on 'ER+ Breast cancer (Combined Oncoarray; iCOGS; GWAS meta analys

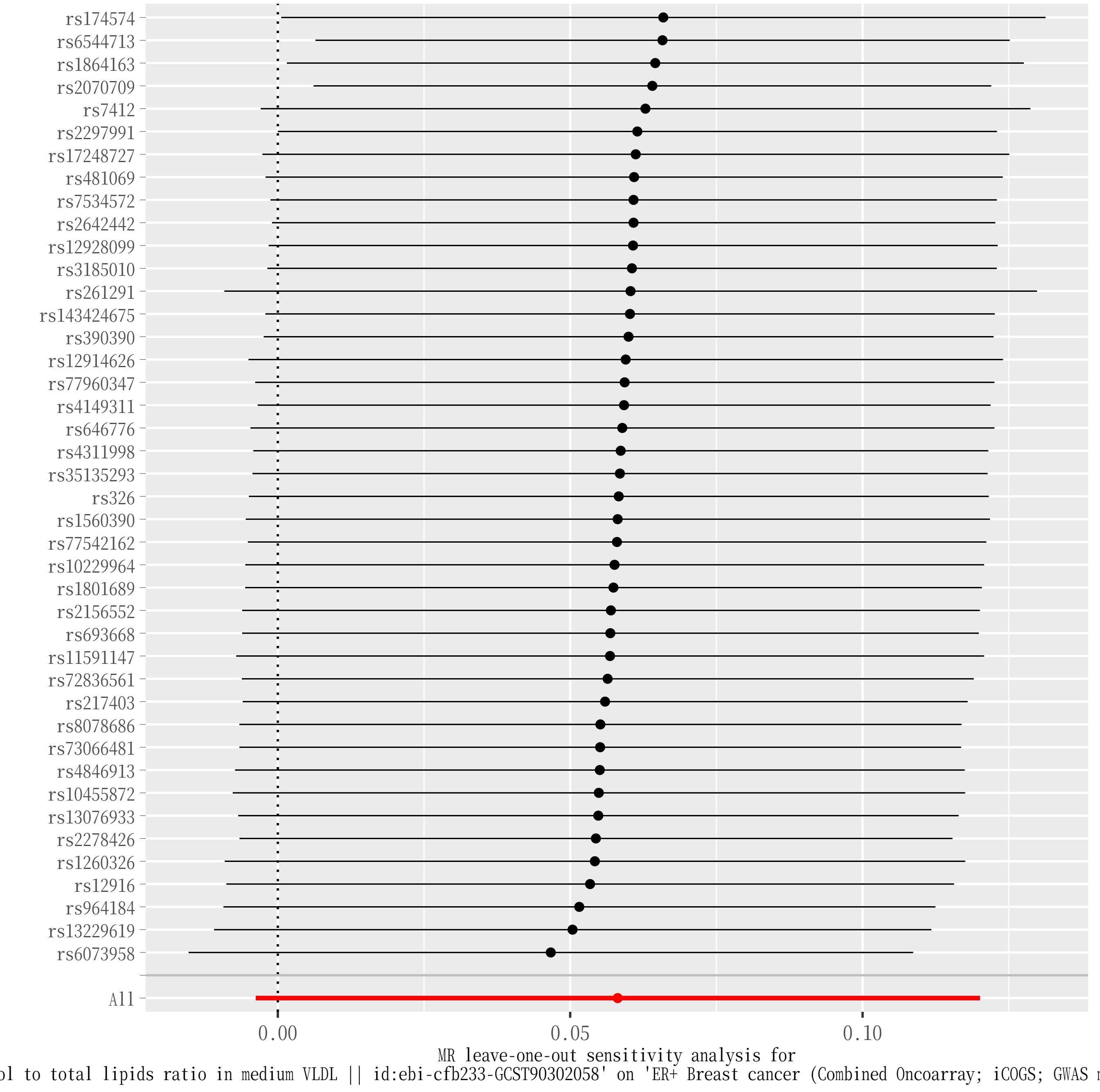
rs174574
rs6544713
rs1864163
rs2070709
rs7412
rs2297991
rs17248727
rs481069
rs7534572
rs2642442
rs12928099
rs3185010
rs261291
rs143424675
rs390390
rs12914626
rs77960347
rs4149311
rs646776
rs4311998
rs35135293
rs326
rs1560390
rs77542162
rs10229964
rs1801689
rs2156552
rs693668
rs11591147
rs72836561
rs217403
rs8078686
rs73066481
rs4846913
rs10455872
rs13076933
rs2278426
rs1260326
rs12916
rs964184
rs13229619
rs6073958
All
0.00
0.05
0.10
MR leave-one-out sensitivity analysis for
ol to total lipids ratio in medium VLDL || id:ebi-cfb233-GCST90302058' on 'ER+ Breast cancer (Combined Oncoarray; iCOGS; GWAS m

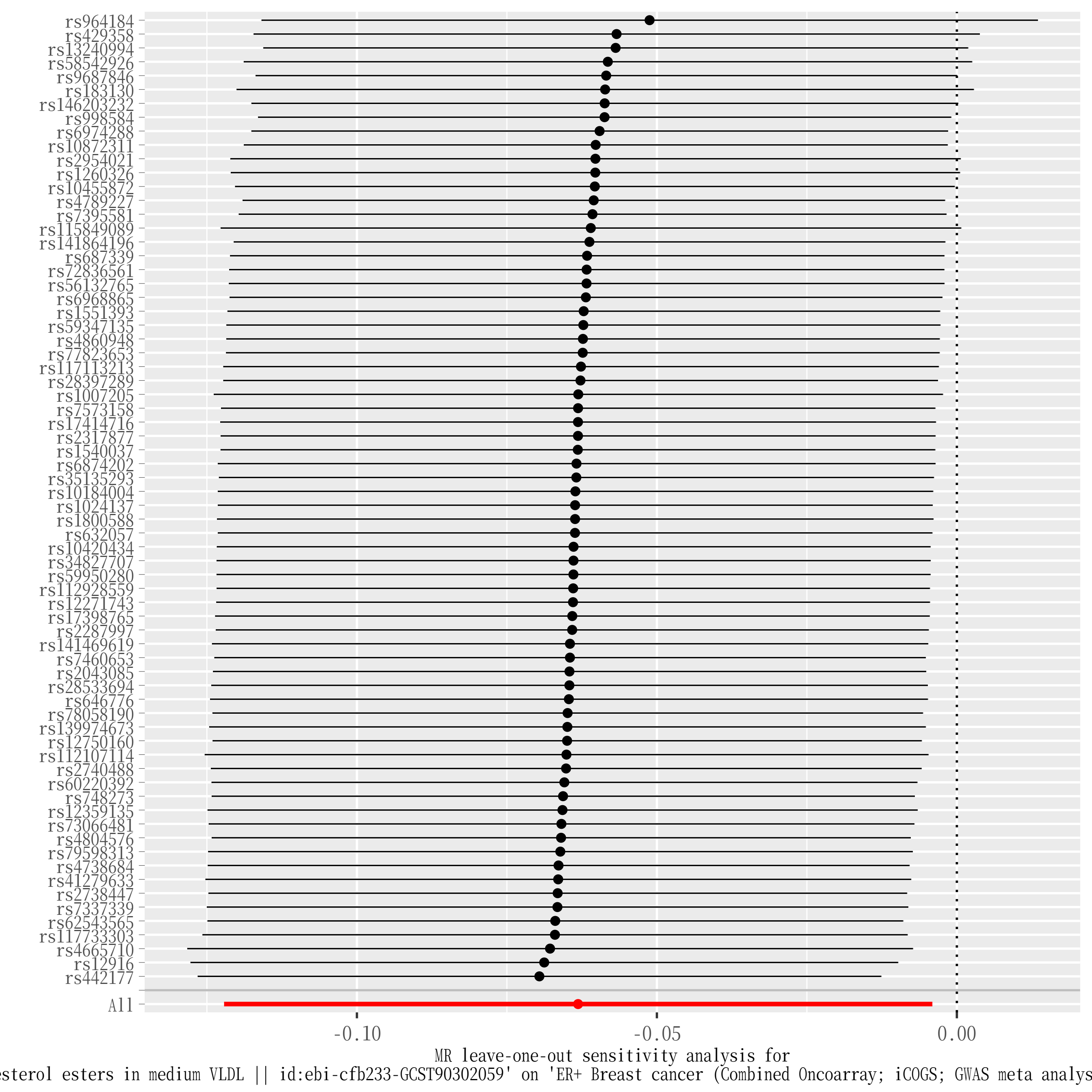
rs964184
rs429358
rs13240994
rs58542926
rs9687846
rs183130
rs146203232
rs998584
rs6974288
rs10872311
rs2954021
rs1260326
rs10455872
rs4789227
rs7395581
rs115849089
rs141864196
rs687339
rs72836561
rs56132765
rs6968865
rs1551393
rs59347135
rs4860948
rs77823653
rs117113213
rs28397289
rs1007205
rs7573158
rs17414716
rs2317877
rs1540037
rs6874202
rs35135293
rs10184004
rs1024137
rs1800588
rs632057
rs10420434
rs34827707
rs59950280
rs112928559
rs12271743
rs17398765
rs2287997
rs141469619
rs7460653
rs2043085
rs28533694
rs646776
rs78058190
rs139974673
rs12750160
rs112107114
rs2740488
rs60220392
rs748273
rs12359135
rs73066481
rs4804576
rs79598313
rs4738684
rs41279633
rs2738447
rs7337339
rs62543565
rs117733303
rs4665710
rs12916
rs442177
All
-0.10
-0.05
0.00
MR leave-one-out sensitivity analysis for
esterol esters in medium VLDL || id:ebi-cfb233-GCST90302059' on 'ER+ Breast cancer (Combined Oncoarray; iCOGS; GWAS meta analys

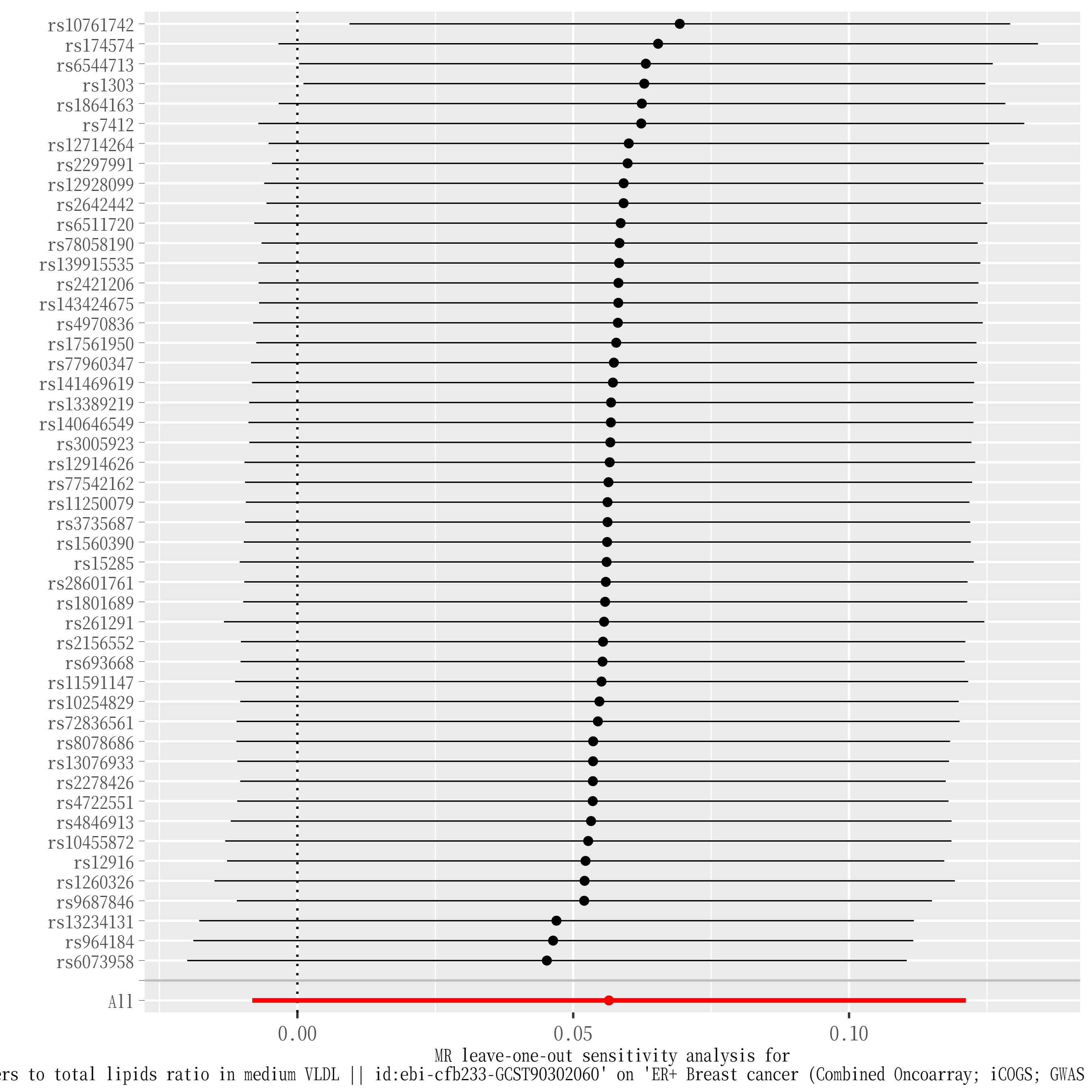
rs10761742
rs174574
rs6544713
rs1303
rs1864163
rs7412
rs12714264
rs2297991
rs12928099
rs2642442
rs6511720
rs78058190
rs139915535
rs2421206
rs143424675
rs4970836
rs17561950
rs77960347
rs141469619
rs13389219
rs140646549
rs3005923
rs12914626
rs77542162
rs11250079
rs3735687
rs1560390
rs15285
rs28601761
rs1801689
rs261291
rs2156552
rs693668
rs11591147
rs10254829
rs72836561
rs8078686
rs13076933
rs2278426
rs4722551
rs4846913
rs10455872
rs12916
rs1260326
rs9687846
rs13234131
rs964184
rs6073958
All
0.00
0.05
0.10
MR leave-one-out sensitivity analysis for
ers to total lipids ratio in medium VLDL || id:ebi-cfb233-GCST90302060' on 'ER+ Breast cancer (Combined Oncoarray; iCOGS; GWAS

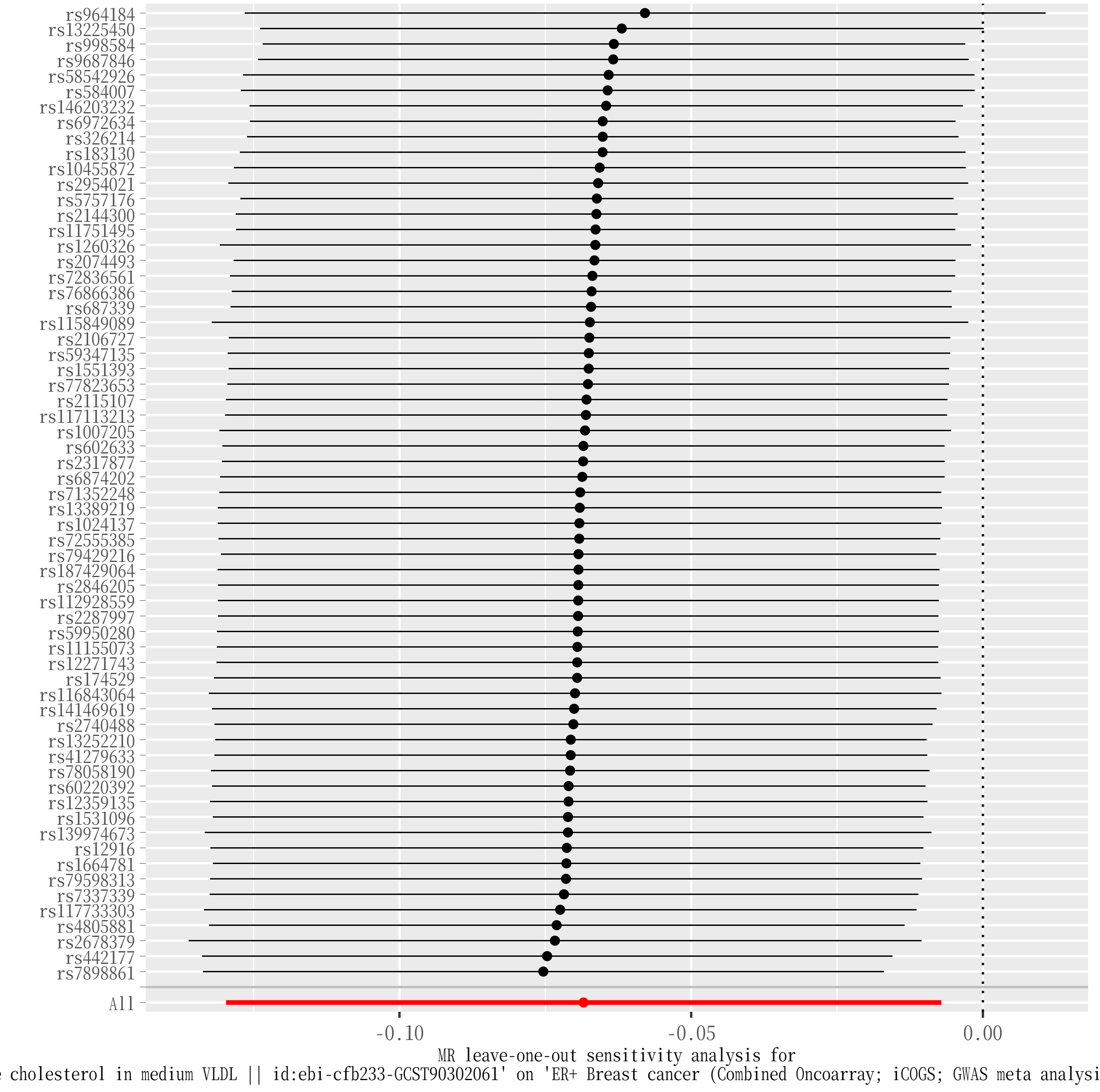

rs964184
rs13225450
rs998584
rs9687846
rs58542926
rs584007
rs146203232
rs6972634
rs326214
rs183130
rs10455872
rs2954021
rs5757176
rs2144300
rs11751495
rs1260326
rs2074493
rs72836561
rs76866386
rs687339
rs115849089
rs2106727
rs59347135
rs1551393
rs77823653
rs2115107
rs117113213
rs1007205
rs602633
rs2317877
rs6874202
rs71352248
rs13389219
rs1024137
rs72555385
rs79429216
rs187429064
rs2846205
rs112928559
rs2287997
rs59950280
rs11155073
rs12271743
rs174529
rs116843064
rs141469619
rs2740488
rs13252210
rs41279633
rs78058190
rs60220392
rs12359135
rs1531096
rs139974673
rs12916
rs1664781
rs79598313
rs7337339
rs117733303
rs4805881
rs2678379
rs442177
rs7898861
All
-0.10
-0.05
0.00
MR leave-one-out sensitivity analysis for
e cholesterol in medium VLDL || id:ebi-cfb233-GCST90302061' on 'ER+ Breast cancer (Combined Oncoarray; iCOGS; GWAS meta analysi

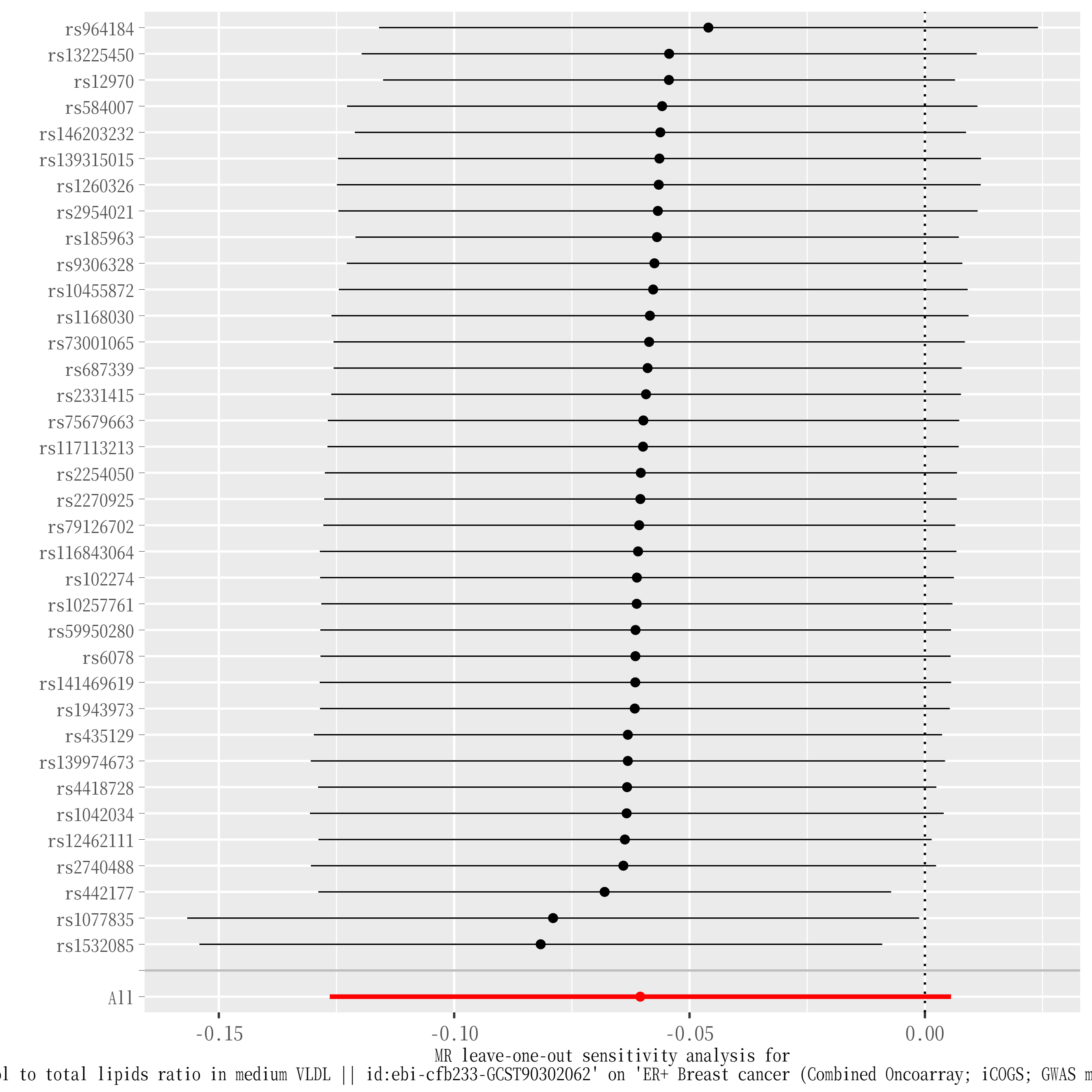
rs964184
rs13225450
rs12970
rs584007
rs146203232
rs139315015
rs1260326
rs2954021
rs185963
rs9306328
rs10455872
rs1168030
rs73001065
rs687339
rs2331415
rs75679663
rs117113213
rs2254050
rs2270925
rs79126702
rs116843064
rs102274
rs10257761
rs59950280
rs6078
rs141469619
rs1943973
rs435129
rs139974673
rs4418728
rs1042034
rs12462111
rs2740488
rs442177
rs1077835
rs1532085
All
-0.15
-0.10
-0.05
0.00
MR leave-one-out sensitivity analysis for
ol to total lipids ratio in medium VLDL || id:ebi-cfb233-GCST90302062' on 'ER+ Breast cancer (Combined Oncoarray; iCOGS; GWAS m

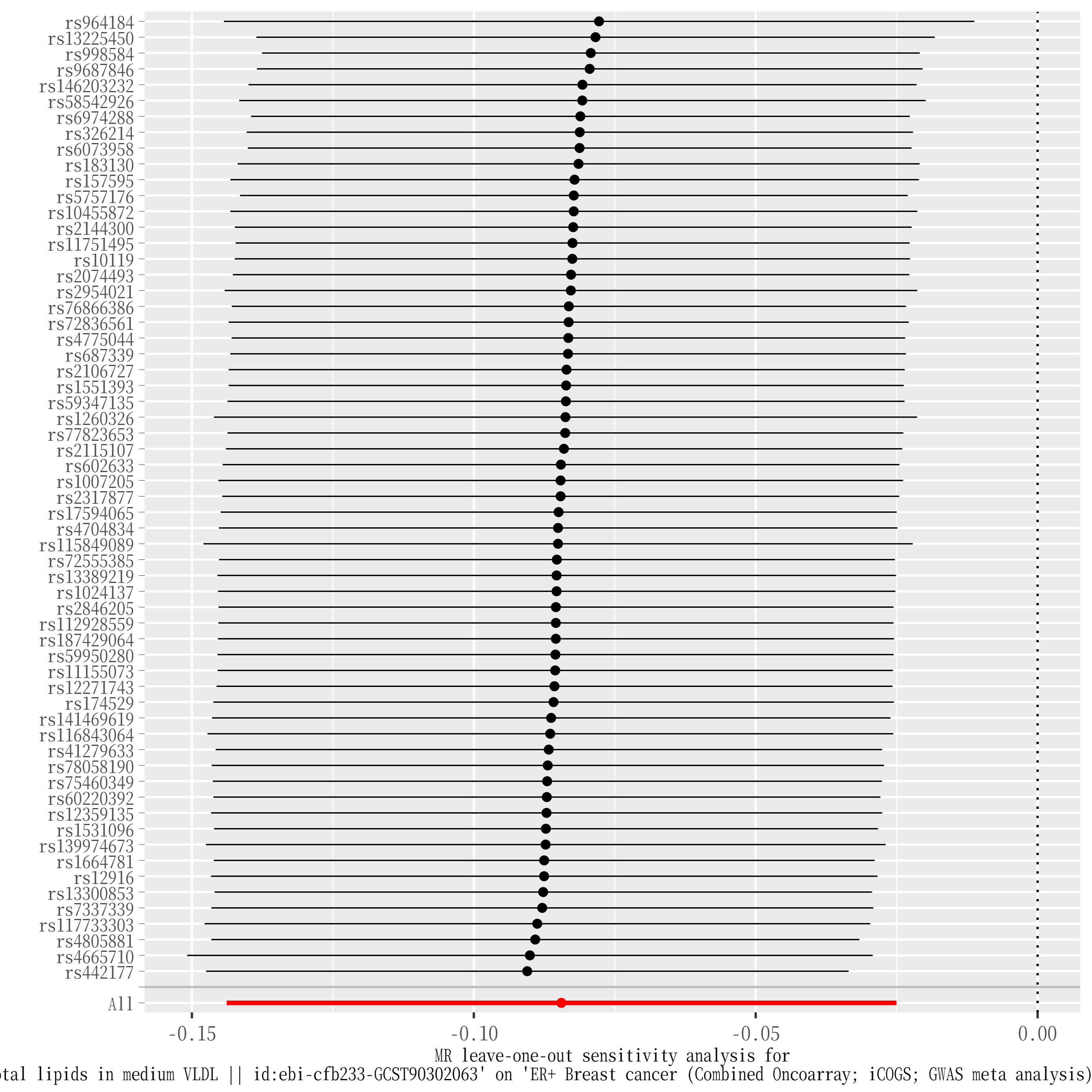
rs964184
rs13225450
rs998584
rs9687846
rs146203232
rs58542926
rs6974288
rs326214
rs6073958
rs183130
rs157595
rs5757176
rs10455872
rs2144300
rs11751495
rs10119
rs2074493
rs2954021
rs76866386
rs72836561
rs4775044
rs687339
rs2106727
rs1551393
rs59347135
rs1260326
rs77823653
rs2115107
rs602633
rs1007205
rs2317877
rs17594065
rs4704834
rs115849089
rs72555385
rs13389219
rs1024137
rs2846205
rs112928559
rs187429064
rs59950280
rs11155073
rs12271743
rs174529
rs141469619
rs116843064
rs41279633
rs78058190
rs75460349
rs60220392
rs12359135
rs1531096
rs139974673
rs1664781
rs12916
rs13300853
rs7337339
rs117733303
rs4805881
rs4665710
rs442177
All
-0.15
-0.10
-0.05
0.00
MR leave-one-out sensitivity analysis for
otal lipids in medium VLDL || id:ebi-cfb233-GCST90302063' on 'ER+ Breast cancer (Combined Oncoarray; iCOGS; GWAS meta analysis)

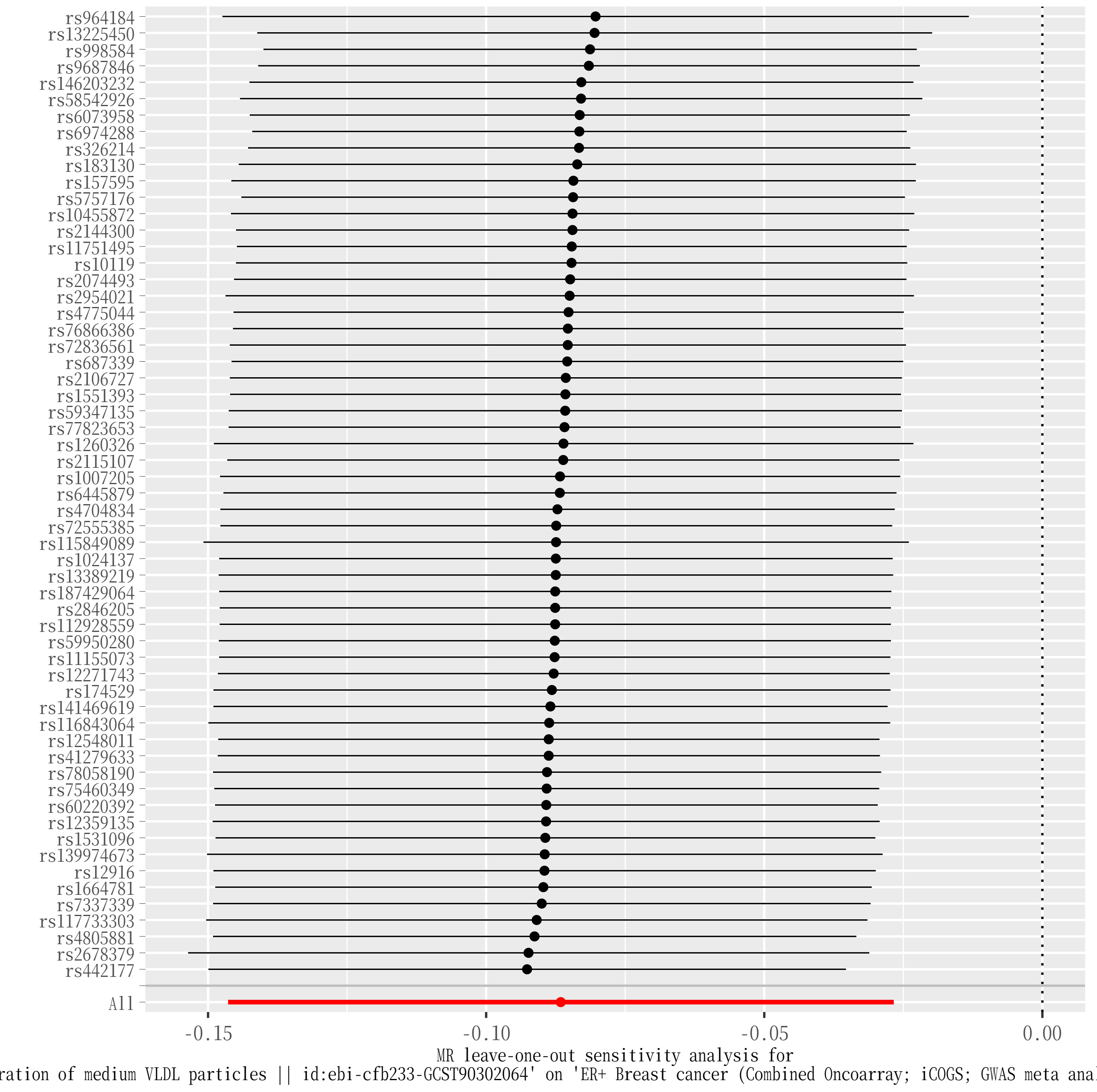
rs964184
rs13225450
rs998584
rs9687846
rs146203232
rs58542926
rs6073958
rs6974288
rs326214
rs183130
rs157595
rs5757176
rs10455872
rs2144300
rs11751495
rs10119
rs2074493
rs2954021
rs4775044
rs76866386
rs72836561
rs687339
rs2106727
rs1551393
rs59347135
rs77823653
rs1260326
rs2115107
rs1007205
rs6445879
rs4704834
rs72555385
rs115849089
rs1024137
rs13389219
rs187429064
rs2846205
rs112928559
rs59950280
rs11155073
rs12271743
rs174529
rs141469619
rs116843064
rs12548011
rs41279633
rs78058190
rs75460349
rs60220392
rs12359135
rs1531096
rs139974673
rs12916
rs1664781
rs7337339
rs117733303
rs4805881
rs2678379
rs442177
All
-0.15
-0.10
-0.05
0.00
MR leave-one-out sensitivity analysis for
ration of medium VLDL particles || id:ebi-cfb233-GCST90302064' on 'ER+ Breast cancer (Combined Oncoarray; iCOGS; GWAS meta anal

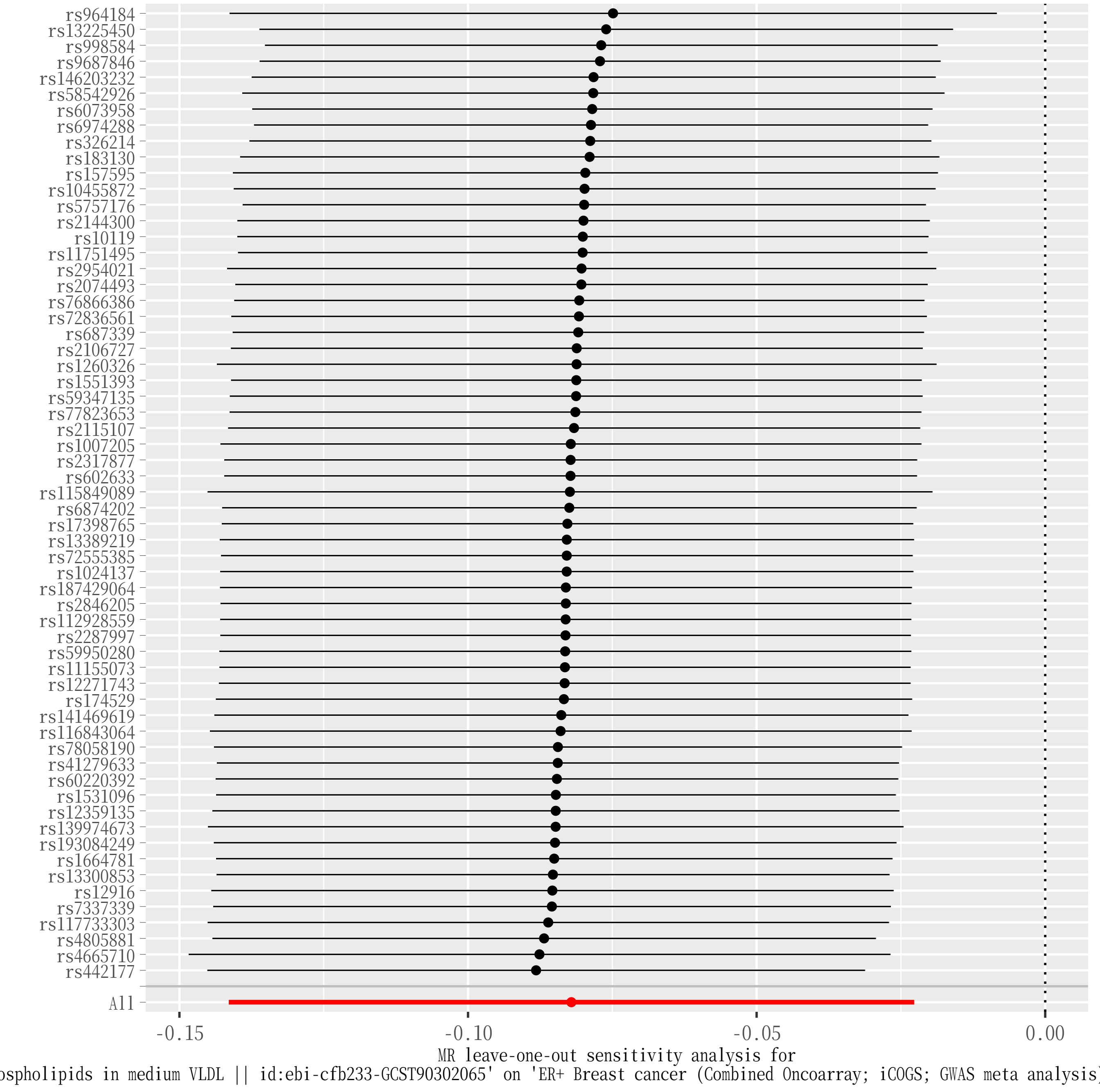
rs964184
rs13225450
rs998584
rs9687846
rs146203232
rs58542926
rs6073958
rs6974288
rs326214
rs183130
rs157595
rs10455872
rs5757176
rs2144300
rs10119
rs11751495
rs2954021
rs2074493
rs76866386
rs72836561
rs687339
rs2106727
rs1260326
rs1551393
rs59347135
rs77823653
rs2115107
rs1007205
rs2317877
rs602633
rs115849089
rs6874202
rs17398765
rs13389219
rs72555385
rs1024137
rs187429064
rs2846205
rs112928559
rs2287997
rs59950280
rs11155073
rs12271743
rs174529
rs141469619
rs116843064
rs78058190
rs41279633
rs60220392
rs1531096
rs12359135
rs139974673
rs193084249
rs1664781
rs13300853
rs12916
rs7337339
rs117733303
rs4805881
rs4665710
rs442177
All
-0.15
-0.10
-0.05
0.00
MR leave-one-out sensitivity analysis for
ospholipids in medium VLDL || id:ebi-cfb233-GCST90302065' on 'ER+ Breast cancer (Combined Oncoarray; iCOGS; GWAS meta analysis)

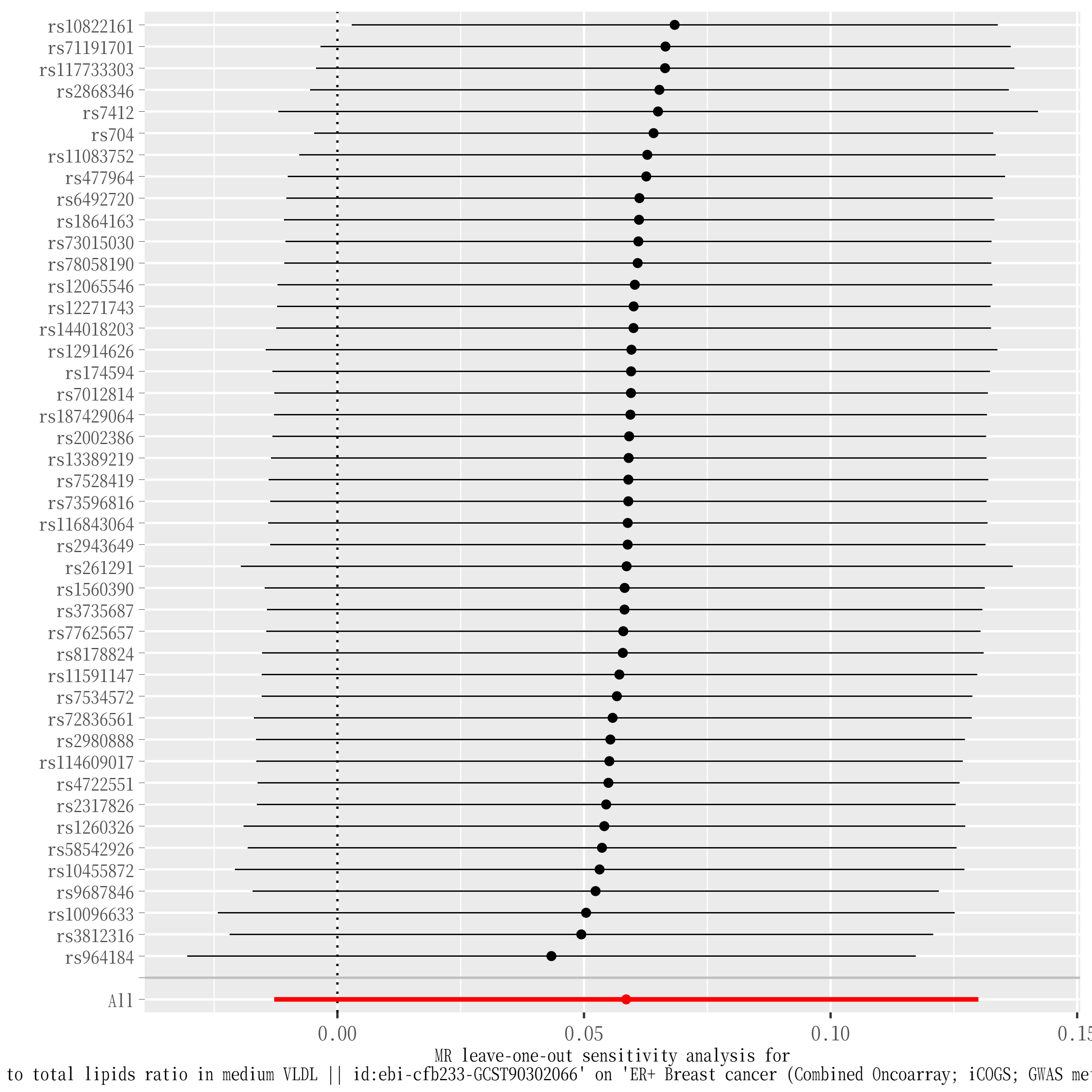

rs10822161
rs71191701
rs117733303
rs2868346
rs7412
rs704
rs11083752
rs477964
rs6492720
rs1864163
rs73015030
rs78058190
rs12065546
rs12271743
rs144018203
rs12914626
rs174594
rs7012814
rs187429064
rs2002386
rs13389219
rs7528419
rs73596816
rs116843064
rs2943649
rs261291
rs1560390
rs3735687
rs77625657
rs8178824
rs11591147
rs7534572
rs72836561
rs2980888
rs114609017
rs4722551
rs2317826
rs1260326
rs58542926
rs10455872
rs9687846
rs10096633
rs3812316
rs964184
All
0.00
0.05
0.10
0.15
MR leave-one-out sensitivity analysis for
to total lipids ratio in medium VLDL || id:ebi-cfb233-GCST90302066' on 'ER+ Breast cancer (Combined Oncoarray; iCOGS; GWAS me

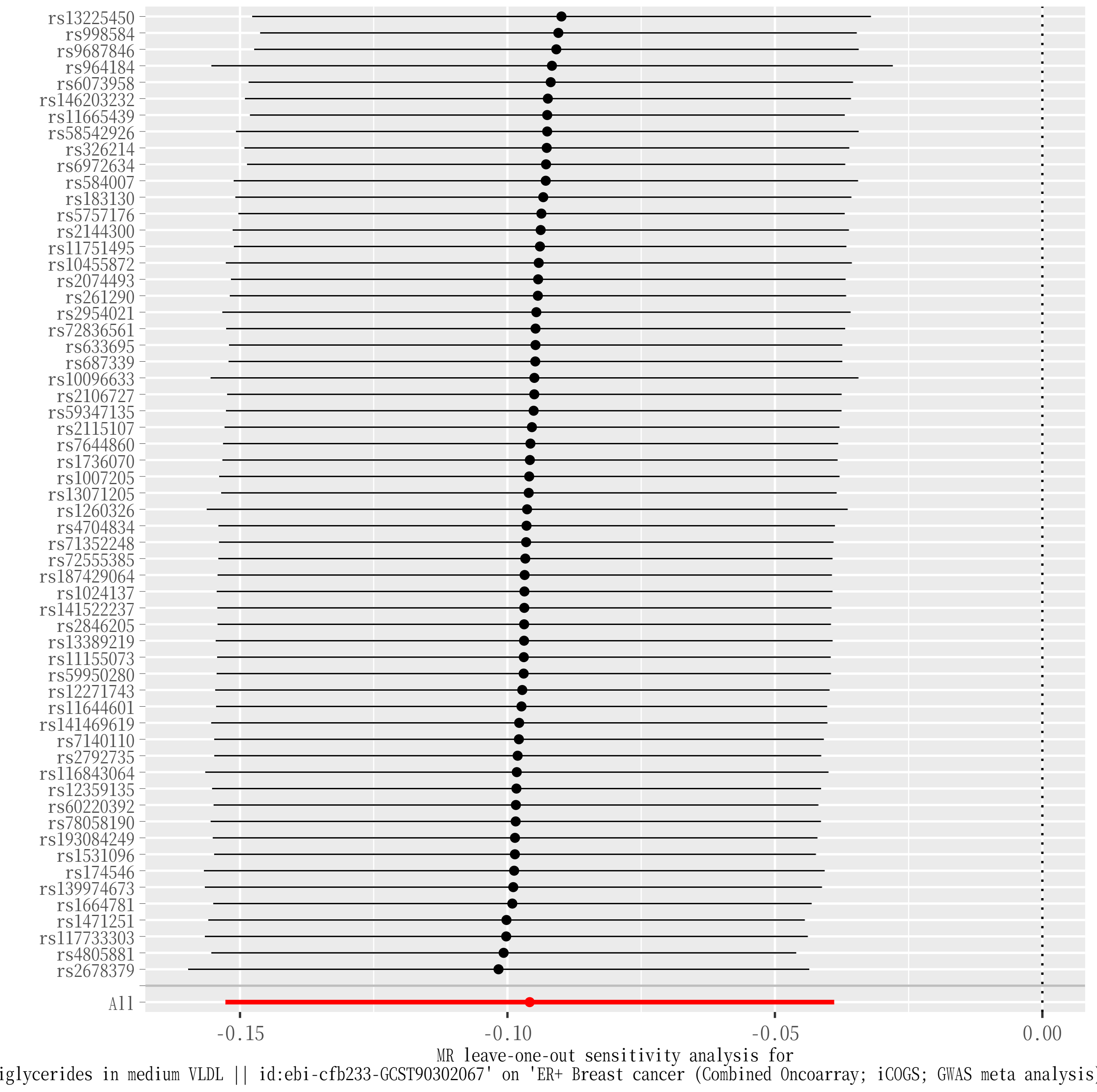

rs13225450
rs998584
rs9687846
rs964184
rs6073958
rs146203232
rs11665439
rs58542926
rs326214
rs6972634
rs584007
rs183130
rs5757176
rs2144300
rs11751495
rs10455872
rs2074493
rs261290
rs2954021
rs72836561
rs633695
rs687339
rs10096633
rs2106727
rs59347135
rs2115107
rs7644860
rs1736070
rs1007205
rs13071205
rs1260326
rs4704834
rs71352248
rs72555385
rs187429064
rs1024137
rs141522237
rs2846205
rs13389219
rs11155073
rs59950280
rs12271743
rs11644601
rs141469619
rs7140110
rs2792735
rs116843064
rs12359135
rs60220392
rs78058190
rs193084249
rs1531096
rs174546
rs139974673
rs1664781
rs1471251
rs117733303
rs4805881
rs2678379
All
-0.15
-0.10
-0.05
0.00
MR leave-one-out sensitivity analysis for
iglycerides in medium VLDL || id:ebi-cfb233-GCST90302067' on 'ER+ Breast cancer (Combined Oncoarray; iCOGS; GWAS meta analysis)

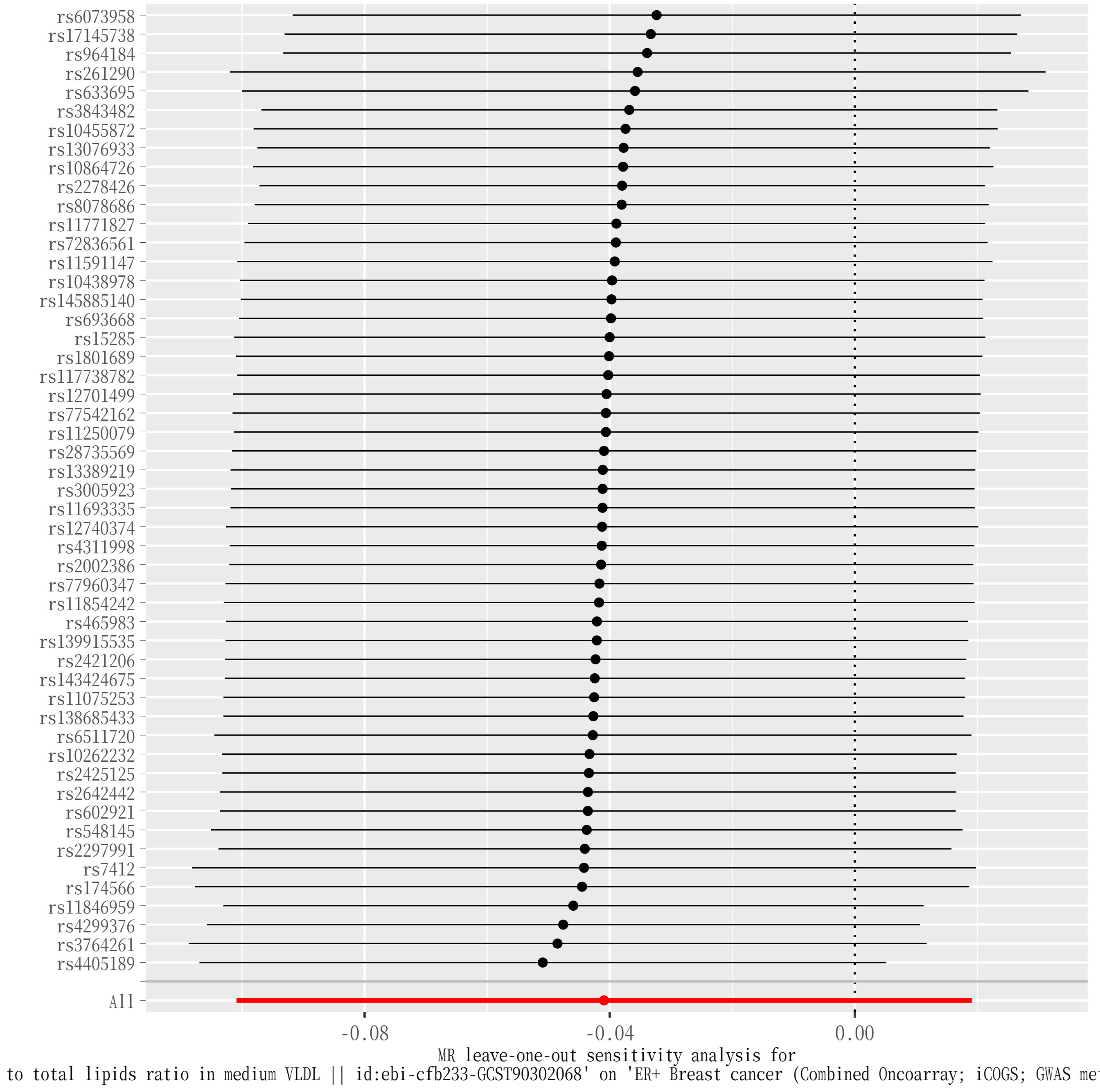
rs6073958
rs17145738
rs964184
rs261290
rs633695
rs3843482
rs10455872
rs13076933
rs10864726
rs2278426
rs8078686
rs11771827
rs72836561
rs11591147
rs10438978
rs145885140
rs693668
rs15285
rs1801689
rs117738782
rs12701499
rs77542162
rs11250079
rs28735569
rs13389219
rs3005923
rs11693335
rs12740374
rs4311998
rs2002386
rs77960347
rs11854242
rs465983
rs139915535
rs2421206
rs143424675
rs11075253
rs138685433
rs6511720
rs10262232
rs2425125
rs2642442
rs602921
rs548145
rs2297991
rs7412
rs174566
rs11846959
rs4299376
rs3764261
rs4405189
All
-0.08
-0.04
0.00
MR leave-one-out sensitivity analysis for
to total lipids ratio in medium VLDL || id:ebi-cfb233-GCST90302068' on 'ER+ Breast cancer (Combined Oncoarray; iCOGS; GWAS me

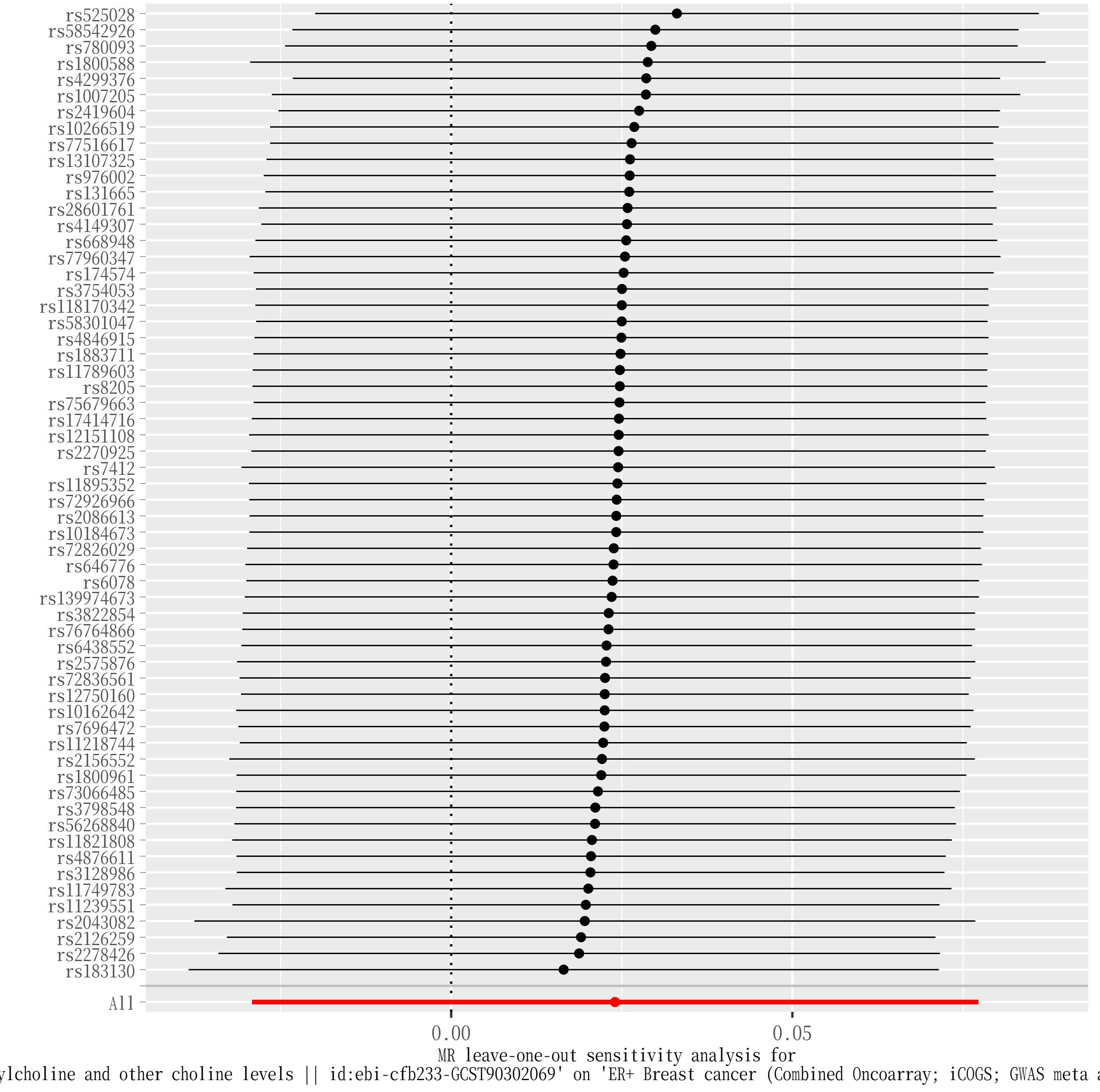

rs525028
rs58542926
rs780093
rs1800588
rs4299376
rs1007205
rs2419604
rs10266519
rs77516617
rs13107325
rs976002
rs131665
rs28601761
rs4149307
rs668948
rs77960347
rs174574
rs3754053
rs118170342
rs58301047
rs4846915
rs1883711
rs11789603
rs8205
rs75679663
rs17414716
rs12151108
rs2270925
rs7412
rs11895352
rs72926966
rs2086613
rs10184673
rs72826029
rs646776
rs6078
rs139974673
rs3822854
rs76764866
rs6438552
rs2575876
rs72836561
rs12750160
rs10162642
rs7696472
rs11218744
rs2156552
rs1800961
rs73066485
rs3798548
rs56268840
rs11821808
rs4876611
rs3128986
rs11749783
rs11239551
rs2043082
rs2126259
rs2278426
rs183130
All
0.00
0.05
MR leave-one-out sensitivity analysis for
ylcholine and other choline levels || id:ebi-cfb233-GCST90302069' on 'ER+ Breast cancer (Combined Oncoarray; iCOGS; GWAS meta a

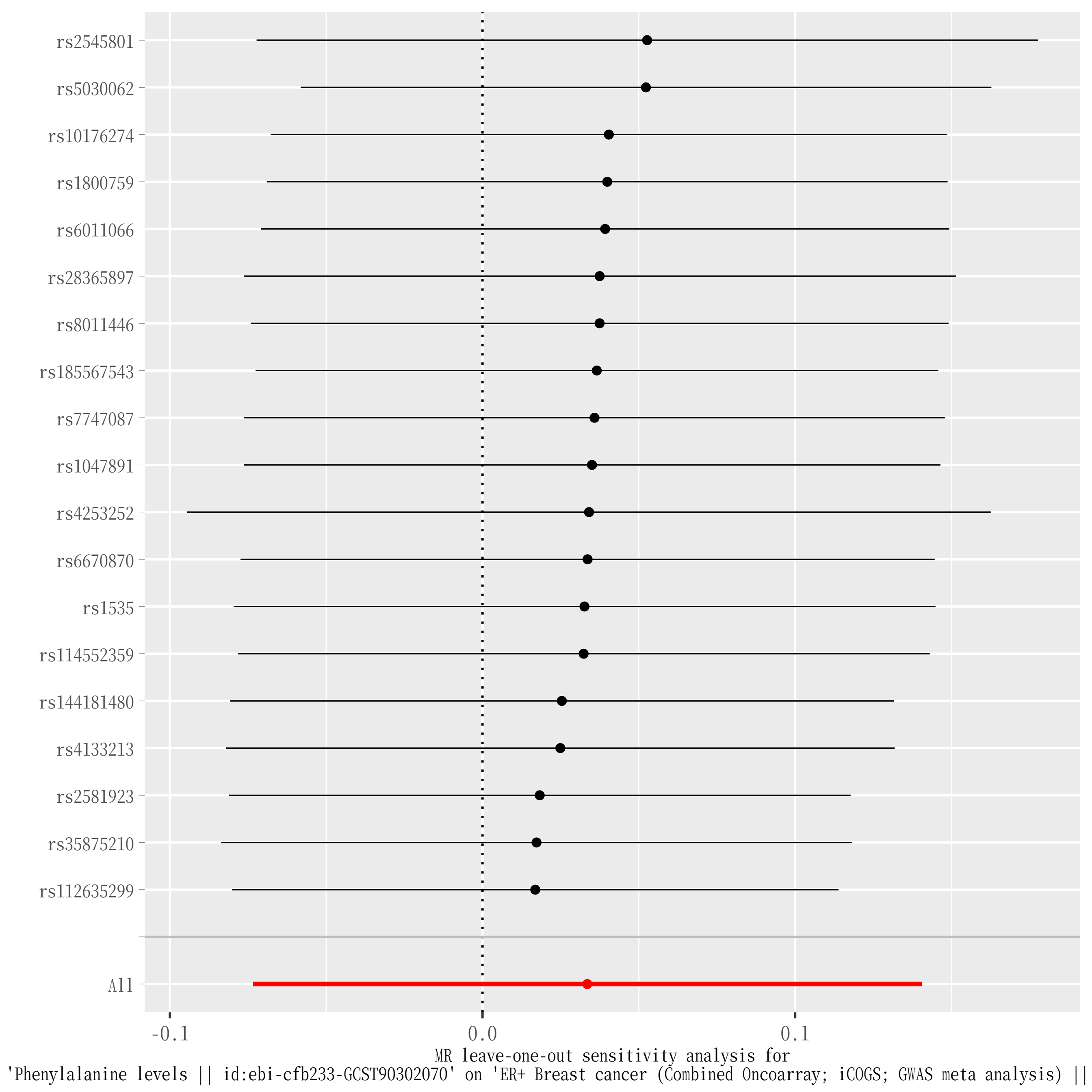
rs2545801
rs5030062
rs10176274
rs1800759
rs6011066
rs28365897
rs8011446
rs185567543
rs7747087
rs1047891
rs4253252
rs6670870
rs1535
rs114552359
rs144181480
rs4133213
rs2581923
rs35875210
rs112635299
All
-0.1
0.0
0.1
MR leave-one-out sensitivity analysis for
'Phenylalanine levels || id:ebi-cfb233-GCST90302070' on 'ER+ Breast cancer (Combined Oncoarray; iCOGS; GWAS meta analysis) ||

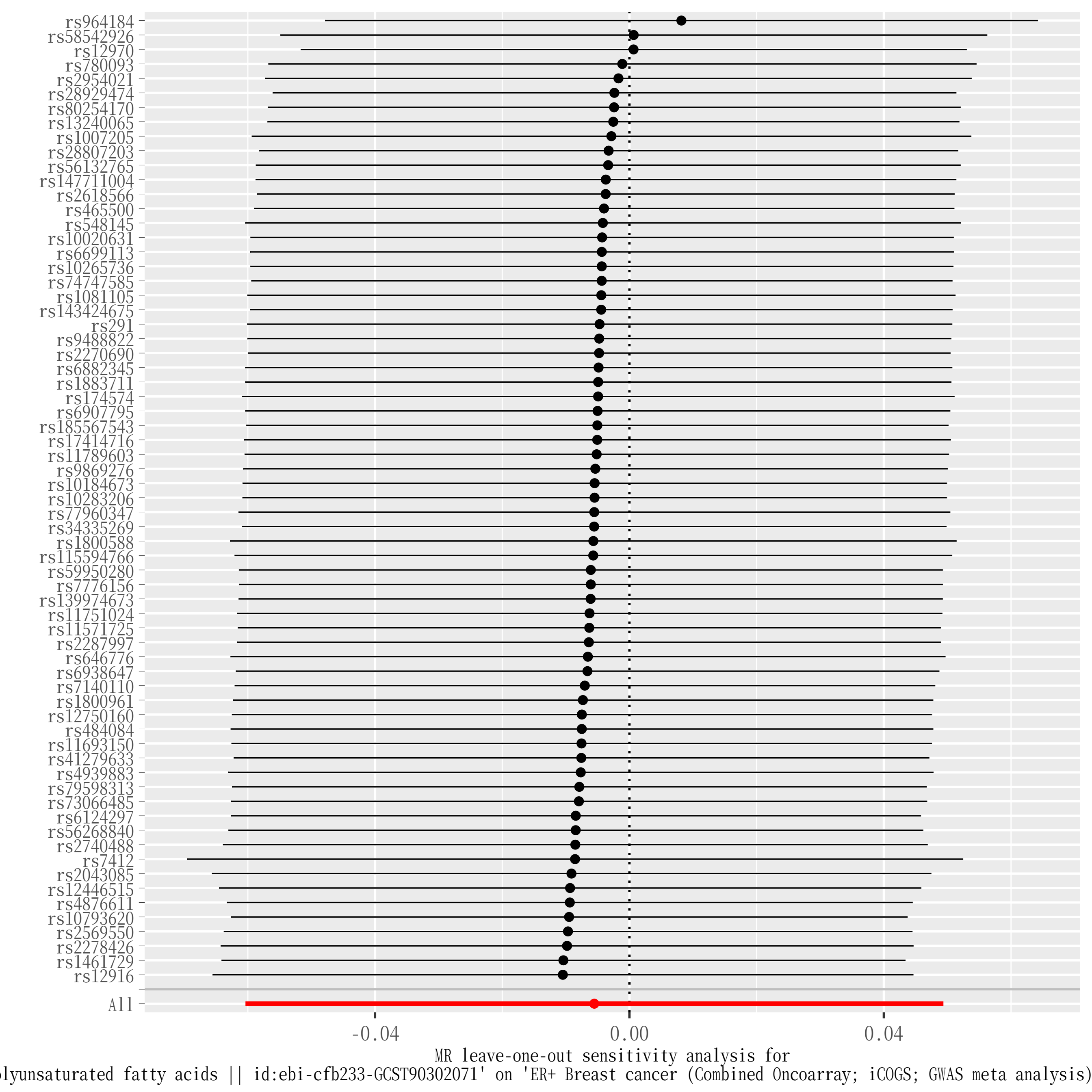
rs964184
rs58542926
rs12970
rs780093
rs2954021
rs28929474
rs80254170
rs13240065
rs1007205
rs28807203
rs56132765
rs147711004
rs2618566
rs465500
rs548145
rs10020631
rs6699113
rs10265736
rs74747585
rs1081105
rs143424675
rs291
rs9488822
rs2270690
rs6882345
rs1883711
rs174574
rs6907795
rs185567543
rs17414716
rs11789603
rs9869276
rs10184673
rs10283206
rs77960347
rs34335269
rs1800588
rs115594766
rs59950280
rs7776156
rs139974673
rs11751024
rs11571725
rs2287997
rs646776
rs6938647
rs7140110
rs1800961
rs12750160
rs484084
rs11693150
rs41279633
rs4939883
rs79598313
rs73066485
rs6124297
rs56268840
rs2740488
rs7412
rs2043085
rs12446515
rs4876611
rs10793620
rs2569550
rs2278426
rs1461729
rs12916
All
-0.04
0.00
0.04
MR leave-one-out sensitivity analysis for
lyunsaturated fatty acids || id:ebi-cfb233-GCST90302071' on 'ER+ Breast cancer (Combined Oncoarray; iCOGS; GWAS meta analysis)

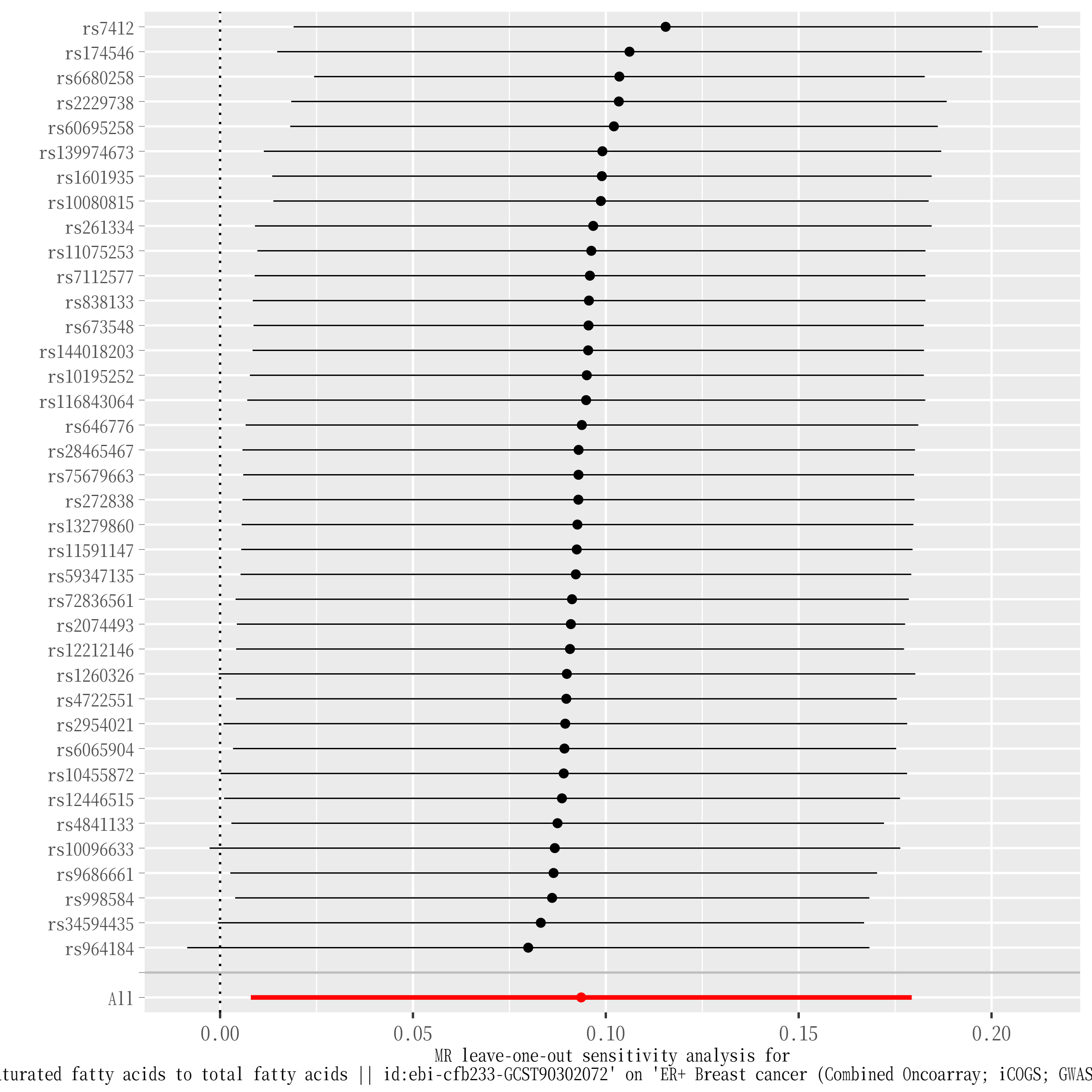
rs7412
rs174546
rs6680258
rs2229738
rs60695258
rs139974673
rs1601935
rs10080815
rs261334
rs11075253
rs7112577
rs838133
rs673548
rs144018203
rs10195252
rs116843064
rs646776
rs28465467
rs75679663
rs272838
rs13279860
rs11591147
rs59347135
rs72836561
rs2074493
rs12212146
rs1260326
rs4722551
rs2954021
rs6065904
rs10455872
rs12446515
rs4841133
rs10096633
rs9686661
rs998584
rs34594435
rs964184
All
0.00
0.05
0.10
0.15
0.20
MR leave-one-out sensitivity analysis for
turated fatty acids to total fatty acids || id:ebi-cfb233-GCST90302072' on 'ER+ Breast cancer (Combined Oncoarray; iCOGS; GWAS

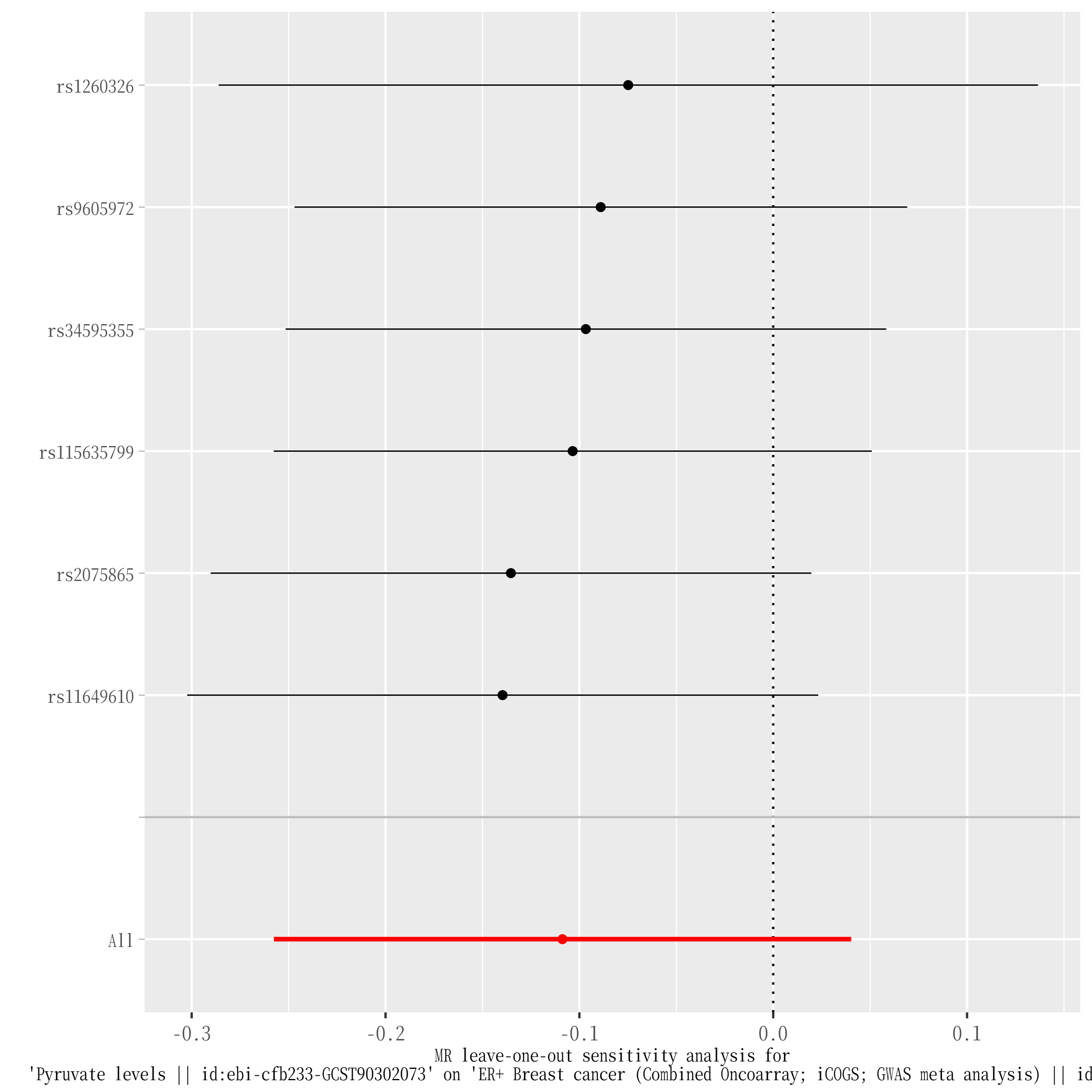
rs1260326
rs9605972
rs34595355
rs115635799
rs2075865
rs11649610
All
-0.3
-0.2
-0.1
0.0
0.1
MR leave-one-out sensitivity analysis for
'Pyruvate levels || id:ebi-cfb233-GCST90302073' on 'ER+ Breast cancer (Combined Oncoarray; iCOGS; GWAS meta analysis) || id

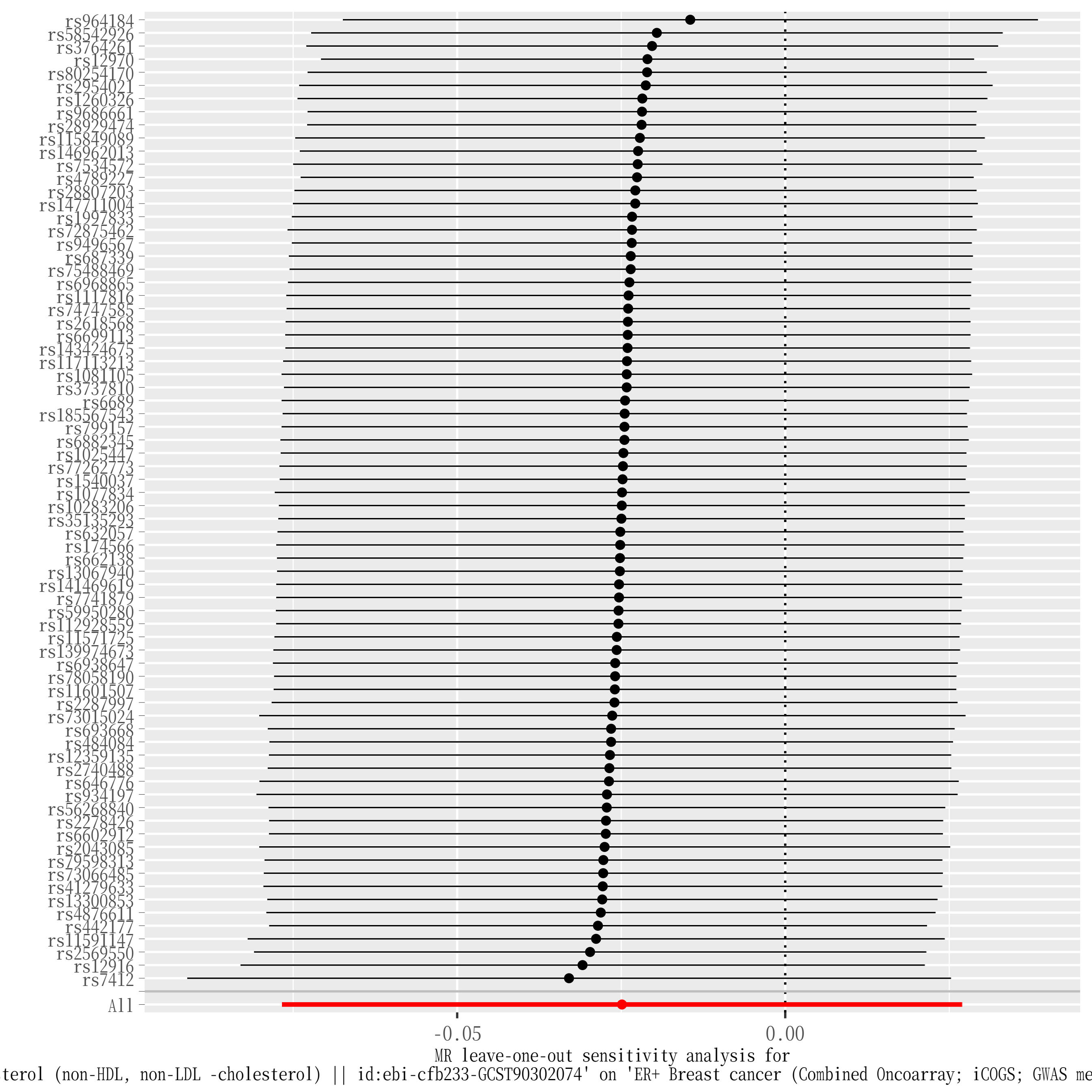
rs964184
rs58542926
rs3764261
rs12970
rs80254170
rs2954021
rs1260326
rs9686661
rs28929474
rs115849089
rs146962013
rs7534572
rs4789227
rs28807203
rs147711004
rs1997833
rs72875462
rs9496567
rs687339
rs75488469
rs6968865
rs1117816
rs74747585
rs2618568
rs6699113
rs143424675
rs117113213
rs1081105
rs3737810
rs6689
rs185567543
rs799157
rs6882345
rs1025447
rs77262773
rs1540037
rs1077834
rs10283206
rs35135293
rs632057
rs174566
rs662138
rs13067940
rs141469619
rs7741879
rs59950280
rs112928559
rs11571725
rs139974673
rs6938647
rs78058190
rs11601507
rs2287997
rs73015024
rs693668
rs484084
rs12359135
rs2740488
rs646776
rs934197
rs56268840
rs2278426
rs6602912
rs2043085
rs79598313
rs73066485
rs41279633
rs13300853
rs4876611
rs442177
rs11591147
rs2569550
rs12916
rs7412
All
-0.05
0.00
MR leave-one-out sensitivity analysis for
terol (non-HDL, non-LDL -cholesterol) || id:ebi-cfb233-GCST90302074' on 'ER+ Breast cancer (Combined Oncoarray; iCOGS; GWAS me

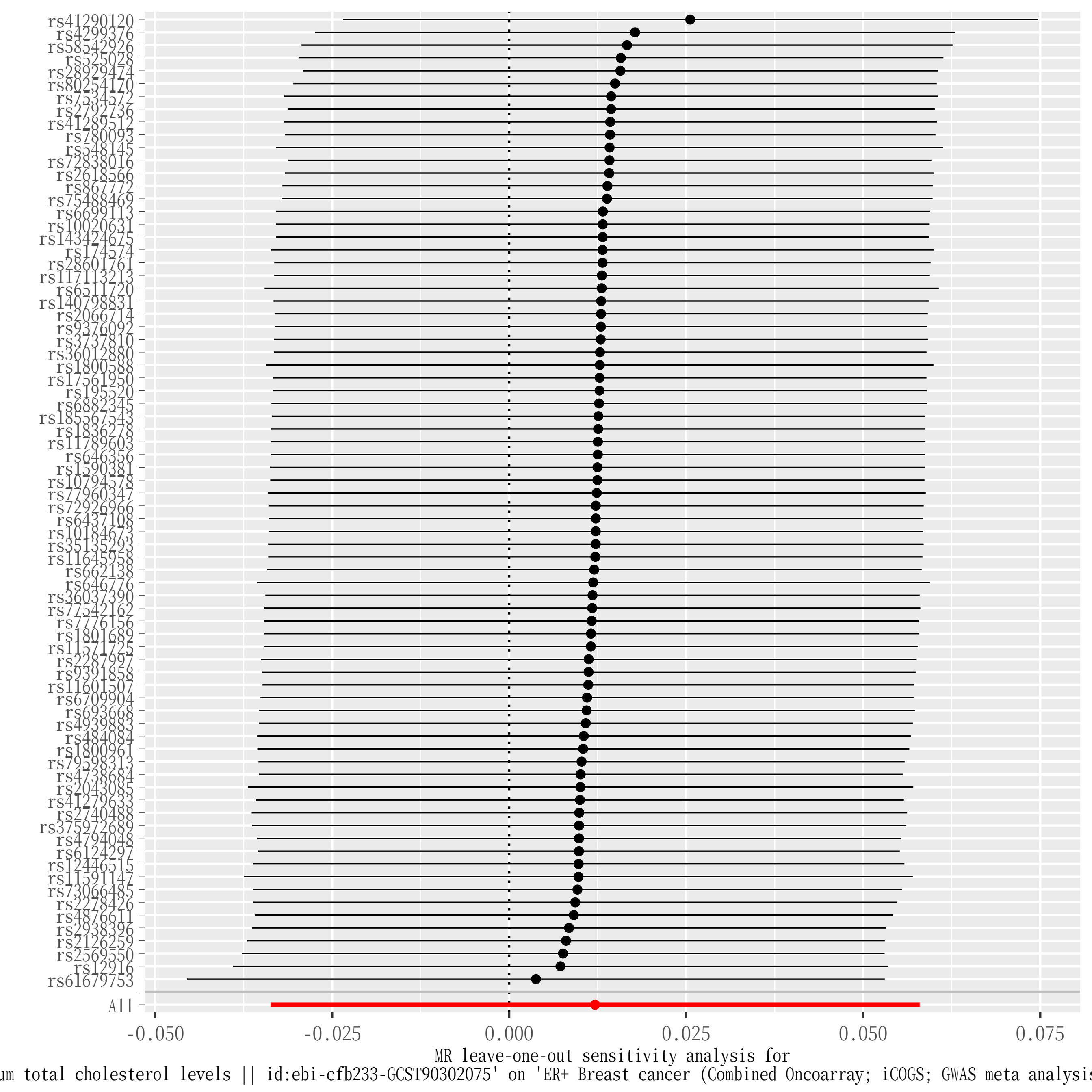

rs41290120
rs4299376
rs58542926
rs525028
rs28929474
rs80254170
rs7534572
rs2792736
rs41289512
rs780093
rs548145
rs72838016
rs2618566
rs867772
rs75488469
rs6699113
rs10020631
rs143424675
rs174574
rs28601761
rs117113213
rs6511720
rs140798831
rs2066714
rs9376092
rs3737810
rs36012880
rs1800588
rs17561950
rs195520
rs6882345
rs185567543
rs1836278
rs11789603
rs646356
rs1590381
rs10794578
rs77960347
rs72926966
rs6437108
rs10184673
rs35135293
rs11645958
rs662138
rs646776
rs36037390
rs77542162
rs7776156
rs1801689
rs11571725
rs2287997
rs9391858
rs11601507
rs6709904
rs693668
rs4939883
rs484084
rs1800961
rs79598313
rs4738684
rs2043085
rs41279633
rs2740488
rs375972689
rs4794048
rs6124297
rs12446515
rs11591147
rs73066485
rs2278426
rs4876611
rs2938396
rs2126259
rs2569550
rs12916
rs61679753
All
-0.050
-0.025
0.000
0.025
0.050
0.075
MR leave-one-out sensitivity analysis for
um total cholesterol levels || id:ebi-cfb233-GCST90302075' on 'ER+ Breast cancer (Combined Oncoarray; iCOGS; GWAS meta analysis

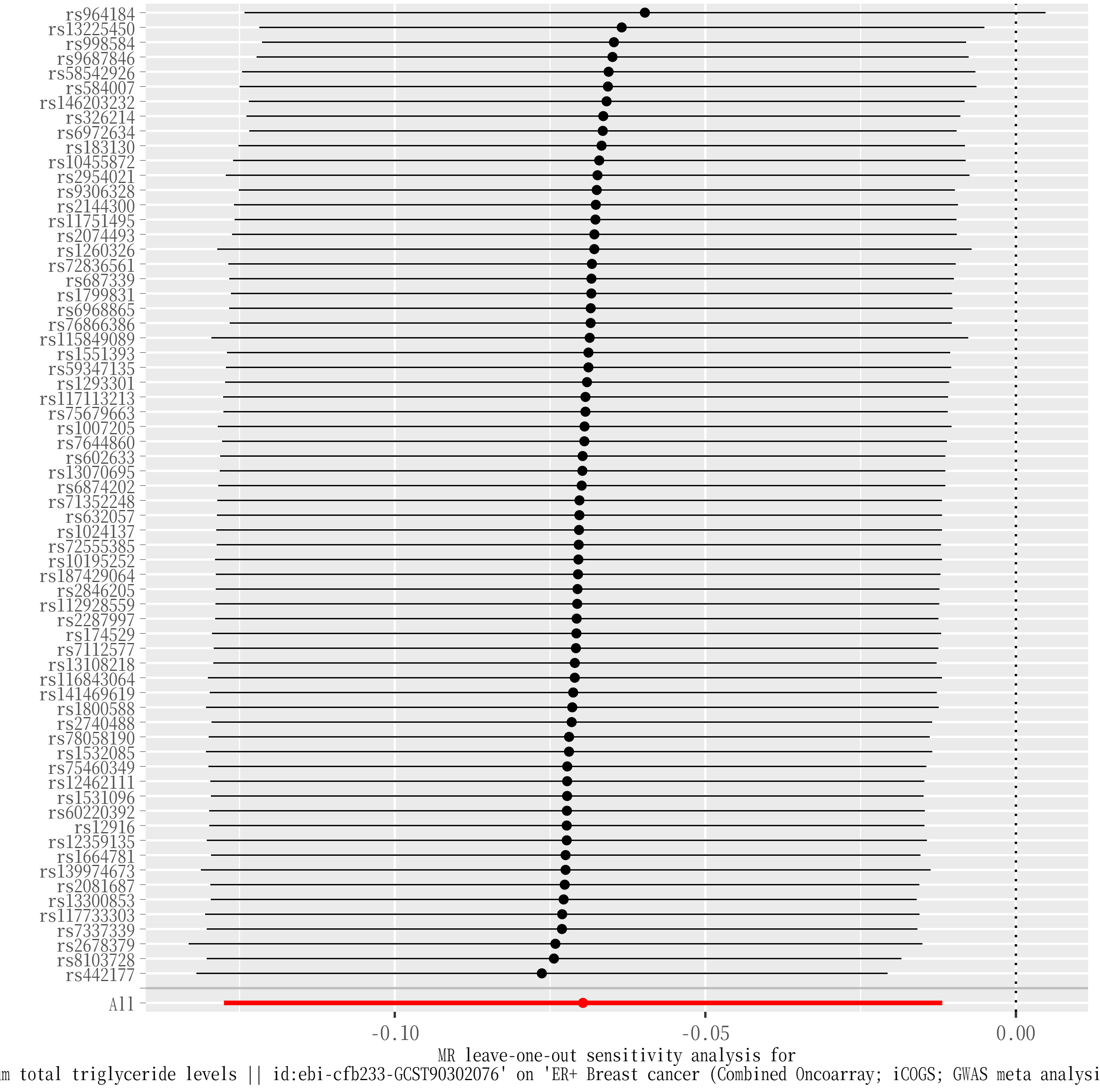
rs964184
rs13225450
rs998584
rs9687846
rs58542926
rs584007
rs146203232
rs326214
rs6972634
rs183130
rs10455872
rs2954021
rs9306328
rs2144300
rs11751495
rs2074493
rs1260326
rs72836561
rs687339
rs1799831
rs6968865
rs76866386
rs115849089
rs1551393
rs59347135
rs1293301
rs117113213
rs75679663
rs1007205
rs7644860
rs602633
rs13070695
rs6874202
rs71352248
rs632057
rs1024137
rs72555385
rs10195252
rs187429064
rs2846205
rs112928559
rs2287997
rs174529
rs7112577
rs13108218
rs116843064
rs141469619
rs1800588
rs2740488
rs78058190
rs1532085
rs75460349
rs12462111
rs1531096
rs60220392
rs12916
rs12359135
rs1664781
rs139974673
rs2081687
rs13300853
rs117733303
rs7337339
rs2678379
rs8103728
rs442177
All
-0.10
-0.05
0.00
MR leave-one-out sensitivity analysis for
m total triglyceride levels || id:ebi-cfb233-GCST90302076' on 'ER+ Breast cancer (Combined Oncoarray; iCOGS; GWAS meta analysi

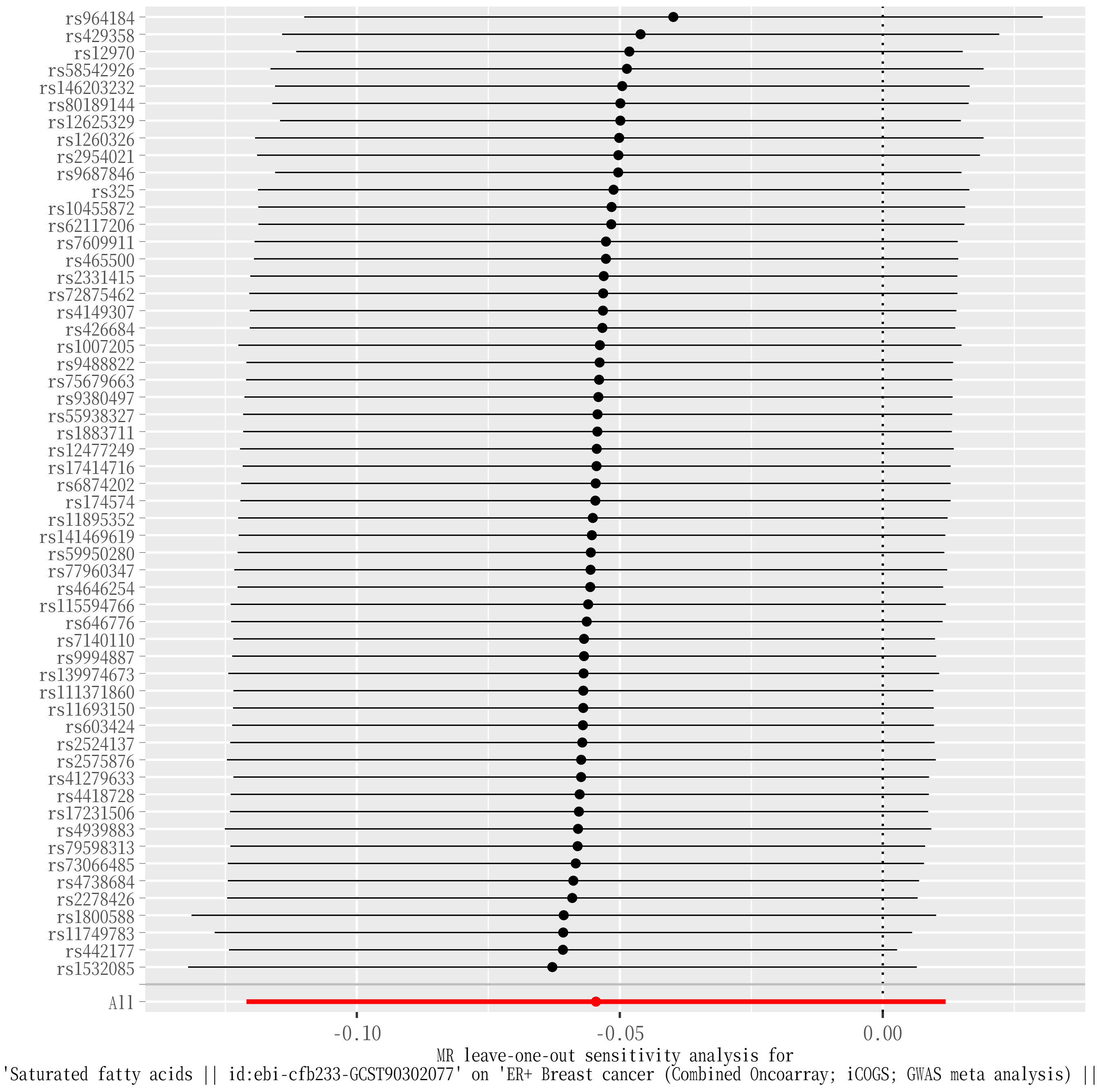

rs964184
rs429358
rs12970
rs58542926
rs146203232
rs80189144
rs12625329
rs1260326
rs2954021
rs9687846
rs325
rs10455872
rs62117206
rs7609911
rs465500
rs2331415
rs72875462
rs4149307
rs426684
rs1007205
rs9488822
rs75679663
rs9380497
rs55938327
rs1883711
rs12477249
rs17414716
rs6874202
rs174574
rs11895352
rs141469619
rs59950280
rs77960347
rs4646254
rs115594766
rs646776
rs7140110
rs9994887
rs139974673
rs111371860
rs11693150
rs603424
rs2524137
rs2575876
rs41279633
rs4418728
rs17231506
rs4939883
rs79598313
rs73066485
rs4738684
rs2278426
rs1800588
rs11749783
rs442177
rs1532085
All
-0.10
-0.05
0.00
MR leave-one-out sensitivity analysis for
'Saturated fatty acids || id:ebi-cfb233-GCST90302077' on 'ER+ Breast cancer (Combined Oncoarray; iCOGS; GWAS meta analysis) ||

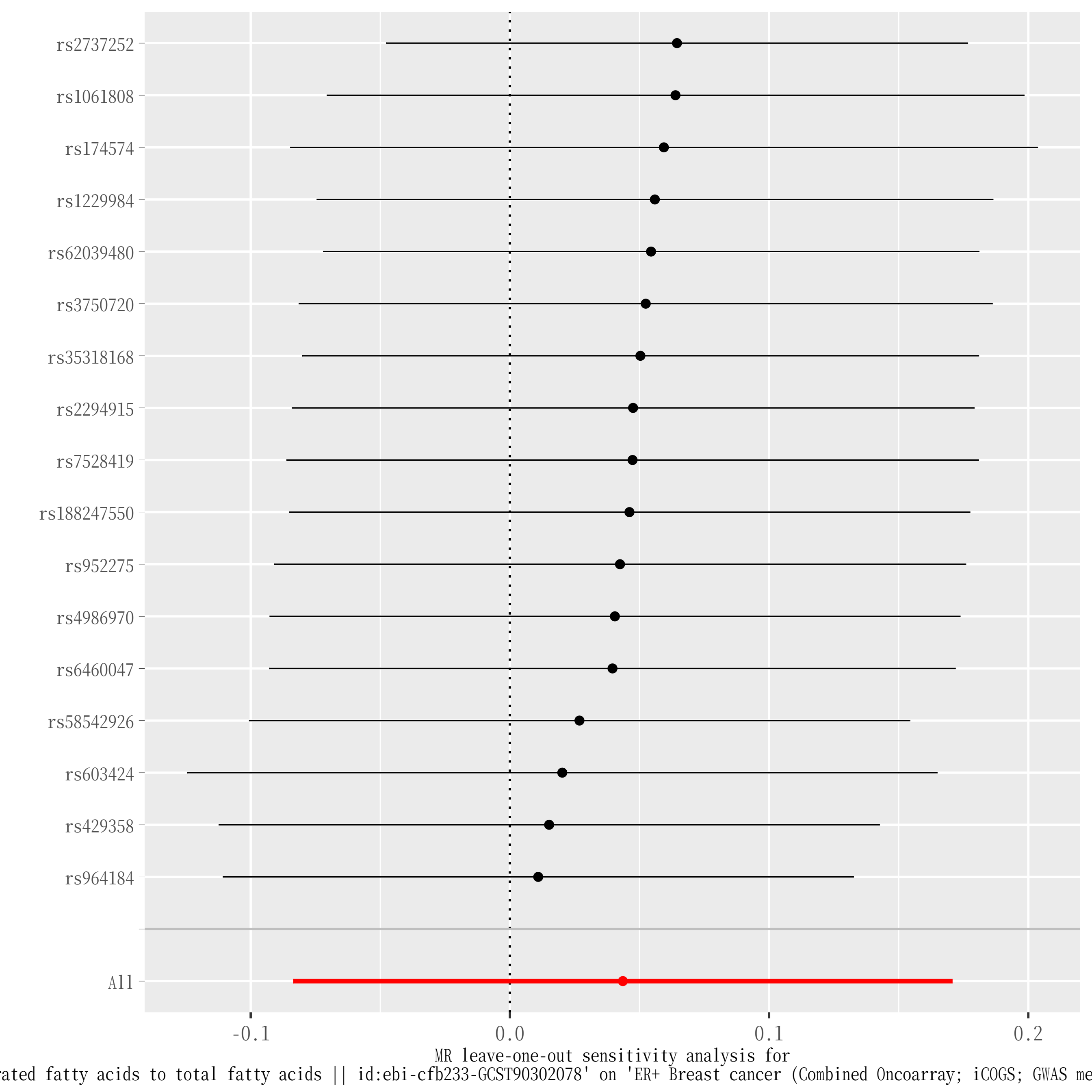
rs2737252
rs1061808
rs174574
rs1229984
rs62039480
rs3750720
rs35318168
rs2294915
rs7528419
rs188247550
rs952275
rs4986970
rs6460047
rs58542926
rs603424
rs429358
rs964184
All
-0.1
0.0
0.1
0.2
MR leave-one-out sensitivity analysis for
rated fatty acids to total fatty acids || id:ebi-cfb233-GCST90302078' on 'ER+ Breast cancer (Combined Oncoarray; iCOGS; GWAS me

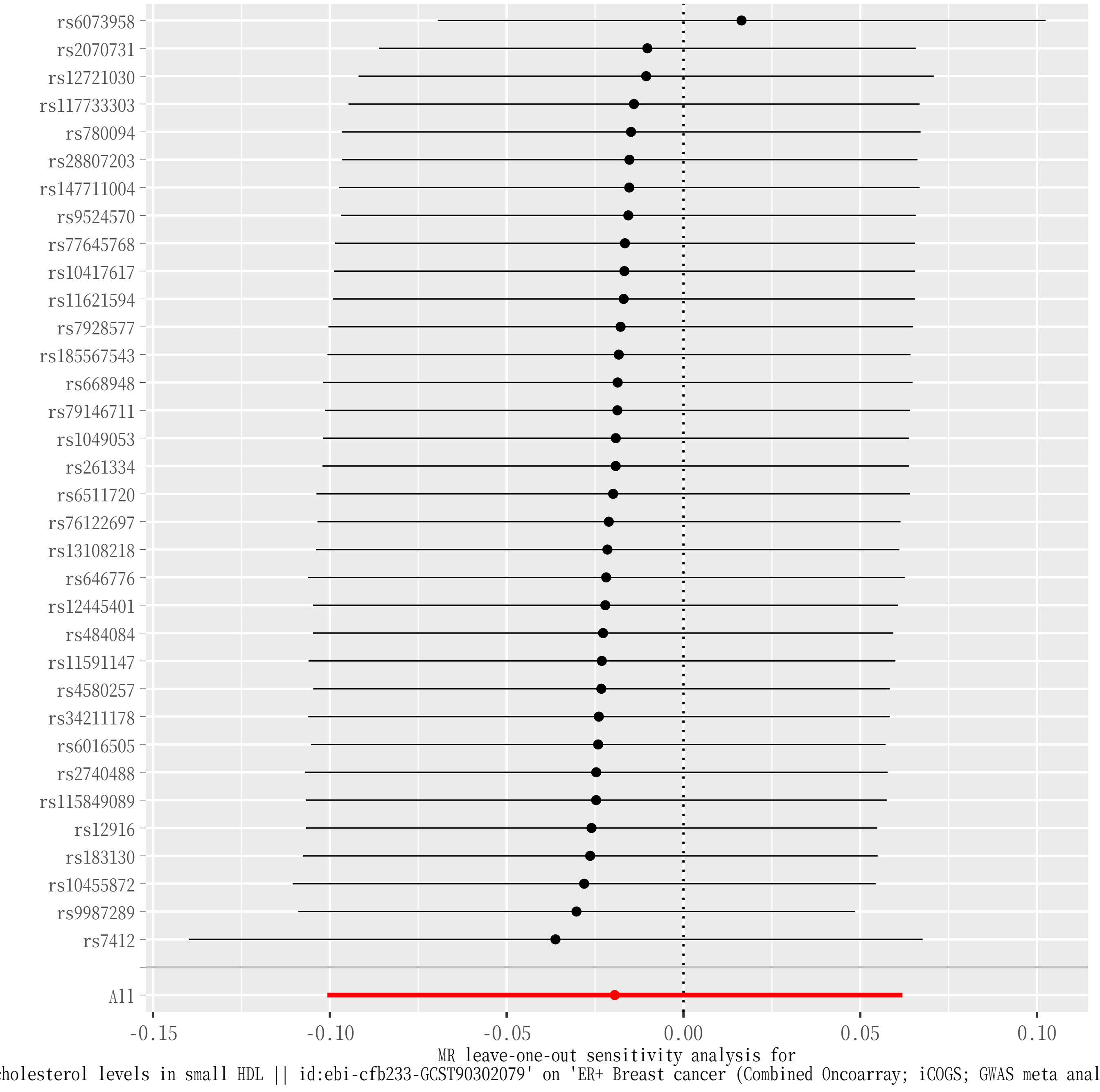
rs6073958
rs2070731
rs12721030
rs117733303
rs780094
rs28807203
rs147711004
rs9524570
rs77645768
rs10417617
rs11621594
rs7928577
rs185567543
rs668948
rs79146711
rs1049053
rs261334
rs6511720
rs76122697
rs13108218
rs646776
rs12445401
rs484084
rs11591147
rs4580257
rs34211178
rs6016505
rs2740488
rs115849089
rs12916
rs183130
rs10455872
rs9987289
rs7412
All
-0.15
-0.10
-0.05
0.00
0.05
0.10
MR leave-one-out sensitivity analysis for
holesterol levels in small HDL || id:ebi-cfb233-GCST90302079' on 'ER+ Breast cancer (Combined Oncoarray; iCOGS; GWAS meta anal

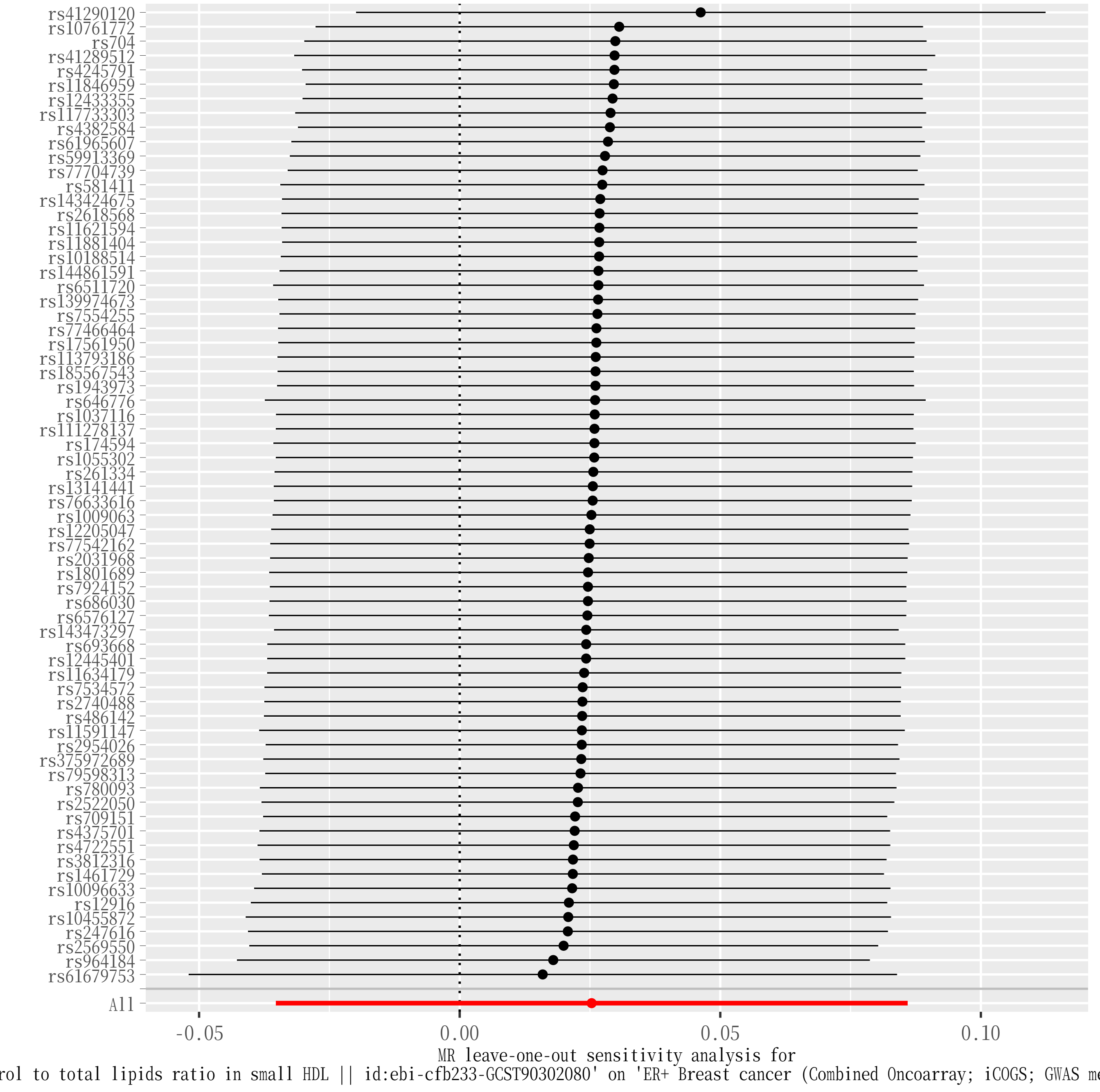
rs41290120
rs10761772
rs704
rs41289512
rs4245791
rs11846959
rs12433355
rs117733303
rs4382584
rs61965607
rs59913369
rs77704739
rs581411
rs143424675
rs2618568
rs11621594
rs11881404
rs10188514
rs144861591
rs6511720
rs139974673
rs7554255
rs77466464
rs17561950
rs113793186
rs185567543
rs1943973
rs646776
rs1037116
rs111278137
rs174594
rs1055302
rs261334
rs13141441
rs76633616
rs1009063
rs12205047
rs77542162
rs2031968
rs1801689
rs7924152
rs686030
rs6576127
rs143473297
rs693668
rs12445401
rs11634179
rs7534572
rs2740488
rs486142
rs11591147
rs2954026
rs375972689
rs79598313
rs780093
rs2522050
rs709151
rs4375701
rs4722551
rs3812316
rs1461729
rs10096633
rs12916
rs10455872
rs247616
rs2569550
rs964184
rs61679753
All
-0.05
0.00
0.05
0.10
MR leave-one-out sensitivity analysis for
rol to total lipids ratio in small HDL || id:ebi-cfb233-GCST90302080' on 'ER+ Breast cancer (Combined Oncoarray; iCOGS; GWAS me

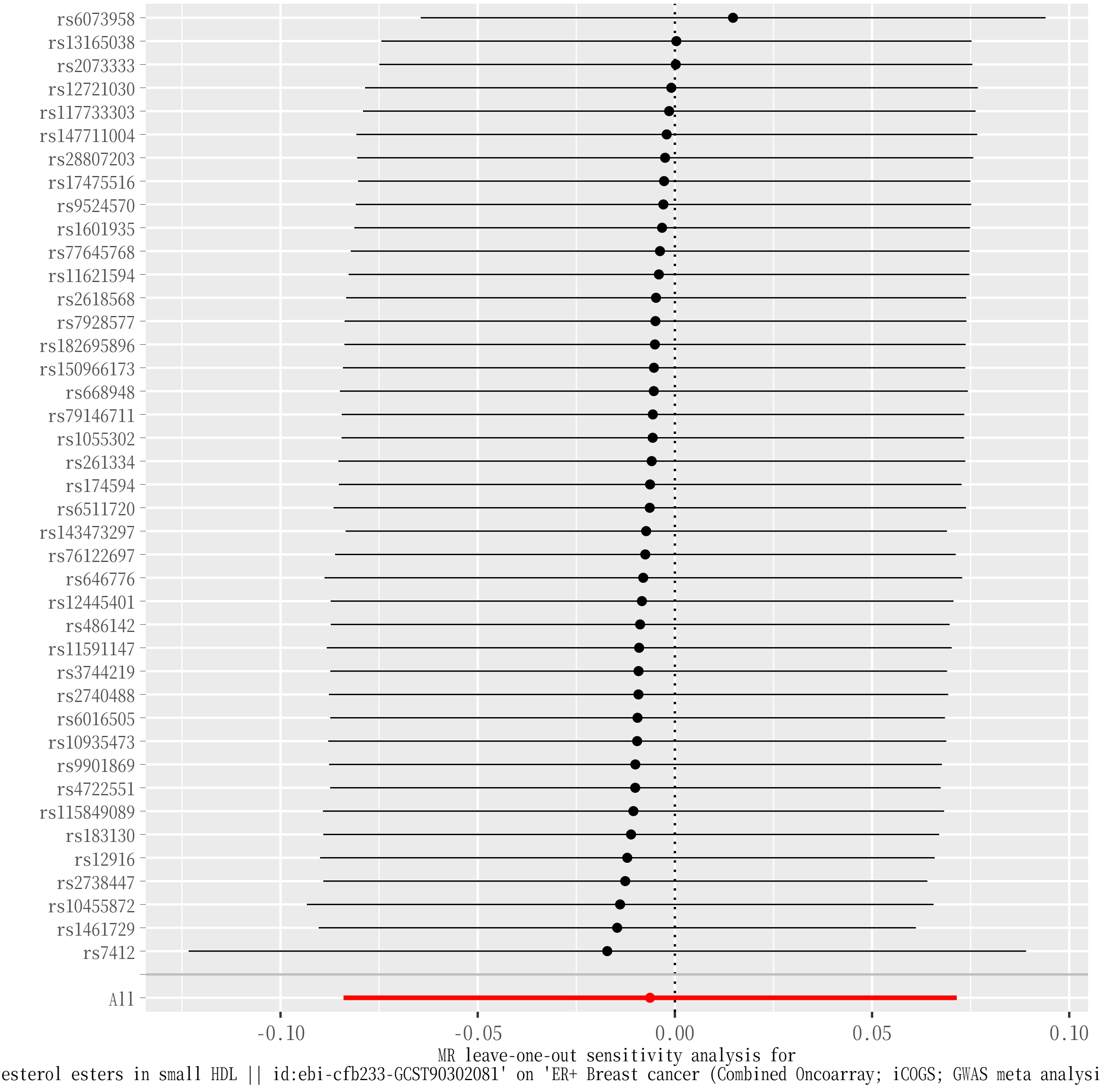

rs6073958
rs13165038
rs2073333
rs12721030
rs117733303
rs147711004
rs28807203
rs17475516
rs9524570
rs1601935
rs77645768
rs11621594
rs2618568
rs7928577
rs182695896
rs150966173
rs668948
rs79146711
rs1055302
rs261334
rs174594
rs6511720
rs143473297
rs76122697
rs646776
rs12445401
rs486142
rs11591147
rs3744219
rs2740488
rs6016505
rs10935473
rs9901869
rs4722551
rs115849089
rs183130
rs12916
rs2738447
rs10455872
rs1461729
rs7412
All
-0.10
-0.05
0.00
0.05
0.10
MR leave-one-out sensitivity analysis for
esterol esters in small HDL || id:ebi-cfb233-GCST90302081' on 'ER+ Breast cancer (Combined Oncoarray; iCOGS; GWAS meta analysi

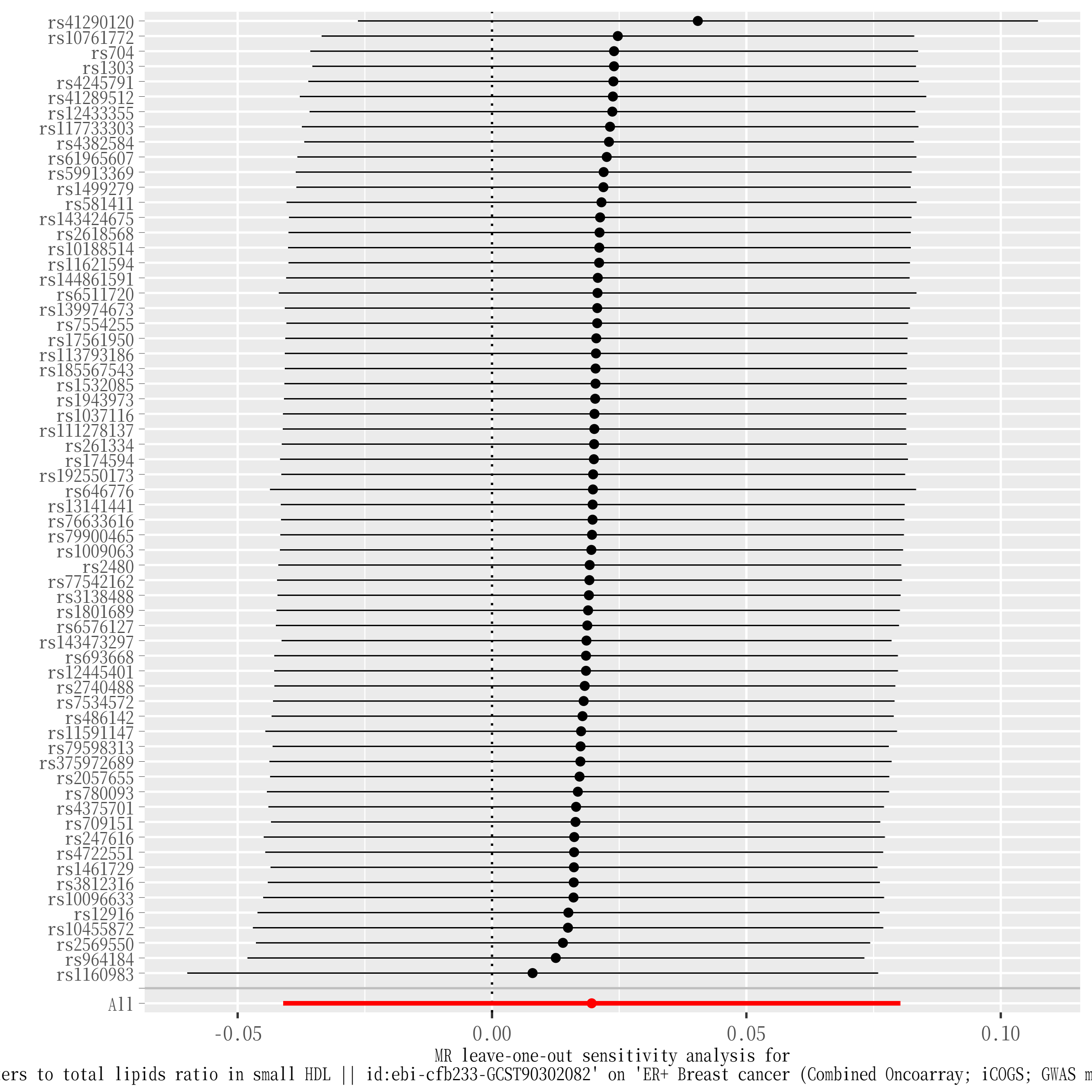

rs41290120
rs10761772
rs704
rs1303
rs4245791
rs41289512
rs12433355
rs117733303
rs4382584
rs61965607
rs59913369
rs1499279
rs581411
rs143424675
rs2618568
rs10188514
rs11621594
rs144861591
rs6511720
rs139974673
rs7554255
rs17561950
rs113793186
rs185567543
rs1532085
rs1943973
rs1037116
rs111278137
rs261334
rs174594
rs192550173
rs646776
rs13141441
rs76633616
rs79900465
rs1009063
rs2480
rs77542162
rs3138488
rs1801689
rs6576127
rs143473297
rs693668
rs12445401
rs2740488
rs7534572
rs486142
rs11591147
rs79598313
rs375972689
rs2057655
rs780093
rs4375701
rs709151
rs247616
rs4722551
rs1461729
rs3812316
rs10096633
rs12916
rs10455872
rs2569550
rs964184
rs1160983
All
-0.05
0.00
0.05
0.10
MR leave-one-out sensitivity analysis for
ers to total lipids ratio in small HDL || id:ebi-cfb233-GCST90302082' on 'ER+ Breast cancer (Combined Oncoarray; iCOGS; GWAS m

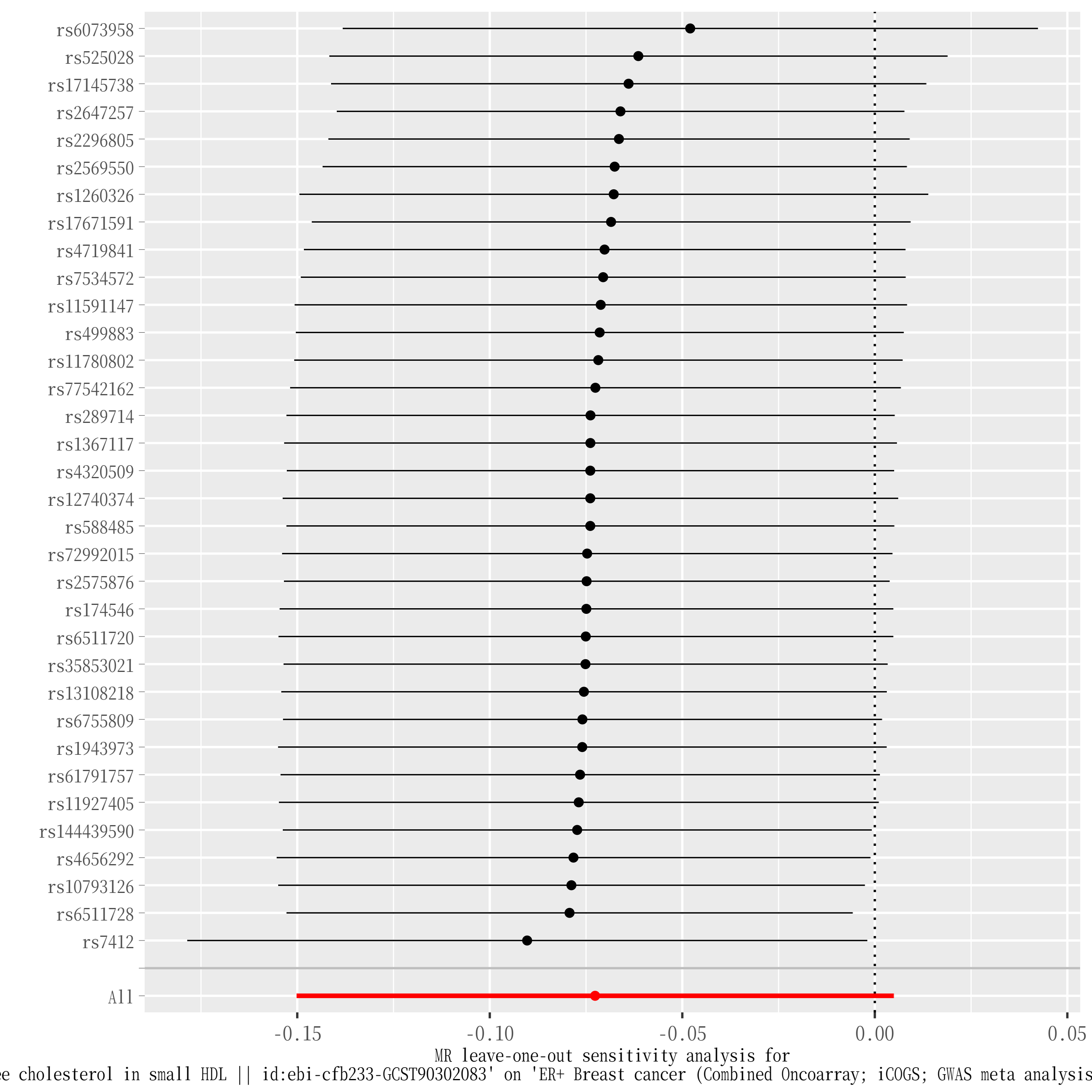
rs6073958
rs525028
rs17145738
rs2647257
rs2296805
rs2569550
rs1260326
rs17671591
rs4719841
rs7534572
rs11591147
rs499883
rs11780802
rs77542162
rs289714
rs1367117
rs4320509
rs12740374
rs588485
rs72992015
rs2575876
rs174546
rs6511720
rs35853021
rs13108218
rs6755809
rs1943973
rs61791757
rs11927405
rs144439590
rs4656292
rs10793126
rs6511728
rs7412
All
-0.15
-0.10
-0.05
0.00
0.05
MR leave-one-out sensitivity analysis for
e cholesterol in small HDL || id:ebi-cfb233-GCST90302083' on 'ER+ Breast cancer (Combined Oncoarray; iCOGS; GWAS meta analysis

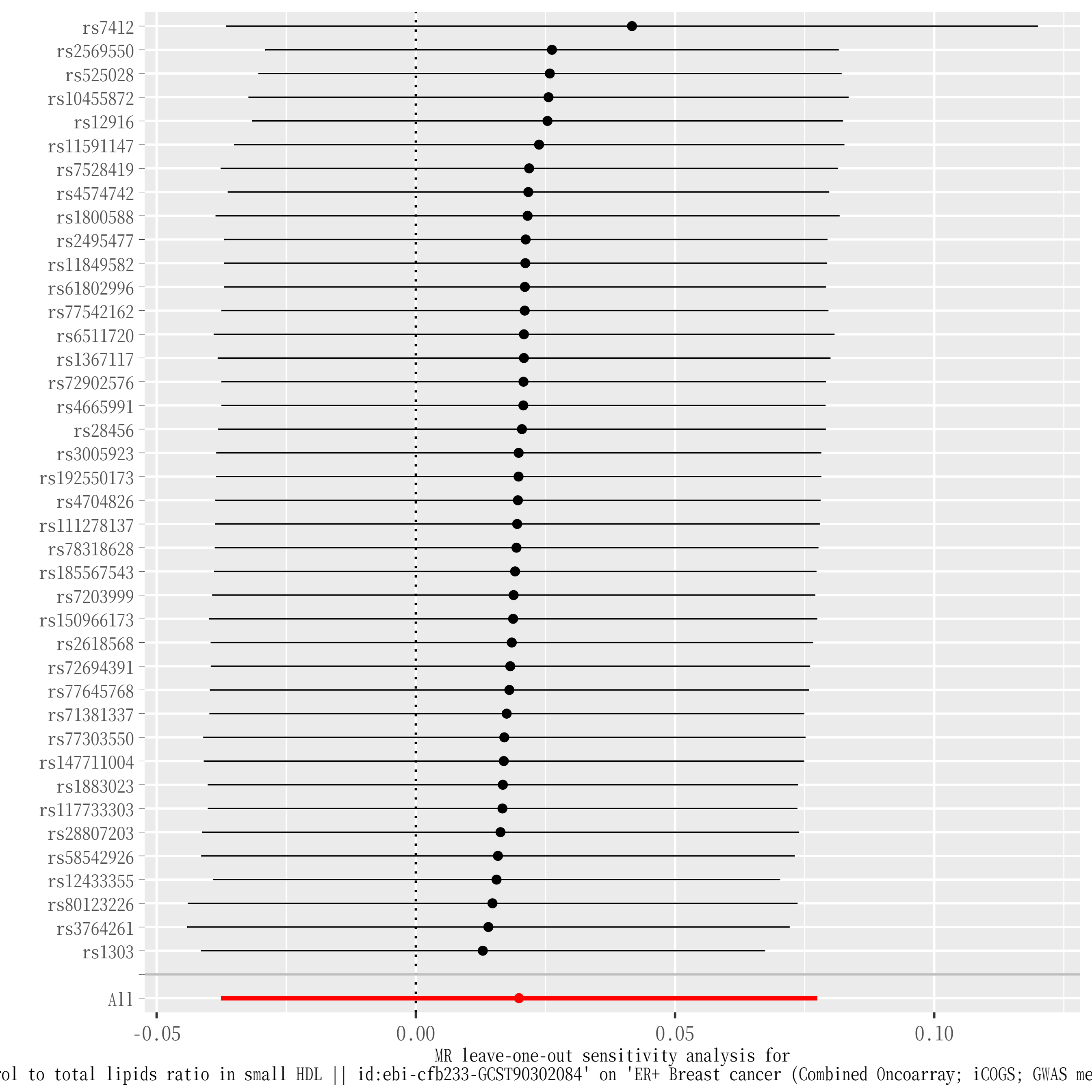
rs7412
rs2569550
rs525028
rs10455872
rs12916
rs11591147
rs7528419
rs4574742
rs1800588
rs2495477
rs11849582
rs61802996
rs77542162
rs6511720
rs1367117
rs72902576
rs4665991
rs28456
rs3005923
rs192550173
rs4704826
rs111278137
rs78318628
rs185567543
rs7203999
rs150966173
rs2618568
rs72694391
rs77645768
rs71381337
rs77303550
rs147711004
rs1883023
rs117733303
rs28807203
rs58542926
rs12433355
rs80123226
rs3764261
rs1303
All
-0.05
0.00
0.05
0.10
MR leave-one-out sensitivity analysis for
rol to total lipids ratio in small HDL || id:ebi-cfb233-GCST90302084' on 'ER+ Breast cancer (Combined Oncoarray; iCOGS; GWAS me

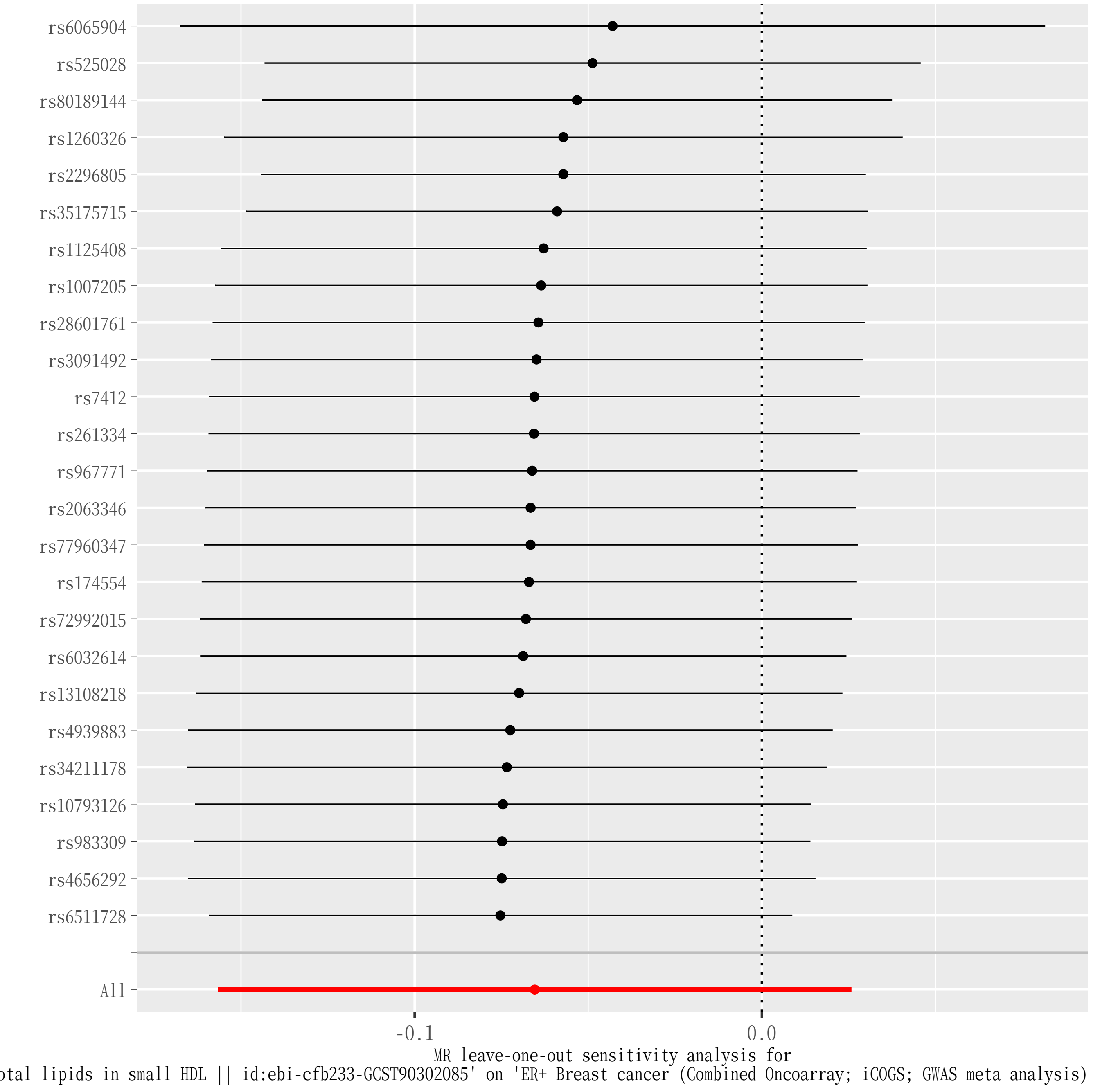
rs6065904
rs525028
rs80189144
rs1260326
rs2296805
rs35175715
rs1125408
rs1007205
rs28601761
rs3091492
rs7412
rs261334
rs967771
rs2063346
rs77960347
rs174554
rs72992015
rs6032614
rs13108218
rs4939883
rs34211178
rs10793126
rs983309
rs4656292
rs6511728
All
-0.1
0.0
MR leave-one-out sensitivity analysis for
otal lipids in small HDL || id:ebi-cfb233-GCST90302085' on 'ER+ Breast cancer (Combined Oncoarray; iCOGS; GWAS meta analysis)

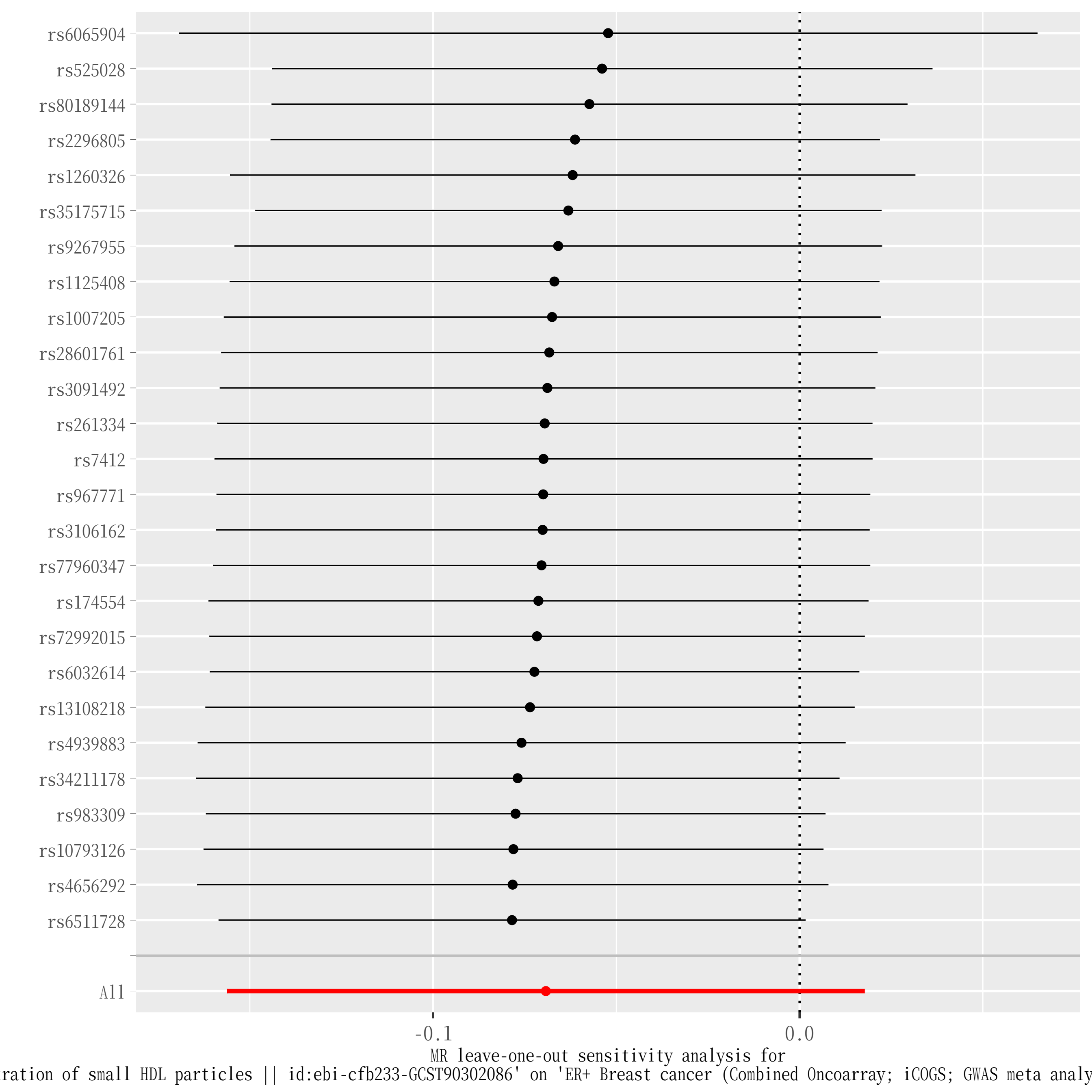
rs6065904
rs525028
rs80189144
rs2296805
rs1260326
rs35175715
rs9267955
rs1125408
rs1007205
rs28601761
rs3091492
rs261334
rs7412
rs967771
rs3106162
rs77960347
rs174554
rs72992015
rs6032614
rs13108218
rs4939883
rs34211178
rs983309
rs10793126
rs4656292
rs6511728
All
-0.1
0.0
MR leave-one-out sensitivity analysis for
ration of small HDL particles || id:ebi-cfb233-GCST90302086' on 'ER+ Breast cancer (Combined Oncoarray; iCOGS; GWAS meta analy

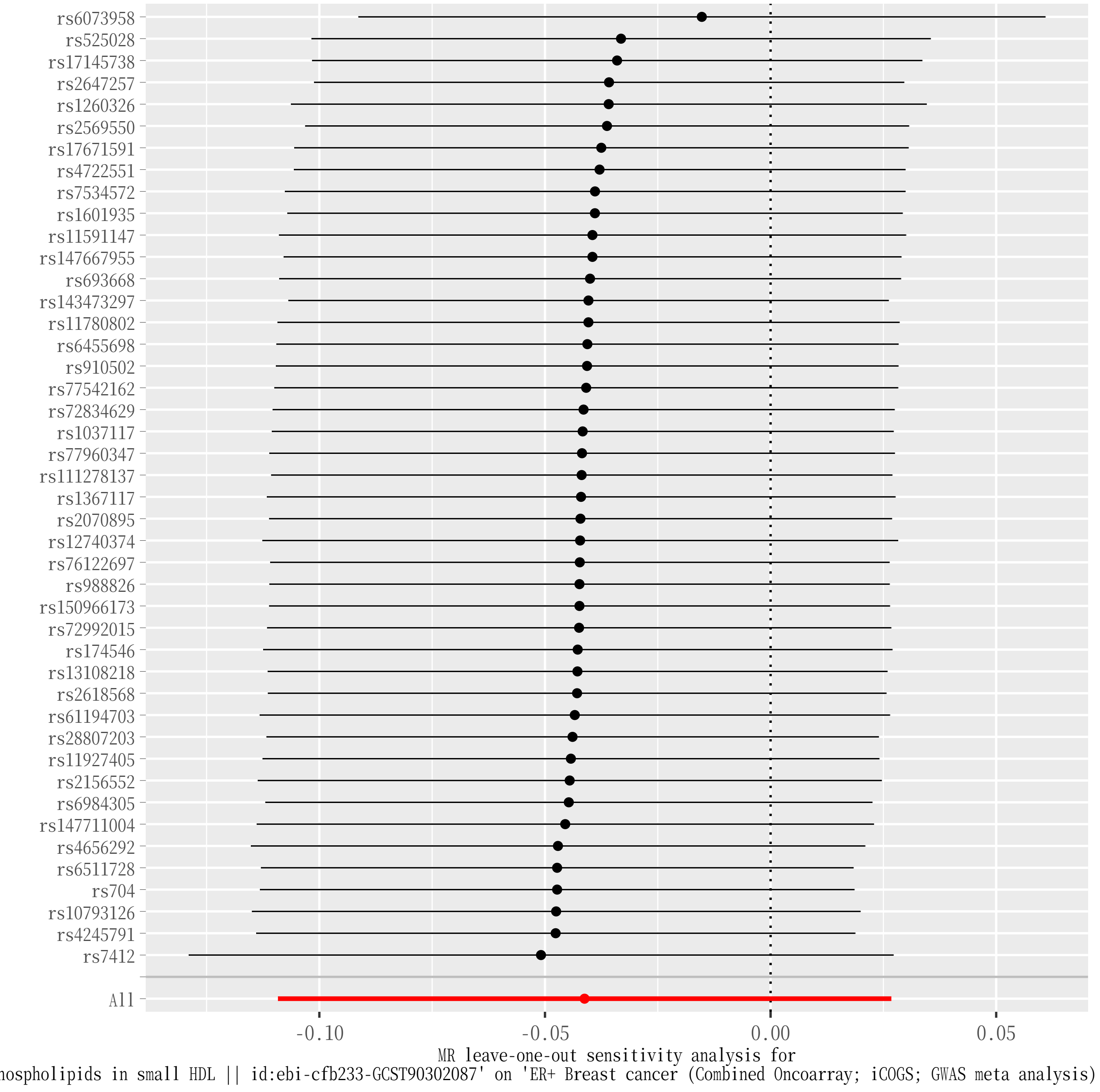
rs6073958
rs525028
rs17145738
rs2647257
rs1260326
rs2569550
rs17671591
rs4722551
rs7534572
rs1601935
rs11591147
rs147667955
rs693668
rs143473297
rs11780802
rs6455698
rs910502
rs77542162
rs72834629
rs1037117
rs77960347
rs111278137
rs1367117
rs2070895
rs12740374
rs76122697
rs988826
rs150966173
rs72992015
rs174546
rs13108218
rs2618568
rs61194703
rs28807203
rs11927405
rs2156552
rs6984305
rs147711004
rs4656292
rs6511728
rs704
rs10793126
rs4245791
rs7412
All
-0.10
-0.05
0.00
0.05
MR leave-one-out sensitivity analysis for
hospholipids in small HDL || id:ebi-cfb233-GCST90302087' on 'ER+ Breast cancer (Combined Oncoarray; iCOGS; GWAS meta analysis)

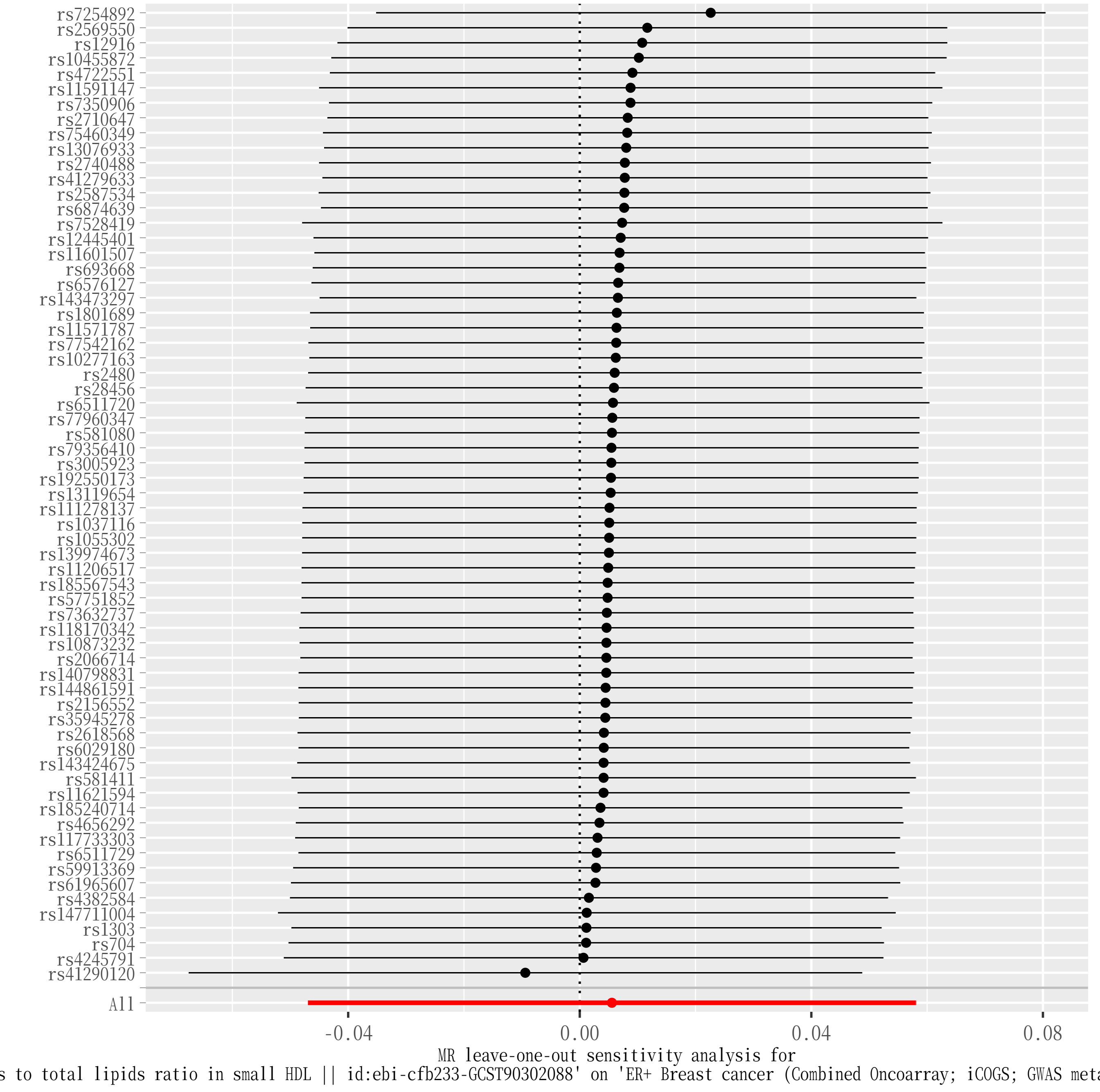
rs7254892
rs2569550
rs12916
rs10455872
rs4722551
rs11591147
rs7350906
rs2710647
rs75460349
rs13076933
rs2740488
rs41279633
rs2587534
rs6874639
rs7528419
rs12445401
rs11601507
rs693668
rs6576127
rs143473297
rs1801689
rs11571787
rs77542162
rs10277163
rs2480
rs28456
rs6511720
rs77960347
rs581080
rs79356410
rs3005923
rs192550173
rs13119654
rs111278137
rs1037116
rs1055302
rs139974673
rs11206517
rs185567543
rs57751852
rs73632737
rs118170342
rs10873232
rs2066714
rs140798831
rs144861591
rs2156552
rs35945278
rs2618568
rs6029180
rs143424675
rs581411
rs11621594
rs185240714
rs4656292
rs117733303
rs6511729
rs59913369
rs61965607
rs4382584
rs147711004
rs1303
rs704
rs4245791
rs41290120
All
-0.04
0.00
0.04
0.08
MR leave-one-out sensitivity analysis for
s to total lipids ratio in small HDL || id:ebi-cfb233-GCST90302088' on 'ER+ Breast cancer (Combined Oncoarray; iCOGS; GWAS meta

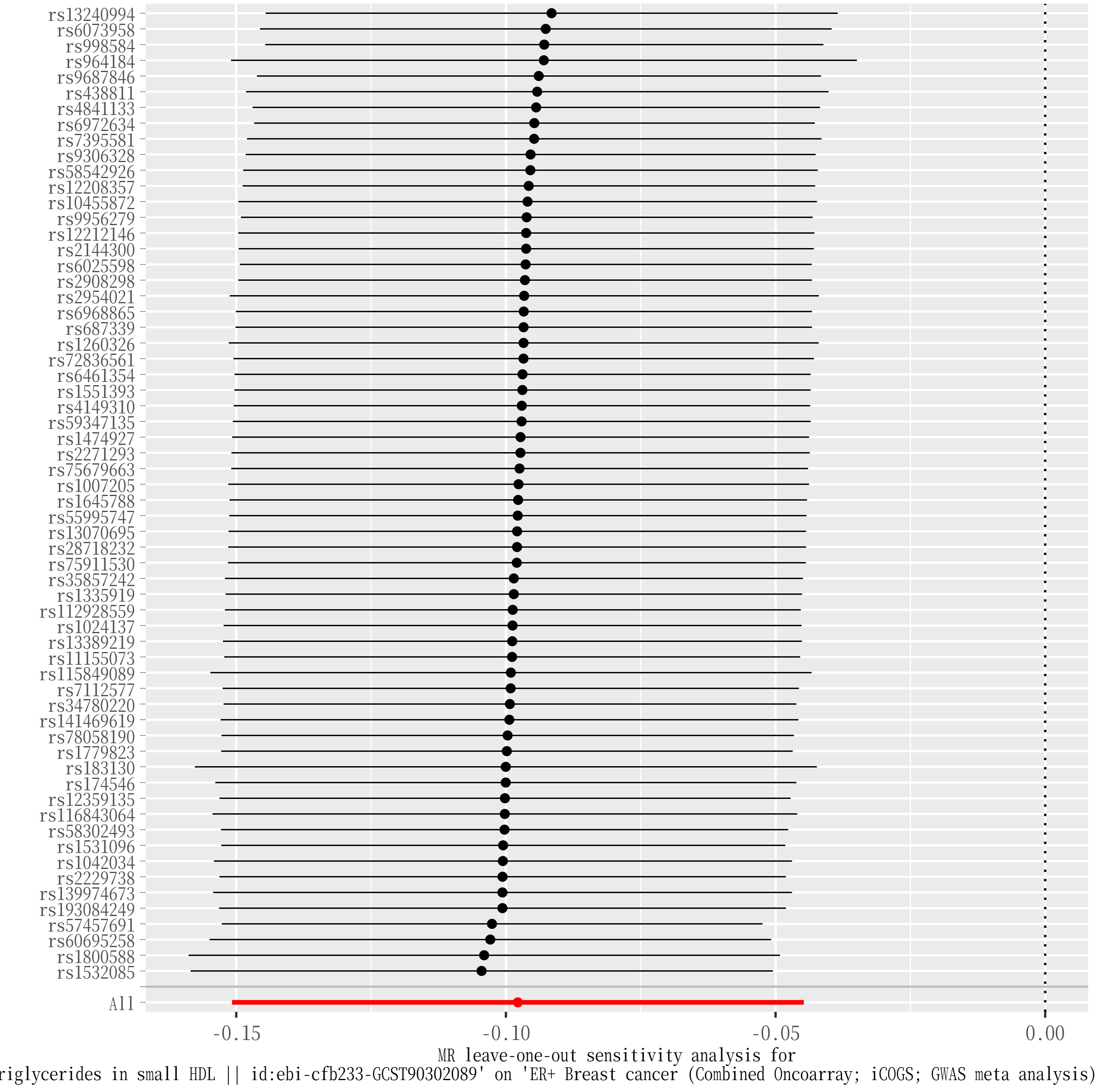
rs13240994
rs6073958
rs998584
rs964184
rs9687846
rs438811
rs4841133
rs6972634
rs7395581
rs9306328
rs58542926
rs12208357
rs10455872
rs9956279
rs12212146
rs2144300
rs6025598
rs2908298
rs2954021
rs6968865
rs687339
rs1260326
rs72836561
rs6461354
rs1551393
rs4149310
rs59347135
rs1474927
rs2271293
rs75679663
rs1007205
rs1645788
rs55995747
rs13070695
rs28718232
rs75911530
rs35857242
rs1335919
rs112928559
rs1024137
rs13389219
rs11155073
rs115849089
rs7112577
rs34780220
rs141469619
rs78058190
rs1779823
rs183130
rs174546
rs12359135
rs116843064
rs58302493
rs1531096
rs1042034
rs2229738
rs139974673
rs193084249
rs57457691
rs60695258
rs1800588
rs1532085
All
-0.15
-0.10
-0.05
0.00
MR leave-one-out sensitivity analysis for
riglycerides in small HDL || id:ebi-cfb233-GCST90302089' on 'ER+ Breast cancer (Combined Oncoarray; iCOGS; GWAS meta analysis)

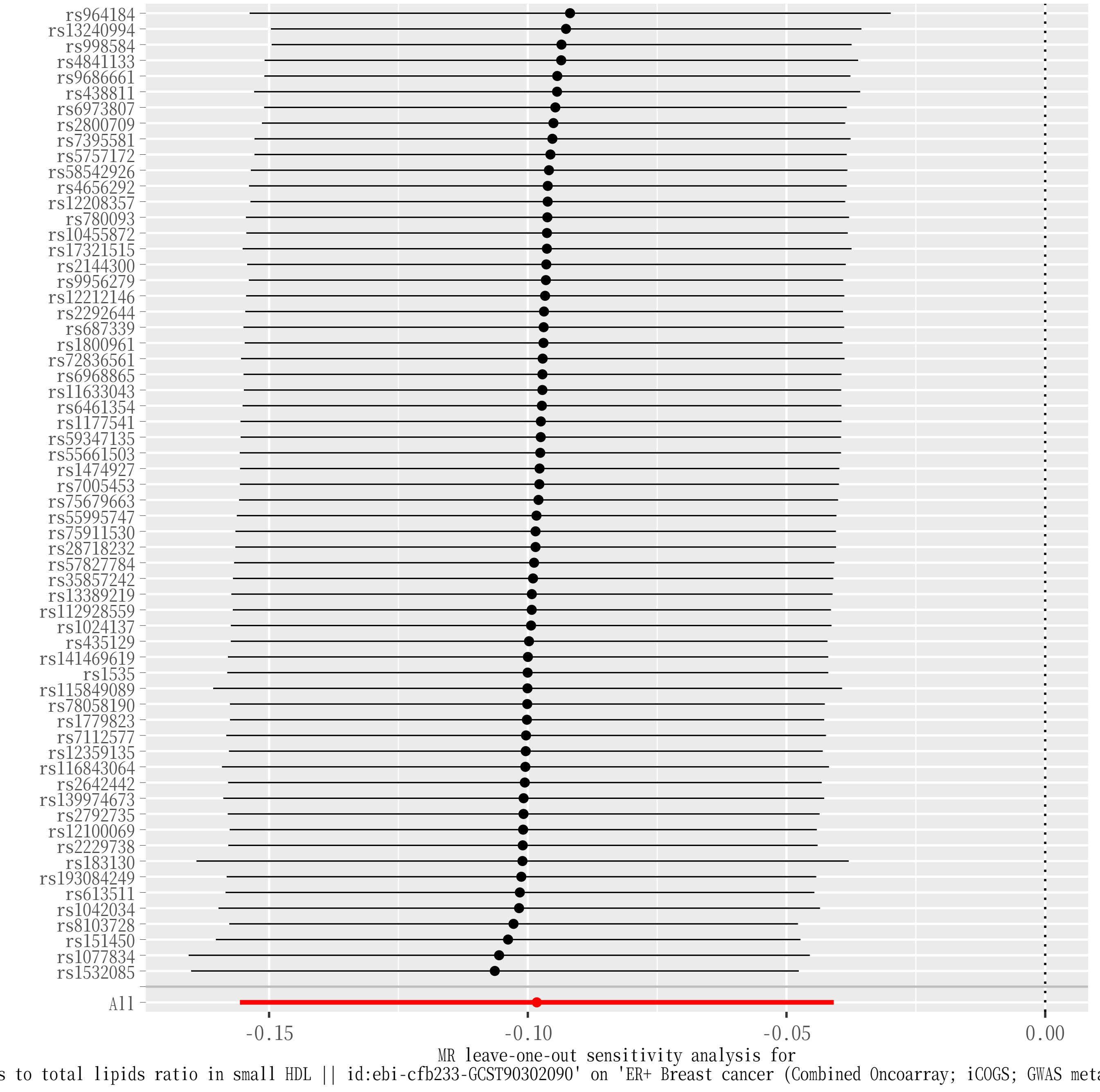
rs964184
rs13240994
rs998584
rs4841133
rs9686661
rs438811
rs6973807
rs2800709
rs7395581
rs5757172
rs58542926
rs4656292
rs12208357
rs780093
rs10455872
rs17321515
rs2144300
rs9956279
rs12212146
rs2292644
rs687339
rs1800961
rs72836561
rs6968865
rs11633043
rs6461354
rs1177541
rs59347135
rs55661503
rs1474927
rs7005453
rs75679663
rs55995747
rs75911530
rs28718232
rs57827784
rs35857242
rs13389219
rs112928559
rs1024137
rs435129
rs141469619
rs1535
rs115849089
rs78058190
rs1779823
rs7112577
rs12359135
rs116843064
rs2642442
rs139974673
rs2792735
rs12100069
rs2229738
rs183130
rs193084249
rs613511
rs1042034
rs8103728
rs151450
rs1077834
rs1532085
All
-0.15
-0.10
-0.05
0.00
MR leave-one-out sensitivity analysis for
s to total lipids ratio in small HDL || id:ebi-cfb233-GCST90302090' on 'ER+ Breast cancer (Combined Oncoarray; iCOGS; GWAS meta

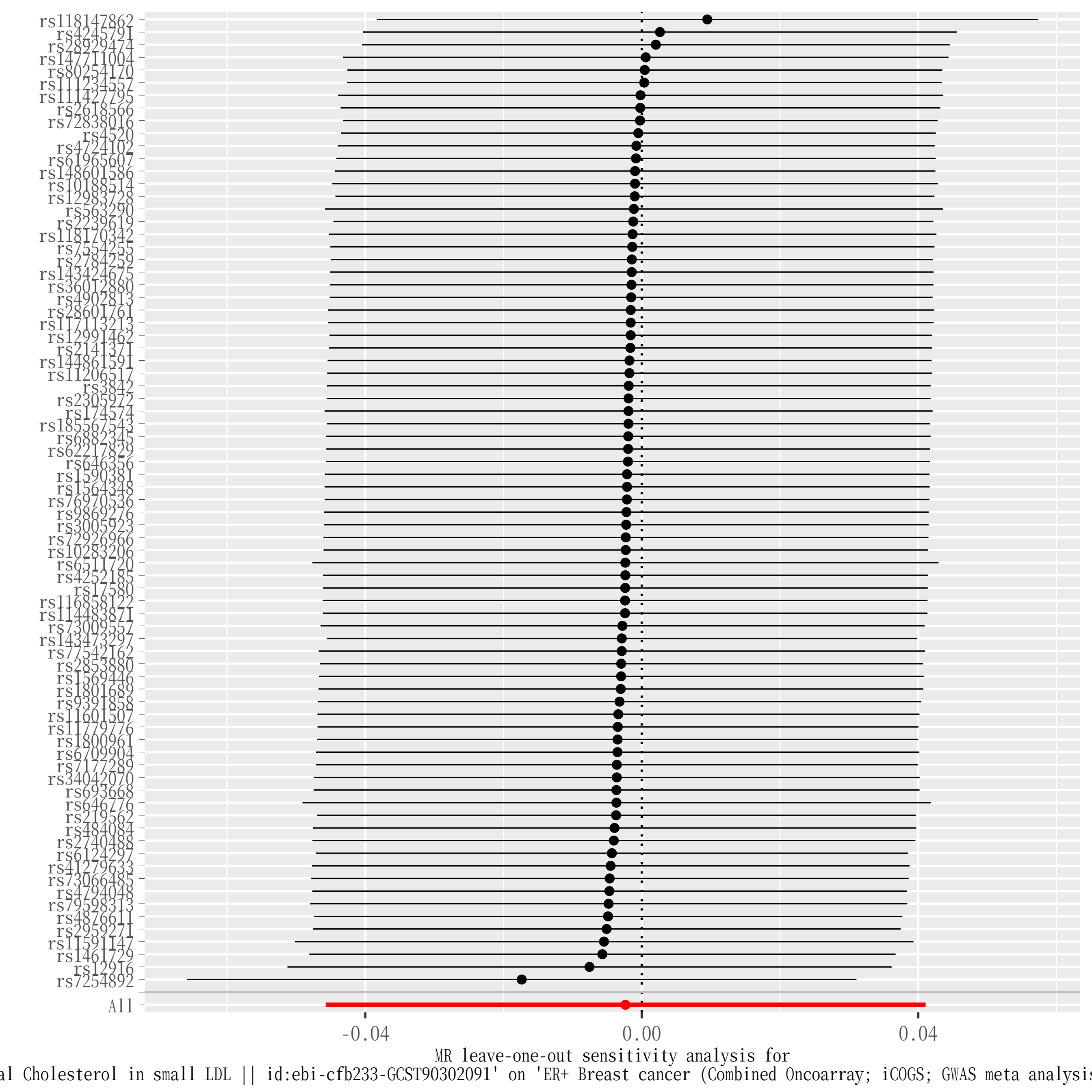
rs118147862
rs4245791
rs28929474
rs147711004
rs80254170
rs111234557
rs111427795
rs2618566
rs72838016
rs4520
rs4724102
rs61965607
rs148601586
rs10188514
rs12983728
rs563290
rs2239619
rs118170342
rs7554255
rs2784259
rs143424675
rs36012880
rs4902813
rs28601761
rs117113213
rs12991462
rs2141371
rs144861591
rs11206517
rs3842
rs2305972
rs174574
rs185567543
rs6882345
rs62217829
rs646356
rs1590381
rs1564348
rs76970536
rs9869276
rs3005923
rs72926966
rs10283206
rs6511720
rs4252185
rs17580
rs116858122
rs114483871
rs73009557
rs143473297
rs77542162
rs2853880
rs1569446
rs1801689
rs9391858
rs11601507
rs11779776
rs1800961
rs6709904
rs7177289
rs34042070
rs693668
rs646776
rs219562
rs484084
rs2740488
rs6124297
rs41279633
rs73066485
rs4794048
rs79598313
rs4876611
rs2959271
rs11591147
rs1461729
rs12916
rs7254892
All
-0.04
0.00
0.04
MR leave-one-out sensitivity analysis for
al Cholesterol in small LDL || id:ebi-cfb233-GCST90302091' on 'ER+ Breast cancer (Combined Oncoarray; iCOGS; GWAS meta analysis

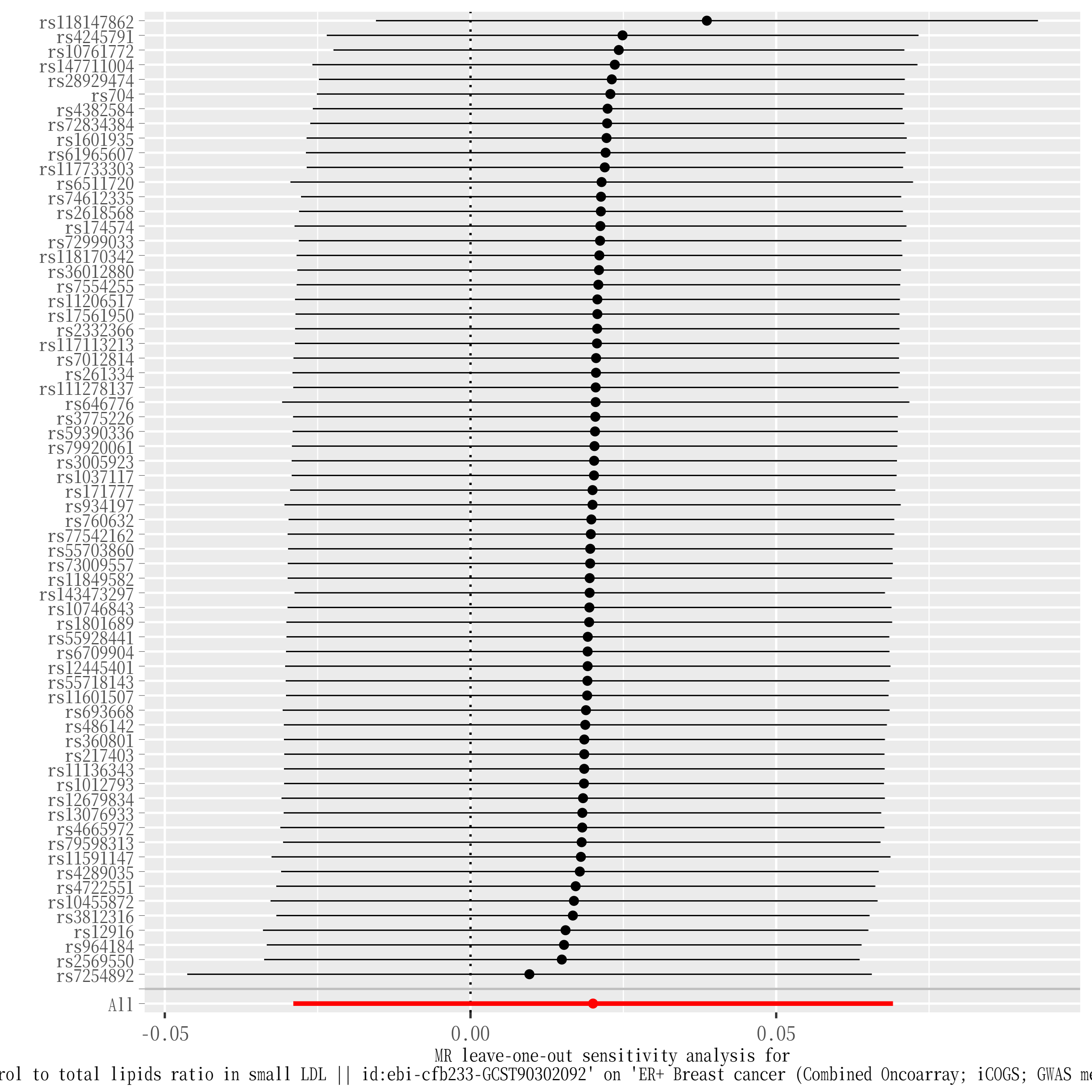
rs118147862
rs4245791
rs10761772
rs147711004
rs28929474
rs704
rs4382584
rs72834384
rs1601935
rs61965607
rs117733303
rs6511720
rs74612335
rs2618568
rs174574
rs72999033
rs118170342
rs36012880
rs7554255
rs11206517
rs17561950
rs2332366
rs117113213
rs7012814
rs261334
rs111278137
rs646776
rs3775226
rs59390336
rs79920061
rs3005923
rs1037117
rs171777
rs934197
rs760632
rs77542162
rs55703860
rs73009557
rs11849582
rs143473297
rs10746843
rs1801689
rs55928441
rs6709904
rs12445401
rs55718143
rs11601507
rs693668
rs486142
rs360801
rs217403
rs11136343
rs1012793
rs12679834
rs13076933
rs4665972
rs79598313
rs11591147
rs4289035
rs4722551
rs10455872
rs3812316
rs12916
rs964184
rs2569550
rs7254892
All
-0.05
0.00
0.05
MR leave-one-out sensitivity analysis for
rol to total lipids ratio in small LDL || id:ebi-cfb233-GCST90302092' on 'ER+ Breast cancer (Combined Oncoarray; iCOGS; GWAS me

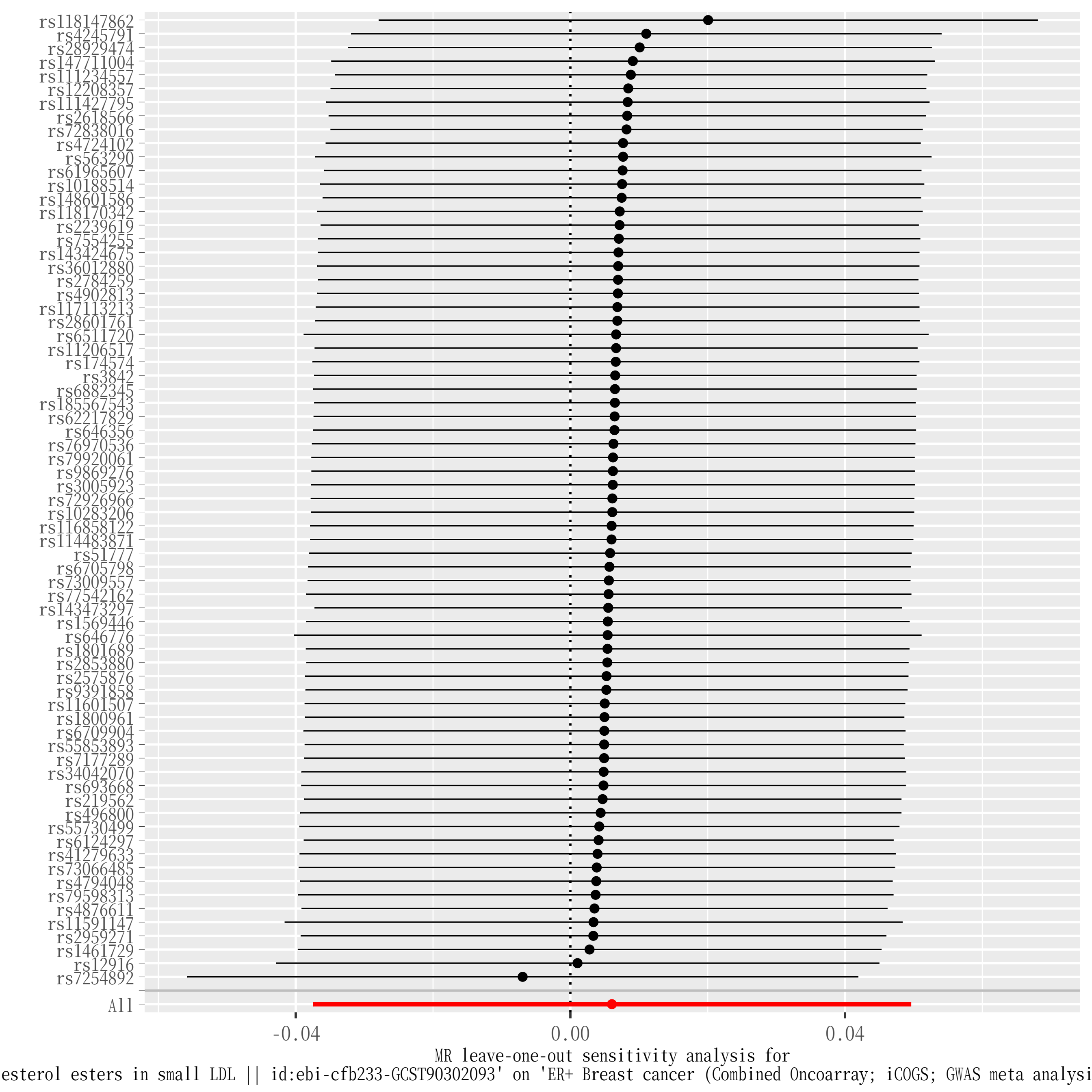
rs118147862
rs4245791
rs28929474
rs147711004
rs111234557
rs12208357
rs111427795
rs2618566
rs72838016
rs4724102
rs563290
rs61965607
rs10188514
rs148601586
rs118170342
rs2239619
rs7554255
rs143424675
rs36012880
rs2784259
rs4902813
rs117113213
rs28601761
rs6511720
rs11206517
rs174574
rs3842
rs6882345
rs185567543
rs62217829
rs646356
rs76970536
rs79920061
rs9869276
rs3005923
rs72926966
rs10283206
rs116858122
rs114483871
rs51777
rs6705798
rs73009557
rs77542162
rs143473297
rs1569446
rs646776
rs1801689
rs2853880
rs2575876
rs9391858
rs11601507
rs1800961
rs6709904
rs55853893
rs7177289
rs34042070
rs693668
rs219562
rs496800
rs55730499
rs6124297
rs41279633
rs73066485
rs4794048
rs79598313
rs4876611
rs11591147
rs2959271
rs1461729
rs12916
rs7254892
All
-0.04
0.00
0.04
MR leave-one-out sensitivity analysis for
esterol esters in small LDL || id:ebi-cfb233-GCST90302093' on 'ER+ Breast cancer (Combined Oncoarray; iCOGS; GWAS meta analysi

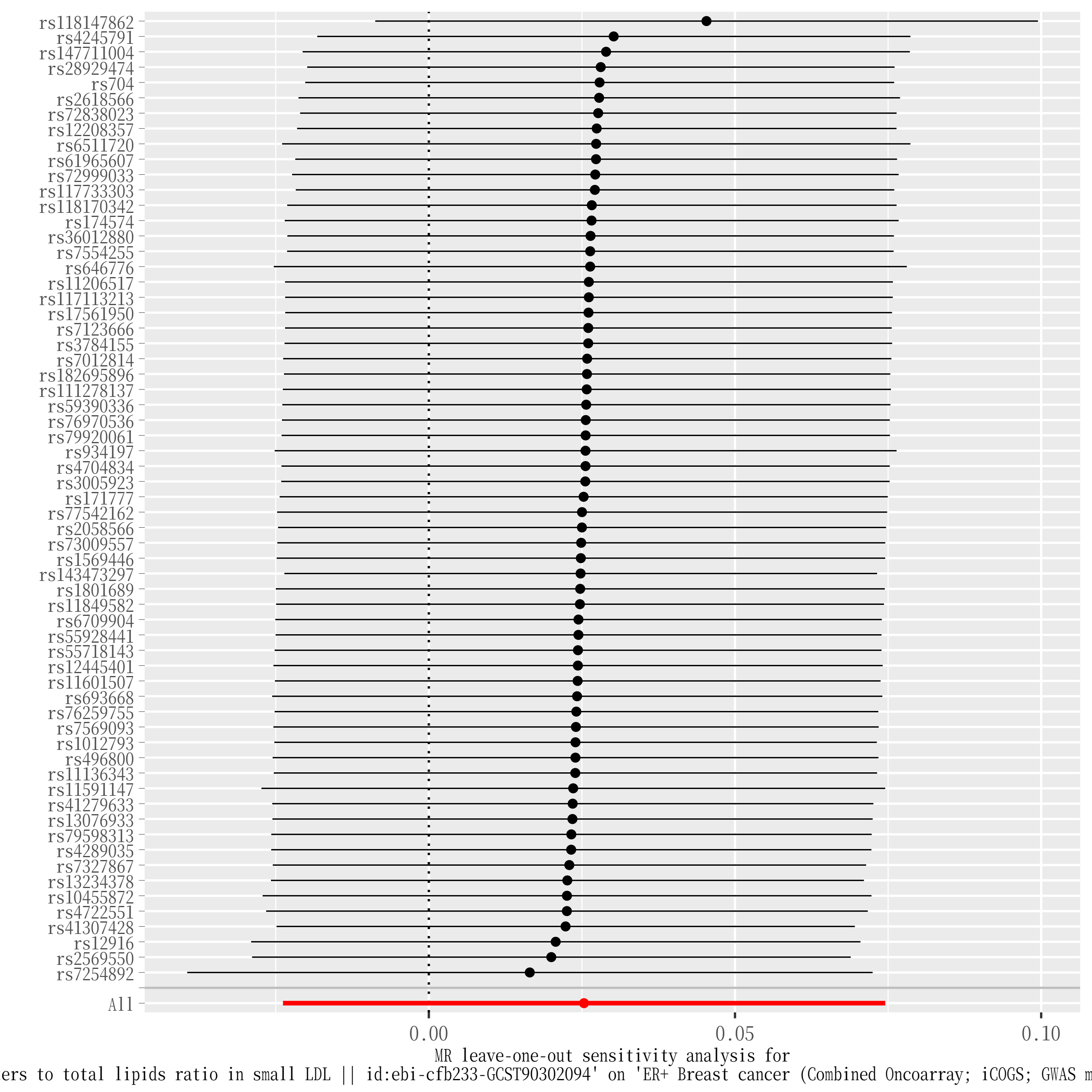

rs118147862
rs4245791
rs147711004
rs28929474
rs704
rs2618566
rs72838023
rs12208357
rs6511720
rs61965607
rs72999033
rs117733303
rs118170342
rs174574
rs36012880
rs7554255
rs646776
rs11206517
rs117113213
rs17561950
rs7123666
rs3784155
rs7012814
rs182695896
rs111278137
rs59390336
rs76970536
rs79920061
rs934197
rs4704834
rs3005923
rs171777
rs77542162
rs2058566
rs73009557
rs1569446
rs143473297
rs1801689
rs11849582
rs6709904
rs55928441
rs55718143
rs12445401
rs11601507
rs693668
rs76259755
rs7569093
rs1012793
rs496800
rs11136343
rs11591147
rs41279633
rs13076933
rs79598313
rs4289035
rs7327867
rs13234378
rs10455872
rs4722551
rs41307428
rs12916
rs2569550
rs7254892
All
0.00
0.05
0.10
MR leave-one-out sensitivity analysis for
ers to total lipids ratio in small LDL || id:ebi-cfb233-GCST90302094' on 'ER+ Breast cancer (Combined Oncoarray; iCOGS; GWAS m

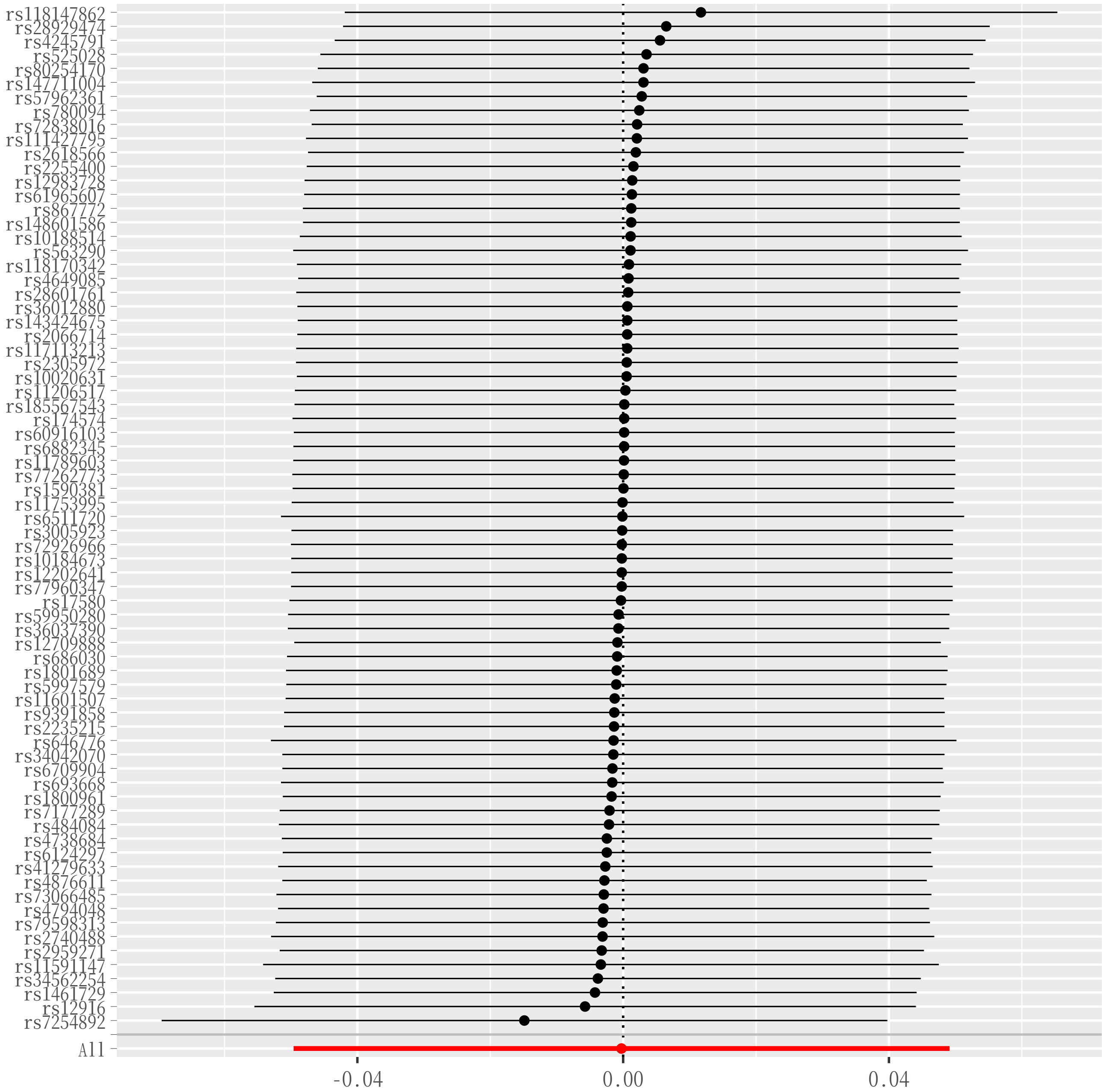

MR leave-one-out sensitivity analysis for
ee cholesterol in small LDL || id:ebi-cfb233-GCST90302095' on 'ER+ Breast cancer (Combined Oncoarray; iCOGS; GWAS meta analysis

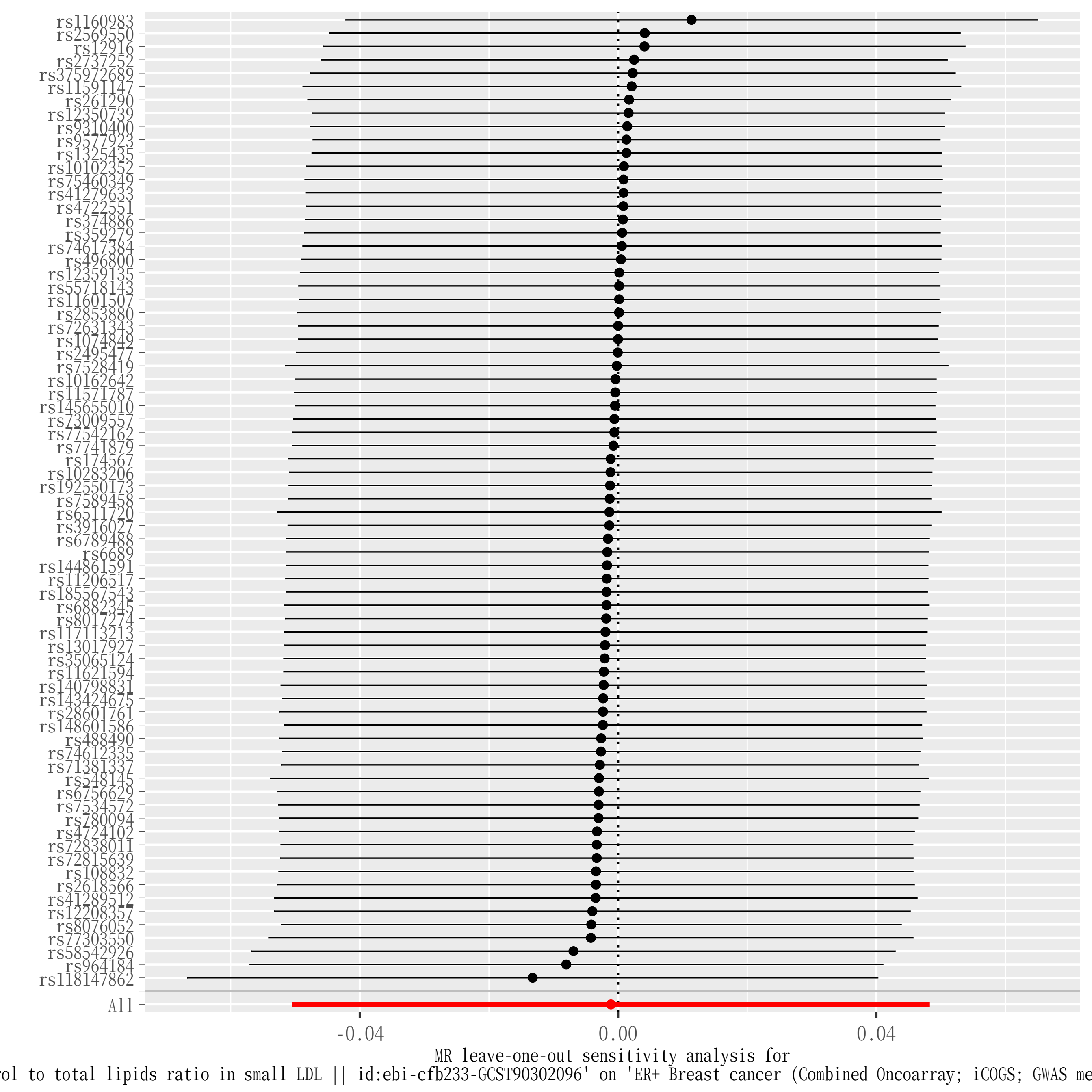

rs1160983
rs2569550
rs12916
rs2737252
rs375972689
rs11591147
rs261290
rs12350739
rs9310400
rs9577923
rs1325435
rs10102352
rs75460349
rs41279633
rs4722551
rs374886
rs359279
rs74617384
rs496800
rs12359135
rs55718143
rs11601507
rs2853880
rs72631343
rs1074849
rs2495477
rs7528419
rs10162642
rs11571787
rs145655010
rs73009557
rs77542162
rs7741879
rs174567
rs10283206
rs192550173
rs7589458
rs6511720
rs3916027
rs6789488
rs6689
rs144861591
rs11206517
rs185567543
rs6882345
rs8017274
rs117113213
rs13017927
rs35065124
rs11621594
rs140798831
rs143424675
rs28601761
rs148601586
rs488490
rs74612335
rs71381337
rs548145
rs6756629
rs7534572
rs780094
rs4724102
rs72838011
rs72815639
rs108832
rs2618566
rs41289512
rs12208357
rs8076052
rs77303550
rs58542926
rs964184
rs118147862
All
-0.04
0.00
0.04
MR leave-one-out sensitivity analysis for
rol to total lipids ratio in small LDL || id:ebi-cfb233-GCST90302096' on 'ER+ Breast cancer (Combined Oncoarray; iCOGS; GWAS me

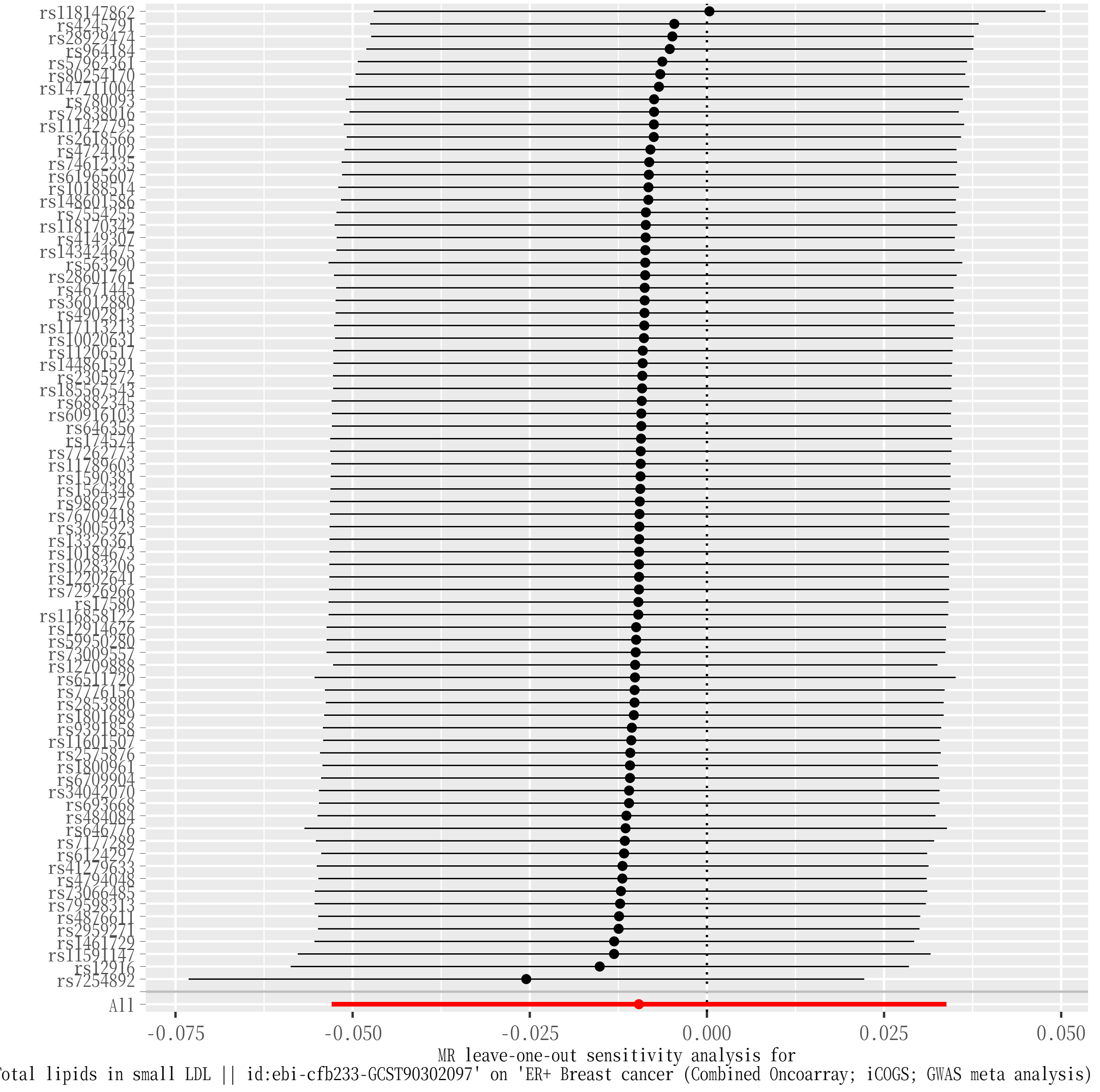
rs118147862
rs4245791
rs28929474
rs964184
rs57962361
rs80254170
rs147711004
rs780093
rs72838016
rs111427795
rs2618566
rs4724102
rs74612335
rs61965607
rs10188514
rs148601586
rs7554255
rs118170342
rs4149307
rs143424675
rs563290
rs28601761
rs4671445
rs36012880
rs4902813
rs117113213
rs10020631
rs11206517
rs144861591
rs2305972
rs185567543
rs6882345
rs60916103
rs646356
rs174574
rs77262773
rs11789603
rs1590381
rs1564348
rs9869276
rs76709418
rs3005923
rs13326361
rs10184673
rs10283206
rs12202641
rs72926966
rs17580
rs116858122
rs12914626
rs59950280
rs73009557
rs12709888
rs6511720
rs7776156
rs2853880
rs1801689
rs9391858
rs11601507
rs2575876
rs1800961
rs6709904
rs34042070
rs693668
rs484084
rs646776
rs7177289
rs6124297
rs41279633
rs4794048
rs73066485
rs79598313
rs4876611
rs2959271
rs1461729
rs11591147
rs12916
rs7254892
All
-0.075
-0.050
-0.025
0.000
0.025
0.050
MR leave-one-out sensitivity analysis for
'Total lipids in small LDL || id:ebi-cfb233-GCST90302097' on 'ER+ Breast cancer (Combined Oncoarray; iCOGS; GWAS meta analysis)'

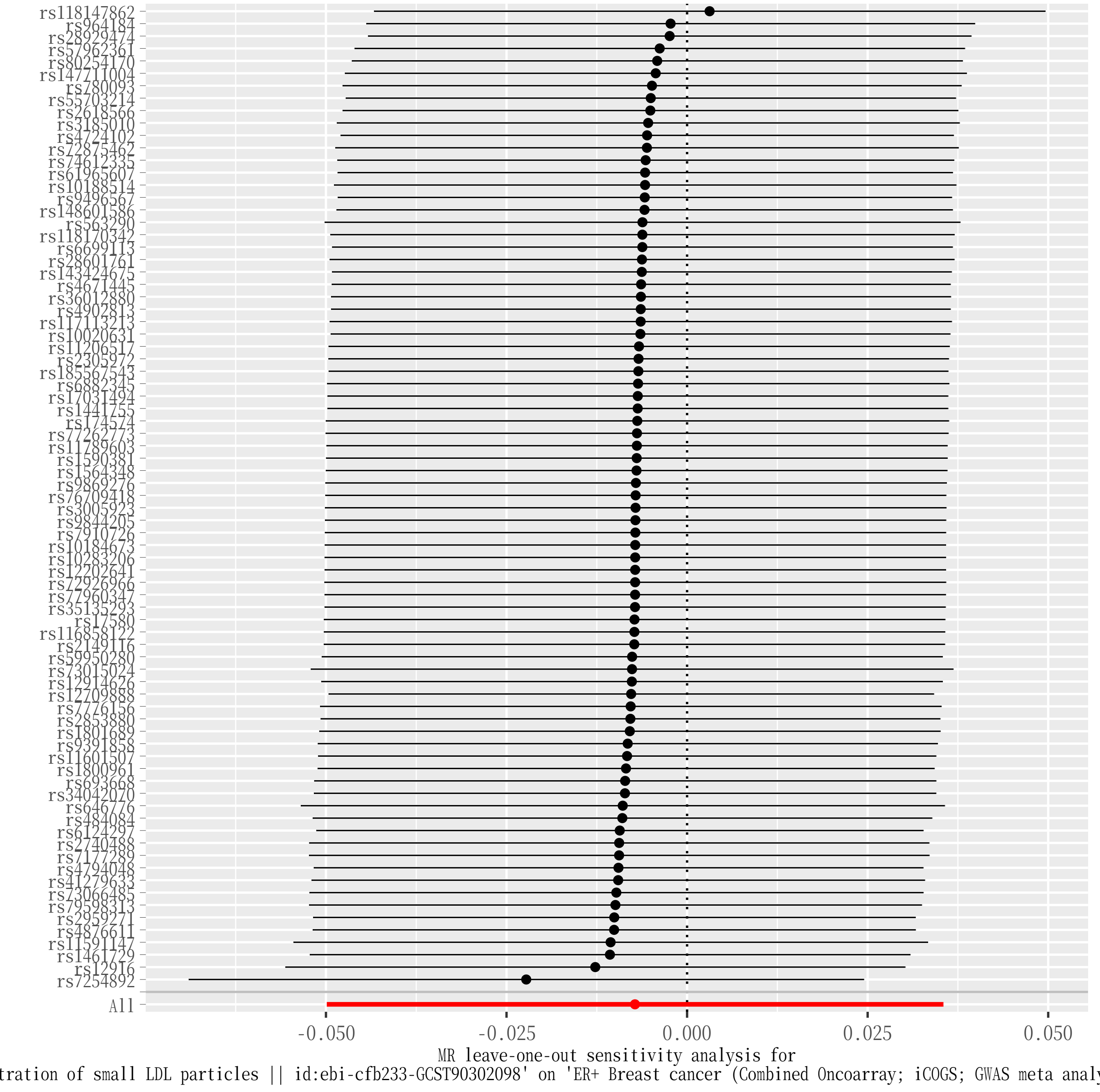
rs118147862
rs964184
rs28929474
rs57962361
rs80254170
rs147711004
rs780093
rs55703214
rs2618566
rs3185010
rs4724102
rs72875462
rs74612335
rs61965607
rs10188514
rs9496567
rs148601586
rs563290
rs118170342
rs6699113
rs28601761
rs143424675
rs4671445
rs36012880
rs4902813
rs117113213
rs10020631
rs11206517
rs2305272
rs185567543
rs6882345
rs17031494
rs1441755
rs174574
rs77262773
rs11789603
rs1590381
rs1564348
rs9869276
rs76709418
rs3005923
rs9844205
rs7910726
rs10184673
rs10283206
rs12202641
rs72926966
rs77960347
rs35135293
rs17580
rs116858122
rs2149116
rs59950280
rs73015024
rs12914626
rs12709888
rs7776156
rs2853880
rs1801689
rs9391858
rs11601507
rs1800961
rs693668
rs34042070
rs646776
rs484084
rs6124297
rs2740488
rs7177289
rs4724048
rs41279633
rs73066485
rs79598313
rs2959271
rs4876611
rs11591147
rs1461729
rs12916
rs7254892
All
-0.050
-0.025
0.000
0.025
0.050
MR leave-one-out sensitivity analysis for
tration of small LDL particles || id:ebi-cfb233-GCST90302098' on 'ER+ Breast cancer (Combined Oncoarray; iCOGS; GWAS meta analy

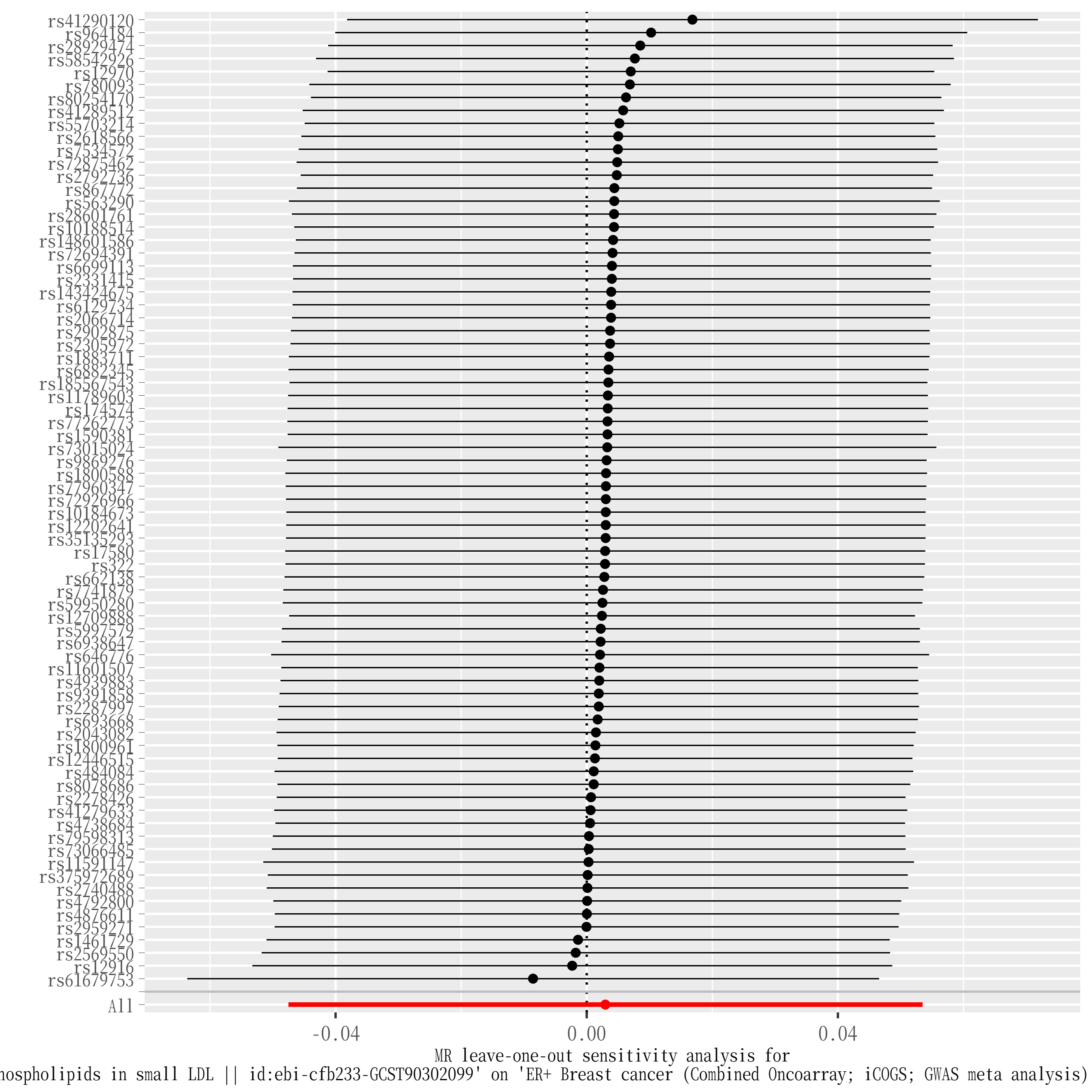
rs41290120
rs964184
rs28929474
rs58542926
rs12970
rs780093
rs80254170
rs41289512
rs55703214
rs2618566
rs7534572
rs72875462
rs2792736
rs867772
rs563290
rs28601761
rs10188514
rs148601586
rs72694391
rs6699113
rs2331415
rs143424675
rs6129734
rs2066714
rs2902875
rs2305972
rs1883711
rs6882345
rs185567543
rs11789603
rs174574
rs77262773
rs1590381
rs73015024
rs9869276
rs1800588
rs77960347
rs72926966
rs10184673
rs12202641
rs35135293
rs17580
rs322
rs662138
rs7741879
rs59950280
rs12709888
rs5997579
rs6938647
rs646776
rs11601507
rs4939883
rs9391858
rs2287997
rs693668
rs2043082
rs1800961
rs12446515
rs484084
rs8078686
rs2278426
rs41279633
rs4738684
rs79598313
rs73066485
rs11591147
rs375972689
rs2740488
rs4792800
rs4876611
rs2959271
rs1461729
rs2569550
rs12916
rs61679753
All
-0.04
0.00
0.04
MR leave-one-out sensitivity analysis for
hospholipids in small LDL || id:ebi-cfb233-GCST90302099' on 'ER+ Breast cancer (Combined Oncoarray; iCOGS; GWAS meta analysis)

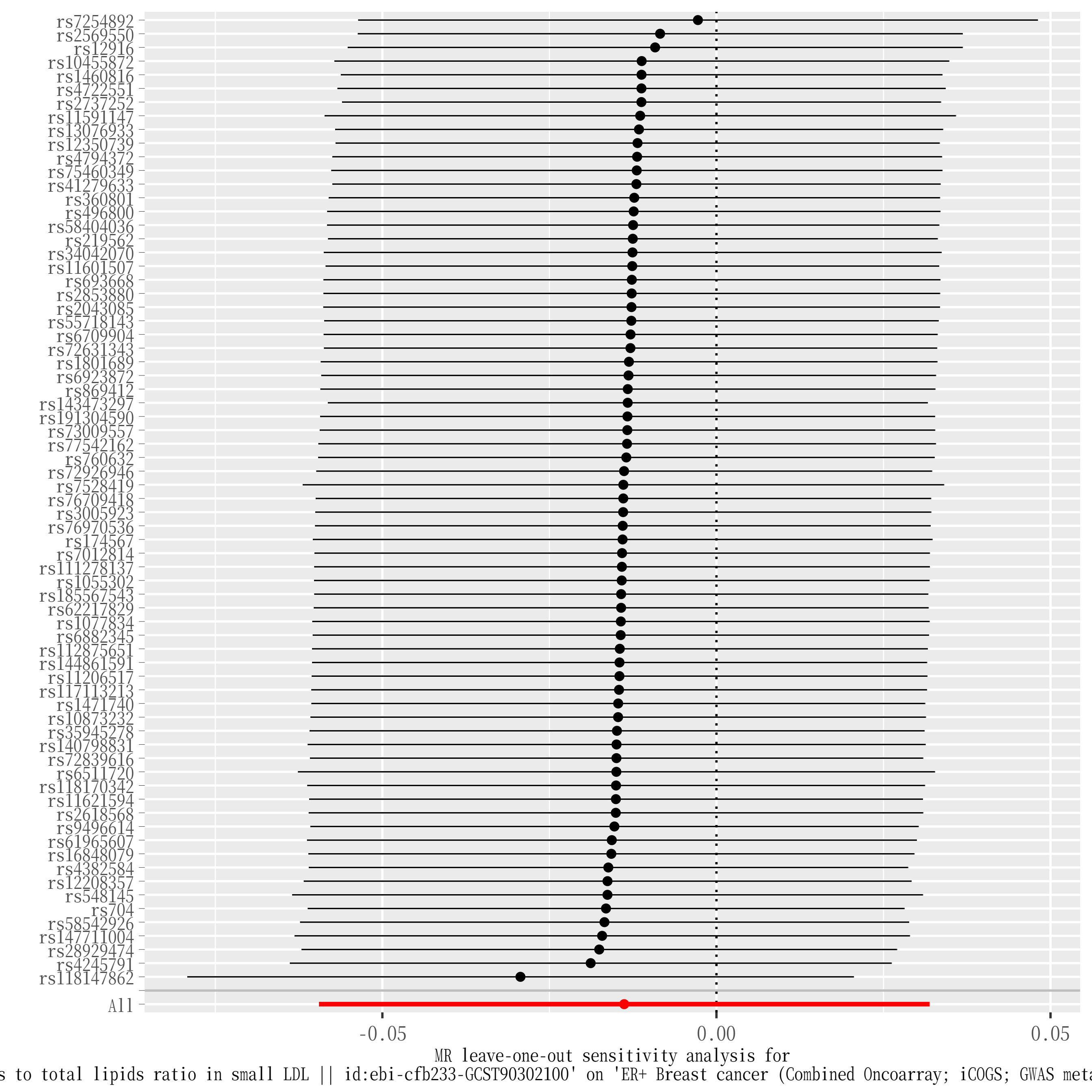

rs7254892
rs2569550
rs12916
rs10455872
rs1460816
rs4722551
rs2737252
rs11591147
rs13076933
rs12350739
rs4794372
rs75460349
rs41279633
rs360801
rs496800
rs58404036
rs219562
rs34042070
rs11601507
rs693668
rs2853880
rs2043085
rs55718143
rs6709904
rs72631343
rs1801689
rs6923872
rs869412
rs143473297
rs191304590
rs73009557
rs77542162
rs760632
rs72926946
rs7528419
rs76709418
rs3005923
rs76970536
rs174567
rs7012814
rs111278137
rs1055302
rs185567543
rs62217829
rs1077834
rs6882345
rs112875651
rs144861591
rs11206517
rs117113213
rs1471740
rs10873232
rs35945278
rs140798831
rs72839616
rs6511720
rs118170342
rs11621594
rs2618568
rs9496614
rs61965607
rs16848079
rs4382584
rs12208357
rs548145
rs704
rs58542926
rs147711004
rs28929474
rs4245791
rs118147862
All
-0.05
0.00
0.05
MR leave-one-out sensitivity analysis for
s to total lipids ratio in small LDL || id:ebi-cfb233-GCST90302100' on 'ER+ Breast cancer (Combined Oncoarray; iCOGS; GWAS meta

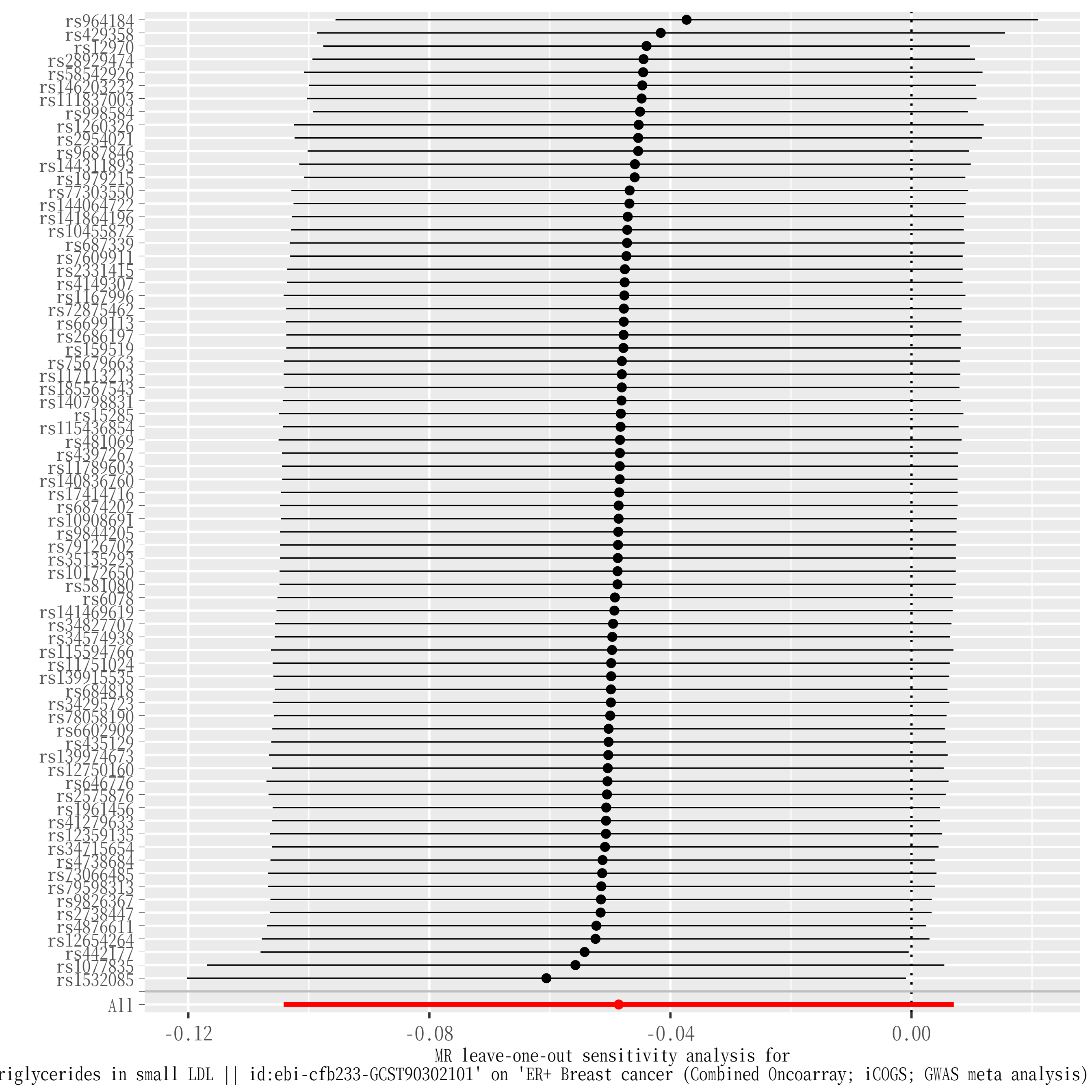
rs964184
rs429358
rs12970
rs28929474
rs58542926
rs146203232
rs111837003
rs998584
rs1260326
rs2954021
rs9687846
rs144311893
rs1979215
rs77303550
rs144064722
rs141864196
rs10455872
rs687339
rs7609911
rs2331415
rs4149307
rs1167996
rs72875462
rs6699113
rs2686197
rs159519
rs75679663
rs117113213
rs185567543
rs140798831
rs15285
rs115436854
rs481069
rs4397267
rs11789603
rs140836760
rs17414716
rs6874202
rs10908691
rs9844205
rs79126702
rs35135293
rs10172650
rs581080
rs6078
rs141469619
rs34827707
rs34574938
rs115594766
rs11751024
rs139915535
rs684818
rs34295723
rs78058190
rs6602909
rs435129
rs139974673
rs12750160
rs646776
rs2575876
rs1961456
rs41279633
rs12359135
rs34715654
rs4738684
rs73066485
rs79598313
rs9826367
rs2738447
rs4876611
rs12654264
rs442177
rs1077835
rs1532085
All
-0.12
-0.08
-0.04
0.00
MR leave-one-out sensitivity analysis for
riglycerides in small LDL || id:ebi-cfb233-GCST90302101' on 'ER+ Breast cancer (Combined Oncoarray; iCOGS; GWAS meta analysis)

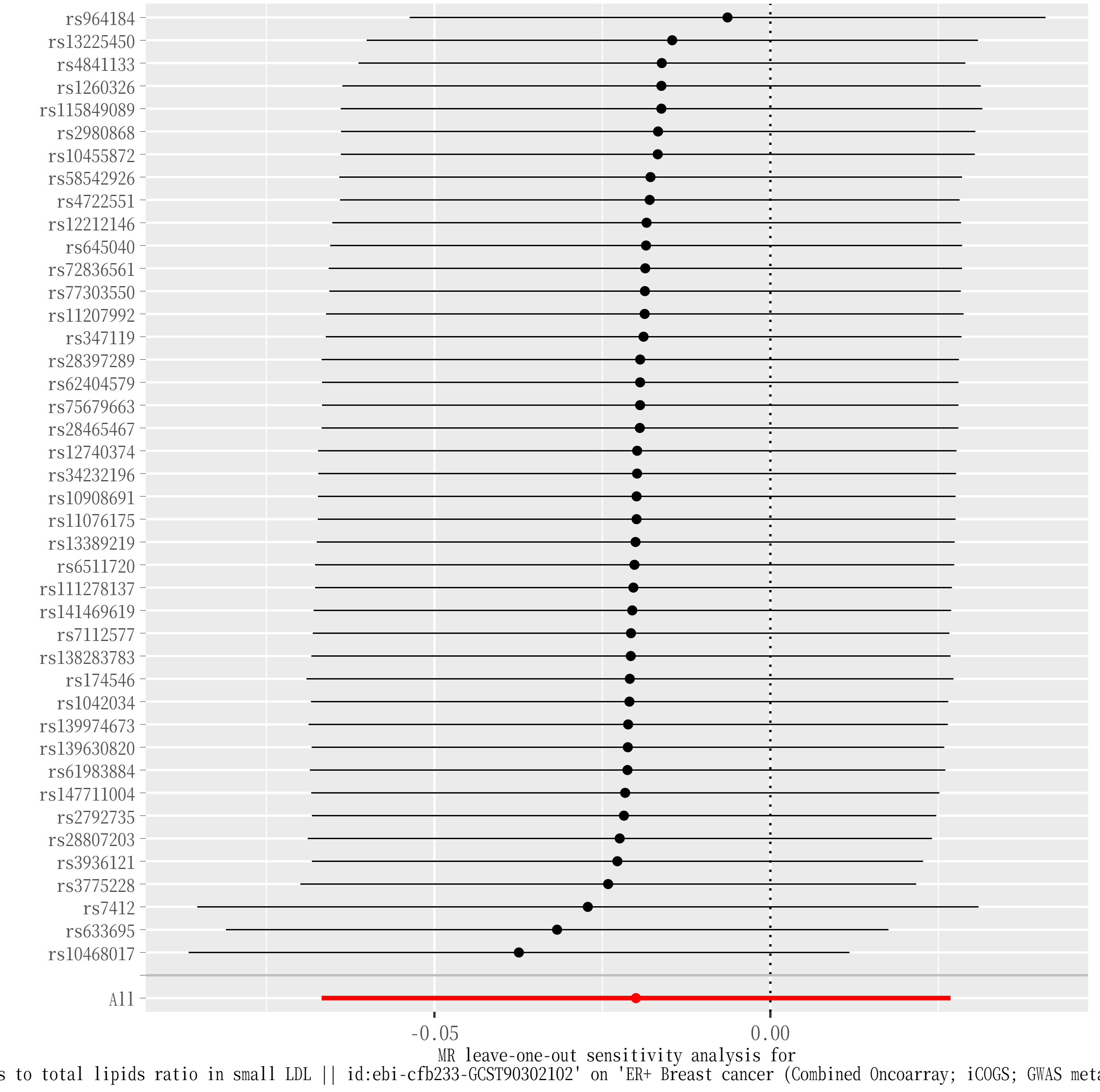
rs964184
rs13225450
rs4841133
rs1260326
rs115849089
rs2980868
rs10455872
rs58542926
rs4722551
rs12212146
rs645040
rs72836561
rs77303550
rs11207992
rs347119
rs28397289
rs62404579
rs75679663
rs28465467
rs12740374
rs34232196
rs10908691
rs11076175
rs13389219
rs6511720
rs111278137
rs141469619
rs7112577
rs138283783
rs174546
rs1042034
rs139974673
rs139630820
rs61983884
rs147711004
rs2792735
rs28807203
rs3936121
rs3775228
rs7412
rs633695
rs10468017
All
-0.05
0.00
MR leave-one-out sensitivity analysis for
s to total lipids ratio in small LDL || id:ebi-cfb233-GCST90302102' on 'ER+ Breast cancer (Combined Oncoarray; iCOGS; GWAS meta

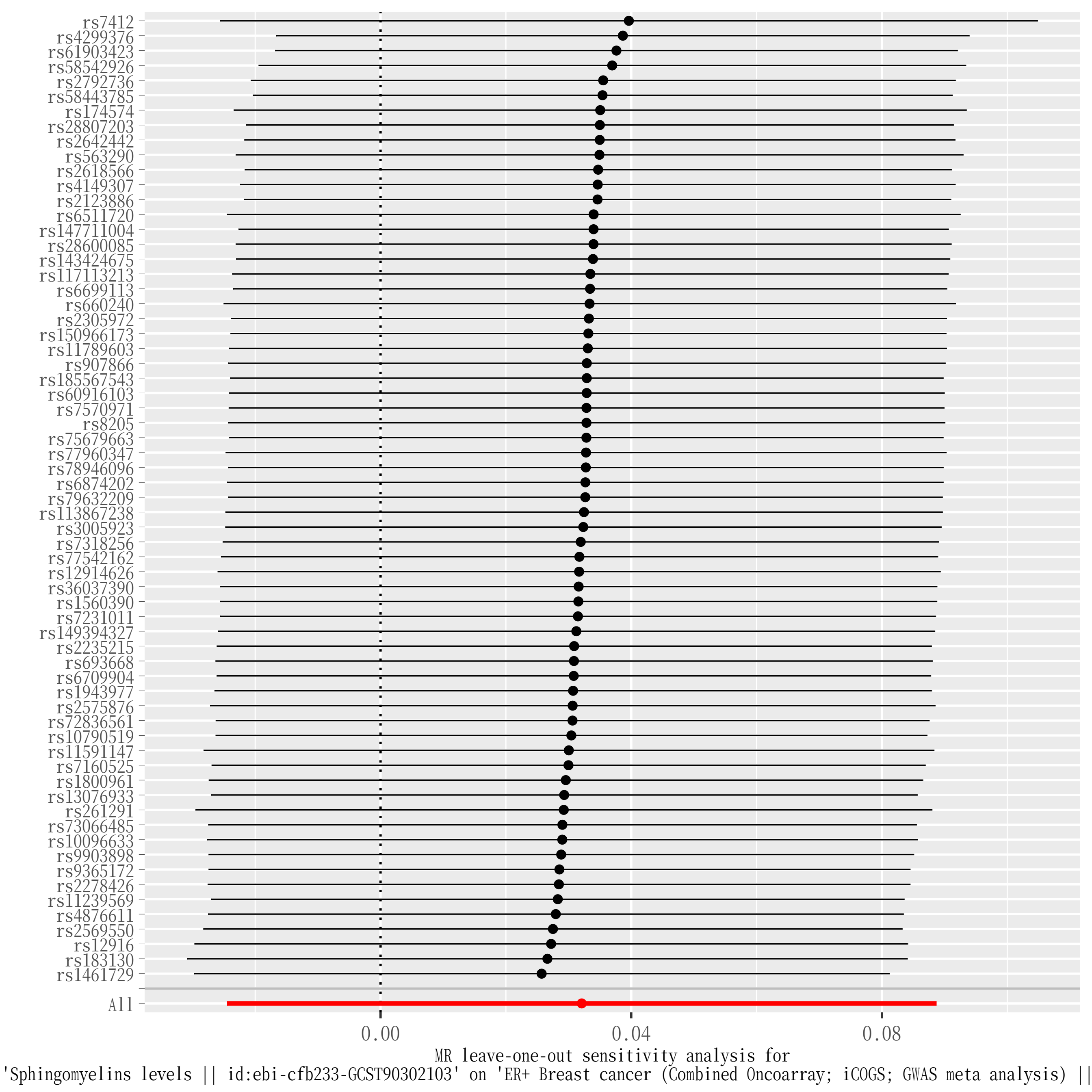
rs7412
rs4299376
rs61903423
rs58542926
rs2792736
rs58443785
rs174574
rs28807203
rs2642442
rs563290
rs2618566
rs4149307
rs2123886
rs6511720
rs147711004
rs28600085
rs143424675
rs117113213
rs6699113
rs660240
rs2305972
rs150966173
rs11789603
rs907866
rs185567543
rs60916103
rs7570971
rs8205
rs75679663
rs77960347
rs78946096
rs6874202
rs79632209
rs113867238
rs3005923
rs7318256
rs77542162
rs12914626
rs36037390
rs1560390
rs7231011
rs149394327
rs2235215
rs693668
rs6709904
rs1943977
rs2575876
rs72836561
rs10790519
rs11591147
rs7160525
rs1800961
rs13076933
rs261291
rs73066485
rs10096633
rs9903898
rs9365172
rs2278426
rs11239569
rs4876611
rs2569550
rs12916
rs183130
rs1461729
All
0.00
0.04
0.08
MR leave-one-out sensitivity analysis for
'Sphingomyelins levels || id:ebi-cfb233-GCST90302103' on 'ER+ Breast cancer (Combined Oncoarray; iCOGS; GWAS meta analysis) ||

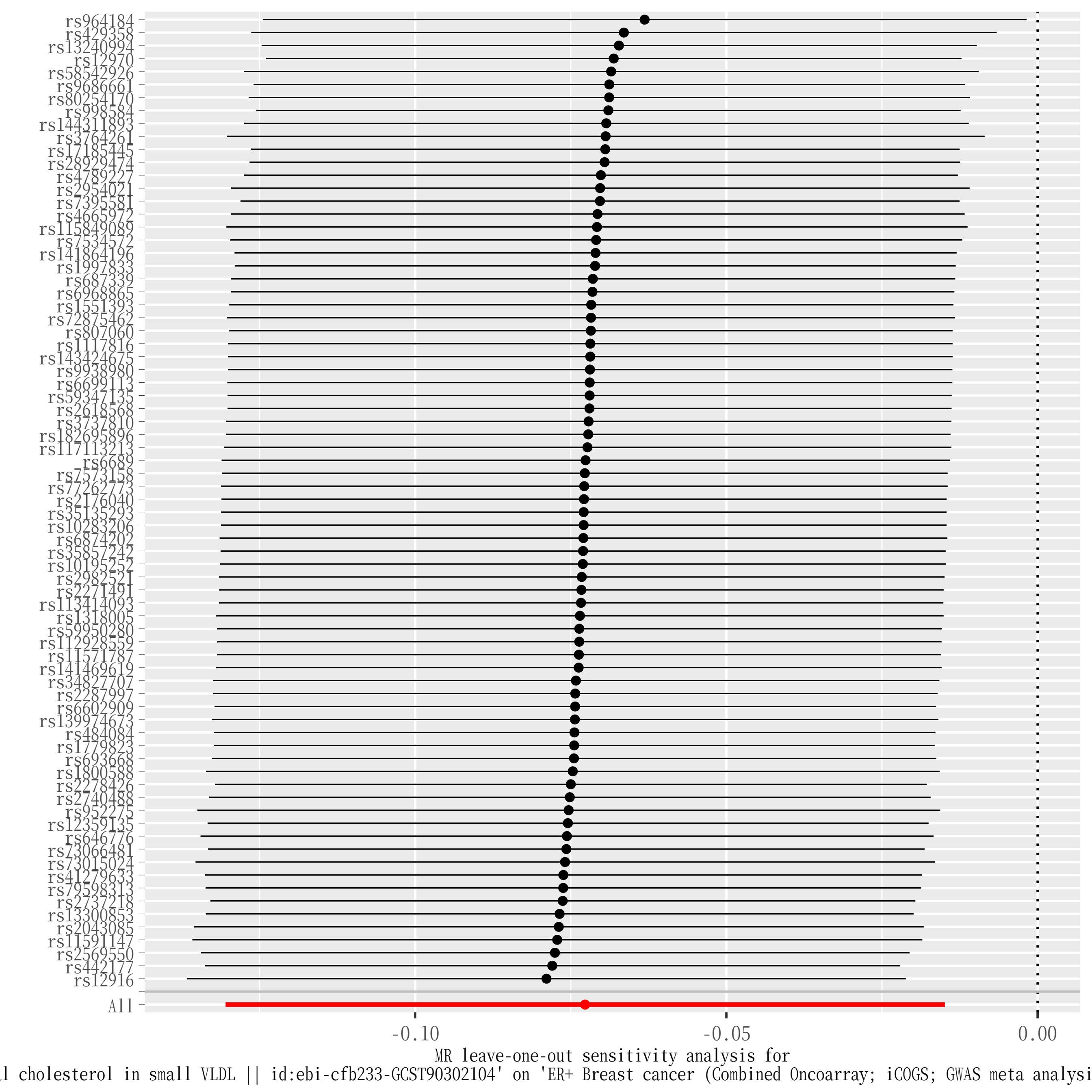
rs964184
rs429358
rs13240994
rs12970
rs58542926
rs9686661
rs80254170
rs998584
rs144311893
rs3764261
rs17185445
rs28929474
rs4789227
rs2954021
rs7395581
rs4665972
rs115849089
rs7534572
rs141864196
rs1997833
rs687339
rs6968865
rs1551393
rs72875462
rs807060
rs1117816
rs143424675
rs9938980
rs6699113
rs59347135
rs2618568
rs3737810
rs182695896
rs117113213
rs6689
rs7573158
rs77262773
rs2176040
rs35135293
rs10283206
rs6874202
rs35857242
rs10195252
rs2982521
rs2271491
rs113414093
rs1318005
rs59950280
rs112928559
rs11571787
rs141469619
rs34827707
rs2287997
rs6602909
rs139974673
rs484084
rs1779823
rs693668
rs1800588
rs2278426
rs2740488
rs952275
rs12359135
rs646776
rs73066481
rs73015024
rs41279633
rs79598313
rs2737218
rs13300853
rs2043085
rs11591147
rs2569550
rs442177
rs12916
All
-0.10
-0.05
0.00
MR leave-one-out sensitivity analysis for
l cholesterol in small VLDL || id:ebi-cfb233-GCST90302104' on 'ER+ Breast cancer (Combined Oncoarray; iCOGS; GWAS meta analysi

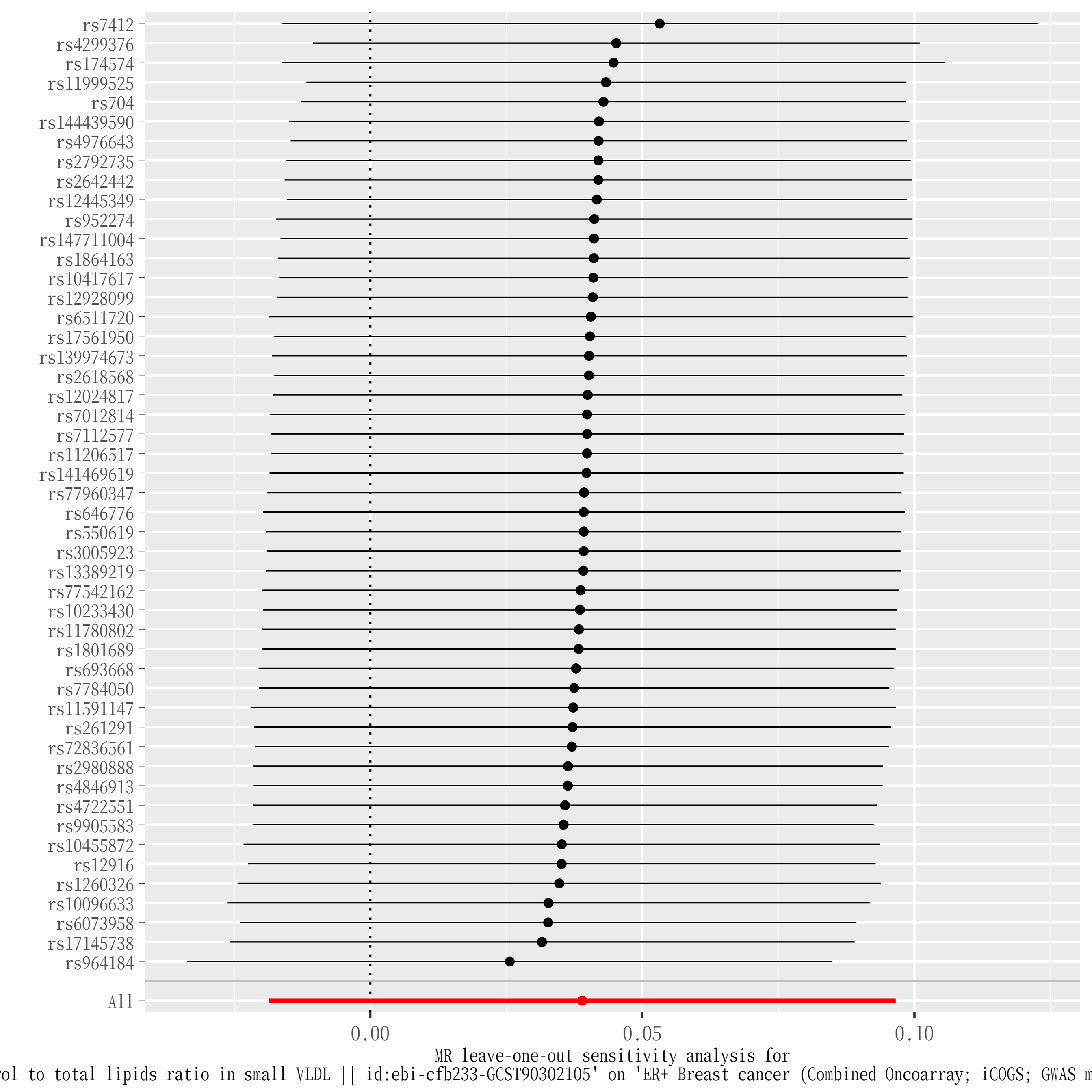
rs7412
rs4299376
rs174574
rs11999525
rs704
rs144439590
rs4976643
rs2792735
rs2642442
rs12445349
rs952274
rs147711004
rs1864163
rs10417617
rs12928099
rs6511720
rs17561950
rs139974673
rs2618568
rs12024817
rs7012814
rs7112577
rs11206517
rs141469619
rs77960347
rs646776
rs550619
rs3005923
rs13389219
rs77542162
rs10233430
rs11780802
rs1801689
rs693668
rs7784050
rs11591147
rs261291
rs72836561
rs2980888
rs4846913
rs4722551
rs9905583
rs10455872
rs12916
rs1260326
rs10096633
rs6073958
rs17145738
rs964184
All
0.00
0.05
0.10
MR leave-one-out sensitivity analysis for
rol to total lipids ratio in small VLDL || id:ebi-cfb233-GCST90302105' on 'ER+ Breast cancer (Combined Oncoarray; iCOGS; GWAS m

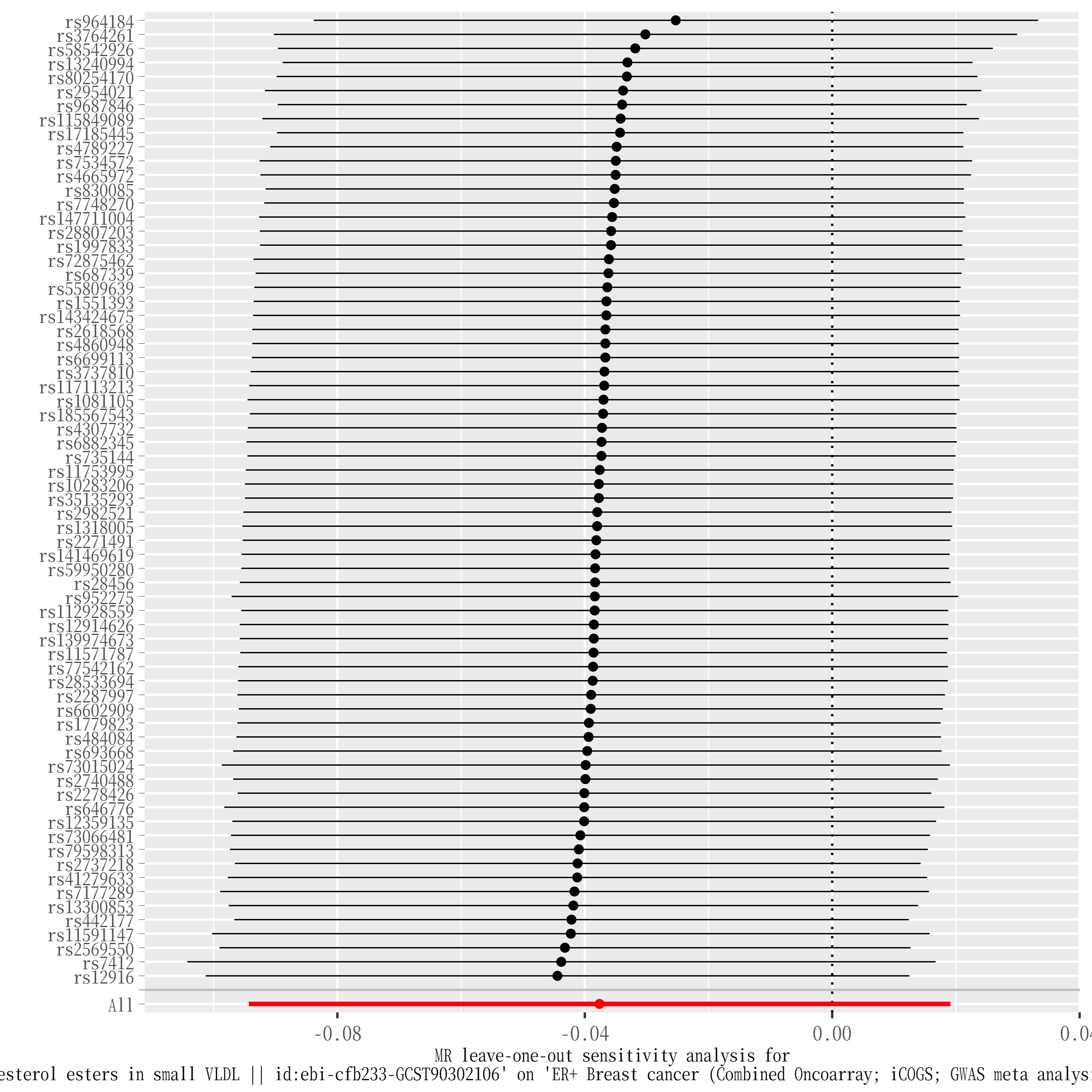
rs964184
rs3764261
rs58542926
rs13240994
rs80254170
rs2954021
rs9687846
rs115849089
rs17185445
rs4789227
rs7534572
rs4665972
rs830085
rs7748270
rs147711004
rs28807203
rs1997833
rs72875462
rs687339
rs55809639
rs1551393
rs143424675
rs2618568
rs4860948
rs6699113
rs3737810
rs117113213
rs1081105
rs185567543
rs4307732
rs6882345
rs735144
rs11753995
rs10283206
rs35135293
rs2982521
rs1318005
rs2271491
rs141469619
rs59950280
rs28456
rs952275
rs112928559
rs12914626
rs139974673
rs11571787
rs77542162
rs28533694
rs2287997
rs6602909
rs1779823
rs484084
rs693668
rs73015024
rs2740488
rs2278426
rs646776
rs12359135
rs73066481
rs79598313
rs2737218
rs41279633
rs7177289
rs13300853
rs442177
rs11591147
rs2569550
rs7412
rs12916
All
-0.08
-0.04
0.00
0.04
MR leave-one-out sensitivity analysis for
esterol esters in small VLDL || id:ebi-cfb233-GCST90302106' on 'ER+ Breast cancer (Combined Oncoarray; iCOGS; GWAS meta analysis

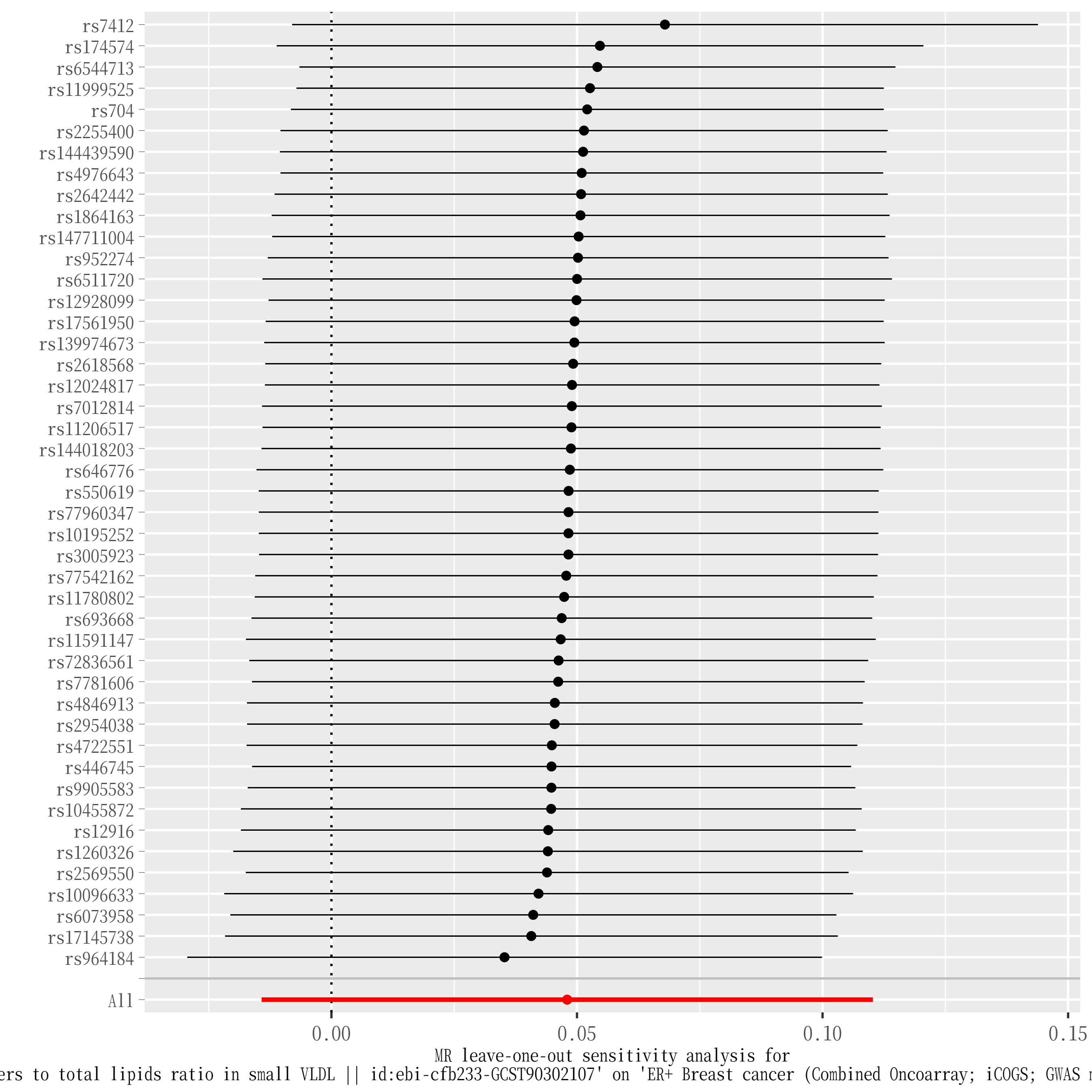

rs7412
rs174574
rs6544713
rs11999525
rs704
rs2255400
rs144439590
rs4976643
rs2642442
rs1864163
rs147711004
rs952274
rs6511720
rs12928099
rs17561950
rs139974673
rs2618568
rs12024817
rs7012814
rs11206517
rs144018203
rs646776
rs550619
rs77960347
rs10195252
rs3005923
rs77542162
rs11780802
rs693668
rs11591147
rs72836561
rs7781606
rs4846913
rs2954038
rs4722551
rs446745
rs9905583
rs10455872
rs12916
rs1260326
rs2569550
rs10096633
rs6073958
rs17145738
rs964184
All
0.00
0.05
0.10
0.15
MR leave-one-out sensitivity analysis for
ers to total lipids ratio in small VLDL || id:ebi-cfb233-GCST90302107' on 'ER+ Breast cancer (Combined Oncoarray; iCOGS; GWAS m

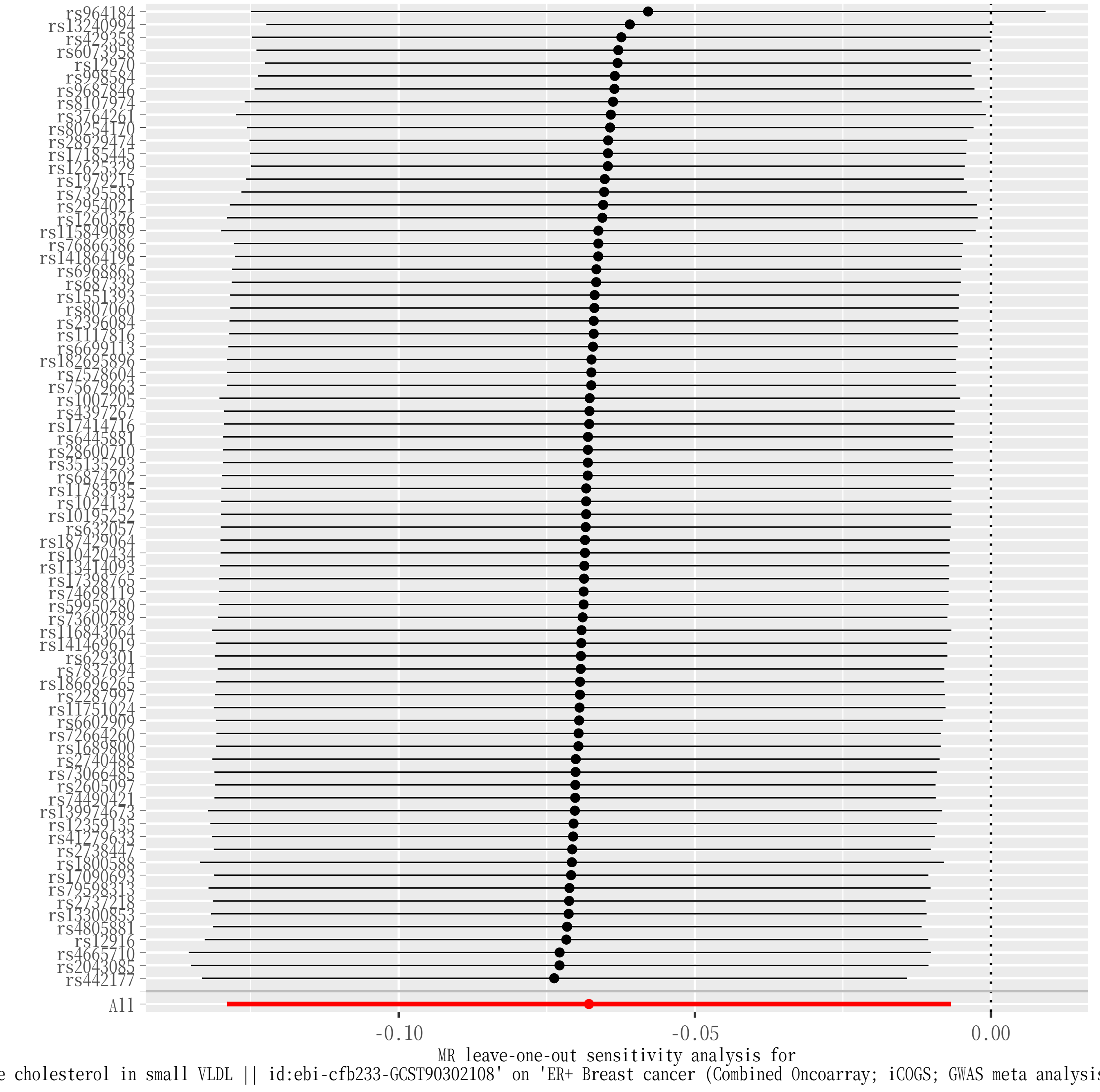
rs964184
rs13240994
rs429358
rs6073958
rs12970
rs998584
rs9687846
rs8107974
rs3764261
rs80254170
rs28929474
rs17185445
rs12625329
rs1979215
rs7395581
rs2954021
rs1260326
rs115849089
rs76866386
rs141864196
rs6968865
rs687339
rs1551393
rs807060
rs2396084
rs1117816
rs6699113
rs182695896
rs7578604
rs75679663
rs1007205
rs4397267
rs17414716
rs6445881
rs28600710
rs35135293
rs6874202
rs11783935
rs1024137
rs10195252
rs632057
rs187429064
rs10420434
rs113414093
rs17398765
rs74698119
rs59950280
rs73600289
rs116843064
rs141469619
rs629301
rs7837694
rs186696265
rs2287997
rs11751024
rs6602909
rs72664260
rs1689800
rs2740488
rs73066485
rs2605097
rs74490421
rs139974673
rs12359135
rs41279633
rs2738447
rs1800588
rs17090693
rs79598313
rs2737218
rs13300853
rs4805881
rs12916
rs4665710
rs2043085
rs442177
All
-0.10
-0.05
0.00
MR leave-one-out sensitivity analysis for
e cholesterol in small VLDL || id:ebi-cfb233-GCST90302108' on 'ER+ Breast cancer (Combined Oncoarray; iCOGS; GWAS meta analysis

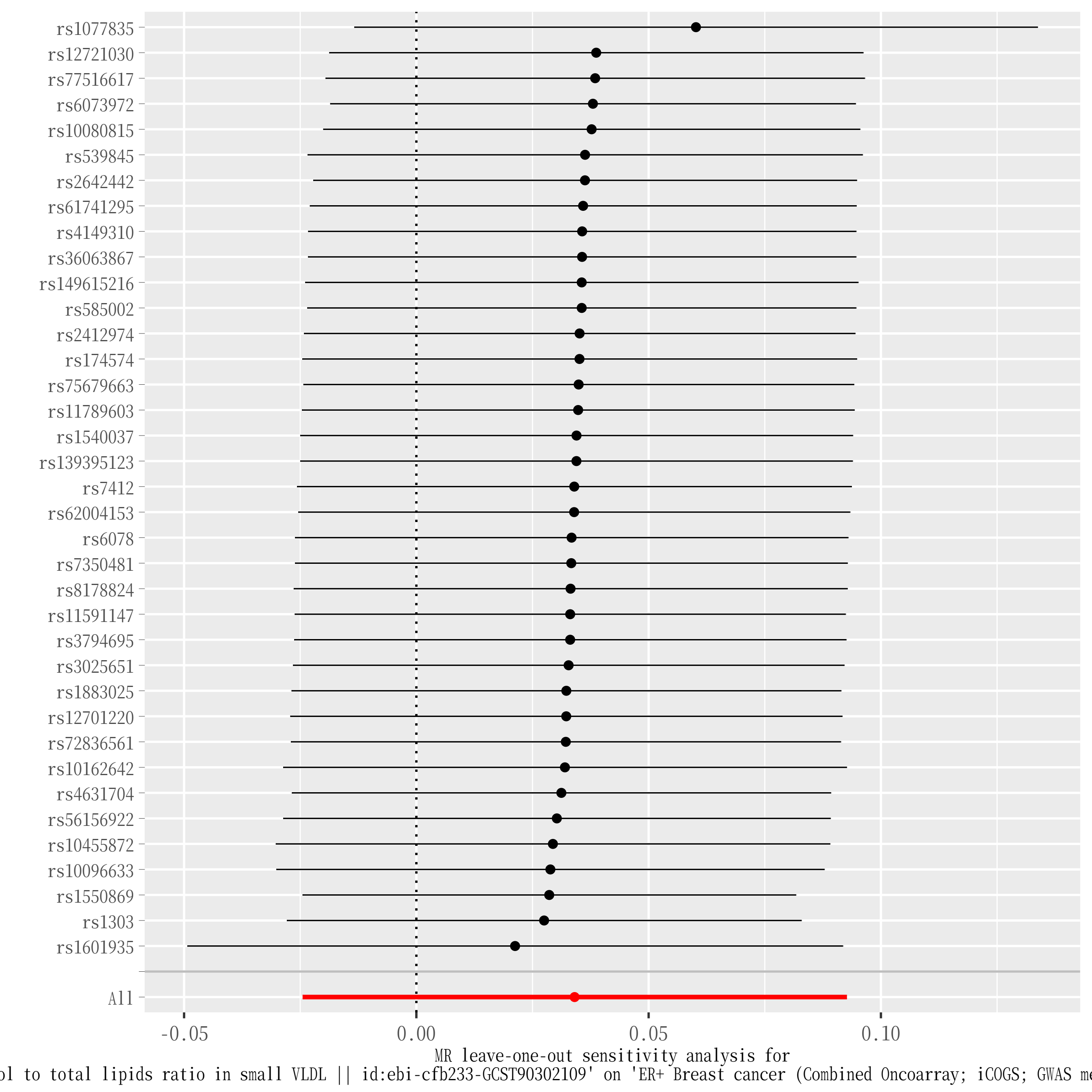
rs1077835
rs12721030
rs77516617
rs6073972
rs10080815
rs539845
rs2642442
rs61741295
rs4149310
rs36063867
rs149615216
rs585002
rs2412974
rs174574
rs75679663
rs11789603
rs1540037
rs139395123
rs7412
rs62004153
rs6078
rs7350481
rs8178824
rs11591147
rs3794695
rs3025651
rs1883025
rs12701220
rs72836561
rs10162642
rs4631704
rs56156922
rs10455872
rs10096633
rs1550869
rs1303
rs1601935
All
-0.05
0.00
0.05
0.10
MR leave-one-out sensitivity analysis for
ol to total lipids ratio in small VLDL || id:ebi-cfb233-GCST90302109' on 'ER+ Breast cancer (Combined Oncoarray; iCOGS; GWAS me

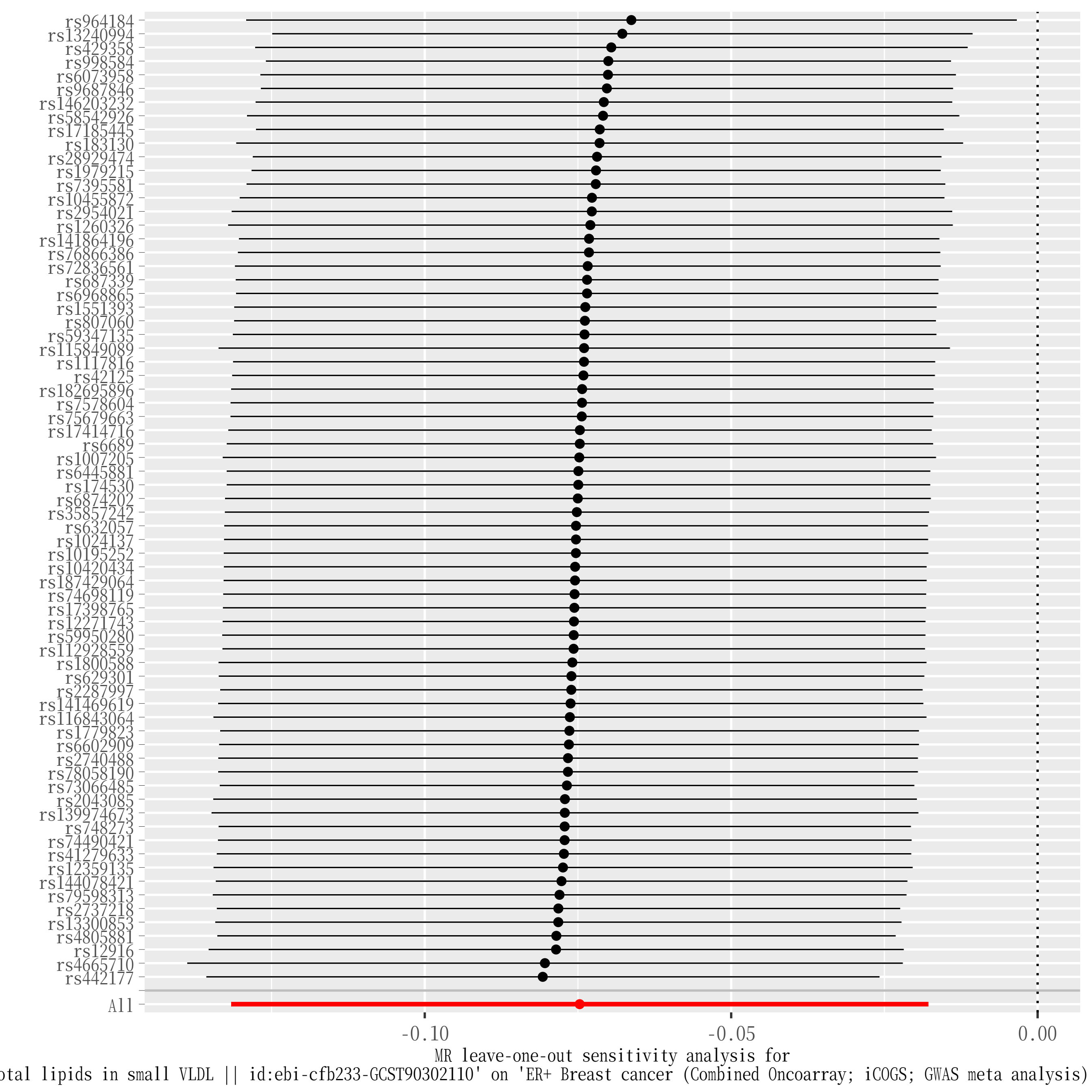

rs964184
rs13240994
rs429358
rs998584
rs6073958
rs9687846
rs146203232
rs58542926
rs17185445
rs183130
rs28929474
rs1979215
rs7395581
rs10455872
rs2954021
rs1260326
rs141864196
rs76866386
rs72836561
rs687339
rs6968865
rs1551393
rs807060
rs59347135
rs115849089
rs1117816
rs42125
rs182695896
rs7578604
rs75679663
rs17414716
rs6689
rs1007205
rs6445881
rs174530
rs6874202
rs35857242
rs632057
rs1024137
rs10195252
rs10420434
rs187429064
rs74698119
rs17398765
rs12271743
rs59950280
rs112928559
rs1800588
rs629301
rs2287997
rs141469619
rs116843064
rs1779823
rs6602909
rs2740488
rs78058190
rs73066485
rs2043085
rs139974673
rs748273
rs74490421
rs41279633
rs12359135
rs144078421
rs79598313
rs2737218
rs13300853
rs4805881
rs12916
rs4665710
rs442177
All
-0.10
-0.05
0.00
MR leave-one-out sensitivity analysis for
otal lipids in small VLDL || id:ebi-cfb233-GCST90302110' on 'ER+ Breast cancer (Combined Oncoarray; iCOGS; GWAS meta analysis)

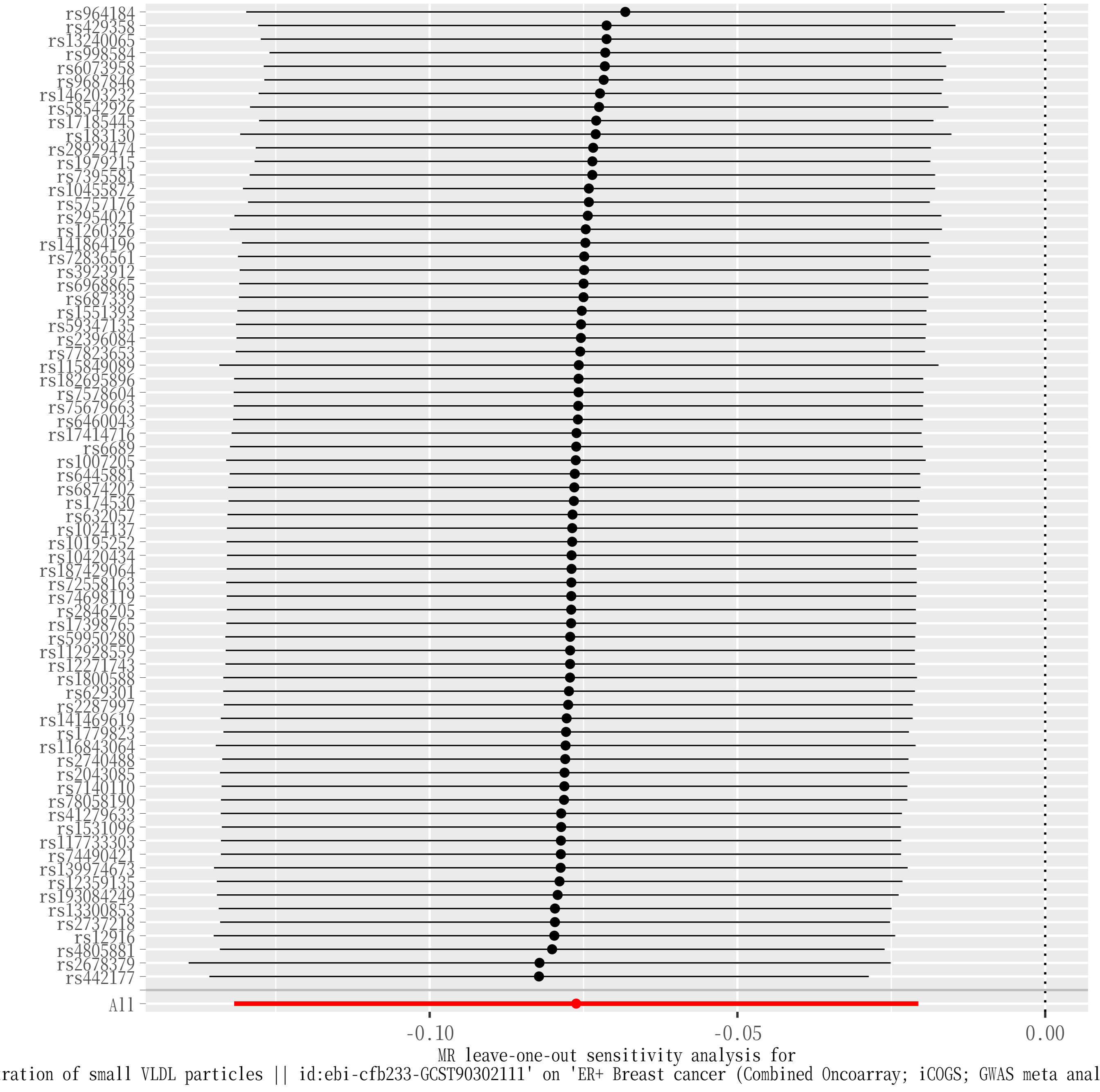

rs964184
rs429358
rs13240065
rs998584
rs6073958
rs9687846
rs146203232
rs58542926
rs17185445
rs183130
rs28929474
rs1979215
rs7395581
rs10455872
rs5757176
rs2954021
rs1260326
rs141864196
rs72836561
rs3923912
rs6968865
rs687339
rs1551393
rs59347135
rs2396084
rs77823653
rs115849089
rs182695896
rs7578604
rs75679663
rs6460043
rs17414716
rs6689
rs1007205
rs6445881
rs6874202
rs174530
rs632057
rs1024137
rs10195252
rs10420434
rs187429064
rs72558163
rs74698119
rs2846205
rs17398765
rs59950280
rs112928559
rs12271743
rs1800588
rs629301
rs2287997
rs141469619
rs1779823
rs116843064
rs2740488
rs2043085
rs7140110
rs78058190
rs41279633
rs1531096
rs117733303
rs74490421
rs139974673
rs12359135
rs193084249
rs13300853
rs2737218
rs12916
rs4805881
rs2678379
rs442177
All
-0.10
-0.05
0.00
MR leave-one-out sensitivity analysis for
ration of small VLDL particles || id:ebi-cfb233-GCST90302111' on 'ER+ Breast cancer (Combined Oncoarray; iCOGS; GWAS meta anal

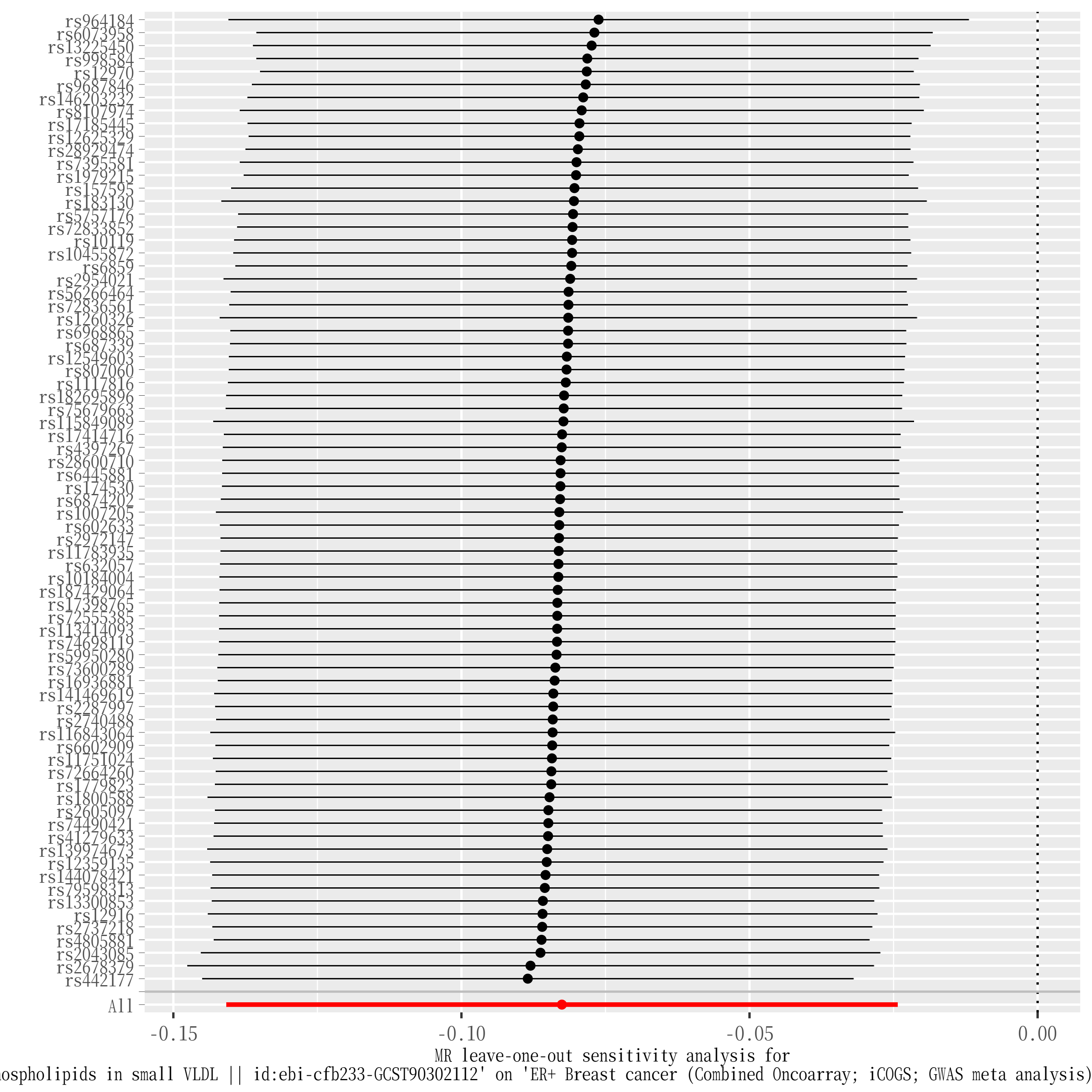
rs964184
rs6073958
rs13225450
rs998584
rs12970
rs9687846
rs146203232
rs8107974
rs17185445
rs12625329
rs28929474
rs7395581
rs1979215
rs157595
rs183130
rs5757176
rs72833852
rs10119
rs10455872
rs6859
rs2954021
rs56266464
rs72836561
rs1260326
rs6968865
rs687339
rs12549603
rs807060
rs1117816
rs182695896
rs75679663
rs115849089
rs17414716
rs4397267
rs28600710
rs6445881
rs174530
rs6874202
rs1007205
rs602633
rs2972147
rs11783935
rs632057
rs10184004
rs187429064
rs17398765
rs72555385
rs113414093
rs74698119
rs59950280
rs73600289
rs16936881
rs141469619
rs2287997
rs2740488
rs116843064
rs6602909
rs11751024
rs72664260
rs1779823
rs1800588
rs2605097
rs74490421
rs41279633
rs139974673
rs12359135
rs144078421
rs79598313
rs13300853
rs12916
rs2737218
rs4805881
rs2043085
rs2678379
rs442177
All
-0.15
-0.10
-0.05
0.00
MR leave-one-out sensitivity analysis for
hospholipids in small VLDL || id:ebi-cfb233-GCST90302112' on 'ER+ Breast cancer (Combined Oncoarray; iCOGS; GWAS meta analysis)

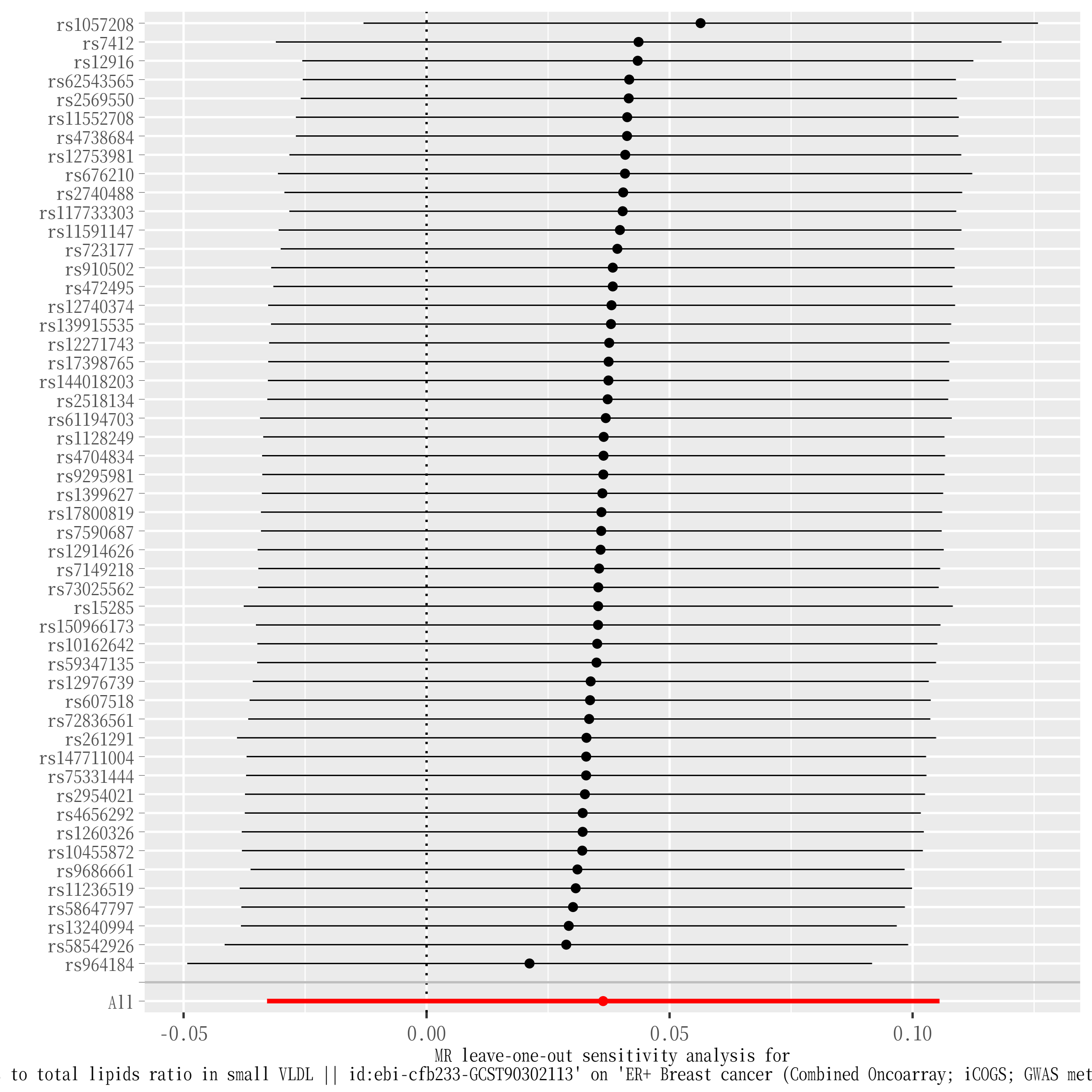
rs1057208
rs7412
rs12916
rs62543565
rs2569550
rs11552708
rs4738684
rs12753981
rs676210
rs2740488
rs117733303
rs11591147
rs723177
rs910502
rs472495
rs12740374
rs139915535
rs12271743
rs17398765
rs144018203
rs2518134
rs61194703
rs1128249
rs4704834
rs9295981
rs1399627
rs17800819
rs7590687
rs12914626
rs7149218
rs73025562
rs15285
rs150966173
rs10162642
rs59347135
rs12976739
rs607518
rs72836561
rs261291
rs147711004
rs75331444
rs2954021
rs4656292
rs1260326
rs10455872
rs9686661
rs11236519
rs58647797
rs13240994
rs58542926
rs964184
All
-0.05
0.00
0.05
0.10
MR leave-one-out sensitivity analysis for
to total lipids ratio in small VLDL || id:ebi-cfb233-GCST90302113' on 'ER+ Breast cancer (Combined Oncoarray; iCOGS; GWAS met

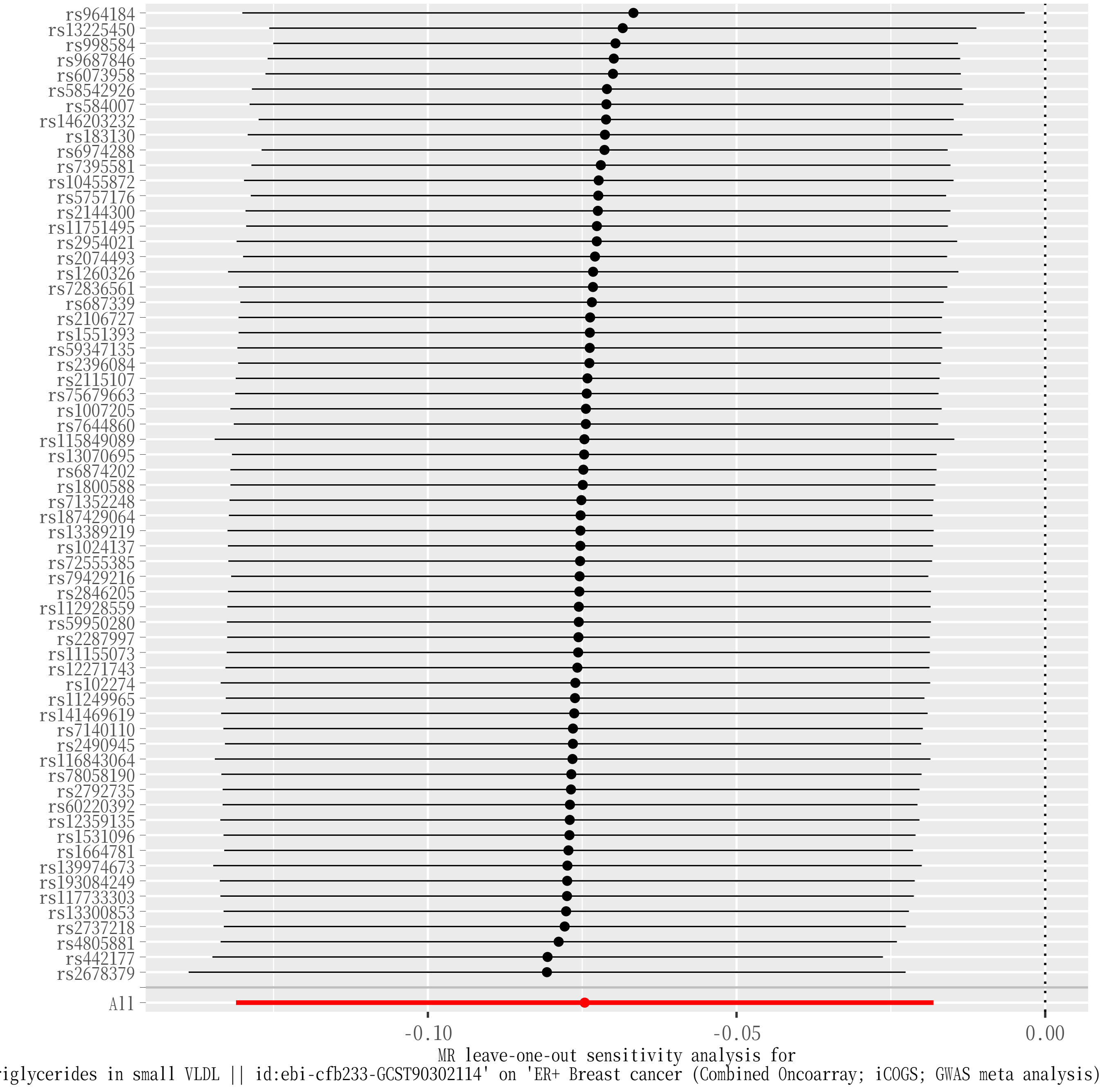
rs964184
rs13225450
rs998584
rs9687846
rs6073958
rs58542926
rs584007
rs146203232
rs183130
rs6974288
rs7395581
rs10455872
rs5757176
rs2144300
rs11751495
rs2954021
rs2074493
rs1260326
rs72836561
rs687339
rs2106727
rs1551393
rs59347135
rs2396084
rs2115107
rs75679663
rs1007205
rs7644860
rs115849089
rs13070695
rs6874202
rs1800588
rs71352248
rs187429064
rs13389219
rs1024137
rs72555385
rs79429216
rs2846205
rs112928559
rs59950280
rs2287997
rs11155073
rs12271743
rs102274
rs11249965
rs141469619
rs7140110
rs2490945
rs116843064
rs78058190
rs2792735
rs60220392
rs12359135
rs1531096
rs1664781
rs139974673
rs193084249
rs117733303
rs13300853
rs2737218
rs4805881
rs442177
rs2678379
All
-0.10
-0.05
0.00
MR leave-one-out sensitivity analysis for
riglycerides in small VLDL || id:ebi-cfb233-GCST90302114' on 'ER+ Breast cancer (Combined Oncoarray; iCOGS; GWAS meta analysis)

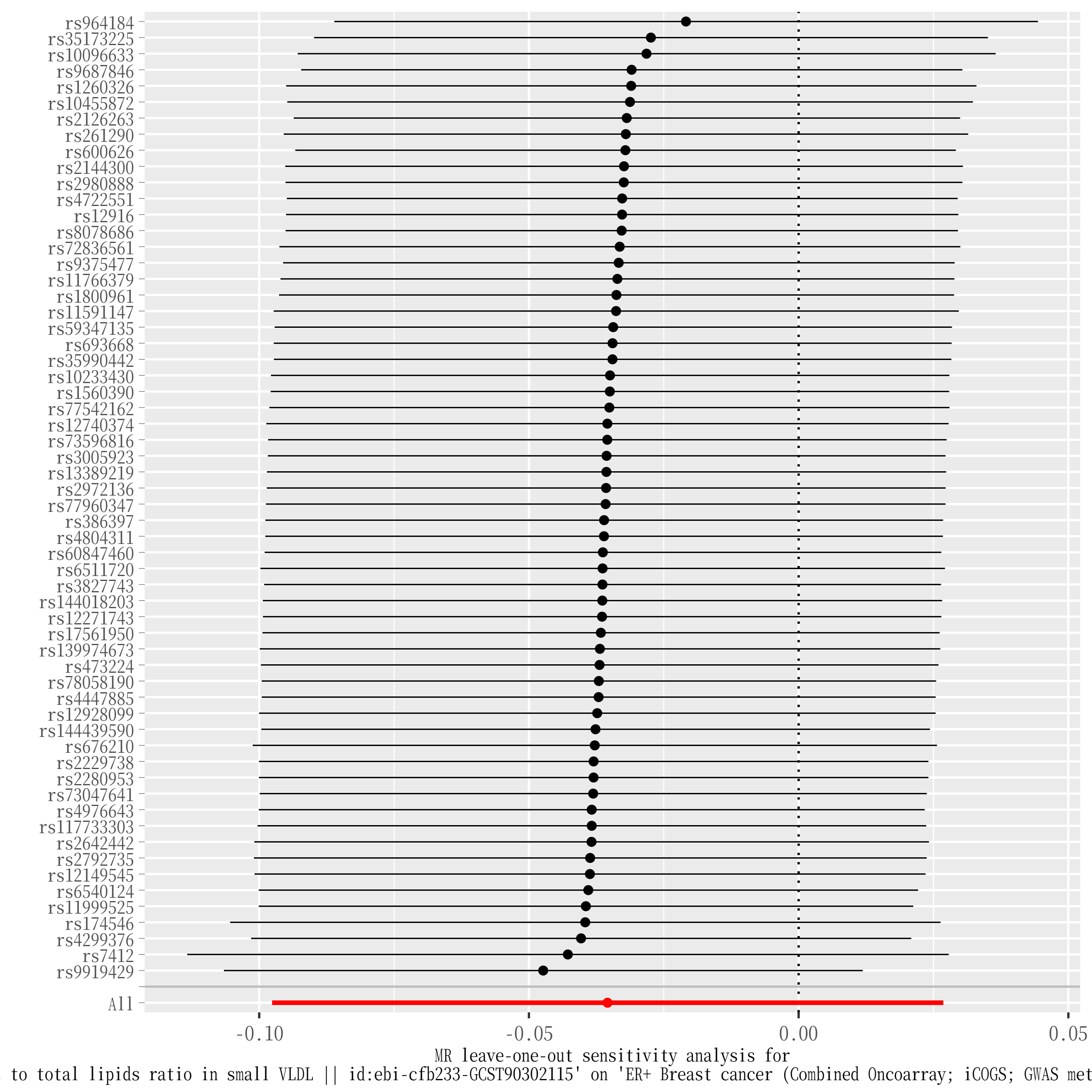
rs964184
rs35173225
rs10096633
rs9687846
rs1260326
rs10455872
rs2126263
rs261290
rs600626
rs2144300
rs2980888
rs4722551
rs12916
rs8078686
rs72836561
rs9375477
rs11766379
rs1800961
rs11591147
rs59347135
rs693668
rs35990442
rs10233430
rs1560390
rs77542162
rs12740374
rs73596816
rs3005923
rs13389219
rs2972136
rs77960347
rs386397
rs4804311
rs60847460
rs6511720
rs3827743
rs144018203
rs12271743
rs17561950
rs139974673
rs473224
rs78058190
rs4447885
rs12928099
rs144439590
rs676210
rs2229738
rs2280953
rs73047641
rs4976643
rs117733303
rs2642442
rs2792735
rs12149545
rs6540124
rs11999525
rs174546
rs4299376
rs7412
rs9919429
All
-0.10
-0.05
0.00
0.05
MR leave-one-out sensitivity analysis for
to total lipids ratio in small VLDL || id:ebi-cfb233-GCST90302115' on 'ER+ Breast cancer (Combined Oncoarray; iCOGS; GWAS met

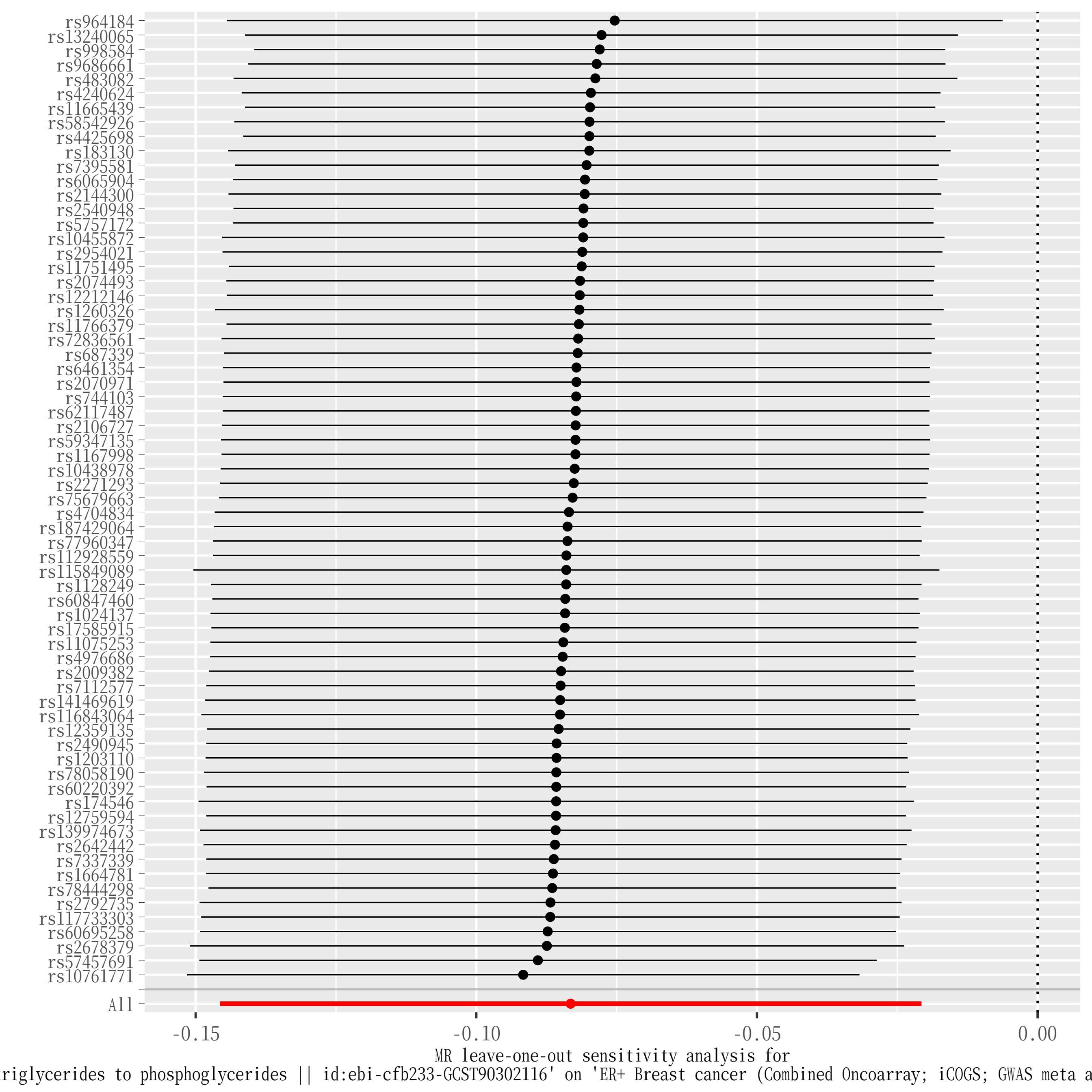
rs964184
rs13240065
rs998584
rs9686661
rs483082
rs4240624
rs11665439
rs58542926
rs4425698
rs183130
rs7395581
rs6065904
rs2144300
rs2540948
rs5757172
rs10455872
rs2954021
rs11751495
rs2074493
rs12212146
rs1260326
rs11766379
rs72836561
rs687339
rs6461354
rs2070971
rs744103
rs62117487
rs2106727
rs59347135
rs1167998
rs10438978
rs2271293
rs75679663
rs4704834
rs187429064
rs77960347
rs112928559
rs115849089
rs1128249
rs60847460
rs1024137
rs17585915
rs11075253
rs4976686
rs2009382
rs7112577
rs141469619
rs116843064
rs12359135
rs2490945
rs1203110
rs78058190
rs60220392
rs174546
rs12759594
rs139974673
rs2642442
rs7337339
rs1664781
rs78444298
rs2792735
rs117733303
rs60695258
rs2678379
rs57457691
rs10761771
All
-0.15
-0.10
-0.05
0.00
MR leave-one-out sensitivity analysis for
riglycerides to phosphoglycerides || id:ebi-cfb233-GCST90302116' on 'ER+ Breast cancer (Combined Oncoarray; iCOGS; GWAS meta a

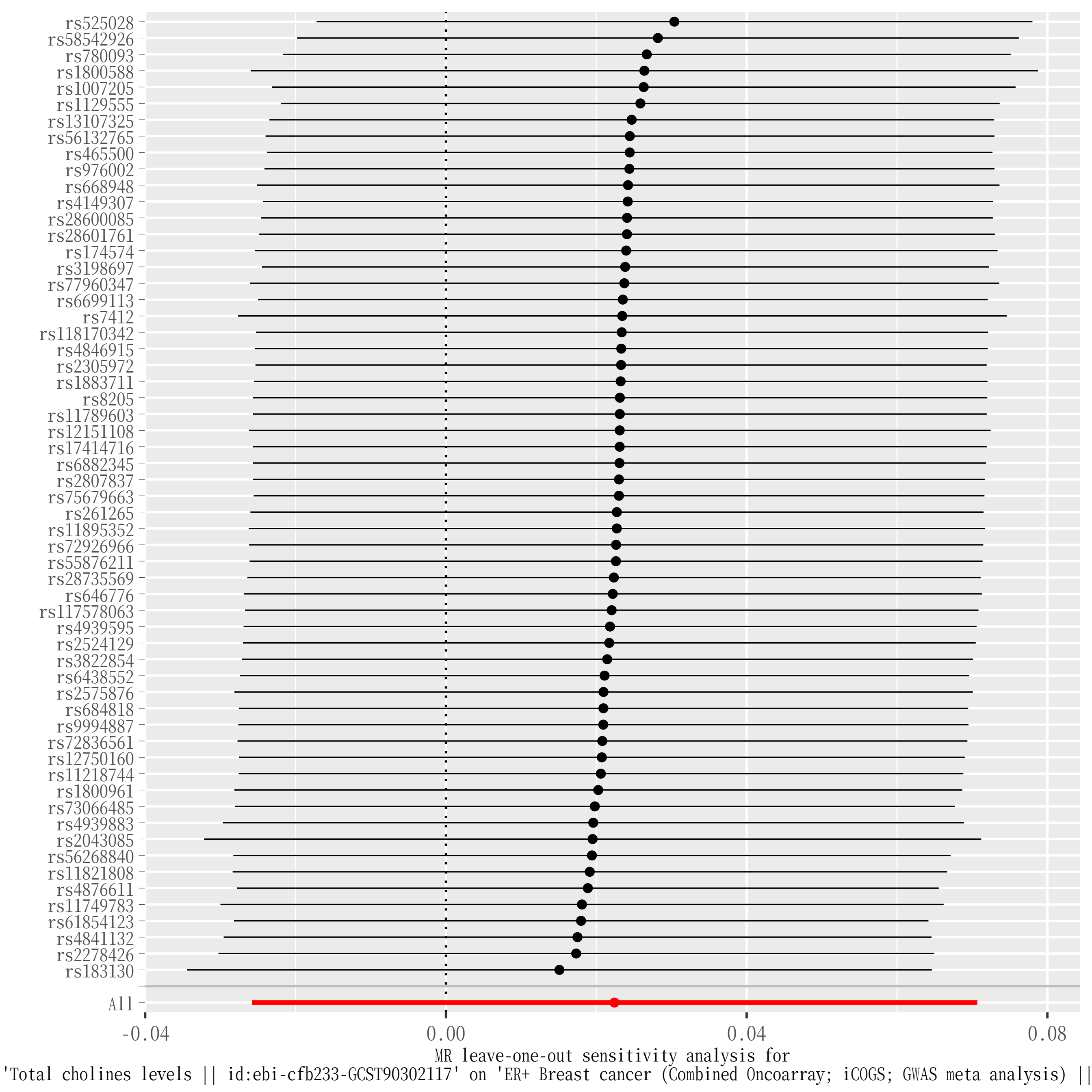
rs525028
rs58542926
rs780093
rs1800588
rs1007205
rs1129555
rs13107325
rs56132765
rs465500
rs976002
rs668948
rs4149307
rs28600085
rs28601761
rs174574
rs3198697
rs77960347
rs6699113
rs7412
rs118170342
rs4846915
rs2305972
rs1883711
rs8205
rs11789603
rs12151108
rs17414716
rs6882345
rs2807837
rs75679663
rs261265
rs11895352
rs72926966
rs55876211
rs28735569
rs646776
rs117578063
rs4939595
rs2524129
rs3822854
rs6438552
rs2575876
rs684818
rs9994887
rs72836561
rs12750160
rs11218744
rs1800961
rs73066485
rs4939883
rs2043085
rs56268840
rs11821808
rs4876611
rs11749783
rs61854123
rs4841132
rs2278426
rs183130
All
-0.04
0.00
0.04
0.08
MR leave-one-out sensitivity analysis for
'Total cholines levels || id:ebi-cfb233-GCST90302117' on 'ER+ Breast cancer (Combined Oncoarray; iCOGS; GWAS meta analysis) ||

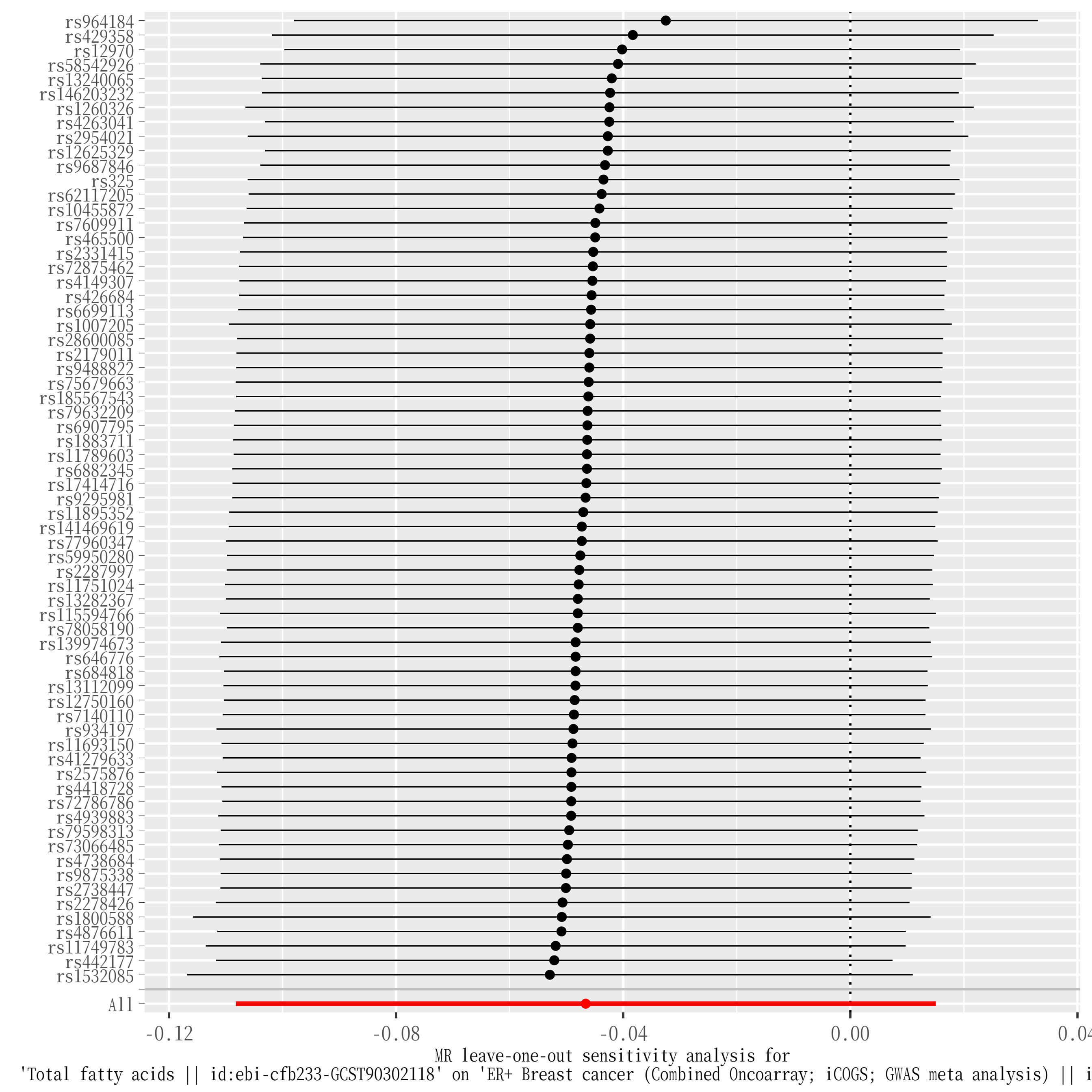
rs964184
rs429358
rs12970
rs58542926
rs13240065
rs146203232
rs1260326
rs4263041
rs2954021
rs12625329
rs9687846
rs325
rs62117205
rs10455872
rs7609911
rs465500
rs2331415
rs72875462
rs4149307
rs426684
rs6699113
rs1007205
rs28600085
rs2179011
rs9488822
rs75679663
rs185567543
rs79632209
rs6907795
rs1883711
rs11789603
rs6882345
rs17414716
rs9295981
rs11895352
rs141469619
rs77960347
rs59950280
rs2287997
rs11751024
rs13282367
rs115594766
rs78058190
rs139974673
rs646776
rs684818
rs13112099
rs12750160
rs7140110
rs934197
rs11693150
rs41279633
rs2575876
rs4418728
rs72786786
rs4939883
rs79598313
rs73066485
rs4738684
rs9875338
rs2738447
rs2278426
rs1800588
rs4876611
rs11749783
rs442177
rs1532085
All
-0.12
-0.08
-0.04
0.00
MR leave-one-out sensitivity analysis for
'Total fatty acids || id:ebi-cfb233-GCST90302118' on 'ER+ Breast cancer (Combined Oncoarray; iCOGS; GWAS meta analysis) || i

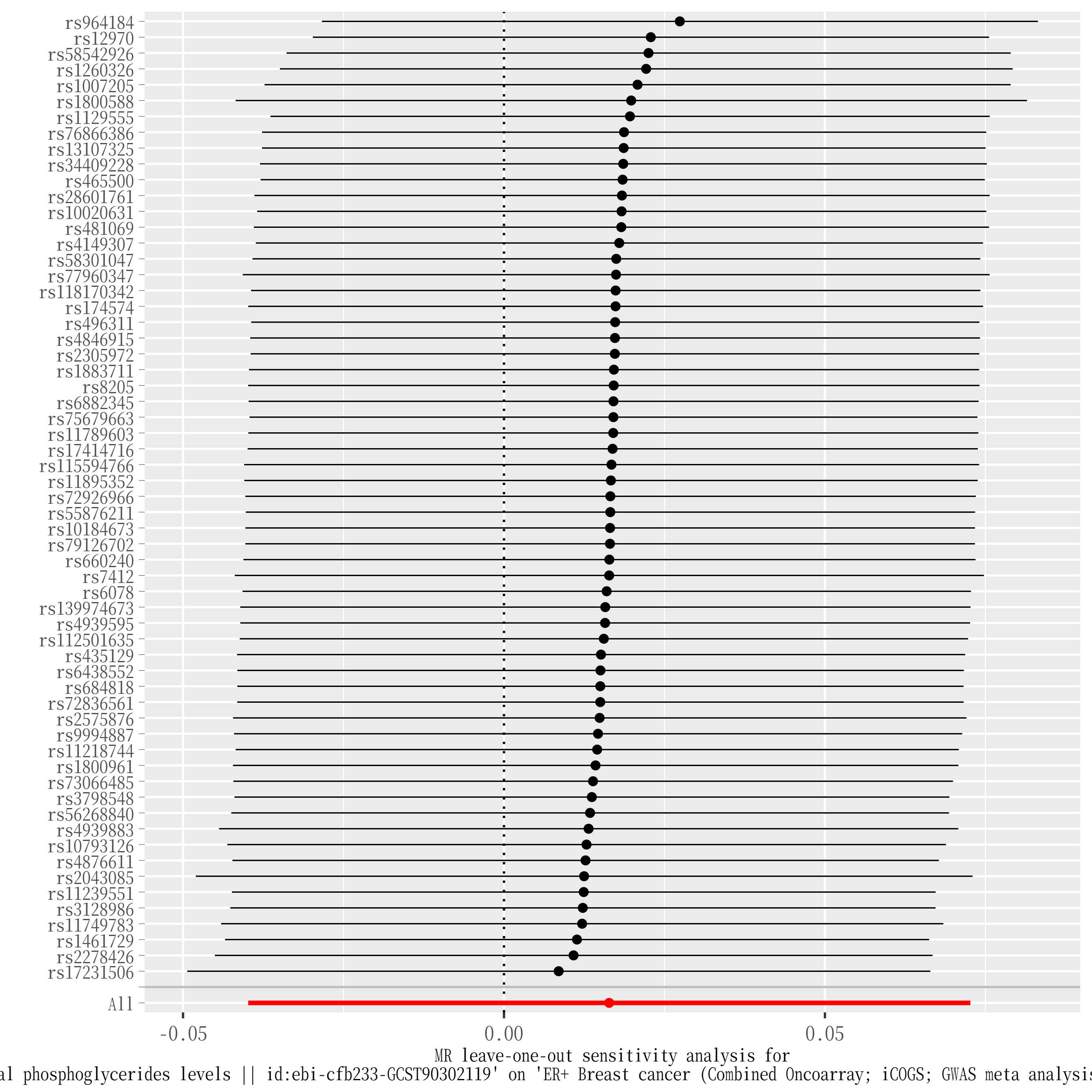

rs964184
rs12970
rs58542926
rs1260326
rs1007205
rs1800588
rs1129555
rs76866386
rs13107325
rs34409228
rs465500
rs28601761
rs10020631
rs481069
rs4149307
rs58301047
rs77960347
rs118170342
rs174574
rs496311
rs4846915
rs2305972
rs1883711
rs8205
rs6882345
rs75679663
rs11789603
rs17414716
rs115594766
rs11895352
rs72926966
rs55876211
rs10184673
rs79126702
rs660240
rs7412
rs6078
rs139974673
rs4939595
rs112501635
rs435129
rs6438552
rs684818
rs72836561
rs2575876
rs9994887
rs11218744
rs1800961
rs73066485
rs3798548
rs56268840
rs4939883
rs10793126
rs4876611
rs2043085
rs11239551
rs3128986
rs11749783
rs1461729
rs2278426
rs17231506
All
-0.05
0.00
0.05
MR leave-one-out sensitivity analysis for
al phosphoglycerides levels || id:ebi-cfb233-GCST90302119' on 'ER+ Breast cancer (Combined Oncoarray; iCOGS; GWAS meta analysis

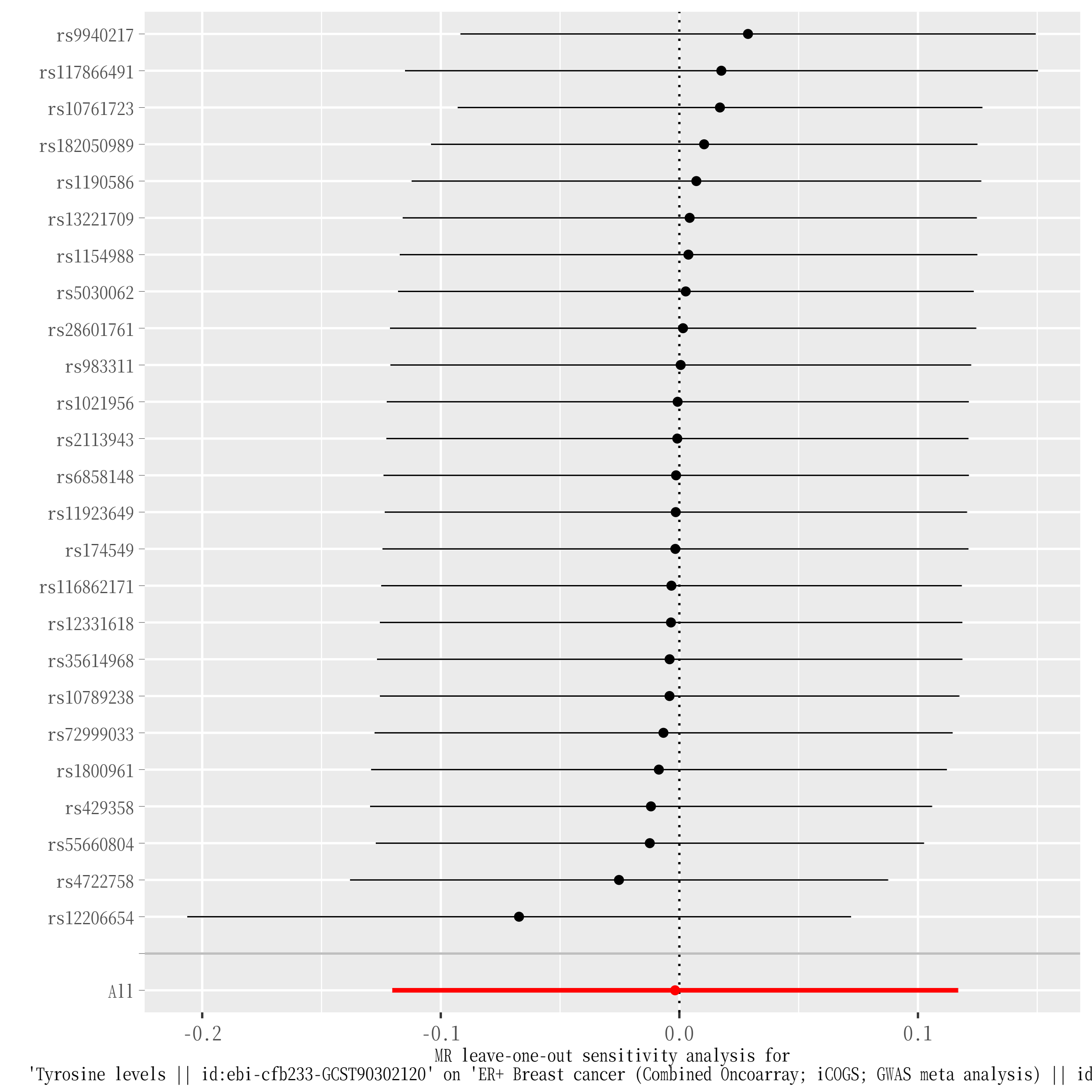
rs9940217
rs117866491
rs10761723
rs182050989
rs1190586
rs13221709
rs1154988
rs5030062
rs28601761
rs983311
rs1021956
rs2113943
rs6858148
rs11923649
rs174549
rs116862171
rs12331618
rs35614968
rs10789238
rs72999033
rs1800961
rs429358
rs55660804
rs4722758
rs12206654
All
-0.2
-0.1
0.0
0.1
MR leave-one-out sensitivity analysis for
'Tyrosine levels || id:ebi-cfb233-GCST90302120' on 'ER+ Breast cancer (Combined Oncoarray; iCOGS; GWAS meta analysis) || id

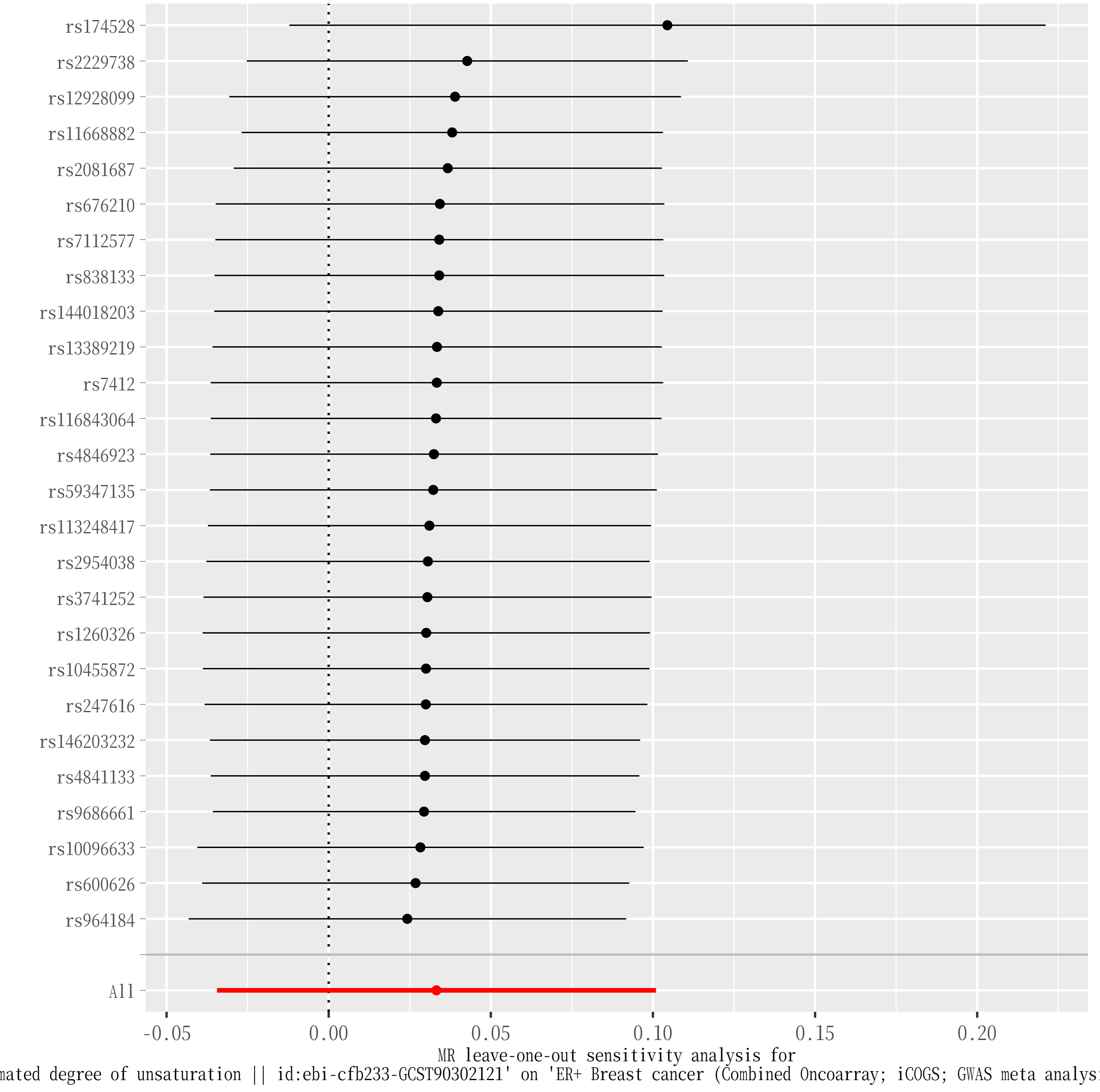
rs174528
rs2229738
rs12928099
rs11668882
rs2081687
rs676210
rs7112577
rs838133
rs144018203
rs13389219
rs7412
rs116843064
rs4846923
rs59347135
rs113248417
rs2954038
rs3741252
rs1260326
rs10455872
rs247616
rs146203232
rs4841133
rs9686661
rs10096633
rs600626
rs964184
All
-0.05
0.00
0.05
0.10
0.15
0.20
MR leave-one-out sensitivity analysis for
mated degree of unsaturation || id:ebi-cfb233-GCST90302121' on 'ER+ Breast cancer (Combined Oncoarray; iCOGS; GWAS meta analys

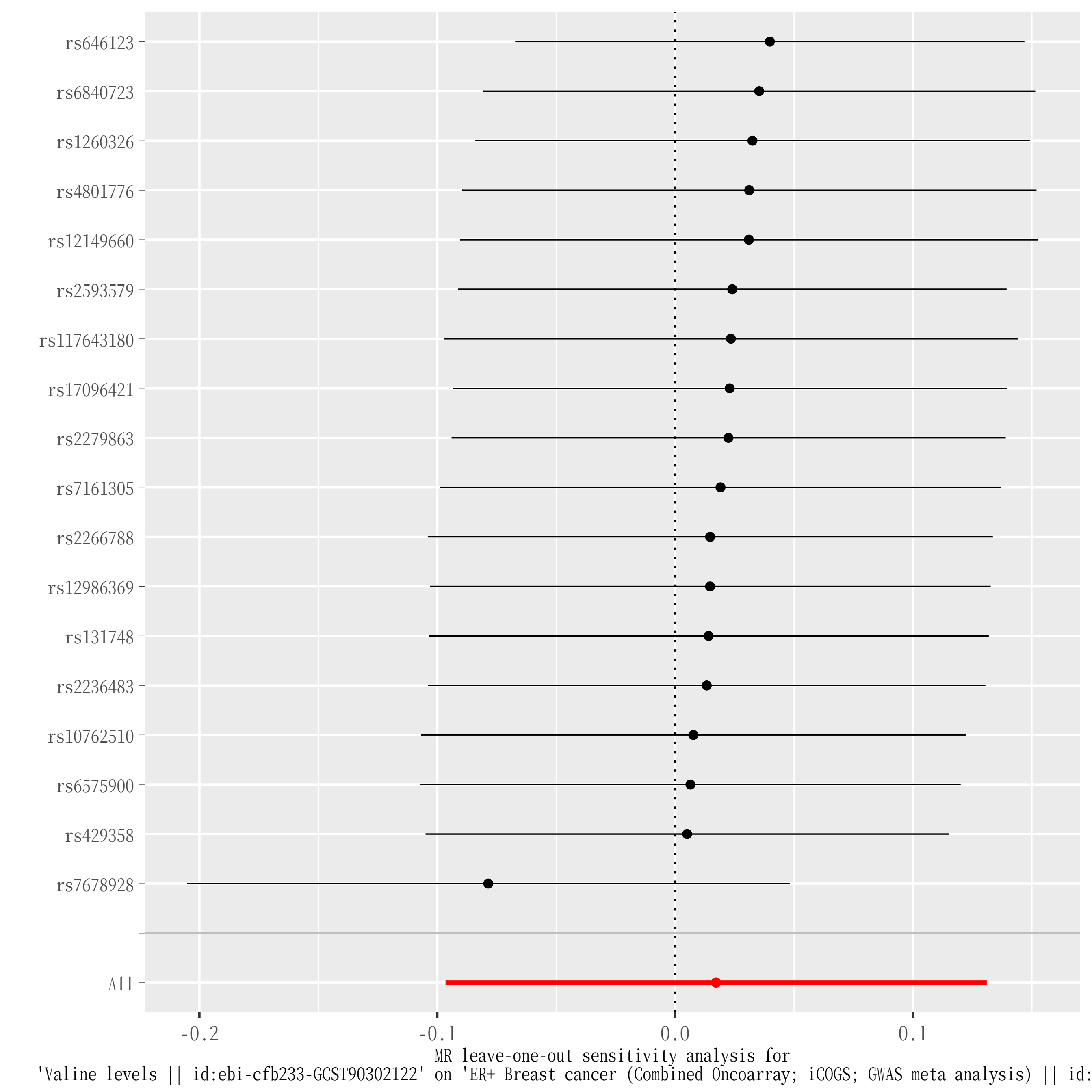

rs646123
rs6840723
rs1260326
rs4801776
rs12149660
rs2593579
rs117643180
rs17096421
rs2279863
rs7161305
rs2266788
rs12986369
rs131748
rs2236483
rs10762510
rs6575900
rs429358
rs7678928
All
-0.2
-0.1
0.0
0.1
MR leave-one-out sensitivity analysis for
'Valine levels || id:ebi-cfb233-GCST90302122' on 'ER+ Breast cancer (Combined Oncoarray; iCOGS; GWAS meta analysis) || id:

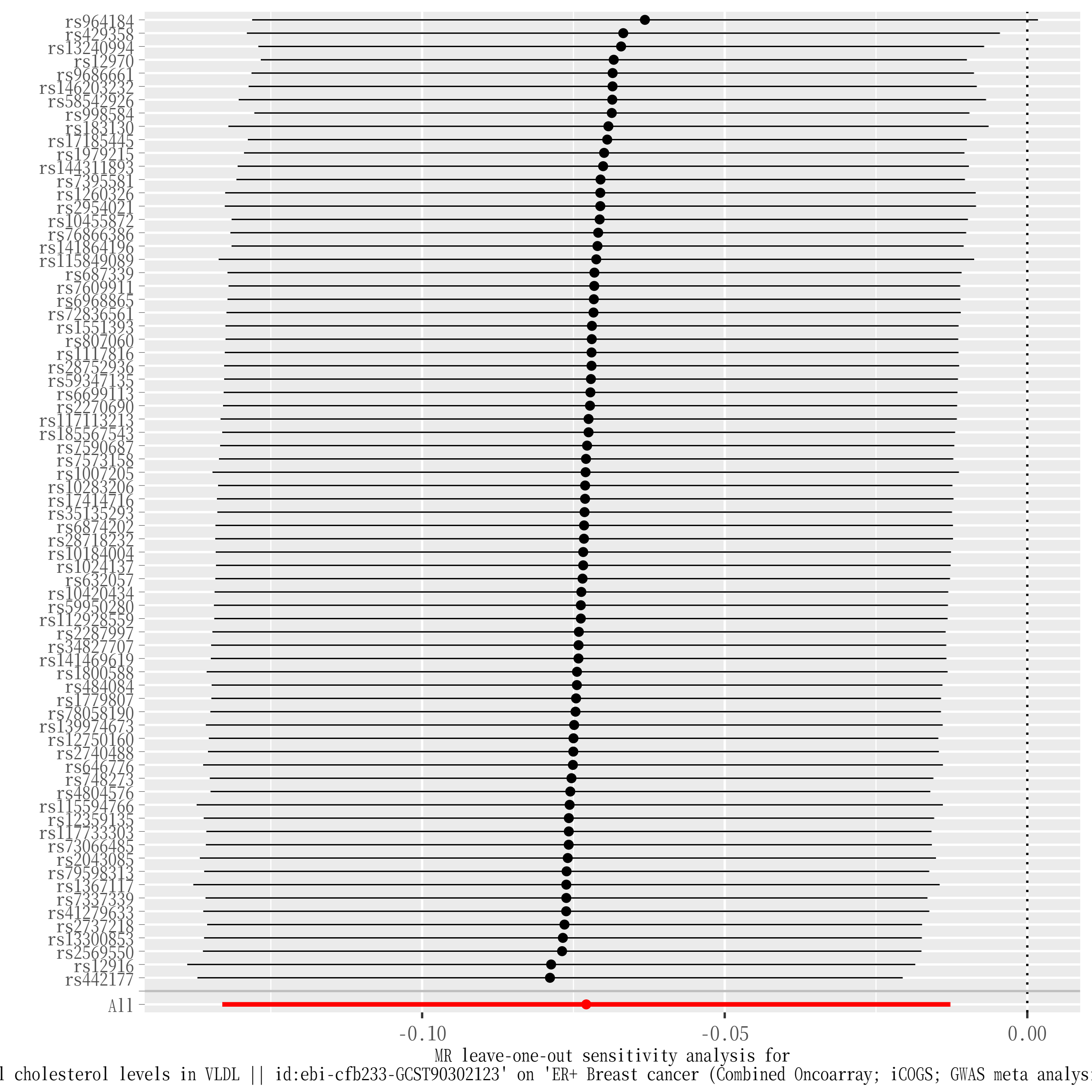
rs964184
rs429358
rs13240994
rs12970
rs9686661
rs146203232
rs58542926
rs998584
rs183130
rs17185445
rs1979215
rs144311893
rs7395581
rs1260326
rs2954021
rs10455872
rs76866386
rs141864196
rs115849089
rs687339
rs7609911
rs6968865
rs72836561
rs1551393
rs807060
rs1117816
rs28752936
rs59347135
rs6699113
rs2270690
rs117113213
rs185567543
rs7590687
rs7573158
rs1007205
rs10283206
rs17414716
rs35135293
rs6874202
rs28718232
rs10184004
rs1024137
rs632057
rs10420434
rs59950280
rs112928559
rs2287997
rs34827707
rs141469619
rs1800588
rs484084
rs1779807
rs78058190
rs139974673
rs12750160
rs2740488
rs646776
rs748273
rs4804576
rs115594766
rs12359135
rs117733303
rs73066485
rs2043085
rs79598313
rs1367117
rs7337339
rs41279633
rs2737218
rs13300853
rs2569550
rs12916
rs442177
All
-0.10
-0.05
0.00
MR leave-one-out sensitivity analysis for
l cholesterol levels in VLDL || id:ebi-cfb233-GCST90302123' on 'ER+ Breast cancer (Combined Oncoarray; iCOGS; GWAS meta analys

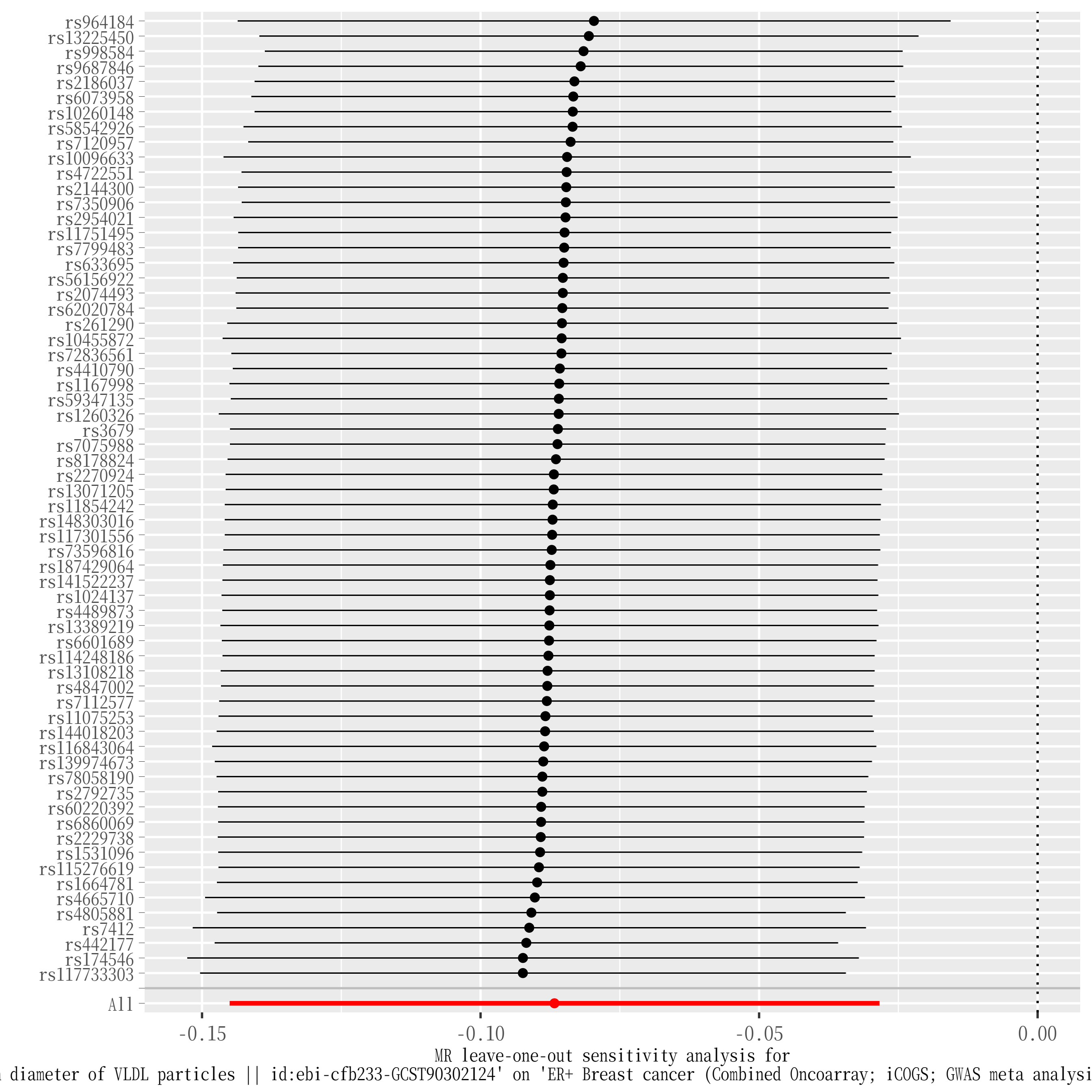
rs964184
rs13225450
rs998584
rs9687846
rs2186037
rs6073958
rs10260148
rs58542926
rs7120957
rs10096633
rs4722551
rs2144300
rs7350906
rs2954021
rs11751495
rs7799483
rs633695
rs56156922
rs2074493
rs62020784
rs261290
rs10455872
rs72836561
rs4410790
rs1167998
rs59347135
rs1260326
rs3679
rs7075988
rs8178824
rs2270924
rs13071205
rs11854242
rs148303016
rs117301556
rs73596816
rs187429064
rs141522237
rs1024137
rs4489873
rs13389219
rs6601689
rs114248186
rs13108218
rs4847002
rs7112577
rs11075253
rs144018203
rs116843064
rs139974673
rs78058190
rs2792735
rs60220392
rs6860069
rs2229738
rs1531096
rs115276619
rs1664781
rs4665710
rs4805881
rs7412
rs442177
rs174546
rs117733303
All
-0.15
-0.10
-0.05
0.00
MR leave-one-out sensitivity analysis for
n diameter of VLDL particles || id:ebi-cfb233-GCST90302124' on 'ER+ Breast cancer (Combined Oncoarray; iCOGS; GWAS meta analysi

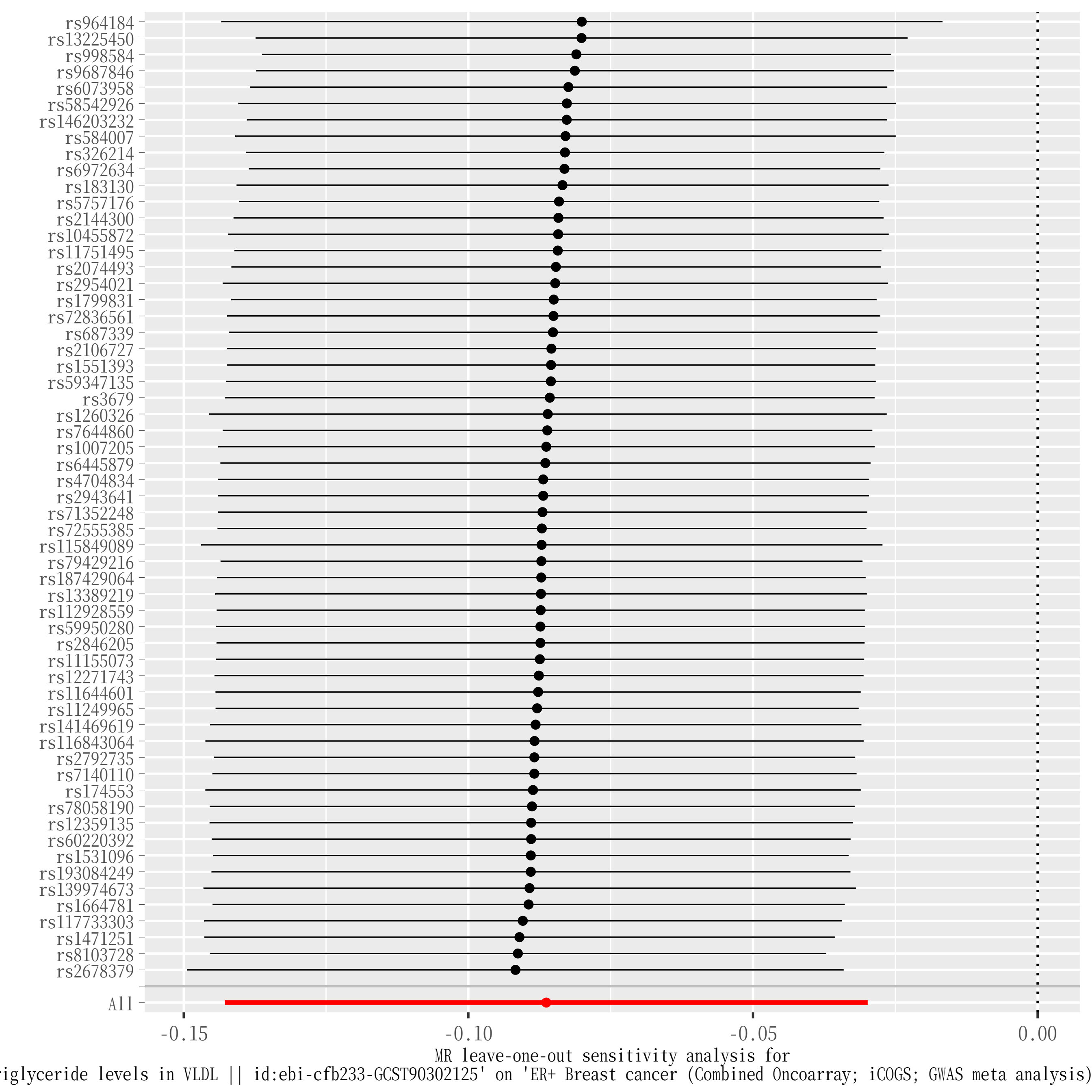
rs964184
rs13225450
rs998584
rs9687846
rs6073958
rs58542926
rs146203232
rs584007
rs326214
rs6972634
rs183130
rs5757176
rs2144300
rs10455872
rs11751495
rs2074493
rs2954021
rs1799831
rs72836561
rs687339
rs2106727
rs1551393
rs59347135
rs3679
rs1260326
rs7644860
rs1007205
rs6445879
rs4704834
rs2943641
rs71352248
rs72555385
rs115849089
rs79429216
rs187429064
rs13389219
rs112928559
rs59950280
rs2846205
rs11155073
rs12271743
rs11644601
rs11249965
rs141469619
rs116843064
rs2792735
rs7140110
rs174553
rs78058190
rs12359135
rs60220392
rs1531096
rs193084249
rs139974673
rs1664781
rs117733303
rs1471251
rs8103728
rs2678379
All
-0.15
-0.10
-0.05
0.00
MR leave-one-out sensitivity analysis for
riglyceride levels in VLDL || id:ebi-cfb233-GCST90302125' on 'ER+ Breast cancer (Combined Oncoarray; iCOGS; GWAS meta analysis)

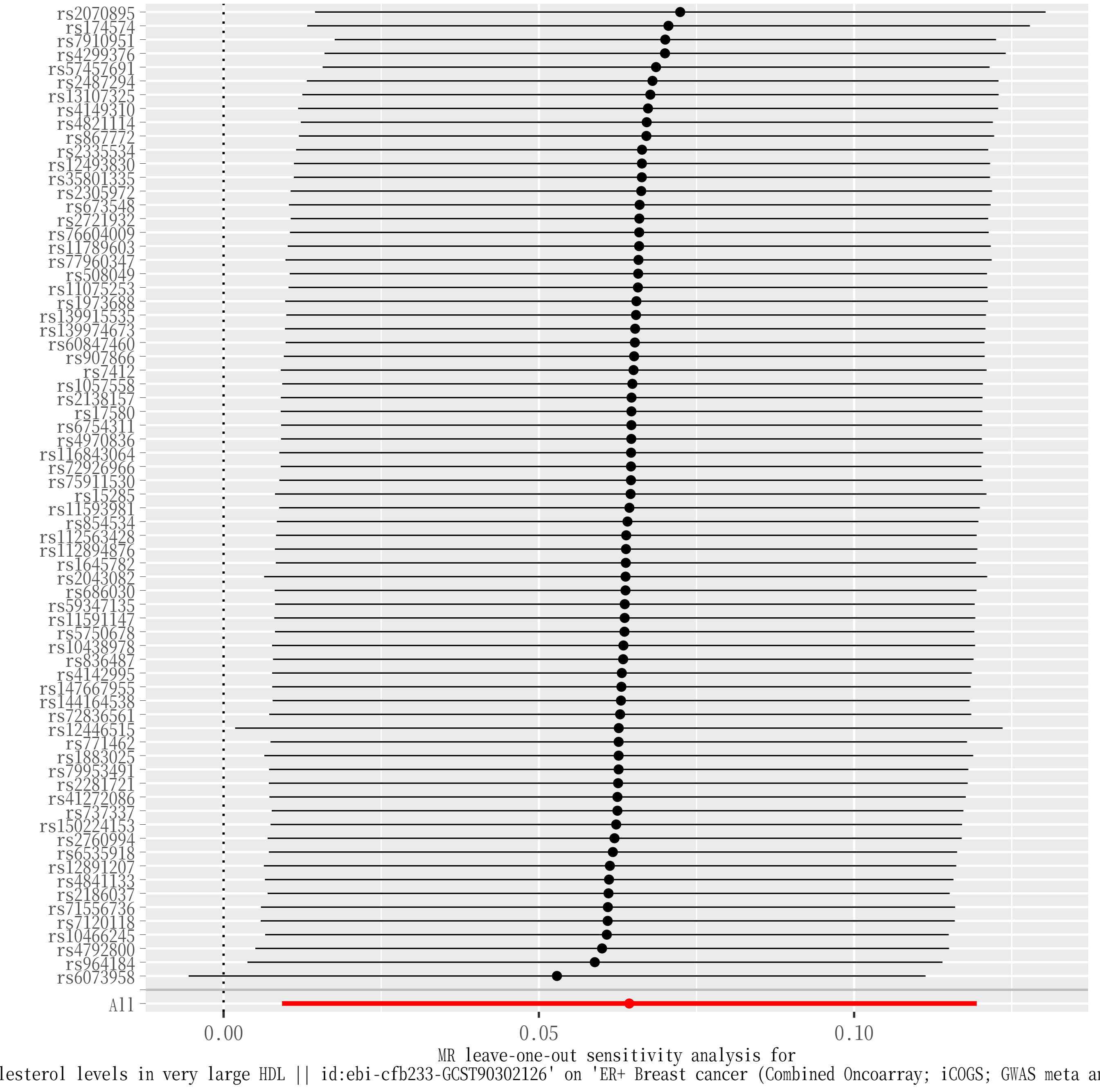
rs2070895
rs174574
rs7910951
rs4299376
rs57457691
rs2487294
rs13107325
rs4149310
rs4821114
rs867772
rs2335534
rs12493830
rs35801335
rs2305972
rs673548
rs2721932
rs76604009
rs11789603
rs77960347
rs508049
rs11075253
rs1973688
rs139915535
rs139974673
rs60847460
rs907866
rs7412
rs1057558
rs2138157
rs17580
rs6754311
rs4970836
rs116843064
rs72926966
rs75911530
rs15285
rs11593981
rs854534
rs112563428
rs112894876
rs1645782
rs2043082
rs686030
rs59347135
rs11591147
rs5750678
rs10438978
rs836487
rs4142995
rs147667955
rs144164538
rs72836561
rs12446515
rs771462
rs1883025
rs79953491
rs2281721
rs41272086
rs737337
rs150224153
rs2760994
rs6535918
rs12891207
rs4841133
rs2186037
rs71556736
rs7120118
rs10466245
rs4792800
rs964184
rs6073958
All
0.00
0.05
0.10
MR leave-one-out sensitivity analysis for
lesterol levels in very large HDL || id:ebi-cfb233-GCST90302126' on 'ER+ Breast cancer (Combined Oncoarray; iCOGS; GWAS meta an

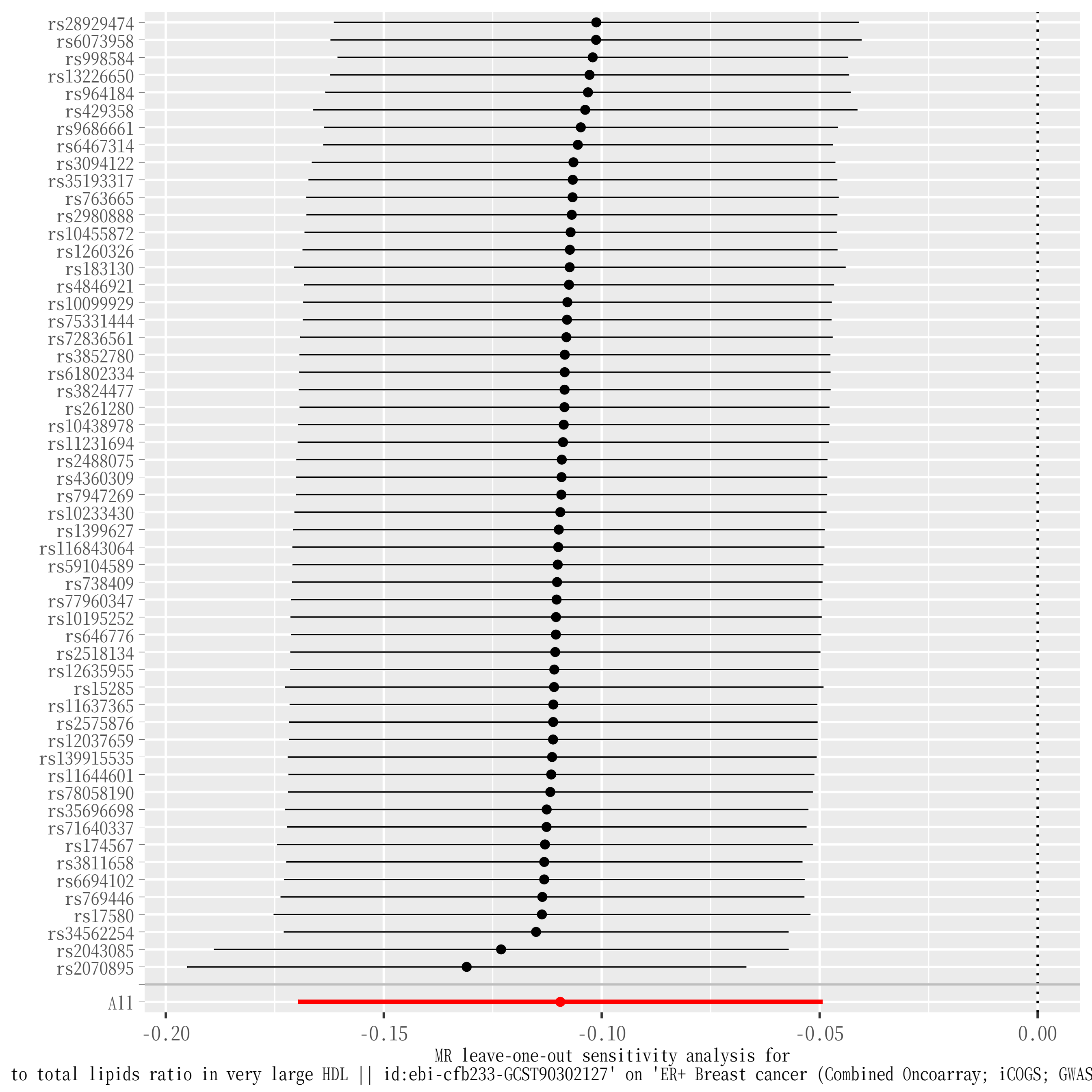
rs28929474
rs6073958
rs998584
rs13226650
rs964184
rs429358
rs9686661
rs6467314
rs3094122
rs35193317
rs763665
rs2980888
rs10455872
rs1260326
rs183130
rs4846921
rs10099929
rs75331444
rs72836561
rs3852780
rs61802334
rs3824477
rs261280
rs10438978
rs11231694
rs2488075
rs4360309
rs7947269
rs10233430
rs1399627
rs116843064
rs59104589
rs738409
rs77960347
rs10195252
rs646776
rs2518134
rs12635955
rs15285
rs11637365
rs2575876
rs12037659
rs139915535
rs11644601
rs78058190
rs35696698
rs71640337
rs174567
rs3811658
rs6694102
rs769446
rs17580
rs34562254
rs2043085
rs2070895
All
-0.20
-0.15
-0.10
-0.05
0.00
MR leave-one-out sensitivity analysis for
to total lipids ratio in very large HDL || id:ebi-cfb233-GCST90302127' on 'ER+ Breast cancer (Combined Oncoarray; iCOGS; GWAS

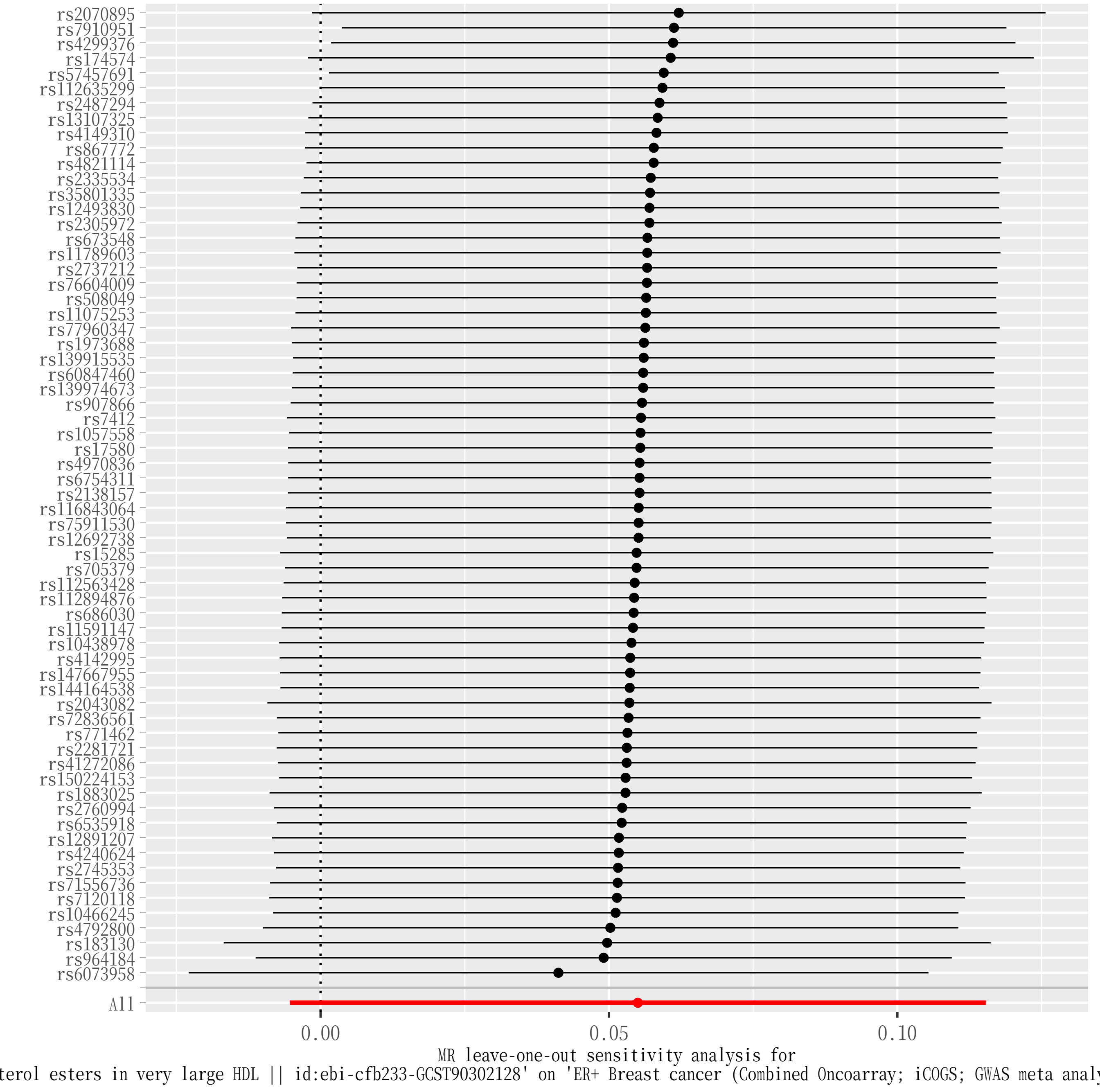
rs2070895
rs7910951
rs4299376
rs174574
rs57457691
rs112635299
rs2487294
rs13107325
rs4149310
rs867772
rs4821114
rs2335534
rs35801335
rs12493830
rs2305972
rs673548
rs11789603
rs2737212
rs76604009
rs508049
rs11075253
rs77960347
rs1973688
rs139915535
rs60847460
rs139974673
rs907866
rs7412
rs1057558
rs17580
rs4970836
rs6754311
rs2138157
rs116843064
rs75911530
rs12692738
rs15285
rs705379
rs112563428
rs112894876
rs686030
rs11591147
rs10438978
rs4142995
rs147667955
rs144164538
rs2043082
rs72836561
rs771462
rs2281721
rs41272086
rs150224153
rs1883025
rs2760994
rs6535918
rs12891207
rs4240624
rs2745353
rs71556736
rs7120118
rs10466245
rs4792800
rs183130
rs964184
rs6073958
All
0.00
0.05
0.10
MR leave-one-out sensitivity analysis for
terol esters in very large HDL || id:ebi-cfb233-GCST90302128' on 'ER+ Breast cancer (Combined Oncoarray; iCOGS; GWAS meta analy

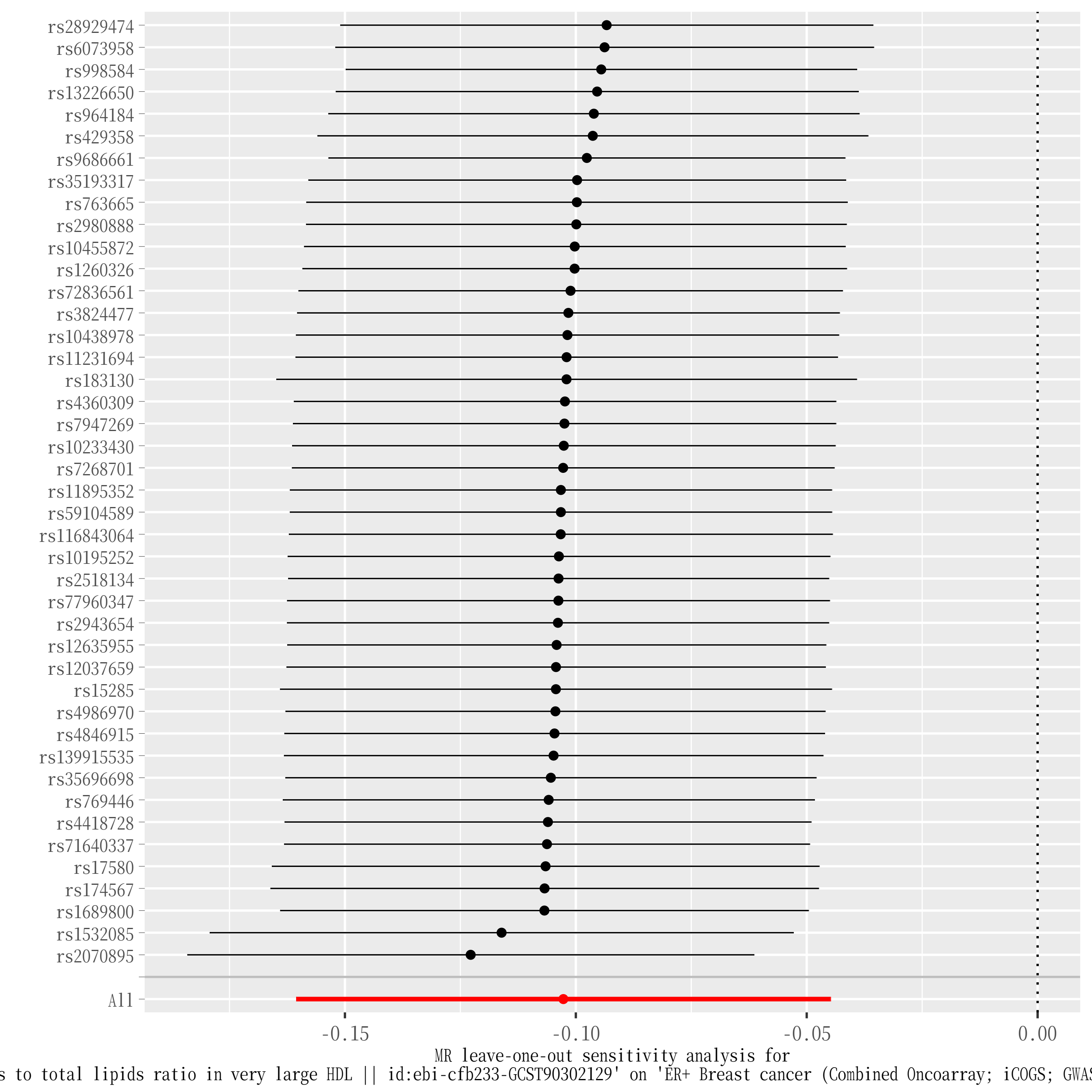
rs28929474
rs6073958
rs998584
rs13226650
rs964184
rs429358
rs9686661
rs35193317
rs763665
rs2980888
rs10455872
rs1260326
rs72836561
rs3824477
rs10438978
rs11231694
rs183130
rs4360309
rs7947269
rs10233430
rs7268701
rs11895352
rs59104589
rs116843064
rs10195252
rs2518134
rs77960347
rs2943654
rs12635955
rs12037659
rs15285
rs4986970
rs4846915
rs139915535
rs35696698
rs769446
rs4418728
rs71640337
rs17580
rs174567
rs1689800
rs1532085
rs2070895
All
-0.15
-0.10
-0.05
0.00
MR leave-one-out sensitivity analysis for
s to total lipids ratio in very large HDL || id:ebi-cfb233-GCST90302129' on 'ER+ Breast cancer (Combined Oncoarray; iCOGS; GWAS

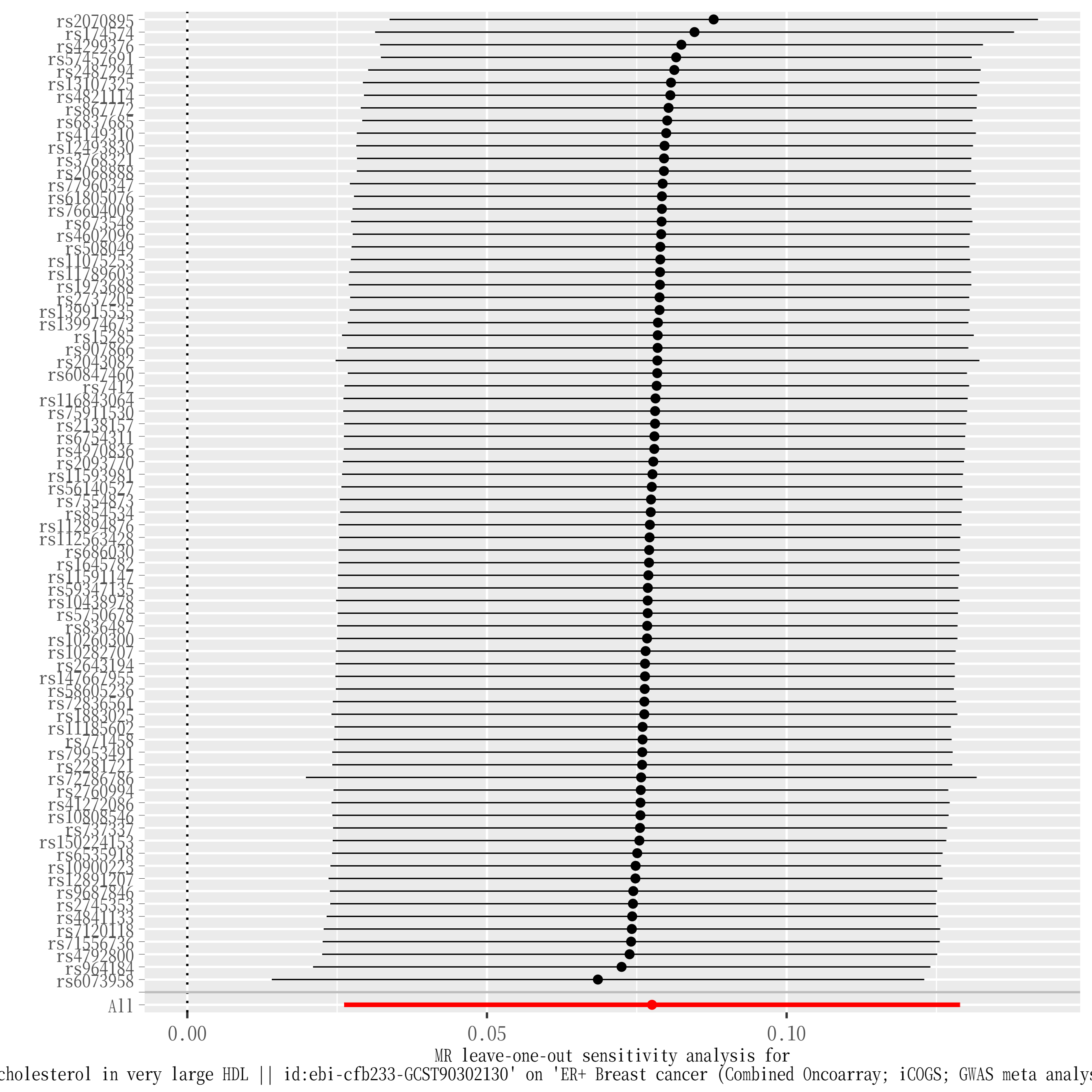
rs2070895
rs174574
rs4299376
rs57457691
rs2487294
rs13107325
rs4821114
rs867772
rs6837685
rs4149310
rs12493830
rs3768321
rs2068888
rs77960347
rs61805076
rs76604009
rs673548
rs4602096
rs508049
rs11075253
rs11789603
rs1273688
rs2737205
rs139915535
rs139974673
rs15285
rs907866
rs2043082
rs60847460
rs7412
rs116843064
rs75911530
rs2138157
rs6754311
rs4970836
rs2093770
rs11593981
rs56140527
rs7554873
rs854534
rs112894876
rs112563428
rs686030
rs1645782
rs11591147
rs59347135
rs10438978
rs5750678
rs836487
rs10260300
rs10282707
rs2643194
rs147667955
rs58605236
rs72836561
rs1883025
rs11185602
rs771458
rs79953491
rs2281721
rs72786786
rs2760994
rs41272086
rs10808546
rs737337
rs150224153
rs6535918
rs10900223
rs12891207
rs9687846
rs2745353
rs4841133
rs7120118
rs71556736
rs4792800
rs964184
rs6073958
All
0.00
0.05
0.10
MR leave-one-out sensitivity analysis for
cholesterol in very large HDL || id:ebi-cfb233-GCST90302130' on 'ER+ Breast cancer (Combined Oncoarray; iCOGS; GWAS meta analys

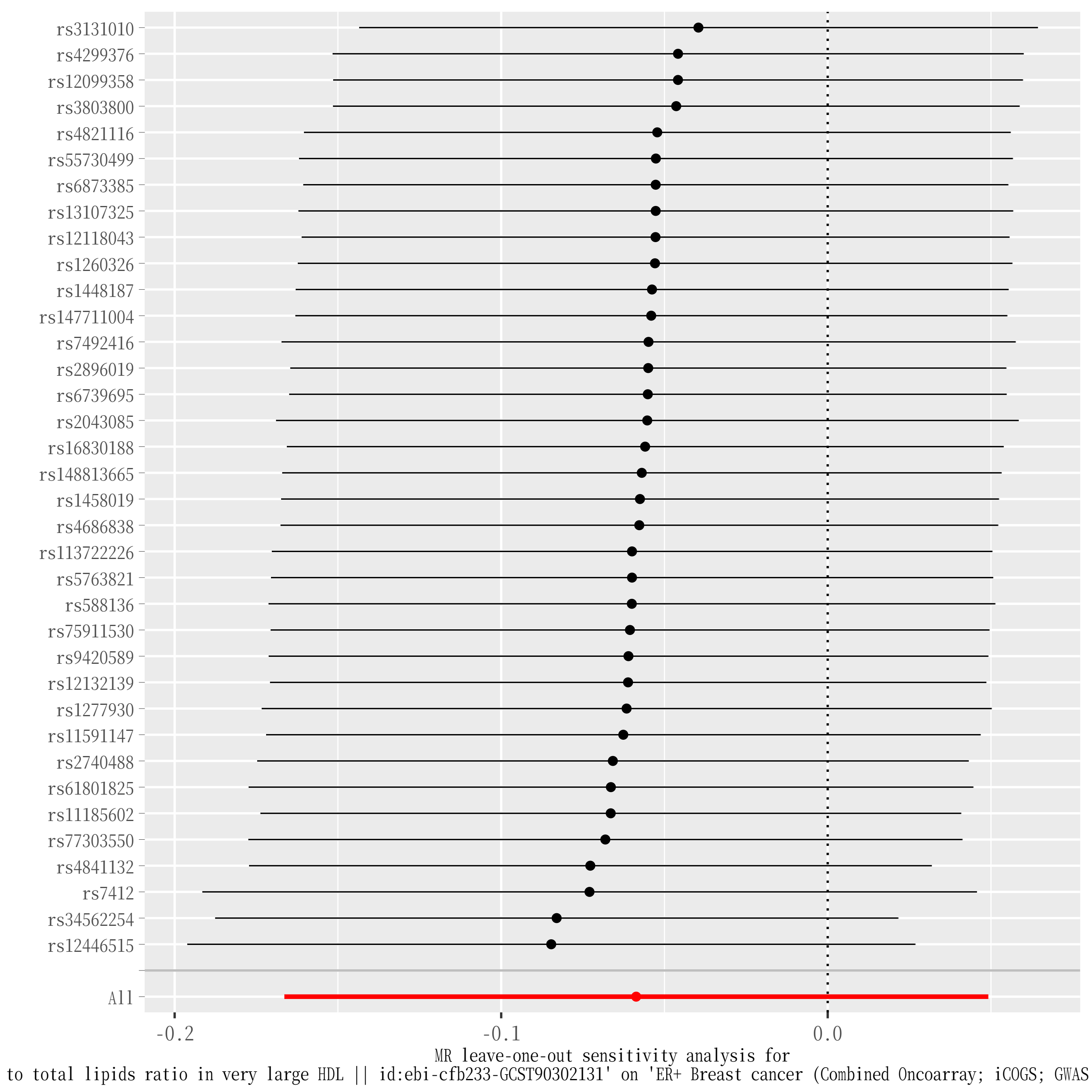

rs3131010
rs4299376
rs12099358
rs3803800
rs4821116
rs55730499
rs6873385
rs13107325
rs12118043
rs1260326
rs1448187
rs147711004
rs7492416
rs2896019
rs6739695
rs2043085
rs16830188
rs148813665
rs1458019
rs4686838
rs113722226
rs5763821
rs588136
rs75911530
rs9420589
rs12132139
rs1277930
rs11591147
rs2740488
rs61801825
rs11185602
rs77303550
rs4841132
rs7412
rs34562254
rs12446515
All
-0.2
-0.1
0.0
MR leave-one-out sensitivity analysis for
to total lipids ratio in very large HDL || id:ebi-cfb233-GCST90302131' on 'ER+ Breast cancer (Combined Oncoarray; iCOGS; GWAS

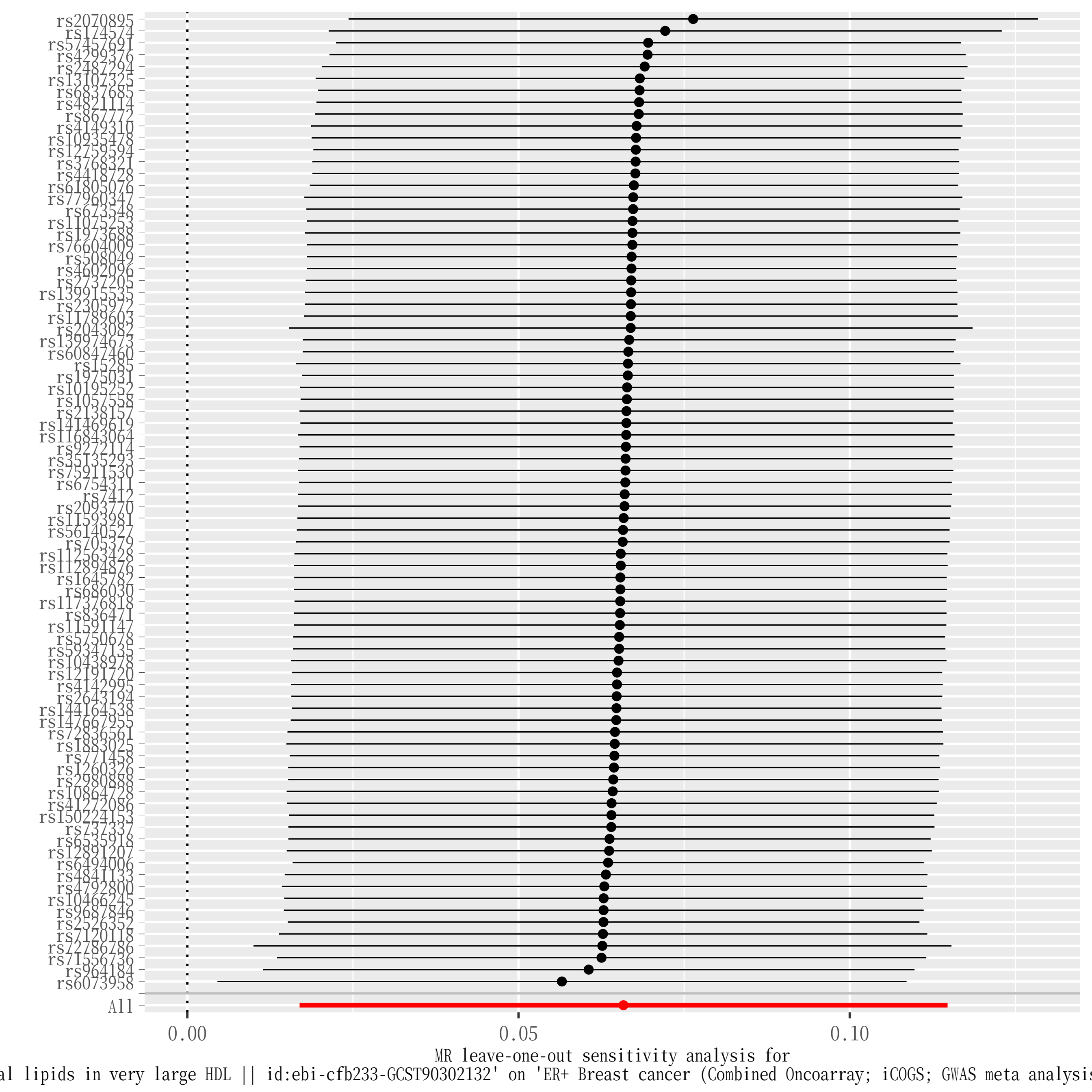

rs2070895
rs174574
rs57457691
rs4299376
rs2487294
rs13107325
rs6837685
rs4821114
rs867772
rs4149310
rs10935478
rs12259594
rs3768321
rs4418728
rs61805076
rs77960347
rs673548
rs11075253
rs1973688
rs76604009
rs508049
rs4602096
rs2737205
rs139915535
rs2305972
rs11789603
rs2043082
rs139974673
rs60847460
rs15285
rs1975031
rs10195252
rs1057558
rs2138157
rs141469619
rs116843064
rs9272114
rs35135293
rs75911530
rs6754311
rs7412
rs2093770
rs11593981
rs56140527
rs705379
rs112563428
rs112894876
rs1645782
rs686030
rs117376818
rs836471
rs11591147
rs5750678
rs59347135
rs10438978
rs12191720
rs4142995
rs2643194
rs144164538
rs147667255
rs72836561
rs1883025
rs771458
rs1260326
rs2980888
rs10864728
rs41272086
rs150224153
rs737337
rs6535918
rs12891207
rs6494006
rs4841133
rs4792800
rs10466245
rs9687846
rs2526352
rs7120118
rs72786786
rs71556736
rs964184
rs6073958
All
0.00
0.05
0.10
MR leave-one-out sensitivity analysis for
al lipids in very large HDL || id:ebi-cfb233-GCST90302132' on 'ER+ Breast cancer (Combined Oncoarray; iCOGS; GWAS meta analysis

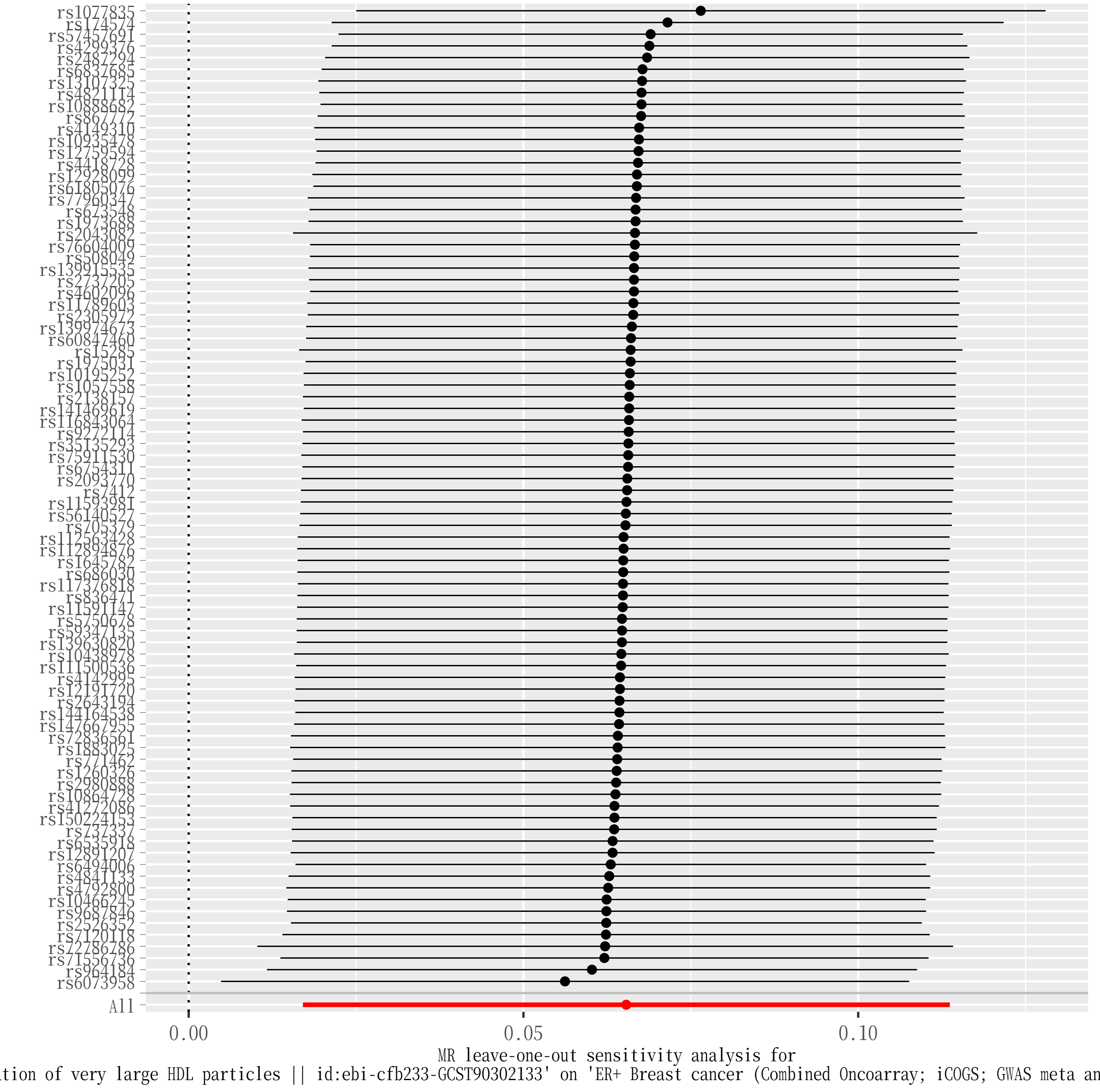

rs1077835
rs174574
rs57457691
rs4299376
rs2487294
rs6837685
rs13107325
rs4821114
rs10888682
rs867772
rs4149310
rs10935478
rs12759594
rs4418728
rs12928099
rs61805076
rs77960347
rs673548
rs1973688
rs2043082
rs76604009
rs508049
rs139915535
rs2737205
rs4602096
rs11782603
rs2305972
rs139974673
rs60847460
rs15285
rs1975031
rs10195252
rs1057558
rs2138157
rs141469619
rs116843064
rs9272114
rs35135293
rs75911530
rs6754311
rs2093770
rs7412
rs11593981
rs56140527
rs705379
rs112563428
rs112894876
rs1645782
rs686030
rs117376818
rs836471
rs11591147
rs5750678
rs59347135
rs139630820
rs10438978
rs111500536
rs4142995
rs12191720
rs2643194
rs144164538
rs147667955
rs72836561
rs1883025
rs771462
rs1260326
rs2980888
rs10864728
rs41272086
rs150224153
rs737337
rs6535918
rs12891207
rs6494006
rs4841133
rs4792800
rs10466245
rs9687846
rs2526352
rs7120118
rs72786786
rs71556736
rs964184
rs6073958
All
0.00
0.05
0.10
MR leave-one-out sensitivity analysis for
ition of very large HDL particles || id:ebi-cfb233-GCST90302133' on 'ER+ Breast cancer (Combined Oncoarray; iCOGS; GWAS meta an

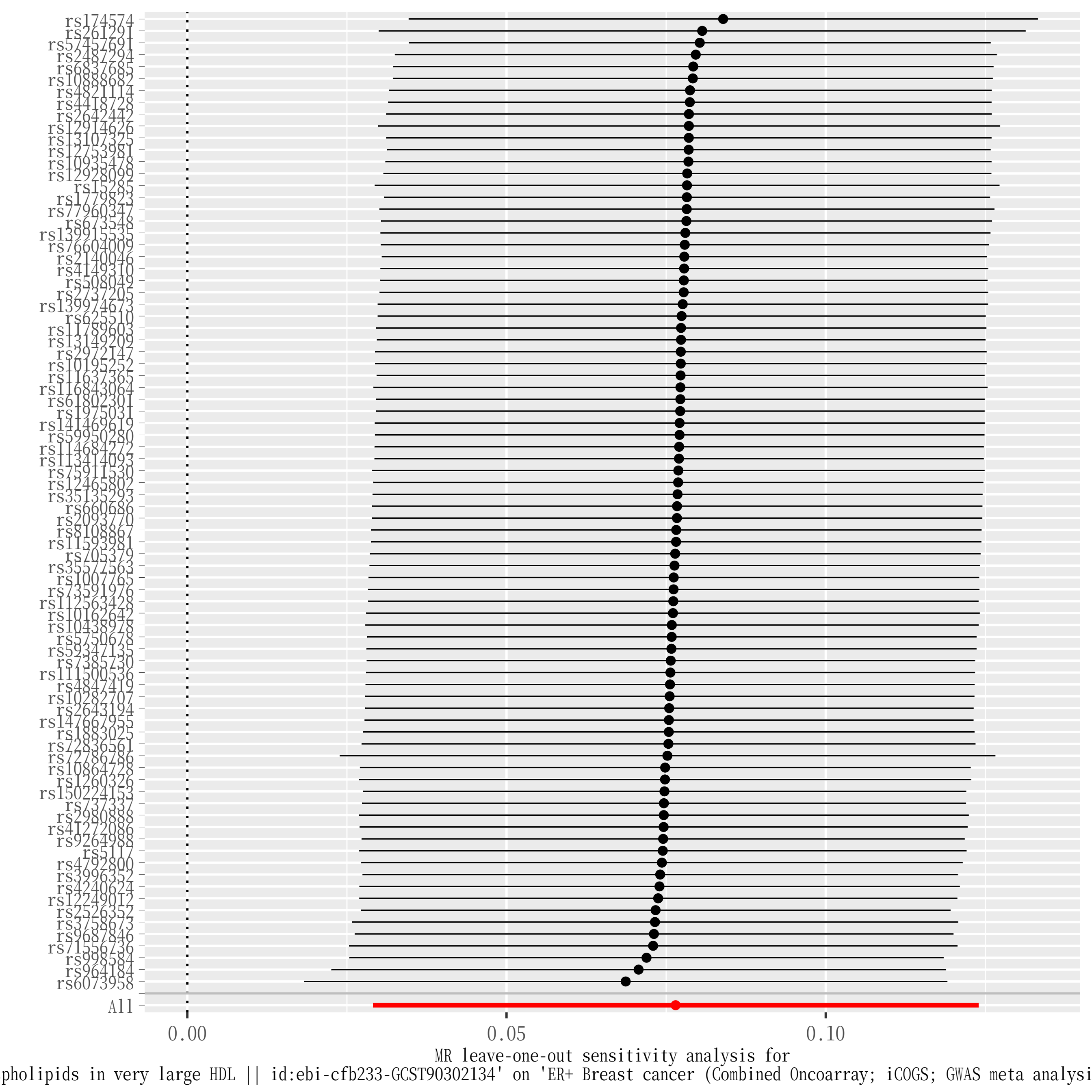
rs174574
rs261291
rs57457691
rs2487294
rs6837685
rs10888682
rs4821114
rs4418728
rs2642442
rs12914626
rs13107325
rs12753981
rs10935478
rs12928099
rs15285
rs1779823
rs77960347
rs673548
rs139915535
rs76604009
rs2140046
rs4149310
rs508049
rs2737205
rs139974673
rs625510
rs11789603
rs13149209
rs2972147
rs10195252
rs11637365
rs116843064
rs61802301
rs1975031
rs141469619
rs59950280
rs114684272
rs113414093
rs75911530
rs12465802
rs35135293
rs660686
rs2093770
rs8108867
rs11593981
rs705379
rs35577563
rs1007765
rs73591976
rs112563428
rs10162642
rs10438978
rs5750678
rs52347135
rs7385730
rs111500536
rs4847419
rs10282707
rs2643194
rs147667955
rs1883025
rs72836561
rs72786786
rs10864728
rs1260326
rs150224153
rs737337
rs2980888
rs41272086
rs9264988
rs5117
rs4792800
rs3996352
rs4240624
rs12249012
rs2526352
rs3758673
rs9687846
rs71556736
rs998584
rs964184
rs6073958
All
0.00
0.05
0.10
MR leave-one-out sensitivity analysis for
pholipids in very large HDL || id:ebi-cfb233-GCST90302134' on 'ER+ Breast cancer (Combined Oncoarray; iCOGS; GWAS meta analysi

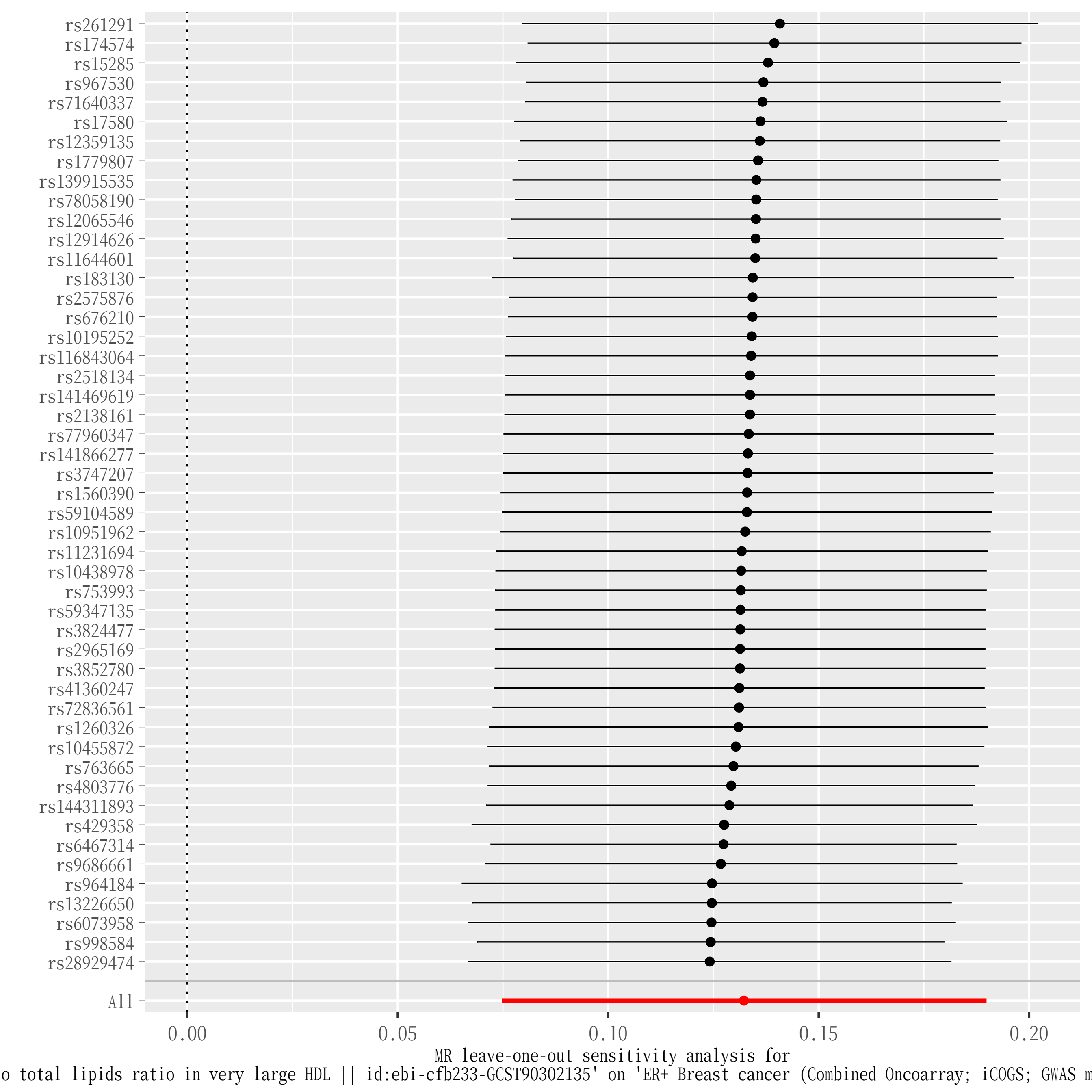
rs261291
rs174574
rs15285
rs967530
rs71640337
rs17580
rs12359135
rs1779807
rs139915535
rs78058190
rs12065546
rs12914626
rs11644601
rs183130
rs2575876
rs676210
rs10195252
rs116843064
rs2518134
rs141469619
rs2138161
rs77960347
rs141866277
rs3747207
rs1560390
rs59104589
rs10951962
rs11231694
rs10438978
rs753993
rs59347135
rs3824477
rs2965169
rs3852780
rs41360247
rs72836561
rs1260326
rs10455872
rs763665
rs4803776
rs144311893
rs429358
rs6467314
rs9686661
rs964184
rs13226650
rs6073958
rs998584
rs28929474
All
0.00
0.05
0.10
0.15
0.20
MR leave-one-out sensitivity analysis for
o total lipids ratio in very large HDL || id:ebi-cfb233-GCST90302135' on 'ER+ Breast cancer (Combined Oncoarray; iCOGS; GWAS m

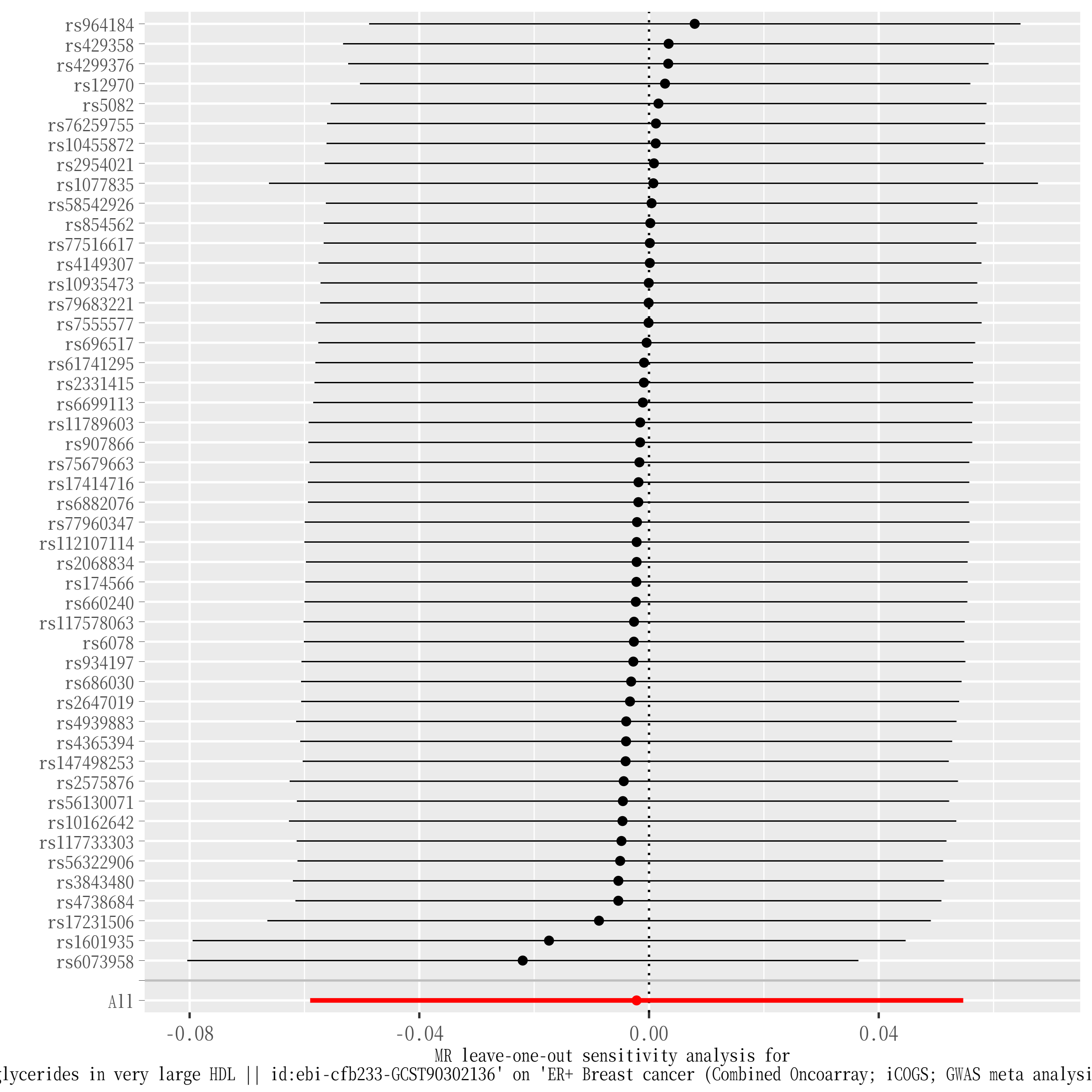
rs964184
rs429358
rs4299376
rs12970
rs5082
rs76259755
rs10455872
rs2954021
rs1077835
rs58542926
rs854562
rs77516617
rs4149307
rs10935473
rs79683221
rs7555577
rs696517
rs61741295
rs2331415
rs6699113
rs11789603
rs907866
rs75679663
rs17414716
rs6882076
rs77960347
rs112107114
rs2068834
rs174566
rs660240
rs117578063
rs6078
rs934197
rs686030
rs2647019
rs4939883
rs4365394
rs147498253
rs2575876
rs56130071
rs10162642
rs117733303
rs56322906
rs3843480
rs4738684
rs17231506
rs1601935
rs6073958
All
-0.08
-0.04
0.00
0.04
MR leave-one-out sensitivity analysis for
lycerides in very large HDL || id:ebi-cfb233-GCST90302136' on 'ER+ Breast cancer (Combined Oncoarray; iCOGS; GWAS meta analysi

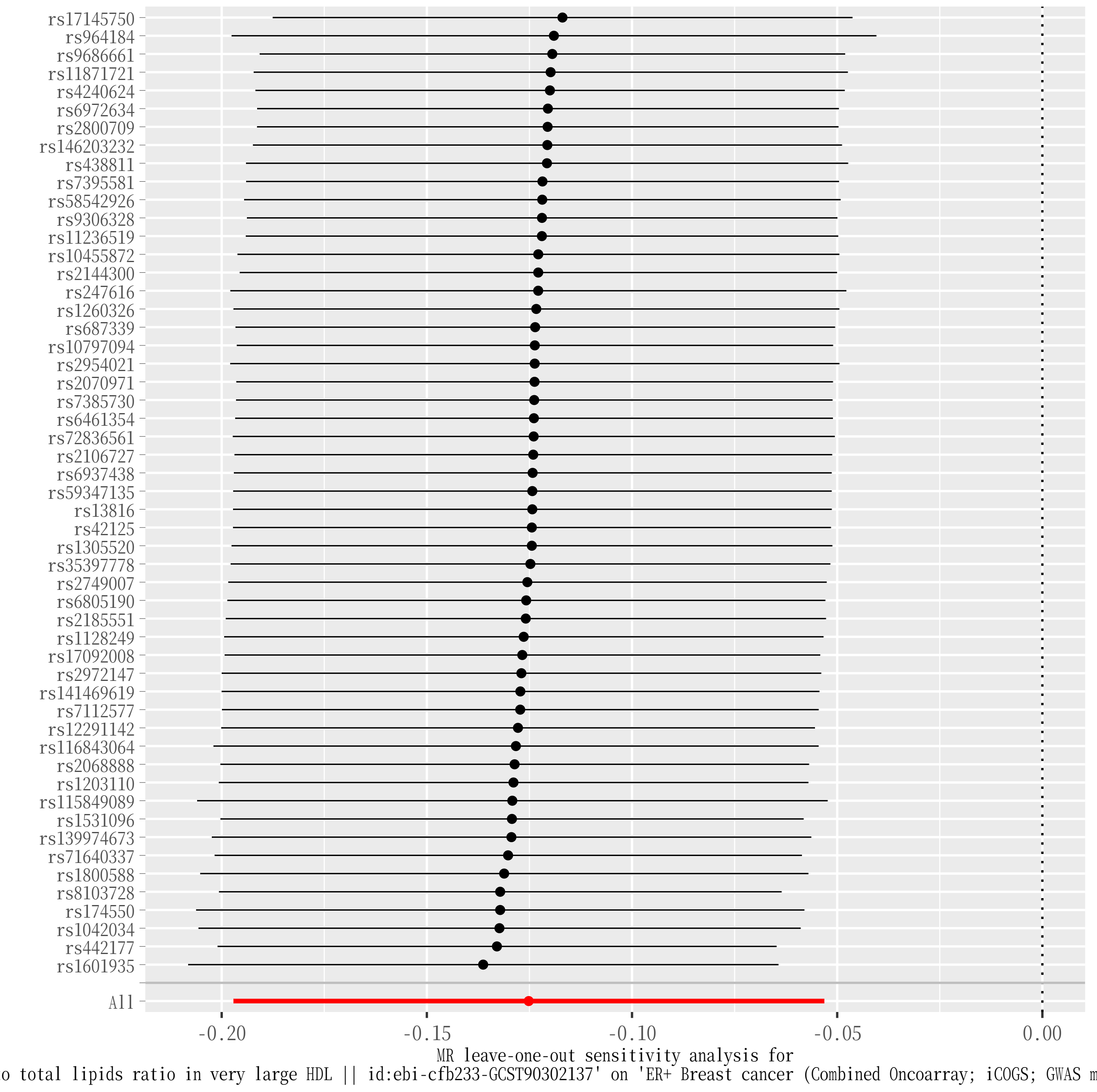
rs17145750
rs964184
rs9686661
rs11871721
rs4240624
rs6972634
rs2800709
rs146203232
rs438811
rs7395581
rs58542926
rs9306328
rs11236519
rs10455872
rs2144300
rs247616
rs1260326
rs687339
rs10797094
rs2954021
rs2070971
rs7385730
rs6461354
rs72836561
rs2106727
rs6937438
rs59347135
rs13816
rs42125
rs1305520
rs35397778
rs2749007
rs6805190
rs2185551
rs1128249
rs17092008
rs2972147
rs141469619
rs7112577
rs12291142
rs116843064
rs2068888
rs1203110
rs115849089
rs1531096
rs139974673
rs71640337
rs1800588
rs8103728
rs174550
rs1042034
rs442177
rs1601935
All
-0.20
-0.15
-0.10
-0.05
0.00
MR leave-one-out sensitivity analysis for
o total lipids ratio in very large HDL || id:ebi-cfb233-GCST90302137' on 'ER+ Breast cancer (Combined Oncoarray; iCOGS; GWAS m

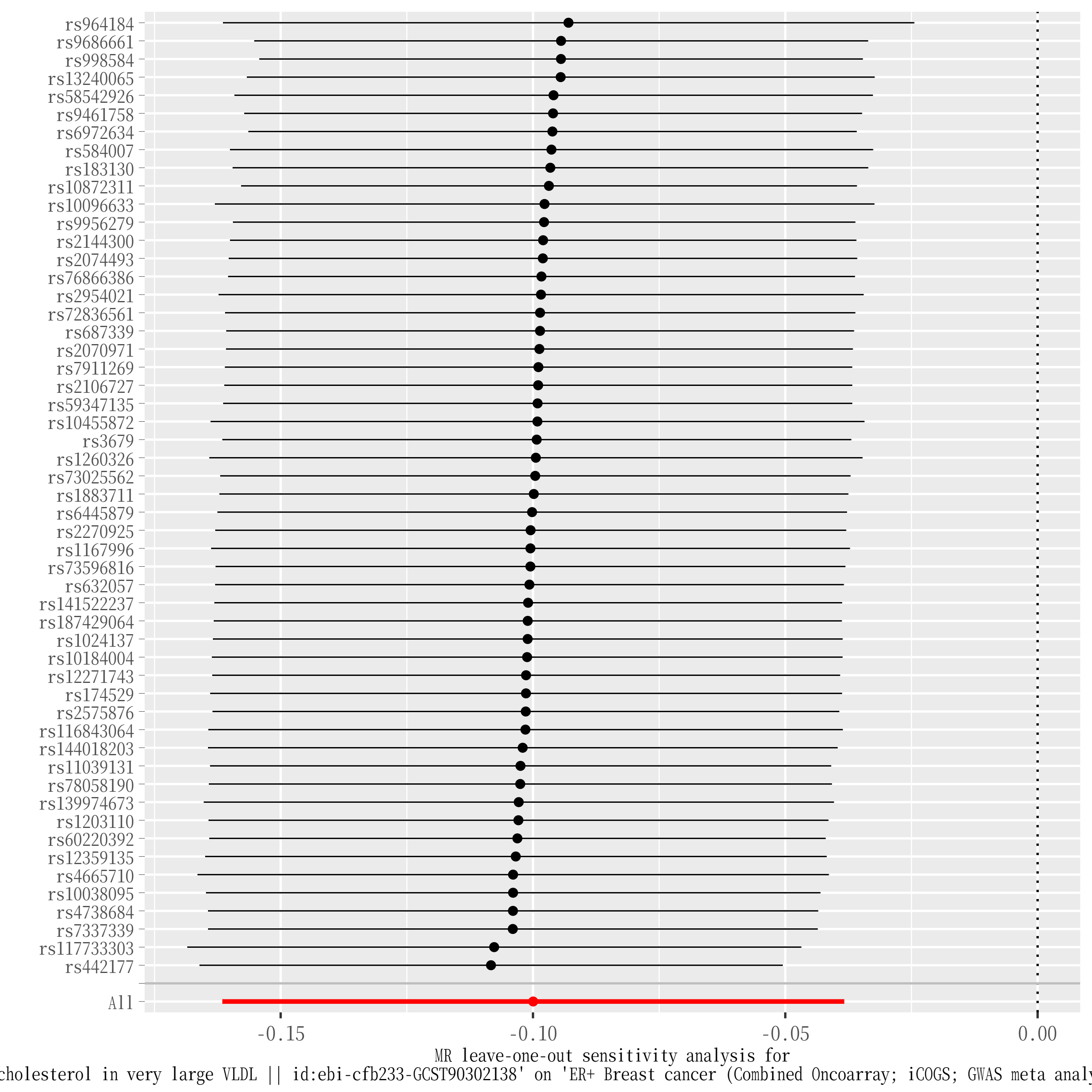

rs964184
rs9686661
rs998584
rs13240065
rs58542926
rs9461758
rs6972634
rs584007
rs183130
rs10872311
rs10096633
rs9956279
rs2144300
rs2074493
rs76866386
rs2954021
rs72836561
rs687339
rs2070971
rs7911269
rs2106727
rs59347135
rs10455872
rs3679
rs1260326
rs73025562
rs1883711
rs6445879
rs2270925
rs1167996
rs73596816
rs632057
rs141522237
rs187429064
rs1024137
rs10184004
rs12271743
rs174529
rs2575876
rs116843064
rs144018203
rs11039131
rs78058190
rs139974673
rs1203110
rs60220392
rs12359135
rs4665710
rs10038095
rs4738684
rs7337339
rs117733303
rs442177
All
-0.15
-0.10
-0.05
0.00
MR leave-one-out sensitivity analysis for
cholesterol in very large VLDL || id:ebi-cfb233-GCST90302138' on 'ER+ Breast cancer (Combined Oncoarray; iCOGS; GWAS meta analy

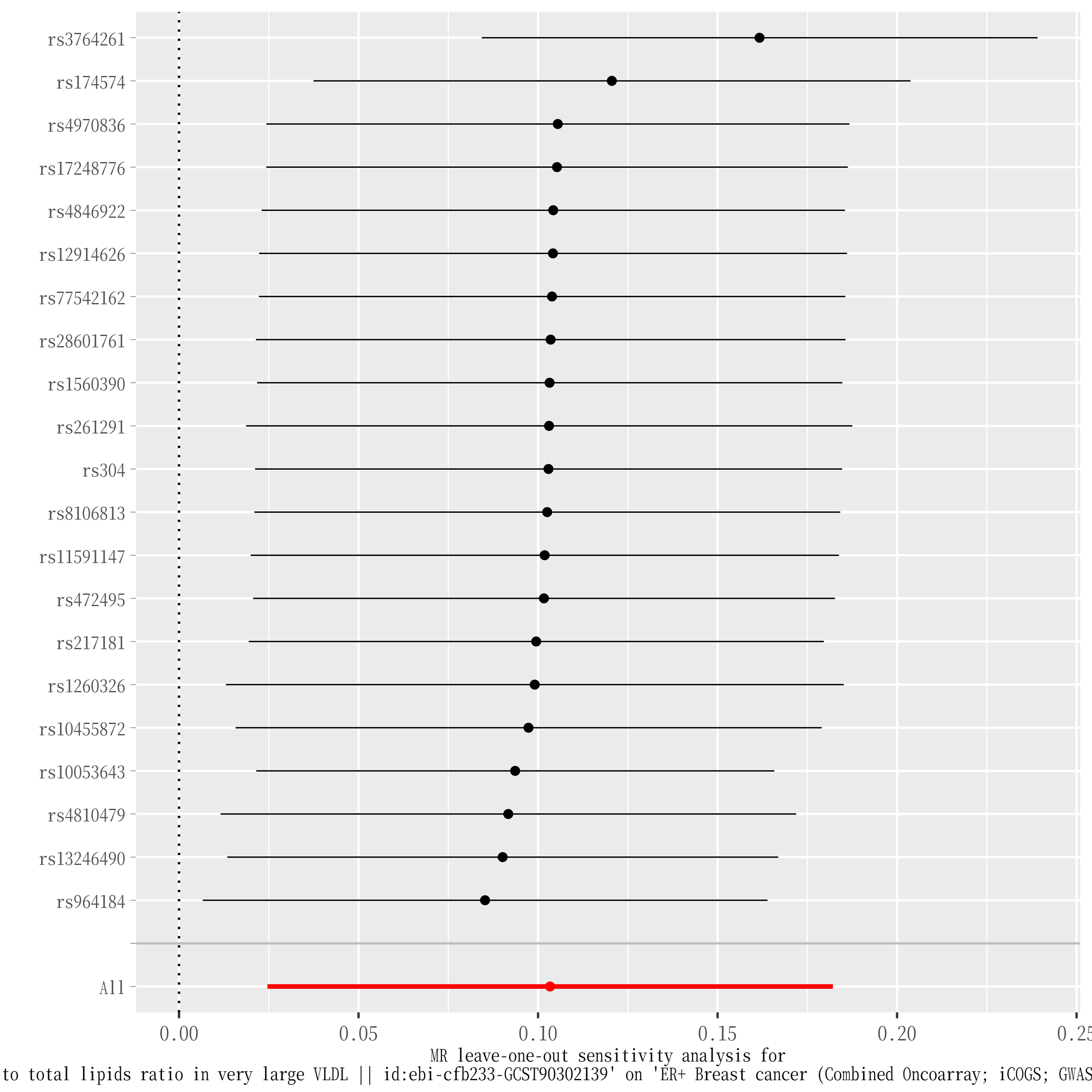
rs3764261
rs174574
rs4970836
rs17248776
rs4846922
rs12914626
rs77542162
rs28601761
rs1560390
rs261291
rs304
rs8106813
rs11591147
rs472495
rs217181
rs1260326
rs10455872
rs10053643
rs4810479
rs13246490
rs964184
All
0.00
0.05
0.10
0.15
0.20
0.25
MR leave-one-out sensitivity analysis for
to total lipids ratio in very large VLDL || id:ebi-cfb233-GCST90302139' on 'ER+ Breast cancer (Combined Oncoarray; iCOGS; GWAS

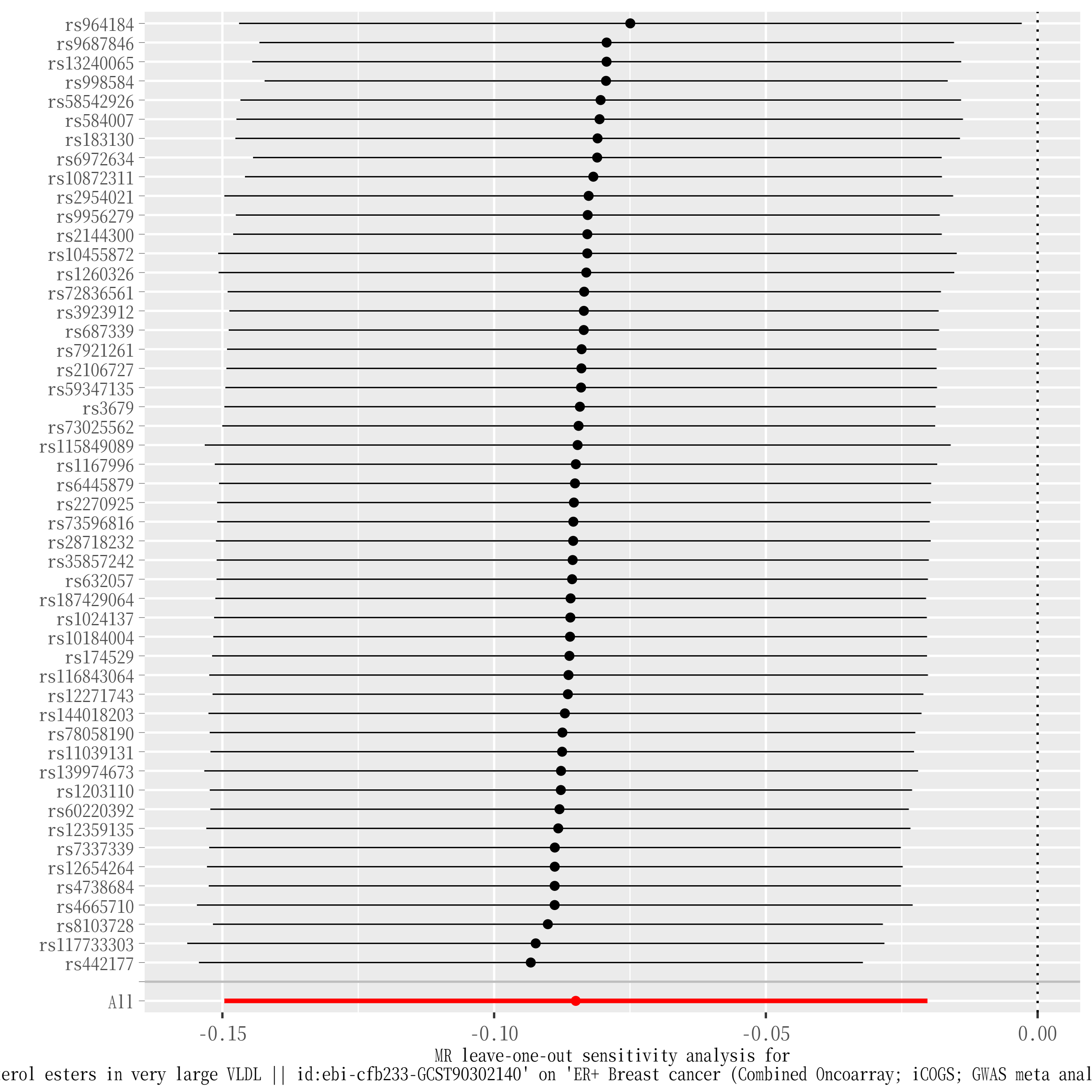
rs964184
rs9687846
rs13240065
rs998584
rs58542926
rs584007
rs183130
rs6972634
rs10872311
rs2954021
rs9956279
rs2144300
rs10455872
rs1260326
rs72836561
rs3923912
rs687339
rs7921261
rs2106727
rs59347135
rs3679
rs73025562
rs115849089
rs1167996
rs6445879
rs2270925
rs73596816
rs28718232
rs35857242
rs632057
rs187429064
rs1024137
rs10184004
rs174529
rs116843064
rs12271743
rs144018203
rs78058190
rs11039131
rs139974673
rs1203110
rs60220392
rs12359135
rs7337339
rs12654264
rs4738684
rs4665710
rs8103728
rs117733303
rs442177
All
-0.15
-0.10
-0.05
0.00
MR leave-one-out sensitivity analysis for
erol esters in very large VLDL || id:ebi-cfb233-GCST90302140' on 'ER+ Breast cancer (Combined Oncoarray; iCOGS; GWAS meta anal

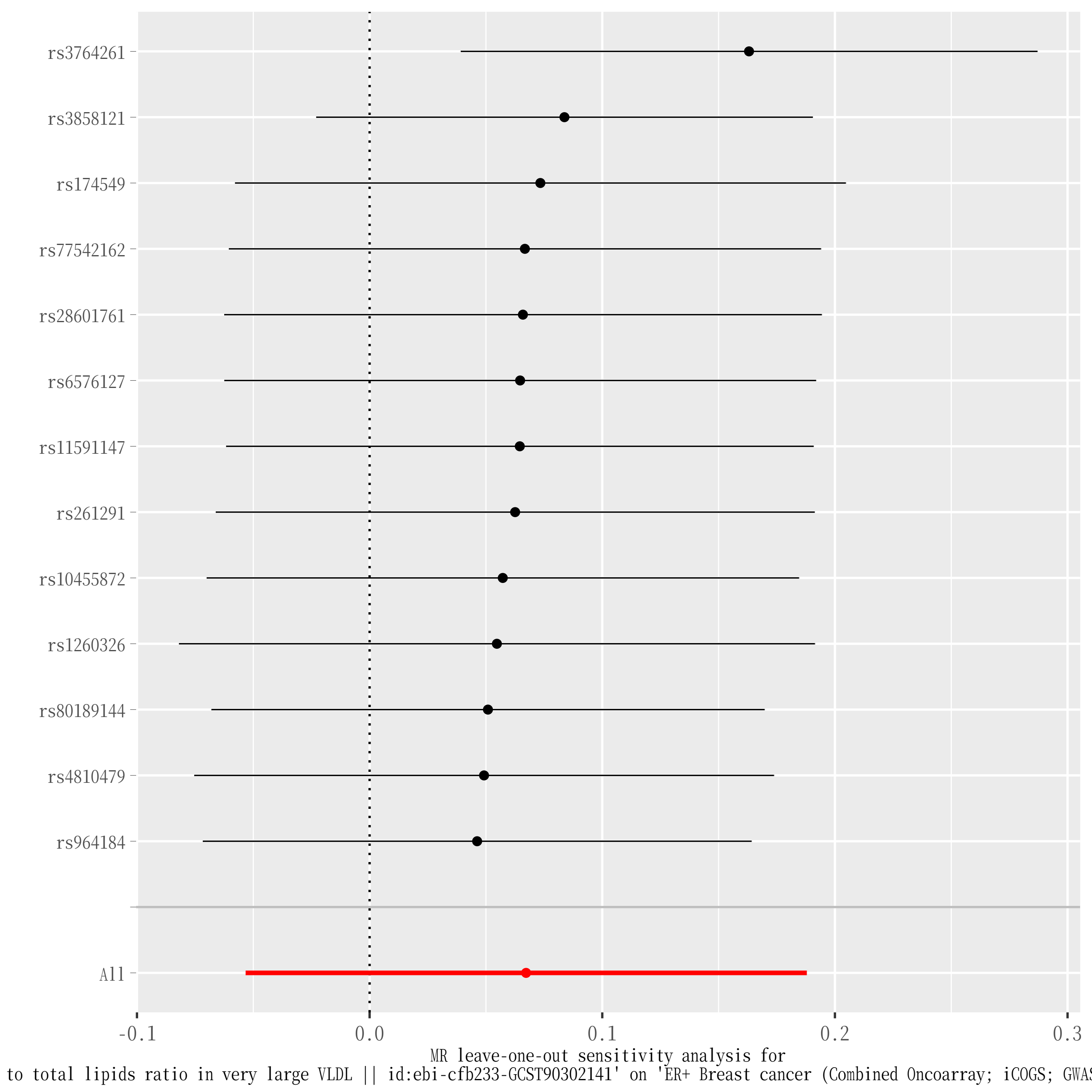
rs3764261
rs3858121
rs174549
rs77542162
rs28601761
rs6576127
rs11591147
rs261291
rs10455872
rs1260326
rs80189144
rs4810479
rs964184
All
-0.1
0.0
0.1
0.2
0.3
MR leave-one-out sensitivity analysis for
to total lipids ratio in very large VLDL || id:ebi-cfb233-GCST90302141' on 'ER+ Breast cancer (Combined Oncoarray; iCOGS; GWAS

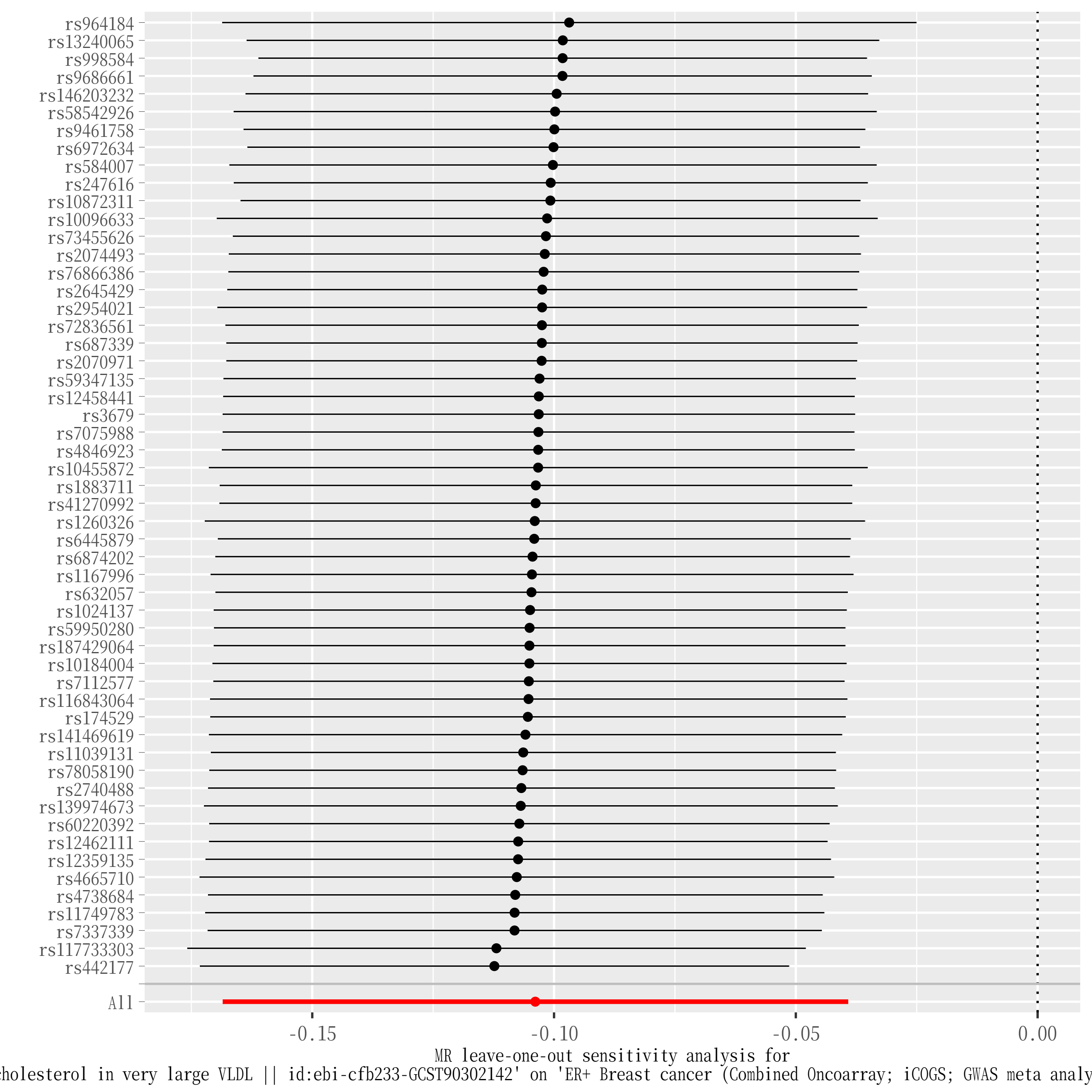
rs964184
rs13240065
rs998584
rs9686661
rs146203232
rs58542926
rs9461758
rs6972634
rs584007
rs247616
rs10872311
rs10096633
rs73455626
rs2074493
rs76866386
rs2645429
rs2954021
rs72836561
rs687339
rs2070971
rs59347135
rs12458441
rs3679
rs7075988
rs4846923
rs10455872
rs1883711
rs41270992
rs1260326
rs6445879
rs6874202
rs1167996
rs632057
rs1024137
rs59950280
rs187429064
rs10184004
rs7112577
rs116843064
rs174529
rs141469619
rs11039131
rs78058190
rs2740488
rs139974673
rs60220392
rs12462111
rs12359135
rs4665710
rs4738684
rs11749783
rs7337339
rs117733303
rs442177
All
-0.15
-0.10
-0.05
0.00
MR leave-one-out sensitivity analysis for
holesterol in very large VLDL || id:ebi-cfb233-GCST90302142' on 'ER+ Breast cancer (Combined Oncoarray; iCOGS; GWAS meta analy

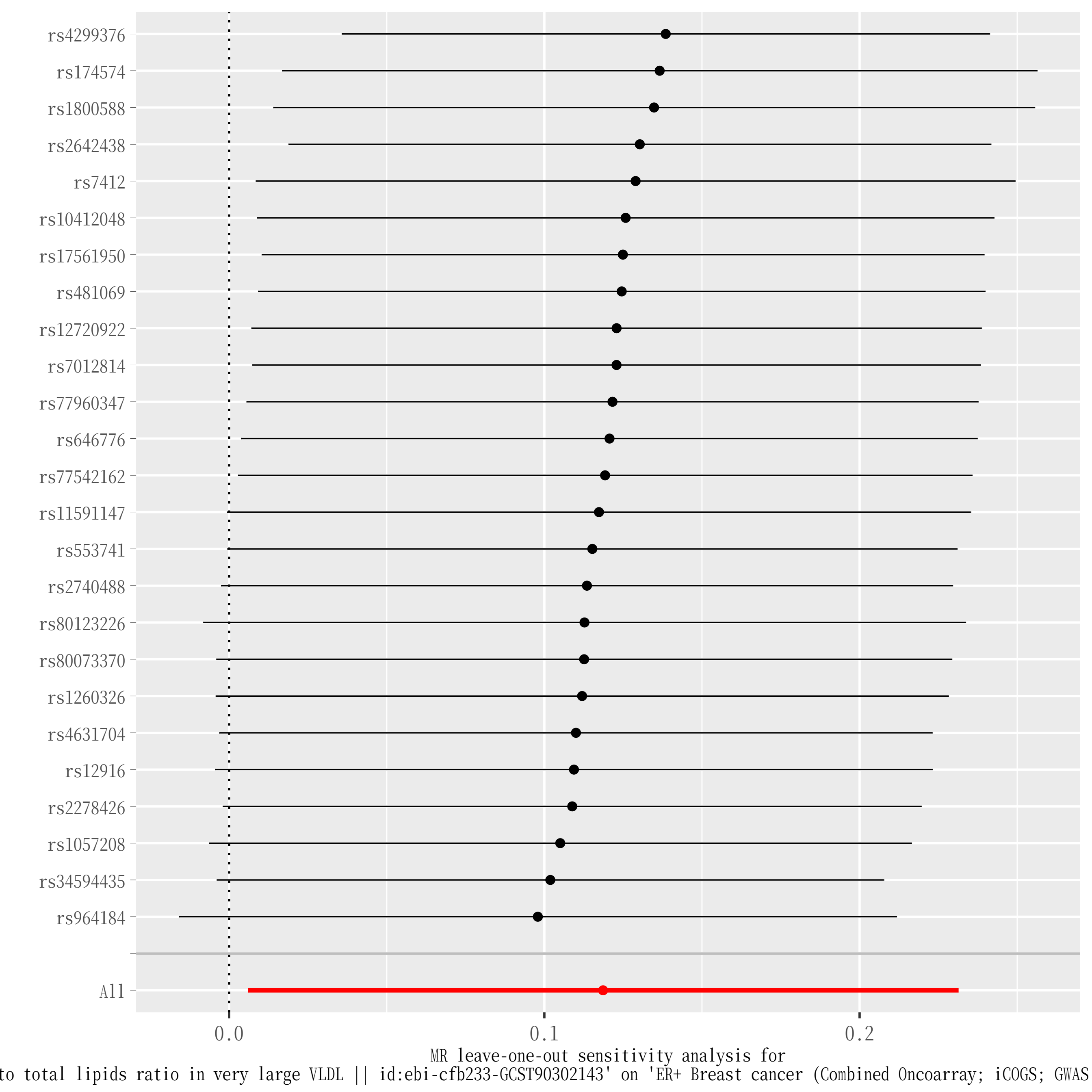
rs4299376
rs174574
rs1800588
rs2642438
rs7412
rs10412048
rs17561950
rs481069
rs12720922
rs7012814
rs77960347
rs646776
rs77542162
rs11591147
rs553741
rs2740488
rs80123226
rs80073370
rs1260326
rs4631704
rs12916
rs2278426
rs1057208
rs34594435
rs964184
All
0.0
0.1
0.2
MR leave-one-out sensitivity analysis for
to total lipids ratio in very large VLDL || id:ebi-cfb233-GCST90302143' on 'ER+ Breast cancer (Combined Oncoarray; iCOGS; GWAS

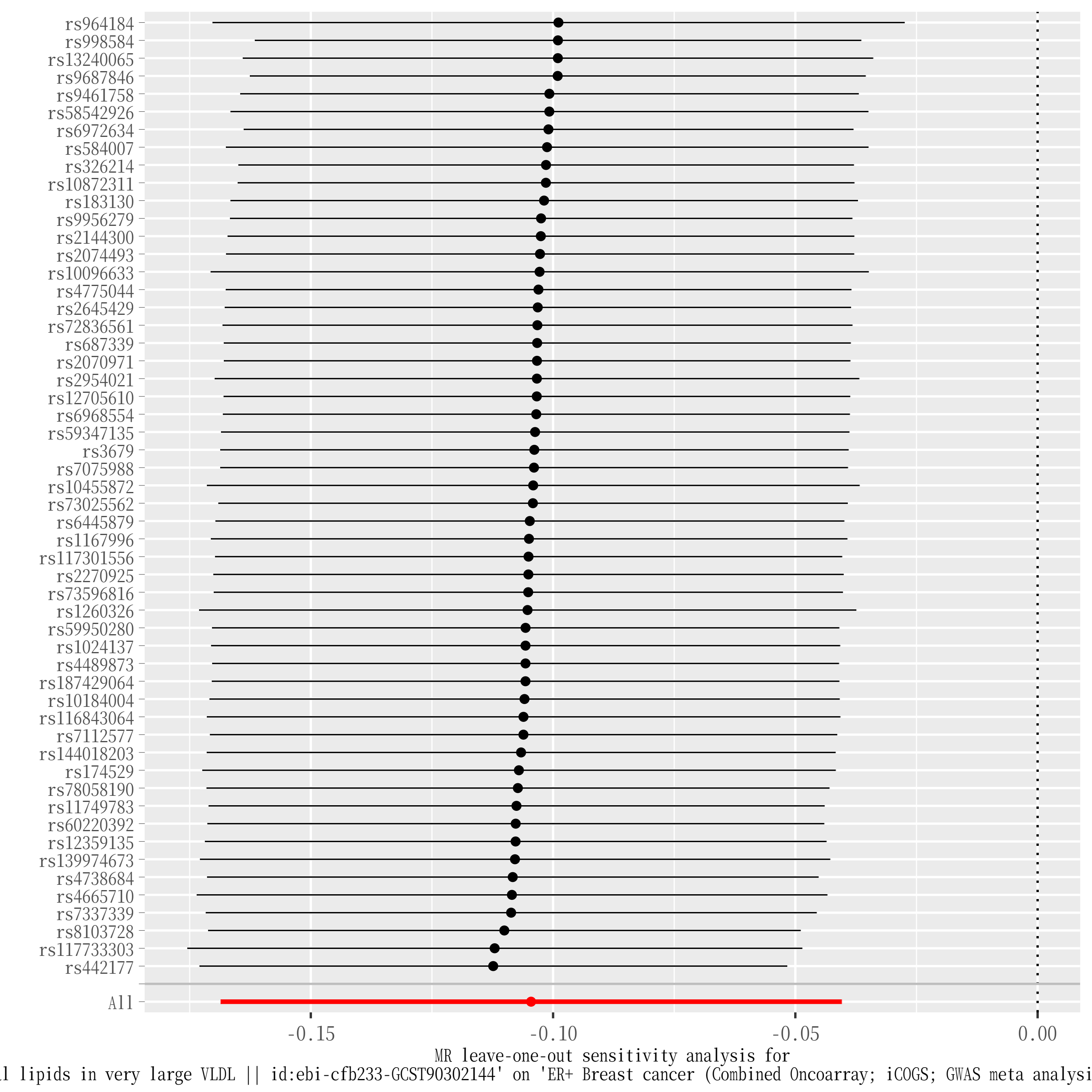
rs964184
rs998584
rs13240065
rs9687846
rs9461758
rs58542926
rs6972634
rs584007
rs326214
rs10872311
rs183130
rs9956279
rs2144300
rs2074493
rs10096633
rs4775044
rs2645429
rs72836561
rs687339
rs2070971
rs2954021
rs12705610
rs6968554
rs59347135
rs3679
rs7075988
rs10455872
rs73025562
rs6445879
rs1167996
rs117301556
rs2270925
rs73596816
rs1260326
rs59950280
rs1024137
rs4489873
rs187429064
rs10184004
rs116843064
rs7112577
rs144018203
rs174529
rs78058190
rs11749783
rs60220392
rs12359135
rs139974673
rs4738684
rs4665710
rs7337339
rs8103728
rs117733303
rs442177
All
-0.15
-0.10
-0.05
0.00
MR leave-one-out sensitivity analysis for
l lipids in very large VLDL || id:ebi-cfb233-GCST90302144' on 'ER+ Breast cancer (Combined Oncoarray; iCOGS; GWAS meta analysi

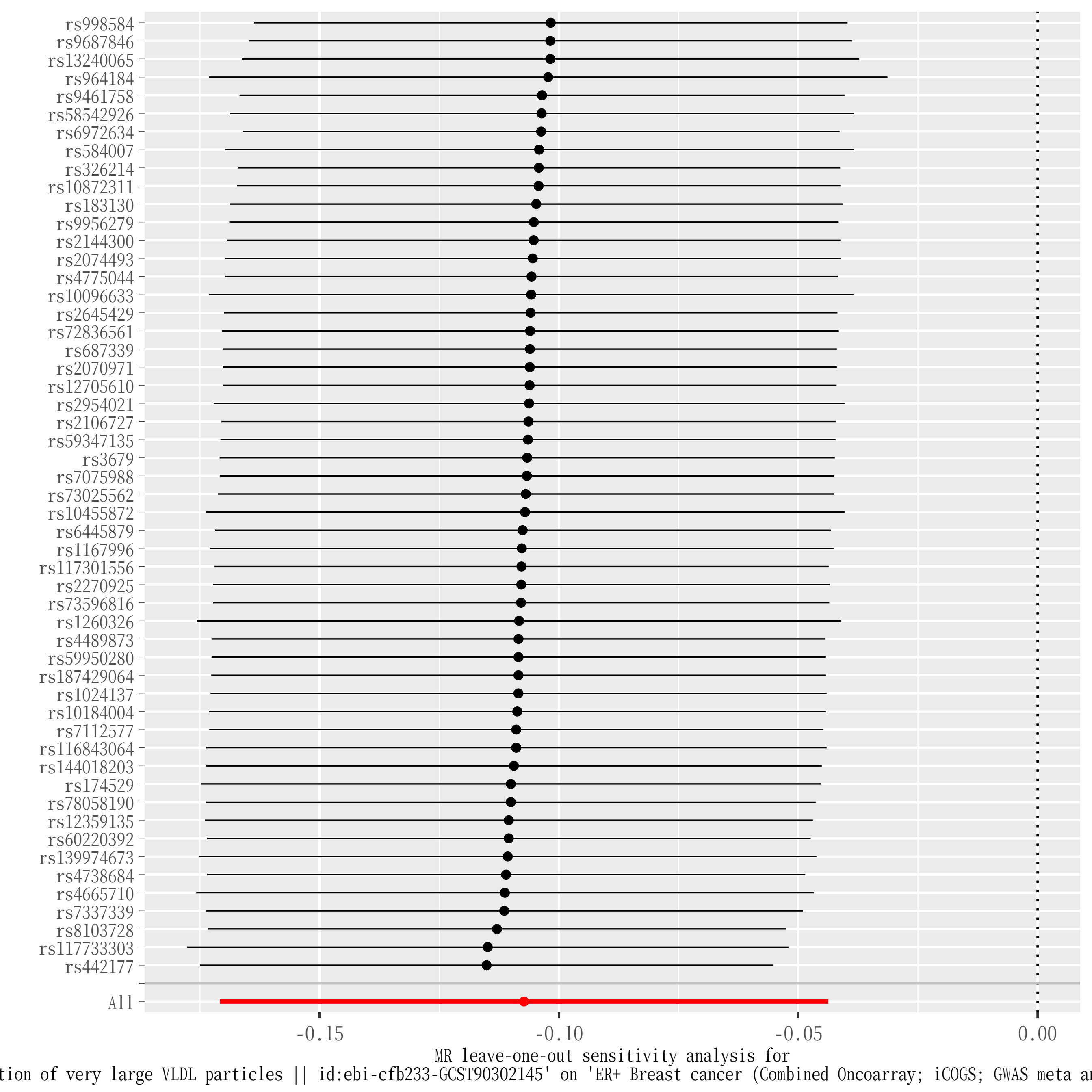
rs998584
rs9687846
rs13240065
rs964184
rs9461758
rs58542926
rs6972634
rs584007
rs326214
rs10872311
rs183130
rs9956279
rs2144300
rs2074493
rs4775044
rs10096633
rs2645429
rs72836561
rs687339
rs2070971
rs12705610
rs2954021
rs2106727
rs59347135
rs3679
rs7075988
rs73025562
rs10455872
rs6445879
rs1167996
rs117301556
rs2270925
rs73596816
rs1260326
rs4489873
rs59950280
rs187429064
rs1024137
rs10184004
rs7112577
rs116843064
rs144018203
rs174529
rs78058190
rs12359135
rs60220392
rs139974673
rs4738684
rs4665710
rs7337339
rs8103728
rs117733303
rs442177
All
-0.15
-0.10
-0.05
0.00
MR leave-one-out sensitivity analysis for
tion of very large VLDL particles || id:ebi-cfb233-GCST90302145' on 'ER+ Breast cancer (Combined Oncoarray; iCOGS; GWAS meta an

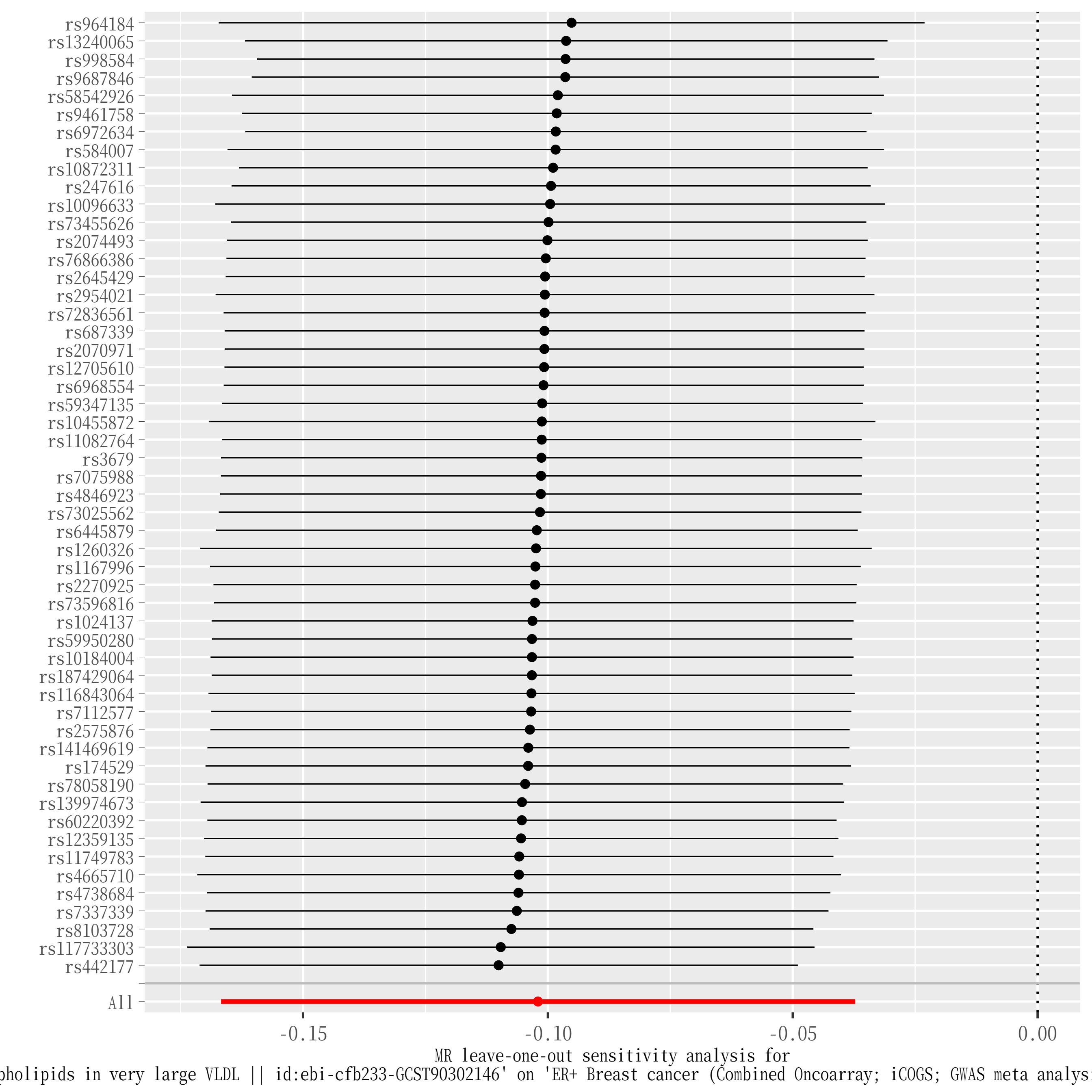
rs964184
rs13240065
rs998584
rs9687846
rs58542926
rs9461758
rs6972634
rs584007
rs10872311
rs247616
rs10096633
rs73455626
rs2074493
rs76866386
rs2645429
rs2954021
rs72836561
rs687339
rs2070971
rs12705610
rs6968554
rs59347135
rs10455872
rs11082764
rs3679
rs7075988
rs4846923
rs73025562
rs6445879
rs1260326
rs1167996
rs2270925
rs73596816
rs1024137
rs59950280
rs10184004
rs187429064
rs116843064
rs7112577
rs2575876
rs141469619
rs174529
rs78058190
rs139974673
rs60220392
rs12359135
rs11749783
rs4665710
rs4738684
rs7337339
rs8103728
rs117733303
rs442177
All
-0.15
-0.10
-0.05
0.00
MR leave-one-out sensitivity analysis for
pholipids in very large VLDL || id:ebi-cfb233-GCST90302146' on 'ER+ Breast cancer (Combined Oncoarray; iCOGS; GWAS meta analys

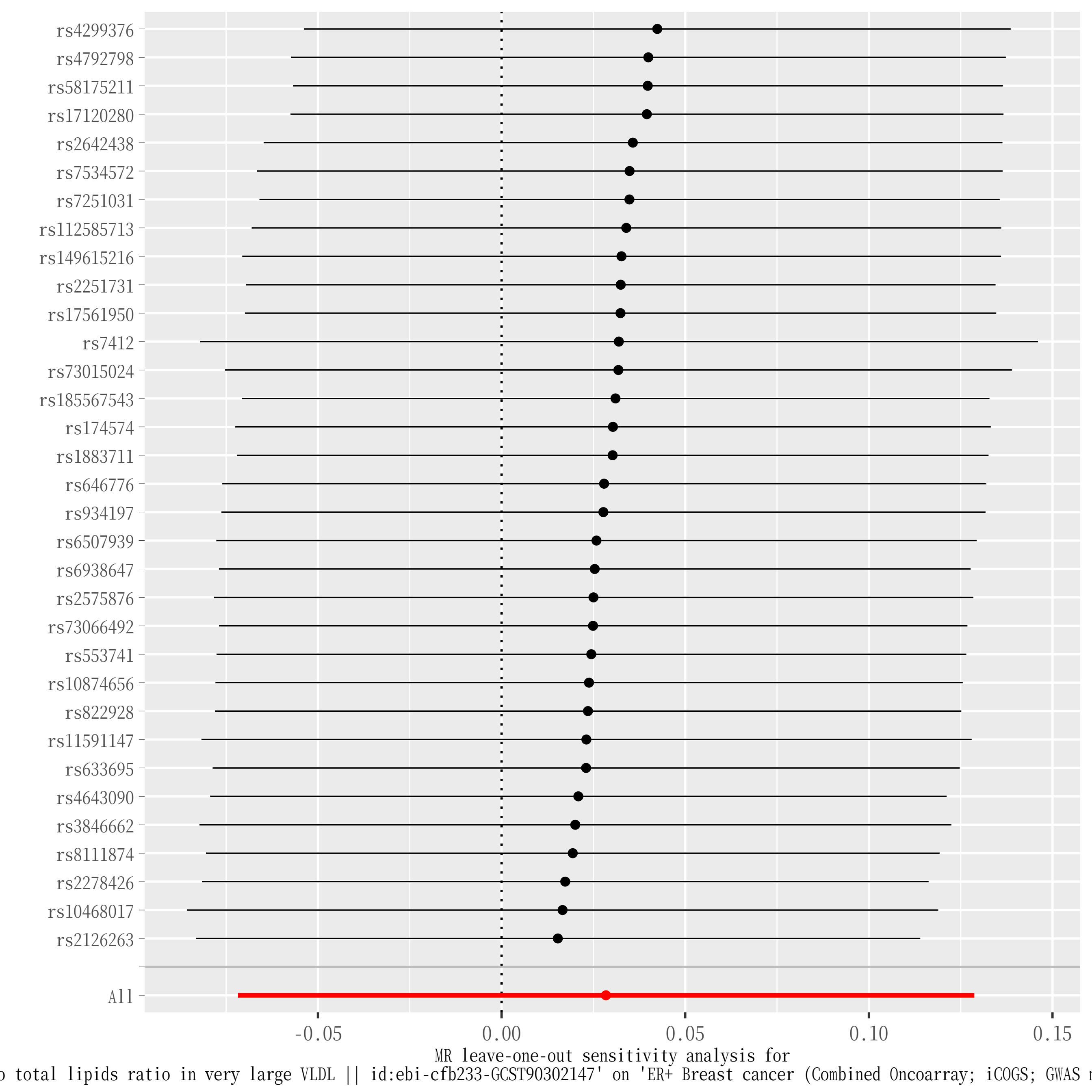
rs4299376
rs4792798
rs58175211
rs17120280
rs2642438
rs7534572
rs7251031
rs112585713
rs149615216
rs2251731
rs17561950
rs7412
rs73015024
rs185567543
rs174574
rs1883711
rs646776
rs934197
rs6507939
rs6938647
rs2575876
rs73066492
rs553741
rs10874656
rs822928
rs11591147
rs633695
rs4643090
rs3846662
rs8111874
rs2278426
rs10468017
rs2126263
All
-0.05
0.00
0.05
0.10
0.15
MR leave-one-out sensitivity analysis for
o total lipids ratio in very large VLDL || id:ebi-cfb233-GCST90302147' on 'ER+ Breast cancer (Combined Oncoarray; iCOGS; GWAS m

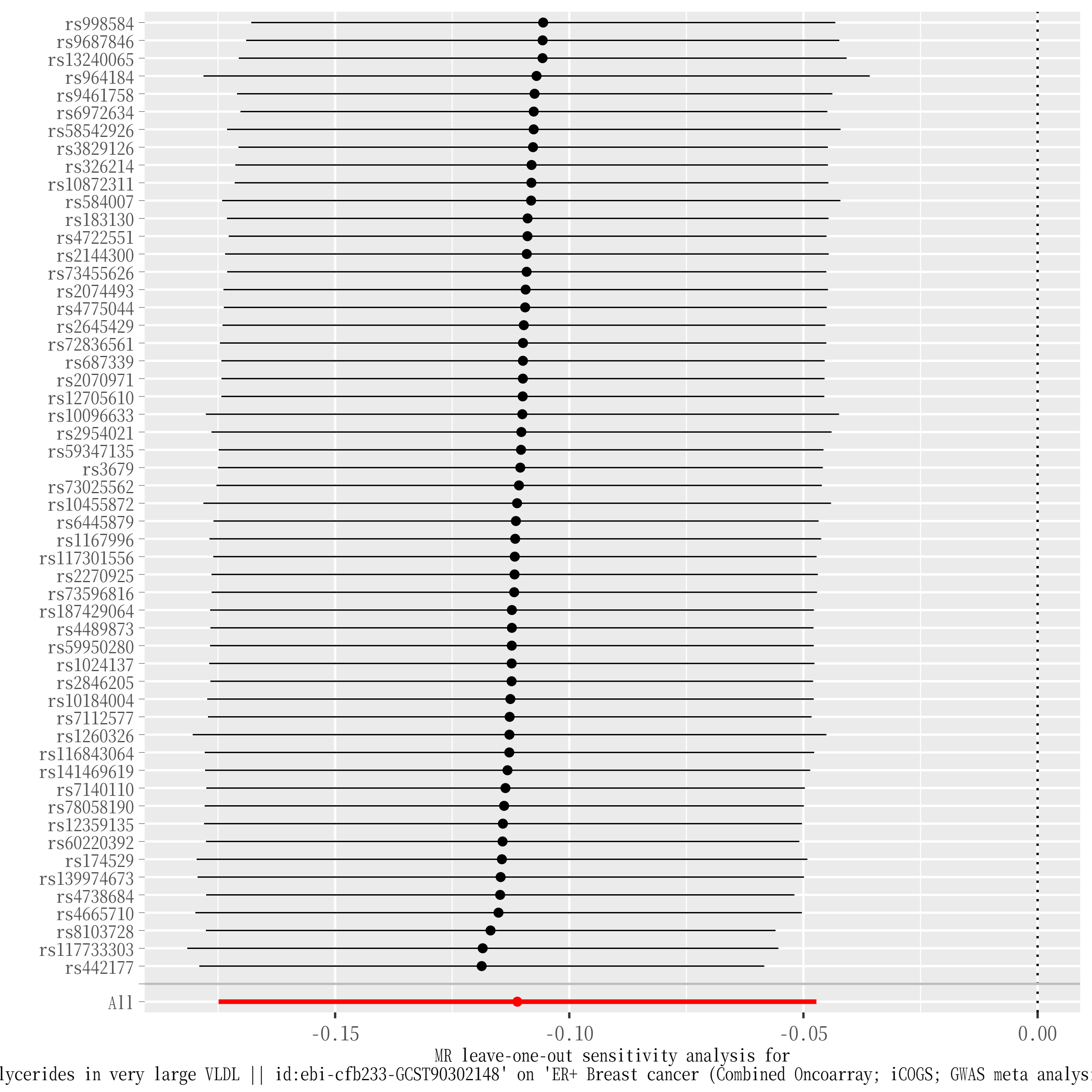
rs998584
rs9687846
rs13240065
rs964184
rs9461758
rs6972634
rs58542926
rs3829126
rs326214
rs10872311
rs584007
rs183130
rs4722551
rs2144300
rs73455626
rs2074493
rs4775044
rs2645429
rs72836561
rs687339
rs2070971
rs12705610
rs10096633
rs2954021
rs59347135
rs3679
rs73025562
rs10455872
rs6445879
rs1167996
rs117301556
rs2270925
rs73596816
rs187429064
rs4489873
rs59950280
rs1024137
rs2846205
rs10184004
rs7112577
rs1260326
rs116843064
rs141469619
rs7140110
rs78058190
rs12359135
rs60220392
rs174529
rs139974673
rs4738684
rs4665710
rs8103728
rs117733303
rs442177
All
-0.15
-0.10
-0.05
0.00
MR leave-one-out sensitivity analysis for
lycerides in very large VLDL || id:ebi-cfb233-GCST90302148' on 'ER+ Breast cancer (Combined Oncoarray; iCOGS; GWAS meta analys

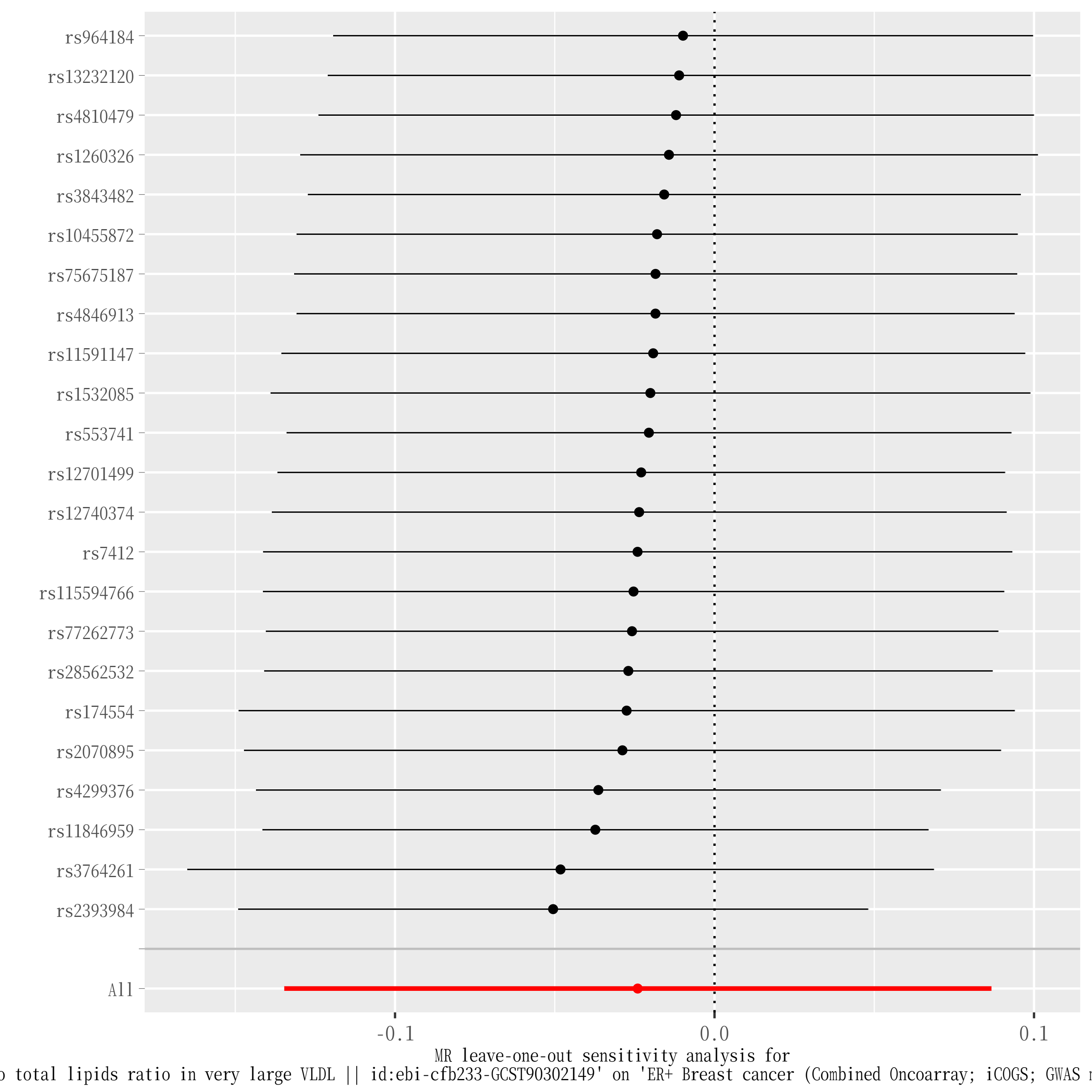

rs964184
rs13232120
rs4810479
rs1260326
rs3843482
rs10455872
rs75675187
rs4846913
rs11591147
rs1532085
rs553741
rs12701499
rs12740374
rs7412
rs115594766
rs77262773
rs28562532
rs174554
rs2070895
rs4299376
rs11846959
rs3764261
rs2393984
All
-0.1
0.0
0.1
MR leave-one-out sensitivity analysis for
o total lipids ratio in very large VLDL || id:ebi-cfb233-GCST90302149' on 'ER+ Breast cancer (Combined Oncoarray; iCOGS; GWAS r

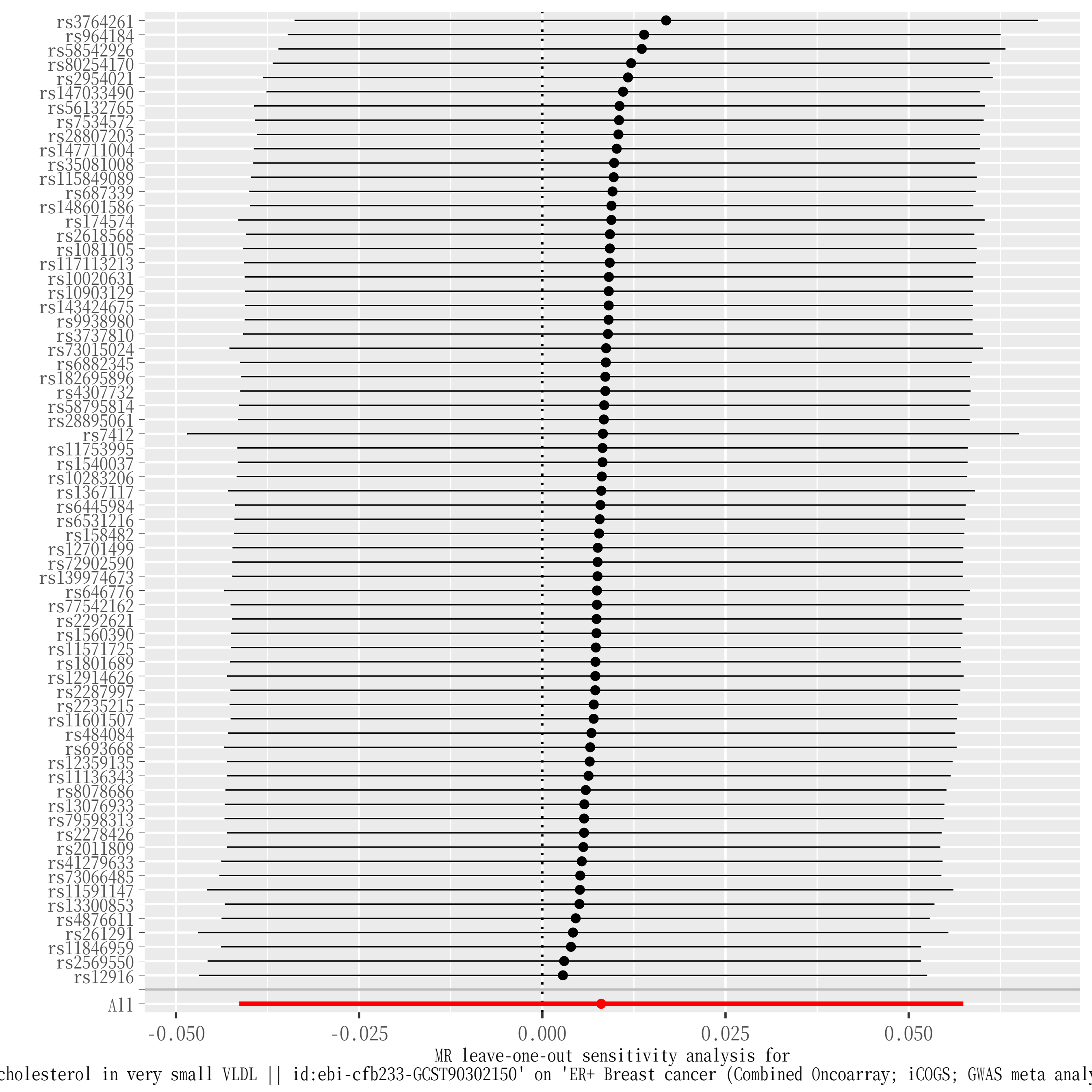
rs3764261
rs964184
rs58542926
rs80254170
rs2954021
rs147033490
rs56132765
rs7534572
rs28807203
rs147711004
rs35081008
rs115849089
rs687339
rs148601586
rs174574
rs2618568
rs1081105
rs117113213
rs10020631
rs10903129
rs143424675
rs9938980
rs3737810
rs73015024
rs6882345
rs182695896
rs4307732
rs58795814
rs28895061
rs7412
rs11753995
rs1540037
rs10283206
rs1367117
rs6445984
rs6531216
rs158482
rs12701499
rs72902590
rs139974673
rs646776
rs77542162
rs2292621
rs1560390
rs11571725
rs1801689
rs12914626
rs2287997
rs2235215
rs11601507
rs484084
rs693668
rs12359135
rs11136343
rs8078686
rs13076933
rs79598313
rs2278426
rs2011809
rs41279633
rs73066485
rs11591147
rs13300853
rs4876611
rs261291
rs11846959
rs2569550
rs12916
All
-0.050
-0.025
0.000
0.025
0.050
MR leave-one-out sensitivity analysis for
cholesterol in very small VLDL || id:ebi-cfb233-GCST90302150' on 'ER+ Breast cancer (Combined Oncoarray; iCOGS; GWAS meta analy

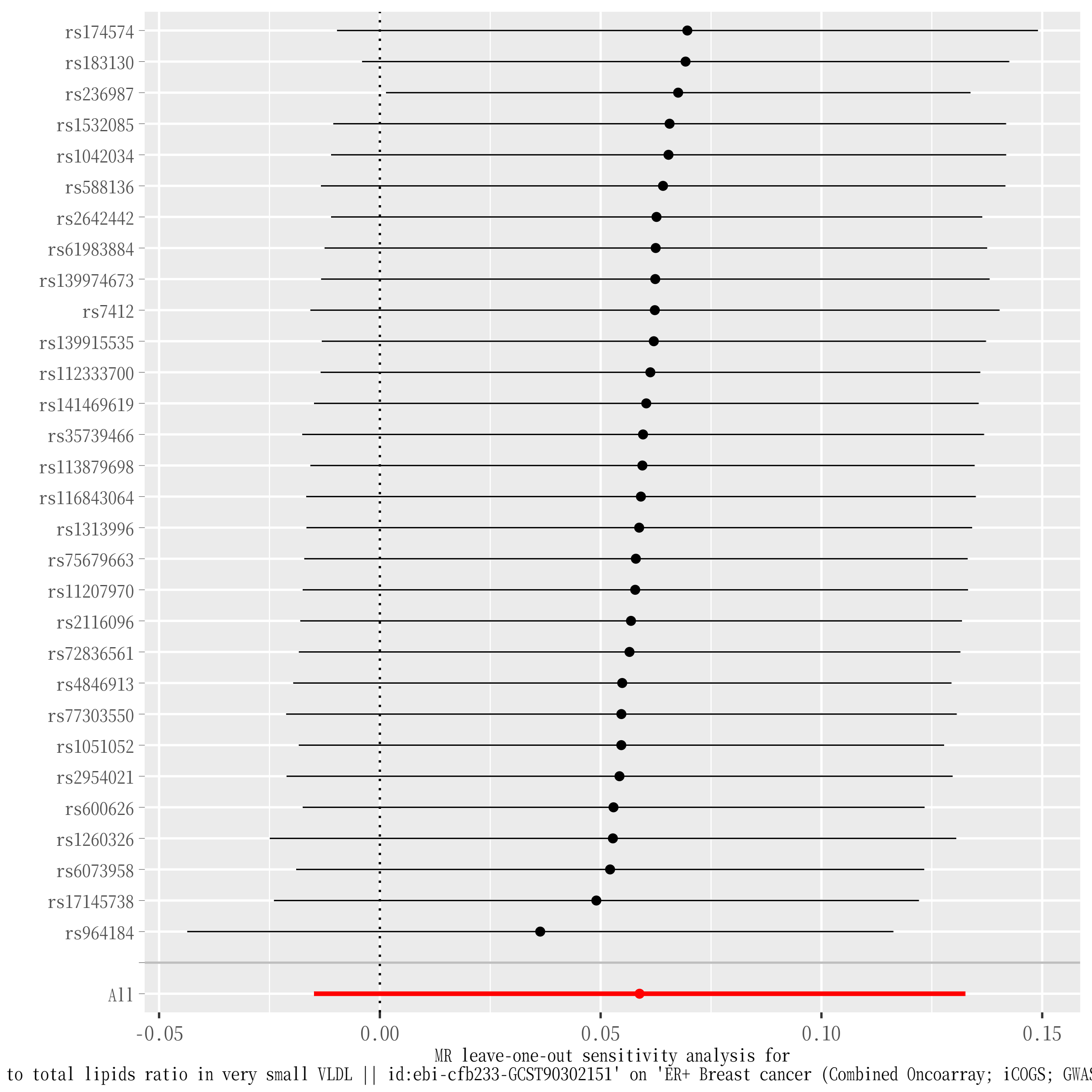
rs174574
rs183130
rs236987
rs1532085
rs1042034
rs588136
rs2642442
rs61983884
rs139974673
rs7412
rs139915535
rs112333700
rs141469619
rs35739466
rs113879698
rs116843064
rs1313996
rs75679663
rs11207970
rs2116096
rs72836561
rs4846913
rs77303550
rs1051052
rs2954021
rs600626
rs1260326
rs6073958
rs17145738
rs964184
All
-0.05
0.00
0.05
0.10
0.15
MR leave-one-out sensitivity analysis for
to total lipids ratio in very small VLDL || id:ebi-cfb233-GCST90302151' on 'ER+ Breast cancer (Combined Oncoarray; iCOGS; GWAS

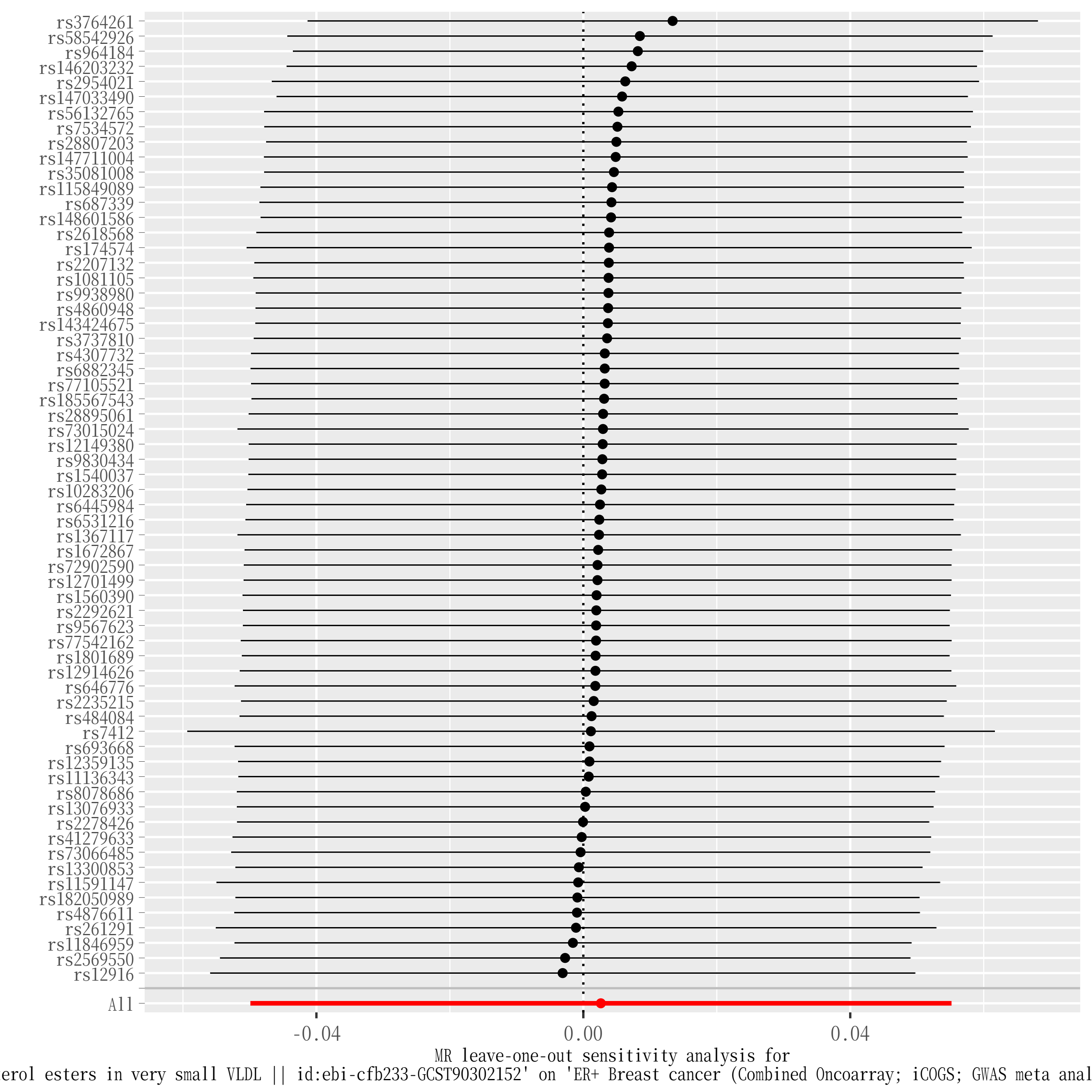

rs3764261
rs58542926
rs964184
rs146203232
rs2954021
rs147033490
rs56132765
rs7534572
rs28807203
rs147711004
rs35081008
rs115849089
rs687339
rs148601586
rs2618568
rs174574
rs2207132
rs1081105
rs9938980
rs4860948
rs143424675
rs3737810
rs4307732
rs6882345
rs77105521
rs185567543
rs28895061
rs73015024
rs12149380
rs9830434
rs1540037
rs10283206
rs6445984
rs6531216
rs1367117
rs1672867
rs72902590
rs12701499
rs1560390
rs2292621
rs9567623
rs77542162
rs1801689
rs12914626
rs646776
rs2235215
rs484084
rs7412
rs693668
rs12359135
rs11136343
rs8078686
rs13076933
rs2278426
rs41279633
rs73066485
rs13300853
rs11591147
rs182050989
rs4876611
rs261291
rs11846959
rs2569550
rs12916
All
-0.04
0.00
0.04
MR leave-one-out sensitivity analysis for
erol esters in very small VLDL || id:ebi-cfb233-GCST90302152' on 'ER+ Breast cancer (Combined Oncoarray; iCOGS; GWAS meta anal

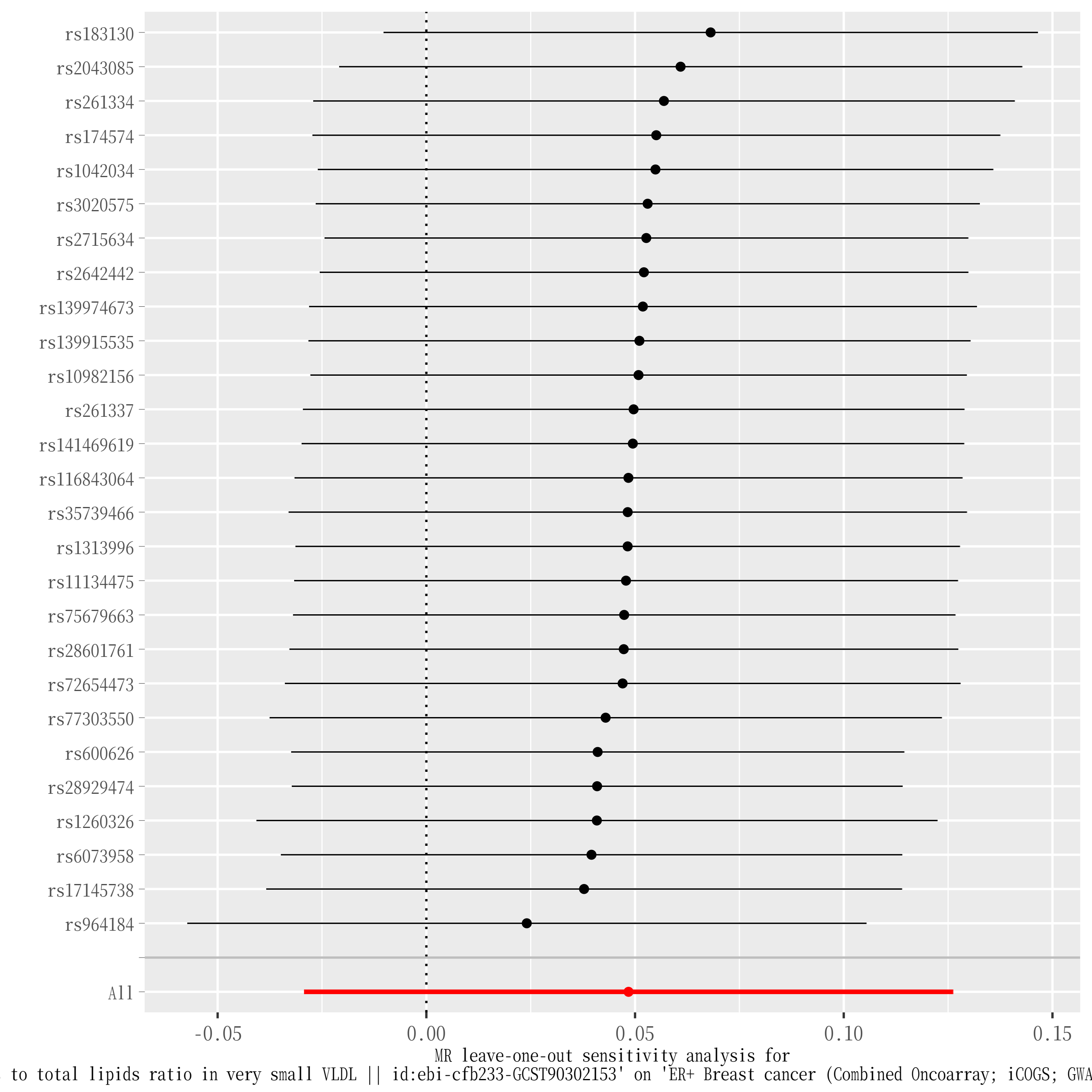
rs183130
rs2043085
rs261334
rs174574
rs1042034
rs3020575
rs2715634
rs2642442
rs139974673
rs139915535
rs10982156
rs261337
rs141469619
rs116843064
rs35739466
rs1313996
rs11134475
rs75679663
rs28601761
rs72654473
rs77303550
rs600626
rs28929474
rs1260326
rs6073958
rs17145738
rs964184
All
-0.05
0.00
0.05
0.10
0.15
MR leave-one-out sensitivity analysis for
to total lipids ratio in very small VLDL || id:ebi-cfb233-GCST90302153' on 'ER+ Breast cancer (Combined Oncoarray; iCOGS; GWA

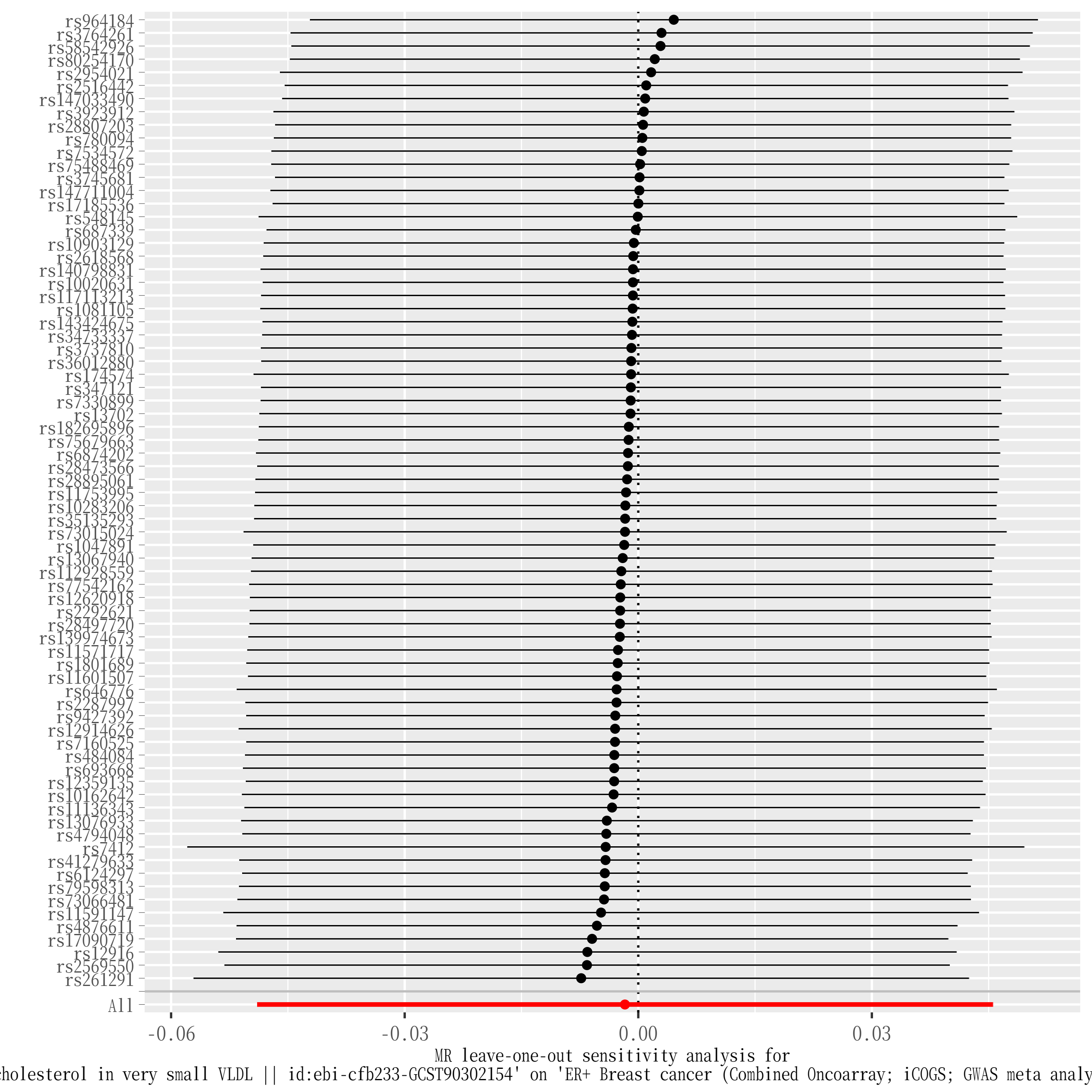
rs964184
rs3764261
rs58542926
rs80254170
rs2954021
rs2516442
rs147033490
rs3923912
rs28807203
rs780094
rs7534572
rs75488469
rs3745681
rs147711004
rs17185536
rs548145
rs687339
rs10903129
rs2618568
rs140798831
rs10020631
rs117113213
rs1081105
rs143424675
rs34733337
rs3737810
rs36012880
rs174574
rs347121
rs7330899
rs13702
rs182695896
rs75679663
rs6874202
rs28473566
rs28895061
rs11753995
rs10283206
rs35135293
rs73015024
rs1047891
rs13067940
rs112928559
rs77542162
rs12620918
rs2292621
rs28497720
rs139974673
rs11571717
rs1801689
rs11601507
rs646776
rs2287997
rs9427392
rs12914626
rs7160525
rs484084
rs693668
rs12359135
rs10162642
rs11136343
rs13076933
rs4794048
rs7412
rs41279633
rs6124297
rs79598313
rs73066481
rs11591147
rs4876611
rs17090719
rs12916
rs2569550
rs261291
All
-0.06
-0.03
0.00
0.03
MR leave-one-out sensitivity analysis for
cholesterol in very small VLDL || id:ebi-cfb233-GCST90302154' on 'ER+ Breast cancer (Combined Oncoarray; iCOGS; GWAS meta analy

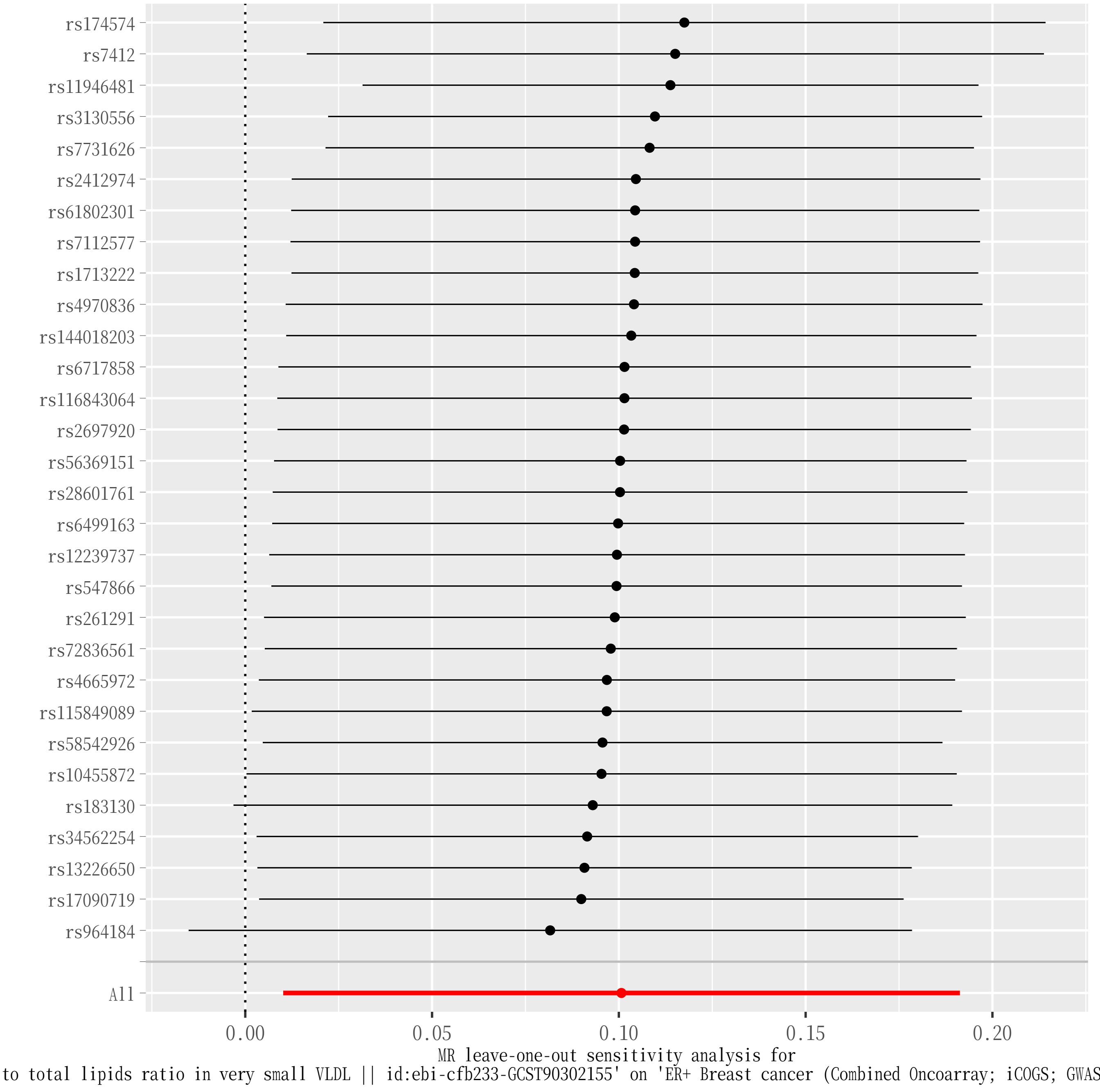
rs174574
rs7412
rs11946481
rs3130556
rs7731626
rs2412974
rs61802301
rs7112577
rs1713222
rs4970836
rs144018203
rs6717858
rs116843064
rs2697920
rs56369151
rs28601761
rs6499163
rs12239737
rs547866
rs261291
rs72836561
rs4665972
rs115849089
rs58542926
rs10455872
rs183130
rs34562254
rs13226650
rs17090719
rs964184
All
0.00
0.05
0.10
0.15
0.20
MR leave-one-out sensitivity analysis for
to total lipids ratio in very small VLDL || id:ebi-cfb233-GCST90302155' on 'ER+ Breast cancer (Combined Oncoarray; iCOGS; GWAS

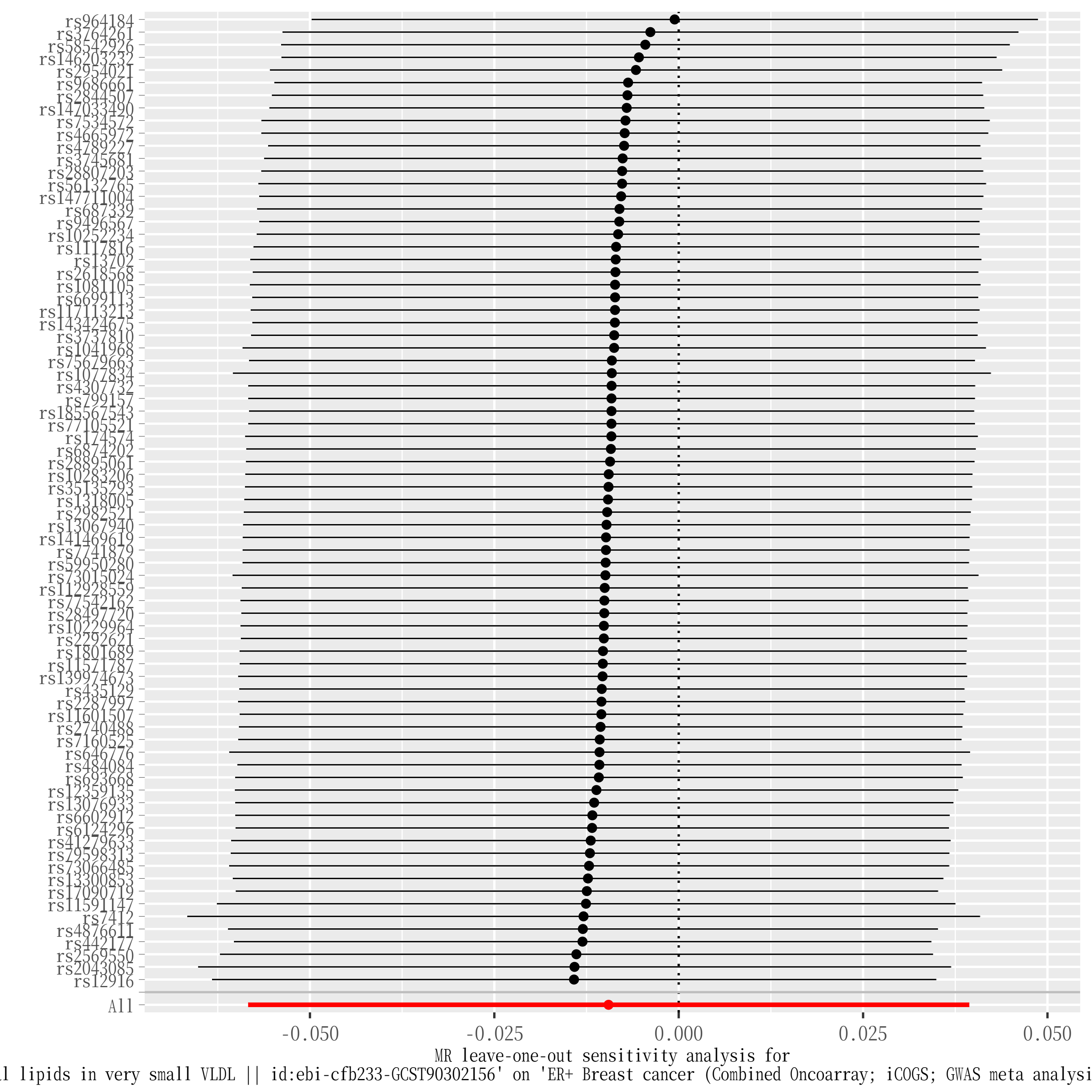
rs964184
rs3764261
rs58542926
rs146203232
rs2954021
rs9686661
rs2844507
rs147033490
rs7534572
rs4665972
rs4789227
rs3745681
rs28807203
rs56132765
rs147711004
rs687339
rs9496567
rs10252234
rs1117816
rs13702
rs2618568
rs1081105
rs6699113
rs117113213
rs143424675
rs3737810
rs1041968
rs75679663
rs1077834
rs4307732
rs799157
rs185567543
rs77105521
rs174574
rs6874202
rs28895061
rs10283206
rs35135293
rs1318005
rs2982521
rs13067940
rs141469619
rs7741879
rs59950280
rs73015024
rs112928559
rs77542162
rs28497720
rs10229964
rs2292621
rs1801689
rs11571787
rs139974673
rs435129
rs2287997
rs11601507
rs2740488
rs7160525
rs646776
rs484084
rs693668
rs12359135
rs13076933
rs6602912
rs6124296
rs41279633
rs79598313
rs73066485
rs13300853
rs17090719
rs11591147
rs7412
rs4876611
rs442177
rs2569550
rs2043085
rs12916
All
-0.050
-0.025
0.000
0.025
0.050
MR leave-one-out sensitivity analysis for
l lipids in very small VLDL || id:ebi-cfb233-GCST90302156' on 'ER+ Breast cancer (Combined Oncoarray; iCOGS; GWAS meta analysi

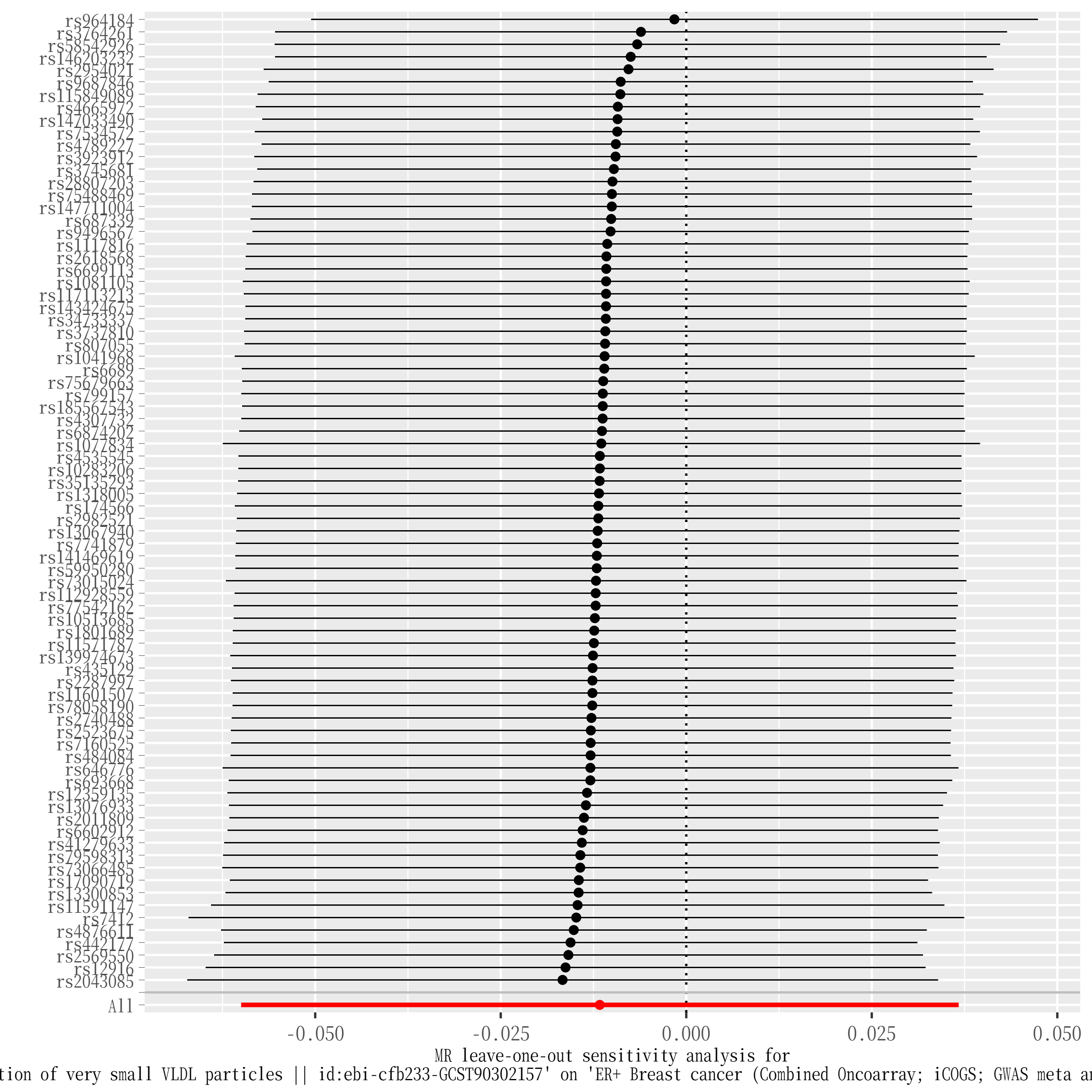

rs964184
rs3764261
rs58542926
rs146203232
rs2954021
rs9687846
rs115849089
rs4665972
rs147033490
rs7534572
rs4789227
rs3923912
rs3745681
rs28807203
rs75488469
rs147711004
rs687339
rs9496567
rs1117816
rs2618568
rs6699113
rs1081105
rs117113213
rs143424675
rs34733337
rs3737810
rs807055
rs1041968
rs6689
rs75679663
rs799157
rs185567543
rs4307732
rs6874202
rs1077834
rs4535545
rs10283206
rs35135293
rs1318005
rs174566
rs2982521
rs13067940
rs7741879
rs141469619
rs59950280
rs73015024
rs112928559
rs77542162
rs10513685
rs1801689
rs11571787
rs139974673
rs435129
rs2287997
rs11601507
rs78058190
rs2740488
rs2523675
rs7160525
rs484084
rs646776
rs693668
rs12359135
rs13076933
rs2011809
rs6602912
rs41279633
rs79598313
rs73066485
rs17090719
rs13300853
rs11591147
rs7412
rs4876611
rs442177
rs2569550
rs12916
rs2043085
All
-0.050
-0.025
0.000
0.025
0.050
MR leave-one-out sensitivity analysis for
tion of very small VLDL particles || id:ebi-cfb233-GCST90302157' on 'ER+ Breast cancer (Combined Oncoarray; iCOGS; GWAS meta an

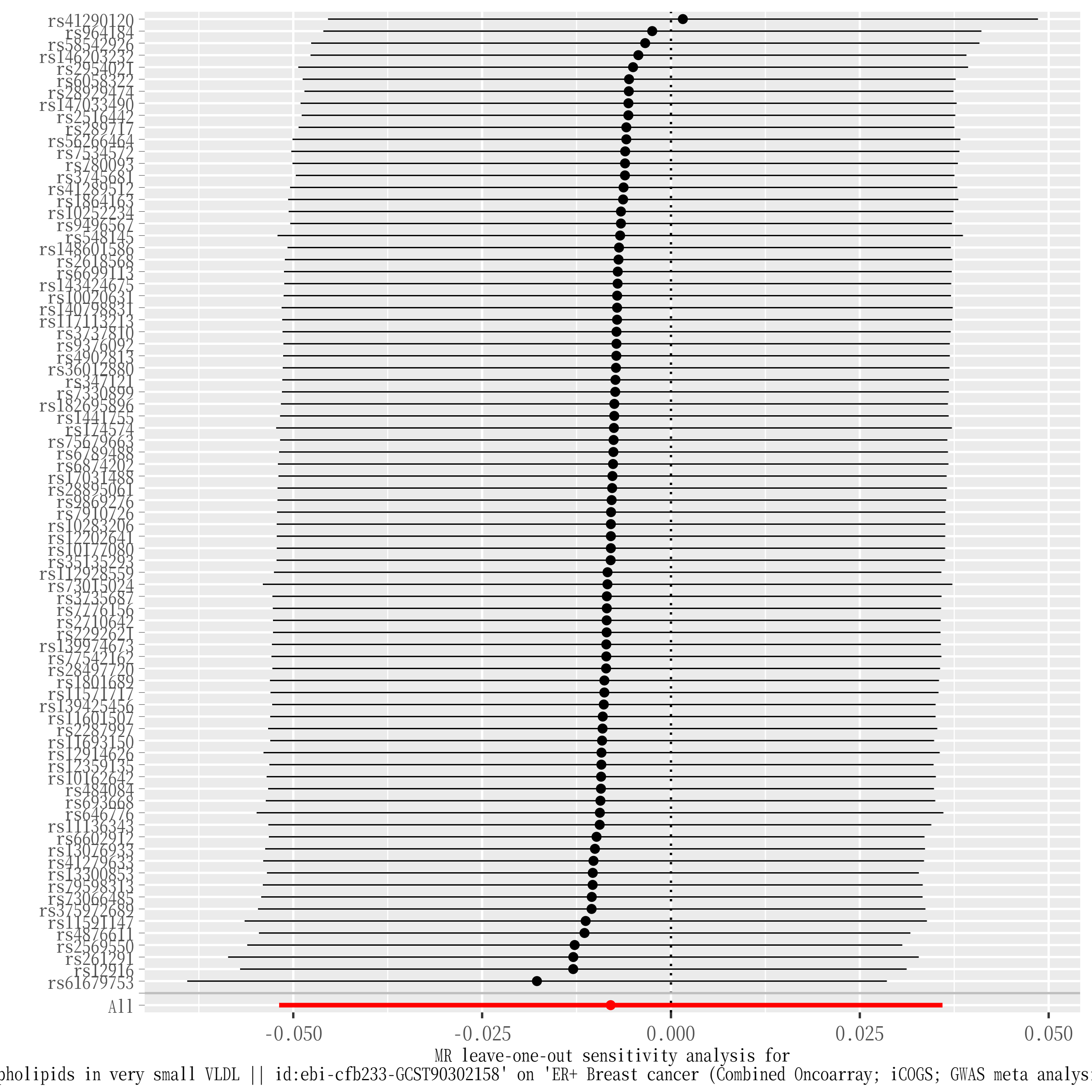
rs41290120
rs964184
rs58542926
rs146203232
rs2954021
rs6058322
rs28929474
rs147033490
rs2516442
rs289717
rs56266464
rs7534572
rs780093
rs3745681
rs41289512
rs1864163
rs10252234
rs9496567
rs548145
rs148601586
rs2618568
rs6699113
rs143424675
rs10020631
rs140798831
rs117113213
rs3737810
rs9376092
rs4902813
rs36012880
rs347121
rs7330899
rs182695826
rs1441755
rs174574
rs75679663
rs6789488
rs6874202
rs17031488
rs28895061
rs9869276
rs7910726
rs10283206
rs12202641
rs10177080
rs35135293
rs112928559
rs73015024
rs3735687
rs7776156
rs2710642
rs2292621
rs139974673
rs77542162
rs28497720
rs1801689
rs11571717
rs139425456
rs11601507
rs2287927
rs11693150
rs12914626
rs12359135
rs10162642
rs484084
rs693668
rs646776
rs11136343
rs6602912
rs13076933
rs41279633
rs13300853
rs79598313
rs73066485
rs375972682
rs115911147
rs4876611
rs2569550
rs261291
rs12916
rs61679753
All
-0.050
-0.025
0.000
0.025
0.050
MR leave-one-out sensitivity analysis for
pholipids in very small VLDL || id:ebi-cfb233-GCST90302158' on 'ER+ Breast cancer (Combined Oncoarray; iCOGS; GWAS meta analys

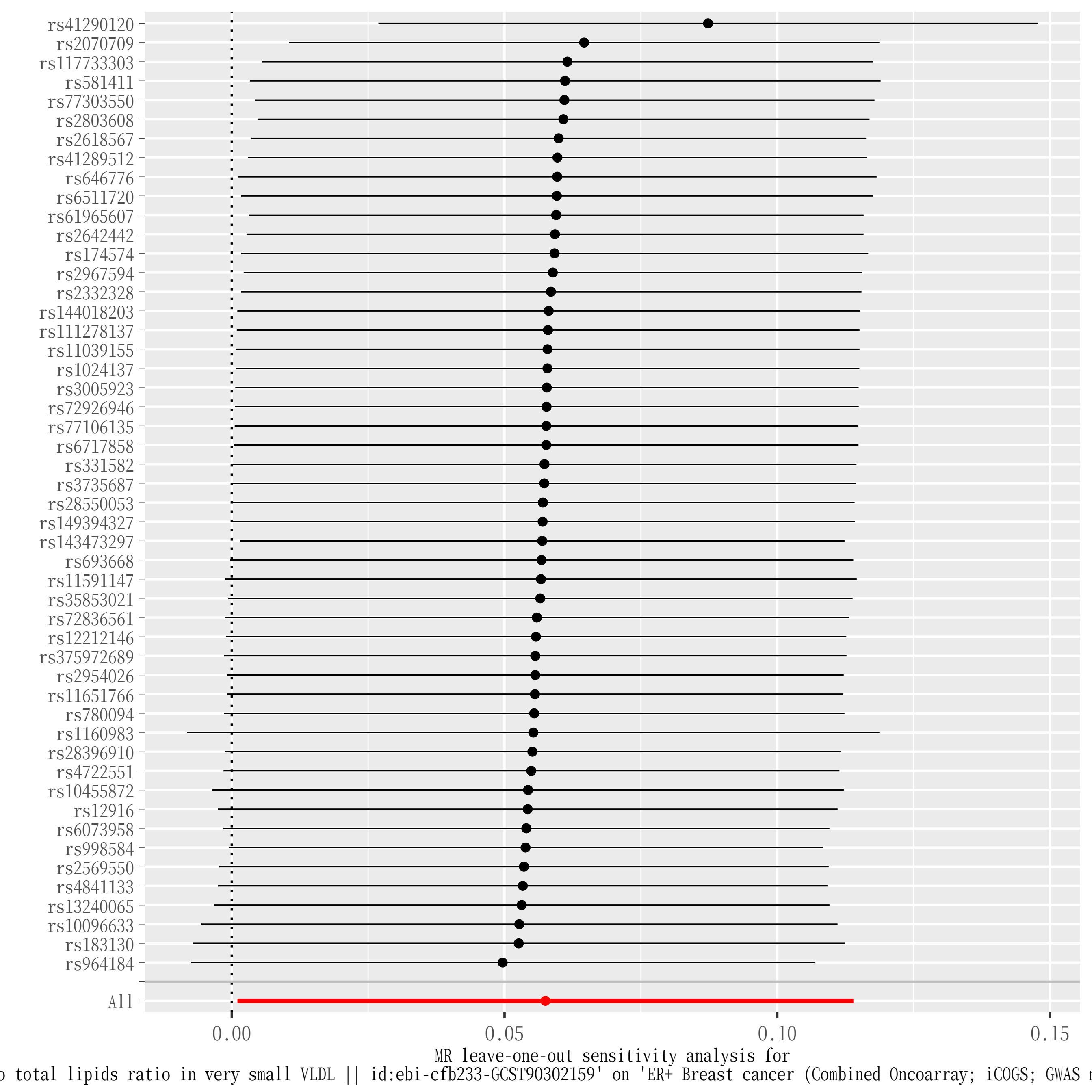
rs41290120
rs2070709
rs117733303
rs581411
rs77303550
rs2803608
rs2618567
rs41289512
rs646776
rs6511720
rs61965607
rs2642442
rs174574
rs2967594
rs2332328
rs144018203
rs111278137
rs11039155
rs1024137
rs3005923
rs72926946
rs77106135
rs6717858
rs331582
rs3735687
rs28550053
rs149394327
rs143473297
rs693668
rs11591147
rs35853021
rs72836561
rs12212146
rs375972689
rs2954026
rs11651766
rs780094
rs1160983
rs28396910
rs4722551
rs10455872
rs12916
rs6073958
rs998584
rs2569550
rs4841133
rs13240065
rs10096633
rs183130
rs964184
All
0.00
0.05
0.10
0.15
MR leave-one-out sensitivity analysis for
o total lipids ratio in very small VLDL || id:ebi-cfb233-GCST90302159' on 'ER+ Breast cancer (Combined Oncoarray; iCOGS; GWAS m

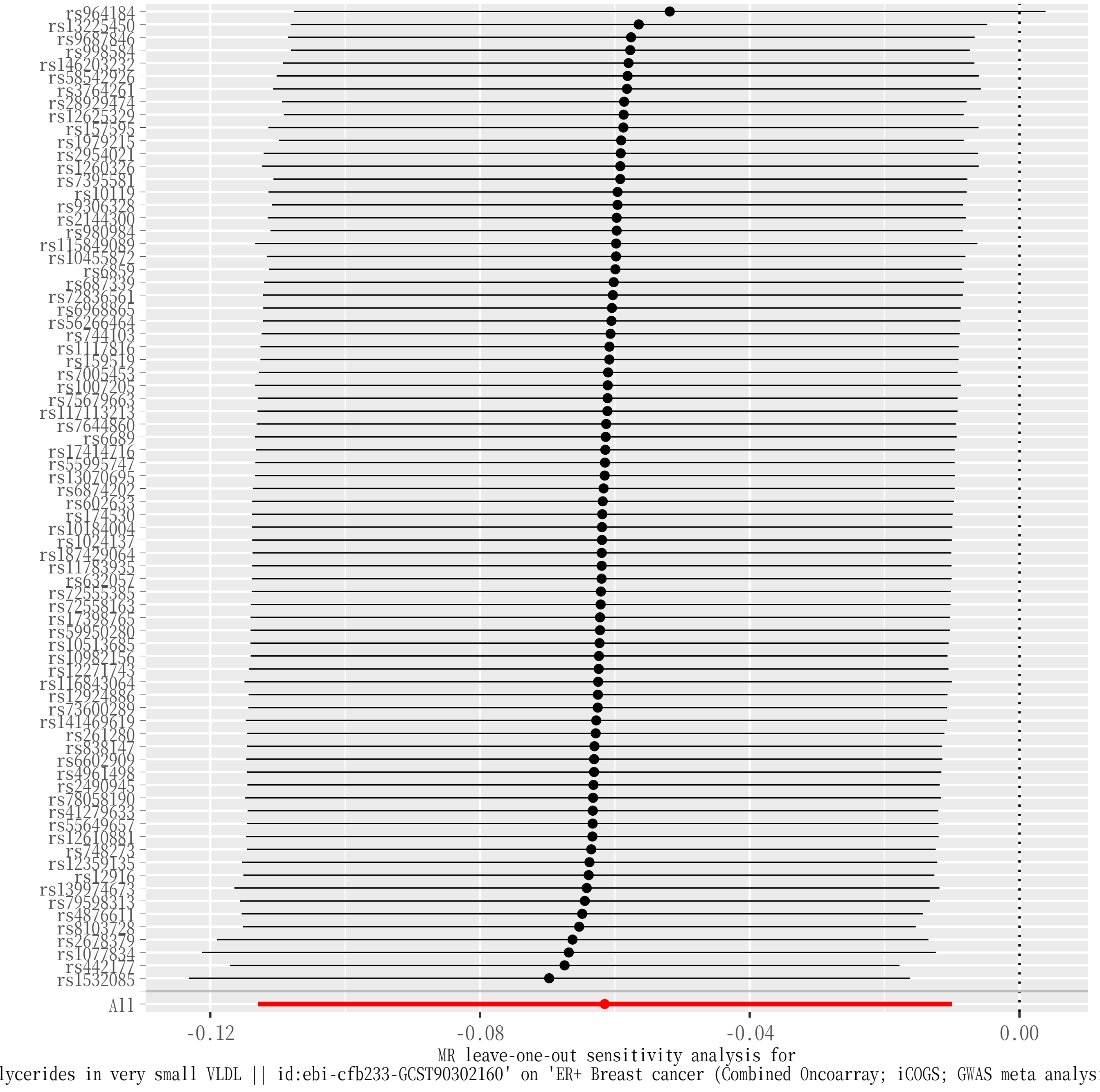
rs964184
rs13225450
rs9687846
rs998584
rs146203232
rs58542926
rs3764261
rs28929474
rs12625329
rs157595
rs1979215
rs2954021
rs1260326
rs7395581
rs10119
rs9306328
rs2144300
rs980984
rs115849089
rs10455872
rs6859
rs687339
rs72836561
rs6968865
rs56266464
rs744103
rs1117816
rs159519
rs7005453
rs1007205
rs75679663
rs117113213
rs7644860
rs6689
rs17414716
rs55995747
rs13070695
rs6874202
rs602633
rs174530
rs10184004
rs1024137
rs187429064
rs11783935
rs632057
rs72555385
rs72558163
rs17398765
rs59950280
rs10513685
rs10982156
rs12271743
rs116843064
rs12924886
rs73600289
rs141469619
rs261280
rs838147
rs6602909
rs4961498
rs2490945
rs78058190
rs41279633
rs55649657
rs12610881
rs748273
rs12359135
rs12916
rs139974673
rs79598313
rs4876611
rs8103728
rs2678379
rs1077834
rs442177
rs1532085
All
-0.12
-0.08
-0.04
0.00
MR leave-one-out sensitivity analysis for
lycerides in very small VLDL || id:ebi-cfb233-GCST90302160' on 'ER+ Breast cancer (Combined Oncoarray; iCOGS; GWAS meta analys

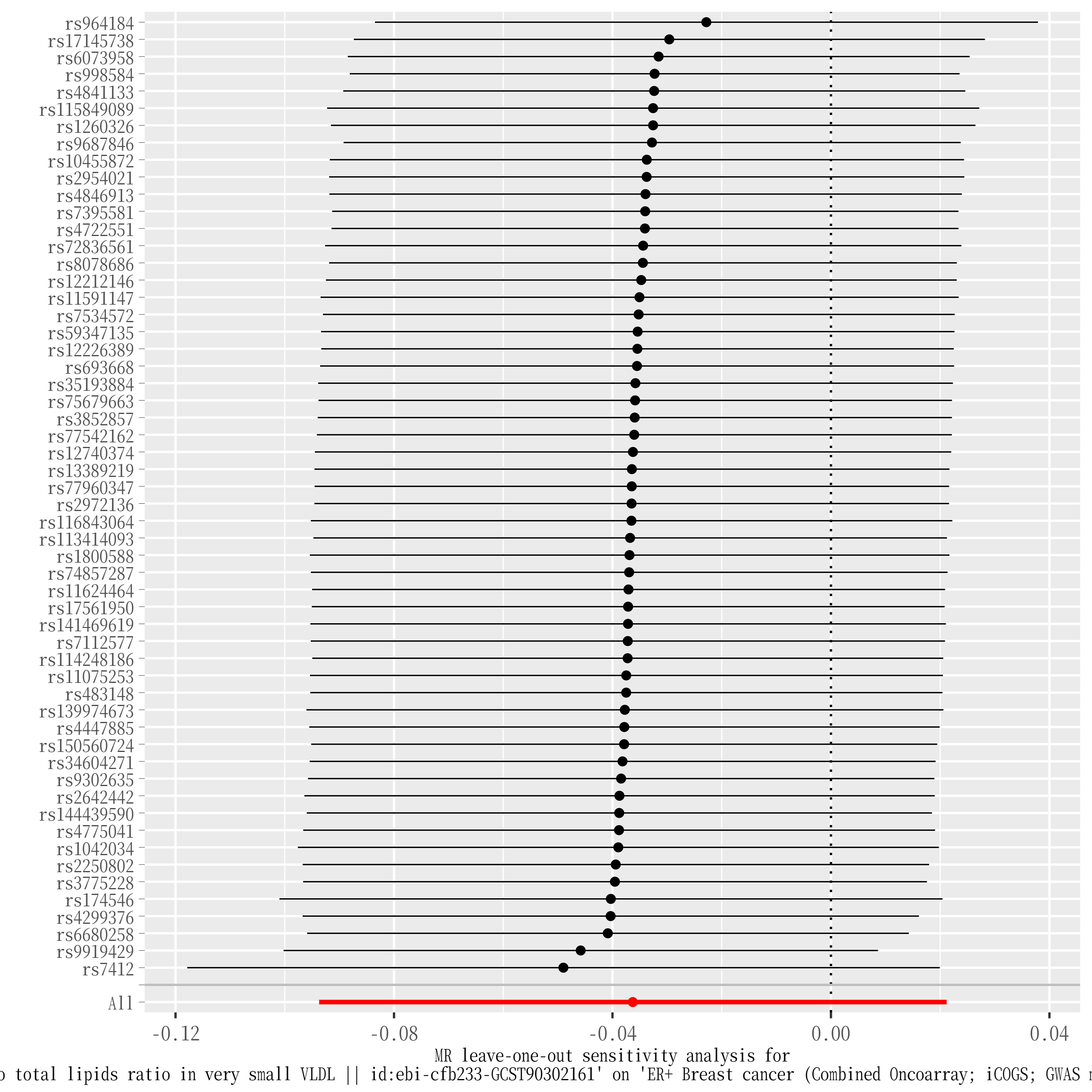

rs964184
rs17145738
rs6073958
rs998584
rs4841133
rs115849089
rs1260326
rs9687846
rs10455872
rs2954021
rs4846913
rs7395581
rs4722551
rs72836561
rs8078686
rs12212146
rs11591147
rs7534572
rs59347135
rs12226389
rs693668
rs35193884
rs75679663
rs3852857
rs77542162
rs12740374
rs13389219
rs77960347
rs2972136
rs116843064
rs113414093
rs1800588
rs74857287
rs11624464
rs17561950
rs141469619
rs7112577
rs114248186
rs11075253
rs483148
rs139974673
rs4447885
rs150560724
rs34604271
rs9302635
rs2642442
rs144439590
rs4775041
rs1042034
rs2250802
rs3775228
rs174546
rs4299376
rs6680258
rs9919429
rs7412
All
-0.12
-0.08
-0.04
0.00
0.04
MR leave-one-out sensitivity analysis for
o total lipids ratio in very small VLDL || id:ebi-cfb233-GCST90302161' on 'ER+ Breast cancer (Combined Oncoarray; iCOGS; GWAS m

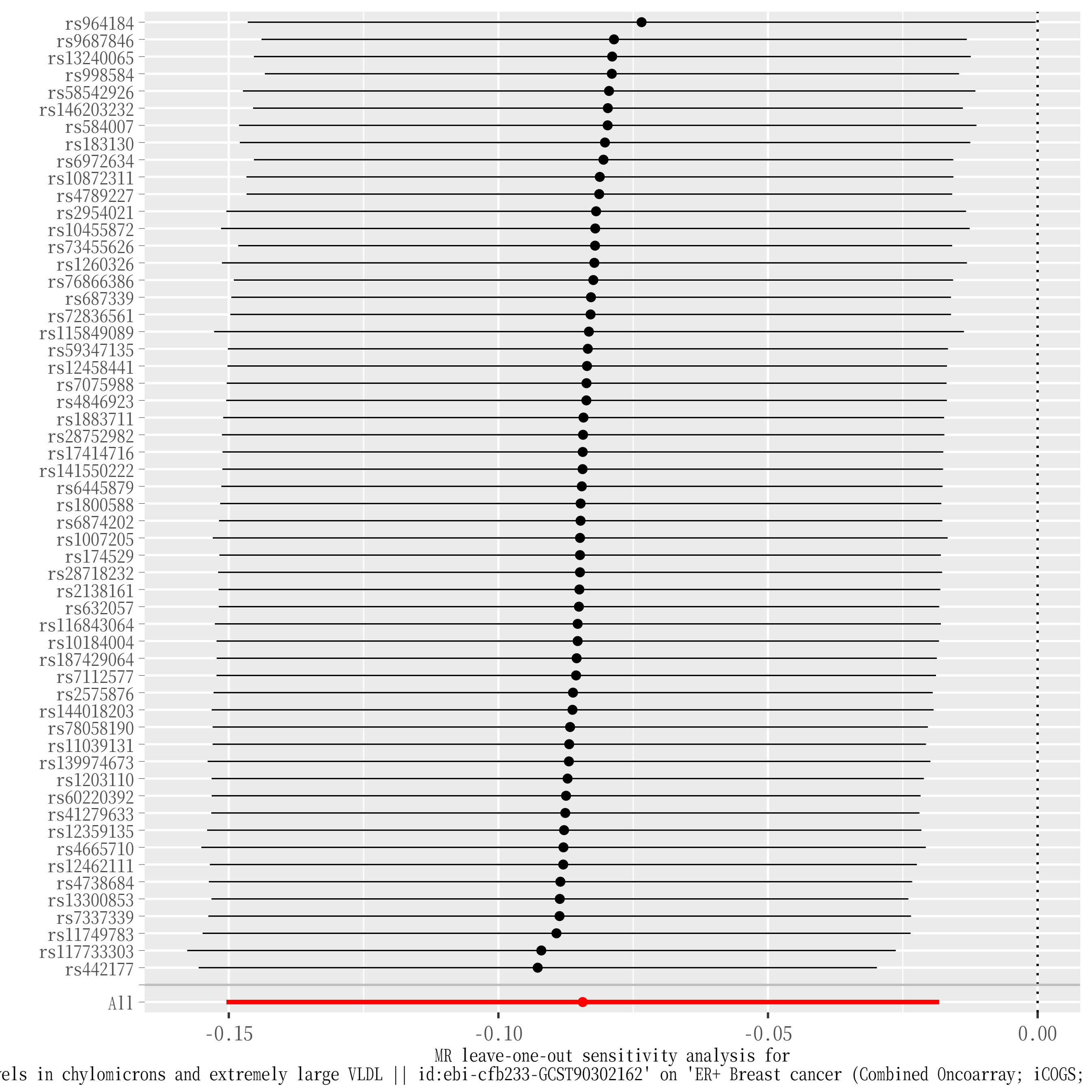
rs964184
rs9687846
rs13240065
rs998584
rs58542926
rs146203232
rs584007
rs183130
rs6972634
rs10872311
rs4789227
rs2954021
rs10455872
rs73455626
rs1260326
rs76866386
rs687339
rs72836561
rs115849089
rs59347135
rs12458441
rs7075988
rs4846923
rs1883711
rs28752982
rs17414716
rs141550222
rs6445879
rs1800588
rs6874202
rs1007205
rs174529
rs28718232
rs2138161
rs632057
rs116843064
rs10184004
rs187429064
rs7112577
rs2575876
rs144018203
rs78058190
rs11039131
rs139974673
rs1203110
rs60220392
rs41279633
rs12359135
rs4665710
rs12462111
rs4738684
rs13300853
rs7337339
rs11749783
rs117733303
rs442177
All
-0.15
-0.10
-0.05
0.00
MR leave-one-out sensitivity analysis for
els in chylomicrons and extremely large VLDL || id:ebi-cfb233-GCST90302162' on 'ER+ Breast cancer (Combined Oncoarray; iCOGS;

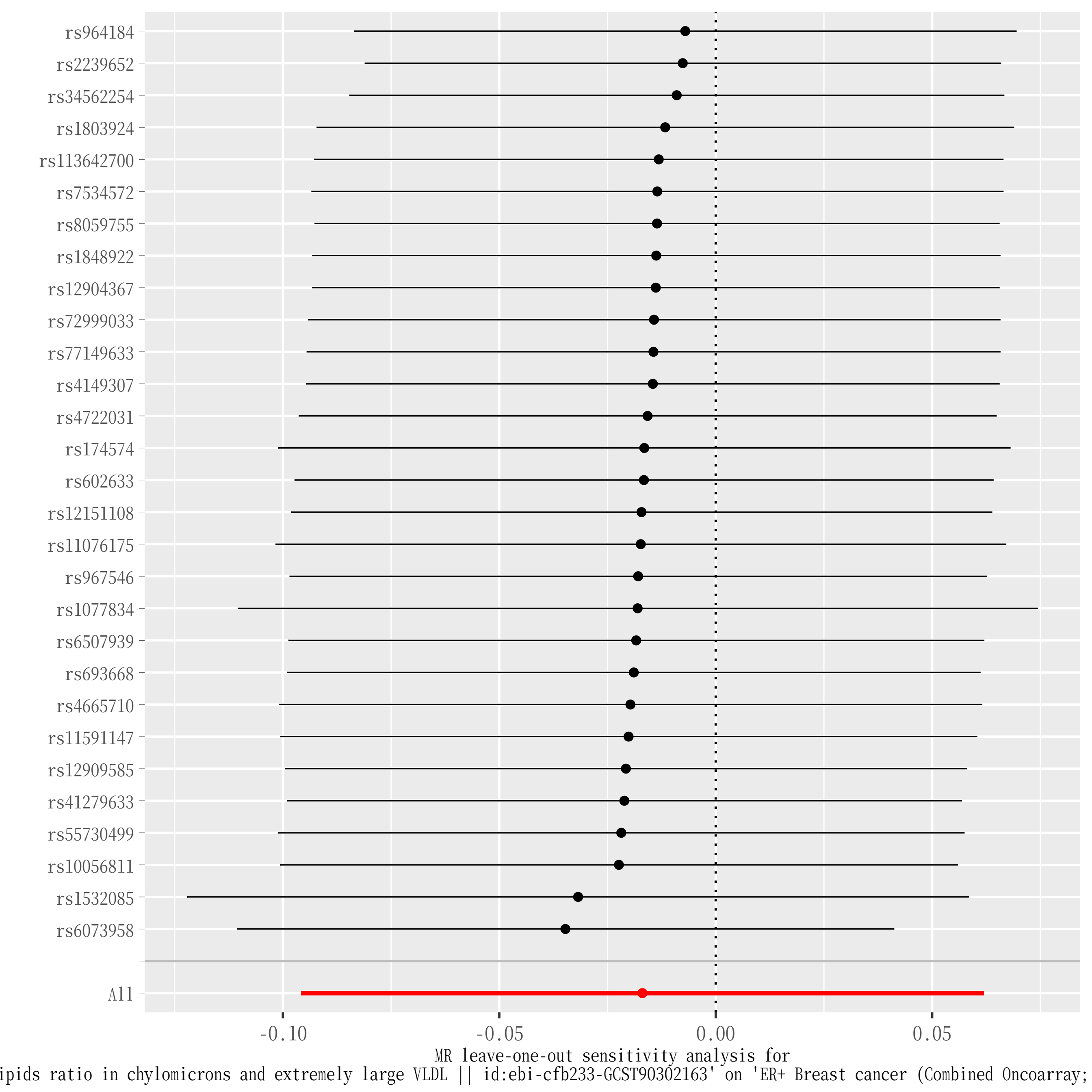
rs964184
rs2239652
rs34562254
rs1803924
rs113642700
rs7534572
rs8059755
rs1848922
rs12904367
rs72999033
rs77149633
rs4149307
rs4722031
rs174574
rs602633
rs12151108
rs11076175
rs967546
rs1077834
rs6507939
rs693668
rs4665710
rs11591147
rs12909585
rs41279633
rs55730499
rs10056811
rs1532085
rs6073958
All
-0.10
-0.05
0.00
0.05
MR leave-one-out sensitivity analysis for
ipids ratio in chylomicrons and extremely large VLDL || id:ebi-cfb233-GCST90302163' on 'ER+ Breast cancer (Combined Oncoarray;

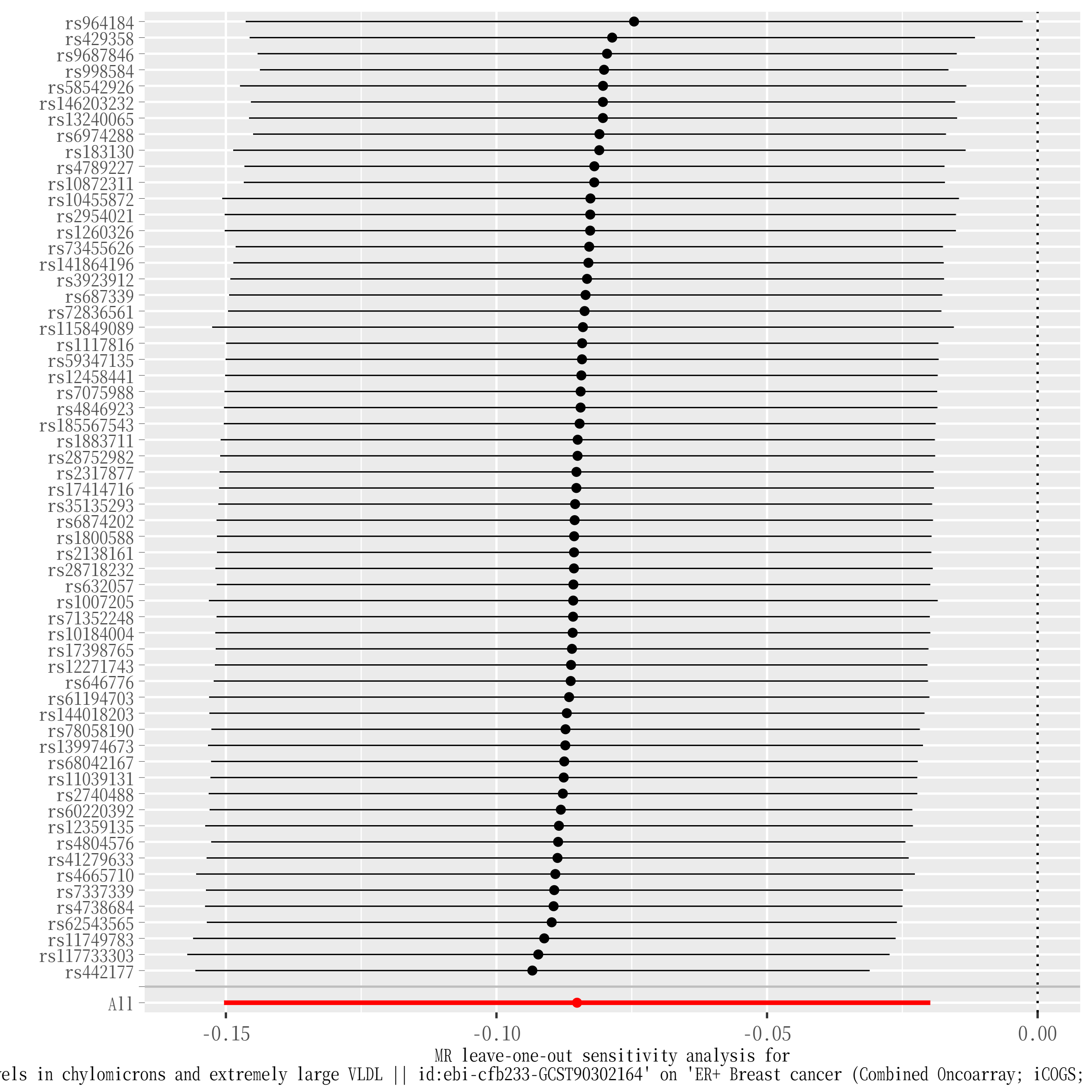
rs964184
rs429358
rs9687846
rs998584
rs58542926
rs146203232
rs13240065
rs6974288
rs183130
rs4789227
rs10872311
rs10455872
rs2954021
rs1260326
rs73455626
rs141864196
rs3923912
rs687339
rs72836561
rs115849089
rs1117816
rs59347135
rs12458441
rs7075988
rs4846923
rs185567543
rs1883711
rs28752982
rs2317877
rs17414716
rs35135293
rs6874202
rs1800588
rs2138161
rs28718232
rs632057
rs1007205
rs71352248
rs10184004
rs17398765
rs12271743
rs646776
rs61194703
rs144018203
rs78058190
rs139974673
rs68042167
rs11039131
rs2740488
rs60220392
rs12359135
rs4804576
rs41279633
rs4665710
rs7337339
rs4738684
rs62543565
rs11749783
rs117733303
rs442177
All
-0.15
-0.10
-0.05
0.00
MR leave-one-out sensitivity analysis for
rels in chylomicrons and extremely large VLDL || id:ebi-cfb233-GCST90302164' on 'ER+ Breast cancer (Combined Oncoarray; iCOGS;

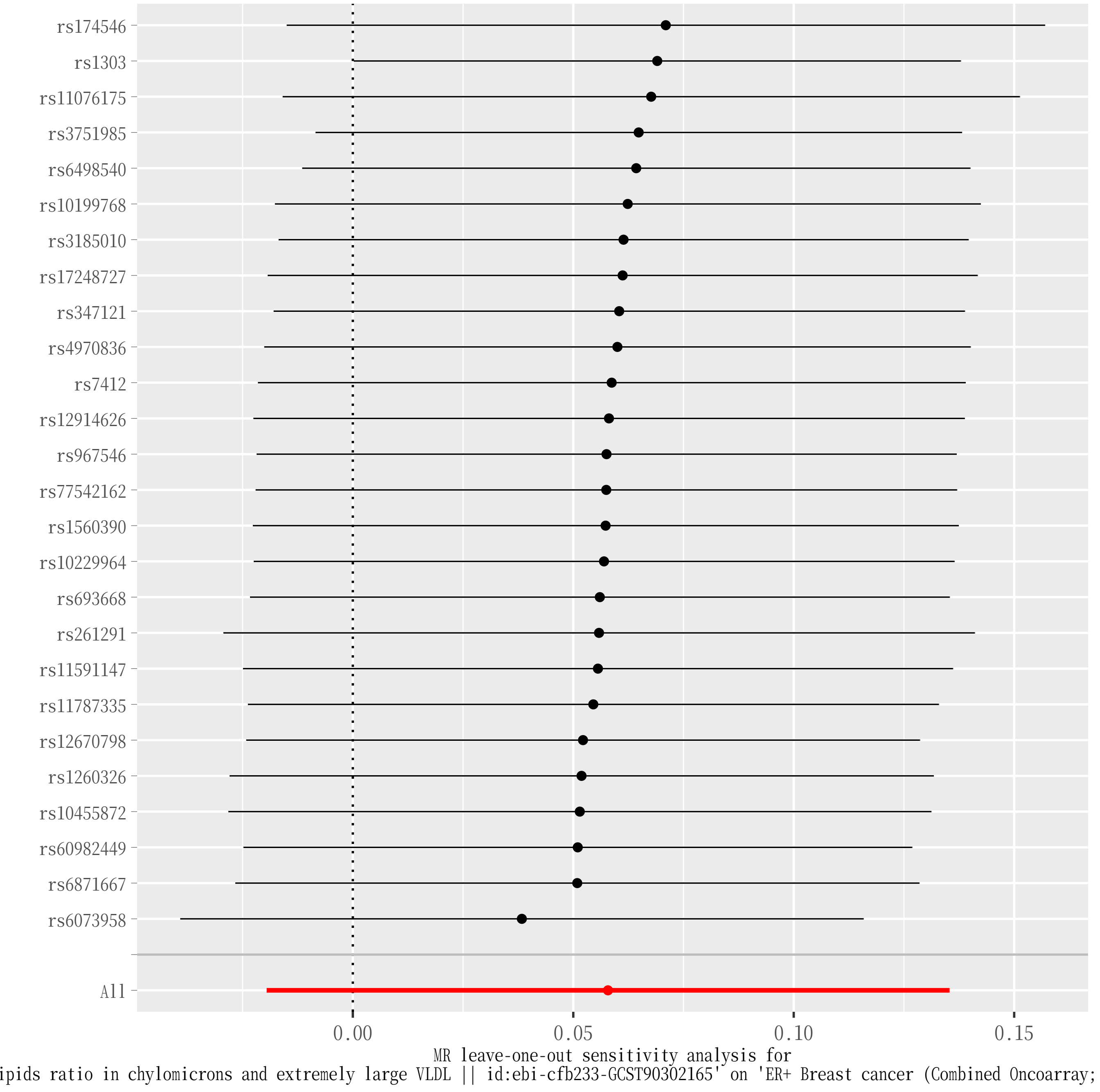
rs174546
rs1303
rs11076175
rs3751985
rs6498540
rs10199768
rs3185010
rs17248727
rs347121
rs4970836
rs7412
rs12914626
rs967546
rs77542162
rs1560390
rs10229964
rs693668
rs261291
rs11591147
rs11787335
rs12670798
rs1260326
rs10455872
rs60982449
rs6871667
rs6073958
All
0.00
0.05
0.10
0.15
MR leave-one-out sensitivity analysis for
ipids ratio in chylomicrons and extremely large VLDL || id:ebi-cfb233-GCST90302165' on 'ER+ Breast cancer (Combined Oncoarray;

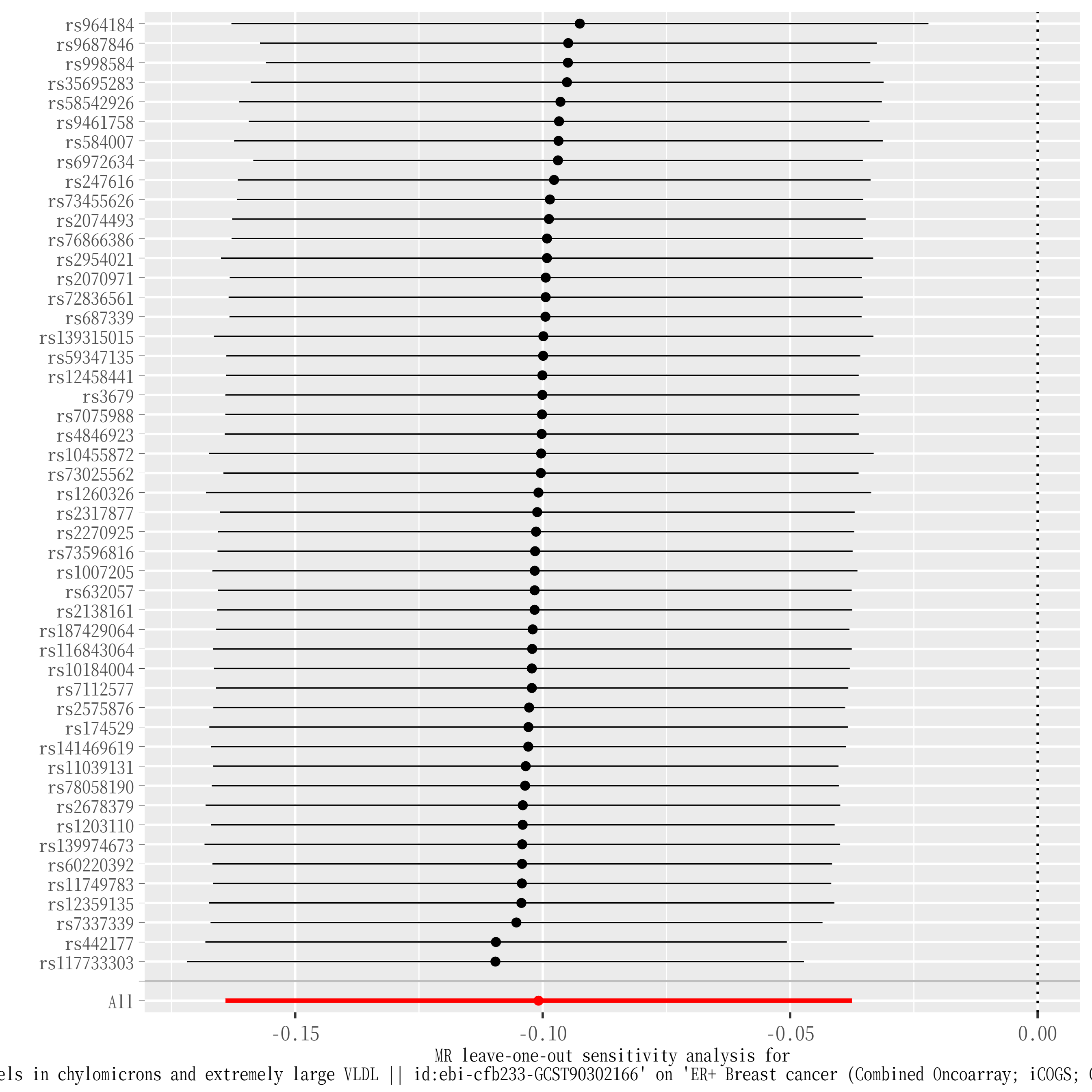
rs964184
rs9687846
rs998584
rs35695283
rs58542926
rs9461758
rs584007
rs6972634
rs247616
rs73455626
rs2074493
rs76866386
rs2954021
rs2070971
rs72836561
rs687339
rs139315015
rs59347135
rs12458441
rs3679
rs7075988
rs4846923
rs10455872
rs73025562
rs1260326
rs2317877
rs2270925
rs73596816
rs1007205
rs632057
rs2138161
rs187429064
rs116843064
rs10184004
rs7112577
rs2575876
rs174529
rs141469619
rs11039131
rs78058190
rs2678379
rs1203110
rs139974673
rs60220392
rs11749783
rs12359135
rs7337339
rs442177
rs117733303
All
-0.15
-0.10
-0.05
0.00
MR leave-one-out sensitivity analysis for
els in chylomicrons and extremely large VLDL || id:ebi-cfb233-GCST90302166' on 'ER+ Breast cancer (Combined Oncoarray; iCOGS; G

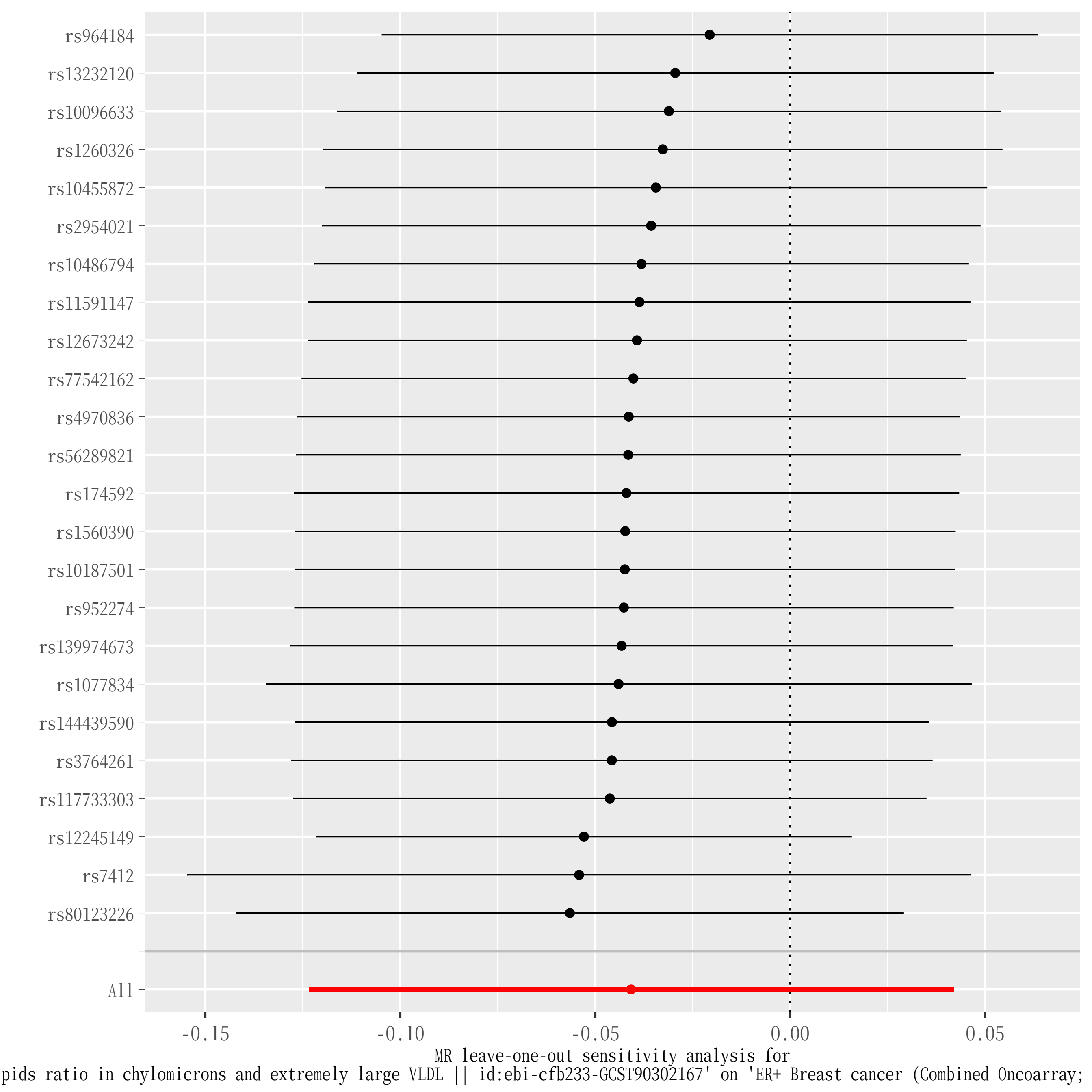
rs964184
rs13232120
rs10096633
rs1260326
rs10455872
rs2954021
rs10486794
rs11591147
rs12673242
rs77542162
rs4970836
rs56289821
rs174592
rs1560390
rs10187501
rs952274
rs139974673
rs1077834
rs144439590
rs3764261
rs117733303
rs12245149
rs7412
rs80123226
All
-0.15
-0.10
-0.05
0.00
0.05
MR leave-one-out sensitivity analysis for
pids ratio in chylomicrons and extremely large VLDL || id:ebi-cfb233-GCST90302167' on 'ER+ Breast cancer (Combined Oncoarray;

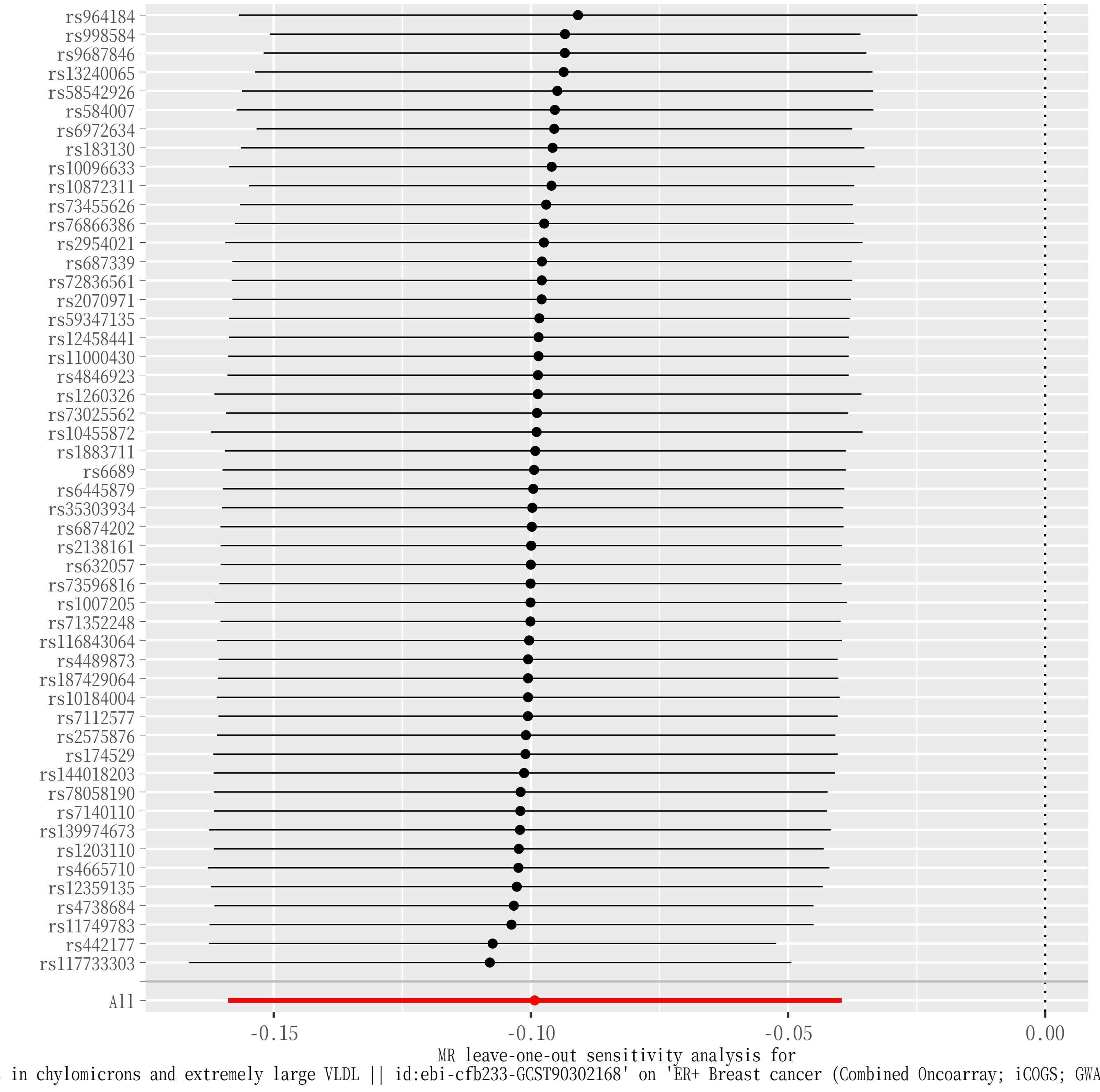
rs964184
rs998584
rs9687846
rs13240065
rs58542926
rs584007
rs6972634
rs183130
rs10096633
rs10872311
rs73455626
rs76866386
rs2954021
rs687339
rs72836561
rs2070971
rs59347135
rs12458441
rs11000430
rs4846923
rs1260326
rs73025562
rs10455872
rs1883711
rs6689
rs6445879
rs35303934
rs6874202
rs2138161
rs632057
rs73596816
rs1007205
rs71352248
rs116843064
rs4489873
rs187429064
rs10184004
rs7112577
rs2575876
rs174529
rs144018203
rs78058190
rs7140110
rs139974673
rs1203110
rs4665710
rs12359135
rs4738684
rs11749783
rs442177
rs117733303
All
-0.15
-0.10
-0.05
0.00
MR leave-one-out sensitivity analysis for
in chylomicrons and extremely large VLDL || id:ebi-cfb233-GCST90302168' on 'ER+ Breast cancer (Combined Oncoarray; iCOGS; GWA

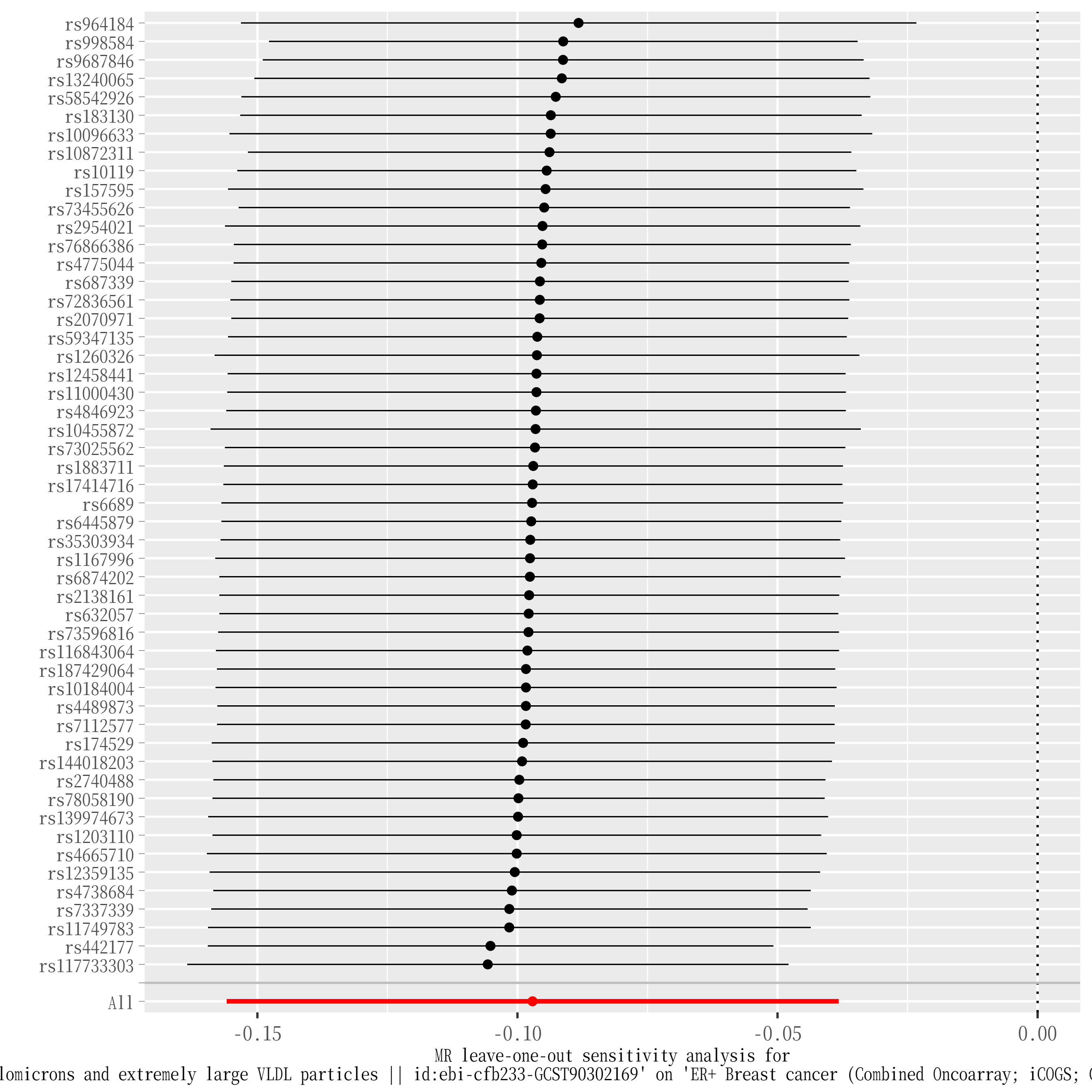

rs964184
rs998584
rs9687846
rs13240065
rs58542926
rs183130
rs10096633
rs10872311
rs10119
rs157595
rs73455626
rs2954021
rs76866386
rs4775044
rs687339
rs72836561
rs2070971
rs59347135
rs1260326
rs12458441
rs11000430
rs4846923
rs10455872
rs73025562
rs1883711
rs17414716
rs6689
rs6445879
rs35303934
rs1167996
rs6874202
rs2138161
rs632057
rs73596816
rs116843064
rs187429064
rs10184004
rs4489873
rs7112577
rs174529
rs144018203
rs2740488
rs78058190
rs139974673
rs1203110
rs4665710
rs12359135
rs4738684
rs7337339
rs11749783
rs442177
rs117733303
All
-0.15
-0.10
-0.05
0.00
MR leave-one-out sensitivity analysis for
lomicrons and extremely large VLDL particles || id:ebi-cfb233-GCST90302169' on 'ER+ Breast cancer (Combined Oncoarray; iCOGS; (

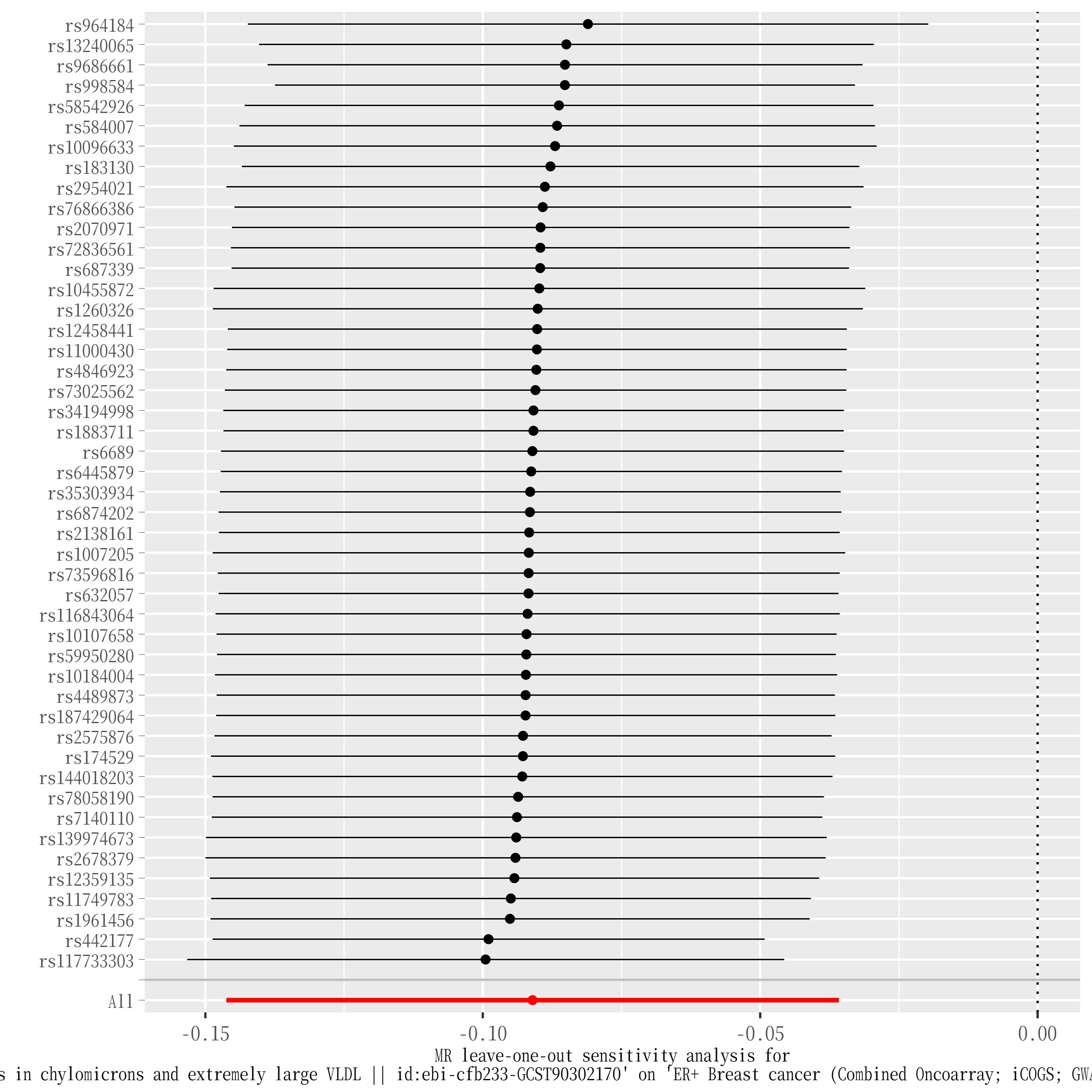
rs964184
rs13240065
rs9686661
rs998584
rs58542926
rs584007
rs10096633
rs183130
rs2954021
rs76866386
rs2070971
rs72836561
rs687339
rs10455872
rs1260326
rs12458441
rs11000430
rs4846923
rs73025562
rs34194998
rs1883711
rs6689
rs6445879
rs35303934
rs6874202
rs2138161
rs1007205
rs73596816
rs632057
rs116843064
rs10107658
rs59950280
rs10184004
rs4489873
rs187429064
rs2575876
rs174529
rs144018203
rs78058190
rs7140110
rs139974673
rs2678379
rs12359135
rs11749783
rs1961456
rs442177
rs117733303
All
-0.15
-0.10
-0.05
0.00
MR leave-one-out sensitivity analysis for
s in chylomicrons and extremely large VLDL || id:ebi-cfb233-GCST90302170' on 'ER+ Breast cancer (Combined Oncoarray; iCOGS; GW

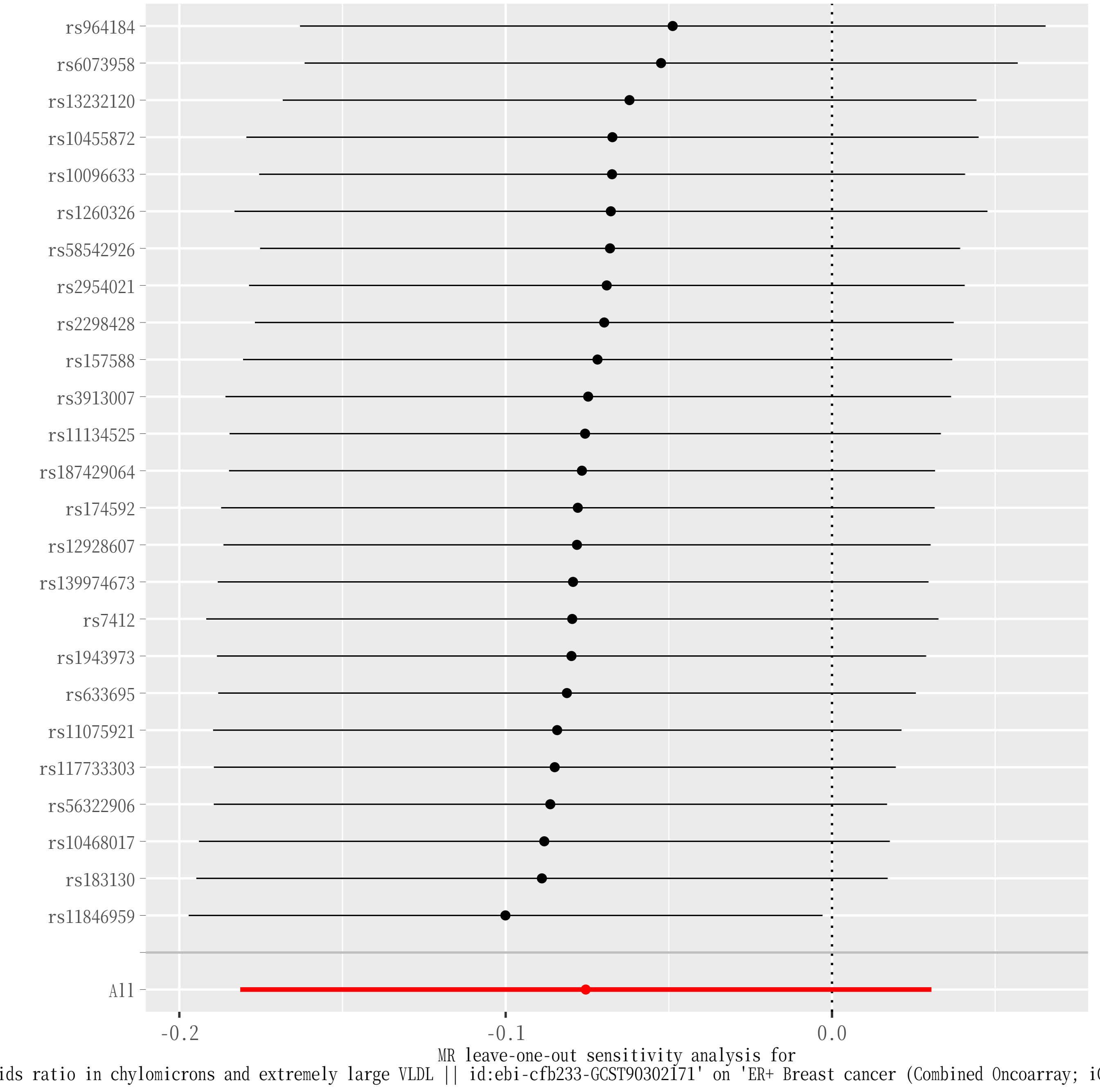
rs964184
rs6073958
rs13232120
rs10455872
rs10096633
rs1260326
rs58542926
rs2954021
rs2298428
rs157588
rs3913007
rs11134525
rs187429064
rs174592
rs12928607
rs139974673
rs7412
rs1943973
rs633695
rs11075921
rs117733303
rs56322906
rs10468017
rs183130
rs11846959
All
-0.2
-0.1
0.0
MR leave-one-out sensitivity analysis for
ids ratio in chylomicrons and extremely large VLDL || id:ebi-cfb233-GCST90302171' on 'ER+ Breast cancer (Combined Oncoarray; i

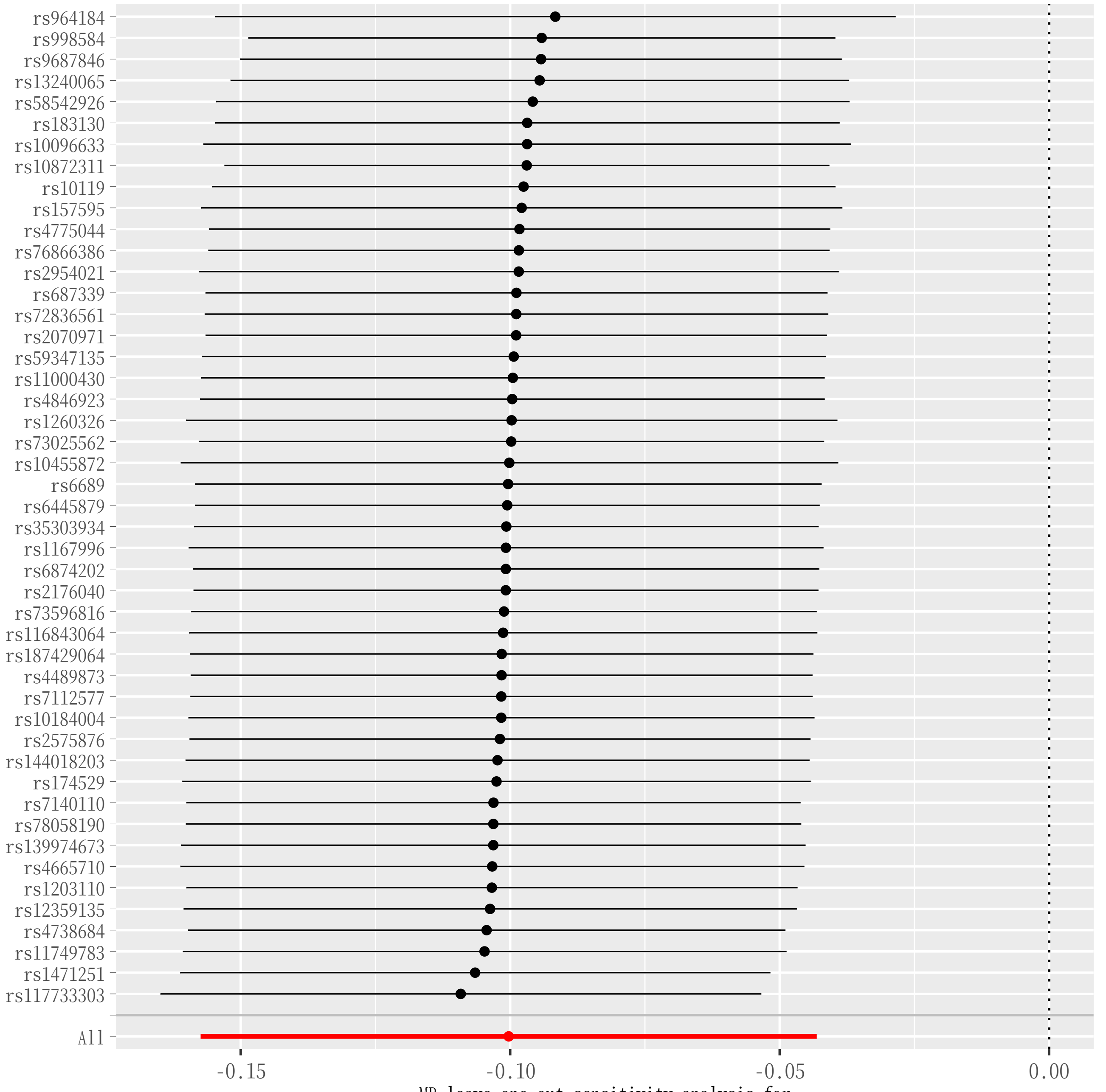

MR leave-one-out sensitivity analysis for
s in chylomicrons and extremely large VLDL || id:ebi-cfb233-GCST90302172' on 'ER+ Breast cancer (Combined Oncoarray; iCOGS; GW

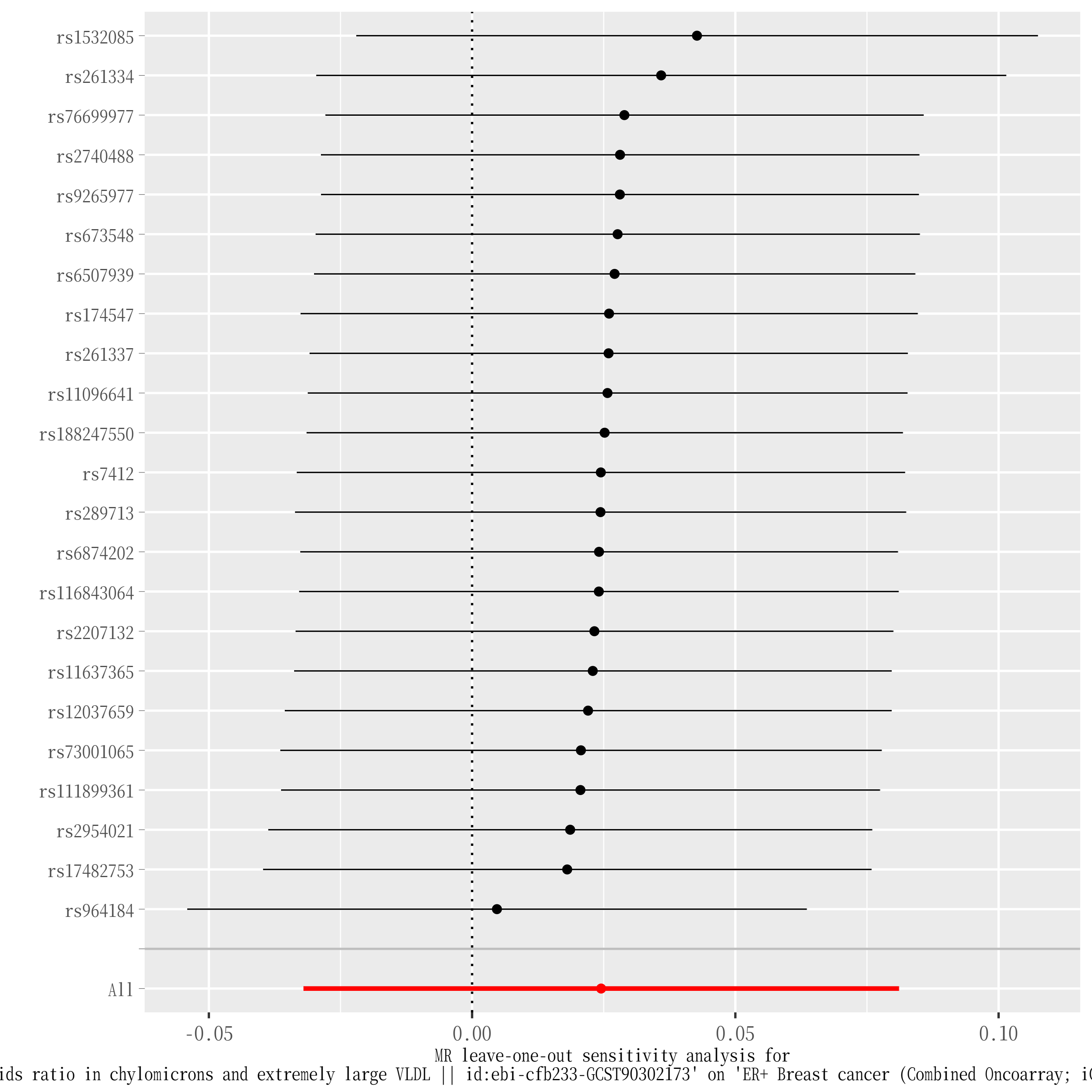
rs1532085
rs261334
rs76699977
rs2740488
rs9265977
rs673548
rs6507939
rs174547
rs261337
rs11096641
rs188247550
rs7412
rs289713
rs6874202
rs116843064
rs2207132
rs11637365
rs12037659
rs73001065
rs111899361
rs2954021
rs17482753
rs964184
All
-0.05
0.00
0.05
0.10
MR leave-one-out sensitivity analysis for
ids ratio in chylomicrons and extremely large VLDL || id:ebi-cfb233-GCST90302173' on 'ER+ Breast cancer (Combined Oncoarray; iC
