# Supplementary material for: Assessing the causal relationships between circulating metabolic biomarkers and breast cancer by using mendelian randomization
Source: Front Genet. 2024 Dec 18;15:1448748. doi: 10.3389/fgene.2024.1448748 (PMC11688392; doi:10.3389/fgene.2024.1448748)
Supplement: Supplementary file 13 [file DataSheet12.pdf]

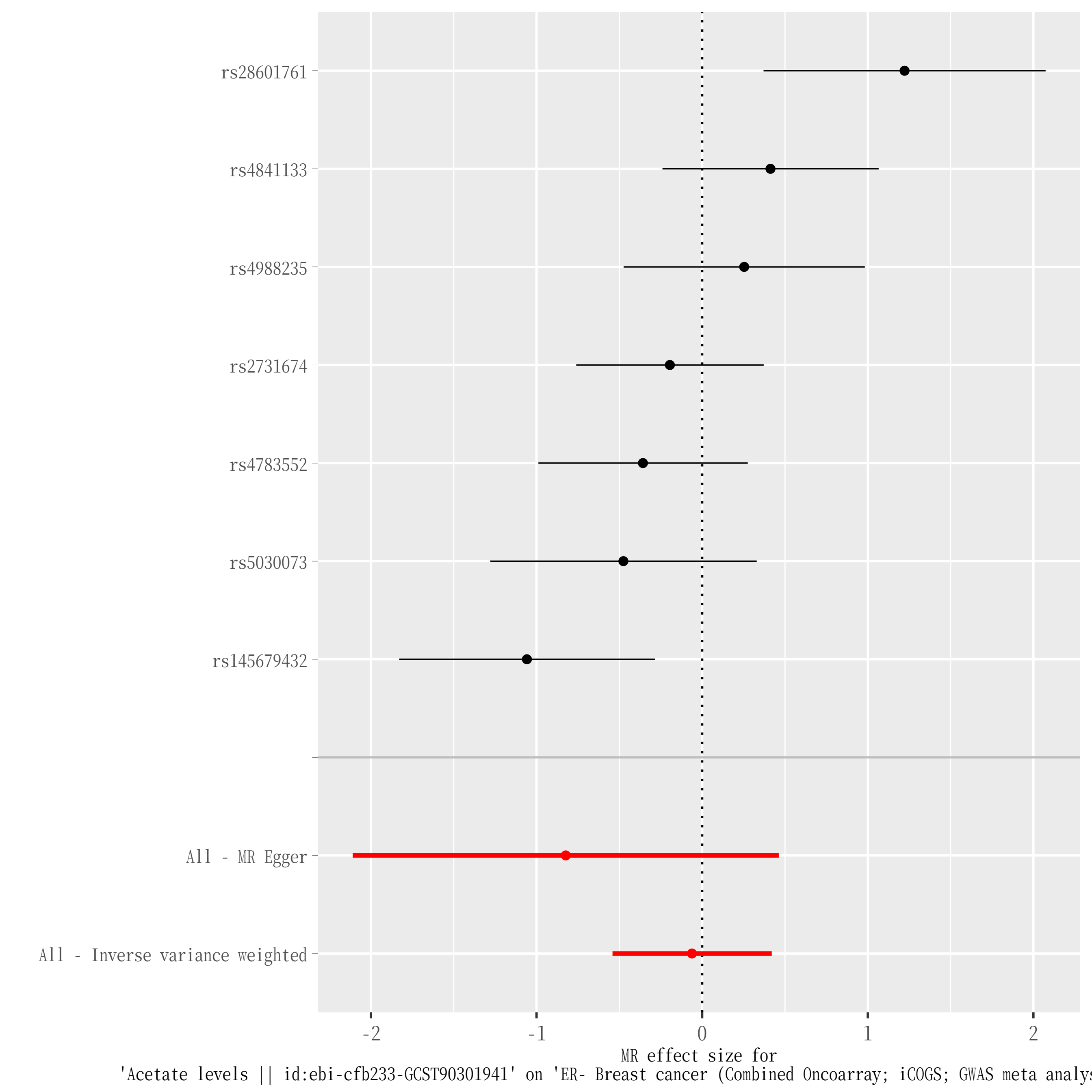

rs28601761
rs4841133
rs4988235
rs2731674
rs4783552
rs5030073
rs145679432
All - MR Egger
All - Inverse variance weighted
-2
-1
0
1
2
MR effect size for
'Acetate levels || id:ebi-cfb233-GCST90301941' on 'ER- Breast cancer (Combined Oncoarray; iCOGS; GWAS meta analys

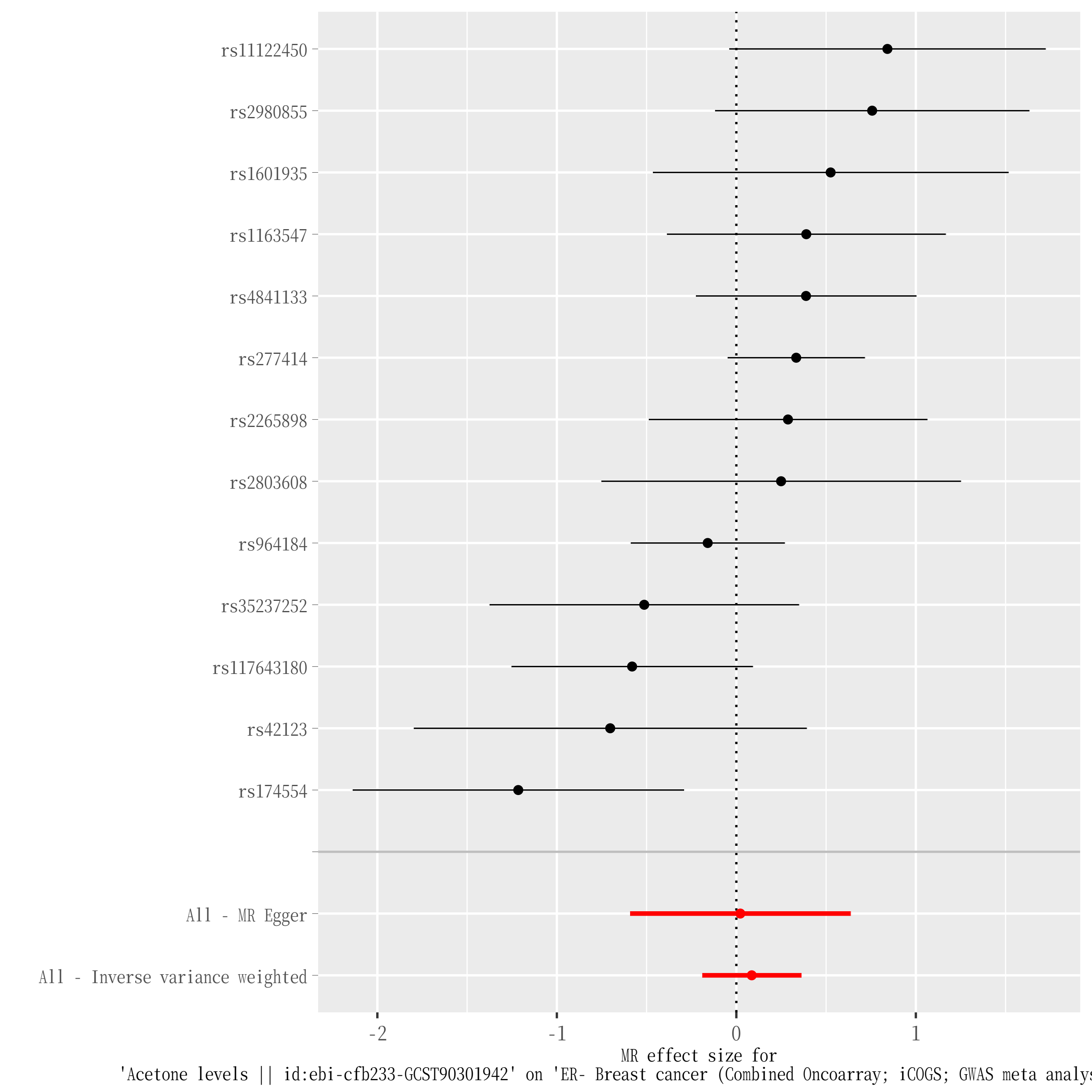
rs11122450
rs2980855
rs1601935
rs1163547
rs4841133
rs277414
rs2265898
rs2803608
rs964184
rs35237252
rs117643180
rs42123
rs174554
All - MR Egger
All - Inverse variance weighted
-2
-1
0
1
MR effect size for
'Acetone levels || id:ebi-cfb233-GCST90301942' on 'ER- Breast cancer (Combined Oncoarray; iCOGS; GWAS meta analys

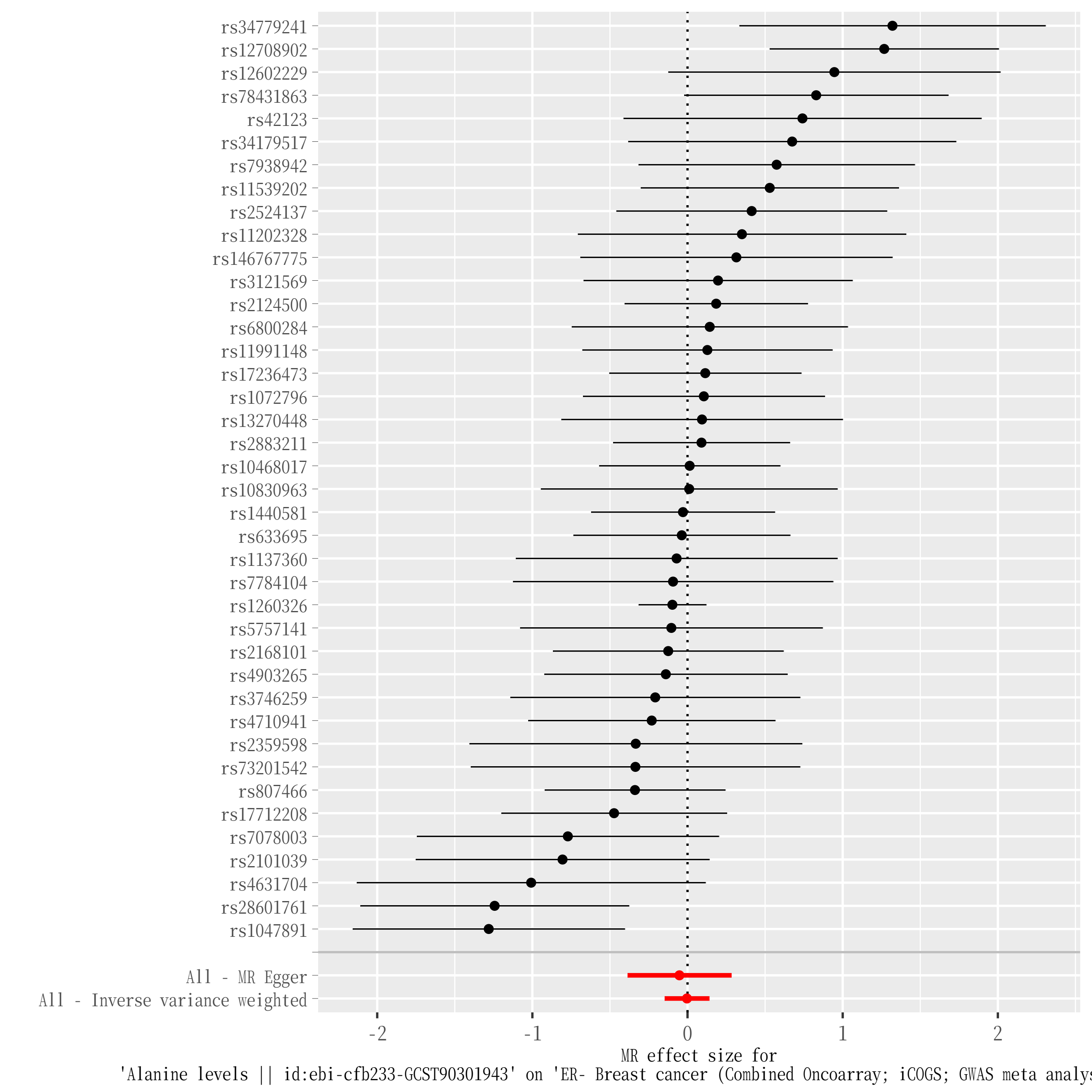
rs34779241
rs12708902
rs12602229
rs78431863
rs42123
rs34179517
rs7938942
rs11539202
rs2524137
rs11202328
rs146767775
rs3121569
rs2124500
rs6800284
rs11991148
rs17236473
rs1072796
rs13270448
rs2883211
rs10468017
rs10830963
rs1440581
rs633695
rs1137360
rs7784104
rs1260326
rs5757141
rs2168101
rs4903265
rs3746259
rs4710941
rs2359598
rs73201542
rs807466
rs17712208
rs7078003
rs2101039
rs4631704
rs28601761
rs1047891
All - MR Egger
All - Inverse variance weighted
-2
-1
0
1
2
MR effect size for
'Alanine levels || id:ebi-cfb233-GCST90301943' on 'ER- Breast cancer (Combined Oncoarray; iCOGS; GWAS meta analys

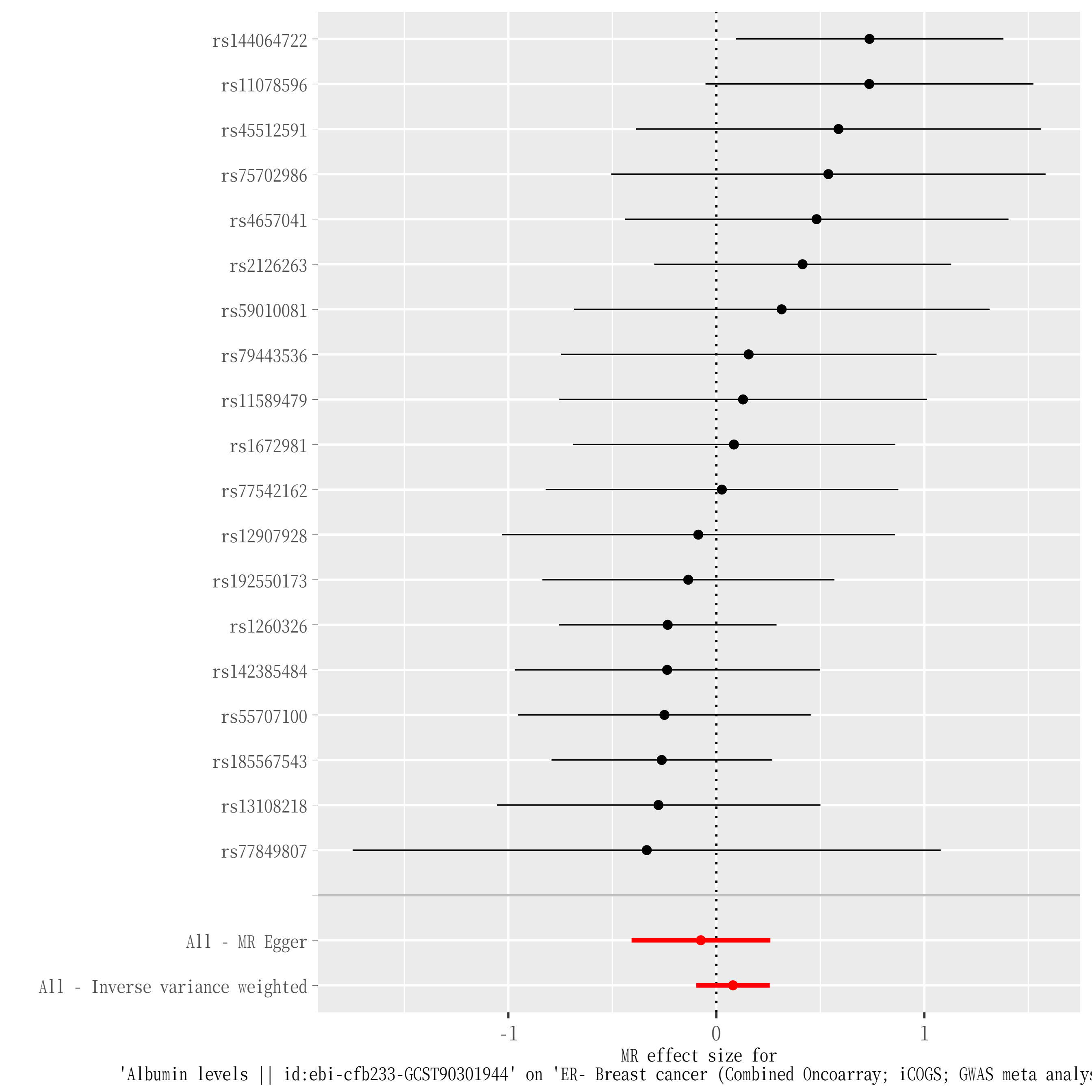

rs144064722
rs11078596
rs45512591
rs75702986
rs4657041
rs2126263
rs59010081
rs79443536
rs11589479
rs1672981
rs77542162
rs12907928
rs192550173
rs1260326
rs142385484
rs55707100
rs185567543
rs13108218
rs77849807
All - MR Egger
All - Inverse variance weighted
-1
0
1
MR effect size for
'Albumin levels || id:ebi-cfb233-GCST90301944' on 'ER- Breast cancer (Combined Oncoarray; iCOGS; GWAS meta analys

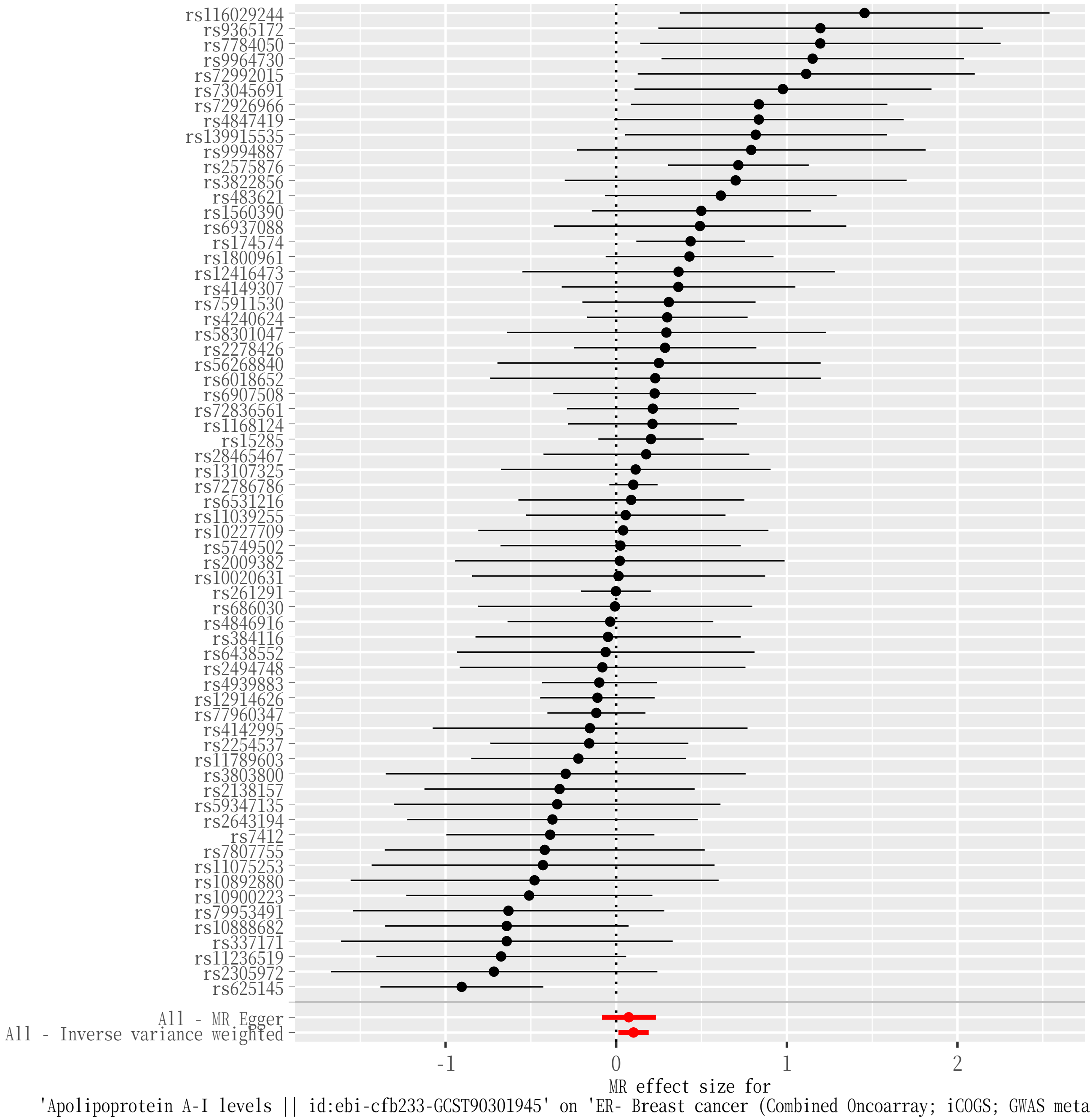
rs116029244
rs9365172
rs7784050
rs9964730
rs72992015
rs73045691
rs72926966
rs4847419
rs139915535
rs9994887
rs2575876
rs3822856
rs483621
rs1560390
rs6937088
rs174574
rs1800961
rs12416473
rs4149307
rs75911530
rs4240624
rs58301047
rs2278426
rs56268840
rs6018652
rs6907508
rs72836561
rs1168124
rs15285
rs28465467
rs13107325
rs72786786
rs6531216
rs11039255
rs10227709
rs5749502
rs2009382
rs10020631
rs261291
rs686030
rs4846916
rs384116
rs6438552
rs2494748
rs4939883
rs12914626
rs77960347
rs4142995
rs2254537
rs11789603
rs3803800
rs2138157
rs59347135
rs2643194
rs7412
rs7807755
rs11075253
rs10892880
rs10900223
rs79953491
rs10888682
rs337171
rs11236519
rs2305972
rs625145
All - MR Egger
All - Inverse variance weighted
-1
0
1
2
MR effect size for
'Apolipoprotein A-I levels || id:ebi-cfb233-GCST90301945' on 'ER- Breast cancer (Combined Oncoarray; iCOGS; GWAS meta

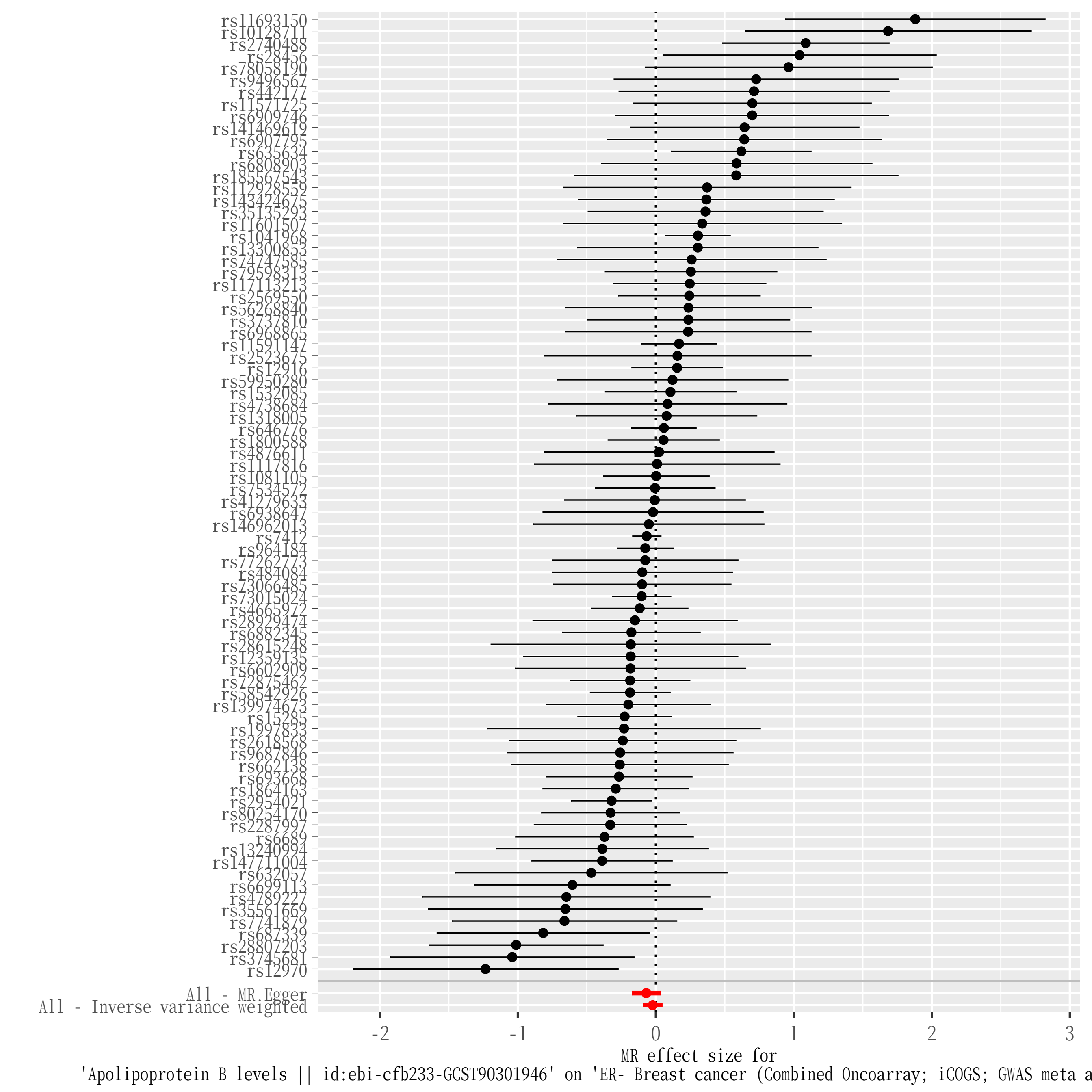

rs11693150
rs10128711
rs2740488
rs28456
rs78058190
rs9496567
rs442177
rs11571725
rs6909746
rs141469619
rs6907795
rs635634
rs6808903
rs185567543
rs112928559
rs143424675
rs35135293
rs11601507
rs1041968
rs13300853
rs74747585
rs79598313
rs117113213
rs2569550
rs56268840
rs3737810
rs6268865
rs11591147
rs2523675
rs12916
rs59950280
rs1532085
rs4738684
rs1318005
rs646776
rs1800588
rs4876611
rs1117816
rs1081105
rs7534572
rs41279633
rs6938647
rs146962013
rs7412
rs964184
rs77262773
rs484084
rs73066485
rs73015024
rs4665972
rs28929474
rs6882345
rs28615248
rs12359135
rs6602909
rs72875462
rs58542926
rs139974673
rs15285
rs1997833
rs2618568
rs9687846
rs662138
rs693668
rs1864163
rs2954021
rs80254170
rs2287997
rs6689
rs13240994
rs147711004
rs632057
rs6699113
rs4789227
rs35561669
rs7741879
rs687339
rs28807203
rs3745681
rs12970
All - MR Egger
All - Inverse variance weighted
-2
-1
0
1
2
3
MR effect size for
'Apolipoprotein B levels || id:ebi-cfb233-GCST90301946' on 'ER- Breast cancer (Combined Oncoarray; iCOGS; GWAS meta a

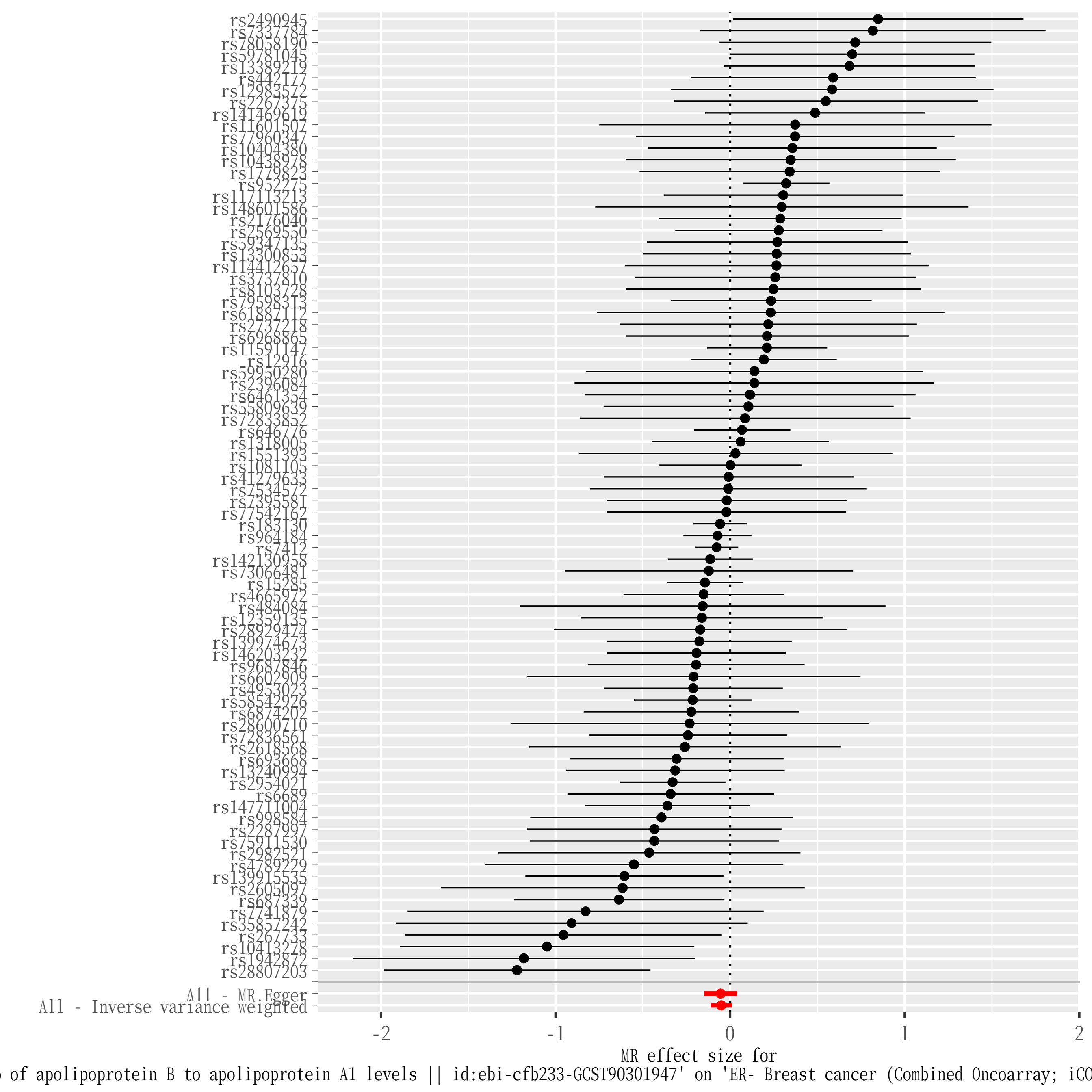
rs2490945
rs7337784
rs78058190
rs59781045
rs13389219
rs442177
rs12983572
rs2267375
rs141469619
rs11601507
rs77960347
rs10404380
rs10438978
rs1779823
rs952275
rs117113213
rs148601586
rs2176040
rs2569550
rs59347135
rs13300853
rs114412657
rs3737810
rs8103728
rs79598313
rs61887112
rs2737218
rs6968865
rs11591147
rs12916
rs59950280
rs2396084
rs6461354
rs55809639
rs72833852
rs646776
rs1318005
rs1551393
rs1081105
rs41279633
rs7534572
rs7395581
rs77542162
rs183130
rs964184
rs7412
rs142130958
rs73066481
rs15285
rs4665972
rs484084
rs12359135
rs28929474
rs139974673
rs146203232
rs9687846
rs6602909
rs4953023
rs58542926
rs6874202
rs28600710
rs72836561
rs2618568
rs693668
rs13240994
rs2954021
rs6689
rs147711004
rs998584
rs2287997
rs75911530
rs2982521
rs4789229
rs139915535
rs2605097
rs687339
rs7741879
rs35857242
rs267733
rs10413278
rs1942872
rs28807203
All - MR Egger
All - Inverse variance weighted
-2
-1
0
1
2
MR effect size for
o of apolipoprotein B to apolipoprotein A1 levels || id:ebi-cfb233-GCST90301947' on 'ER- Breast cancer (Combined Oncoarray; iC0

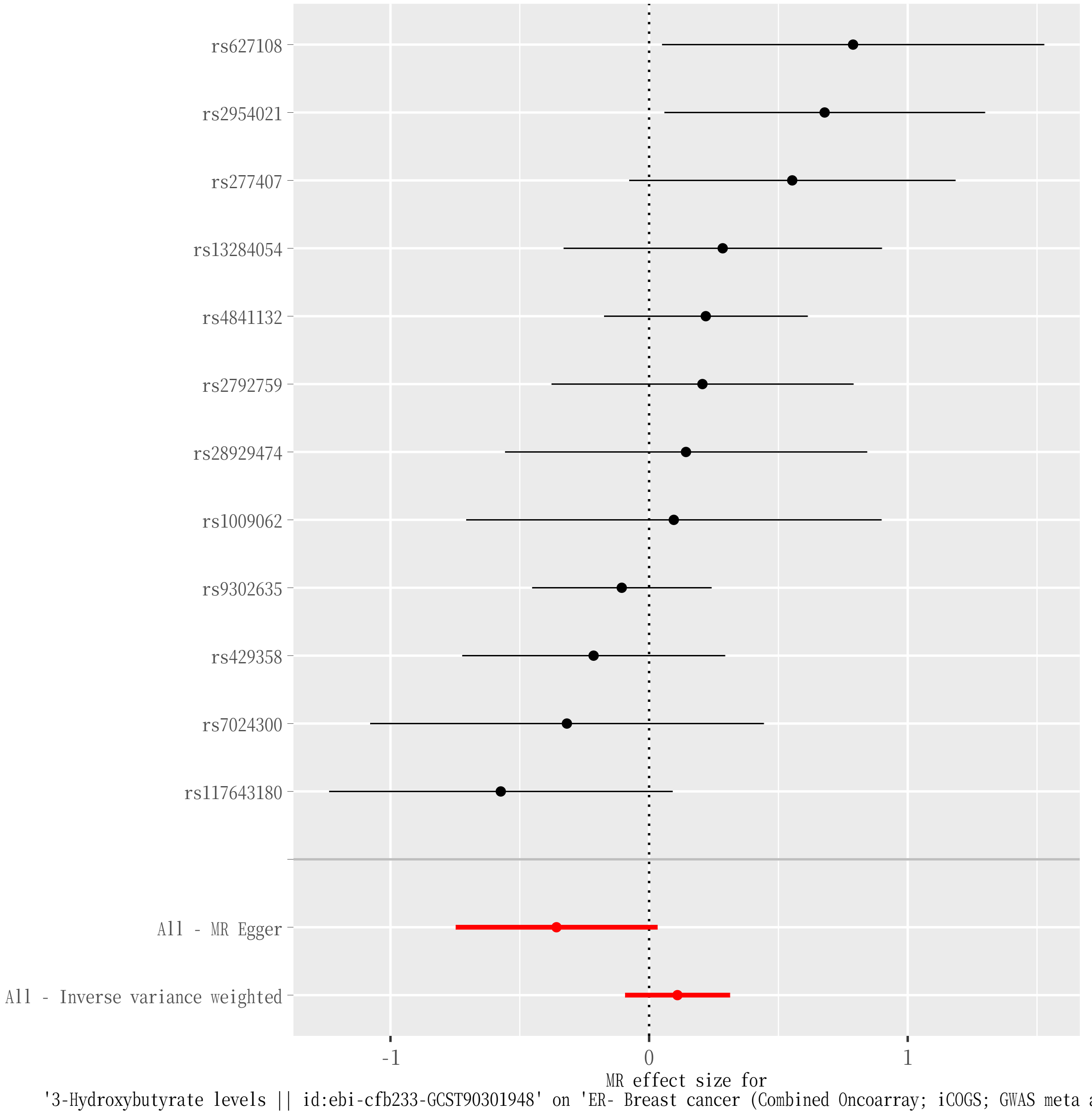

rs627108
rs2954021
rs277407
rs13284054
rs4841132
rs2792759
rs28929474
rs1009062
rs9302635
rs429358
rs7024300
rs117643180
All - MR Egger
All - Inverse variance weighted
-1
0
1
MR effect size for
'3-Hydroxybutyrate levels || id:ebi-cfb233-GCST90301948' on 'ER- Breast cancer (Combined Oncoarray; iCOGS; GWAS meta a

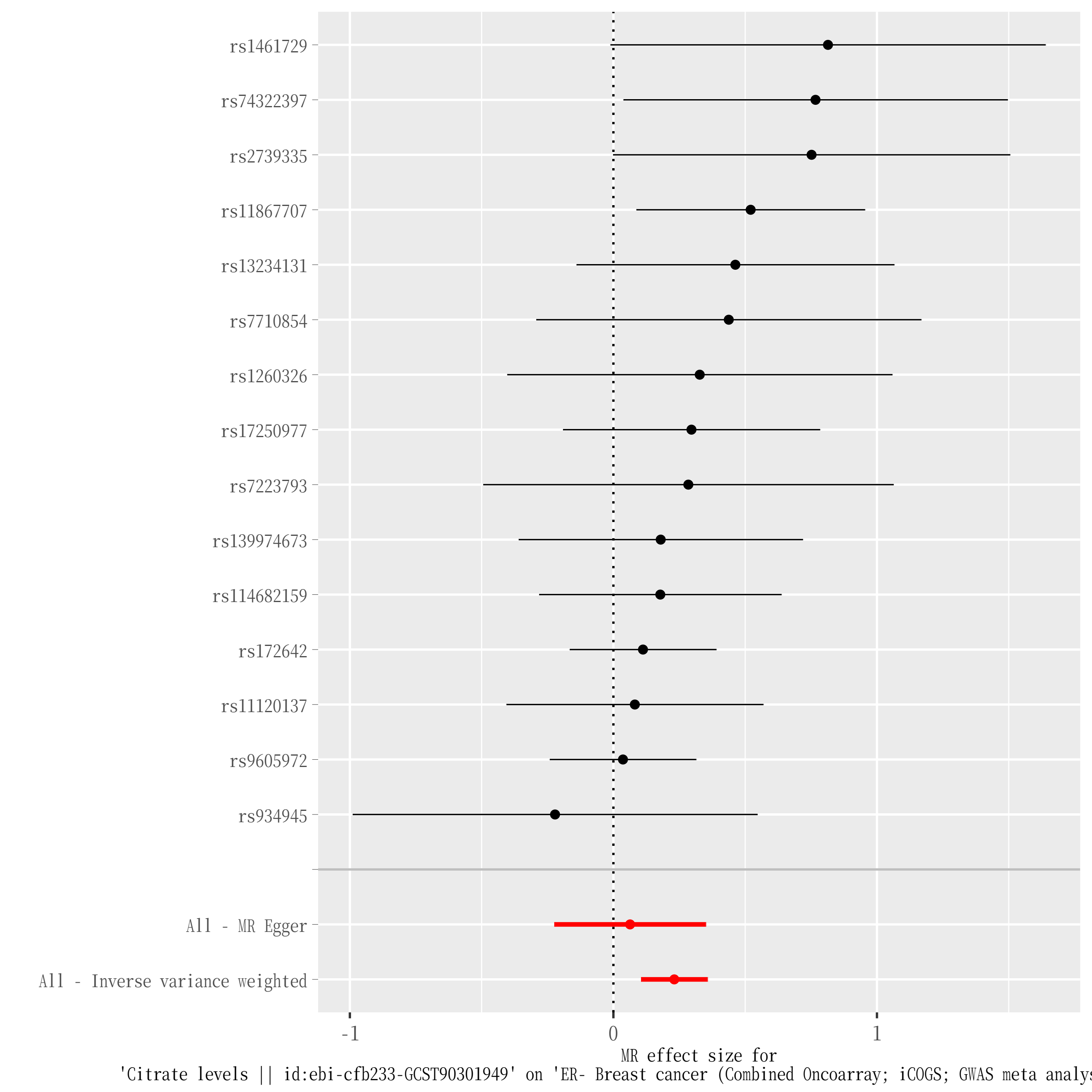

rs1461729
rs74322397
rs2739335
rs11867707
rs13234131
rs7710854
rs1260326
rs17250977
rs7223793
rs139974673
rs114682159
rs172642
rs11120137
rs9605972
rs934945
All - MR Egger
All - Inverse variance weighted
-1
0
1
MR effect size for
'Citrate levels || id:ebi-cfb233-GCST90301949' on 'ER- Breast cancer (Combined Oncoarray; iCOGS; GWAS meta analys

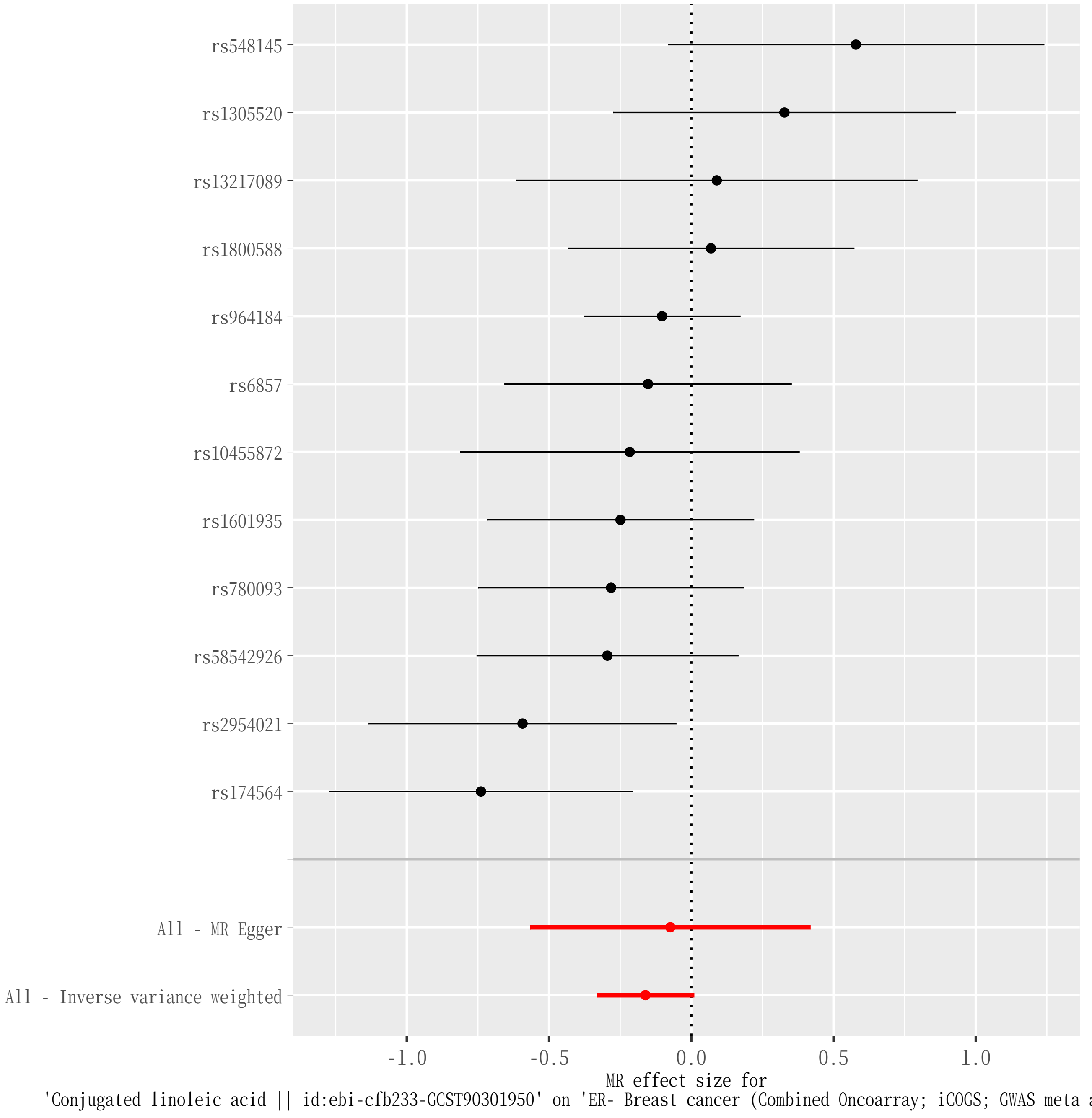
rs548145
rs1305520
rs13217089
rs1800588
rs964184
rs6857
rs10455872
rs1601935
rs780093
rs58542926
rs2954021
rs174564
All - MR Egger
All - Inverse variance weighted
-1.0
-0.5
0.0
0.5
1.0
MR effect size for
'Conjugated linoleic acid || id:ebi-cfb233-GCST90301950' on 'ER- Breast cancer (Combined Oncoarray; iCOGS; GWAS meta a

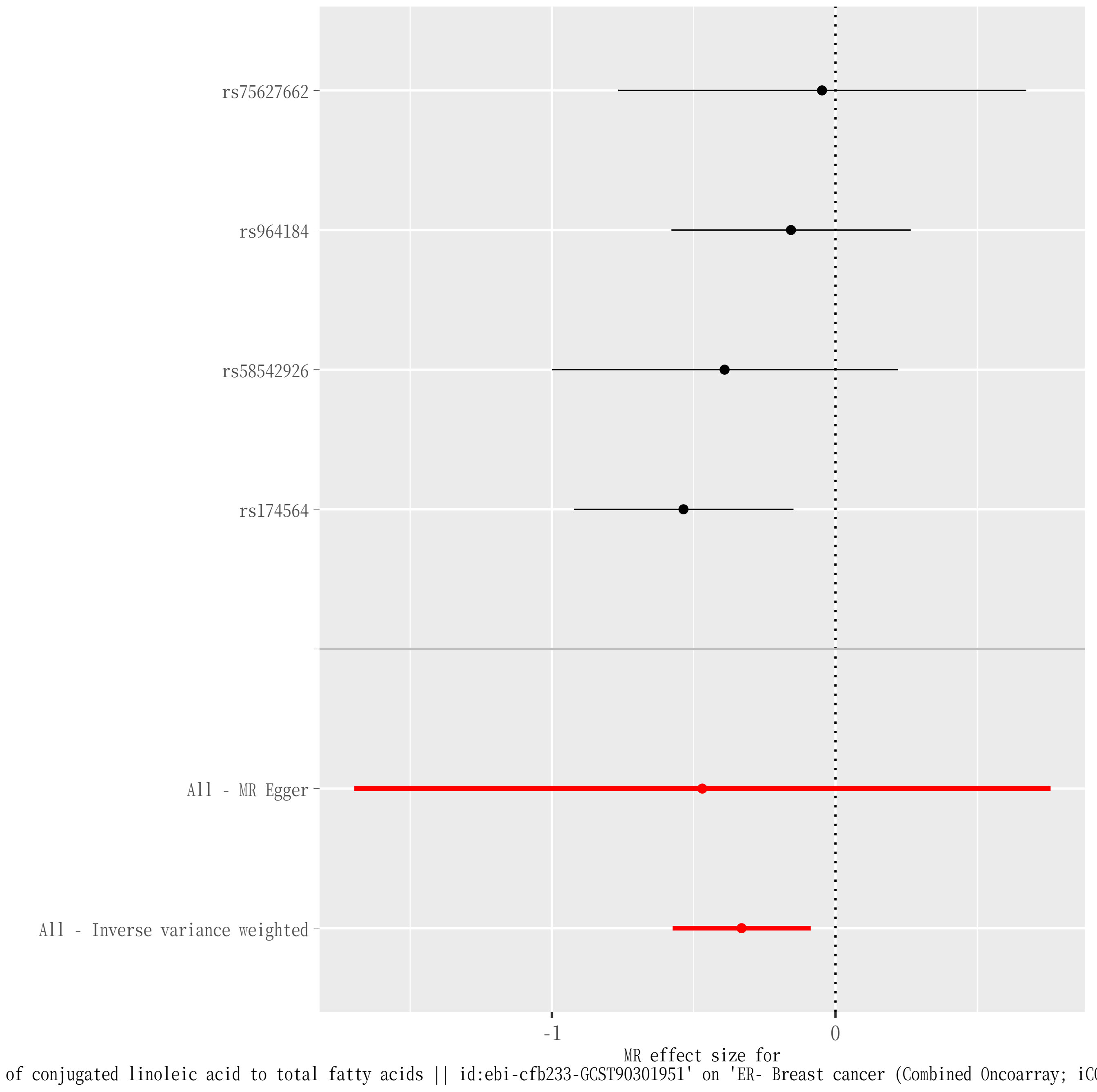

rs75627662
rs964184
rs58542926
rs174564
All - MR Egger
All - Inverse variance weighted
-1
0
MR effect size for
of conjugated linoleic acid to total fatty acids || id:ebi-cfb233-GCST90301951' on 'ER- Breast cancer (Combined Oncoarray; iC

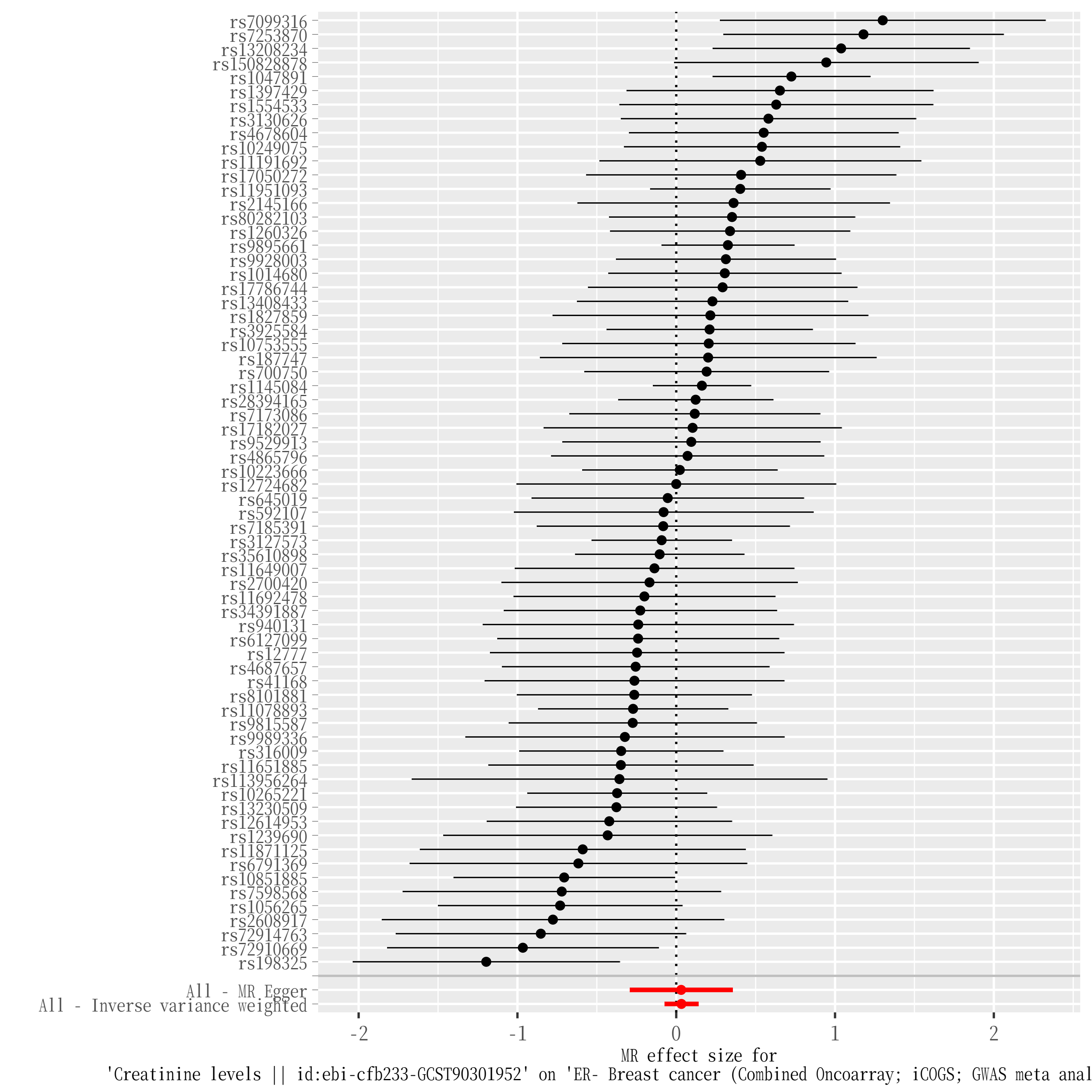

rs7099316
rs7253870
rs13208234
rs150828878
rs1047891
rs1397429
rs1554533
rs3130626
rs4678604
rs10249075
rs11191692
rs17050272
rs11951093
rs2145166
rs80282103
rs1260326
rs9895661
rs9928003
rs1014680
rs17786744
rs13408433
rs1827859
rs3925584
rs10753555
rs187747
rs700750
rs1145084
rs28394165
rs7173086
rs17182027
rs9529913
rs4865796
rs10223666
rs12724682
rs645019
rs592107
rs7185391
rs3127573
rs35610898
rs11649007
rs2700420
rs11692478
rs34391887
rs940131
rs6127099
rs12777
rs4687657
rs41168
rs8101881
rs11078893
rs9815587
rs9989336
rs316009
rs11651885
rs113956264
rs10265221
rs13230509
rs12614953
rs1239690
rs11871125
rs6791369
rs10851885
rs7598568
rs1056265
rs2608917
rs72914763
rs72910669
rs198325
All - MR Egger
All - Inverse variance weighted
-2
-1
0
1
2
MR effect size for
'Creatinine levels || id:ebi-cfb233-GCST90301952' on 'ER- Breast cancer (Combined Oncoarray; iCOGS; GWAS meta anal

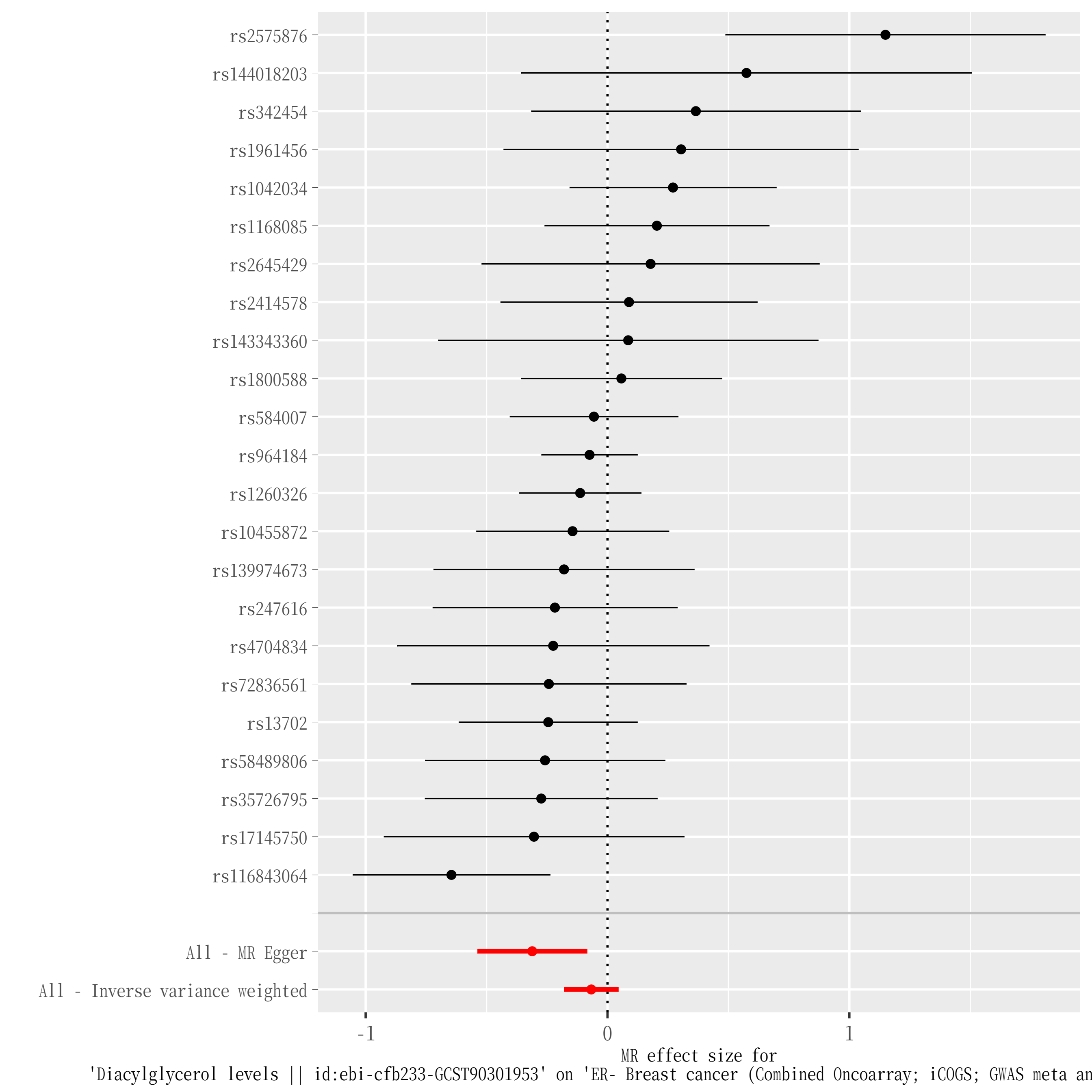
rs2575876
rs144018203
rs342454
rs1961456
rs1042034
rs1168085
rs2645429
rs2414578
rs143343360
rs1800588
rs584007
rs964184
rs1260326
rs10455872
rs139974673
rs247616
rs4704834
rs72836561
rs13702
rs58489806
rs35726795
rs17145750
rs116843064
All - MR Egger
All - Inverse variance weighted
-1
0
1
MR effect size for
'Diacylglycerol levels || id:ebi-cfb233-GCST90301953' on 'ER- Breast cancer (Combined Oncoarray; iCOGS; GWAS meta an

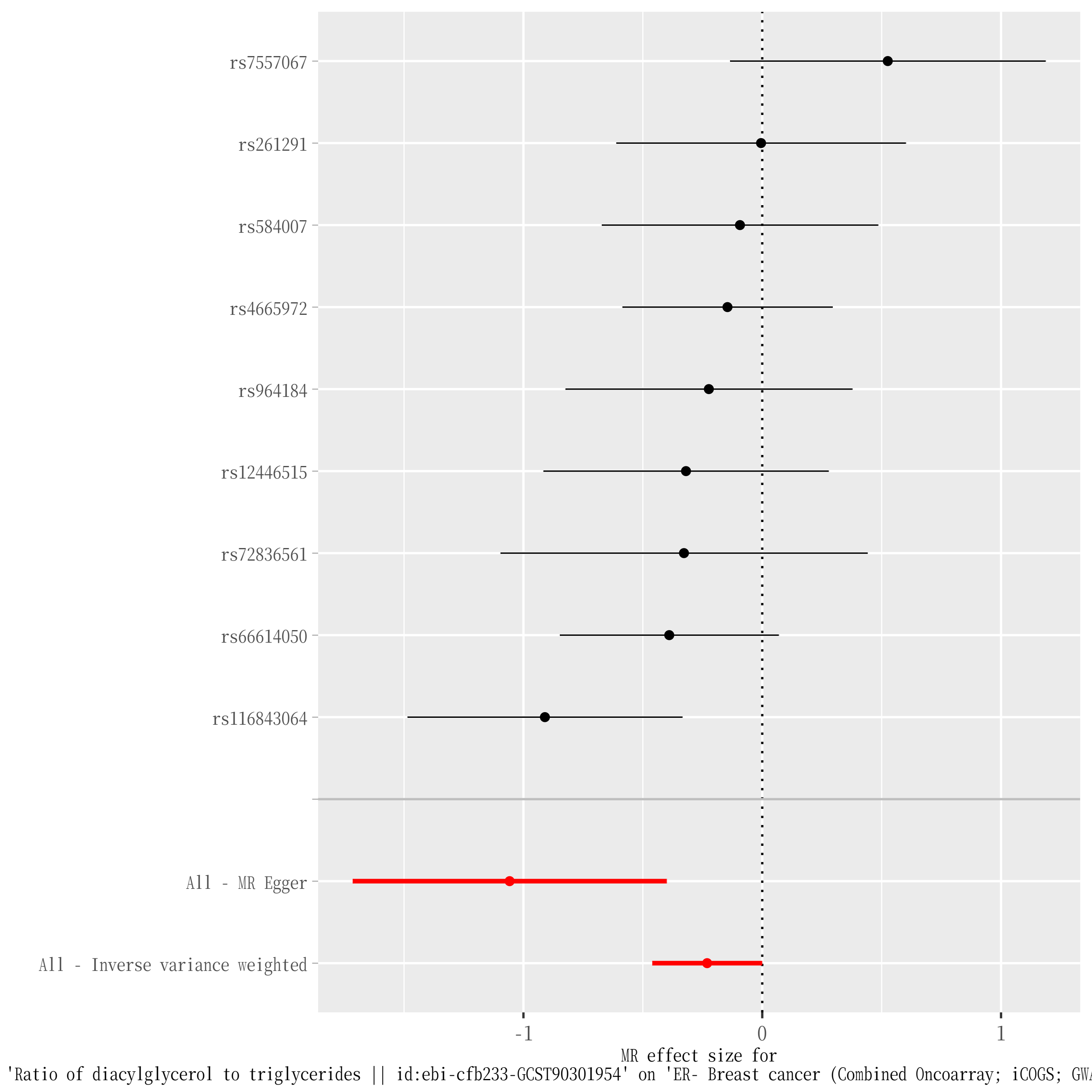
rs7557067
rs261291
rs584007
rs4665972
rs964184
rs12446515
rs72836561
rs66614050
rs116843064
All - MR Egger
All - Inverse variance weighted
-1
0
1
MR effect size for
'Ratio of diacylglycerol to triglycerides || id:ebi-cfb233-GCST90301954' on 'ER- Breast cancer (Combined Oncoarray; iCOGS; GW

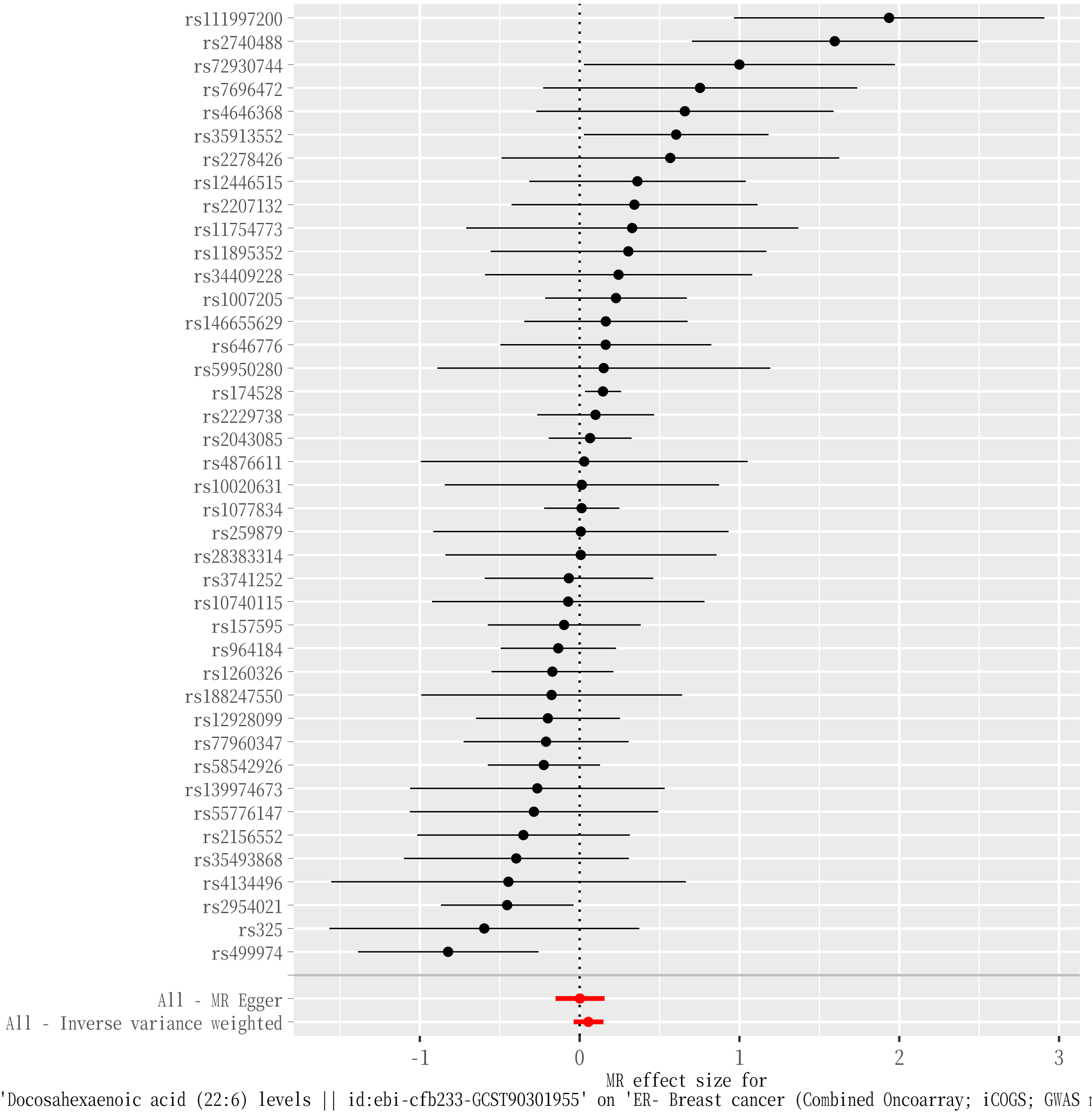
rs111997200
rs2740488
rs72930744
rs7696472
rs4646368
rs35913552
rs2278426
rs12446515
rs2207132
rs11754773
rs11895352
rs34409228
rs1007205
rs146655629
rs646776
rs59950280
rs174528
rs2229738
rs2043085
rs4876611
rs10020631
rs1077834
rs259879
rs28383314
rs3741252
rs10740115
rs157595
rs964184
rs1260326
rs188247550
rs12928099
rs77960347
rs58542926
rs139974673
rs55776147
rs2156552
rs35493868
rs4134496
rs2954021
rs325
rs499974
All - MR Egger
All - Inverse variance weighted
-1
0
1
2
3
MR effect size for
'Docosahexaenoic acid (22:6) levels || id:ebi-cfb233-GCST90301955' on 'ER- Breast cancer (Combined Oncoarray; iCOGS; GWAS m

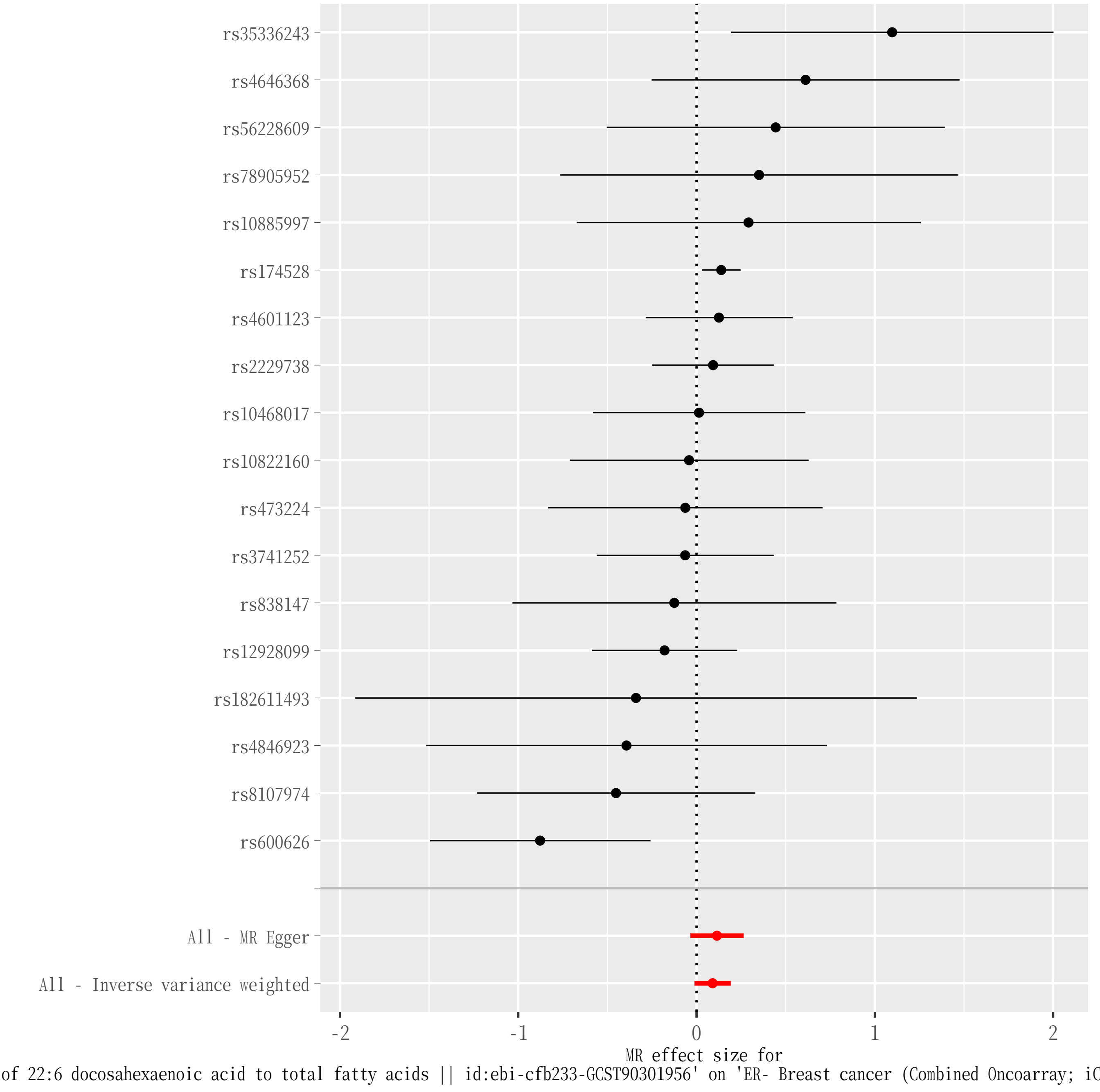

rs35336243
rs4646368
rs56228609
rs78905952
rs10885997
rs174528
rs4601123
rs2229738
rs10468017
rs10822160
rs473224
rs3741252
rs838147
rs12928099
rs182611493
rs4846923
rs8107974
rs600626
All - MR Egger
All - Inverse variance weighted
-2
-1
0
1
2
MR effect size for
of 22:6 docosahexaenoic acid to total fatty acids || id:ebi-cfb233-GCST90301956' on 'ER- Breast cancer (Combined Oncoarray; iC

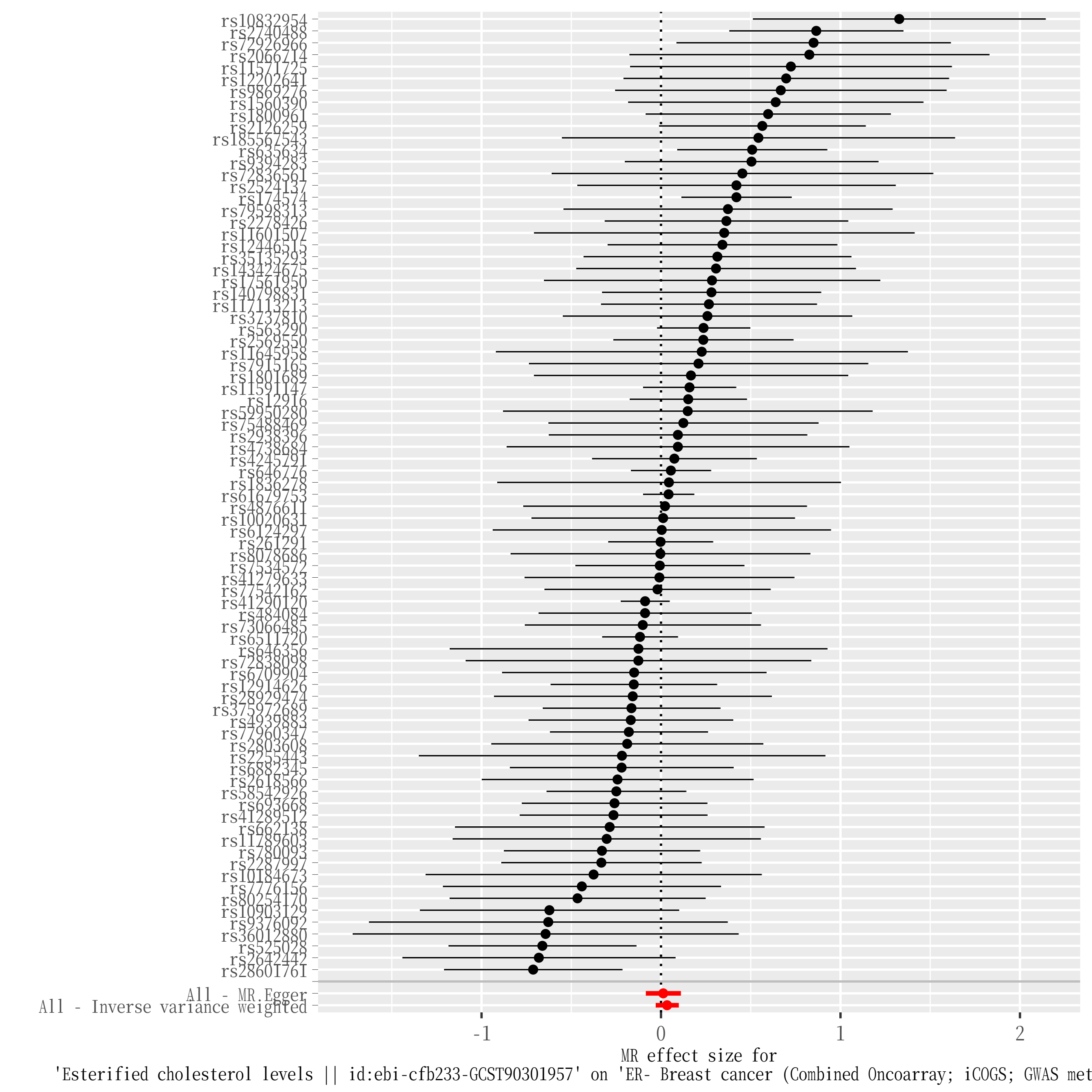
rs10832954
rs2740488
rs72926966
rs2066714
rs11571725
rs12202641
rs9869276
rs1560390
rs1800961
rs2126259
rs185567543
rs635634
rs9394283
rs72836561
rs2524137
rs174574
rs79598313
rs2278426
rs11601507
rs12446515
rs35135293
rs143424675
rs17561950
rs140798831
rs117113213
rs3737810
rs563290
rs2562550
rs11645958
rs7915165
rs1801689
rs11591147
rs12916
rs59950280
rs75488469
rs2238396
rs4738684
rs4245791
rs646776
rs1836278
rs61679753
rs4876611
rs10020631
rs6124297
rs261291
rs8078686
rs7534572
rs41279633
rs77542162
rs41290120
rs484084
rs73066485
rs6511720
rs646356
rs72838098
rs6709904
rs12914626
rs28929474
rs375972689
rs4939883
rs77960347
rs2803608
rs2255443
rs6882345
rs2618566
rs58542926
rs693668
rs41289512
rs662138
rs11789603
rs780093
rs2287927
rs10184673
rs7776156
rs80254170
rs10903129
rs9376092
rs36012880
rs525028
rs2642442
rs28601761
All - MR Egger
All - Inverse variance weighted
-1
0
1
2
MR effect size for
'Esterified cholesterol levels || id:ebi-cfb233-GCST90301957' on 'ER- Breast cancer (Combined Oncoarray; iCOGS; GWAS met

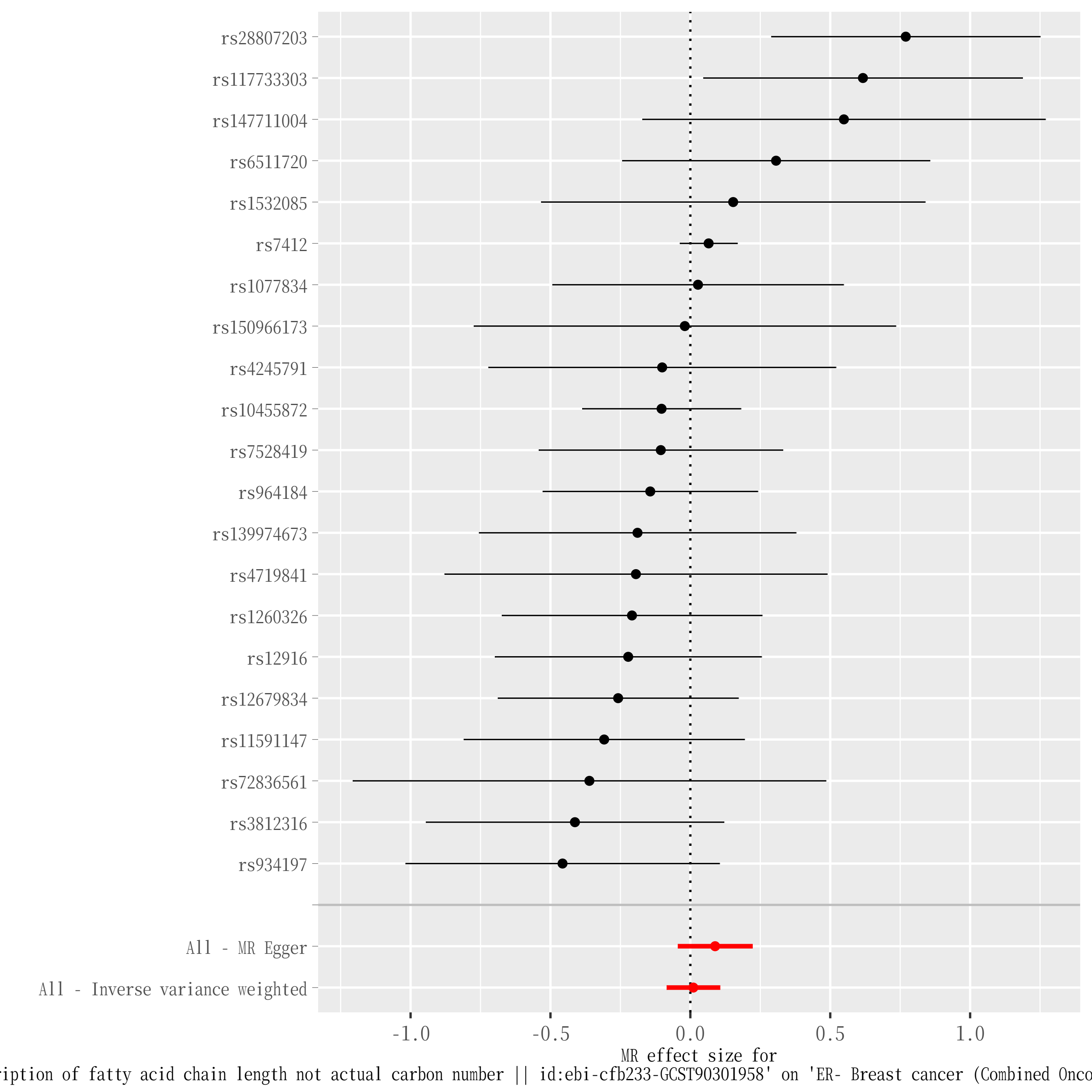

rs28807203
rs117733303
rs147711004
rs6511720
rs1532085
rs7412
rs1077834
rs150966173
rs4245791
rs10455872
rs7528419
rs964184
rs139974673
rs4719841
rs1260326
rs12916
rs12679834
rs11591147
rs72836561
rs3812316
rs934197
All - MR Egger
All - Inverse variance weighted
-1.0
-0.5
0.0
0.5
1.0
MR effect size for
ription of fatty acid chain length not actual carbon number || id:ebi-cfb233-GCST90301958' on 'ER- Breast cancer (Combined Onco

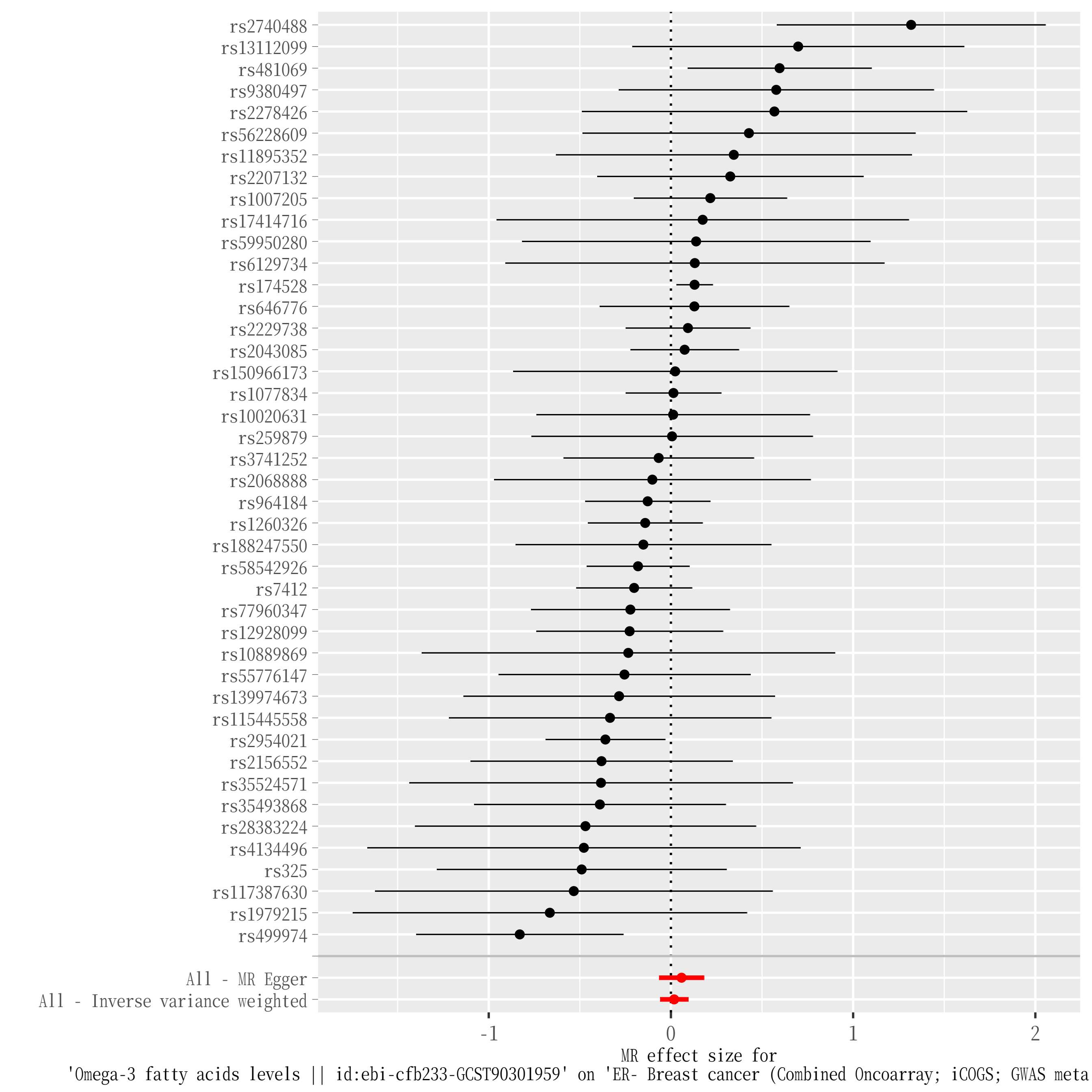
rs2740488
rs13112099
rs481069
rs9380497
rs2278426
rs56228609
rs11895352
rs2207132
rs1007205
rs17414716
rs59950280
rs6129734
rs174528
rs646776
rs2229738
rs2043085
rs150966173
rs1077834
rs10020631
rs259879
rs3741252
rs2068888
rs964184
rs1260326
rs188247550
rs58542926
rs7412
rs77960347
rs12928099
rs10889869
rs55776147
rs139974673
rs115445558
rs2954021
rs2156552
rs35524571
rs35493868
rs28383224
rs4134496
rs325
rs117387630
rs1979215
rs499974
All - MR Egger
All - Inverse variance weighted
-1
0
1
2
MR effect size for
'Omega-3 fatty acids levels || id:ebi-cfb233-GCST90301959' on 'ER- Breast cancer (Combined Oncoarray; iCOGS; GWAS meta

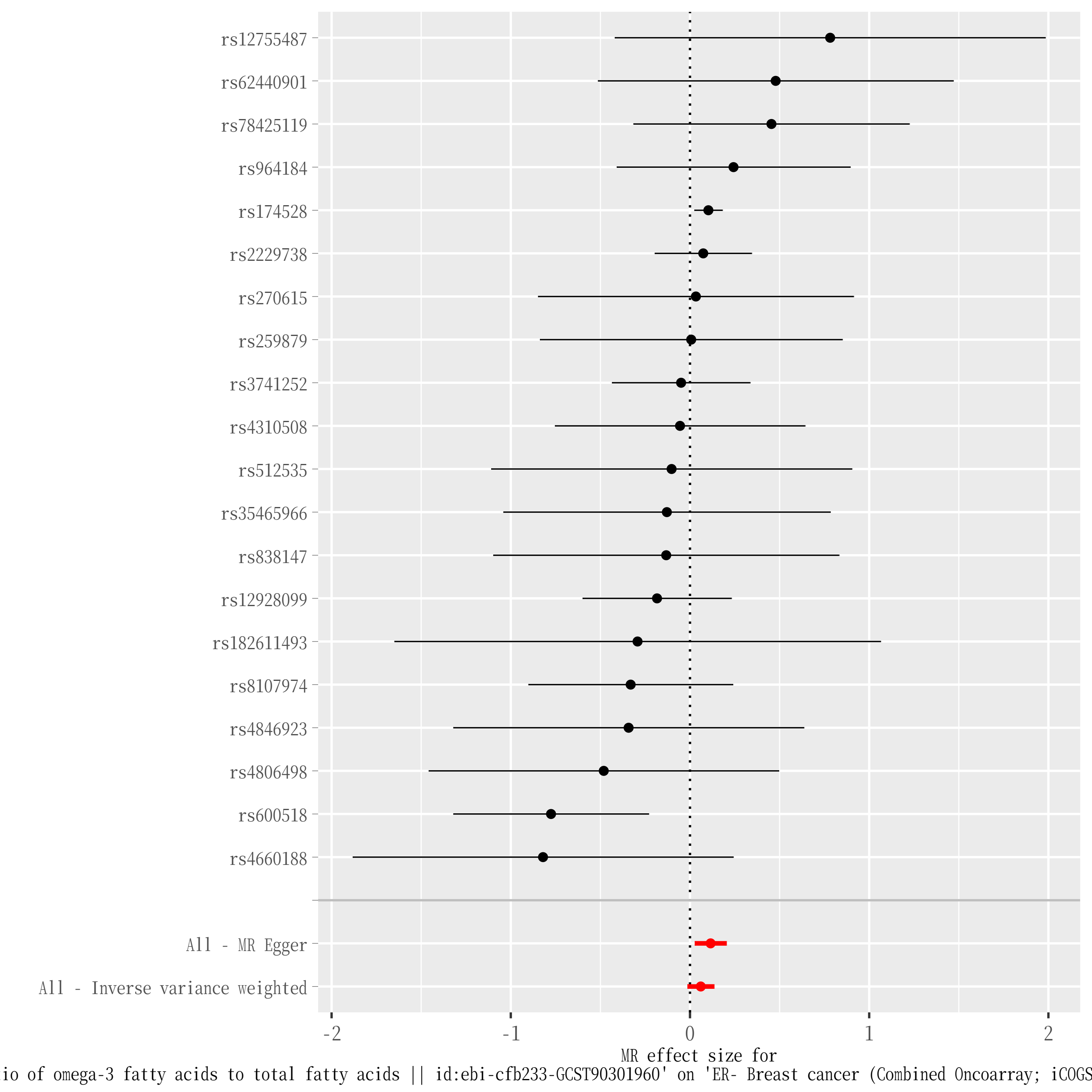
rs12755487
rs62440901
rs78425119
rs964184
rs174528
rs2229738
rs270615
rs259879
rs3741252
rs4310508
rs512535
rs35465966
rs838147
rs12928099
rs182611493
rs8107974
rs4846923
rs4806498
rs600518
rs4660188
All - MR Egger
All - Inverse variance weighted
-2
-1
0
1
2
MR effect size for
io of omega-3 fatty acids to total fatty acids || id:ebi-cfb233-GCST90301960' on 'ER- Breast cancer (Combined Oncoarray; iCOGS

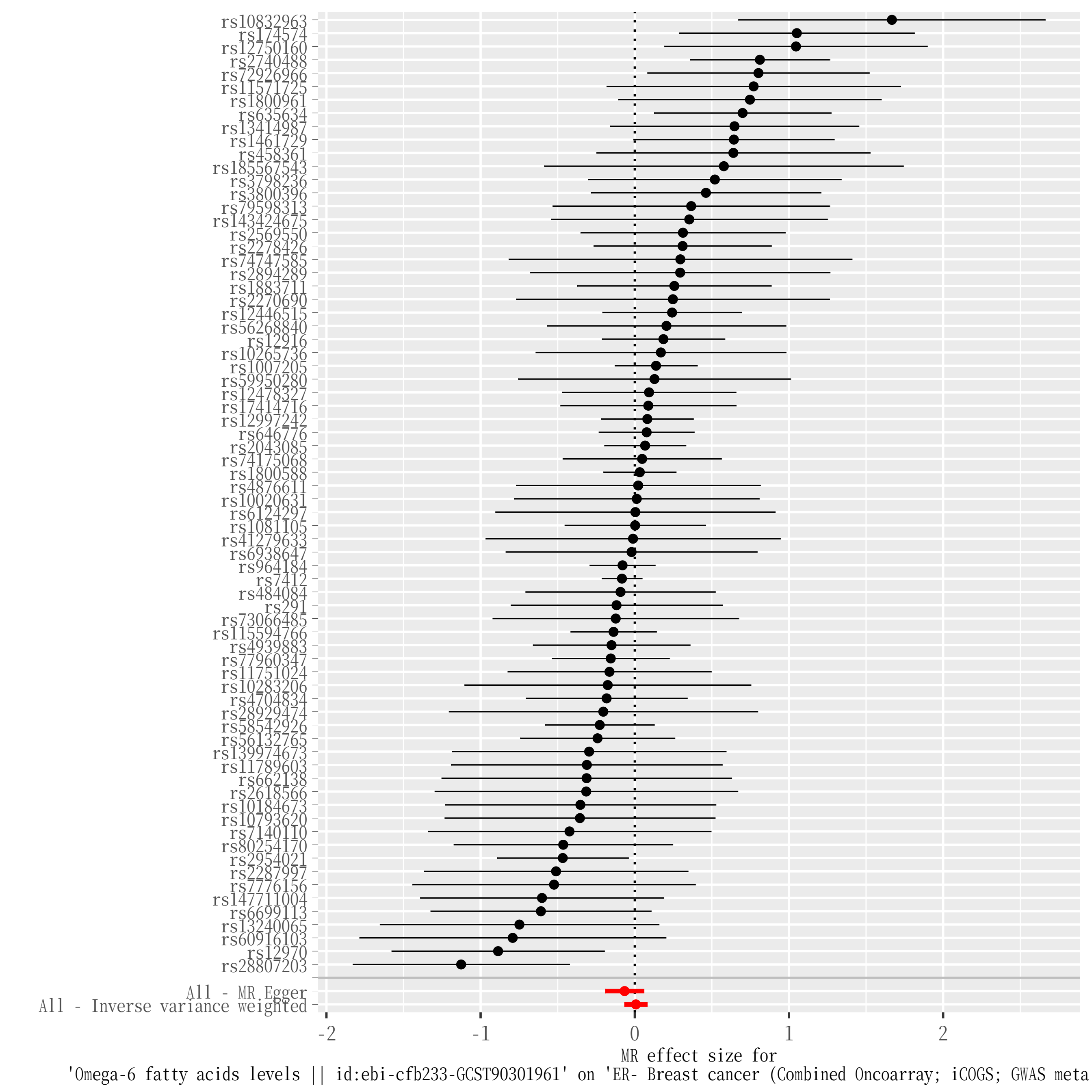
rs10832963
rs174574
rs12750160
rs2740488
rs72926966
rs11571725
rs1800961
rs635634
rs13414987
rs1461729
rs458361
rs185567543
rs3798236
rs3800396
rs79598313
rs143424675
rs2569550
rs2278426
rs74747585
rs2894289
rs1883711
rs2270690
rs12446515
rs56268840
rs12916
rs10265736
rs1007205
rs59950280
rs12478327
rs17414716
rs12997242
rs646776
rs2043085
rs74175068
rs1800588
rs4876611
rs10020631
rs6124297
rs1081105
rs41279633
rs6938647
rs964184
rs7412
rs484084
rs291
rs73066485
rs115594766
rs4939883
rs77960347
rs11751024
rs10283206
rs4704834
rs28929474
rs58542926
rs56132765
rs139974673
rs11789603
rs662138
rs2618566
rs10184673
rs10793620
rs7140110
rs80254170
rs2954021
rs2287997
rs7776156
rs147711004
rs6699113
rs13240065
rs60916103
rs12970
rs28807203
All - MR Egger
All - Inverse variance weighted
-2
-1
0
1
2
MR effect size for
'Omega-6 fatty acids levels || id:ebi-cfb233-GCST90301961' on 'ER- Breast cancer (Combined Oncoarray; iCOGS; GWAS meta

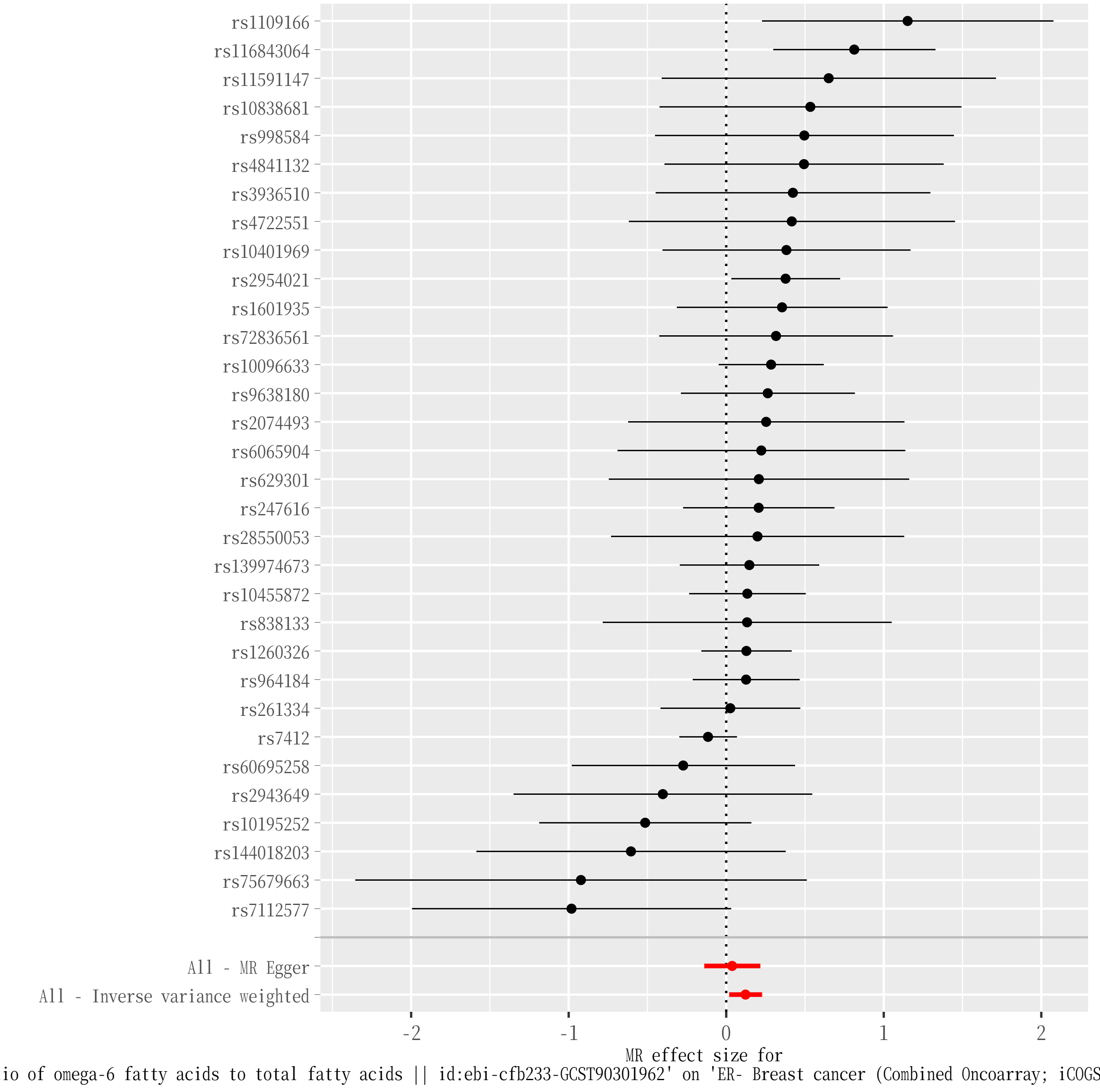
rs1109166
rs116843064
rs11591147
rs10838681
rs998584
rs4841132
rs3936510
rs4722551
rs10401969
rs2954021
rs1601935
rs72836561
rs10096633
rs9638180
rs2074493
rs6065904
rs629301
rs247616
rs28550053
rs139974673
rs10455872
rs838133
rs1260326
rs964184
rs261334
rs7412
rs60695258
rs2943649
rs10195252
rs144018203
rs75679663
rs7112577
All - MR Egger
All - Inverse variance weighted
-2
-1
0
1
2
MR effect size for
io of omega-6 fatty acids to total fatty acids || id:ebi-cfb233-GCST90301962' on 'ER- Breast cancer (Combined Oncoarray; iCOGS

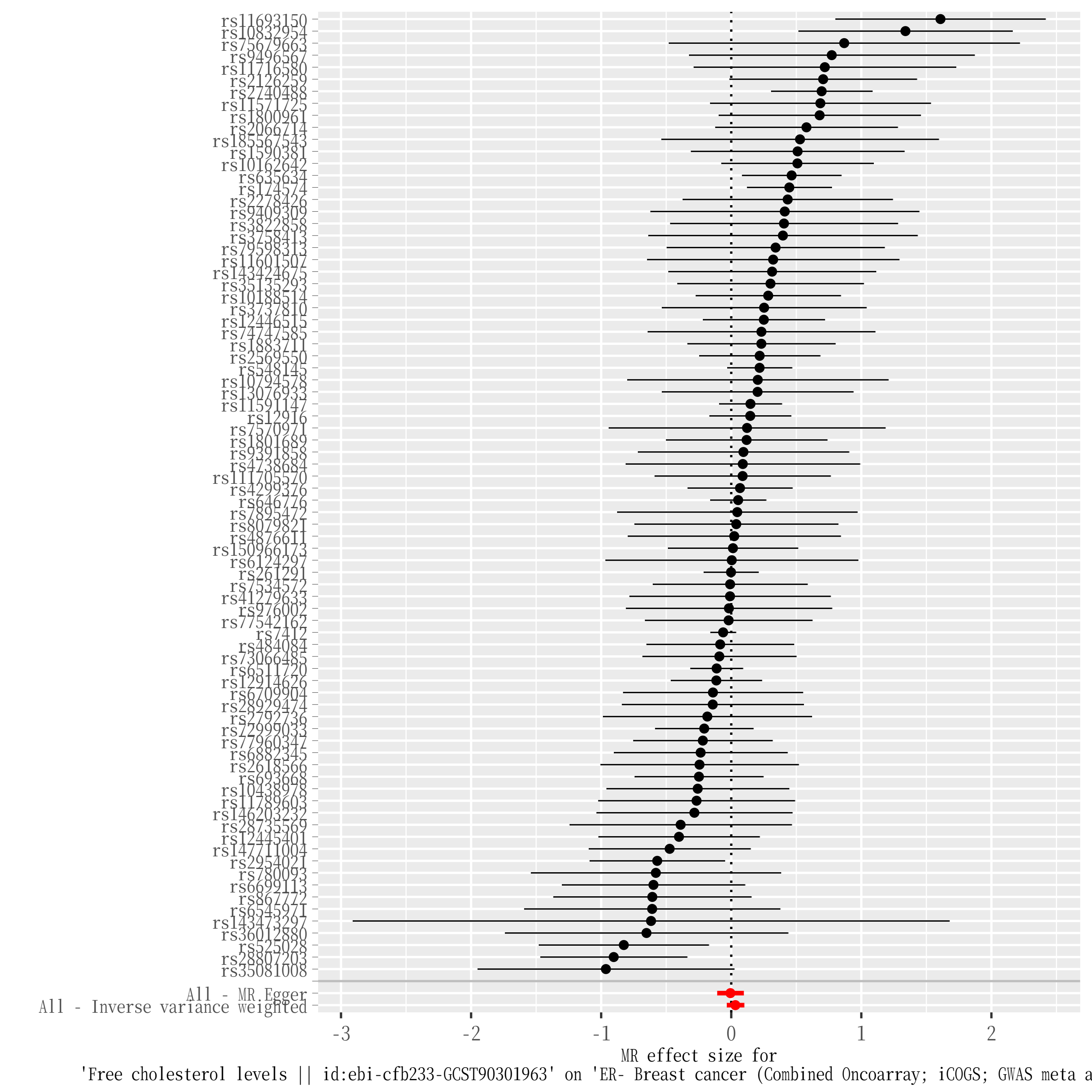
rs11693150
rs10832954
rs75679663
rs9496567
rs11716580
rs2126259
rs2740488
rs11571725
rs1800961
rs2066714
rs185567543
rs1590381
rs10162642
rs635634
rs174574
rs2278426
rs9409309
rs3822858
rs3758413
rs79598313
rs11601507
rs143424675
rs35135293
rs10188514
rs3737810
rs12446515
rs74747585
rs1883711
rs2569550
rs548145
rs10794578
rs13076933
rs11591147
rs12916
rs7570971
rs1801689
rs9391858
rs4738684
rs111705570
rs4299376
rs646776
rs7895472
rs8079821
rs4876611
rs150966173
rs6124297
rs261291
rs7534572
rs41279633
rs976002
rs77542162
rs7412
rs484084
rs73066485
rs6511720
rs12914626
rs6709904
rs28929474
rs2792736
rs72999033
rs77960347
rs6882345
rs2618566
rs693668
rs10438978
rs11789603
rs146203232
rs28735569
rs12445401
rs147711004
rs2954021
rs780093
rs6699113
rs867772
rs6545971
rs143473297
rs36012880
rs525028
rs28807203
rs35081008
All - MR Egger
All - Inverse variance weighted
-3
-2
-1
0
1
2
MR effect size for
'Free cholesterol levels || id:ebi-cfb233-GCST90301963' on 'ER- Breast cancer (Combined Oncoarray; iCOGS; GWAS meta a

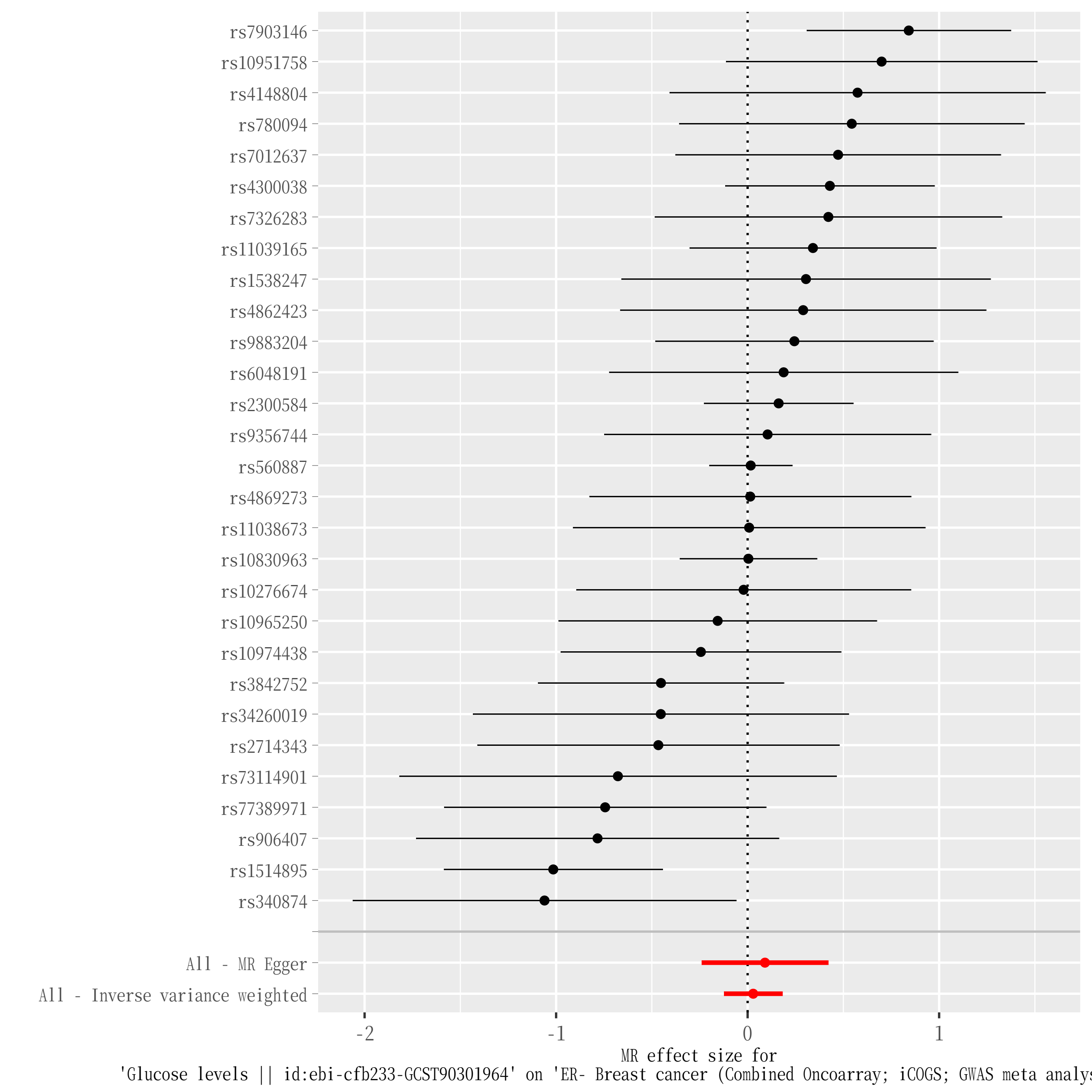
rs7903146
rs10951758
rs4148804
rs780094
rs7012637
rs4300038
rs7326283
rs11039165
rs1538247
rs4862423
rs9883204
rs6048191
rs2300584
rs9356744
rs560887
rs4869273
rs11038673
rs10830963
rs10276674
rs10965250
rs10974438
rs3842752
rs34260019
rs2714343
rs73114901
rs77389971
rs906407
rs1514895
rs340874
All - MR Egger
All - Inverse variance weighted
-2
-1
0
1
MR effect size for
'Glucose levels || id:ebi-cfb233-GCST90301964' on 'ER- Breast cancer (Combined Oncoarray; iCOGS; GWAS meta analys

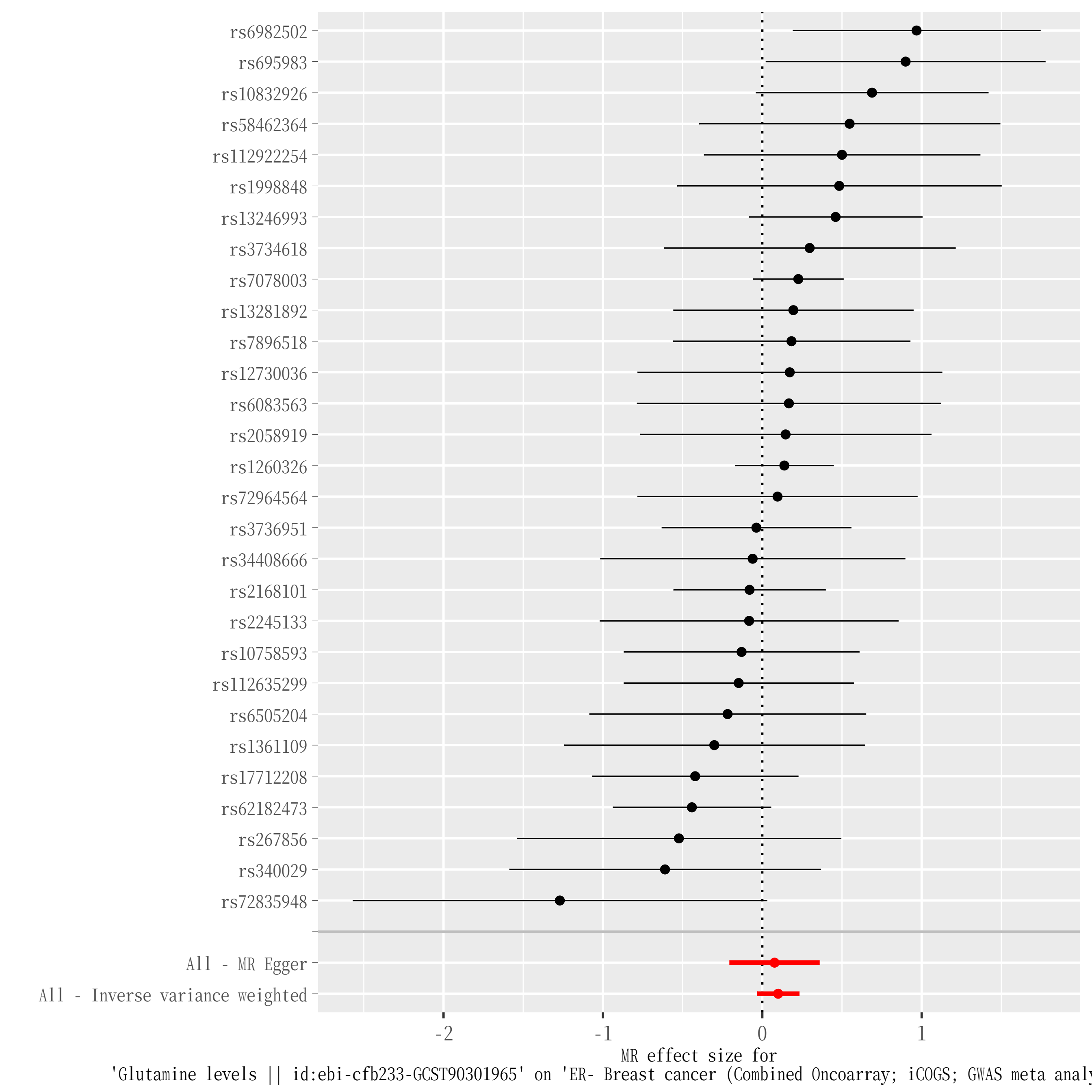
rs6982502
rs695983
rs10832926
rs58462364
rs112922254
rs1998848
rs13246993
rs3734618
rs7078003
rs13281892
rs7896518
rs12730036
rs6083563
rs2058919
rs1260326
rs72964564
rs3736951
rs34408666
rs2168101
rs2245133
rs10758593
rs112635299
rs6505204
rs1361109
rs17712208
rs62182473
rs267856
rs340029
rs72835948
All - MR Egger
All - Inverse variance weighted
-2
-1
0
1
MR effect size for
'Glutamine levels || id:ebi-cfb233-GCST90301965' on 'ER- Breast cancer (Combined Oncoarray; iCOGS; GWAS meta analy

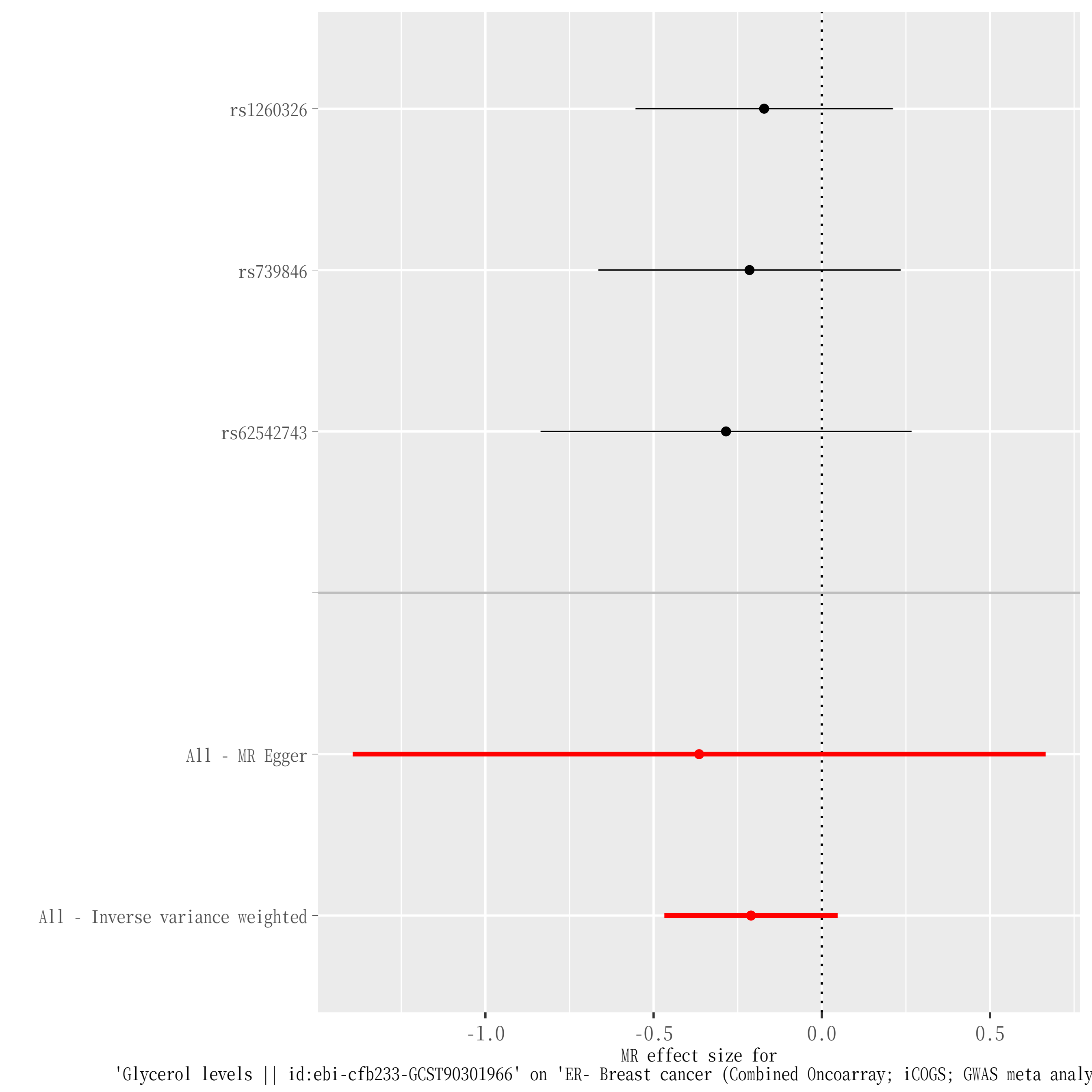
rs1260326
rs739846
rs62542743
All - MR Egger
All - Inverse variance weighted
-1.0
-0.5
0.0
0.5
MR effect size for
'Glycerol levels || id:ebi-cfb233-GCST90301966' on 'ER- Breast cancer (Combined Oncoarray; iCOGS; GWAS meta analy

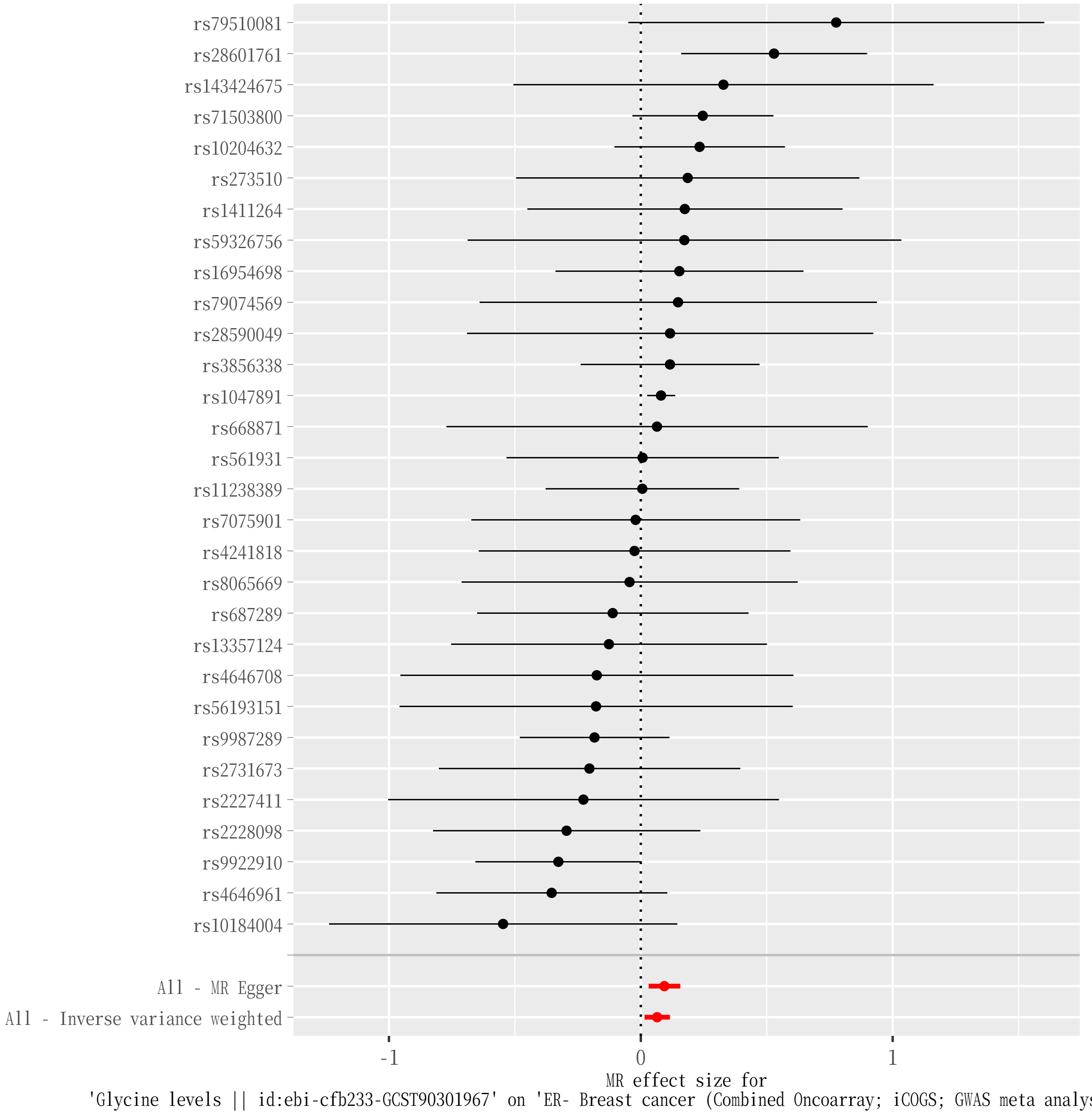
rs79510081
rs28601761
rs143424675
rs71503800
rs10204632
rs273510
rs1411264
rs59326756
rs16954698
rs79074569
rs28590049
rs3856338
rs1047891
rs668871
rs561931
rs11238389
rs7075901
rs4241818
rs8065669
rs687289
rs13357124
rs4646708
rs56193151
rs9987289
rs2731673
rs2227411
rs2228098
rs9922910
rs4646961
rs10184004
All - MR Egger
All - Inverse variance weighted
-1
0
1
MR effect size for
'Glycine levels || id:ebi-cfb233-GCST90301967' on 'ER- Breast cancer (Combined Oncoarray; iCOGS; GWAS meta analy

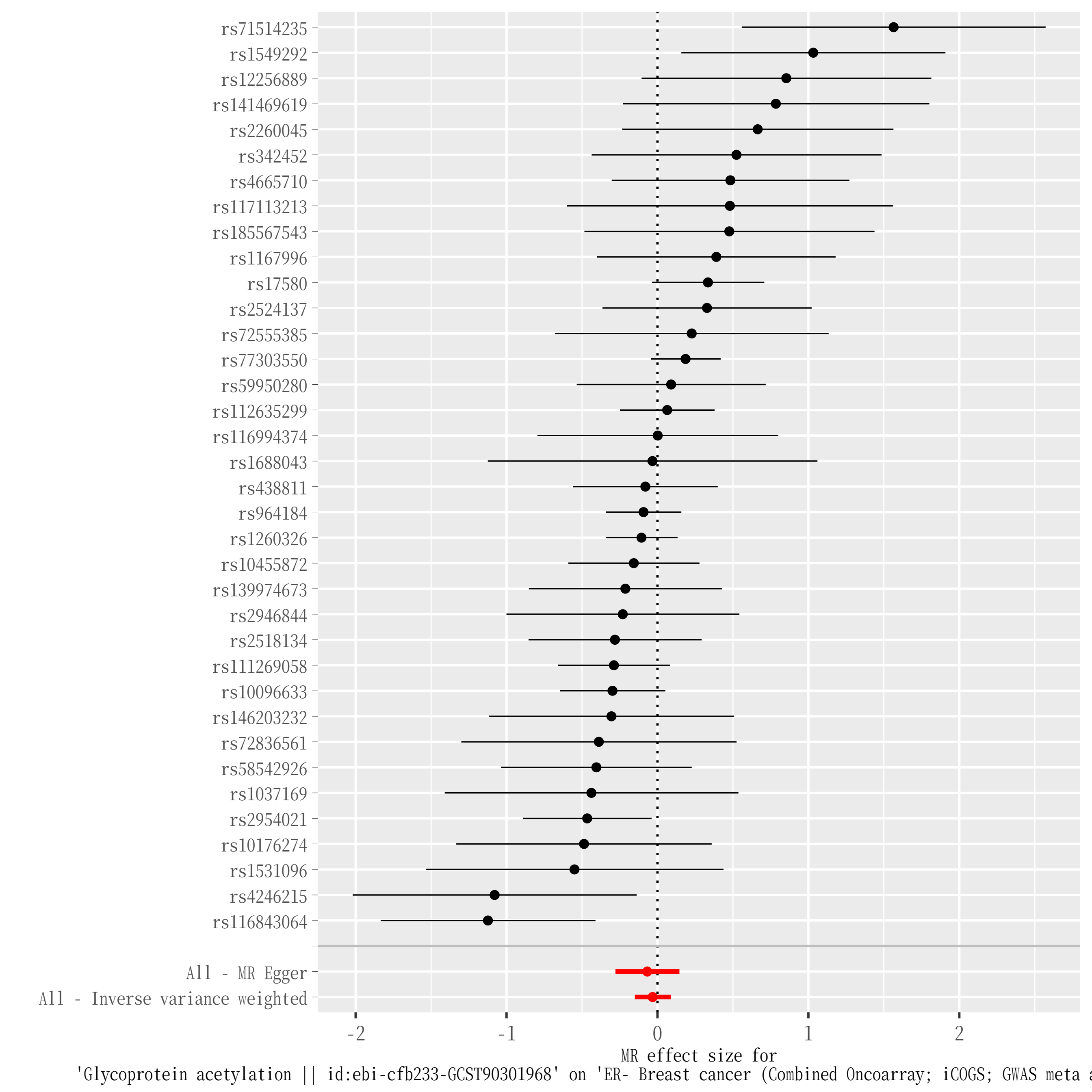
rs71514235
rs1549292
rs12256889
rs141469619
rs2260045
rs342452
rs4665710
rs117113213
rs185567543
rs1167996
rs17580
rs2524137
rs72555385
rs77303550
rs59950280
rs112635299
rs116994374
rs1688043
rs438811
rs964184
rs1260326
rs10455872
rs139974673
rs2946844
rs2518134
rs111269058
rs10096633
rs146203232
rs72836561
rs58542926
rs1037169
rs2954021
rs10176274
rs1531096
rs4246215
rs116843064
All - MR Egger
All - Inverse variance weighted
-2
-1
0
1
2
MR effect size for
'Glycoprotein acetylation || id:ebi-cfb233-GCST90301968' on 'ER- Breast cancer (Combined Oncoarray; iCOGS; GWAS meta a

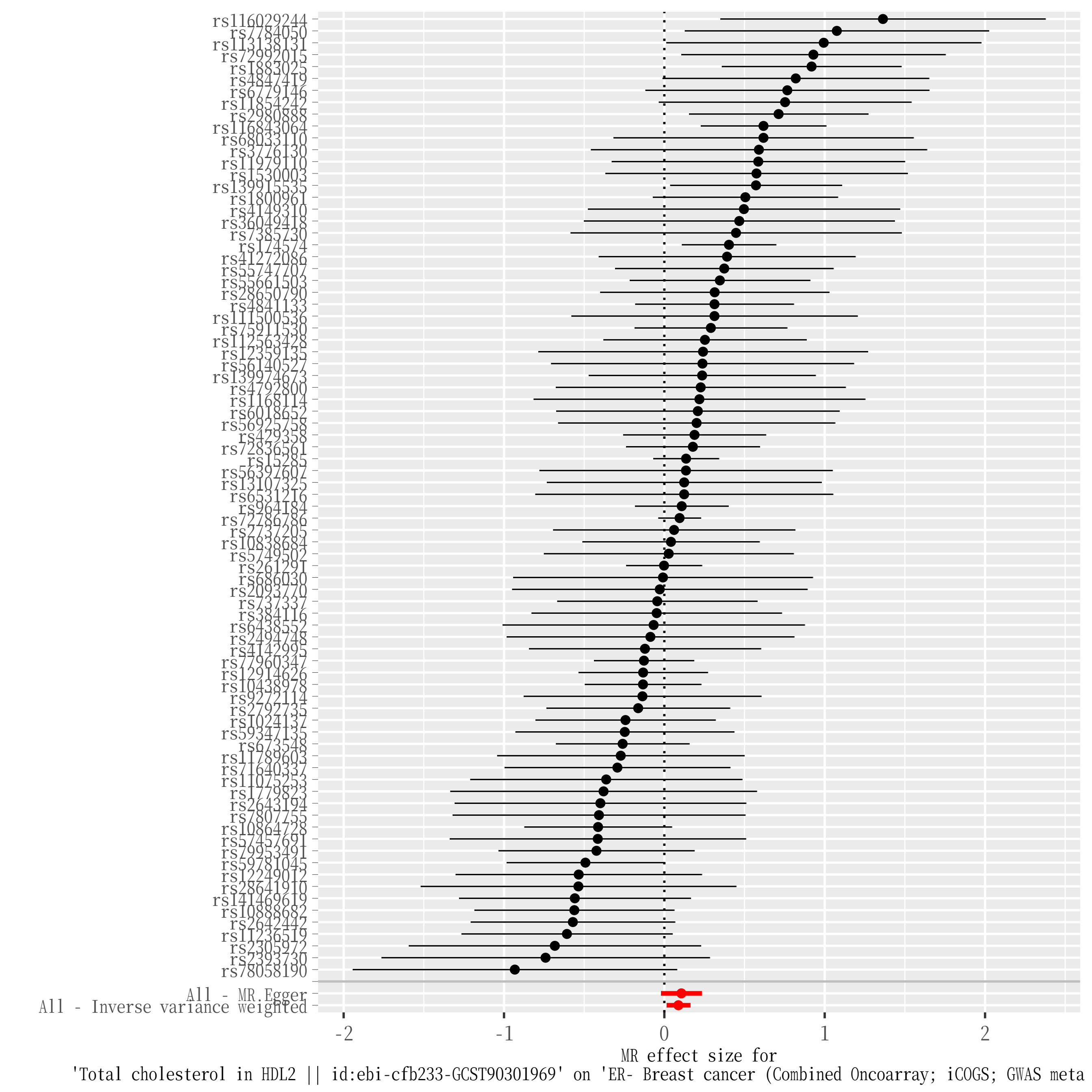
rs116029244
rs7784050
rs113138131
rs72992015
rs1883025
rs4847419
rs6779146
rs11854242
rs2980888
rs116843064
rs68033110
rs3776130
rs11979110
rs1530003
rs139915535
rs1800961
rs4149310
rs36042418
rs7385730
rs174574
rs41272086
rs55747707
rs55661503
rs28650790
rs4841133
rs111500536
rs75911530
rs112563428
rs12359135
rs56140527
rs139974673
rs4792800
rs1168114
rs6018652
rs56925758
rs429358
rs72836561
rs15285
rs56397607
rs13107325
rs6531216
rs964184
rs72786786
rs2737205
rs10838684
rs5749502
rs261291
rs686030
rs2093770
rs737337
rs384116
rs6438552
rs2494748
rs4142995
rs77960347
rs12914626
rs10438978
rs9272114
rs2792735
rs1024137
rs59347135
rs673548
rs11789603
rs71640337
rs11075253
rs1779823
rs2643194
rs7807755
rs10864728
rs57457691
rs79953491
rs59781045
rs12249012
rs28641910
rs141469619
rs10888682
rs2642442
rs11236519
rs2305972
rs2393730
rs78058190
All - MR Egger
All - Inverse variance weighted
-2
-1
0
1
2
MR effect size for
'Total cholesterol in HDL2 || id:ebi-cfb233-GCST90301969' on 'ER- Breast cancer (Combined Oncoarray; iCOGS; GWAS meta

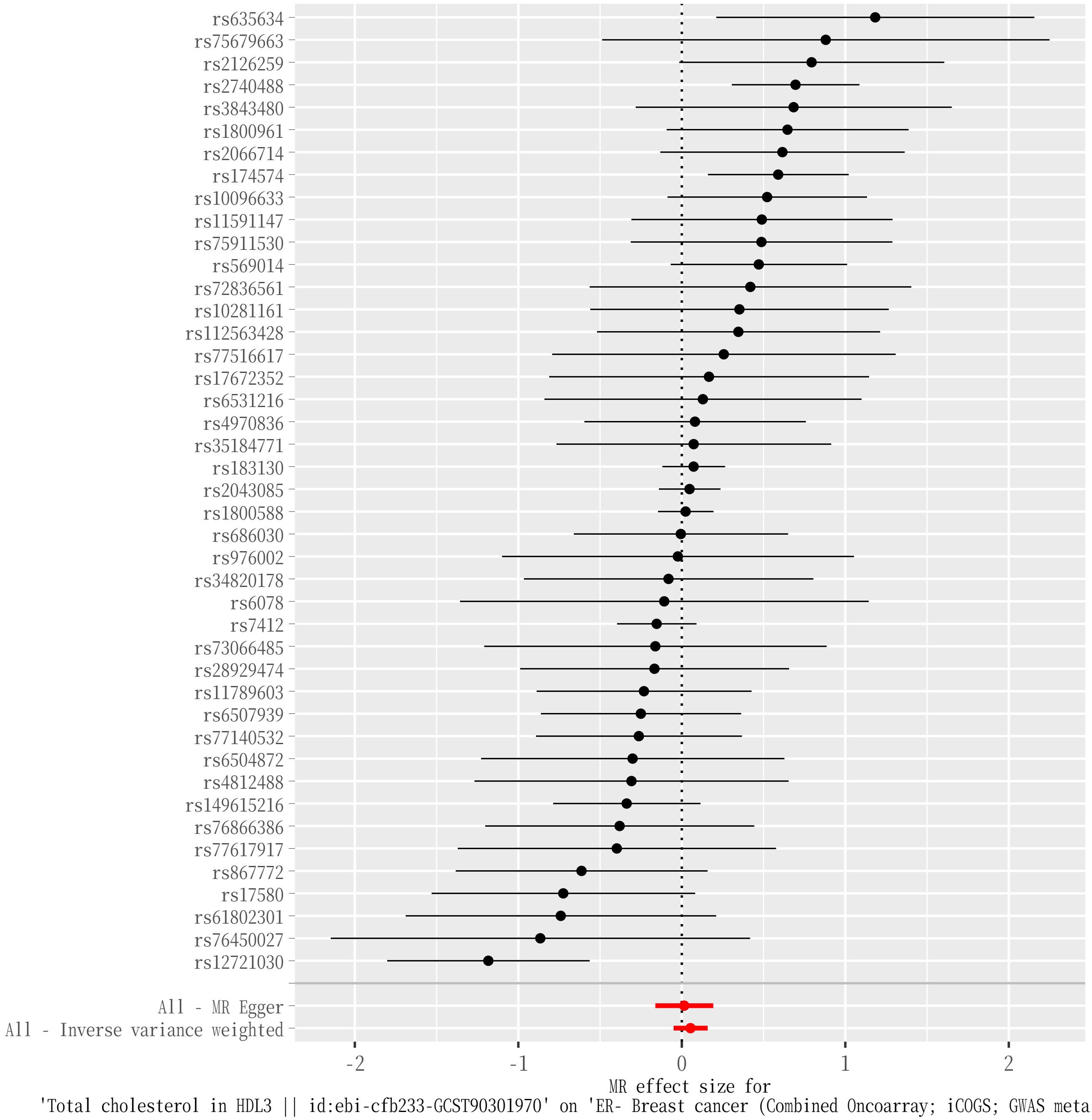
rs635634
rs75679663
rs2126259
rs2740488
rs3843480
rs1800961
rs2066714
rs174574
rs10096633
rs11591147
rs75911530
rs569014
rs72836561
rs10281161
rs112563428
rs77516617
rs17672352
rs6531216
rs4970836
rs35184771
rs183130
rs2043085
rs1800588
rs686030
rs976002
rs34820178
rs6078
rs7412
rs73066485
rs28929474
rs11789603
rs6507939
rs77140532
rs6504872
rs4812488
rs149615216
rs76866386
rs77617917
rs867772
rs17580
rs61802301
rs76450027
rs12721030
All - MR Egger
All - Inverse variance weighted
-2
-1
0
1
2
MR effect size for
'Total cholesterol in HDL3 || id:ebi-cfb233-GCST90301970' on 'ER- Breast cancer (Combined Oncoarray; iCOGS; GWAS meta

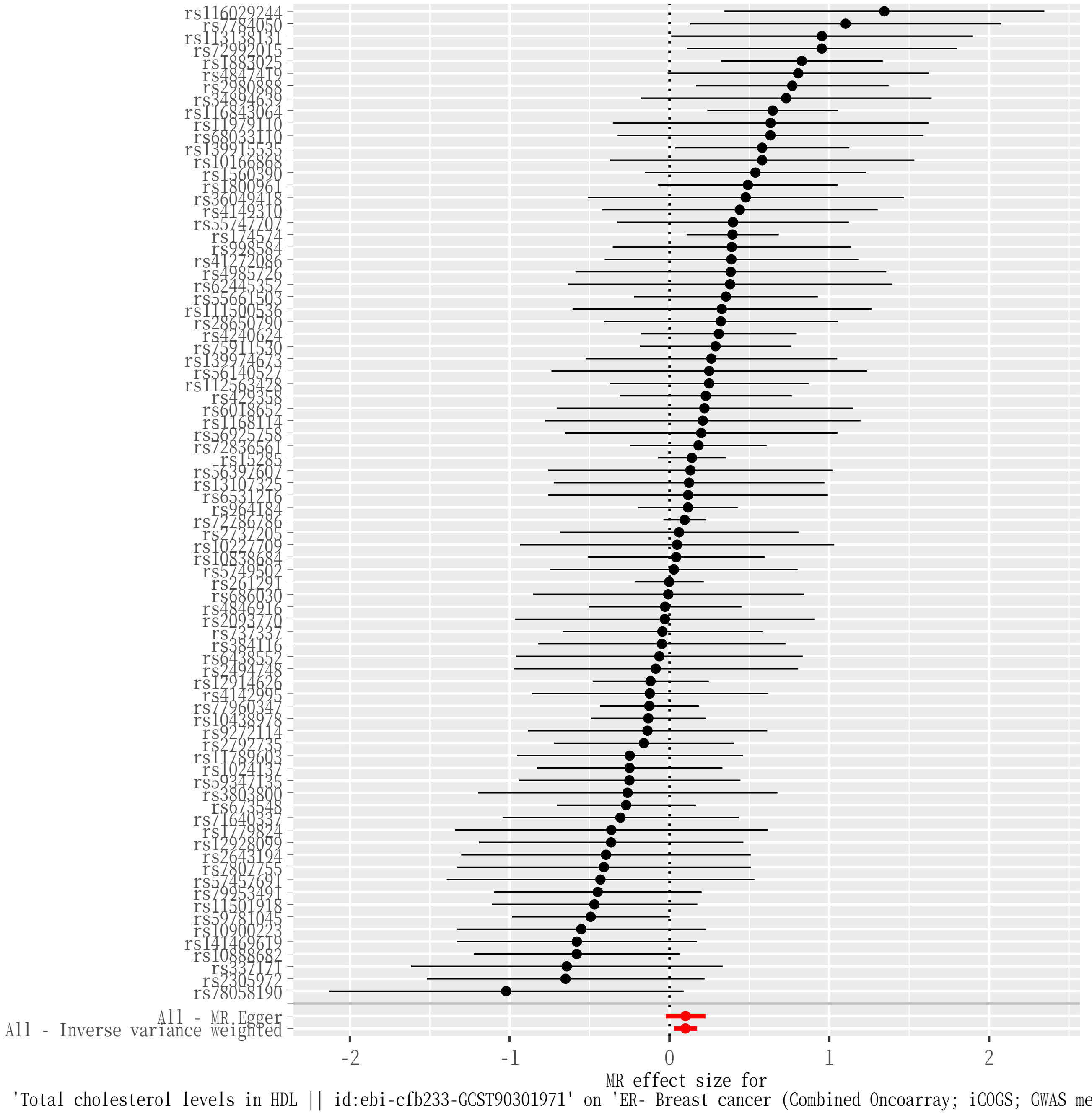
rs116029244
rs7784050
rs113138131
rs72992015
rs1883025
rs4847419
rs2980888
rs34894639
rs116843064
rs11979110
rs68033110
rs139915535
rs10166868
rs1560390
rs1800961
rs36049418
rs4149310
rs55747707
rs174574
rs998584
rs41272086
rs4985726
rs62445352
rs55661503
rs111500536
rs28650790
rs4240624
rs75911530
rs139974673
rs56140527
rs112563428
rs429358
rs6018652
rs1168114
rs56925758
rs72836561
rs15285
rs56397607
rs13107325
rs6531216
rs964184
rs72786786
rs2737205
rs10227709
rs10838684
rs5749502
rs261291
rs686030
rs4846916
rs2093770
rs737337
rs384116
rs6438552
rs2494748
rs12914626
rs4142995
rs77960347
rs10438978
rs9272114
rs2792735
rs11789603
rs1024137
rs59347135
rs3803800
rs673548
rs71640337
rs1779824
rs12928099
rs2643194
rs7807755
rs57457691
rs79953491
rs11501918
rs59781045
rs10900223
rs141469619
rs10888682
rs337171
rs2305972
rs78058190
All - MR Egger
All - Inverse variance weighted
-2
-1
0
1
2
MR effect size for
'Total cholesterol levels in HDL || id:ebi-cfb233-GCST90301971' on 'ER- Breast cancer (Combined Oncoarray; iCOGS; GWAS me

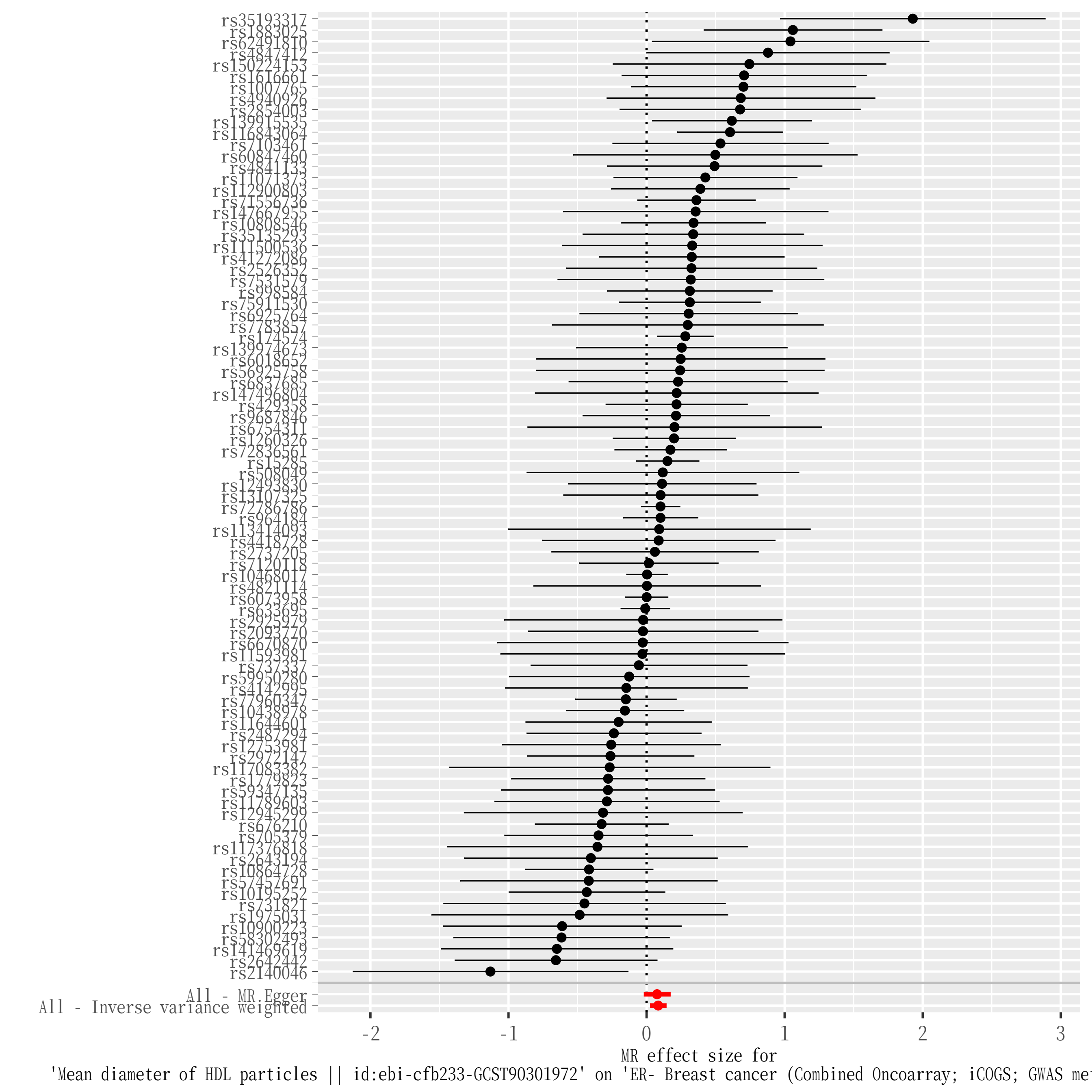
rs35193317
rs1883025
rs62491810
rs4847412
rs150224153
rs1616661
rs1007765
rs4940926
rs2854003
rs139915535
rs116843064
rs7103461
rs60847460
rs4841133
rs11071373
rs112900803
rs71556736
rs147667955
rs10808546
rs35135293
rs111500536
rs41272086
rs2526352
rs7531579
rs998584
rs75911530
rs6925764
rs7783857
rs174574
rs139974673
rs6018652
rs56925758
rs6837685
rs147496804
rs429358
rs9687846
rs6754311
rs1260326
rs72836561
rs15285
rs508049
rs12493830
rs13107325
rs72786786
rs964184
rs113414093
rs4418728
rs2737205
rs7120118
rs10468017
rs4821114
rs6073958
rs633695
rs2925979
rs2093770
rs6670870
rs11593981
rs737337
rs59950280
rs4142995
rs77960347
rs10438978
rs11644601
rs2487294
rs12753981
rs2972147
rs117083382
rs1779823
rs59347135
rs11789603
rs12945299
rs676210
rs705379
rs117376818
rs2643194
rs10864728
rs57457691
rs10195252
rs731821
rs1975031
rs10900223
rs58302493
rs141469619
rs2642442
rs2140046
All - MR Egger
All - Inverse variance weighted
-2
-1
0
1
2
3
MR effect size for
'Mean diameter of HDL particles || id:ebi-cfb233-GCST90301972' on 'ER- Breast cancer (Combined Oncoarray; iCOGS; GWAS me

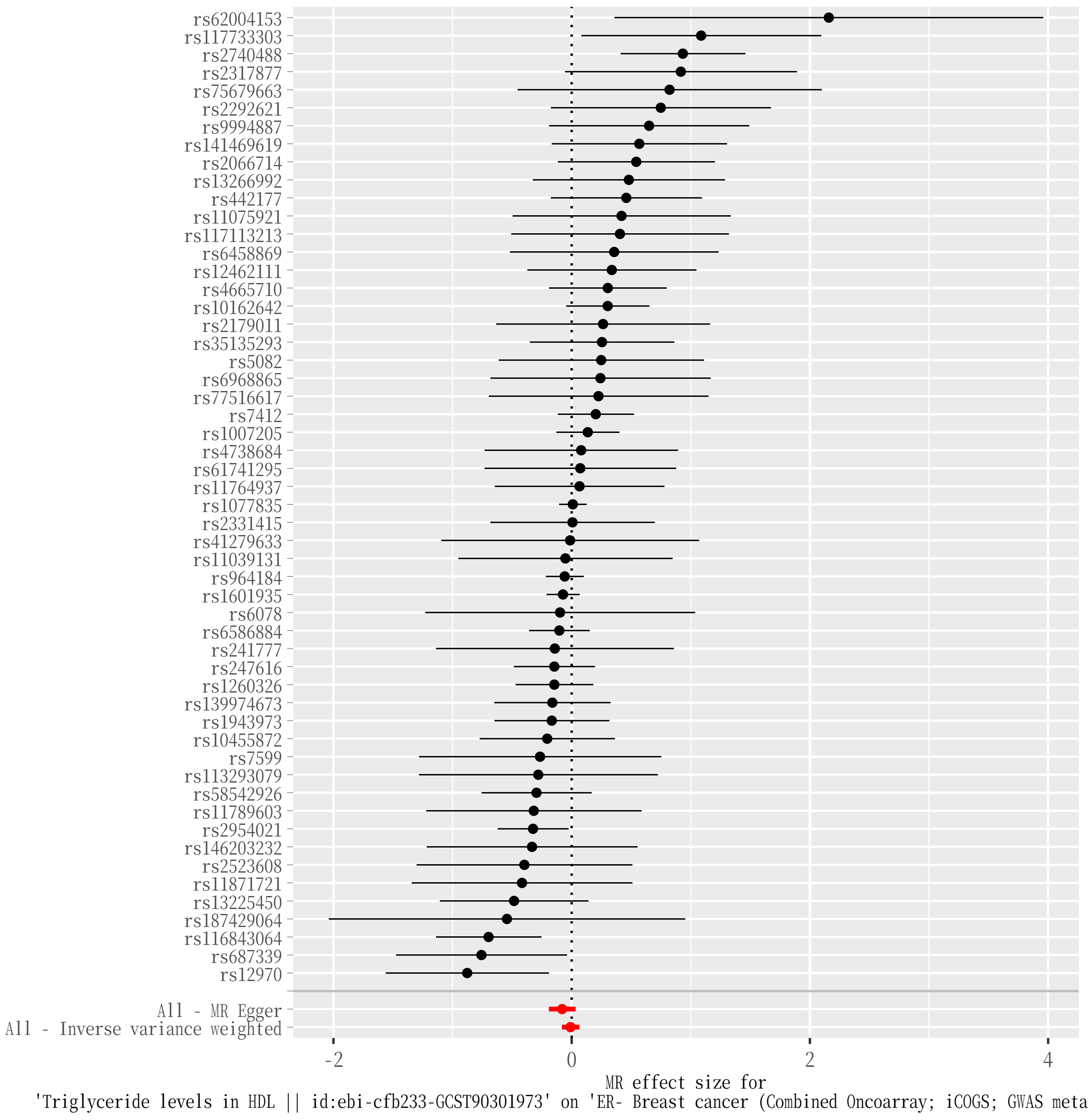
rs62004153
rs117733303
rs2740488
rs2317877
rs75679663
rs2292621
rs9994887
rs141469619
rs2066714
rs13266992
rs442177
rs11075921
rs117113213
rs6458869
rs12462111
rs4665710
rs10162642
rs2179011
rs35135293
rs5082
rs6968865
rs77516617
rs7412
rs1007205
rs4738684
rs61741295
rs11764937
rs1077835
rs2331415
rs41279633
rs11039131
rs964184
rs1601935
rs6078
rs6586884
rs241777
rs247616
rs1260326
rs139974673
rs1943973
rs10455872
rs7599
rs113293079
rs58542926
rs11789603
rs2954021
rs146203232
rs2523608
rs11871721
rs13225450
rs187429064
rs116843064
rs687339
rs12970
All - MR Egger
All - Inverse variance weighted
-2
0
2
4
MR effect size for
'Triglyceride levels in HDL || id:ebi-cfb233-GCST90301973' on 'ER- Breast cancer (Combined Oncoarray; iCOGS; GWAS meta

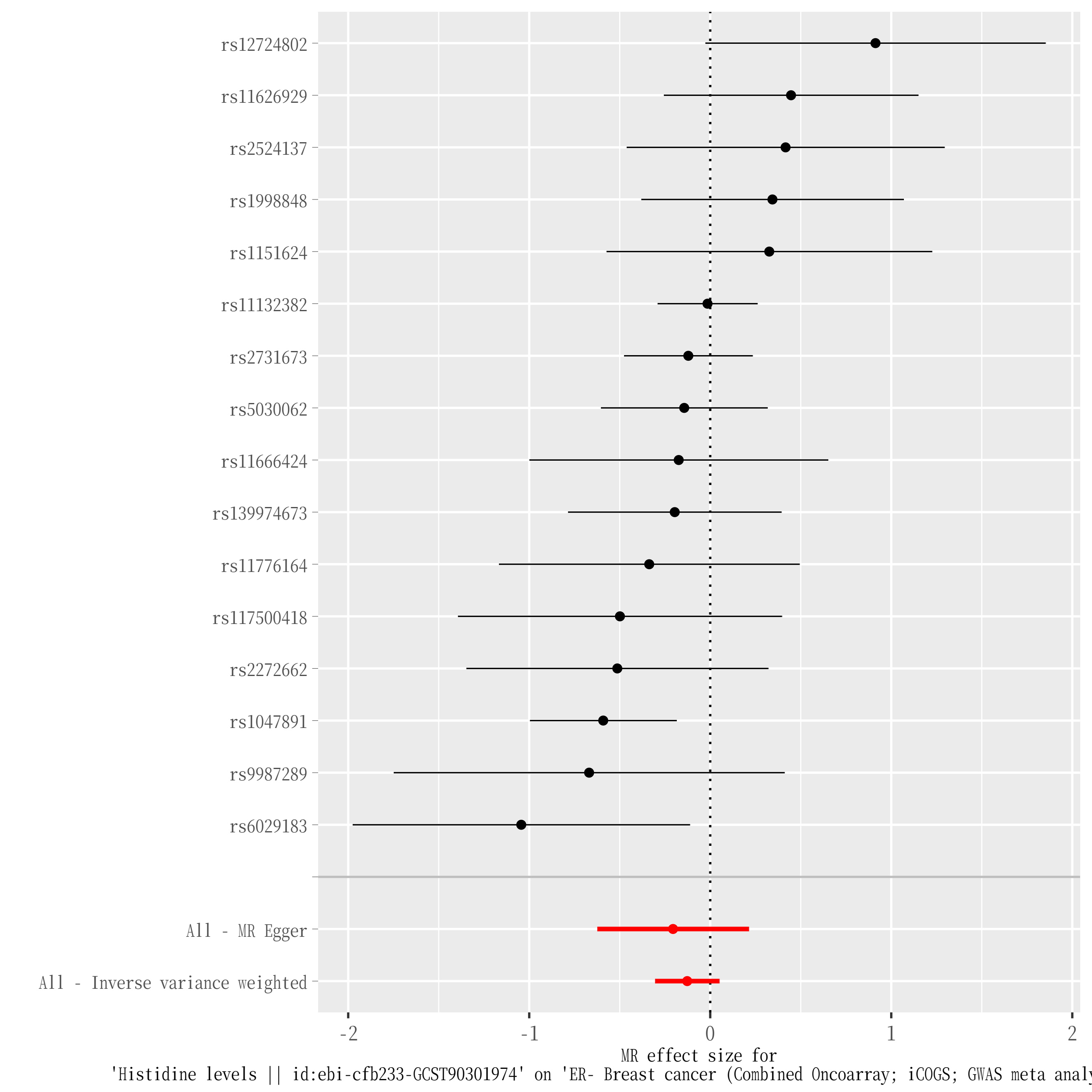
rs12724802
rs11626929
rs2524137
rs1998848
rs1151624
rs11132382
rs2731673
rs5030062
rs11666424
rs139974673
rs11776164
rs117500418
rs2272662
rs1047891
rs9987289
rs6029183
All - MR Egger
All - Inverse variance weighted
-2
-1
0
1
2
MR effect size for
'Histidine levels || id:ebi-cfb233-GCST90301974' on 'ER- Breast cancer (Combined Oncoarray; iCOGS; GWAS meta analy

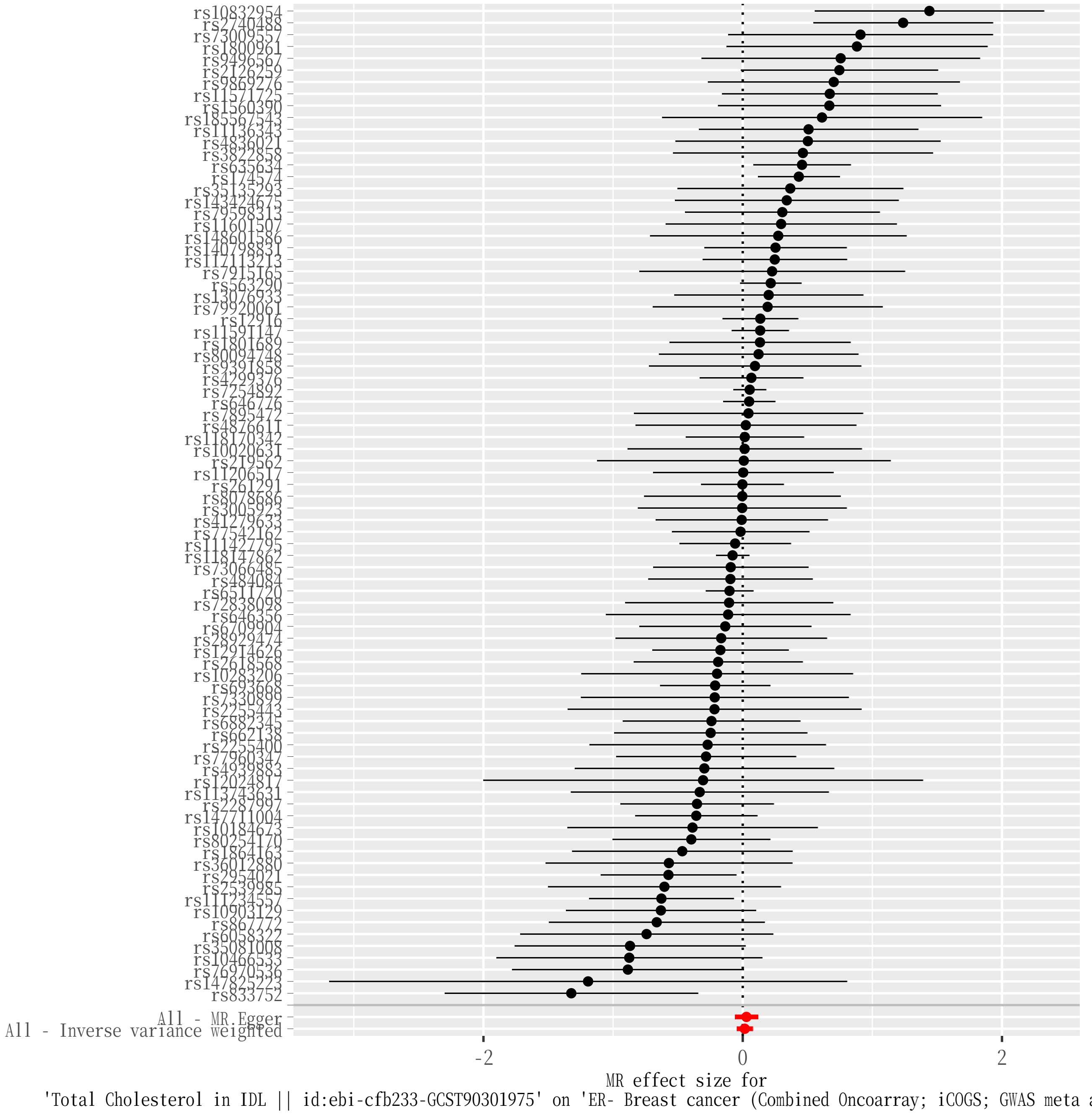

rs10832954
rs2740488
rs73009557
rs1800961
rs9496567
rs2126259
rs9869276
rs11571725
rs1560390
rs185567543
rs11136343
rs4836021
rs3822858
rs635634
rs174574
rs35135293
rs143424675
rs79598313
rs11601507
rs148601586
rs140798831
rs112113213
rs7915165
rs563290
rs13076933
rs79920061
rs12916
rs11591147
rs1801689
rs80094748
rs9391858
rs4299376
rs7254892
rs646776
rs7895472
rs4876611
rs118170342
rs10020631
rs219562
rs11206517
rs261291
rs8078686
rs3005923
rs41279633
rs77542162
rs111427795
rs118147862
rs73066485
rs484084
rs6511720
rs72838098
rs646356
rs6709904
rs28929474
rs12914626
rs2618568
rs10283206
rs693668
rs7330899
rs2255443
rs6882345
rs662138
rs2255400
rs7760347
rs4939883
rs12024817
rs113743631
rs2287997
rs147711004
rs10184673
rs80254170
rs1864163
rs36012880
rs2954021
rs2539985
rs111234557
rs10903129
rs867772
rs6058322
rs35081008
rs10466533
rs76970536
rs147825223
rs833752
All - MR Egger
All - Inverse variance weighted
-2
0
2
MR effect size for
'Total Cholesterol in IDL || id:ebi-cfb233-GCST90301975' on 'ER- Breast cancer (Combined Oncoarray; iCOGS; GWAS meta a

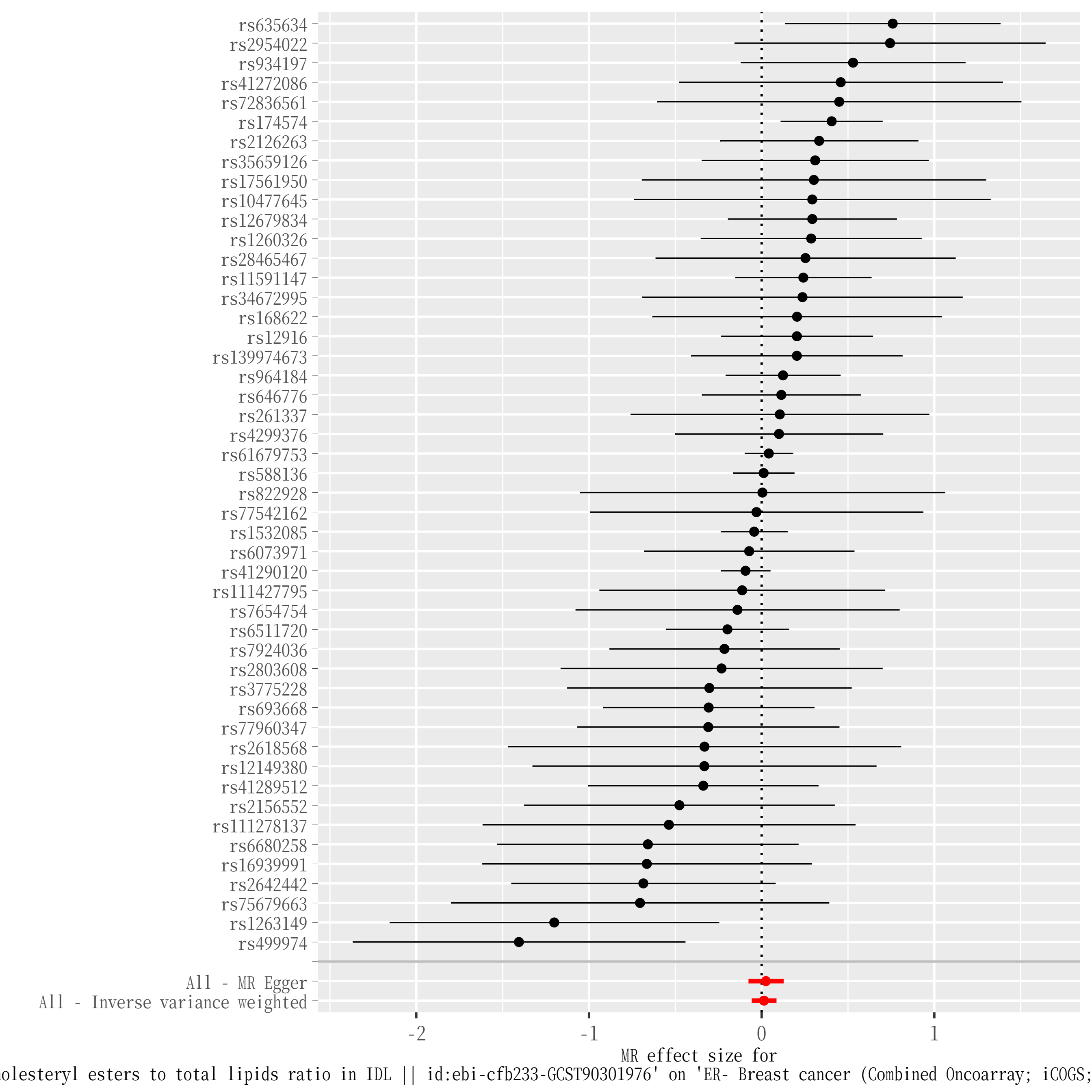

rs635634
rs2954022
rs934197
rs41272086
rs72836561
rs174574
rs2126263
rs35659126
rs17561950
rs10477645
rs12679834
rs1260326
rs28465467
rs11591147
rs34672995
rs168622
rs12916
rs139974673
rs964184
rs646776
rs261337
rs4299376
rs61679753
rs588136
rs822928
rs77542162
rs1532085
rs6073971
rs41290120
rs111427795
rs7654754
rs6511720
rs7924036
rs2803608
rs3775228
rs693668
rs77960347
rs2618568
rs12149380
rs41289512
rs2156552
rs111278137
rs6680258
rs16939991
rs2642442
rs75679663
rs1263149
rs499974
All - MR Egger
All - Inverse variance weighted
-2
-1
0
1
MR effect size for
holesteryl esters to total lipids ratio in IDL || id:ebi-cfb233-GCST90301976' on 'ER- Breast cancer (Combined Oncoarray; iCOGS;

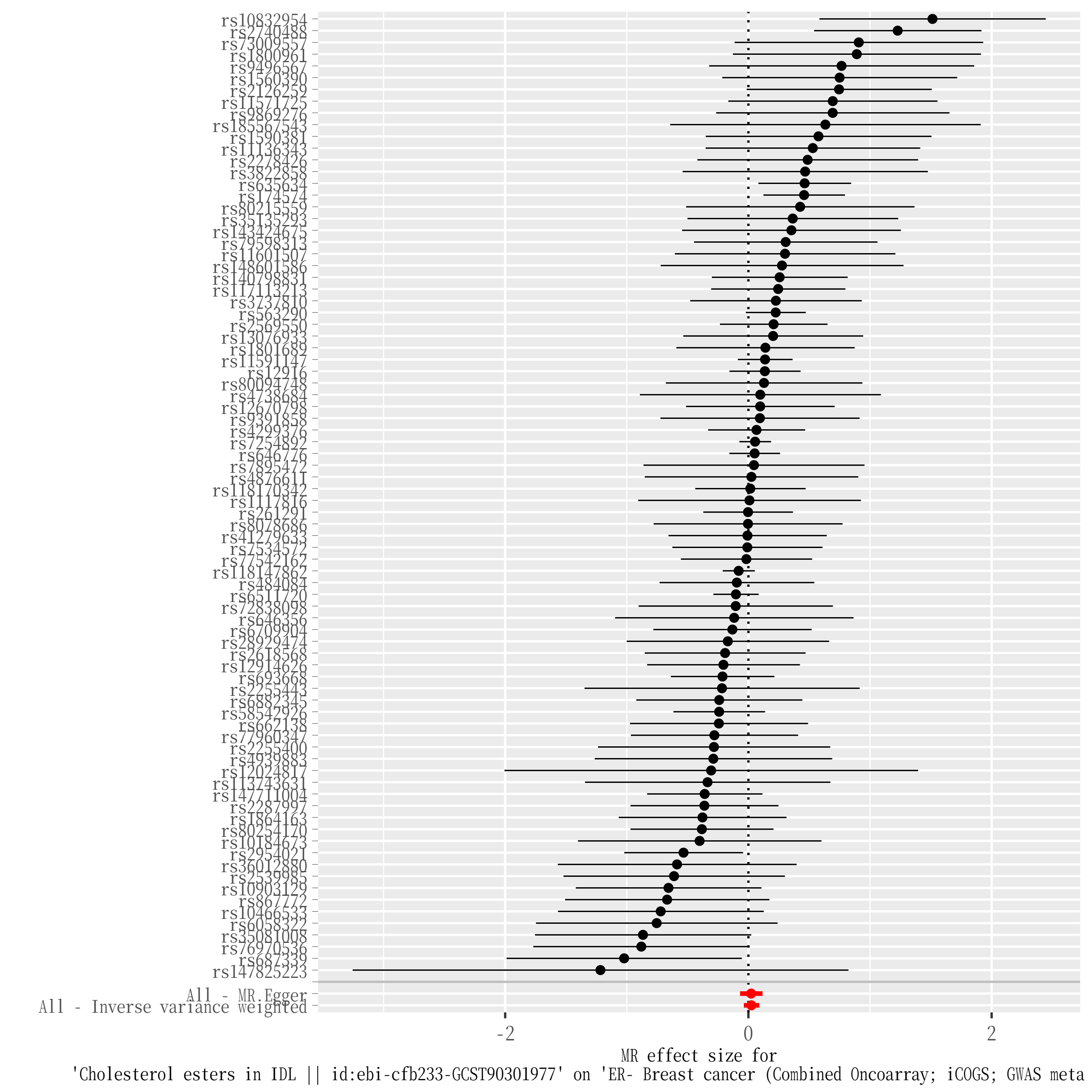
rs10832954
rs2740488
rs73009557
rs1800961
rs9496567
rs1560320
rs2126259
rs11571725
rs9869276
rs185567543
rs1590381
rs11136343
rs2278426
rs3822858
rs635634
rs174574
rs80215559
rs35135293
rs143424675
rs79598313
rs11601507
rs148601586
rs140798831
rs117113213
rs3737810
rs563290
rs2562550
rs13076933
rs1801689
rs11591147
rs12916
rs80094748
rs4738684
rs12670798
rs9391858
rs4299376
rs7254892
rs646776
rs7895472
rs4876611
rs118170342
rs1117816
rs261291
rs8078686
rs41279633
rs7534572
rs77542162
rs118147862
rs484084
rs6511720
rs72838098
rs646356
rs6709904
rs28929474
rs2618568
rs12914626
rs693668
rs2255443
rs6882345
rs58542926
rs662138
rs77960347
rs2255400
rs4939883
rs12024817
rs113743631
rs147711004
rs2287997
rs1864163
rs80254170
rs10184673
rs2954021
rs36012880
rs2539985
rs10903129
rs867772
rs10466533
rs6058322
rs35081008
rs76970536
rs687339
rs147825223
All - MR Egger
All - Inverse variance weighted
-2
0
2
MR effect size for
'Cholesterol esters in IDL || id:ebi-cfb233-GCST90301977' on 'ER- Breast cancer (Combined Oncoarray; iCOGS; GWAS meta

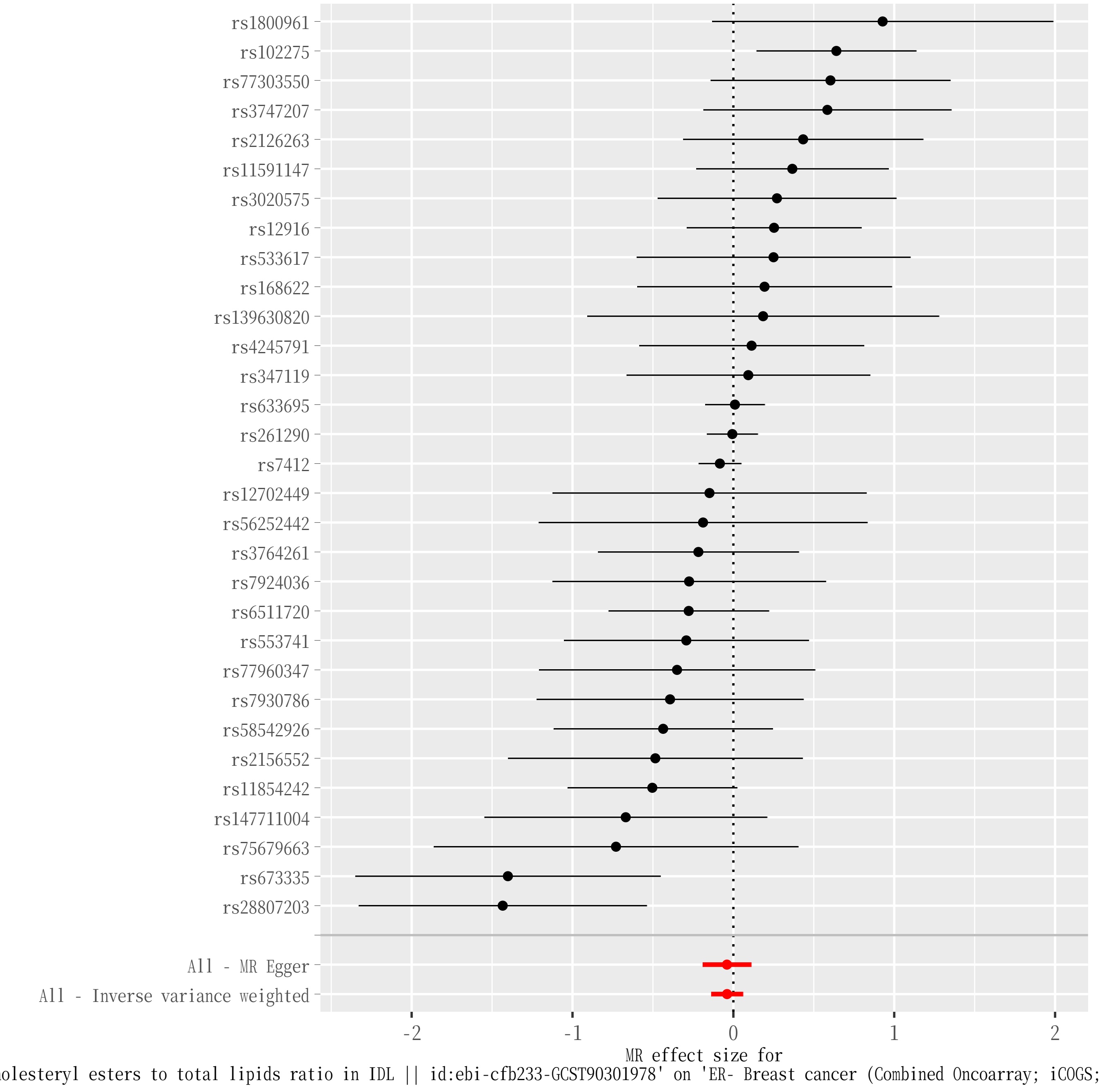
rs1800961
rs102275
rs77303550
rs3747207
rs2126263
rs11591147
rs3020575
rs12916
rs533617
rs168622
rs139630820
rs4245791
rs347119
rs633695
rs261290
rs7412
rs12702449
rs56252442
rs3764261
rs7924036
rs6511720
rs553741
rs77960347
rs7930786
rs58542926
rs2156552
rs11854242
rs147711004
rs75679663
rs673335
rs28807203
All - MR Egger
All - Inverse variance weighted
-2
-1
0
1
2
MR effect size for
holesteryl esters to total lipids ratio in IDL || id:ebi-cfb233-GCST90301978' on 'ER- Breast cancer (Combined Oncoarray; iCOGS;

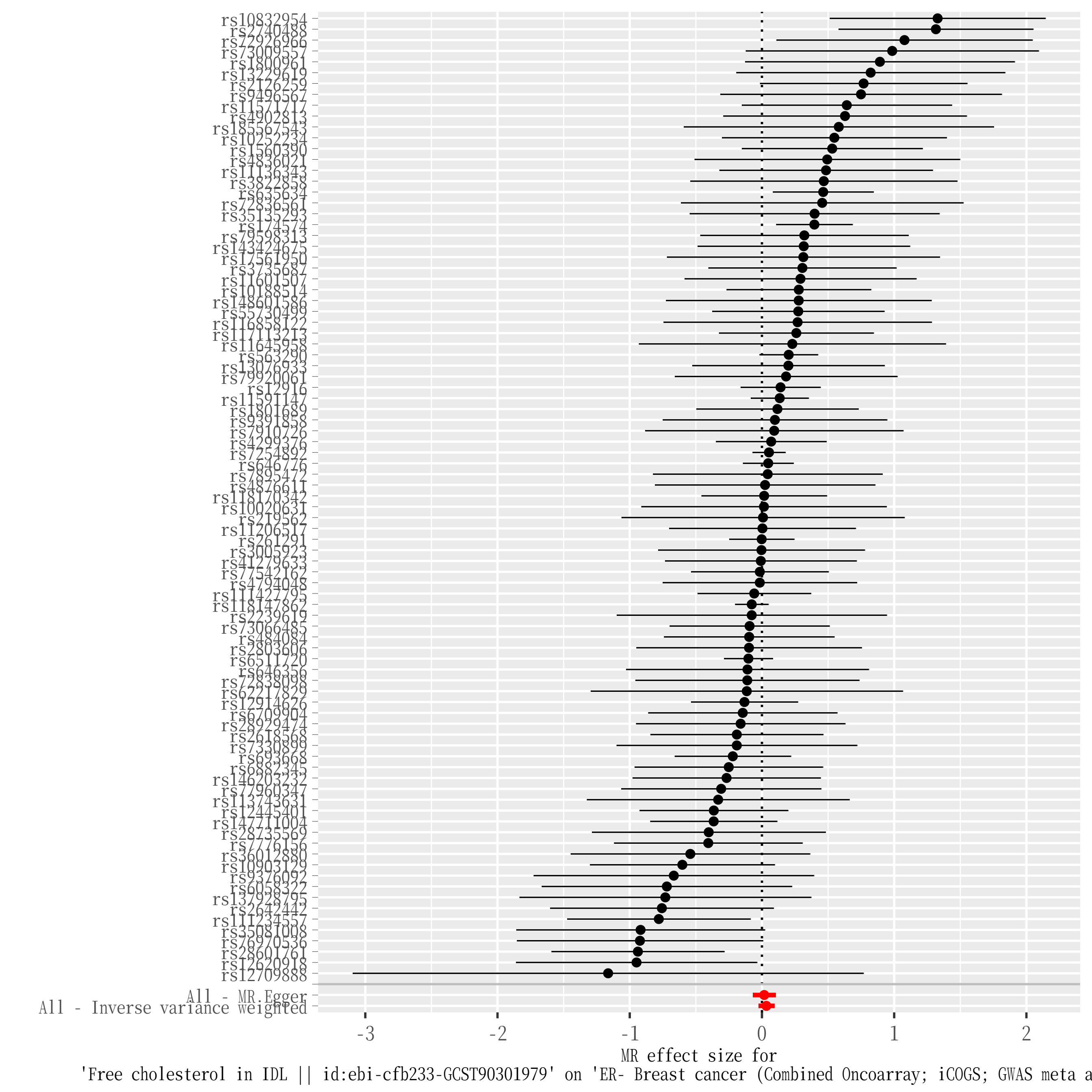
rs10832954
rs2740488
rs72926966
rs73009557
rs1800961
rs13229619
rs2126259
rs9496567
rs11571717
rs4902813
rs185567543
rs10252234
rs1560390
rs4836021
rs11136343
rs3822858
rs635634
rs72836561
rs35135293
rs174574
rs79598313
rs143424675
rs17561950
rs3735687
rs11601507
rs10188514
rs148601586
rs55730499
rs116858122
rs117113213
rs11645958
rs563290
rs13076933
rs79920061
rs12916
rs11591147
rs1801689
rs9391858
rs7910726
rs4299376
rs7254892
rs646776
rs7895472
rs4876611
rs118170342
rs10020631
rs219562
rs11206517
rs261291
rs3005923
rs41279633
rs77542162
rs4794048
rs11142795
rs118147862
rs2239619
rs73066485
rs484084
rs2803606
rs6511720
rs646356
rs72838028
rs62217829
rs12914626
rs6709904
rs28929474
rs2618566
rs7330899
rs693668
rs6882345
rs146203232
rs7960347
rs113743631
rs12445401
rs147711004
rs28735569
rs7776156
rs36012880
rs10903129
rs9376092
rs6058322
rs137928795
rs2642442
rs111234557
rs35081008
rs76270536
rs28601761
rs12620918
rs12709888
All - MR Egger
All - Inverse variance weighted
-3
-2
-1
0
1
2
MR effect size for
'Free cholesterol in IDL || id:ebi-cfb233-GCST90301979' on 'ER- Breast cancer (Combined Oncoarray; iCOGS; GWAS meta a

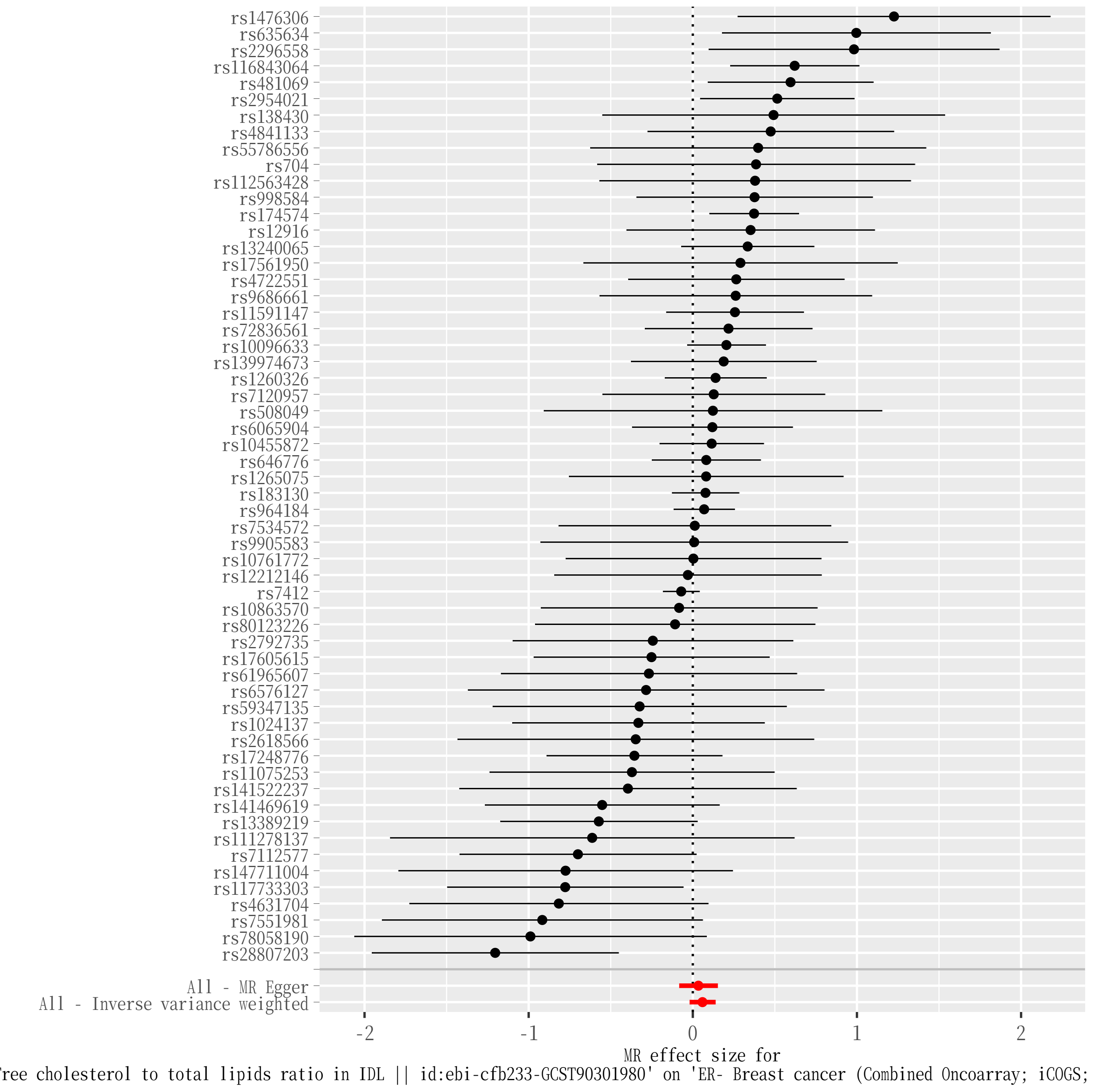
rs1476306
rs635634
rs2296558
rs116843064
rs481069
rs2954021
rs138430
rs4841133
rs55786556
rs704
rs112563428
rs998584
rs174574
rs12916
rs13240065
rs17561950
rs4722551
rs9686661
rs11591147
rs72836561
rs10096633
rs139974673
rs1260326
rs7120957
rs508049
rs6065904
rs10455872
rs646776
rs1265075
rs183130
rs964184
rs7534572
rs9905583
rs10761772
rs12212146
rs7412
rs10863570
rs80123226
rs2792735
rs17605615
rs61965607
rs6576127
rs59347135
rs1024137
rs2618566
rs17248776
rs11075253
rs141522237
rs141469619
rs13389219
rs111278137
rs7112577
rs147711004
rs117733303
rs4631704
rs7551981
rs78058190
rs28807203
All - MR Egger
All - Inverse variance weighted
-2
-1
0
1
2
MR effect size for
'Free cholesterol to total lipids ratio in IDL || id:ebi-cfb233-GCST90301980' on 'ER- Breast cancer (Combined Oncoarray; iCOGS;

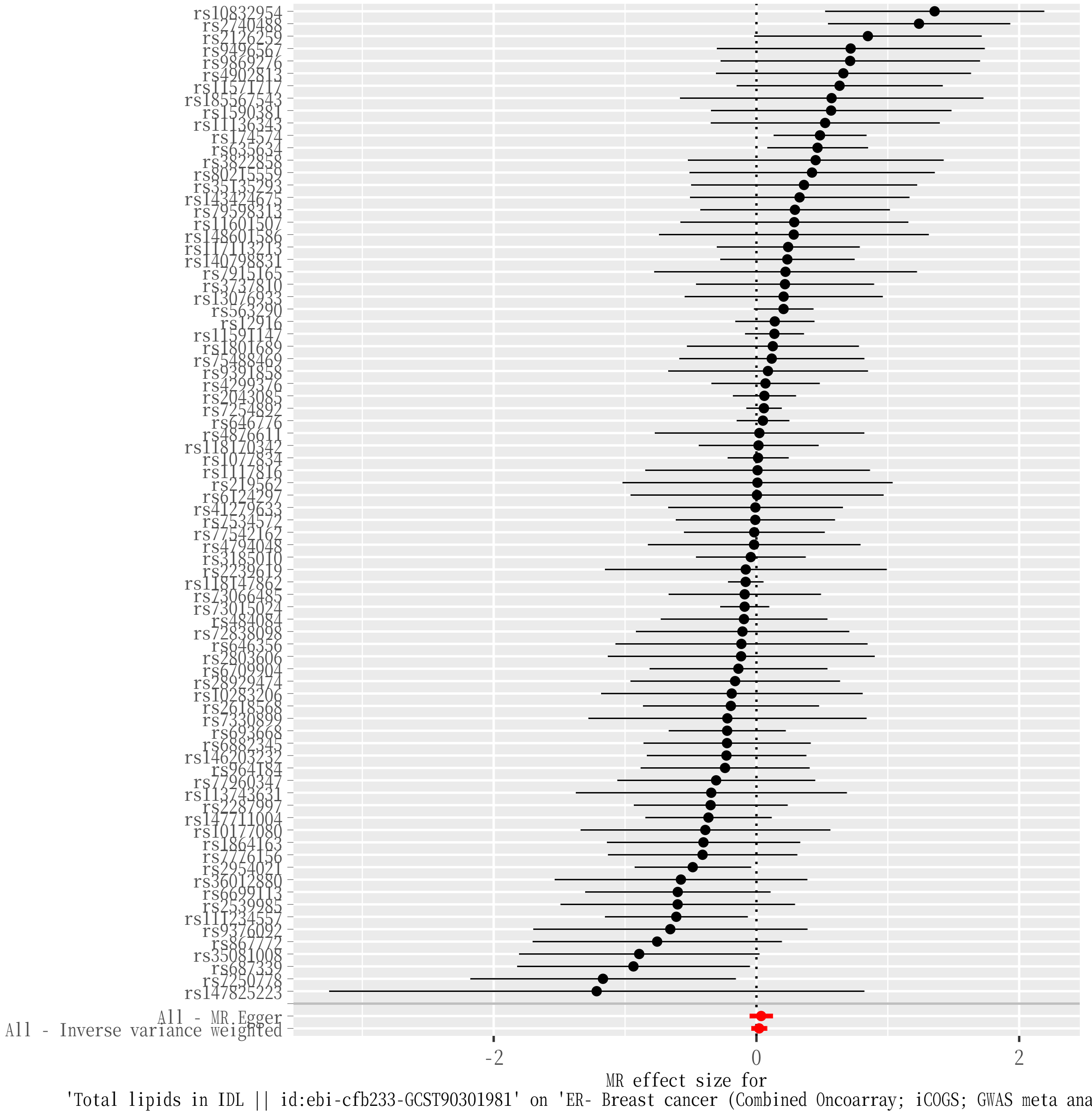
rs10832954
rs2740488
rs2126259
rs9496567
rs9869276
rs4902813
rs11571717
rs185567543
rs1590381
rs11136343
rs174574
rs635634
rs3822858
rs80215559
rs35135293
rs143424675
rs79598313
rs11601507
rs148601586
rs117113213
rs140798831
rs7915165
rs3737810
rs13076933
rs563290
rs12916
rs11591147
rs1801689
rs75488469
rs9391858
rs4299376
rs2043085
rs7254892
rs646776
rs4876611
rs118170342
rs1077834
rs1117816
rs219562
rs6124297
rs41279633
rs7534572
rs77542162
rs4794048
rs3185010
rs2239619
rs118147862
rs73066485
rs73015024
rs484084
rs72838098
rs646356
rs2803606
rs6709904
rs28929474
rs10283206
rs2618568
rs7330899
rs693668
rs6882345
rs146203232
rs964184
rs77960347
rs113743631
rs2287997
rs147711004
rs10177080
rs1864163
rs7776156
rs2954021
rs36012880
rs6699113
rs2539985
rs111234557
rs9376092
rs867772
rs35081008
rs687339
rs2250778
rs147825223
All - MR Egger
All - Inverse variance weighted
-2
0
2
MR effect size for
'Total lipids in IDL || id:ebi-cfb233-GCST90301981' on 'ER- Breast cancer (Combined Oncoarray; iCOGS; GWAS meta ana

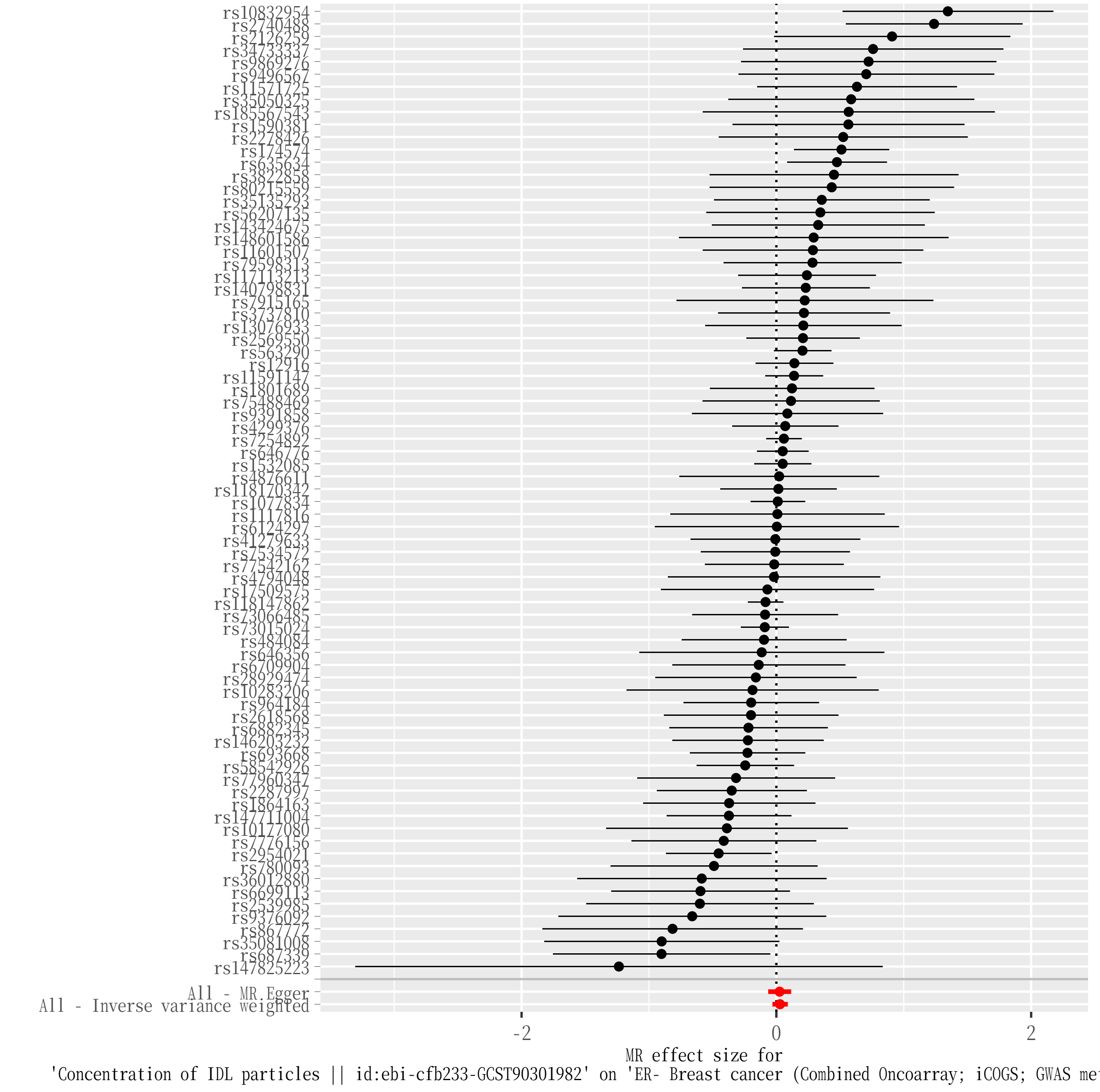
rs10832954
rs2740488
rs2126259
rs34733337
rs9869276
rs9496567
rs11571725
rs35050325
rs185567543
rs1590381
rs2278426
rs174574
rs635634
rs3822858
rs80215559
rs35135293
rs56207135
rs143424675
rs148601586
rs11601507
rs79598313
rs117113213
rs140798831
rs7915165
rs3737810
rs13076933
rs2569550
rs563290
rs12916
rs11591147
rs1801689
rs75488469
rs9391858
rs4299376
rs7254892
rs646776
rs1532085
rs4876611
rs118170342
rs1077834
rs1117816
rs6124297
rs41279633
rs7534572
rs77542162
rs4794048
rs17509575
rs118147862
rs73066485
rs73015024
rs484084
rs646356
rs6709904
rs28929474
rs10283206
rs964184
rs2618568
rs6882345
rs146203232
rs693668
rs58542926
rs77960347
rs2287997
rs1864163
rs147711004
rs10177080
rs7776156
rs2954021
rs780093
rs36012880
rs6699113
rs2539985
rs9376092
rs867772
rs35081008
rs687339
rs147825223
All - MR Egger
All - Inverse variance weighted
-2
0
2
MR effect size for
'Concentration of IDL particles || id:ebi-cfb233-GCST90301982' on 'ER- Breast cancer (Combined Oncoarray; iCOGS; GWAS me

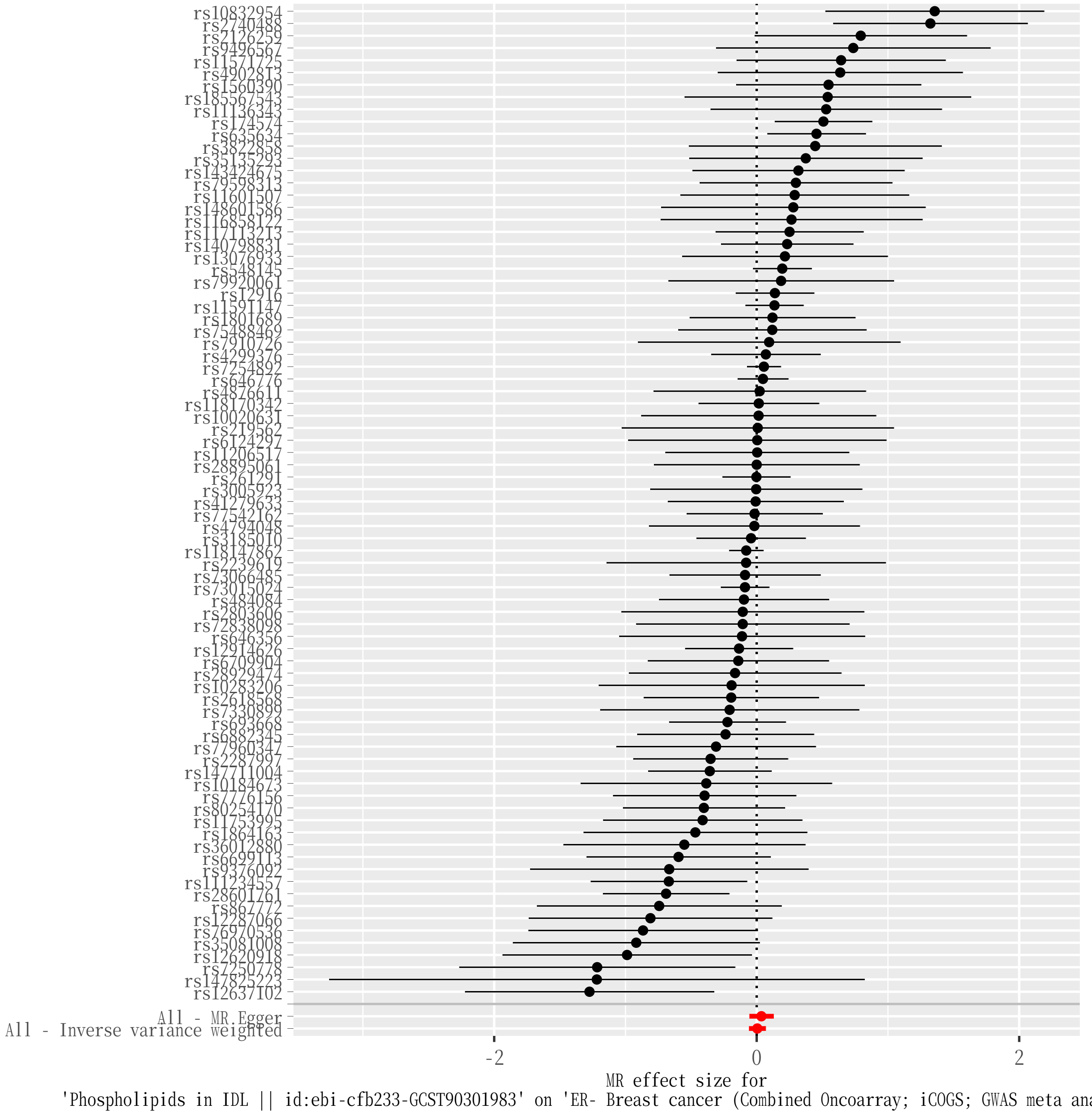
rs10832954
rs2740488
rs2126259
rs9496567
rs11571725
rs4902813
rs1560390
rs185567543
rs11136343
rs174574
rs635634
rs3822858
rs35135293
rs143424675
rs79598313
rs11601507
rs148601586
rs116858122
rs117113213
rs140798831
rs13076933
rs548145
rs79920061
rs12916
rs11591147
rs1801689
rs75488469
rs7910726
rs4299376
rs7254892
rs646776
rs4876611
rs118170342
rs10020631
rs219562
rs6124297
rs11206517
rs28895061
rs261291
rs3005923
rs41279633
rs77542162
rs4794048
rs3185010
rs118147862
rs2239619
rs73066485
rs73015024
rs484084
rs2803606
rs72838098
rs646356
rs12914626
rs6709904
rs28929474
rs10283206
rs2618568
rs7330899
rs693668
rs6882345
rs77960347
rs2287997
rs147711004
rs10184673
rs7776156
rs80254170
rs11753995
rs1864163
rs36012880
rs6699113
rs9376092
rs111234557
rs28601761
rs862772
rs12287066
rs76970536
rs35081008
rs12620918
rs7250778
rs147825223
rs12637102
All - MR Egger
All - Inverse variance weighted
-2
0
2
MR effect size for
'Phospholipids in IDL || id:ebi-cfb233-GCST90301983' on 'ER- Breast cancer (Combined Oncoarray; iCOGS; GWAS meta ana

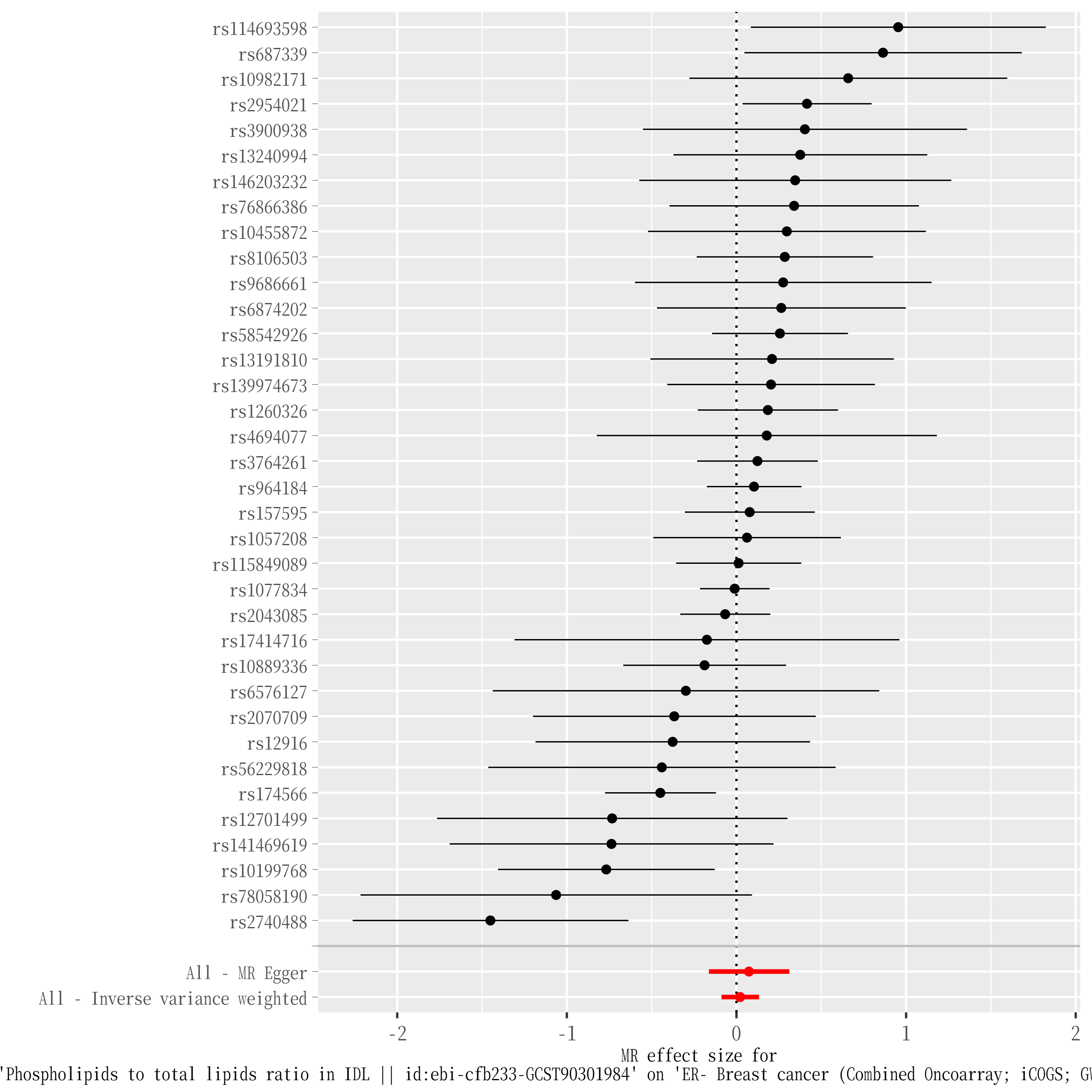
rs114693598
rs687339
rs10982171
rs2954021
rs3900938
rs13240994
rs146203232
rs76866386
rs10455872
rs8106503
rs9686661
rs6874202
rs58542926
rs13191810
rs139974673
rs1260326
rs4694077
rs3764261
rs964184
rs157595
rs1057208
rs115849089
rs1077834
rs2043085
rs17414716
rs10889336
rs6576127
rs2070709
rs12916
rs56229818
rs174566
rs12701499
rs141469619
rs10199768
rs78058190
rs2740488
All - MR Egger
All - Inverse variance weighted
-2
-1
0
1
2
MR effect size for
'Phospholipids to total lipids ratio in IDL || id:ebi-cfb233-GCST90301984' on 'ER- Breast cancer (Combined Oncoarray; iCOGS; GW

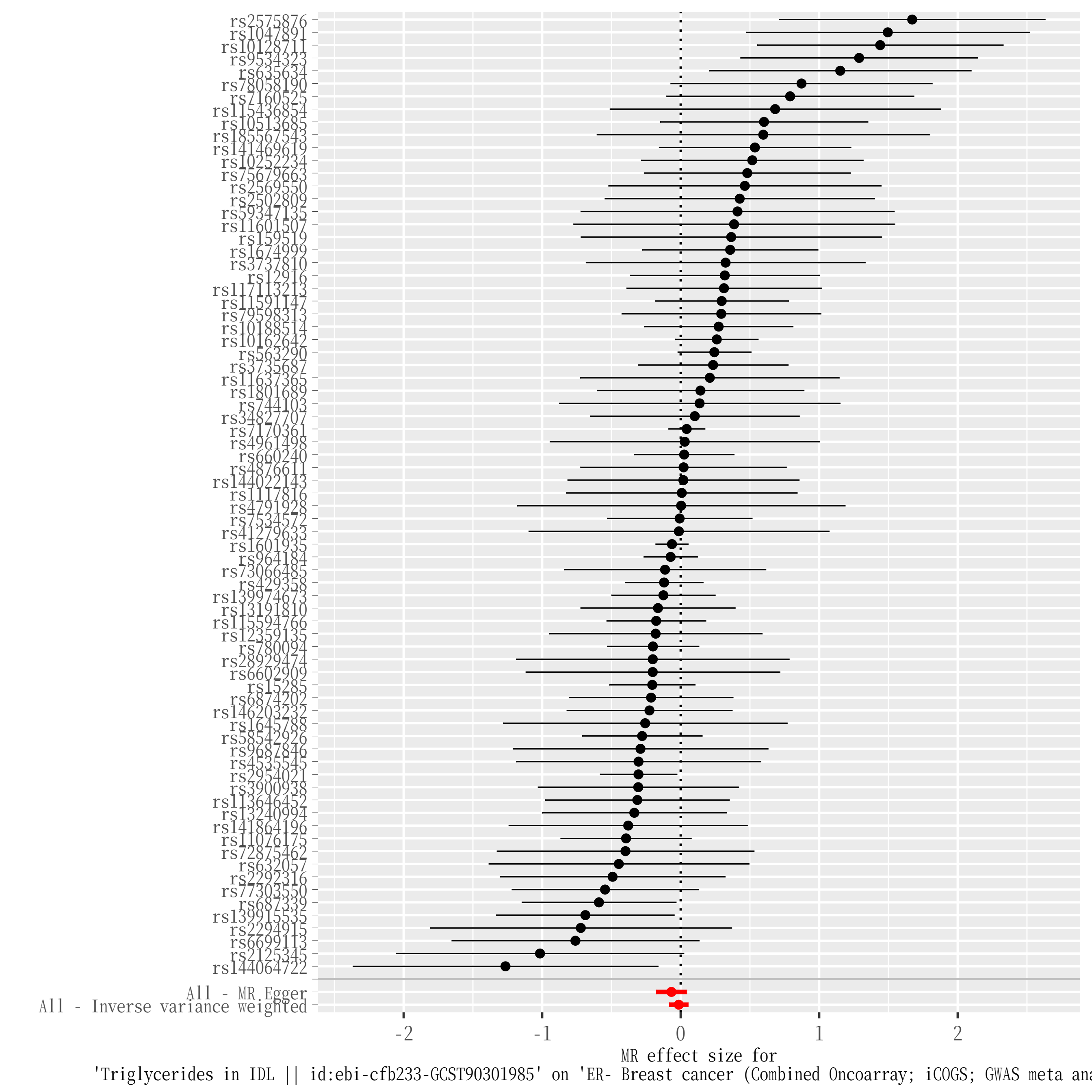
rs2575876
rs1047891
rs10128711
rs9534323
rs635634
rs78058190
rs7160525
rs115436854
rs10513685
rs185567543
rs141469619
rs10252234
rs75679663
rs2569550
rs2502809
rs59347135
rs11601507
rs159519
rs1674999
rs3737810
rs12916
rs117113213
rs11591147
rs79598313
rs10188514
rs10162642
rs563290
rs3735687
rs11637365
rs1801689
rs744103
rs34827707
rs7170361
rs4961498
rs660240
rs4876611
rs144022143
rs1117816
rs4791928
rs7534572
rs41279633
rs1601935
rs964184
rs73066485
rs429358
rs139974673
rs13191810
rs115594766
rs12359135
rs780094
rs28929474
rs6602909
rs15285
rs6874202
rs146203232
rs1645788
rs58542926
rs9687846
rs4535545
rs2954021
rs3900938
rs113646452
rs13240994
rs141864196
rs11076175
rs72875462
rs632057
rs2292316
rs77303550
rs687339
rs139915535
rs2294915
rs6699113
rs2125345
rs144064722
All - MR Egger
All - Inverse variance weighted
-2
-1
0
1
2
MR effect size for
'Triglycerides in IDL || id:ebi-cfb233-GCST90301985' on 'ER- Breast cancer (Combined Oncoarray; iCOGS; GWAS meta ana

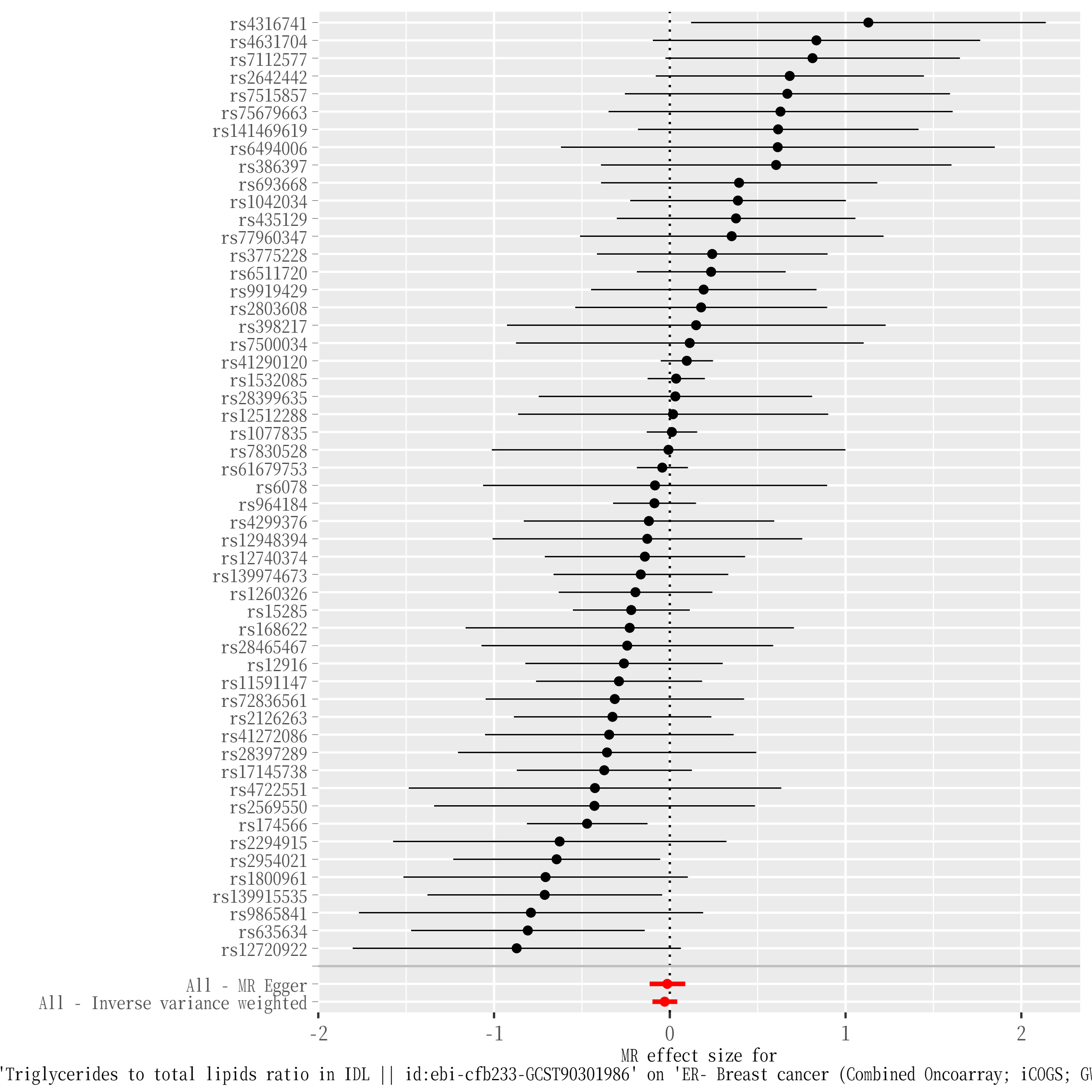
rs4316741
rs4631704
rs7112577
rs2642442
rs7515857
rs75679663
rs141469619
rs6494006
rs386397
rs693668
rs1042034
rs435129
rs77960347
rs3775228
rs6511720
rs9919429
rs2803608
rs398217
rs7500034
rs41290120
rs1532085
rs28399635
rs12512288
rs1077835
rs7830528
rs61679753
rs6078
rs964184
rs4299376
rs12948394
rs12740374
rs139974673
rs1260326
rs15285
rs168622
rs28465467
rs12916
rs11591147
rs72836561
rs2126263
rs41272086
rs28397289
rs17145738
rs4722551
rs2569550
rs174566
rs2294915
rs2954021
rs1800961
rs139915535
rs9865841
rs635634
rs12720922
All - MR Egger
All - Inverse variance weighted
-2
-1
0
1
2
MR effect size for
'Triglycerides to total lipids ratio in IDL || id:ebi-cfb233-GCST90301986' on 'ER- Breast cancer (Combined Oncoarray; iCOGS; G

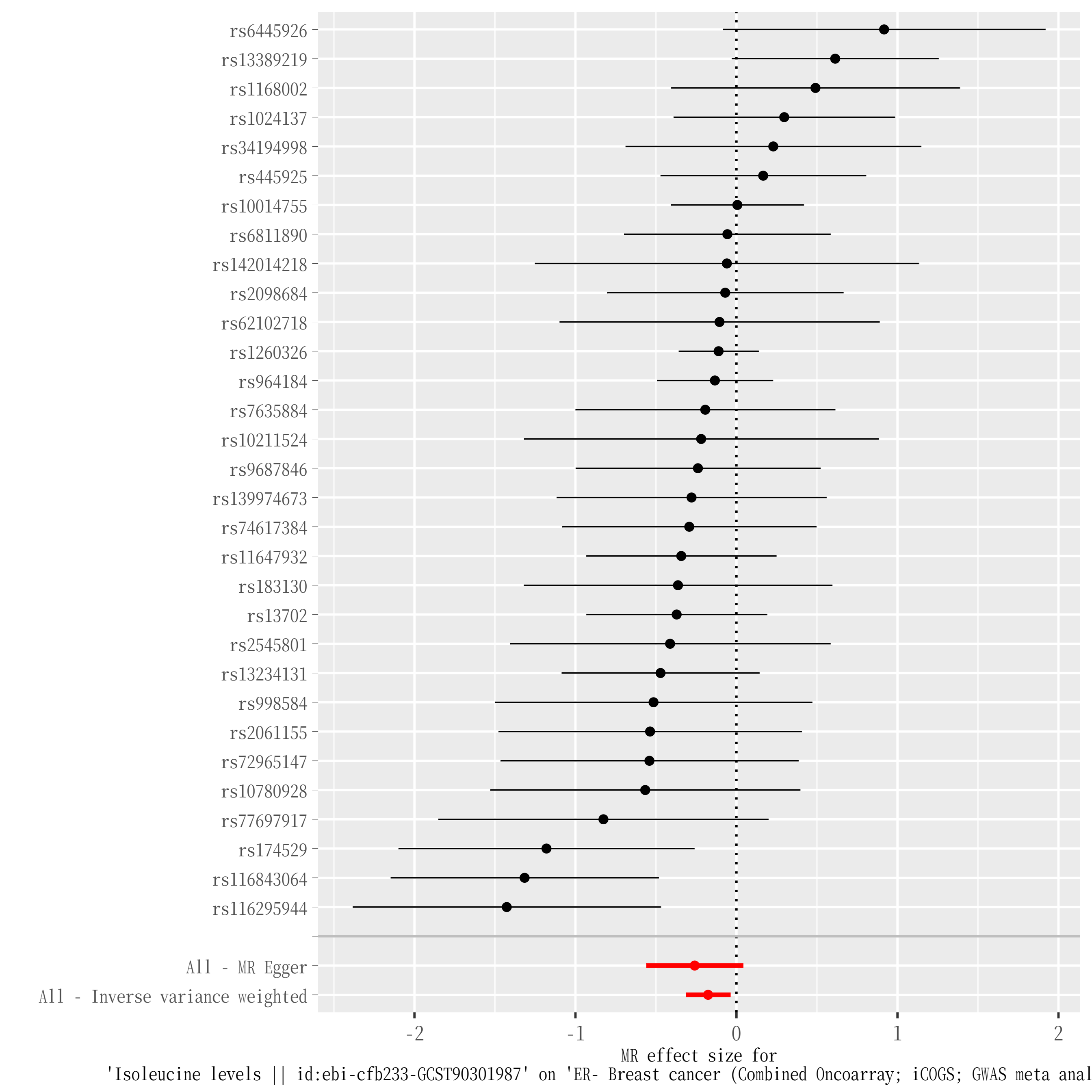
rs6445926
rs13389219
rs1168002
rs1024137
rs34194998
rs445925
rs10014755
rs6811890
rs142014218
rs2098684
rs62102718
rs1260326
rs964184
rs7635884
rs10211524
rs9687846
rs139974673
rs74617384
rs11647932
rs183130
rs13702
rs2545801
rs13234131
rs998584
rs2061155
rs72965147
rs10780928
rs77697917
rs174529
rs116843064
rs116295944
All - MR Egger
All - Inverse variance weighted
-2
-1
0
1
2
MR effect size for
'Isoleucine levels || id:ebi-cfb233-GCST90301987' on 'ER- Breast cancer (Combined Oncoarray; iCOGS; GWAS meta anal

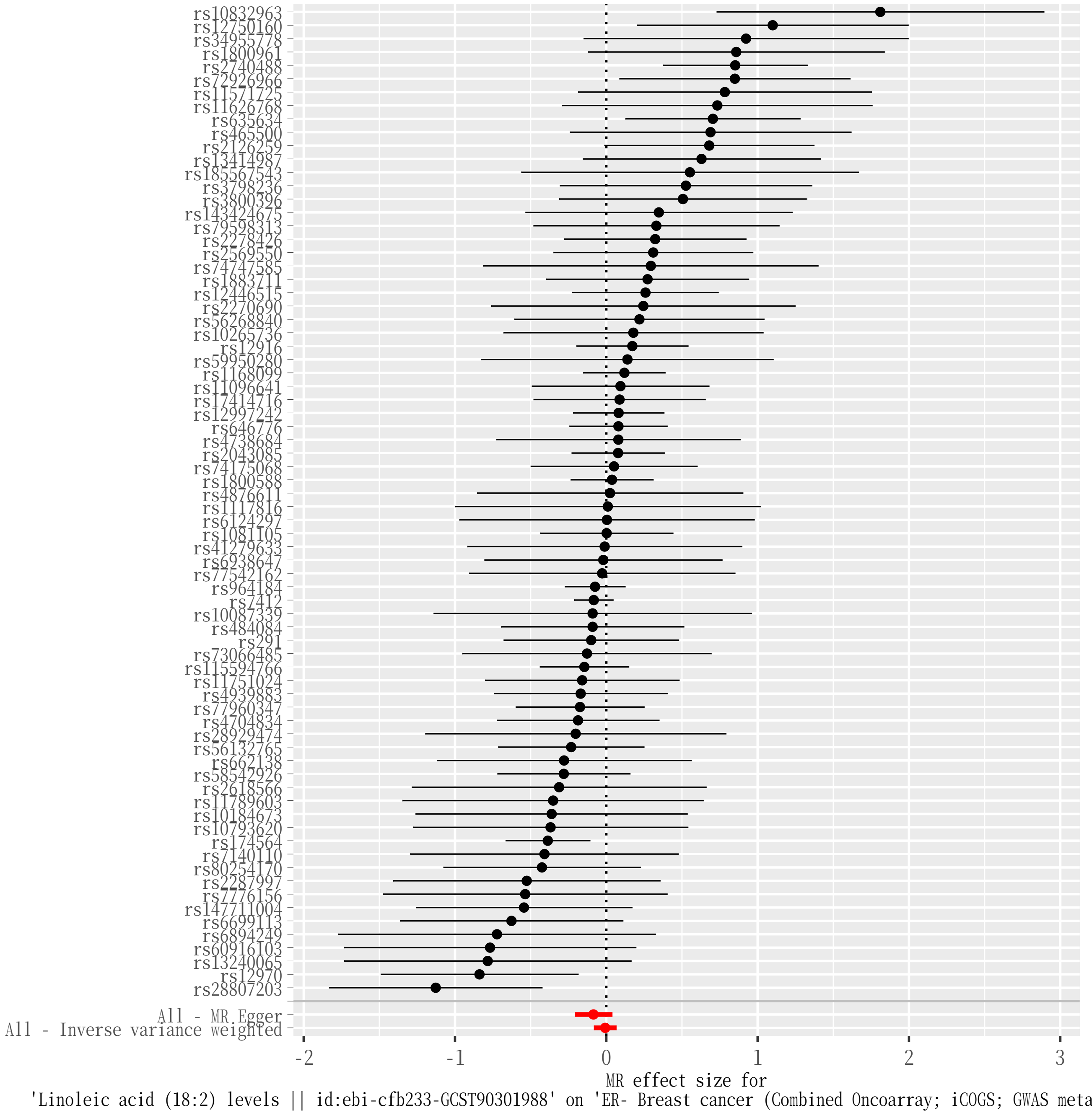
rs10832963
rs12750160
rs34955778
rs1800961
rs2740488
rs72926966
rs11571725
rs11626768
rs635634
rs465500
rs2126259
rs13414987
rs185567543
rs3798236
rs3800396
rs143424675
rs79598313
rs2278426
rs2569550
rs74747585
rs1883711
rs12446515
rs2270690
rs56268840
rs10265736
rs12916
rs59950280
rs1168099
rs11096641
rs17414716
rs12997242
rs646776
rs4738684
rs2043085
rs74175068
rs1800588
rs4876611
rs1117816
rs6124297
rs1081105
rs41279633
rs6938647
rs77542162
rs964184
rs7412
rs10087339
rs484084
rs291
rs73066485
rs115594766
rs11751024
rs4939883
rs77960347
rs4704834
rs28929474
rs56132765
rs662138
rs58542926
rs2618566
rs11789603
rs10184673
rs10793620
rs174564
rs7140110
rs80254170
rs2287997
rs7776156
rs147711004
rs6699113
rs6894249
rs60916103
rs13240065
rs12970
rs28807203
All - MR Egger
All - Inverse variance weighted
-2
-1
0
1
2
3
MR effect size for
'Linoleic acid (18:2) levels || id:ebi-cfb233-GCST90301988' on 'ER- Breast cancer (Combined Oncoarray; iCOGS; GWAS meta

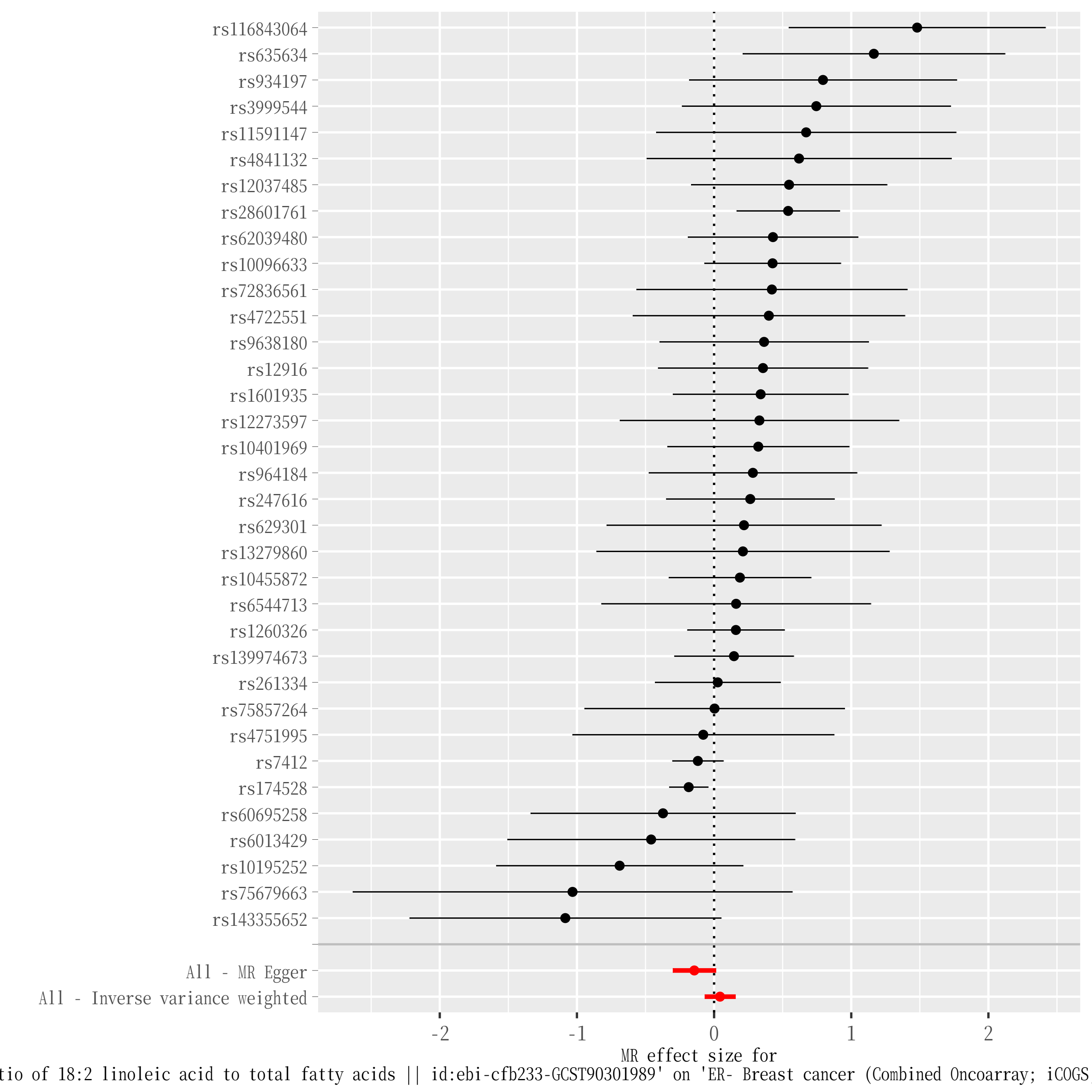
rs116843064
rs635634
rs934197
rs3999544
rs11591147
rs4841132
rs12037485
rs28601761
rs62039480
rs10096633
rs72836561
rs4722551
rs9638180
rs12916
rs1601935
rs12273597
rs10401969
rs964184
rs247616
rs629301
rs13279860
rs10455872
rs6544713
rs1260326
rs139974673
rs261334
rs75857264
rs4751995
rs7412
rs174528
rs60695258
rs6013429
rs10195252
rs75679663
rs143355652
All - MR Egger
All - Inverse variance weighted
-2
-1
0
1
2
MR effect size for
tio of 18:2 linoleic acid to total fatty acids || id:ebi-cfb233-GCST90301989' on 'ER- Breast cancer (Combined Oncoarray; iCOGS

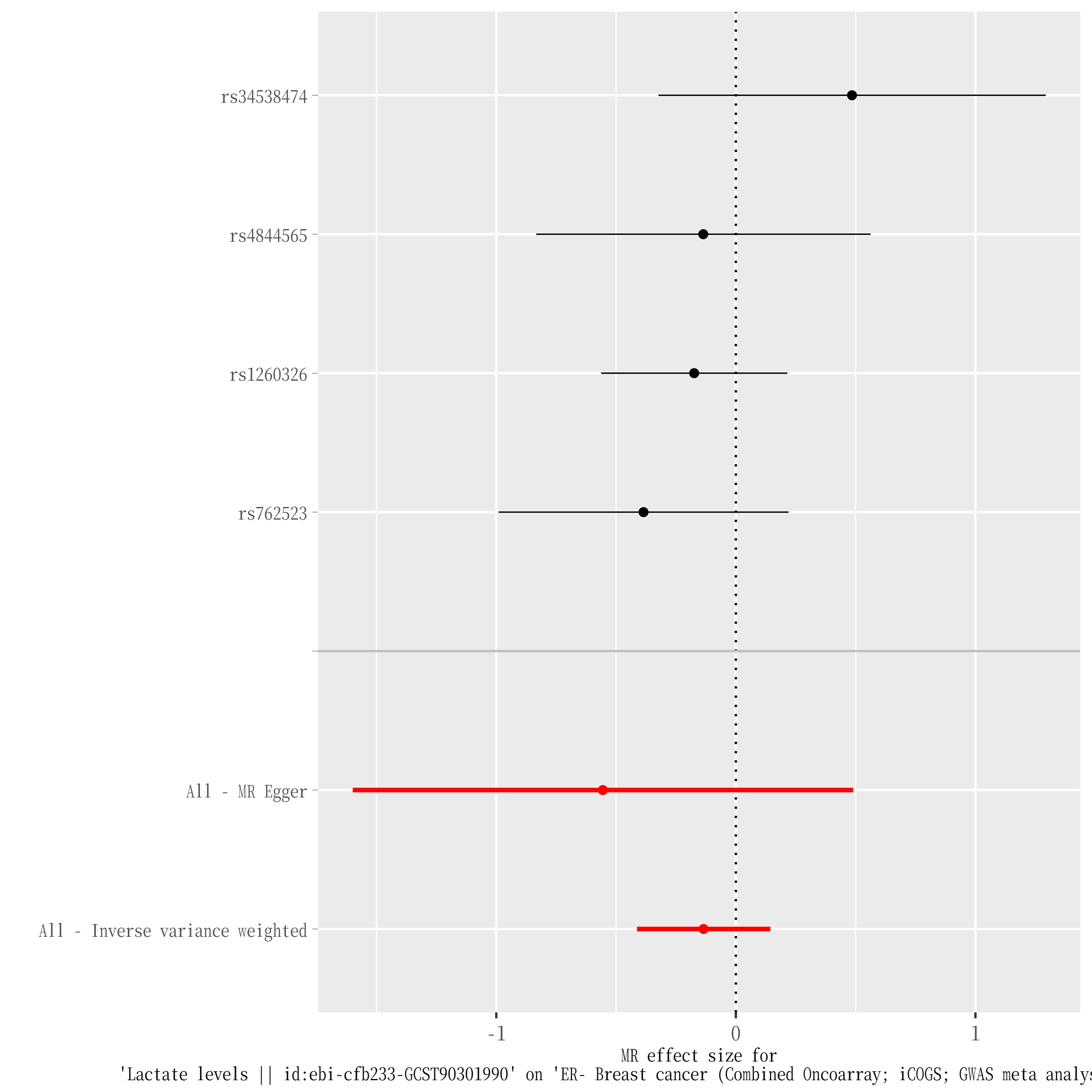
rs34538474
rs4844565
rs1260326
rs762523
All - MR Egger
All - Inverse variance weighted
-1
0
1
MR effect size for
'Lactate levels || id:ebi-cfb233-GCST90301990' on 'ER- Breast cancer (Combined Oncoarray; iCOGS; GWAS meta analys

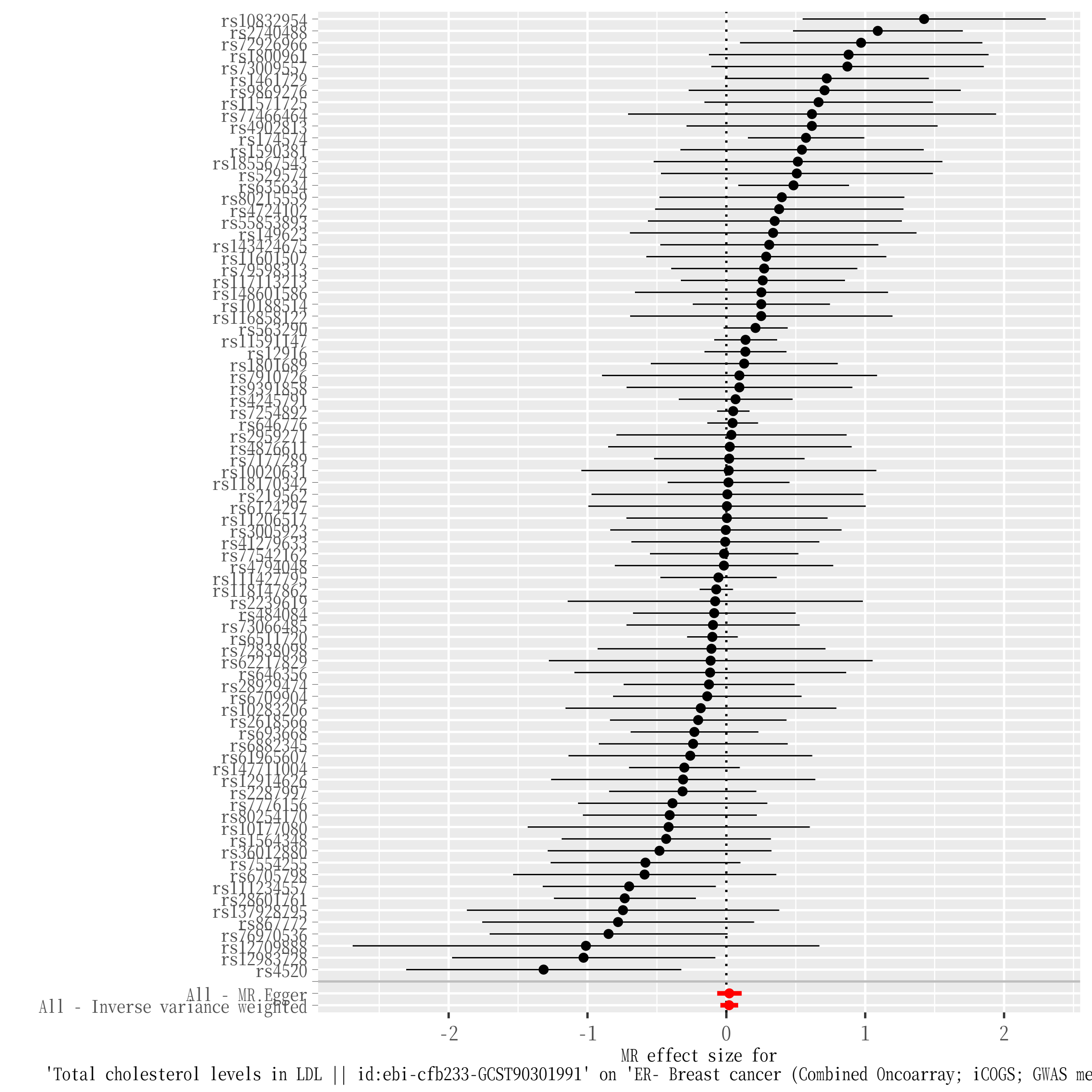
rs10832954
rs2740488
rs72926966
rs1800961
rs73009557
rs1461729
rs9869276
rs11571725
rs77466464
rs4902813
rs174574
rs1590381
rs185567543
rs522574
rs635634
rs80215559
rs4724102
rs55853893
rs149623
rs143424675
rs11601507
rs79598313
rs117113213
rs148601586
rs10188514
rs116858122
rs563290
rs11591147
rs12916
rs1801689
rs7910726
rs9391858
rs4245791
rs7254892
rs646776
rs2959271
rs4876611
rs7177289
rs10020631
rs118170342
rs219562
rs6124297
rs11206517
rs3005923
rs41279633
rs77542162
rs4794048
rs11142795
rs118147862
rs2239619
rs484084
rs73066485
rs6511720
rs72838098
rs62217829
rs646356
rs28929474
rs6709904
rs10283206
rs2618566
rs693668
rs6882345
rs61965607
rs147711004
rs12914626
rs2287997
rs7776156
rs80254170
rs10177080
rs1564348
rs36012880
rs7554255
rs6705798
rs111234557
rs28601761
rs137928795
rs867772
rs76970536
rs12709888
rs12983728
rs4520
All - MR Egger
All - Inverse variance weighted
-2
-1
0
1
2
MR effect size for
'Total cholesterol levels in LDL || id:ebi-cfb233-GCST90301991' on 'ER- Breast cancer (Combined Oncoarray; iCOGS; GWAS me

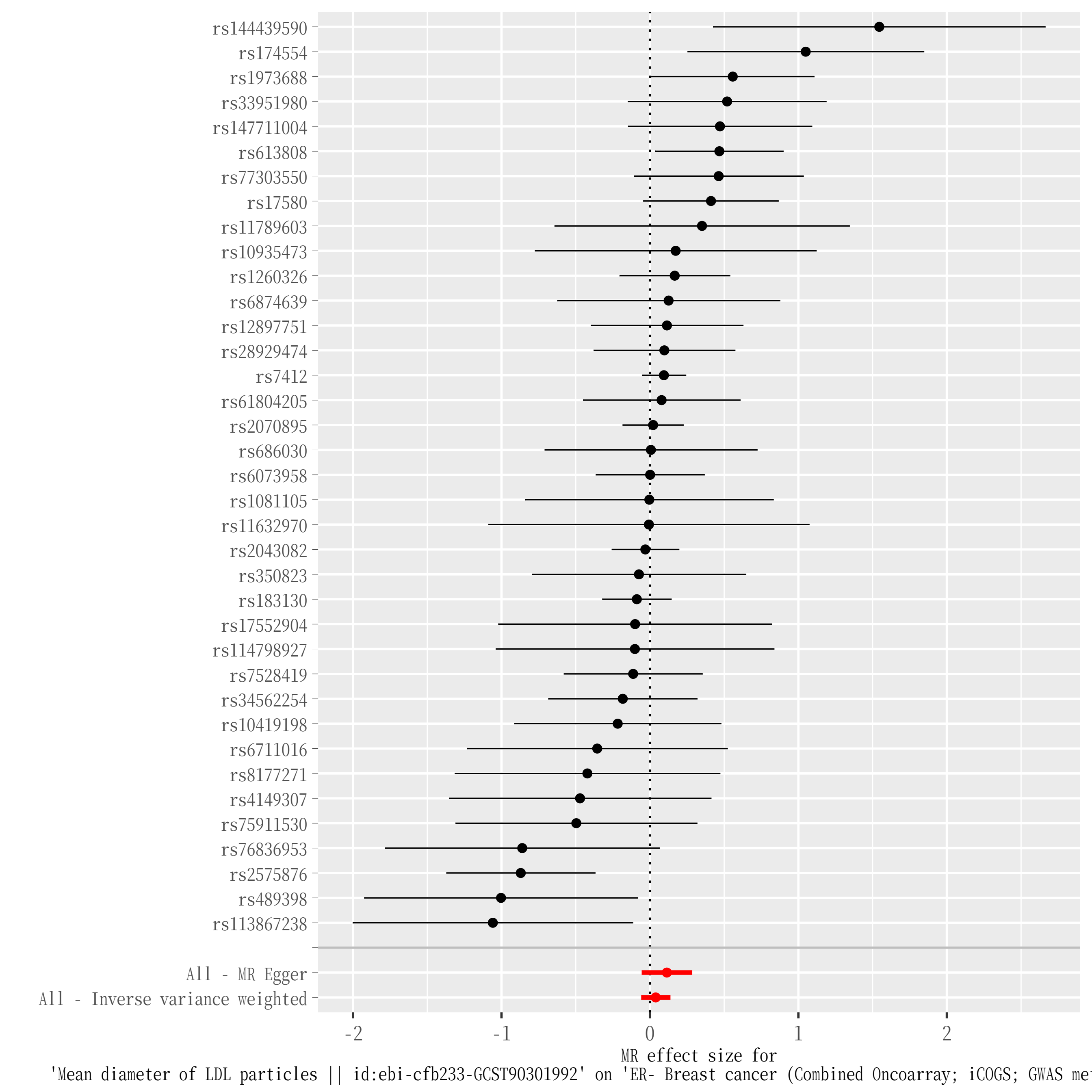
rs144439590
rs174554
rs1973688
rs33951980
rs147711004
rs613808
rs77303550
rs17580
rs11789603
rs10935473
rs1260326
rs6874639
rs12897751
rs28929474
rs7412
rs61804205
rs2070895
rs686030
rs6073958
rs1081105
rs11632970
rs2043082
rs350823
rs183130
rs17552904
rs114798927
rs7528419
rs34562254
rs10419198
rs6711016
rs8177271
rs4149307
rs75911530
rs76836953
rs2575876
rs489398
rs113867238
All - MR Egger
All - Inverse variance weighted
-2
-1
0
1
2
MR effect size for
'Mean diameter of LDL particles || id:ebi-cfb233-GCST90301992' on 'ER- Breast cancer (Combined Oncoarray; iCOGS; GWAS me

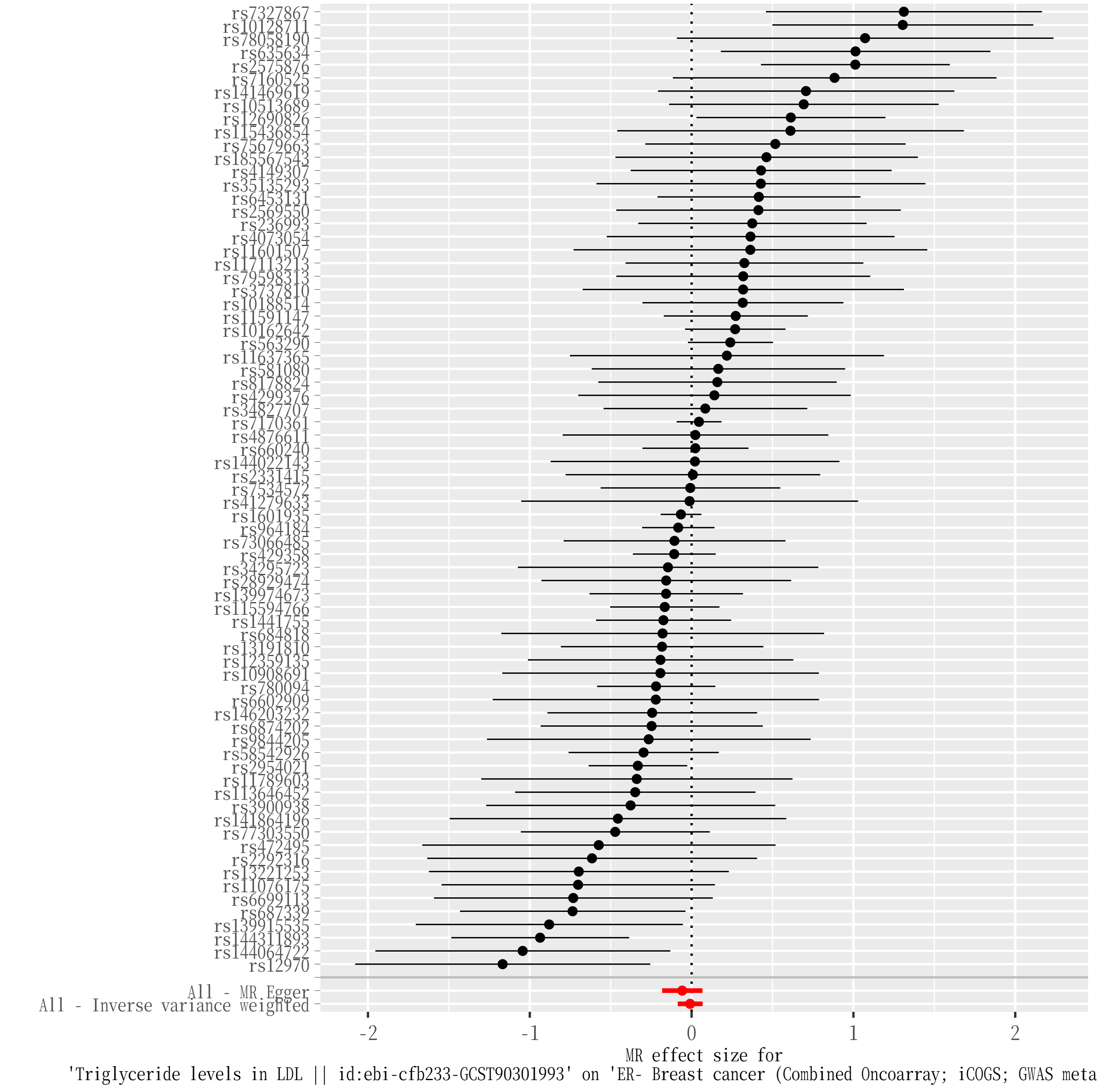
rs7327867
rs10128711
rs78058190
rs635634
rs2575876
rs7160525
rs141469619
rs10513689
rs12690826
rs115436854
rs75679663
rs185567543
rs4149307
rs35135293
rs6453131
rs2569550
rs236993
rs4073054
rs11601507
rs117113213
rs79598313
rs3737810
rs10188514
rs11591147
rs10162642
rs563290
rs11637365
rs581080
rs8178824
rs4299376
rs34827707
rs7170361
rs4876611
rs660240
rs144022143
rs2331415
rs7534572
rs41279633
rs1601935
rs964184
rs73066485
rs429358
rs34295723
rs28929474
rs139974673
rs115594766
rs1441755
rs684818
rs13191810
rs12359135
rs10908691
rs780094
rs6602909
rs146203232
rs6874202
rs9844205
rs58542926
rs2954021
rs11789603
rs113646452
rs3900938
rs141864196
rs77303550
rs472495
rs2292316
rs13221253
rs11076175
rs6699113
rs687339
rs139915535
rs144311893
rs144064722
rs12970
All - MR Egger
All - Inverse variance weighted
-2
-1
0
1
2
MR effect size for
'Triglyceride levels in LDL || id:ebi-cfb233-GCST90301993' on 'ER- Breast cancer (Combined Oncoarray; iCOGS; GWAS meta

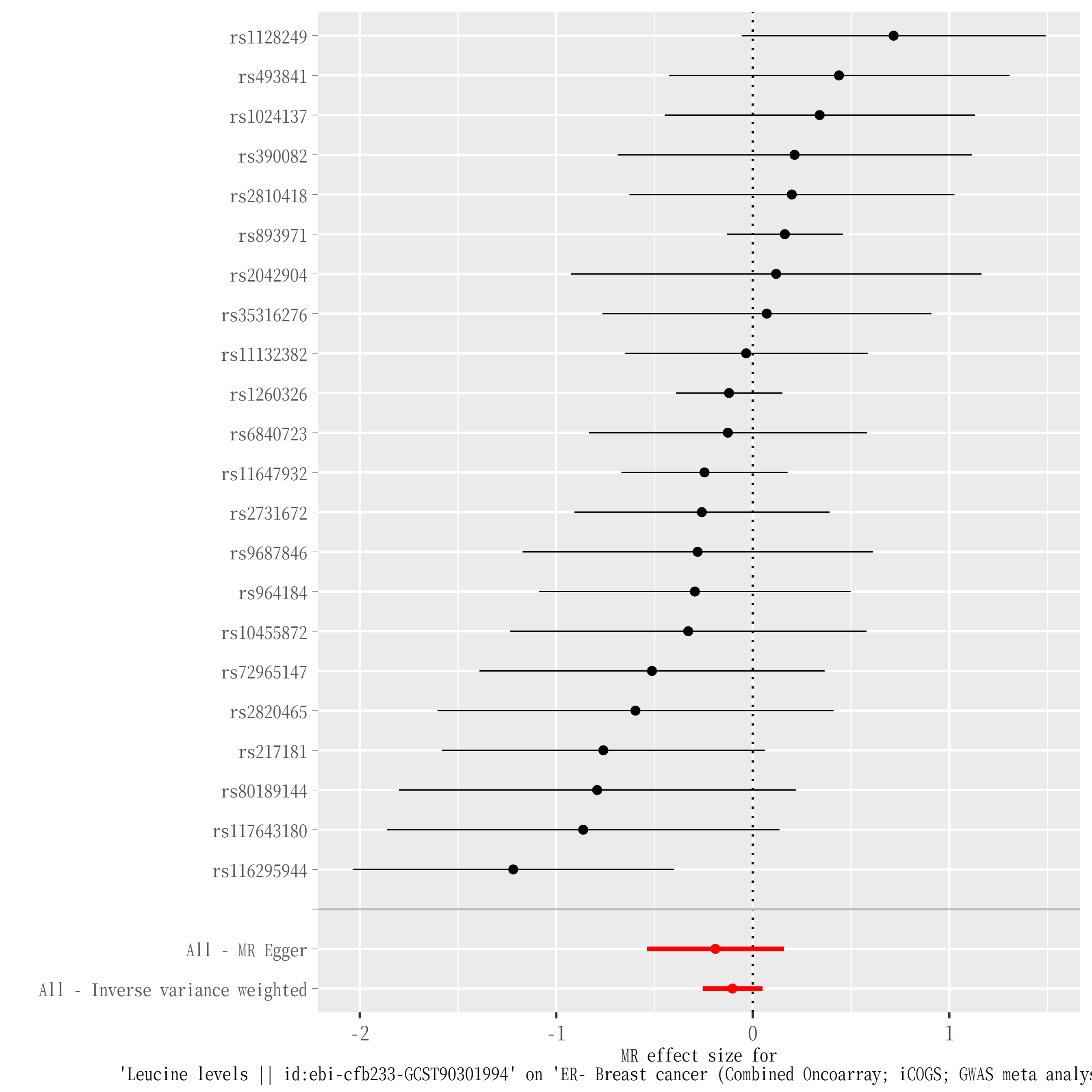
rs1128249
rs493841
rs1024137
rs390082
rs2810418
rs893971
rs2042904
rs35316276
rs11132382
rs1260326
rs6840723
rs11647932
rs2731672
rs9687846
rs964184
rs10455872
rs72965147
rs2820465
rs217181
rs80189144
rs117643180
rs116295944
All - MR Egger
All - Inverse variance weighted
-2
-1
0
1
MR effect size for
'Leucine levels || id:ebi-cfb233-GCST90301994' on 'ER- Breast cancer (Combined Oncoarray; iCOGS; GWAS meta analys

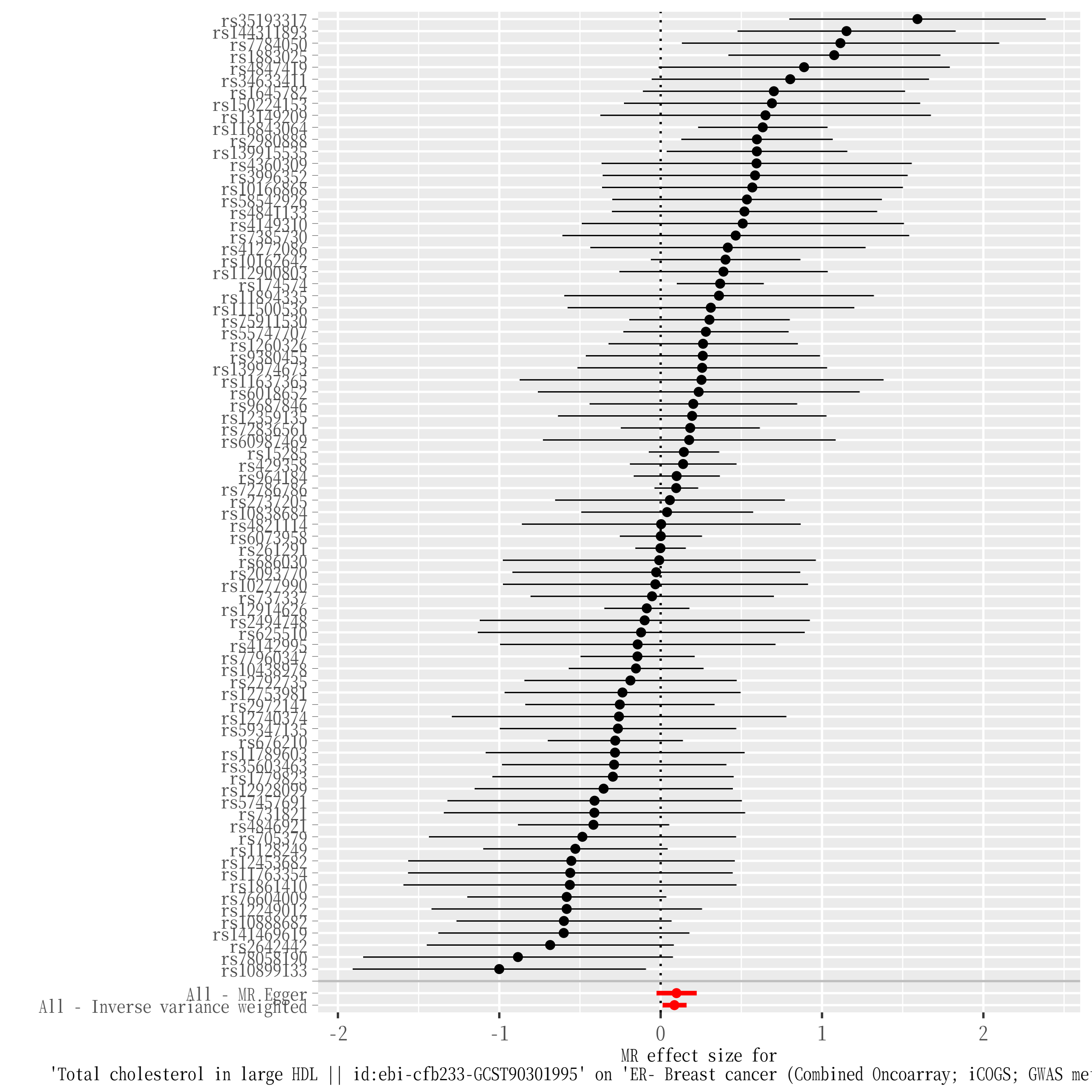
rs35193317
rs144311893
rs7784050
rs1883025
rs4847419
rs34633411
rs1645782
rs150224153
rs13149209
rs116843064
rs2980888
rs139915535
rs4360309
rs3996352
rs10166868
rs58542926
rs4841133
rs4149310
rs7385730
rs41272086
rs10162642
rs112900803
rs174574
rs11894335
rs111500536
rs75911530
rs55747707
rs1260326
rs9380455
rs139974673
rs11637365
rs6018652
rs9687846
rs12359135
rs72836561
rs60987469
rs15285
rs429358
rs964184
rs72786786
rs2737205
rs10838684
rs4821114
rs6073958
rs261291
rs686030
rs2093770
rs10277990
rs737337
rs12914626
rs2494748
rs625510
rs4142995
rs77960347
rs10438978
rs2792735
rs12753981
rs2972147
rs12740374
rs59347135
rs676210
rs11789603
rs35603463
rs1779823
rs12928099
rs57457691
rs731821
rs4846921
rs705379
rs1128249
rs12453682
rs11763354
rs1861410
rs76604009
rs12249012
rs10888682
rs141469619
rs2642442
rs78058190
rs10899133
All - MR Egger
All - Inverse variance weighted
-2
-1
0
1
2
MR effect size for
'Total cholesterol in large HDL || id:ebi-cfb233-GCST90301995' on 'ER- Breast cancer (Combined Oncoarray; iCOGS; GWAS me

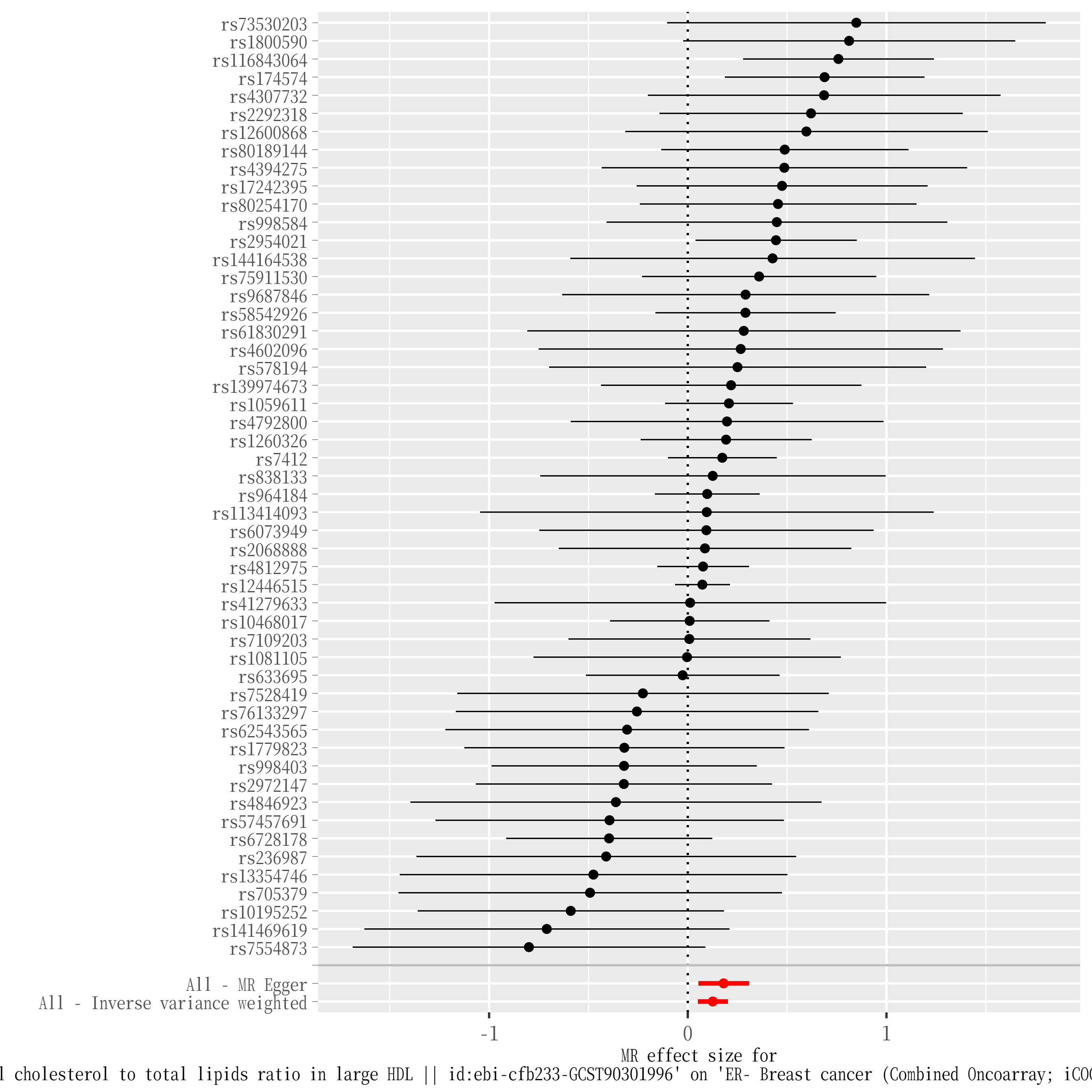

rs73530203
rs1800590
rs116843064
rs174574
rs4307732
rs2292318
rs12600868
rs80189144
rs4394275
rs17242395
rs80254170
rs998584
rs2954021
rs144164538
rs75911530
rs9687846
rs58542926
rs61830291
rs4602096
rs578194
rs139974673
rs1059611
rs4792800
rs1260326
rs7412
rs838133
rs964184
rs113414093
rs6073949
rs2068888
rs4812975
rs12446515
rs41279633
rs10468017
rs7109203
rs1081105
rs633695
rs7528419
rs76133297
rs62543565
rs1779823
rs998403
rs2972147
rs4846923
rs57457691
rs6728178
rs236987
rs13354746
rs705379
rs10195252
rs141469619
rs7554873
All - MR Egger
All - Inverse variance weighted
-1
0
1
MR effect size for
l cholesterol to total lipids ratio in large HDL || id:ebi-cfb233-GCST90301996' on 'ER- Breast cancer (Combined Oncoarray; iCOG

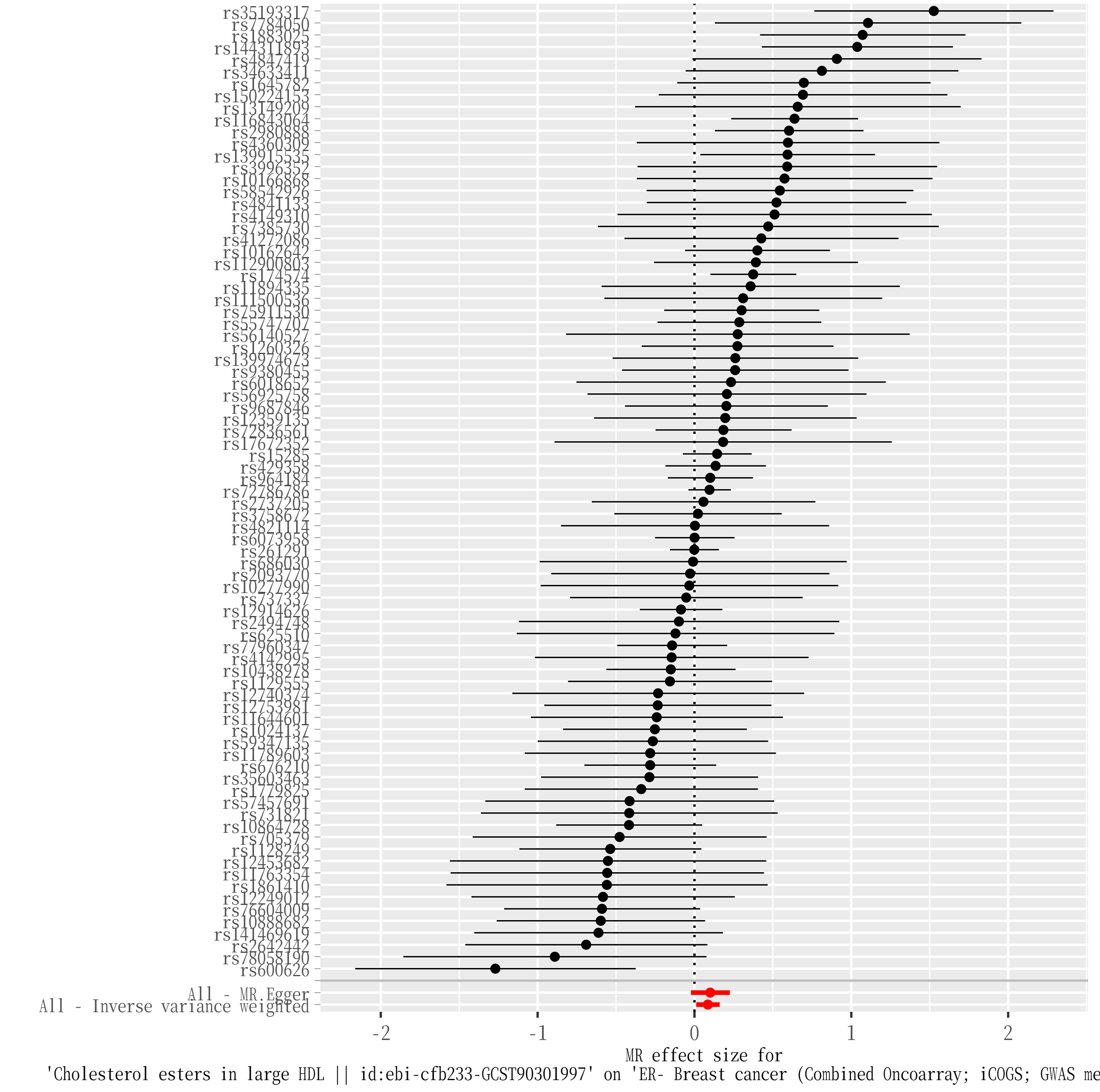
rs35193317
rs7784050
rs1883025
rs144311893
rs4847419
rs34633411
rs1645782
rs150224153
rs13149209
rs116843064
rs2980888
rs4360309
rs139915535
rs3996352
rs10166868
rs58542926
rs4841133
rs4149310
rs7385730
rs41272086
rs10162642
rs112900803
rs174574
rs11894335
rs111500536
rs75911530
rs55747707
rs56140527
rs1260326
rs139974673
rs9380455
rs6018652
rs56925758
rs9687846
rs12359135
rs72836561
rs17672352
rs15285
rs429358
rs264184
rs72786786
rs2737205
rs3758672
rs4821114
rs6073958
rs261291
rs686030
rs2023770
rs10277990
rs737337
rs12914626
rs2494748
rs625510
rs77960347
rs4142995
rs10438978
rs1129555
rs12740374
rs12753981
rs11644601
rs1024137
rs59347135
rs11789603
rs676210
rs35603463
rs1779825
rs57457691
rs731821
rs10864728
rs705379
rs1128249
rs12453682
rs11763354
rs1861410
rs12249012
rs76604009
rs10888682
rs141469619
rs2642442
rs78058190
rs600626
All - MR Egger
All - Inverse variance weighted
-2
-1
0
1
2
MR effect size for
'Cholesterol esters in large HDL || id:ebi-cfb233-GCST90301997' on 'ER- Breast cancer (Combined Oncoarray; iCOGS; GWAS me

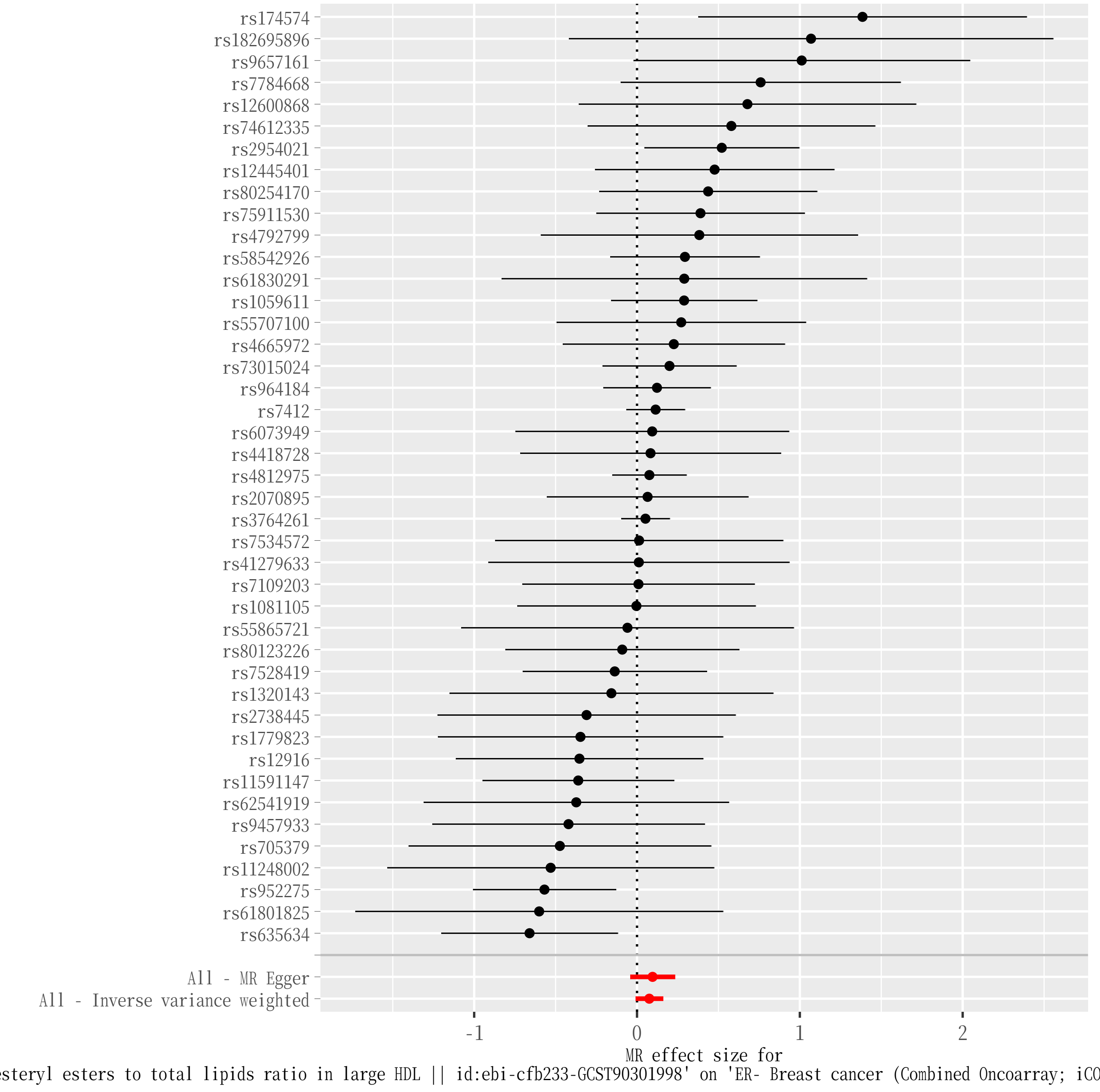

rs174574
rs182695896
rs9657161
rs7784668
rs12600868
rs74612335
rs2954021
rs12445401
rs80254170
rs75911530
rs4792799
rs58542926
rs61830291
rs1059611
rs55707100
rs4665972
rs73015024
rs964184
rs7412
rs6073949
rs4418728
rs4812975
rs2070895
rs3764261
rs7534572
rs41279633
rs7109203
rs1081105
rs55865721
rs80123226
rs7528419
rs1320143
rs2738445
rs1779823
rs12916
rs11591147
rs62541919
rs9457933
rs705379
rs11248002
rs952275
rs61801825
rs635634
All - MR Egger
All - Inverse variance weighted
-1
0
1
2
MR effect size for
esteryl esters to total lipids ratio in large HDL || id:ebi-cfb233-GCST90301998' on 'ER- Breast cancer (Combined Oncoarray; iCO

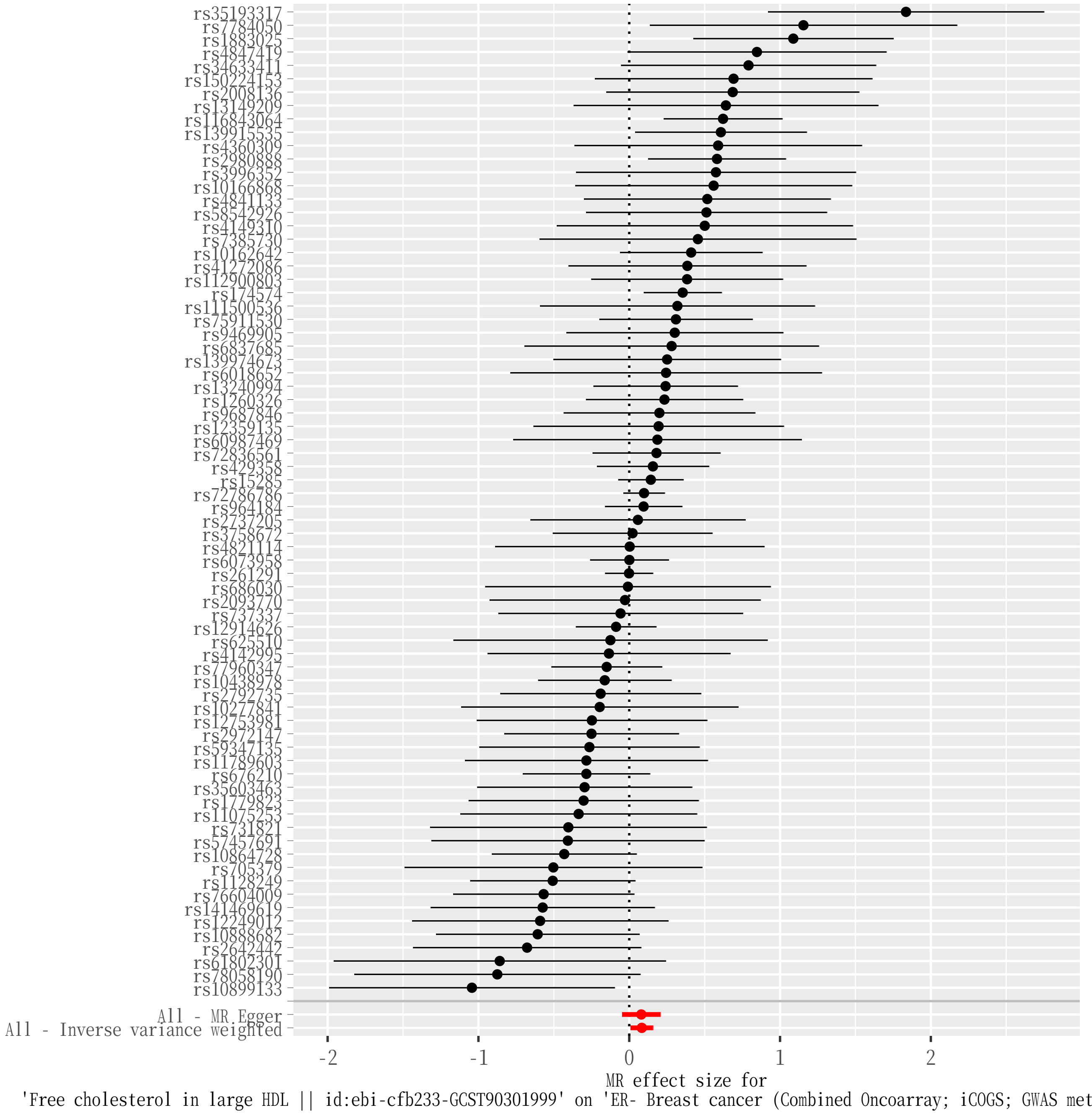

rs35193317
rs7784050
rs1883025
rs4847419
rs34633411
rs150224153
rs2008136
rs13149209
rs116843064
rs139915535
rs4360309
rs2980888
rs3996352
rs10166868
rs4841133
rs58542926
rs4149310
rs7385730
rs10162642
rs41272086
rs112900803
rs174574
rs111500536
rs75911530
rs9469905
rs6837685
rs139974673
rs6018652
rs13240994
rs1260326
rs9687846
rs12359135
rs60987469
rs72836561
rs429358
rs15285
rs72786786
rs964184
rs2737205
rs3758672
rs4821114
rs6073958
rs261291
rs686030
rs2093770
rs737337
rs12914626
rs625510
rs4142995
rs77960347
rs10438978
rs2792735
rs10277841
rs12753981
rs2972147
rs59347135
rs11789603
rs676210
rs35603463
rs1772823
rs11075253
rs731821
rs57457691
rs10864728
rs705379
rs1128249
rs76604009
rs141469619
rs12249012
rs10888682
rs2642442
rs61802301
rs78058190
rs10899133
All - MR Egger
All - Inverse variance weighted
-2
-1
0
1
2
MR effect size for
'Free cholesterol in large HDL || id:ebi-cfb233-GCST90301999' on 'ER- Breast cancer (Combined Oncoarray; iCOGS; GWAS met

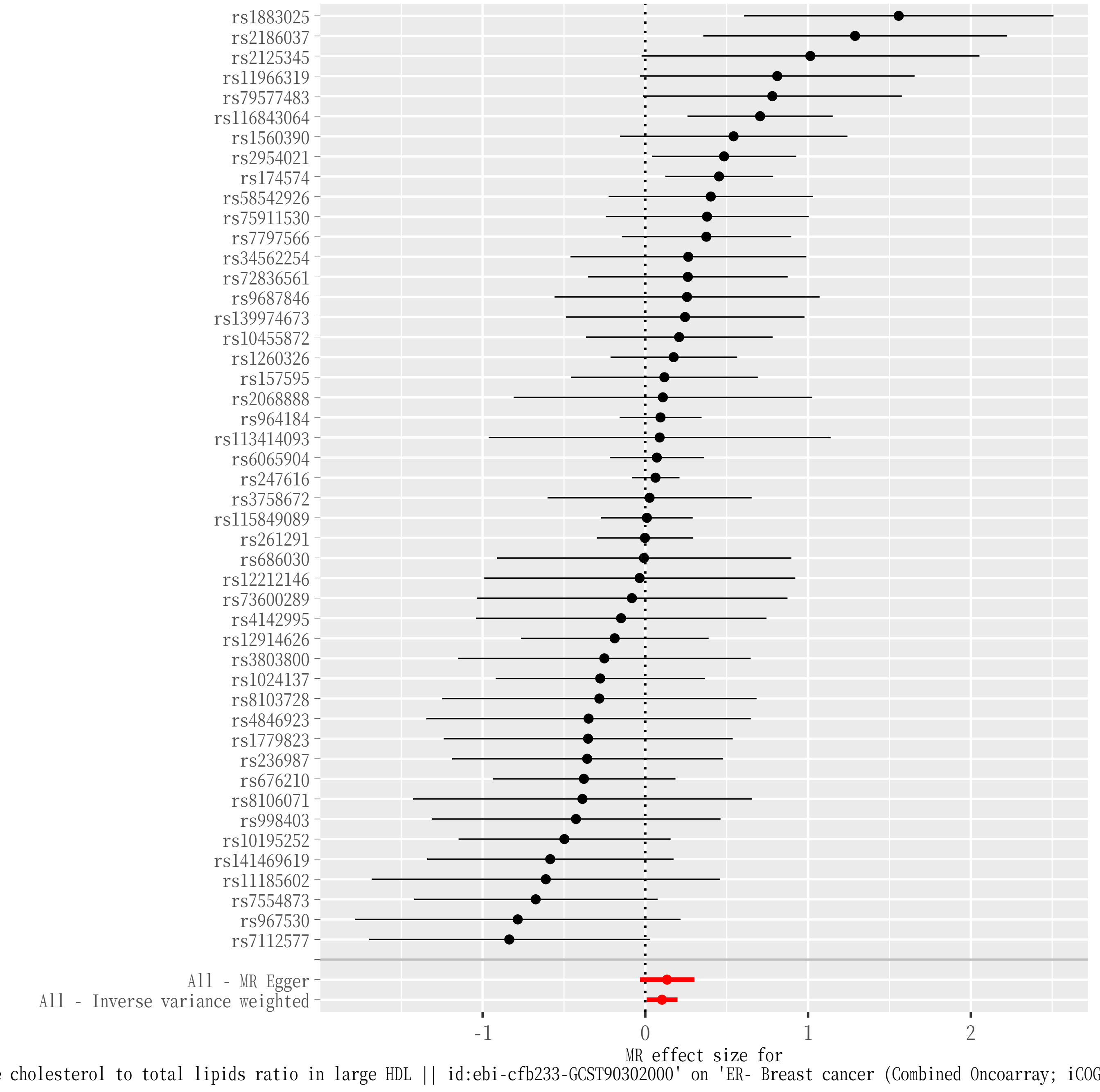
rs1883025
rs2186037
rs2125345
rs11966319
rs79577483
rs116843064
rs1560390
rs2954021
rs174574
rs58542926
rs75911530
rs7797566
rs34562254
rs72836561
rs9687846
rs139974673
rs10455872
rs1260326
rs157595
rs2068888
rs964184
rs113414093
rs6065904
rs247616
rs3758672
rs115849089
rs261291
rs686030
rs12212146
rs73600289
rs4142995
rs12914626
rs3803800
rs1024137
rs8103728
rs4846923
rs1779823
rs236987
rs676210
rs8106071
rs998403
rs10195252
rs141469619
rs11185602
rs7554873
rs967530
rs7112577
All - MR Egger
All - Inverse variance weighted
-1
0
1
2
MR effect size for
e cholesterol to total lipids ratio in large HDL || id:ebi-cfb233-GCST90302000' on 'ER- Breast cancer (Combined Oncoarray; iCOG

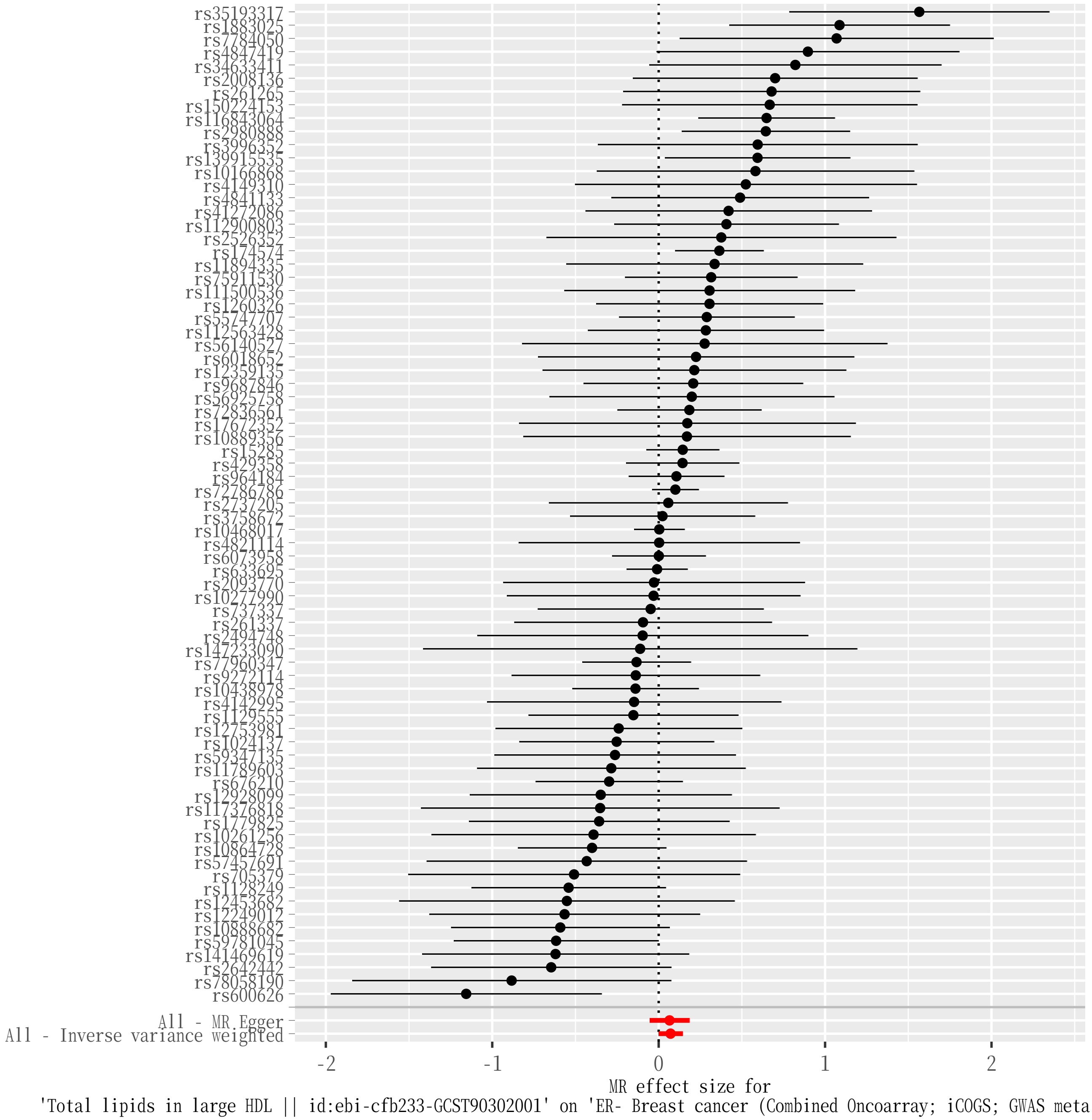
rs35193317
rs1883025
rs7784050
rs4847419
rs34633411
rs2008136
rs261265
rs150224153
rs116843064
rs2980888
rs3996352
rs139915535
rs10166868
rs4149310
rs4841133
rs41272086
rs112900803
rs2526352
rs174574
rs11894335
rs75911530
rs111500536
rs1260326
rs55747707
rs112563428
rs56140527
rs6018652
rs12359135
rs9687846
rs56925758
rs72836561
rs17672352
rs10889356
rs15285
rs429358
rs964184
rs72786786
rs2737205
rs3758672
rs10468017
rs4821114
rs6073958
rs633695
rs2023770
rs10277990
rs737337
rs261337
rs2494748
rs147233090
rs77960347
rs9272114
rs10438978
rs4142995
rs1129555
rs12753981
rs1024137
rs59347135
rs11789603
rs676210
rs12928099
rs117376818
rs1779825
rs10261256
rs10864728
rs57457621
rs705379
rs1128249
rs12453682
rs12249012
rs10888682
rs59781045
rs141469619
rs2642442
rs78058190
rs600626
All - MR Egger
All - Inverse variance weighted
-2
-1
0
1
2
MR effect size for
'Total lipids in large HDL || id:ebi-cfb233-GCST90302001' on 'ER- Breast cancer (Combined Oncoarray; iCOGS; GWAS meta

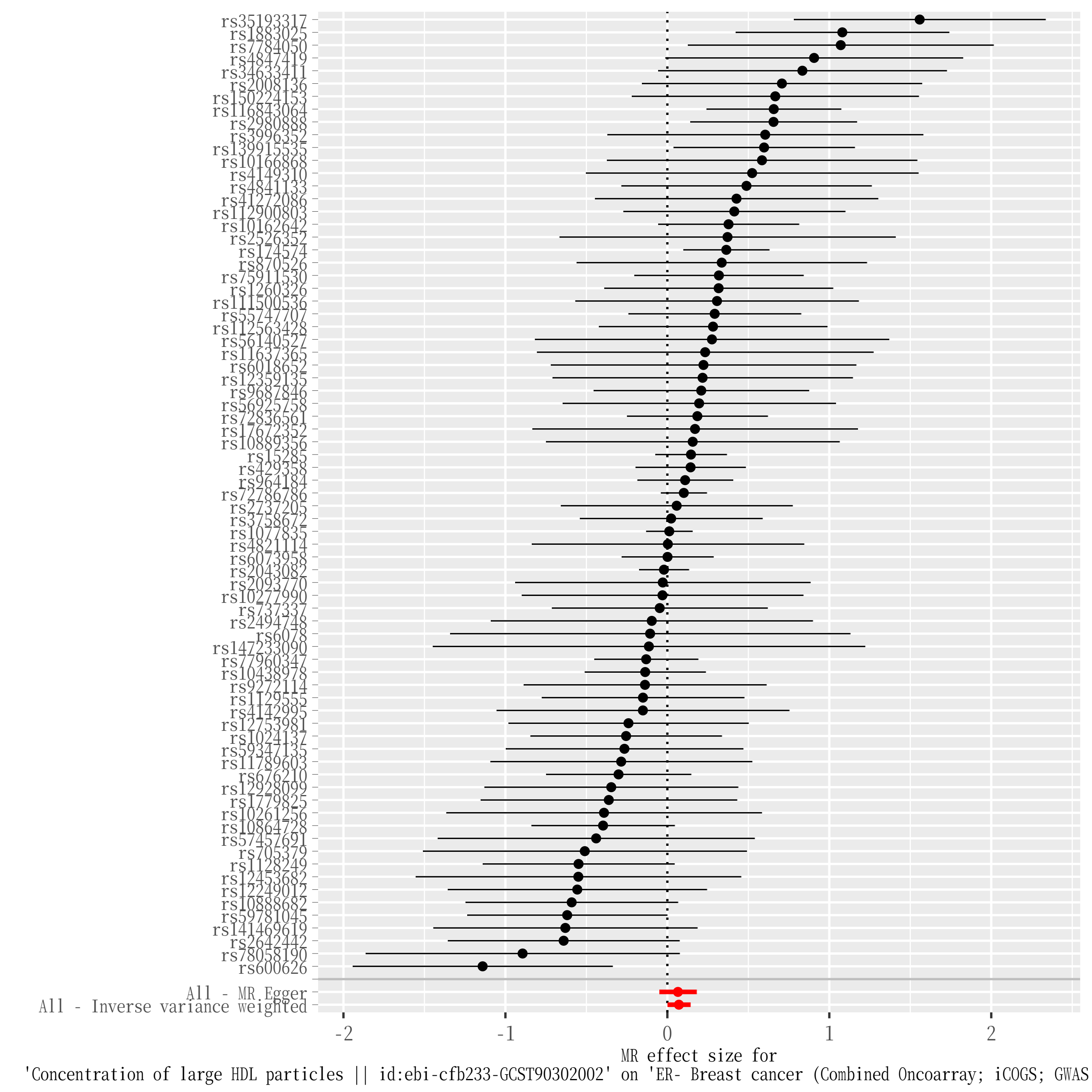
rs35193317
rs1883025
rs7784050
rs4847419
rs34633411
rs2008136
rs150224153
rs116843064
rs2980888
rs3996352
rs139915535
rs10166868
rs4149310
rs4841133
rs41272086
rs112900803
rs10162642
rs2526352
rs174574
rs870526
rs75911530
rs1260326
rs111500536
rs55747707
rs112563428
rs56140527
rs11637365
rs6018652
rs12359135
rs9687846
rs56925758
rs72836561
rs17672352
rs10882356
rs15285
rs429358
rs964184
rs72786786
rs2737205
rs3758672
rs1077835
rs4821114
rs6073958
rs2043082
rs2093770
rs10277990
rs737337
rs2494748
rs6078
rs147233090
rs77960347
rs10438978
rs9272114
rs1129555
rs4142995
rs12753981
rs1024137
rs59347135
rs11789603
rs676210
rs12928099
rs1779825
rs10261256
rs10864728
rs57457691
rs705379
rs1128249
rs12453682
rs12249012
rs10888682
rs59781045
rs141469619
rs2642442
rs78058190
rs600626
All - MR Egger
All - Inverse variance weighted
-2
-1
0
1
2
MR effect size for
'Concentration of large HDL particles || id:ebi-cfb233-GCST90302002' on 'ER- Breast cancer (Combined Oncoarray; iCOGS; GWAS

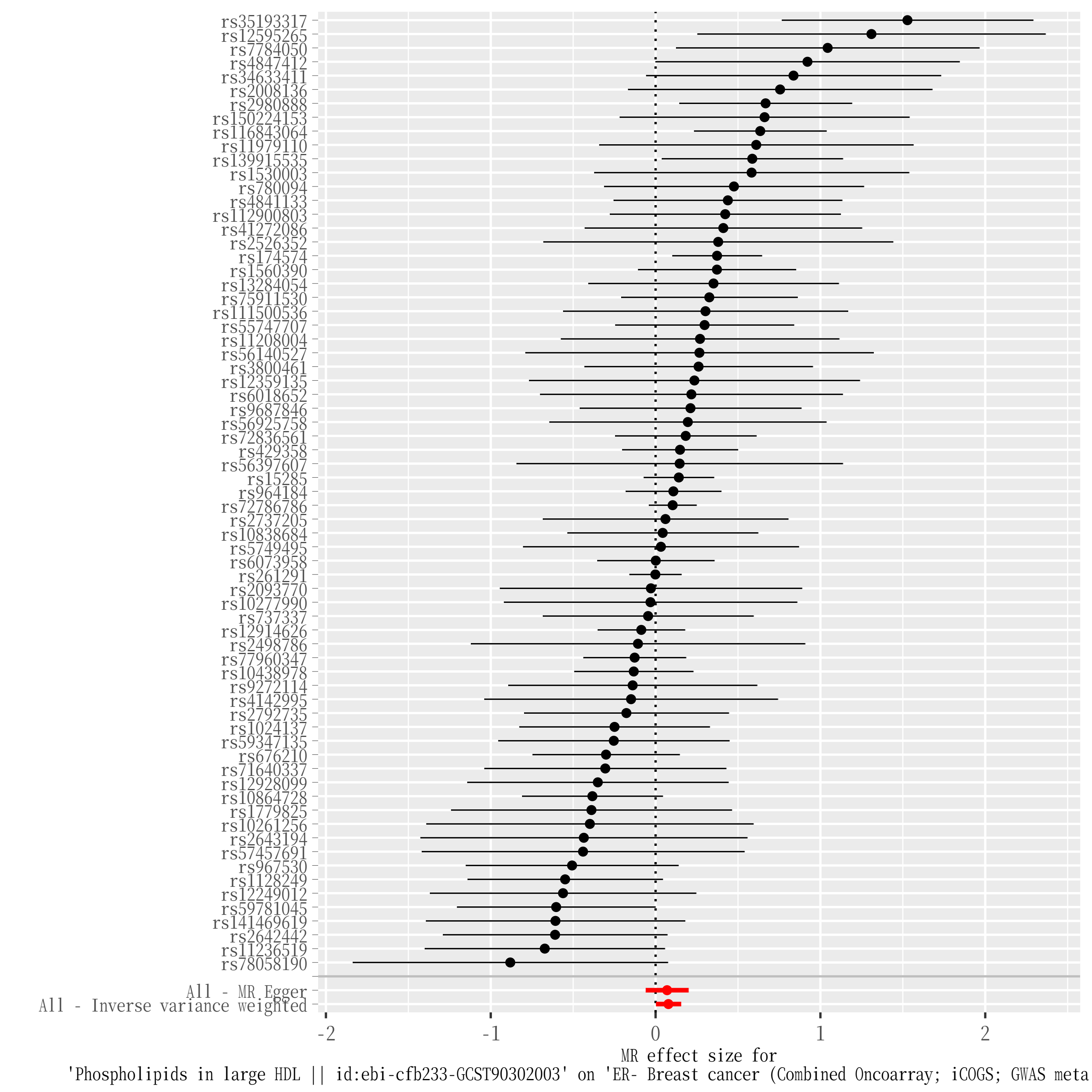
rs35193317
rs12595265
rs7784050
rs4847412
rs34633411
rs2008136
rs2980888
rs150224153
rs116843064
rs11979110
rs139915535
rs1530003
rs780094
rs4841133
rs112900803
rs41272086
rs2526352
rs174574
rs1560390
rs13284054
rs75911530
rs111500536
rs55747707
rs11208004
rs56140527
rs3800461
rs12359135
rs6018652
rs9687846
rs56925758
rs72836561
rs429358
rs56397607
rs15285
rs964184
rs72786786
rs2737205
rs10838684
rs5749495
rs6073958
rs261291
rs2093770
rs10277990
rs737337
rs12914626
rs2498786
rs77960347
rs10438978
rs9272114
rs4142995
rs2792735
rs1024137
rs59347135
rs676210
rs71640337
rs12928099
rs10864728
rs1779825
rs10261256
rs2643194
rs57457691
rs967530
rs1128249
rs12249012
rs59781045
rs141469619
rs2642442
rs11236519
rs78058190
All - MR Egger
All - Inverse variance weighted
-2
-1
0
1
2
MR effect size for
'Phospholipids in large HDL || id:ebi-cfb233-GCST90302003' on 'ER- Breast cancer (Combined Oncoarray; iCOGS; GWAS meta

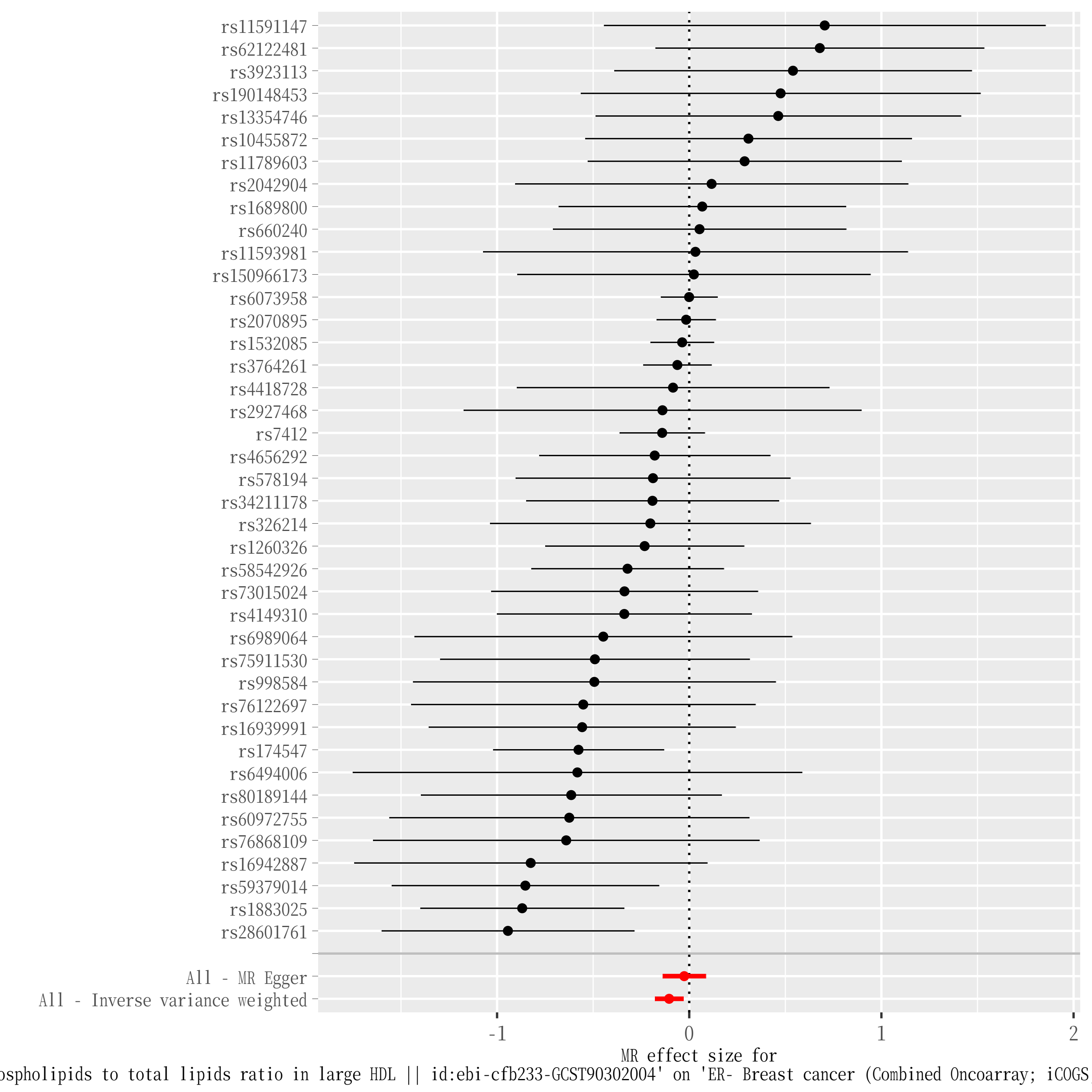
rs11591147
rs62122481
rs3923113
rs190148453
rs13354746
rs10455872
rs11789603
rs2042904
rs1689800
rs660240
rs11593981
rs150966173
rs6073958
rs2070895
rs1532085
rs3764261
rs4418728
rs2927468
rs7412
rs4656292
rs578194
rs34211178
rs326214
rs1260326
rs58542926
rs73015024
rs4149310
rs6989064
rs75911530
rs998584
rs76122697
rs16939991
rs174547
rs6494006
rs80189144
rs60972755
rs76868109
rs16942887
rs59379014
rs1883025
rs28601761
All - MR Egger
All - Inverse variance weighted
-1
0
1
2
MR effect size for
ospholipids to total lipids ratio in large HDL || id:ebi-cfb233-GCST90302004' on 'ER- Breast cancer (Combined Oncoarray; iCOGS

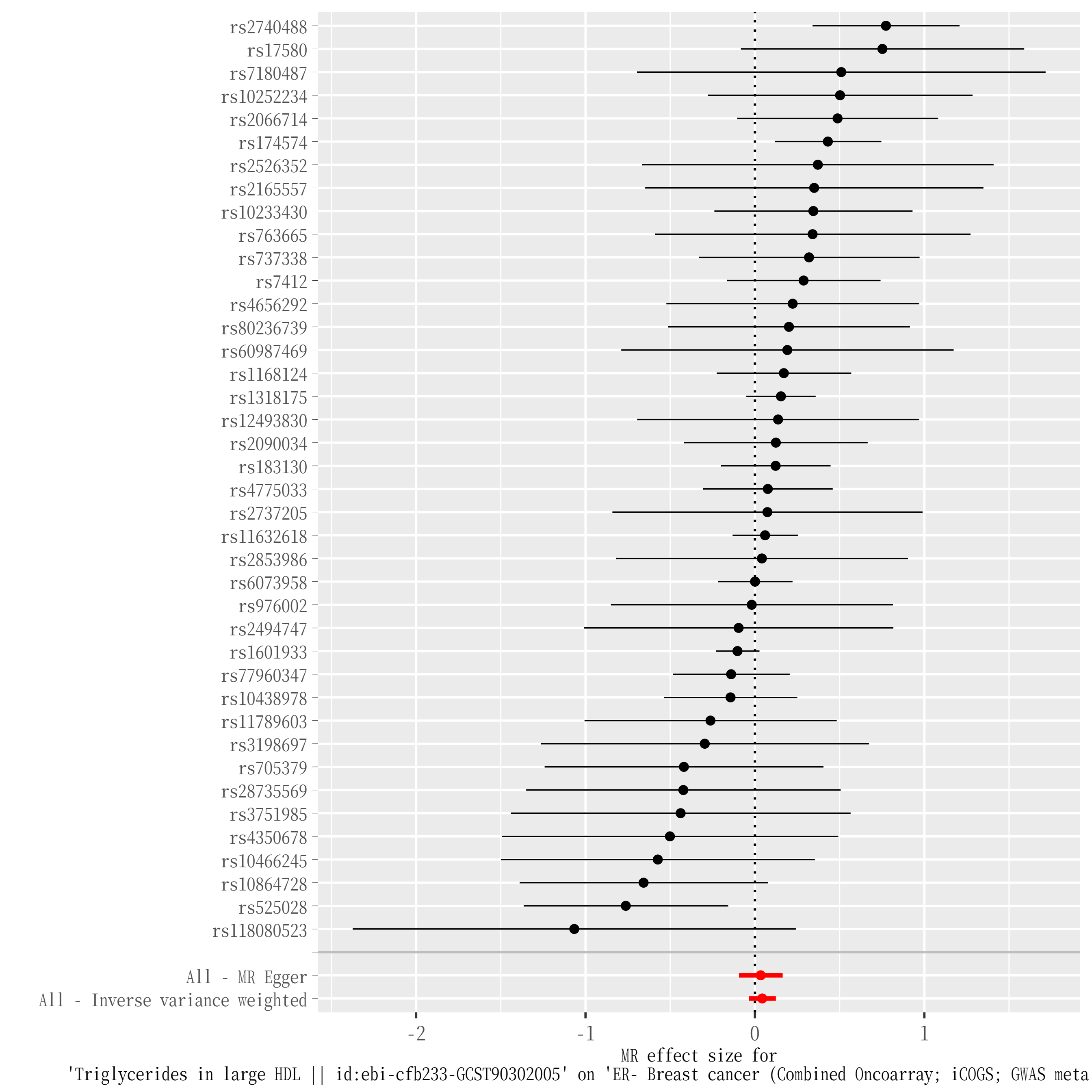
rs2740488
rs17580
rs7180487
rs10252234
rs2066714
rs174574
rs2526352
rs2165557
rs10233430
rs763665
rs737338
rs7412
rs4656292
rs80236739
rs60987469
rs1168124
rs1318175
rs12493830
rs2090034
rs183130
rs4775033
rs2737205
rs11632618
rs2853986
rs6073958
rs976002
rs2494747
rs1601933
rs77960347
rs10438978
rs11789603
rs3198697
rs705379
rs28735569
rs3751985
rs4350678
rs10466245
rs10864728
rs525028
rs118080523
All - MR Egger
All - Inverse variance weighted
-2
-1
0
1
MR effect size for
'Triglycerides in large HDL || id:ebi-cfb233-GCST90302005' on 'ER- Breast cancer (Combined Oncoarray; iCOGS; GWAS meta

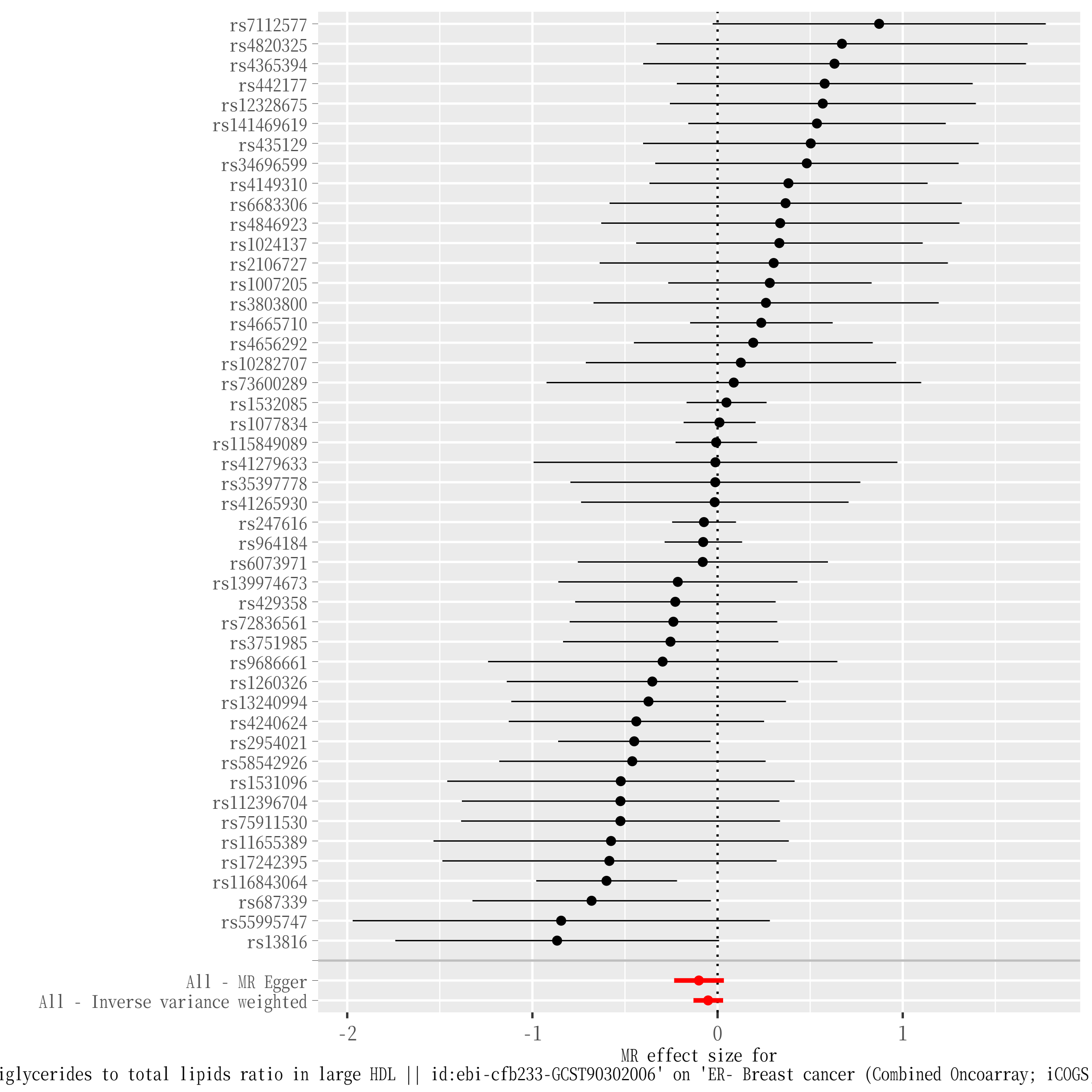

rs7112577
rs4820325
rs4365394
rs442177
rs12328675
rs141469619
rs435129
rs34696599
rs4149310
rs6683306
rs4846923
rs1024137
rs2106727
rs1007205
rs3803800
rs4665710
rs4656292
rs10282707
rs73600289
rs1532085
rs1077834
rs115849089
rs41279633
rs35397778
rs41265930
rs247616
rs964184
rs6073971
rs139974673
rs429358
rs72836561
rs3751985
rs9686661
rs1260326
rs13240994
rs4240624
rs2954021
rs58542926
rs1531096
rs112396704
rs75911530
rs11655389
rs17242395
rs116843064
rs687339
rs55995747
rs13816
All - MR Egger
All - Inverse variance weighted
-2
-1
0
1
MR effect size for
iglycerides to total lipids ratio in large HDL || id:ebi-cfb233-GCST90302006' on 'ER- Breast cancer (Combined Oncoarray; iCOGS

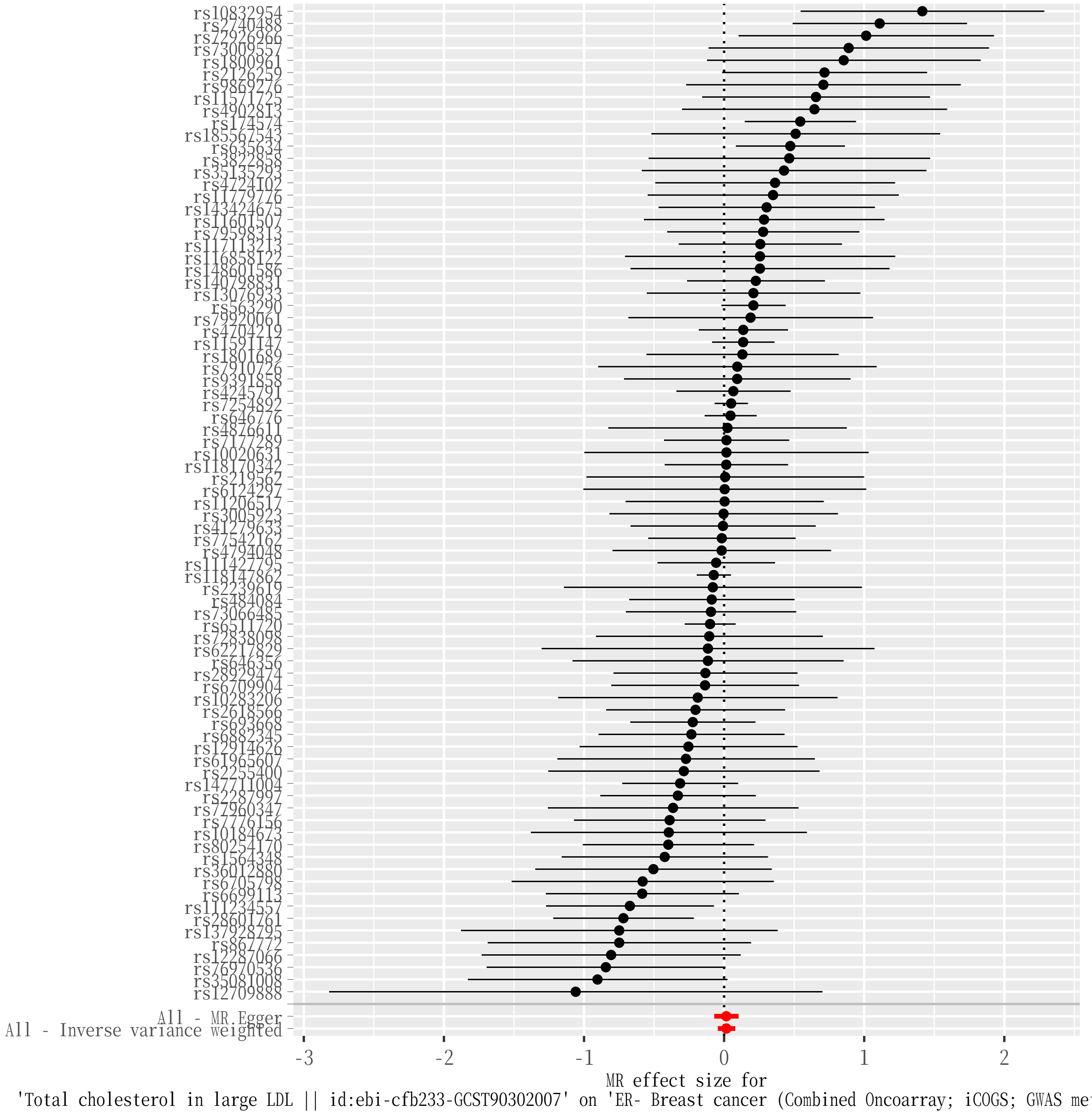
rs10832954
rs2740488
rs72926966
rs73009557
rs1800961
rs2126259
rs9869276
rs11571725
rs4902813
rs174574
rs185567543
rs635634
rs3822858
rs35135293
rs4724102
rs11779776
rs143424675
rs11601507
rs79598313
rs117113213
rs116858122
rs148601586
rs140798831
rs13076933
rs563290
rs7920061
rs4704219
rs11591147
rs1801689
rs7910726
rs9391858
rs4245791
rs7254892
rs646776
rs4876611
rs7177289
rs10020631
rs118170342
rs219562
rs6124297
rs11206517
rs3005923
rs41279633
rs77542162
rs4794048
rs111422795
rs118147862
rs2239619
rs484084
rs73066485
rs6511720
rs72838098
rs62217829
rs646356
rs28929474
rs6709904
rs10283206
rs2618566
rs693668
rs6882345
rs12914626
rs61965607
rs2255400
rs147711004
rs2287997
rs7260347
rs7776156
rs10184673
rs80254170
rs1564348
rs36012880
rs6705798
rs6699113
rs111234557
rs28601761
rs137928795
rs862772
rs12287066
rs76970536
rs35081008
rs12709888
All - MR Egger
All - Inverse variance weighted
-3
-2
-1
0
1
2
MR effect size for
'Total cholesterol in large LDL || id:ebi-cfb233-GCST90302007' on 'ER- Breast cancer (Combined Oncoarray; iCOGS; GWAS me

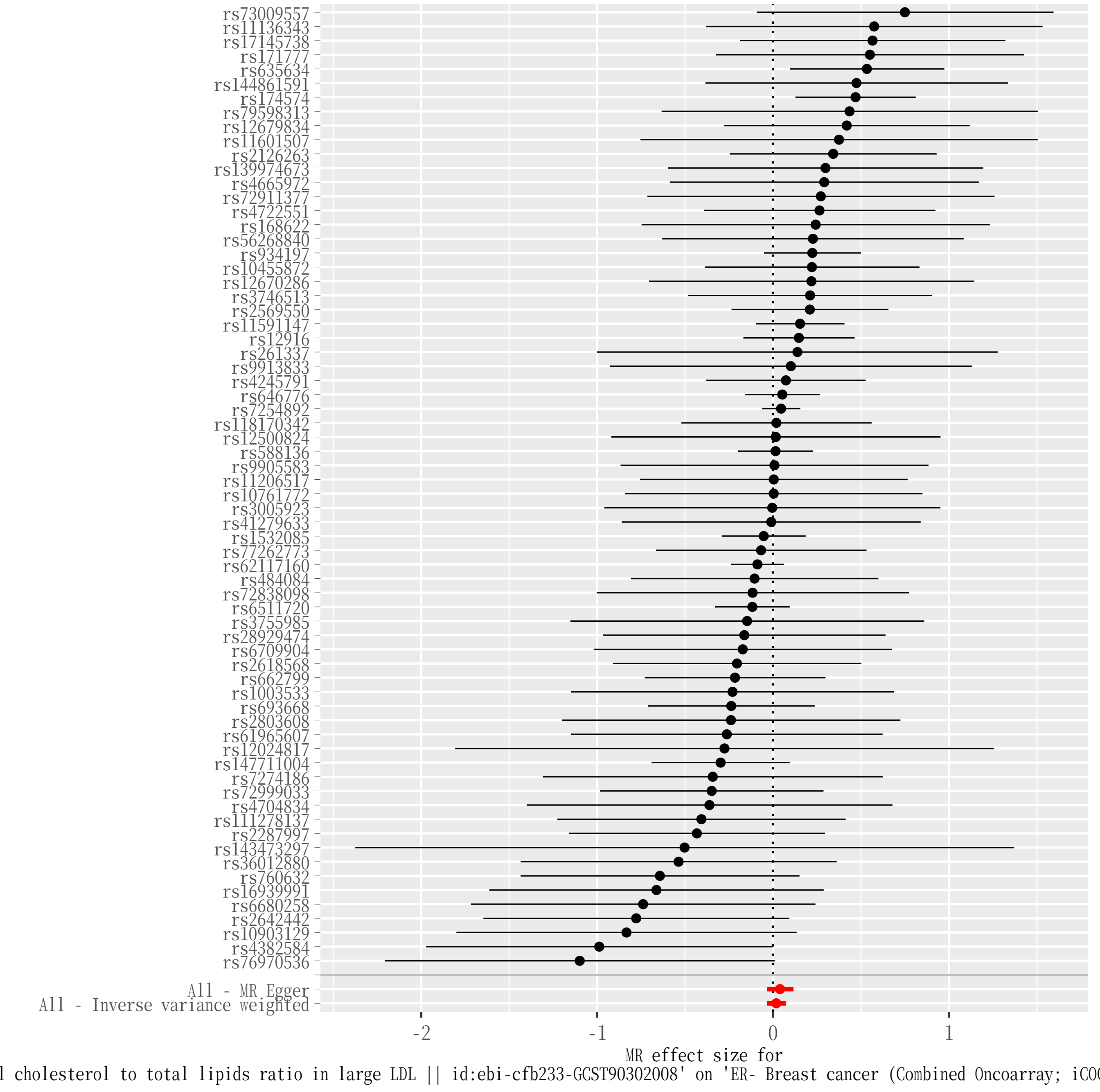

rs73009557
rs11136343
rs17145738
rs171777
rs635634
rs144861591
rs174574
rs79598313
rs12679834
rs11601507
rs2126263
rs139974673
rs4665972
rs72911377
rs4722551
rs168622
rs56268840
rs934197
rs10455872
rs12670286
rs3746513
rs2569550
rs11591147
rs12916
rs261337
rs9913833
rs4245791
rs646776
rs7254892
rs118170342
rs12500824
rs588136
rs9905583
rs11206517
rs10761772
rs3005923
rs41279633
rs1532085
rs77262773
rs62117160
rs484084
rs72838098
rs6511720
rs3755985
rs28929474
rs6709904
rs2618568
rs662799
rs1003533
rs693668
rs2803608
rs61965607
rs12024817
rs147711004
rs7274186
rs72999033
rs4704834
rs111278137
rs2287997
rs143473297
rs36012880
rs760632
rs16939991
rs6680258
rs2642442
rs10903129
rs4382584
rs76970536
All - MR Egger
All - Inverse variance weighted
-2
-1
0
1
MR effect size for
l cholesterol to total lipids ratio in large LDL || id:ebi-cfb233-GCST90302008' on 'ER- Breast cancer (Combined Oncoarray; iCOG

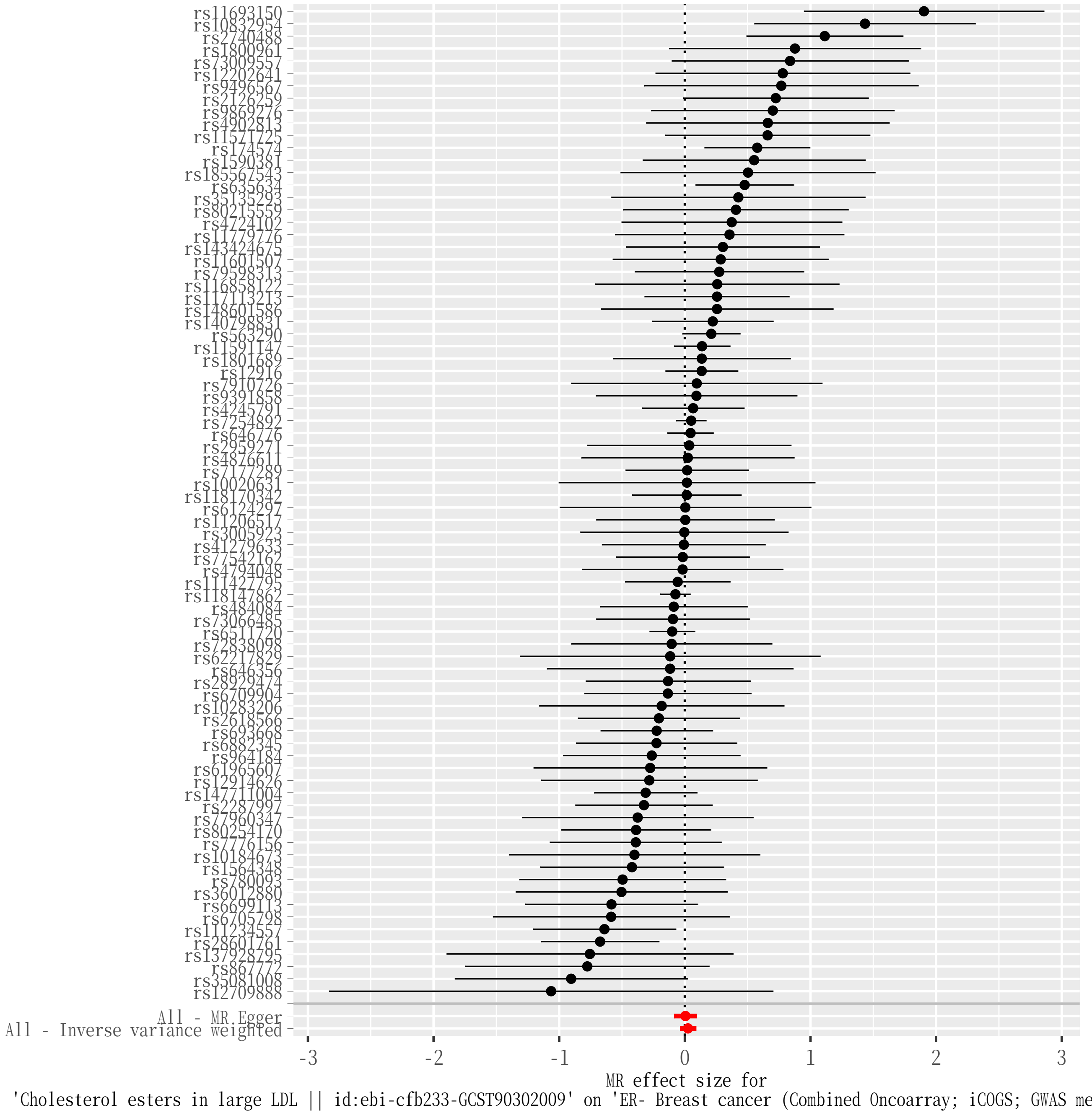

rs11693150
rs10832954
rs2740488
rs1800961
rs73009557
rs12202641
rs9496567
rs2126259
rs9869276
rs4902813
rs11571725
rs174574
rs1590381
rs185567543
rs635634
rs35135293
rs80215559
rs4724102
rs11779776
rs143424675
rs11601507
rs79598313
rs116858122
rs117113213
rs148601586
rs140798831
rs563290
rs11591147
rs1801689
rs12916
rs7910726
rs9391858
rs4245791
rs7254892
rs646776
rs2959271
rs4876611
rs7177289
rs10020631
rs118170342
rs6124297
rs11206517
rs3005923
rs41279633
rs77542162
rs4794048
rs111427795
rs118147862
rs484084
rs73066485
rs6511720
rs72838098
rs62217829
rs646356
rs28929474
rs6709904
rs10283206
rs2618566
rs693668
rs6882345
rs964184
rs61965607
rs12914626
rs147711004
rs2287997
rs77960347
rs80254170
rs7776156
rs10184673
rs1564348
rs780093
rs36012880
rs6699113
rs6705798
rs111234557
rs28601761
rs137928795
rs867772
rs35081008
rs12709888
All - MR Egger
All - Inverse variance weighted
-3
-2
-1
0
1
2
3
MR effect size for
'Cholesterol esters in large LDL || id:ebi-cfb233-GCST90302009' on 'ER- Breast cancer (Combined Oncoarray; iCOGS; GWAS me

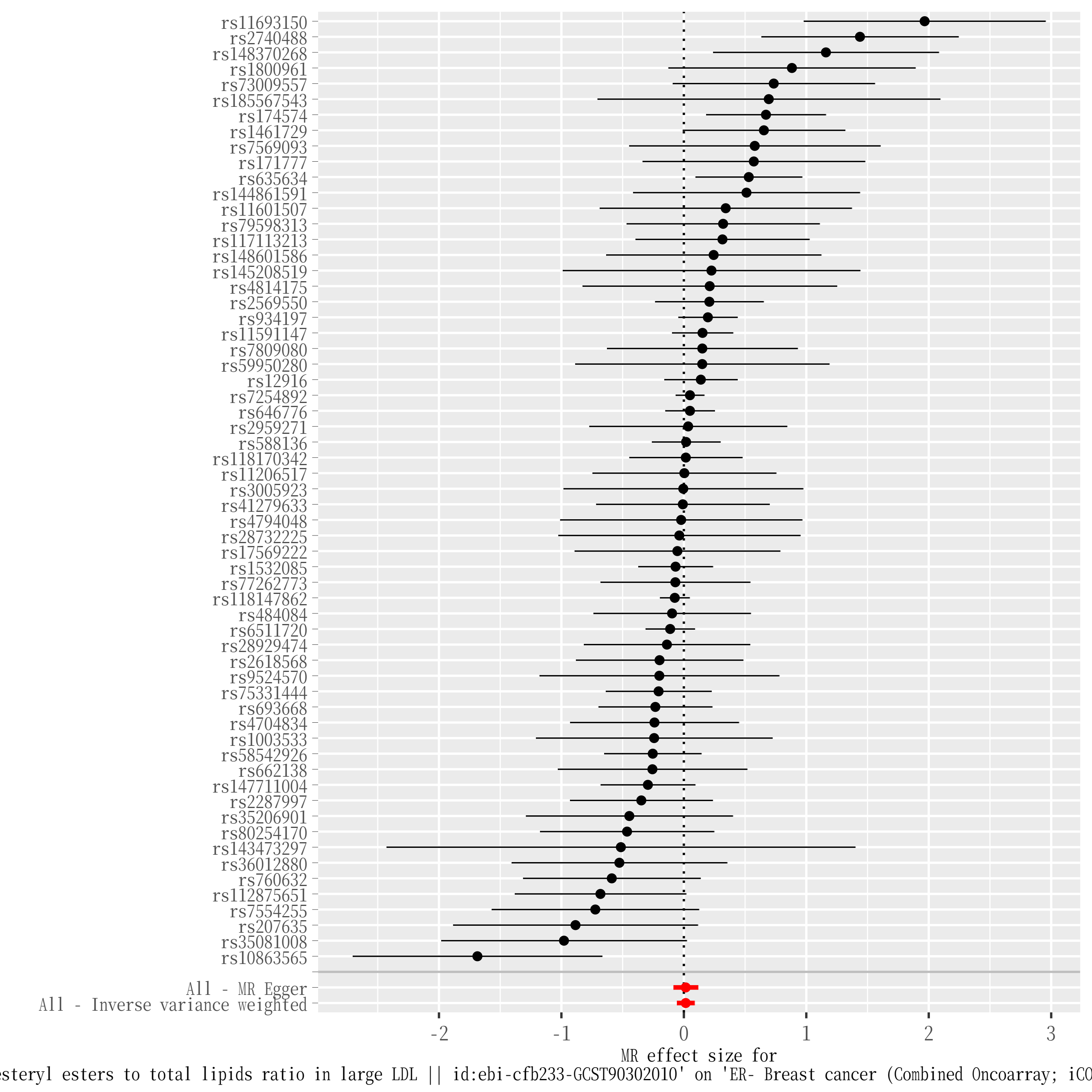
rs11693150
rs2740488
rs148370268
rs1800961
rs73009557
rs185567543
rs174574
rs1461729
rs7569093
rs171777
rs635634
rs144861591
rs11601507
rs79598313
rs117113213
rs148601586
rs145208519
rs4814175
rs2569550
rs934197
rs11591147
rs7809080
rs59950280
rs12916
rs7254892
rs646776
rs2959271
rs588136
rs118170342
rs11206517
rs3005923
rs41279633
rs4794048
rs28732225
rs17569222
rs1532085
rs77262773
rs118147862
rs484084
rs6511720
rs28929474
rs2618568
rs9524570
rs75331444
rs693668
rs4704834
rs1003533
rs58542926
rs662138
rs147711004
rs2287997
rs35206901
rs80254170
rs143473297
rs36012880
rs760632
rs112875651
rs7554255
rs207635
rs35081008
rs10863565
All - MR Egger
All - Inverse variance weighted
-2
-1
0
1
2
3
MR effect size for
esteryl esters to total lipids ratio in large LDL || id:ebi-cfb233-GCST90302010' on 'ER- Breast cancer (Combined Oncoarray; iCO

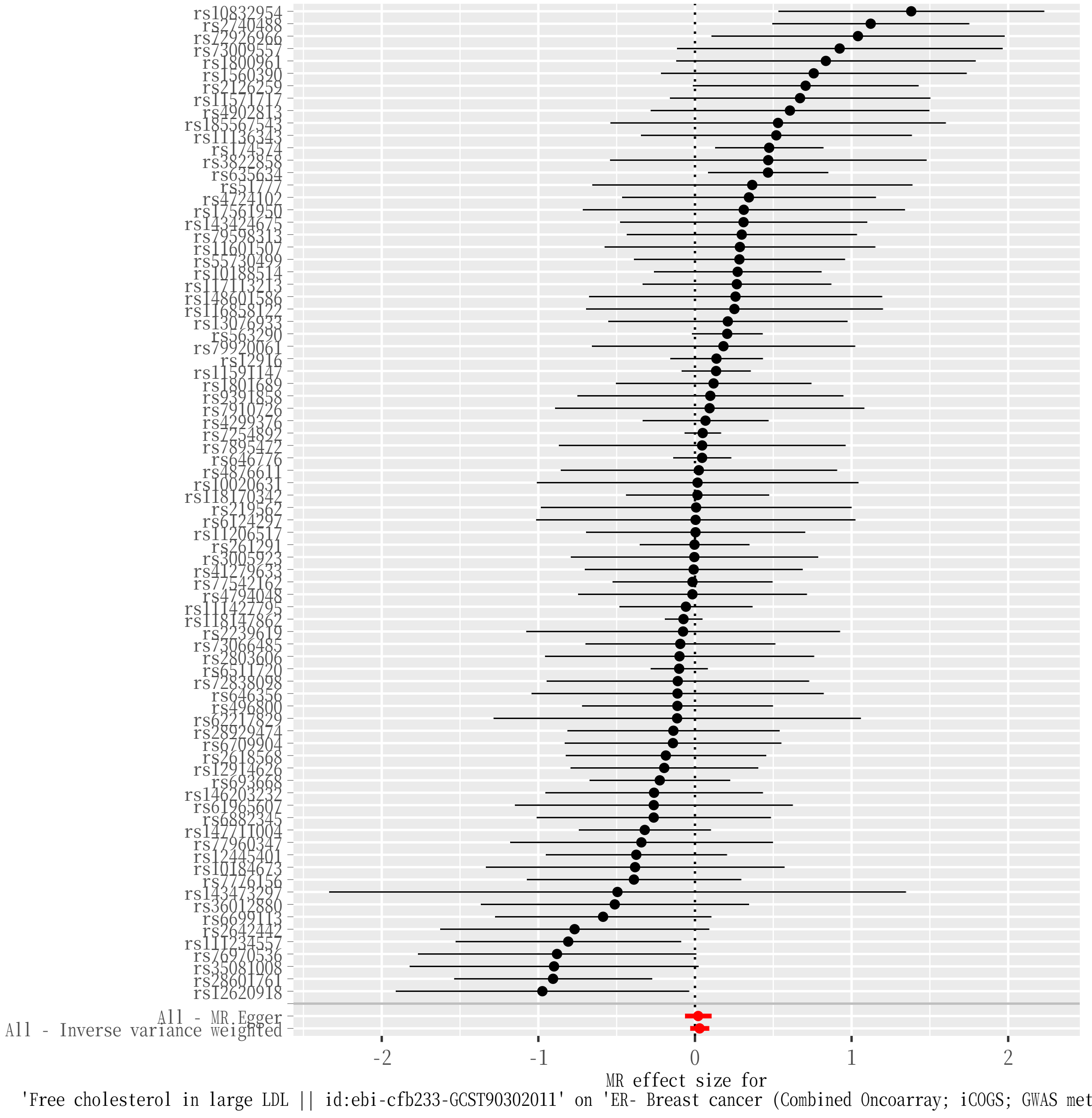

rs10832954
rs2740488
rs72926966
rs73009557
rs1800961
rs1560390
rs2126259
rs11571717
rs4902813
rs185567543
rs1136343
rs174574
rs3822858
rs635634
rs51777
rs4724102
rs17561950
rs143424675
rs79598313
rs11601507
rs55730499
rs10188514
rs117113213
rs148601586
rs116858122
rs13076933
rs563290
rs79920061
rs12916
rs11591147
rs1801689
rs9391858
rs7910726
rs4299376
rs7254892
rs7895472
rs646776
rs4876611
rs10020631
rs118170342
rs219562
rs6124297
rs11206517
rs261291
rs3005923
rs41279633
rs77542162
rs4794048
rs111427795
rs118147862
rs2239619
rs73066485
rs2803606
rs6511720
rs72838098
rs646356
rs496800
rs62217829
rs28929474
rs6709904
rs2618568
rs12914626
rs693668
rs146203232
rs61965607
rs6882345
rs147711004
rs77960347
rs12445401
rs10184673
rs7776156
rs143473297
rs36012880
rs6699113
rs2642442
rs111234557
rs76970536
rs35081008
rs28601761
rs12620918
All - MR Egger
All - Inverse variance weighted
-2
-1
0
1
2
MR effect size for
'Free cholesterol in large LDL || id:ebi-cfb233-GCST90302011' on 'ER- Breast cancer (Combined Oncoarray; iCOGS; GWAS met

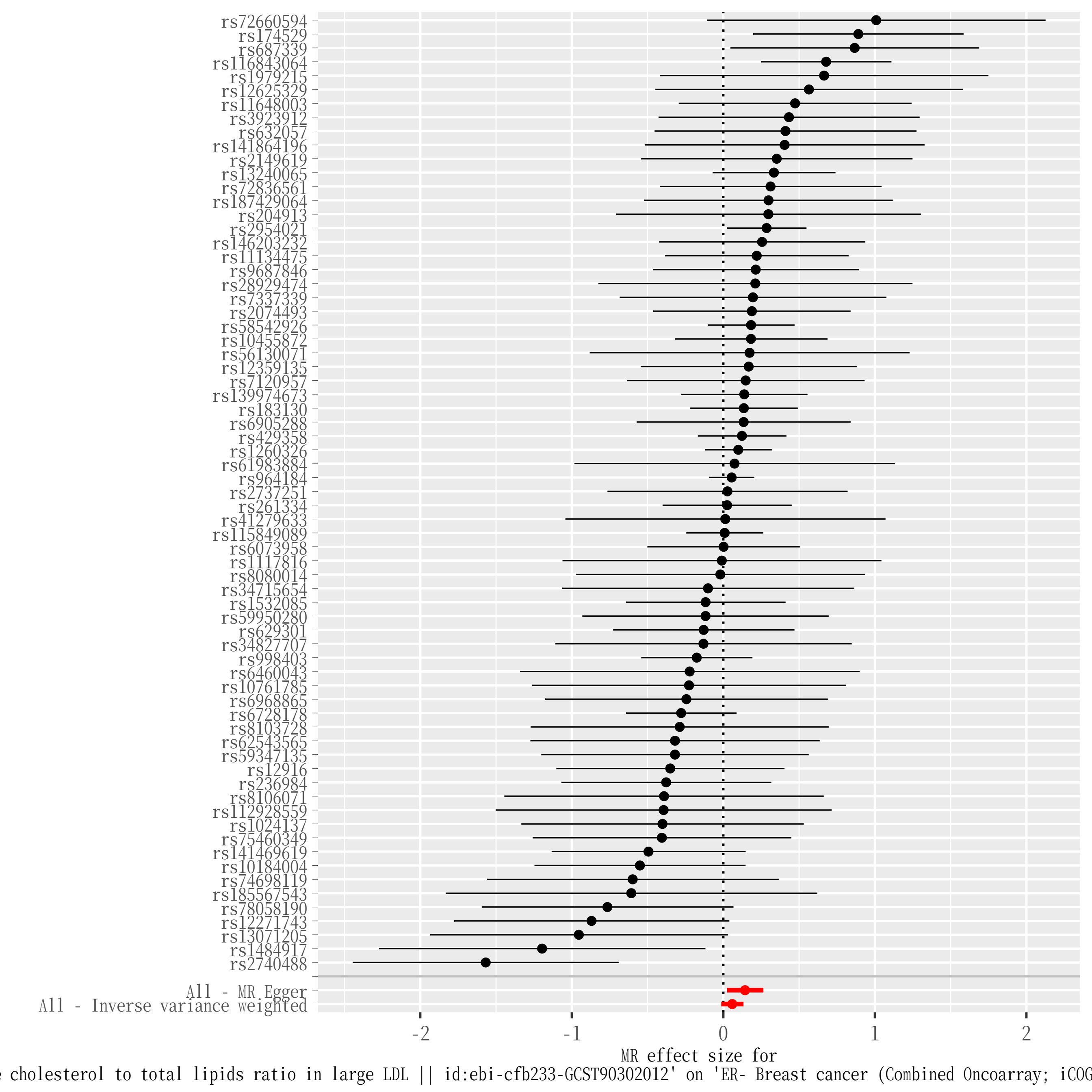
rs72660594
rs174529
rs687339
rs116843064
rs1979215
rs12625329
rs11648003
rs3923912
rs632057
rs141864196
rs2149619
rs13240065
rs72836561
rs187429064
rs204913
rs2954021
rs146203232
rs11134475
rs9687846
rs28929474
rs7337339
rs2074493
rs58542926
rs10455872
rs56130071
rs12359135
rs7120957
rs139974673
rs183130
rs6905288
rs429358
rs1260326
rs61983884
rs964184
rs2737251
rs261334
rs41279633
rs115849089
rs6073958
rs1117816
rs8080014
rs34715654
rs1532085
rs59950280
rs629301
rs34827707
rs998403
rs6460043
rs10761785
rs6968865
rs6728178
rs8103728
rs62543565
rs59347135
rs12916
rs236984
rs8106071
rs112928559
rs1024137
rs75460349
rs141469619
rs10184004
rs74698119
rs185567543
rs78058190
rs12271743
rs13071205
rs1484917
rs2740488
All - MR Egger
All - Inverse variance weighted
-2
-1
0
1
2
MR effect size for
e cholesterol to total lipids ratio in large LDL || id:ebi-cfb233-GCST90302012' on 'ER- Breast cancer (Combined Oncoarray; iCOG

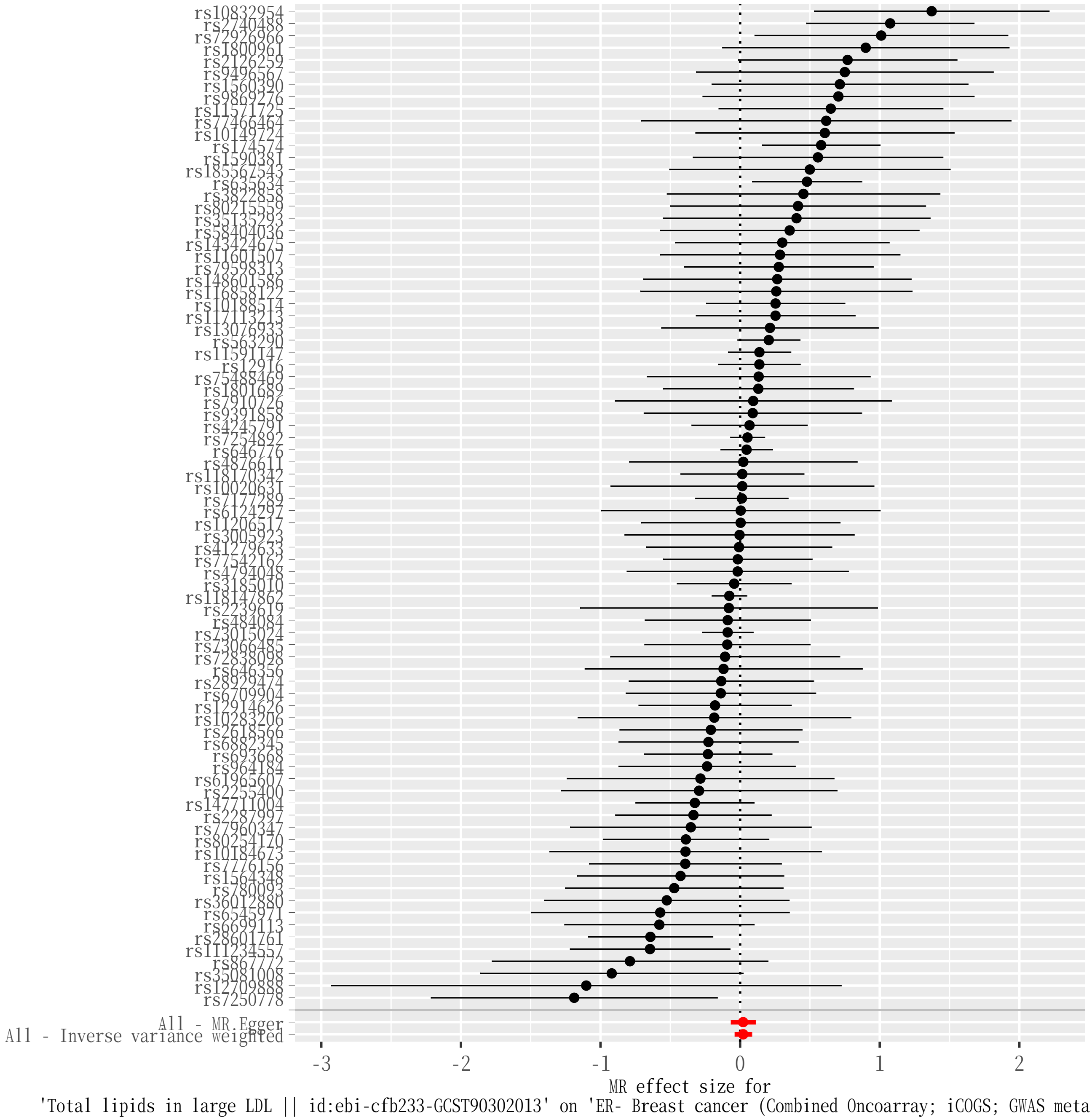
rs10832954
rs2740488
rs72926966
rs1800961
rs2126259
rs9496567
rs1560390
rs9869276
rs11571725
rs77466464
rs10149724
rs174574
rs1590381
rs185567543
rs635634
rs3822858
rs80215559
rs35135293
rs58404036
rs143424675
rs11601507
rs79598313
rs148601586
rs116858122
rs10188514
rs117113213
rs13076933
rs563290
rs11591147
rs12916
rs75488469
rs1801689
rs7910726
rs9391858
rs4245791
rs7254892
rs646776
rs4876611
rs118170342
rs10020631
rs7177289
rs6124297
rs11206517
rs3005923
rs41279633
rs77542162
rs4794048
rs3185010
rs118147862
rs2239619
rs484084
rs73015024
rs73066485
rs72838028
rs646356
rs28929474
rs6709904
rs12914626
rs10283206
rs2618566
rs6882345
rs693668
rs964184
rs61965607
rs2255400
rs147711004
rs2287997
rs77960347
rs80254170
rs10184673
rs7776156
rs1564348
rs780093
rs36012880
rs6545971
rs6699113
rs28601761
rs111234557
rs867772
rs35081008
rs12709888
rs7250778
All - MR Egger
All - Inverse variance weighted
-3
-2
-1
0
1
2
MR effect size for
'Total lipids in large LDL || id:ebi-cfb233-GCST90302013' on 'ER- Breast cancer (Combined Oncoarray; iCOGS; GWAS meta

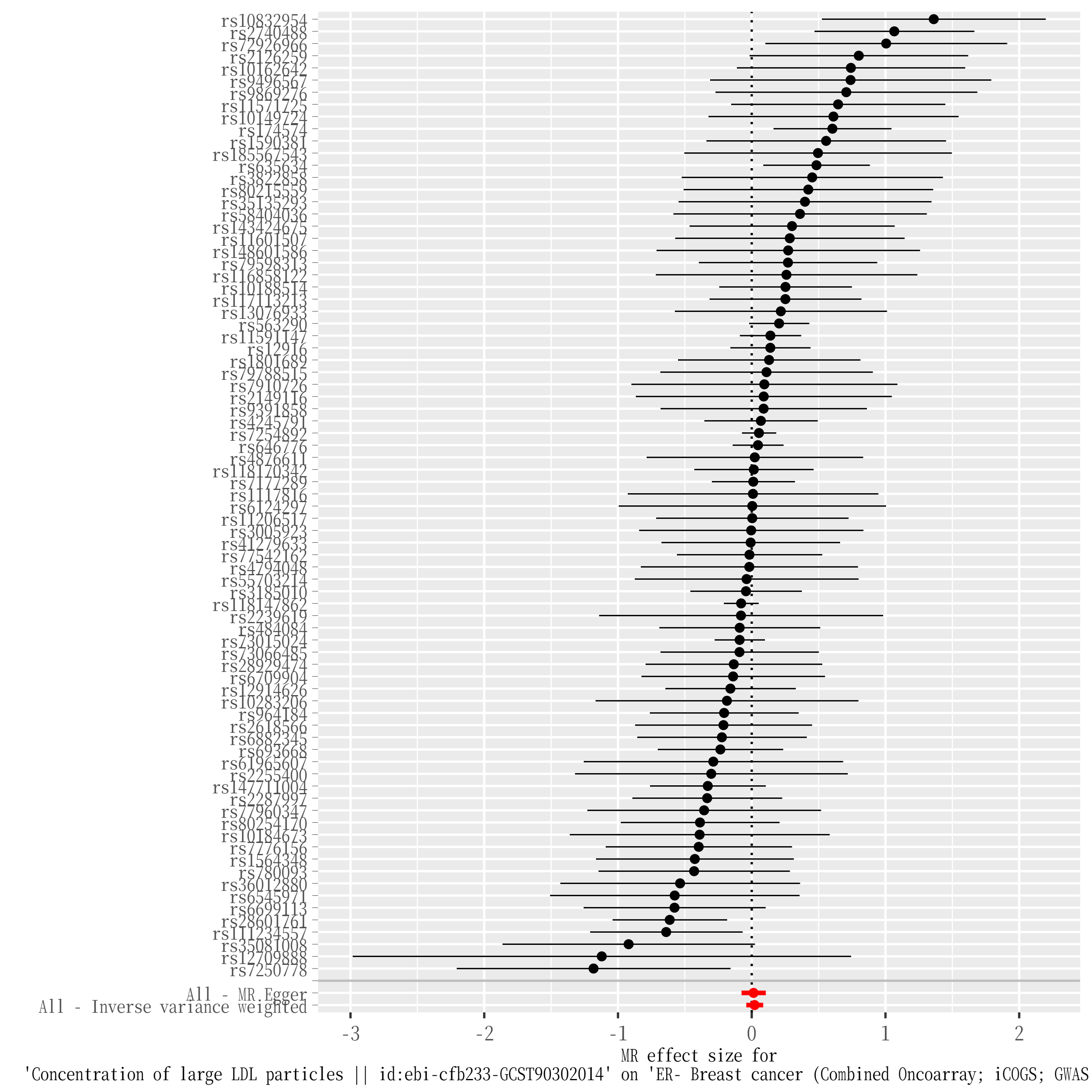
rs10832954
rs2740488
rs72926966
rs2126259
rs10162642
rs9496567
rs9869276
rs11571725
rs10149724
rs174574
rs1590381
rs185567543
rs635634
rs3822858
rs80215559
rs35135293
rs58404036
rs143424675
rs11601507
rs148601586
rs79528313
rs116858122
rs10188514
rs117113213
rs13076933
rs563290
rs11591147
rs12916
rs1801689
rs79788515
rs7910726
rs2149116
rs9391858
rs4245791
rs7254892
rs646776
rs4876611
rs118170342
rs7172289
rs1117816
rs6124297
rs11206517
rs3005923
rs41279633
rs77542162
rs4794048
rs55703214
rs3185010
rs118147862
rs2239619
rs484084
rs73015024
rs73066485
rs28929474
rs6709904
rs12914626
rs10283206
rs964184
rs2618566
rs6882345
rs693668
rs61965607
rs2255400
rs147711004
rs2287997
rs77960347
rs80254170
rs10184673
rs7776156
rs1564348
rs780093
rs36012880
rs6545971
rs6699113
rs28601761
rs111234557
rs35081008
rs12709888
rs7250778
All - MR Egger
All - Inverse variance weighted
-3
-2
-1
0
1
2
MR effect size for
'Concentration of large LDL particles || id:ebi-cfb233-GCST90302014' on 'ER- Breast cancer (Combined Oncoarray; iCOGS; GWAS

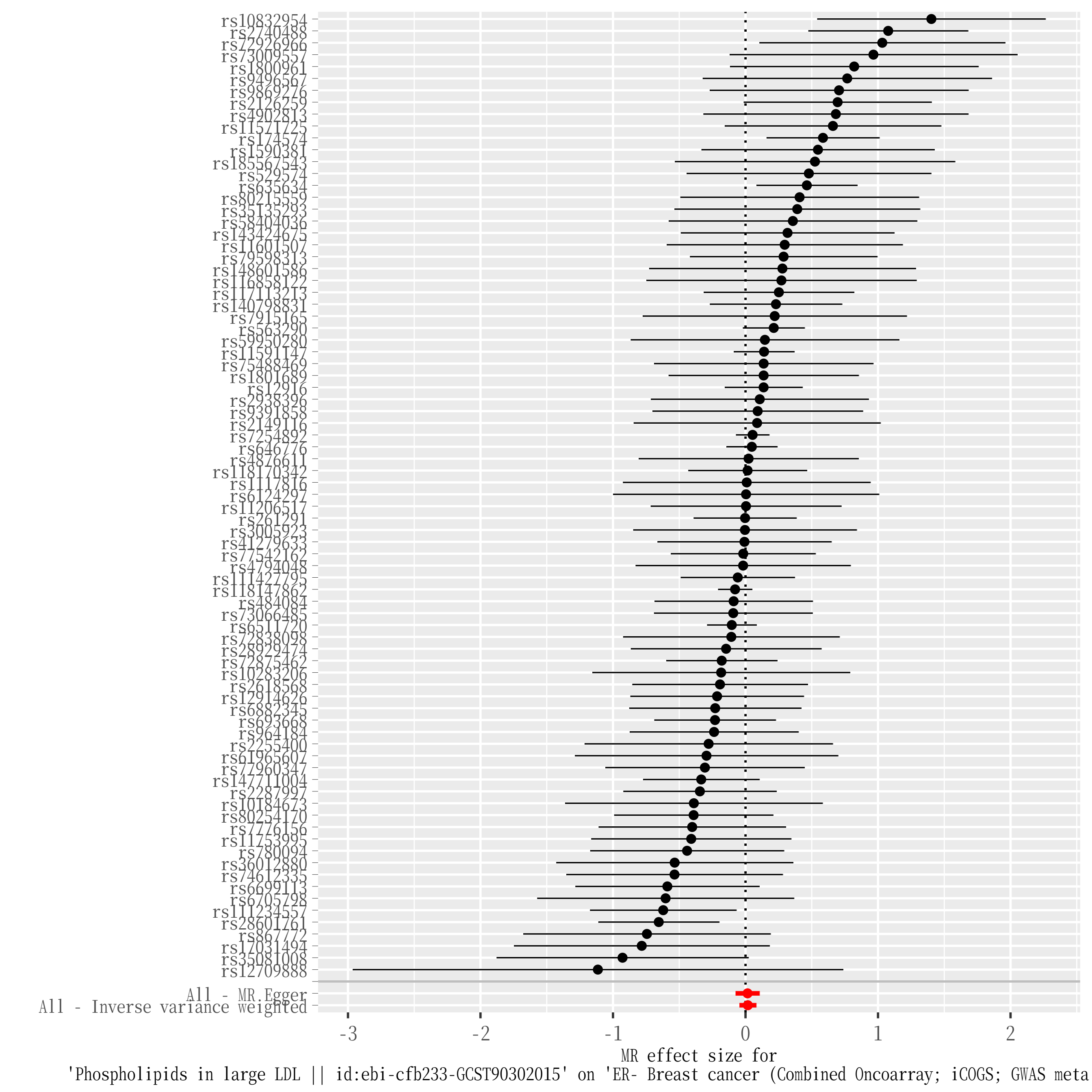
rs10832954
rs2740488
rs72926966
rs73009557
rs1800961
rs9496567
rs9869276
rs2126259
rs4902813
rs11571725
rs174574
rs1590381
rs185567543
rs522574
rs635634
rs80215559
rs35135293
rs58404036
rs143424675
rs11601507
rs79598313
rs148601586
rs116858122
rs117113213
rs140798831
rs7915165
rs563290
rs59950280
rs11591147
rs75488469
rs1801689
rs12916
rs2938396
rs9391858
rs2149116
rs7254892
rs646776
rs4876611
rs118170342
rs1117816
rs6124297
rs11206517
rs261291
rs3005923
rs41279633
rs77542162
rs4794048
rs11422795
rs118147862
rs484084
rs73066485
rs6511720
rs72838098
rs28929474
rs72875462
rs10283206
rs2618568
rs12914626
rs6882345
rs693668
rs964184
rs2255400
rs61965607
rs77260347
rs147711004
rs2287927
rs10184673
rs80254170
rs7776156
rs11753995
rs780094
rs36012880
rs74612335
rs6699113
rs6705798
rs111234557
rs28601761
rs867772
rs17031494
rs35081008
rs12709888
All - MR Egger
All - Inverse variance weighted
-3
-2
-1
0
1
2
MR effect size for
'Phospholipids in large LDL || id:ebi-cfb233-GCST90302015' on 'ER- Breast cancer (Combined Oncoarray; iCOGS; GWAS meta

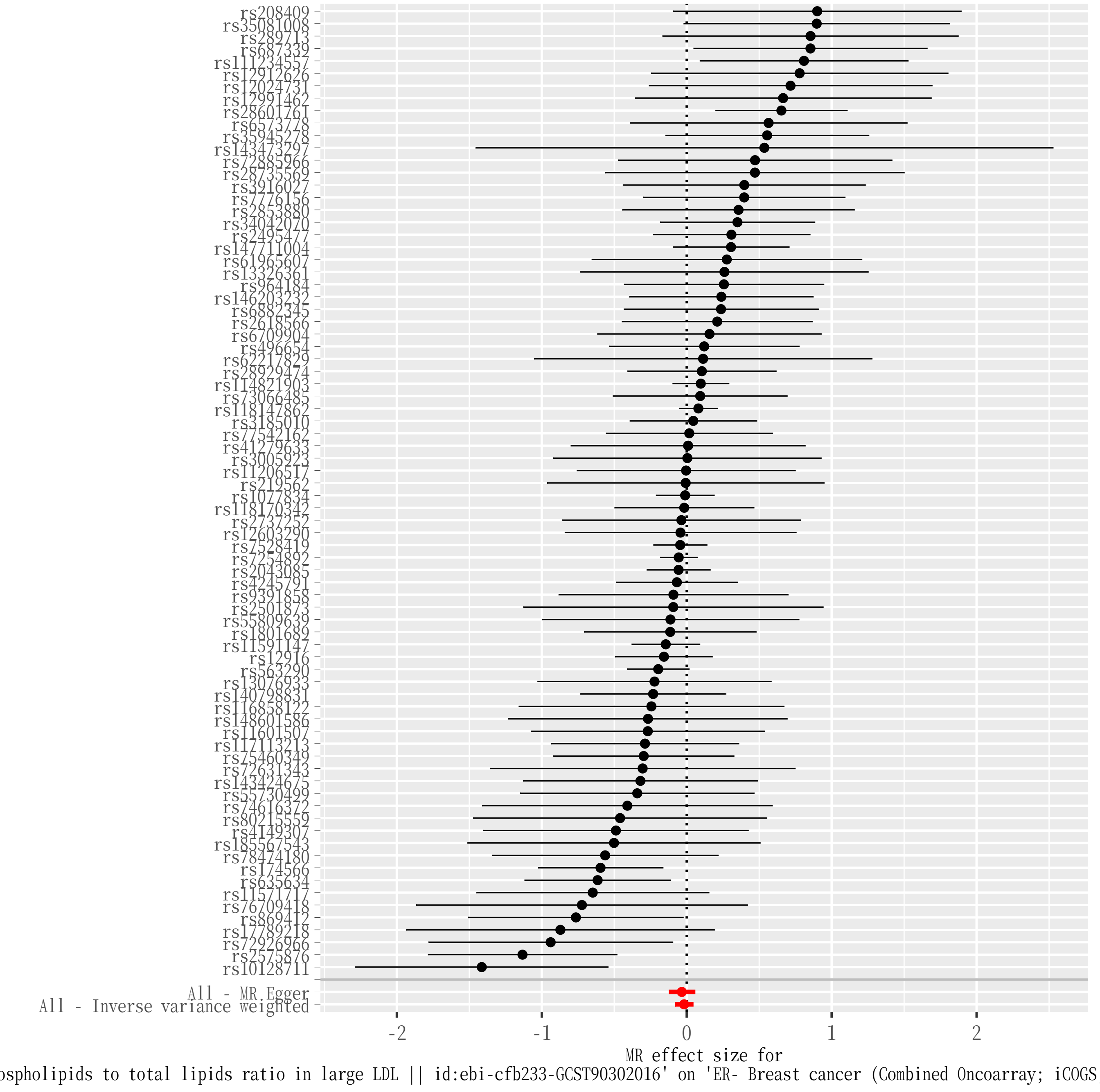

rs208409
rs35081008
rs289713
rs687339
rs111234557
rs12912626
rs12024731
rs12991462
rs28601761
rs6573778
rs35945278
rs143473297
rs72885266
rs28735569
rs3916027
rs7776156
rs2853880
rs34042070
rs2495477
rs147711004
rs61965607
rs13326361
rs964184
rs146203232
rs6882345
rs2618566
rs6709904
rs496654
rs62217829
rs28929474
rs114821903
rs73066485
rs118147862
rs3185010
rs77542162
rs41279633
rs3005923
rs11206517
rs219562
rs1077834
rs118170342
rs2737252
rs12603290
rs7528419
rs7254892
rs2043085
rs4245791
rs9391858
rs2501873
rs55809639
rs1801689
rs11591147
rs12916
rs563290
rs13076933
rs140798831
rs116858122
rs148601586
rs11601507
rs117113213
rs75460349
rs72631343
rs143424675
rs55730499
rs74616372
rs80215559
rs4149307
rs185567543
rs78474180
rs174566
rs635634
rs11571717
rs76709418
rs869412
rs17789218
rs72926966
rs2575876
rs10128711
All - MR Egger
All - Inverse variance weighted
-2
-1
0
1
2
MR effect size for
ospholipids to total lipids ratio in large LDL || id:ebi-cfb233-GCST90302016' on 'ER- Breast cancer (Combined Oncoarray; iCOGS

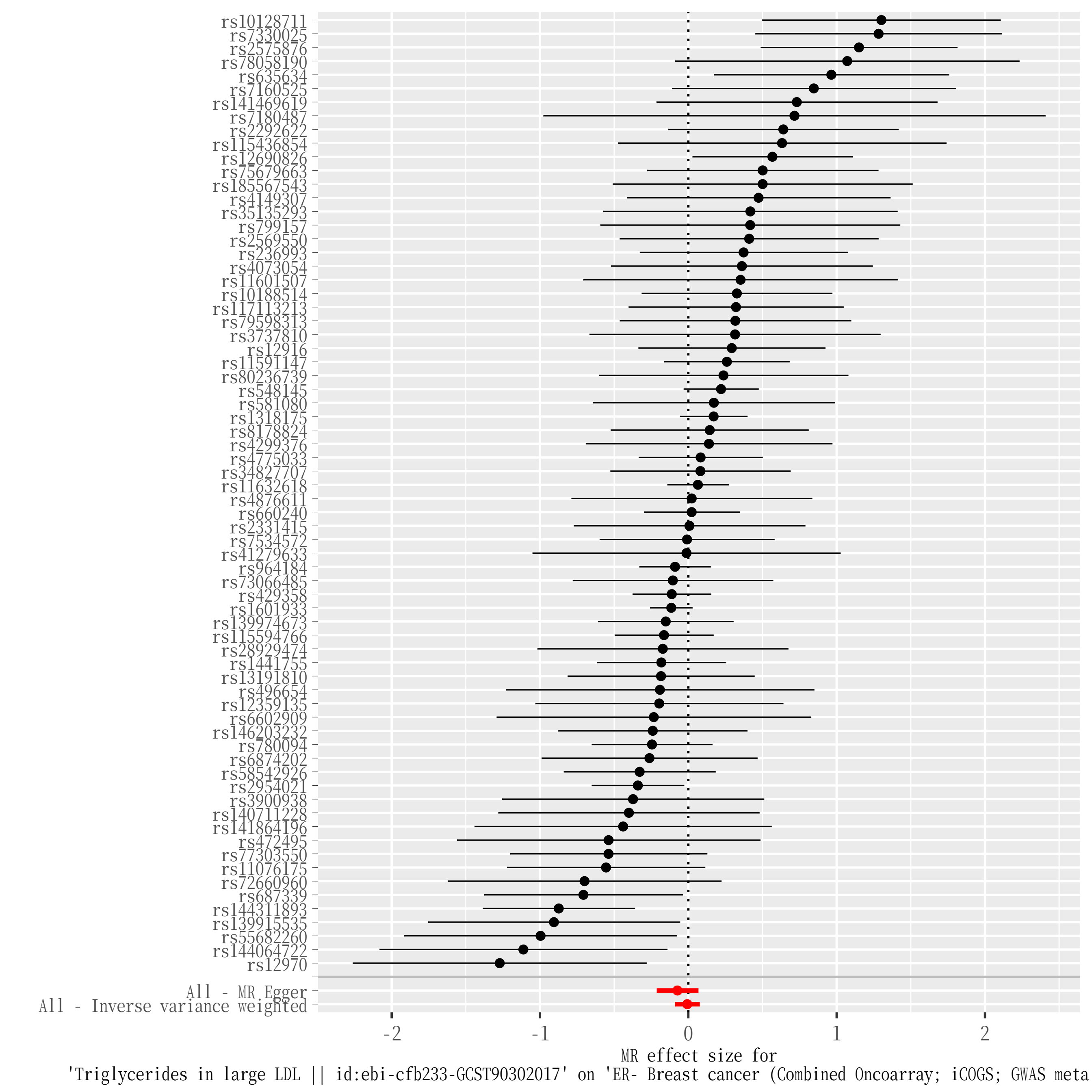
rs10128711
rs7330025
rs2575876
rs78058190
rs635634
rs7160525
rs141469619
rs7180487
rs2292622
rs115436854
rs12690826
rs75679663
rs185567543
rs4149307
rs35135293
rs799157
rs2569550
rs236993
rs4073054
rs11601507
rs10188514
rs117113213
rs79598313
rs3737810
rs12916
rs11591147
rs80236739
rs548145
rs581080
rs1318175
rs8178824
rs4299376
rs4775033
rs34827707
rs11632618
rs4876611
rs660240
rs2331415
rs7534572
rs41279633
rs964184
rs73066485
rs429358
rs1601933
rs139974673
rs115594766
rs28929474
rs1441755
rs13191810
rs496654
rs12359135
rs6602909
rs146203232
rs780094
rs6874202
rs58542926
rs2954021
rs3900938
rs140711228
rs141864196
rs472495
rs77303550
rs11076175
rs72660960
rs687339
rs144311893
rs139915535
rs55682260
rs144064722
rs12970
All - MR Egger
All - Inverse variance weighted
-2
-1
0
1
2
MR effect size for
'Triglycerides in large LDL || id:ebi-cfb233-GCST90302017' on 'ER- Breast cancer (Combined Oncoarray; iCOGS; GWAS meta

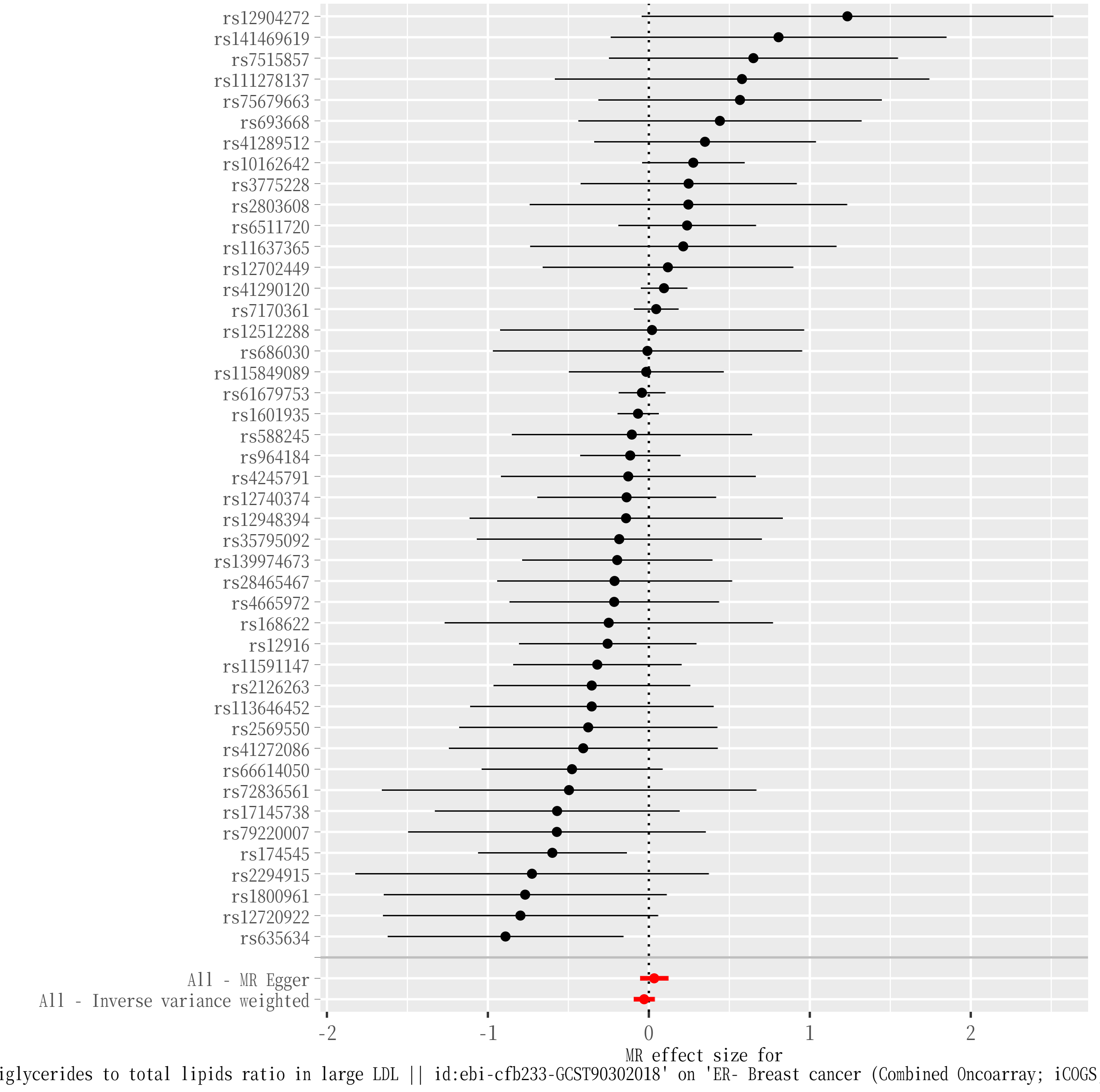
rs12904272
rs141469619
rs7515857
rs111278137
rs75679663
rs693668
rs41289512
rs10162642
rs3775228
rs2803608
rs6511720
rs11637365
rs12702449
rs41290120
rs7170361
rs12512288
rs686030
rs115849089
rs61679753
rs1601935
rs588245
rs964184
rs4245791
rs12740374
rs12948394
rs35795092
rs139974673
rs28465467
rs4665972
rs168622
rs12916
rs11591147
rs2126263
rs113646452
rs2569550
rs41272086
rs66614050
rs72836561
rs17145738
rs79220007
rs174545
rs2294915
rs1800961
rs12720922
rs635634
All - MR Egger
All - Inverse variance weighted
-2
-1
0
1
2
MR effect size for
iglycerides to total lipids ratio in large LDL || id:ebi-cfb233-GCST90302018' on 'ER- Breast cancer (Combined Oncoarray; iCOGS

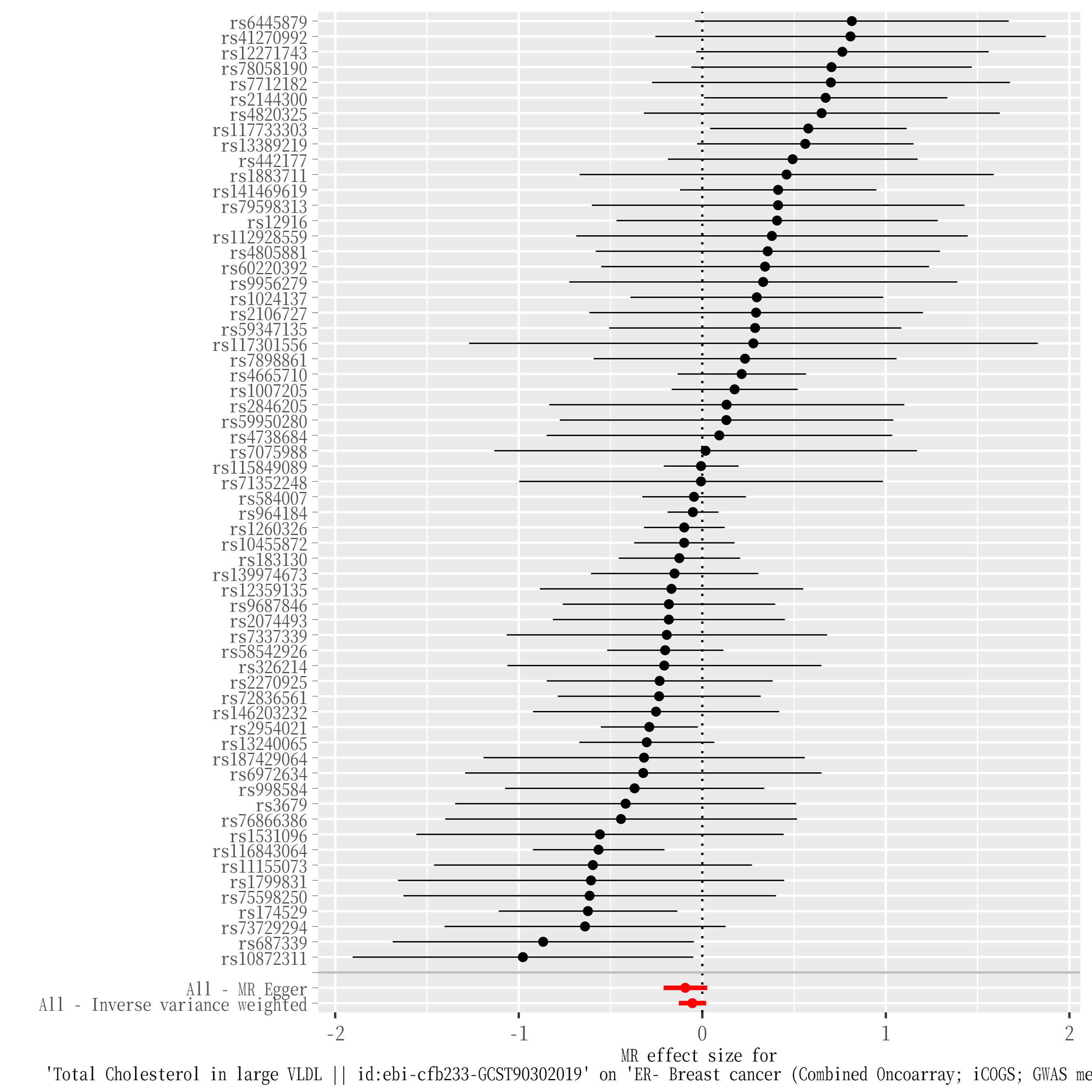
rs6445879
rs41270992
rs12271743
rs78058190
rs7712182
rs2144300
rs4820325
rs117733303
rs13389219
rs442177
rs1883711
rs141469619
rs79598313
rs12916
rs112928559
rs4805881
rs60220392
rs9956279
rs1024137
rs2106727
rs59347135
rs117301556
rs7898861
rs4665710
rs1007205
rs2846205
rs59950280
rs4738684
rs7075988
rs115849089
rs71352248
rs584007
rs964184
rs1260326
rs10455872
rs183130
rs139974673
rs12359135
rs9687846
rs2074493
rs7337339
rs58542926
rs326214
rs2270925
rs72836561
rs146203232
rs2954021
rs13240065
rs187429064
rs6972634
rs998584
rs3679
rs76866386
rs1531096
rs116843064
rs11155073
rs1799831
rs75598250
rs174529
rs73729294
rs687339
rs10872311
All - MR Egger
All - Inverse variance weighted
-2
-1
0
1
2
MR effect size for
'Total Cholesterol in large VLDL || id:ebi-cfb233-GCST90302019' on 'ER- Breast cancer (Combined Oncoarray; iCOGS; GWAS me

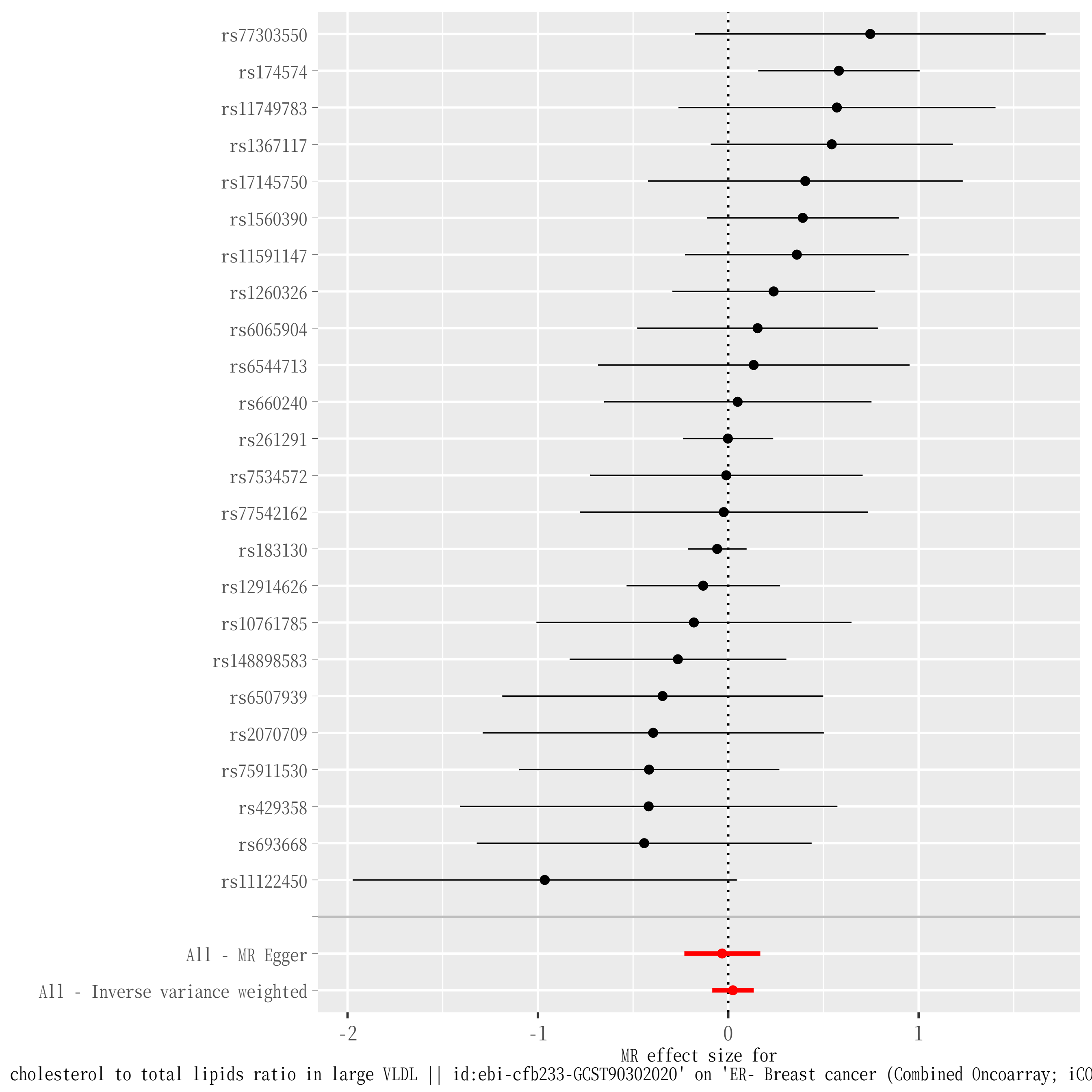
rs77303550
rs174574
rs11749783
rs1367117
rs17145750
rs1560390
rs11591147
rs1260326
rs6065904
rs6544713
rs660240
rs261291
rs7534572
rs77542162
rs183130
rs12914626
rs10761785
rs148898583
rs6507939
rs2070709
rs75911530
rs429358
rs693668
rs11122450
All - MR Egger
All - Inverse variance weighted
-2
-1
0
1
MR effect size for
cholesterol to total lipids ratio in large VLDL || id:ebi-cfb233-GCST90302020' on 'ER- Breast cancer (Combined Oncoarray; iCO

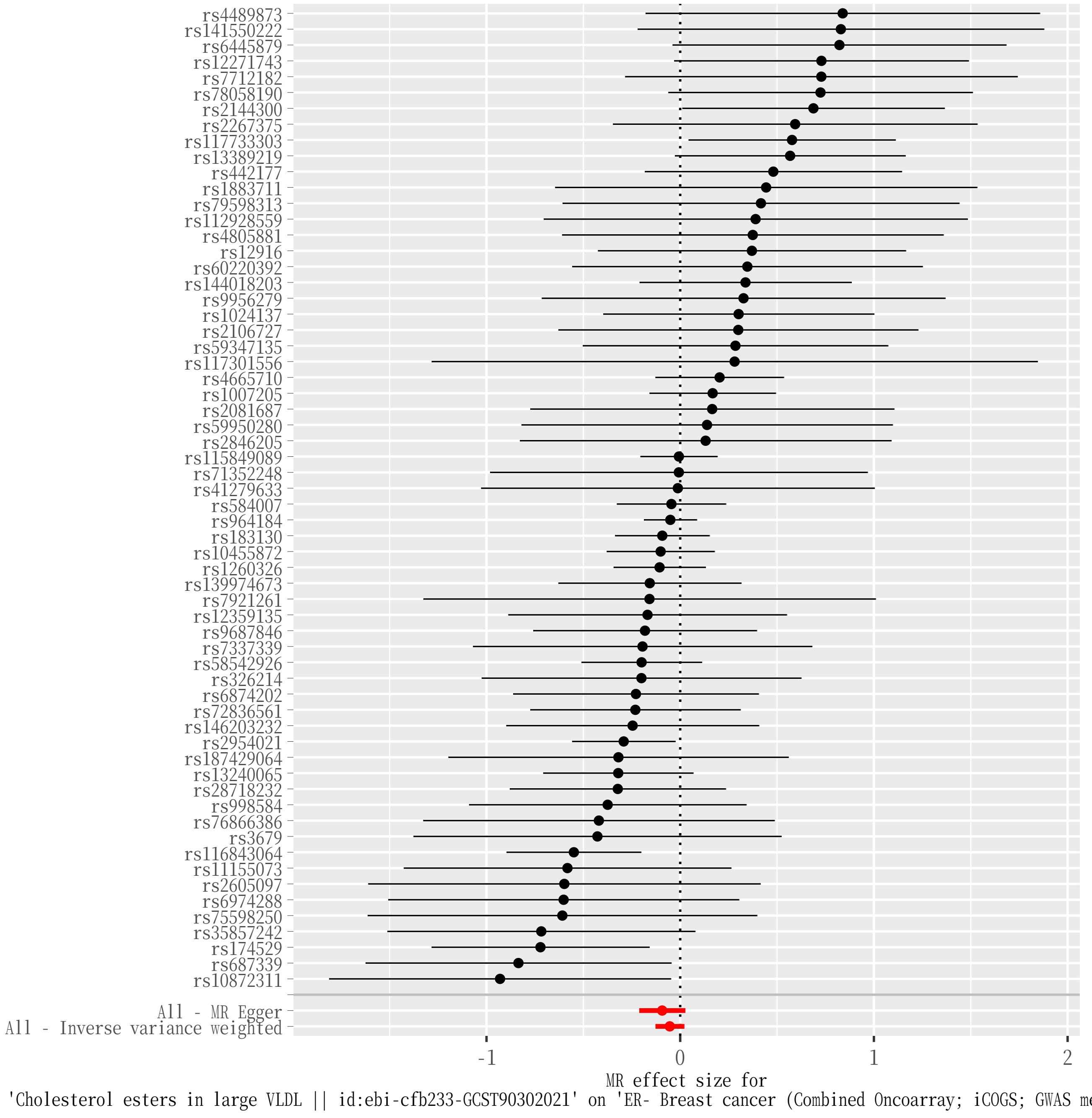
rs4489873
rs141550222
rs6445879
rs12271743
rs7712182
rs78058190
rs2144300
rs2267375
rs117733303
rs13389219
rs442177
rs1883711
rs79598313
rs112928559
rs4805881
rs12916
rs60220392
rs144018203
rs9956279
rs1024137
rs2106727
rs59347135
rs117301556
rs4665710
rs1007205
rs2081687
rs59950280
rs2846205
rs115849089
rs71352248
rs41279633
rs584007
rs964184
rs183130
rs10455872
rs1260326
rs139974673
rs7921261
rs12359135
rs9687846
rs7337339
rs58542926
rs326214
rs6874202
rs72836561
rs146203232
rs2954021
rs187429064
rs13240065
rs28718232
rs998584
rs76866386
rs3679
rs116843064
rs11155073
rs2605097
rs6974288
rs75598250
rs35857242
rs174529
rs687339
rs10872311
All - MR Egger
All - Inverse variance weighted
-1
0
1
2
MR effect size for
'Cholesterol esters in large VLDL || id:ebi-cfb233-GCST90302021' on 'ER- Breast cancer (Combined Oncoarray; iCOGS; GWAS me

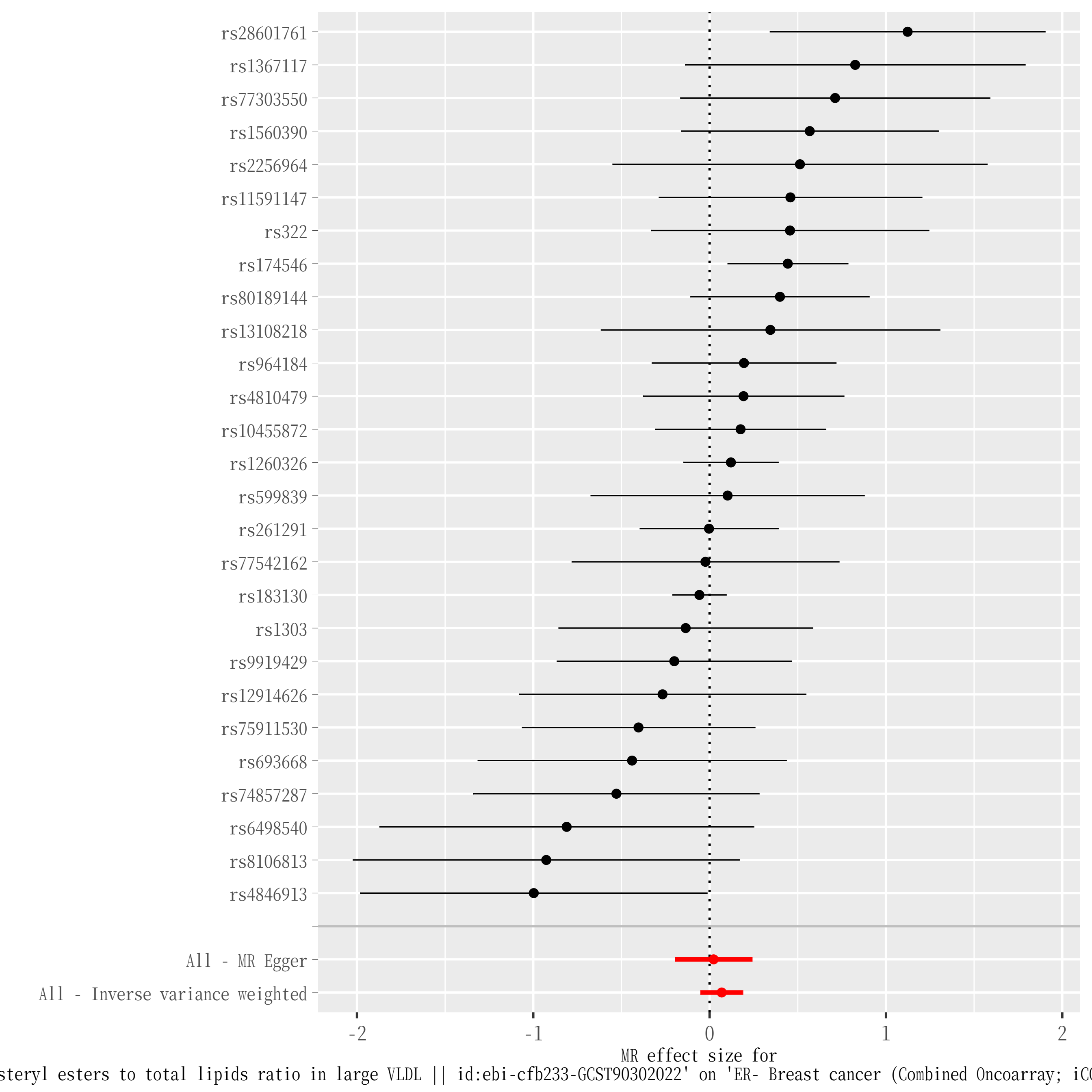

rs28601761
rs1367117
rs77303550
rs1560390
rs2256964
rs11591147
rs322
rs174546
rs80189144
rs13108218
rs964184
rs4810479
rs10455872
rs1260326
rs599839
rs261291
rs77542162
rs183130
rs1303
rs9919429
rs12914626
rs75911530
rs693668
rs74857287
rs6498540
rs8106813
rs4846913
All - MR Egger
All - Inverse variance weighted
-2
-1
0
1
2
MR effect size for
steryl esters to total lipids ratio in large VLDL || id:ebi-cfb233-GCST90302022' on 'ER- Breast cancer (Combined Oncoarray; iC

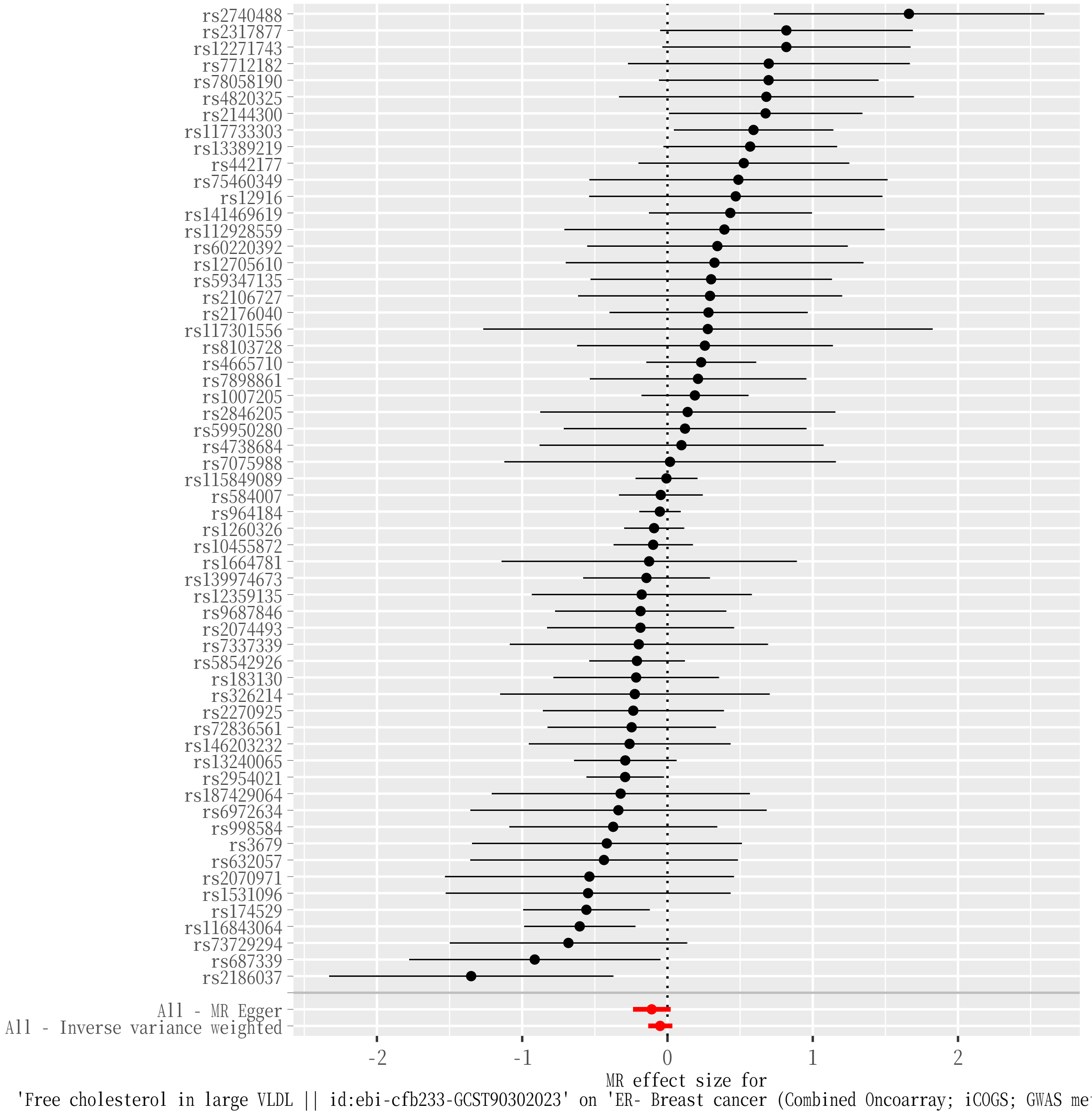

rs2740488
rs2317877
rs12271743
rs7712182
rs78058190
rs4820325
rs2144300
rs117733303
rs13389219
rs442177
rs75460349
rs12916
rs141469619
rs112928559
rs60220392
rs12705610
rs59347135
rs2106727
rs2176040
rs117301556
rs8103728
rs4665710
rs7898861
rs1007205
rs2846205
rs59950280
rs4738684
rs7075988
rs115849089
rs584007
rs964184
rs1260326
rs10455872
rs1664781
rs139974673
rs12359135
rs9687846
rs2074493
rs7337339
rs58542926
rs183130
rs326214
rs2270925
rs72836561
rs146203232
rs13240065
rs2954021
rs187429064
rs6972634
rs998584
rs3679
rs632057
rs2070971
rs1531096
rs174529
rs116843064
rs73729294
rs687339
rs2186037
All - MR Egger
All - Inverse variance weighted
-2
-1
0
1
2
MR effect size for
'Free cholesterol in large VLDL || id:ebi-cfb233-GCST90302023' on 'ER- Breast cancer (Combined Oncoarray; iCOGS; GWAS me

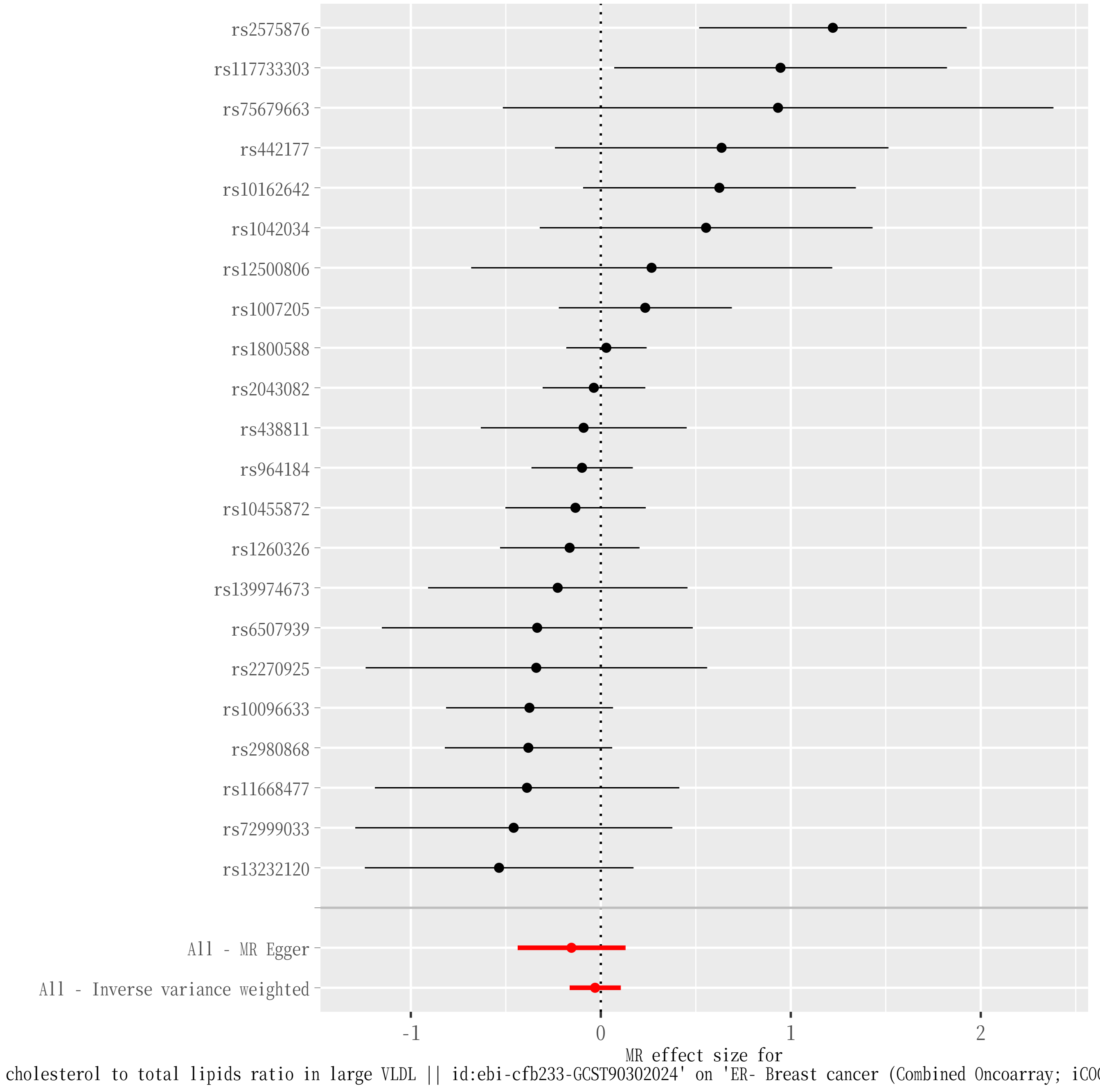
rs2575876
rs117733303
rs75679663
rs442177
rs10162642
rs1042034
rs12500806
rs1007205
rs1800588
rs2043082
rs438811
rs964184
rs10455872
rs1260326
rs139974673
rs6507939
rs2270925
rs10096633
rs2980868
rs11668477
rs72999033
rs13232120
All - MR Egger
All - Inverse variance weighted
-1
0
1
2
MR effect size for
cholesterol to total lipids ratio in large VLDL || id:ebi-cfb233-GCST90302024' on 'ER- Breast cancer (Combined Oncoarray; iCO

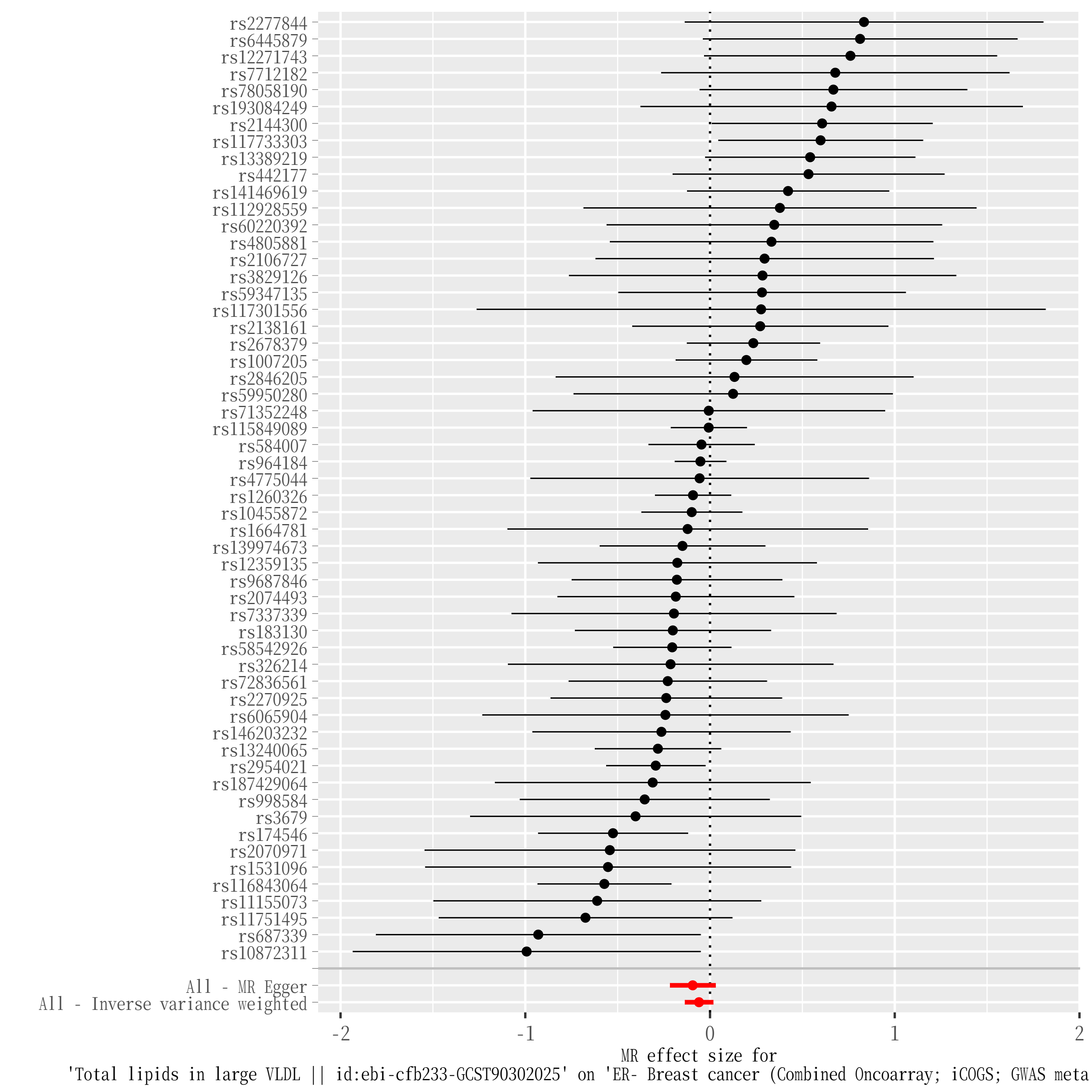
rs2277844
rs6445879
rs12271743
rs7712182
rs78058190
rs193084249
rs2144300
rs117733303
rs13389219
rs442177
rs141469619
rs112928559
rs60220392
rs4805881
rs2106727
rs3829126
rs59347135
rs117301556
rs2138161
rs2678379
rs1007205
rs2846205
rs59950280
rs71352248
rs115849089
rs584007
rs964184
rs4775044
rs1260326
rs10455872
rs1664781
rs139974673
rs12359135
rs9687846
rs2074493
rs7337339
rs183130
rs58542926
rs326214
rs72836561
rs2270925
rs6065904
rs146203232
rs13240065
rs2954021
rs187429064
rs998584
rs3679
rs174546
rs2070971
rs1531096
rs116843064
rs11155073
rs11751495
rs687339
rs10872311
All - MR Egger
All - Inverse variance weighted
-2
-1
0
1
2
MR effect size for
'Total lipids in large VLDL || id:ebi-cfb233-GCST90302025' on 'ER- Breast cancer (Combined Oncoarray; iCOGS; GWAS meta

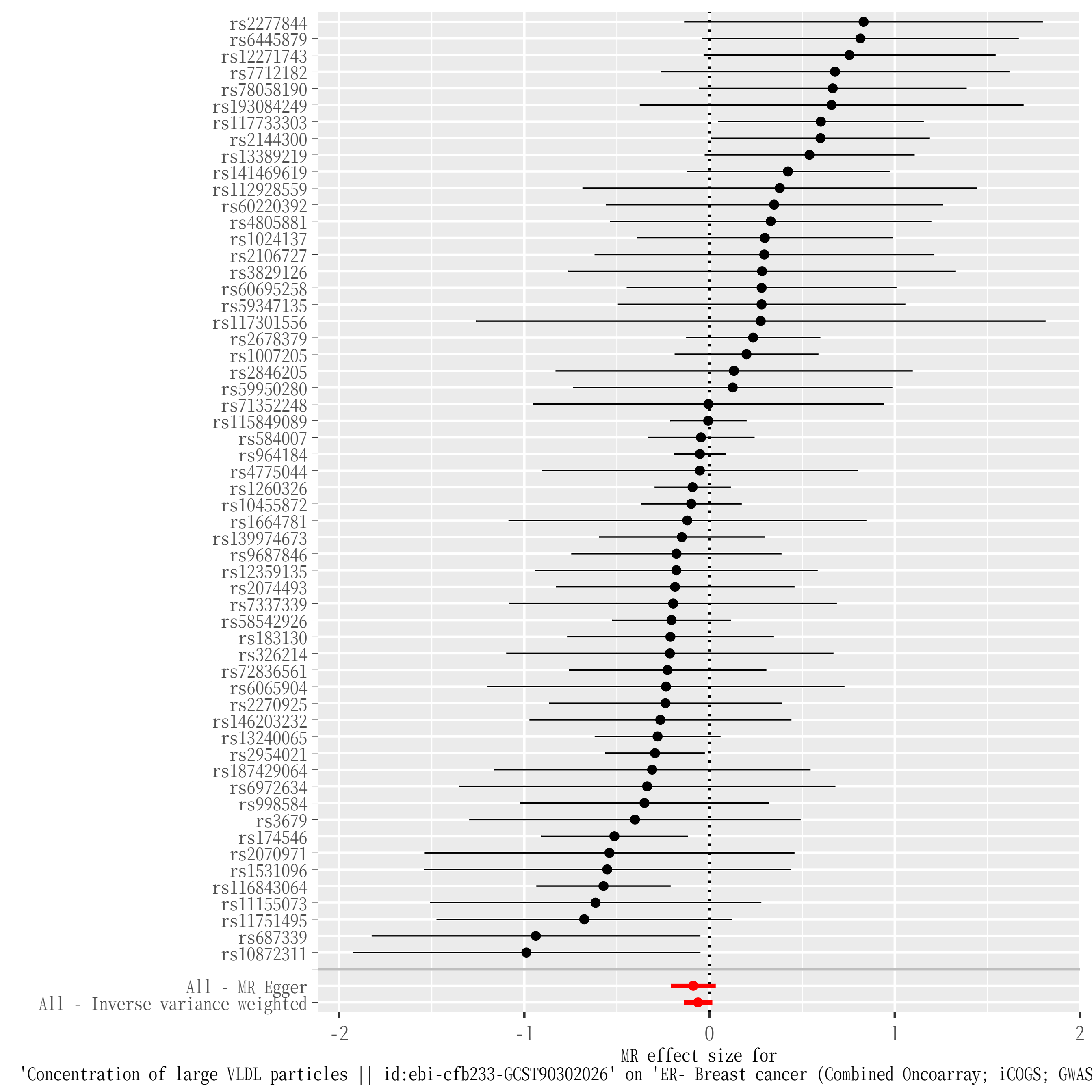

rs2277844
rs6445879
rs12271743
rs7712182
rs78058190
rs193084249
rs117733303
rs2144300
rs13389219
rs141469619
rs112928559
rs60220392
rs4805881
rs1024137
rs2106727
rs3829126
rs60695258
rs59347135
rs117301556
rs2678379
rs1007205
rs2846205
rs59950280
rs71352248
rs115849089
rs584007
rs964184
rs4775044
rs1260326
rs10455872
rs1664781
rs139974673
rs9687846
rs12359135
rs2074493
rs7337339
rs58542926
rs183130
rs326214
rs72836561
rs6065904
rs2270925
rs146203232
rs13240065
rs2954021
rs187429064
rs6972634
rs998584
rs3679
rs174546
rs2070971
rs1531096
rs116843064
rs11155073
rs11751495
rs687339
rs10872311
All - MR Egger
All - Inverse variance weighted
-2
-1
0
1
2
MR effect size for
'Concentration of large VLDL particles || id:ebi-cfb233-GCST90302026' on 'ER- Breast cancer (Combined Oncoarray; iCOGS; GWAS

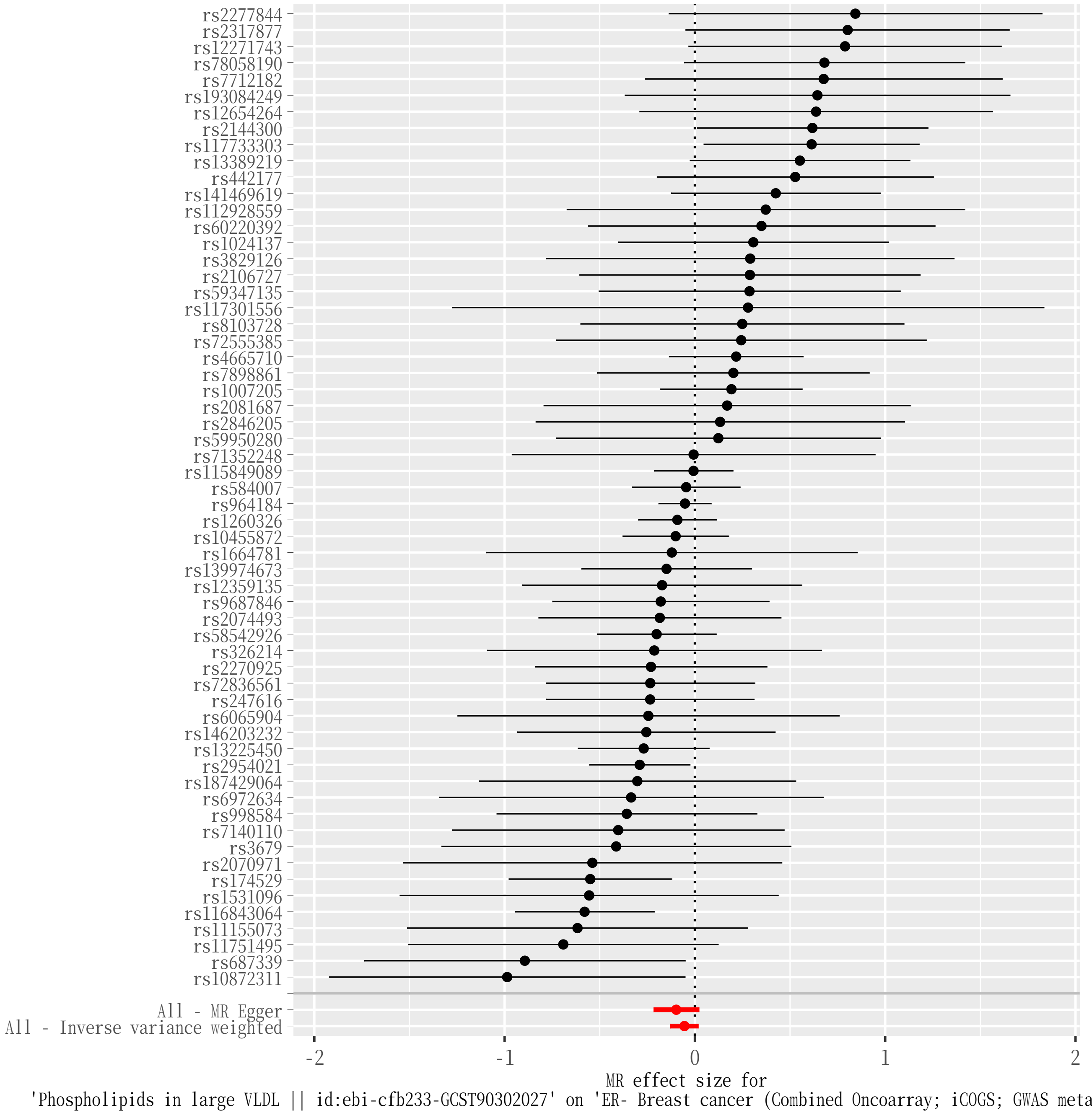
rs2277844
rs2317877
rs12271743
rs78058190
rs7712182
rs193084249
rs12654264
rs2144300
rs117733303
rs13389219
rs442177
rs141469619
rs112928559
rs60220392
rs1024137
rs3829126
rs2106727
rs59347135
rs117301556
rs8103728
rs72555385
rs4665710
rs7898861
rs1007205
rs2081687
rs2846205
rs59950280
rs71352248
rs115849089
rs584007
rs964184
rs1260326
rs10455872
rs1664781
rs139974673
rs12359135
rs9687846
rs2074493
rs58542926
rs326214
rs2270925
rs72836561
rs247616
rs6065904
rs146203232
rs13225450
rs2954021
rs187429064
rs6972634
rs998584
rs7140110
rs3679
rs2070971
rs174529
rs1531096
rs116843064
rs11155073
rs11751495
rs687339
rs10872311
All - MR Egger
All - Inverse variance weighted
-2
-1
0
1
2
MR effect size for
'Phospholipids in large VLDL || id:ebi-cfb233-GCST90302027' on 'ER- Breast cancer (Combined Oncoarray; iCOGS; GWAS meta

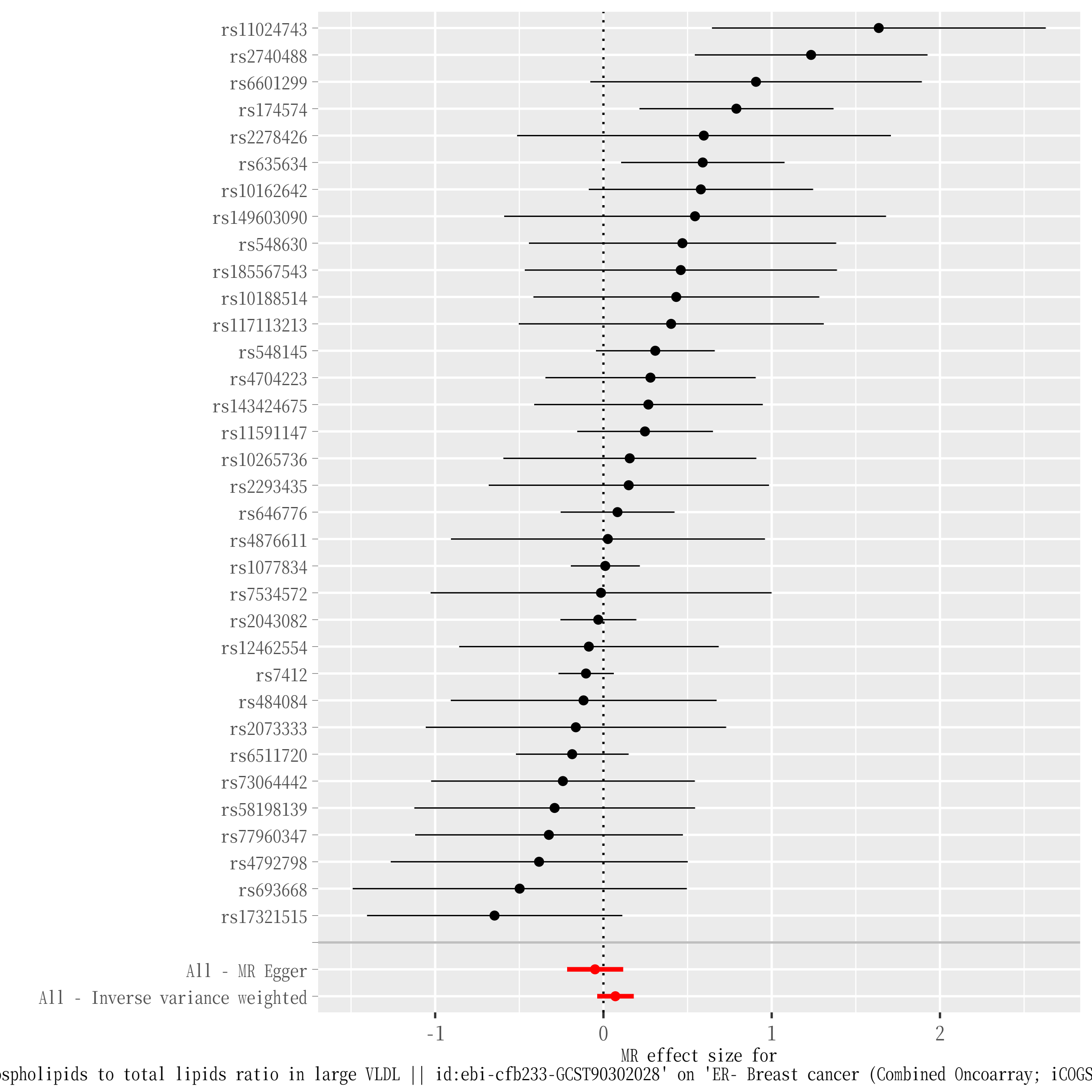
rs11024743
rs2740488
rs6601299
rs174574
rs2278426
rs635634
rs10162642
rs149603090
rs548630
rs185567543
rs10188514
rs117113213
rs548145
rs4704223
rs143424675
rs11591147
rs10265736
rs2293435
rs646776
rs4876611
rs1077834
rs7534572
rs2043082
rs12462554
rs7412
rs484084
rs2073333
rs6511720
rs73064442
rs58198139
rs77960347
rs4792798
rs693668
rs17321515
All - MR Egger
All - Inverse variance weighted
-1
0
1
2
MR effect size for
ospholipids to total lipids ratio in large VLDL || id:ebi-cfb233-GCST90302028' on 'ER- Breast cancer (Combined Oncoarray; iCOGS

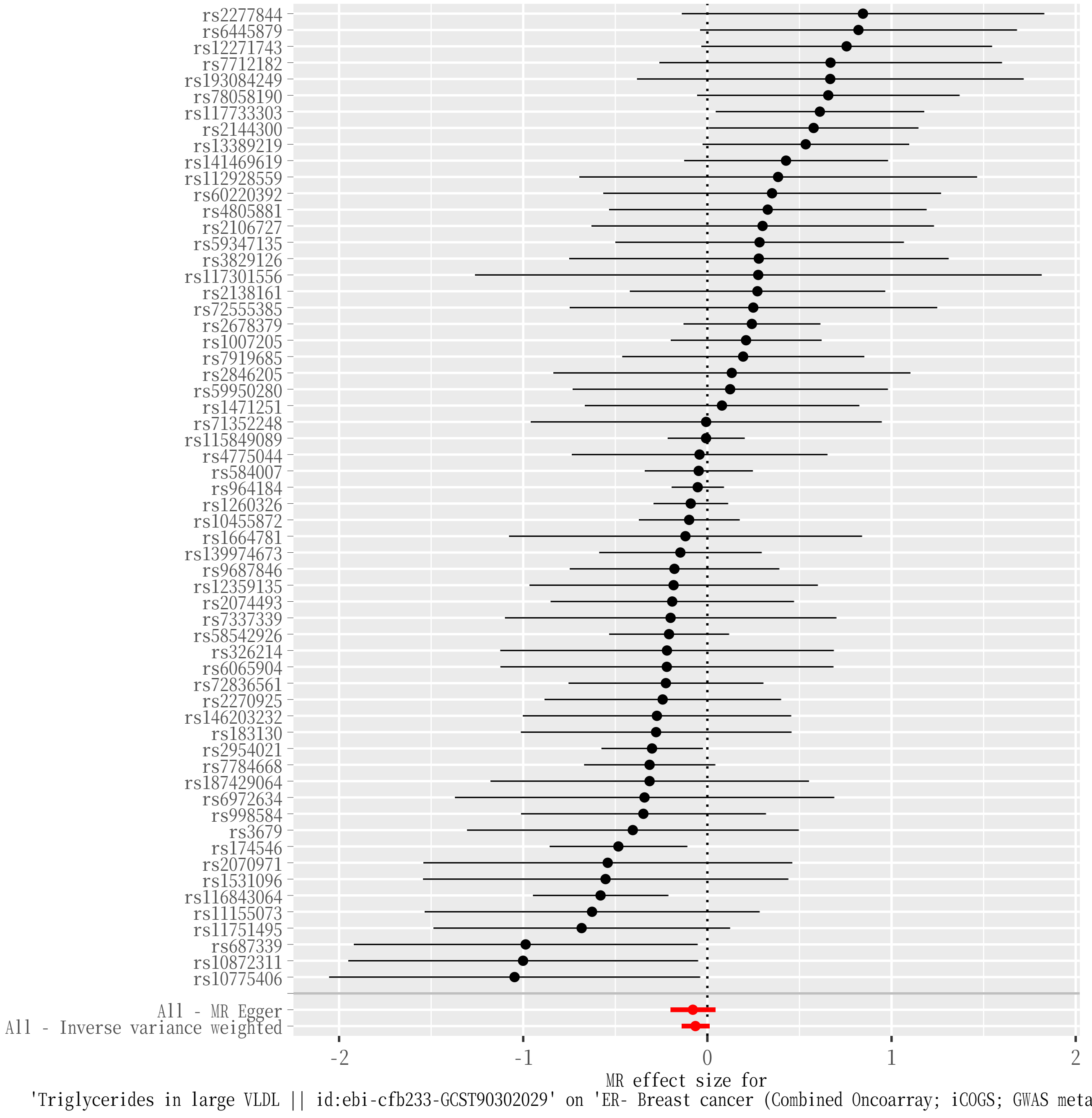
rs2277844
rs6445879
rs12271743
rs7712182
rs193084249
rs78058190
rs117733303
rs2144300
rs13389219
rs141469619
rs112928559
rs60220392
rs4805881
rs2106727
rs59347135
rs3829126
rs117301556
rs2138161
rs72555385
rs2678379
rs1007205
rs7919685
rs2846205
rs59950280
rs1471251
rs71352248
rs115849089
rs4775044
rs584007
rs964184
rs1260326
rs10455872
rs1664781
rs139974673
rs9687846
rs12359135
rs2074493
rs7337339
rs58542926
rs326214
rs6065904
rs72836561
rs2270925
rs146203232
rs183130
rs2954021
rs7784668
rs187429064
rs6972634
rs998584
rs3679
rs174546
rs2070971
rs1531096
rs116843064
rs11155073
rs11751495
rs687339
rs10872311
rs10775406
All - MR Egger
All - Inverse variance weighted
-2
-1
0
1
2
MR effect size for
'Triglycerides in large VLDL || id:ebi-cfb233-GCST90302029' on 'ER- Breast cancer (Combined Oncoarray; iCOGS; GWAS meta

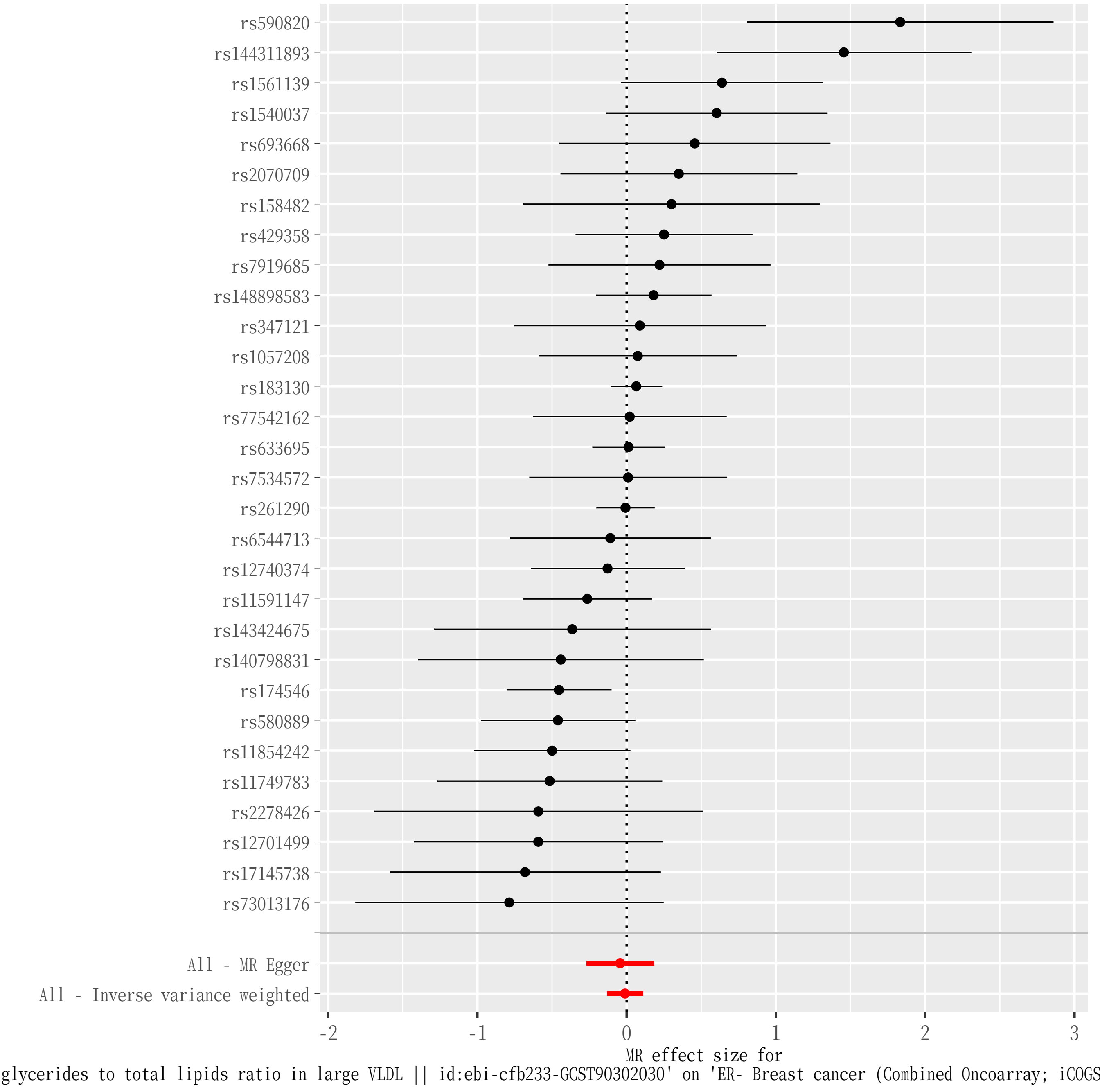

rs590820
rs144311893
rs1561139
rs1540037
rs693668
rs2070709
rs158482
rs429358
rs7919685
rs148898583
rs347121
rs1057208
rs183130
rs77542162
rs633695
rs7534572
rs261290
rs6544713
rs12740374
rs11591147
rs143424675
rs140798831
rs174546
rs580889
rs11854242
rs11749783
rs2278426
rs12701499
rs17145738
rs73013176
All - MR Egger
All - Inverse variance weighted
-2
-1
0
1
2
3
MR effect size for
glycerides to total lipids ratio in large VLDL || id:ebi-cfb233-GCST90302030' on 'ER- Breast cancer (Combined Oncoarray; iCOGS

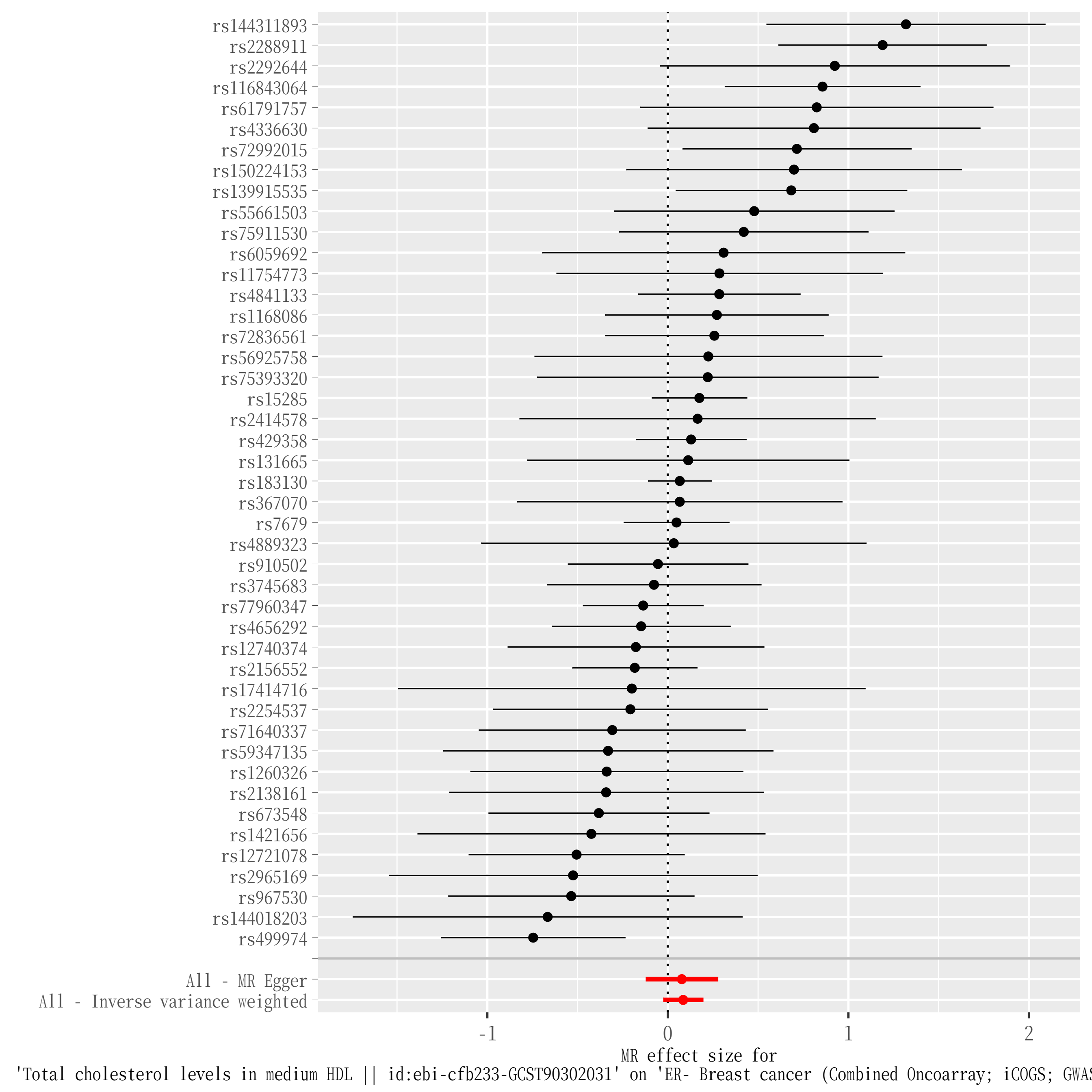
rs144311893
rs2288911
rs2292644
rs116843064
rs61791757
rs4336630
rs72992015
rs150224153
rs139915535
rs55661503
rs75911530
rs6059692
rs11754773
rs4841133
rs1168086
rs72836561
rs56925758
rs75393320
rs15285
rs2414578
rs429358
rs131665
rs183130
rs367070
rs7679
rs4889323
rs910502
rs3745683
rs77960347
rs4656292
rs12740374
rs2156552
rs17414716
rs2254537
rs71640337
rs59347135
rs1260326
rs2138161
rs673548
rs1421656
rs12721078
rs2965169
rs967530
rs144018203
rs499974
All - MR Egger
All - Inverse variance weighted
-1
0
1
2
MR effect size for
'Total cholesterol levels in medium HDL || id:ebi-cfb233-GCST90302031' on 'ER- Breast cancer (Combined Oncoarray; iCOGS; GWAS

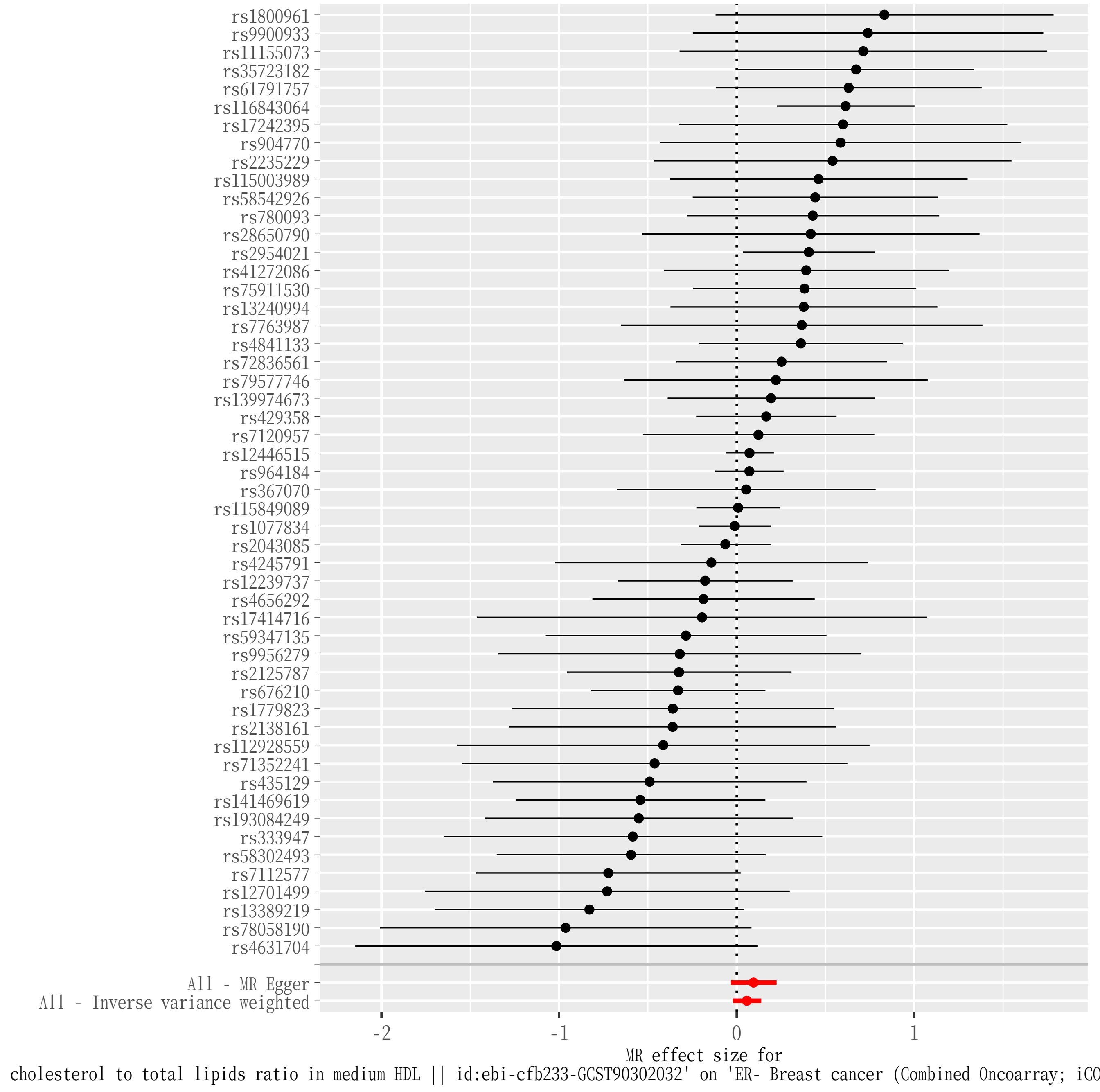

rs1800961
rs9900933
rs11155073
rs35723182
rs61791757
rs116843064
rs17242395
rs904770
rs2235229
rs115003989
rs58542926
rs780093
rs28650790
rs2954021
rs41272086
rs75911530
rs13240994
rs7763987
rs4841133
rs72836561
rs79577746
rs139974673
rs429358
rs7120957
rs12446515
rs964184
rs367070
rs115849089
rs1077834
rs2043085
rs4245791
rs12239737
rs4656292
rs17414716
rs59347135
rs9956279
rs2125787
rs676210
rs1779823
rs2138161
rs112928559
rs71352241
rs435129
rs141469619
rs193084249
rs333947
rs58302493
rs7112577
rs12701499
rs13389219
rs78058190
rs4631704
All - MR Egger
All - Inverse variance weighted
-2
-1
0
1
MR effect size for
cholesterol to total lipids ratio in medium HDL || id:ebi-cfb233-GCST90302032' on 'ER- Breast cancer (Combined Oncoarray; iCO

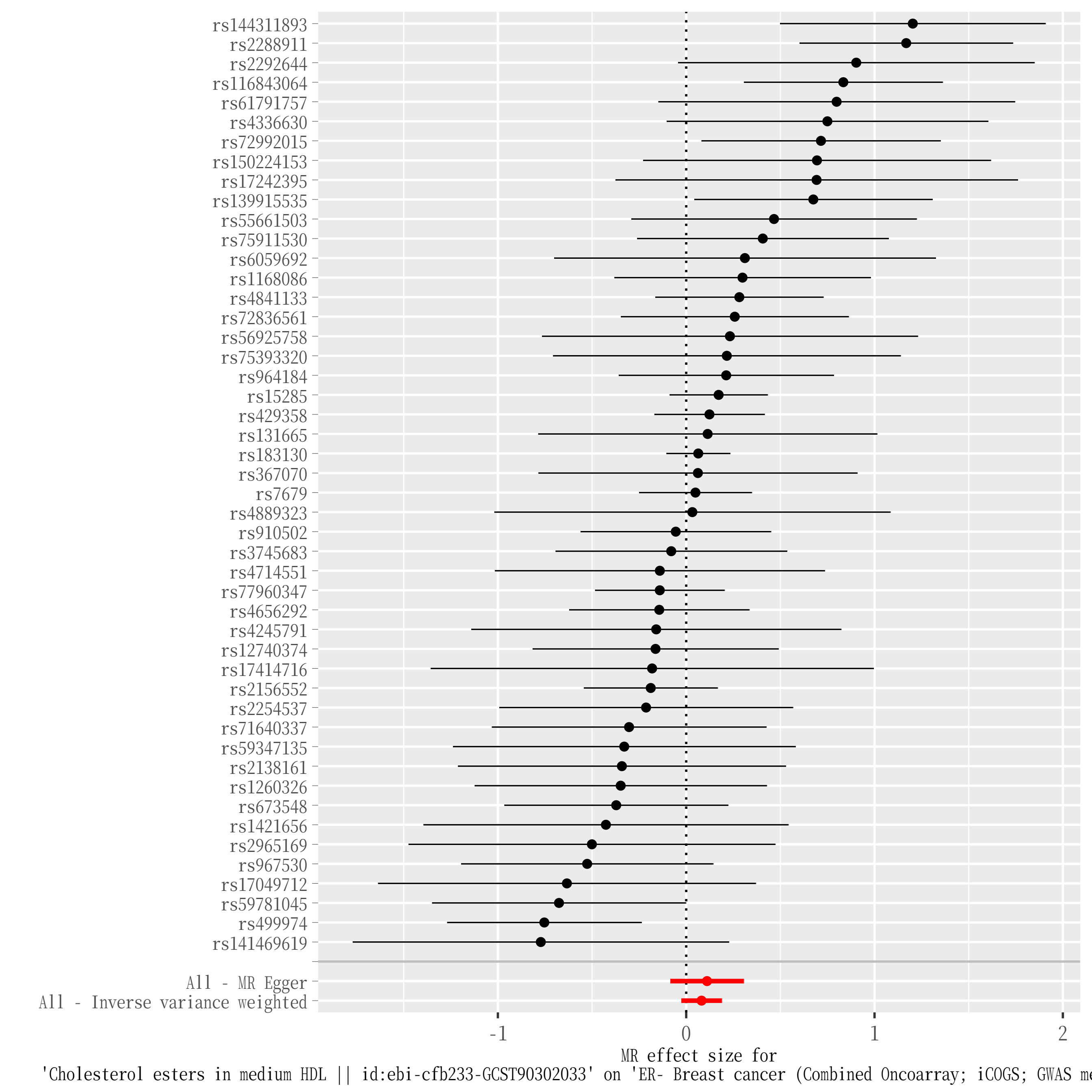
rs144311893
rs2288911
rs2292644
rs116843064
rs61791757
rs4336630
rs72992015
rs150224153
rs17242395
rs139915535
rs55661503
rs75911530
rs6059692
rs1168086
rs4841133
rs72836561
rs56925758
rs75393320
rs964184
rs15285
rs429358
rs131665
rs183130
rs367070
rs7679
rs4889323
rs910502
rs3745683
rs4714551
rs77960347
rs4656292
rs4245791
rs12740374
rs17414716
rs2156552
rs2254537
rs71640337
rs59347135
rs2138161
rs1260326
rs673548
rs1421656
rs2965169
rs967530
rs17049712
rs59781045
rs499974
rs141469619
All - MR Egger
All - Inverse variance weighted
-1
0
1
2
MR effect size for
'Cholesterol esters in medium HDL || id:ebi-cfb233-GCST90302033' on 'ER- Breast cancer (Combined Oncoarray; iCOGS; GWAS me

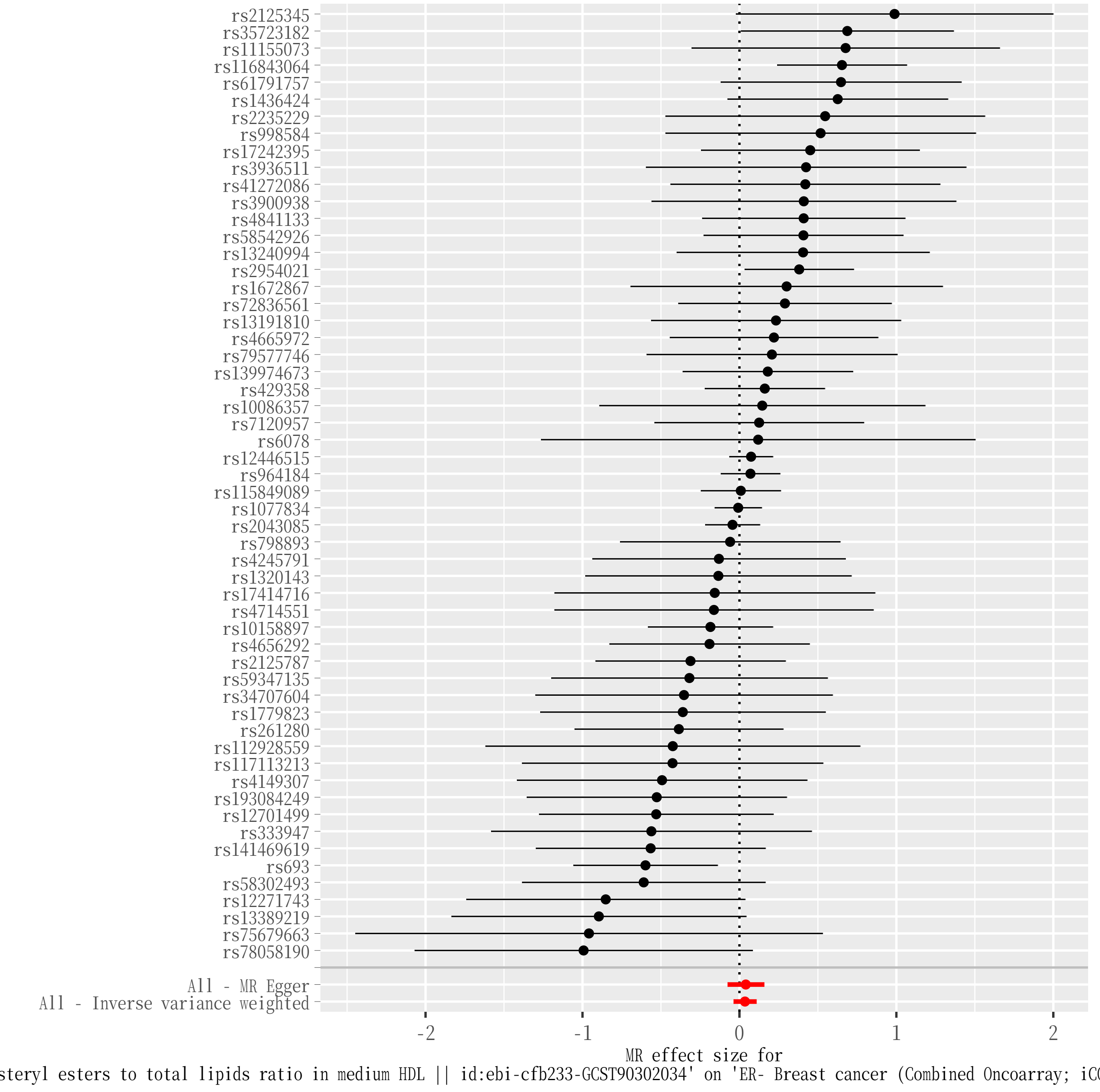

rs2125345
rs35723182
rs11155073
rs116843064
rs61791757
rs1436424
rs2235229
rs998584
rs17242395
rs3936511
rs41272086
rs3900938
rs4841133
rs58542926
rs13240994
rs2954021
rs1672867
rs72836561
rs13191810
rs4665972
rs79577746
rs139974673
rs429358
rs10086357
rs7120957
rs6078
rs12446515
rs964184
rs115849089
rs1077834
rs2043085
rs798893
rs4245791
rs1320143
rs17414716
rs4714551
rs10158897
rs4656292
rs2125787
rs59347135
rs34707604
rs1779823
rs261280
rs112928559
rs117113213
rs4149307
rs193084249
rs12701499
rs333947
rs141469619
rs693
rs58302493
rs12271743
rs13389219
rs75679663
rs78058190
All - MR Egger
All - Inverse variance weighted
-2
-1
0
1
2
MR effect size for
steryl esters to total lipids ratio in medium HDL || id:ebi-cfb233-GCST90302034' on 'ER- Breast cancer (Combined Oncoarray; iC

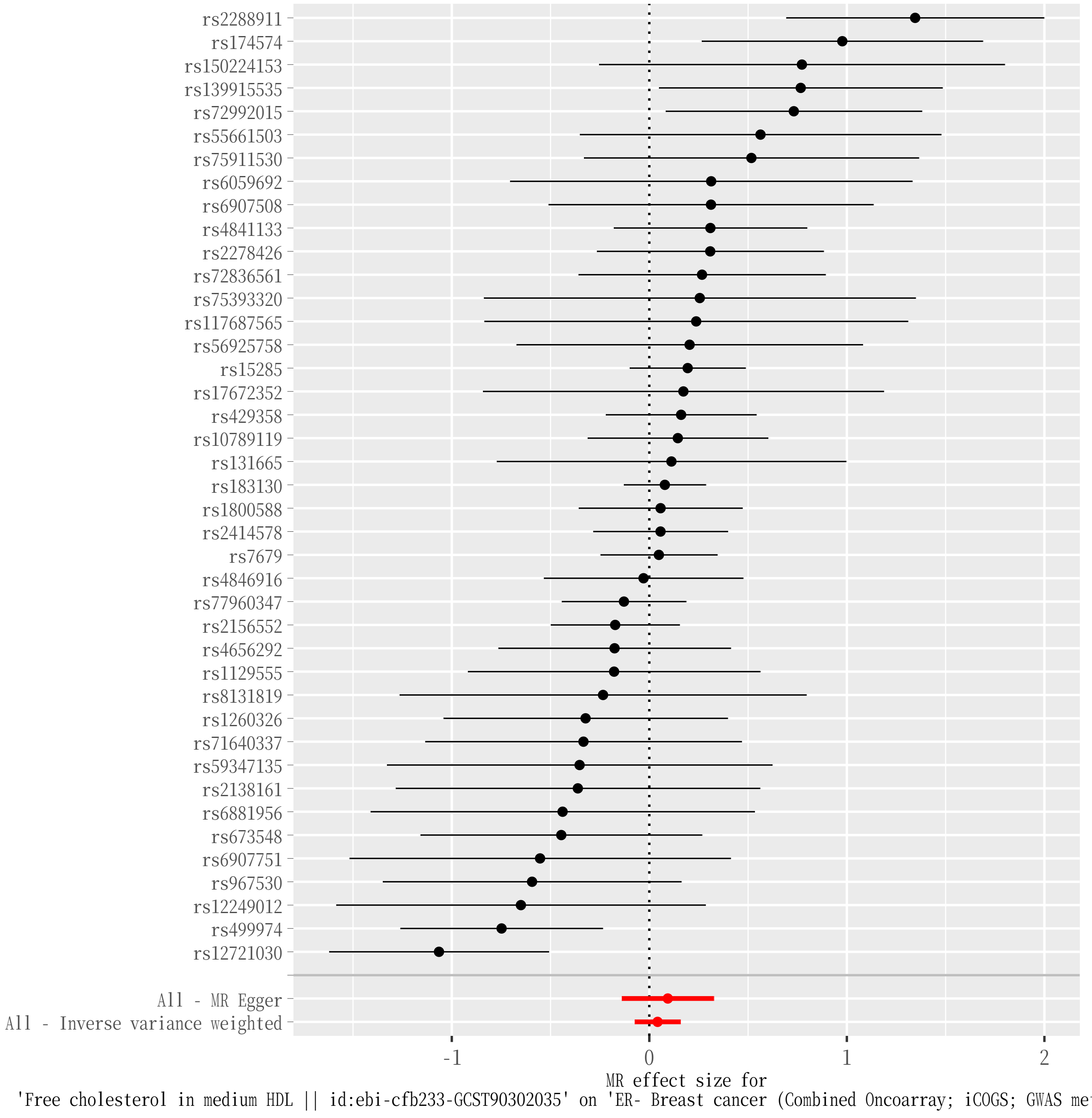

rs2288911
rs174574
rs150224153
rs139915535
rs72992015
rs55661503
rs75911530
rs6059692
rs6907508
rs4841133
rs2278426
rs72836561
rs75393320
rs117687565
rs56925758
rs15285
rs17672352
rs429358
rs10789119
rs131665
rs183130
rs1800588
rs2414578
rs7679
rs4846916
rs77960347
rs2156552
rs4656292
rs1129555
rs8131819
rs1260326
rs71640337
rs59347135
rs2138161
rs6881956
rs673548
rs6907751
rs967530
rs12249012
rs499974
rs12721030
All - MR Egger
All - Inverse variance weighted
-1
0
1
2
MR effect size for
'Free cholesterol in medium HDL || id:ebi-cfb233-GCST90302035' on 'ER- Breast cancer (Combined Oncoarray; iCOGS; GWAS me

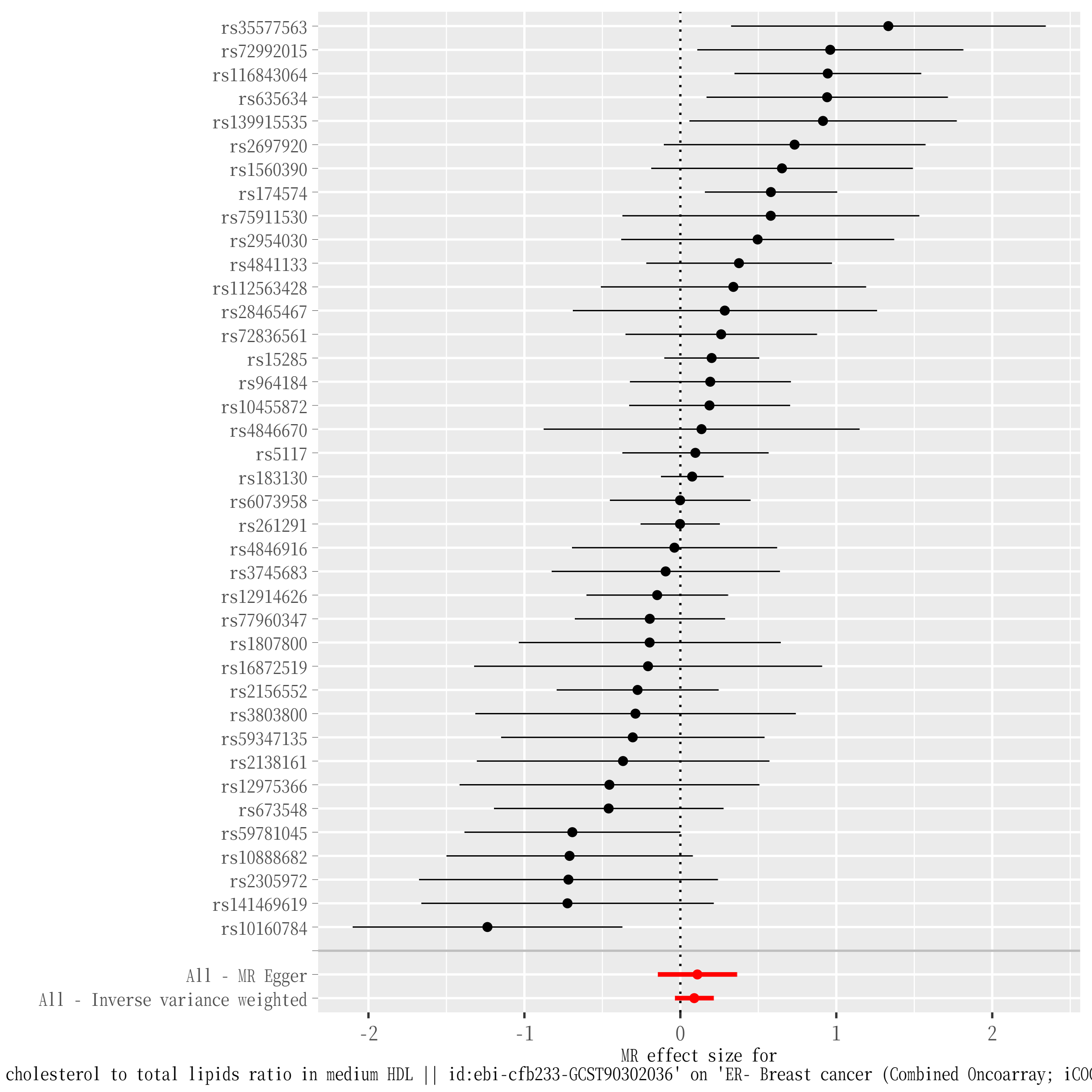
rs35577563
rs72992015
rs116843064
rs635634
rs139915535
rs2697920
rs1560390
rs174574
rs75911530
rs2954030
rs4841133
rs112563428
rs28465467
rs72836561
rs15285
rs964184
rs10455872
rs4846670
rs5117
rs183130
rs6073958
rs261291
rs4846916
rs3745683
rs12914626
rs77960347
rs1807800
rs16872519
rs2156552
rs3803800
rs59347135
rs2138161
rs12975366
rs673548
rs59781045
rs10888682
rs2305972
rs141469619
rs10160784
All - MR Egger
All - Inverse variance weighted
-2
-1
0
1
2
MR effect size for
cholesterol to total lipids ratio in medium HDL || id:ebi-cfb233-GCST90302036' on 'ER- Breast cancer (Combined Oncoarray; iCO

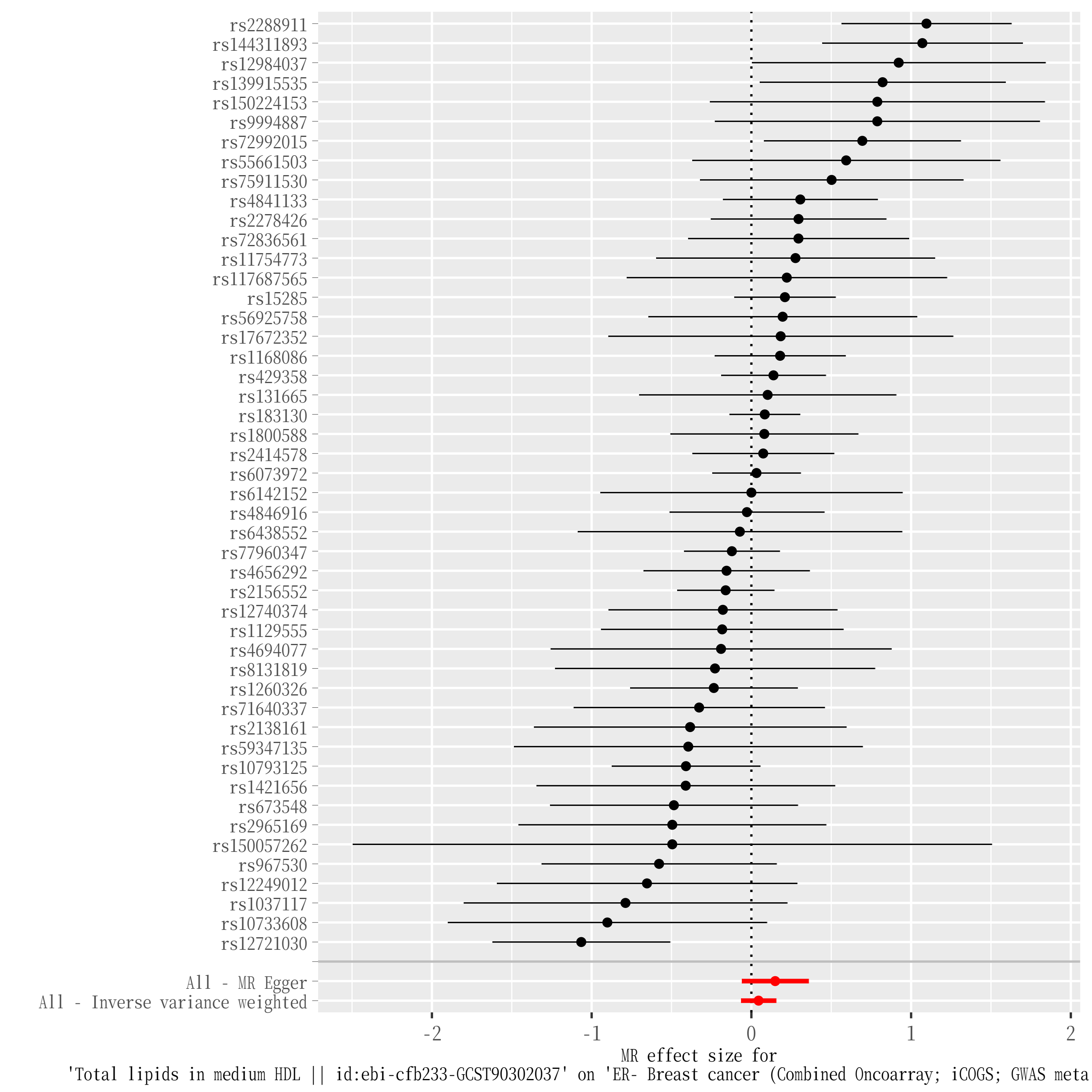

rs2288911
rs144311893
rs12984037
rs139915535
rs150224153
rs9994887
rs72992015
rs55661503
rs75911530
rs4841133
rs2278426
rs72836561
rs11754773
rs117687565
rs15285
rs56925758
rs17672352
rs1168086
rs429358
rs131665
rs183130
rs1800588
rs2414578
rs6073972
rs6142152
rs4846916
rs6438552
rs77960347
rs4656292
rs2156552
rs12740374
rs1129555
rs4694077
rs8131819
rs1260326
rs71640337
rs2138161
rs59347135
rs10793125
rs1421656
rs673548
rs2965169
rs150057262
rs967530
rs12249012
rs1037117
rs10733608
rs12721030
All - MR Egger
All - Inverse variance weighted
-2
-1
0
1
2
MR effect size for
'Total lipids in medium HDL || id:ebi-cfb233-GCST90302037' on 'ER- Breast cancer (Combined Oncoarray; iCOGS; GWAS meta

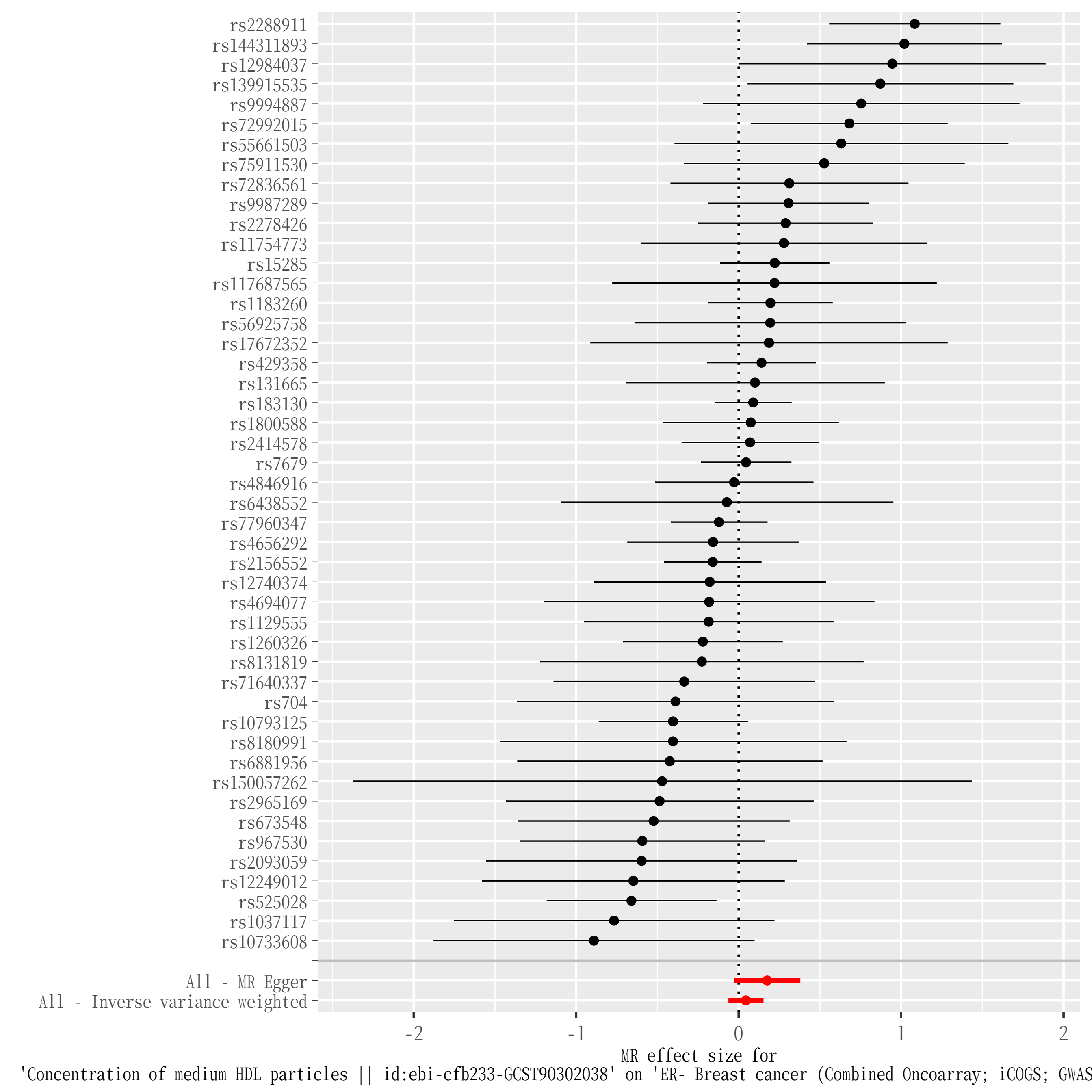
rs2288911
rs144311893
rs12984037
rs139915535
rs9994887
rs72992015
rs55661503
rs75911530
rs72836561
rs9987289
rs2278426
rs11754773
rs15285
rs117687565
rs1183260
rs56925758
rs17672352
rs429358
rs131665
rs183130
rs1800588
rs2414578
rs7679
rs4846916
rs6438552
rs77960347
rs4656292
rs2156552
rs12740374
rs4694077
rs1129555
rs1260326
rs8131819
rs71640337
rs704
rs10793125
rs8180991
rs6881956
rs150057262
rs2965169
rs673548
rs967530
rs2093059
rs12249012
rs525028
rs1037117
rs10733608
All - MR Egger
All - Inverse variance weighted
-2
-1
0
1
2
MR effect size for
'Concentration of medium HDL particles || id:ebi-cfb233-GCST90302038' on 'ER- Breast cancer (Combined Oncoarray; iCOGS; GWAS

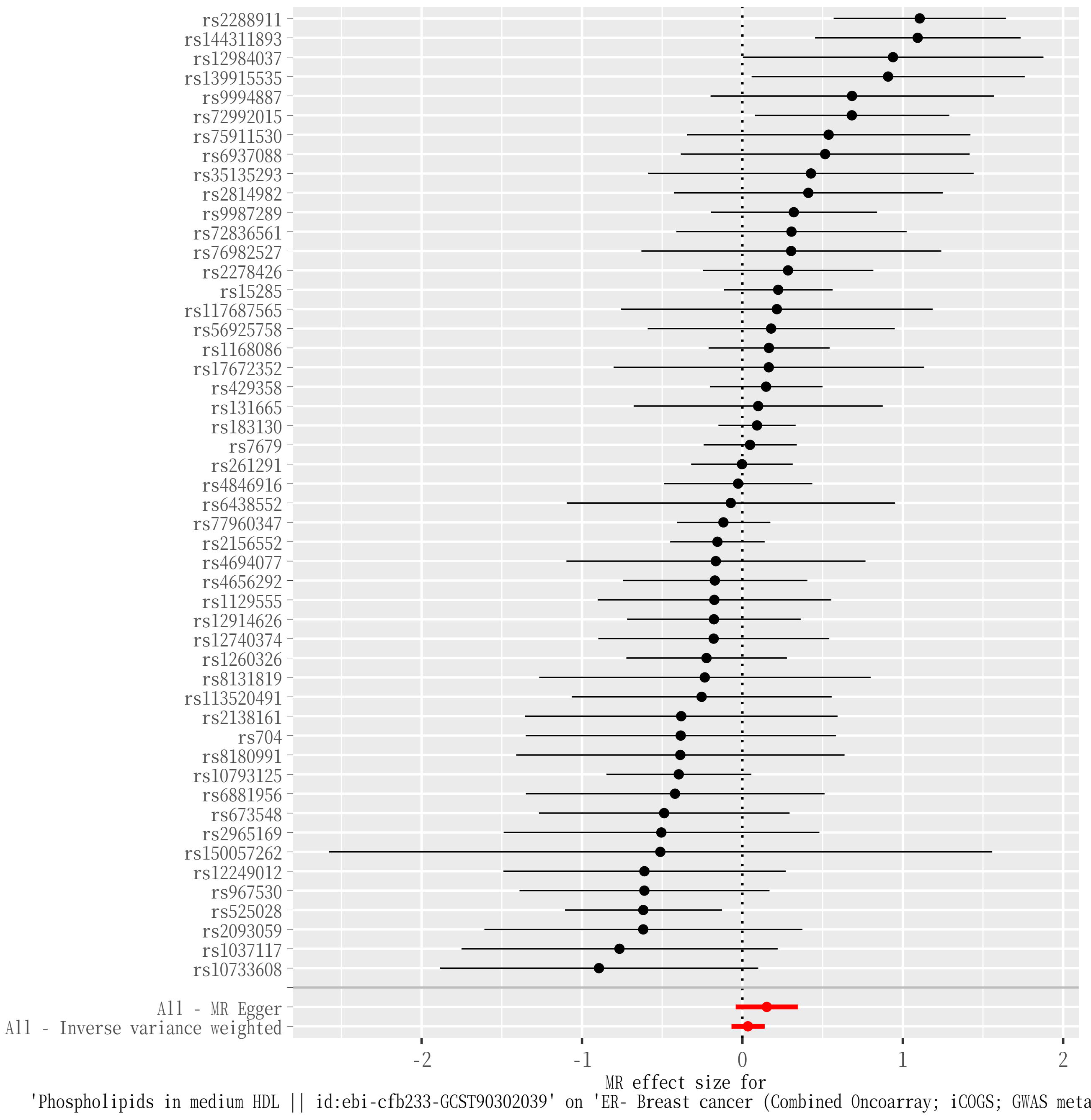

rs2288911
rs144311893
rs12984037
rs139915535
rs9994887
rs72992015
rs75911530
rs6937088
rs35135293
rs2814982
rs9987289
rs72836561
rs76982527
rs2278426
rs15285
rs117687565
rs56925758
rs1168086
rs17672352
rs429358
rs131665
rs183130
rs7679
rs261291
rs4846916
rs6438552
rs77960347
rs2156552
rs4694077
rs4656292
rs1129555
rs12914626
rs12740374
rs1260326
rs8131819
rs113520491
rs2138161
rs704
rs8180991
rs10793125
rs6881956
rs673548
rs2965169
rs150057262
rs12249012
rs967530
rs525028
rs2093059
rs1037117
rs10733608
All - MR Egger
All - Inverse variance weighted
-2
-1
0
1
2
MR effect size for
'Phospholipids in medium HDL || id:ebi-cfb233-GCST90302039' on 'ER- Breast cancer (Combined Oncoarray; iCOGS; GWAS meta

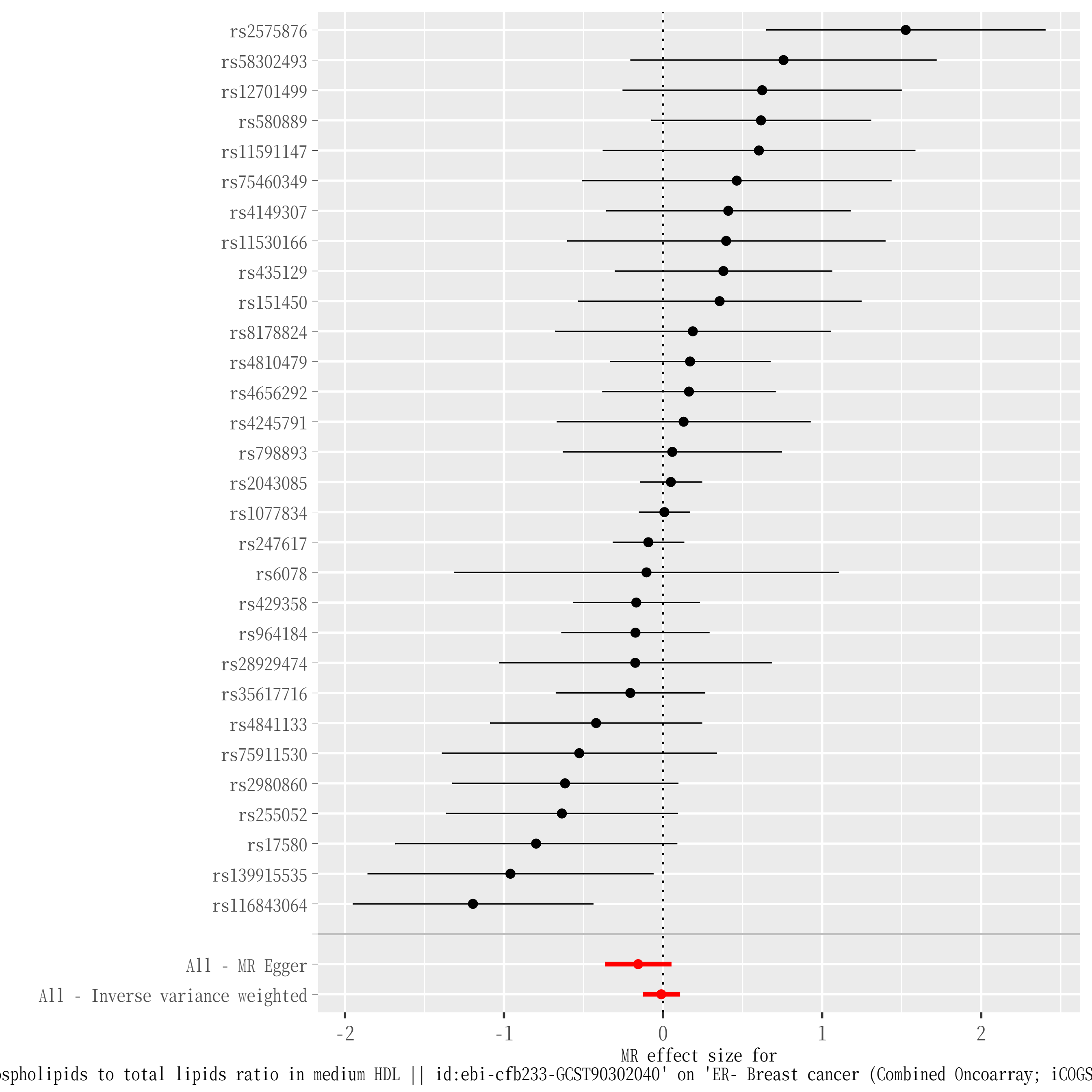
rs2575876
rs58302493
rs12701499
rs580889
rs11591147
rs75460349
rs4149307
rs11530166
rs435129
rs151450
rs8178824
rs4810479
rs4656292
rs4245791
rs798893
rs2043085
rs1077834
rs247617
rs6078
rs429358
rs964184
rs28929474
rs35617716
rs4841133
rs75911530
rs2980860
rs255052
rs17580
rs139915535
rs116843064
All - MR Egger
All - Inverse variance weighted
-2
-1
0
1
2
MR effect size for
ospholipids to total lipids ratio in medium HDL || id:ebi-cfb233-GCST90302040' on 'ER- Breast cancer (Combined Oncoarray; iCOGS

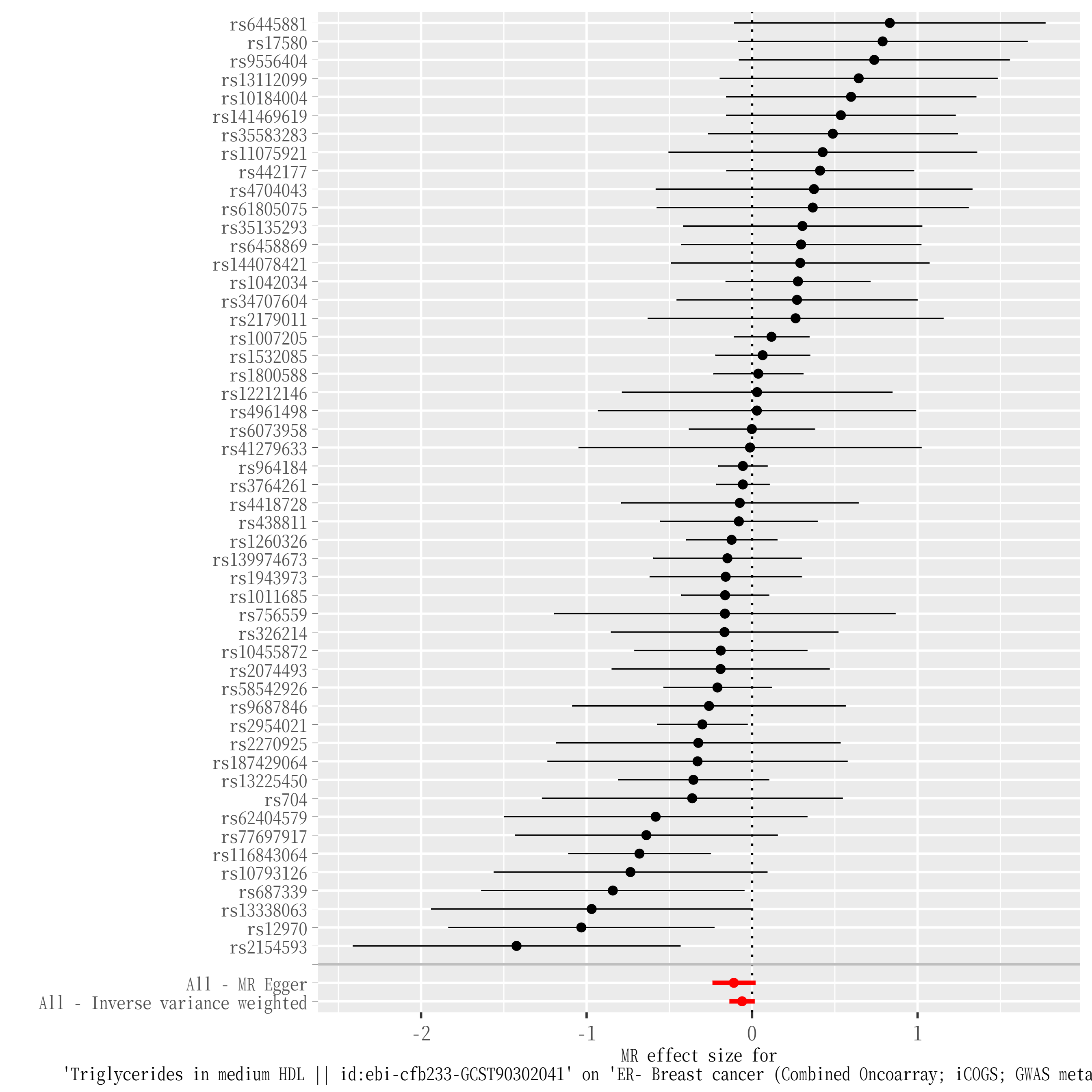
rs6445881
rs17580
rs9556404
rs13112099
rs10184004
rs141469619
rs35583283
rs11075921
rs442177
rs4704043
rs61805075
rs35135293
rs6458869
rs144078421
rs1042034
rs34707604
rs2179011
rs1007205
rs1532085
rs1800588
rs12212146
rs4961498
rs6073958
rs41279633
rs964184
rs3764261
rs4418728
rs438811
rs1260326
rs139974673
rs1943973
rs1011685
rs756559
rs326214
rs10455872
rs2074493
rs58542926
rs9687846
rs2954021
rs2270925
rs187429064
rs13225450
rs704
rs62404579
rs77697917
rs116843064
rs10793126
rs687339
rs13338063
rs12970
rs2154593
All - MR Egger
All - Inverse variance weighted
-2
-1
0
1
MR effect size for
'Triglycerides in medium HDL || id:ebi-cfb233-GCST90302041' on 'ER- Breast cancer (Combined Oncoarray; iCOGS; GWAS meta

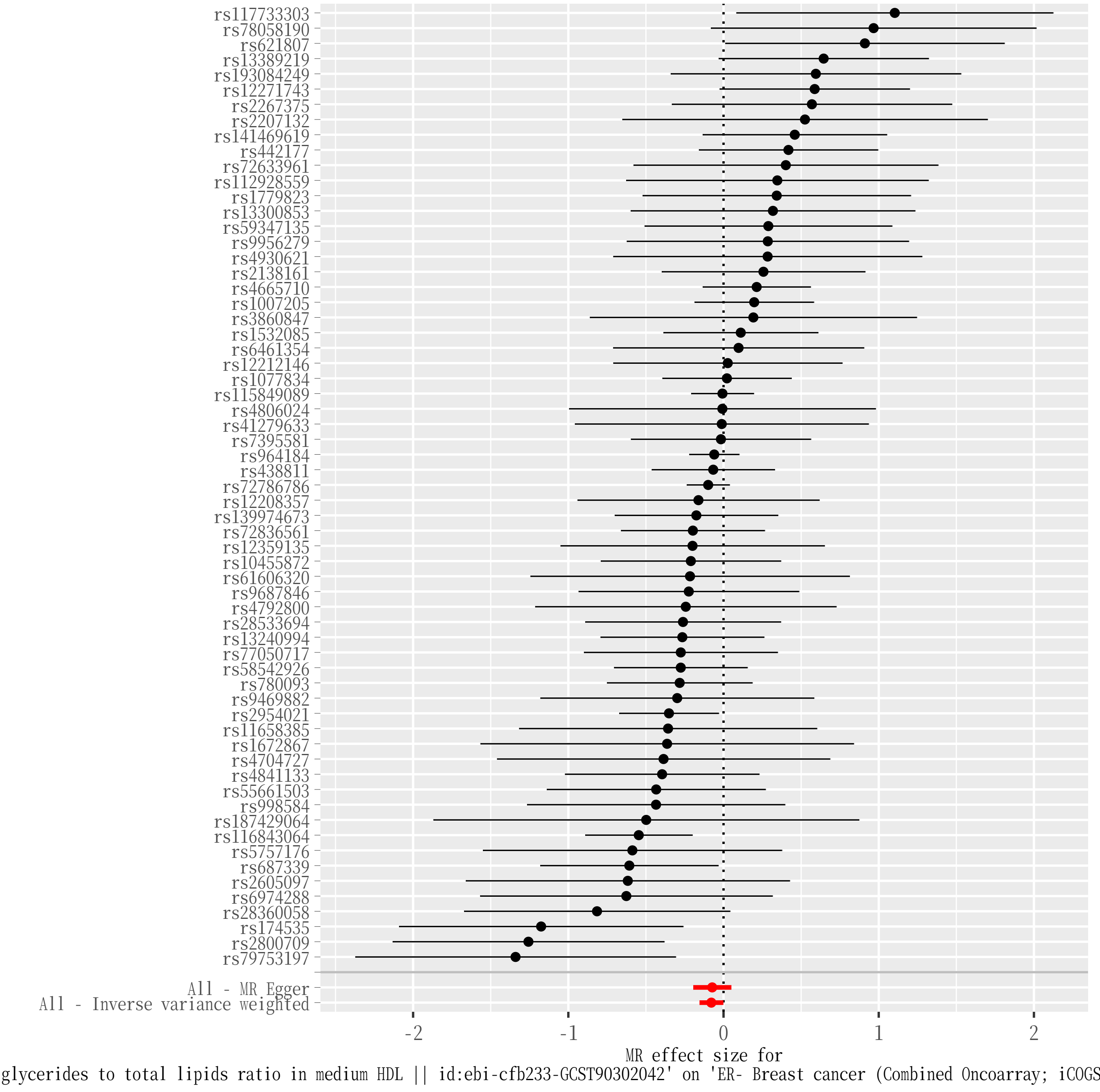

rs117733303
rs78058190
rs621807
rs13389219
rs193084249
rs12271743
rs2267375
rs2207132
rs141469619
rs442177
rs72633961
rs112928559
rs1779823
rs13300853
rs59347135
rs9956279
rs4930621
rs2138161
rs4665710
rs1007205
rs3860847
rs1532085
rs6461354
rs12212146
rs1077834
rs115849089
rs4806024
rs41279633
rs7395581
rs964184
rs438811
rs72786786
rs12208357
rs139974673
rs72836561
rs12359135
rs10455872
rs61606320
rs9687846
rs4792800
rs28533694
rs13240994
rs77050717
rs58542926
rs780093
rs9469882
rs2954021
rs11658385
rs1672867
rs4704727
rs4841133
rs55661503
rs998584
rs187429064
rs116843064
rs5757176
rs687339
rs2605097
rs6974288
rs28360058
rs174535
rs2800709
rs79753197
All - MR Egger
All - Inverse variance weighted
-2
-1
0
1
2
MR effect size for
glycerides to total lipids ratio in medium HDL || id:ebi-cfb233-GCST90302042' on 'ER- Breast cancer (Combined Oncoarray; iCOGS

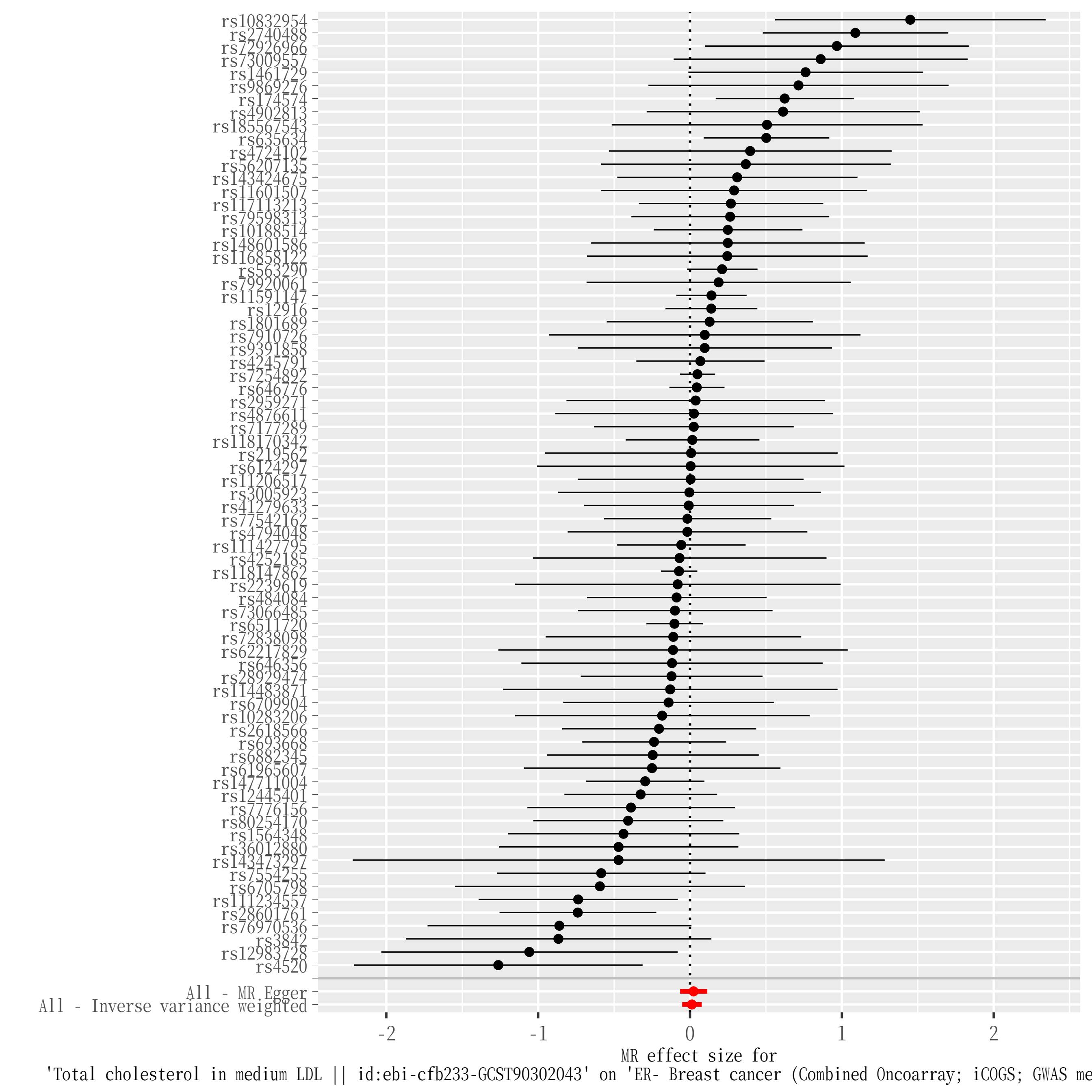
rs10832954
rs2740488
rs72926966
rs73009557
rs1461729
rs9869276
rs174574
rs4902813
rs185567543
rs635634
rs4724102
rs56207135
rs143424675
rs11601507
rs117113213
rs79598313
rs10188514
rs148601586
rs116858122
rs563290
rs79920061
rs11591147
rs12916
rs1801689
rs7910726
rs9391858
rs4245791
rs7254892
rs646776
rs2959271
rs4876611
rs7177289
rs118170342
rs219562
rs6124297
rs11206517
rs3005923
rs41279633
rs77542162
rs4794048
rs111427795
rs4252185
rs118147862
rs2239619
rs484084
rs73066485
rs6511720
rs72838098
rs62217829
rs646356
rs28929474
rs114483871
rs6709904
rs10283206
rs2618566
rs693668
rs6882345
rs61965607
rs147711004
rs12445401
rs7776156
rs80254170
rs1564348
rs36012880
rs143473297
rs7554255
rs6705798
rs111234557
rs28601761
rs76970536
rs3842
rs12983728
rs4520
All - MR Egger
All - Inverse variance weighted
-2
-1
0
1
2
MR effect size for
'Total cholesterol in medium LDL || id:ebi-cfb233-GCST90302043' on 'ER- Breast cancer (Combined Oncoarray; iCOGS; GWAS me

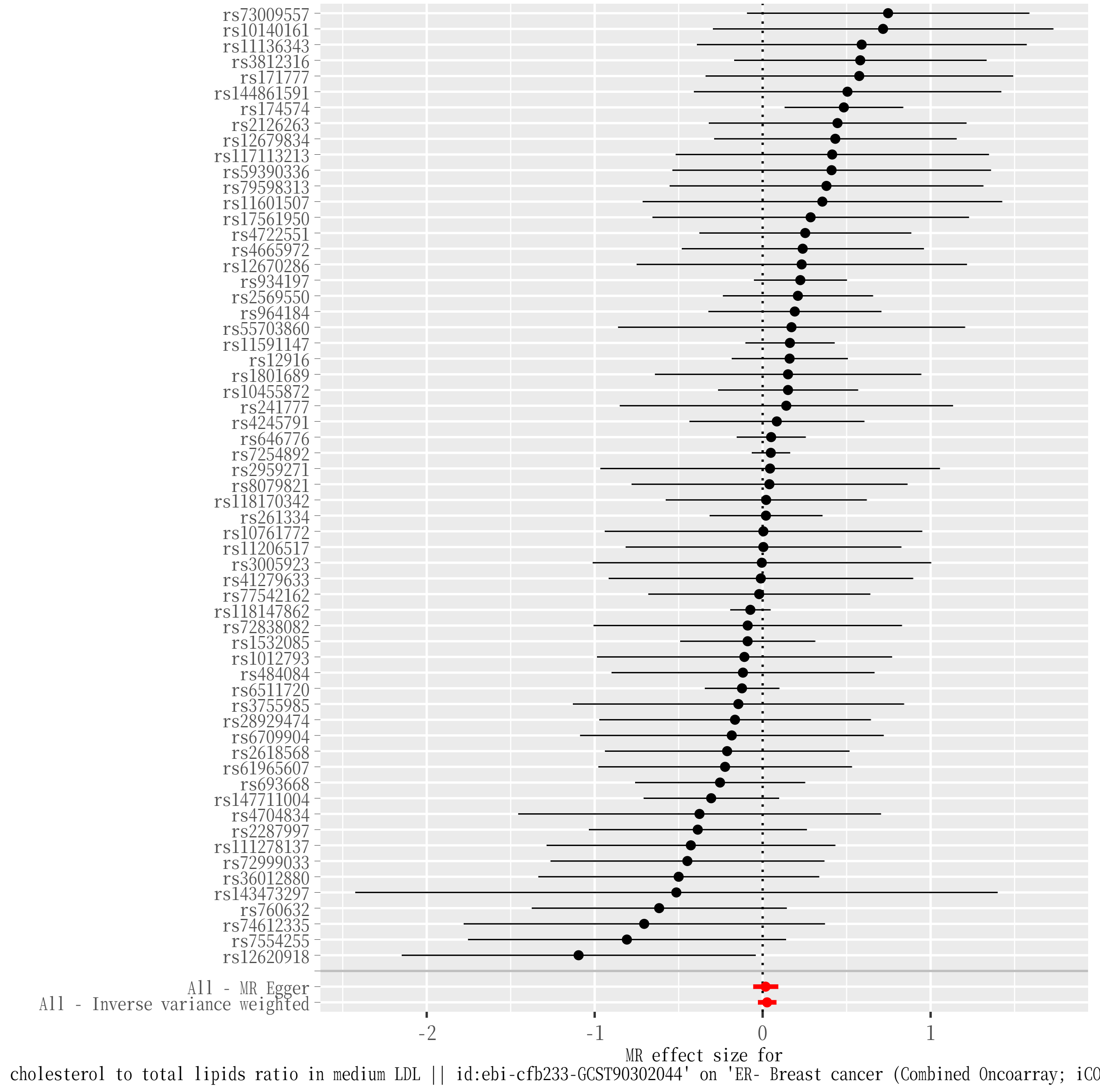

rs73009557
rs10140161
rs11136343
rs3812316
rs171777
rs144861591
rs174574
rs2126263
rs12679834
rs117113213
rs59390336
rs79598313
rs11601507
rs17561950
rs4722551
rs4665972
rs12670286
rs934197
rs2569550
rs964184
rs55703860
rs11591147
rs12916
rs1801689
rs10455872
rs241777
rs4245791
rs646776
rs7254892
rs2959271
rs8079821
rs118170342
rs261334
rs10761772
rs11206517
rs3005923
rs41279633
rs77542162
rs118147862
rs72838082
rs1532085
rs1012793
rs484084
rs6511720
rs3755985
rs28929474
rs6709904
rs2618568
rs61965607
rs693668
rs147711004
rs4704834
rs2287997
rs111278137
rs72999033
rs36012880
rs143473297
rs760632
rs74612335
rs7554255
rs12620918
All - MR Egger
All - Inverse variance weighted
-2
-1
0
1
MR effect size for
cholesterol to total lipids ratio in medium LDL || id:ebi-cfb233-GCST90302044' on 'ER- Breast cancer (Combined Oncoarray; iCO

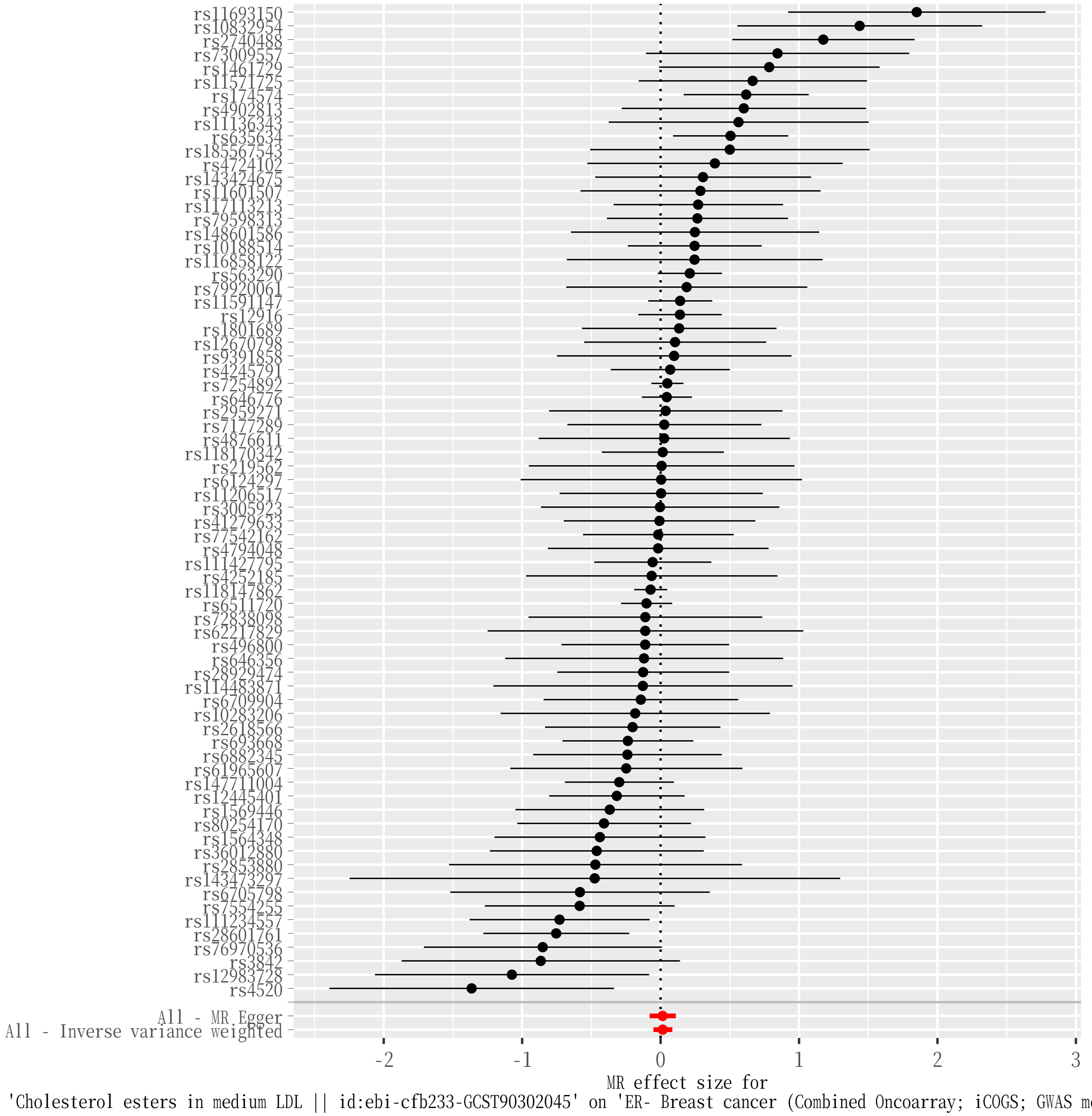
rs11693150
rs10832954
rs2740488
rs73009557
rs1461729
rs11571725
rs174574
rs4902813
rs11136343
rs635634
rs185567543
rs4724102
rs143424675
rs11601507
rs117113213
rs79598313
rs148601586
rs10188514
rs116858122
rs563290
rs79920061
rs11591147
rs12916
rs1801689
rs12670798
rs9391858
rs4245791
rs7254892
rs646776
rs2959271
rs7177289
rs4876611
rs118170342
rs219562
rs6124297
rs11206517
rs3005923
rs41279633
rs77542162
rs4794048
rs111427795
rs4252185
rs118147862
rs6511720
rs72838098
rs62217829
rs496800
rs646356
rs28929474
rs114483871
rs6709904
rs10283206
rs2618566
rs693668
rs6882345
rs61965607
rs147711004
rs12445401
rs1569446
rs80254170
rs1564348
rs36012880
rs2853880
rs143473297
rs6705798
rs7554255
rs111234557
rs28601761
rs76970536
rs3842
rs12983728
rs4520
All - MR Egger
All - Inverse variance weighted
-2
-1
0
1
2
3
MR effect size for
'Cholesterol esters in medium LDL || id:ebi-cfb233-GCST90302045' on 'ER- Breast cancer (Combined Oncoarray; iCOGS; GWAS me

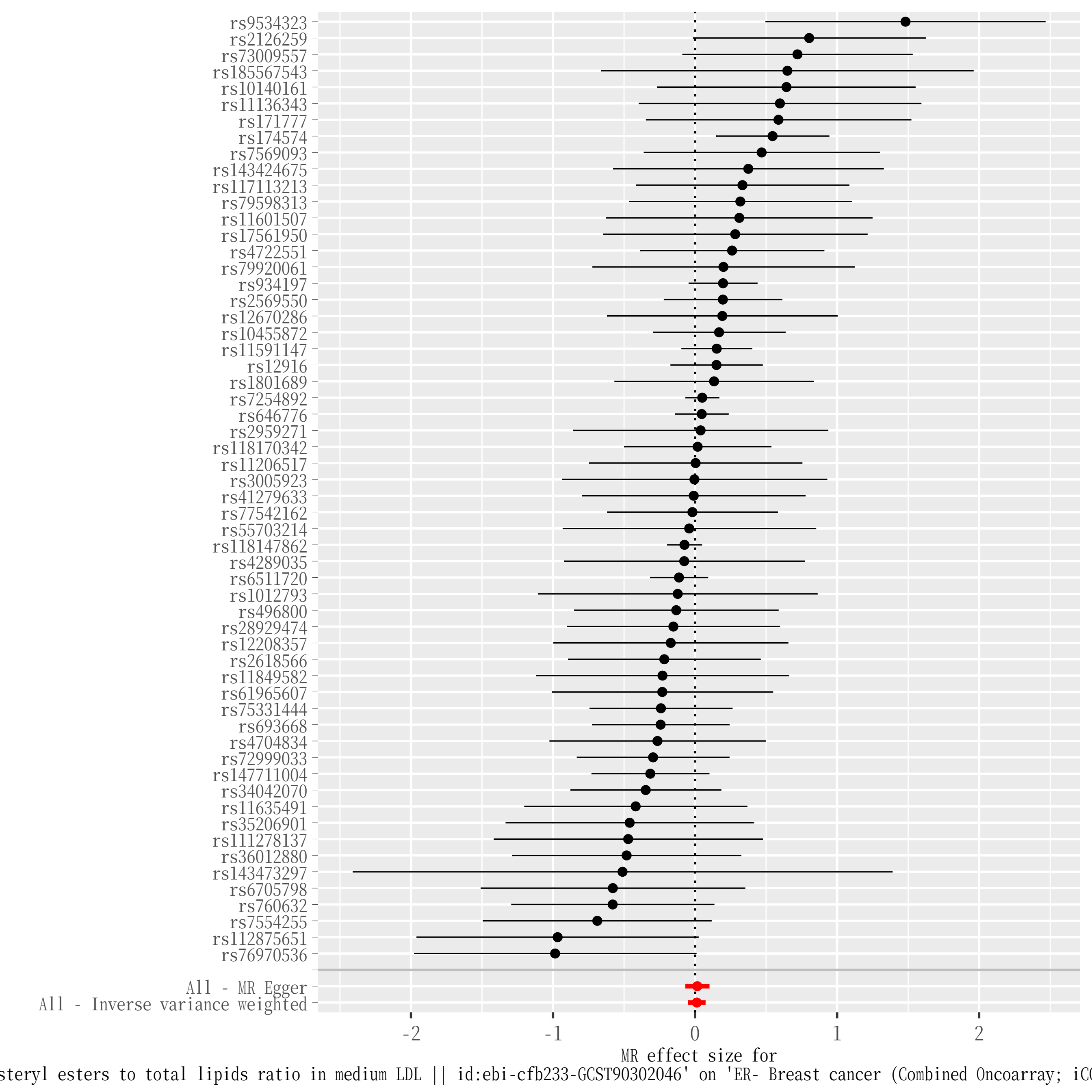
rs9534323
rs2126259
rs73009557
rs185567543
rs10140161
rs11136343
rs171777
rs174574
rs7569093
rs143424675
rs117113213
rs79598313
rs11601507
rs17561950
rs4722551
rs79920061
rs934197
rs2569550
rs12670286
rs10455872
rs11591147
rs12916
rs1801689
rs7254892
rs646776
rs2959271
rs118170342
rs11206517
rs3005923
rs41279633
rs77542162
rs55703214
rs118147862
rs4289035
rs6511720
rs1012793
rs496800
rs28929474
rs12208357
rs2618566
rs11849582
rs61965607
rs75331444
rs693668
rs4704834
rs72999033
rs147711004
rs34042070
rs11635491
rs35206901
rs111278137
rs36012880
rs143473297
rs6705798
rs760632
rs7554255
rs112875651
rs76970536
All - MR Egger
All - Inverse variance weighted
-2
-1
0
1
2
MR effect size for
steryl esters to total lipids ratio in medium LDL || id:ebi-cfb233-GCST90302046' on 'ER- Breast cancer (Combined Oncoarray; iC

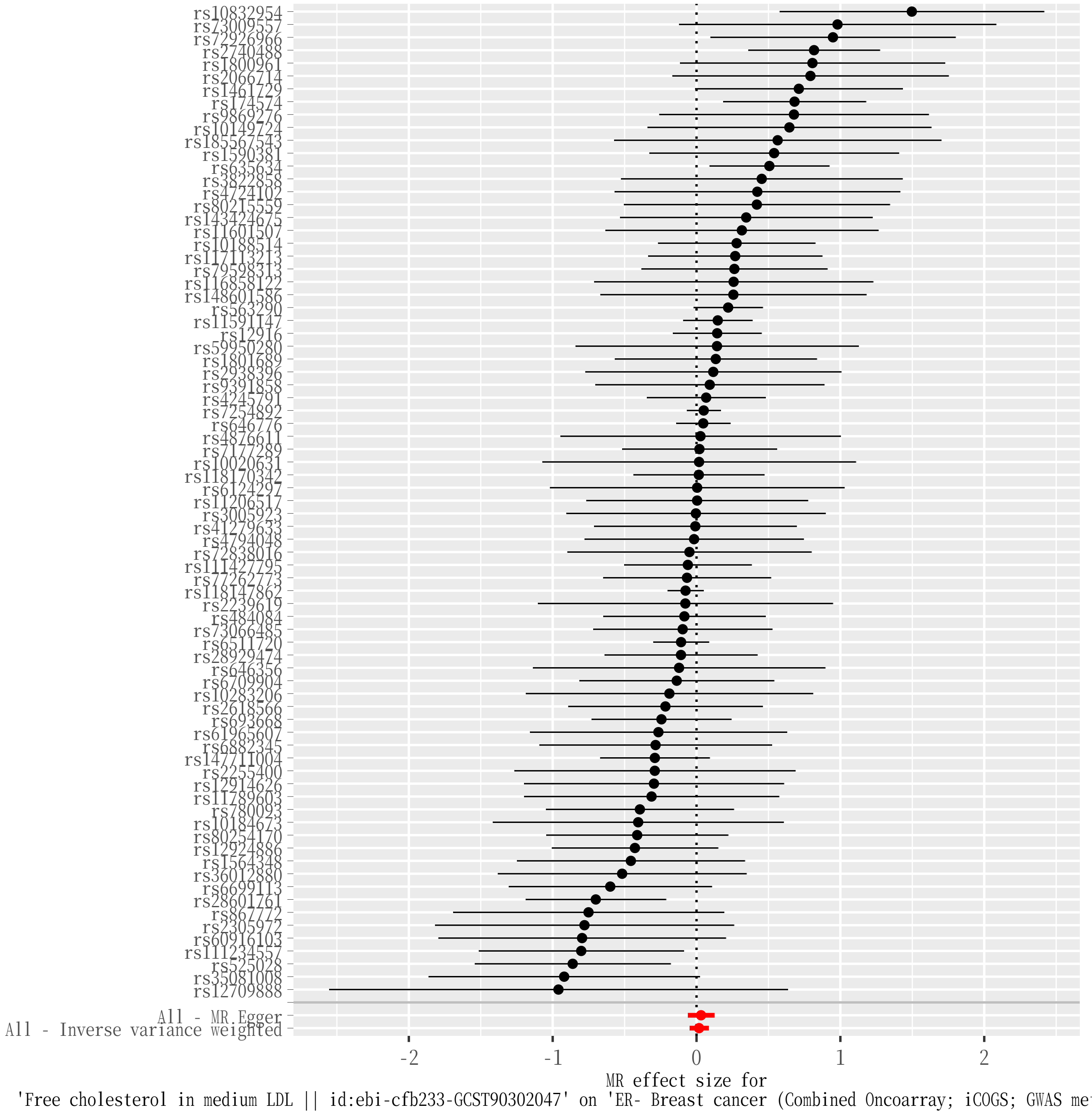
rs10832954
rs73009557
rs72926966
rs2740488
rs1800961
rs2066714
rs1461729
rs174574
rs9869276
rs10149724
rs185567543
rs1590381
rs635634
rs3822858
rs4724102
rs80215559
rs143424675
rs11601507
rs10188514
rs117113213
rs79598313
rs116858122
rs148601586
rs563290
rs11591147
rs12916
rs59950280
rs1801689
rs2938396
rs9391858
rs4245791
rs7254892
rs646776
rs4876611
rs7177289
rs10020631
rs118170342
rs6124297
rs11206517
rs3005923
rs41279633
rs4794048
rs72838016
rs111427795
rs77262773
rs118147862
rs2239619
rs484084
rs73066485
rs6511720
rs28929474
rs646356
rs6709904
rs10283206
rs2618566
rs693668
rs61965607
rs6882345
rs147711004
rs2255400
rs12914626
rs11789603
rs780093
rs10184673
rs80254170
rs12224886
rs1564348
rs36012880
rs6699113
rs28601761
rs867772
rs2305972
rs60916103
rs111234557
rs525028
rs35081008
rs12709888
All - MR Egger
All - Inverse variance weighted
-2
-1
0
1
2
MR effect size for
'Free cholesterol in medium LDL || id:ebi-cfb233-GCST90302047' on 'ER- Breast cancer (Combined Oncoarray; iCOGS; GWAS me

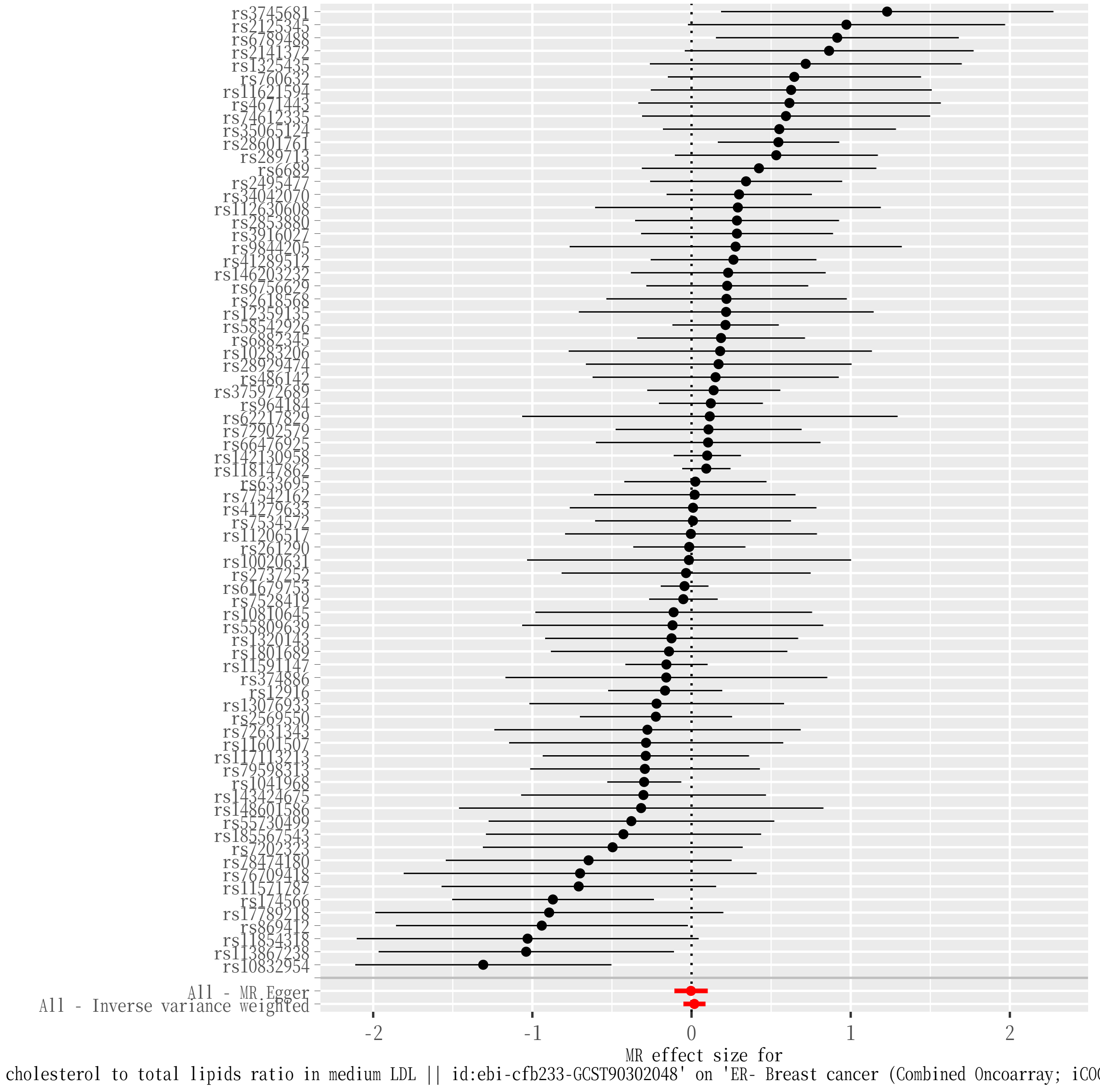

rs3745681
rs2125345
rs6789488
rs2141372
rs1325435
rs760632
rs11621594
rs4671443
rs74612335
rs35065124
rs28601761
rs289713
rs6689
rs2495477
rs34042070
rs112630608
rs2853880
rs3916027
rs9844205
rs41289512
rs146203232
rs6756629
rs2618568
rs12359135
rs58542926
rs6882345
rs10283206
rs28929474
rs486142
rs375972689
rs964184
rs62217829
rs72902579
rs66476925
rs142130958
rs118147862
rs633695
rs77542162
rs41279633
rs7534572
rs11206517
rs261290
rs10020631
rs2737252
rs61679753
rs7528419
rs10810645
rs55809639
rs1320143
rs1801689
rs11591147
rs374886
rs12916
rs13076933
rs2569550
rs72631343
rs11601507
rs117113213
rs79598313
rs1041968
rs143424675
rs148601586
rs55730499
rs185567543
rs7202323
rs78474180
rs76709418
rs11571787
rs174566
rs17789218
rs869412
rs11854318
rs113867238
rs10832954
All - MR Egger
All - Inverse variance weighted
-2
-1
0
1
2
MR effect size for
cholesterol to total lipids ratio in medium LDL || id:ebi-cfb233-GCST90302048' on 'ER- Breast cancer (Combined Oncoarray; iCO

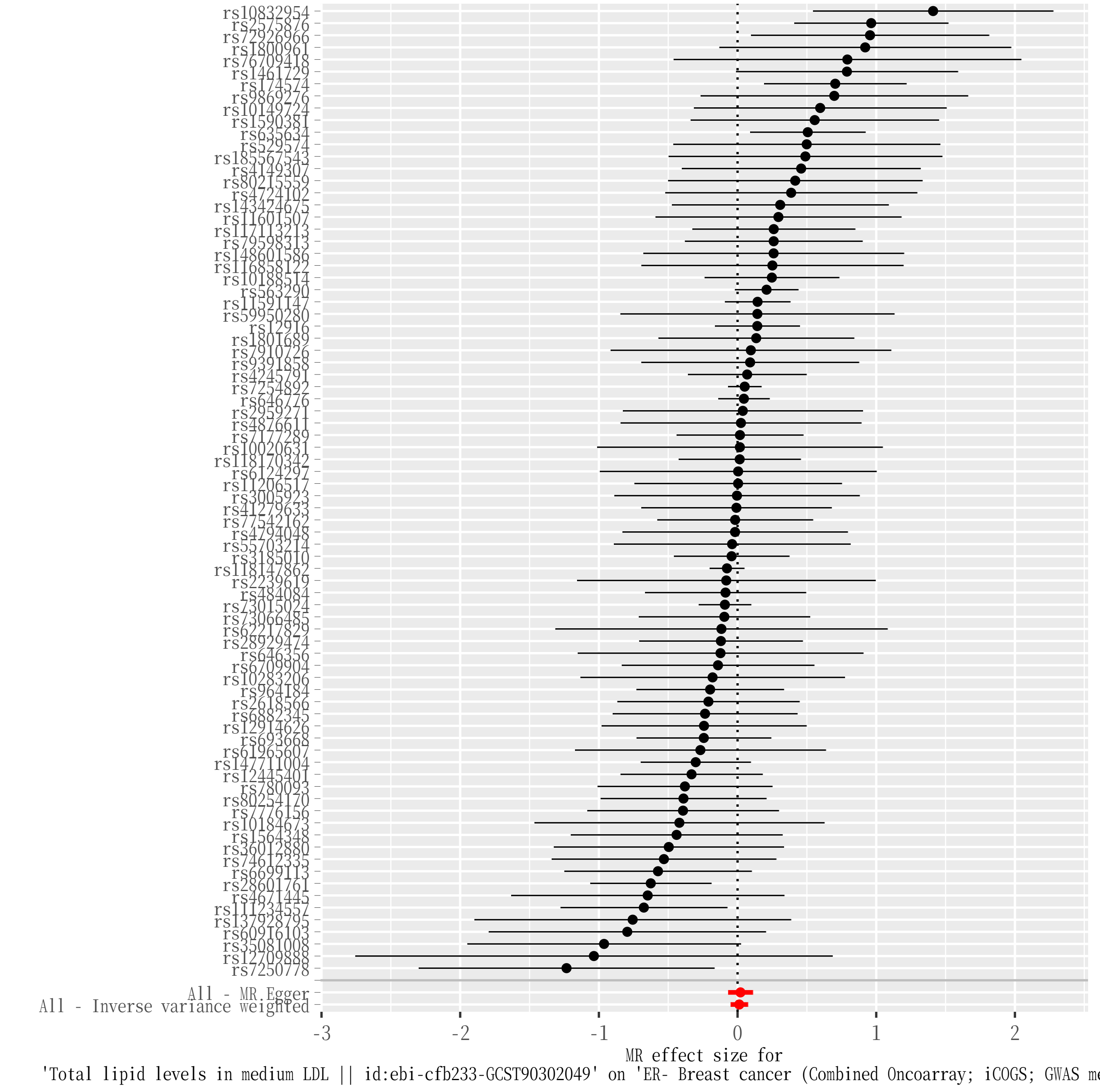
rs10832954
rs2575876
rs72926966
rs1800961
rs76709418
rs1461729
rs174574
rs9869276
rs10149724
rs1590381
rs635634
rs529574
rs185567543
rs4149307
rs80215559
rs4724102
rs143424675
rs11601507
rs117113213
rs79598313
rs148601586
rs116858122
rs10188514
rs563290
rs11591147
rs59950280
rs12916
rs1801689
rs7910726
rs9391858
rs4245791
rs7254892
rs646776
rs2959271
rs4876611
rs7177289
rs10020631
rs118170342
rs6124297
rs11206517
rs3005923
rs41279633
rs77542162
rs4794048
rs55703214
rs3185010
rs118147862
rs2239619
rs484084
rs73015024
rs73066485
rs62217829
rs28929474
rs646356
rs6709904
rs10283206
rs964184
rs2618566
rs6882345
rs12914626
rs693668
rs61965607
rs147711004
rs12445401
rs780093
rs80254170
rs7776156
rs10184673
rs1564348
rs36012880
rs74612335
rs6699113
rs28601761
rs4671445
rs111234557
rs137928795
rs60916103
rs35081008
rs12709888
rs7250778
All - MR Egger
All - Inverse variance weighted
-3
-2
-1
0
1
2
MR effect size for
'Total lipid levels in medium LDL || id:ebi-cfb233-GCST90302049' on 'ER- Breast cancer (Combined Oncoarray; iCOGS; GWAS me

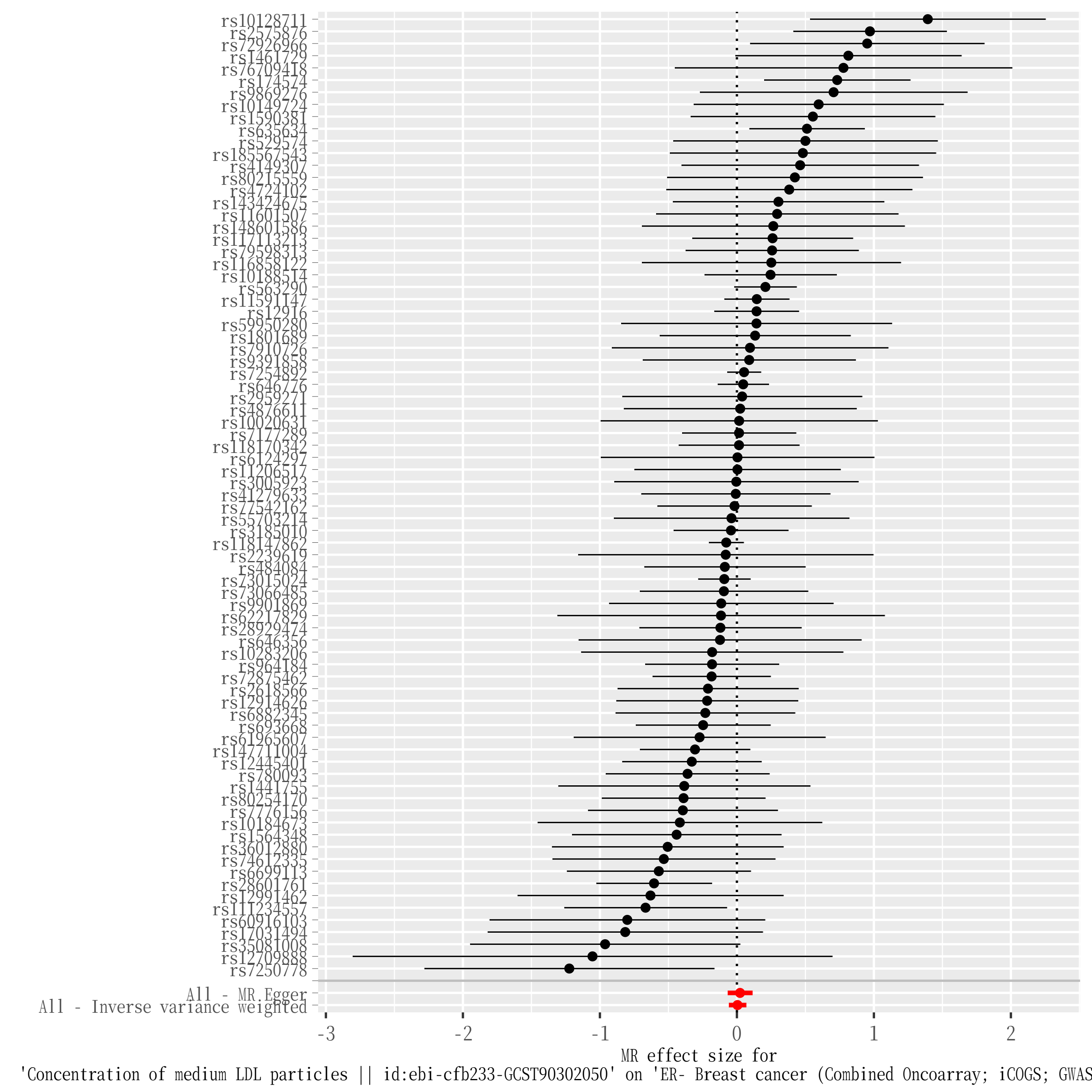
rs10128711
rs2575876
rs72926966
rs1461729
rs76709418
rs174574
rs9869276
rs10149724
rs1590381
rs635634
rs529574
rs185567543
rs4149307
rs80215559
rs4724102
rs143424675
rs11601507
rs148601586
rs117113213
rs79598313
rs116858122
rs10188514
rs563290
rs11591147
rs12916
rs5950280
rs1801689
rs7910726
rs9391858
rs7254892
rs646776
rs2959271
rs4876611
rs10020631
rs7177289
rs118170342
rs6124297
rs11206517
rs3005923
rs41279633
rs77542162
rs55703214
rs3185010
rs118147862
rs2239619
rs484084
rs73015024
rs73066485
rs9901869
rs62217829
rs28929474
rs646356
rs10283206
rs964184
rs72875462
rs2618566
rs12914626
rs6882345
rs693668
rs61965607
rs147711004
rs12445401
rs780093
rs1441755
rs80254170
rs7776156
rs10184673
rs1564348
rs36012880
rs74612335
rs6699113
rs28601761
rs12991462
rs111234557
rs60916103
rs17031494
rs35081008
rs12709888
rs7250778
All - MR Egger
All - Inverse variance weighted
-3
-2
-1
0
1
2
MR effect size for
'Concentration of medium LDL particles || id:ebi-cfb233-GCST90302050' on 'ER- Breast cancer (Combined Oncoarray; iCOGS; GWAS

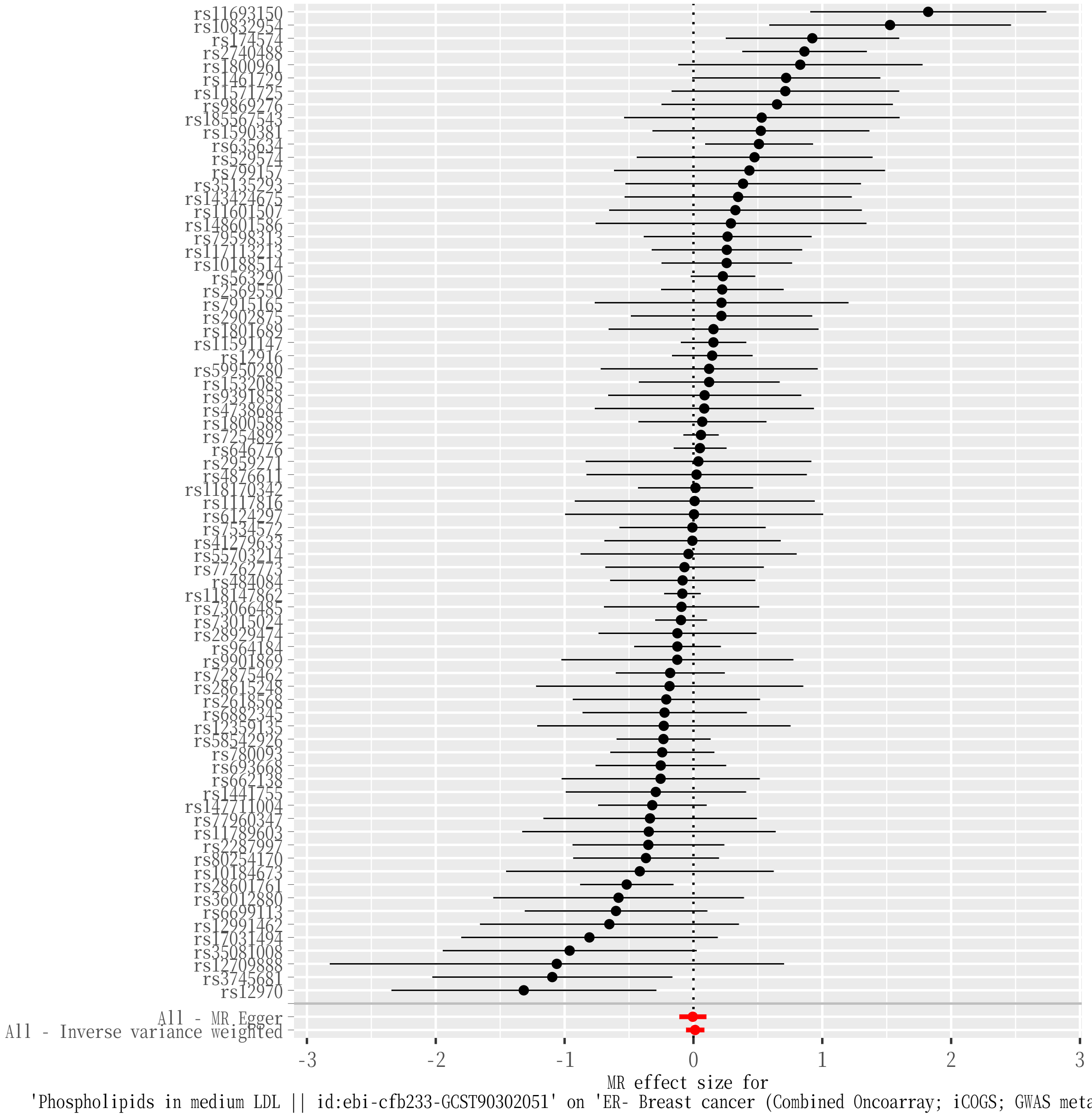
rs11693150
rs10832954
rs174574
rs2740488
rs1800961
rs1461729
rs11571725
rs9869276
rs185567543
rs1590381
rs635634
rs529574
rs799157
rs35135293
rs143424675
rs11601507
rs148601586
rs72598313
rs117113213
rs10188514
rs563290
rs2569550
rs7915165
rs2902875
rs1801689
rs11591147
rs12916
rs59950280
rs1532085
rs9391858
rs4738684
rs1800588
rs7254892
rs646776
rs2959271
rs4876611
rs118170342
rs1117816
rs6124297
rs7534572
rs41279633
rs55703214
rs77262773
rs484084
rs118147862
rs73066485
rs73015024
rs28929474
rs964184
rs9901869
rs72875462
rs28615248
rs2618568
rs6882345
rs12359135
rs58542926
rs780093
rs693668
rs662138
rs1441755
rs147711004
rs77960347
rs11789603
rs2287997
rs80254170
rs10184673
rs28601761
rs36012880
rs6699113
rs12991462
rs17031494
rs35081008
rs12709888
rs3745681
rs12970
All - MR Egger
All - Inverse variance weighted
-3
-2
-1
0
1
2
3
MR effect size for
'Phospholipids in medium LDL || id:ebi-cfb233-GCST90302051' on 'ER- Breast cancer (Combined Oncoarray; iCOGS; GWAS meta

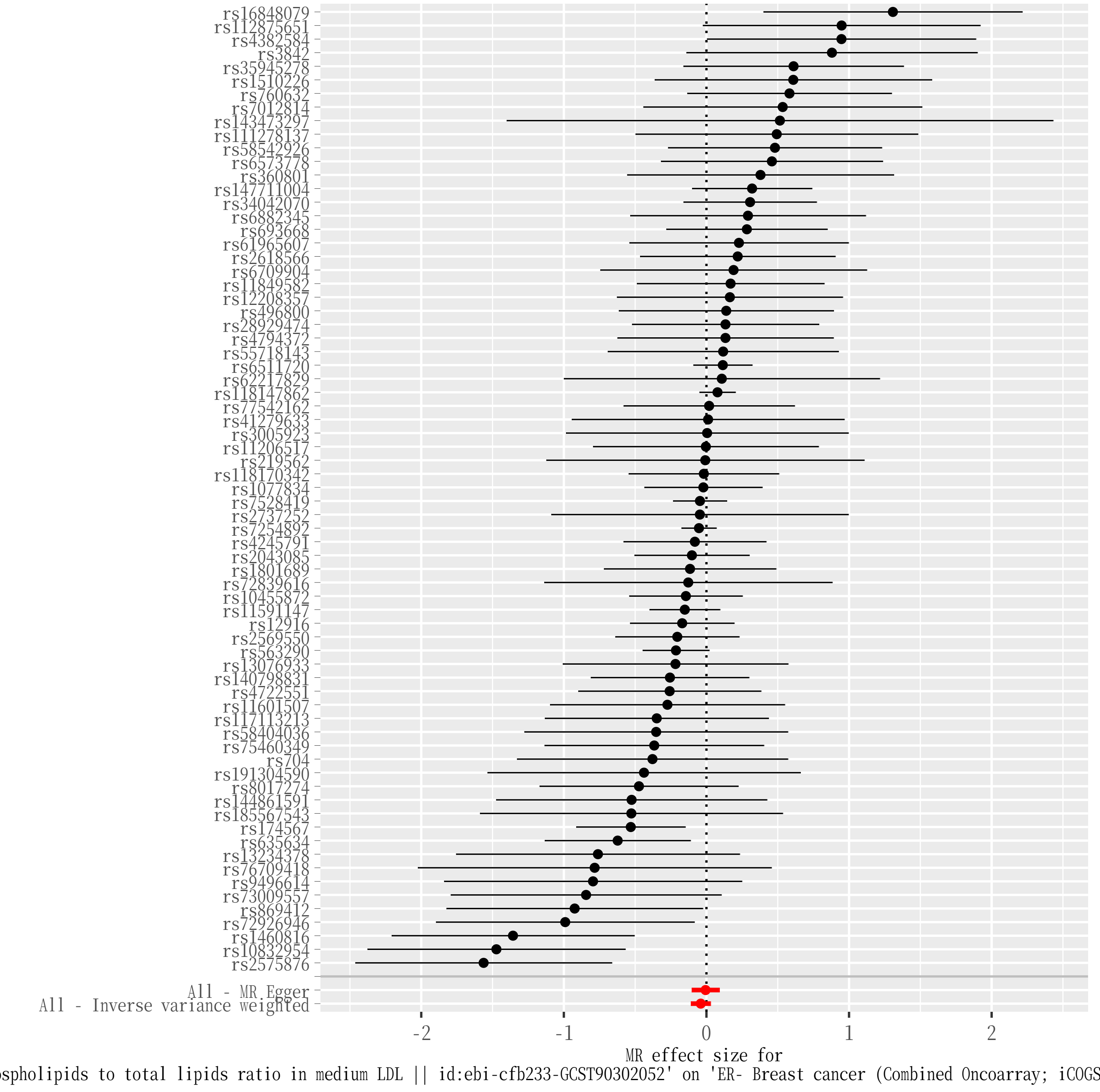
rs16848079
rs112875651
rs4382584
rs3842
rs35945278
rs1510226
rs760632
rs7012814
rs143473297
rs111278137
rs58542926
rs6573778
rs360801
rs147711004
rs34042070
rs6882345
rs693668
rs61965607
rs2618566
rs6709904
rs11849582
rs12208357
rs496800
rs28929474
rs4794372
rs55718143
rs6511720
rs62217829
rs118147862
rs77542162
rs41279633
rs3005923
rs11206517
rs219562
rs118170342
rs1077834
rs7528419
rs2737252
rs7254892
rs4245791
rs2043085
rs1801689
rs72839616
rs10455872
rs11591147
rs12916
rs2569550
rs563290
rs13076933
rs140798831
rs4722551
rs11601507
rs117113213
rs58404036
rs75460349
rs704
rs191304590
rs8017274
rs144861591
rs185567543
rs174567
rs635634
rs13234378
rs76709418
rs9496614
rs73009557
rs869412
rs72926946
rs1460816
rs10832954
rs2575876
All - MR Egger
All - Inverse variance weighted
-2
-1
0
1
2
MR effect size for
ospholipids to total lipids ratio in medium LDL || id:ebi-cfb233-GCST90302052' on 'ER- Breast cancer (Combined Oncoarray; iCOGS

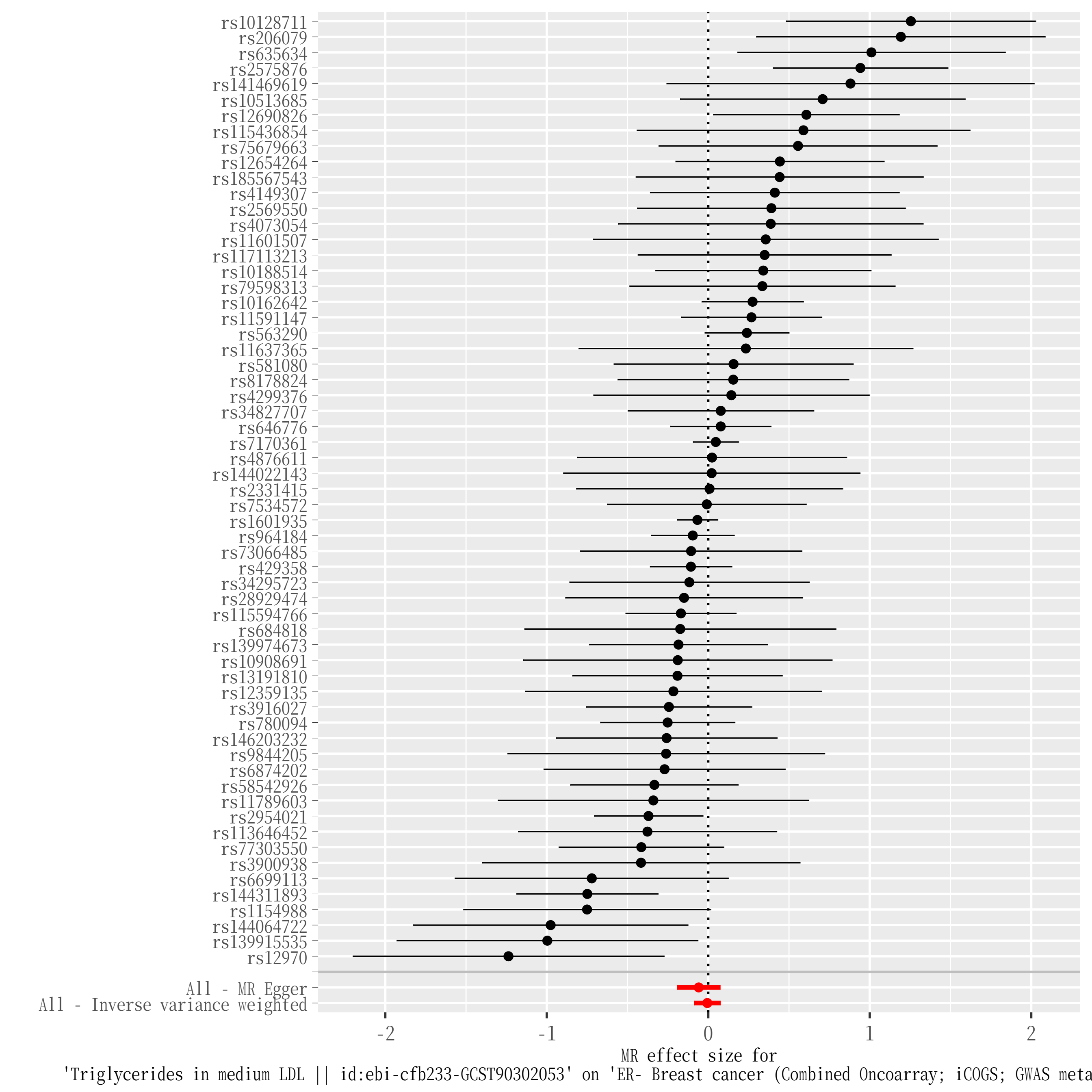

rs10128711
rs206079
rs635634
rs2575876
rs141469619
rs10513685
rs12690826
rs115436854
rs75679663
rs12654264
rs185567543
rs4149307
rs2569550
rs4073054
rs11601507
rs117113213
rs10188514
rs79598313
rs10162642
rs11591147
rs563290
rs11637365
rs581080
rs8178824
rs4299376
rs34827707
rs646776
rs7170361
rs4876611
rs144022143
rs2331415
rs7534572
rs1601935
rs964184
rs73066485
rs429358
rs34295723
rs28929474
rs115594766
rs684818
rs139974673
rs10908691
rs13191810
rs12359135
rs3916027
rs780094
rs146203232
rs9844205
rs6874202
rs58542926
rs11789603
rs2954021
rs113646452
rs77303550
rs3900938
rs6699113
rs144311893
rs1154988
rs144064722
rs139915535
rs12970
All - MR Egger
All - Inverse variance weighted
-2
-1
0
1
2
MR effect size for
'Triglycerides in medium LDL || id:ebi-cfb233-GCST90302053' on 'ER- Breast cancer (Combined Oncoarray; iCOGS; GWAS meta

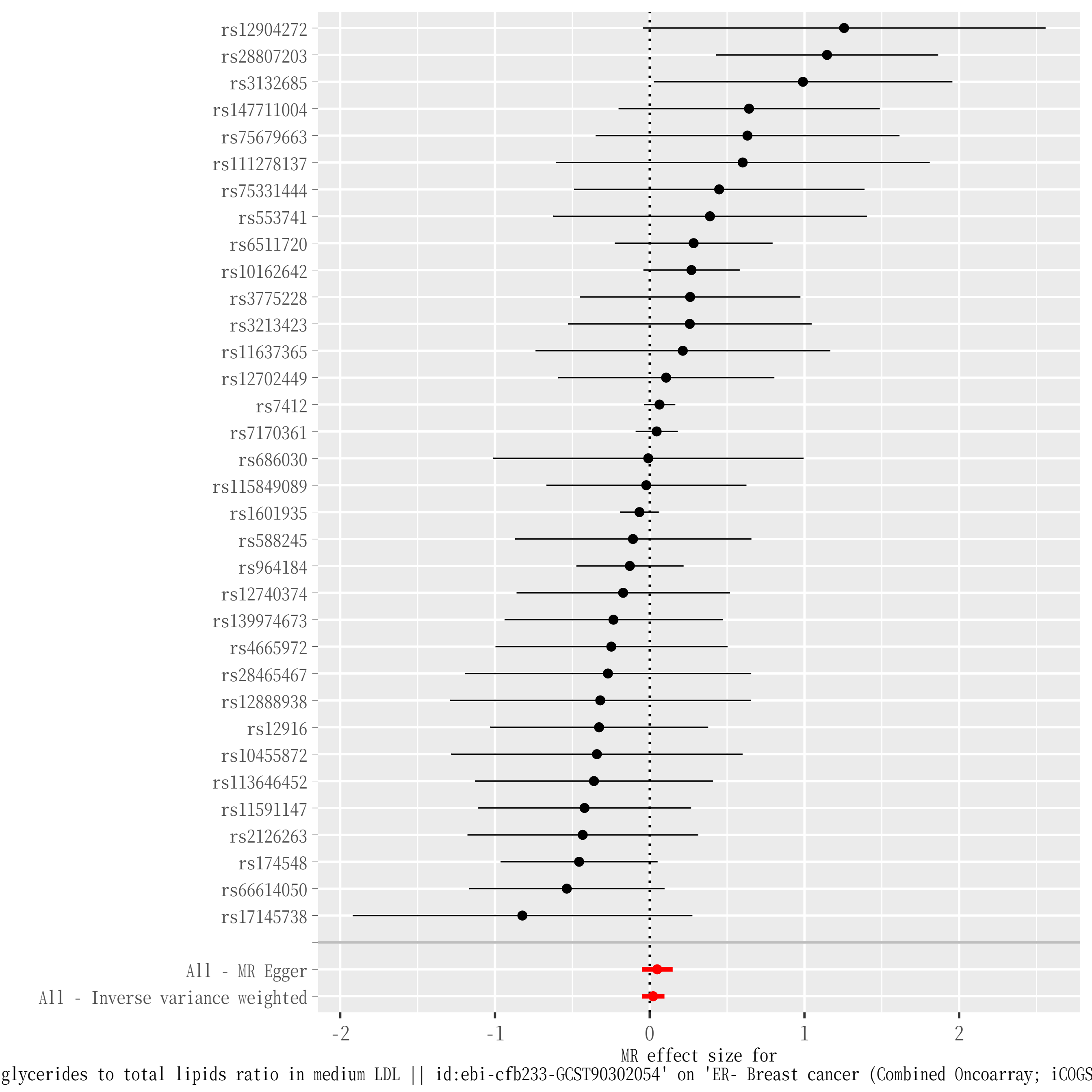
rs12904272
rs28807203
rs3132685
rs147711004
rs75679663
rs111278137
rs75331444
rs553741
rs6511720
rs10162642
rs3775228
rs3213423
rs11637365
rs12702449
rs7412
rs7170361
rs686030
rs115849089
rs1601935
rs588245
rs964184
rs12740374
rs139974673
rs4665972
rs28465467
rs12888938
rs12916
rs10455872
rs113646452
rs11591147
rs2126263
rs174548
rs66614050
rs17145738
All - MR Egger
All - Inverse variance weighted
-2
-1
0
1
2
MR effect size for
glycerides to total lipids ratio in medium LDL || id:ebi-cfb233-GCST90302054' on 'ER- Breast cancer (Combined Oncoarray; iCOGS

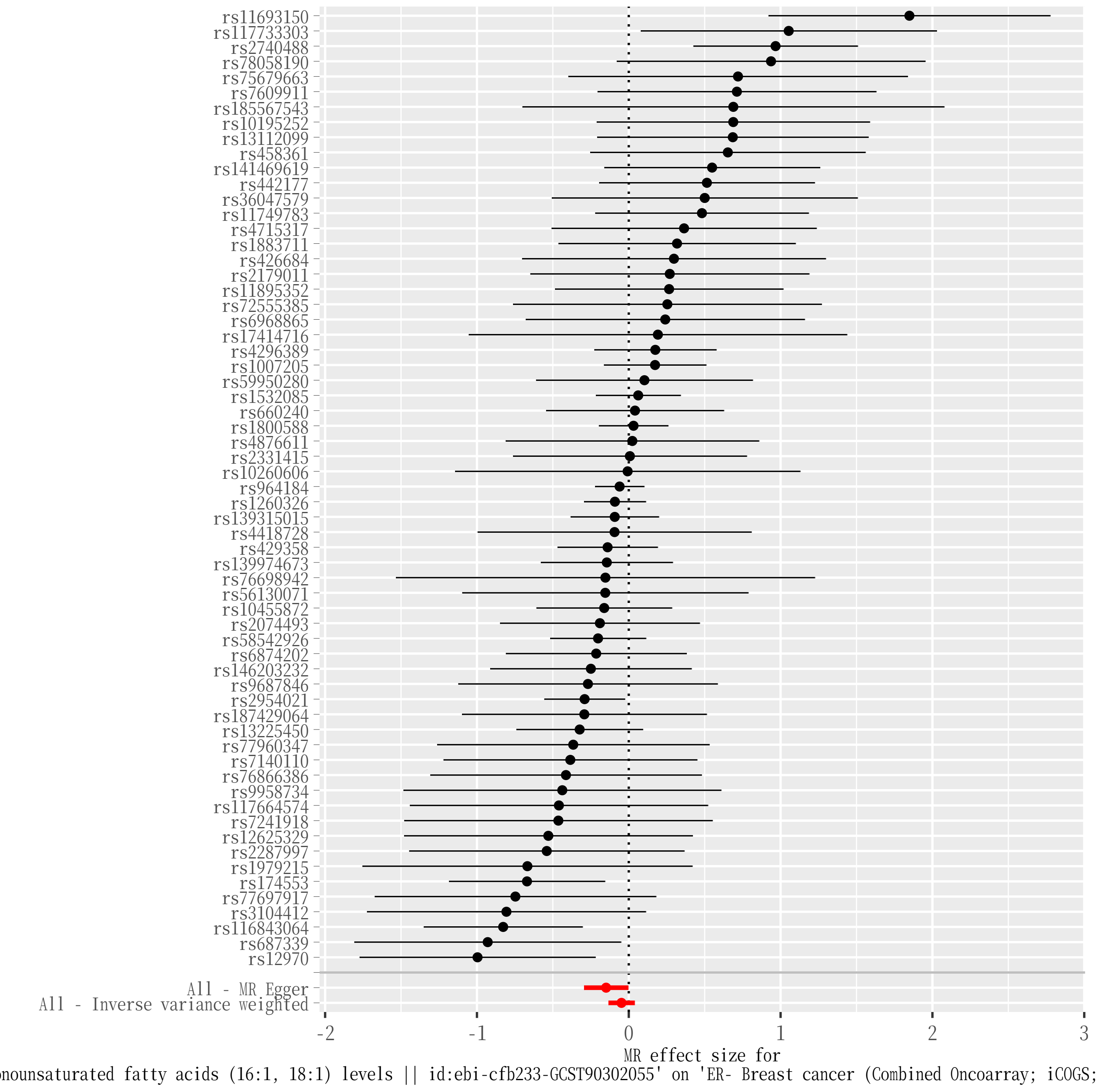
rs11693150
rs117733303
rs2740488
rs78058190
rs75679663
rs7609911
rs185567543
rs10195252
rs13112099
rs458361
rs141469619
rs442177
rs36047579
rs11749783
rs4715317
rs1883711
rs426684
rs2179011
rs11895352
rs72555385
rs6968865
rs17414716
rs4296389
rs1007205
rs59950280
rs1532085
rs660240
rs1800588
rs4876611
rs2331415
rs10260606
rs964184
rs1260326
rs139315015
rs4418728
rs429358
rs139974673
rs76698942
rs56130071
rs10455872
rs2074493
rs58542926
rs6874202
rs146203232
rs9687846
rs2954021
rs187429064
rs13225450
rs77960347
rs7140110
rs76866386
rs9958734
rs117664574
rs7241918
rs12625329
rs2287997
rs1979215
rs174553
rs77697917
rs3104412
rs116843064
rs687339
rs12970
All - MR Egger
All - Inverse variance weighted
-2
-1
0
1
2
3
MR effect size for
onounsaturated fatty acids (16:1, 18:1) levels || id:ebi-cfb233-GCST90302055' on 'ER- Breast cancer (Combined Oncoarray; iCOGS;

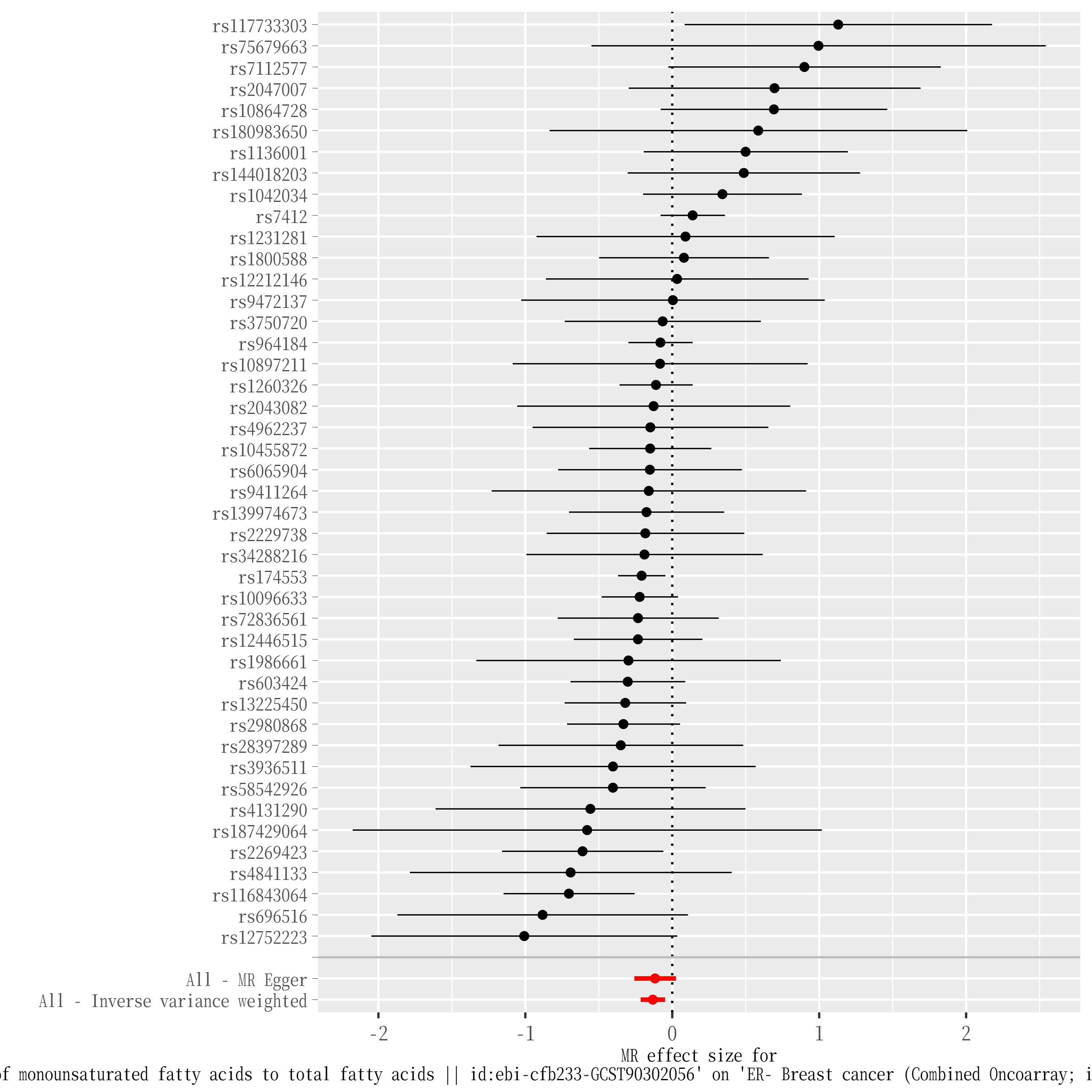
rs117733303
rs75679663
rs7112577
rs2047007
rs10864728
rs180983650
rs1136001
rs144018203
rs1042034
rs7412
rs1231281
rs1800588
rs12212146
rs9472137
rs3750720
rs964184
rs10897211
rs1260326
rs2043082
rs4962237
rs10455872
rs6065904
rs9411264
rs139974673
rs2229738
rs34288216
rs174553
rs10096633
rs72836561
rs12446515
rs1986661
rs603424
rs13225450
rs2980868
rs28397289
rs3936511
rs58542926
rs4131290
rs187429064
rs2269423
rs4841133
rs116843064
rs696516
rs12752223
All - MR Egger
All - Inverse variance weighted
-2
-1
0
1
2
MR effect size for
of monounsaturated fatty acids to total fatty acids || id:ebi-cfb233-GCST90302056' on 'ER- Breast cancer (Combined Oncoarray; i

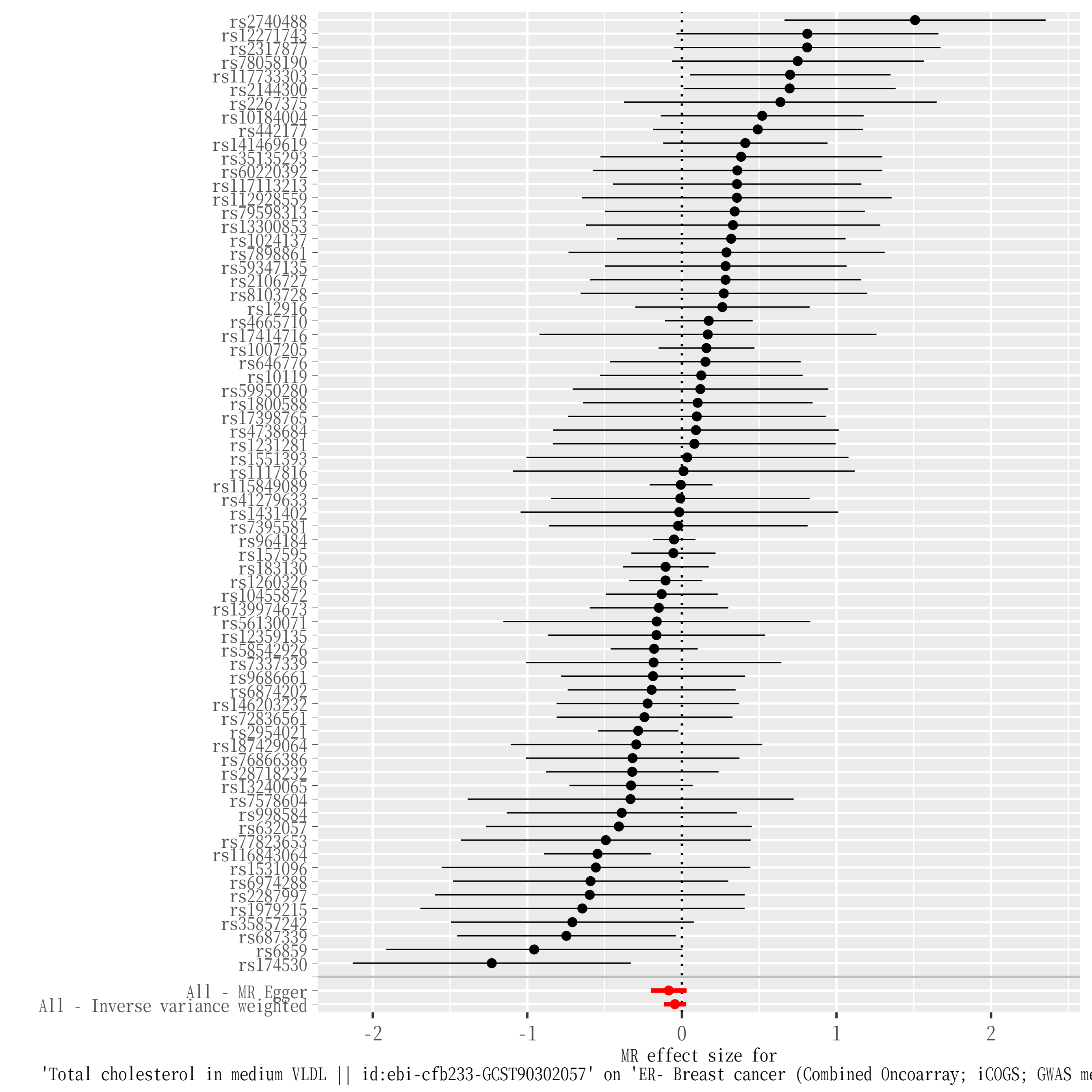
rs2740488
rs12271743
rs2317877
rs78058190
rs117733303
rs2144300
rs2267375
rs10184004
rs442177
rs141469619
rs35135293
rs60220392
rs117113213
rs112928559
rs79598313
rs13300853
rs1024137
rs7898861
rs59347135
rs2106727
rs8103728
rs12916
rs4665710
rs17414716
rs1007205
rs646776
rs10119
rs59950280
rs1800588
rs17398765
rs4738684
rs1231281
rs1551393
rs1117816
rs115849089
rs41279633
rs1431402
rs7395581
rs964184
rs157595
rs183130
rs1260326
rs10455872
rs139974673
rs56130071
rs12359135
rs58542926
rs7337339
rs9686661
rs6874202
rs146203232
rs72836561
rs2954021
rs187429064
rs76866386
rs28718232
rs13240065
rs7578604
rs998584
rs632057
rs77823653
rs116843064
rs1531096
rs6974288
rs2287997
rs1979215
rs35857242
rs687339
rs6859
rs174530
All - MR Egger
All - Inverse variance weighted
-2
-1
0
1
2
MR effect size for
'Total cholesterol in medium VLDL || id:ebi-cfb233-GCST90302057' on 'ER- Breast cancer (Combined Oncoarray; iCOGS; GWAS me

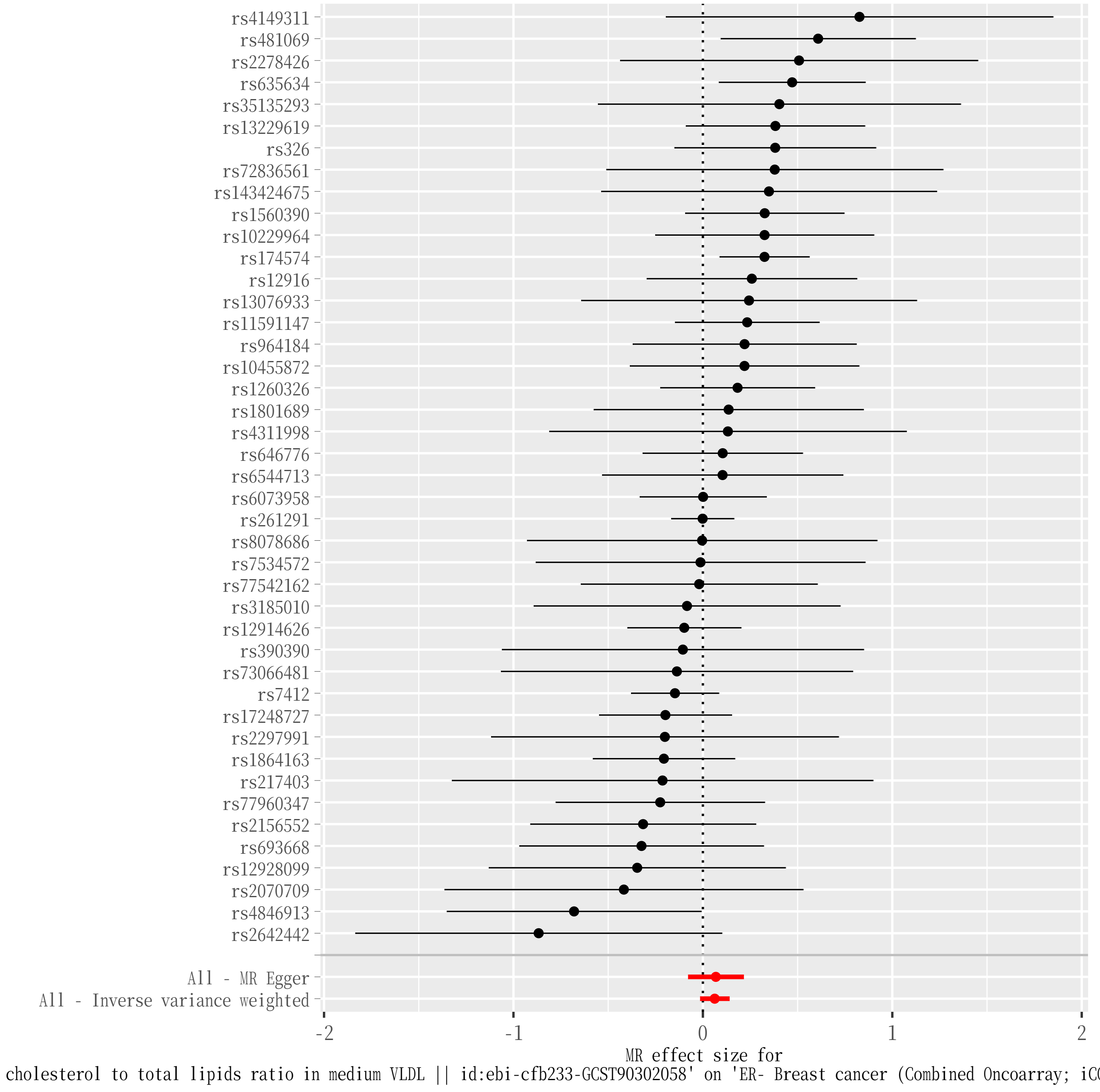
rs4149311
rs481069
rs2278426
rs635634
rs35135293
rs13229619
rs326
rs72836561
rs143424675
rs1560390
rs10229964
rs174574
rs12916
rs13076933
rs11591147
rs964184
rs10455872
rs1260326
rs1801689
rs4311998
rs646776
rs6544713
rs6073958
rs261291
rs8078686
rs7534572
rs77542162
rs3185010
rs12914626
rs390390
rs73066481
rs7412
rs17248727
rs2297991
rs1864163
rs217403
rs77960347
rs2156552
rs693668
rs12928099
rs2070709
rs4846913
rs2642442
All - MR Egger
All - Inverse variance weighted
-2
-1
0
1
2
MR effect size for
cholesterol to total lipids ratio in medium VLDL || id:ebi-cfb233-GCST90302058' on 'ER- Breast cancer (Combined Oncoarray; iC

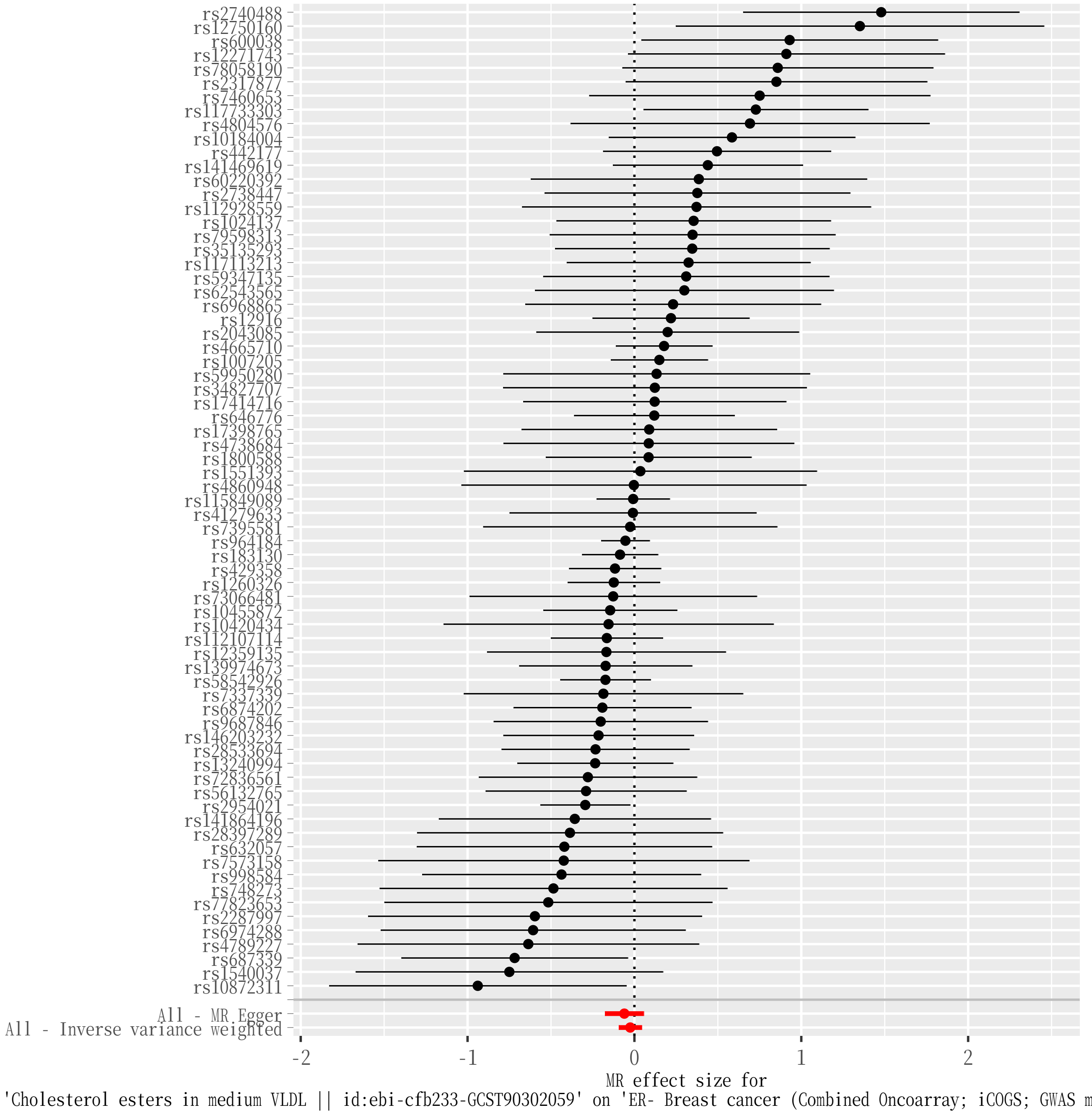
rs2740488
rs12750160
rs600038
rs12271743
rs78058190
rs2317877
rs7460653
rs117733303
rs4804576
rs10184004
rs442177
rs141469619
rs60220392
rs2738447
rs112928559
rs1024137
rs79598313
rs35135293
rs117113213
rs59347135
rs62543565
rs6968865
rs12916
rs2043085
rs4665710
rs1007205
rs59950280
rs34827707
rs17414716
rs646776
rs17398765
rs4738684
rs1800588
rs1551393
rs4860948
rs115849089
rs41279633
rs7395581
rs964184
rs183130
rs429358
rs1260326
rs73066481
rs10455872
rs10420434
rs112107114
rs12359135
rs139974673
rs58542926
rs7337339
rs6874202
rs9687846
rs146203232
rs28533694
rs13240994
rs72836561
rs56132765
rs2954021
rs141864196
rs28397289
rs632057
rs7573158
rs998584
rs748273
rs77823653
rs2287997
rs6974288
rs4789227
rs687339
rs1540037
rs10872311
All - MR Egger
All - Inverse variance weighted
-2
-1
0
1
2
MR effect size for
'Cholesterol esters in medium VLDL || id:ebi-cfb233-GCST90302059' on 'ER- Breast cancer (Combined Oncoarray; iCOGS; GWAS m

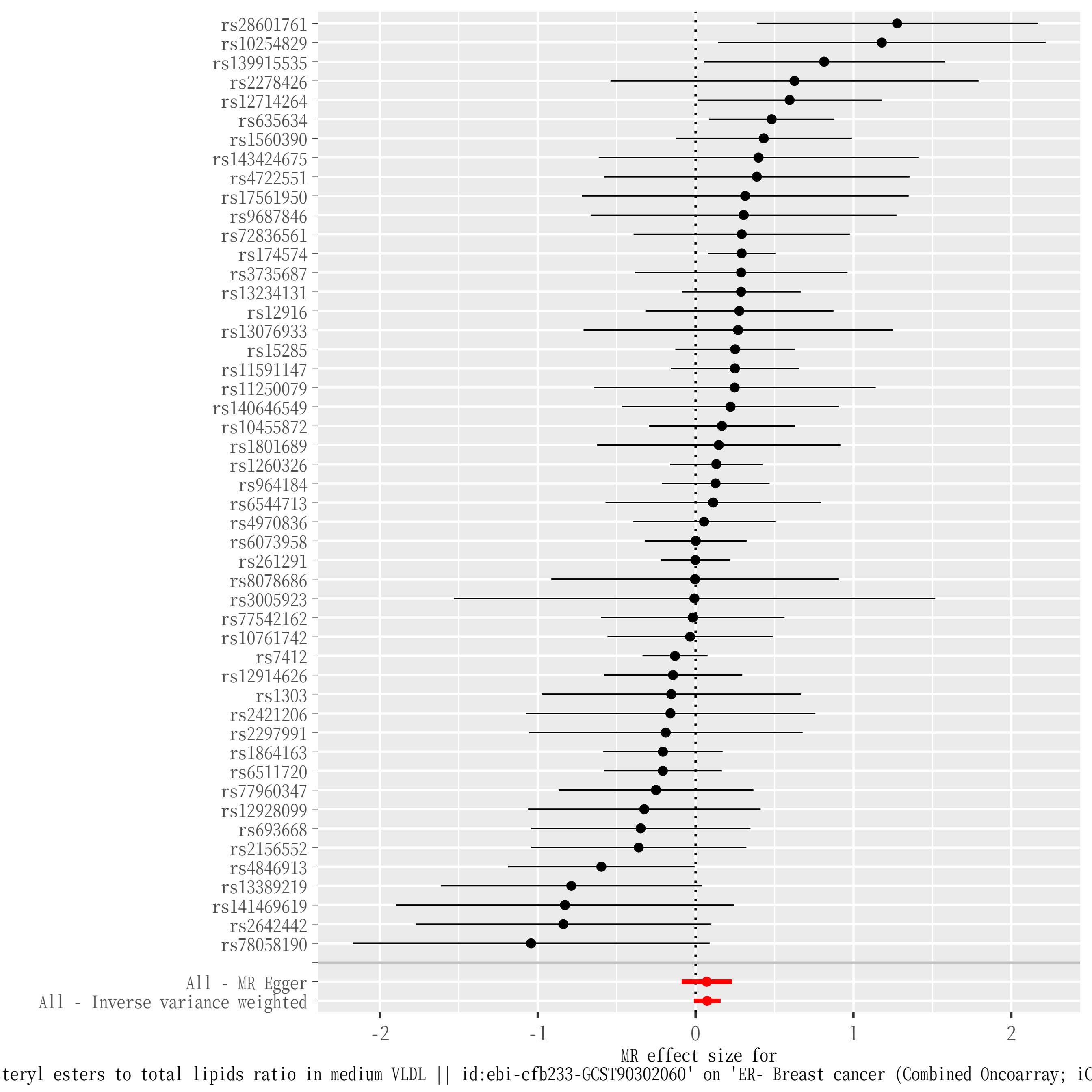
rs28601761
rs10254829
rs139915535
rs2278426
rs12714264
rs635634
rs1560390
rs143424675
rs4722551
rs17561950
rs9687846
rs72836561
rs174574
rs3735687
rs13234131
rs12916
rs13076933
rs15285
rs11591147
rs11250079
rs140646549
rs10455872
rs1801689
rs1260326
rs964184
rs6544713
rs4970836
rs6073958
rs261291
rs8078686
rs3005923
rs77542162
rs10761742
rs7412
rs12914626
rs1303
rs2421206
rs2297991
rs1864163
rs6511720
rs77960347
rs12928099
rs693668
rs2156552
rs4846913
rs13389219
rs141469619
rs2642442
rs78058190
All - MR Egger
All - Inverse variance weighted
-2
-1
0
1
2
MR effect size for
teryl esters to total lipids ratio in medium VLDL || id:ebi-cfb233-GCST90302060' on 'ER- Breast cancer (Combined Oncoarray; iC

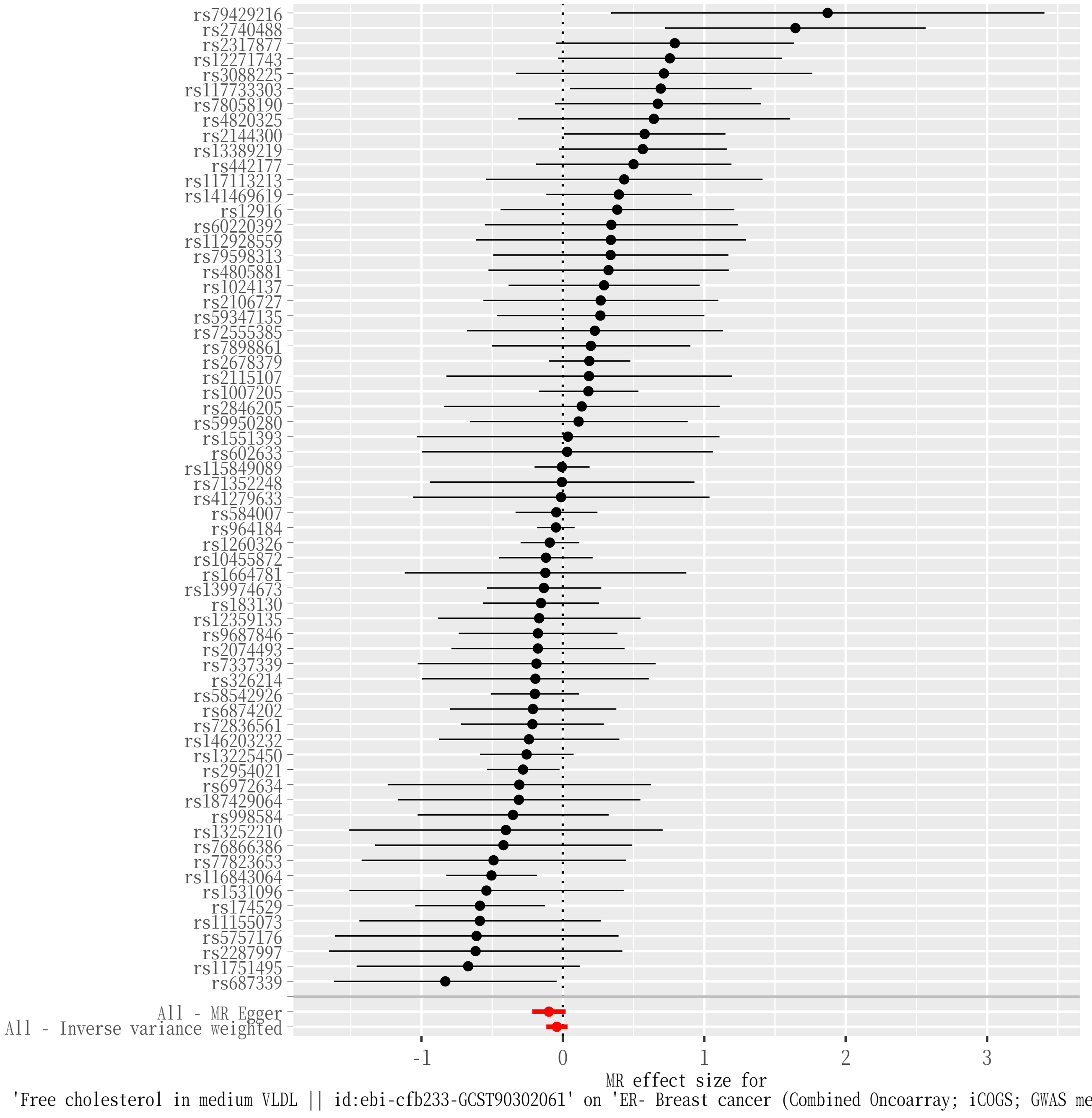
rs79429216
rs2740488
rs2317877
rs12271743
rs3088225
rs117733303
rs78058190
rs4820325
rs2144300
rs13389219
rs442177
rs117113213
rs141469619
rs12916
rs60220392
rs112928559
rs79598313
rs4805881
rs1024137
rs2106727
rs59347135
rs72555385
rs7898861
rs2678379
rs2115107
rs1007205
rs2846205
rs59950280
rs1551393
rs602633
rs115849089
rs71352248
rs41279633
rs584007
rs964184
rs1260326
rs10455872
rs1664781
rs139974673
rs183130
rs12359135
rs9687846
rs2074493
rs7337339
rs326214
rs58542926
rs6874202
rs72836561
rs146203232
rs13225450
rs2954021
rs6972634
rs187429064
rs998584
rs13252210
rs76866386
rs77823653
rs116843064
rs1531096
rs174529
rs11155073
rs5757176
rs2287997
rs11751495
rs687339
All - MR Egger
All - Inverse variance weighted
-1
0
1
2
3
MR effect size for
'Free cholesterol in medium VLDL || id:ebi-cfb233-GCST90302061' on 'ER- Breast cancer (Combined Oncoarray; iCOGS; GWAS me

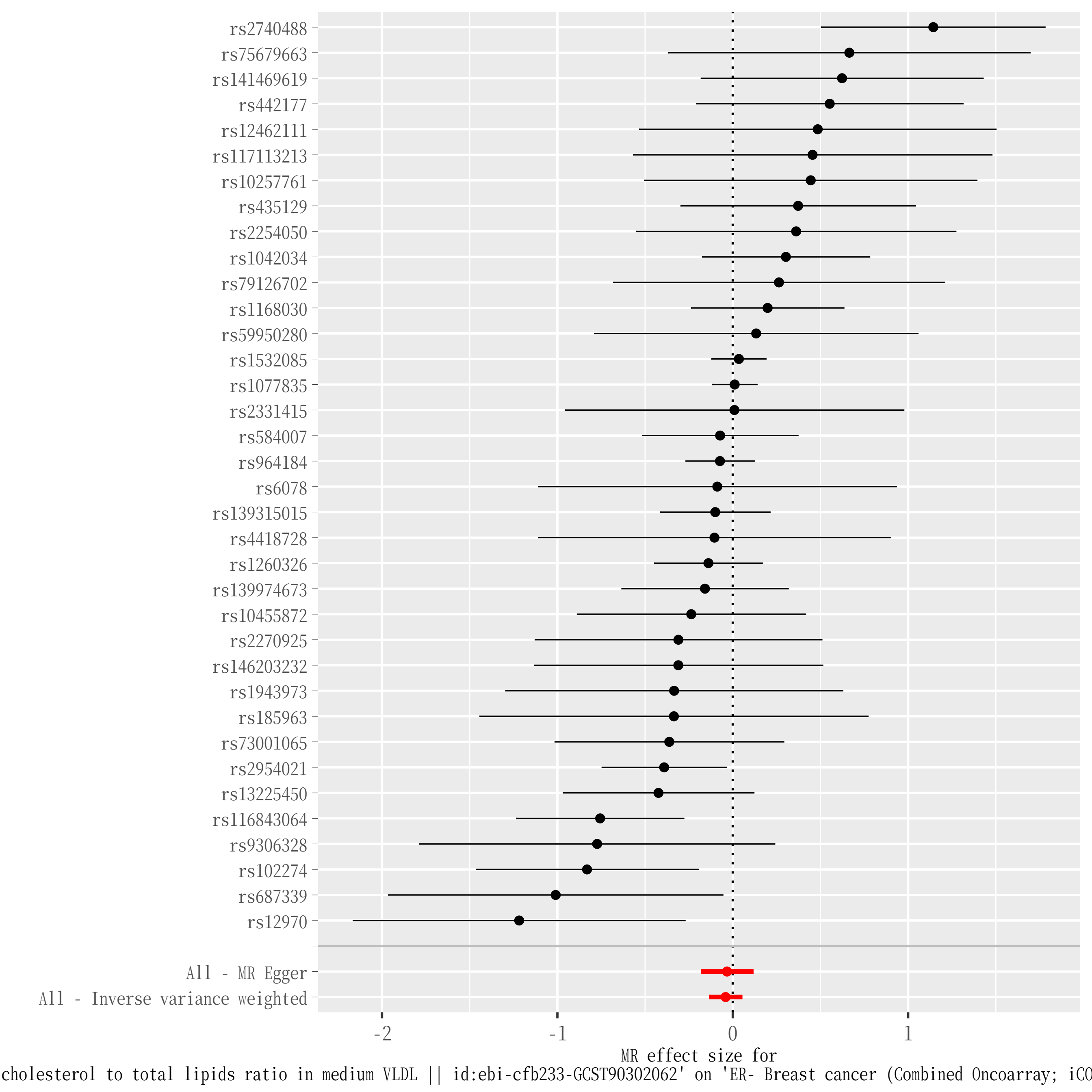

rs2740488
rs75679663
rs141469619
rs442177
rs12462111
rs117113213
rs10257761
rs435129
rs2254050
rs1042034
rs79126702
rs1168030
rs59950280
rs1532085
rs1077835
rs2331415
rs584007
rs964184
rs6078
rs139315015
rs4418728
rs1260326
rs139974673
rs10455872
rs2270925
rs146203232
rs1943973
rs185963
rs73001065
rs2954021
rs13225450
rs116843064
rs9306328
rs102274
rs687339
rs12970
All - MR Egger
All - Inverse variance weighted
-2
-1
0
1
MR effect size for
cholesterol to total lipids ratio in medium VLDL || id:ebi-cfb233-GCST90302062' on 'ER- Breast cancer (Combined Oncoarray; iCO

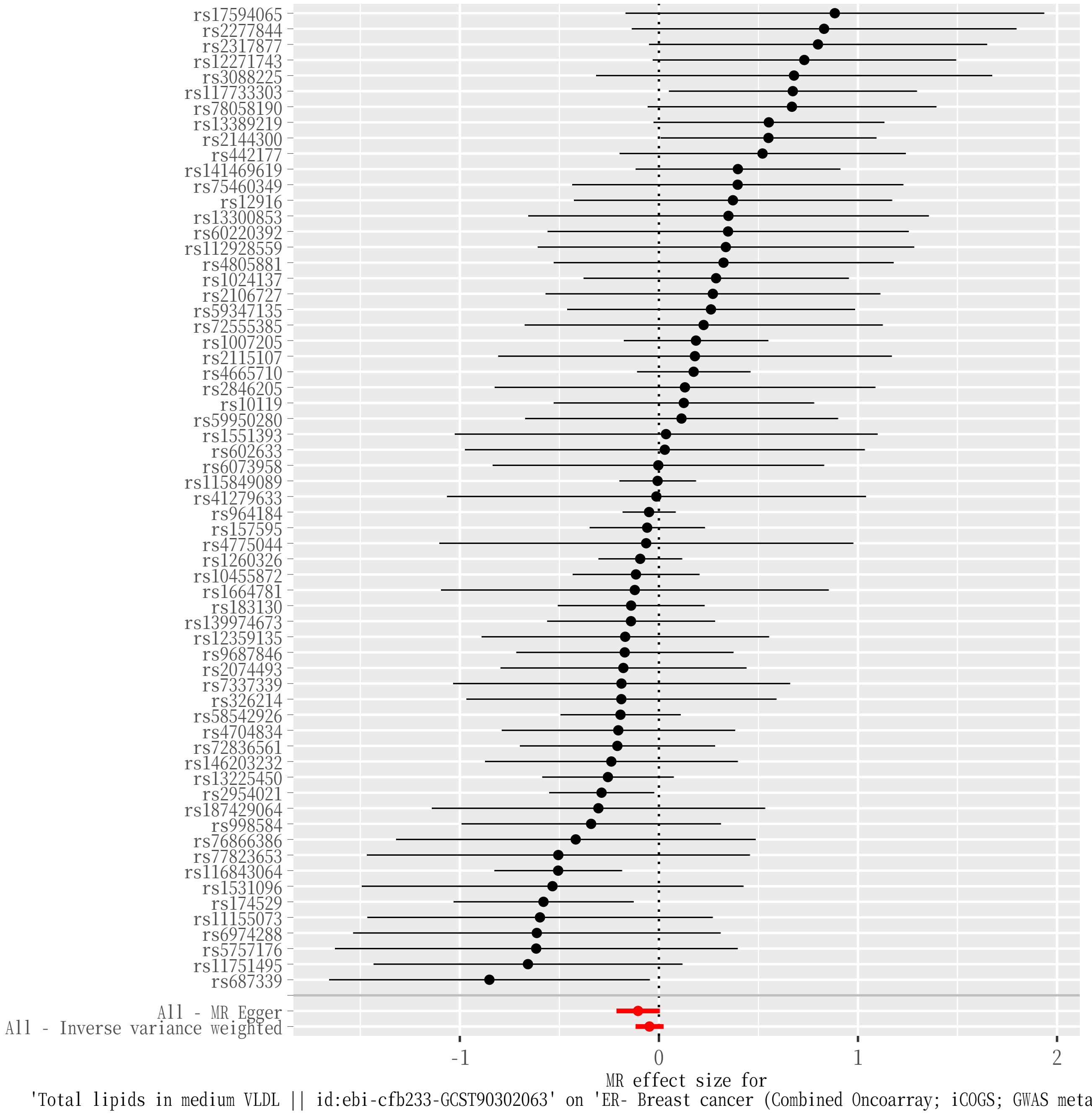
rs17594065
rs2277844
rs2317877
rs12271743
rs3088225
rs117733303
rs78058190
rs13389219
rs2144300
rs442177
rs141469619
rs75460349
rs12916
rs13300853
rs60220392
rs112928559
rs4805881
rs1024137
rs2106727
rs59347135
rs72555385
rs1007205
rs2115107
rs4665710
rs2846205
rs10119
rs59950280
rs1551393
rs602633
rs6073958
rs115849089
rs41279633
rs964184
rs157595
rs4775044
rs1260326
rs10455872
rs1664781
rs183130
rs139974673
rs12359135
rs9687846
rs2074493
rs7337339
rs326214
rs58542926
rs4704834
rs72836561
rs146203232
rs13225450
rs2954021
rs187429064
rs998584
rs76866386
rs77823653
rs116843064
rs1531096
rs174529
rs11155073
rs6974288
rs5757176
rs11751495
rs687339
All - MR Egger
All - Inverse variance weighted
-1
0
1
2
MR effect size for
'Total lipids in medium VLDL || id:ebi-cfb233-GCST90302063' on 'ER- Breast cancer (Combined Oncoarray; iCOGS; GWAS meta

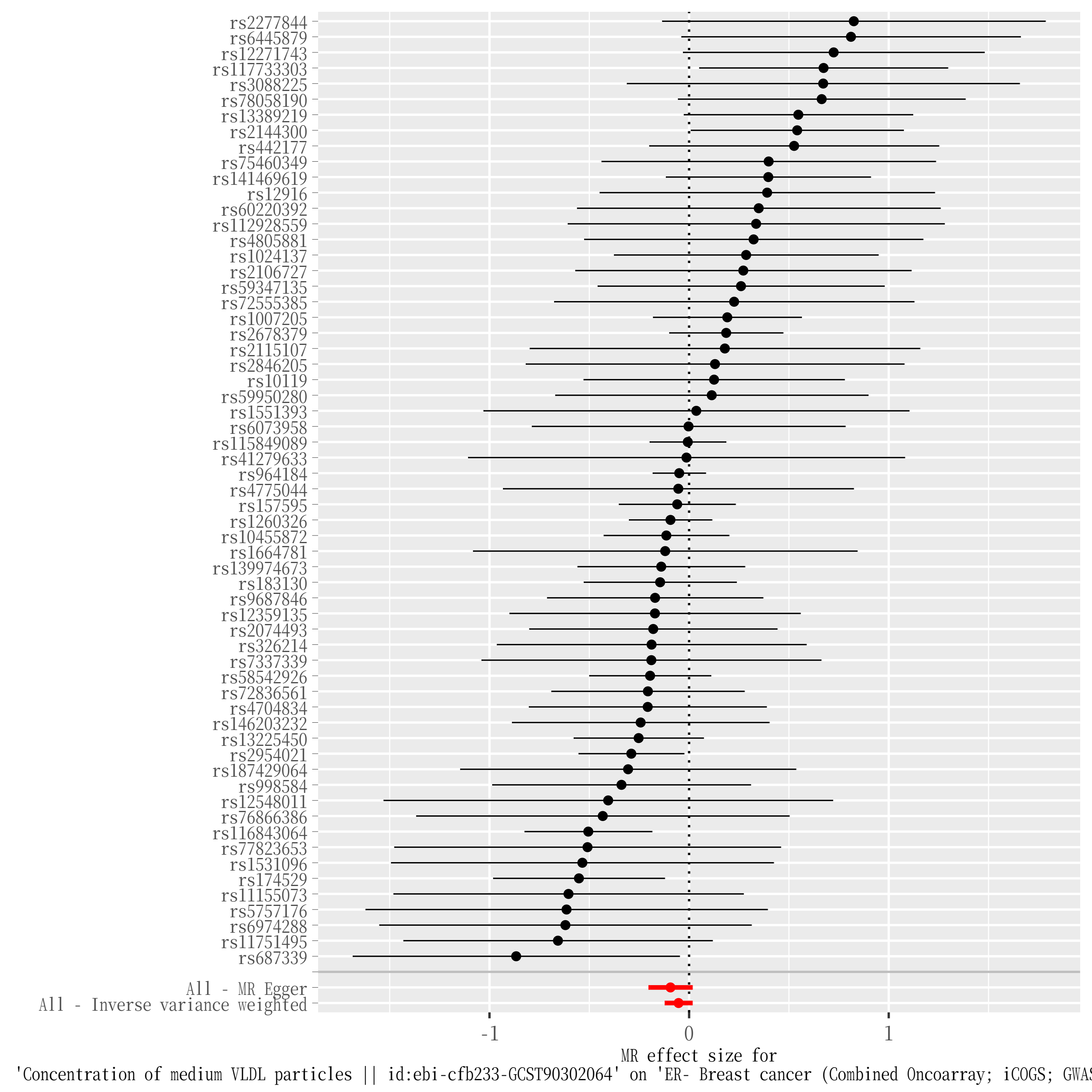
rs2277844
rs6445879
rs12271743
rs117733303
rs3088225
rs78058190
rs13389219
rs2144300
rs442177
rs75460349
rs141469619
rs12916
rs60220392
rs112928559
rs4805881
rs1024137
rs2106727
rs59347135
rs72555385
rs1007205
rs2678379
rs2115107
rs2846205
rs10119
rs59950280
rs1551393
rs6073958
rs115849089
rs41279633
rs964184
rs4775044
rs157595
rs1260326
rs10455872
rs1664781
rs139974673
rs183130
rs9687846
rs12359135
rs2074493
rs326214
rs7337339
rs58542926
rs72836561
rs4704834
rs146203232
rs13225450
rs2954021
rs187429064
rs998584
rs12548011
rs76866386
rs116843064
rs77823653
rs1531096
rs174529
rs11155073
rs5757176
rs6974288
rs11751495
rs687339
All - MR Egger
All - Inverse variance weighted
-1
0
1
MR effect size for
'Concentration of medium VLDL particles || id:ebi-cfb233-GCST90302064' on 'ER- Breast cancer (Combined Oncoarray; iCOGS; GWAS

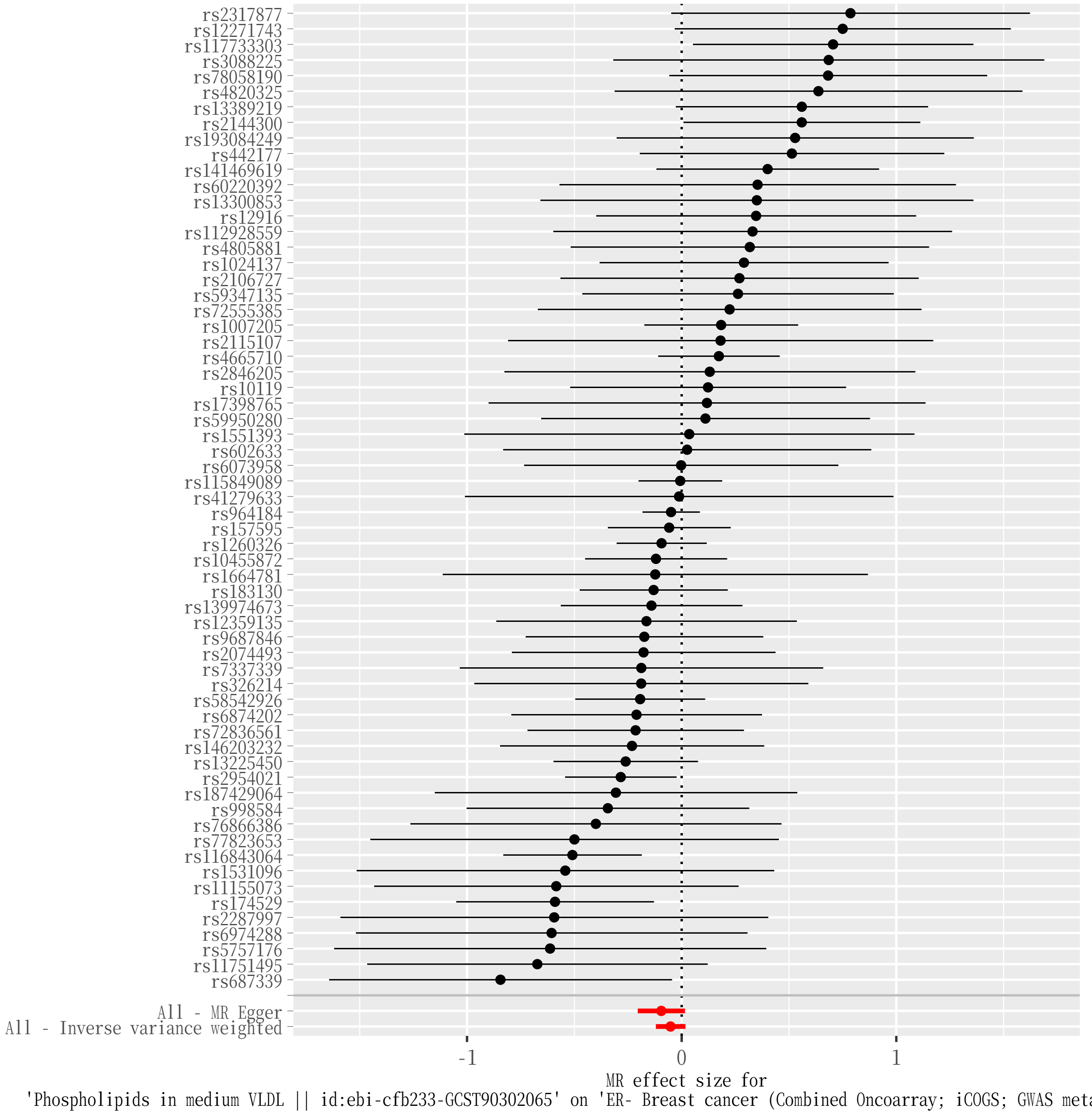
rs2317877
rs12271743
rs117733303
rs3088225
rs78058190
rs4820325
rs13389219
rs2144300
rs193084249
rs442177
rs141469619
rs60220392
rs13300853
rs12916
rs112928559
rs4805881
rs1024137
rs2106727
rs59347135
rs72555385
rs1007205
rs2115107
rs4665710
rs2846205
rs10119
rs17398765
rs59950280
rs1551393
rs602633
rs6073958
rs115849089
rs41279633
rs964184
rs157595
rs1260326
rs10455872
rs1664781
rs183130
rs139974673
rs12359135
rs9687846
rs2074493
rs7337339
rs326214
rs58542926
rs6874202
rs72836561
rs146203232
rs13225450
rs2954021
rs187429064
rs998584
rs76866386
rs77823653
rs116843064
rs1531096
rs11155073
rs174529
rs2287997
rs6974288
rs5757176
rs11751495
rs687339
All - MR Egger
All - Inverse variance weighted
-1
0
1
MR effect size for
'Phospholipids in medium VLDL || id:ebi-cfb233-GCST90302065' on 'ER- Breast cancer (Combined Oncoarray; iCOGS; GWAS meta

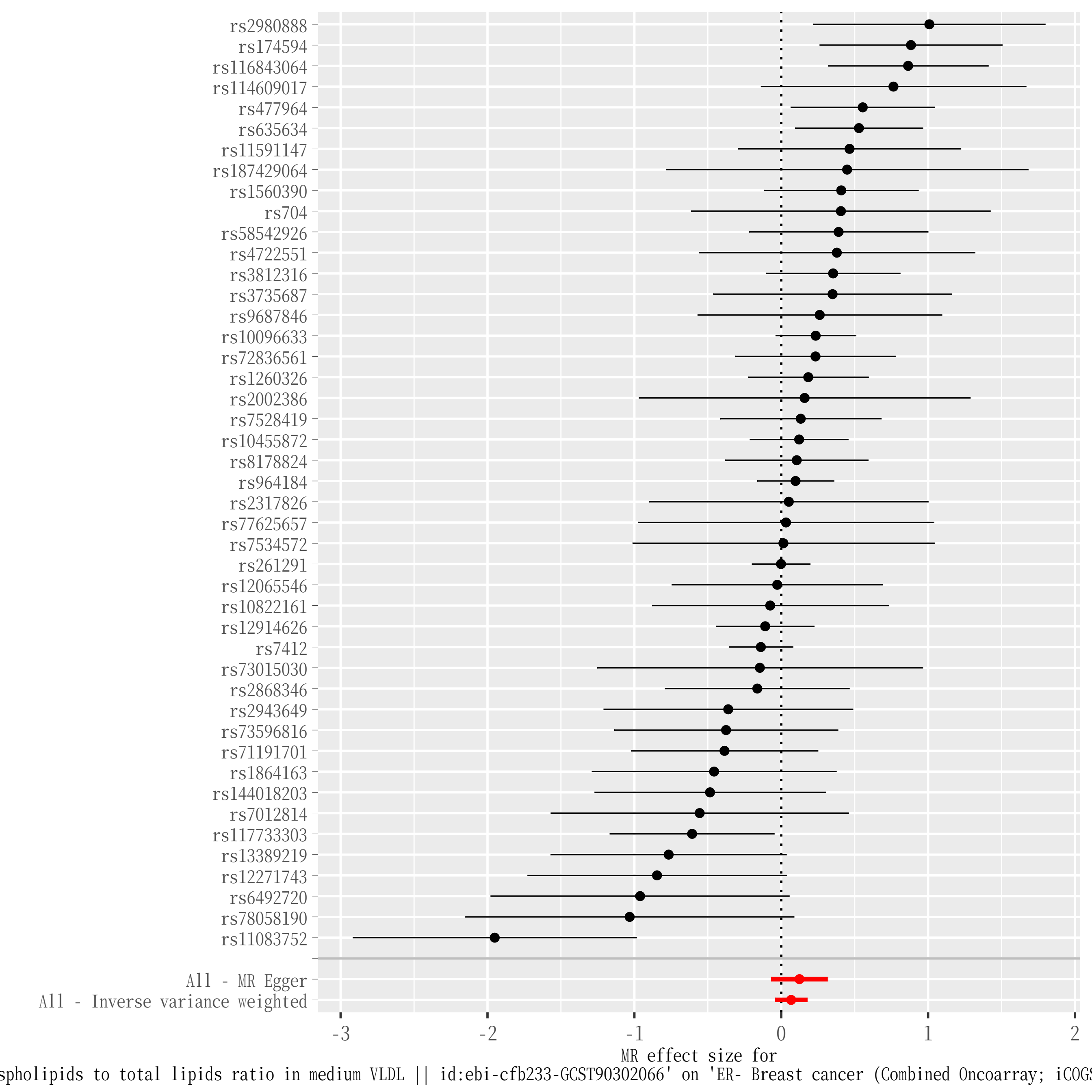

rs2980888
rs174594
rs116843064
rs114609017
rs477964
rs635634
rs11591147
rs187429064
rs1560390
rs704
rs58542926
rs4722551
rs3812316
rs3735687
rs9687846
rs10096633
rs72836561
rs1260326
rs2002386
rs7528419
rs10455872
rs8178824
rs964184
rs2317826
rs77625657
rs7534572
rs261291
rs12065546
rs10822161
rs12914626
rs7412
rs73015030
rs2868346
rs2943649
rs73596816
rs71191701
rs1864163
rs144018203
rs7012814
rs117733303
rs13389219
rs12271743
rs6492720
rs78058190
rs11083752
All - MR Egger
All - Inverse variance weighted
-3
-2
-1
0
1
2
MR effect size for
spholipids to total lipids ratio in medium VLDL || id:ebi-cfb233-GCST90302066' on 'ER- Breast cancer (Combined Oncoarray; iCOGS

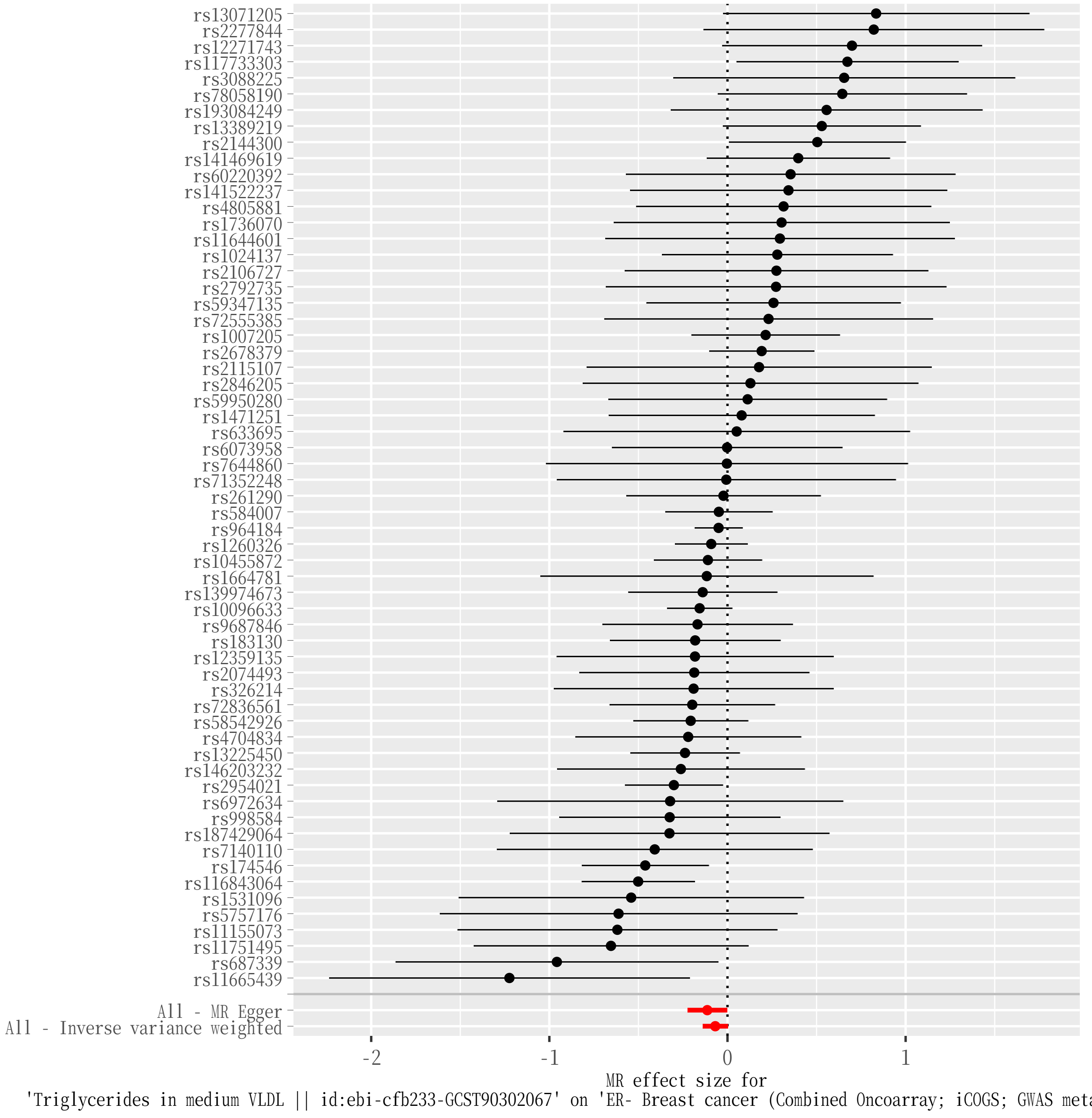

rs13071205
rs2277844
rs12271743
rs117733303
rs3088225
rs78058190
rs193084249
rs13389219
rs2144300
rs141469619
rs60220392
rs141522237
rs4805881
rs1736070
rs11644601
rs1024137
rs2106727
rs2792735
rs59347135
rs72555385
rs1007205
rs2678379
rs2115107
rs2846205
rs59950280
rs1471251
rs633695
rs6073958
rs7644860
rs71352248
rs261290
rs584007
rs964184
rs1260326
rs10455872
rs1664781
rs139974673
rs10096633
rs9687846
rs183130
rs12359135
rs2074493
rs326214
rs72836561
rs58542926
rs4704834
rs13225450
rs146203232
rs2954021
rs6972634
rs998584
rs187429064
rs7140110
rs174546
rs116843064
rs1531096
rs5757176
rs11155073
rs11751495
rs687339
rs11665439
All - MR Egger
All - Inverse variance weighted
-2
-1
0
1
MR effect size for
'Triglycerides in medium VLDL || id:ebi-cfb233-GCST90302067' on 'ER- Breast cancer (Combined Oncoarray; iCOGS; GWAS meta

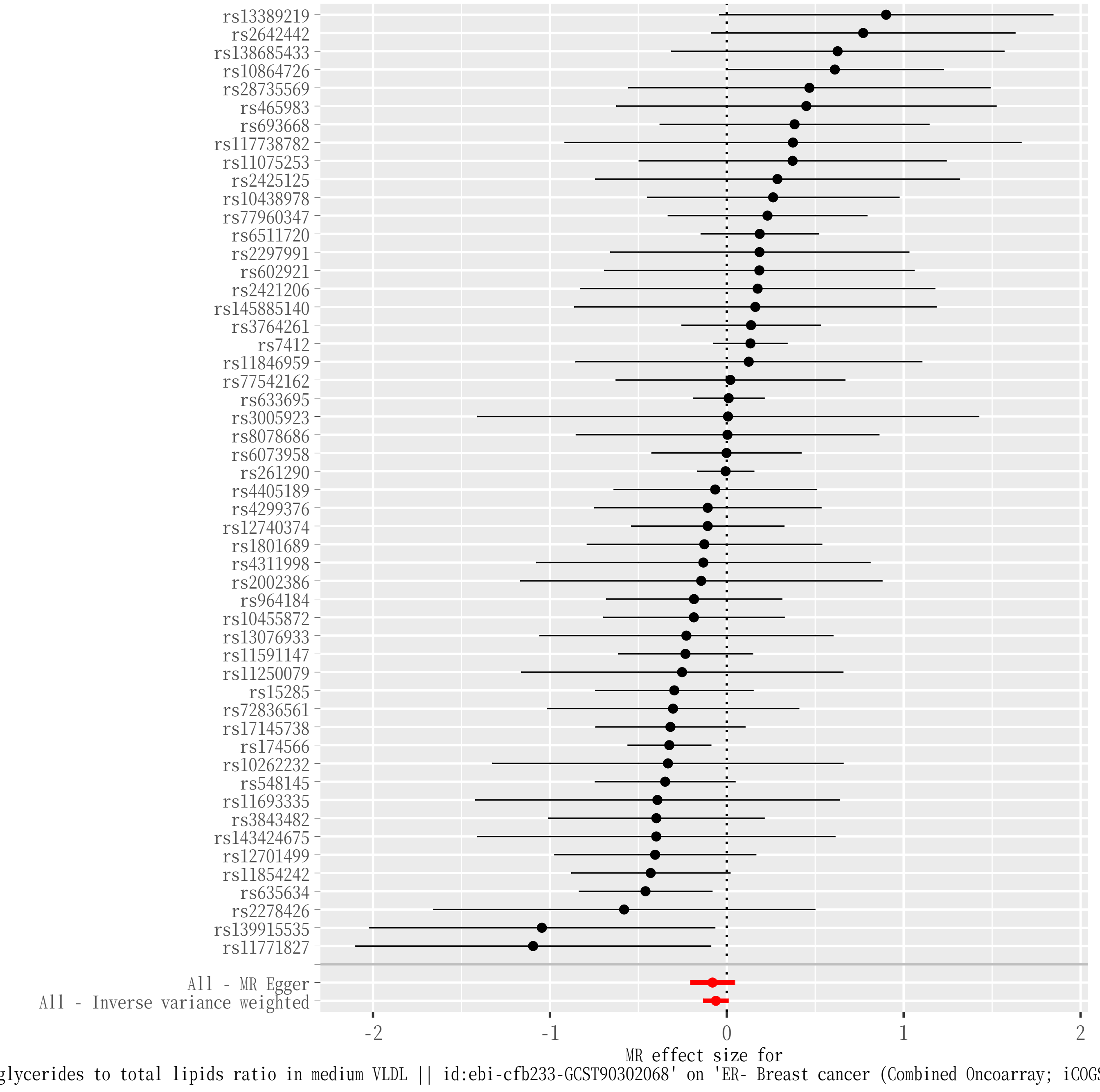
rs13389219
rs2642442
rs138685433
rs10864726
rs28735569
rs465983
rs693668
rs117738782
rs11075253
rs2425125
rs10438978
rs77960347
rs6511720
rs2297991
rs602921
rs2421206
rs145885140
rs3764261
rs7412
rs11846959
rs77542162
rs633695
rs3005923
rs8078686
rs6073958
rs261290
rs4405189
rs4299376
rs12740374
rs1801689
rs4311998
rs2002386
rs964184
rs10455872
rs13076933
rs11591147
rs11250079
rs15285
rs72836561
rs17145738
rs174566
rs10262232
rs548145
rs11693335
rs3843482
rs143424675
rs12701499
rs11854242
rs635634
rs2278426
rs139915535
rs11771827
All - MR Egger
All - Inverse variance weighted
-2
-1
0
1
2
MR effect size for
glycerides to total lipids ratio in medium VLDL || id:ebi-cfb233-GCST90302068' on 'ER- Breast cancer (Combined Oncoarray; iCOGS

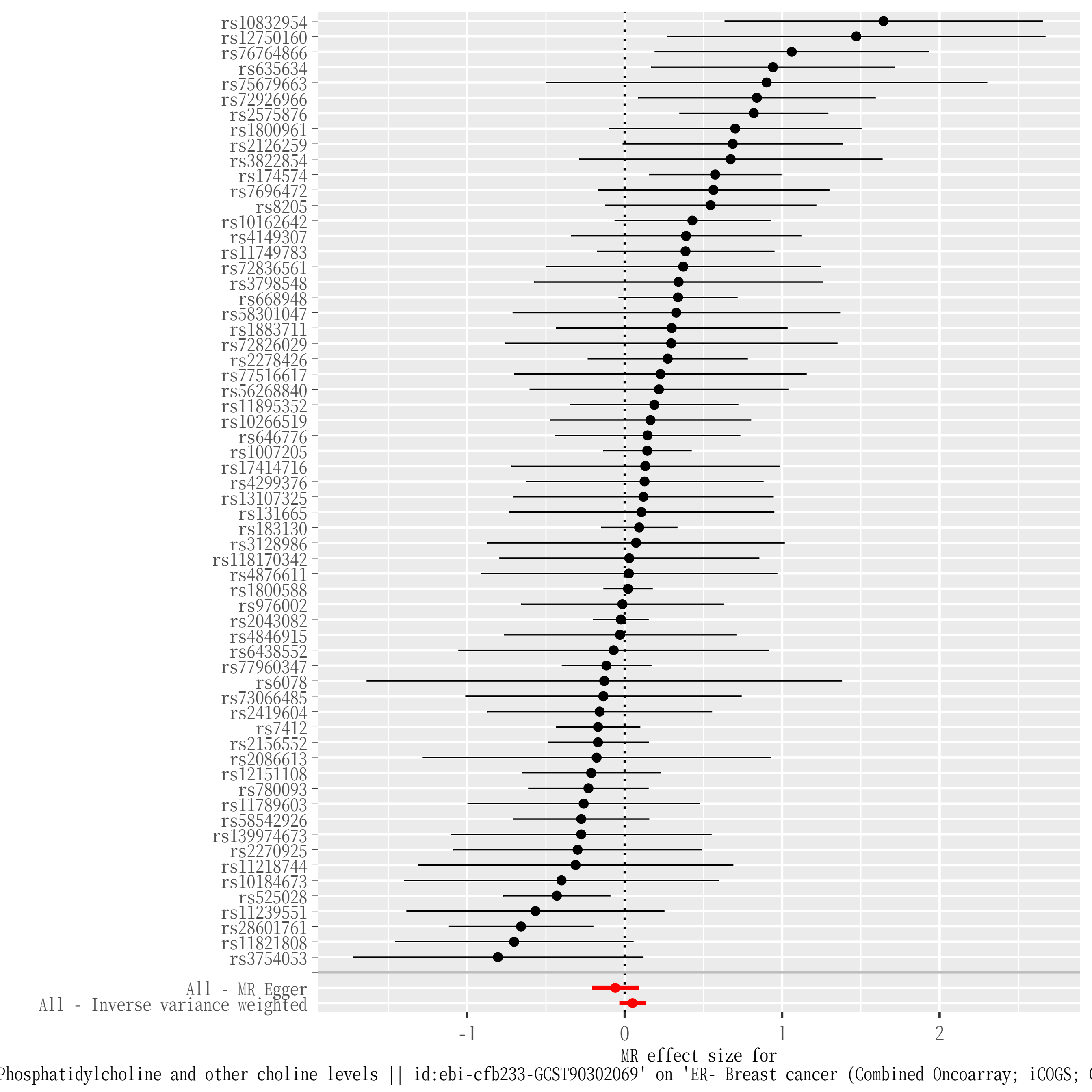

rs10832954
rs12750160
rs76764866
rs635634
rs75679663
rs72926966
rs2575876
rs1800961
rs2126259
rs3822854
rs174574
rs7696472
rs8205
rs10162642
rs4149307
rs11749783
rs72836561
rs3798548
rs668948
rs58301047
rs1883711
rs72826029
rs2278426
rs77516617
rs56268840
rs11895352
rs10266519
rs646776
rs1007205
rs17414716
rs4299376
rs13107325
rs131665
rs183130
rs3128986
rs118170342
rs4876611
rs1800588
rs976002
rs2043082
rs4846915
rs6438552
rs77960347
rs6078
rs73066485
rs2419604
rs7412
rs2156552
rs2086613
rs12151108
rs780093
rs11789603
rs58542926
rs139974673
rs2270925
rs11218744
rs10184673
rs525028
rs11239551
rs28601761
rs11821808
rs3754053
All - MR Egger
All - Inverse variance weighted
-1
0
1
2
MR effect size for
Phosphatidylcholine and other choline levels || id:ebi-cfb233-GCST90302069' on 'ER- Breast cancer (Combined Oncoarray; iCOGS; (

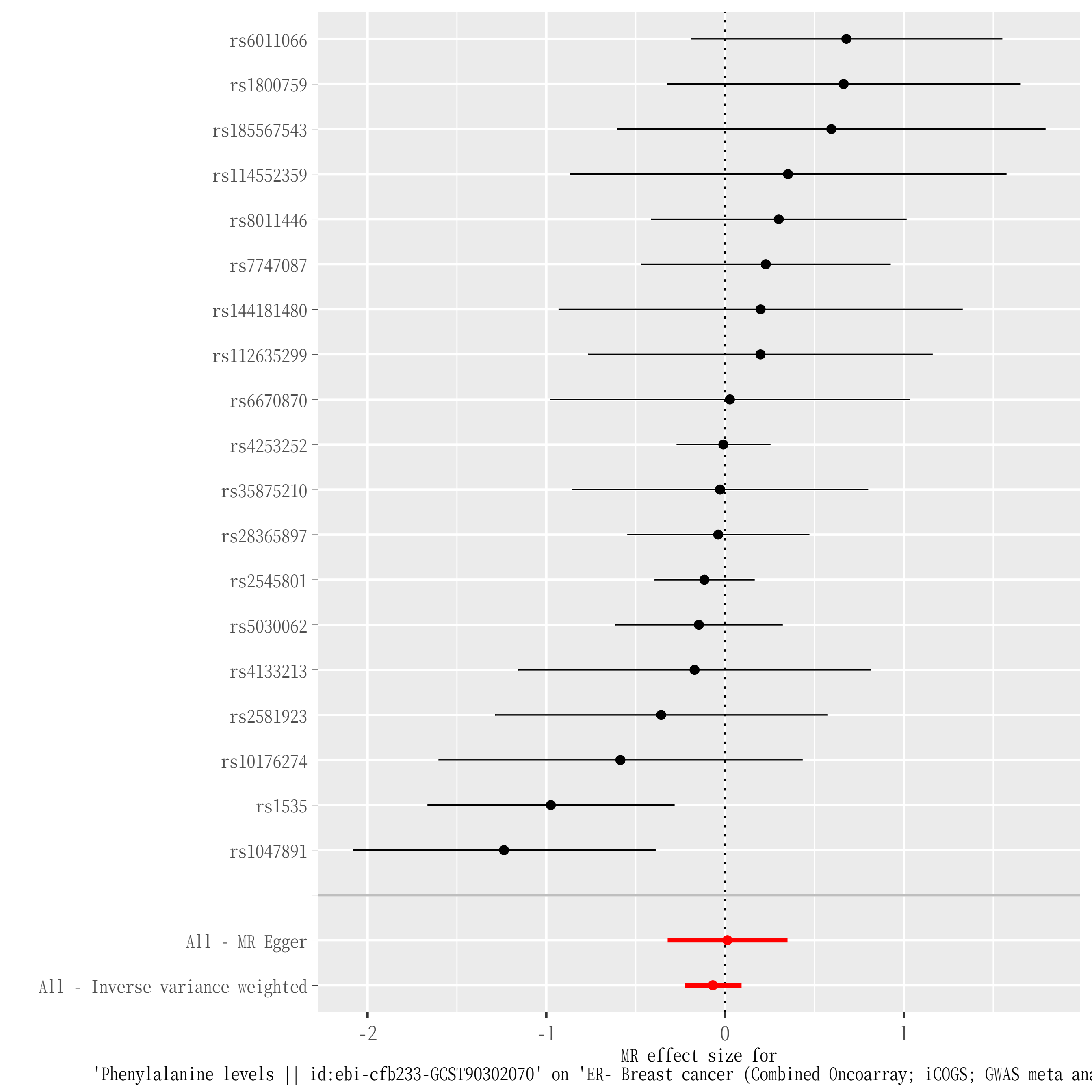
rs6011066
rs1800759
rs185567543
rs114552359
rs8011446
rs7747087
rs144181480
rs112635299
rs6670870
rs4253252
rs35875210
rs28365897
rs2545801
rs5030062
rs4133213
rs2581923
rs10176274
rs1535
rs1047891
All - MR Egger
All - Inverse variance weighted
-2
-1
0
1
MR effect size for
'Phenylalanine levels || id:ebi-cfb233-GCST90302070' on 'ER- Breast cancer (Combined Oncoarray; iCOGS; GWAS meta ana

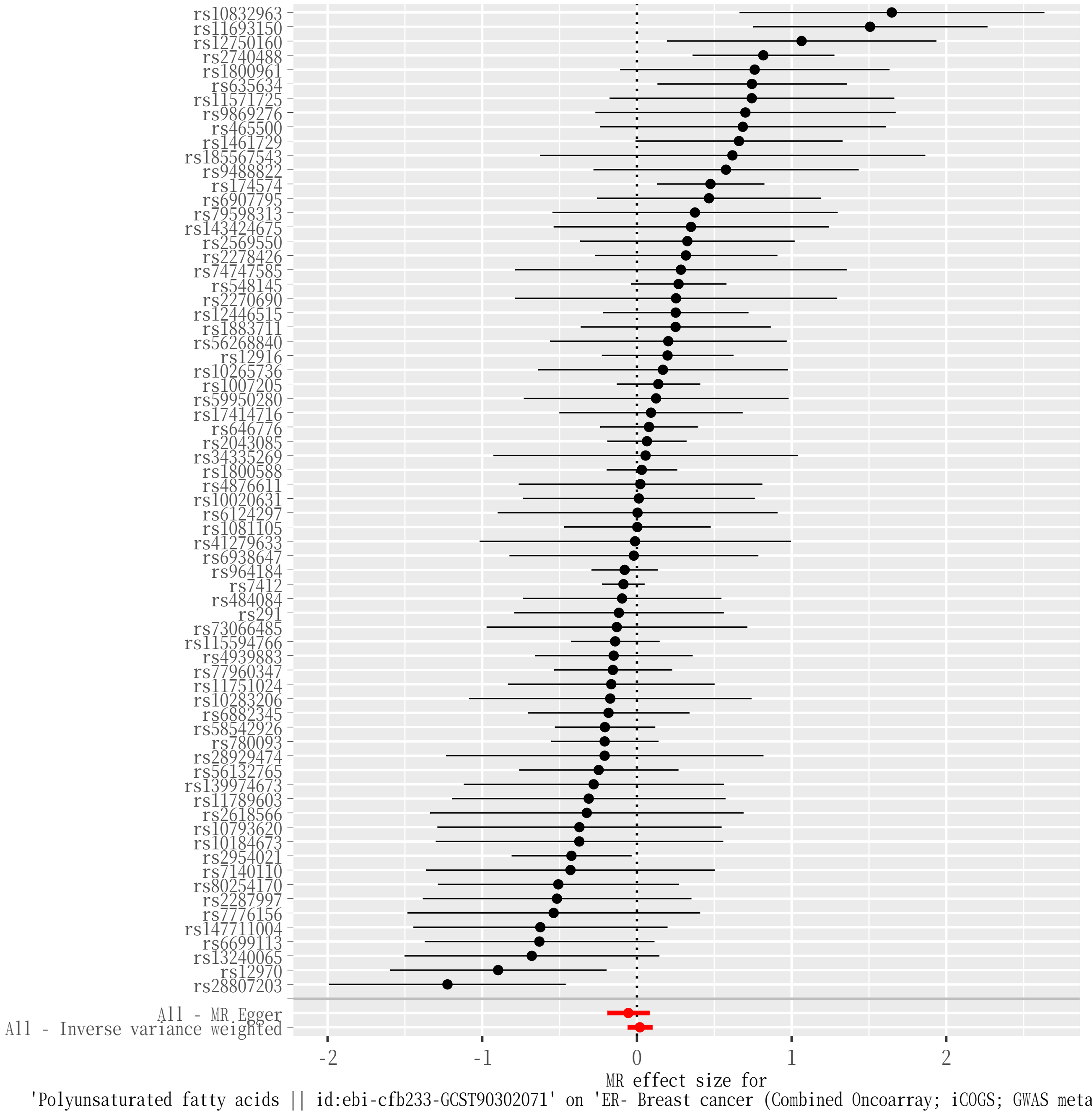
rs10832963
rs11693150
rs12750160
rs2740488
rs1800961
rs635634
rs11571725
rs9869276
rs465500
rs1461729
rs185567543
rs9488822
rs174574
rs6907795
rs79598313
rs143424675
rs2569550
rs2278426
rs74747585
rs548145
rs2270690
rs12446515
rs1883711
rs56268840
rs12916
rs10265736
rs1007205
rs59950280
rs17414716
rs646776
rs2043085
rs34335269
rs1800588
rs4876611
rs10020631
rs6124297
rs1081105
rs41279633
rs6938647
rs964184
rs7412
rs484084
rs291
rs73066485
rs115594766
rs4939883
rs77960347
rs11751024
rs10283206
rs6882345
rs58542926
rs780093
rs28929474
rs56132765
rs139974673
rs11789603
rs2618566
rs10793620
rs10184673
rs2954021
rs7140110
rs80254170
rs2287997
rs7776156
rs147711004
rs6699113
rs13240065
rs12970
rs28807203
All - MR Egger
All - Inverse variance weighted
-2
-1
0
1
2
MR effect size for
'Polyunsaturated fatty acids || id:ebi-cfb233-GCST90302071' on 'ER- Breast cancer (Combined Oncoarray; iCOGS; GWAS meta

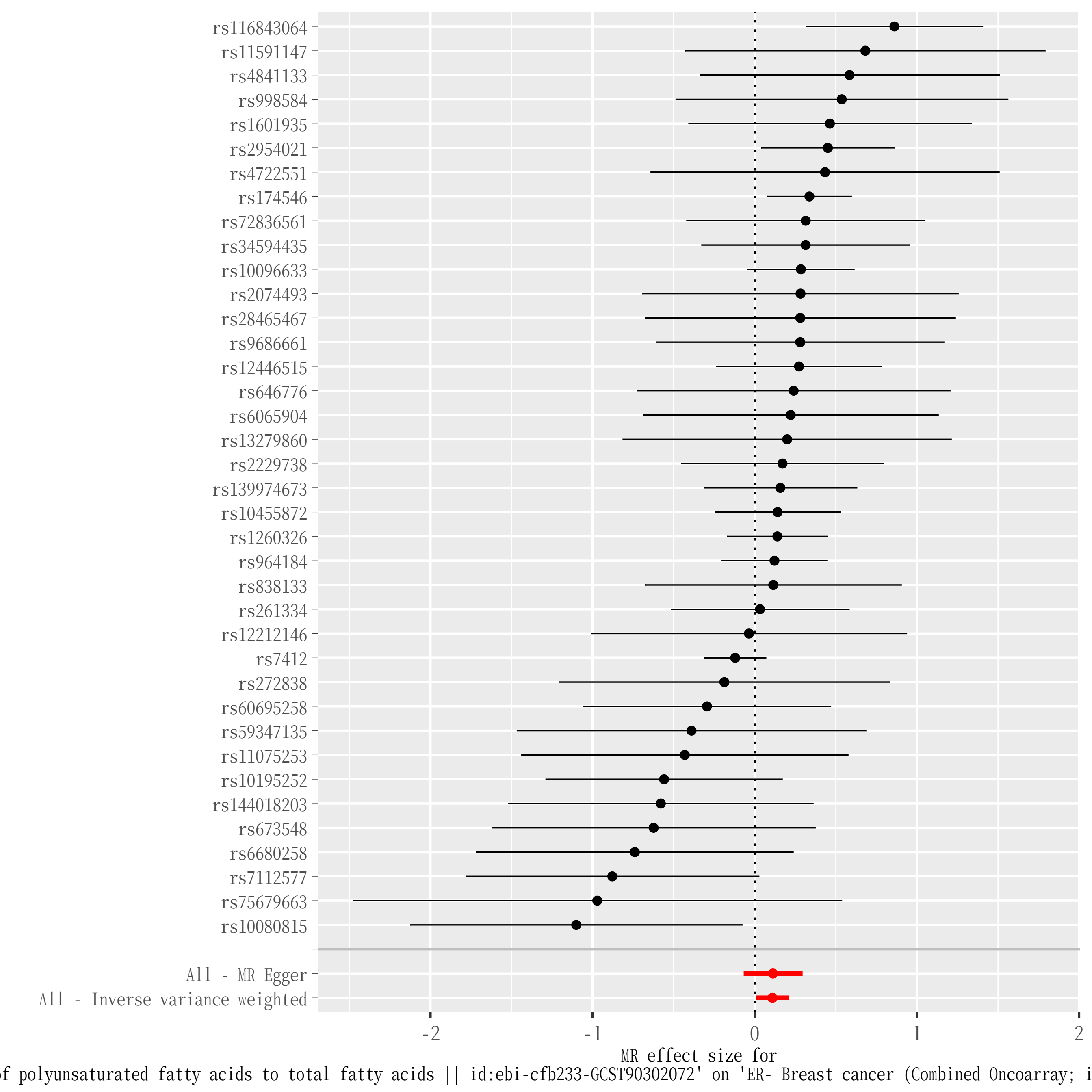
rs116843064
rs11591147
rs4841133
rs998584
rs1601935
rs2954021
rs4722551
rs174546
rs72836561
rs34594435
rs10096633
rs2074493
rs28465467
rs9686661
rs12446515
rs646776
rs6065904
rs13279860
rs2229738
rs139974673
rs10455872
rs1260326
rs964184
rs838133
rs261334
rs12212146
rs7412
rs272838
rs60695258
rs59347135
rs11075253
rs10195252
rs144018203
rs673548
rs6680258
rs7112577
rs75679663
rs10080815
All - MR Egger
All - Inverse variance weighted
-2
-1
0
1
2
MR effect size for
of polyunsaturated fatty acids to total fatty acids || id:ebi-cfb233-GCST90302072' on 'ER- Breast cancer (Combined Oncoarray; i

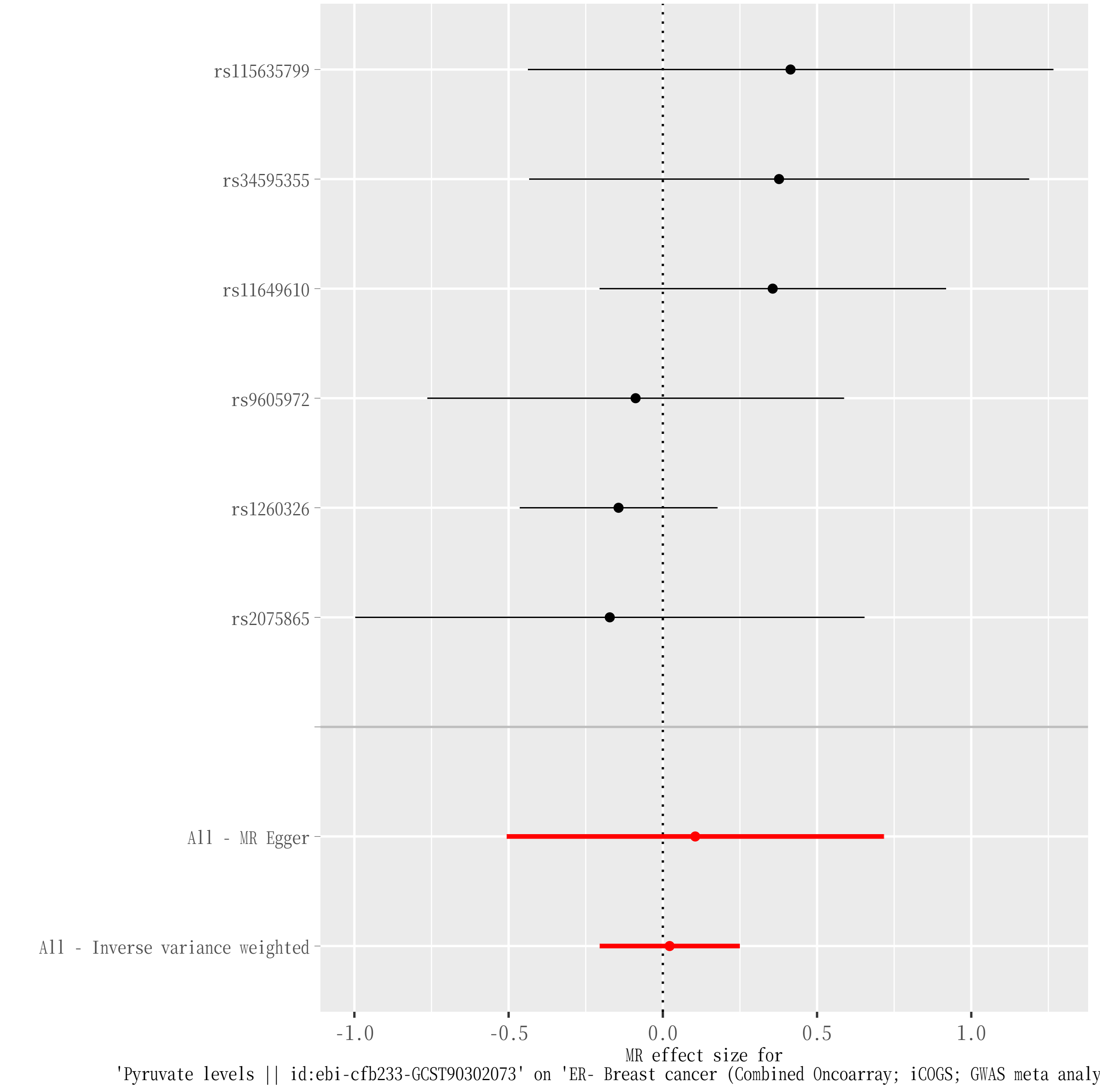
rs115635799
rs34595355
rs11649610
rs9605972
rs1260326
rs2075865
All - MR Egger
All - Inverse variance weighted
-1.0
-0.5
0.0
0.5
1.0
MR effect size for
'Pyruvate levels || id:ebi-cfb233-GCST90302073' on 'ER- Breast cancer (Combined Oncoarray; iCOGS; GWAS meta analy

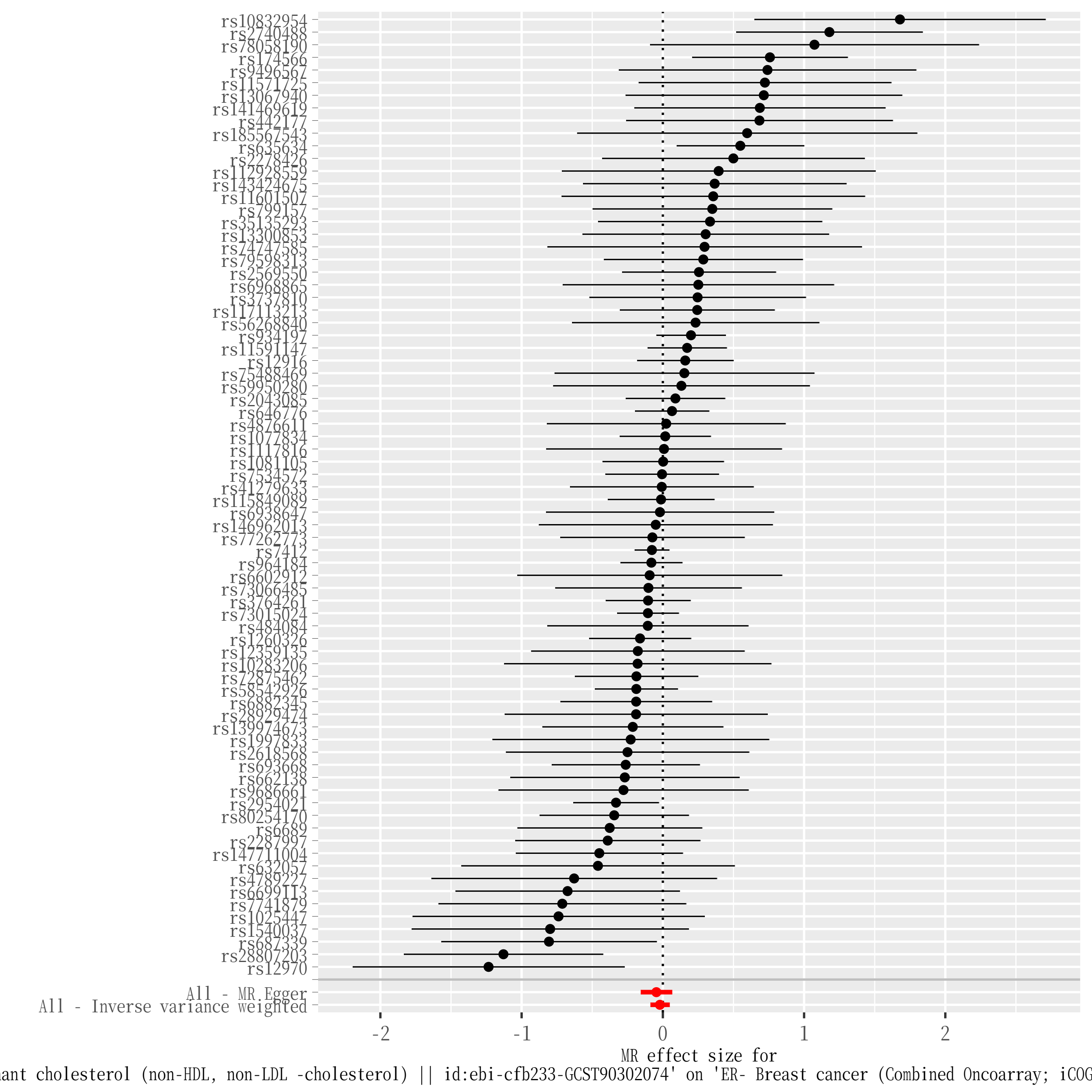

rs10832954
rs2740488
rs78058190
rs174566
rs9496567
rs11571725
rs13067940
rs141469619
rs442177
rs185567543
rs635634
rs2278426
rs112928559
rs143424675
rs11601507
rs799157
rs35135293
rs13300853
rs74747585
rs79598313
rs2569550
rs6968865
rs3737810
rs117113213
rs56268840
rs934197
rs11591147
rs12916
rs75488469
rs59950280
rs2043085
rs646776
rs4876611
rs1077834
rs1117816
rs1081105
rs7534572
rs41279633
rs115849089
rs6938647
rs146962013
rs77262773
rs7412
rs964184
rs6602912
rs73066485
rs3764261
rs73015024
rs484084
rs1260326
rs12359135
rs10283206
rs72875462
rs58542926
rs6882345
rs28929474
rs139974673
rs1997833
rs2618568
rs693668
rs662138
rs9686661
rs2954021
rs80254170
rs6689
rs2287997
rs147711004
rs632057
rs4789227
rs6699113
rs7741879
rs1025447
rs1540037
rs687339
rs28807203
rs12970
All - MR Egger
All - Inverse variance weighted
-2
-1
0
1
2
MR effect size for
ant cholesterol (non-HDL, non-LDL -cholesterol) || id:ebi-cfb233-GCST90302074' on 'ER- Breast cancer (Combined Oncoarray; iCOG

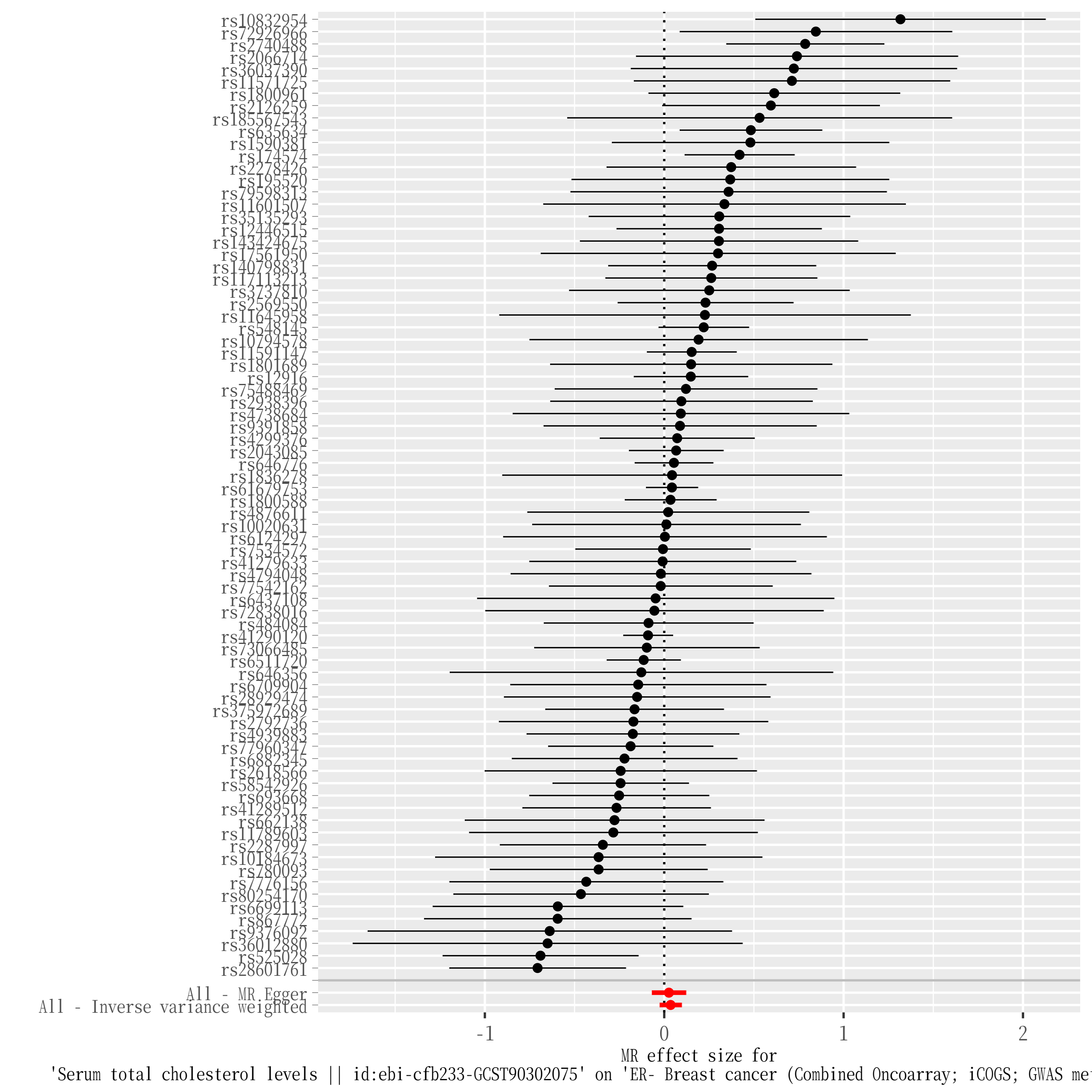
rs10832954
rs72926966
rs2740488
rs2066714
rs36037390
rs11571725
rs1800961
rs2126259
rs185567543
rs635634
rs1520381
rs174574
rs2278426
rs195520
rs79598313
rs11601507
rs35135293
rs12446515
rs143424675
rs17561950
rs140798831
rs117113213
rs3737810
rs2569550
rs11645958
rs548145
rs10794578
rs11591147
rs1801689
rs12916
rs75488469
rs2938396
rs4738684
rs9391858
rs4299376
rs2043085
rs646776
rs1836278
rs61679753
rs1800588
rs4876611
rs10020631
rs6124297
rs7534572
rs41279633
rs4794048
rs77542162
rs6437108
rs72838016
rs484084
rs41290120
rs73066485
rs6511720
rs646356
rs6709904
rs28929474
rs375972689
rs2792736
rs4939883
rs77960347
rs6882345
rs2618566
rs58542926
rs693668
rs41289512
rs662138
rs11789603
rs2287927
rs10184673
rs780093
rs7776156
rs80254170
rs6699113
rs867772
rs9376092
rs36012880
rs525028
rs28601761
All - MR Egger
All - Inverse variance weighted
-1
0
1
2
MR effect size for
'Serum total cholesterol levels || id:ebi-cfb233-GCST90302075' on 'ER- Breast cancer (Combined Oncoarray; iCOGS; GWAS me

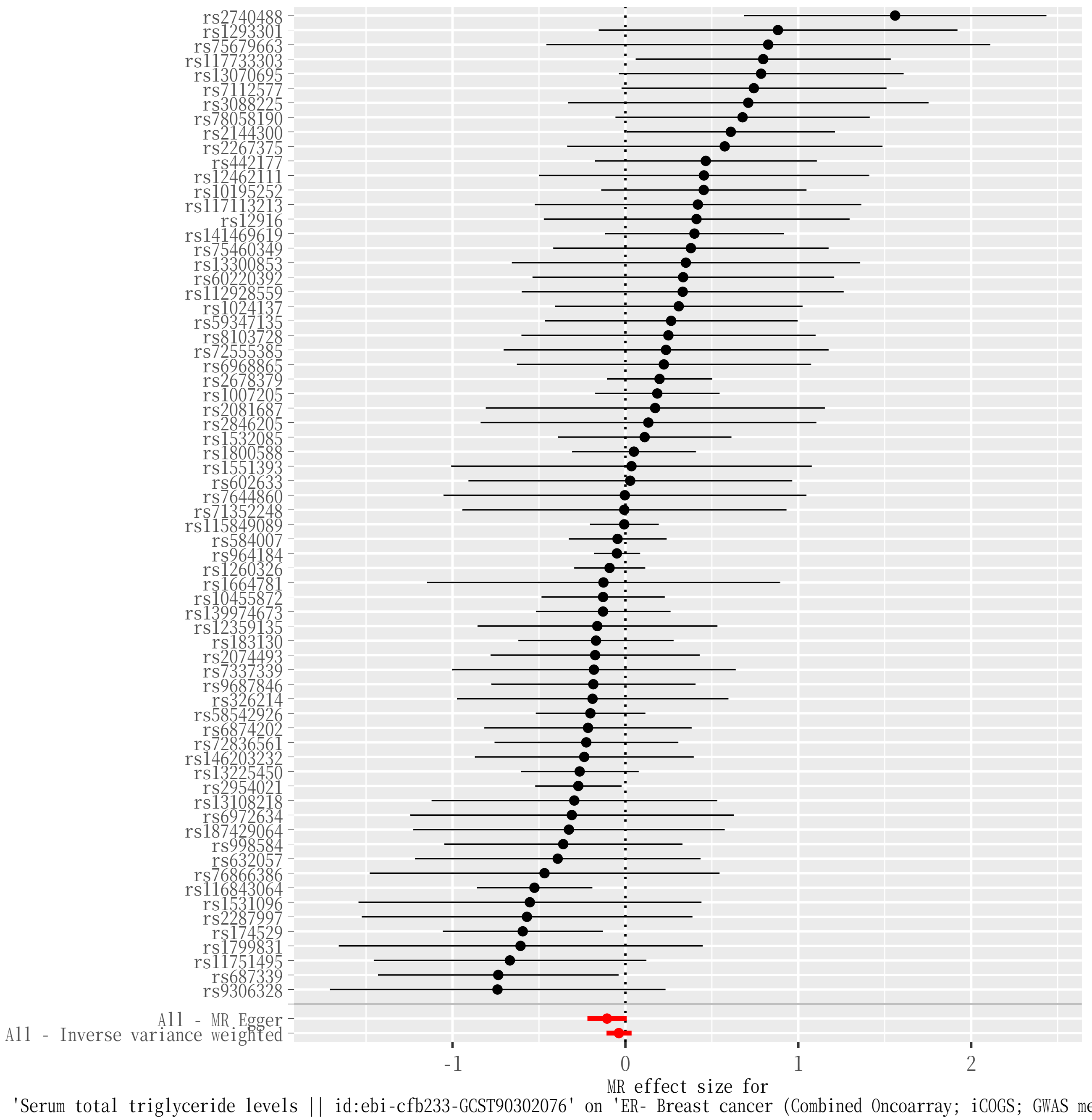
rs2740488
rs1293301
rs75679663
rs117733303
rs13070695
rs7112577
rs3088225
rs78058190
rs2144300
rs2267375
rs442177
rs12462111
rs10195252
rs117113213
rs12916
rs141469619
rs75460349
rs13300853
rs60220392
rs112928559
rs1024137
rs59347135
rs8103728
rs72555385
rs6968865
rs2678379
rs1007205
rs2081687
rs2846205
rs1532085
rs1800588
rs1551393
rs602633
rs7644860
rs71352248
rs115849089
rs584007
rs964184
rs1260326
rs1664781
rs10455872
rs139974673
rs12359135
rs183130
rs2074493
rs7337339
rs9687846
rs326214
rs58542926
rs6874202
rs72836561
rs146203232
rs13225450
rs2954021
rs13108218
rs6972634
rs187429064
rs998584
rs632057
rs76866386
rs116843064
rs1531096
rs2287997
rs174529
rs1799831
rs11751495
rs687339
rs9306328
All - MR Egger
All - Inverse variance weighted
-1
0
1
2
MR effect size for
'Serum total triglyceride levels || id:ebi-cfb233-GCST90302076' on 'ER- Breast cancer (Combined Oncoarray; iCOGS; GWAS me

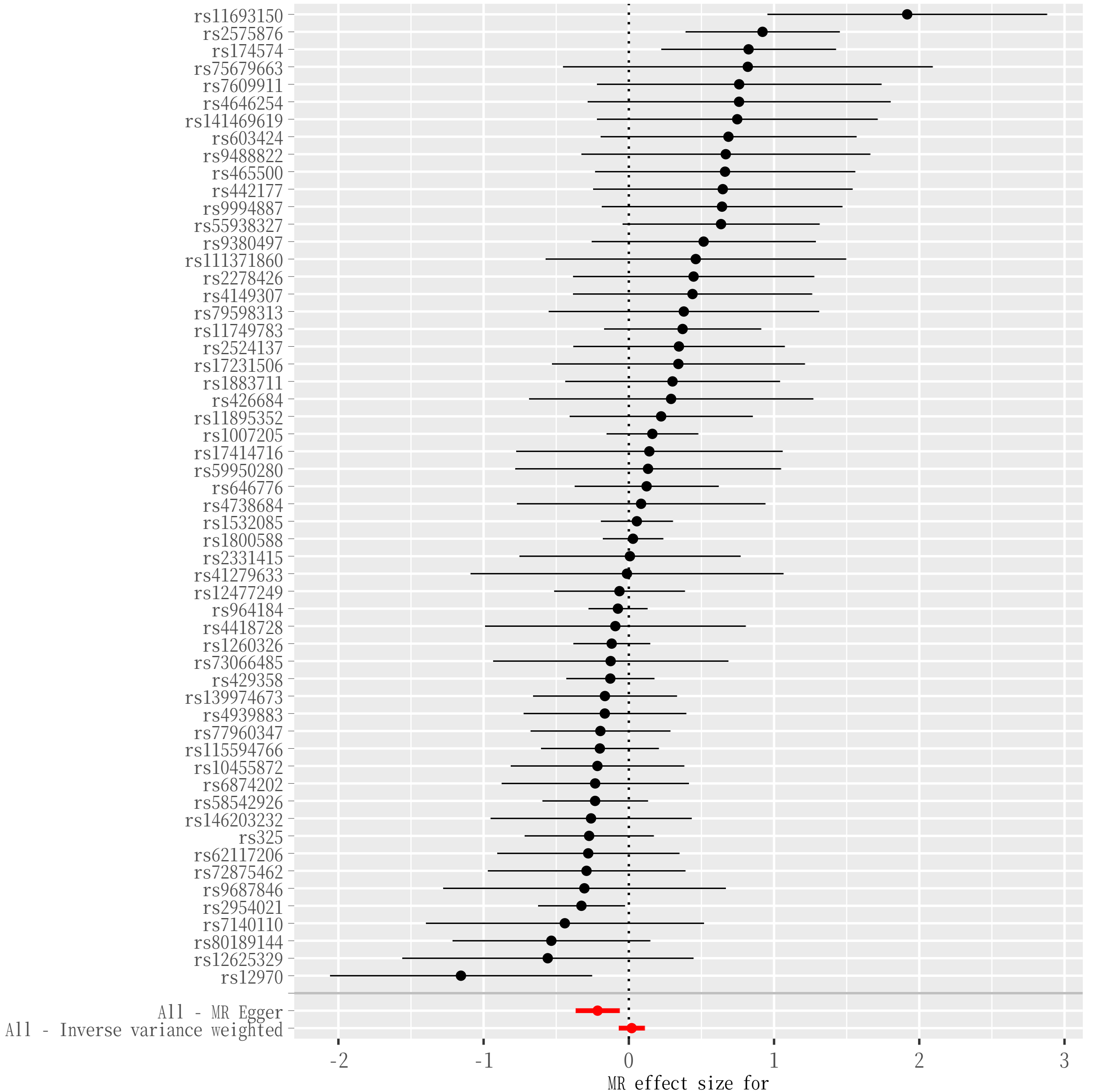

'Saturated fatty acids || id:ebi-cfb233-GCST90302077' on 'ER- Breast cancer (Combined Oncoarray; iCOGS; GWAS meta an

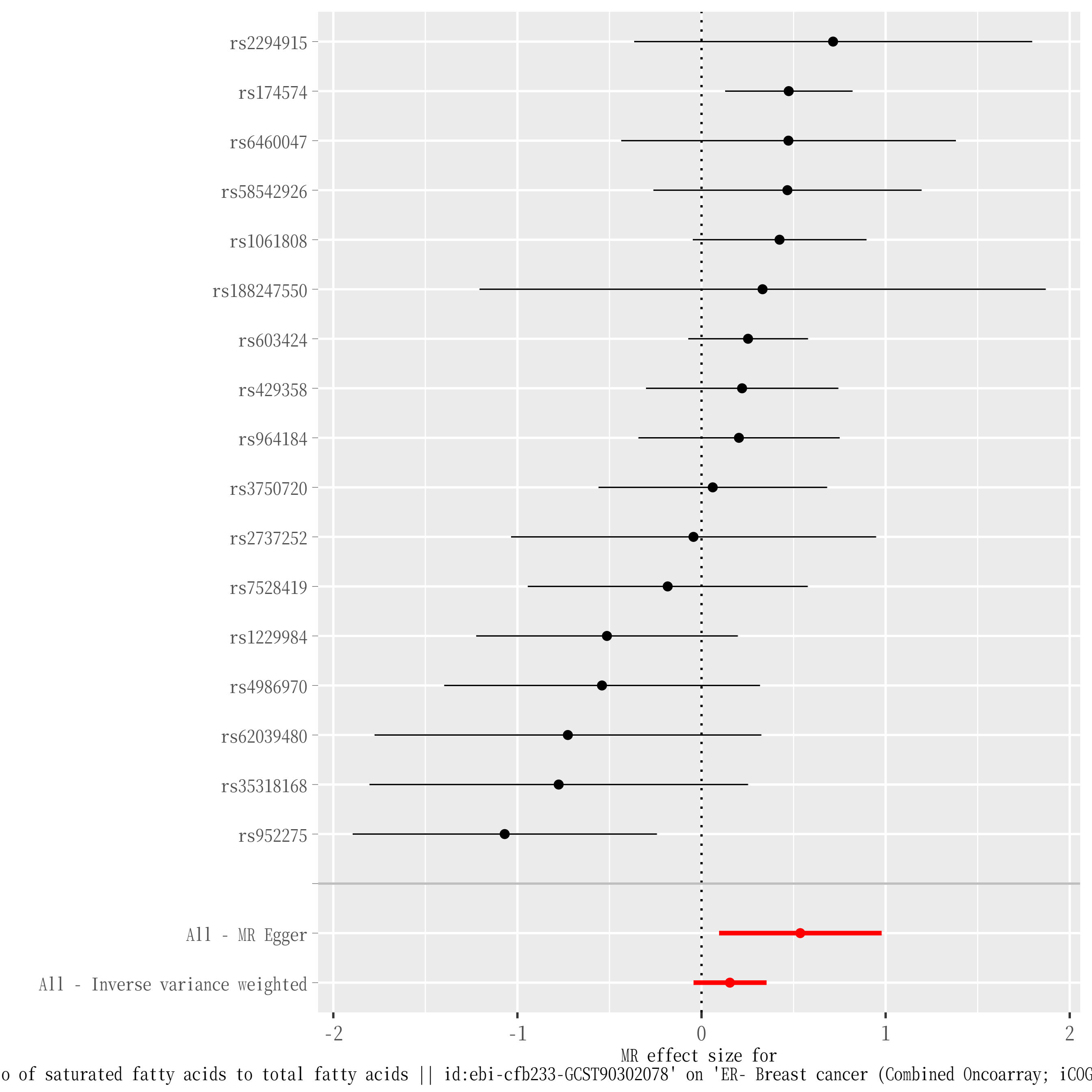
rs2294915
rs174574
rs6460047
rs58542926
rs1061808
rs188247550
rs603424
rs429358
rs964184
rs3750720
rs2737252
rs7528419
rs1229984
rs4986970
rs62039480
rs35318168
rs952275
All - MR Egger
All - Inverse variance weighted
-2
-1
0
1
2
MR effect size for
o of saturated fatty acids to total fatty acids || id:ebi-cfb233-GCST90302078' on 'ER- Breast cancer (Combined Oncoarray; iCOG

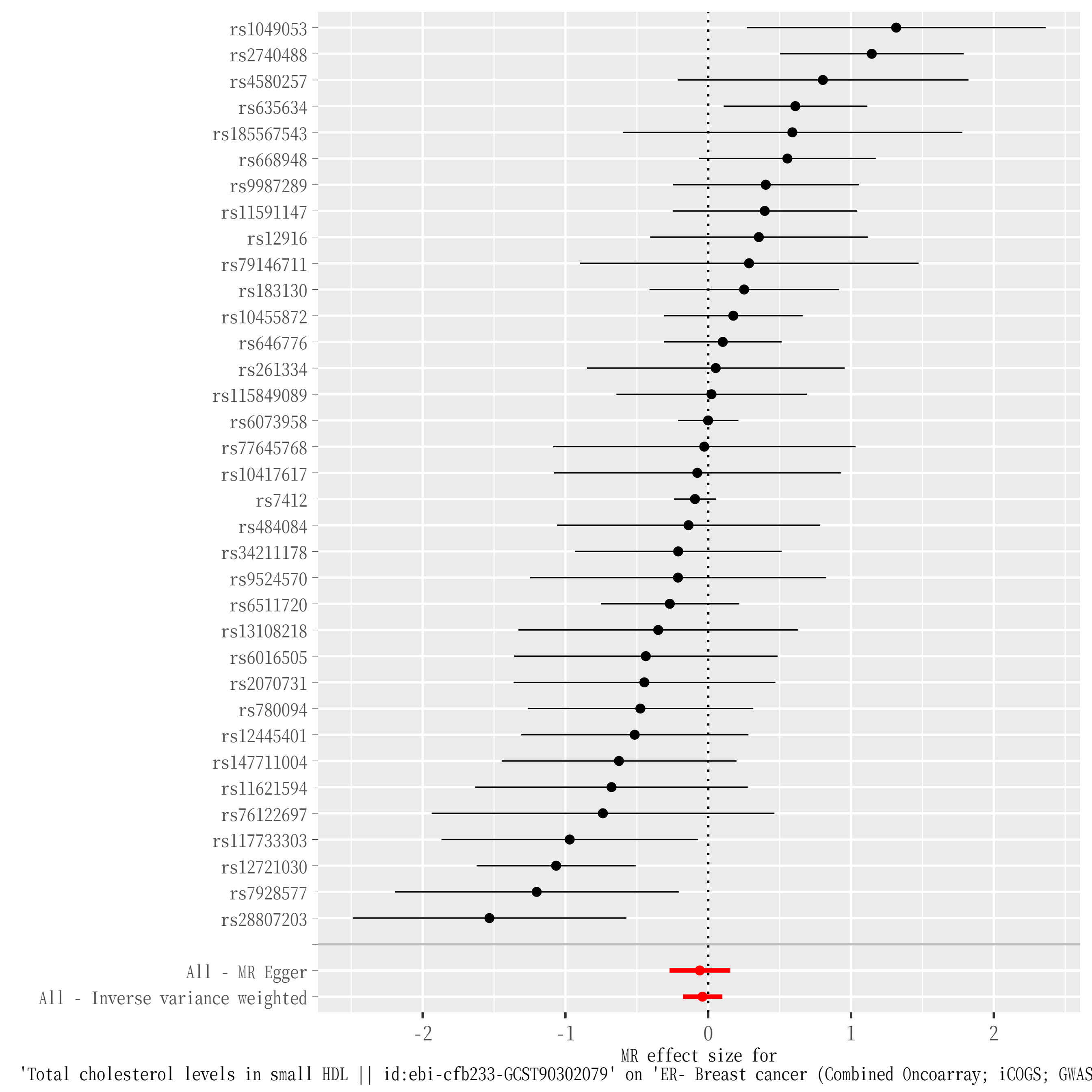

rs1049053
rs2740488
rs4580257
rs635634
rs185567543
rs668948
rs9987289
rs11591147
rs12916
rs79146711
rs183130
rs10455872
rs646776
rs261334
rs115849089
rs6073958
rs77645768
rs10417617
rs7412
rs484084
rs34211178
rs9524570
rs6511720
rs13108218
rs6016505
rs2070731
rs780094
rs12445401
rs147711004
rs11621594
rs76122697
rs117733303
rs12721030
rs7928577
rs28807203
All - MR Egger
All - Inverse variance weighted
-2
-1
0
1
2
MR effect size for
'Total cholesterol levels in small HDL || id:ebi-cfb233-GCST90302079' on 'ER- Breast cancer (Combined Oncoarray; iCOGS; GWAS

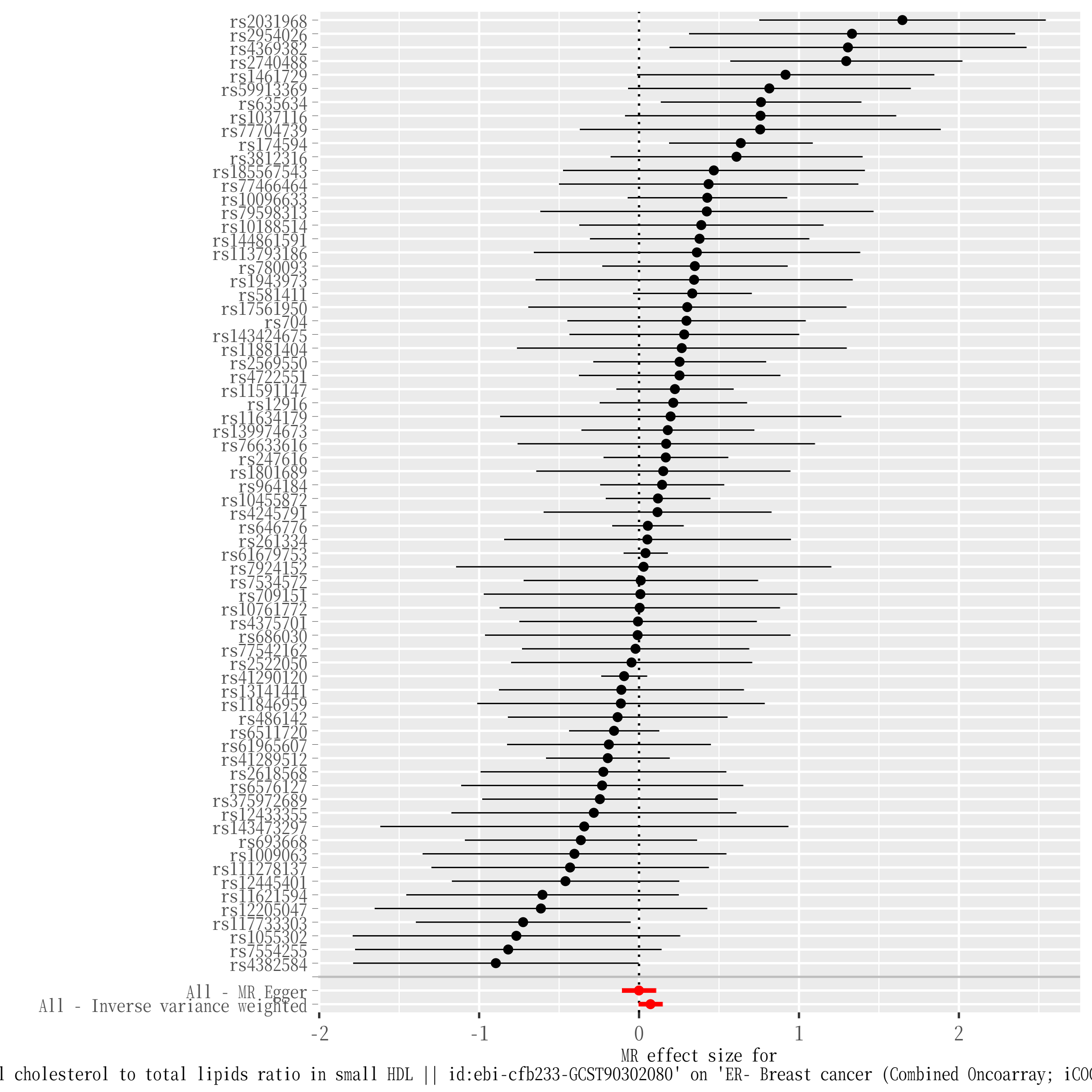

rs2031968
rs2954026
rs4369382
rs2740488
rs1461729
rs59913369
rs635634
rs1037116
rs77704739
rs174594
rs3812316
rs185567543
rs77466464
rs10096633
rs79598313
rs10188514
rs144861591
rs113793186
rs780093
rs1943973
rs581411
rs17561950
rs704
rs143424675
rs11881404
rs2569550
rs4722551
rs11591147
rs12916
rs11634179
rs139974673
rs76633616
rs247616
rs1801689
rs964184
rs10455872
rs4245791
rs646776
rs261334
rs61679753
rs7924152
rs7534572
rs709151
rs10761772
rs4375701
rs686030
rs77542162
rs2522050
rs41290120
rs13141441
rs11846959
rs486142
rs6511720
rs61965607
rs41289512
rs2618568
rs6576127
rs375972689
rs12433355
rs143473297
rs693668
rs1009063
rs111278137
rs12445401
rs11621594
rs12205047
rs117733303
rs1055302
rs7554255
rs4382584
All - MR Egger
All - Inverse variance weighted
-2
-1
0
1
2
MR effect size for
l cholesterol to total lipids ratio in small HDL || id:ebi-cfb233-GCST90302080' on 'ER- Breast cancer (Combined Oncoarray; iCO

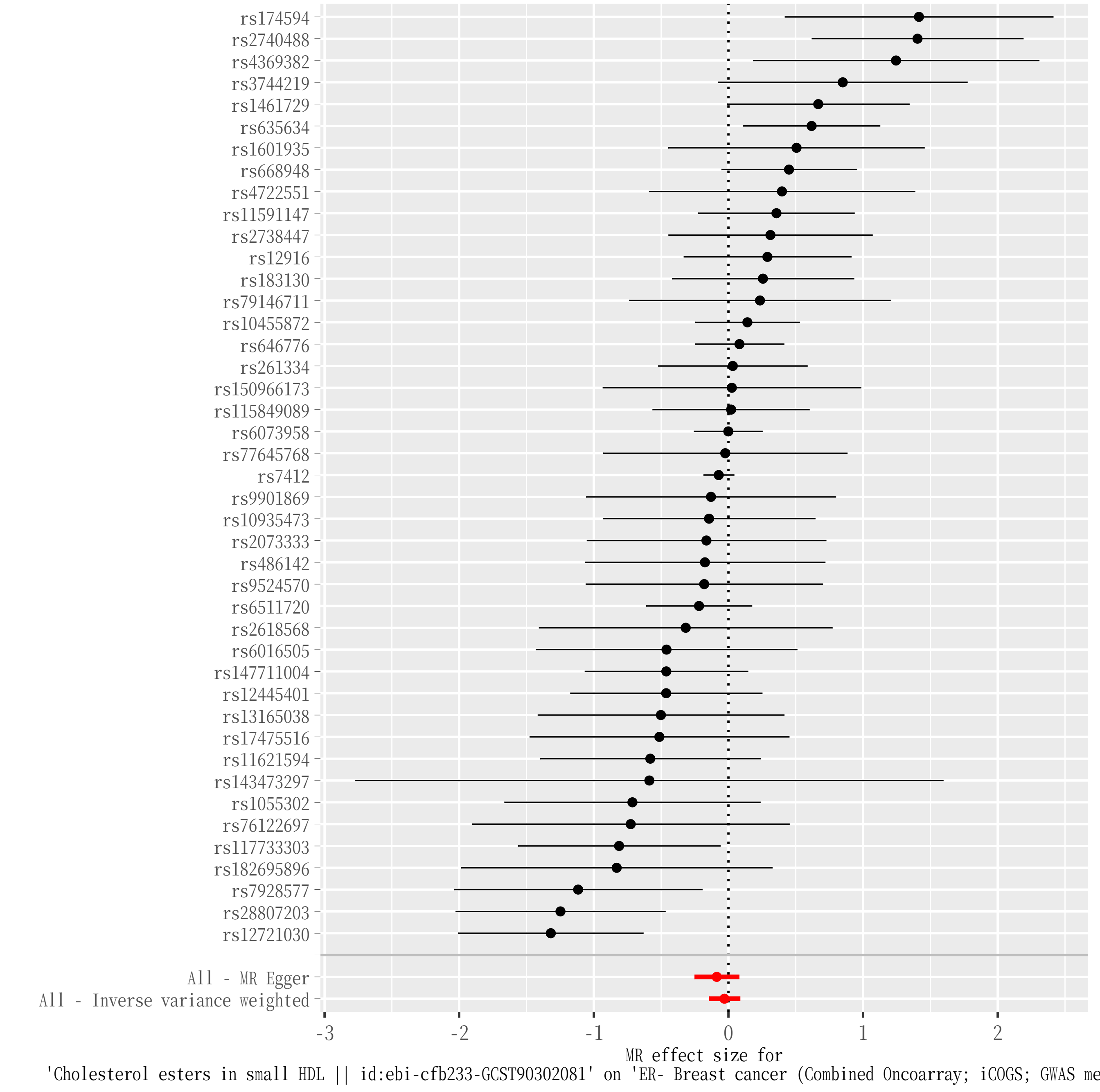
rs174594
rs2740488
rs4369382
rs3744219
rs1461729
rs635634
rs1601935
rs668948
rs4722551
rs11591147
rs2738447
rs12916
rs183130
rs79146711
rs10455872
rs646776
rs261334
rs150966173
rs115849089
rs6073958
rs77645768
rs7412
rs9901869
rs10935473
rs2073333
rs486142
rs9524570
rs6511720
rs2618568
rs6016505
rs147711004
rs12445401
rs13165038
rs17475516
rs11621594
rs143473297
rs1055302
rs76122697
rs117733303
rs182695896
rs7928577
rs28807203
rs12721030
All - MR Egger
All - Inverse variance weighted
-3
-2
-1
0
1
2
MR effect size for
'Cholesterol esters in small HDL || id:ebi-cfb233-GCST90302081' on 'ER- Breast cancer (Combined Oncoarray; iCOGS; GWAS me

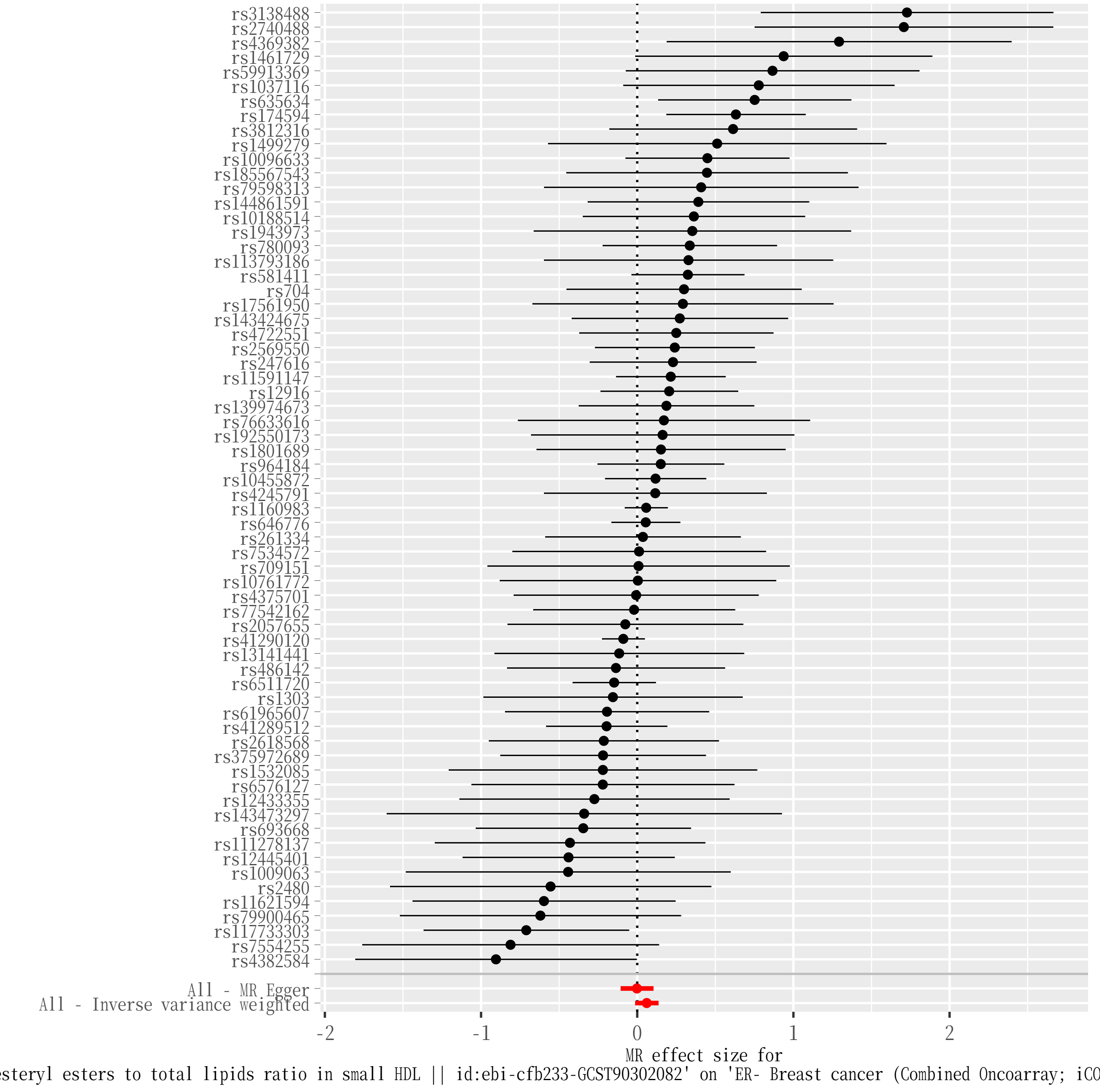
rs3138488
rs2740488
rs4369382
rs1461729
rs59913369
rs1037116
rs635634
rs174594
rs3812316
rs1499279
rs10096633
rs185567543
rs79598313
rs144861591
rs10188514
rs1943973
rs780093
rs113793186
rs581411
rs704
rs17561950
rs143424675
rs4722551
rs2569550
rs247616
rs11591147
rs12916
rs139974673
rs76633616
rs192550173
rs1801689
rs964184
rs10455872
rs4245791
rs1160983
rs646776
rs261334
rs7534572
rs709151
rs10761772
rs4375701
rs77542162
rs2057655
rs41290120
rs13141441
rs486142
rs6511720
rs1303
rs61965607
rs41289512
rs2618568
rs375972689
rs1532085
rs6576127
rs12433355
rs143473297
rs693668
rs111278137
rs12445401
rs1009063
rs2480
rs11621594
rs79900465
rs117733303
rs7554255
rs4382584
All - MR Egger
All - Inverse variance weighted
-2
-1
0
1
2
MR effect size for
esteryl esters to total lipids ratio in small HDL || id:ebi-cfb233-GCST90302082' on 'ER- Breast cancer (Combined Oncoarray; iCO

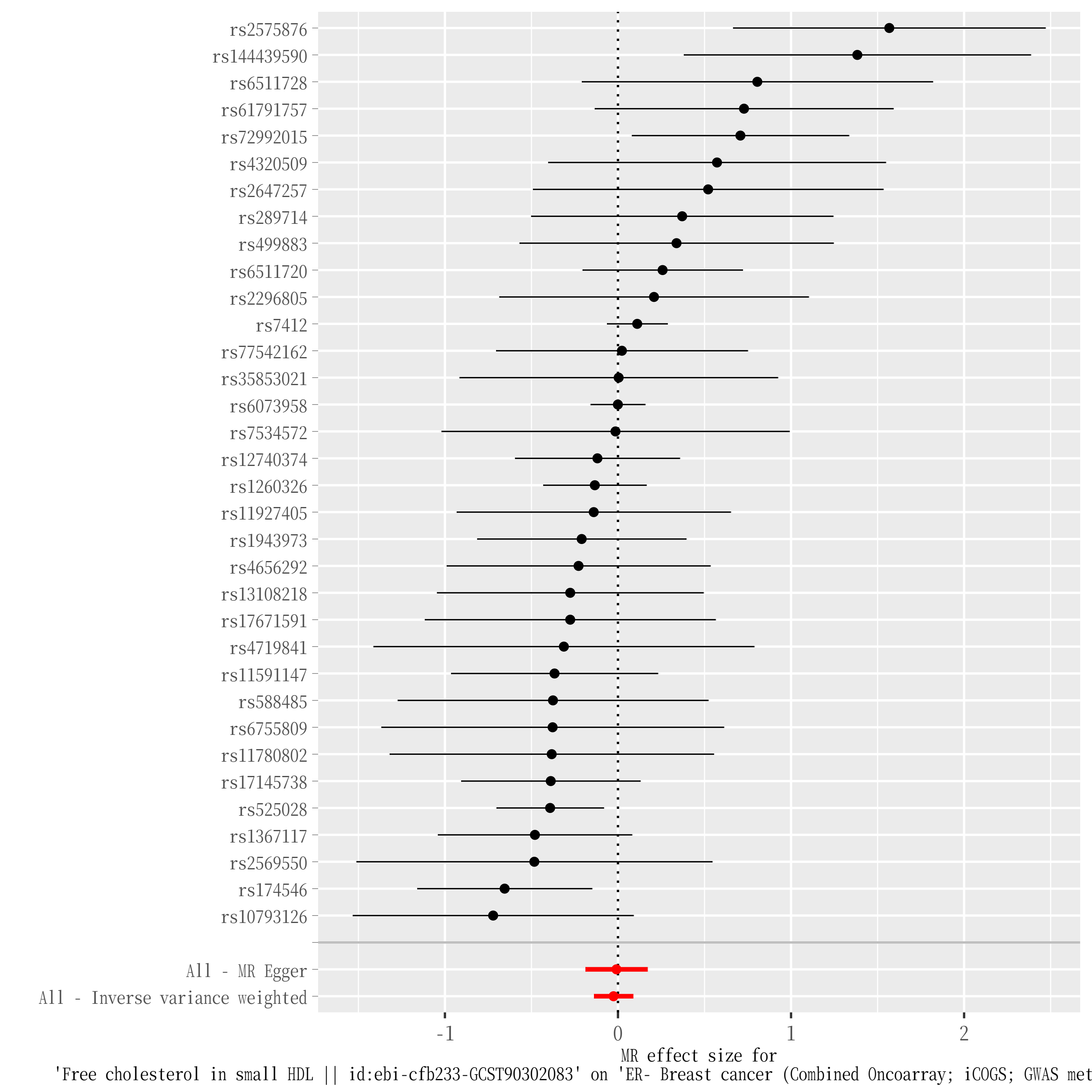
rs2575876
rs144439590
rs6511728
rs61791757
rs72992015
rs4320509
rs2647257
rs289714
rs499883
rs6511720
rs2296805
rs7412
rs77542162
rs35853021
rs6073958
rs7534572
rs12740374
rs1260326
rs11927405
rs1943973
rs4656292
rs13108218
rs17671591
rs4719841
rs11591147
rs588485
rs6755809
rs11780802
rs17145738
rs525028
rs1367117
rs2569550
rs174546
rs10793126
All - MR Egger
All - Inverse variance weighted
-1
0
1
2
MR effect size for
'Free cholesterol in small HDL || id:ebi-cfb233-GCST90302083' on 'ER- Breast cancer (Combined Oncoarray; iCOGS; GWAS met

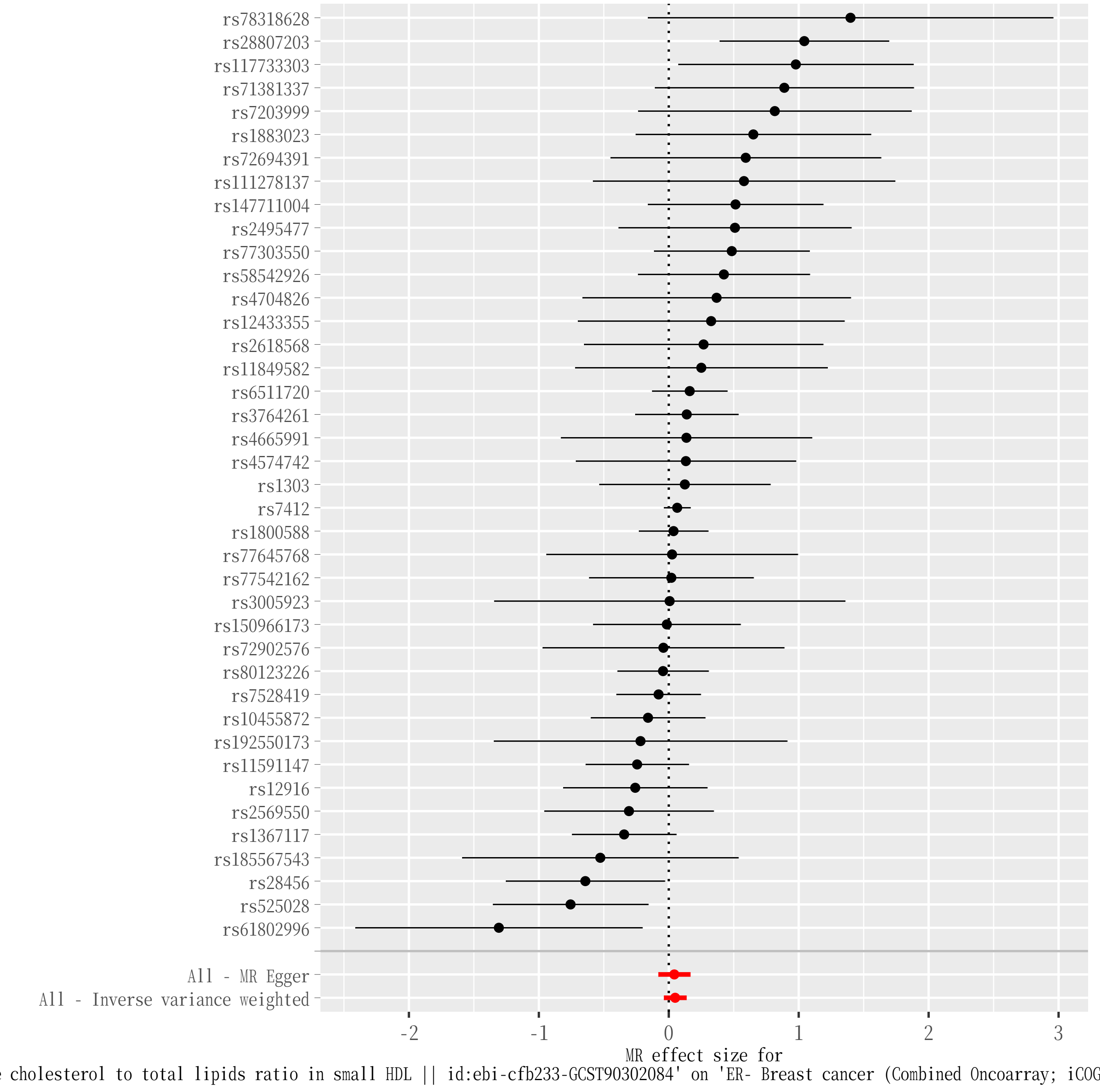

rs78318628
rs28807203
rs117733303
rs71381337
rs7203999
rs1883023
rs72694391
rs111278137
rs147711004
rs2495477
rs77303550
rs58542926
rs4704826
rs12433355
rs2618568
rs11849582
rs6511720
rs3764261
rs4665991
rs4574742
rs1303
rs7412
rs1800588
rs77645768
rs77542162
rs3005923
rs150966173
rs72902576
rs80123226
rs7528419
rs10455872
rs192550173
rs11591147
rs12916
rs2569550
rs1367117
rs185567543
rs28456
rs525028
rs61802996
All - MR Egger
All - Inverse variance weighted
-2
-1
0
1
2
3
MR effect size for
e cholesterol to total lipids ratio in small HDL || id:ebi-cfb233-GCST90302084' on 'ER- Breast cancer (Combined Oncoarray; iCOG

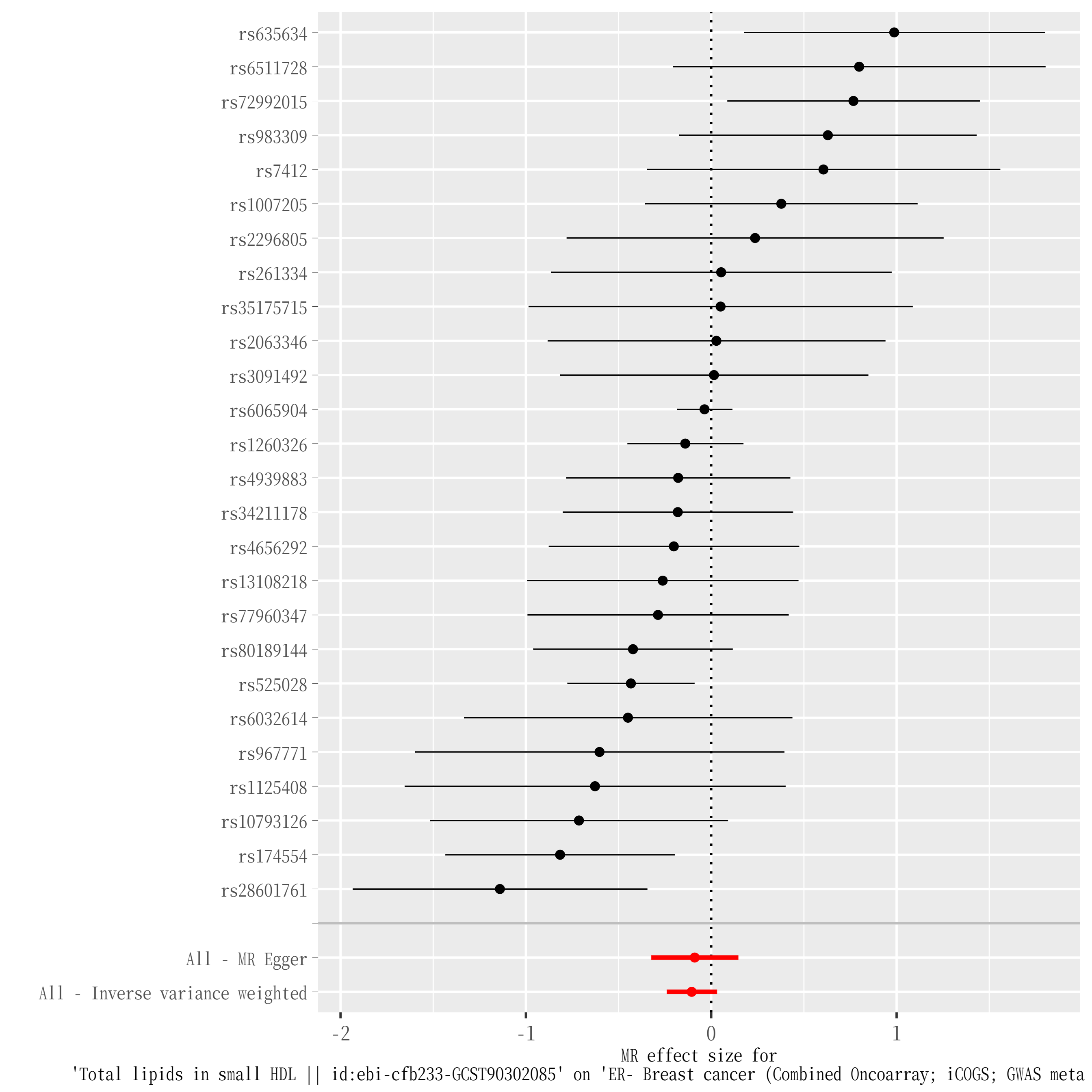
rs635634
rs6511728
rs72992015
rs983309
rs7412
rs1007205
rs2296805
rs261334
rs35175715
rs2063346
rs3091492
rs6065904
rs1260326
rs4939883
rs34211178
rs4656292
rs13108218
rs77960347
rs80189144
rs525028
rs6032614
rs967771
rs1125408
rs10793126
rs174554
rs28601761
All - MR Egger
All - Inverse variance weighted
-2
-1
0
1
MR effect size for
'Total lipids in small HDL || id:ebi-cfb233-GCST90302085' on 'ER- Breast cancer (Combined Oncoarray; iCOGS; GWAS meta

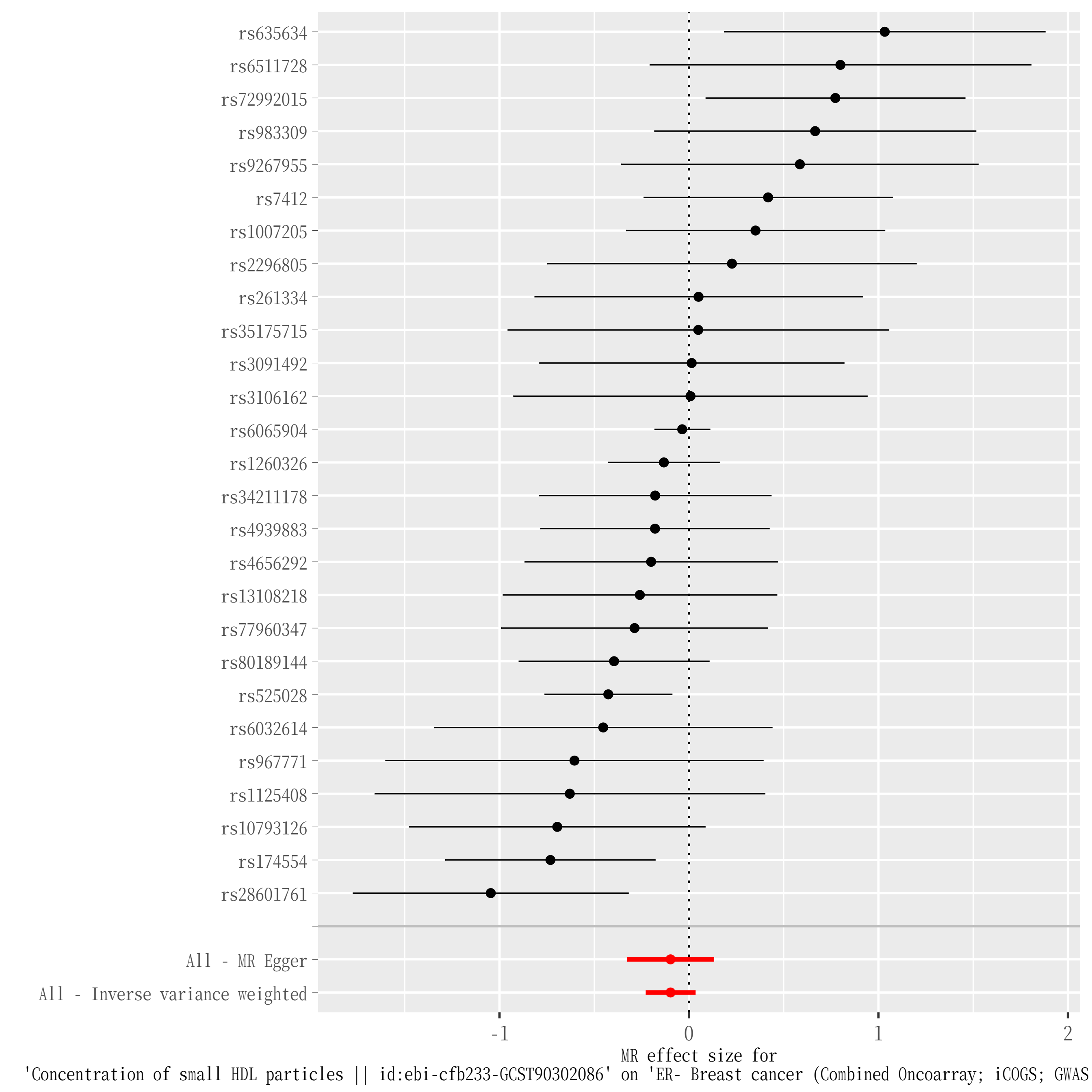
rs635634
rs6511728
rs72992015
rs983309
rs9267955
rs7412
rs1007205
rs2296805
rs261334
rs35175715
rs3091492
rs3106162
rs6065904
rs1260326
rs34211178
rs4939883
rs4656292
rs13108218
rs77960347
rs80189144
rs525028
rs6032614
rs967771
rs1125408
rs10793126
rs174554
rs28601761
All - MR Egger
All - Inverse variance weighted
-1
0
1
2
MR effect size for
'Concentration of small HDL particles || id:ebi-cfb233-GCST90302086' on 'ER- Breast cancer (Combined Oncoarray; iCOGS; GWAS

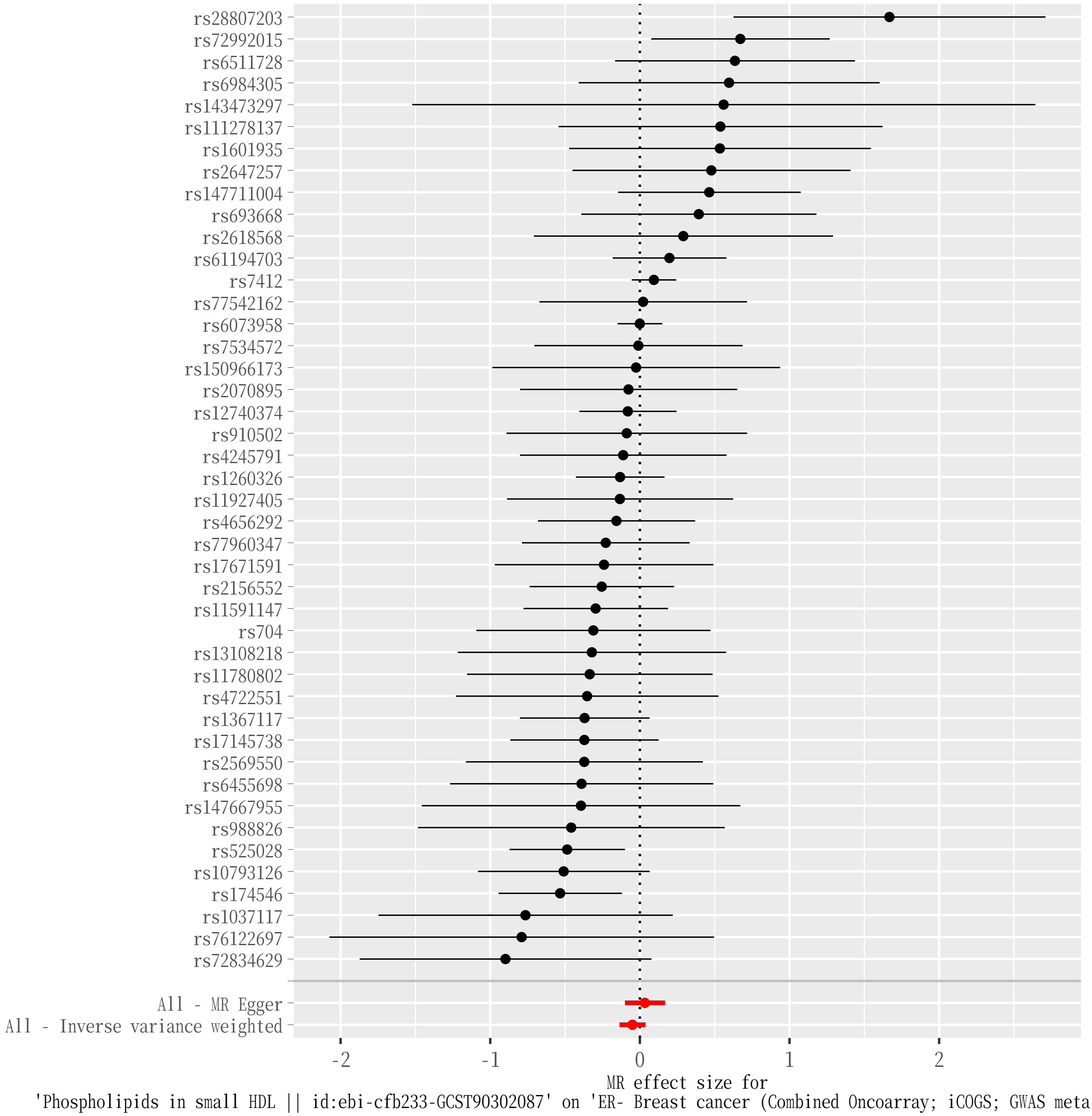
rs28807203
rs72992015
rs6511728
rs6984305
rs143473297
rs111278137
rs1601935
rs2647257
rs147711004
rs693668
rs2618568
rs61194703
rs7412
rs77542162
rs6073958
rs7534572
rs150966173
rs2070895
rs12740374
rs910502
rs4245791
rs1260326
rs11927405
rs4656292
rs77960347
rs17671591
rs2156552
rs11591147
rs704
rs13108218
rs11780802
rs4722551
rs1367117
rs17145738
rs2569550
rs6455698
rs147667955
rs988826
rs525028
rs10793126
rs174546
rs1037117
rs76122697
rs72834629
All - MR Egger
All - Inverse variance weighted
-2
-1
0
1
2
MR effect size for
'Phospholipids in small HDL || id:ebi-cfb233-GCST90302087' on 'ER- Breast cancer (Combined Oncoarray; iCOGS; GWAS meta

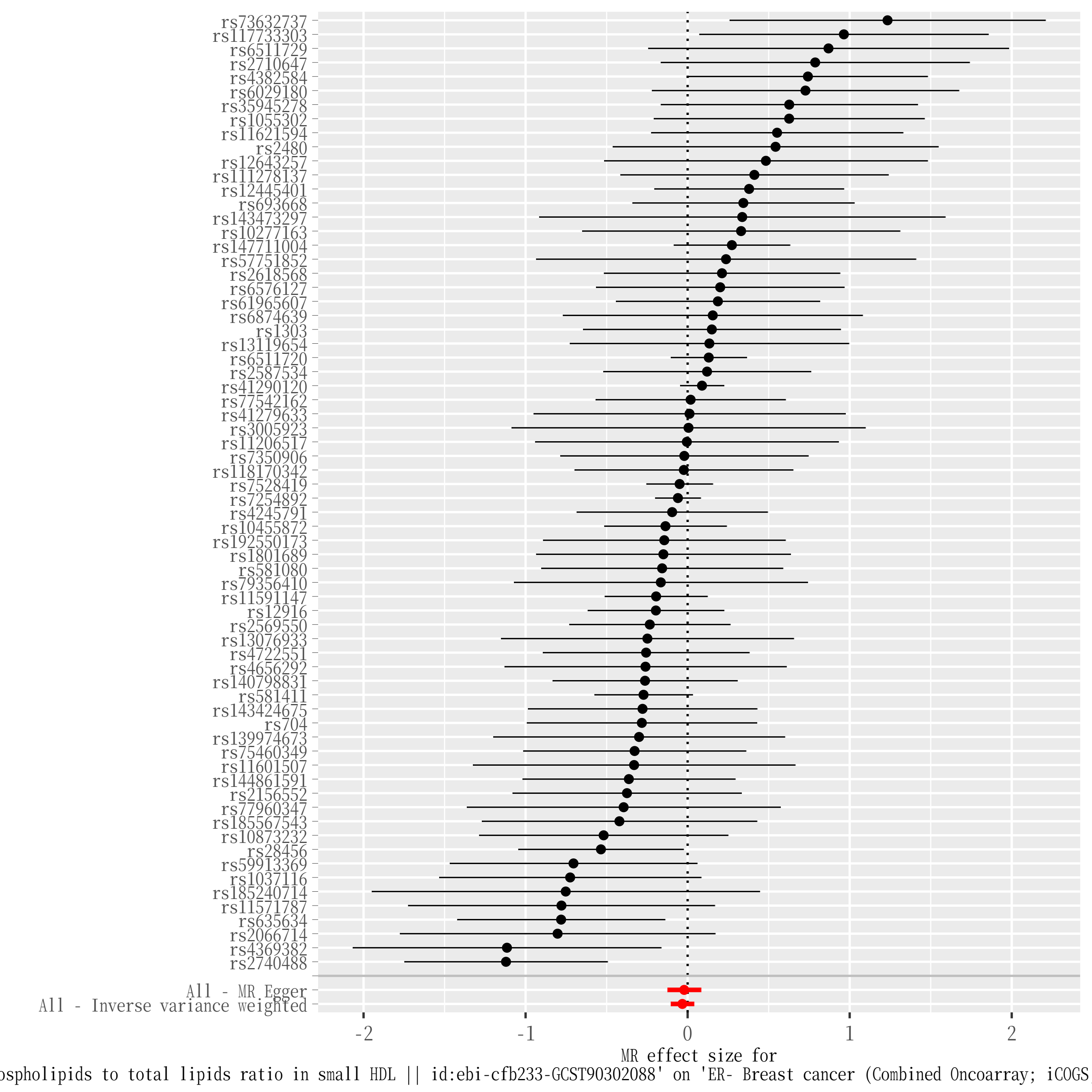
rs73632737
rs117733303
rs6511729
rs2710647
rs4382584
rs6029180
rs35945278
rs1055302
rs11621594
rs2480
rs12643257
rs111278137
rs12445401
rs693668
rs143473297
rs10277163
rs147711004
rs57751852
rs2618568
rs6576127
rs61965607
rs6874639
rs1303
rs13119654
rs6511720
rs2587534
rs41290120
rs77542162
rs41279633
rs3005923
rs11206517
rs7350906
rs118170342
rs7528419
rs7254892
rs4245791
rs10455872
rs192550173
rs1801689
rs581080
rs79356410
rs11591147
rs12916
rs2569550
rs13076933
rs4722551
rs4656292
rs140798831
rs581411
rs143424675
rs704
rs139974673
rs75460349
rs11601507
rs144861591
rs2156552
rs77960347
rs185567543
rs10873232
rs28456
rs59913369
rs1037116
rs185240714
rs11571787
rs635634
rs2066714
rs4369382
rs2740488
All - MR Egger
All - Inverse variance weighted
-2
-1
0
1
2
MR effect size for
ospholipids to total lipids ratio in small HDL || id:ebi-cfb233-GCST90302088' on 'ER- Breast cancer (Combined Oncoarray; iCOGS

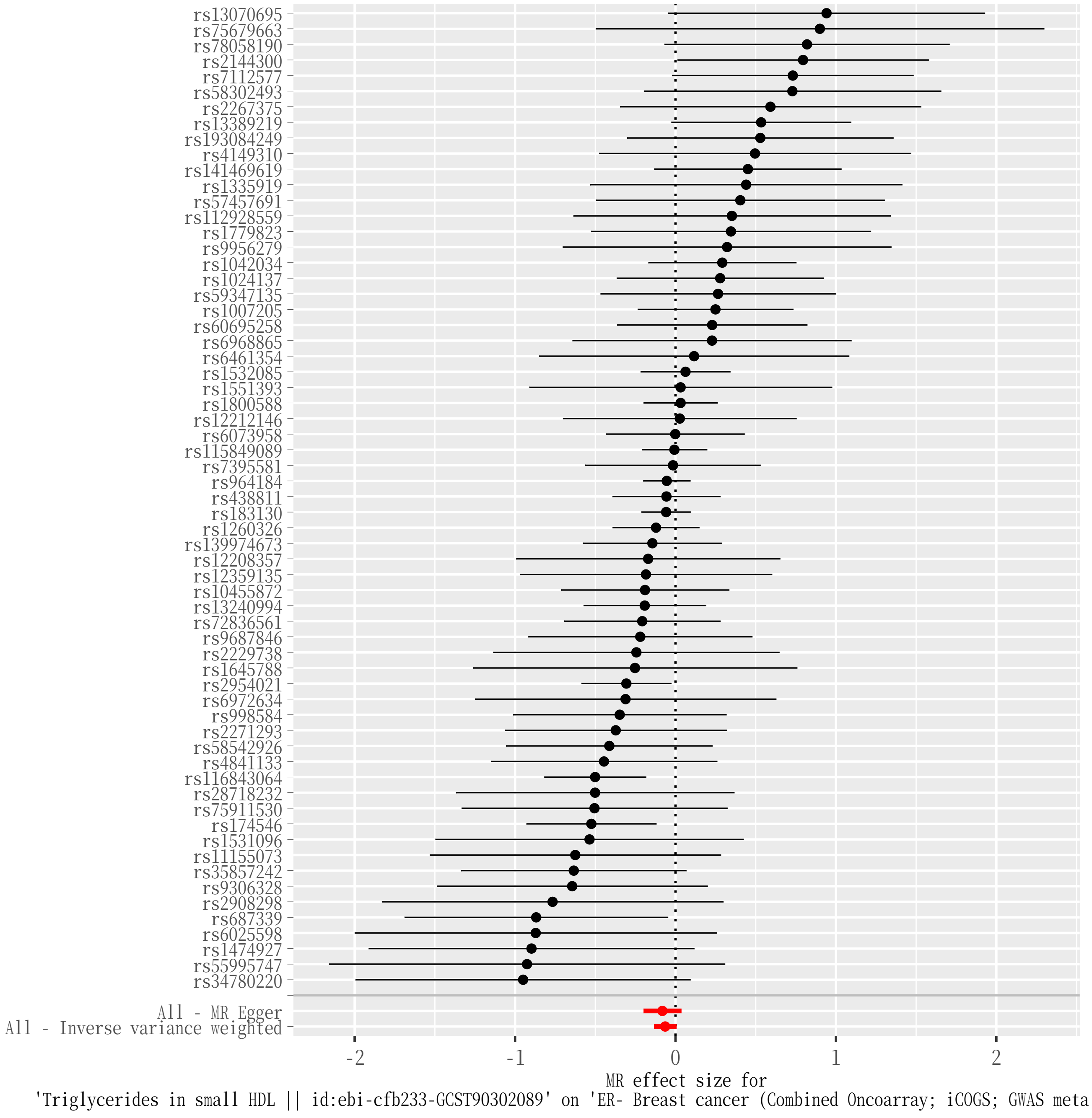

rs13070695
rs75679663
rs78058190
rs2144300
rs7112577
rs58302493
rs2267375
rs13389219
rs193084249
rs4149310
rs141469619
rs1335919
rs57457691
rs112928559
rs1779823
rs9956279
rs1042034
rs1024137
rs59347135
rs1007205
rs60695258
rs6968865
rs6461354
rs1532085
rs1551393
rs1800588
rs12212146
rs6073958
rs115849089
rs7395581
rs964184
rs438811
rs183130
rs1260326
rs139974673
rs12208357
rs12359135
rs10455872
rs13240994
rs72836561
rs9687846
rs2229738
rs1645788
rs2954021
rs6972634
rs998584
rs2271293
rs58542926
rs4841133
rs116843064
rs28718232
rs75911530
rs174546
rs1531096
rs11155073
rs35857242
rs9306328
rs2908298
rs687339
rs6025598
rs1474927
rs55995747
rs34780220
All - MR Egger
All - Inverse variance weighted
-2
-1
0
1
2
MR effect size for
'Triglycerides in small HDL || id:ebi-cfb233-GCST90302089' on 'ER- Breast cancer (Combined Oncoarray; iCOGS; GWAS meta

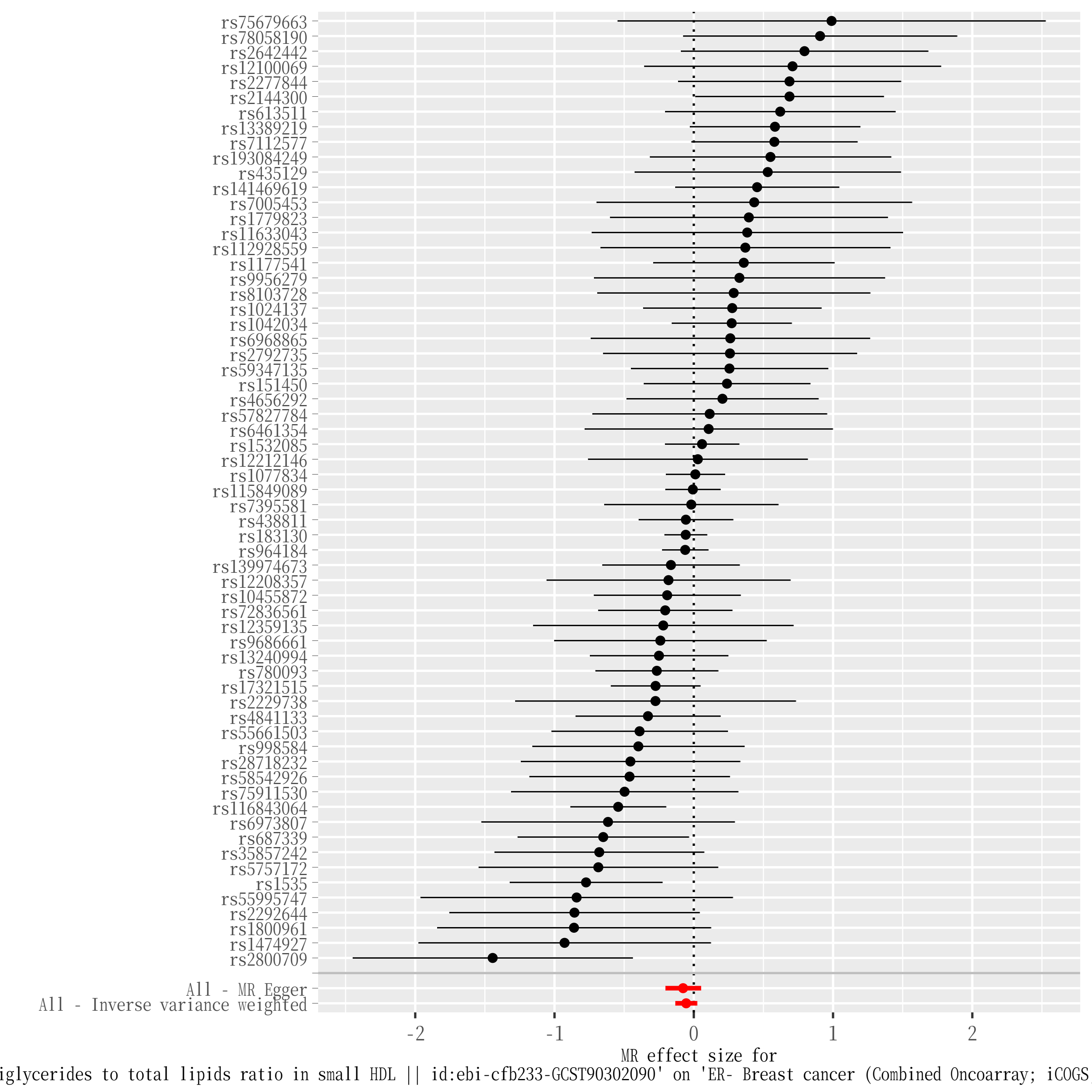
rs75679663
rs78058190
rs2642442
rs12100069
rs2277844
rs2144300
rs613511
rs13389219
rs7112577
rs193084249
rs435129
rs141469619
rs7005453
rs1779823
rs11633043
rs112928559
rs1177541
rs9956279
rs8103728
rs1024137
rs1042034
rs6968865
rs2792735
rs59347135
rs151450
rs4656292
rs57827784
rs6461354
rs1532085
rs12212146
rs1077834
rs115849089
rs7395581
rs438811
rs183130
rs964184
rs139974673
rs12208357
rs10455872
rs72836561
rs12359135
rs9686661
rs13240994
rs780093
rs17321515
rs2229738
rs4841133
rs55661503
rs998584
rs28718232
rs58542926
rs75911530
rs116843064
rs6973807
rs687339
rs35857242
rs5757172
rs1535
rs55995747
rs2292644
rs1800961
rs1474927
rs2800709
All - MR Egger
All - Inverse variance weighted
-2
-1
0
1
2
MR effect size for
iglycerides to total lipids ratio in small HDL || id:ebi-cfb233-GCST90302090' on 'ER- Breast cancer (Combined Oncoarray; iCOGS

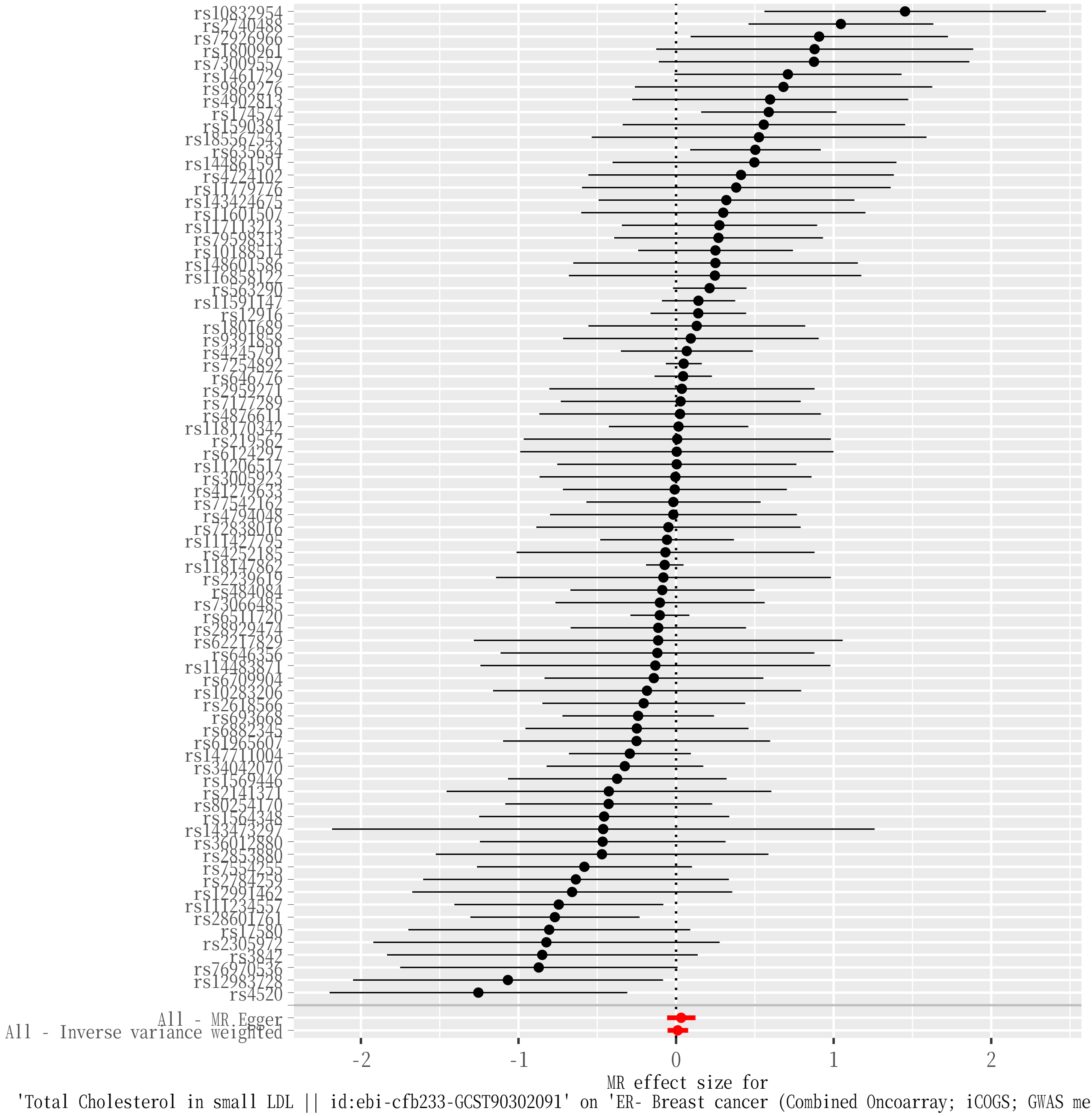
rs10832954
rs2740488
rs72926966
rs1800961
rs73009557
rs1461729
rs9869276
rs4902813
rs174574
rs1590381
rs185567543
rs635634
rs144861591
rs4724102
rs11779776
rs143424675
rs11601507
rs117113213
rs79598313
rs10188514
rs148601586
rs116858122
rs563290
rs11591147
rs12916
rs1801689
rs9391858
rs4245791
rs7254892
rs646776
rs2959271
rs7177289
rs4876611
rs118170342
rs219562
rs6124297
rs11206517
rs3005923
rs41279633
rs77542162
rs4794048
rs72838016
rs111427795
rs4252185
rs118147862
rs2239619
rs484084
rs73066485
rs6511720
rs28929474
rs62217829
rs646356
rs114483871
rs6709904
rs10283206
rs2618566
rs693668
rs6882345
rs61965607
rs147711004
rs34042070
rs1569446
rs2141371
rs80254170
rs1564348
rs143473297
rs36012880
rs2853880
rs7554255
rs2784259
rs12991462
rs111234557
rs28601761
rs17580
rs2305972
rs3842
rs76970536
rs12983728
rs4520
All - MR Egger
All - Inverse variance weighted
-2
-1
0
1
2
MR effect size for
'Total Cholesterol in small LDL || id:ebi-cfb233-GCST90302091' on 'ER- Breast cancer (Combined Oncoarray; iCOGS; GWAS me

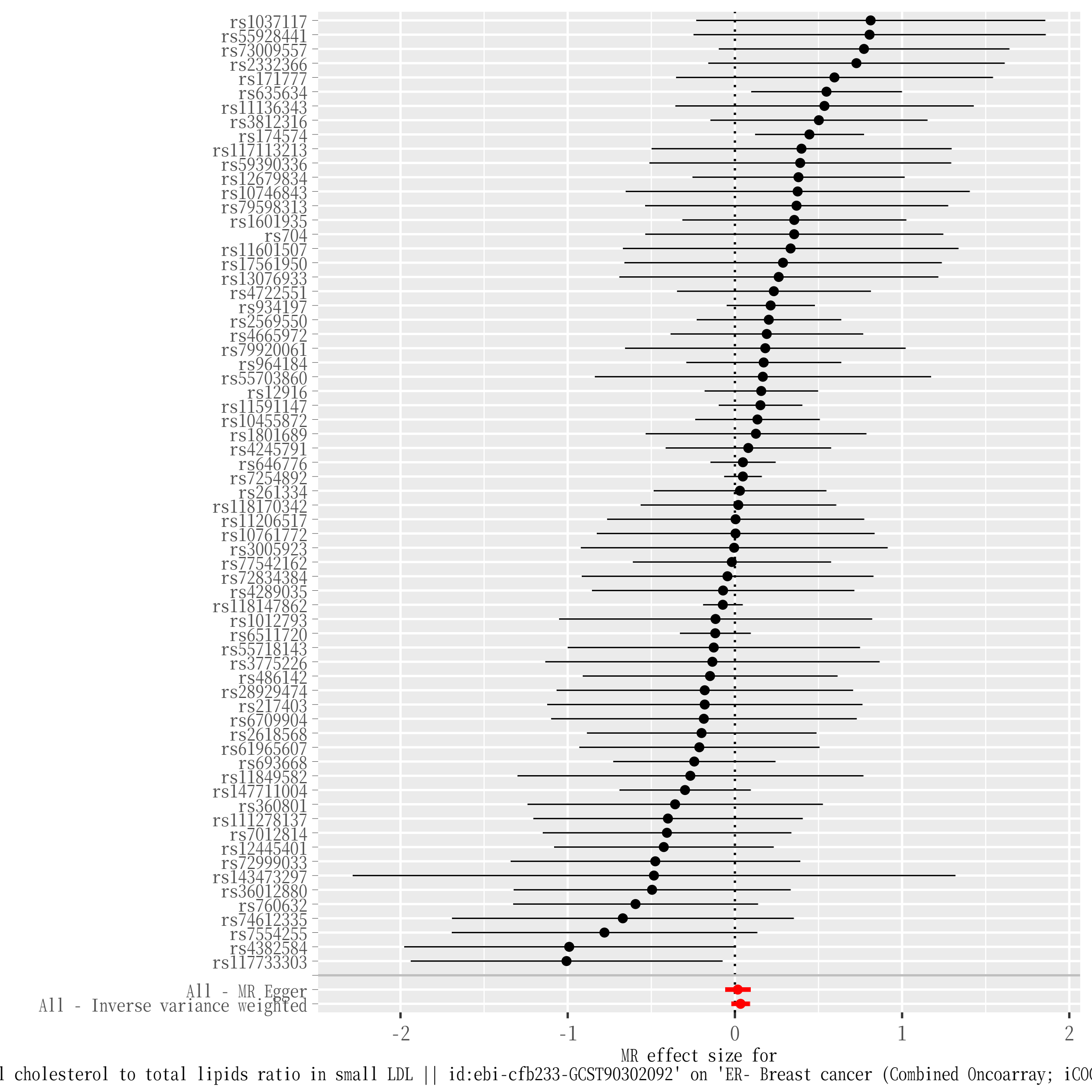
rs1037117
rs55928441
rs73009557
rs2332366
rs171777
rs635634
rs11136343
rs3812316
rs174574
rs117113213
rs59390336
rs12679834
rs10746843
rs79598313
rs1601935
rs704
rs11601507
rs17561950
rs13076933
rs4722551
rs934197
rs2569550
rs4665972
rs79920061
rs964184
rs55703860
rs12916
rs11591147
rs10455872
rs1801689
rs4245791
rs646776
rs7254892
rs261334
rs118170342
rs11206517
rs10761772
rs3005923
rs77542162
rs72834384
rs4289035
rs118147862
rs1012793
rs6511720
rs55718143
rs3775226
rs486142
rs28929474
rs217403
rs6709904
rs2618568
rs61965607
rs693668
rs11849582
rs147711004
rs360801
rs111278137
rs7012814
rs12445401
rs72999033
rs143473297
rs36012880
rs760632
rs74612335
rs7554255
rs4382584
rs117733303
All - MR Egger
All - Inverse variance weighted
-2
-1
0
1
2
MR effect size for
l cholesterol to total lipids ratio in small LDL || id:ebi-cfb233-GCST90302092' on 'ER- Breast cancer (Combined Oncoarray; iCO

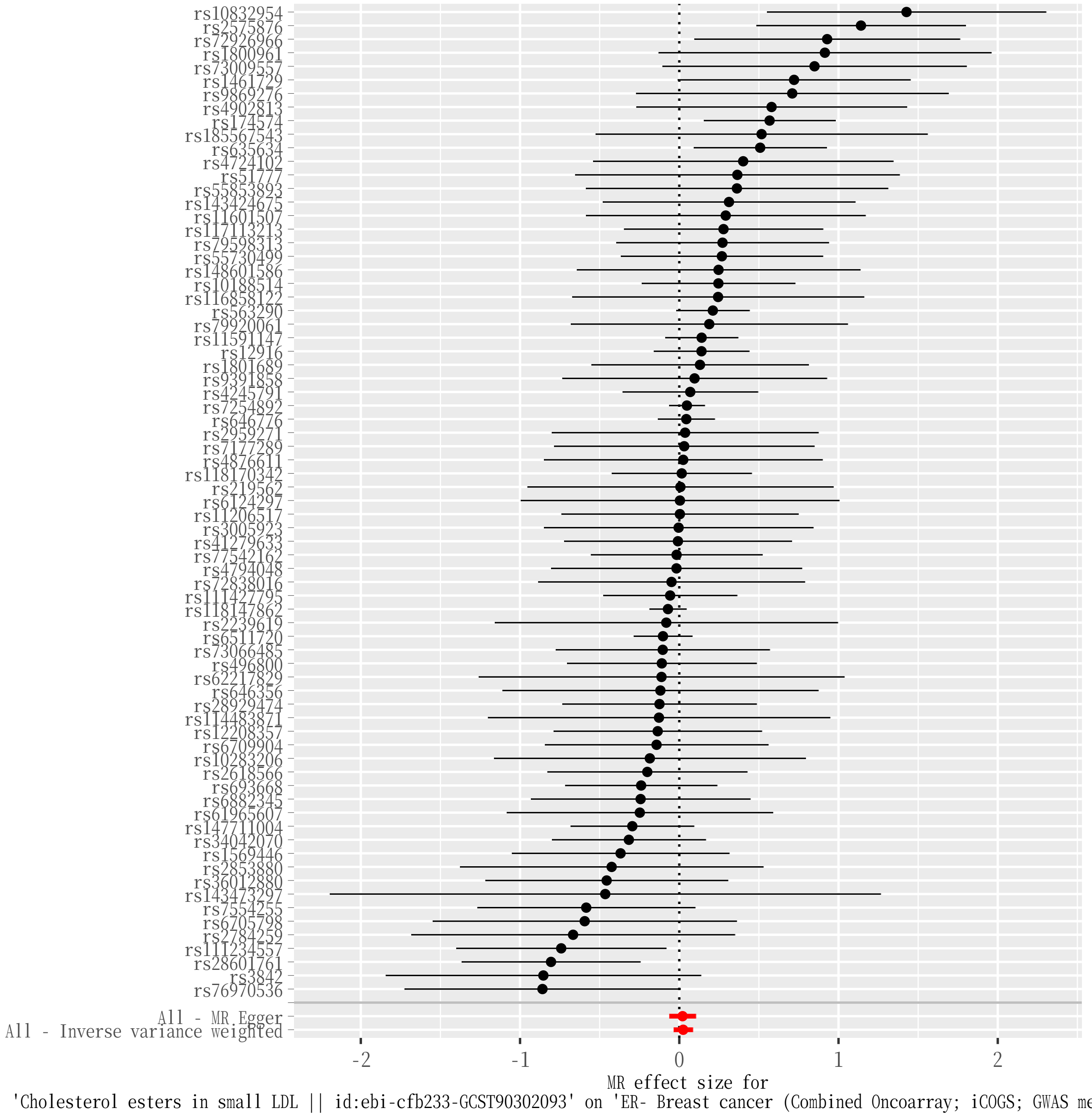
rs10832954
rs2575876
rs72926966
rs1800961
rs73009557
rs1461729
rs9869276
rs4902813
rs174574
rs185567543
rs635634
rs4724102
rs51777
rs55853893
rs143424675
rs11601507
rs117113213
rs79598313
rs55730499
rs148601586
rs10188514
rs116858122
rs563290
rs79920061
rs11591147
rs12916
rs1801689
rs9391858
rs4245791
rs7254892
rs646776
rs2959271
rs7177289
rs4876611
rs118170342
rs219562
rs6124297
rs11206517
rs3005923
rs41279633
rs77542162
rs4794048
rs72838016
rs111427795
rs118147862
rs2239619
rs6511720
rs73066485
rs496800
rs62217829
rs646356
rs28929474
rs114483871
rs12208357
rs6709904
rs10283206
rs2618566
rs693668
rs6882345
rs61965607
rs147711004
rs34042070
rs1569446
rs2853880
rs36012880
rs143473297
rs7554255
rs6705798
rs2784259
rs111234557
rs28601761
rs3842
rs76970536
All - MR Egger
All - Inverse variance weighted
-2
-1
0
1
2
MR effect size for
'Cholesterol esters in small LDL || id:ebi-cfb233-GCST90302093' on 'ER- Breast cancer (Combined Oncoarray; iCOGS; GWAS me

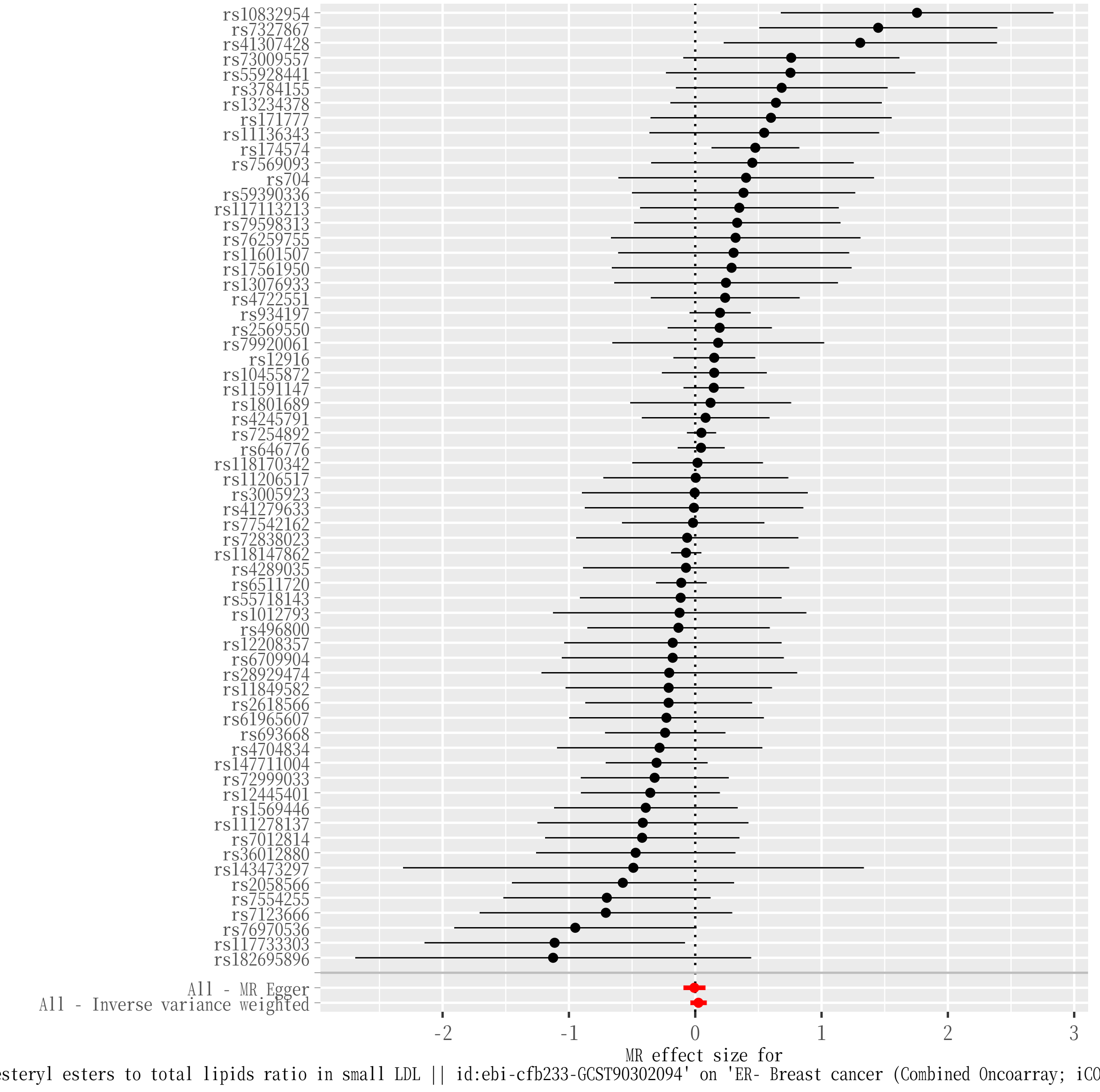
rs10832954
rs7327867
rs41307428
rs73009557
rs55928441
rs3784155
rs13234378
rs171777
rs11136343
rs174574
rs7569093
rs704
rs59390336
rs117113213
rs79598313
rs76259755
rs11601507
rs17561950
rs13076933
rs4722551
rs934197
rs2569550
rs79920061
rs12916
rs10455872
rs11591147
rs1801689
rs4245791
rs7254892
rs646776
rs118170342
rs11206517
rs3005923
rs41279633
rs77542162
rs72838023
rs118147862
rs4289035
rs6511720
rs55718143
rs1012793
rs496800
rs12208357
rs6709904
rs28929474
rs11849582
rs2618566
rs61965607
rs693668
rs4704834
rs147711004
rs72999033
rs12445401
rs1569446
rs111278137
rs7012814
rs36012880
rs143473297
rs2058566
rs7554255
rs7123666
rs76970536
rs117733303
rs182695896
All - MR Egger
All - Inverse variance weighted
-2
-1
0
1
2
3
MR effect size for
esteryl esters to total lipids ratio in small LDL || id:ebi-cfb233-GCST90302094' on 'ER- Breast cancer (Combined Oncoarray; iC0

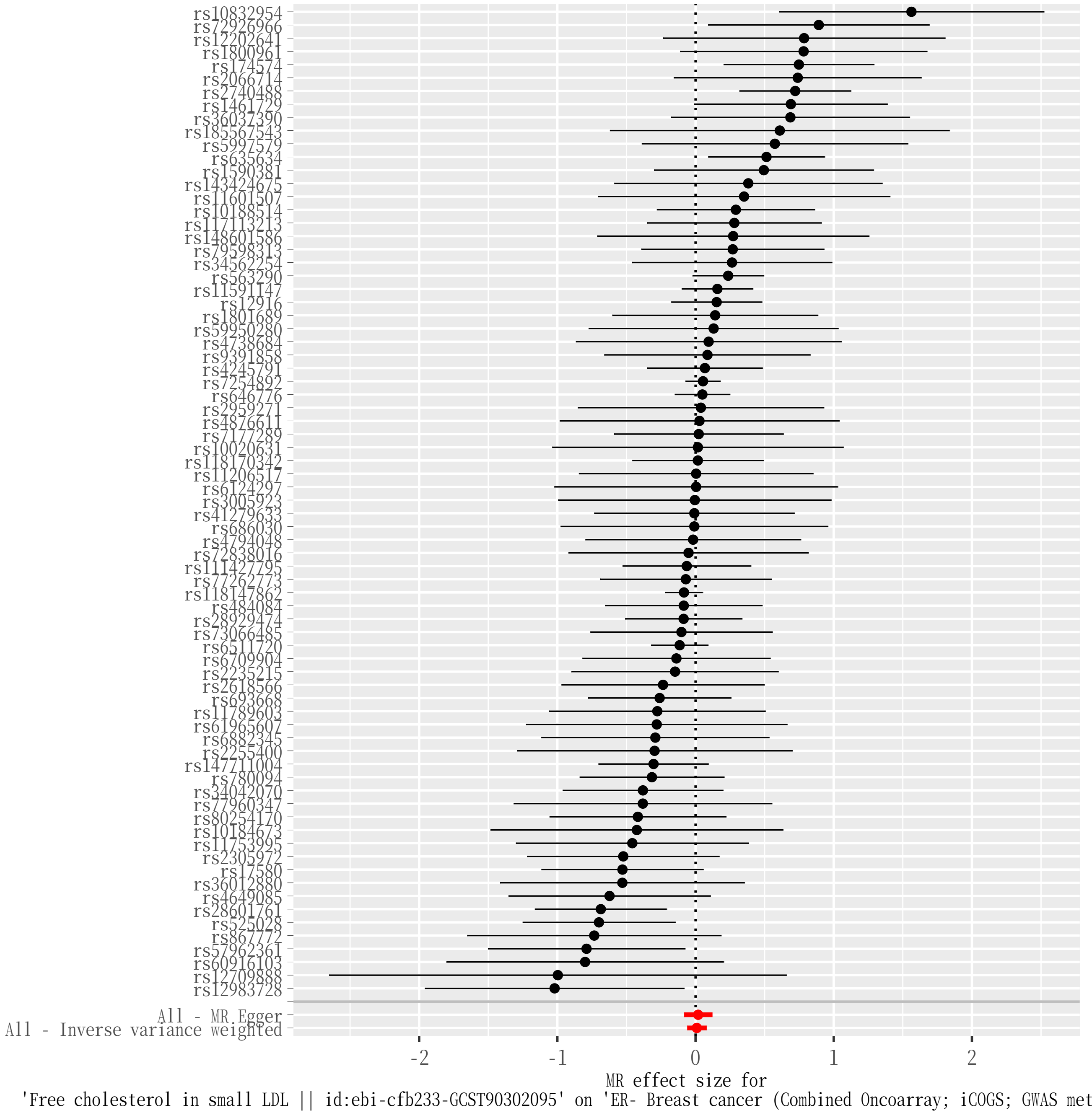
rs10832954
rs72926966
rs12202641
rs1800961
rs174574
rs2066714
rs2740488
rs1461729
rs36037390
rs185567543
rs5997579
rs635634
rs1590381
rs143424675
rs11601507
rs10188514
rs117113213
rs148601586
rs79598313
rs34562254
rs563290
rs11591147
rs12916
rs1801689
rs59950280
rs4738684
rs9391858
rs4245791
rs7254892
rs646776
rs2959271
rs4876611
rs7177289
rs10020631
rs118170342
rs11206517
rs6124297
rs3005923
rs41279633
rs686030
rs4794048
rs72838016
rs111427795
rs77262773
rs118147862
rs484084
rs28929474
rs73066485
rs6511720
rs6709904
rs2235215
rs2618566
rs693668
rs11789603
rs61965607
rs6882345
rs2255400
rs147711004
rs780094
rs34042070
rs77960347
rs80254170
rs10184673
rs11753995
rs2305972
rs17580
rs36012880
rs4649085
rs28601761
rs525028
rs867772
rs57962361
rs60916103
rs12709888
rs12983728
All - MR Egger
All - Inverse variance weighted
-2
-1
0
1
2
MR effect size for
'Free cholesterol in small LDL || id:ebi-cfb233-GCST90302095' on 'ER- Breast cancer (Combined Oncoarray; iCOGS; GWAS met

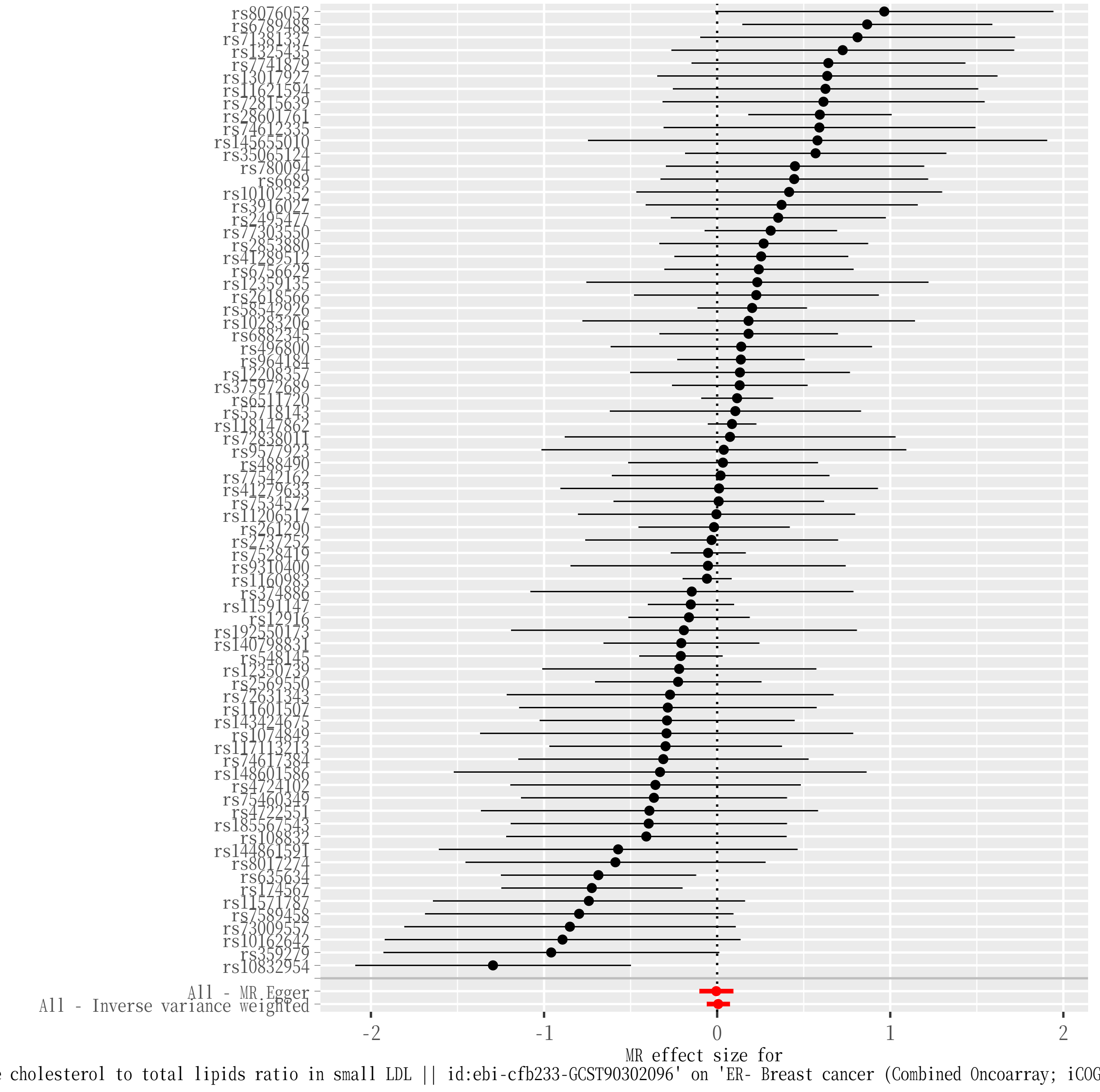
rs8076052
rs6789488
rs71381337
rs1325435
rs7741879
rs13017927
rs11621594
rs72815639
rs28601761
rs74612335
rs145655010
rs35065124
rs780094
rs6689
rs10102352
rs3916027
rs2495477
rs77303550
rs2853880
rs41289512
rs6756629
rs12359135
rs2618566
rs58542926
rs10283206
rs6882345
rs496800
rs964184
rs12208357
rs375972689
rs6511720
rs55718143
rs118147862
rs72838011
rs9577923
rs488490
rs77542162
rs41279633
rs7534572
rs11206517
rs261290
rs2737252
rs7528419
rs9310400
rs1160983
rs374886
rs11591147
rs12916
rs192550173
rs140798831
rs548145
rs12350739
rs2569550
rs72631343
rs11601507
rs143424675
rs1074849
rs117113213
rs74617384
rs148601586
rs4724102
rs75460349
rs4722551
rs185567543
rs108832
rs144861591
rs8017274
rs635634
rs174567
rs11571787
rs7589458
rs73009557
rs10162642
rs359279
rs10832954
All - MR Egger
All - Inverse variance weighted
-2
-1
0
1
2
MR effect size for
e cholesterol to total lipids ratio in small LDL || id:ebi-cfb233-GCST90302096' on 'ER- Breast cancer (Combined Oncoarray; iCOG

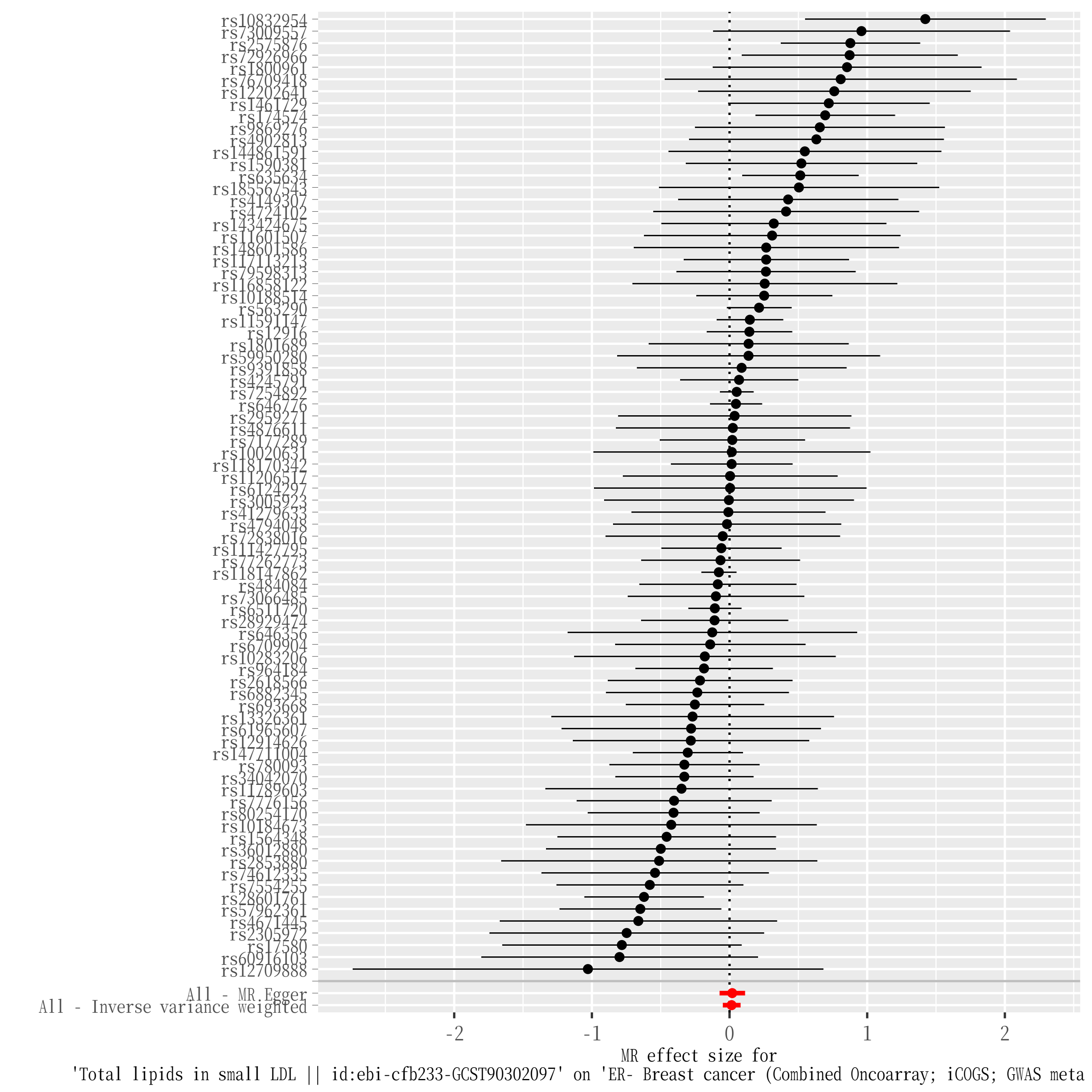
rs10832954
rs73002557
rs2575876
rs72926966
rs1800961
rs76709418
rs12202641
rs1461729
rs174574
rs9869276
rs4902813
rs144861591
rs1590381
rs635634
rs185567543
rs4149307
rs4724102
rs143424675
rs11601507
rs148601586
rs117113213
rs79598313
rs116858122
rs10188514
rs563290
rs11591147
rs12916
rs1801689
rs59950280
rs9391858
rs4245791
rs7254892
rs646776
rs2959271
rs4826611
rs7177289
rs10020631
rs118170342
rs11206517
rs6124297
rs3005923
rs41279633
rs4794048
rs72838016
rs111427795
rs7262773
rs118147862
rs484084
rs73066485
rs6511720
rs28929474
rs646356
rs6709904
rs10283206
rs964184
rs2618566
rs6882345
rs693668
rs13326361
rs61965607
rs12914626
rs147211004
rs780093
rs34042070
rs11789603
rs7776156
rs80254170
rs10184673
rs1564348
rs36012880
rs2853880
rs74612335
rs7554255
rs28601761
rs57962361
rs4671445
rs2305972
rs17580
rs60916103
rs12709888
All - MR Egger
All - Inverse variance weighted
-2
-1
0
1
2
MR effect size for
'Total lipids in small LDL || id:ebi-cfb233-GCST90302097' on 'ER- Breast cancer (Combined Oncoarray; iCOGS; GWAS meta

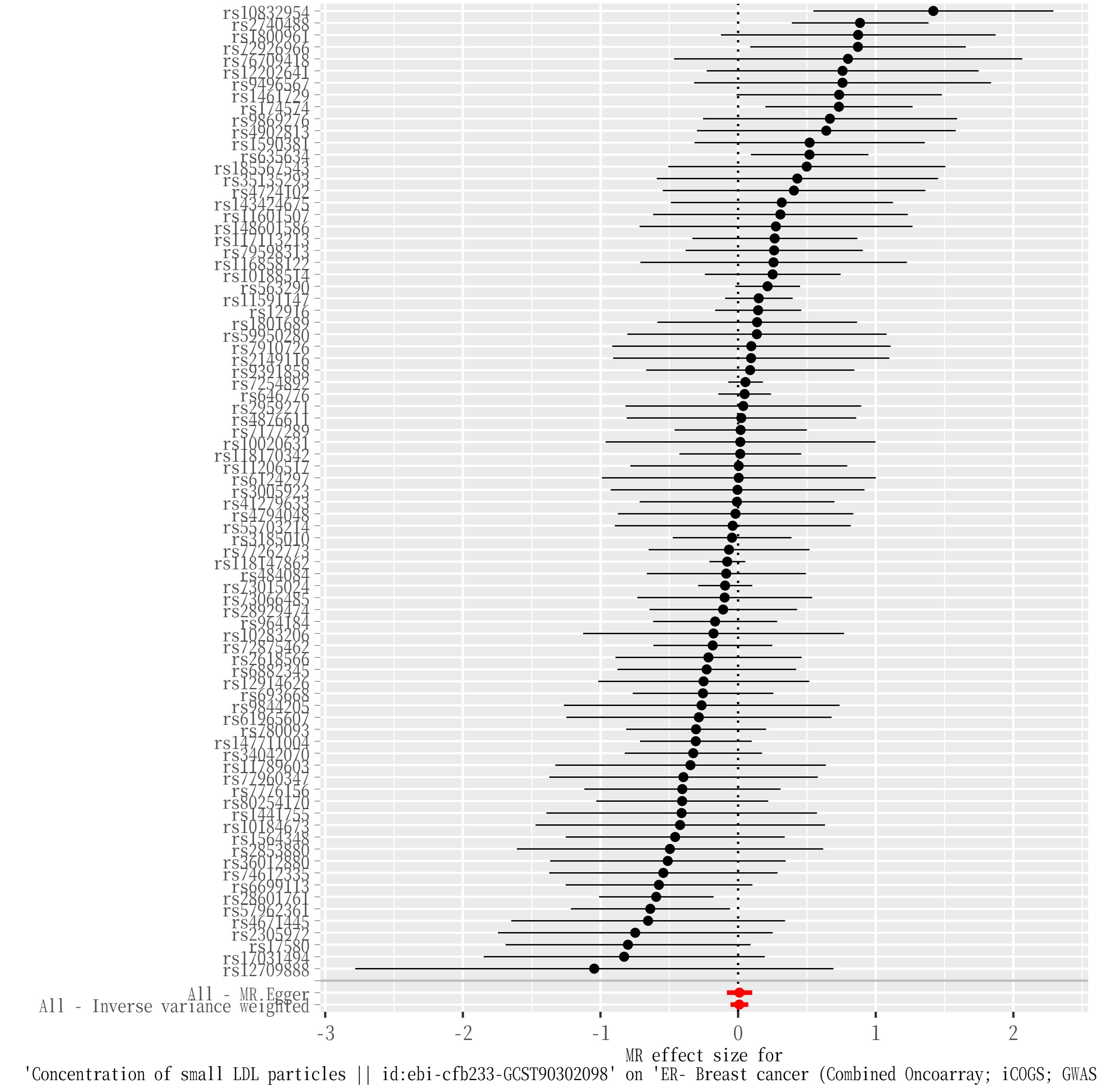

rs10832954
rs2740488
rs1800961
rs72926966
rs76709418
rs12202641
rs9496567
rs1461729
rs174574
rs9869276
rs4902813
rs1590381
rs635634
rs185567543
rs35135293
rs4724102
rs143424675
rs11601507
rs148601586
rs117113213
rs79598313
rs116858122
rs10188514
rs563290
rs11591147
rs12916
rs1801689
rs59950280
rs7910726
rs2149116
rs9391858
rs7254892
rs646776
rs2959271
rs4876611
rs7177289
rs10020631
rs118170342
rs11206517
rs6124297
rs3005923
rs41279633
rs4794048
rs55703214
rs3185010
rs77262773
rs118147862
rs484084
rs73015024
rs73066485
rs28929474
rs964184
rs10283206
rs72875462
rs2618566
rs6882345
rs12914626
rs693668
rs9844205
rs61265607
rs780093
rs147711004
rs34042070
rs11789603
rs77960347
rs7776156
rs80254170
rs1441755
rs10184673
rs1564348
rs2853880
rs36012880
rs74612335
rs6699113
rs28601761
rs57962361
rs4671445
rs2305972
rs17580
rs17031494
rs12709888
All - MR Egger
All - Inverse variance weighted
-3
-2
-1
0
1
2
MR effect size for
'Concentration of small LDL particles || id:ebi-cfb233-GCST90302098' on 'ER- Breast cancer (Combined Oncoarray; iCOGS; GWAS

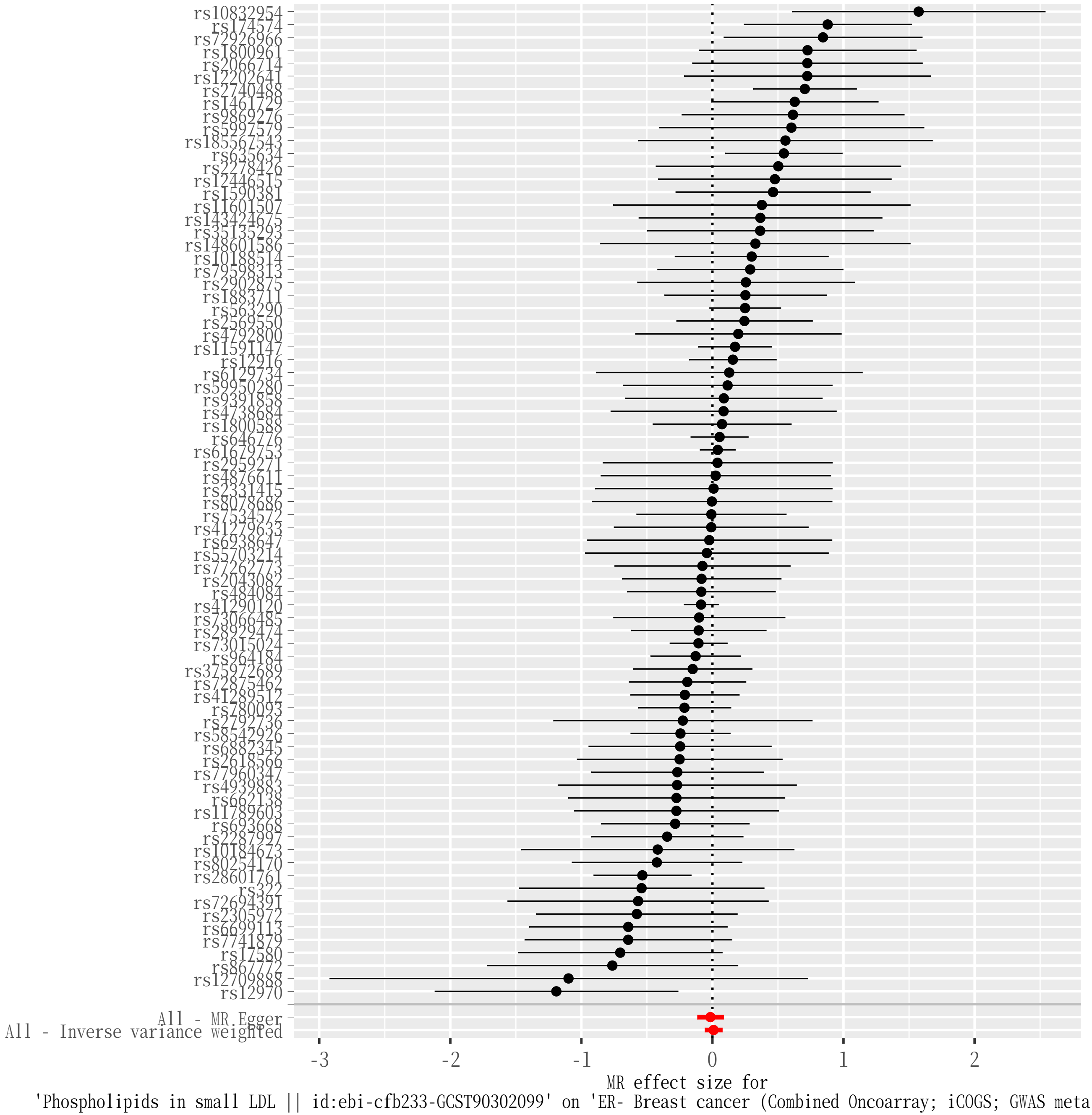
rs10832954
rs174574
rs72926966
rs1800961
rs2066714
rs12202641
rs2740488
rs1461729
rs9869276
rs5997579
rs185567543
rs635634
rs2278426
rs12446515
rs1590381
rs11601507
rs143424675
rs35135293
rs148601586
rs10188514
rs79598313
rs2902875
rs1883711
rs563290
rs2569550
rs4792800
rs11591147
rs12916
rs6129734
rs59950280
rs9391858
rs4738684
rs1800588
rs646776
rs61679753
rs2959271
rs4876611
rs2331415
rs8078686
rs7534572
rs41279633
rs6938647
rs55703214
rs77262773
rs2043082
rs484084
rs41290120
rs73066485
rs28929474
rs73015024
rs964184
rs375972689
rs72875462
rs41289512
rs780093
rs2792736
rs58542926
rs6882345
rs2618566
rs77960347
rs4939883
rs662138
rs11789603
rs693668
rs2287997
rs10184673
rs80254170
rs28601761
rs322
rs72694391
rs2305972
rs6699113
rs7741879
rs17580
rs867772
rs12709888
rs12970
All - MR Egger
All - Inverse variance weighted
-3
-2
-1
0
1
2
MR effect size for
'Phospholipids in small LDL || id:ebi-cfb233-GCST90302099' on 'ER- Breast cancer (Combined Oncoarray; iCOGS; GWAS meta

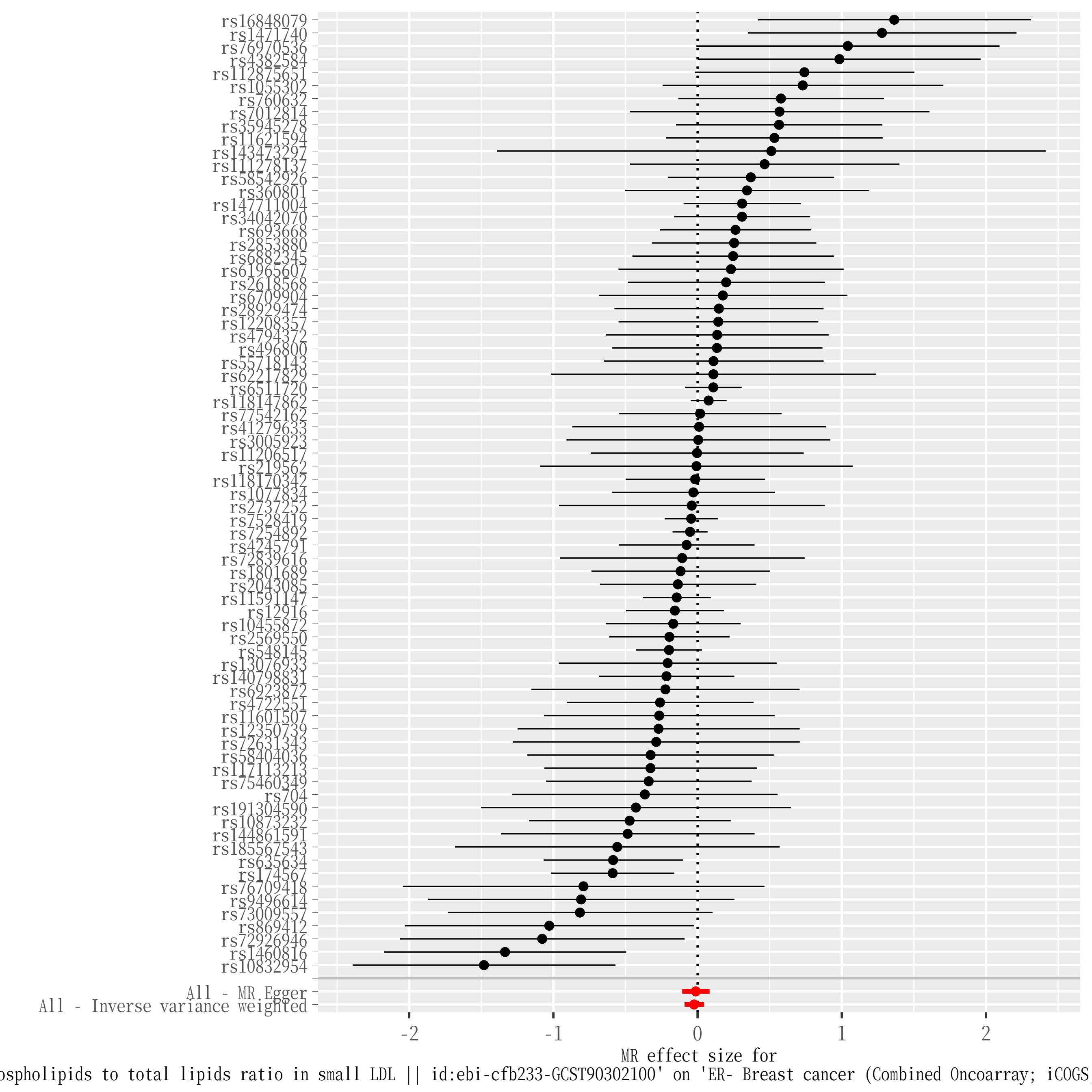

rs16848079
rs1471740
rs76970536
rs4382584
rs112875651
rs1055302
rs760632
rs7012814
rs35945278
rs11621594
rs143473297
rs111278137
rs58542926
rs360801
rs147711004
rs34042070
rs693668
rs2853880
rs6882345
rs61965607
rs2618568
rs6709904
rs28929474
rs12208357
rs4794372
rs496800
rs55718143
rs62217829
rs6511720
rs118147862
rs77542162
rs41279633
rs3005923
rs11206517
rs219562
rs118170342
rs1077834
rs2737252
rs7528419
rs7254892
rs4245791
rs72839616
rs1801689
rs2043085
rs11591147
rs12916
rs10455872
rs2569550
rs548145
rs13076933
rs140798831
rs6923872
rs4722551
rs11601507
rs12350739
rs72631343
rs58404036
rs117113213
rs75460349
rs704
rs191304590
rs10873232
rs144861591
rs185567543
rs635634
rs174567
rs76709418
rs9496614
rs73009557
rs869412
rs72926946
rs1460816
rs10832954
All - MR Egger
All - Inverse variance weighted
-2
-1
0
1
2
MR effect size for
ospholipids to total lipids ratio in small LDL || id:ebi-cfb233-GCST90302100' on 'ER- Breast cancer (Combined Oncoarray; iCOGS

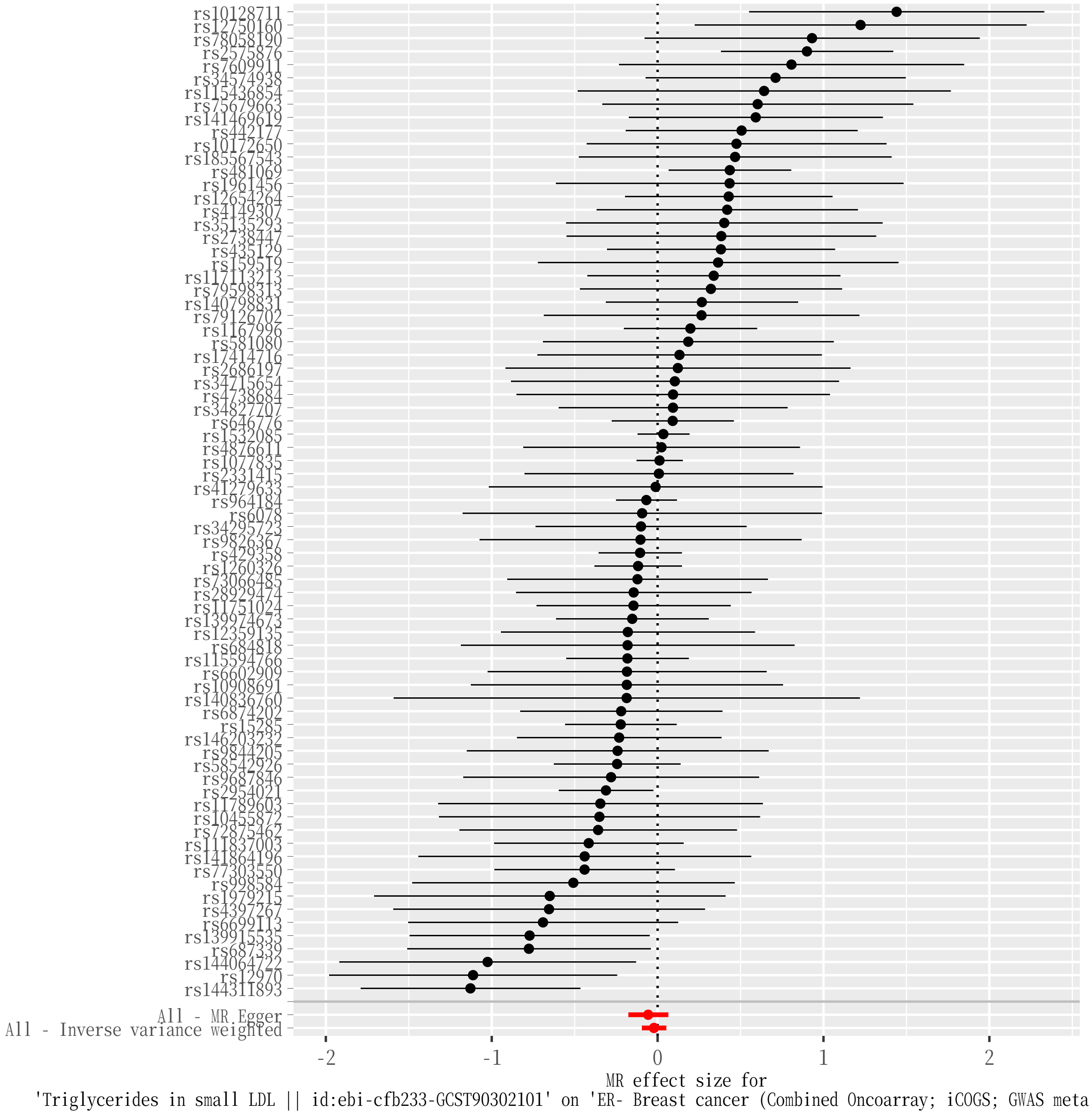
rs10128711
rs12750160
rs78058190
rs2575876
rs7609911
rs34574938
rs115436854
rs75679663
rs141469619
rs442177
rs10172650
rs185567543
rs481069
rs1961456
rs12654264
rs4149307
rs35135293
rs2738447
rs435129
rs159519
rs117113213
rs79598313
rs140798831
rs79126702
rs1167996
rs581080
rs17414716
rs2686197
rs34715654
rs4738684
rs34827707
rs646776
rs1532085
rs4876611
rs1077835
rs2331415
rs41279633
rs964184
rs6078
rs34295723
rs9826367
rs429358
rs1260326
rs73066485
rs28929474
rs11751024
rs139974673
rs12359135
rs684818
rs115594766
rs6602909
rs10908691
rs140836760
rs6874202
rs15285
rs146203232
rs9844205
rs58542926
rs9687846
rs2954021
rs11789603
rs10455872
rs72875462
rs111837003
rs141864126
rs77303550
rs998584
rs1979215
rs4397267
rs6692113
rs139915535
rs687339
rs144064722
rs12970
rs144311893
All - MR Egger
All - Inverse variance weighted
-2
-1
0
1
2
MR effect size for
'Triglycerides in small LDL || id:ebi-cfb233-GCST90302101' on 'ER- Breast cancer (Combined Oncoarray; iCOGS; GWAS meta

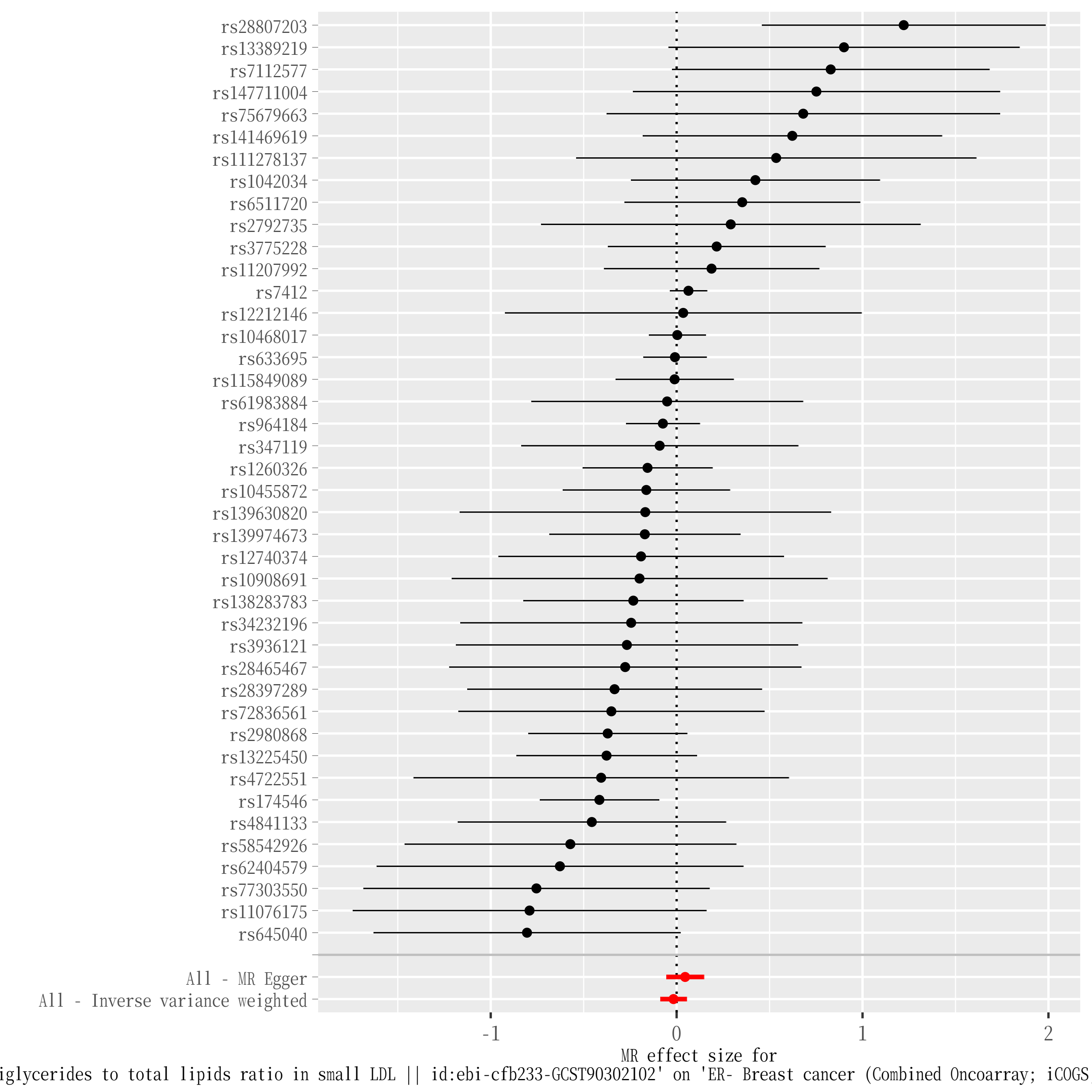
rs28807203
rs13389219
rs7112577
rs147711004
rs75679663
rs141469619
rs111278137
rs1042034
rs6511720
rs2792735
rs3775228
rs11207992
rs7412
rs12212146
rs10468017
rs633695
rs115849089
rs61983884
rs964184
rs347119
rs1260326
rs10455872
rs139630820
rs139974673
rs12740374
rs10908691
rs138283783
rs34232196
rs3936121
rs28465467
rs28397289
rs72836561
rs2980868
rs13225450
rs4722551
rs174546
rs4841133
rs58542926
rs62404579
rs77303550
rs11076175
rs645040
All - MR Egger
All - Inverse variance weighted
-1
0
1
2
MR effect size for
iglycerides to total lipids ratio in small LDL || id:ebi-cfb233-GCST90302102' on 'ER- Breast cancer (Combined Oncoarray; iCOGS

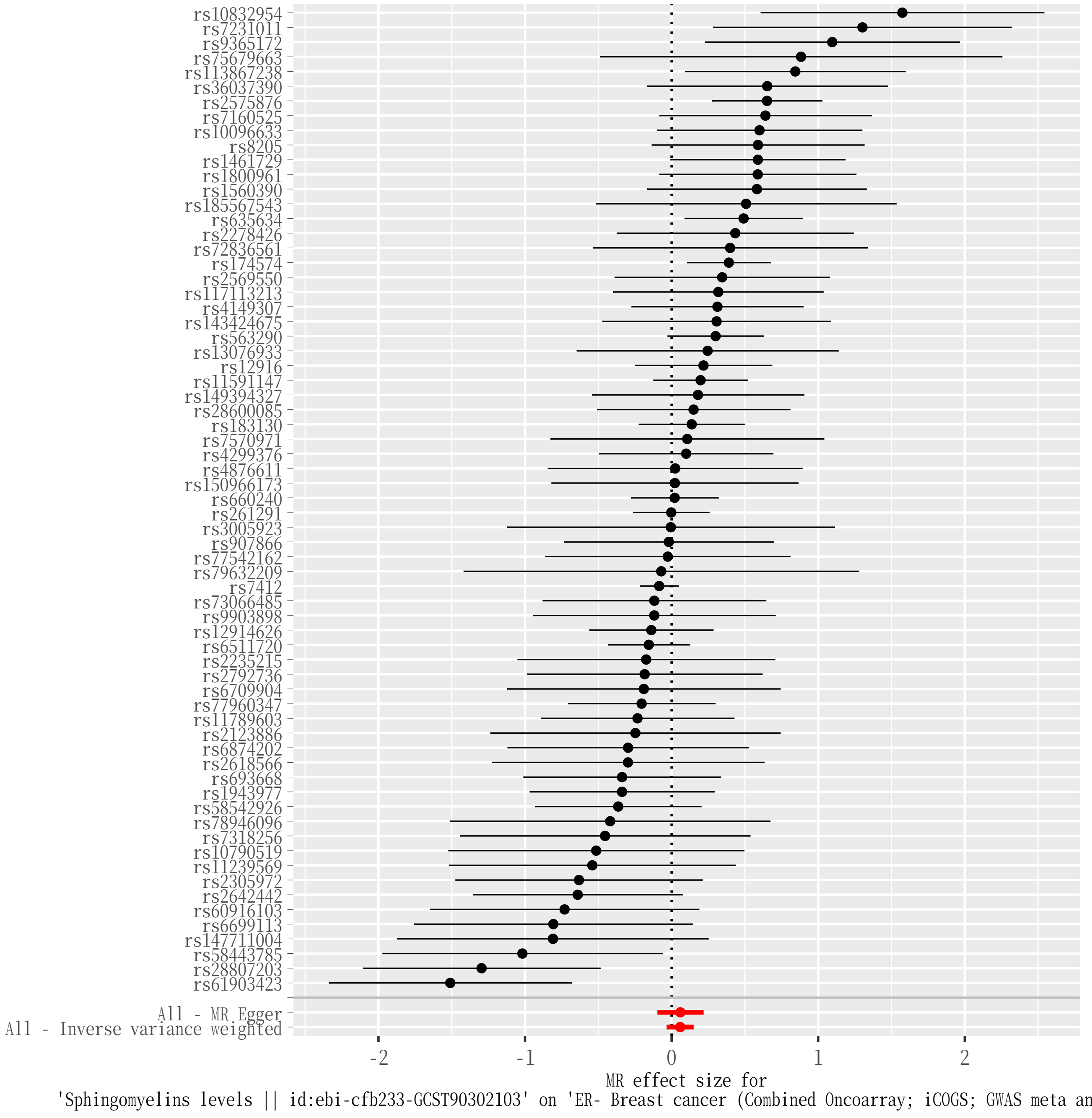
rs10832954
rs7231011
rs9365172
rs75679663
rs113867238
rs36037390
rs2575876
rs7160525
rs10096633
rs8205
rs1461729
rs1800961
rs1560390
rs185567543
rs635634
rs2278426
rs72836561
rs174574
rs2569550
rs117113213
rs4149307
rs143424675
rs563290
rs13076933
rs12916
rs11591147
rs149394327
rs28600085
rs183130
rs7570971
rs4299376
rs4876611
rs150966173
rs660240
rs261291
rs3005923
rs907866
rs77542162
rs79632209
rs7412
rs73066485
rs9903898
rs12914626
rs6511720
rs2235215
rs2792736
rs6709904
rs77960347
rs11789603
rs2123886
rs6874202
rs2618566
rs693668
rs1943977
rs58542926
rs78946096
rs7318256
rs10790519
rs11239569
rs2305972
rs2642442
rs60916103
rs6699113
rs147711004
rs58443785
rs28807203
rs61903423
All - MR Egger
All - Inverse variance weighted
-2
-1
0
1
2
MR effect size for
'Sphingomyelins levels || id:ebi-cfb233-GCST90302103' on 'ER- Breast cancer (Combined Oncoarray; iCOGS; GWAS meta an

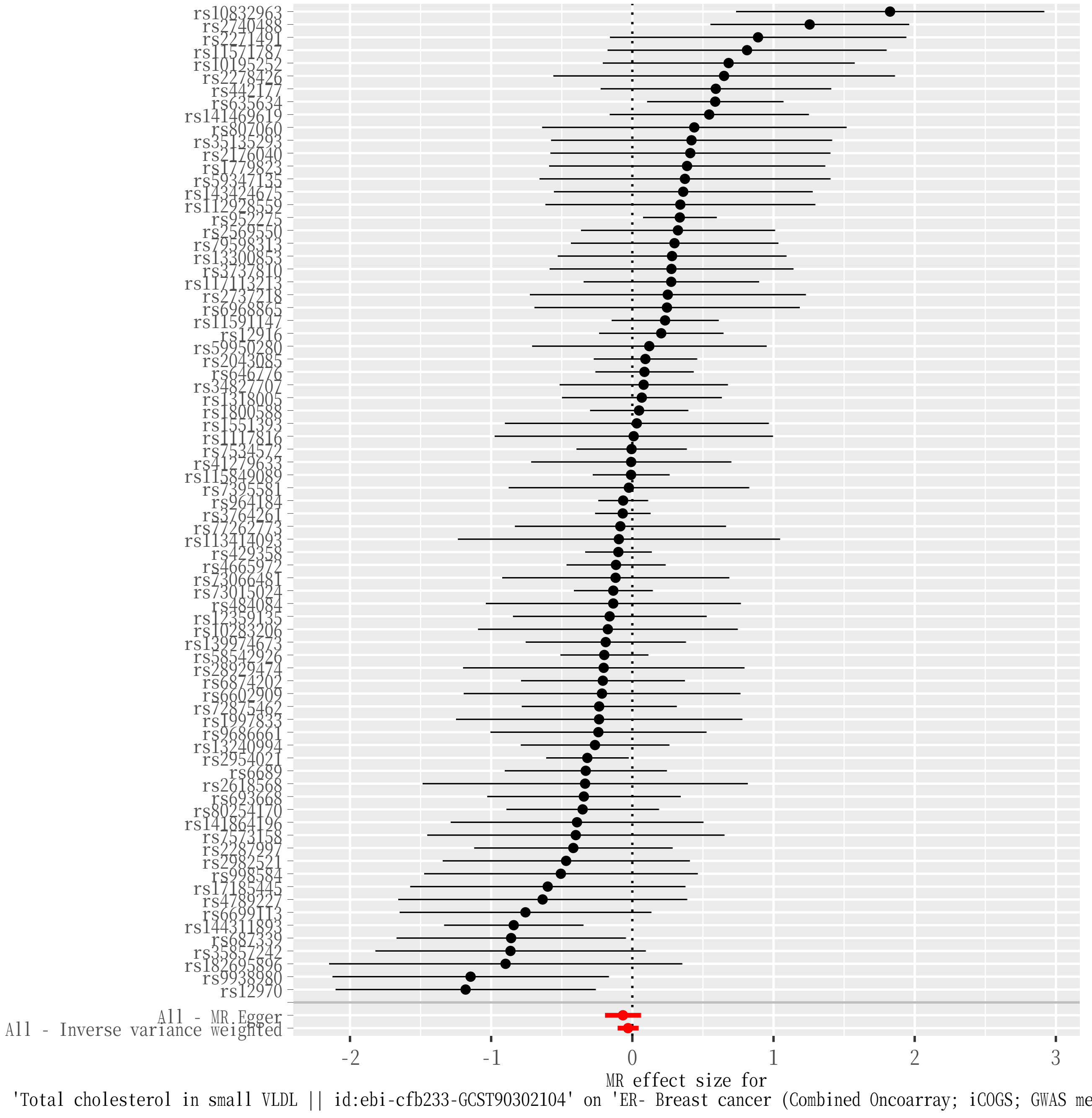

rs10832963
rs2740488
rs2271491
rs11571787
rs10195252
rs2278426
rs442177
rs635634
rs141469619
rs807060
rs35135293
rs2176040
rs1779823
rs59347135
rs143424675
rs112928559
rs952275
rs2569550
rs79598313
rs13300853
rs3737810
rs117113213
rs2737218
rs6968865
rs11591147
rs12916
rs59950280
rs2043085
rs646776
rs34827707
rs1318005
rs1800588
rs1551393
rs1117816
rs7534572
rs41279633
rs115849089
rs7395581
rs964184
rs3764261
rs77262773
rs113414093
rs429358
rs4665972
rs73066481
rs73015024
rs484084
rs12359135
rs10283206
rs139974673
rs58542926
rs28929474
rs6874202
rs6602909
rs72875462
rs1997833
rs9686661
rs13240994
rs2954021
rs6689
rs2618568
rs693668
rs80254170
rs141864196
rs7573158
rs2287997
rs2982521
rs998584
rs17185445
rs4789227
rs6699113
rs144311893
rs682339
rs35857242
rs182695896
rs9938980
rs12970
All - MR Egger
All - Inverse variance weighted
-2
-1
0
1
2
3
MR effect size for
'Total cholesterol in small VLDL || id:ebi-cfb233-GCST90302104' on 'ER- Breast cancer (Combined Oncoarray; iCOGS; GWAS me

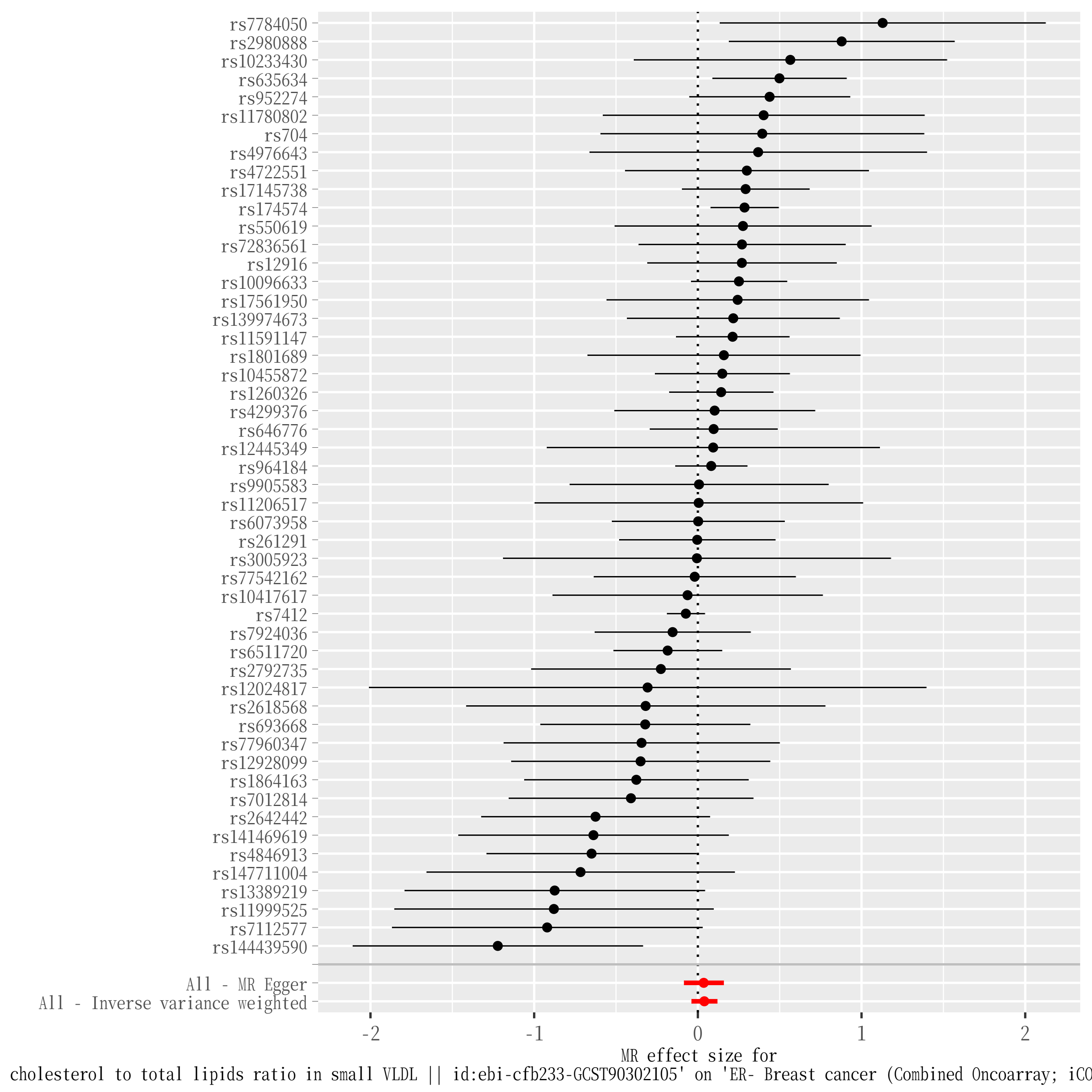

rs7784050
rs2980888
rs10233430
rs635634
rs952274
rs11780802
rs704
rs4976643
rs4722551
rs17145738
rs174574
rs550619
rs72836561
rs12916
rs10096633
rs17561950
rs139974673
rs11591147
rs1801689
rs10455872
rs1260326
rs4299376
rs646776
rs12445349
rs964184
rs9905583
rs11206517
rs6073958
rs261291
rs3005923
rs77542162
rs10417617
rs7412
rs7924036
rs6511720
rs2792735
rs12024817
rs2618568
rs693668
rs77960347
rs12928099
rs1864163
rs7012814
rs2642442
rs141469619
rs4846913
rs147711004
rs13389219
rs11999525
rs7112577
rs144439590
All - MR Egger
All - Inverse variance weighted
-2
-1
0
1
2
MR effect size for
cholesterol to total lipids ratio in small VLDL || id:ebi-cfb233-GCST90302105' on 'ER- Breast cancer (Combined Oncoarray; iC0

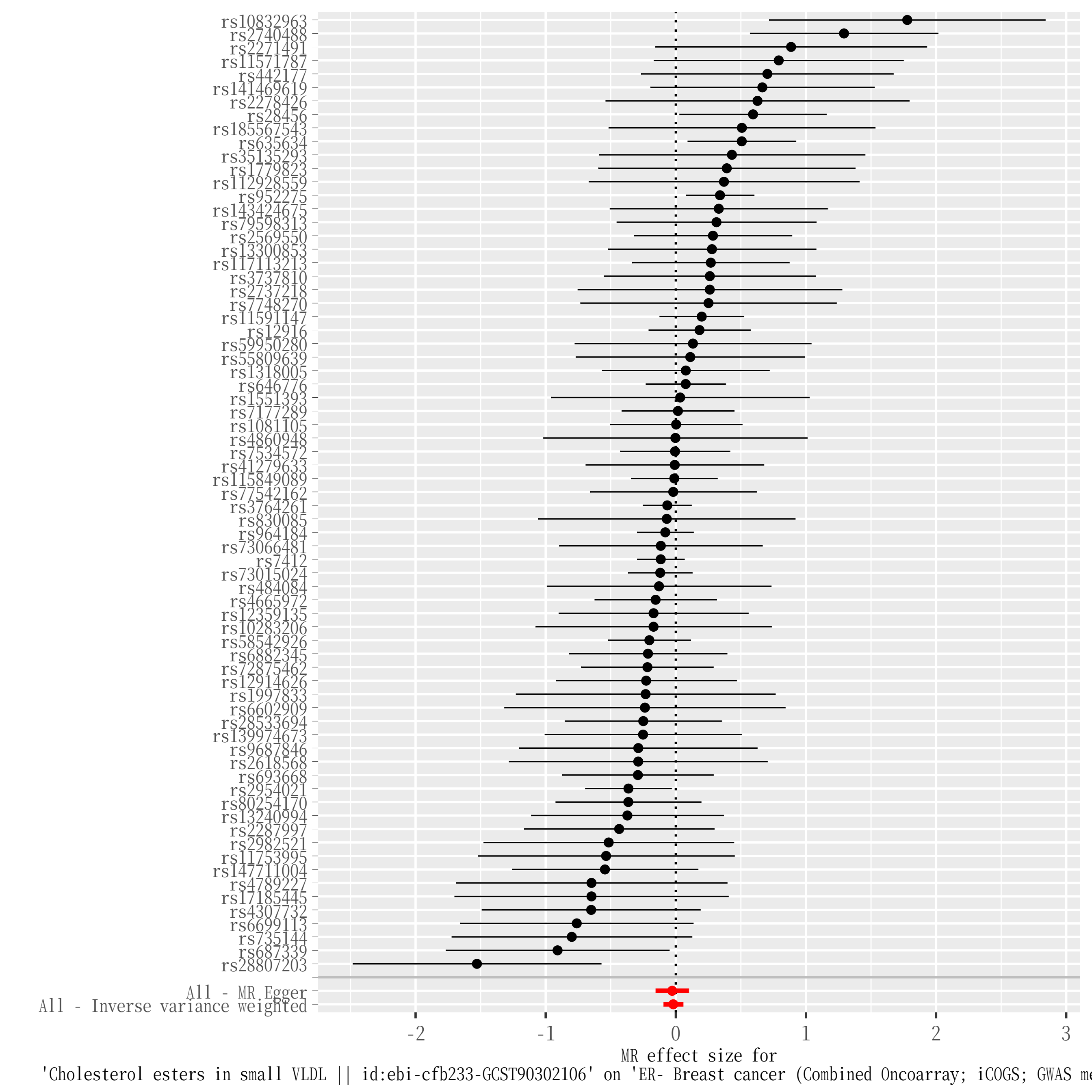
rs10832963
rs2740488
rs2271491
rs11571787
rs442177
rs141469619
rs2278426
rs28456
rs185567543
rs635634
rs35135293
rs1779823
rs112928559
rs952275
rs143424675
rs79598313
rs2569550
rs13300853
rs117113213
rs3737810
rs2737218
rs7748270
rs11591147
rs12916
rs59950280
rs55809639
rs1318005
rs646776
rs1551393
rs7177289
rs1081105
rs4860948
rs7534572
rs41279633
rs115849089
rs77542162
rs3764261
rs830085
rs964184
rs73066481
rs7412
rs73015024
rs484084
rs4665972
rs12359135
rs10283206
rs58542926
rs6882345
rs72875462
rs12914626
rs1997833
rs6602909
rs28533694
rs139974673
rs9687846
rs2618568
rs693668
rs2954021
rs80254170
rs13240994
rs2287997
rs2982521
rs11753995
rs147711004
rs4789227
rs17185445
rs4307732
rs6699113
rs735144
rs687339
rs28807203
All - MR Egger
All - Inverse variance weighted
-2
-1
0
1
2
3
MR effect size for
'Cholesterol esters in small VLDL || id:ebi-cfb233-GCST90302106' on 'ER- Breast cancer (Combined Oncoarray; iCOGS; GWAS me

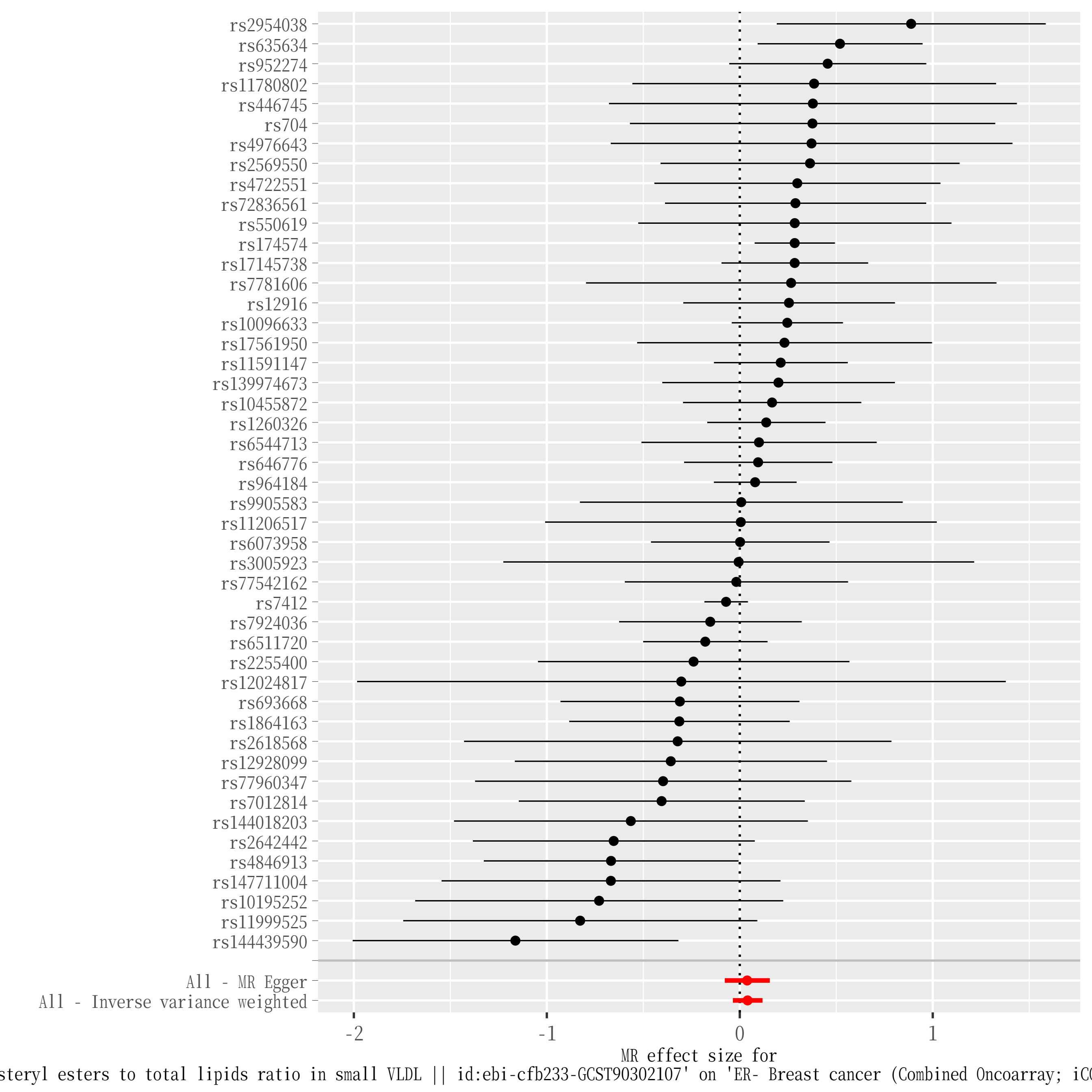

rs2954038
rs635634
rs952274
rs11780802
rs446745
rs704
rs4976643
rs2569550
rs4722551
rs72836561
rs550619
rs174574
rs17145738
rs7781606
rs12916
rs10096633
rs17561950
rs11591147
rs139974673
rs10455872
rs1260326
rs6544713
rs646776
rs964184
rs9905583
rs11206517
rs6073958
rs3005923
rs77542162
rs7412
rs7924036
rs6511720
rs2255400
rs12024817
rs693668
rs1864163
rs2618568
rs12928099
rs77960347
rs7012814
rs144018203
rs2642442
rs4846913
rs147711004
rs10195252
rs11999525
rs144439590
All - MR Egger
All - Inverse variance weighted
-2
-1
0
1
MR effect size for
steryl esters to total lipids ratio in small VLDL || id:ebi-cfb233-GCST90302107' on 'ER- Breast cancer (Combined Oncoarray; iC

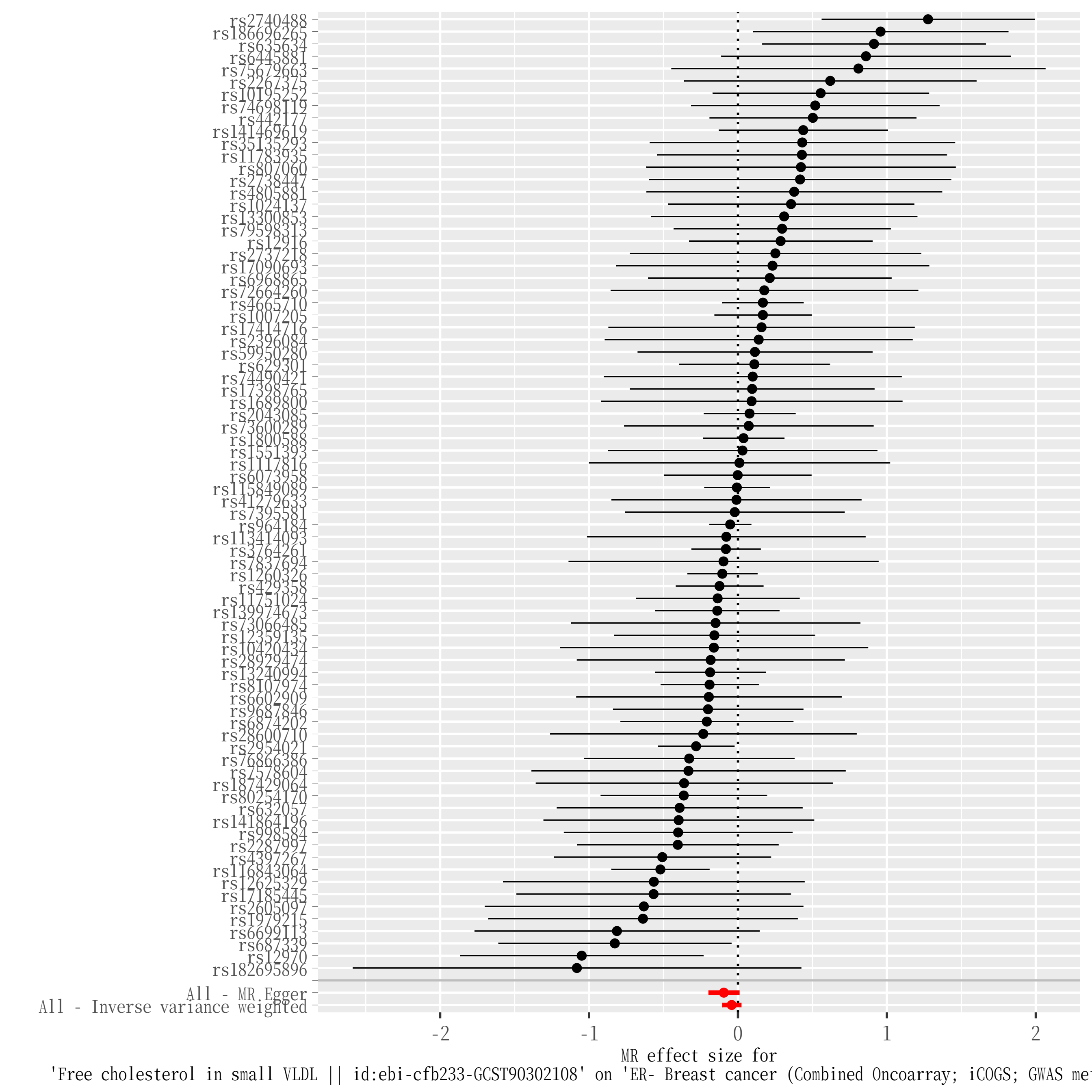

rs2740488
rs186696265
rs635634
rs6445881
rs75679663
rs2267375
rs10195252
rs74698119
rs442177
rs141469619
rs35135293
rs11783935
rs807060
rs2738447
rs4805881
rs1024137
rs13300853
rs79598313
rs12916
rs2737218
rs17090693
rs6968865
rs72664260
rs4665710
rs1007205
rs17414716
rs2396084
rs59950280
rs629301
rs74490421
rs17398765
rs1689800
rs2043085
rs73600289
rs1800588
rs1551393
rs1117816
rs6073958
rs115849089
rs41279633
rs7395581
rs964184
rs113414093
rs3764261
rs7837694
rs1260326
rs429358
rs11751024
rs139974673
rs7306485
rs12359135
rs10420434
rs28929474
rs1324094
rs8107974
rs6602909
rs9687846
rs6874202
rs28600710
rs2954021
rs76866386
rs7578604
rs187429064
rs80254170
rs632057
rs141864196
rs998584
rs2287997
rs4397267
rs116843064
rs12625329
rs17185445
rs2605097
rs1979215
rs6699113
rs687339
rs12970
rs182695896
All - MR Egger
All - Inverse variance weighted
-2
-1
0
1
2
MR effect size for
'Free cholesterol in small VLDL || id:ebi-cfb233-GCST90302108' on 'ER- Breast cancer (Combined Oncoarray; iCOGS; GWAS me

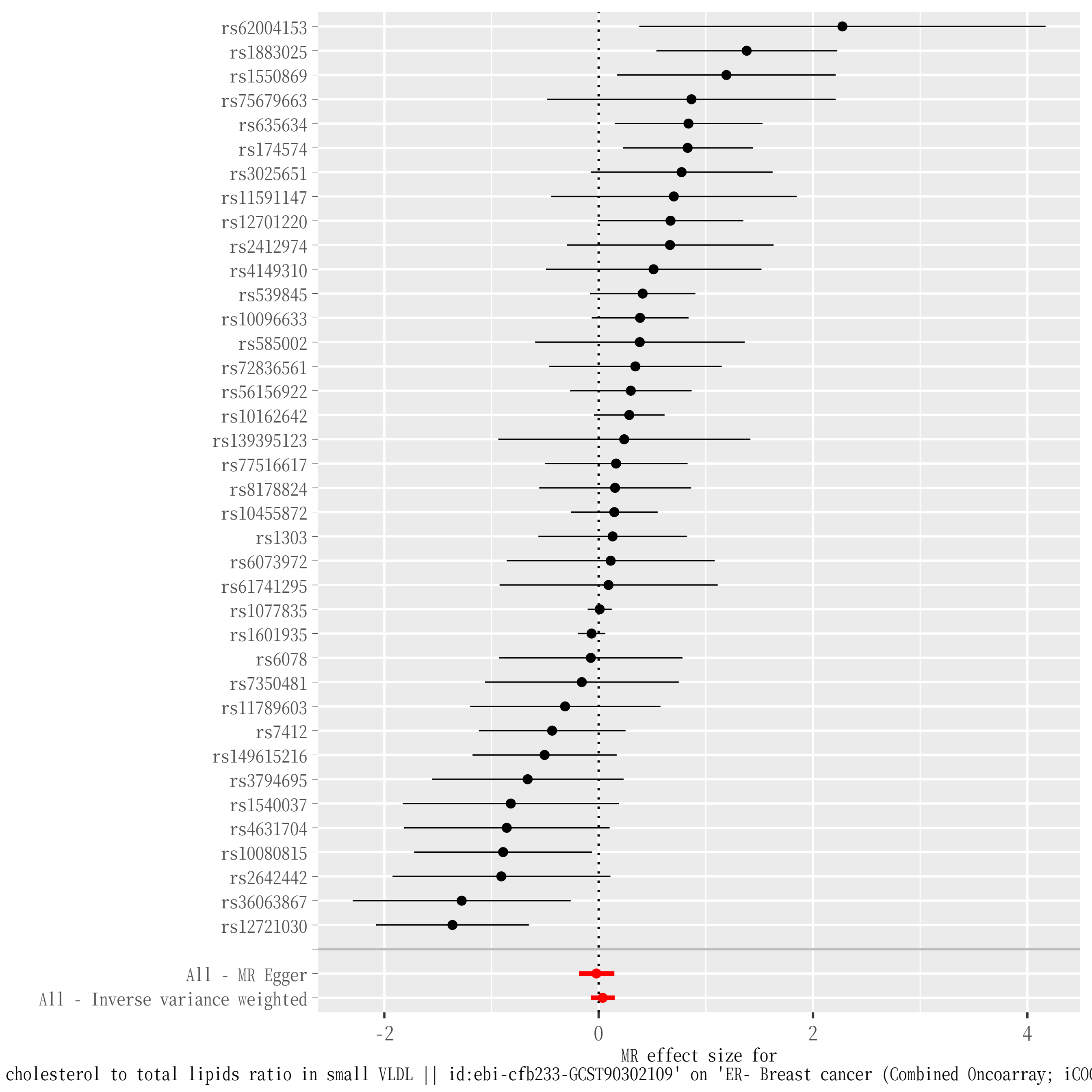

rs62004153
rs1883025
rs1550869
rs75679663
rs635634
rs174574
rs3025651
rs11591147
rs12701220
rs2412974
rs4149310
rs539845
rs10096633
rs585002
rs72836561
rs56156922
rs10162642
rs139395123
rs77516617
rs8178824
rs10455872
rs1303
rs6073972
rs61741295
rs1077835
rs1601935
rs6078
rs7350481
rs11789603
rs7412
rs149615216
rs3794695
rs1540037
rs4631704
rs10080815
rs2642442
rs36063867
rs12721030
All - MR Egger
All - Inverse variance weighted
-2
0
2
4
MR effect size for
cholesterol to total lipids ratio in small VLDL || id:ebi-cfb233-GCST90302109' on 'ER- Breast cancer (Combined Oncoarray; iCO

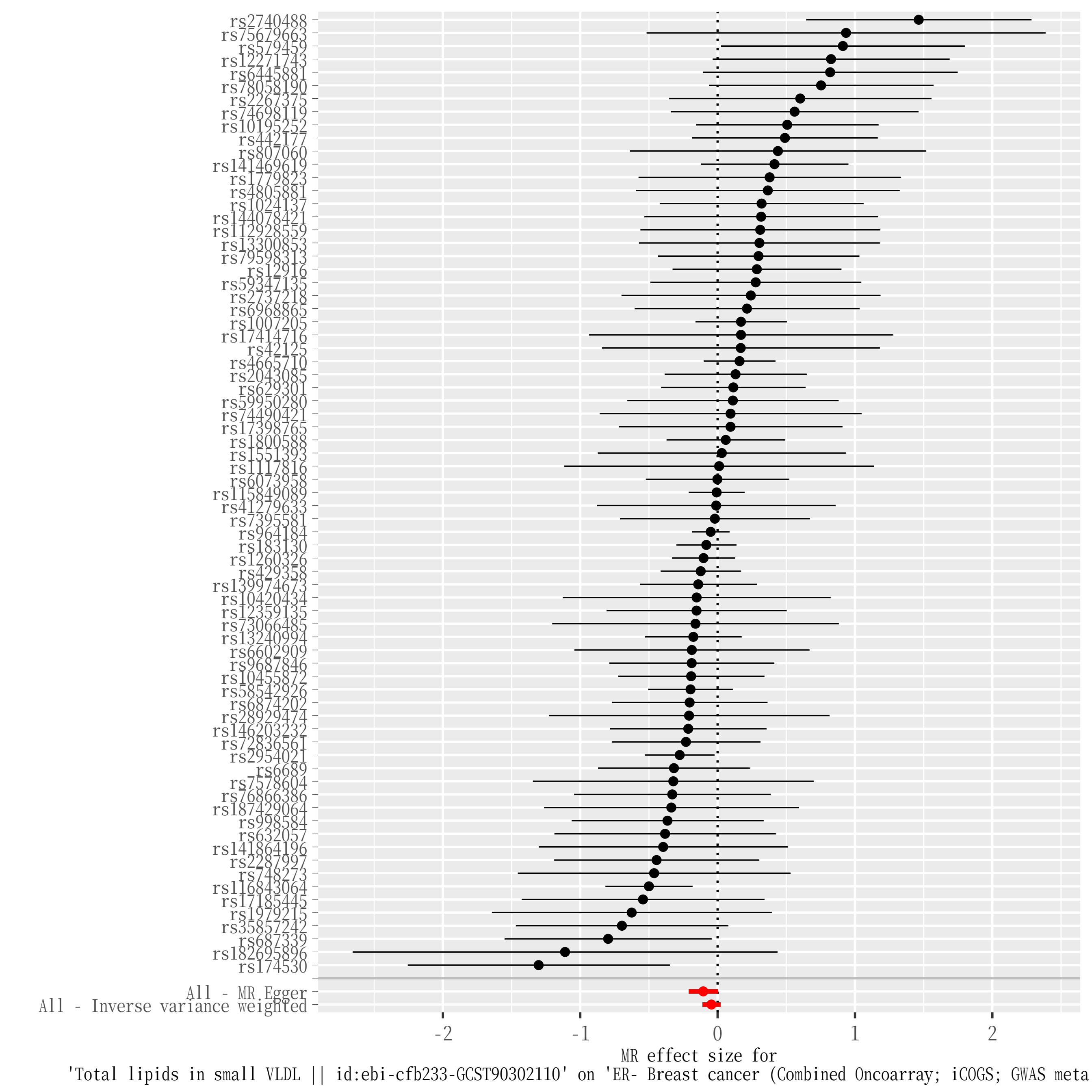
rs2740488
rs75679663
rs579459
rs12271743
rs6445881
rs78058190
rs2267375
rs74698119
rs10195252
rs442177
rs807060
rs141469619
rs1779823
rs4805881
rs1024137
rs144078421
rs112928559
rs13300853
rs79598313
rs12916
rs59347135
rs2737218
rs6968865
rs1007205
rs17414716
rs42125
rs4665710
rs2043085
rs629301
rs59950280
rs74490421
rs17398765
rs1800588
rs1551393
rs1117816
rs6073958
rs115849089
rs41279633
rs7395581
rs964184
rs183130
rs1260326
rs429358
rs139974673
rs10420434
rs12359135
rs73066485
rs13240994
rs6602909
rs9687846
rs10455872
rs58542926
rs6874202
rs28929474
rs146203232
rs72836561
rs2954021
rs6689
rs7578604
rs76866386
rs187429064
rs998584
rs632057
rs141864196
rs2287997
rs748273
rs116843064
rs17185445
rs1979215
rs35857242
rs687339
rs182695896
rs174530
All - MR Egger
All - Inverse variance weighted
-2
-1
0
1
2
MR effect size for
'Total lipids in small VLDL || id:ebi-cfb233-GCST90302110' on 'ER- Breast cancer (Combined Oncoarray; iCOGS; GWAS meta

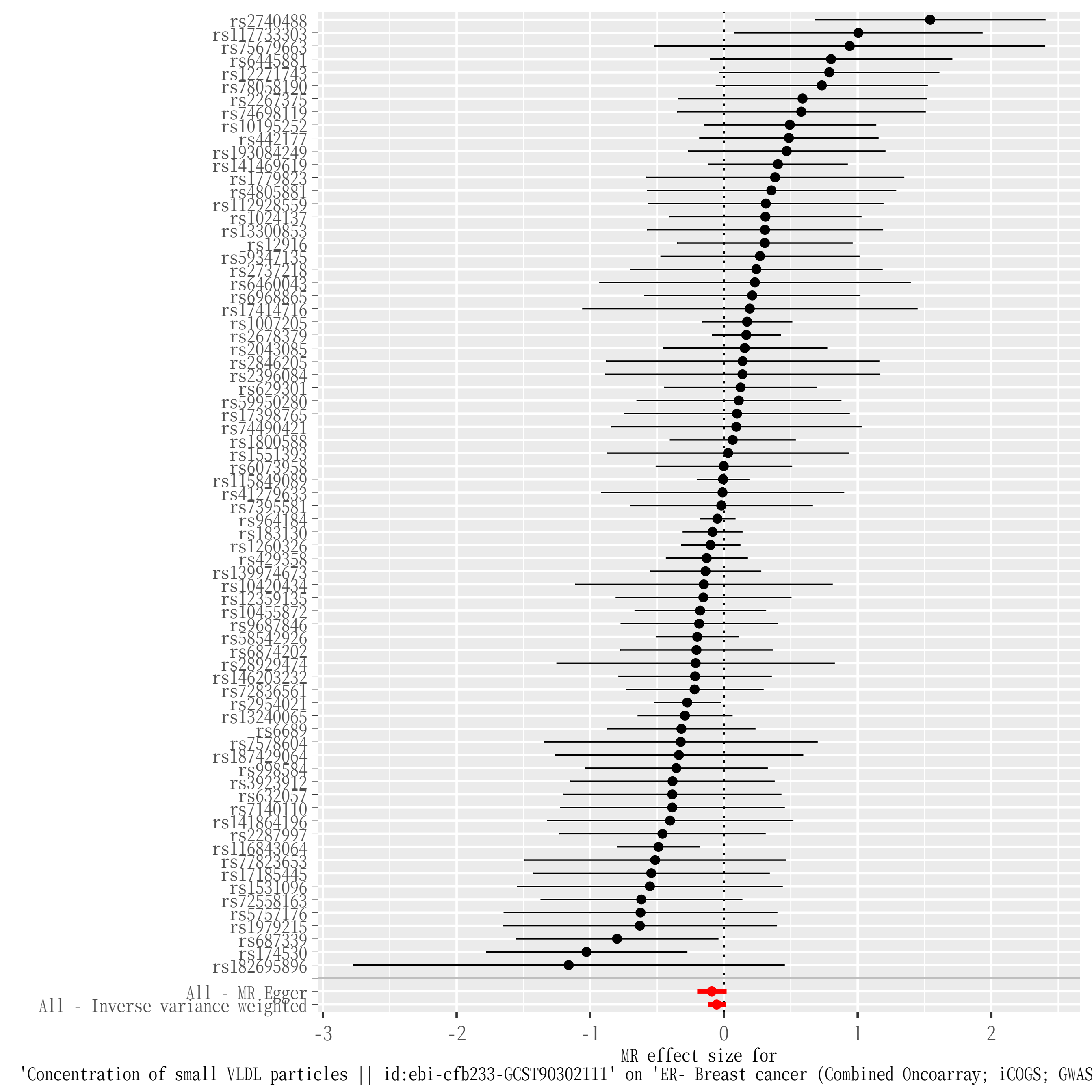
rs2740488
rs117733303
rs75679663
rs6445881
rs12271743
rs78058190
rs2267375
rs74698119
rs10195252
rs442177
rs193084249
rs141469619
rs1779823
rs4805881
rs112928559
rs1024137
rs13300853
rs12916
rs59347135
rs2737218
rs6460043
rs6968865
rs17414716
rs1007205
rs2678379
rs2043085
rs2846205
rs2396084
rs629301
rs59950280
rs17398765
rs74490421
rs1800588
rs1551393
rs6073958
rs115849089
rs41279633
rs7395581
rs964184
rs183130
rs1260326
rs429358
rs139974673
rs10420434
rs12359135
rs10455872
rs9687846
rs58542926
rs6874202
rs28929474
rs146203232
rs72836561
rs2954021
rs13240065
rs6689
rs7578604
rs187429064
rs998584
rs3923912
rs632057
rs7140110
rs141864196
rs2287997
rs116843064
rs77823653
rs17185445
rs1531096
rs72558163
rs5757176
rs1979215
rs687339
rs174530
rs182695896
All - MR Egger
All - Inverse variance weighted
-3
-2
-1
0
1
2
MR effect size for
'Concentration of small VLDL particles || id:ebi-cfb233-GCST90302111' on 'ER- Breast cancer (Combined Oncoarray; iCOGS; GWAS

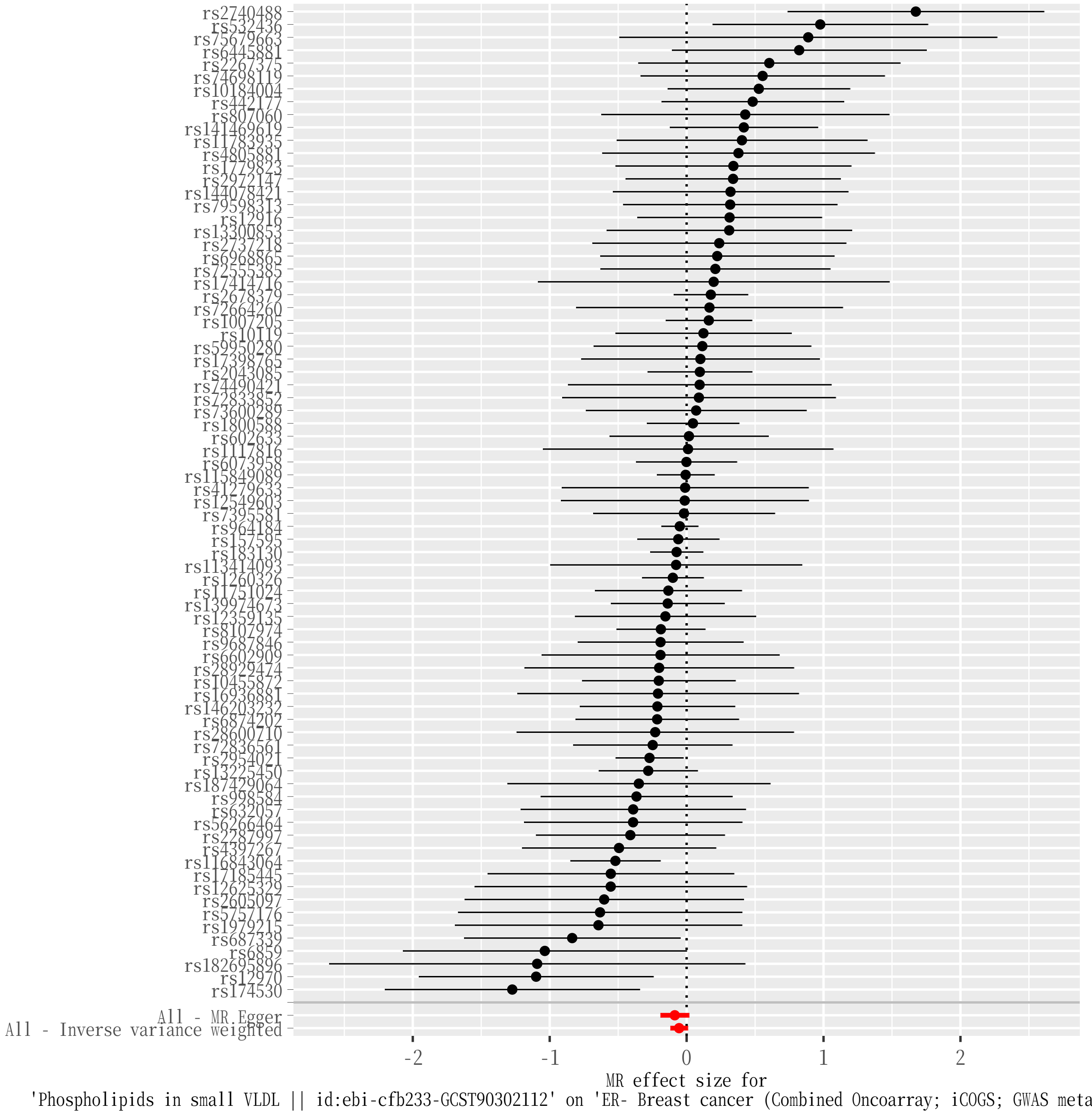

rs2740488
rs532436
rs75679663
rs6445881
rs2267375
rs74698119
rs10184004
rs442177
rs807060
rs141469619
rs11783935
rs4805881
rs1779823
rs2972147
rs144078421
rs79598313
rs12916
rs13300853
rs2737218
rs6968865
rs72555385
rs17414716
rs2678379
rs72664260
rs1007205
rs10119
rs59950280
rs17398765
rs2043085
rs74490421
rs72833852
rs73600289
rs1800588
rs602633
rs1117816
rs6073958
rs115849089
rs41279633
rs12549603
rs7395581
rs964184
rs157595
rs183130
rs113414093
rs1260326
rs11751024
rs139974673
rs12359135
rs8107974
rs9687846
rs6602909
rs28929474
rs10455872
rs16936881
rs146203232
rs6874202
rs28600710
rs72836561
rs2954021
rs13225450
rs187429064
rs998584
rs632057
rs56266464
rs2287997
rs4397267
rs116843064
rs17185445
rs12625329
rs2605097
rs5757176
rs1979215
rs687339
rs6859
rs182695896
rs12970
rs174530
All - MR Egger
All - Inverse variance weighted
-2
-1
0
1
2
MR effect size for
'Phospholipids in small VLDL || id:ebi-cfb233-GCST90302112' on 'ER- Breast cancer (Combined Oncoarray; iCOGS; GWAS meta

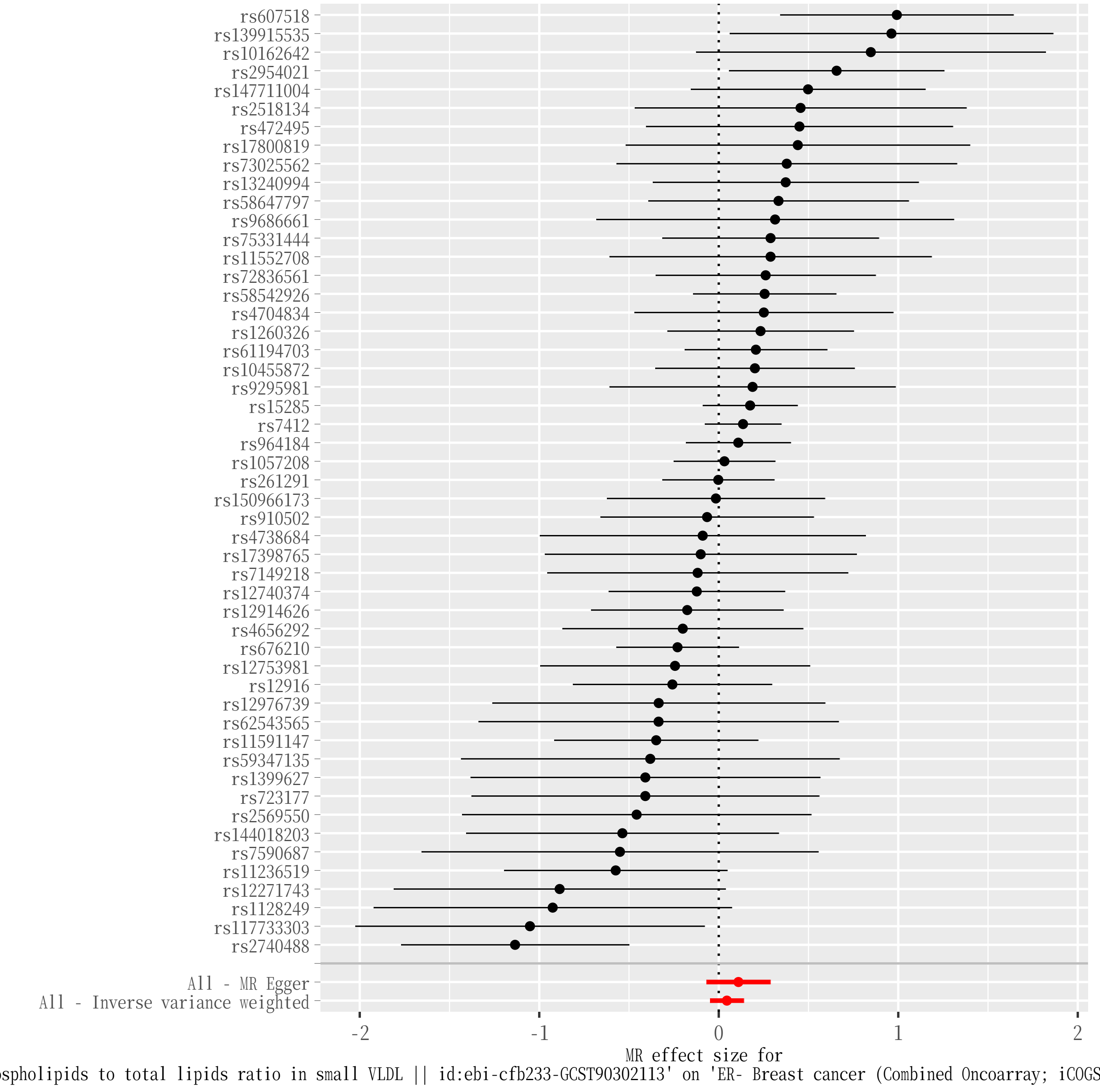
rs607518
rs139915535
rs10162642
rs2954021
rs147711004
rs2518134
rs472495
rs17800819
rs73025562
rs13240994
rs58647797
rs9686661
rs75331444
rs11552708
rs72836561
rs58542926
rs4704834
rs1260326
rs61194703
rs10455872
rs9295981
rs15285
rs7412
rs964184
rs1057208
rs261291
rs150966173
rs910502
rs4738684
rs17398765
rs7149218
rs12740374
rs12914626
rs4656292
rs676210
rs12753981
rs12916
rs12976739
rs62543565
rs11591147
rs59347135
rs1399627
rs723177
rs2569550
rs144018203
rs7590687
rs11236519
rs12271743
rs1128249
rs117733303
rs2740488
All - MR Egger
All - Inverse variance weighted
-2
-1
0
1
2
MR effect size for
ospholipids to total lipids ratio in small VLDL || id:ebi-cfb233-GCST90302113' on 'ER- Breast cancer (Combined Oncoarray; iCOGS

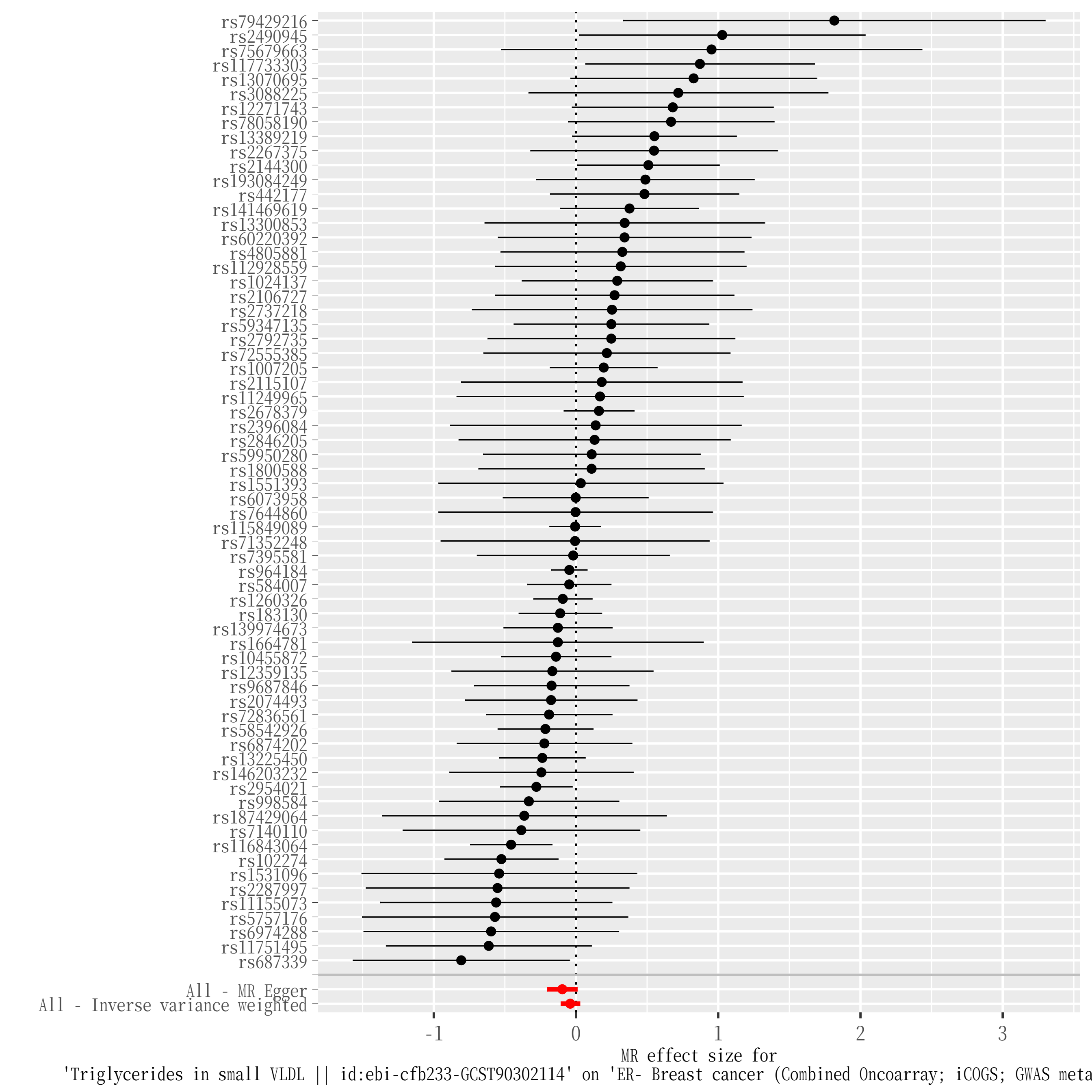
rs79429216
rs2490945
rs75679663
rs117733303
rs13070695
rs3088225
rs12271743
rs78058190
rs13389219
rs2267375
rs2144300
rs193084249
rs442177
rs141469619
rs13300853
rs60220392
rs4805881
rs112928559
rs1024137
rs2106727
rs2737218
rs59347135
rs2792735
rs72555385
rs1007205
rs2115107
rs11249965
rs2678379
rs2396084
rs2846205
rs59950280
rs1800588
rs1551393
rs6073958
rs7644860
rs115849089
rs71352248
rs7395581
rs964184
rs584007
rs1260326
rs183130
rs139974673
rs1664781
rs10455872
rs12359135
rs9687846
rs2074493
rs72836561
rs58542926
rs6874202
rs13225450
rs146203232
rs2954021
rs998584
rs187429064
rs7140110
rs116843064
rs102274
rs1531096
rs2287997
rs11155073
rs5757176
rs6974288
rs11751495
rs687339
All - MR Egger
All - Inverse variance weighted
-1
0
1
2
3
MR effect size for
'Triglycerides in small VLDL || id:ebi-cfb233-GCST90302114' on 'ER- Breast cancer (Combined Oncoarray; iCOGS; GWAS meta

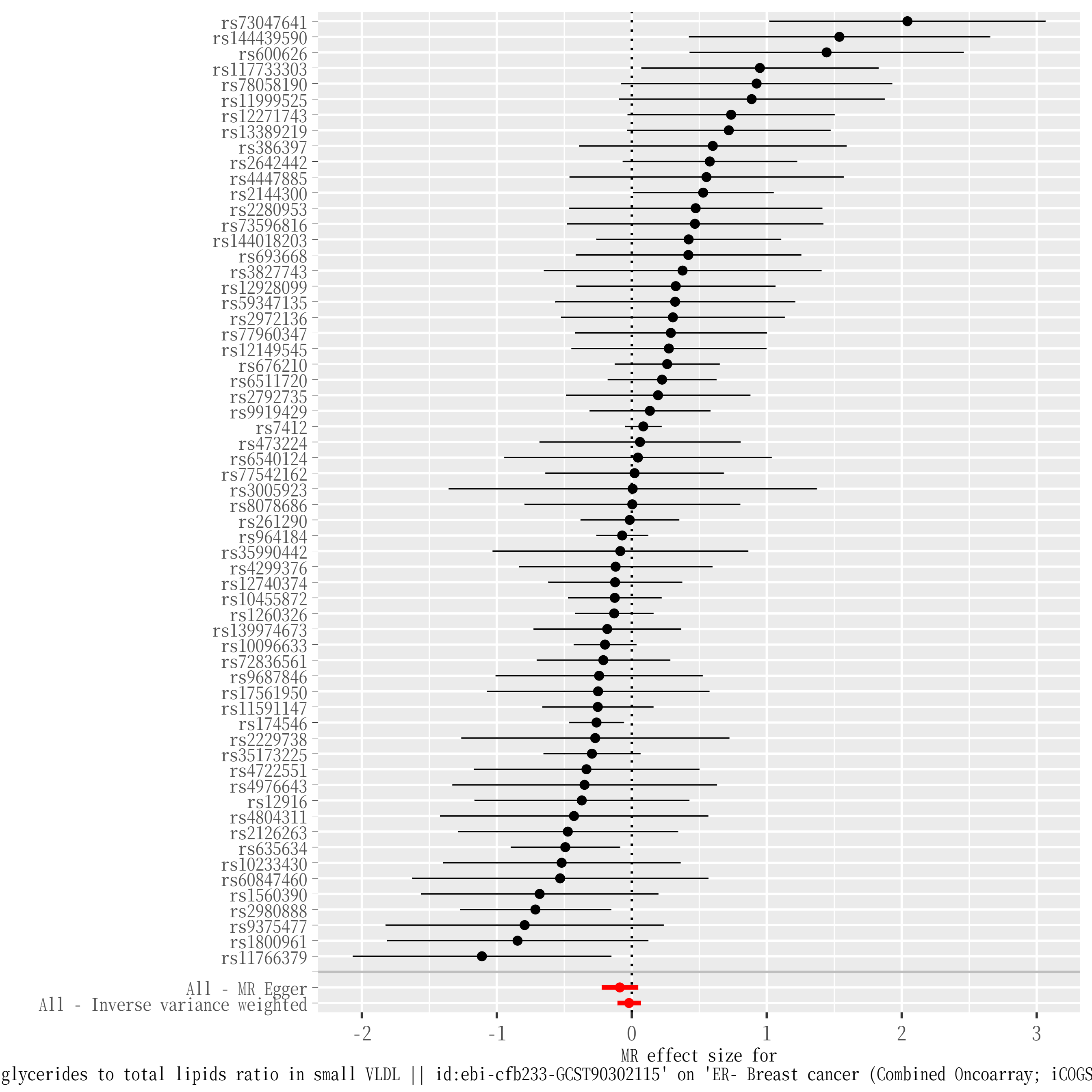

rs73047641
rs144439590
rs600626
rs117733303
rs78058190
rs11999525
rs12271743
rs13389219
rs386397
rs2642442
rs4447885
rs2144300
rs2280953
rs73596816
rs144018203
rs693668
rs3827743
rs12928099
rs59347135
rs2972136
rs77960347
rs12149545
rs676210
rs6511720
rs2792735
rs9919429
rs7412
rs473224
rs6540124
rs77542162
rs3005923
rs8078686
rs261290
rs964184
rs35990442
rs4299376
rs12740374
rs10455872
rs1260326
rs139974673
rs10096633
rs72836561
rs9687846
rs17561950
rs11591147
rs174546
rs2229738
rs35173225
rs4722551
rs4976643
rs12916
rs4804311
rs2126263
rs635634
rs10233430
rs60847460
rs1560390
rs2980888
rs9375477
rs1800961
rs11766379
All - MR Egger
All - Inverse variance weighted
-2
-1
0
1
2
3
MR effect size for
glycerides to total lipids ratio in small VLDL || id:ebi-cfb233-GCST90302115' on 'ER- Breast cancer (Combined Oncoarray; iCOGS

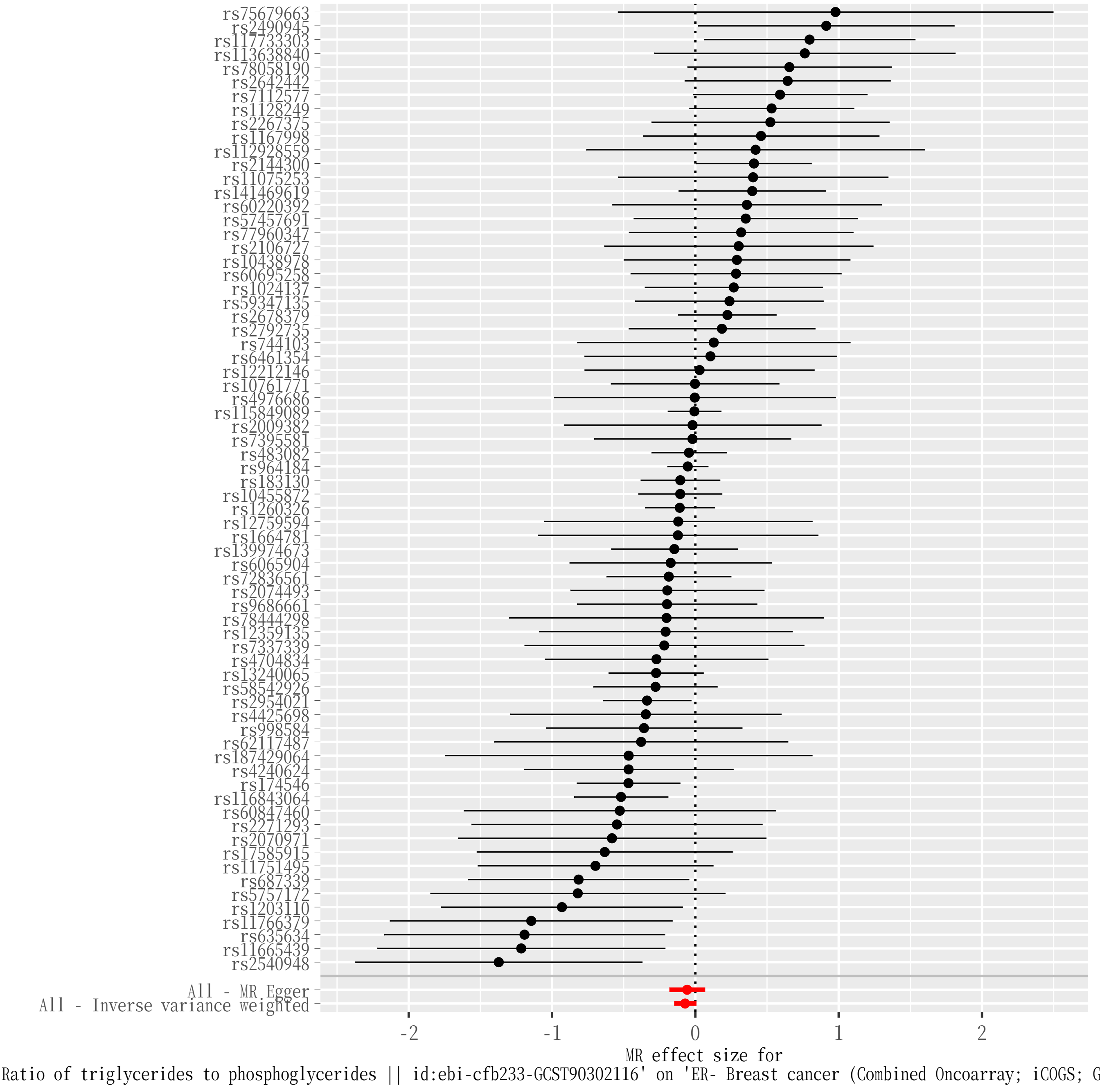
rs75679663
rs2490945
rs117733303
rs113638840
rs78058190
rs2642442
rs7112577
rs1128249
rs2267375
rs1167998
rs112928559
rs2144300
rs11075253
rs141469619
rs60220392
rs57457691
rs77960347
rs2106727
rs10438978
rs60695258
rs1024137
rs59347135
rs2678379
rs2792735
rs744103
rs6461354
rs12212146
rs10761771
rs4976686
rs115849089
rs2009382
rs7395581
rs483082
rs964184
rs183130
rs10455872
rs1260326
rs12759594
rs1664781
rs139974673
rs6065904
rs72836561
rs2074493
rs9686661
rs78444298
rs12359135
rs7337339
rs4704834
rs13240065
rs58542926
rs2954021
rs4425698
rs998584
rs62117487
rs187429064
rs4240624
rs174546
rs116843064
rs60847460
rs2271293
rs2070971
rs17585915
rs11751495
rs687339
rs5757172
rs1203110
rs11766379
rs635634
rs11665439
rs2540948
All - MR Egger
All - Inverse variance weighted
-2
-1
0
1
2
MR effect size for
'Ratio of triglycerides to phosphoglycerides || id:ebi-cfb233-GCST90302116' on 'ER- Breast cancer (Combined Oncoarray; iCOGS; G

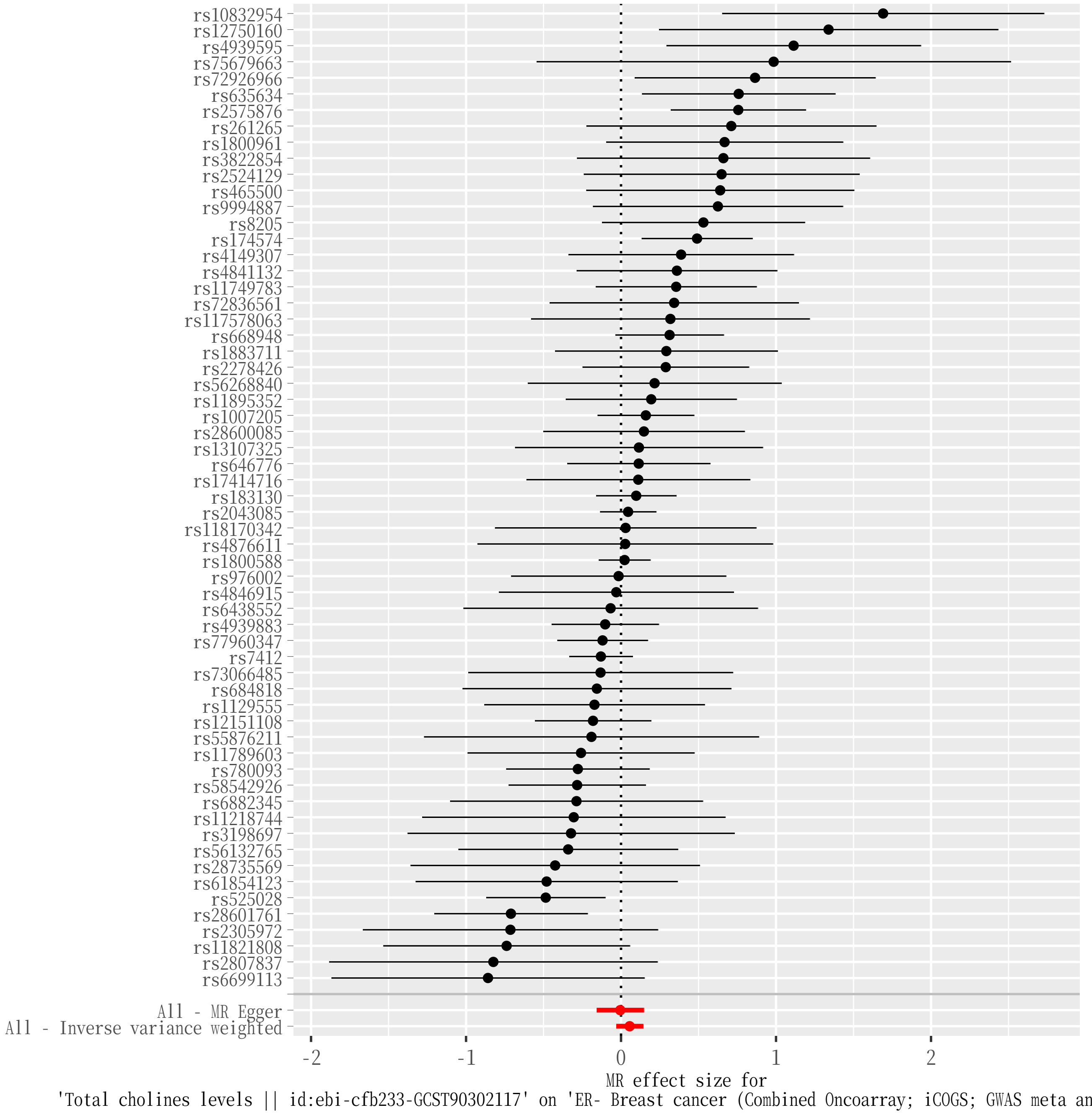

rs10832954
rs12750160
rs4939595
rs75679663
rs72926966
rs635634
rs2575876
rs261265
rs1800961
rs3822854
rs2524129
rs465500
rs9994887
rs8205
rs174574
rs4149307
rs4841132
rs11749783
rs72836561
rs117578063
rs668948
rs1883711
rs2278426
rs56268840
rs11895352
rs1007205
rs28600085
rs13107325
rs646776
rs17414716
rs183130
rs2043085
rs118170342
rs4876611
rs1800588
rs976002
rs4846915
rs6438552
rs4939883
rs77960347
rs7412
rs73066485
rs684818
rs1129555
rs12151108
rs55876211
rs11789603
rs780093
rs58542926
rs6882345
rs11218744
rs3198697
rs56132765
rs28735569
rs61854123
rs525028
rs28601761
rs2305972
rs11821808
rs2807837
rs6699113
All - MR Egger
All - Inverse variance weighted
-2
-1
0
1
2
MR effect size for
'Total cholines levels || id:ebi-cfb233-GCST90302117' on 'ER- Breast cancer (Combined Oncoarray; iCOGS; GWAS meta an

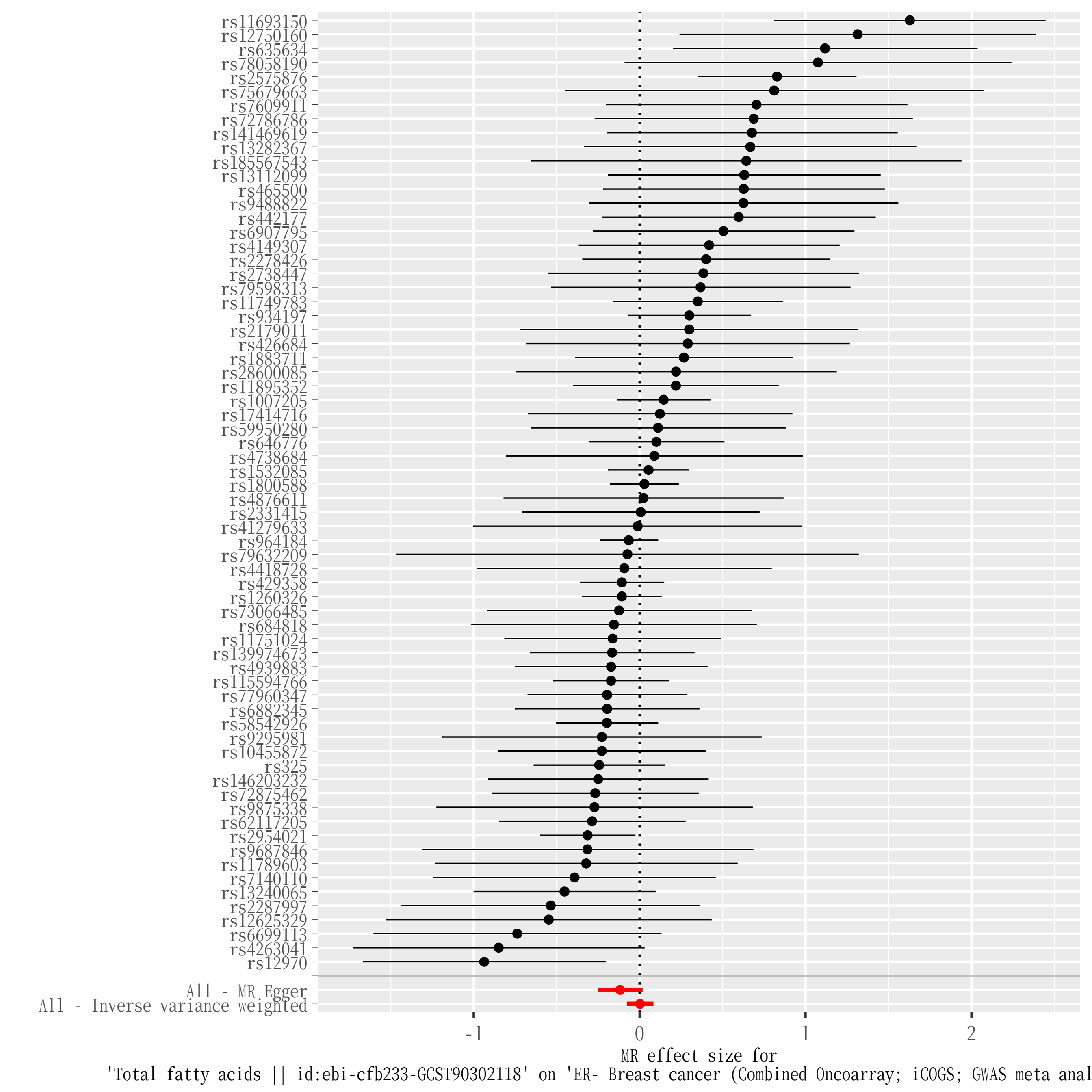
rs11693150
rs12750160
rs635634
rs78058190
rs2575876
rs75679663
rs7609911
rs72786786
rs141469619
rs13282367
rs185567543
rs13112099
rs465500
rs9488822
rs442177
rs6907795
rs4149307
rs2278426
rs2738447
rs79598313
rs11749783
rs934197
rs2179011
rs426684
rs1883711
rs28600085
rs11895352
rs1007205
rs17414716
rs59950280
rs646776
rs4738684
rs1532085
rs1800588
rs4876611
rs2331415
rs41279633
rs964184
rs79632209
rs4418728
rs429358
rs1260326
rs73066485
rs684818
rs11751024
rs139974673
rs4939883
rs115594766
rs77960347
rs6882345
rs58542926
rs9295981
rs10455872
rs325
rs146203232
rs72875462
rs9875338
rs62117205
rs2954021
rs9687846
rs11789603
rs7140110
rs13240065
rs2287997
rs12625329
rs6699113
rs4263041
rs12970
All - MR Egger
All - Inverse variance weighted
-1
0
1
2
MR effect size for
'Total fatty acids || id:ebi-cfb233-GCST90302118' on 'ER- Breast cancer (Combined Oncoarray; iCOGS; GWAS meta anal

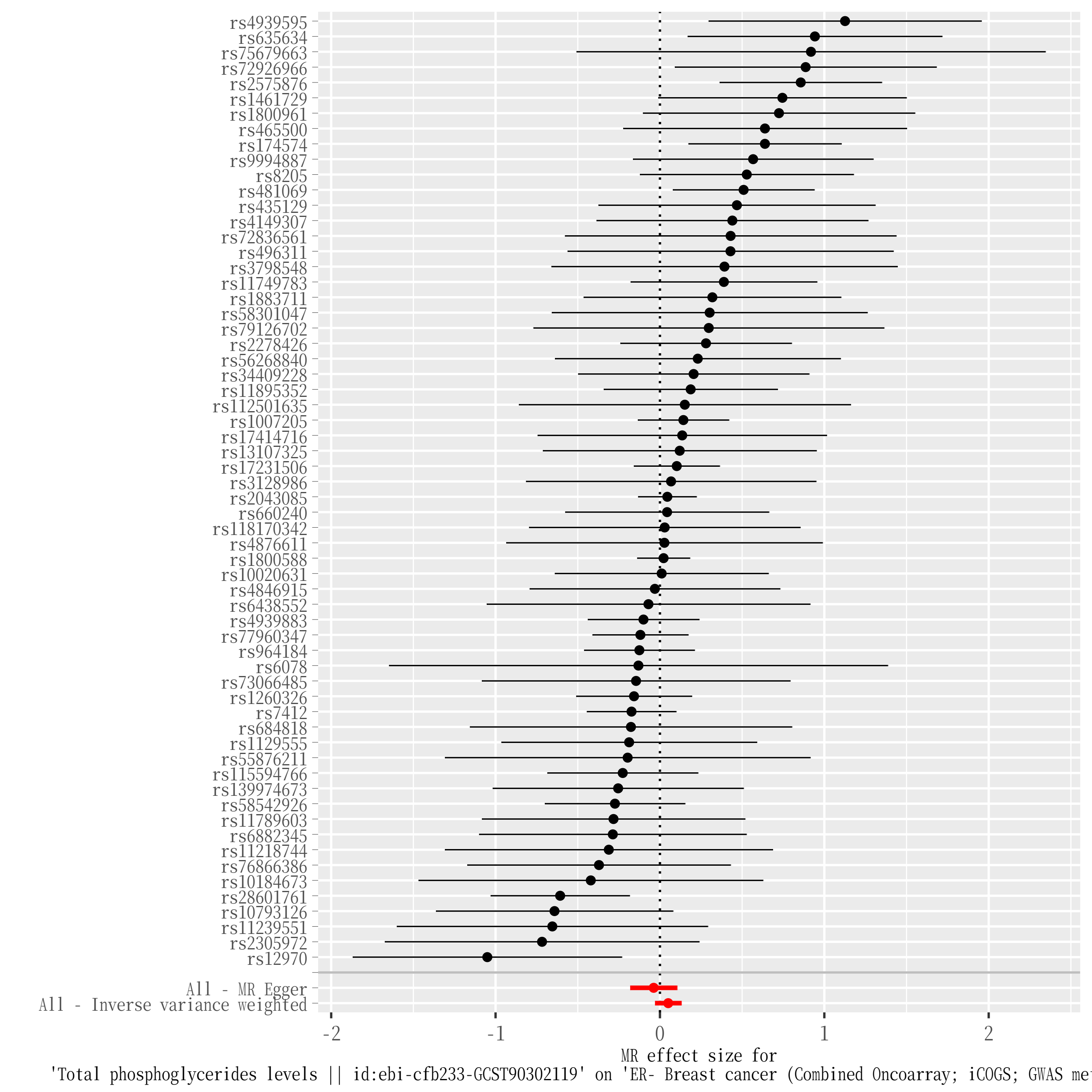
rs4939595
rs635634
rs75679663
rs72926966
rs2575876
rs1461729
rs1800961
rs465500
rs174574
rs9994887
rs8205
rs481069
rs435129
rs4149307
rs72836561
rs496311
rs3798548
rs11749783
rs1883711
rs58301047
rs79126702
rs2278426
rs56268840
rs34409228
rs11895352
rs112501635
rs1007205
rs17414716
rs13107325
rs17231506
rs3128986
rs2043085
rs660240
rs118170342
rs4876611
rs1800588
rs10020631
rs4846915
rs6438552
rs4939883
rs77960347
rs964184
rs6078
rs73066485
rs1260326
rs7412
rs684818
rs1129555
rs55876211
rs115594766
rs139974673
rs58542926
rs11789603
rs6882345
rs11218744
rs76866386
rs10184673
rs28601761
rs10793126
rs11239551
rs2305972
rs12970
All - MR Egger
All - Inverse variance weighted
-2
-1
0
1
2
MR effect size for
'Total phosphoglycerides levels || id:ebi-cfb233-GCST90302119' on 'ER- Breast cancer (Combined Oncoarray; iCOGS; GWAS me

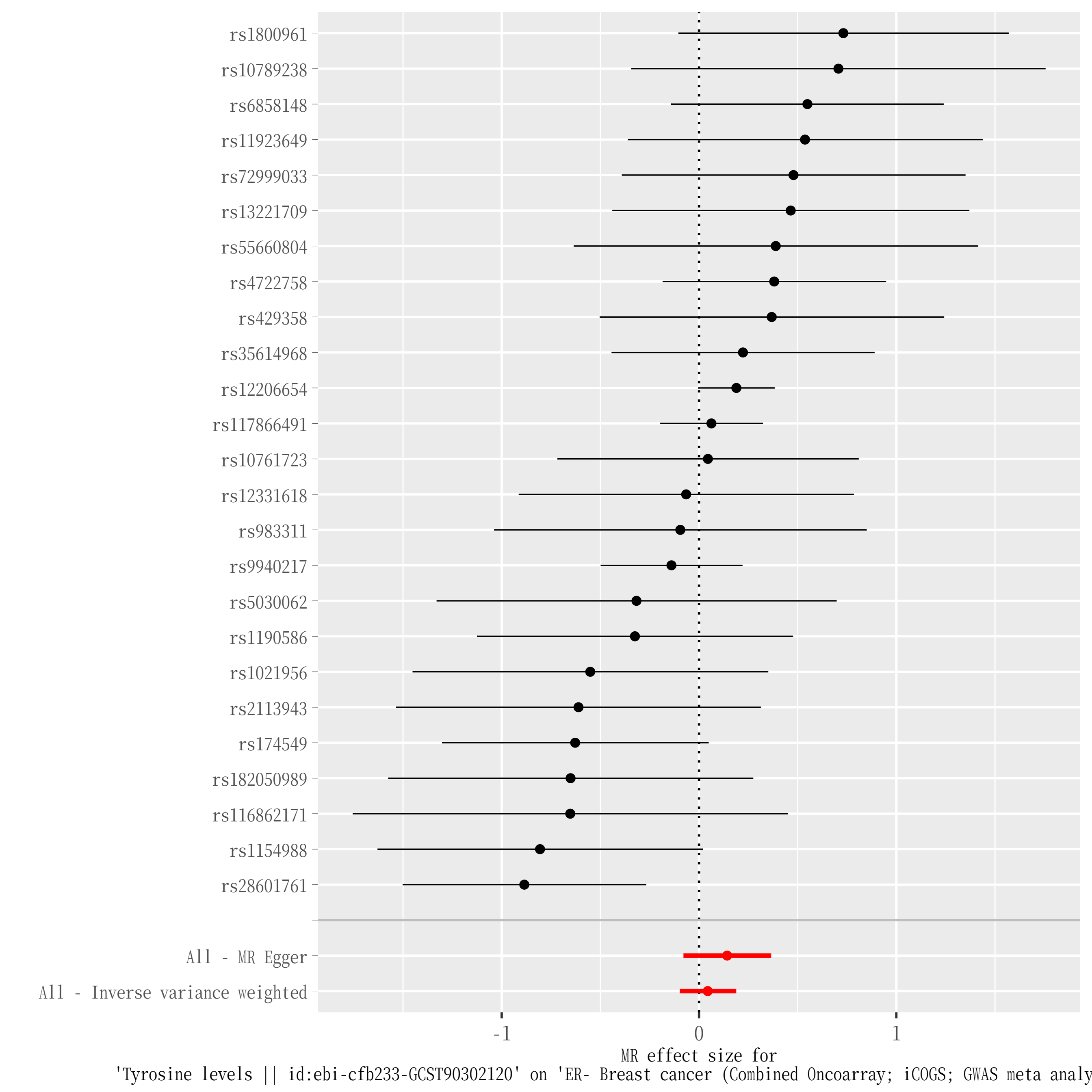
rs1800961
rs10789238
rs6858148
rs11923649
rs72999033
rs13221709
rs55660804
rs4722758
rs429358
rs35614968
rs12206654
rs117866491
rs10761723
rs12331618
rs983311
rs9940217
rs5030062
rs1190586
rs1021956
rs2113943
rs174549
rs182050989
rs116862171
rs1154988
rs28601761
All - MR Egger
All - Inverse variance weighted
-1
0
1
MR effect size for
'Tyrosine levels || id:ebi-cfb233-GCST90302120' on 'ER- Breast cancer (Combined Oncoarray; iCOGS; GWAS meta analy

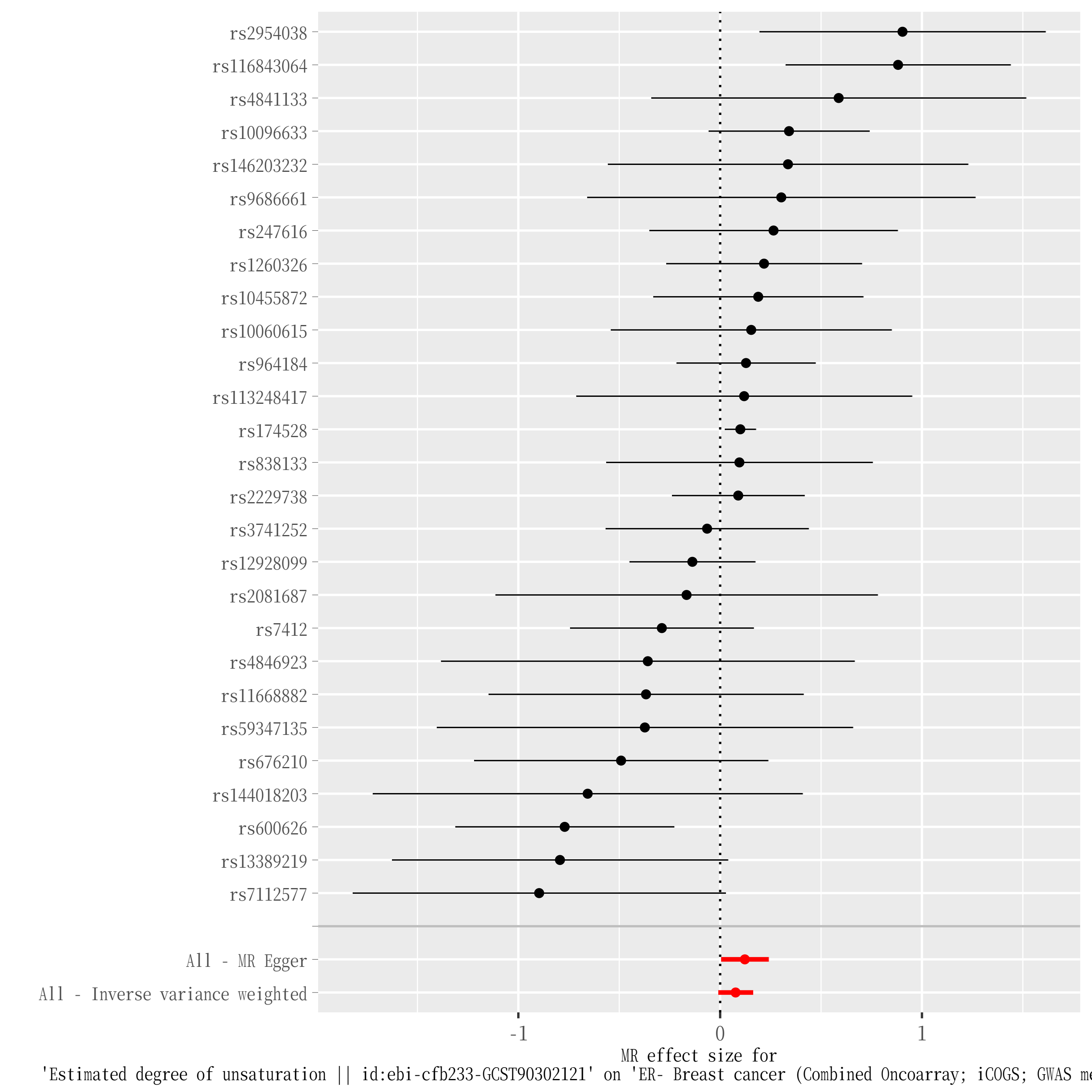

rs2954038
rs116843064
rs4841133
rs10096633
rs146203232
rs9686661
rs247616
rs1260326
rs10455872
rs10060615
rs964184
rs113248417
rs174528
rs838133
rs2229738
rs3741252
rs12928099
rs2081687
rs7412
rs4846923
rs11668882
rs59347135
rs676210
rs144018203
rs600626
rs13389219
rs7112577
All - MR Egger
All - Inverse variance weighted
-1
0
1
MR effect size for
'Estimated degree of unsaturation || id:ebi-cfb233-GCST90302121' on 'ER- Breast cancer (Combined Oncoarray; iCOGS; GWAS me

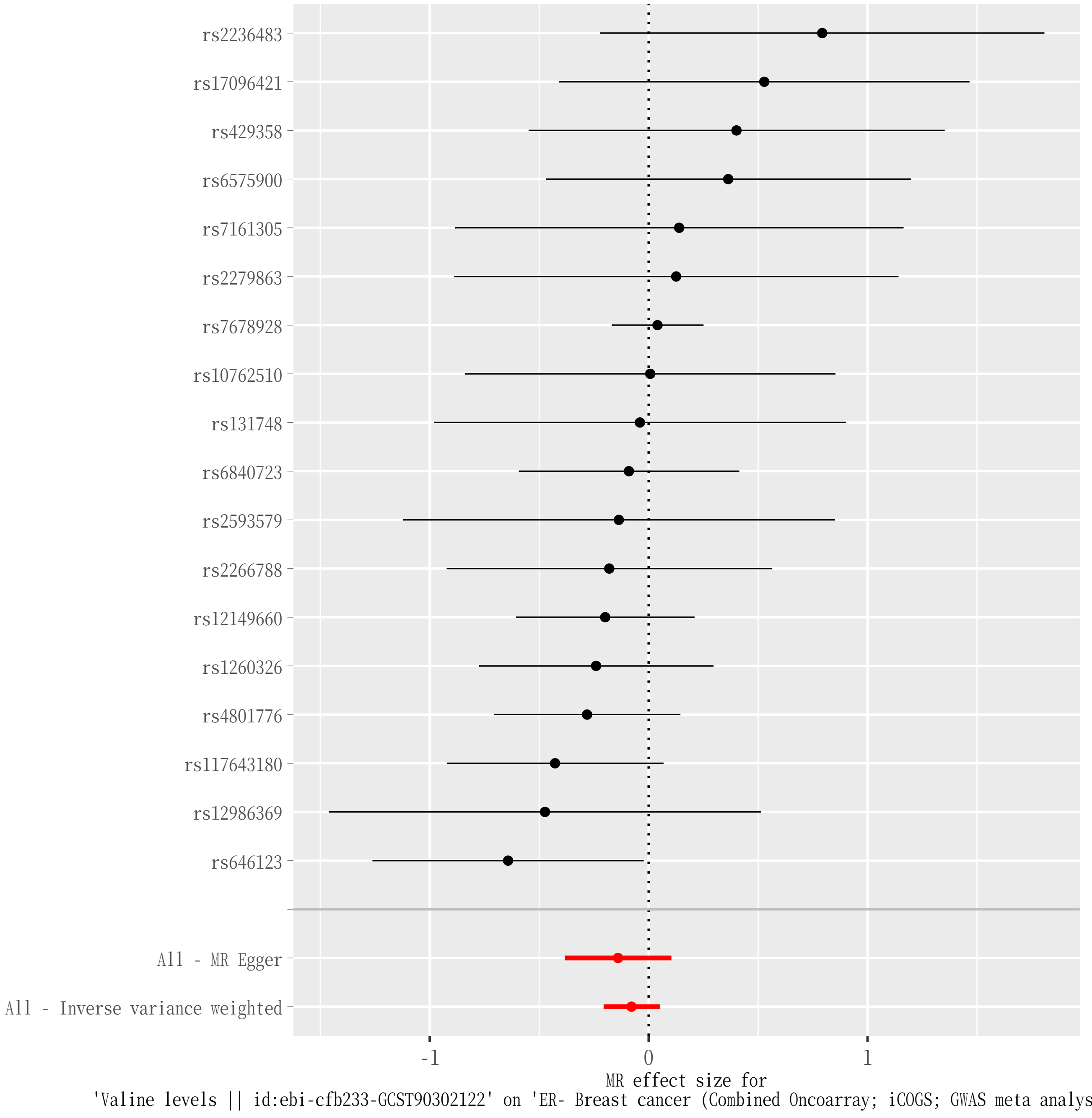

rs2236483
rs17096421
rs429358
rs6575900
rs7161305
rs2279863
rs7678928
rs10762510
rs131748
rs6840723
rs2593579
rs2266788
rs12149660
rs1260326
rs4801776
rs117643180
rs12986369
rs646123
All - MR Egger
All - Inverse variance weighted
-1
0
1
MR effect size for
'Valine levels || id:ebi-cfb233-GCST90302122' on 'ER- Breast cancer (Combined Oncoarray; iCOGS; GWAS meta analys

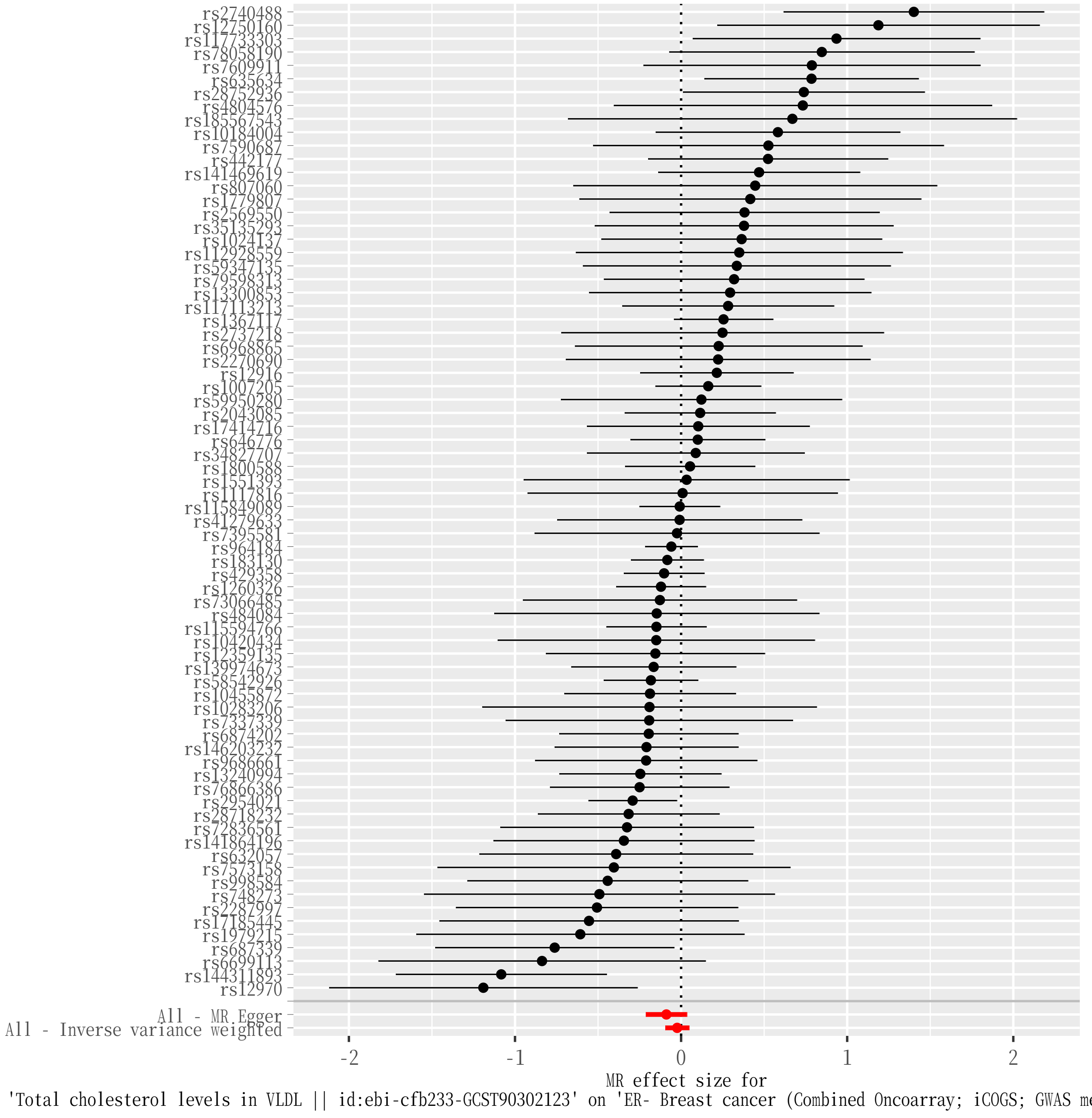

rs2740488
rs12750160
rs117733303
rs78058190
rs7609911
rs635634
rs28752936
rs4804576
rs185567543
rs10184004
rs7590687
rs442177
rs141469619
rs807060
rs1779807
rs2569550
rs35135293
rs1024137
rs112928559
rs59347135
rs79598313
rs13300853
rs117113213
rs1367117
rs2737218
rs6968865
rs2270690
rs12916
rs1007205
rs59950280
rs2043085
rs17414716
rs646776
rs34827707
rs1800588
rs1551393
rs1117816
rs115849089
rs41279633
rs7395581
rs964184
rs183130
rs429358
rs1260326
rs73066485
rs484084
rs115594766
rs10420434
rs12359135
rs139974673
rs58542926
rs10455872
rs10283206
rs7337339
rs6874202
rs146203232
rs9686661
rs13240994
rs76866386
rs2954021
rs28718232
rs72836561
rs141864196
rs632057
rs7573158
rs998584
rs748273
rs2287997
rs17185445
rs1979215
rs687339
rs6699113
rs144311893
rs12970
All - MR Egger
All - Inverse variance weighted
-2
-1
0
1
2
MR effect size for
'Total cholesterol levels in VLDL || id:ebi-cfb233-GCST90302123' on 'ER- Breast cancer (Combined Oncoarray; iCOGS; GWAS me

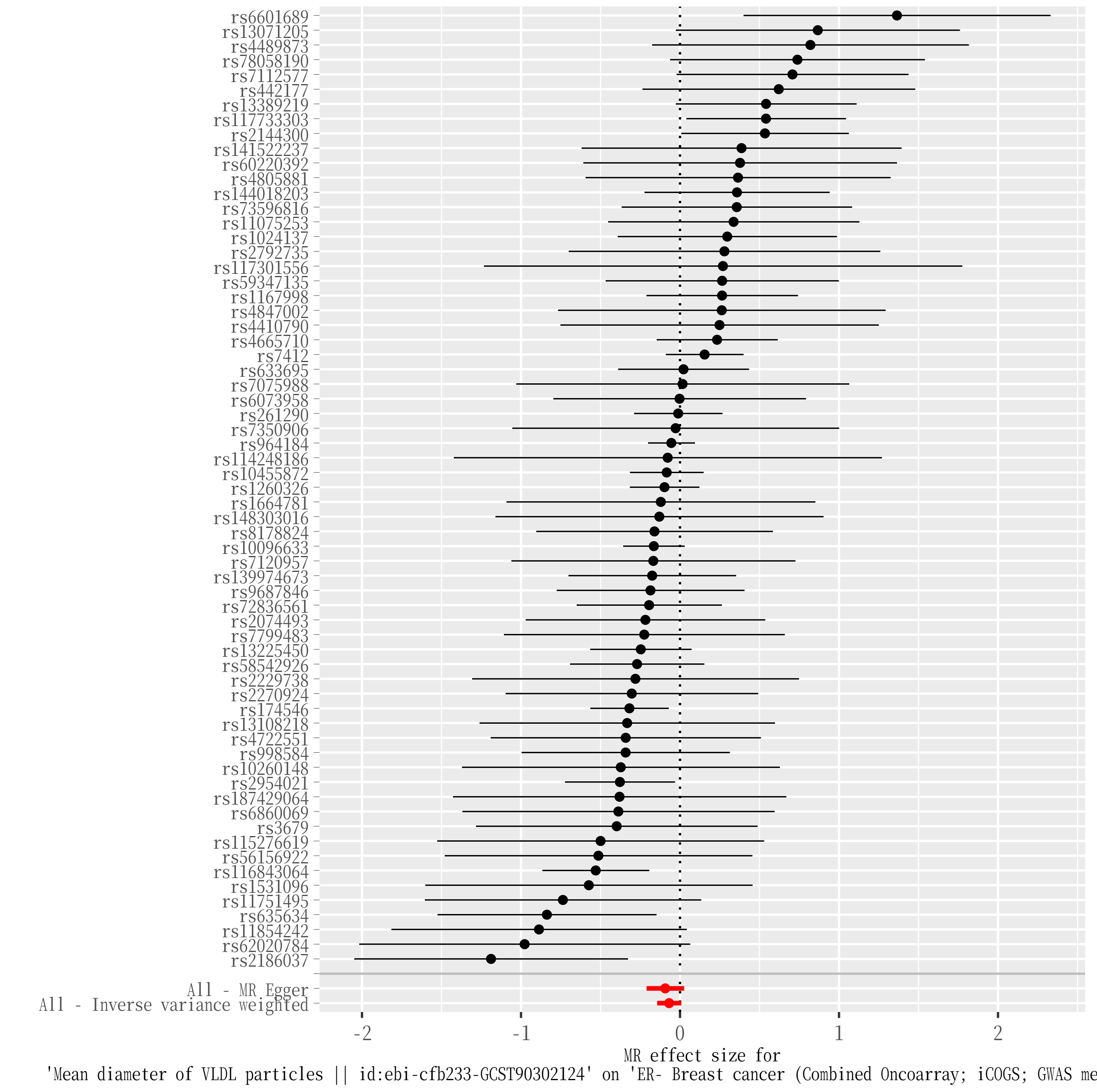

rs6601689
rs13071205
rs4489873
rs78058190
rs7112577
rs442177
rs13389219
rs117733303
rs2144300
rs141522237
rs60220392
rs4805881
rs144018203
rs73596816
rs11075253
rs1024137
rs2792735
rs117301556
rs59347135
rs1167998
rs4847002
rs4410790
rs4665710
rs7412
rs633695
rs7075988
rs6073958
rs261290
rs7350906
rs964184
rs114248186
rs10455872
rs1260326
rs1664781
rs148303016
rs8178824
rs10096633
rs7120957
rs139974673
rs9687846
rs72836561
rs2074493
rs7799483
rs13225450
rs58542926
rs2229738
rs2270924
rs174546
rs13108218
rs4722551
rs998584
rs10260148
rs2954021
rs187429064
rs6860069
rs3679
rs115276619
rs56156922
rs116843064
rs1531096
rs11751495
rs635634
rs11854242
rs62020784
rs2186037
All - MR Egger
All - Inverse variance weighted
-2
-1
0
1
2
MR effect size for
'Mean diameter of VLDL particles || id:ebi-cfb233-GCST90302124' on 'ER- Breast cancer (Combined Oncoarray; iCOGS; GWAS me

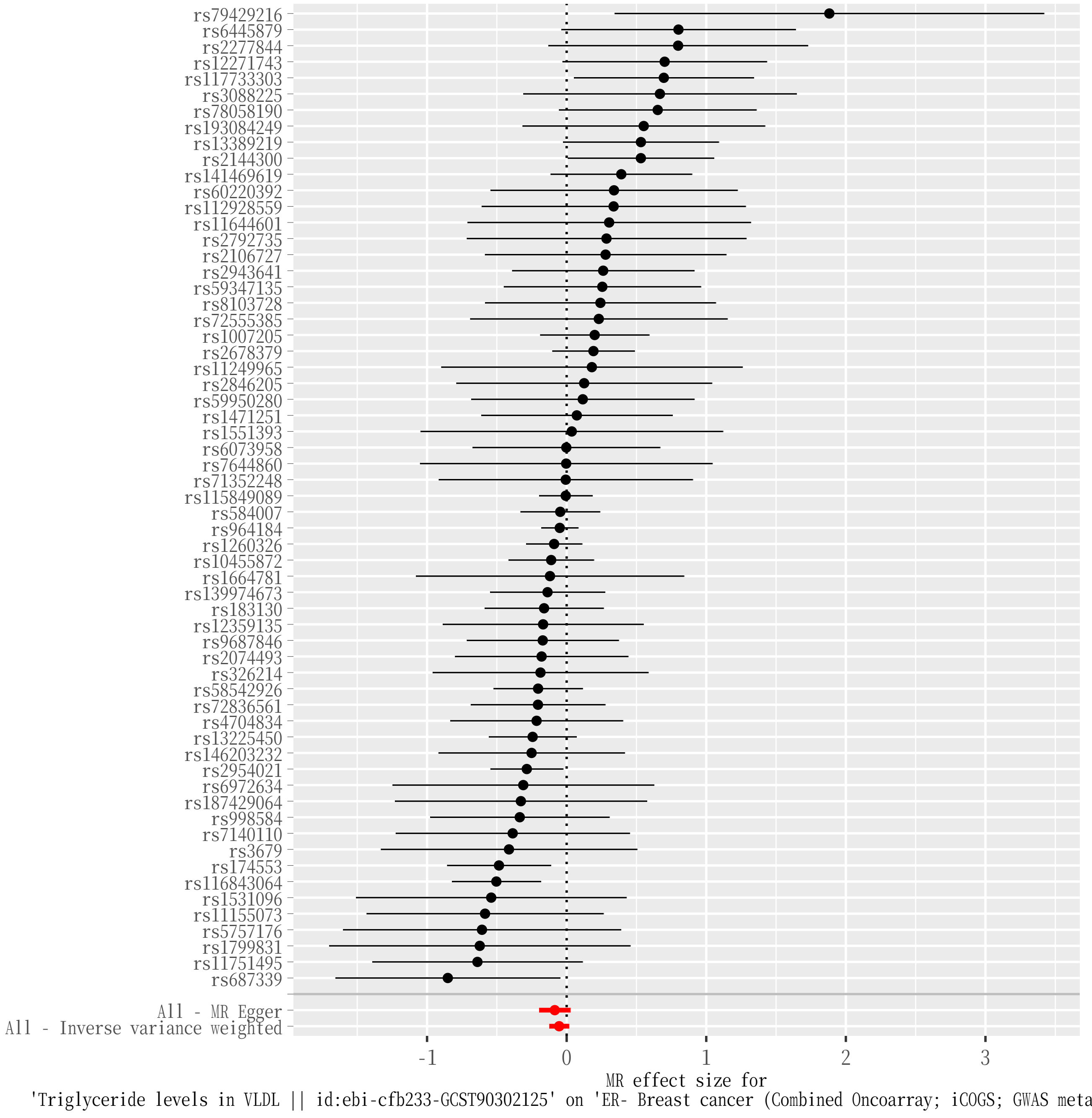
rs79429216
rs6445879
rs2277844
rs12271743
rs117733303
rs3088225
rs78058190
rs193084249
rs13389219
rs2144300
rs141469619
rs60220392
rs112928559
rs11644601
rs2792735
rs2106727
rs2943641
rs59347135
rs8103728
rs72555385
rs1007205
rs2678379
rs11249965
rs2846205
rs59950280
rs1471251
rs1551393
rs6073958
rs7644860
rs71352248
rs115849089
rs584007
rs964184
rs1260326
rs10455872
rs1664781
rs139974673
rs183130
rs12359135
rs9687846
rs2074493
rs326214
rs58542926
rs72836561
rs4704834
rs13225450
rs146203232
rs2954021
rs6972634
rs187429064
rs998584
rs7140110
rs3679
rs174553
rs116843064
rs1531096
rs11155073
rs5757176
rs1799831
rs11751495
rs687339
All - MR Egger
All - Inverse variance weighted
-1
0
1
2
3
MR effect size for
'Triglyceride levels in VLDL || id:ebi-cfb233-GCST90302125' on 'ER- Breast cancer (Combined Oncoarray; iCOGS; GWAS meta

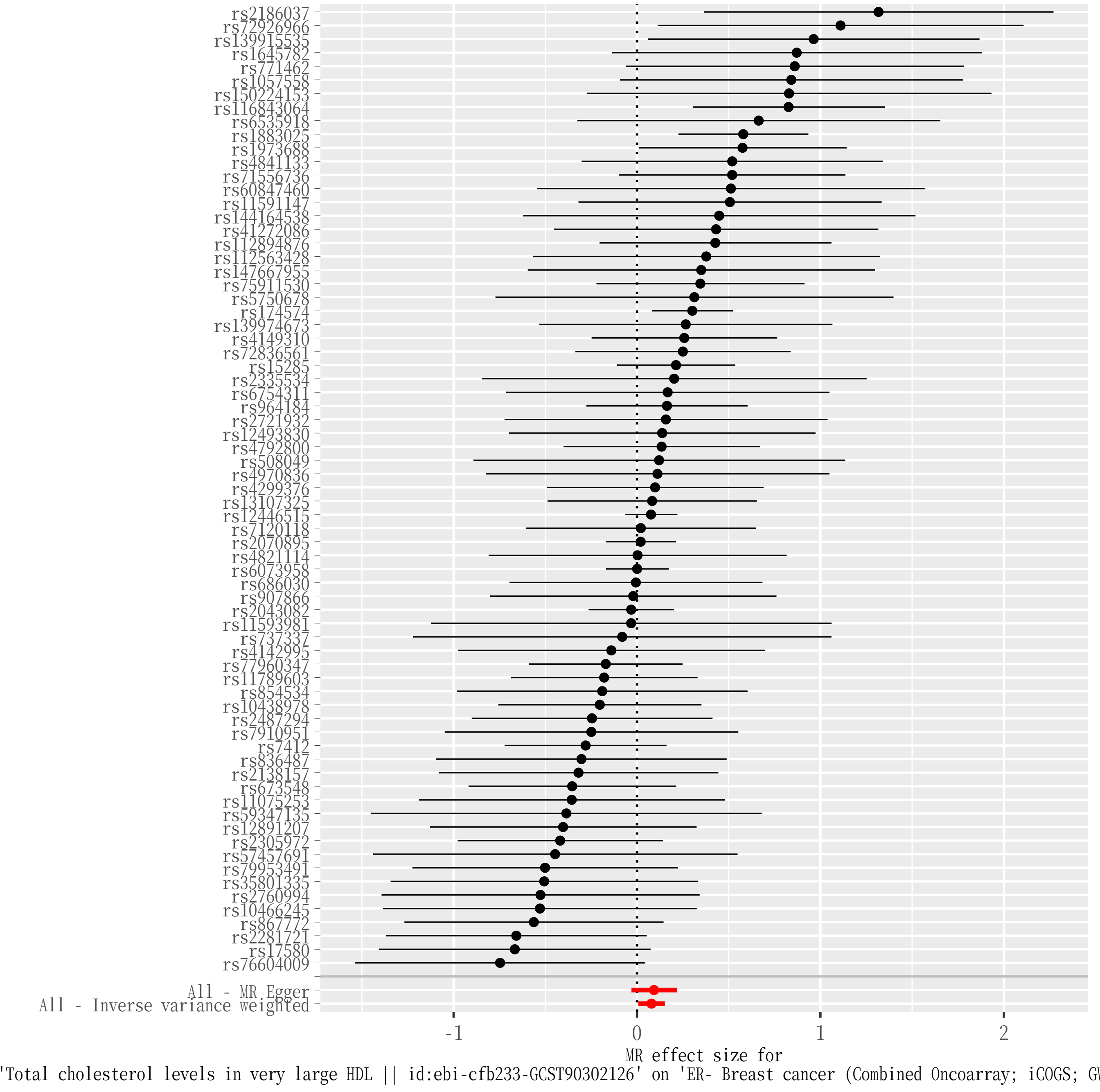

rs2186037
rs72926966
rs139915535
rs1645782
rs771462
rs1057558
rs150224153
rs116843064
rs6535918
rs1883025
rs1973688
rs4841133
rs71556736
rs60847460
rs11591147
rs144164538
rs41272086
rs112894876
rs112563428
rs147667955
rs75911530
rs5750678
rs174574
rs139974673
rs4149310
rs72836561
rs15285
rs2335534
rs6754311
rs964184
rs2721932
rs12493830
rs4792800
rs508049
rs4970836
rs4299376
rs13107325
rs12446515
rs7120118
rs2070895
rs4821114
rs6073958
rs686030
rs907866
rs2043082
rs11593981
rs737337
rs4142995
rs77960347
rs11789603
rs854534
rs10438978
rs2487294
rs7910951
rs7412
rs836487
rs2138157
rs673548
rs11075253
rs59347135
rs12891207
rs2305972
rs57457691
rs79953491
rs35801335
rs2760994
rs10466245
rs867772
rs2281721
rs17580
rs76604009
All - MR Egger
All - Inverse variance weighted
-1
0
1
2
MR effect size for
'Total cholesterol levels in very large HDL || id:ebi-cfb233-GCST90302126' on 'ER- Breast cancer (Combined Oncoarray; iCOGS; GV

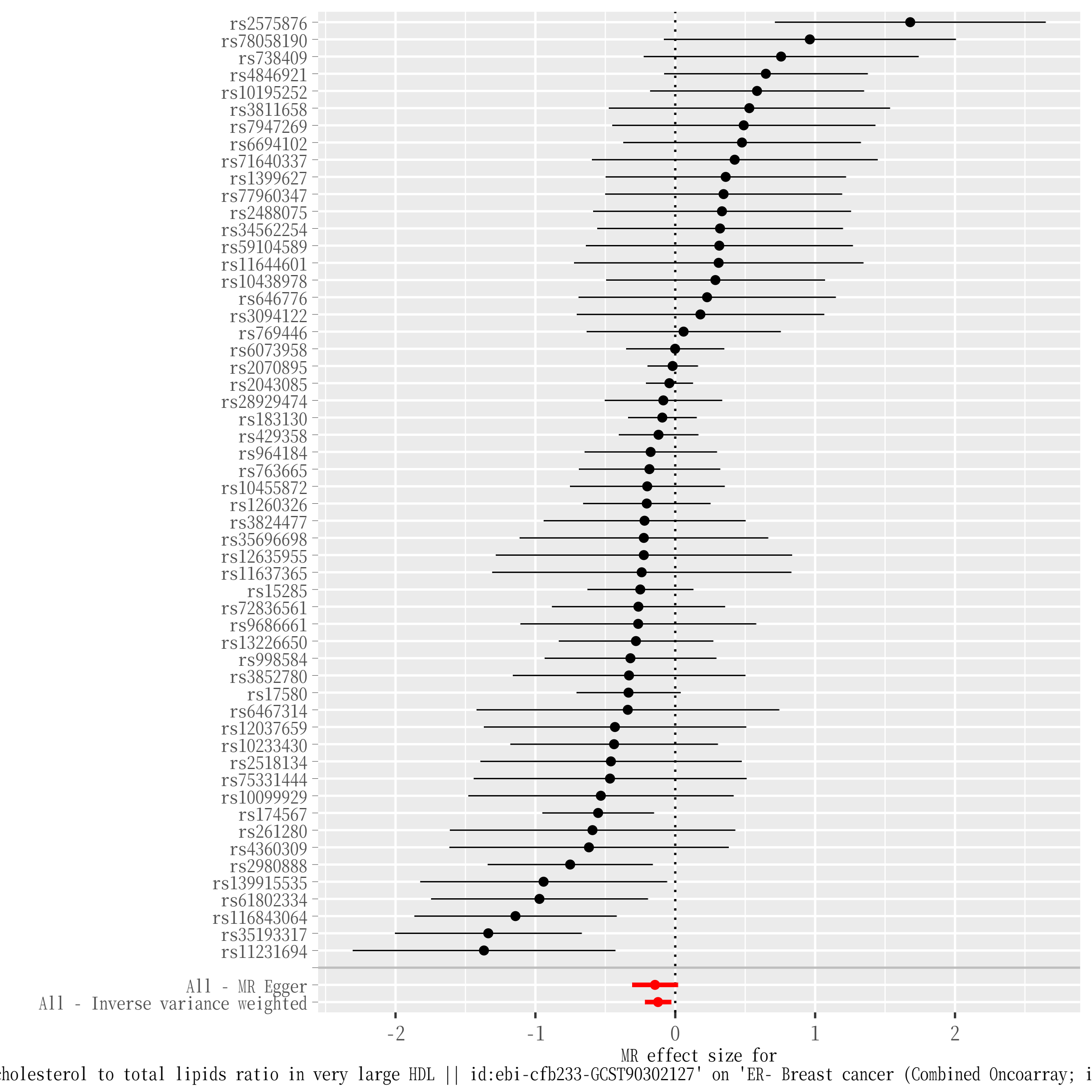

rs2575876
rs78058190
rs738409
rs4846921
rs10195252
rs3811658
rs7947269
rs6694102
rs71640337
rs1399627
rs77960347
rs2488075
rs34562254
rs59104589
rs11644601
rs10438978
rs646776
rs3094122
rs769446
rs6073958
rs2070895
rs2043085
rs28929474
rs183130
rs429358
rs964184
rs763665
rs10455872
rs1260326
rs3824477
rs35696698
rs12635955
rs11637365
rs15285
rs72836561
rs9686661
rs13226650
rs998584
rs3852780
rs17580
rs6467314
rs12037659
rs10233430
rs2518134
rs75331444
rs10099929
rs174567
rs261280
rs4360309
rs2980888
rs139915535
rs61802334
rs116843064
rs35193317
rs11231694
All - MR Egger
All - Inverse variance weighted
-2
-1
0
1
2
MR effect size for
cholesterol to total lipids ratio in very large HDL || id:ebi-cfb233-GCST90302127' on 'ER- Breast cancer (Combined Oncoarray; i

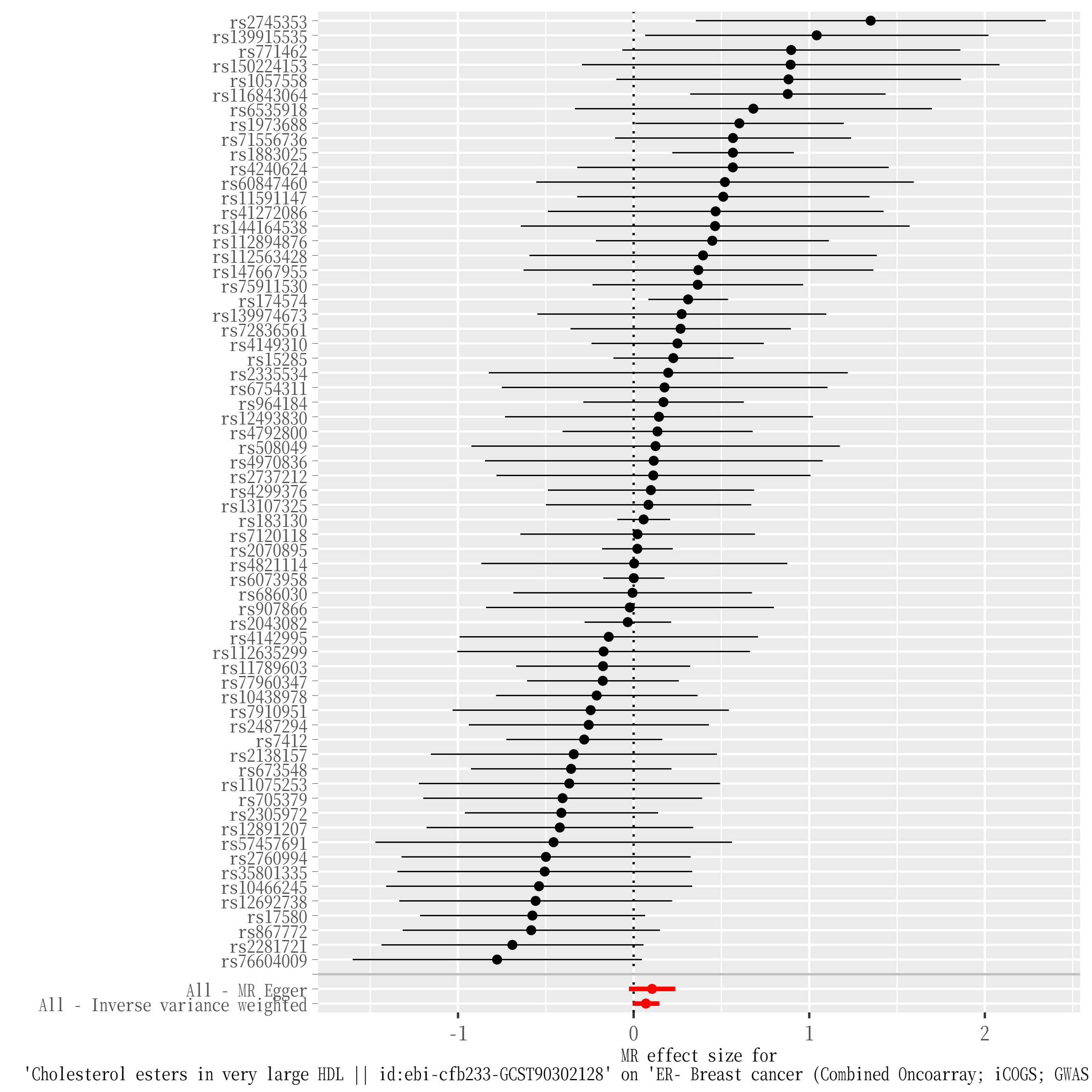
rs2745353
rs139915535
rs771462
rs150224153
rs1057558
rs116843064
rs6535918
rs1973688
rs71556736
rs1883025
rs4240624
rs60847460
rs11591147
rs41272086
rs144164538
rs112894876
rs112563428
rs147667955
rs75911530
rs174574
rs139974673
rs72836561
rs4149310
rs15285
rs2335534
rs6754311
rs964184
rs12493830
rs4792800
rs508049
rs4970836
rs2737212
rs4299376
rs13107325
rs183130
rs7120118
rs2070895
rs4821114
rs6073958
rs686030
rs907866
rs2043082
rs4142995
rs112635299
rs11789603
rs77960347
rs10438978
rs7910951
rs2487294
rs7412
rs2138157
rs673548
rs11075253
rs705379
rs2305972
rs12891207
rs57457691
rs2760994
rs35801335
rs10466245
rs12692738
rs17580
rs867772
rs2281721
rs76604009
All - MR Egger
All - Inverse variance weighted
-1
0
1
2
MR effect size for
'Cholesterol esters in very large HDL || id:ebi-cfb233-GCST90302128' on 'ER- Breast cancer (Combined Oncoarray; iCOGS; GWAS

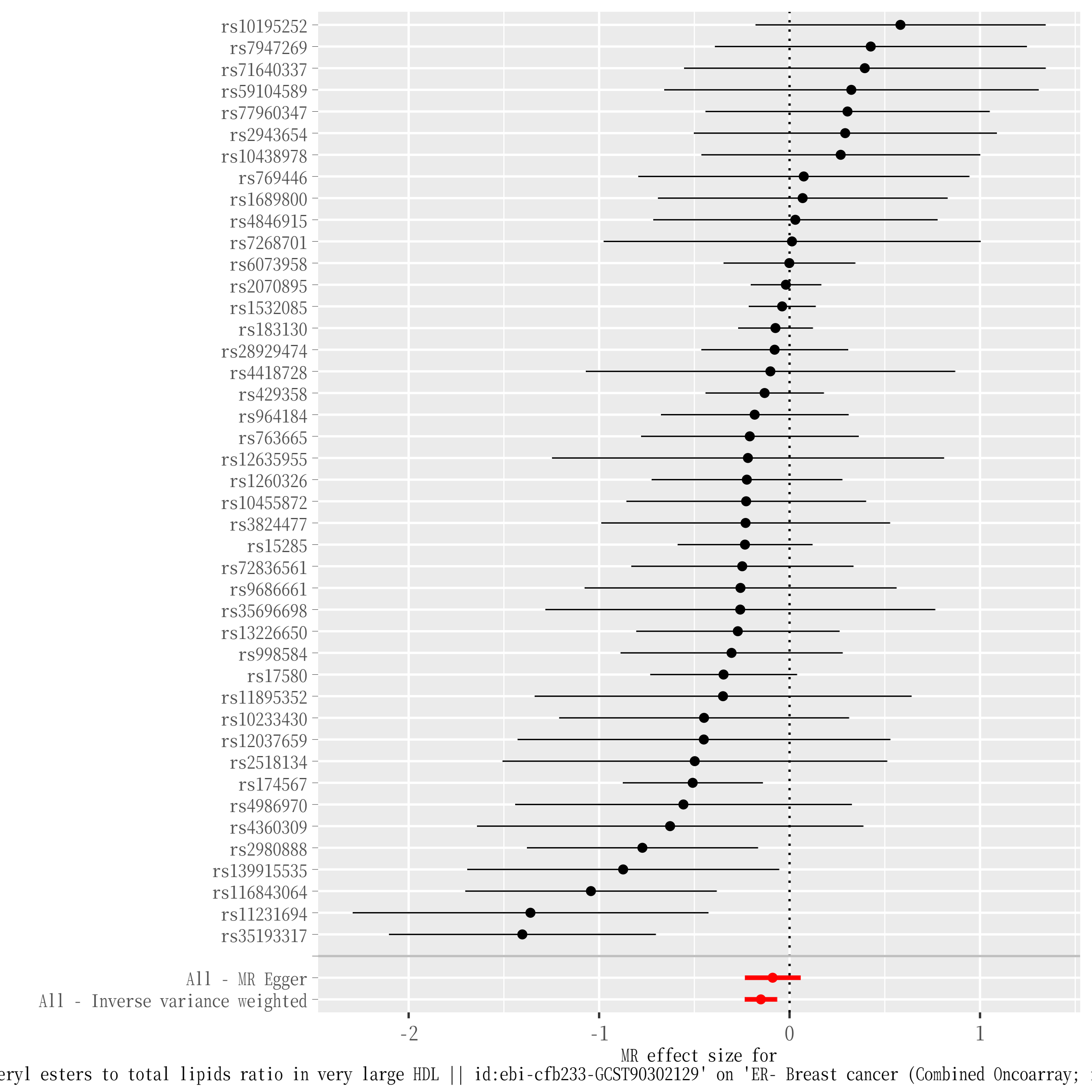

rs10195252
rs7947269
rs71640337
rs59104589
rs77960347
rs2943654
rs10438978
rs769446
rs1689800
rs4846915
rs7268701
rs6073958
rs2070895
rs1532085
rs183130
rs28929474
rs4418728
rs429358
rs964184
rs763665
rs12635955
rs1260326
rs10455872
rs3824477
rs15285
rs72836561
rs9686661
rs35696698
rs13226650
rs998584
rs17580
rs11895352
rs10233430
rs12037659
rs2518134
rs174567
rs4986970
rs4360309
rs2980888
rs139915535
rs116843064
rs11231694
rs35193317
All - MR Egger
All - Inverse variance weighted
-2
-1
0
1
MR effect size for
eryl esters to total lipids ratio in very large HDL || id:ebi-cfb233-GCST90302129' on 'ER- Breast cancer (Combined Oncoarray; i

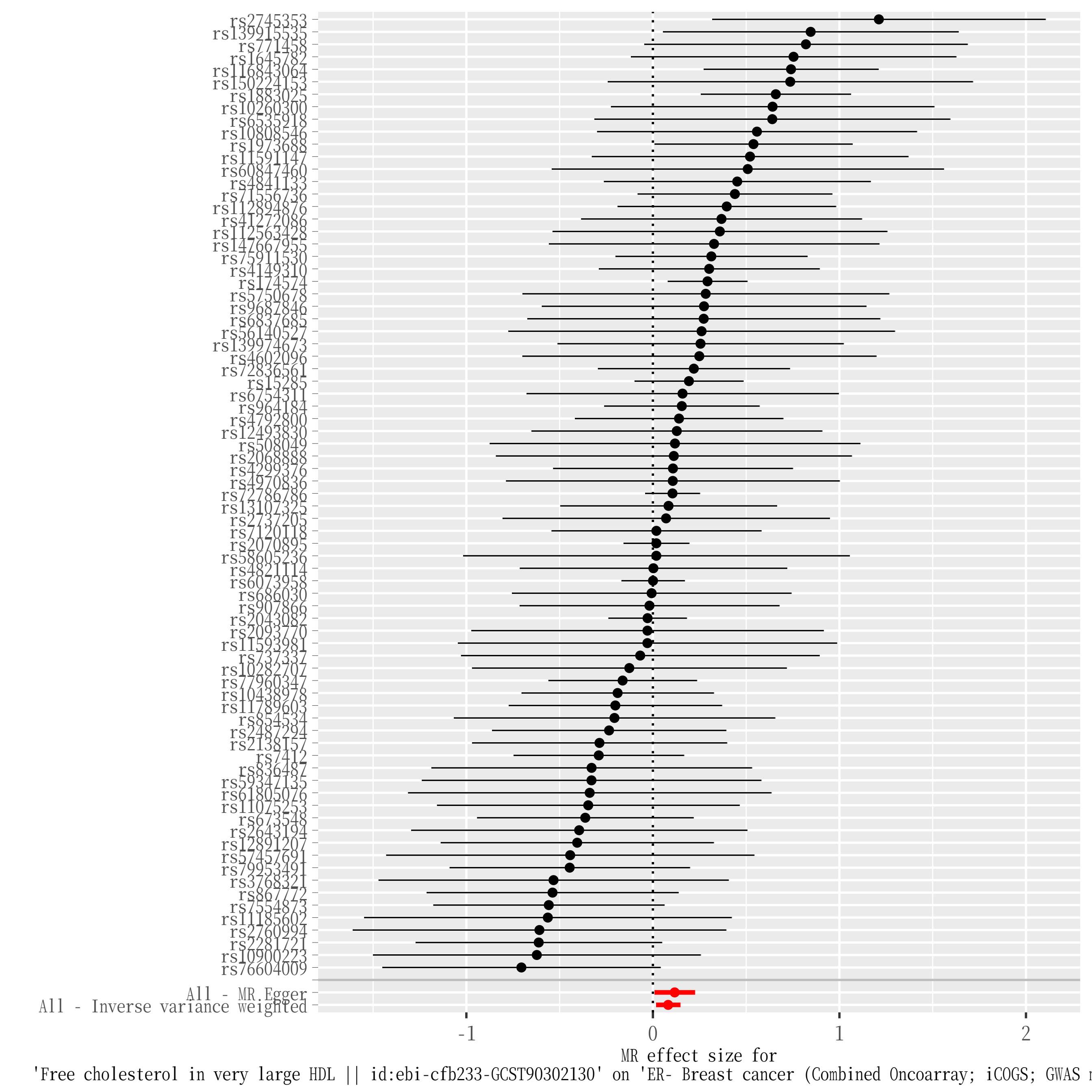
rs2745353
rs139215535
rs771458
rs1645782
rs116843064
rs150224153
rs1883025
rs10260300
rs6535918
rs10808546
rs1973688
rs11591147
rs60847460
rs4841133
rs71556736
rs112894876
rs41272086
rs112563428
rs147667955
rs75911530
rs4149310
rs174574
rs5750678
rs9687846
rs6837685
rs56140527
rs139974673
rs4602096
rs72836561
rs15285
rs6754311
rs964184
rs4792800
rs12493830
rs508049
rs2068888
rs4299376
rs4970836
rs72786786
rs13107325
rs2737205
rs7120118
rs2070895
rs58605236
rs4821114
rs6073958
rs686030
rs907866
rs2043082
rs2093770
rs11593981
rs737337
rs10282707
rs77960347
rs10438978
rs11789603
rs854534
rs2487294
rs2138157
rs7412
rs836487
rs59347135
rs61805076
rs11075253
rs673548
rs2643194
rs12891207
rs57457691
rs7995491
rs3768321
rs867772
rs7554873
rs11185602
rs2760994
rs2281721
rs10900223
rs76604009
All - MR Egger
All - Inverse variance weighted
-1
0
1
2
MR effect size for
'Free cholesterol in very large HDL || id:ebi-cfb233-GCST90302130' on 'ER- Breast cancer (Combined Oncoarray; iCOGS; GWAS

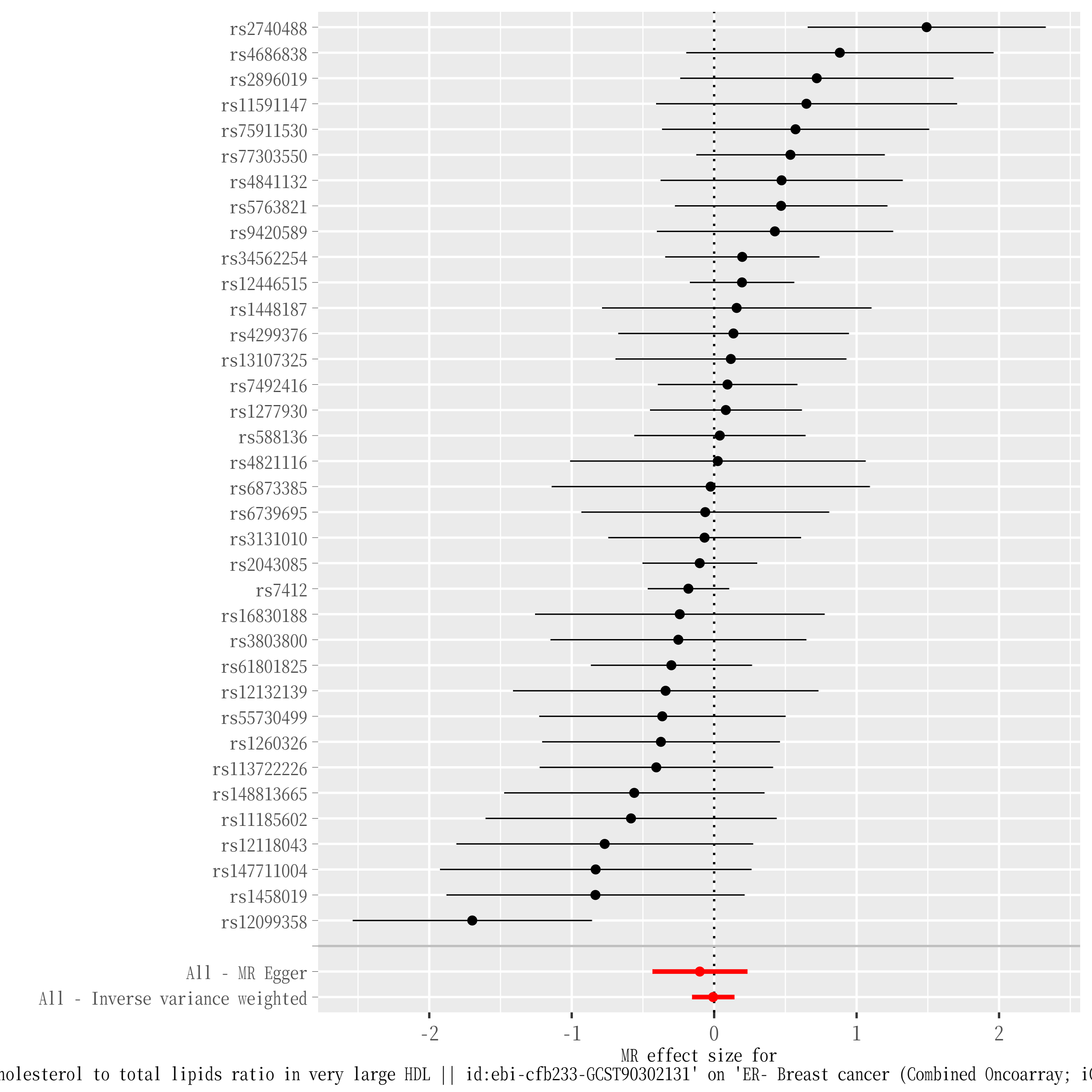
rs2740488
rs4686838
rs2896019
rs11591147
rs75911530
rs77303550
rs4841132
rs5763821
rs9420589
rs34562254
rs12446515
rs1448187
rs4299376
rs13107325
rs7492416
rs1277930
rs588136
rs4821116
rs6873385
rs6739695
rs3131010
rs2043085
rs7412
rs16830188
rs3803800
rs61801825
rs12132139
rs55730499
rs1260326
rs113722226
rs148813665
rs11185602
rs12118043
rs147711004
rs1458019
rs12099358
All - MR Egger
All - Inverse variance weighted
-2
-1
0
1
2
MR effect size for
holesterol to total lipids ratio in very large HDL || id:ebi-cfb233-GCST90302131' on 'ER- Breast cancer (Combined Oncoarray; i

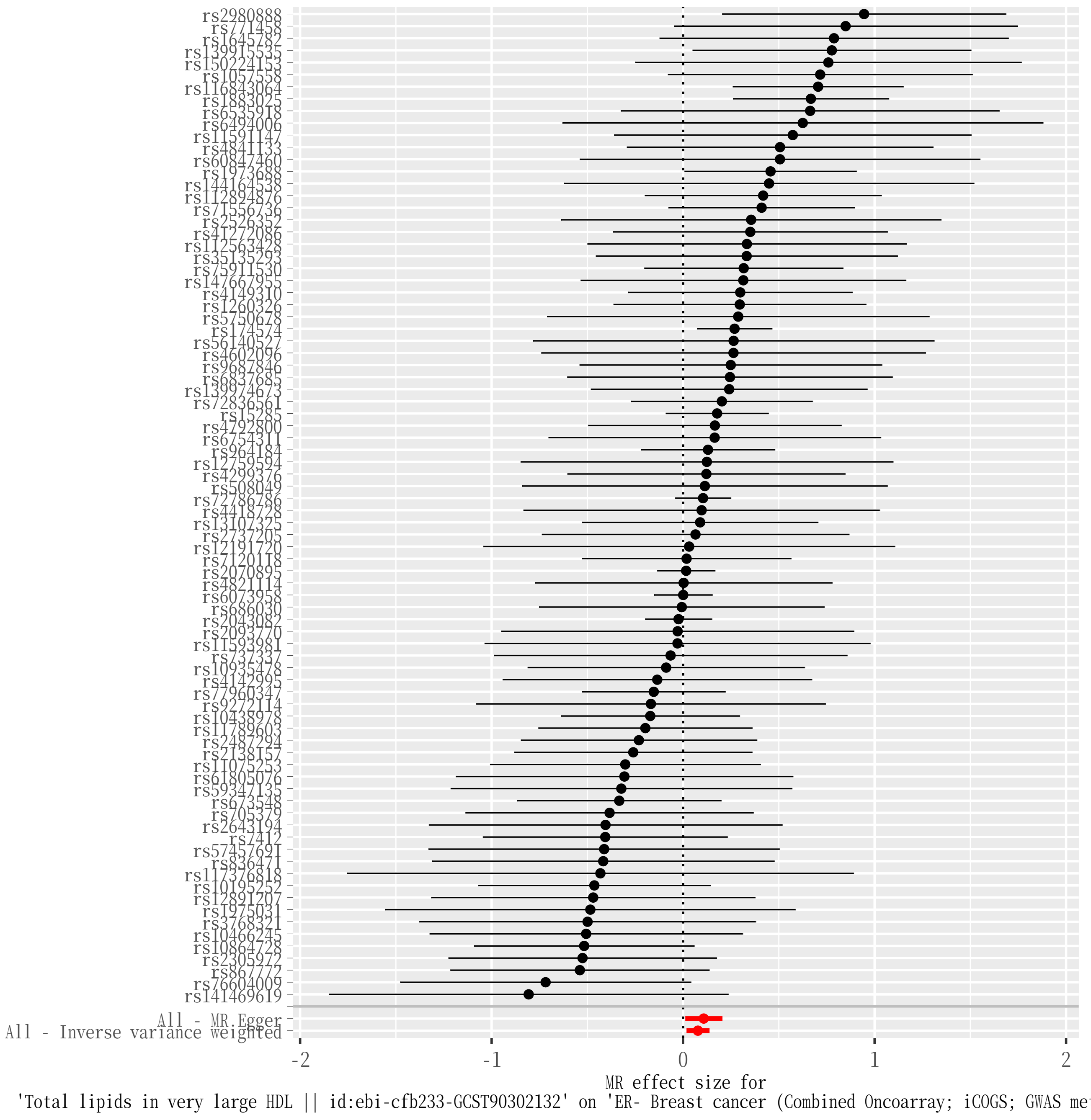

rs2980888
rs771458
rs1645782
rs139915535
rs150224153
rs1057558
rs116843064
rs1883025
rs6535918
rs6494006
rs11591147
rs4841133
rs60847460
rs1973688
rs144164538
rs112894876
rs71556736
rs2526352
rs41272086
rs112563428
rs35135293
rs75911530
rs147667955
rs4149310
rs1260326
rs5750678
rs174574
rs56140527
rs4602096
rs9687846
rs6837685
rs139974673
rs72836561
rs15285
rs4722800
rs6754311
rs964184
rs12759594
rs4299376
rs508049
rs72786786
rs4418728
rs13107325
rs2737205
rs12191720
rs7120118
rs2070895
rs4821114
rs6073958
rs686030
rs2043082
rs2093770
rs11593981
rs737337
rs10935478
rs4142995
rs77960347
rs9272114
rs10438978
rs11789603
rs2487294
rs2138157
rs11075253
rs61805076
rs59347135
rs673548
rs705379
rs2643194
rs7412
rs57457691
rs836471
rs117376818
rs10195252
rs12891207
rs1975031
rs3768321
rs10466245
rs10864728
rs2305972
rs867772
rs76604009
rs141469619
All - MR Egger
All - Inverse variance weighted
-2
-1
0
1
2
MR effect size for
'Total lipids in very large HDL || id:ebi-cfb233-GCST90302132' on 'ER- Breast cancer (Combined Oncoarray; iCOGS; GWAS me

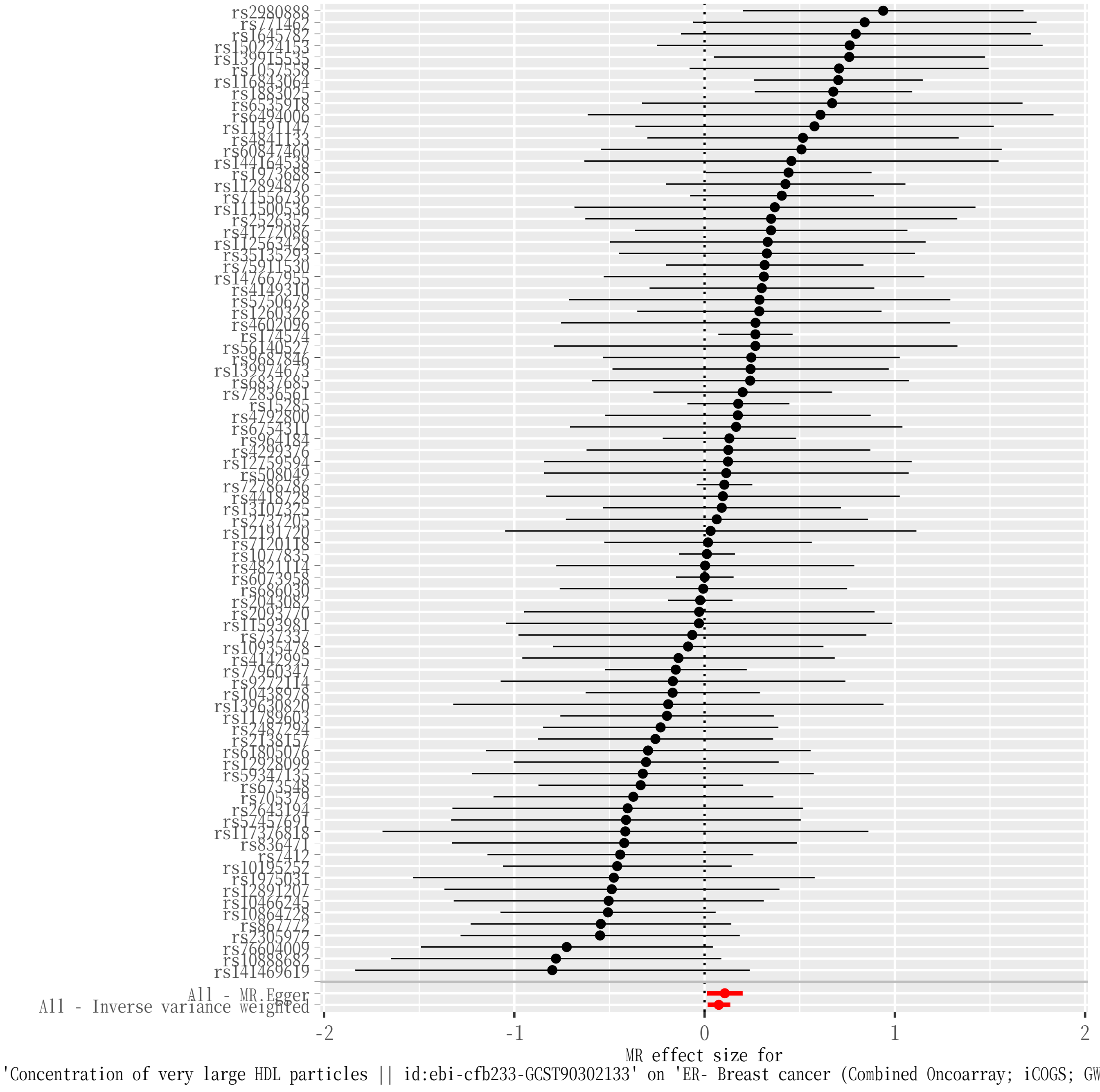

rs2980888
rs771462
rs1645782
rs150224153
rs139915535
rs1057558
rs116843064
rs1883025
rs6535918
rs6494006
rs11591147
rs4841133
rs60847460
rs144164538
rs1973688
rs112894876
rs71556736
rs111500536
rs2526352
rs41272086
rs112563428
rs35135293
rs75911530
rs147667955
rs4149310
rs5750678
rs1260326
rs4602096
rs174574
rs56140527
rs9687846
rs139974673
rs6837685
rs72836561
rs15285
rs4792800
rs6754311
rs964184
rs4299376
rs12759594
rs508049
rs72786786
rs4418728
rs13107325
rs2737205
rs12191720
rs7120118
rs1077835
rs4821114
rs6073958
rs686030
rs2043082
rs2093770
rs11593981
rs737337
rs10935478
rs4142995
rs77960347
rs9272114
rs10438978
rs139630820
rs11789603
rs2487294
rs2138157
rs61805076
rs12928099
rs59347135
rs673548
rs705379
rs2643194
rs57457691
rs117376818
rs836471
rs7412
rs10195252
rs1975031
rs12891207
rs10466245
rs10864728
rs867772
rs2305972
rs76604009
rs10888682
rs141469619
All - MR Egger
All - Inverse variance weighted
-2
-1
0
1
2
MR effect size for
'Concentration of very large HDL particles || id:ebi-cfb233-GCST90302133' on 'ER- Breast cancer (Combined Oncoarray; iCOGS; GW

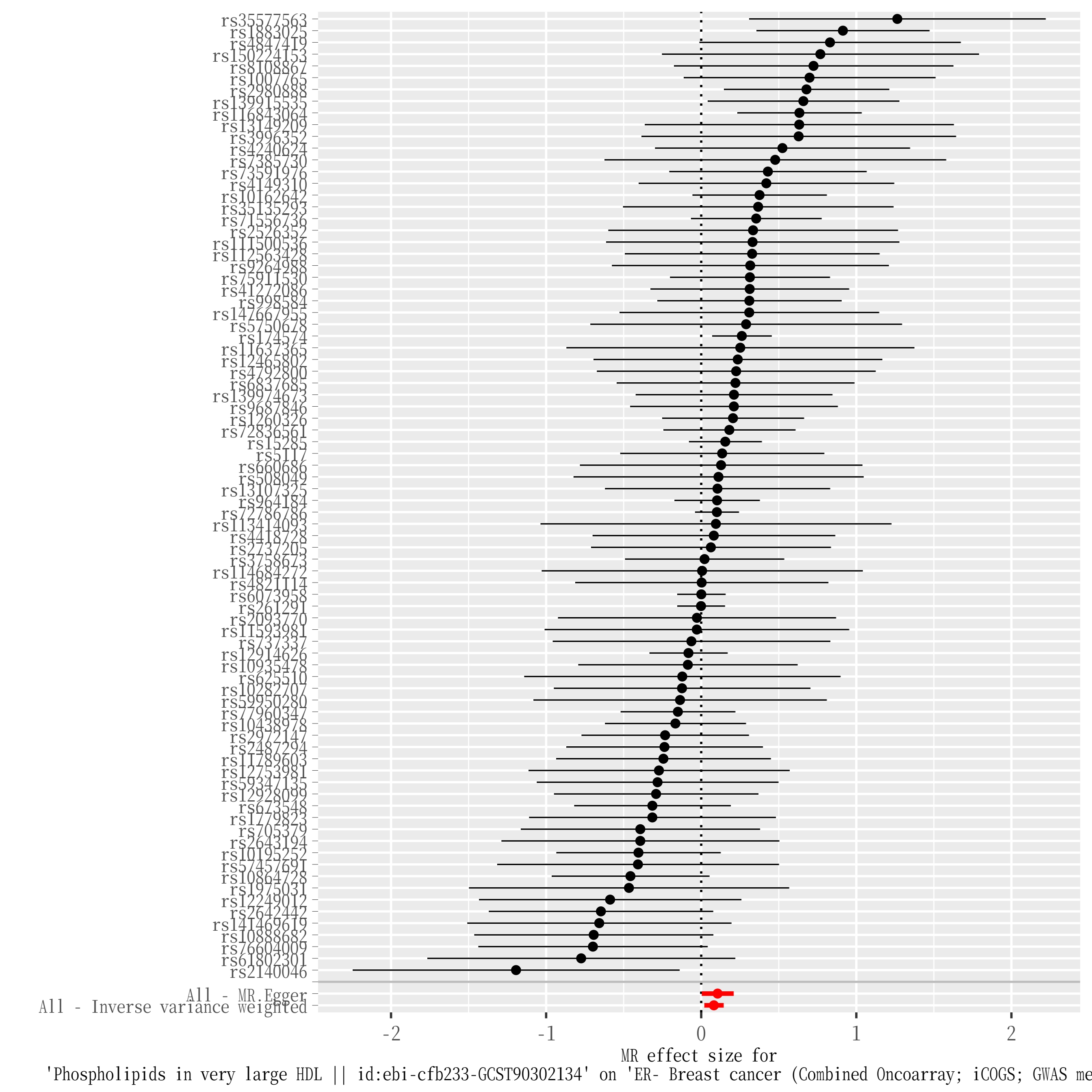
rs35577563
rs1883025
rs4847419
rs150224153
rs8108867
rs1007765
rs2980888
rs139915535
rs116843064
rs13149209
rs3996352
rs4240624
rs7385730
rs73591976
rs4149310
rs10162642
rs35135293
rs71556736
rs2526352
rs111500536
rs112563428
rs9264988
rs75911530
rs41272086
rs998584
rs147667955
rs5750678
rs174574
rs11637365
rs12465802
rs4792800
rs6837685
rs139974673
rs9687846
rs1260326
rs72836561
rs15285
rs5117
rs660686
rs508049
rs13107325
rs964184
rs72786786
rs113414093
rs4418728
rs2737205
rs3758673
rs114684272
rs4821114
rs6073958
rs261291
rs2093770
rs11593981
rs737337
rs12914626
rs10935478
rs625510
rs10282707
rs59950280
rs77960347
rs10438978
rs2972147
rs2487294
rs11789603
rs12753981
rs59347135
rs12928099
rs673548
rs1279823
rs705379
rs2643194
rs10195252
rs57457691
rs10864728
rs1975031
rs12249012
rs2642442
rs141469619
rs10888682
rs76604009
rs61802301
rs2140046
All - MR Egger
All - Inverse variance weighted
-2
-1
0
1
2
MR effect size for
'Phospholipids in very large HDL || id:ebi-cfb233-GCST90302134' on 'ER- Breast cancer (Combined Oncoarray; iCOGS; GWAS me

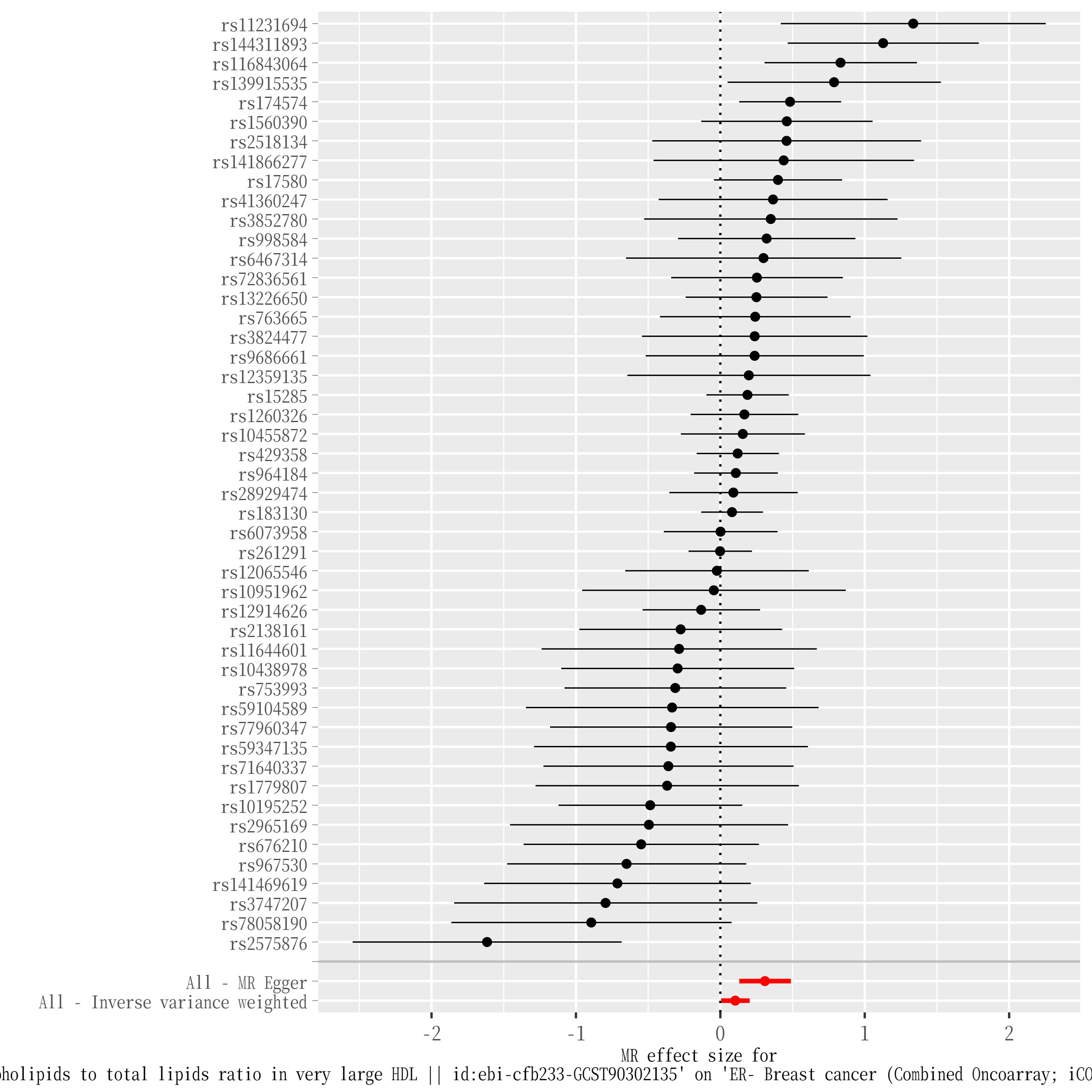

rs11231694
rs144311893
rs116843064
rs139915535
rs174574
rs1560390
rs2518134
rs141866277
rs17580
rs41360247
rs3852780
rs998584
rs6467314
rs72836561
rs13226650
rs763665
rs3824477
rs9686661
rs12359135
rs15285
rs1260326
rs10455872
rs429358
rs964184
rs28929474
rs183130
rs6073958
rs261291
rs12065546
rs10951962
rs12914626
rs2138161
rs11644601
rs10438978
rs753993
rs59104589
rs77960347
rs59347135
rs71640337
rs1779807
rs10195252
rs2965169
rs676210
rs967530
rs141469619
rs3747207
rs78058190
rs2575876
All - MR Egger
All - Inverse variance weighted
-2
-1
0
1
2
MR effect size for
pholipids to total lipids ratio in very large HDL || id:ebi-cfb233-GCST90302135' on 'ER- Breast cancer (Combined Oncoarray; iCO

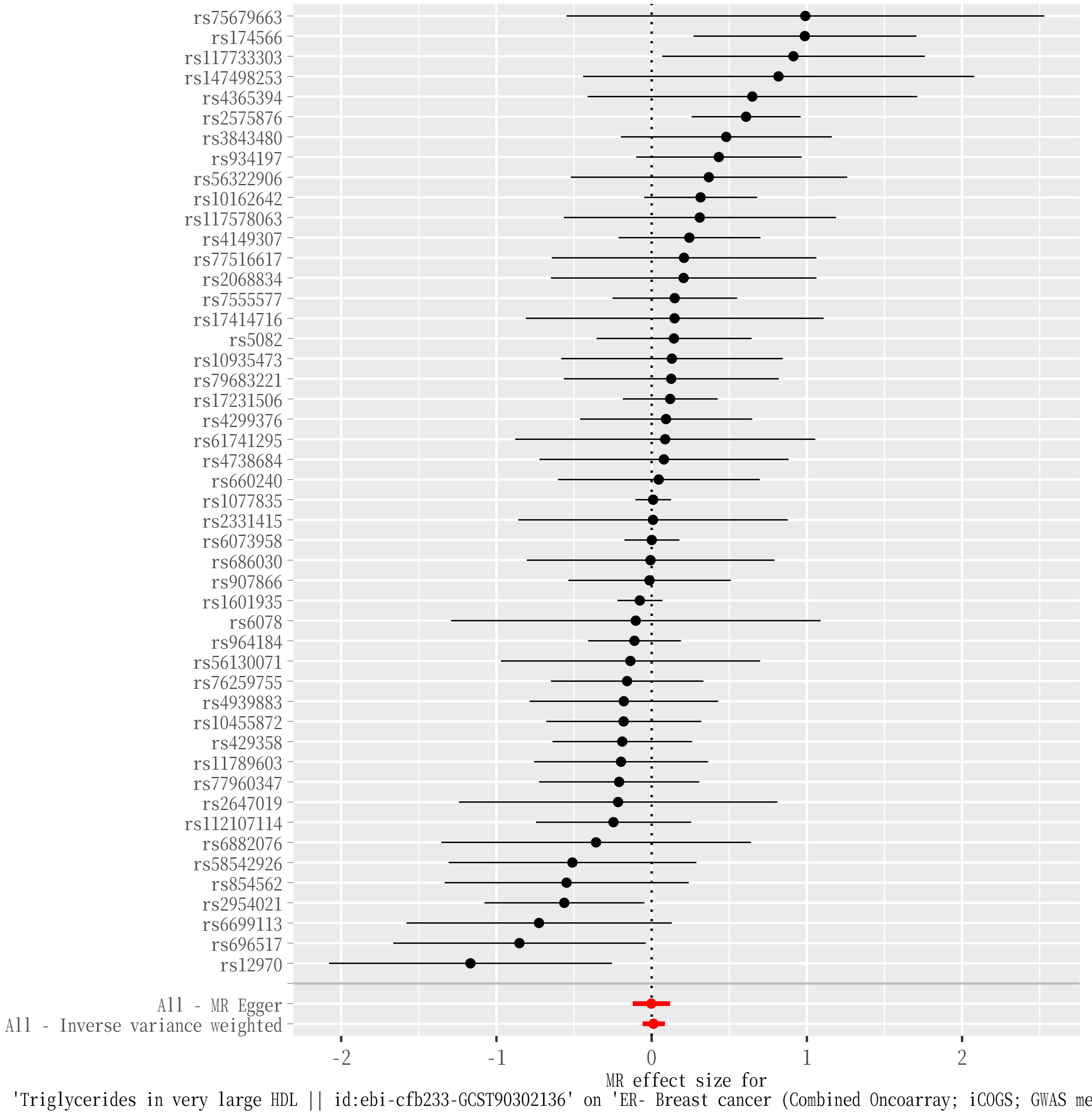
rs75679663
rs174566
rs117733303
rs147498253
rs4365394
rs2575876
rs3843480
rs934197
rs56322906
rs10162642
rs117578063
rs4149307
rs77516617
rs2068834
rs7555577
rs17414716
rs5082
rs10935473
rs79683221
rs17231506
rs4299376
rs61741295
rs4738684
rs660240
rs1077835
rs2331415
rs6073958
rs686030
rs907866
rs1601935
rs6078
rs964184
rs56130071
rs76259755
rs4939883
rs10455872
rs429358
rs11789603
rs77960347
rs2647019
rs112107114
rs6882076
rs58542926
rs854562
rs2954021
rs6699113
rs696517
rs12970
All - MR Egger
All - Inverse variance weighted
-2
-1
0
1
2
MR effect size for
'Triglycerides in very large HDL || id:ebi-cfb233-GCST90302136' on 'ER- Breast cancer (Combined Oncoarray; iCOGS; GWAS me

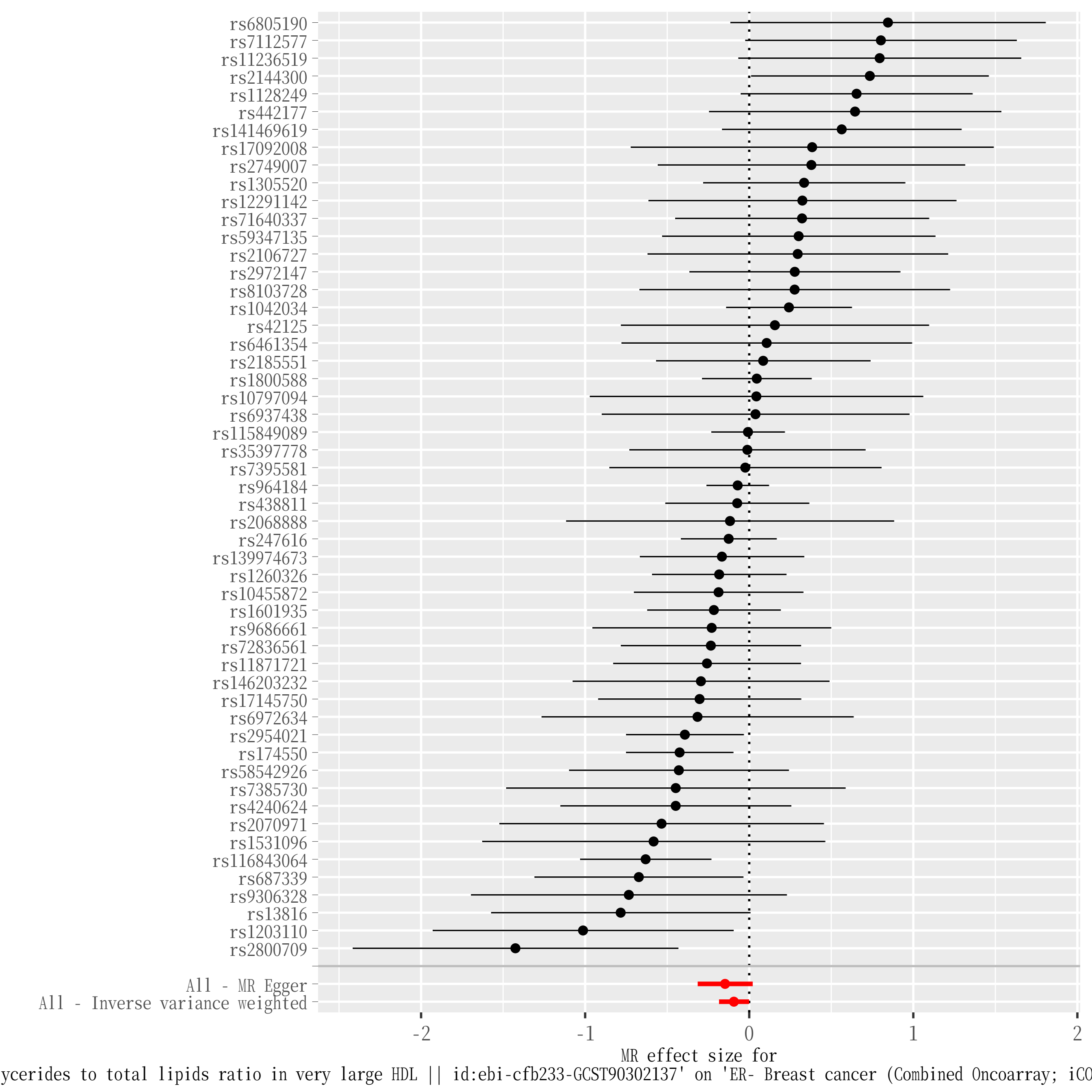
rs6805190
rs7112577
rs11236519
rs2144300
rs1128249
rs442177
rs141469619
rs17092008
rs2749007
rs1305520
rs12291142
rs71640337
rs59347135
rs2106727
rs2972147
rs8103728
rs1042034
rs42125
rs6461354
rs2185551
rs1800588
rs10797094
rs6937438
rs115849089
rs35397778
rs7395581
rs964184
rs438811
rs2068888
rs247616
rs139974673
rs1260326
rs10455872
rs1601935
rs9686661
rs72836561
rs11871721
rs146203232
rs17145750
rs6972634
rs2954021
rs174550
rs58542926
rs7385730
rs4240624
rs2070971
rs1531096
rs116843064
rs687339
rs9306328
rs13816
rs1203110
rs2800709
All - MR Egger
All - Inverse variance weighted
-2
-1
0
1
2
MR effect size for
ycerides to total lipids ratio in very large HDL || id:ebi-cfb233-GCST90302137' on 'ER- Breast cancer (Combined Oncoarray; iCO

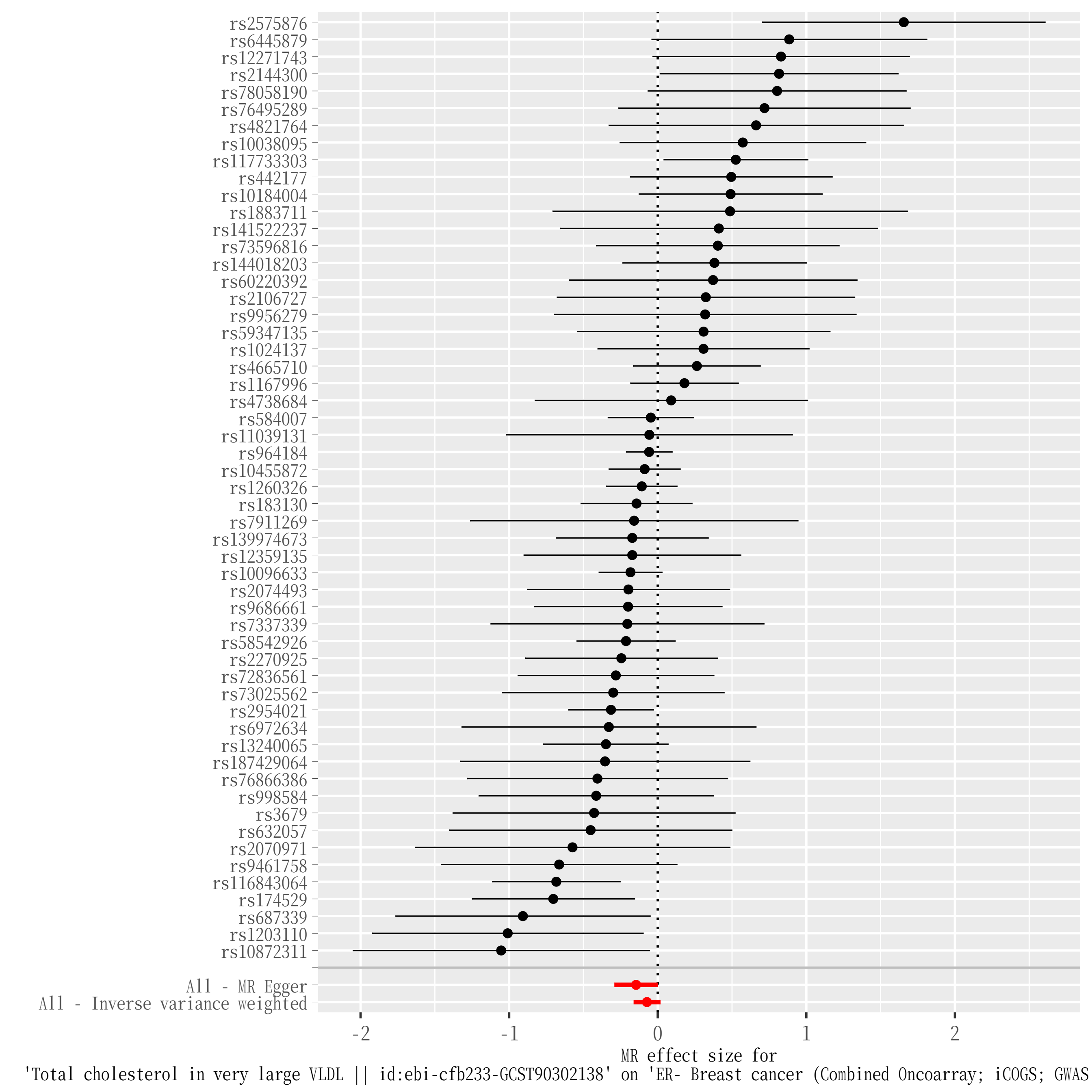

rs2575876
rs6445879
rs12271743
rs2144300
rs78058190
rs76495289
rs4821764
rs10038095
rs117733303
rs442177
rs10184004
rs1883711
rs141522237
rs73596816
rs144018203
rs60220392
rs2106727
rs9956279
rs59347135
rs1024137
rs4665710
rs1167996
rs4738684
rs584007
rs11039131
rs964184
rs10455872
rs1260326
rs183130
rs7911269
rs139974673
rs12359135
rs10096633
rs2074493
rs9686661
rs7337339
rs58542926
rs2270925
rs72836561
rs73025562
rs2954021
rs6972634
rs13240065
rs187429064
rs76866386
rs998584
rs3679
rs632057
rs2070971
rs9461758
rs116843064
rs174529
rs687339
rs1203110
rs10872311
All - MR Egger
All - Inverse variance weighted
-2
-1
0
1
2
MR effect size for
'Total cholesterol in very large VLDL || id:ebi-cfb233-GCST90302138' on 'ER- Breast cancer (Combined Oncoarray; iCOGS; GWAS

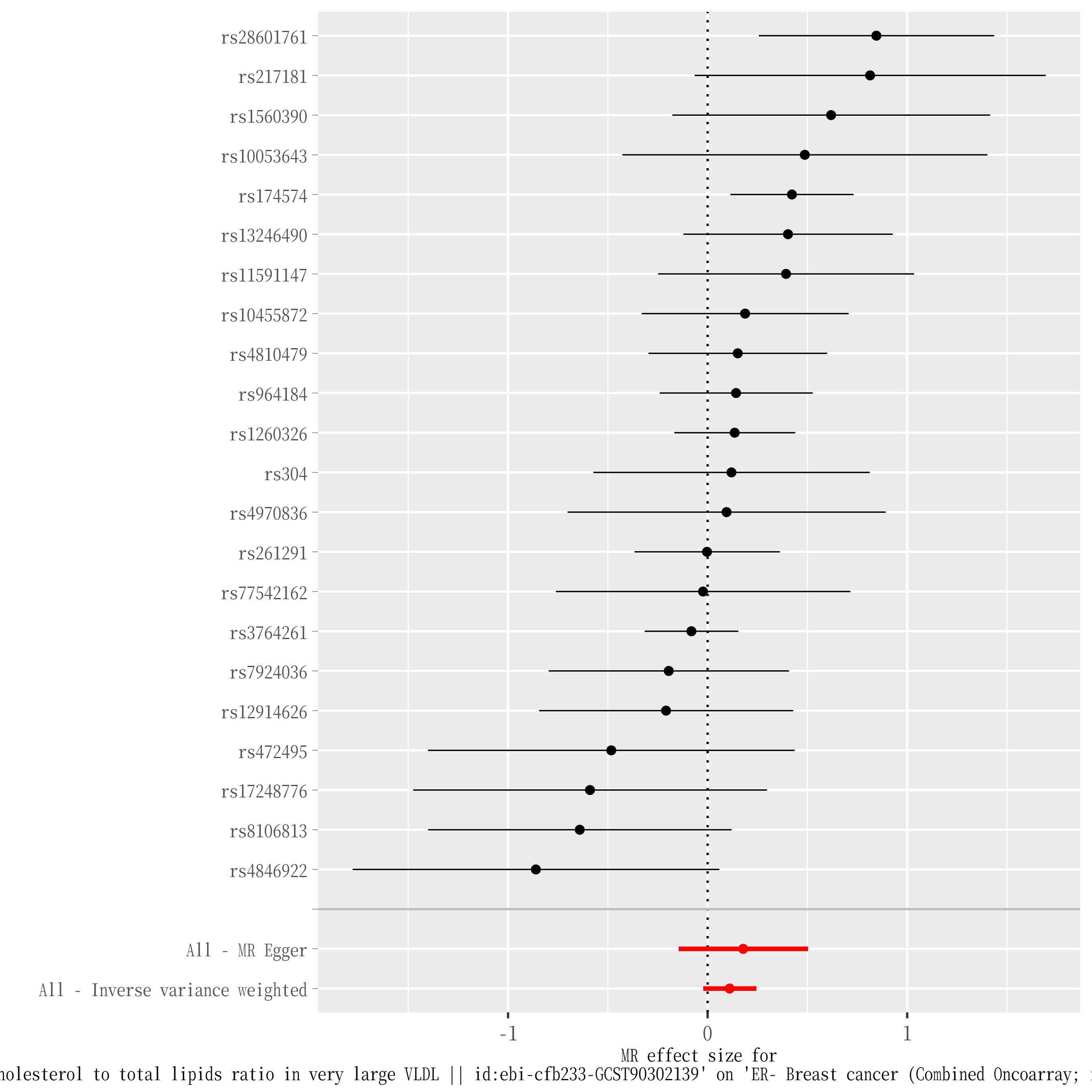

rs28601761
rs217181
rs1560390
rs10053643
rs174574
rs13246490
rs11591147
rs10455872
rs4810479
rs964184
rs1260326
rs304
rs4970836
rs261291
rs77542162
rs3764261
rs7924036
rs12914626
rs472495
rs17248776
rs8106813
rs4846922
All - MR Egger
All - Inverse variance weighted
-1
0
1
MR effect size for
holesterol to total lipids ratio in very large VLDL || id:ebi-cfb233-GCST90302139' on 'ER- Breast cancer (Combined Oncoarray;

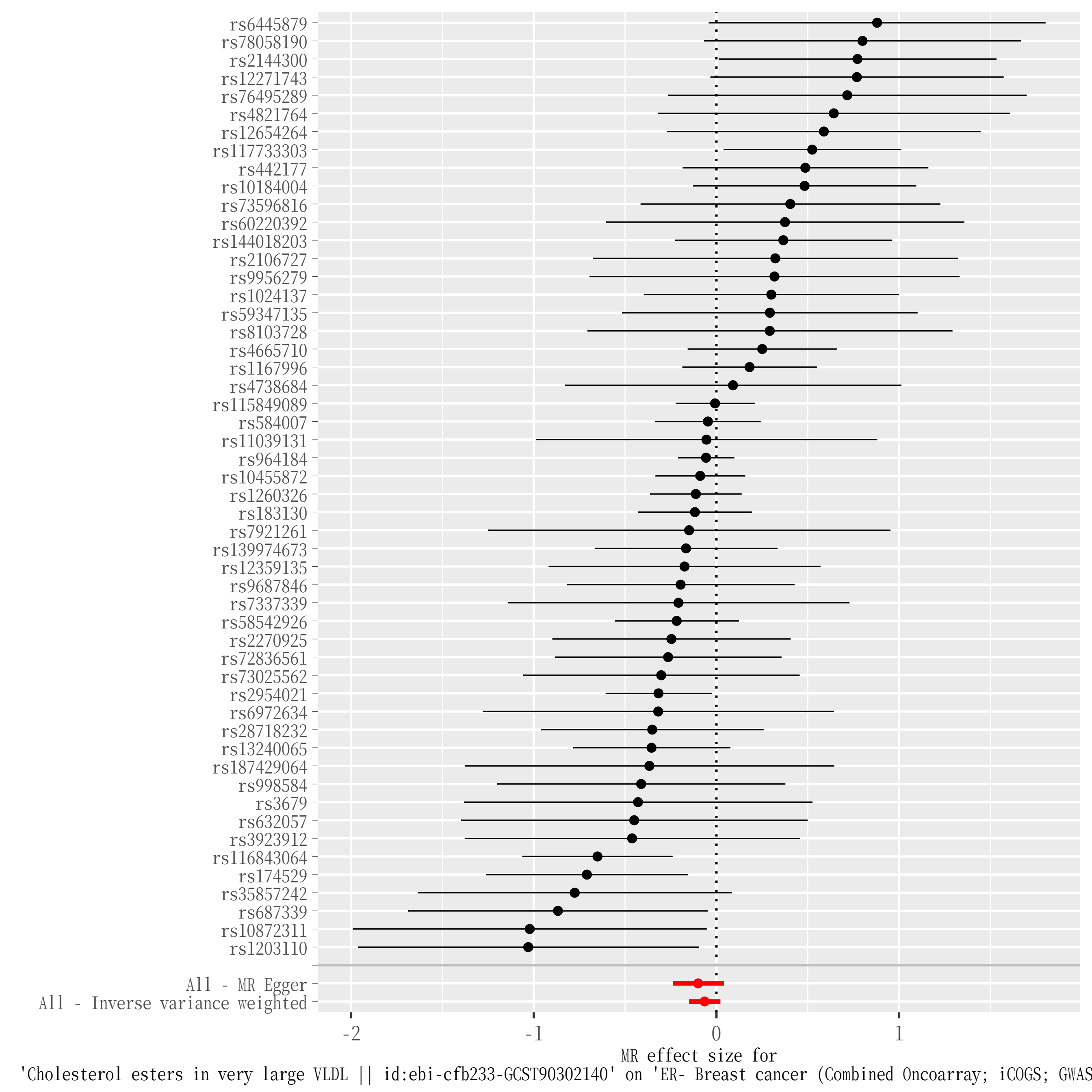
rs6445879
rs78058190
rs2144300
rs12271743
rs76495289
rs4821764
rs12654264
rs117733303
rs442177
rs10184004
rs73596816
rs60220392
rs144018203
rs2106727
rs9956279
rs1024137
rs59347135
rs8103728
rs4665710
rs1167996
rs4738684
rs115849089
rs584007
rs11039131
rs964184
rs10455872
rs1260326
rs183130
rs7921261
rs139974673
rs12359135
rs9687846
rs7337339
rs58542926
rs2270925
rs72836561
rs73025562
rs2954021
rs6972634
rs28718232
rs13240065
rs187429064
rs998584
rs3679
rs632057
rs3923912
rs116843064
rs174529
rs35857242
rs687339
rs10872311
rs1203110
All - MR Egger
All - Inverse variance weighted
-2
-1
0
1
MR effect size for
'Cholesterol esters in very large VLDL || id:ebi-cfb233-GCST90302140' on 'ER- Breast cancer (Combined Oncoarray; iCOGS; GWAS

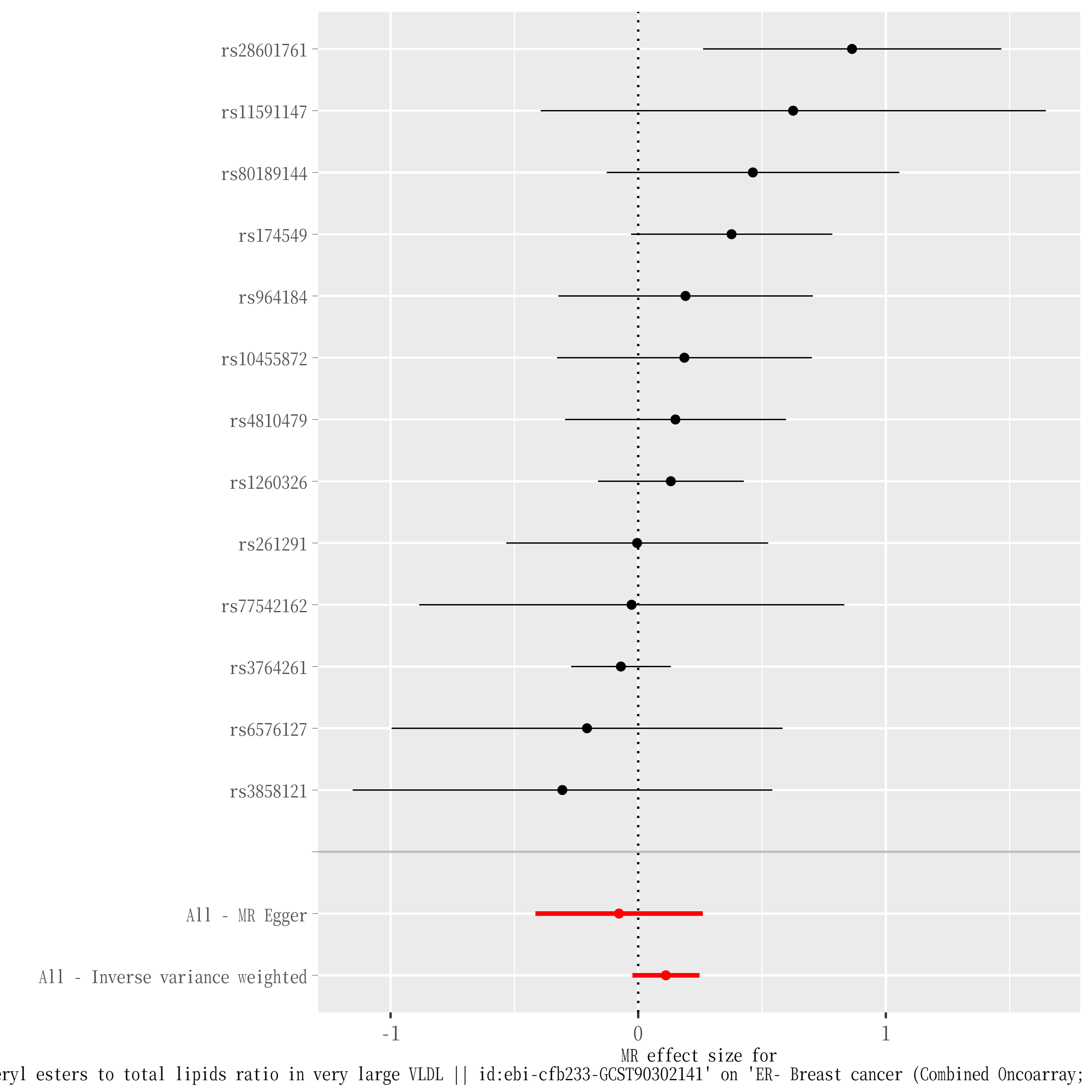
rs28601761
rs11591147
rs80189144
rs174549
rs964184
rs10455872
rs4810479
rs1260326
rs261291
rs77542162
rs3764261
rs6576127
rs3858121
All - MR Egger
All - Inverse variance weighted
-1
0
1
MR effect size for
eryl esters to total lipids ratio in very large VLDL || id:ebi-cfb233-GCST90302141' on 'ER- Breast cancer (Combined Oncoarray;

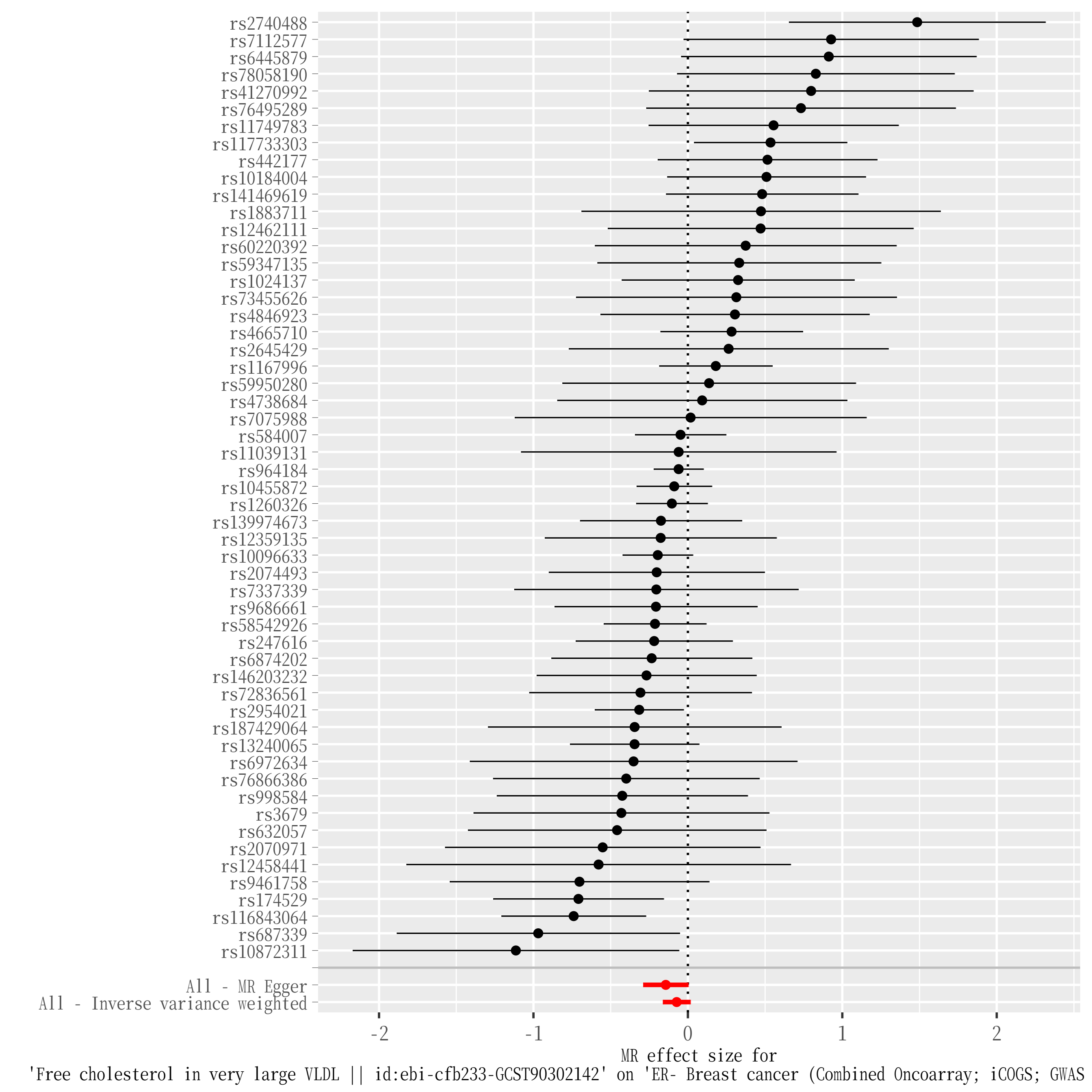
rs2740488
rs7112577
rs6445879
rs78058190
rs41270992
rs76495289
rs11749783
rs117733303
rs442177
rs10184004
rs141469619
rs1883711
rs12462111
rs60220392
rs59347135
rs1024137
rs73455626
rs4846923
rs4665710
rs2645429
rs1167996
rs59950280
rs4738684
rs7075988
rs584007
rs11039131
rs964184
rs10455872
rs1260326
rs139974673
rs12359135
rs10096633
rs2074493
rs7337339
rs9686661
rs58542926
rs247616
rs6874202
rs146203232
rs72836561
rs2954021
rs187429064
rs13240065
rs6972634
rs76866386
rs998584
rs3679
rs632057
rs2070971
rs12458441
rs9461758
rs174529
rs116843064
rs687339
rs10872311
All - MR Egger
All - Inverse variance weighted
-2
-1
0
1
2
MR effect size for
'Free cholesterol in very large VLDL || id:ebi-cfb233-GCST90302142' on 'ER- Breast cancer (Combined Oncoarray; iCOGS; GWAS

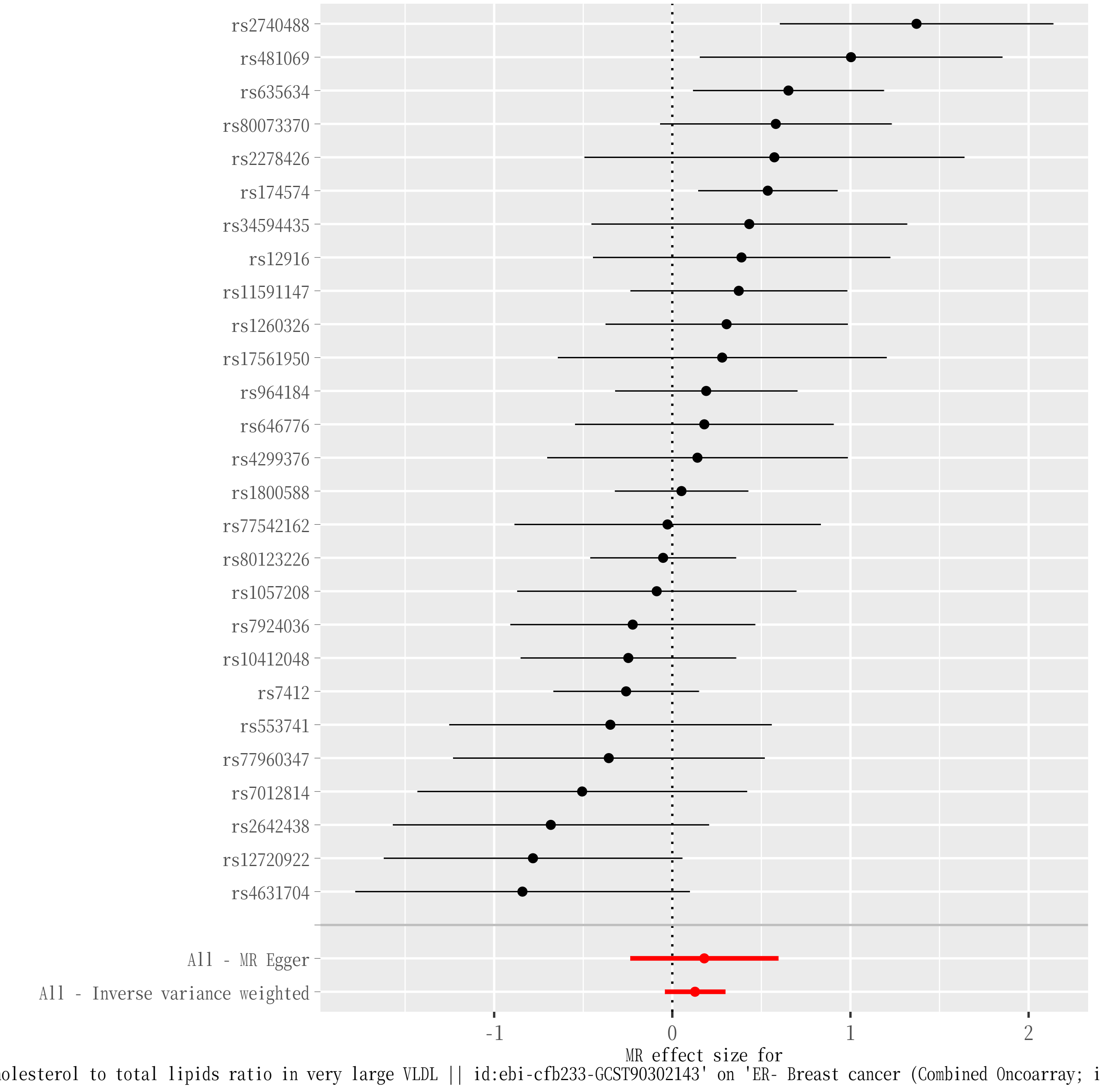

rs2740488
rs481069
rs635634
rs80073370
rs2278426
rs174574
rs34594435
rs12916
rs11591147
rs1260326
rs17561950
rs964184
rs646776
rs4299376
rs1800588
rs77542162
rs80123226
rs1057208
rs7924036
rs10412048
rs7412
rs553741
rs77960347
rs7012814
rs2642438
rs12720922
rs4631704
All - MR Egger
All - Inverse variance weighted
-1
0
1
2
MR effect size for
holesterol to total lipids ratio in very large VLDL || id:ebi-cfb233-GCST90302143' on 'ER- Breast cancer (Combined Oncoarray; i

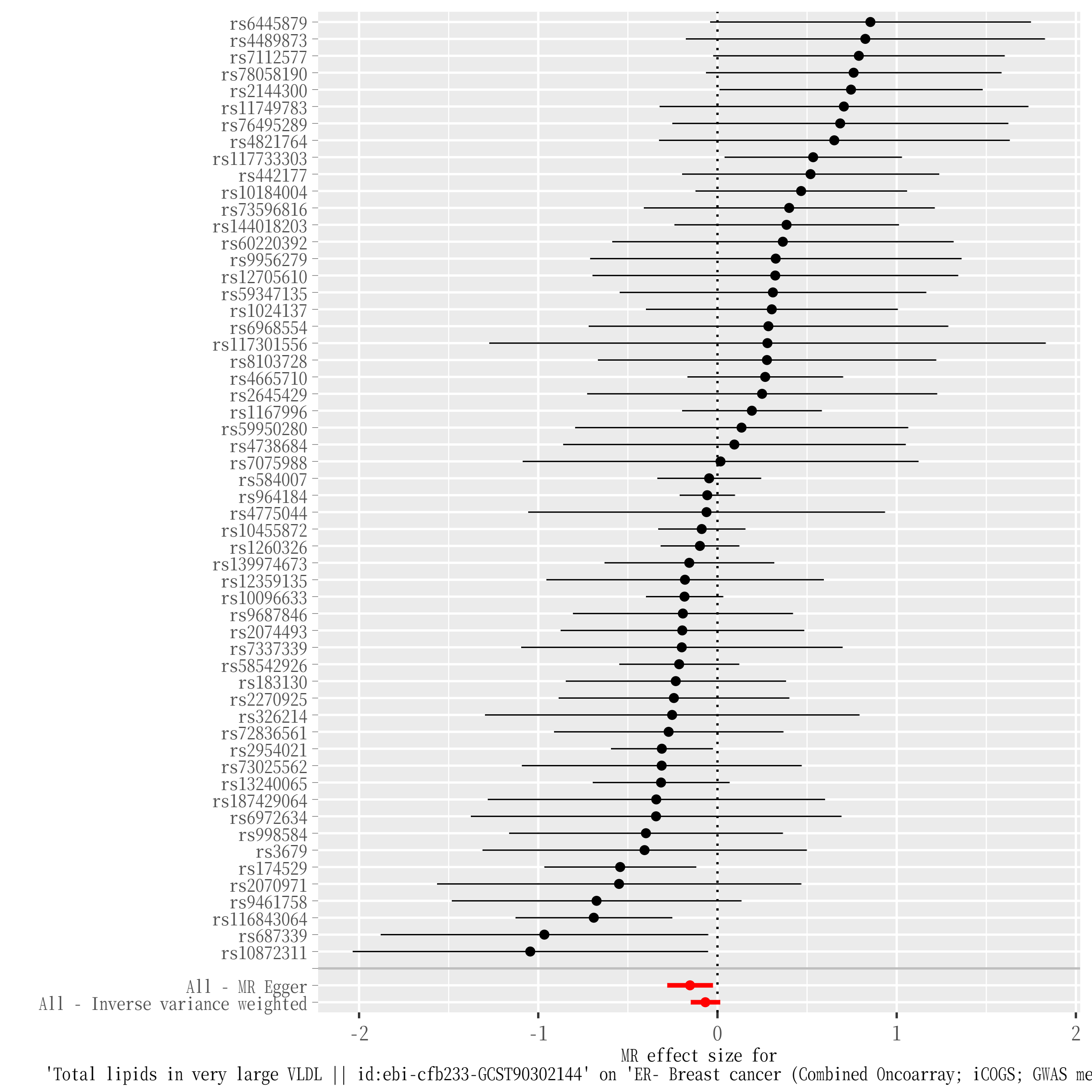

rs6445879
rs4489873
rs7112577
rs78058190
rs2144300
rs11749783
rs76495289
rs4821764
rs117733303
rs442177
rs10184004
rs73596816
rs144018203
rs60220392
rs9956279
rs12705610
rs59347135
rs1024137
rs6968554
rs117301556
rs8103728
rs4665710
rs2645429
rs1167996
rs59950280
rs4738684
rs7075988
rs584007
rs964184
rs4775044
rs10455872
rs1260326
rs139974673
rs12359135
rs10096633
rs9687846
rs2074493
rs7337339
rs58542926
rs183130
rs2270925
rs326214
rs72836561
rs2954021
rs73025562
rs13240065
rs187429064
rs6972634
rs998584
rs3679
rs174529
rs2070971
rs9461758
rs116843064
rs687339
rs10872311
All - MR Egger
All - Inverse variance weighted
-2
-1
0
1
2
MR effect size for
'Total lipids in very large VLDL || id:ebi-cfb233-GCST90302144' on 'ER- Breast cancer (Combined Oncoarray; iCOGS; GWAS me

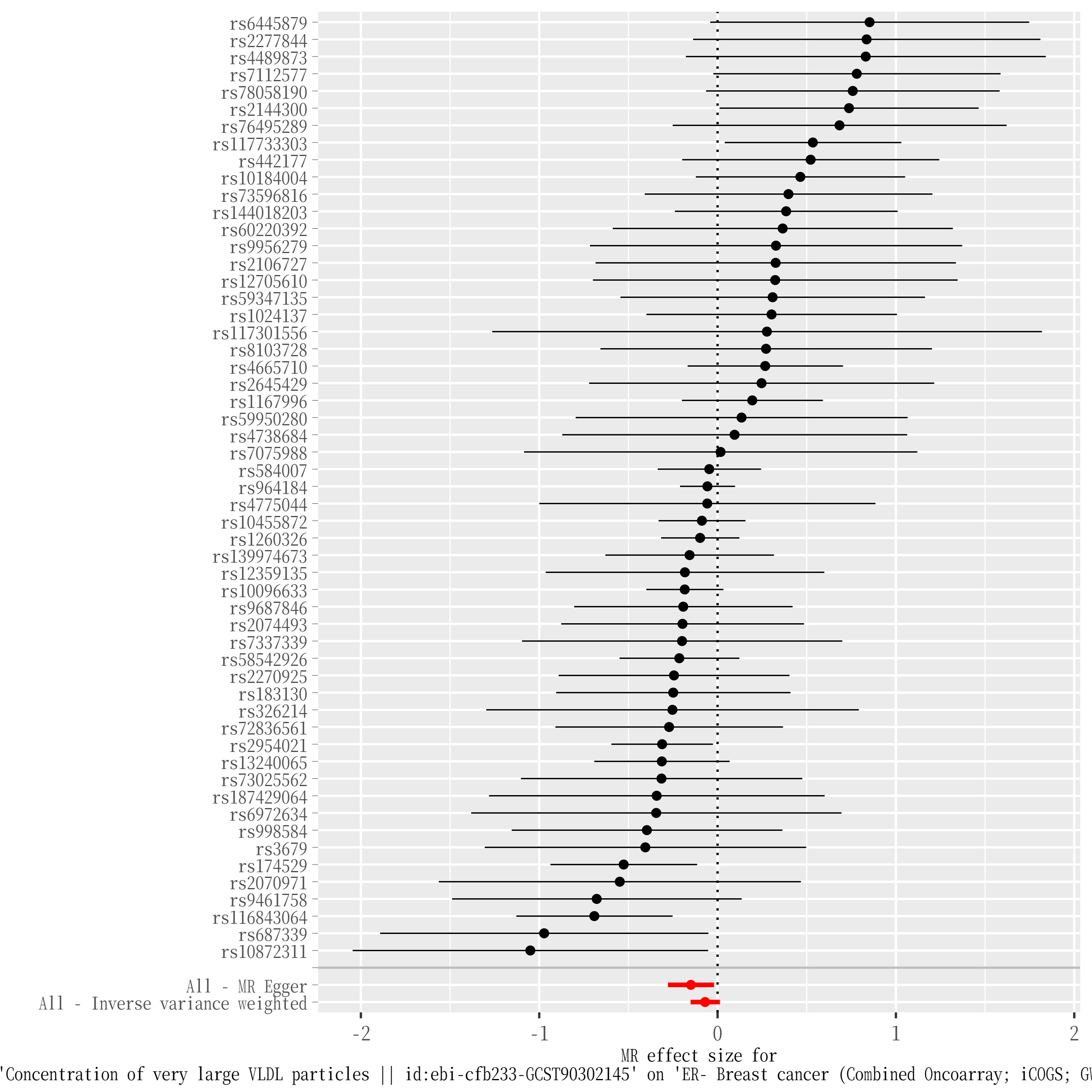
rs6445879
rs2277844
rs4489873
rs7112577
rs78058190
rs2144300
rs76495289
rs117733303
rs442177
rs10184004
rs73596816
rs144018203
rs60220392
rs9956279
rs2106727
rs12705610
rs59347135
rs1024137
rs117301556
rs8103728
rs4665710
rs2645429
rs1167996
rs59950280
rs4738684
rs7075988
rs584007
rs964184
rs4775044
rs10455872
rs1260326
rs139974673
rs12359135
rs10096633
rs9687846
rs2074493
rs7337339
rs58542926
rs2270925
rs183130
rs326214
rs72836561
rs2954021
rs13240065
rs73025562
rs187429064
rs6972634
rs998584
rs3679
rs174529
rs2070971
rs9461758
rs116843064
rs687339
rs10872311
All - MR Egger
All - Inverse variance weighted
-2
-1
0
1
2
MR effect size for
'Concentration of very large VLDL particles || id:ebi-cfb233-GCST90302145' on 'ER- Breast cancer (Combined Oncoarray; iCOGS; G

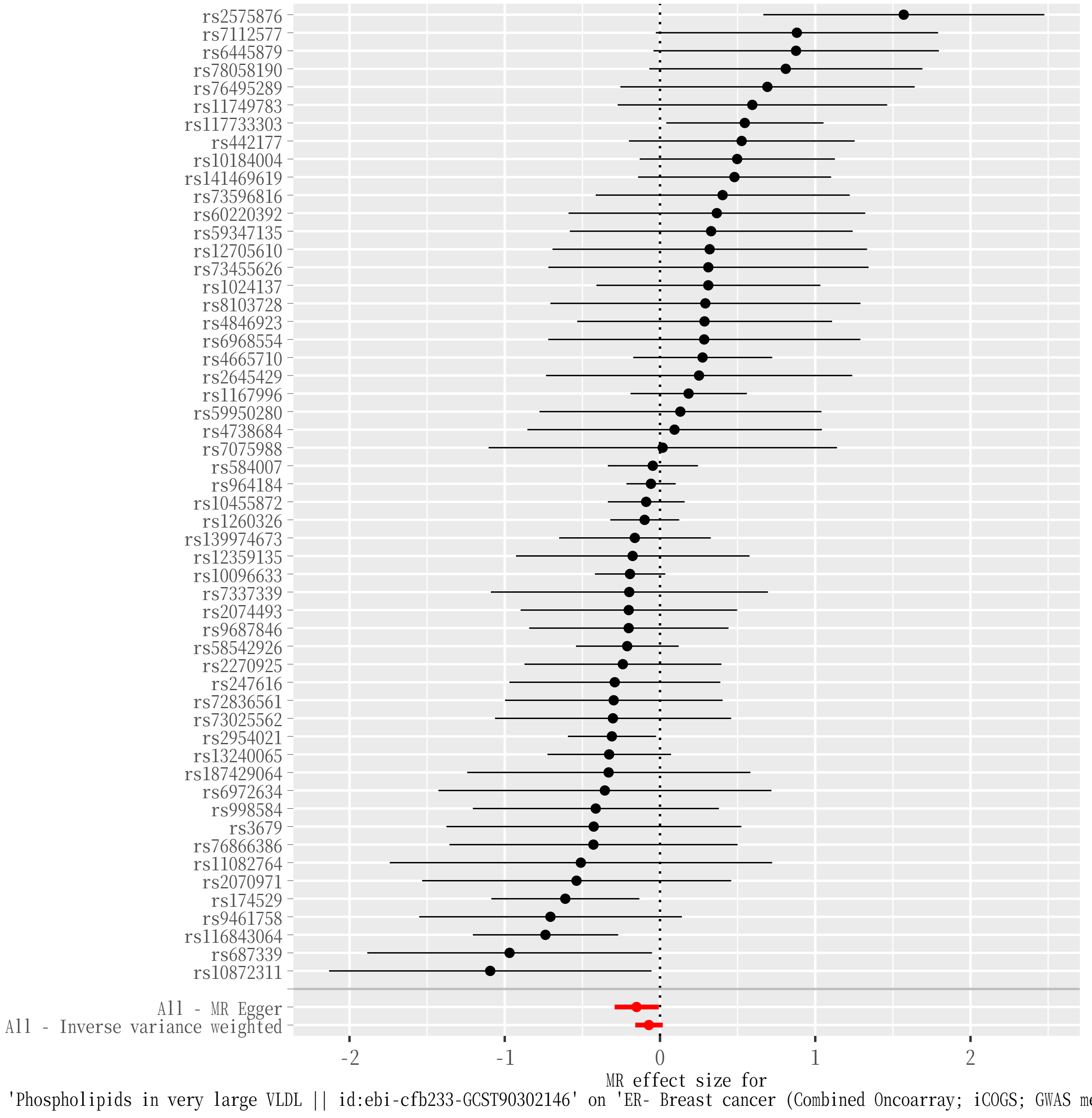
rs2575876
rs7112577
rs6445879
rs78058190
rs76495289
rs11749783
rs117733303
rs442177
rs10184004
rs141469619
rs73596816
rs60220392
rs59347135
rs12705610
rs73455626
rs1024137
rs8103728
rs4846923
rs6968554
rs4665710
rs2645429
rs1167996
rs59950280
rs4738684
rs7075988
rs584007
rs964184
rs10455872
rs1260326
rs139974673
rs12359135
rs10096633
rs7337339
rs2074493
rs9687846
rs58542926
rs2270925
rs247616
rs72836561
rs73025562
rs2954021
rs13240065
rs187429064
rs6972634
rs998584
rs3679
rs76866386
rs11082764
rs2070971
rs174529
rs9461758
rs116843064
rs687339
rs10872311
All - MR Egger
All - Inverse variance weighted
-2
-1
0
1
2
MR effect size for
'Phospholipids in very large VLDL || id:ebi-cfb233-GCST90302146' on 'ER- Breast cancer (Combined Oncoarray; iCOGS; GWAS me

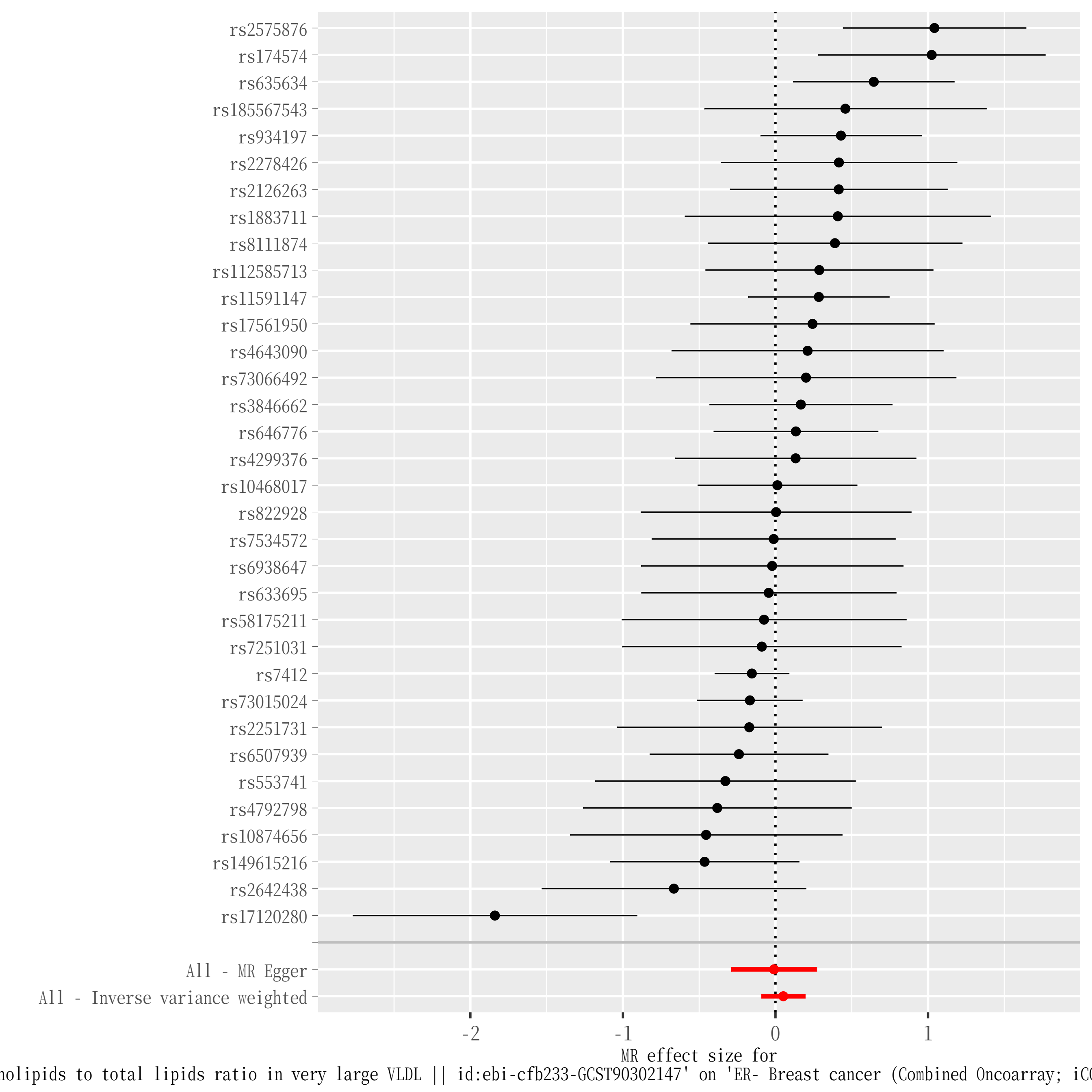

rs2575876
rs174574
rs635634
rs185567543
rs934197
rs2278426
rs2126263
rs1883711
rs8111874
rs112585713
rs11591147
rs17561950
rs4643090
rs73066492
rs3846662
rs646776
rs4299376
rs10468017
rs822928
rs7534572
rs6938647
rs633695
rs58175211
rs7251031
rs7412
rs73015024
rs2251731
rs6507939
rs553741
rs4792798
rs10874656
rs149615216
rs2642438
rs17120280
All - MR Egger
All - Inverse variance weighted
-2
-1
0
1
MR effect size for
holipids to total lipids ratio in very large VLDL || id:ebi-cfb233-GCST90302147' on 'ER- Breast cancer (Combined Oncoarray; iC

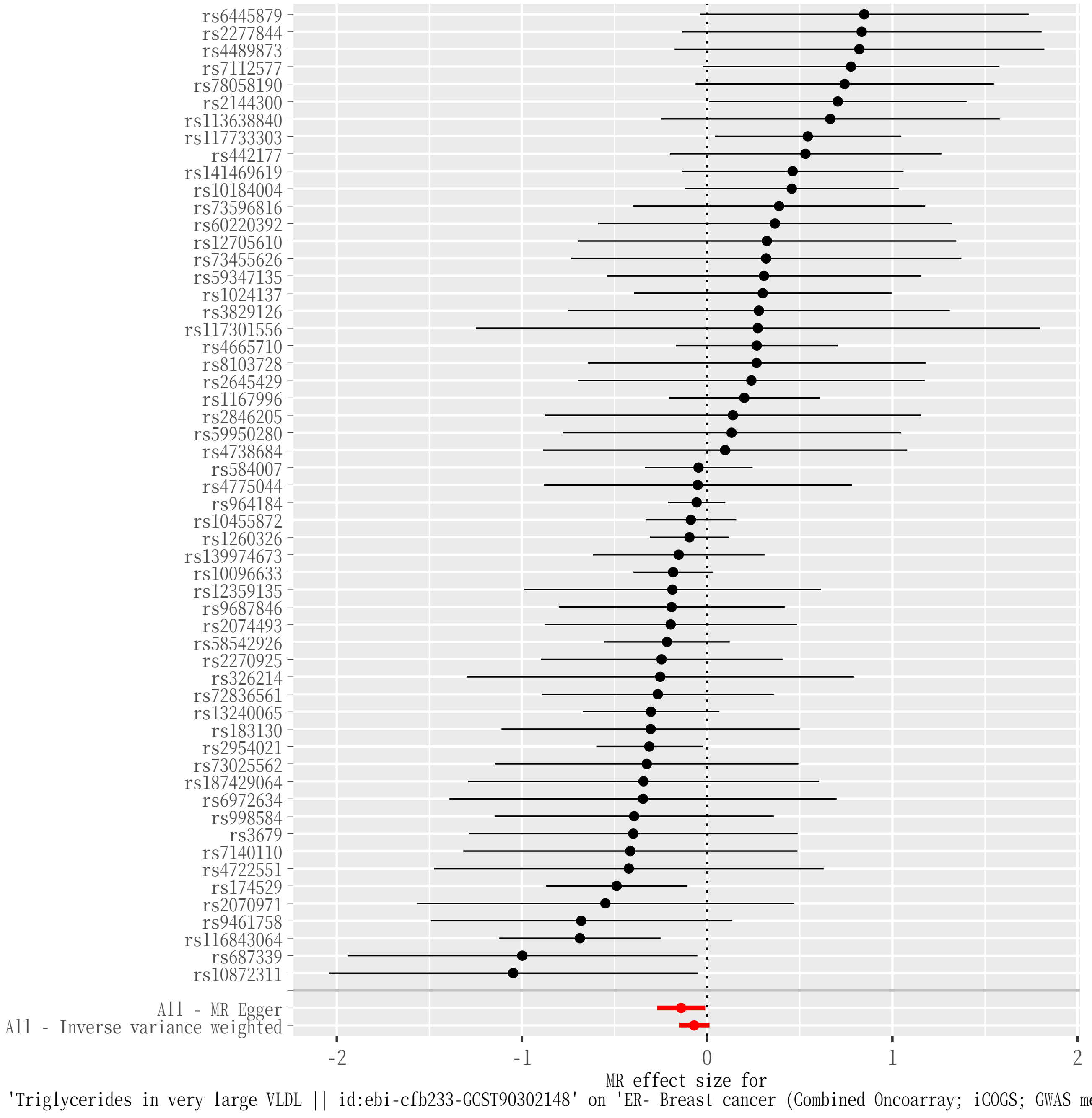
rs6445879
rs2277844
rs4489873
rs7112577
rs78058190
rs2144300
rs113638840
rs117733303
rs442177
rs141469619
rs10184004
rs73596816
rs60220392
rs12705610
rs73455626
rs59347135
rs1024137
rs3829126
rs117301556
rs4665710
rs8103728
rs2645429
rs1167996
rs2846205
rs59950280
rs4738684
rs584007
rs4775044
rs964184
rs10455872
rs1260326
rs139974673
rs10096633
rs12359135
rs9687846
rs2074493
rs58542926
rs2270925
rs326214
rs72836561
rs13240065
rs183130
rs2954021
rs73025562
rs187429064
rs6972634
rs998584
rs3679
rs7140110
rs4722551
rs174529
rs2070971
rs9461758
rs116843064
rs687339
rs10872311
All - MR Egger
All - Inverse variance weighted
-2
-1
0
1
2
MR effect size for
'Triglycerides in very large VLDL || id:ebi-cfb233-GCST90302148' on 'ER- Breast cancer (Combined Oncoarray; iCOGS; GWAS me

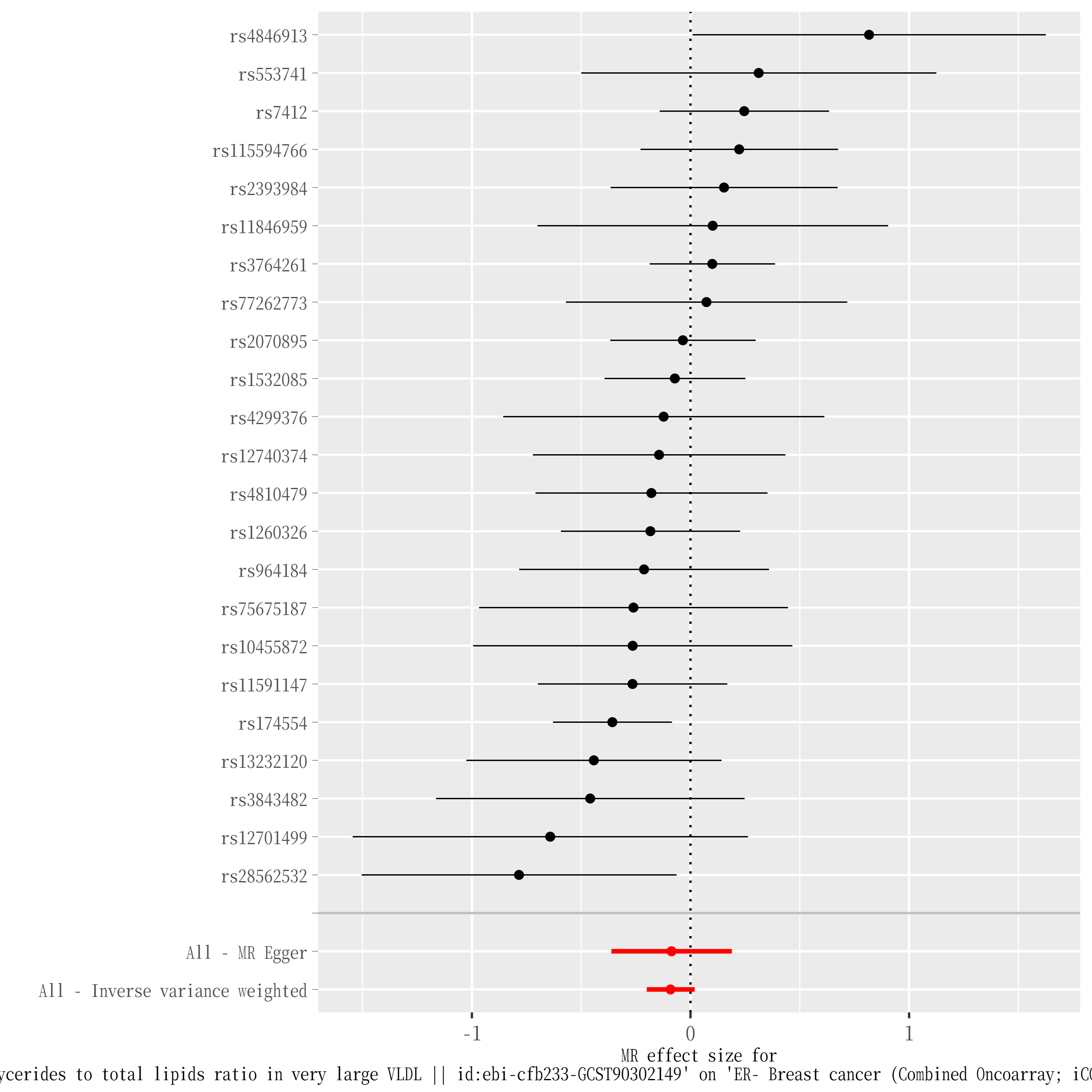
rs4846913
rs553741
rs7412
rs115594766
rs2393984
rs11846959
rs3764261
rs77262773
rs2070895
rs1532085
rs4299376
rs12740374
rs4810479
rs1260326
rs964184
rs75675187
rs10455872
rs11591147
rs174554
rs13232120
rs3843482
rs12701499
rs28562532
All - MR Egger
All - Inverse variance weighted
-1
0
1
MR effect size for
ycerides to total lipids ratio in very large VLDL || id:ebi-cfb233-GCST90302149' on 'ER- Breast cancer (Combined Oncoarray; iC

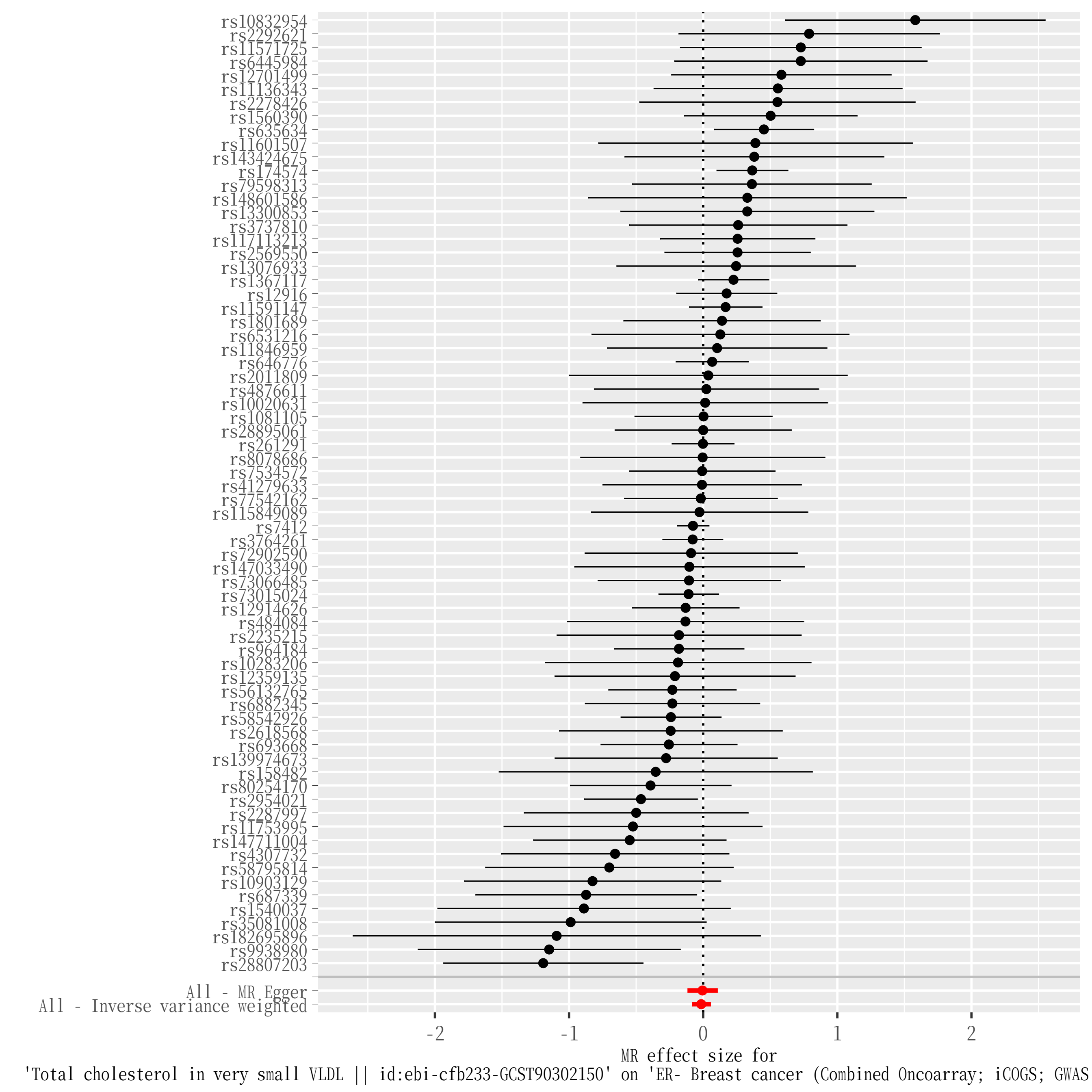
rs10832954
rs2292621
rs11571725
rs6445984
rs12701499
rs11136343
rs2278426
rs1560390
rs635634
rs11601507
rs143424675
rs174574
rs79598313
rs148601586
rs13300853
rs3737810
rs117113213
rs2569550
rs13076933
rs1367117
rs12916
rs11591147
rs1801689
rs6531216
rs11846959
rs646776
rs2011809
rs4876611
rs10020631
rs1081105
rs28895061
rs261291
rs8078686
rs7534572
rs41279633
rs77542162
rs115849089
rs7412
rs3764261
rs72902590
rs147033490
rs73066485
rs73015024
rs12914626
rs484084
rs2235215
rs964184
rs10283206
rs12359135
rs56132765
rs6882345
rs58542926
rs2618568
rs693668
rs139974673
rs158482
rs80254170
rs2954021
rs2287997
rs11753995
rs147711004
rs4307732
rs58795814
rs10903129
rs687339
rs1540037
rs35081008
rs182695896
rs9938980
rs28807203
All - MR Egger
All - Inverse variance weighted
-2
-1
0
1
2
MR effect size for
'Total cholesterol in very small VLDL || id:ebi-cfb233-GCST90302150' on 'ER- Breast cancer (Combined Oncoarray; iCOGS; GWAS

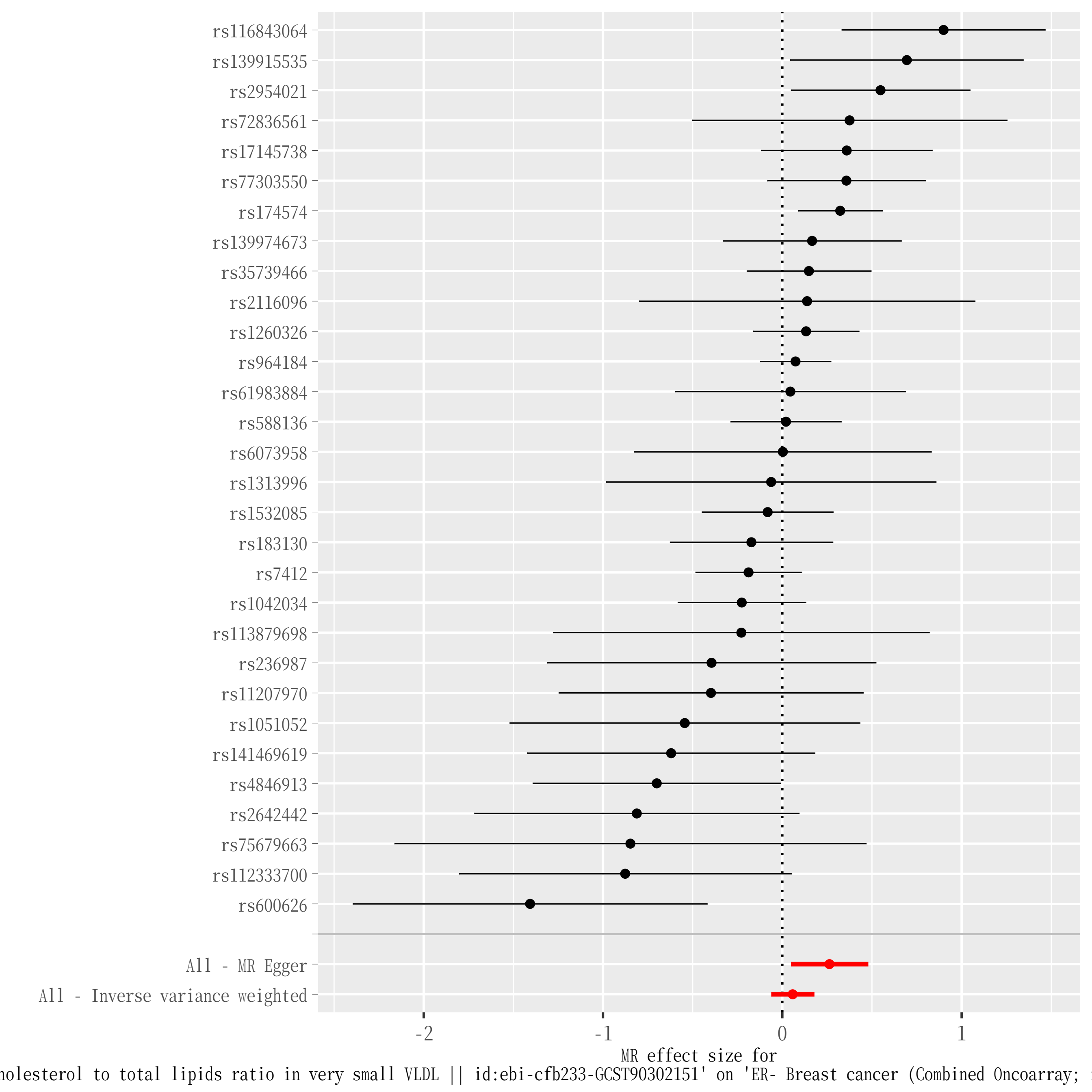
rs116843064
rs139915535
rs2954021
rs72836561
rs17145738
rs77303550
rs174574
rs139974673
rs35739466
rs2116096
rs1260326
rs964184
rs61983884
rs588136
rs6073958
rs1313996
rs1532085
rs183130
rs7412
rs1042034
rs113879698
rs236987
rs11207970
rs1051052
rs141469619
rs4846913
rs2642442
rs75679663
rs112333700
rs600626
All - MR Egger
All - Inverse variance weighted
-2
-1
0
1
MR effect size for
holesterol to total lipids ratio in very small VLDL || id:ebi-cfb233-GCST90302151' on 'ER- Breast cancer (Combined Oncoarray;

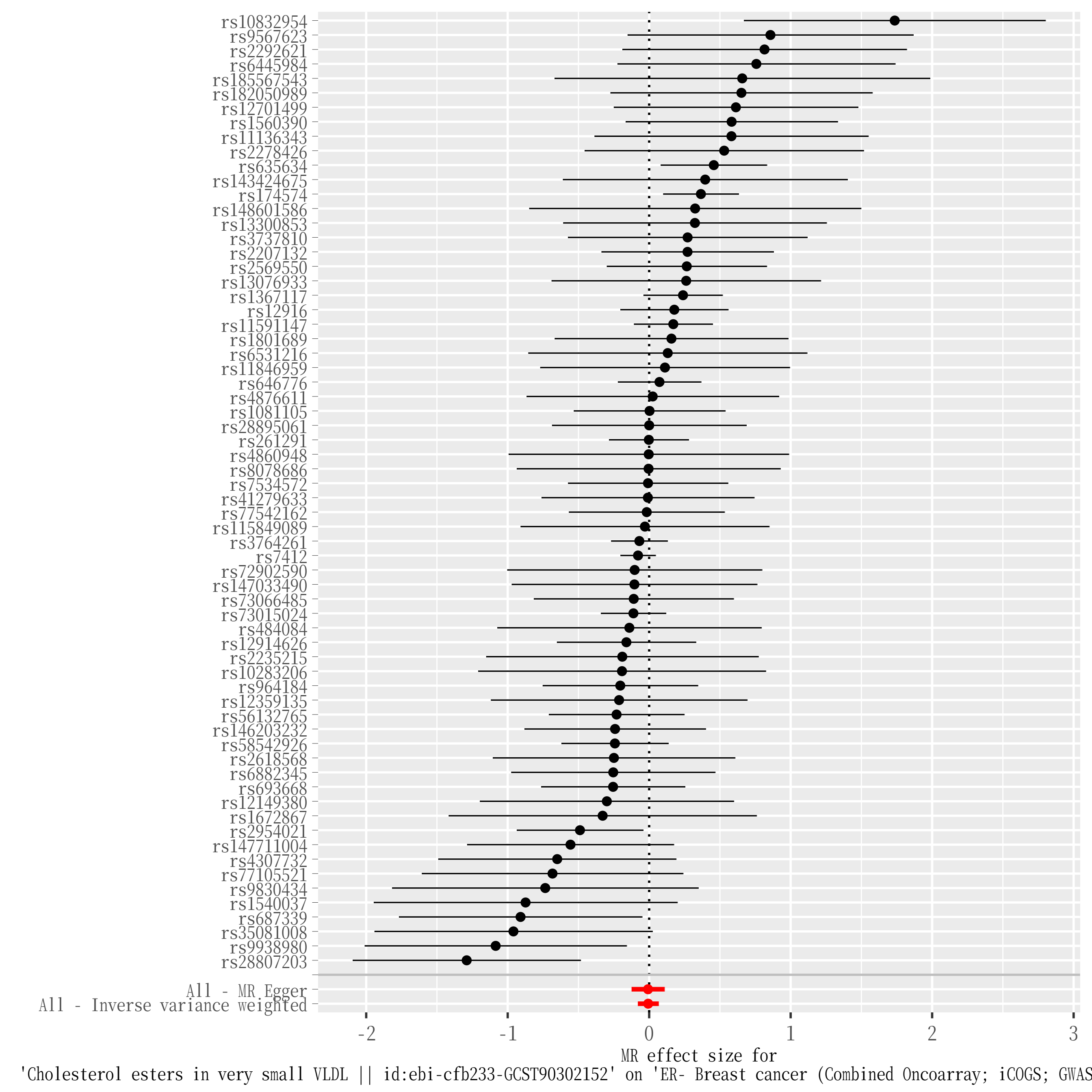
rs10832954
rs9567623
rs2292621
rs6445984
rs185567543
rs182050989
rs12701499
rs1560390
rs11136343
rs2278426
rs635634
rs143424675
rs174574
rs148601586
rs13300853
rs3737810
rs2207132
rs2569550
rs13076933
rs1367117
rs12916
rs11591147
rs1801689
rs6531216
rs11846959
rs646776
rs4876611
rs1081105
rs28895061
rs261291
rs4860948
rs8078686
rs7534572
rs41279633
rs77542162
rs115849089
rs3764261
rs7412
rs72902590
rs147033490
rs73066485
rs73015024
rs484084
rs12914626
rs2235215
rs10283206
rs964184
rs12359135
rs56132765
rs146203232
rs58542926
rs2618568
rs6882345
rs693668
rs12149380
rs1672867
rs2954021
rs147711004
rs4307732
rs77105521
rs9830434
rs1540037
rs687339
rs35081008
rs9938980
rs28807203
All - MR Egger
All - Inverse variance weighted
-2
-1
0
1
2
3
MR effect size for
'Cholesterol esters in very small VLDL || id:ebi-cfb233-GCST90302152' on 'ER- Breast cancer (Combined Oncoarray; iCOGS; GWAS

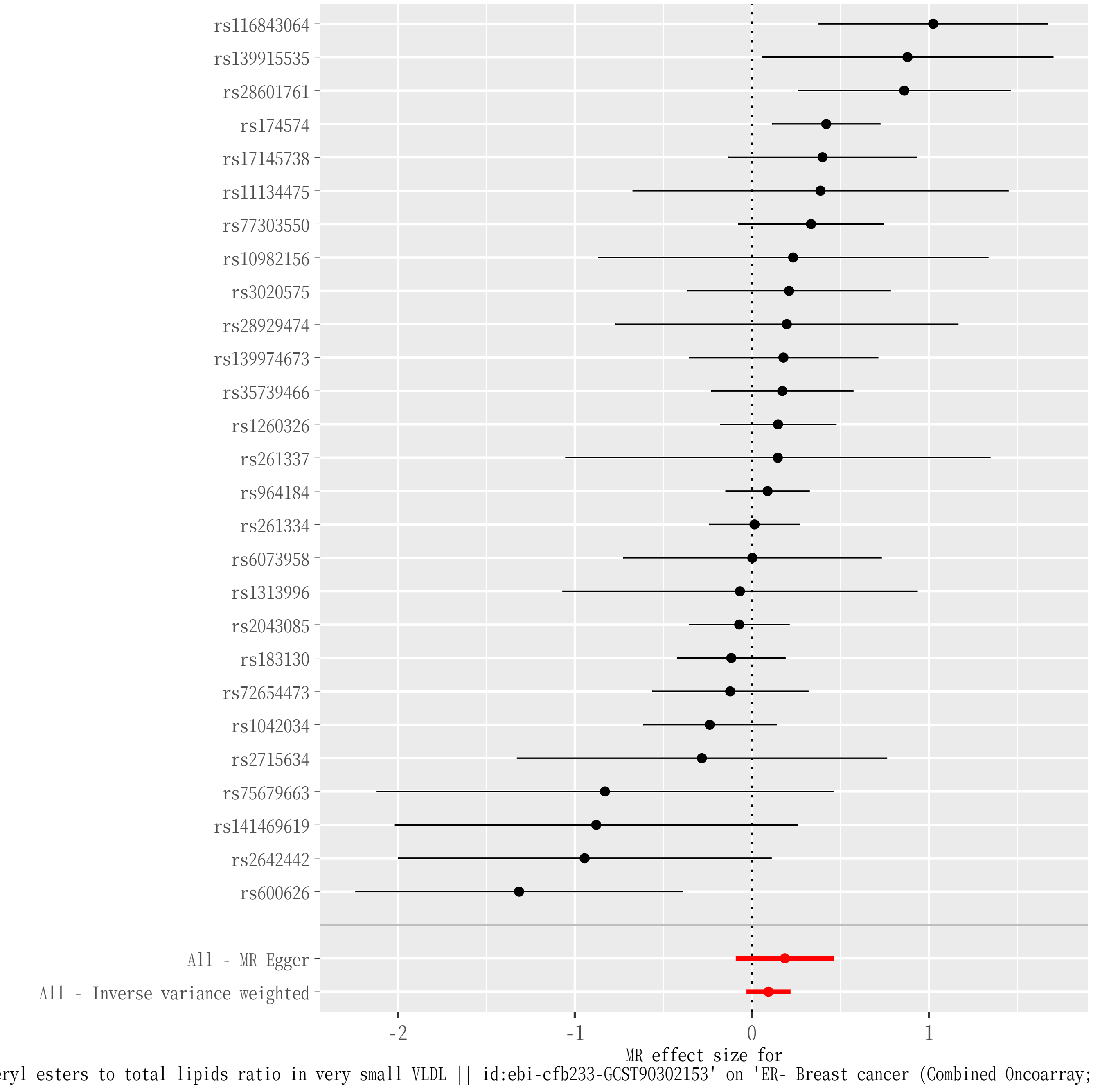
rs116843064
rs139915535
rs28601761
rs174574
rs17145738
rs11134475
rs77303550
rs10982156
rs3020575
rs28929474
rs139974673
rs35739466
rs1260326
rs261337
rs964184
rs261334
rs6073958
rs1313996
rs2043085
rs183130
rs72654473
rs1042034
rs2715634
rs75679663
rs141469619
rs2642442
rs600626
All - MR Egger
All - Inverse variance weighted
-2
-1
0
1
MR effect size for
eryl esters to total lipids ratio in very small VLDL || id:ebi-cfb233-GCST90302153' on 'ER- Breast cancer (Combined Oncoarray;

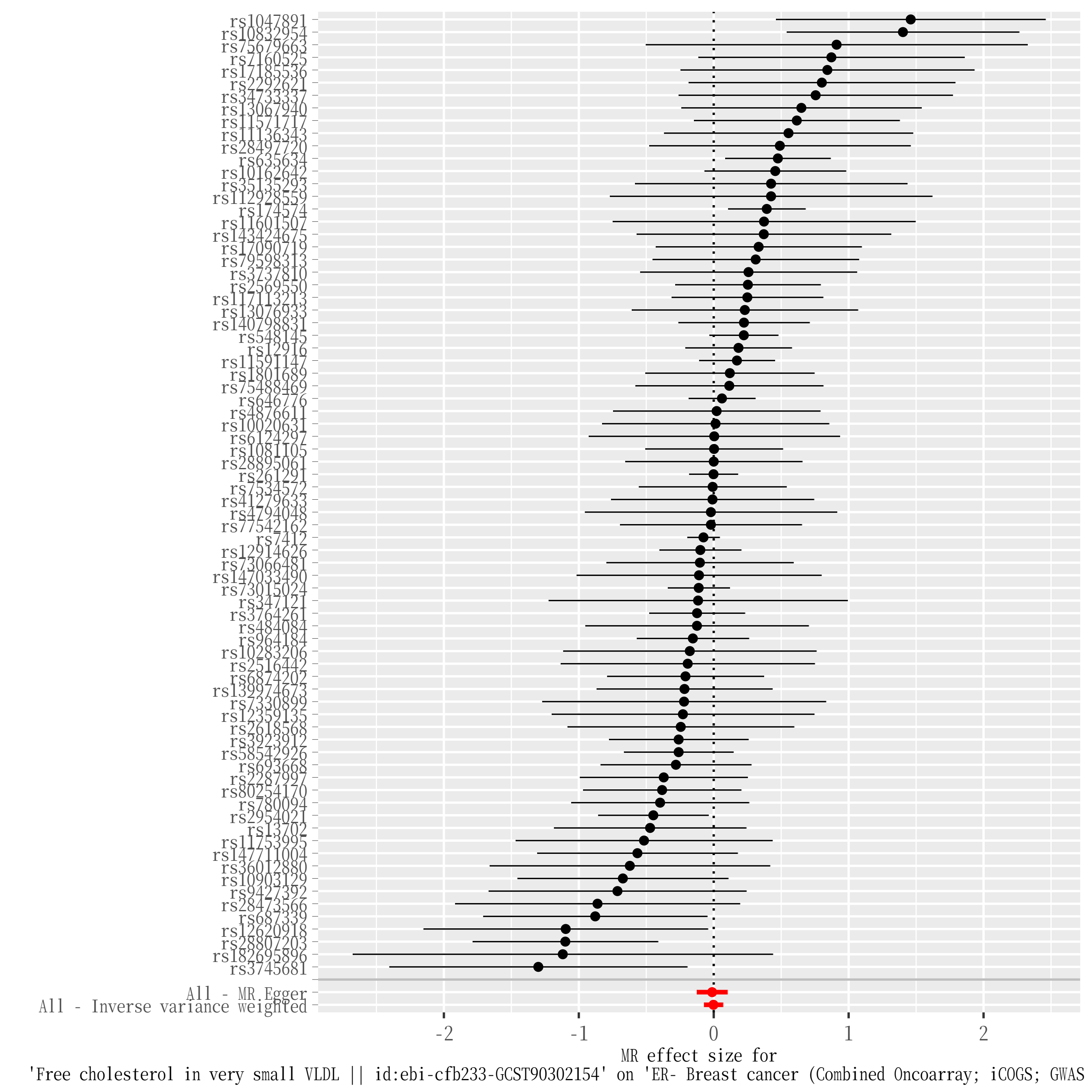
rs1047891
rs10832954
rs75679663
rs7160525
rs17185536
rs2292621
rs34733337
rs13067940
rs11571717
rs11136343
rs28497720
rs635634
rs10162642
rs35135293
rs112928559
rs174574
rs11601507
rs143424675
rs17090719
rs79598313
rs3737810
rs2569550
rs117113213
rs13076933
rs140798831
rs548145
rs12916
rs11591147
rs1801689
rs75488469
rs646776
rs4876611
rs10020631
rs6124297
rs1081105
rs28895061
rs261291
rs7534572
rs41279633
rs4794048
rs77542162
rs7412
rs12914626
rs73066481
rs147033490
rs73015024
rs347121
rs3764261
rs484084
rs964184
rs10283206
rs2516442
rs6874202
rs139974673
rs7330899
rs12359135
rs2618568
rs3923912
rs58542926
rs693668
rs2287997
rs80254170
rs780094
rs2954021
rs13702
rs11753995
rs147711004
rs36012880
rs10903129
rs9427392
rs28473566
rs687339
rs12620918
rs28807203
rs182695896
rs3745681
All - MR Egger
All - Inverse variance weighted
-2
-1
0
1
2
MR effect size for
'Free cholesterol in very small VLDL || id:ebi-cfb233-GCST90302154' on 'ER- Breast cancer (Combined Oncoarray; iCOGS; GWAS

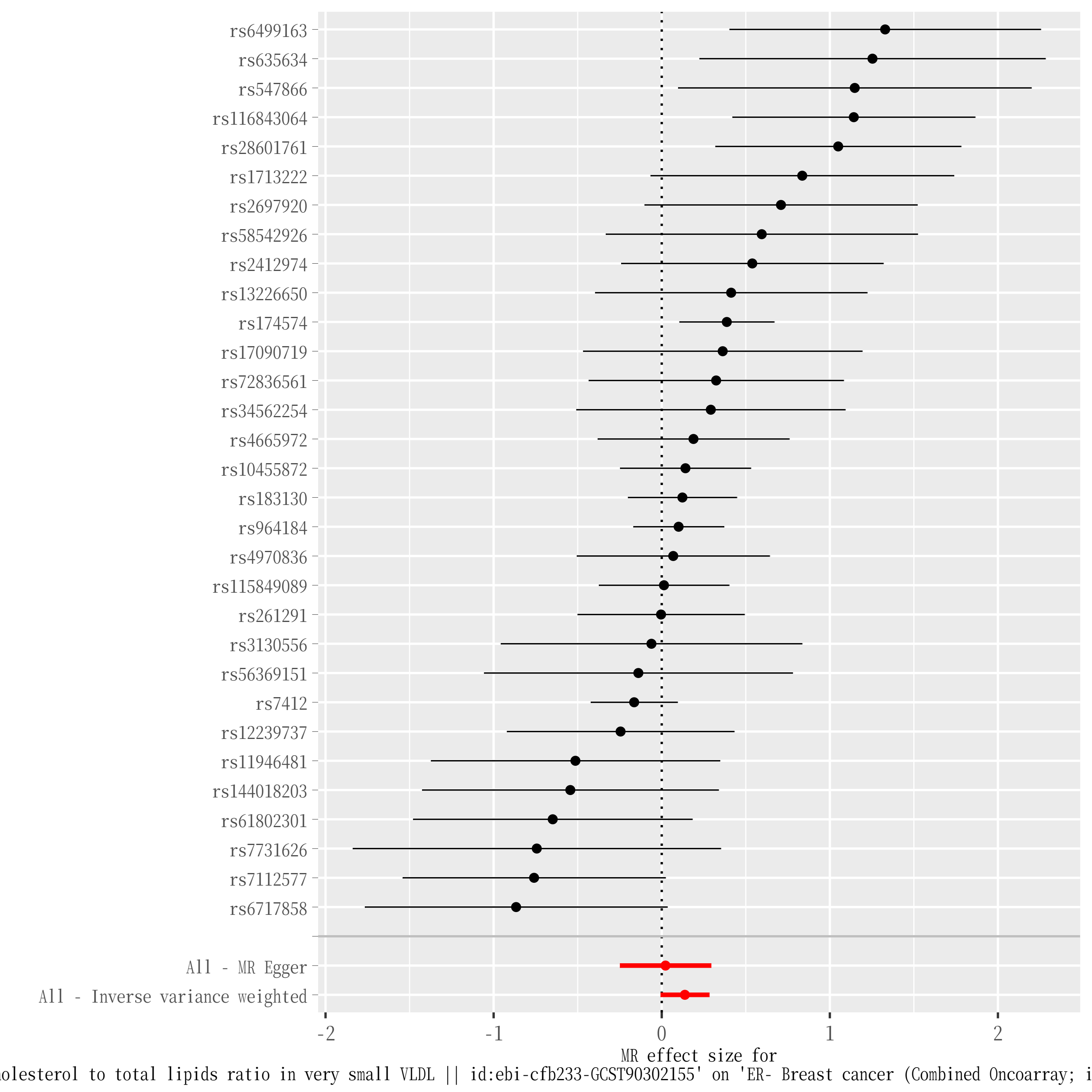
rs6499163
rs635634
rs547866
rs116843064
rs28601761
rs1713222
rs2697920
rs58542926
rs2412974
rs13226650
rs174574
rs17090719
rs72836561
rs34562254
rs4665972
rs10455872
rs183130
rs964184
rs4970836
rs115849089
rs261291
rs3130556
rs56369151
rs7412
rs12239737
rs11946481
rs144018203
rs61802301
rs7731626
rs7112577
rs6717858
All - MR Egger
All - Inverse variance weighted
-2
-1
0
1
2
MR effect size for
holesterol to total lipids ratio in very small VLDL || id:ebi-cfb233-GCST90302155' on 'ER- Breast cancer (Combined Oncoarray; i

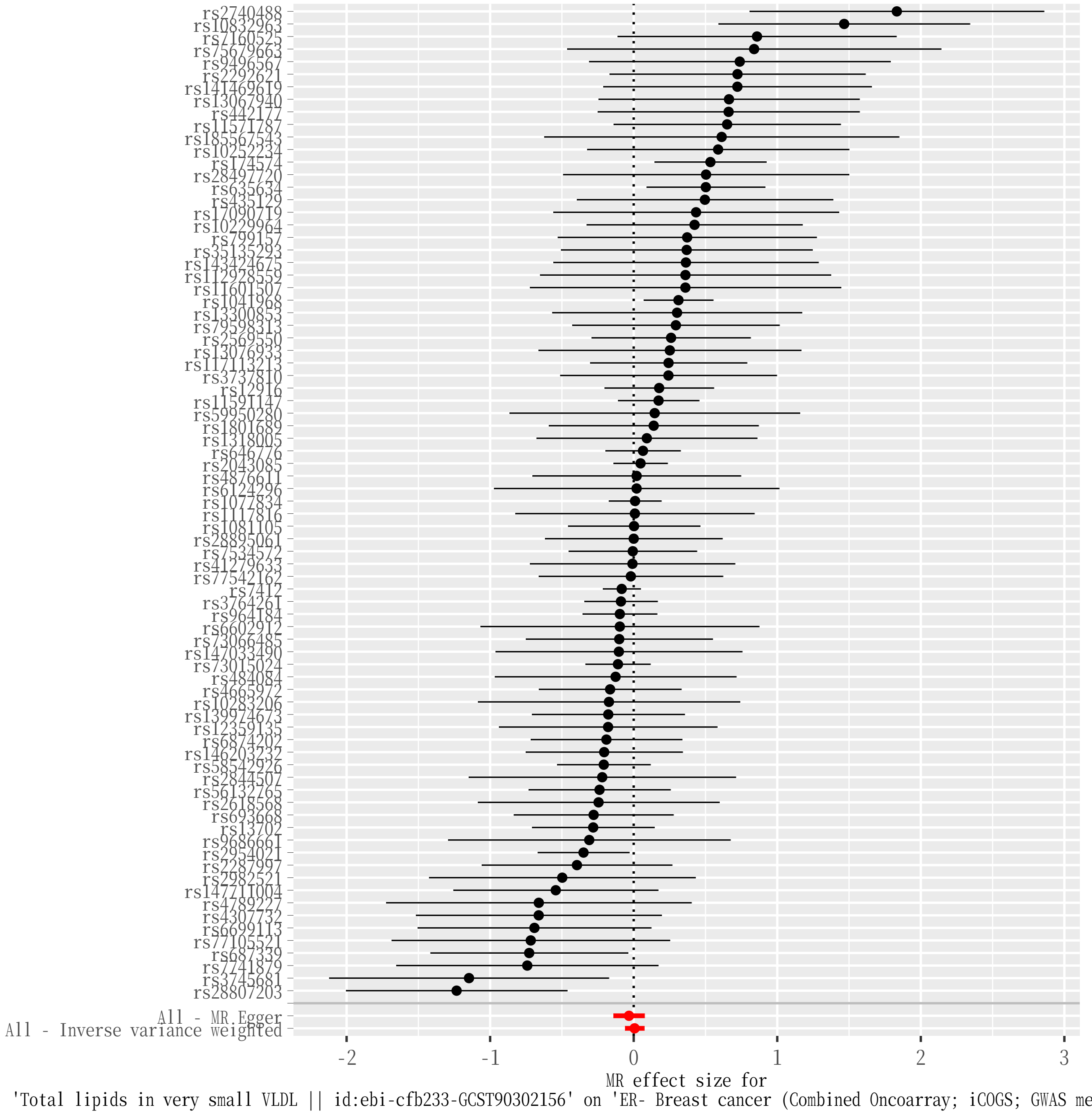

rs2740488
rs10832963
rs7160525
rs75679663
rs9496567
rs2292621
rs141469619
rs13067940
rs442177
rs11571787
rs185567543
rs10252234
rs174574
rs28497720
rs635634
rs435129
rs17090719
rs10229964
rs792157
rs35135293
rs143424675
rs112928559
rs11601507
rs1041968
rs13300853
rs79598313
rs2569550
rs13076933
rs117113213
rs3737810
rs12916
rs11591147
rs59950280
rs1801689
rs1318005
rs646776
rs2043085
rs4876611
rs6124296
rs1077834
rs1117816
rs1081105
rs28895061
rs7534572
rs41279633
rs77542162
rs7412
rs3764261
rs964184
rs6602912
rs73066485
rs147033490
rs73015024
rs484084
rs4665972
rs10283206
rs139974673
rs12359135
rs6874202
rs146203232
rs58542926
rs2844507
rs56132765
rs2618568
rs693668
rs13702
rs9686661
rs2954021
rs2287927
rs2982521
rs147711004
rs4789227
rs4307732
rs6699113
rs77105521
rs687339
rs7741879
rs3745681
rs28807203
All - MR Egger
All - Inverse variance weighted
-2
-1
0
1
2
3
MR effect size for
'Total lipids in very small VLDL || id:ebi-cfb233-GCST90302156' on 'ER- Breast cancer (Combined Oncoarray; iCOGS; GWAS me

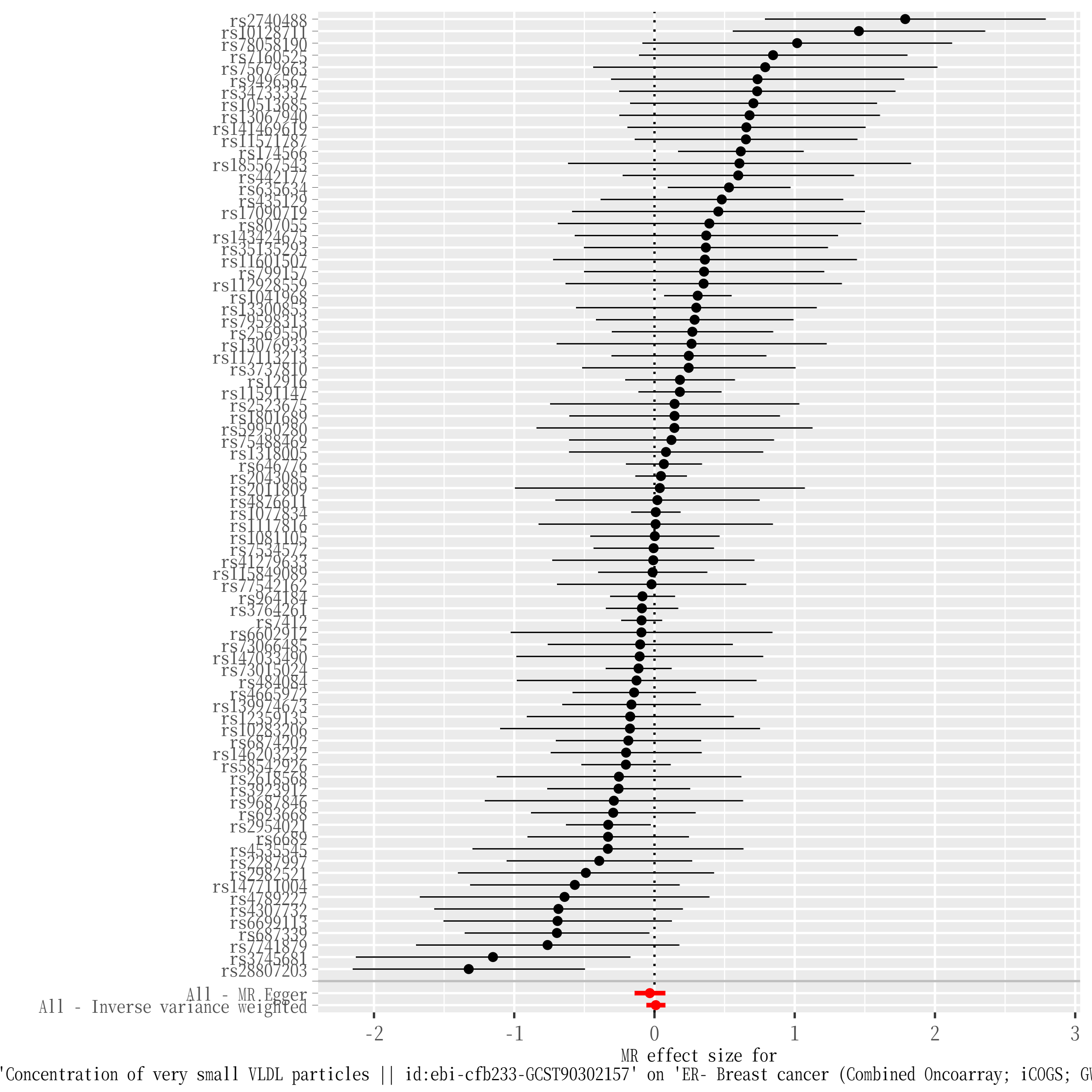

rs2740488
rs10128711
rs78058190
rs7160525
rs75679663
rs9496567
rs34733337
rs10513685
rs13067940
rs141469619
rs11571787
rs174566
rs185567543
rs442177
rs635634
rs435129
rs17090719
rs807055
rs143424675
rs35135293
rs11601502
rs799157
rs112928559
rs1041968
rs13300853
rs79598313
rs2569550
rs13076933
rs117113213
rs3737810
rs12916
rs11591147
rs2523675
rs1801689
rs59950280
rs75488469
rs1318005
rs646776
rs2043085
rs2011809
rs4876611
rs1077834
rs1117816
rs1081105
rs7534572
rs41279633
rs115849089
rs77542162
rs964184
rs3764261
rs7412
rs6602912
rs73066485
rs147033490
rs73015024
rs484084
rs4665972
rs139974673
rs12359135
rs10283206
rs6874202
rs146203232
rs58542926
rs2618568
rs3923912
rs9687846
rs693668
rs2954021
rs6689
rs4535545
rs2287997
rs2982521
rs147711004
rs4789227
rs4307732
rs6699113
rs687339
rs7741879
rs3745681
rs28807203
All - MR Egger
All - Inverse variance weighted
-2
-1
0
1
2
3
MR effect size for
'Concentration of very small VLDL particles || id:ebi-cfb233-GCST90302157' on 'ER- Breast cancer (Combined Oncoarray; iCOGS; G

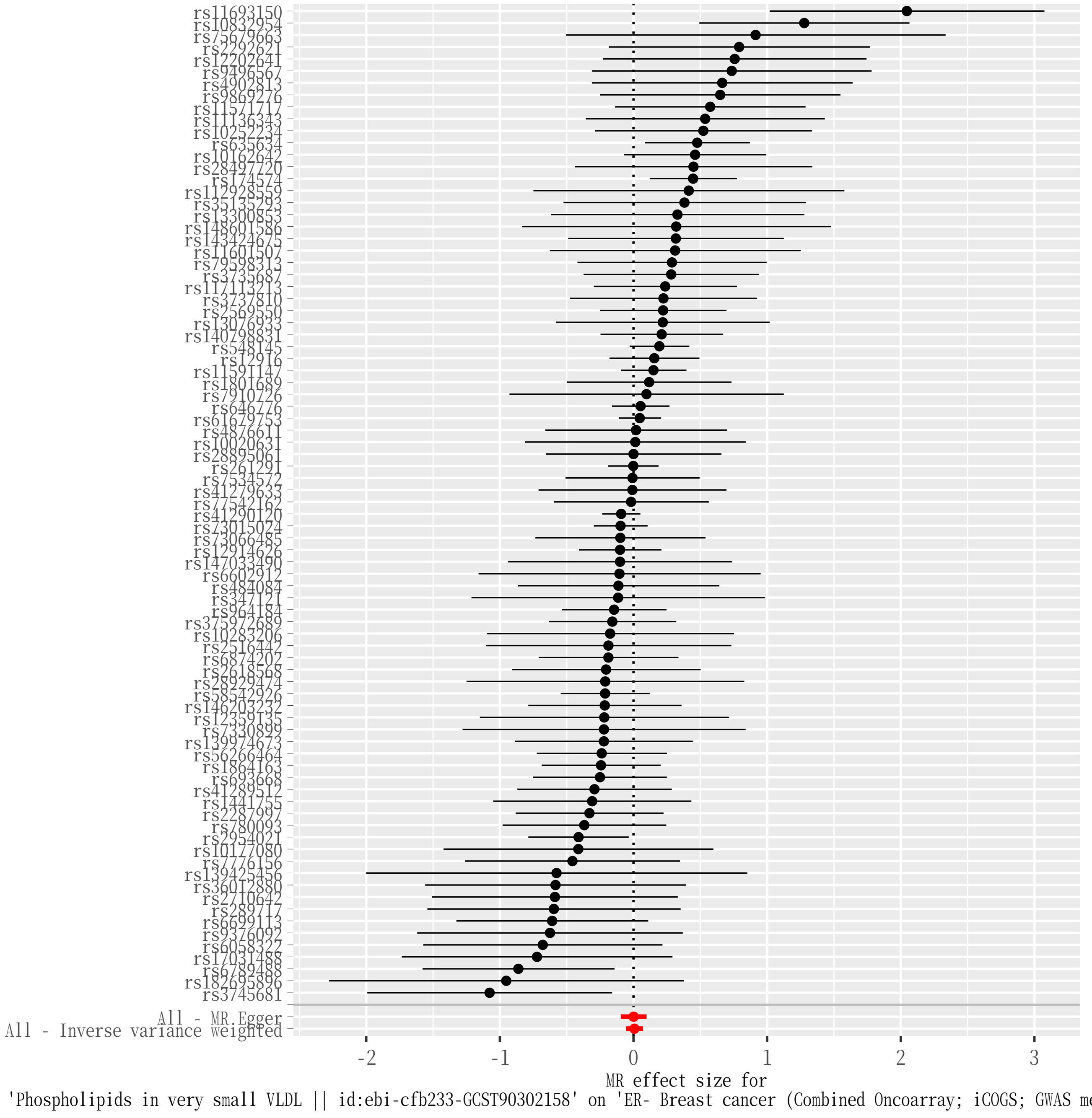

rs11693150
rs10832954
rs75679663
rs2292621
rs12202641
rs9496567
rs4902813
rs9869276
rs11571717
rs11136343
rs10252234
rs635634
rs10162642
rs28497720
rs174574
rs112928559
rs35135293
rs13300853
rs148601586
rs143424675
rs11601507
rs79598313
rs3735687
rs117113213
rs3737810
rs2569550
rs13076933
rs140798831
rs548145
rs12916
rs11591147
rs1801689
rs7910726
rs646776
rs61679753
rs4876611
rs10020631
rs28895061
rs261291
rs7534572
rs41279633
rs77542162
rs41290120
rs73015024
rs73066485
rs12914626
rs147033490
rs6602912
rs484084
rs347121
rs964184
rs375972689
rs10283206
rs2516442
rs6874202
rs2618568
rs28929474
rs58542926
rs146203232
rs12359135
rs7330899
rs139974673
rs56266464
rs1864163
rs693668
rs41289512
rs1441755
rs2287997
rs780093
rs2954021
rs10177080
rs7776156
rs139425456
rs36012880
rs2710642
rs289717
rs6699113
rs9376092
rs6058322
rs17031488
rs6789488
rs182695896
rs3745681
All - MR Egger
All - Inverse variance weighted
-2
-1
0
1
2
3
MR effect size for
'Phospholipids in very small VLDL || id:ebi-cfb233-GCST90302158' on 'ER- Breast cancer (Combined Oncoarray; iCOGS; GWAS me

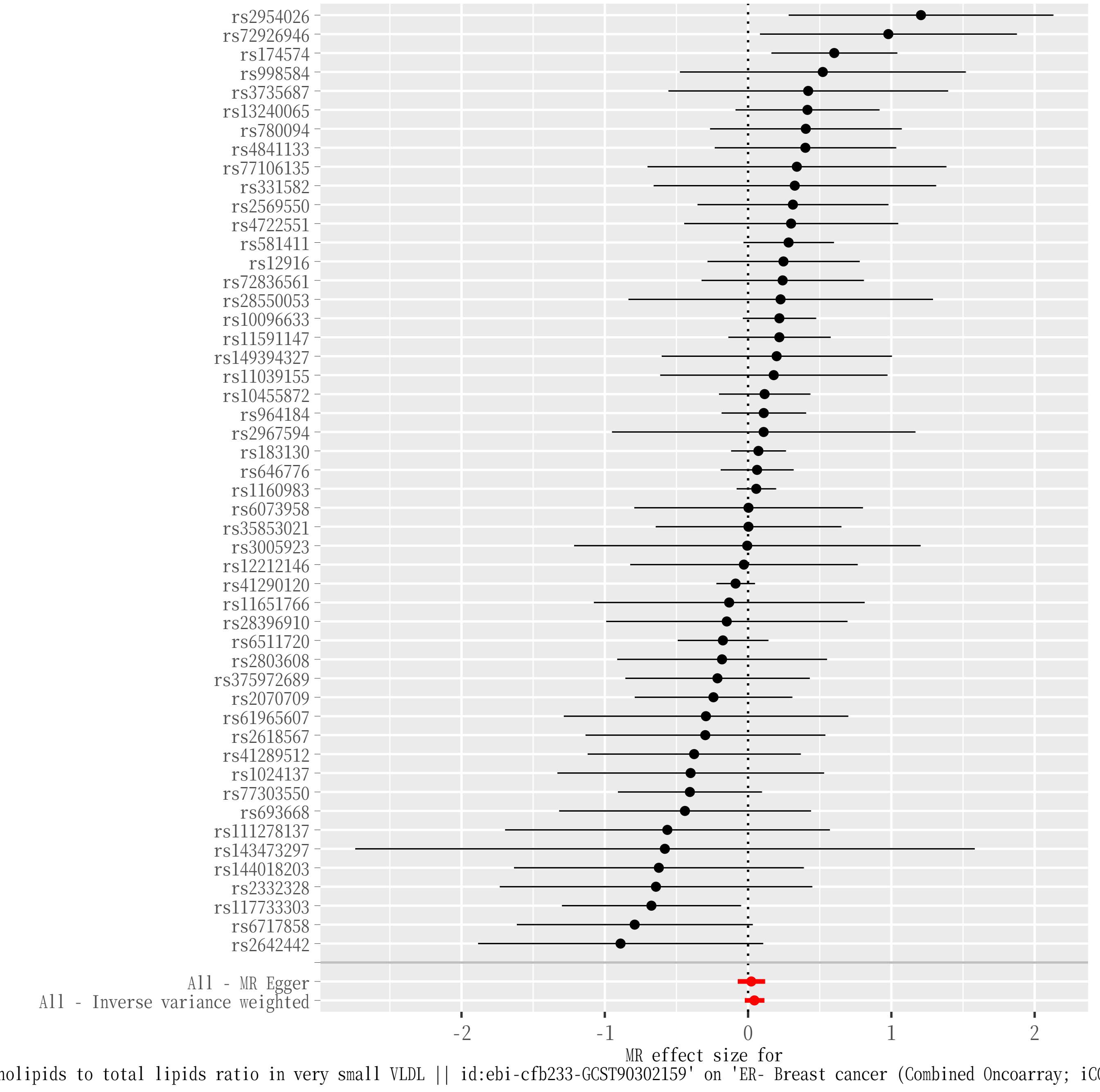
rs2954026
rs72926946
rs174574
rs998584
rs3735687
rs13240065
rs780094
rs4841133
rs77106135
rs331582
rs2569550
rs4722551
rs581411
rs12916
rs72836561
rs28550053
rs10096633
rs11591147
rs149394327
rs11039155
rs10455872
rs964184
rs2967594
rs183130
rs646776
rs1160983
rs6073958
rs35853021
rs3005923
rs12212146
rs41290120
rs11651766
rs28396910
rs6511720
rs2803608
rs375972689
rs2070709
rs61965607
rs2618567
rs41289512
rs1024137
rs77303550
rs693668
rs111278137
rs143473297
rs144018203
rs2332328
rs117733303
rs6717858
rs2642442
All - MR Egger
All - Inverse variance weighted
-2
-1
0
1
2
MR effect size for
holipids to total lipids ratio in very small VLDL || id:ebi-cfb233-GCST90302159' on 'ER- Breast cancer (Combined Oncoarray; iC

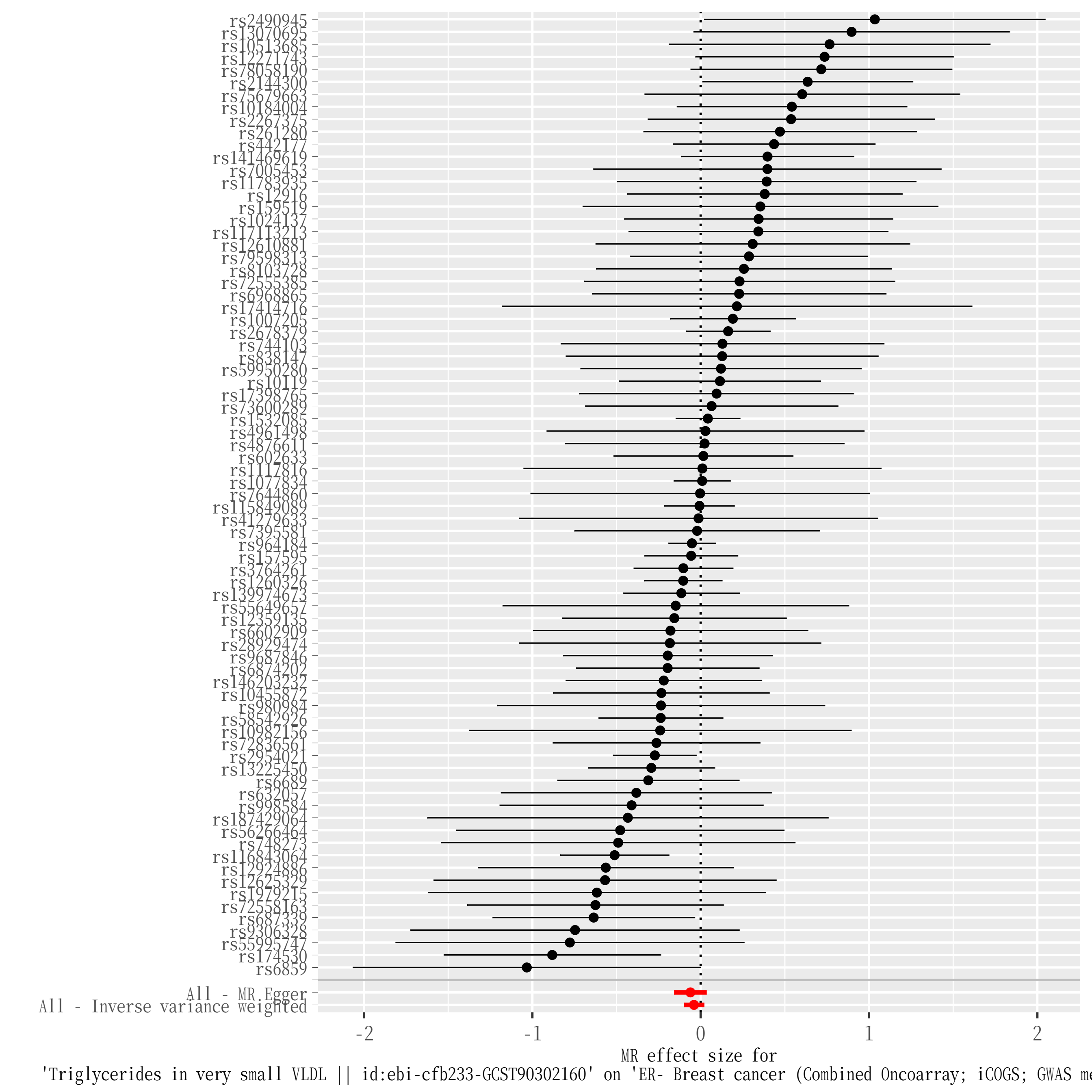
rs2490945
rs13070695
rs10513685
rs12271743
rs78058190
rs2144300
rs75679663
rs10184004
rs2267375
rs261280
rs442177
rs141469619
rs7005453
rs11783935
rs12916
rs159519
rs1024137
rs117113213
rs12610881
rs79598313
rs8103728
rs72555385
rs6968865
rs17414716
rs1007205
rs2678379
rs744103
rs838147
rs59950280
rs10119
rs17398765
rs73600289
rs1532085
rs4961498
rs4876611
rs602633
rs1117816
rs1077834
rs7644860
rs115849089
rs41279633
rs7395581
rs964184
rs157595
rs3764261
rs1260326
rs139974673
rs55649657
rs12359135
rs6602909
rs28929474
rs9687846
rs6874202
rs146203232
rs10455872
rs980984
rs58542926
rs10982156
rs72836561
rs2954021
rs13225450
rs6689
rs632057
rs998584
rs187429064
rs56266464
rs748273
rs116843064
rs12924886
rs12625329
rs1979215
rs72558163
rs687339
rs9306328
rs55925747
rs174530
rs6859
All - MR Egger
All - Inverse variance weighted
-2
-1
0
1
2
MR effect size for
'Triglycerides in very small VLDL || id:ebi-cfb233-GCST90302160' on 'ER- Breast cancer (Combined Oncoarray; iCOGS; GWAS me

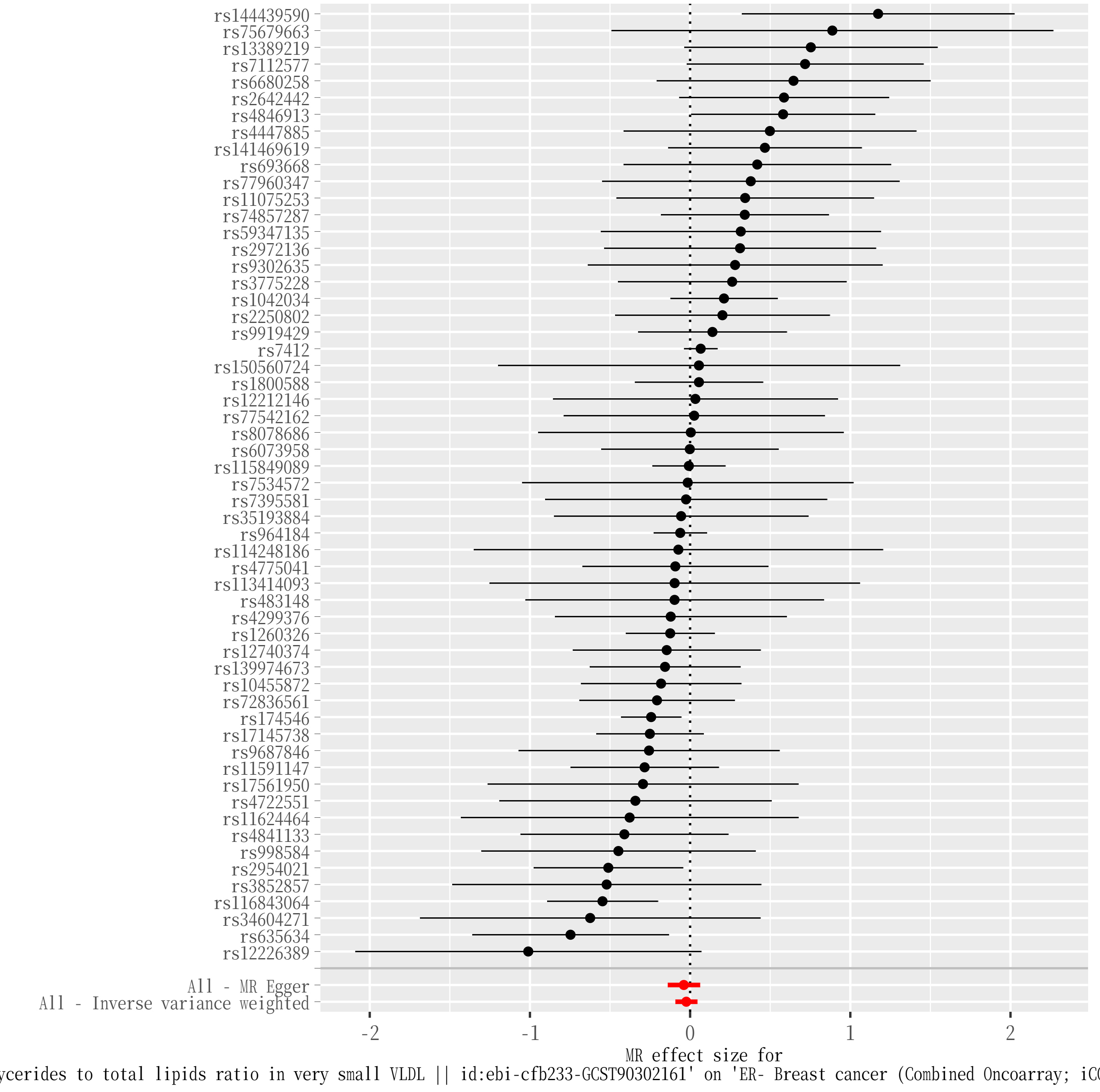

rs144439590
rs75679663
rs13389219
rs7112577
rs6680258
rs2642442
rs4846913
rs4447885
rs141469619
rs693668
rs77960347
rs11075253
rs74857287
rs59347135
rs2972136
rs9302635
rs3775228
rs1042034
rs2250802
rs9919429
rs7412
rs150560724
rs1800588
rs12212146
rs77542162
rs8078686
rs6073958
rs115849089
rs7534572
rs7395581
rs35193884
rs964184
rs114248186
rs4775041
rs113414093
rs483148
rs4299376
rs1260326
rs12740374
rs139974673
rs10455872
rs72836561
rs174546
rs17145738
rs9687846
rs11591147
rs17561950
rs4722551
rs11624464
rs4841133
rs998584
rs2954021
rs3852857
rs116843064
rs34604271
rs635634
rs12226389
All - MR Egger
All - Inverse variance weighted
-2
-1
0
1
2
MR effect size for
ycerides to total lipids ratio in very small VLDL || id:ebi-cfb233-GCST90302161' on 'ER- Breast cancer (Combined Oncoarray; iC

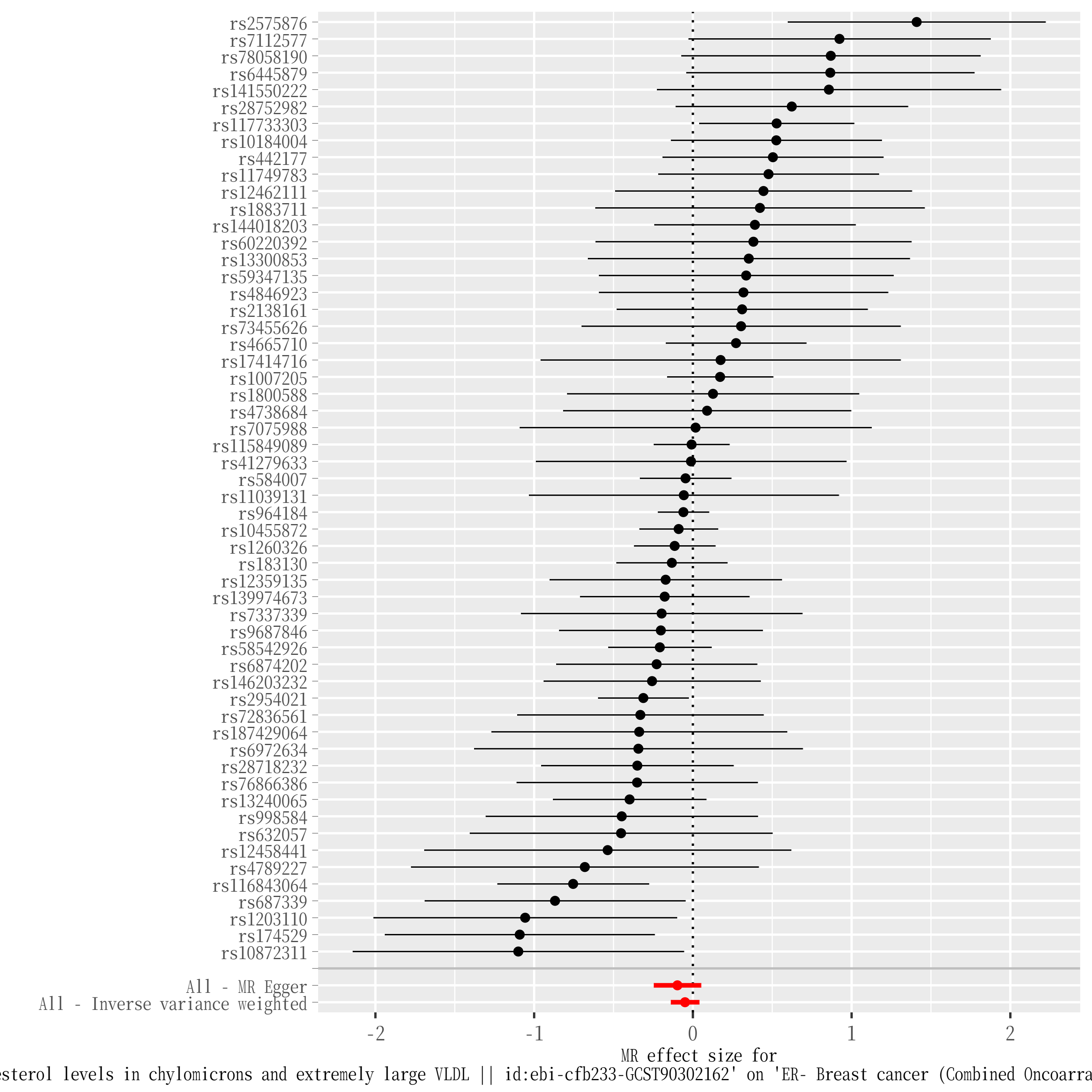

rs2575876
rs7112577
rs78058190
rs6445879
rs141550222
rs28752982
rs117733303
rs10184004
rs442177
rs11749783
rs12462111
rs1883711
rs144018203
rs60220392
rs13300853
rs59347135
rs4846923
rs2138161
rs73455626
rs4665710
rs17414716
rs1007205
rs1800588
rs4738684
rs7075988
rs115849089
rs41279633
rs584007
rs11039131
rs964184
rs10455872
rs1260326
rs183130
rs12359135
rs139974673
rs7337339
rs9687846
rs58542926
rs6874202
rs146203232
rs2954021
rs72836561
rs187429064
rs6972634
rs28718232
rs76866386
rs13240065
rs998584
rs632057
rs12458441
rs4789227
rs116843064
rs687339
rs1203110
rs174529
rs10872311
All - MR Egger
All - Inverse variance weighted
-2
-1
0
1
2
MR effect size for
esterol levels in chylomicrons and extremely large VLDL || id:ebi-cfb233-GCST90302162' on 'ER- Breast cancer (Combined Oncoarra

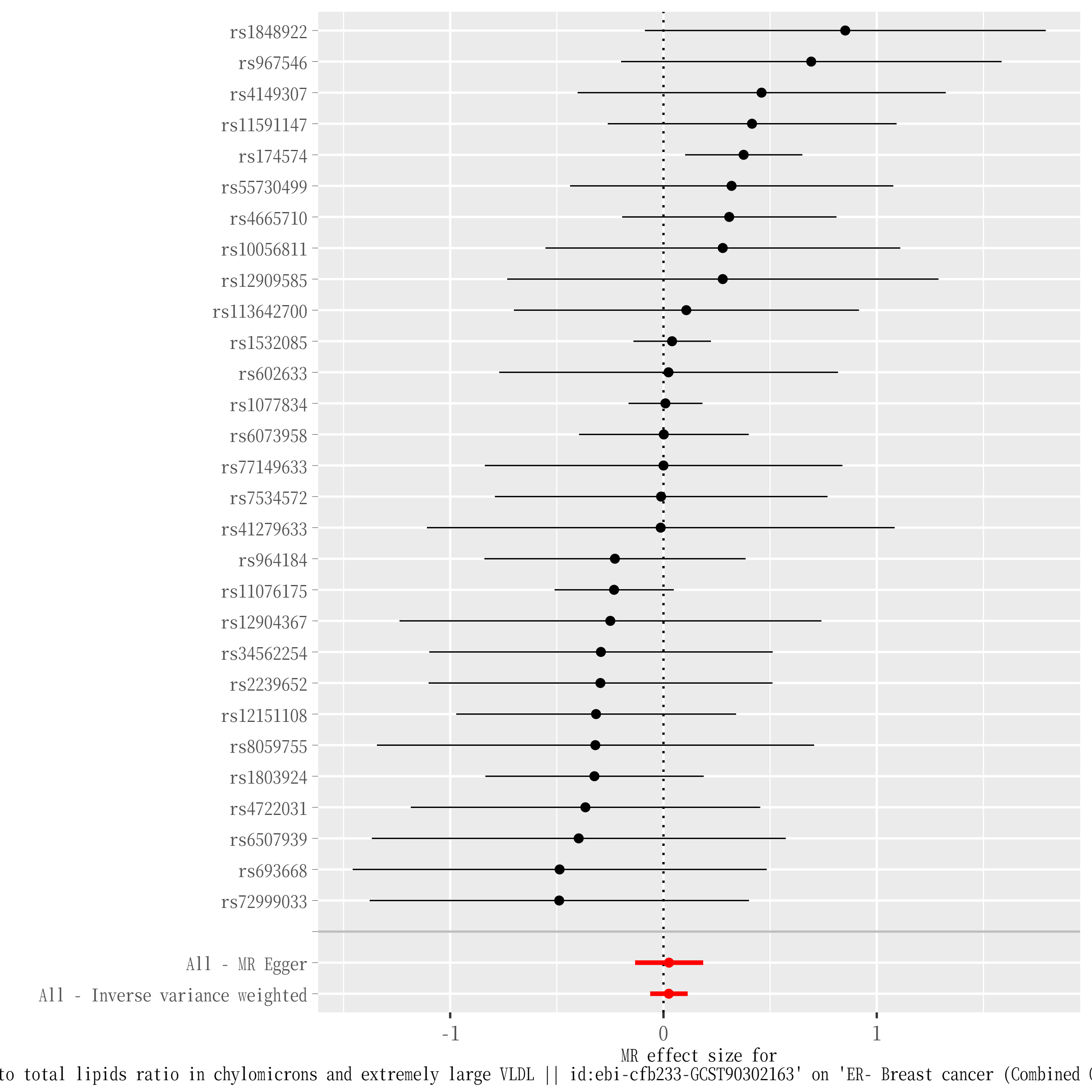
rs1848922
rs967546
rs4149307
rs11591147
rs174574
rs55730499
rs4665710
rs10056811
rs12909585
rs113642700
rs1532085
rs602633
rs1077834
rs6073958
rs77149633
rs7534572
rs41279633
rs964184
rs11076175
rs12904367
rs34562254
rs2239652
rs12151108
rs8059755
rs1803924
rs4722031
rs6507939
rs693668
rs72999033
All - MR Egger
All - Inverse variance weighted
-1
0
1
MR effect size for
to total lipids ratio in chylomicrons and extremely large VLDL || id:ebi-cfb233-GCST90302163' on 'ER- Breast cancer (Combined O

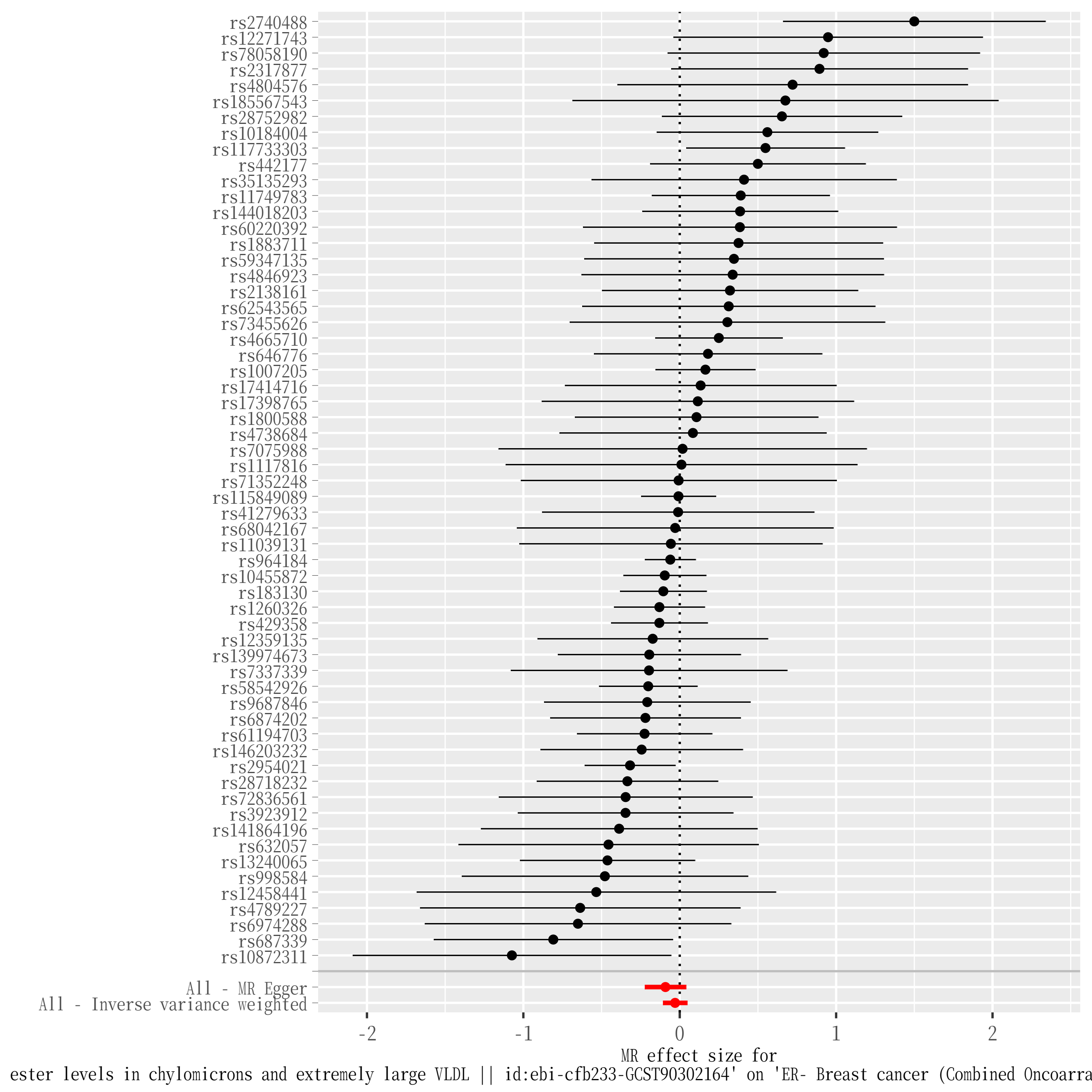
rs2740488
rs12271743
rs78058190
rs2317877
rs4804576
rs185567543
rs28752982
rs10184004
rs117733303
rs442177
rs35135293
rs11749783
rs144018203
rs60220392
rs1883711
rs59347135
rs4846923
rs2138161
rs62543565
rs73455626
rs4665710
rs646776
rs1007205
rs17414716
rs17398765
rs1800588
rs4738684
rs7075988
rs1117816
rs71352248
rs115849089
rs41279633
rs68042167
rs11039131
rs964184
rs10455872
rs183130
rs1260326
rs429358
rs12359135
rs139974673
rs7337339
rs58542926
rs9687846
rs6874202
rs61194703
rs146203232
rs2954021
rs28718232
rs72836561
rs3923912
rs141864196
rs632057
rs13240065
rs998584
rs12458441
rs4789227
rs6974288
rs687339
rs10872311
All - MR Egger
All - Inverse variance weighted
-2
-1
0
1
2
MR effect size for
ester levels in chylomicrons and extremely large VLDL || id:ebi-cfb233-GCST90302164' on 'ER- Breast cancer (Combined Oncoarra

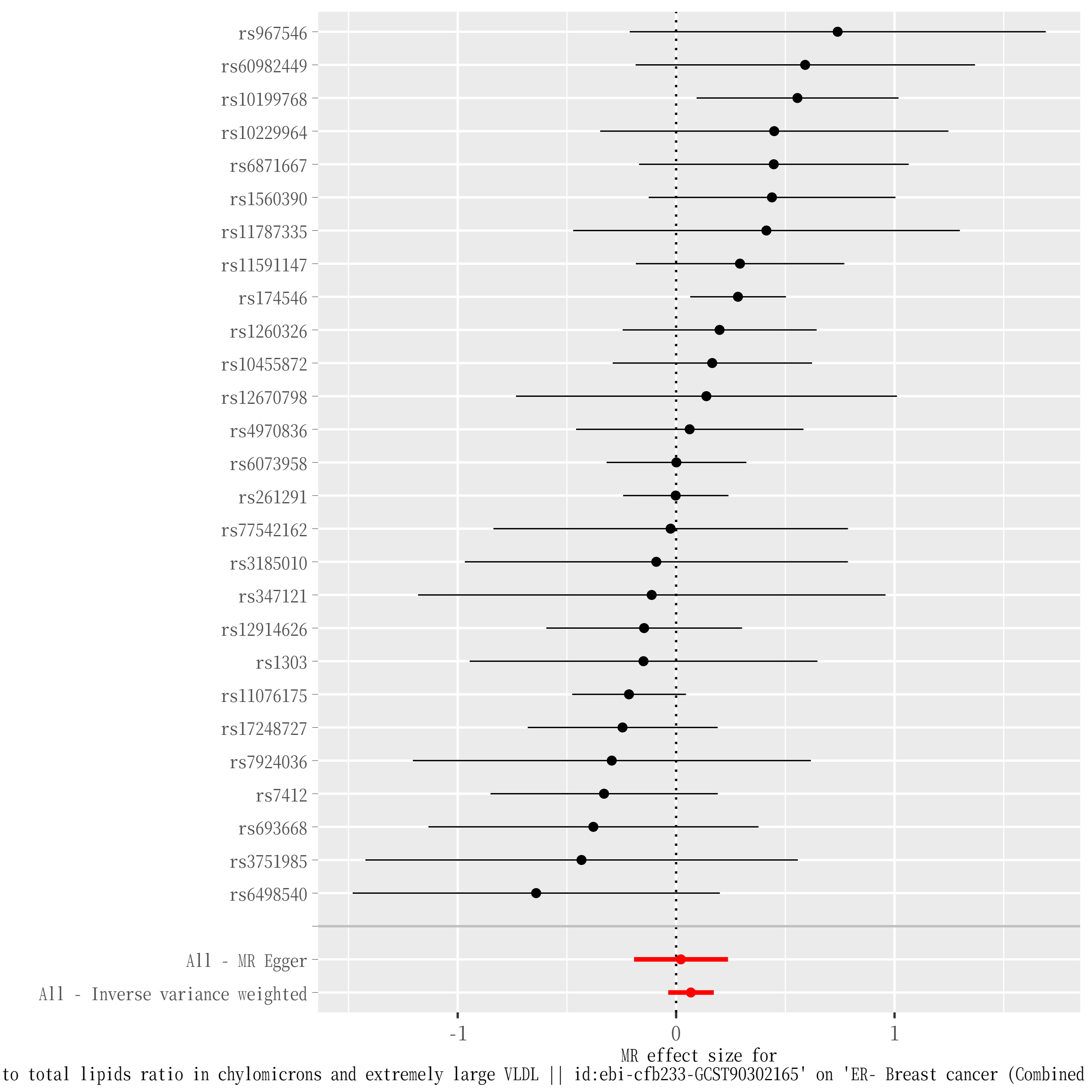
rs967546
rs60982449
rs10199768
rs10229964
rs6871667
rs1560390
rs11787335
rs11591147
rs174546
rs1260326
rs10455872
rs12670798
rs4970836
rs6073958
rs261291
rs77542162
rs3185010
rs347121
rs12914626
rs1303
rs11076175
rs17248727
rs7924036
rs7412
rs693668
rs3751985
rs6498540
All - MR Egger
All - Inverse variance weighted
-1
0
1
MR effect size for
to total lipids ratio in chylomicrons and extremely large VLDL || id:ebi-cfb233-GCST90302165' on 'ER- Breast cancer (Combined

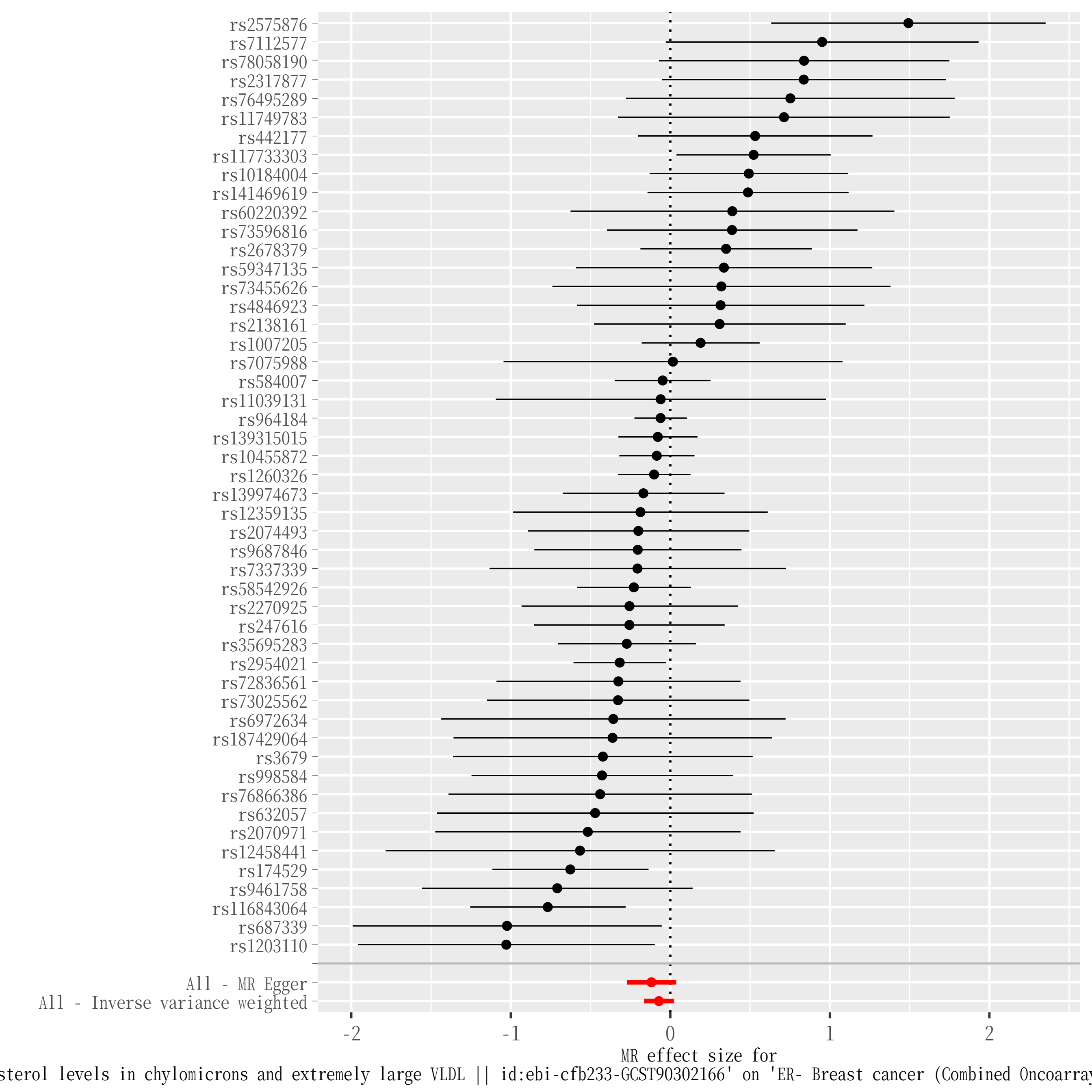
rs2575876
rs7112577
rs78058190
rs2317877
rs76495289
rs11749783
rs442177
rs117733303
rs10184004
rs141469619
rs60220392
rs73596816
rs2678379
rs59347135
rs73455626
rs4846923
rs2138161
rs1007205
rs7075988
rs584007
rs11039131
rs964184
rs139315015
rs10455872
rs1260326
rs139974673
rs12359135
rs2074493
rs9687846
rs7337339
rs58542926
rs2270925
rs247616
rs35695283
rs2954021
rs72836561
rs73025562
rs6972634
rs187429064
rs3679
rs998584
rs76866386
rs632057
rs2070971
rs12458441
rs174529
rs9461758
rs116843064
rs687339
rs1203110
All - MR Egger
All - Inverse variance weighted
-2
-1
0
1
2
MR effect size for
sterol levels in chylomicrons and extremely large VLDL || id:ebi-cfb233-GCST90302166' on 'ER- Breast cancer (Combined Oncoarray

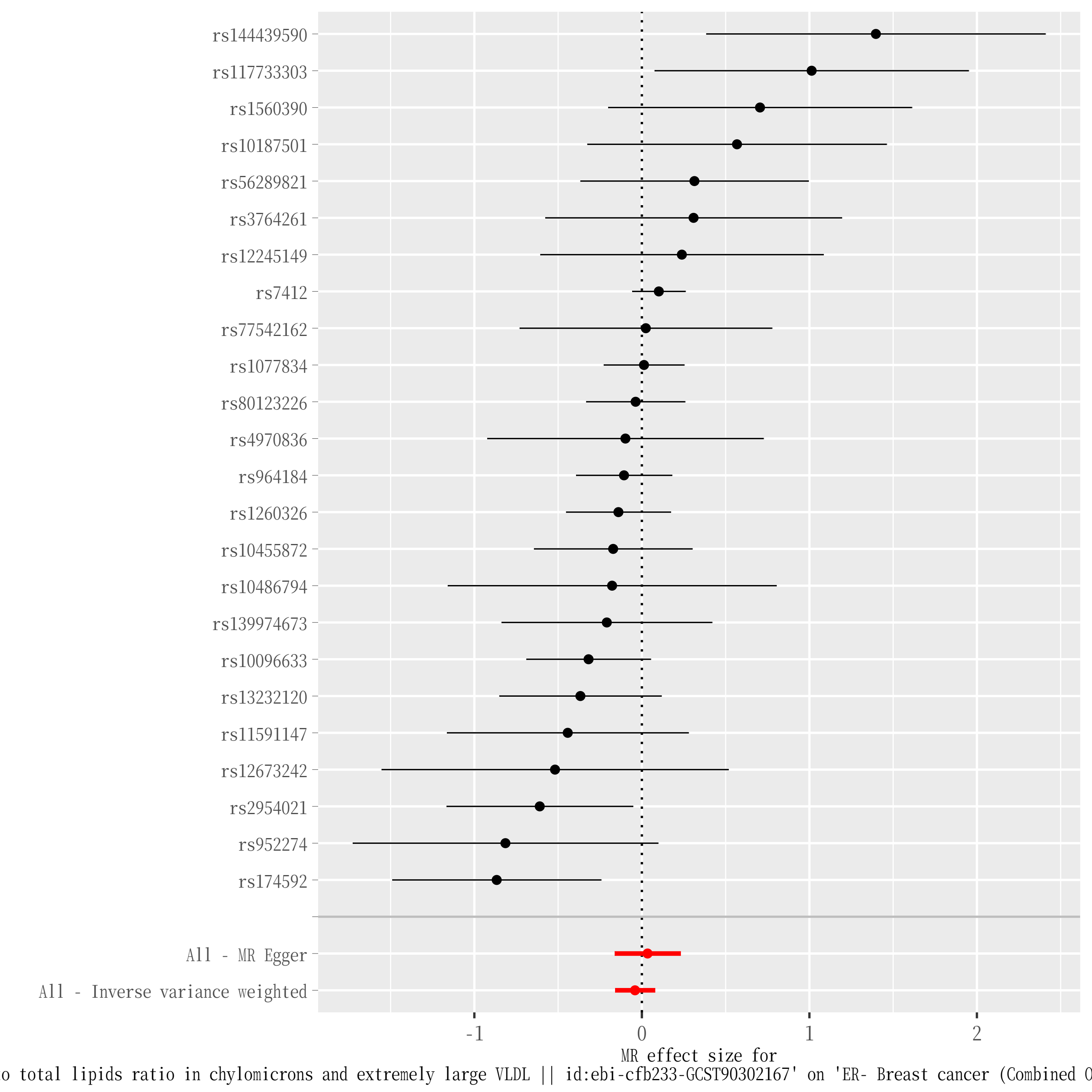

rs144439590
rs117733303
rs1560390
rs10187501
rs56289821
rs3764261
rs12245149
rs7412
rs77542162
rs1077834
rs80123226
rs4970836
rs964184
rs1260326
rs10455872
rs10486794
rs139974673
rs10096633
rs13232120
rs11591147
rs12673242
rs2954021
rs952274
rs174592
All - MR Egger
All - Inverse variance weighted
-1
0
1
2
MR effect size for
o total lipids ratio in chylomicrons and extremely large VLDL || id:ebi-cfb233-GCST90302167' on 'ER- Breast cancer (Combined O

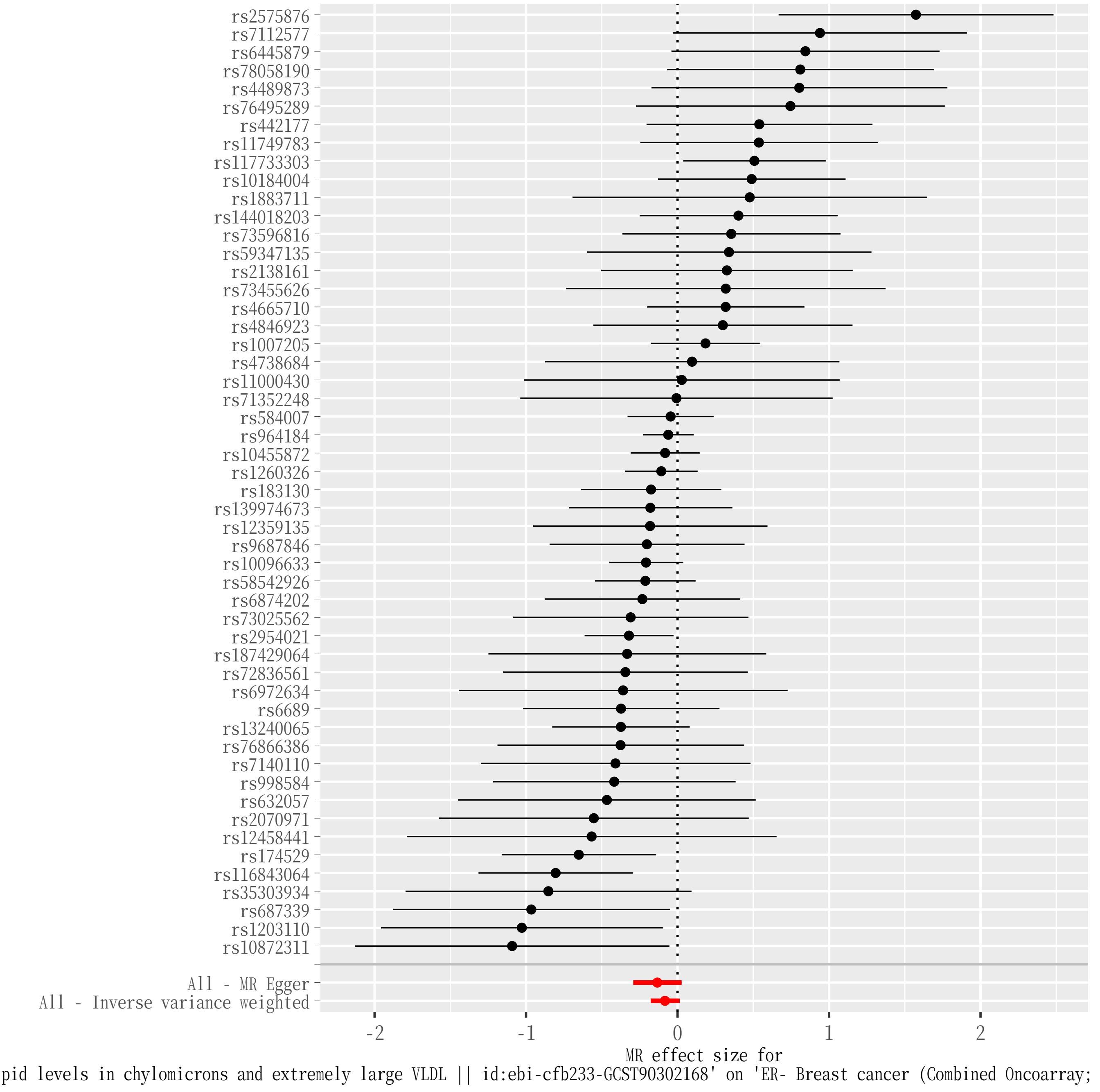
rs2575876
rs7112577
rs6445879
rs78058190
rs4489873
rs76495289
rs442177
rs11749783
rs117733303
rs10184004
rs1883711
rs144018203
rs73596816
rs59347135
rs2138161
rs73455626
rs4665710
rs4846923
rs1007205
rs4738684
rs11000430
rs71352248
rs584007
rs964184
rs10455872
rs1260326
rs183130
rs139974673
rs12359135
rs9687846
rs10096633
rs58542926
rs6874202
rs73025562
rs2954021
rs187429064
rs72836561
rs6972634
rs6689
rs13240065
rs76866386
rs7140110
rs998584
rs632057
rs2070971
rs12458441
rs174529
rs116843064
rs35303934
rs687339
rs1203110
rs10872311
All - MR Egger
All - Inverse variance weighted
-2
-1
0
1
2
MR effect size for
pid levels in chylomicrons and extremely large VLDL || id:ebi-cfb233-GCST90302168' on 'ER- Breast cancer (Combined Oncoarray;

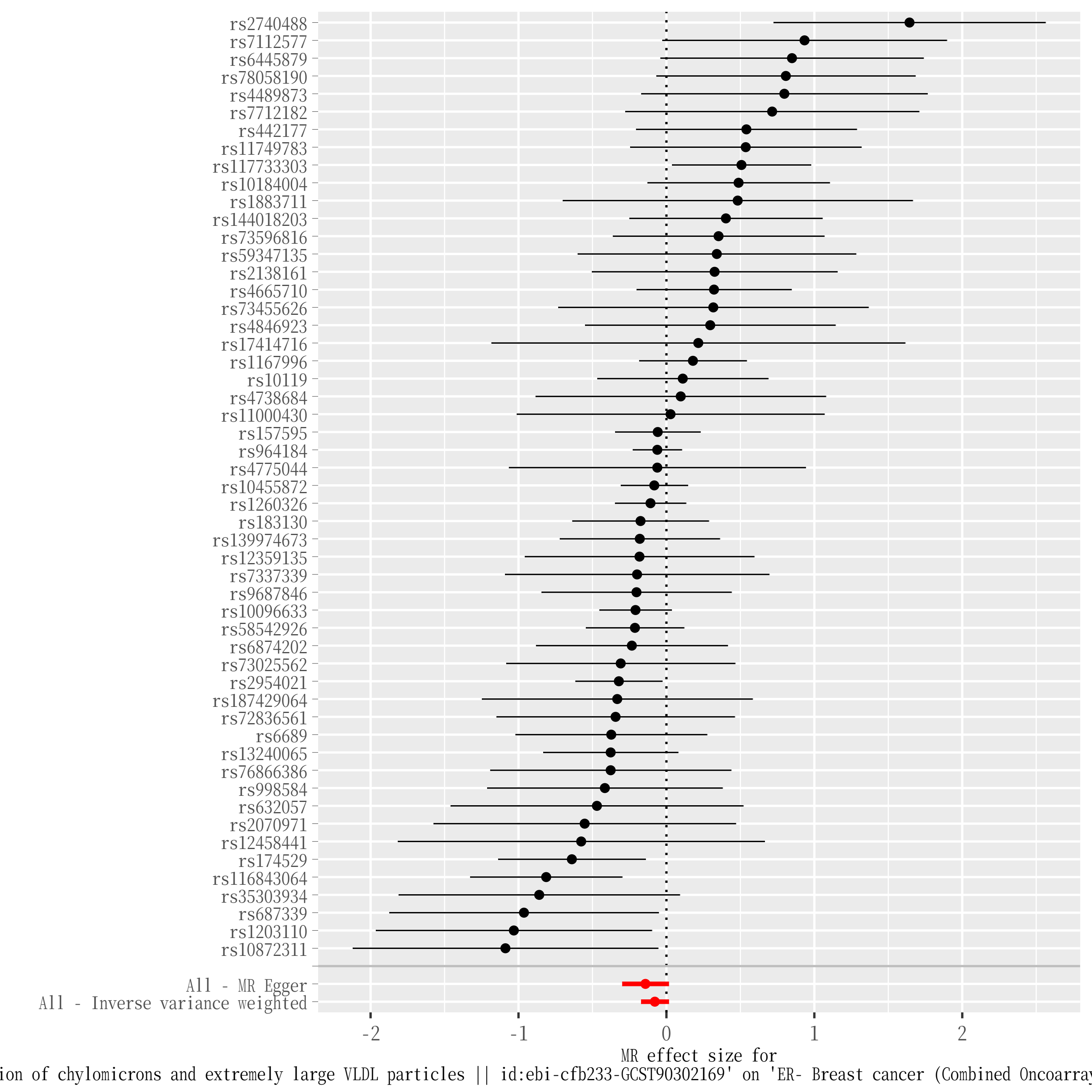

rs2740488
rs7112577
rs6445879
rs78058190
rs4489873
rs7712182
rs442177
rs11749783
rs117733303
rs10184004
rs1883711
rs144018203
rs73596816
rs59347135
rs2138161
rs4665710
rs73455626
rs4846923
rs17414716
rs1167996
rs10119
rs4738684
rs11000430
rs157595
rs964184
rs4775044
rs10455872
rs1260326
rs183130
rs139974673
rs12359135
rs7337339
rs9687846
rs10096633
rs58542926
rs6874202
rs73025562
rs2954021
rs187429064
rs72836561
rs6689
rs13240065
rs76866386
rs998584
rs632057
rs2070971
rs12458441
rs174529
rs116843064
rs35303934
rs687339
rs1203110
rs10872311
All - MR Egger
All - Inverse variance weighted
-2
-1
0
1
2
MR effect size for
ion of chylomicrons and extremely large VLDL particles || id:ebi-cfb233-GCST90302169' on 'ER- Breast cancer (Combined Oncoarray

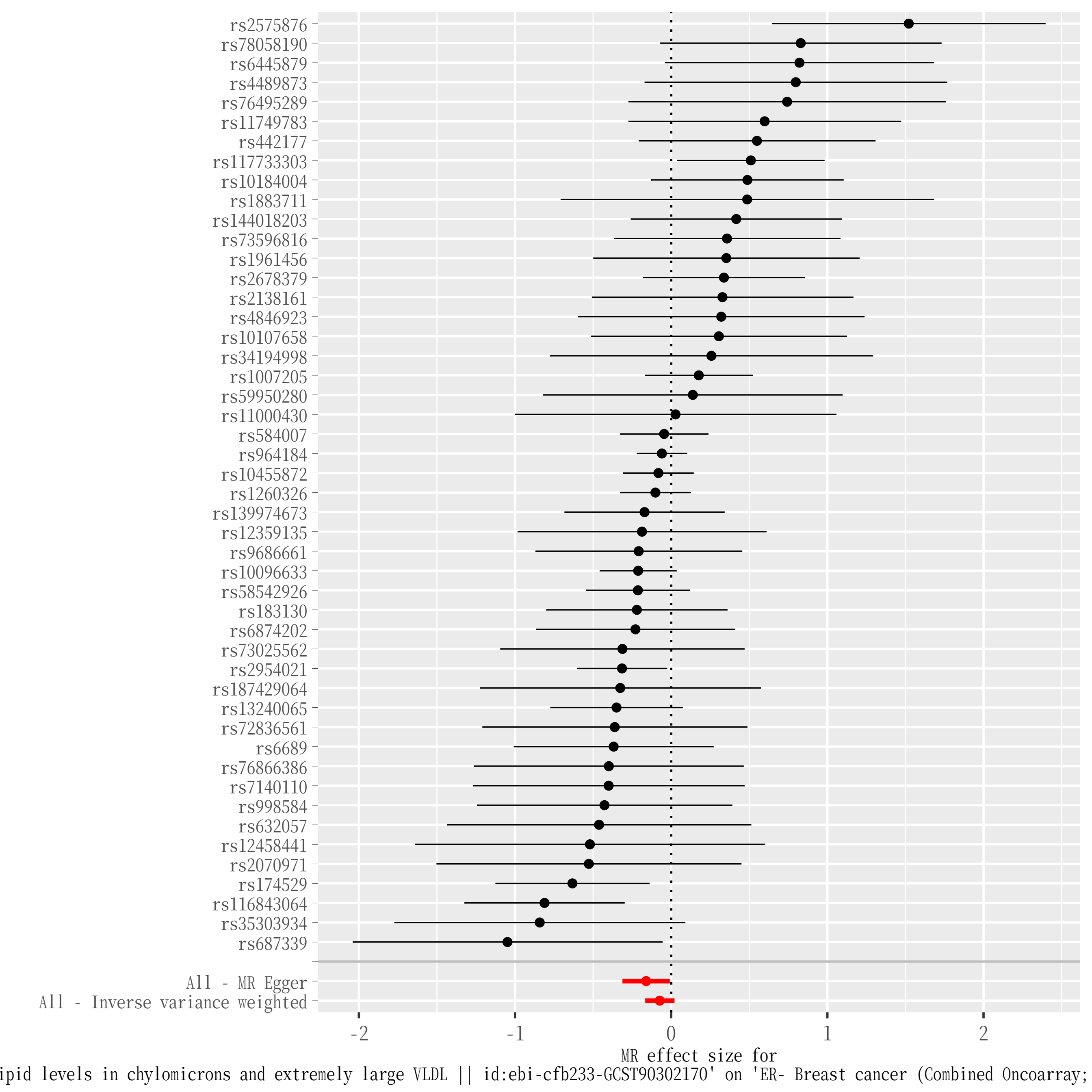
rs2575876
rs78058190
rs6445879
rs4489873
rs76495289
rs11749783
rs442177
rs117733303
rs10184004
rs1883711
rs144018203
rs73596816
rs1961456
rs2678379
rs2138161
rs4846923
rs10107658
rs34194998
rs1007205
rs59950280
rs11000430
rs584007
rs964184
rs10455872
rs1260326
rs139974673
rs12359135
rs9686661
rs10096633
rs58542926
rs183130
rs6874202
rs73025562
rs2954021
rs187429064
rs13240065
rs72836561
rs6689
rs76866386
rs7140110
rs998584
rs632057
rs12458441
rs2070971
rs174529
rs116843064
rs35303934
rs687339
All - MR Egger
All - Inverse variance weighted
-2
-1
0
1
2
MR effect size for
ipid levels in chylomicrons and extremely large VLDL || id:ebi-cfb233-GCST90302170' on 'ER- Breast cancer (Combined Oncoarray;

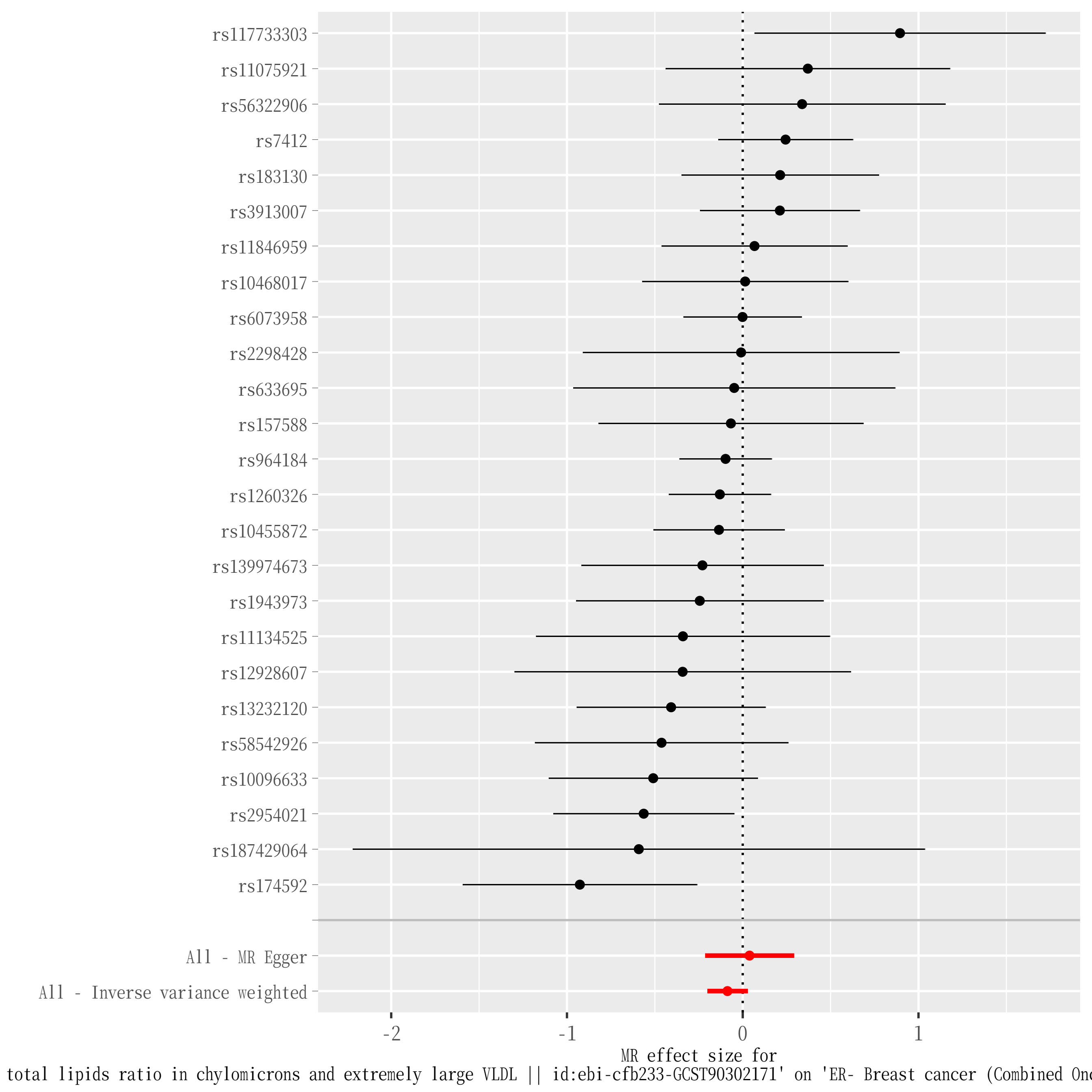
rs117733303
rs11075921
rs56322906
rs7412
rs183130
rs3913007
rs11846959
rs10468017
rs6073958
rs2298428
rs633695
rs157588
rs964184
rs1260326
rs10455872
rs139974673
rs1943973
rs11134525
rs12928607
rs13232120
rs58542926
rs10096633
rs2954021
rs187429064
rs174592
All - MR Egger
All - Inverse variance weighted
-2
-1
0
1
MR effect size for
total lipids ratio in chylomicrons and extremely large VLDL || id:ebi-cfb233-GCST90302171' on 'ER- Breast cancer (Combined On

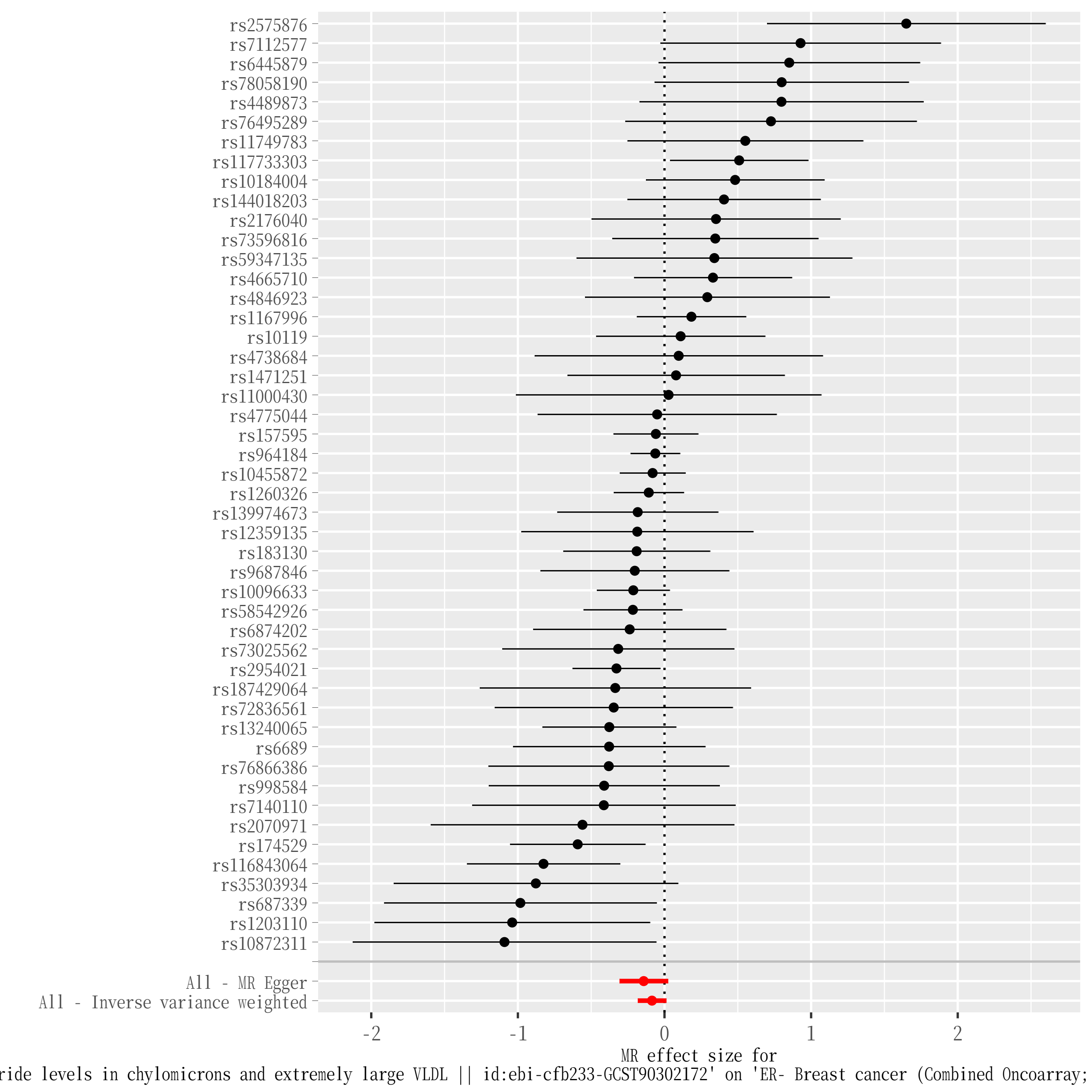
rs2575876
rs7112577
rs6445879
rs78058190
rs4489873
rs76495289
rs11749783
rs117733303
rs10184004
rs144018203
rs2176040
rs73596816
rs59347135
rs4665710
rs4846923
rs1167996
rs10119
rs4738684
rs1471251
rs11000430
rs4775044
rs157595
rs964184
rs10455872
rs1260326
rs139974673
rs12359135
rs183130
rs9687846
rs10096633
rs58542926
rs6874202
rs73025562
rs2954021
rs187429064
rs72836561
rs13240065
rs6689
rs76866386
rs998584
rs7140110
rs2070971
rs174529
rs116843064
rs35303934
rs687339
rs1203110
rs10872311
All - MR Egger
All - Inverse variance weighted
-2
-1
0
1
2
MR effect size for
ride levels in chylomicrons and extremely large VLDL || id:ebi-cfb233-GCST90302172' on 'ER- Breast cancer (Combined Oncoarray;

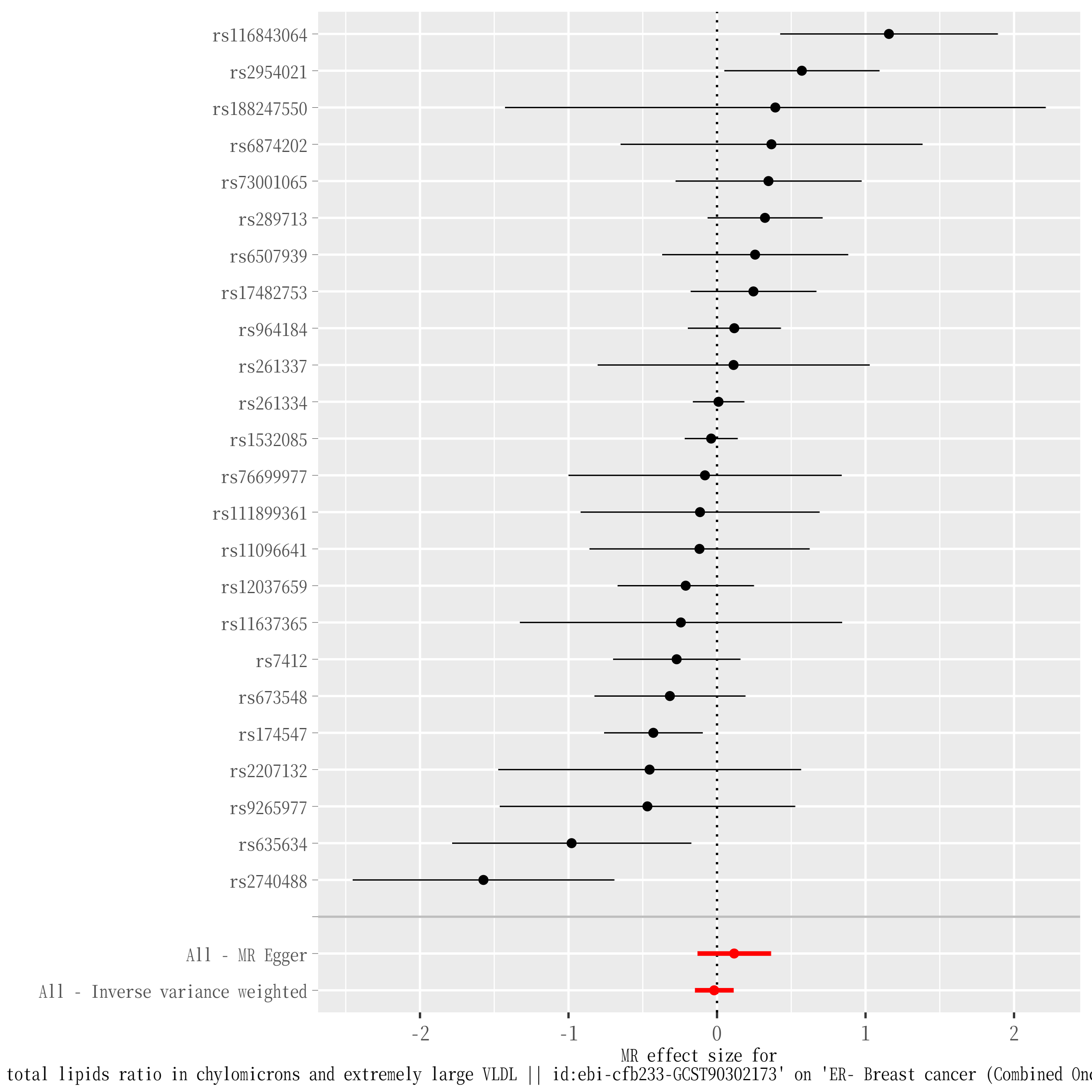
rs116843064
rs2954021
rs188247550
rs6874202
rs73001065
rs289713
rs6507939
rs17482753
rs964184
rs261337
rs261334
rs1532085
rs76699977
rs111899361
rs11096641
rs12037659
rs11637365
rs7412
rs673548
rs174547
rs2207132
rs9265977
rs635634
rs2740488
All - MR Egger
All - Inverse variance weighted
-2
-1
0
1
2
MR effect size for
total lipids ratio in chylomicrons and extremely large VLDL || id:ebi-cfb233-GCST90302173' on 'ER- Breast cancer (Combined On
