# Supplementary material for: Assessing the causal relationships between circulating metabolic biomarkers and breast cancer by using mendelian randomization
Source: Front Genet. 2024 Dec 18;15:1448748. doi: 10.3389/fgene.2024.1448748 (PMC11688392; doi:10.3389/fgene.2024.1448748)
Supplement: Supplementary file 15 [file DataSheet10.pdf]

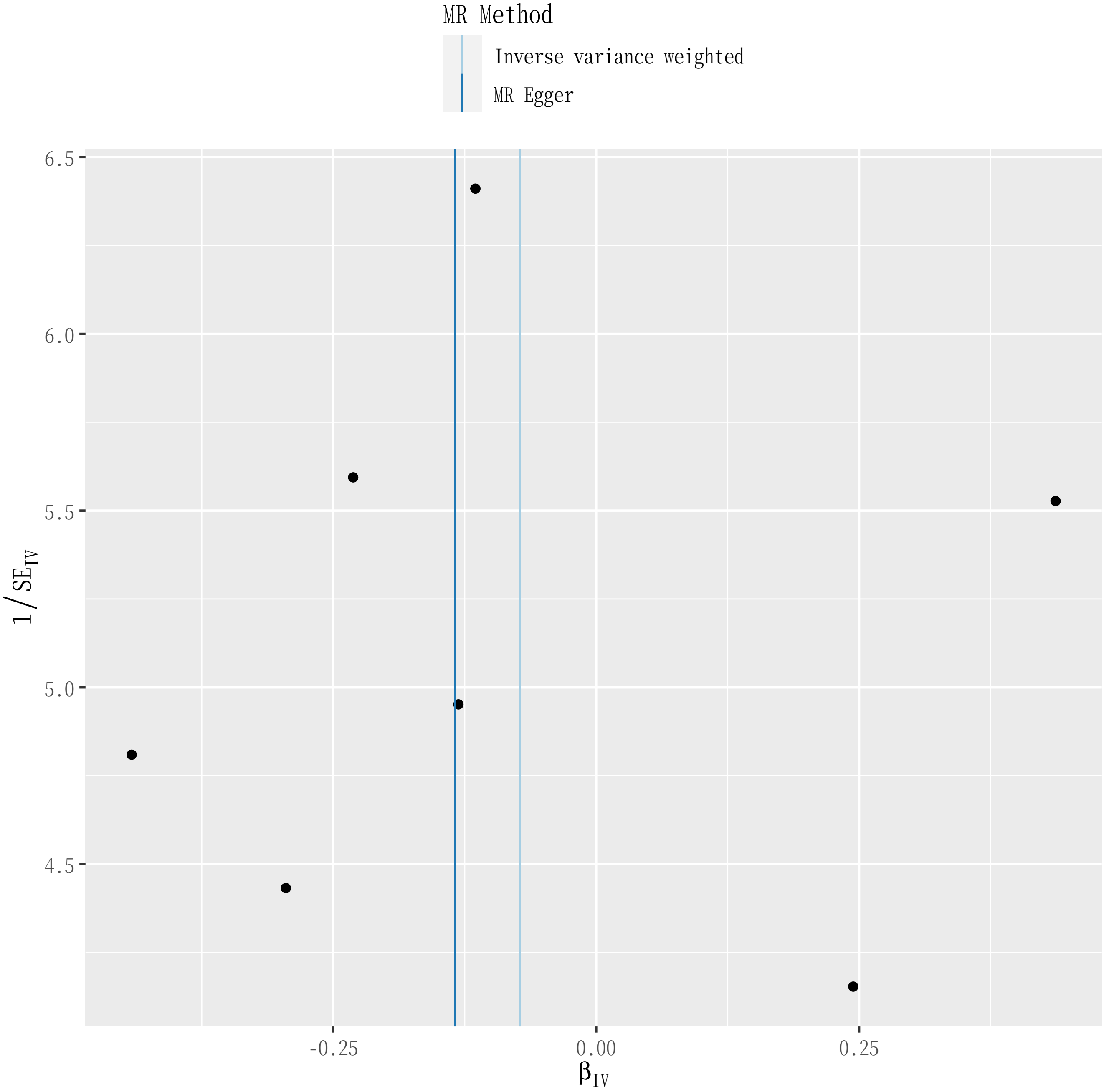
MR Method
Inverse variance weighted
MR Egger
6.5
6.0
5.5
5.0
4.5
$1/SE_{IV}$
-0.25
0.00
0.25
β_{IV}

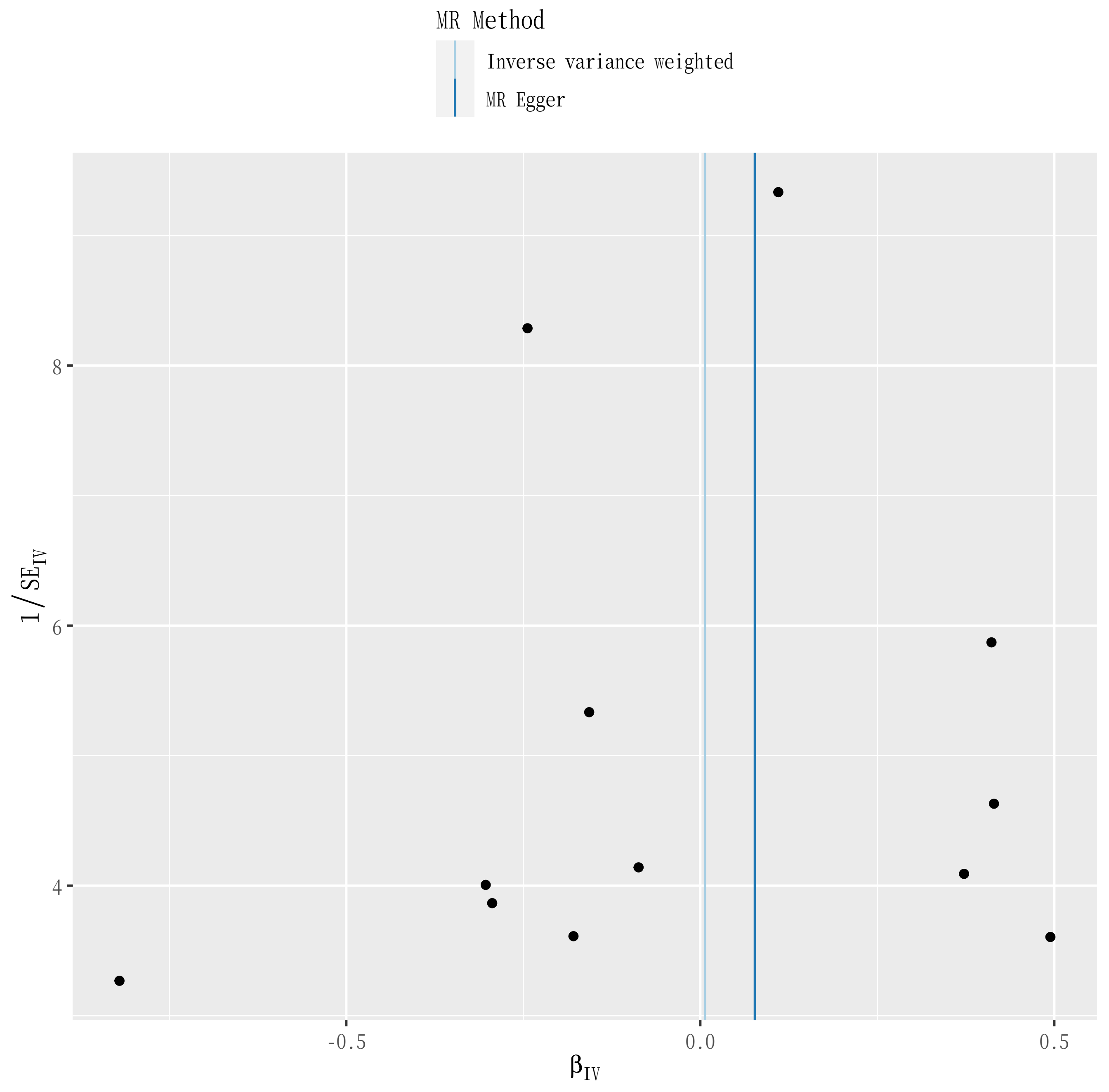
MR Method
Inverse variance weighted
MR Egger
8
6
4
$1/SE_{IV}$
-0.5
0.0
0.5
β_{IV}

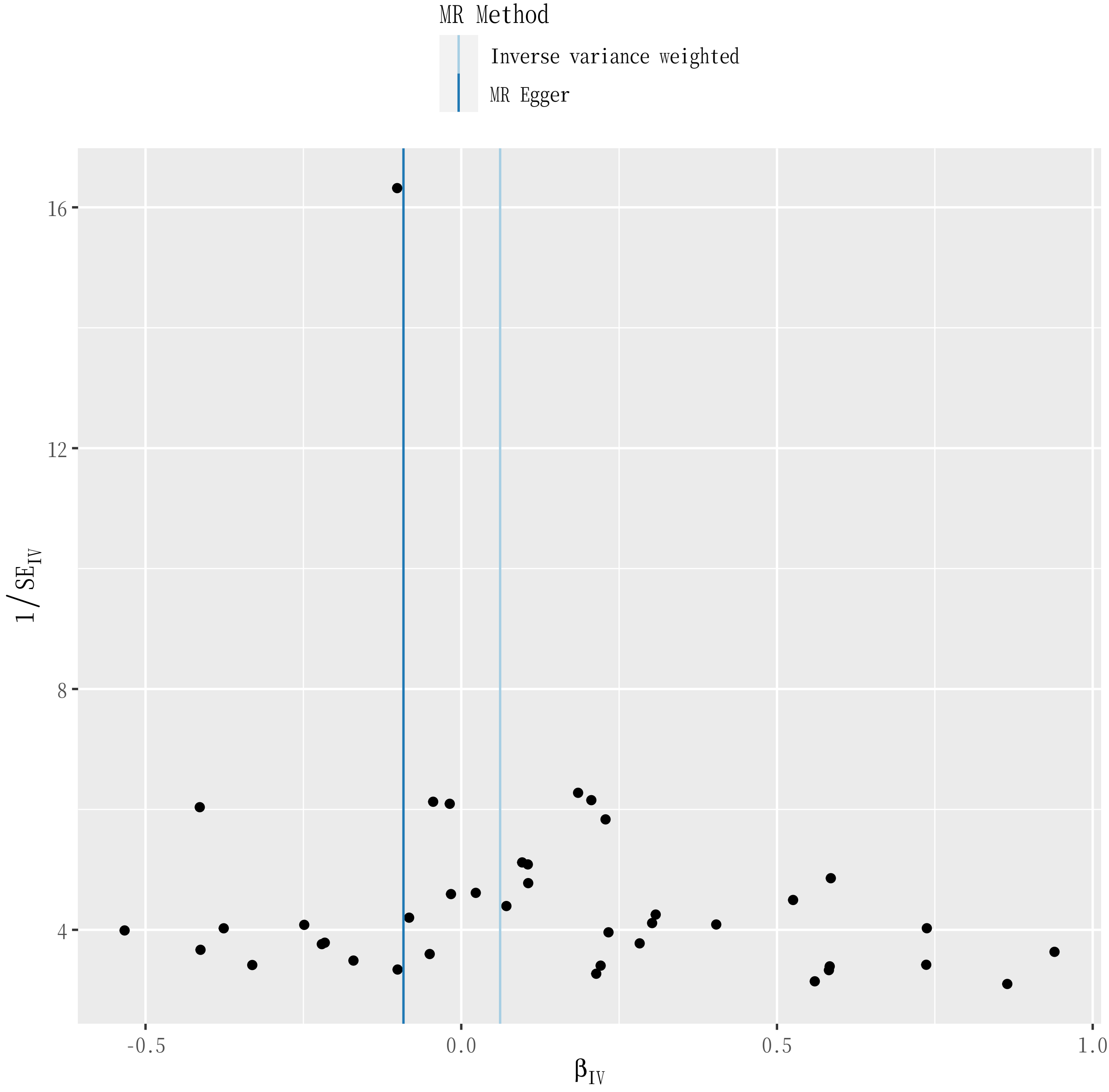
MR Method
Inverse variance weighted
MR Egger
16
12
8
4
$1/SE_{IV}$
-0.5
0.0
0.5
1.0
β_{IV}

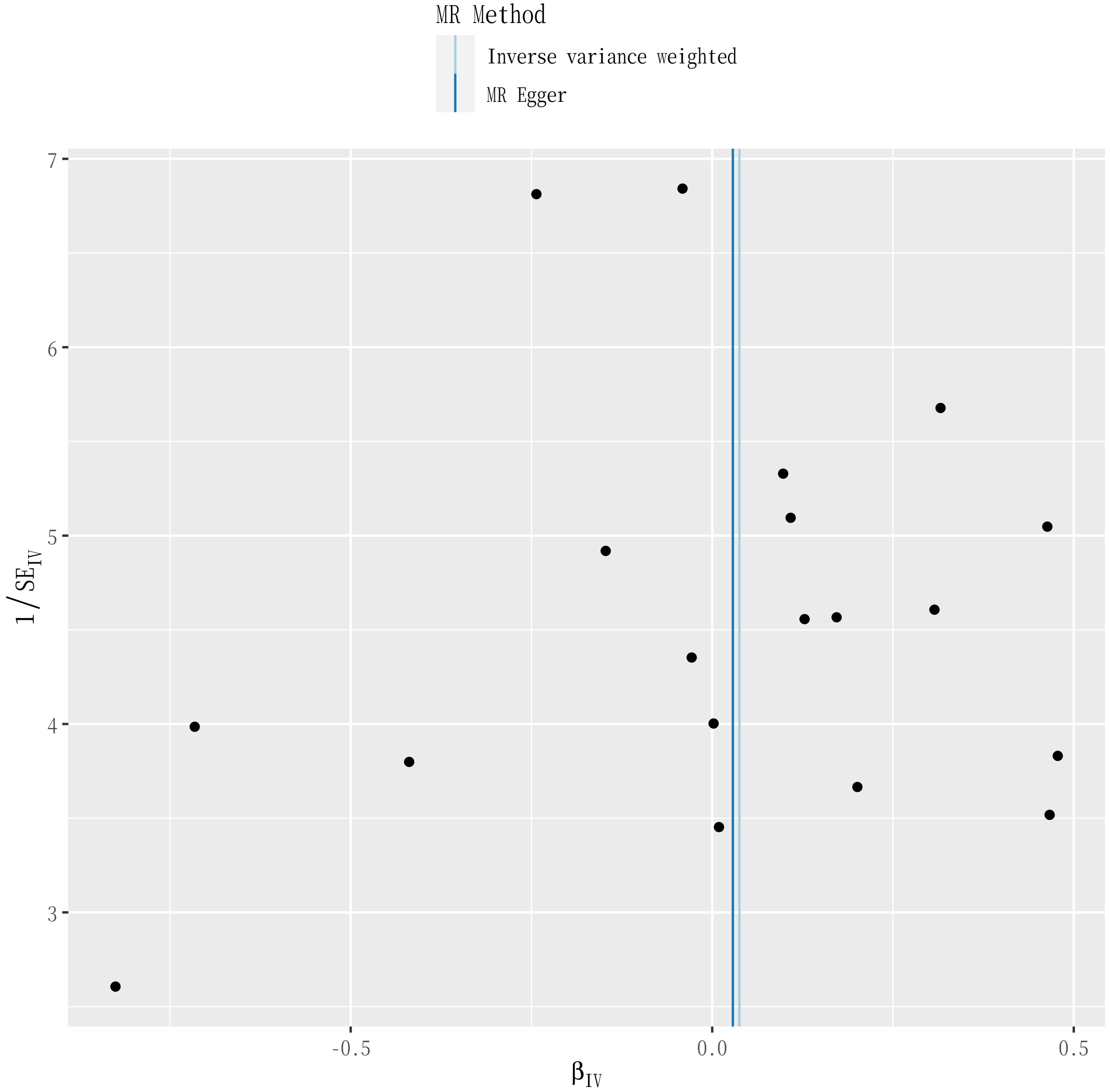

MR Method
Inverse variance weighted
MR Egger
7
6
5
4
3
$1/SE_{IV}$
-0.5
0.0
0.5
β_{IV}

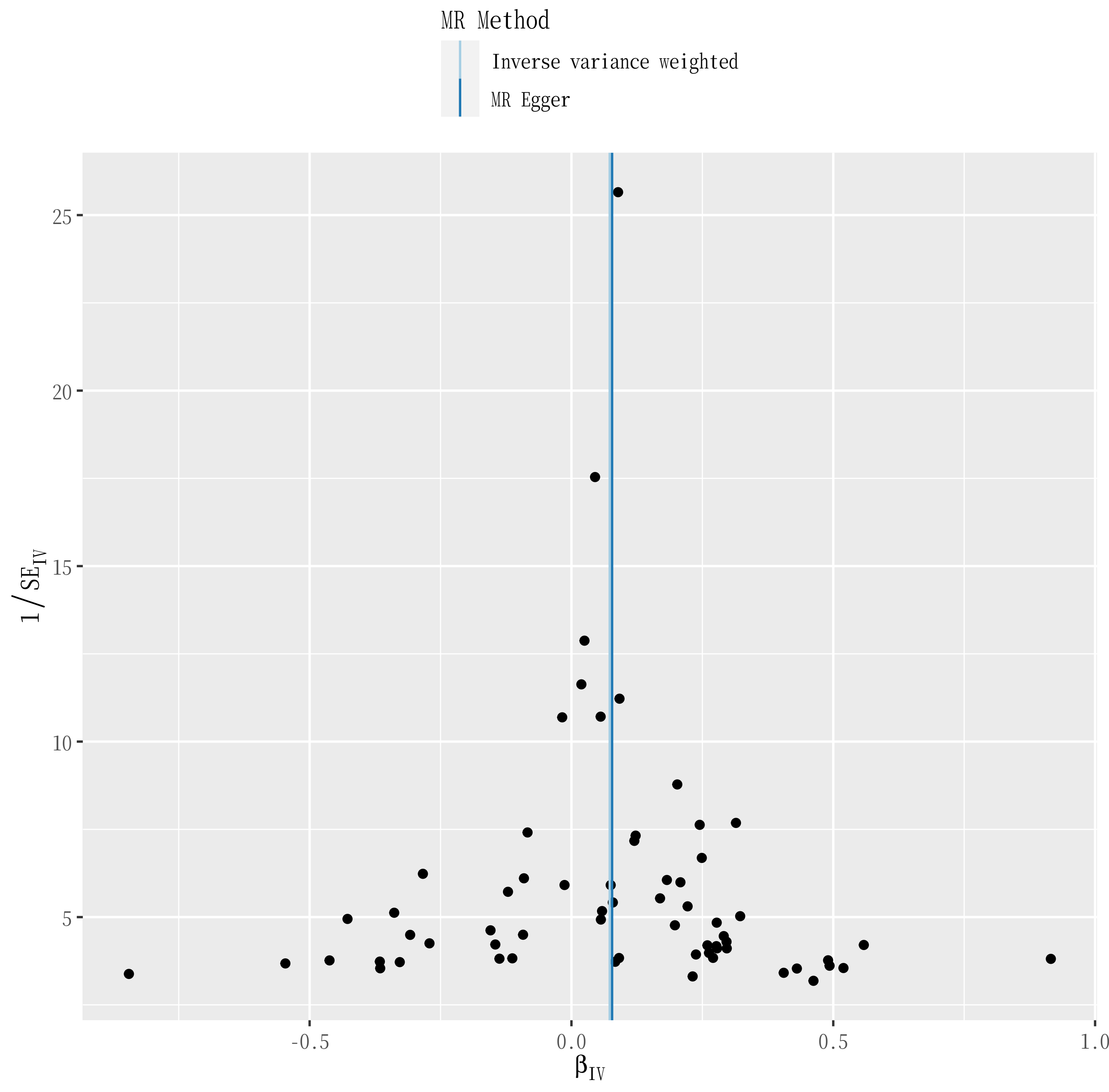
MR Method
Inverse variance weighted
MR Egger
25
20
15
10
5
$1/SE_{IV}$
-0.5
0.0
0.5
1.0
β_{IV}

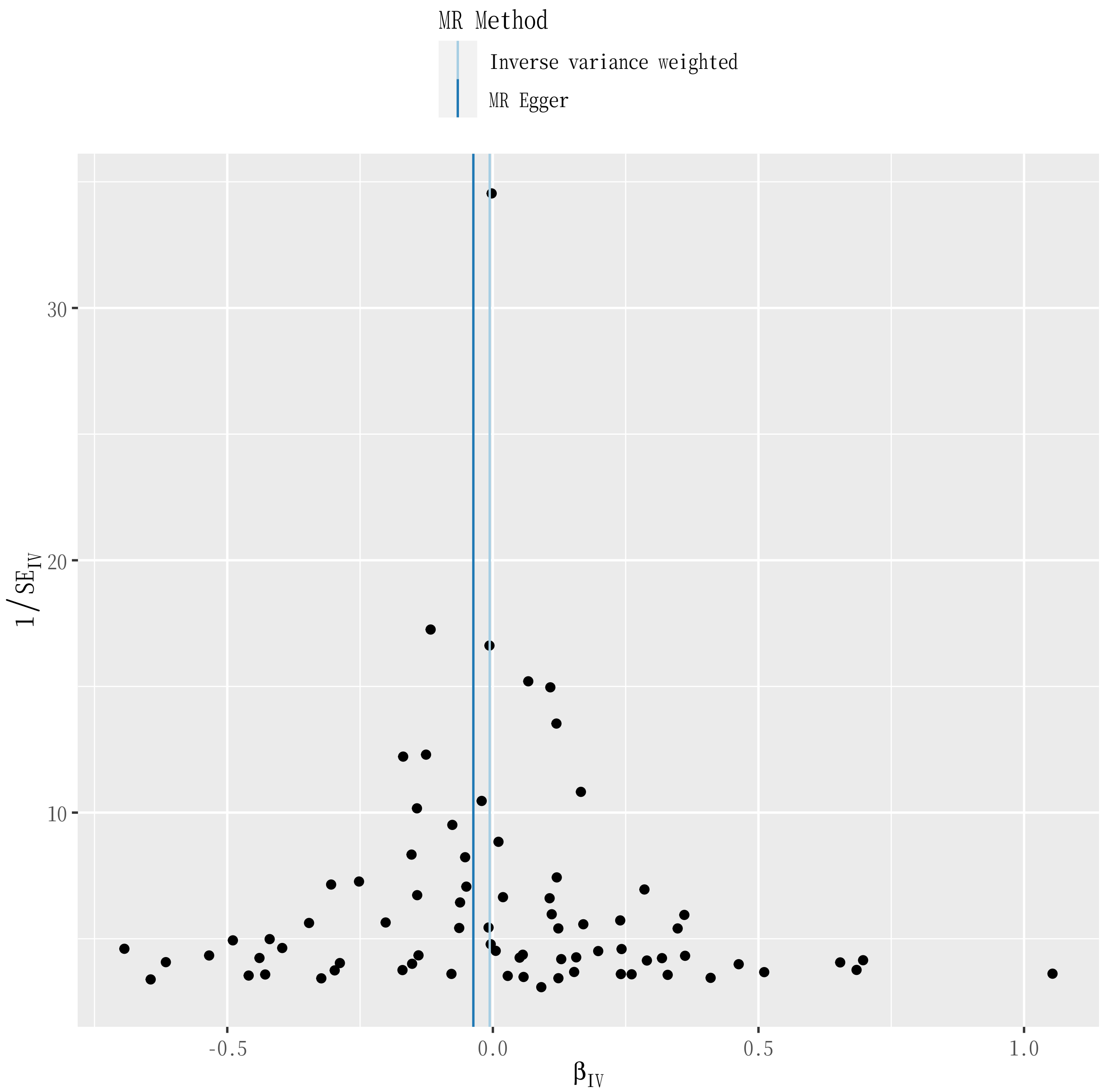
MR Method
Inverse variance weighted
MR Egger
30
20
10
$1/SE_{IV}$
-0.5
0.0
0.5
1.0
β_{IV}

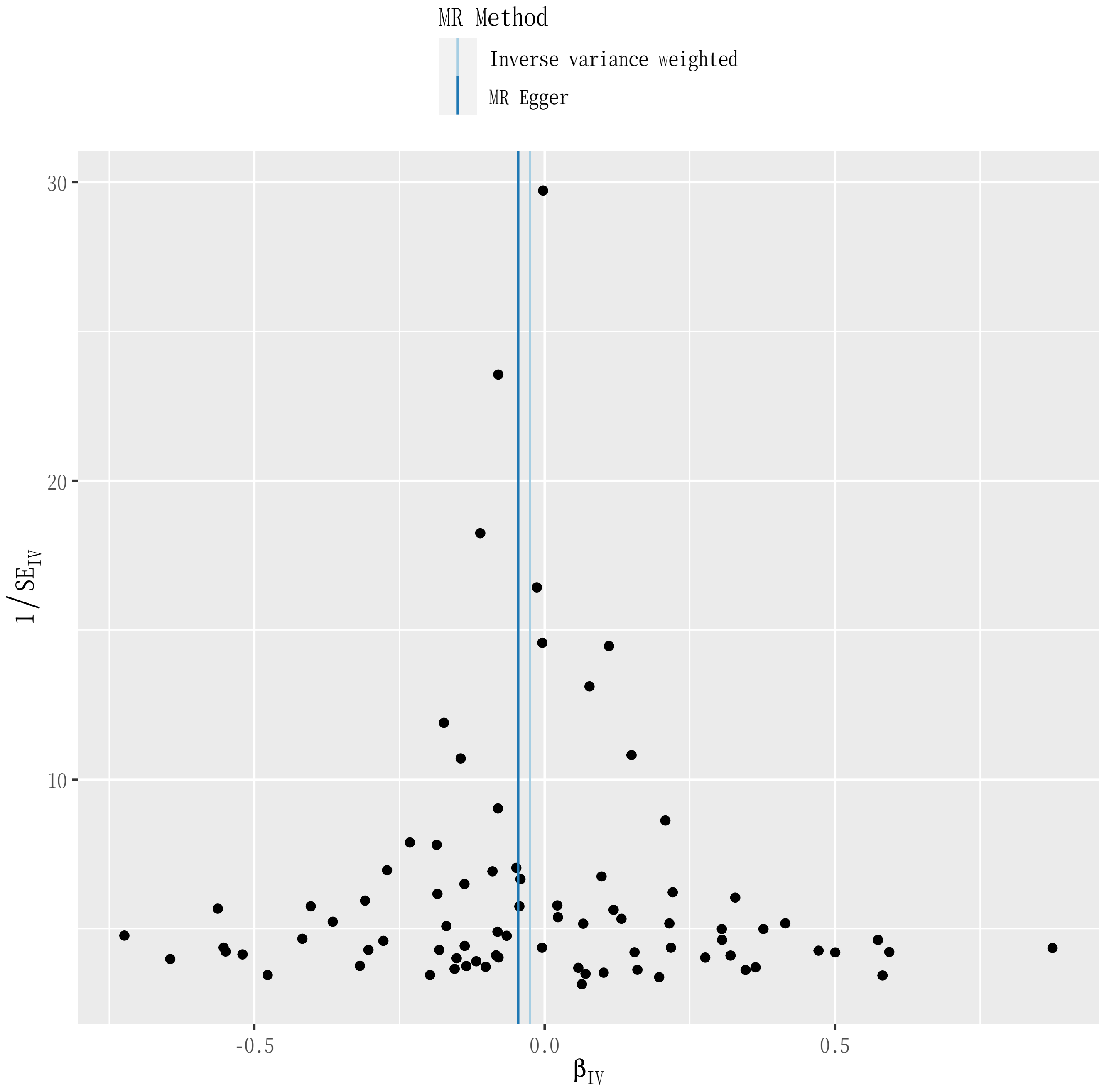

MR Method
Inverse variance weighted
MR Egger
30
20
10
-0.5
0.0
0.5
1/SE_IV
β_IV

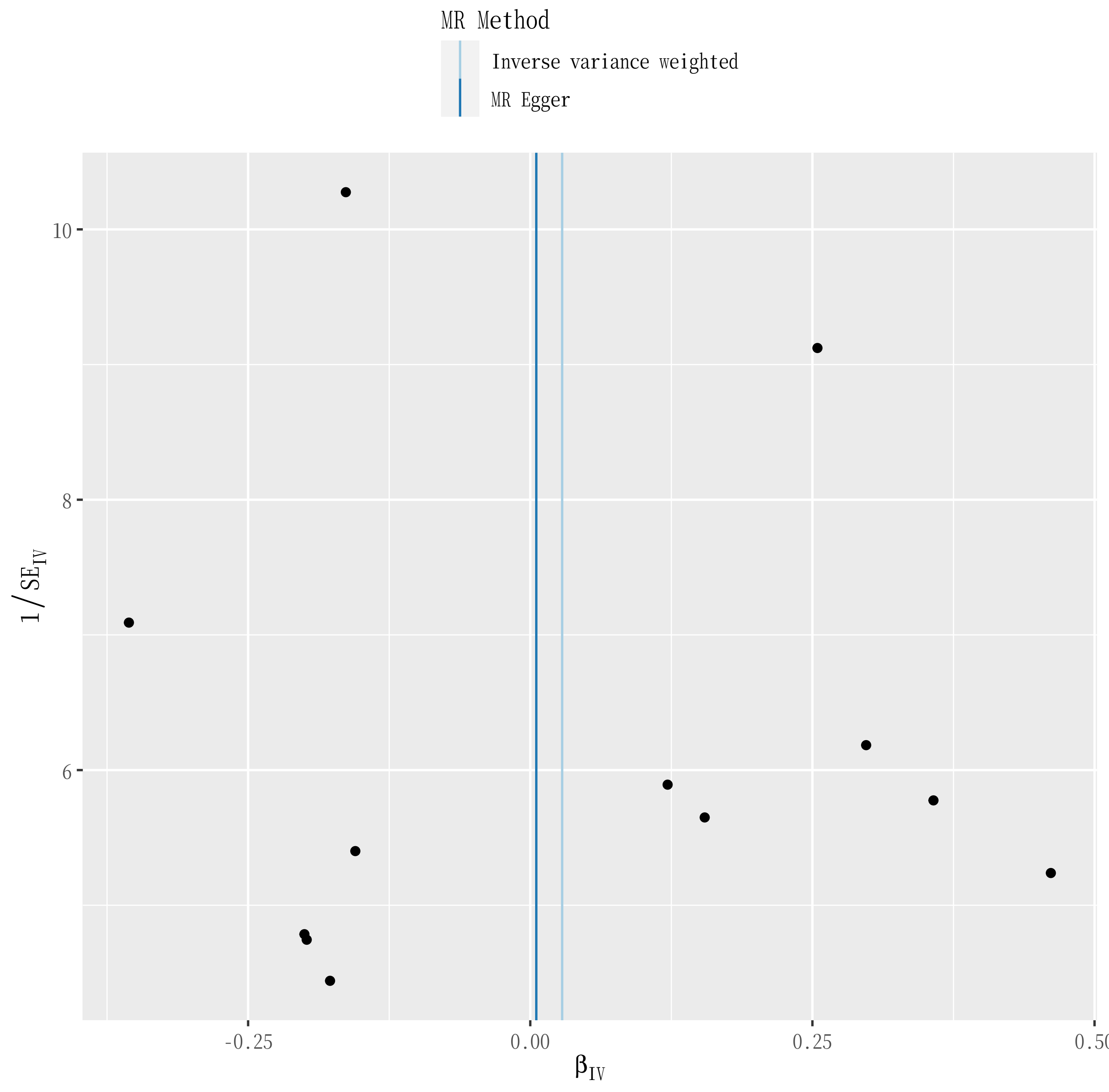
MR Method
Inverse variance weighted
MR Egger
10
8
6
$1/SE_{IV}$
-0.25
0.00
0.25
0.50
β_{IV}

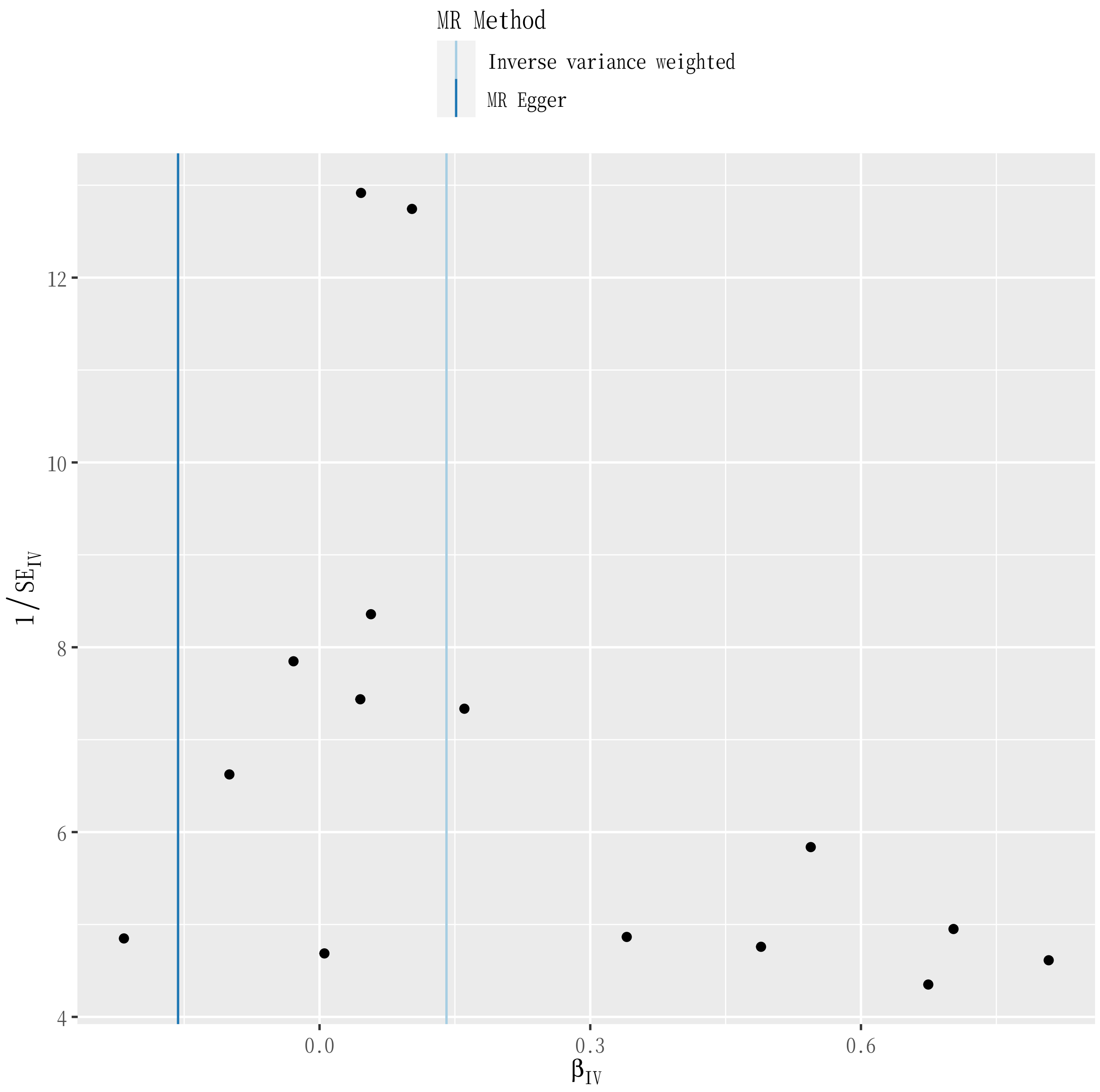

MR Method
Inverse variance weighted
MR Egger
12
10
8
6
4
$1/SE_{IV}$
0.0
0.3
0.6
β_{IV}

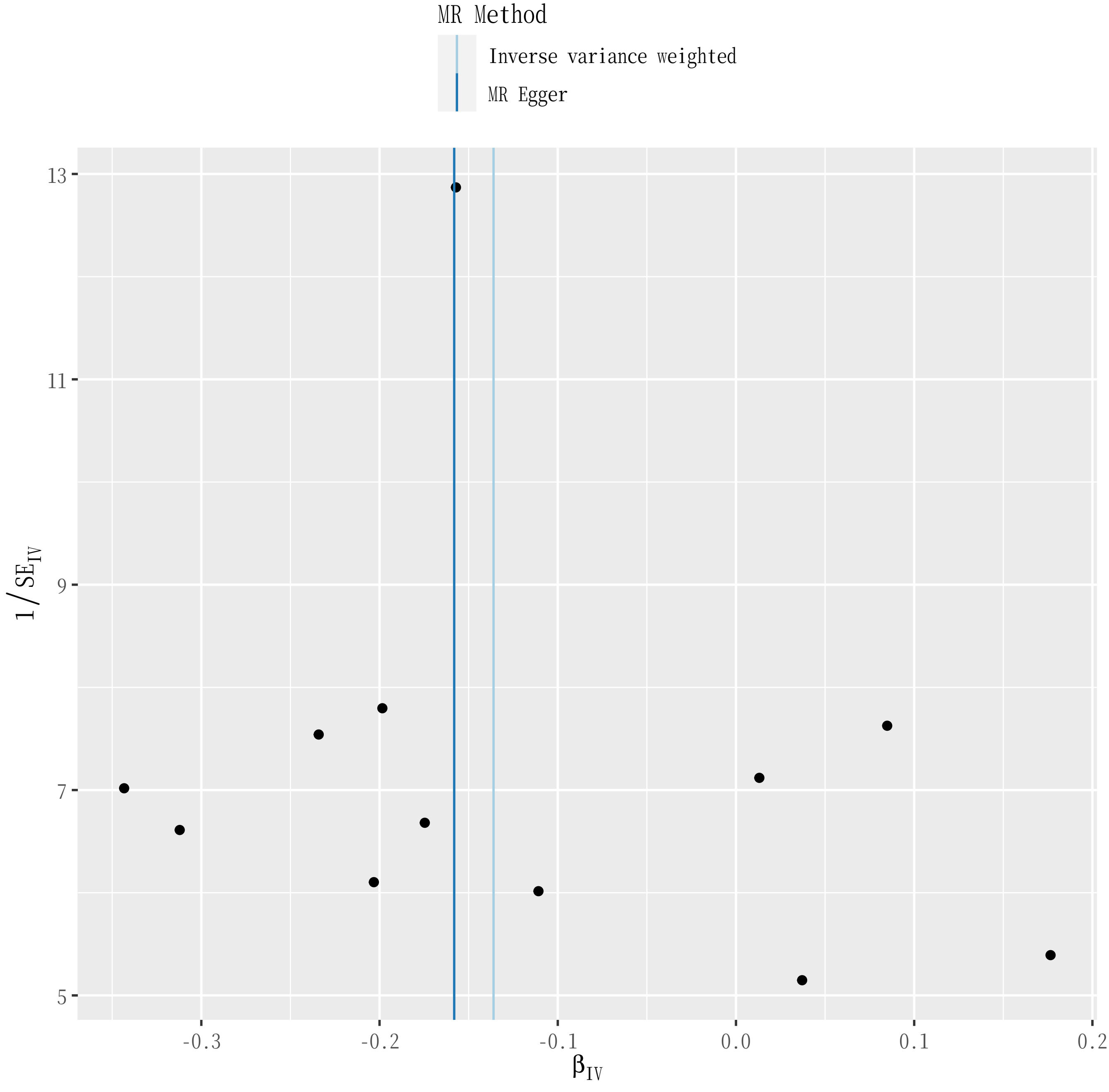
MR Method
Inverse variance weighted
MR Egger
13
11
9
7
5
$1/SE_{IV}$
-0.3
-0.2
-0.1
0.0
0.1
0.2
β_{IV}

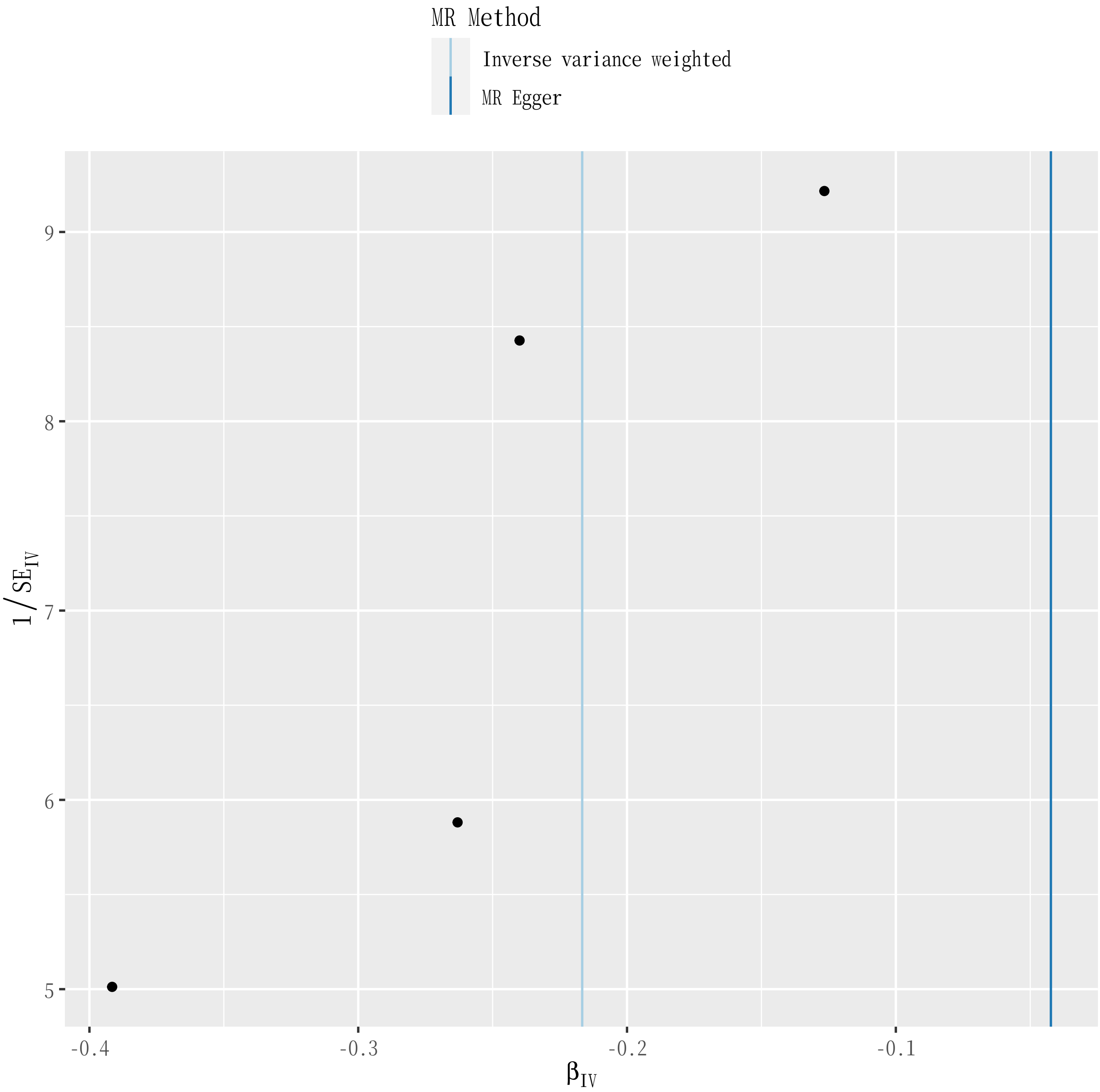
MR Method
Inverse variance weighted
MR Egger
1/SE_IV
9
8
7
6
5
-0.4
-0.3
-0.2
-0.1
β_IV

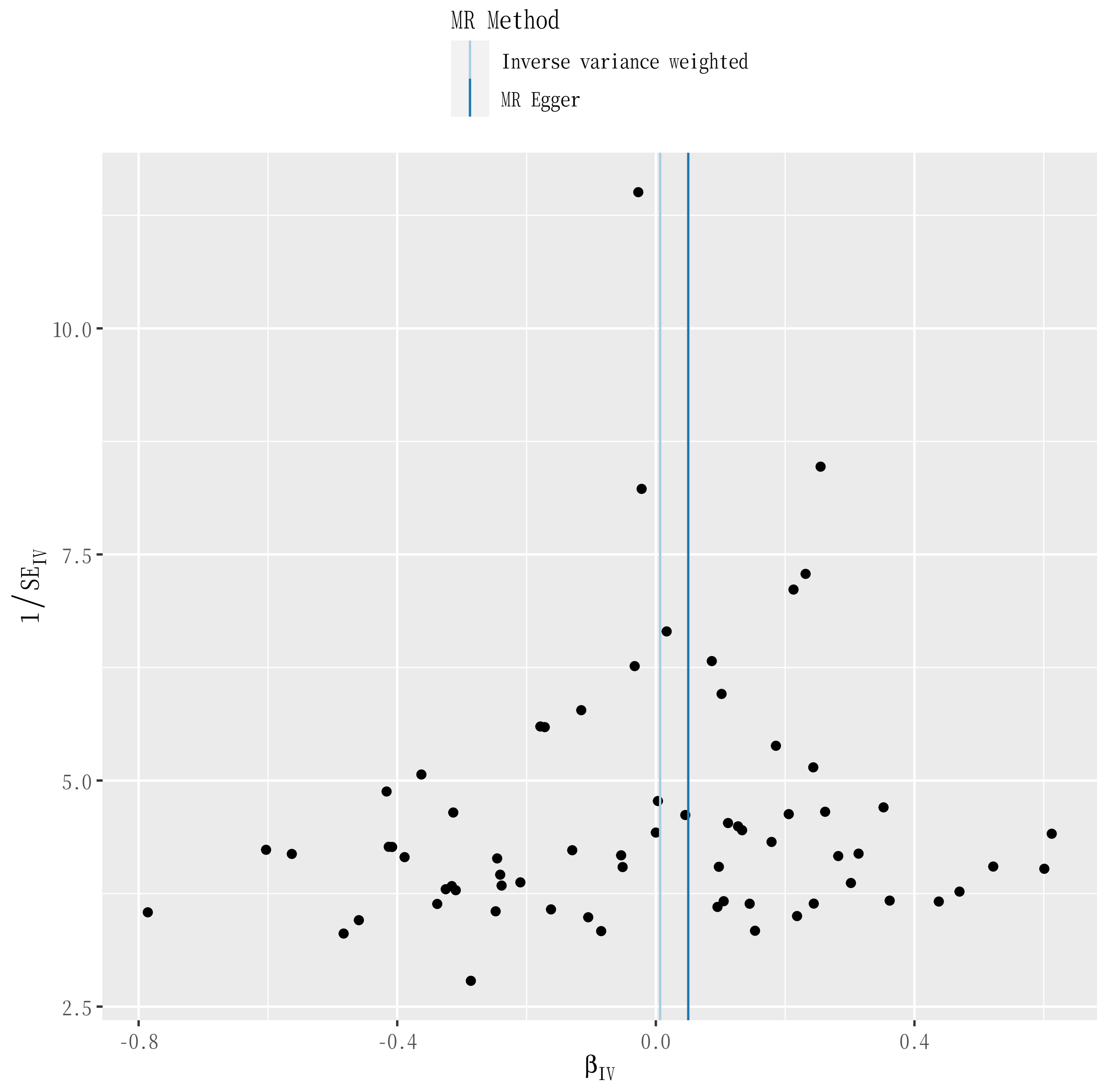
MR Method
Inverse variance weighted
MR Egger
10.0
7.5
5.0
2.5
$1/SE_{IV}$
-0.8
-0.4
0.0
0.4
β_{IV}

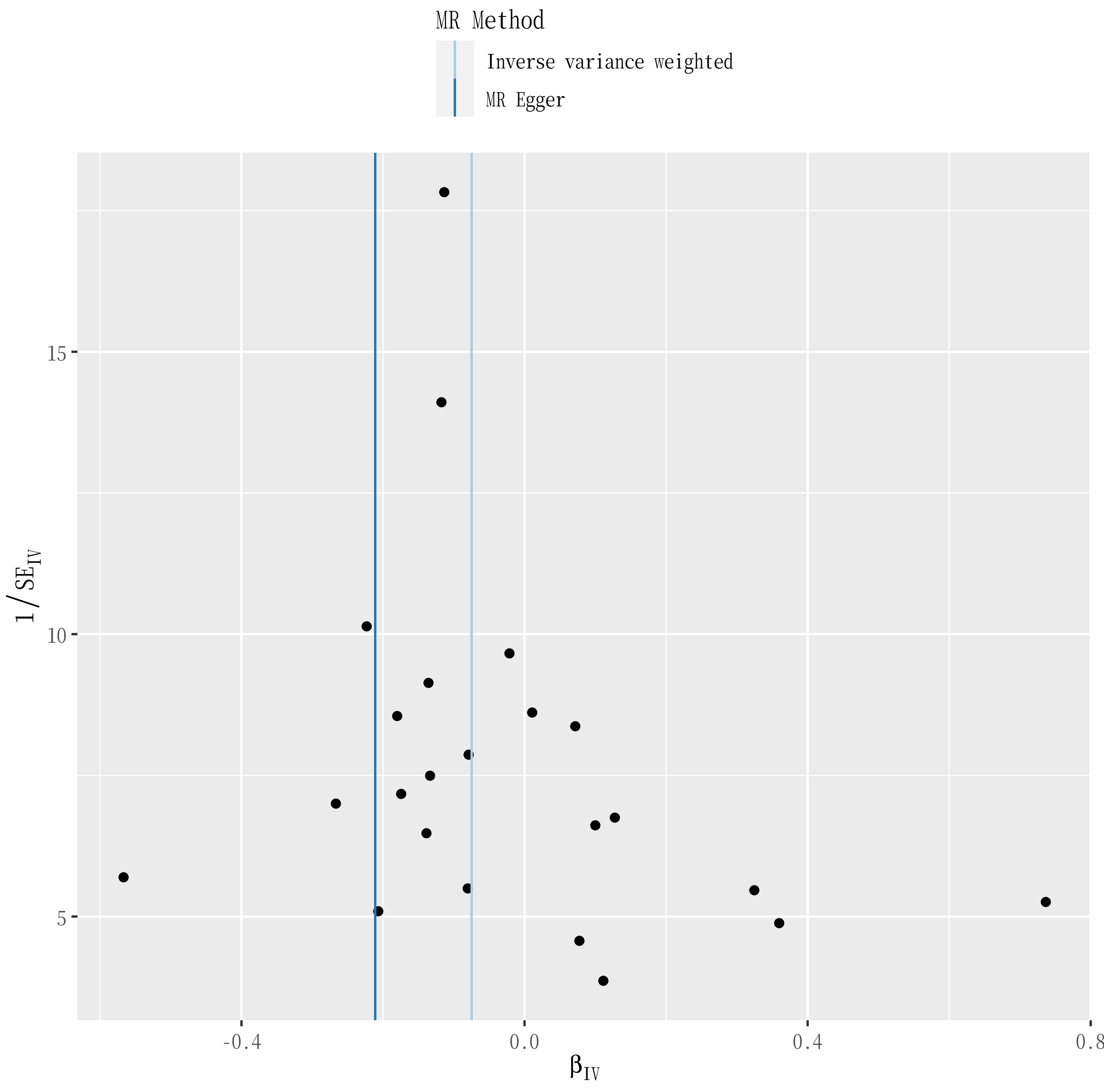

MR Method
Inverse variance weighted
MR Egger
15
10
5
-0.4
0.0
0.4
0.8
$1/SE_{IV}$
β_{IV}

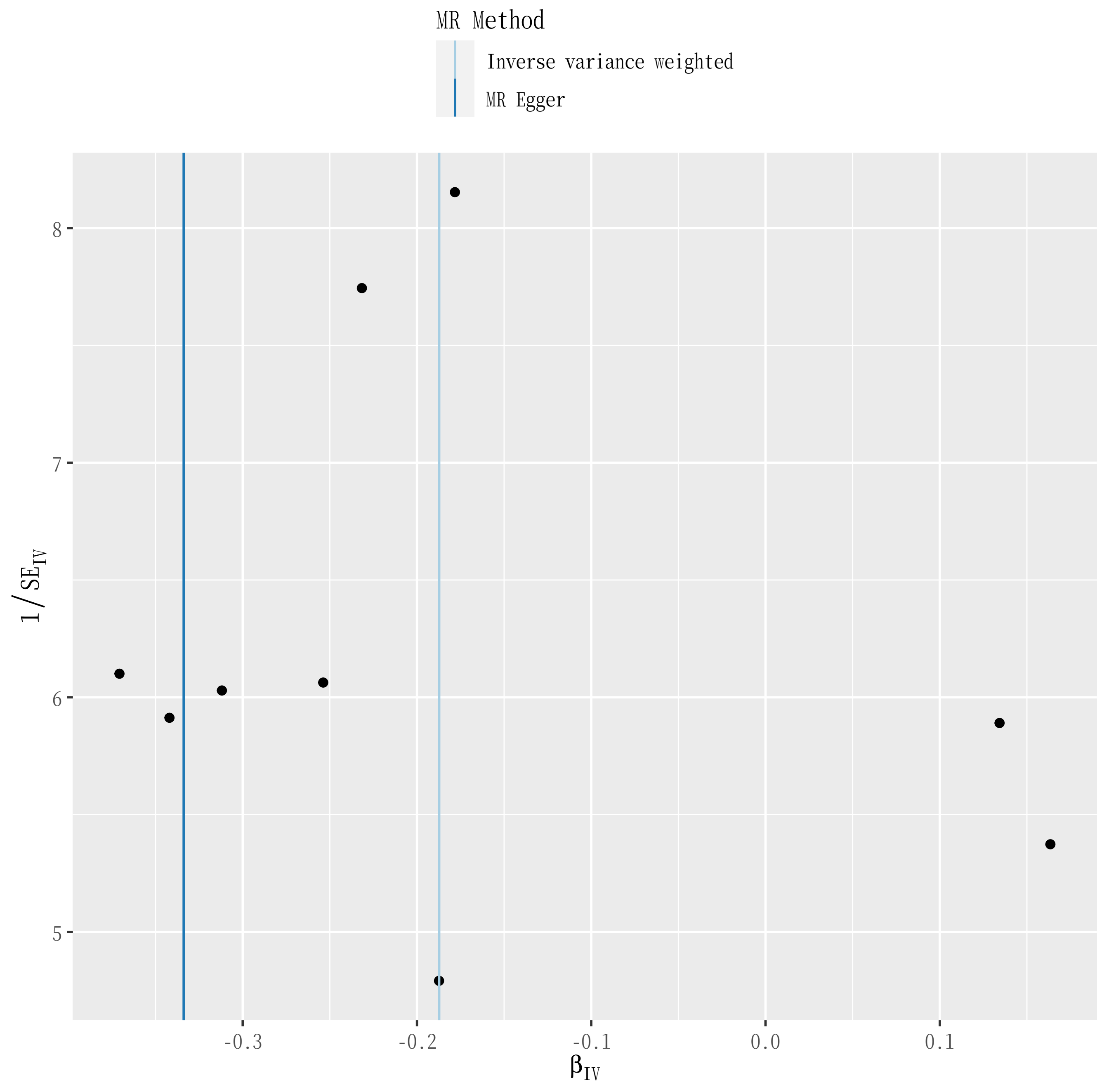
MR Method
Inverse variance weighted
MR Egger
8
7
6
5
-0.3
-0.2
-0.1
0.0
0.1
1/SE_IV
β_IV

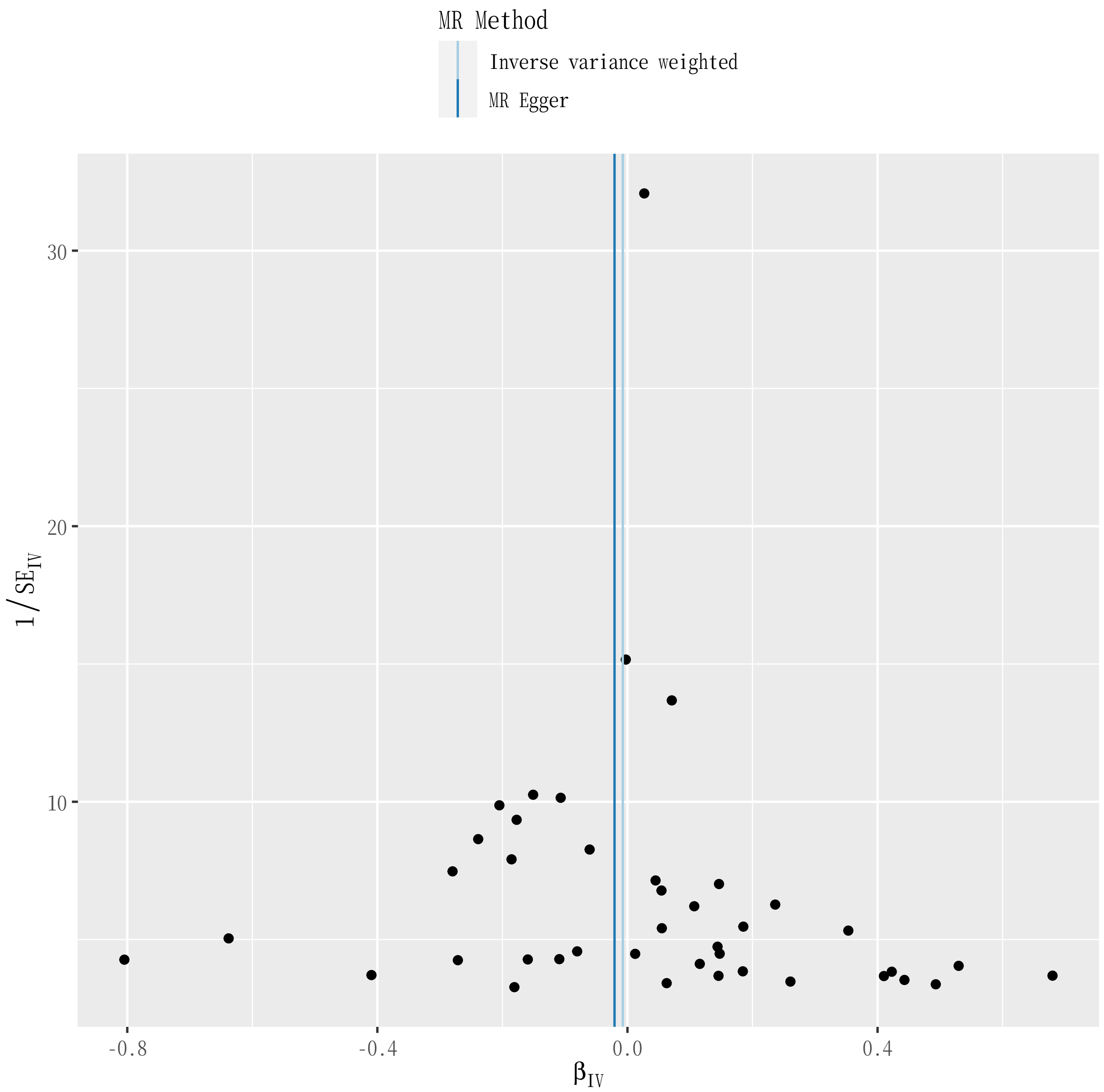
MR Method
Inverse variance weighted
MR Egger
30
20
10
$1/SE_{IV}$
-0.8
-0.4
0.0
0.4
β_{IV}

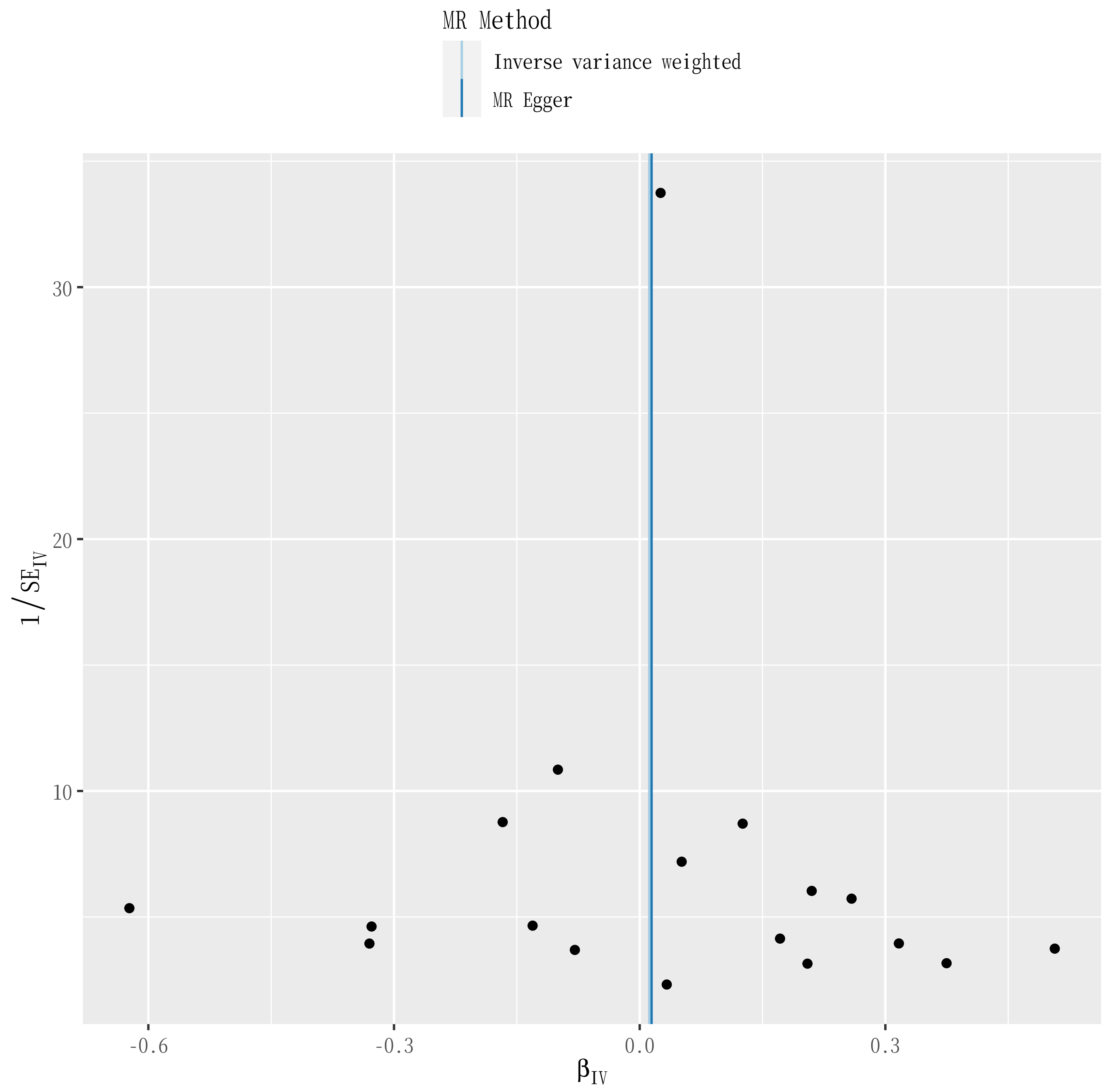
MR Method
Inverse variance weighted
MR Egger
30
20
10
$1/SE_{IV}$
-0.6
-0.3
0.0
0.3
β_{IV}

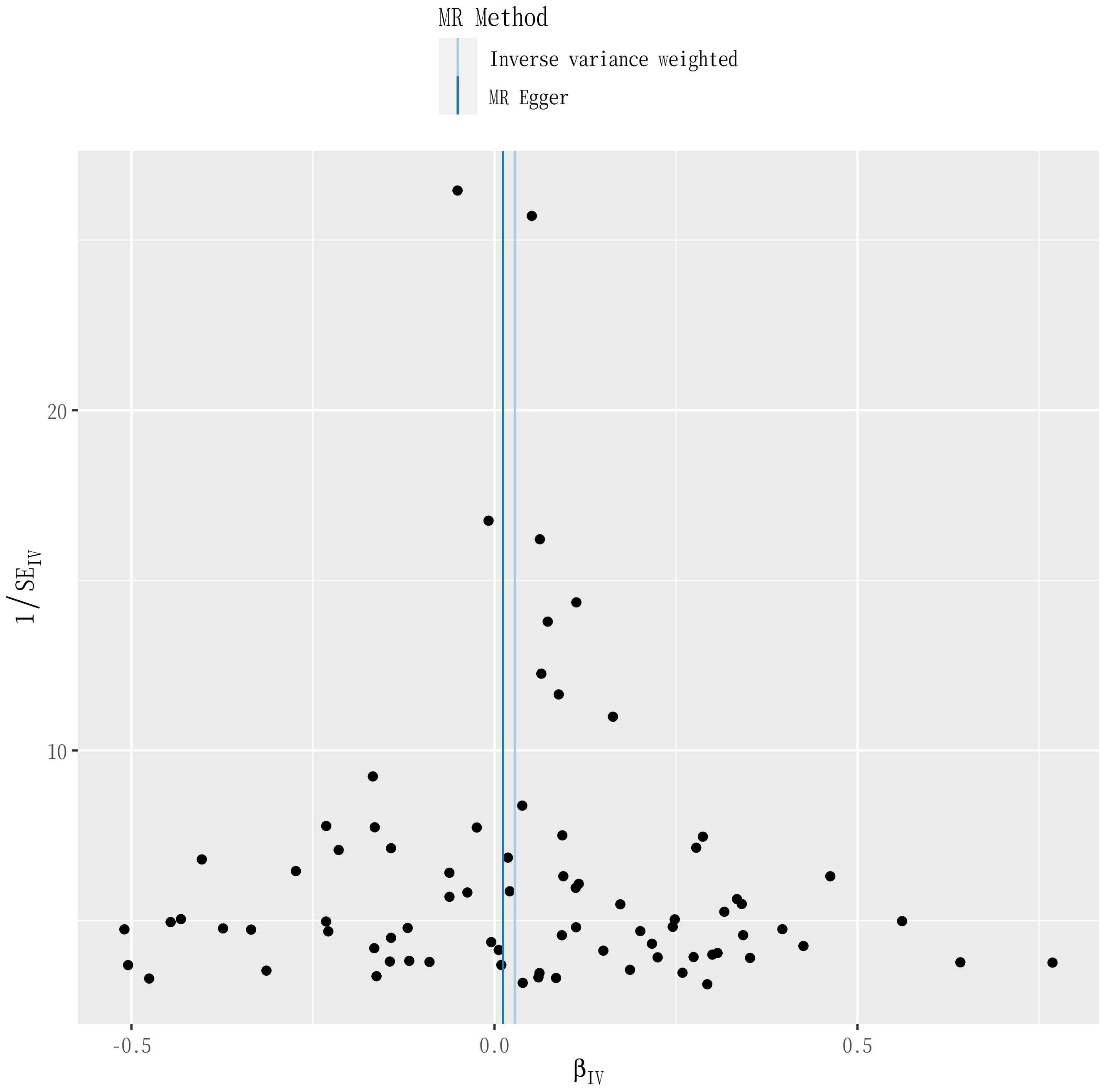

MR Method
Inverse variance weighted
MR Egger
20
10
-0.5
0.0
0.5
$1/SE_{IV}$
β_{IV}

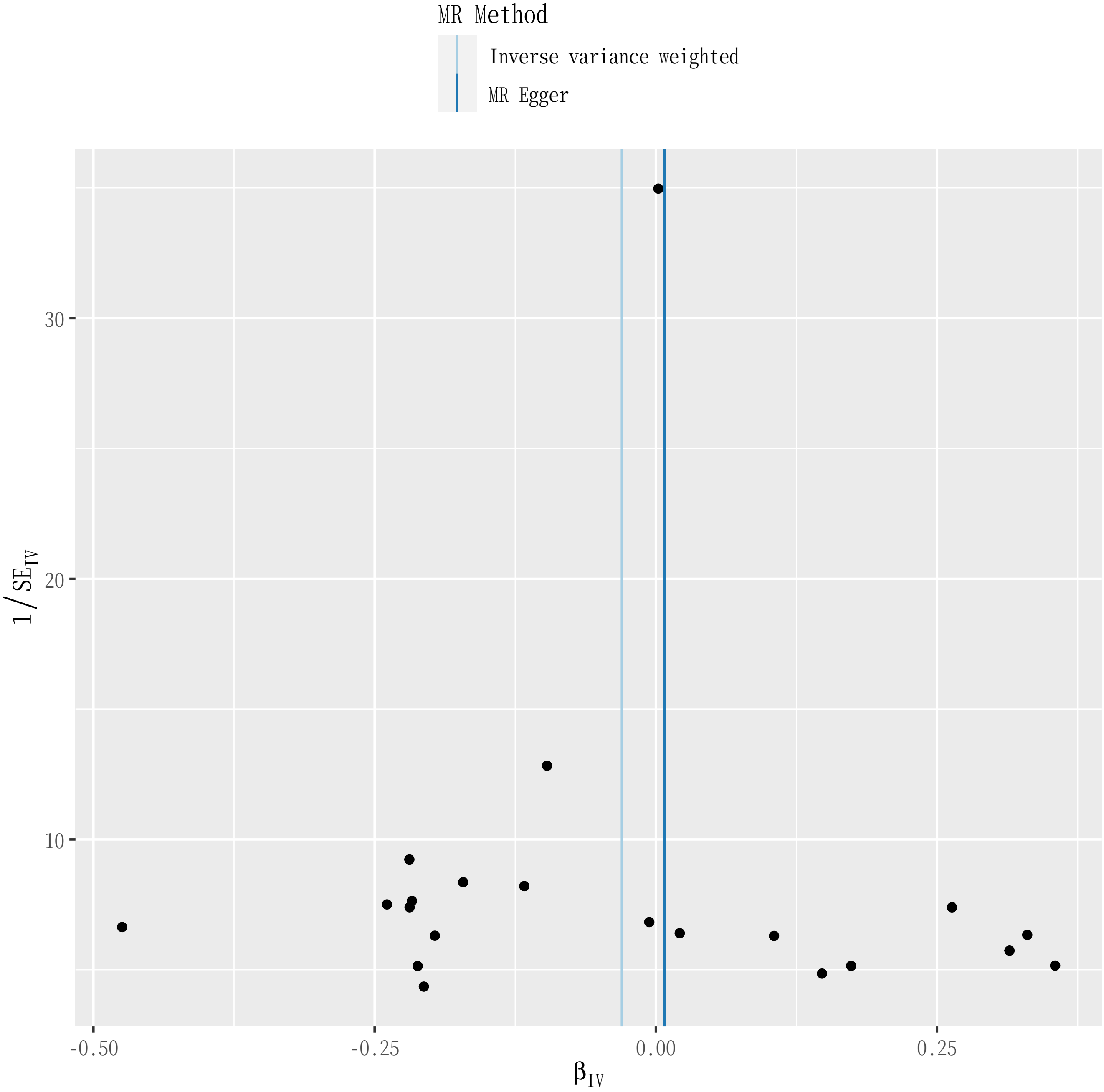
MR Method
Inverse variance weighted
MR Egger
30
20
10
1/SE_IV
-0.50
-0.25
0.00
0.25
β_IV

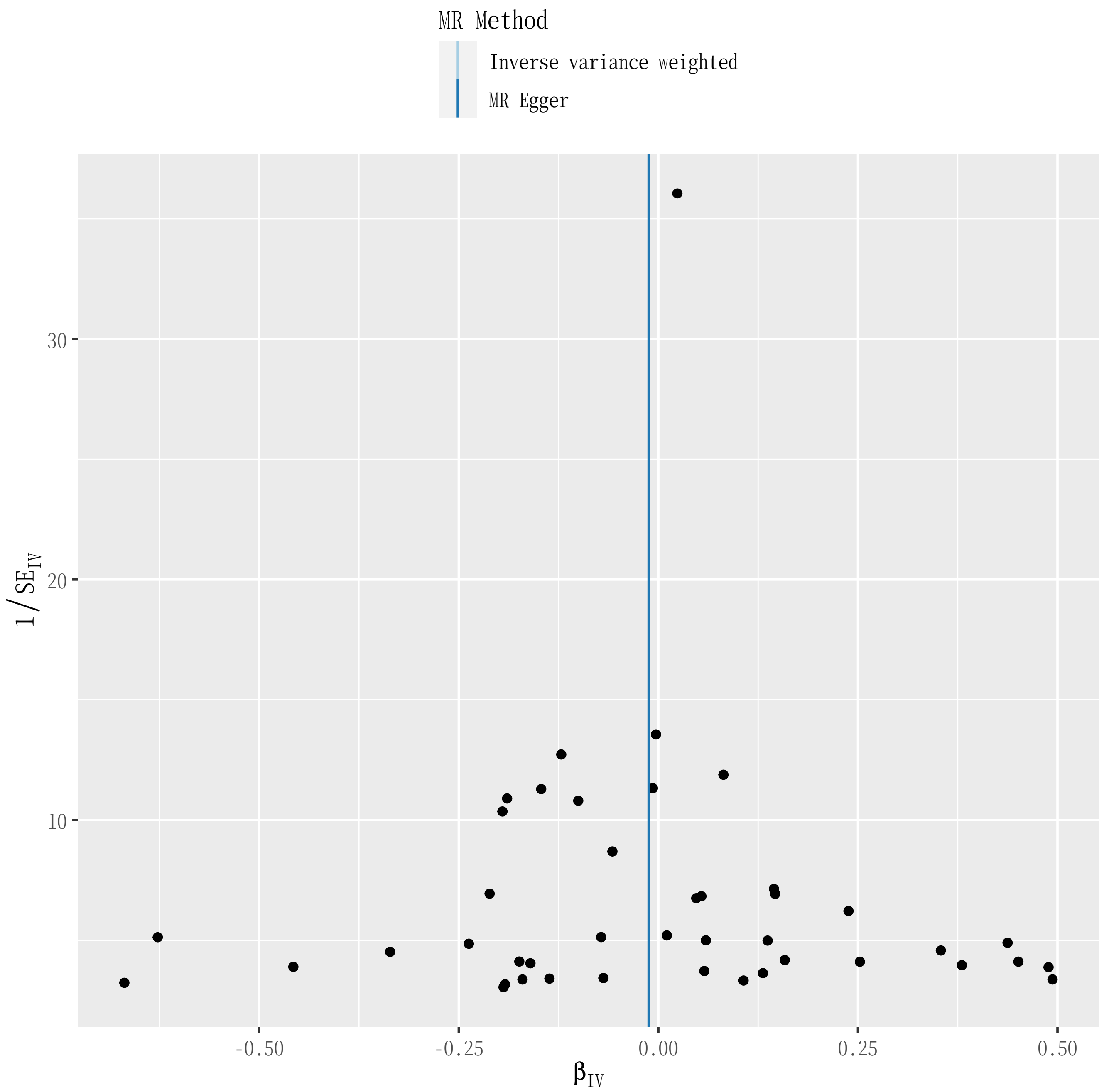
MR Method
Inverse variance weighted
MR Egger
30
20
10
-0.50
-0.25
0.00
0.25
0.50
$1/SE_{IV}$
β_{IV}

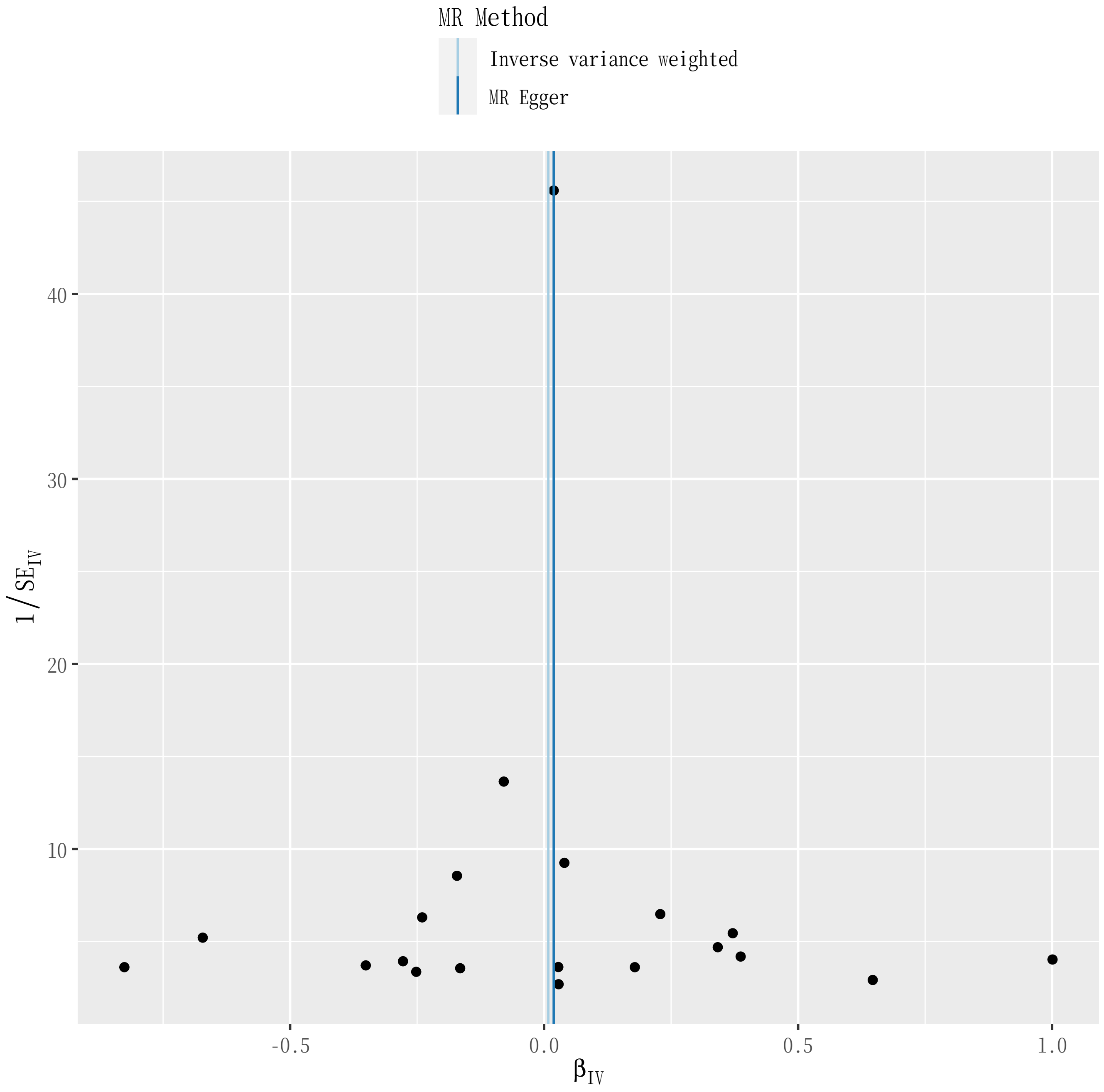
MR Method
Inverse variance weighted
MR Egger
40
30
20
10
-0.5
0.0
0.5
1.0
1/SE_IV
β_IV

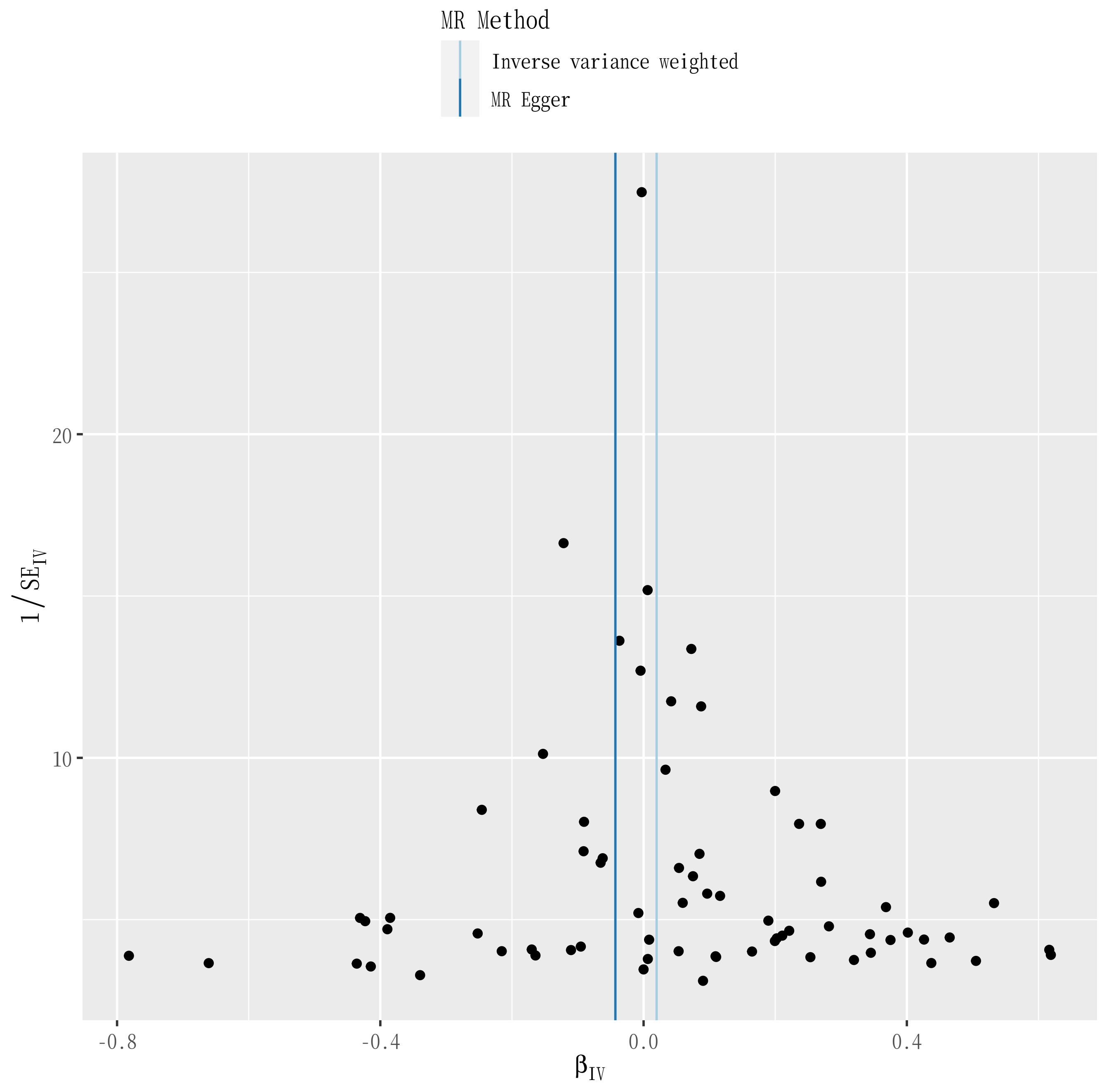
MR Method
Inverse variance weighted
MR Egger
20
10
-0.8
-0.4
0.0
0.4
$1/SE_{IV}$
β_{IV}

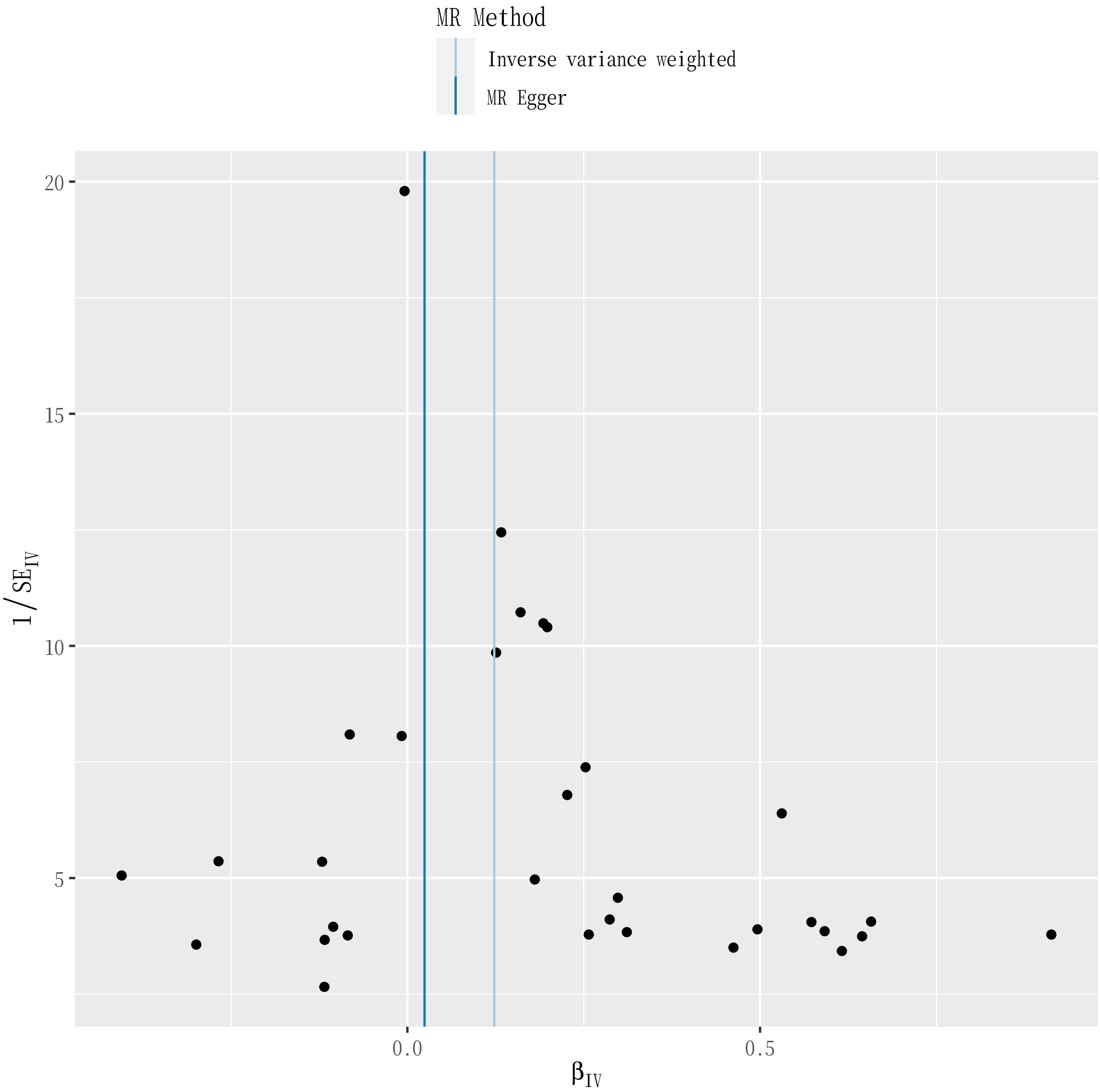

MR Method
Inverse variance weighted
MR Egger
20
15
10
5
0.0
0.5
$1/SE_{IV}$
β_{IV}

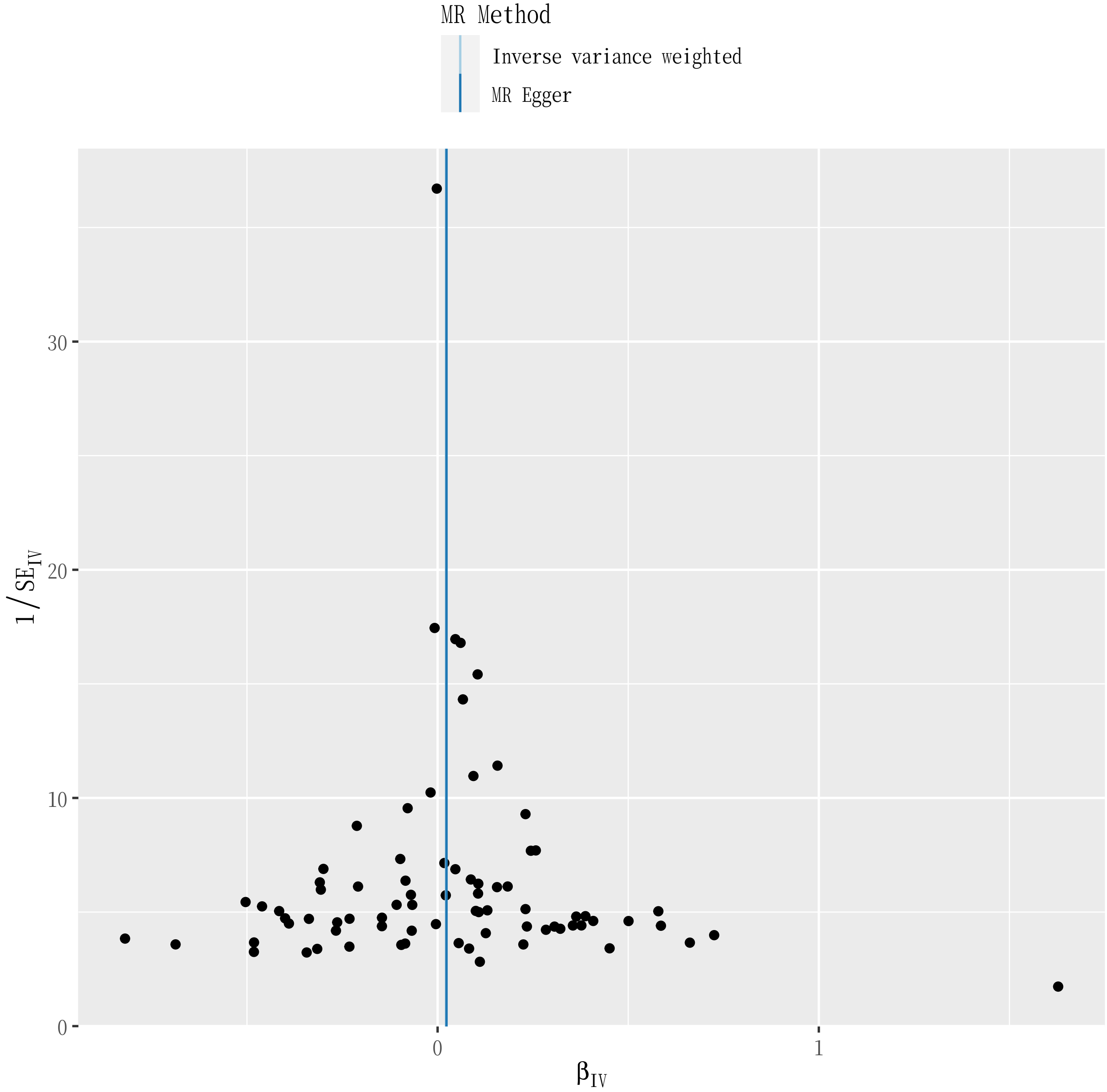

MR Method
Inverse variance weighted
MR Egger
30
20
10
0
0
1
$1/SE_{IV}$
β_{IV}

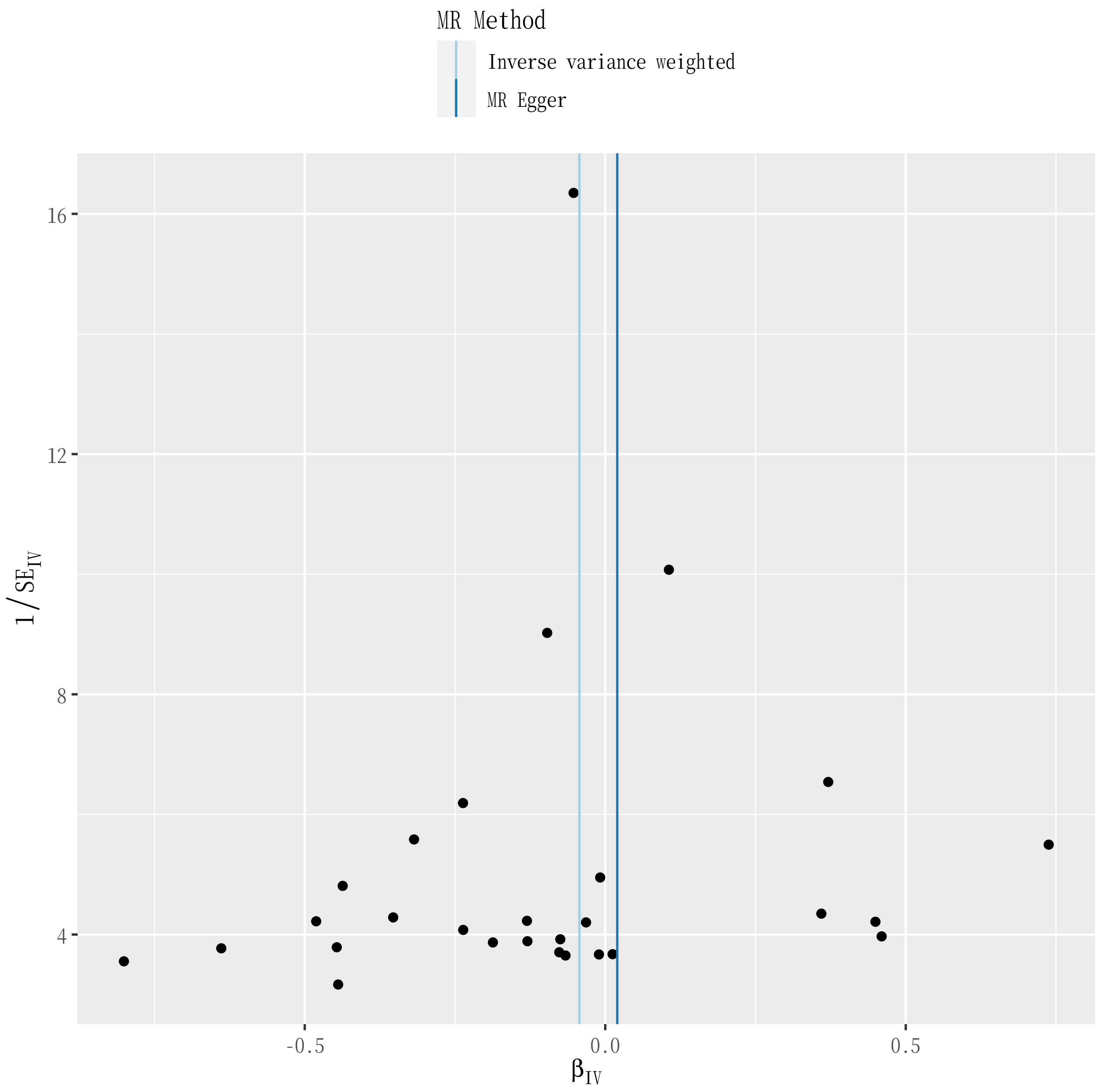

MR Method
Inverse variance weighted
MR Egger
16
12
8
4
1/SE_IV
-0.5
0.0
0.5
β_IV

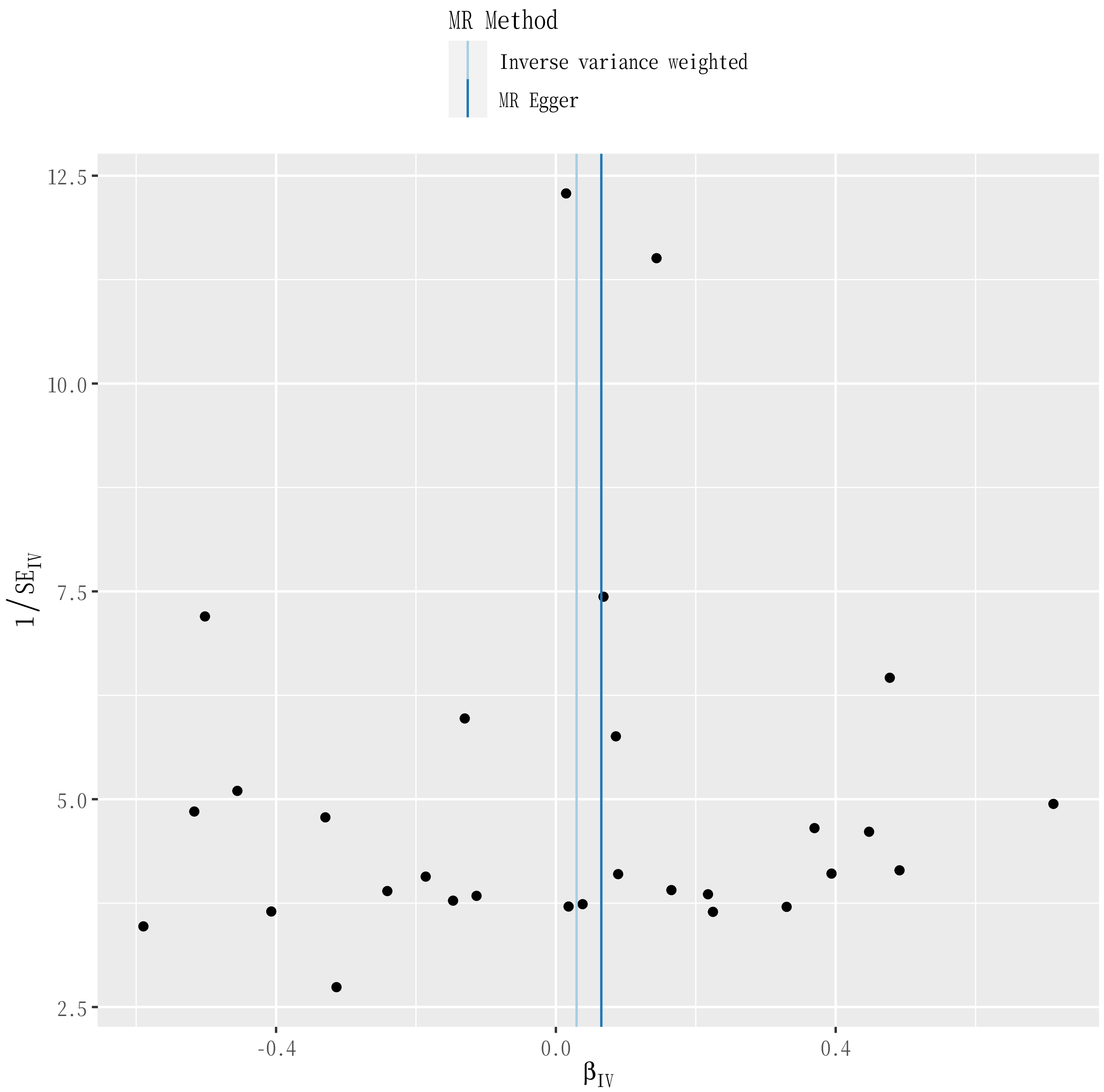

MR Method
Inverse variance weighted
MR Egger
12.5
10.0
7.5
5.0
2.5
$1/SE_{IV}$
-0.4
0.0
0.4
β_{IV}

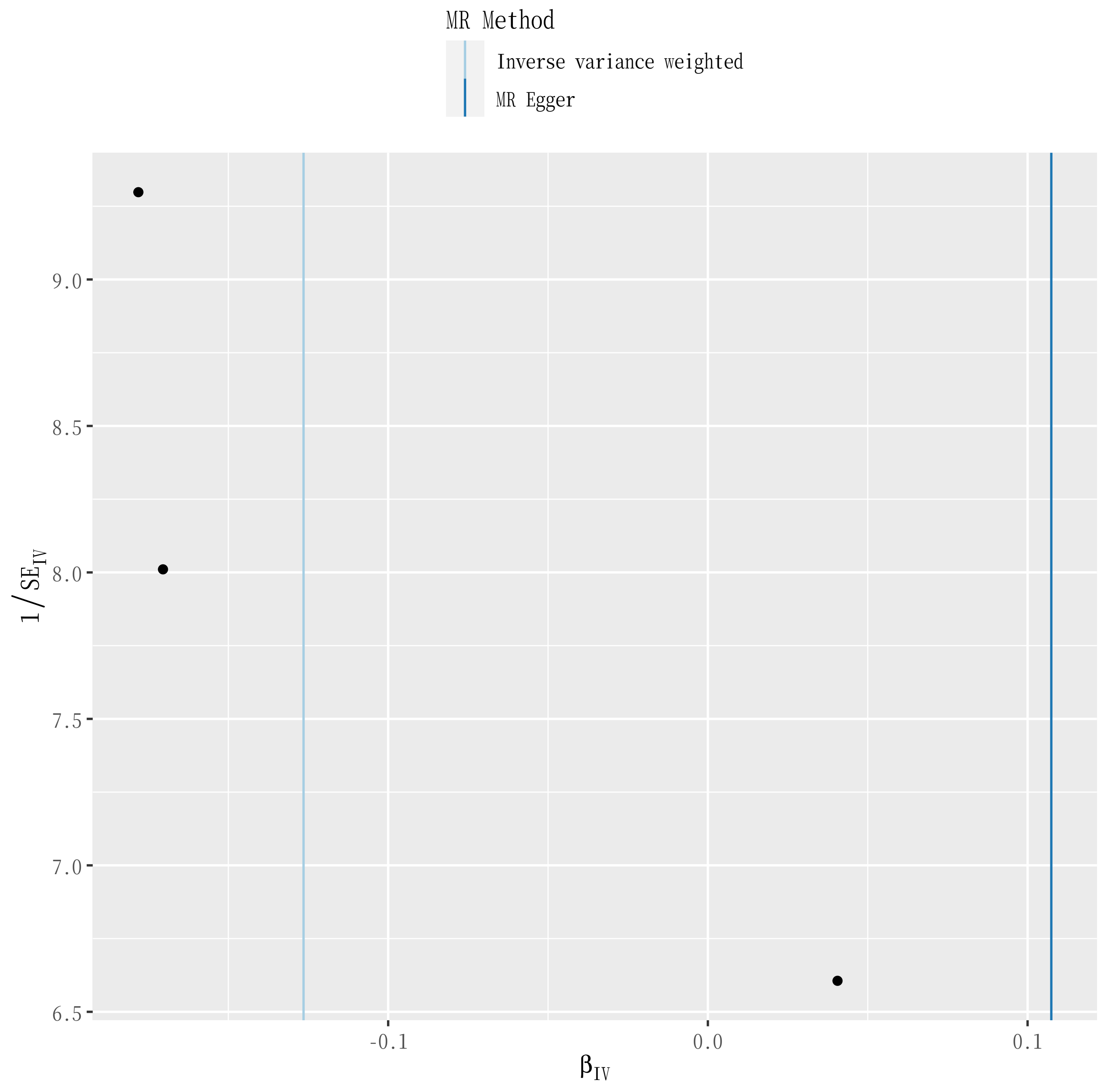
MR Method
Inverse variance weighted
MR Egger
9.0
8.5
8.0
7.5
7.0
6.5
-0.1
0.0
0.1
$1/SE_{IV}$
β_{IV}

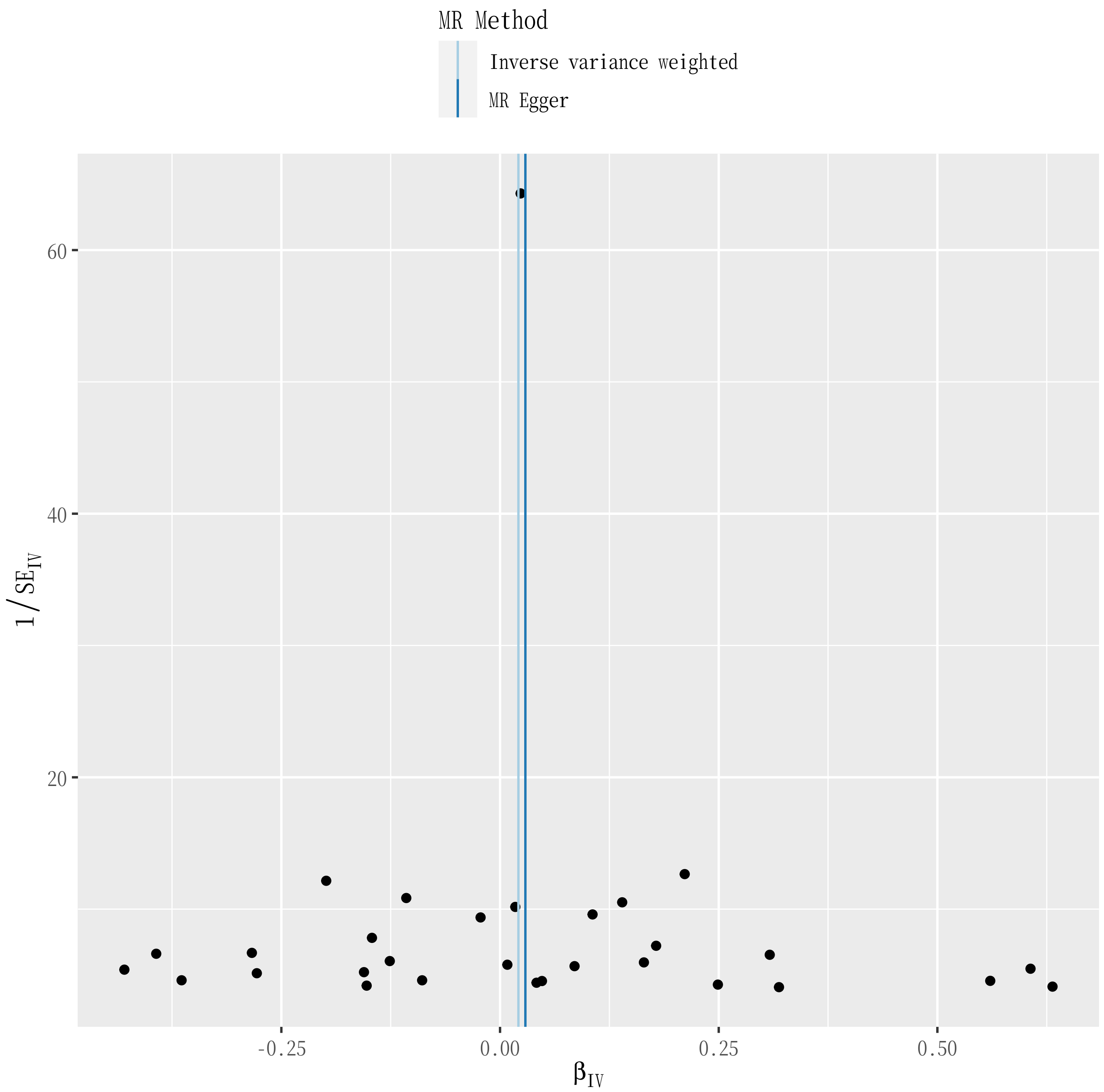
MR Method
Inverse variance weighted
MR Egger
60
40
20
1/SE_IV
-0.25
0.00
0.25
0.50
β_IV

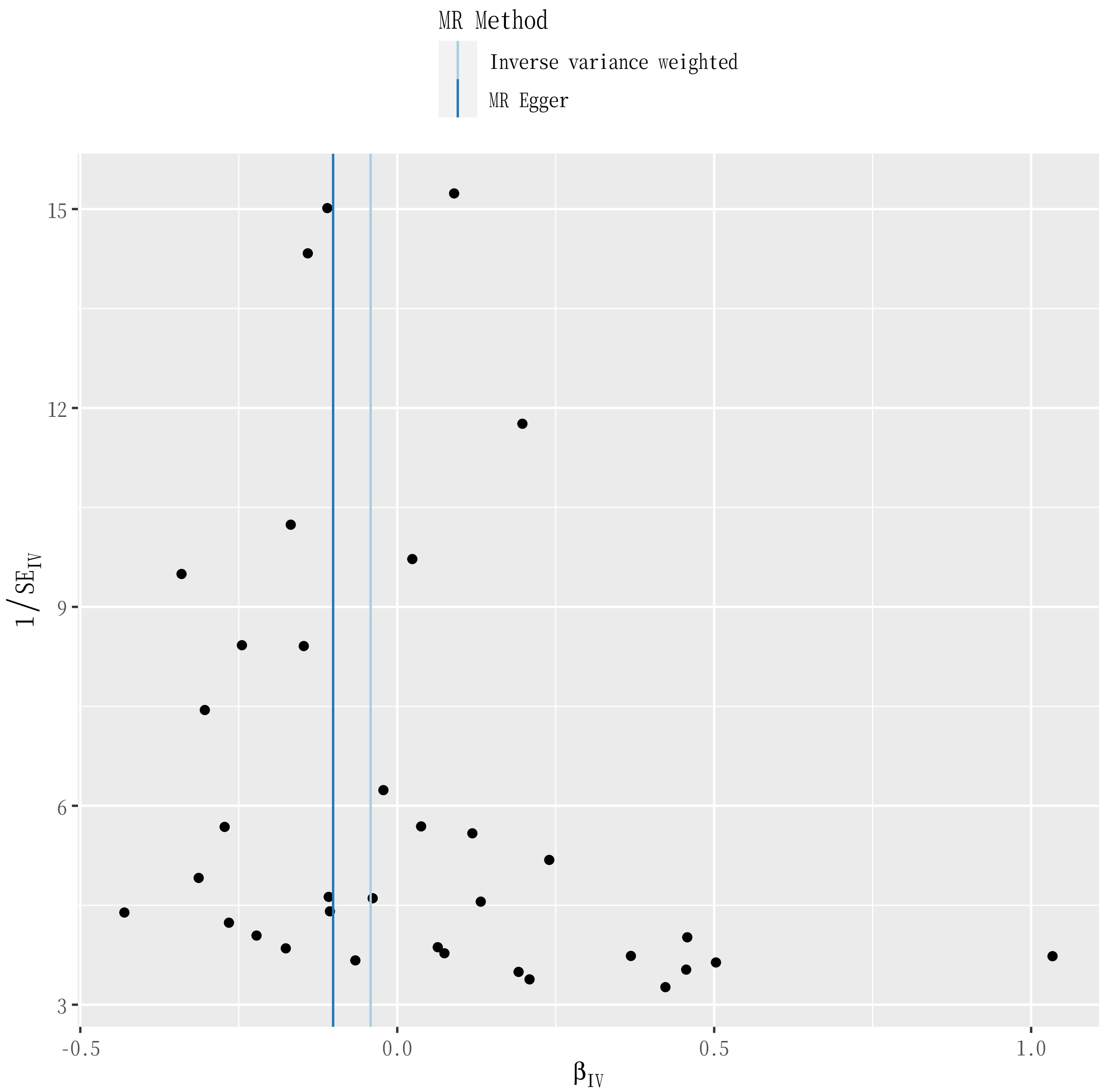
MR Method
Inverse variance weighted
MR Egger
15
12
9
6
3
1/SE_IV
-0.5
0.0
0.5
1.0
β_IV

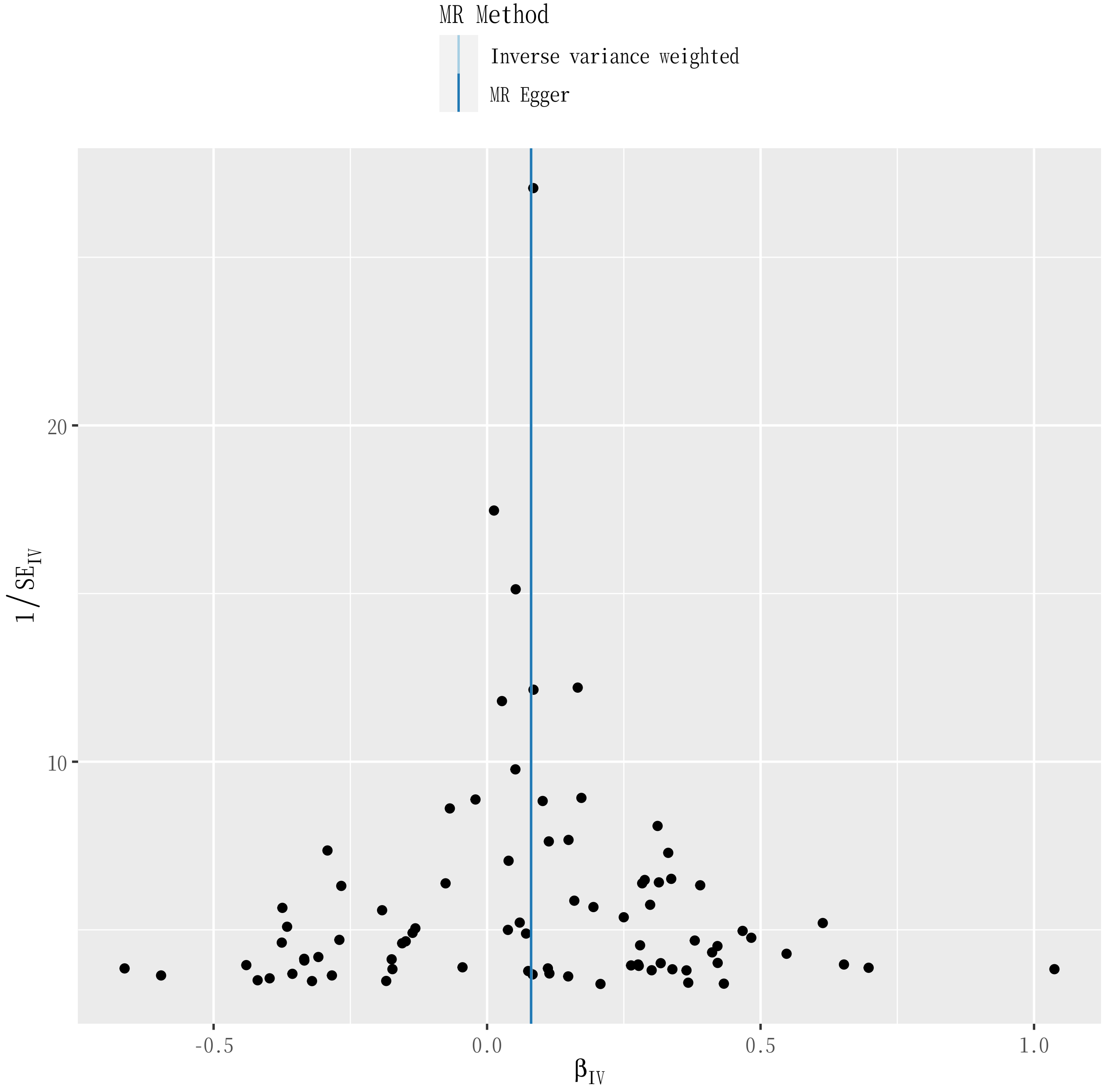

MR Method
Inverse variance weighted
MR Egger
20
10
-0.5
0.0
0.5
1.0
$1/SE_{IV}$
β_{IV}

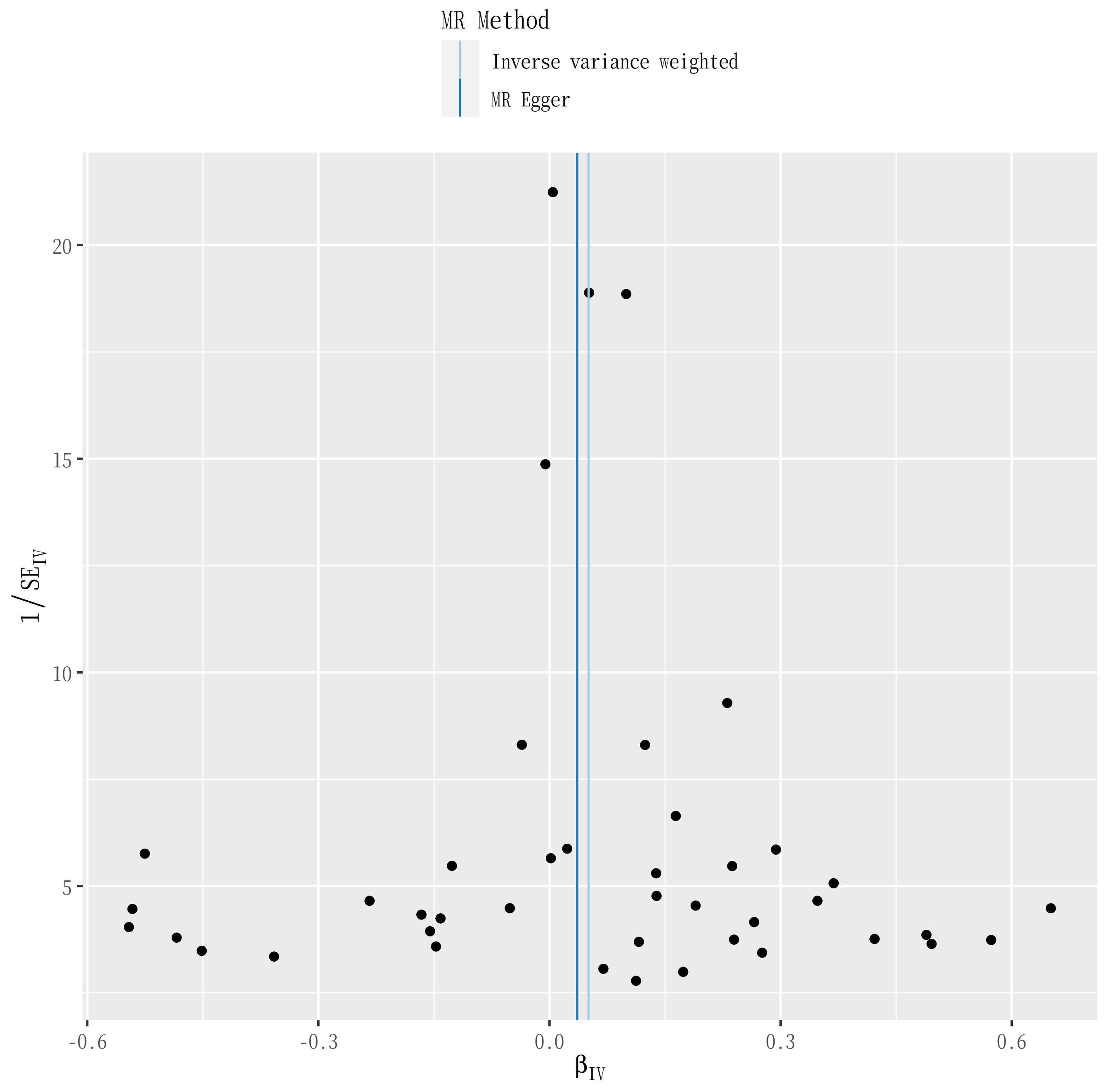
MR Method
Inverse variance weighted
MR Egger
1/SE_IV
20
15
10
5
-0.6
-0.3
0.0
0.3
0.6
β_IV

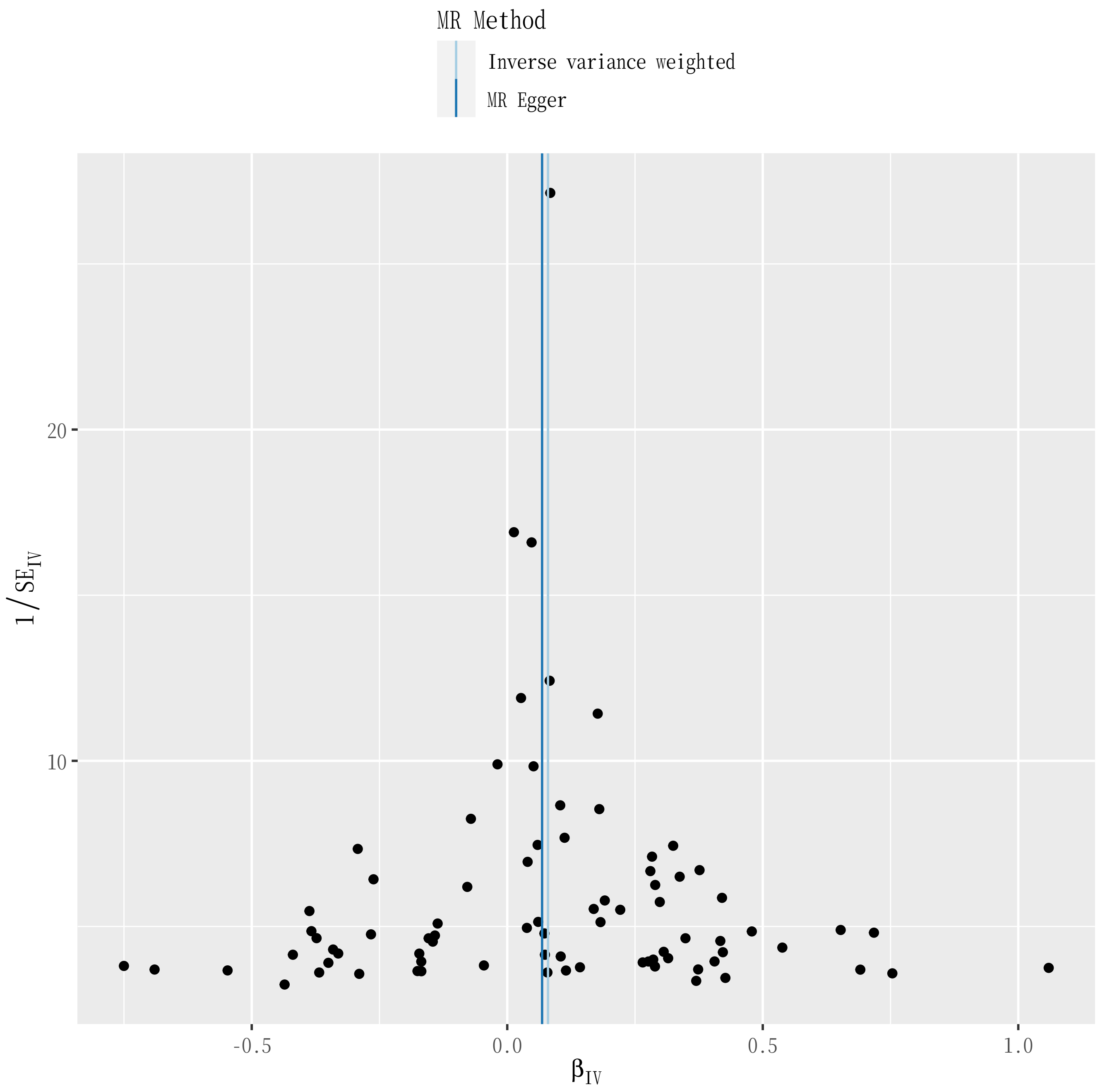
MR Method
Inverse variance weighted
MR Egger
20
10
$1/SE_{IV}$
-0.5
0.0
0.5
1.0
β_{IV}

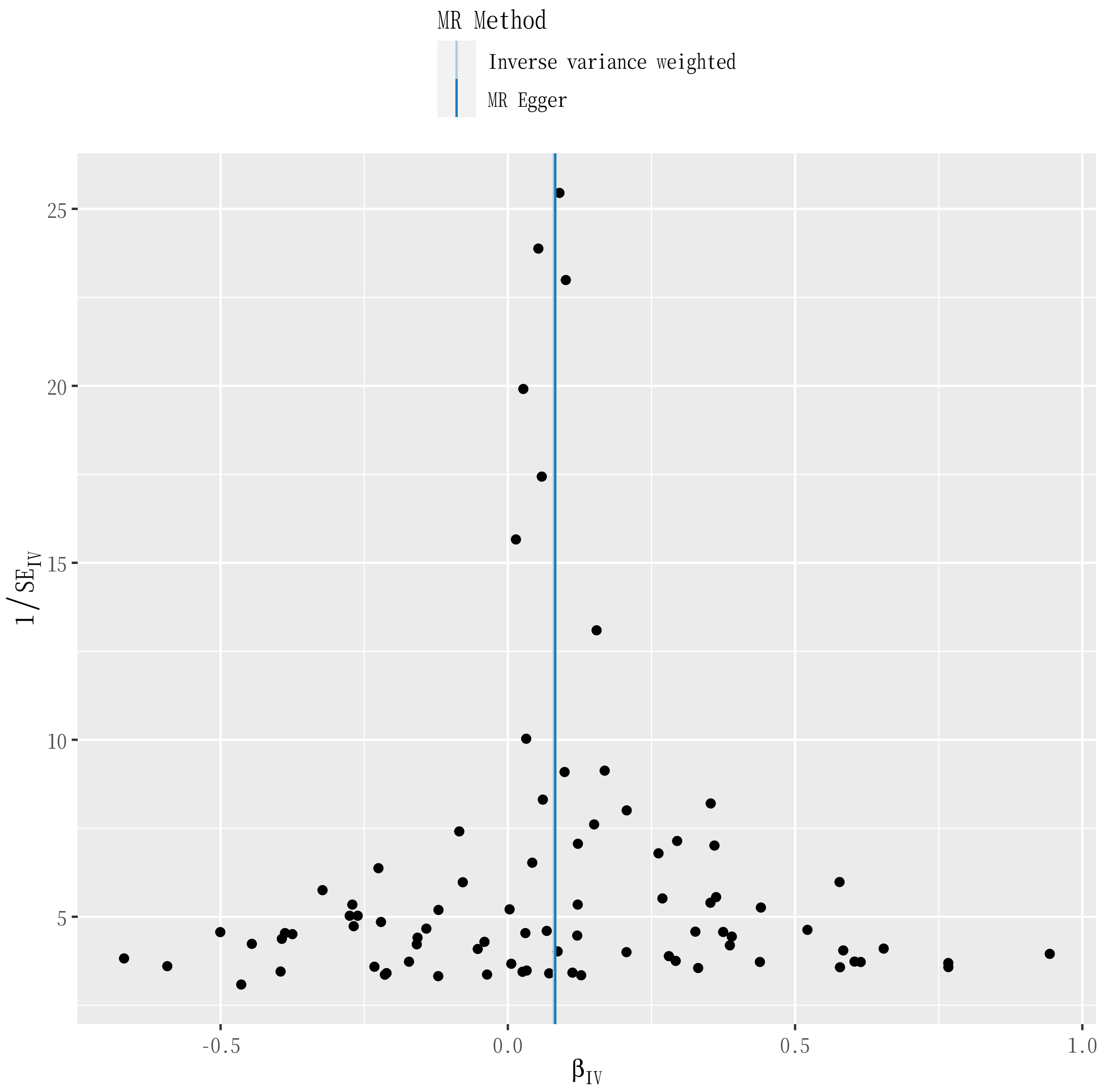
MR Method
Inverse variance weighted
MR Egger
25
20
15
10
5
$1/SE_{IV}$
-0.5
0.0
0.5
1.0
β_{IV}

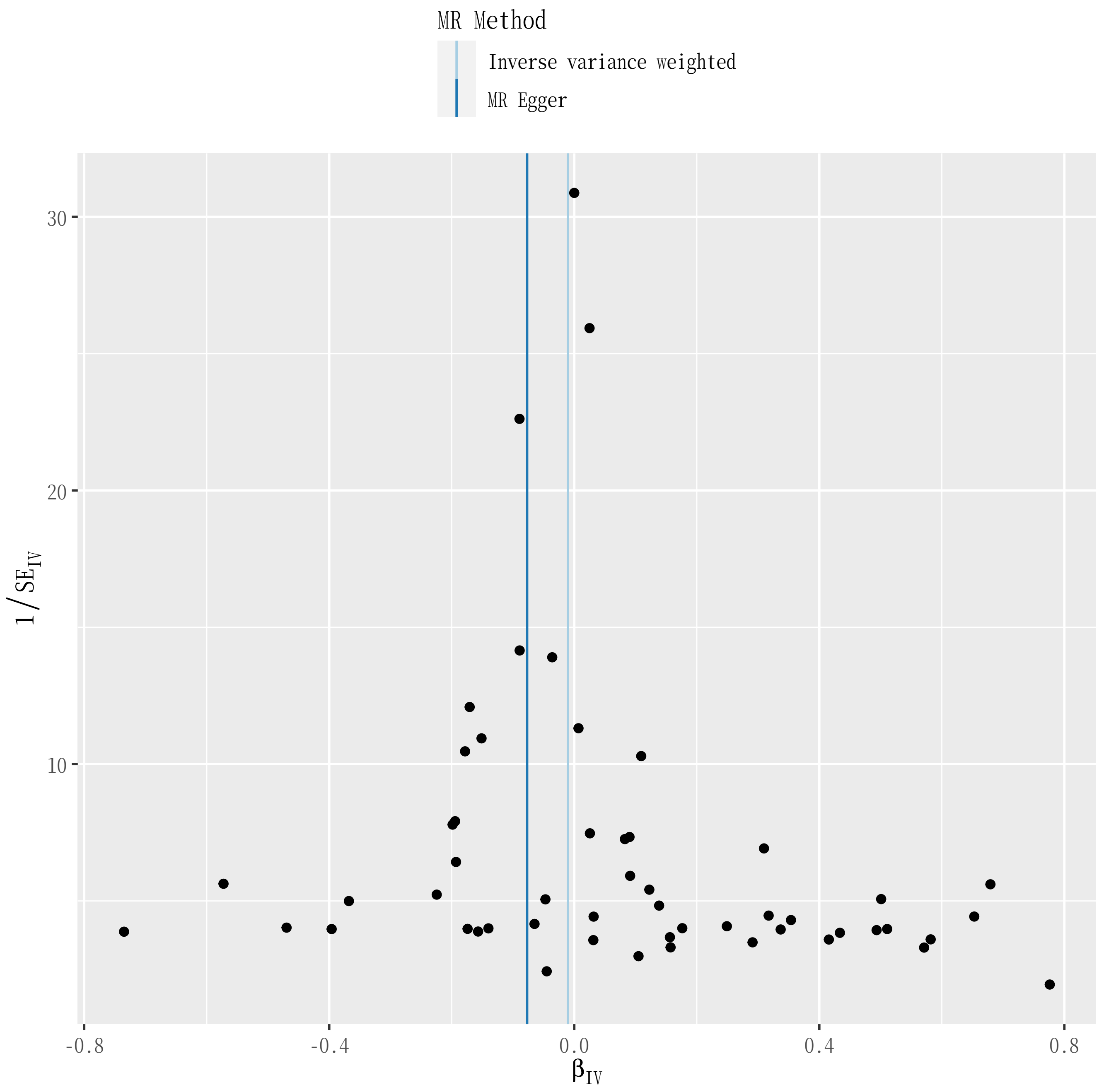

MR Method
Inverse variance weighted
MR Egger
30
20
10
$1/SE_{IV}$
-0.8
-0.4
0.0
0.4
0.8
β_{IV}

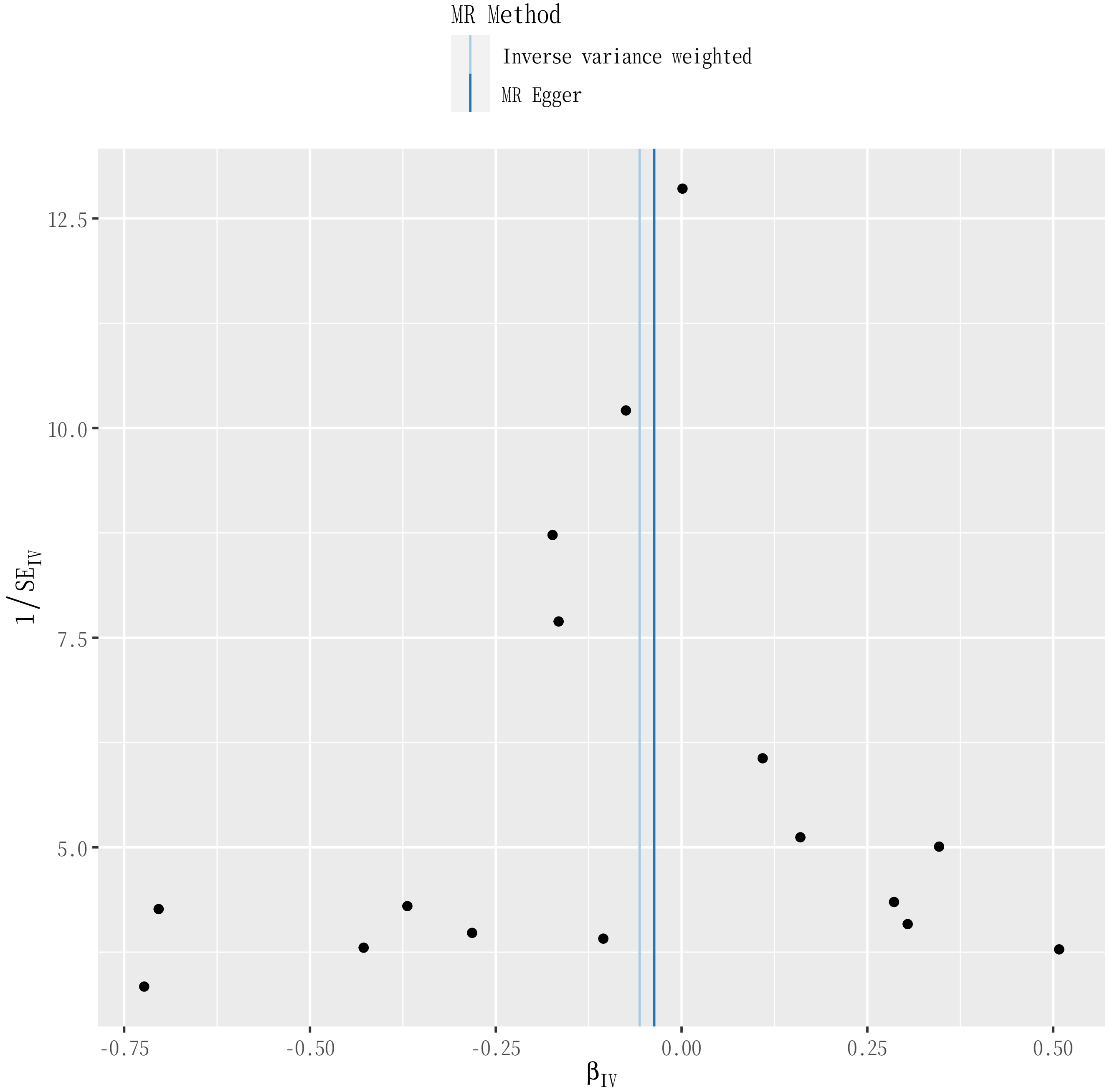
MR Method
Inverse variance weighted
MR Egger
12.5
10.0
7.5
5.0
$1/SE_{IV}$
-0.75
-0.50
-0.25
0.00
0.25
0.50
β_{IV}

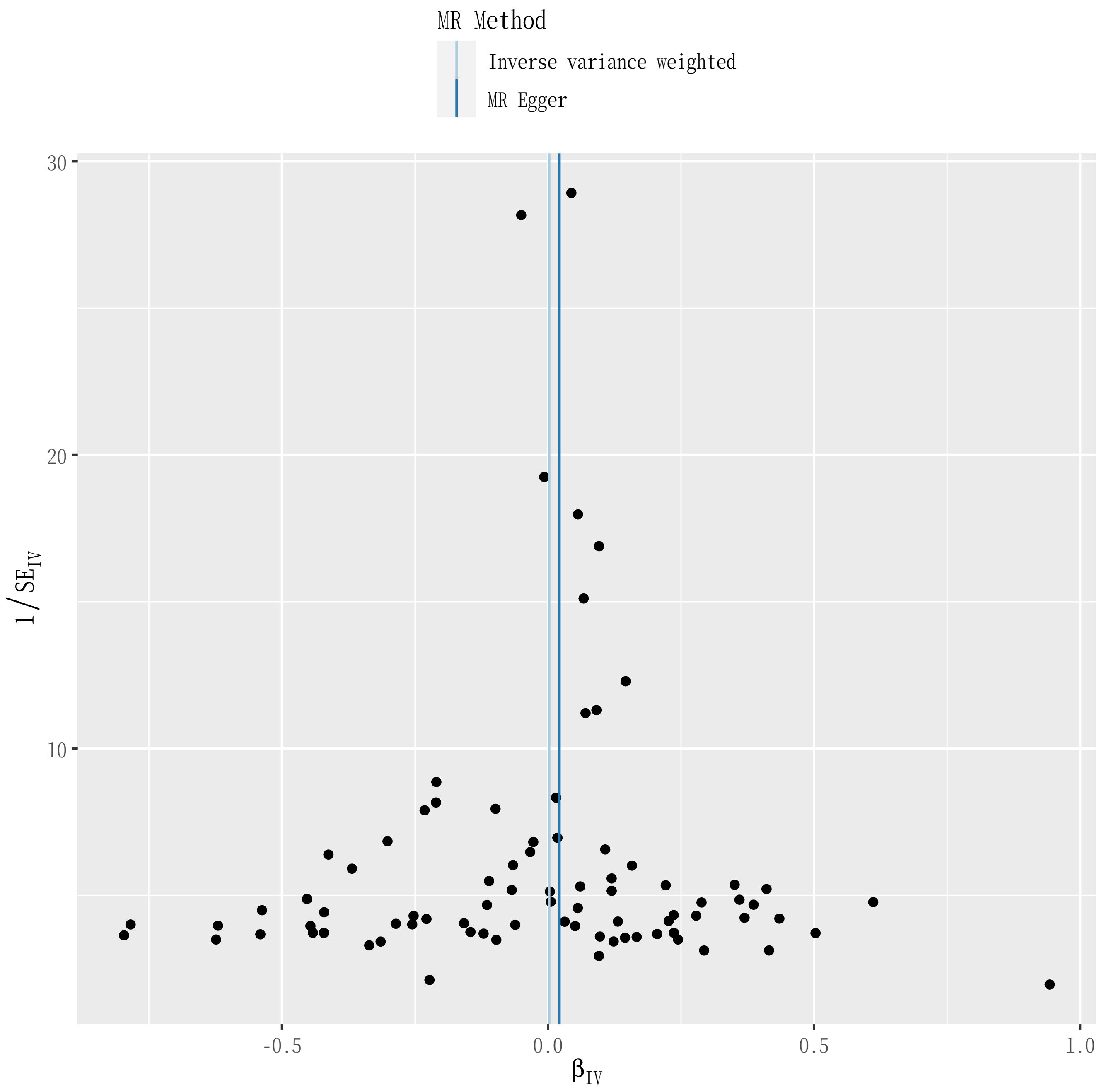
MR Method
Inverse variance weighted
MR Egger
30
20
10
1/SE_IV
-0.5
0.0
0.5
1.0
β_IV

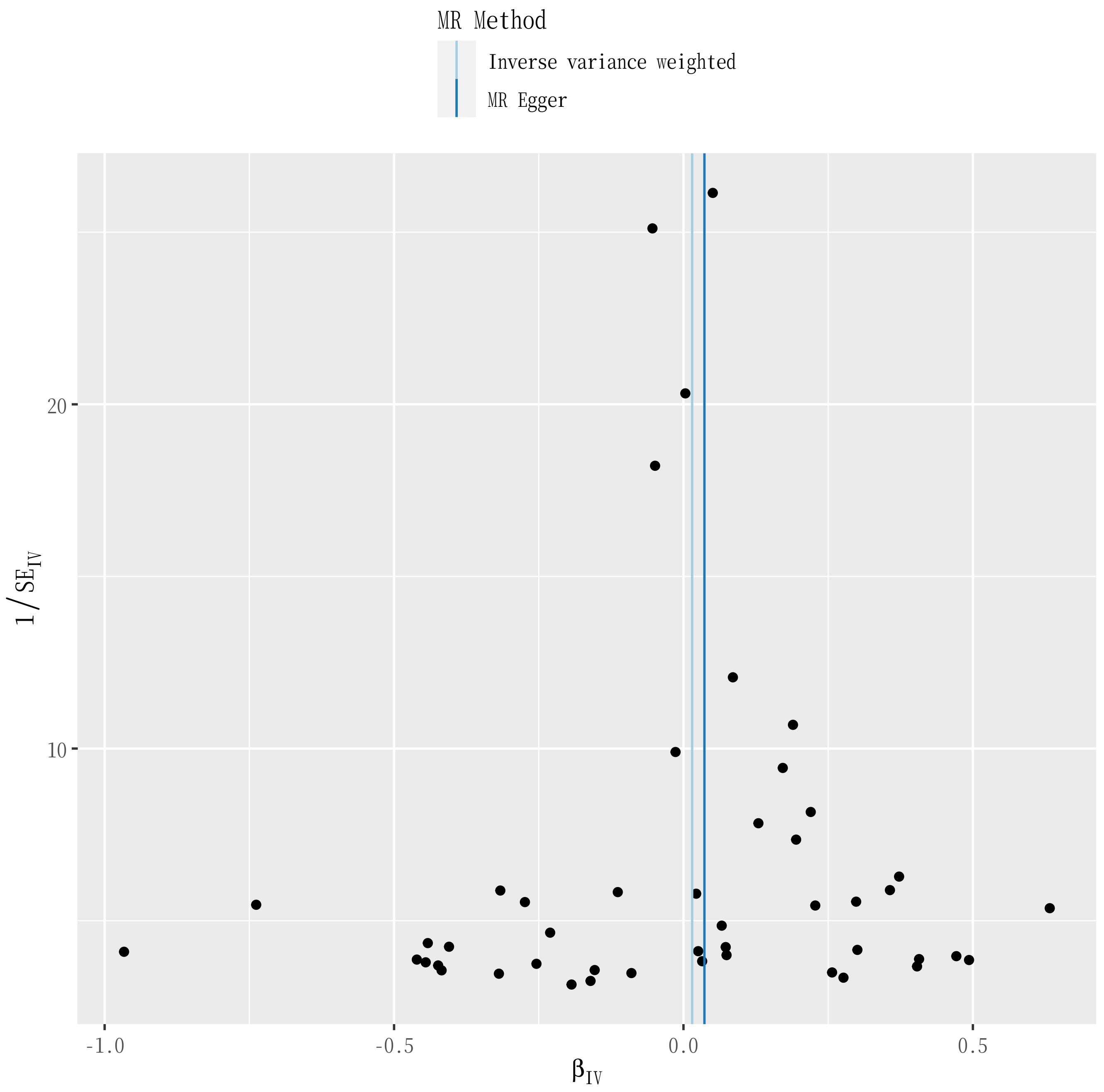
MR Method
Inverse variance weighted
MR Egger
20
10
$1/SE_{IV}$
-1.0
-0.5
0.0
0.5
β_{IV}

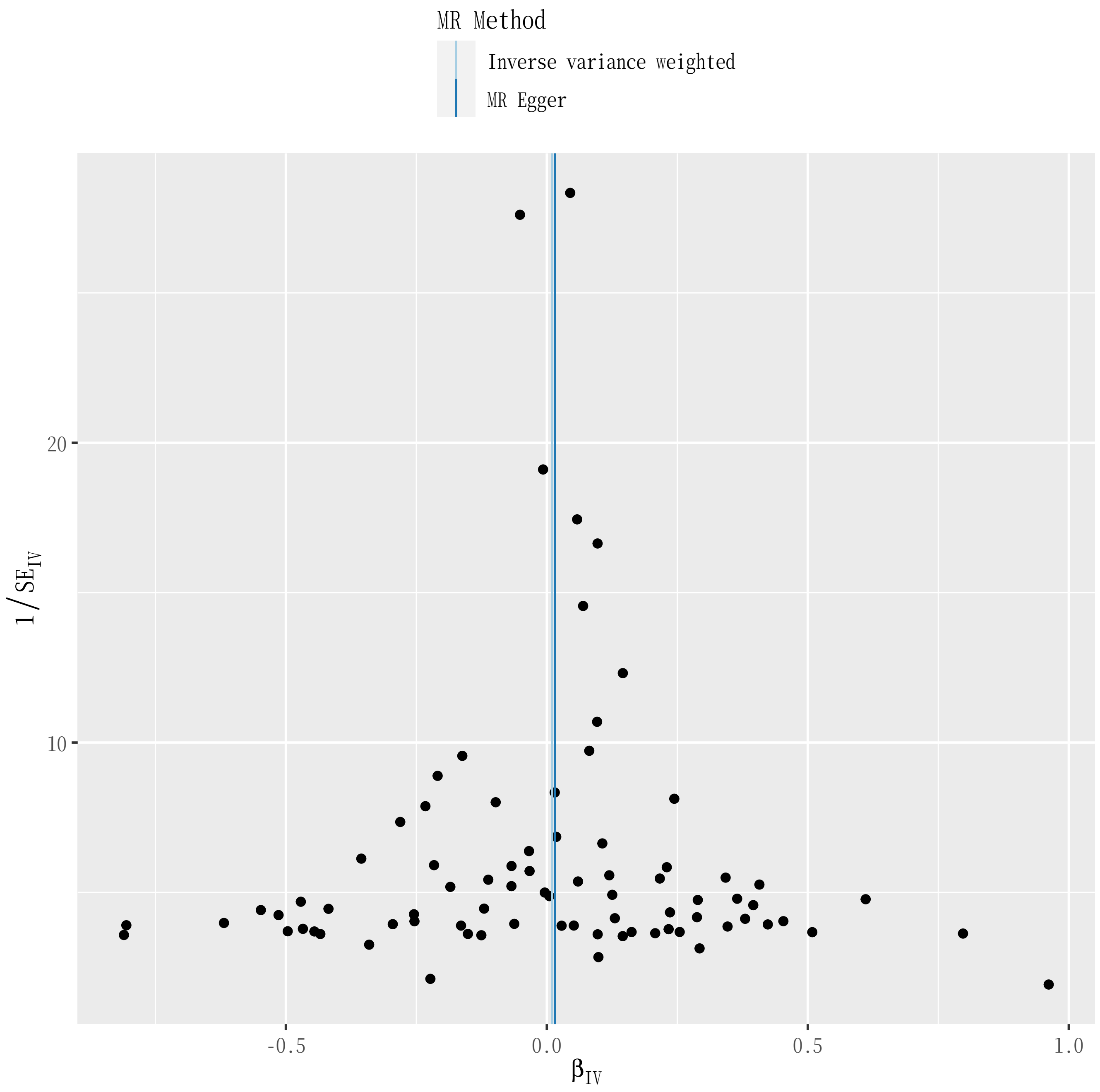
MR Method
Inverse variance weighted
MR Egger
1/SE_IV
20
10
-0.5
0.0
0.5
1.0
β_IV

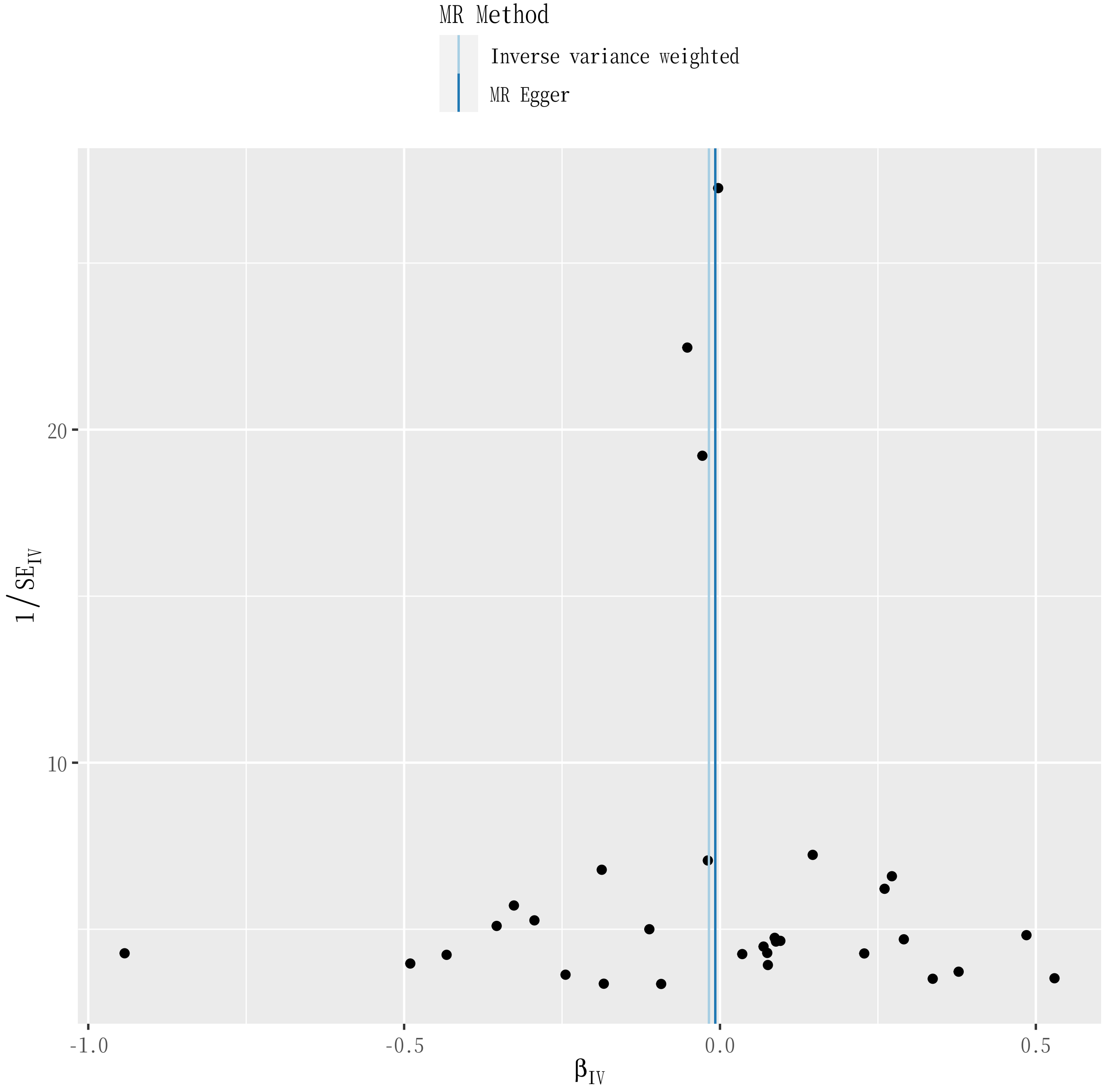

MR Method
Inverse variance weighted
MR Egger
20
10
-1.0
-0.5
0.0
0.5
$1/SE_{IV}$
β_{IV}

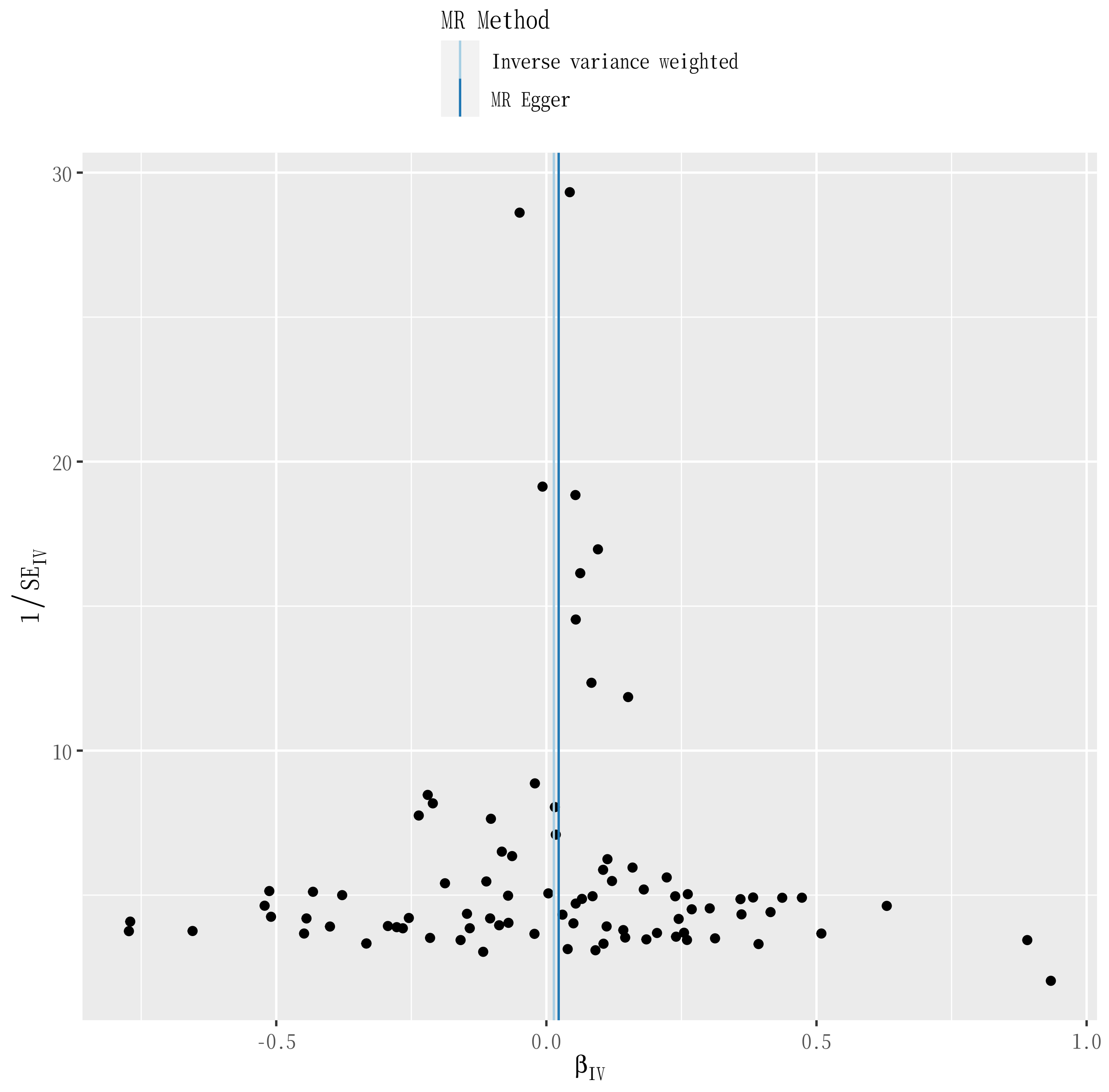
MR Method
Inverse variance weighted
MR Egger
30
20
10
-0.5
0.0
0.5
1.0
$1/SE_{IV}$
β_{IV}

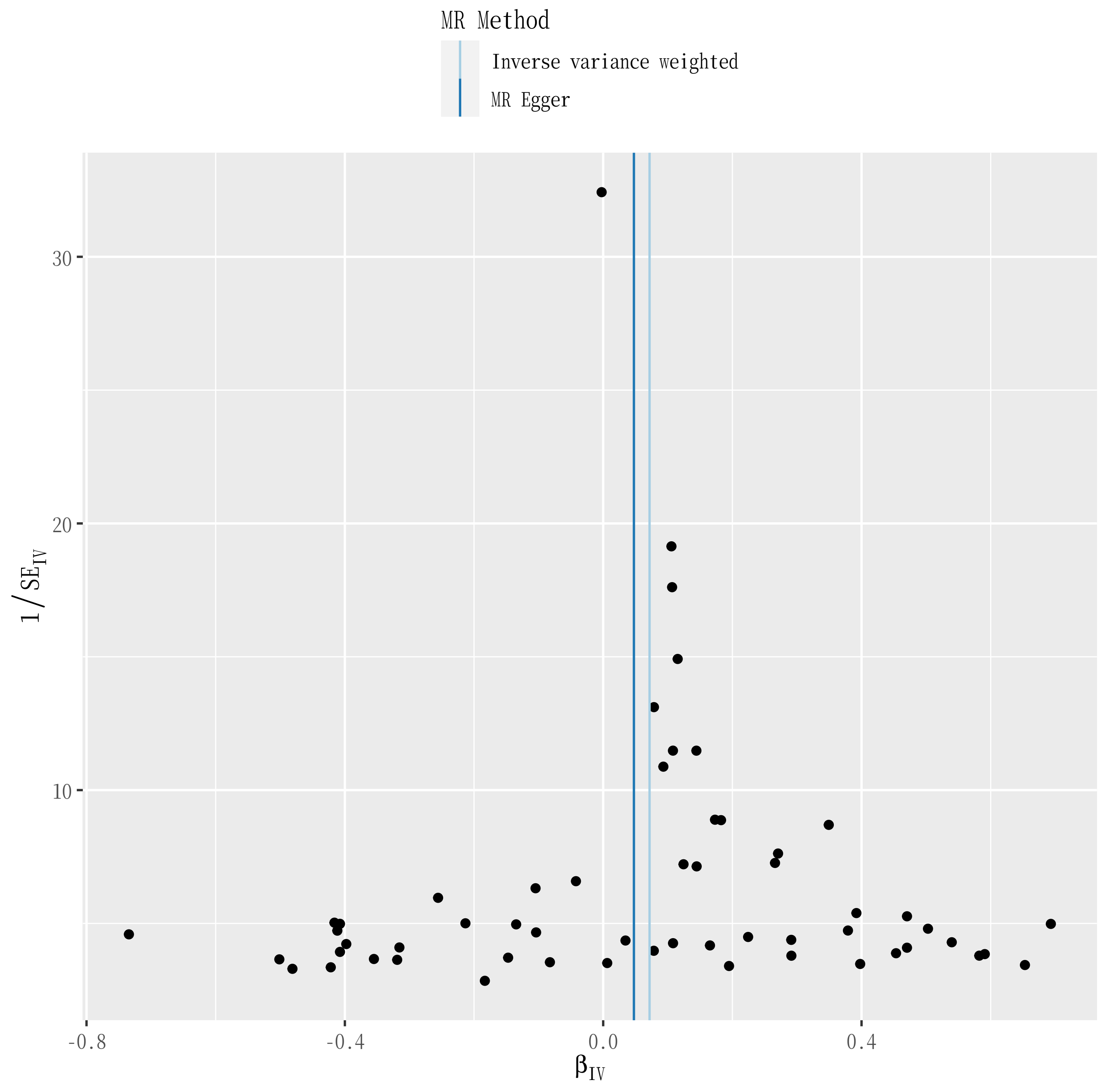

MR Method
Inverse variance weighted
MR Egger
30
20
10
1/SE_IV
-0.8
-0.4
0.0
0.4
β_IV

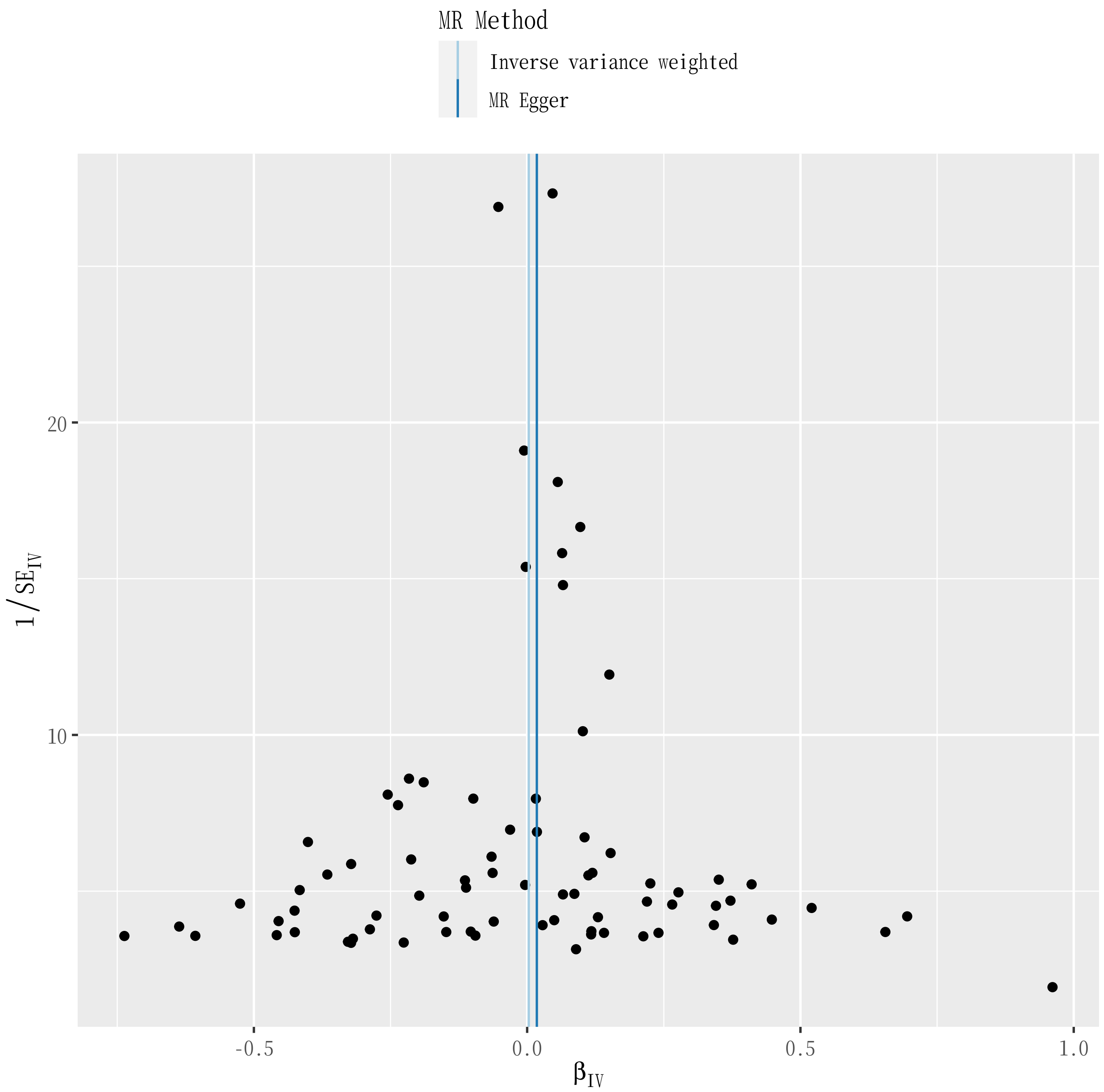
MR Method
Inverse variance weighted
MR Egger
20
10
-0.5
0.0
0.5
1.0
1/SE_IV
β_IV

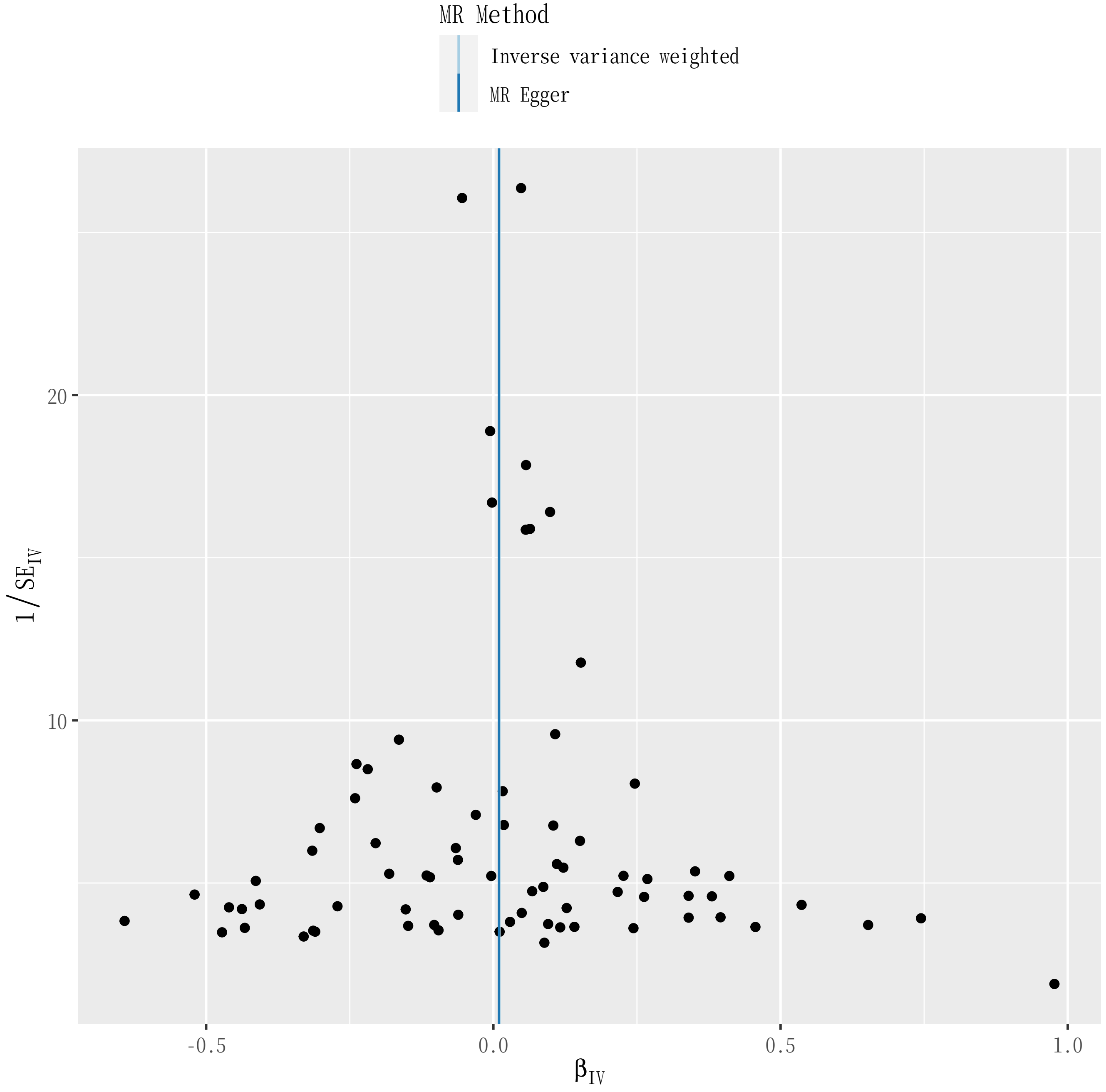
MR Method
Inverse variance weighted
MR Egger
20
10
$1/SE_{IV}$
-0.5
0.0
0.5
1.0
β_{IV}

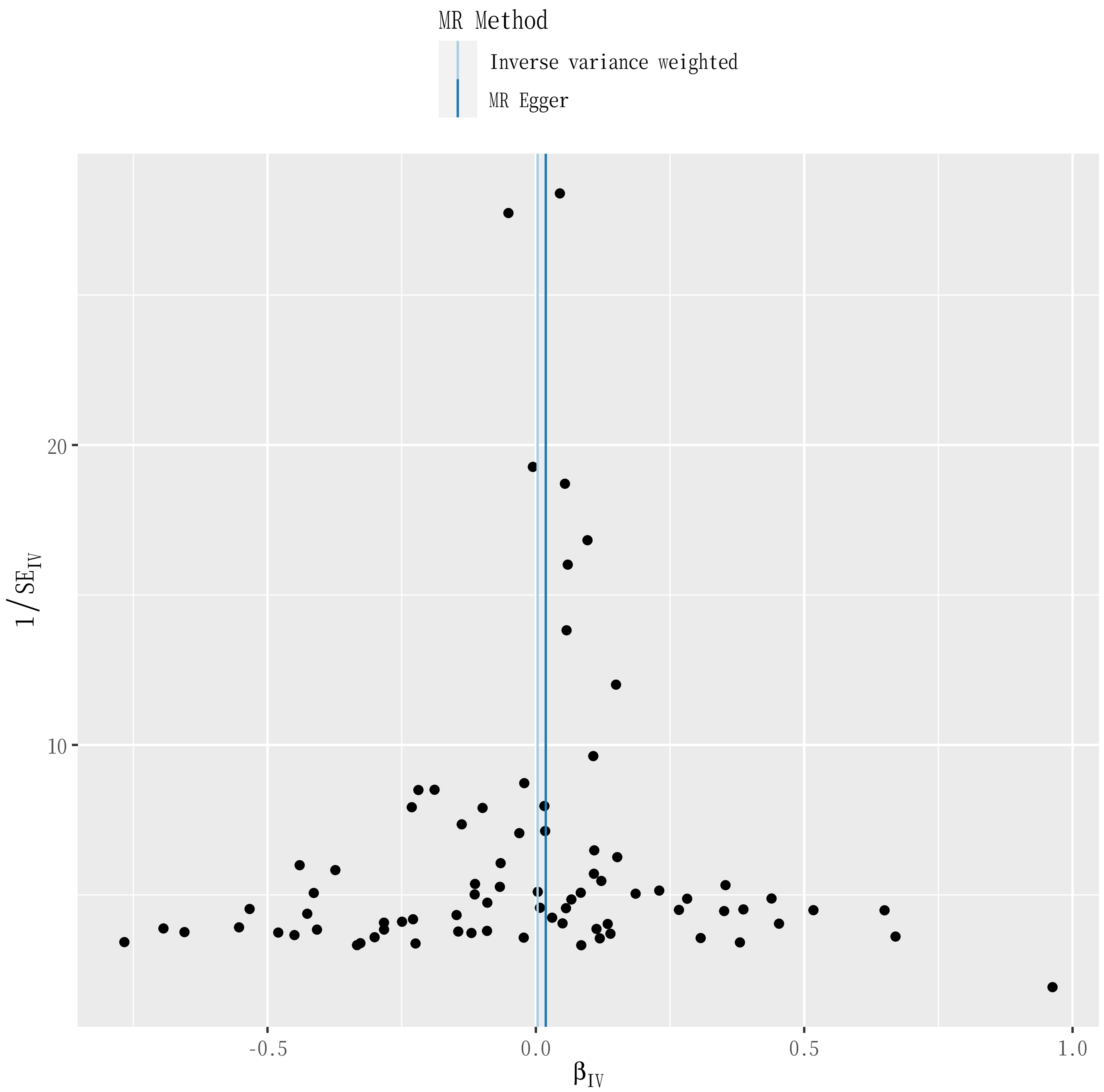

MR Method
Inverse variance weighted
MR Egger
20
10
-0.5
0.0
0.5
1.0
$1/SE_{IV}$
β_{IV}

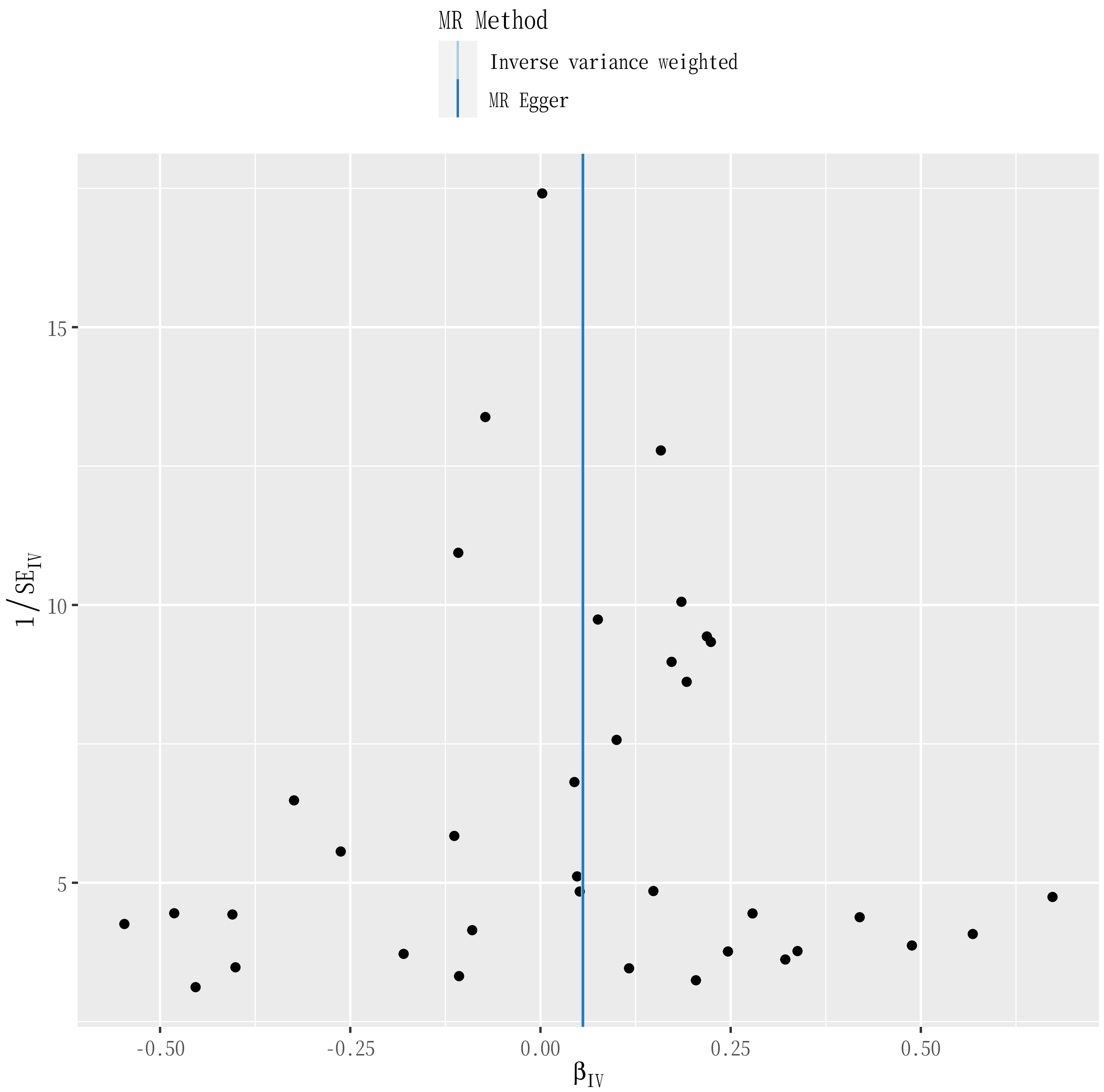
MR Method
Inverse variance weighted
MR Egger
15
10
5
$1/SE_{IV}$
-0.50
-0.25
0.00
0.25
0.50
β_{IV}

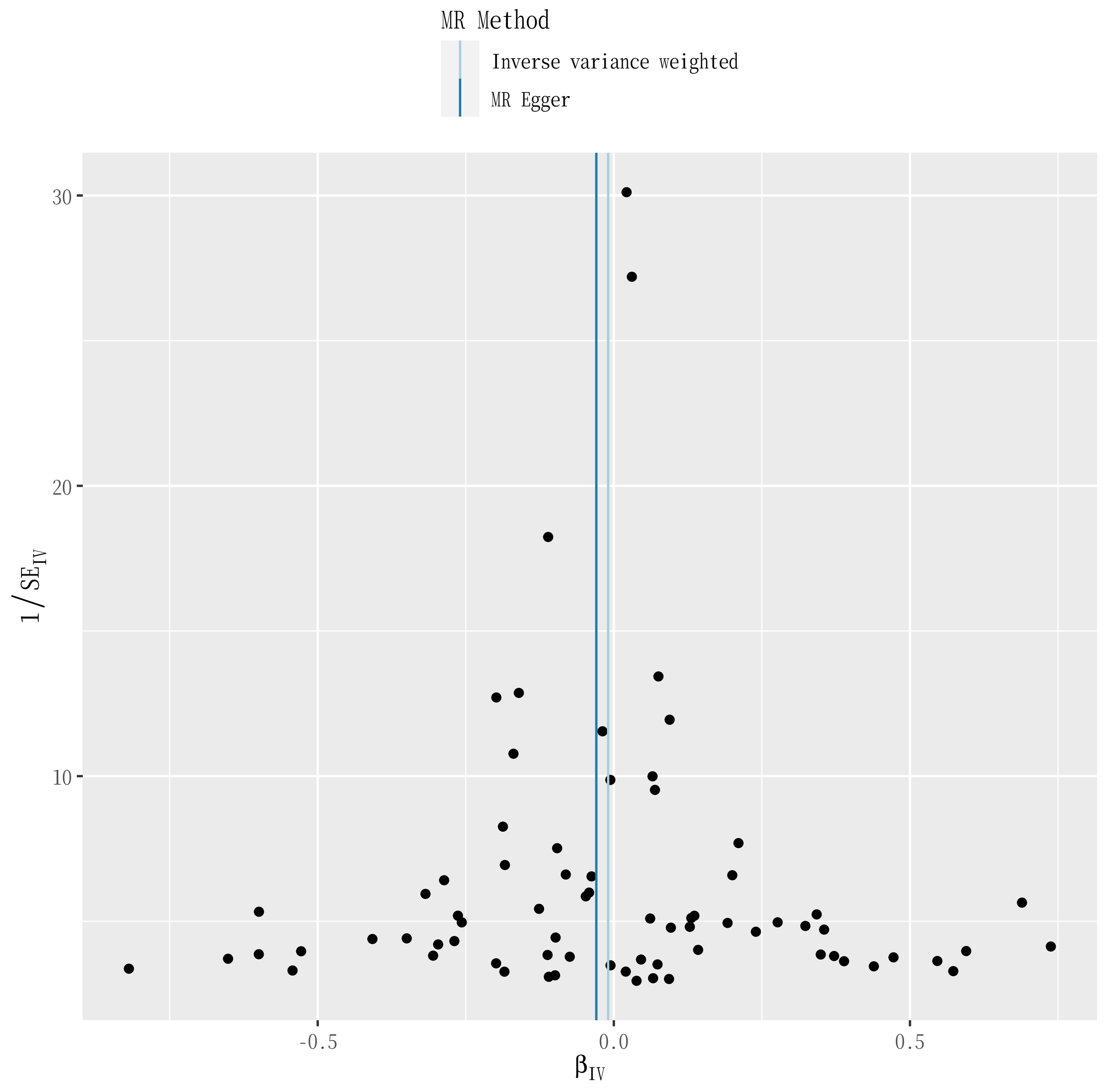

MR Method
Inverse variance weighted
MR Egger
30
20
10
$1/SE_{IV}$
-0.5
0.0
0.5
β_{IV}

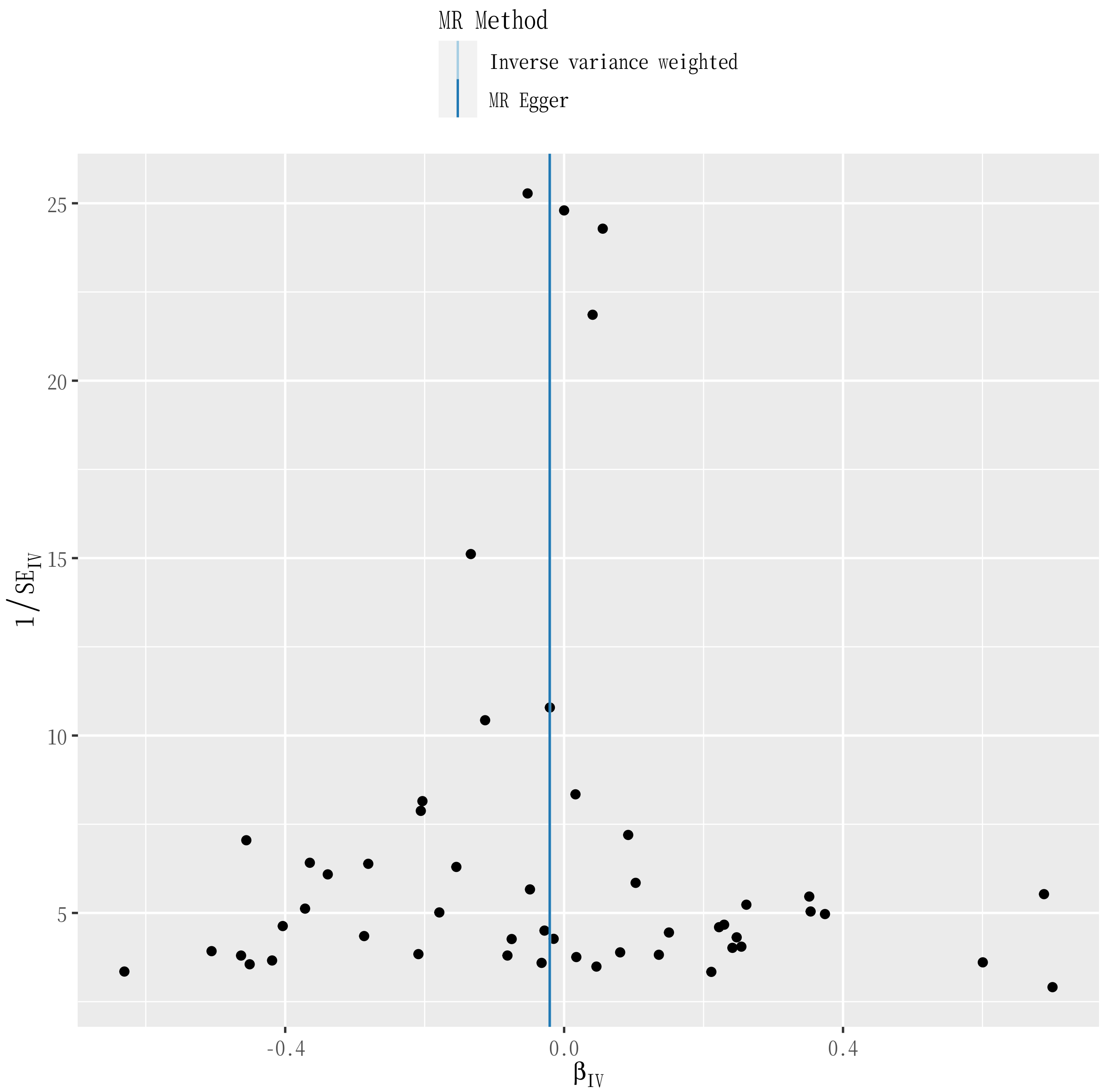
MR Method
Inverse variance weighted
MR Egger
25
20
15
10
5
1/SE_IV
-0.4
0.0
0.4
β_IV

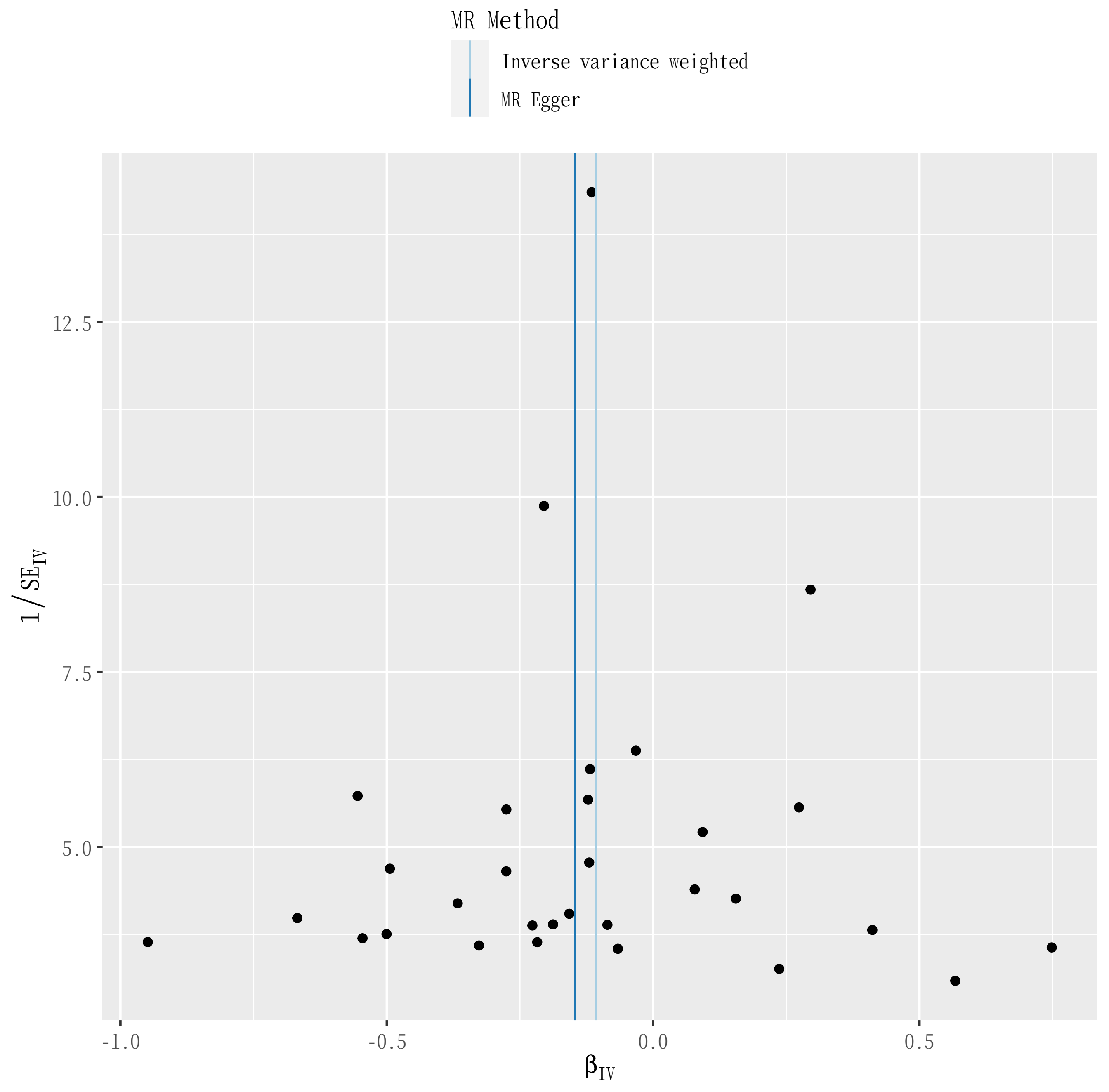

MR Method
Inverse variance weighted
MR Egger
12.5
10.0
7.5
5.0
1/SE_IV
-1.0
-0.5
0.0
0.5
β_IV

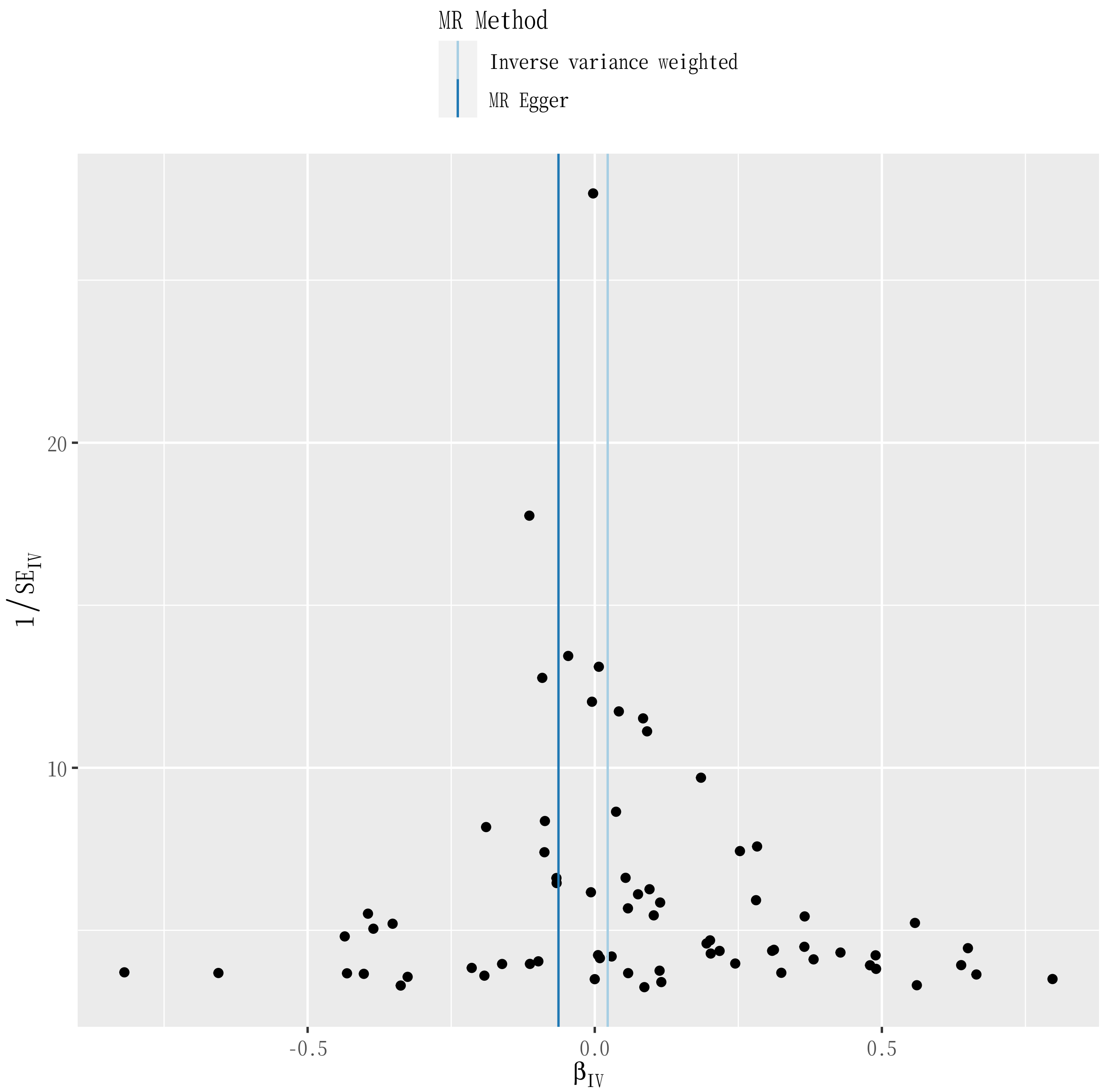
MR Method
Inverse variance weighted
MR Egger
20
10
1/SE_IV
-0.5
0.0
0.5
β_IV

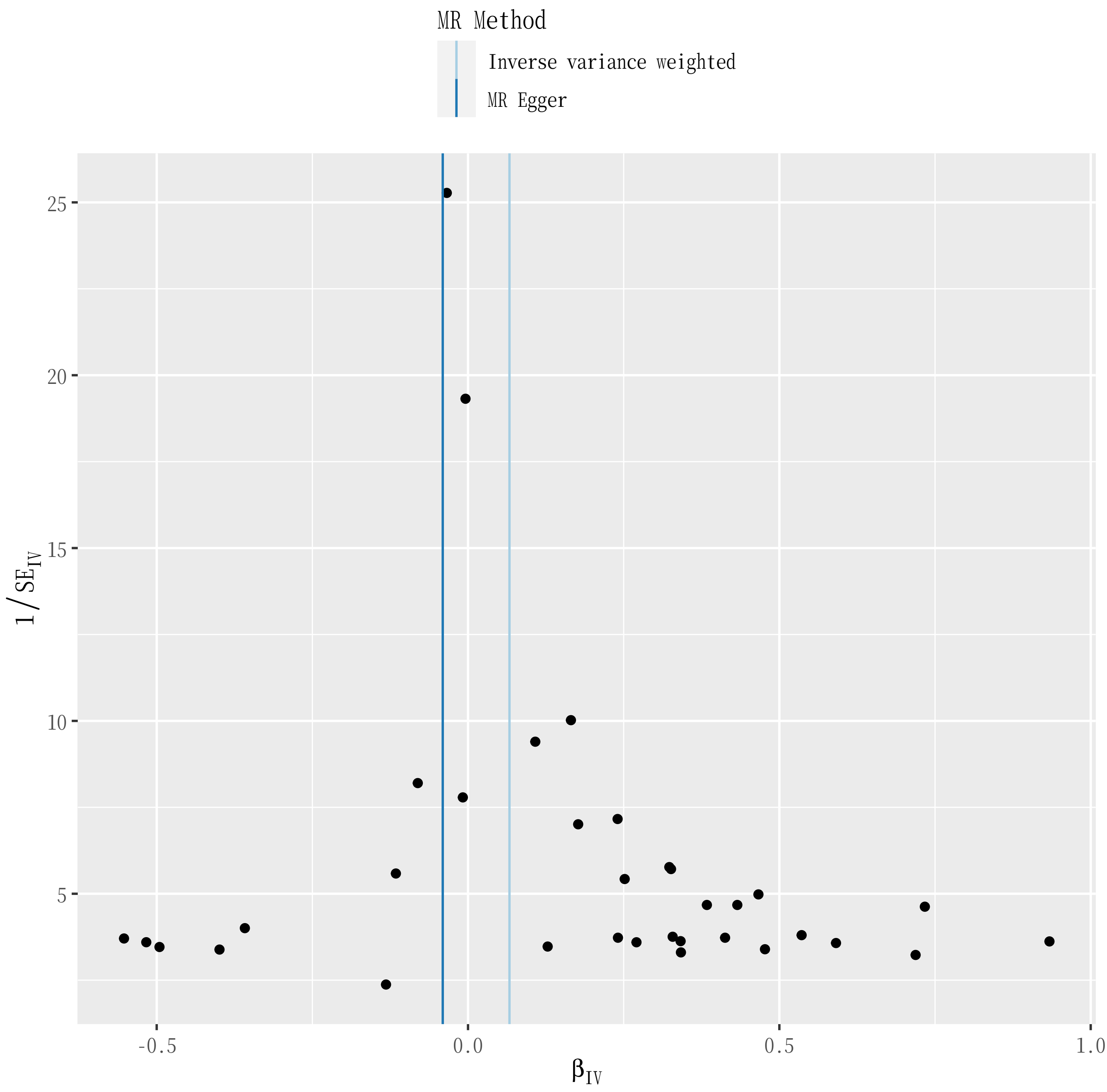

MR Method
Inverse variance weighted
MR Egger
25
20
15
10
5
-0.5
0.0
0.5
1.0
$1/SE_{IV}$
β_{IV}

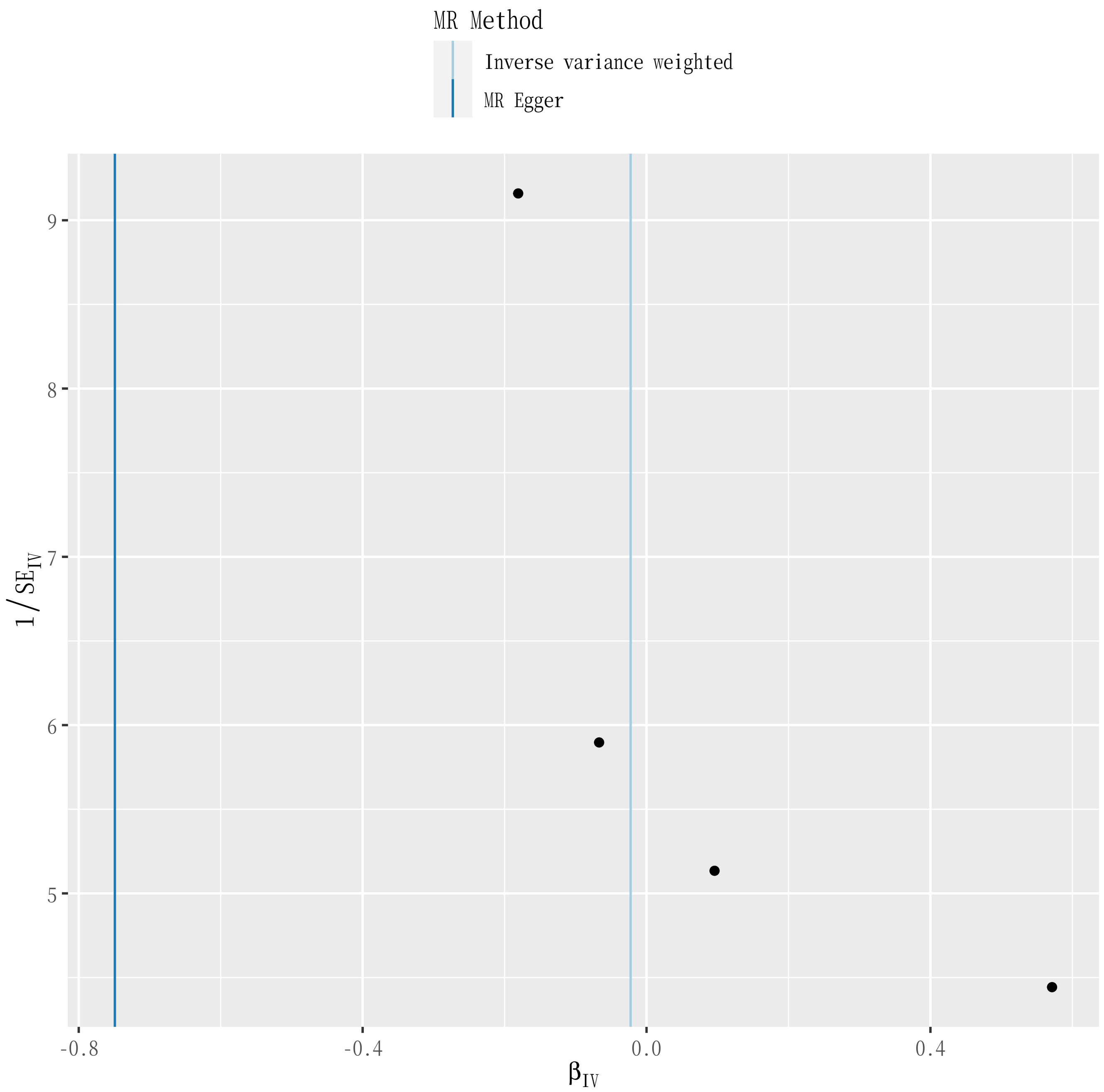
MR Method
Inverse variance weighted
MR Egger
1/SE_IV
9
8
7
6
5
-0.8
-0.4
0.0
0.4
β_IV

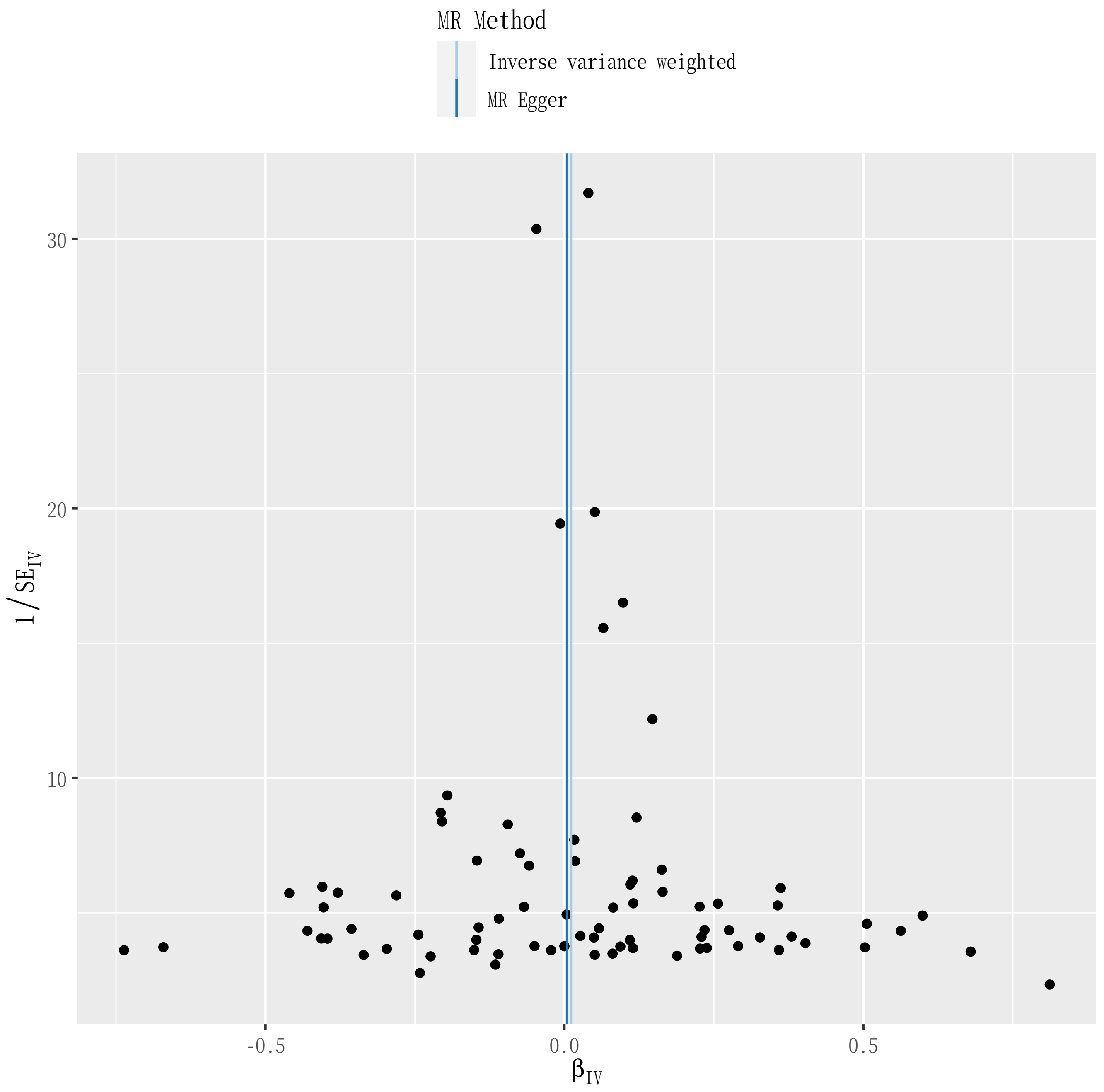

MR Method
Inverse variance weighted
MR Egger
30
20
10
1/SE_IV
-0.5
0.0
0.5
β_IV

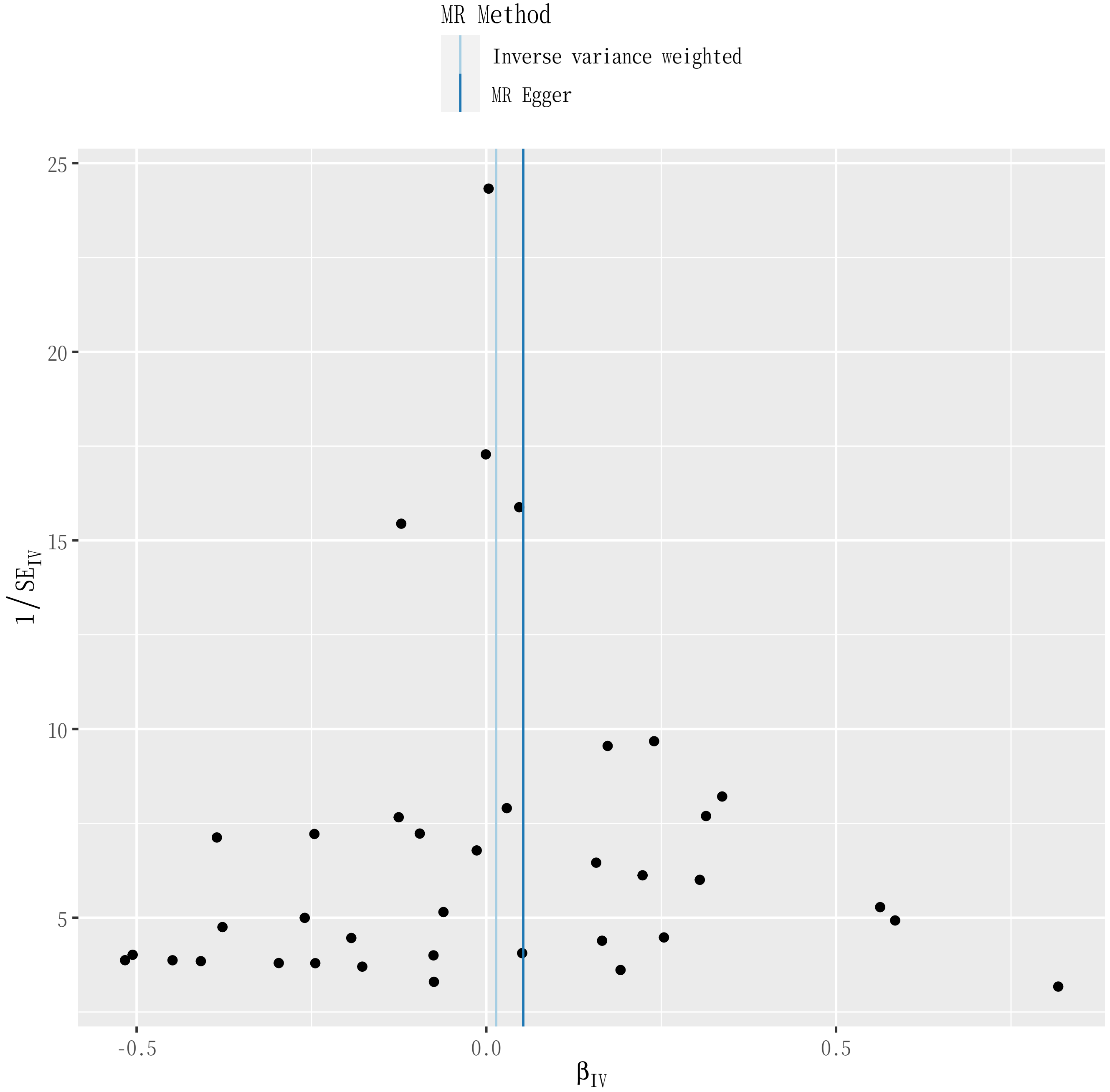

MR Method
Inverse variance weighted
MR Egger
25
20
15
10
5
1/SE_IV
-0.5
0.0
0.5
β_IV

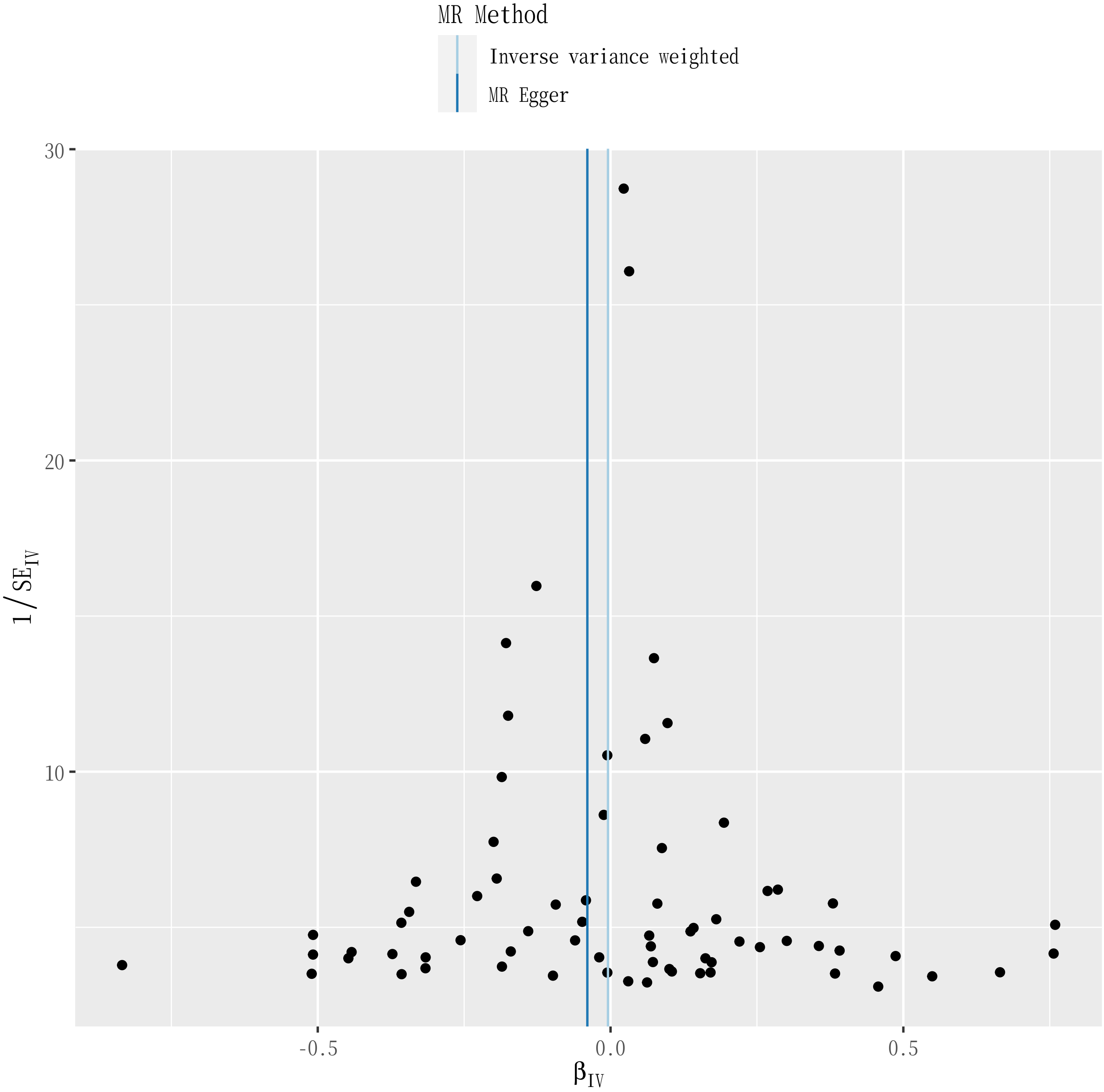

MR Method
Inverse variance weighted
MR Egger
30
20
10
1/SE_IV
-0.5
0.0
0.5
β_IV

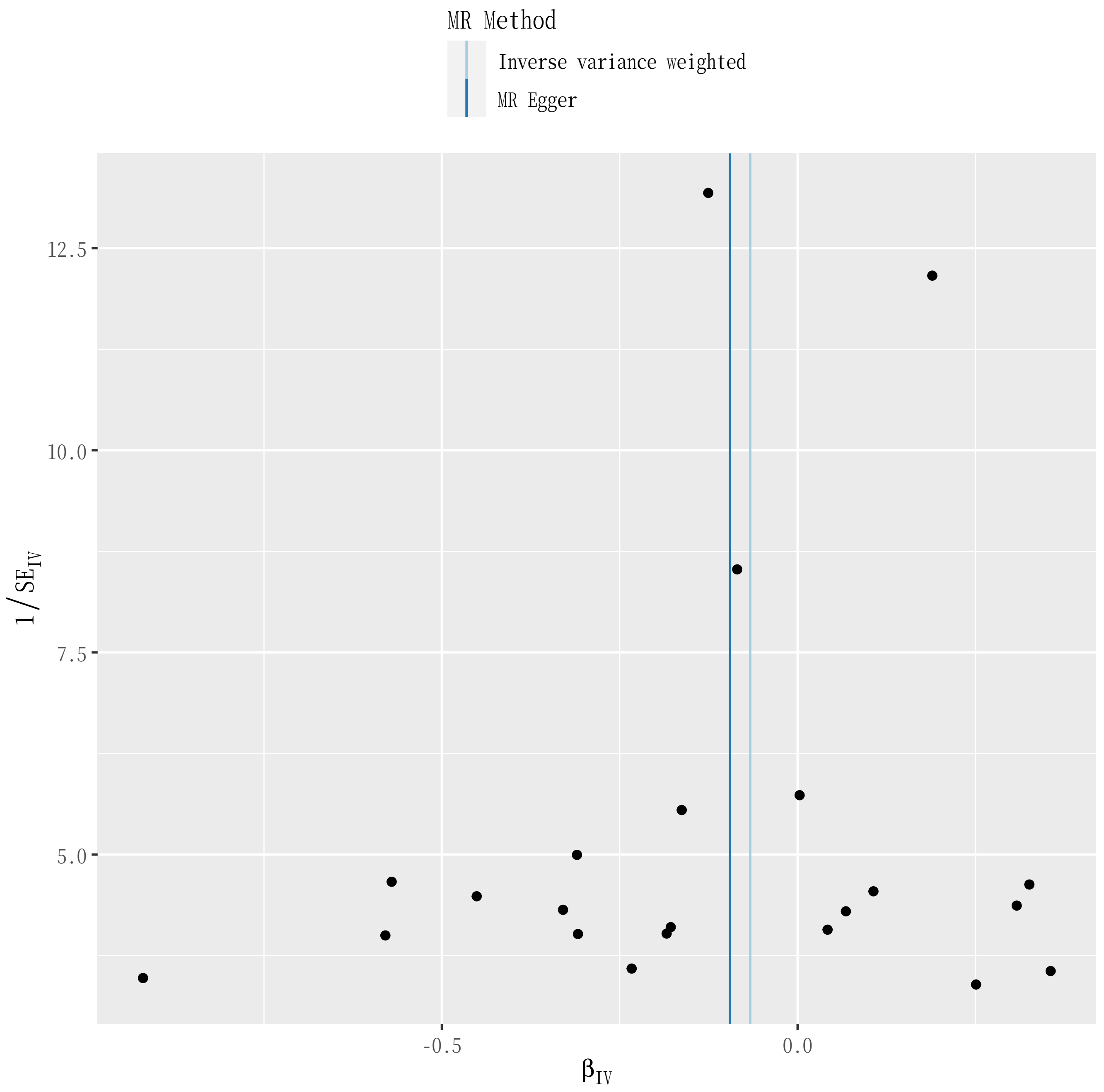

MR Method
Inverse variance weighted
MR Egger
12.5
10.0
7.5
5.0
$1/SE_{IV}$
-0.5
0.0
β_{IV}

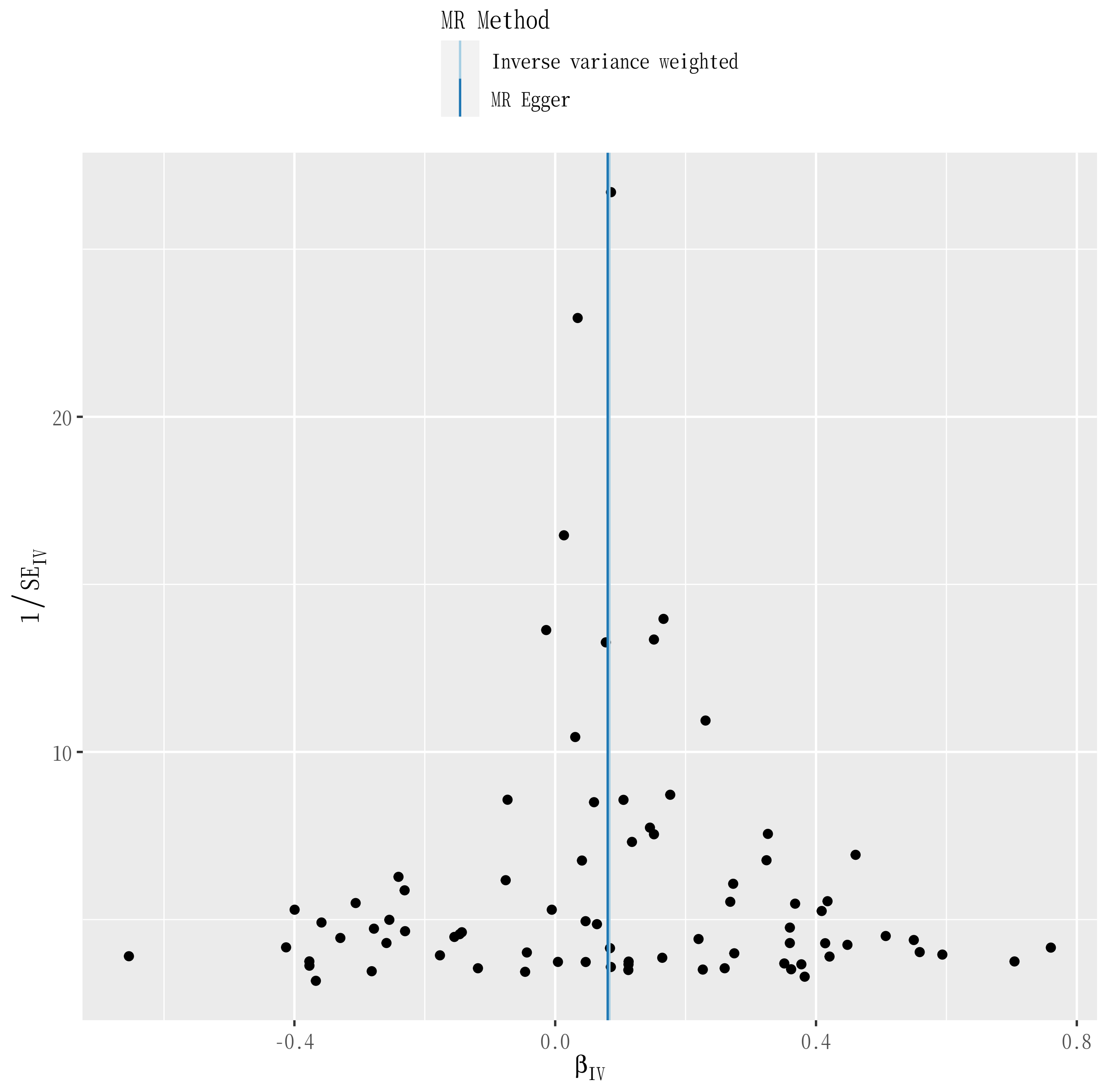

MR Method
Inverse variance weighted
MR Egger
20
10
-0.4
0.0
0.4
0.8
$1/SE_{IV}$
β_{IV}

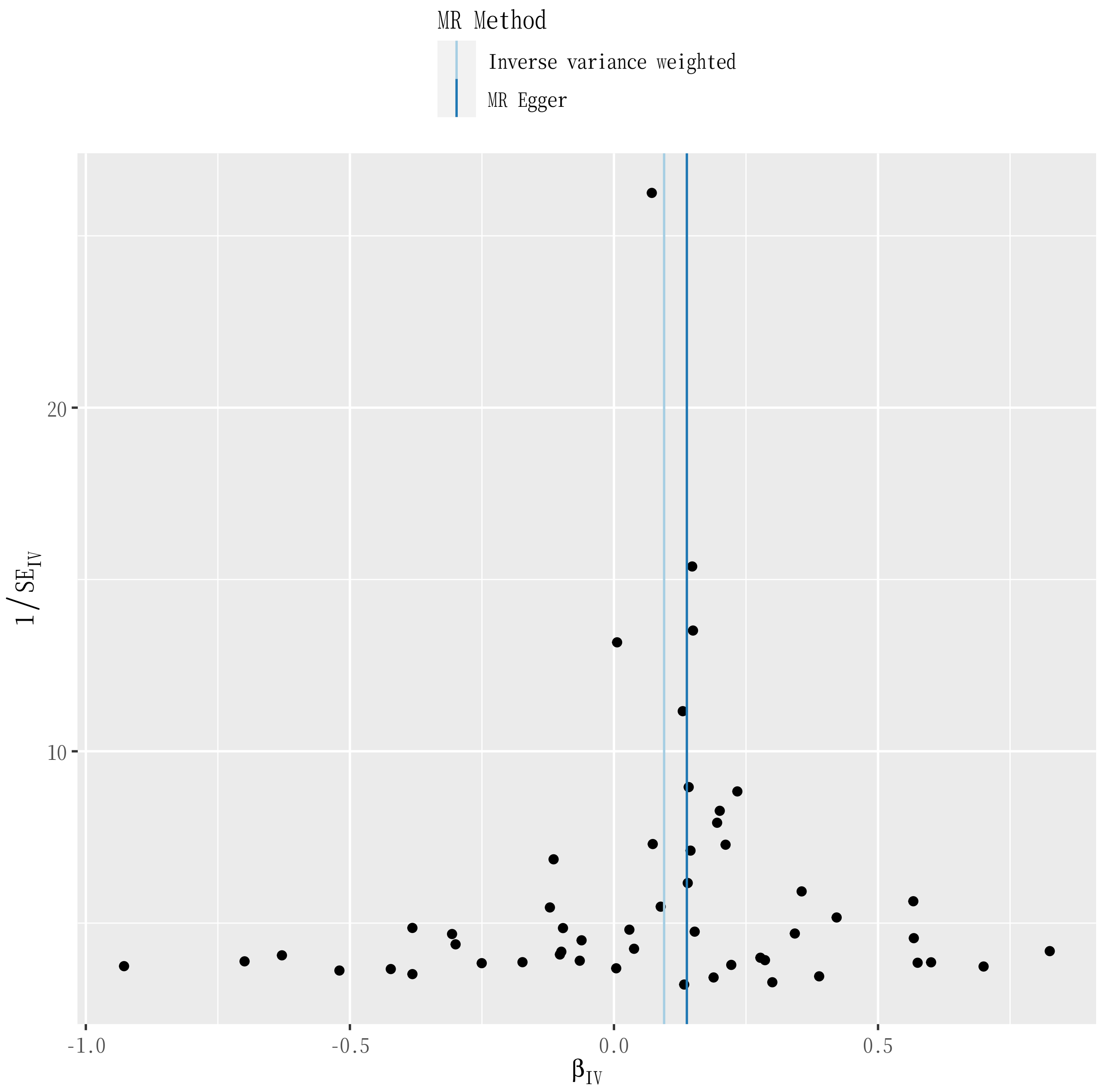
MR Method
Inverse variance weighted
MR Egger
20
10
1/SE_IV
-1.0
-0.5
0.0
0.5
β_IV

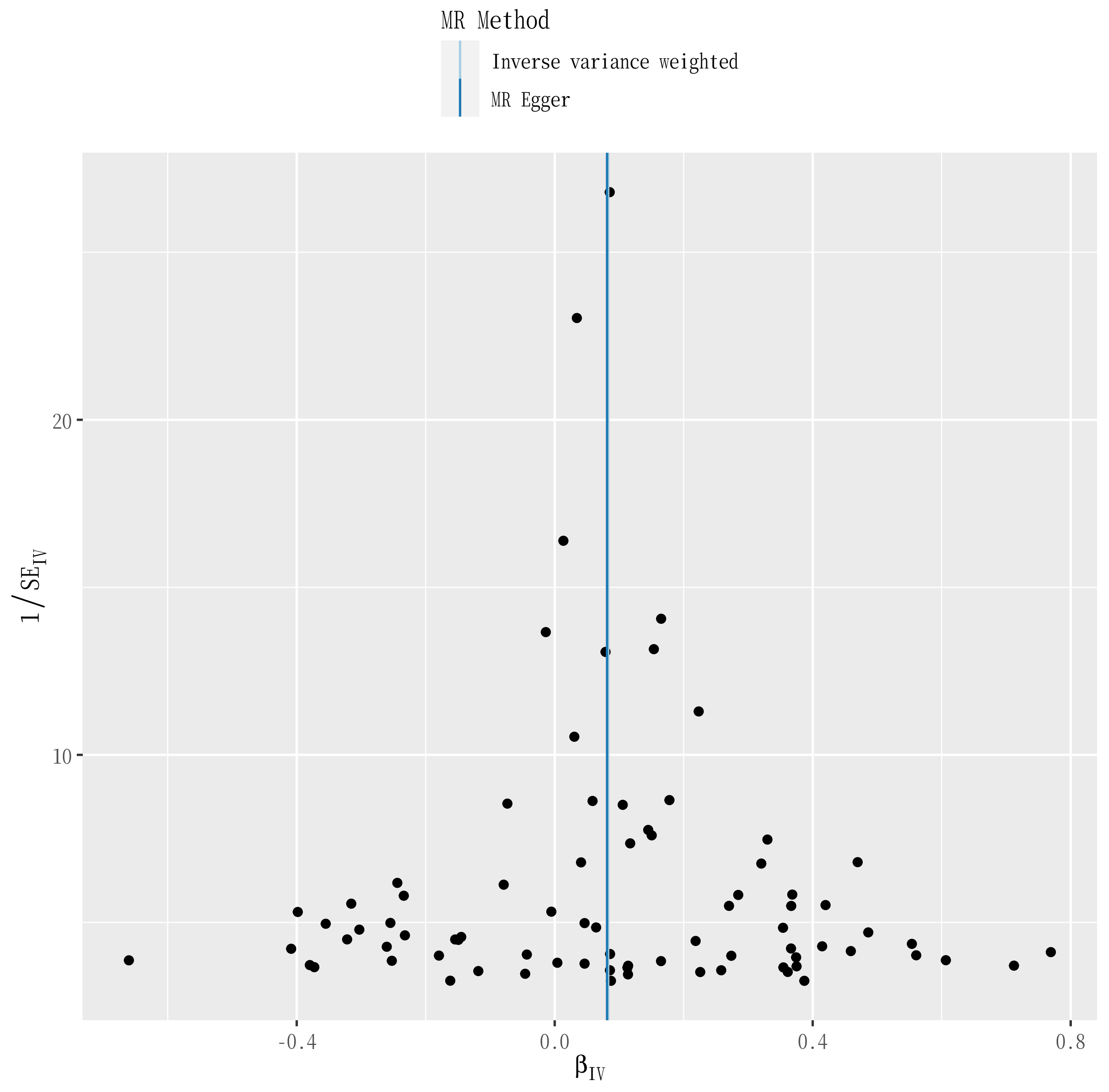
MR Method
Inverse variance weighted
MR Egger
20
10
$1/SE_{IV}$
-0.4
0.0
0.4
0.8
β_{IV}

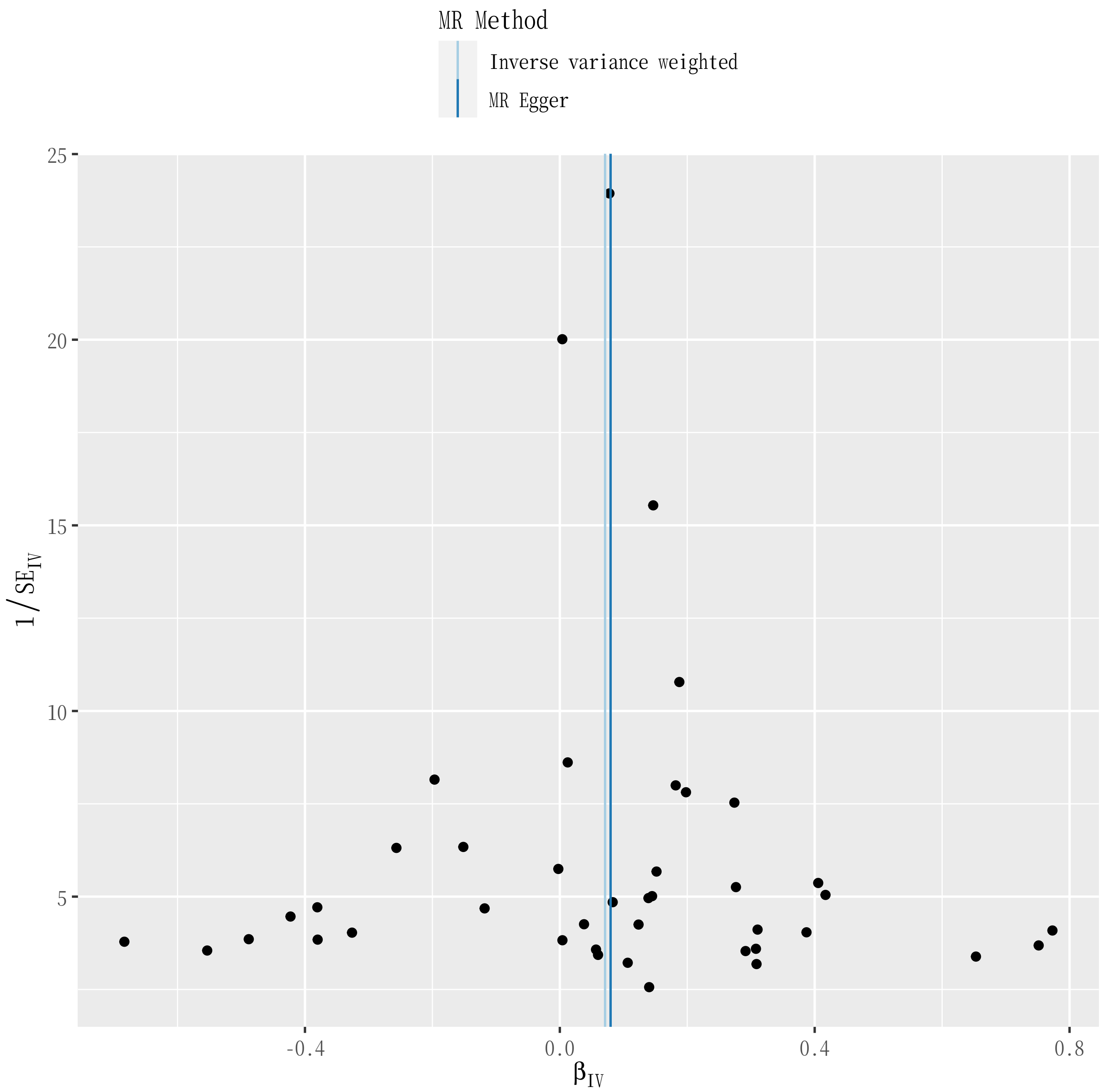
MR Method
Inverse variance weighted
MR Egger
25
20
15
10
5
1/SE_IV
-0.4
0.0
0.4
0.8
β_IV

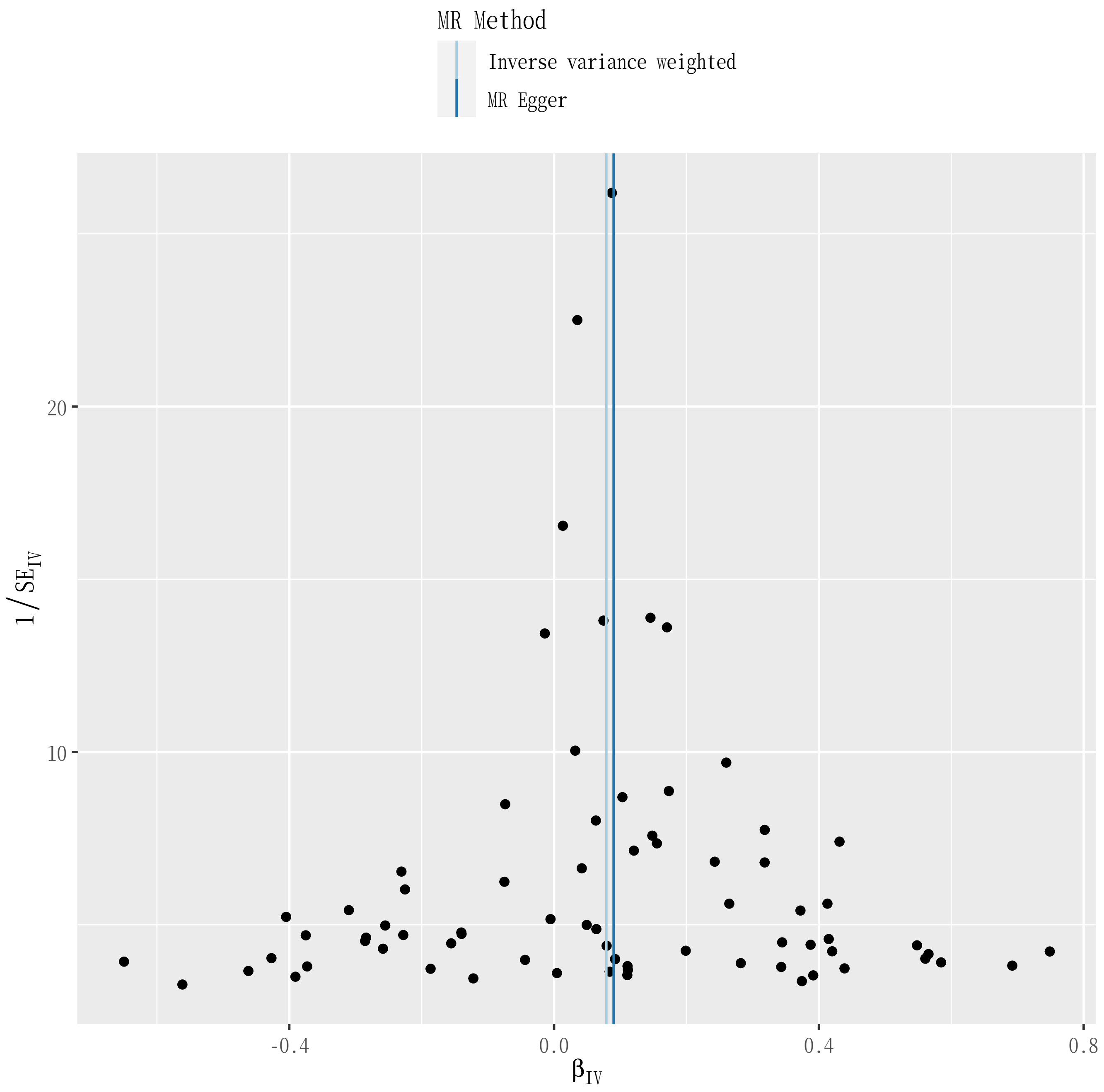

MR Method
Inverse variance weighted
MR Egger
20
10
$1/SE_{IV}$
-0.4
0.0
0.4
0.8
β_{IV}

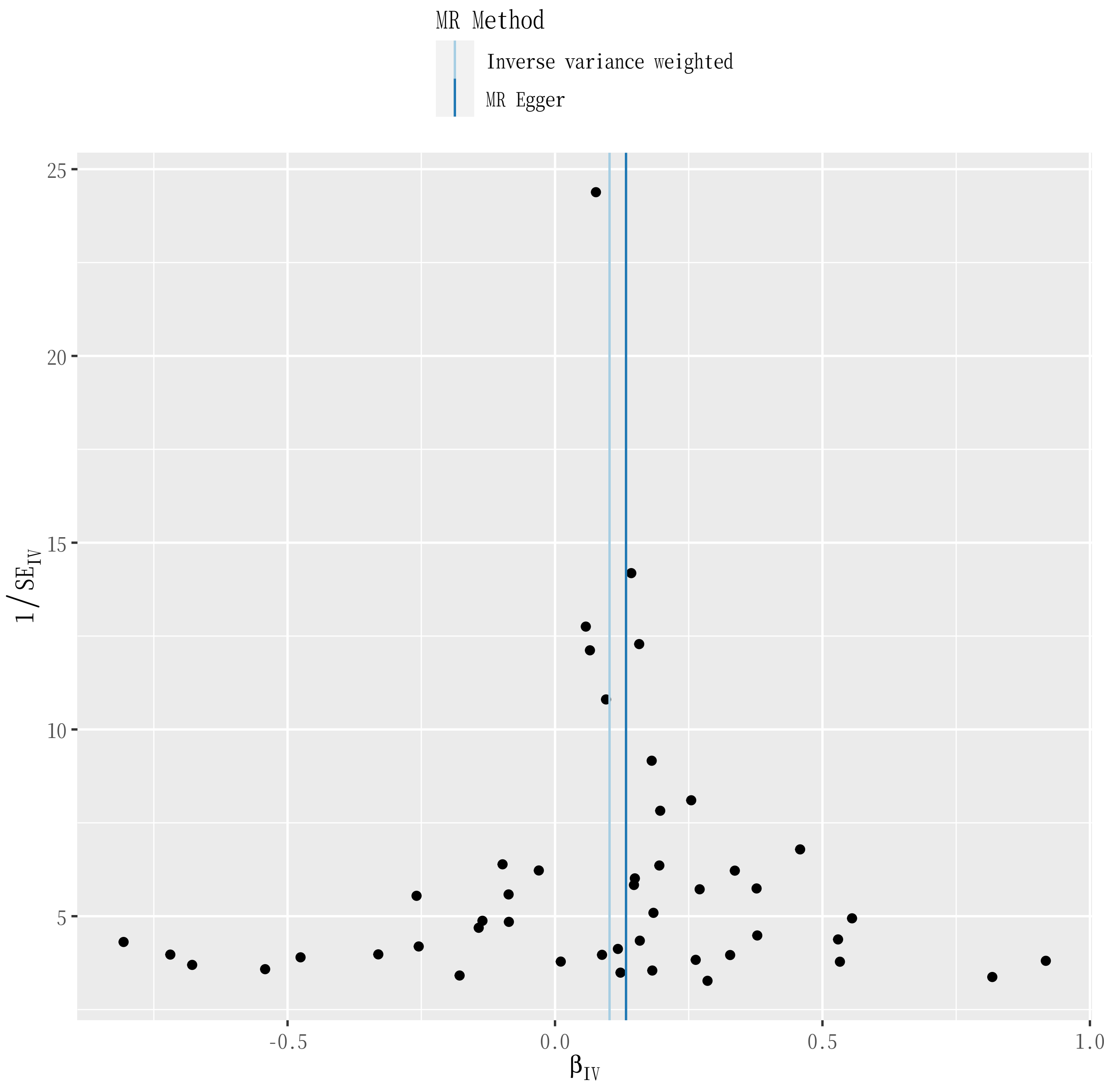
MR Method
Inverse variance weighted
MR Egger
25
20
15
10
5
$1/SE_{IV}$
-0.5
0.0
0.5
1.0
β_{IV}

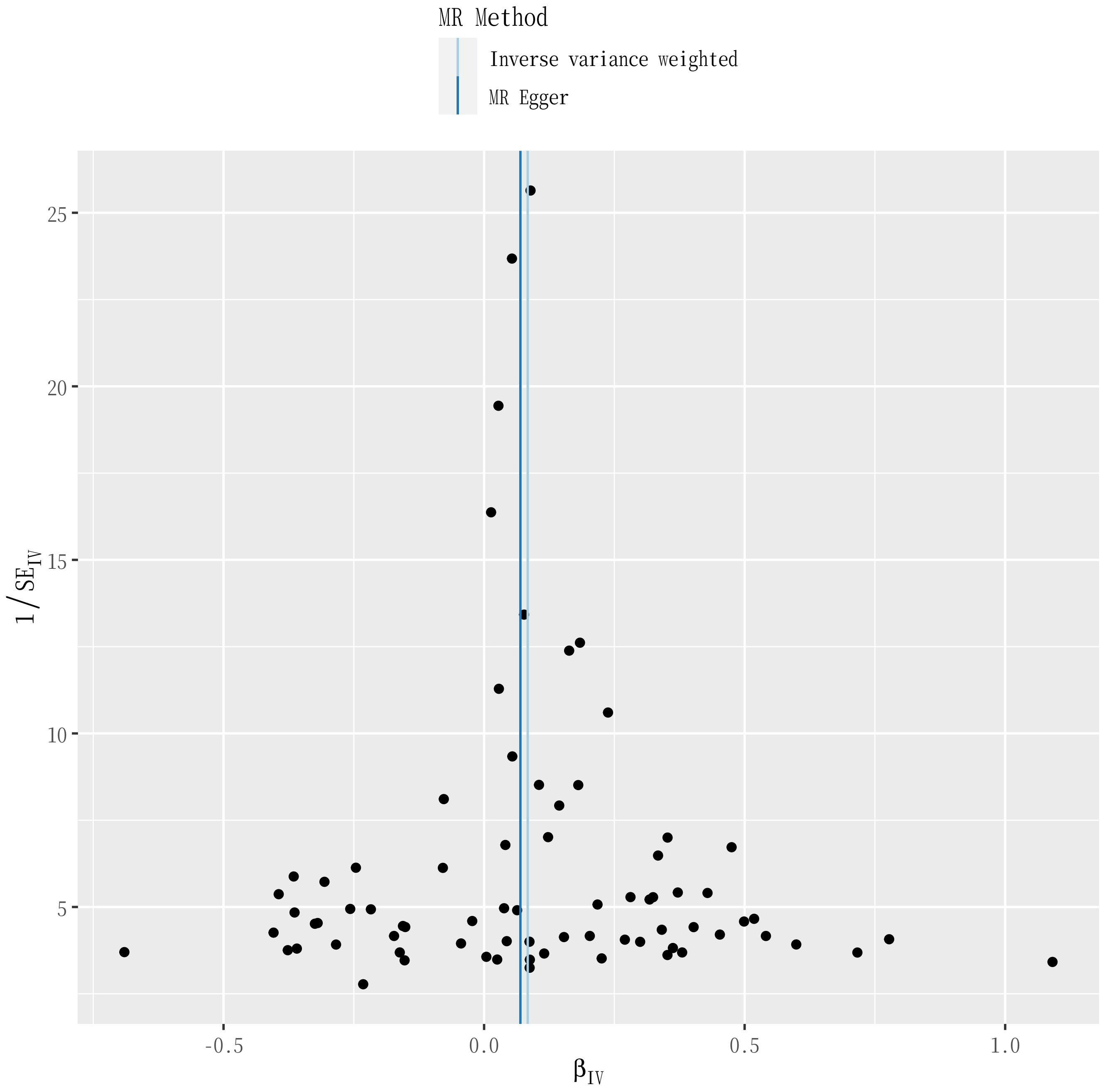

MR Method
Inverse variance weighted
MR Egger
25
20
15
10
5
$1/SE_{IV}$
-0.5
0.0
0.5
1.0
β_{IV}

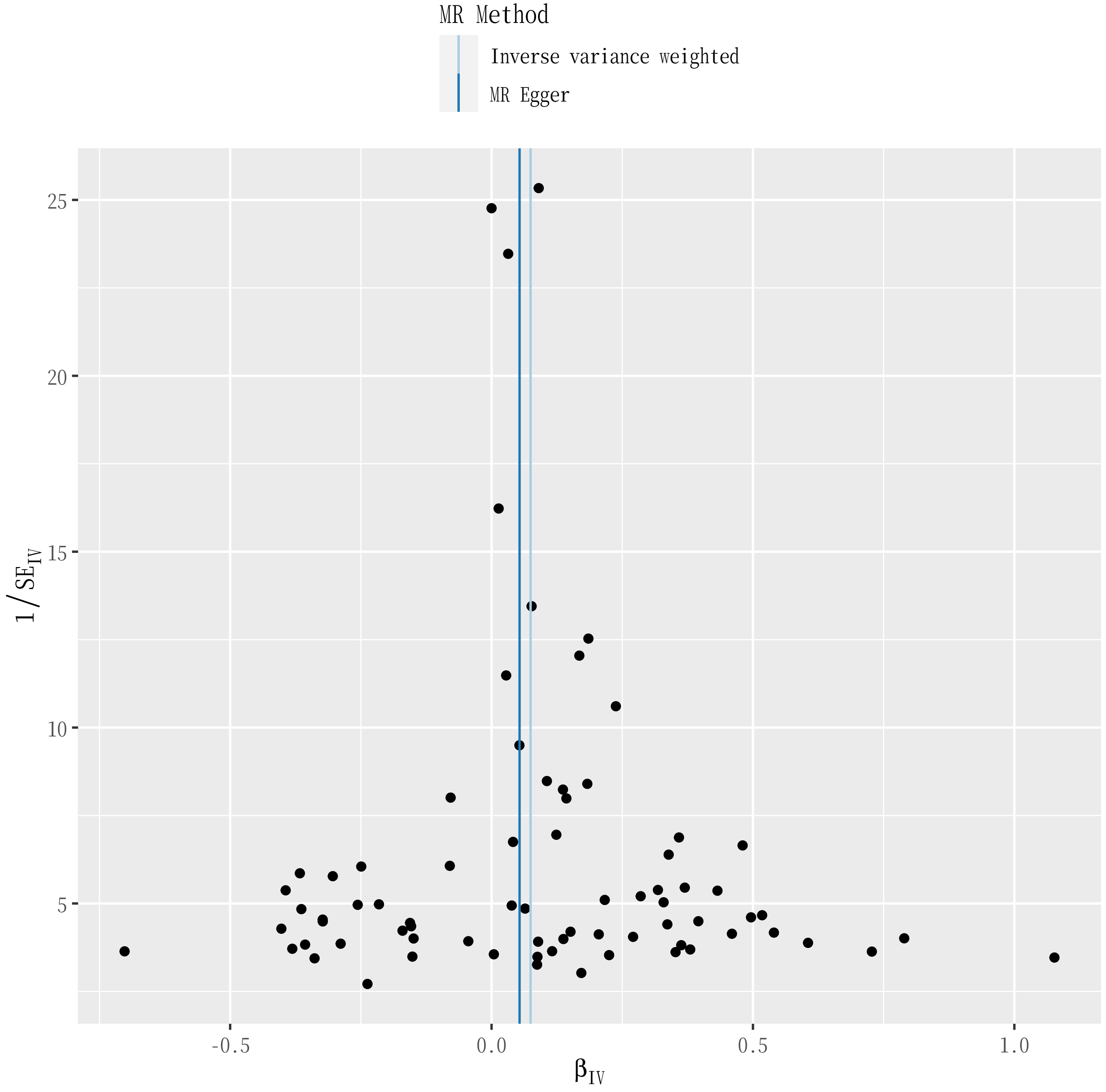

MR Method
Inverse variance weighted
MR Egger
25
20
15
10
5
-0.5
0.0
0.5
1.0
$1/SE_{IV}$
β_{IV}

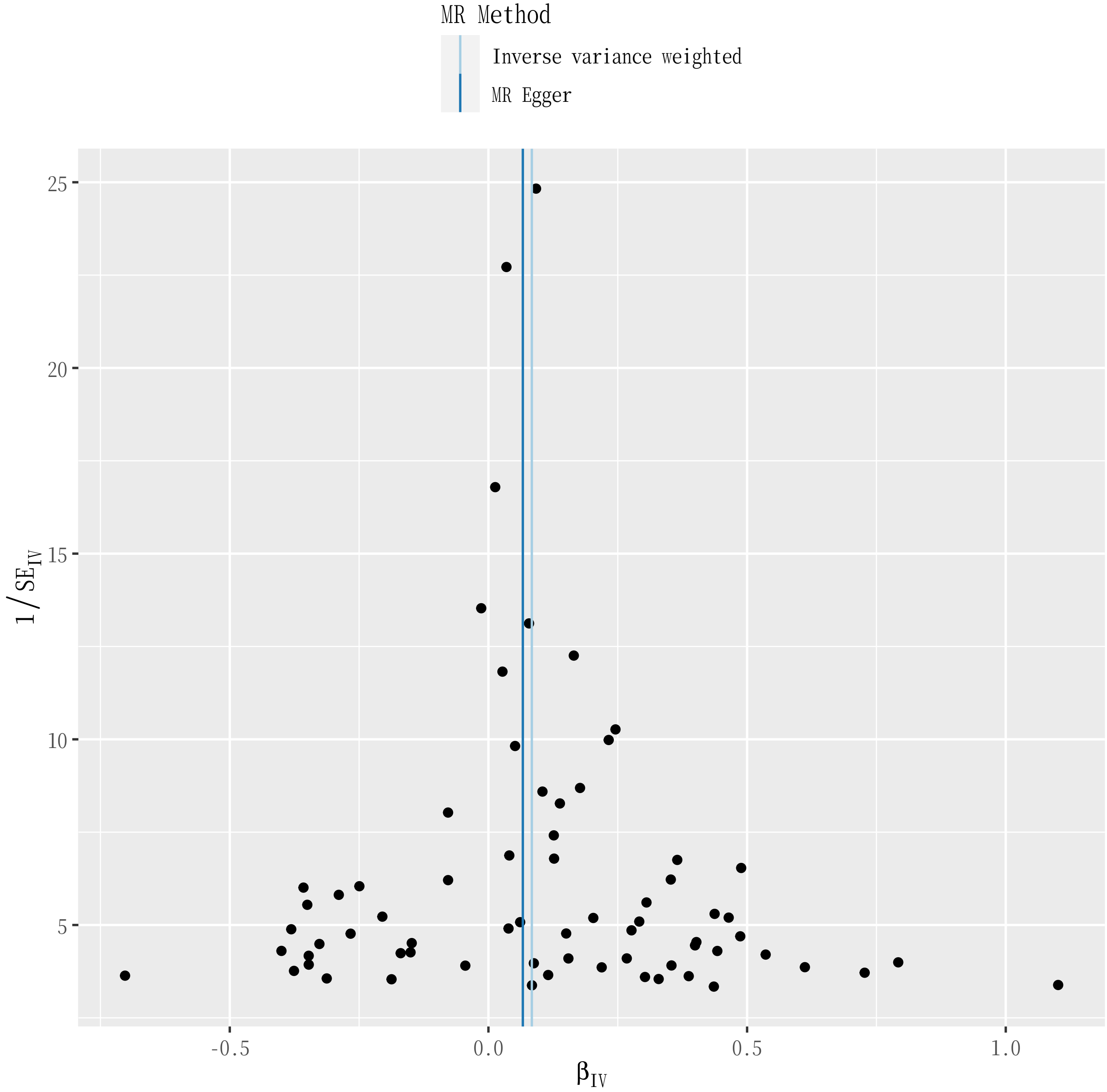
MR Method
Inverse variance weighted
MR Egger
25
20
15
10
5
1/SE_IV
-0.5
0.0
0.5
1.0
β_IV

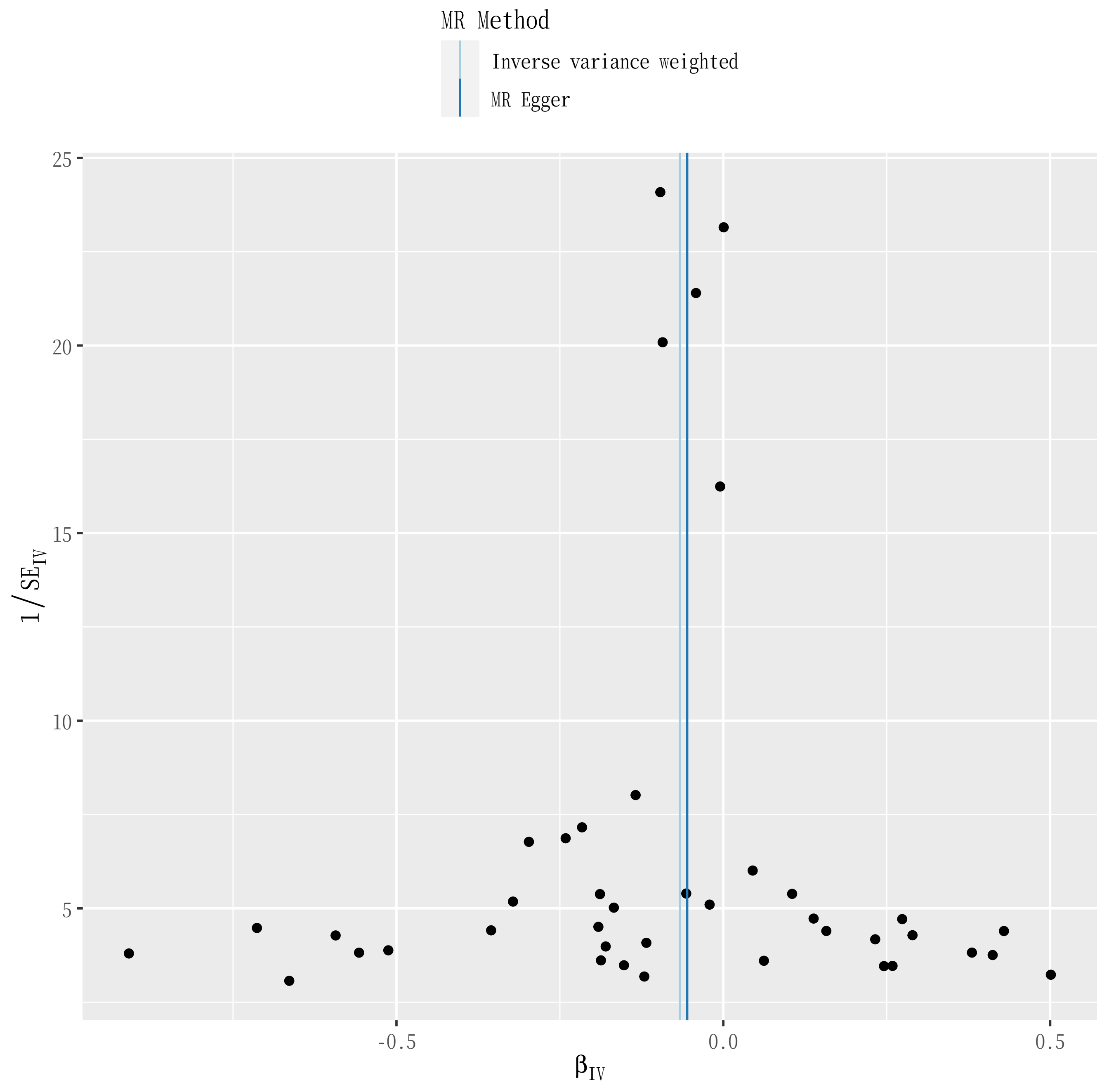
MR Method
Inverse variance weighted
MR Egger
25
20
15
10
5
1/SE_IV
-0.5
0.0
0.5
β_IV

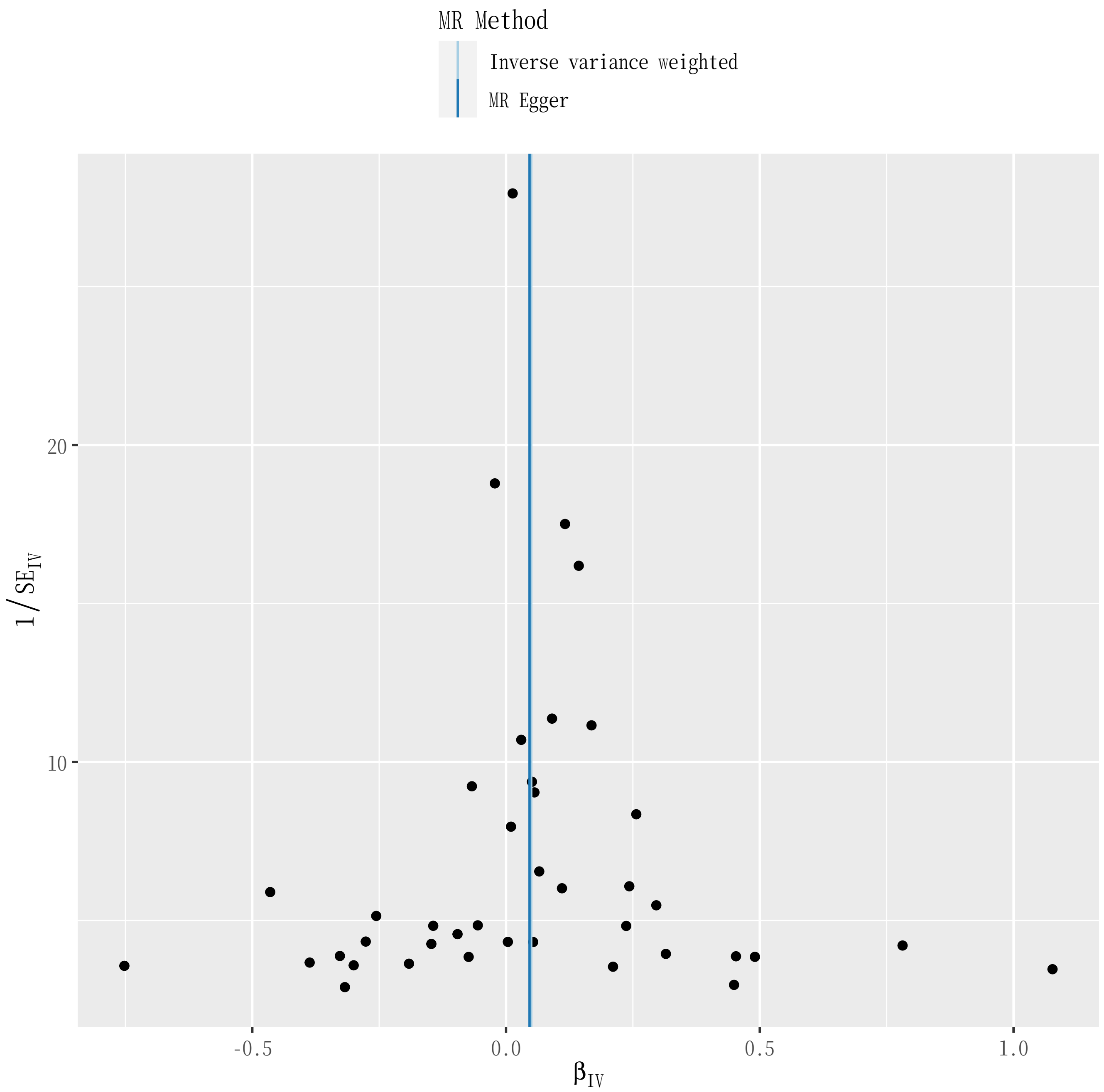
MR Method
Inverse variance weighted
MR Egger
20
10
-0.5
0.0
0.5
1.0
$1/SE_{IV}$
β_{IV}

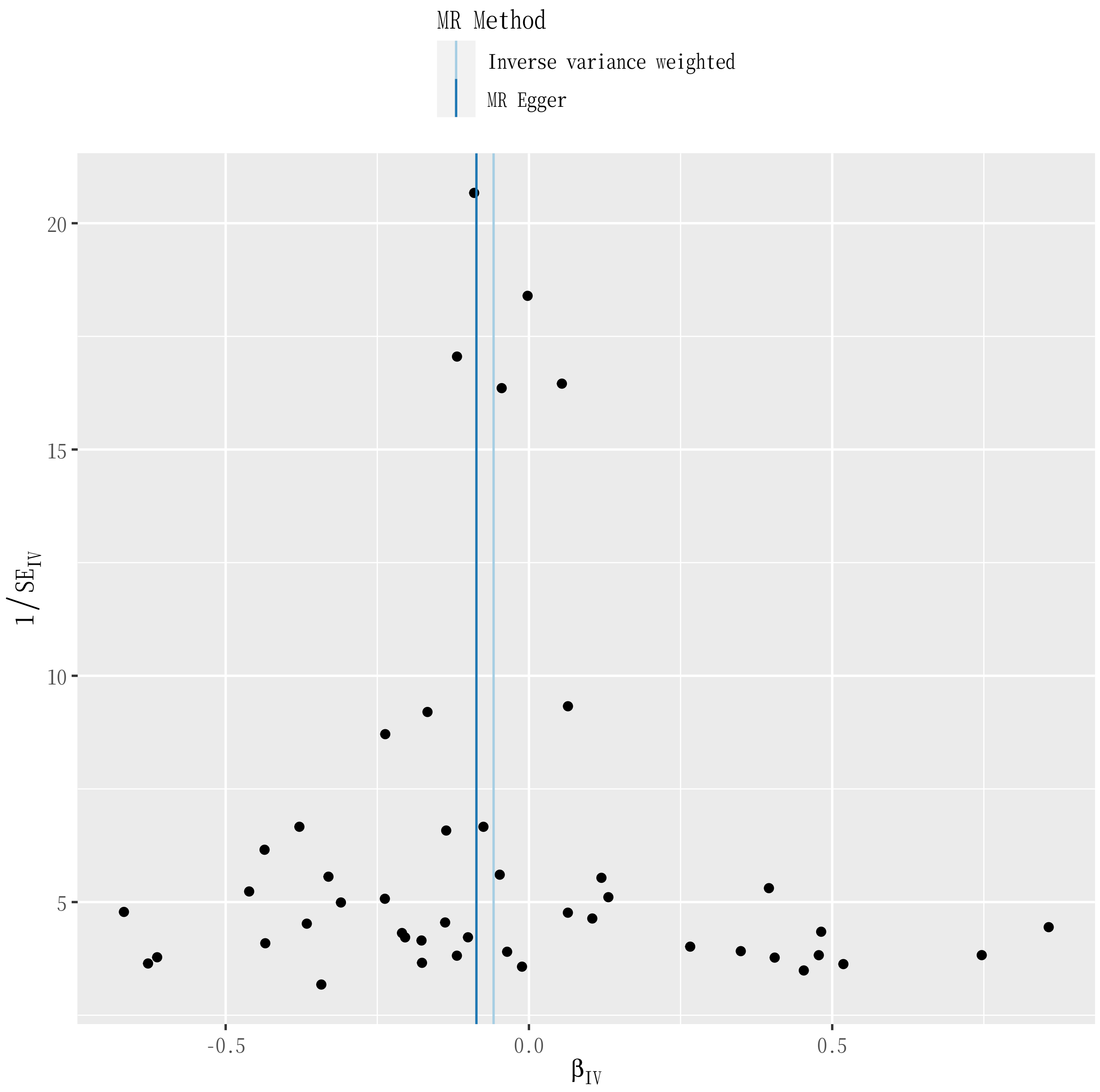
MR Method
Inverse variance weighted
MR Egger
20
15
10
5
1/SE_IV
-0.5
0.0
0.5
β_IV

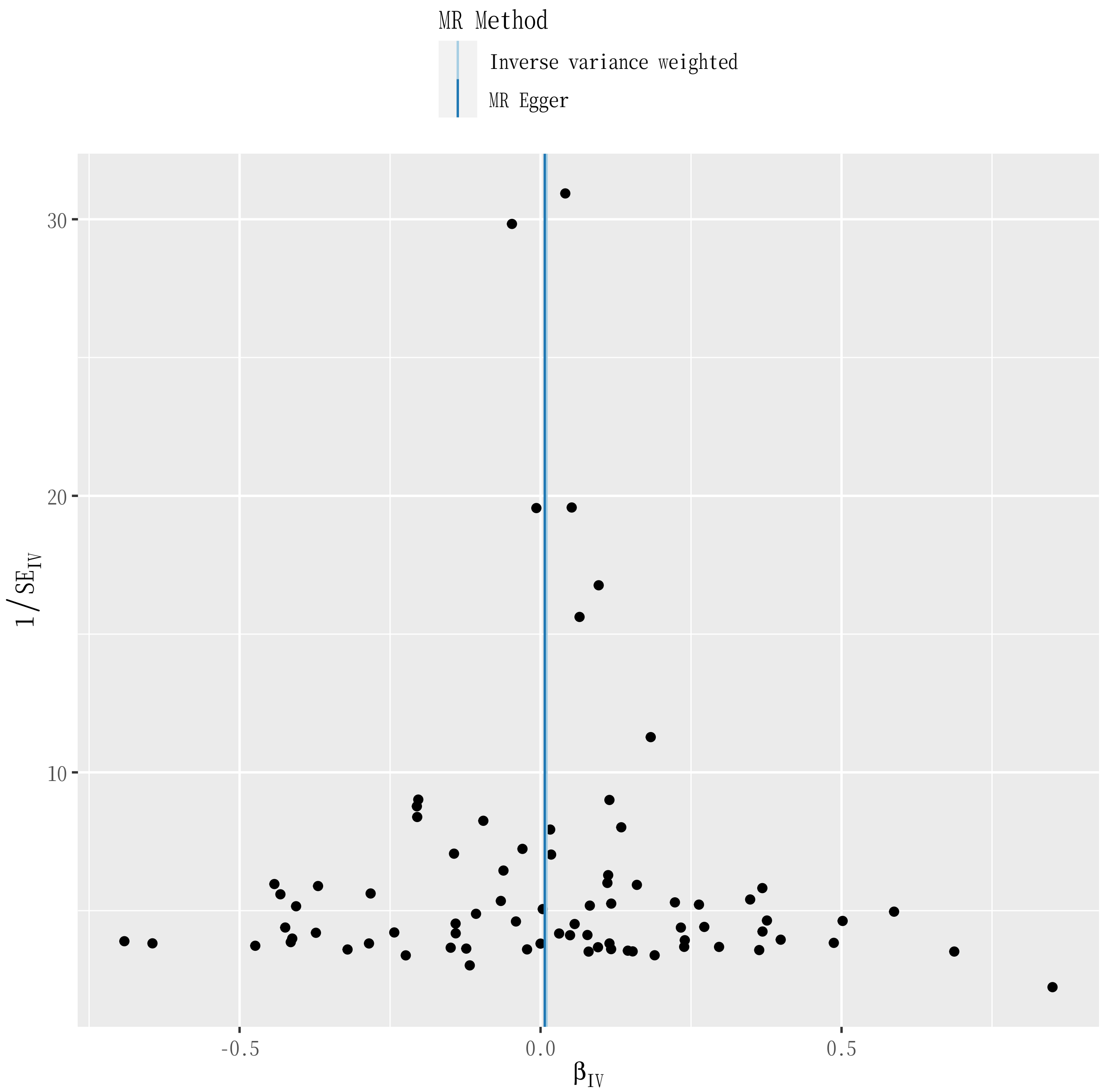
MR Method
Inverse variance weighted
MR Egger
30
20
10
$1/SE_{IV}$
-0.5
0.0
0.5
β_{IV}

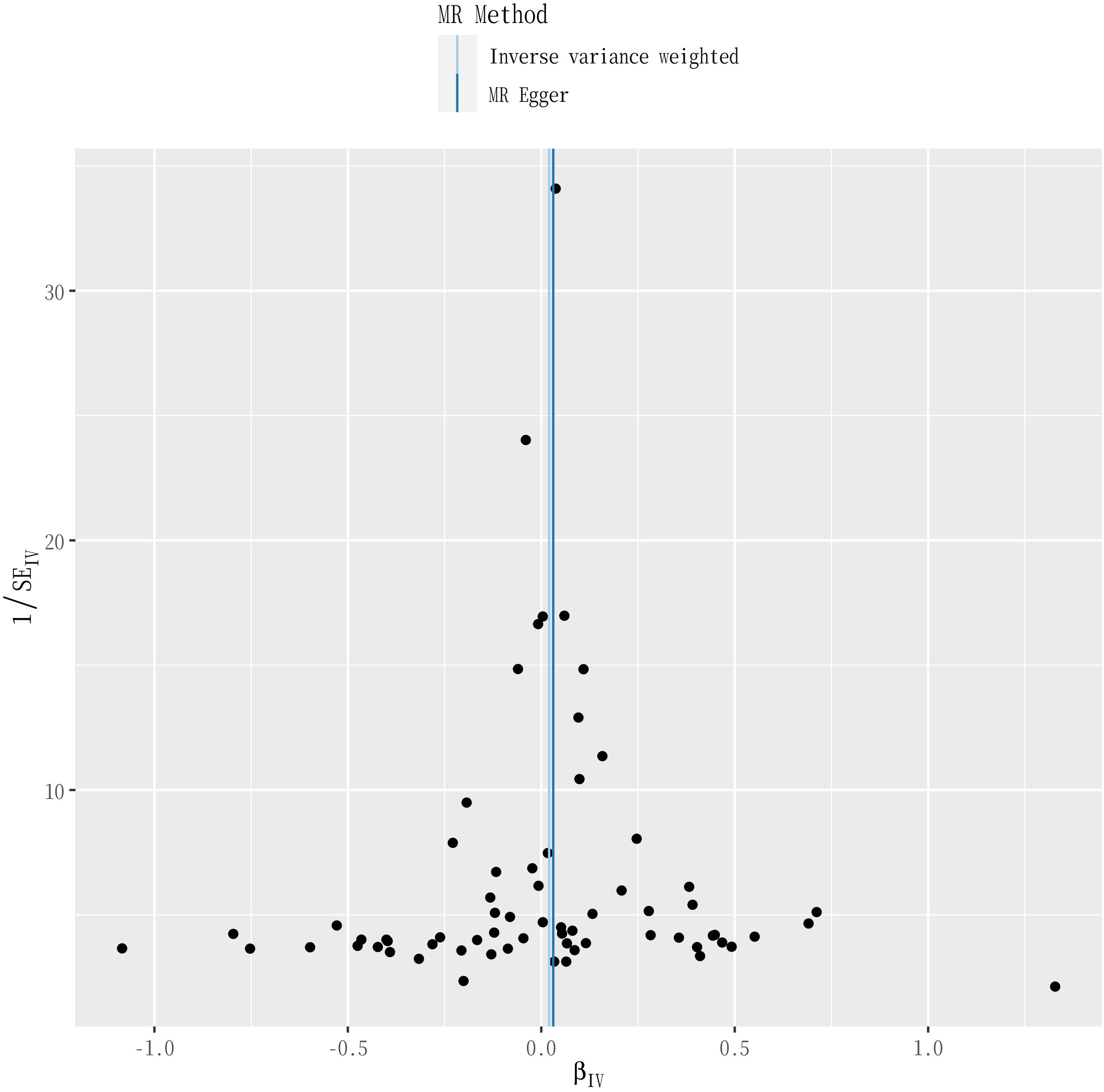
MR Method
Inverse variance weighted
MR Egger
30
20
10
-1.0
-0.5
0.0
0.5
1.0
$1/SE_{IV}$
β_{IV}

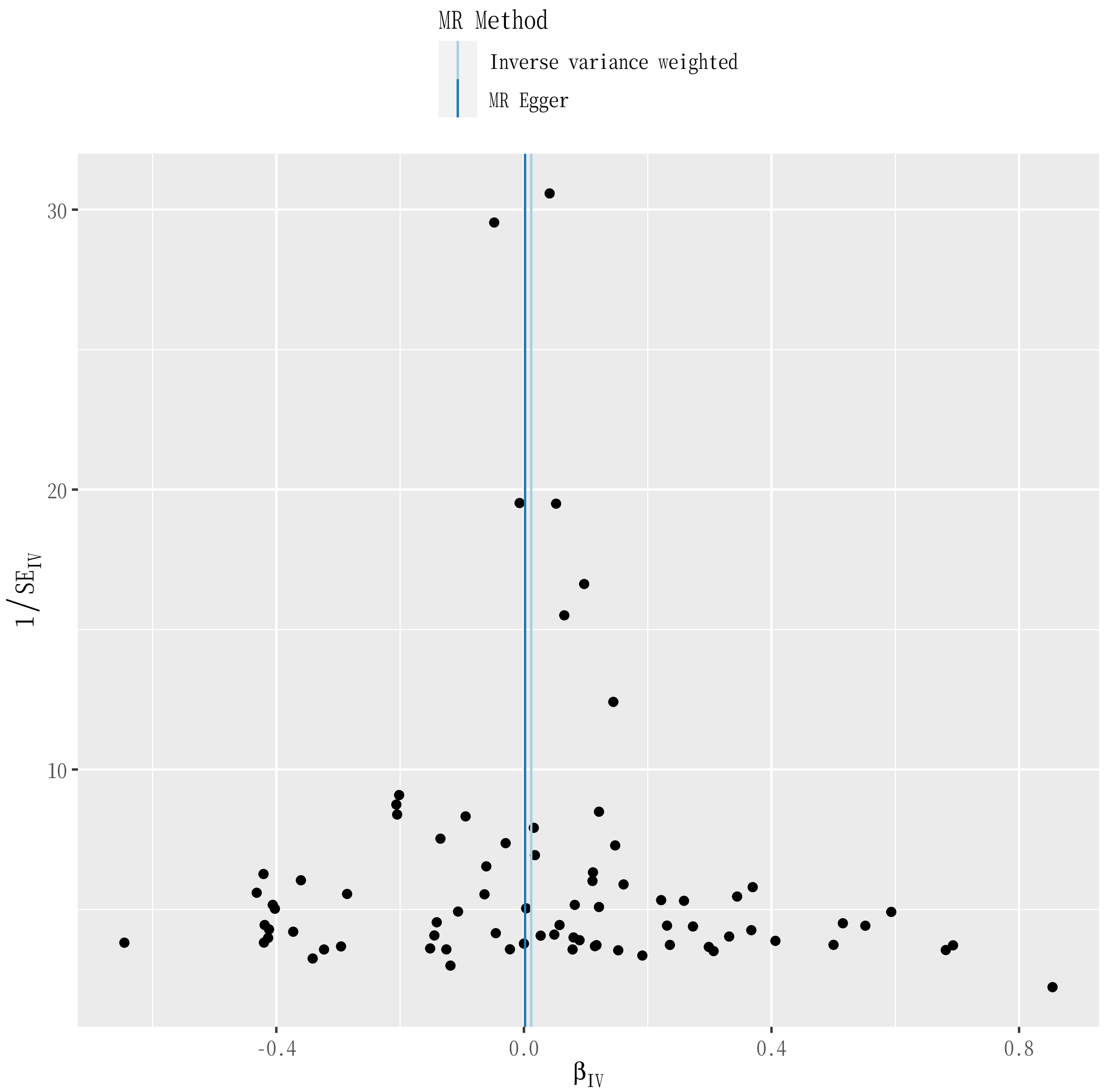

MR Method
Inverse variance weighted
MR Egger
30
20
10
$1/SE_{IV}$
-0.4
0.0
0.4
0.8
β_{IV}

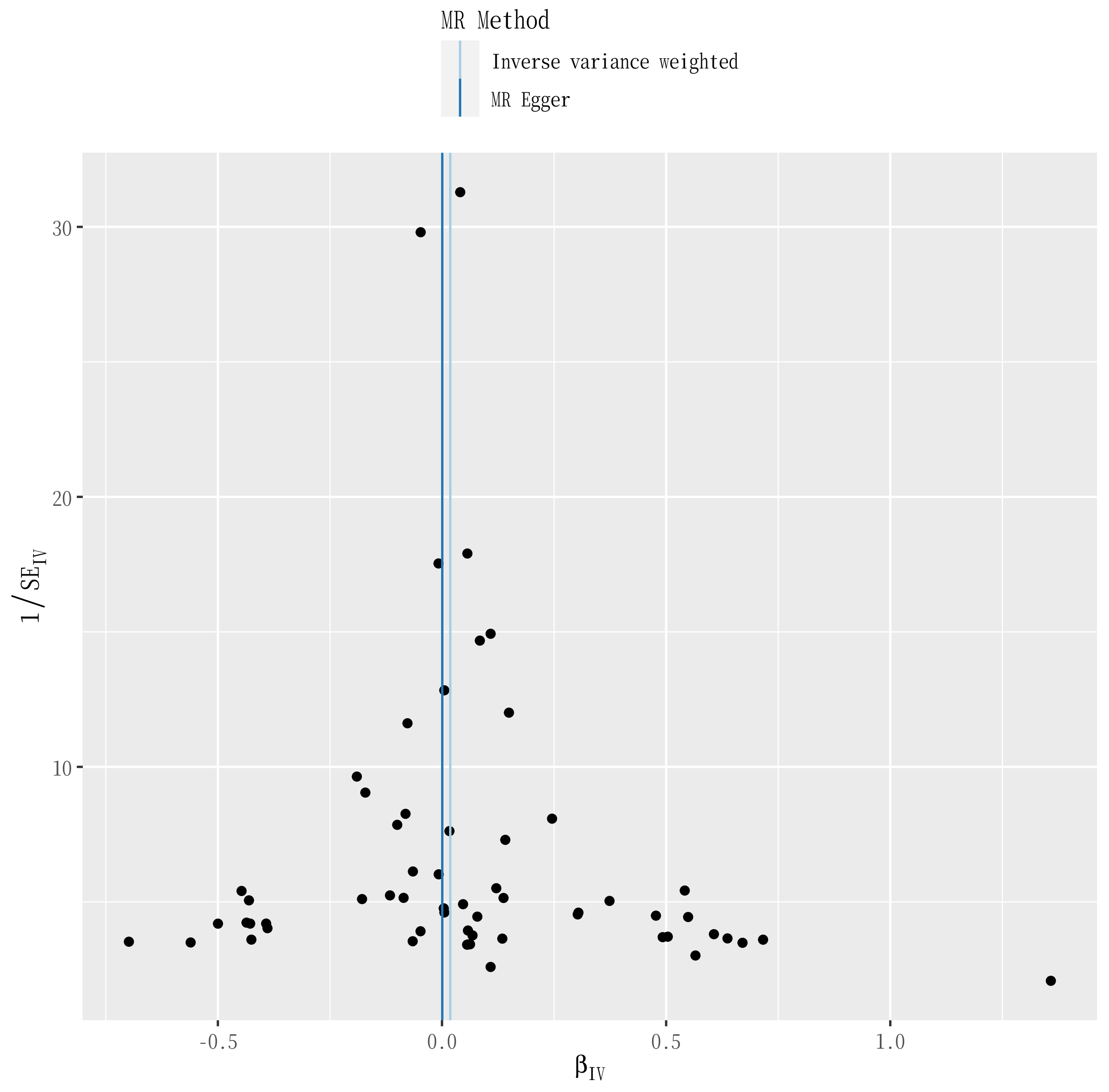

MR Method
Inverse variance weighted
MR Egger
30
20
10
-0.5
0.0
0.5
1.0
$1/SE_{IV}$
β_{IV}

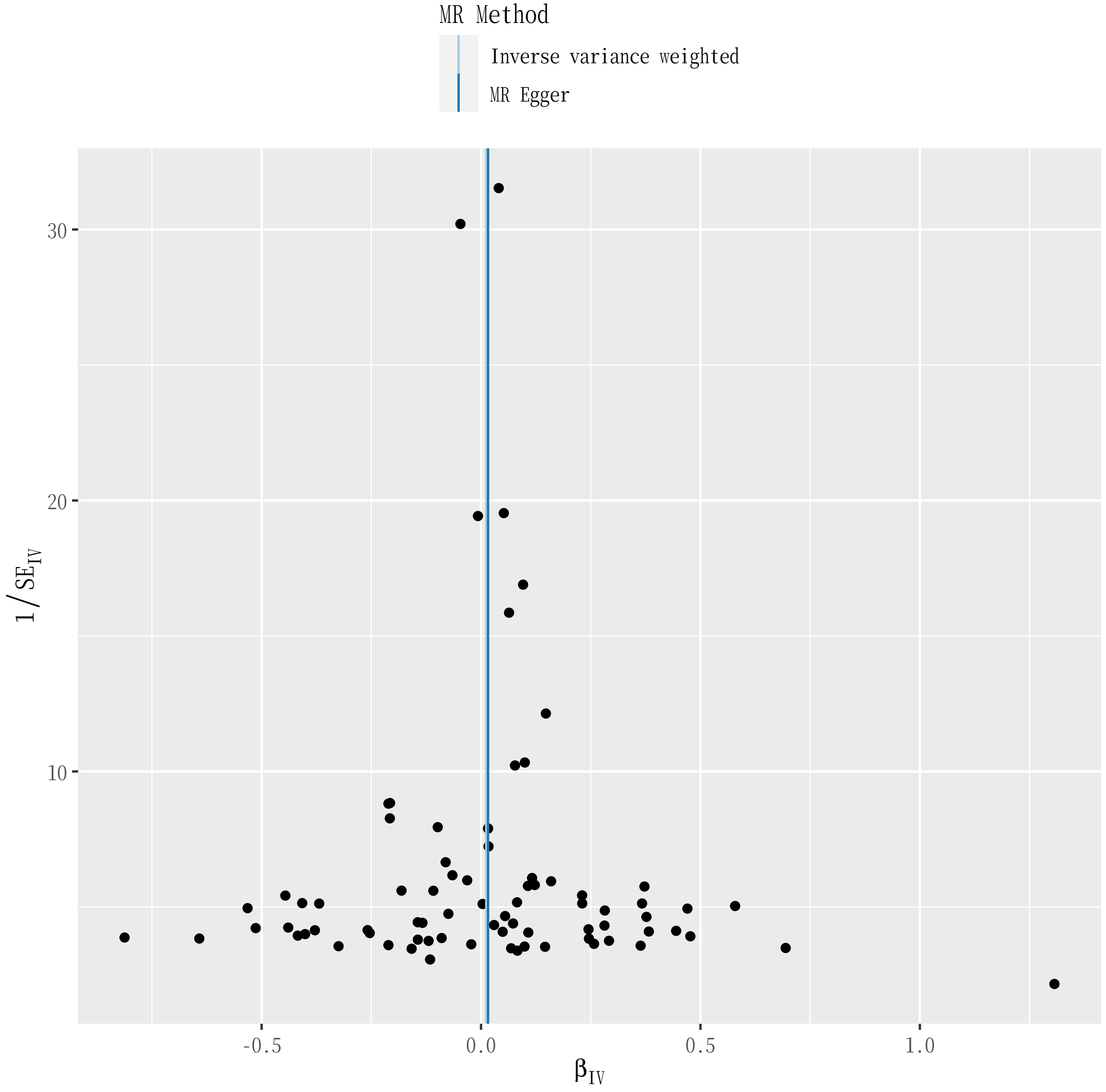
MR Method
Inverse variance weighted
MR Egger
30
20
10
-0.5
0.0
0.5
1.0
1/SE_IV
β_IV

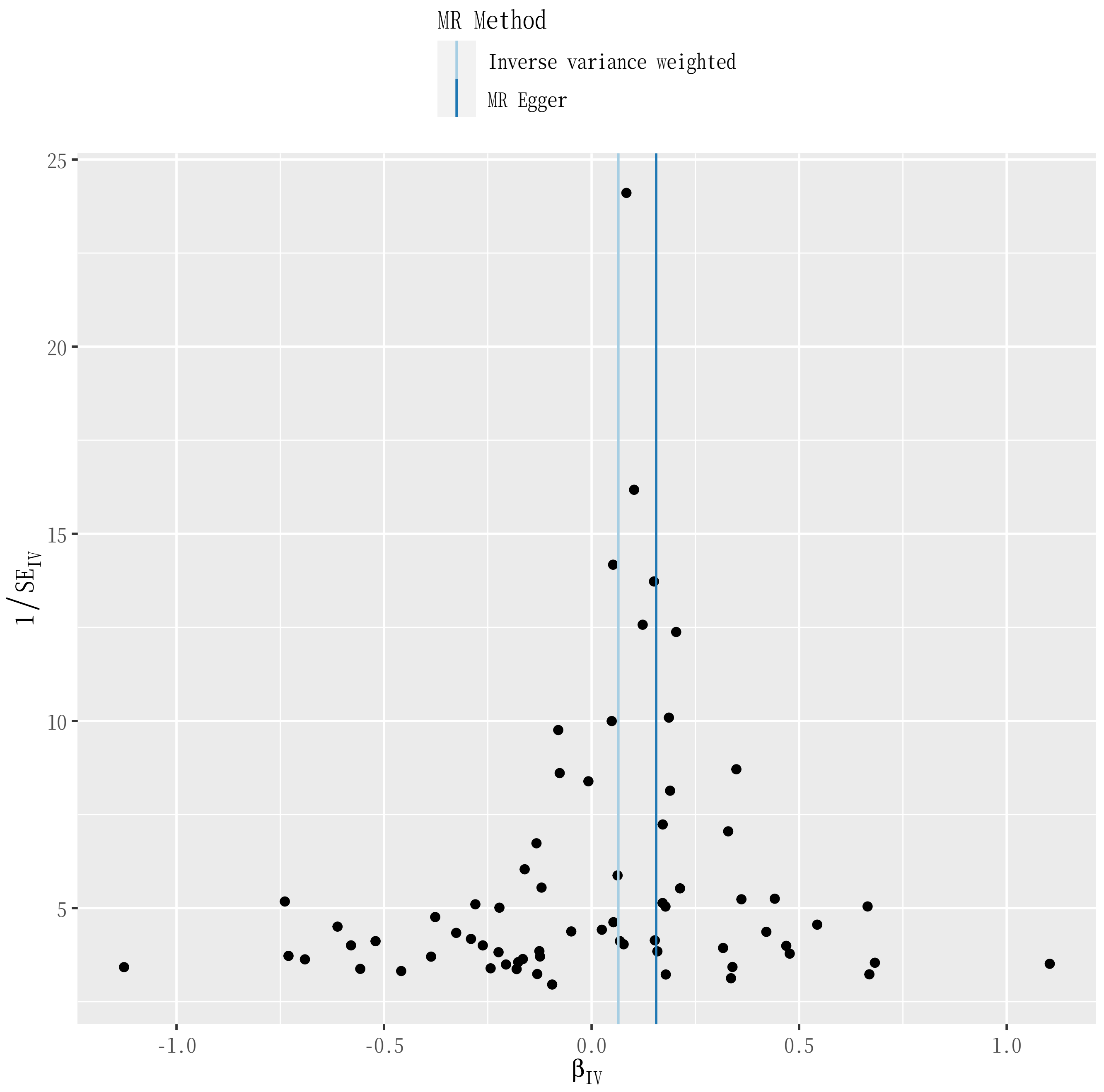

MR Method
Inverse variance weighted
MR Egger
25
20
15
10
5
1/SE_IV
-1.0
-0.5
0.0
0.5
1.0
β_IV

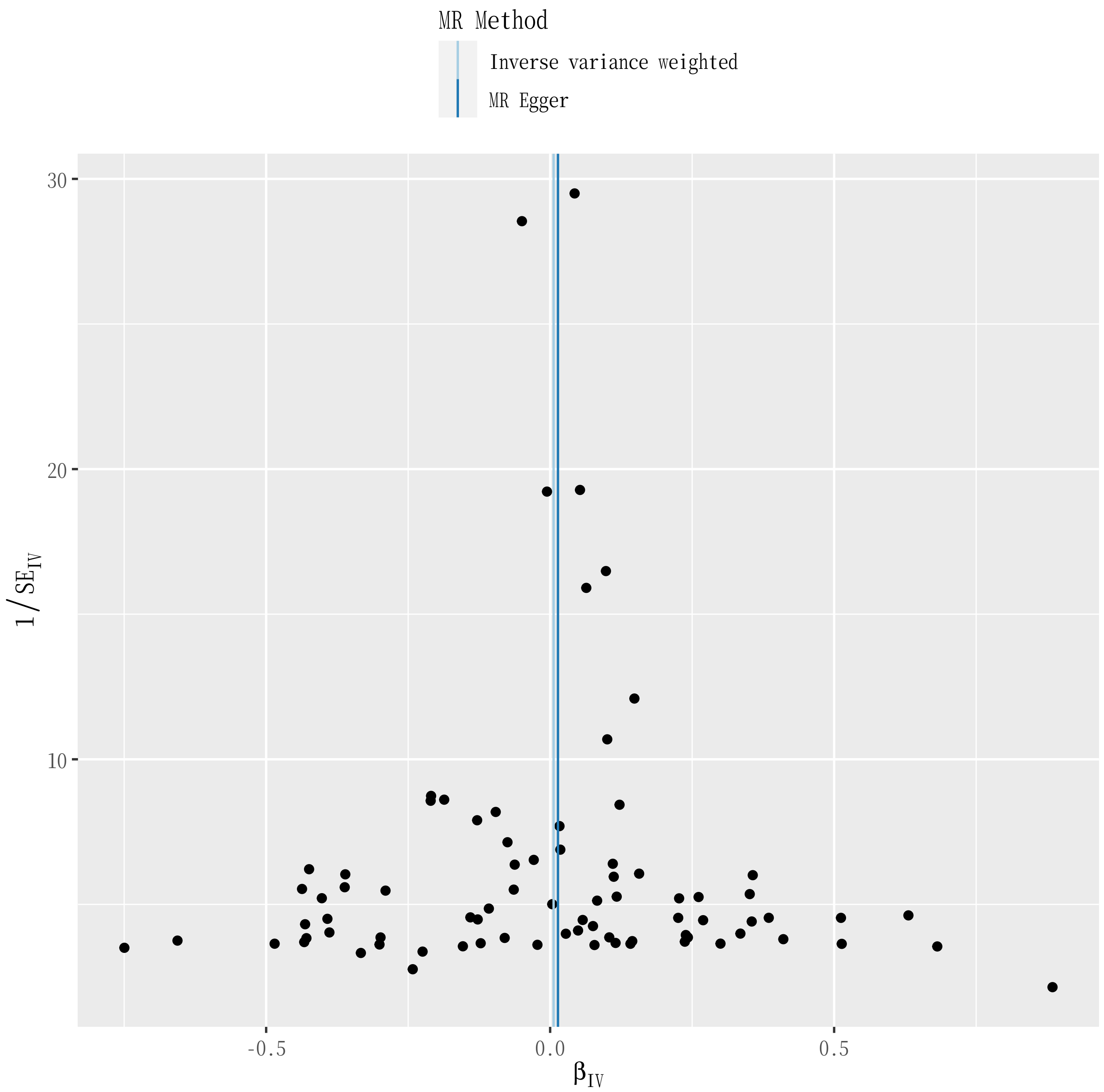

MR Method
Inverse variance weighted
MR Egger
30
20
10
1/SE_IV
-0.5
0.0
0.5
β_IV

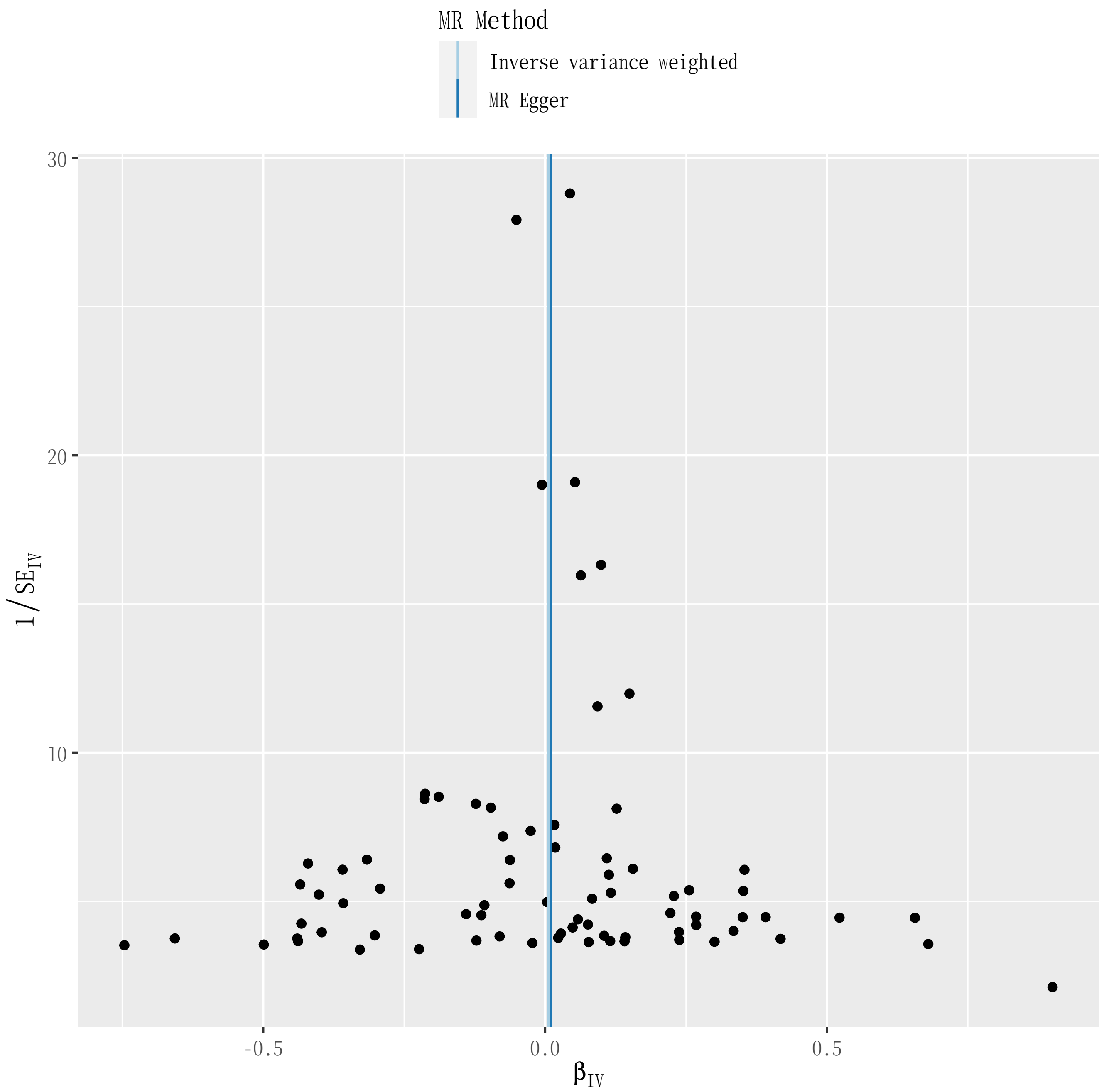
MR Method
Inverse variance weighted
MR Egger
30
20
10
1/SE_IV
-0.5
0.0
0.5
β_IV

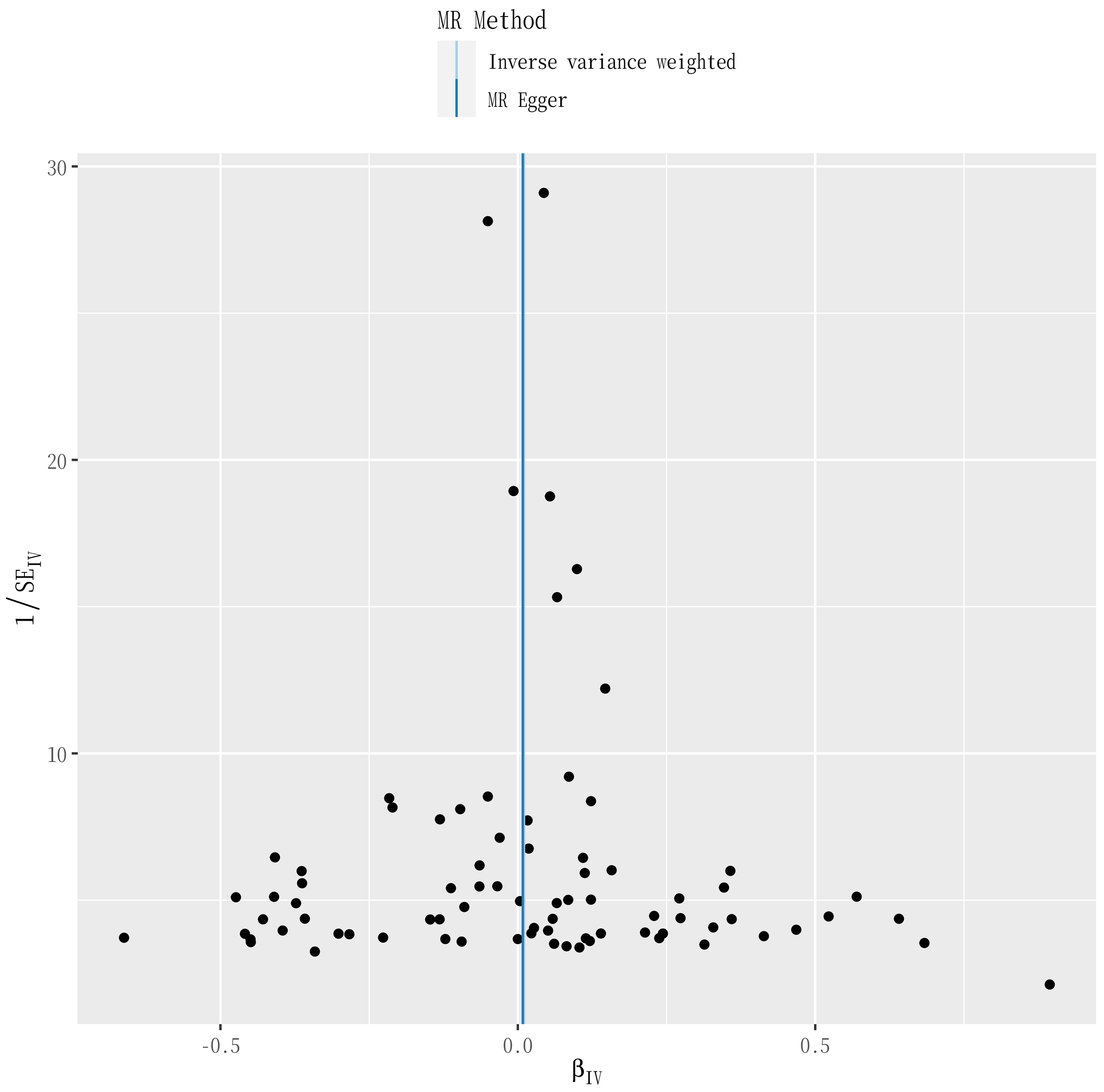
MR Method
Inverse variance weighted
MR Egger
30
20
10
-0.5
0.0
0.5
$1/SE_{IV}$
β_{IV}

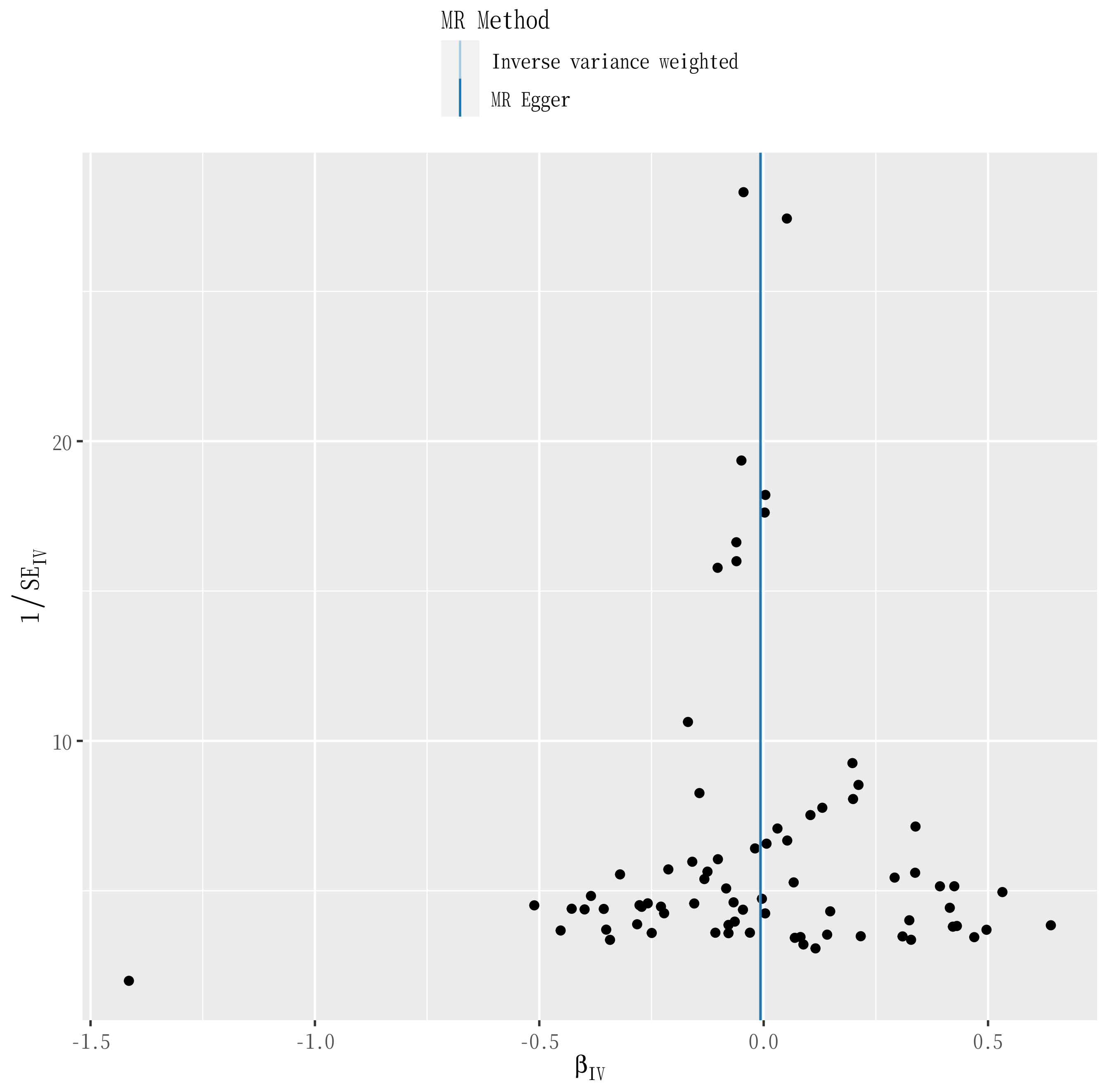

MR Method
Inverse variance weighted
MR Egger
20
10
1/SE_{IV}
-1.5
-1.0
-0.5
0.0
0.5
β_{IV}

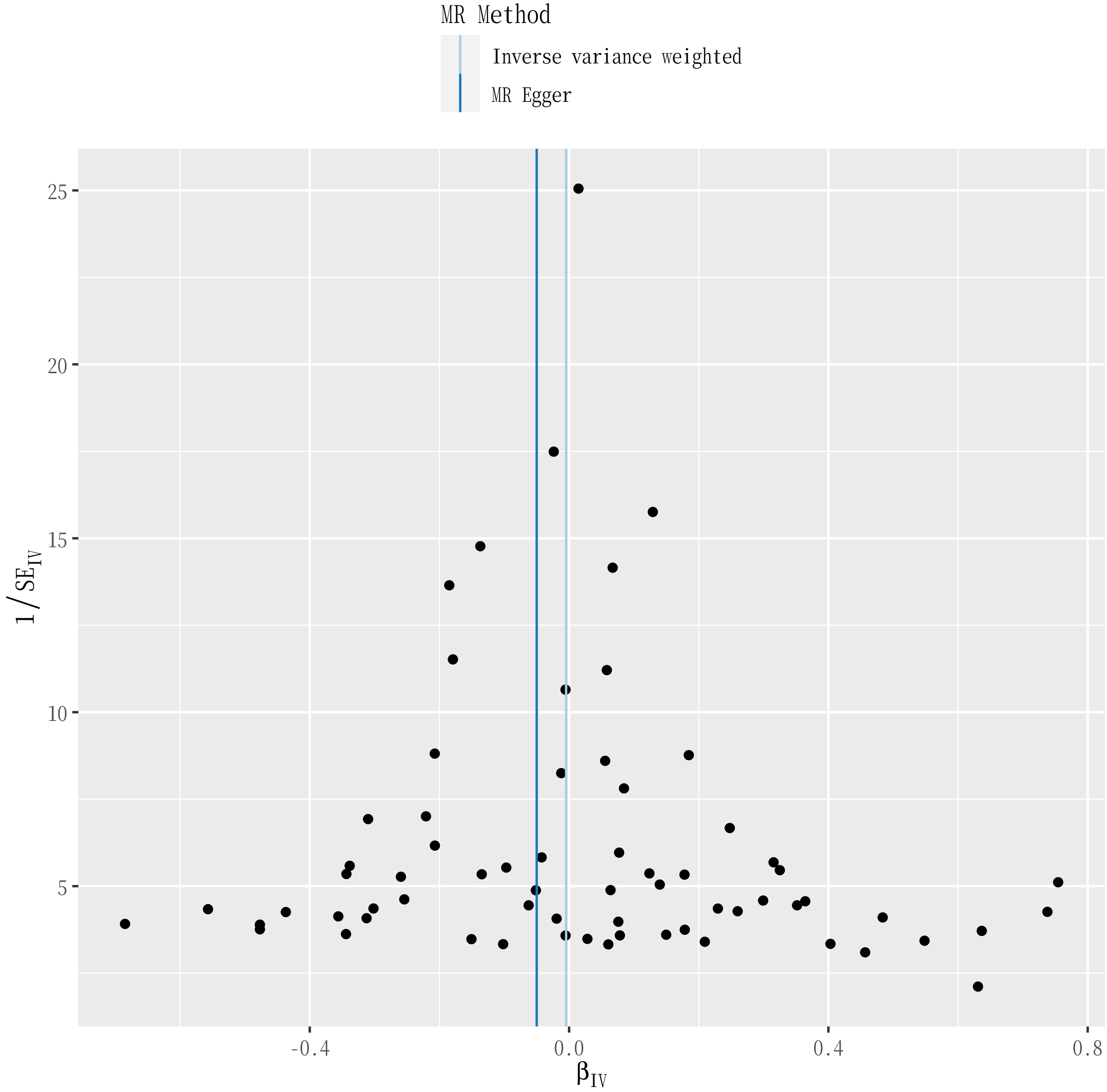

MR Method
Inverse variance weighted
MR Egger
25
20
15
10
5
1/SE_IV
-0.4
0.0
0.4
0.8
β_IV

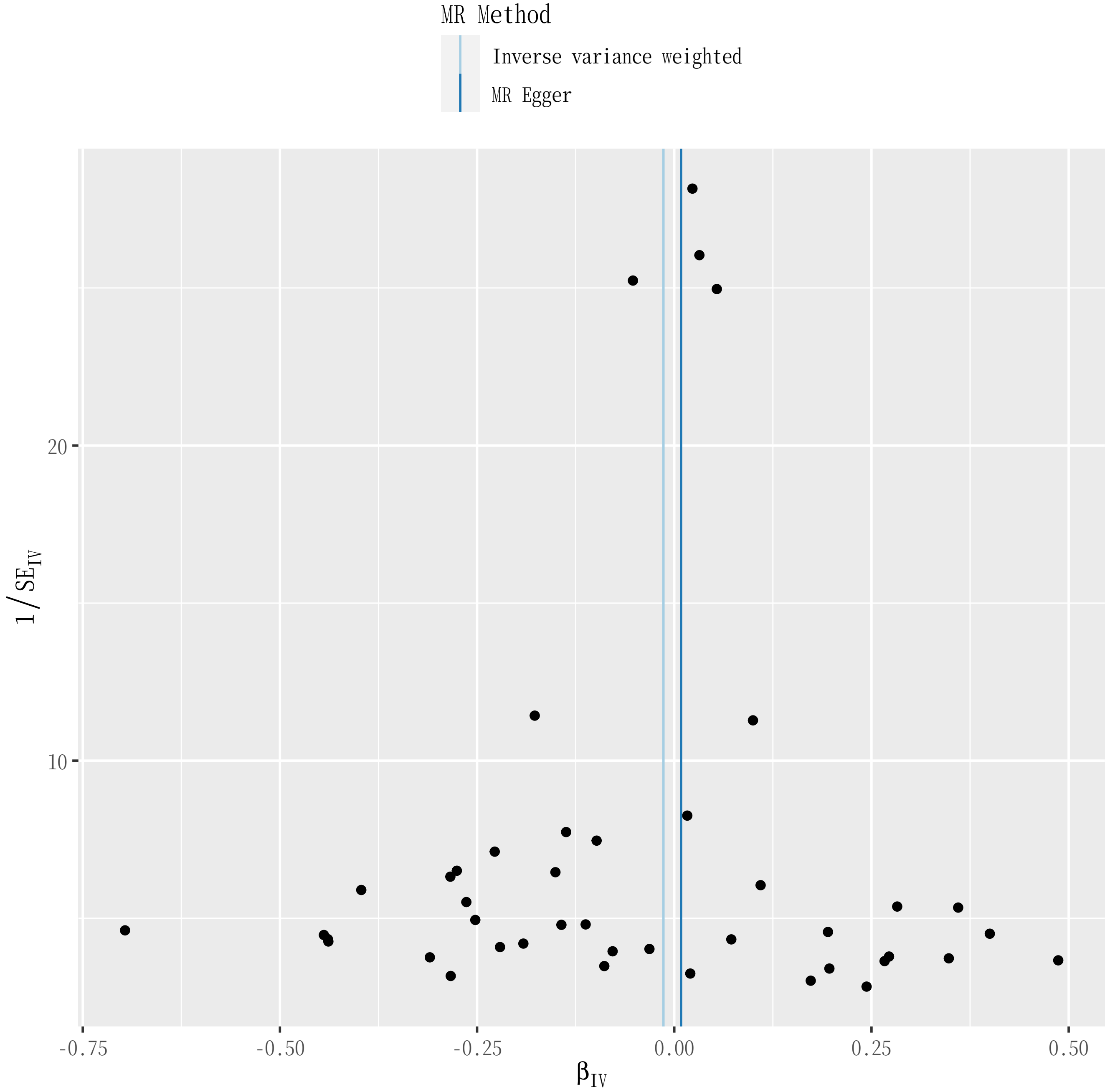
MR Method
Inverse variance weighted
MR Egger
$1/SE_{IV}$
20
10
-0.75
-0.50
-0.25
0.00
0.25
0.50
β_{IV}

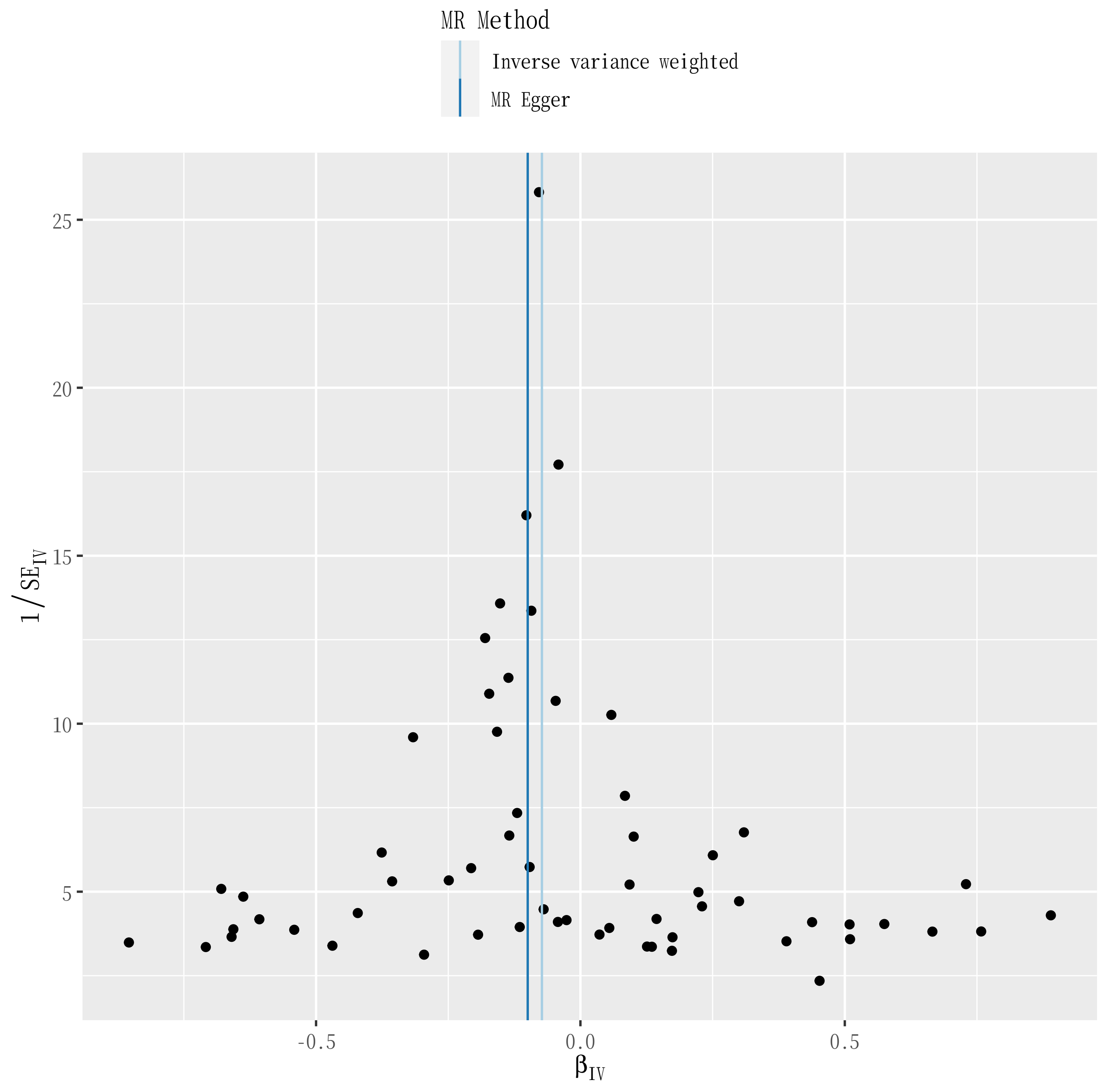
MR Method
Inverse variance weighted
MR Egger
25
20
15
10
5
1/SE_IV
-0.5
0.0
0.5
β_IV

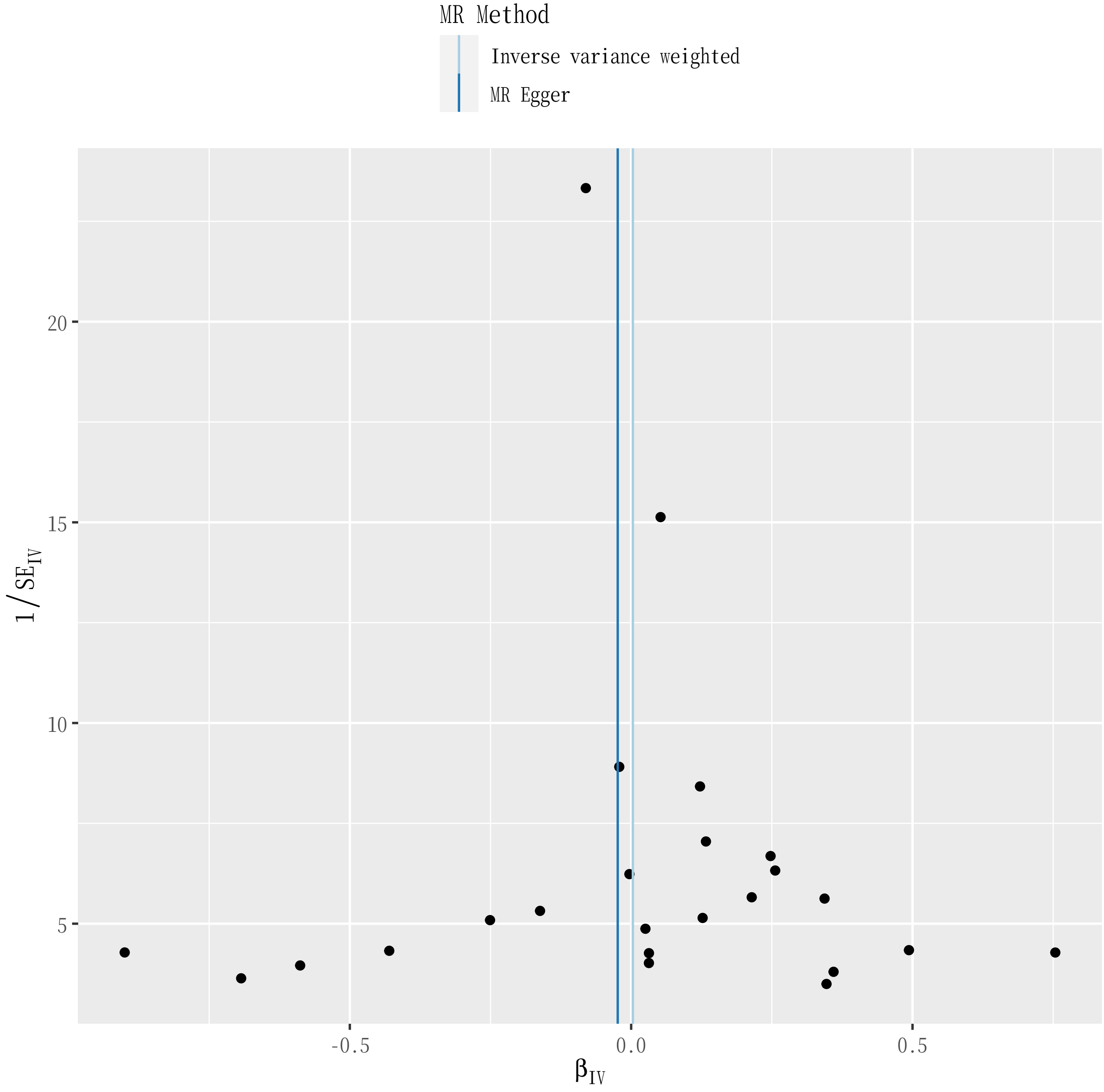
MR Method
Inverse variance weighted
MR Egger
1/SE_IV
20
15
10
5
-0.5
0.0
0.5
β_IV

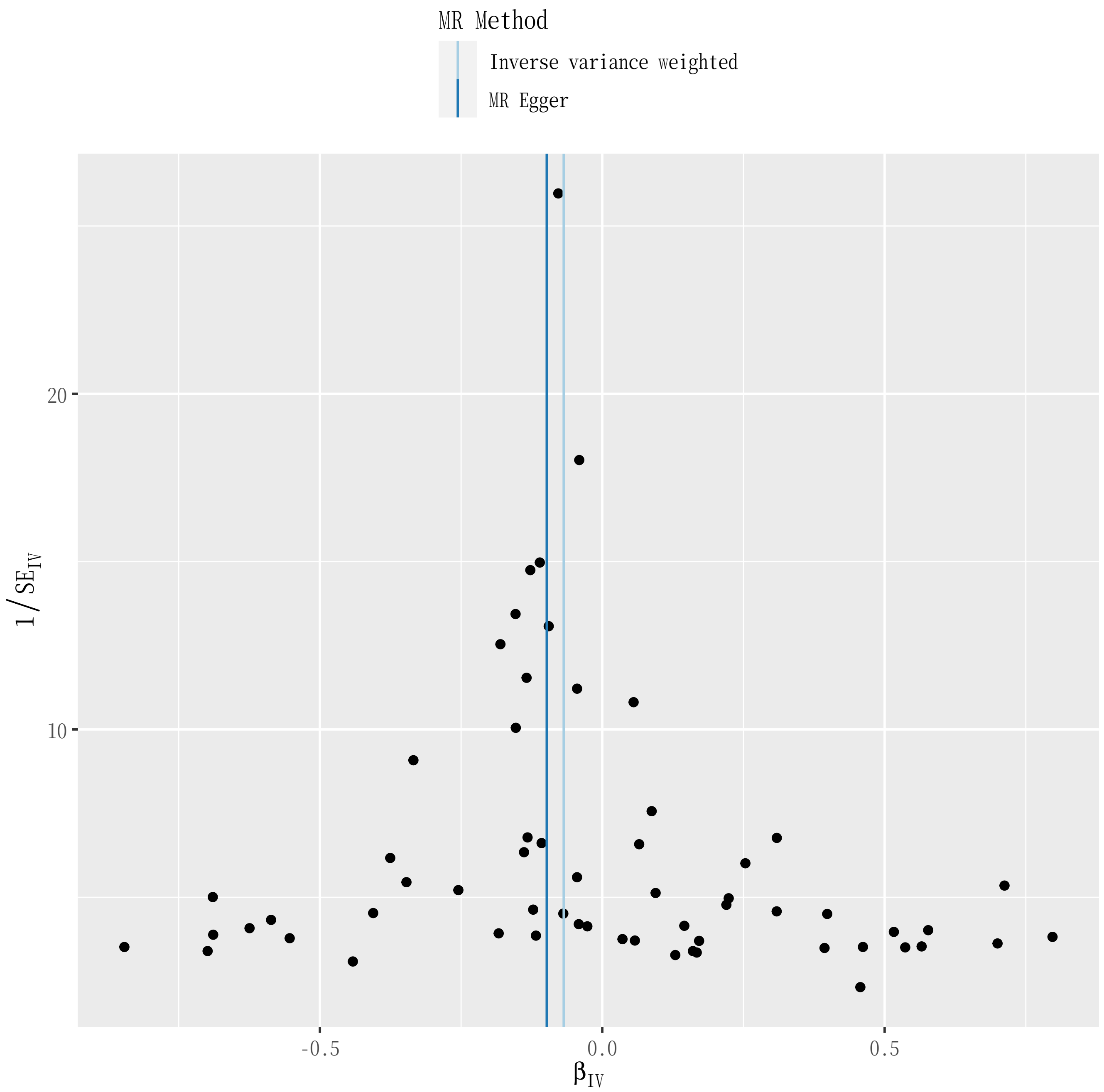
MR Method
Inverse variance weighted
MR Egger
20
10
-0.5
0.0
0.5
$1/SE_{IV}$
β_{IV}

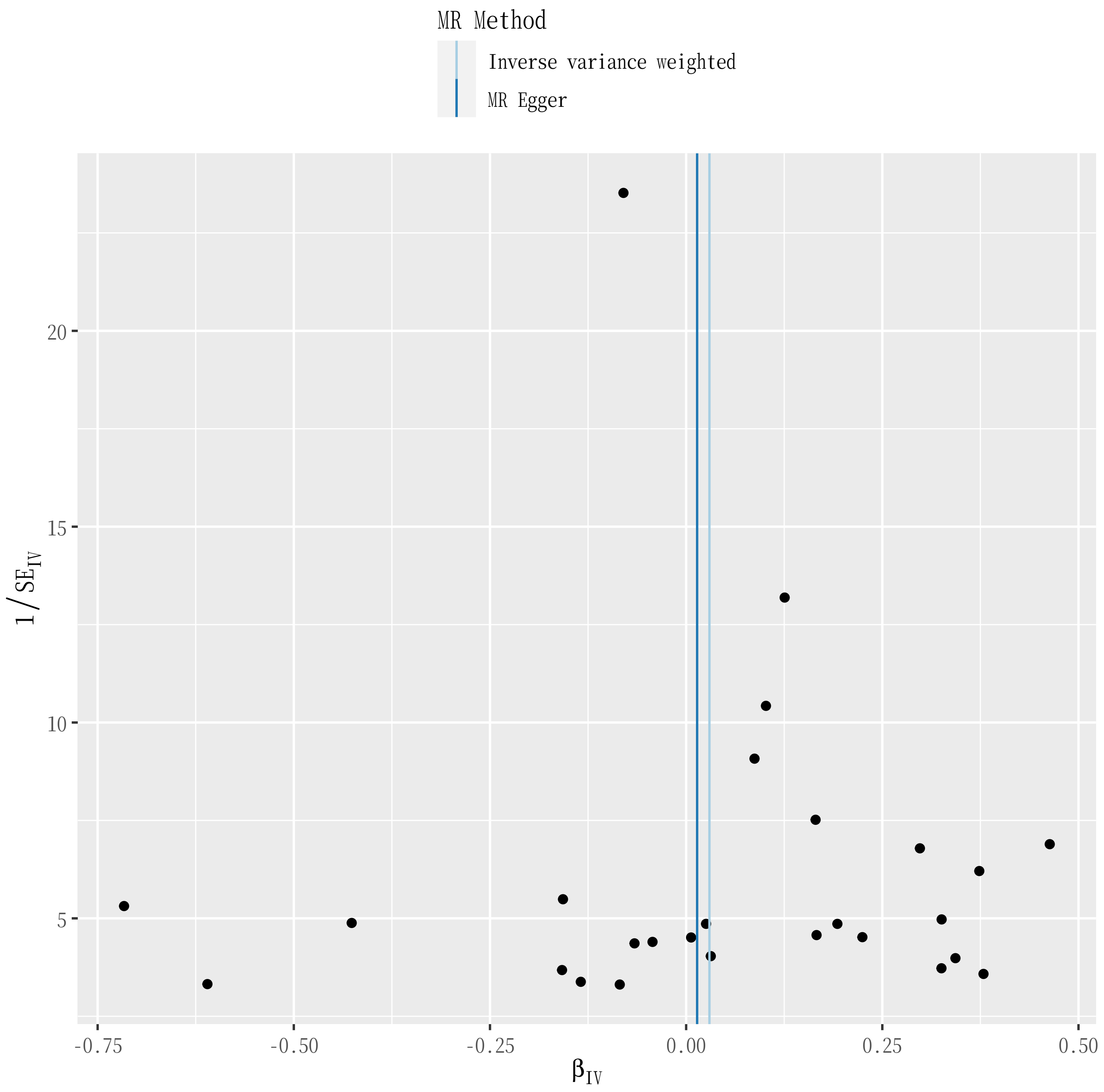
MR Method
Inverse variance weighted
MR Egger
$1/SE_{IV}$
20
15
10
5
-0.75
-0.50
-0.25
0.00
0.25
0.50
β_{IV}

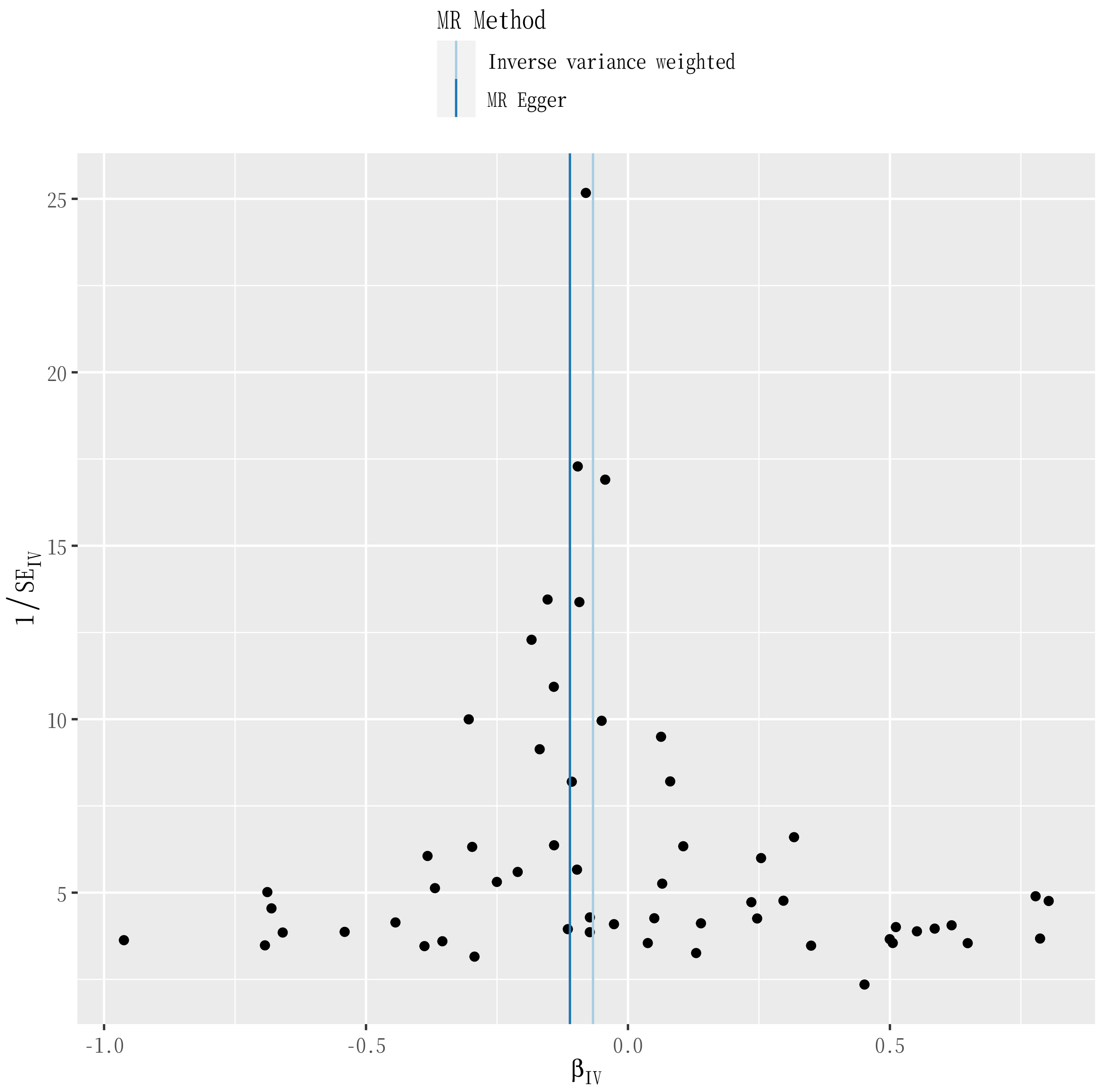

MR Method
Inverse variance weighted
MR Egger
25
20
15
10
5
1/SE_IV
-1.0
-0.5
0.0
0.5
β_IV

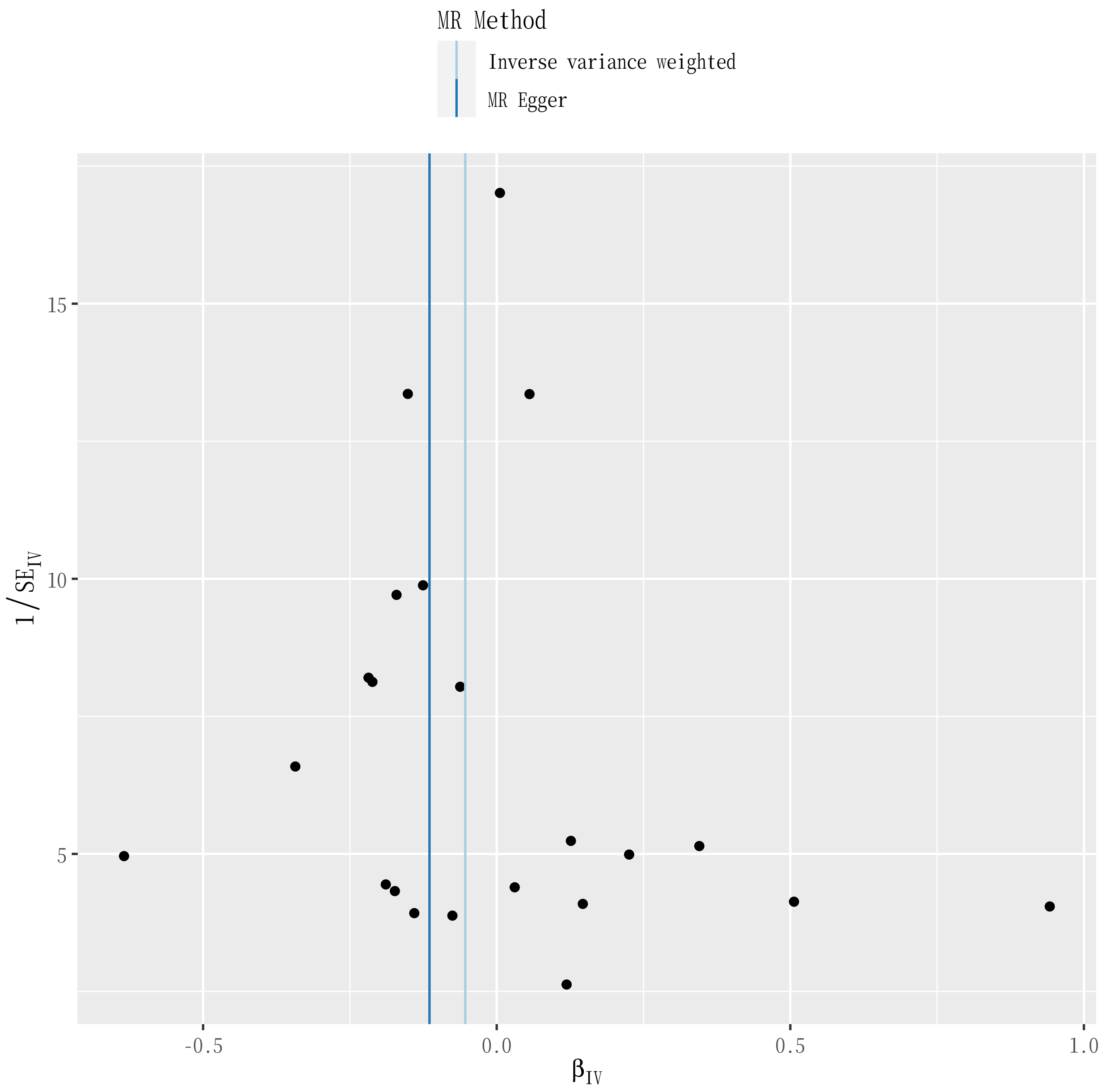

MR Method
Inverse variance weighted
MR Egger
15
10
5
$1/SE_{IV}$
-0.5
0.0
0.5
1.0
β_{IV}

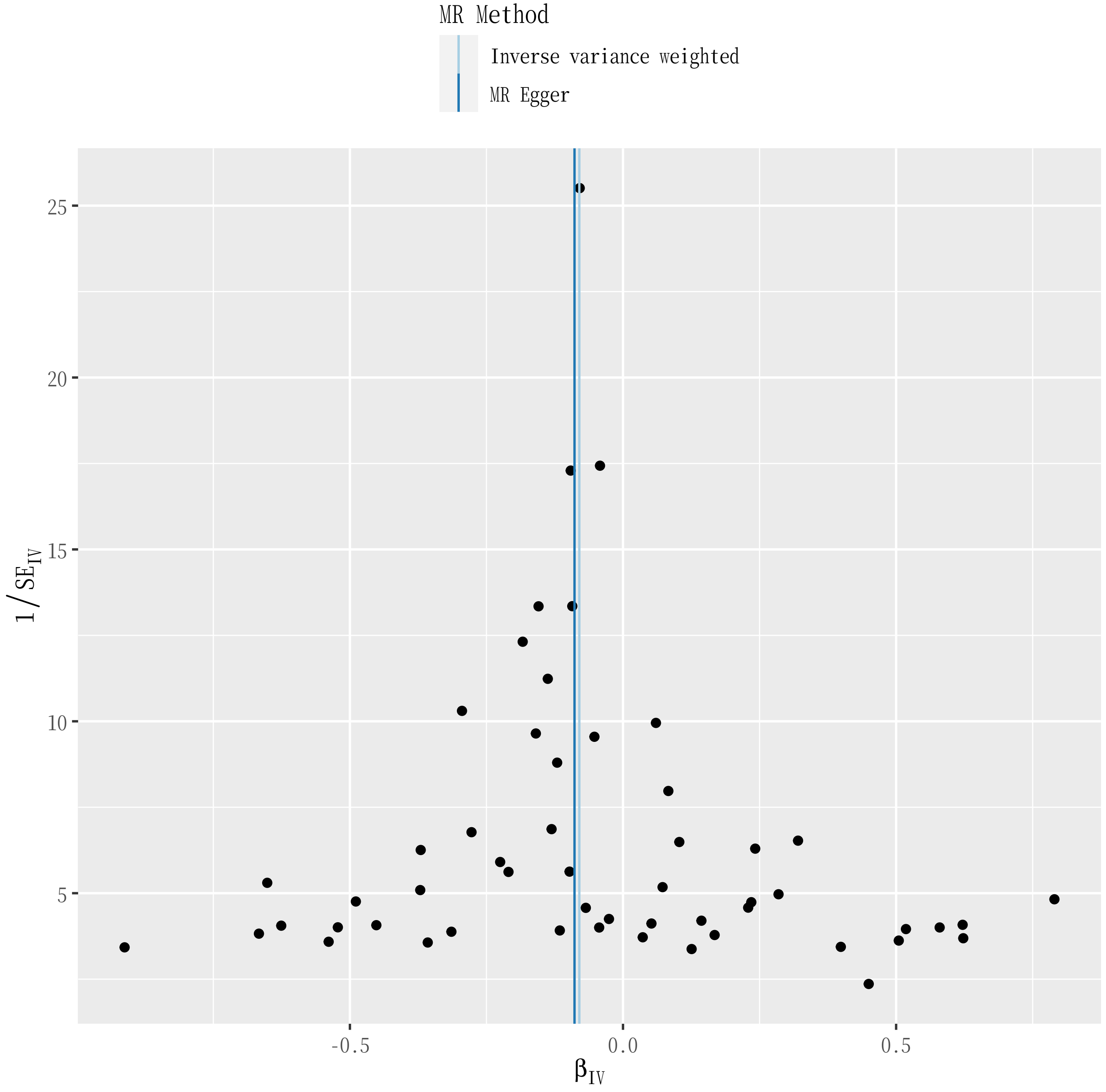

MR Method
Inverse variance weighted
MR Egger
25
20
15
10
5
$1/SE_{IV}$
-0.5
0.0
0.5
β_{IV}

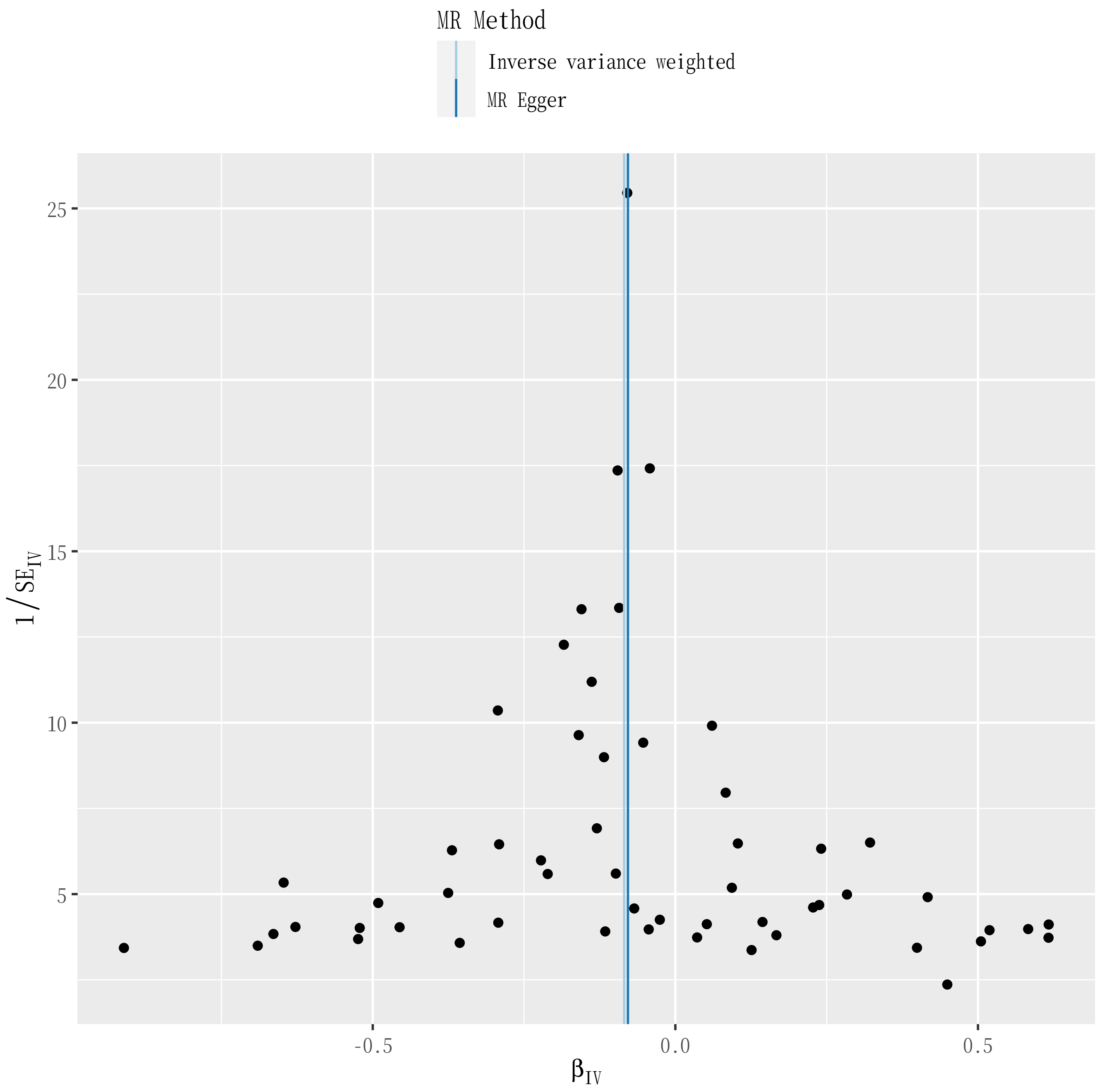

MR Method
Inverse variance weighted
MR Egger
25
20
15
10
5
1/SE_IV
-0.5
0.0
0.5
β_IV

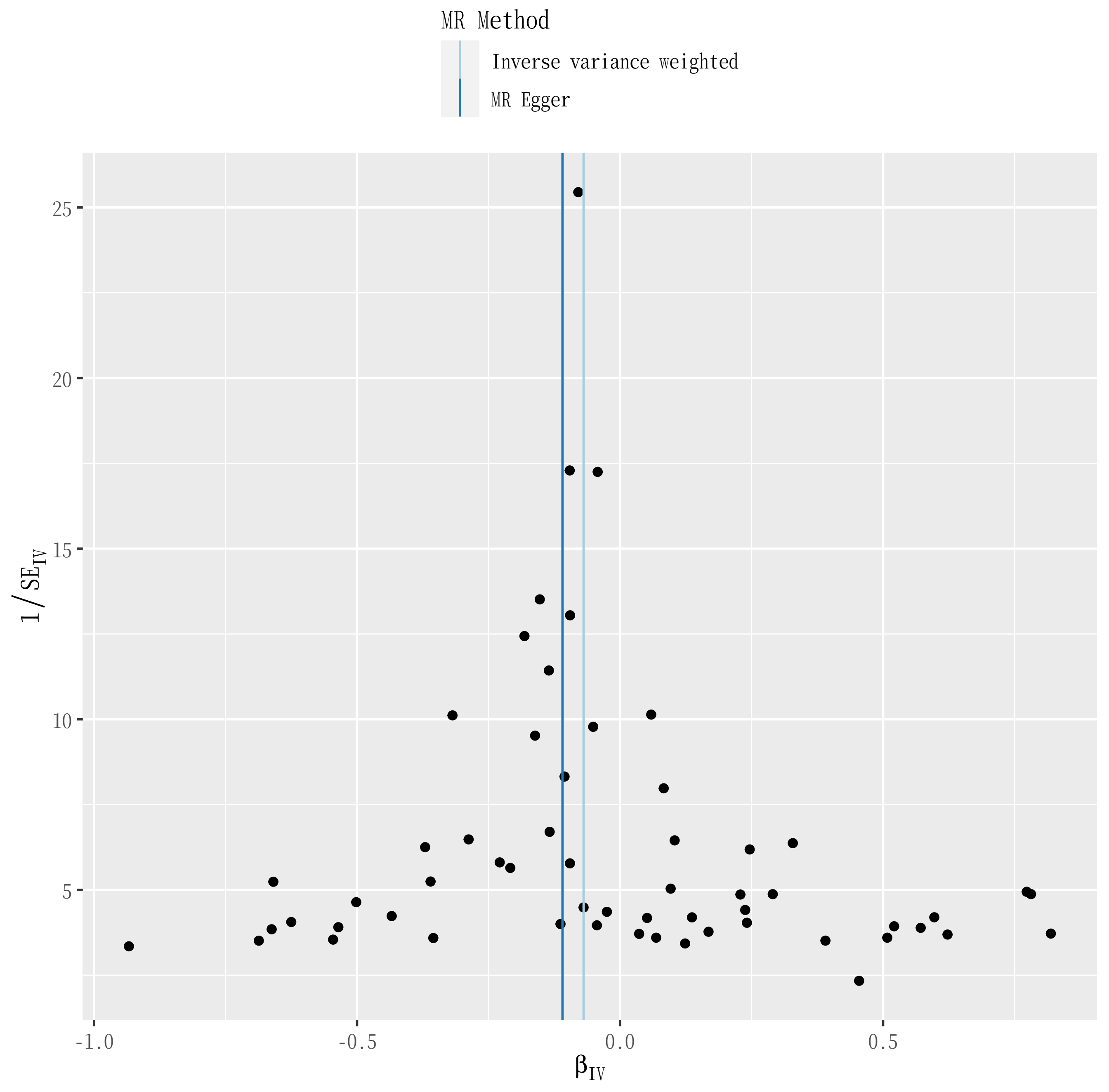
MR Method
Inverse variance weighted
MR Egger
25
20
15
10
5
1/SE_IV
-1.0
-0.5
0.0
0.5
β_IV

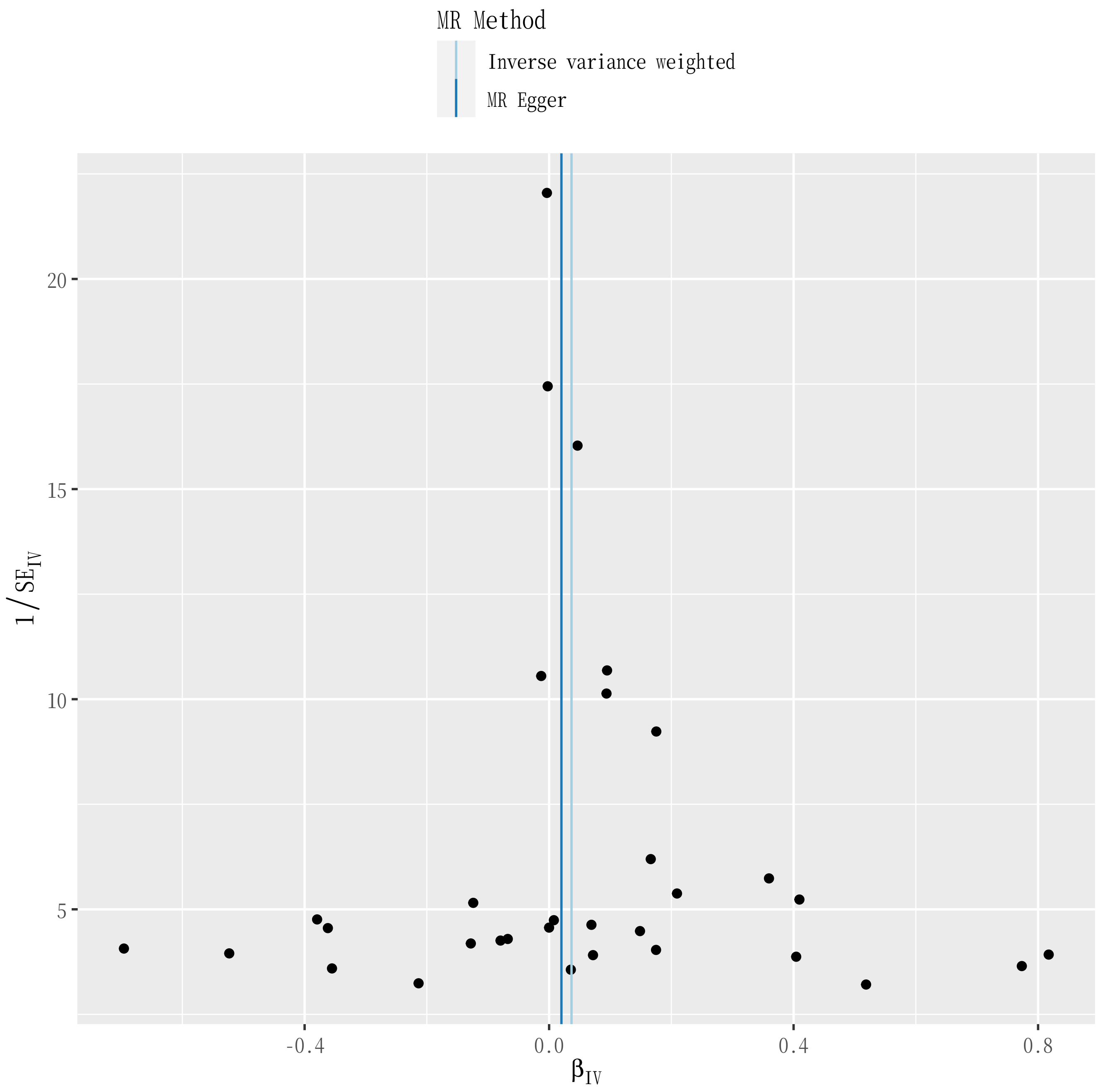
MR Method
Inverse variance weighted
MR Egger
20
15
10
5
$1/SE_{IV}$
-0.4
0.0
0.4
0.8
β_{IV}

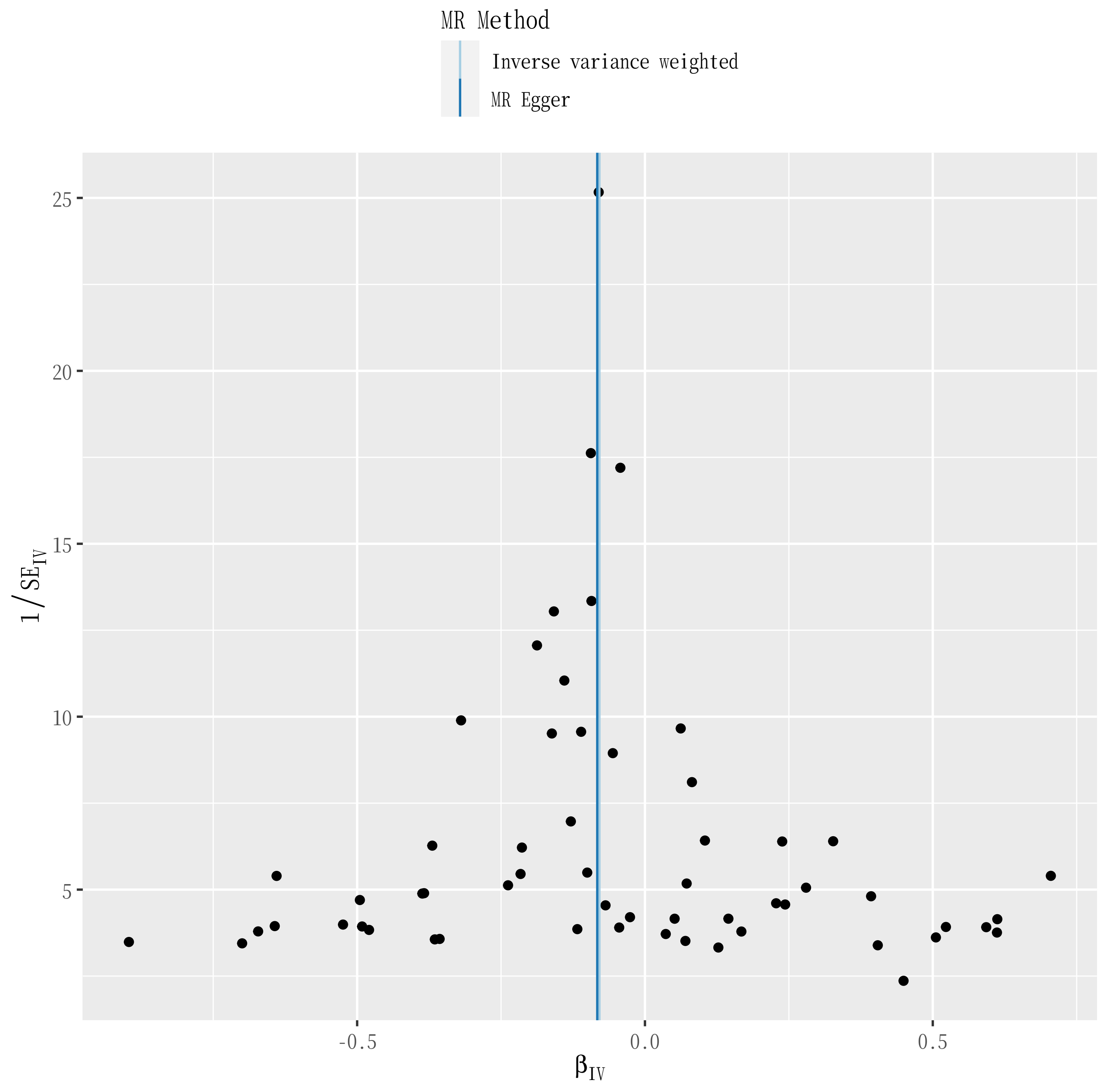
MR Method
Inverse variance weighted
MR Egger
25
20
15
10
5
1/SE_IV
-0.5
0.0
0.5
β_IV

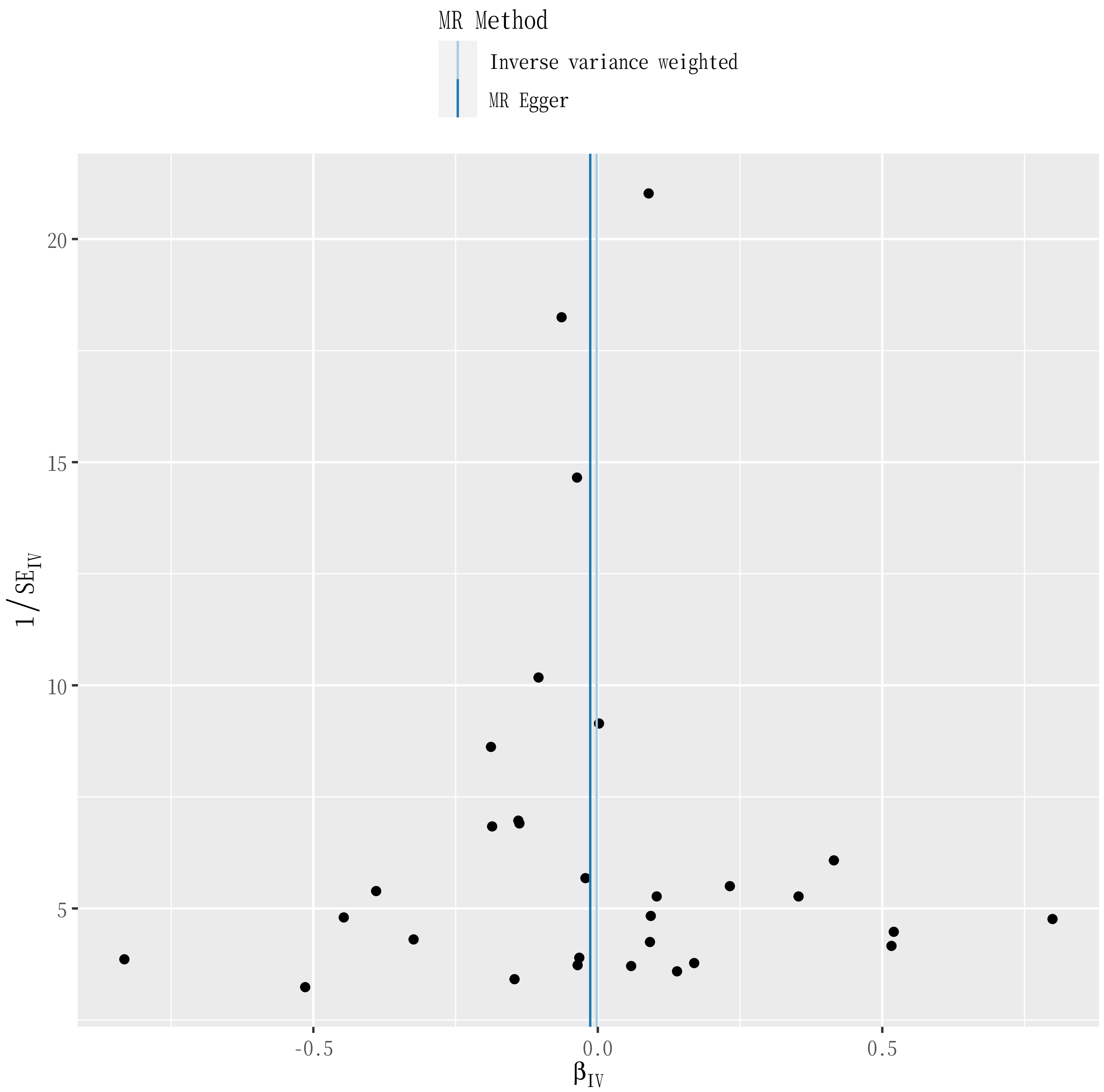
MR Method
Inverse variance weighted
MR Egger
20
15
10
5
-0.5
0.0
0.5
$1/SE_{IV}$
β_{IV}

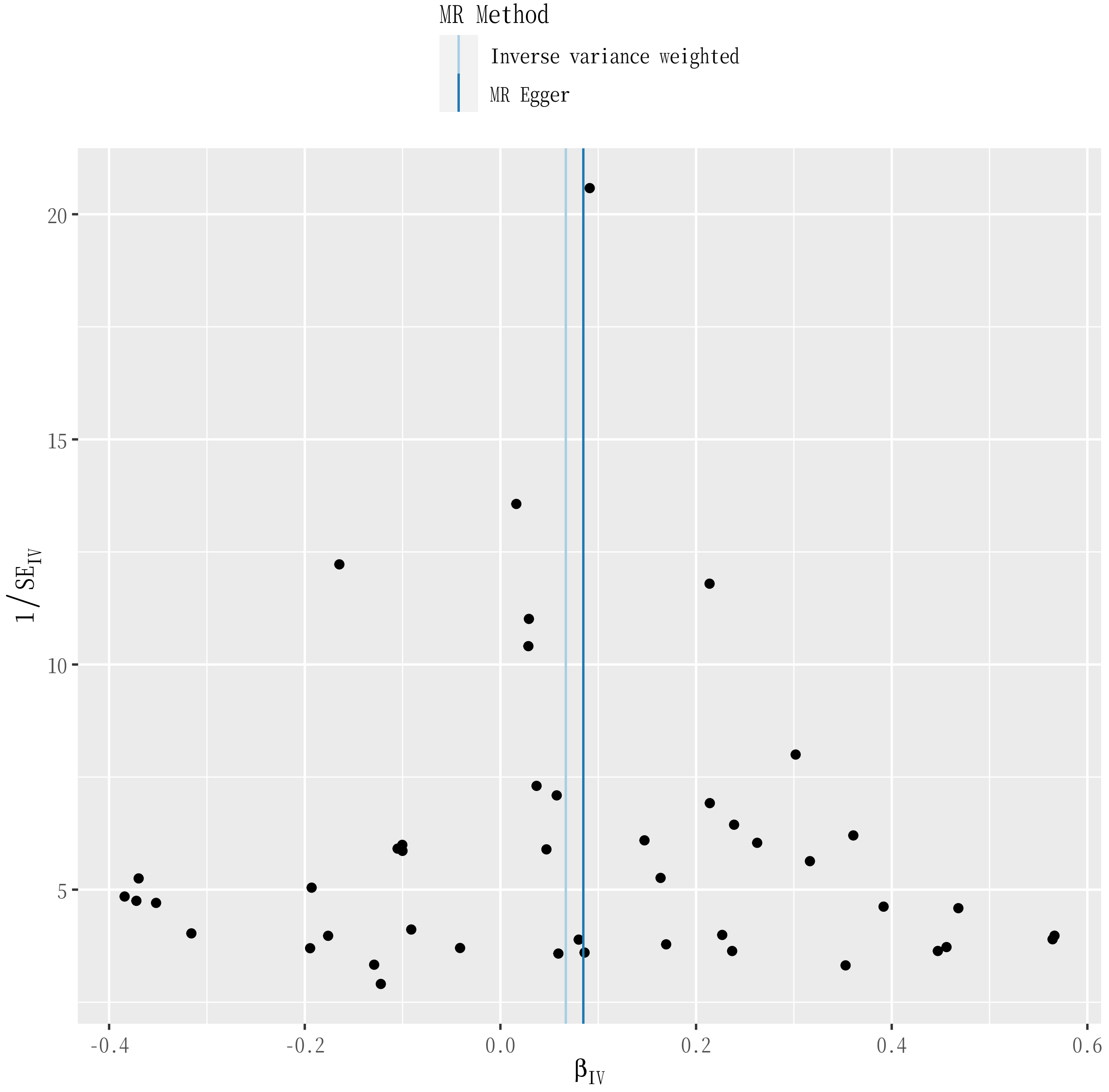
MR Method
Inverse variance weighted
MR Egger
20
15
10
5
$1/SE_{IV}$
-0.4
-0.2
0.0
0.2
0.4
0.6
β_{IV}

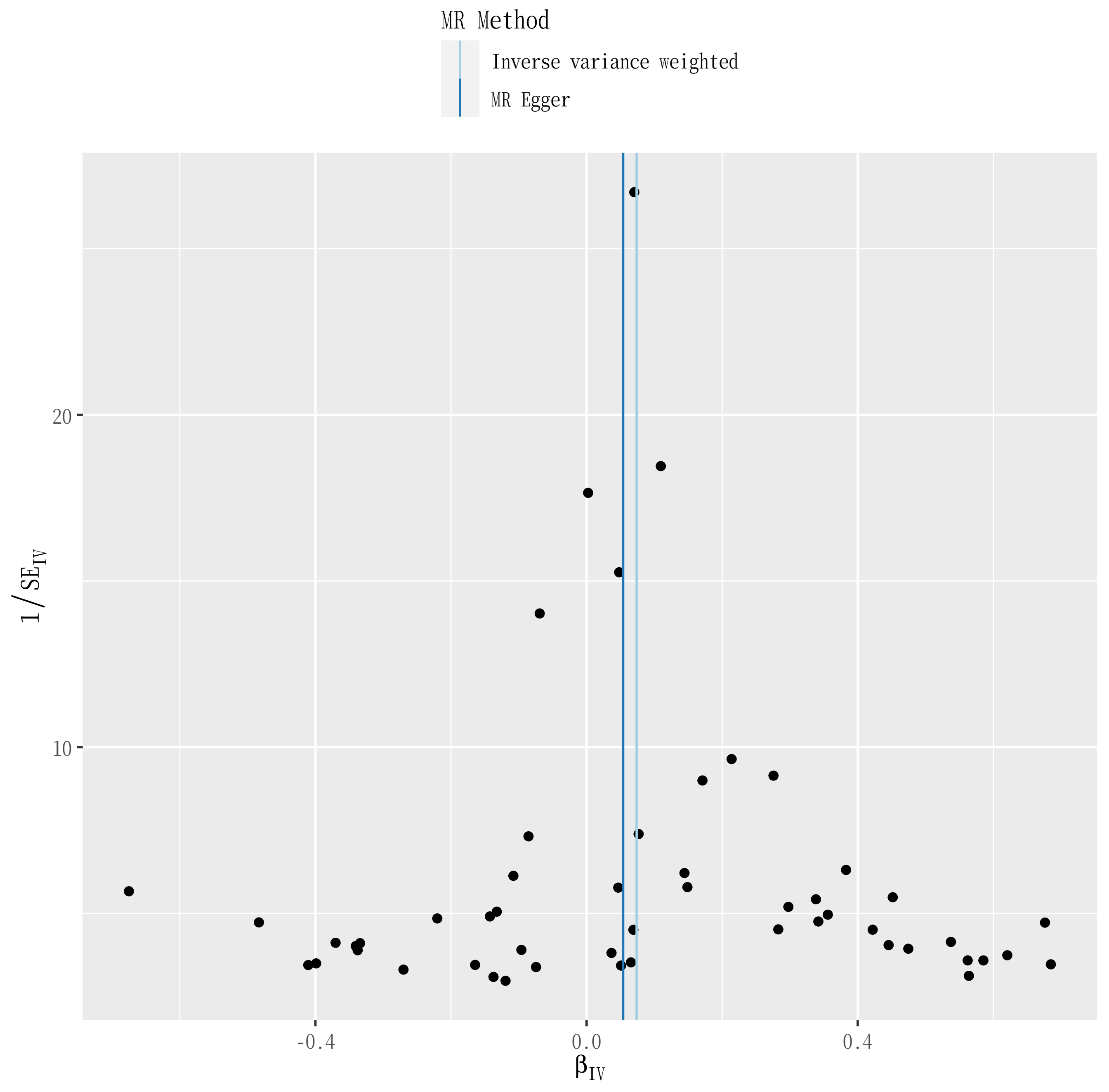
MR Method
Inverse variance weighted
MR Egger
20
10
1/SE_IV
-0.4
0.0
0.4
β_IV

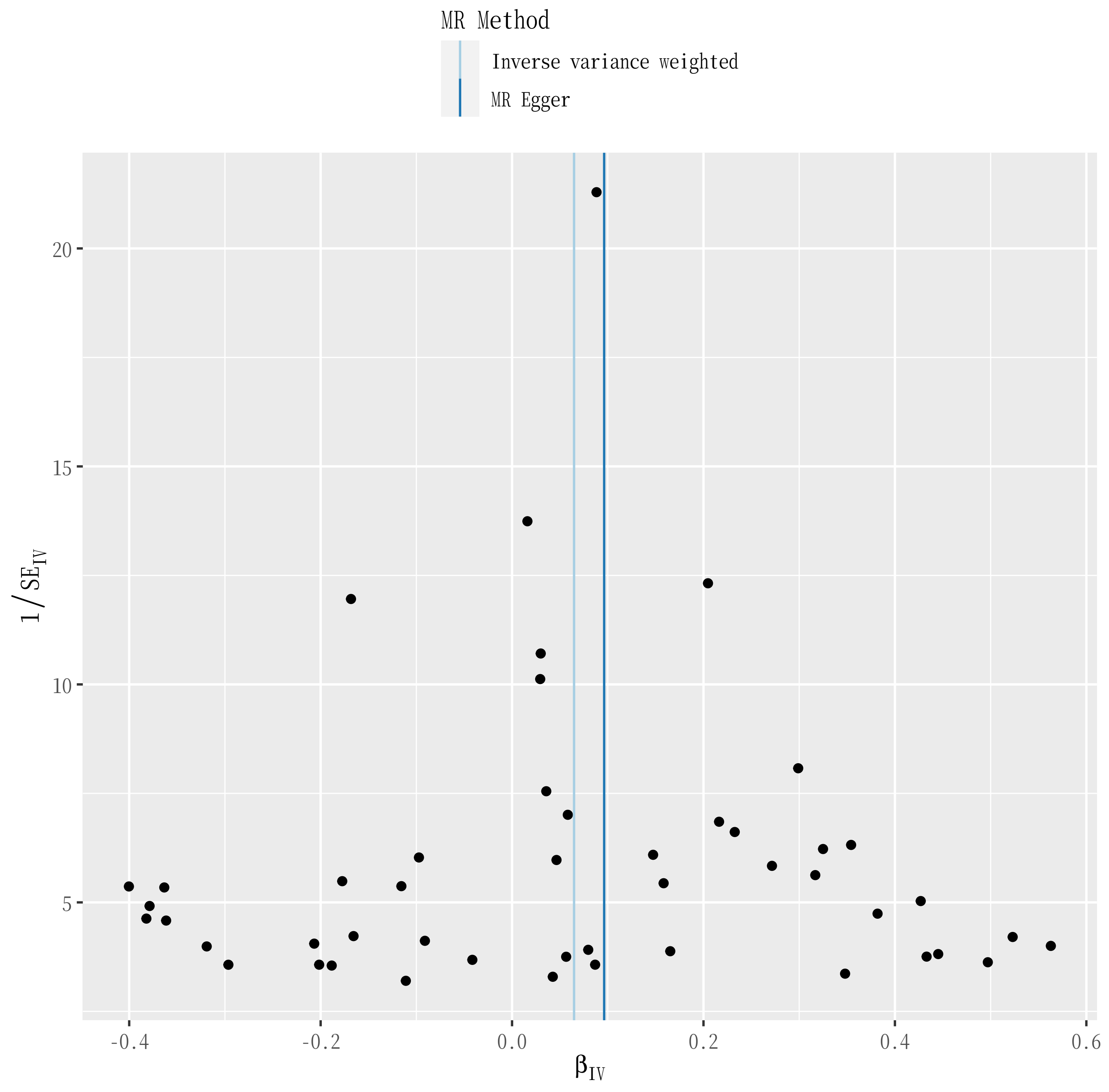
MR Method
Inverse variance weighted
MR Egger
20
15
10
5
-0.4
-0.2
0.0
0.2
0.4
0.6
$1/SE_{IV}$
β_{IV}

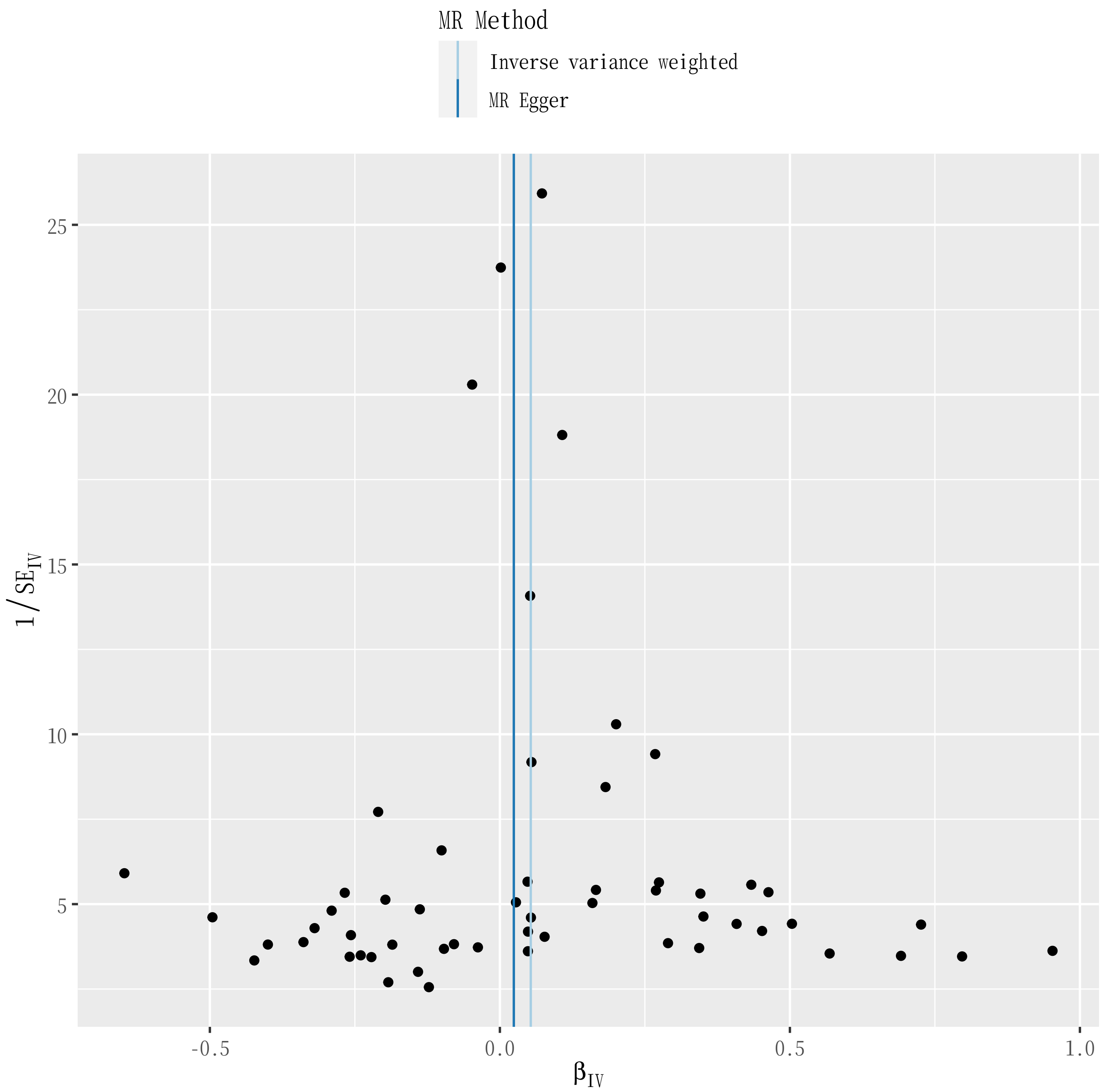
MR Method
Inverse variance weighted
MR Egger
25
20
15
10
5
-0.5
0.0
0.5
1.0
$1/SE_{IV}$
β_{IV}

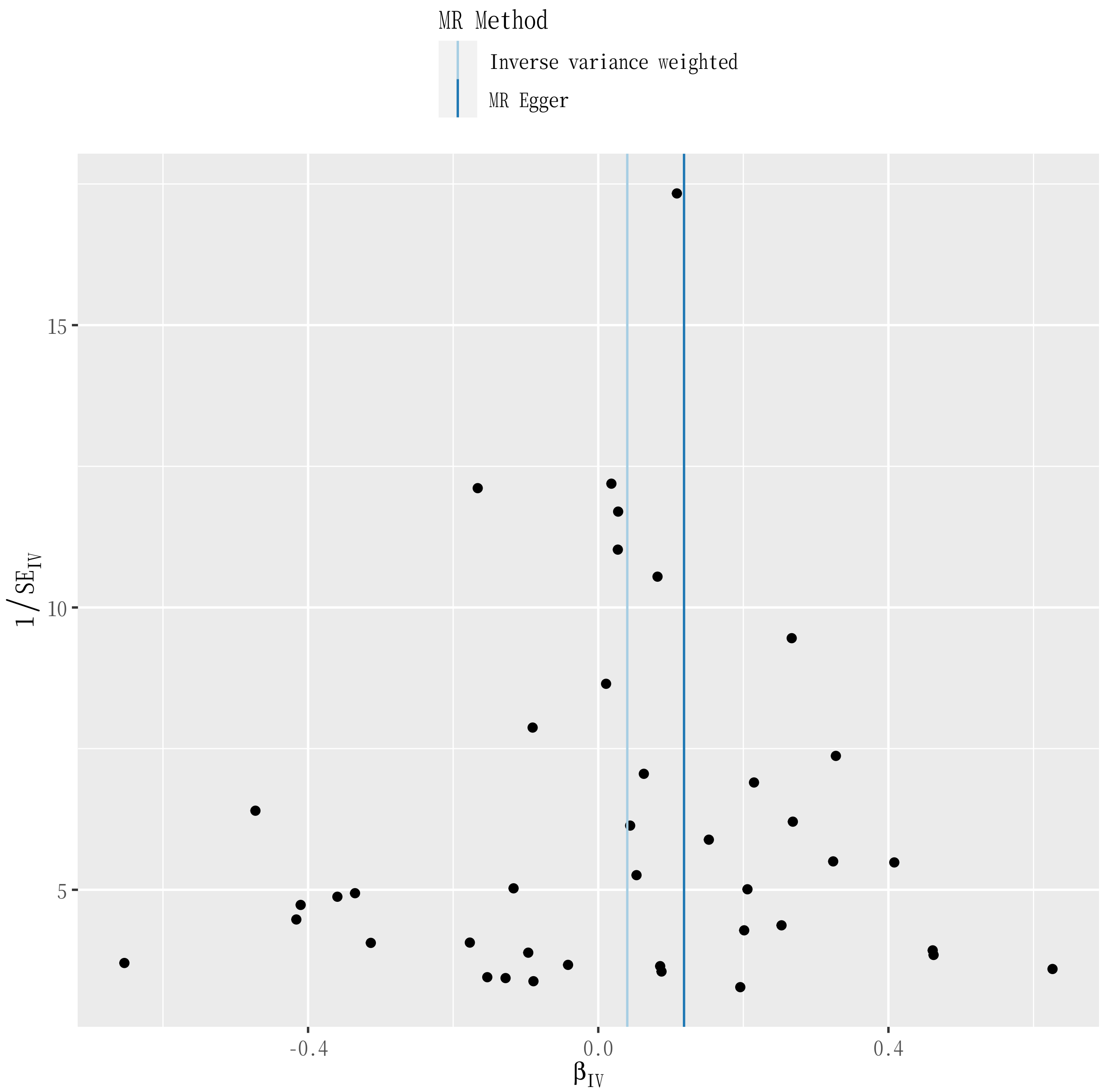
MR Method
Inverse variance weighted
MR Egger
15
10
5
-0.4
0.0
0.4
$1/SE_{IV}$
β_{IV}

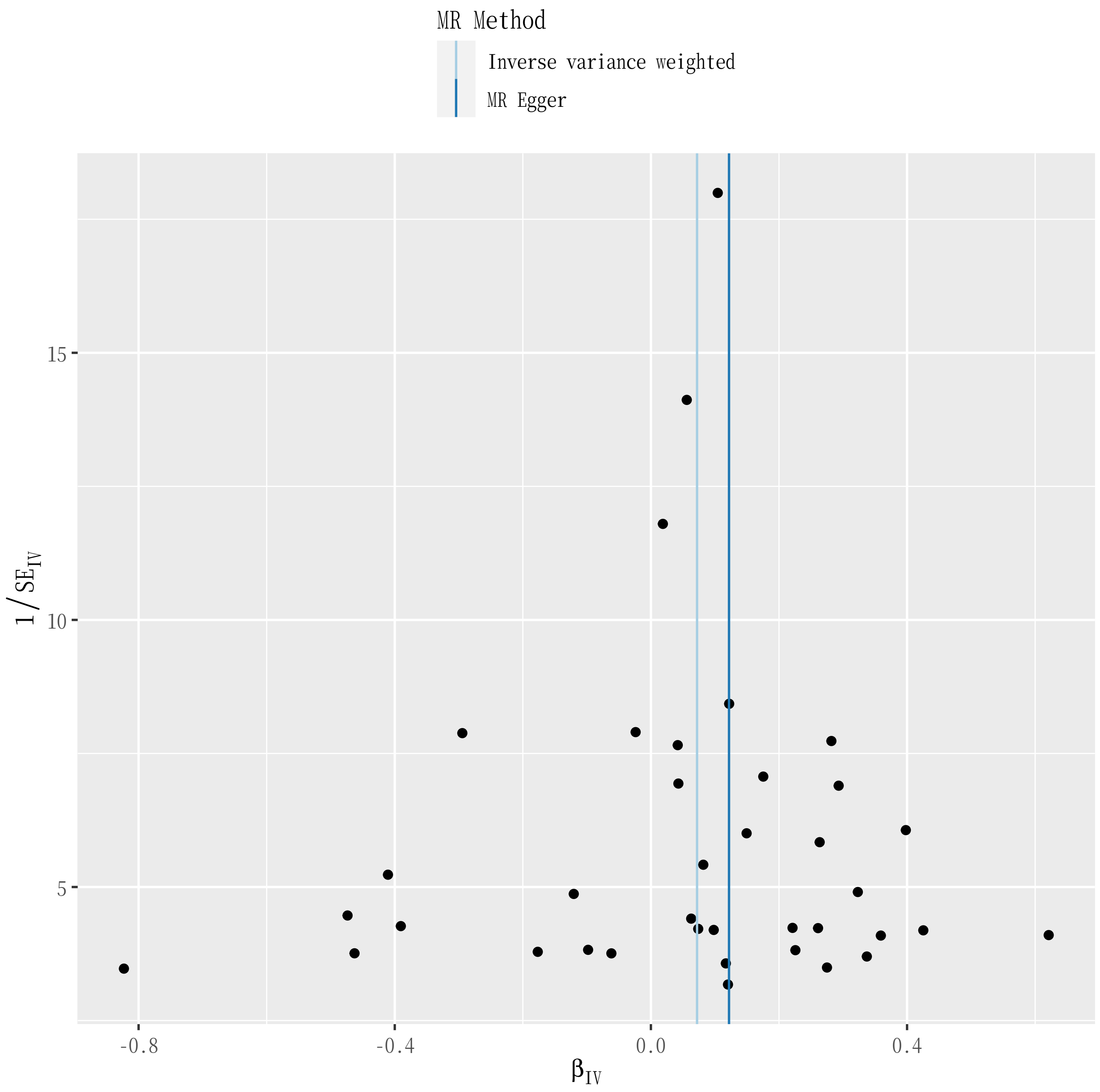

MR Method
Inverse variance weighted
MR Egger
15
10
5
1/SE_IV
-0.8
-0.4
0.0
0.4
β_IV

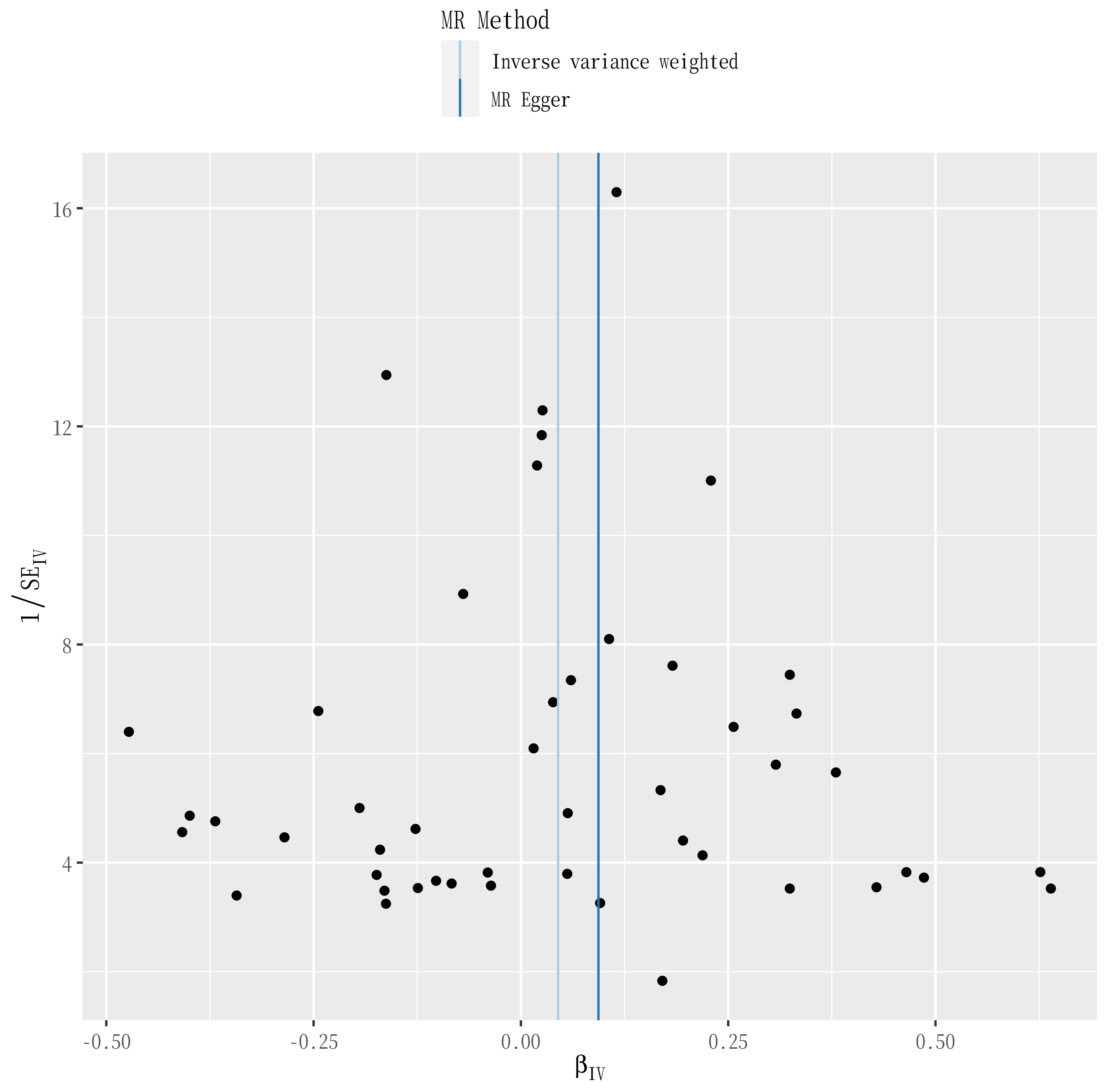

MR Method
Inverse variance weighted
MR Egger
16
12
8
4
1/SE_IV
-0.50
-0.25
0.00
0.25
0.50
β_IV

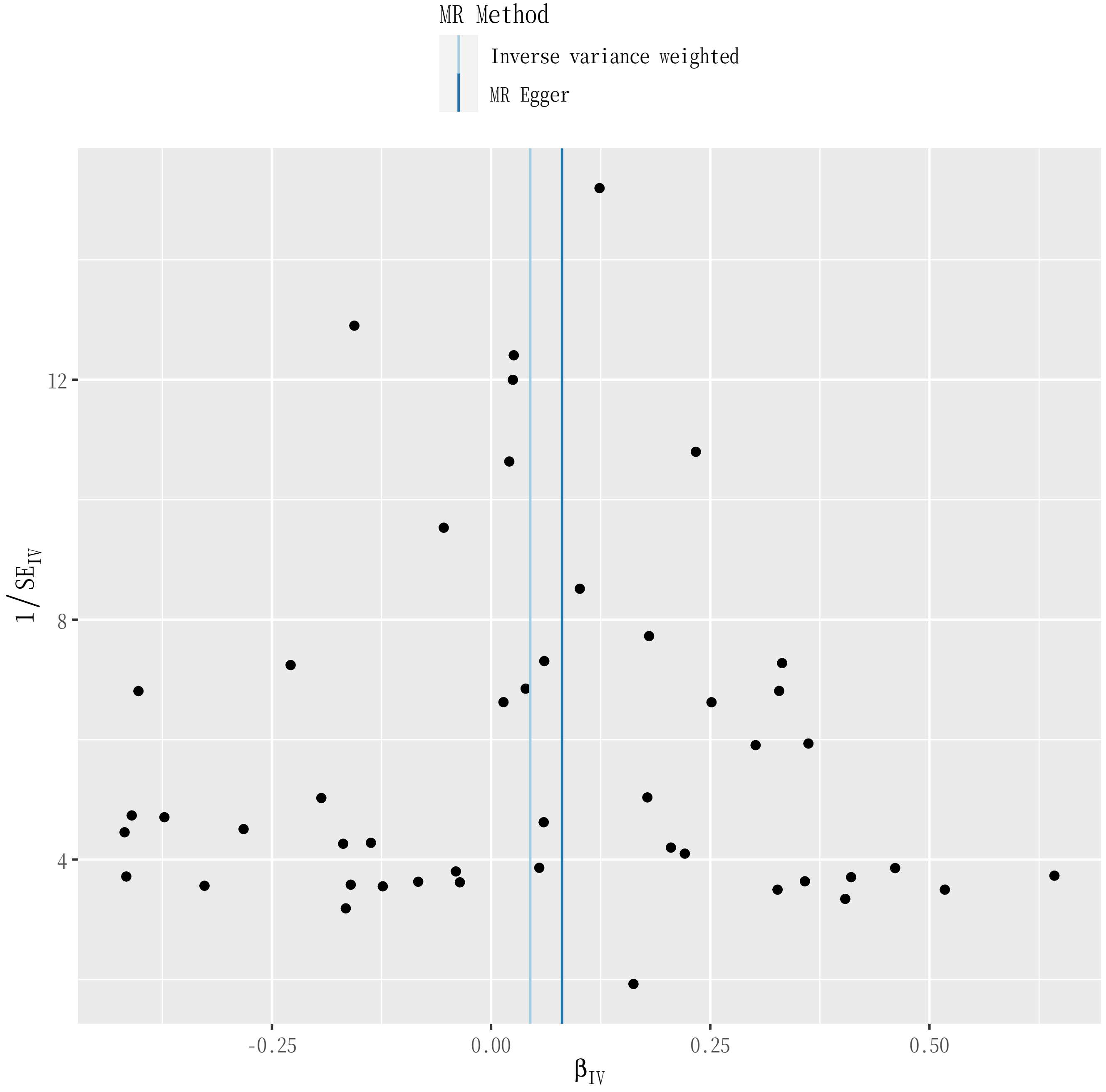
MR Method
Inverse variance weighted
MR Egger
1/SE_IV
12
8
4
-0.25
0.00
0.25
0.50
β_IV

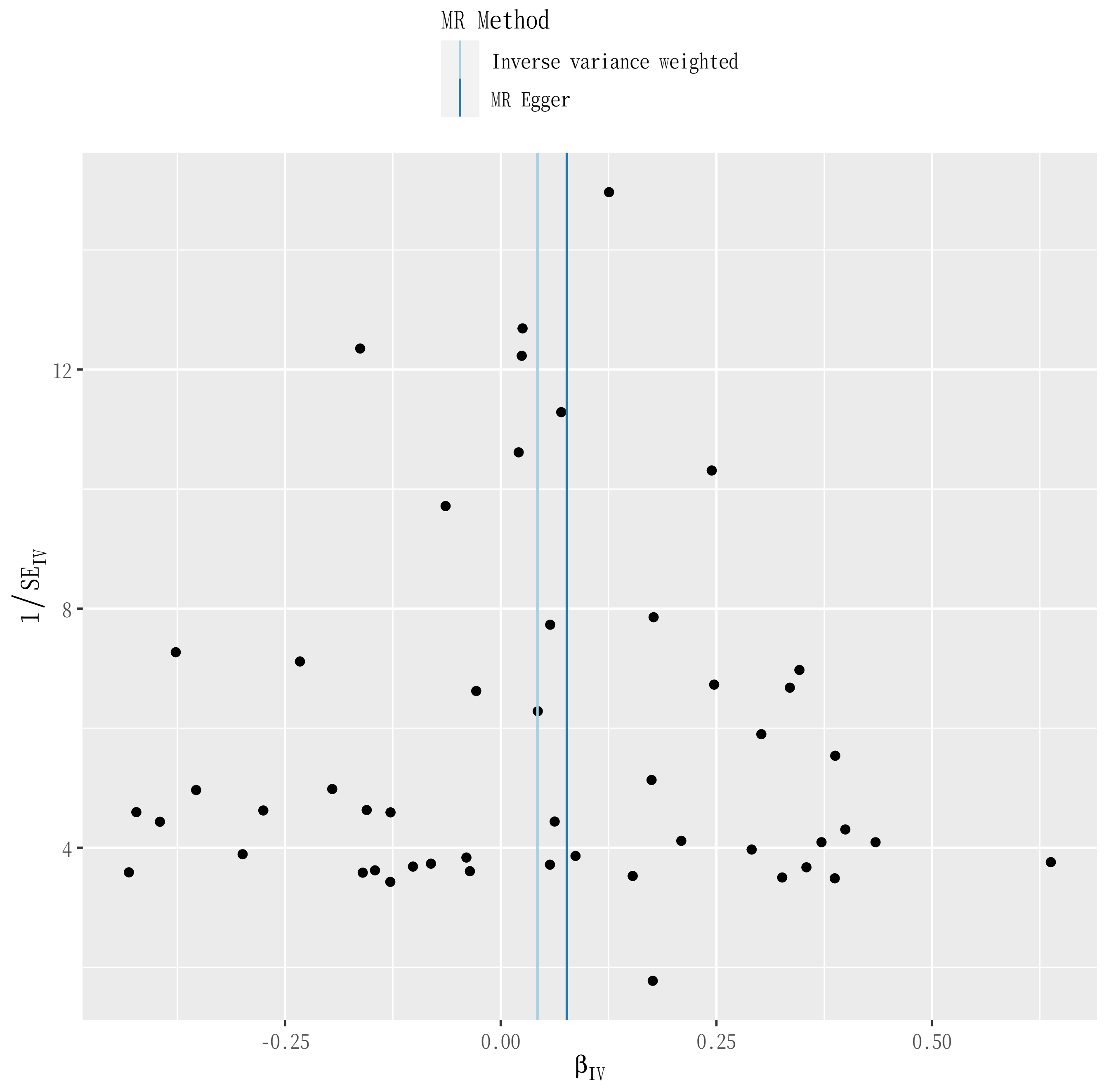
MR Method
Inverse variance weighted
MR Egger
12
8
4
$1/SE_{IV}$
-0.25
0.00
0.25
0.50
β_{IV}

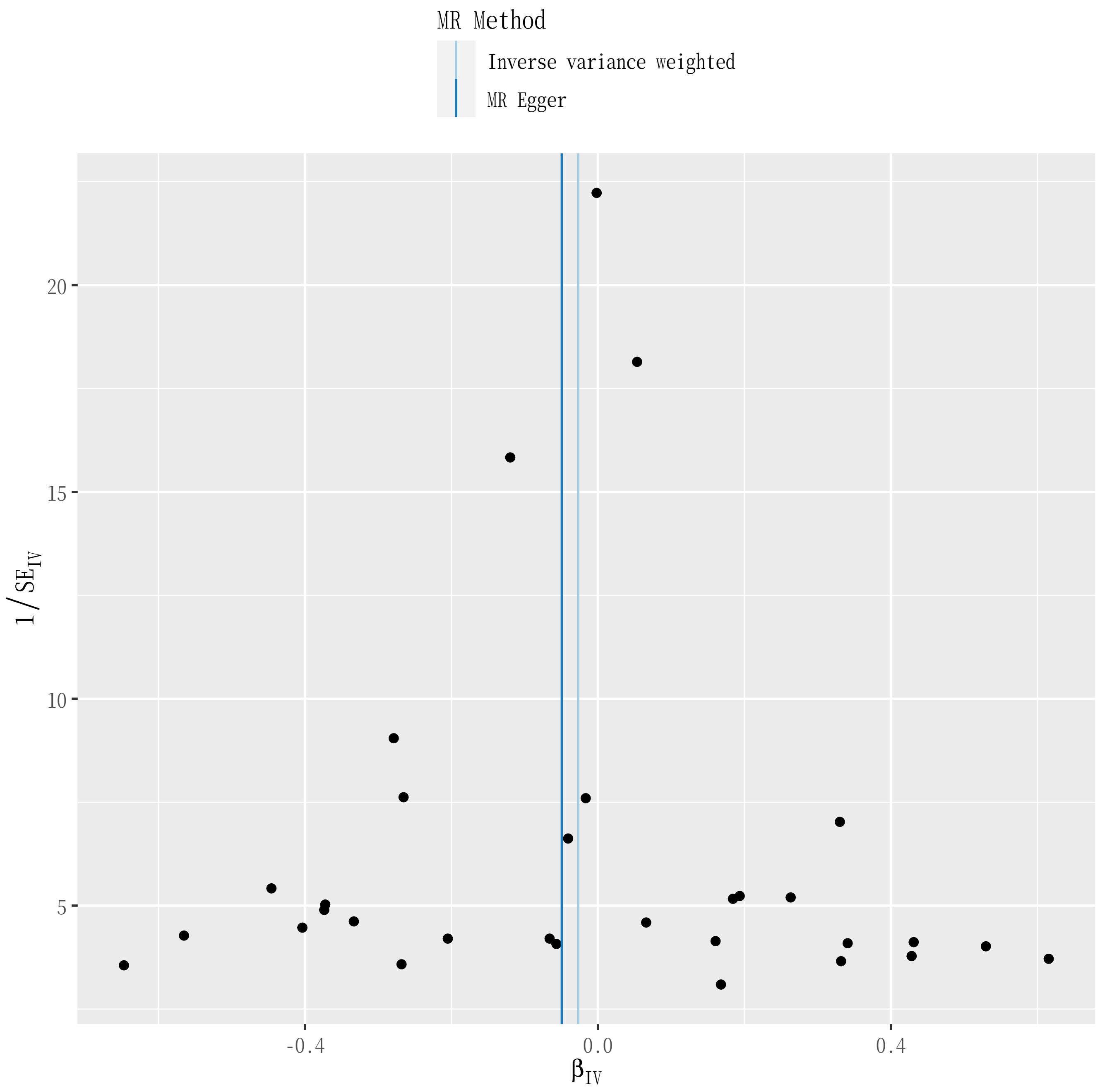
MR Method
Inverse variance weighted
MR Egger
20
15
10
5
$1/SE_{IV}$
-0.4
0.0
0.4
β_{IV}

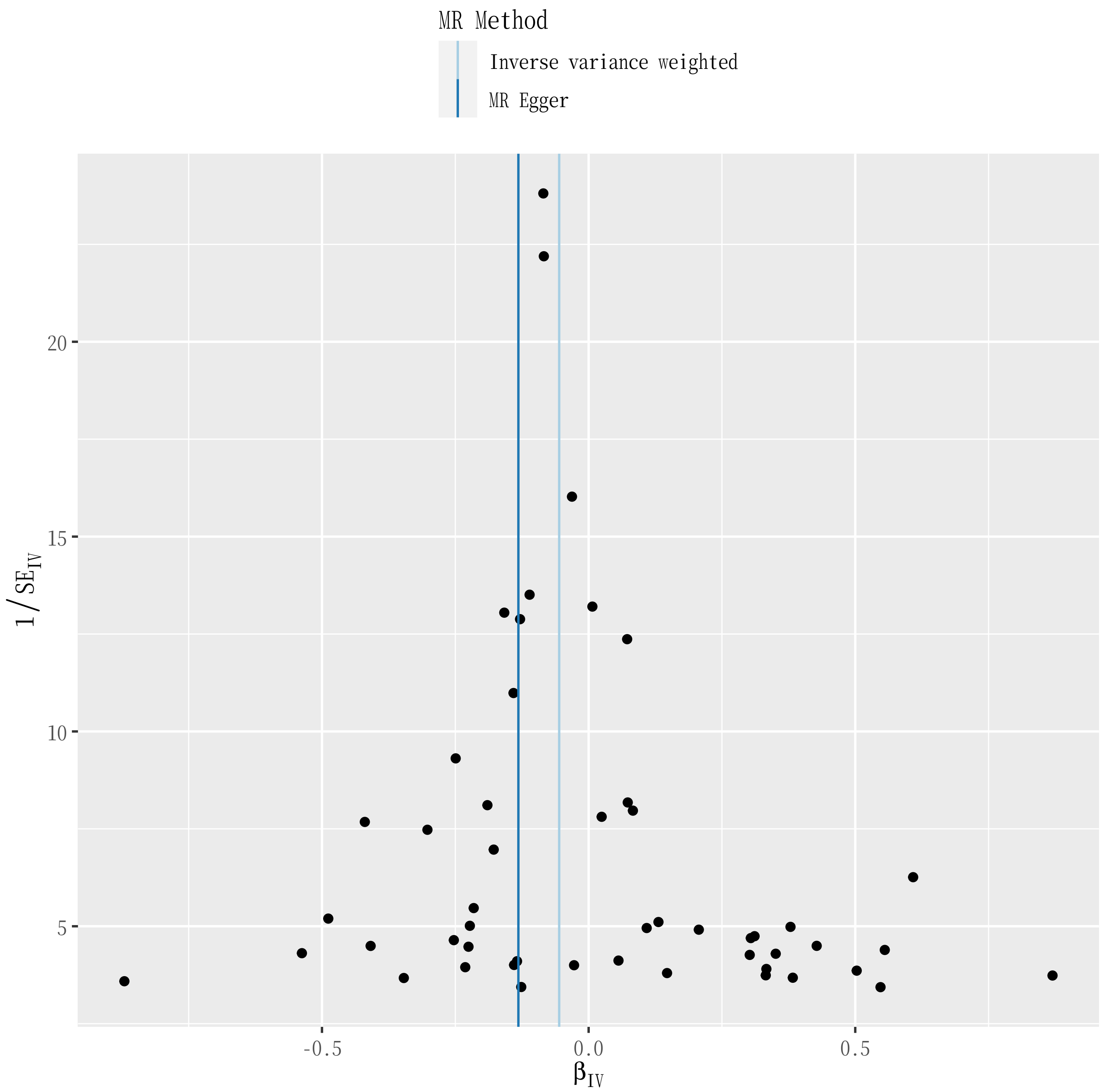

MR Method
Inverse variance weighted
MR Egger
20
15
10
5
$1/SE_{IV}$
-0.5
0.0
0.5
β_{IV}

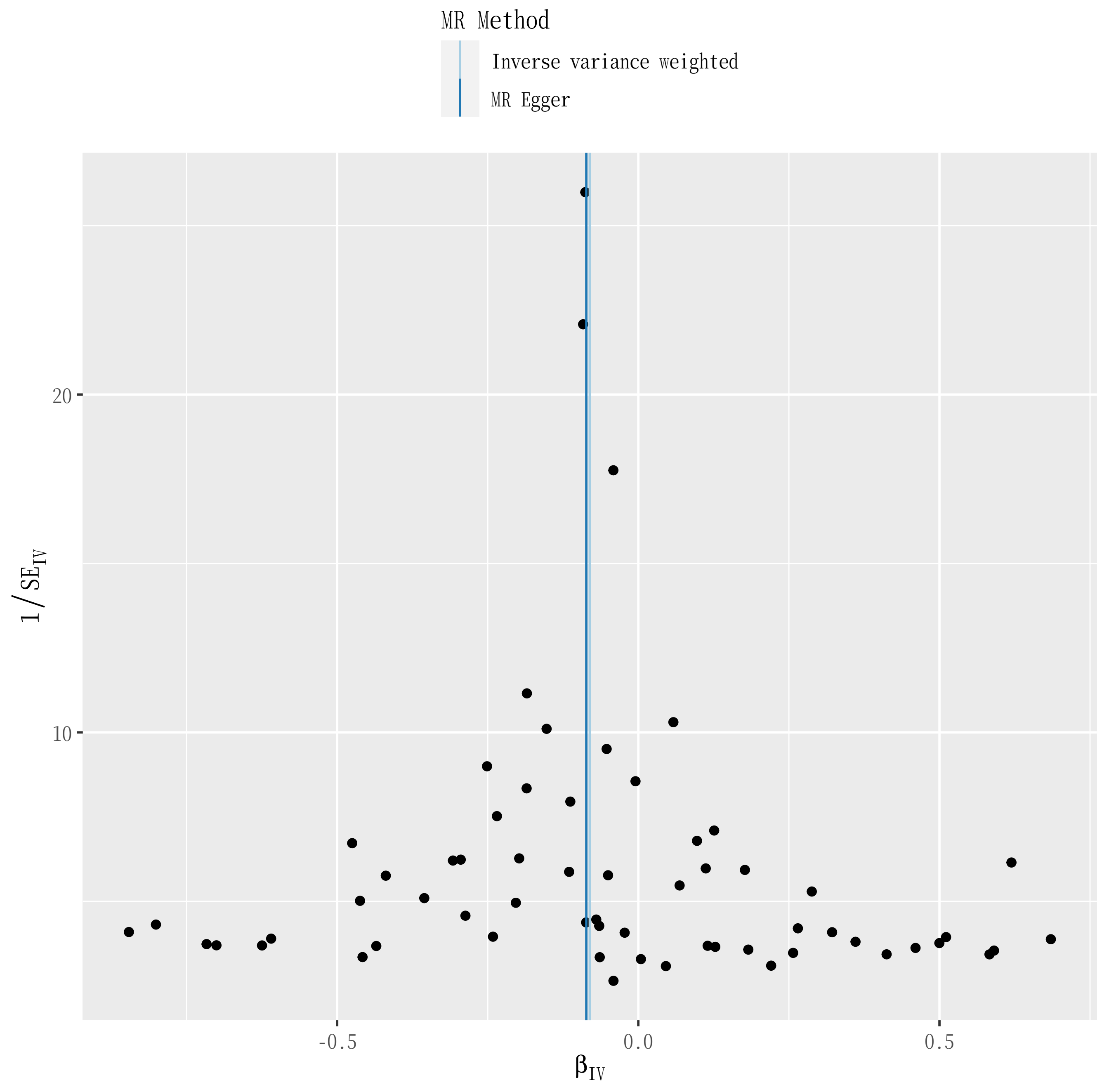
MR Method
Inverse variance weighted
MR Egger
20
10
$1/SE_{IV}$
-0.5
0.0
0.5
β_{IV}

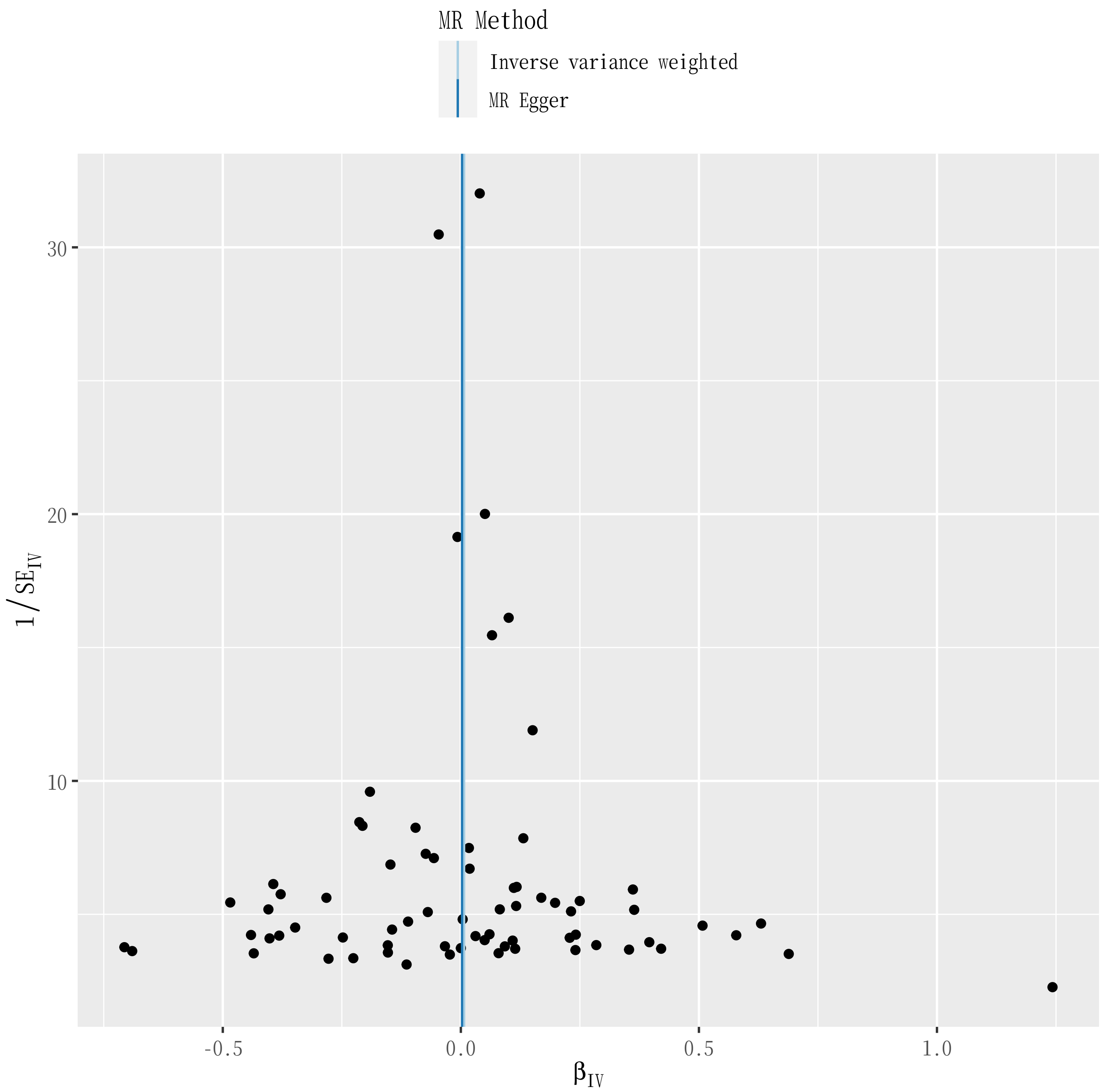
MR Method
Inverse variance weighted
MR Egger
30
20
10
-0.5
0.0
0.5
1.0
$1/SE_{IV}$
β_{IV}

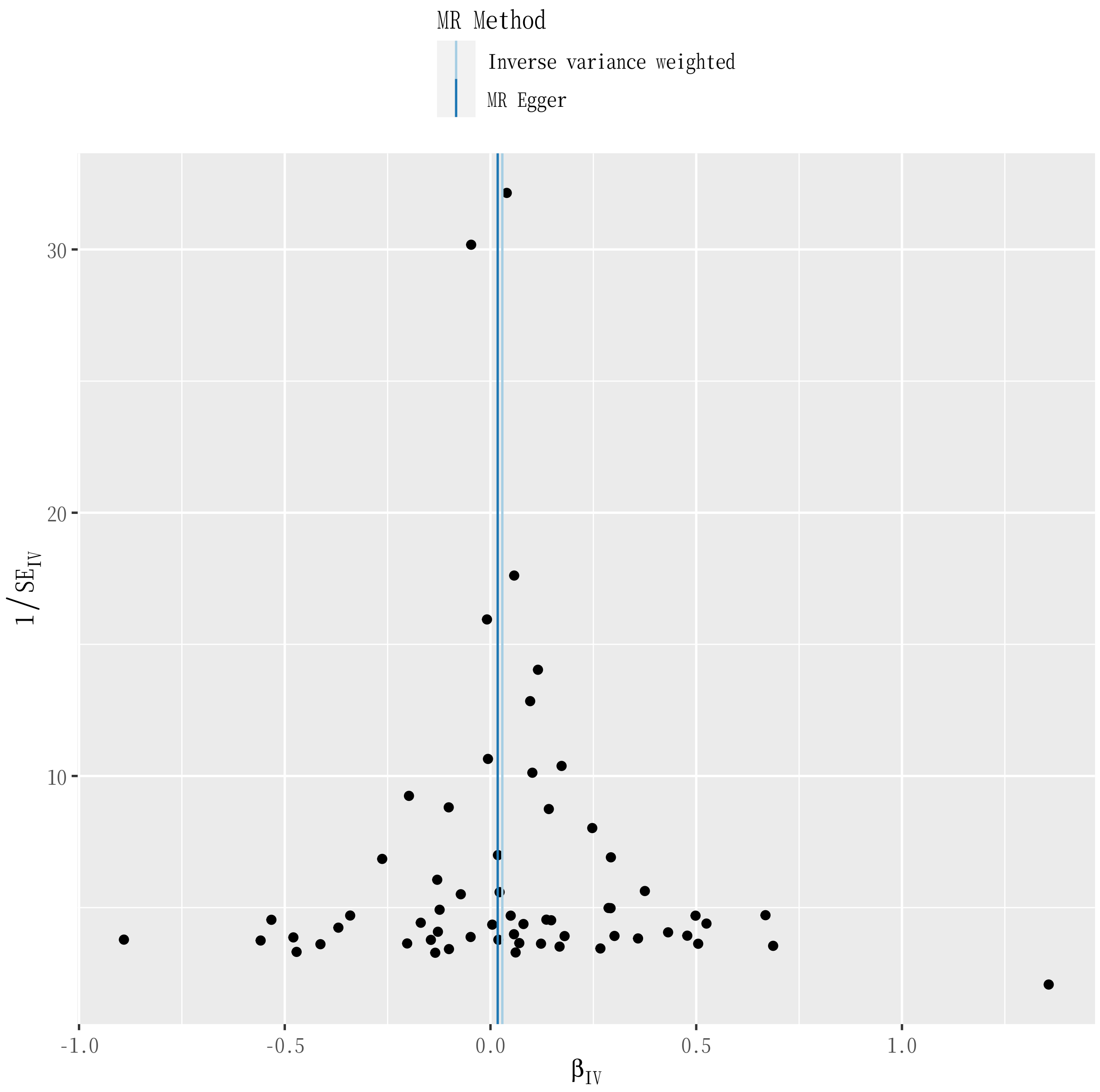
MR Method
Inverse variance weighted
MR Egger
30
20
10
$1/SE_{IV}$
-1.0
-0.5
0.0
0.5
1.0
β_{IV}

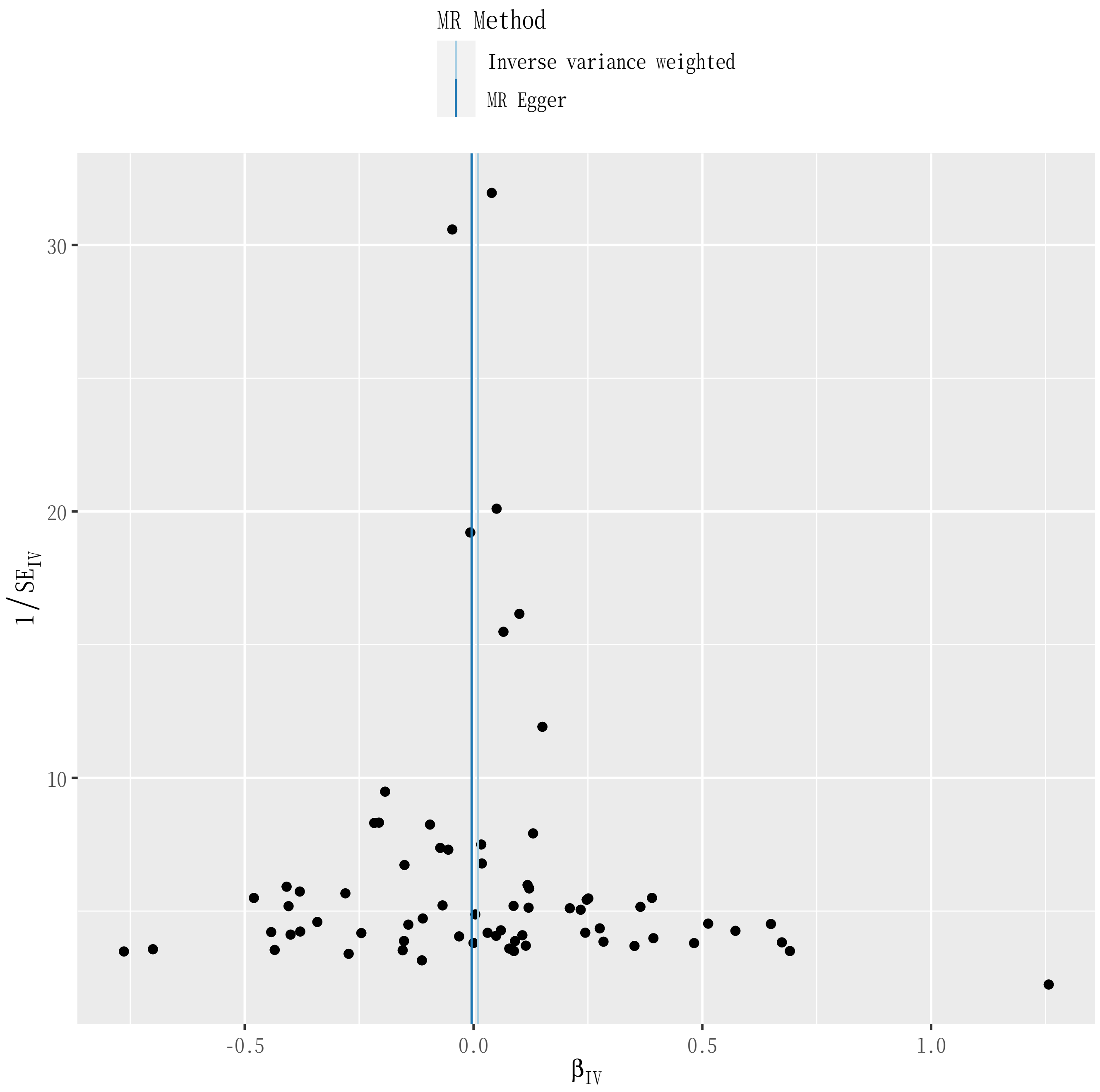

MR Method
Inverse variance weighted
MR Egger
30
20
10
1/SE_IV
-0.5
0.0
0.5
1.0
β_IV

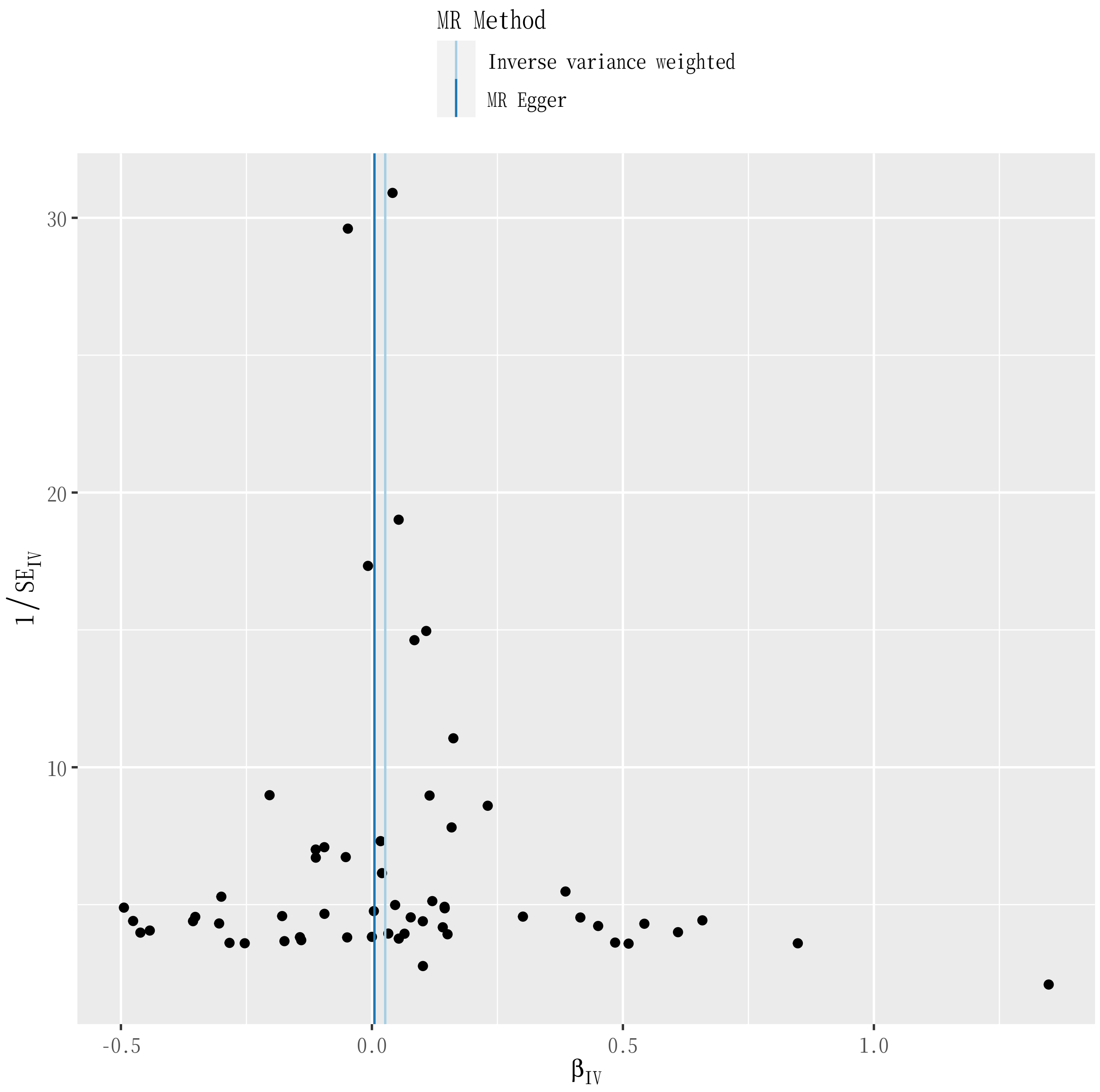

MR Method
Inverse variance weighted
MR Egger
30
20
10
$1/SE_{IV}$
-0.5
0.0
0.5
1.0
β_{IV}

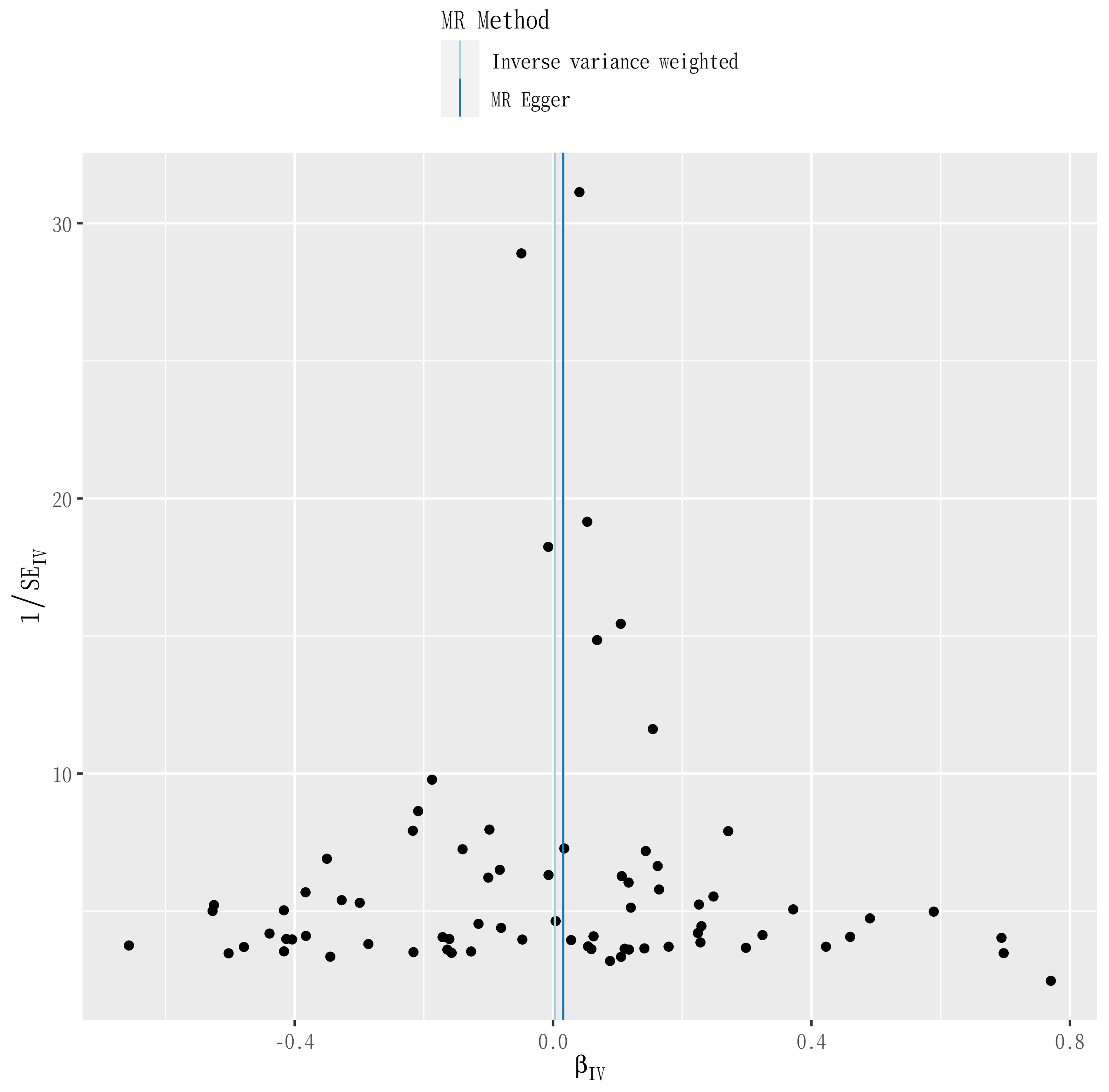

MR Method
Inverse variance weighted
MR Egger
30
20
10
-0.4
0.0
0.4
0.8
$1/SE_{IV}$
β_{IV}

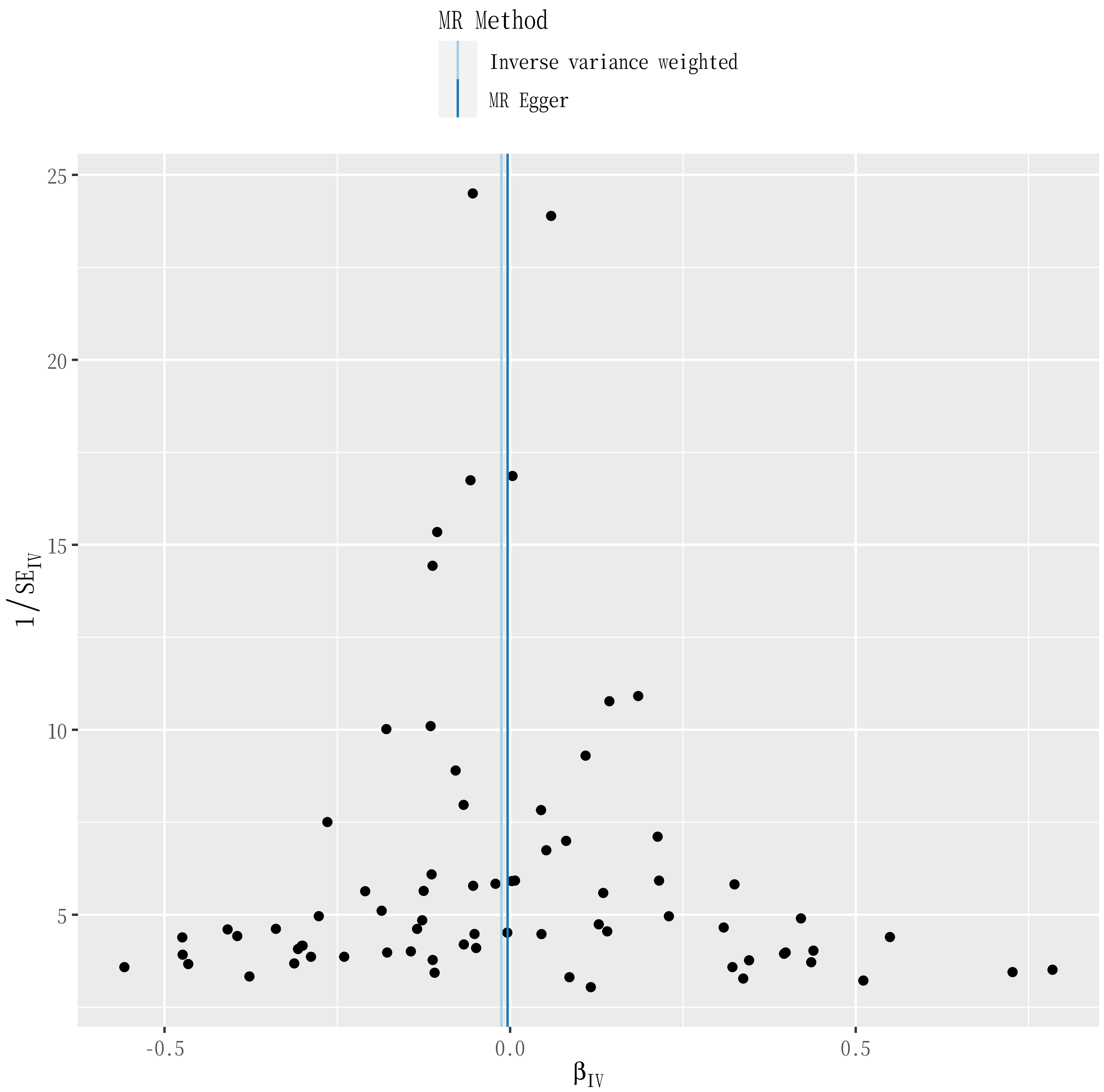
MR Method
Inverse variance weighted
MR Egger
25
20
15
10
5
$1/SE_{IV}$
-0.5
0.0
0.5
β_{IV}

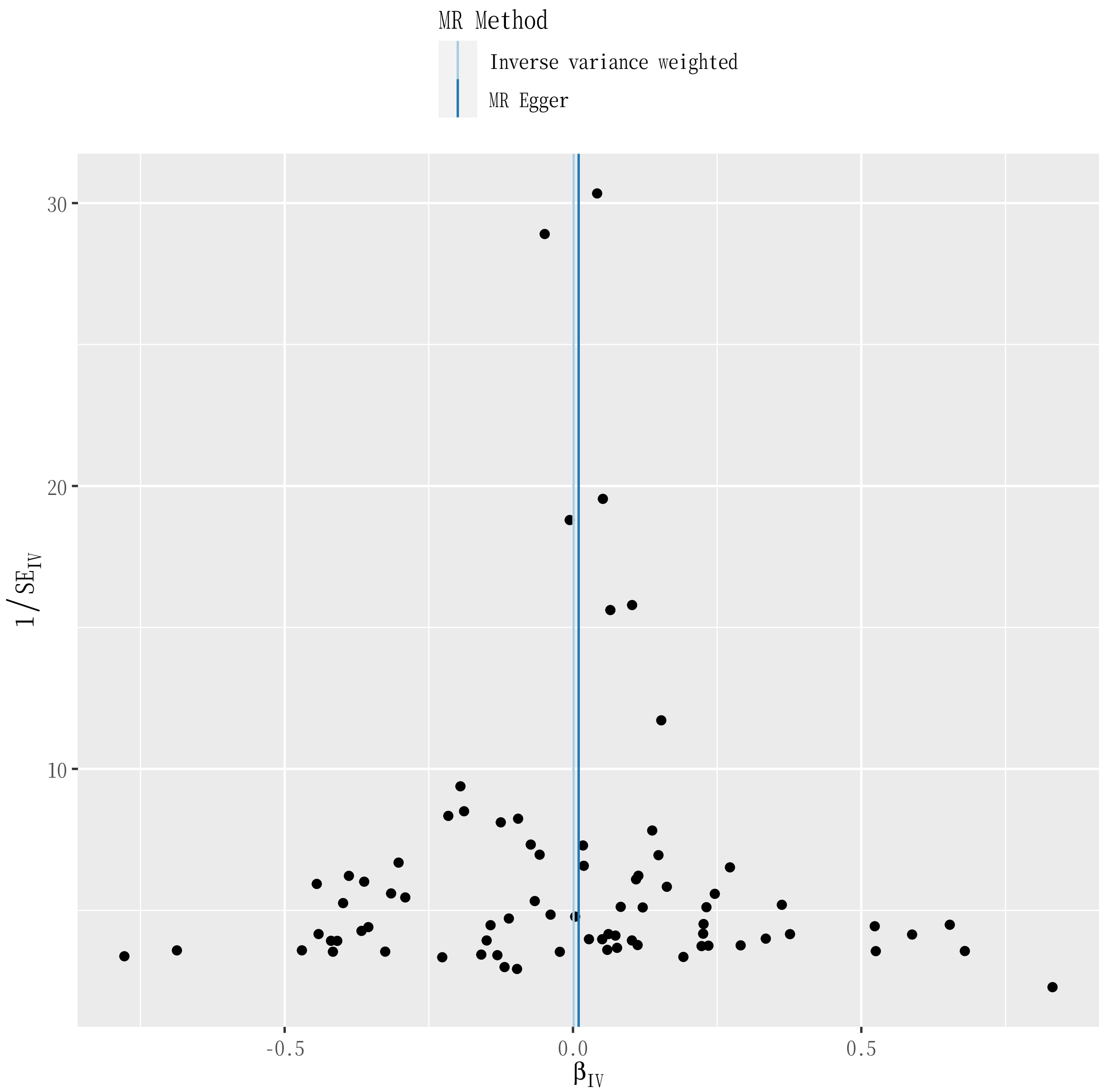
MR Method
Inverse variance weighted
MR Egger
30
20
10
1/SE_IV
-0.5
0.0
0.5
β_IV

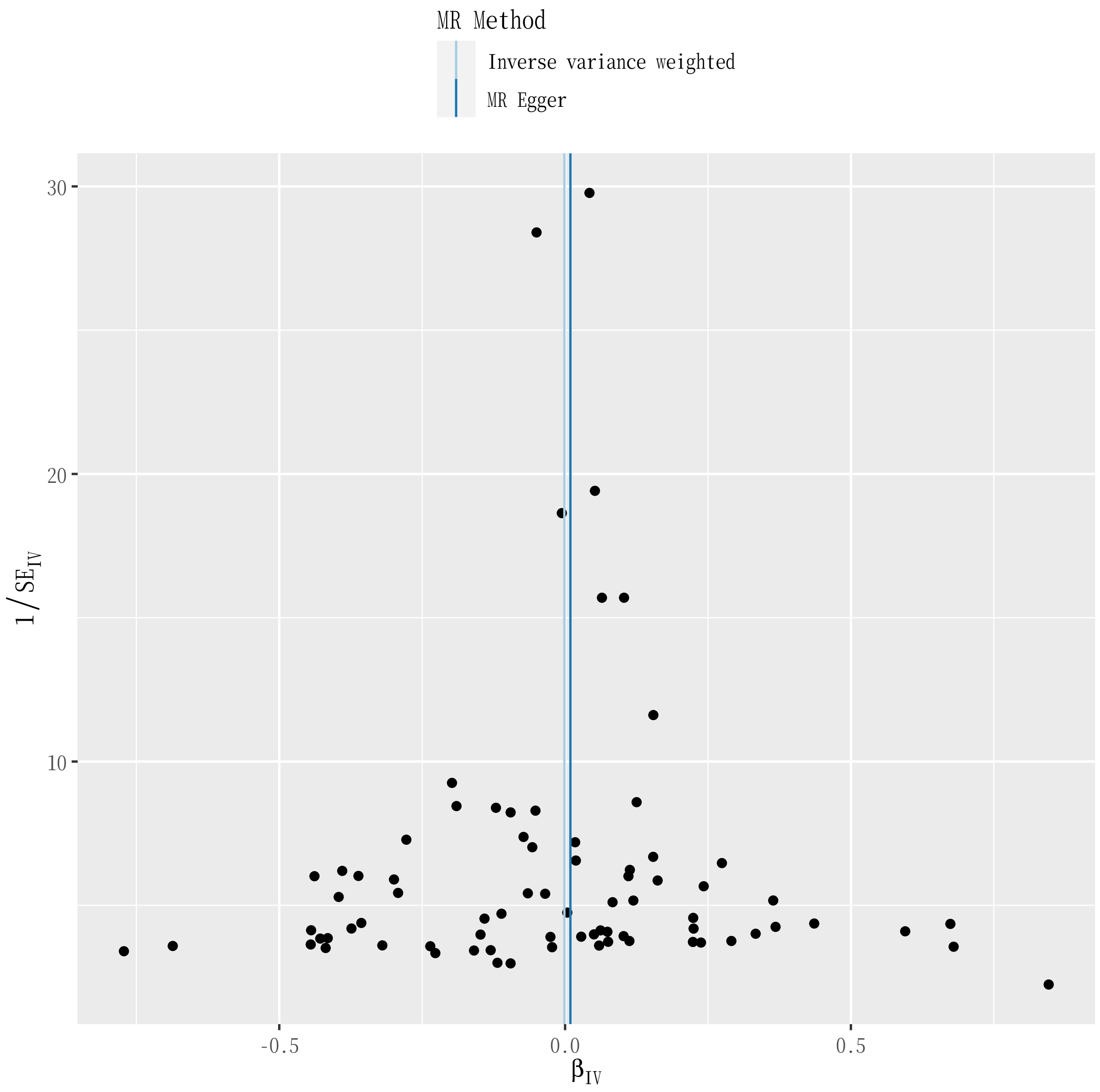
MR Method
Inverse variance weighted
MR Egger
30
20
10
1/SE_{IV}
-0.5
0.0
0.5
$β_{IV}$

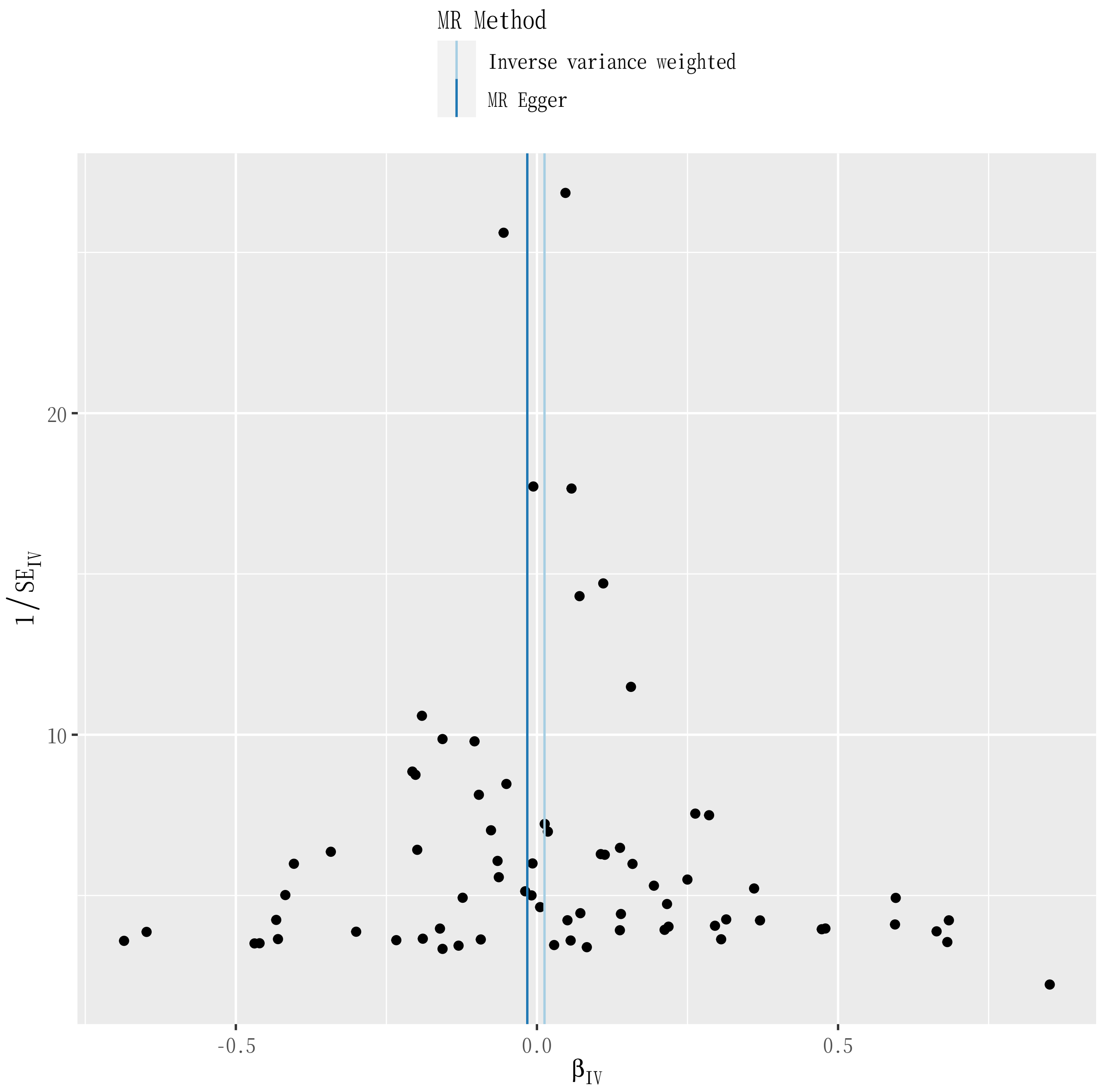

MR Method
Inverse variance weighted
MR Egger
20
10
$1/SE_{IV}$
-0.5
0.0
0.5
β_{IV}

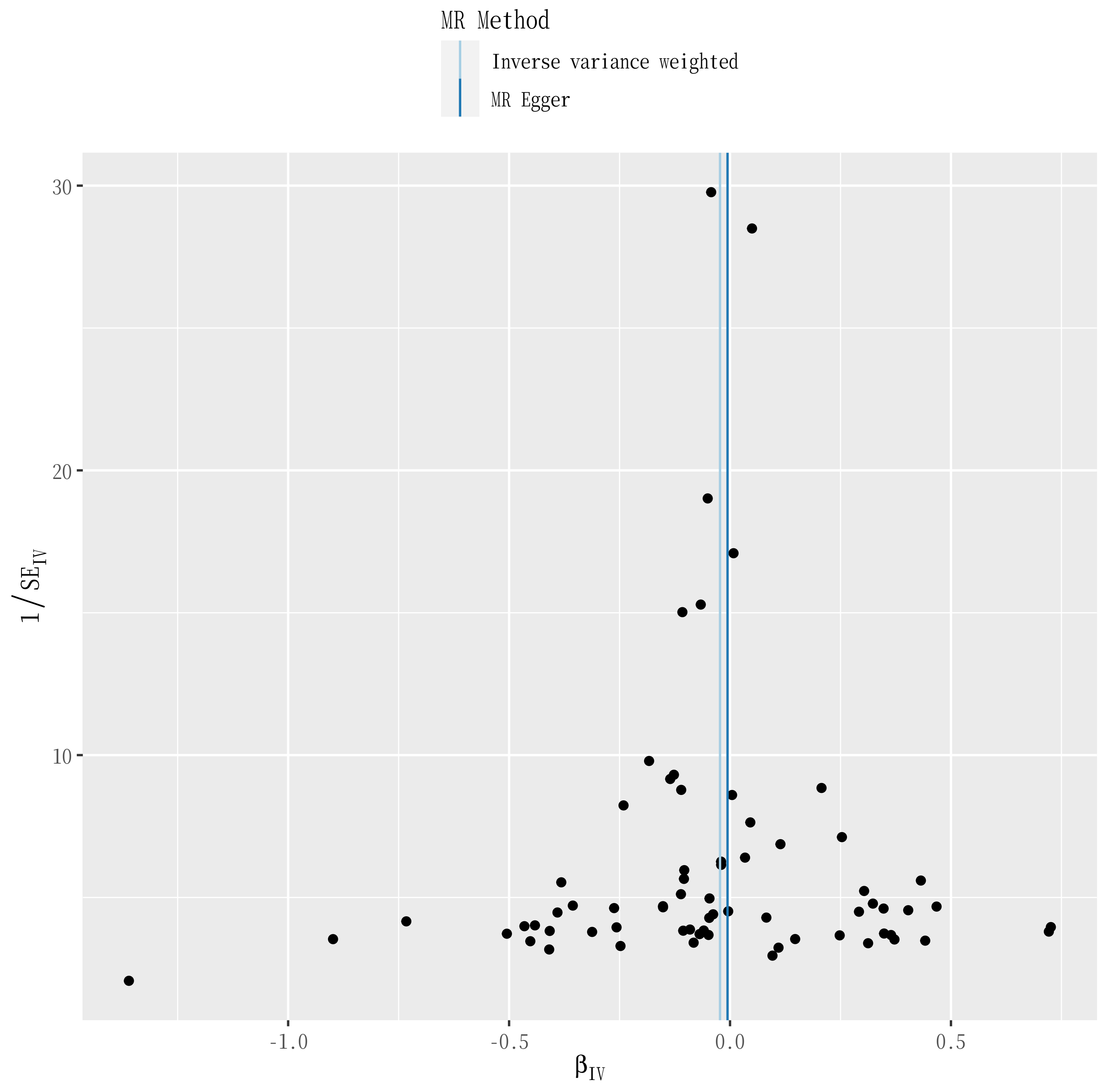

MR Method
Inverse variance weighted
MR Egger
30
20
10
1/SE_IV
-1.0
-0.5
0.0
0.5
β_IV

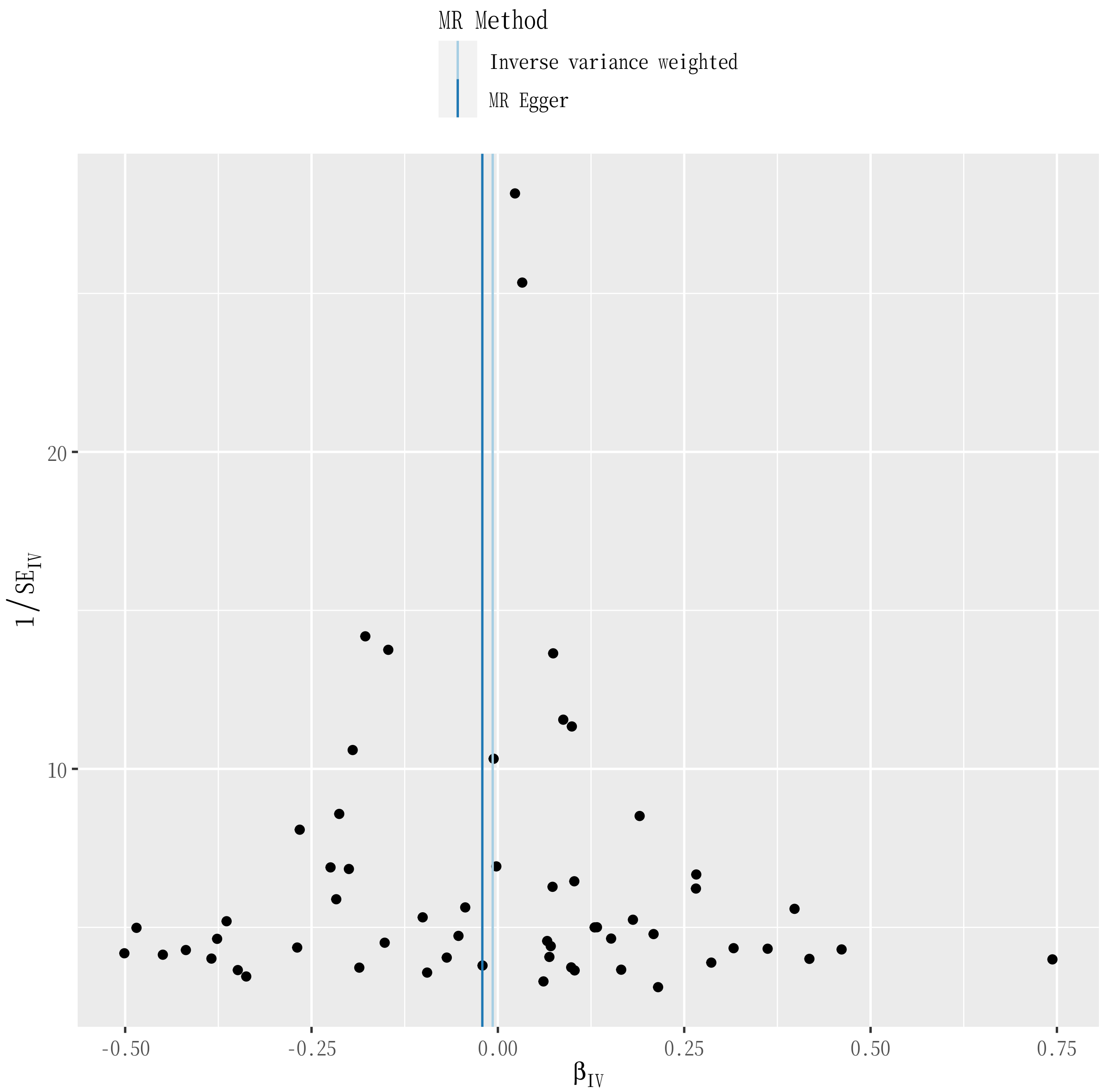

MR Method
Inverse variance weighted
MR Egger
20
10
1/SE_IV
-0.50
-0.25
0.00
0.25
0.50
0.75
β_IV

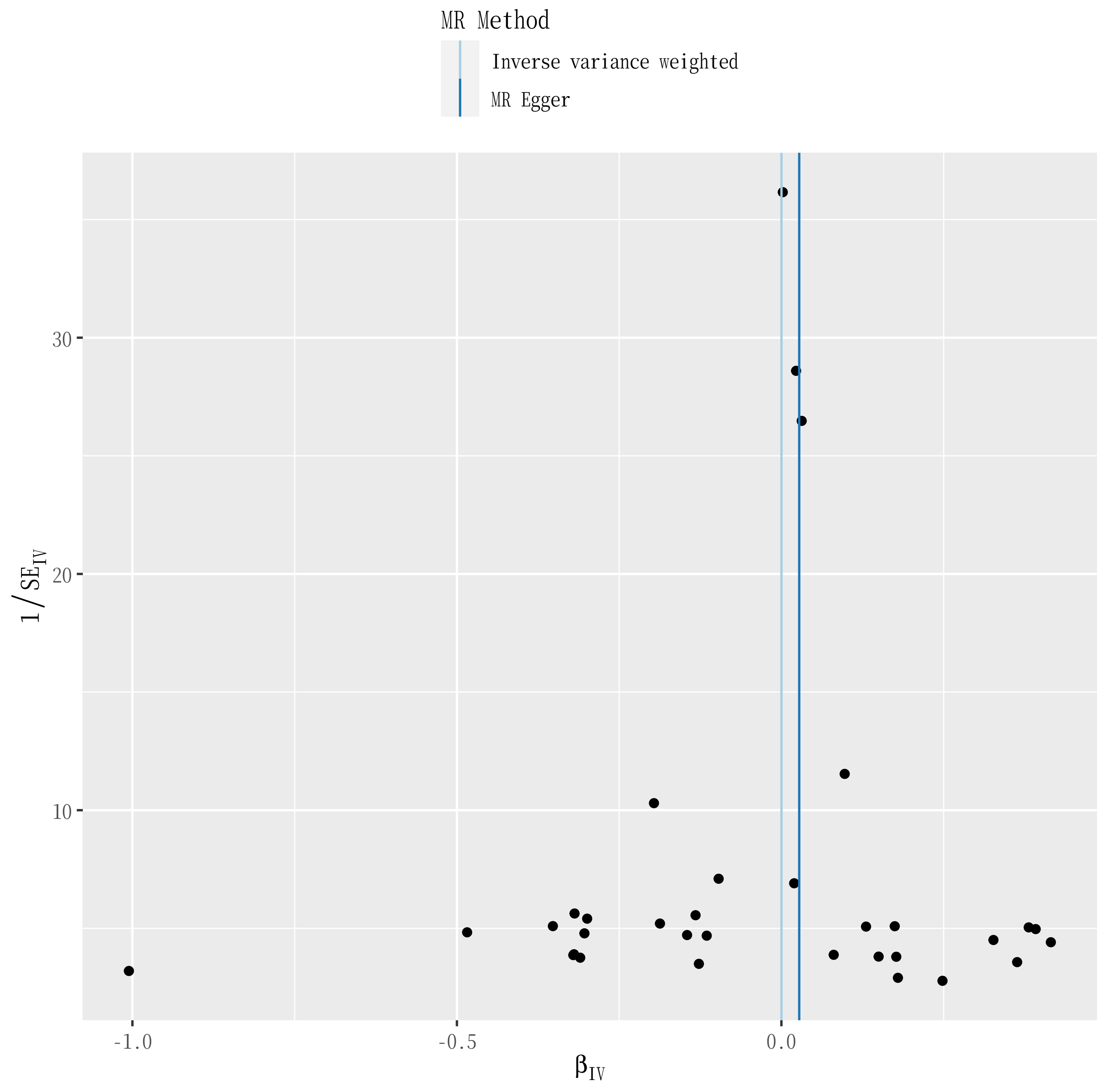
MR Method
Inverse variance weighted
MR Egger
30
20
10
1/SE_IV
-1.0
-0.5
0.0
β_IV

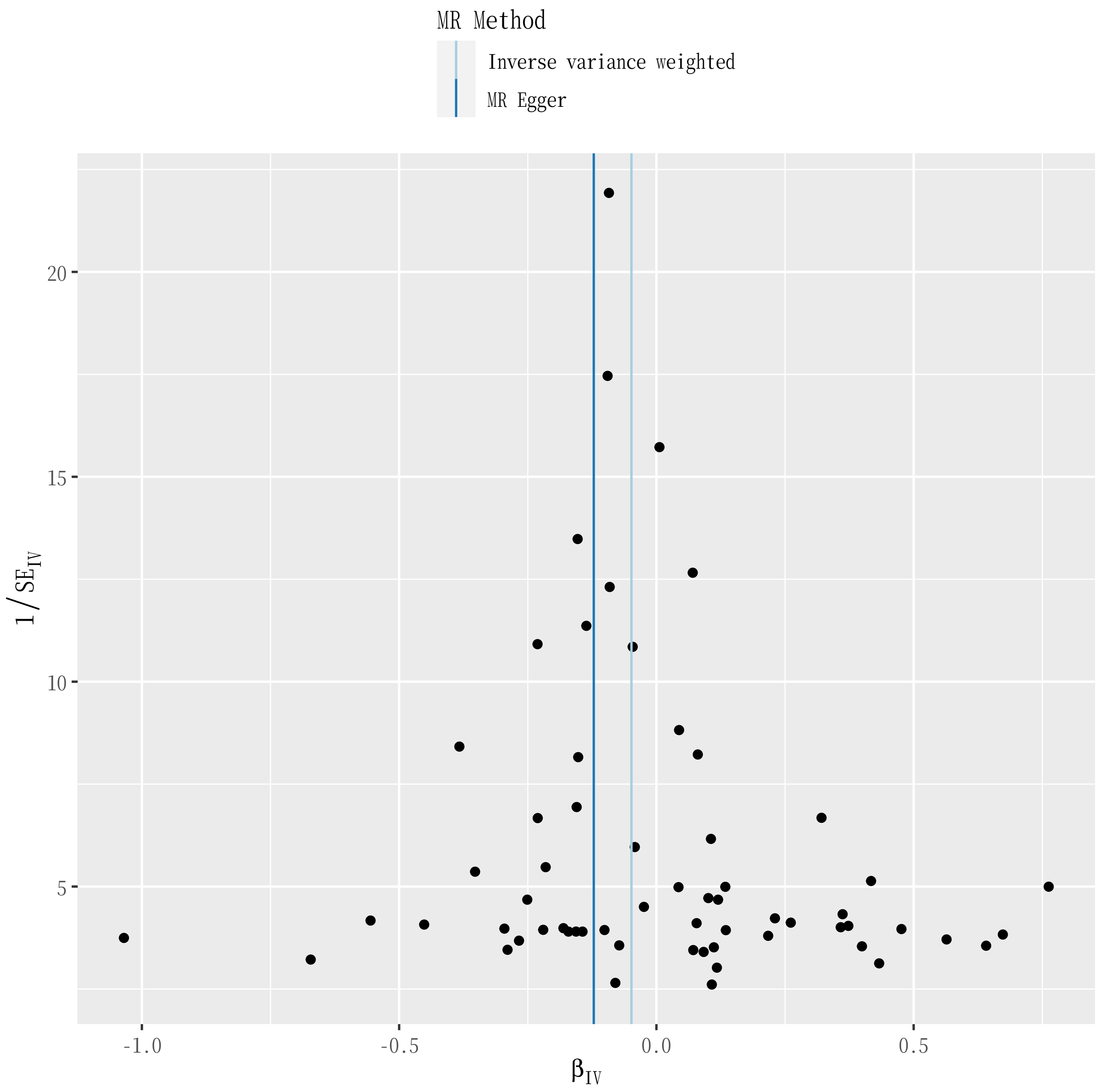
MR Method
Inverse variance weighted
MR Egger
20
15
10
5
-1.0
-0.5
0.0
0.5
$1/SE_{IV}$
β_{IV}

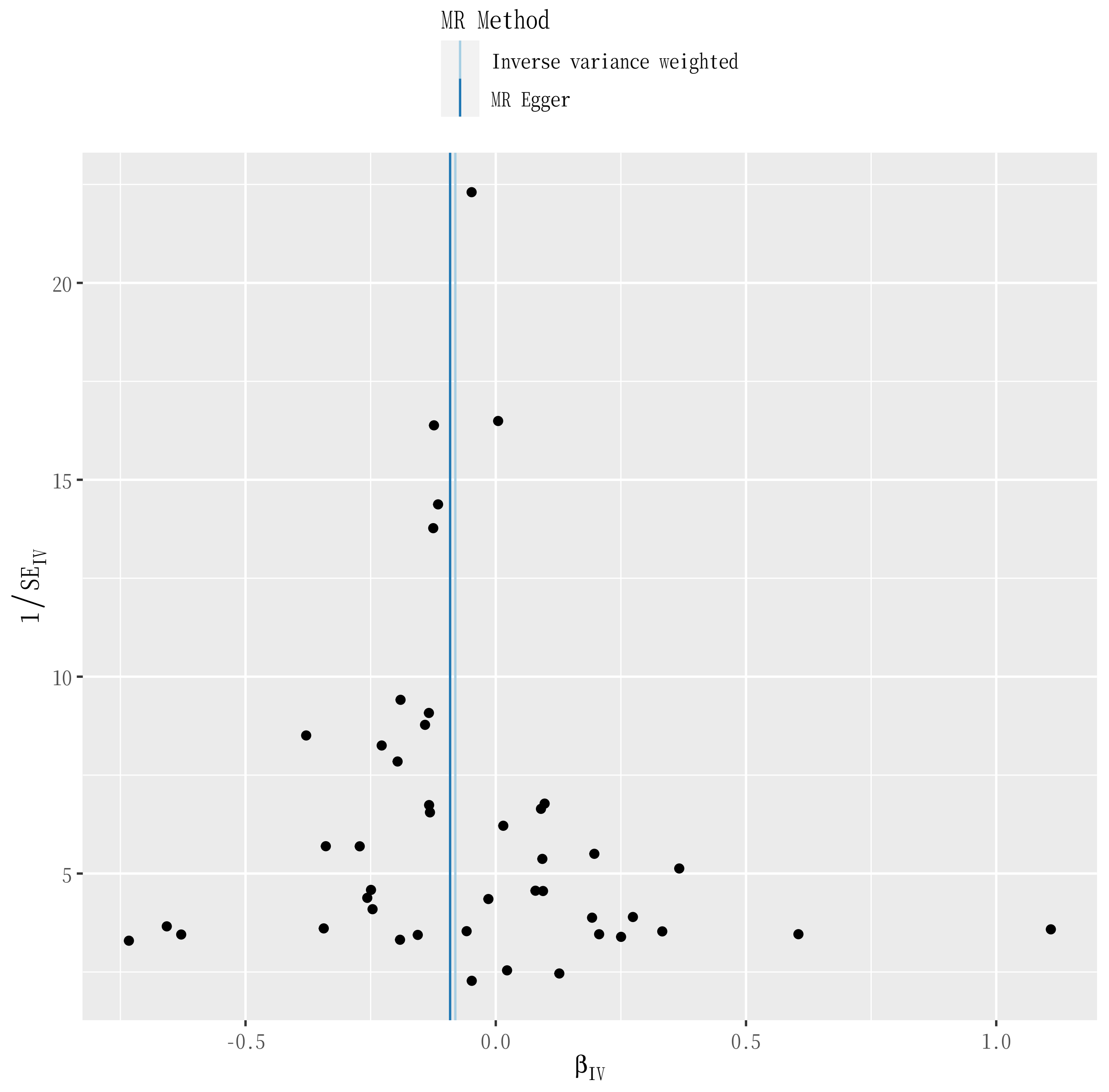

MR Method
Inverse variance weighted
MR Egger
20
15
10
5
1/SE_IV
-0.5
0.0
0.5
1.0
β_IV

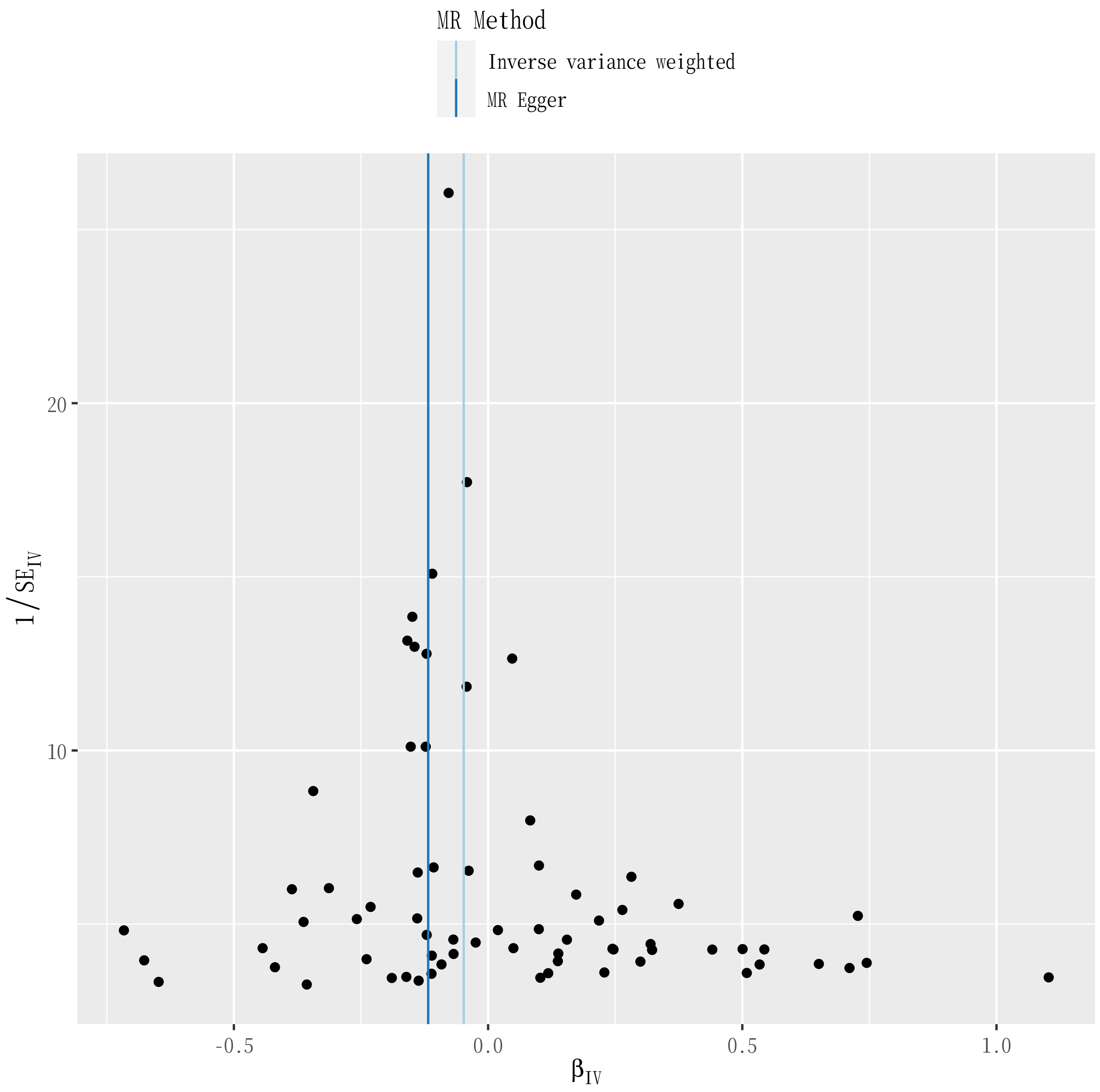

MR Method
Inverse variance weighted
MR Egger
20
10
1/SE_IV
-0.5
0.0
0.5
1.0
β_IV

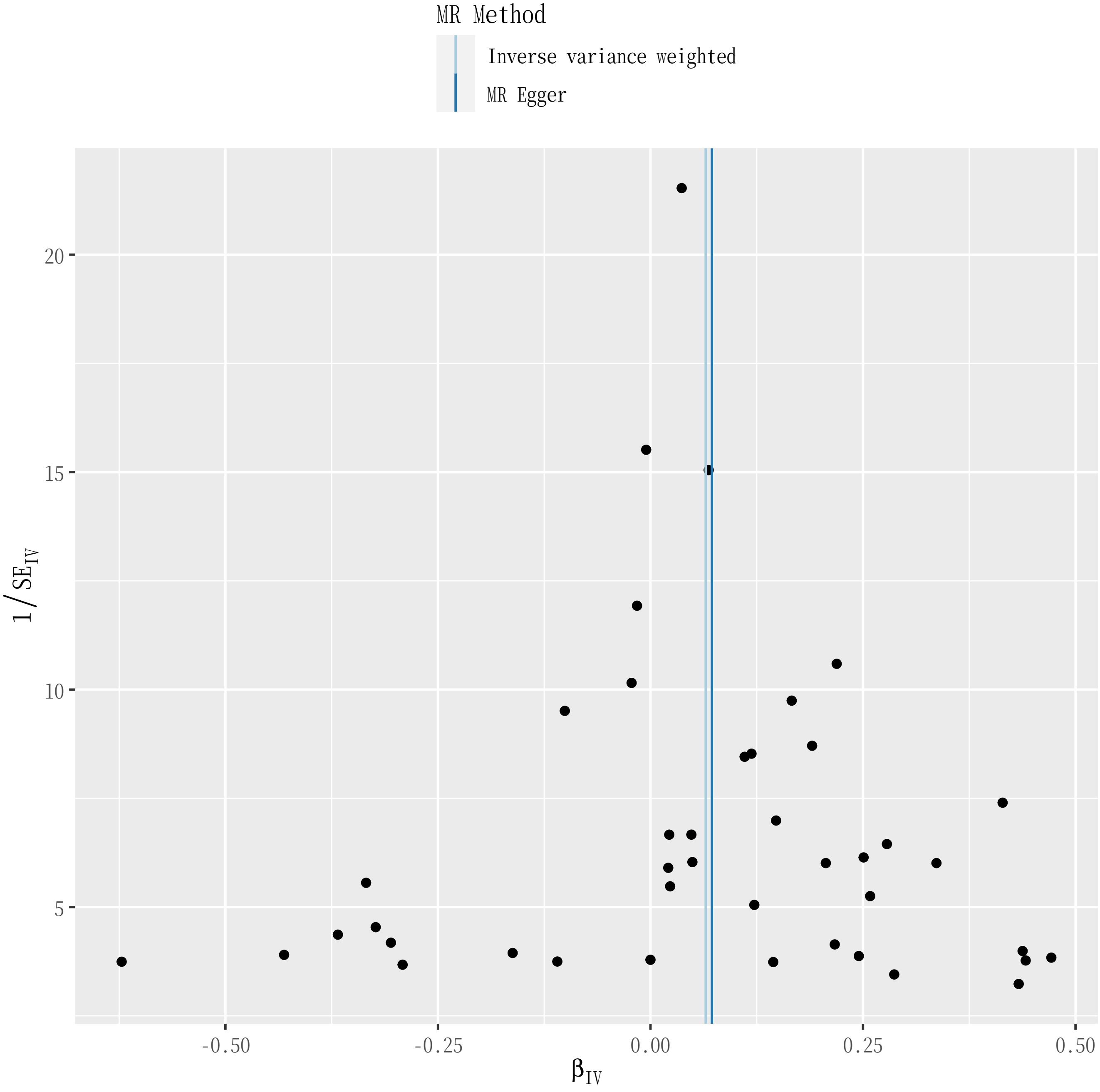

MR Method
Inverse variance weighted
MR Egger
20
15
10
5
1/SE$_{IV}$
-0.50
-0.25
0.00
0.25
0.50
β$_{IV}$

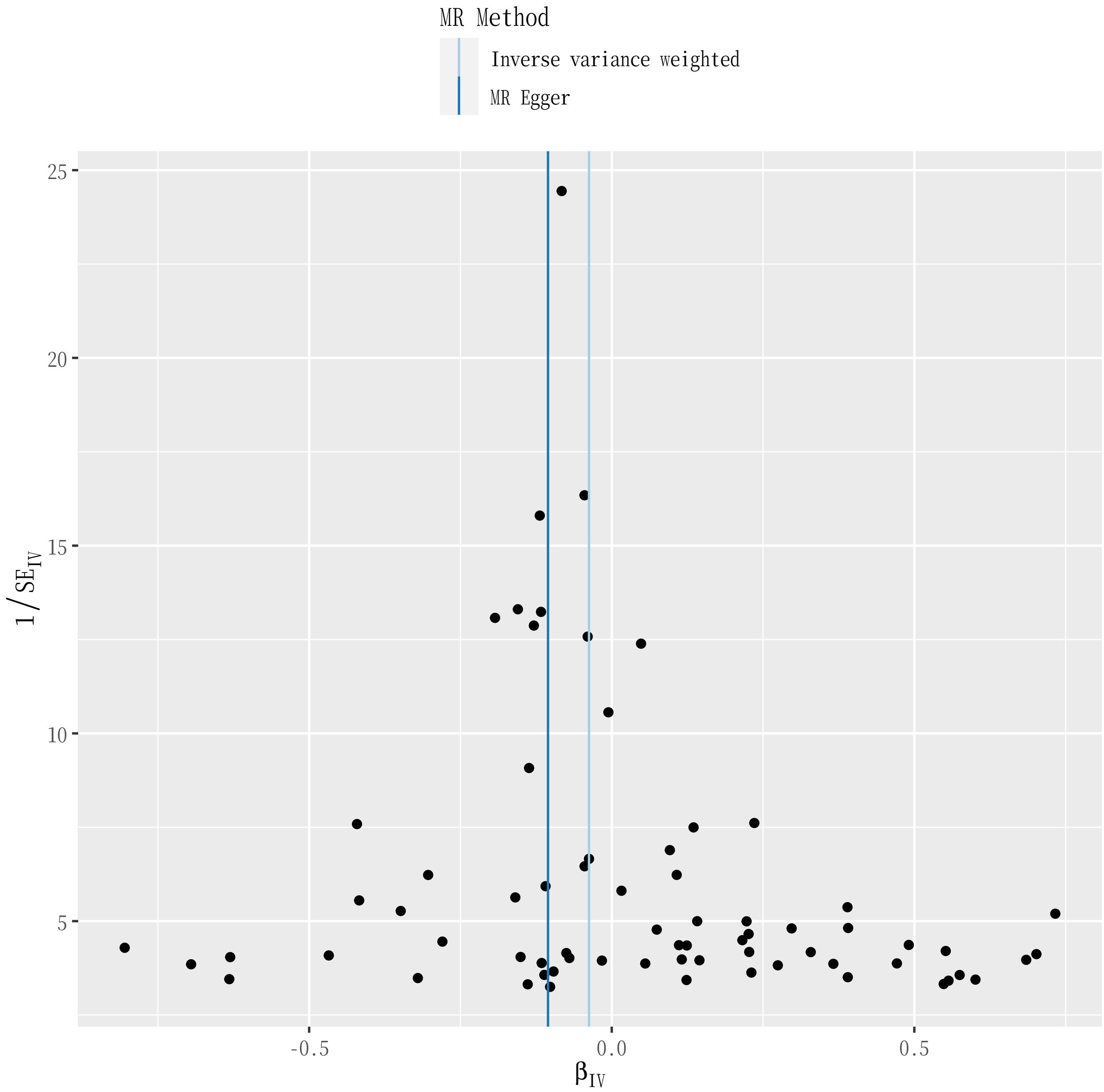
MR Method
Inverse variance weighted
MR Egger
25
20
15
10
5
-0.5
0.0
0.5
1/SE_IV
β_IV

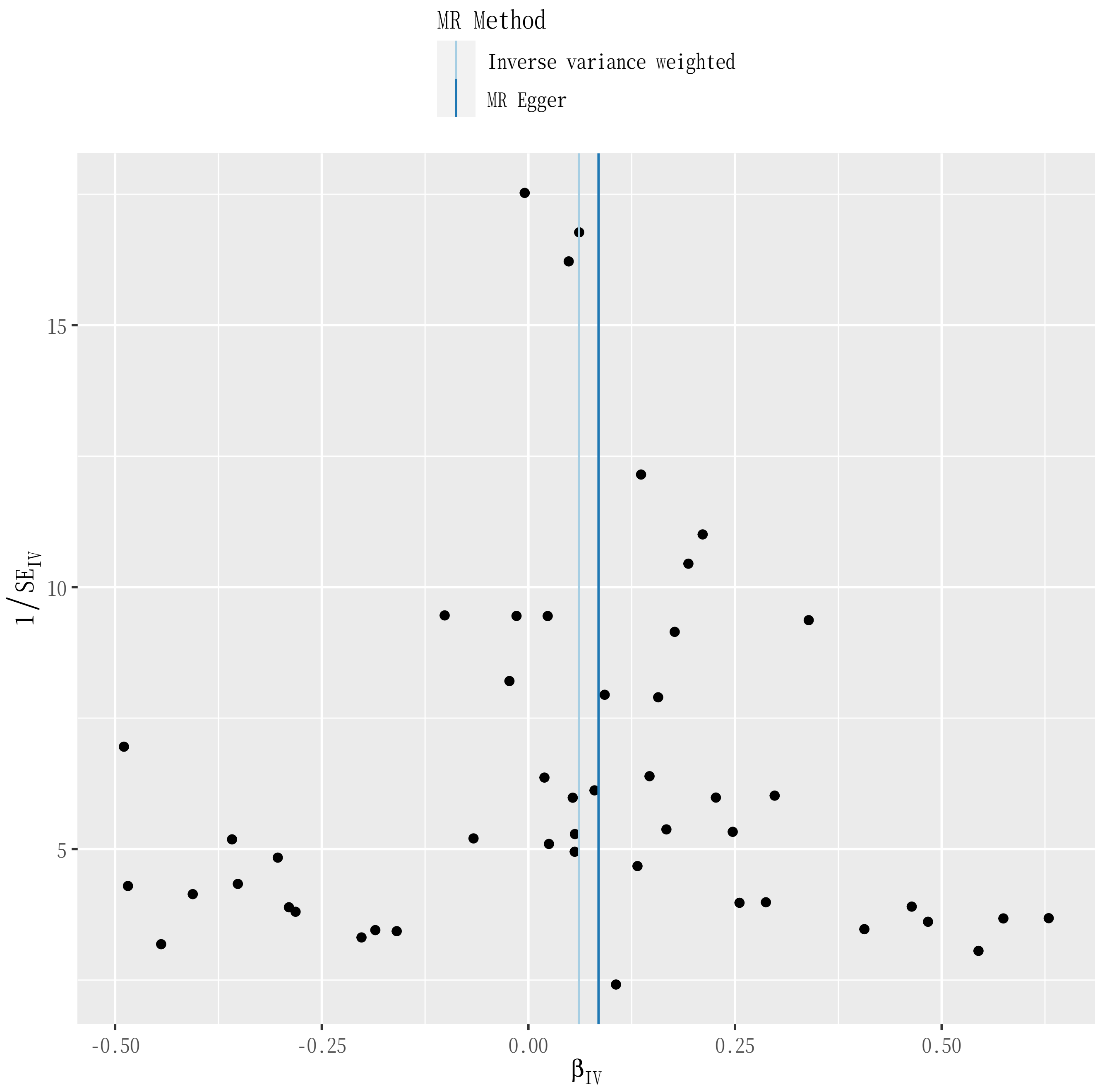

MR Method
Inverse variance weighted
MR Egger
15
10
5
1/SE_IV
-0.50
-0.25
0.00
0.25
0.50
β_IV

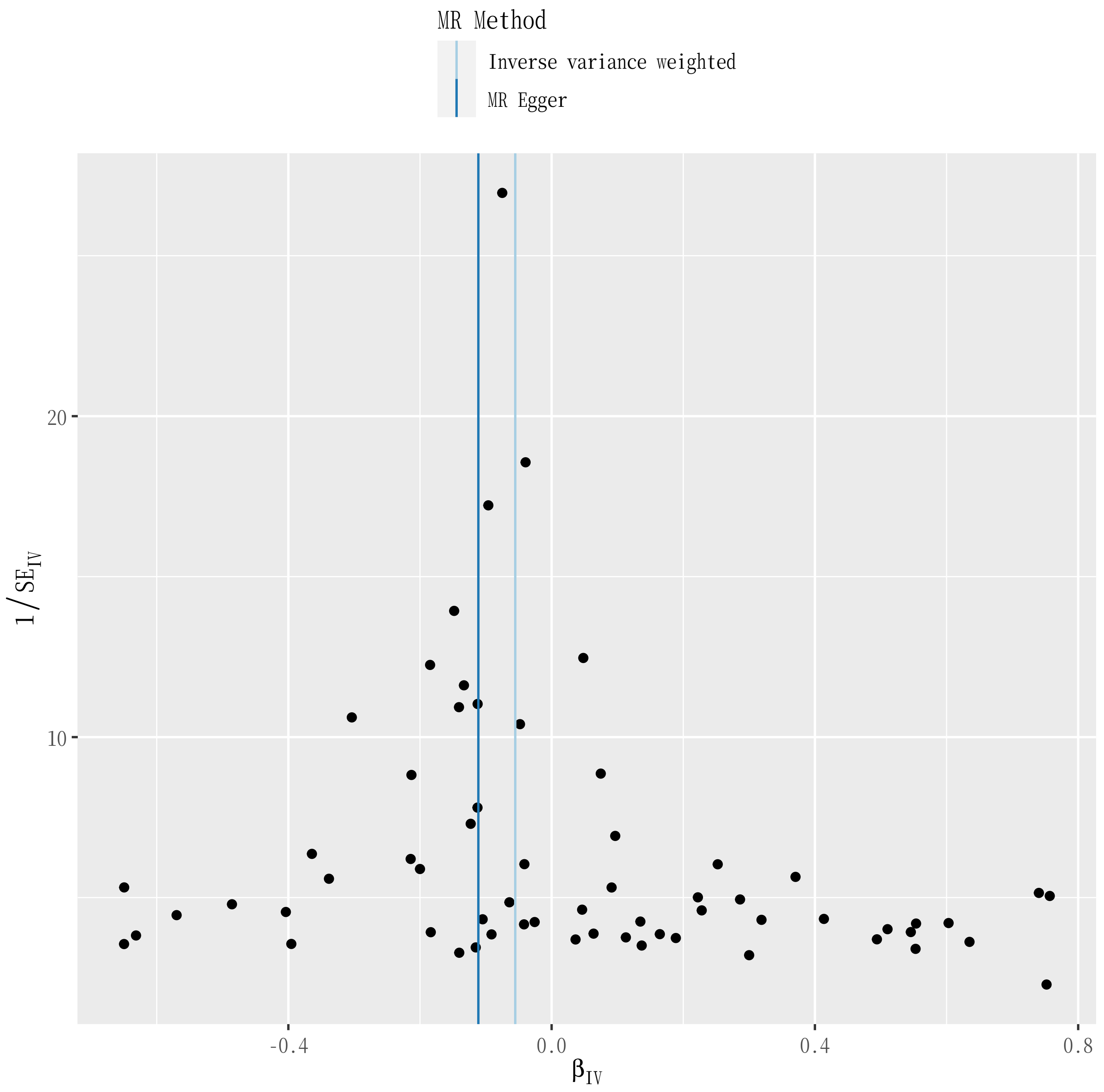
MR Method
Inverse variance weighted
MR Egger
20
10
1/SE_IV
-0.4
0.0
0.4
0.8
β_IV

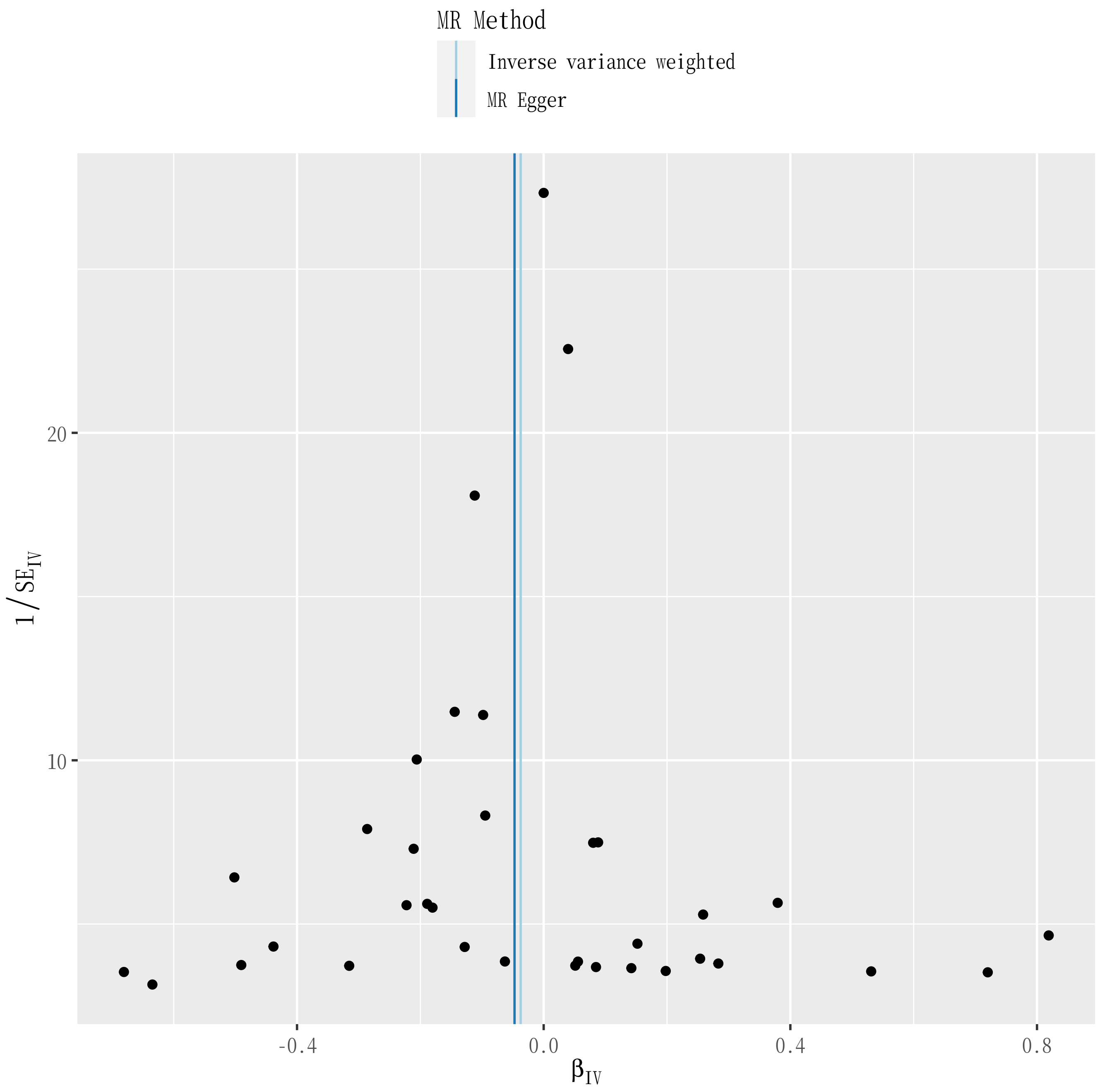

MR Method
Inverse variance weighted
MR Egger
20
10
1/SE$_{IV}$
-0.4
0.0
0.4
0.8
β_{IV}

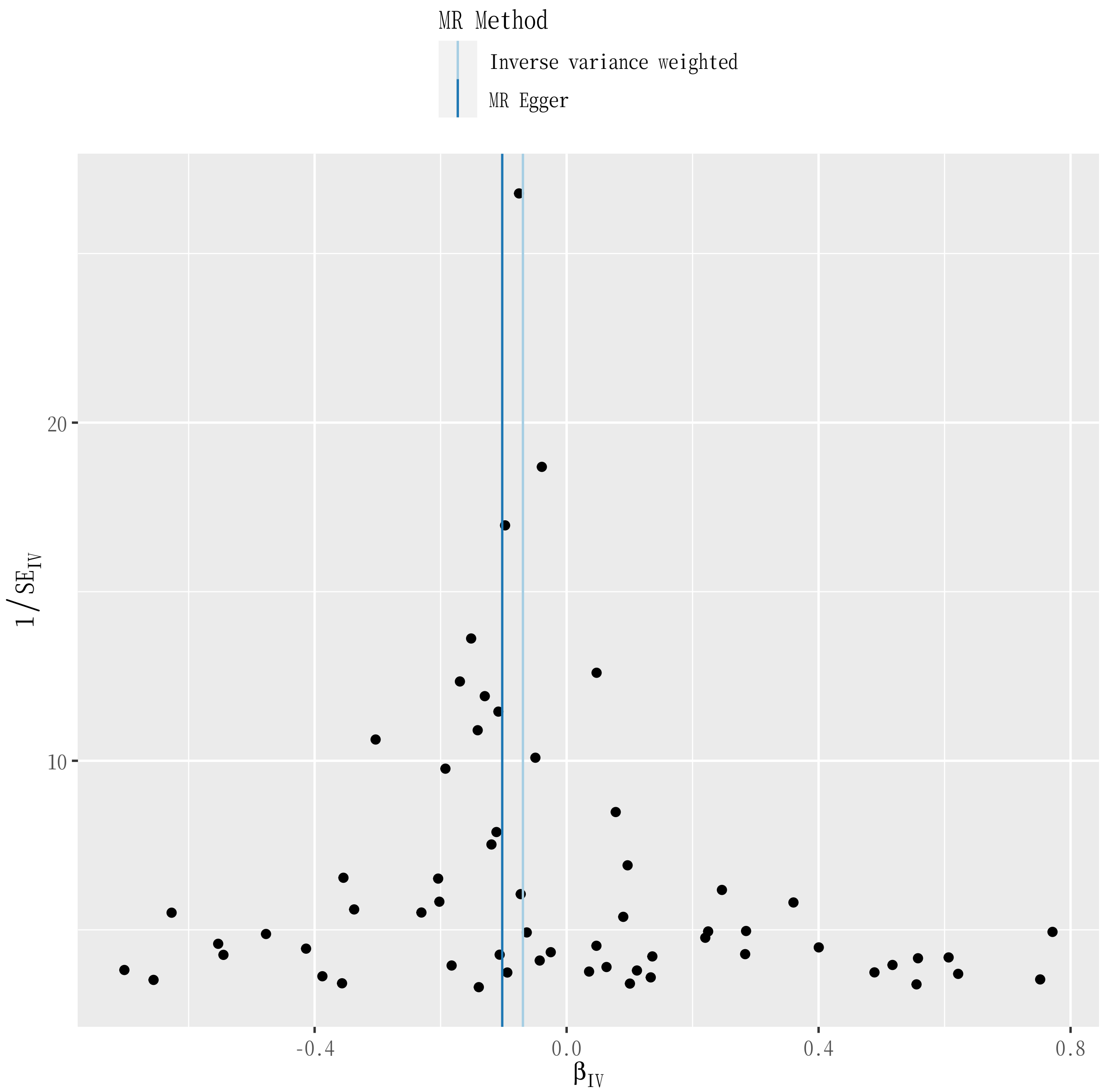

MR Method
Inverse variance weighted
MR Egger
20
10
$1/SE_{IV}$
-0.4
0.0
0.4
0.8
β_{IV}

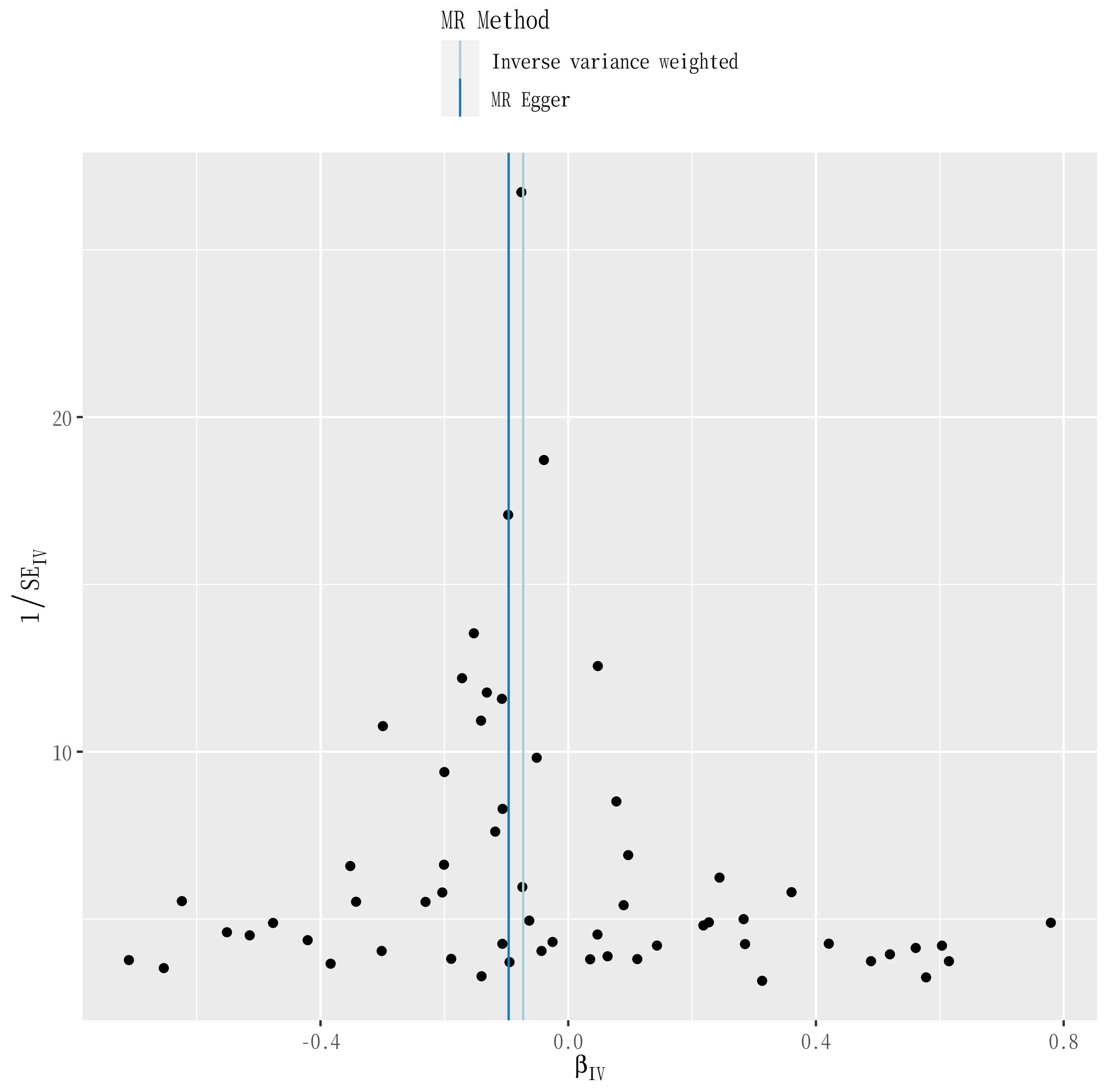

MR Method
Inverse variance weighted
MR Egger
20
10
$1/SE_{IV}$
-0.4
0.0
0.4
0.8
β_{IV}

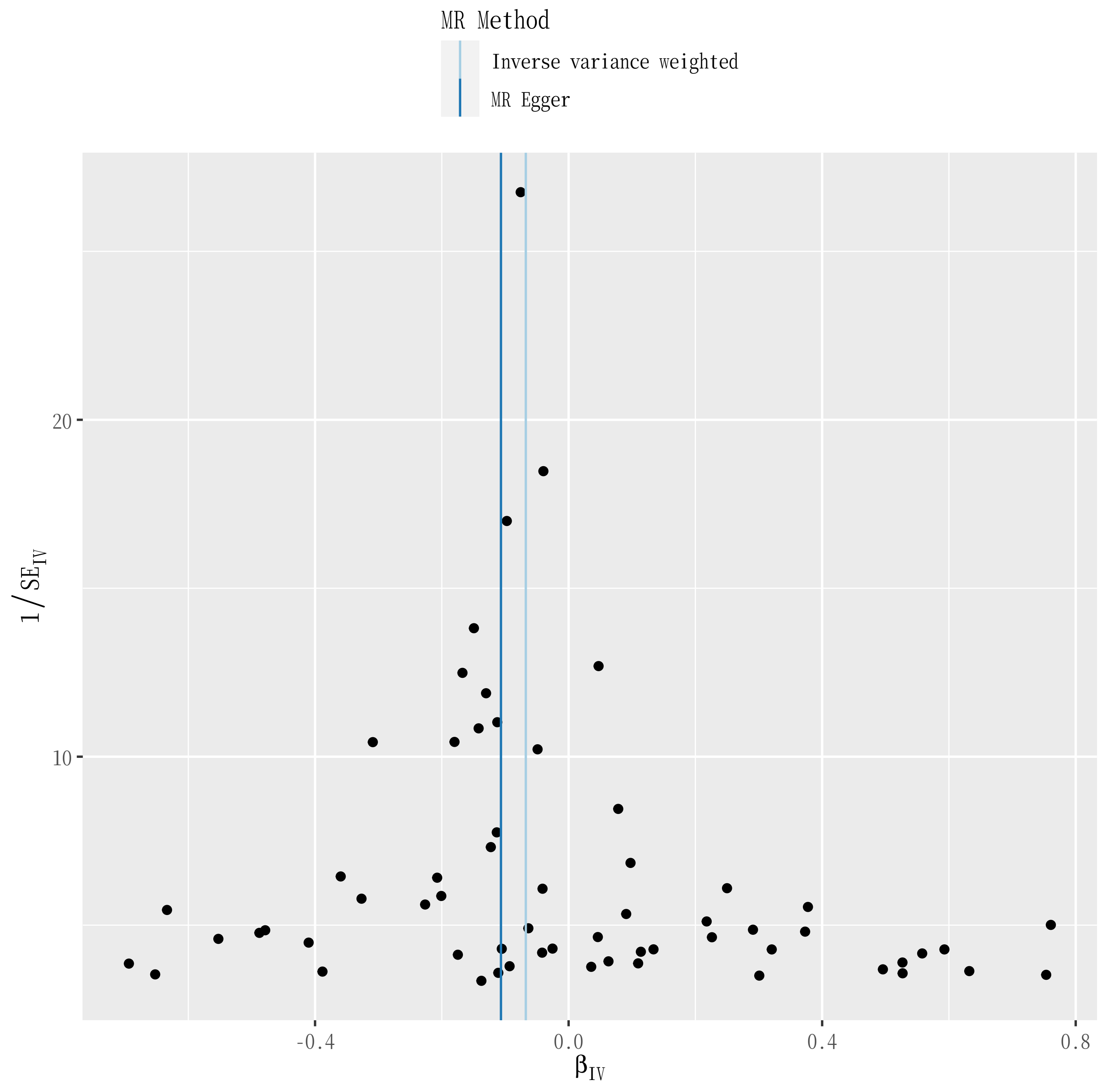

MR Method
Inverse variance weighted
MR Egger
20
10
$1/SE_{IV}$
-0.4
0.0
0.4
0.8
β_{IV}

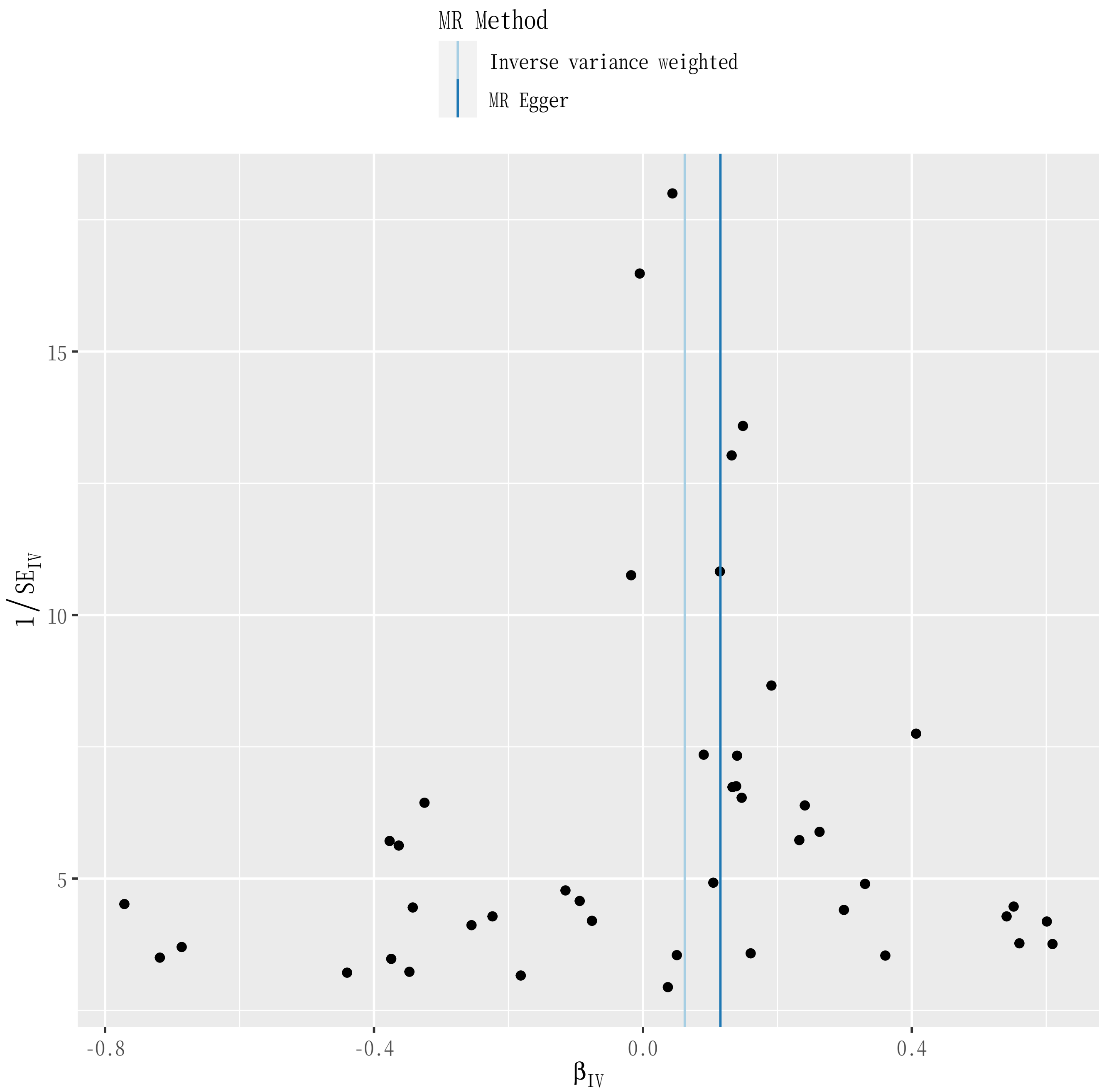

MR Method
Inverse variance weighted
MR Egger
15
10
5
1/SE_IV
-0.8
-0.4
0.0
0.4
β_IV

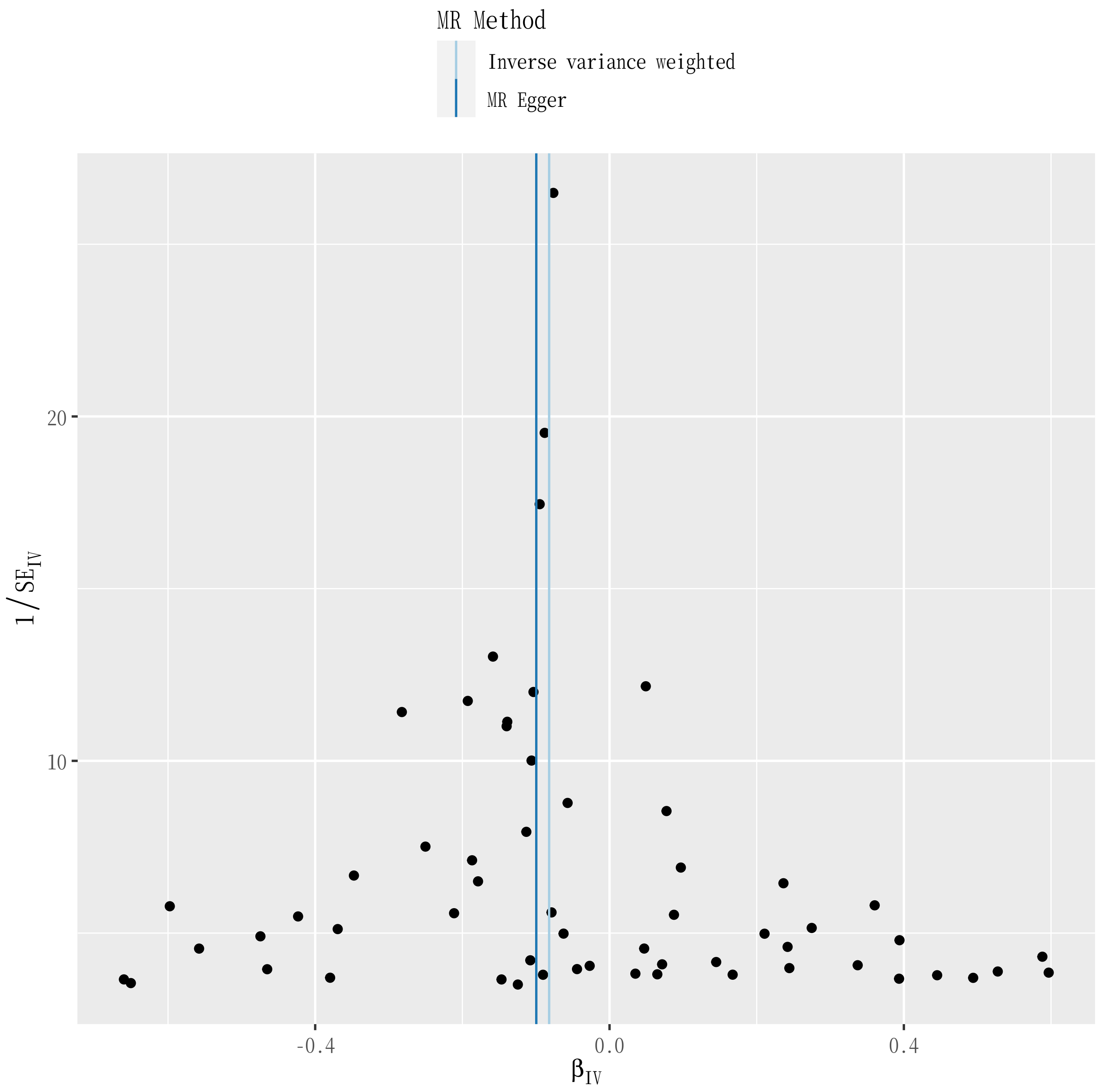
MR Method
Inverse variance weighted
MR Egger
20
10
1/SE_IV
-0.4
0.0
0.4
β_IV

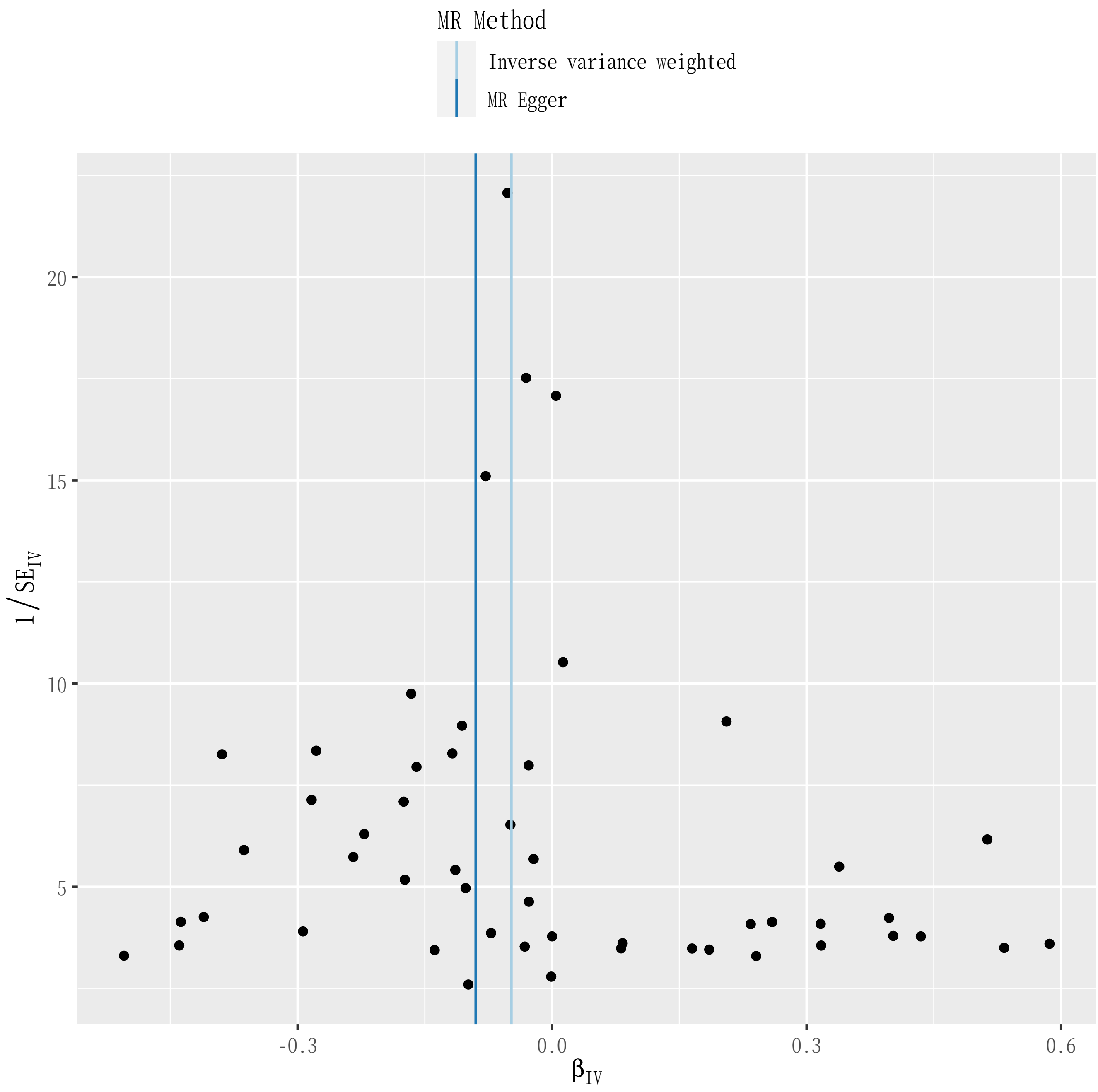
MR Method
Inverse variance weighted
MR Egger
20
15
10
5
-0.3
0.0
0.3
0.6
1/SE_IV
β_IV

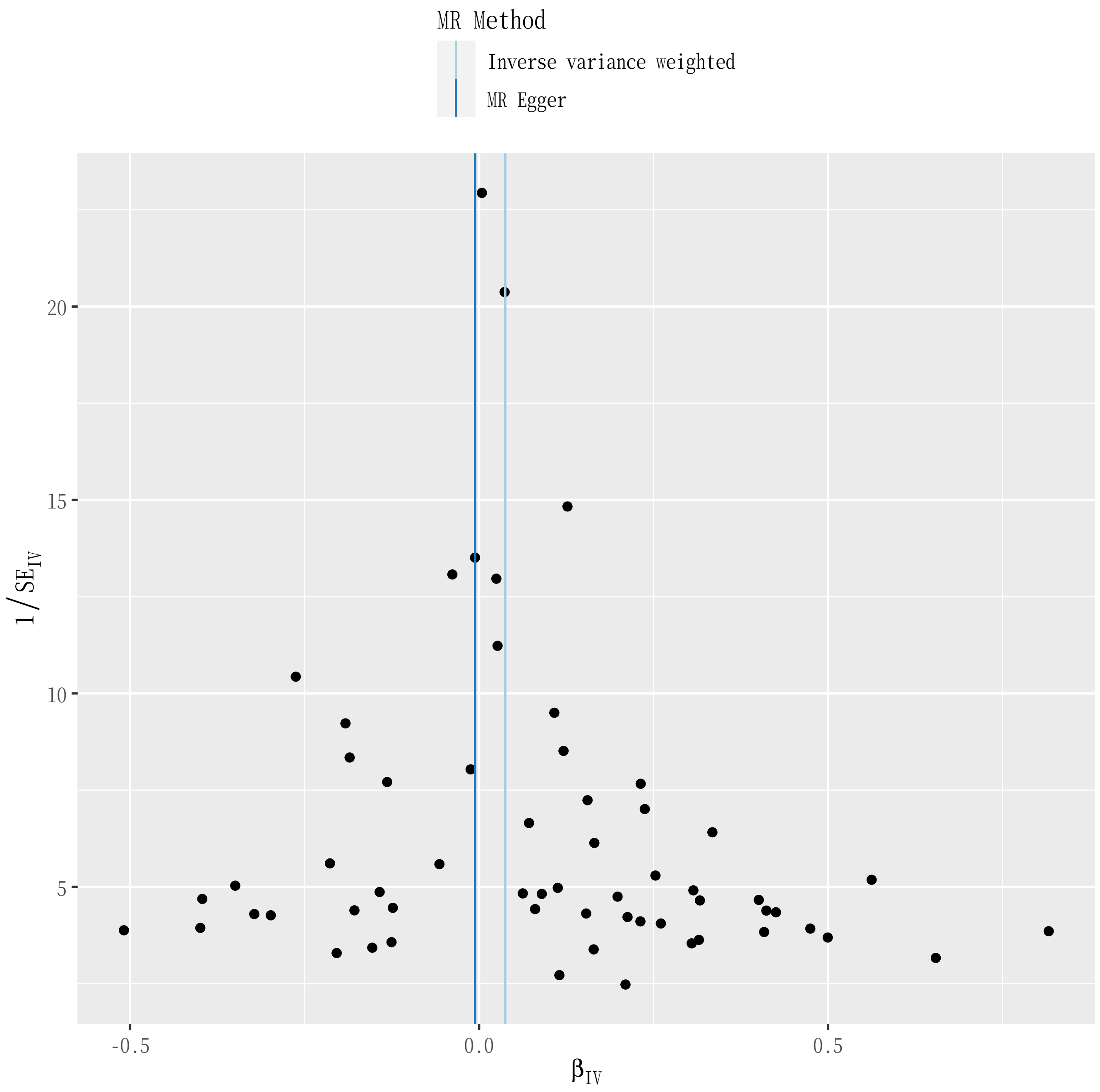
MR Method
Inverse variance weighted
MR Egger
20
15
10
5
1/SE_IV
-0.5
0.0
0.5
β_IV

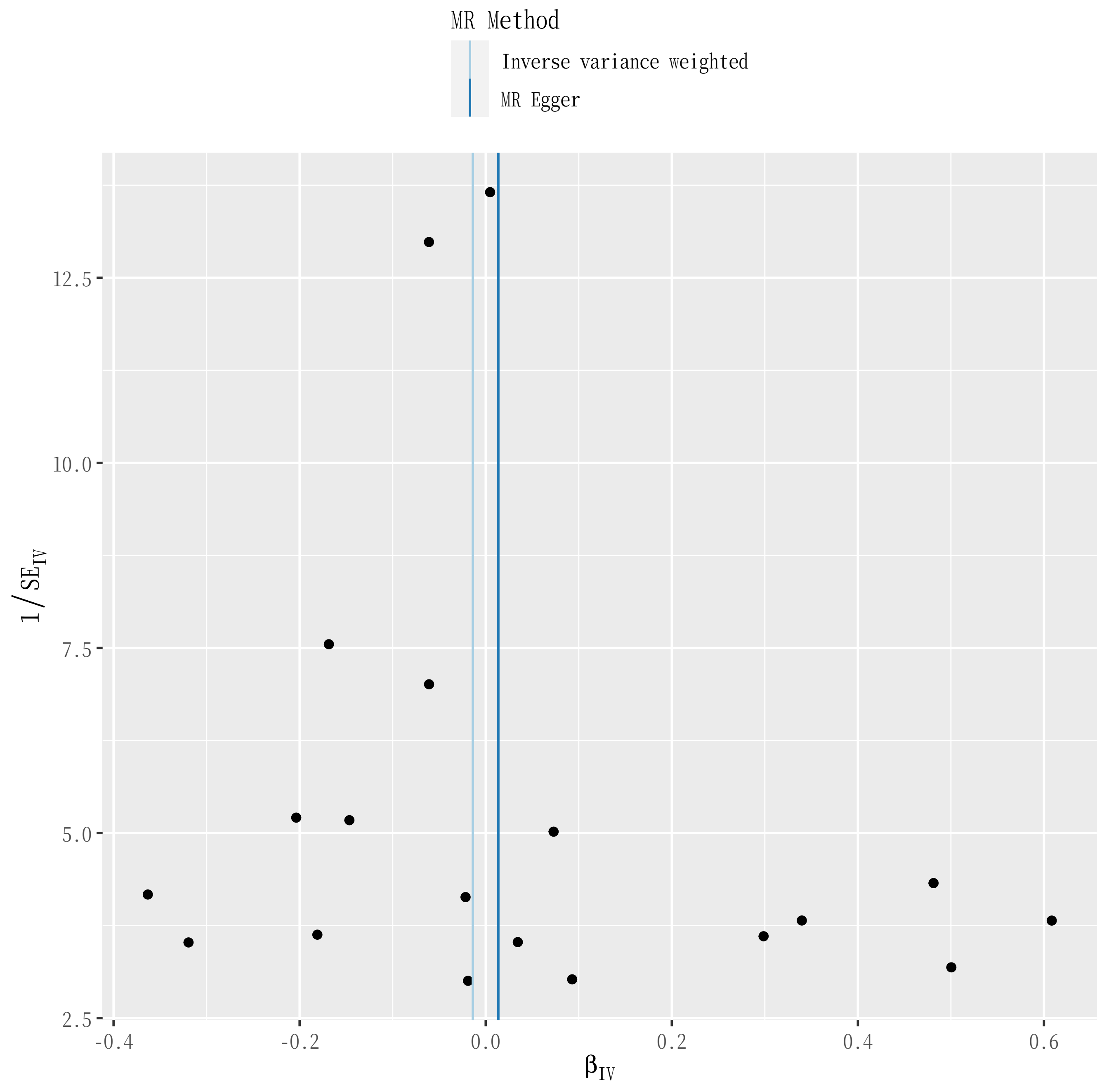
MR Method
Inverse variance weighted
MR Egger
12.5
10.0
7.5
5.0
2.5
$1/SE_{IV}$
-0.4
-0.2
0.0
0.2
0.4
0.6
β_{IV}

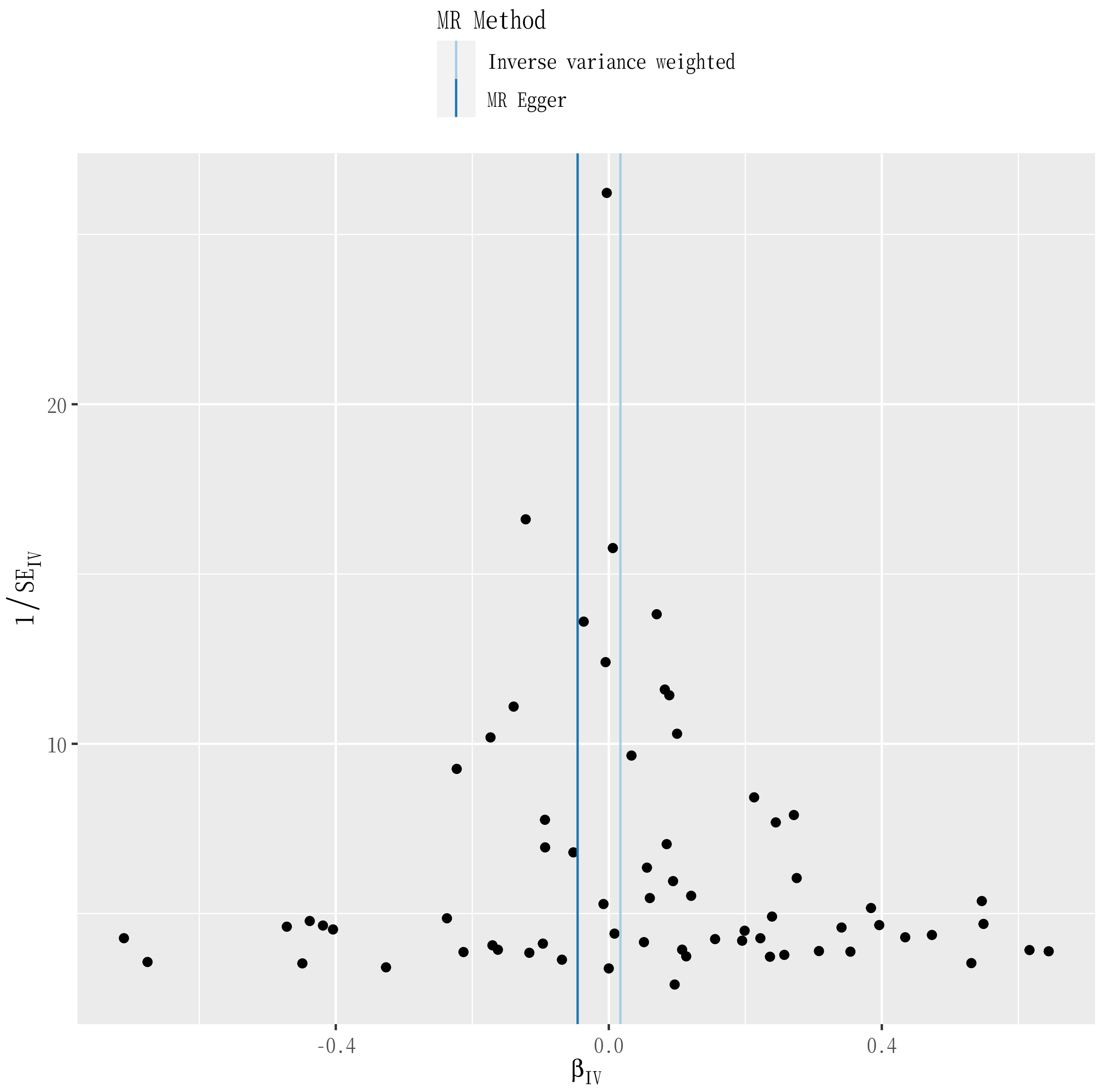

MR Method
Inverse variance weighted
MR Egger
20
10
-0.4
0.0
0.4
1/SE_IV
β_IV

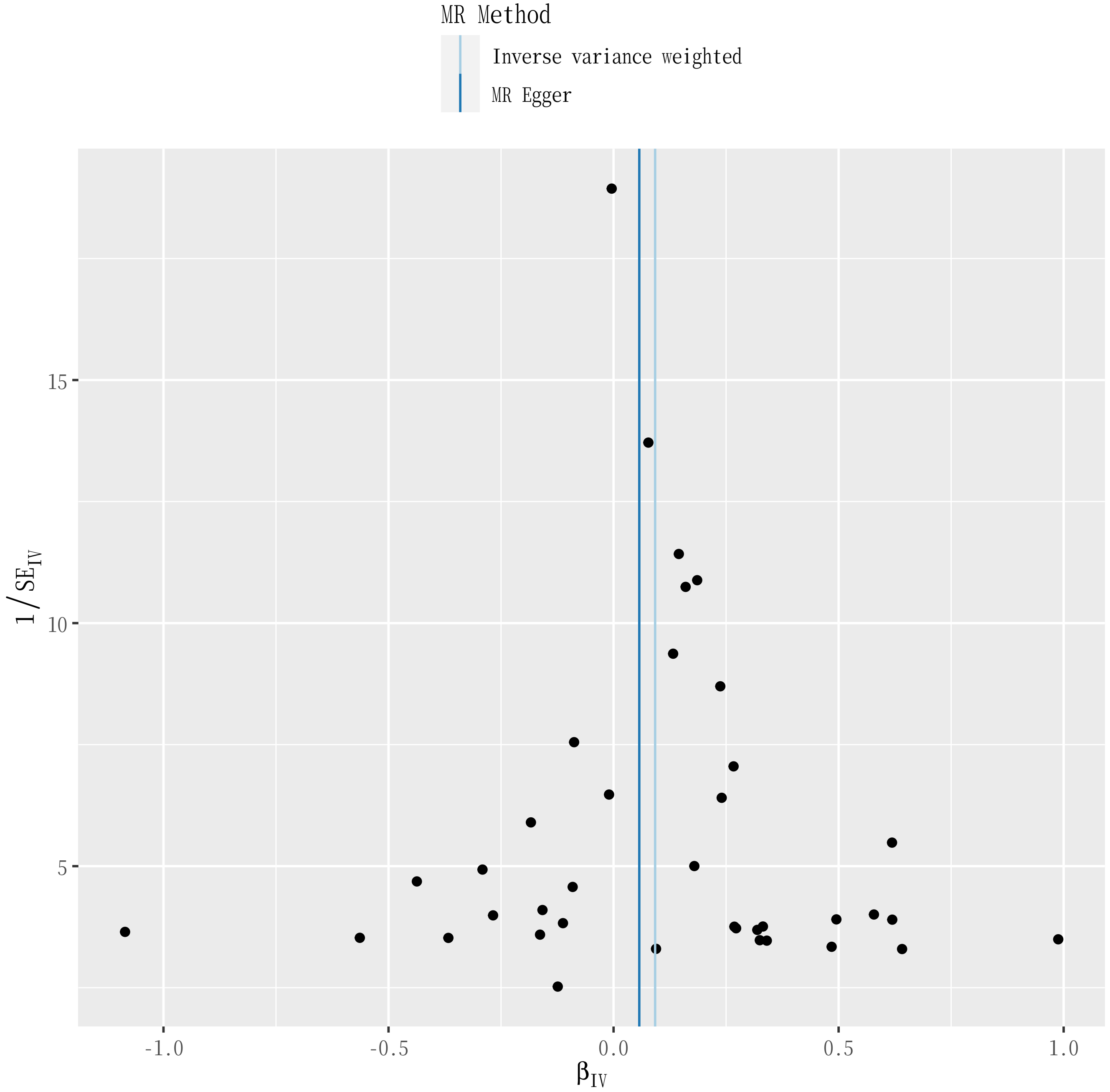

MR Method
Inverse variance weighted
MR Egger
15
10
5
1/SE_IV
-1.0
-0.5
0.0
0.5
1.0
β_IV

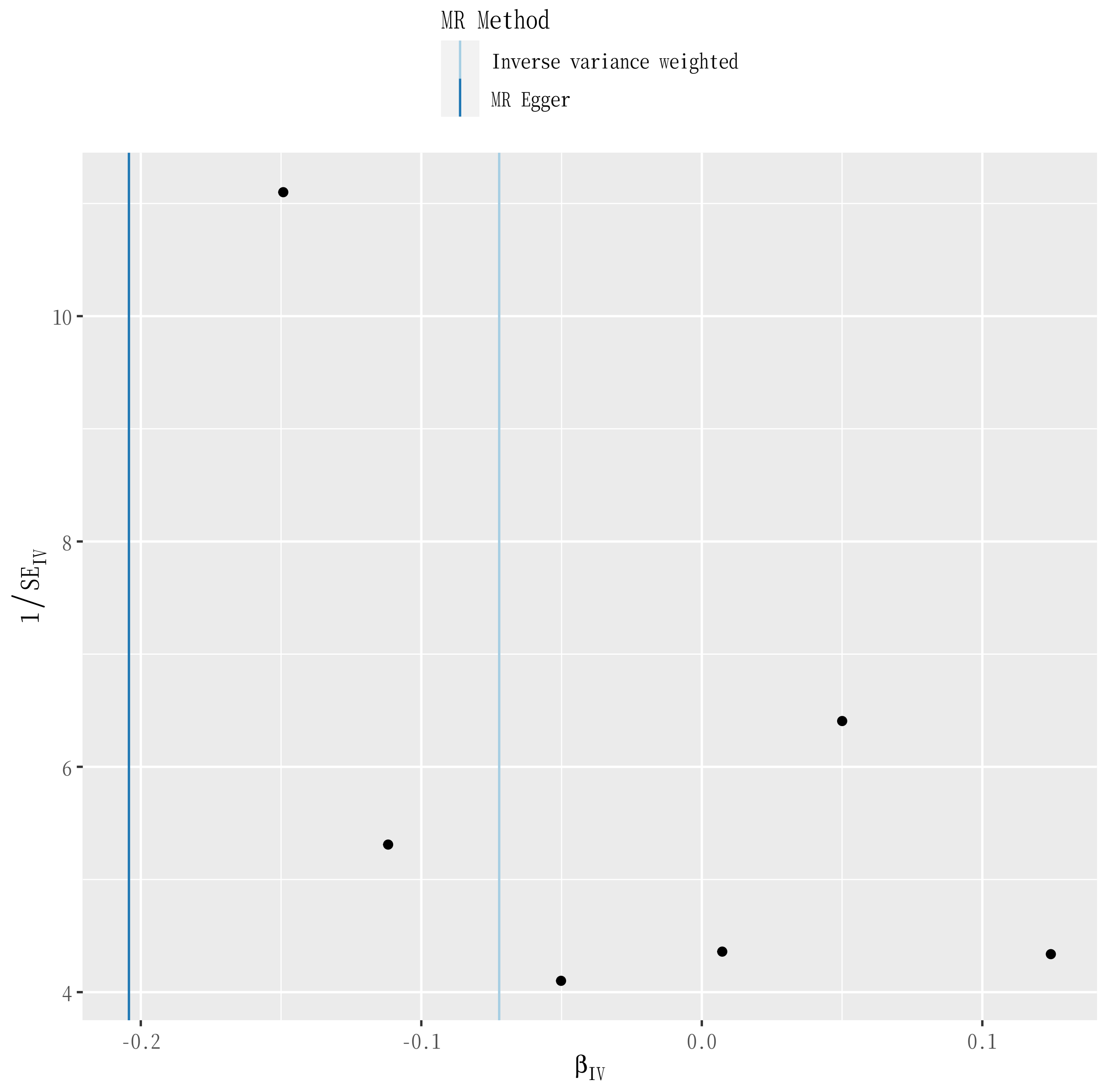

MR Method
Inverse variance weighted
MR Egger
1/SE_IV
10
8
6
4
-0.2
-0.1
0.0
0.1
β_IV

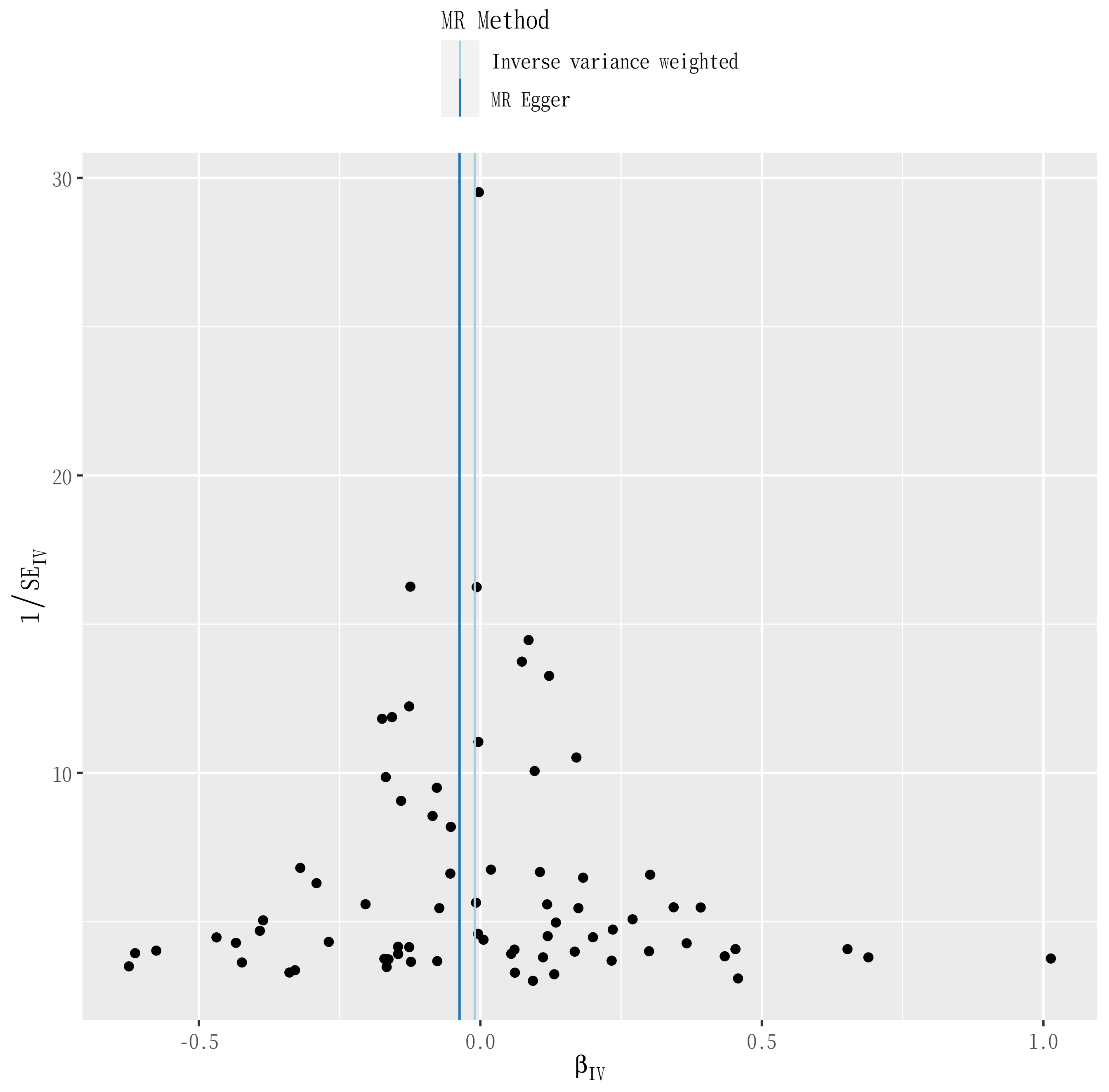

MR Method
Inverse variance weighted
MR Egger
30
20
10
$1/SE_{IV}$
-0.5
0.0
0.5
1.0
β_{IV}

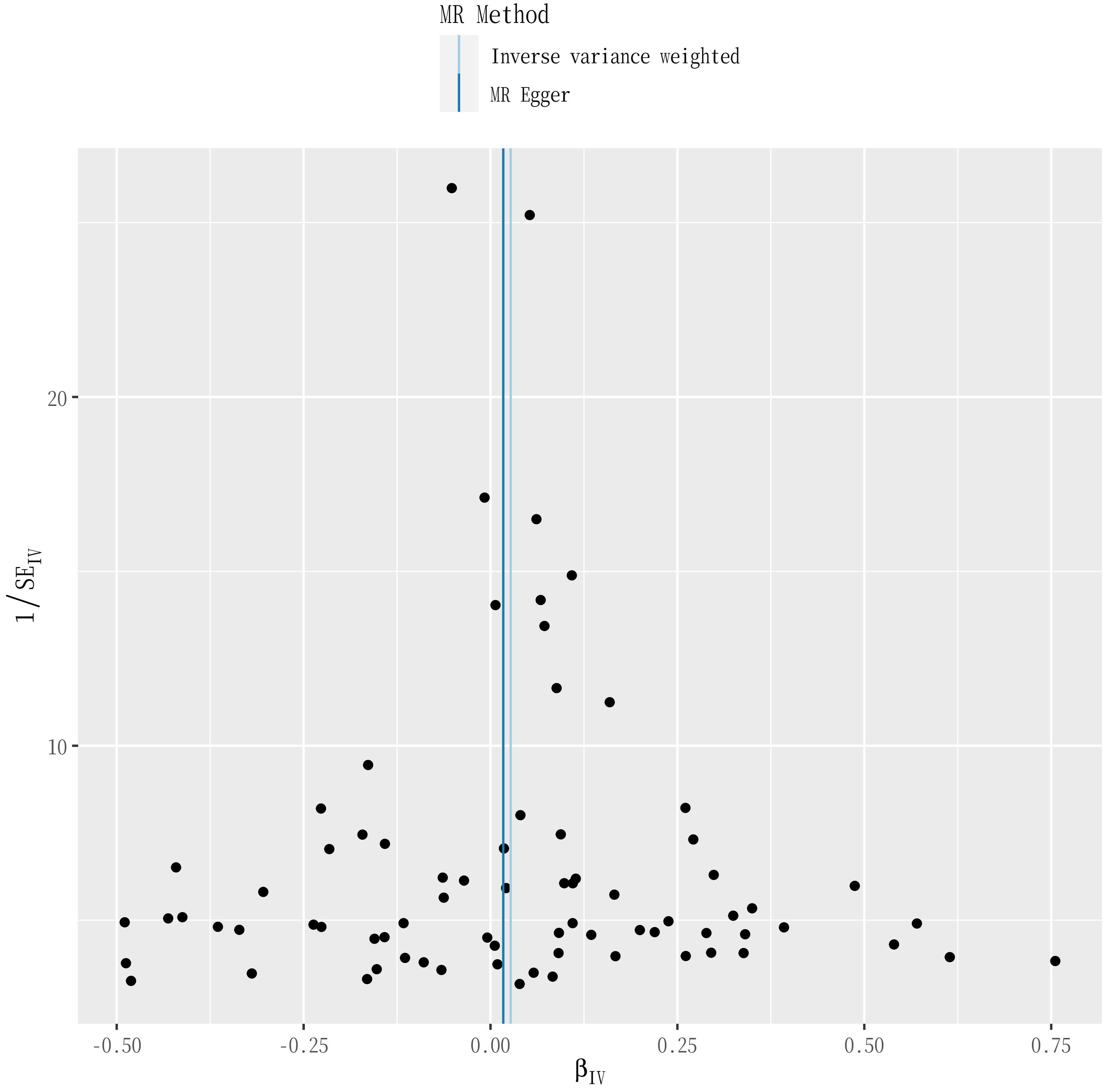
MR Method
Inverse variance weighted
MR Egger
20
10
-0.50
-0.25
0.00
0.25
0.50
0.75
$1/SE_{IV}$
β_{IV}

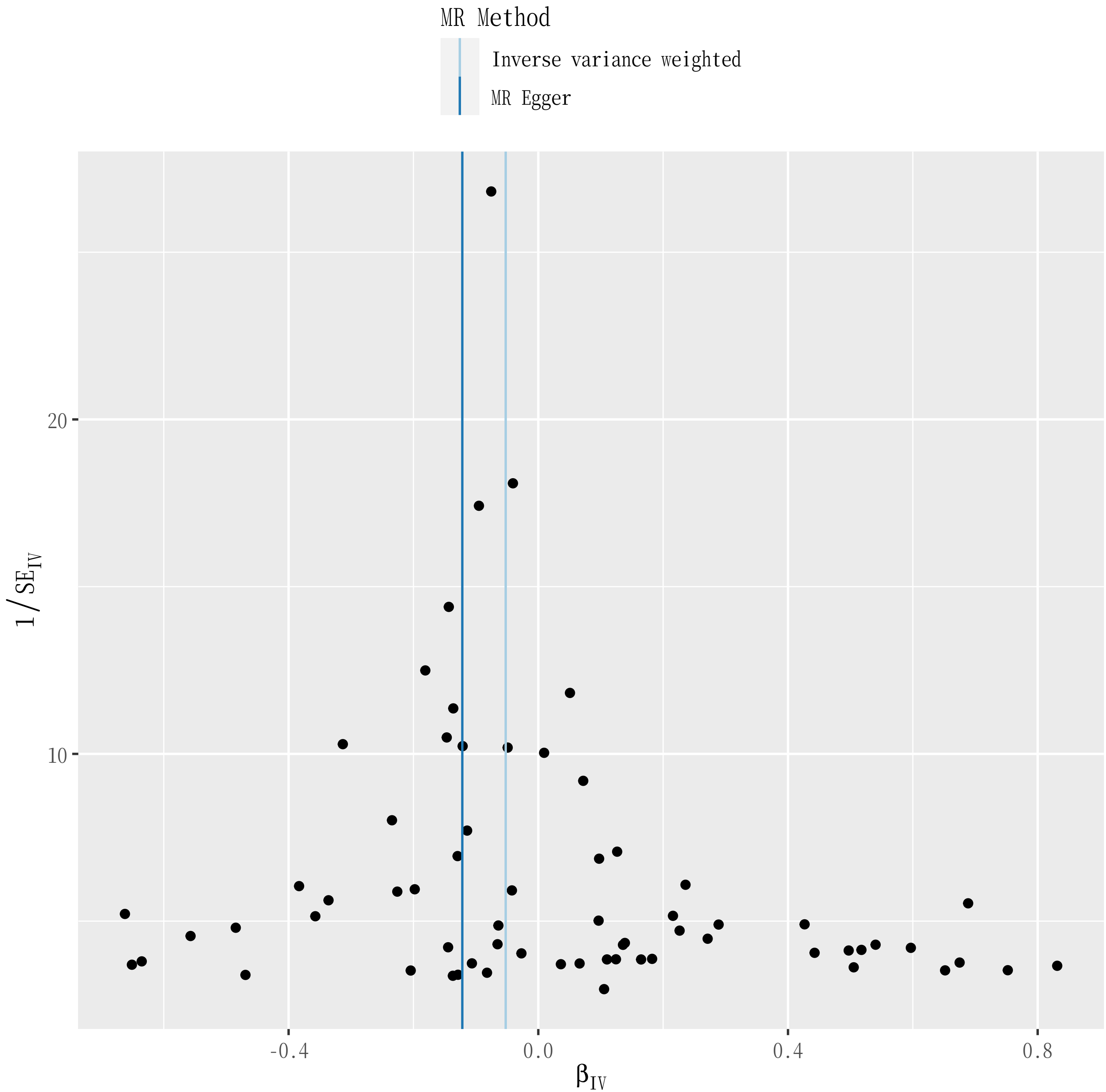
MR Method
Inverse variance weighted
MR Egger
20
10
1/SE_IV
-0.4
0.0
0.4
0.8
β_IV

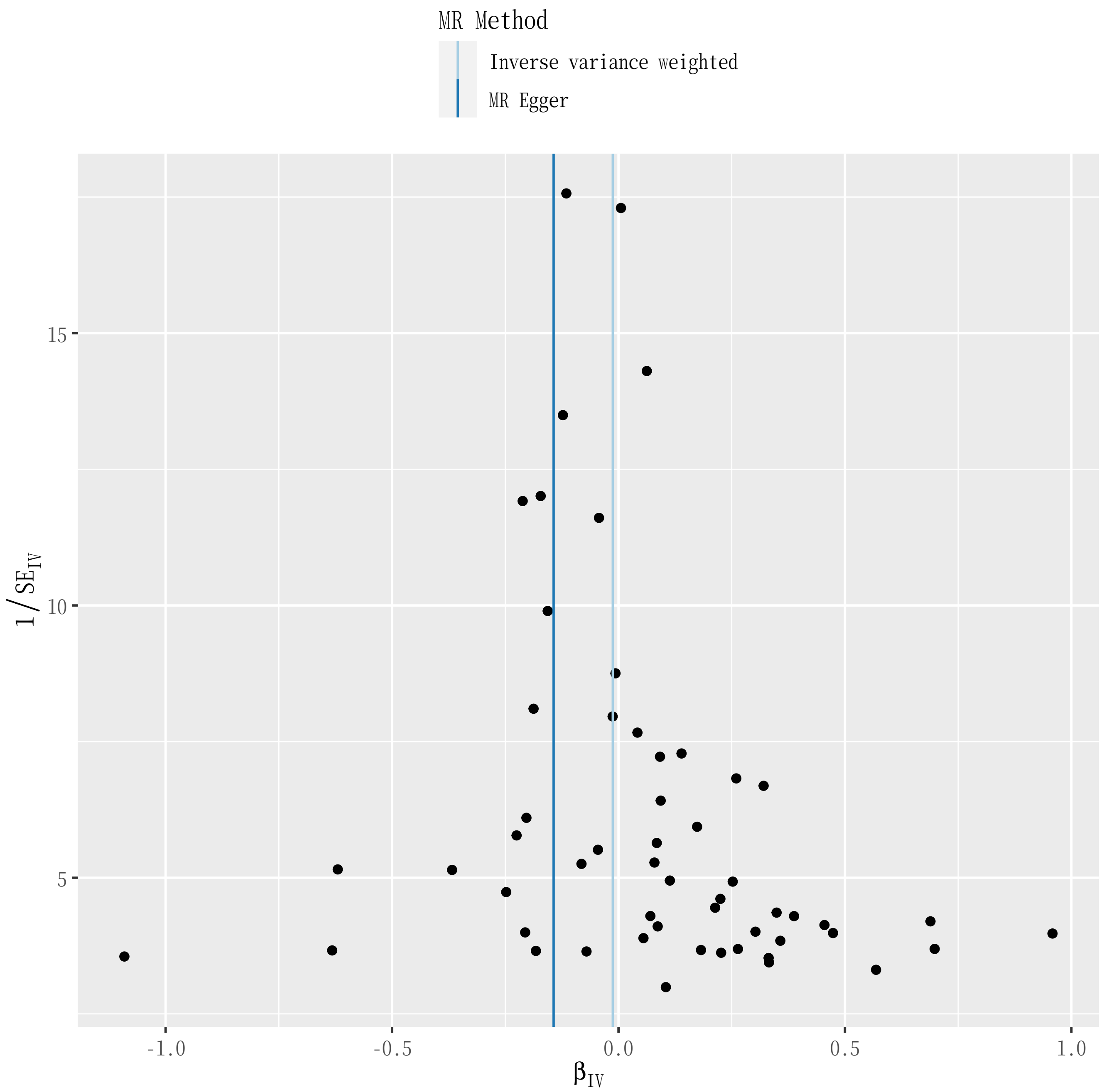
MR Method
Inverse variance weighted
MR Egger
15
10
5
$1/SE_{IV}$
-1.0
-0.5
0.0
0.5
1.0
β_{IV}

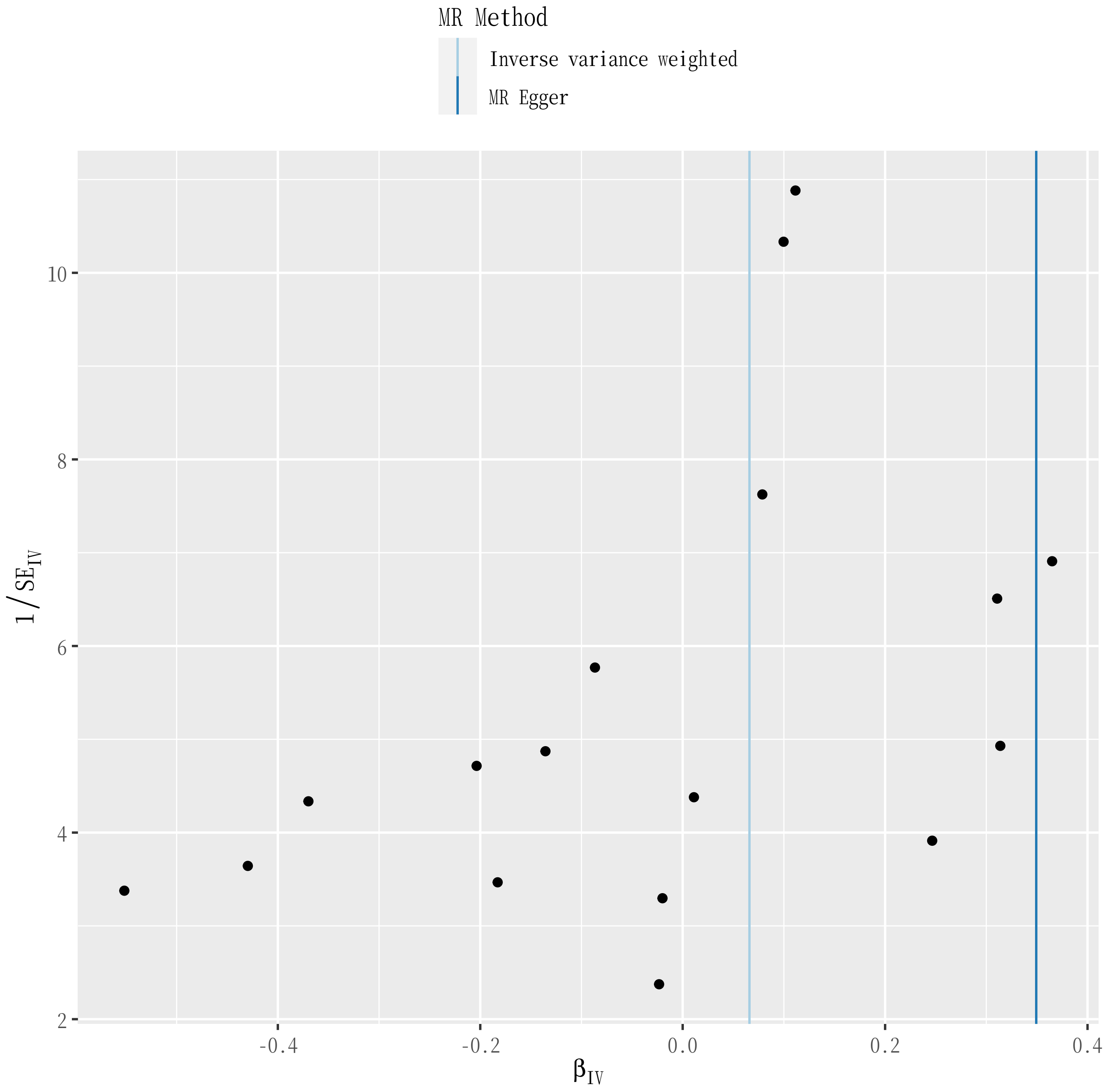

MR Method
Inverse variance weighted
MR Egger
10
8
6
4
2
-0.4
-0.2
0.0
0.2
0.4
$1/SE_{IV}$
β_{IV}

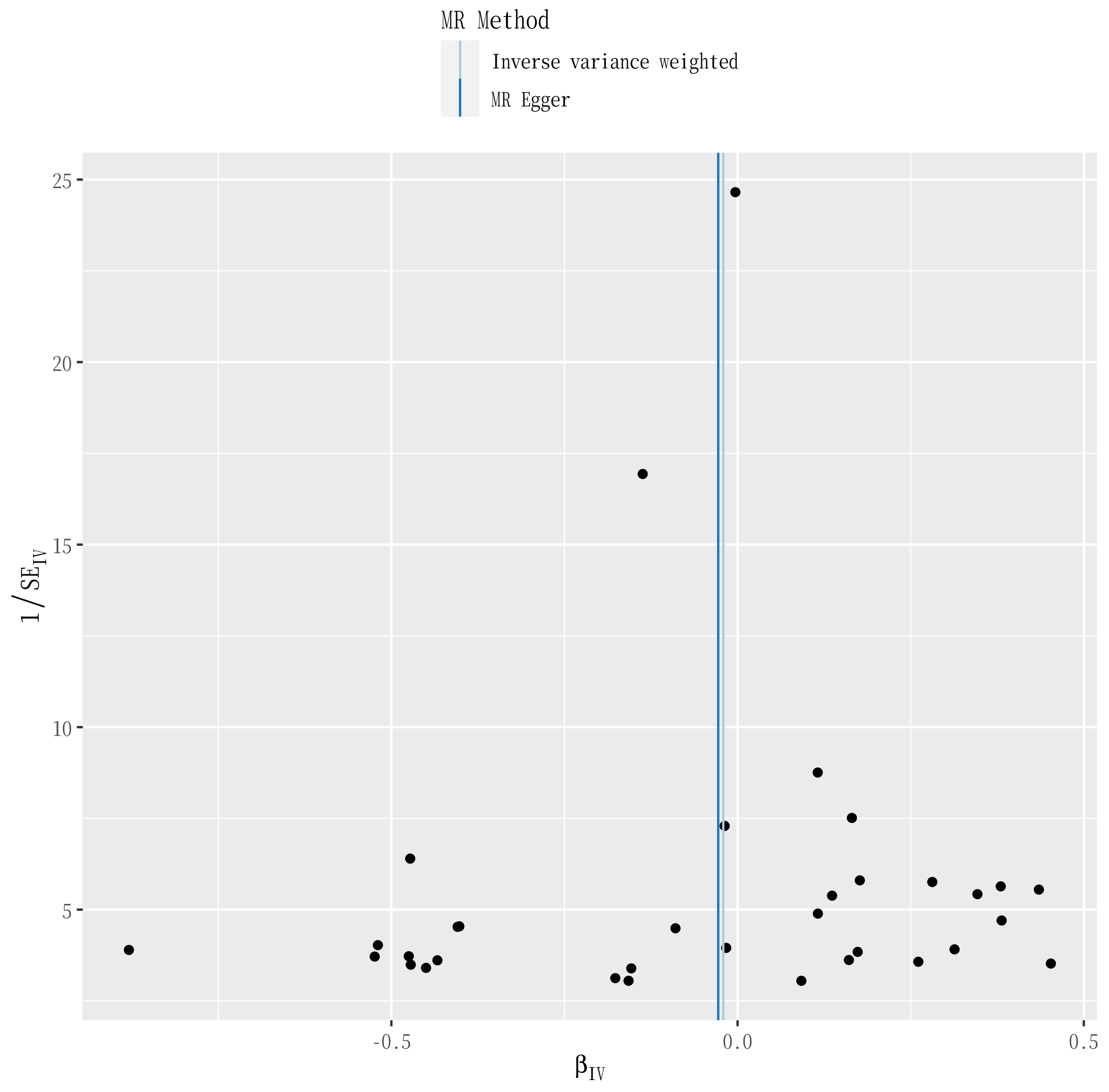
MR Method
Inverse variance weighted
MR Egger
25
20
15
10
5
1/SE_IV
-0.5
0.0
0.5
β_IV

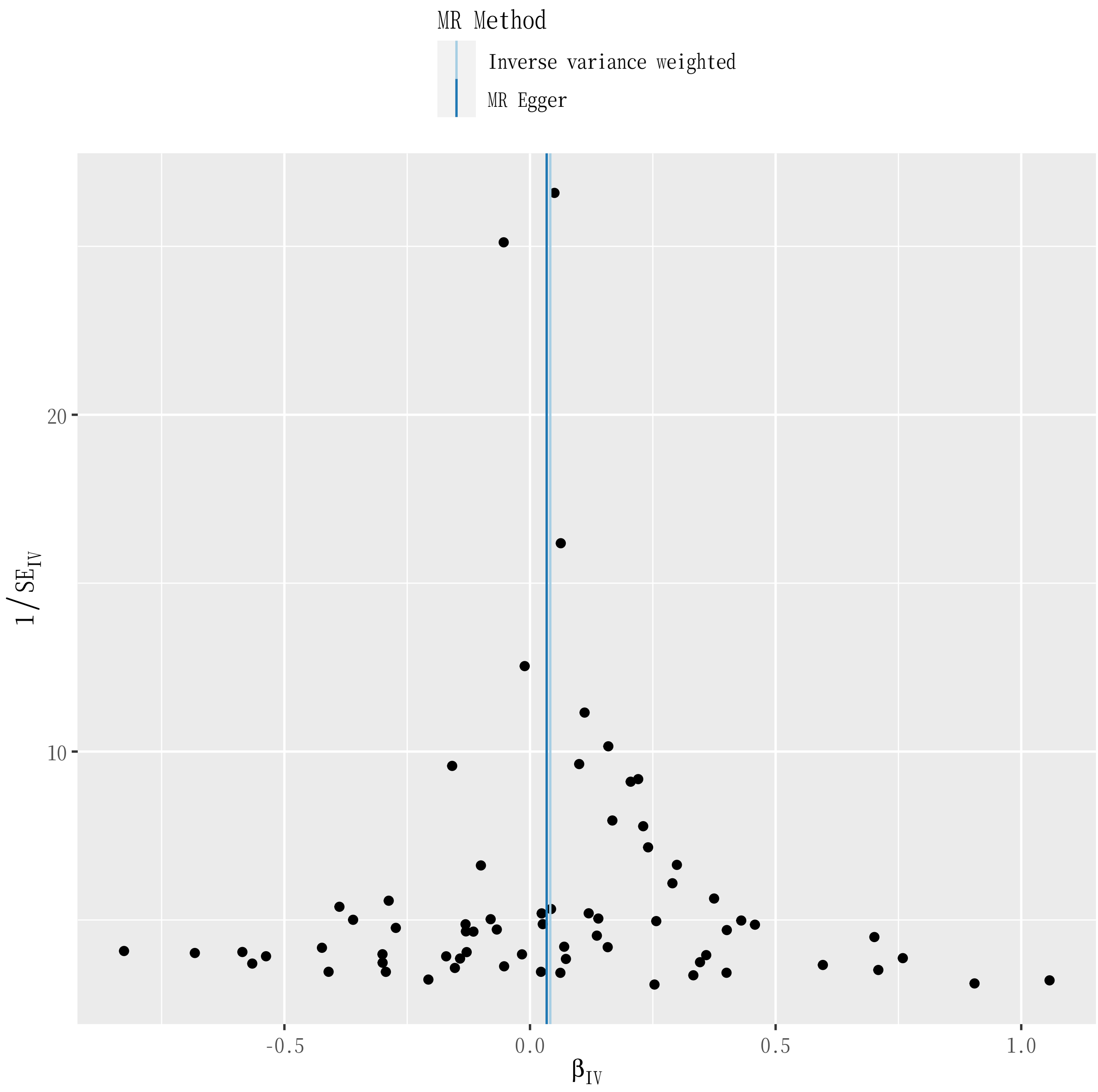
MR Method
Inverse variance weighted
MR Egger
20
10
$1/SE_{IV}$
-0.5
0.0
0.5
1.0
β_{IV}

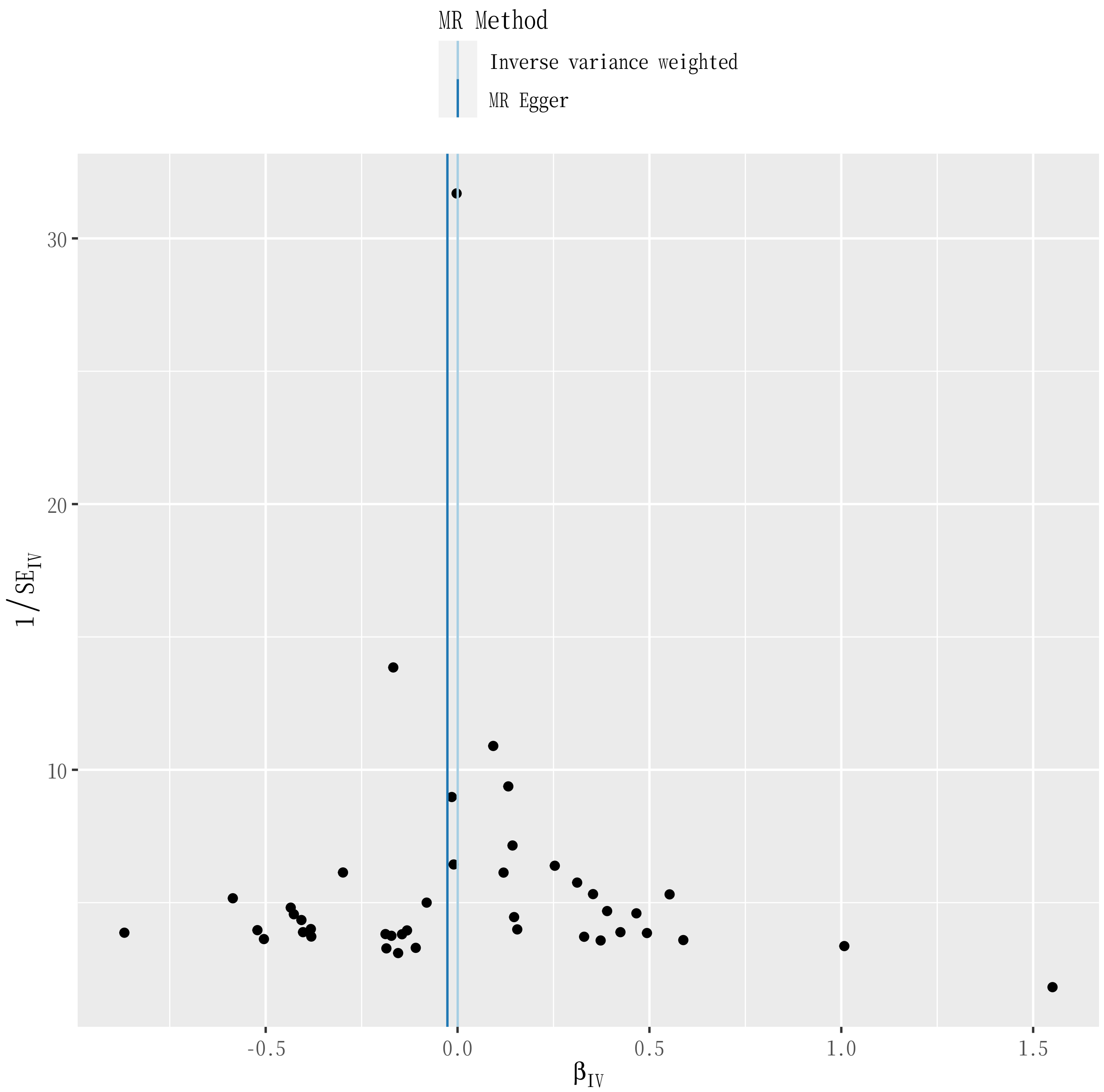

MR Method
Inverse variance weighted
MR Egger
30
20
10
1/SE_IV
-0.5
0.0
0.5
1.0
1.5
β_IV

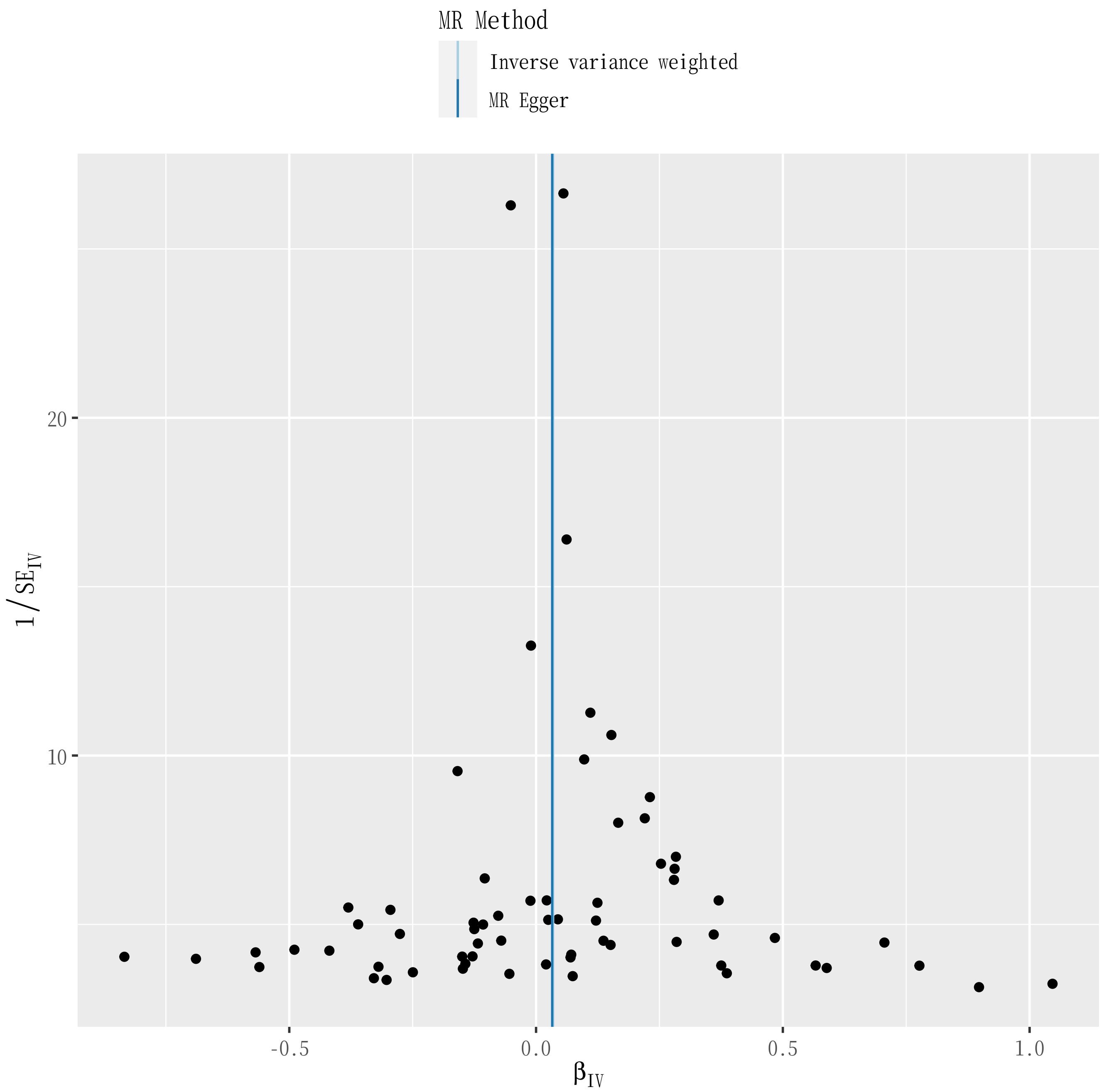
MR Method
Inverse variance weighted
MR Egger
20
10
-0.5
0.0
0.5
1.0
$1/SE_{IV}$
β_{IV}

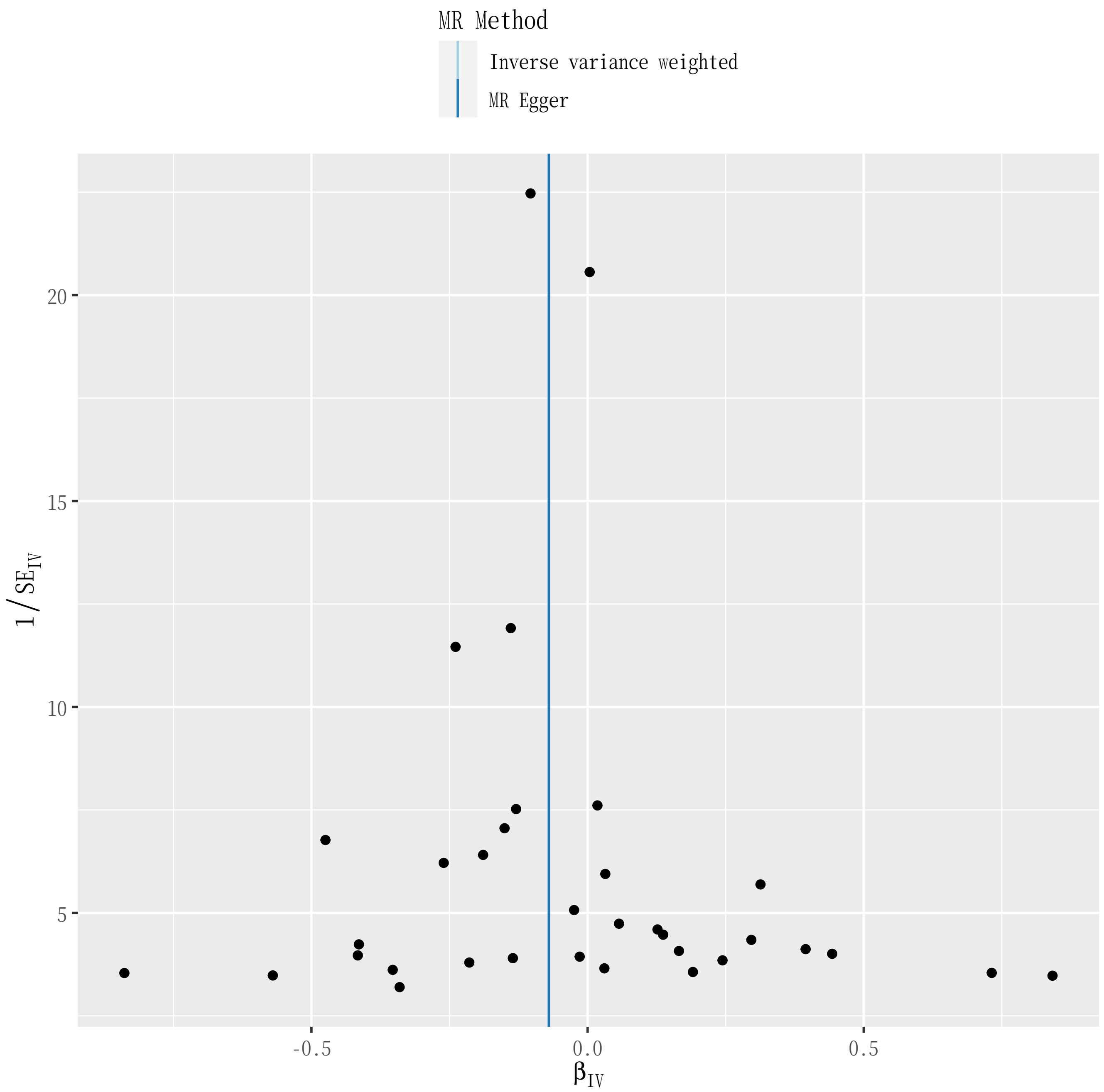
MR Method
Inverse variance weighted
MR Egger
1/SE_IV
20
15
10
5
-0.5
0.0
0.5
β_IV

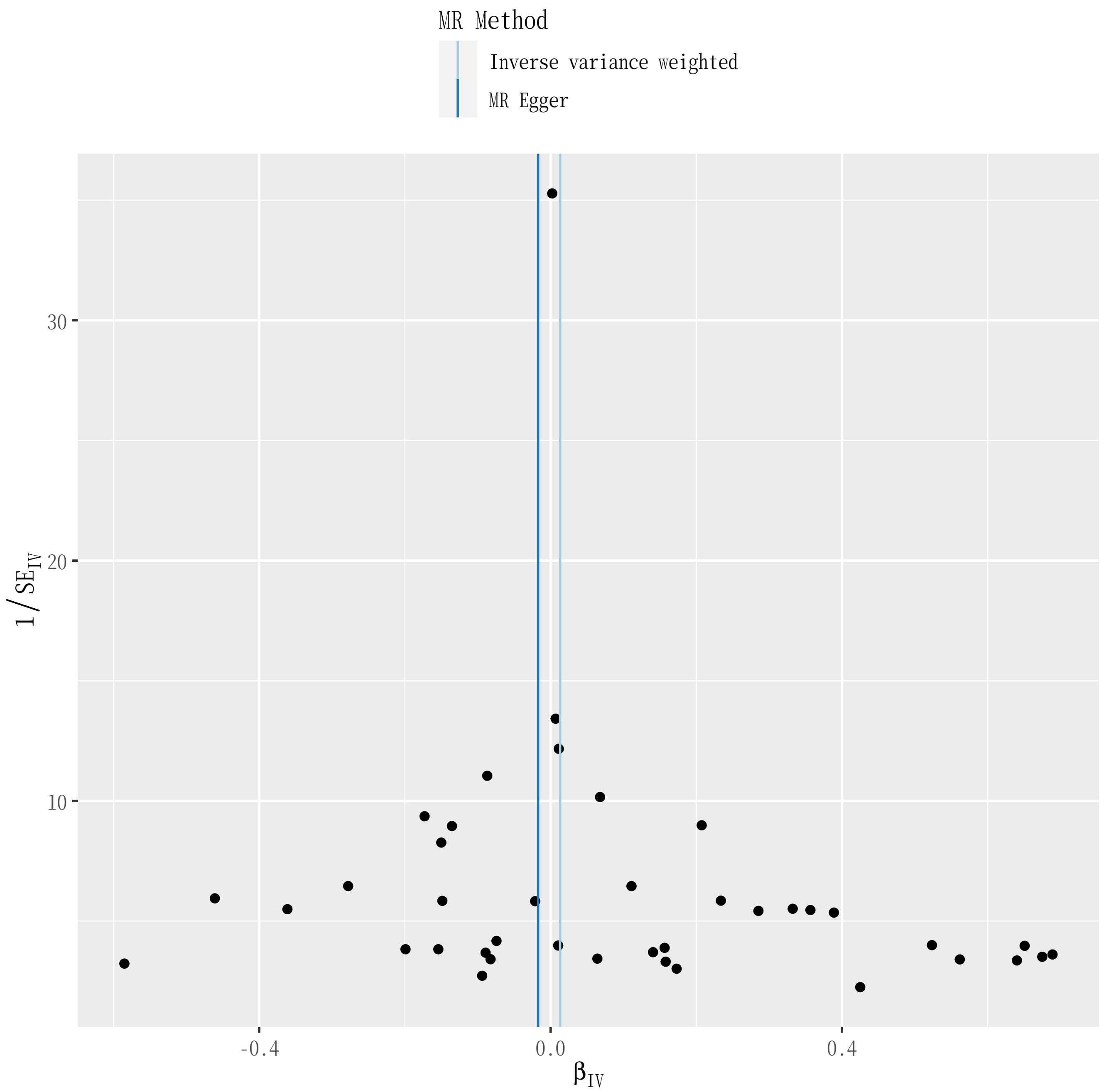

MR Method
Inverse variance weighted
MR Egger
30
20
10
1/SE_IV
-0.4
0.0
0.4
β_IV

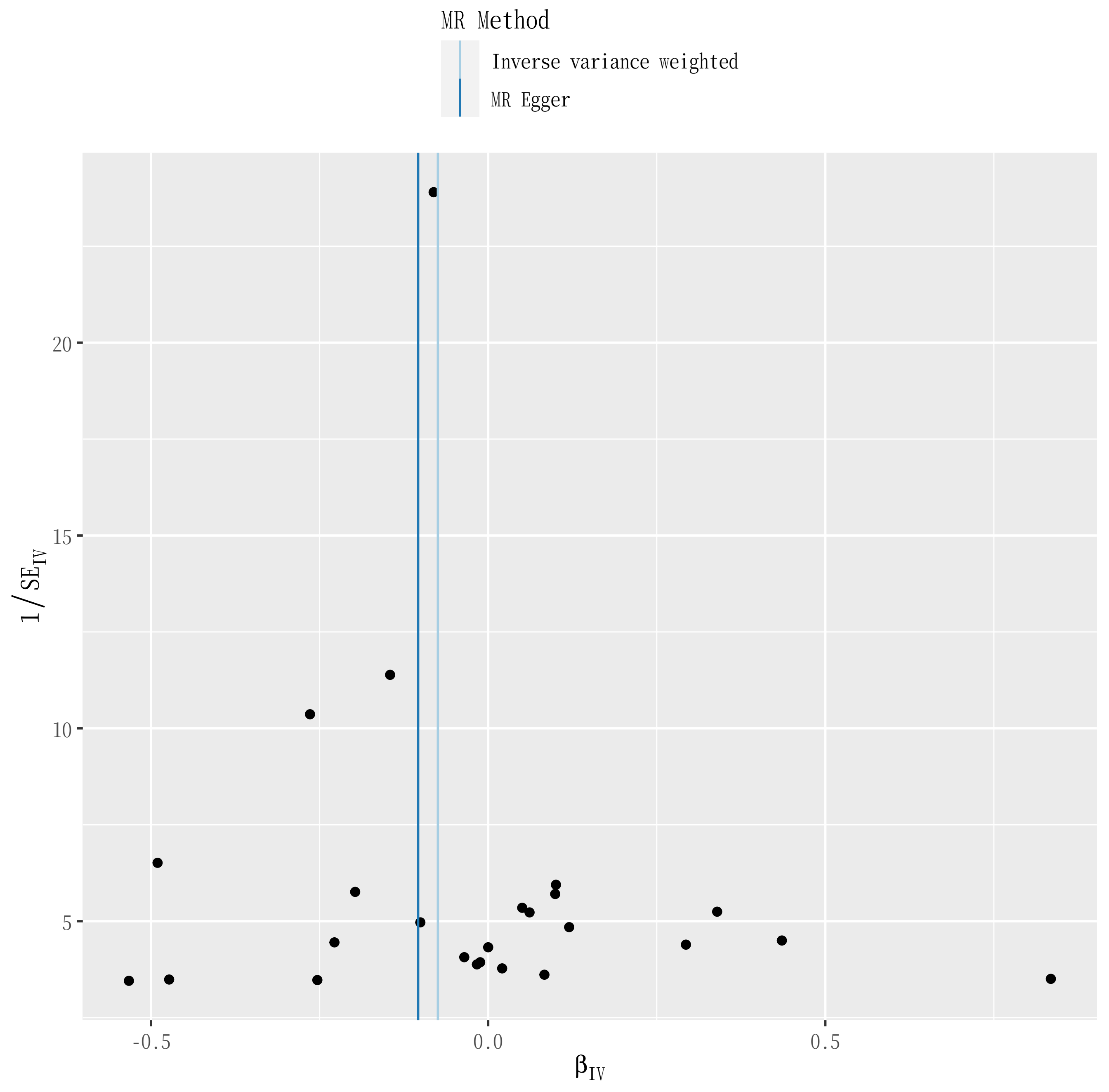

MR Method
Inverse variance weighted
MR Egger
20
15
10
5
-0.5
0.0
0.5
1/SE_IV
β_IV

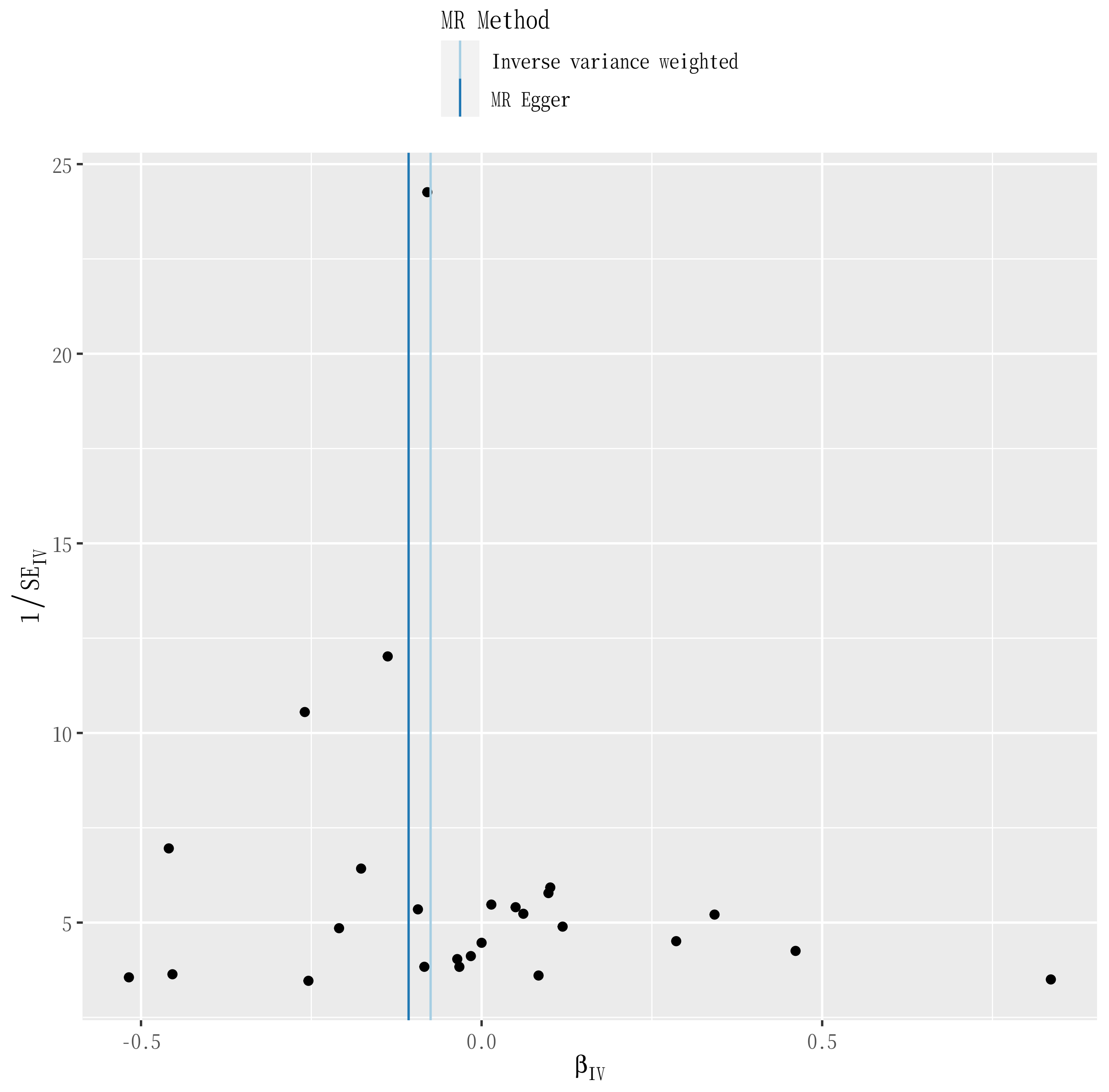

MR Method
Inverse variance weighted
MR Egger
25
20
15
10
5
1/SE_IV
-0.5
0.0
0.5
β_IV

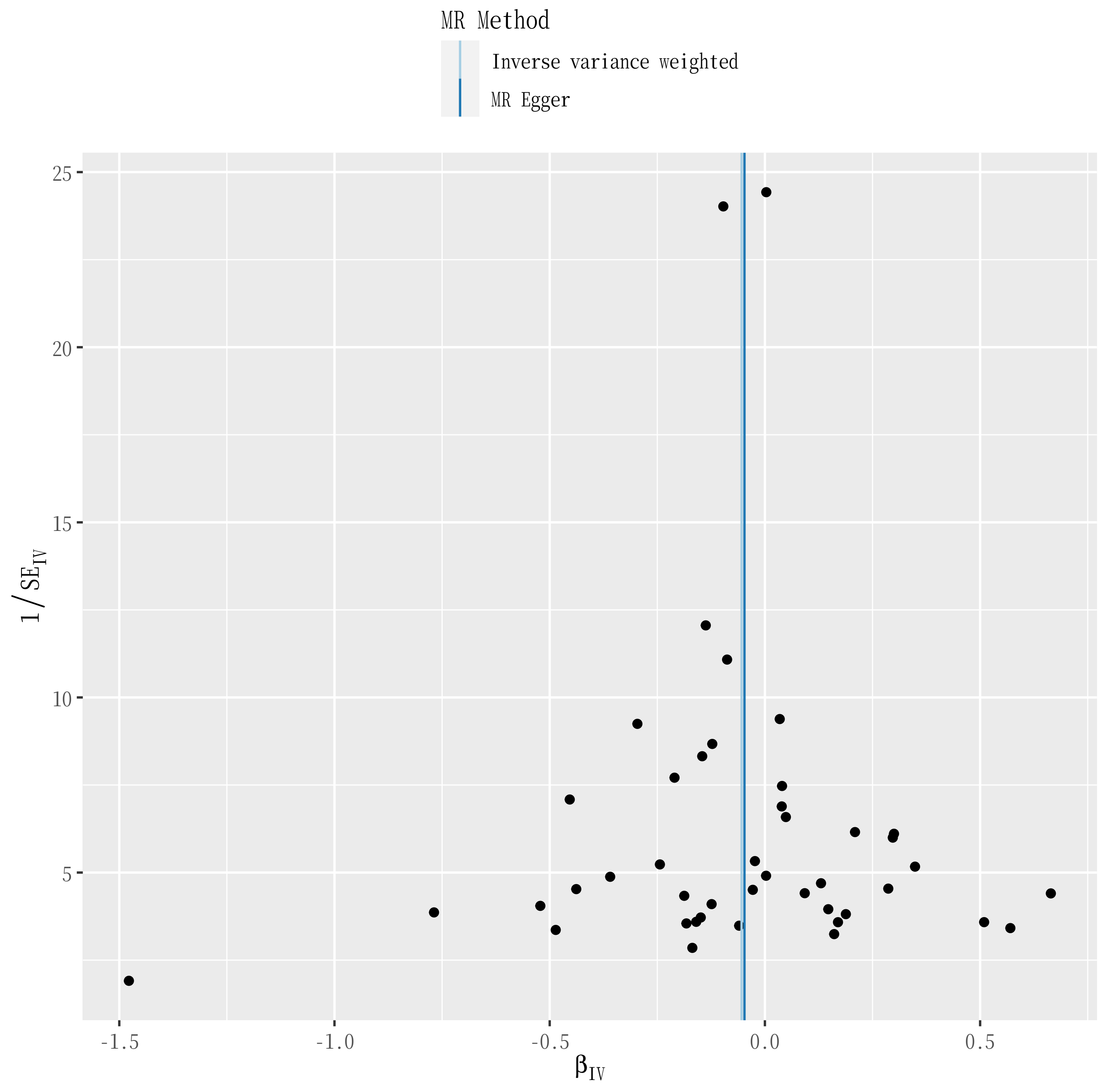
MR Method
Inverse variance weighted
MR Egger
25
20
15
10
5
$1/SE_{IV}$
-1.5
-1.0
-0.5
0.0
0.5
β_{IV}

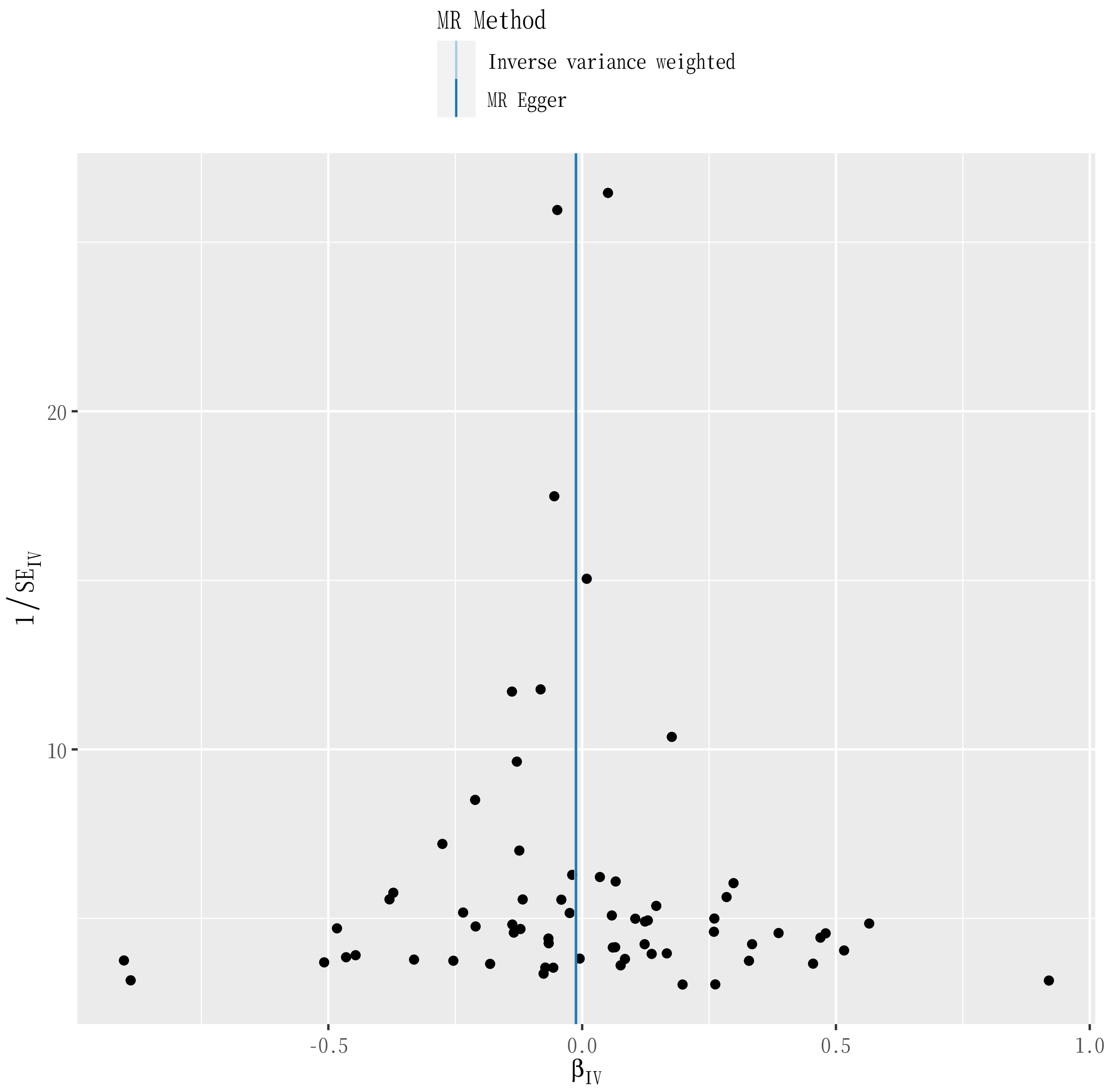

MR Method
Inverse variance weighted
MR Egger
20
10
-0.5
0.0
0.5
1.0
$1/SE_{IV}$
β_{IV}

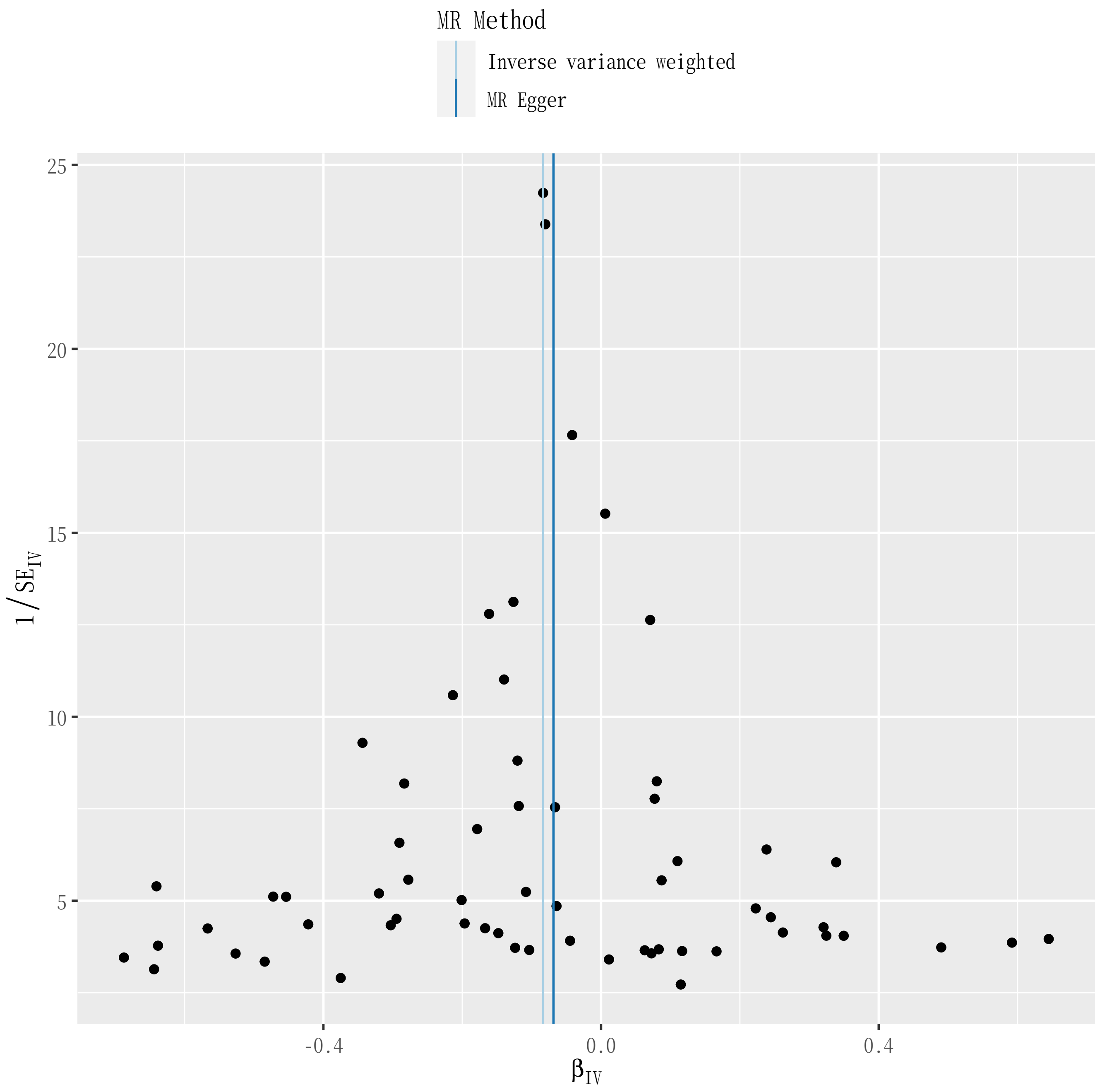

MR Method
Inverse variance weighted
MR Egger
25
20
15
10
5
$1/SE_{IV}$
-0.4
0.0
0.4
β_{IV}

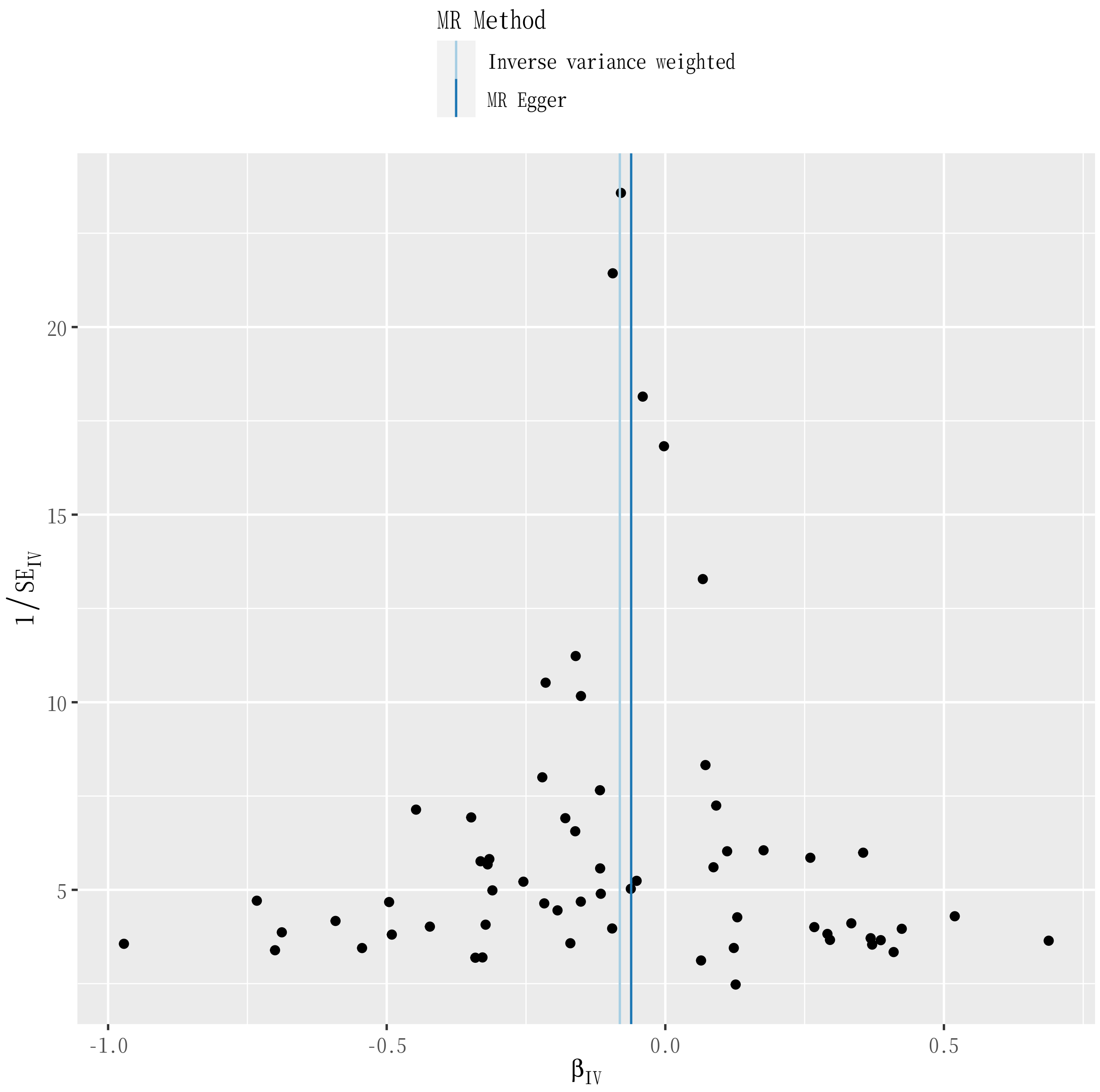

MR Method
Inverse variance weighted
MR Egger
20
15
10
5
-1.0
-0.5
0.0
0.5
$1/SE_{IV}$
β_{IV}

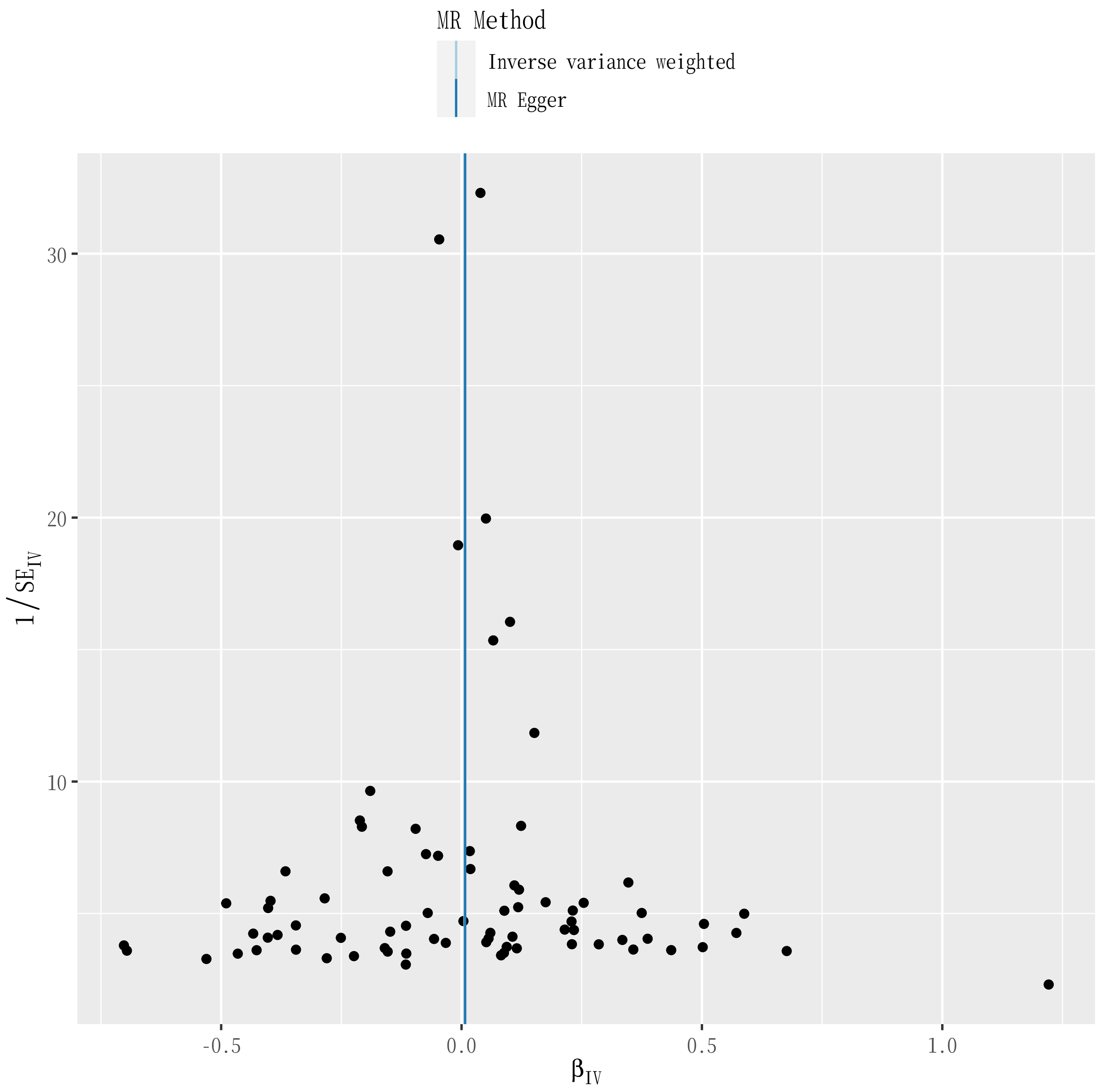
MR Method
Inverse variance weighted
MR Egger
30
20
10
1/SE$_{IV}$
-0.5
0.0
0.5
1.0
β$_{IV}$

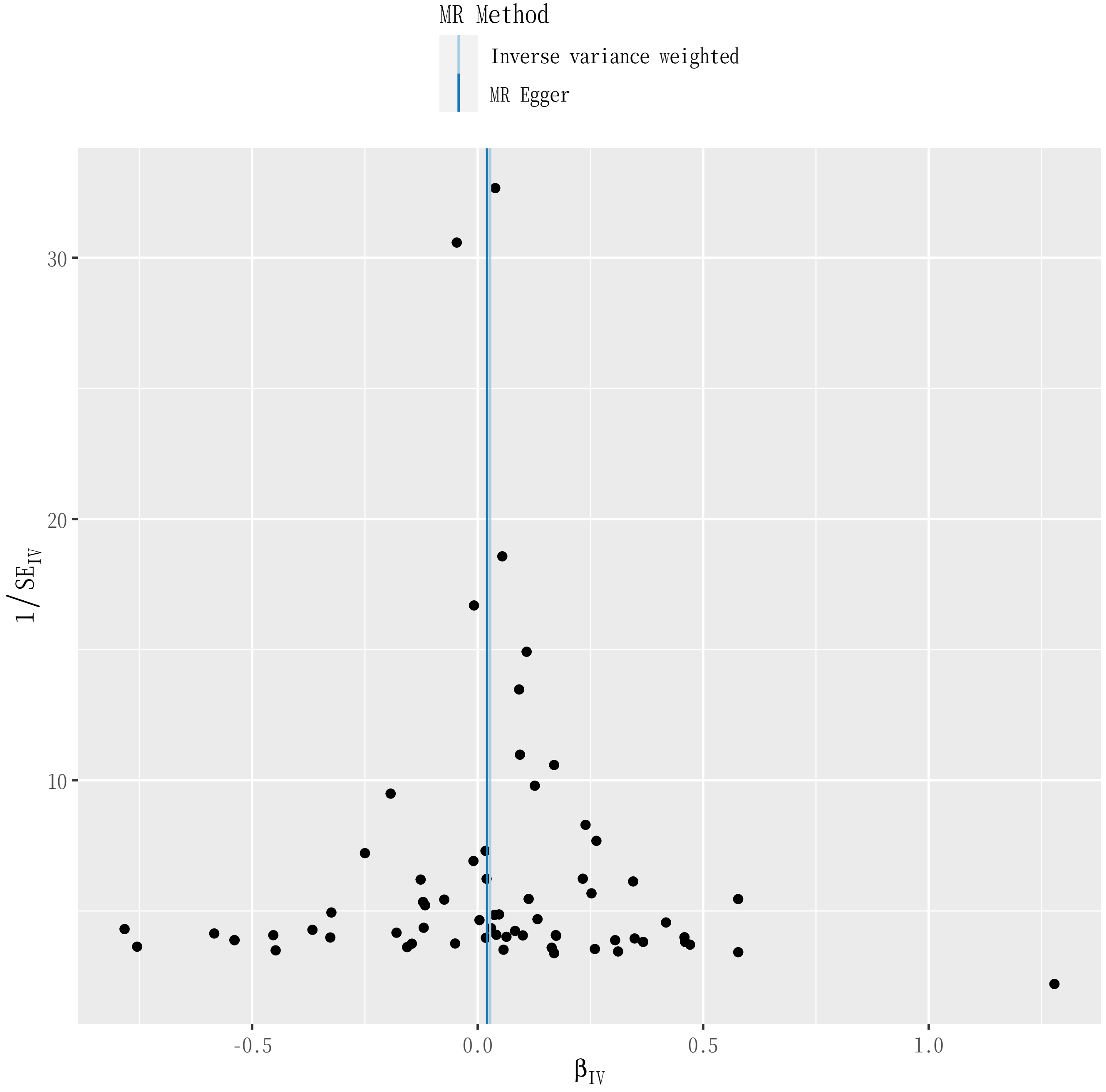
MR Method
Inverse variance weighted
MR Egger
30
20
10
1/SE_IV
-0.5
0.0
0.5
1.0
β_IV

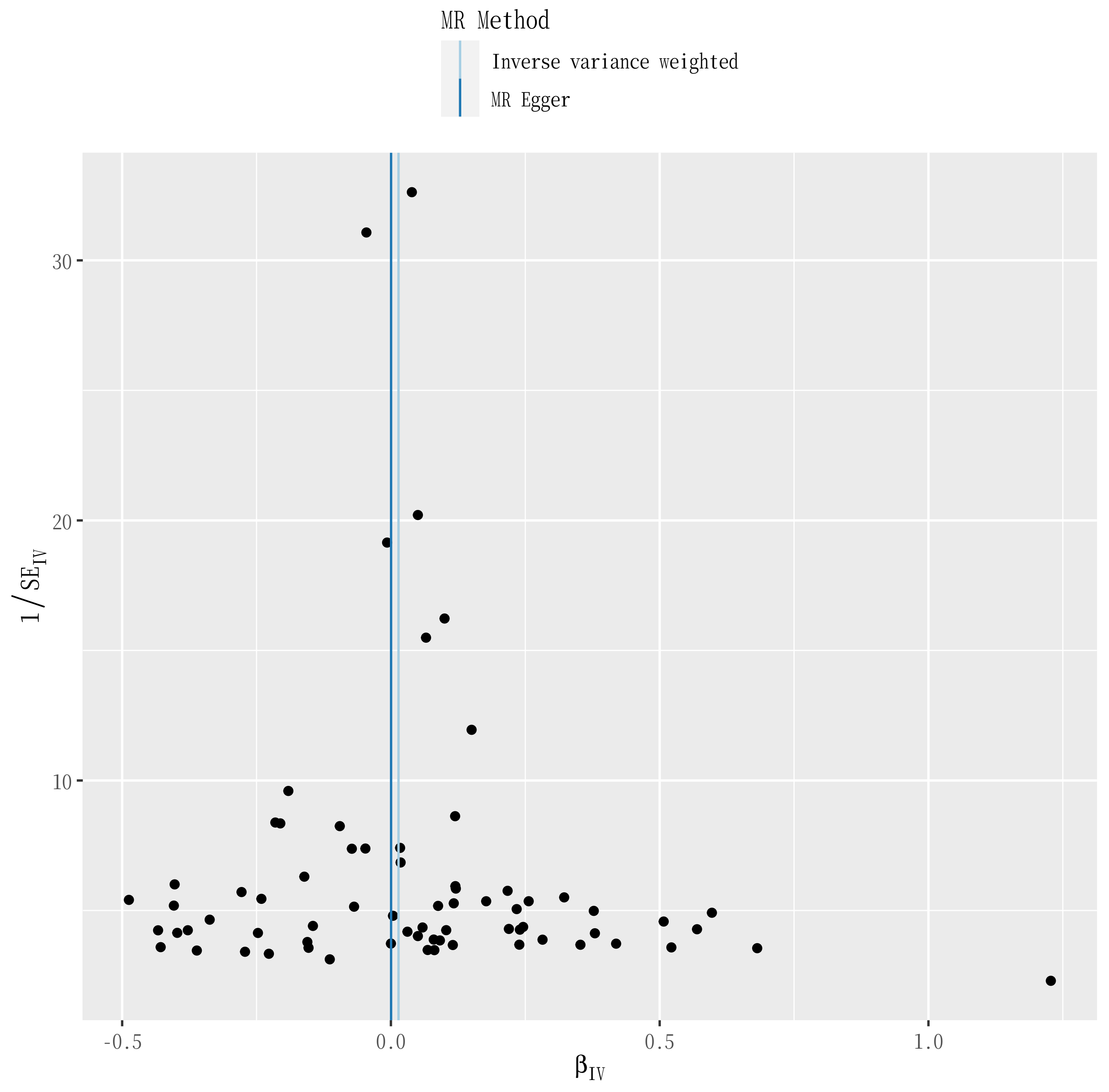
MR Method
Inverse variance weighted
MR Egger
30
20
10
1/SE_IV
-0.5
0.0
0.5
1.0
β_IV

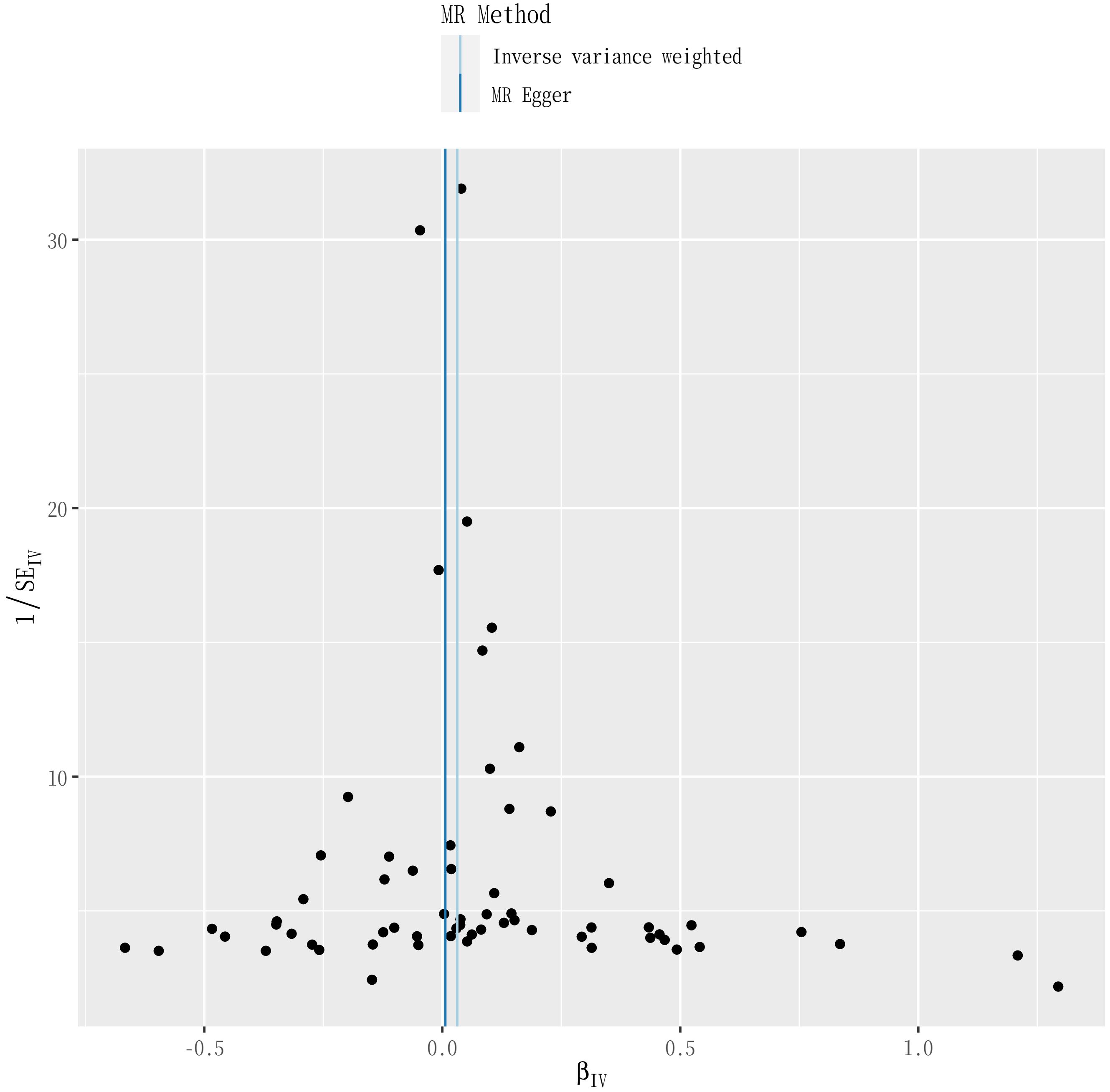

MR Method
Inverse variance weighted
MR Egger
30
20
10
1/SE_IV
-0.5
0.0
0.5
1.0
β_IV

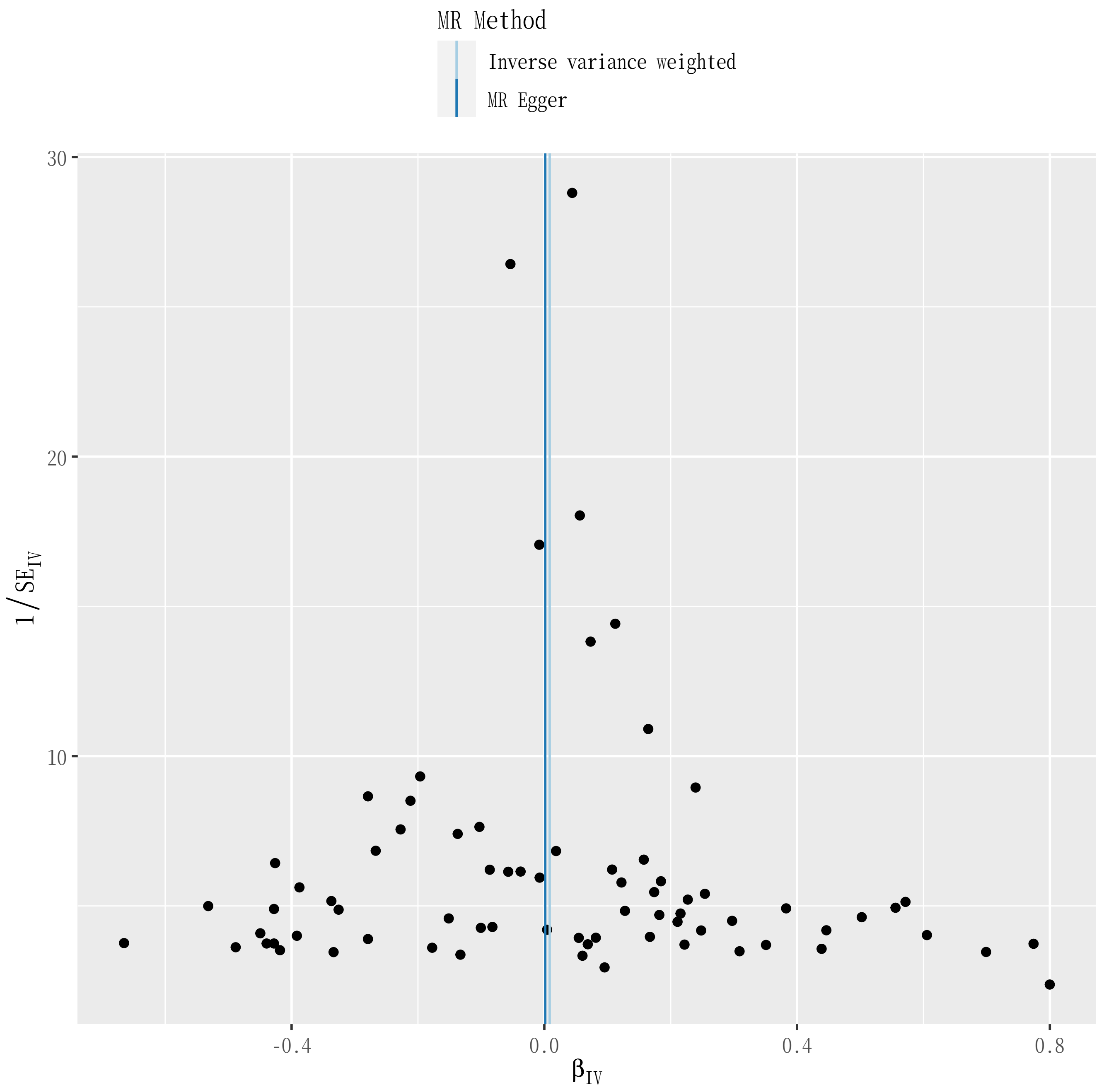
MR Method
Inverse variance weighted
MR Egger
30
20
10
1/SE_IV
-0.4
0.0
0.4
0.8
β_IV

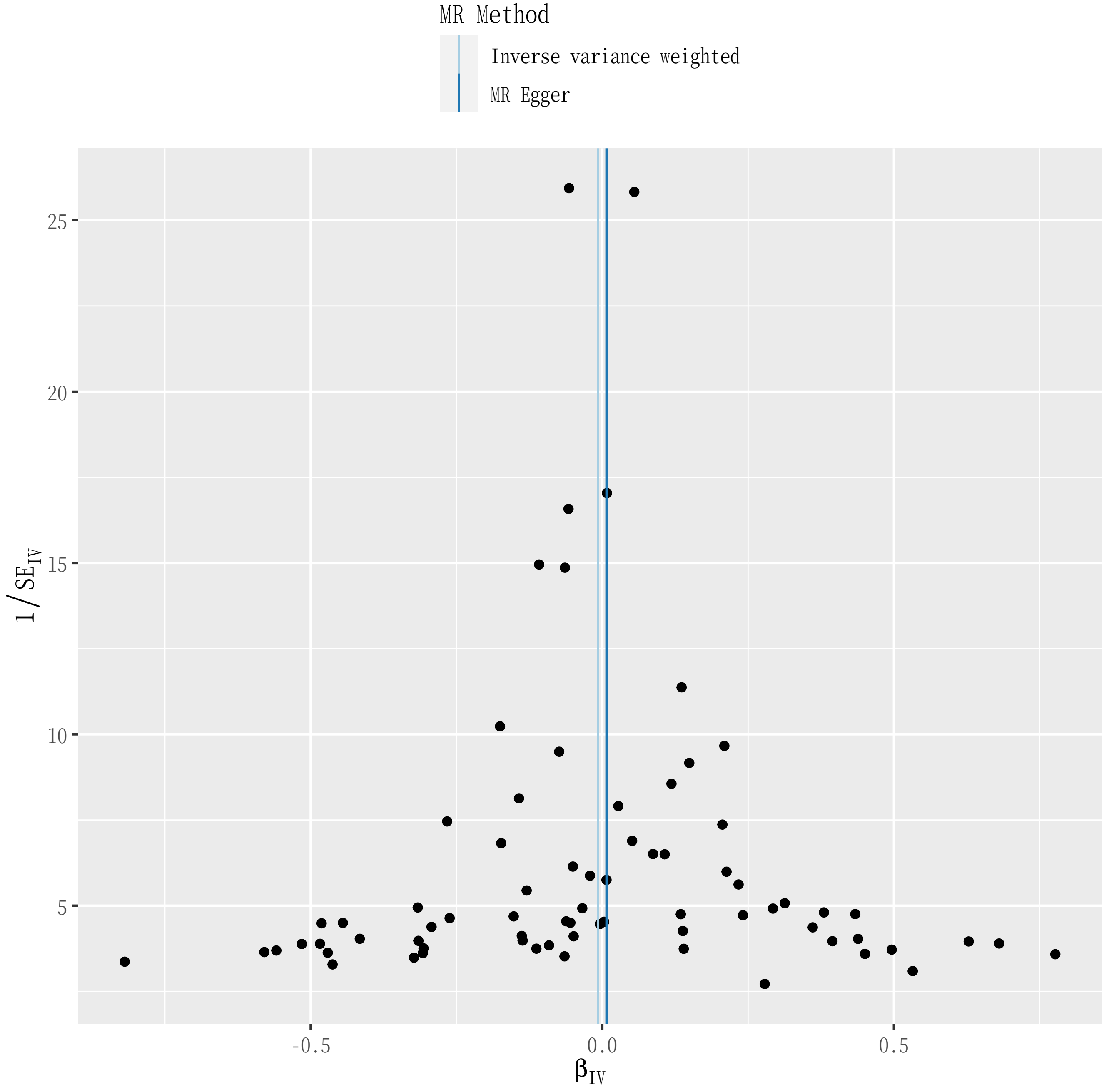

MR Method
Inverse variance weighted
MR Egger
25
20
15
10
5
$1/SE_{IV}$
-0.5
0.0
0.5
β_{IV}

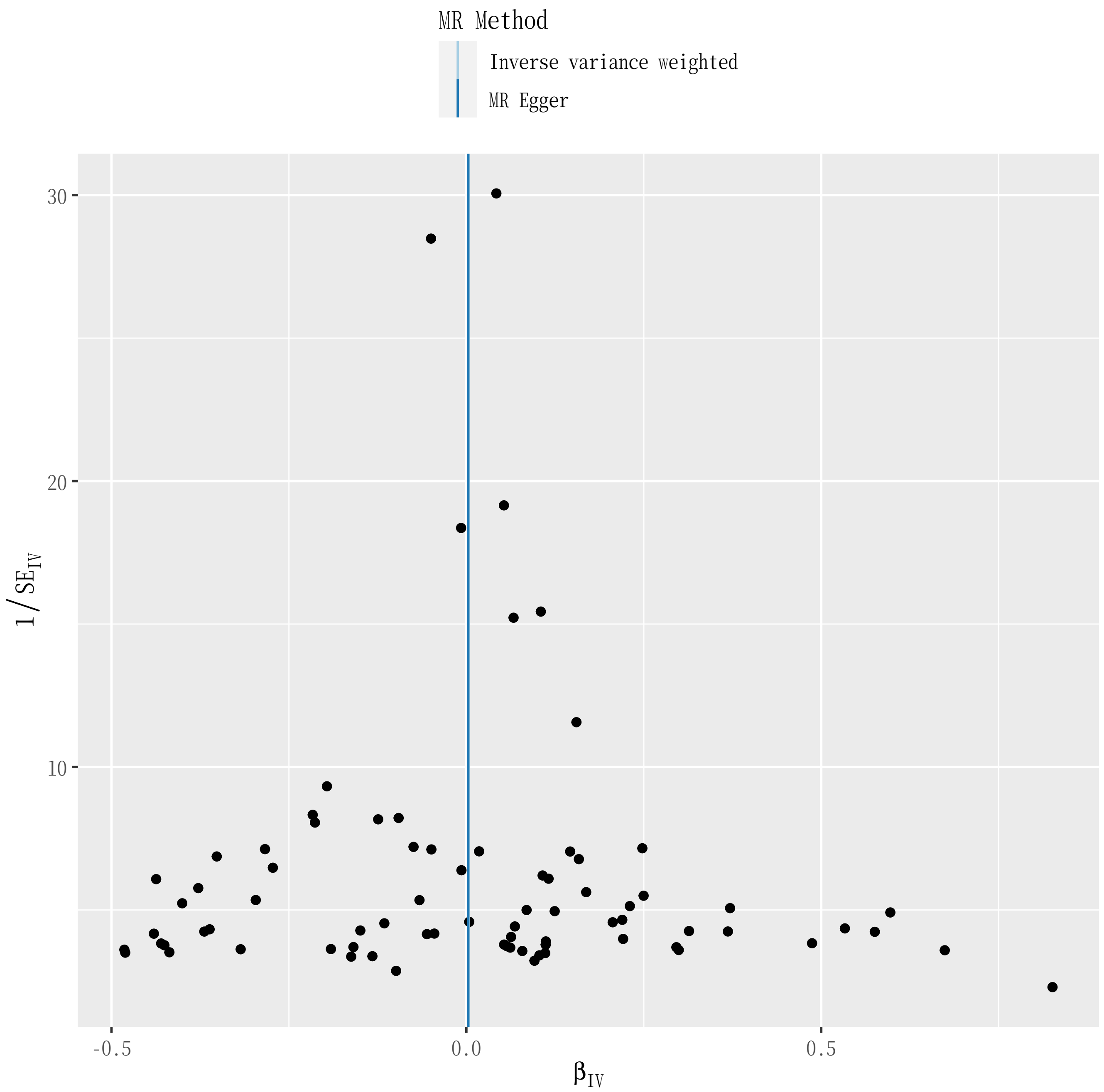

MR Method
Inverse variance weighted
MR Egger
30
20
10
1/SE_IV
-0.5
0.0
0.5
β_IV

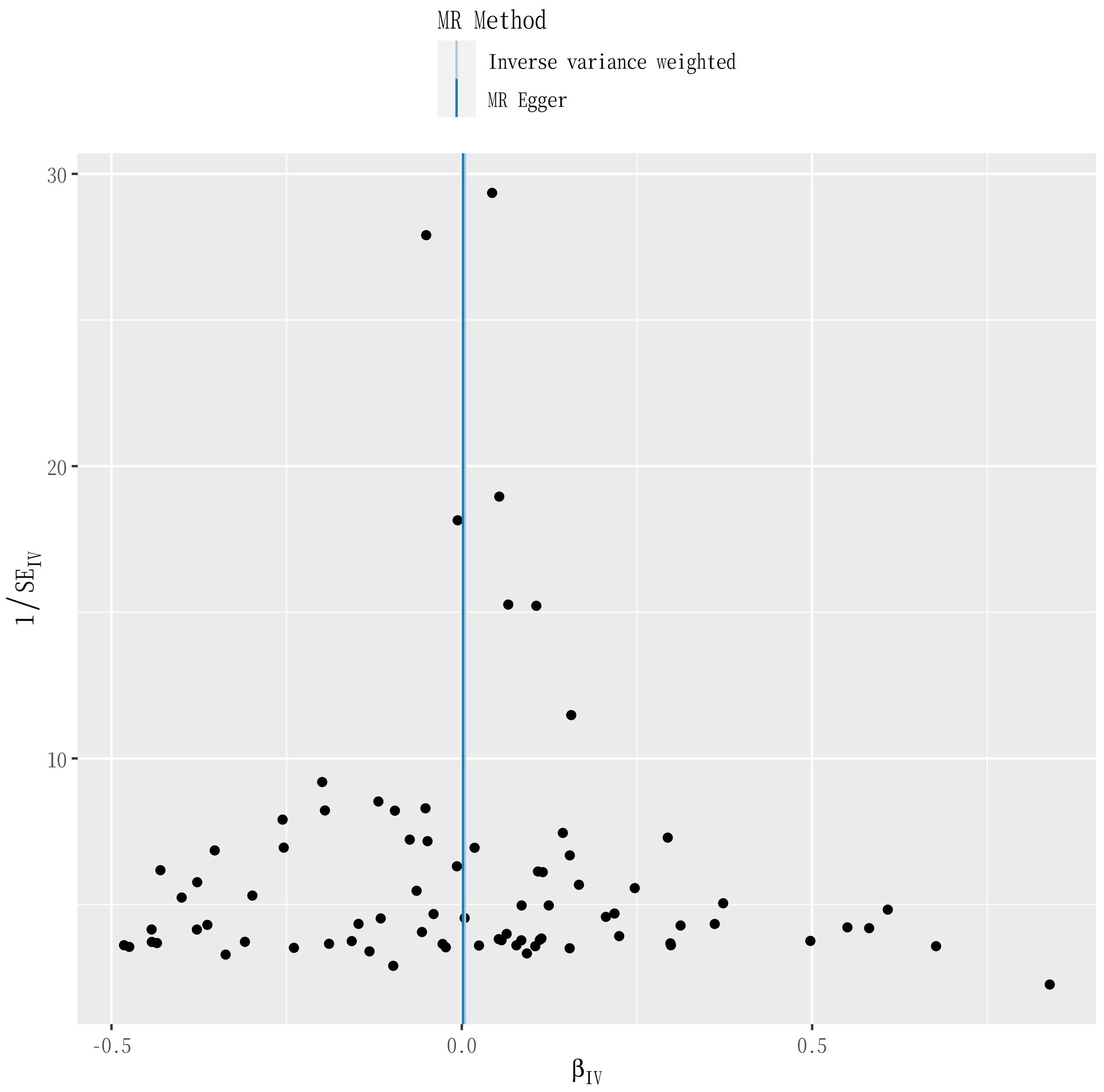

MR Method
Inverse variance weighted
MR Egger
30
20
10
1/SE_IV
-0.5
0.0
0.5
β_IV

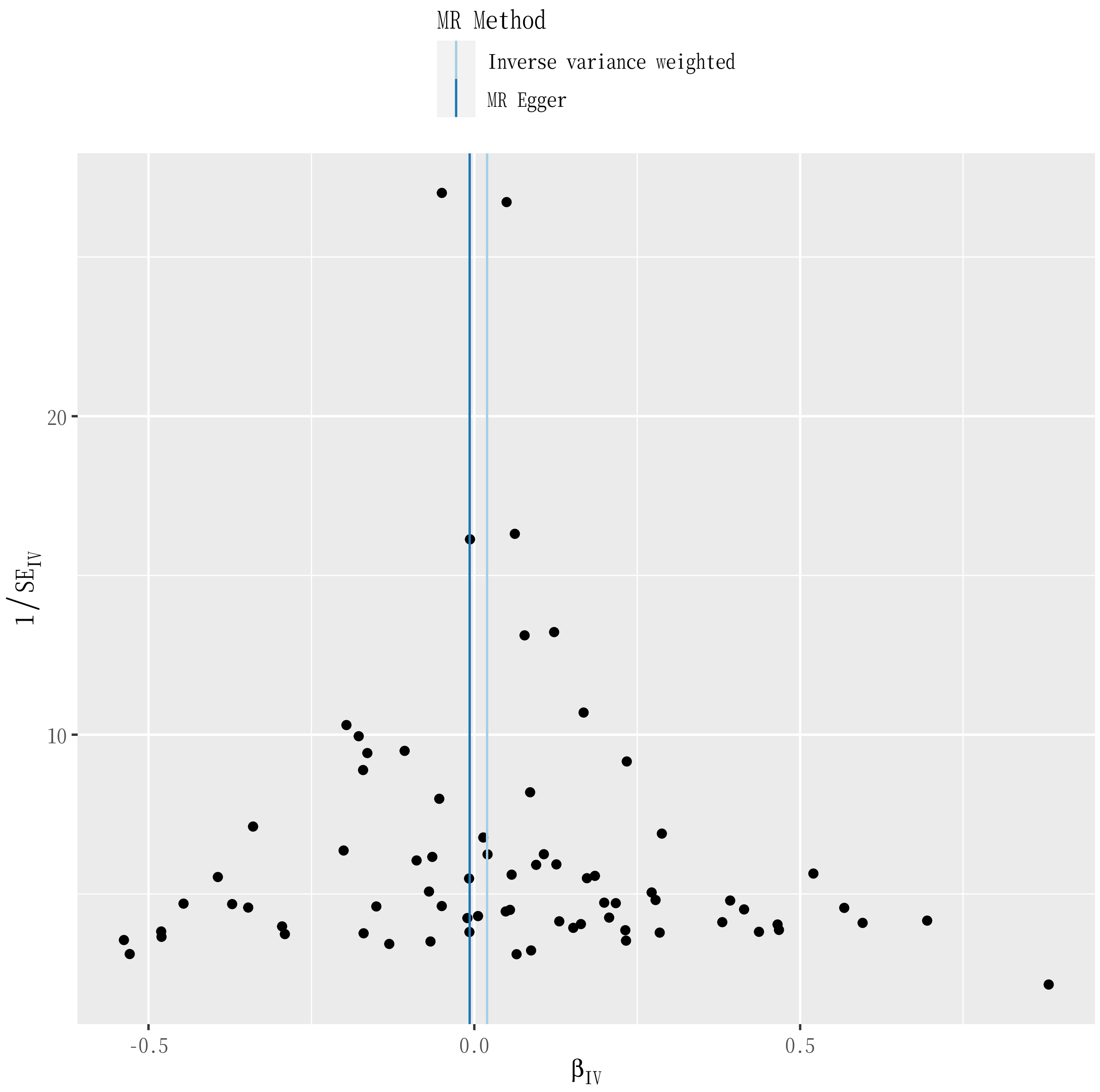

MR Method
Inverse variance weighted
MR Egger
20
10
$1/SE_{IV}$
-0.5
0.0
0.5
β_{IV}

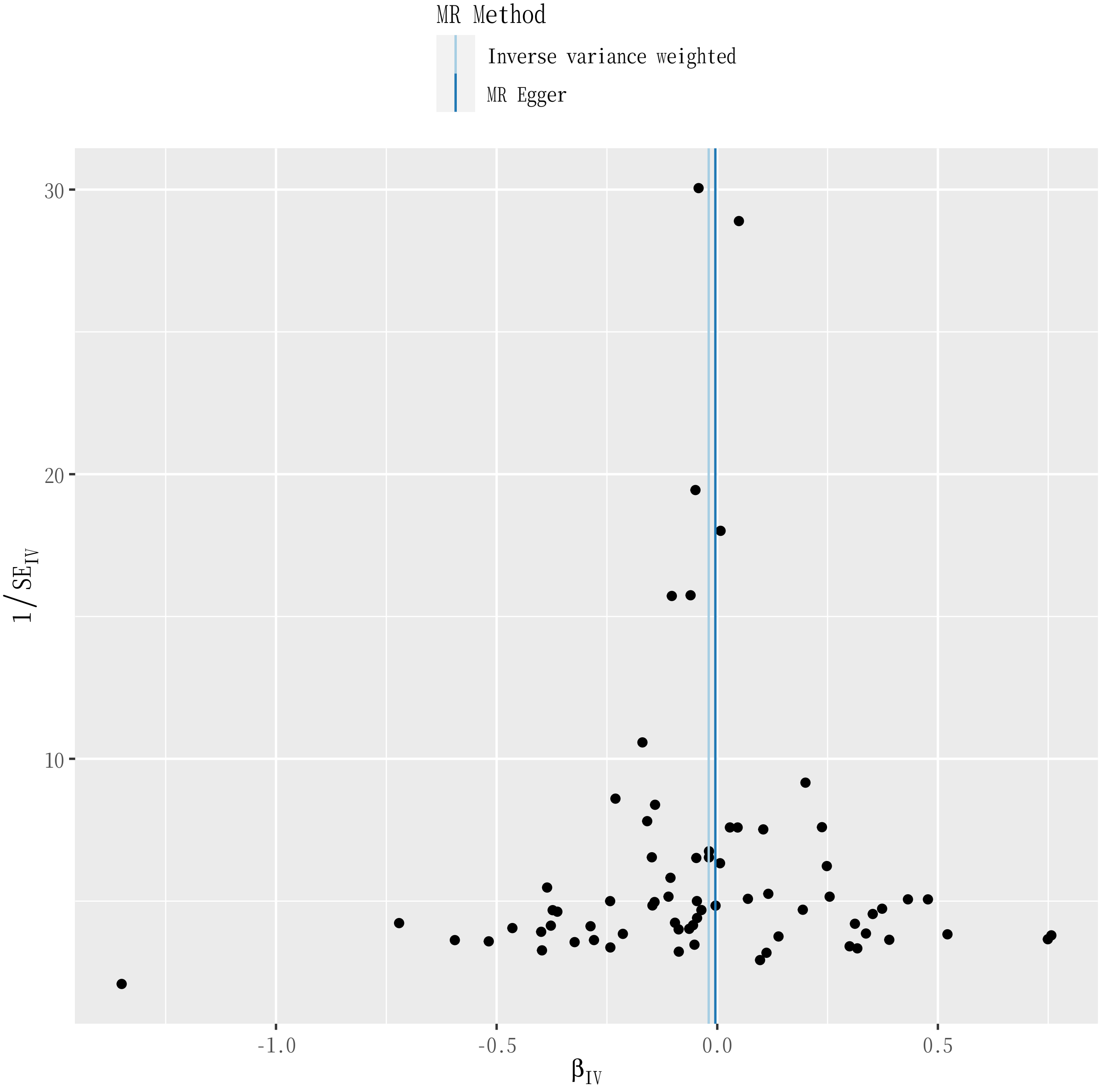
MR Method
Inverse variance weighted
MR Egger
30
20
10
-1.0
-0.5
0.0
0.5
$1/SE_{IV}$
β_{IV}

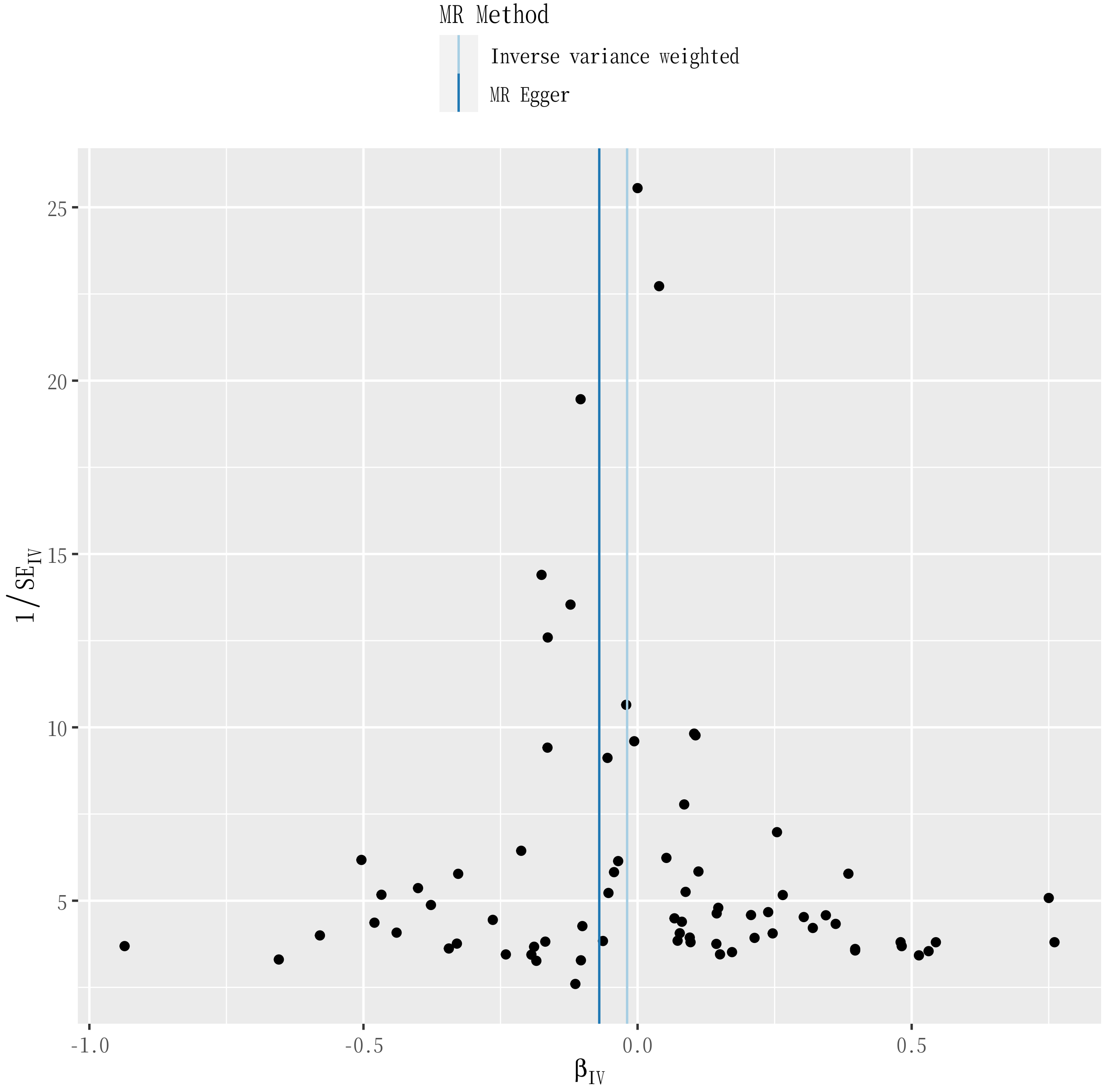

MR Method
Inverse variance weighted
MR Egger
25
20
15
10
5
1/SE_IV
-1.0
-0.5
0.0
0.5
β_IV

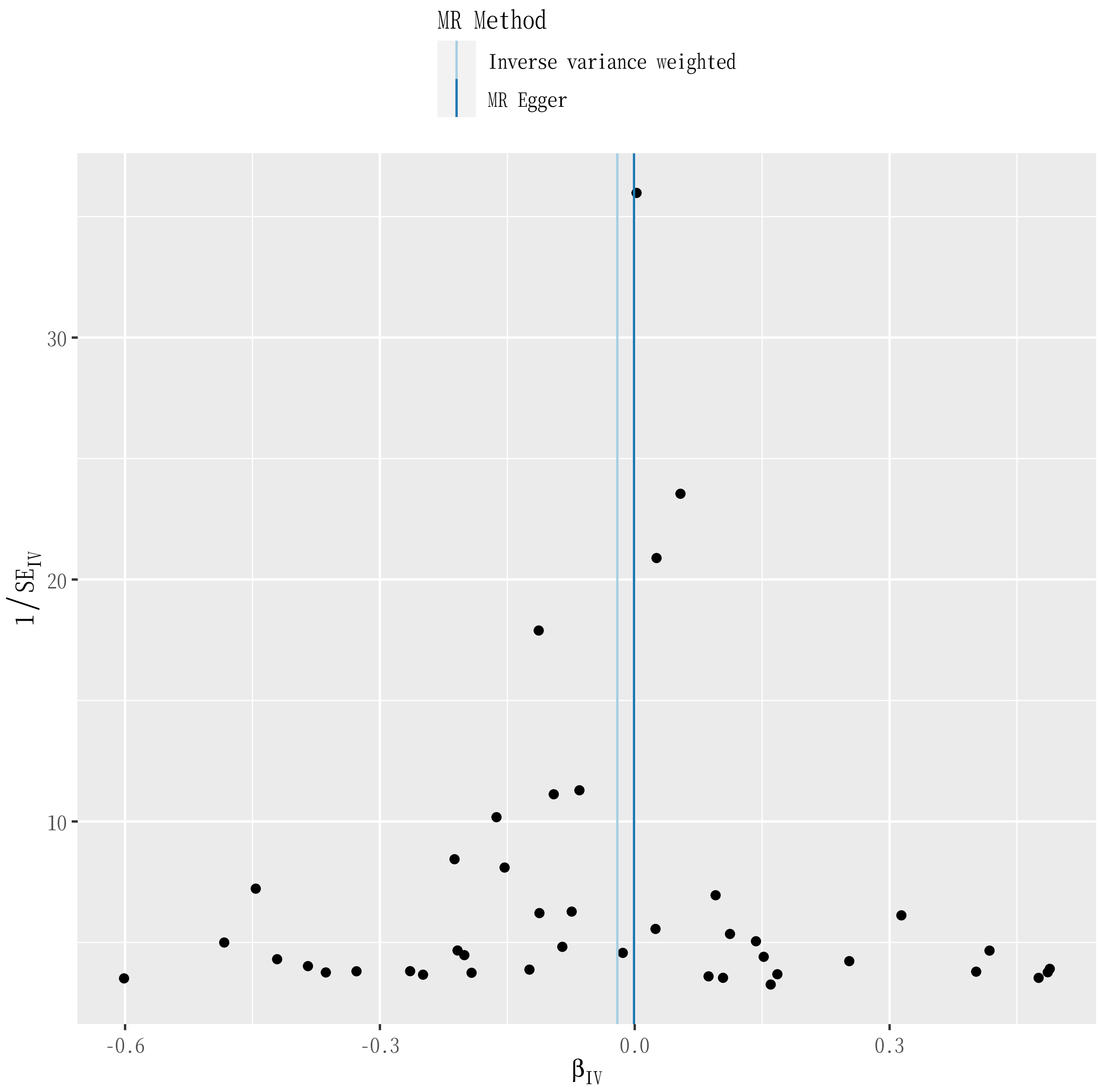
MR Method
Inverse variance weighted
MR Egger
30
20
10
-0.6
-0.3
0.0
0.3
$1/SE_{IV}$
β_{IV}

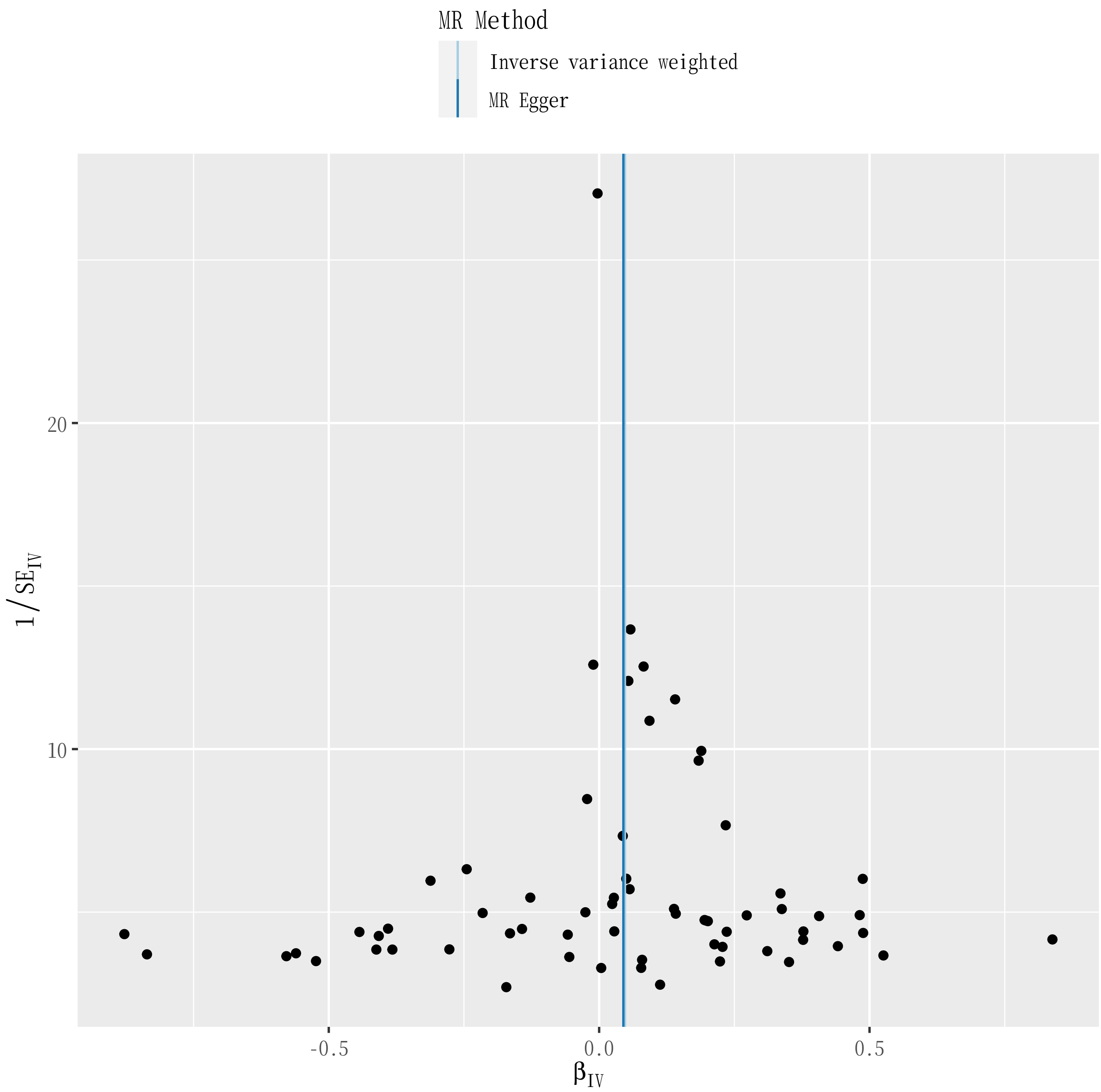
MR Method
Inverse variance weighted
MR Egger
20
10
1/SE_IV
-0.5
0.0
0.5
β_IV

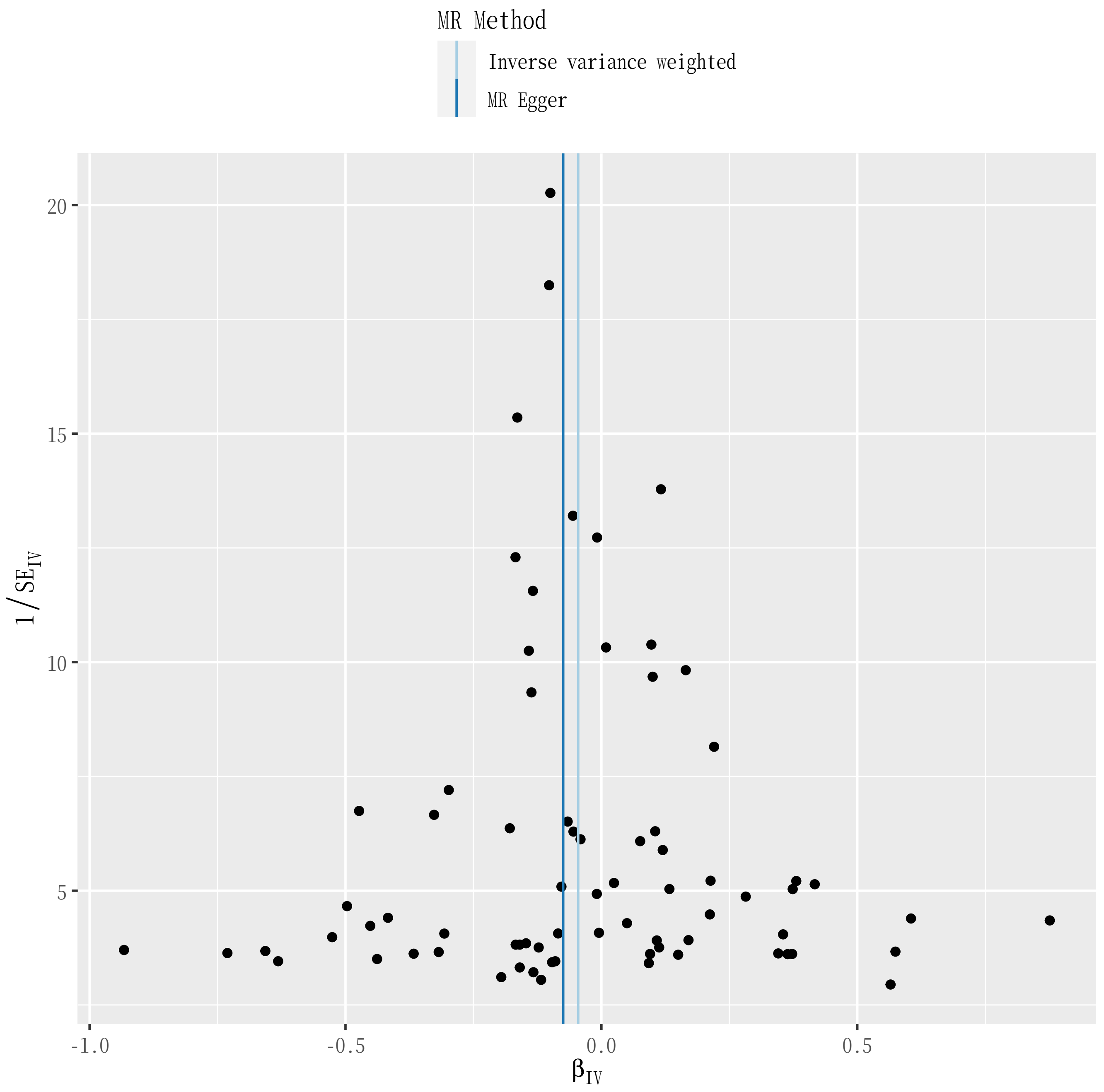
MR Method
Inverse variance weighted
MR Egger
$1/SE_{IV}$
20
15
10
5
-1.0
-0.5
0.0
0.5
β_{IV}

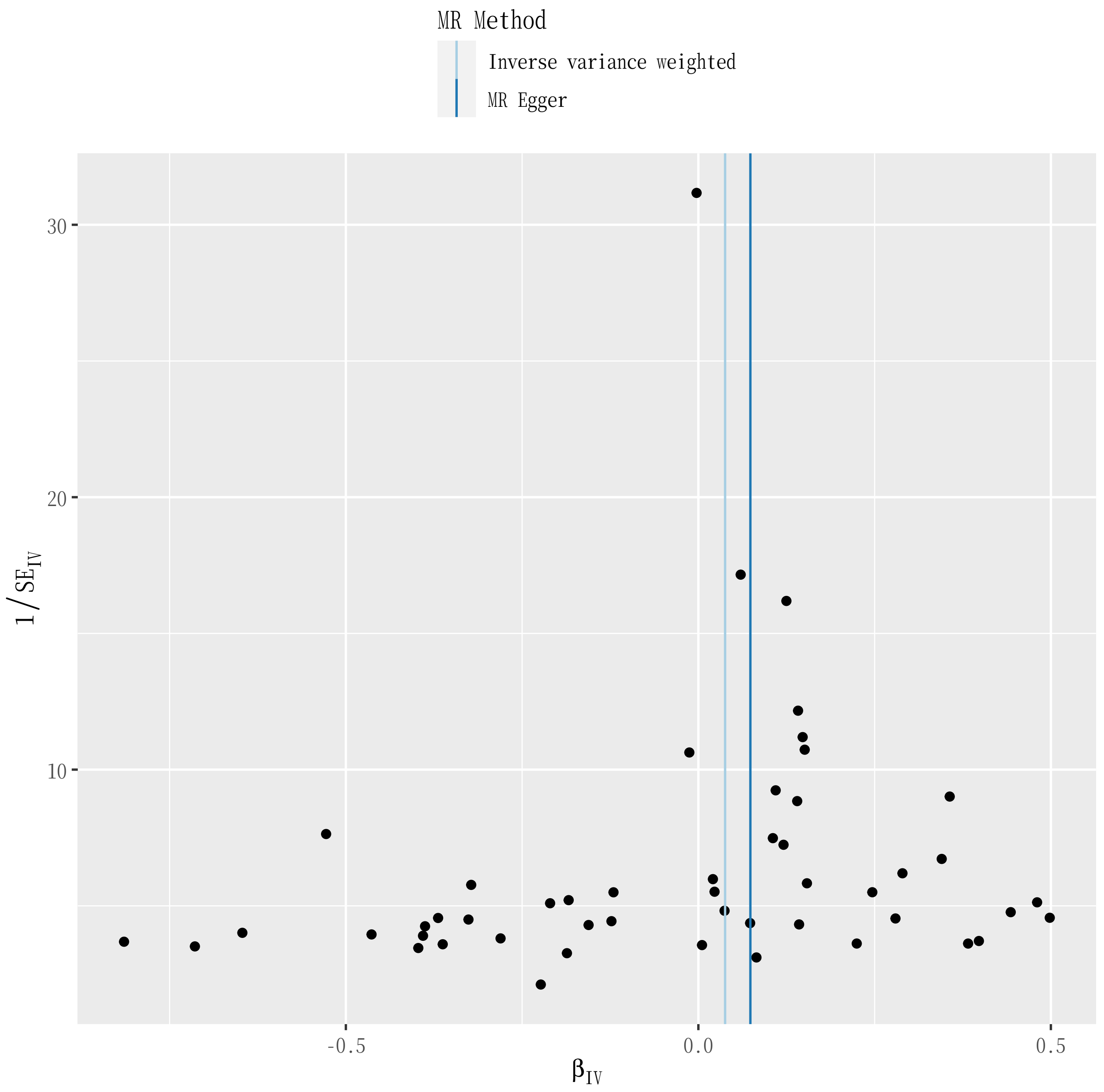

MR Method
Inverse variance weighted
MR Egger
30
20
10
$1/SE_{IV}$
-0.5
0.0
0.5
β_{IV}

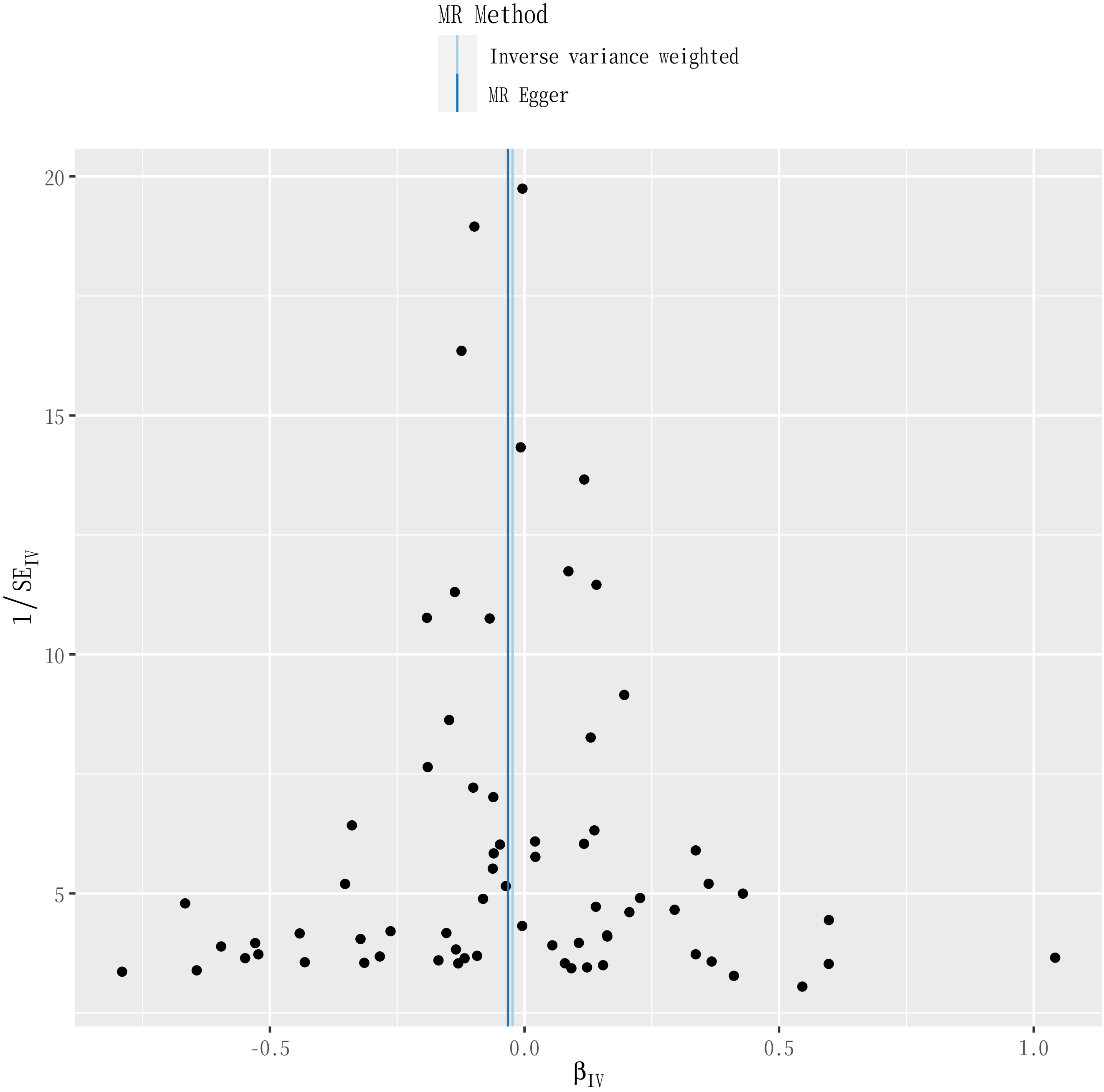

MR Method
Inverse variance weighted
MR Egger
20
15
10
5
$1/SE_{IV}$
-0.5
0.0
0.5
1.0
β_{IV}

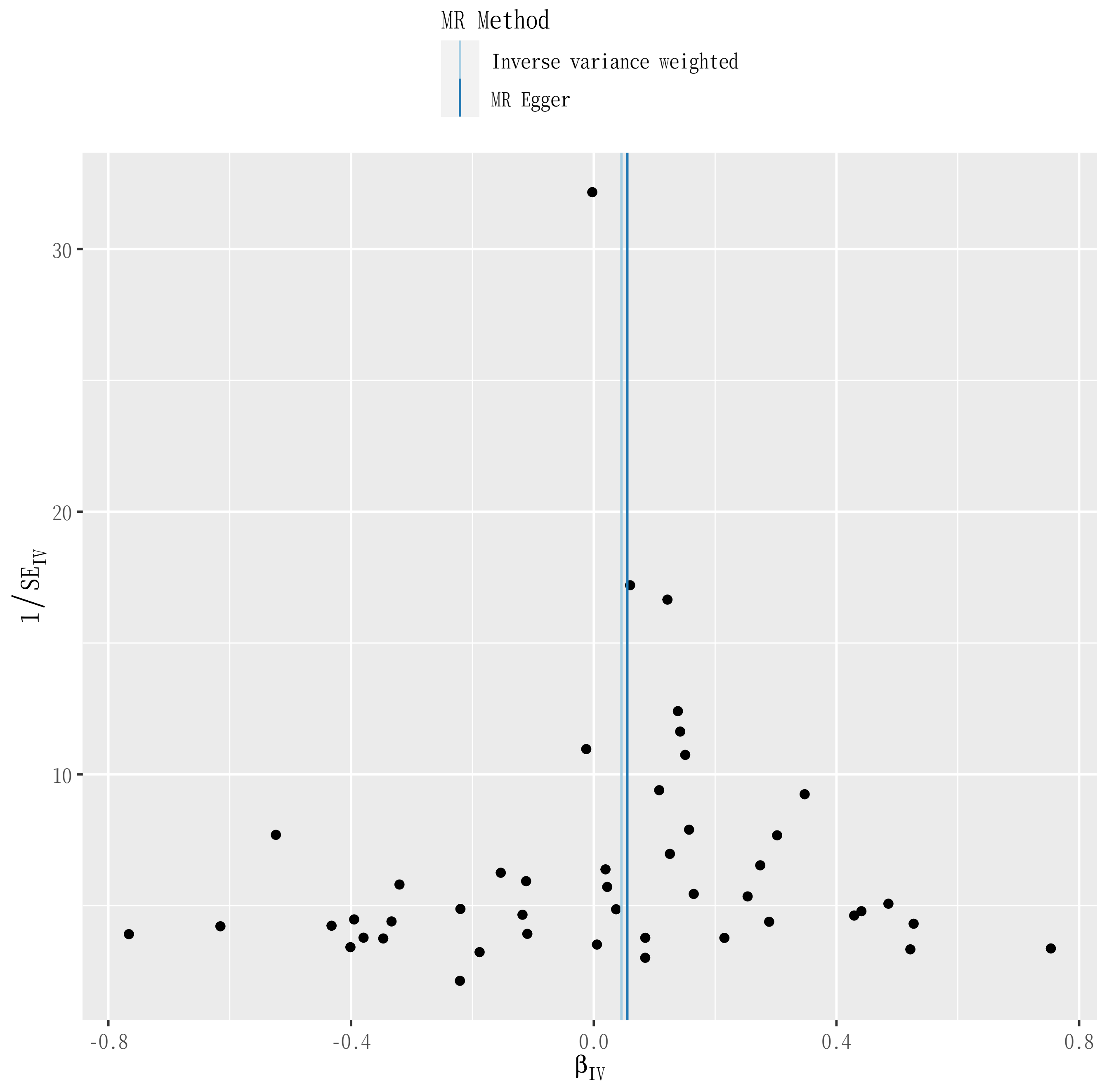

MR Method
Inverse variance weighted
MR Egger
30
20
10
$1/SE_{IV}$
-0.8
-0.4
0.0
0.4
0.8
β_{IV}

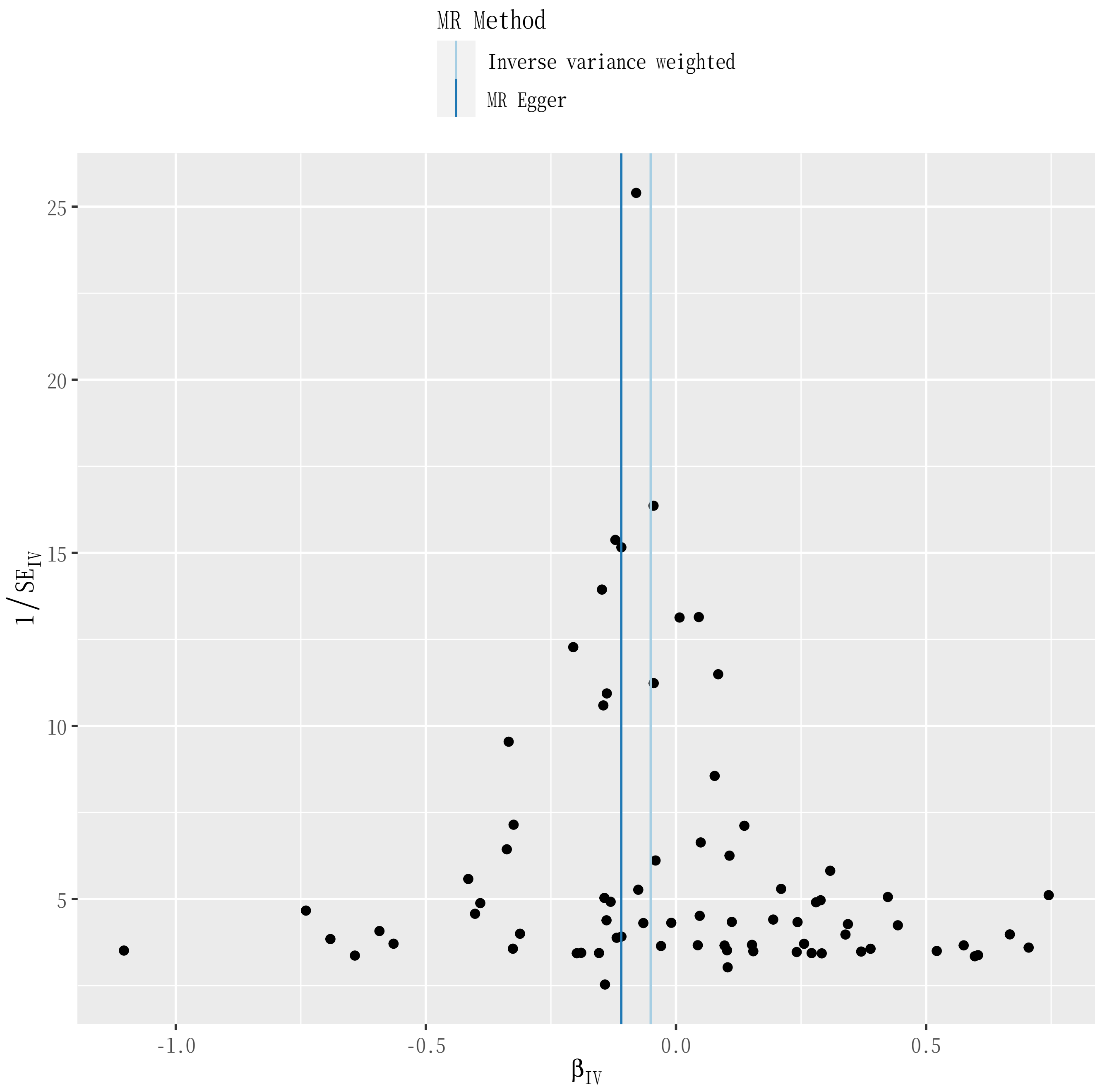

MR Method
Inverse variance weighted
MR Egger
25
20
15
10
5
1/SE_IV
-1.0
-0.5
0.0
0.5
β_IV

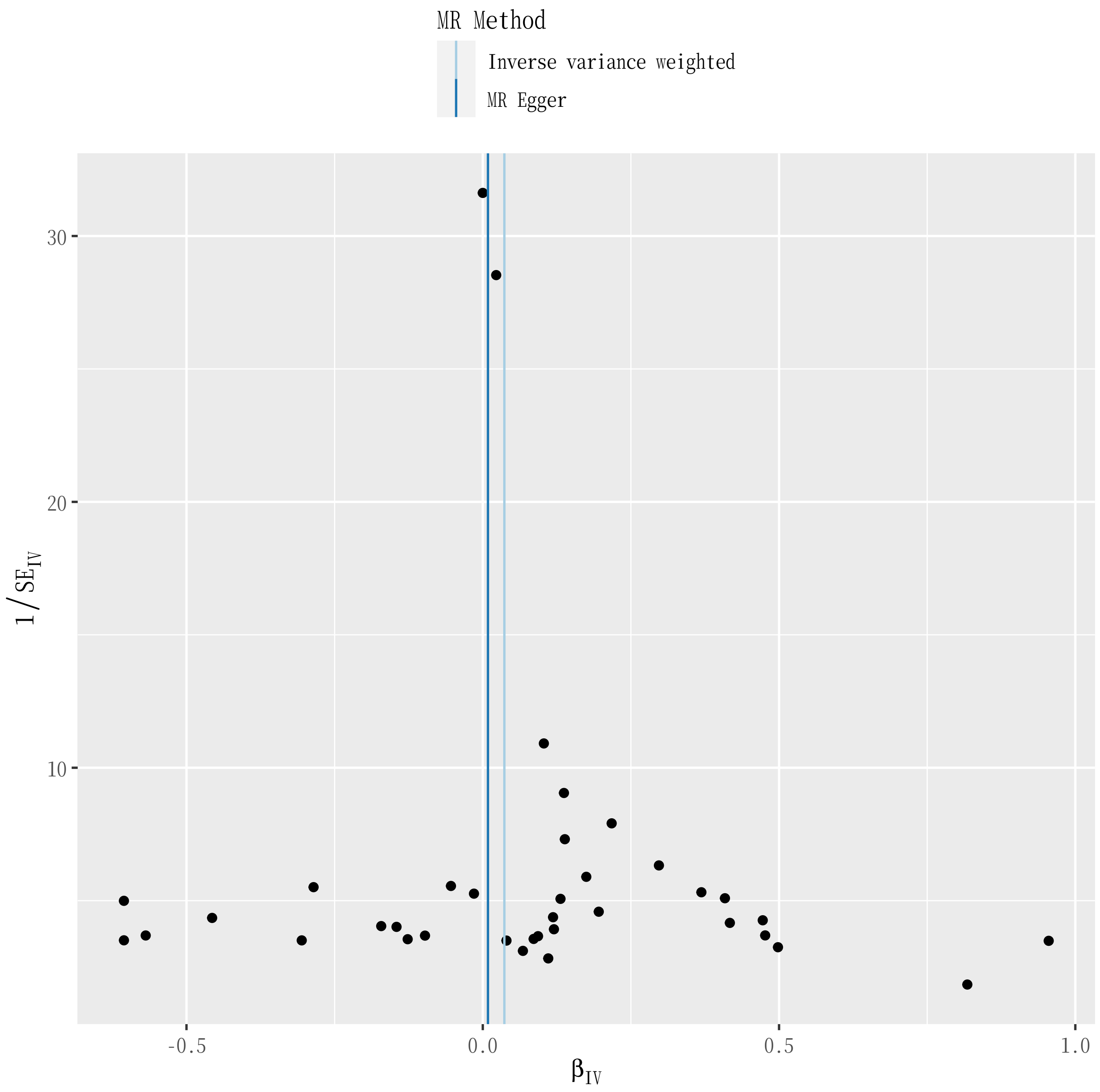

MR Method
Inverse variance weighted
MR Egger
30
20
10
1/SE_IV
-0.5
0.0
0.5
1.0
β_IV

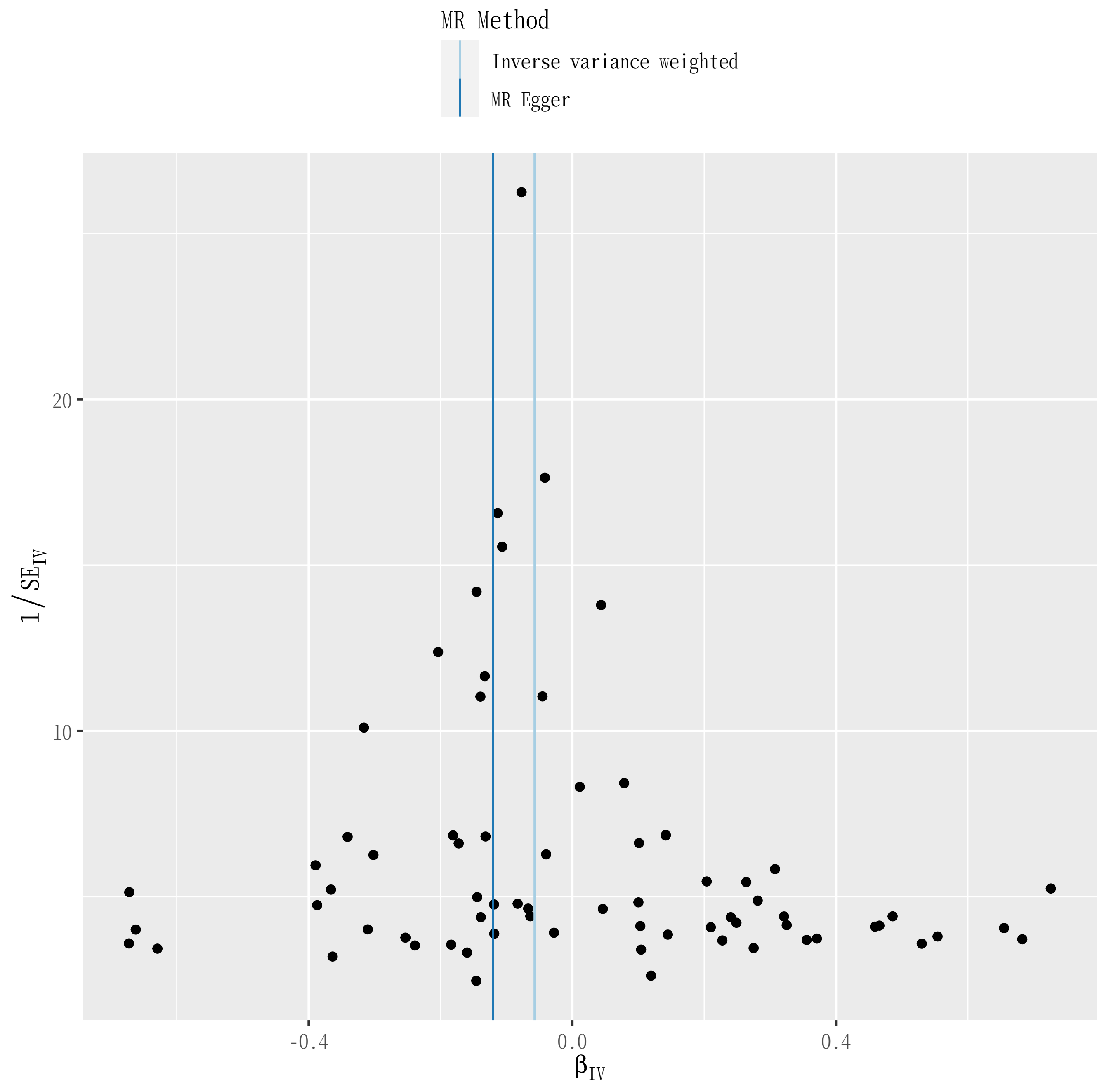
MR Method
Inverse variance weighted
MR Egger
20
10
1/SE$_{IV}$
-0.4
0.0
0.4
β$_{IV}$

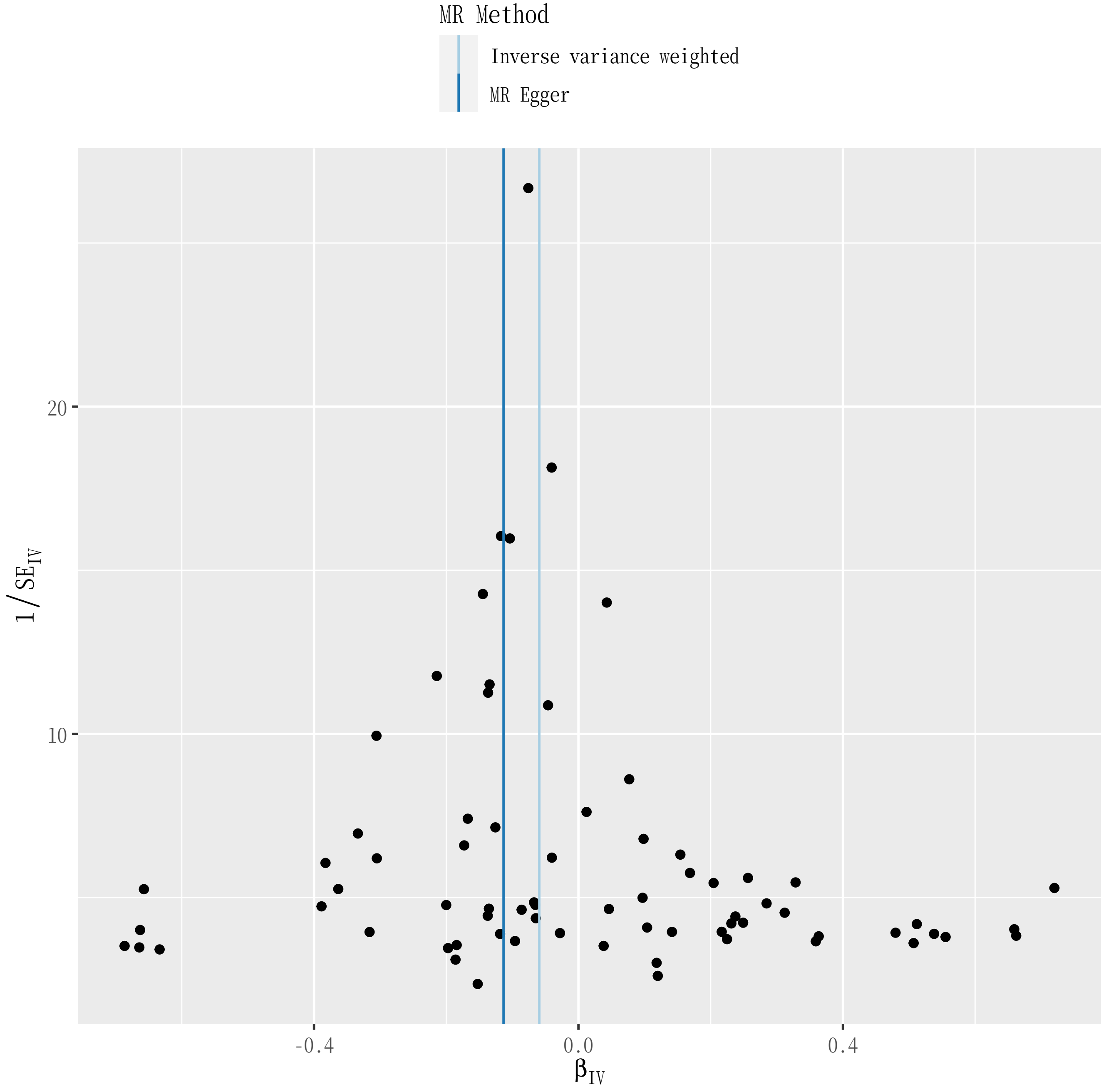
MR Method
Inverse variance weighted
MR Egger
1/SE_IV
20
10
-0.4
0.0
0.4
β_IV

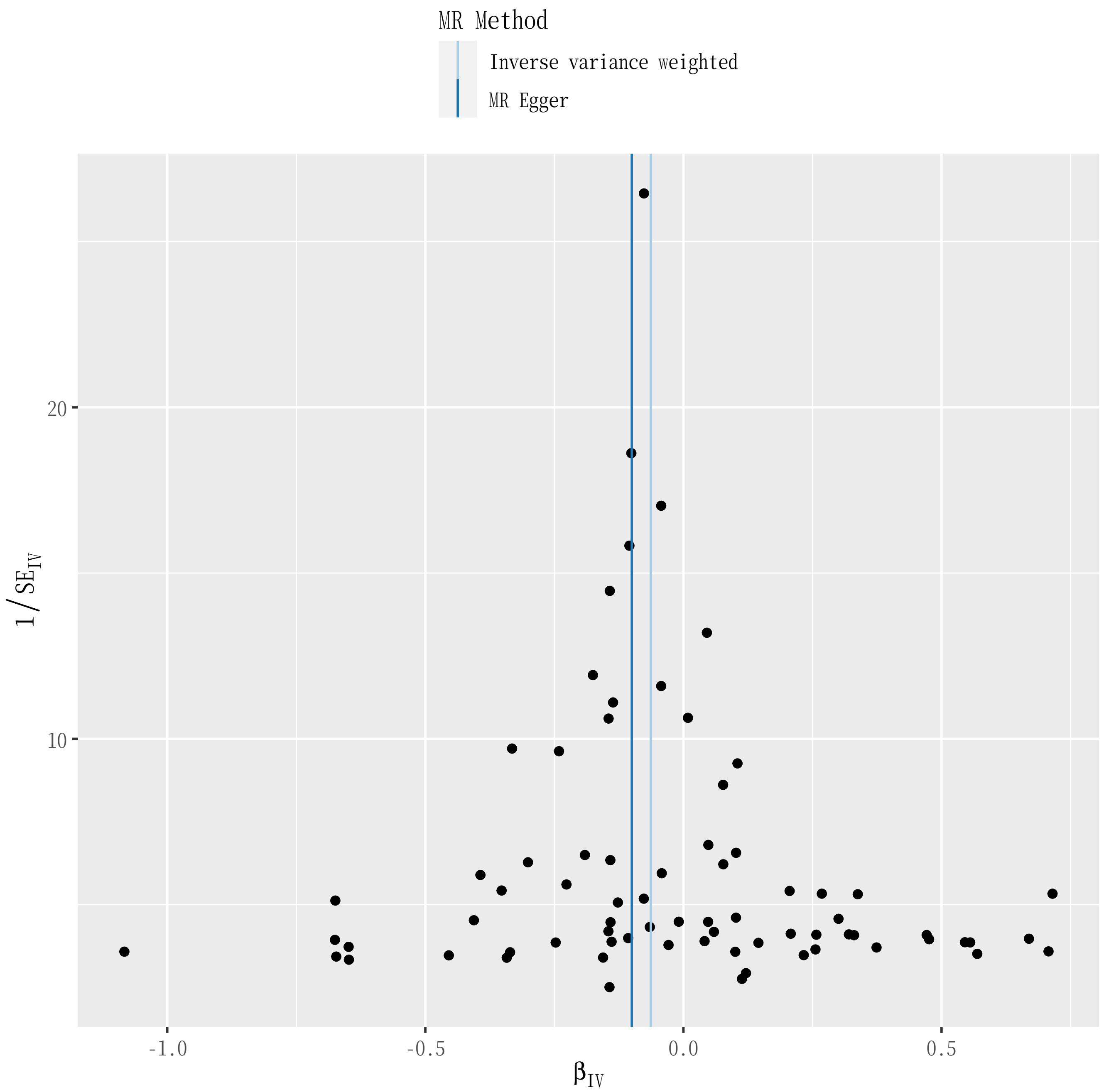
MR Method
Inverse variance weighted
MR Egger
20
10
1/SE_IV
-1.0
-0.5
0.0
0.5
β_IV

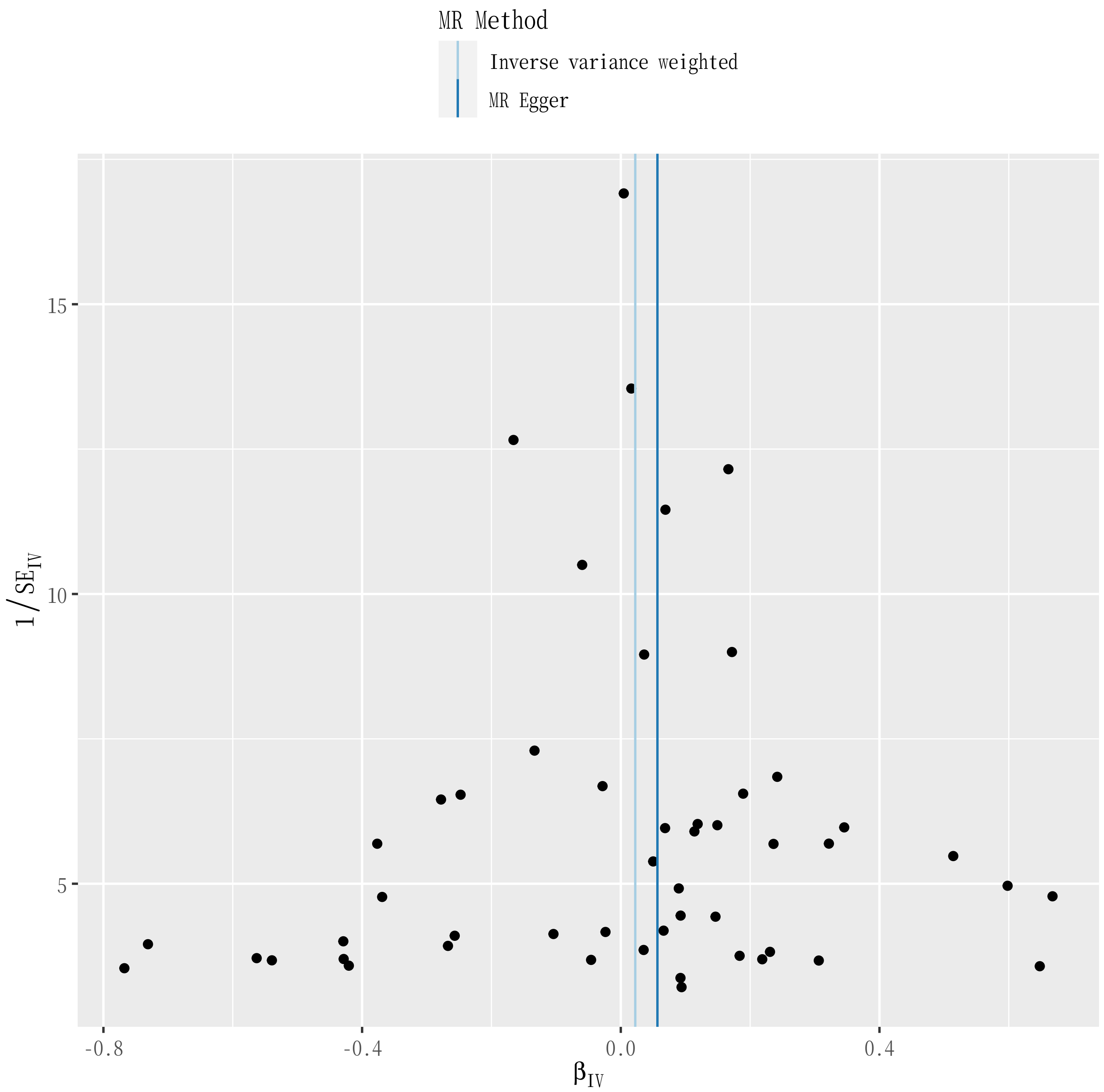
MR Method
Inverse variance weighted
MR Egger
15
10
5
-0.8
-0.4
0.0
0.4
$1/SE_{IV}$
β_{IV}

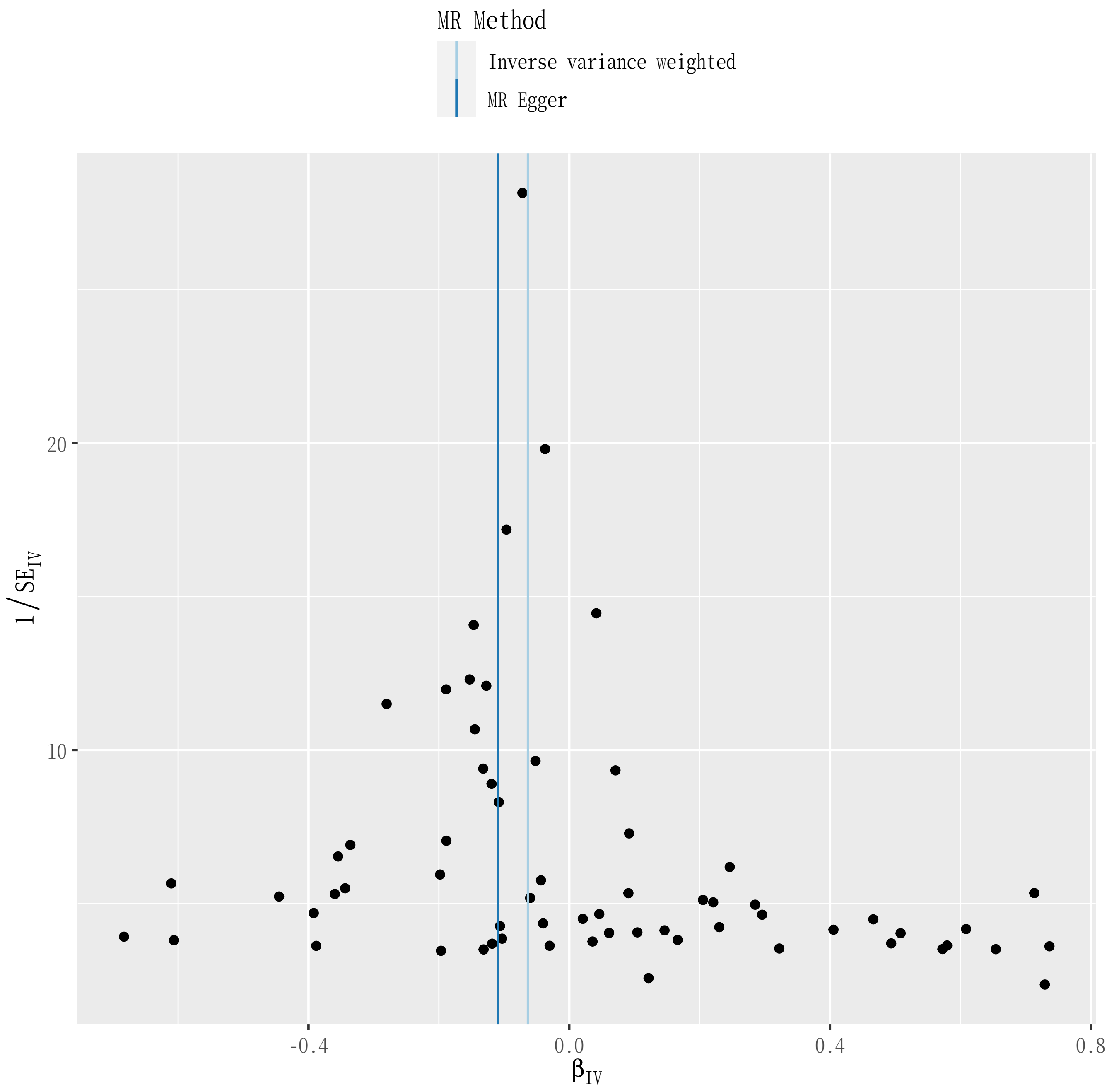
MR Method
Inverse variance weighted
MR Egger
20
10
1/SE_IV
-0.4
0.0
0.4
0.8
β_IV

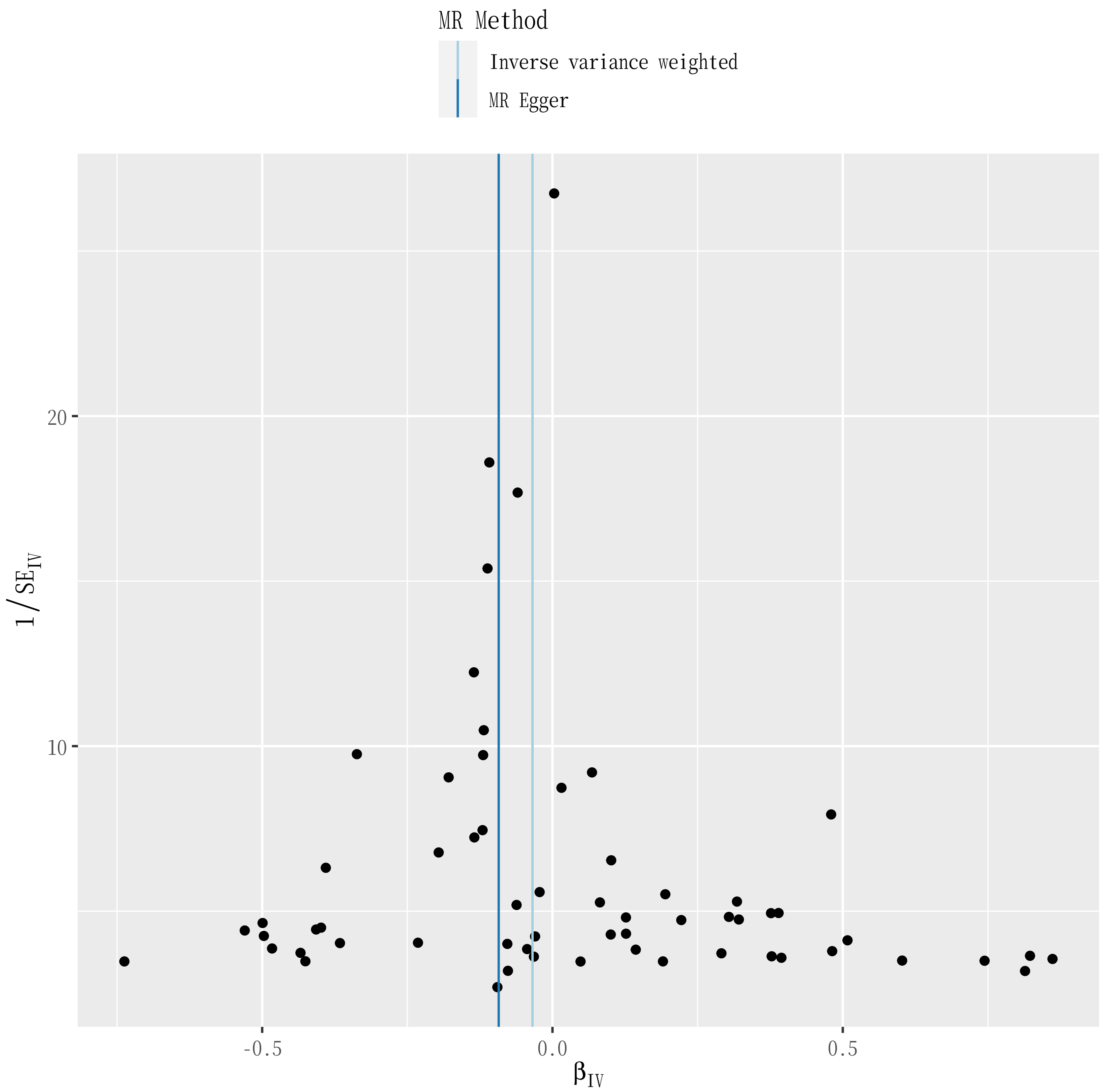
MR Method
Inverse variance weighted
MR Egger
20
10
-0.5
0.0
0.5
$1/SE_{IV}$
β_{IV}

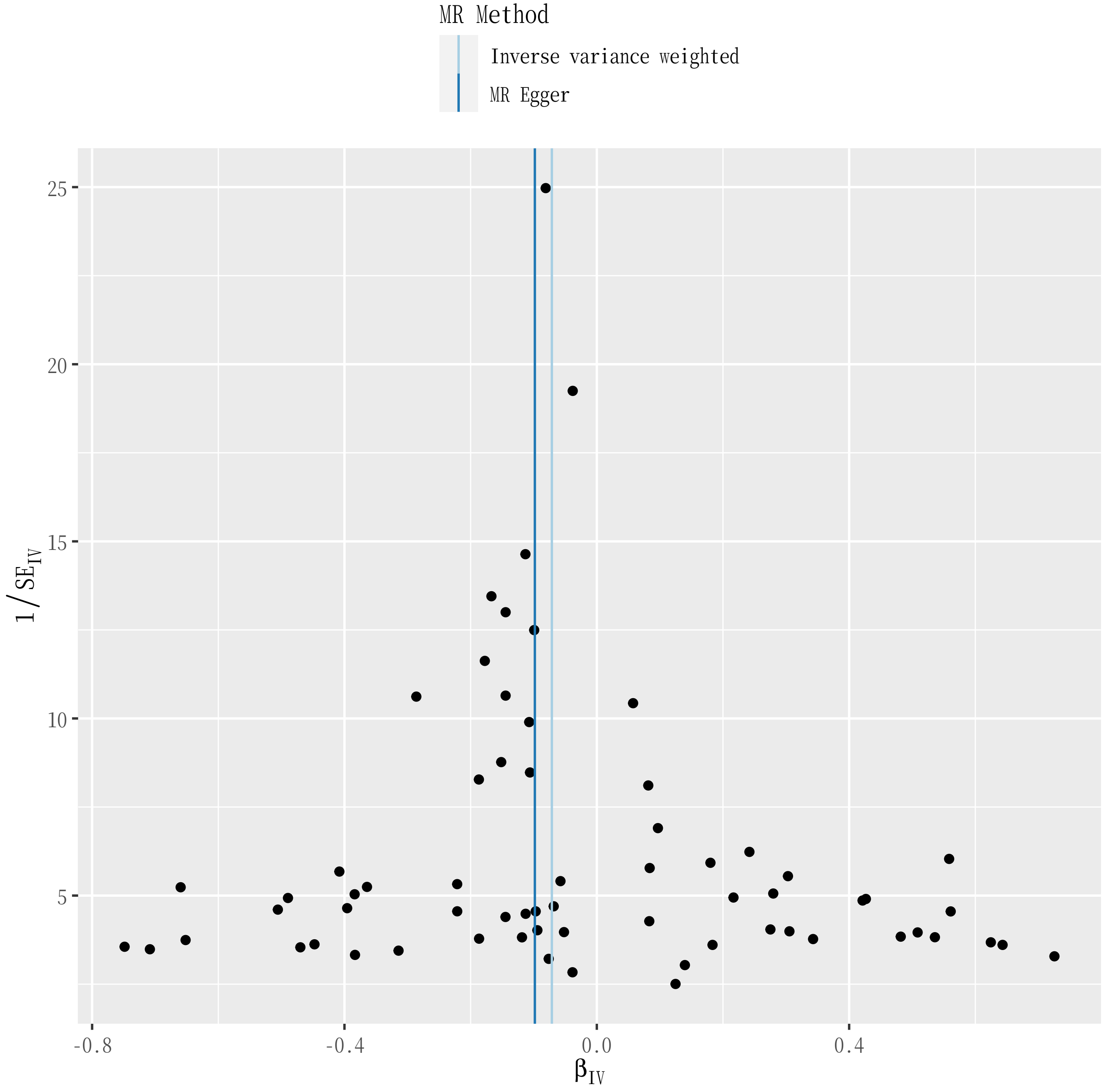

MR Method
Inverse variance weighted
MR Egger
1/SE_IV
β_IV
25
20
15
10
5
-0.8
-0.4
0.0
0.4

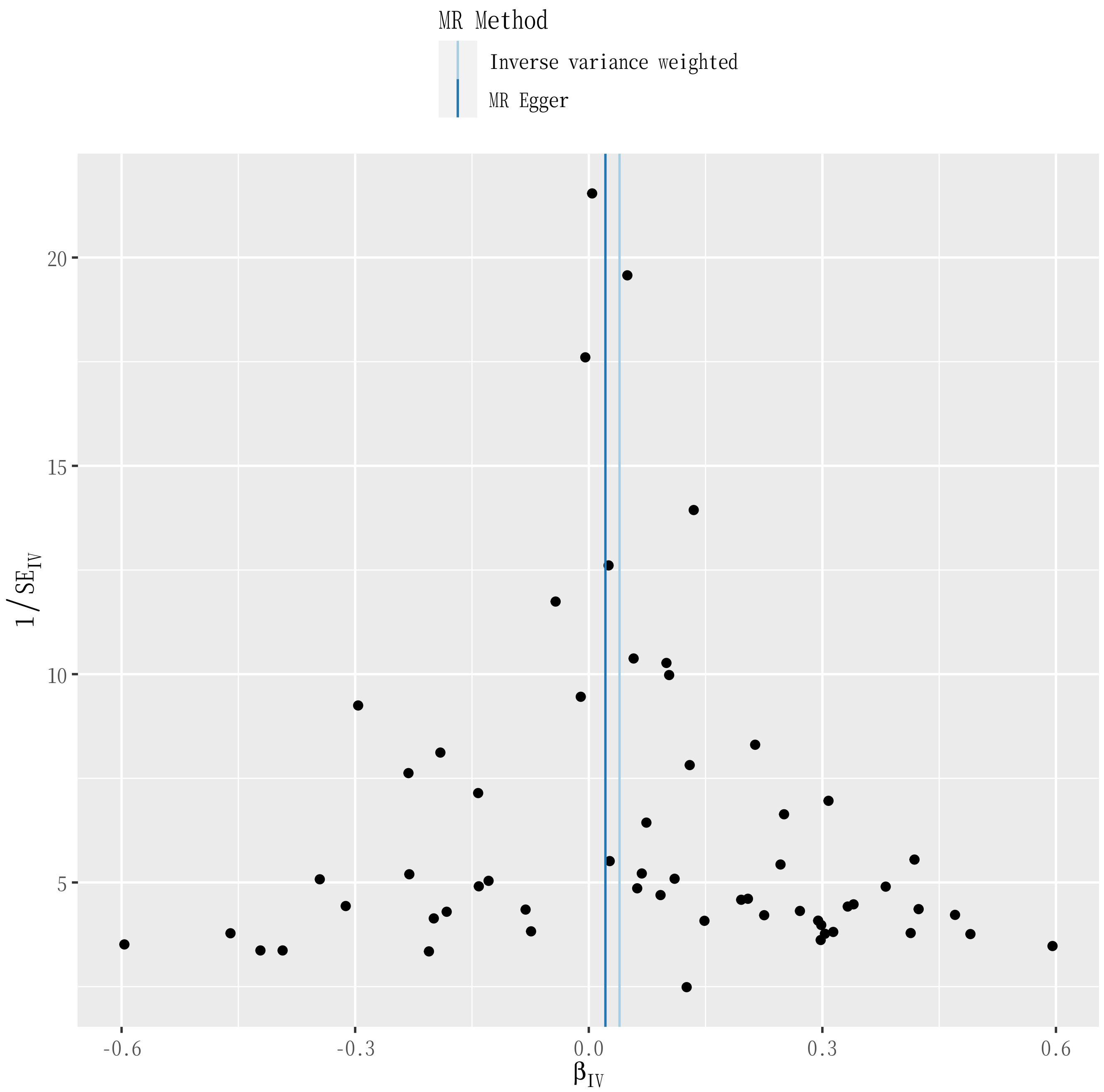
MR Method
Inverse variance weighted
MR Egger
20
15
10
5
1/SE_IV
-0.6
-0.3
0.0
0.3
0.6
β_IV

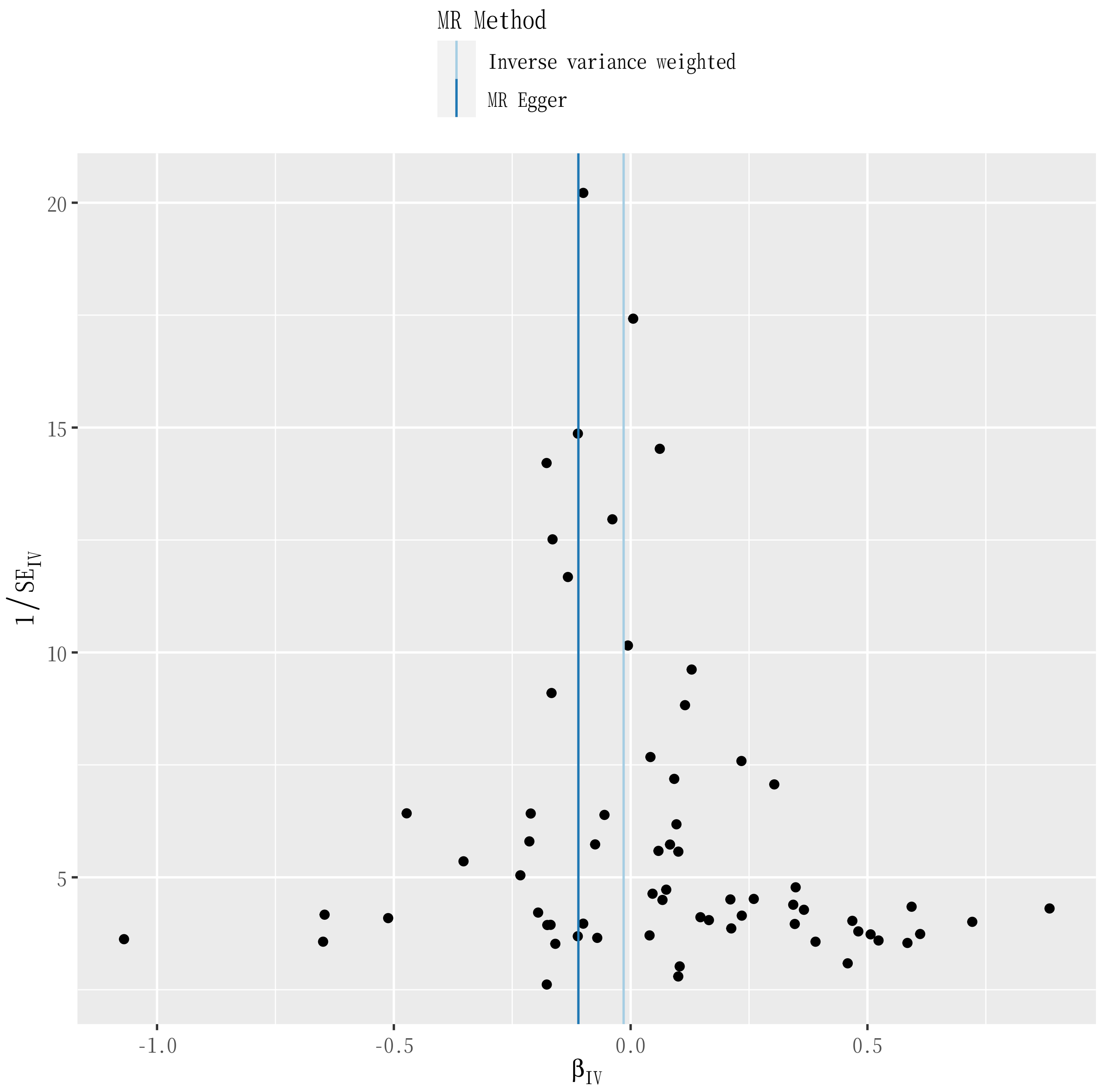
MR Method
Inverse variance weighted
MR Egger
20
15
10
5
1/SE_IV
-1.0
-0.5
0.0
0.5
β_IV

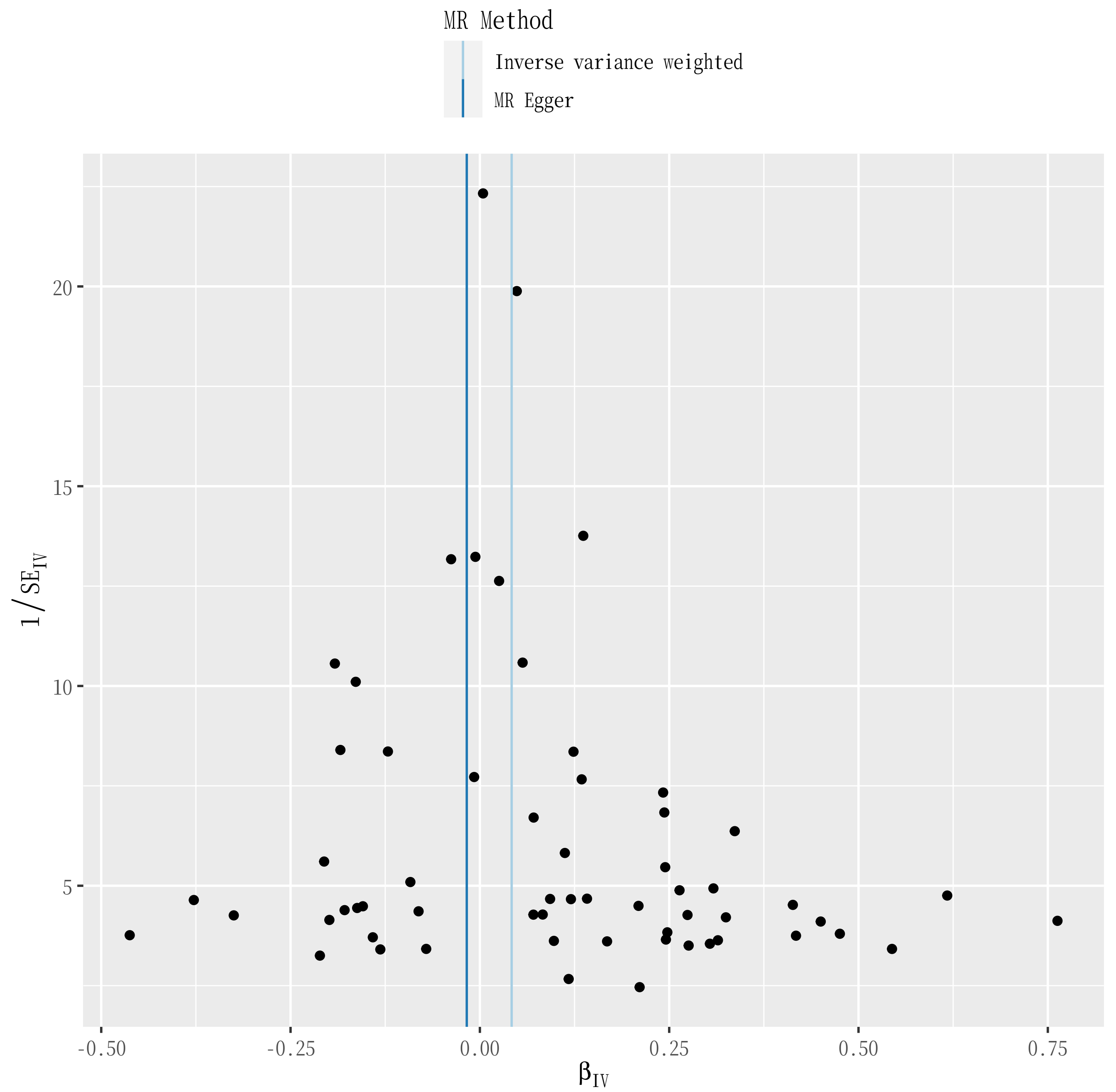

MR Method
Inverse variance weighted
MR Egger
20
15
10
5
-0.50
-0.25
0.00
0.25
0.50
0.75
βIV
1/SEIV

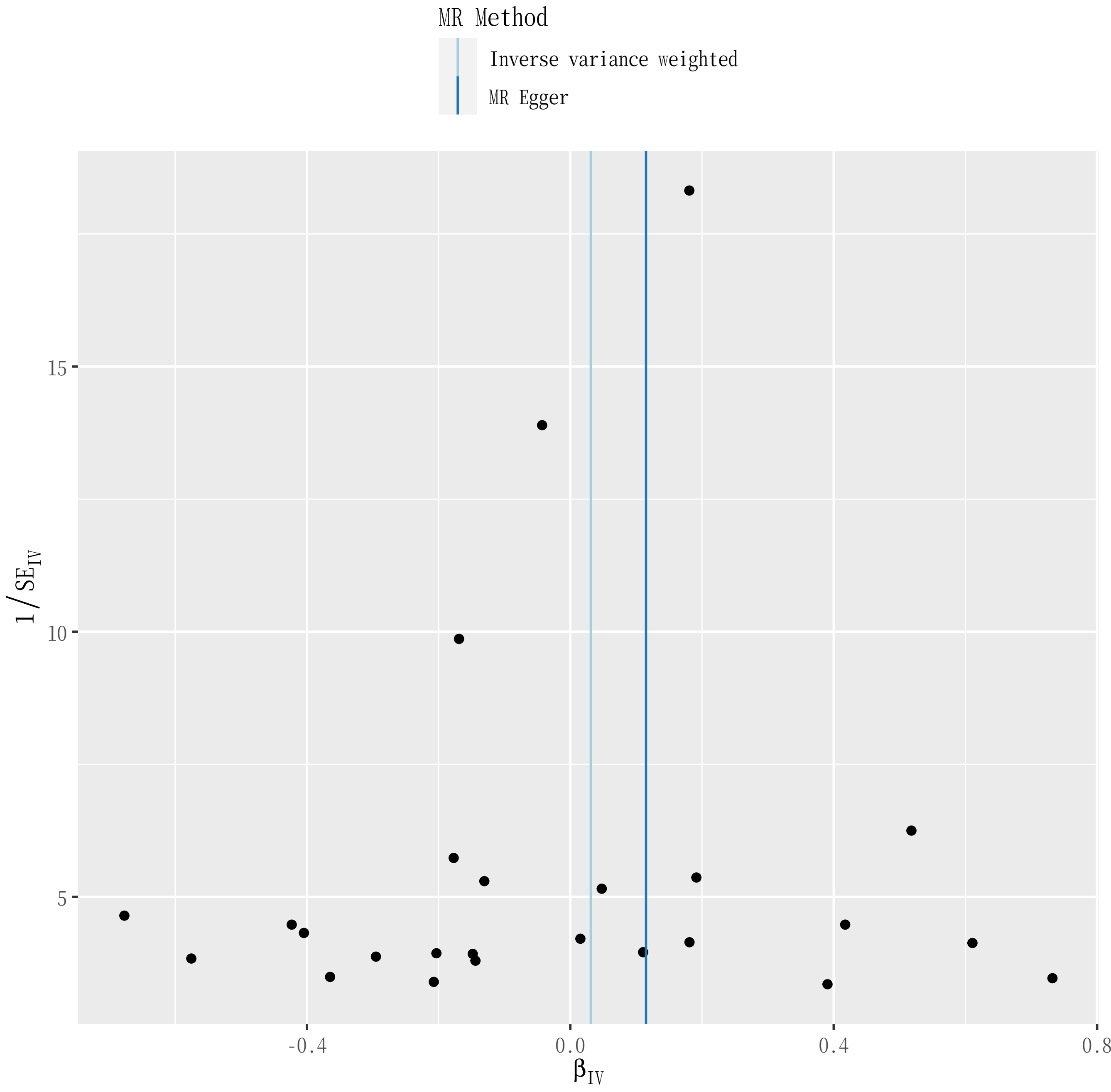

MR Method
Inverse variance weighted
MR Egger
15
10
5
$1/SE_{IV}$
-0.4
0.0
0.4
0.8
β_{IV}

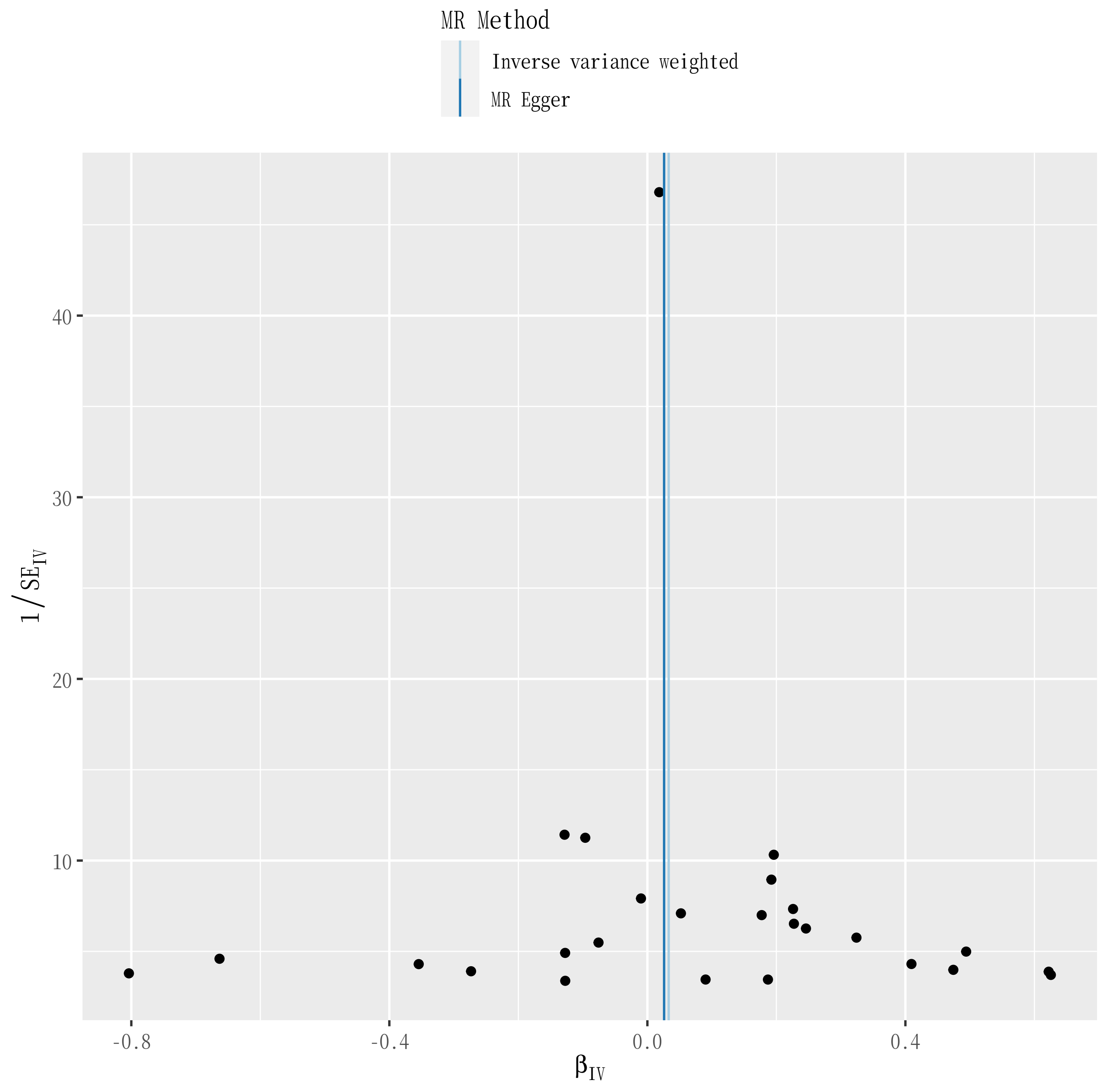

MR Method
Inverse variance weighted
MR Egger
40
30
20
10
1/SE_IV
-0.8
-0.4
0.0
0.4
β_IV

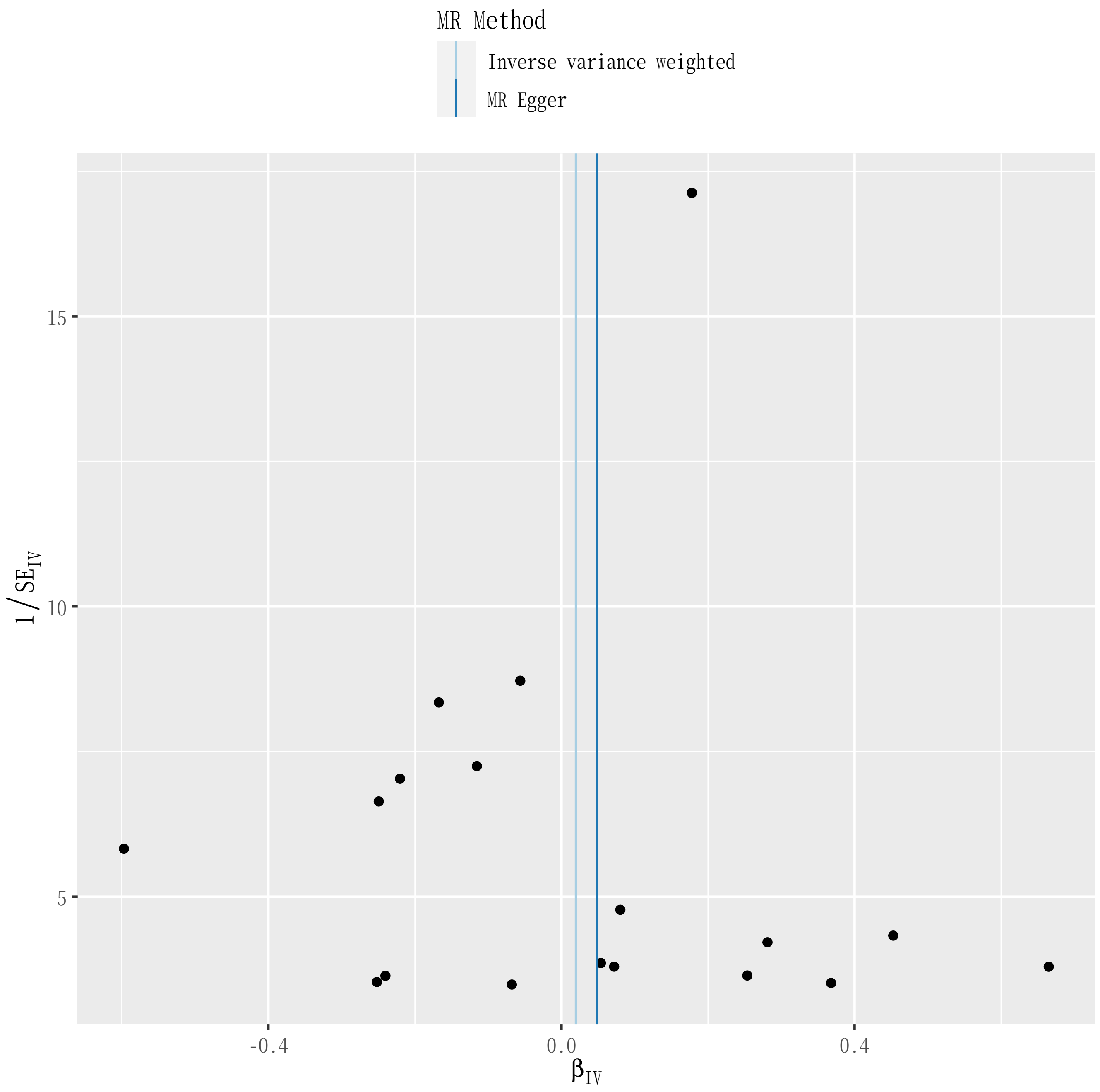

MR Method
Inverse variance weighted
MR Egger
15
10
5
1/SE_IV
-0.4
0.0
0.4
β_IV

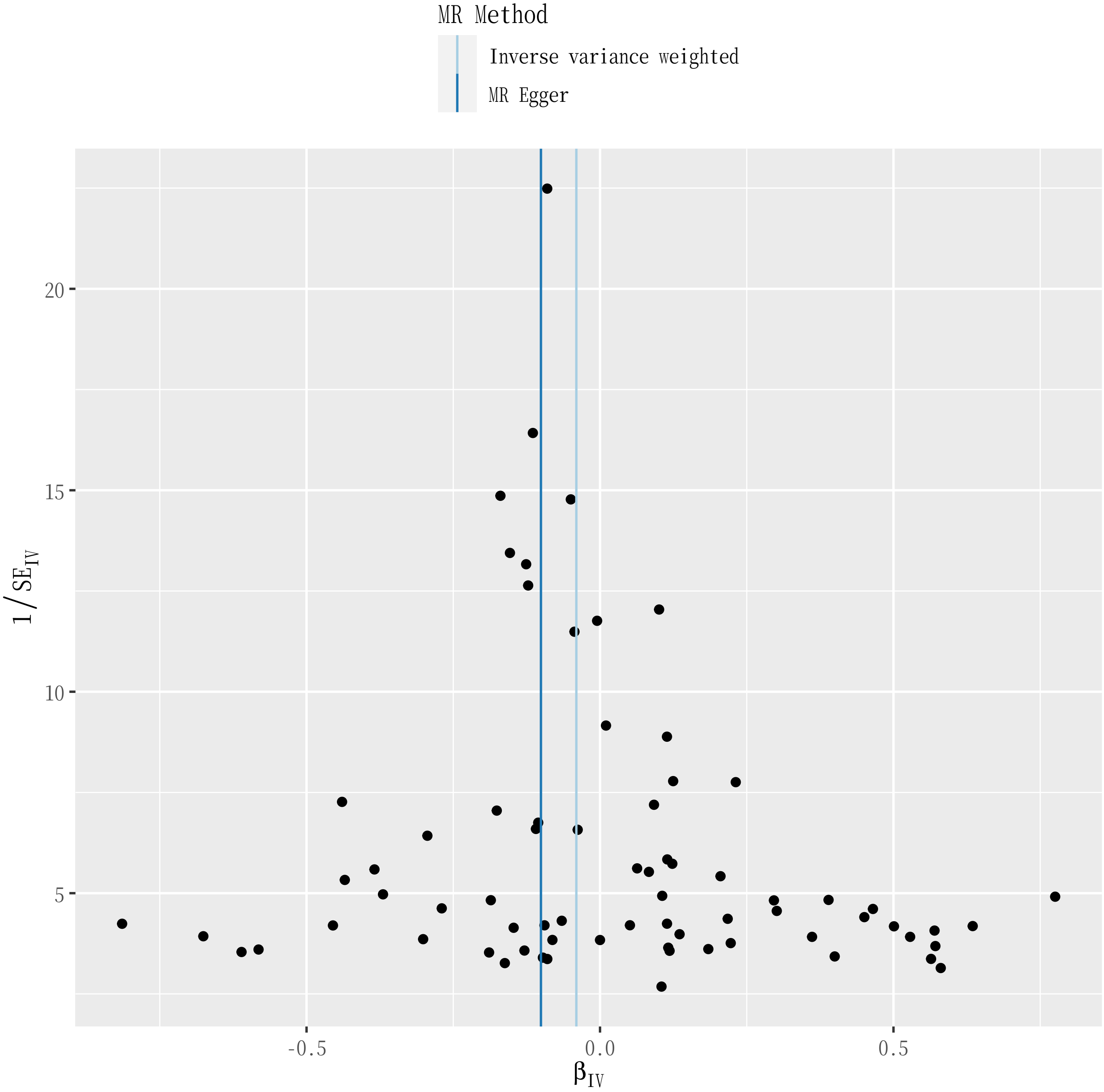
MR Method
Inverse variance weighted
MR Egger
20
15
10
5
1/SE$_{IV}$
-0.5
0.0
0.5
β$_{IV}$

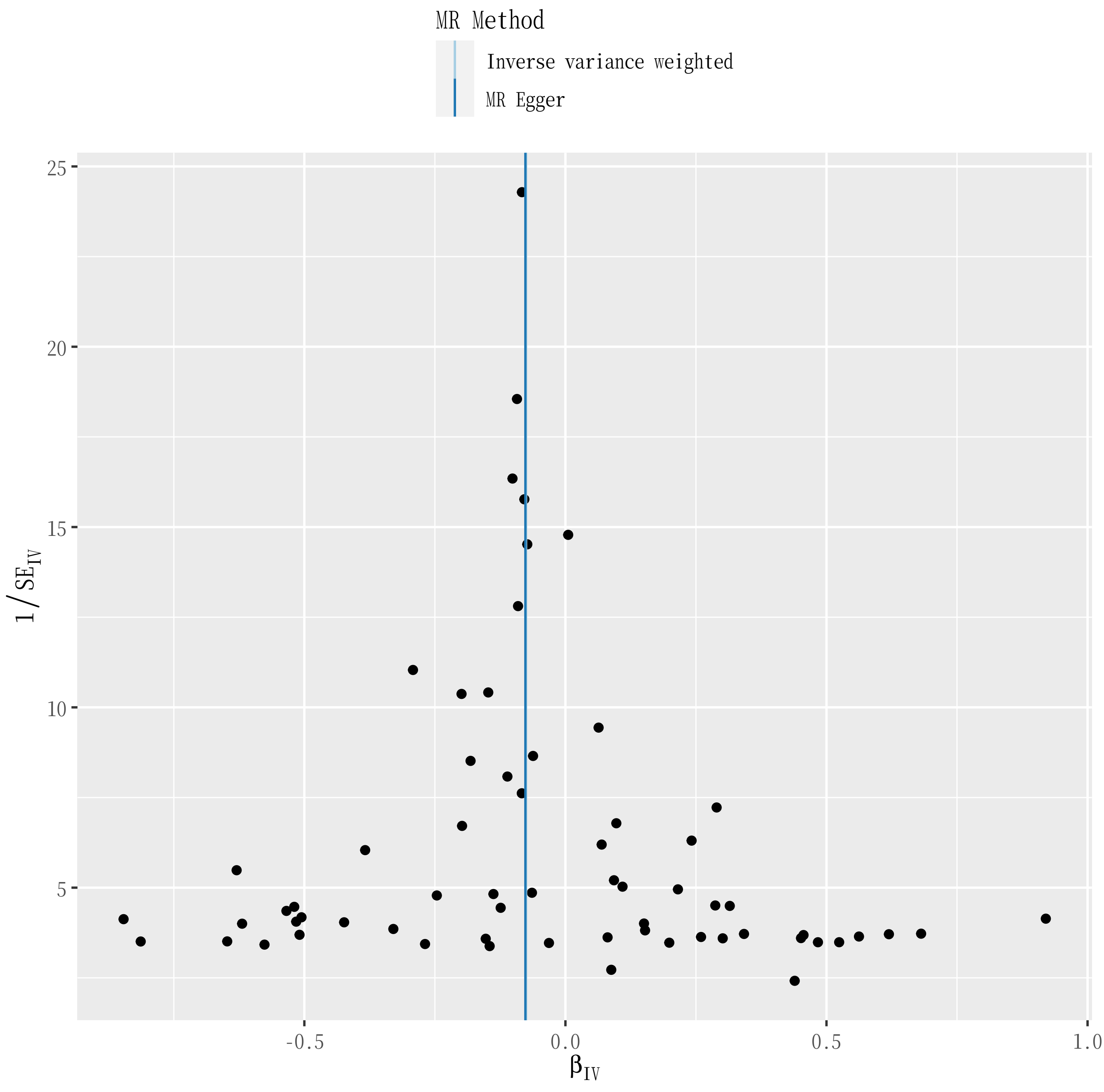
MR Method
Inverse variance weighted
MR Egger
25
20
15
10
5
1/SE_IV
-0.5
0.0
0.5
1.0
β_IV

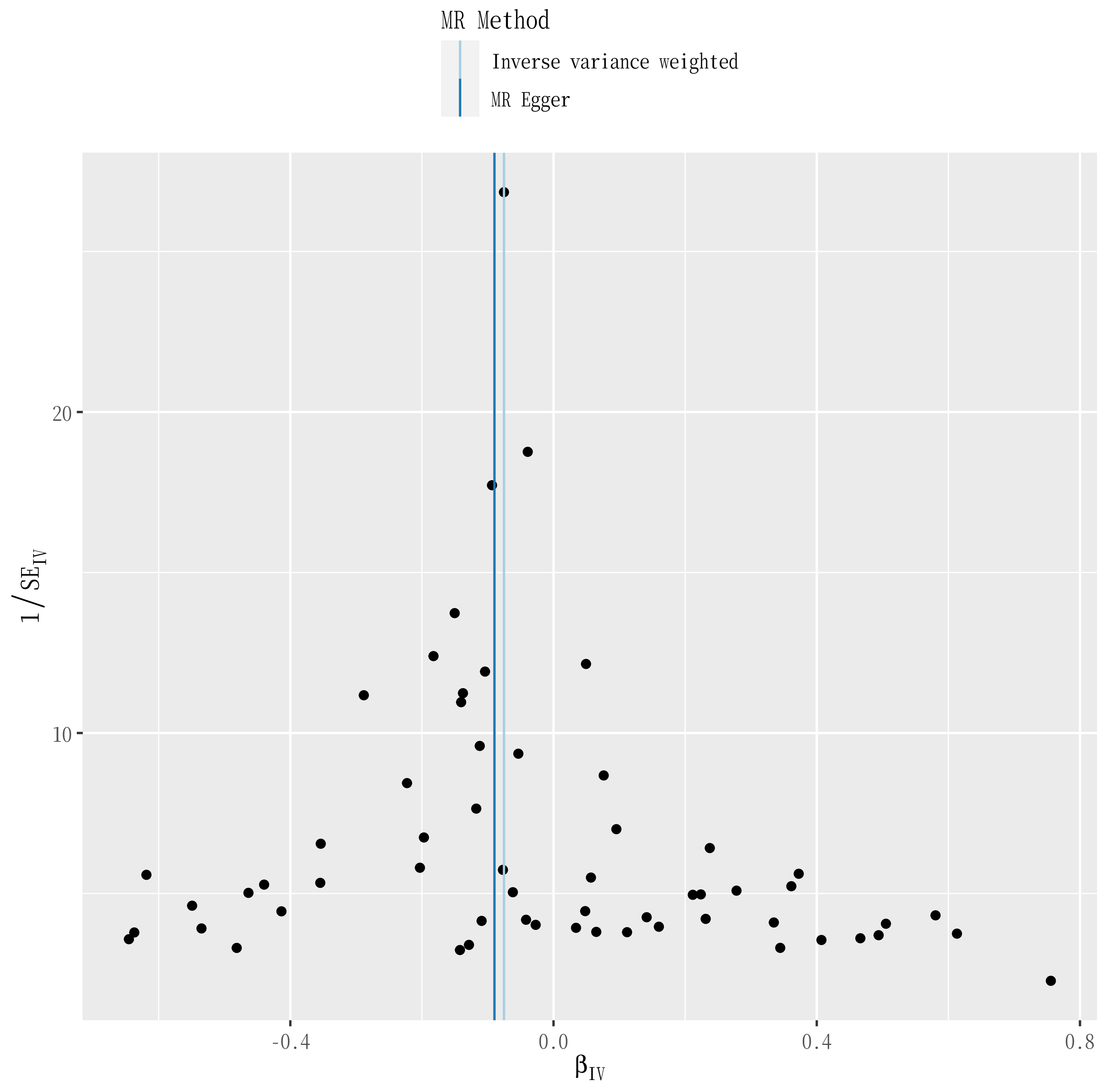
MR Method
Inverse variance weighted
MR Egger
20
10
1/SE_IV
-0.4
0.0
0.4
0.8
β_IV

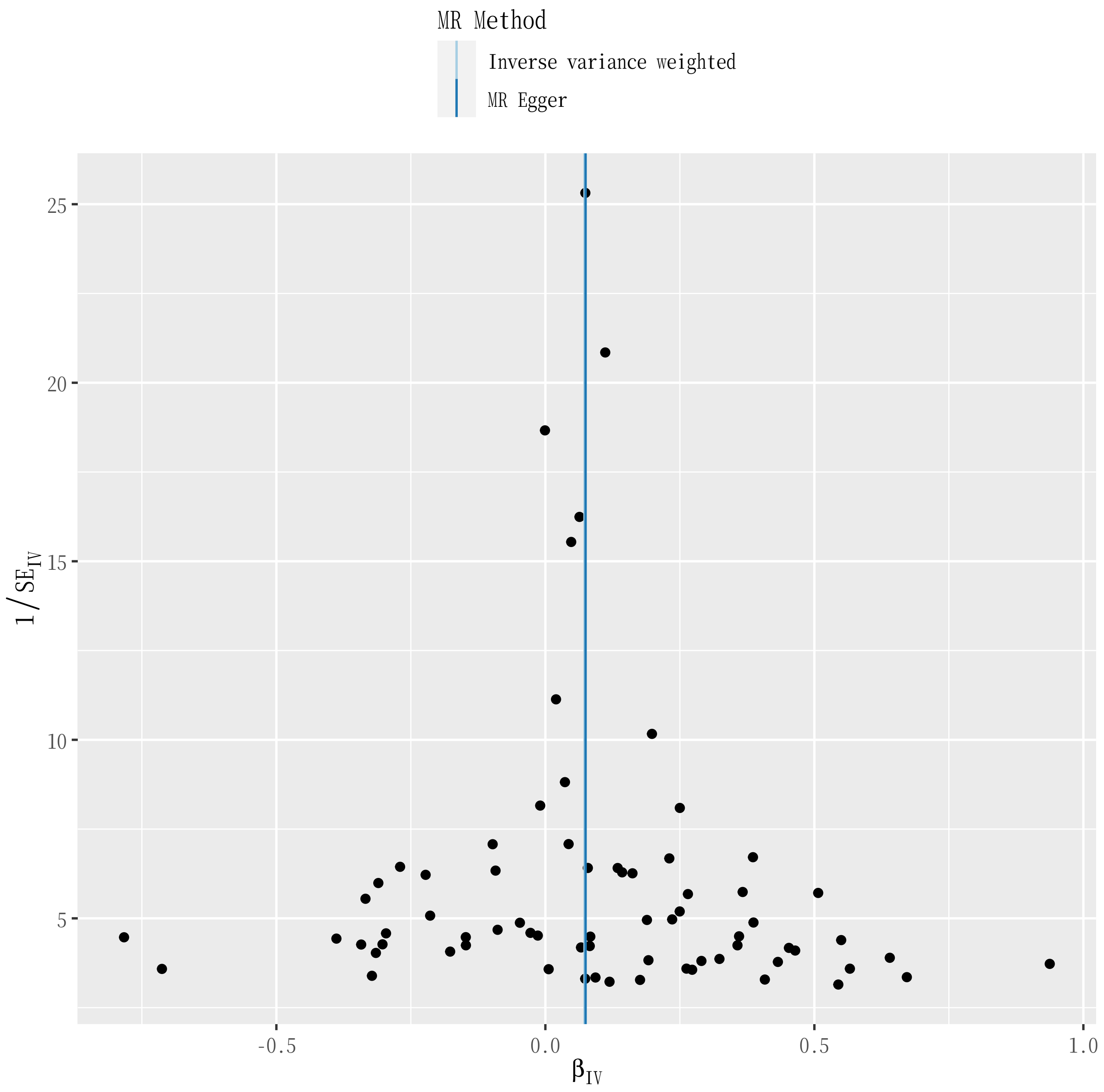
MR Method
Inverse variance weighted
MR Egger
25
20
15
10
5
1/SE_IV
-0.5
0.0
0.5
1.0
β_IV

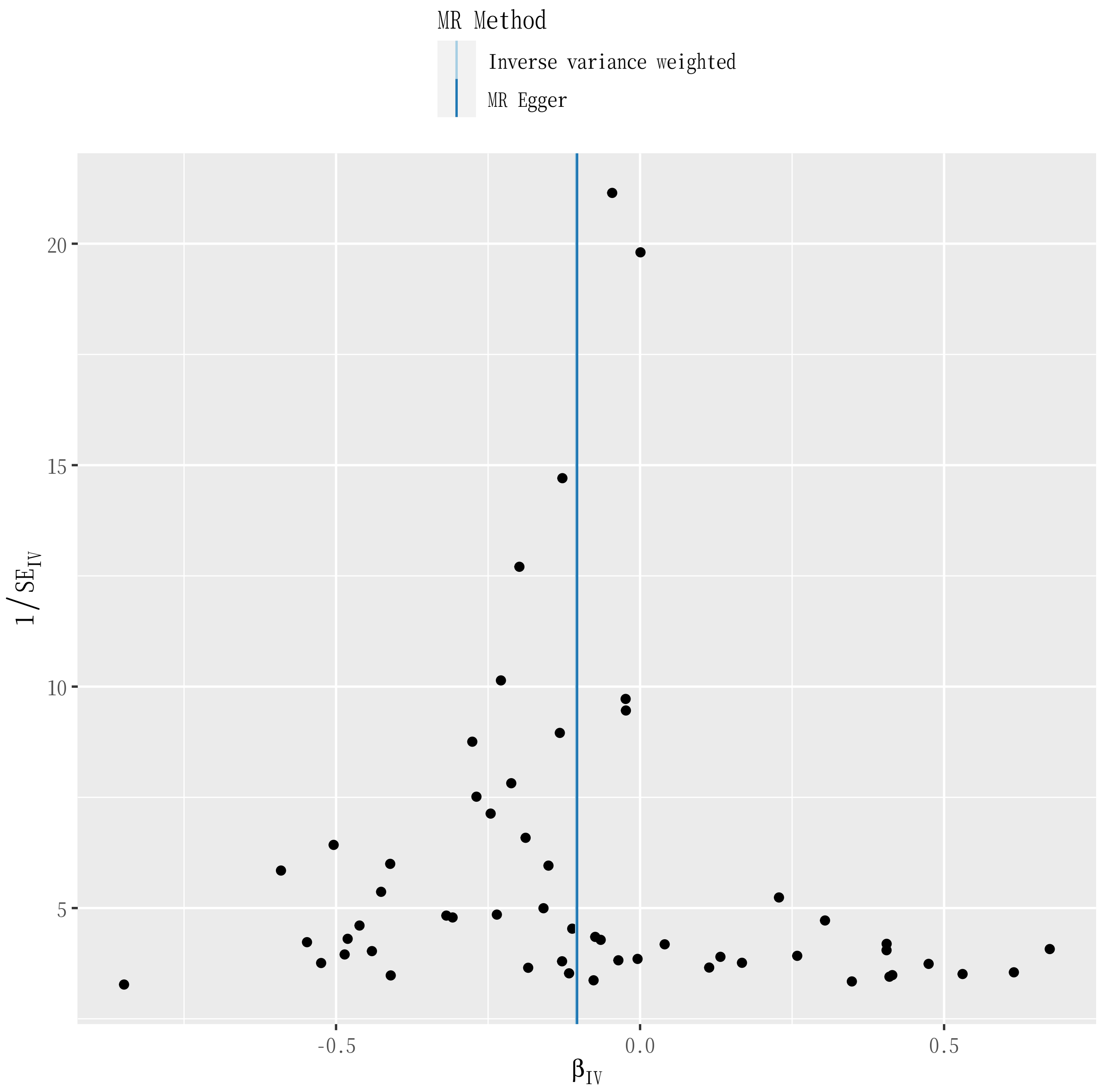
MR Method
Inverse variance weighted
MR Egger
20
15
10
5
$1/SE_{IV}$
-0.5
0.0
0.5
β_{IV}

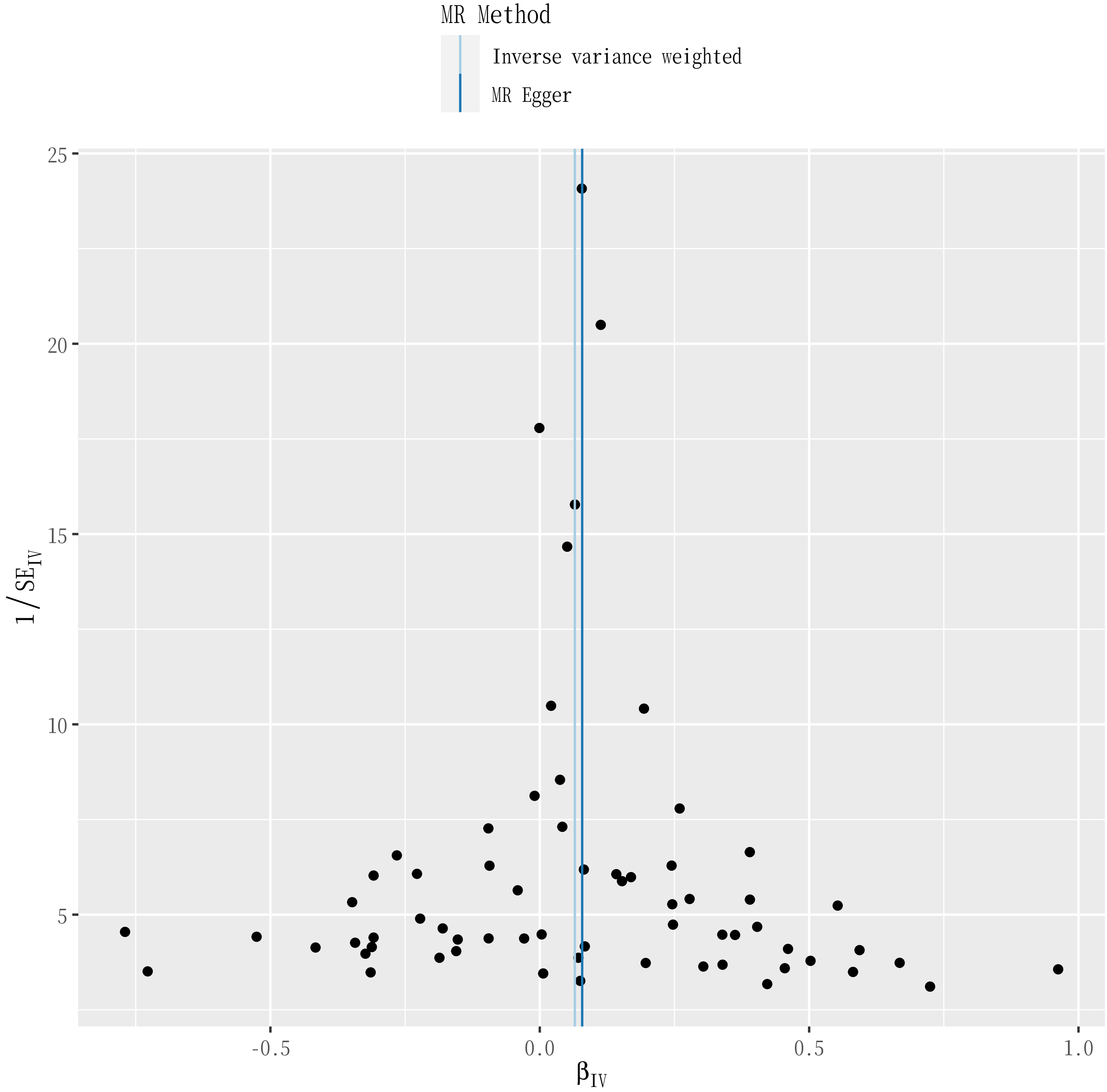
MR Method
Inverse variance weighted
MR Egger
25
20
15
10
5
1/SE_IV
-0.5
0.0
0.5
1.0
β_IV

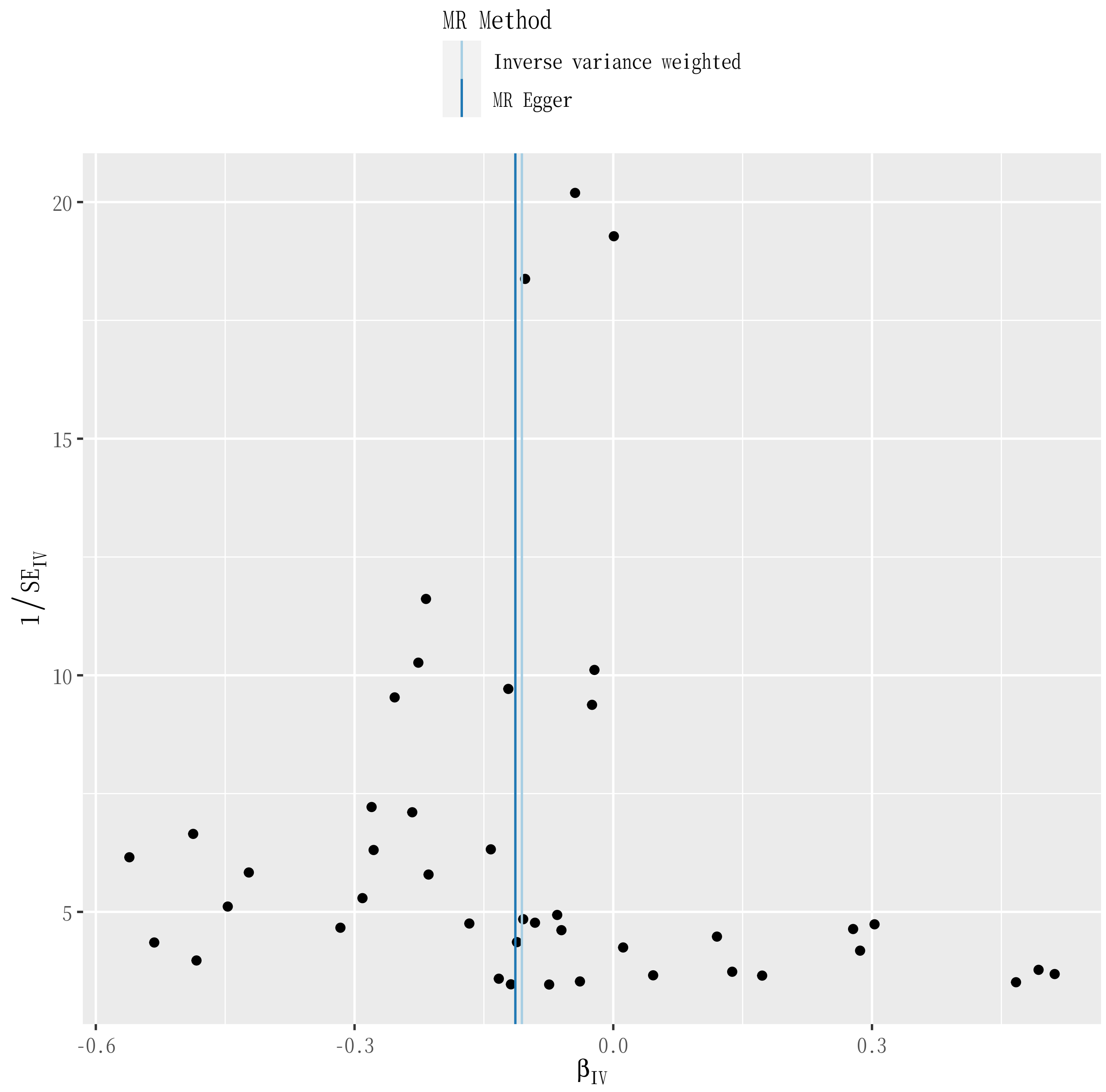

MR Method
Inverse variance weighted
MR Egger
1/SE_IV
20
15
10
5
-0.6
-0.3
0.0
0.3
β_IV

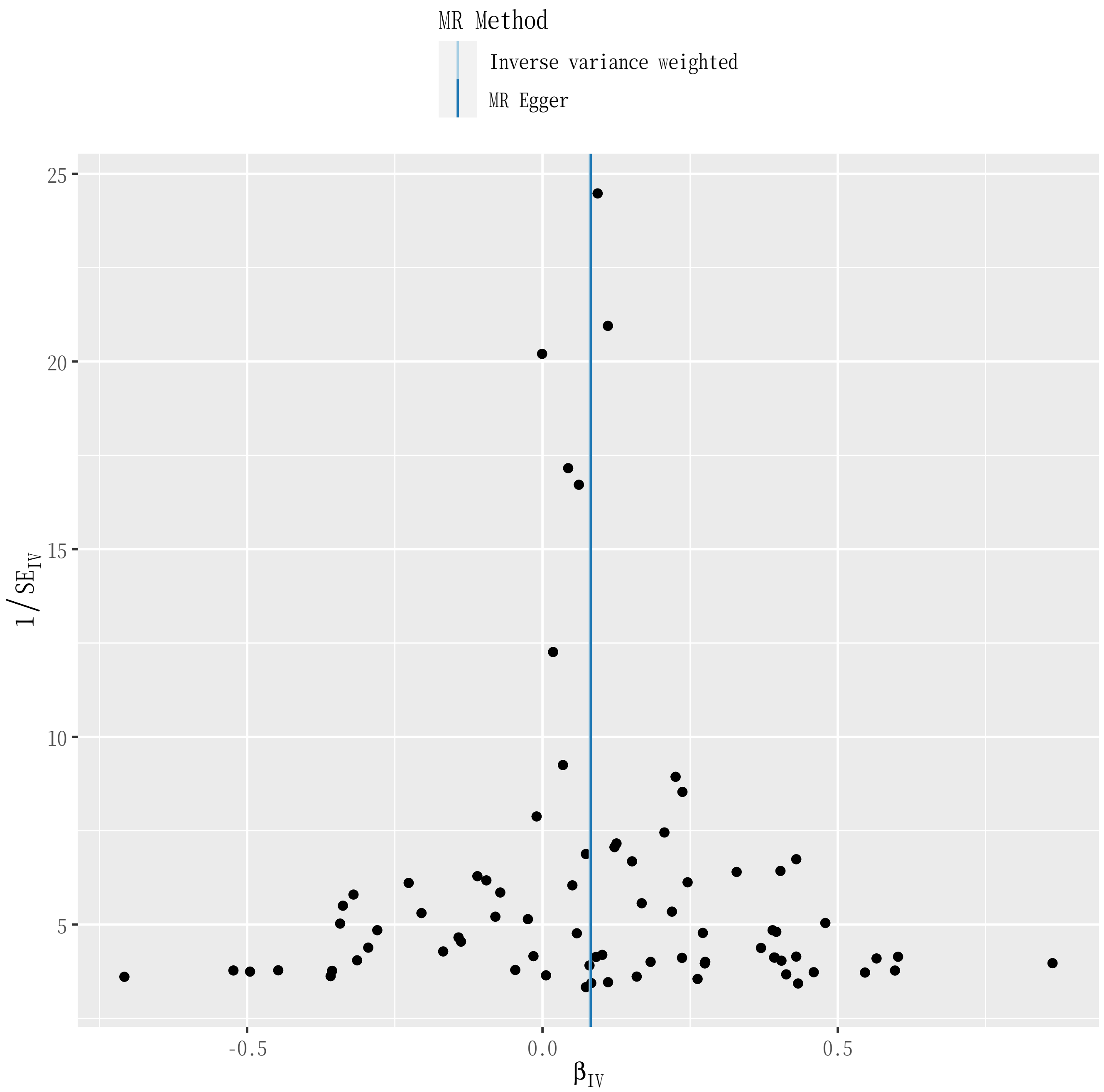

MR Method
Inverse variance weighted
MR Egger
25
20
15
10
5
-0.5
0.0
0.5
1/SE_IV
β_IV

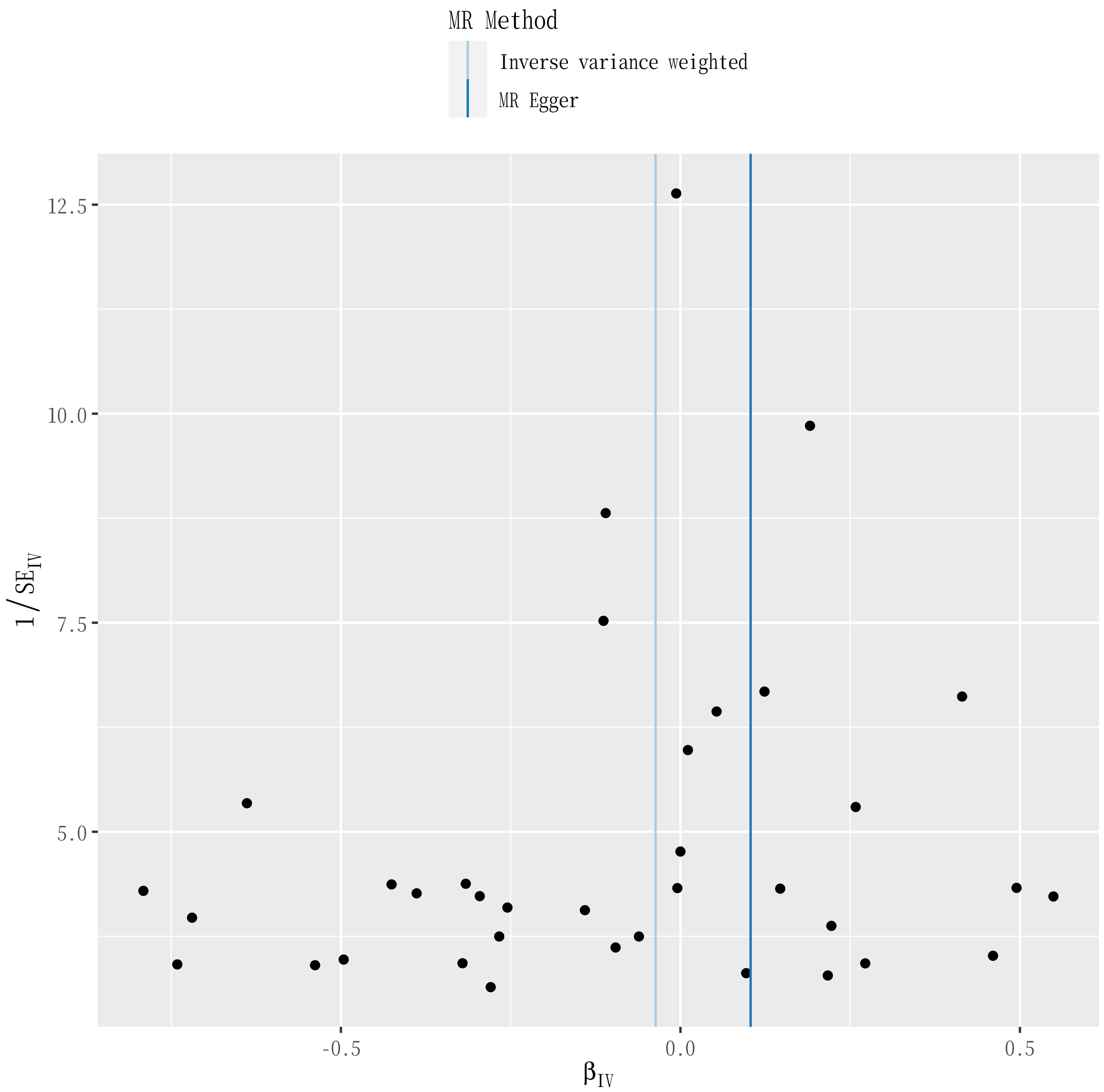

MR Method
Inverse variance weighted
MR Egger
12.5
10.0
7.5
5.0
1/SE_IV
-0.5
0.0
0.5
β_IV

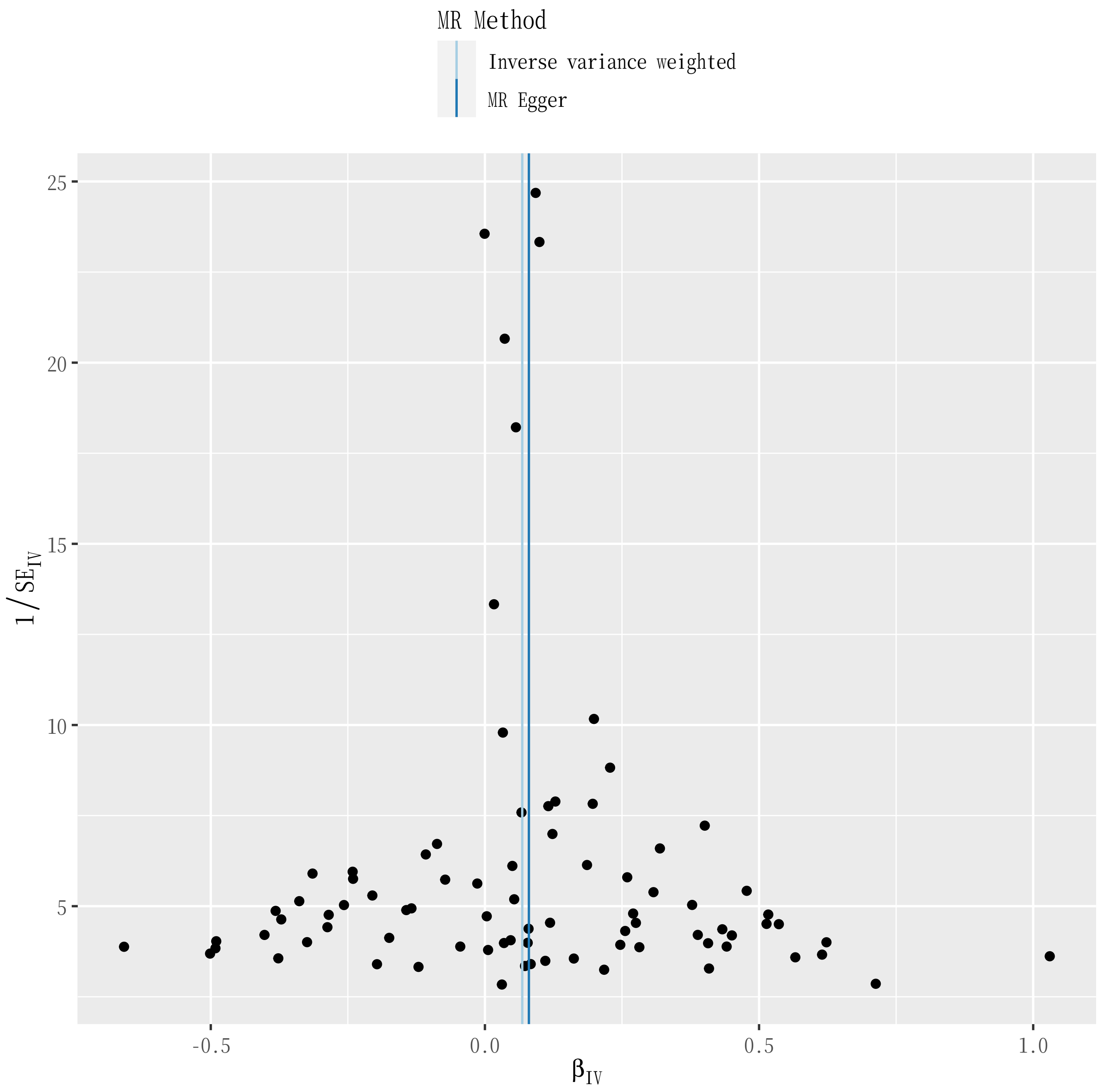

MR Method
Inverse variance weighted
MR Egger
25
20
15
10
5
1/SE_IV
-0.5
0.0
0.5
1.0
β_IV

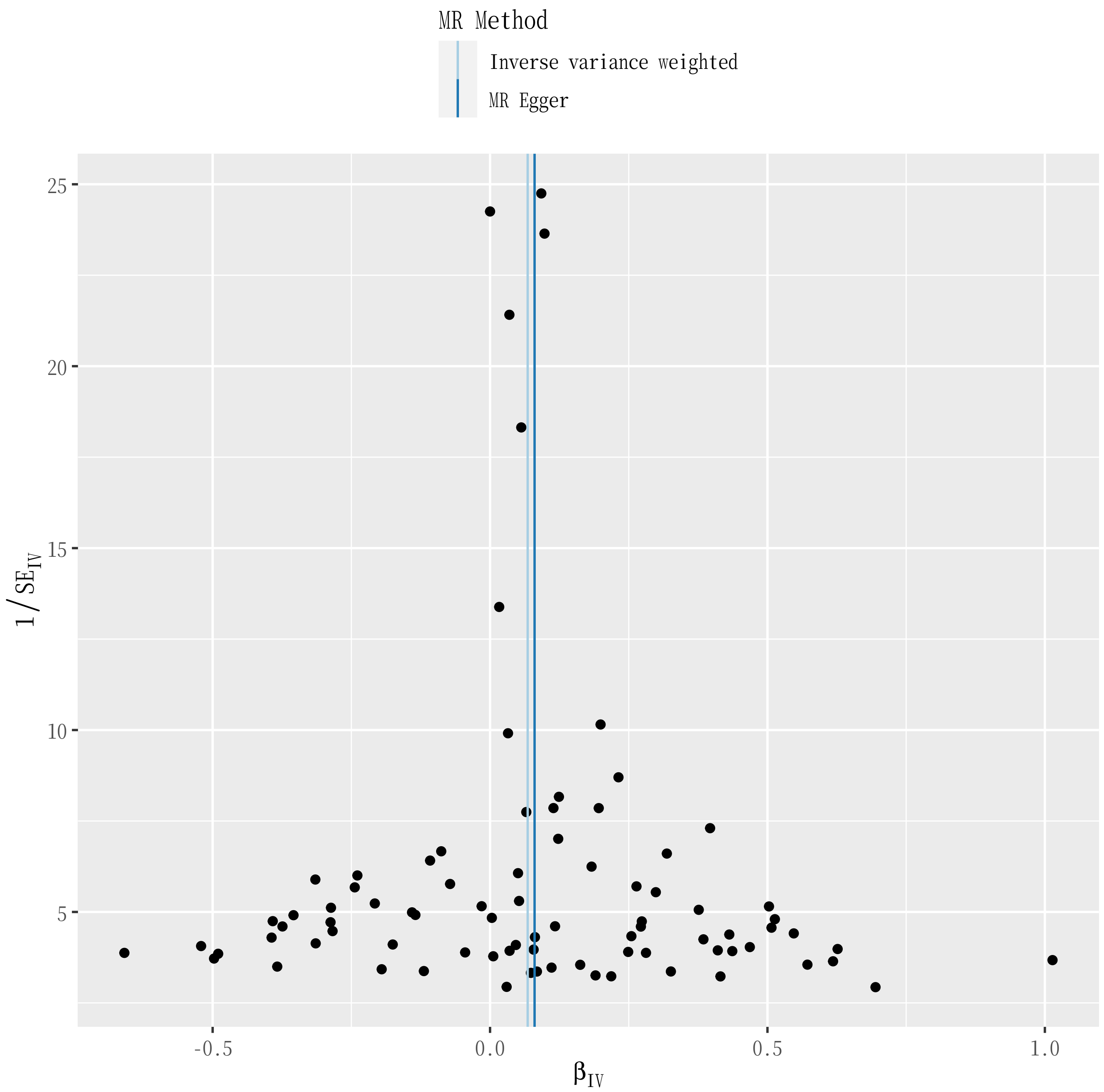

MR Method
Inverse variance weighted
MR Egger
25
20
15
10
5
$1/SE_{IV}$
-0.5
0.0
0.5
1.0
β_{IV}

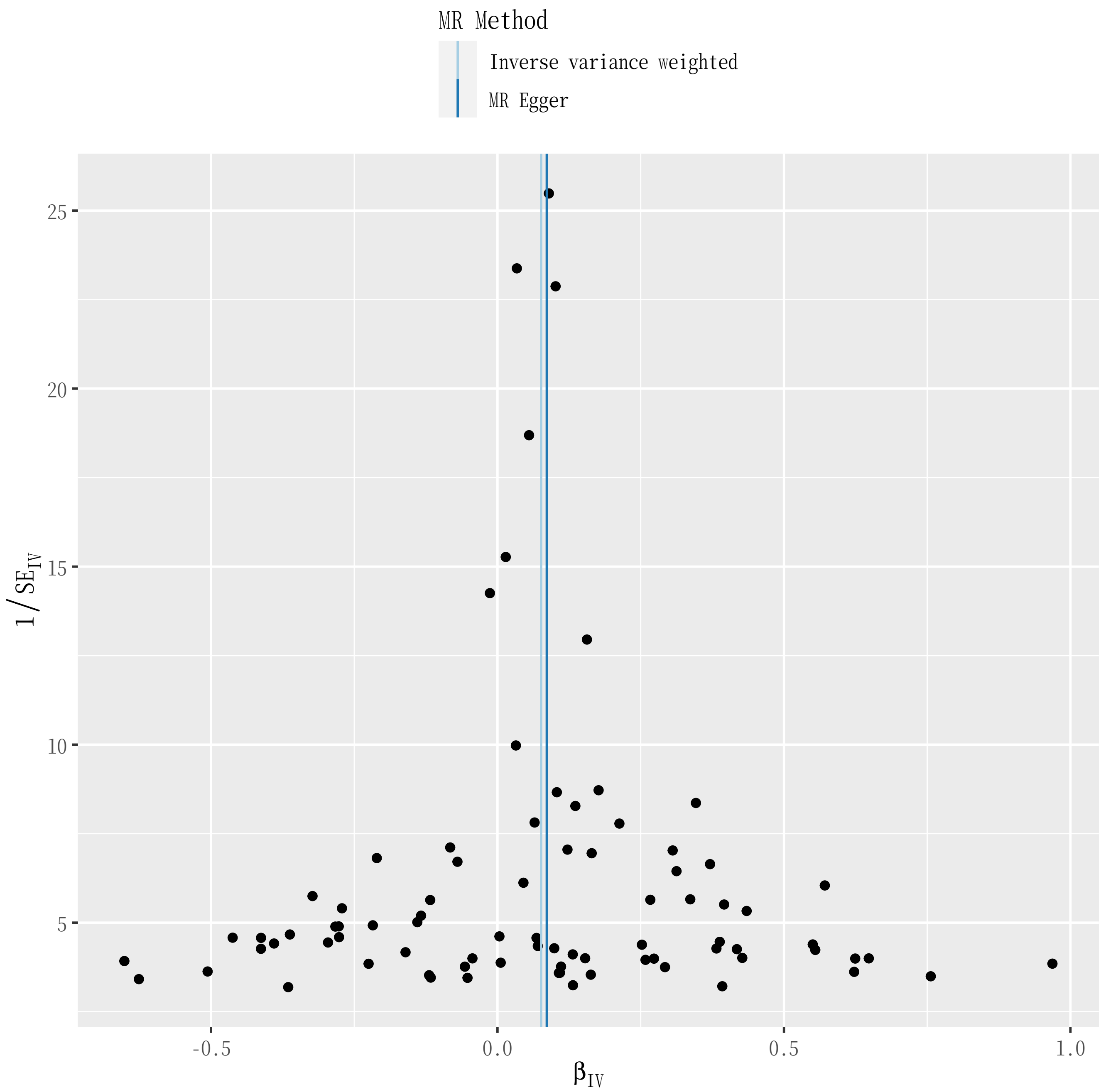

MR Method
Inverse variance weighted
MR Egger
25
20
15
10
5
$1/SE_{IV}$
-0.5
0.0
0.5
1.0
β_{IV}

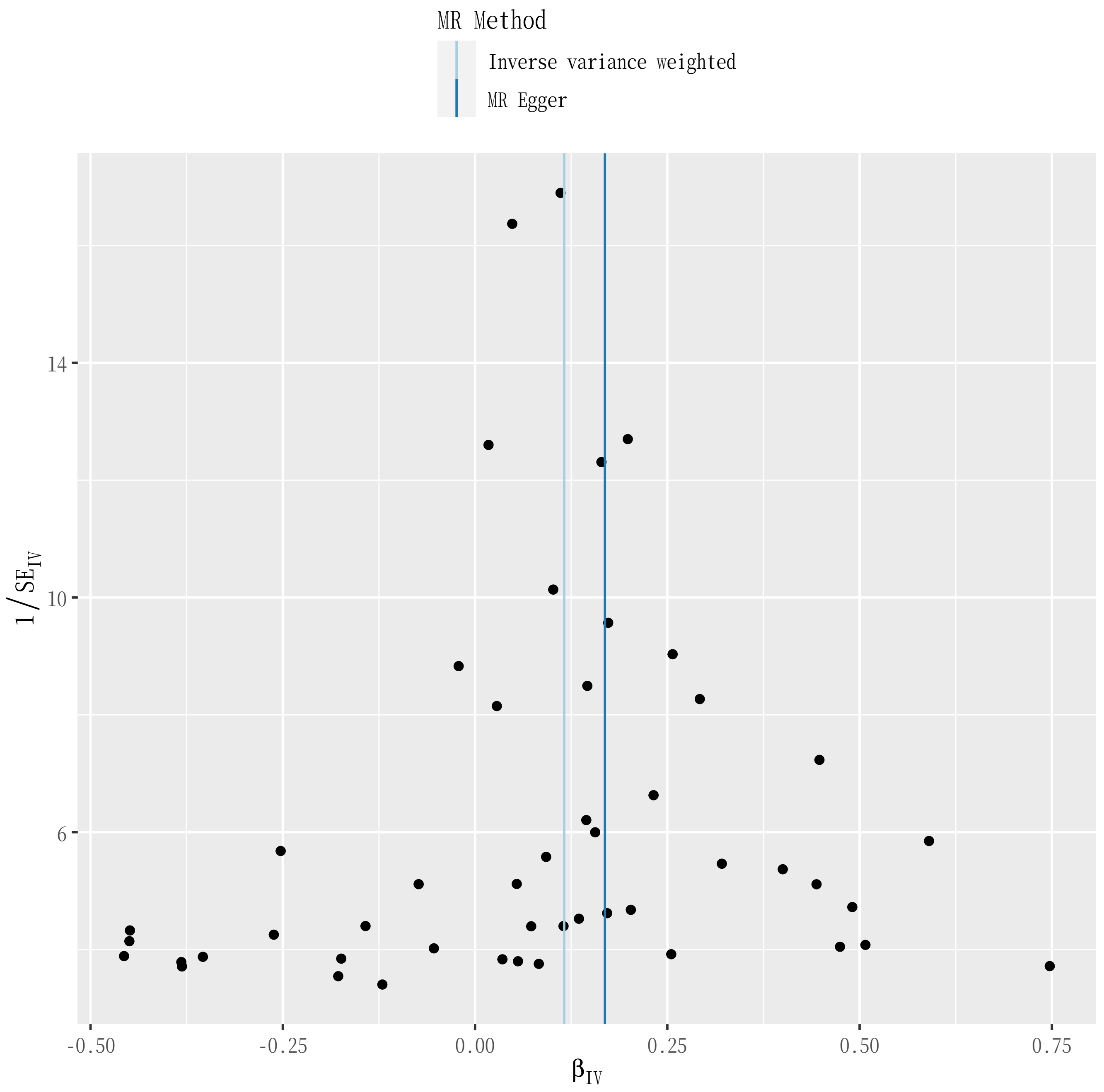

MR Method
Inverse variance weighted
MR Egger
14
10
6
-0.50
-0.25
0.00
0.25
0.50
0.75
$1/SE_{IV}$
β_{IV}

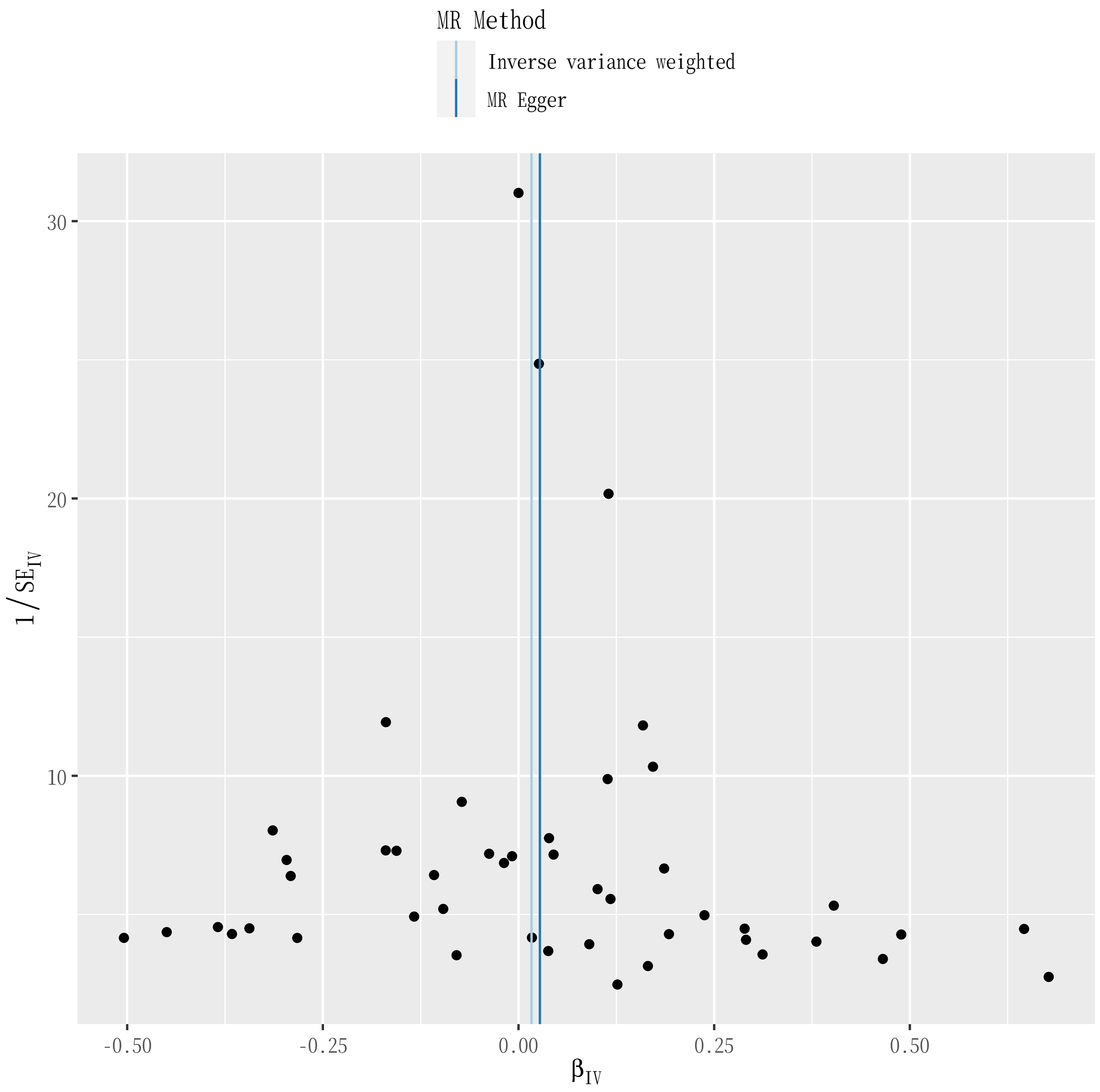
MR Method
Inverse variance weighted
MR Egger
30
20
10
1/SE_IV
-0.50
-0.25
0.00
0.25
0.50
β_IV

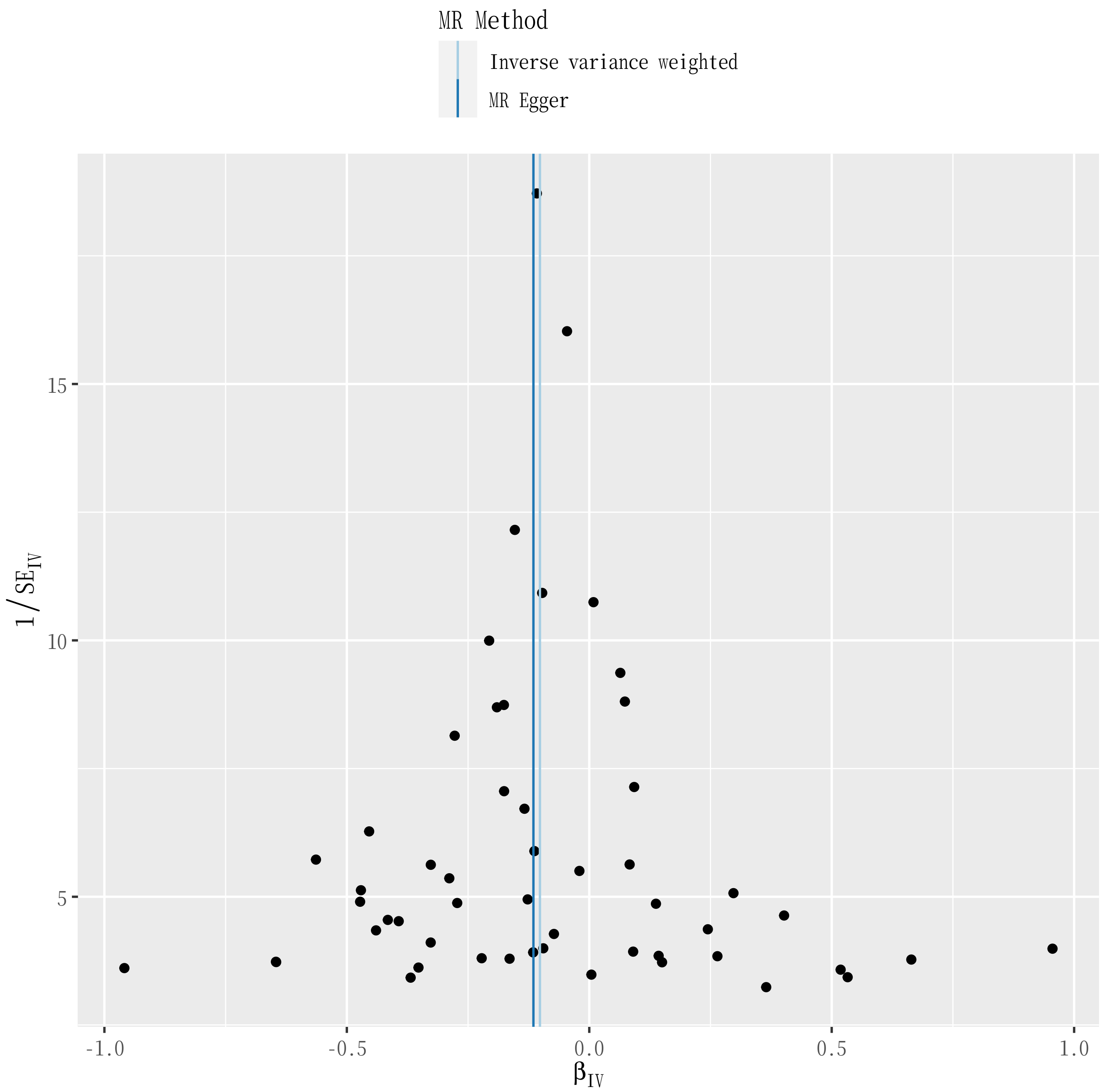

MR Method
Inverse variance weighted
MR Egger
15
10
5
$1/SE_{IV}$
-1.0
-0.5
0.0
0.5
1.0
β_{IV}

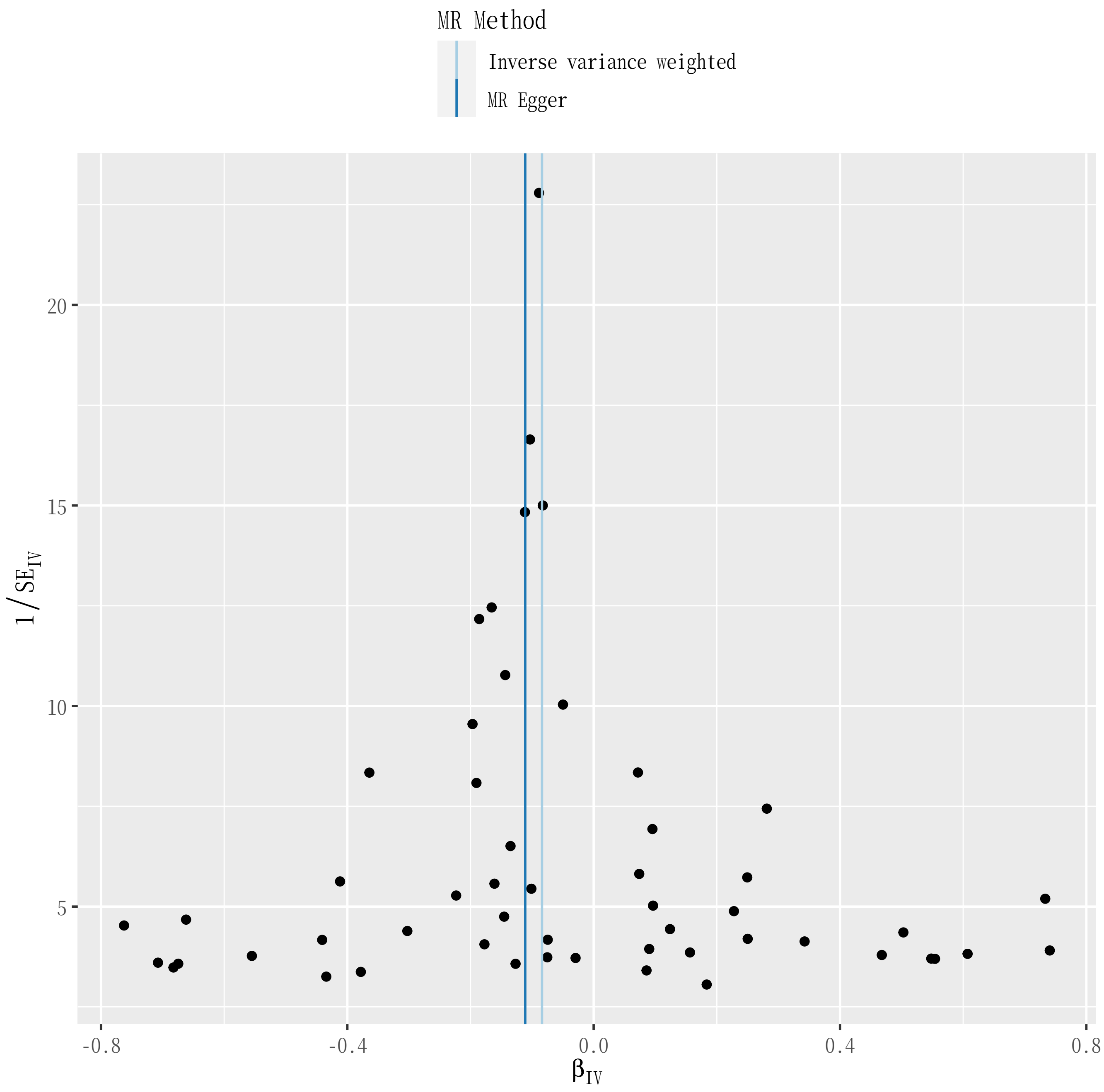

MR Method
Inverse variance weighted
MR Egger
20
15
10
5
$1/SE_{IV}$
-0.8
-0.4
0.0
0.4
0.8
β_{IV}

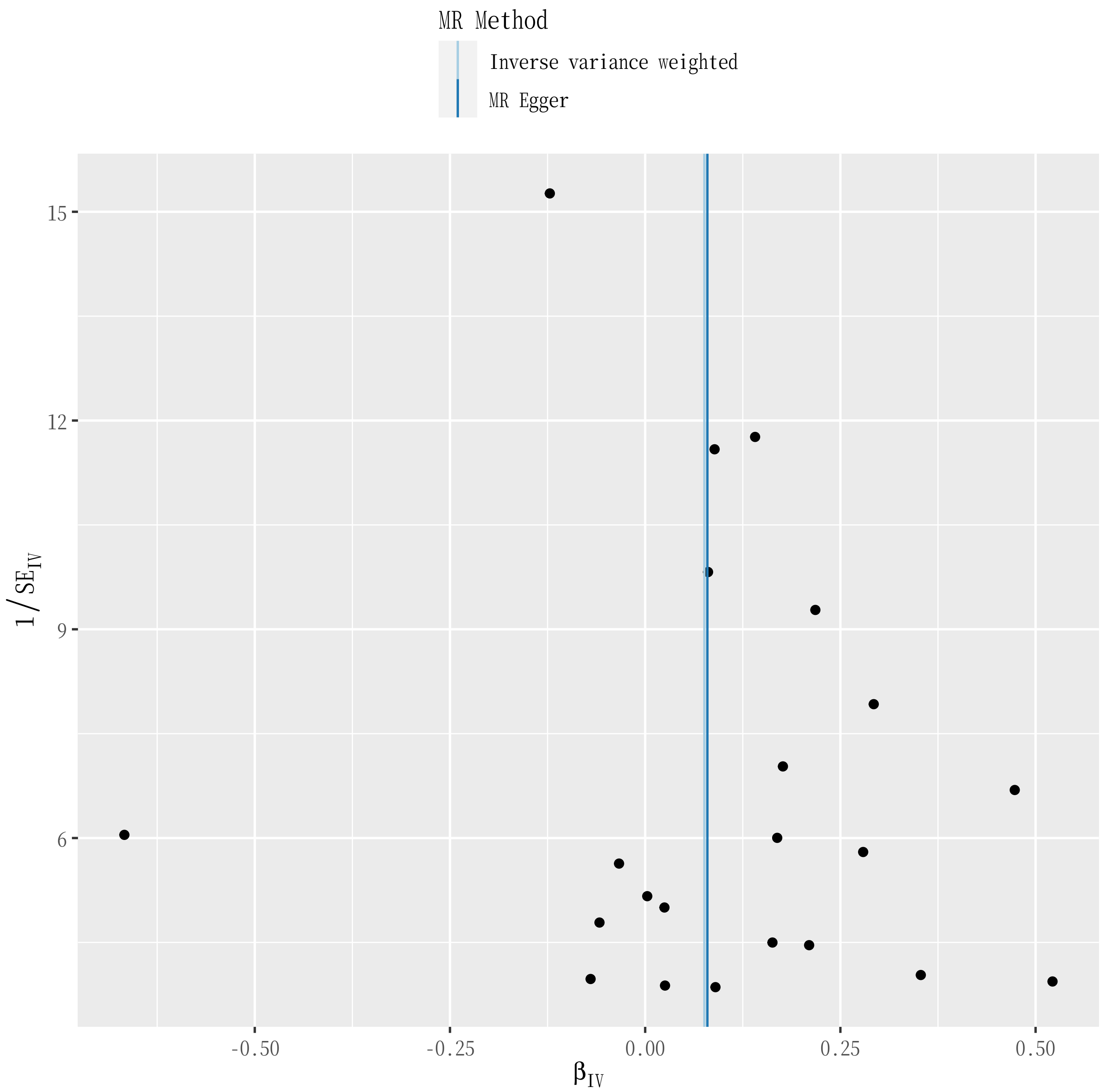

MR Method
Inverse variance weighted
MR Egger
15
12
9
6
-0.50
-0.25
0.00
0.25
0.50
$1/SE_{IV}$
β_{IV}

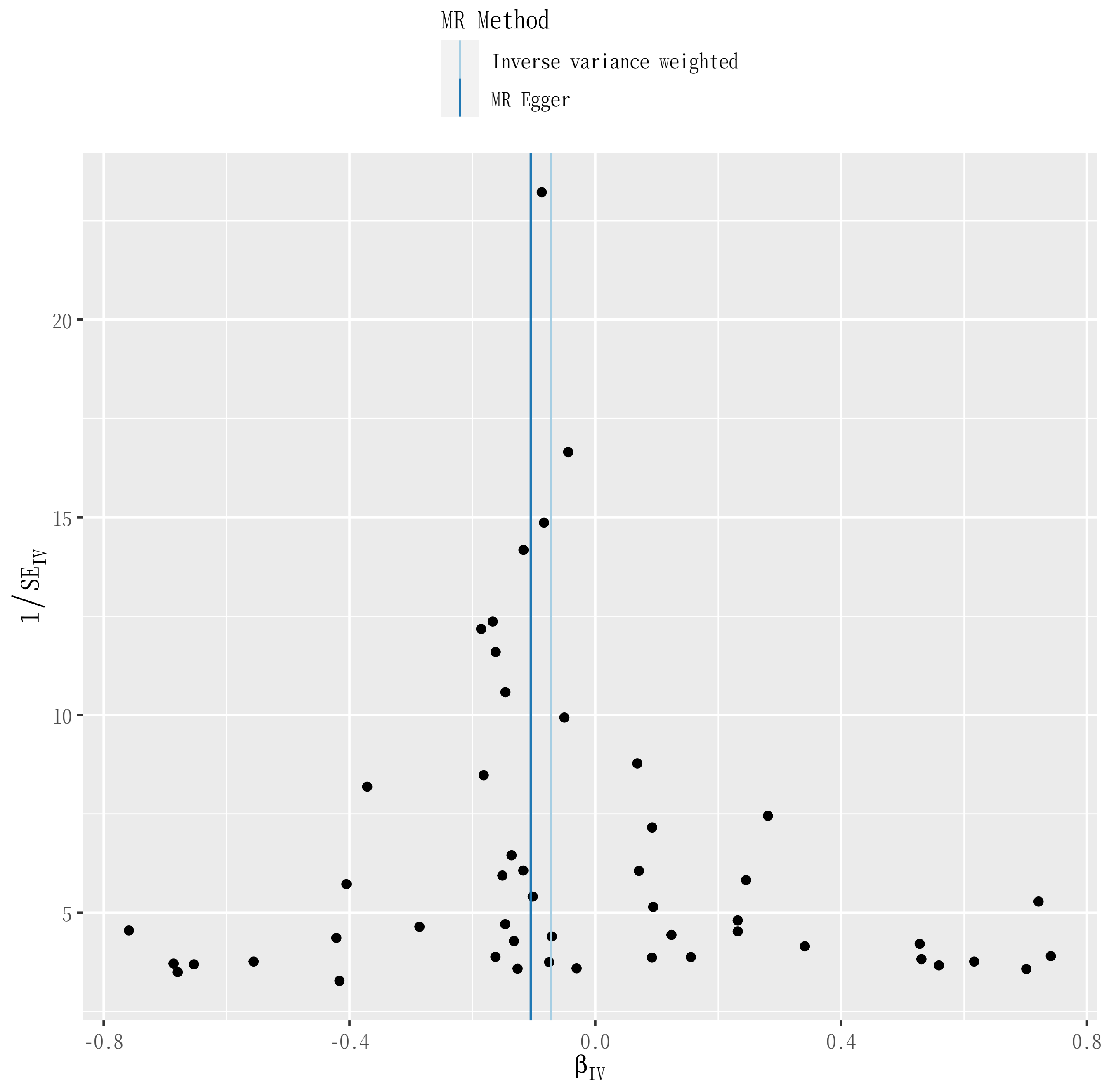

MR Method
Inverse variance weighted
MR Egger
20
15
10
5
1/SE_IV
-0.8
-0.4
0.0
0.4
0.8
β_IV

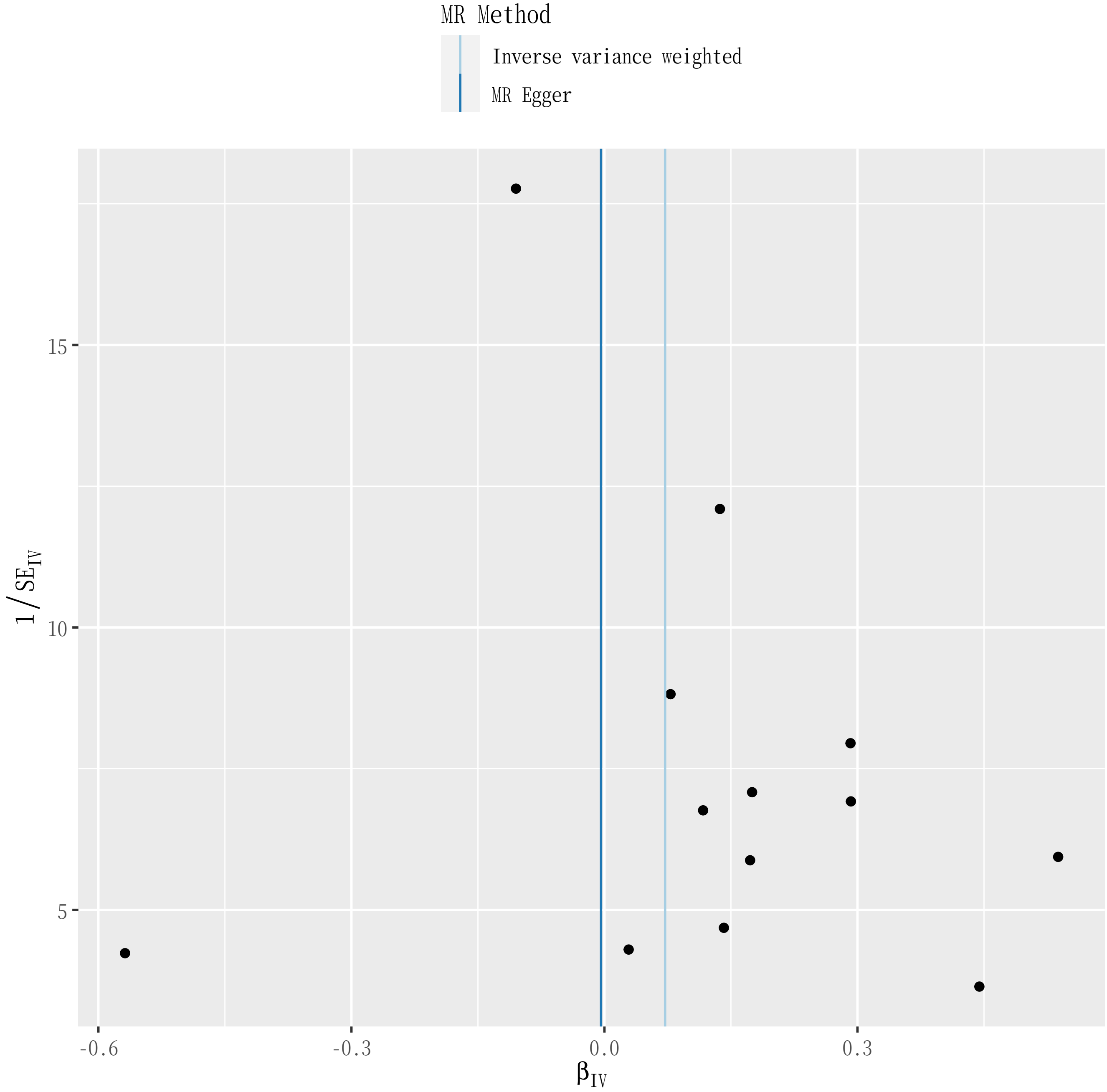

MR Method
Inverse variance weighted
MR Egger
15
10
5
$1/SE_{IV}$
-0.6
-0.3
0.0
0.3
β_{IV}

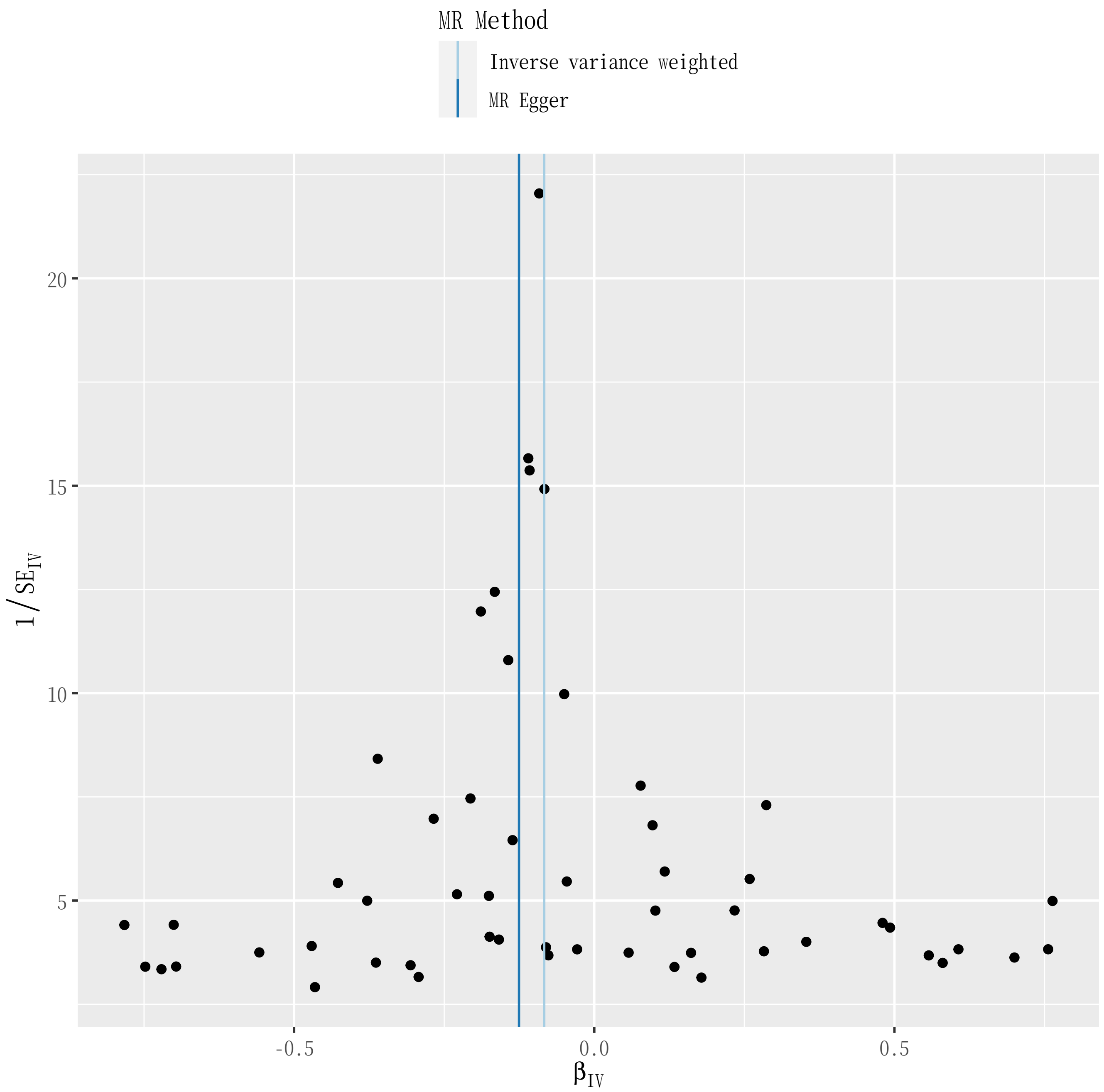
MR Method
Inverse variance weighted
MR Egger
20
15
10
5
-0.5
0.0
0.5
$1/SE_{IV}$
β_{IV}

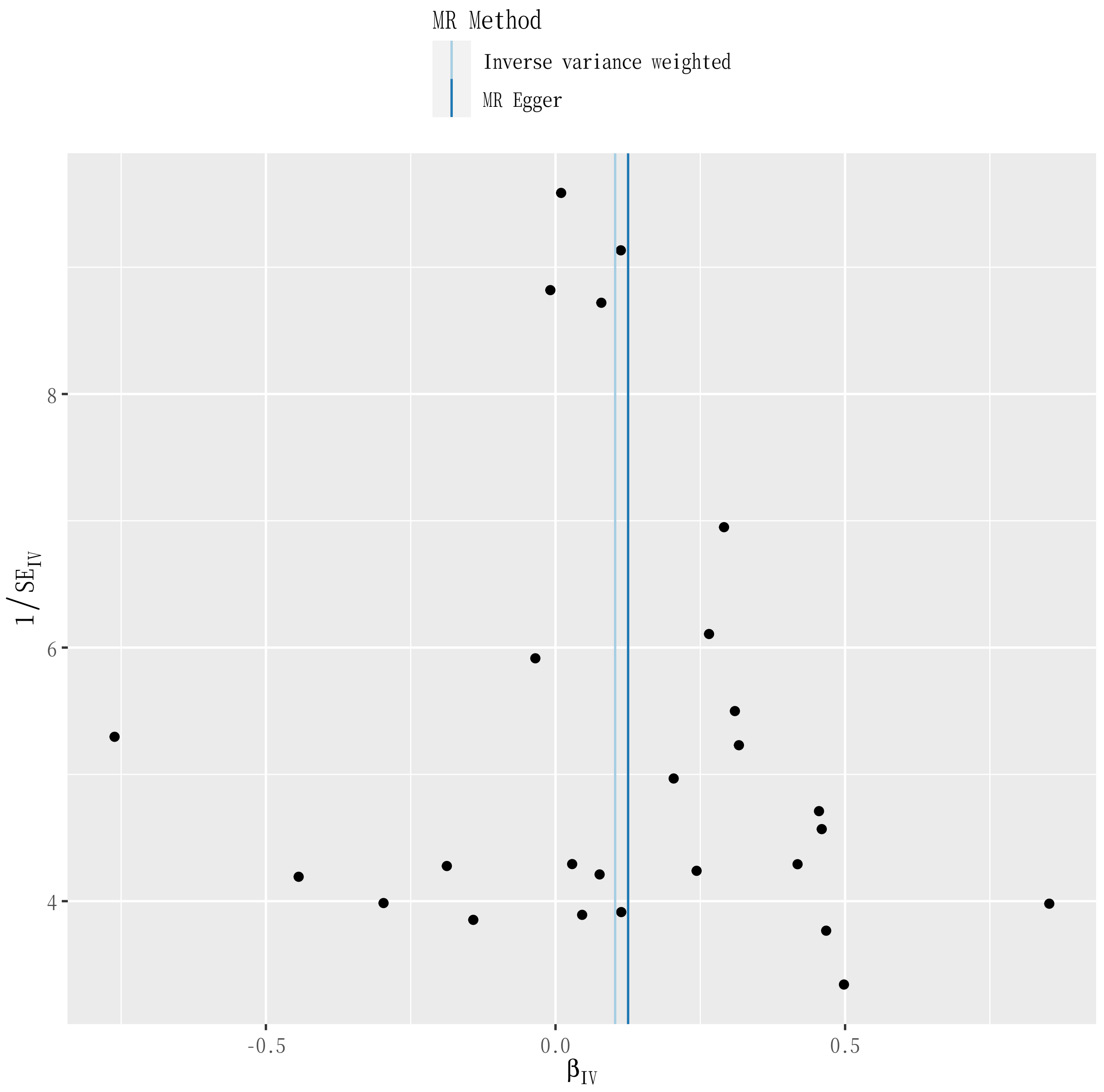

MR Method
Inverse variance weighted
MR Egger
8
6
4
$1/SE_{IV}$
-0.5
0.0
0.5
β_{IV}

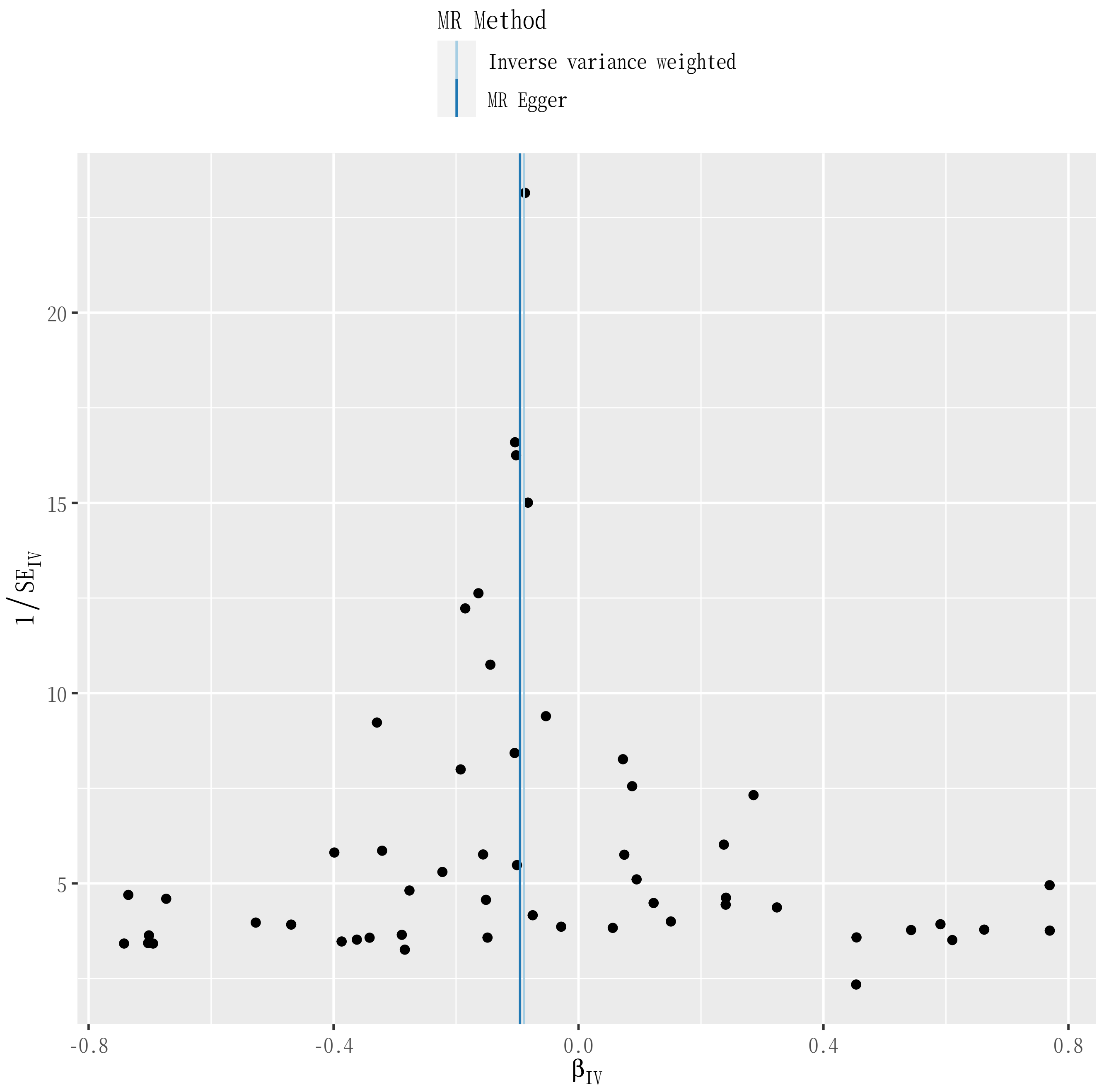

MR Method
Inverse variance weighted
MR Egger
20
15
10
5
1/SE_IV
-0.8
-0.4
0.0
0.4
0.8
β_IV

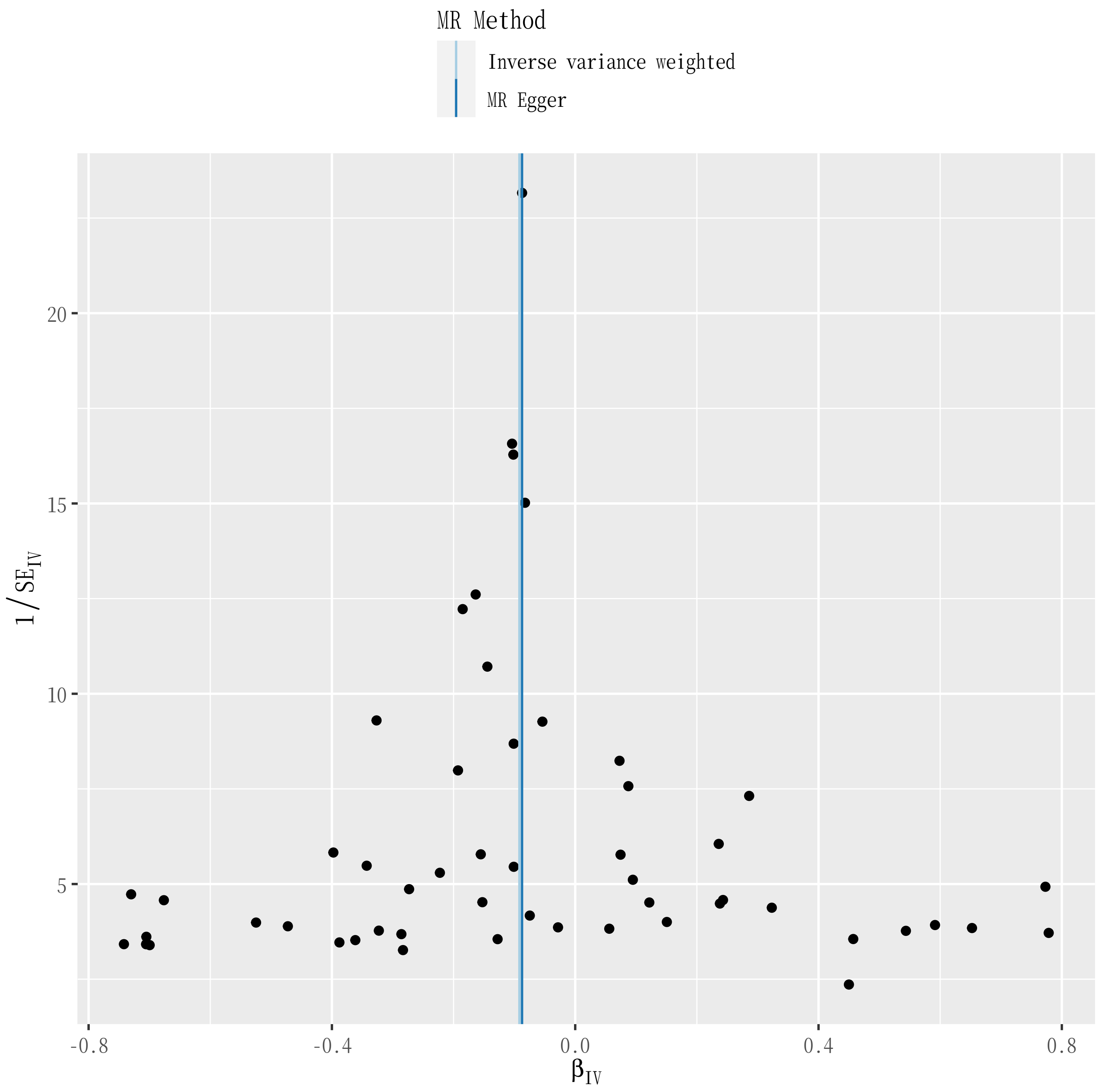
MR Method
Inverse variance weighted
MR Egger
20
15
10
5
$1/SE_{IV}$
-0.8
-0.4
0.0
0.4
0.8
β_{IV}

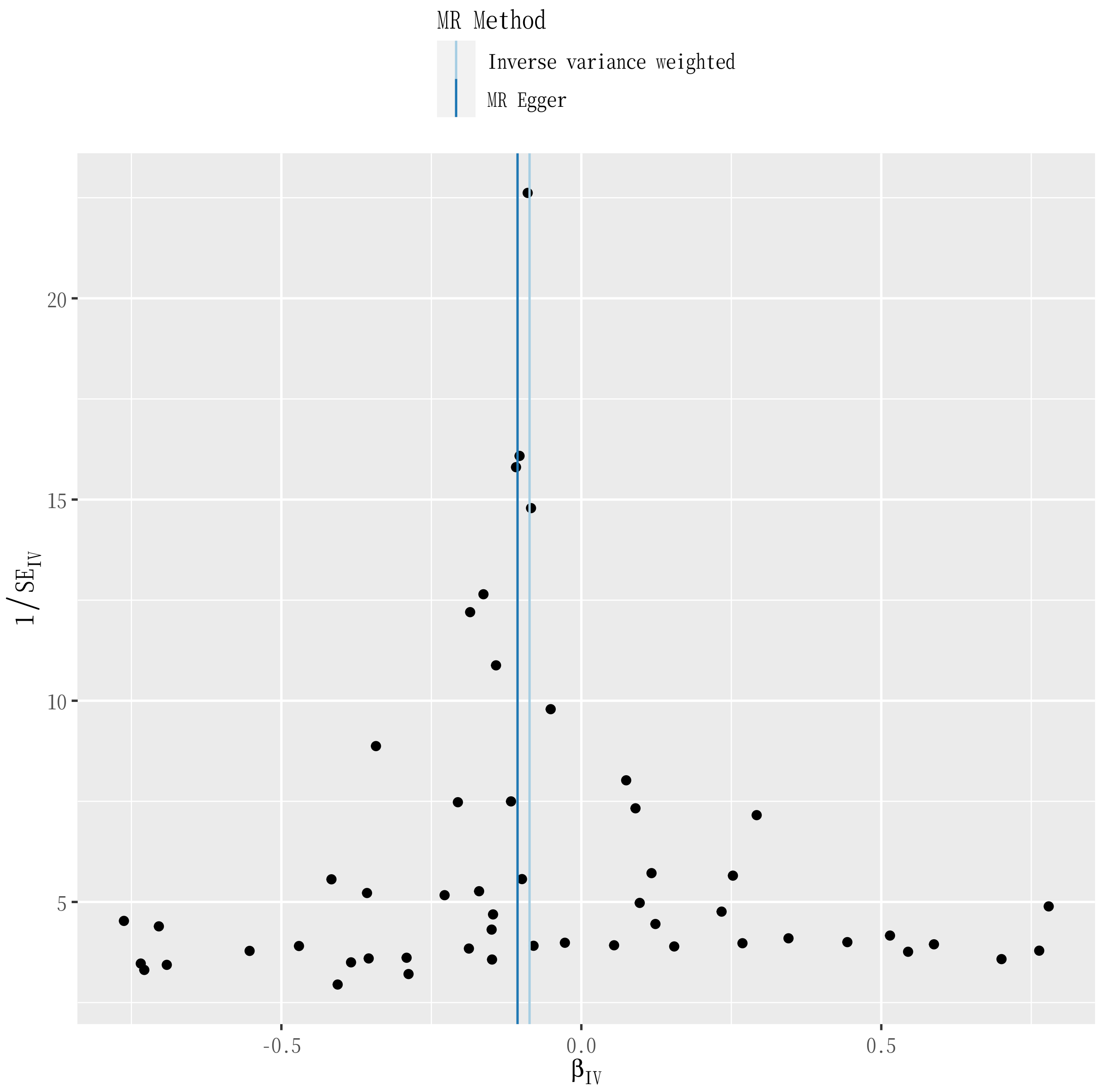
MR Method
Inverse variance weighted
MR Egger
20
15
10
5
-0.5
0.0
0.5
$1/SE_{IV}$
β_{IV}

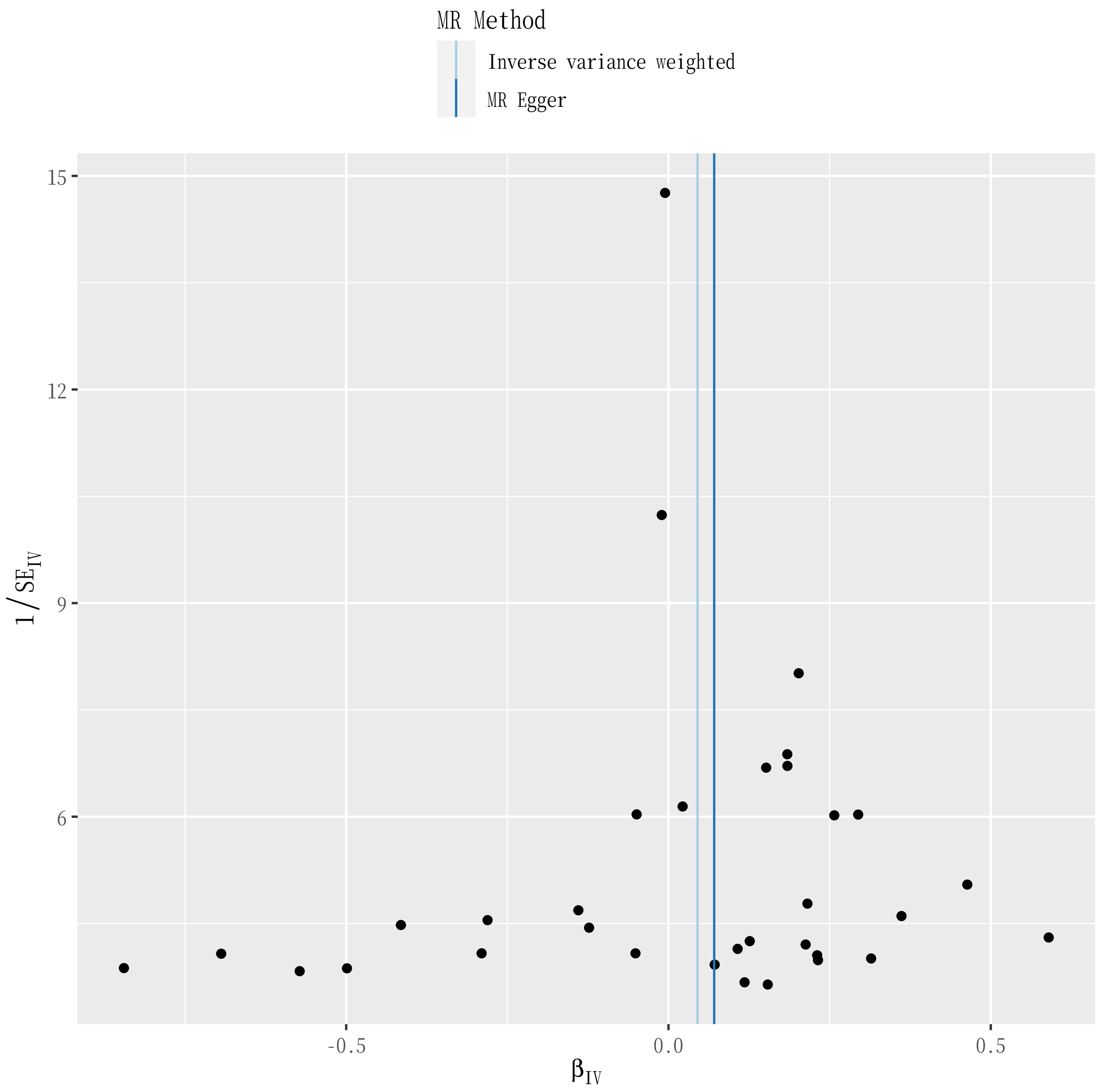
MR Method
Inverse variance weighted
MR Egger
15
12
9
6
$1/SE_{IV}$
-0.5
0.0
0.5
β_{IV}

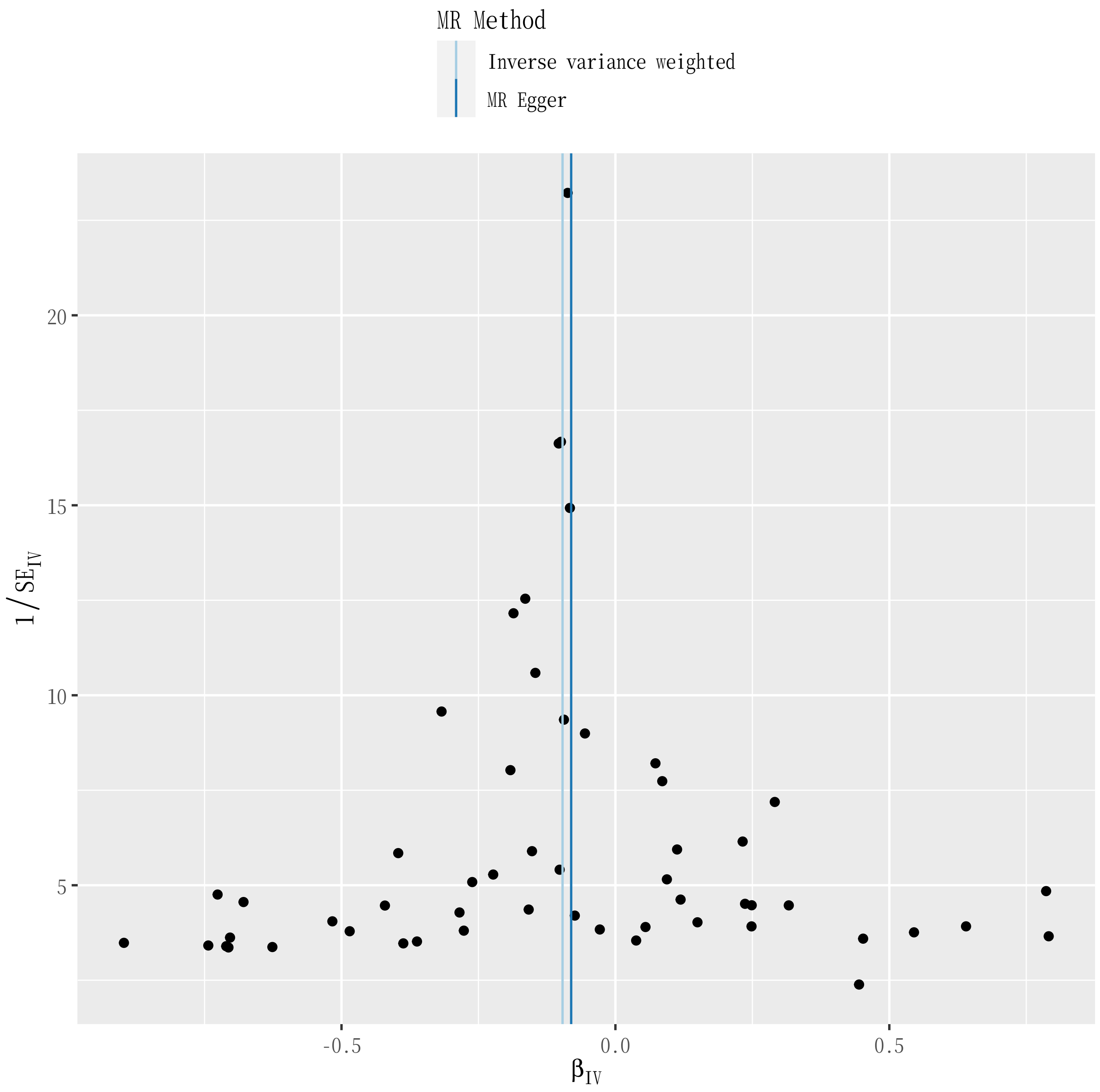
MR Method
Inverse variance weighted
MR Egger
20
15
10
5
$1/SE_{IV}$
-0.5
0.0
0.5
β_{IV}

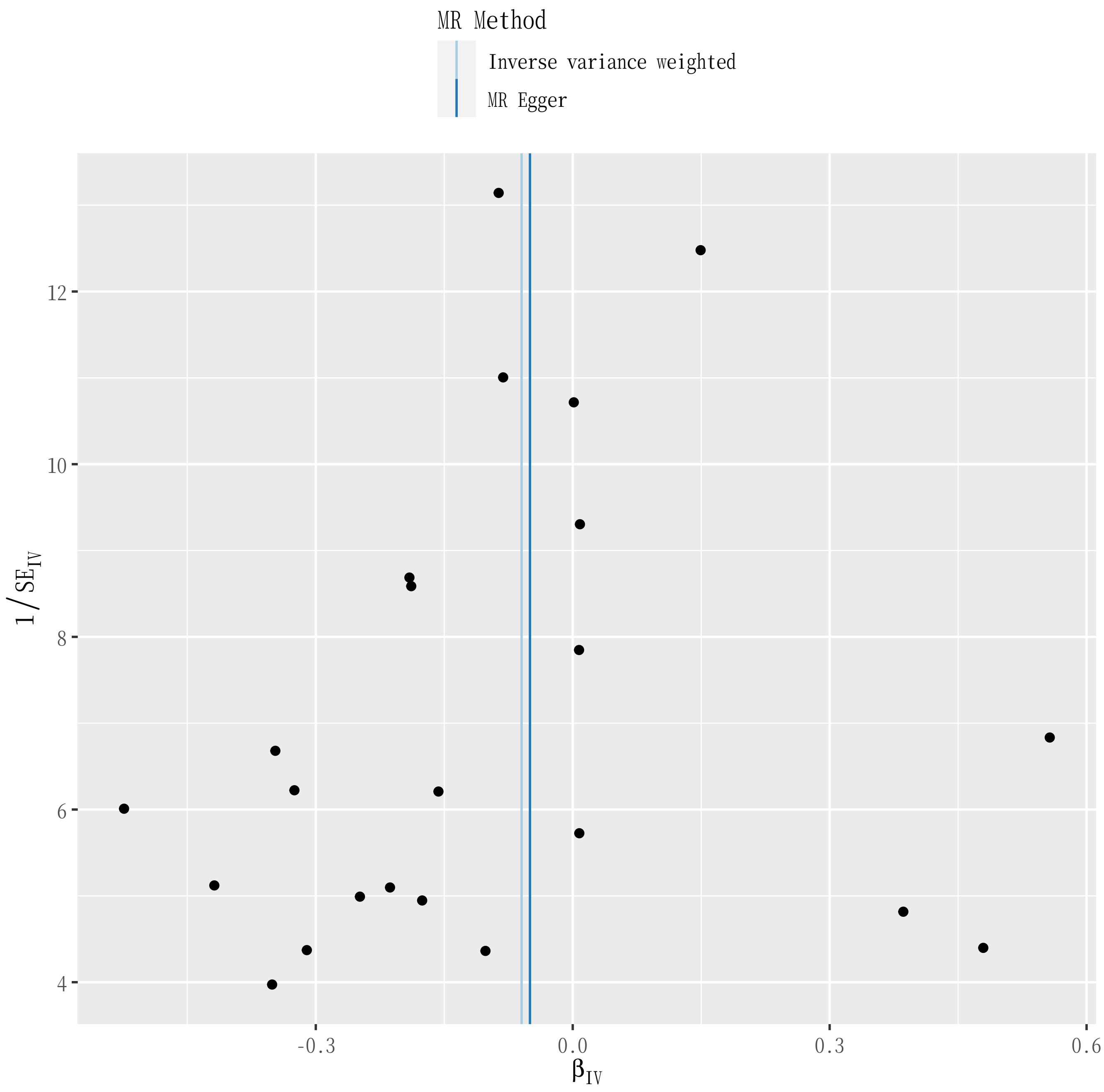
MR Method
Inverse variance weighted
MR Egger
12
10
8
6
4
1/SE_IV
-0.3
0.0
0.3
0.6
β_IV

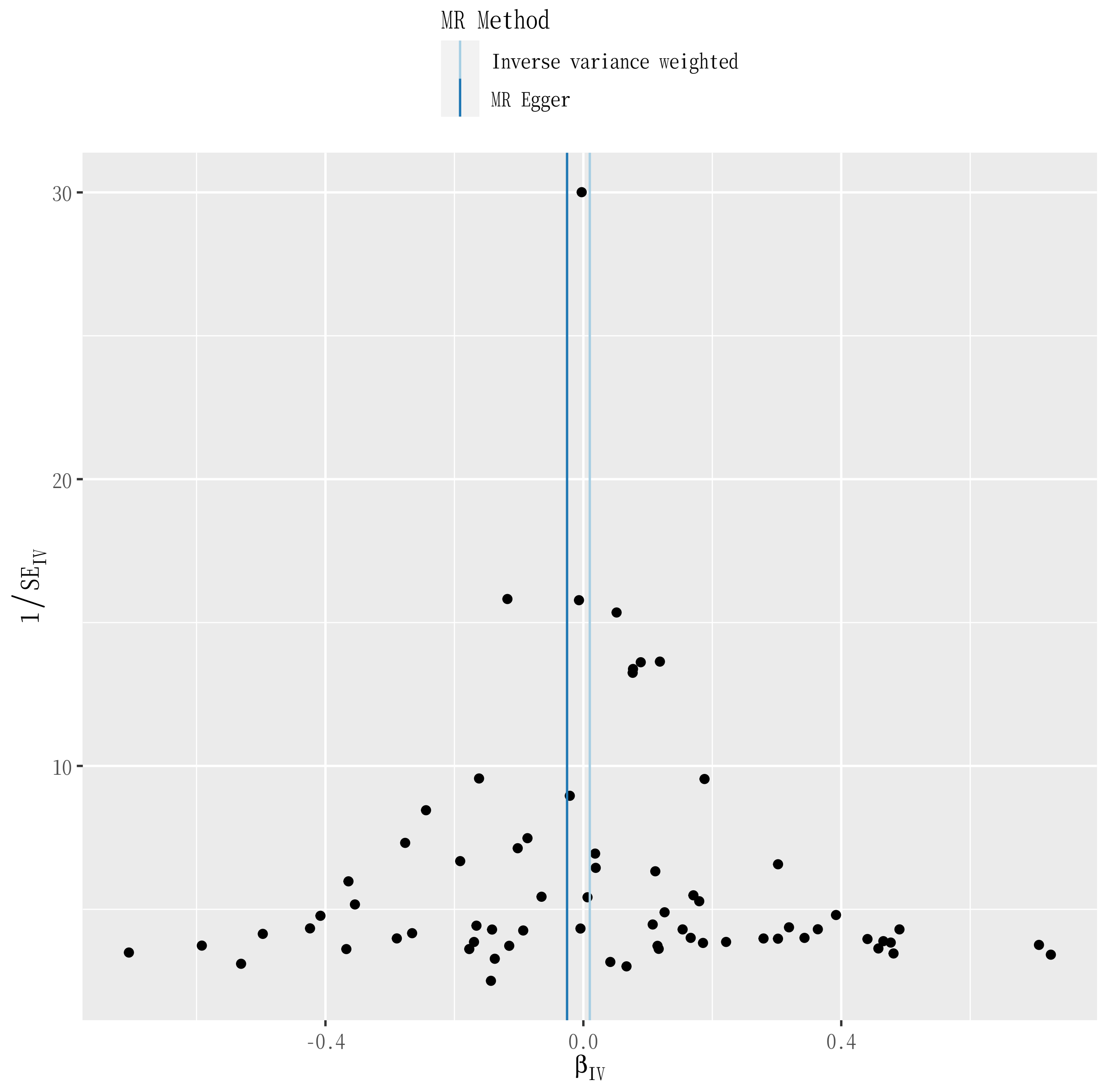

MR Method
Inverse variance weighted
MR Egger
30
20
10
-0.4
0.0
0.4
1/SE_IV
β_IV

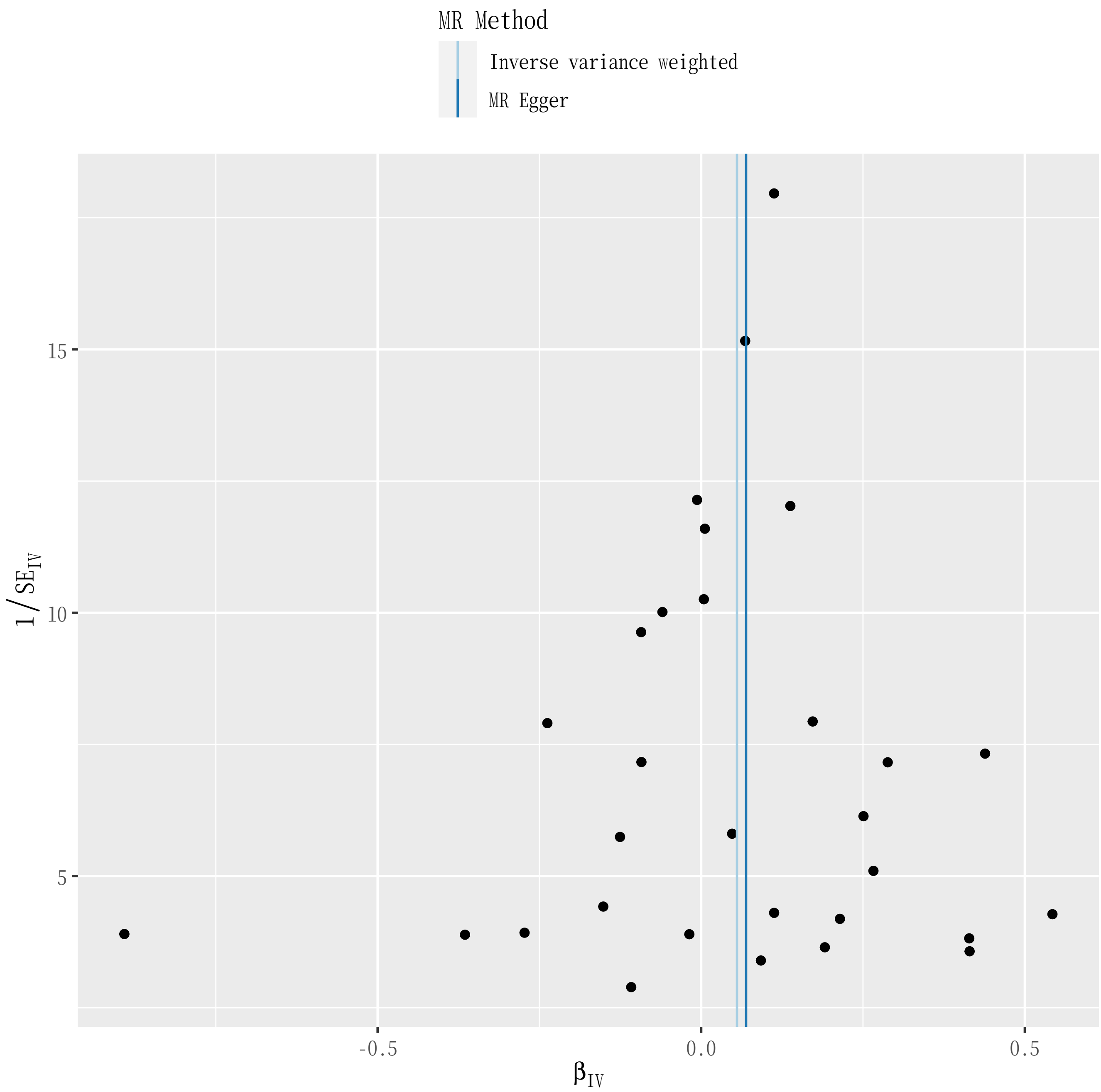

MR Method
Inverse variance weighted
MR Egger
15
10
5
-0.5
0.0
0.5
$1/SE_{IV}$
β_{IV}

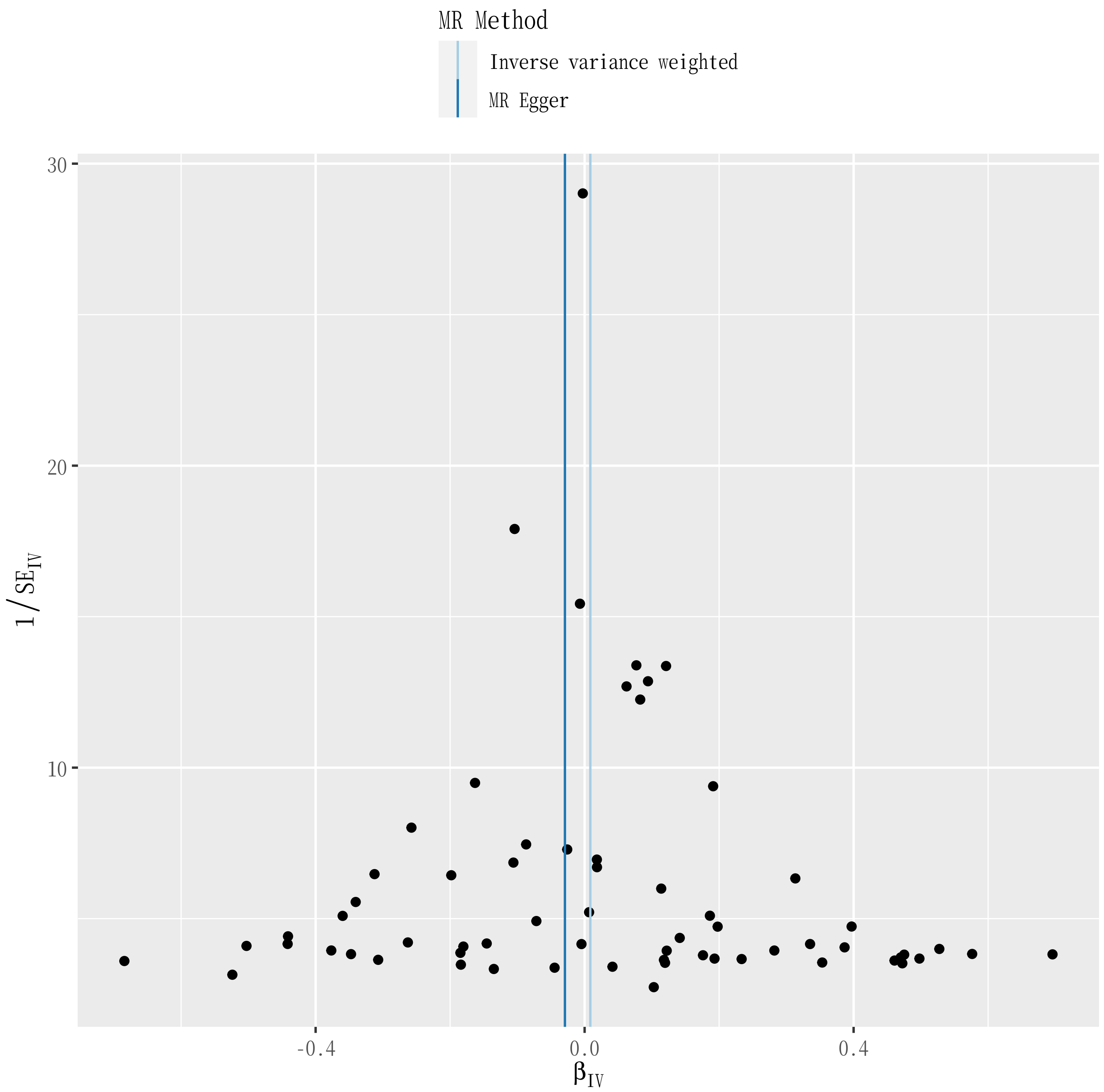
MR Method
Inverse variance weighted
MR Egger
30
20
10
-0.4
0.0
0.4
β_{IV}
$1/SE_{IV}$

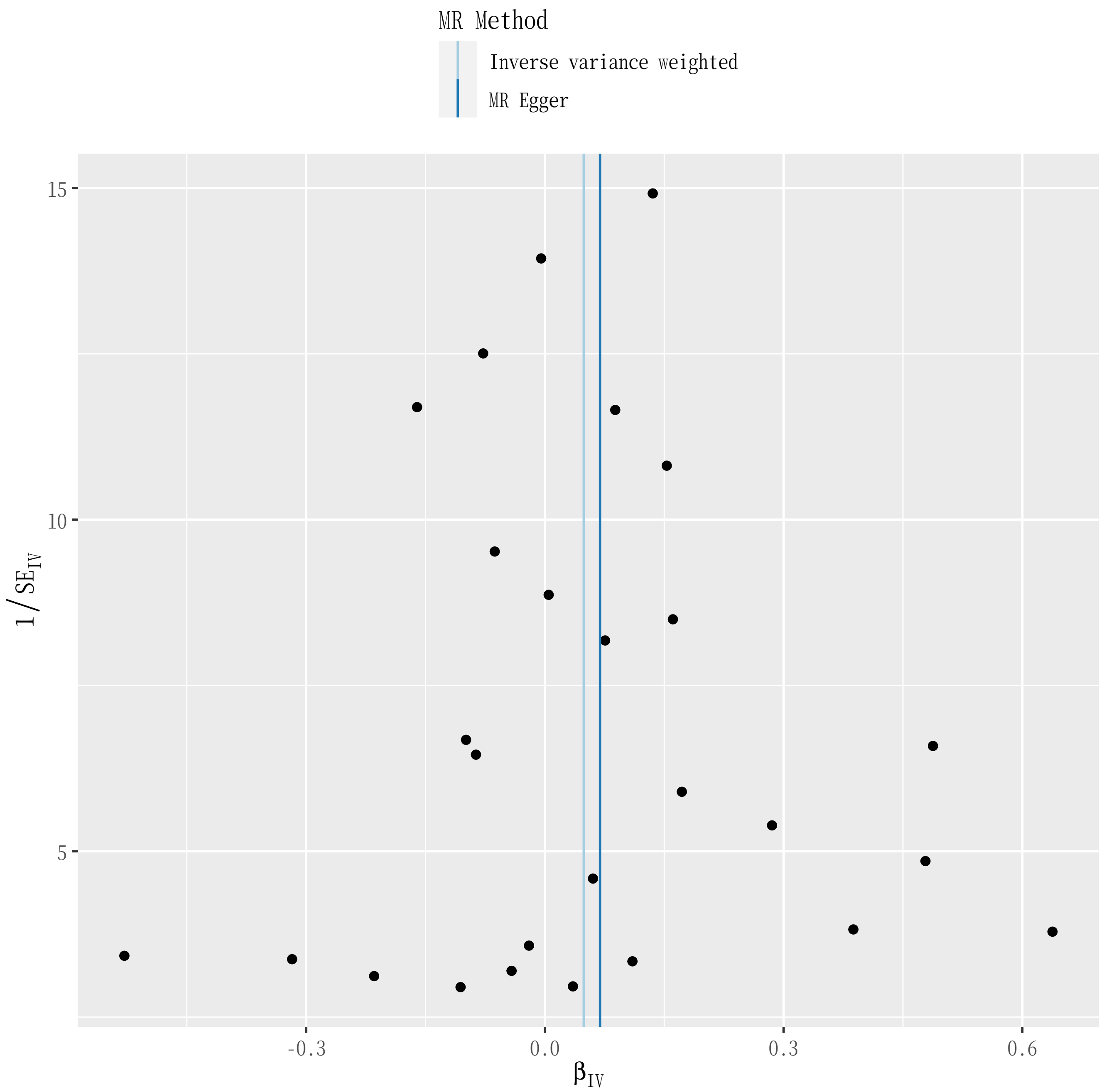
MR Method
Inverse variance weighted
MR Egger
15
10
5
1/SE_IV
-0.3
0.0
0.3
0.6
β_IV

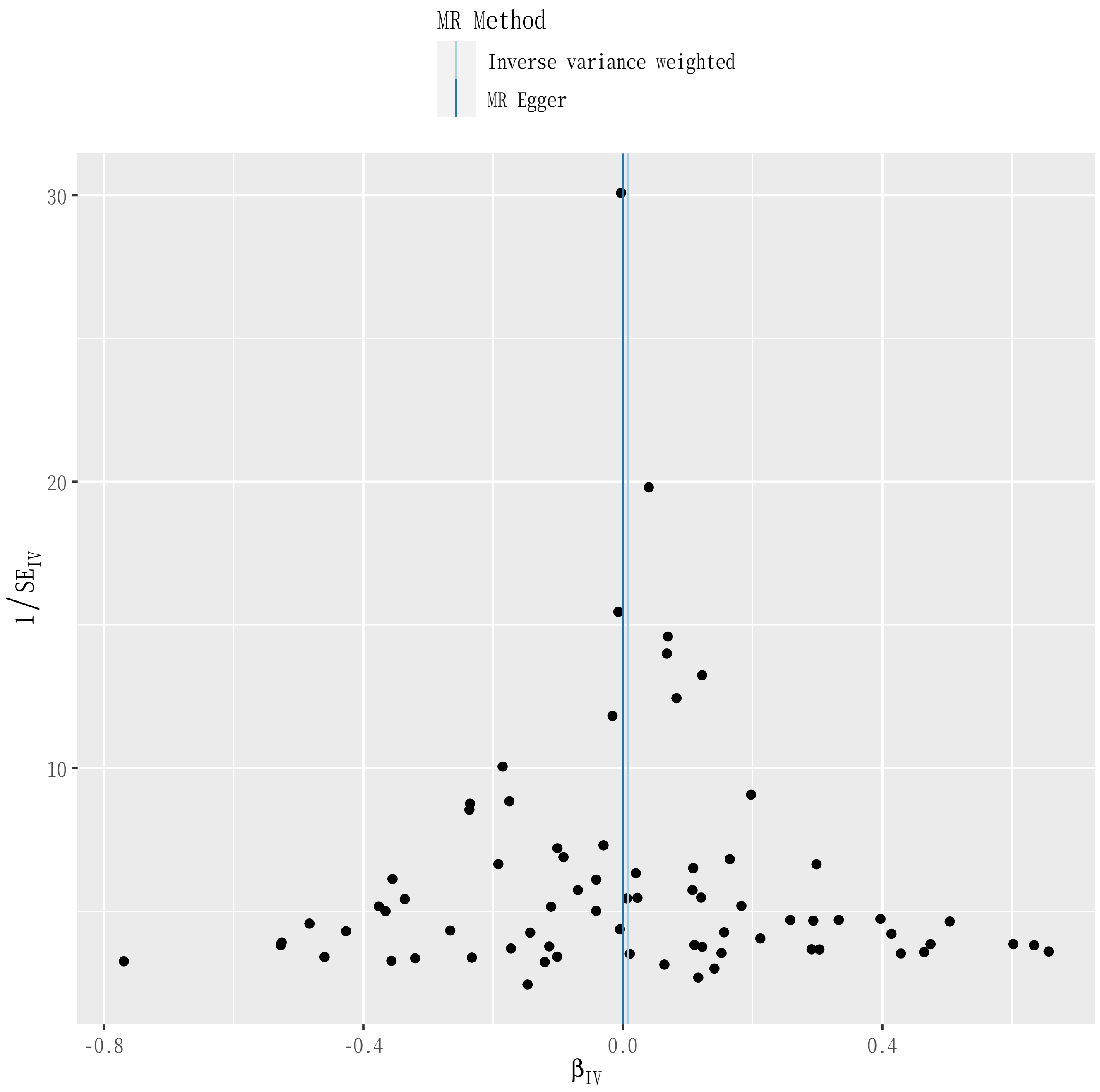

MR Method
Inverse variance weighted
MR Egger
30
20
10
-0.8
-0.4
0.0
0.4
$1/SE_{IV}$
β_{IV}

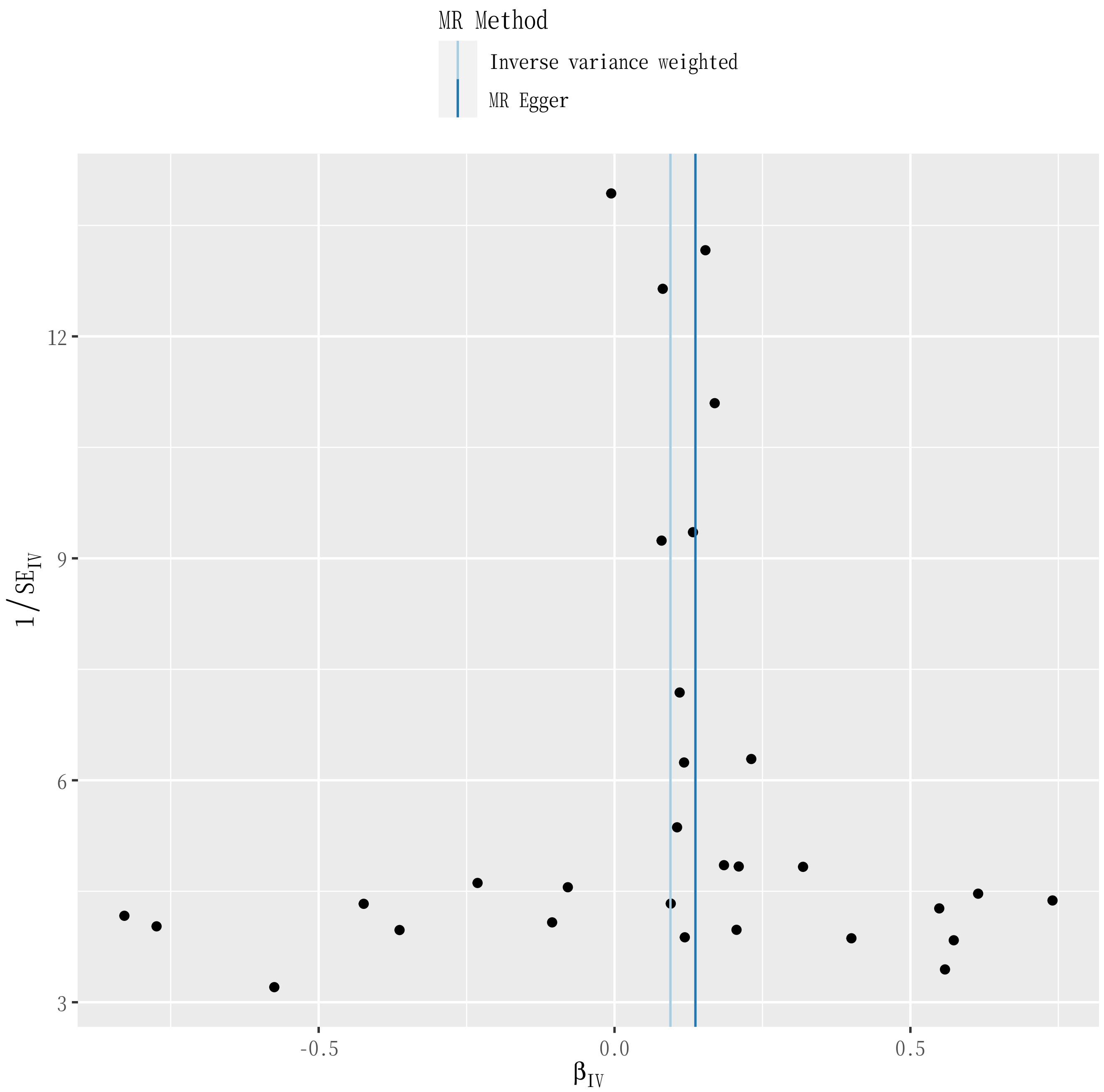
MR Method
Inverse variance weighted
MR Egger
12
9
6
3
1/SE_IV
-0.5
0.0
0.5
β_IV

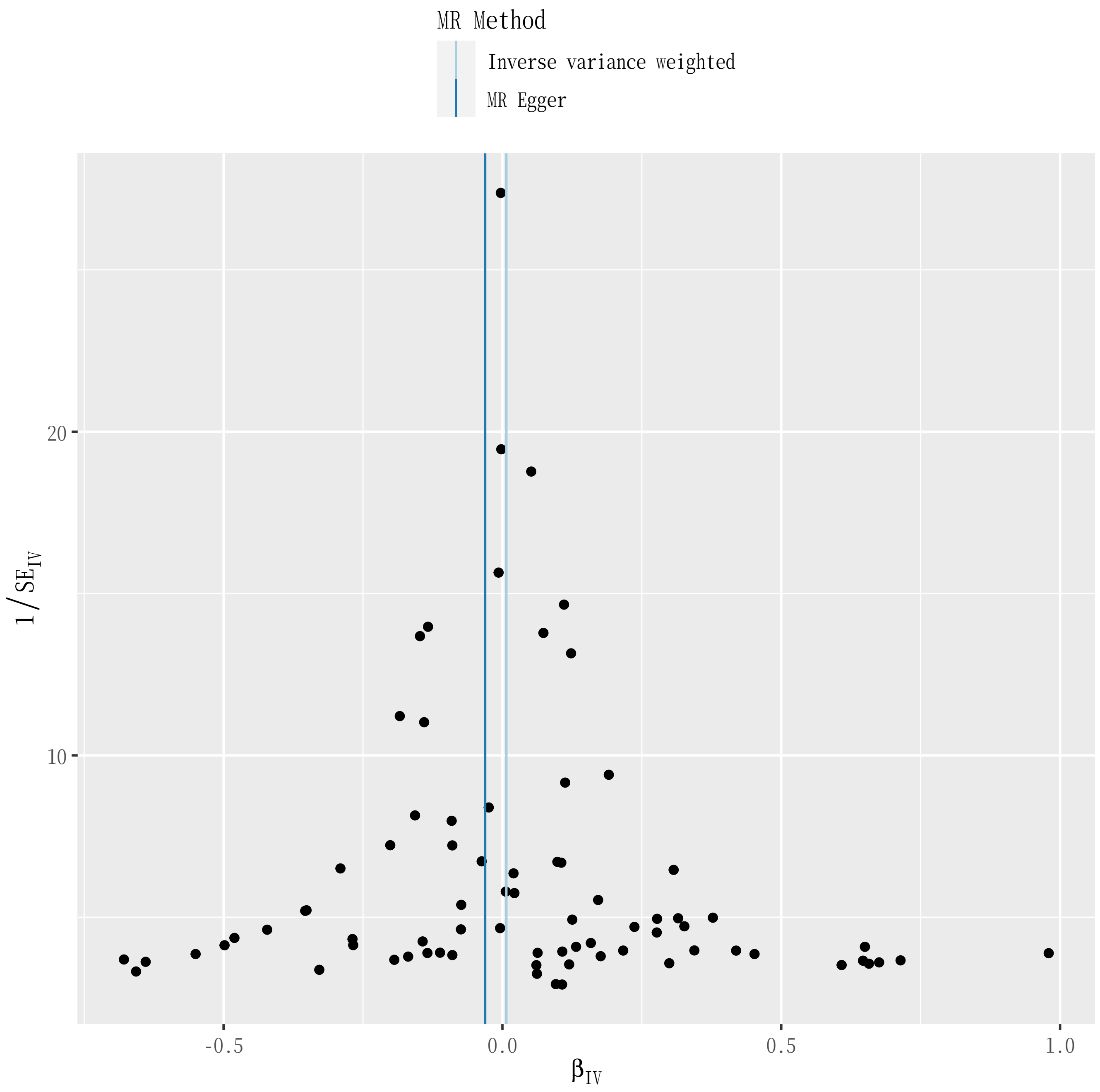
MR Method
Inverse variance weighted
MR Egger
1/SE_IV
20
10
-0.5
0.0
0.5
1.0
β_IV

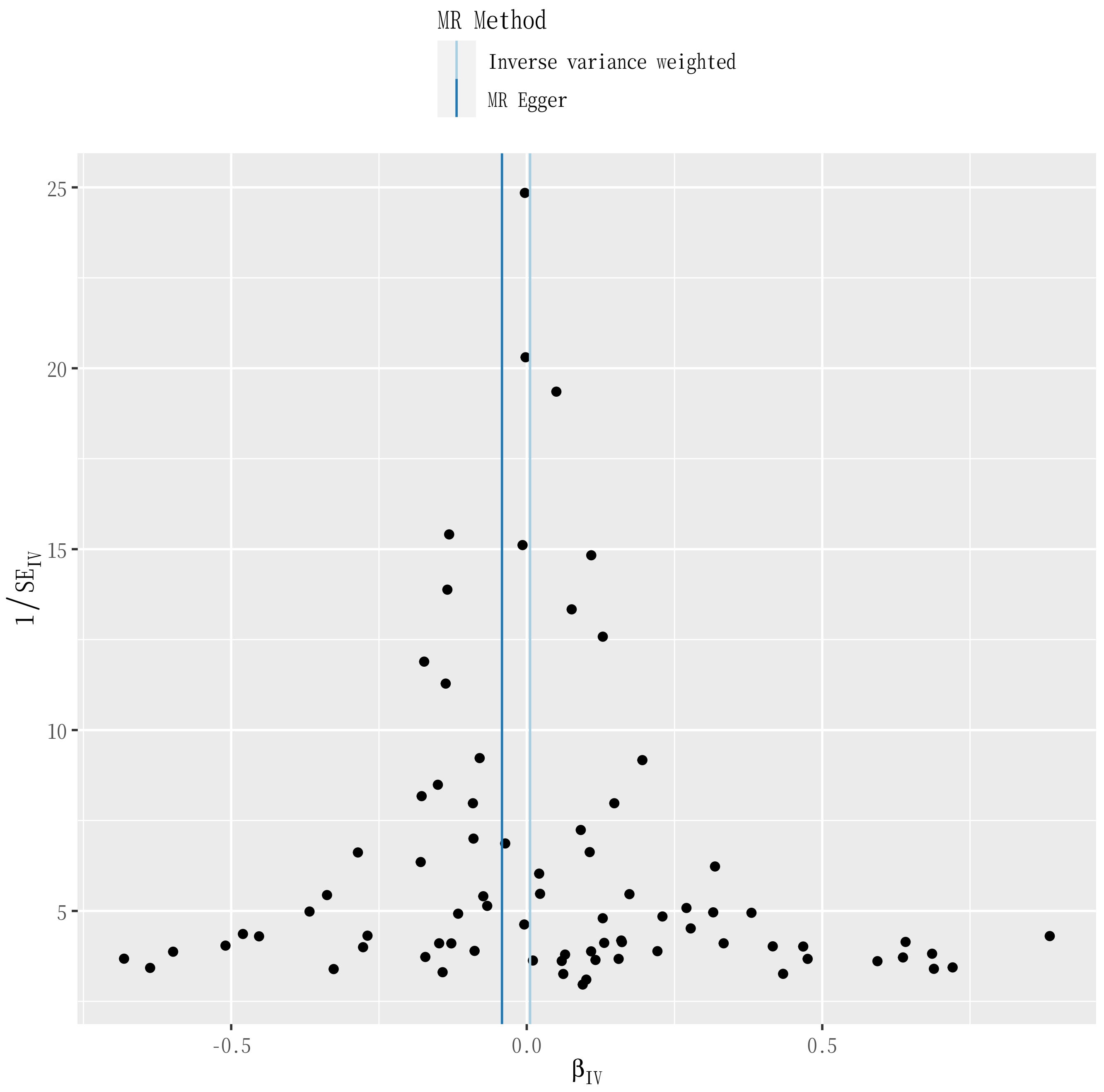

MR Method
Inverse variance weighted
MR Egger
25
20
15
10
5
$1/SE_{IV}$
-0.5
0.0
0.5
β_{IV}

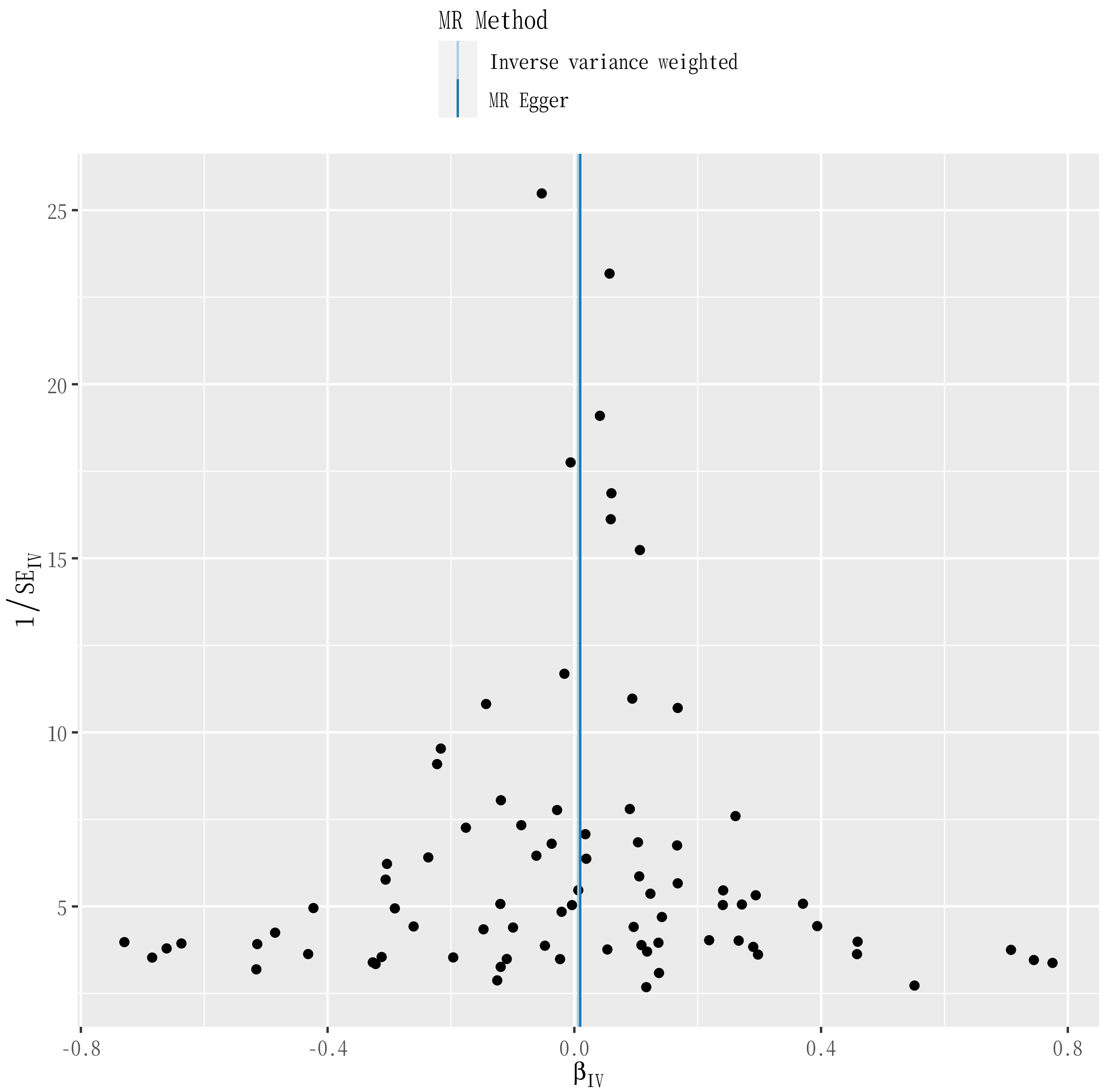

MR Method
Inverse variance weighted
MR Egger
25
20
15
10
5
$1/SE_{IV}$
-0.8
-0.4
0.0
0.4
0.8
β_{IV}

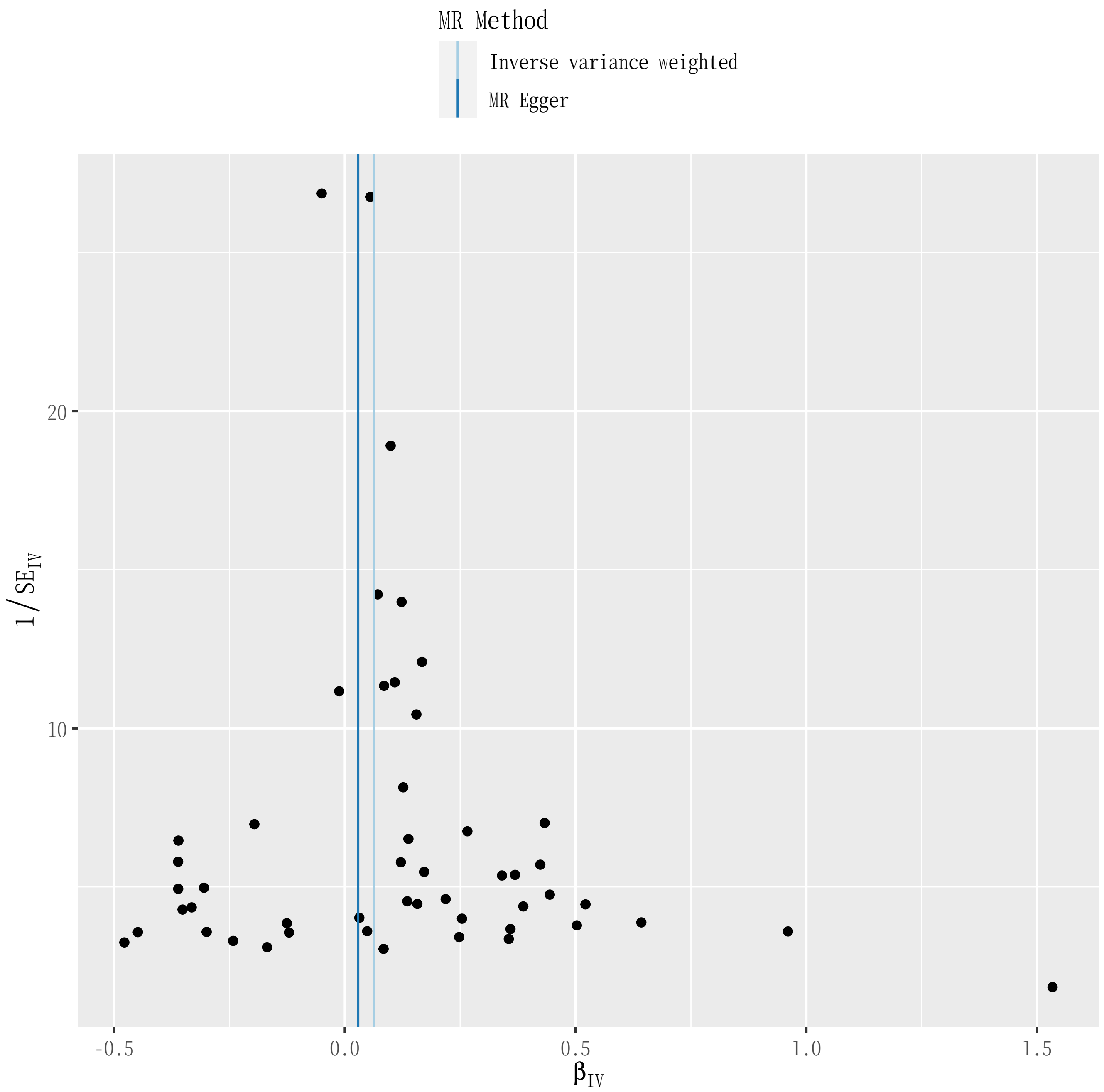

MR Method
Inverse variance weighted
MR Egger
20
10
1/SE_IV
-0.5
0.0
0.5
1.0
1.5
β_IV

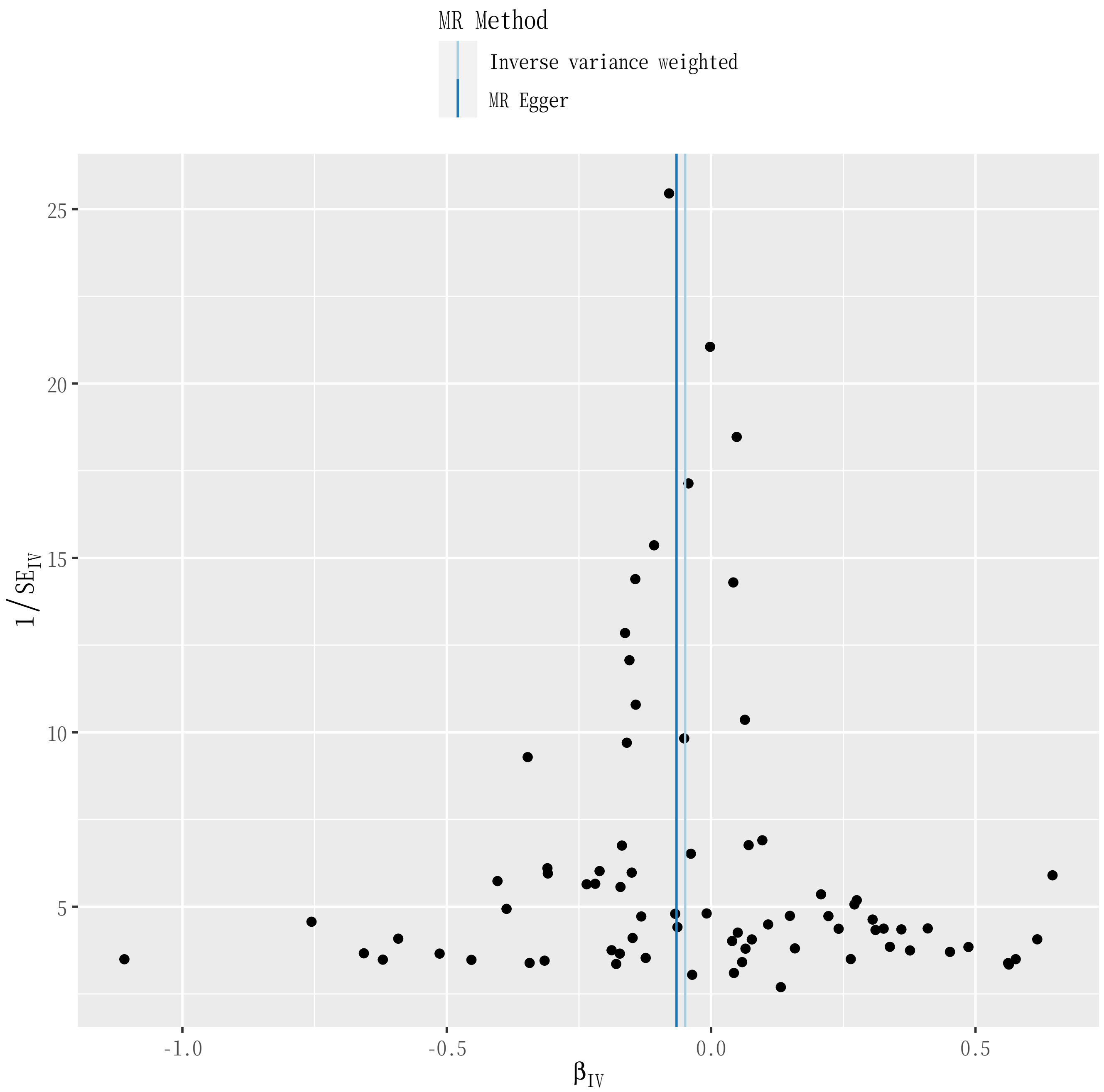
MR Method
Inverse variance weighted
MR Egger
25
20
15
10
5
1/SE_IV
-1.0
-0.5
0.0
0.5
β_IV

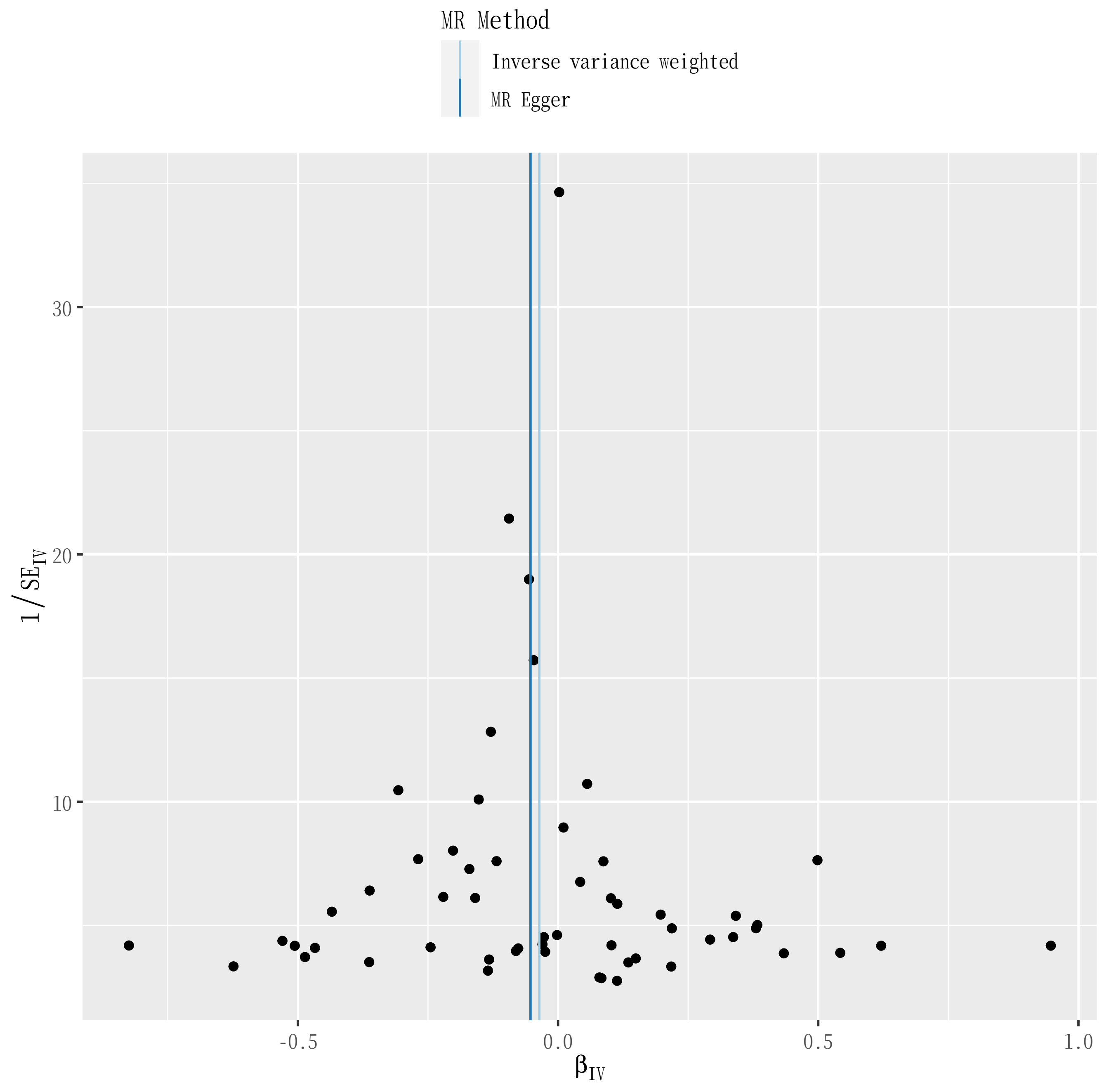

MR Method
Inverse variance weighted
MR Egger
30
20
10
$1/SE_{IV}$
-0.5
0.0
0.5
1.0
β_{IV}

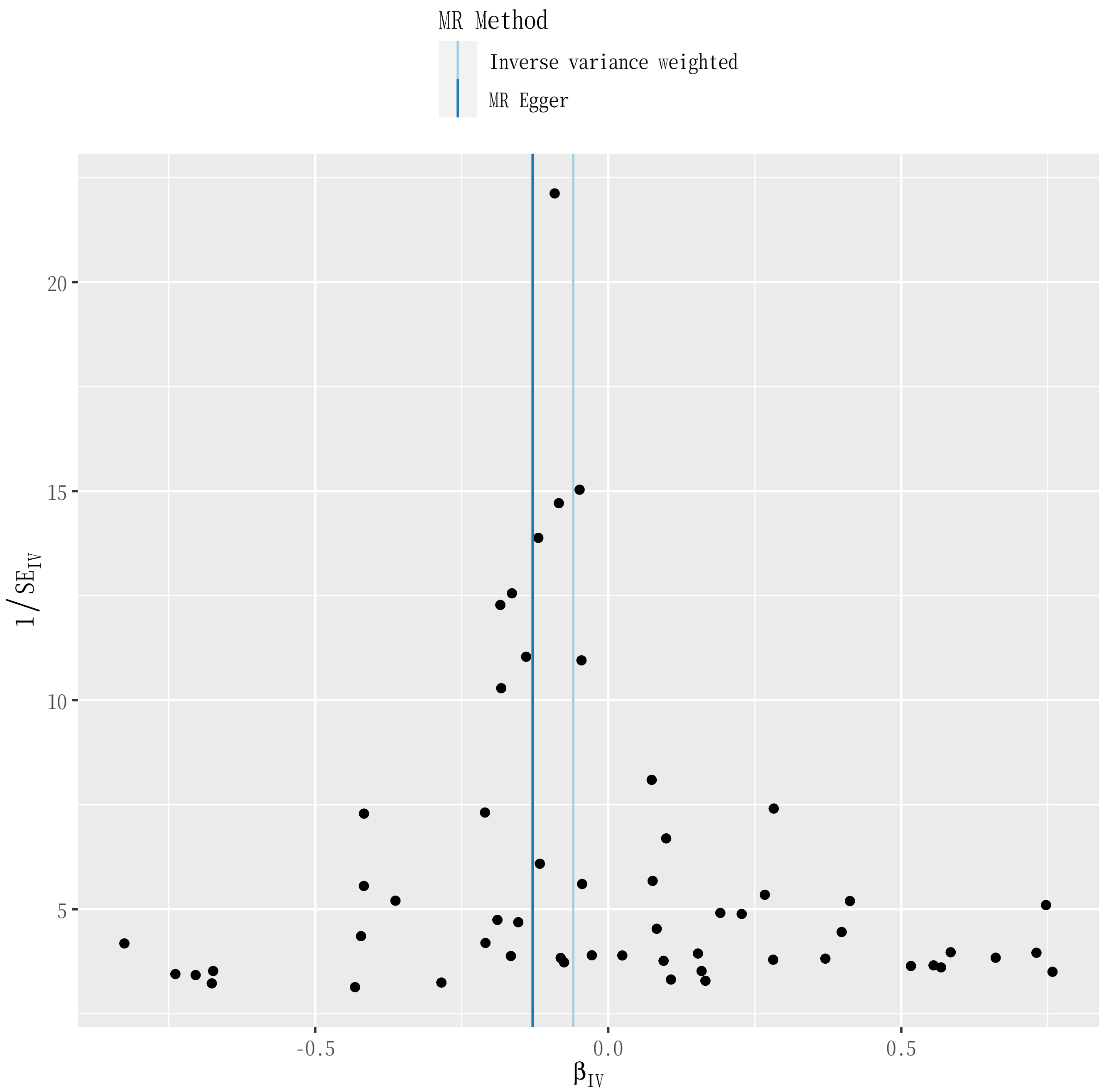

MR Method
Inverse variance weighted
MR Egger
20
15
10
5
$1/SE_{IV}$
-0.5
0.0
0.5
β_{IV}

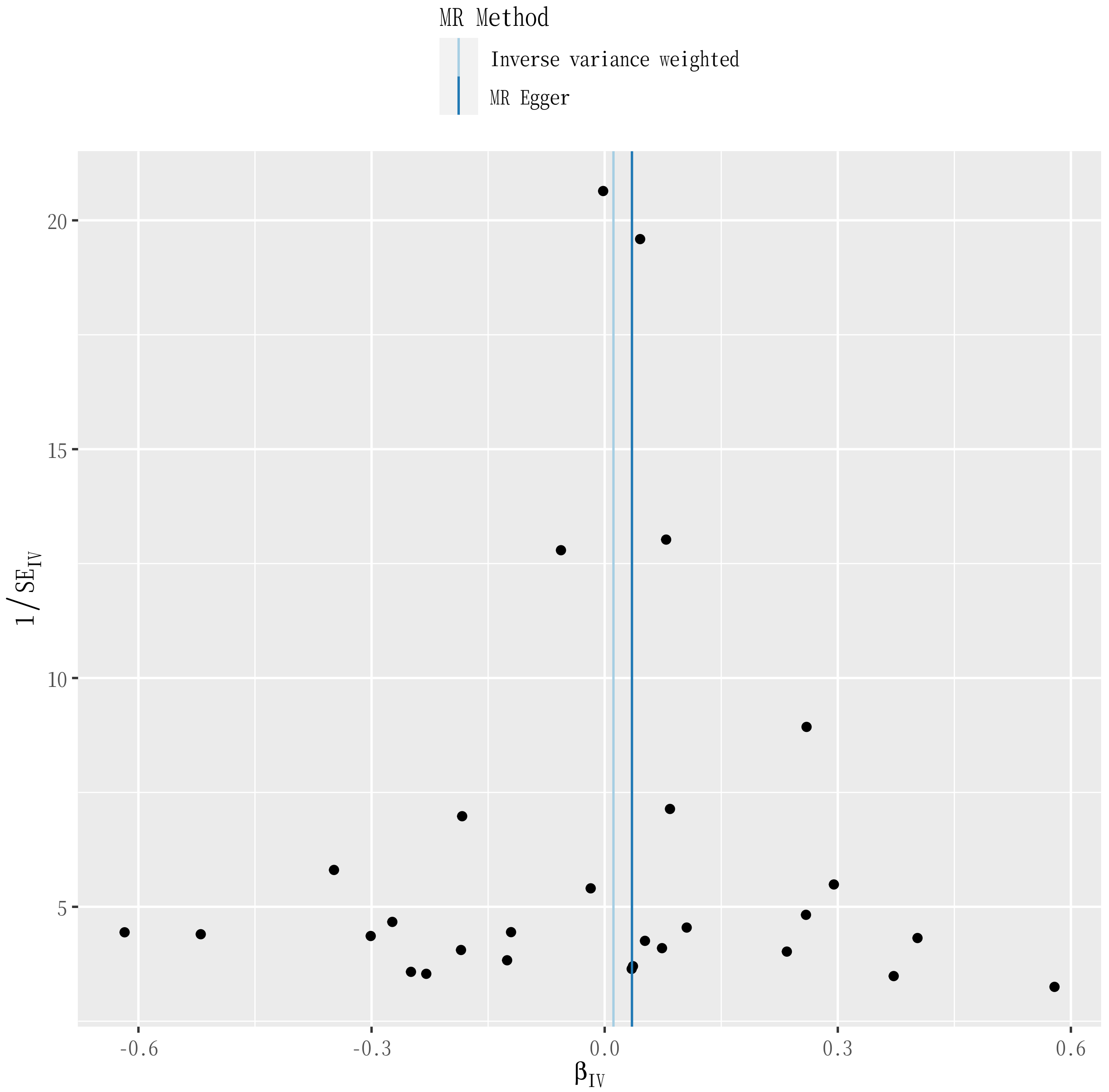

MR Method
Inverse variance weighted
MR Egger
20
15
10
5
$1/SE_{IV}$
-0.6
-0.3
0.0
0.3
0.6
β_{IV}

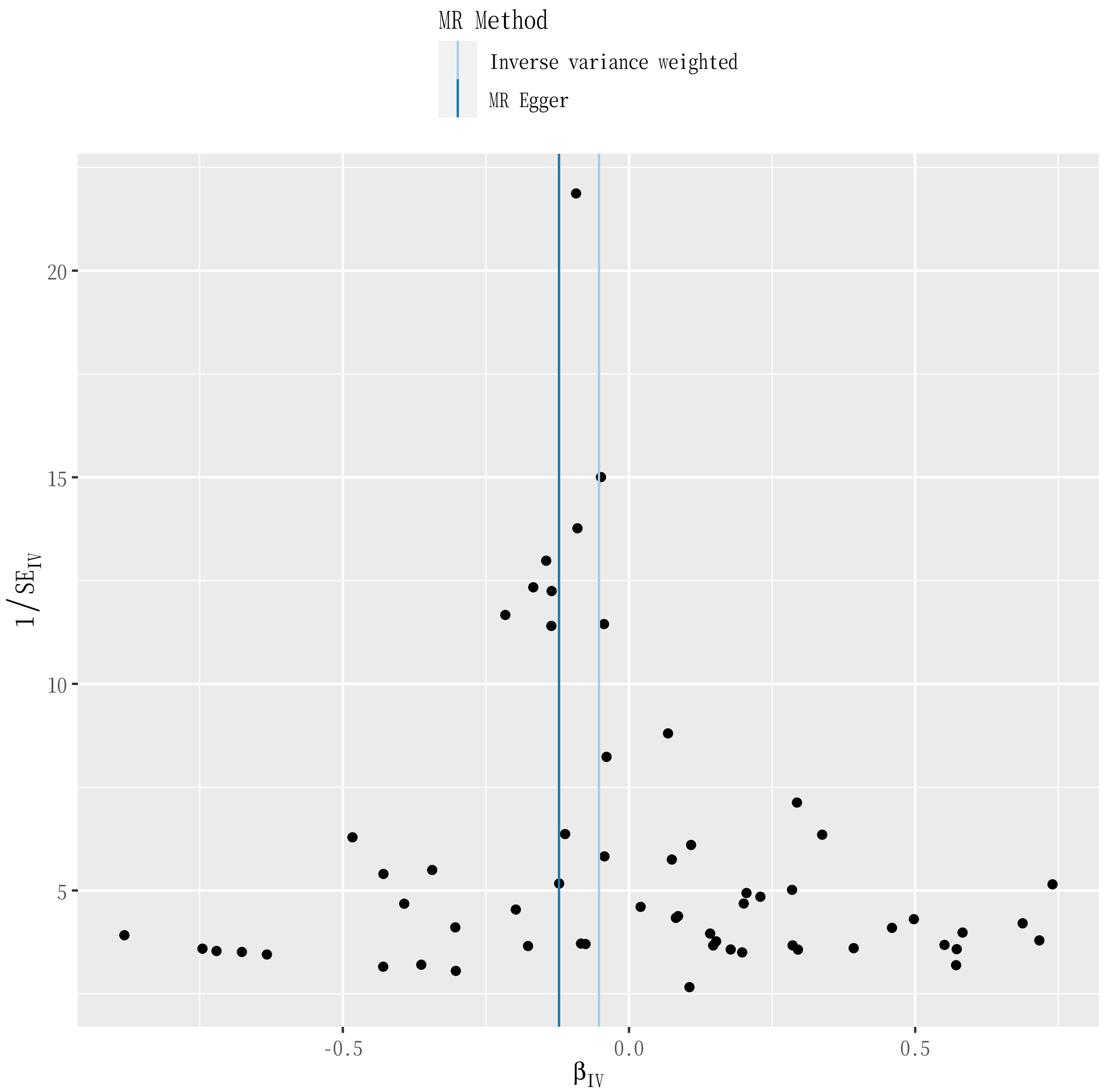
MR Method
Inverse variance weighted
MR Egger
20
15
10
5
1/SE$_{IV}$
-0.5
0.0
0.5
β$_{IV}$

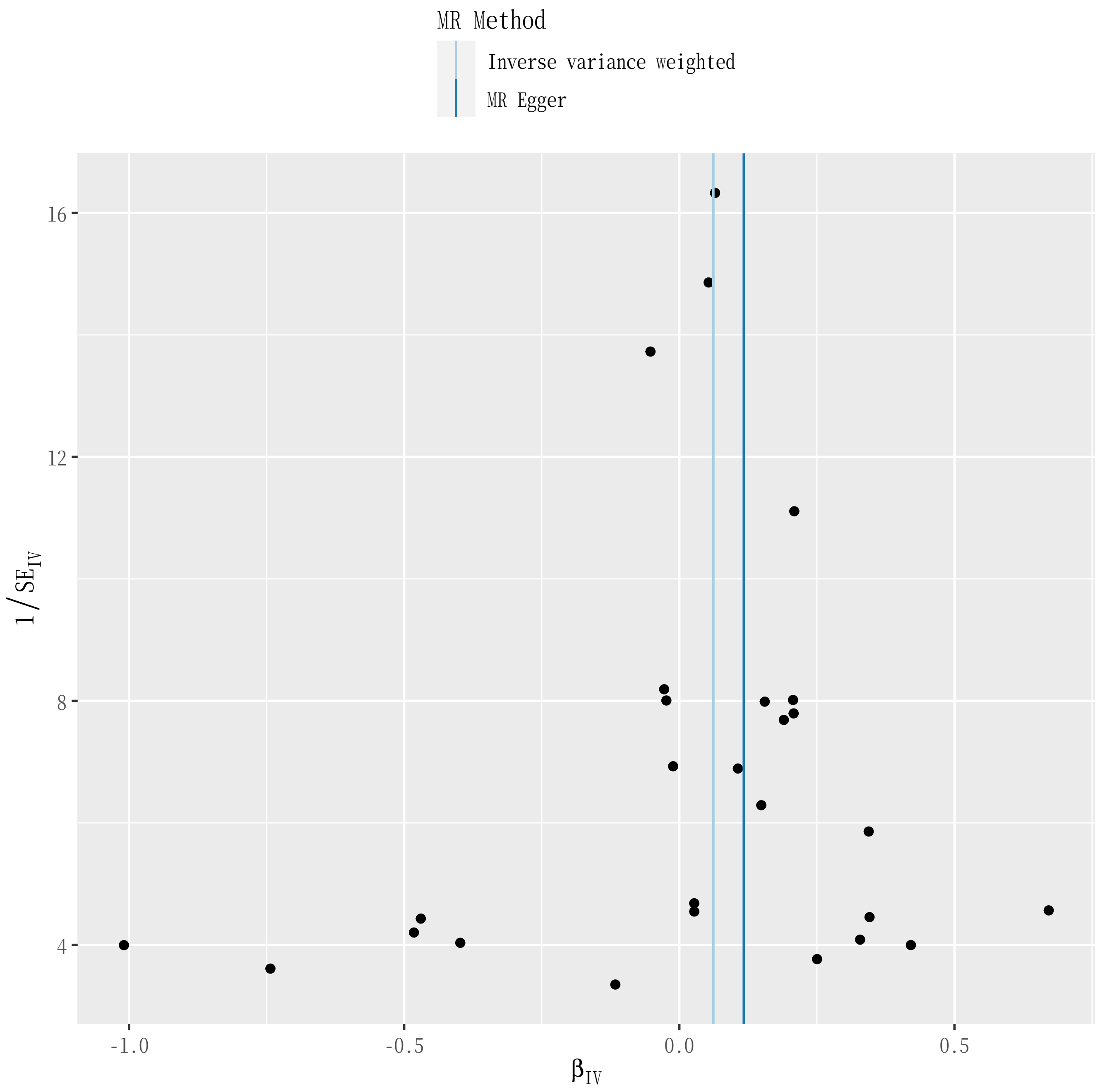
MR Method
Inverse variance weighted
MR Egger
16
12
8
4
1/SE_IV
-1.0
-0.5
0.0
0.5
β_IV

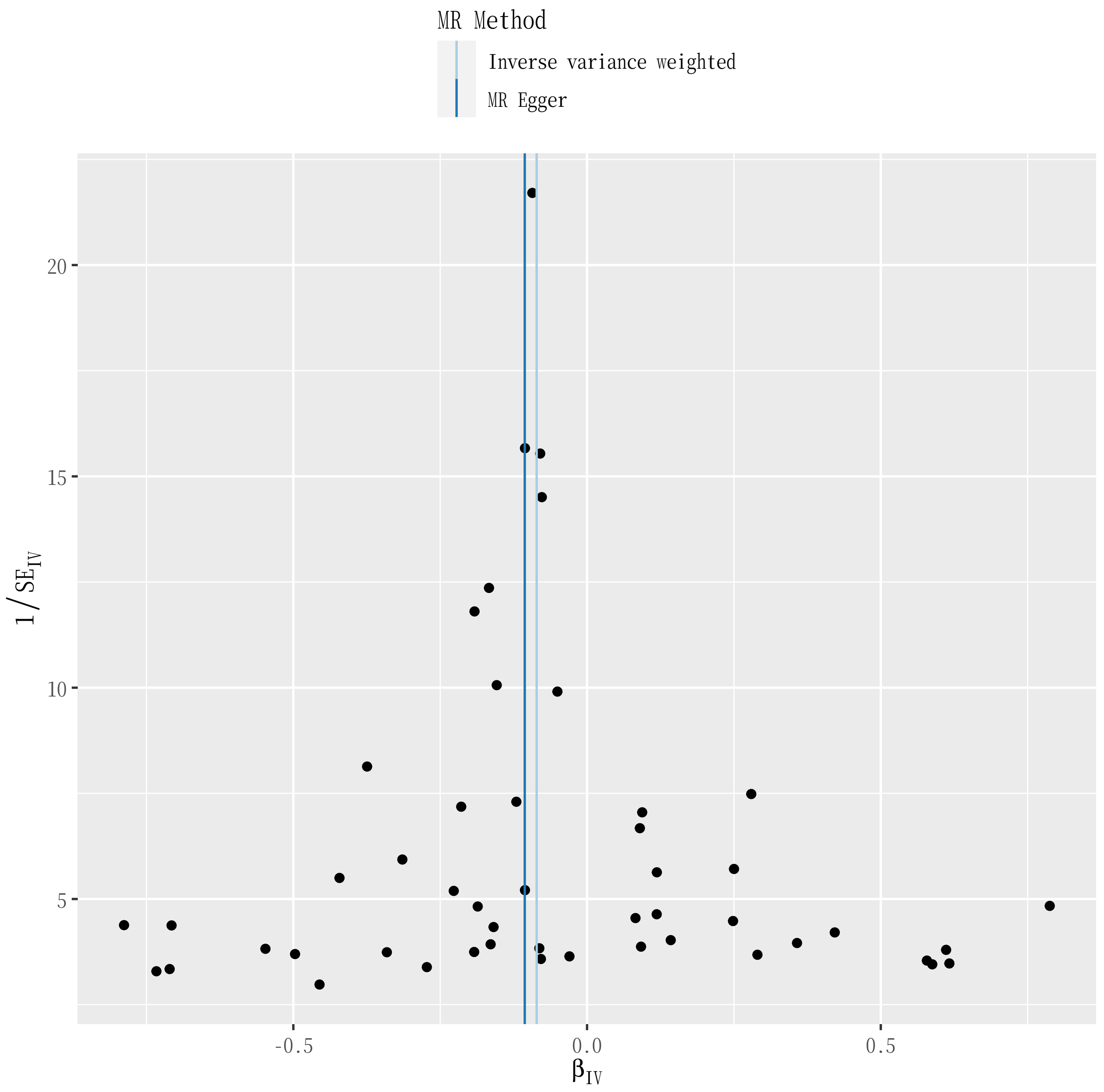
MR Method
Inverse variance weighted
MR Egger
20
15
10
5
-0.5
0.0
0.5
$1/SE_{IV}$
β_{IV}

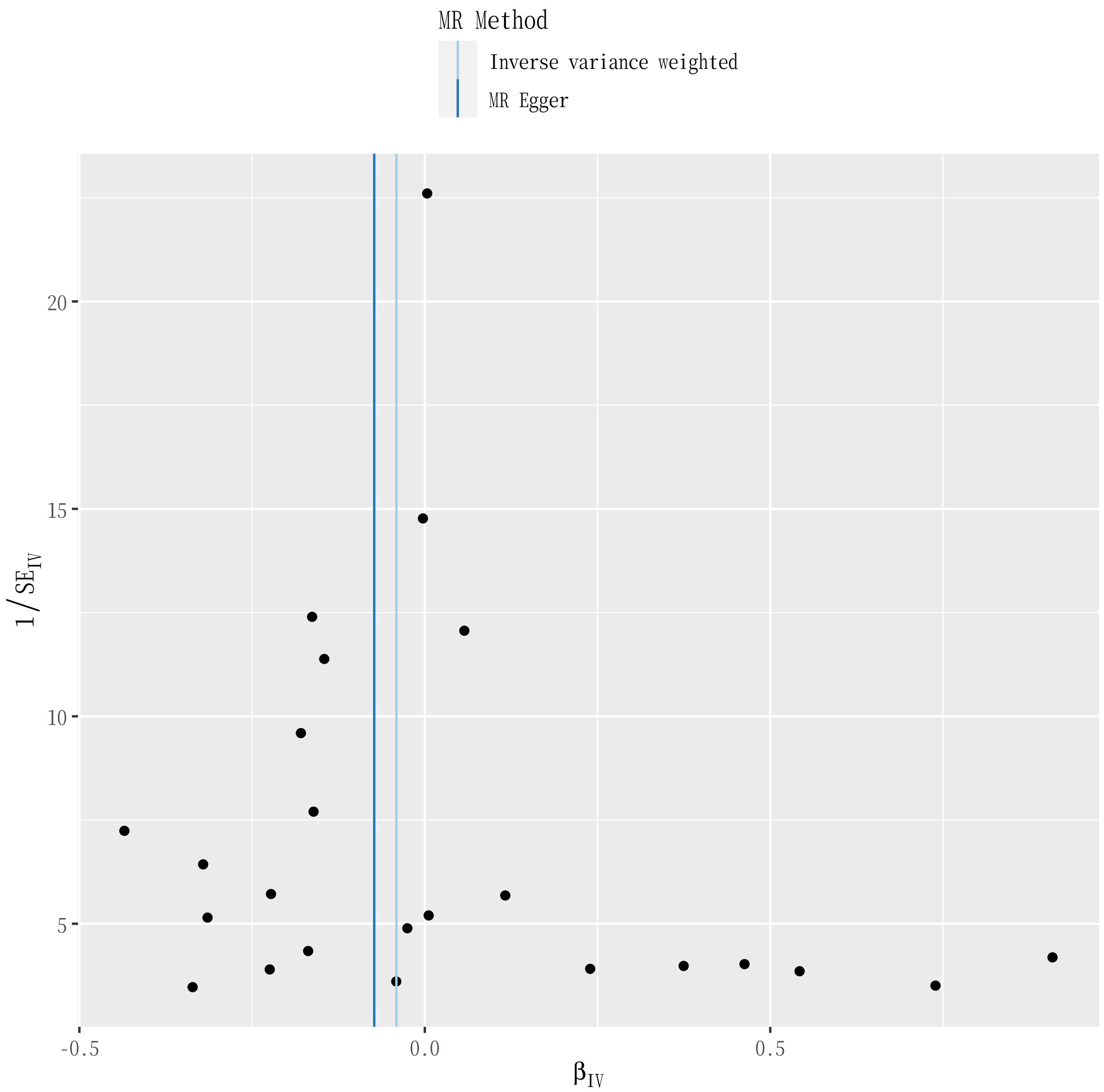
MR Method
Inverse variance weighted
MR Egger
20
15
10
5
1/SE_IV
-0.5
0.0
0.5
β_IV

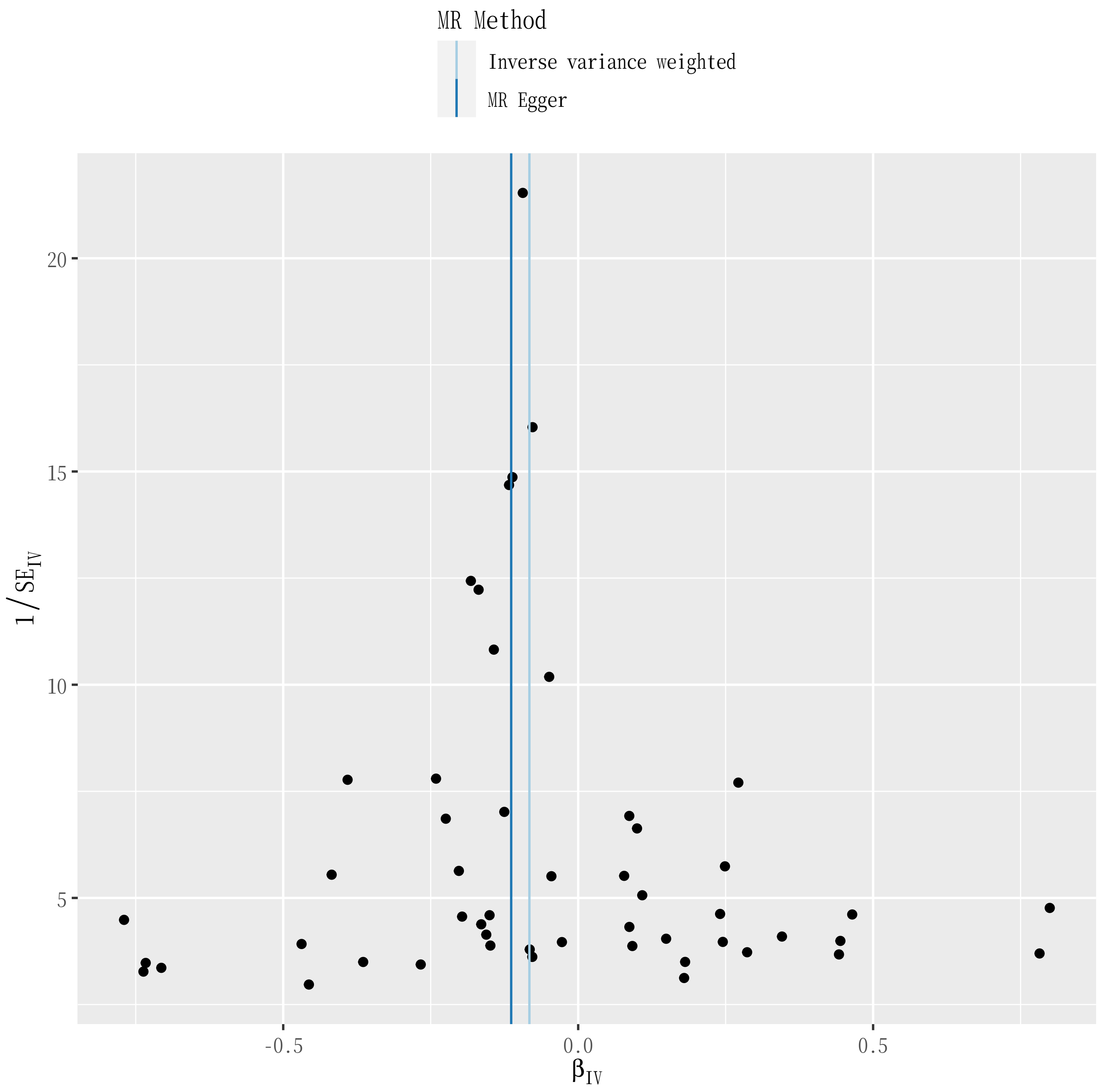

MR Method
Inverse variance weighted
MR Egger
20
15
10
5
1/SE_IV
-0.5
0.0
0.5
β_IV

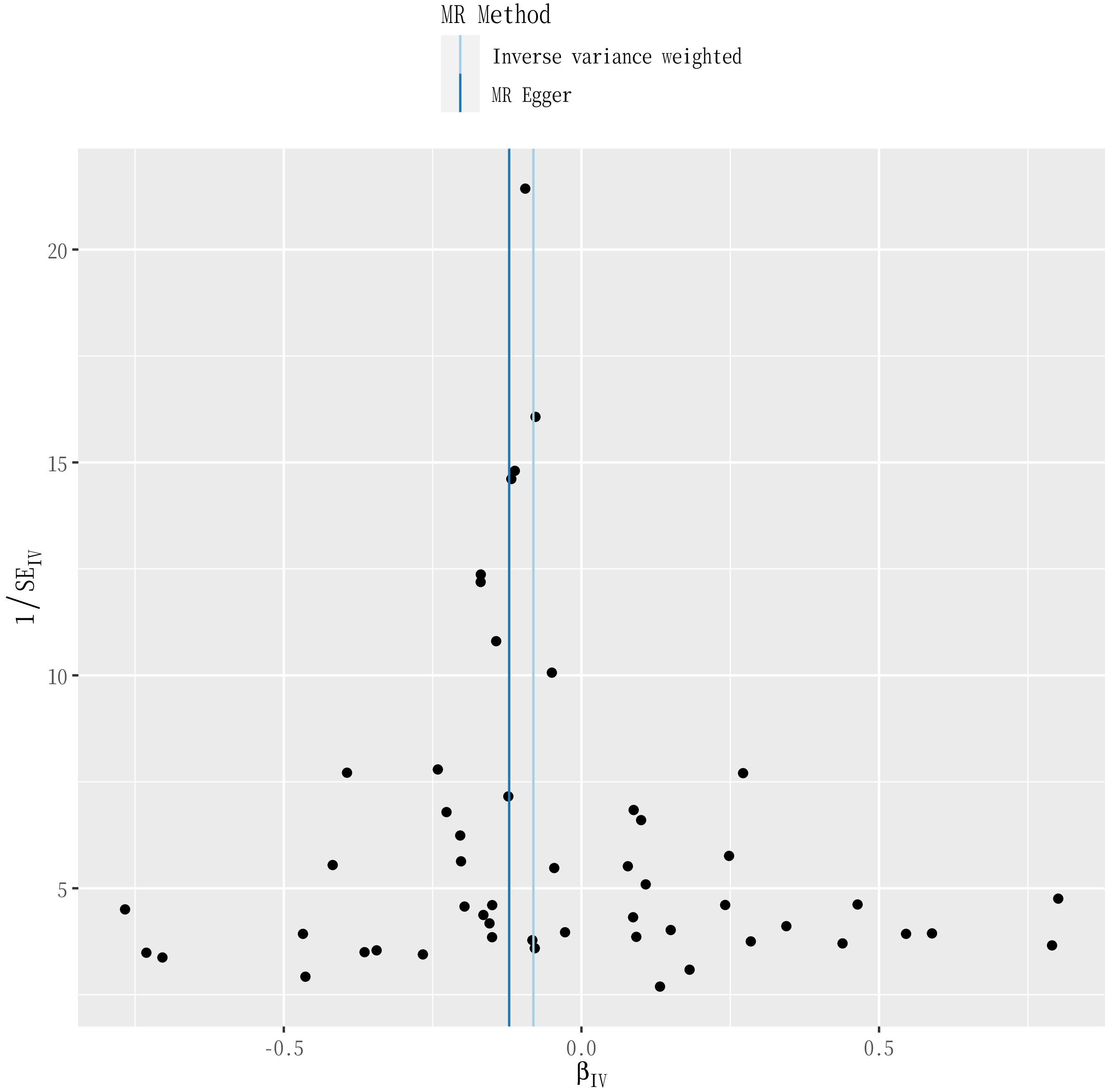
MR Method
Inverse variance weighted
MR Egger
20
15
10
5
$1/SE_{IV}$
-0.5
0.0
0.5
β_{IV}

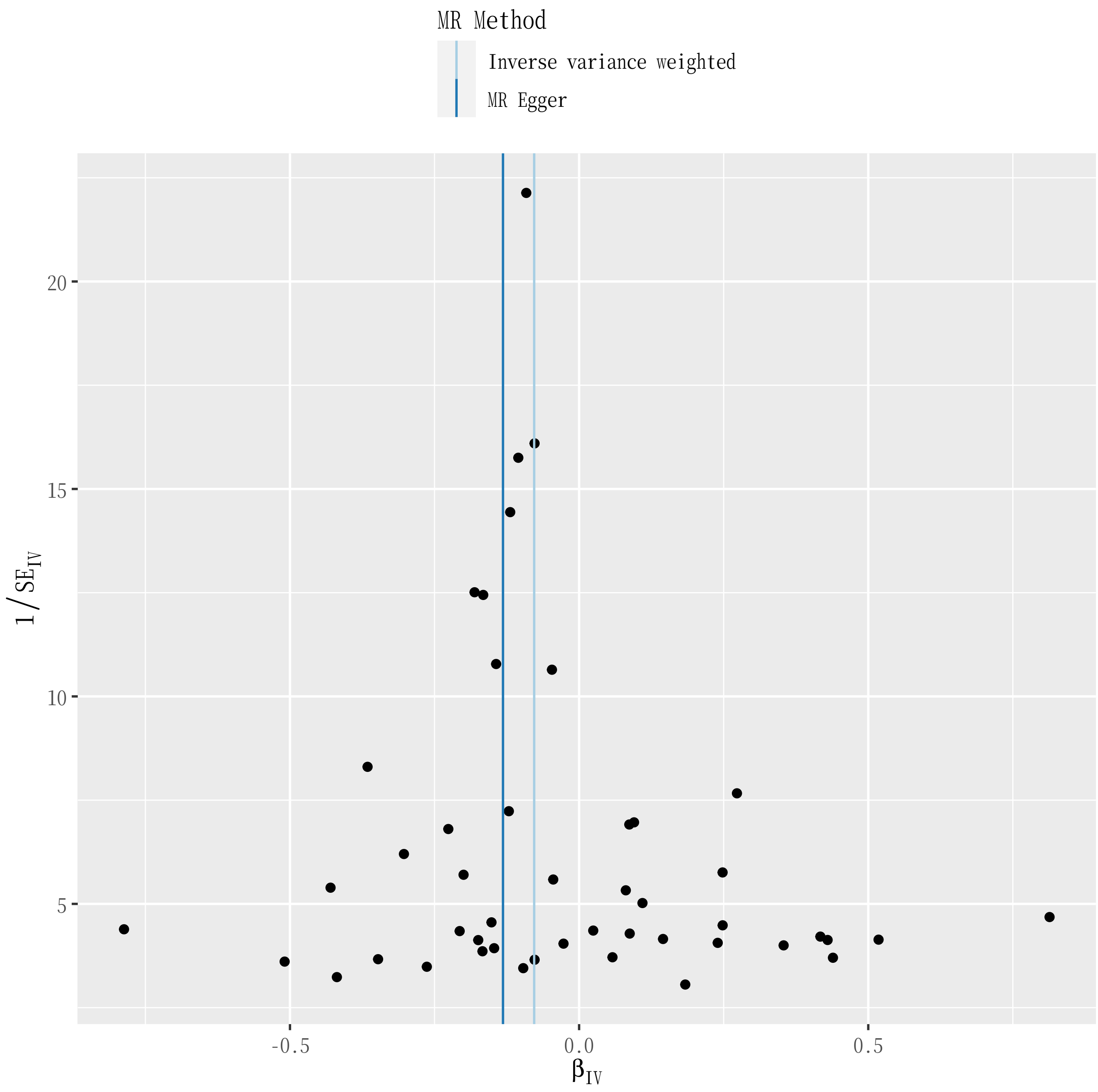

MR Method
Inverse variance weighted
MR Egger
20
15
10
5
-0.5
0.0
0.5
$1/SE_{IV}$
β_{IV}

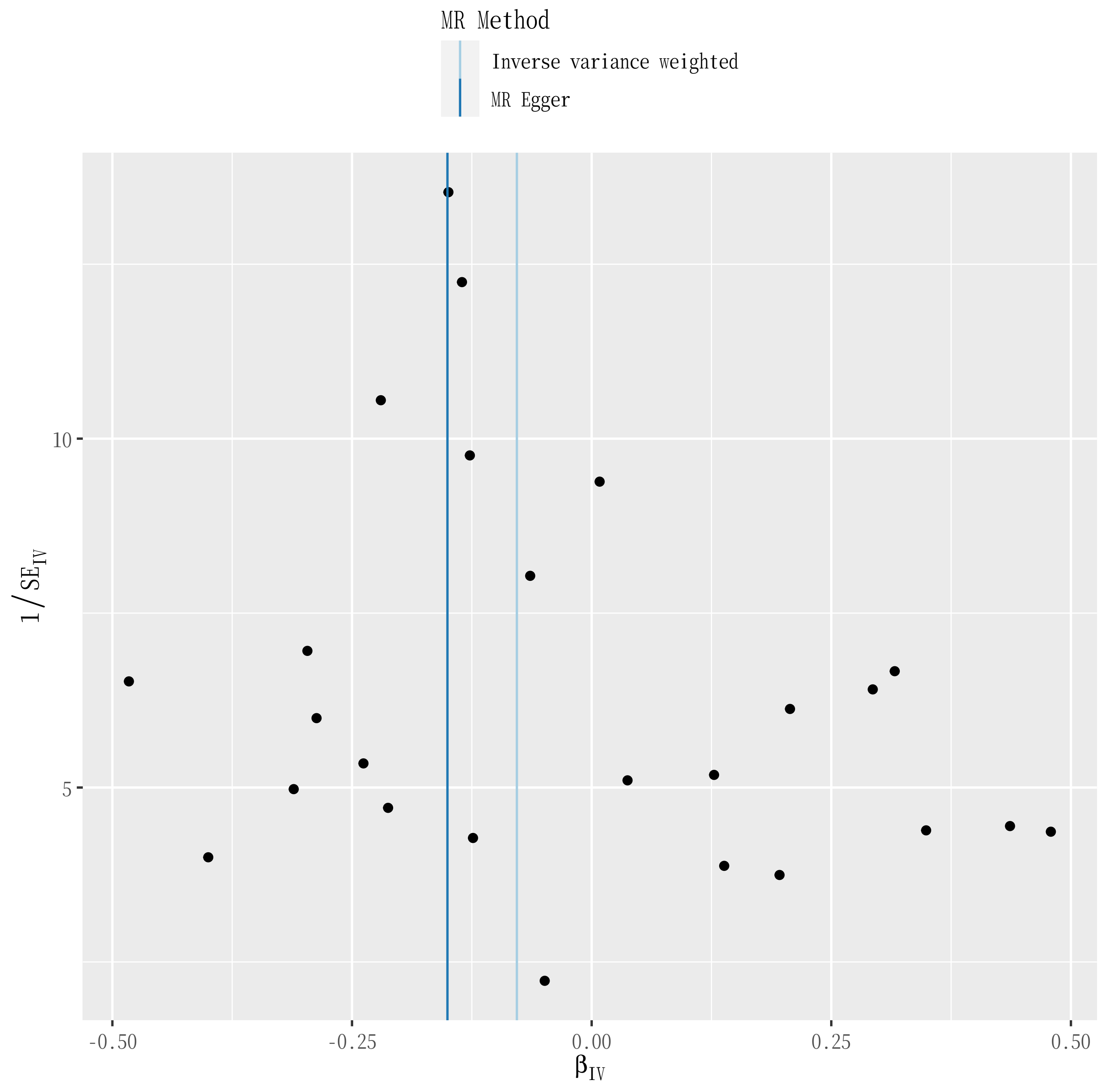

MR Method
Inverse variance weighted
MR Egger
10
5
-0.50
-0.25
0.00
0.25
0.50
$1/SE_{IV}$
β_{IV}

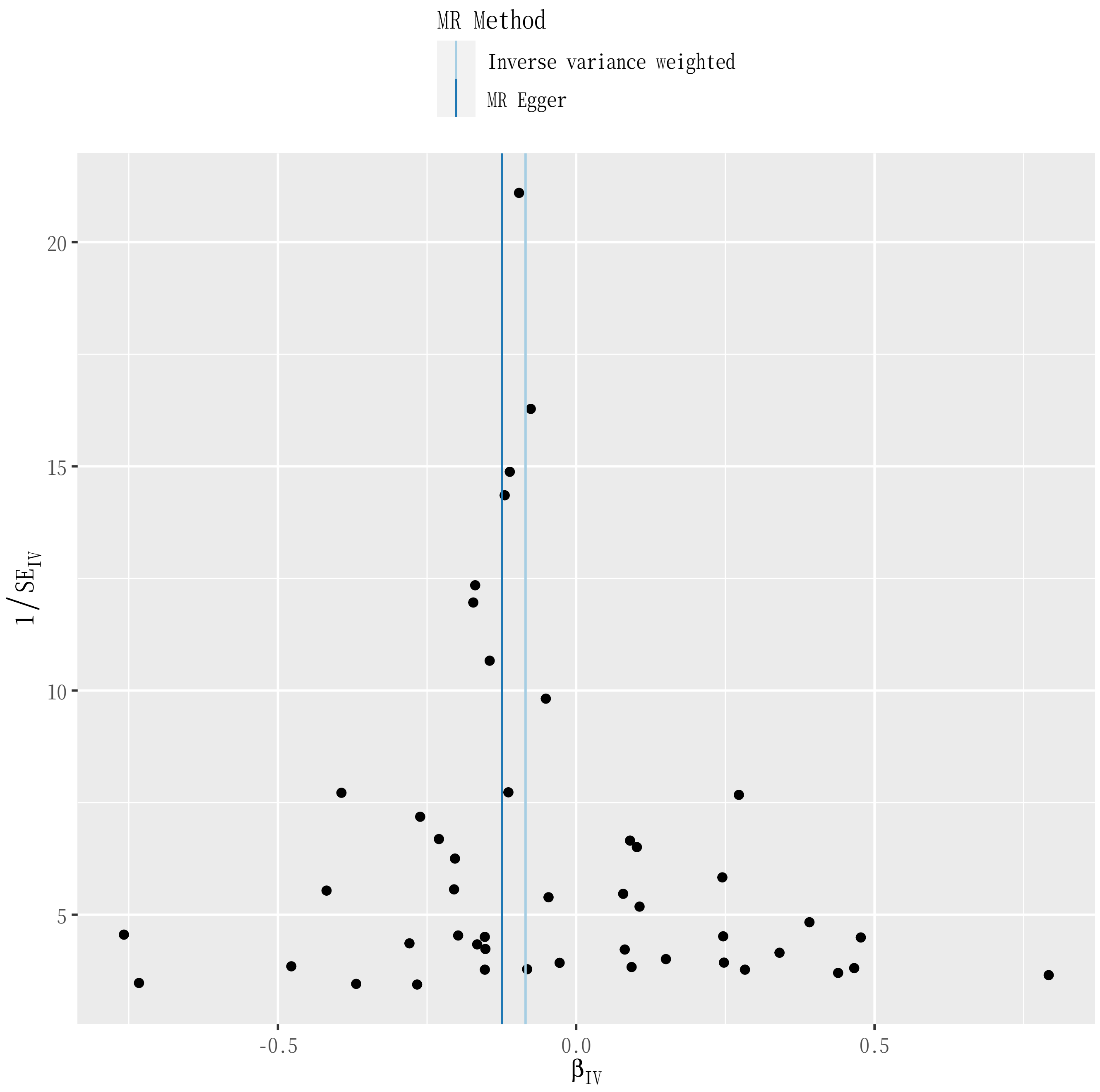

MR Method
Inverse variance weighted
MR Egger
20
15
10
5
-0.5
0.0
0.5
$1/SE_{IV}$
β_{IV}

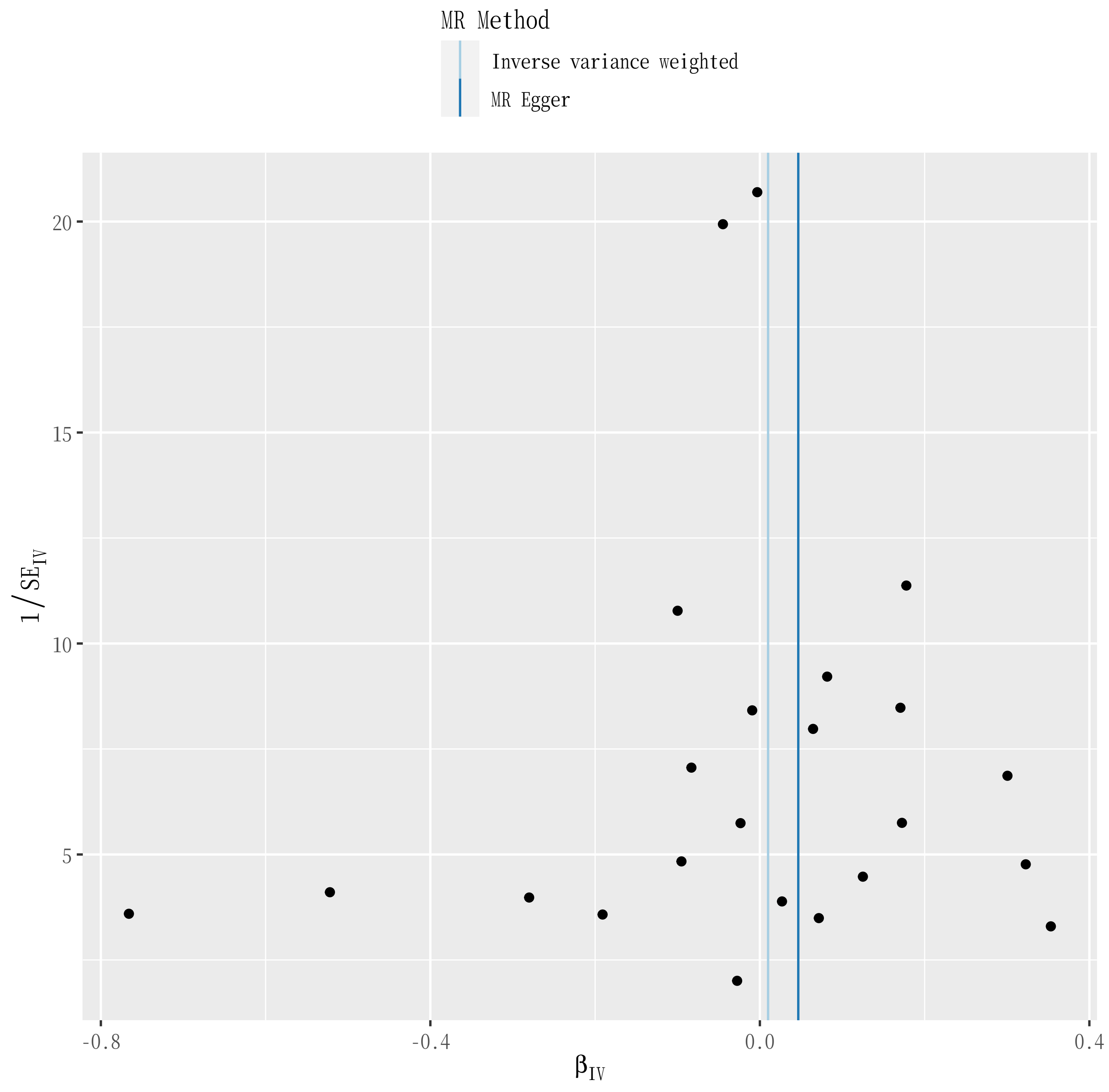
MR Method
Inverse variance weighted
MR Egger
20
15
10
5
-0.8
-0.4
0.0
0.4
$1/SE_{IV}$
β_{IV}
